# Tracking Climate Change through the Spatiotemporal Dynamics of the Teletherms, the Statistically Hottest and Coldest Days of the Year

**DOI:** 10.1371/journal.pone.0154184

**Published:** 2016-05-11

**Authors:** Peter Sheridan Dodds, Lewis Mitchell, Andrew J. Reagan, Christopher M. Danforth

**Affiliations:** 1 Department of Mathematics and Statistics, University of Vermont, Burlington, VT 05401, United States of America; 2 Vermont Center for Complex Systems, University of Vermont, Burlington, VT 05401, United States of America; 3 Computational Story Lab, University of Vermont, Burlington, VT 05401, United States of America; 4 Vermont Advanced Computing Core, University of Vermont, Burlington, VT 05401, United States of America; 5 School of Mathematical Sciences, North Terrace Campus, The University of Adelaide, SA 5005, Australia; Columbia University, UNITED STATES

## Abstract

Instabilities and long term shifts in seasons, whether induced by natural drivers or human activities, pose great disruptive threats to ecological, agricultural, and social systems. Here, we propose, measure, and explore two fundamental markers of location-sensitive seasonal variations: the Summer and Winter Teletherms—the on-average annual dates of the hottest and coldest days of the year. We analyse daily temperature extremes recorded at 1218 stations across the contiguous United States from 1853–2012, and observe large regional variation with the Summer Teletherm falling up to 90 days after the Summer Solstice, and 50 days for the Winter Teletherm after the Winter Solstice. We show that Teletherm temporal dynamics are substantive with clear and in some cases dramatic shifts reflective of system bifurcations. We also compare recorded daily temperature extremes with output from two regional climate models finding considerable though relatively unbiased error. Our work demonstrates that Teletherms are an intuitive, powerful, and statistically sound measure of local climate change, and that they pose detailed, stringent challenges for future theoretical and computational models.

## Introduction

Day length and temperature are two of the most important driving factors for life on Earth and for human culture. While evidently strongly coupled, their relationship is not a simple one in detail.

Due to the regularity of celestial and planetary motion and the relative ease with which sun position can be recorded, the Solstices and Equinoxes have been determined and commemorated by cultures around the world for thousands of years (e.g., Stonehenge), long before being scientifically understood. We thus know with great precision when the longest and shortest day of the year will be, but what about the on-average hottest and coldest days?

Temperature behaves stochastically with highs and lows on a specific date potentially differing greatly relative to surrounding dates and across years. Compounding temperature’s unevenness is that reliable measurement has only been realized in the last few hundred years. Indeed, widespread, systematic recording in the United States, which we study here, only began in the late 1800s. We are only now in a position to capitalize on sufficiently large data sets to give a reasonably solid answer to our question.

We propose to call the dates of on-average extreme temperature the *Teletherms*, using the Greek roots *tele* for distant and *therm* for heat. This construction is patterned after the Latin origin of Solstice with *sol* for sun and *stit* for stationary.

As we will find, the Teletherms and their temperatures are not fixed but vary in both space and time. In particular, we will show that across the United States, the dynamics of the Teletherms are locally coherent but overall highly variable, revealing intricate patterns including bifurcations in dates and both warming and cooling. For many regions, we will also demonstrate that the Teletherm is more appropriately acknowledged as occurring over a range of dates rather than a single one. We will therefore also speak both of each location’s single day Teletherm and its *Teletherm Period* which we define below in a pragmatic fashion.

Our conception of the Teletherm is related to but differs from existing meteorological quantities drawn from stations around the United States. The National Oceanic and Atmospheric Administration (NOAA) captures ‘climate normals’: 30 year averages at day, month, season, and year resolutions for a range of quantities including mean, maximum, and minimum temperatures; precipitation; and snowfall [1]. Climate normals are made available to and used broadly by the public. For example, monthly averages are of great use to people traveling to new areas. NOAA and the National Weather Service provide the Local Climate Analysis Tool (LCAT) at http://nws.weather.gov/lcat/home for people to explore historical and recent climate dynamics. As we explain below, we estimate the Teletherms’ aspects—date, temperature, extent, and period—from a daily maximum and minimum temperature data set, and as such our contribution can be seen as building a new lens for the United States’ rich meteorological data set. We accompany our paper with an interactive site at http://panometer.org/instruments/teletherms to enable those interested to examine climate dynamics through the Teletherms. If the notion of the Teletherm becomes standard, we would hope a version of this site might eventually be incorporated into the LCAT.

Despite the evident imperative of quantifying climate change, the task has proven to be both scientifically complex [2–5] and politically fraught and controversial [6, 7]. Tied as they are to the changing of the seasons [8–12], Teletherm dynamics matter for ecological stability, agriculture, the Earth’s water cycle, the livability of cities [13, 14], and cultural and religious observances.

By formalizing these annual turning points in temperature we hope to help advance our collective understanding of and ability to discern climate change. While we will make a number of general observations regarding Teletherm dynamics, the central objective of our present work is the introduction of a statistically sound quantification of these two fundamental aspects of the annual climate cycle, with the hope of both expanding and challenging future work on climate dynamics.

We structure our paper as follows. We first make some basic observations about the historical weather data set which we build our analysis around, along with a few details about our approach. We then present our main findings, describing and testing our approach to determining Teletherms and Teletherm Periods at specific locations, highlighting a few of the extreme locations such as the hottest Summer Teletherm and coldest Winter Teletherm. Moving out from individual stations, we then examine a range of results for the contiguous United States. We first show that the Winter and Summer Teletherms vary strongly according to geographic location. We then explore the temporal dynamics of regional Teletherms, and discuss their relationship to climate change. Finally, we compare empirical Teletherm dates with those produced by two Regional Climate Models (RCMs). To close, we put forward a few concluding remarks, contemplating future directions.

We provide a complete set of figures and code as part of the paper’s online appendices at http://compstorylab.org/share/papers/dodds2015c.

## Data

We consider daily records of maximum and minimum temperatures for 1218 stations distributed across the contiguous United States for the time period 1853–2012. We draw on the United States Historical Climatology Network (USCHN) data set (version 2.5 through 2012) [15]. Each station is identified with a U.S. Cooperative Observer Network station identification code which we will denote as Station ID.

**Fig 1:**
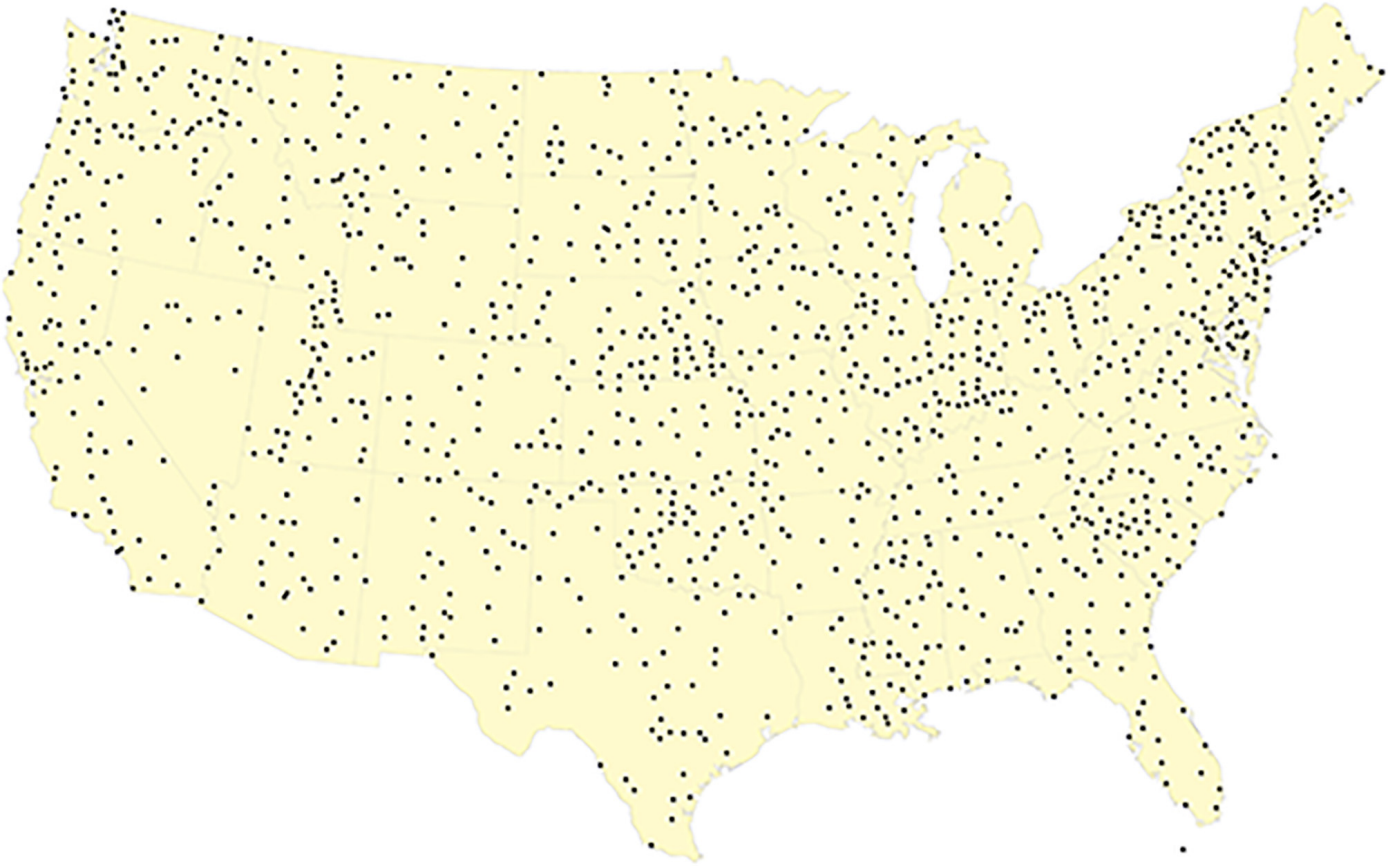
Locations of 1218 weather stations represented in the United States Historical Climatology Network (USCHN) data set (version 2.5 through 2012) [15]. The distribution indicates relatively uniform coverage of the 48 contiguous states.

The scatter plot in Fig 1, along with all maps that follow, demonstrate that the geographic coverage afforded by the stations is fairly uniform with some minor clustering around populous areas.

The temperature records in our data set are not, however, temporally uniform. Stations have different lifespans—the oldest starting in 1853 (Camden 3 W, South Carolina; Station ID: 381310) and the youngest in 1998 (Md Sci Ctr Baltimore, Maryland; Station ID: 185718). Some stations have gaps in their records, a complication which we will deal with as needed in our various analyses. For example, Yellowstone Park in Mammoth, Wyoming (Station ID: 489905) has records for 1894–1903 and 1941–2012, missing a period of 37 years. We will also ignore any datum for which a potential source of error is indicated.

In our analyses, we will use overall day number of the year for maximum temperature starting at January 1. For the minimum temperature, we wrap the calendar and consider days counting forwards from July 1 and running through to June 30 in the following year, thereby roughly centering the low point to better accommodate statistical treatment. To present our results, we presume a 365 day year meaning an adjustment of a day will be needed for a leap year.

Finally, to create a reference tying the Teletherms to the solar cycle, we standardize the Summer and Winter Solstices as falling on June 21 and December 21 (day numbers 172 and 355).

## Analysis and Results

### Teletherms of Individual stations

Our goal is to identify the Teletherms and Teletherm Periods and their respective dynamics in as straightforward a fashion as possible. Because of the stochasticity of temperature, our analysis necessarily involves several steps.

We first compute the mean maximum and minimum temperature for each day of the year at each station. We average over all error-free data points, acknowledging the variability of both length and completeness of each station’s temperature time series. In the following section on Teletherm maps, we will only include averages for stations for which we have data for at least 80% of the dates within a given window.

**Fig 2:**
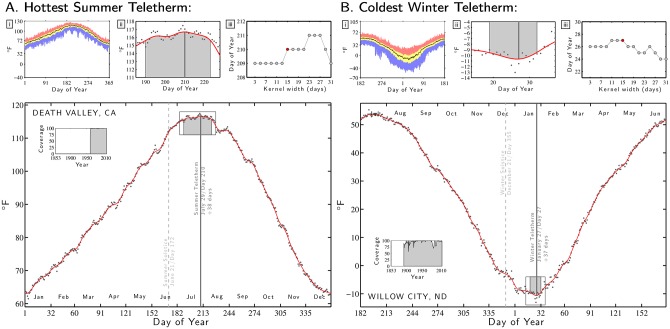
Plots establishing Teletherm date and Teletherm Periods for the examples of A: the hottest summer Teletherm (Death Valley, California) and B: the coldest winter Teletherm (Willow City, North Dakota). The main plots in **A** and **B** show the average daily maximum and minimum temperature (black dots) along with a smoothed curve formed using a Gaussian Kernel (solid red). For all minimum temperature analyses, we wrap the year from July 1 to June 30. The main plots’ insets show the fraction of error-free recording for each year. Subplot **i:** Representation of the spectrum of maximum/minimum temperatures per day of the year. The black curve indicates the median, the blue area indicates lowest to first quartile, yellow the inter-quartile range, and red the fourth quartile. Subplot **ii:** Expansion of the inset around the Teletherm in the main plot. The dark gray vertical line indicates the Teletherm and the lighter gray region the Teletherm Period which we define as the days for which the smoothed maximum/minimum temperature curve is within 2% of the Teletherm’s temperature, relative to the dynamic range of the smoothed curve over the entire 365 days. Subplot **iii:** Robustness diagnostic showing how the Teletherm date varies as a function of Kernel width. We use 15 days, marked in red. See the main text for further details. See Fig 3 for four more extreme Teletherm examples. We provide Teletherm plots for the maximum and minimum temperatures for all 1218 stations in the Supporting Information (S1 and S2 Files) and in the paper’s online appendices at http://compstorylab.org/share/papers/dodds2015c.

**Fig 3:**
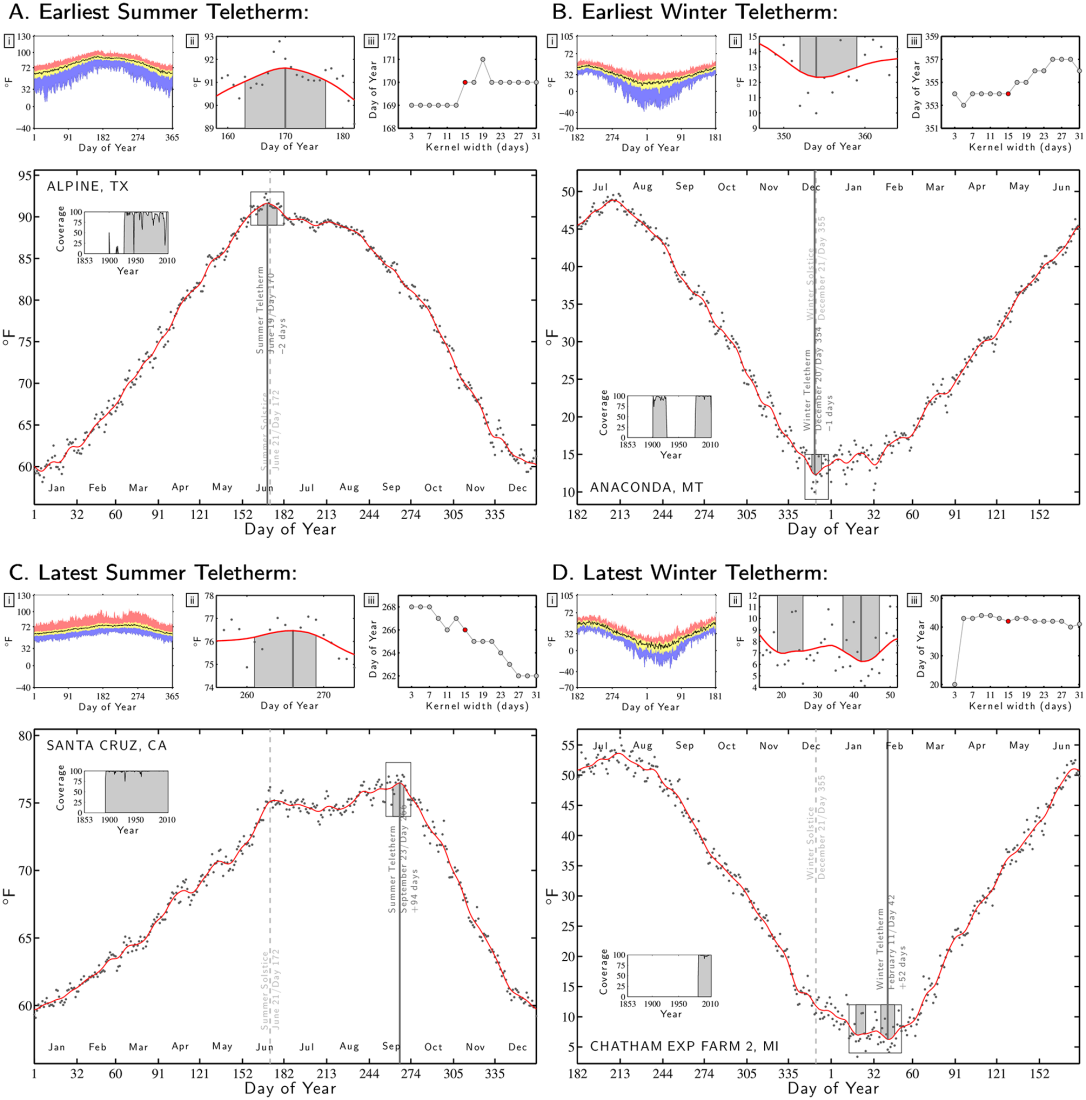
Teletherm plots for four extremes for the contiguous U.S.: the earliest Summer and Winter Teletherms and the latest Summer and Winter Teletherms. See the caption of Fig 2 for full details.

To enable us to illustrate and explain our treatment in full, we will use a selection of six extreme Teletherm locations in the contiguous U.S. In Figs 2A and 2B and 3A–3D, we present diagnostic plots for the following specific Teletherms:

- Fig 2A. Hottest Summer Teletherm: Death Valley, California, (Station ID: 042319).
- Fig 2B. Coldest Winter Teletherm: Willow City, North Dakota, (Station ID: 329445).
- Fig 3A. Earliest Summer Teletherm: Alpine, Texas (Station ID: 410174).
- Fig 3B. Earliest Winter Teletherm: Anaconda, Montana (Station ID: 240199).
- Fig 3C. Latest Summer Teletherm: Santa Cruz, California. (Station ID: 047916).
- Fig 3D. Latest Winter Teletherm: Chatham Exp Farm 2, Michigan (Station ID: 201486).

See S1 and S2 Files for Summer and Winter Teletherm diagnostic plots for all 1218 stations.

Each figure has the same format: a main plot showing average and smoothed maximum or minimum temperature (explained below), and three subplots across the top. We will address the main plots first.

Taking the example of the Death Valley station, in the main plot in Fig 2A, the black dots represent the average maximum temperature for each day of the year. We smooth these points by convolving the average maximum temperature time series with a Gaussian kernel of width 15 days, resulting in the red curve, and we elaborate on this choice below.

After smoothing the data, we assign the day of the most extreme value of the resultant curve as the Teletherm for that station. In all plots, we indicate Teletherms with a gray vertical line and for reference, we locate the Summer or Winter Solstice with a dashed gray vertical line.

The left inset in each main plot shows the fraction of days with error-free data as a function of year. In the case of Death Valley, we see the data set contains records from 1961 on, and that these are fairly complete. For Willow City in Fig 2B, the period of record begins before 1900 but shows an imperfect collection rate; we generally see that winter temperatures, especially minima, are (unsurprisingly) more error prone.

Turning to the Teletherms themselves, for Death Valley, we estimate that the Summer Teletherm occurs on July 29 (day 210), a considerable 38 days after the Summer Solstice (Fig 2A). The coldest Winter Teletherm occurs on January 27 in Willow City, North Dakota, a similarly lengthy 37 days after the Winter Solstice (Fig 2B). While we define Teletherms as the date, each one has of course an associated effective temperature arising from our analysis. For Death Valley, this temperature is 117°*F* (47°*C*) and for Willow City, we find −11°*F* (−24°*C*). Death Valley also has the maximum temperature recorded in the data set: 129°*F* (54°*C*).

The earliest Teletherms occur in Alpine, Texas for the summer (Fig 3A) and Anaconda, Montana for the winter (Fig 3B). These Teletherms precede the adjacent Solstice by two days and one day respectively following a long linear change in temperature, and both display an initially slow return afterwards.

The Teletherms occurring latest in the year have different stories. For the summer, Santa Cruz’s Teletherm is experienced extremely late on September 23—essentially the Autumnal Equinox—around three months (94 days) after the Summer Solstice. As Fig 3C shows, the average maximum temperature for Santa Cruz rises to a false peak (a localized Teletherm) at the Summer Solstice, drops slightly and then climbs again to the true Teletherm. We find similar behavior for stations along the west coast but not to any extent inland, a feature we examine further in the following section.

We estimate that the latest winter Teletherm takes place on February 11—a remarkable 52 days after the Winter Solstice and 9 days after Groundhog Day—at the Chatham Exp Farm 2 station in Michigan’s Upper Peninsula (Fig 3D).

We note that many of the smoothed average maximum and minimum temperature curves we observe exhibit a small periodic behavior as they climb and fall. Not being a focus of our present work, we suggest a more detailed analysis may uncover the source, if any, of these apparent pulsings in the time series.

Continuing with our explanation of our analysis, we move to the three diagnostic subplots marked **i**, **ii**, and **iii** in each of Figs 2A and 2B and 3A–3D. The first subplot **i** summarizes the distribution of maximum or minimum temperature for each station. The black curve gives the median for each day of the year, the yellow region represents the inter-quartile range, and the blue and red regions show the rest of the range. For example, the top of the red region for a Summer Teletherm figure indicates the hottest maximum temperatures, the bottom of the blue the lowest maximum temperatures. The stochasticity of the extreme temperatures measured at the levels of day is readily apparent in these subplots.

The second subplot **ii** is an expanded and rescaled match of the inset in the main plot around the Teletherm. As for the main plot, the black dots show the average maximum or minimum temperature for each day of the year, and the red curve the smoothed version. The gray shaded region shows the full Teletherm Period for a station which we describe below.

The third subplot **iii** shows how the Teletherm varies as a function of the width of Gaussian kernel, providing a measure of robustness. To smooth the data, we used the Matlab command gausswin with Kernel width *W* and standard deviation *σ* = (*W*−1)/4. For the examples in Figs 2A and 2B and 3A–2D, we see that the estimated date of the Teletherm varies relatively little—typically 2 to 4 days—for Kernel widths ranging from 7 to 31.

Our choice of a Gaussian kernel with a width of 15 is a defensible, reasonable, and practical one, well within what is a range of widths producing similar outputs and interpretable as spanning a week to the side of each date. We observe that very narrow kernels may however give quite different results as for the station Chatham Exp Farm 2 in Fig 3D. Such jumps may occur when two or more localized Teletherms are present which we address in the next section.

### Teletherm Periods for Individual stations

In looking more closely at the behavior of average maximum and minimum temperatures, we are obliged to augment our definition of Teletherms beyond single days of the year. Being able to assign one date to a location makes for a simple story but we must acknowledge three aspects: (1) We are working with a statistically speaking small number of samples for each station; (2) The choices we have made in our statistical analysis mean that the specific Teletherm date is subject to minor error; and (3) Fundamentally, some locations undergo on-average maximum or minimum temperatures that hold over a range of dates.

We define the Teletherm Period for a location to be the range of dates, possibly non-contiguous, for which the smoothed maximum/minimum temperature curve lies within 2% of the Teletherm’s temperature as measured with respect to the dynamic range of the smoothed curve. We chose 2% as a cutoff, asserting that the human-experienced temperature would be roughly similar to that of the Teletherm. An alternate approach would be to use an absolute difference (e.g., within 1°*F*); the results will not differ substantially.

Returning to Figs 2A and 2B and 3A–3D, we now identify the gray shaded region in the inset around the Teletherm in the main plot (reproduced in the subplot **ii**) as the Teletherm Period. Across all stations, we see substantial variation in duration and continuity of Teletherm Periods. For Death Valley (Fig 2A) the Teletherm Period lasts an unpleasant 34 days with a smoothed maximum temperature of at least 115.6°*F* (46.4°*C*) (July 9th to August 11th, day numbers 190 to 223). The Winter Teletherm Period for Anaconda, Montana is comparatively brief running 8 days with smoothed minimum temperatures below 13.0°*F* (−10.6°*C*) (December 18th to 25th, numbers 352 to 359).

The station Chatham Exp Farm 2 in Michigan (Fig 2D) shows how our definition may lead to two or more Teletherm Periods surrounding minor cooling or warming periods. In looking across all stations, we see that Winter Teletherms for stations in the Northeast may present a statistically sound early spring thaw, and Burlington WSO AP, Vermont (Station ID: 431081) is another clear example (see Supporting Information S1 and S2 Files and http://compstorylab.org/share/papers/dodds2015c). Evidently, if we used a threshold of, say, 5%, some separated Teletherm Periods would coalesce, but we believe the threshold should be suitably strict.

**Fig 4:**
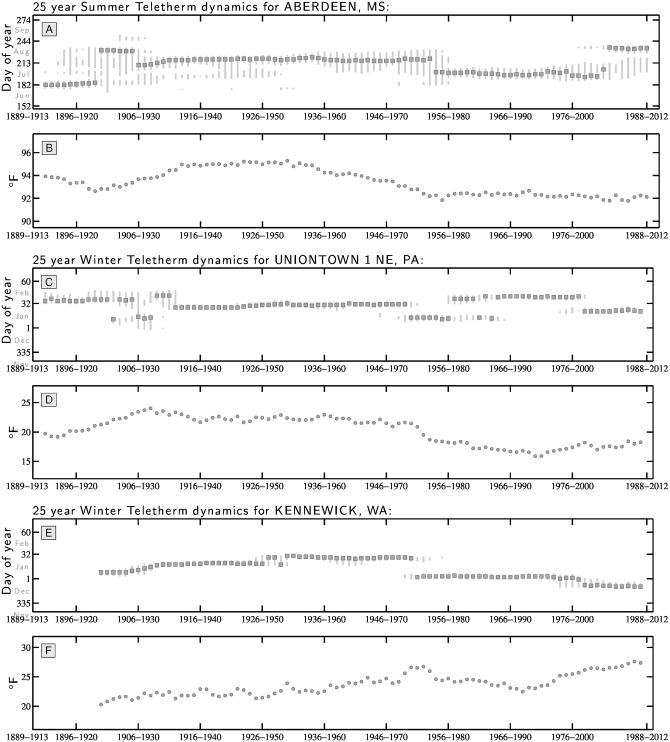
Dynamics of 25 year Teletherm dates, periods, extents, and temperatures for three example locations displaying abrupt switchings in time and gradual increases and decreases of temperature. We provide sets of these plots for 50, 25, and 10 year Teletherms for all 1218 stations in the Supporting Information (S3–S5 Files). The same plots are also available at http://compstorylab.org/share/papers/dodds2015c/places.html.

For the whole data set, we observe considerable though locally coherent variation in dynamics with temperatures rising and falling, and Teletherm periods expanding, dividing, and coalescing, and Teletherm dates switching. In Fig 4, we show example behavior for 25 year Teletherms for Aberdeen, MS (Summer), Uniontown, PA (Winter), and Kennewick, WA (Winter). For Kennewick, we see the 25 year Winter Teletherm moves sharply to an early date around the middle of the 20th century. See Supporting Information S3–S5 Files for the complete set of stations for 50, 25, and 10 year Teletherms.

### Teletherm maps

**Fig 5:**
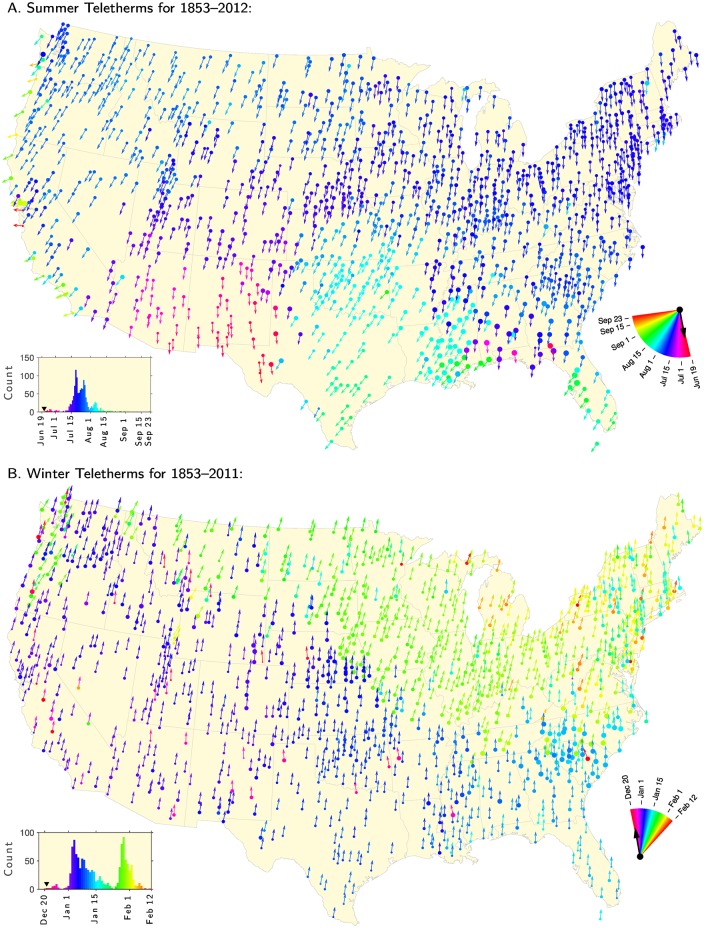
A: Summer Teletherms and B: Winter Teletherms across the contiguous United States based on all data recorded from 1853 to 2012. Arrows point in the direction of the Teletherm’s day of year mapped into angles traveling clockwise with December 31st aligned upwards. The sizes of the markers (discs) represent the duration of a location’s Teletherm Period. In the case of multiple Teletherm Periods, sizes correspond to the full extent. Colors map to Teletherm dates as indicated by the partial color wheel in the bottom right corner of each map. The black arrows in the color wheels show the location of the Solstices. The histograms shows the distributions of the Summer and Winter Teletherm, using the same colors.

We move now to exploring how the Teletherms vary across the contiguous U.S. through maps. Once again drawing on the full data set, we plot the Summer Teletherms in Fig 5A and the Winter Teletherms in Fig 5B. We present accompanying maps of the Teletherm temperatures in S1 and S2 Figs, and a map showing the number of days separating the two Teletherms at each station in S3 Fig.

On all maps here and in the Supplementary Information, we indicate the Teletherm’s day of year by an arrow on a clock. We orient the angle 0 radians upwards and assign days of the standard year to multiples of 1/365 × 2*π* (December 31st then corresponds to angle 0). To reinforce the visibility of variation, we color points and arrows per the color wheel in the bottom right corner of all maps. The black arrows in these color wheels mark the Summer and Winter Solstices as appropriate.

We visually supply information about the Teletherm Period by linearly scaling the size of the marker for each location. For stations with multiple Teletherm Periods, we use what we call the Teletherm Extent—the number of days from the start of the first Teletherm period to the end of the last one (inclusive).

We provide a histogram of the Teletherm days of the year in the bottom left corner of each map, again using the same color scheme. The inverted black triangle identifies the relevant Solstice.

A number of observations stand out. For the Summer Teletherm, we see considerable but largely smooth variation. From Fig 2A and 2C, we had identified that the range of dates for the Summer Teletherm spans 96 days (June 19 in Alpine, Texas to September 23 in Santa Cruz, California), but we now see that the bulk of Teletherms fall between July 15 and August 1 (dark blue). These second-half-of-July Teletherms hold in the north of the contiguous U.S. and extend down into California on the west and Georgia on the east.

The variant Summer Teletherms span several regions. The earliest summer Teletherms occur in Arizona, New Mexico, and the west of Texas (purple/red). In moving from west to east, we see a longitudinal discontinuity in Texas with a switch to relatively late Summer Teletherms, which remain apparent in Oklahoma, Arkansas, Louisiana, Mississippi, and over to Florida. These August Teletherms form a noticeable minor peak in the histogram (light blue). The gulf coast shows some irregularity in the Teletherm but more clearly exhibits the longest Teletherm Extents.

Stations along the west coast show how exposure to the Pacific and incoming weather patterns make them break strongly with the nearby inland Teletherm “directions”, moving to generally later in the year as per example of Santa Cruz we examined earlier (Fig 3C). By contrast, stations along the east coast are consistently aligned with their inland counterparts.

For the Winter Teletherm, we see a different overall pattern with the contiguous U.S. dividing into two regions: the west, midwest, and south with largely early January Winter Teletherms (blue), and the mid-north and northeast showing Teletherms in late January and early February (green). In the northeast’s winter, the temperature continues to fall well beyond the shortest day of the year in the northeast, typically 5 to 6 weeks. We venture that a possible source of this clear regional separation might lie in the jet stream’s dynamics across North America, with snowfall leading to increased albedo in the northern section coupled with a continental-scale shadow of the Rockies. Beyond the scope of the present analysis, future modeling would be needed to properly test such an hypothesis.

### Teletherm dynamics

In order to discern Teletherm dynamics and their potential value in quantifying and studying climate change, we carry out the same smoothing we have performed for the full time range for sliding windows of 50 years in duration. We also now make our data requirements more stringent and estimate the Teletherm and the Teletherm Period(s) for only those time ranges for which we have 80% of all temperatures recorded.

Here, we present and discuss shifts in Summer and Winter Teletherm dates for two example consecutive 50 year periods: 1913–1962 and 1963–2012 (the six month offset leads to references to one year earlier for the Winter Teletherm). We include all related Teletherm maps in the Supporting Information in the form of PDF flipbooks (S6–S9 Files). We also provide interactive visualizations of Teletherm dynamics at http://compstorylab.org/share/papers/dodds2015c and http://panometer.org/instruments/teletherms.

**Fig 6:**
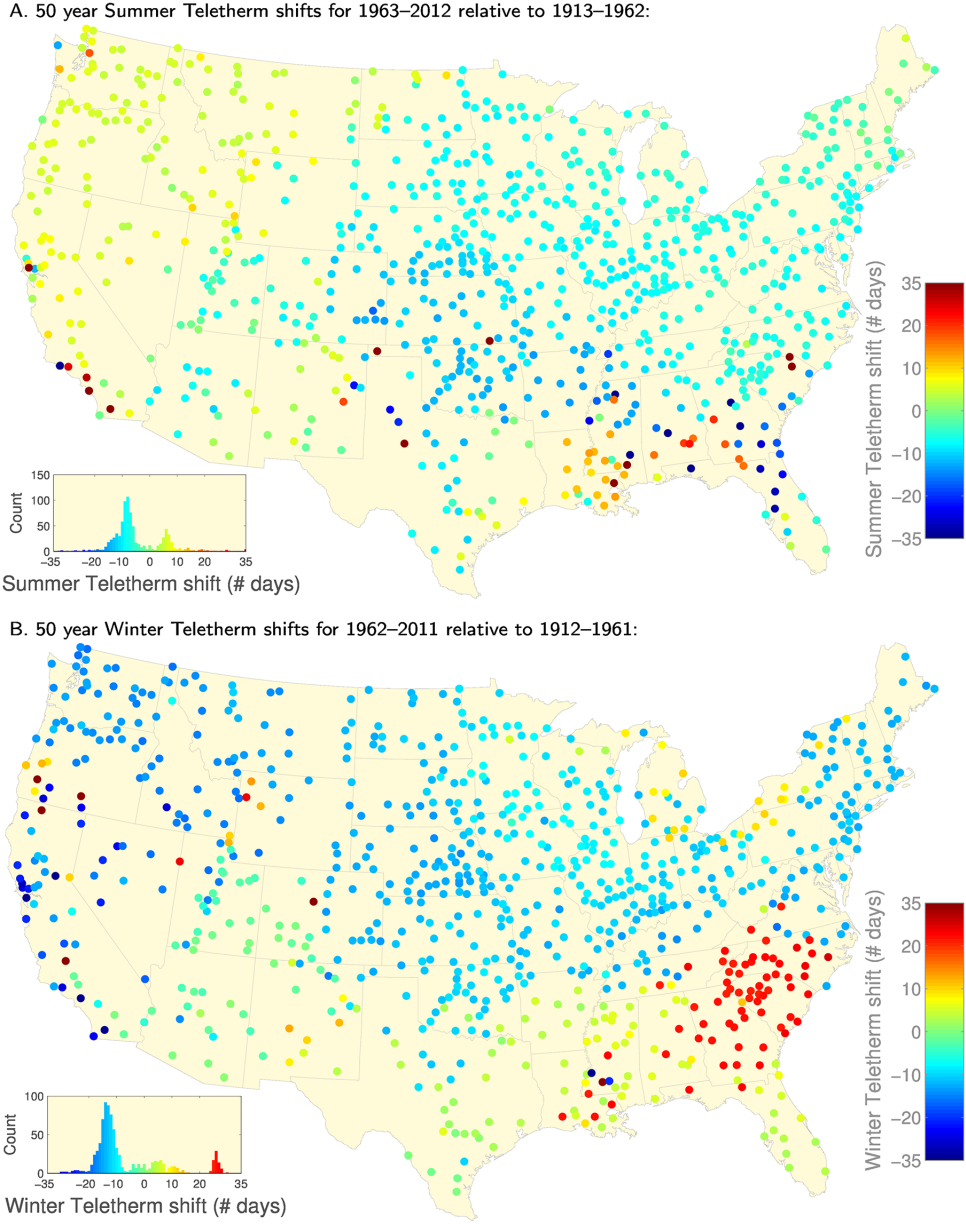
A: Summer Teletherm shifts comparing the 50 year period 1963–2012 relative to 1913–1962. We show a total of 837 out of 1218 (68.7%) which have ≥ 80% error-free data in both 50 year spans. See S4 and S5 Figs for maps of the Summer Teletherms for each period. **B:** Winter Teletherm shifts comparing 1962/1963–2011/2012 relative to 1912/1913–1961/1962. A total of 835 out of 1218, 68.6%, stations have ≥ 80% error-free data. See S6 and S7 Figs for maps of the Winter Teletherms for each period.

In Fig 6A, we show how the Summer Teletherm has moved between these two half century time periods. We compute the “Teletherm shift” in days (see S4 and S5 Figs for plots of the respective Summer Teletherms) and use a color map to present the results. The lower left histogram in Fig 6A represents the distribution of shifts. Now, if change was random, we would expect to see a normal distribution centered around a shift of 0. We instead find two peaks separated from zero shift, meaning very few locations experienced no change. The larger peak (blue) means the Summer Teletherm has moved to earlier in the year, connecting more strongly with the Solstice, and corresponds generally to the northeast extending across and down into the midwest and south. Stations in the west are reflected in the histogram’s smaller peak (green/yellow) indicating the Summer Teletherm has moved to later in the year for that area’s stations.

We show shifts in the Winter Teletherm in Fig 6B (see S6 and S7 Figs for the Teletherms themselves). We again find a texture different to that of the Summer Teletherm. The dominant change is that the Winter Teletherm has advanced to earlier dates in the year across the northern half of the contiguous U.S., and down along the west coast (blue). As the histogram shows, the spread of forward shifts peaks in the range 10 to 20 days. Going in the other direction, we see that the Winter Teletherms for the states of Georgia and the two Carolinas have experienced a delay of around 25 days (red). For the rest of the contiguous U.S., from New Mexico across to Florida, the Winter Teletherm has remained fairly constant. Comparing the histograms for the Winter Teletherm dates in S6 and S7 Figs, we see that three distinct peaks have merged into one grouping over time. In sum, the Winter Teletherm has become more homogeneous in the eastern half of the contiguous U.S., with both early and late Winter Teletherms moving into the first half of January, while largely moving to earlier dates in the west.

In S8A and S8B and S9A and S9B Figs, we present the shifts in Teletherm Temperatures and Extents for the same pair of 50 year periods. The changes in temperature are milder for the Summer Teletherm (± 5°*F*, S8A Fig) than for the Winter (± 10°*F*, S9A Fig). The Extents however have maximally increased or decreased by 30 to 40 days, with the Summer Teletherm seeing the most flux. (S8B and S9B Figs). Some of the other trends we see are that (1) the Summer Teletherm Temperature has dropped in the middle of the contiguous United States while remaining neutral or increasing elsewhere; (2) Summer Teletherm Extents have increased most strongly throughout the south; (3) The Winter Teletherm Temperature has lowered in the South East and increased in the central and western areas of the north; and (4) Winter Teletherm Extents have decreased in the south and increased in areas around the Great Lakes.

Finally, we observe that the transitions in Teletherm features between these two adjacent 50 year periods is not linear, and that window length matters [16]. To show this, we break the same century (1913–2012) into four 25 year periods. First, we see a strengthened version of the same general overall changes to the Teletherm dates as for the 50 year analysis in comparing the last 25 years to the first 25 years (1988–2012 relative to 1913–1937) (S10 Fig). The three transitions between the four 25 year periods show accelerations, stasis, and reversals (see S11, S12 and S13 Figs). For example, in Louisiana and Mississippi, the Summer Teletherm has shifted to later dates but through an advance, retreat, advance movement (Plot A in each of S11, S12 and S13 Figs). Much of the shift toward an earlier Winter Teletherm across the north occurred in the 50 year period 1937–1986 (Plot B in S12 Fig), and the southeast first saw the Winter Teletherm advance and then start to fall back to later dates (Plot B in each of S11, S12 and S13 Figs).

We show the corresponding maps for shifts in Teletherm temperatures and extents in S14–S21 Figs. The transition from the first 25 years to the last 25 years of 1913–2012 sees an average drop in the 25 year Summer Teletherm temperature (mainly due the interior states) but an increase in the Winter Teletherm temperature (concentrated more along the north and down into Utah, Colorado, and Arizona). Of many notable details, we see a dropping of the Winter Teletherm’s temperature, in the eastern half of the contiguous U.S. between 1937–1961 and 1962–1986, followed by a reverse swing upwards over the next 25 years (Plot B in S16 and S17 Figs).

Interpreting the dynamics of the Teletherms is not an easy task and we limit our assertions in this initial work. We might suspect the jet stream may have played a part in the transition of the Winter Teletherm in the southeast. Even without a clear understanding, we can see that impact of these changes is potentially dramatic. The movement of the Winter Teletherm for example alters the local advent of spring, a strong driver of ecological systems.

## Comparison to models

We end our analysis with a comparison of estimated Teletherm data to output from two Regional Climate Models (RCMs) from the North American Regional Climate Change Assessment Program (NARCCAP) [7]. Specifically, we analyze output of the WRF model nested within both the Community Climate Systems Model (CCSM) (version 3) [4] and the National Centers for Environmental Prediction Climate Forecast System Model (NCEP) [18–20]. Details on the data can be found at https://www.earthsystemgrid.org/project/narccap.html.

We use daily temperature extremes from both model systems at all 1218 station locations to compute Summer and Winter Teletherms for the time periods covered by the models: 1968 to 1999 (CCSM) and 1979 to 2004 (NCEP) (see [21] for related work on climate models).

**Fig 7:**
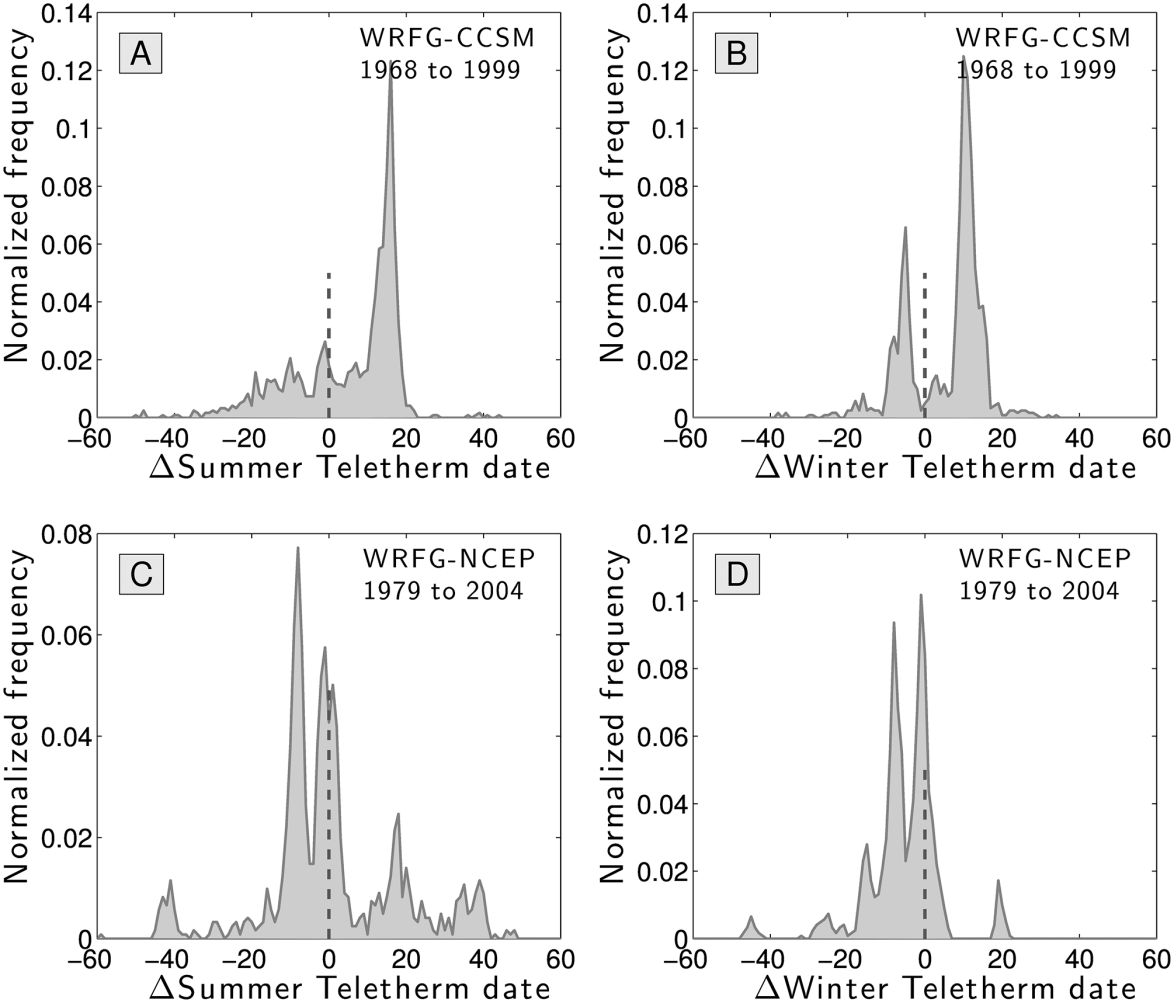
Distributions of errors in days for the Summer and Winter Teletherms at all stations when comparing measured temperatures to the Community Climate Systems Model (CCSM) (version 3) [17] and the National Centers for Environmental Prediction Climate Forecast System Model (NCEP) [18, 19]. The differences are to be interpreted as how many days the models are “off” from the real data. A positive *Δ* means the model’s Teletherm occurs later in the year than the measured one.

Using our historical data set, we also determined the Teletherms for these same time periods. We then found the difference between the models’ Teletherms and the measured Teletherms at each location, and we show the histogram of these differences in Fig 7. In these plots, a positive difference means a model’s Teletherm occurs later in the year than the Teletherm we estimated based on real data.

For both models and for both Winter and Summer Teletherms, we see evidence of characteristic, irregular kinds of errors. For the CCSM, the single peak in Fig 7A shows that the model produces Summer Teletherms that occur 10 to 20 days later in the year than those observed. For the Winter Teletherm, two peaks reflect regional systematic errors. The NCEP model fares somewhat better with a peak around a difference of 0 for both Teletherms, though a second peak of similar size indicates a prediction of earlier Teletherms for a commensurate swathe of stations.

We find the average absolute error in estimating the Summer and Winter Teletherm are 12.88 and 10.05 days for the CCSM, and 12.24 and 7.57 days for the NCEP model. Spearman correlations are mixed with a best value of 0.85 for the CCSM’s Winter Teletherm (*p*-value effectively 0) and a worst case of 0.059 for CCSM’s Summer Teletherm (*p*-value 0.039). At the level of stations, the worst errors for both models are for the Summer Teletherm with spans 78 and 59 days too early and 44 and 48 too late for the CCSM and the NCEP model respectively.

**Fig 8:**
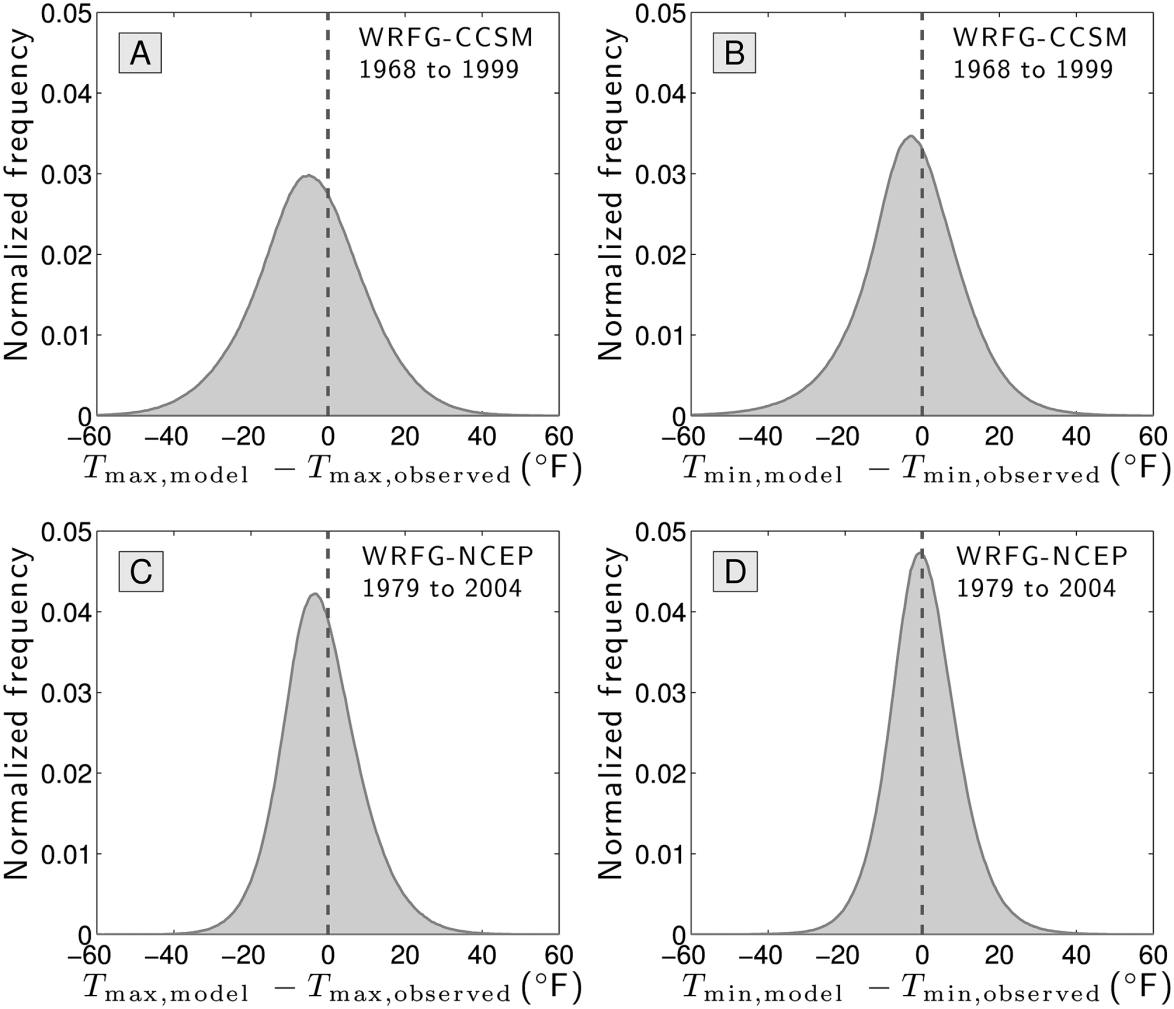
Comparison between predicted daily maximum and minimum temperatures generated by two climate models (CCSM and NCEP) relative to real measurements for all stations.

In Fig 8, we step back from Teletherms, and plot the distribution of errors at the day level between the output of both models and measured maximum and minimum temperatures. This is an exacting test: how does a model fair with predicting the maximum temperature, say, in Death Valley on March 3, 1982, along with all other stations and all other dates over several decades? With approximately 10,000 points per panel, we see a much smoother distribution and the form is now Gaussian-like.

We find that the Spearman correlations between the models’ outputs and measured daily temperature extremes are good, ranging from 0.796 (CCSM, daily minimum temperature) to 0.875 (NCEP, daily maximum temperature) (*p*-values effectively 0). The NCEP model is on average more accurate with an average difference for the daily minimum of 0.53°*F*. The average absolute error varies from 7.32 (NCEP, daily minimum temperature) to 12.1 (CCSM, daily maximum temperature). The potential for wild inaccuracies remain with CCSM’s worst prediction being 95°*F* below the real measurement of a minimum temperature.

In testing these climate models for Teletherm timing and daily temperature extremes, we are certainly asking for more than they have been intended to deliver. Indeed, if these models were integrating in Numerical Weather Prediction mode, with initial values updated through data assimilation, the errors would be much smaller. Nevertheless, understanding the successes and limitations of any model, whether aimed for or not, should be of benefit to future refinements [22].

## Concluding remarks

We were initially motivated by the simple question of when should we expect the on-average warmest and coldest day of the year to occur at a given location. In the northeast of the U.S. for example, the Winter Solstice passes and as the days lengthen, the cold deepens and people begin to wonder when will the winter end. Traditionally, prognosticators have used diverse methods to divine the length of winter such as, famously, how certain species of rodents react to their umbra. And in general, people look for signs of all the seasons arriving such as the emergence of daffodils in spring or the first leaves turning to their autumnal colors. We realized however that a data-driven, less poetic path could be assayed.

While the analysis promised to be initially straightforward (as is often believed to be the case), we soon found that we had to move beyond a single day version of the Teletherm to a Teletherm Period. Overall, we believe we have shown the spatiotemporal variability of the Teletherms and the surrounding Teletherm Periods to be considerable, informative, and of general interest. Importantly, we have seen that the variations in Teletherm characteristics are not a reflection of random noise but rather linear movements, periods of stasis, and switching reminiscent of bifurcations in dynamical systems. Teletherms seem therefore to present a real facet of climate change, whatever the origin.

A number of future directions are possible. Where data is available, our analysis could readily be carried out for other regions around the world. Beyond local interest, such efforts could lead to an effort to patch together a global picture of the Teletherms. Online displays of Teletherms could also eventually include the ability to adjust time frames for the analysis and to show the likelihood that the warmest or coldest day has occurred as a function of day of the year. A global map would also afford more opportunities to test models and hypotheses regarding climate dynamics. For example, does the temporal behavior of Teletherms correlate in an way to changes or stationarity of average annual temperature [14]? The stochastic nature of temperature could also be of value in our collective general education regarding prediction for noisy systems.

We close by venturing that a region’s Teletherm may also be acknowledged annually (using, say, the most recent 50 years), potentially with a set of associated food-based rituals or celebrations.

## Supporting Information

S1 Fig Summer Teletherm temperatures for the full data set (1853–2012). Teletherm temperatures are determined by smoothing the average daily maximum and minimum temperatures; see main text for details.
(TIFF)

S2 Fig Winter Teletherm temperatures for the full data set (1853–2012). Teletherm temperatures are determined by smoothing the average daily maximum and minimum temperatures; see main text for details.
(TIFF)

S3 Fig Number of days from the Winter to the Summer Teletherm. The vertical gray line in the histogram indicates half of a standard 365 day year. The variation is substantial with the northeast showing as short a span as just over 5 months and the west coast as much as 9 months.
(TIFF)

S4 Fig Map of the Summer Teletherms and Teletherm Extents estimated for the 50 year range 1913–1962, to be compared with the equivalent map for 1963–2012 in S5 Fig. Fig 6A in the main text maps the changes in Summer Teletherms between these two periods. Relatively few Summer Teletherms have remained stable with the majority shifting to an earlier date. In the bottom left histograms, the gray horizontal line shows the interquartile range and the inverted triangle the median.
(TIFF)

S5 Fig Map of the Summer Teletherms and Teletherm Extents estimated for the year ranges 1963–2012, to be compared with the preceding map in S4 Fig. Fig 6A in the main text maps the changes in the Summer Teletherm between these two periods.
(TIFF)

S6 Fig Map of the Winter Teletherms and Teletherm Extents estimated for the 50 year range 1912–1961, to be compared with the equivalent map for 1962–2011 in S7 Fig. Fig 6B in the main text maps the changes in Winter Teletherms between these two periods. In the bottom left histogram, the gray horizontal line shows the interquartile range and the inverted triangle the median.
(TIFF)

S7 Fig Map of the Winter Teletherms and Teletherm Extents estimated for the year ranges 1962–2011, to be compared with the preceding map in S6 Fig. See Fig 6B for a map of the changes.
(TIFF)

S8 Fig Shifts for the Summer Teletherm for A: Temperature and B: Extent derived from S4 and S5 Figs. (TIFF)

S9 Fig Shifts for the Winter Teletherm for A: Temperature and B: Extent derived from S6 and S7 Figs. (TIFF)

S10 Fig Teletherm shifts comparing the quarter centuries at the ends of the 1912 to 2012. **A:** Summer Teletherm shifts comparing the 25 year periods 1988–2012 relative to 1912–1937. Out of all 1218 stations, 716 (58.8%) have ≥ 80% error-free data in both 25 year spans. **B:** Winter Teletherm shifts comparing 1987/1988–2011/2012 relative to 1912/1913–1936/1937. A total of 725 out of 1218, 59.5%, stations have ≥ 80% error-free data. The overall patterns are consistent with those observed for the changes between the consecutive 50 year periods spanning the same 100 years, as displayed in Fig 6 in the main text. For both Teletherms, S11, S12 and S13 Figs show the transitions between consecutive 25 year periods.
(TIFF)

S11 Fig Teletherm shifts comparing the quarter centuries at the ends of the 1937 to 2012. **A:** Summer Teletherm shifts comparing the 25 year period 1938–1962 relative to 1912–1937 (837 out of 1218, 68.72%, stations have acceptable data). **B:** Winter Teletherm shifts comparing 1937/1938–1962/1963 relative to 1912/1913–1936/1937 (838 out of 1218, 68.80%, stations have acceptable data).
(TIFF)

S12 Fig Teletherm shifts comparing the quarter centuries at the ends of the 1937 to 1987. **A:** Summer Teletherm shifts comparing the 25 year period 1963–1987 relative to 1938–1962 (1001 out of 1218, 82.18%, stations have acceptable data). **B:** Winter Teletherm shifts comparing 1961/1962–1985/1986 relative to 1937/1938–1961/1962 (1000 out of 1218, 82.10%, stations have acceptable data).
(TIFF)

S13 Fig Teletherm shifts comparing the quarter centuries at the ends of the 1962 to 2012. **A:** Summer Teletherm shifts comparing the 25 year period 1988–2012 relative to 1963–1987 (941 out of 1218, 77.26%, stations have acceptable data). **B:** Winter Teletherm shifts comparing 1987/1988–2011/2012 relative to 1962/1963–1986/1987 (950 out of 1218, 78.00%, stations have acceptable data).
(TIFF)

S14 Fig Teletherm temperature shifts comparing the quarter centuries at the ends of the 1912 to 2012. **A:** Summer Teletherm temperature shifts comparing the 25 year periods 1988–2012 relative to 1912–1937. **B:** Winter Teletherm temperature shifts comparing 1987/1988–2011/2012 relative to 1912/1913–1936/1937.
(TIFF)

S15 Fig Teletherm temperature shifts comparing the quarter centuries at the ends of the 1912 to 1963. **A:** Summer Teletherm temperature shifts comparing the 25 year period 1938–1962 relative to 1912–1937. **B:** Winter Teletherm temperature shifts comparing 1937/1938–1962/1963 relative to 1912/1913–1936/1937.
(TIFF)

S16 Fig Teletherm temperature shifts comparing the quarter centuries at the ends of the 1937 to 1987. **A:** Summer Teletherm temperature shifts comparing the 25 year period 1963–1987 relative to 1938–1962. **B:** Winter Teletherm temperature shifts comparing 1961/1962–1985/1986 relative to 1937/1938–1961/1962.
(TIFF)

S17 Fig Teletherm temperature shifts comparing the quarter centuries at the ends of the 1962 to 2012. **A:** Summer Teletherm temperature shifts comparing the 25 year period 1988–2012 relative to 1963–1987. **B:** Winter Teletherm temperature shifts comparing 1987/1988–2011/2012 relative to 1962/1963–1986/1987.
(TIFF)

S18 Fig Teletherm extent shifts comparing the quarter centuries at the ends of the 1912 to 2012. **A:** Summer Teletherm extent shifts comparing the 25 year periods 1988–2012 relative to 1912–1937. **B:** Winter Teletherm extent shifts comparing 1987/1988–2011/2012 relative to 1912/1913–1936/1937.
(TIFF)

S19 Fig Teletherm extent shifts comparing the quarter centuries at the ends of the 1912 to 1963. **A:** Summer Teletherm extent shifts comparing the 25 year period 1938–1962 relative to 1912–1937. **B:** Winter Teletherm extent shifts comparing 1937/1938–1962/1963 relative to 1912/1913–1936/1937.
(TIFF)

S20 Fig Teletherm extent shifts comparing the quarter centuries at the ends of the 1937 to 1987. **A:** Summer Teletherm extent shifts comparing the 25 year period 1963–1987 relative to 1938–1962. **B:** Winter Teletherm extent shifts comparing 1961/1962–1985/1986 relative to 1937/1938–1961/1962.
(TIFF)

S21 Fig Teletherm extent shifts comparing the quarter centuries at the ends of the 1962 to 2012. **A:** Summer Teletherm extent shifts comparing the 25 year period 1988–2012 relative to 1963–1987. **B:** Winter Teletherm extent shifts comparing 1987/1988–2011/2012 relative to 1962/1963–1986/1987.
(TIFF)

S1 File Summer Teletherm diagnostic plots for all 1218 stations. (PDF)

S2 File Winter Teletherm diagnostic plots for all 1218 stations. (PDF)

S3 File 50 year Teletherm time lines for all 1218 stations. (PDF)

S4 File 25 year Teletherm time lines for all 1218 stations. (PDF)

S5 File 10 year Teletherm time lines for all 1218 stations. (PDF)

S6 File 50 year Summer Teletherm flipbook. (PDF)

S7 File 50 year Winter Teletherm flipbook. (PDF)

S8 File 25 year Summer Teletherm flipbook. (PDF)

S9 File 25 year Winter Teletherm flipbook. (PDF)
