# Supplementary figures and images for: Tracking Climate Change through the Spatiotemporal Dynamics of the Teletherms, the Statistically Hottest and Coldest Days of the Year
Source: PLoS One. 2016 May 11;11(5):e0154184. doi: 10.1371/journal.pone.0154184 (PMC4864332; doi:10.1371/journal.pone.0154184)

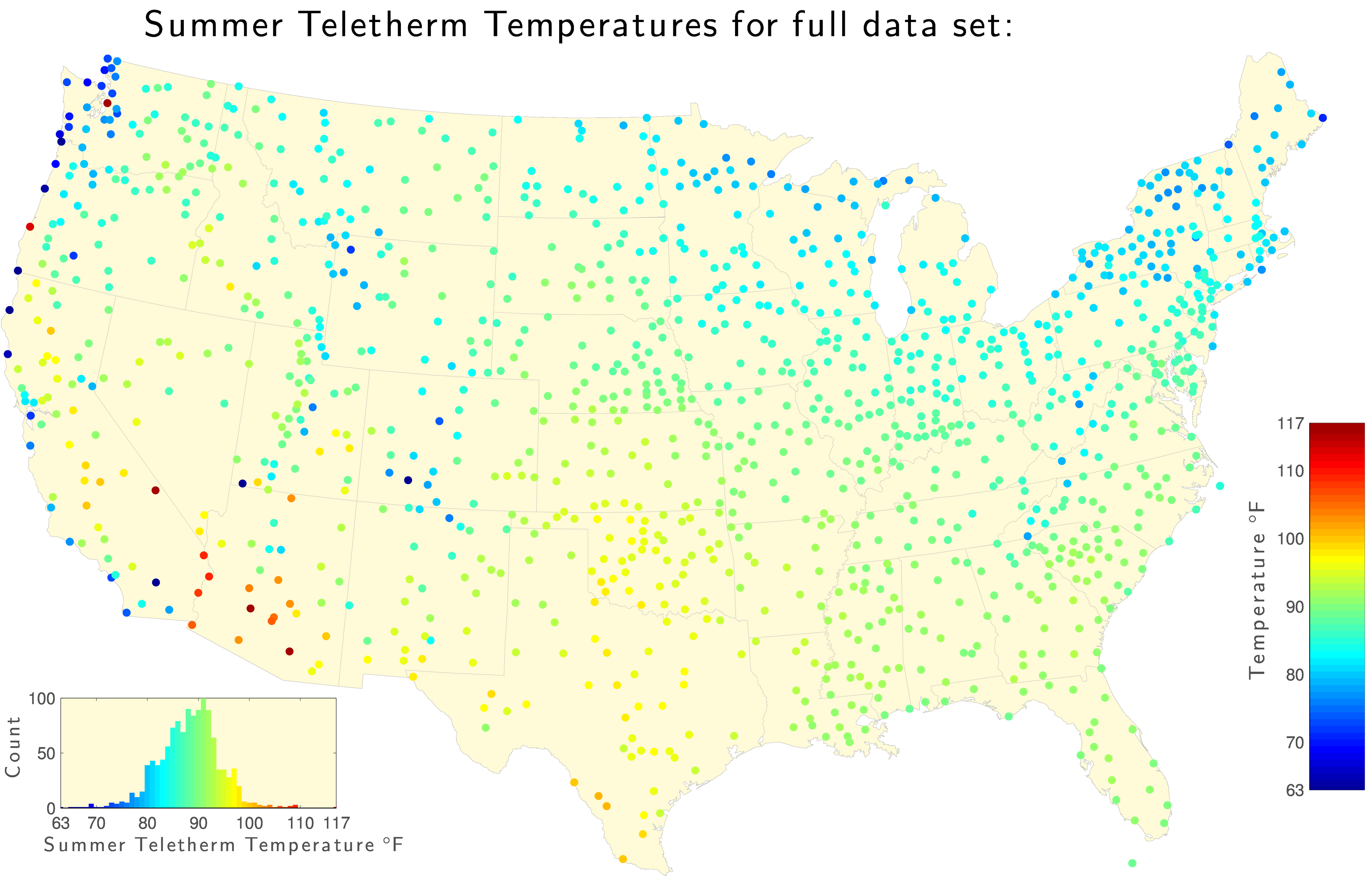

Supplement: S1 Fig — Teletherm temperatures are determined by smoothing the average daily maximum and minimum temperatures; see main text for details. (TIFF) [file pone.0154184.s001.tiff]

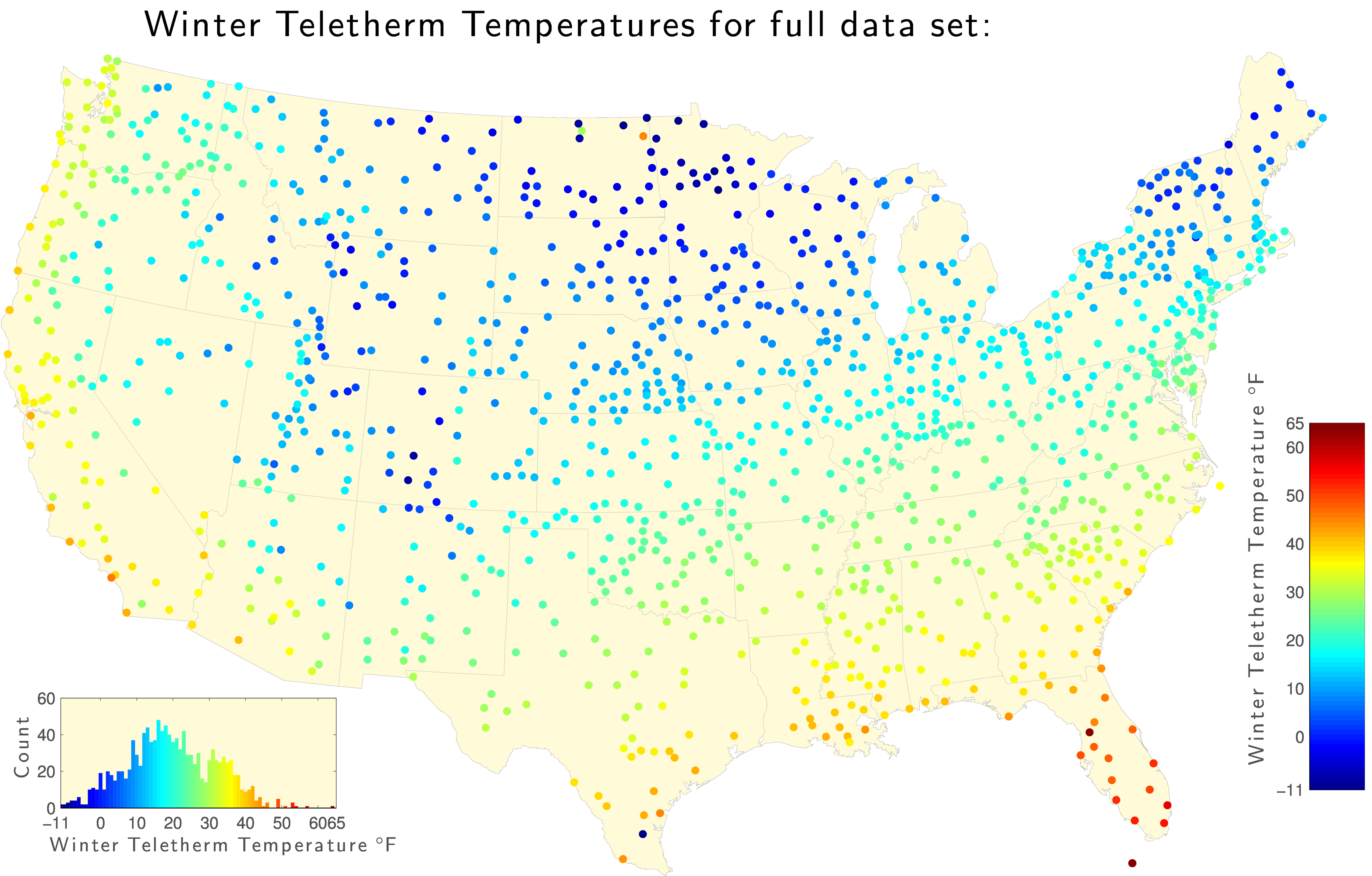

Supplement: S2 Fig — Teletherm temperatures are determined by smoothing the average daily maximum and minimum temperatures; see main text for details. (TIFF) [file pone.0154184.s002.tiff]

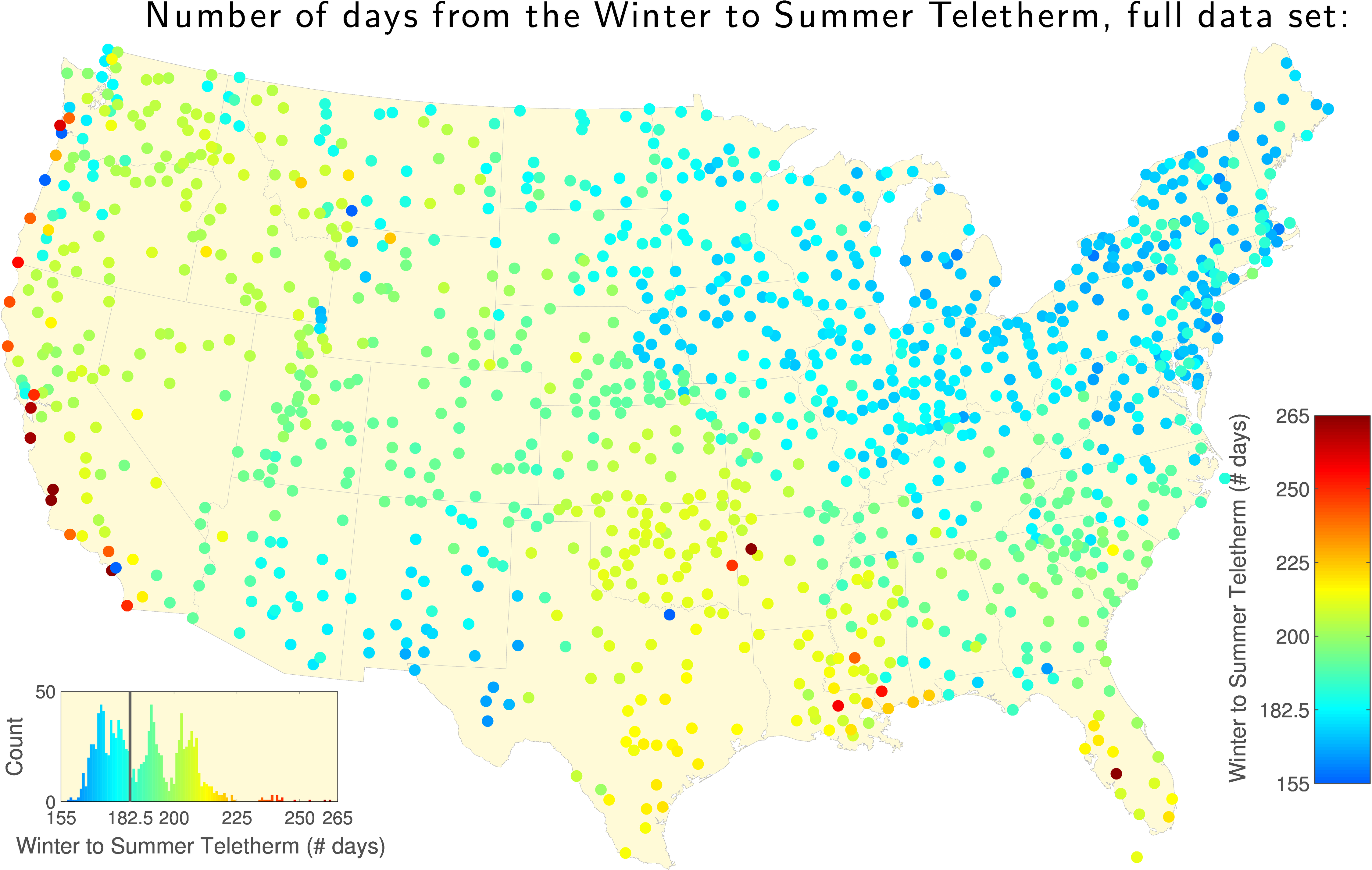

Supplement: S3 Fig — The vertical gray line in the histogram indicates half of a standard 365 day year. The variation is substantial with the northeast showing as short a span as just over 5 months and the west coast as much as 9 months. (TIFF) [file pone.0154184.s003.tiff]

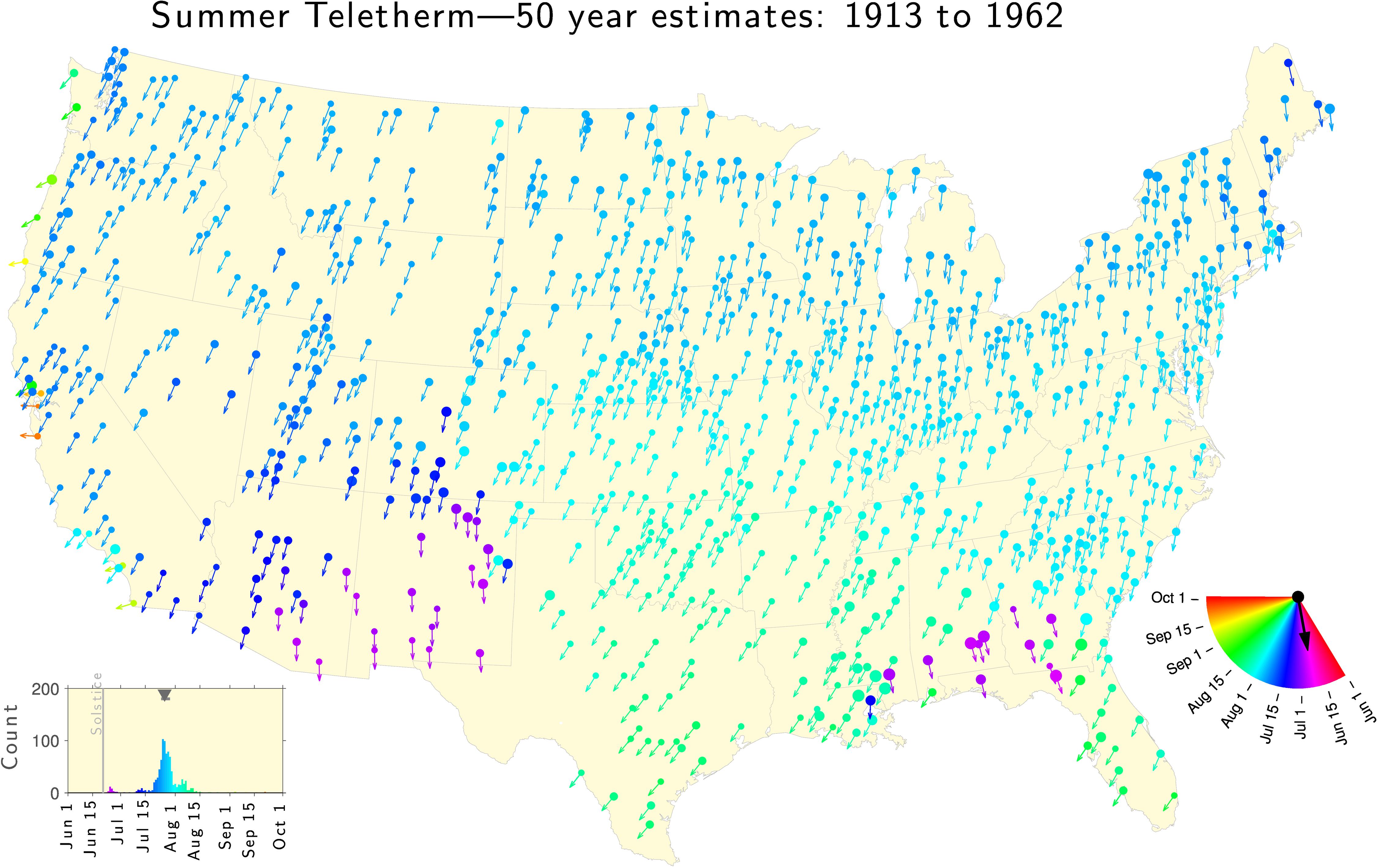

Supplement: S4 Fig — Fig 6A in the main text maps the changes in Summer Teletherms between these two periods. Relatively few Summer Teletherms have remained stable with the majority shifting to an earlier date. In the bottom left histograms, the gray horizontal line shows the interquartile range and the inverted triangle the median. (TIFF) [file pone.0154184.s004.tiff]

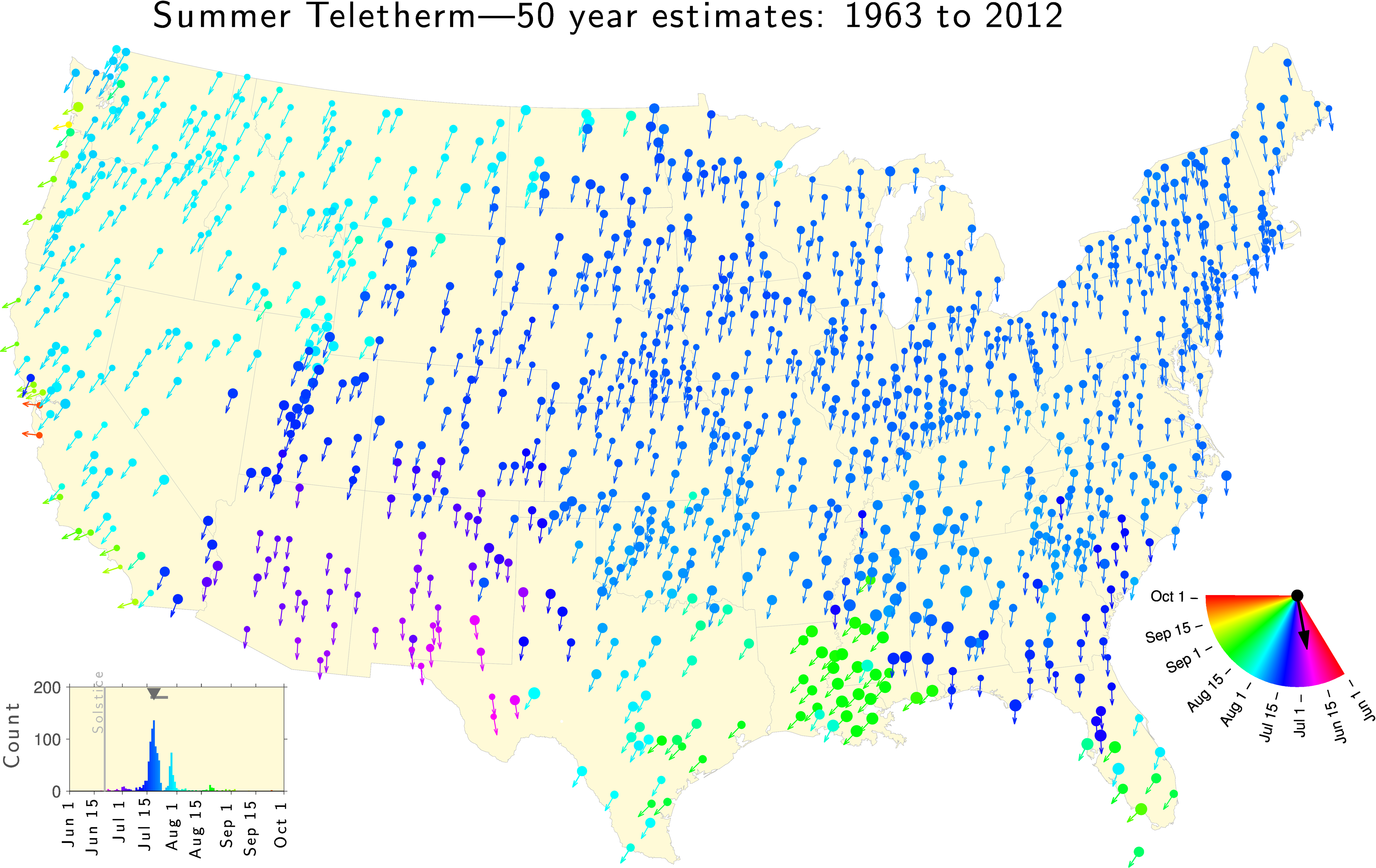

Supplement: S5 Fig — Fig 6A in the main text maps the changes in the Summer Teletherm between these two periods. (TIFF) [file pone.0154184.s005.tiff]

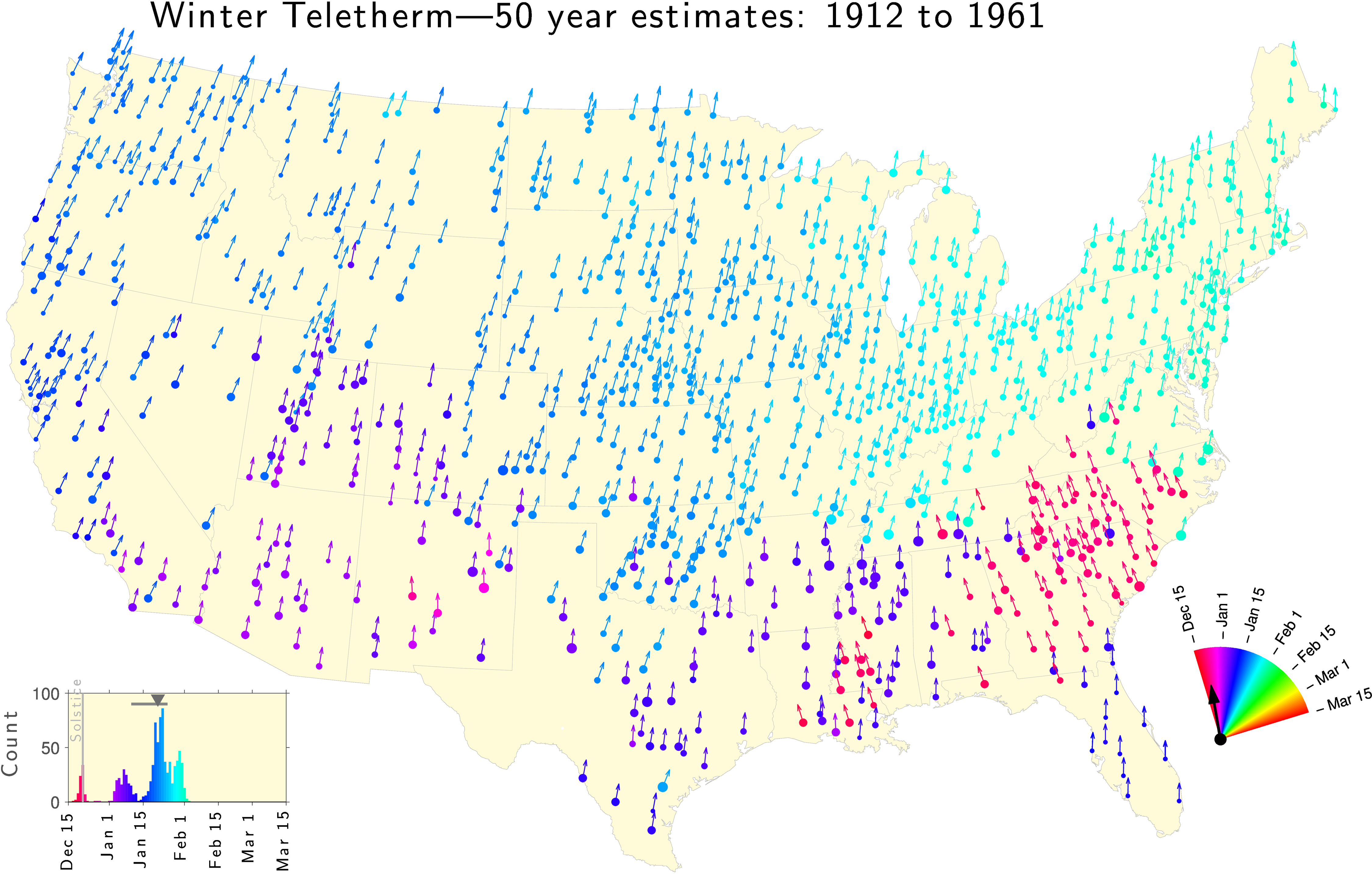

Supplement: S6 Fig — Fig 6B in the main text maps the changes in Winter Teletherms between these two periods. In the bottom left histogram, the gray horizontal line shows the interquartile range and the inverted triangle the median. (TIFF) [file pone.0154184.s006.tiff]

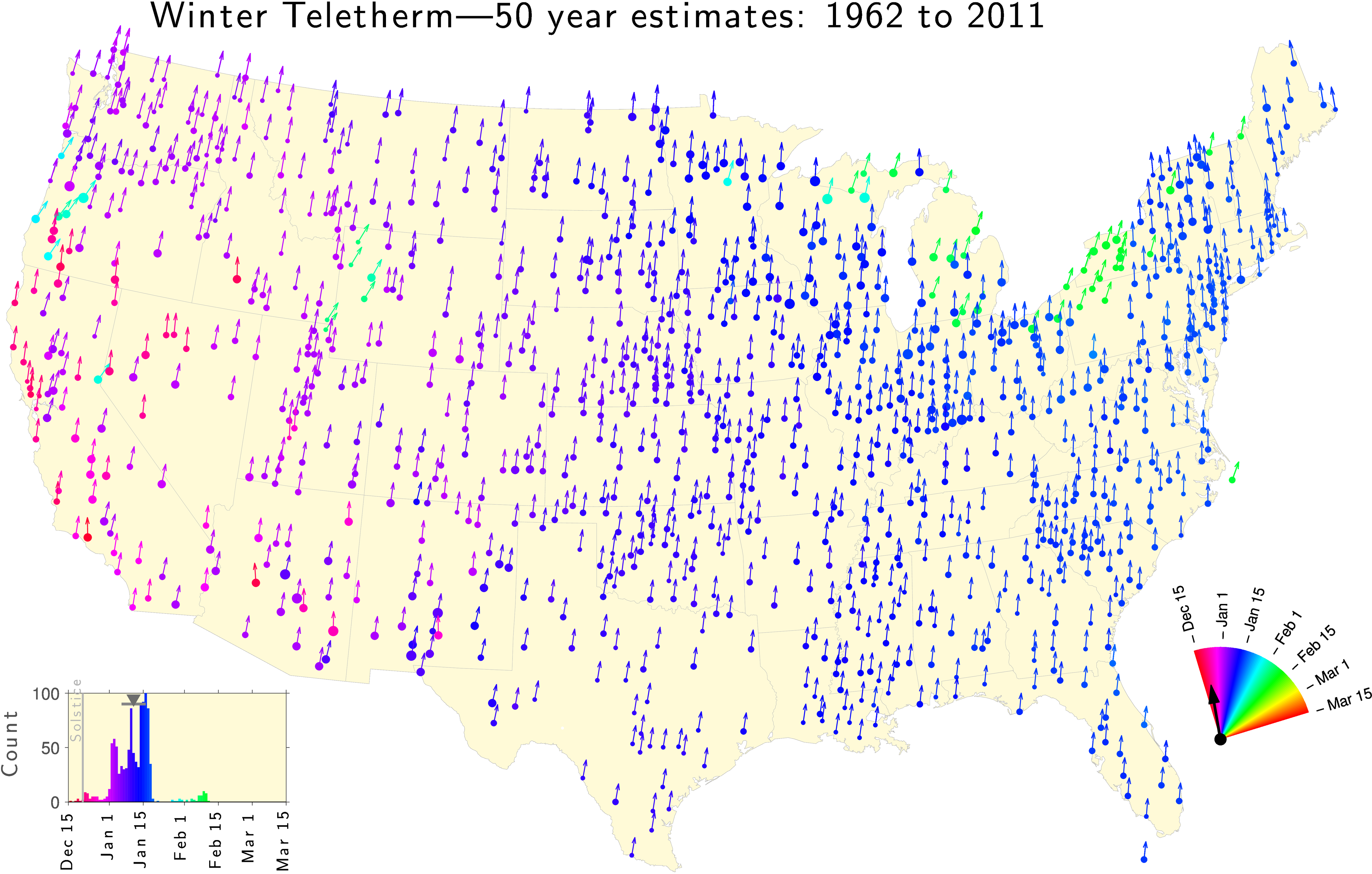

Supplement: S7 Fig — See Fig 6B for a map of the changes. (TIFF) [file pone.0154184.s007.tiff]

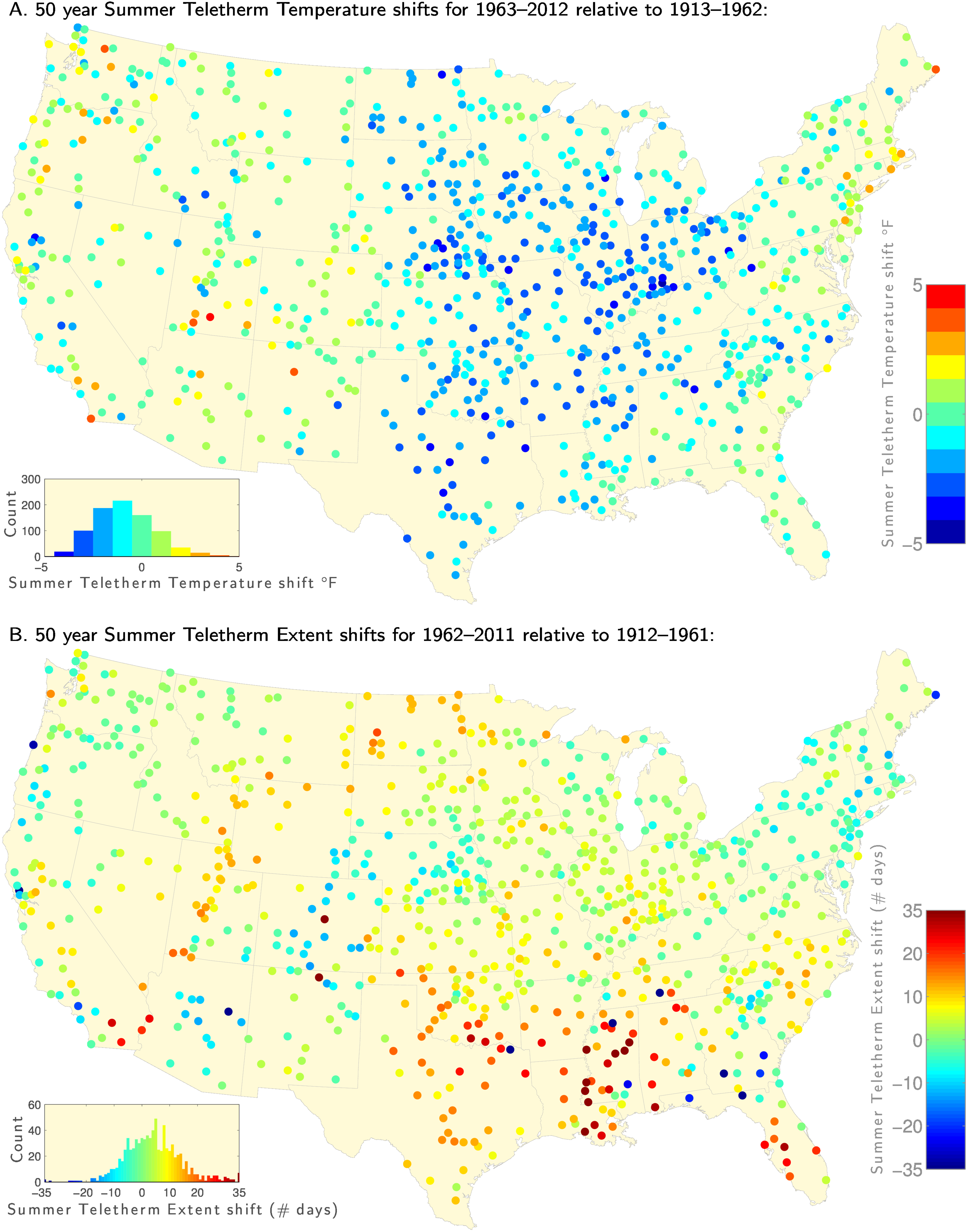

Supplement: S8 Fig — (TIFF) [file pone.0154184.s008.tiff]

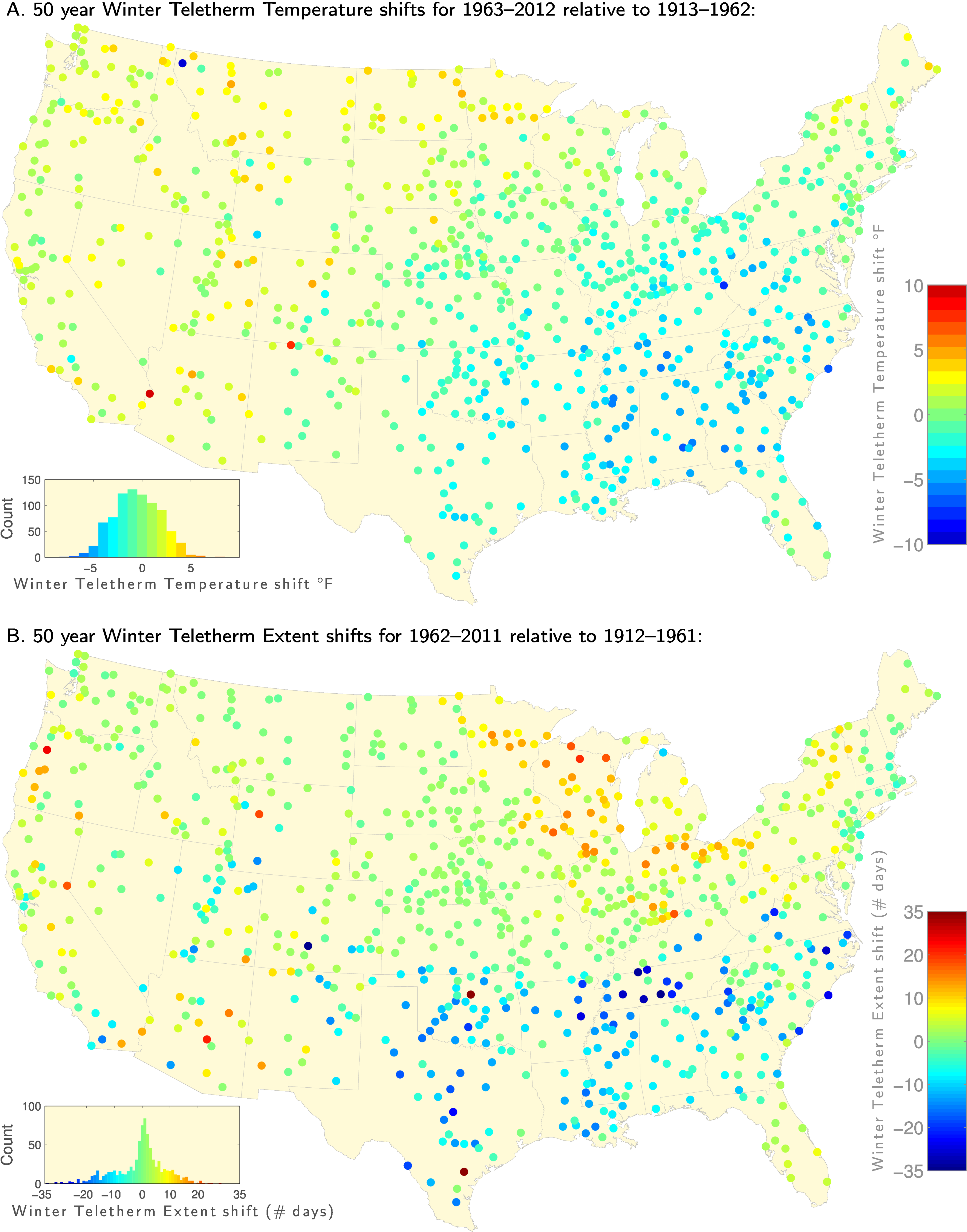

Supplement: S9 Fig — (TIFF) [file pone.0154184.s009.tiff]

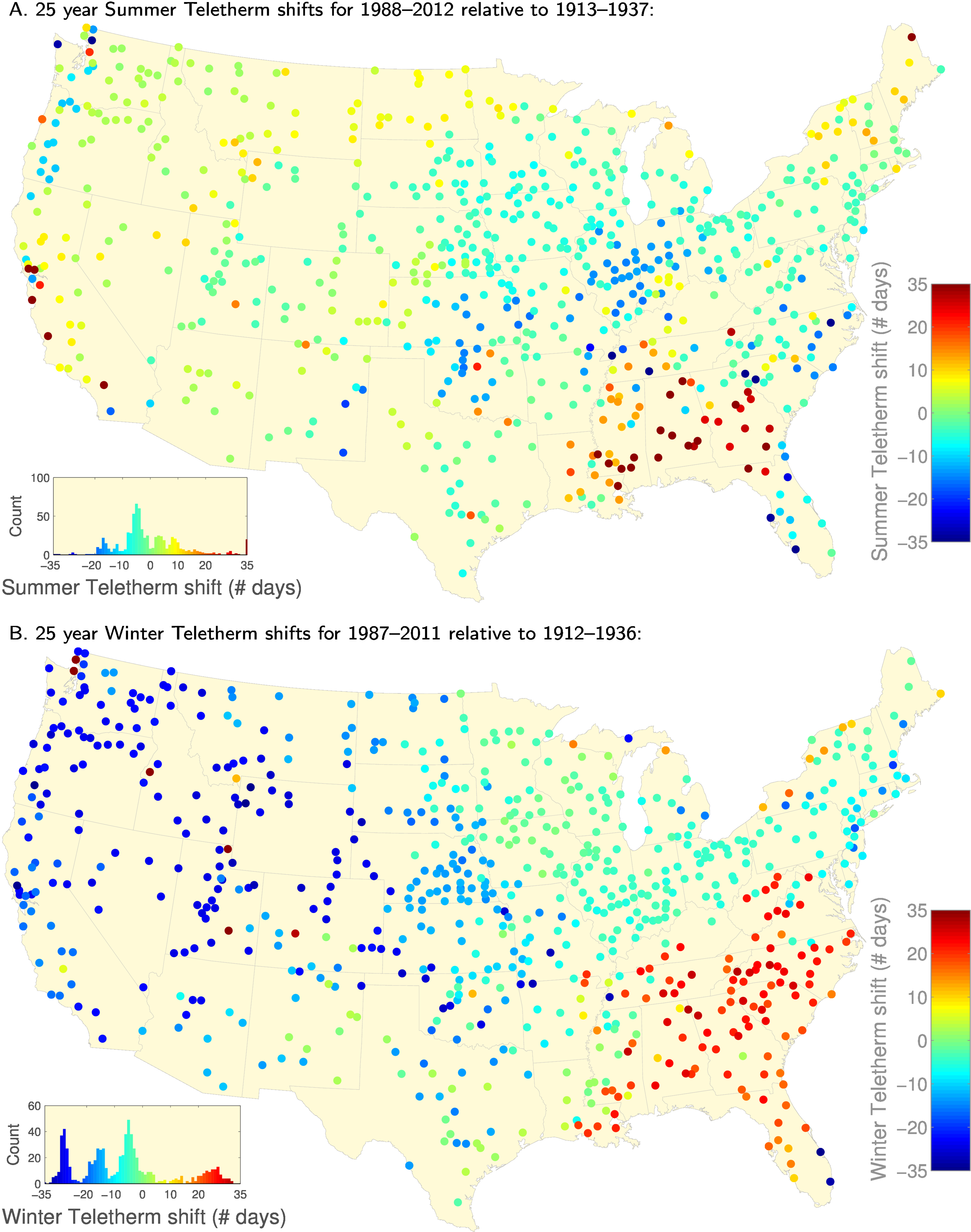

Supplement: S10 Fig — A: Summer Teletherm shifts comparing the 25 year periods 1988–2012 relative to 1912–1937. Out of all 1218 stations, 716 (58.8%) have ≥ 80% error-free data in both 25 year spans. B: Winter Teletherm shifts comparing 1987/1988–2011/2012 relative to 1912/1913–1936/1937. A total of 725 out of 1218, 59.5%, stations have ≥ 80% error-free data. The overall patterns are consistent with those observed for the changes between the consecutive 50 year periods spanning the same 100 years, as displayed in Fig 6 in the main text. For both Teletherms, S11, S12 and S13 Figs show the transitions between consecutive 25 year periods. (TIFF) [file pone.0154184.s010.tiff]

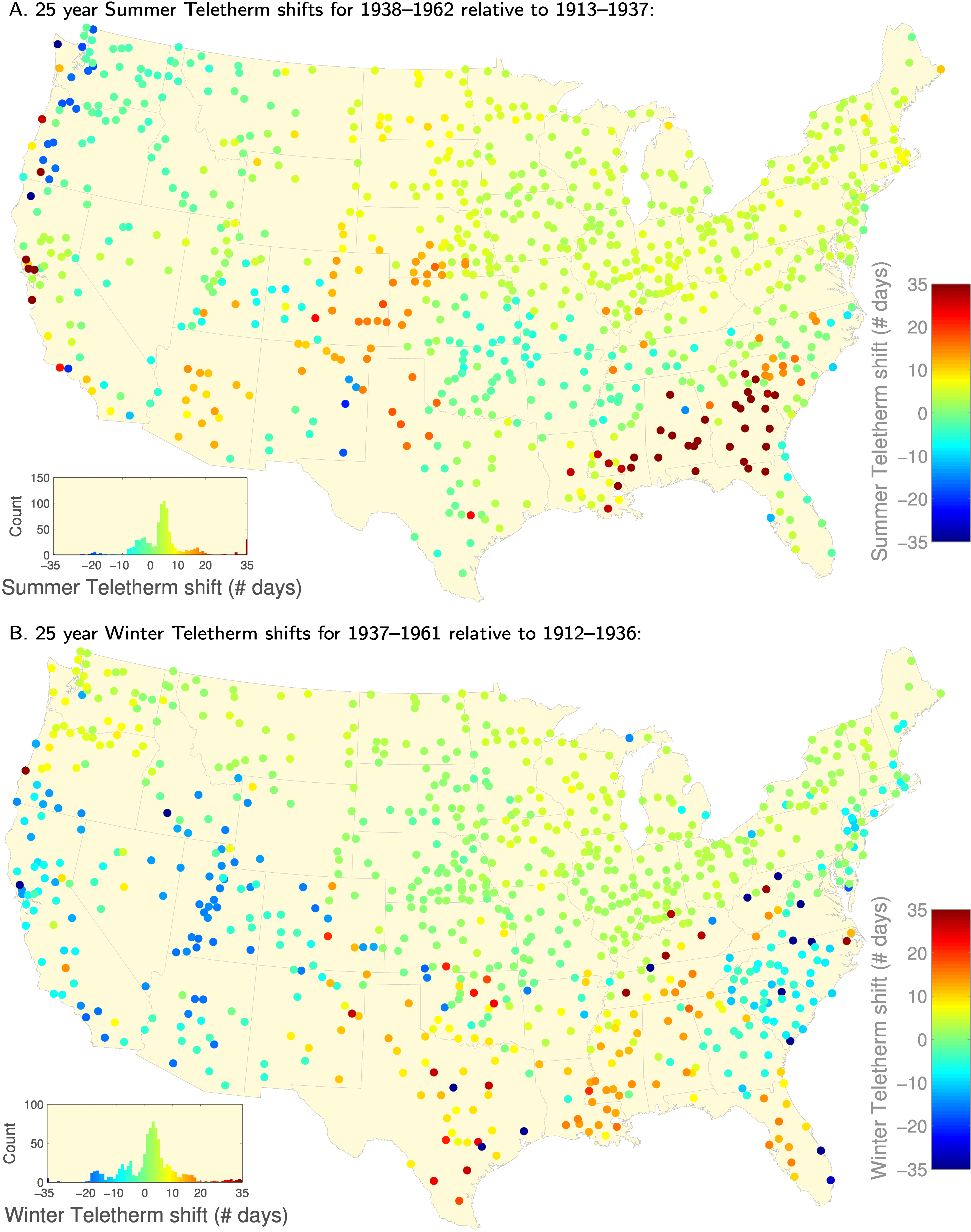

Supplement: S11 Fig — A: Summer Teletherm shifts comparing the 25 year period 1938–1962 relative to 1912–1937 (837 out of 1218, 68.72%, stations have acceptable data). B: Winter Teletherm shifts comparing 1937/1938–1962/1963 relative to 1912/1913–1936/1937 (838 out of 1218, 68.80%, stations have acceptable data). (TIFF) [file pone.0154184.s011.tiff]

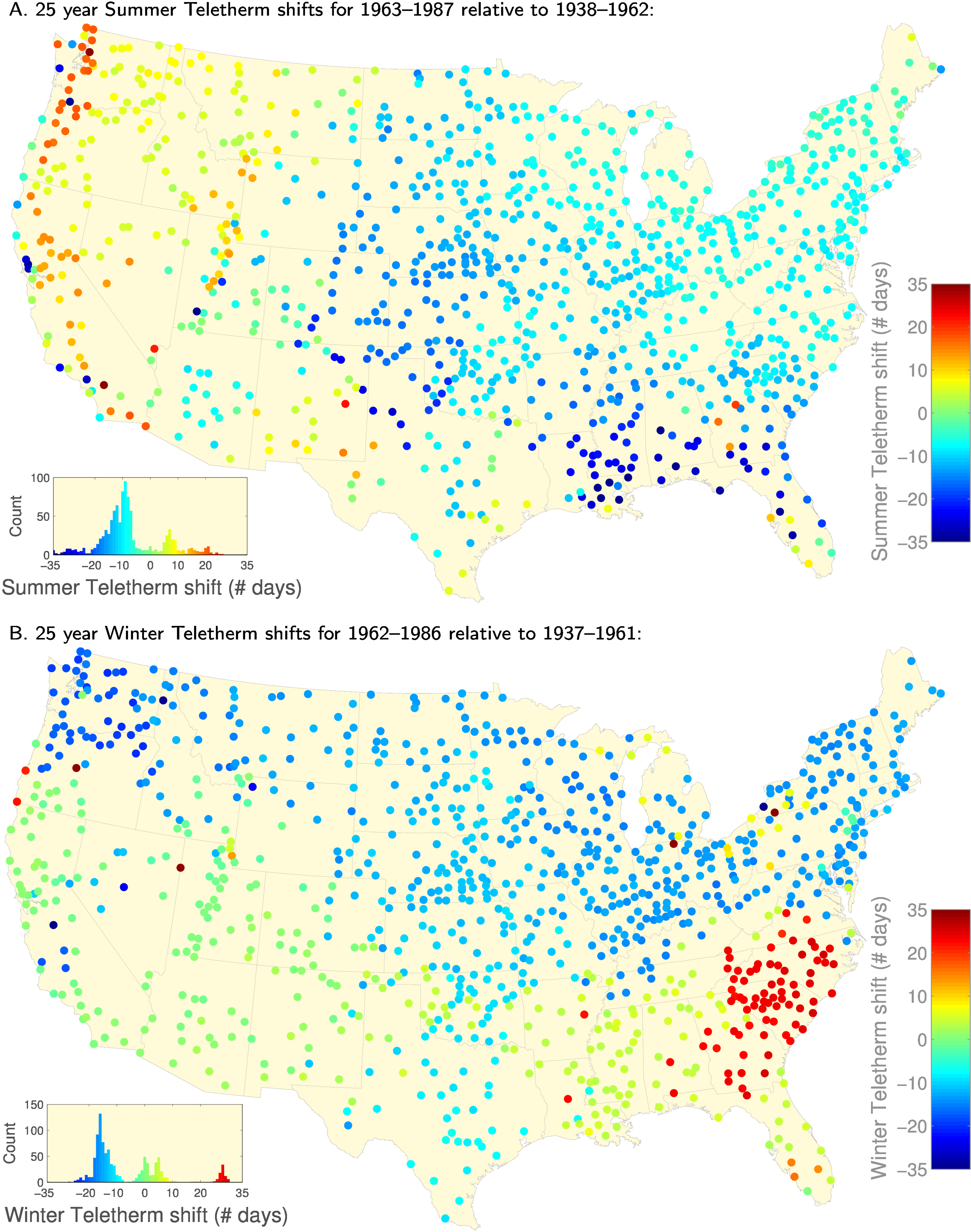

Supplement: S12 Fig — A: Summer Teletherm shifts comparing the 25 year period 1963–1987 relative to 1938–1962 (1001 out of 1218, 82.18%, stations have acceptable data). B: Winter Teletherm shifts comparing 1961/1962–1985/1986 relative to 1937/1938–1961/1962 (1000 out of 1218, 82.10%, stations have acceptable data). (TIFF) [file pone.0154184.s012.tiff]

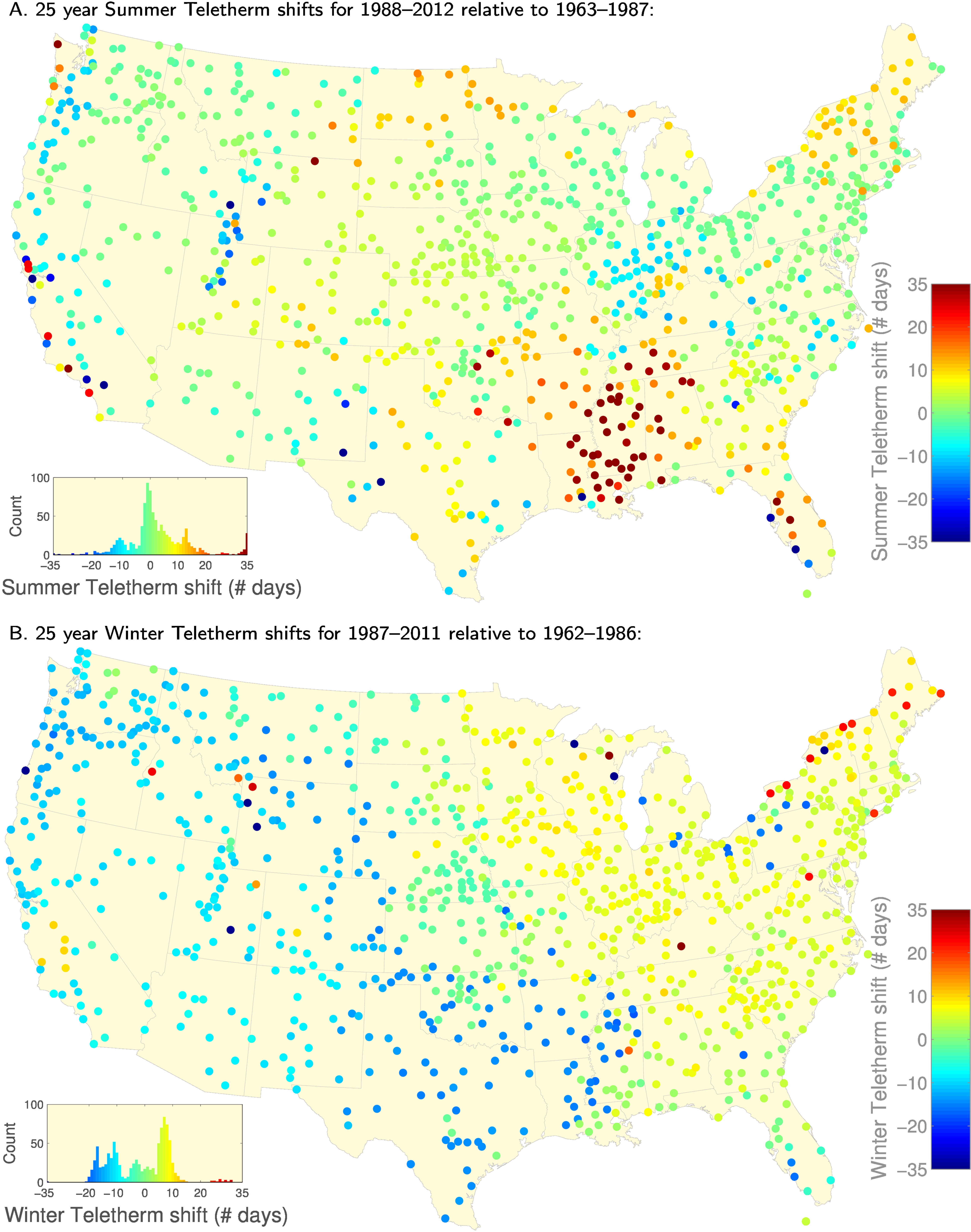

Supplement: S13 Fig — A: Summer Teletherm shifts comparing the 25 year period 1988–2012 relative to 1963–1987 (941 out of 1218, 77.26%, stations have acceptable data). B: Winter Teletherm shifts comparing 1987/1988–2011/2012 relative to 1962/1963–1986/1987 (950 out of 1218, 78.00%, stations have acceptable data). (TIFF) [file pone.0154184.s013.tiff]

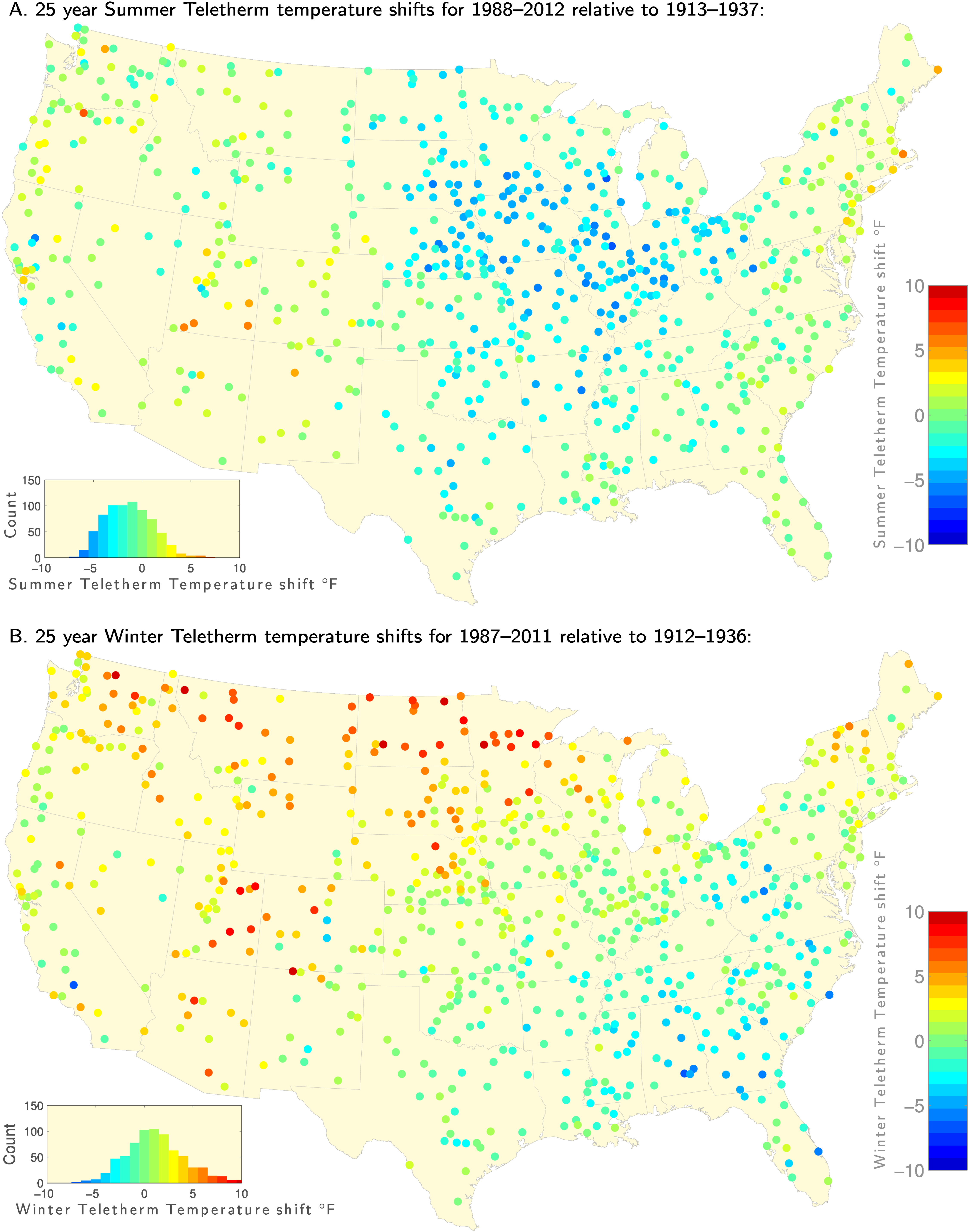

Supplement: S14 Fig — A: Summer Teletherm temperature shifts comparing the 25 year periods 1988–2012 relative to 1912–1937. B: Winter Teletherm temperature shifts comparing 1987/1988–2011/2012 relative to 1912/1913–1936/1937. (TIFF) [file pone.0154184.s014.tiff]

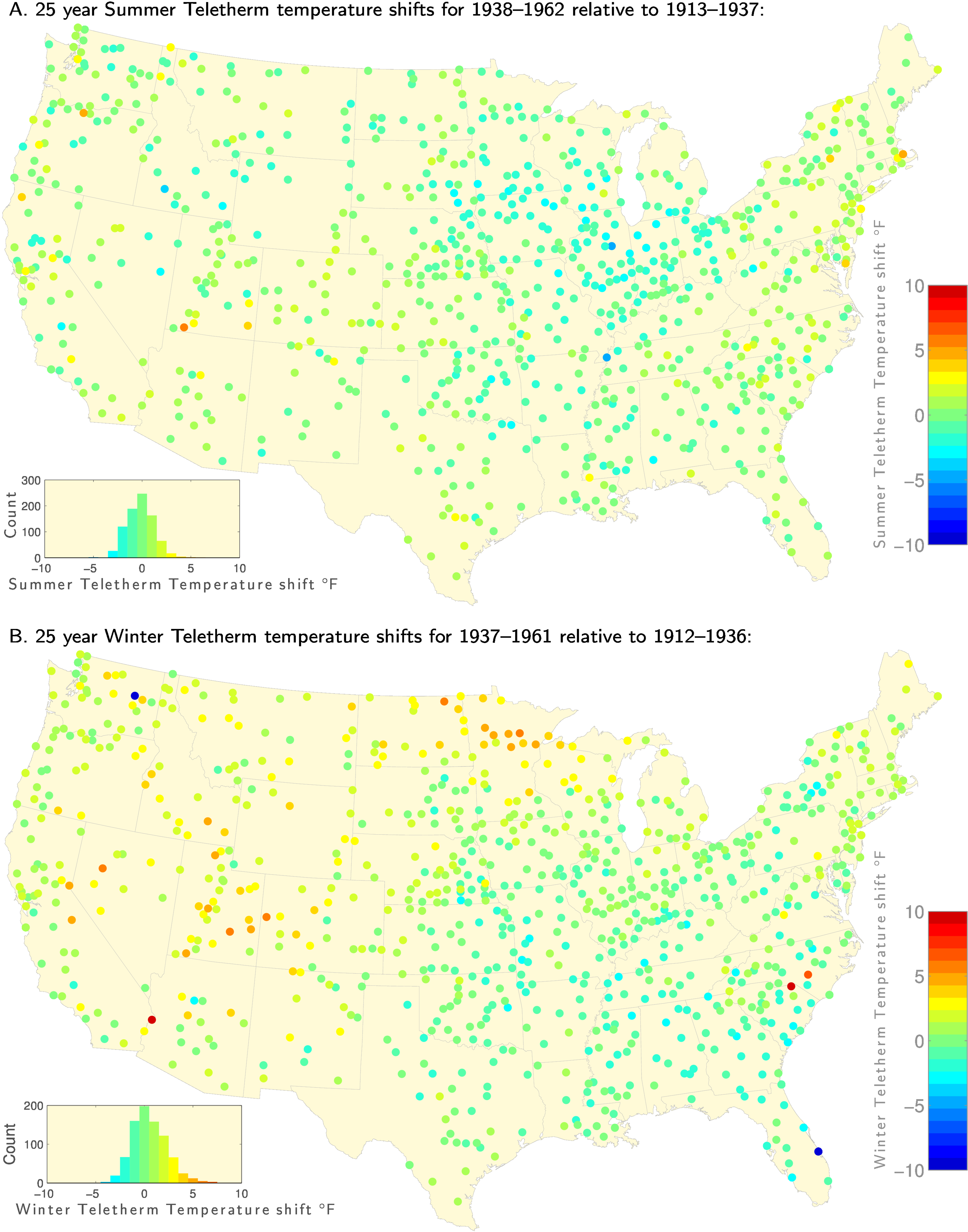

Supplement: S15 Fig — A: Summer Teletherm temperature shifts comparing the 25 year period 1938–1962 relative to 1912–1937. B: Winter Teletherm temperature shifts comparing 1937/1938–1962/1963 relative to 1912/1913–1936/1937. (TIFF) [file pone.0154184.s015.tiff]

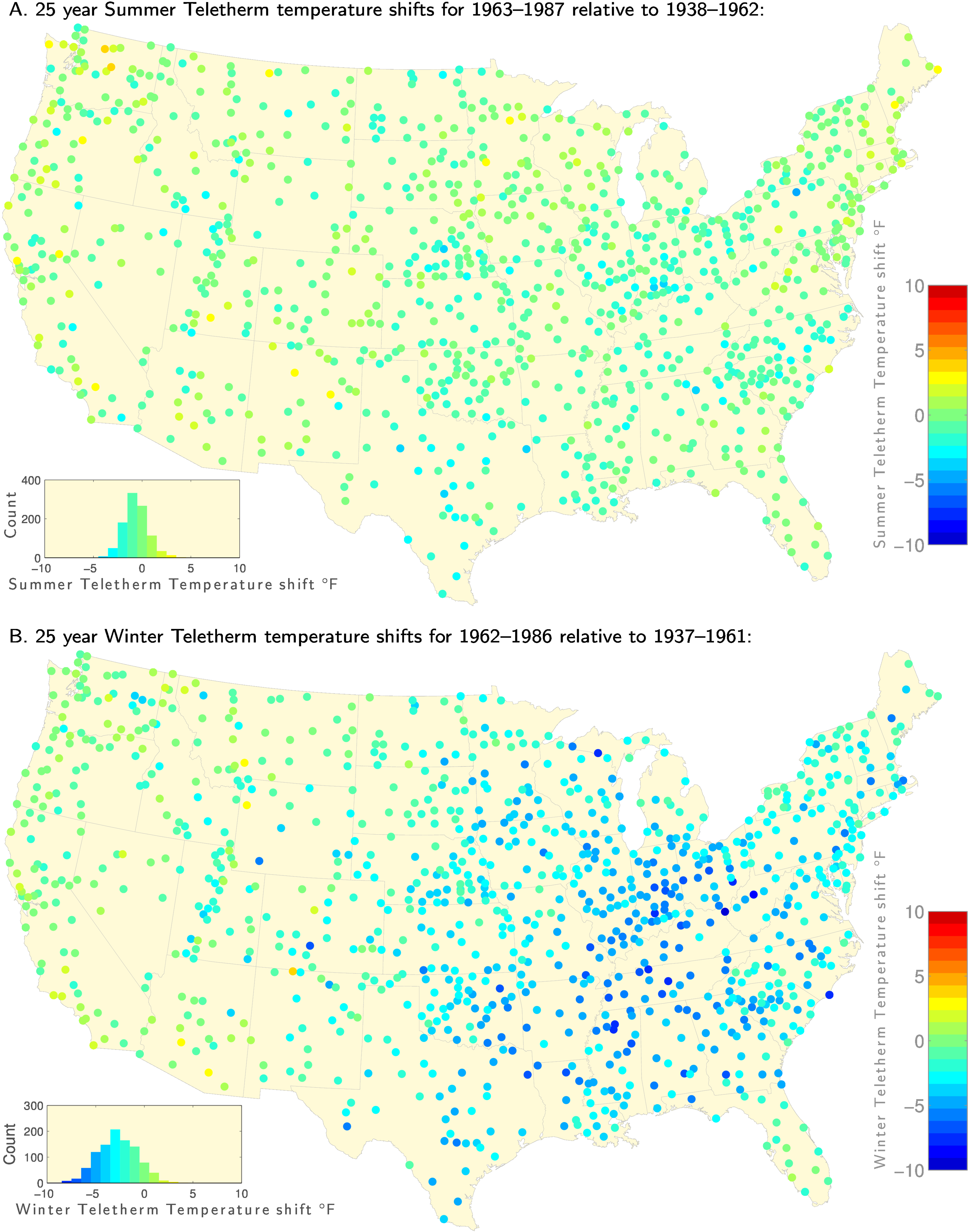

Supplement: S16 Fig — A: Summer Teletherm temperature shifts comparing the 25 year period 1963–1987 relative to 1938–1962. B: Winter Teletherm temperature shifts comparing 1961/1962–1985/1986 relative to 1937/1938–1961/1962. (TIFF) [file pone.0154184.s016.tiff]

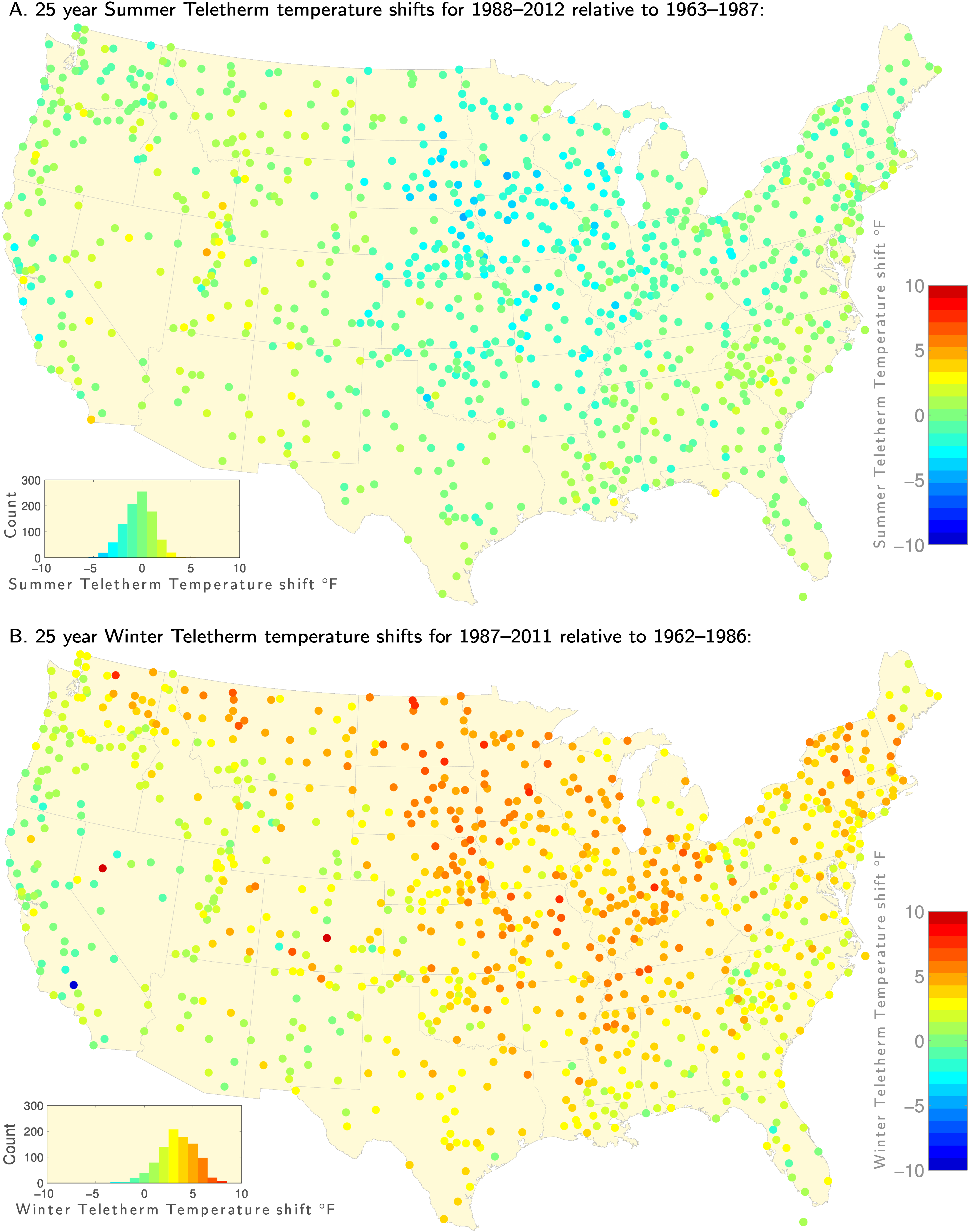

Supplement: S17 Fig — A: Summer Teletherm temperature shifts comparing the 25 year period 1988–2012 relative to 1963–1987. B: Winter Teletherm temperature shifts comparing 1987/1988–2011/2012 relative to 1962/1963–1986/1987. (TIFF) [file pone.0154184.s017.tiff]

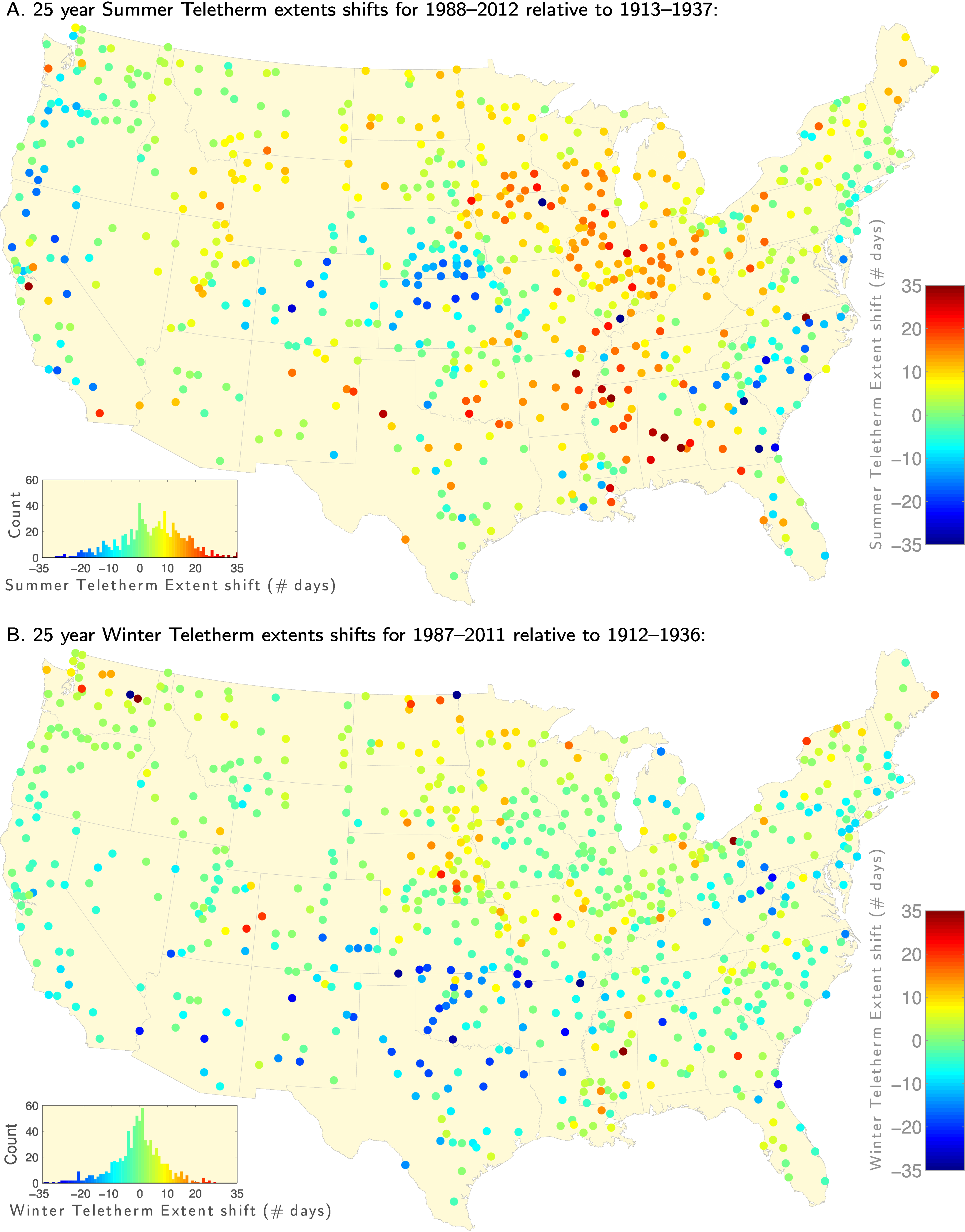

Supplement: S18 Fig — A: Summer Teletherm extent shifts comparing the 25 year periods 1988–2012 relative to 1912–1937. B: Winter Teletherm extent shifts comparing 1987/1988–2011/2012 relative to 1912/1913–1936/1937. (TIFF) [file pone.0154184.s018.tiff]

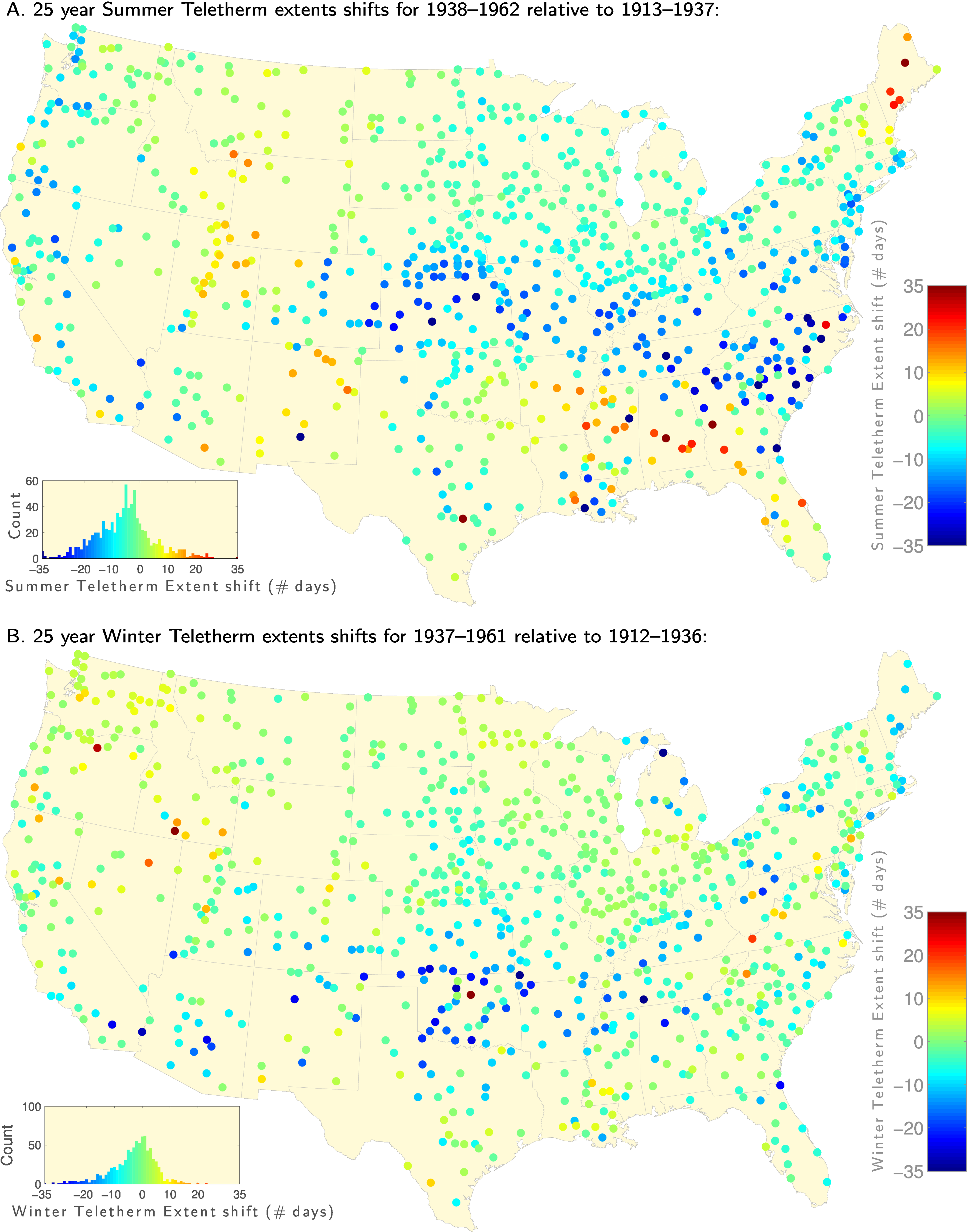

Supplement: S19 Fig — A: Summer Teletherm extent shifts comparing the 25 year period 1938–1962 relative to 1912–1937. B: Winter Teletherm extent shifts comparing 1937/1938–1962/1963 relative to 1912/1913–1936/1937. (TIFF) [file pone.0154184.s019.tiff]

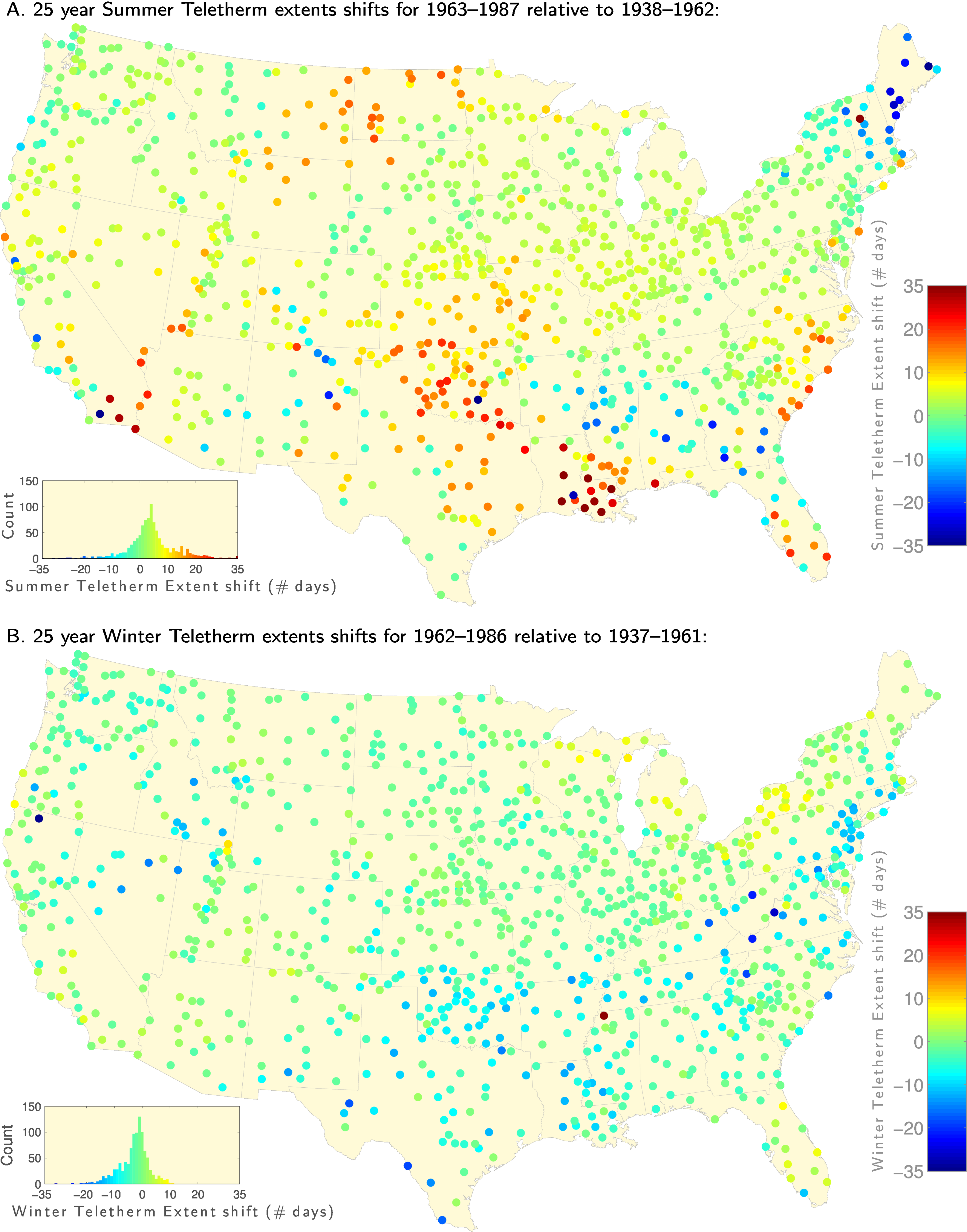

Supplement: S20 Fig — A: Summer Teletherm extent shifts comparing the 25 year period 1963–1987 relative to 1938–1962. B: Winter Teletherm extent shifts comparing 1961/1962–1985/1986 relative to 1937/1938–1961/1962. (TIFF) [file pone.0154184.s020.tiff]

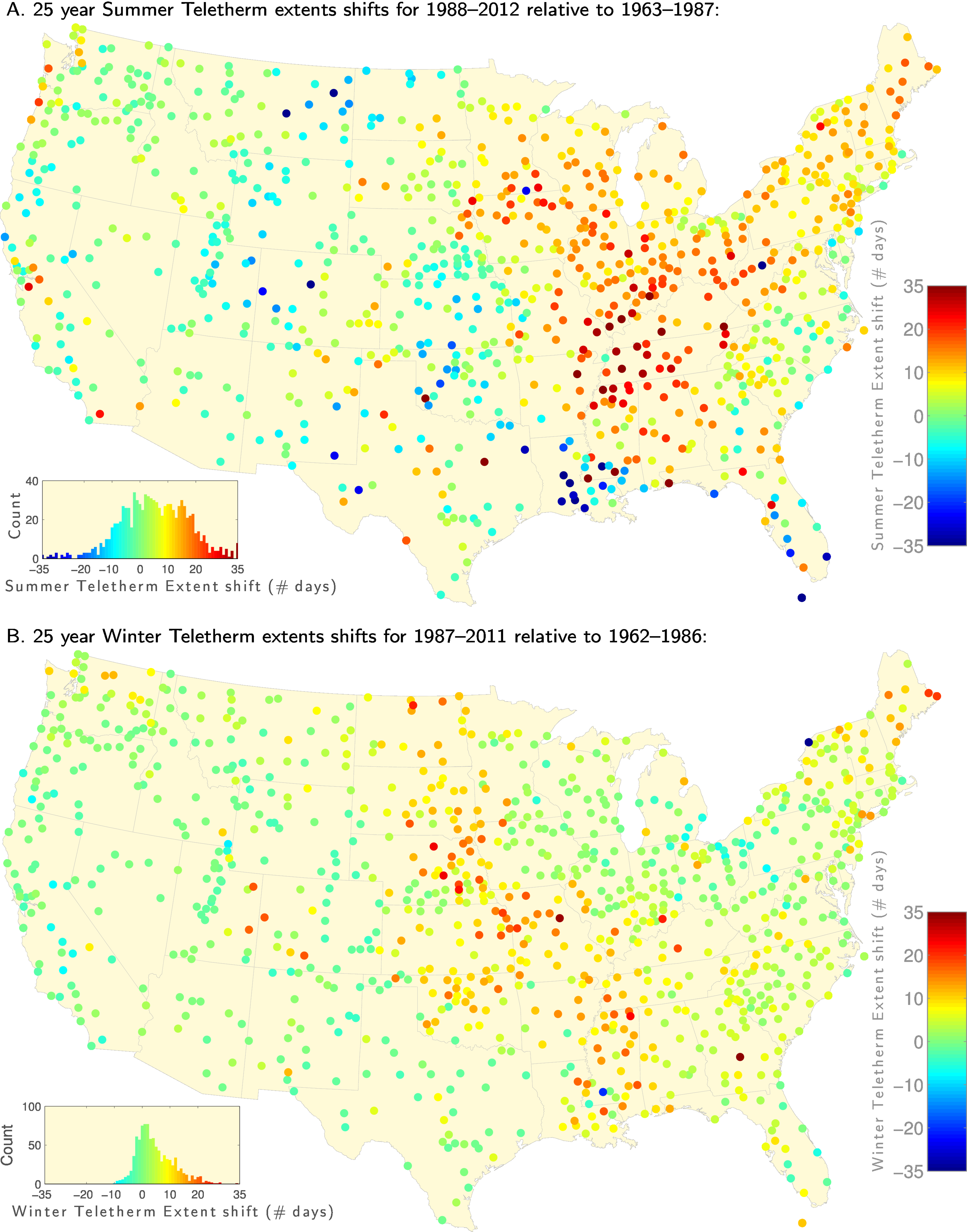

Supplement: S21 Fig — A: Summer Teletherm extent shifts comparing the 25 year period 1988–2012 relative to 1963–1987. B: Winter Teletherm extent shifts comparing 1987/1988–2011/2012 relative to 1962/1963–1986/1987. (TIFF) [file pone.0154184.s021.tiff]
