# Supplementary material for: Tracking Climate Change through the Spatiotemporal Dynamics of the Teletherms, the Statistically Hottest and Coldest Days of the Year
Source: PLoS One. 2016 May 11;11(5):e0154184. doi: 10.1371/journal.pone.0154184 (PMC4864332; doi:10.1371/journal.pone.0154184)

Winter Teletherm—50 year estimates: 1912 to 1961

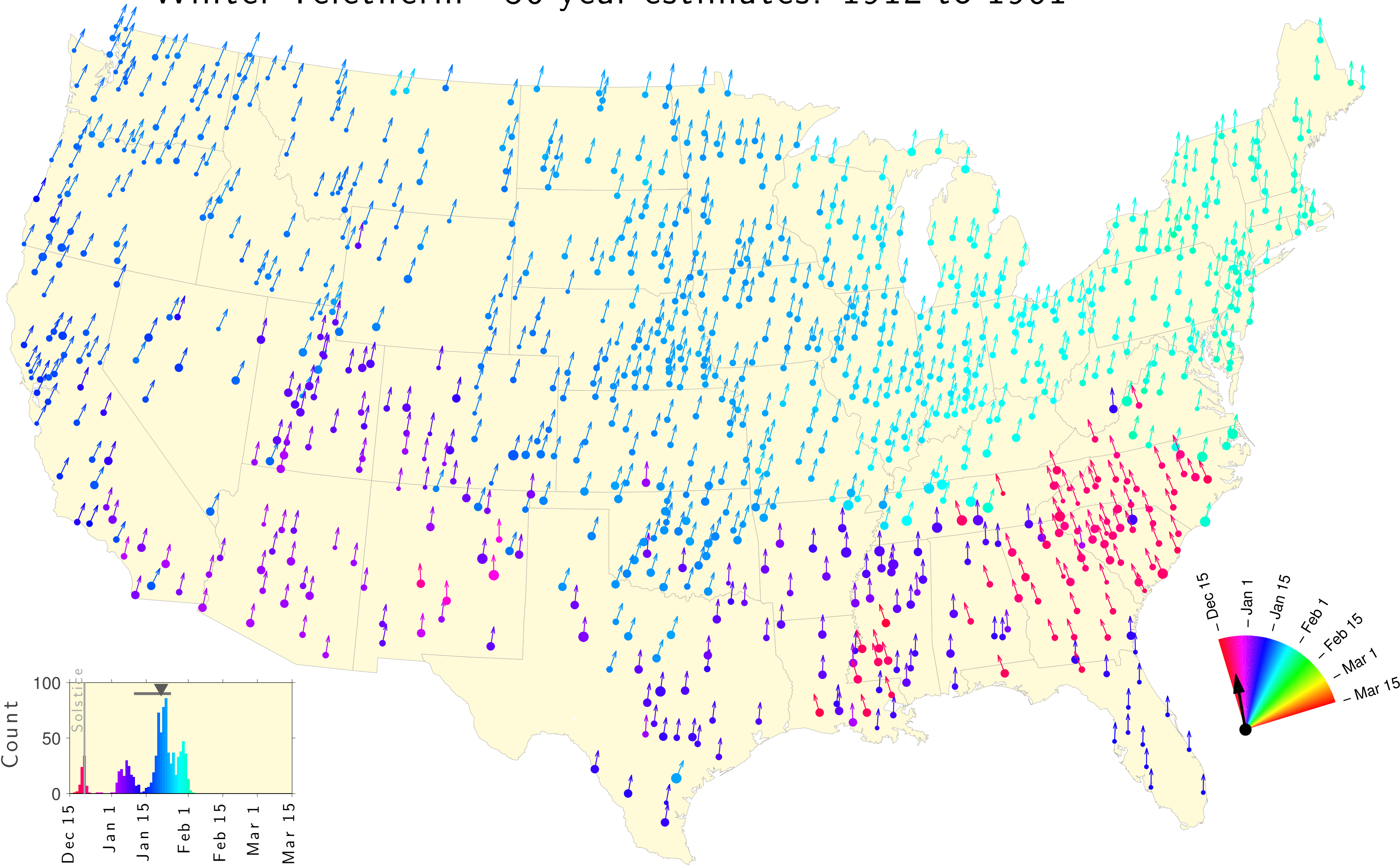

Winter Teletherm—50 year estimates: 1913 to 1962

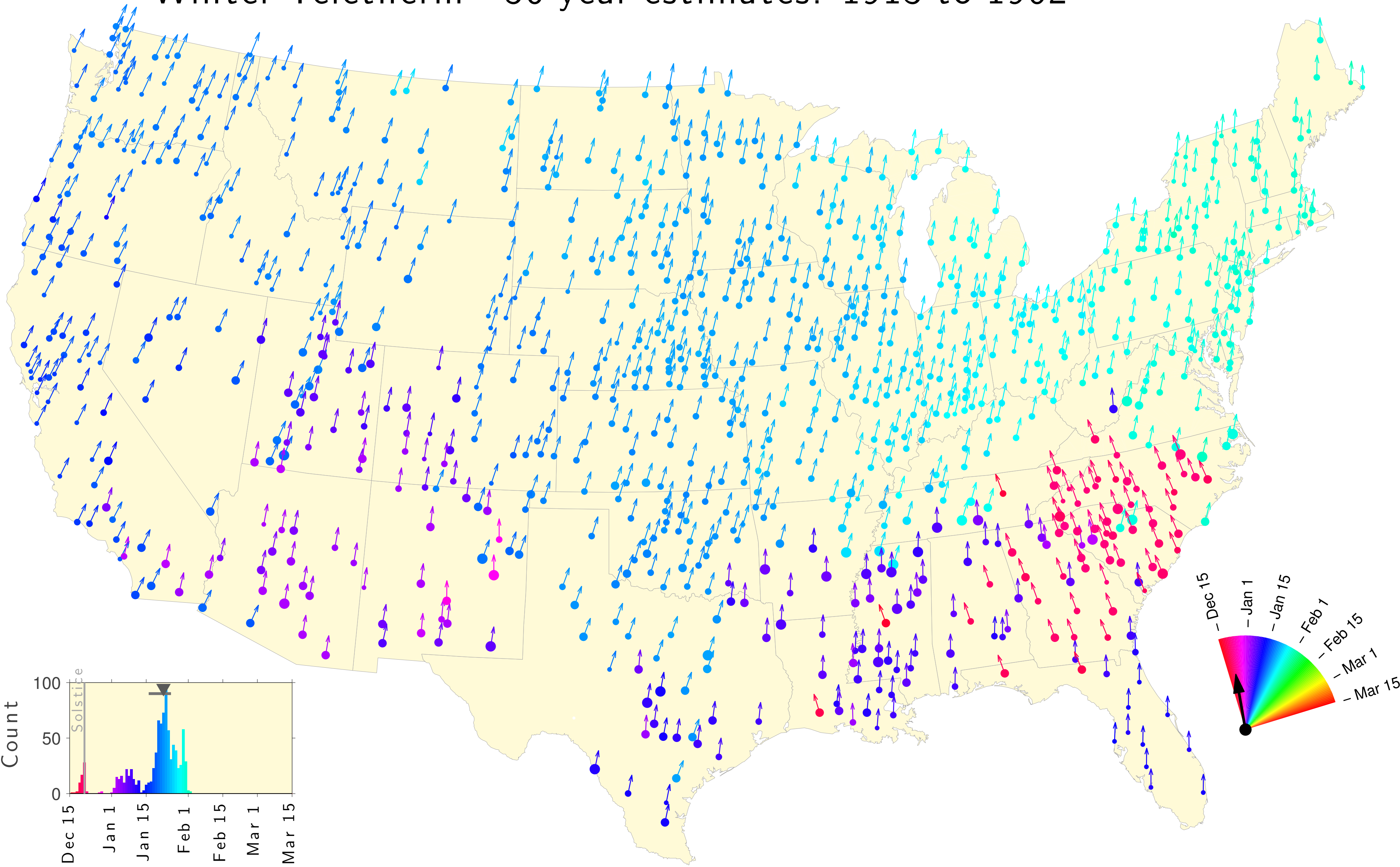

Winter Teletherm—50 year estimates: 1914 to 1963

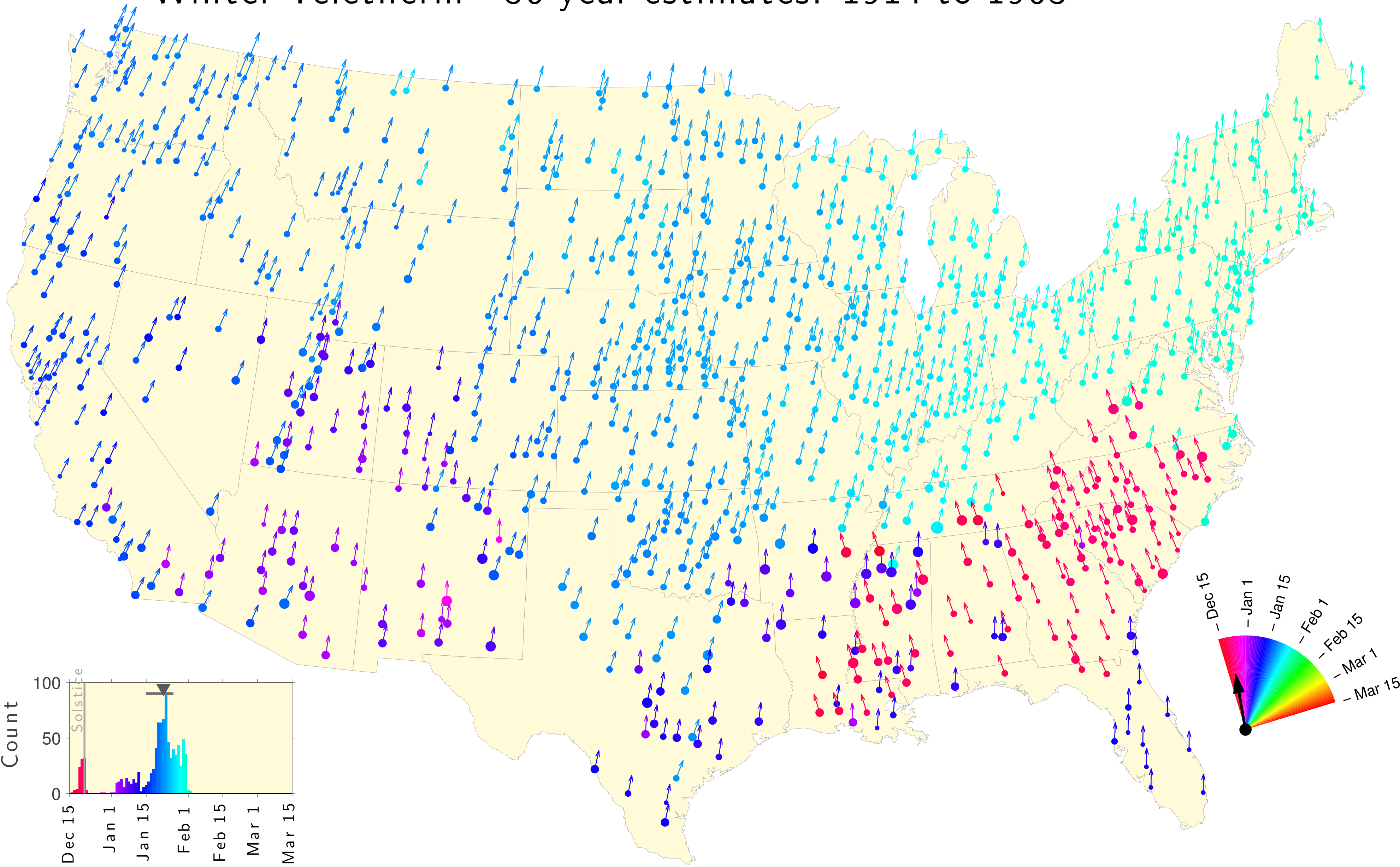

Winter Teletherm—50 year estimates: 1915 to 1964

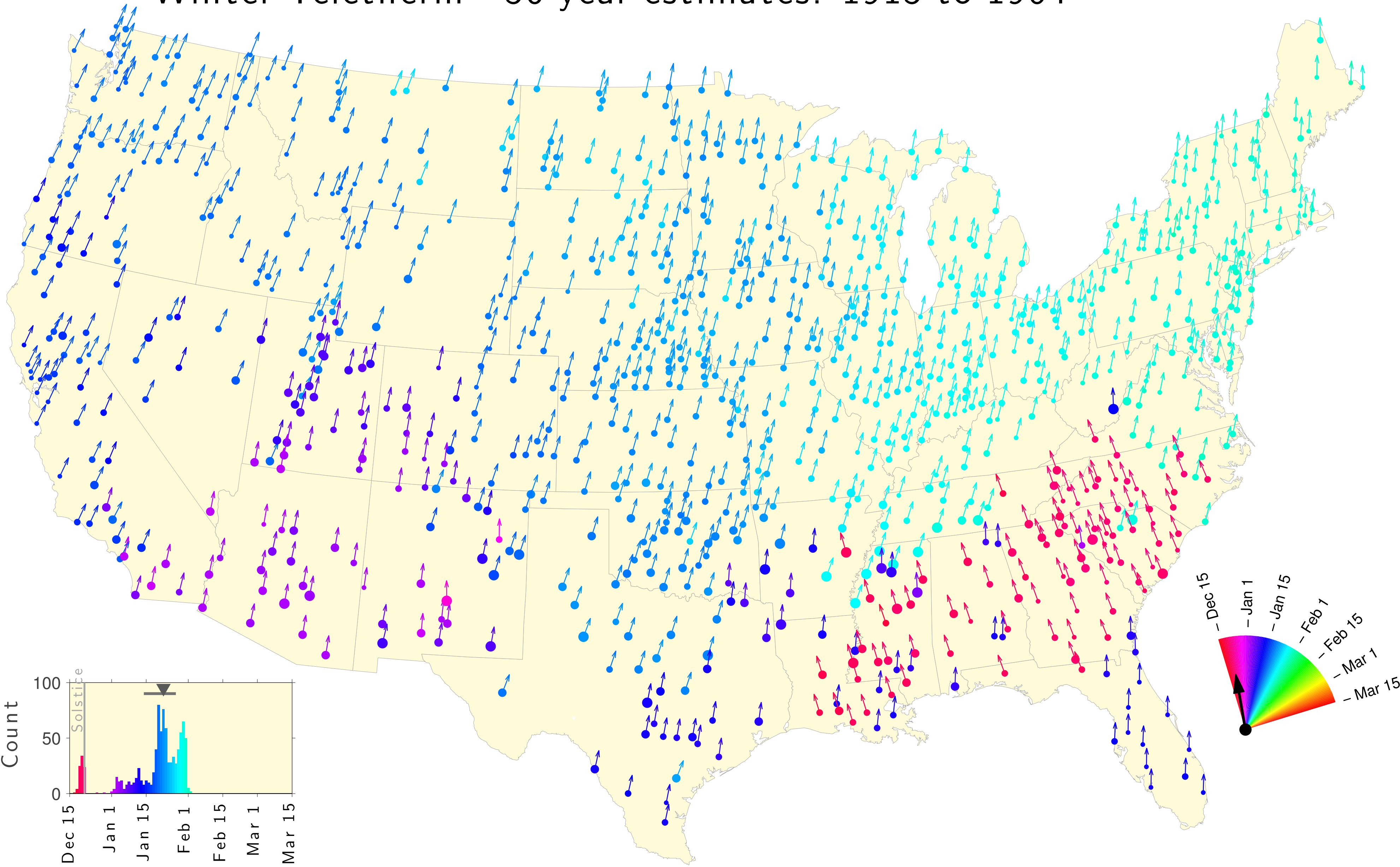

Winter Teletherm—50 year estimates: 1916 to 1965

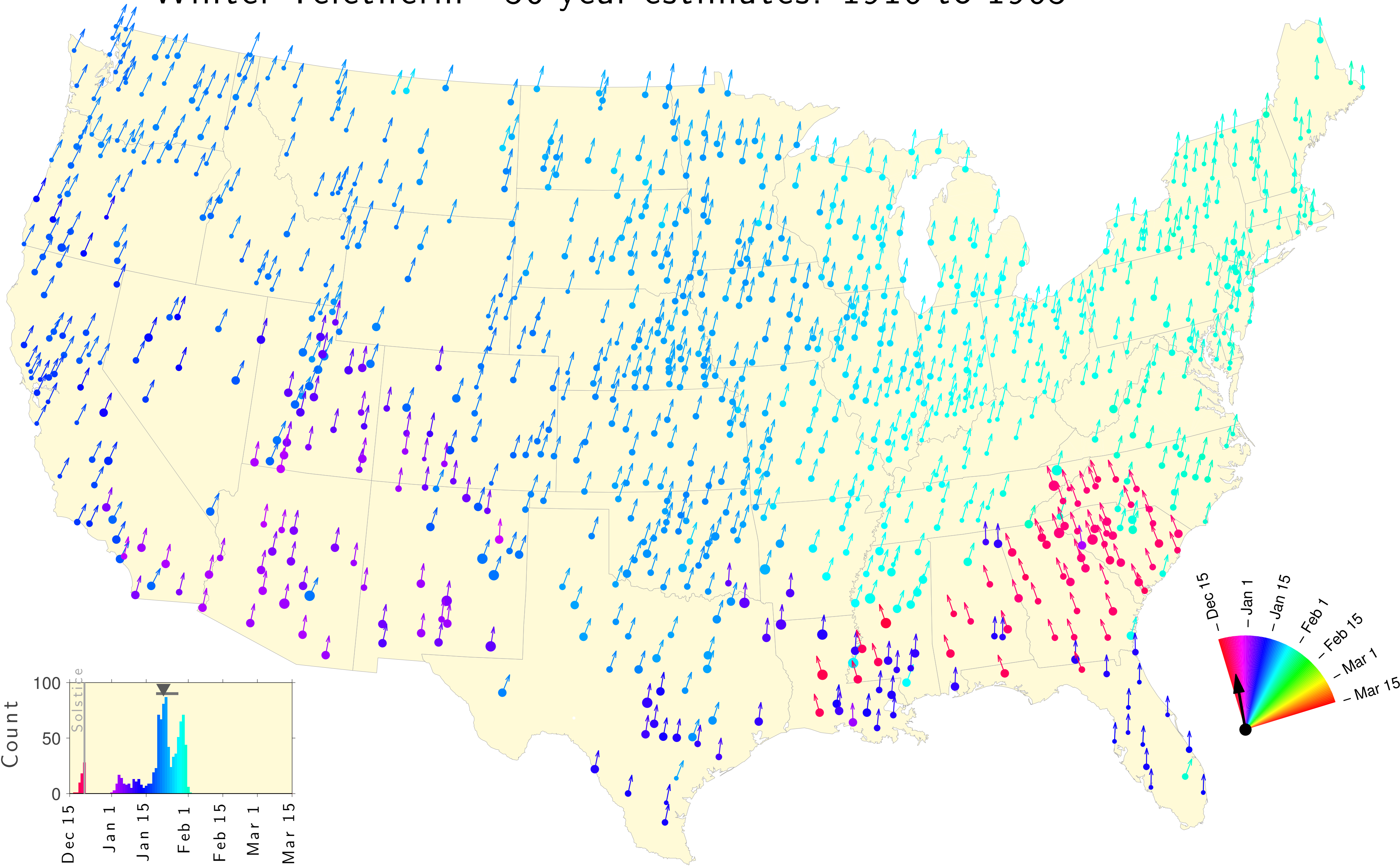

Winter Teletherm—50 year estimates: 1917 to 1966

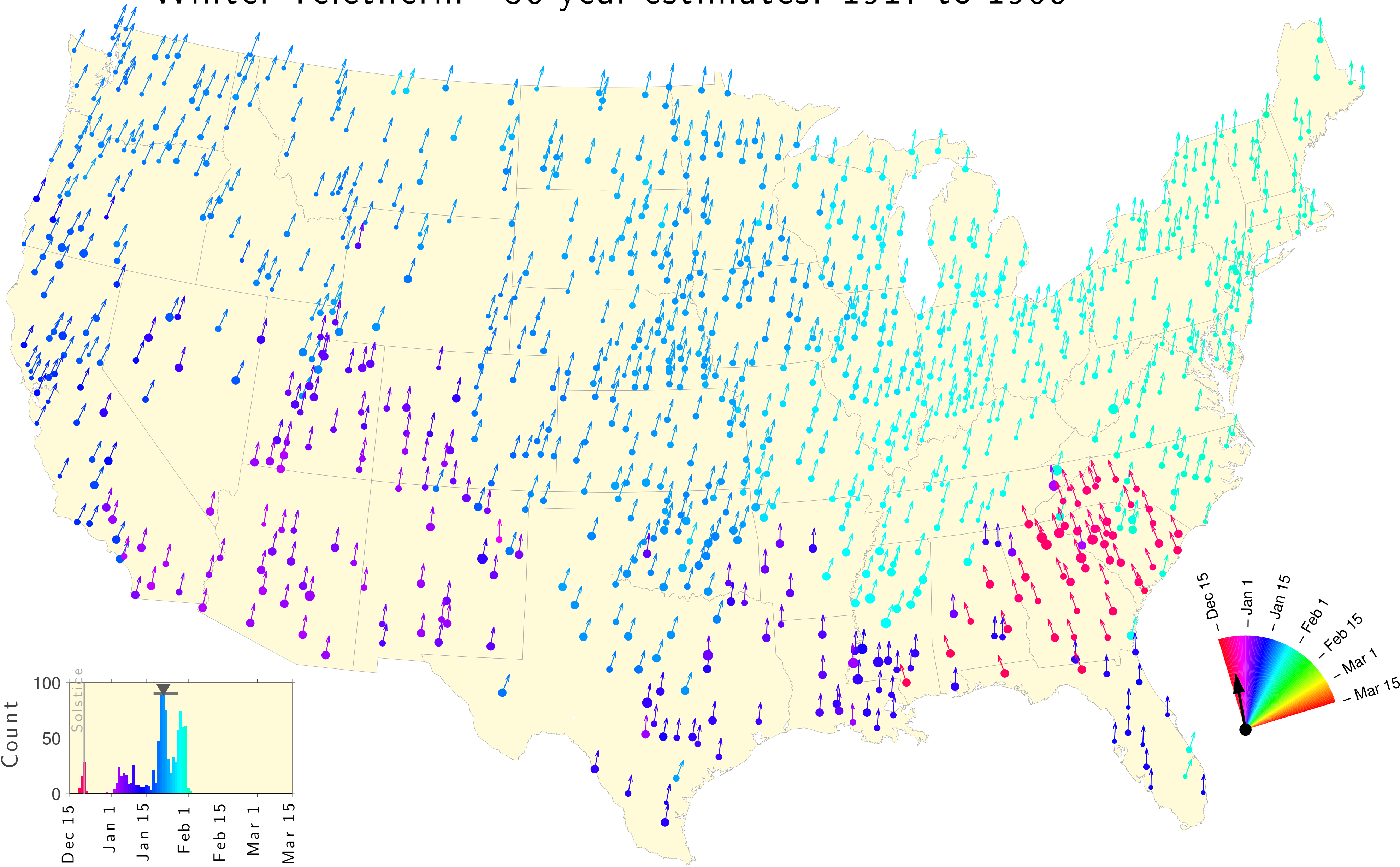

Winter Teletherm—50 year estimates: 1918 to 1967

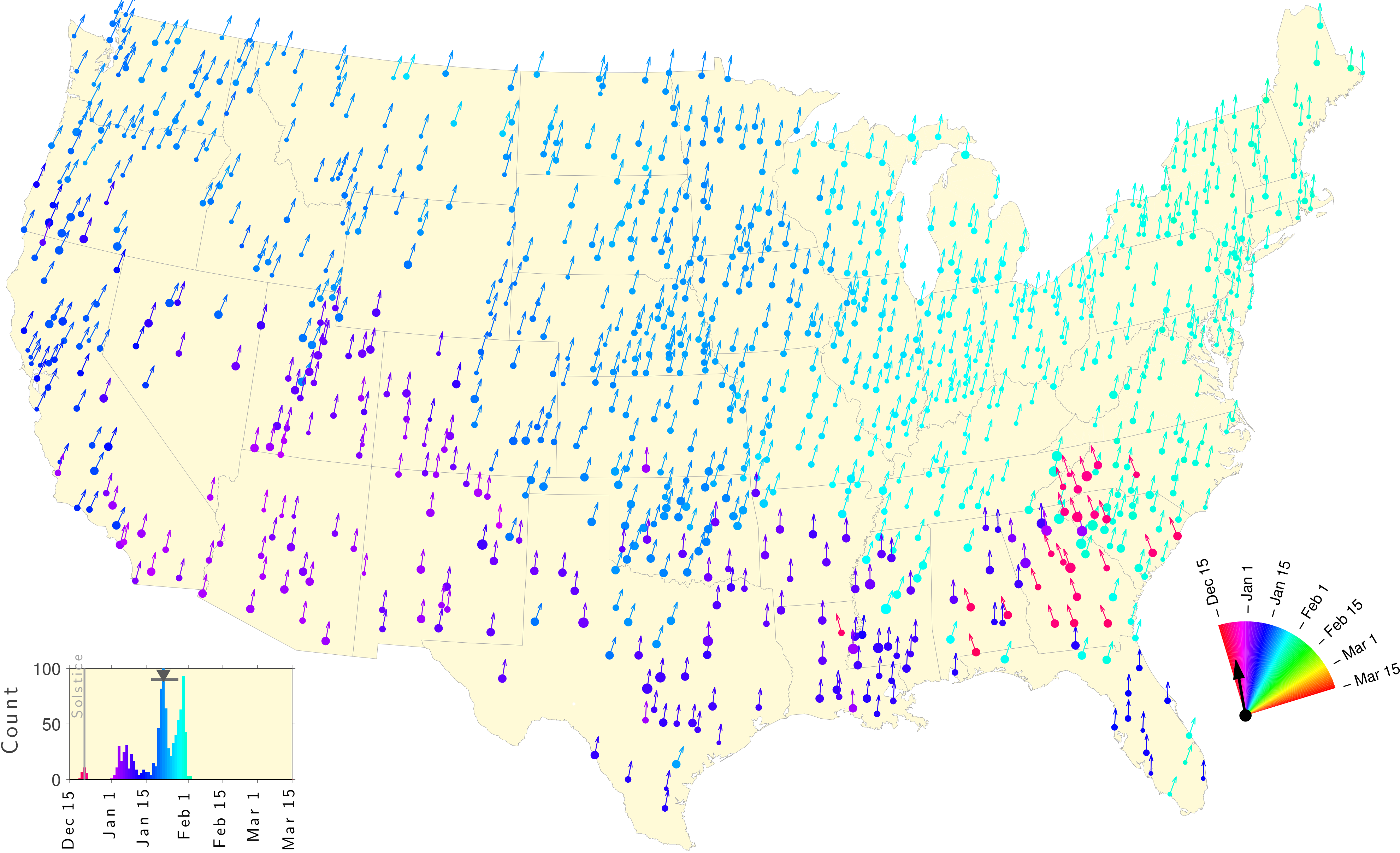

Winter Teletherm—50 year estimates: 1919 to 1968

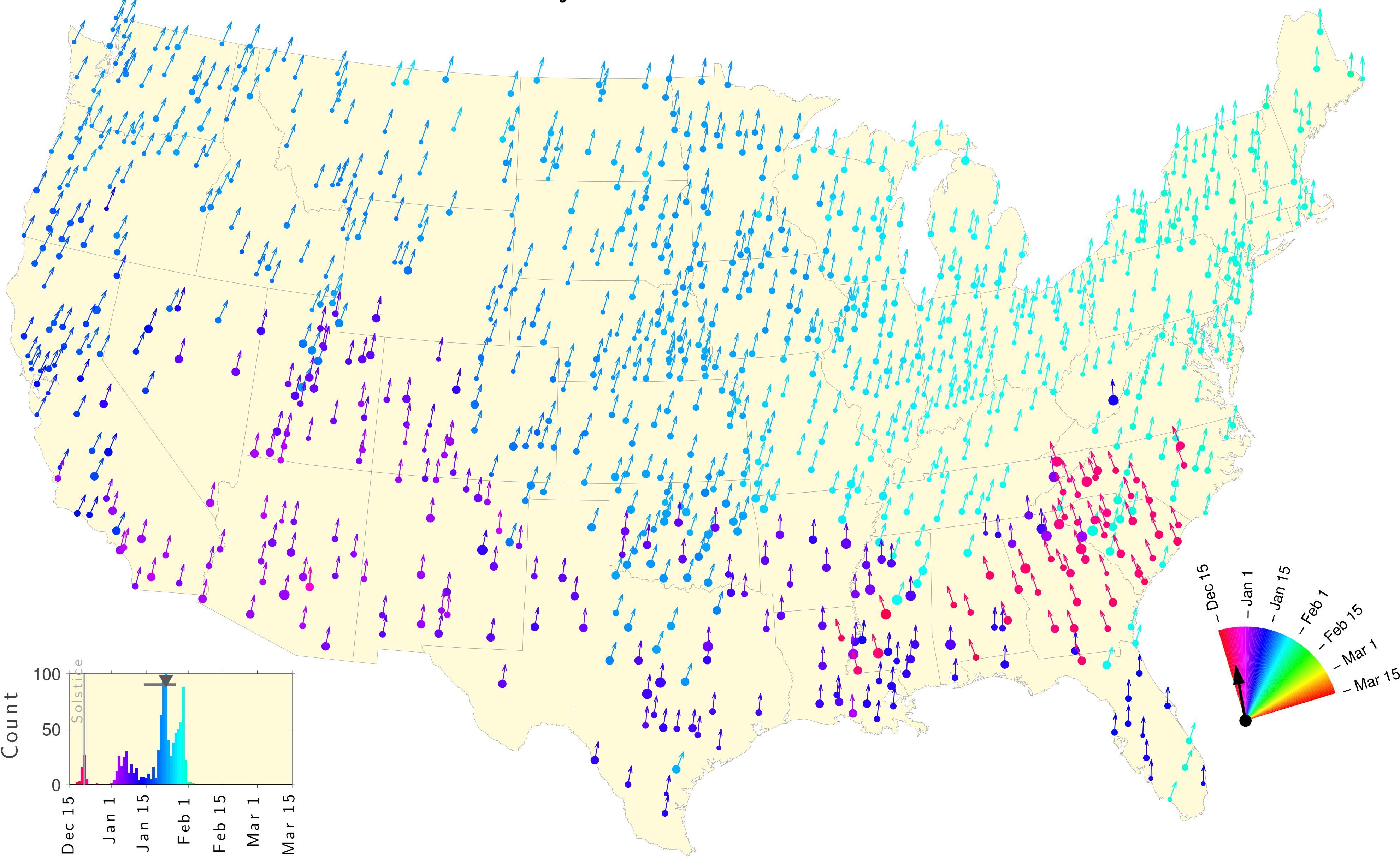

Winter Teletherm—50 year estimates: 1920 to 1969

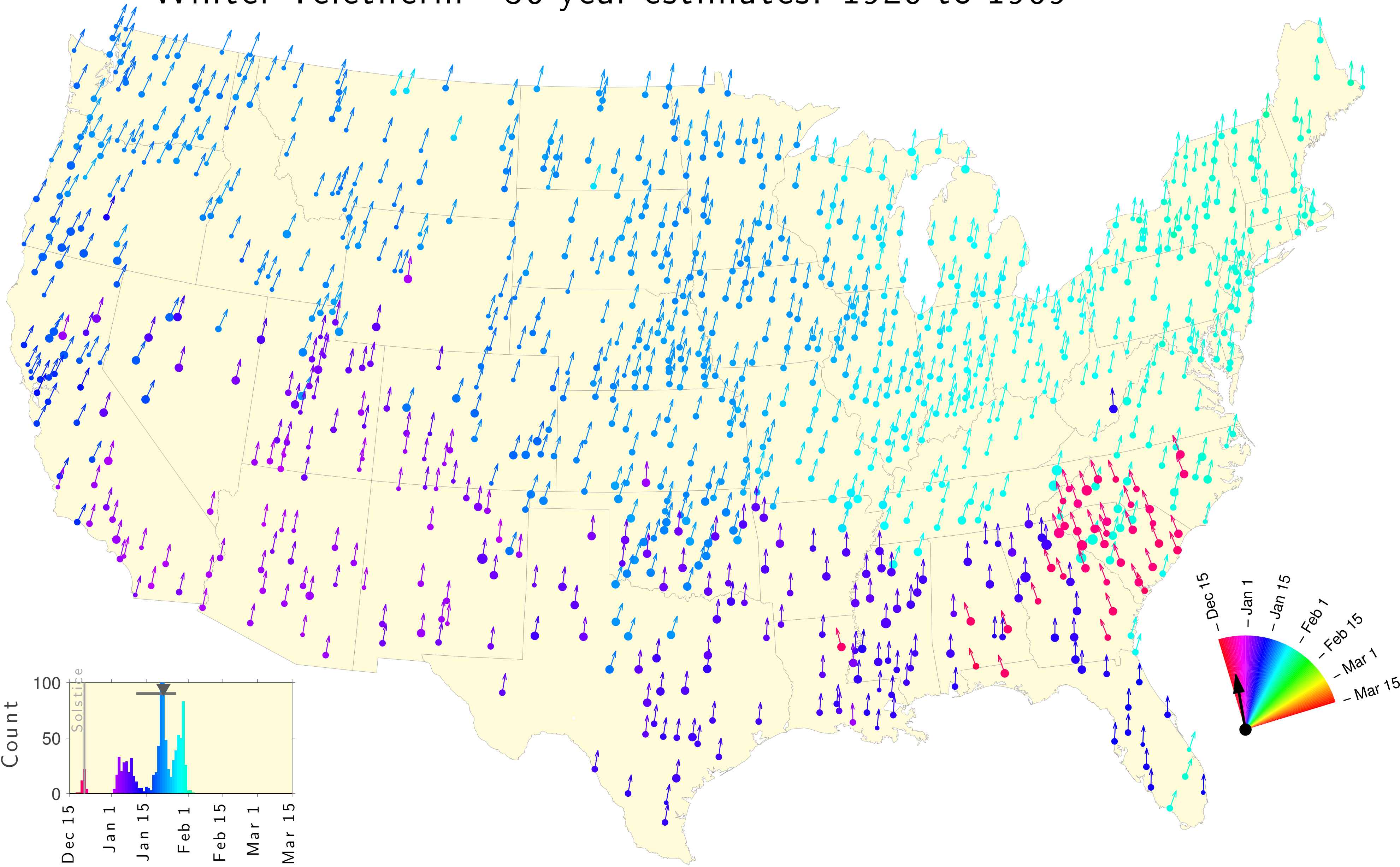

Winter Teletherm—50 year estimates: 1921 to 1970

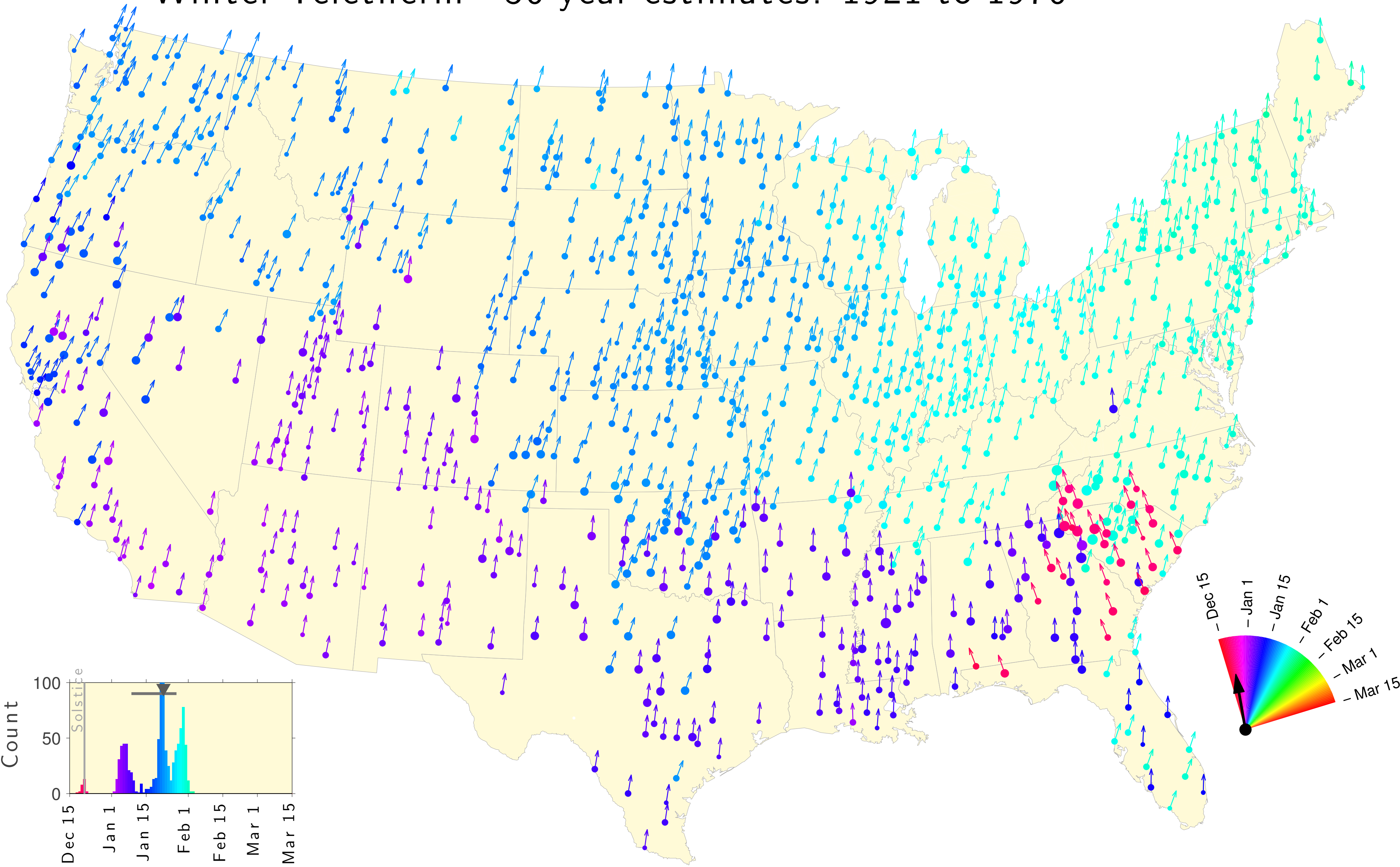

Winter Teletherm—50 year estimates: 1922 to 1971

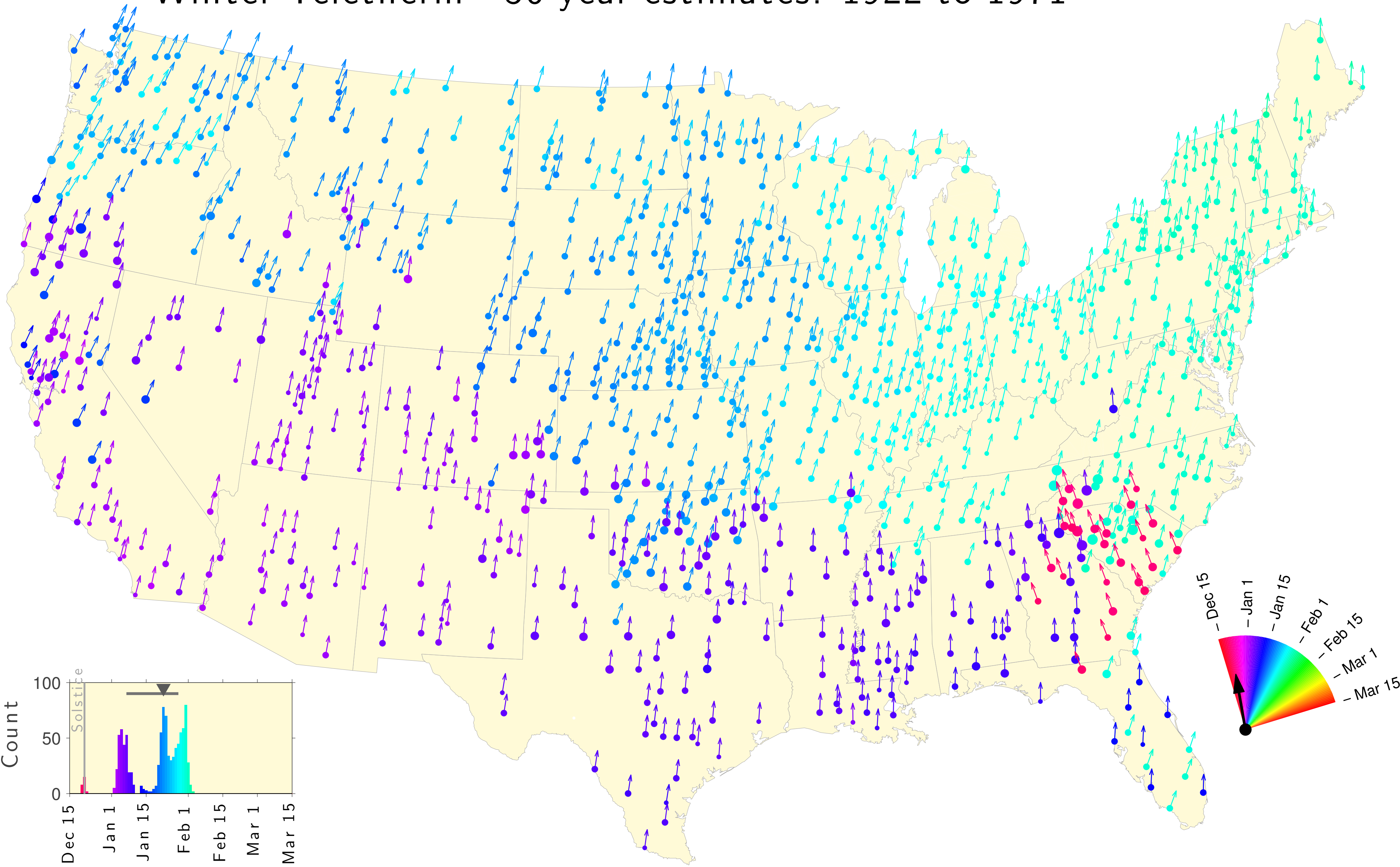

Winter Teletherm—50 year estimates: 1923 to 1972

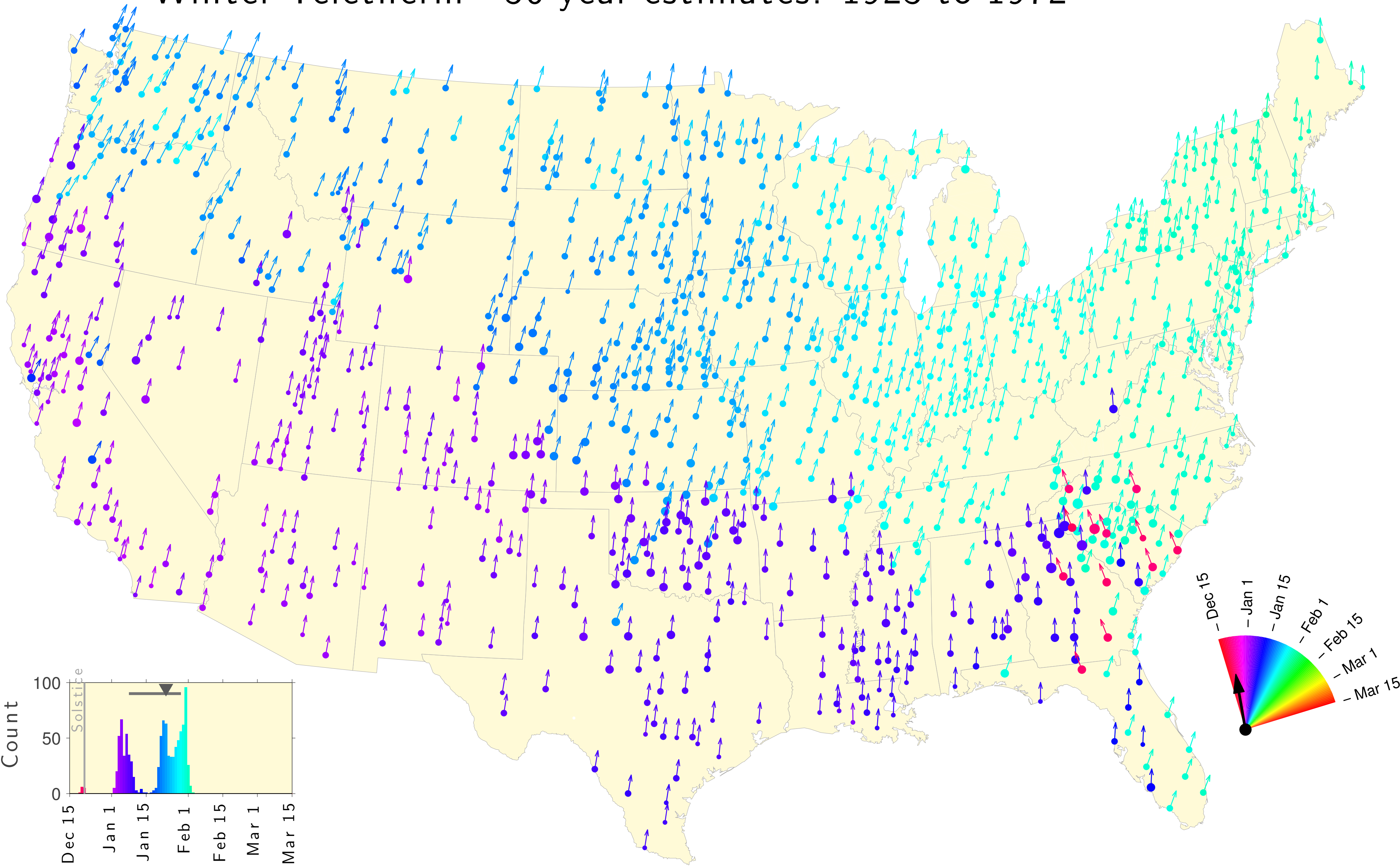

Winter Teletherm—50 year estimates: 1924 to 1973

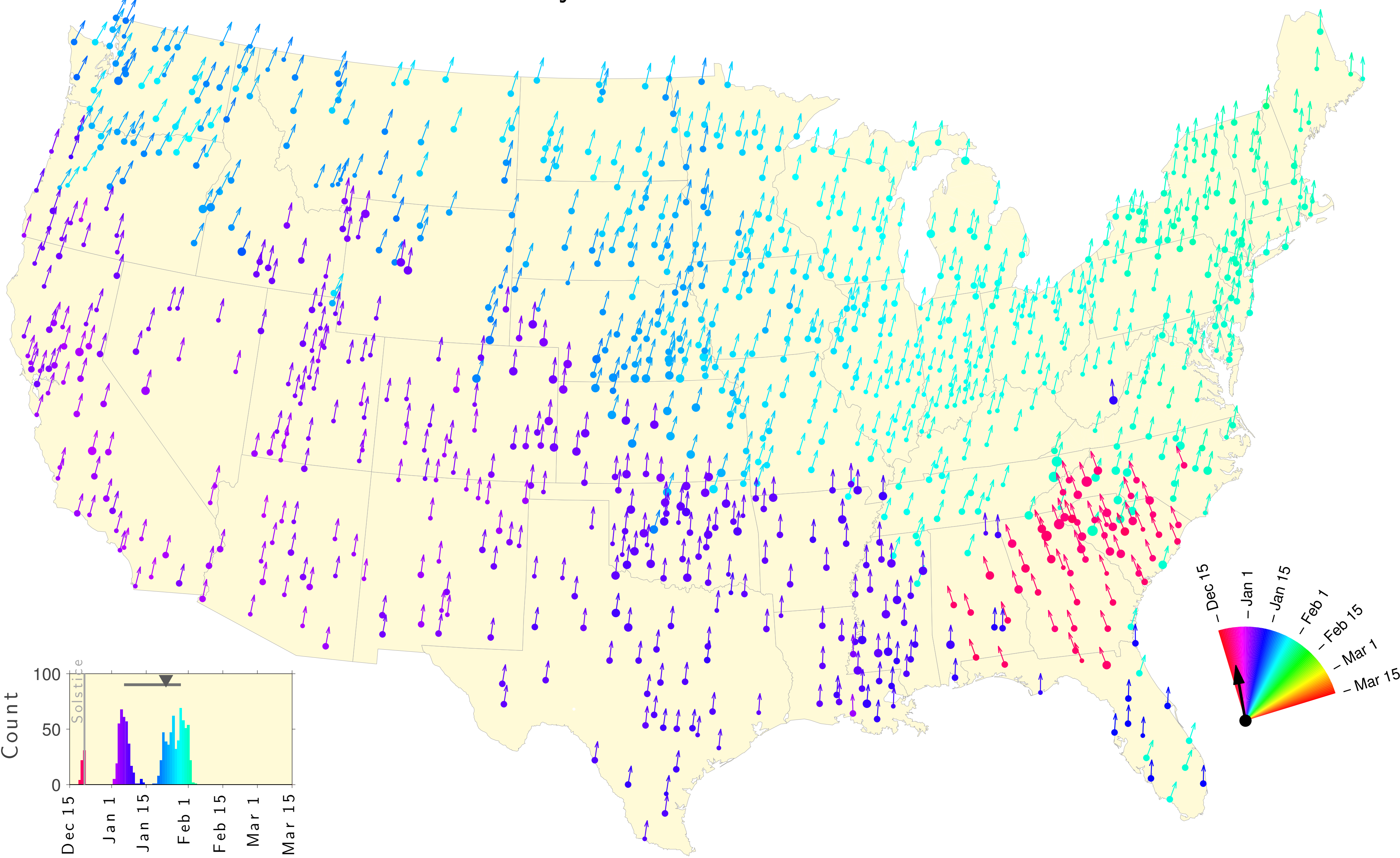

Winter Teletherm—50 year estimates: 1925 to 1974

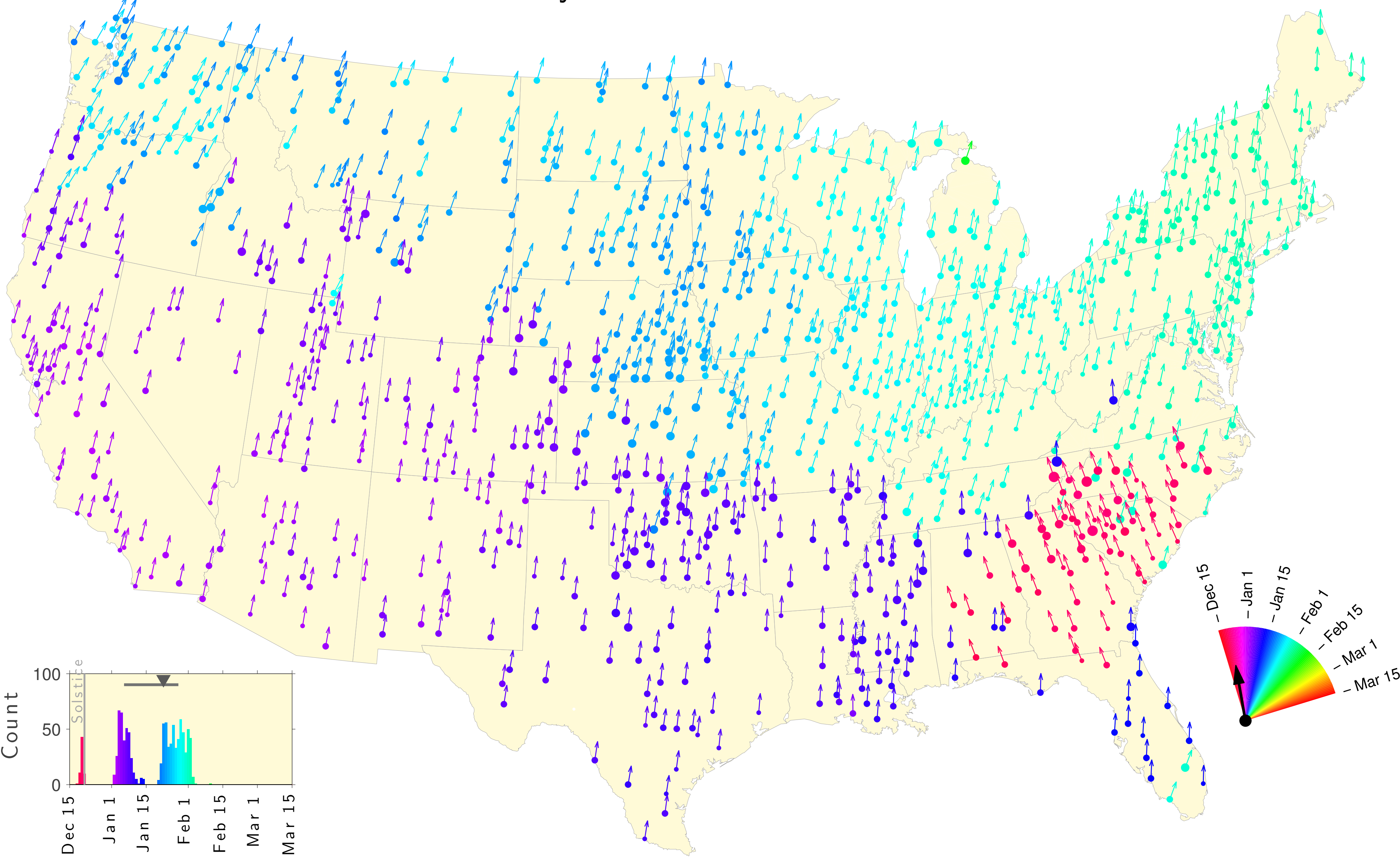

Winter Teletherm—50 year estimates: 1926 to 1975

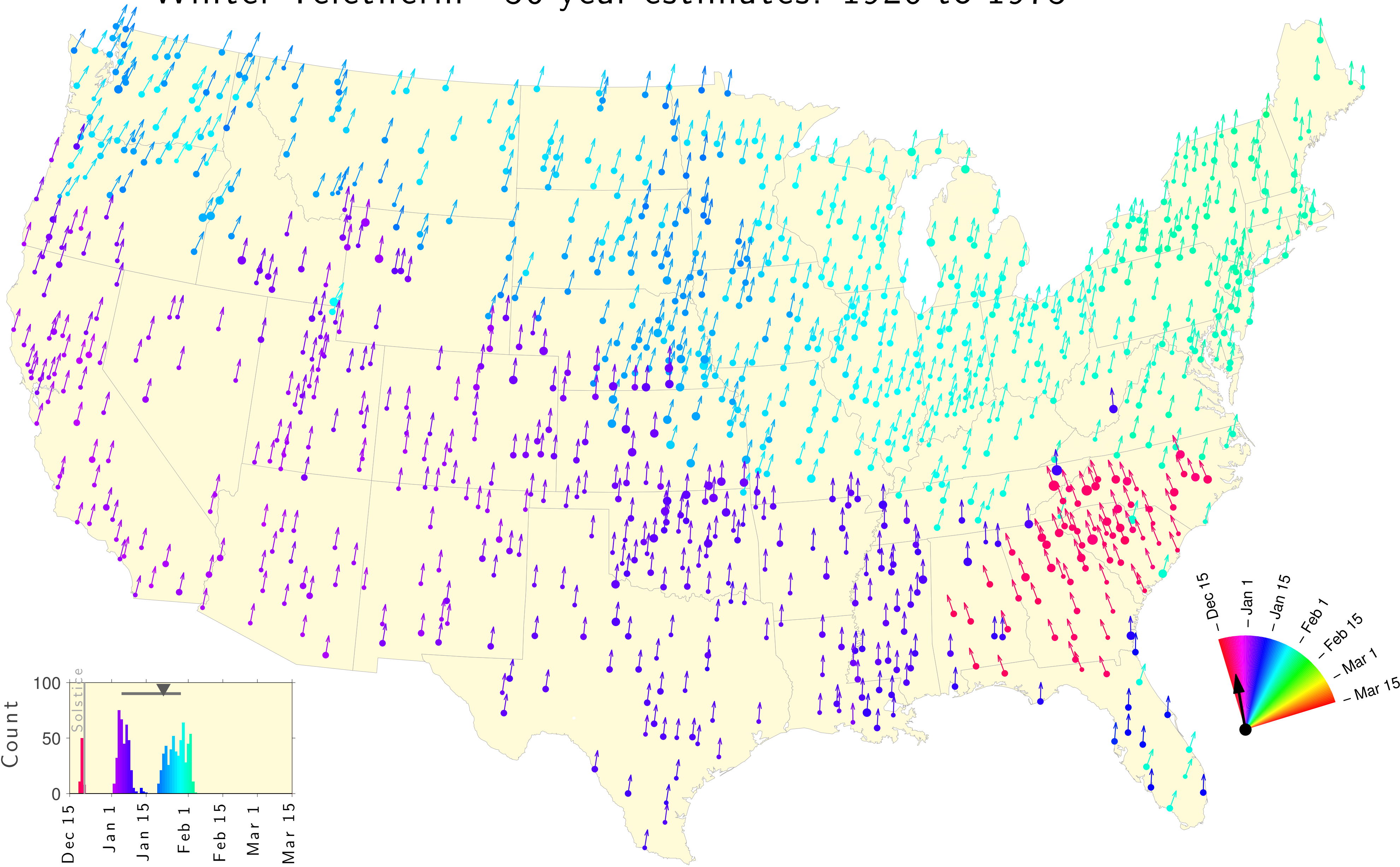

Winter Teletherm—50 year estimates: 1927 to 1976

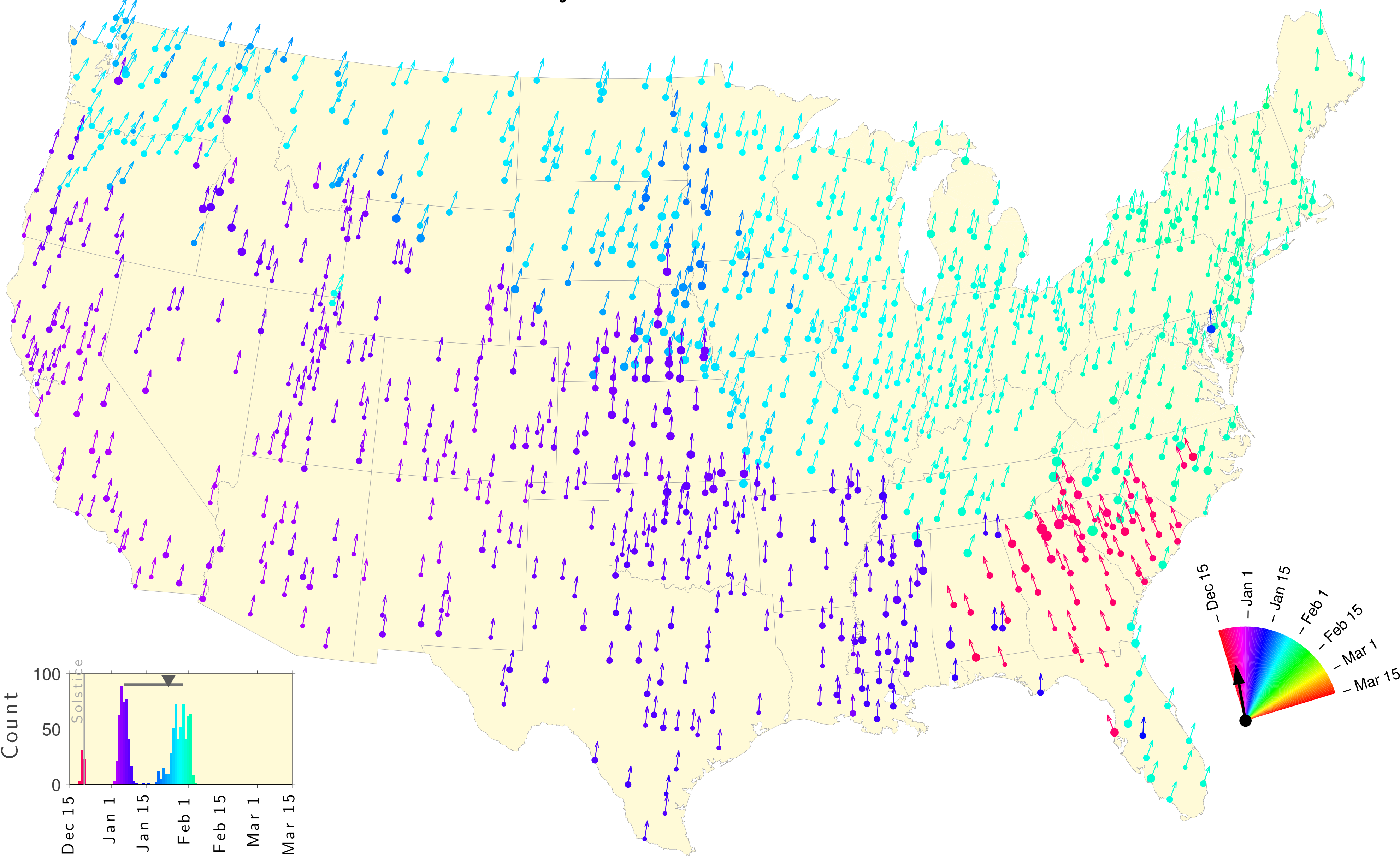

Winter Teletherm—50 year estimates: 1928 to 1977

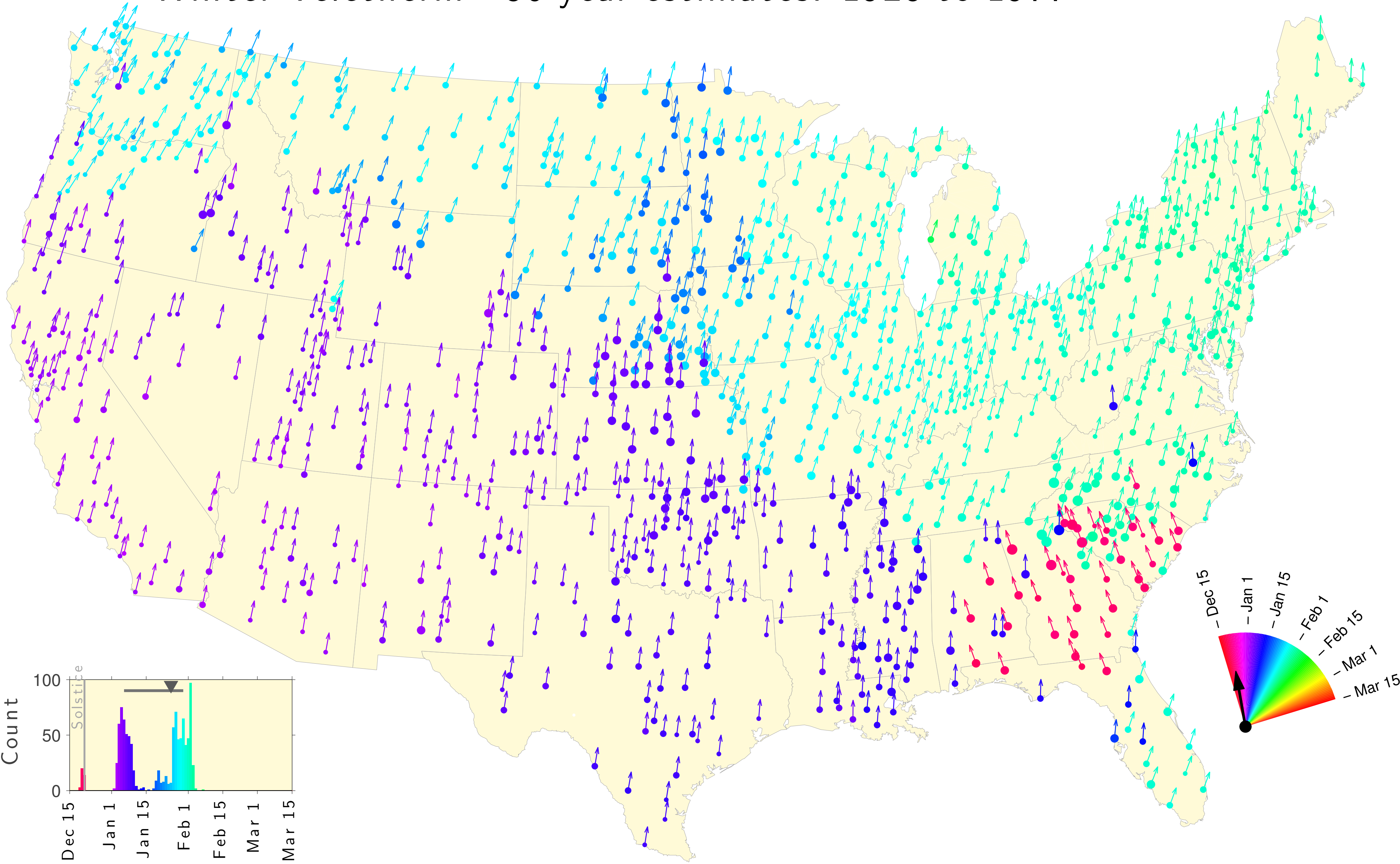

Winter Teletherm—50 year estimates: 1929 to 1978

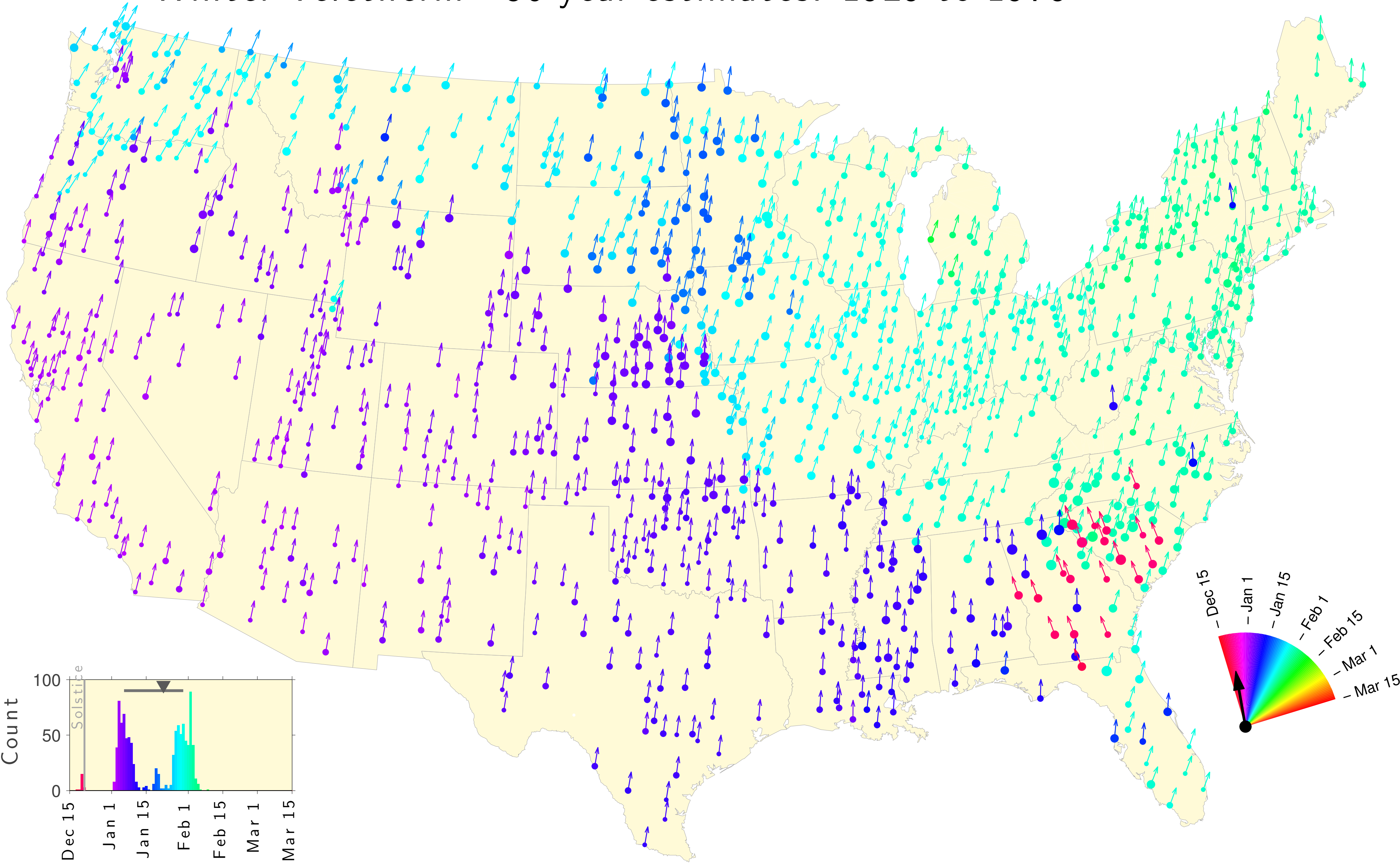

Winter Teletherm—50 year estimates: 1930 to 1979

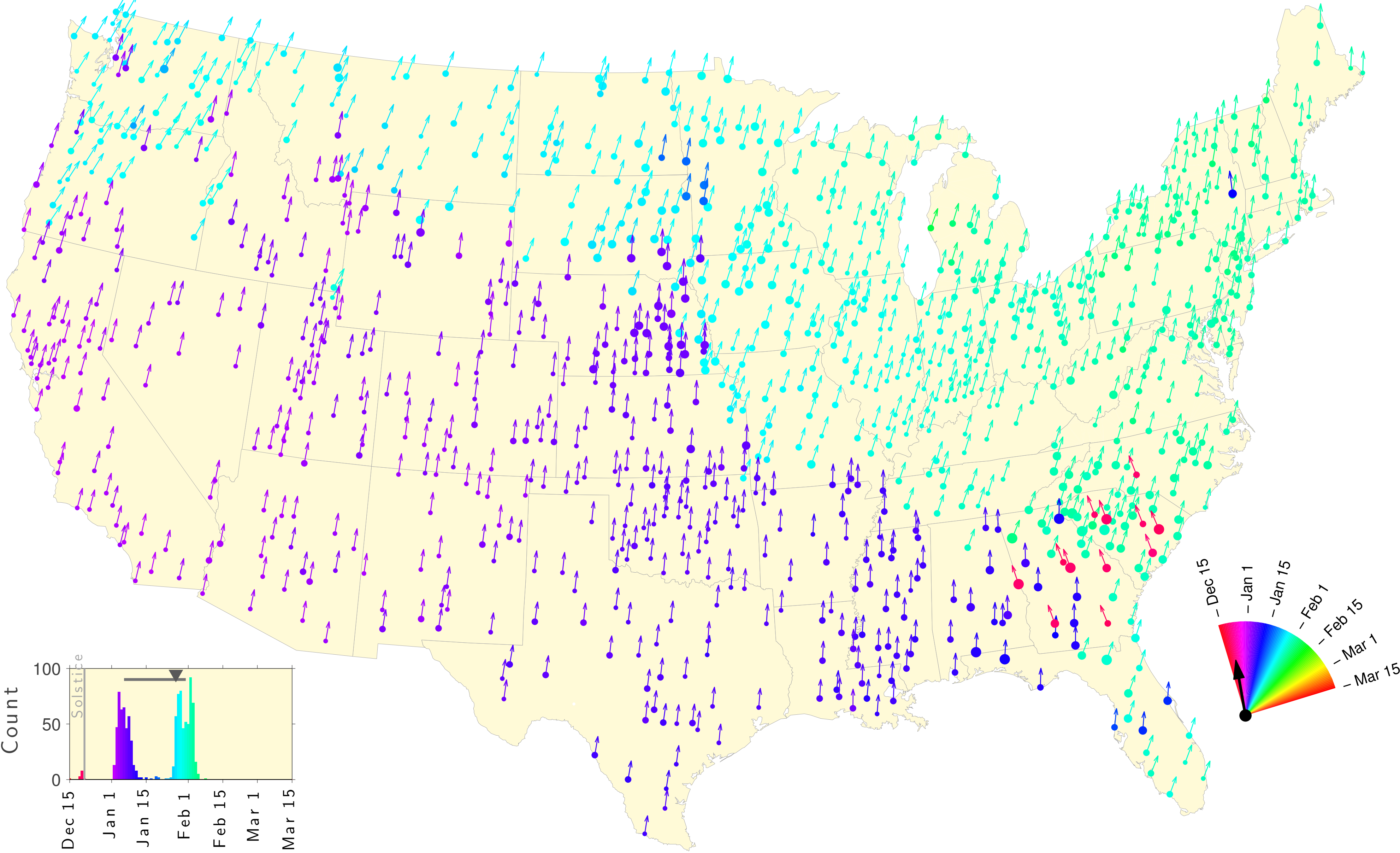

Winter Teletherm—50 year estimates: 1931 to 1980

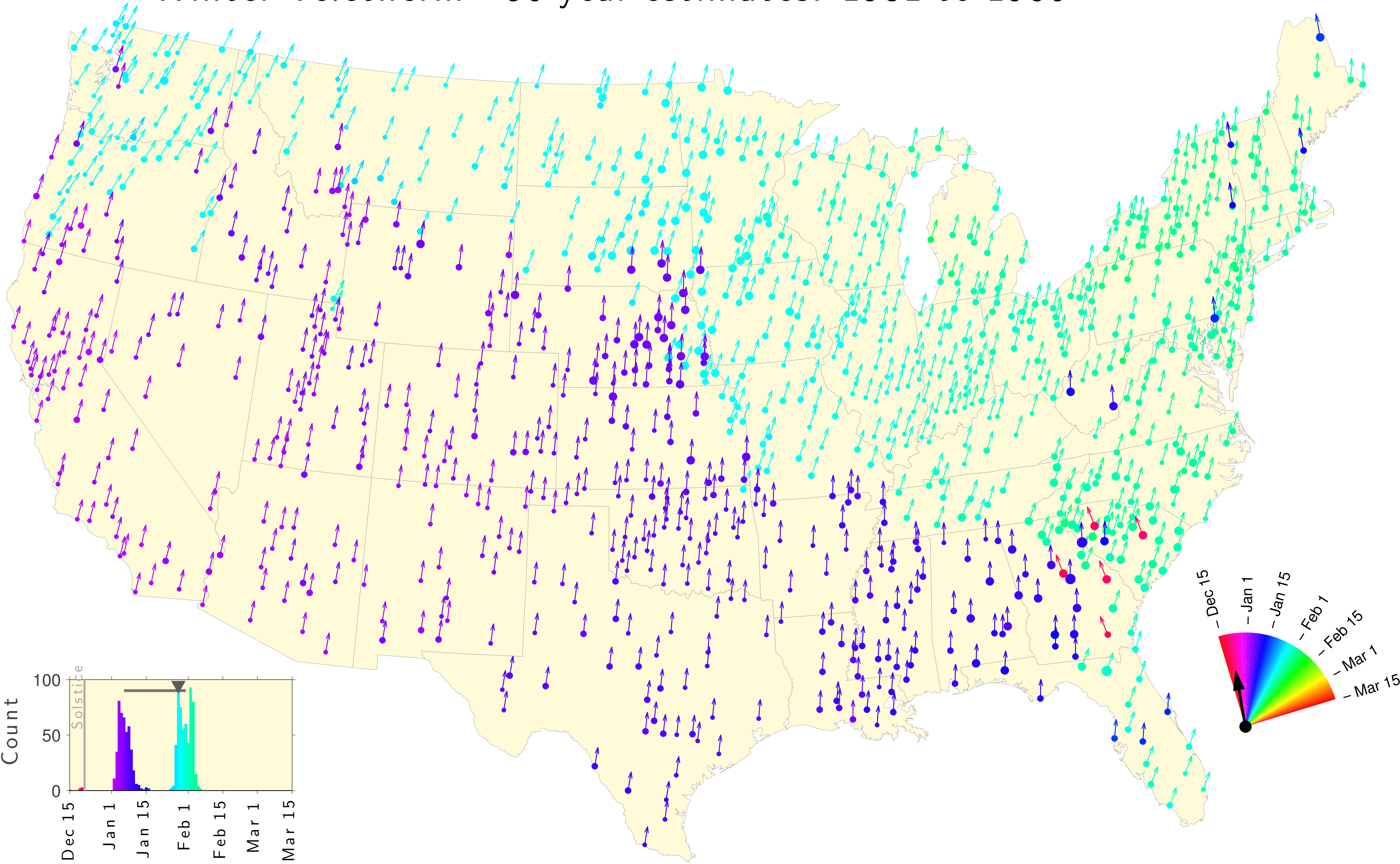

Winter Teletherm—50 year estimates: 1932 to 1981

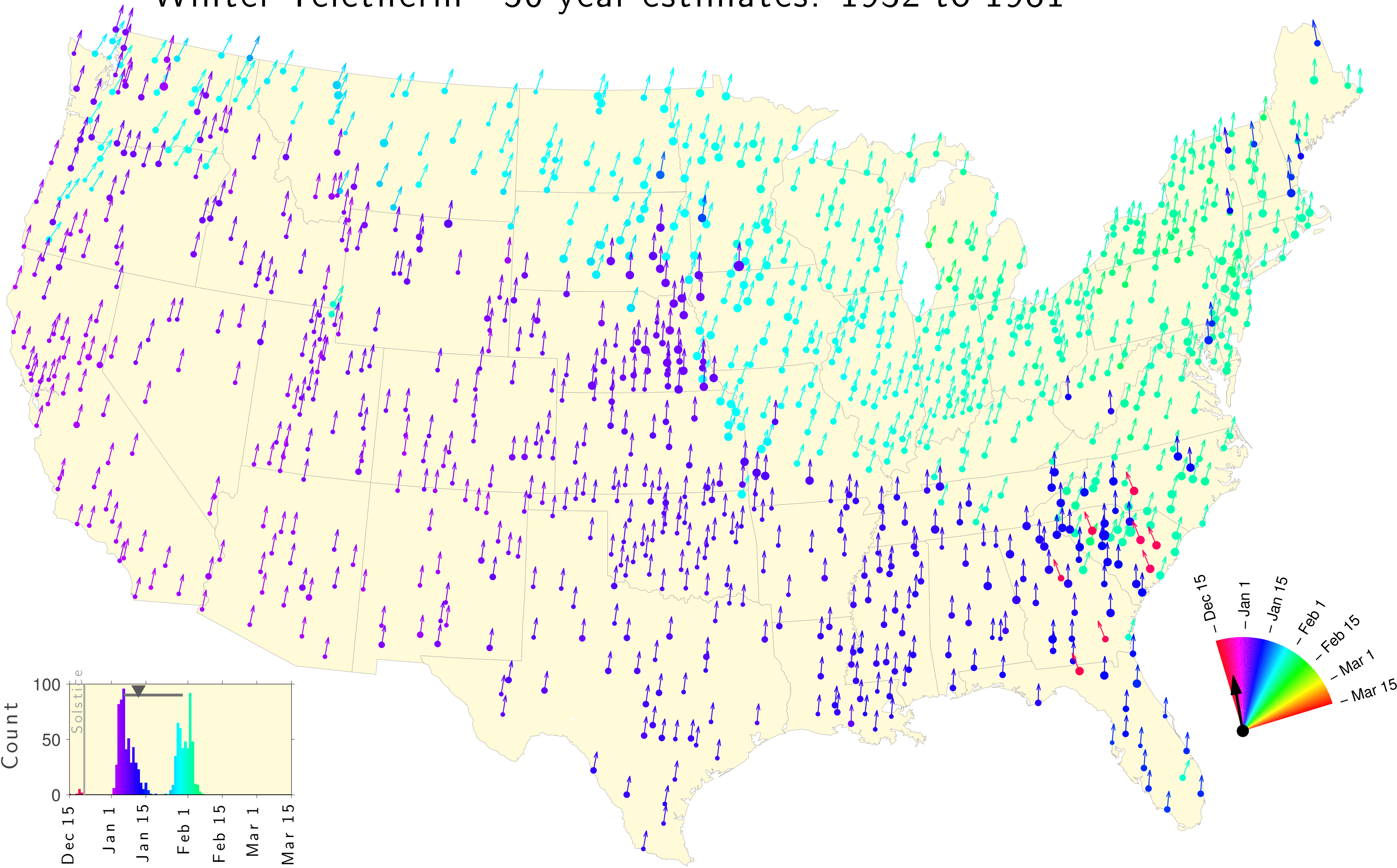

Winter Teletherm—50 year estimates: 1933 to 1982

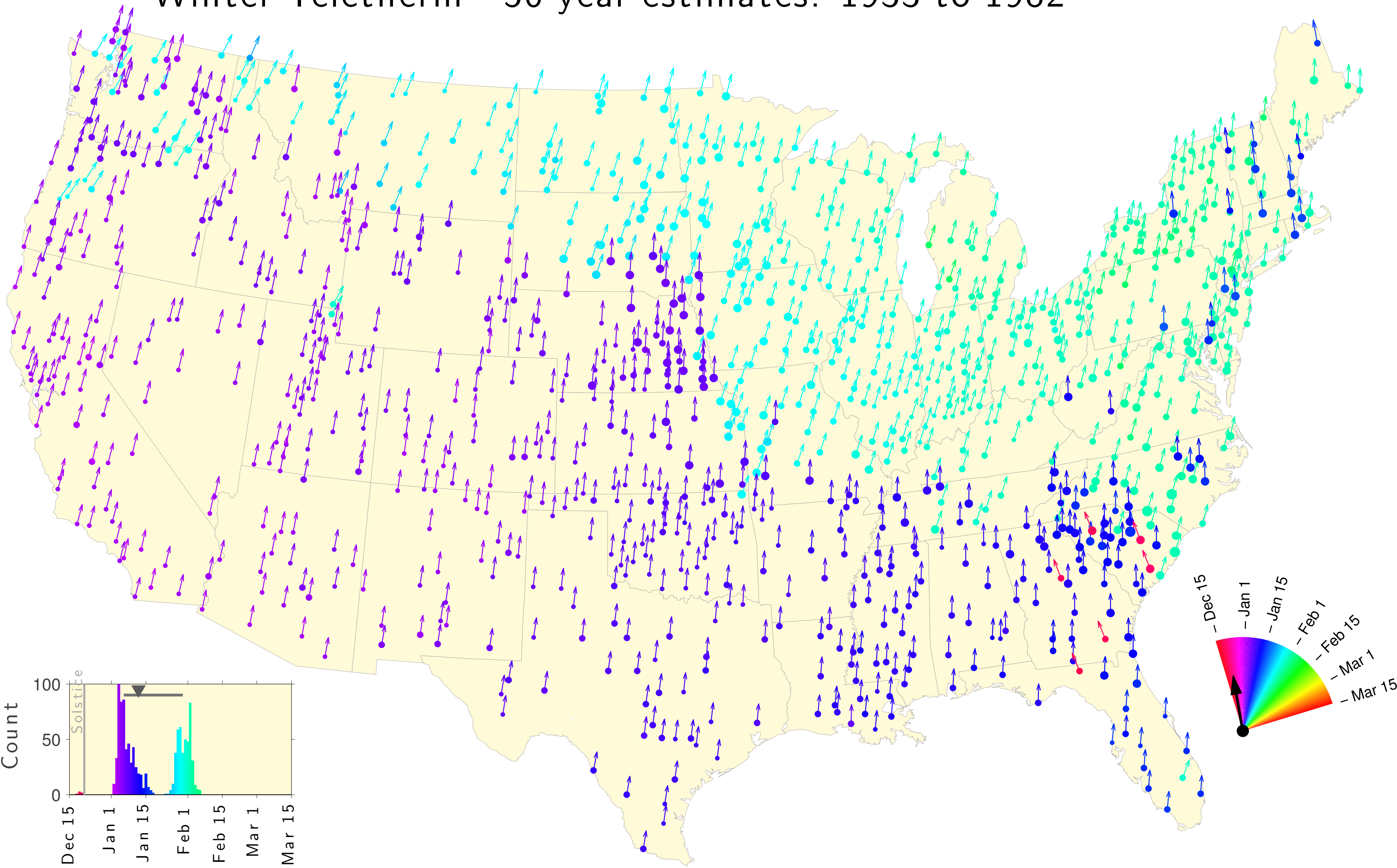

Winter Teletherm—50 year estimates: 1934 to 1983

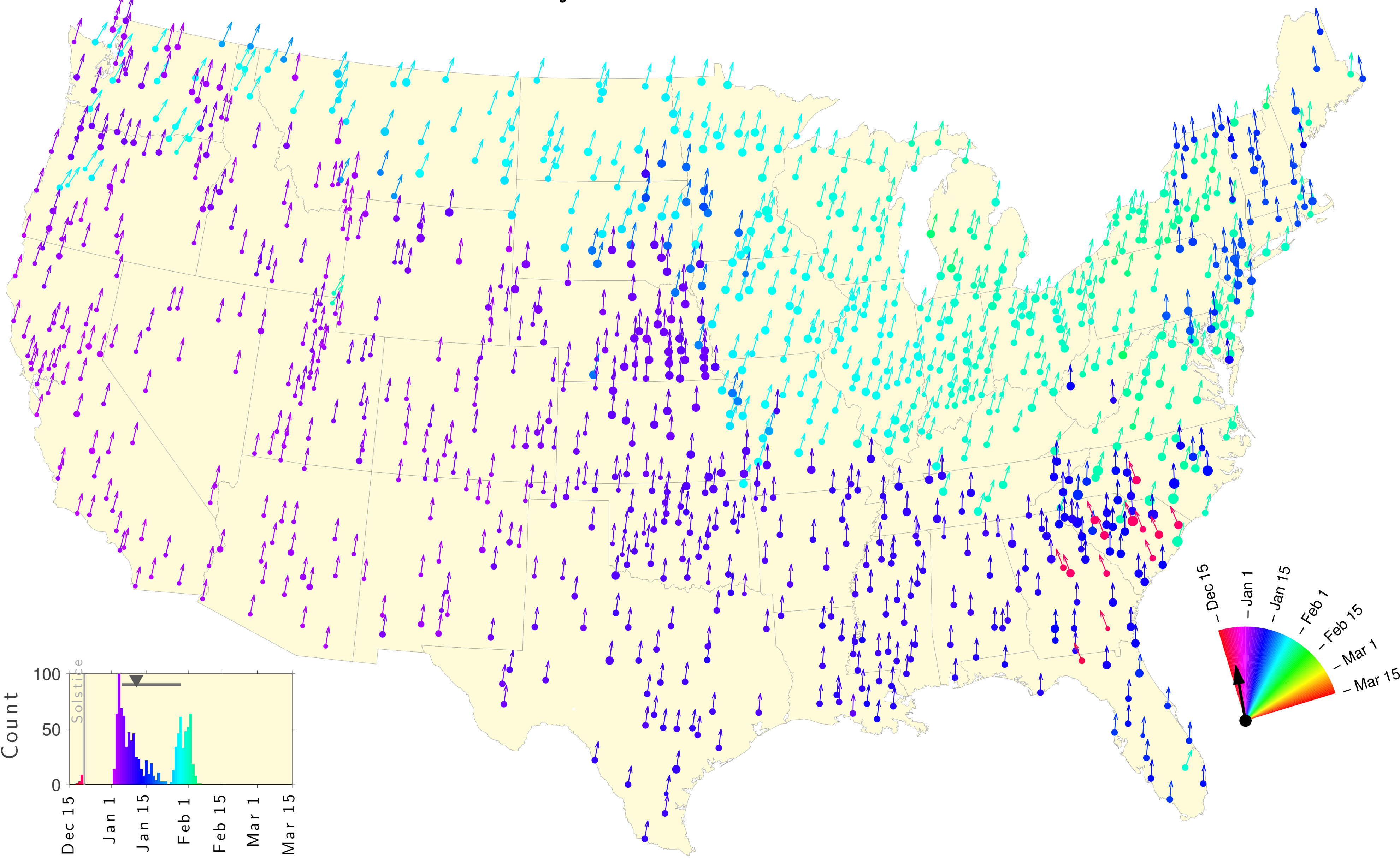

Winter Teletherm—50 year estimates: 1935 to 1984

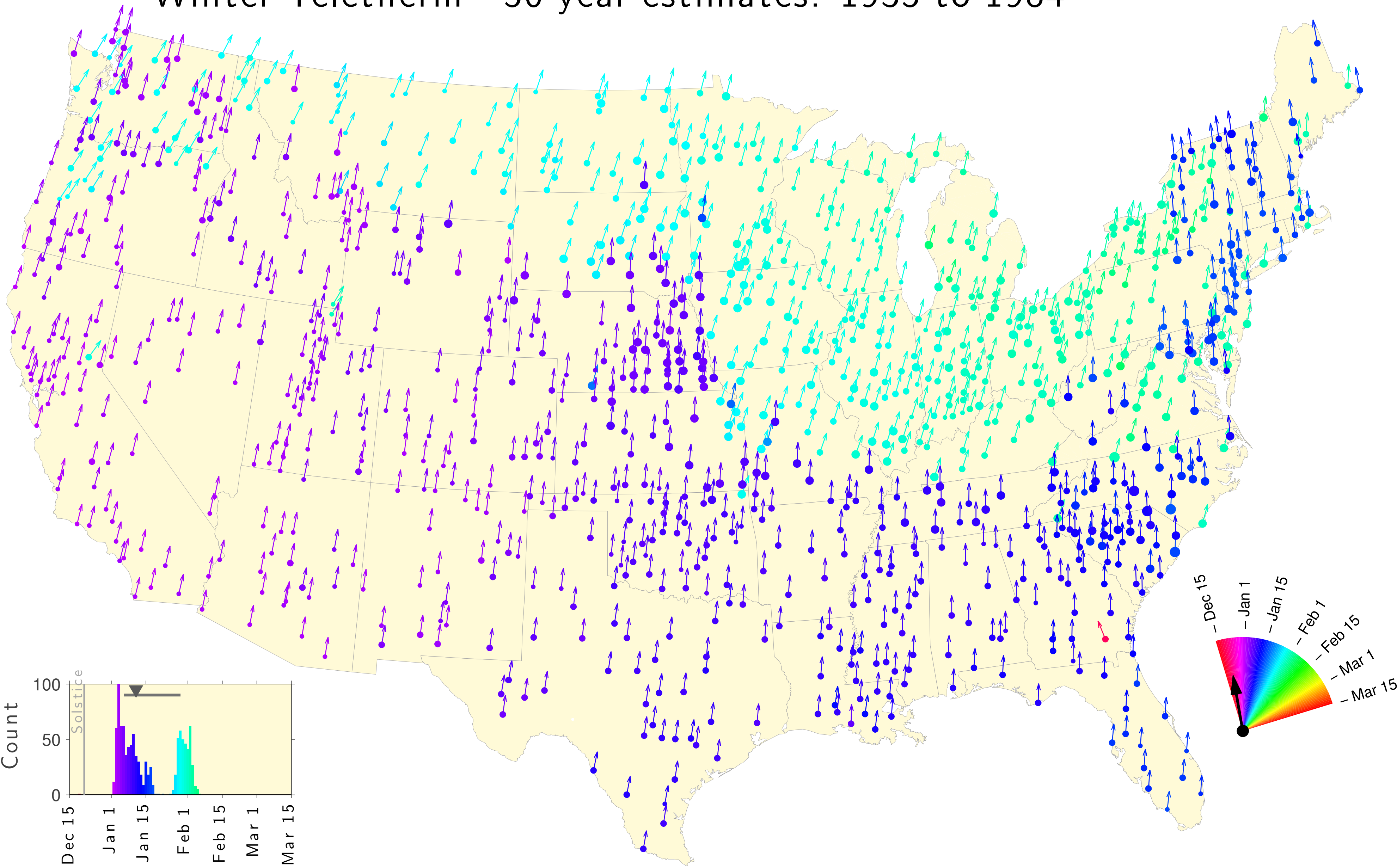

Winter Teletherm—50 year estimates: 1936 to 1985

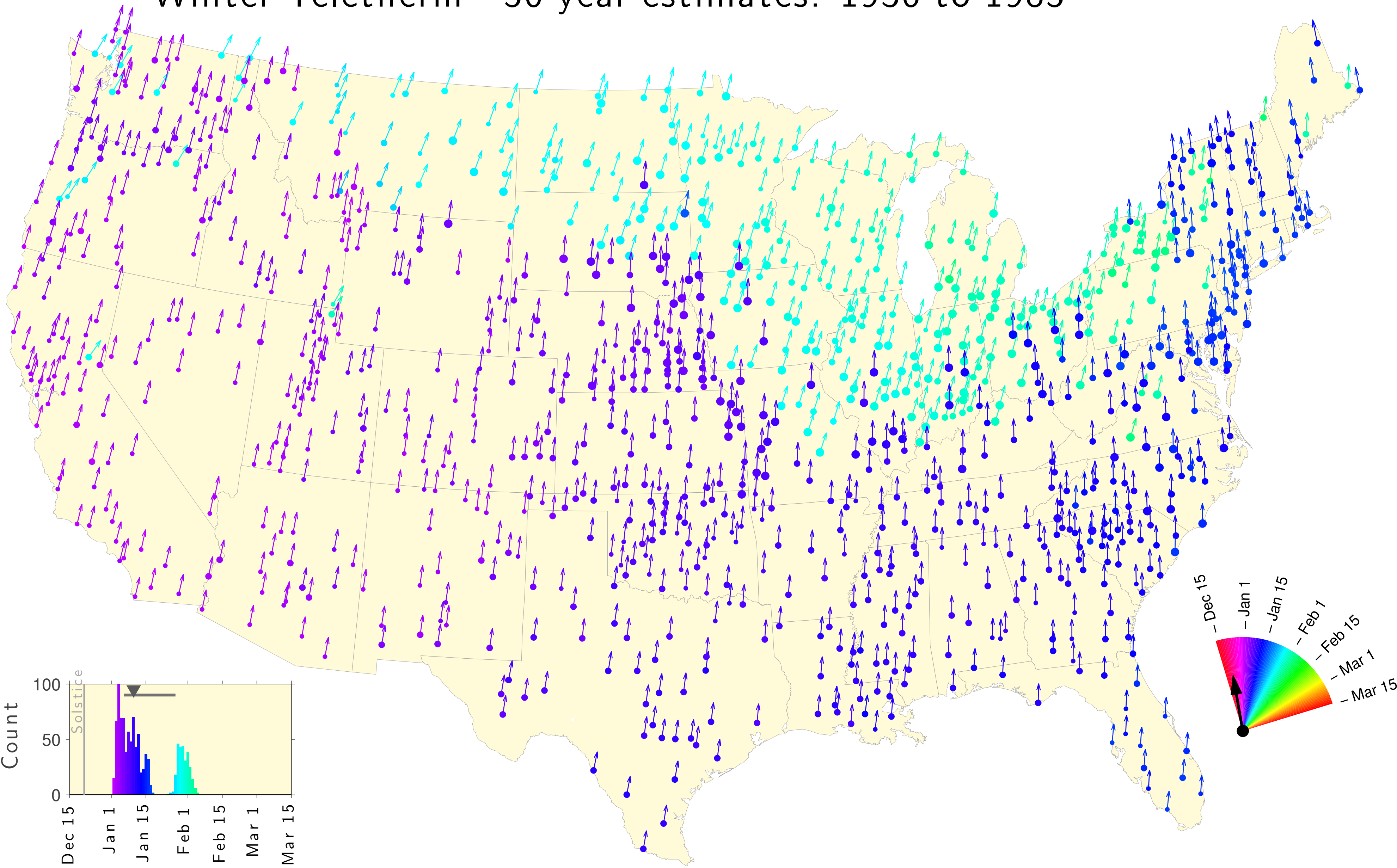

Winter Teletherm—50 year estimates: 1937 to 1986

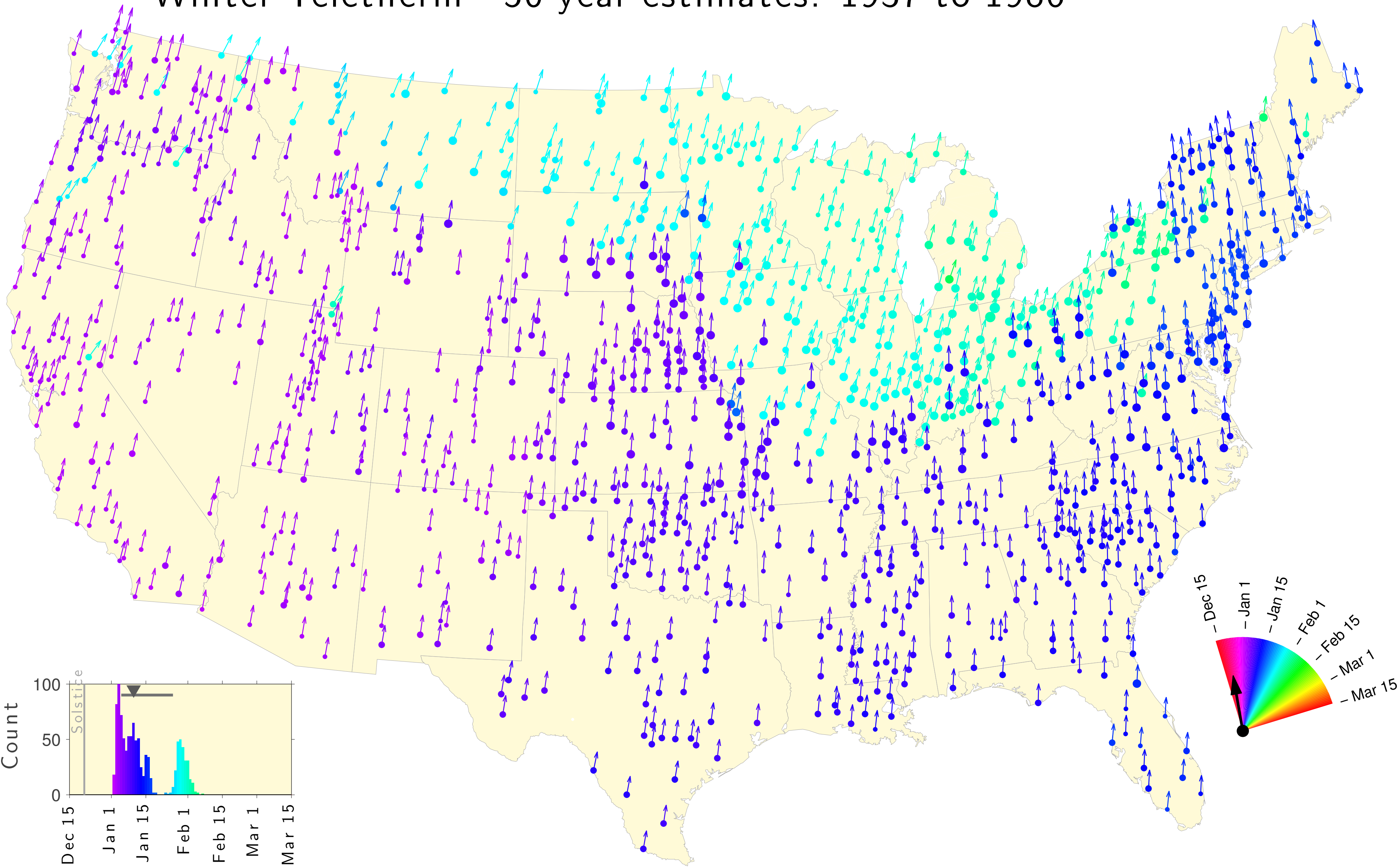

Winter Teletherm—50 year estimates: 1938 to 1987

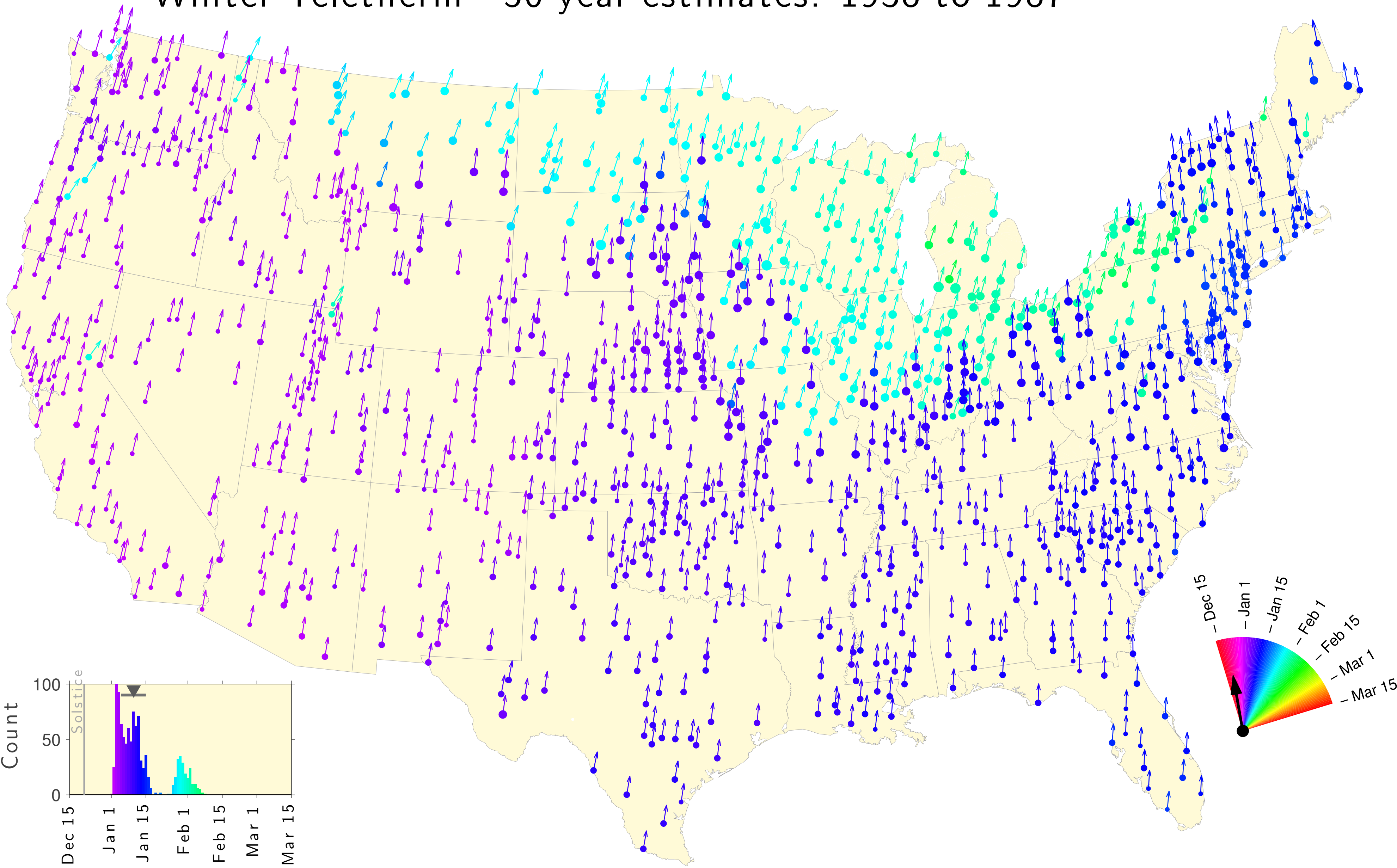

Winter Teletherm—50 year estimates: 1939 to 1988

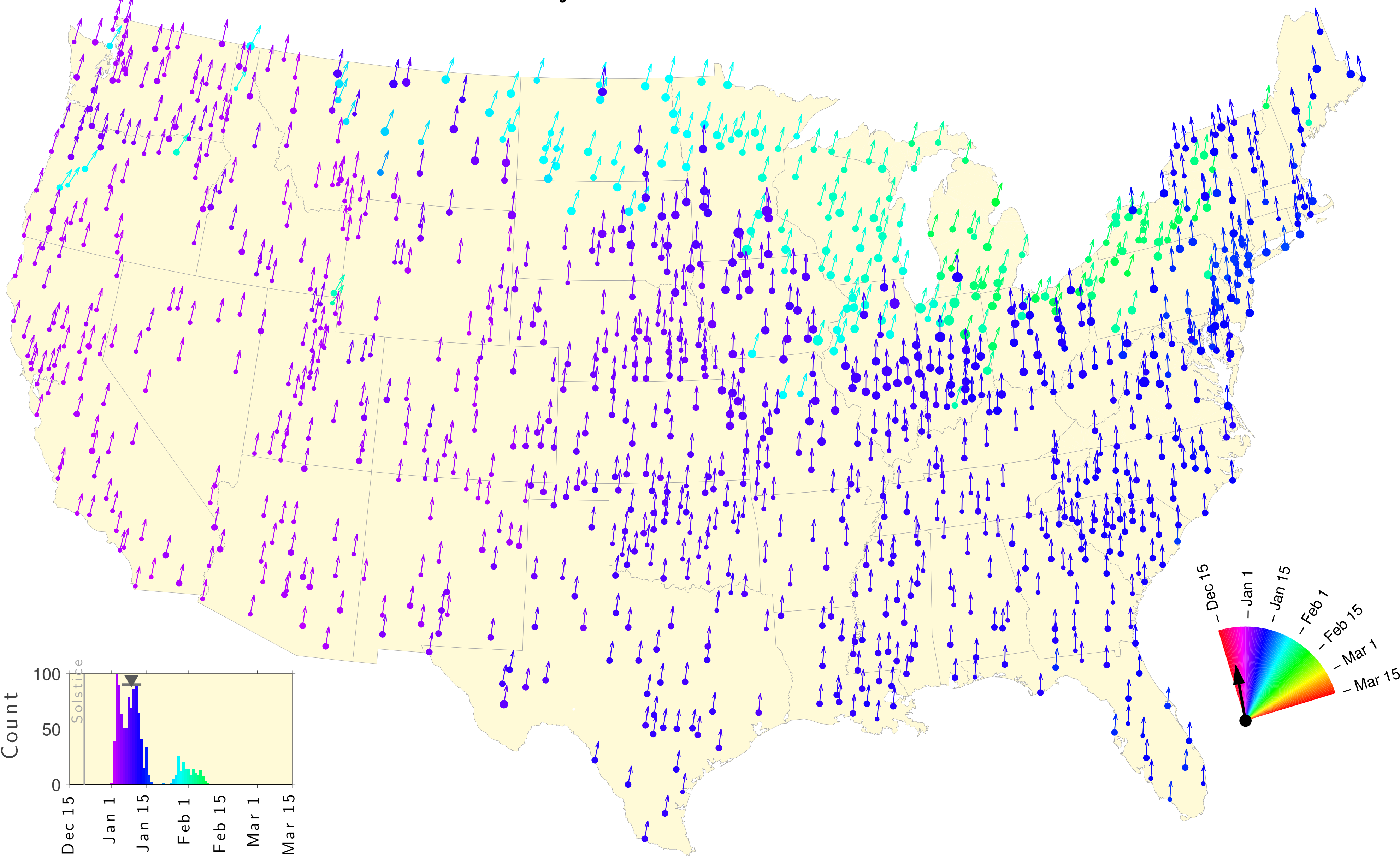

Winter Teletherm—50 year estimates: 1940 to 1989

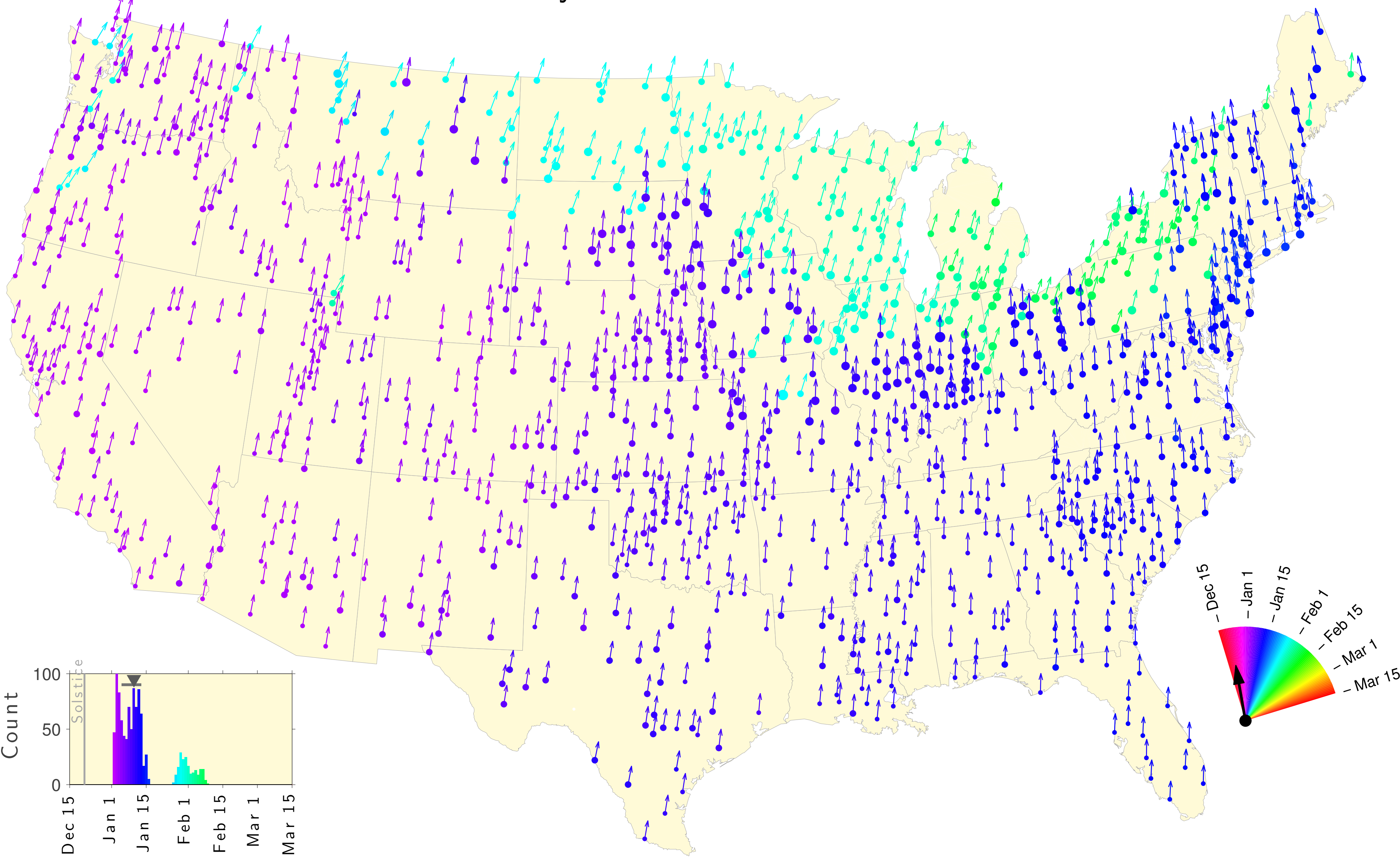

Winter Teletherm—50 year estimates: 1941 to 1990

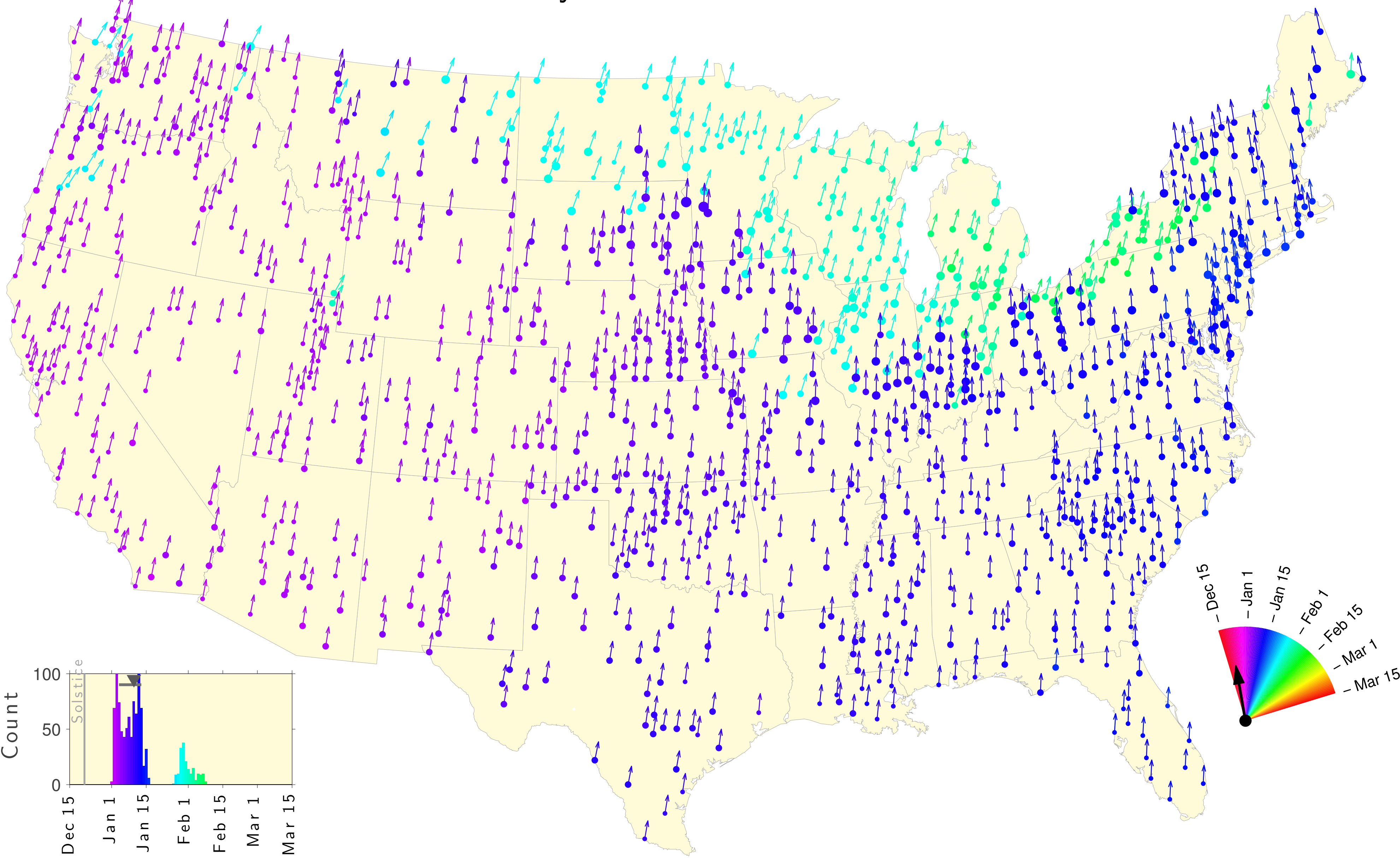

Winter Teletherm—50 year estimates: 1942 to 1991

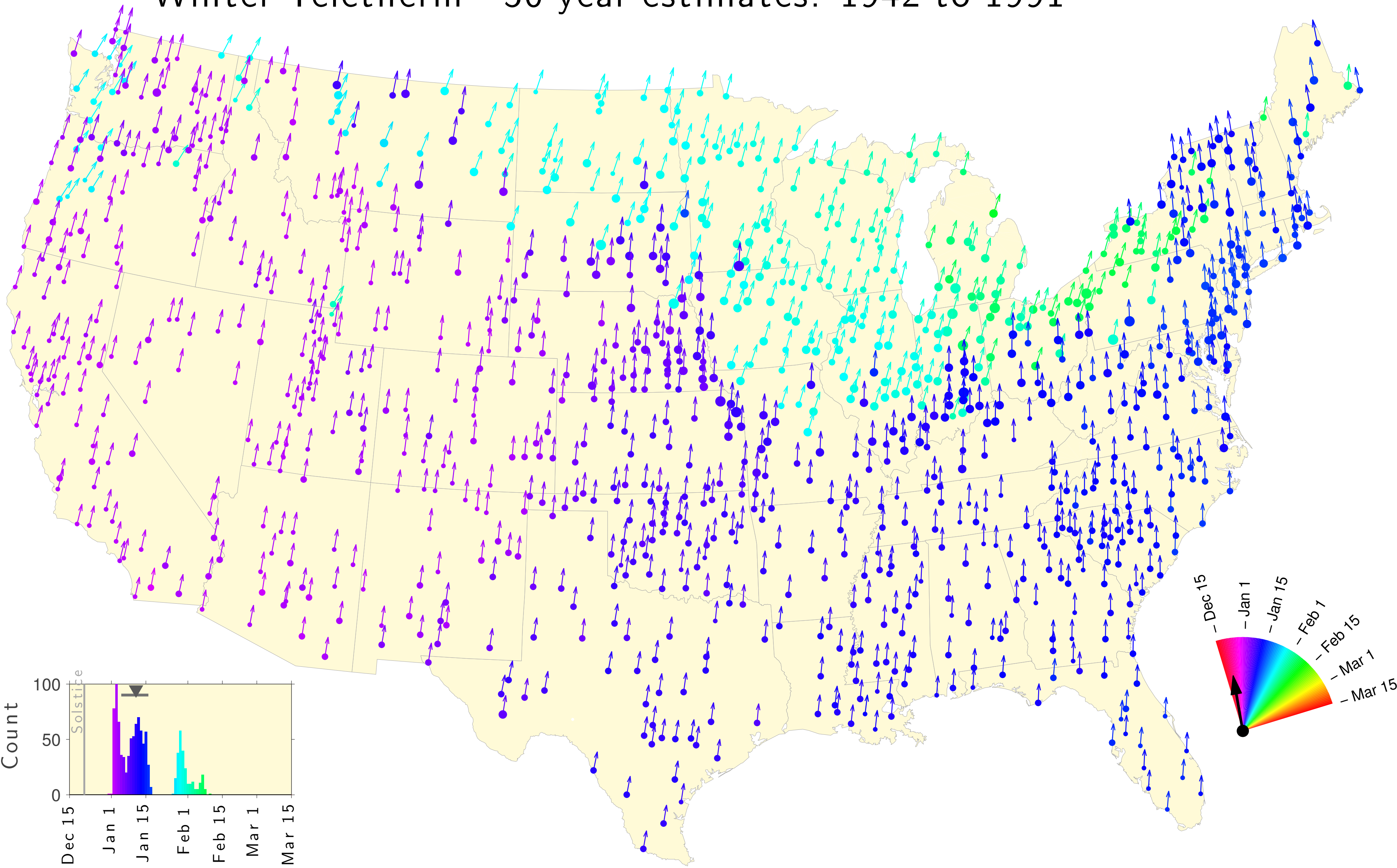

Winter Teletherm—50 year estimates: 1943 to 1992

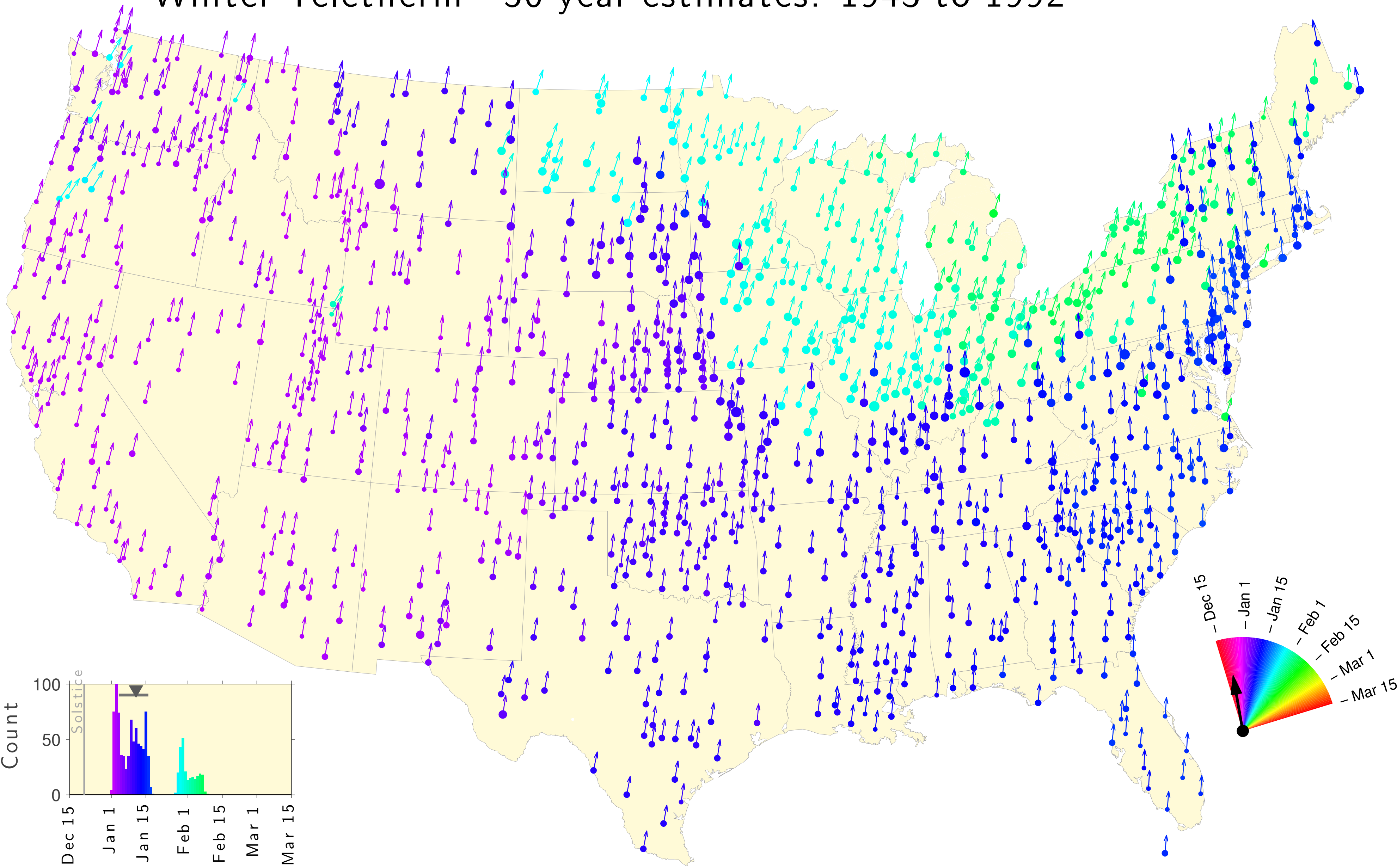

Winter Teletherm—50 year estimates: 1944 to 1993

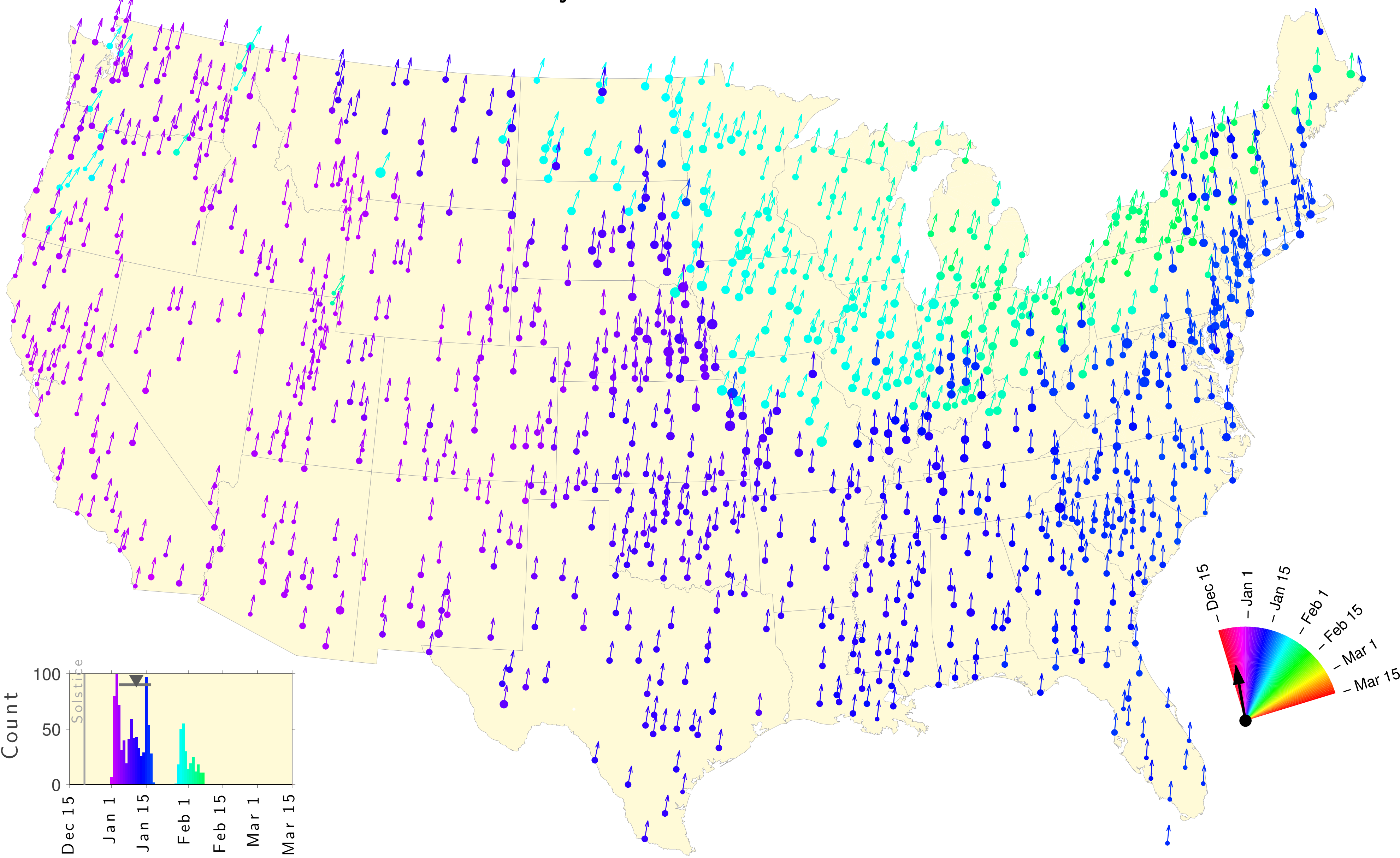

Winter Teletherm—50 year estimates: 1945 to 1994

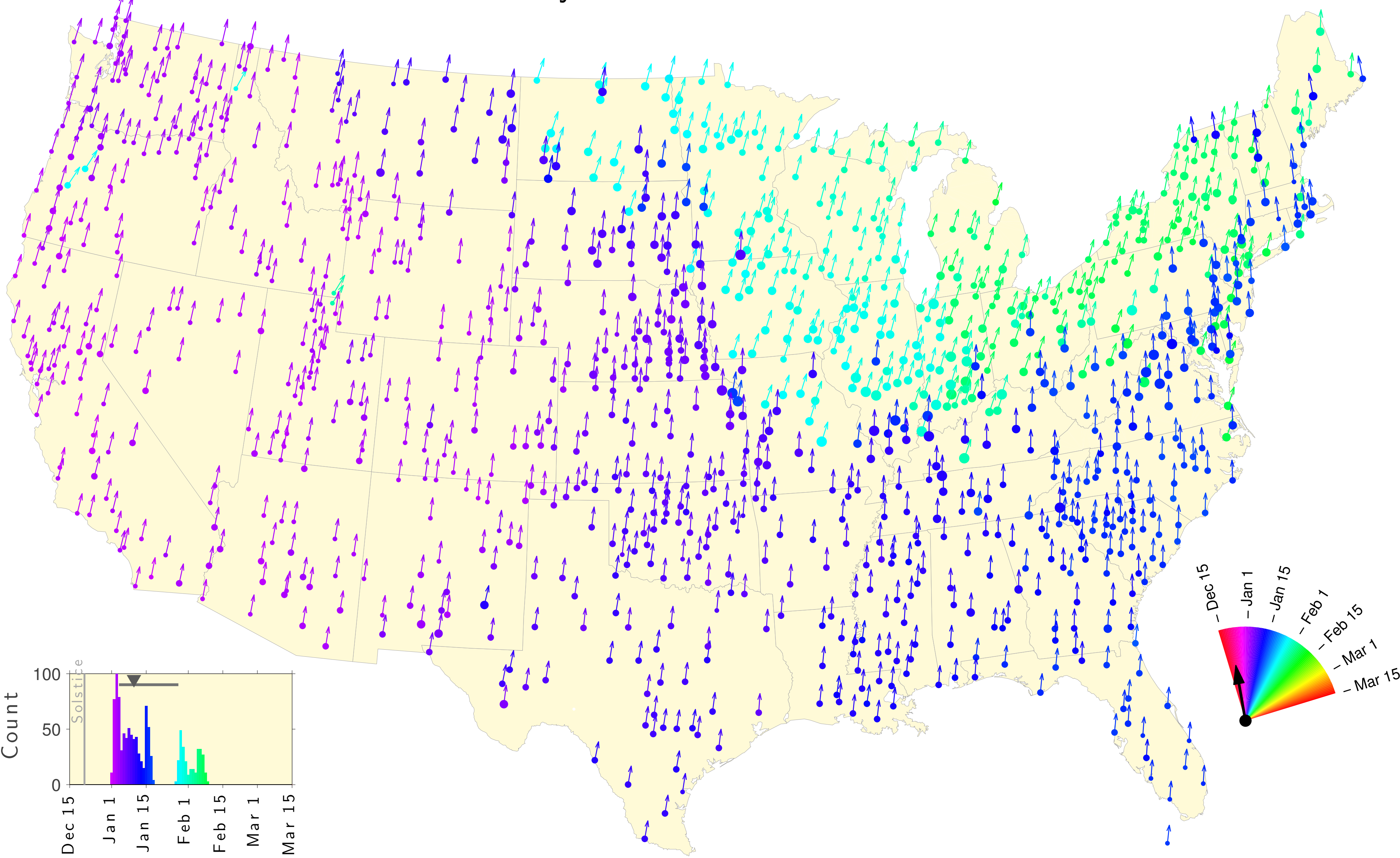

Winter Teletherm—50 year estimates: 1946 to 1995

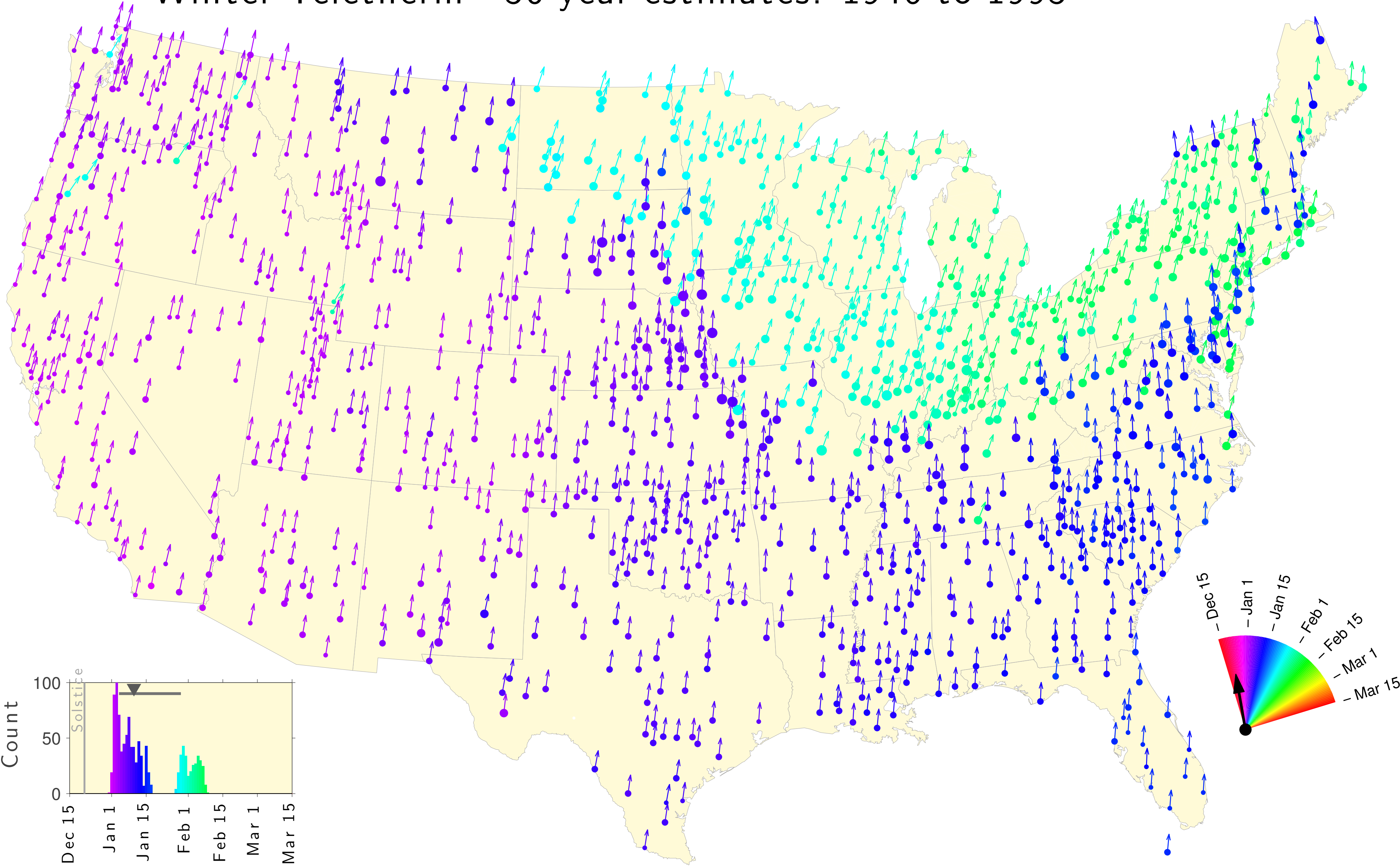

Winter Teletherm—50 year estimates: 1947 to 1996

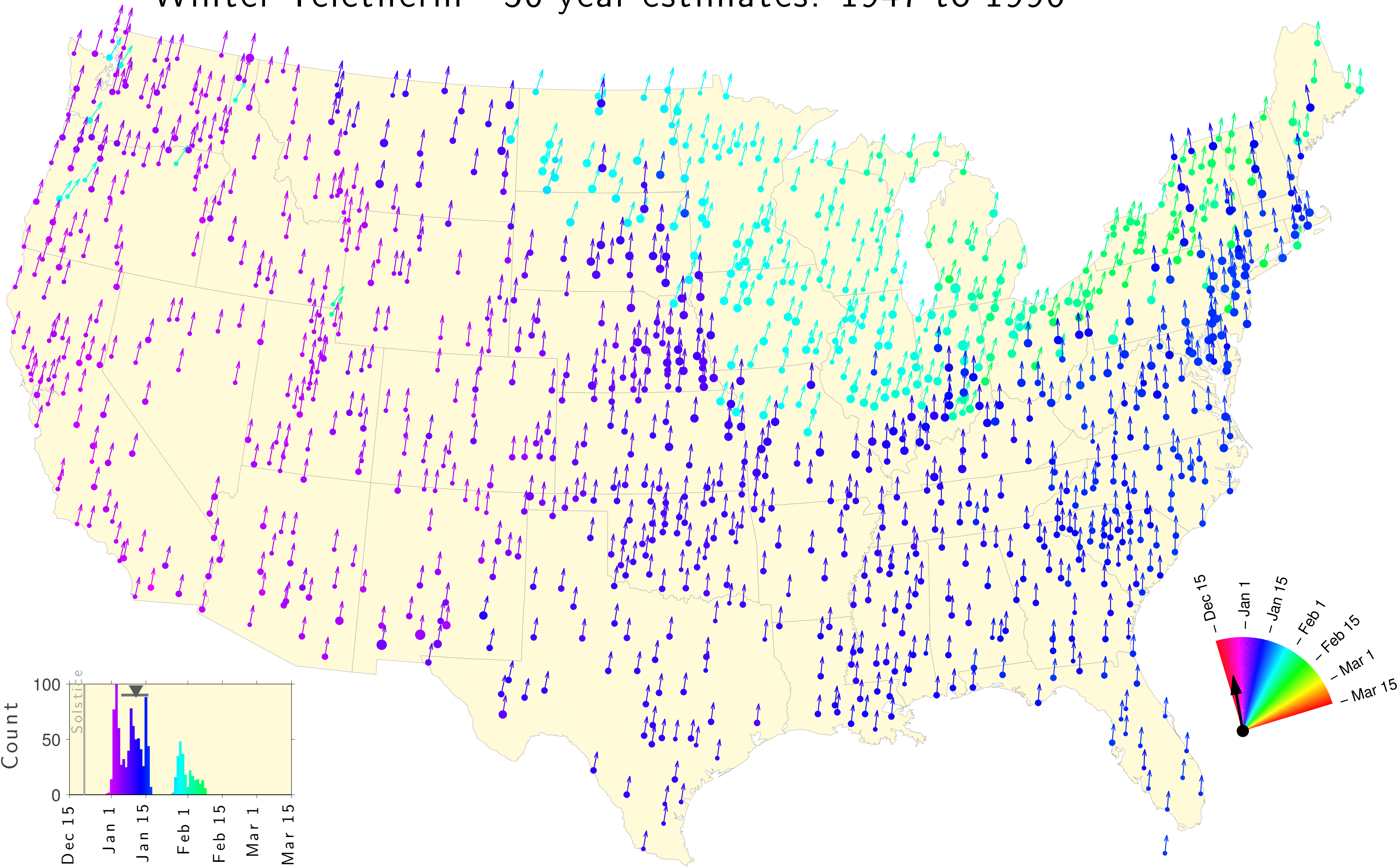

Winter Teletherm—50 year estimates: 1948 to 1997

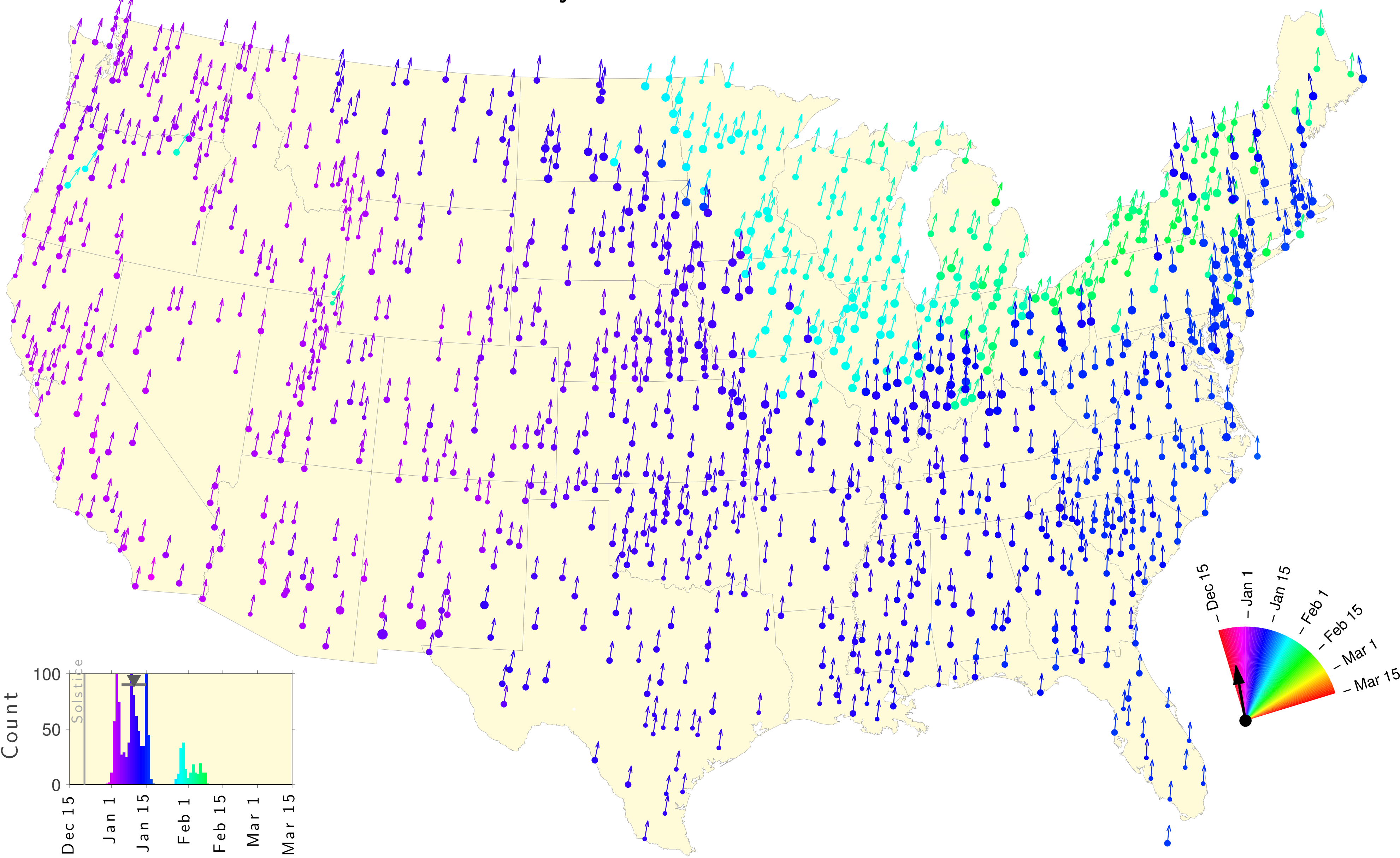

Winter Teletherm—50 year estimates: 1949 to 1998

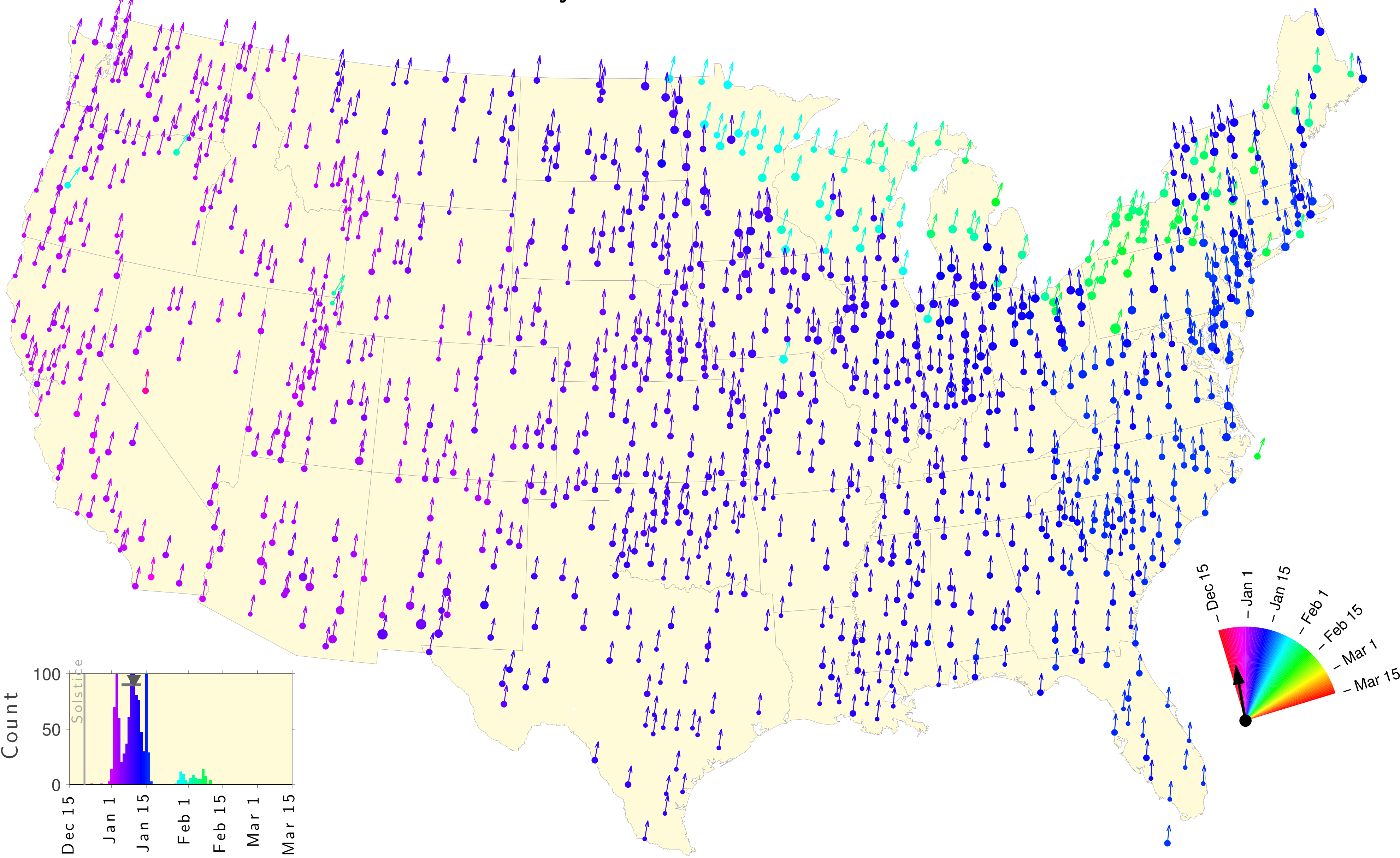

Winter Teletherm—50 year estimates: 1950 to 1999

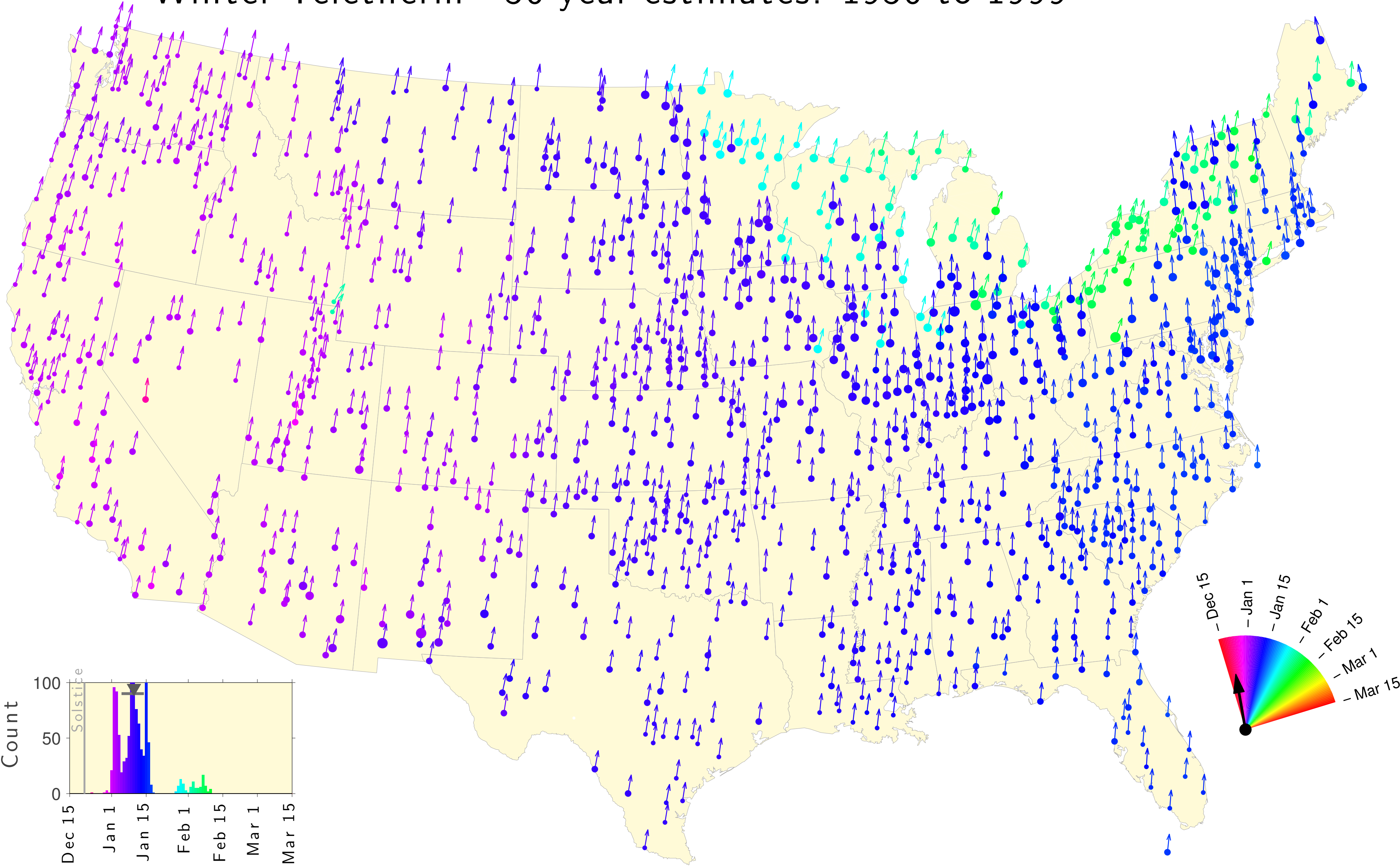

Winter Teletherm—50 year estimates: 1951 to 2000

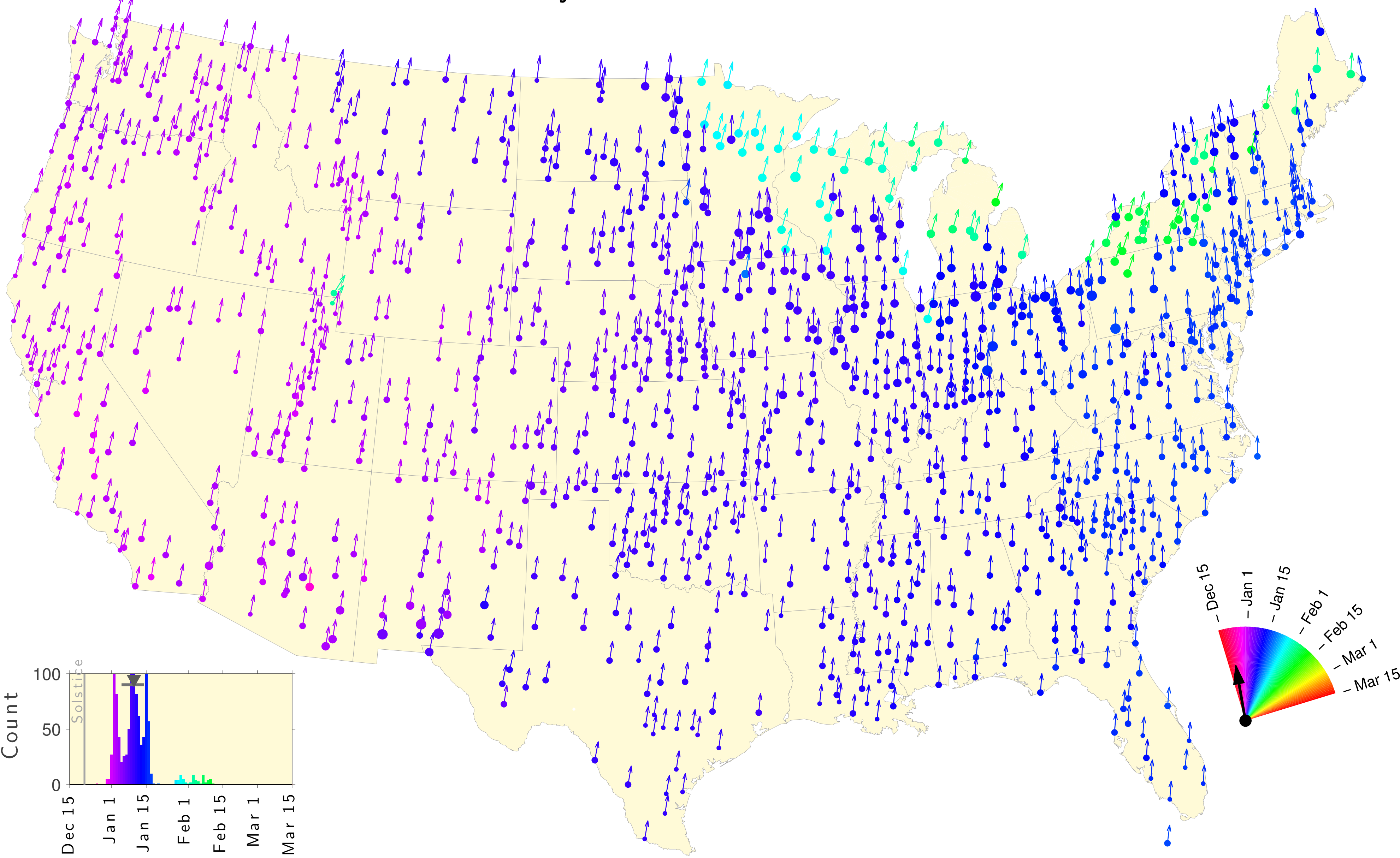

Winter Teletherm—50 year estimates: 1952 to 2001

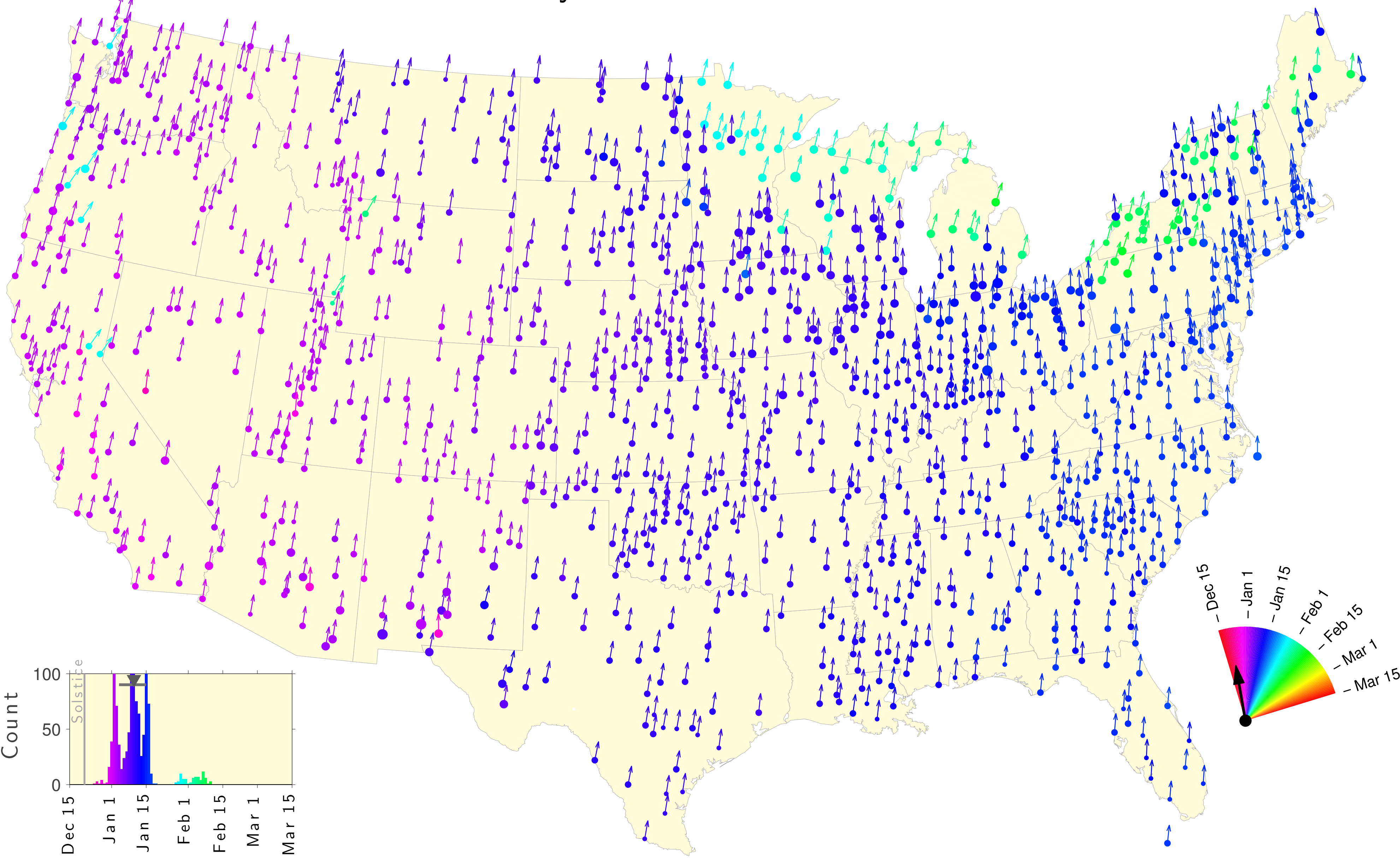

Winter Teletherm—50 year estimates: 1953 to 2002

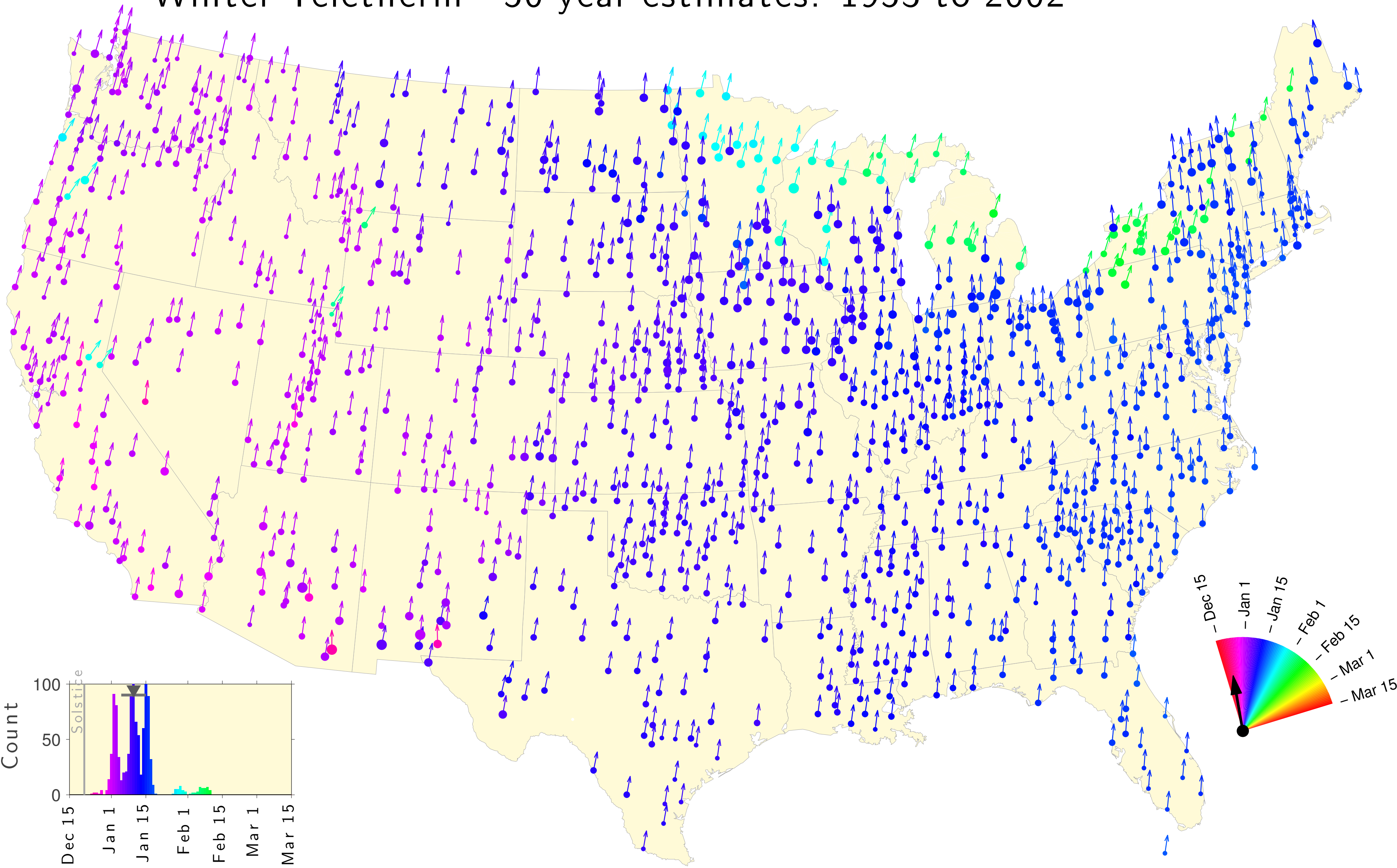

Winter Teletherm—50 year estimates: 1954 to 2003

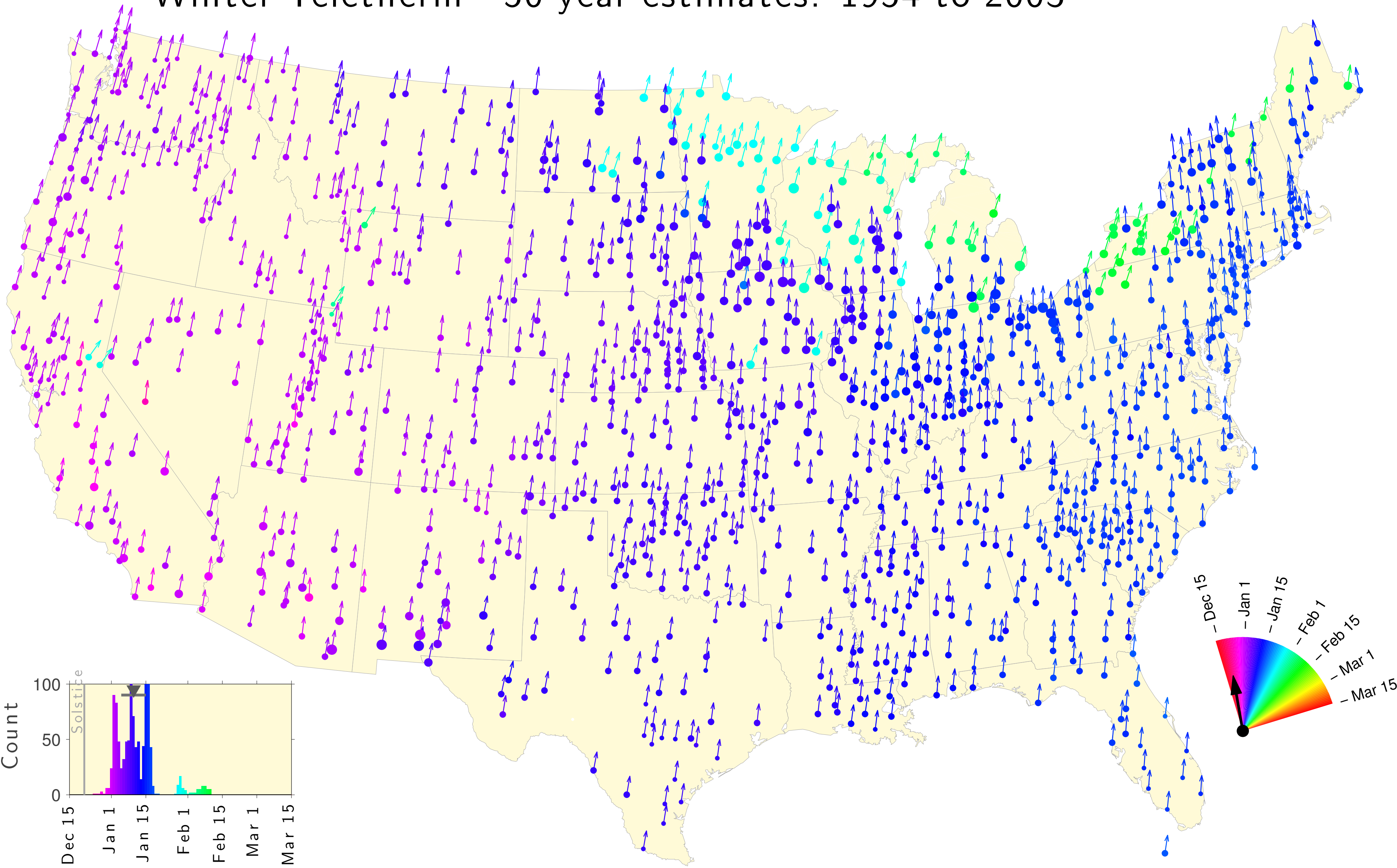

Winter Teletherm—50 year estimates: 1955 to 2004

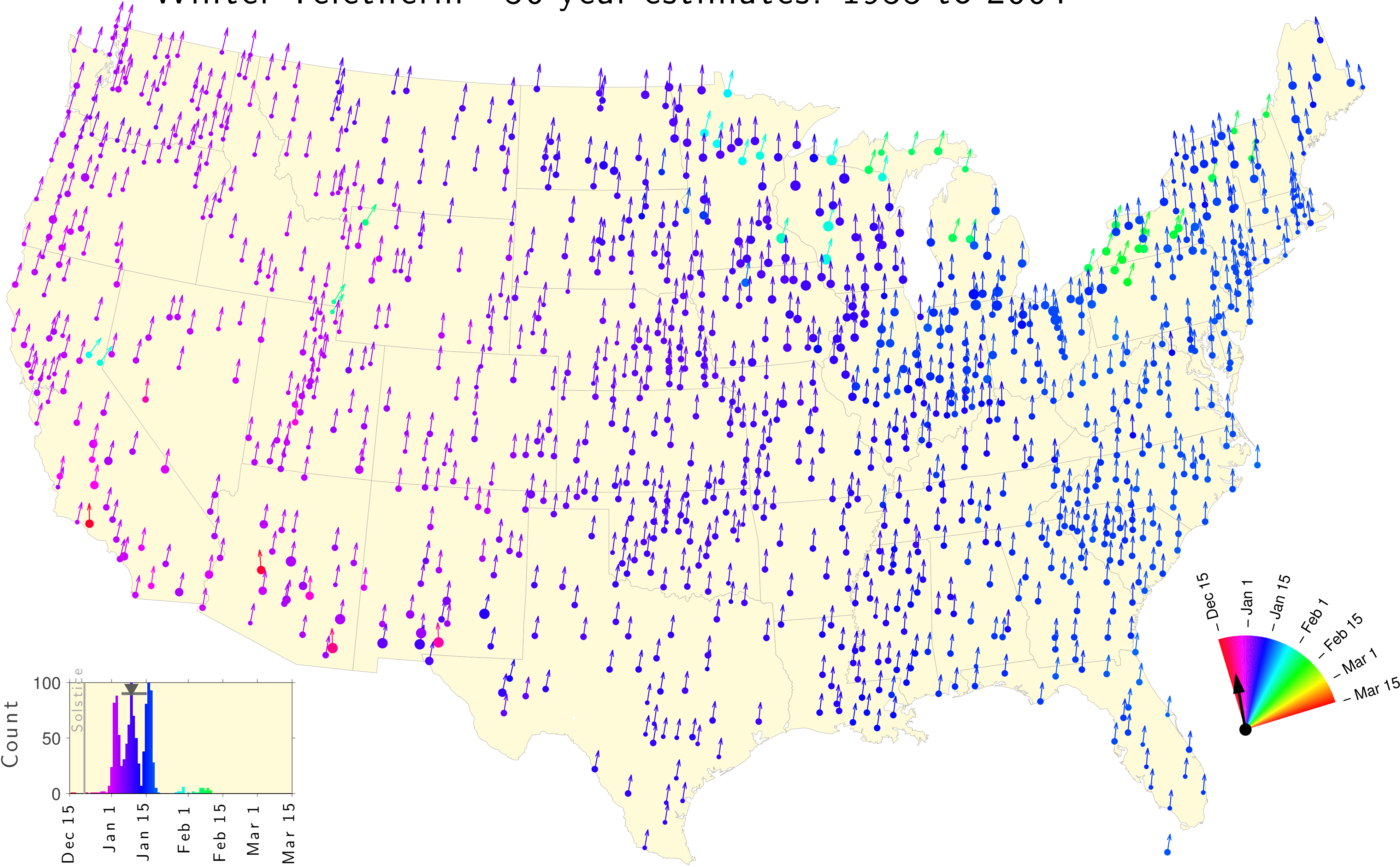

Winter Teletherm—50 year estimates: 1956 to 2005

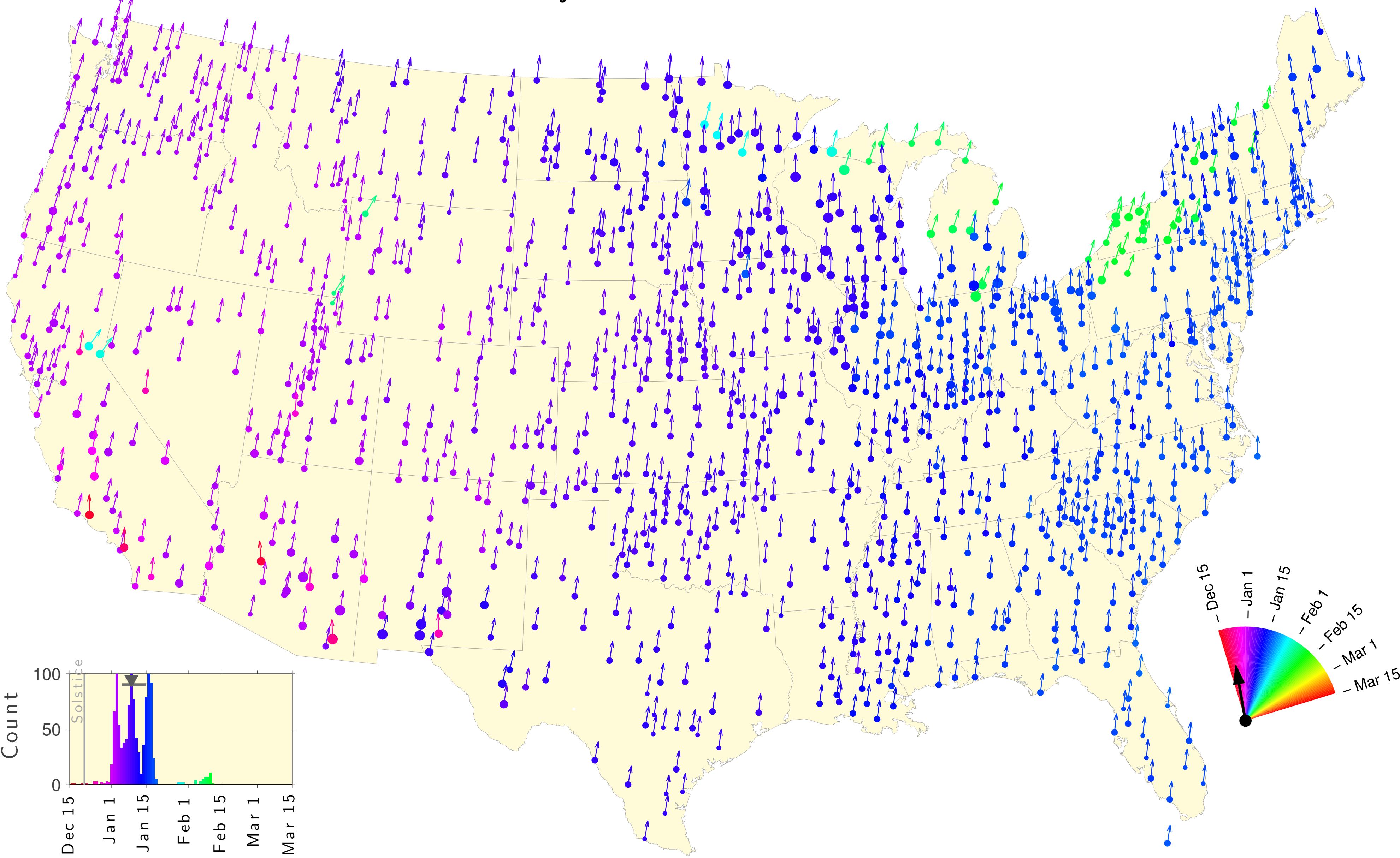

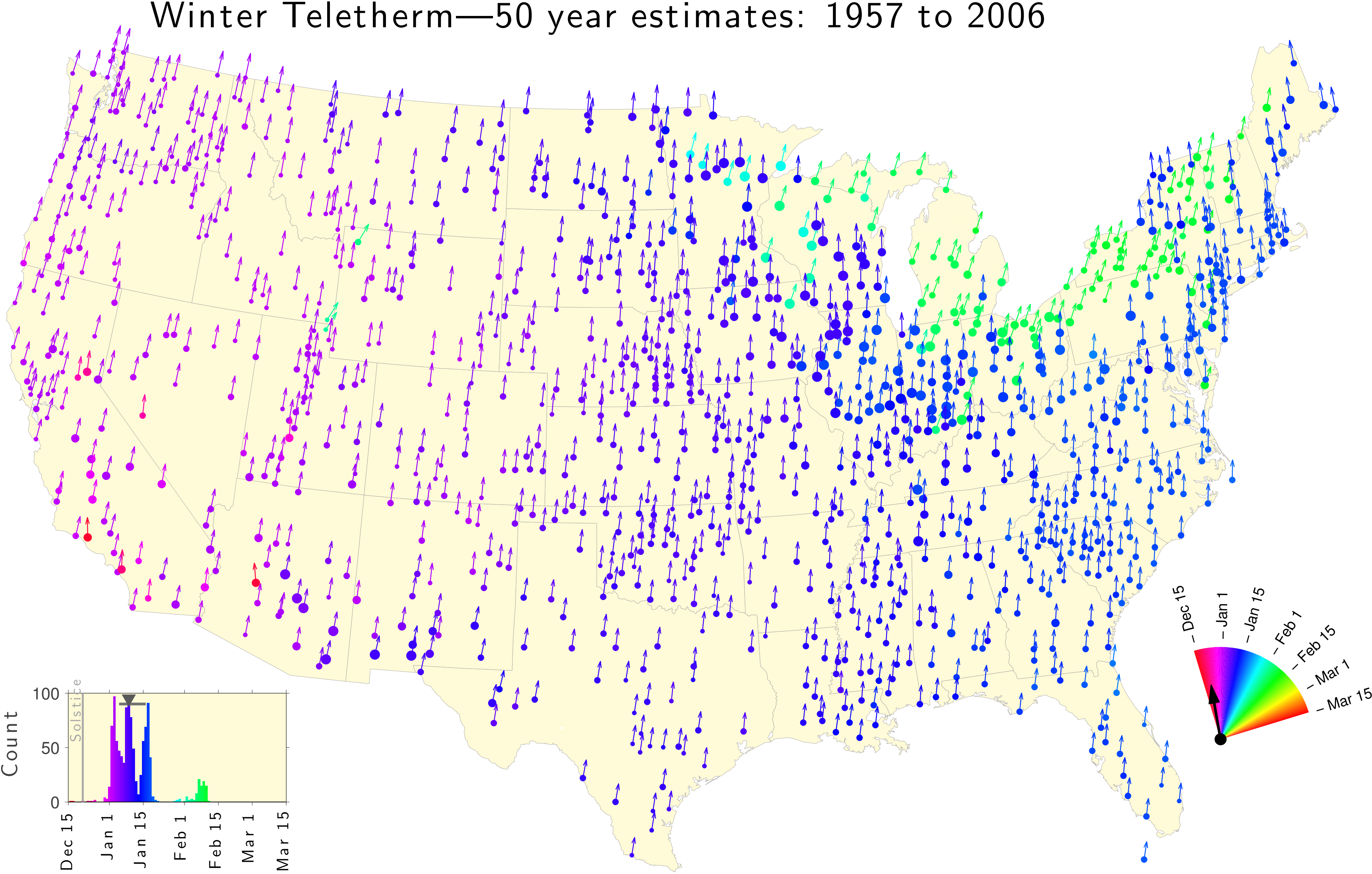
Winter Teletherm—50 year estimates: 1957 to 2006
Count
100
50
0
Solstice
Dec 15
Jan 1
Jan 15
Feb 1
Feb 15
Mar 1
Mar 15
Dec 15
Jan 1
Jan 15
Feb 1
Feb 15
Mar 1
Mar 15

Winter Teletherm—50 year estimates: 1958 to 2007

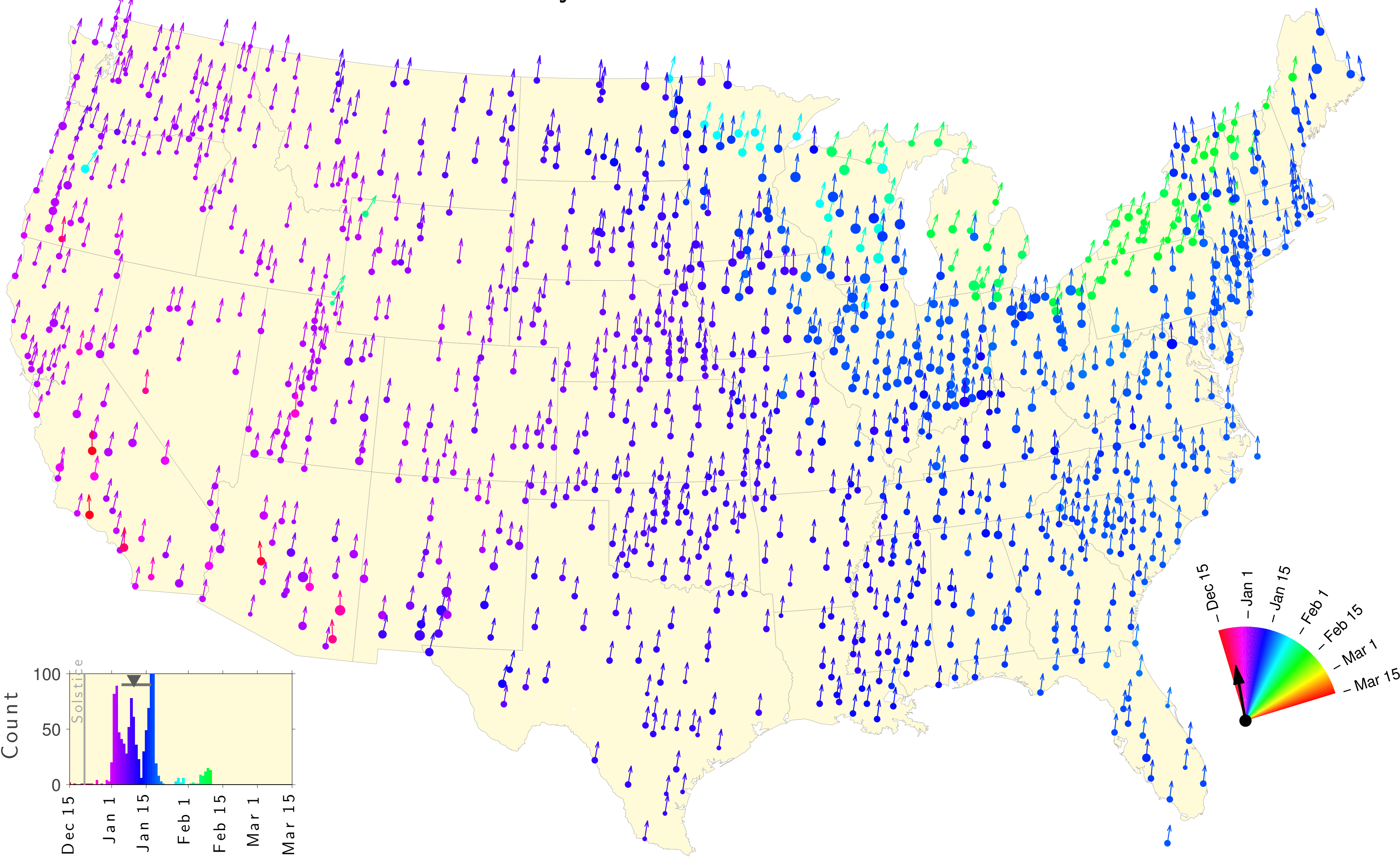

Winter Teletherm—50 year estimates: 1959 to 2008

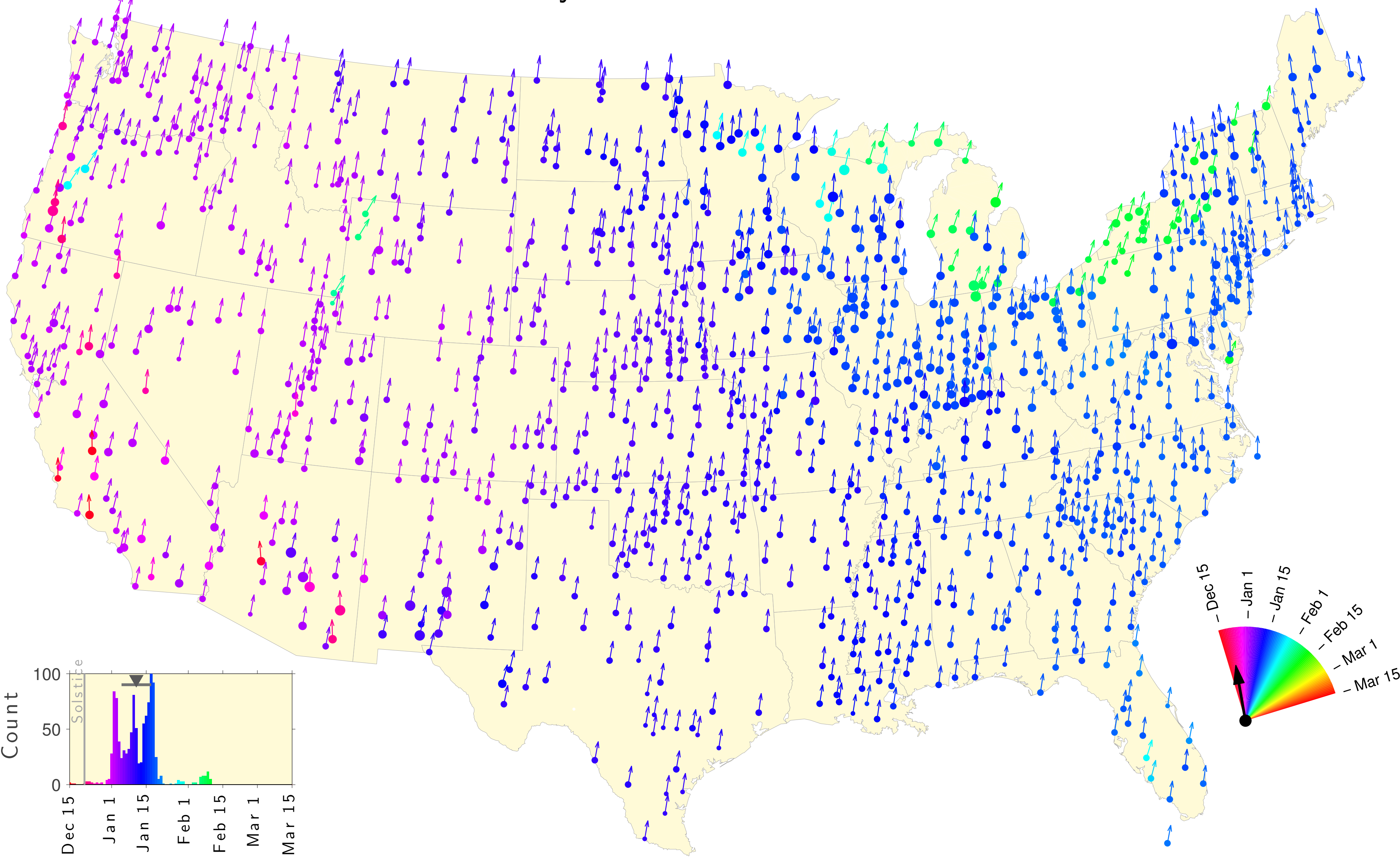

Winter Teletherm—50 year estimates: 1960 to 2009

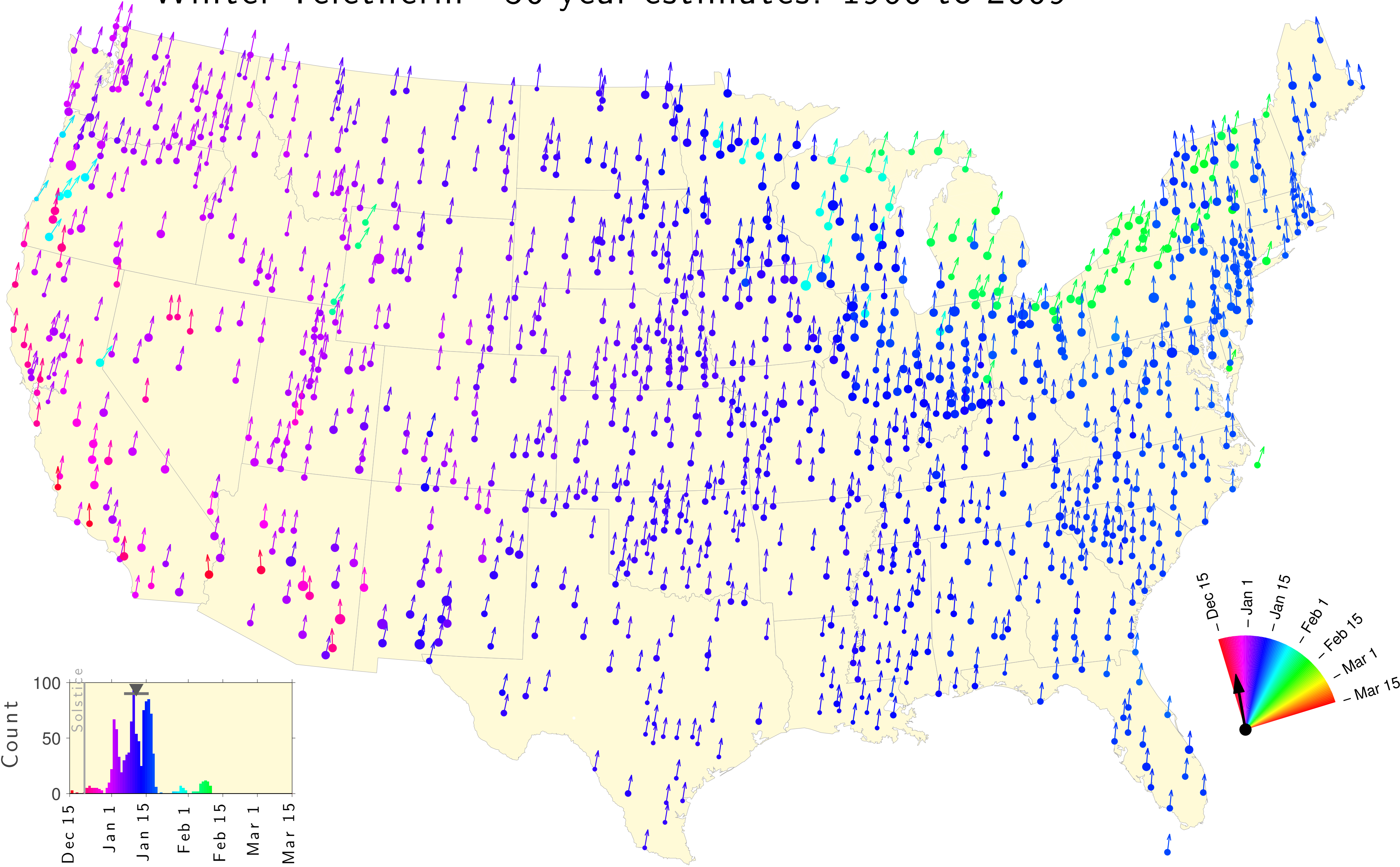

Winter Teletherm—50 year estimates: 1961 to 2010

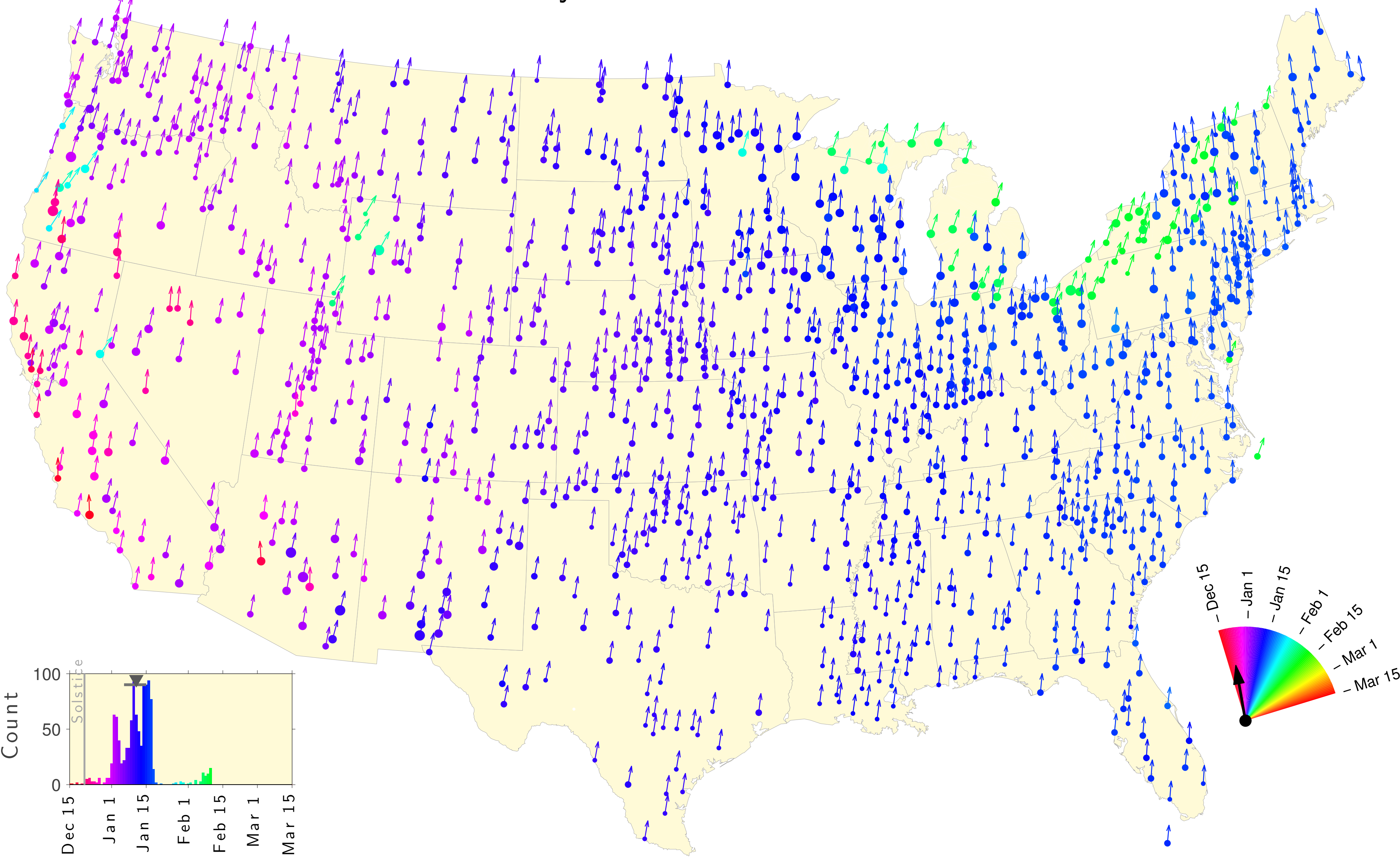

Winter Teletherm—50 year estimates: 1962 to 2011

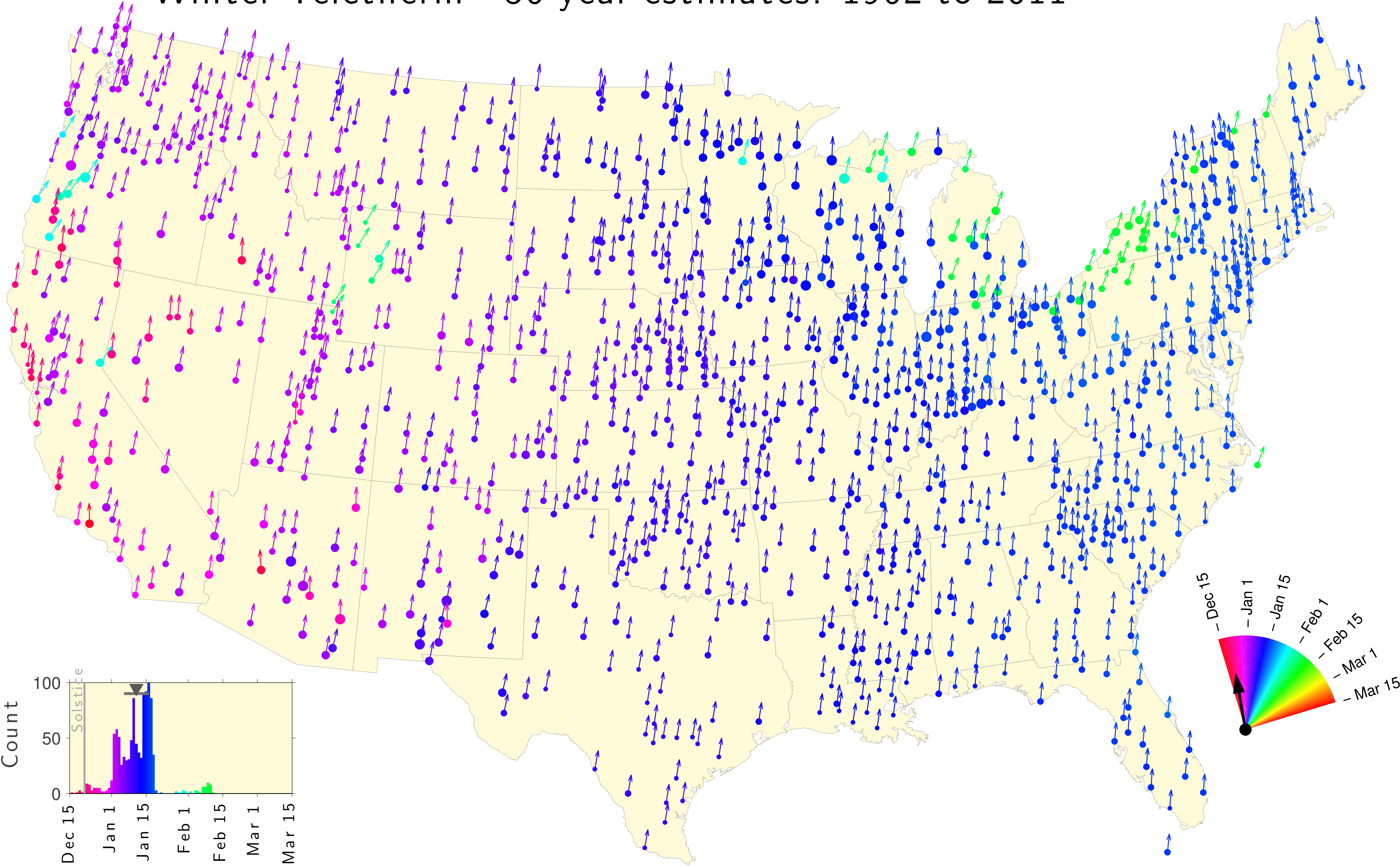

Supplement: S7 File — (PDF) [file pone.0154184.s028.pdf]
