# Supplementary material for: Tracking Climate Change through the Spatiotemporal Dynamics of the Teletherms, the Statistically Hottest and Coldest Days of the Year
Source: PLoS One. 2016 May 11;11(5):e0154184. doi: 10.1371/journal.pone.0154184 (PMC4864332; doi:10.1371/journal.pone.0154184)
Supplement: S1 File — (PDF) [file pone.0154184.s022.pdf]

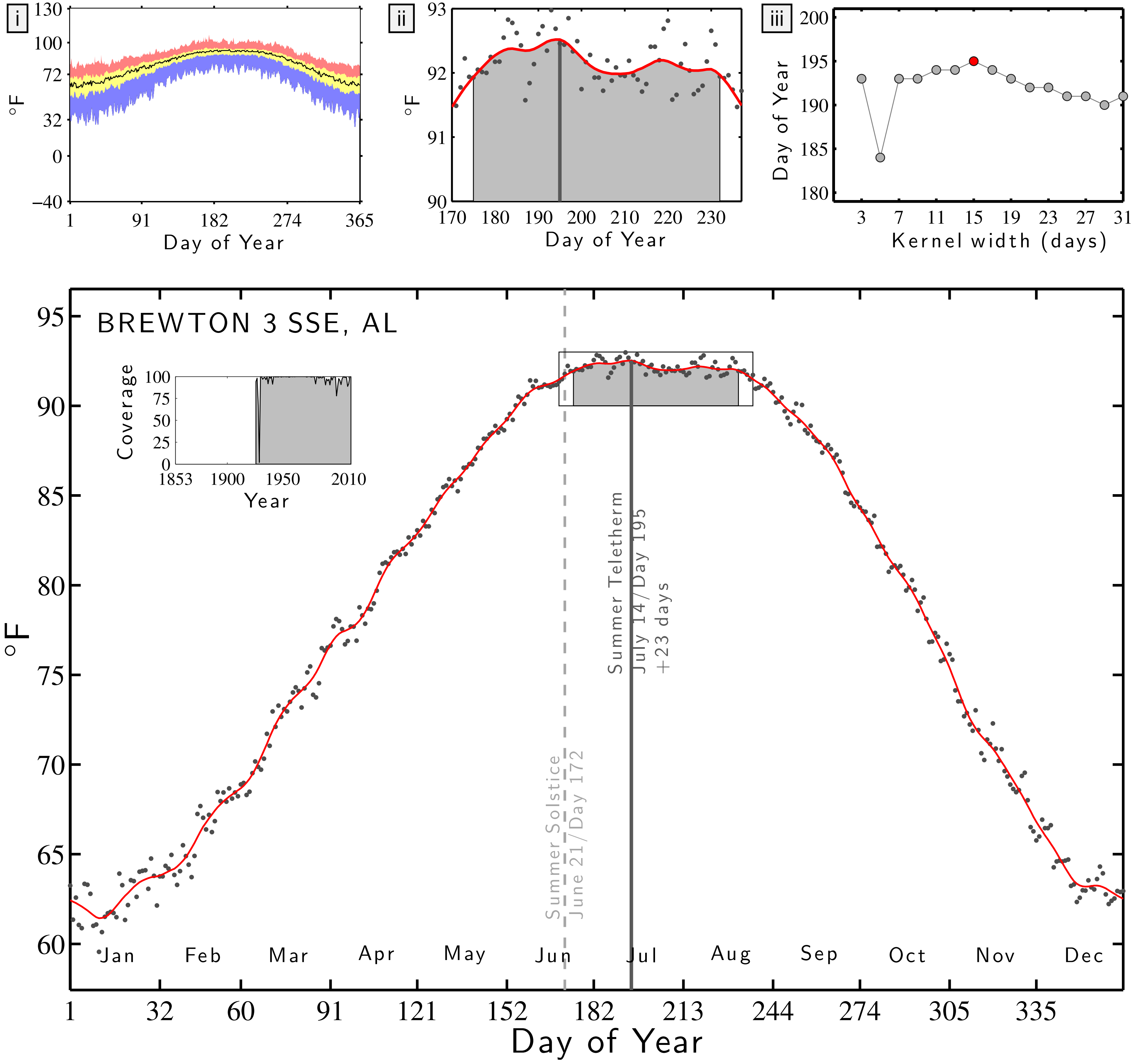

i
°F
Day of Year
ii
°F
Day of Year
iii
Day of Year
Kernel width (days)
BREWTON 3 SSE, AL
Coverage
Year
Summer Solstice
June 21/Day 172
Summer Teletherm
July 14/Day 195
+23 days
Jan
Feb
Mar
Apr
May
Jun
Jul
Aug
Sep
Oct
Nov
Dec
°F
Day of Year

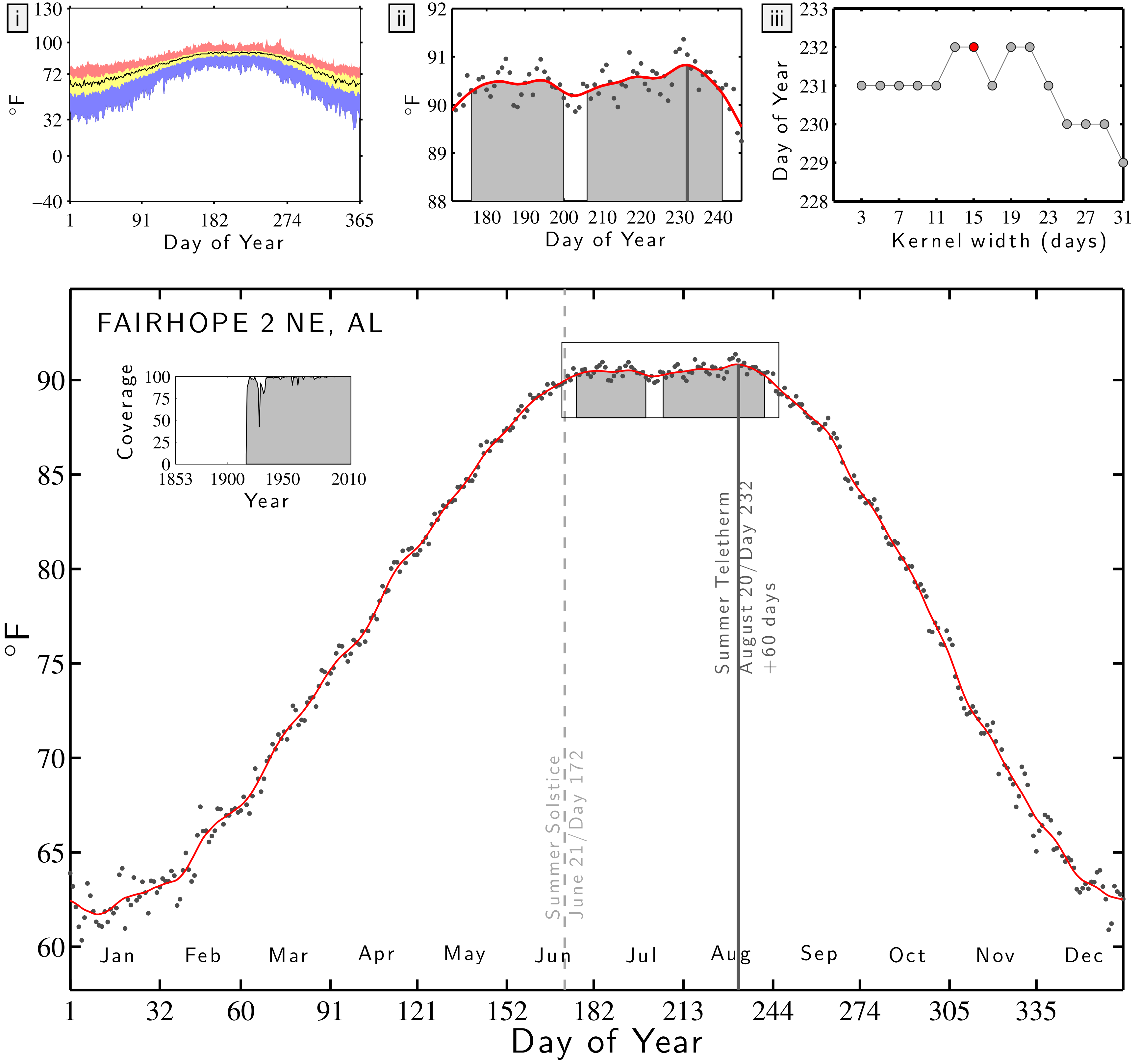

i
°F
Day of Year
ii
°F
Day of Year
iii
Day of Year
Kernel width (days)
FAIRHOPE 2 NE, AL
Coverage
Year
Summer Solstice
June 21 / Day 172
Summer Teletherm
August 20 / Day 232
+60 days
Jan
Feb
Mar
Apr
May
Jun
Jul
Aug
Sep
Oct
Nov
Dec
°F
Day of Year

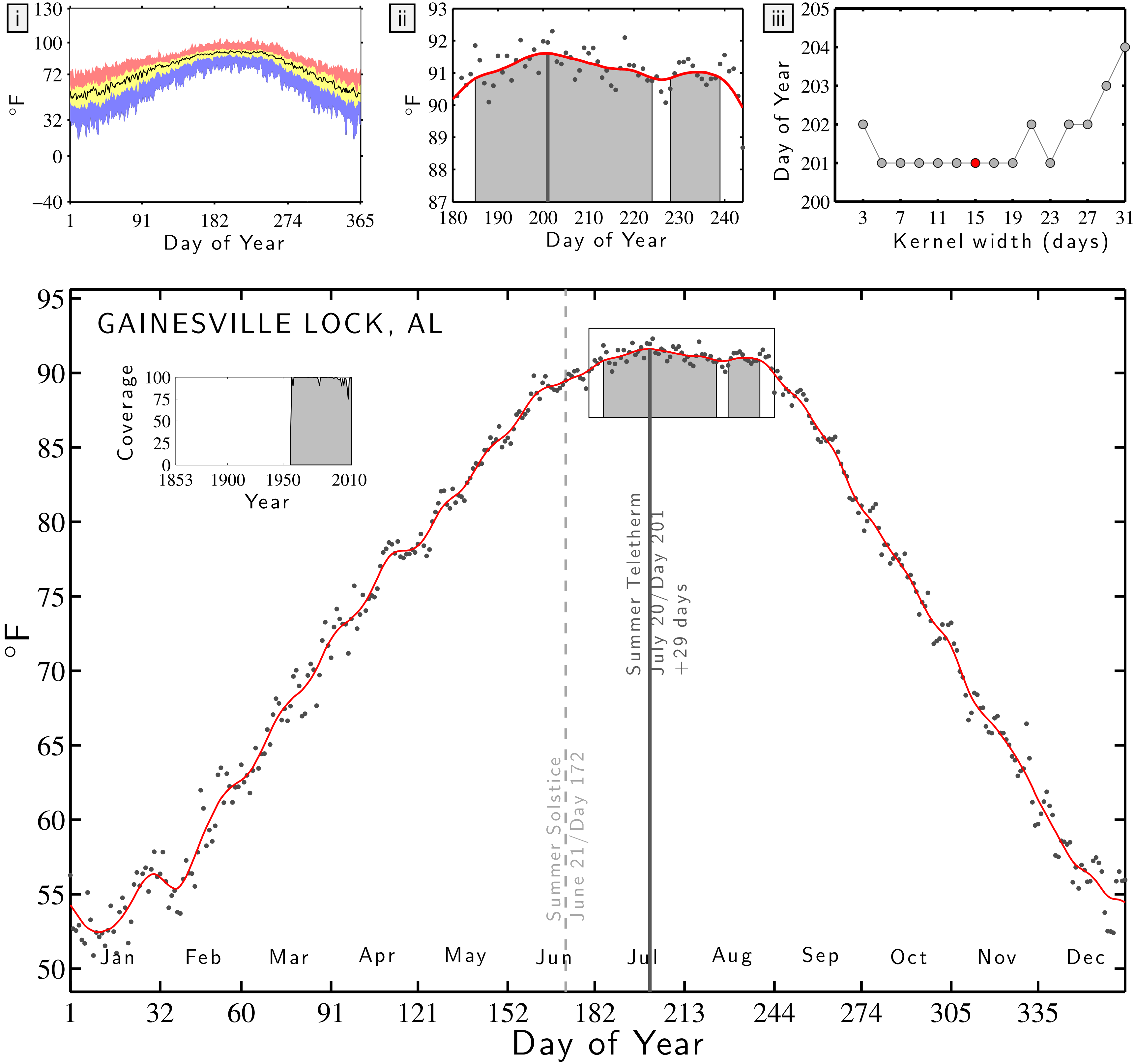

i
°F
Day of Year
ii
°F
Day of Year
iii
Day of Year
Kernel width (days)
GAINESVILLE LOCK, AL
Coverage
Year
Summer Solstice
June 21/Day 172
Summer Teletherm
July 20/Day 201
+29 days
Jan
Feb
Mar
Apr
May
Jun
Jul
Aug
Sep
Oct
Nov
Dec
°F
Day of Year

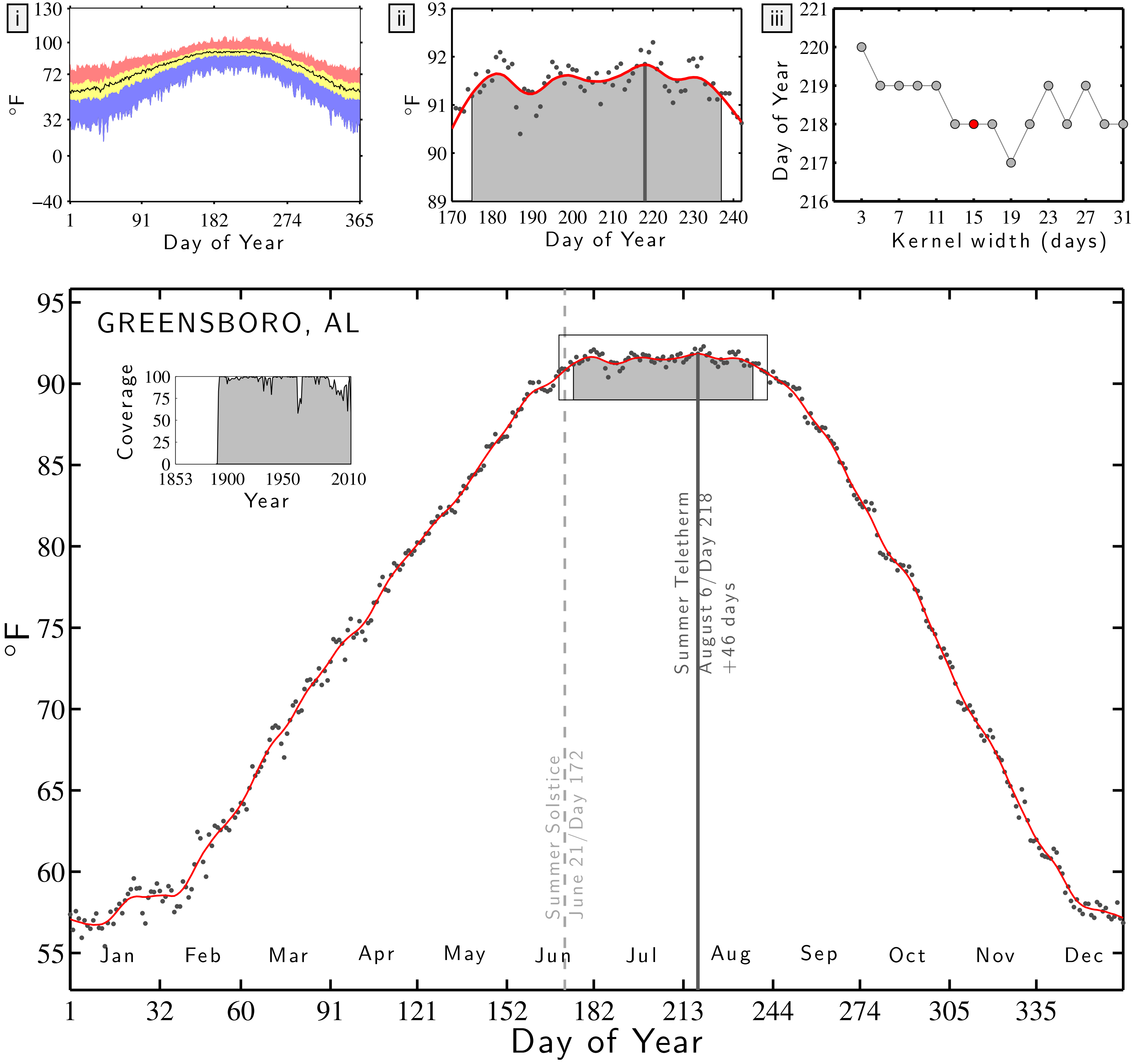

i
°F
Day of Year
ii
Day of Year
iii
Day of Year
Kernel width (days)
GREENSBORO, AL
Coverage
Year
Summer Solstice
June 21/Day 172
Summer Teletherm
August 6/Day 218
+46 days
Jan
Feb
Mar
Apr
May
Jun
Jul
Aug
Sep
Oct
Nov
Dec
Day of Year

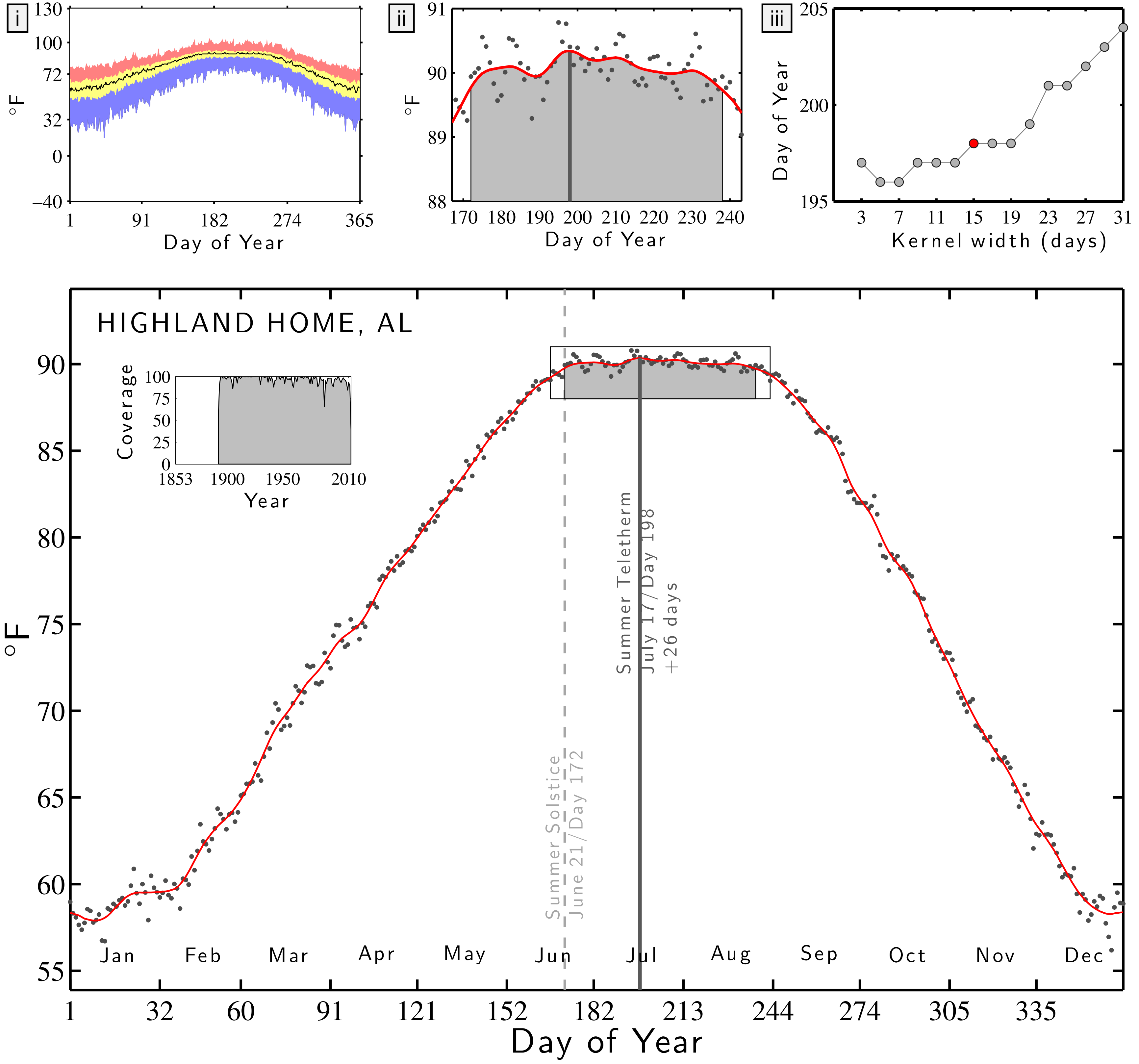

i
°F
Day of Year
ii
°F
Day of Year
iii
Day of Year
Kernel width (days)
HIGHLAND HOME, AL
Coverage
Year
Summer Solstice
June 21/Day 172
Summer Teletherm
July 17/Day 198
+26 days
Jan
Feb
Mar
Apr
May
Jun
Jul
Aug
Sep
Oct
Nov
Dec
°F
Day of Year

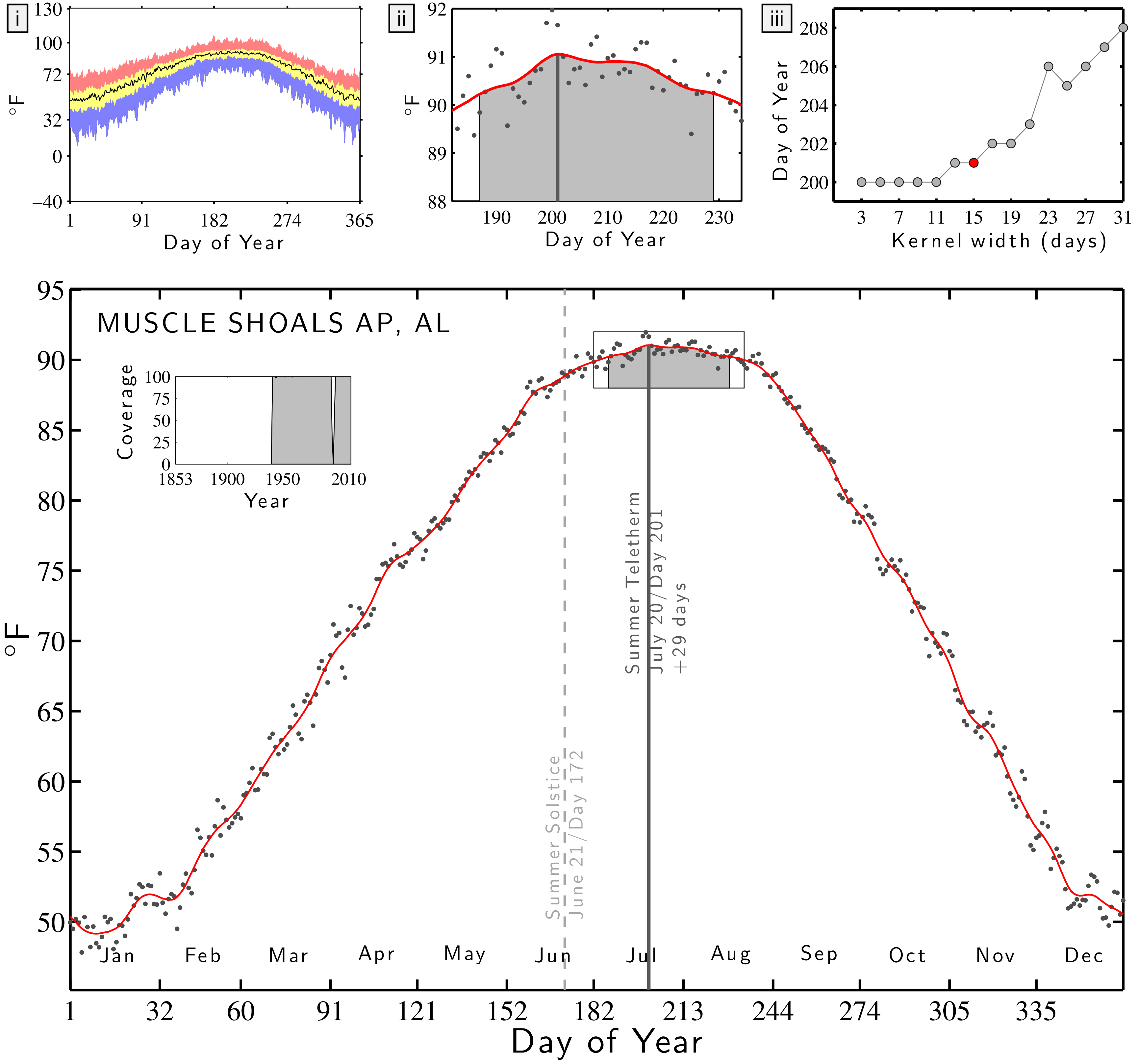

i
°F
130
100
72
32
0
−40
1
91
182
274
365
Day of Year
ii
92
91
90
89
88
190
200
210
220
230
Day of Year
iii
Day of Year
208
206
204
202
200
3
7
11
15
19
23
27
31
Kernel width (days)
MUSCLE SHOALS AP, AL
Coverage
100
75
50
25
0
1853
1900
1950
2010
Year
Summer Solstice
June 21 / Day 172
Summer Teletherm
July 20 / Day 201
+29 days
°F
95
90
85
80
75
70
65
60
55
50
Jan
Feb
Mar
Apr
May
Jun
Jul
Aug
Sep
Oct
Nov
Dec
1
32
60
91
121
152
182
213
244
274
305
335
Day of Year

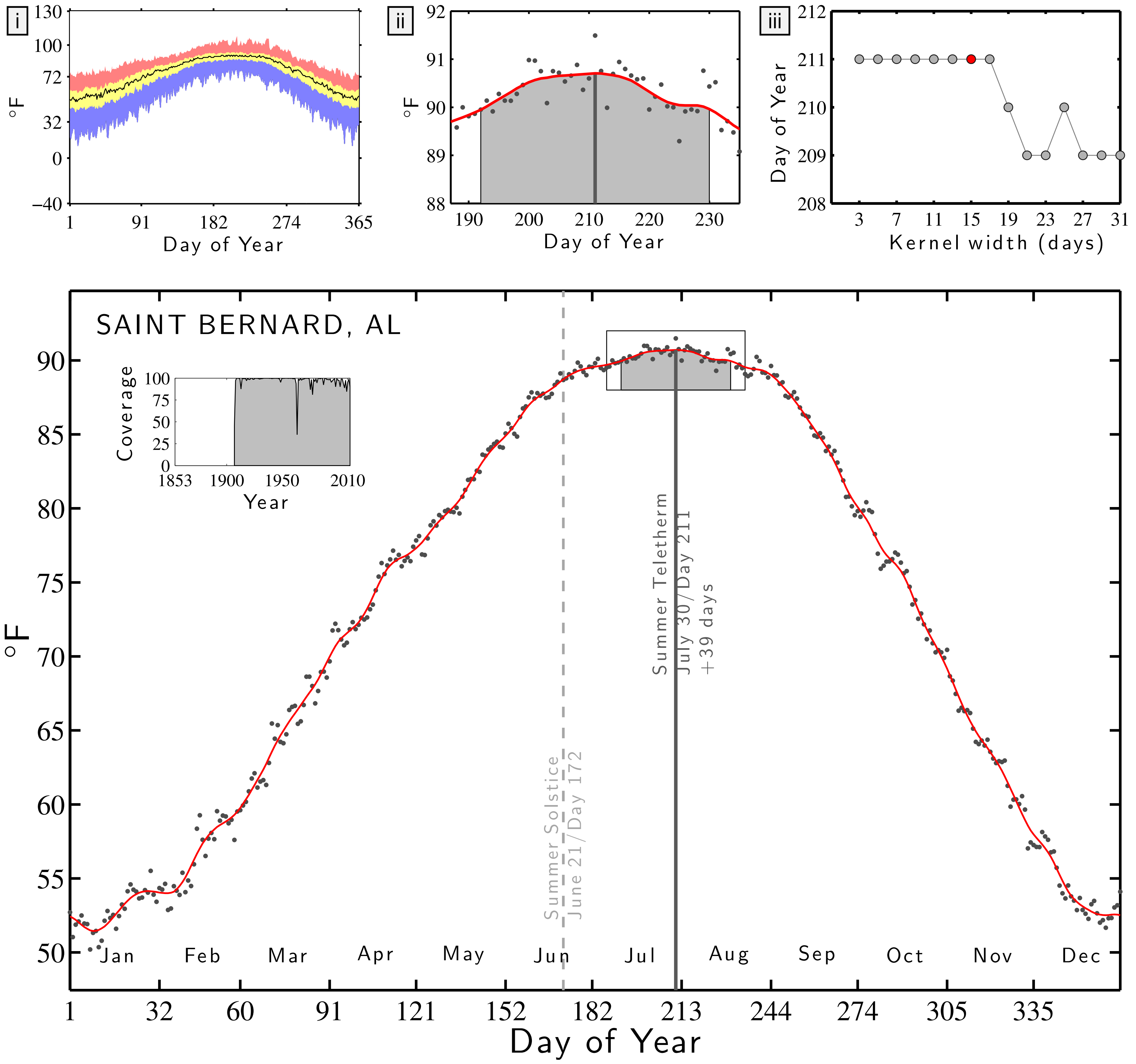

i
ii
iii
Day of Year
°F
Kernel width (days)
SAINT BERNARD, AL
Coverage
Year
Summer Solstice
June 21/Day 172
Summer Teletherm
July 30/Day 211
+39 days
Jan
Feb
Mar
Apr
May
Jun
Jul
Aug
Sep
Oct
Nov
Dec

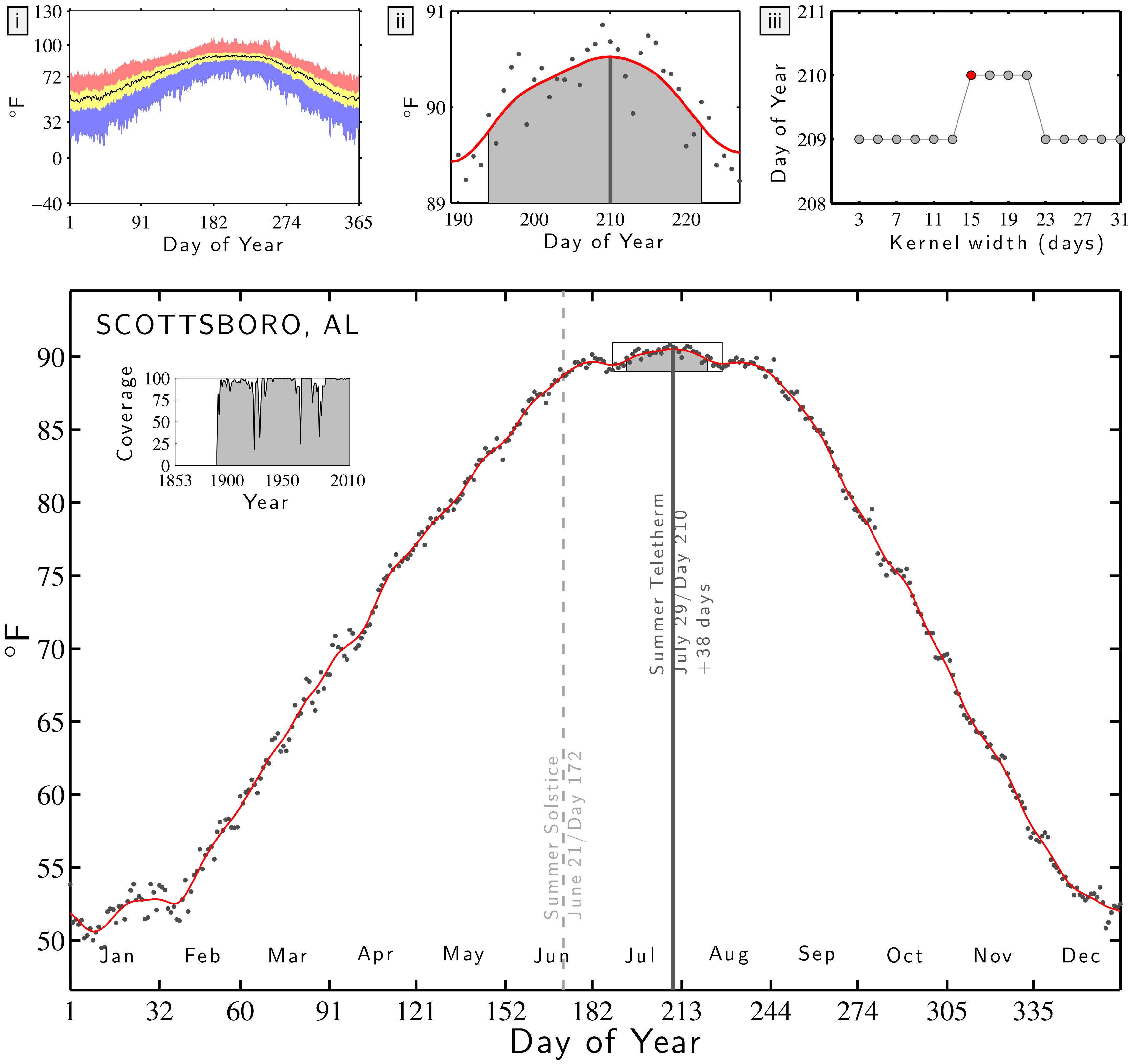

i
°F
Day of Year
ii
°F
Day of Year
iii
Day of Year
Kernel width (days)
SCOTTSBORO, AL
Coverage
Year
1853
1900
1950
2010
Summer Solstice
June 21/Day 172
Summer Teletherm
July 29/Day 210
+38 days
Jan
Feb
Mar
Apr
May
Jun
Jul
Aug
Sep
Oct
Nov
Dec
°F
Day of Year

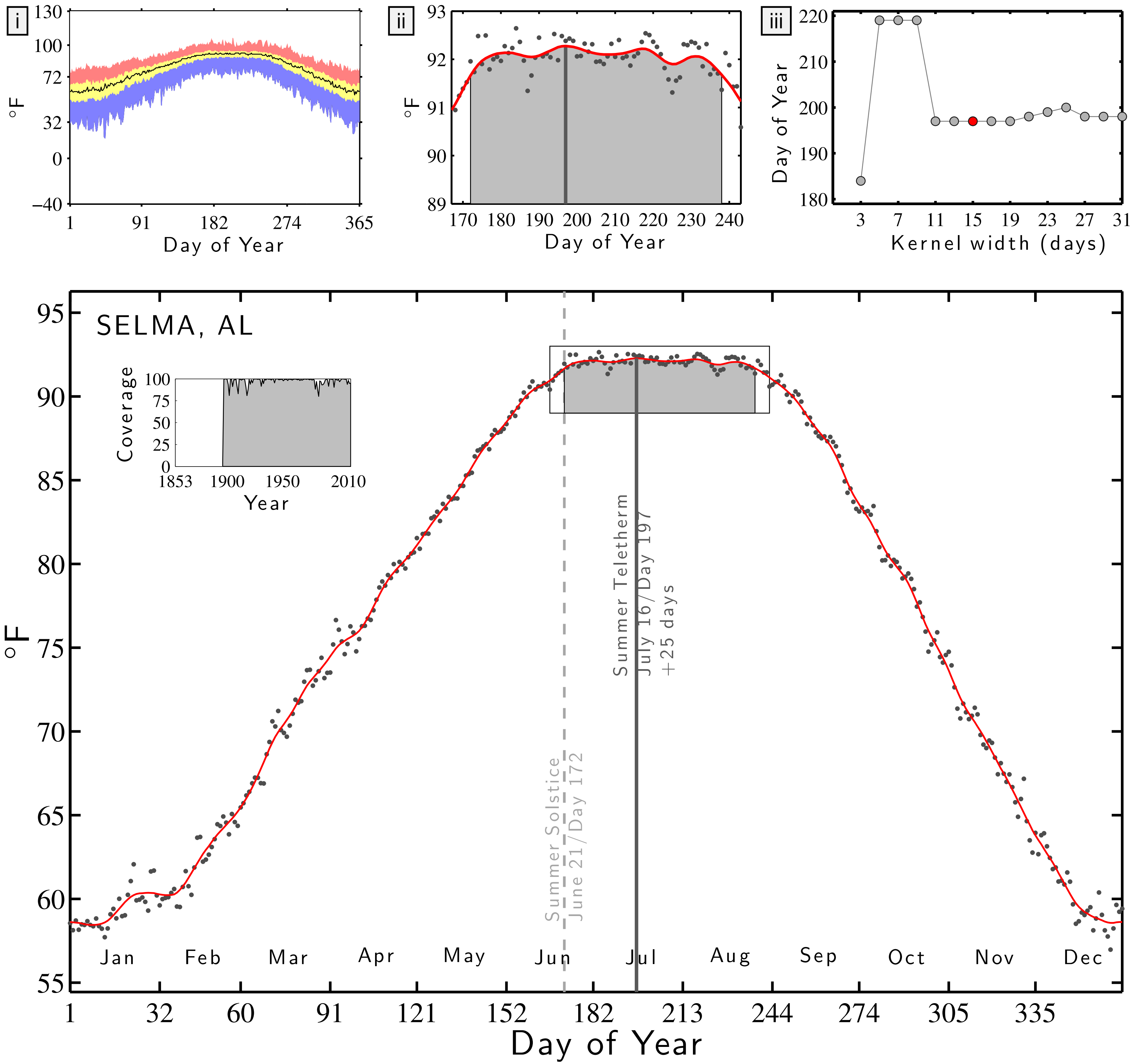

i
ii
iii
Day of Year
°F
Kernel width (days)
SELMA, AL
Coverage
Year
Summer Solstice
June 21/Day 172
Summer Teletherm
July 16/Day 197
+25 days
Jan
Feb
Mar
Apr
May
Jun
Jul
Aug
Sep
Oct
Nov
Dec

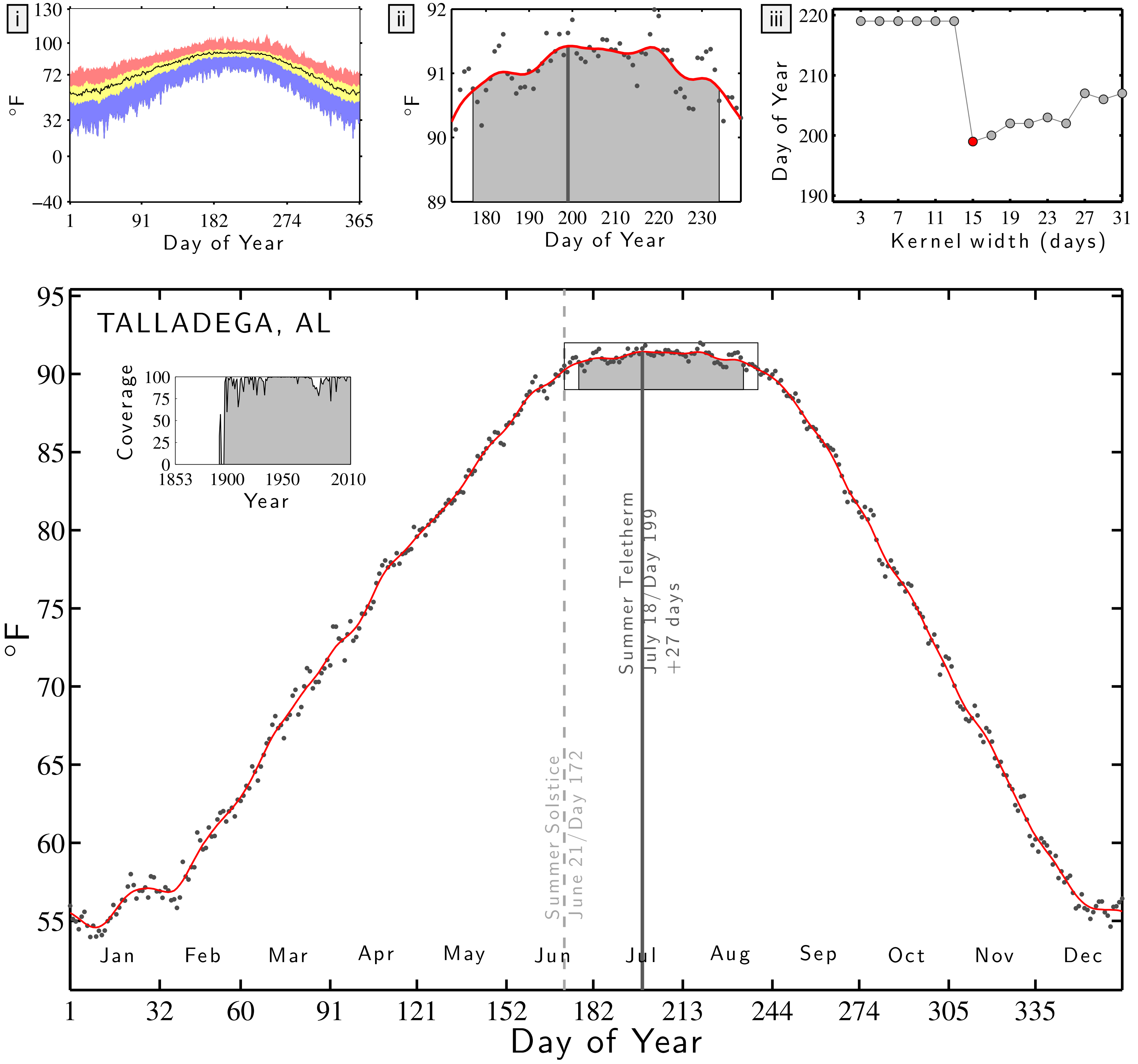

i
ii
iii
°F
Day of Year
Kernel width (days)
TALLADEGA, AL
Coverage
Year
Summer Solstice
June 21 / Day 172
Summer Teletherm
July 18 / Day 199
+27 days
Jan
Feb
Mar
Apr
May
Jun
Jul
Aug
Sep
Oct
Nov
Dec

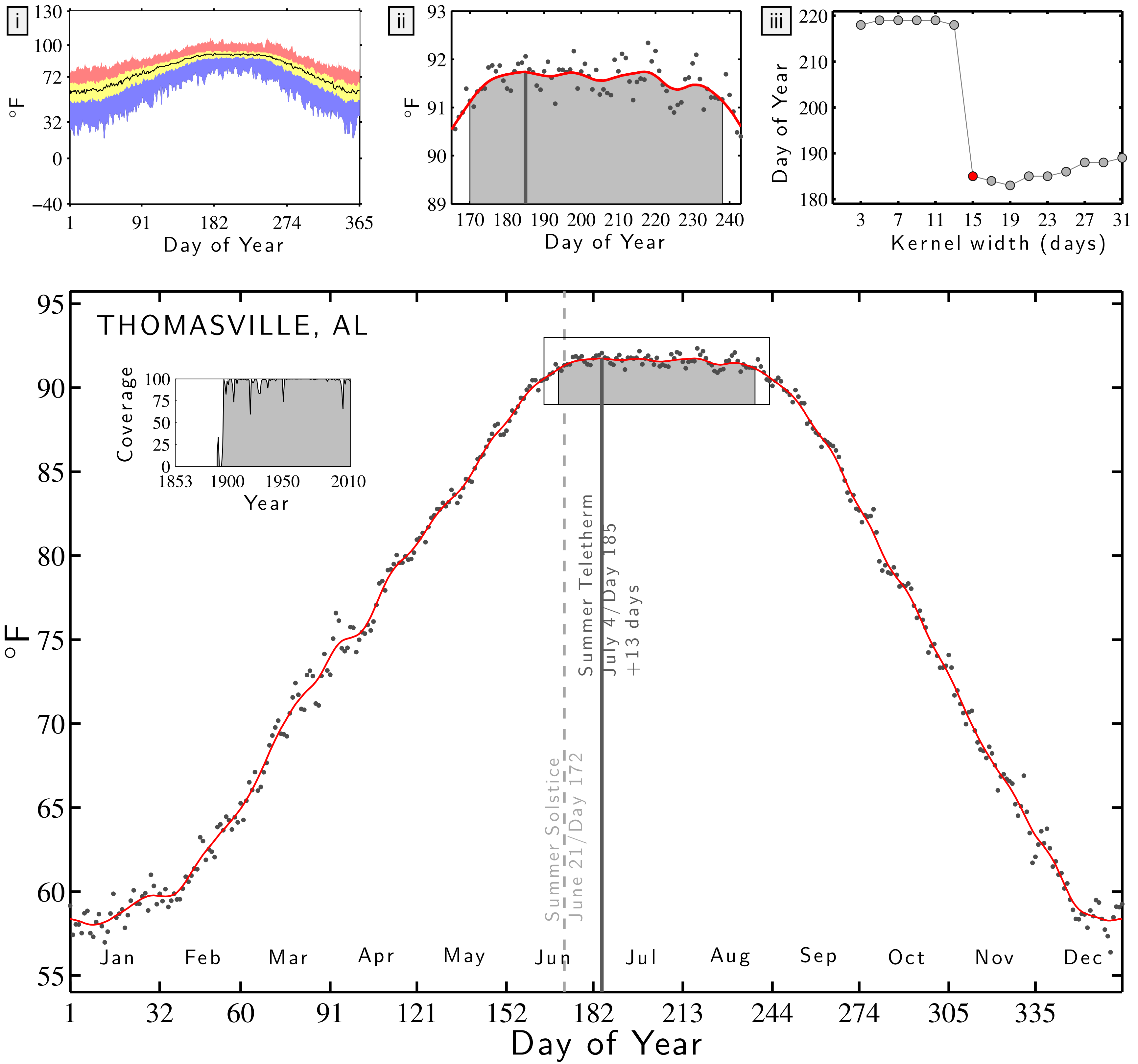

i
ii
iii
°F
Day of Year
Kernel width (days)
THOMASVILLE, AL
Coverage
Year
Summer Solstice
June 21 / Day 172
Summer Teletherm
July 4 / Day 185
+13 days
Jan
Feb
Mar
Apr
May
Jun
Jul
Aug
Sep
Oct
Nov
Dec

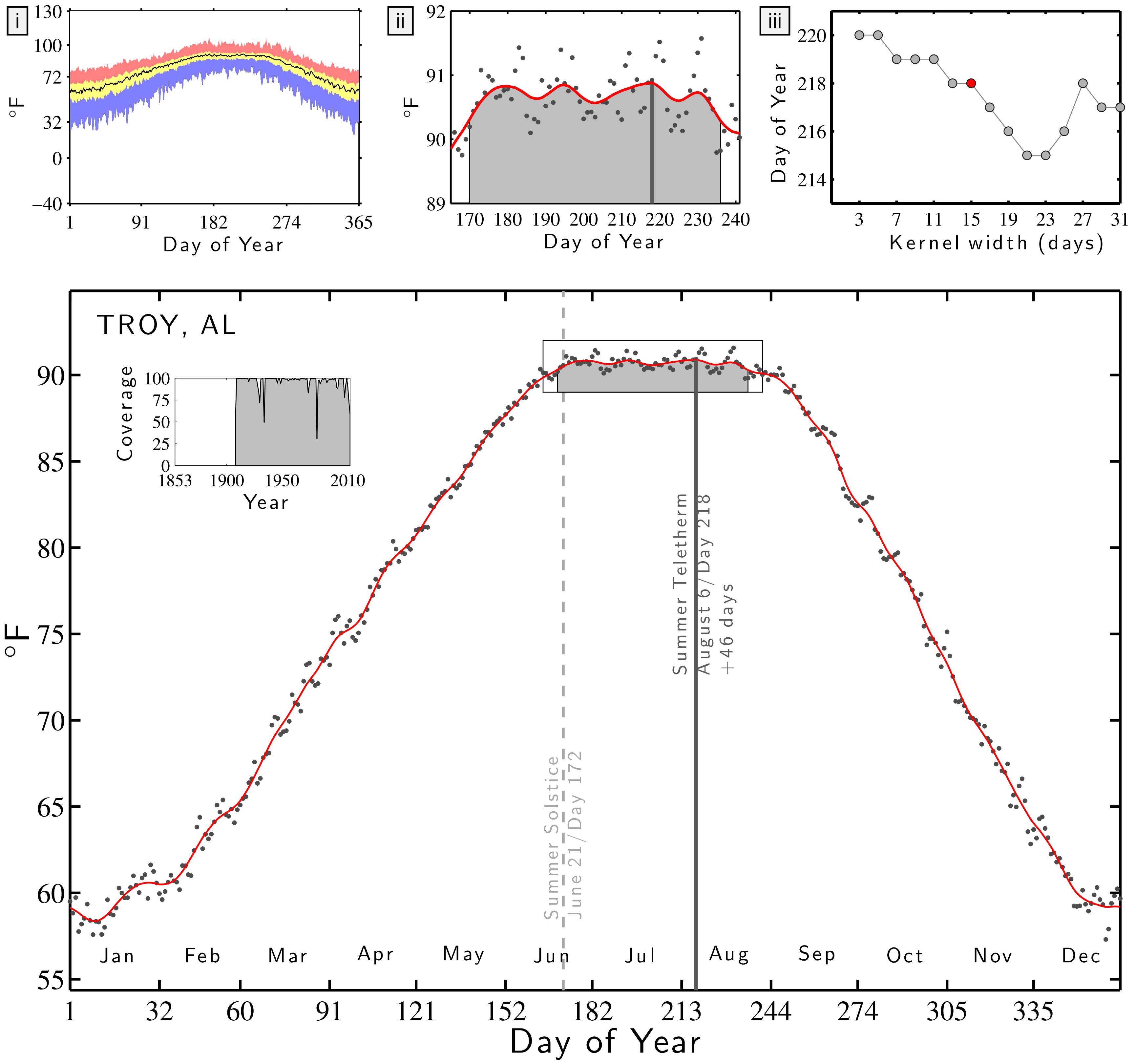

i
ii
iii
°F
Day of Year
Day of Year
Kernel width (days)
TROY, AL
Coverage
Year
Summer Solstice
June 21/Day 172
Summer Teletherm
August 6/Day 218
+46 days
Jan
Feb
Mar
Apr
May
Jun
Jul
Aug
Sep
Oct
Nov
Dec

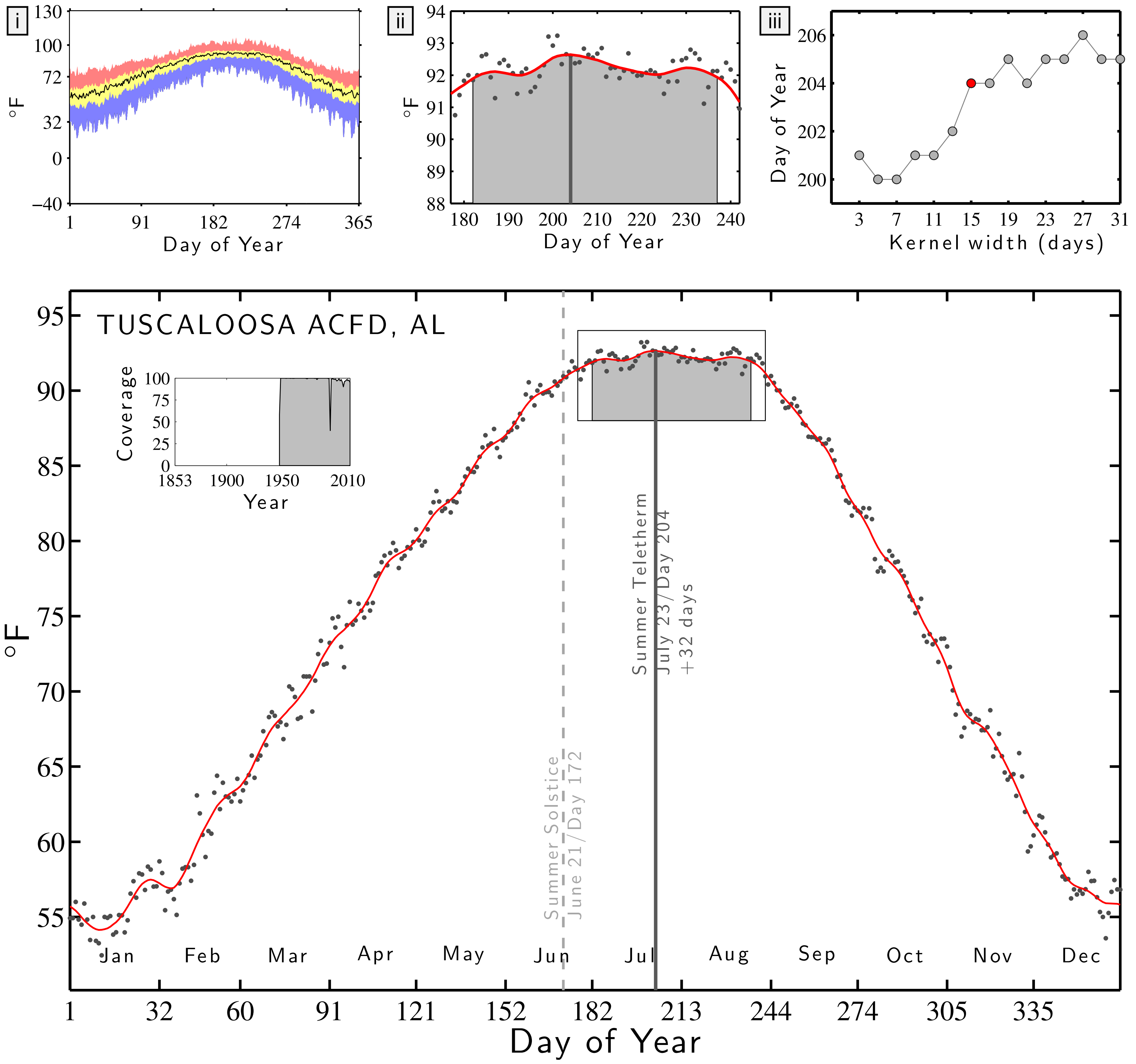

i
130
100
72
32
0
−40
°F
1
91
182
274
365
Day of Year
ii
94
93
92
91
90
89
88
°F
180
190
200
210
220
230
240
Day of Year
iii
206
204
202
200
Day of Year
3
7
11
15
19
23
27
31
Kernel width (days)
TUSCALOOSA ACFD, AL
Coverage
100
75
50
25
0
1853
1900
1950
2010
Year
Summer Solstice
June 21 / Day 172
Summer Teletherm
July 23 / Day 204
+32 days
95
90
85
80
75
70
65
60
55
°F
Jan
Feb
Mar
Apr
May
Jun
Jul
Aug
Sep
Oct
Nov
Dec
1
32
60
91
121
152
182
213
244
274
305
335
Day of Year

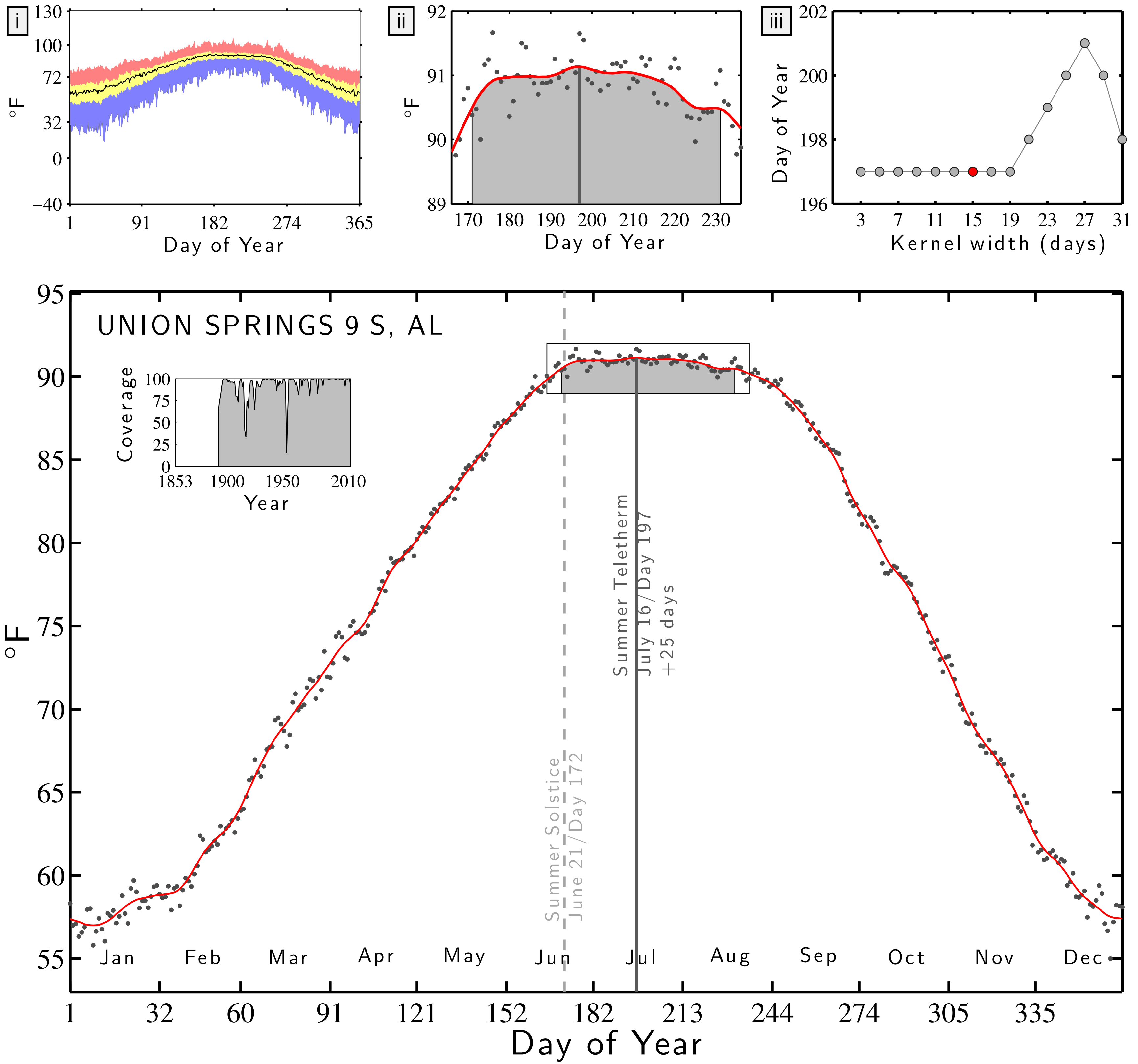

i
°F
Day of Year
ii
°F
Day of Year
iii
Day of Year
Kernel width (days)
UNION SPRINGS 9 S, AL
Coverage
Year
Summer Solstice
June 21/Day 172
Summer Teletherm
July 16/Day 197
+25 days
Jan Feb Mar Apr May Jun Jul Aug Sep Oct Nov Dec
°F
Day of Year

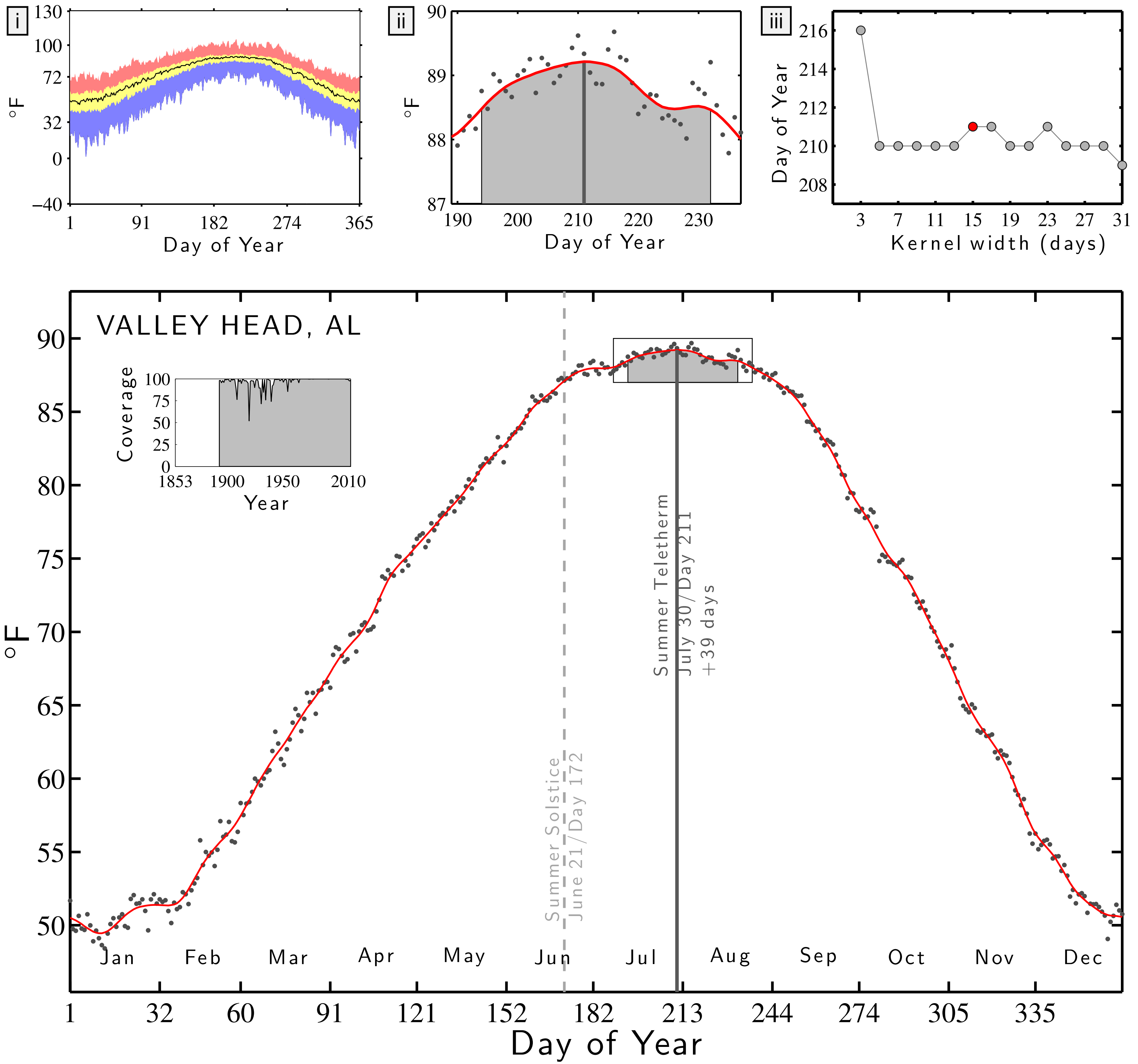
i
ii
iii
°F
Day of Year
Day of Year
Kernel width (days)
VALLEY HEAD, AL
Coverage
Year
Summer Solstice
June 21/Day 172
Summer Teletherm
July 30/Day 211
+39 days
Jan
Feb
Mar
Apr
May
Jun
Jul
Aug
Sep
Oct
Nov
Dec
Day of Year

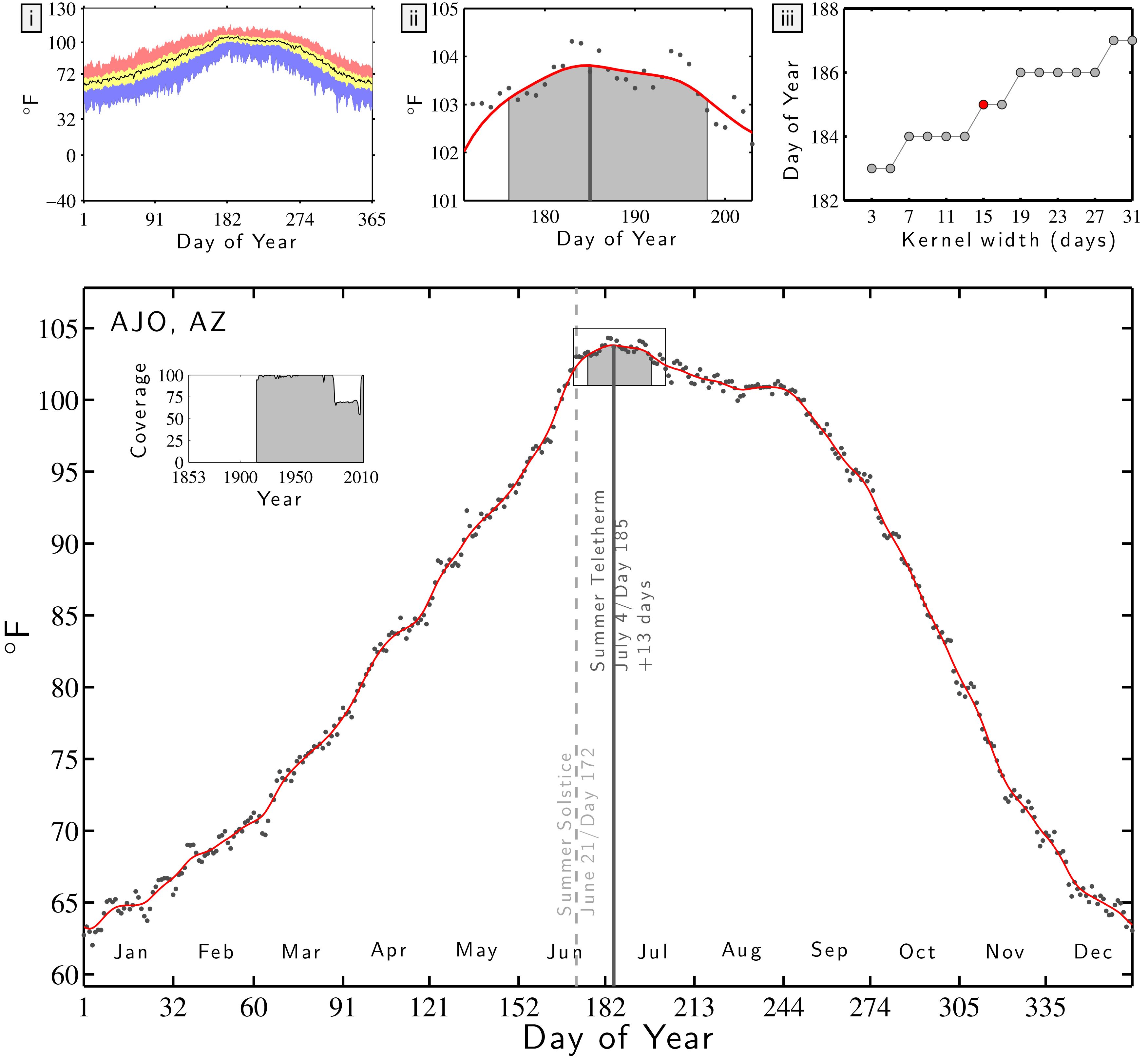

i
°F
Day of Year
ii
°F
Day of Year
iii
Day of Year
Kernel width (days)
AJO, AZ
Coverage
Year
Summer Solstice
June 21/Day 172
Summer Teletherm
July 4/Day 185
+13 days
Jan
Feb
Mar
Apr
May
Jun
Jul
Aug
Sep
Oct
Nov
Dec
°F
Day of Year

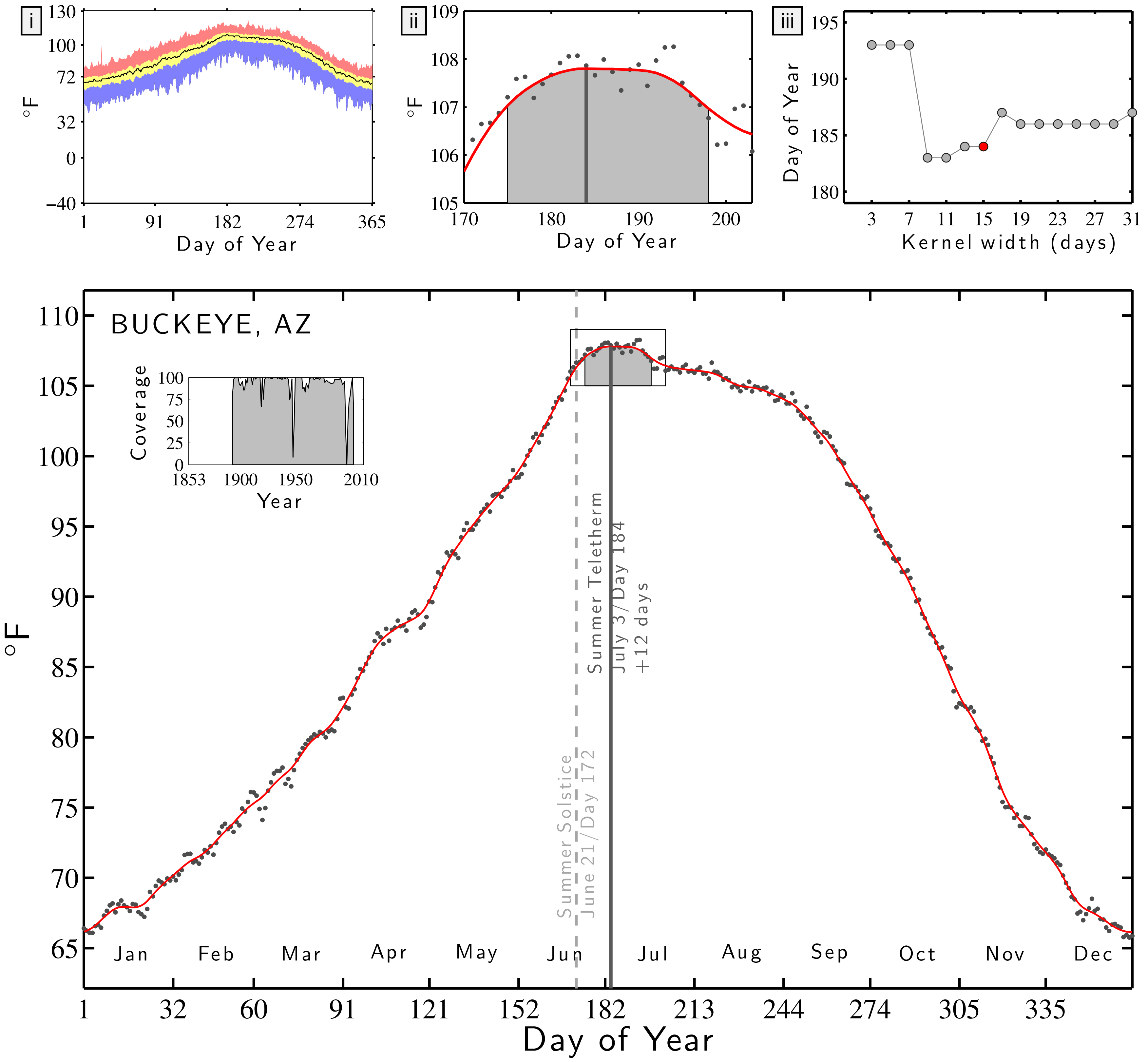

i
ii
iii
°F
Day of Year
Kernel width (days)
BUCKEYE, AZ
Coverage
Year
Summer Teletherm
July 3/Day 184
+12 days
Summer Solstice
June 21/Day 172
Jan
Feb
Mar
Apr
May
Jun
Jul
Aug
Sep
Oct
Nov
Dec

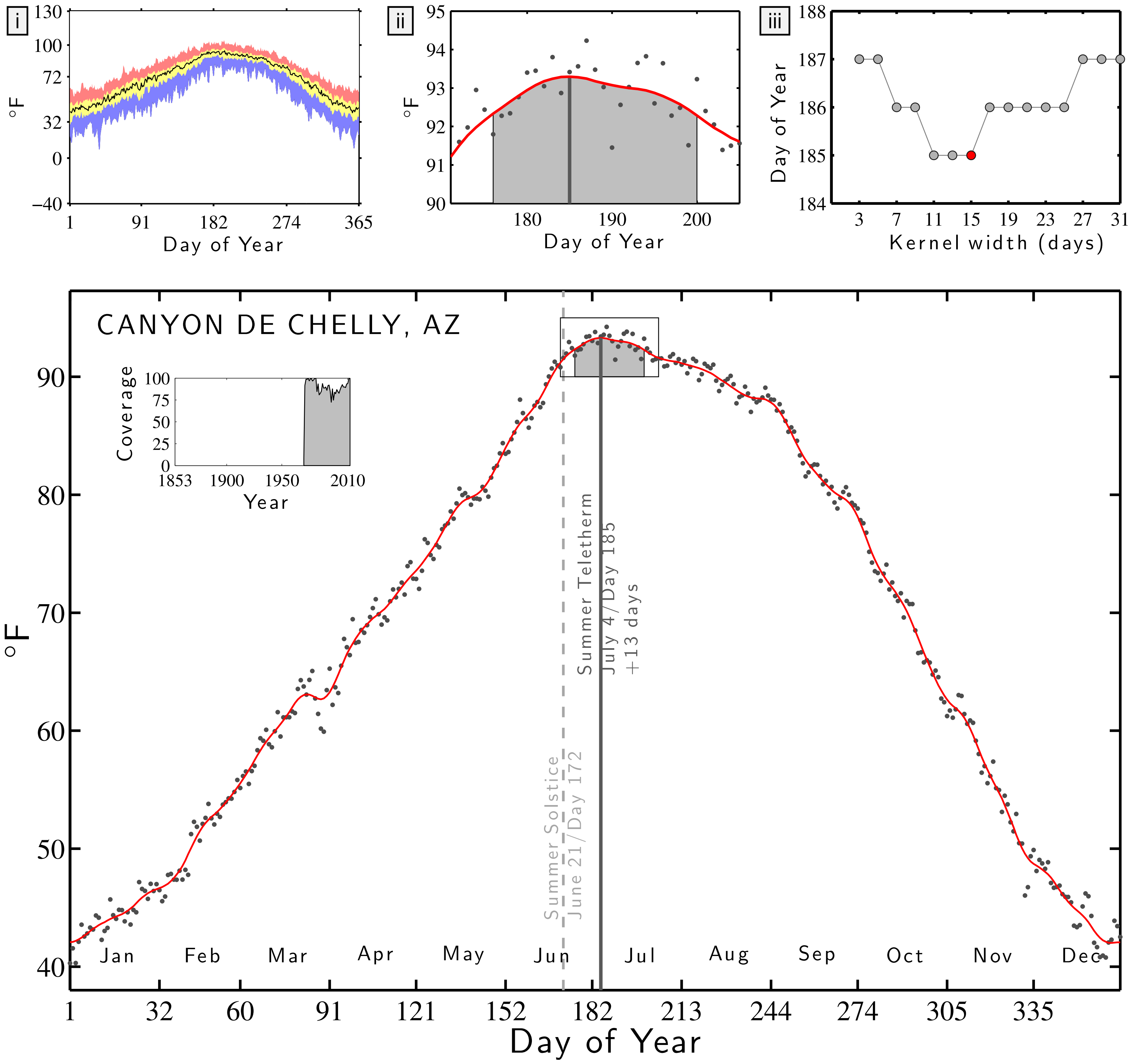

i
ii
iii
°F
Day of Year
Kernel width (days)
CANYON DE CHELLY, AZ
Coverage
Year
Summer Solstice
June 21 / Day 172
Summer Teletherm
July 4 / Day 185
+13 days
Jan
Feb
Mar
Apr
May
Jun
Jul
Aug
Sep
Oct
Nov
Dec

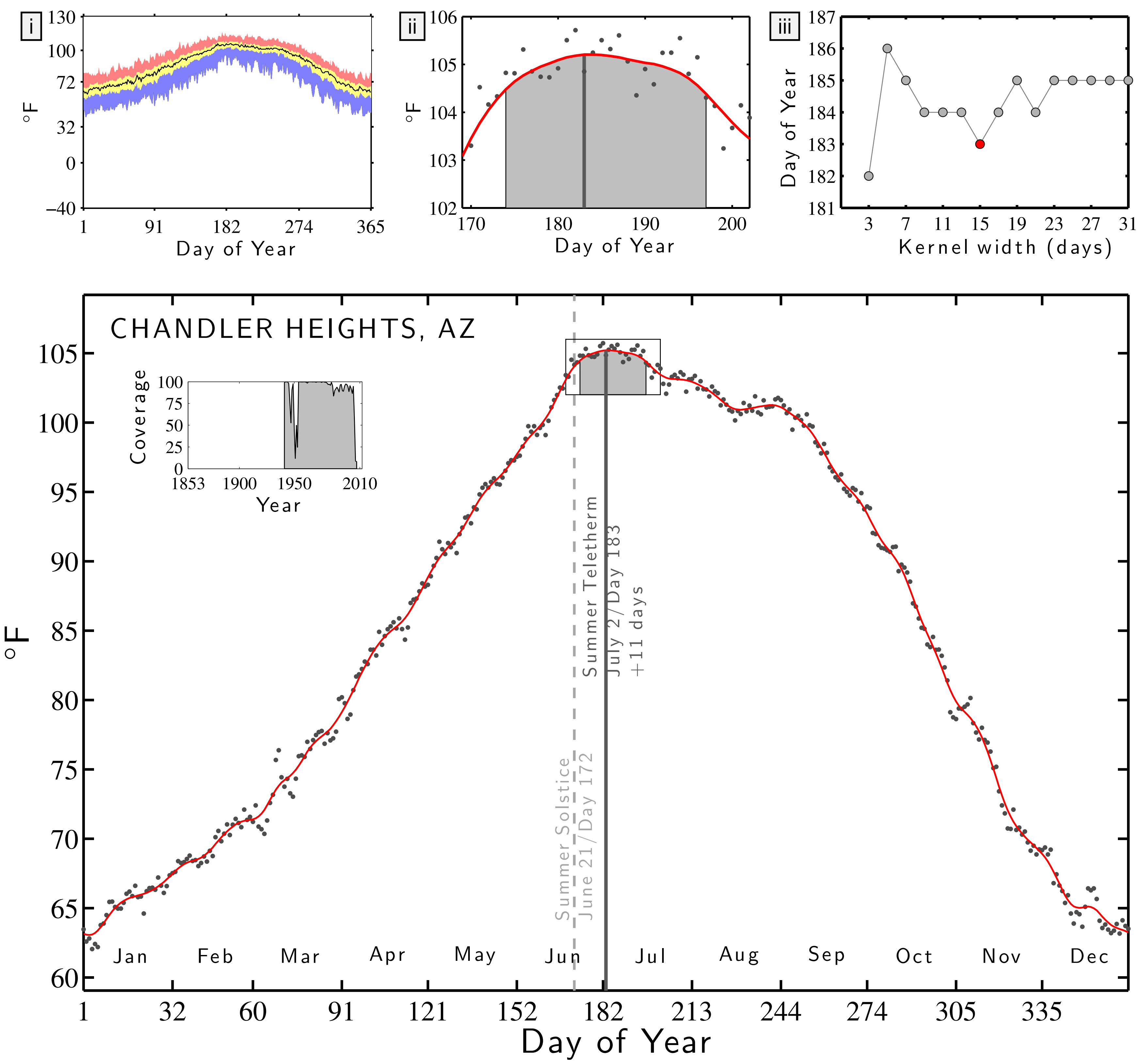
i
ii
iii
Day of Year
°F
Kernel width (days)
CHANDLER HEIGHTS, AZ
Coverage
Year
Summer Teletherm
July 2/Day 183
+11 days
Summer Solstice
June 21/Day 172
Jan
Feb
Mar
Apr
May
Jun
Jul
Aug
Sep
Oct
Nov
Dec

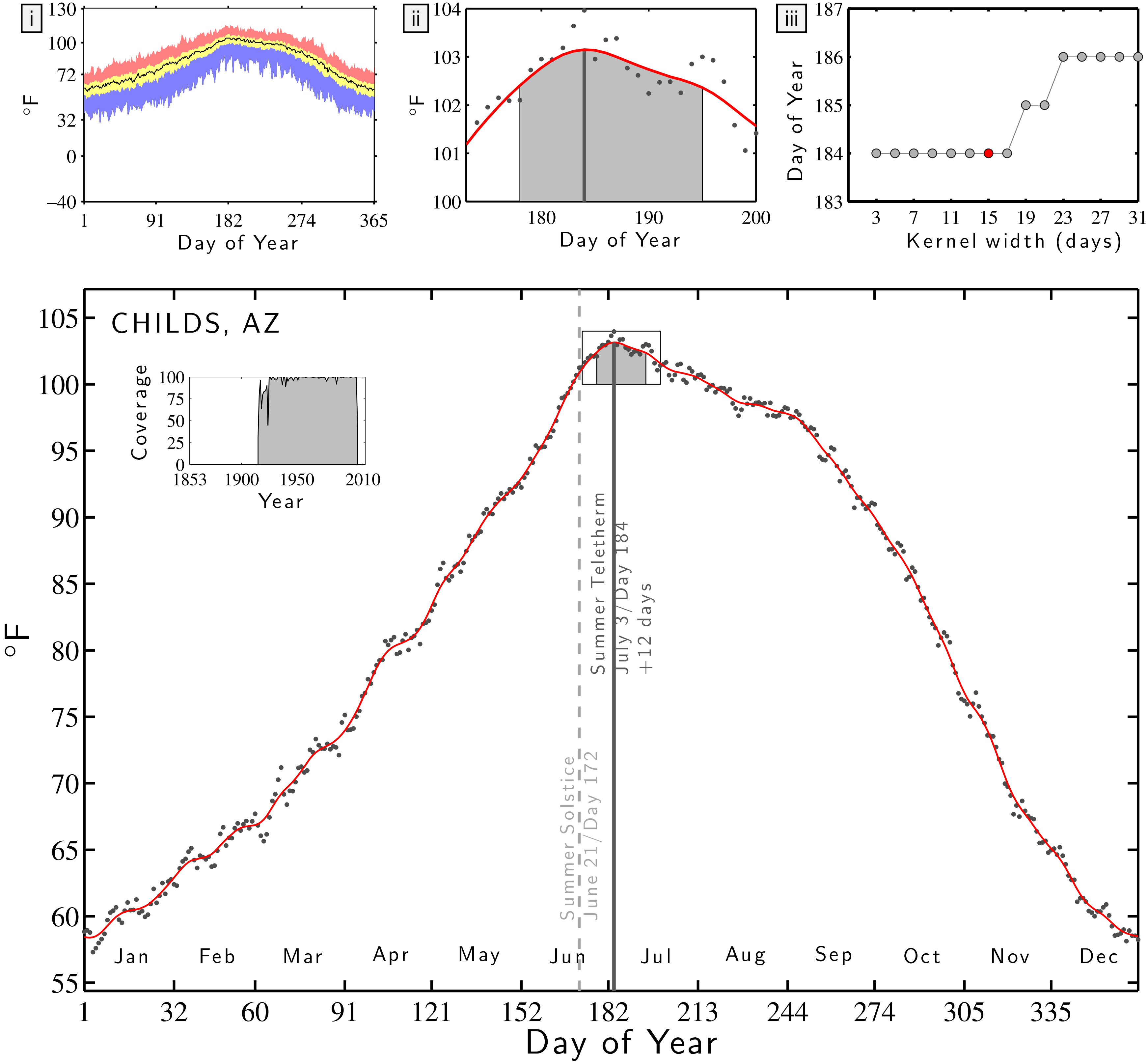
i
ii
iii
°F
Day of Year
Day of Year
Kernel width (days)
CHILDS, AZ
Coverage
Year
Summer Teletherm
July 3/Day 184
+12 days
Summer Solstice
June 21/Day 172
Jan
Feb
Mar
Apr
May
Jun
Jul
Aug
Sep
Oct
Nov
Dec
°F
Day of Year

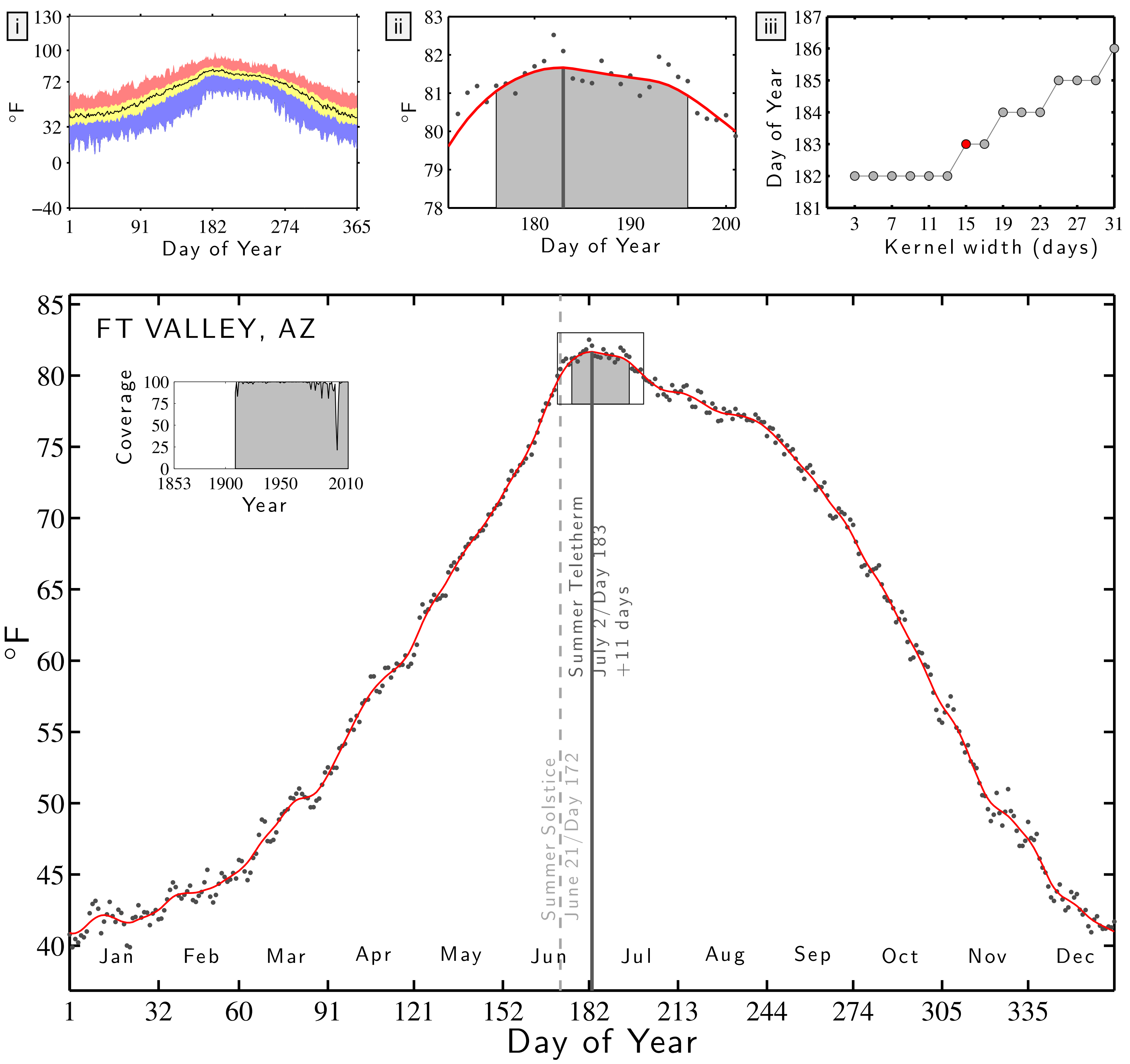

i
ii
iii
°F
Day of Year
Kernel width (days)
FT VALLEY, AZ
Coverage
Year
Summer Solstice
June 21/Day 172
Summer Teletherm
July 2/Day 183
+11 days
Jan
Feb
Mar
Apr
May
Jun
Jul
Aug
Sep
Oct
Nov
Dec

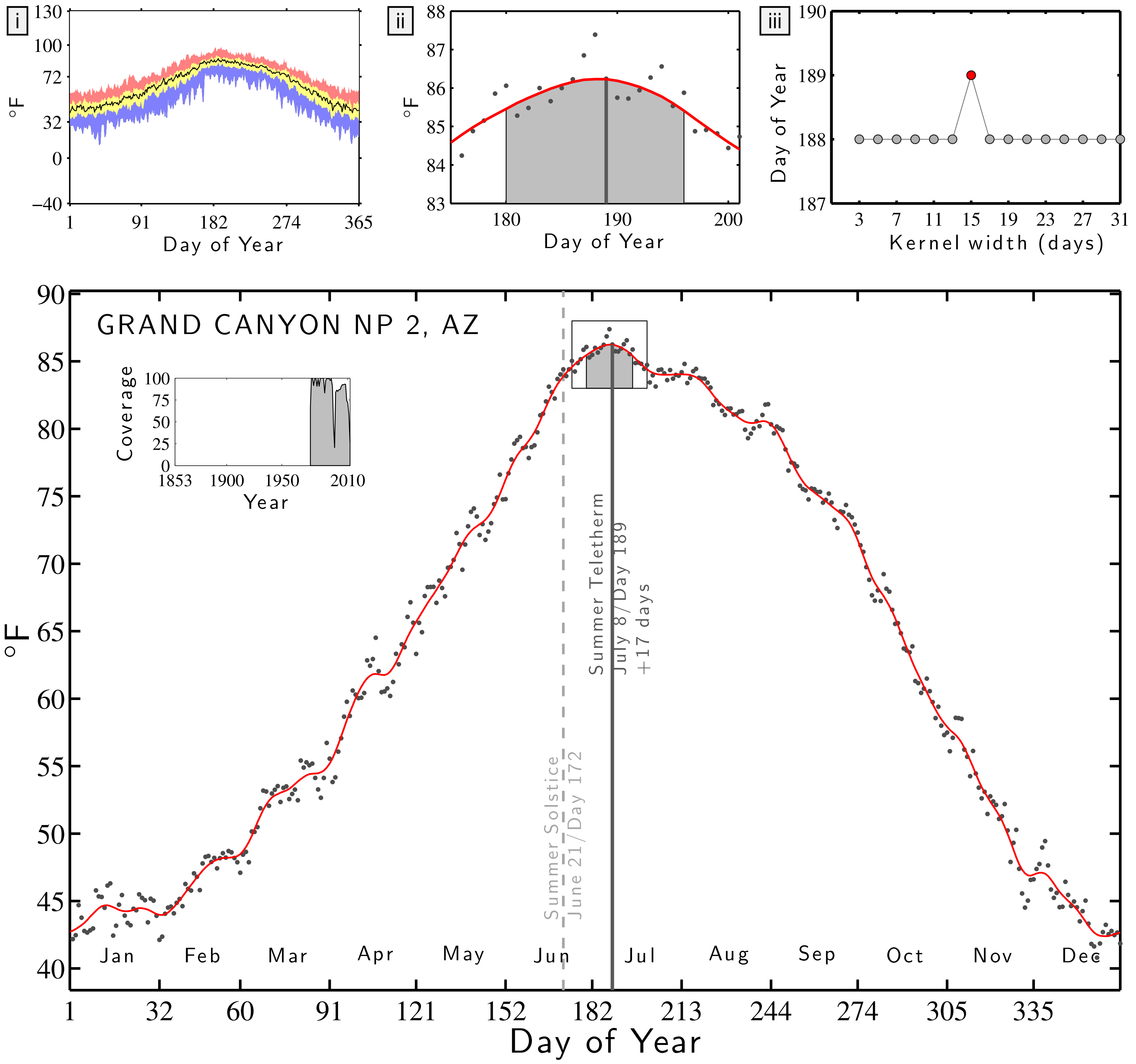

i
ii
iii
°F
Day of Year
Day of Year
Kernel width (days)
GRAND CANYON NP 2, AZ
Coverage
Year
Summer Solstice
June 21/Day 172
Summer Teletherm
July 8/Day 189
+17 days
Jan
Feb
Mar
Apr
May
Jun
Jul
Aug
Sep
Oct
Nov
Dec
Day of Year

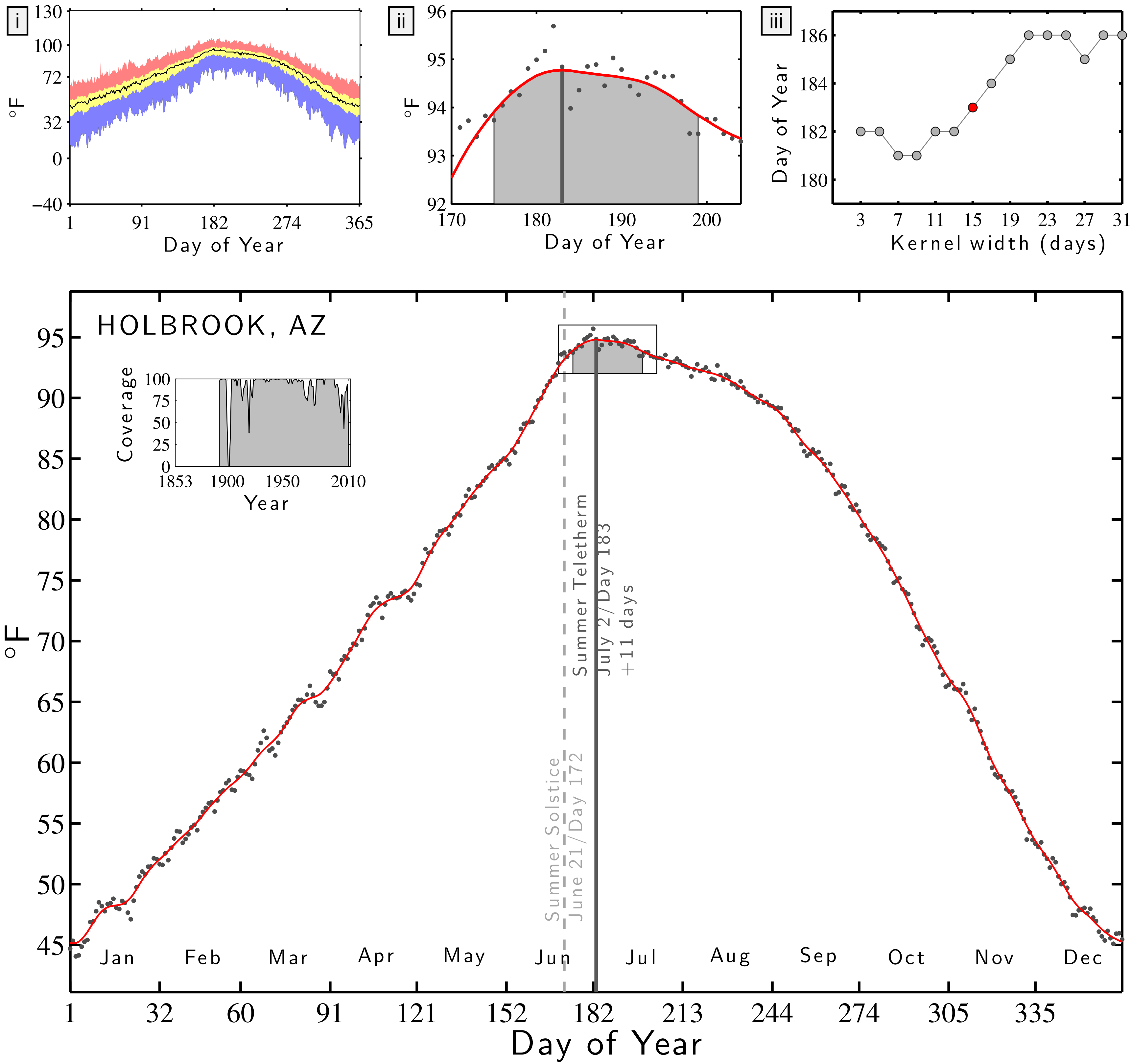

i
ii
iii
°F
Day of Year
Day of Year
Kernel width (days)
HOLBROOK, AZ
Coverage
Year
Summer Solstice
June 21/Day 172
Summer Teletherm
July 2/Day 183
+11 days
Jan
Feb
Mar
Apr
May
Jun
Jul
Aug
Sep
Oct
Nov
Dec

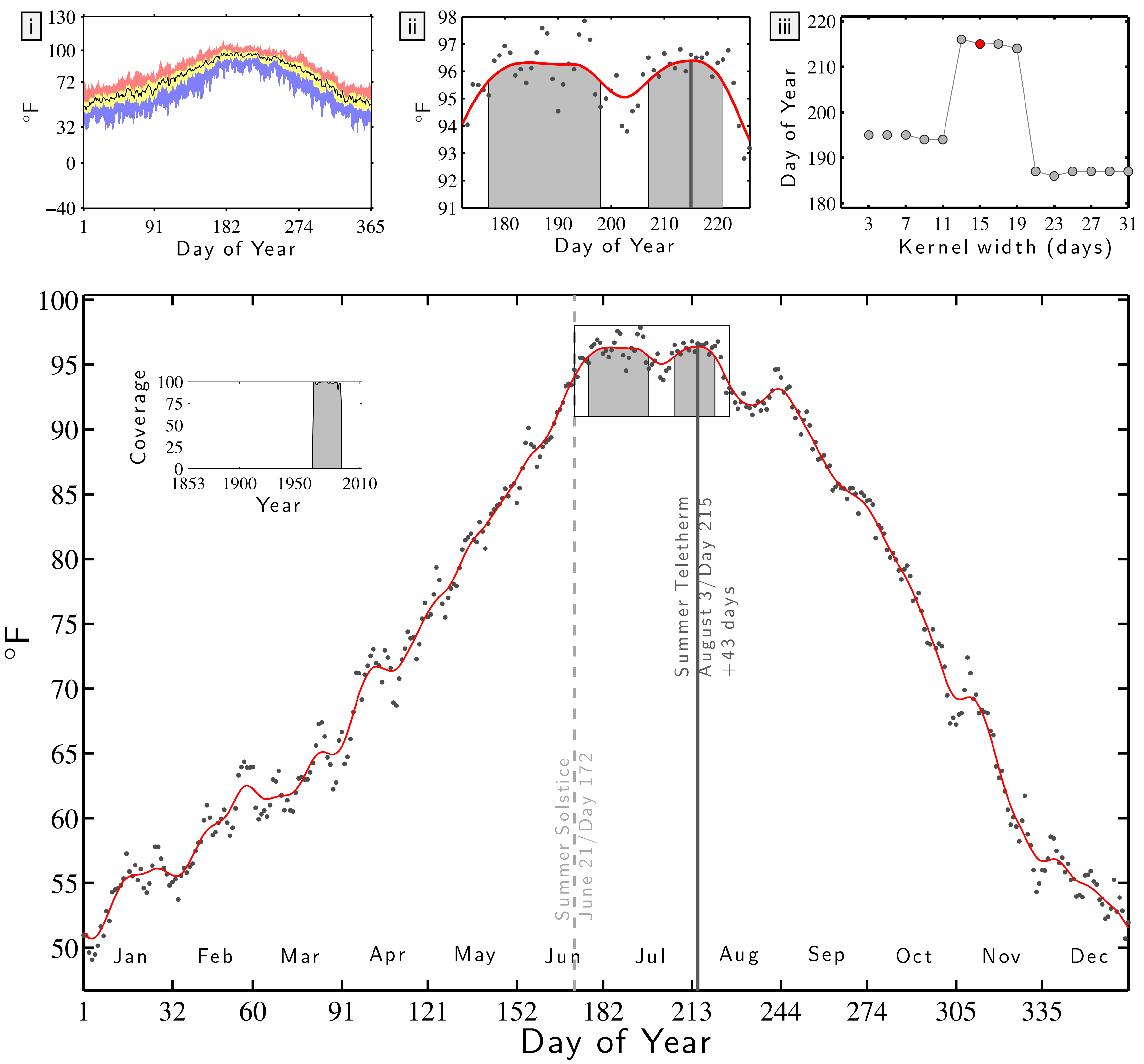

i
°F
Day of Year
ii
°F
Day of Year
iii
Day of Year
Kernel width (days)
Coverage
Year
Summer Solstice
June 21/Day 172
Summer Teletherm
August 3/Day 215
+43 days
Jan
Feb
Mar
Apr
May
Jun
Jul
Aug
Sep
Oct
Nov
Dec
°F
Day of Year

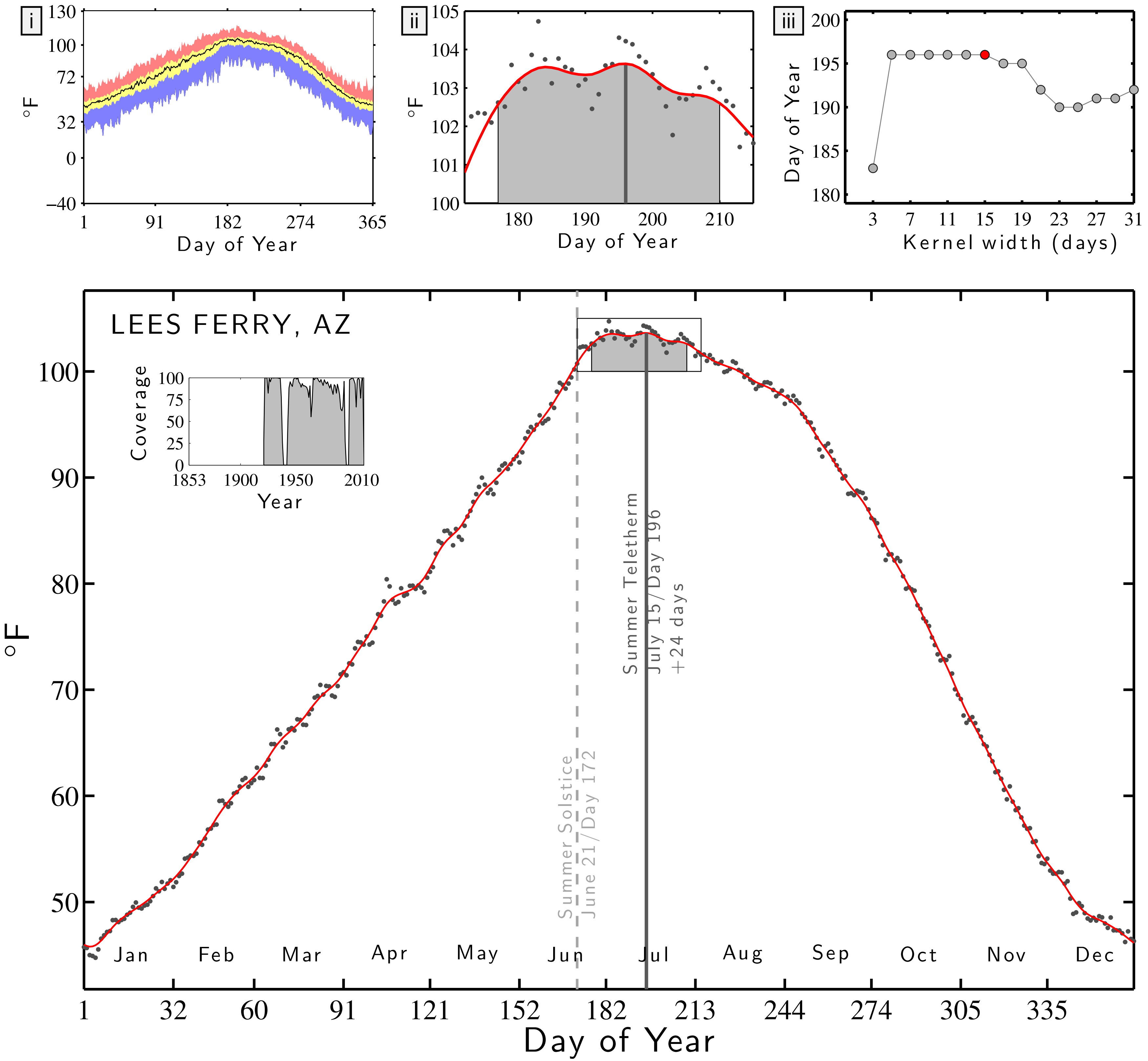
i
ii
iii
°F
Day of Year
Day of Year
Kernel width (days)
LEES FERRY, AZ
Coverage
Year
Summer Solstice
June 21/Day 172
Summer Teletherm
July 15/Day 196
+24 days
Jan
Feb
Mar
Apr
May
Jun
Jul
Aug
Sep
Oct
Nov
Dec
Day of Year

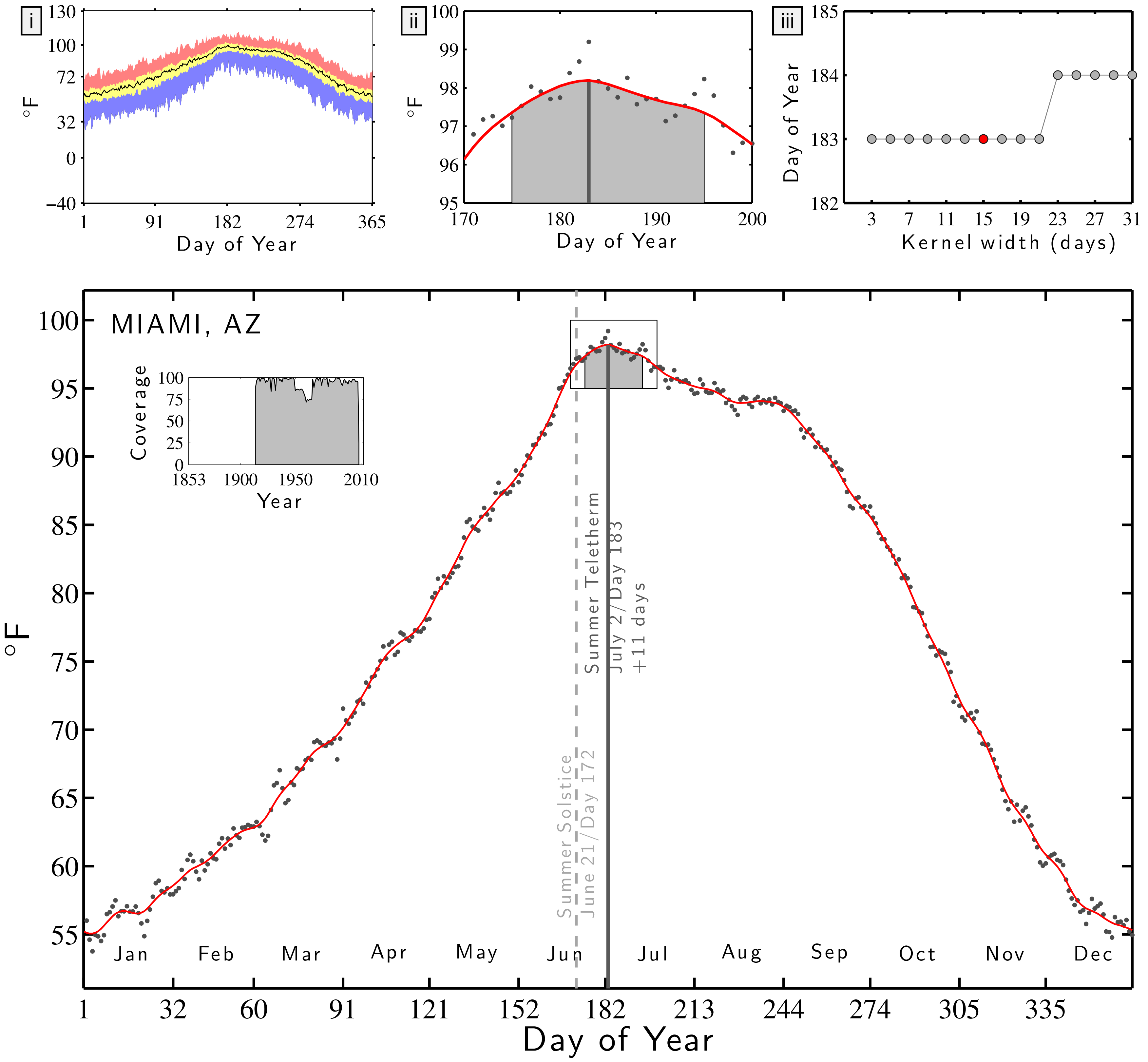

i
°F
Day of Year
ii
°F
Day of Year
iii
Day of Year
Kernel width (days)
MIAMI, AZ
Coverage
Year
Summer Solstice
June 21/Day 172
Summer Teletherm
July 2/Day 183
+11 days
Jan
Feb
Mar
Apr
May
Jun
Jul
Aug
Sep
Oct
Nov
Dec
°F
Day of Year

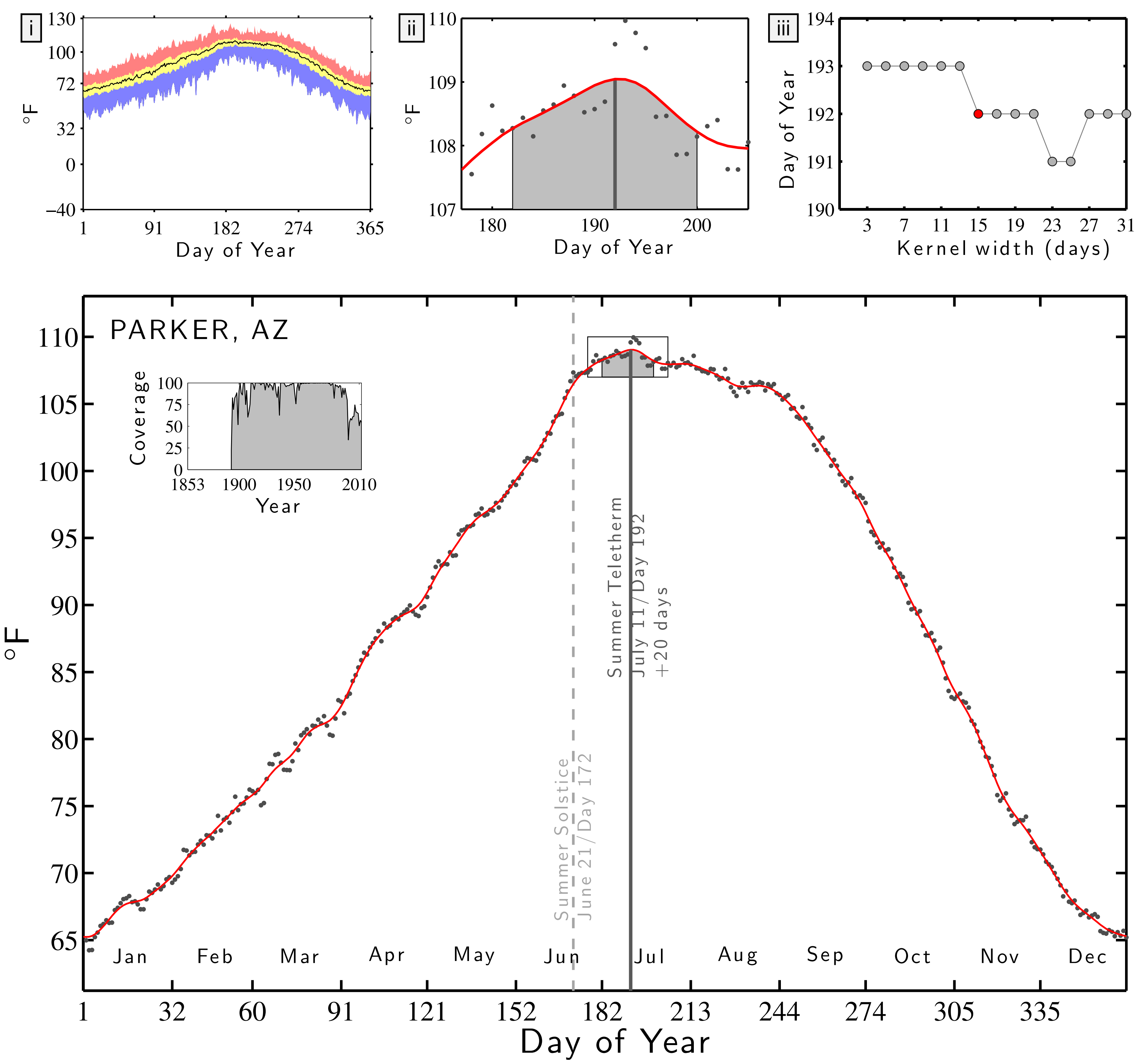

i
ii
iii
Day of Year
Kernel width (days)
°F
Day of Year
PARKER, AZ
Coverage
Year
Summer Solstice
June 21/Day 172
Summer Teletherm
July 11/Day 192
+20 days
Jan
Feb
Mar
Apr
May
Jun
Jul
Aug
Sep
Oct
Nov
Dec

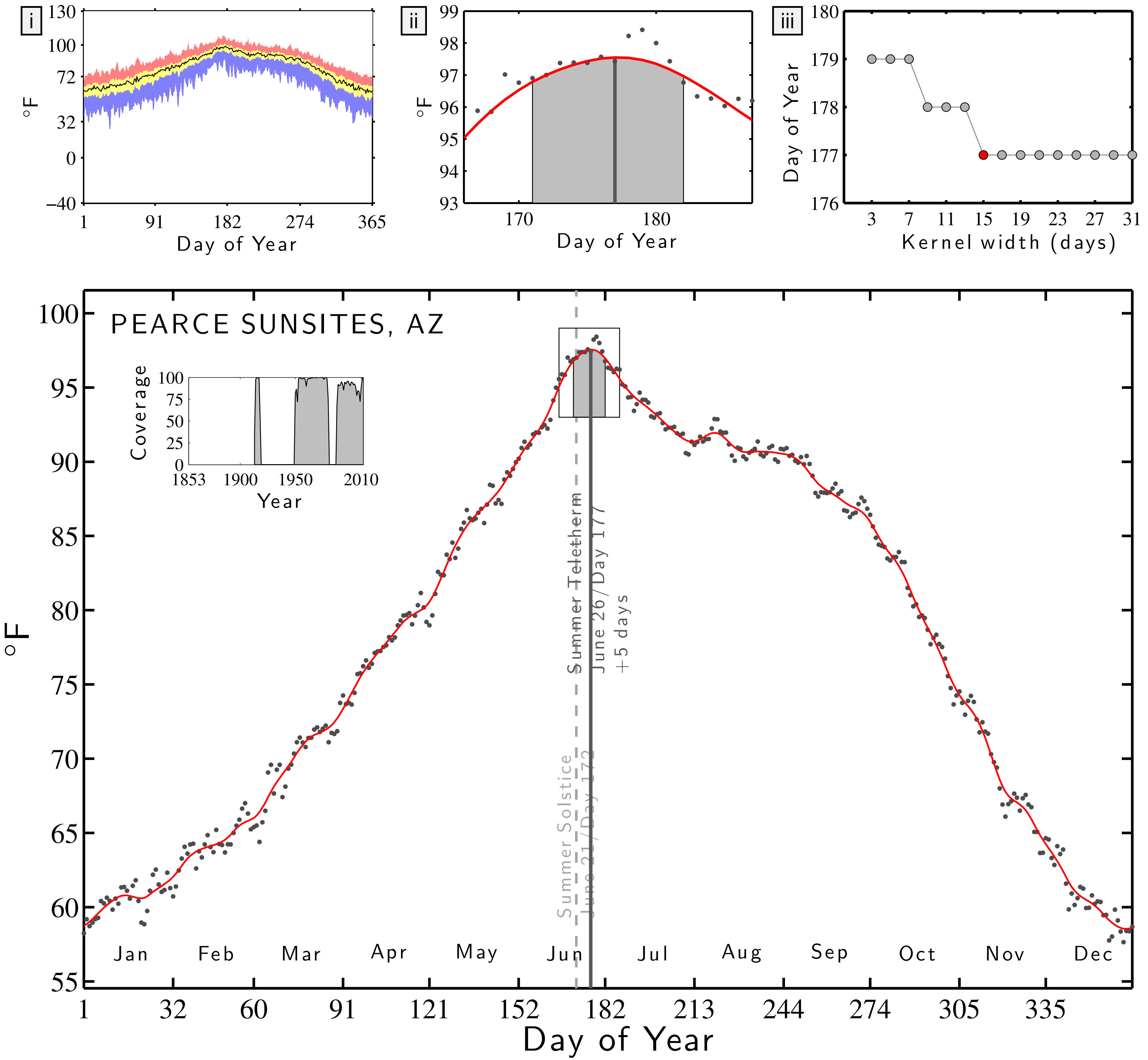

i
ii
iii
PEARCE SUNSITES, AZ
Coverage
Year
Summer Teletherm
June 26/Day 177
+5 days
Summer Solstice
June 21/Day 172
Jan
Feb
Mar
Apr
May
Jun
Jul
Aug
Sep
Oct
Nov
Dec
Day of Year
°F
Kernel width (days)

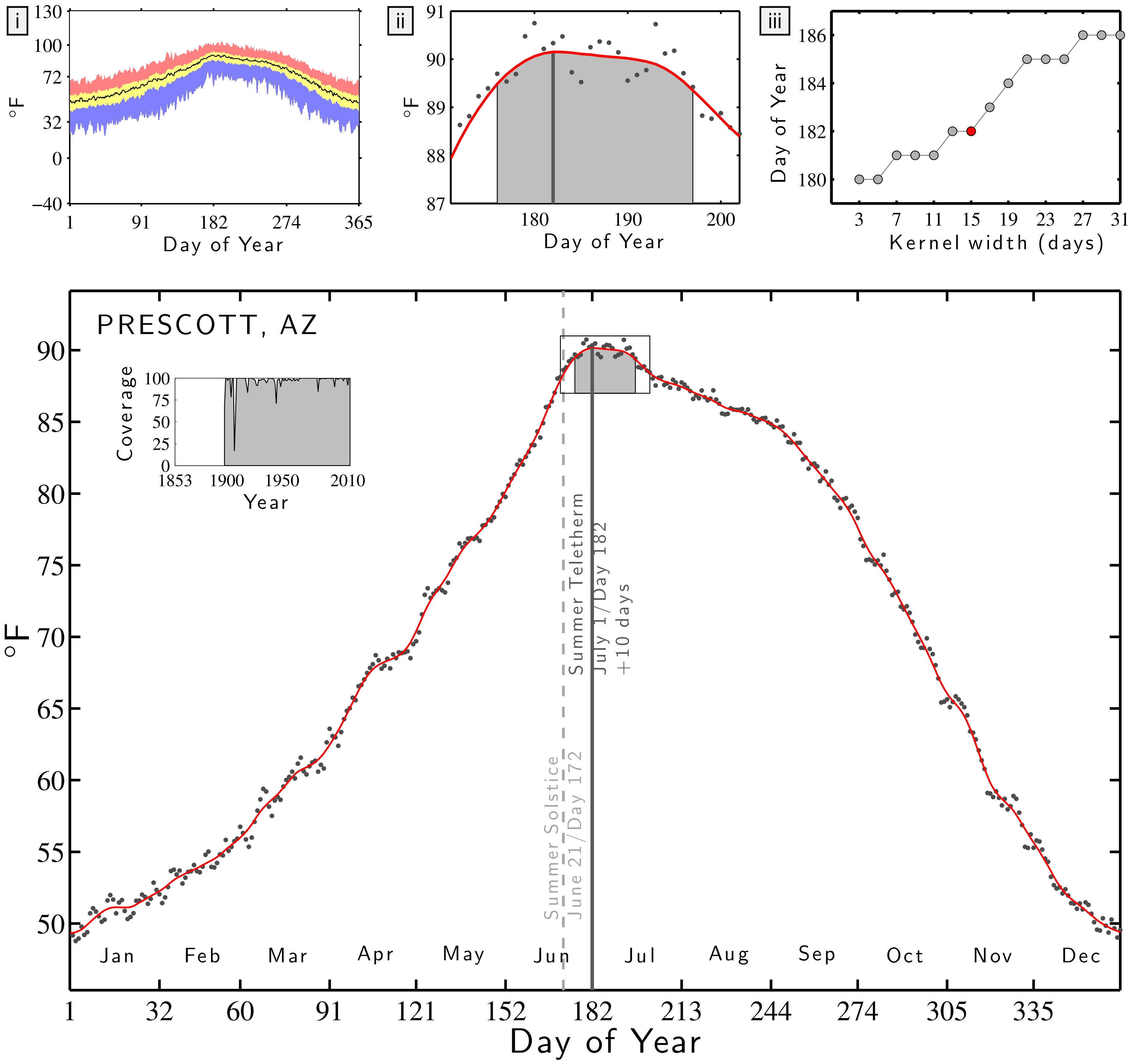

i
ii
iii
°F
Day of Year
Day of Year
Kernel width (days)
PRESCOTT, AZ
Coverage
Year
Summer Solstice
June 21/Day 172
Summer Teletherm
July 1/Day 182
+10 days
Jan
Feb
Mar
Apr
May
Jun
Jul
Aug
Sep
Oct
Nov
Dec

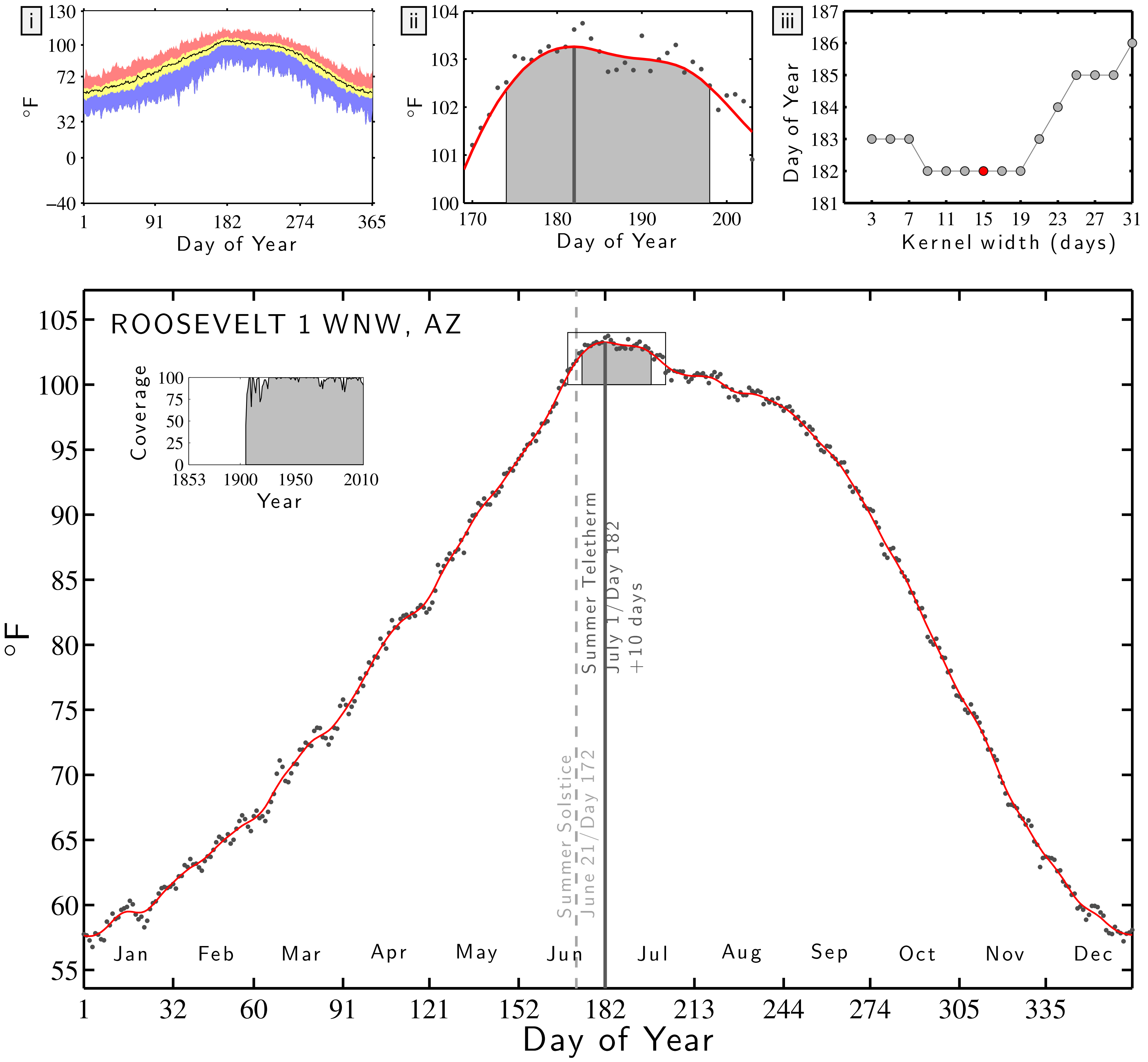

i
°F
Day of Year
ii
Day of Year
iii
Day of Year
Kernel width (days)
ROOSEVELT 1 WNW, AZ
Coverage
Year
Summer Solstice
June 21/Day 172
Summer Teletherm
July 1/Day 182
+10 days
Jan
Feb
Mar
Apr
May
Jun
Jul
Aug
Sep
Oct
Nov
Dec
Day of Year

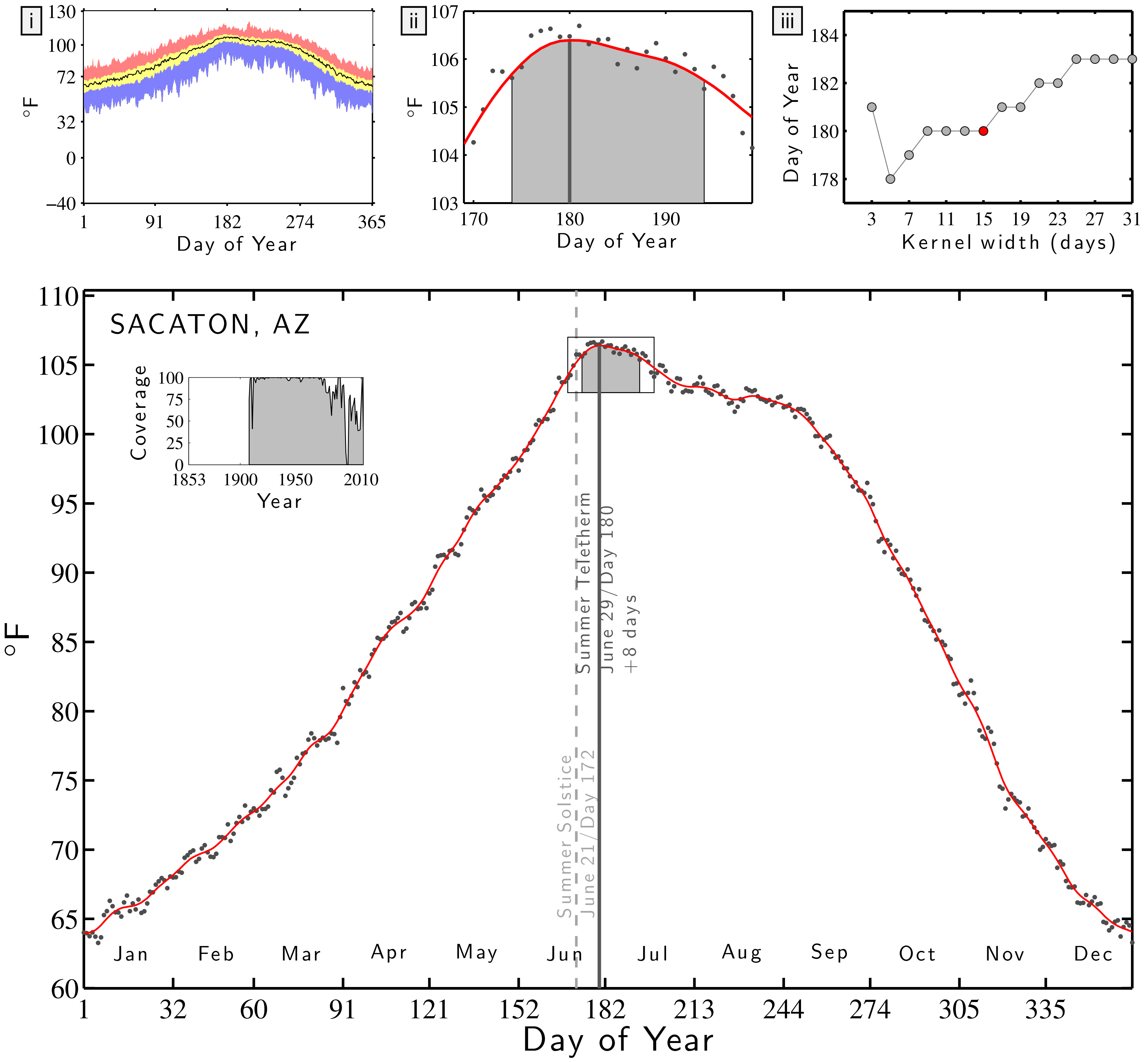

i
ii
iii
Day of Year
°F
Kernel width (days)
Day of Year
SACATON, AZ
Coverage
Year
Summer Solstice
June 21/Day 172
Summer Teletherm
June 29/Day 180
+8 days
Jan
Feb
Mar
Apr
May
Jun
Jul
Aug
Sep
Oct
Nov
Dec

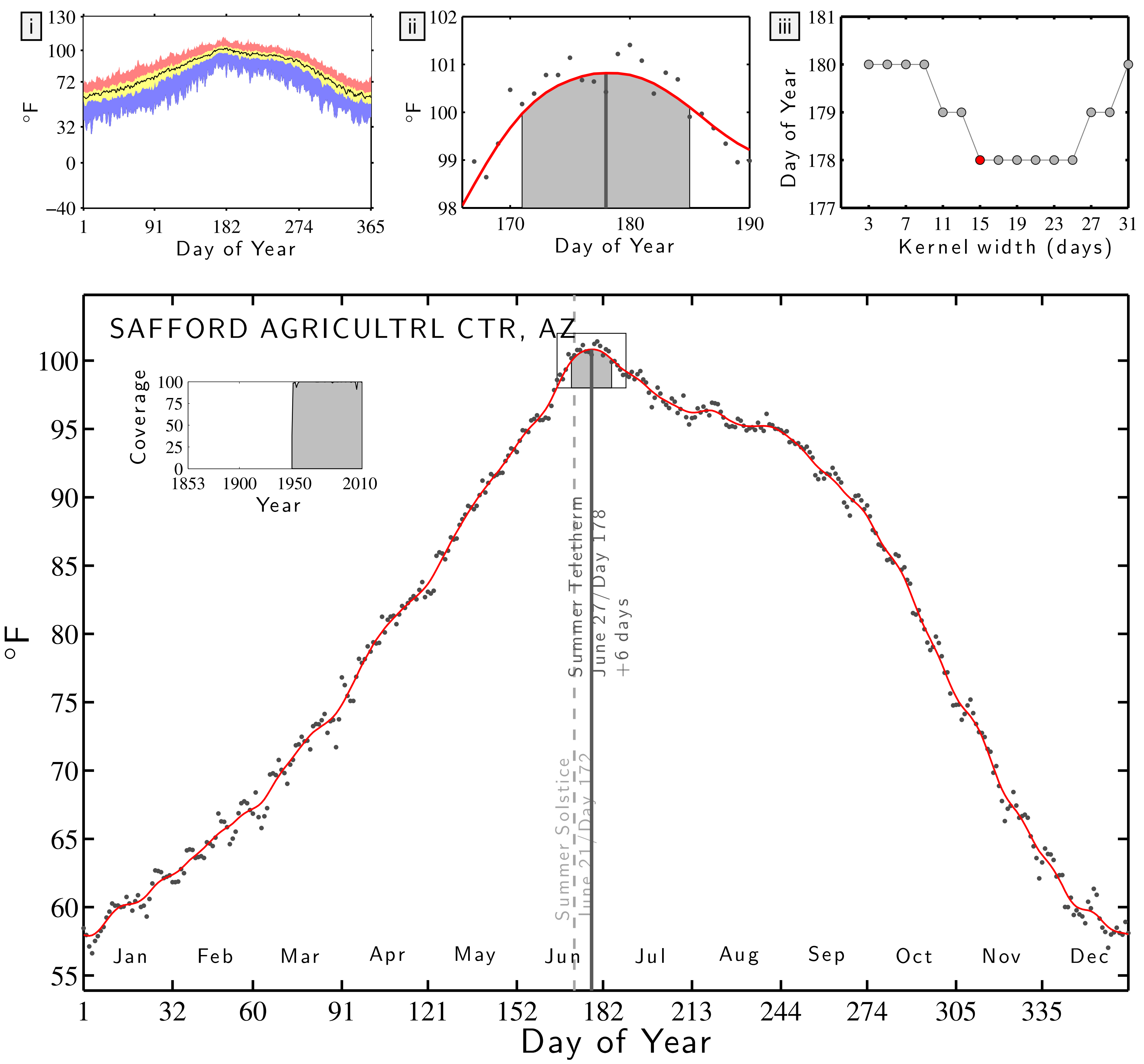

i
ii
iii
°F
Day of Year
Kernel width (days)
SAFFORD AGRICULTRL CTR, AZ
Coverage
Year
Summer Solstice
June 21/Day 172
Summer Teletherm
June 27/Day 178
+6 days
Jan
Feb
Mar
Apr
May
Jun
Jul
Aug
Sep
Oct
Nov
Dec

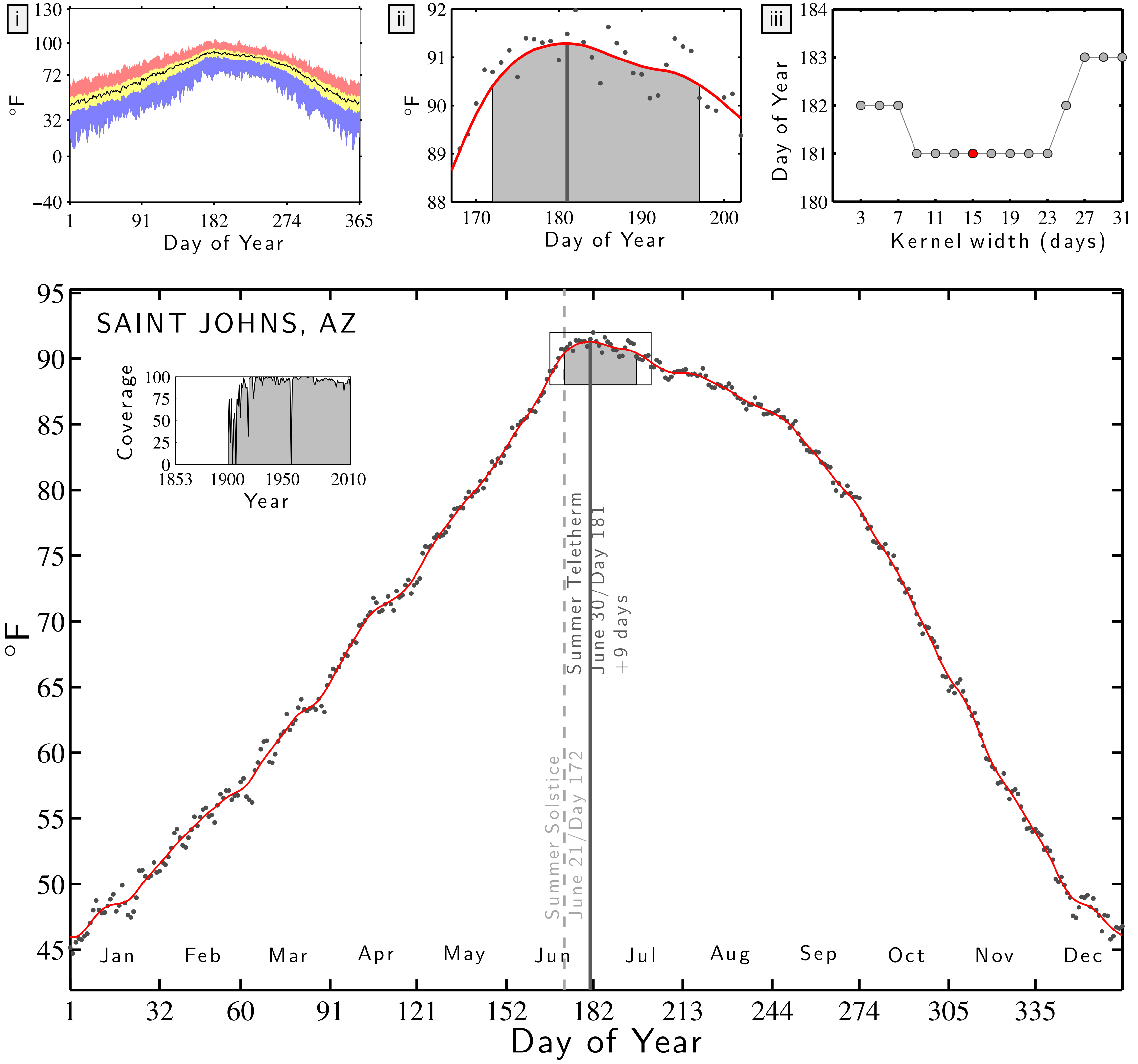

i
°F
Day of Year
ii
°F
Day of Year
iii
Day of Year
Kernel width (days)
SAINT JOHNS, AZ
Coverage
Year
Summer Solstice
June 21/Day 172
Summer Teletherm
June 30/Day 181
+9 days
Jan
Feb
Mar
Apr
May
Jun
Jul
Aug
Sep
Oct
Nov
Dec
°F
Day of Year

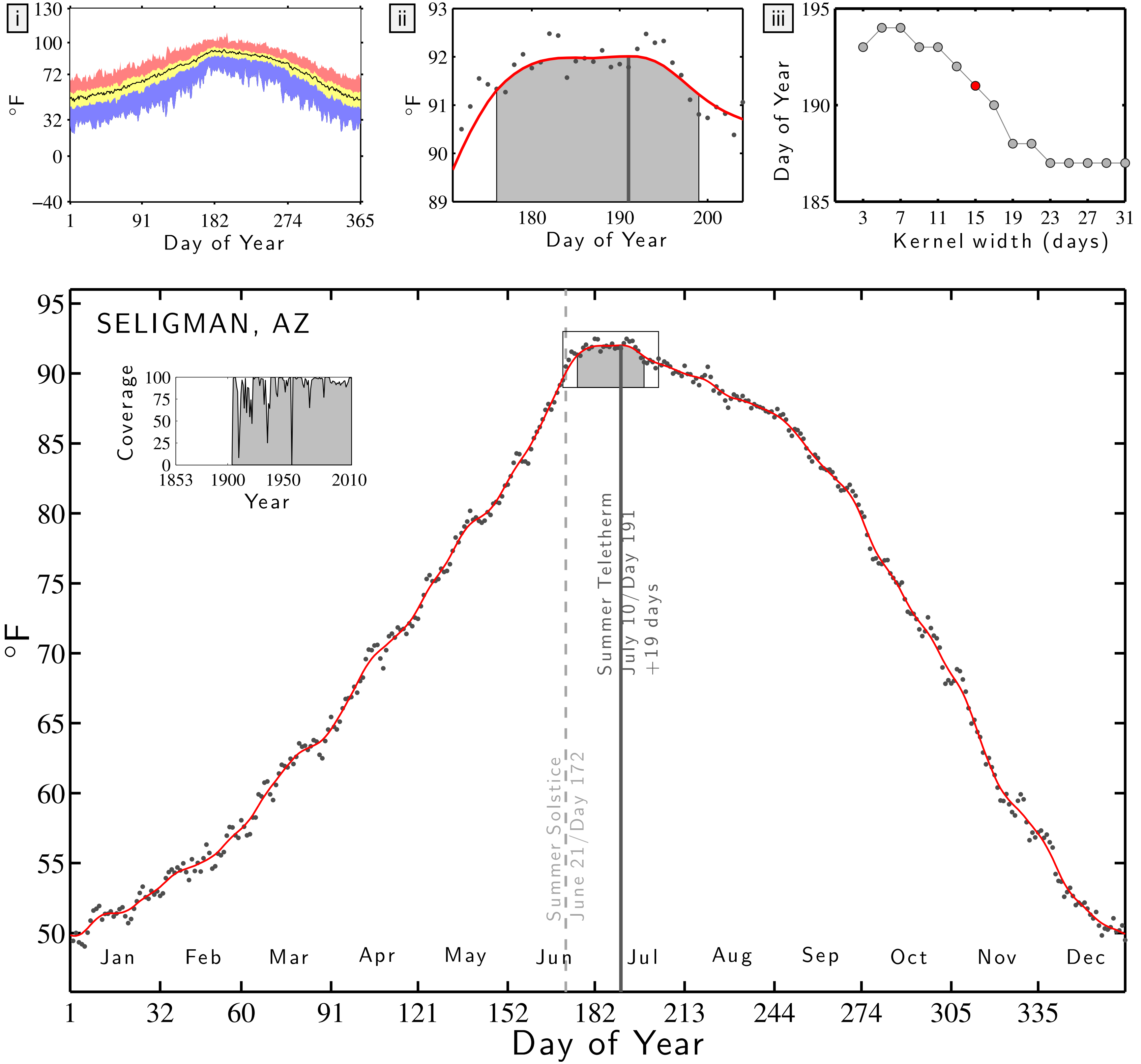
i
°F
Day of Year
ii
°F
Day of Year
iii
Day of Year
Kernel width (days)
SELIGMAN, AZ
Coverage
Year
Summer Solstice
June 21/Day 172
Summer Teletherm
July 10/Day 191
+19 days
Jan
Feb
Mar
Apr
May
Jun
Jul
Aug
Sep
Oct
Nov
Dec
°F
Day of Year

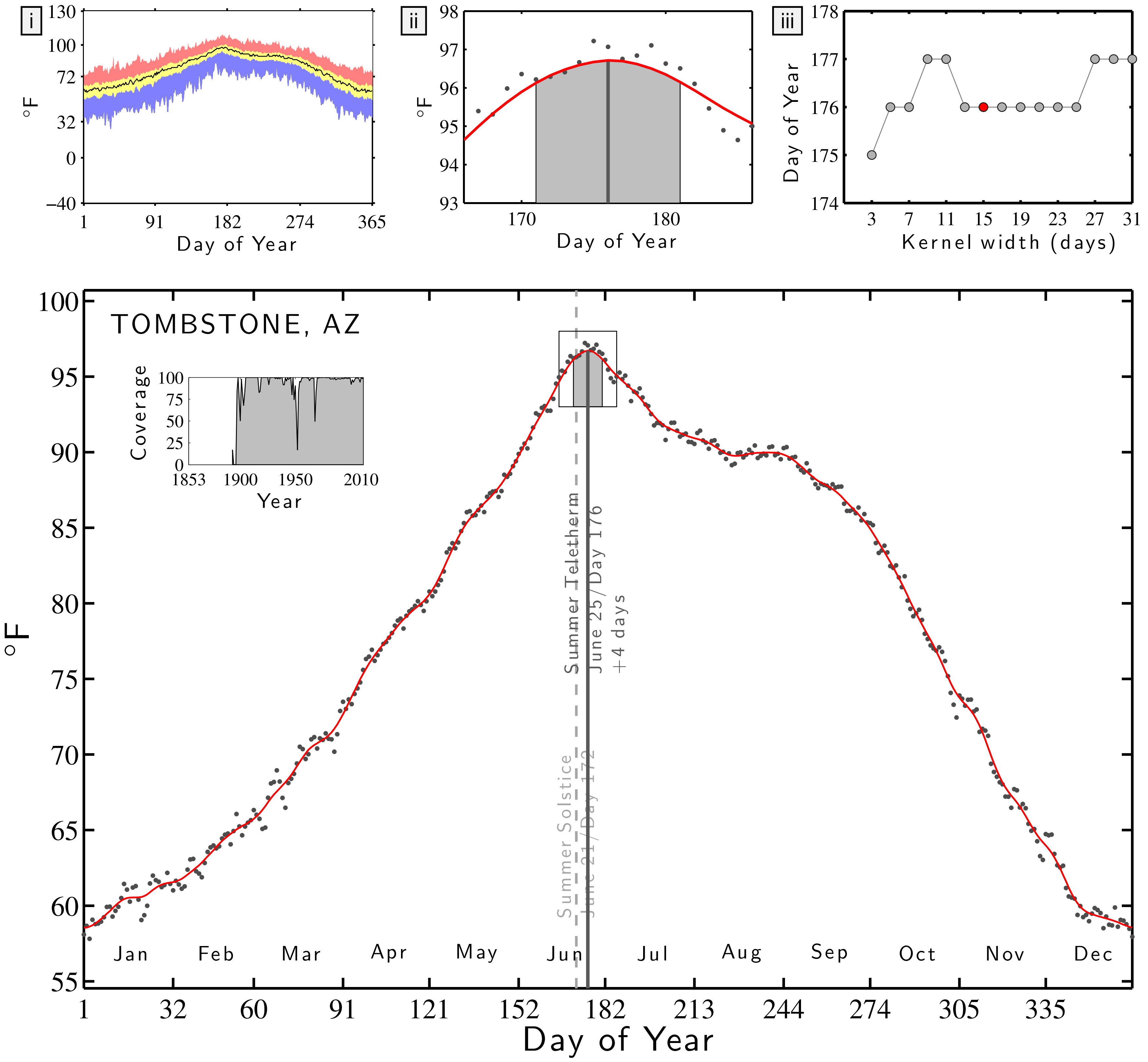

i
°F
Day of Year
ii
°F
Day of Year
iii
Day of Year
Kernel width (days)
TOMBSTONE, AZ
Coverage
Year
Summer Teletherm
June 25/Day 176
+4 days
Summer Solstice
June 21/Day 172
Jan
Feb
Mar
Apr
May
Jun
Jul
Aug
Sep
Oct
Nov
Dec
°F
Day of Year

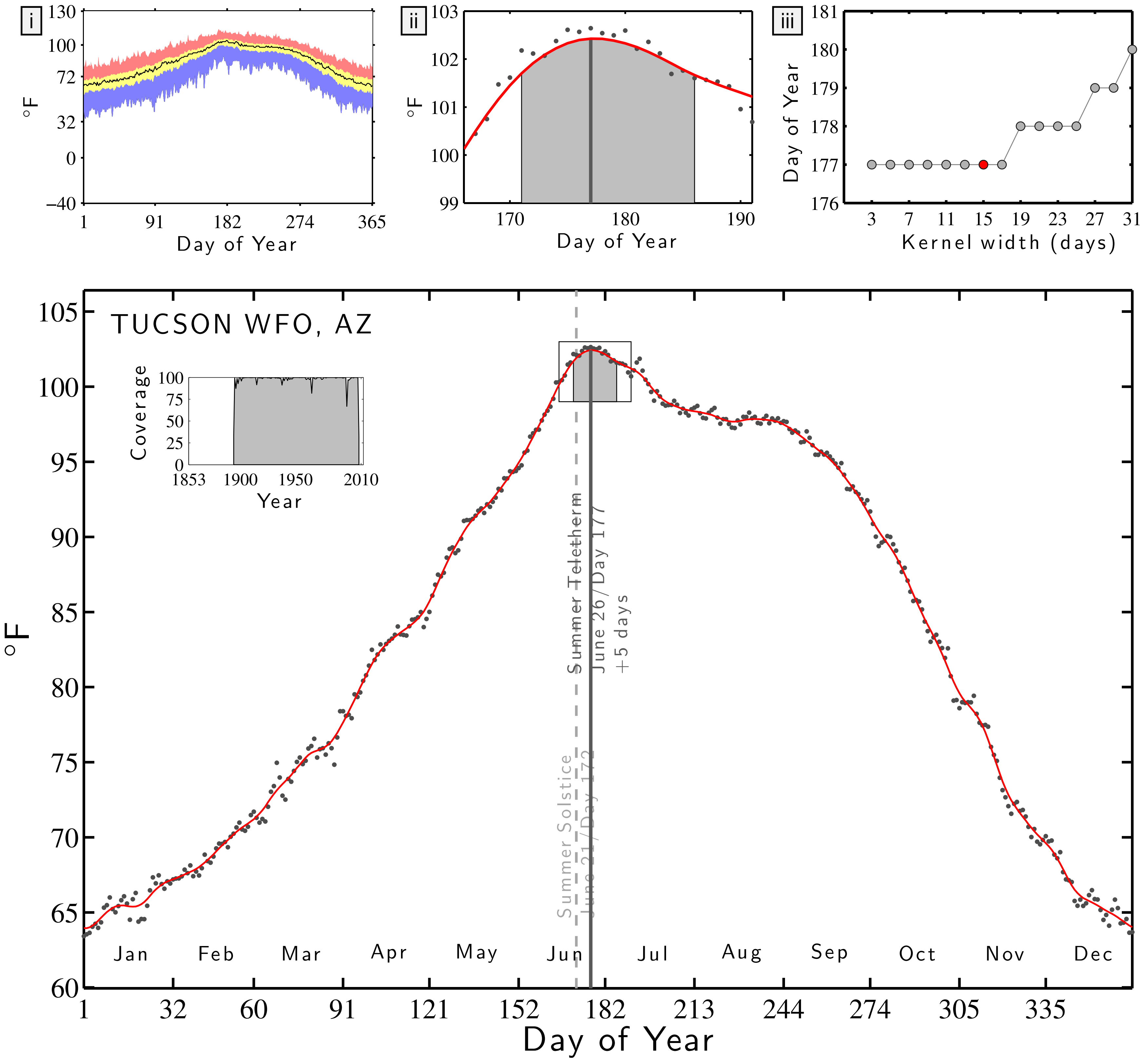
i
°F
Day of Year
ii
°F
Day of Year
iii
Day of Year
Kernel width (days)
TUCSON WFO, AZ
Coverage
Year
Summer Teletherm
June 26/Day 177
+5 days
Summer Solstice
June 21/Day 172
Jan
Feb
Mar
Apr
May
Jun
Jul
Aug
Sep
Oct
Nov
Dec
°F
Day of Year

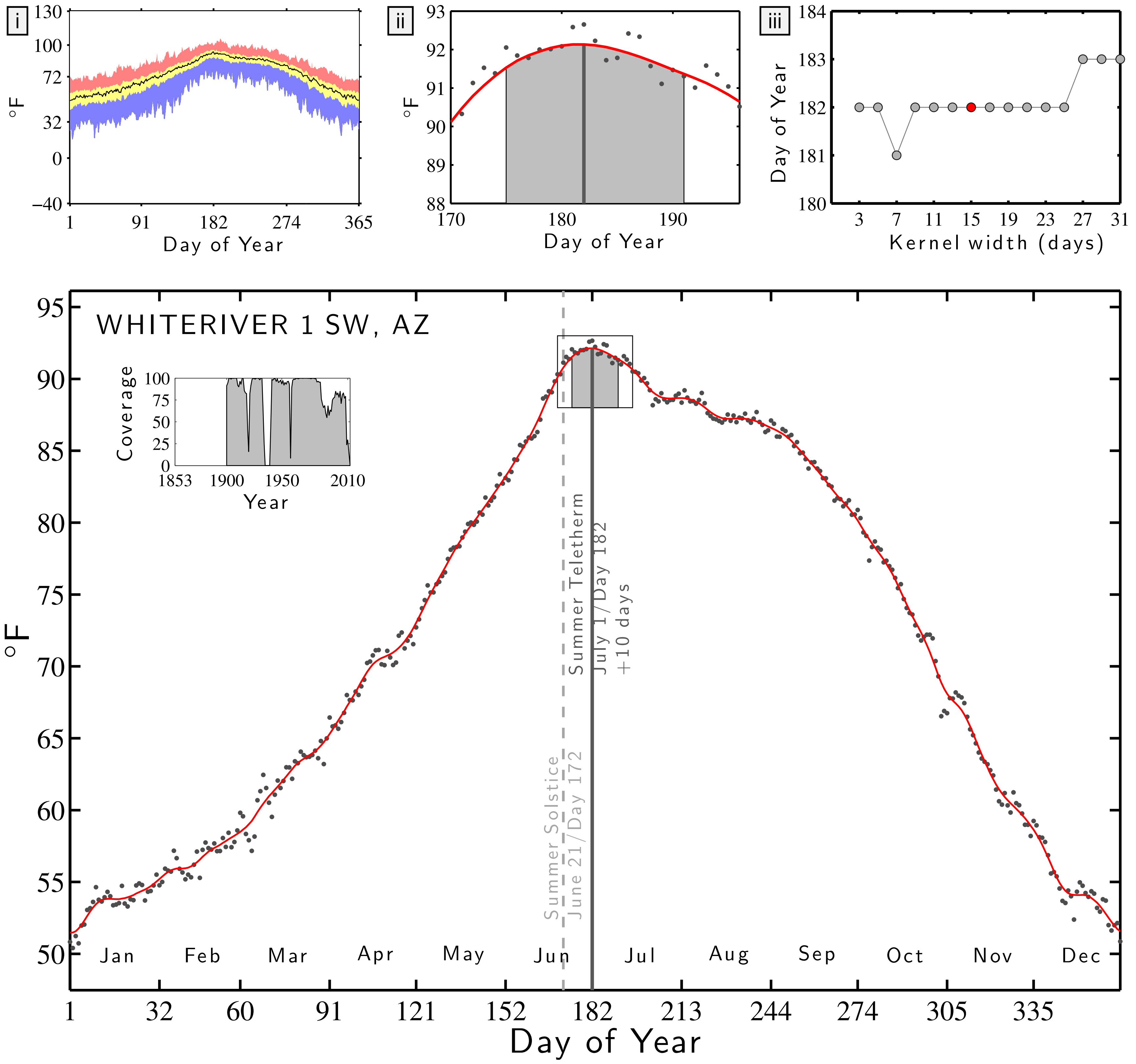

i
°F
Day of Year
ii
°F
Day of Year
iii
Day of Year
Kernel width (days)
WHITERIVER 1 SW, AZ
Coverage
Year
Summer Solstice
June 21/Day 172
Summer Teletherm
July 1/Day 182
+10 days
Jan
Feb
Mar
Apr
May
Jun
Jul
Aug
Sep
Oct
Nov
Dec
°F
Day of Year

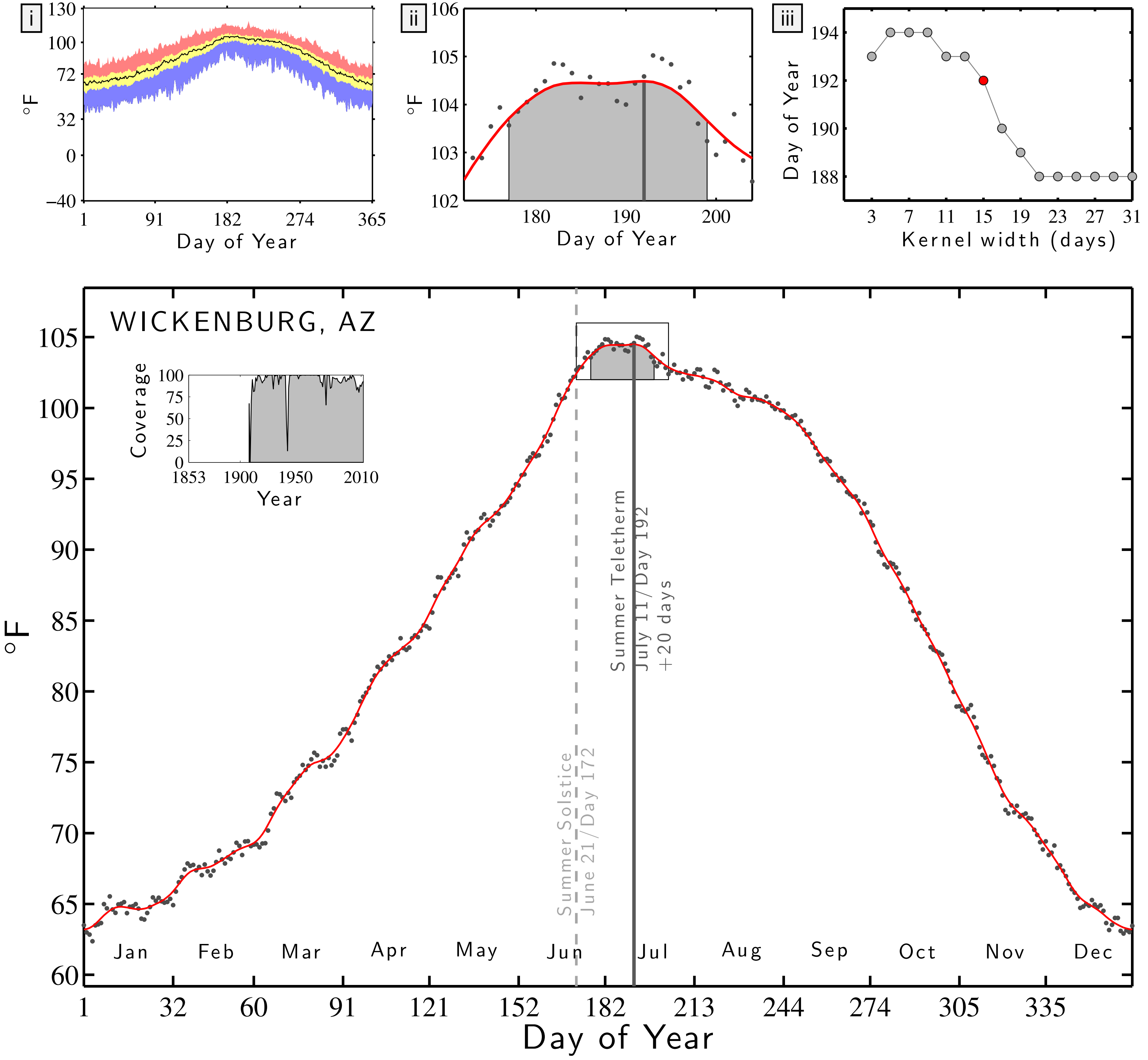

i
ii
iii
°F
Day of Year
Day of Year
Kernel width (days)
WICKENBURG, AZ
Coverage
Year
Summer Solstice
June 21/Day 172
Summer Teletherm
July 11/Day 192
+20 days
Jan
Feb
Mar
Apr
May
Jun
Jul
Aug
Sep
Oct
Nov
Dec
°F
Day of Year

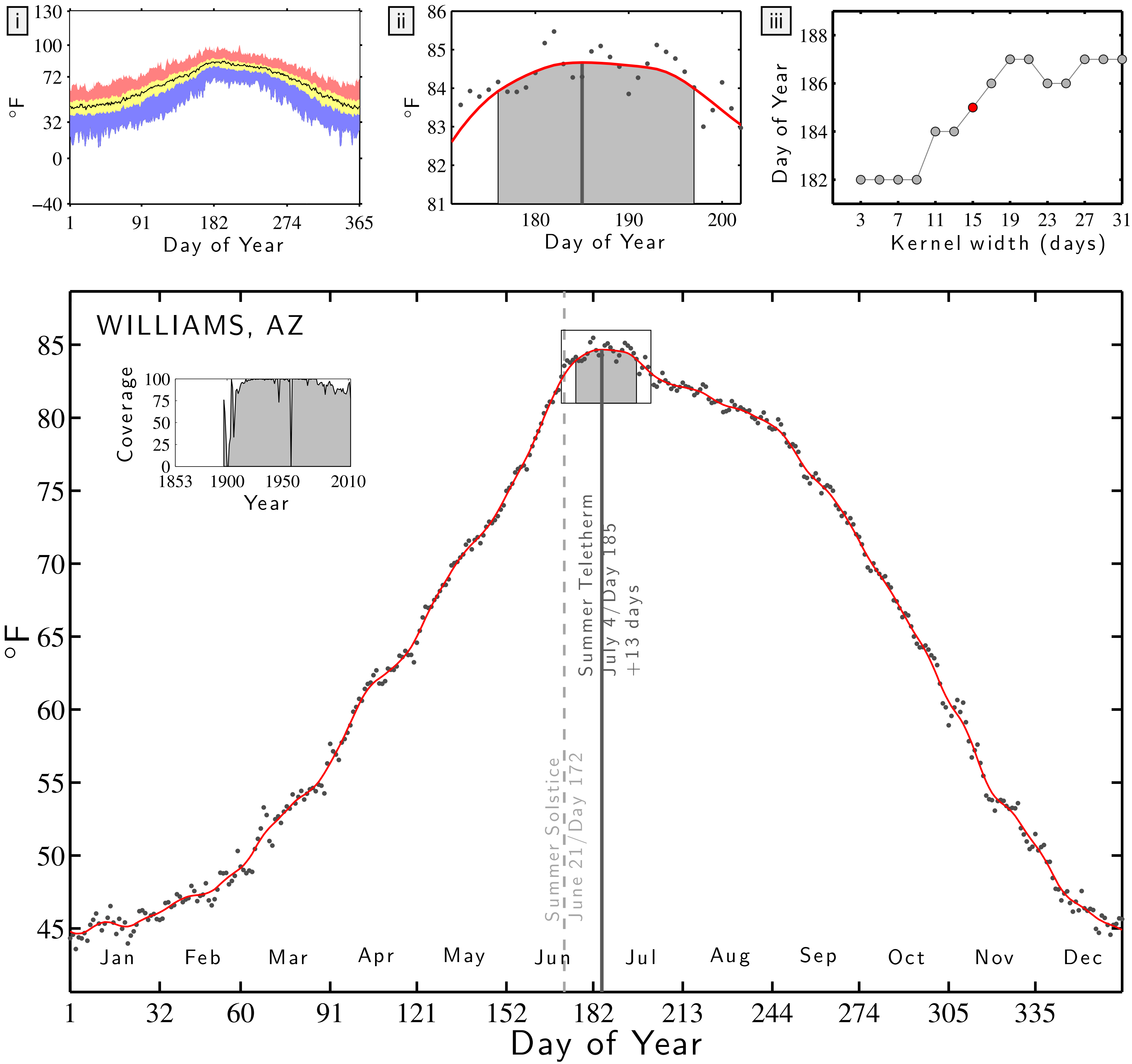

i
ii
iii
°F
Day of Year
Day of Year
Kernel width (days)
WILLIAMS, AZ
Coverage
Year
Summer Solstice
June 21 / Day 172
Summer Teletherm
July 4 / Day 185
+13 days
Jan
Feb
Mar
Apr
May
Jun
Jul
Aug
Sep
Oct
Nov
Dec
Day of Year
°F

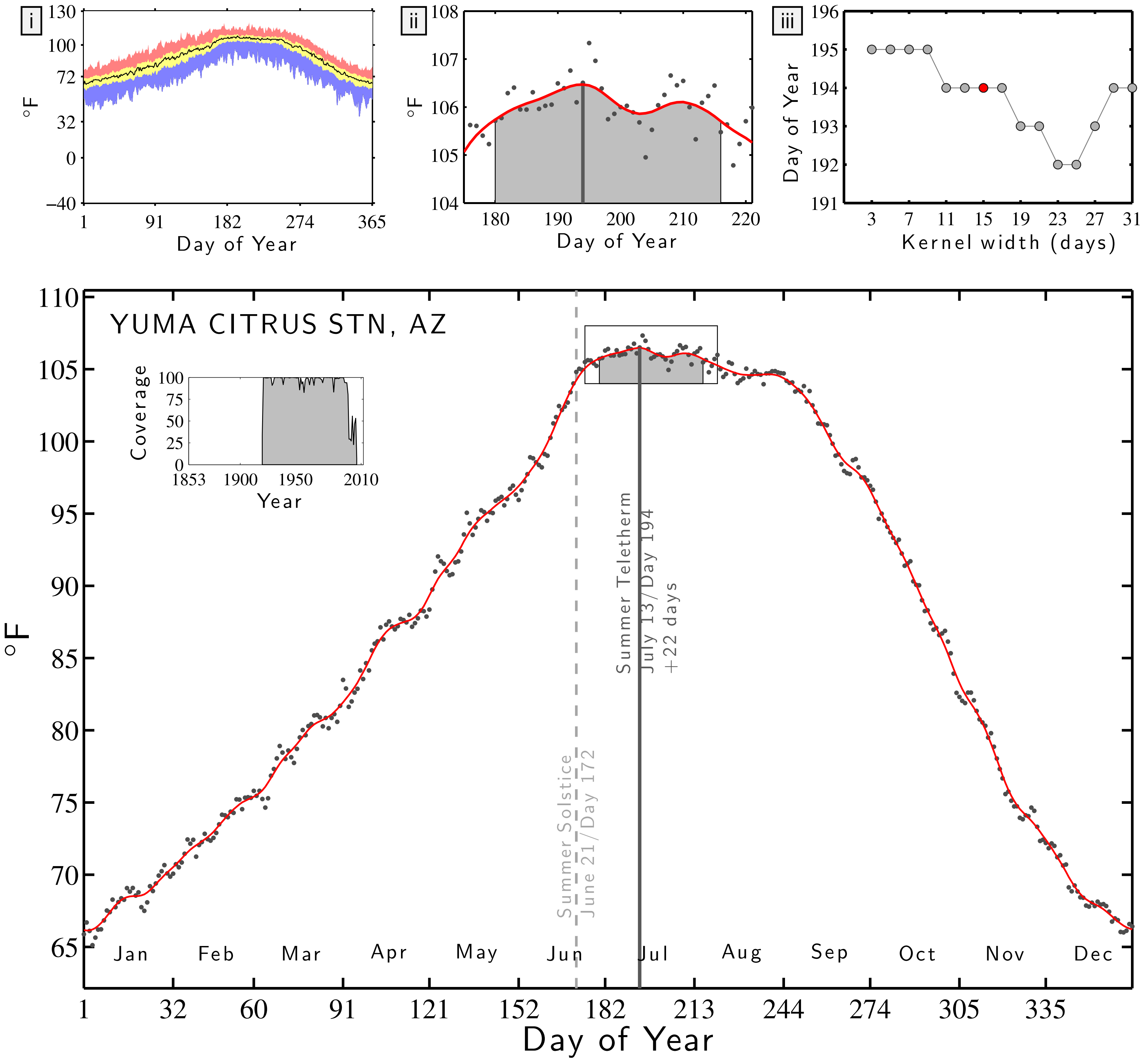

i
°F
Day of Year
ii
°F
Day of Year
iii
Day of Year
Kernel width (days)
YUMA CITRUS STN, AZ
Coverage
Year
Summer Solstice
June 21/Day 172
Summer Teletherm
July 13/Day 194
+22 days
Jan
Feb
Mar
Apr
May
Jun
Jul
Aug
Sep
Oct
Nov
Dec
°F
Day of Year

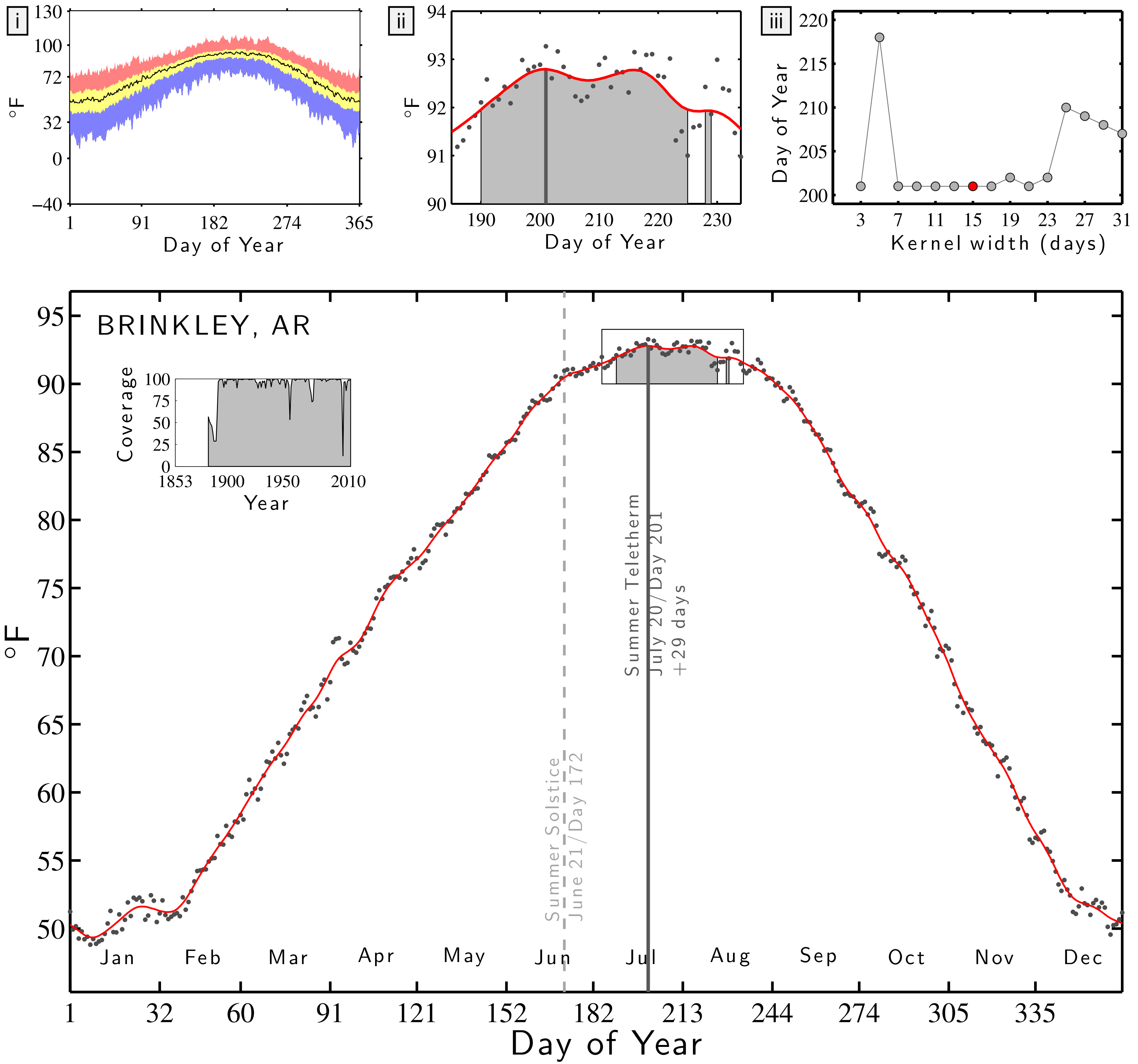

i
ii
iii
°F
Day of Year
Kernel width (days)
BRINKLEY, AR
Coverage
Year
Summer Solstice
June 21/Day 172
Summer Teletherm
July 20/Day 201
+29 days
Jan
Feb
Mar
Apr
May
Jun
Jul
Aug
Sep
Oct
Nov
Dec

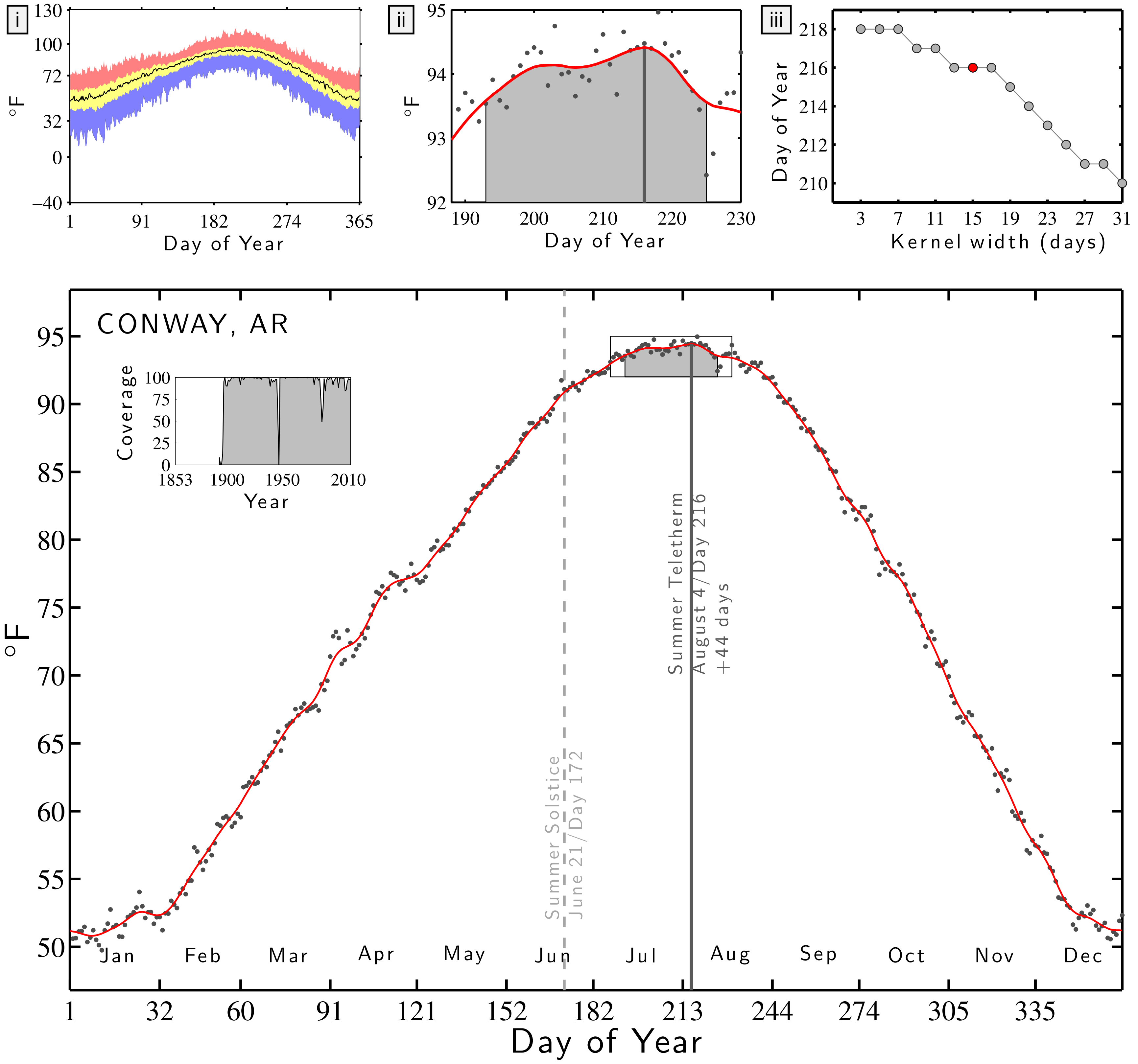

i
°F
Day of Year
ii
°F
Day of Year
iii
Day of Year
Kernel width (days)
CONWAY, AR
Coverage
Year
Summer Solstice
June 21 / Day 172
Summer Teletherm
August 4 / Day 216
+44 days
Jan
Feb
Mar
Apr
May
Jun
Jul
Aug
Sep
Oct
Nov
Dec
°F
Day of Year

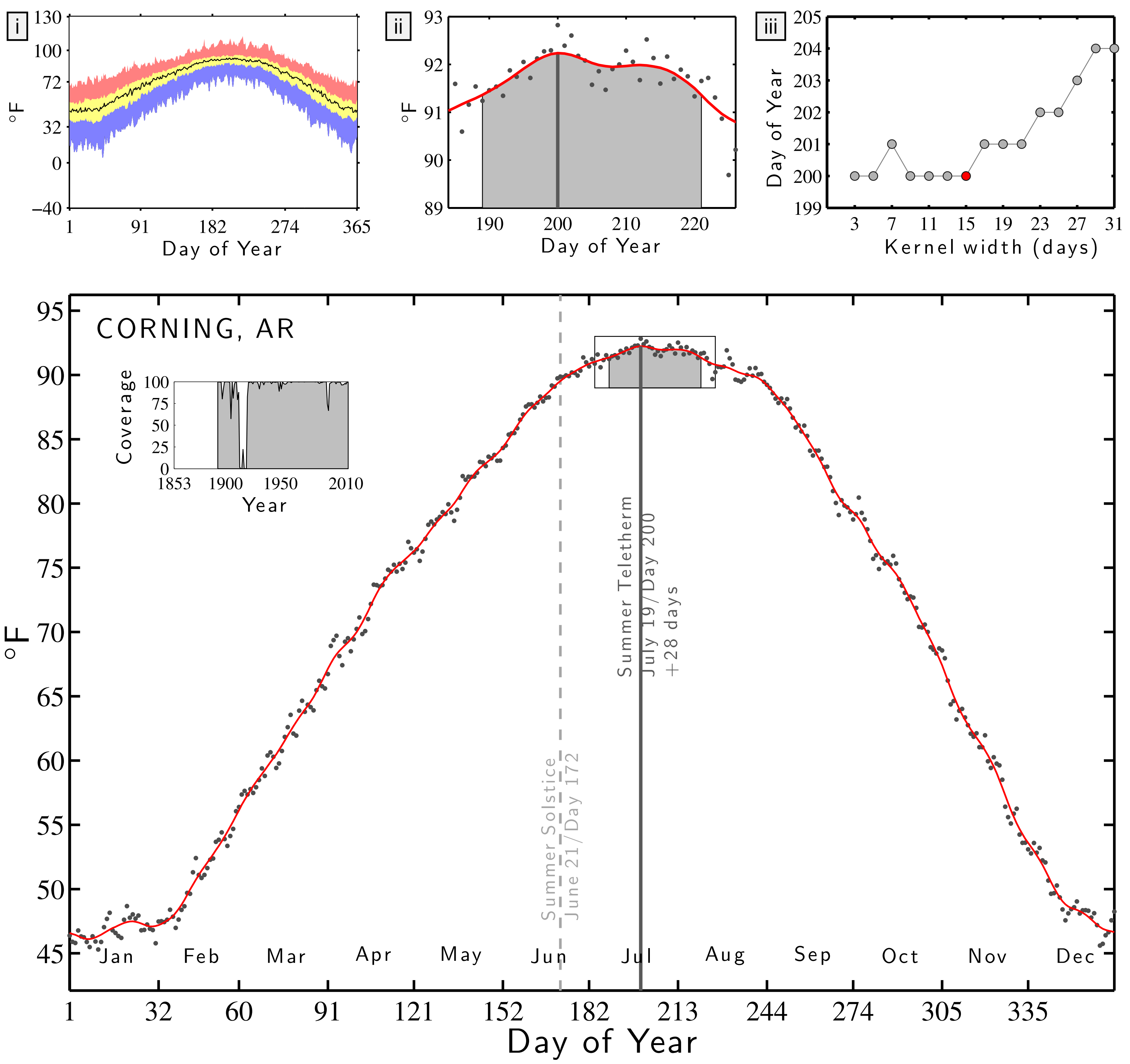

i
ii
iii
°F
Day of Year
Day of Year
Kernel width (days)
CORNING, AR
Coverage
Year
Summer Solstice
June 21/Day 172
Summer Teletherm
July 19/Day 200
+28 days
Jan
Feb
Mar
Apr
May
Jun
Jul
Aug
Sep
Oct
Nov
Dec

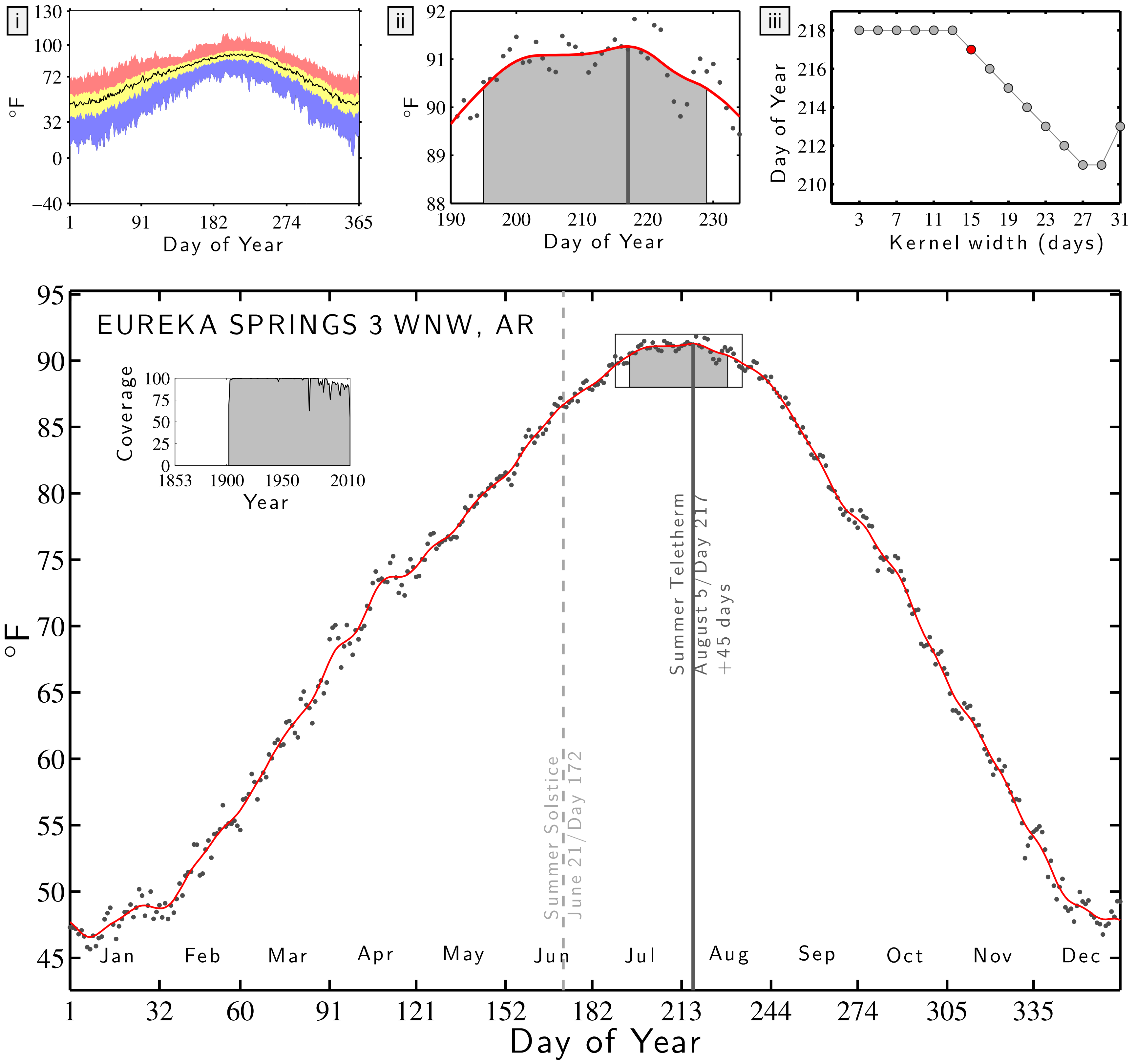

i
ii
iii
°F
Day of Year
Kernel width (days)
EUREKA SPRINGS 3 WNW, AR
Coverage
Year
Summer Solstice
June 21/Day 172
Summer Teletherm
August 5/Day 217
+45 days
Jan
Feb
Mar
Apr
May
Jun
Jul
Aug
Sep
Oct
Nov
Dec

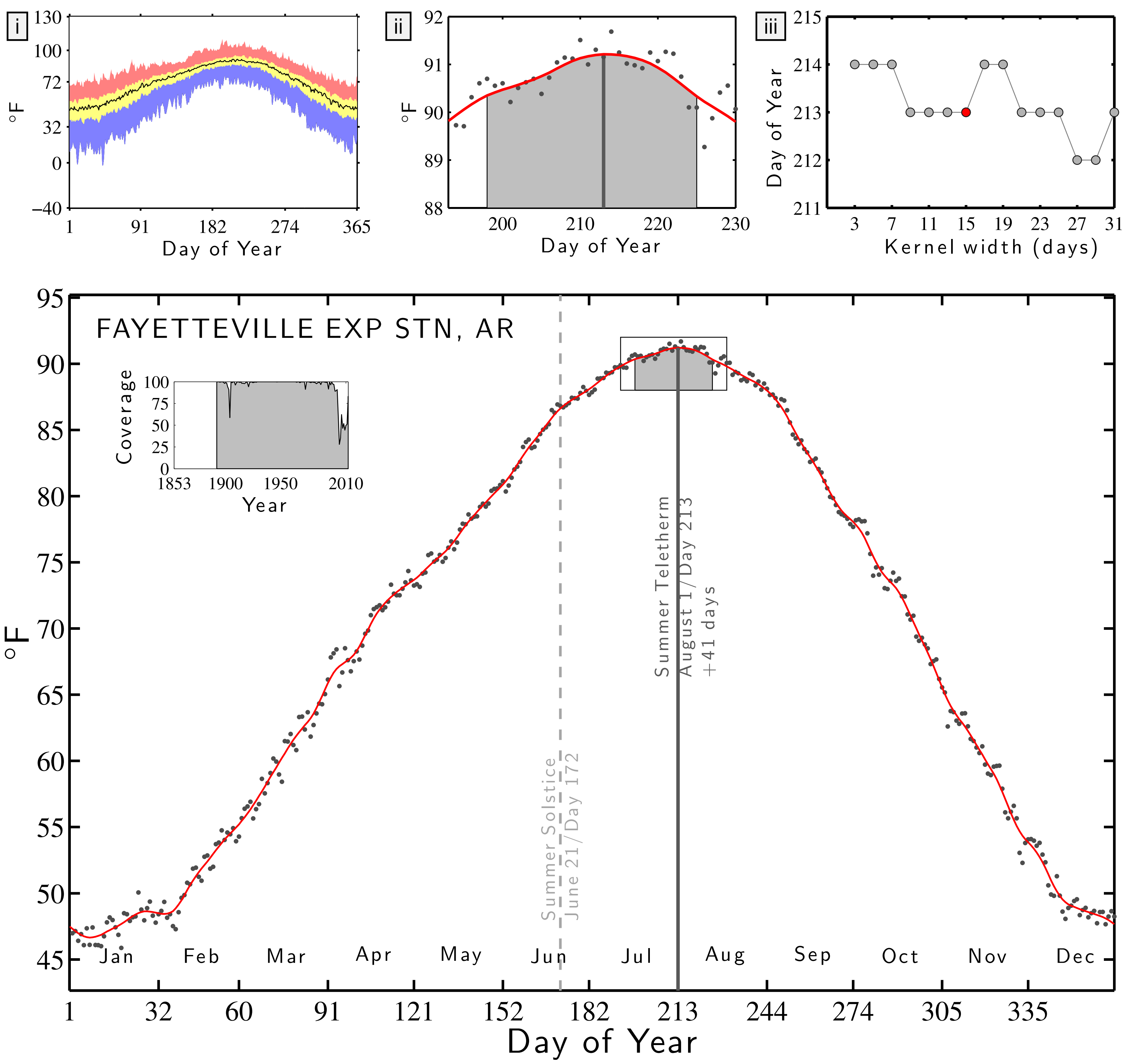

i
ii
iii
°F
Day of Year
Kernel width (days)
FAYETTEVILLE EXP STN, AR
Coverage
Year
Summer Solstice
June 21 / Day 172
Summer Teletherm
August 1 / Day 213
+41 days
Jan
Feb
Mar
Apr
May
Jun
Jul
Aug
Sep
Oct
Nov
Dec

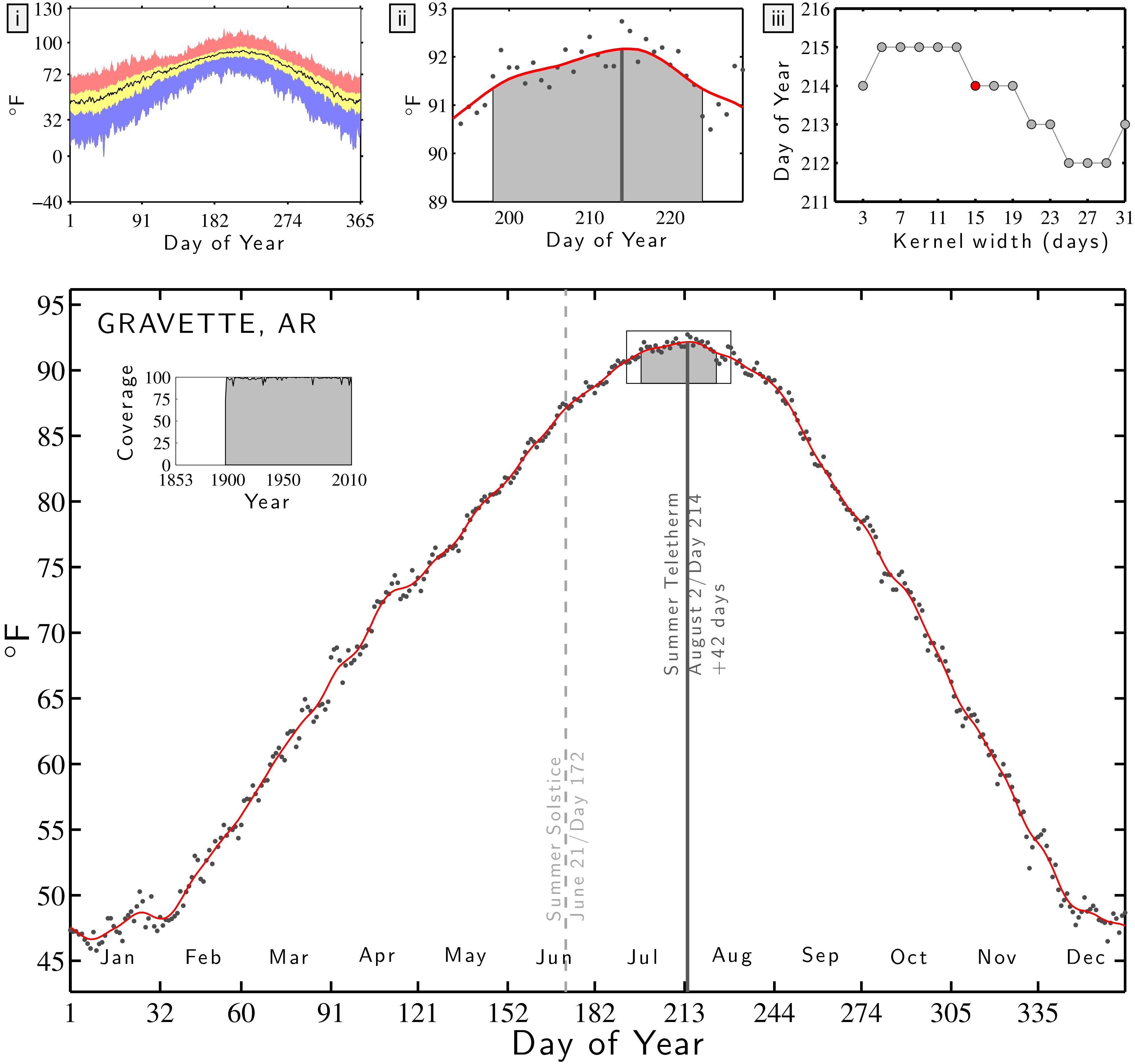

i
ii
iii
°F
Day of Year
Day of Year
Kernel width (days)
GRAVETTE, AR
Coverage
Year
Summer Solstice
June 21/Day 172
Summer Teletherm
August 2/Day 214
+42 days
Jan
Feb
Mar
Apr
May
Jun
Jul
Aug
Sep
Oct
Nov
Dec
Day of Year

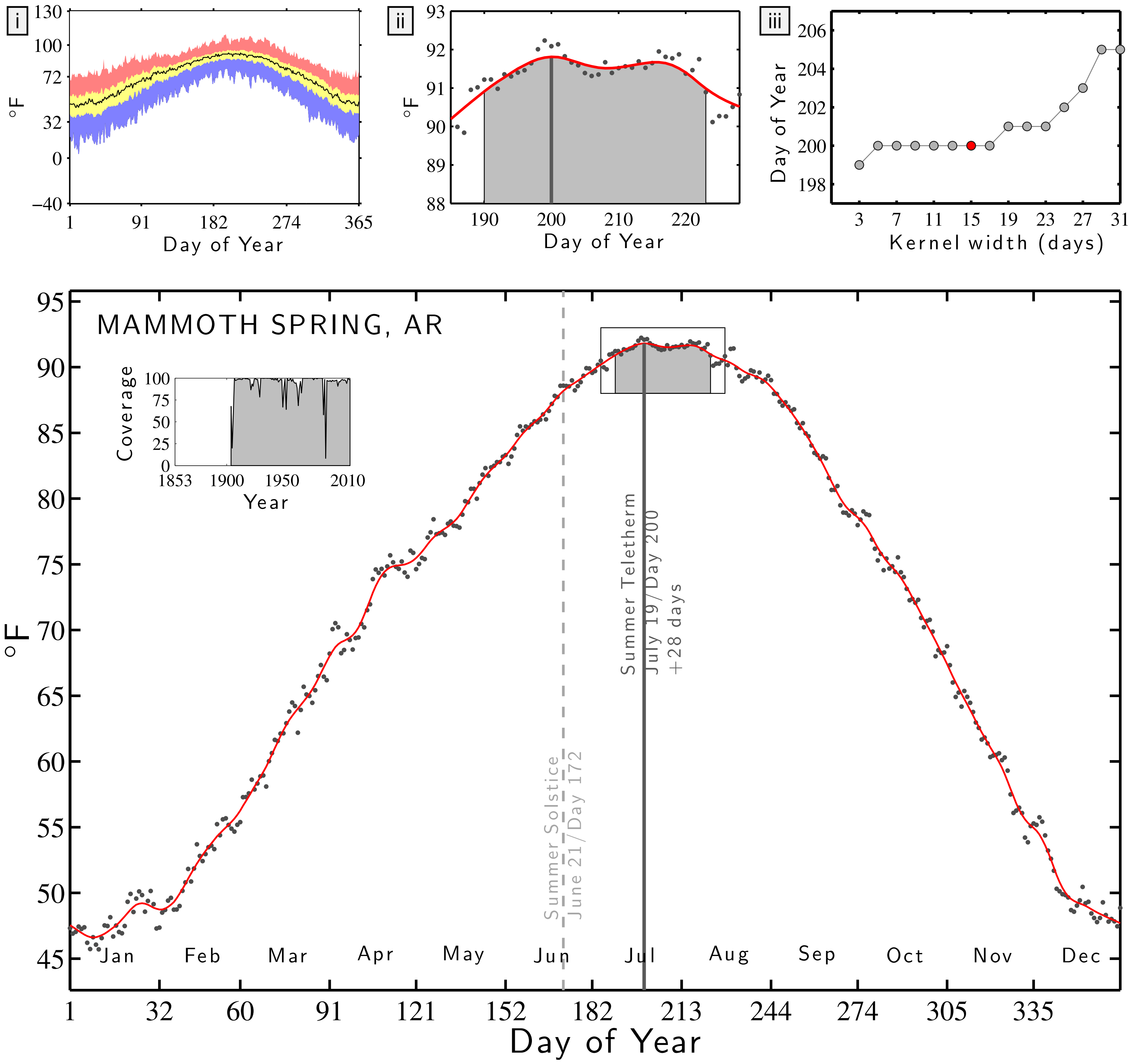

i
ii
iii
°F
Day of Year
Day of Year
Kernel width (days)
MAMMOTH SPRING, AR
Coverage
Year
Summer Solstice
June 21/Day 172
Summer Teletherm
July 19/Day 200
+28 days
Jan
Feb
Mar
Apr
May
Jun
Jul
Aug
Sep
Oct
Nov
Dec
Day of Year

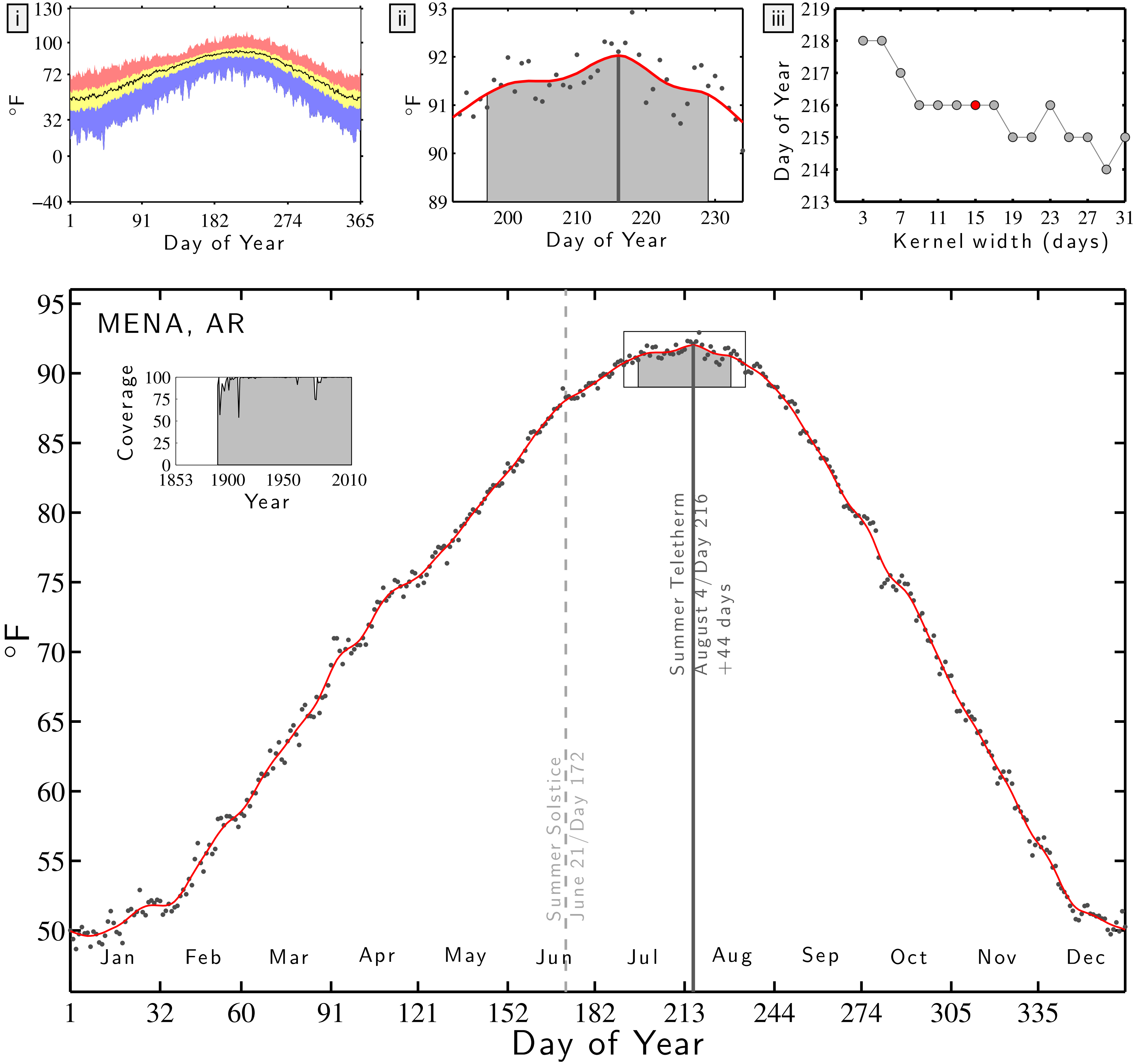

i
°F
Day of Year
ii
Day of Year
iii
Day of Year
Kernel width (days)
MENA, AR
Coverage
Year
Summer Solstice
June 21/Day 172
Summer Teletherm
August 4/Day 216
+44 days
Jan
Feb
Mar
Apr
May
Jun
Jul
Aug
Sep
Oct
Nov
Dec
°F
Day of Year

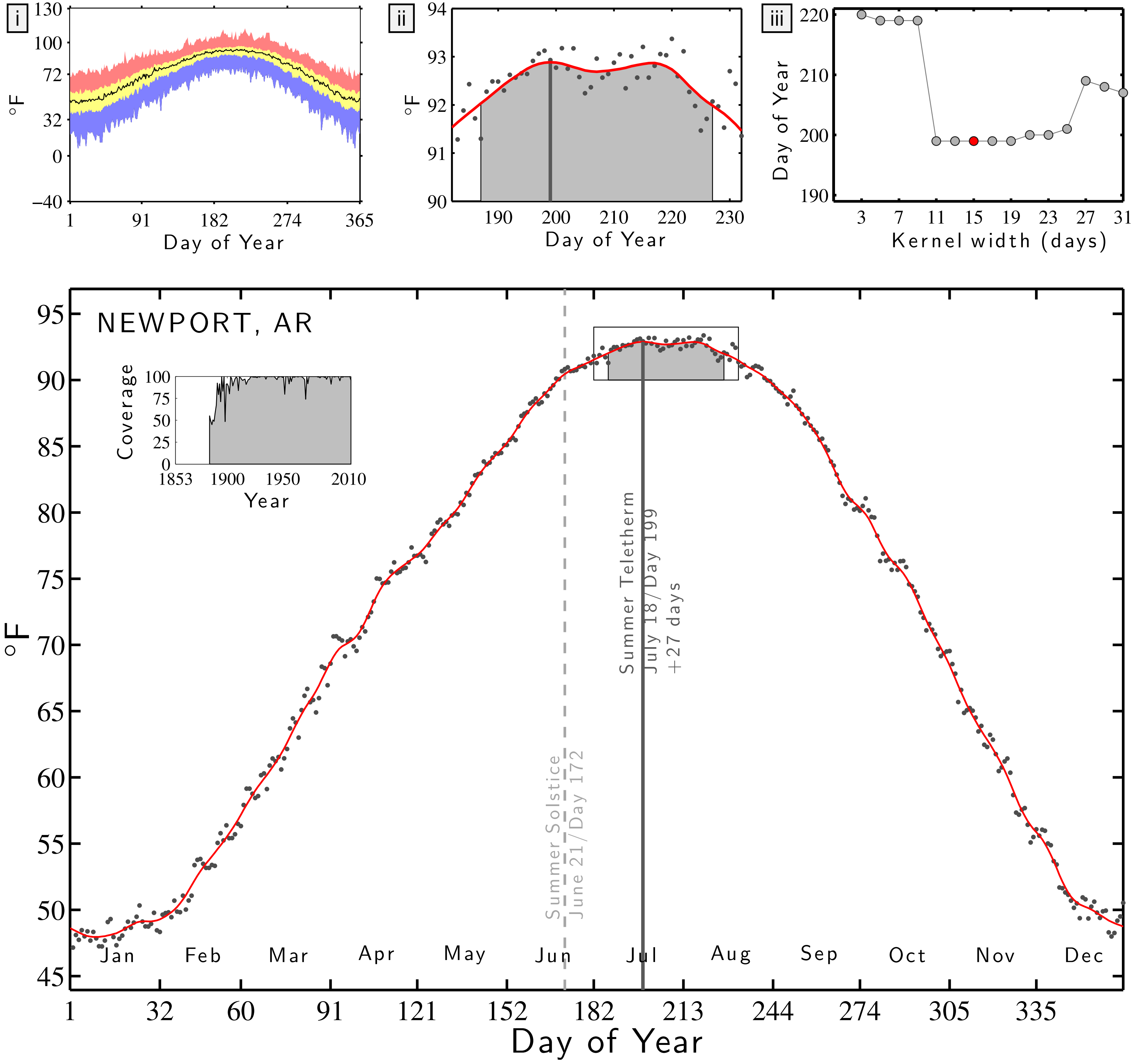

i
ii
iii
°F
Day of Year
Day of Year
Kernel width (days)
NEWPORT, AR
Coverage
Year
Summer Solstice
June 21/Day 172
Summer Teletherm
July 18/Day 199
+27 days
Jan
Feb
Mar
Apr
May
Jun
Jul
Aug
Sep
Oct
Nov
Dec

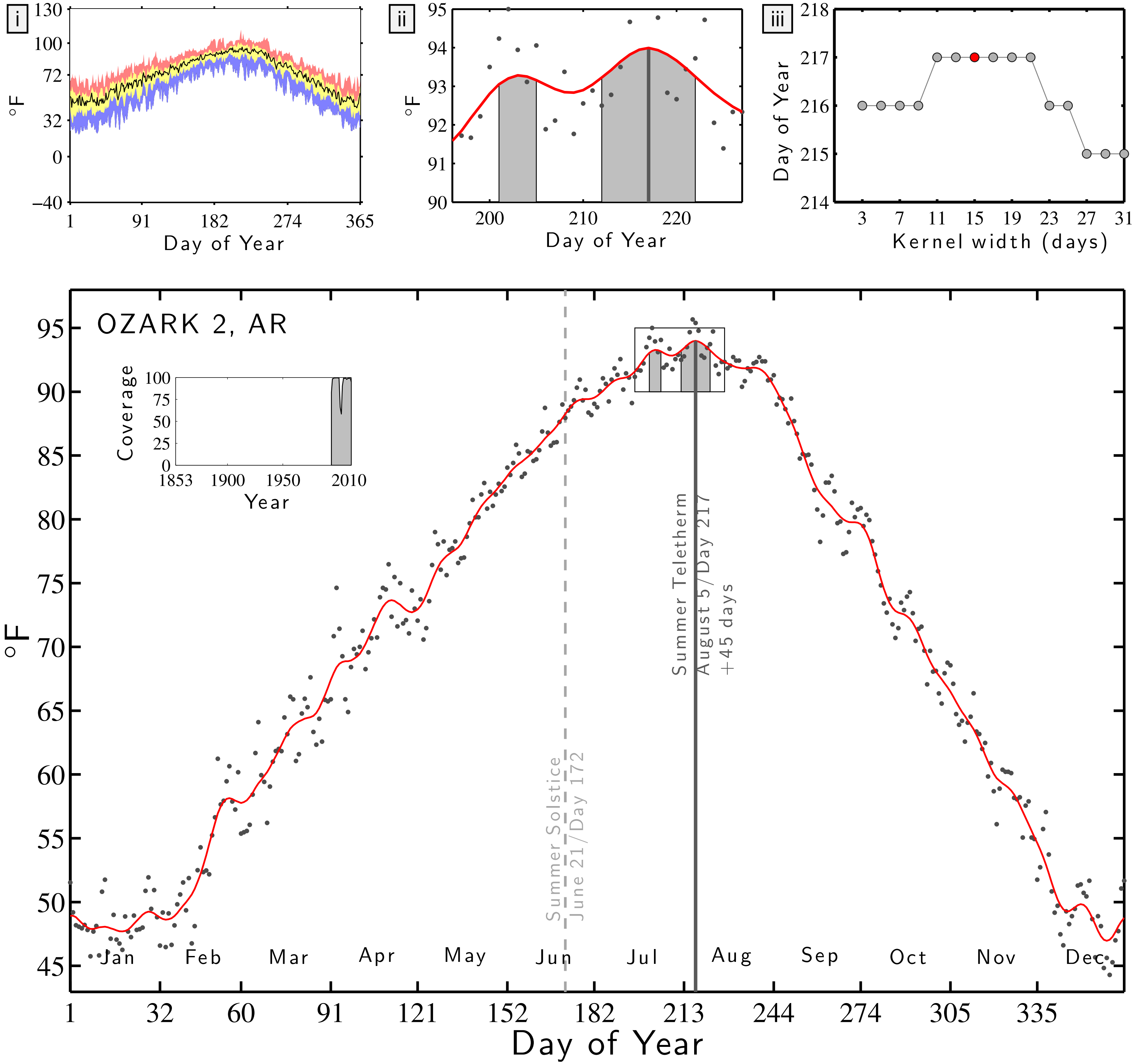

i
°F
Day of Year
ii
°F
Day of Year
iii
Day of Year
Kernel width (days)
OZARK 2, AR
Coverage
Year
Summer Solstice
June 21 / Day 172
Summer Teletherm
August 5 / Day 217
+45 days
Jan
Feb
Mar
Apr
May
Jun
Jul
Aug
Sep
Oct
Nov
Dec
°F
Day of Year

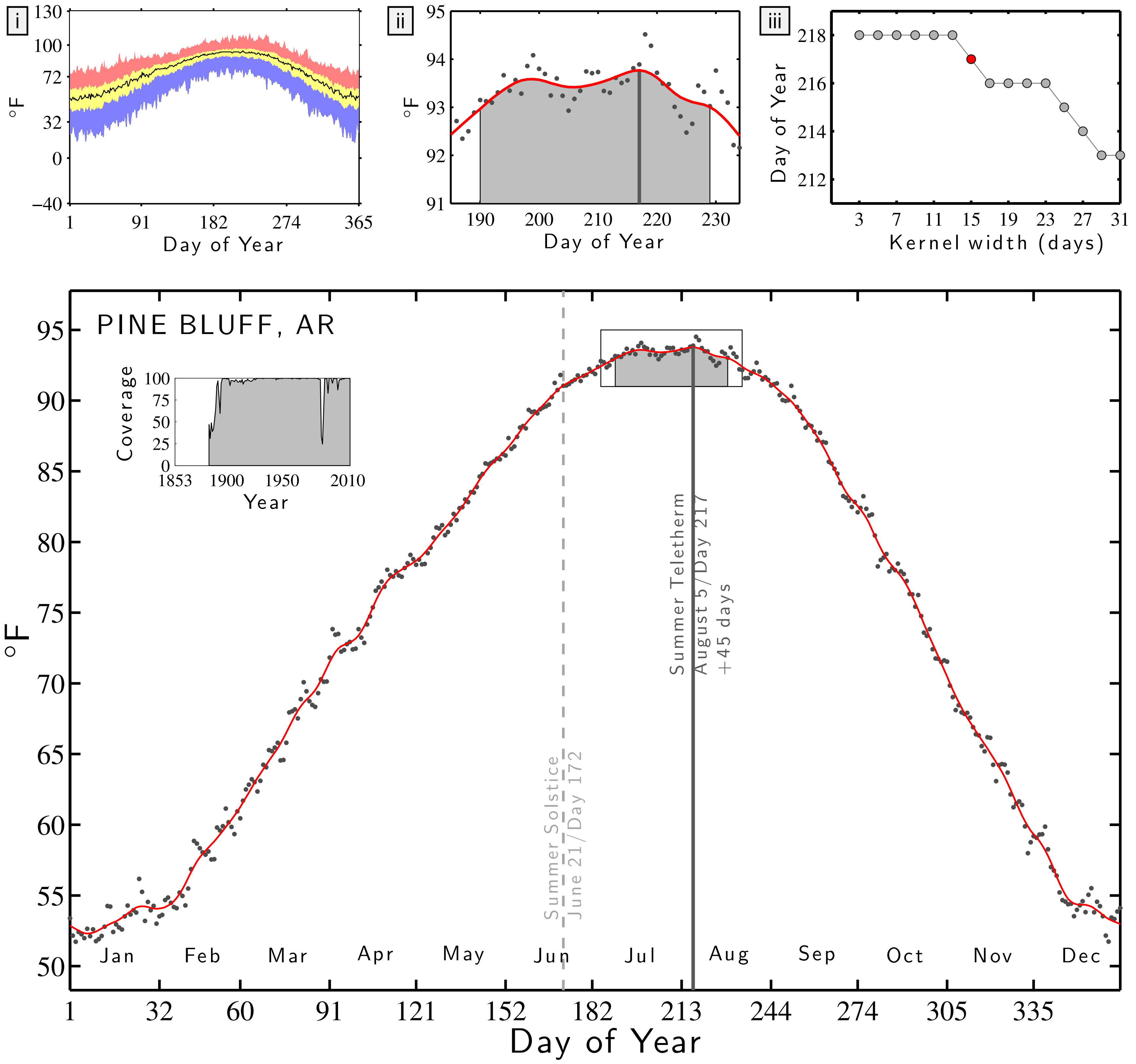

i
ii
iii
°F
Day of Year
Day of Year
Kernel width (days)
PINE BLUFF, AR
Coverage
Year
Summer Solstice
June 21/Day 172
Summer Teletherm
August 5/Day 217
+45 days
Jan
Feb
Mar
Apr
May
Jun
Jul
Aug
Sep
Oct
Nov
Dec

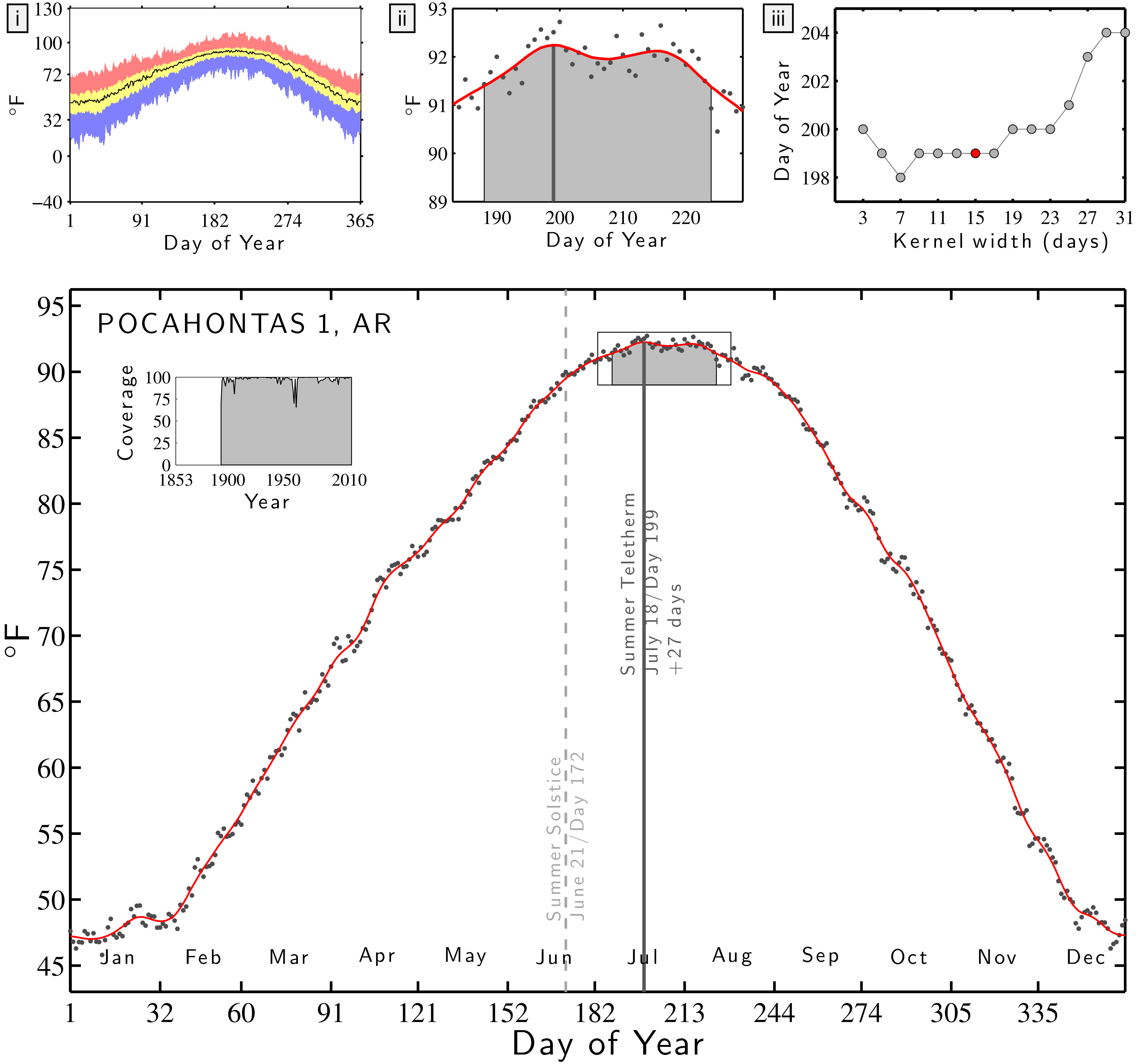

i
ii
iii
POCAHONTAS 1, AR
Summer Solstice
June 21/Day 172
Summer Teletherm
July 18/Day 199
+27 days
Coverage
Year
Day of Year
Kernel width (days)
°F
Jan
Feb
Mar
Apr
May
Jun
Jul
Aug
Sep
Oct
Nov
Dec

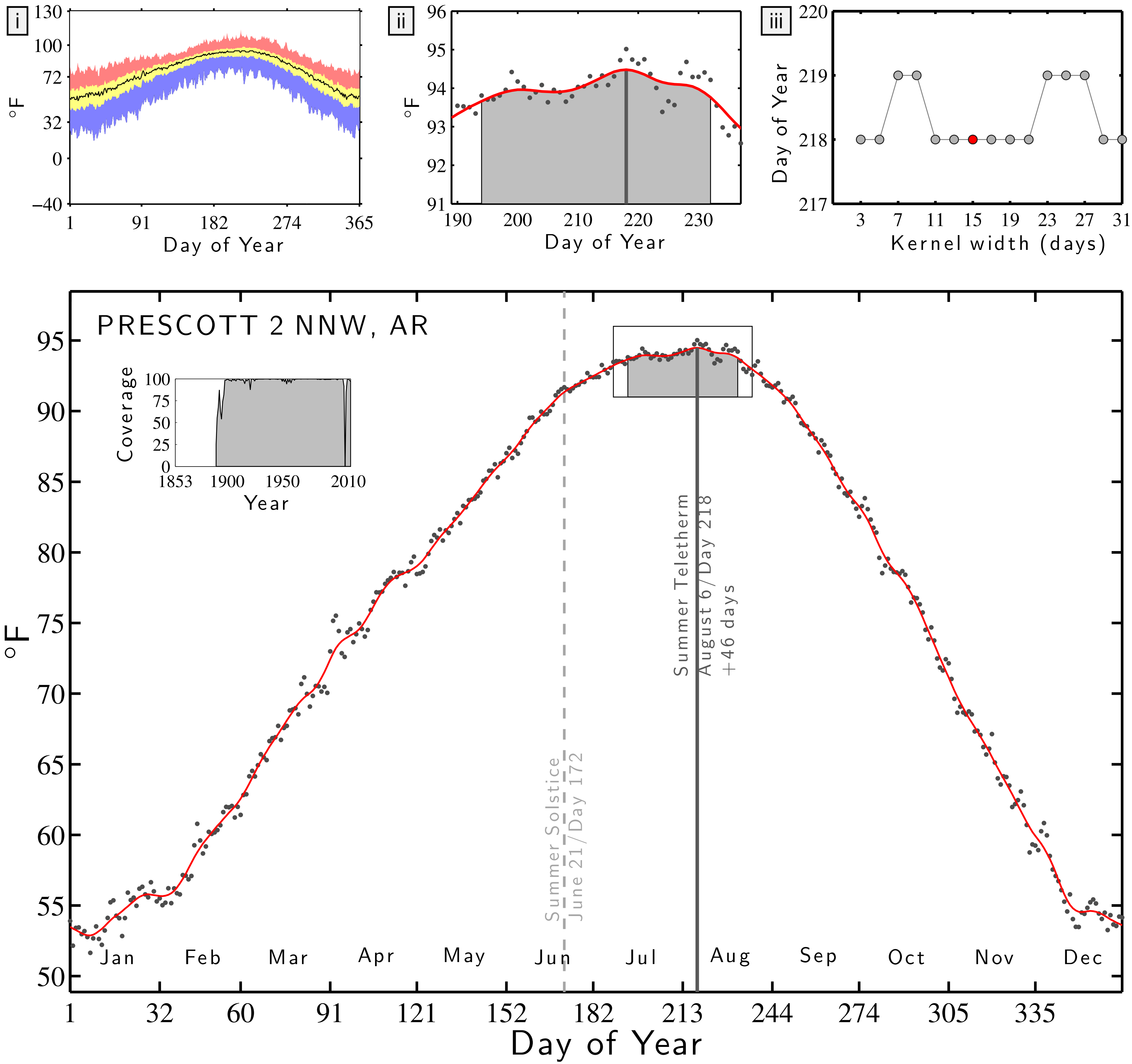

i
°F
Day of Year
ii
°F
Day of Year
iii
Day of Year
Kernel width (days)
PRESCOTT 2 NNW, AR
Coverage
Year
Summer Solstice
June 21 / Day 172
Summer Teletherm
August 6 / Day 218
+46 days
Jan
Feb
Mar
Apr
May
Jun
Jul
Aug
Sep
Oct
Nov
Dec
°F
Day of Year

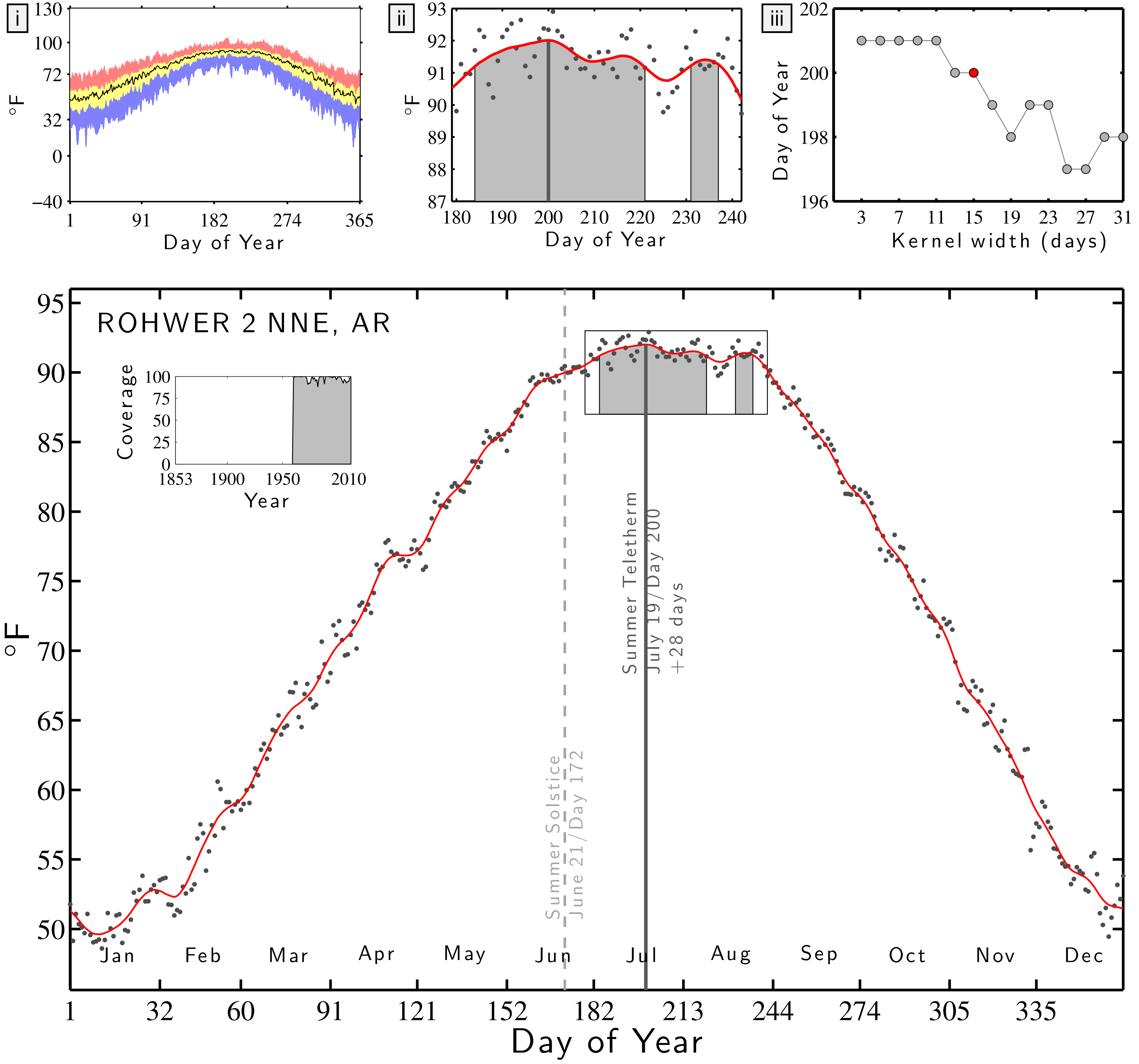

i
°F
130
100
72
32
0
−40
1
91
182
274
365
Day of Year
ii
°F
93
92
91
90
89
88
87
180
190
200
210
220
230
240
Day of Year
iii
Day of Year
202
200
198
196
3
7
11
15
19
23
27
31
Kernel width (days)
ROHWER 2 NNE, AR
Coverage
100
75
50
25
0
1853
1900
1950
2010
Year
Summer Solstice
June 21/Day 172
Summer Teletherm
July 19/Day 200
+28 days
°F
95
90
85
80
75
70
65
60
55
50
Jan
Feb
Mar
Apr
May
Jun
Jul
Aug
Sep
Oct
Nov
Dec
1
32
60
91
121
152
182
213
244
274
305
335
Day of Year

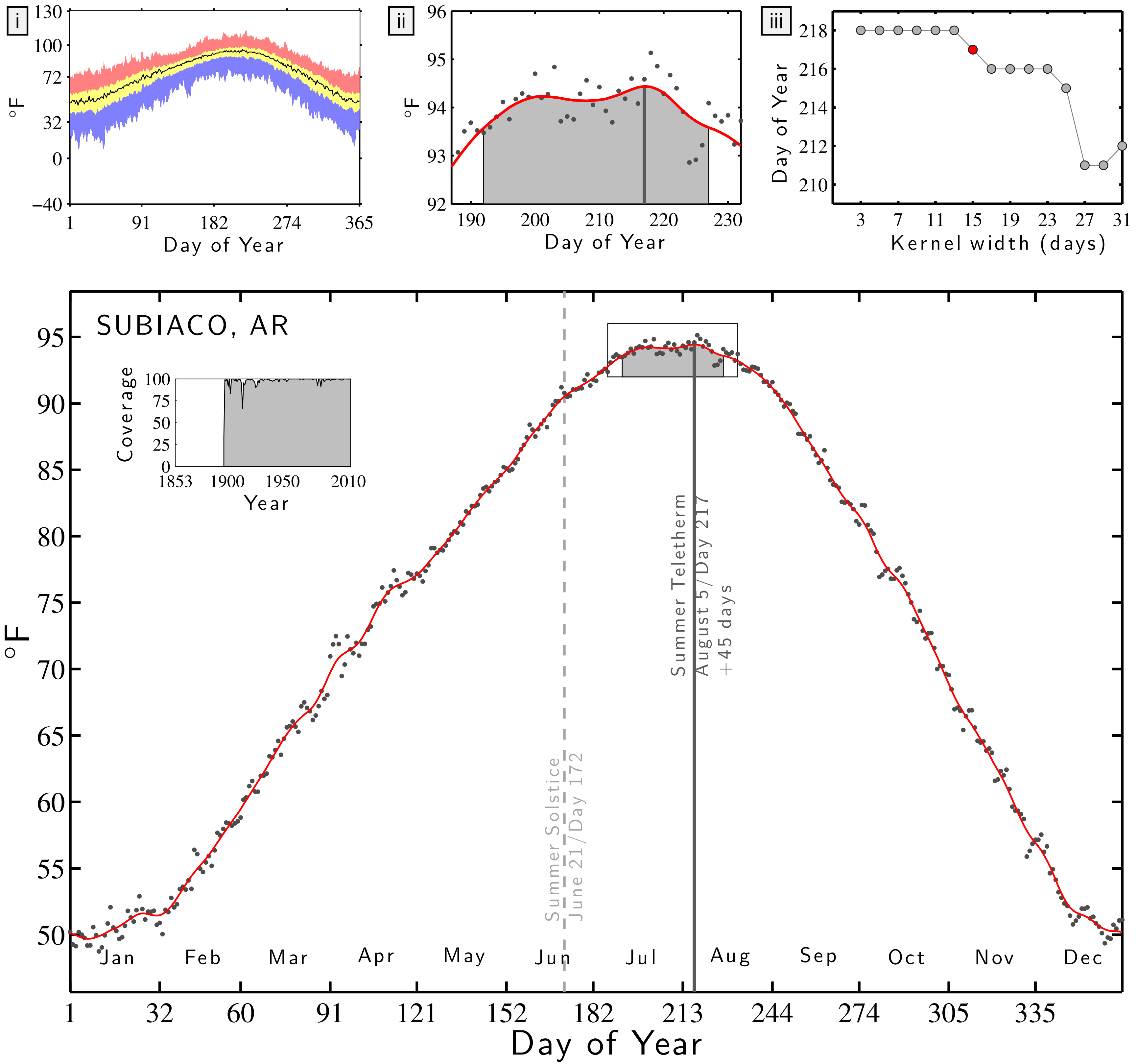

i
ii
iii
°F
Day of Year
Day of Year
Kernel width (days)
SUBIACO, AR
Coverage
Year
Summer Solstice
June 21/Day 172
Summer Teletherm
August 5/Day 217
+45 days
Jan
Feb
Mar
Apr
May
Jun
Jul
Aug
Sep
Oct
Nov
Dec

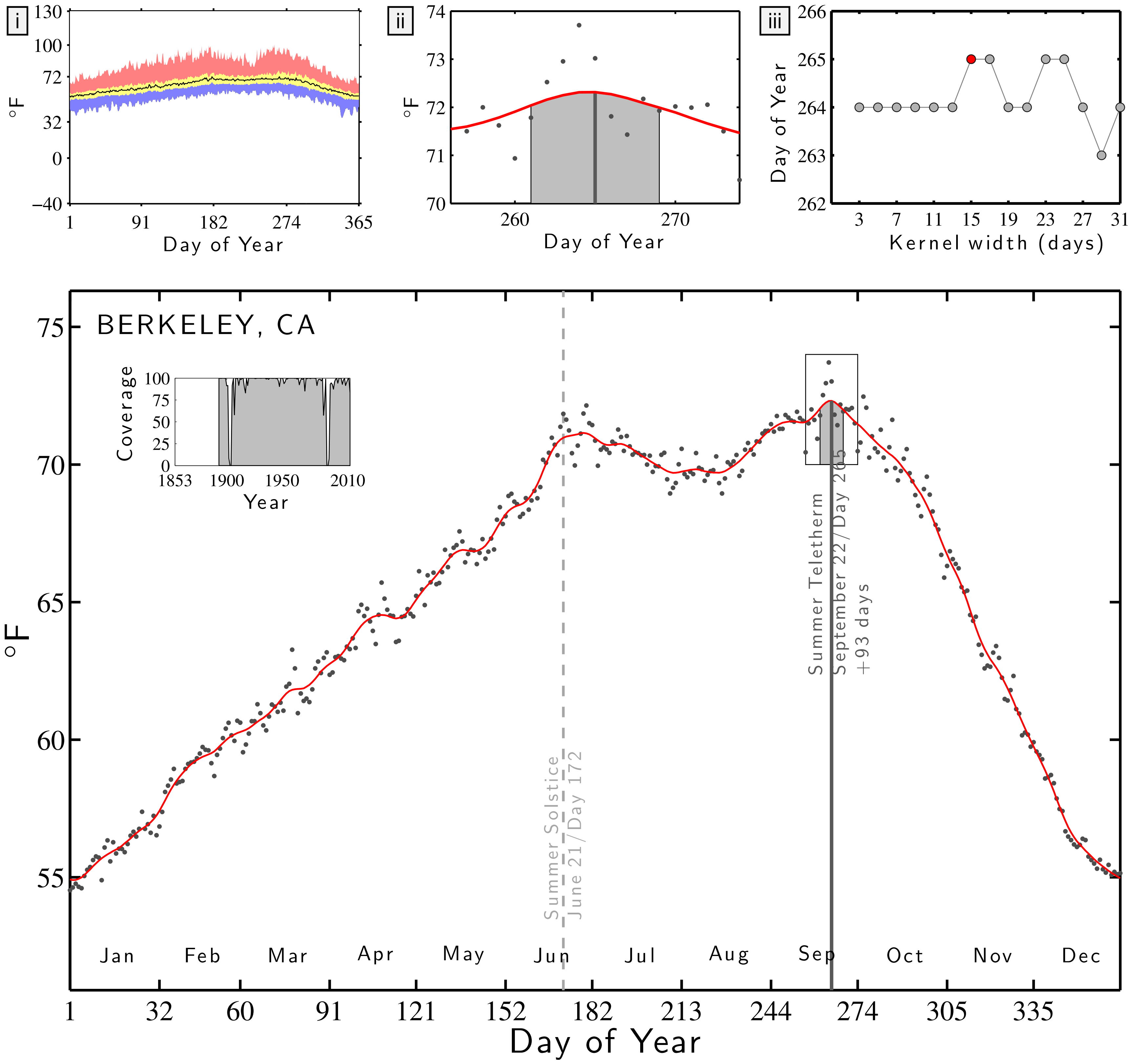

i
°F
Day of Year
ii
°F
Day of Year
iii
Day of Year
Kernel width (days)
BERKELEY, CA
Coverage
Year
Summer Solstice
June 21 / Day 172
Summer Teletherm
September 22 / Day 265
+93 days
Jan
Feb
Mar
Apr
May
Jun
Jul
Aug
Sep
Oct
Nov
Dec
°F
Day of Year

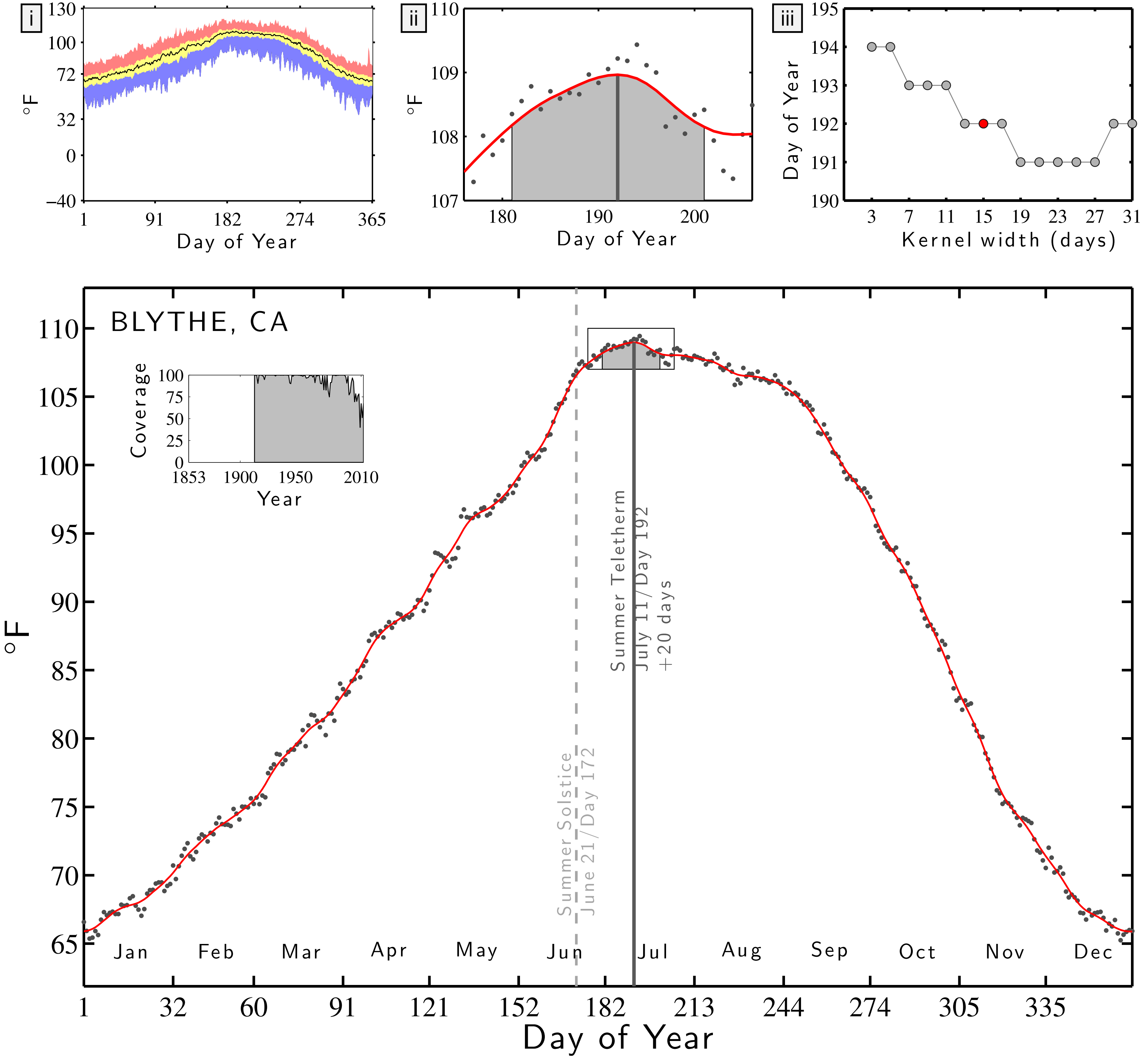

i
°F
Day of Year
ii
°F
Day of Year
iii
Day of Year
Kernel width (days)
BLYTHE, CA
Coverage
Year
Summer Solstice
June 21/Day 172
Summer Teletherm
July 11/Day 192
+20 days
Jan
Feb
Mar
Apr
May
Jun
Jul
Aug
Sep
Oct
Nov
Dec
°F
Day of Year

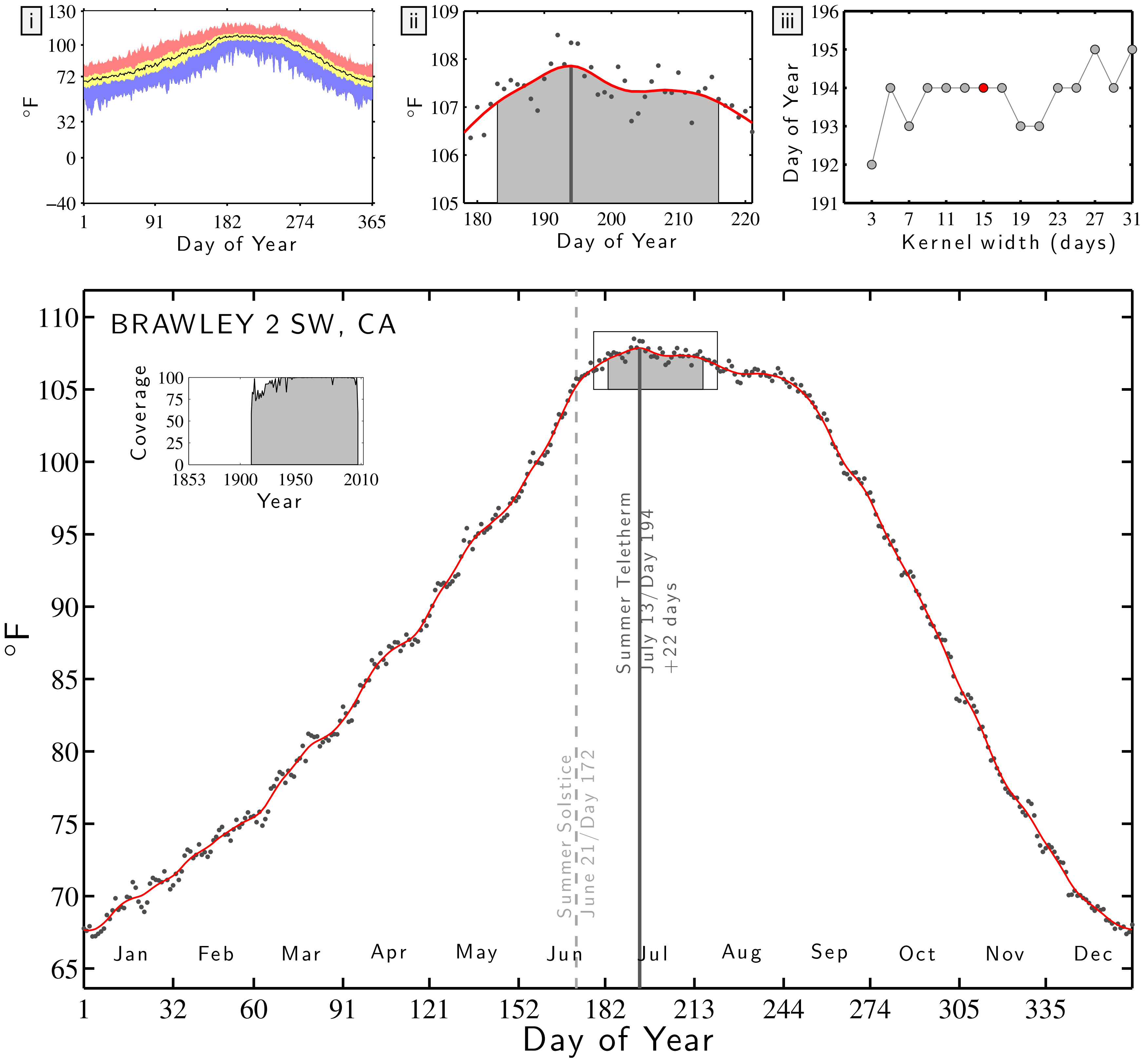

i
°F
Day of Year
ii
°F
Day of Year
iii
Day of Year
Kernel width (days)
BRAWLEY 2 SW, CA
Coverage
Year
Summer Solstice
June 21/Day 172
Summer Teletherm
July 13/Day 194
+22 days
Jan
Feb
Mar
Apr
May
Jun
Jul
Aug
Sep
Oct
Nov
Dec
°F
Day of Year

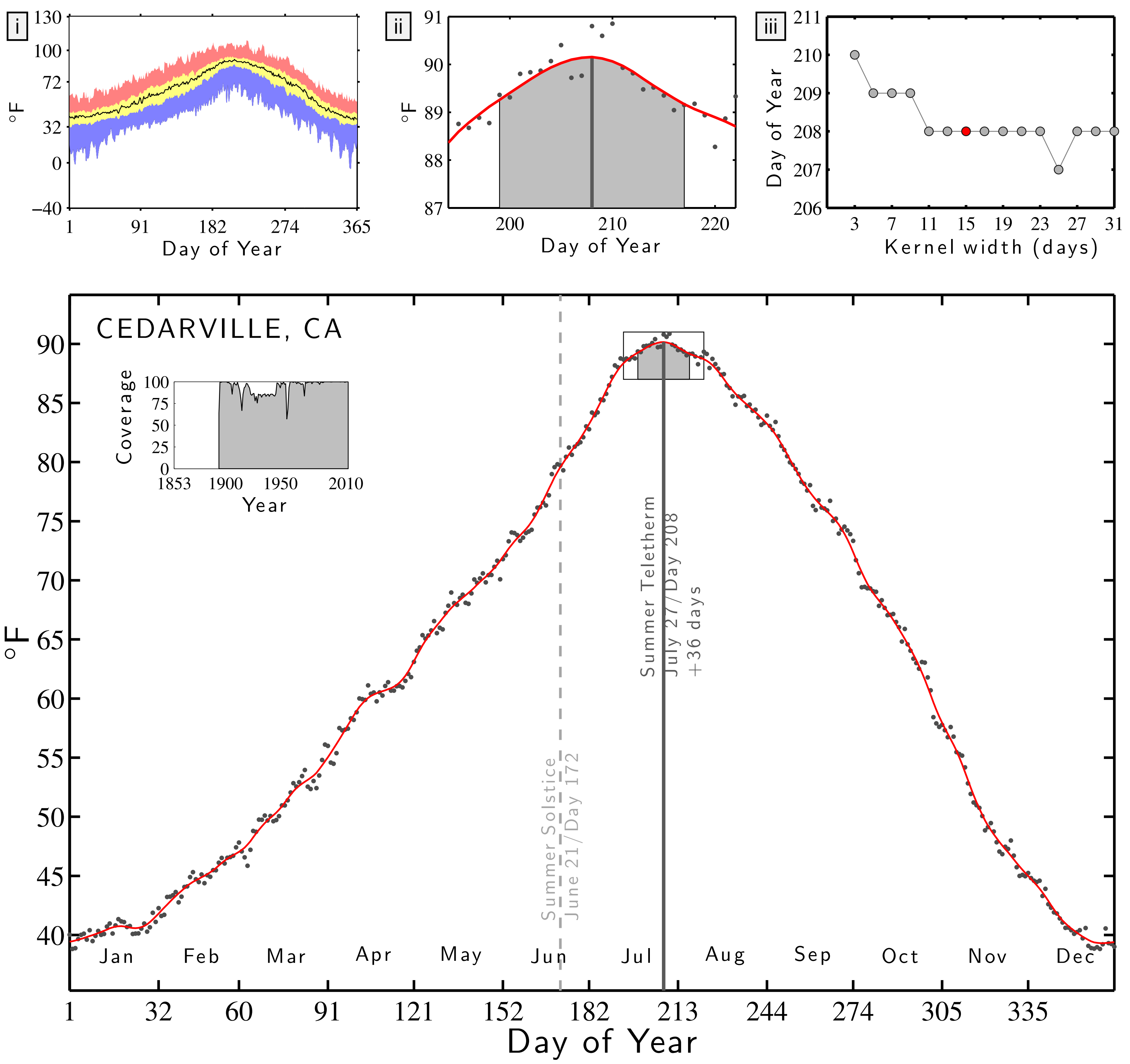
i
°F
Day of Year
ii
°F
Day of Year
iii
Day of Year
Kernel width (days)
CEDARVILLE, CA
Coverage
Year
Summer Solstice
June 21/Day 172
Summer Teletherm
July 27/Day 208
+36 days
Jan Feb Mar Apr May Jun Jul Aug Sep Oct Nov Dec
°F
Day of Year

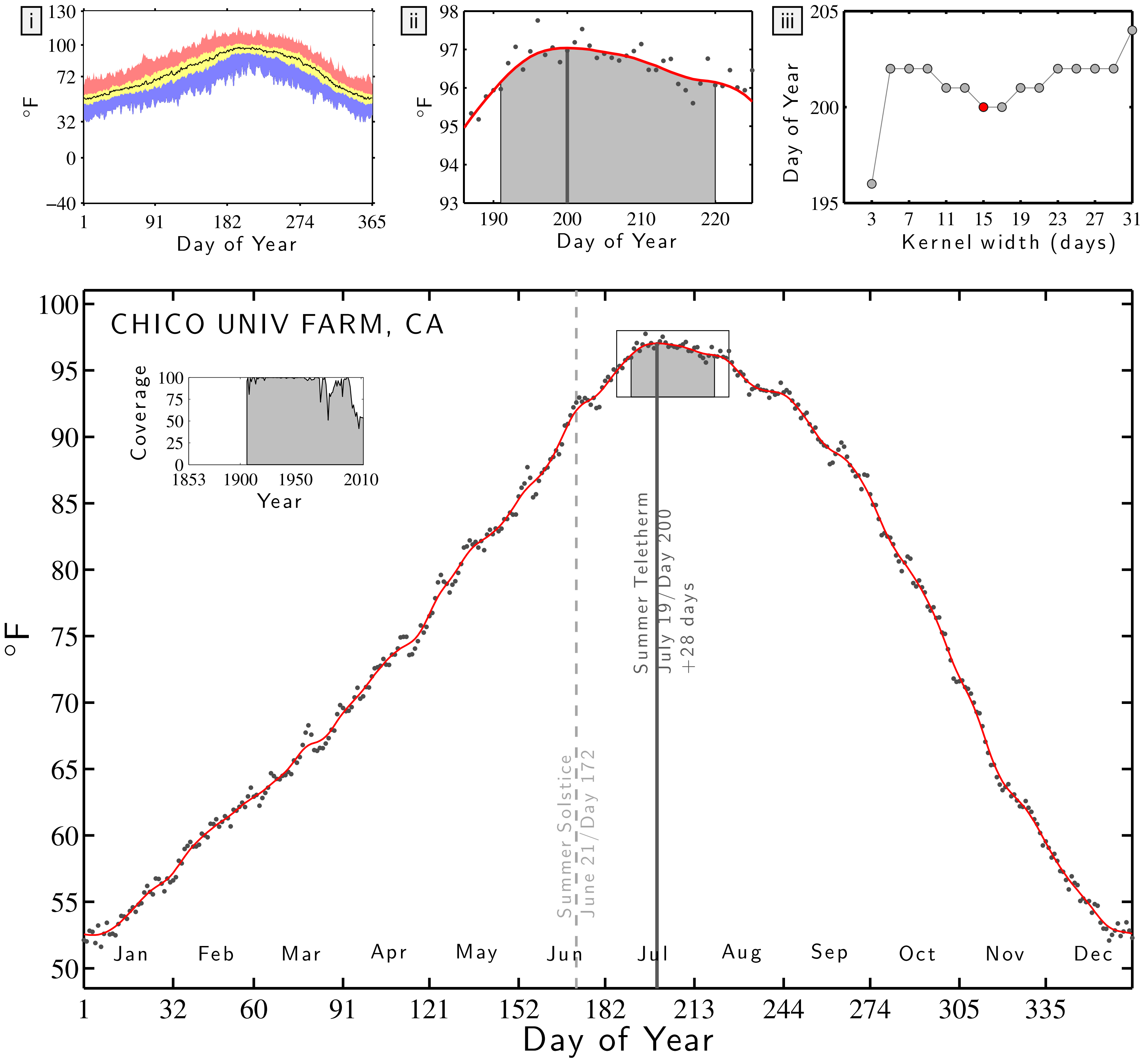

i
°F
Day of Year
ii
°F
Day of Year
iii
Day of Year
Kernel width (days)
CHICO UNIV FARM, CA
Coverage
Year
Summer Solstice
June 21/Day 172
Summer Teletherm
July 19/Day 200
+28 days
Jan
Feb
Mar
Apr
May
Jun
Jul
Aug
Sep
Oct
Nov
Dec
°F
Day of Year

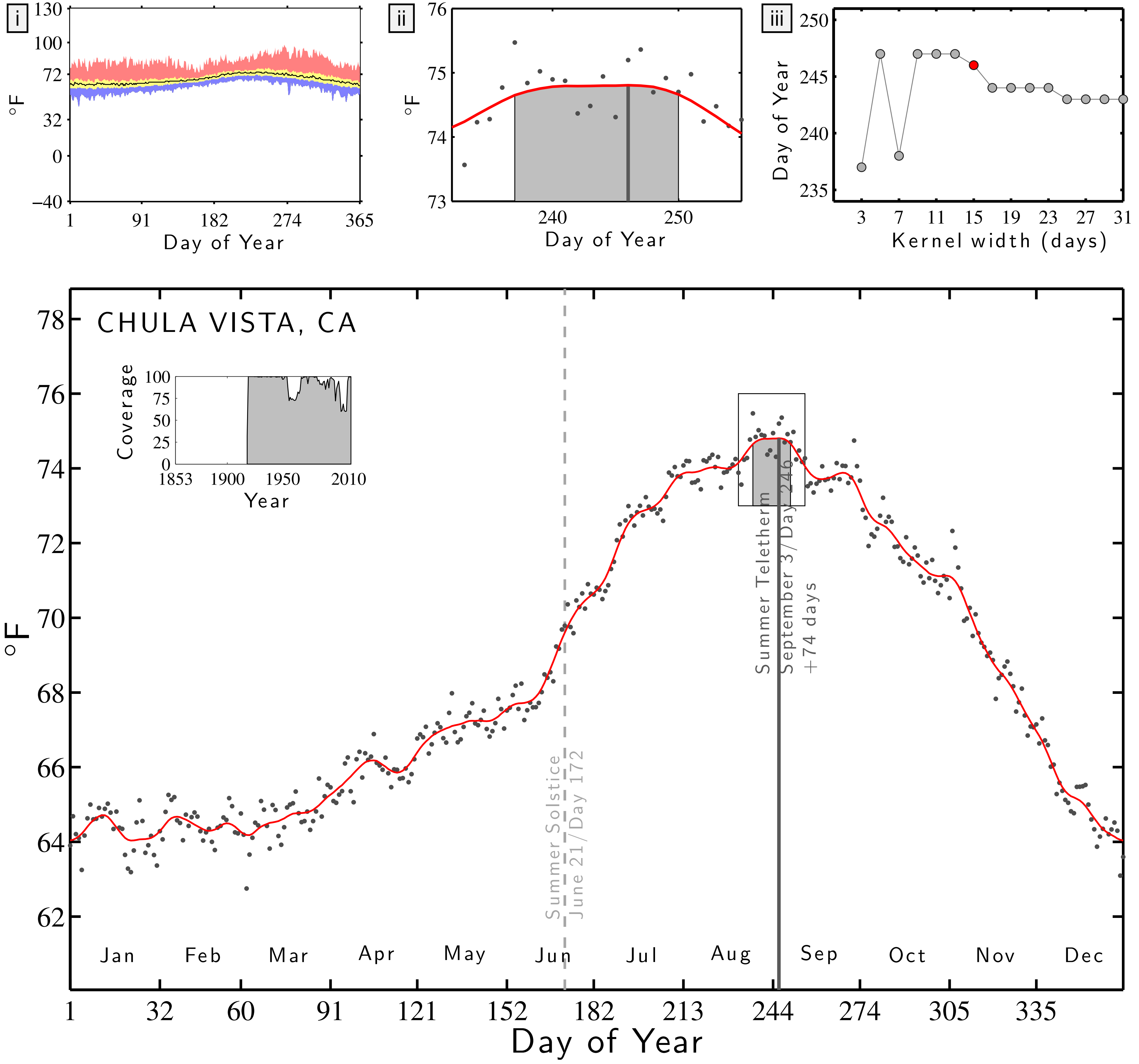

i
130
100
72
32
0
−40
°F
1
91
182
274
365
Day of Year
ii
76
75
74
73
°F
240
250
Day of Year
iii
250
245
240
235
Day of Year
3
7
11
15
19
23
27
31
Kernel width (days)
CHULA VISTA, CA
Coverage
100
75
50
25
0
1853
1900
1950
2010
Year
Summer Solstice
June 21 / Day 172
Summer Teletherm
September 3 / Day 246
+74 days
78
76
74
72
70
68
66
64
62
°F
Jan
Feb
Mar
Apr
May
Jun
Jul
Aug
Sep
Oct
Nov
Dec
1
32
60
91
121
152
182
213
244
274
305
335
Day of Year

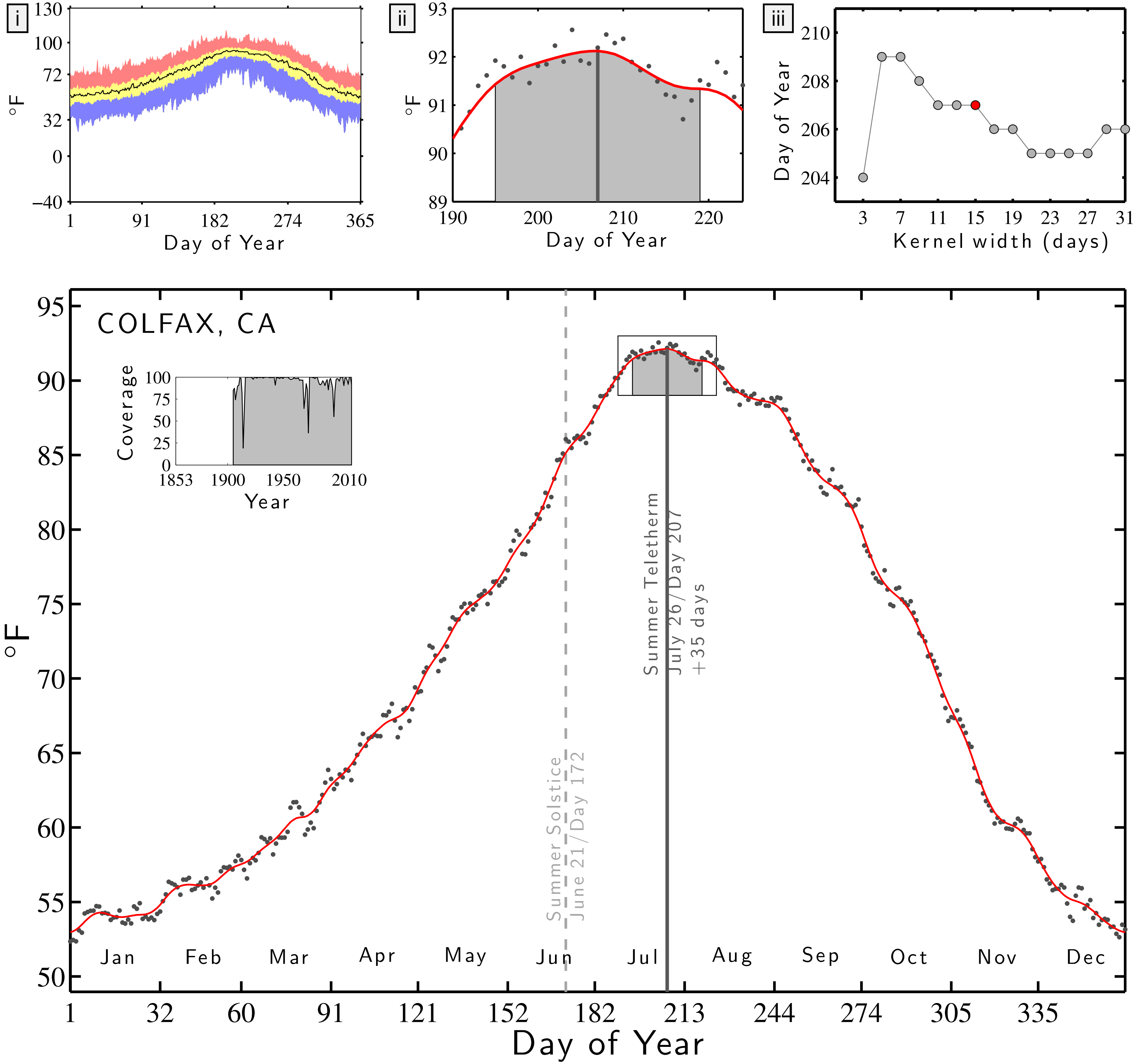

i
ii
iii
°F
Day of Year
Day of Year
Kernel width (days)
COLFAX, CA
Coverage
Year
Summer Solstice
June 21 / Day 172
Summer Teletherm
July 26 / Day 207
+35 days
Jan
Feb
Mar
Apr
May
Jun
Jul
Aug
Sep
Oct
Nov
Dec
Day of Year

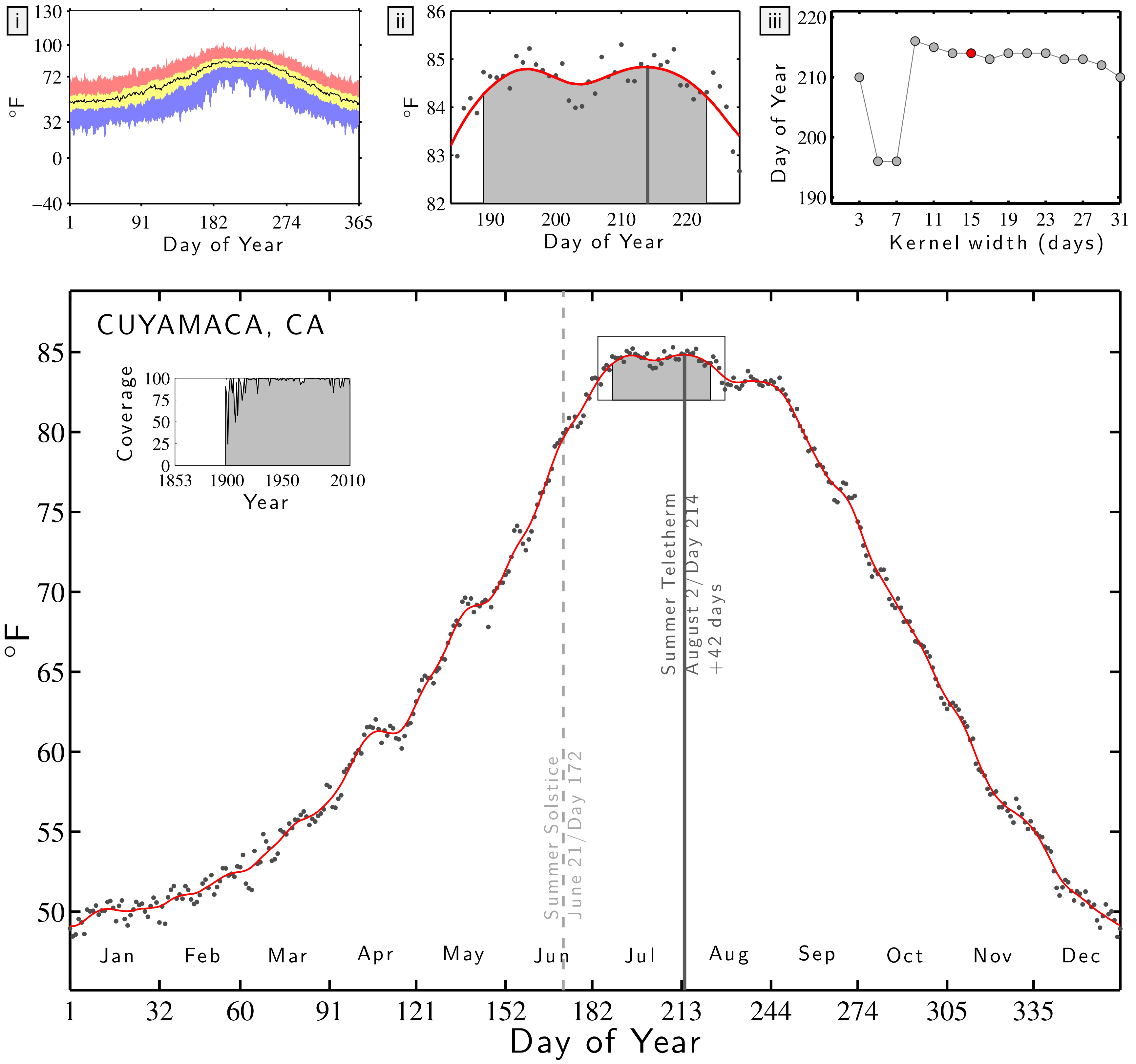

i
°F
Day of Year
ii
°F
Day of Year
iii
Day of Year
Kernel width (days)
CUYAMACA, CA
Coverage
Year
Summer Solstice
June 21/Day 172
Summer Teletherm
August 2/Day 214
+42 days
Jan
Feb
Mar
Apr
May
Jun
Jul
Aug
Sep
Oct
Nov
Dec
°F
Day of Year

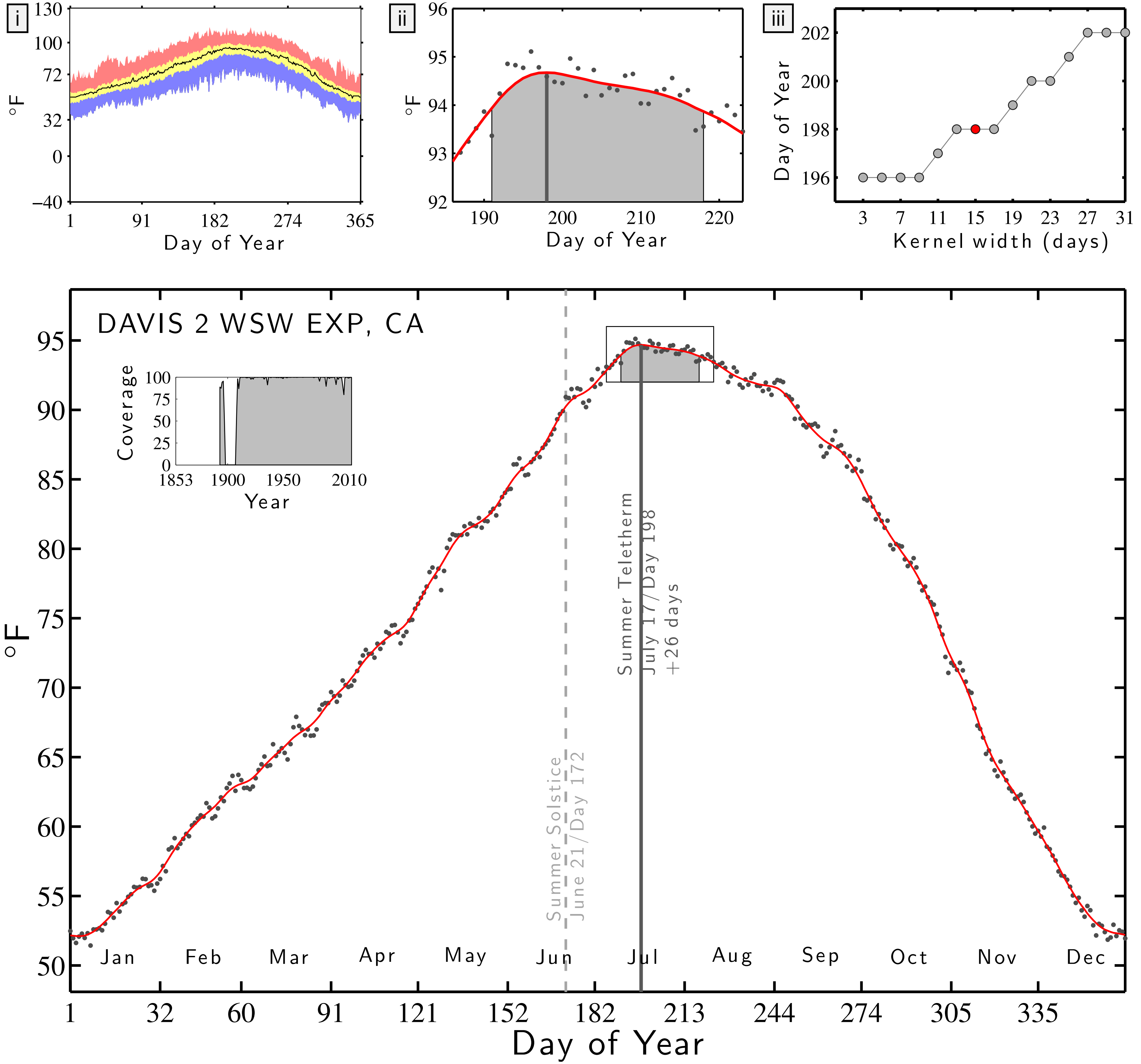

i
ii
iii
°F
Day of Year
Kernel width (days)
Day of Year
DAVIS 2 WSW EXP, CA
Coverage
Year
Summer Solstice
June 21/Day 172
Summer Teletherm
July 17/Day 198
+26 days
Jan
Feb
Mar
Apr
May
Jun
Jul
Aug
Sep
Oct
Nov
Dec
Day of Year

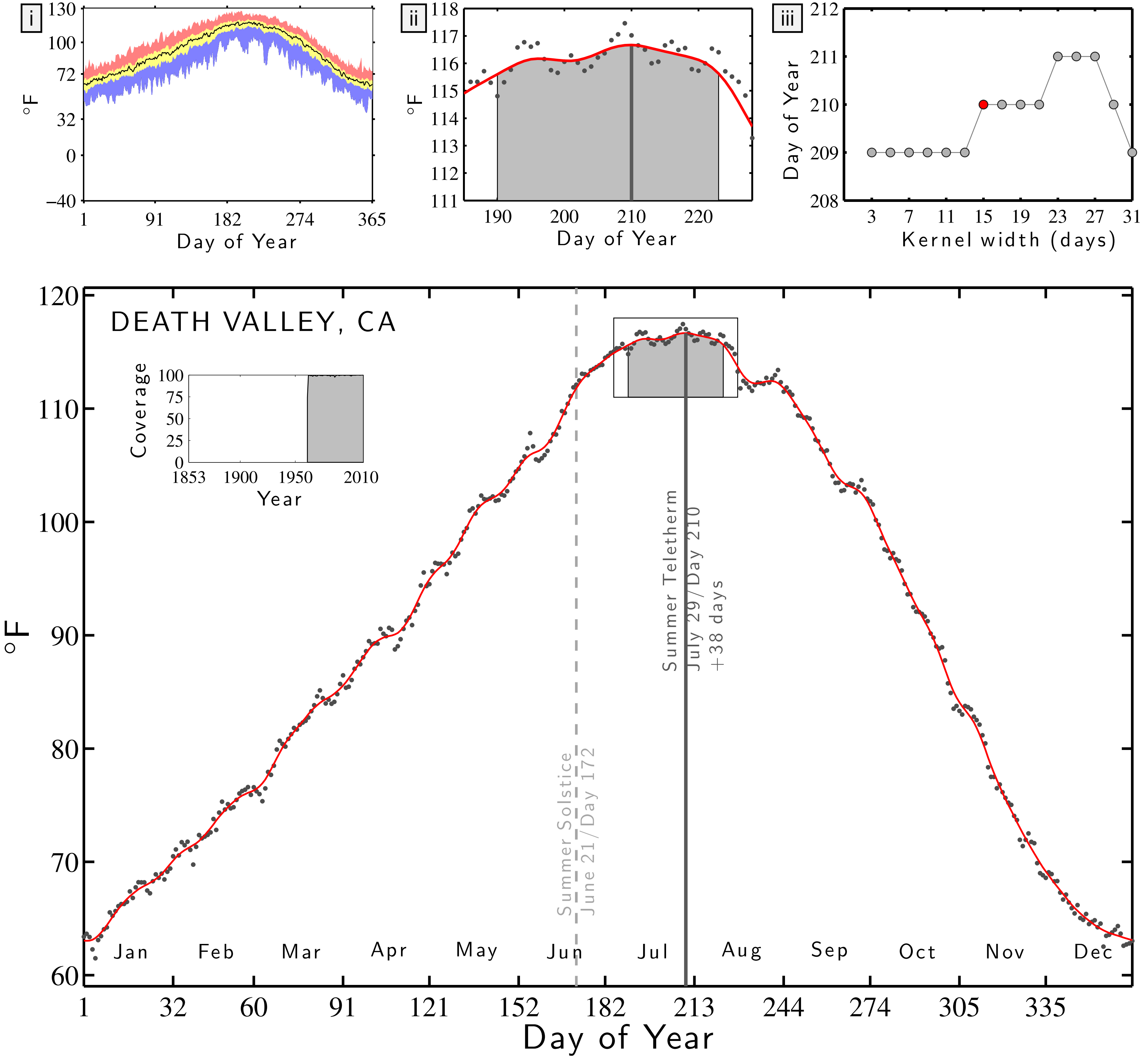

i
130
100
72
32
0
−40
°F
1
91
182
274
365
Day of Year
ii
118
117
116
115
114
113
112
111
°F
190
200
210
220
Day of Year
iii
212
211
210
209
208
Day of Year
3
7
11
15
19
23
27
31
Kernel width (days)
DEATH VALLEY, CA
Coverage
100
75
50
25
0
1853
1900
1950
2010
Year
Summer Solstice
June 21/Day 172
Summer Teletherm
July 29/Day 210
+38 days
120
110
100
90
80
70
60
°F
Jan
Feb
Mar
Apr
May
Jun
Jul
Aug
Sep
Oct
Nov
Dec
1
32
60
91
121
152
182
213
244
274
305
335
Day of Year

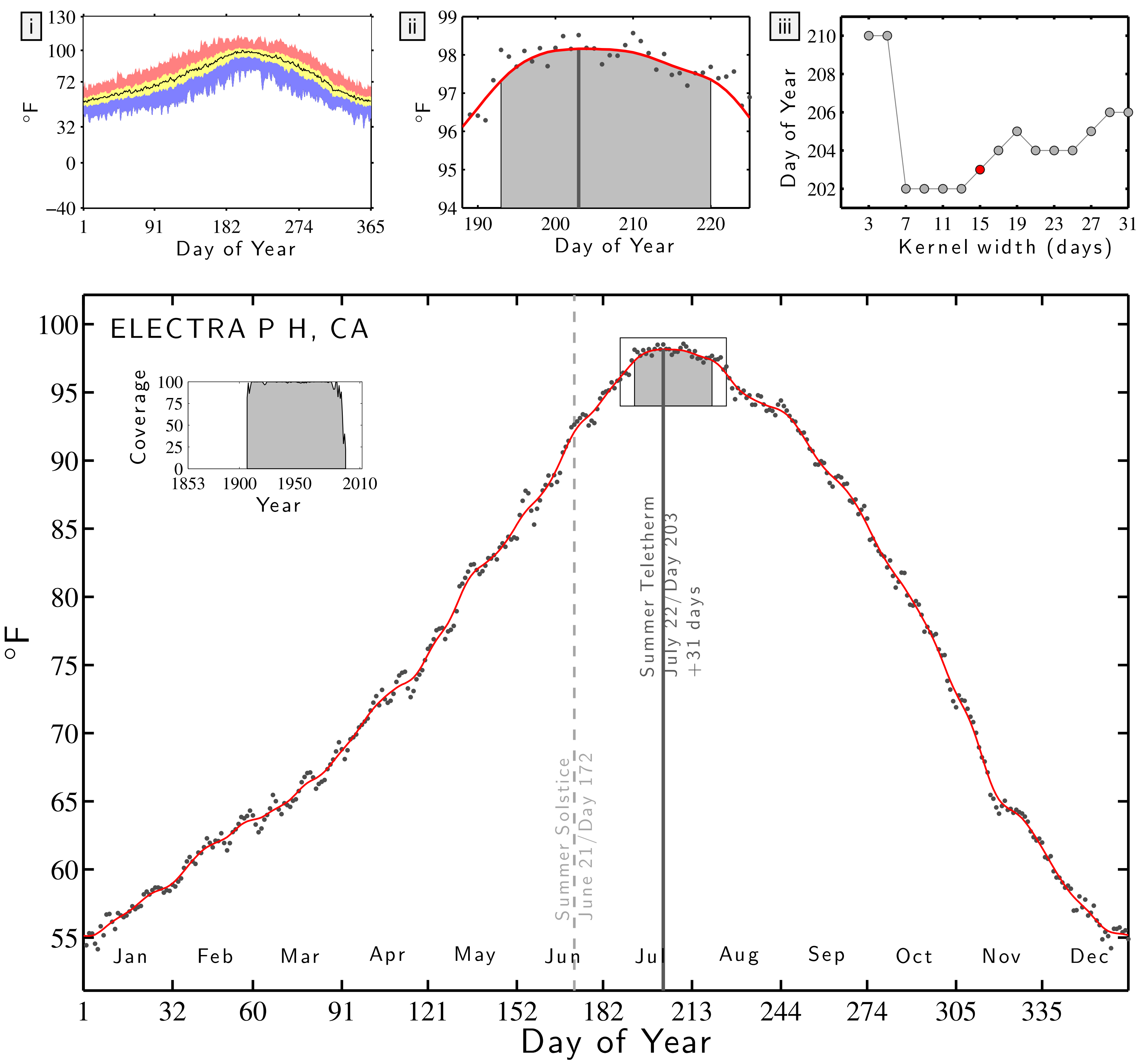
i
ii
iii
Day of Year
°F
Kernel width (days)
ELECTRA P H, CA
Coverage
Year
Summer Solstice
June 21/Day 172
Summer Teletherm
July 22/Day 203
+31 days
Jan
Feb
Mar
Apr
May
Jun
Jul
Aug
Sep
Oct
Nov
Dec

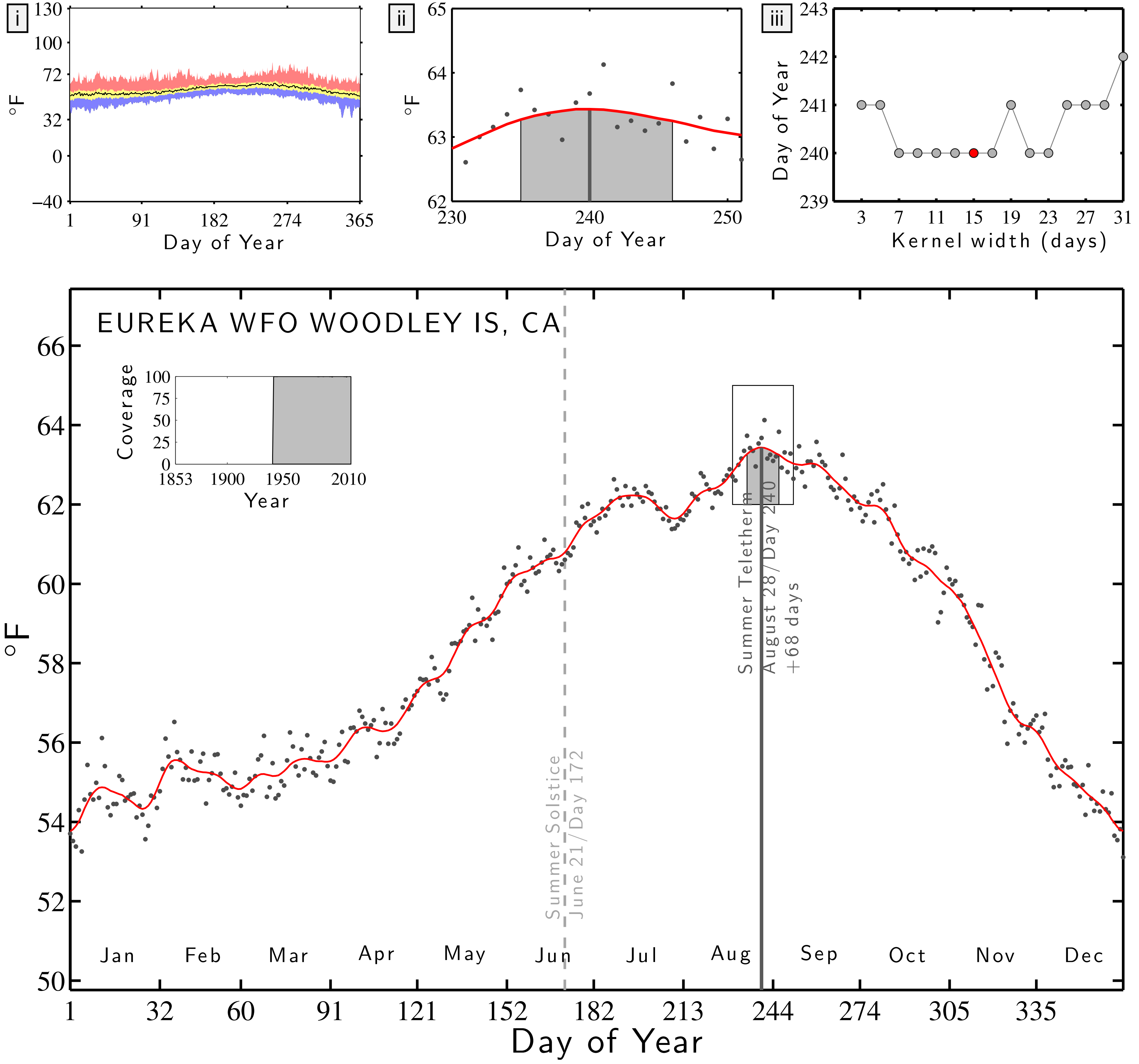

i
ii
iii
Day of Year
°F
Kernel width (days)
EUREKA WFO WOODLEY IS, CA
Coverage
Year
Summer Solstice
June 21 / Day 172
Summer Teletherm
August 28 / Day 240
+68 days
Jan
Feb
Mar
Apr
May
Jun
Jul
Aug
Sep
Oct
Nov
Dec
Day of Year

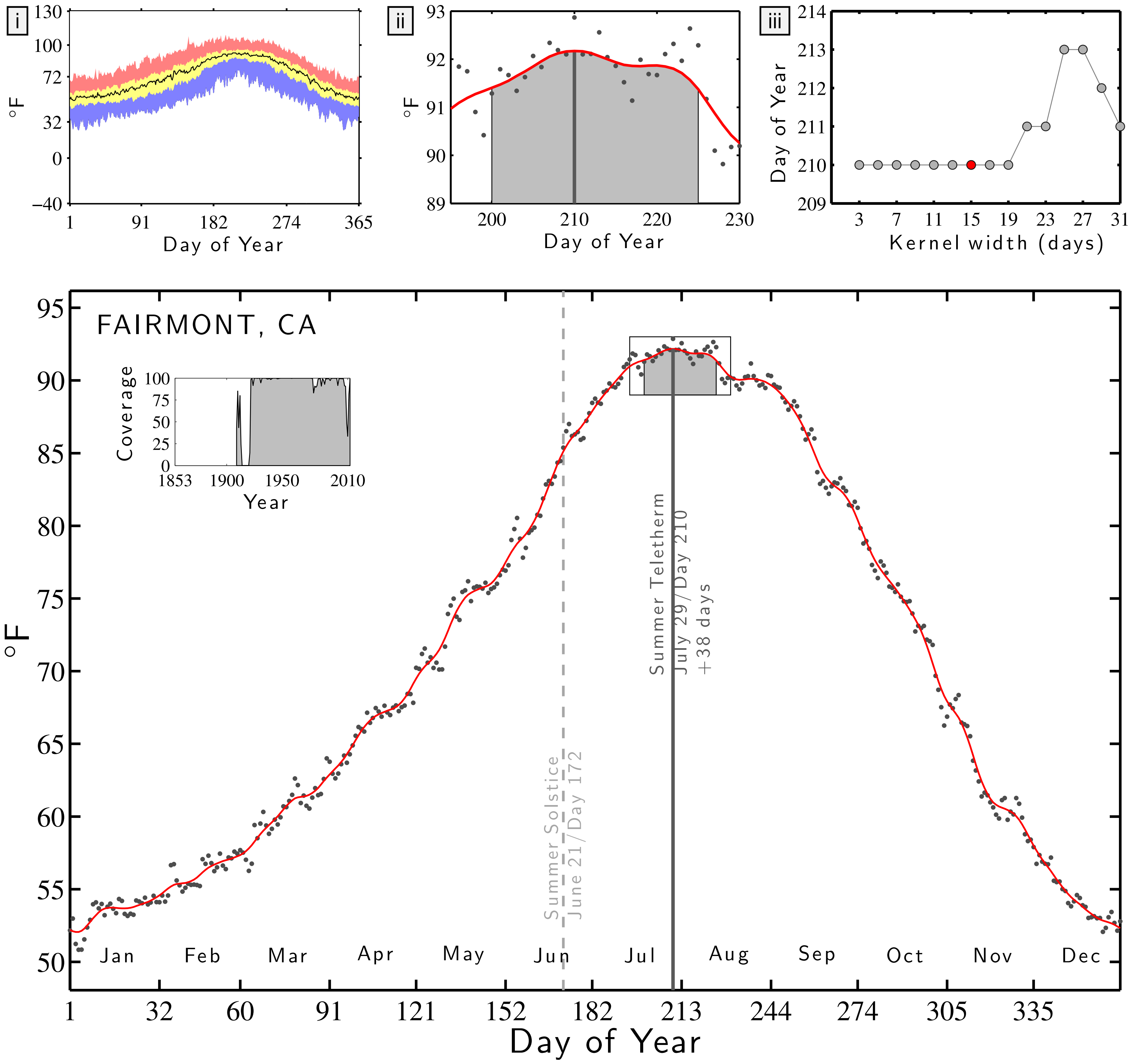

i
ii
iii
°F
Day of Year
Kernel width (days)
FAIRMONT, CA
Coverage
Year
Summer Solstice
June 21 / Day 172
Summer Teletherm
July 29 / Day 210
+38 days
Jan
Feb
Mar
Apr
May
Jun
Jul
Aug
Sep
Oct
Nov
Dec

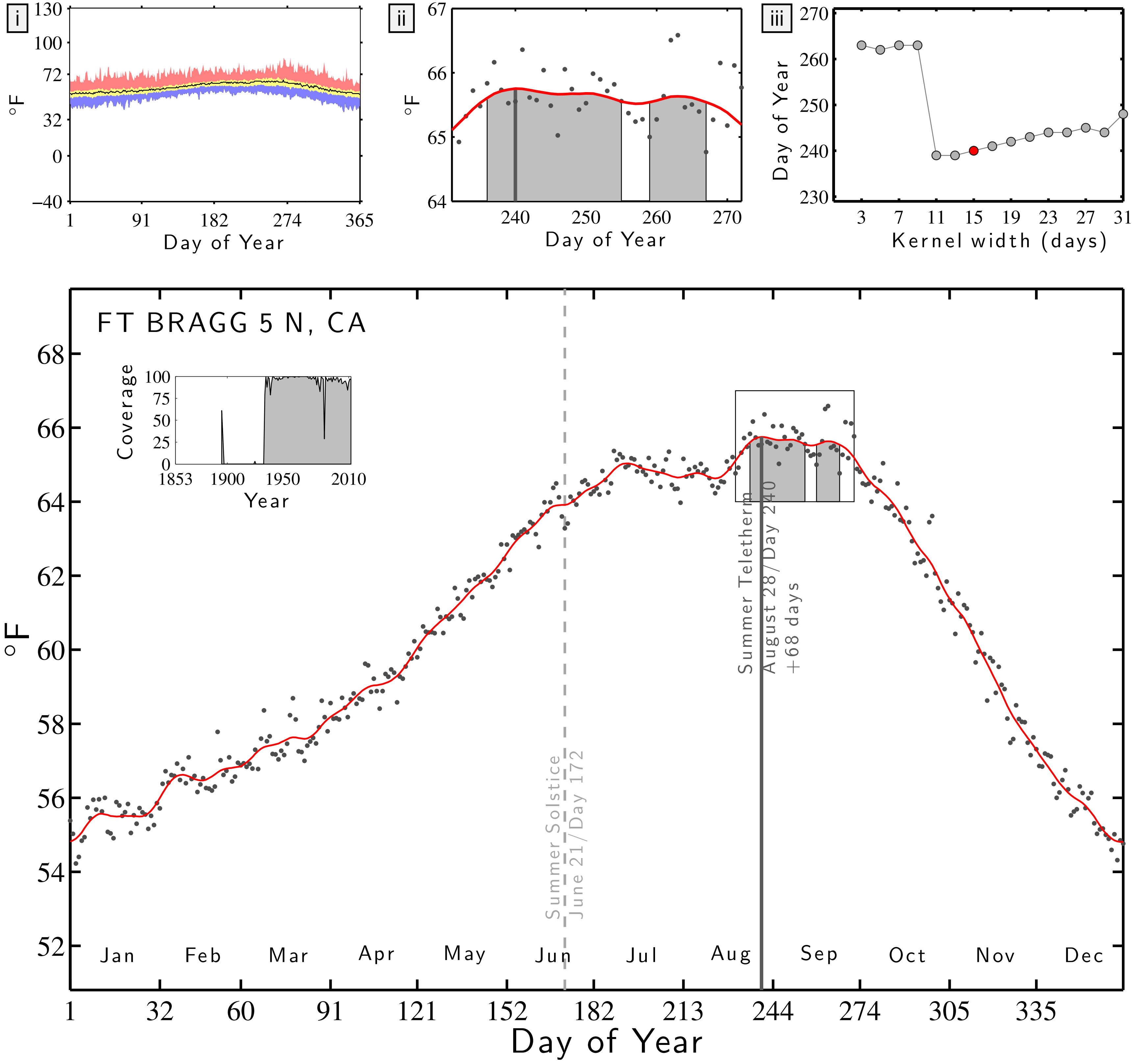

i
°F
130
100
72
32
0
−40
1
91
182
274
365
Day of Year
ii
67
66
65
64
240
250
260
270
Day of Year
iii
Day of Year
270
260
250
240
230
3
7
11
15
19
23
27
31
Kernel width (days)
FT BRAGG 5 N, CA
Coverage
100
75
50
25
0
1853
1900
1950
2010
Year
68
66
64
62
60
58
56
54
52
Summer Solstice
June 21 / Day 172
Summer Teletherm
August 28 / Day 240
+68 days
Jan
Feb
Mar
Apr
May
Jun
Jul
Aug
Sep
Oct
Nov
Dec
1
32
60
91
121
152
182
213
244
274
305
335
Day of Year

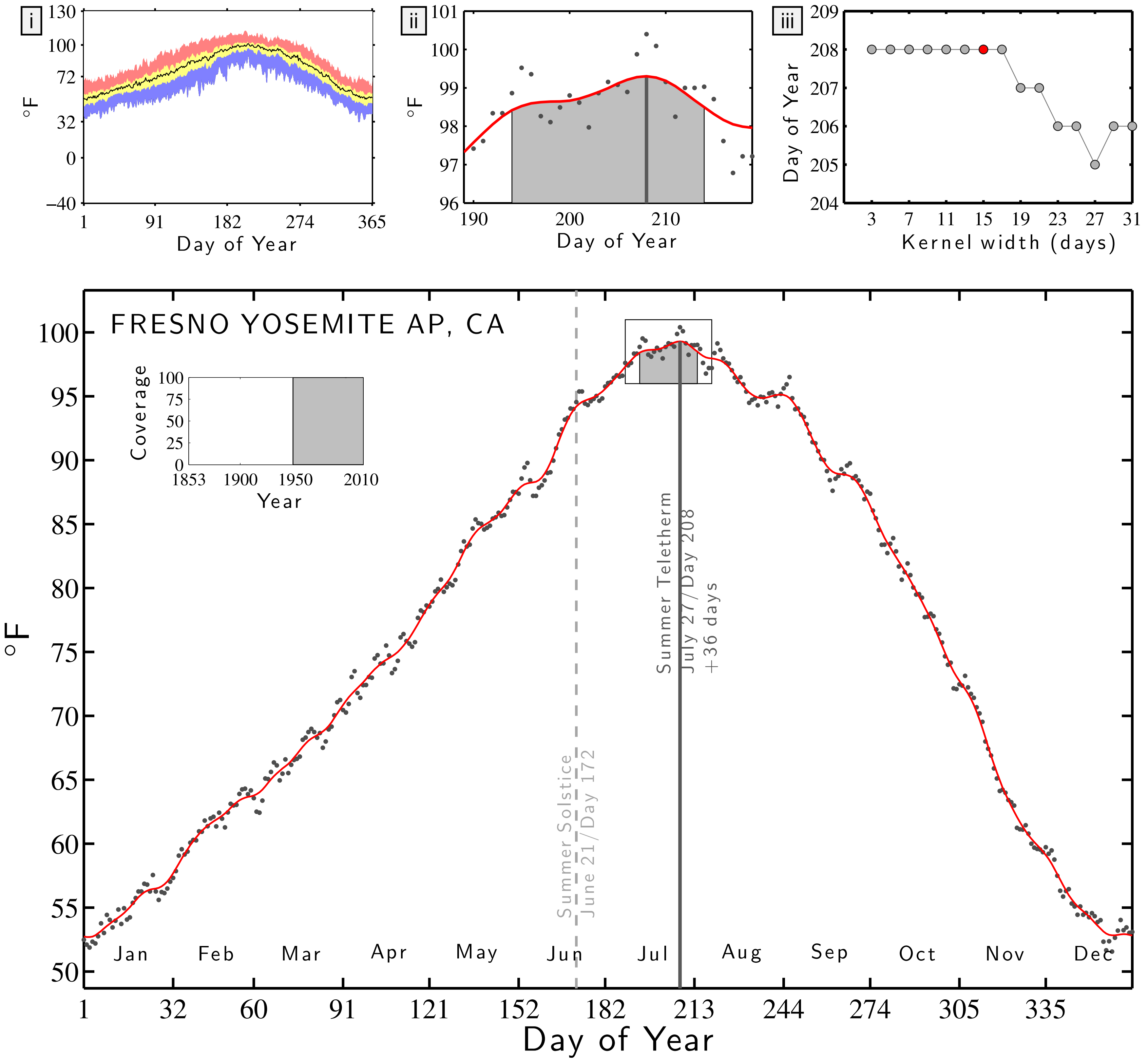

i
ii
iii
°F
Day of Year
Day of Year
Kernel width (days)
FRESNO YOSEMITE AP, CA
Coverage
Year
Summer Solstice
June 21/Day 172
Summer Teletherm
July 27/Day 208
+36 days
Jan
Feb
Mar
Apr
May
Jun
Jul
Aug
Sep
Oct
Nov
Dec

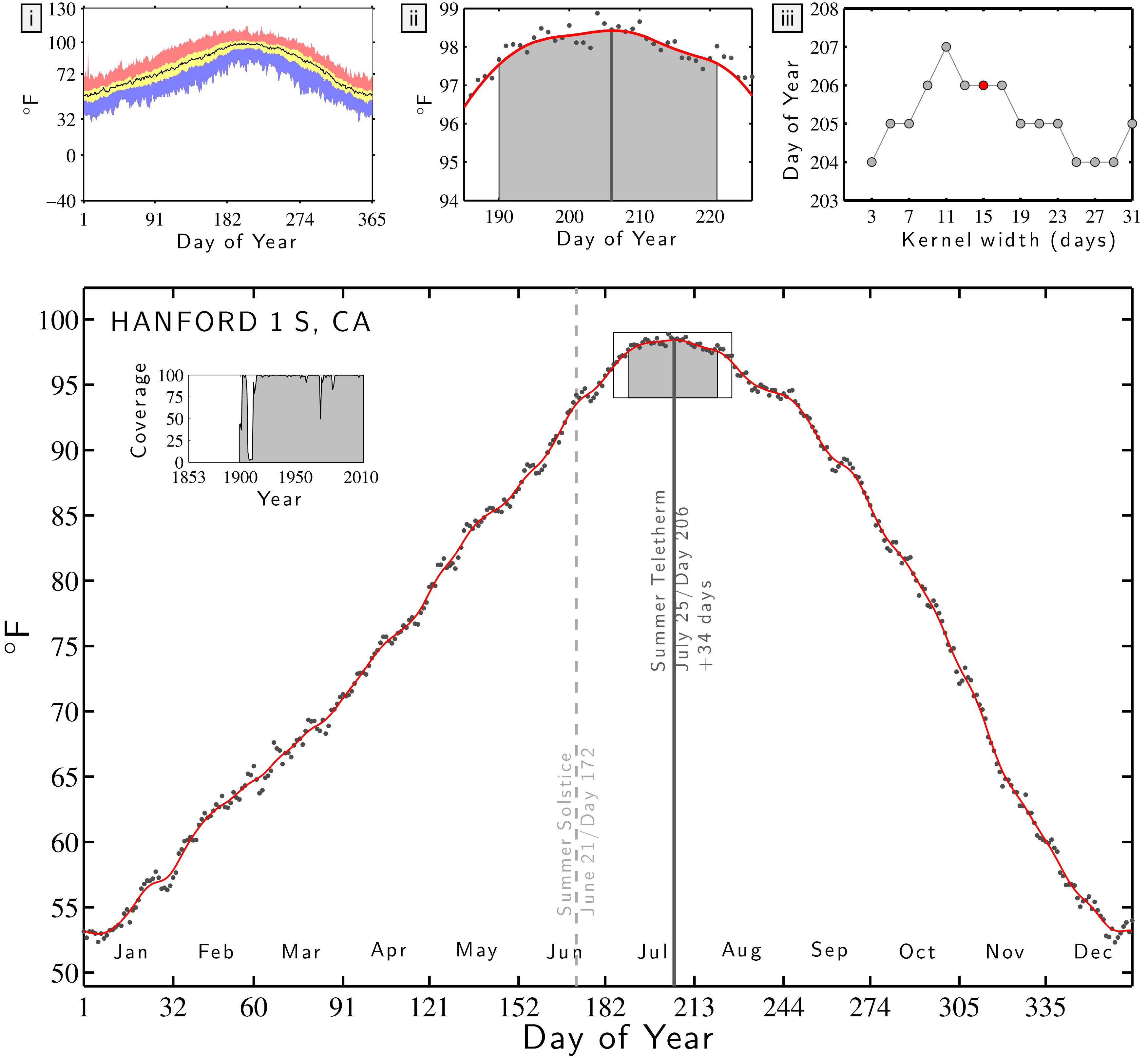

i
°F
130
100
72
32
0
−40
1
91
182
274
365
Day of Year
ii
99
98
97
96
95
94
190
200
210
220
Day of Year
iii
Day of Year
208
207
206
205
204
203
3
7
11
15
19
23
27
31
Kernel width (days)
HANFORD 1 S, CA
Coverage
100
75
50
25
0
1853
1900
1950
2010
Year
Summer Solstice
June 21/Day 172
Summer Teletherm
July 25/Day 206
+34 days
Jan
Feb
Mar
Apr
May
Jun
Jul
Aug
Sep
Oct
Nov
Dec
100
95
90
85
80
75
70
65
60
55
50
1
32
60
91
121
152
182
213
244
274
305
335
Day of Year

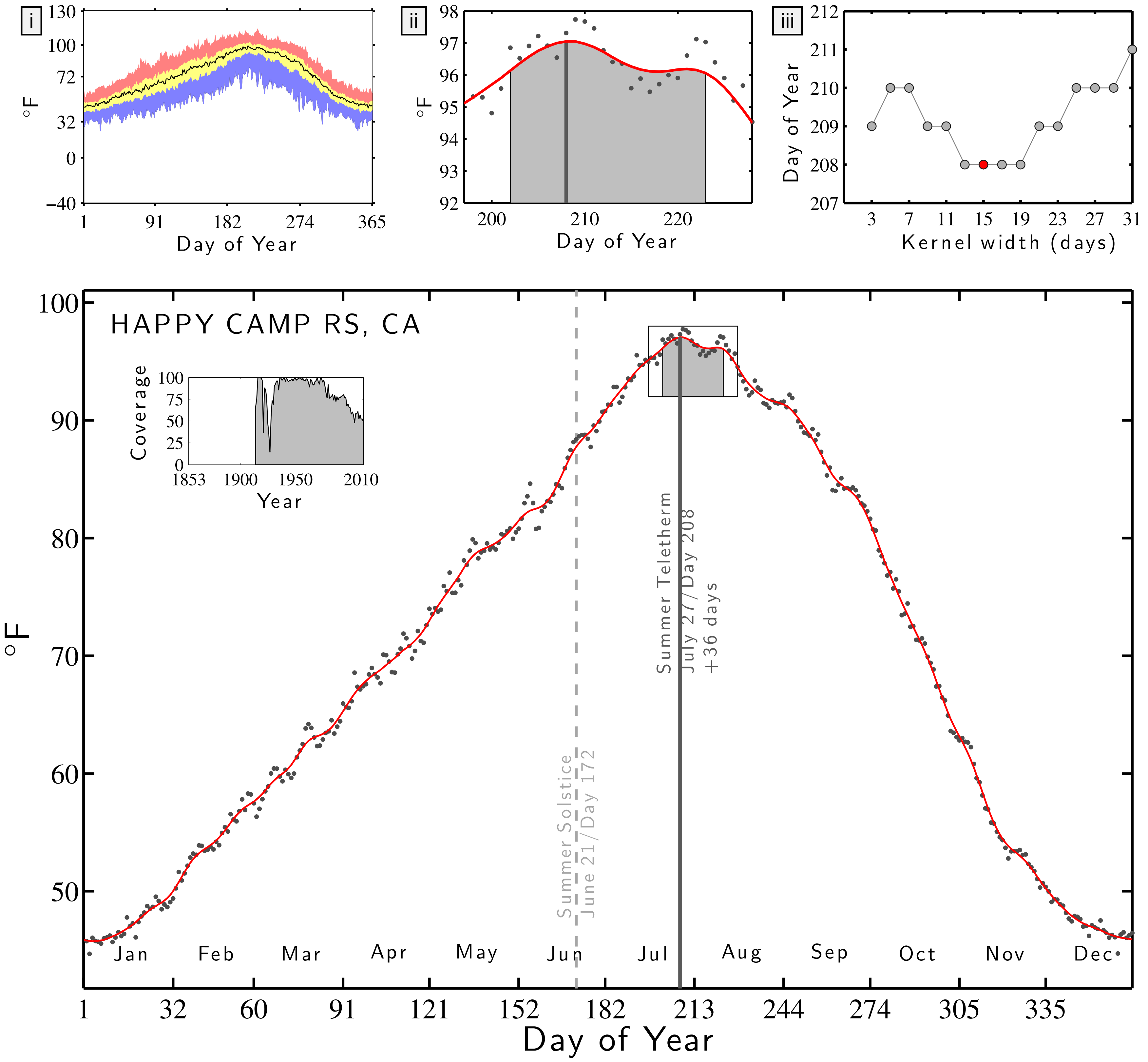

i
ii
iii
Day of Year
Kernel width (days)
HAPPY CAMP RS, CA
Coverage
Year
Summer Solstice
June 21/Day 172
Summer Teletherm
July 27/Day 208
+36 days
Jan
Feb
Mar
Apr
May
Jun
Jul
Aug
Sep
Oct
Nov
Dec
°F
Day of Year

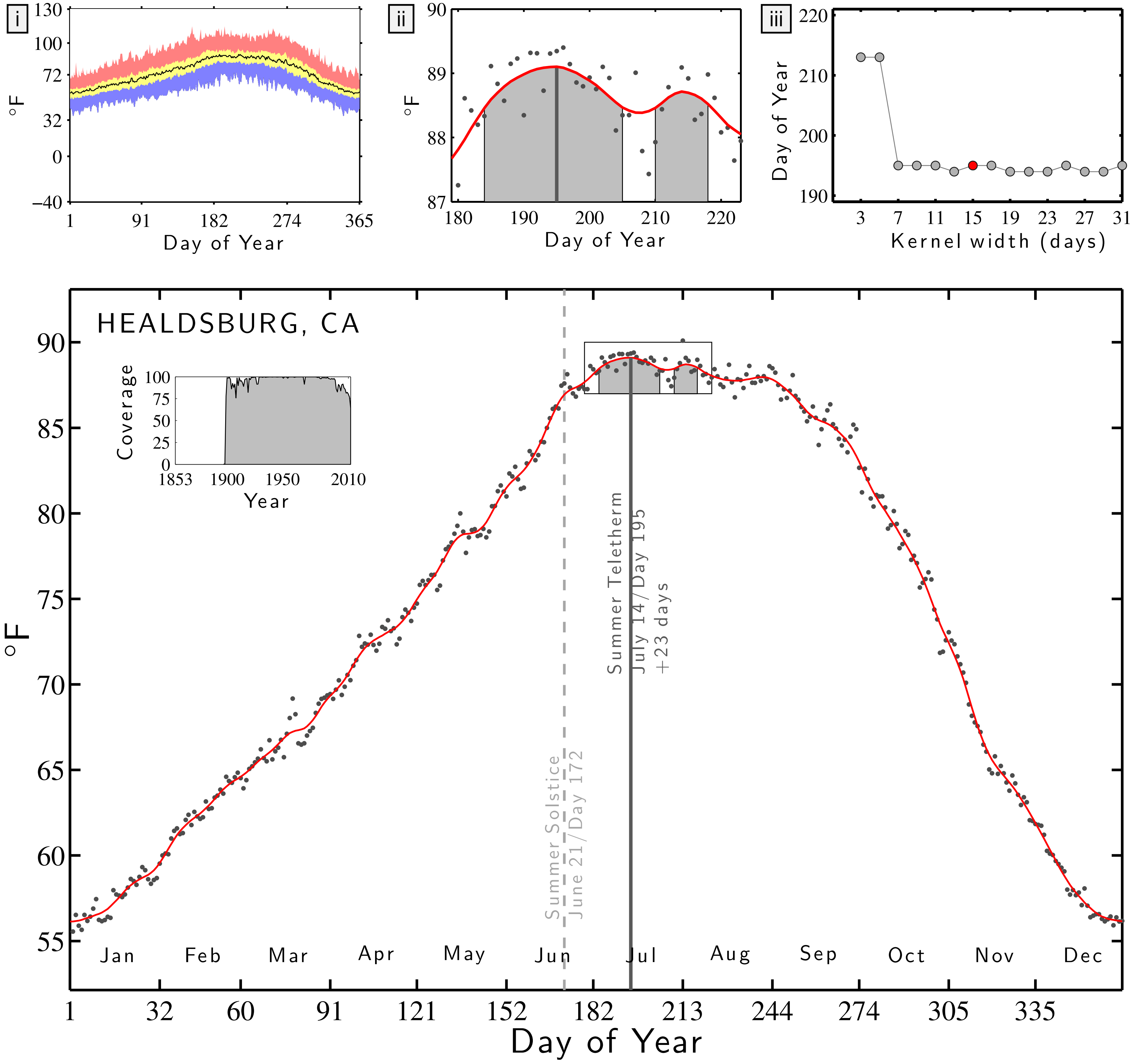

i
°F
Day of Year
ii
°F
Day of Year
iii
Day of Year
Kernel width (days)
HEALDSBURG, CA
Coverage
Year
Summer Solstice
June 21/Day 172
Summer Teletherm
July 14/Day 195
+23 days
Jan
Feb
Mar
Apr
May
Jun
Jul
Aug
Sep
Oct
Nov
Dec
°F
Day of Year

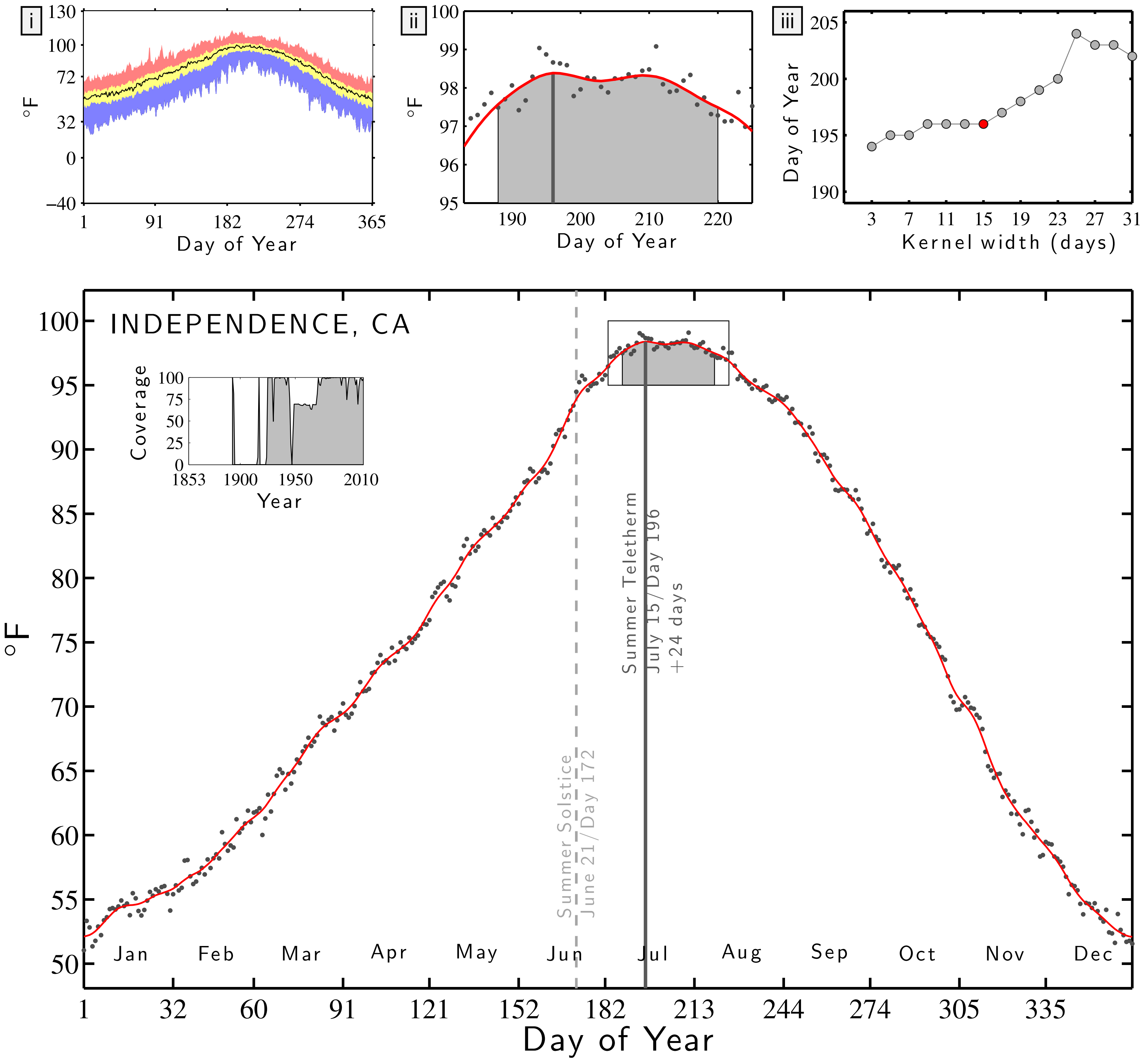

i
°F
Day of Year
ii
Day of Year
iii
Day of Year
Kernel width (days)
INDEPENDENCE, CA
Coverage
Year
Summer Solstice
June 21/Day 172
Summer Teletherm
July 15/Day 196
+24 days
Jan
Feb
Mar
Apr
May
Jun
Jul
Aug
Sep
Oct
Nov
Dec
Day of Year

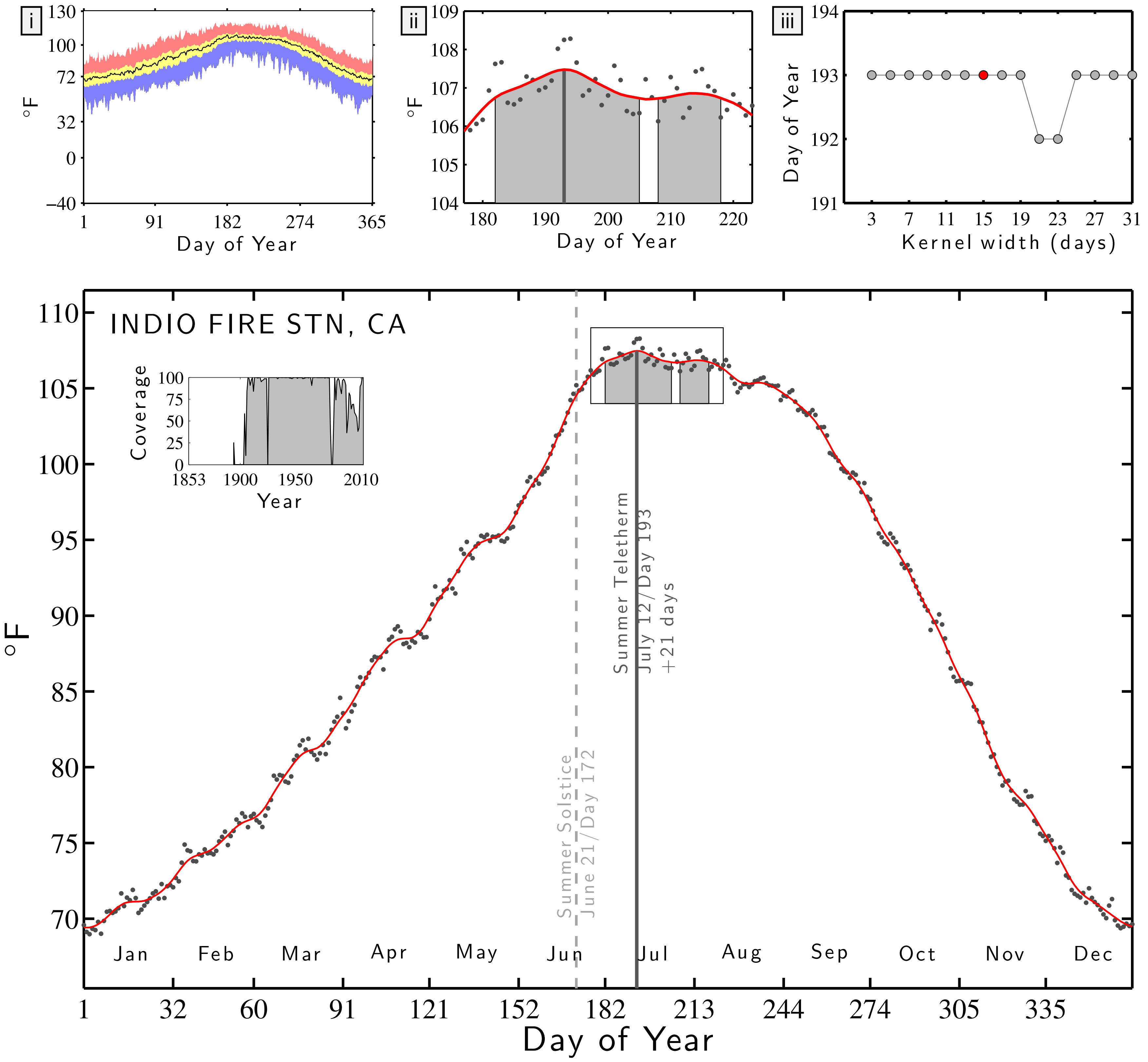
i
°F
Day of Year
ii
°F
Day of Year
iii
Day of Year
Kernel width (days)
INDIO FIRE STN, CA
Coverage
Year
Summer Solstice
June 21/Day 172
Summer Teletherm
July 12/Day 193
+21 days
Jan
Feb
Mar
Apr
May
Jun
Jul
Aug
Sep
Oct
Nov
Dec
°F
Day of Year

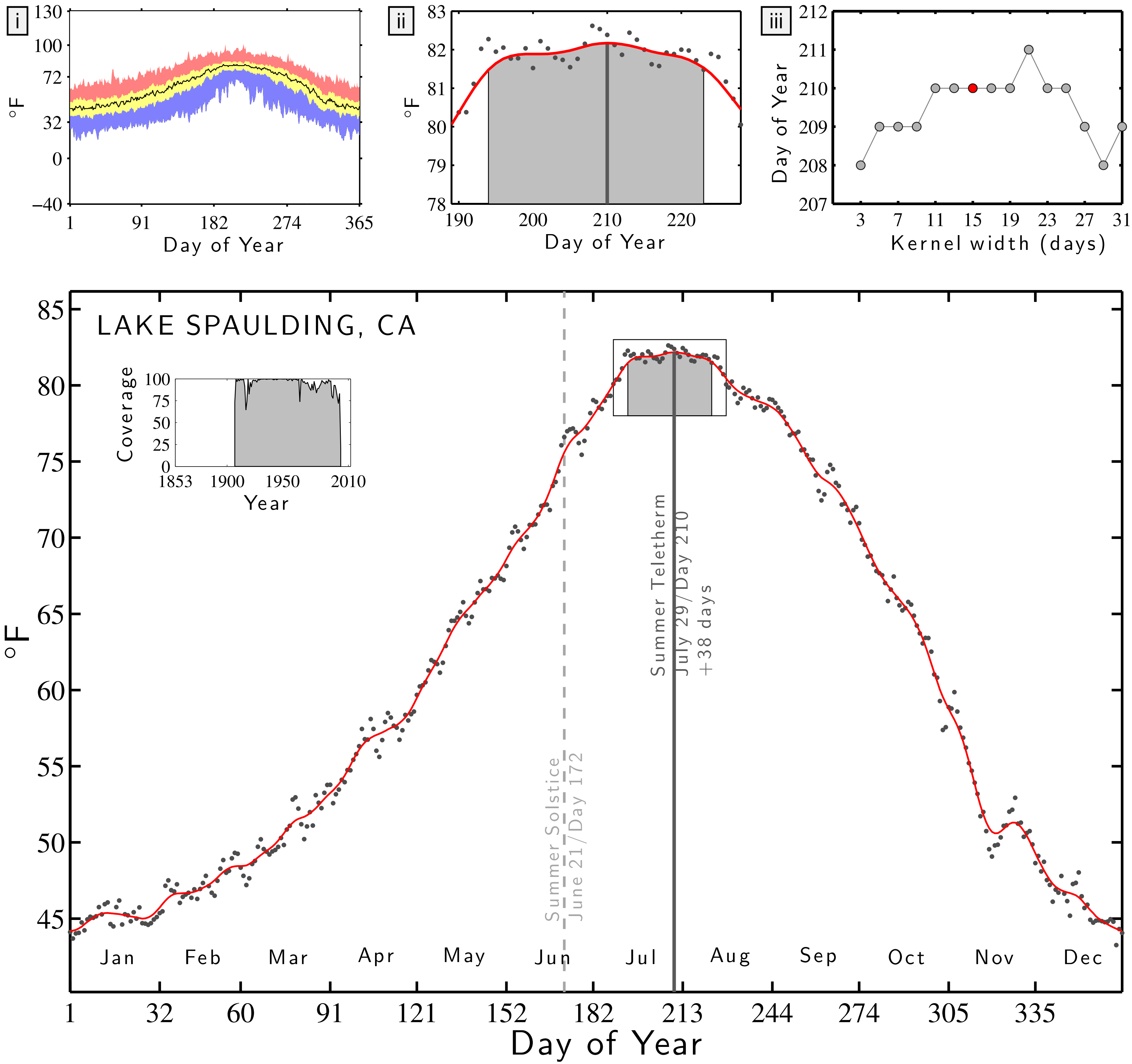

i
°F
Day of Year
ii
°F
Day of Year
iii
Day of Year
Kernel width (days)
LAKE SPAULDING, CA
Coverage
Year
Summer Solstice
June 21 / Day 172
Summer Teletherm
July 29 / Day 210
+38 days
Jan
Feb
Mar
Apr
May
Jun
Jul
Aug
Sep
Oct
Nov
Dec
°F
Day of Year

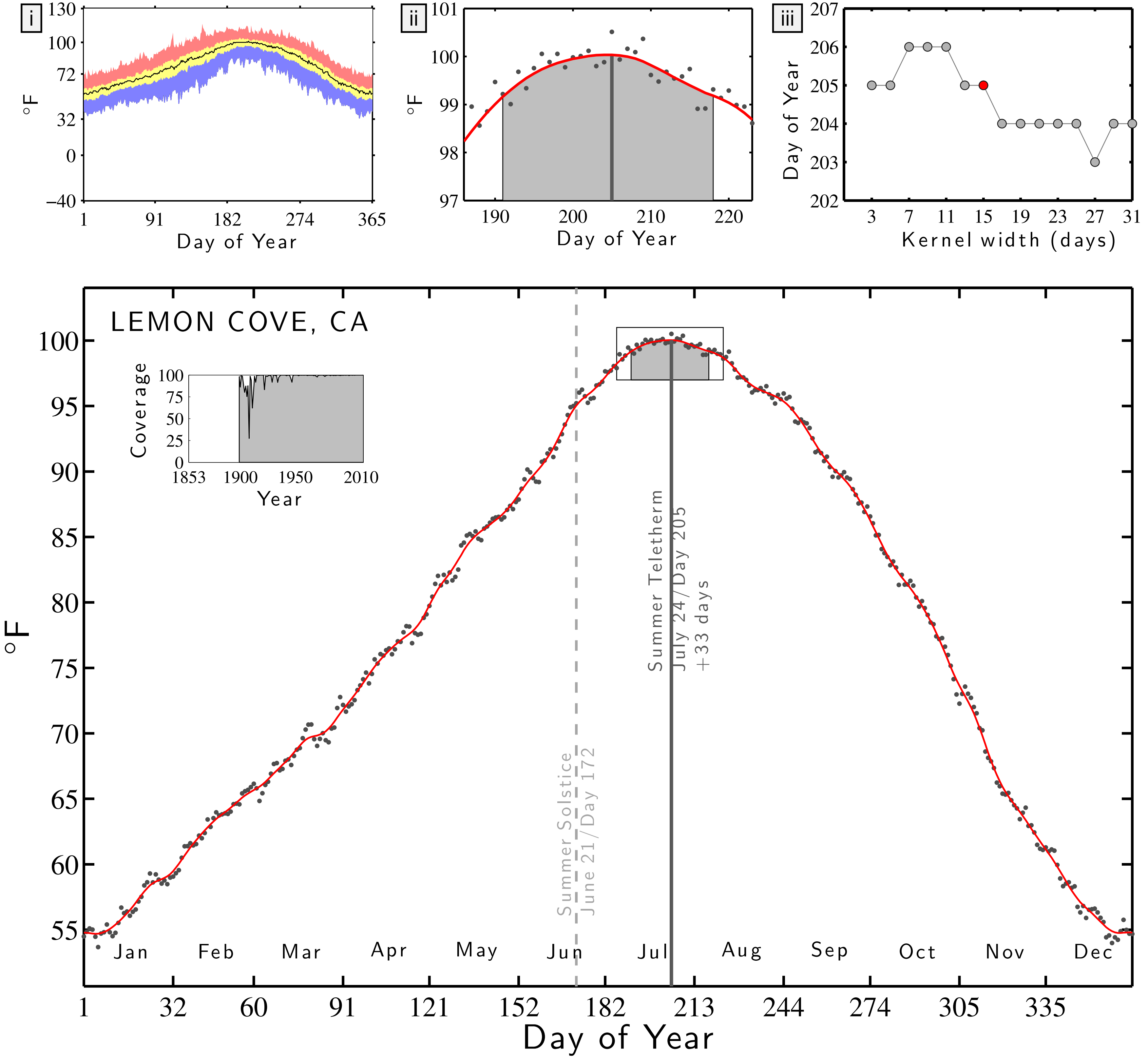

i
°F
Day of Year
ii
°F
Day of Year
iii
Day of Year
Kernel width (days)
LEMON COVE, CA
Coverage
Year
Summer Solstice
June 21/Day 172
Summer Teletherm
July 24/Day 205
+33 days
Jan
Feb
Mar
Apr
May
Jun
Jul
Aug
Sep
Oct
Nov
Dec
°F
Day of Year

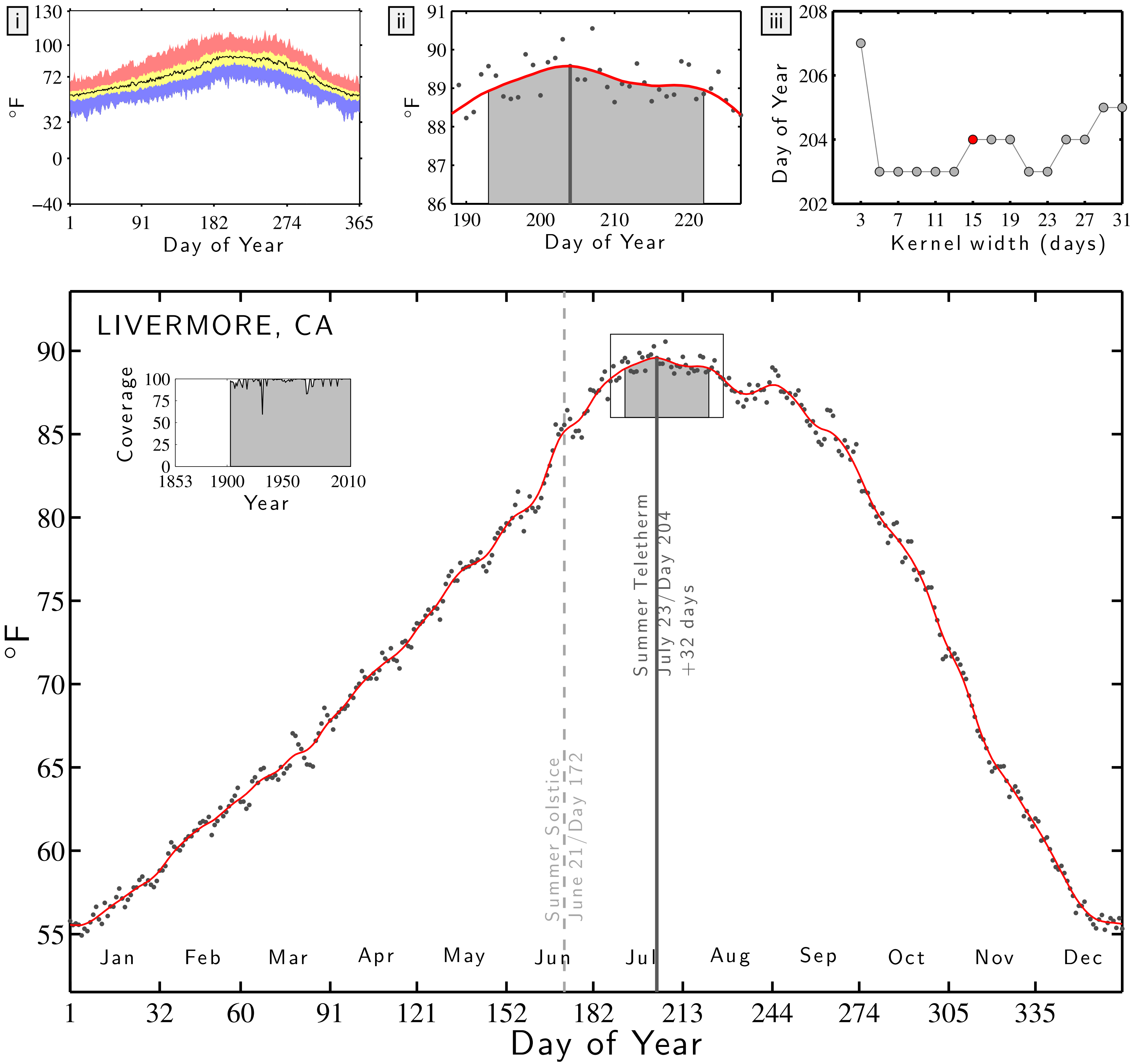

i
°F
Day of Year
ii
°F
Day of Year
iii
Day of Year
Kernel width (days)
LIVERMORE, CA
Coverage
Year
Summer Solstice
June 21 / Day 172
Summer Teletherm
July 23 / Day 204
+32 days
Jan
Feb
Mar
Apr
May
Jun
Jul
Aug
Sep
Oct
Nov
Dec
°F
Day of Year

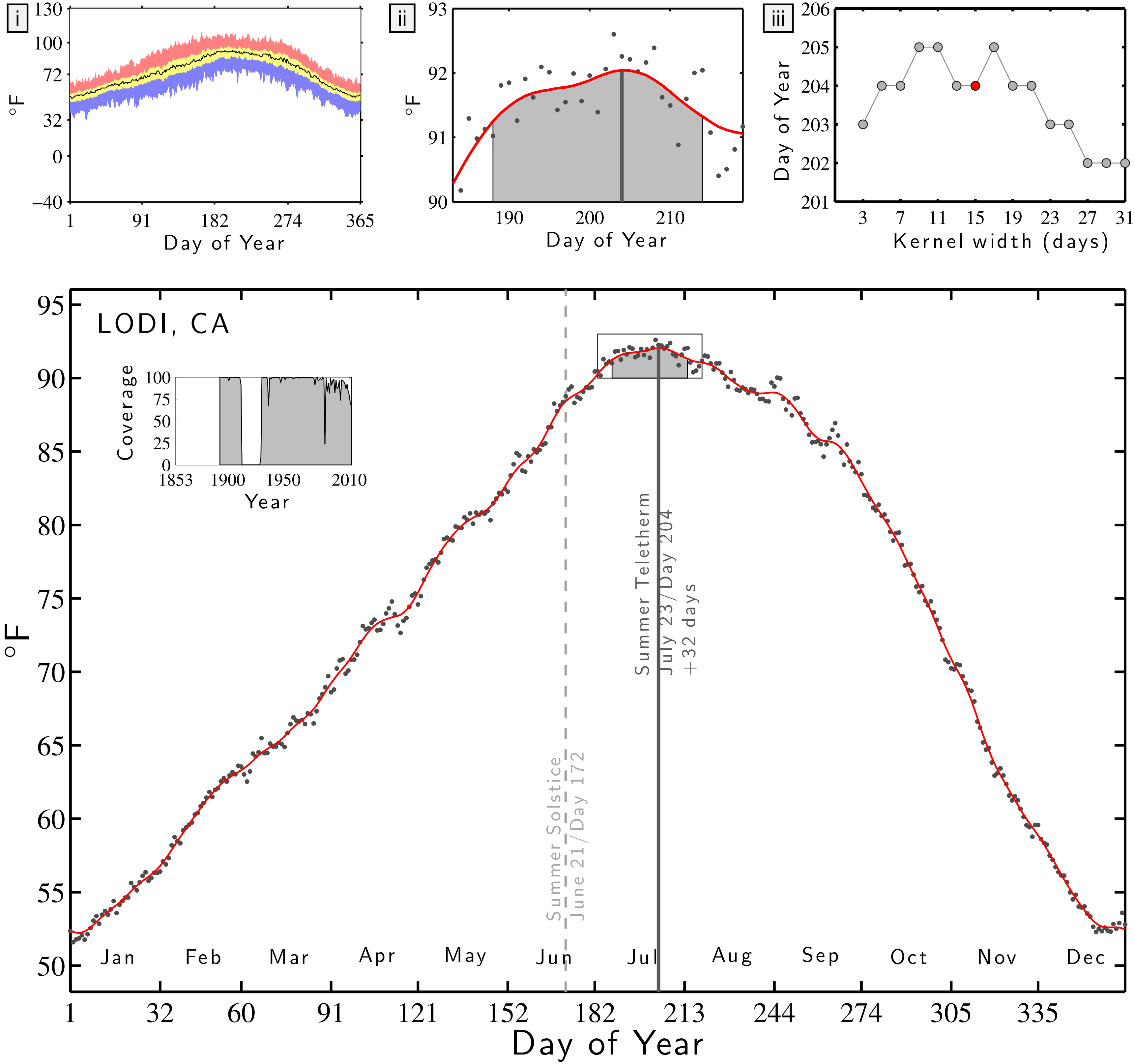

i
°F
Day of Year
ii
°F
Day of Year
iii
Day of Year
Kernel width (days)
LODI, CA
Coverage
Year
Summer Solstice
June 21 / Day 172
Summer Teletherm
July 23 / Day 204
+32 days
Jan
Feb
Mar
Apr
May
Jun
Jul
Aug
Sep
Oct
Nov
Dec
°F
Day of Year

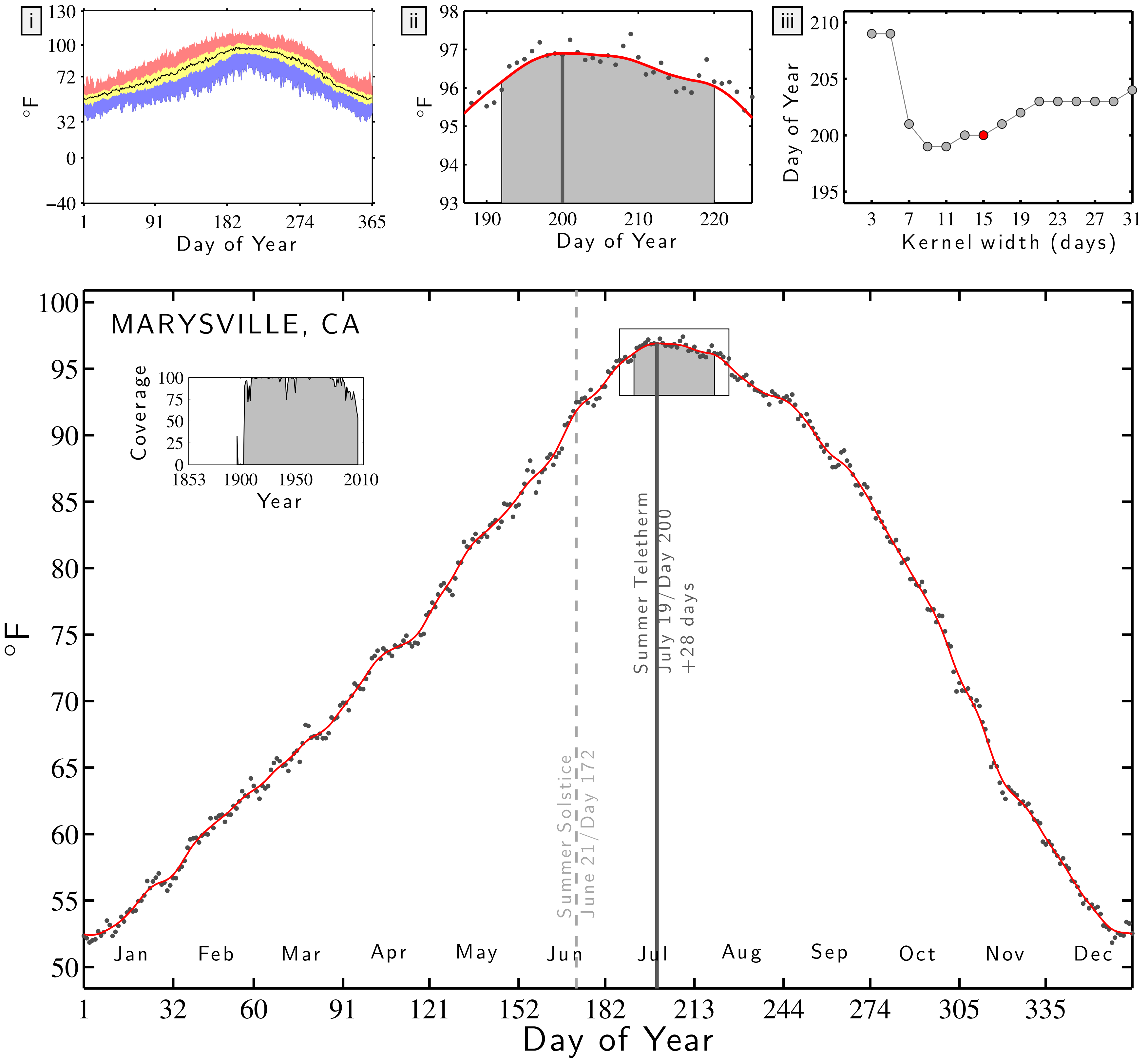

i
°F
Day of Year
ii
°F
Day of Year
iii
Day of Year
Kernel width (days)
MARYSVILLE, CA
Coverage
Year
Summer Solstice
June 21/Day 172
Summer Teletherm
July 19/Day 200
+28 days
Jan
Feb
Mar
Apr
May
Jun
Jul
Aug
Sep
Oct
Nov
Dec
°F
Day of Year

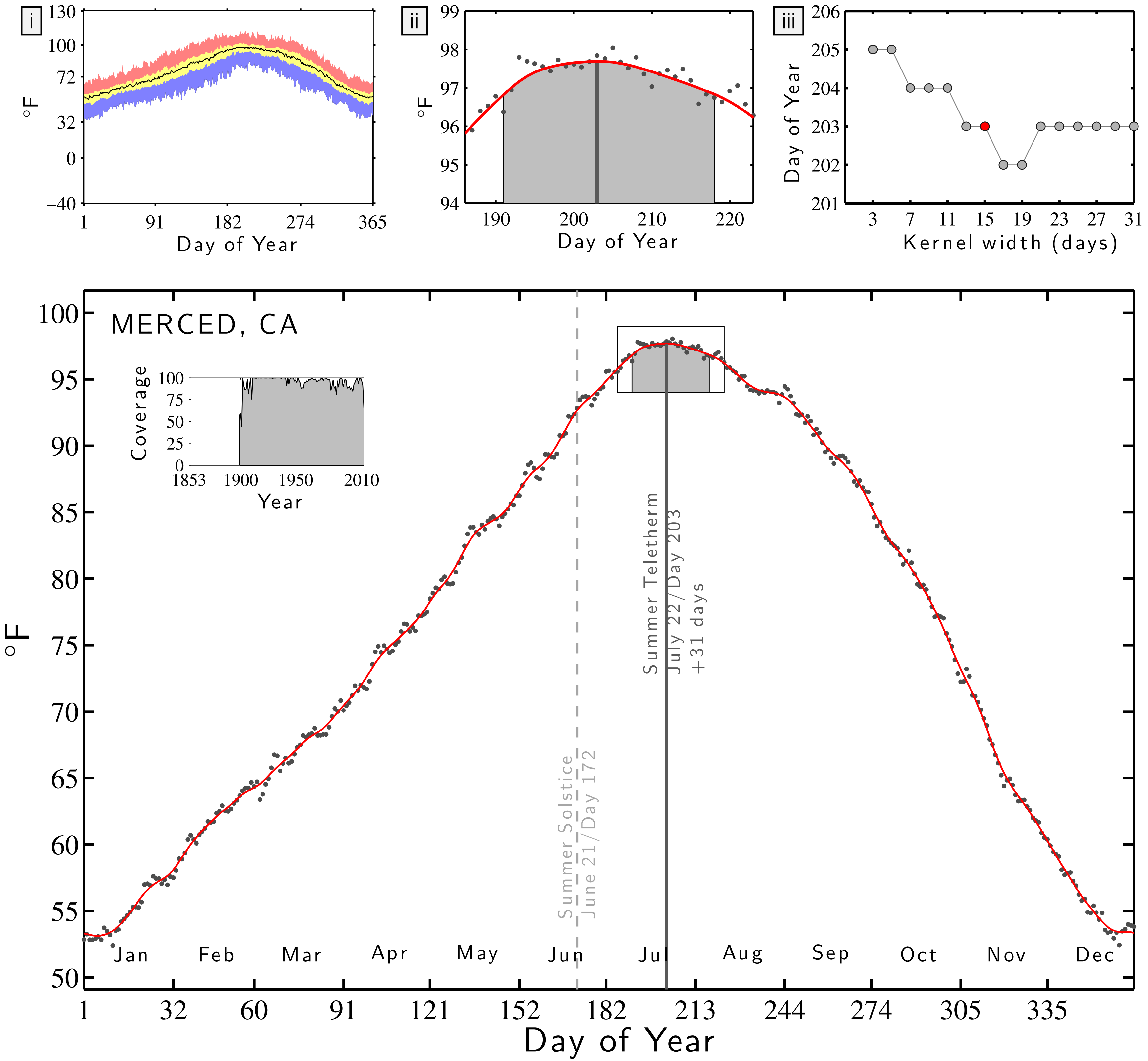

i
°F
Day of Year
ii
°F
Day of Year
iii
Day of Year
Kernel width (days)
MERCED, CA
Coverage
Year
Summer Solstice
June 21/Day 172
Summer Teletherm
July 22/Day 203
+31 days
Jan
Feb
Mar
Apr
May
Jun
Jul
Aug
Sep
Oct
Nov
Dec
°F
Day of Year

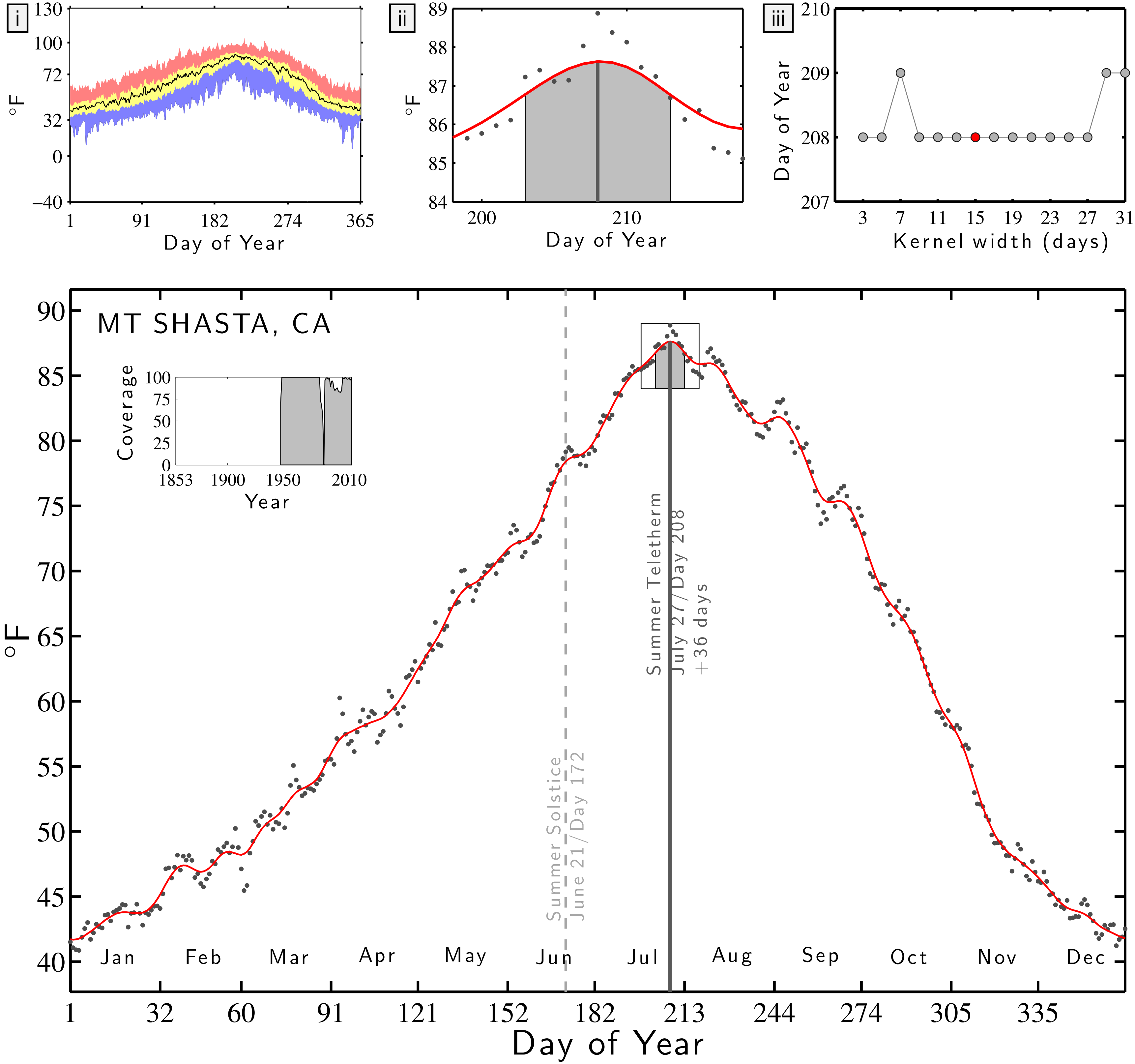

i
°F
Day of Year
ii
°F
Day of Year
iii
Day of Year
Kernel width (days)
MT SHASTA, CA
Coverage
Year
Summer Solstice
June 21/Day 172
Summer Teletherm
July 27/Day 208
+36 days
Jan Feb Mar Apr May Jun Jul Aug Sep Oct Nov Dec
°F
Day of Year

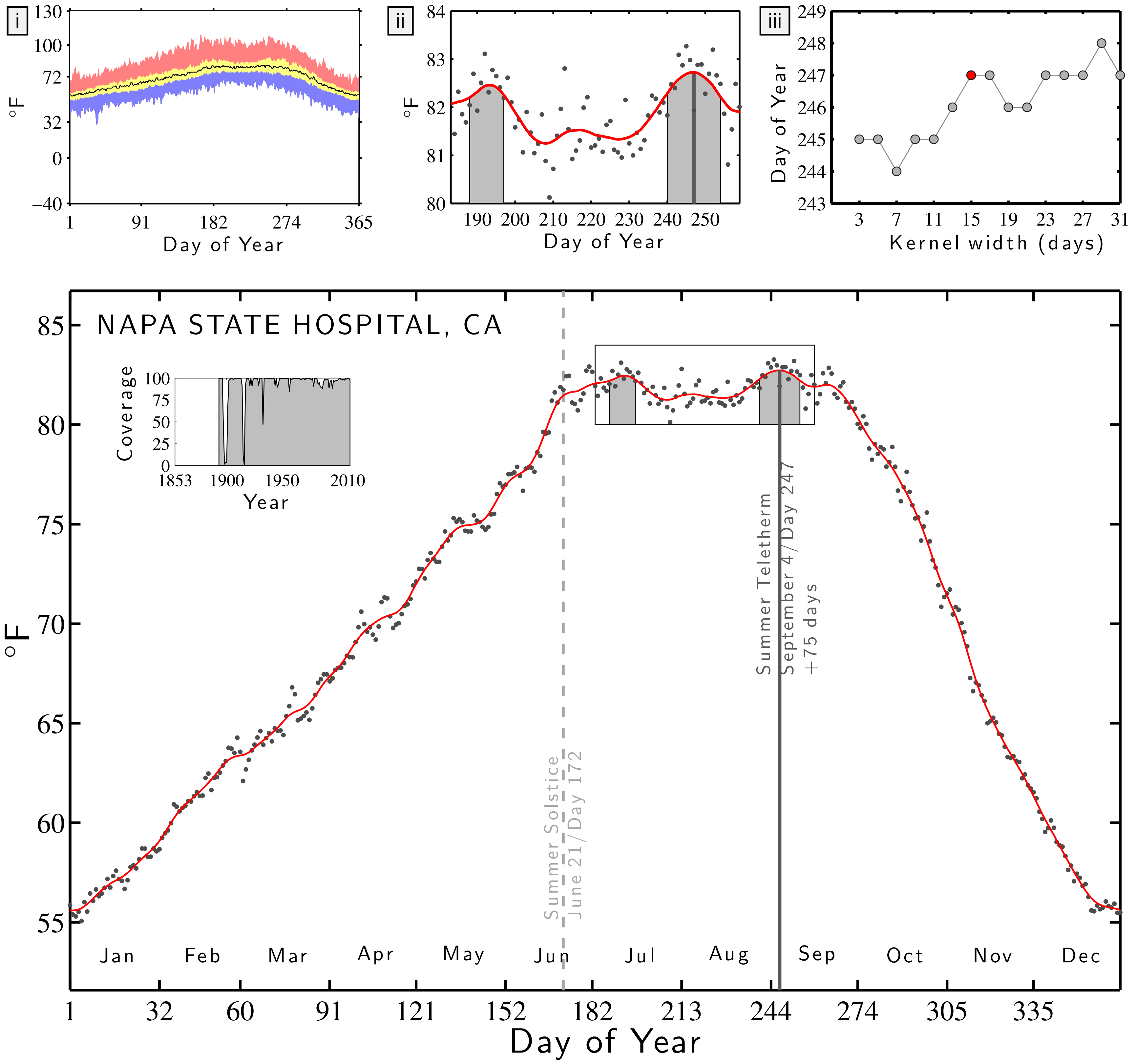

i
°F
Day of Year
ii
Day of Year
iii
Day of Year
Kernel width (days)
NAPA STATE HOSPITAL, CA
Coverage
Year
Summer Solstice
June 21/Day 172
Summer Teletherm
September 4/Day 247
+75 days
Jan
Feb
Mar
Apr
May
Jun
Jul
Aug
Sep
Oct
Nov
Dec
Day of Year

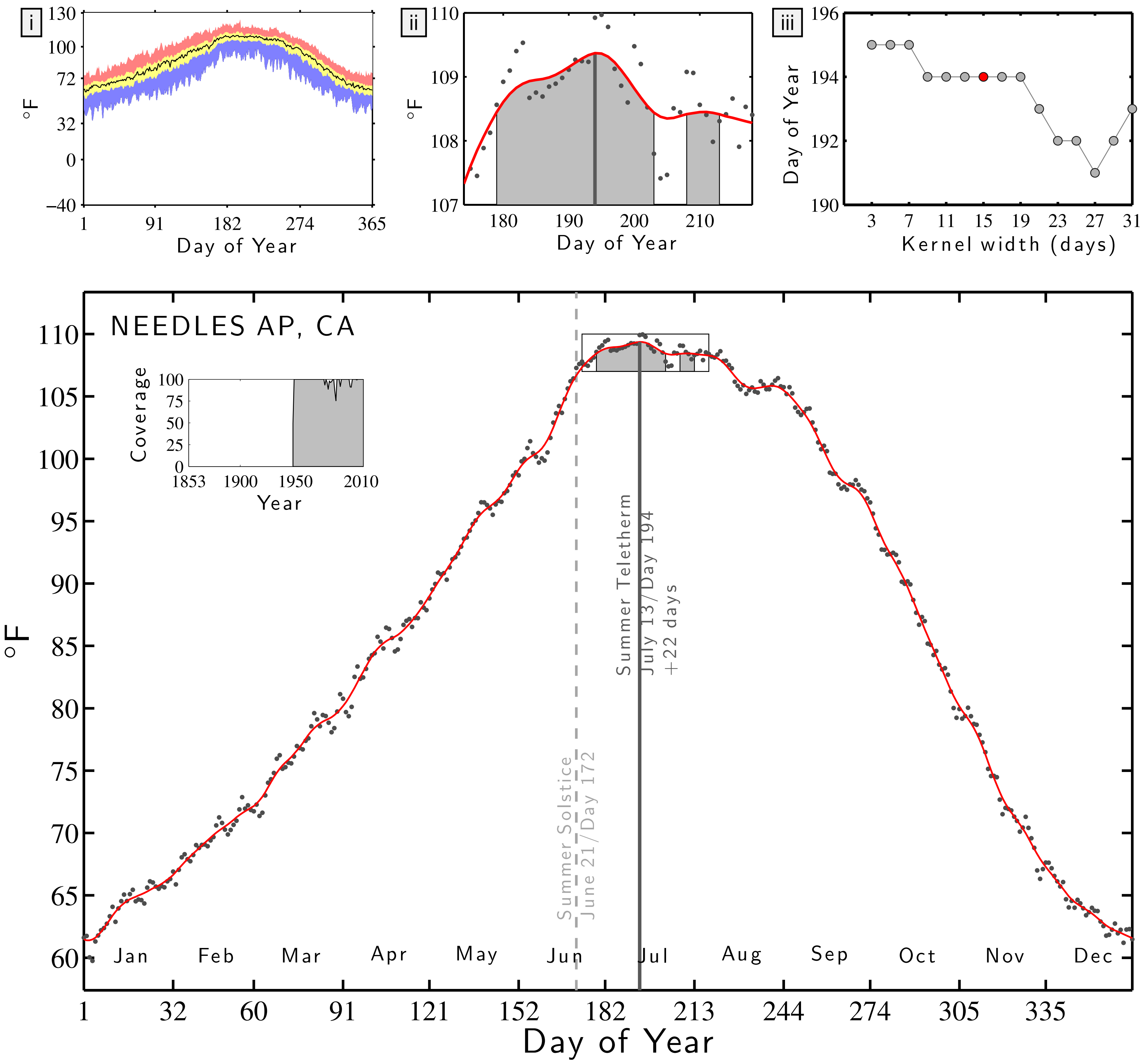

i
ii
iii
°F
Day of Year
Day of Year
Kernel width (days)
NEEDLES AP, CA
Coverage
Year
Summer Solstice
June 21/Day 172
Summer Teletherm
July 13/Day 194
+22 days
Jan
Feb
Mar
Apr
May
Jun
Jul
Aug
Sep
Oct
Nov
Dec

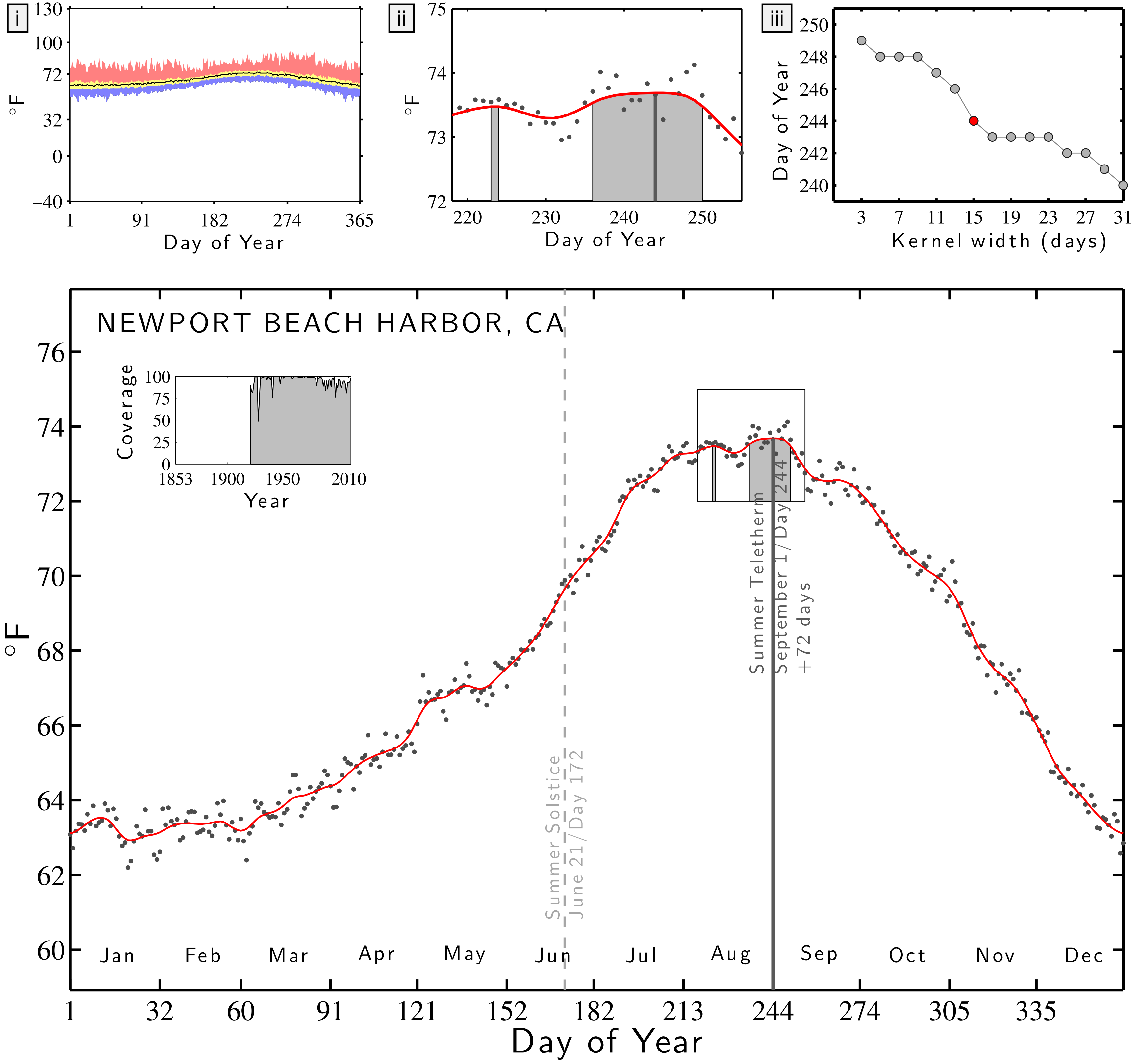

i
°F
130
100
72
32
0
−40
1
91
182
274
365
Day of Year
ii
75
74
73
72
220
230
240
250
Day of Year
iii
Day of Year
250
248
246
244
242
240
3
7
11
15
19
23
27
31
Kernel width (days)
NEWPORT BEACH HARBOR, CA
Coverage
100
75
50
25
0
1853
1900
1950
2010
Year
76
74
72
70
68
66
64
62
60
Summer Solstice
June 21 / Day 172
Summer Teletherm
September 1 / Day 244
+72 days
Jan
Feb
Mar
Apr
May
Jun
Jul
Aug
Sep
Oct
Nov
Dec
1
32
60
91
121
152
182
213
244
274
305
335
Day of Year

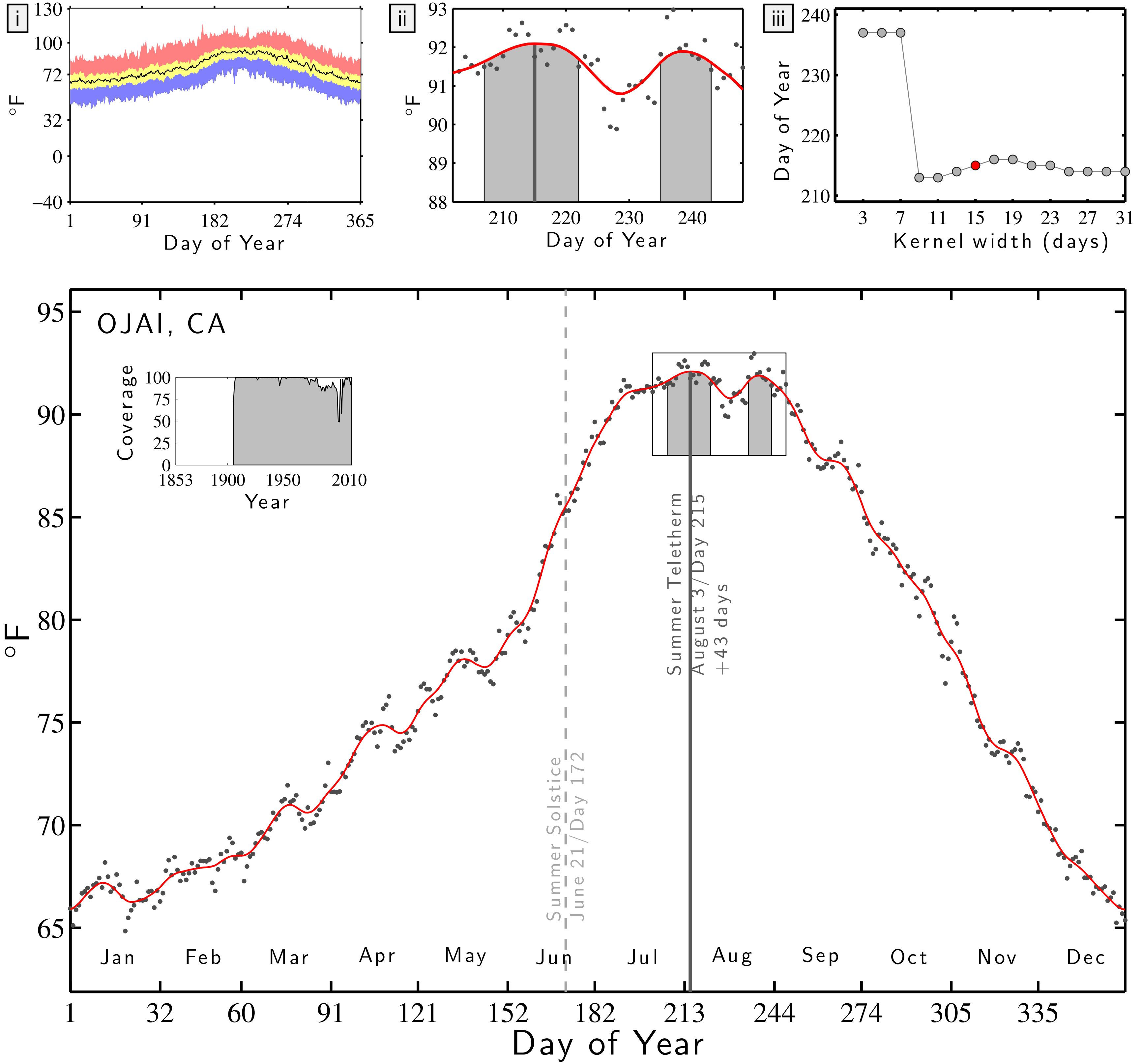

i
ii
iii
°F
Day of Year
Day of Year
Kernel width (days)
OJAI, CA
Coverage
Year
Summer Solstice
June 21 / Day 172
Summer Teletherm
August 3 / Day 215
+43 days
Jan
Feb
Mar
Apr
May
Jun
Jul
Aug
Sep
Oct
Nov
Dec
°F
Day of Year

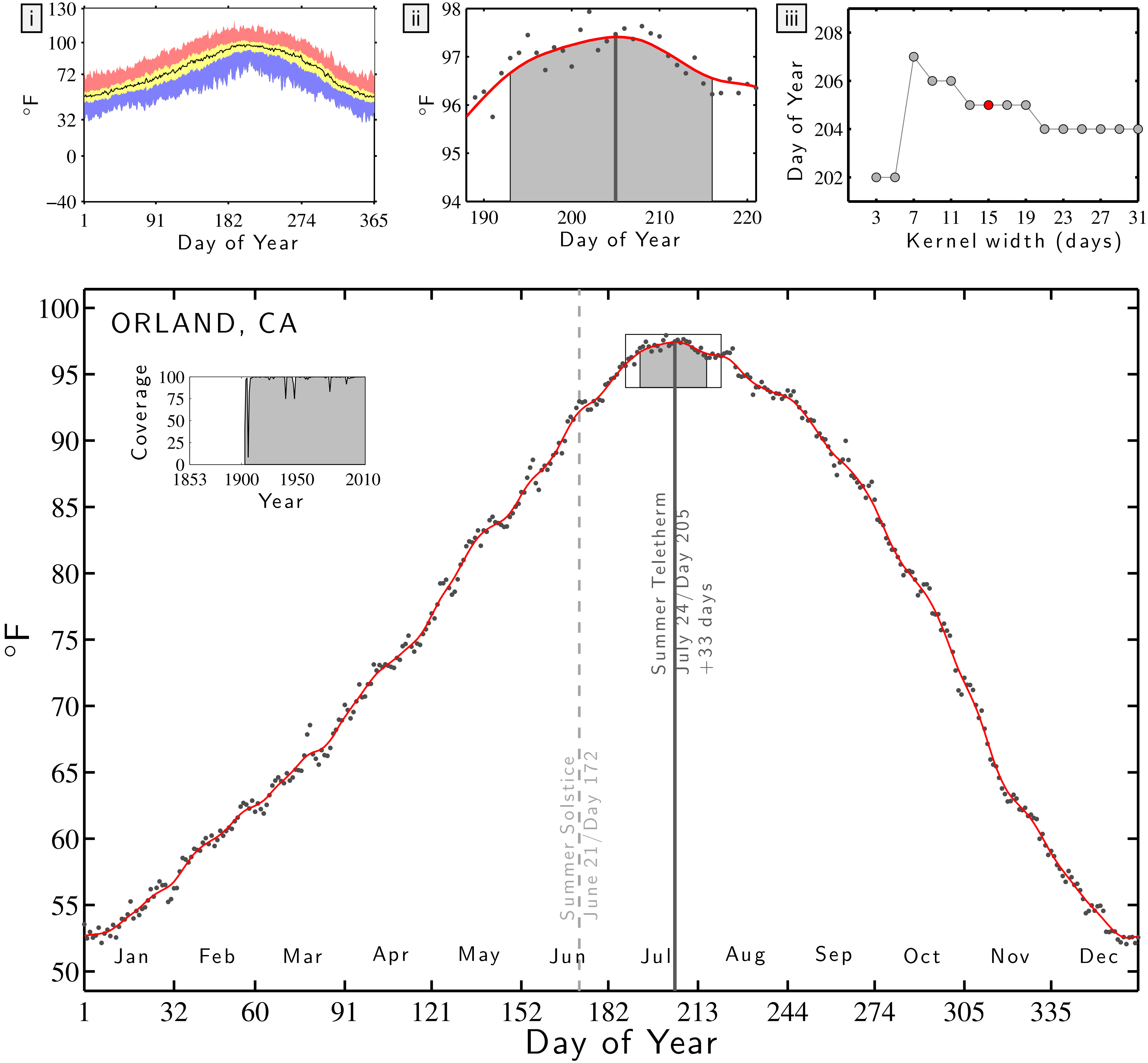

i
°F
Day of Year
ii
°F
Day of Year
iii
Day of Year
Kernel width (days)
ORLAND, CA
Coverage
Year
Summer Solstice
June 21/Day 172
Summer Teletherm
July 24/Day 205
+33 days
Jan
Feb
Mar
Apr
May
Jun
Jul
Aug
Sep
Oct
Nov
Dec
°F
Day of Year

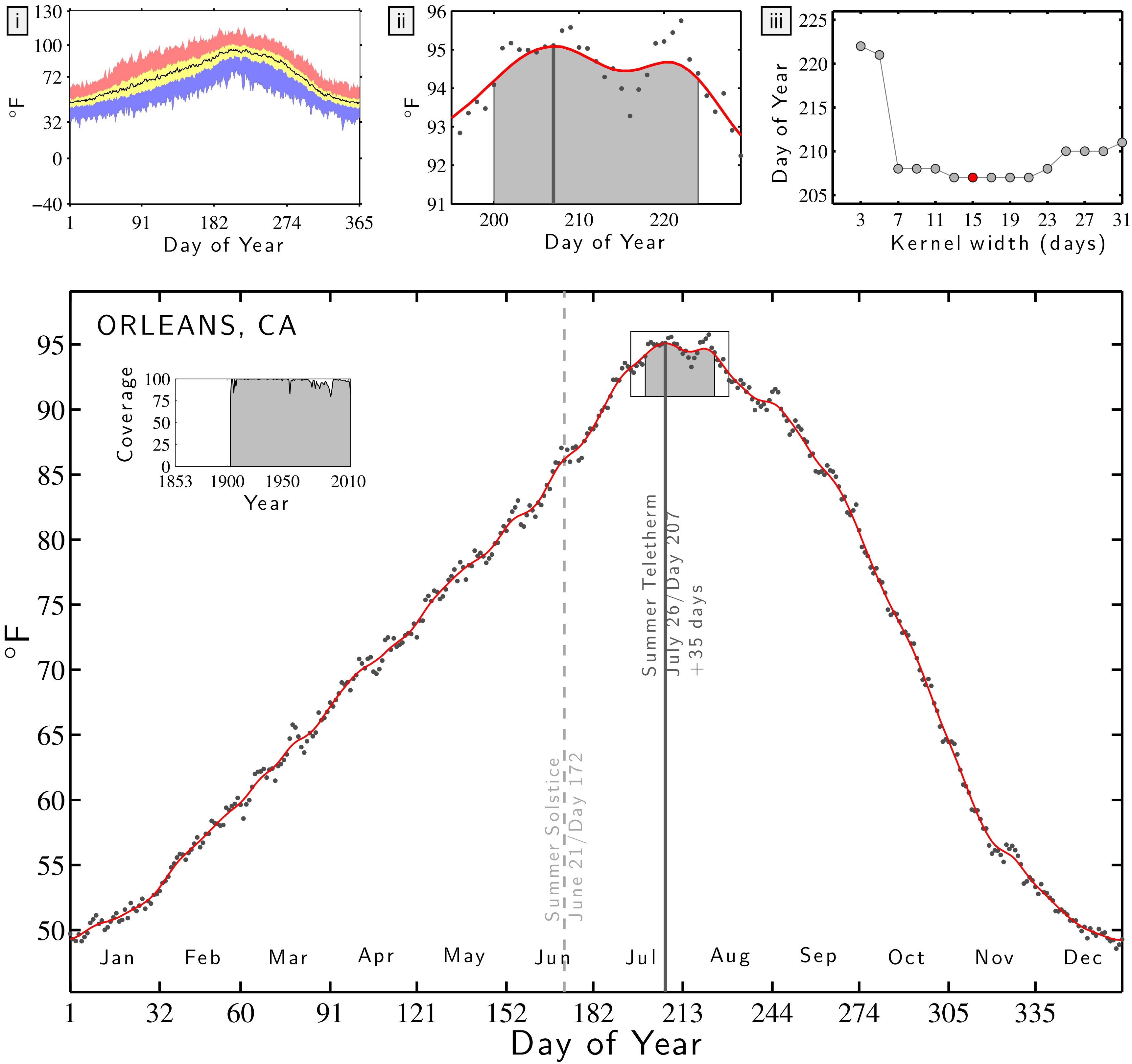

i
°F
Day of Year
ii
°F
Day of Year
iii
Day of Year
Kernel width (days)
ORLEANS, CA
Coverage
Year
Summer Solstice
June 21 / Day 172
Summer Teletherm
July 26 / Day 207
+35 days
Jan
Feb
Mar
Apr
May
Jun
Jul
Aug
Sep
Oct
Nov
Dec
°F
Day of Year

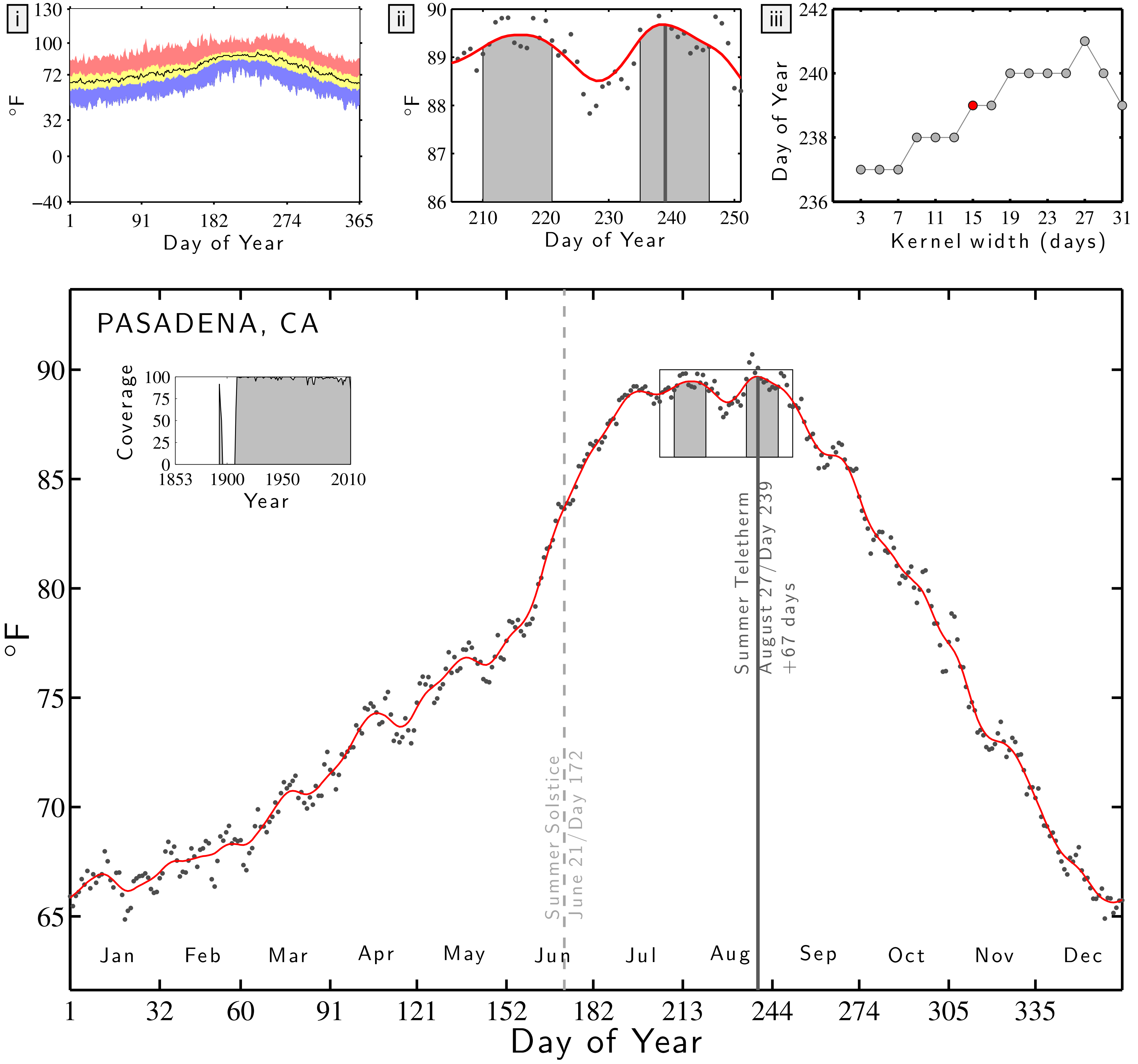

i
°F
Day of Year
ii
°F
Day of Year
iii
Day of Year
Kernel width (days)
PASADENA, CA
Coverage
Year
Summer Solstice
June 21 / Day 172
Summer Teletherm
August 27 / Day 239
+67 days
Jan
Feb
Mar
Apr
May
Jun
Jul
Aug
Sep
Oct
Nov
Dec
°F
Day of Year

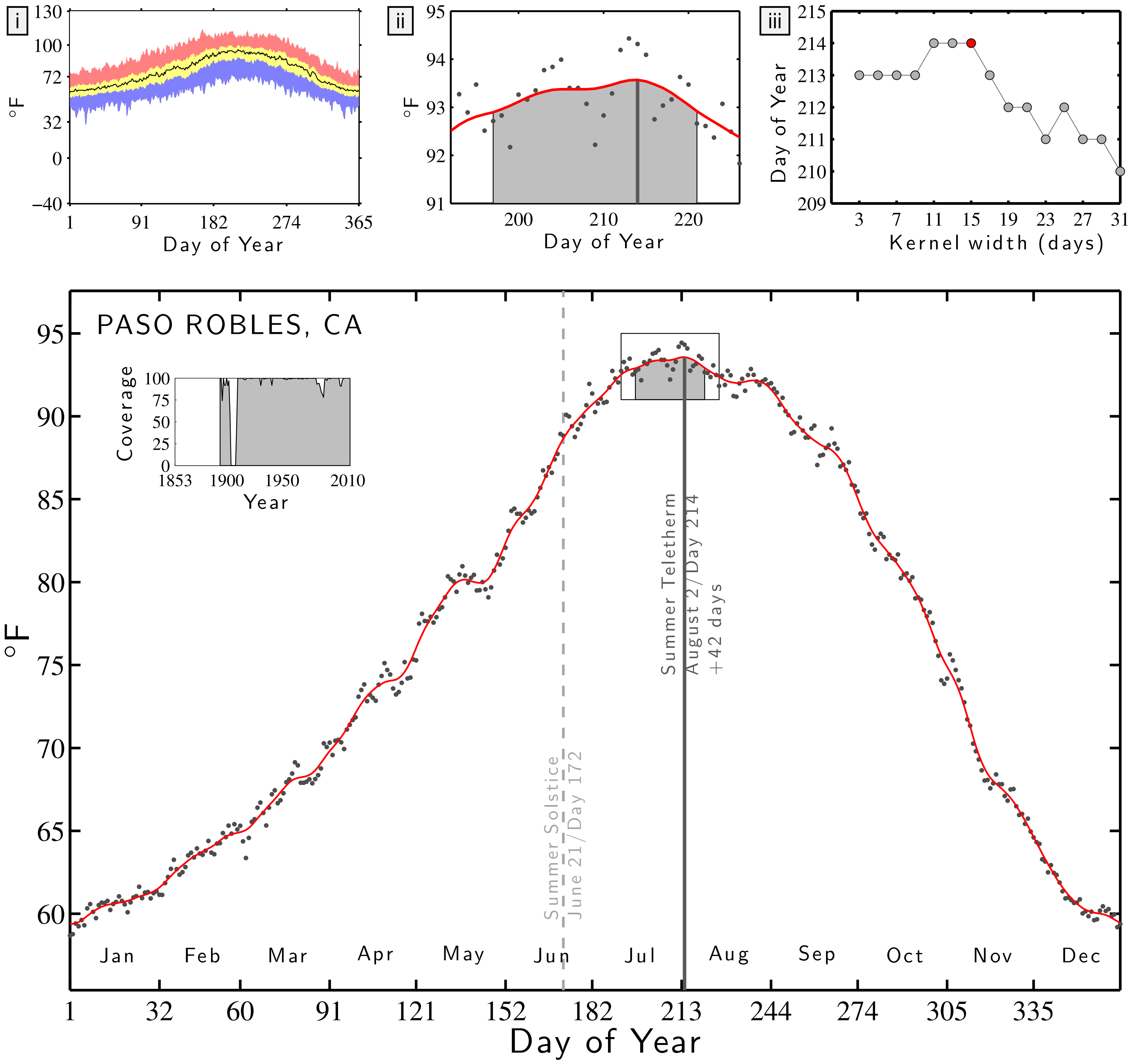

i
°F
Day of Year
ii
°F
Day of Year
iii
Day of Year
Kernel width (days)
PASO ROBLES, CA
Coverage
Year
°F
Summer Solstice
June 21/Day 172
Summer Telethern
August 2/Day 214
+42 days
Jan
Feb
Mar
Apr
May
Jun
Jul
Aug
Sep
Oct
Nov
Dec
Day of Year

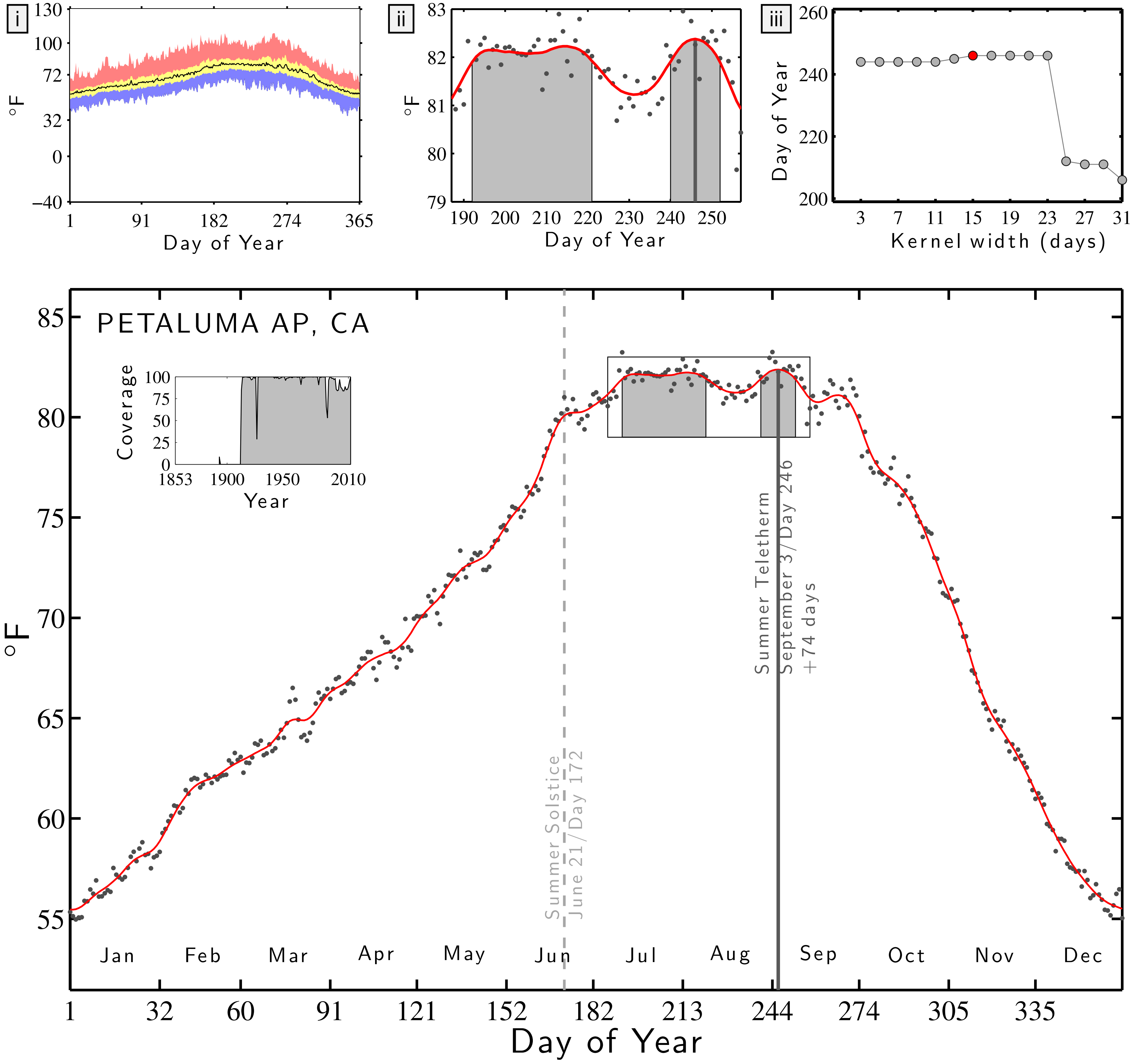

i
ii
iii
PETALUMA AP, CA
Coverage
Year
Summer Solstice
June 21 / Day 172
Summer Teletherm
September 3 / Day 246
+74 days
Kernel width (days)
Day of Year
°F
Jan
Feb
Mar
Apr
May
Jun
Jul
Aug
Sep
Oct
Nov
Dec

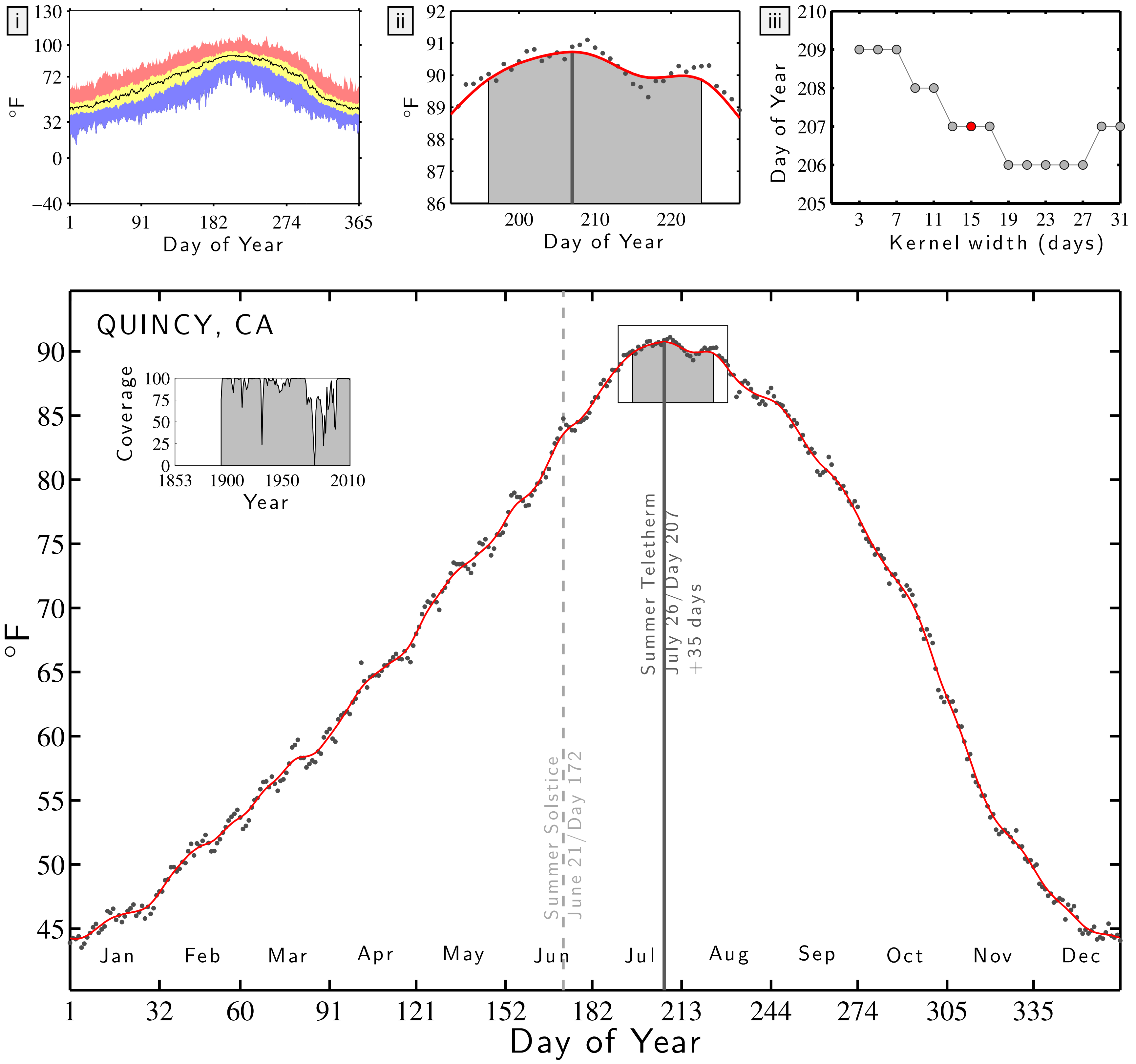

i
ii
iii
°F
Day of Year
Kernel width (days)
QUINCY, CA
Coverage
Year
Summer Solstice
June 21 / Day 172
Summer Teletherm
July 26 / Day 207
+35 days
Jan
Feb
Mar
Apr
May
Jun
Jul
Aug
Sep
Oct
Nov
Dec

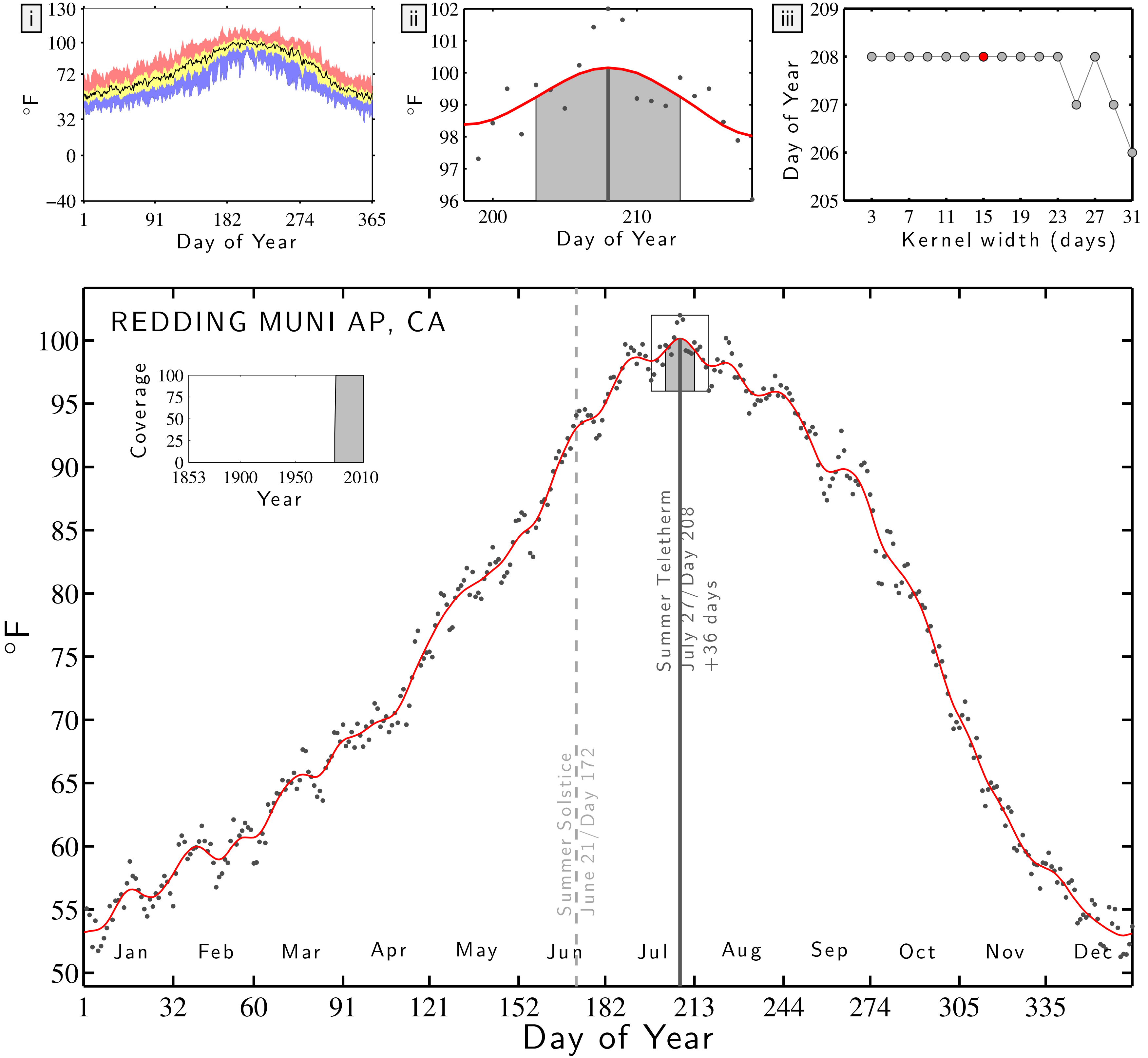

i
ii
iii
Day of Year
Kernel width (days)
°F
REDDING MUNI AP, CA
Coverage
Year
Summer Solstice
June 21 / Day 172
Summer Teletherm
July 27 / Day 208
+36 days
Jan
Feb
Mar
Apr
May
Jun
Jul
Aug
Sep
Oct
Nov
Dec

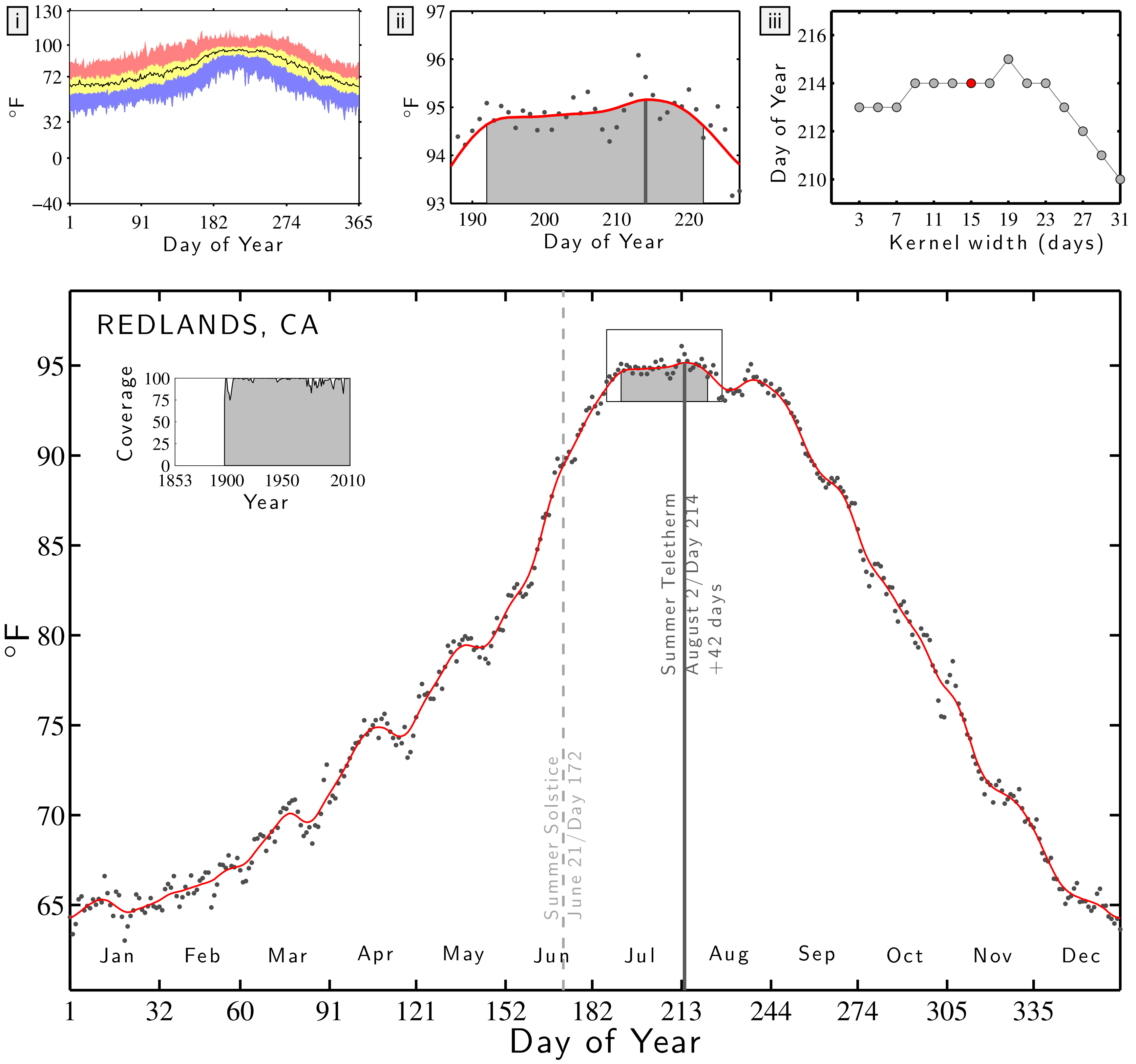
i
°F
Day of Year
ii
°F
Day of Year
iii
Day of Year
Kernel width (days)
REDLANDS, CA
Coverage
Year
Summer Solstice
June 21/Day 172
Summer Teletherm
August 2/Day 214
+42 days
Jan
Feb
Mar
Apr
May
Jun
Jul
Aug
Sep
Oct
Nov
Dec
°F
Day of Year

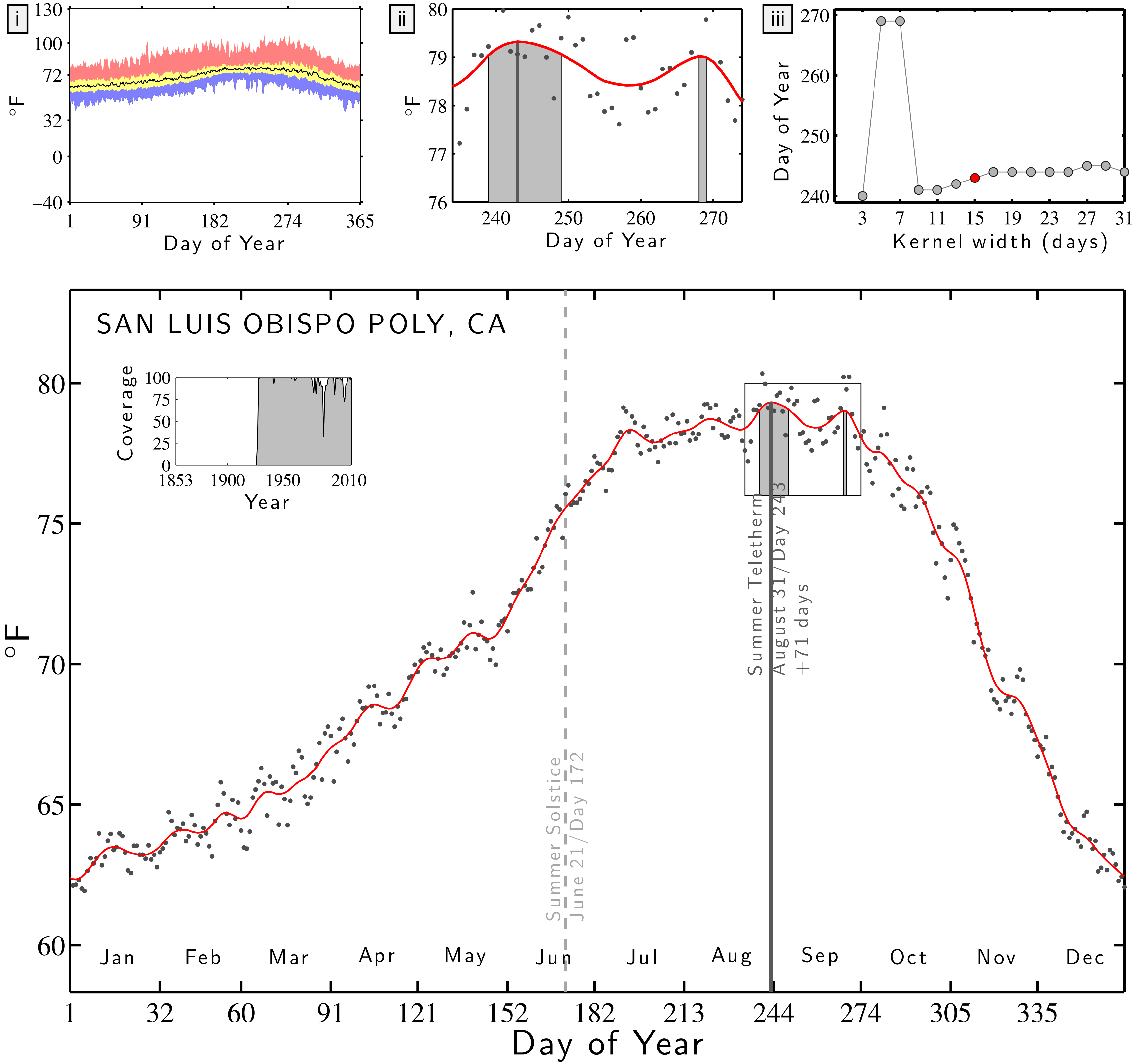

i
ii
iii
Day of Year
°F
Kernel width (days)
SAN LUIS OBISPO POLY, CA
Coverage
Year
Summer Solstice
June 21 / Day 172
Summer Teletherm
August 31 / Day 243
+71 days
Jan
Feb
Mar
Apr
May
Jun
Jul
Aug
Sep
Oct
Nov
Dec

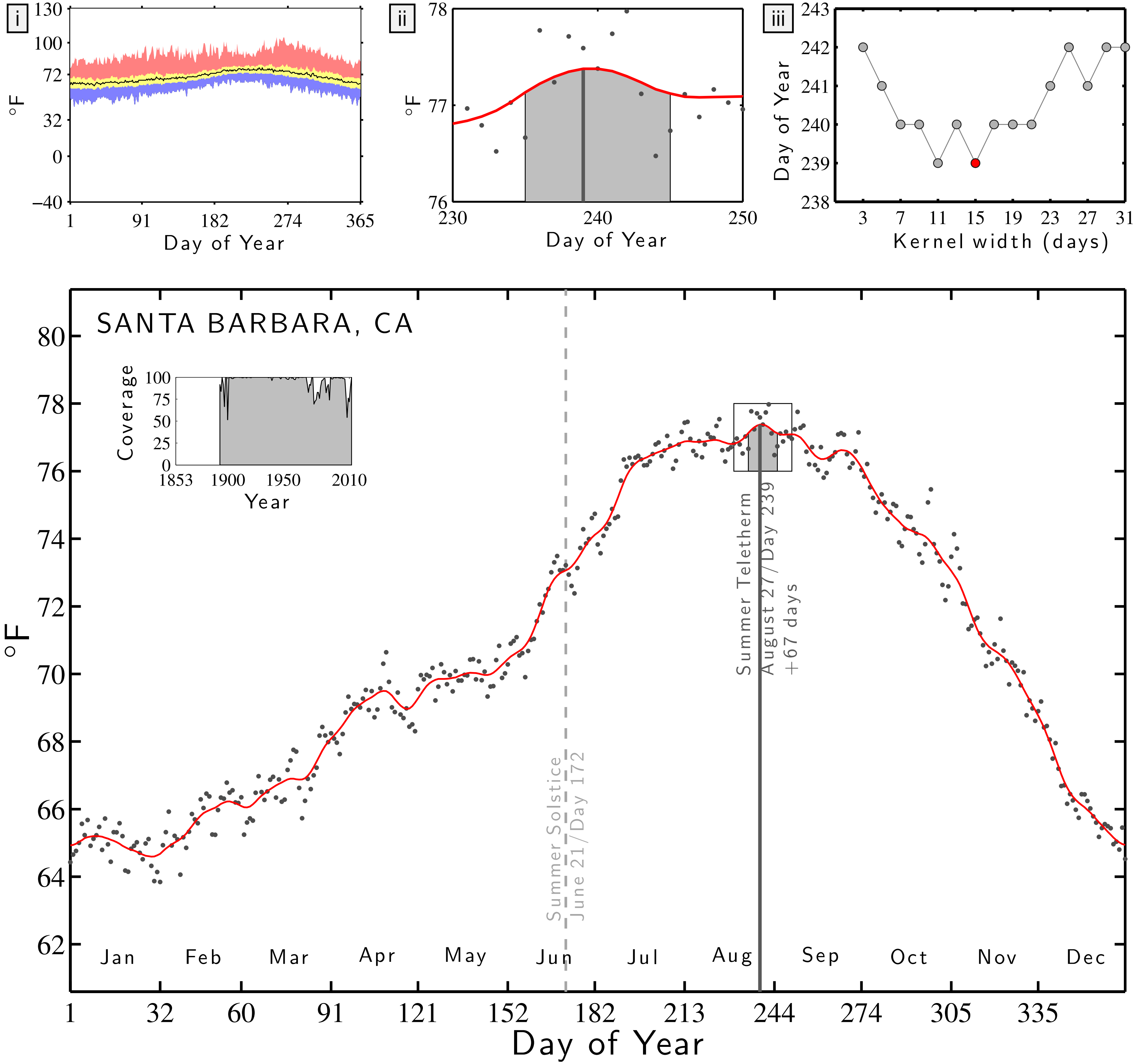

i
ii
iii
°F
Day of Year
Day of Year
Kernel width (days)
SANTA BARBARA, CA
Coverage
Year
Summer Solstice
June 21 / Day 172
Summer Teletherm
August 27 / Day 239
+67 days
Jan
Feb
Mar
Apr
May
Jun
Jul
Aug
Sep
Oct
Nov
Dec

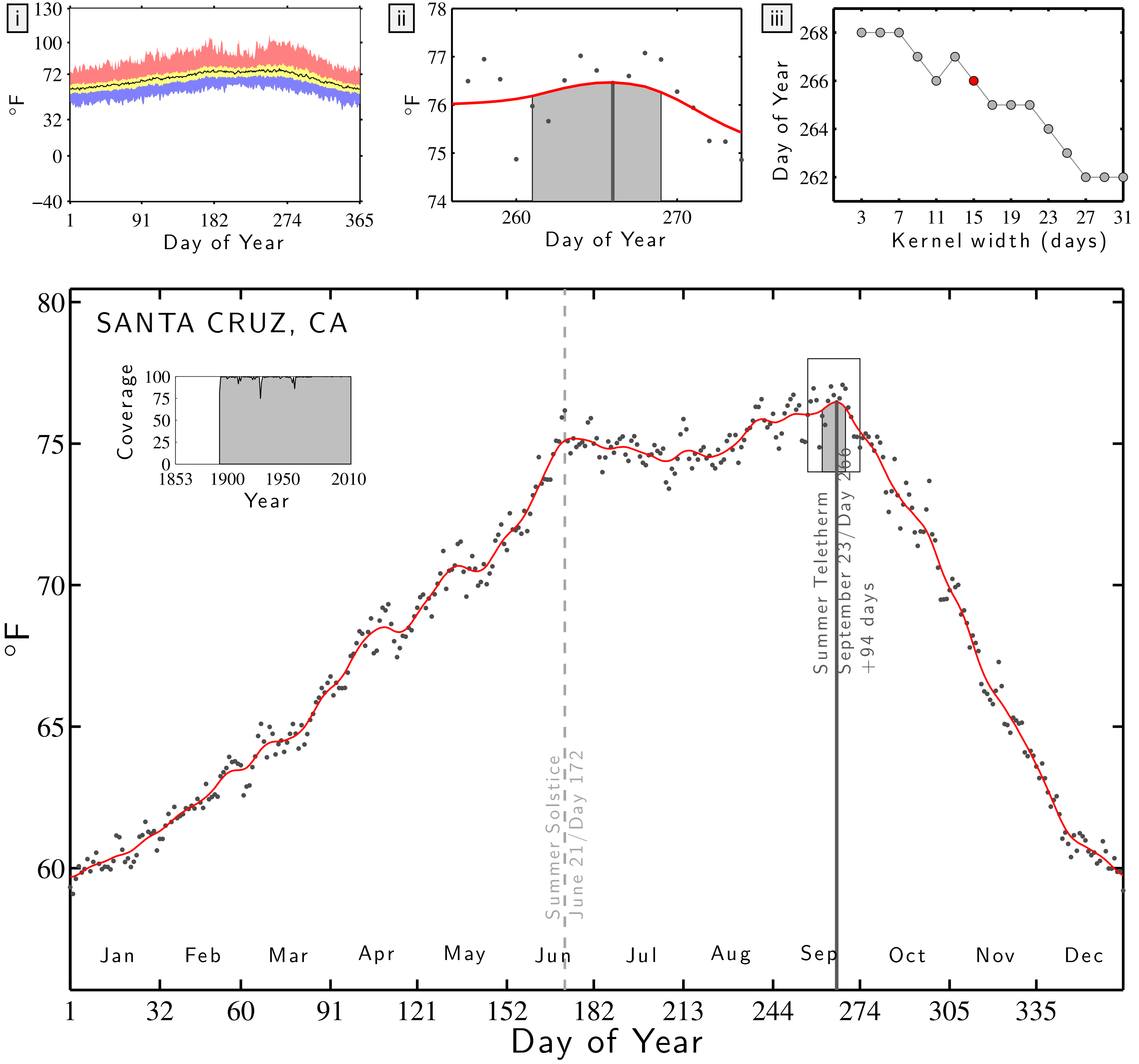

i
°F
Day of Year
ii
°F
Day of Year
iii
Day of Year
Kernel width (days)
SANTA CRUZ, CA
Coverage
Year
Summer Solstice
June 21 / Day 172
Summer Teletherm
September 23 / Day 266
+94 days
Jan Feb Mar Apr May Jun Jul Aug Sep Oct Nov Dec
°F
Day of Year

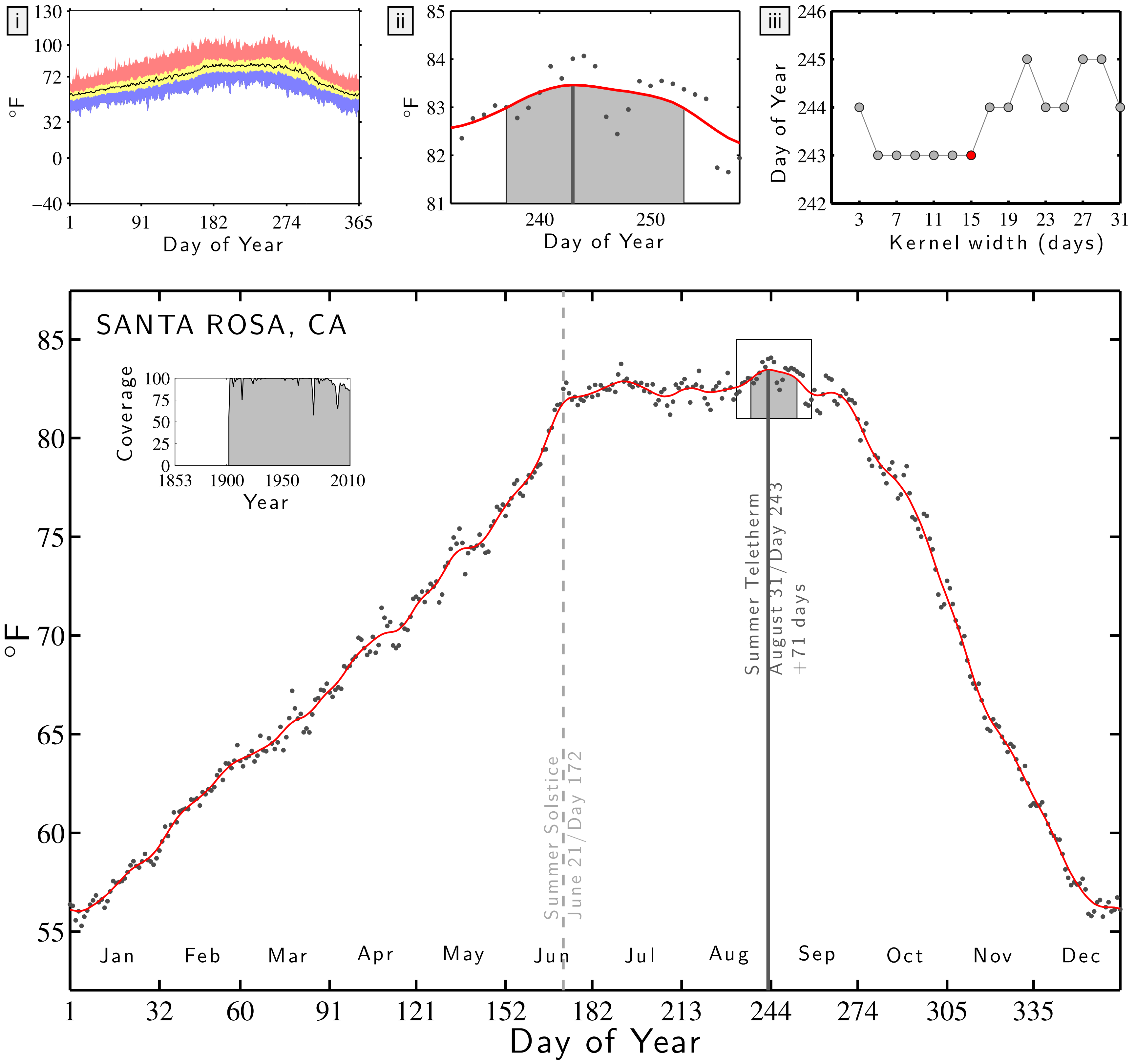

i
°F
Day of Year
ii
iii
Day of Year
Kernel width (days)
SANTA ROSA, CA
Coverage
Year
Summer Solstice
June 21 / Day 172
Summer Teletherm
August 31 / Day 243
+71 days
Jan
Feb
Mar
Apr
May
Jun
Jul
Aug
Sep
Oct
Nov
Dec

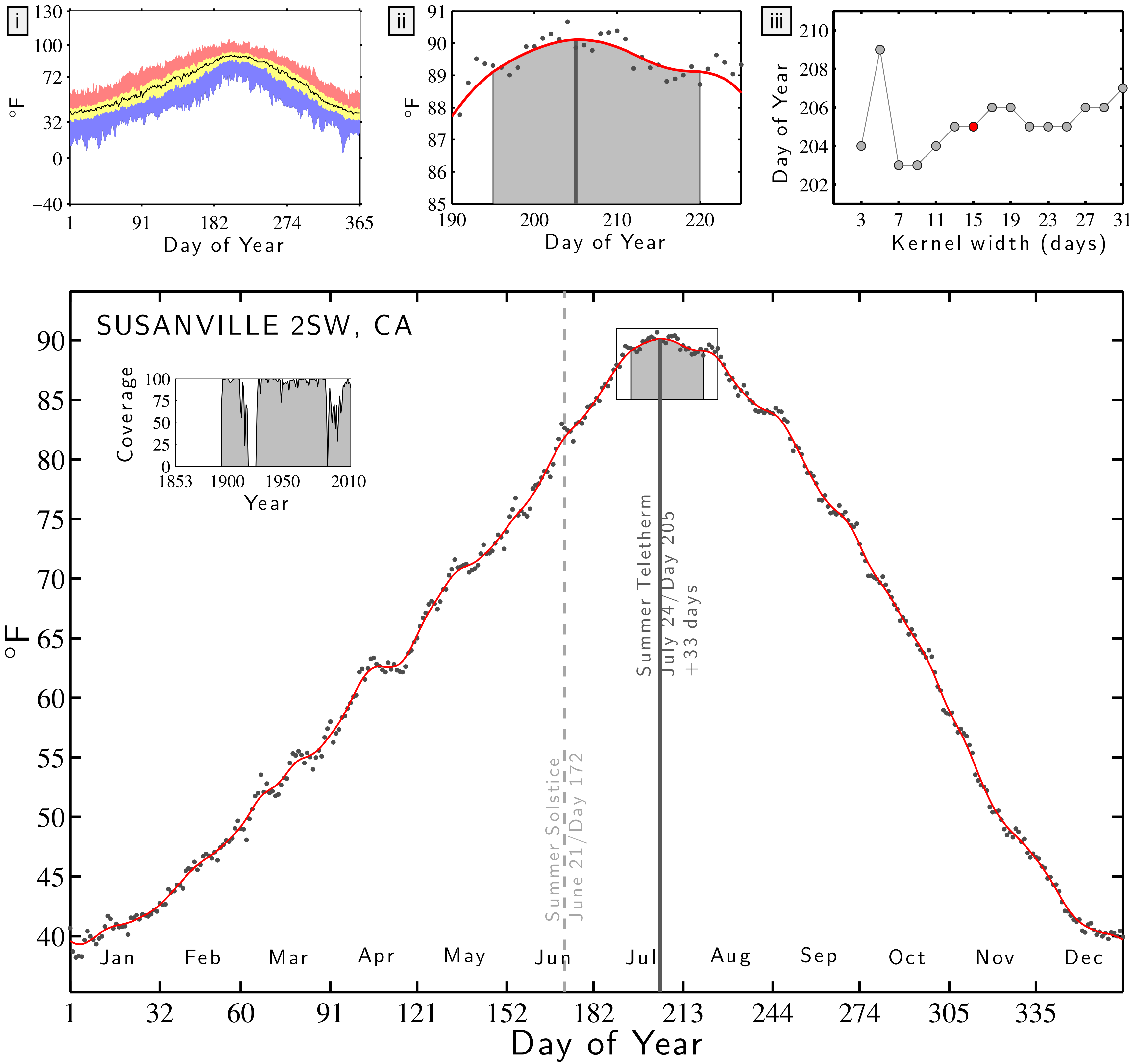

i
ii
iii
°F
Day of Year
Day of Year
Kernel width (days)
SUSANVILLE 2SW, CA
Coverage
Year
Summer Solstice
June 21/Day 172
Summer Teletherm
July 24/Day 205
+33 days
Jan
Feb
Mar
Apr
May
Jun
Jul
Aug
Sep
Oct
Nov
Dec

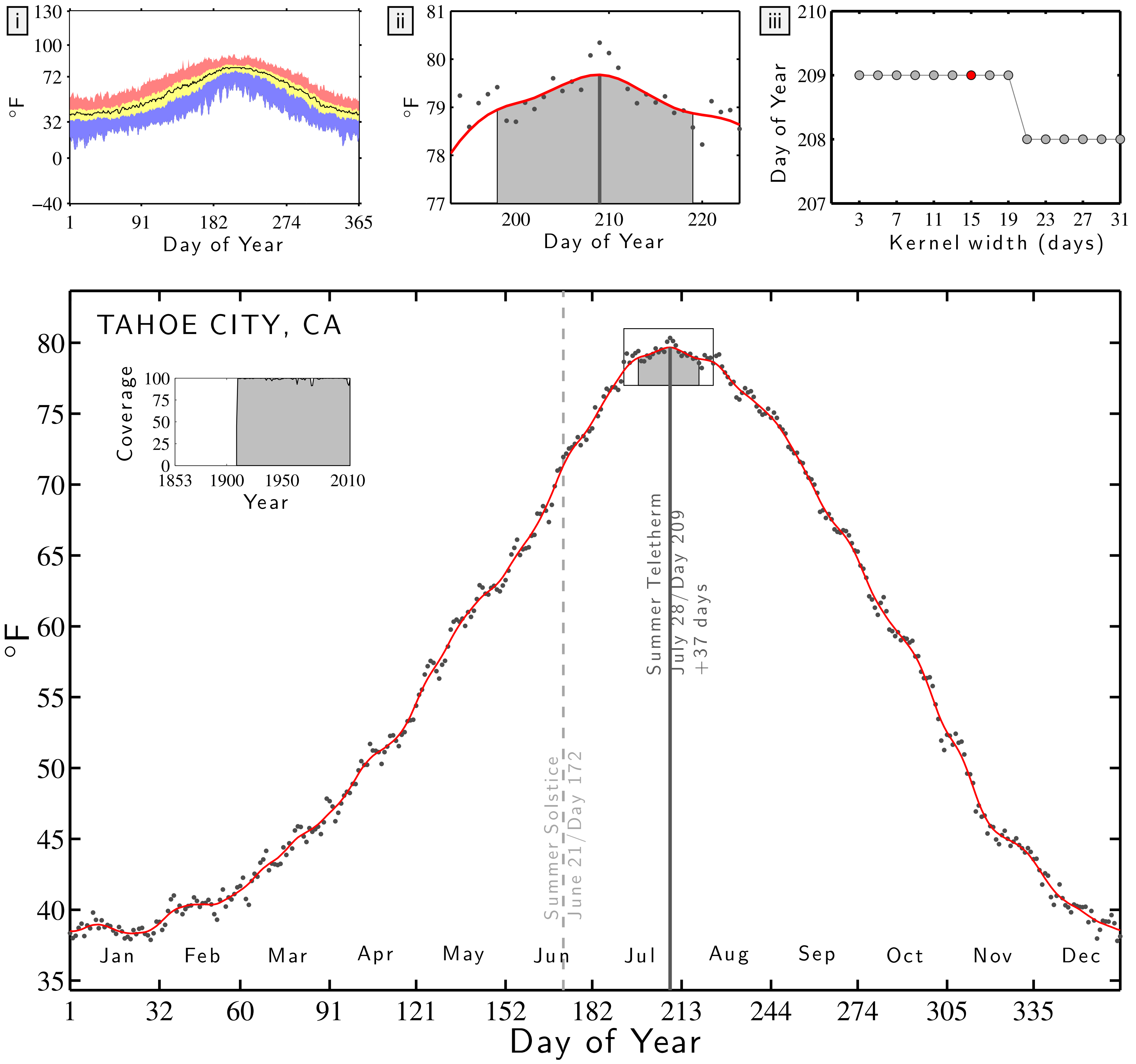

i
°F
Day of Year
ii
°F
Day of Year
iii
Day of Year
Kernel width (days)
TAHOE CITY, CA
Coverage
Year
Summer Solstice
June 21/Day 172
Summer Teletherm
July 28/Day 209
+37 days
Jan
Feb
Mar
Apr
May
Jun
Jul
Aug
Sep
Oct
Nov
Dec
°F
Day of Year

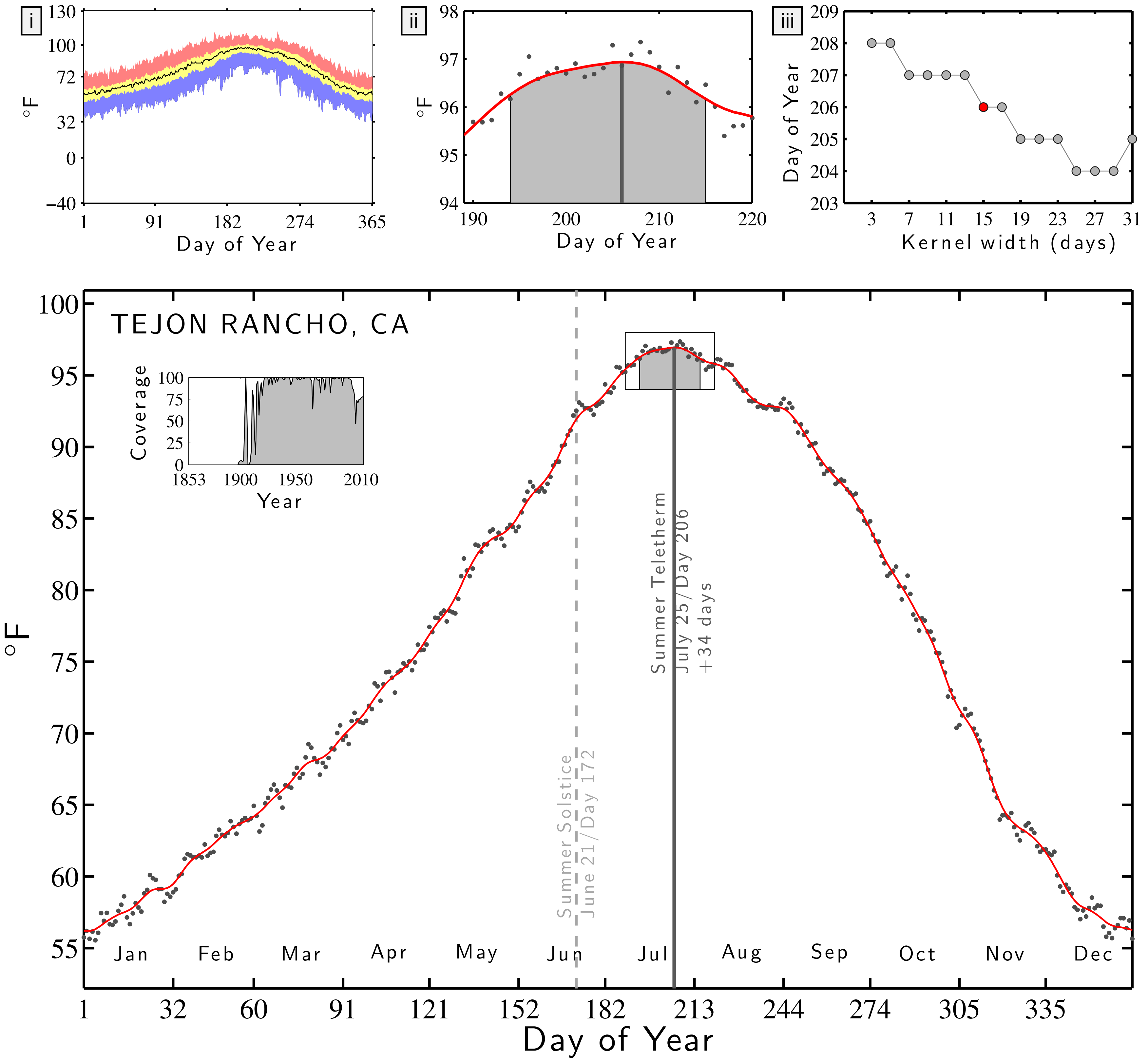

i
130
100
72
32
0
−40
°F
1
91
182
274
365
Day of Year
ii
98
97
96
95
94
°F
190
200
210
220
Day of Year
iii
209
208
207
206
205
204
203
Day of Year
3
7
11
15
19
23
27
31
Kernel width (days)
TEJON RANCHO, CA
Coverage
100
75
50
25
0
1853
1900
1950
2010
Year
Summer Solstice
June 21/Day 172
Summer Teletherm
July 25/Day 206
+34 days
100
95
90
85
80
75
70
65
60
55
°F
Jan
Feb
Mar
Apr
May
Jun
Jul
Aug
Sep
Oct
Nov
Dec
1
32
60
91
121
152
182
213
244
274
305
335
Day of Year

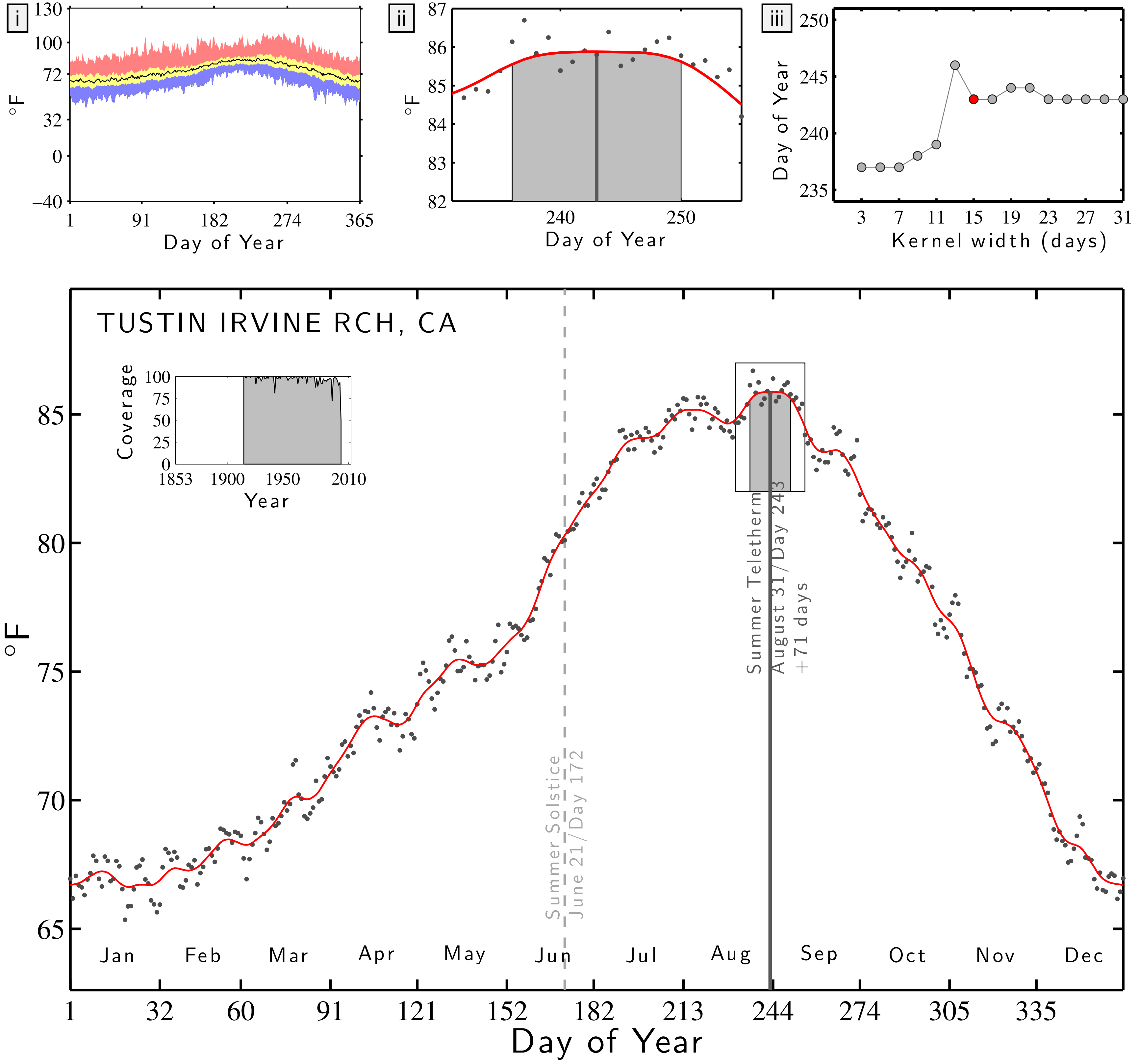

i
ii
iii
°F
Day of Year
Day of Year
Kernel width (days)
TUSTIN IRVINE RCH, CA
Coverage
Year
Summer Solstice
June 21 / Day 172
Summer Teletherm
August 31 / Day 243
+71 days
Jan
Feb
Mar
Apr
May
Jun
Jul
Aug
Sep
Oct
Nov
Dec

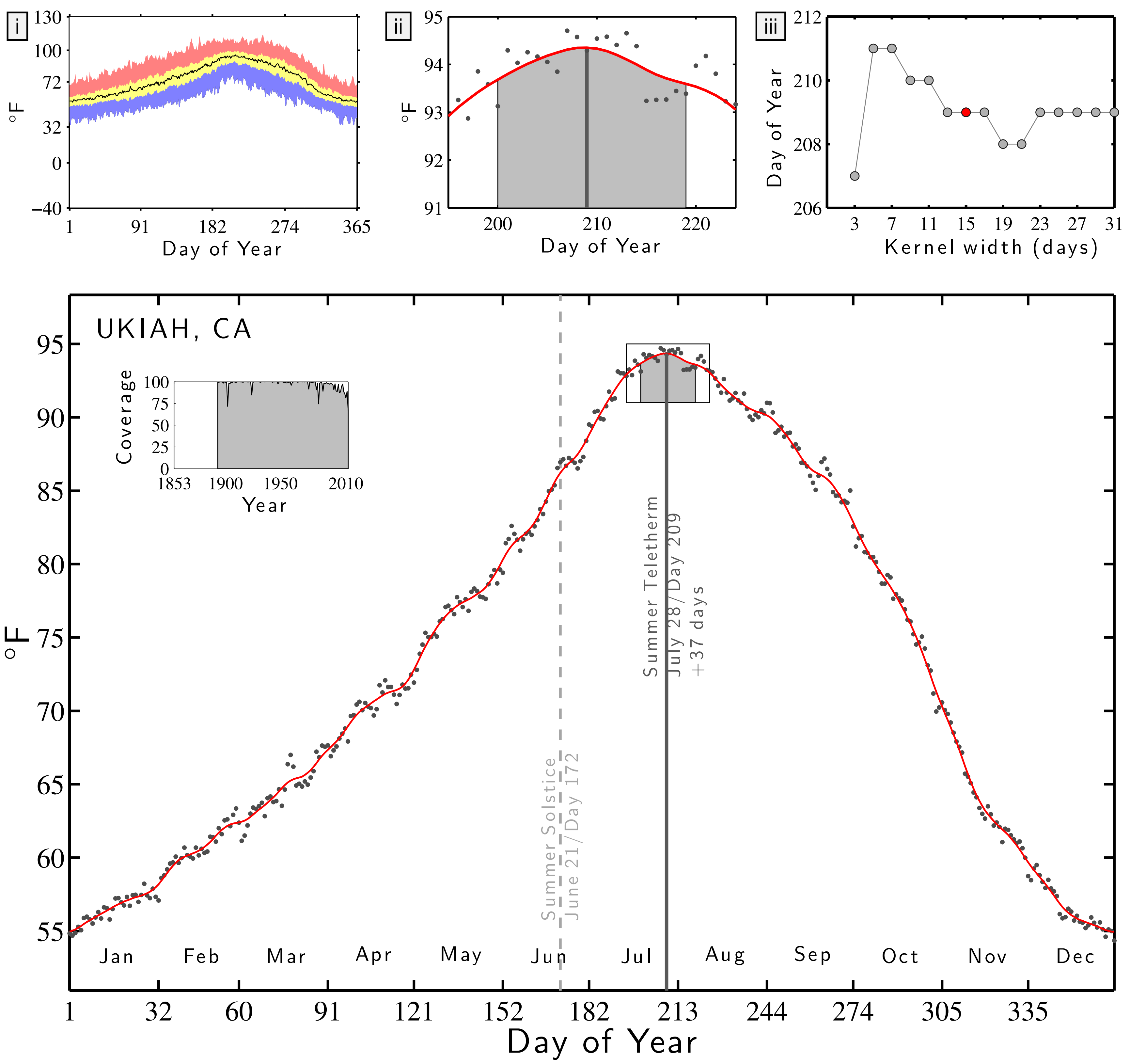

i
130
100
72
32
0
−40
°F
1
91
182
274
365
Day of Year
ii
95
94
93
92
91
°F
200
210
220
Day of Year
iii
212
210
208
206
Day of Year
3
7
11
15
19
23
27
31
Kernel width (days)
UKIAH, CA
Coverage
100
75
50
25
0
1853
1900
1950
2010
Year
95
90
85
80
75
70
65
60
55
°F
Summer Solstice
June 21/Day 172
Summer Teletherm
July 28/Day 209
+37 days
Jan
Feb
Mar
Apr
May
Jun
Jul
Aug
Sep
Oct
Nov
Dec
1
32
60
91
121
152
182
213
244
274
305
335
Day of Year

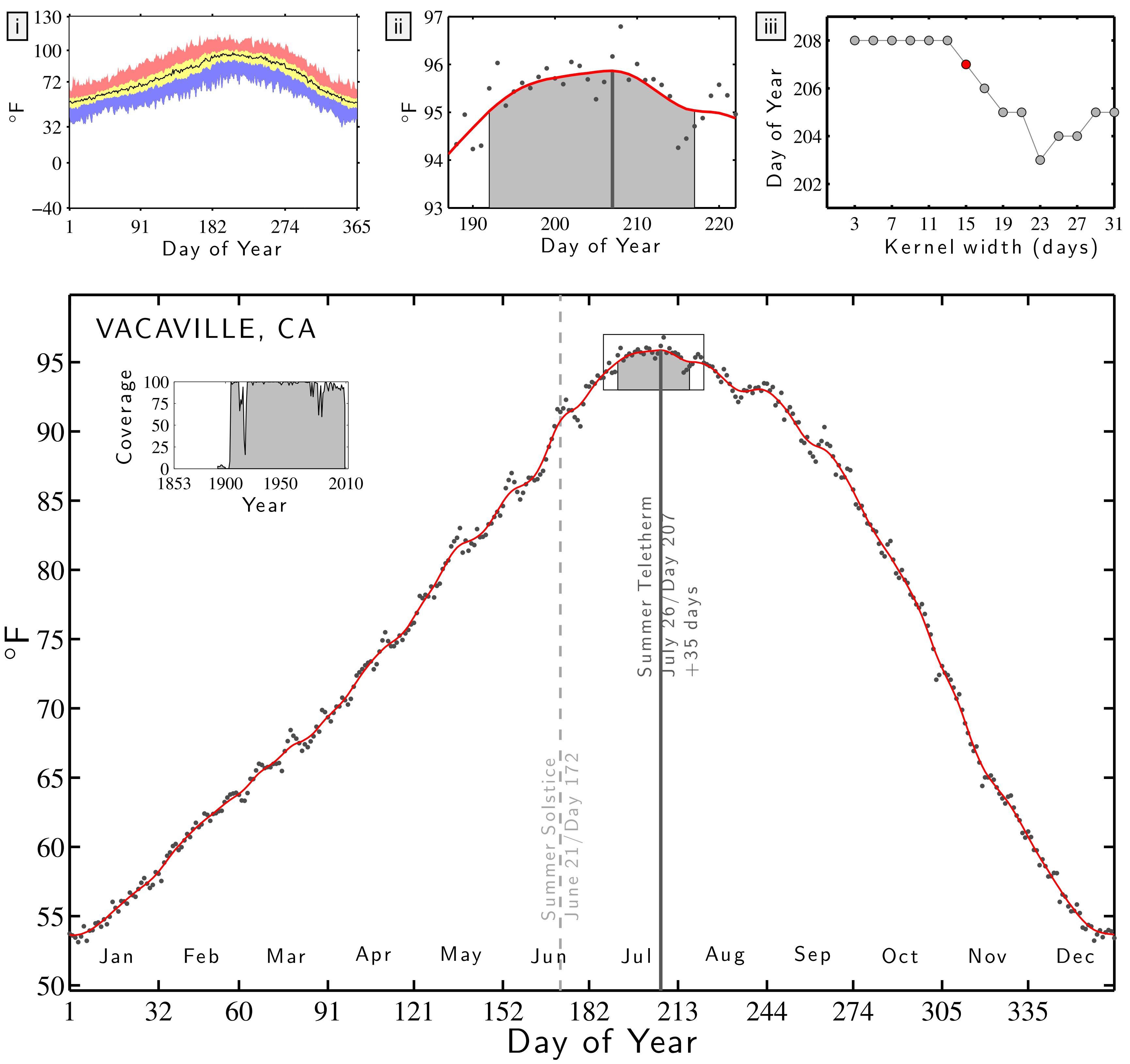

i
ii
iii
Day of Year
°F
Kernel width (days)
VACAVILLE, CA
Coverage
Year
Summer Solstice
June 21 / Day 172
Summer Teletherm
July 26 / Day 207
+35 days
Jan
Feb
Mar
Apr
May
Jun
Jul
Aug
Sep
Oct
Nov
Dec
Day of Year

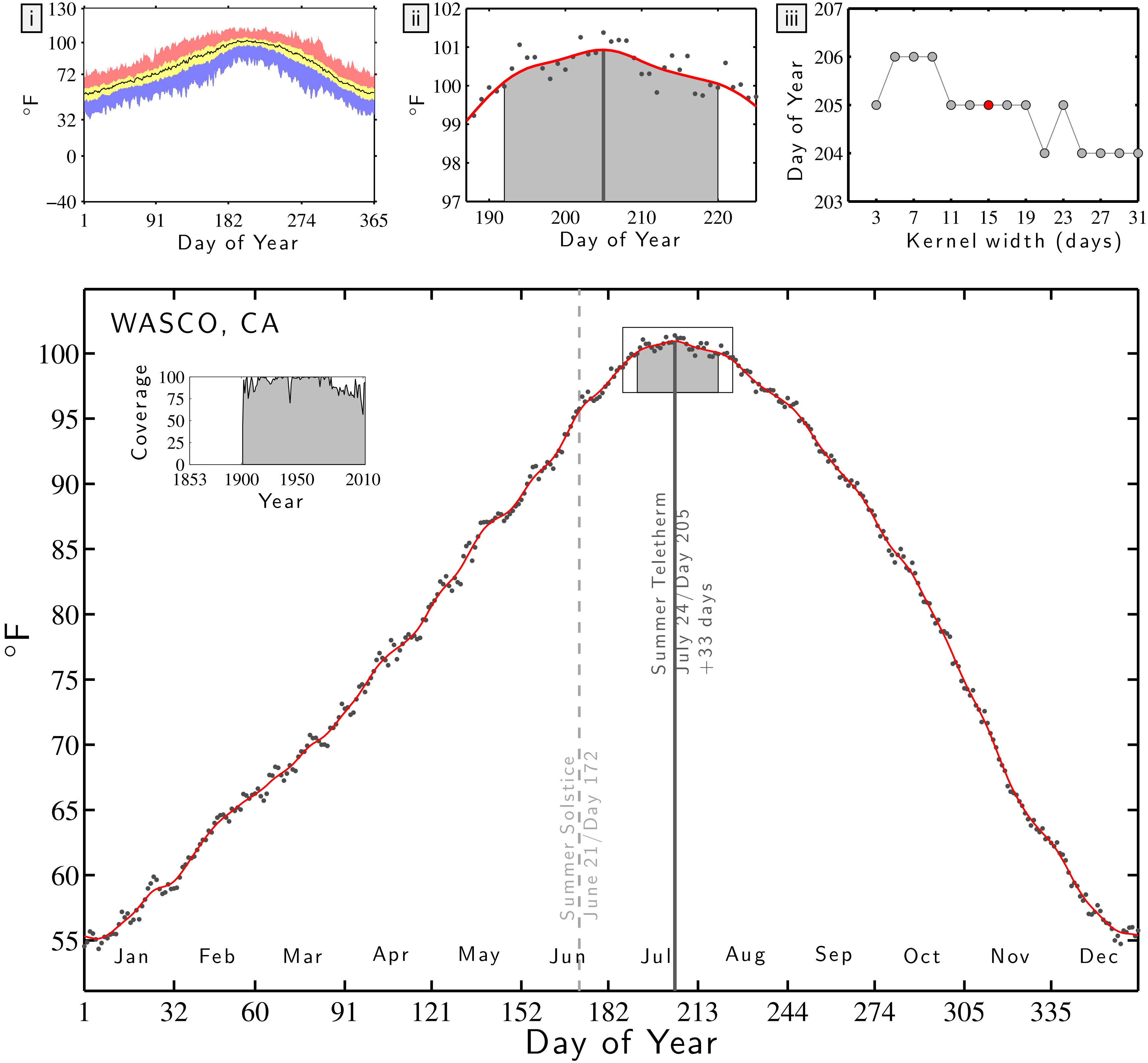

i
°F
Day of Year
ii
°F
Day of Year
iii
Day of Year
Kernel width (days)
WASCO, CA
Coverage
Year
Summer Solstice
June 21/Day 172
Summer Teletherm
July 24/Day 205
+33 days
Jan
Feb
Mar
Apr
May
Jun
Jul
Aug
Sep
Oct
Nov
Dec
°F
Day of Year

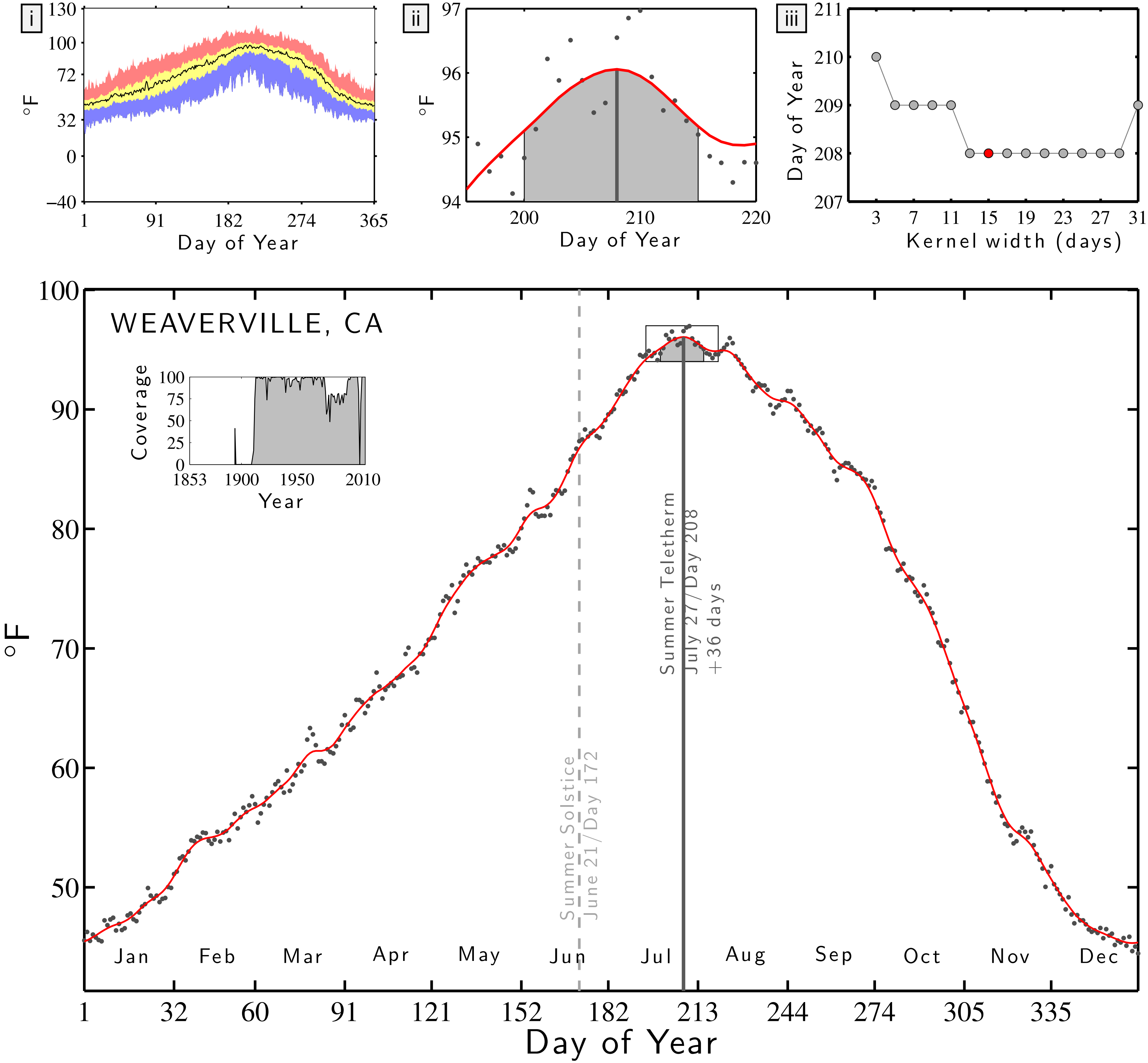

i
°F
130
100
72
32
0
−40
1
91
182
274
365
Day of Year
ii
97
96
95
94
200
210
220
iii
211
210
209
208
207
Day of Year
3
7
11
15
19
23
27
31
Kernel width (days)
WEAVERVILLE, CA
Coverage
100
75
50
25
0
1853
1900
1950
2010
Year
Summer Solstice
June 21 / Day 172
Summer Teletherm
July 27 / Day 208
+36 days
Jan
Feb
Mar
Apr
May
Jun
Jul
Aug
Sep
Oct
Nov
Dec
90
80
70
60
50
1
32
60
91
121
152
182
213
244
274
305
335

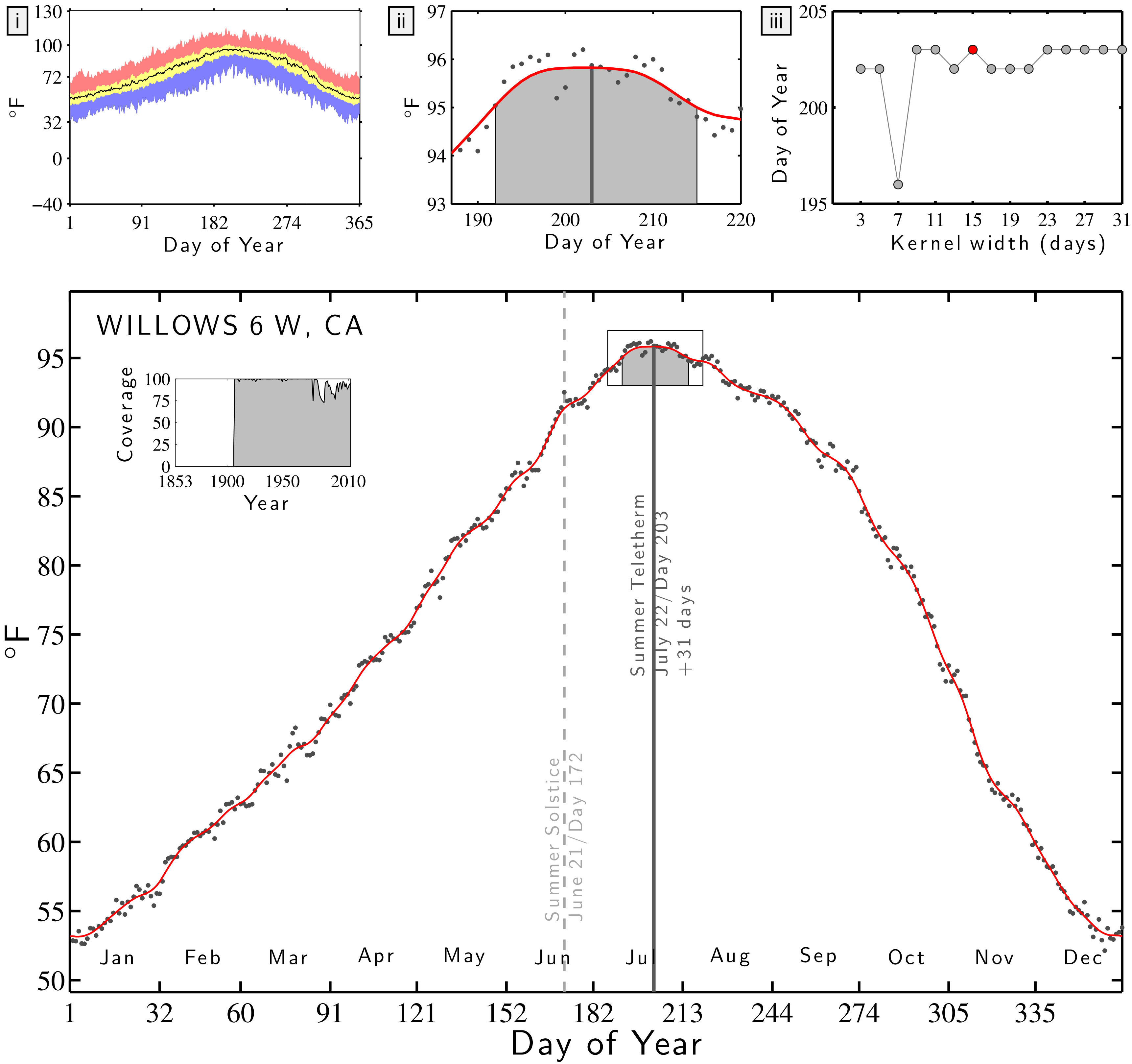

i
°F
Day of Year
ii
°F
Day of Year
iii
Day of Year
Kernel width (days)
WILLOWS 6 W, CA
Coverage
Year
Summer Solstice
June 21/Day 172
Summer Teletherm
July 22/Day 203
+31 days
Jan
Feb
Mar
Apr
May
Jun
Jul
Aug
Sep
Oct
Nov
Dec
°F
Day of Year

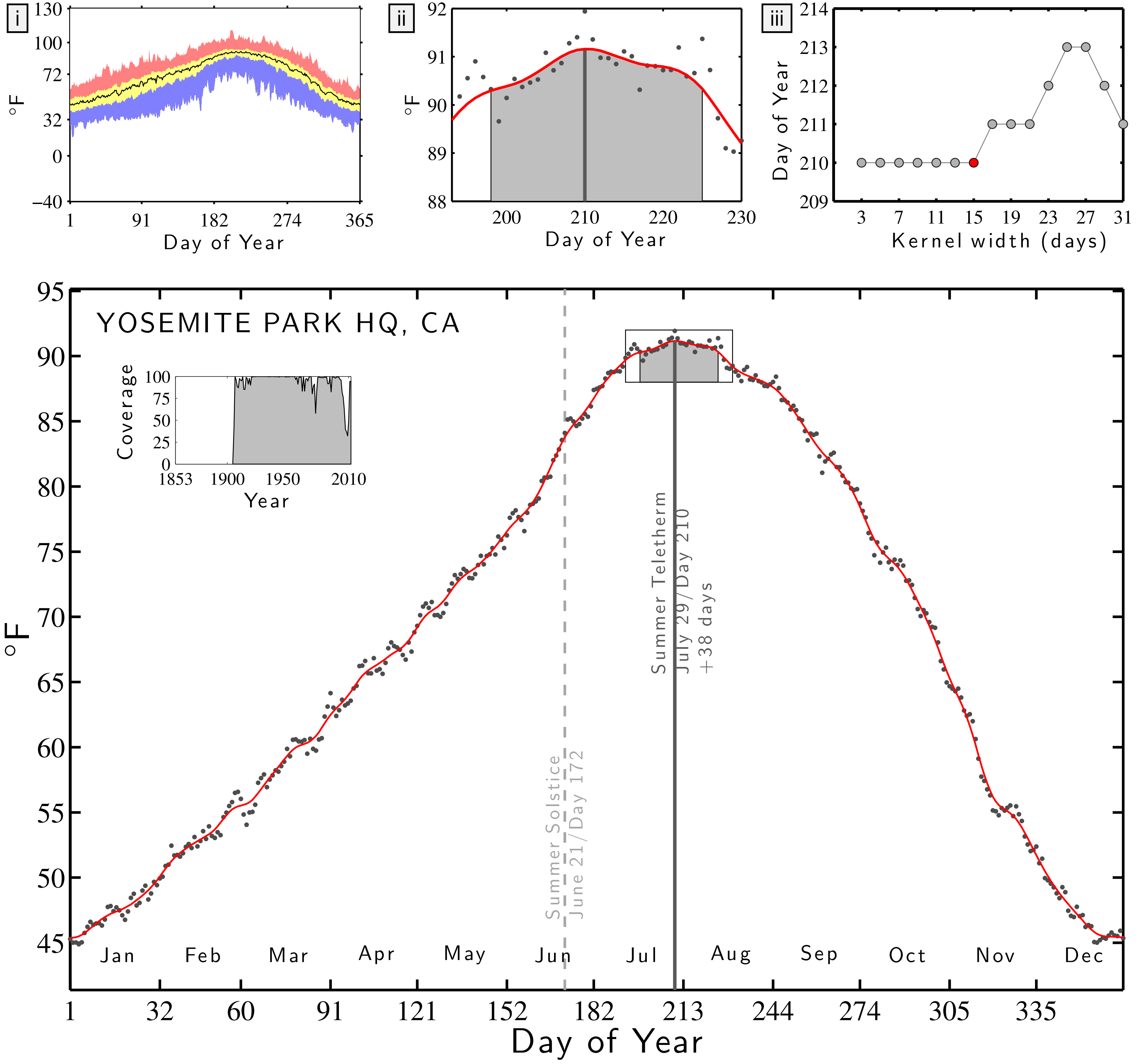

i
ii
iii
°F
Day of Year
Day of Year
Kernel width (days)
YOSEMITE PARK HQ, CA
Coverage
Year
Summer Solstice
June 21 / Day 172
Summer Teletherm
July 29 / Day 210
+38 days
Jan
Feb
Mar
Apr
May
Jun
Jul
Aug
Sep
Oct
Nov
Dec
Day of Year

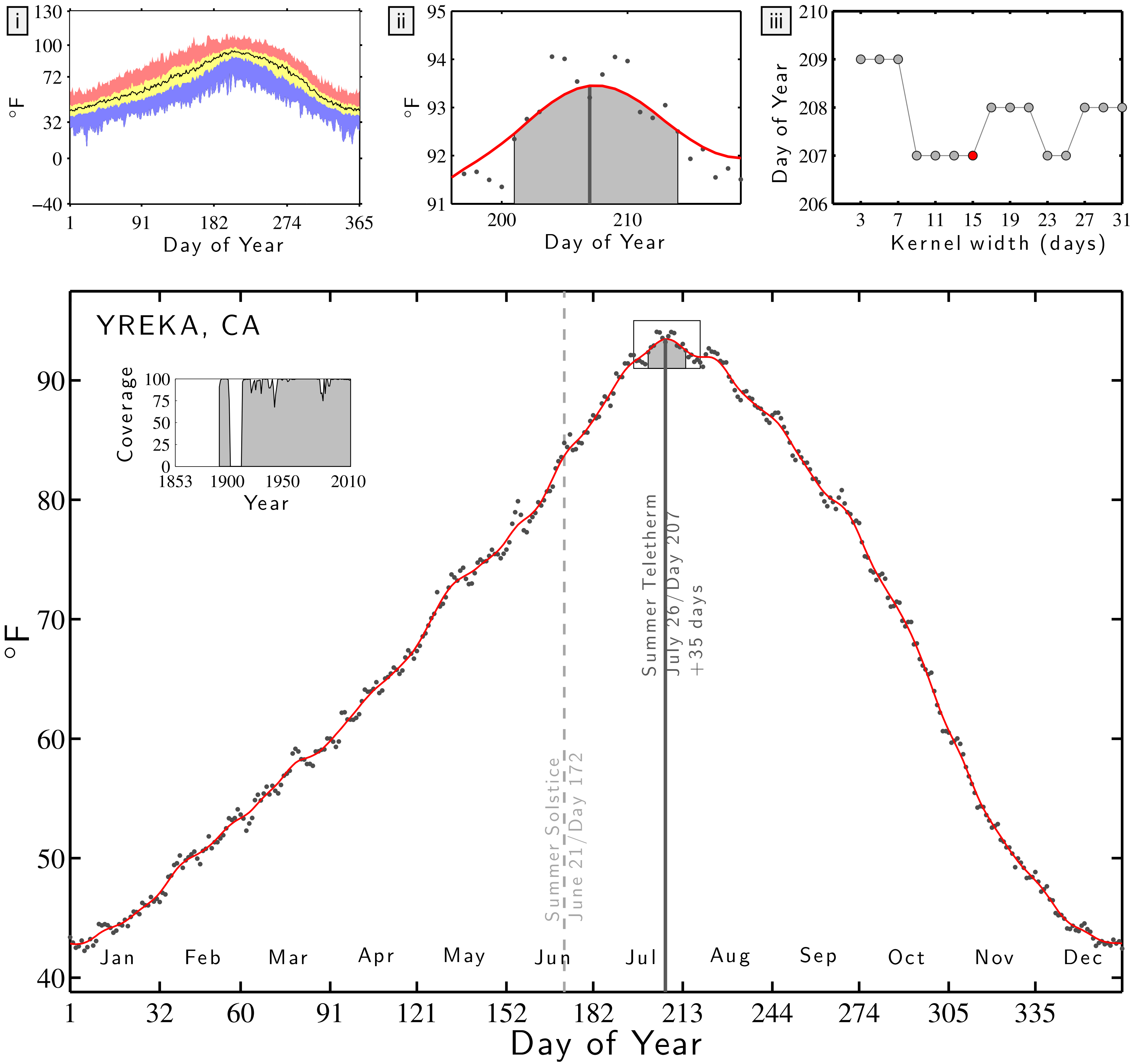

i
°F
Day of Year
ii
°F
Day of Year
iii
Day of Year
Kernel width (days)
YREKA, CA
Coverage
Year
Summer Solstice
June 21 / Day 172
Summer Teletherm
July 26 / Day 207
+35 days
Jan
Feb
Mar
Apr
May
Jun
Jul
Aug
Sep
Oct
Nov
Dec
°F
Day of Year

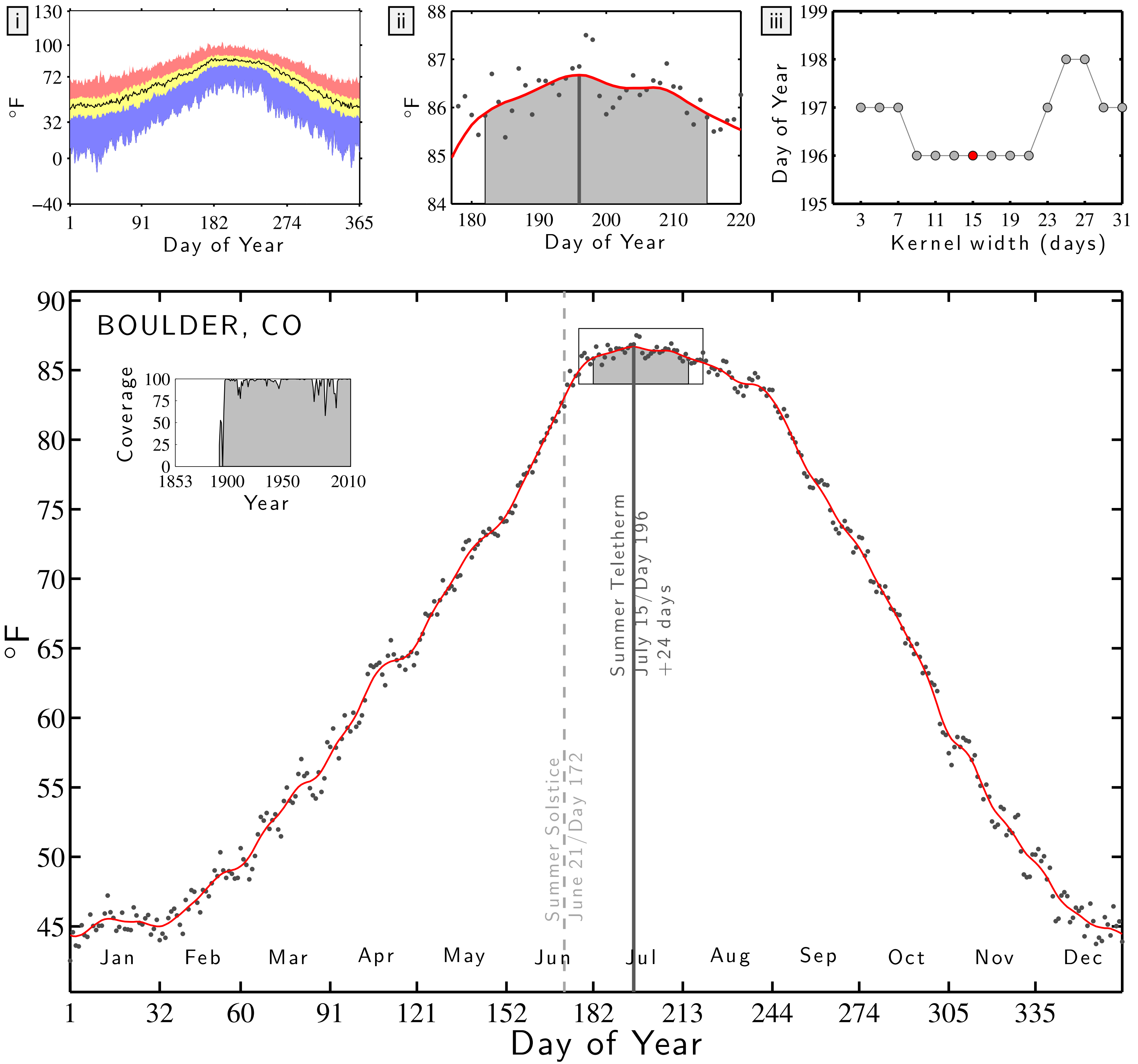

i
ii
iii
°F
Day of Year
Kernel width (days)
BOULDER, CO
Coverage
Year
Summer Solstice
June 21/Day 172
Summer Teletherm
July 15/Day 196
+24 days
Jan
Feb
Mar
Apr
May
Jun
Jul
Aug
Sep
Oct
Nov
Dec

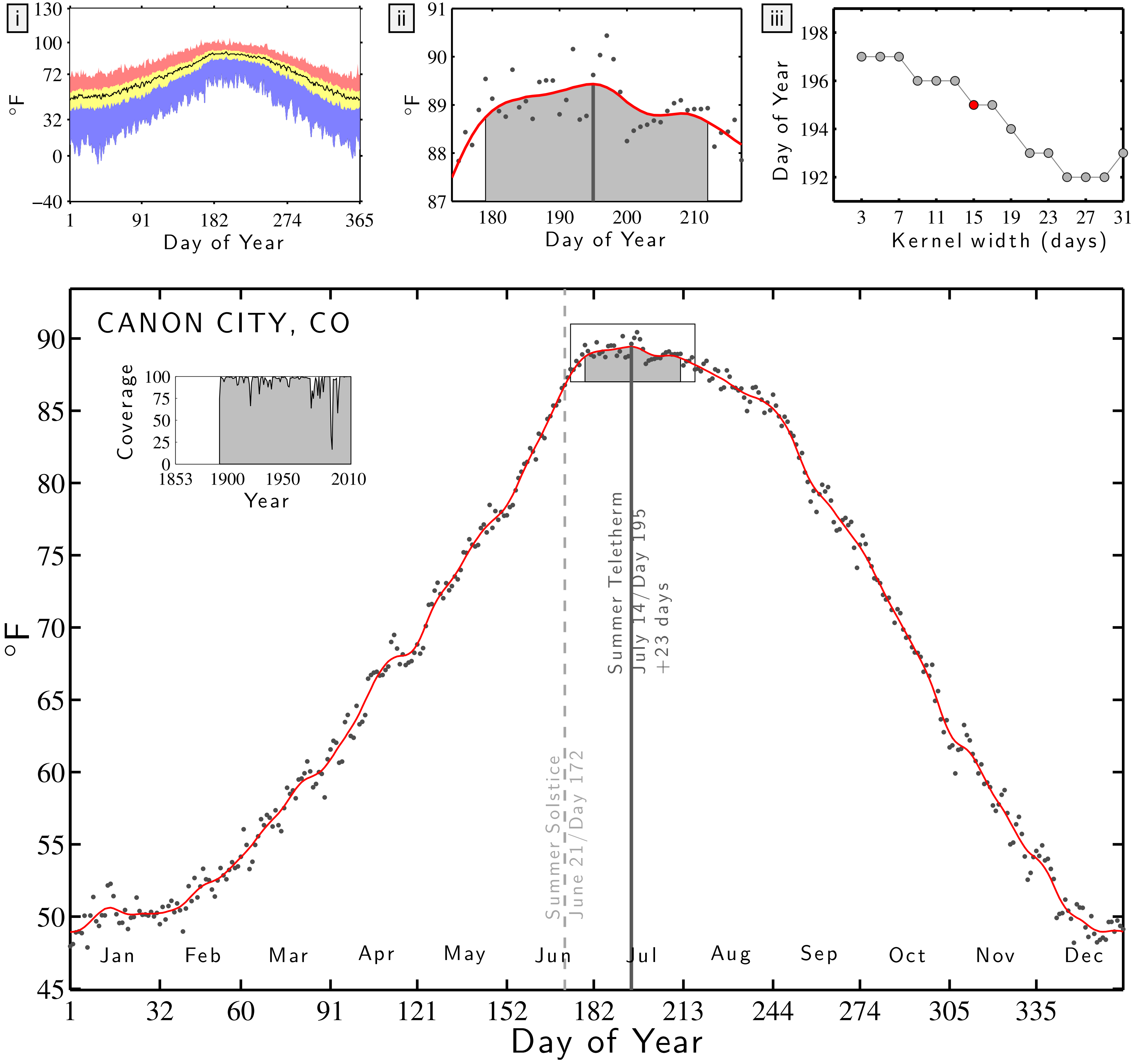

i
°F
Day of Year
ii
°F
Day of Year
iii
Day of Year
Kernel width (days)
CANON CITY, CO
Coverage
Year
Summer Solstice
June 21/Day 172
Summer Teletherm
July 14/Day 195
+23 days
Jan
Feb
Mar
Apr
May
Jun
Jul
Aug
Sep
Oct
Nov
Dec
°F
Day of Year

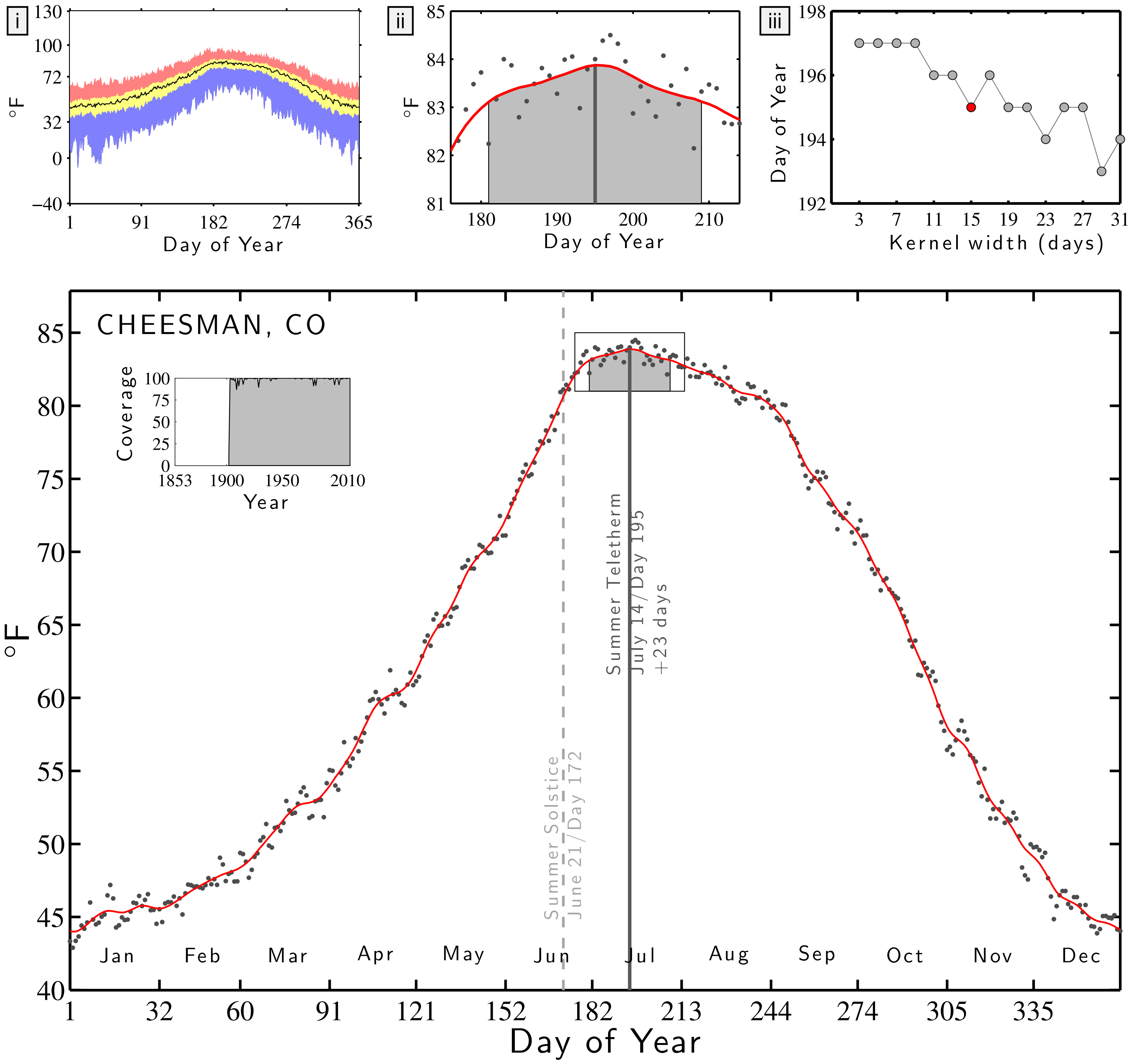

i
°F
Day of Year
ii
°F
Day of Year
iii
Day of Year
Kernel width (days)
CHEESMAN, CO
Coverage
Year
Summer Solstice
June 21/Day 172
Summer Teletherm
July 14/Day 195
+23 days
Jan
Feb
Mar
Apr
May
Jun
Jul
Aug
Sep
Oct
Nov
Dec
°F
Day of Year

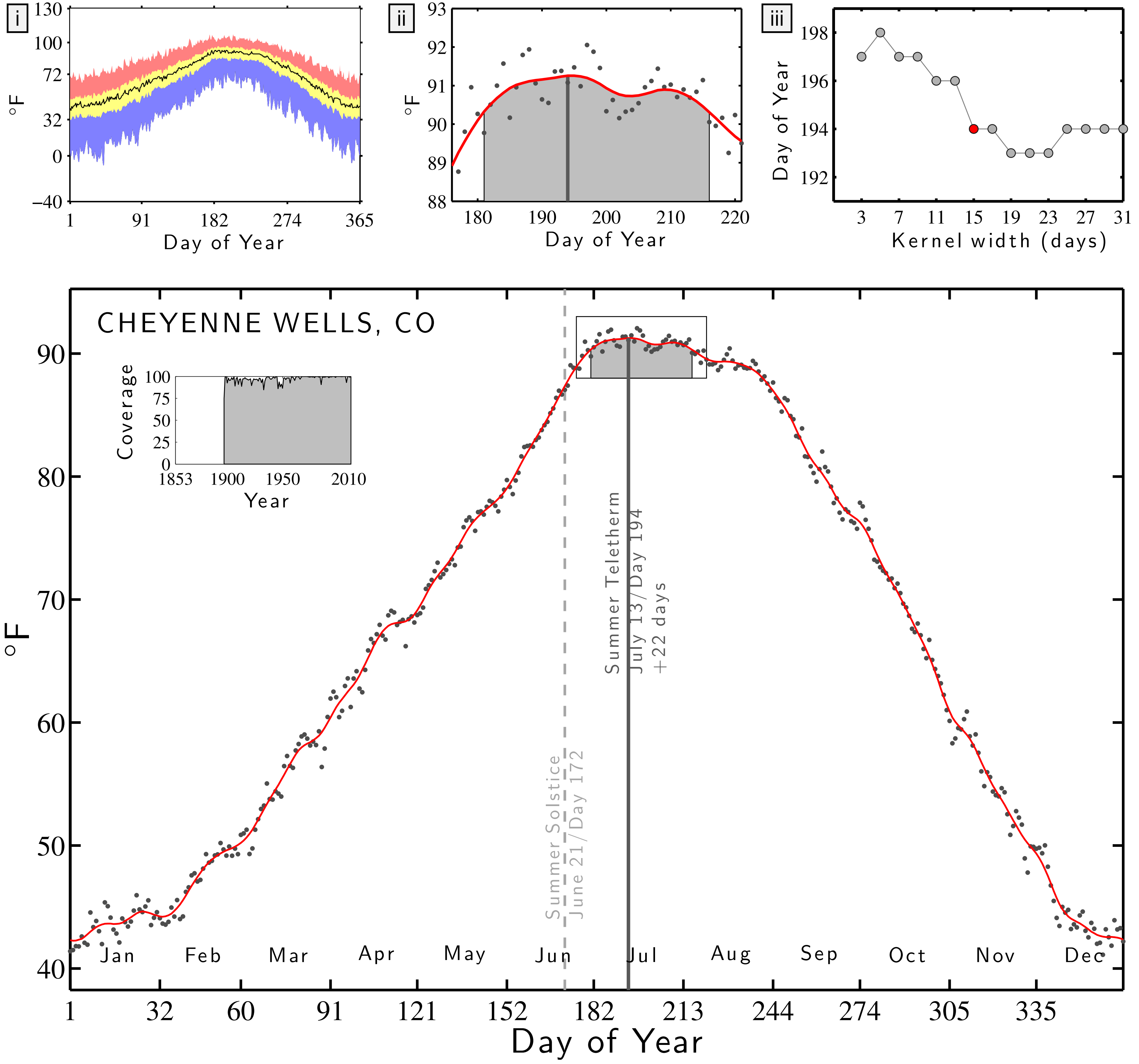
i
ii
iii
°F
Day of Year
Kernel width (days)
CHEYENNE WELLS, CO
Coverage
Year
1853
1900
1950
2010
Summer Solstice
June 21/Day 172
Summer Teletherm
July 13/Day 194
+22 days
Jan
Feb
Mar
Apr
May
Jun
Jul
Aug
Sep
Oct
Nov
Dec

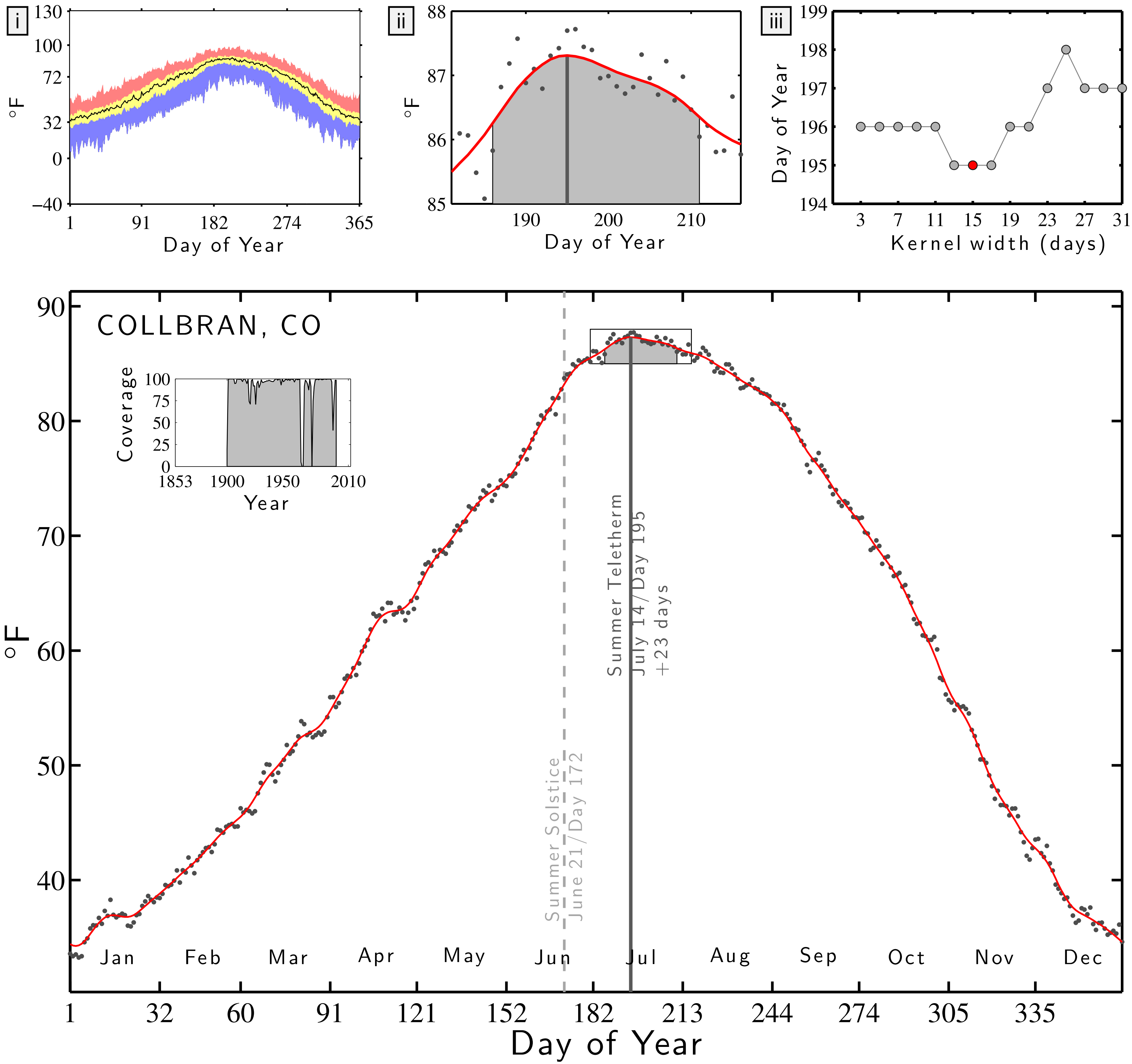

i
°F
130
100
72
32
0
−40
1
91
182
274
365
Day of Year
ii
°F
88
87
86
85
190
200
210
Day of Year
iii
Day of Year
199
198
197
196
195
194
3
7
11
15
19
23
27
31
Kernel width (days)
COLLBRAN, CO
Coverage
100
75
50
25
0
1853
1900
1950
2010
Year
Summer Solstice
June 21/Day 172
Summer Teletherm
July 14/Day 195
+23 days
°F
90
80
70
60
50
40
Jan
Feb
Mar
Apr
May
Jun
Jul
Aug
Sep
Oct
Nov
Dec
1
32
60
91
121
152
182
213
244
274
305
335
Day of Year

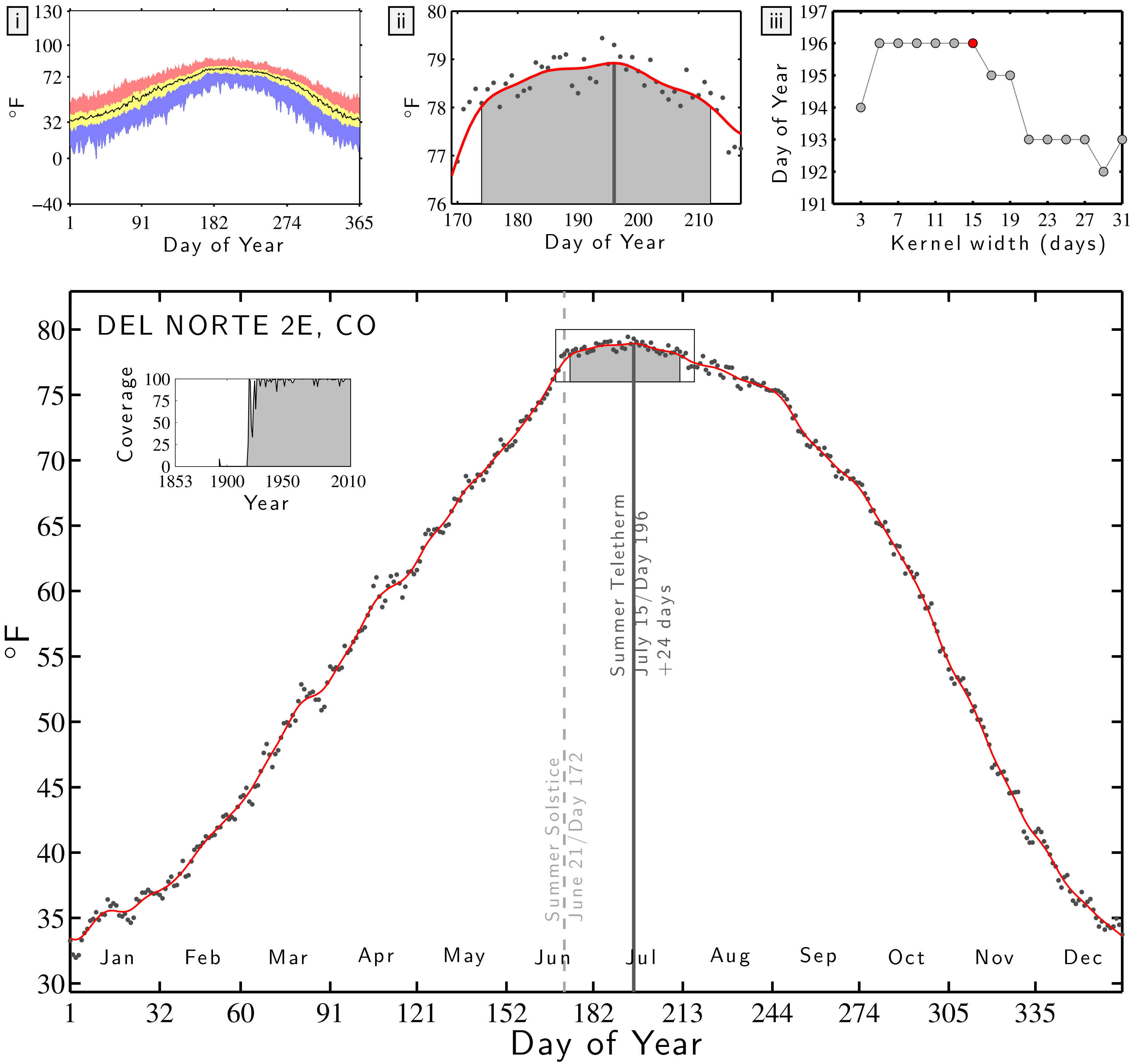
i
°F
130
100
72
32
0
−40
1
91
182
274
365
Day of Year
ii
°F
80
79
78
77
76
170
180
190
200
210
Day of Year
iii
Day of Year
197
196
195
194
193
192
191
3
7
11
15
19
23
27
31
Kernel width (days)
DEL NORTE 2E, CO
Coverage
100
75
50
25
0
1853
1900
1950
2010
Year
Summer Solstice
June 21/Day 172
Summer Teletherm
July 15/Day 196
+24 days
°F
80
75
70
65
60
55
50
45
40
35
30
Jan
Feb
Mar
Apr
May
Jun
Jul
Aug
Sep
Oct
Nov
Dec
1
32
60
91
121
152
182
213
244
274
305
335
Day of Year

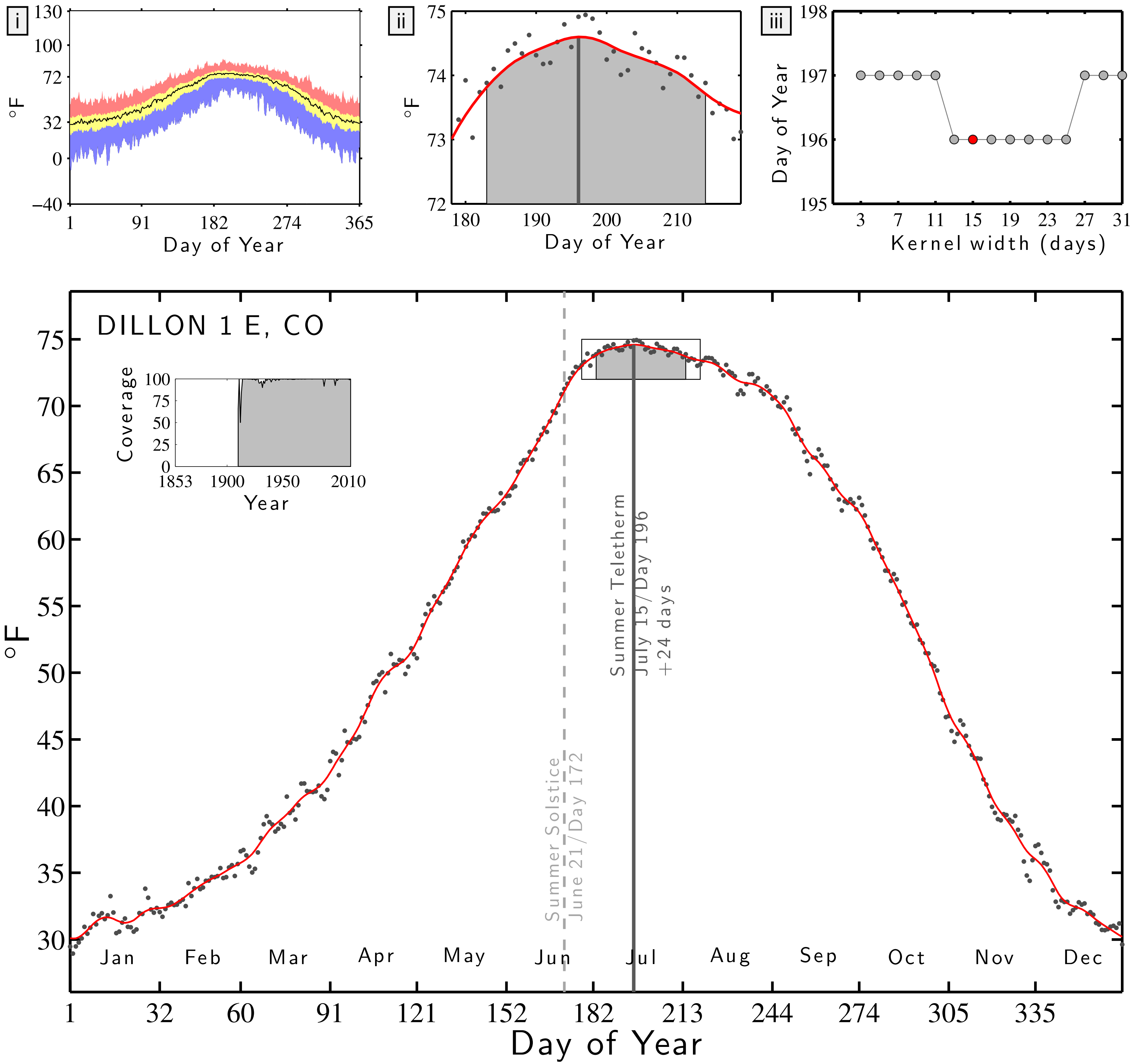

i
°F
Day of Year
ii
°F
Day of Year
iii
Day of Year
Kernel width (days)
DILLON 1 E, CO
Coverage
Year
Summer Solstice
June 21/Day 172
Summer Teletherm
July 15/Day 196
+24 days
Jan
Feb
Mar
Apr
May
Jun
Jul
Aug
Sep
Oct
Nov
Dec
°F
Day of Year

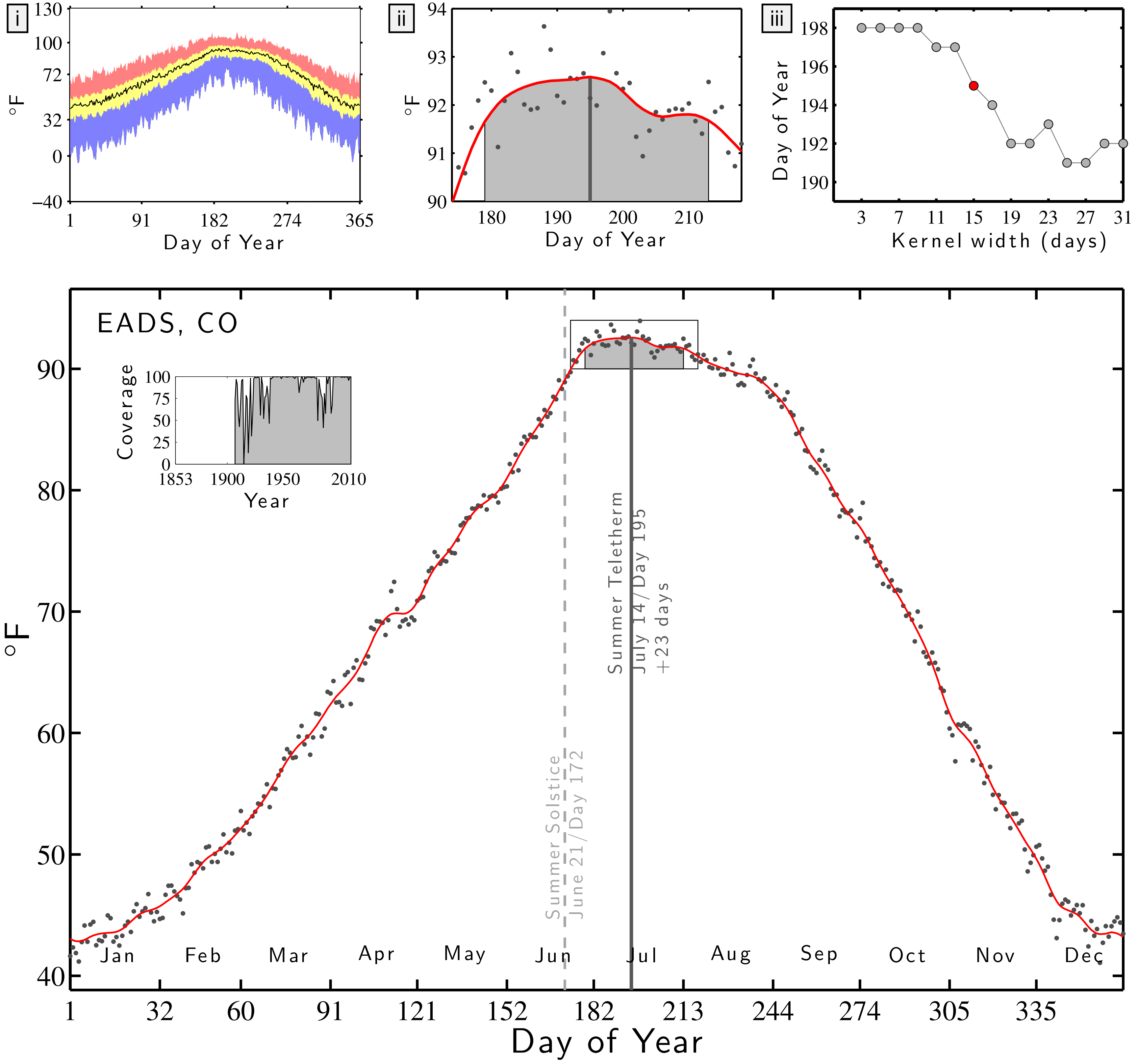

i
ii
iii
°F
Day of Year
Kernel width (days)
EADS, CO
Coverage
Year
Summer Solstice
June 21/Day 172
Summer Teletherm
July 14/Day 195
+23 days
Jan
Feb
Mar
Apr
May
Jun
Jul
Aug
Sep
Oct
Nov
Dec

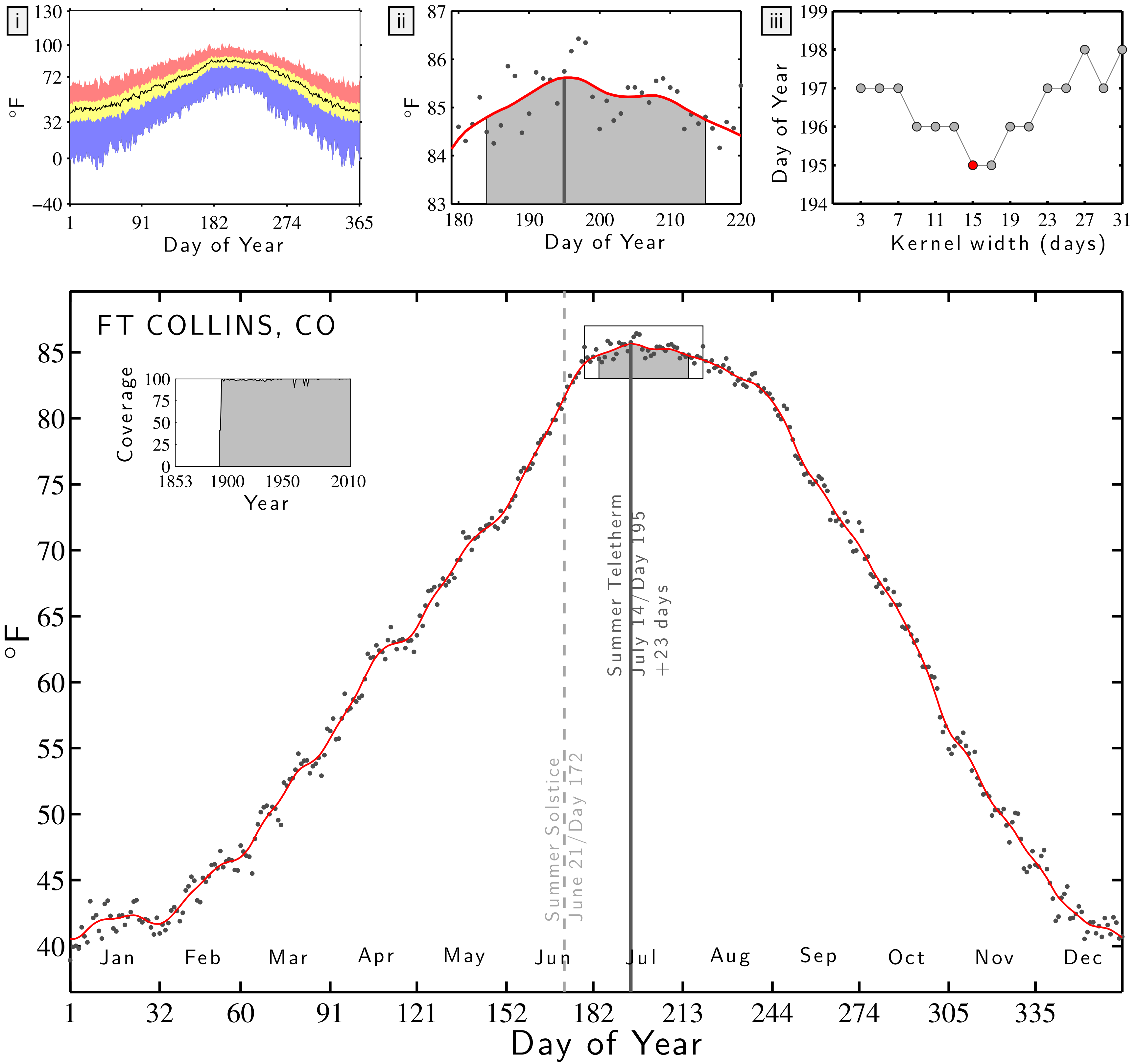

i
ii
iii
Day of Year
Kernel width (days)
°F
FT COLLINS, CO
Coverage
Year
Summer Solstice
June 21/Day 172
Summer Teletherm
July 14/Day 195
+23 days
Jan
Feb
Mar
Apr
May
Jun
Jul
Aug
Sep
Oct
Nov
Dec

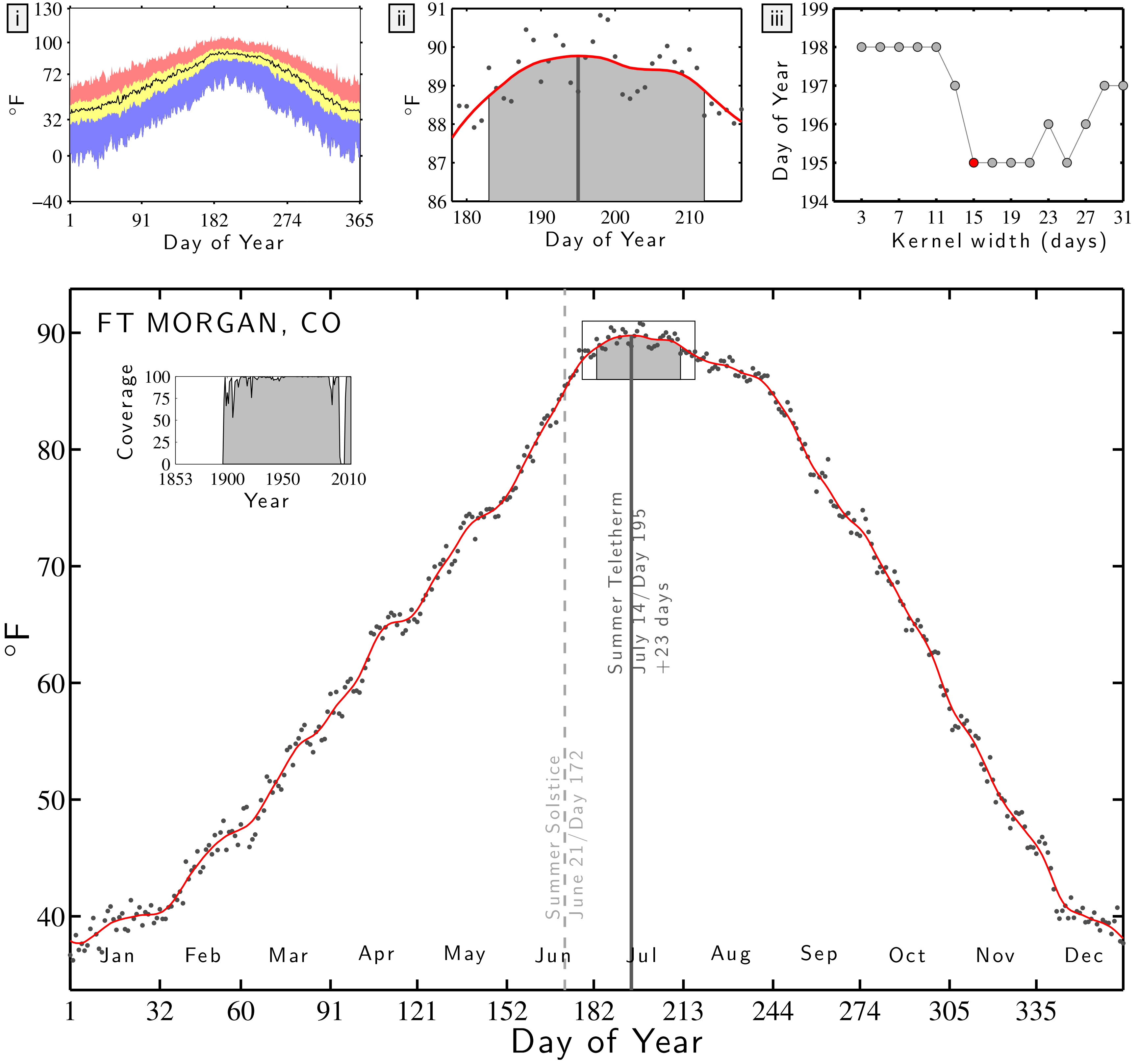

i
°F
Day of Year
ii
°F
Day of Year
iii
Day of Year
Kernel width (days)
FT MORGAN, CO
Coverage
Year
Summer Solstice
June 21/Day 172
Summer Teletherm
July 14/Day 195
+23 days
Jan
Feb
Mar
Apr
May
Jun
Jul
Aug
Sep
Oct
Nov
Dec
°F
Day of Year

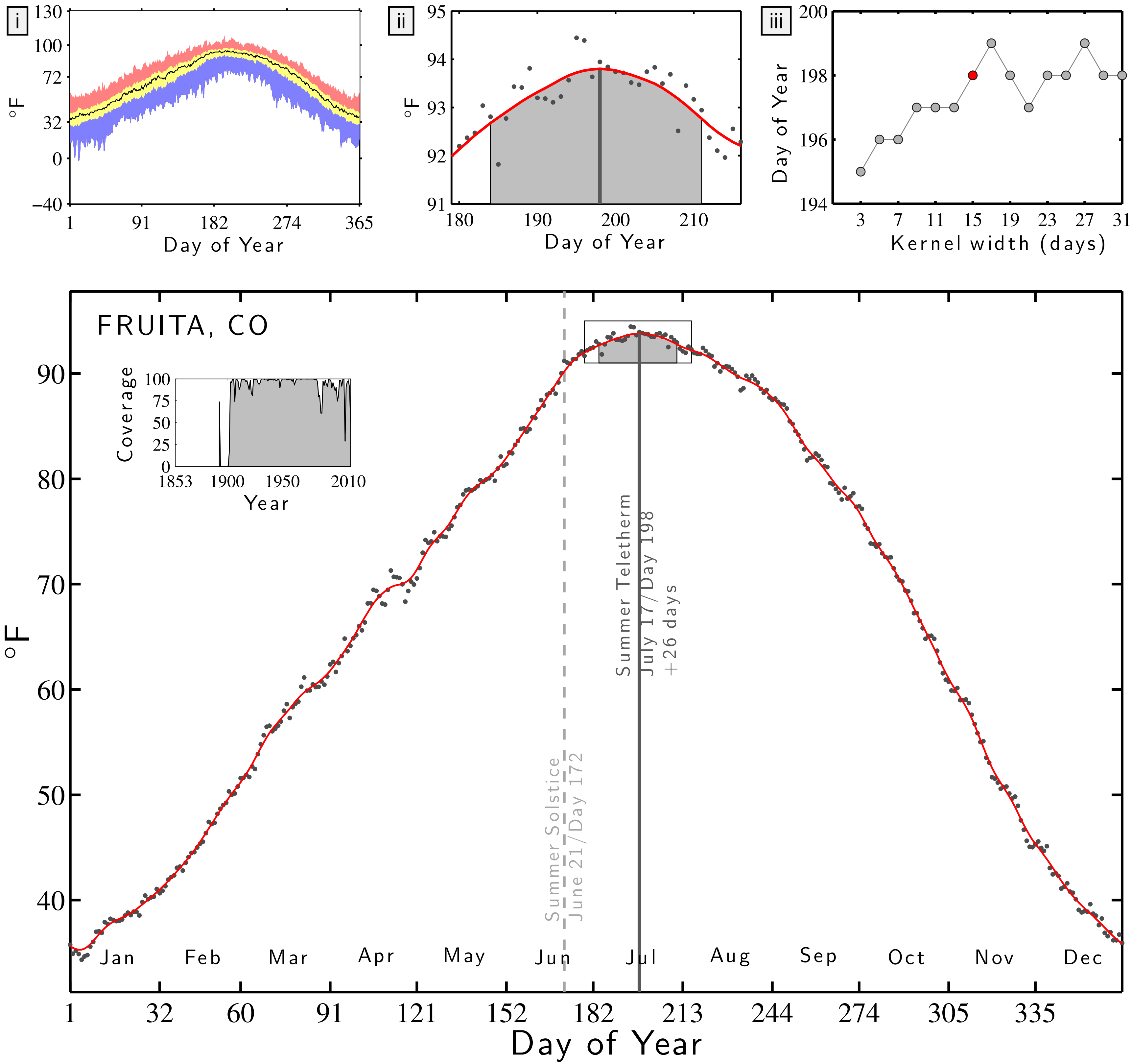

i
°F
Day of Year
ii
°F
Day of Year
iii
Day of Year
Kernel width (days)
FRUITA, CO
Coverage
Year
Summer Solstice
June 21/Day 172
Summer Teletherm
July 17/Day 198
+26 days
Jan
Feb
Mar
Apr
May
Jun
Jul
Aug
Sep
Oct
Nov
Dec
°F
Day of Year

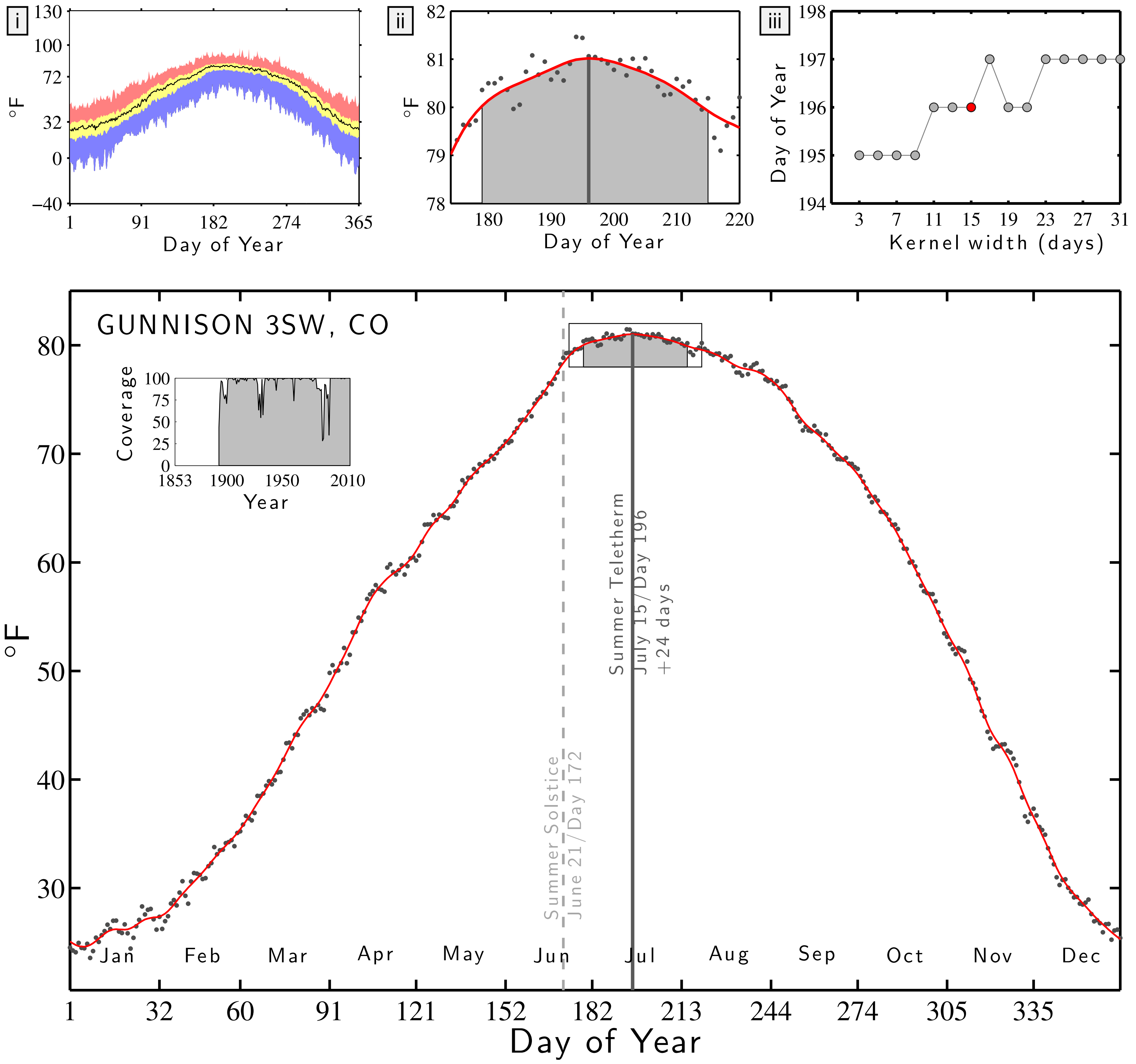
i
°F
Day of Year
ii
°F
Day of Year
iii
Day of Year
Kernel width (days)
GUNNISON 3SW, CO
Coverage
Year
Summer Solstice
June 21/Day 172
Summer Teletherm
July 15/Day 196
+24 days
Jan
Feb
Mar
Apr
May
Jun
Jul
Aug
Sep
Oct
Nov
Dec
°F
Day of Year

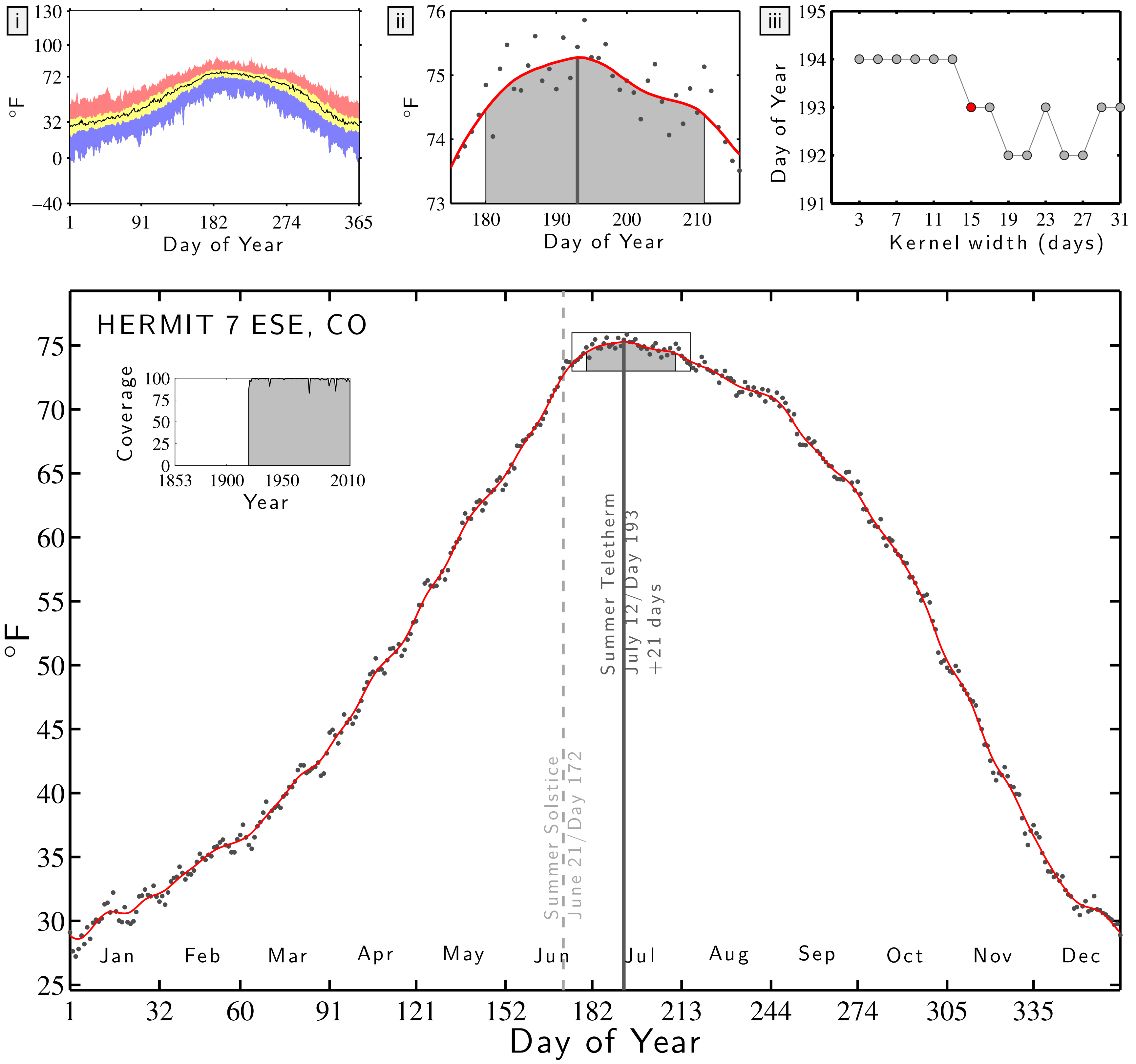

i
130
100
72
32
0
−40
°F
1
91
182
274
365
Day of Year
ii
76
75
74
73
°F
180
190
200
210
Day of Year
iii
195
194
193
192
191
Day of Year
3
7
11
15
19
23
27
31
Kernel width (days)
HERMIT 7 ESE, CO
Coverage
100
75
50
25
0
1853
1900
1950
2010
Year
Summer Solstice
June 21/Day 172
Summer Teletherm
July 12/Day 193
+21 days
75
70
65
60
55
50
45
40
35
30
25
°F
Jan
Feb
Mar
Apr
May
Jun
Jul
Aug
Sep
Oct
Nov
Dec
1
32
60
91
121
152
182
213
244
274
305
335
Day of Year

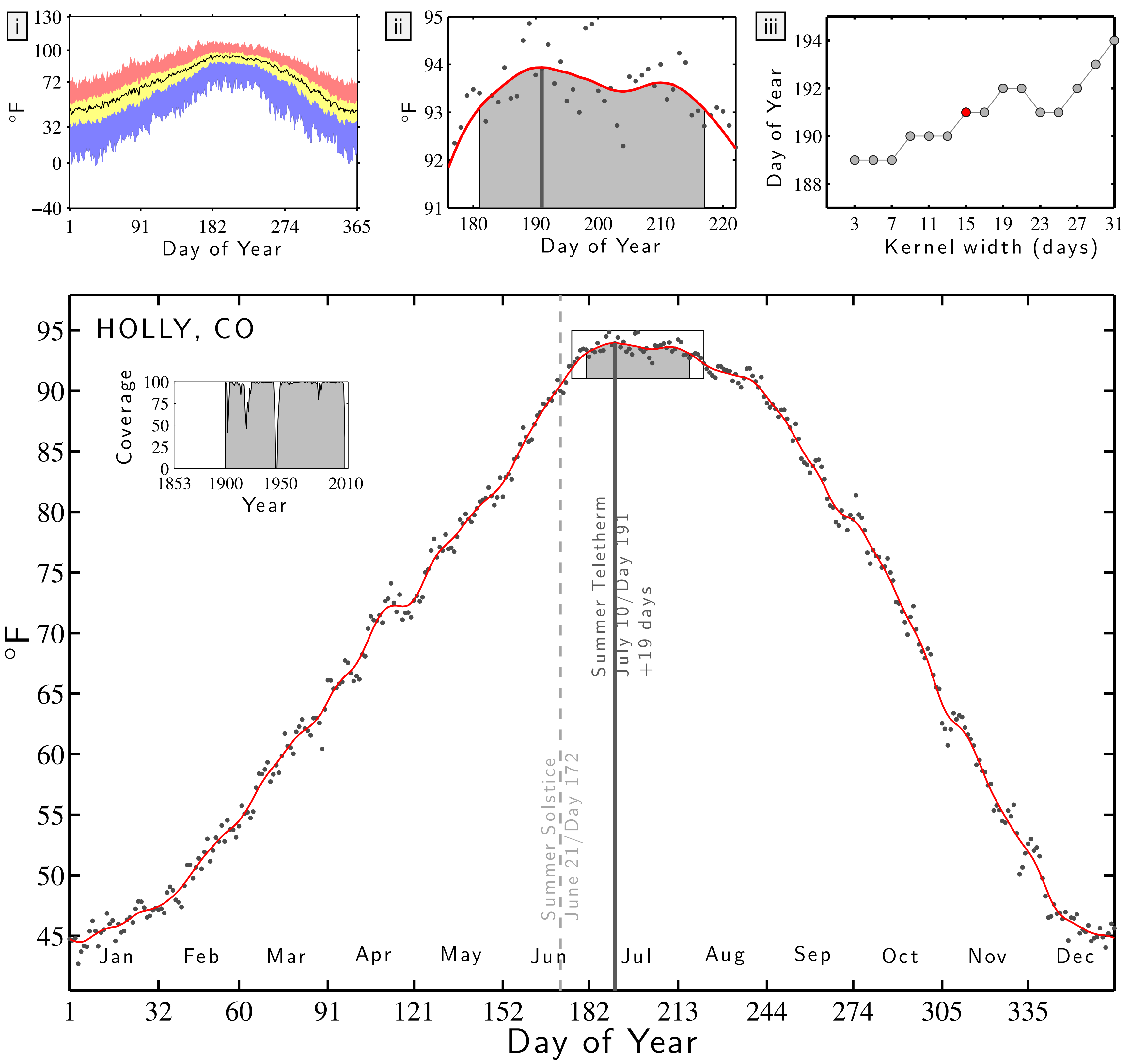

i
°F
Day of Year
ii
°F
Day of Year
iii
Day of Year
Kernel width (days)
HOLLY, CO
Coverage
Year
Summer Solstice
June 21/Day 172
Summer Teletherm
July 10/Day 191
+19 days
Jan
Feb
Mar
Apr
May
Jun
Jul
Aug
Sep
Oct
Nov
Dec
°F
Day of Year

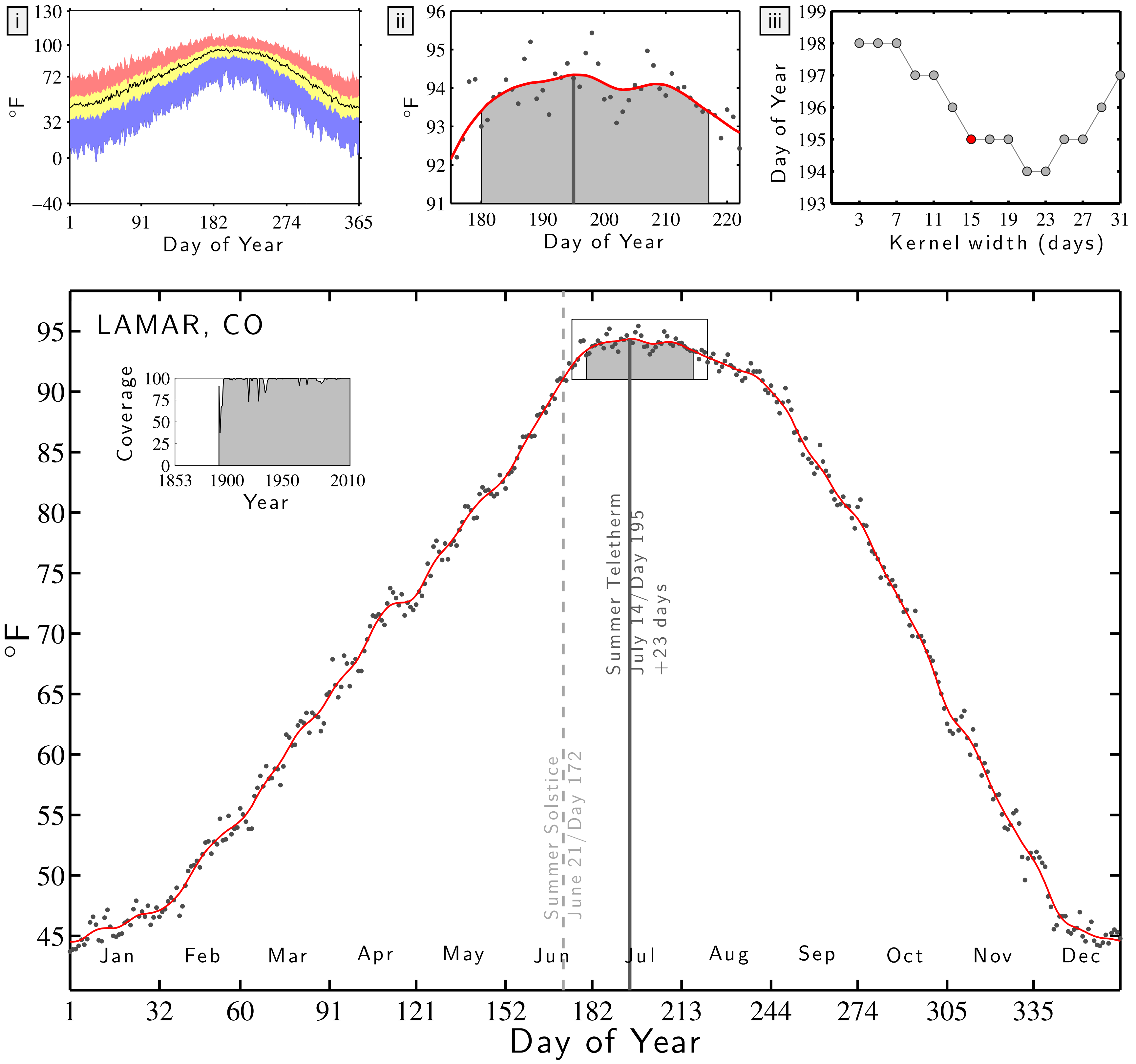

i
°F
Day of Year
ii
°F
Day of Year
iii
Day of Year
Kernel width (days)
LAMAR, CO
Coverage
Year
Summer Solstice
June 21/Day 172
Summer Teletherm
July 14/Day 195
+23 days
Jan
Feb
Mar
Apr
May
Jun
Jul
Aug
Sep
Oct
Nov
Dec
°F
Day of Year

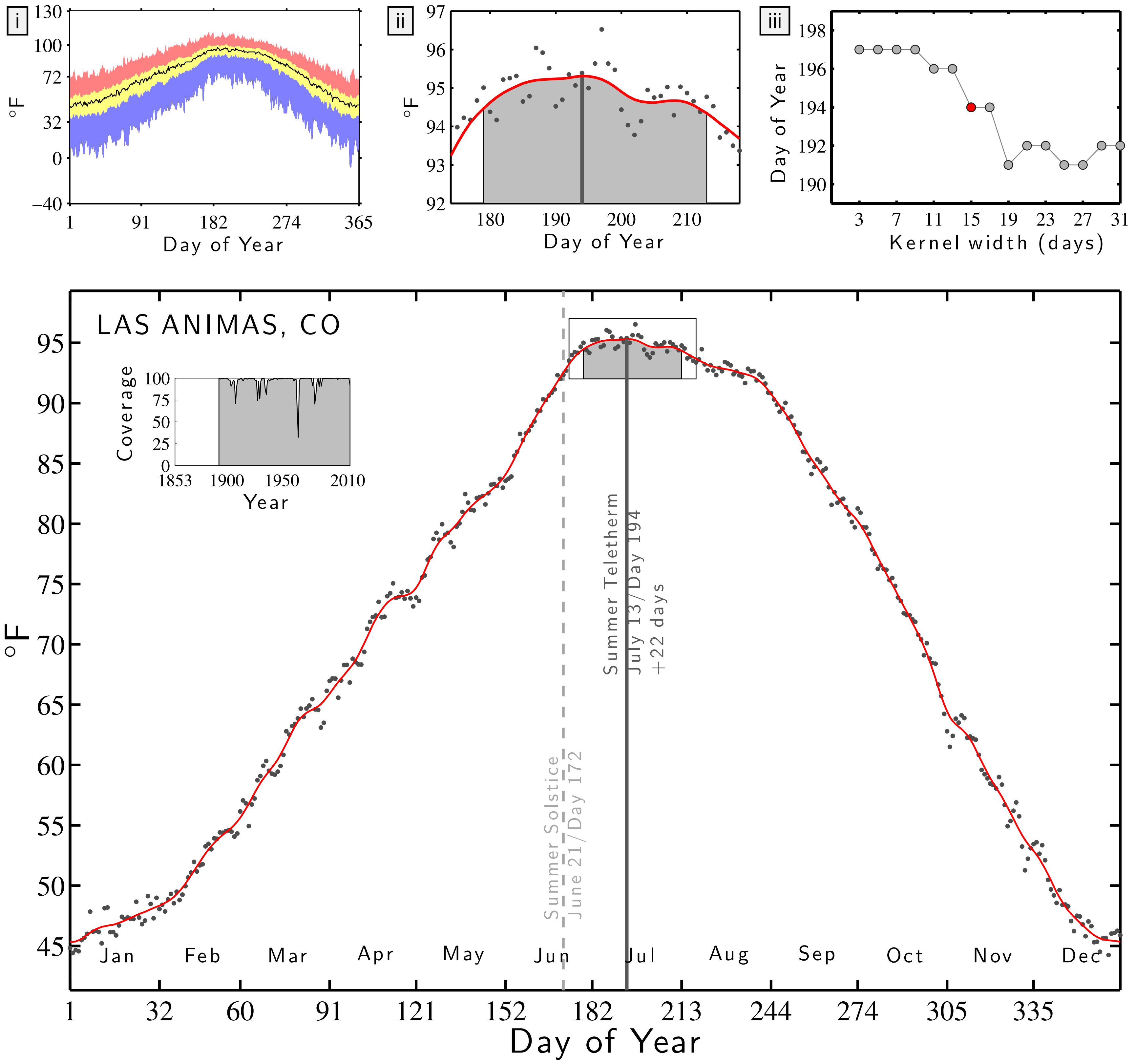

i
ii
iii
°F
Day of Year
Kernel width (days)
LAS ANIMAS, CO
Coverage
Year
Summer Solstice
June 21/Day 172
Summer Teletherm
July 13/Day 194
+22 days
Jan
Feb
Mar
Apr
May
Jun
Jul
Aug
Sep
Oct
Nov
Dec

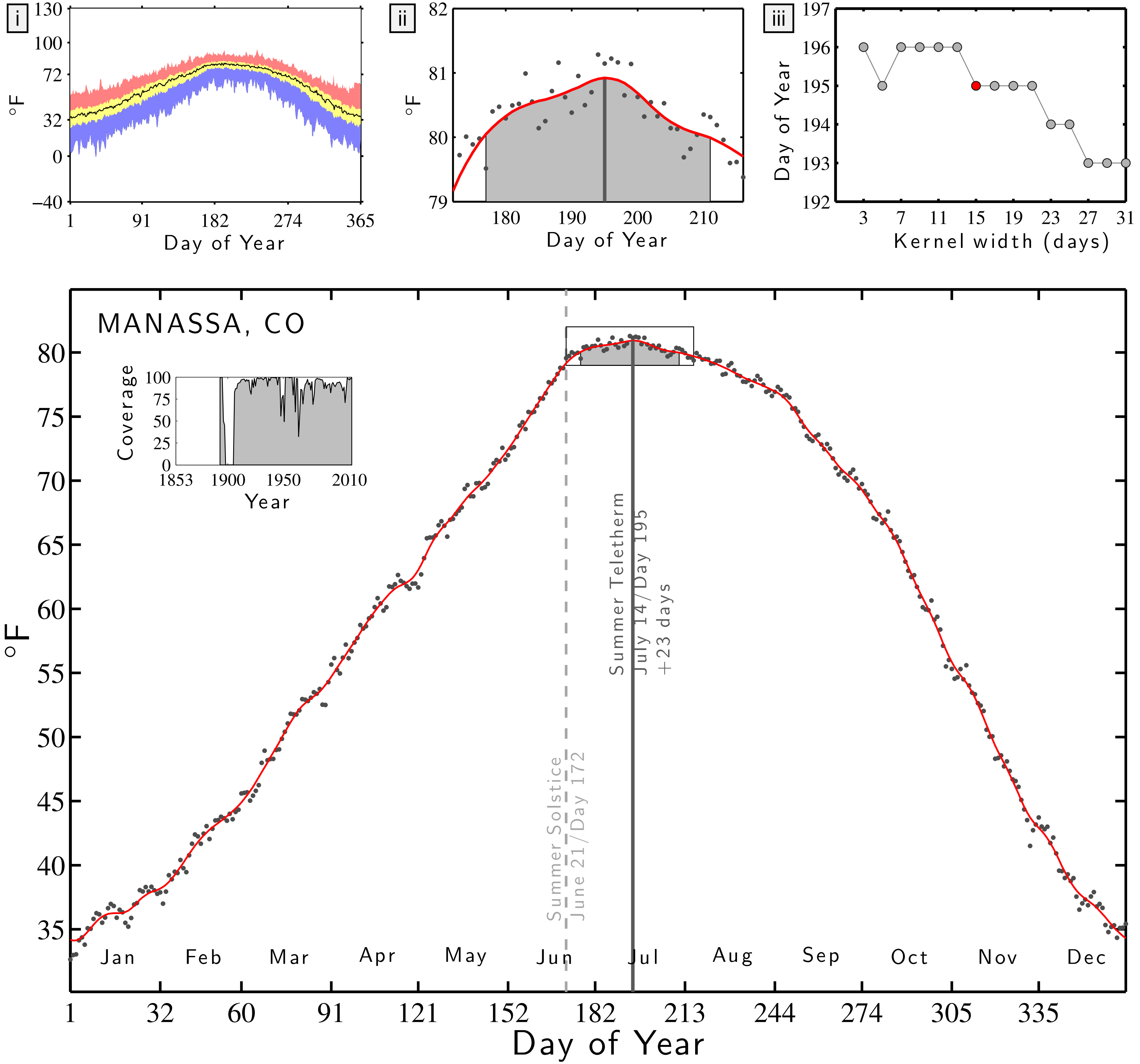

i
°F
Day of Year
ii
°F
Day of Year
iii
Day of Year
Kernel width (days)
MANASSA, CO
Coverage
Year
Summer Solstice
June 21/Day 172
Summer Teletherm
July 14/Day 195
+23 days
Jan
Feb
Mar
Apr
May
Jun
Jul
Aug
Sep
Oct
Nov
Dec
°F
Day of Year

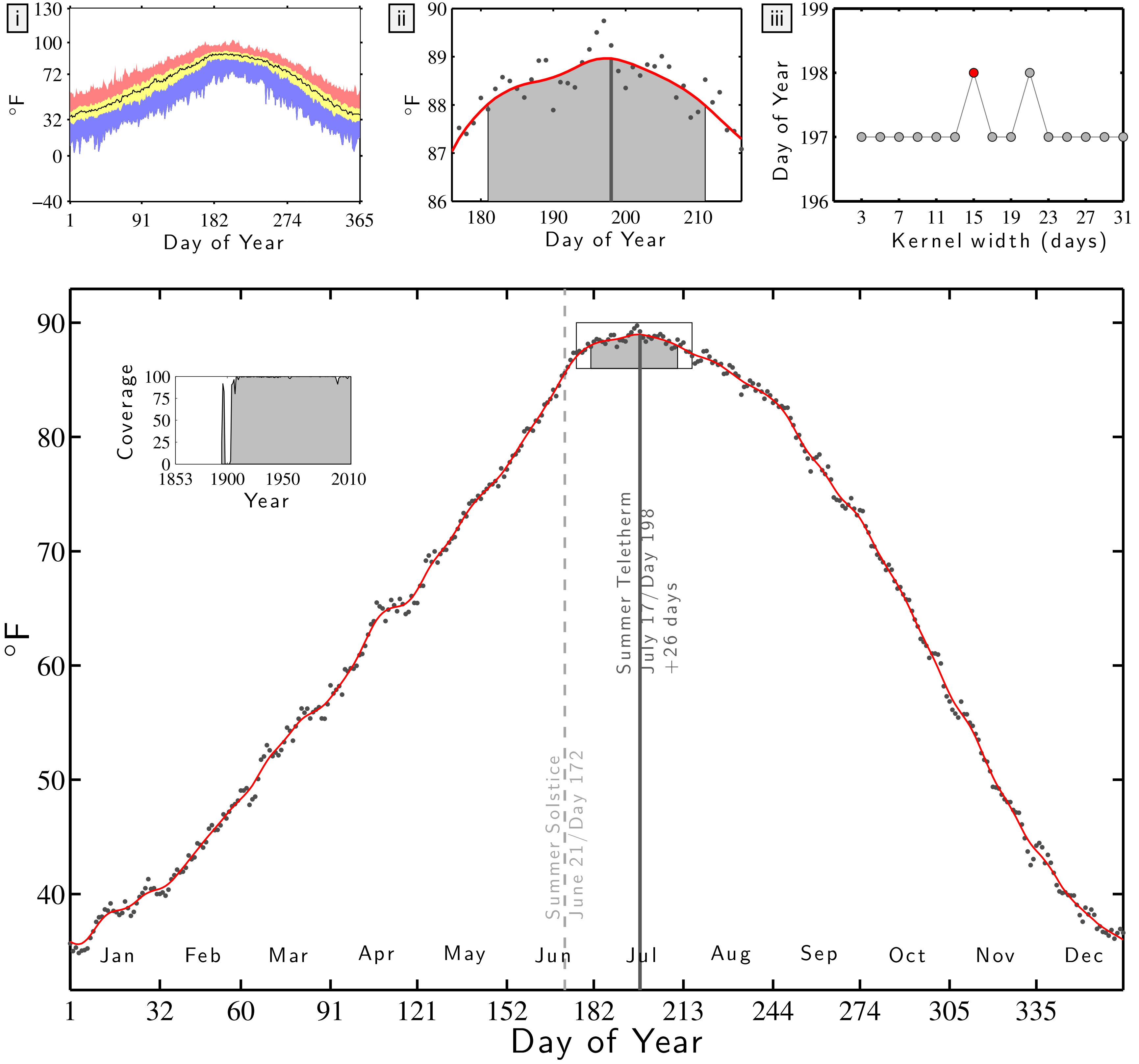

i
°F
130
100
72
32
0
−40
1
91
182
274
365
Day of Year
ii
°F
90
89
88
87
86
180
190
200
210
Day of Year
iii
Day of Year
199
198
197
196
3
7
11
15
19
23
27
31
Kernel width (days)
°F
90
80
70
60
50
40
Coverage
100
75
50
25
0
1853
1900
1950
2010
Year
Summer Solstice
June 21/Day 172
Summer Teletherm
July 17/Day 198
+26 days
Jan
Feb
Mar
Apr
May
Jun
Jul
Aug
Sep
Oct
Nov
Dec
1
32
60
91
121
152
182
213
244
274
305
335
Day of Year

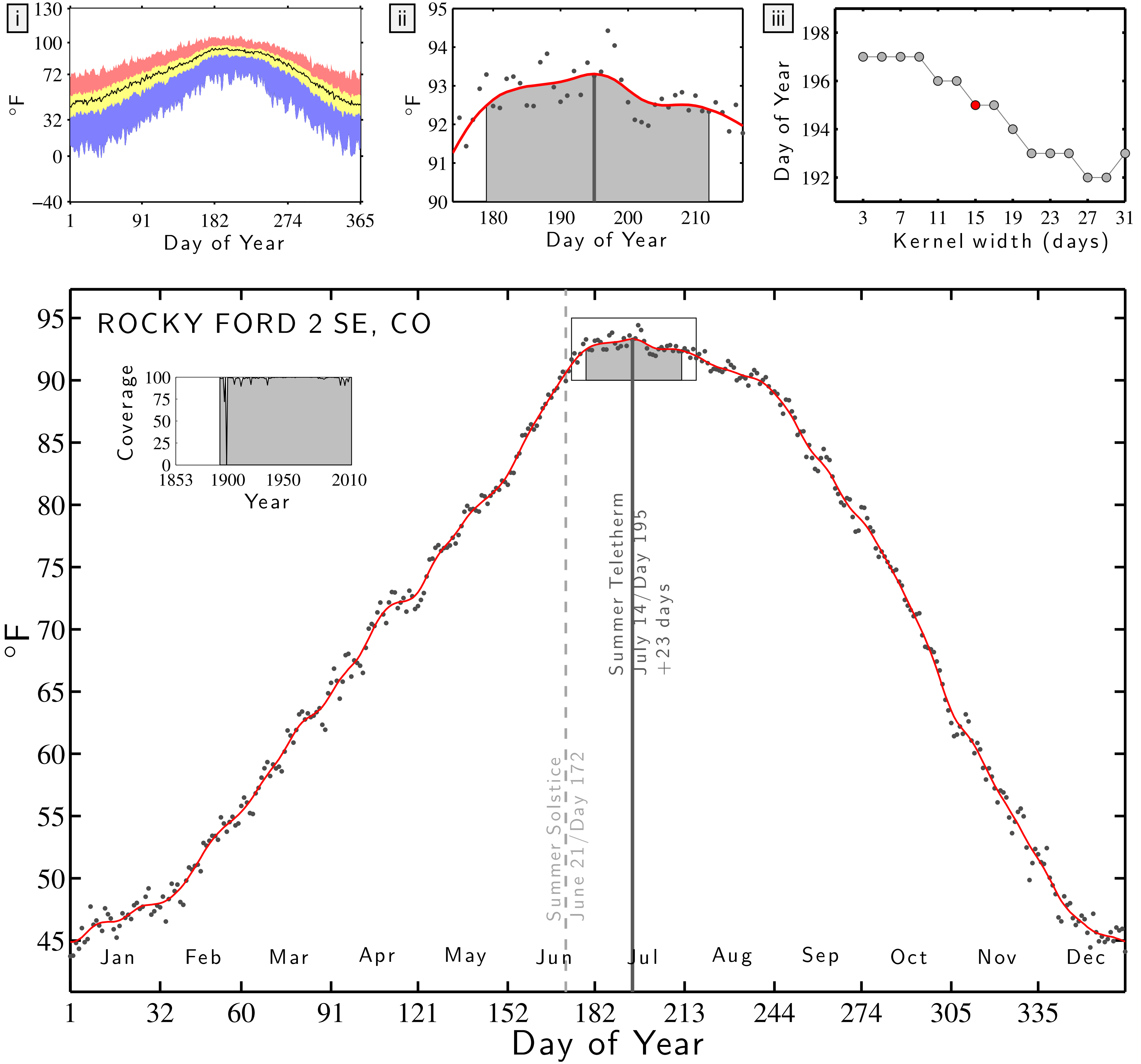

i
ii
iii
Day of Year
Kernel width (days)
ROCKY FORD 2 SE, CO
Coverage
Year
Summer Solstice
June 21/Day 172
Summer Teletherm
July 14/Day 195
+23 days
Jan
Feb
Mar
Apr
May
Jun
Jul
Aug
Sep
Oct
Nov
Dec
°F
Day of Year

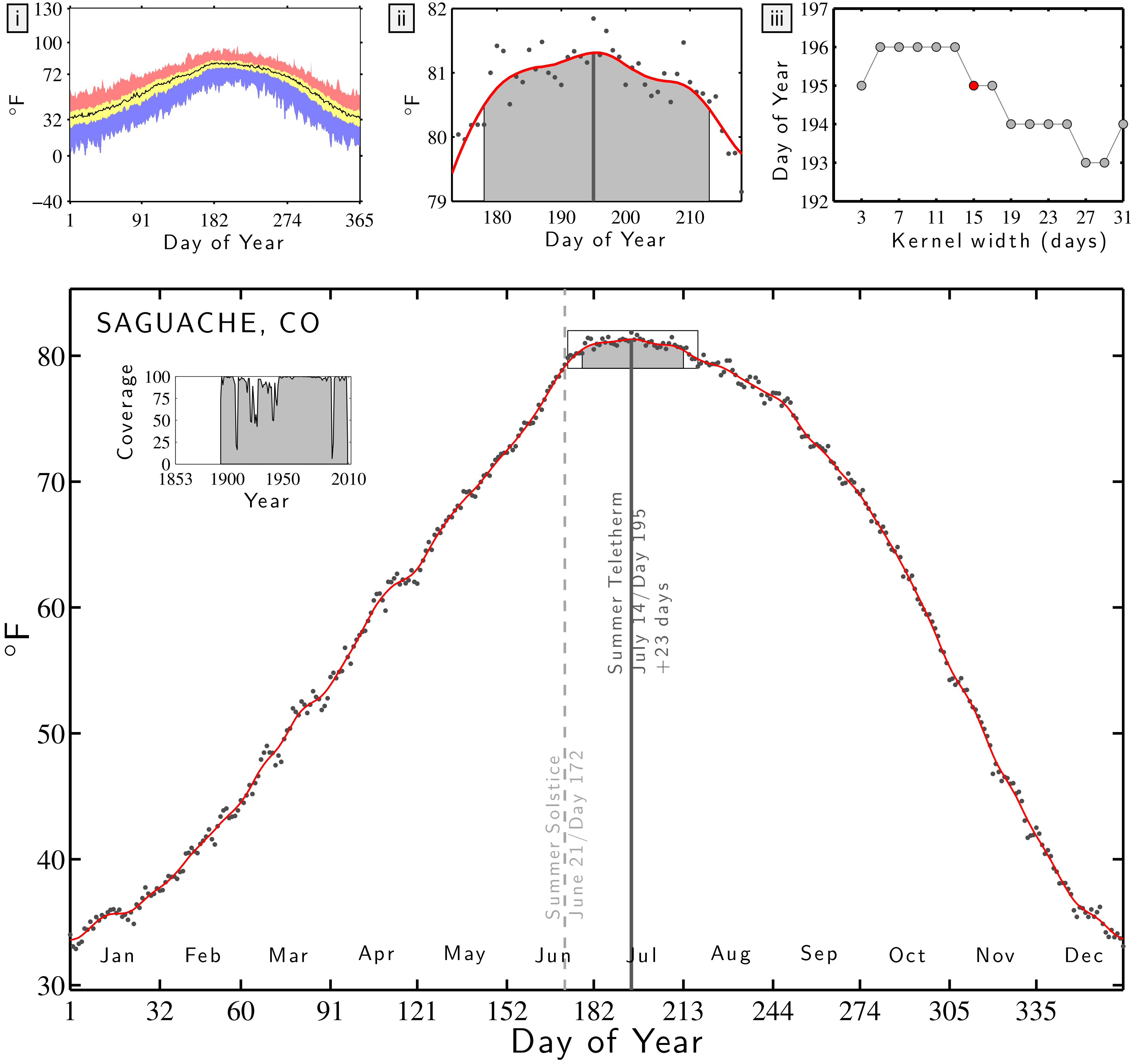

i
°F
Day of Year
ii
°F
Day of Year
iii
Day of Year
Kernel width (days)
SAGUACHE, CO
Coverage
Year
Summer Solstice
June 21/Day 172
Summer Teletherm
July 14/Day 195
+23 days
Jan
Feb
Mar
Apr
May
Jun
Jul
Aug
Sep
Oct
Nov
Dec
°F
Day of Year

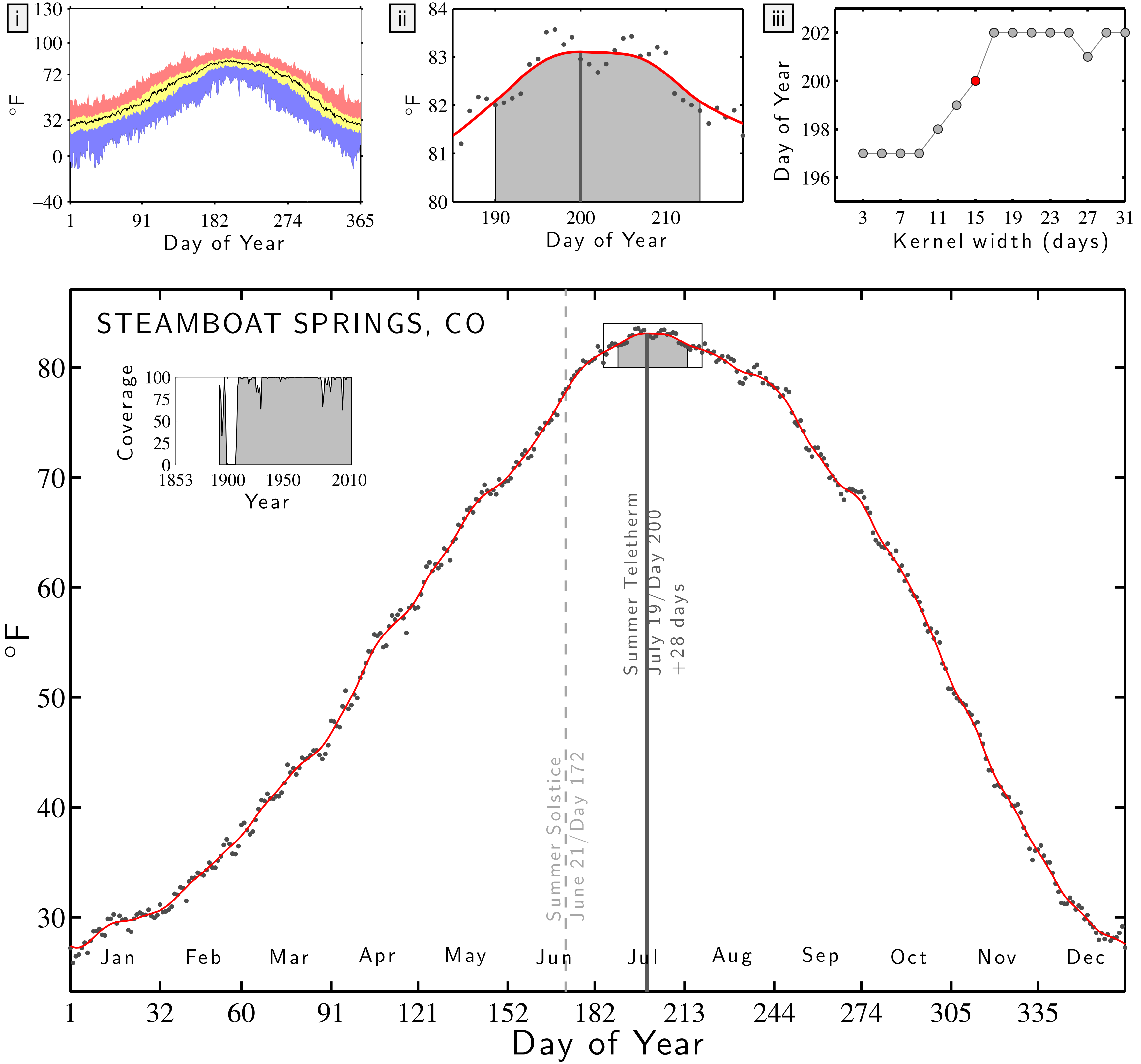

i
°F
Day of Year
ii
°F
Day of Year
iii
Day of Year
Kernel width (days)
STEAMBOAT SPRINGS, CO
Coverage
Year
Summer Solstice
June 21/Day 172
Summer Teletherm
July 19/Day 200
+28 days
Jan
Feb
Mar
Apr
May
Jun
Jul
Aug
Sep
Oct
Nov
Dec
°F
Day of Year

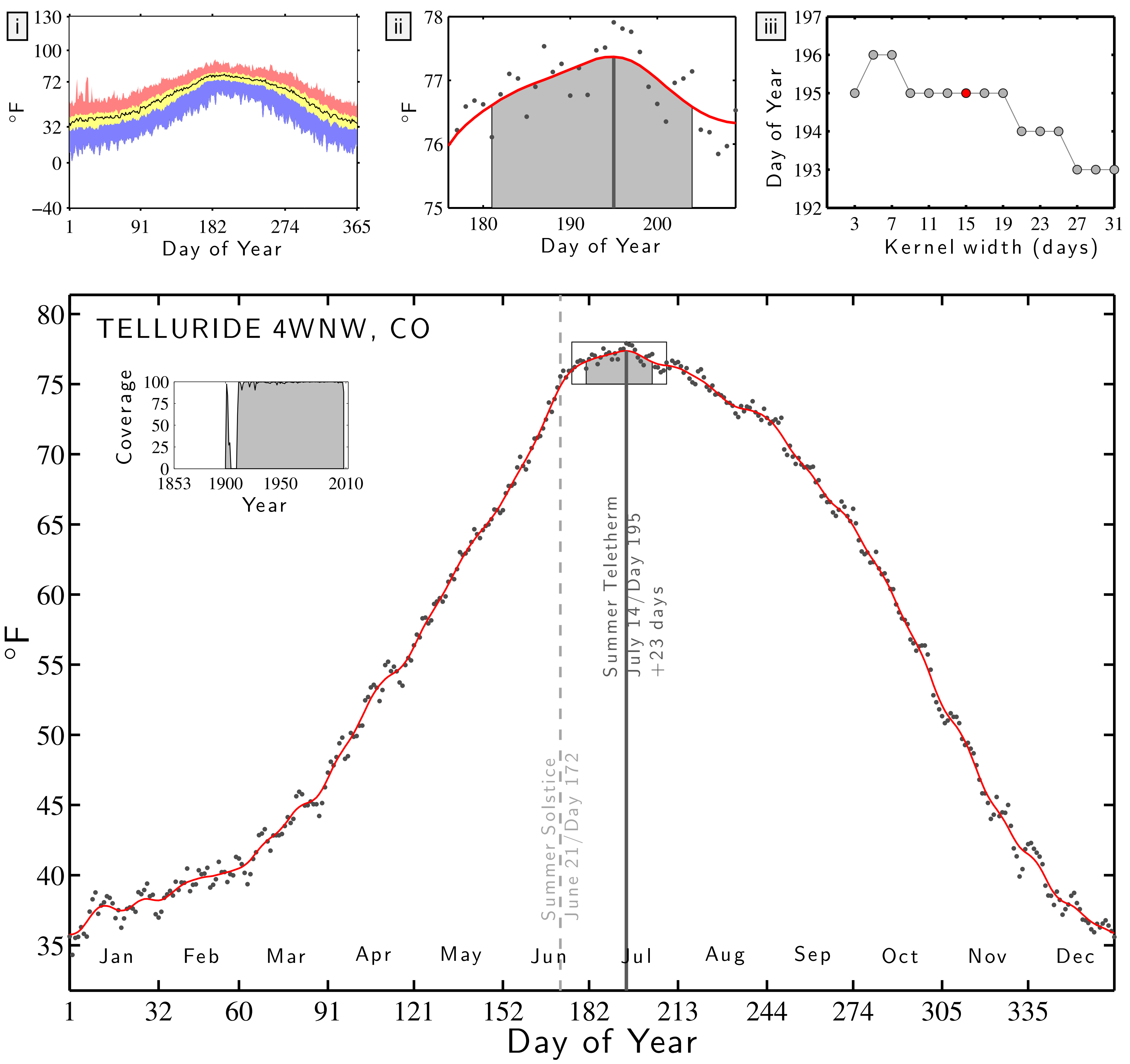

i
°F
130
100
72
32
0
−40
1
91
182
274
365
Day of Year
ii
°F
78
77
76
75
180
190
200
Day of Year
iii
Day of Year
197
196
195
194
193
192
3
7
11
15
19
23
27
31
Kernel width (days)
TELLURIDE 4WNW, CO
Coverage
100
75
50
25
0
1853
1900
1950
2010
Year
Summer Solstice
June 21/Day 172
Summer Teletherm
July 14/Day 195
+23 days
°F
80
75
70
65
60
55
50
45
40
35
Jan
Feb
Mar
Apr
May
Jun
Jul
Aug
Sep
Oct
Nov
Dec
1
32
60
91
121
152
182
213
244
274
305
335
Day of Year

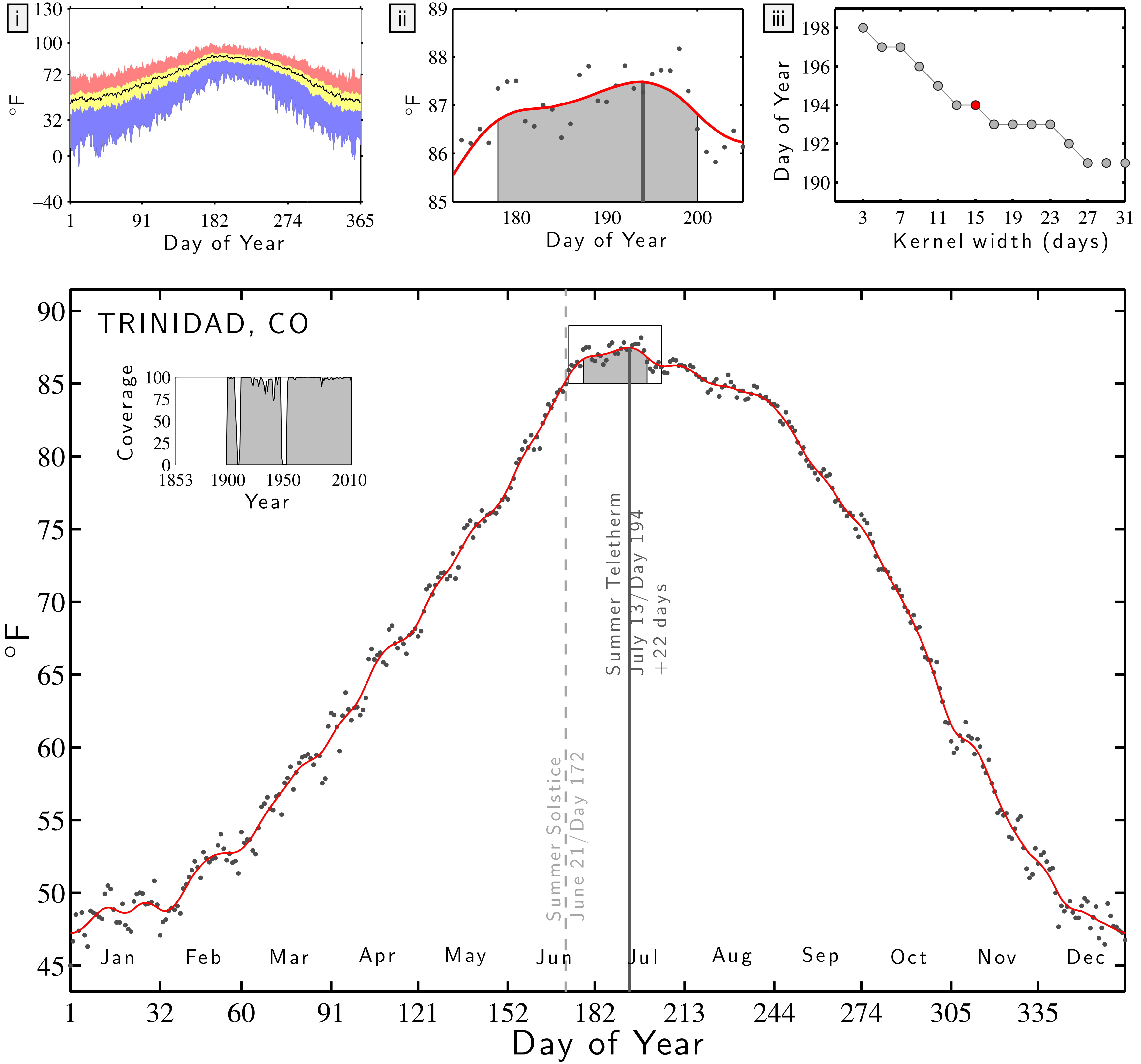
i
°F
130
100
72
32
0
−40
1
91
182
274
365
Day of Year
ii
°F
89
88
87
86
85
180
190
200
Day of Year
iii
Day of Year
198
196
194
192
190
3
7
11
15
19
23
27
31
Kernel width (days)
TRINIDAD, CO
Coverage
100
75
50
25
0
1853
1900
1950
2010
Year
°F
90
85
80
75
70
65
60
55
50
45
Summer Solstice
June 21/Day 172
Summer Teletherm
July 13/Day 194
+22 days
Jan
Feb
Mar
Apr
May
Jun
Jul
Aug
Sep
Oct
Nov
Dec
1
32
60
91
121
152
182
213
244
274
305
335
Day of Year

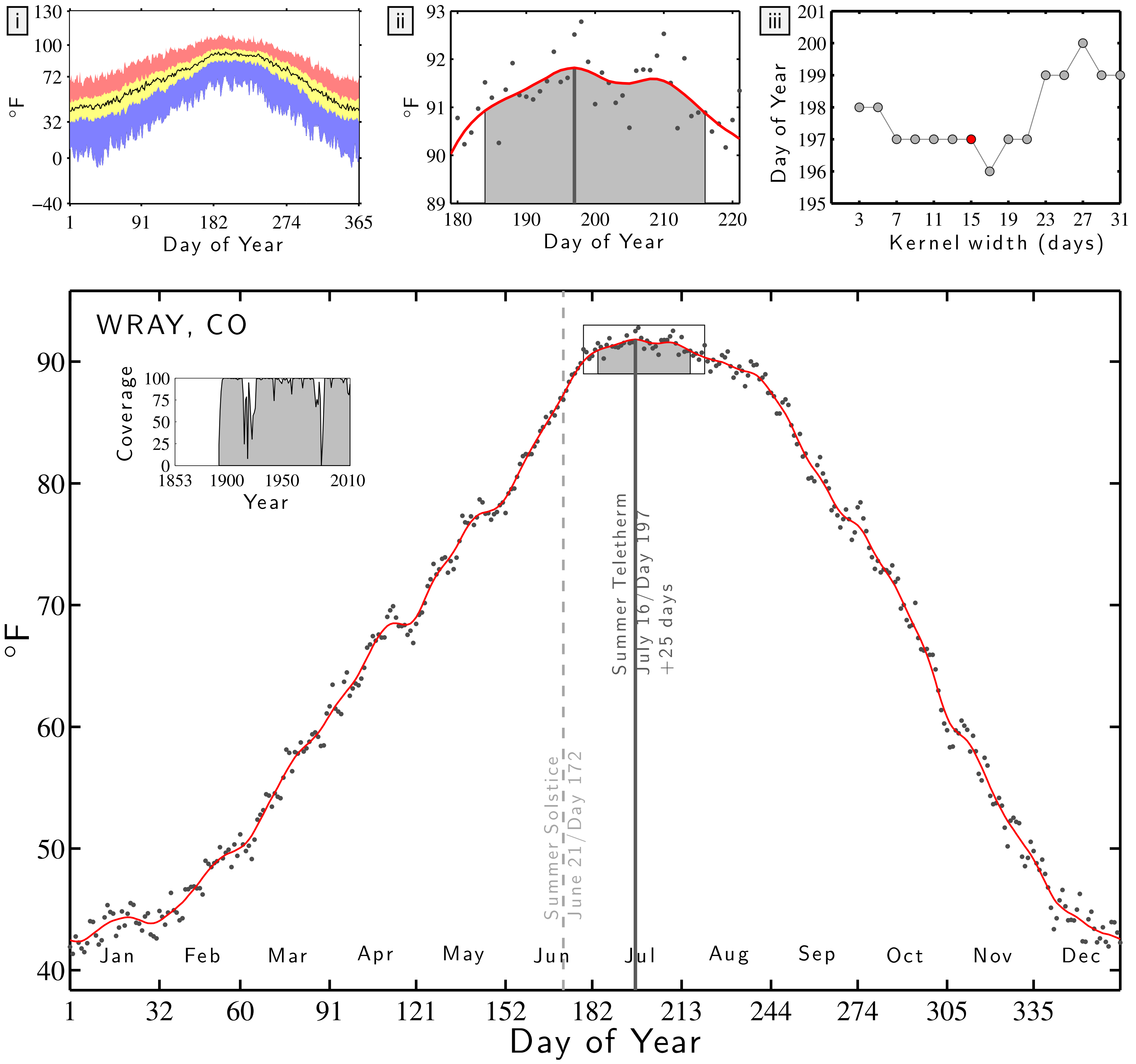

i
°F
Day of Year
ii
°F
Day of Year
iii
Day of Year
Kernel width (days)
WRAY, CO
Coverage
Year
Summer Solstice
June 21/Day 172
Summer Teletherm
July 16/Day 197
+25 days
Jan
Feb
Mar
Apr
May
Jun
Jul
Aug
Sep
Oct
Nov
Dec
°F
Day of Year

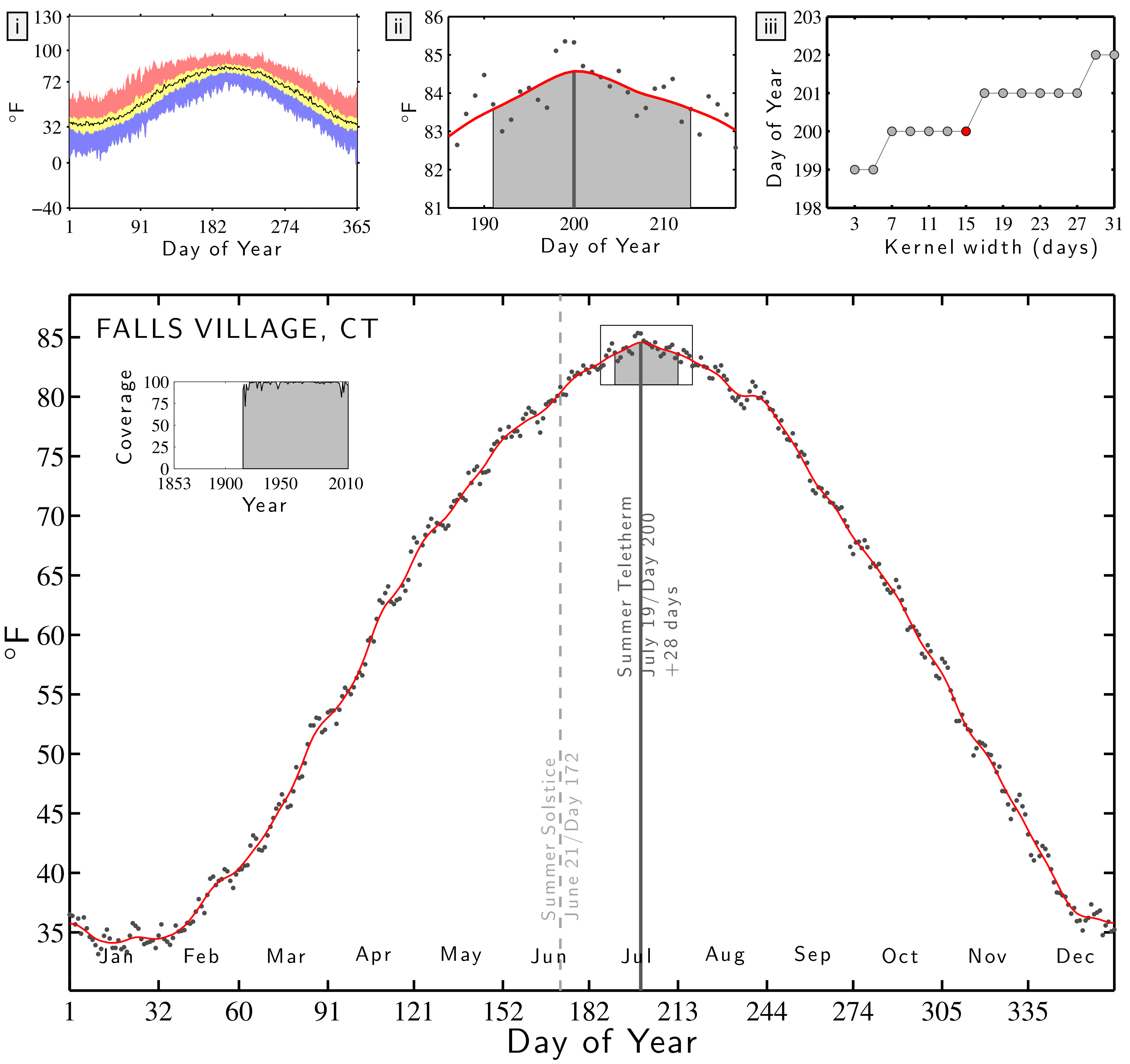

i
ii
iii
°F
Day of Year
Day of Year
Kernel width (days)
FALLS VILLAGE, CT
Coverage
Year
Summer Solstice
June 21/Day 172
Summer Teletherm
July 19/Day 200
+28 days
Jan
Feb
Mar
Apr
May
Jun
Jul
Aug
Sep
Oct
Nov
Dec

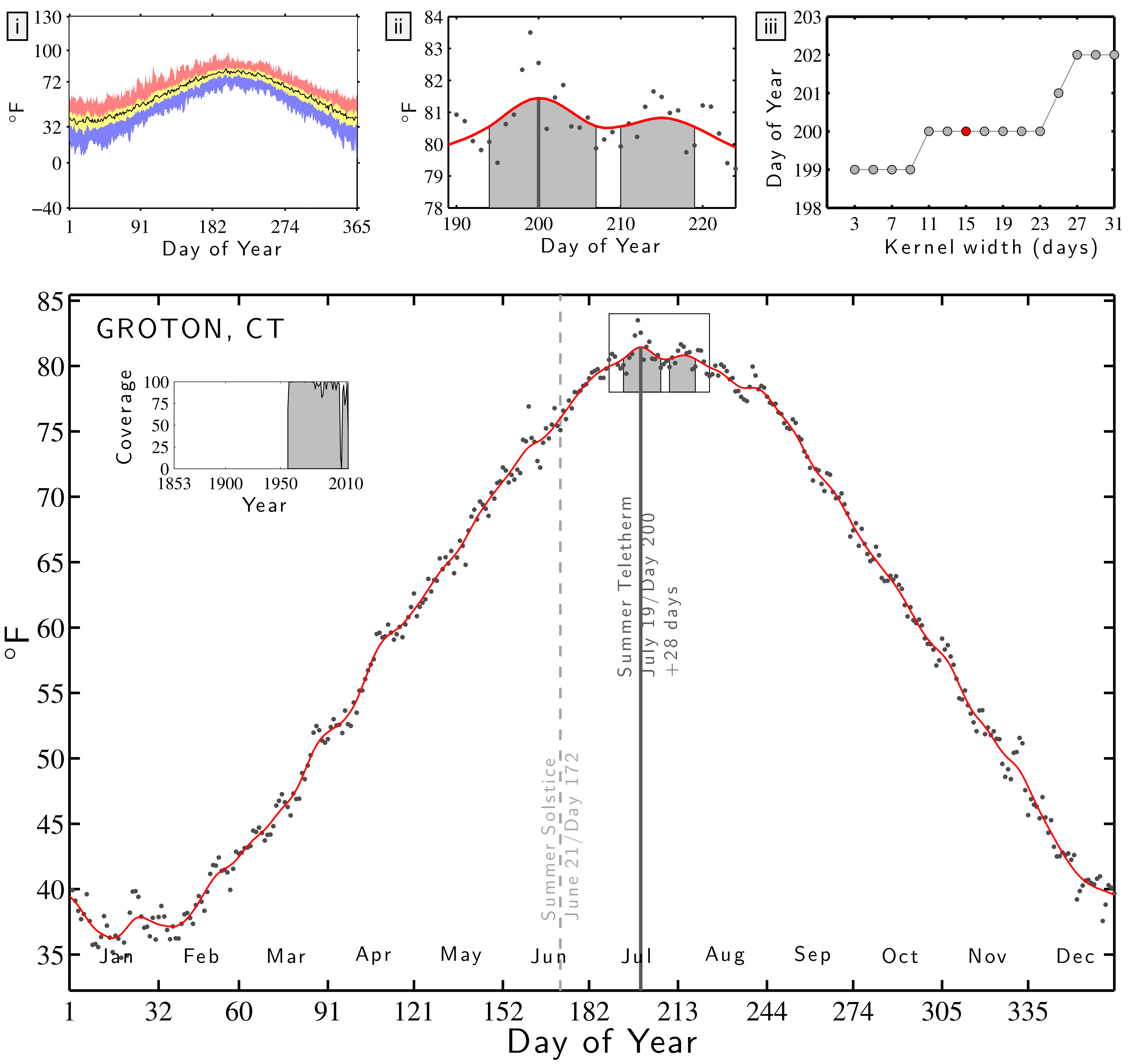

i
ii
iii
Day of Year
Kernel width (days)
°F
GROTON, CT
Coverage
Year
Summer Solstice
June 21/Day 172
Summer Teletherm
July 19/Day 200
+28 days
Jan
Feb
Mar
Apr
May
Jun
Jul
Aug
Sep
Oct
Nov
Dec

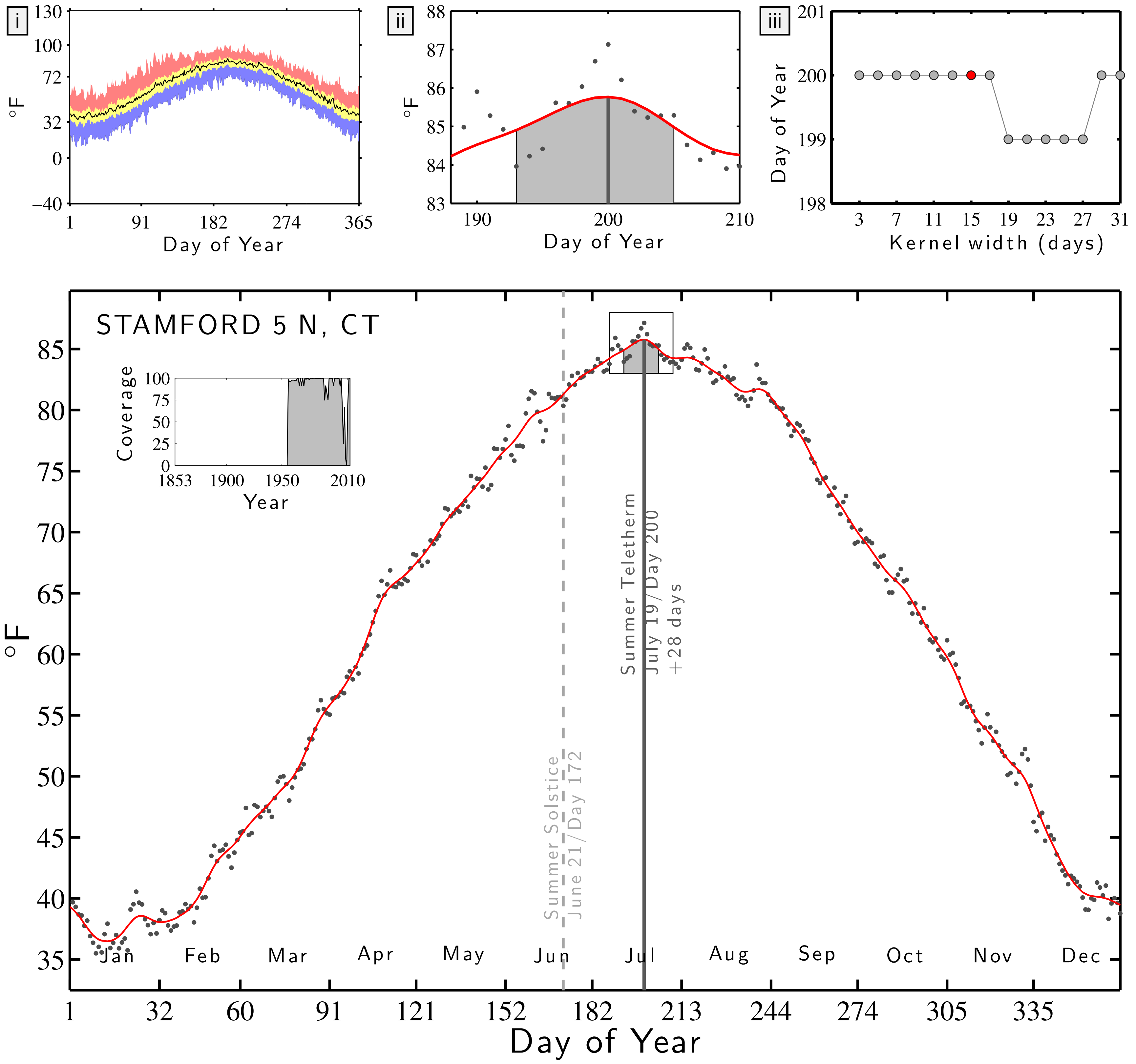

i
°F
Day of Year
ii
°F
Day of Year
iii
Day of Year
Kernel width (days)
STAMFORD 5 N, CT
Coverage
Year
Summer Solstice
June 21/Day 172
Summer Teletherm
July 19/Day 200
+28 days
Jan
Feb
Mar
Apr
May
Jun
Jul
Aug
Sep
Oct
Nov
Dec
°F
Day of Year

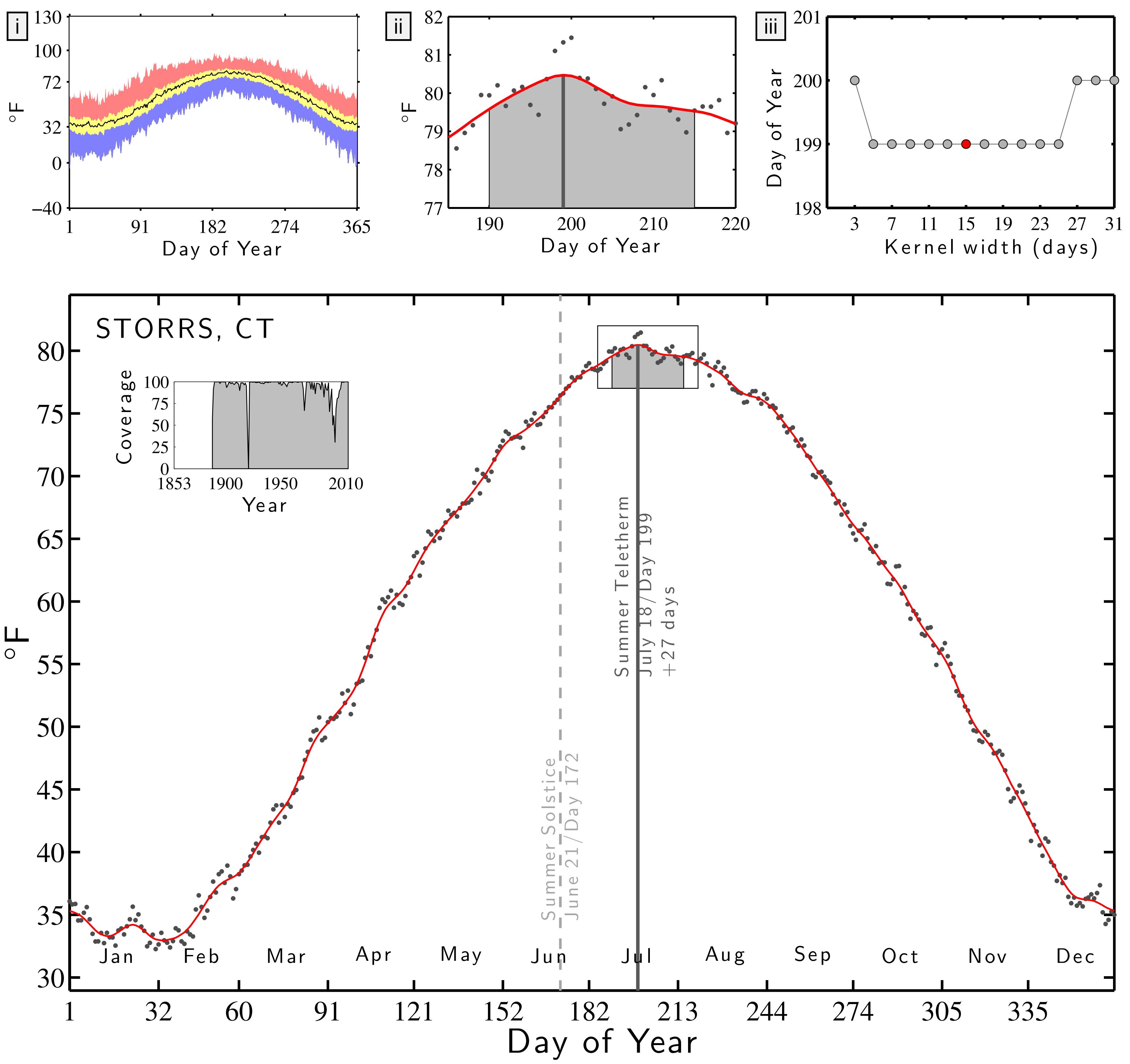

i
ii
iii
°F
Day of Year
Day of Year
Kernel width (days)
STORRS, CT
Coverage
Year
Summer Solstice
June 21/Day 172
Summer Teletherm
July 18/Day 199
+27 days
Jan
Feb
Mar
Apr
May
Jun
Jul
Aug
Sep
Oct
Nov
Dec

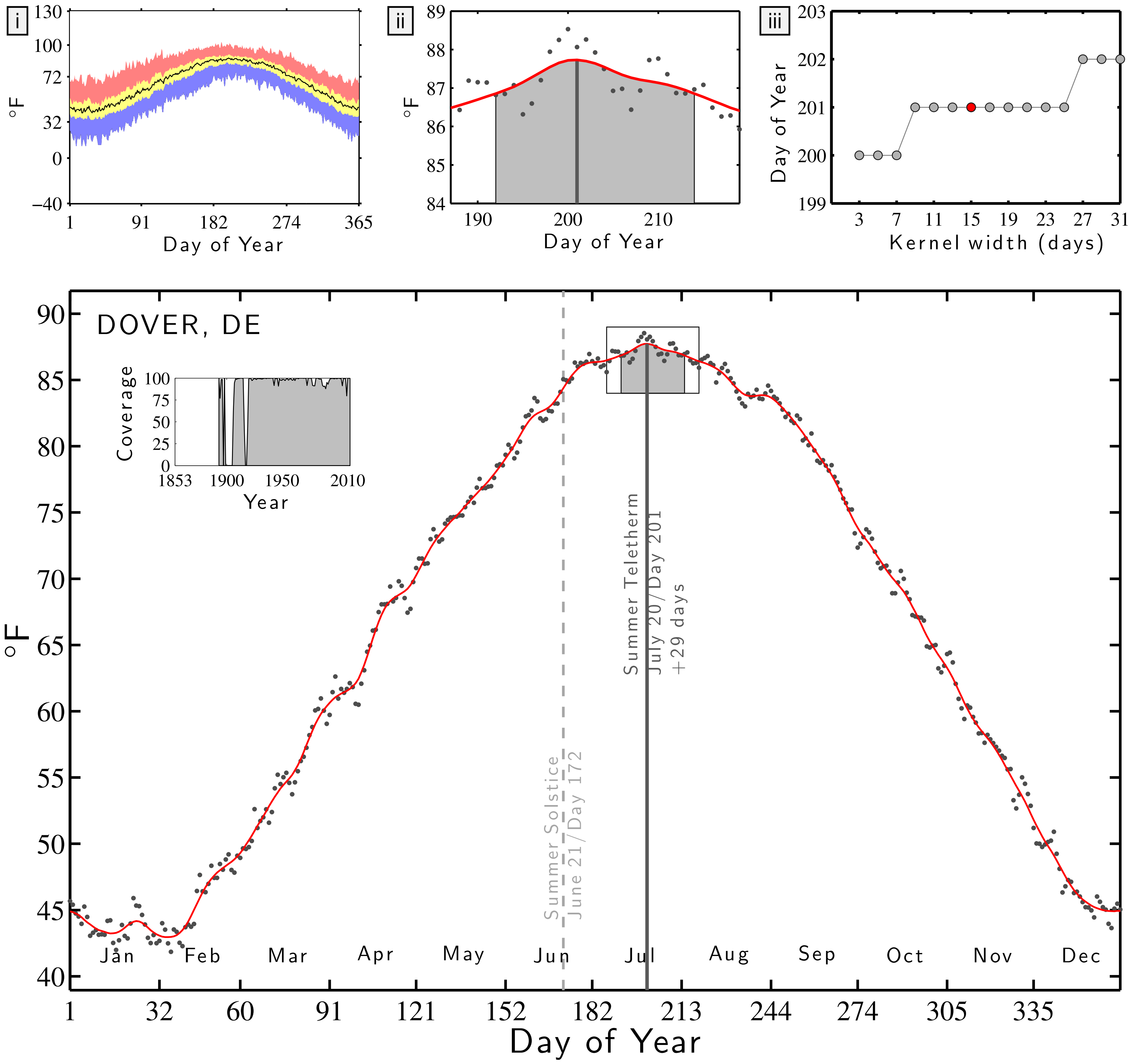
i
°F
Day of Year
ii
°F
Day of Year
iii
Day of Year
Kernel width (days)
DOVER, DE
Coverage
Year
Summer Solstice
June 21/Day 172
Summer Teletherm
July 20/Day 201
+29 days
Jan
Feb
Mar
Apr
May
Jun
Jul
Aug
Sep
Oct
Nov
Dec
°F
Day of Year

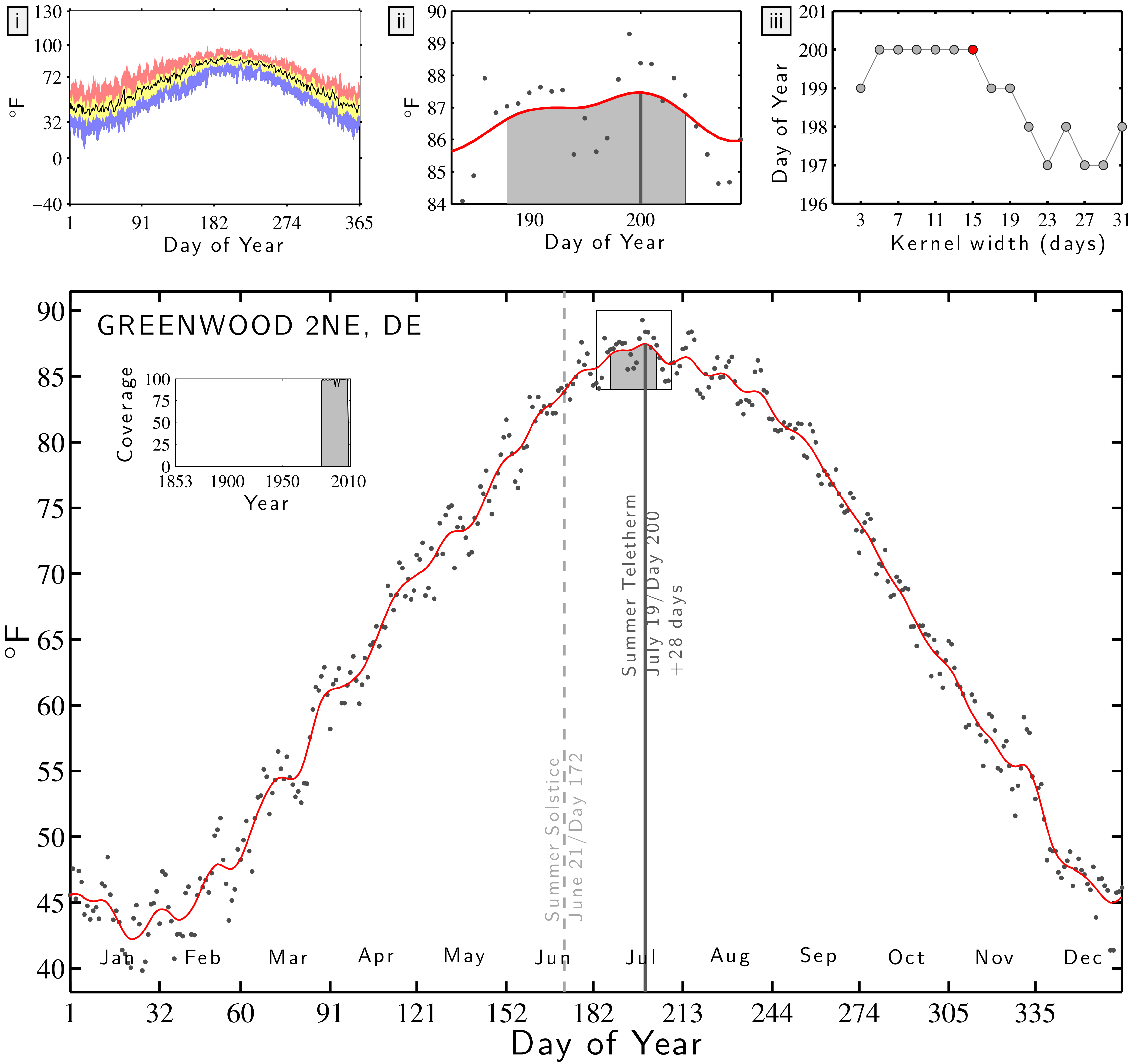

i
°F
Day of Year
ii
°F
Day of Year
iii
Day of Year
Kernel width (days)
GREENWOOD 2NE, DE
Coverage
Year
Summer Solstice
June 21/Day 172
Summer Teletherm
July 19/Day 200
+28 days
Jan
Feb
Mar
Apr
May
Jun
Jul
Aug
Sep
Oct
Nov
Dec
°F
Day of Year

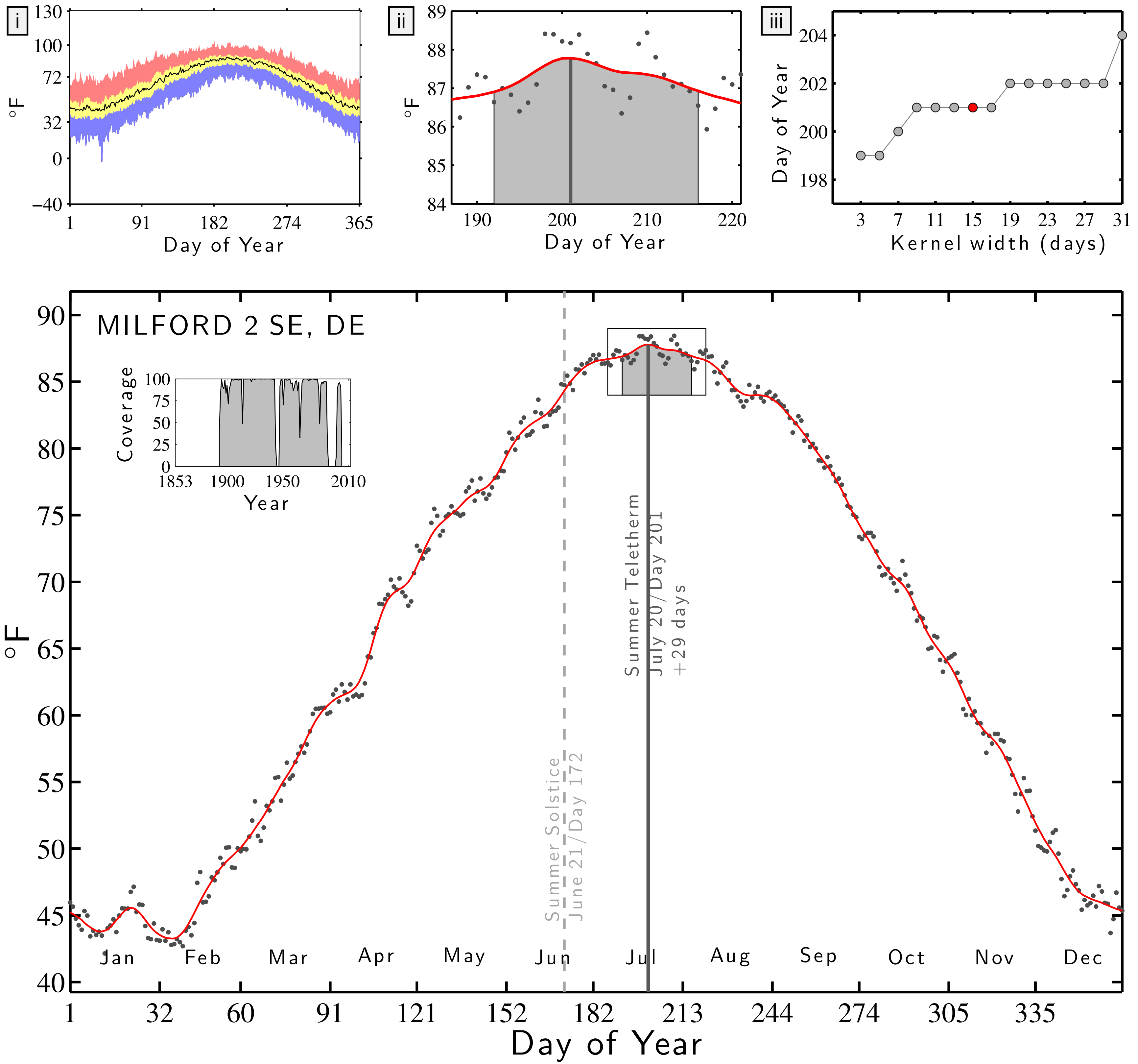

i
°F
Day of Year
ii
°F
Day of Year
iii
Day of Year
Kernel width (days)
MILFORD 2 SE, DE
Coverage
Year
Summer Solstice
June 21/Day 172
Summer Teletherm
July 20/Day 201
+29 days
Jan
Feb
Mar
Apr
May
Jun
Jul
Aug
Sep
Oct
Nov
Dec
°F
Day of Year

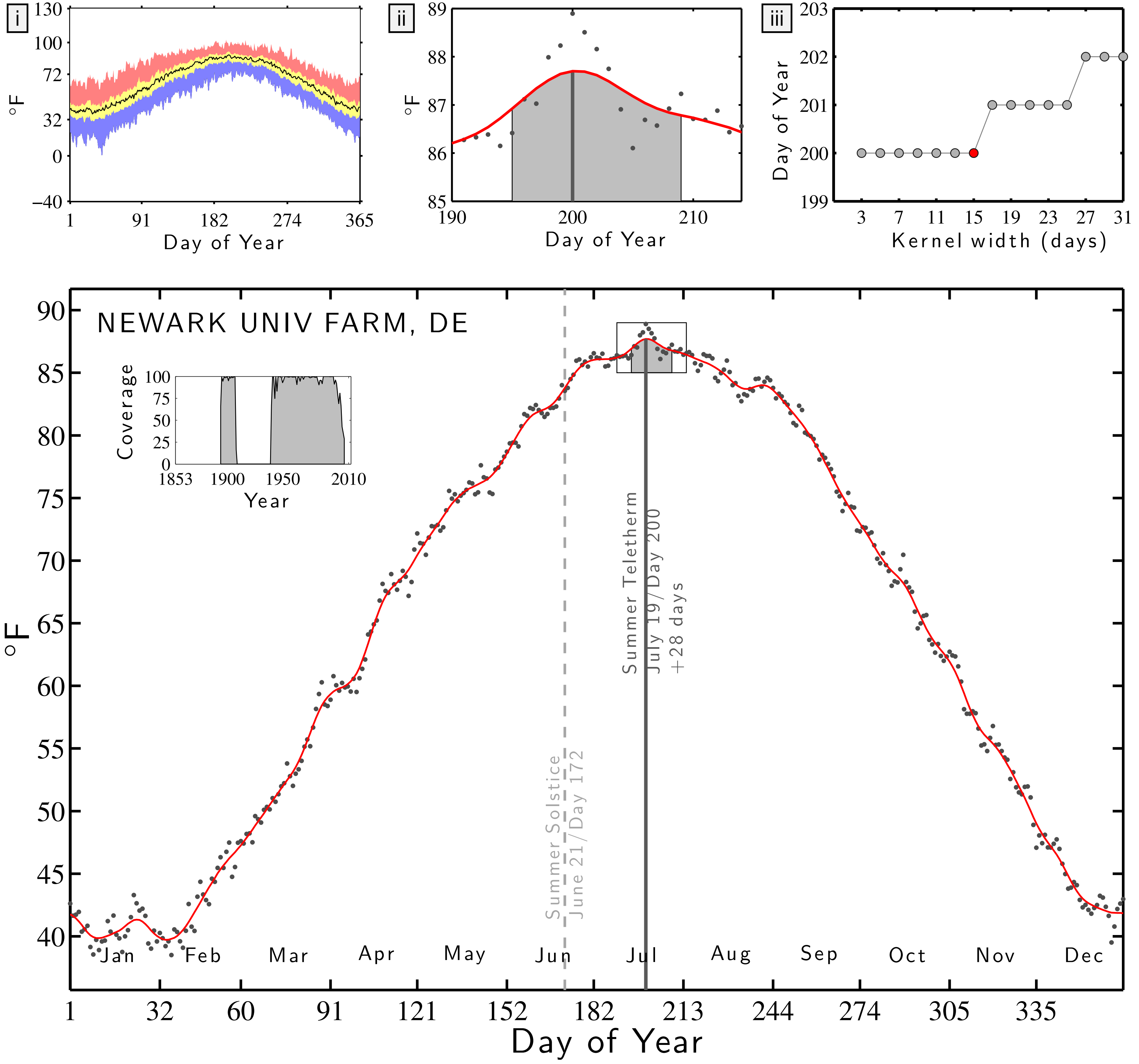

i
ii
iii
°F
Day of Year
Day of Year
Kernel width (days)
NEWARK UNIV FARM, DE
Coverage
Year
Summer Solstice
June 21/Day 172
Summer Teletherm
July 19/Day 200
+28 days
Jan
Feb
Mar
Apr
May
Jun
Jul
Aug
Sep
Oct
Nov
Dec
Day of Year

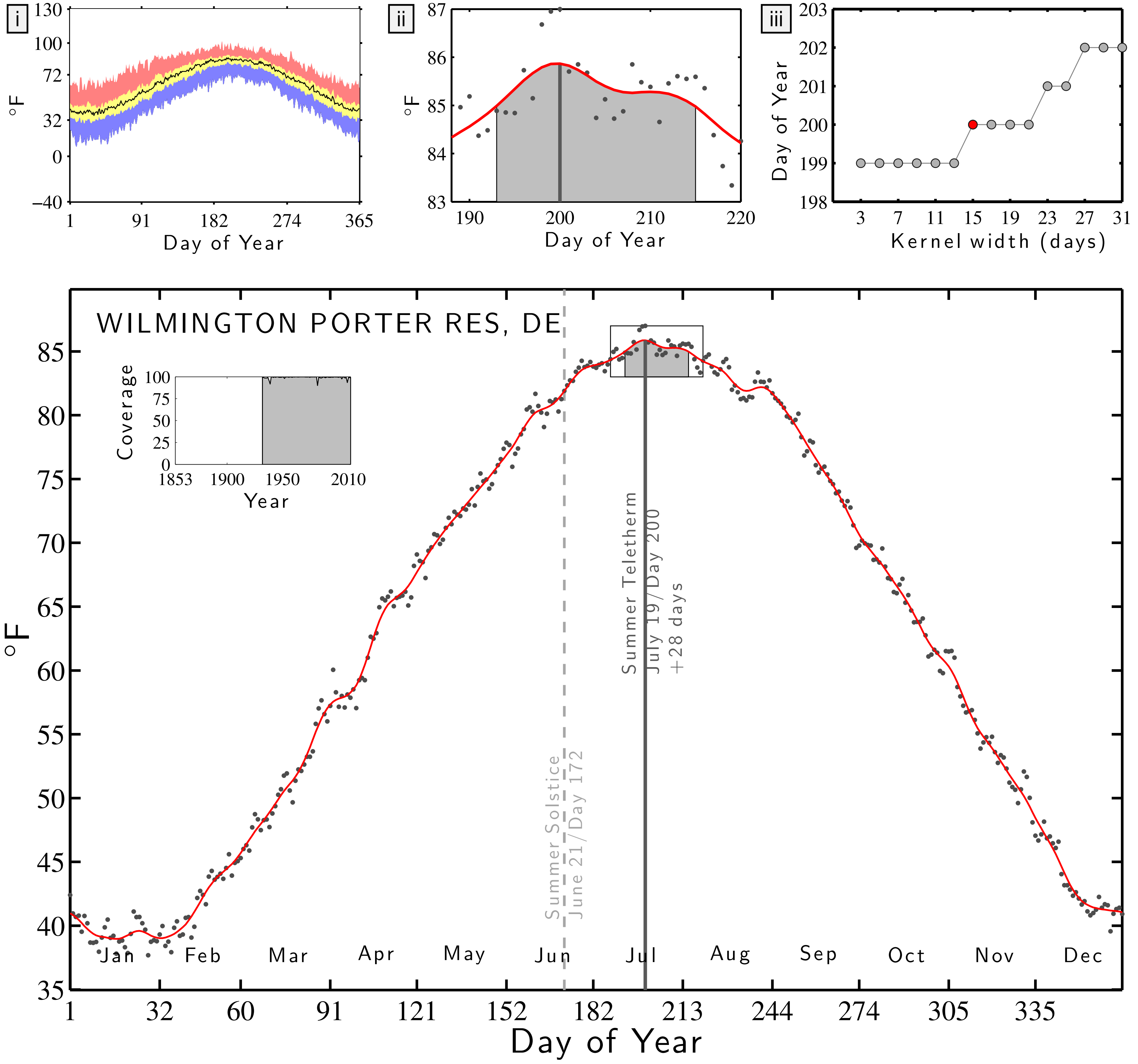

i
°F
Day of Year
ii
°F
Day of Year
iii
Day of Year
Kernel width (days)
WILMINGTON PORTER RES, DE
Coverage
Year
Summer Solstice
June 21 / Day 172
Summer Teletherm
July 19 / Day 200
+28 days
Jan
Feb
Mar
Apr
May
Jun
Jul
Aug
Sep
Oct
Nov
Dec
°F
Day of Year

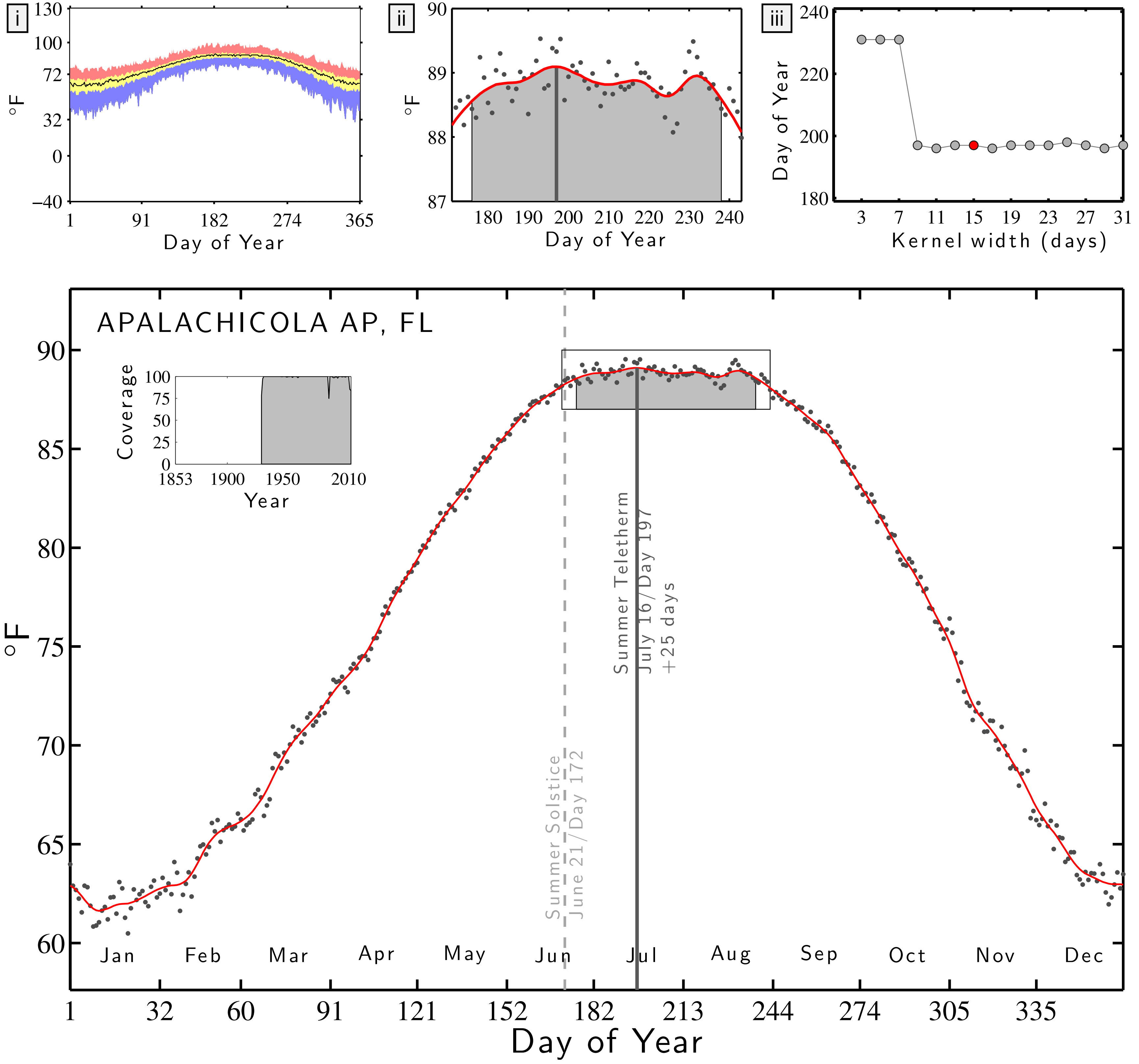

i
ii
iii
Day of Year
°F
Kernel width (days)
APALACHICOLA AP, FL
Coverage
Year
Summer Solstice
June 21/Day 172
Summer Teletherm
July 16/Day 197
+25 days
Jan
Feb
Mar
Apr
May
Jun
Jul
Aug
Sep
Oct
Nov
Dec

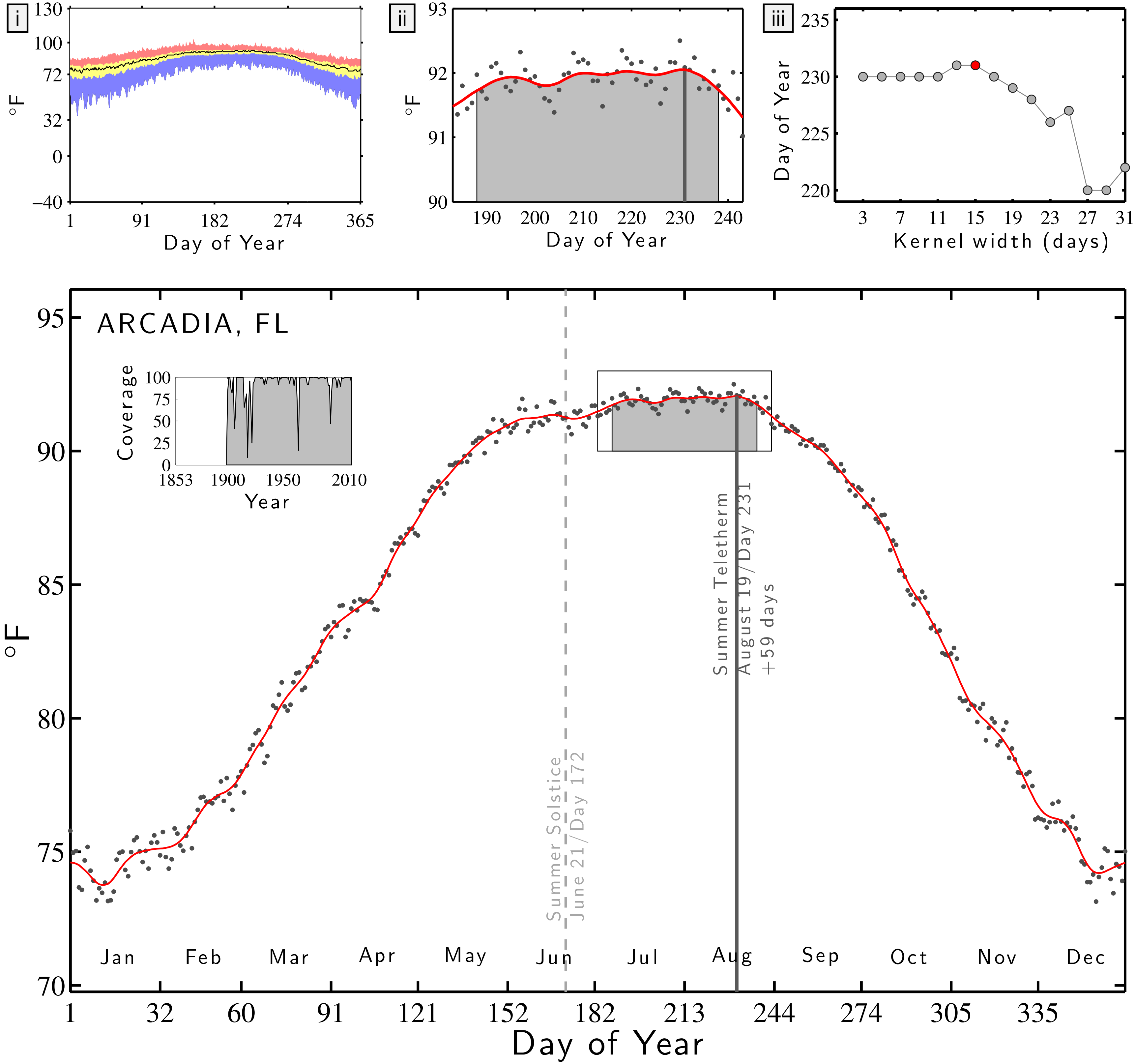
i
°F
Day of Year
ii
°F
Day of Year
iii
Day of Year
Kernel width (days)
ARCADIA, FL
Coverage
Year
Summer Solstice
June 21 / Day 172
Summer Teletherm
August 19 / Day 231
+59 days
Jan
Feb
Mar
Apr
May
Jun
Jul
Aug
Sep
Oct
Nov
Dec
°F
Day of Year

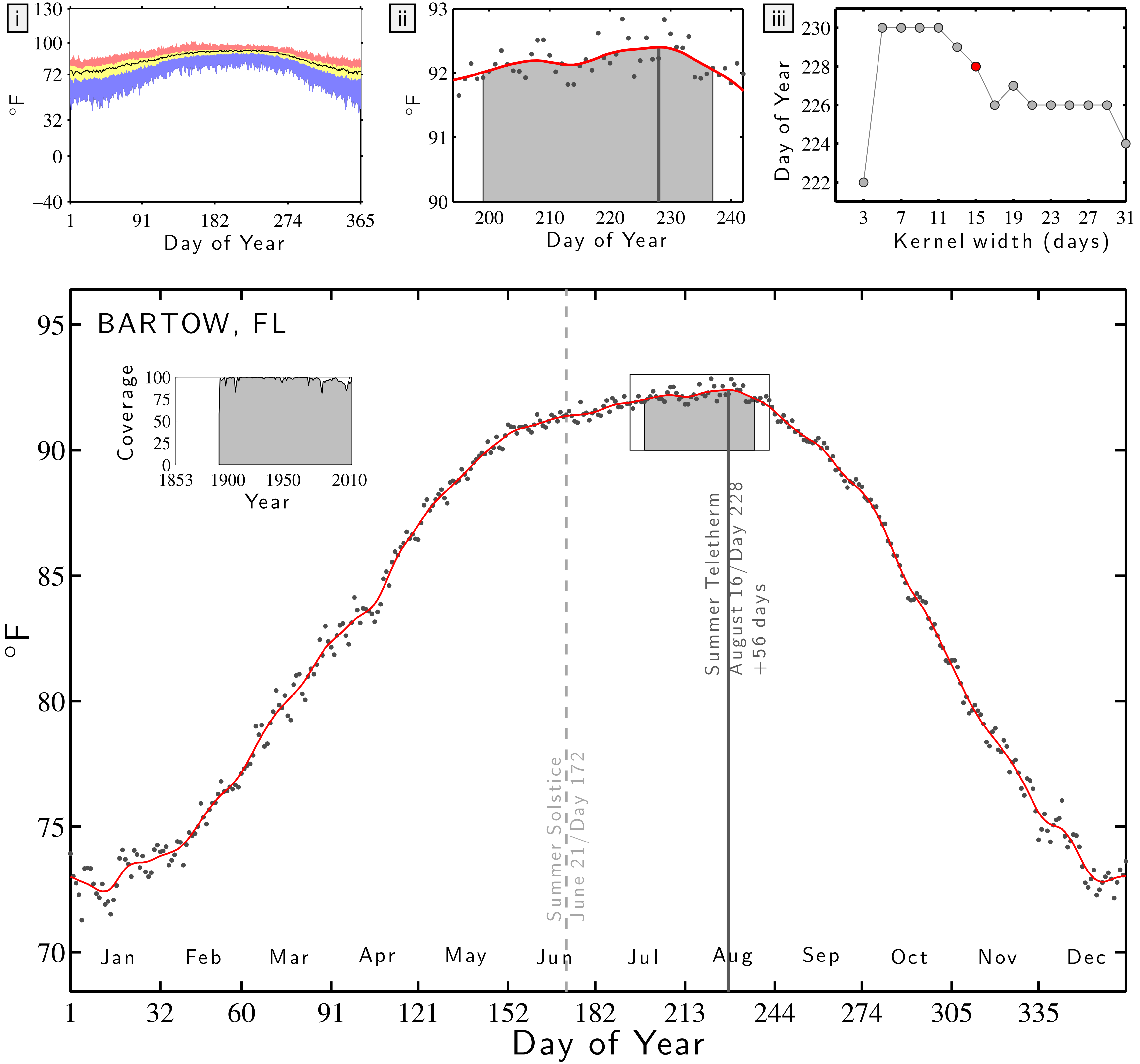

i
ii
iii
Day of Year
°F
Kernel width (days)
BARTOW, FL
Coverage
Year
Summer Solstice
June 21/Day 172
Summer Teletherm
August 16/Day 228
+56 days
Jan
Feb
Mar
Apr
May
Jun
Jul
Aug
Sep
Oct
Nov
Dec

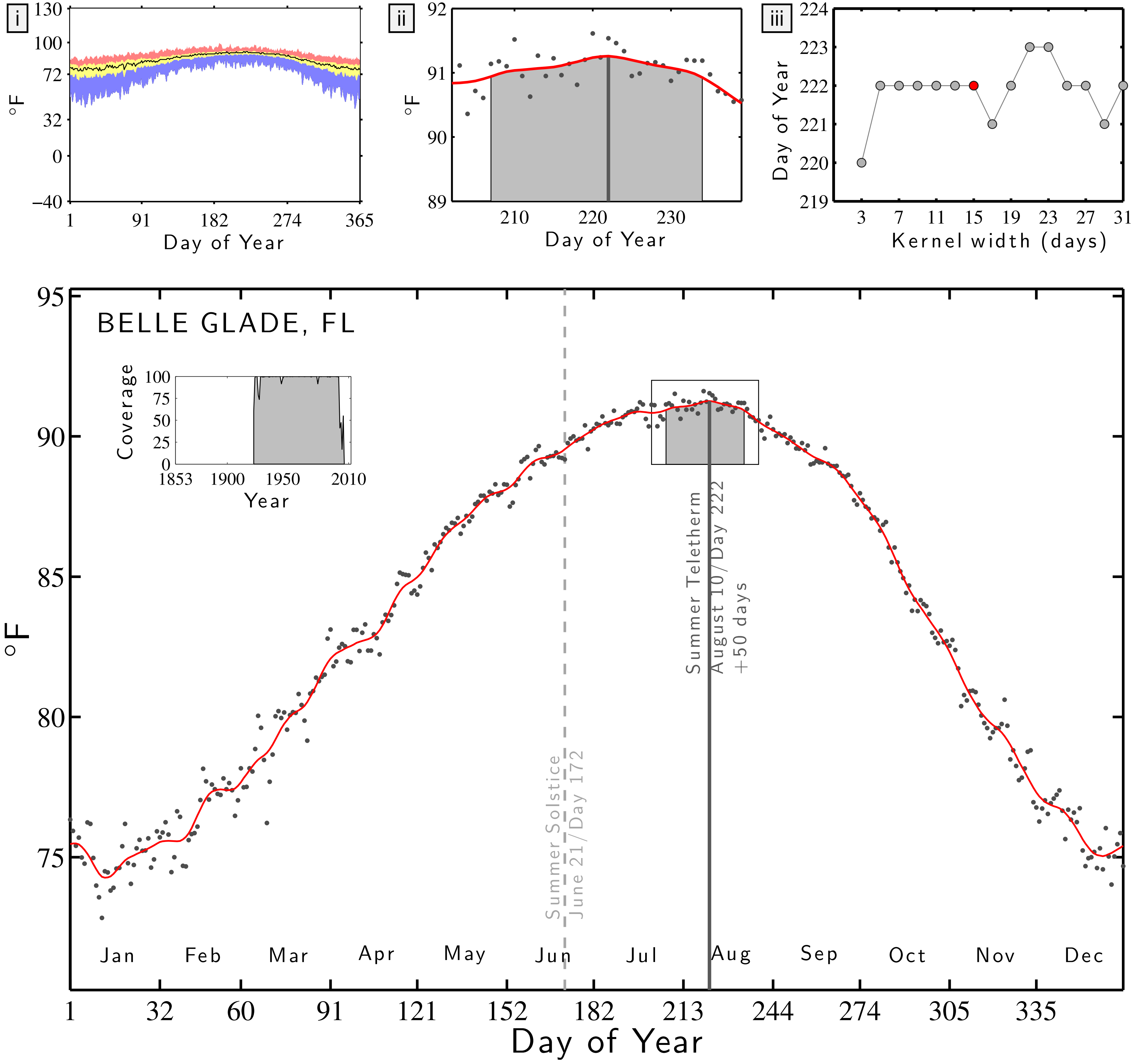

i
°F
130
100
72
32
0
−40
1
91
182
274
365
Day of Year
ii
92
91
90
89
°F
210
220
230
Day of Year
iii
Day of Year
224
223
222
221
220
219
3
7
11
15
19
23
27
31
Kernel width (days)
BELLE GLADE, FL
Coverage
100
75
50
25
0
1853
1900
1950
2010
Year
95
90
85
80
75
°F
Summer Solstice
June 21/Day 172
Summer Teletherm
August 10/Day 222
+50 days
Jan
Feb
Mar
Apr
May
Jun
Jul
Aug
Sep
Oct
Nov
Dec
1
32
60
91
121
152
182
213
244
274
305
335
Day of Year

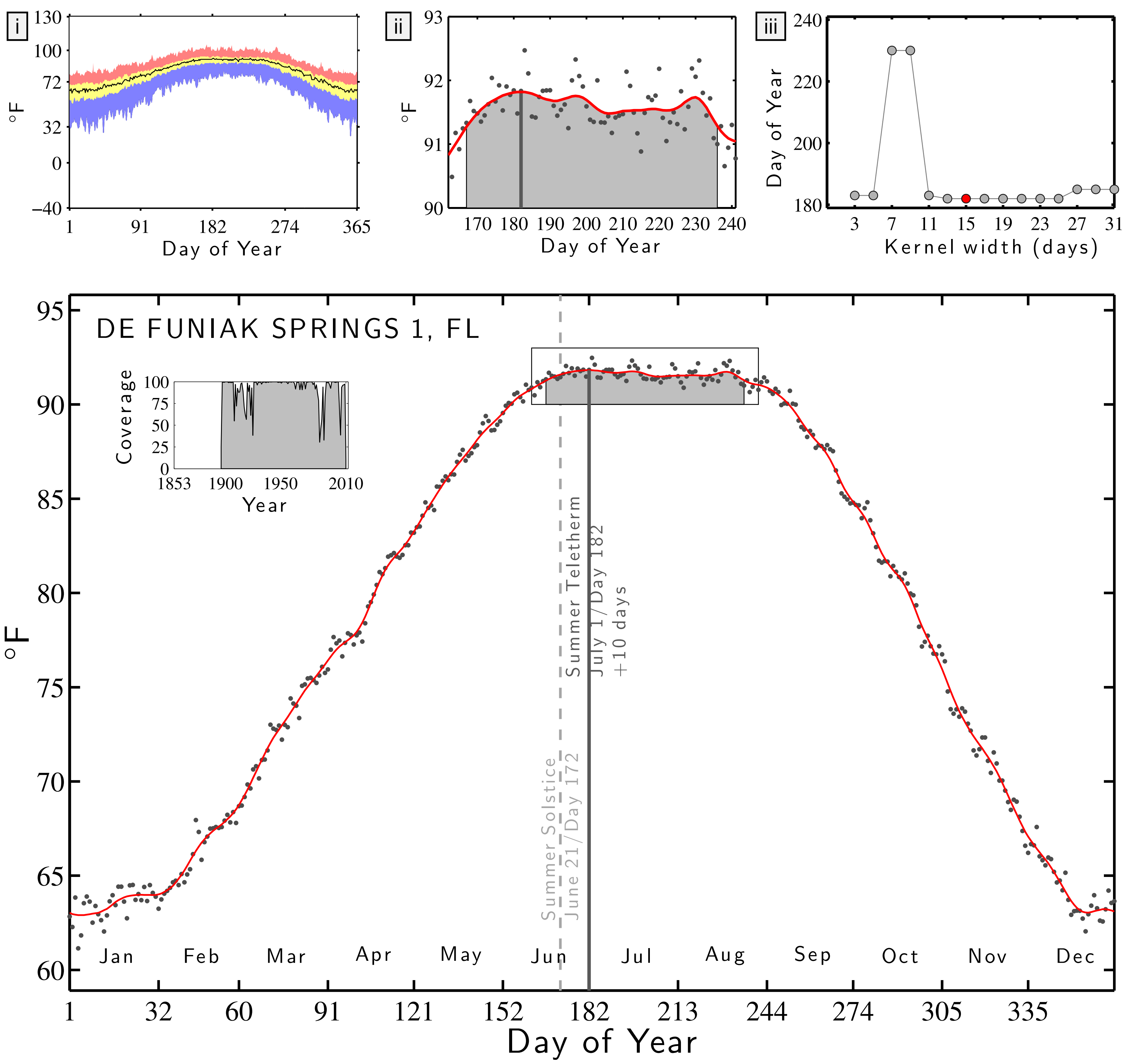

i
Day of Year
°F
ii
Day of Year
°F
iii
Day of Year
Kernel width (days)
DE FUNIAK SPRINGS 1, FL
Coverage
Year
Summer Solstice
June 21/Day 172
Summer Teletherm
July 1/Day 182
+10 days
Jan
Feb
Mar
Apr
May
Jun
Jul
Aug
Sep
Oct
Nov
Dec
°F
Day of Year

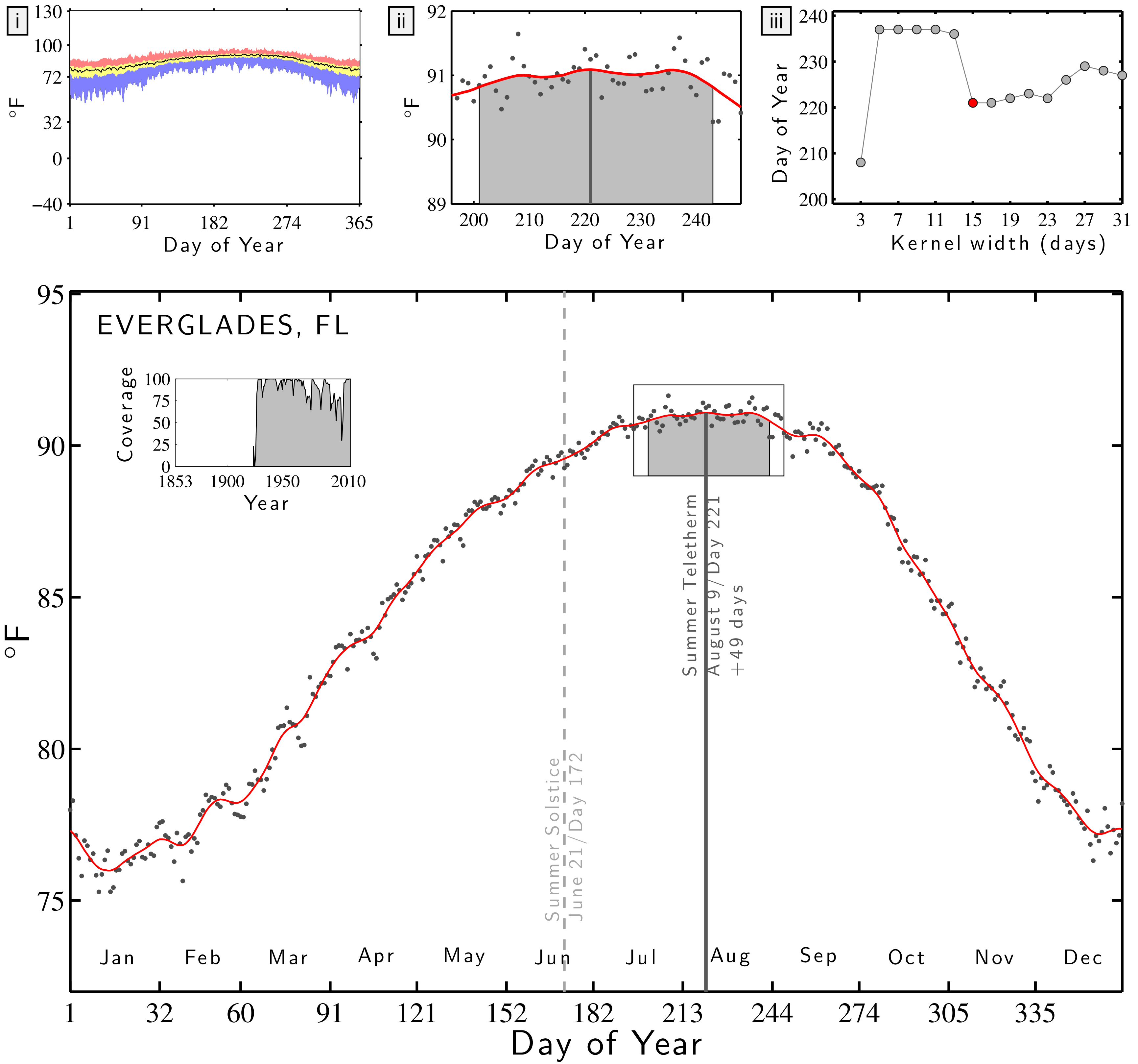

i
°F
Day of Year
ii
°F
Day of Year
iii
Day of Year
Kernel width (days)
EVERGLADES, FL
Coverage
Year
Summer Solstice
June 21/Day 172
Summer Teletherm
August 9/Day 221
+49 days
Jan
Feb
Mar
Apr
May
Jun
Jul
Aug
Sep
Oct
Nov
Dec
°F
Day of Year

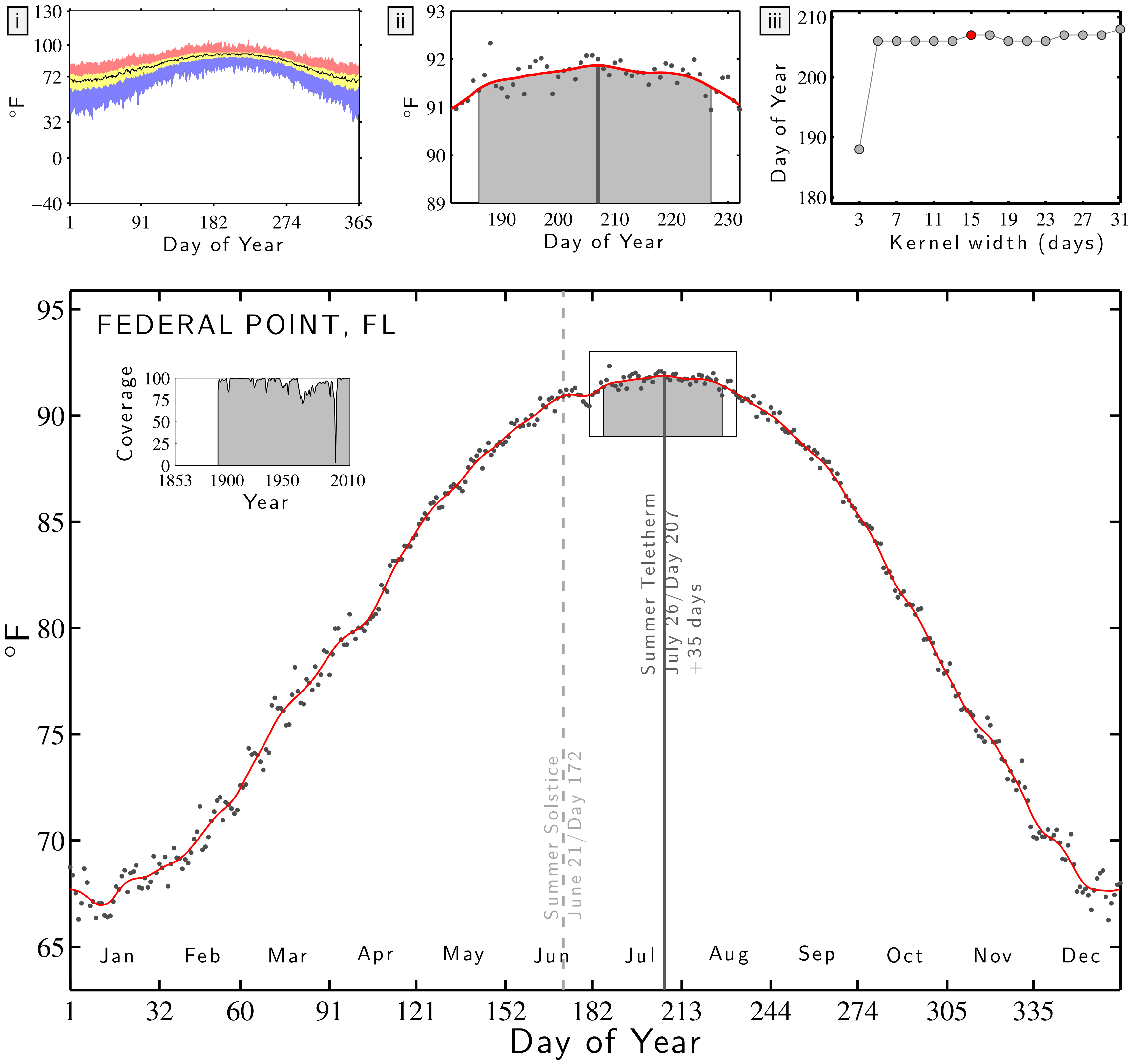
i
130
100
72
32
0
−40
°F
1
91
182
274
365
Day of Year
ii
93
92
91
90
89
°F
190
200
210
220
230
Day of Year
iii
210
200
190
180
Day of Year
3
7
11
15
19
23
27
31
Kernel width (days)
FEDERAL POINT, FL
Coverage
100
75
50
25
0
1853
1900
1950
2010
Year
Summer Solstice
June 21 / Day 172
Summer Teletherm
July 26 / Day 207
+35 days
95
90
85
80
75
70
65
°F
Jan
Feb
Mar
Apr
May
Jun
Jul
Aug
Sep
Oct
Nov
Dec
1
32
60
91
121
152
182
213
244
274
305
335
Day of Year

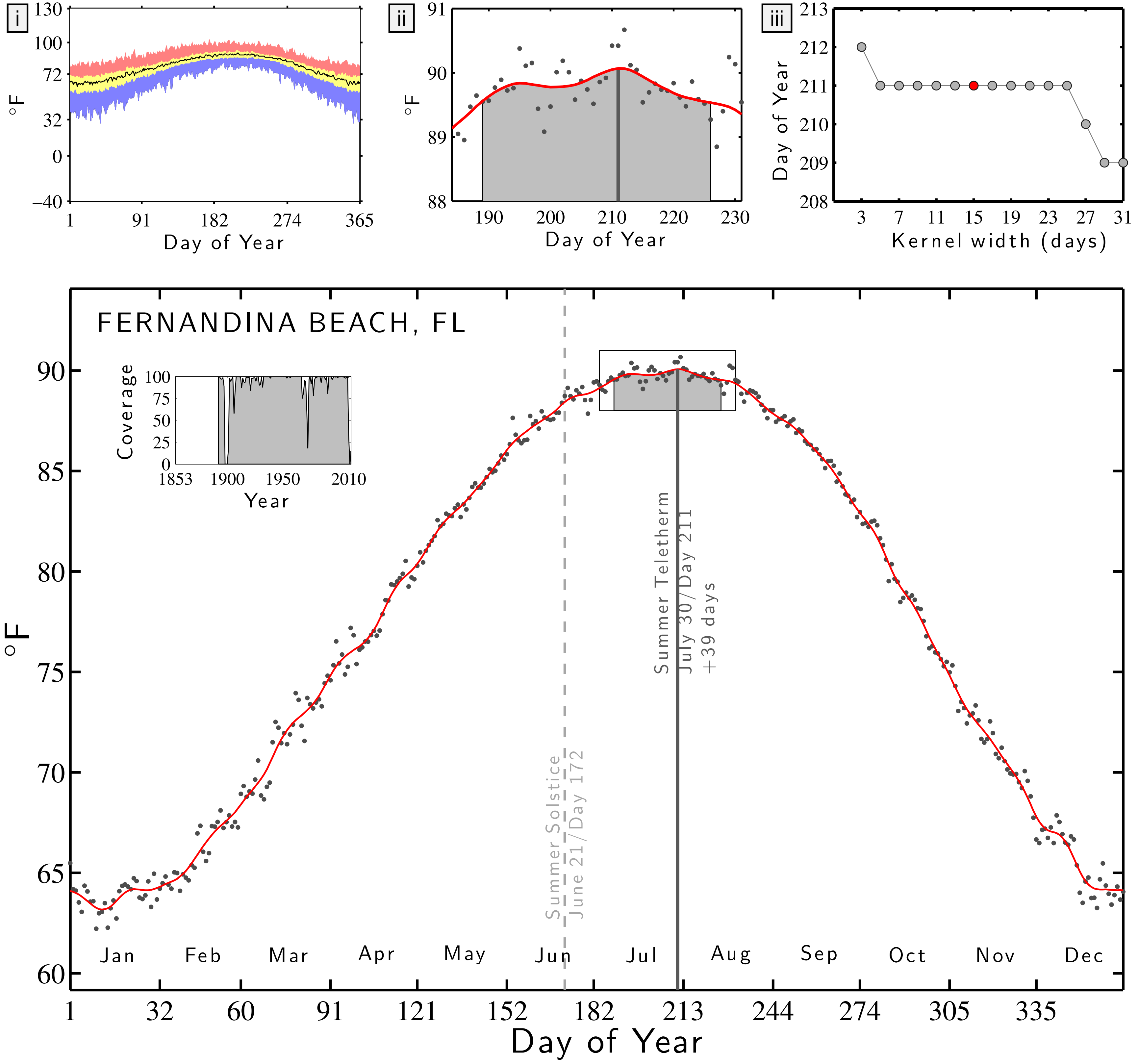

i
ii
iii
°F
Day of Year
Day of Year
Kernel width (days)
FERNANDINA BEACH, FL
Coverage
Year
Summer Solstice
June 21/Day 172
Summer Teletherm
July 30/Day 211
+39 days
Jan
Feb
Mar
Apr
May
Jun
Jul
Aug
Sep
Oct
Nov
Dec

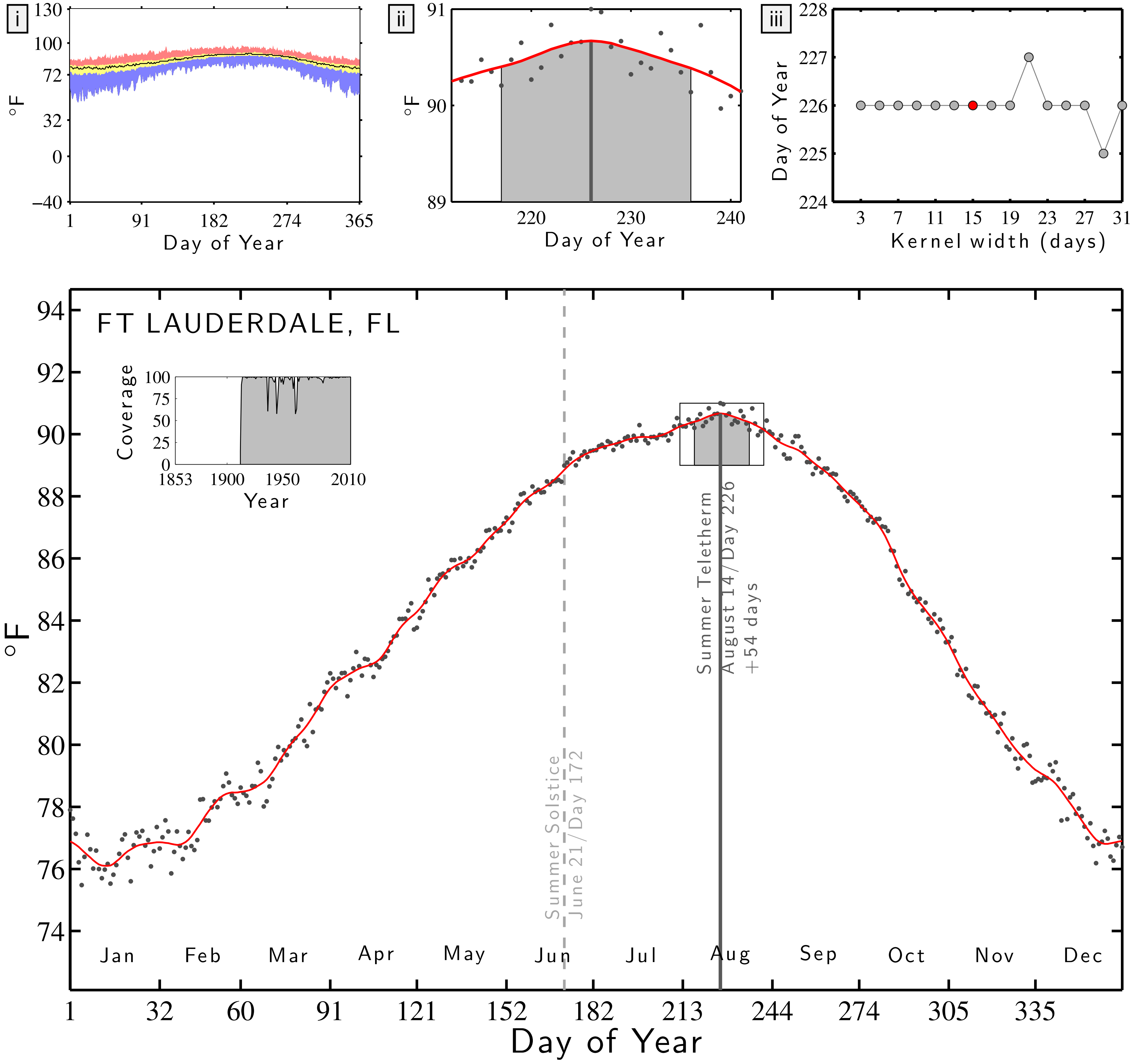

i
°F
Day of Year
ii
°F
Day of Year
iii
Day of Year
Kernel width (days)
FT LAUDERDALE, FL
Coverage
Year
Summer Solstice
June 21 / Day 172
Summer Teletherm
August 14 / Day 226
+54 days
Jan
Feb
Mar
Apr
May
Jun
Jul
Aug
Sep
Oct
Nov
Dec
°F
Day of Year

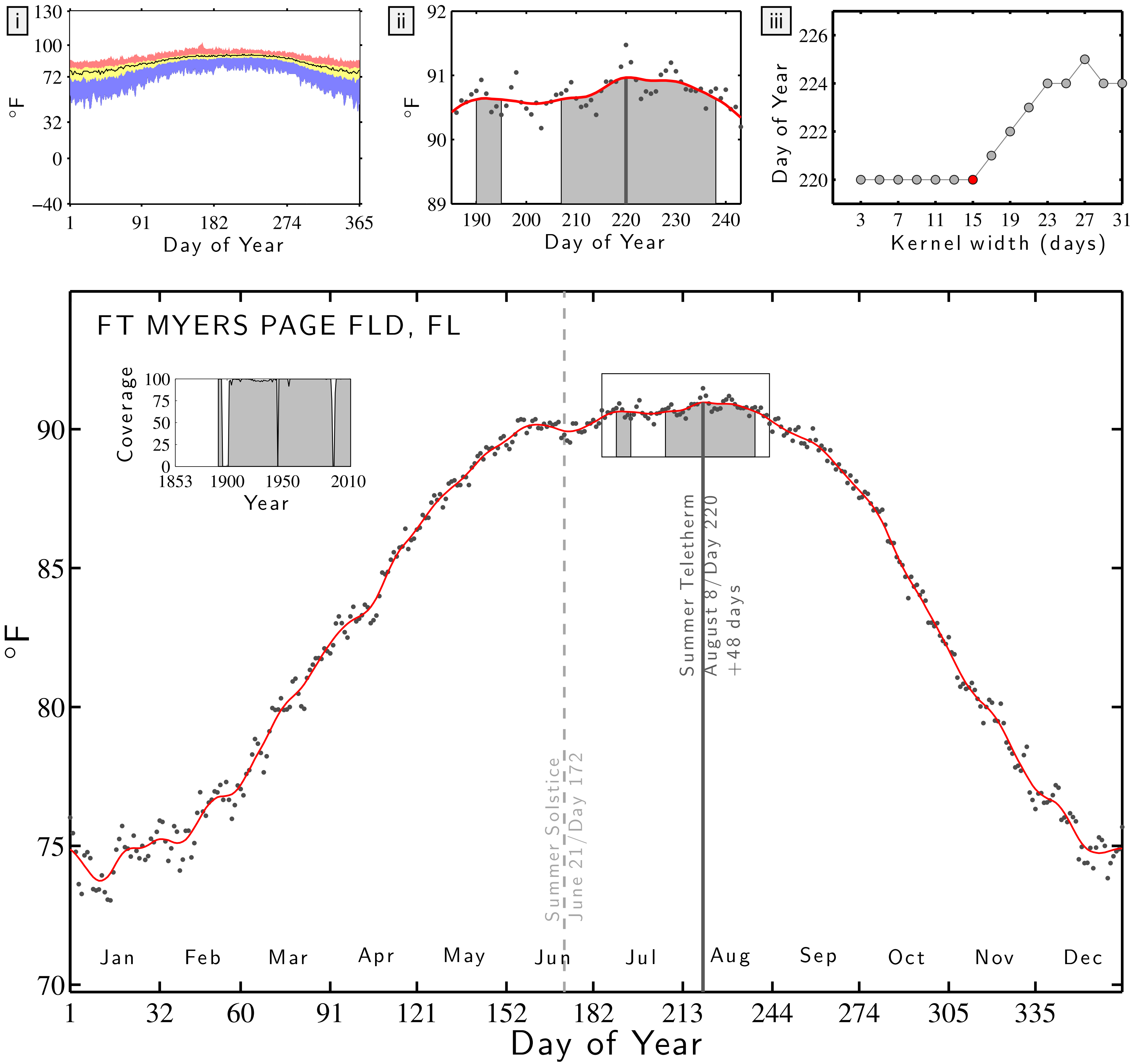

i
°F
Day of Year
ii
iii
Day of Year
Kernel width (days)
FT MYERS PAGE FLD, FL
Coverage
Year
Summer Solstice
June 21/Day 172
Summer Teletherm
August 8/Day 220
+48 days
Jan
Feb
Mar
Apr
May
Jun
Jul
Aug
Sep
Oct
Nov
Dec

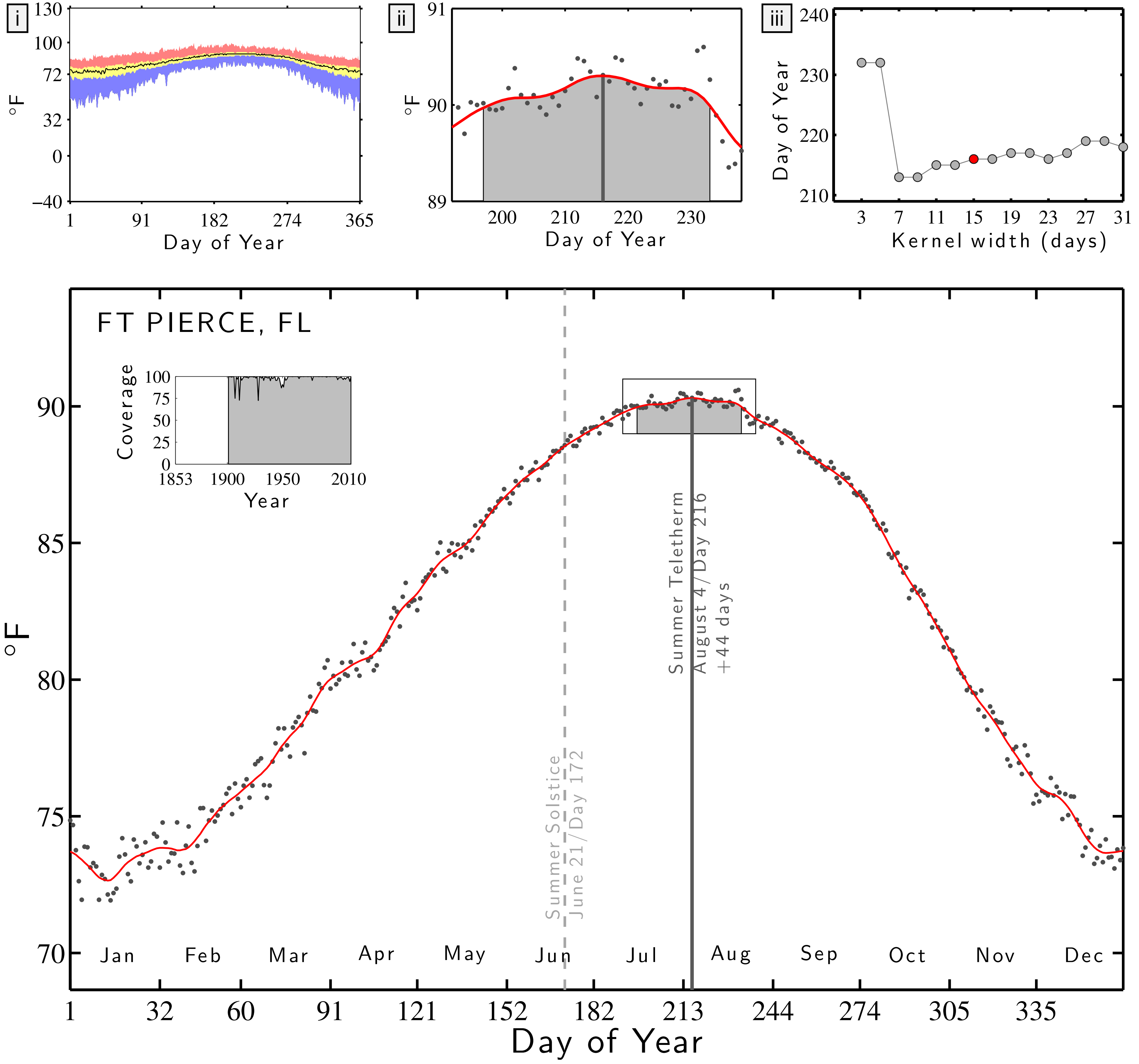

i
ii
iii
°F
Day of Year
Kernel width (days)
FT PIERCE, FL
Coverage
Year
Summer Solstice
June 21 / Day 172
Summer Teletherm
August 4 / Day 216
+44 days
Jan
Feb
Mar
Apr
May
Jun
Jul
Aug
Sep
Oct
Nov
Dec

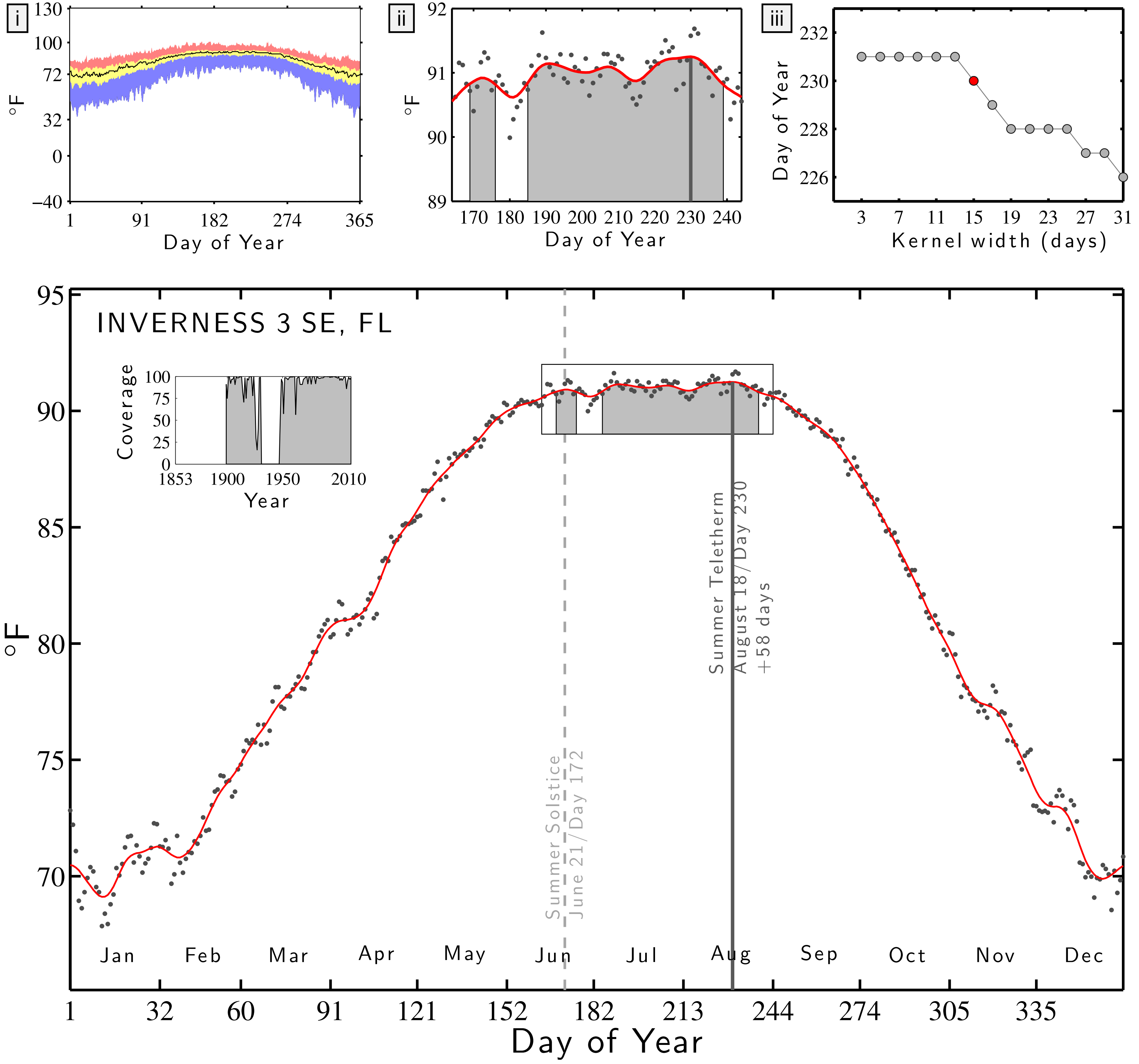

i
°F
Day of Year
ii
°F
Day of Year
iii
Day of Year
Kernel width (days)
INVERNESS 3 SE, FL
Coverage
Year
Summer Solstice
June 21/Day 172
Summer Teletherm
August 18/Day 230
+58 days
Jan
Feb
Mar
Apr
May
Jun
Jul
Aug
Sep
Oct
Nov
Dec
°F
Day of Year

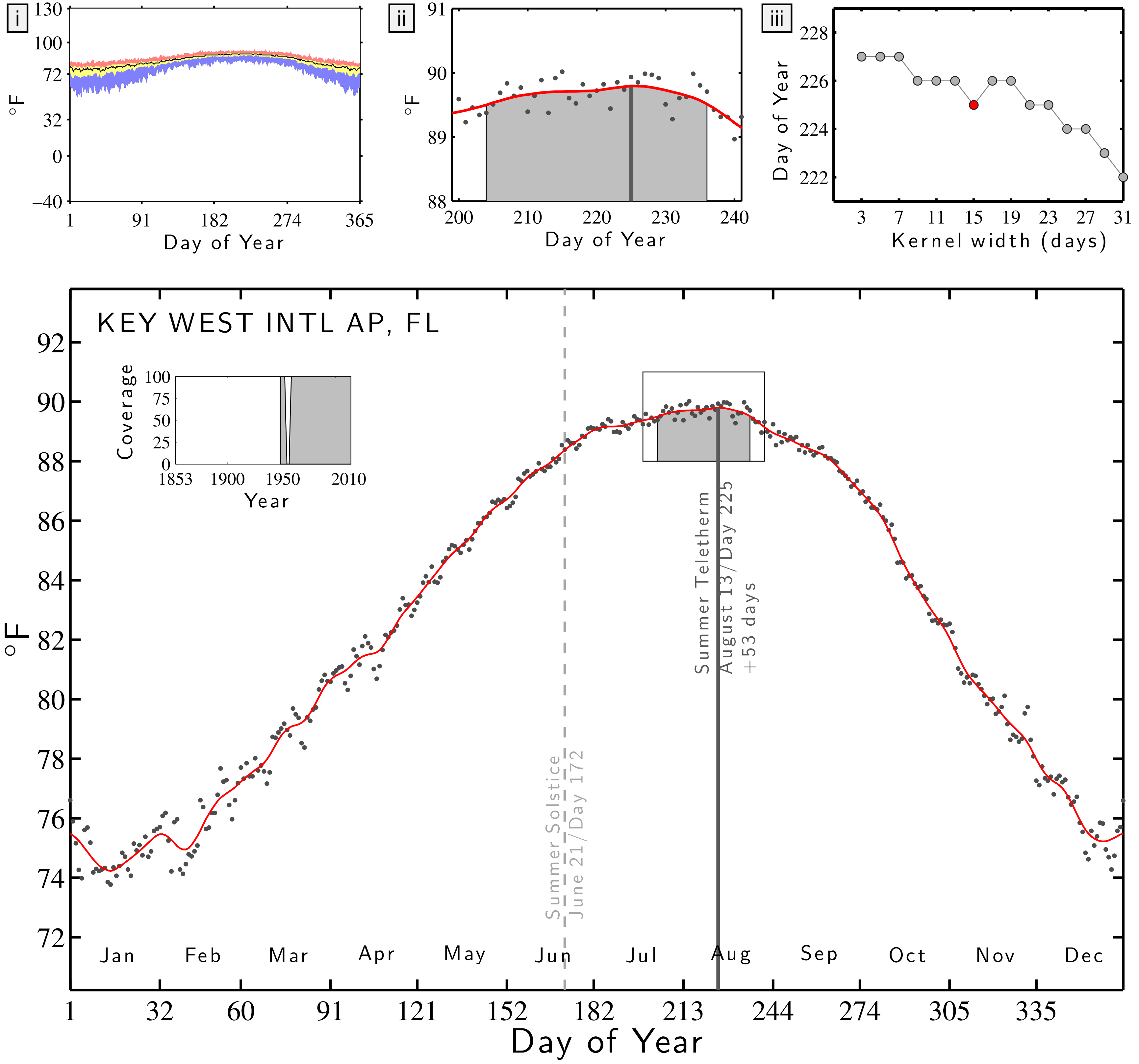

i
°F
Day of Year
ii
°F
Day of Year
iii
Day of Year
Kernel width (days)
KEY WEST INTL AP, FL
Coverage
Year
Summer Solstice
June 21 / Day 172
Summer Teletherm
August 13 / Day 225
+53 days
Jan
Feb
Mar
Apr
May
Jun
Jul
Aug
Sep
Oct
Nov
Dec
°F
Day of Year

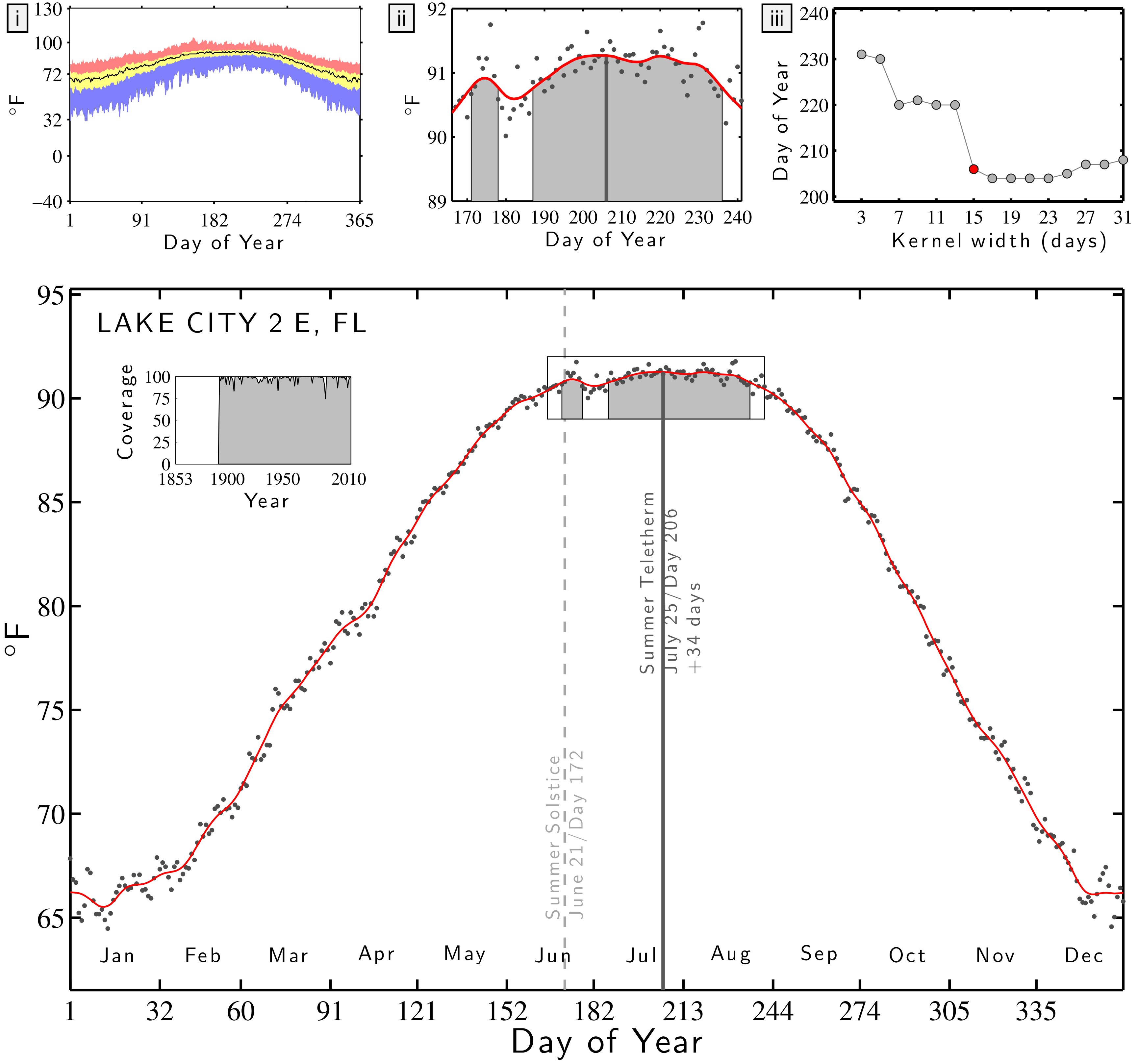

i
°F
Day of Year
ii
°F
Day of Year
iii
Day of Year
Kernel width (days)
LAKE CITY 2 E, FL
Coverage
Year
Summer Solstice
June 21/Day 172
Summer Teletherm
July 25/Day 206
+34 days
Jan
Feb
Mar
Apr
May
Jun
Jul
Aug
Sep
Oct
Nov
Dec
°F
Day of Year

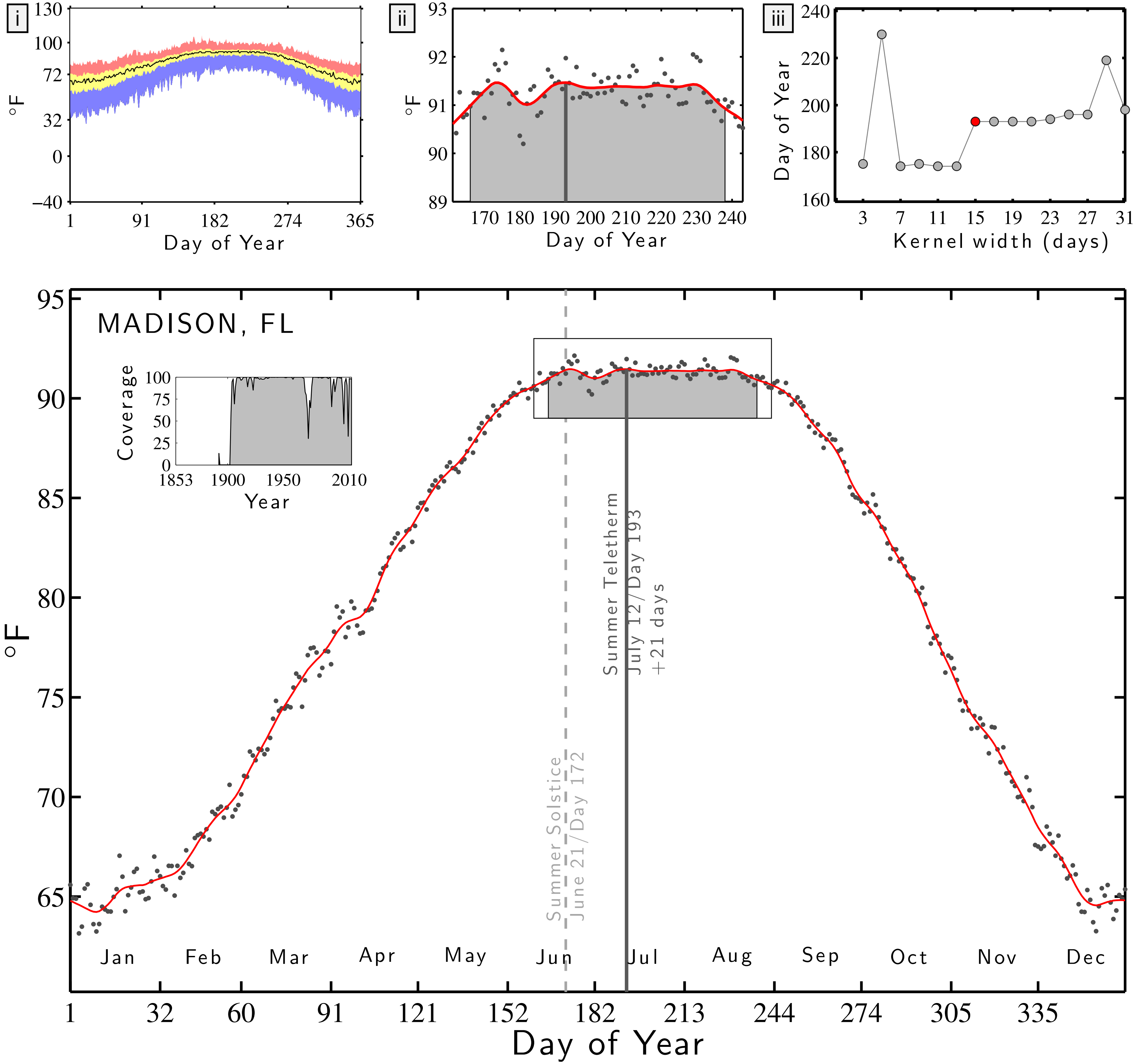

i
°F
Day of Year
ii
°F
Day of Year
iii
Day of Year
Kernel width (days)
MADISON, FL
Coverage
Year
Summer Solstice
June 21/Day 172
Summer Teletherm
July 12/Day 193
+21 days
°F
Jan
Feb
Mar
Apr
May
Jun
Jul
Aug
Sep
Oct
Nov
Dec
Day of Year

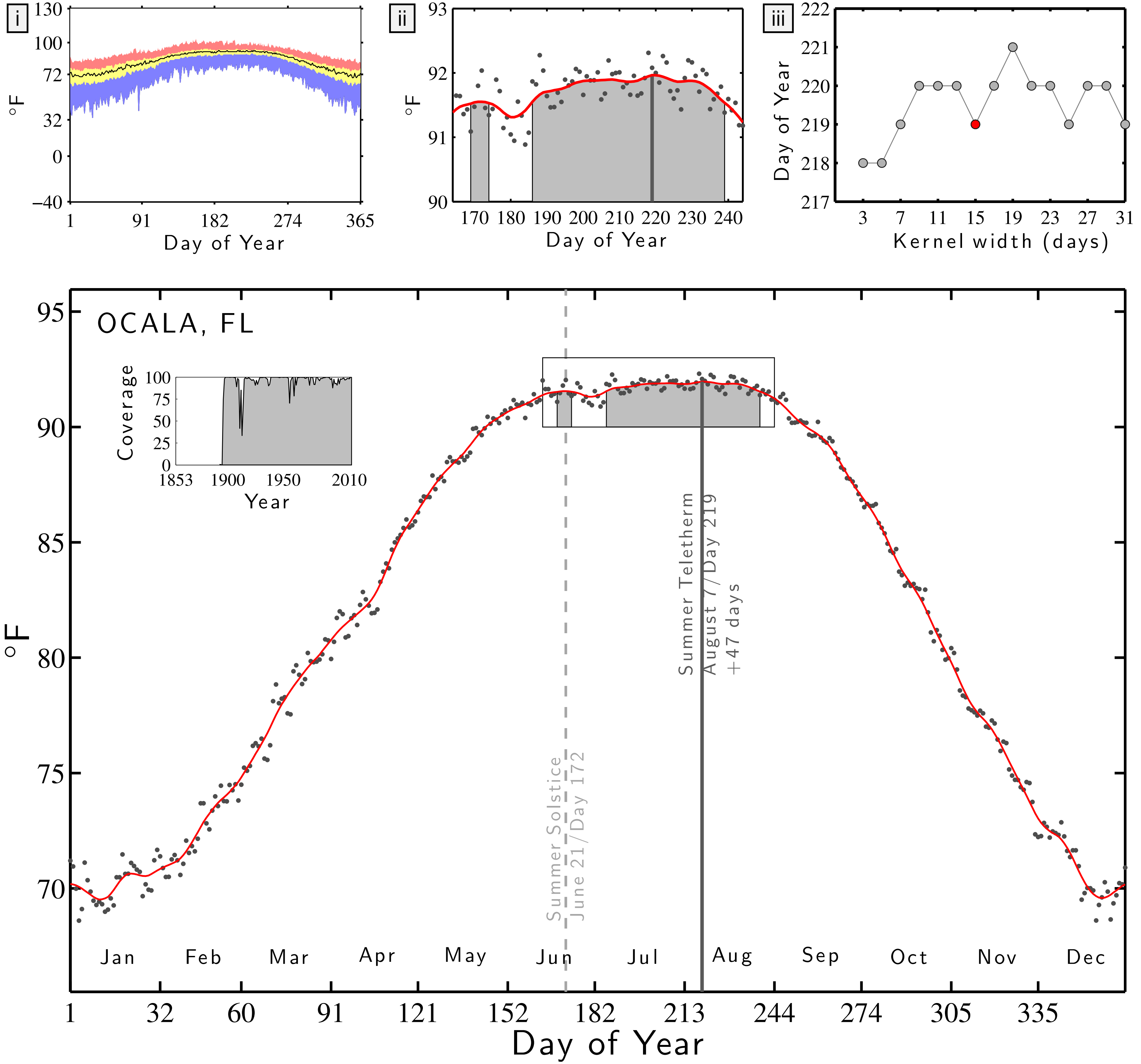

i
°F
Day of Year
ii
°F
Day of Year
iii
Day of Year
Kernel width (days)
OCALA, FL
Coverage
Year
Summer Solstice
June 21 / Day 172
Summer Teletherm
August 7 / Day 219
+47 days
Jan
Feb
Mar
Apr
May
Jun
Jul
Aug
Sep
Oct
Nov
Dec
°F
Day of Year

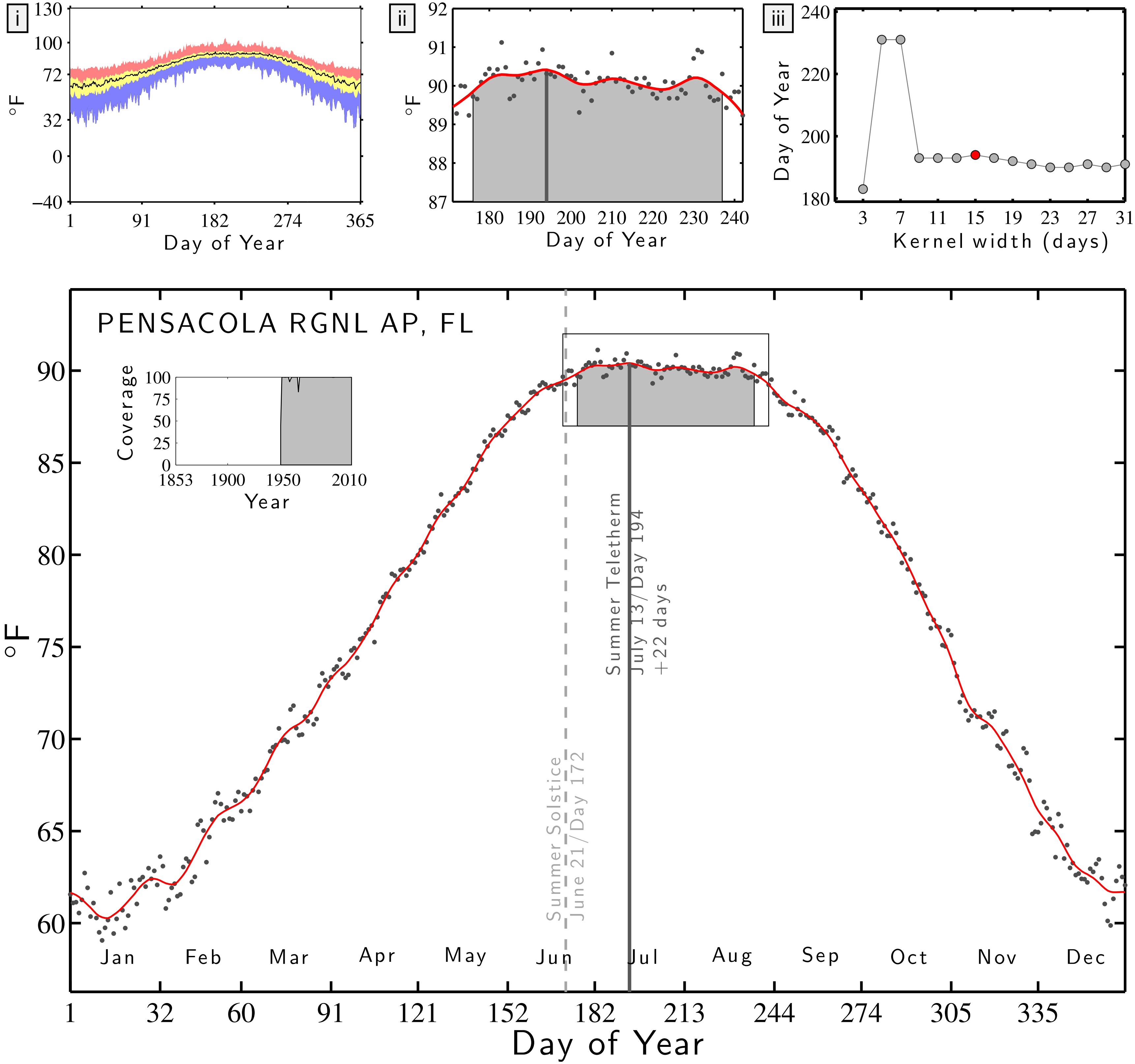

i
ii
iii
°F
Day of Year
Day of Year
Kernel width (days)
PENSACOLA RGNL AP, FL
Coverage
Year
Summer Solstice
June 21/Day 172
Summer Teletherm
July 13/Day 194
+22 days
Jan
Feb
Mar
Apr
May
Jun
Jul
Aug
Sep
Oct
Nov
Dec
Day of Year

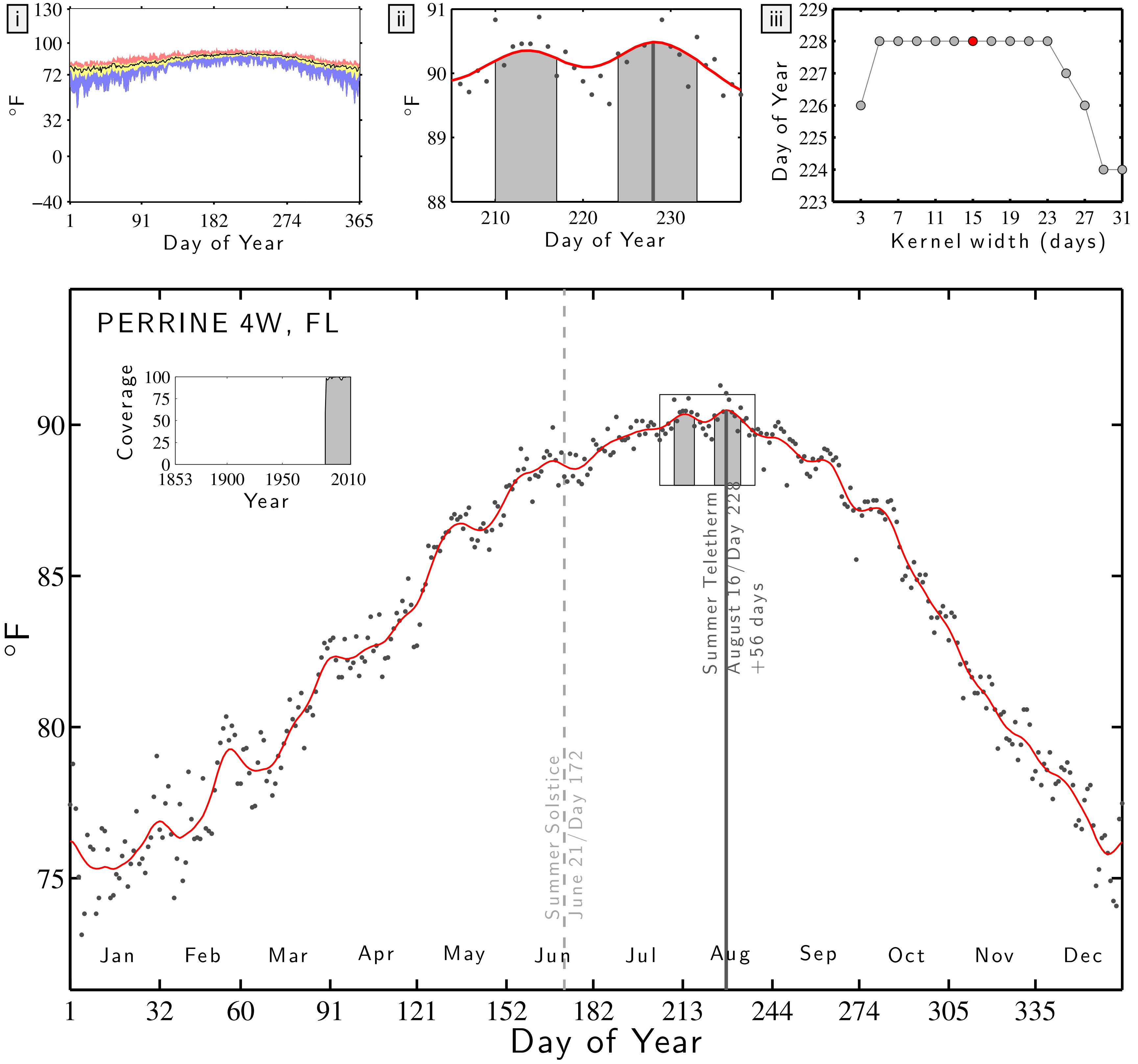
i
ii
iii
Day of Year
°F
Kernel width (days)
PERRINE 4W, FL
Coverage
Year
Summer Solstice
June 21/Day 172
Summer Teletherm
August 16/Day 228
+56 days
Jan
Feb
Mar
Apr
May
Jun
Jul
Aug
Sep
Oct
Nov
Dec

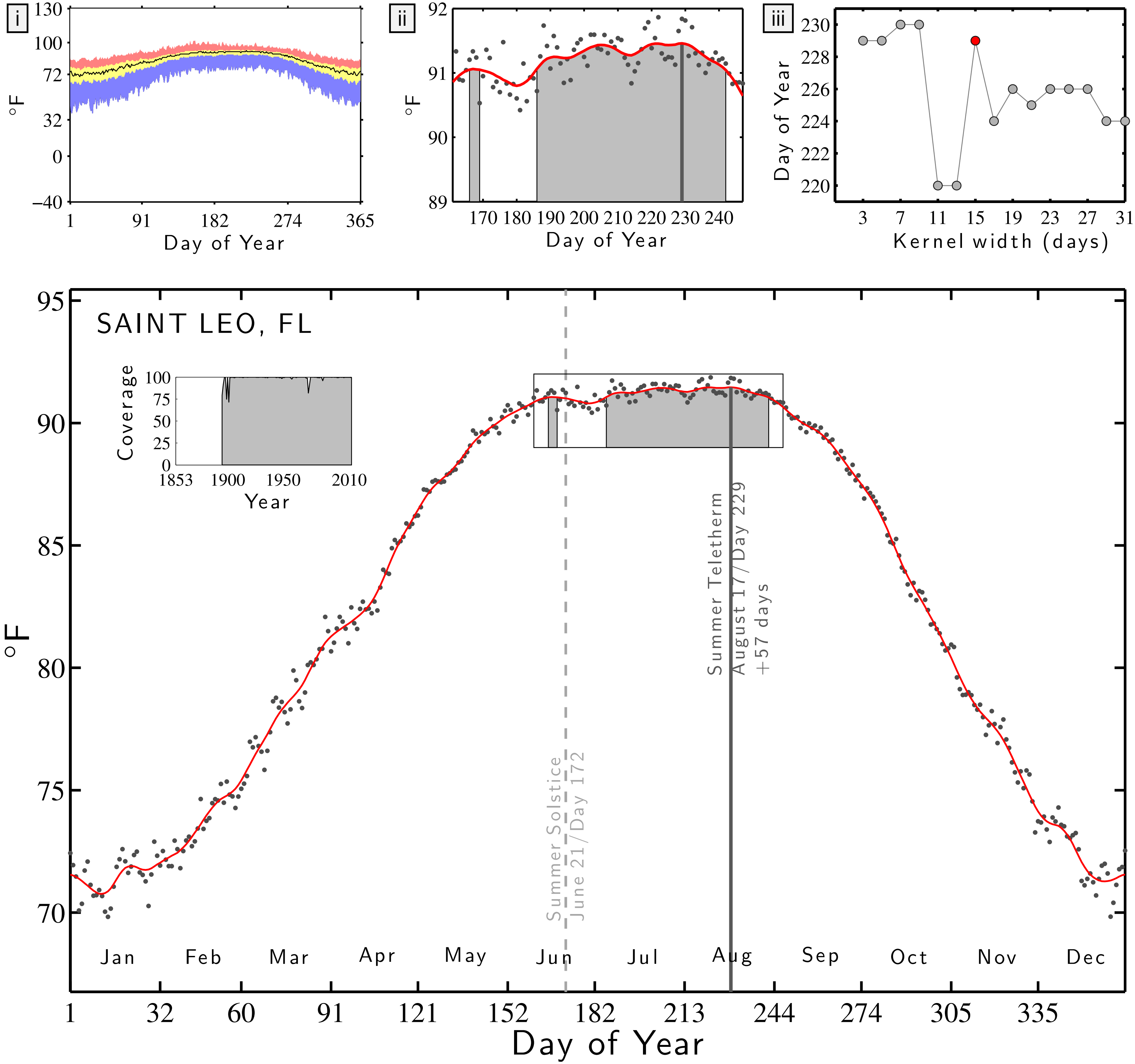

i
130
100
72
32
0
−40
°F
1
91
182
274
365
Day of Year
ii
92
91
90
89
°F
170 180 190 200 210 220 230 240
Day of Year
iii
230
228
226
224
222
220
Day of Year
3
7
11
15
19
23
27
31
Kernel width (days)
SAINT LEO, FL
Coverage
100
75
50
25
0
1853
1900
1950
2010
Year
95
90
85
80
75
70
°F
Summer Solstice
June 21 / Day 172
Summer Teletherm
August 17 / Day 229
+57 days
Jan
Feb
Mar
Apr
May
Jun
Jul
Aug
Sep
Oct
Nov
Dec
1
32
60
91
121
152
182
213
244
274
305
335
Day of Year

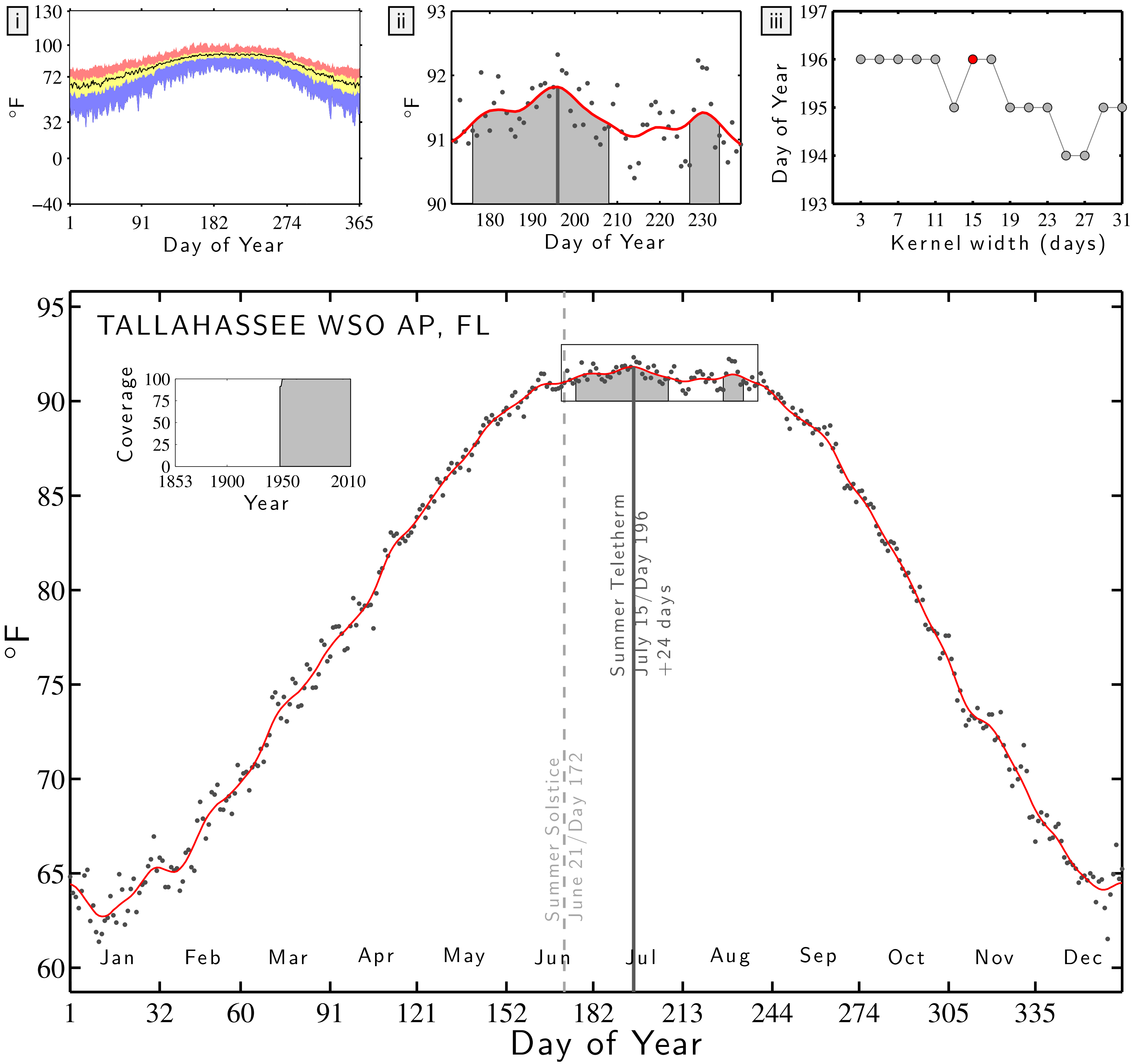

i
ii
iii
°F
Day of Year
Day of Year
Kernel width (days)
TALLAHASSEE WSO AP, FL
Coverage
Year
Summer Solstice
June 21/Day 172
Summer Teletherm
July 15/Day 196
+24 days
Jan Feb Mar Apr May Jun Jul Aug Sep Oct Nov Dec

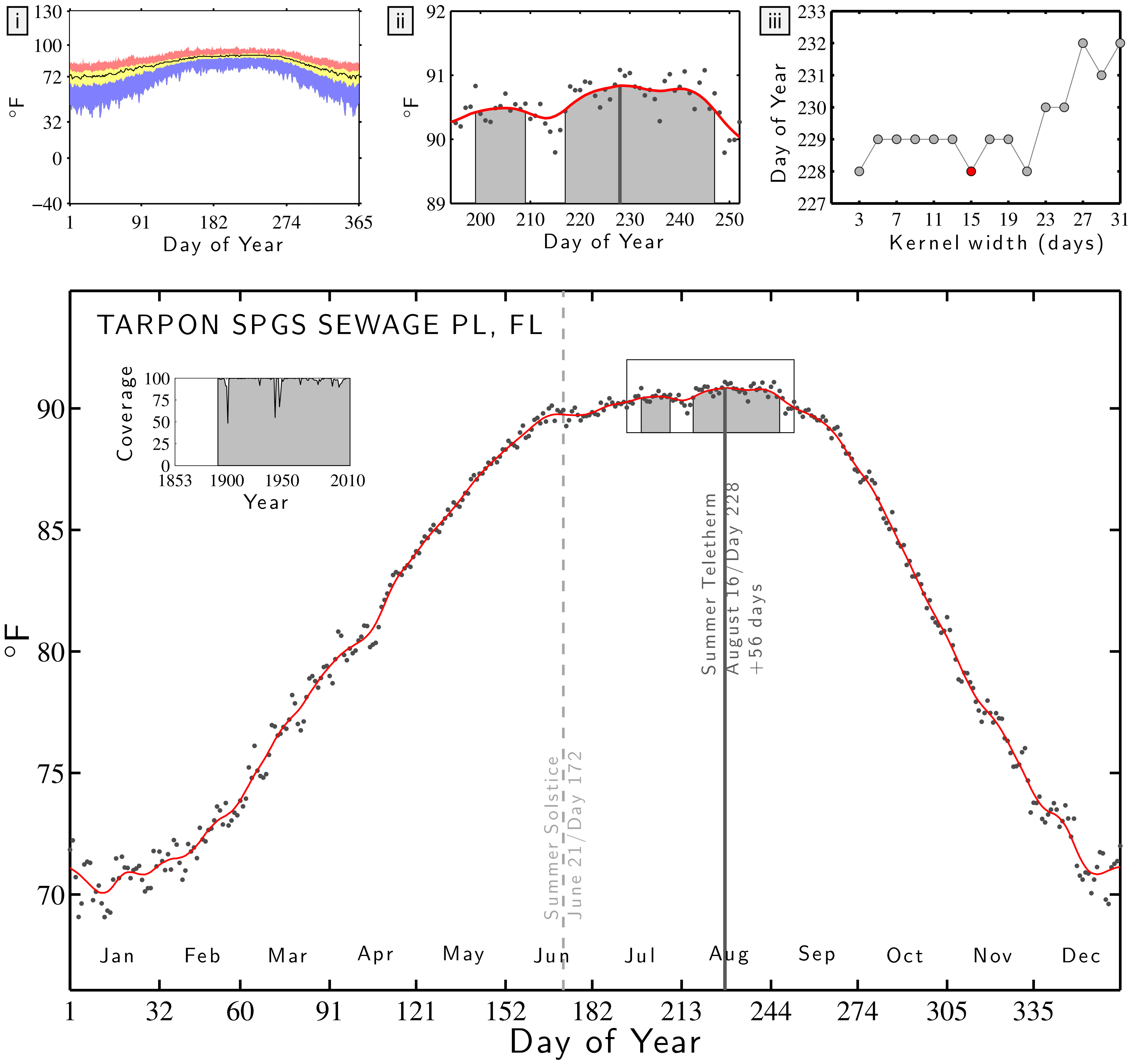

i
ii
iii
°F
Day of Year
Kernel width (days)
TARPON SPGS SEWAGE PL, FL
Coverage
Year
Summer Solstice
June 21/Day 172
Summer Teletherm
August 16/Day 228
+56 days
Jan
Feb
Mar
Apr
May
Jun
Jul
Aug
Sep
Oct
Nov
Dec

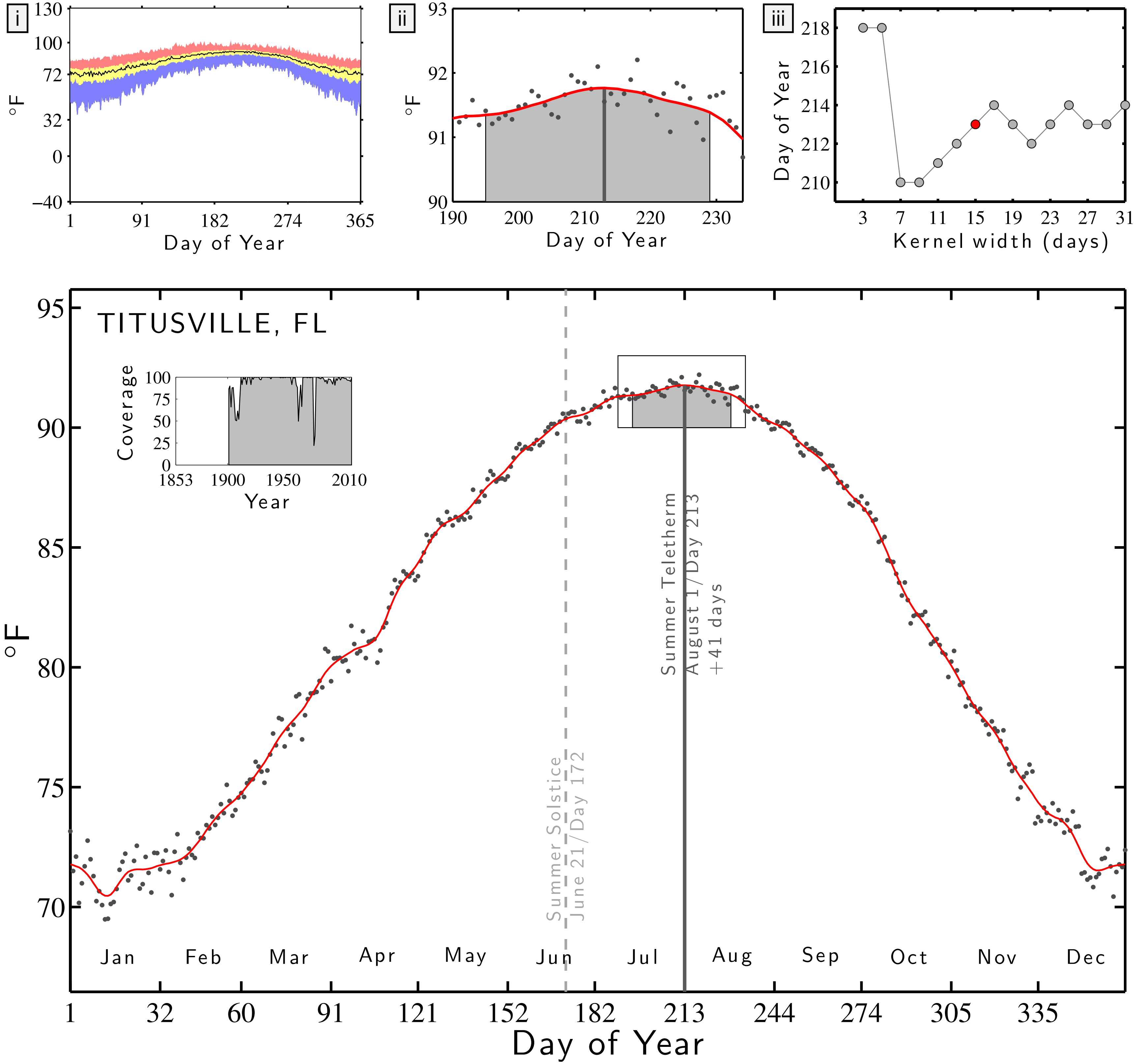

i
°F
130
100
72
32
0
−40
1
91
182
274
365
Day of Year
ii
°F
93
92
91
90
190
200
210
220
230
Day of Year
iii
Day of Year
218
216
214
212
210
3
7
11
15
19
23
27
31
Kernel width (days)
TITUSVILLE, FL
Coverage
100
75
50
25
0
1853
1900
1950
2010
Year
°F
95
90
85
80
75
70
Summer Solstice
June 21 / Day 172
Summer Teletherm
August 1 / Day 213
+41 days
Jan
Feb
Mar
Apr
May
Jun
Jul
Aug
Sep
Oct
Nov
Dec
1
32
60
91
121
152
182
213
244
274
305
335
Day of Year

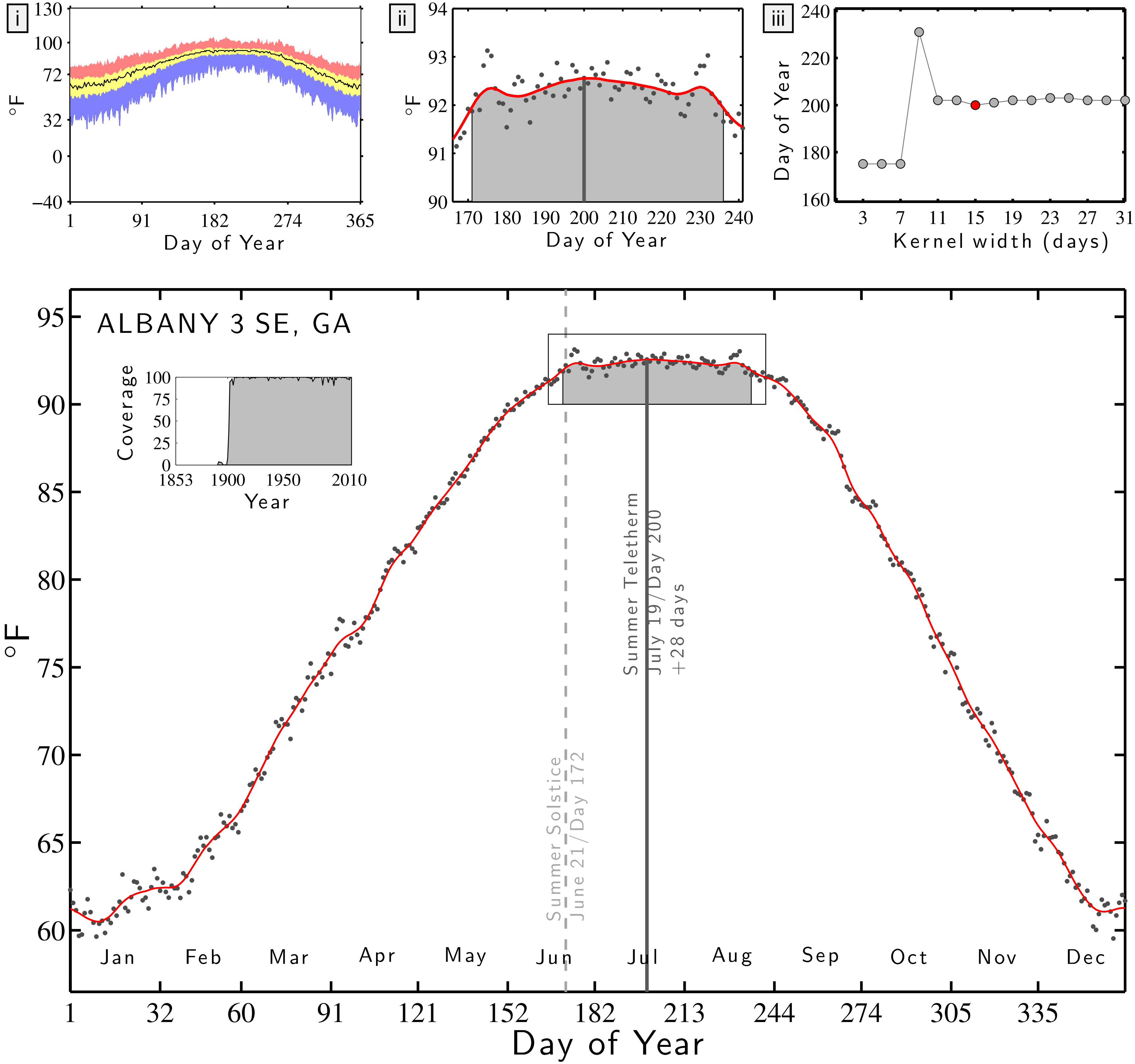

i
ii
iii
°F
Day of Year
Day of Year
Kernel width (days)
ALBANY 3 SE, GA
Coverage
Year
Summer Solstice
June 21/Day 172
Summer Teletherm
July 19/Day 200
+28 days
Jan
Feb
Mar
Apr
May
Jun
Jul
Aug
Sep
Oct
Nov
Dec

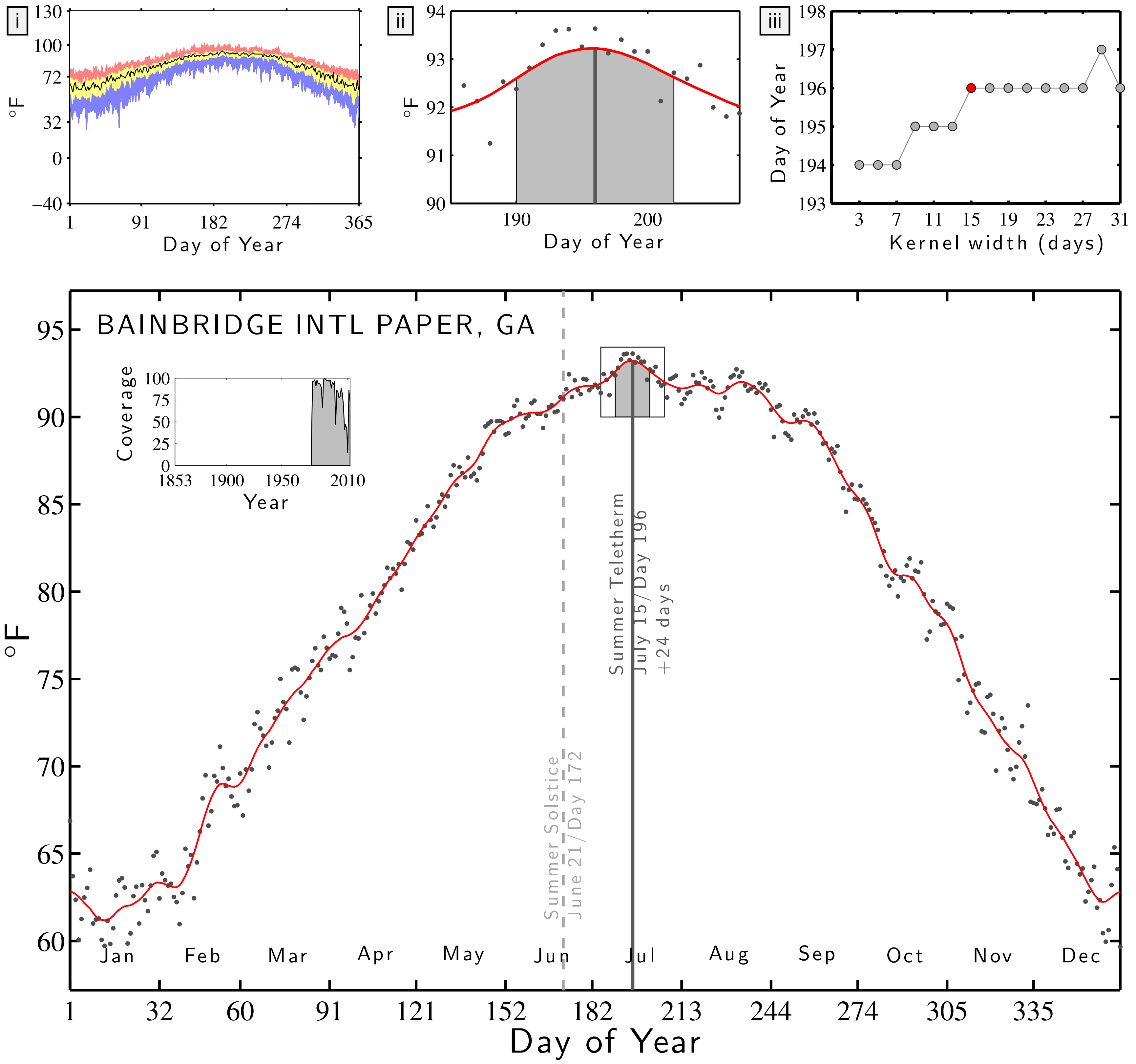

i
ii
iii
°F
Day of Year
Day of Year
Kernel width (days)
BAINBRIDGE INTL PAPER, GA
Coverage
Year
Summer Solstice
June 21 / Day 172
Summer Teletherm
July 15 / Day 196
+24 days
Jan
Feb
Mar
Apr
May
Jun
Jul
Aug
Sep
Oct
Nov
Dec

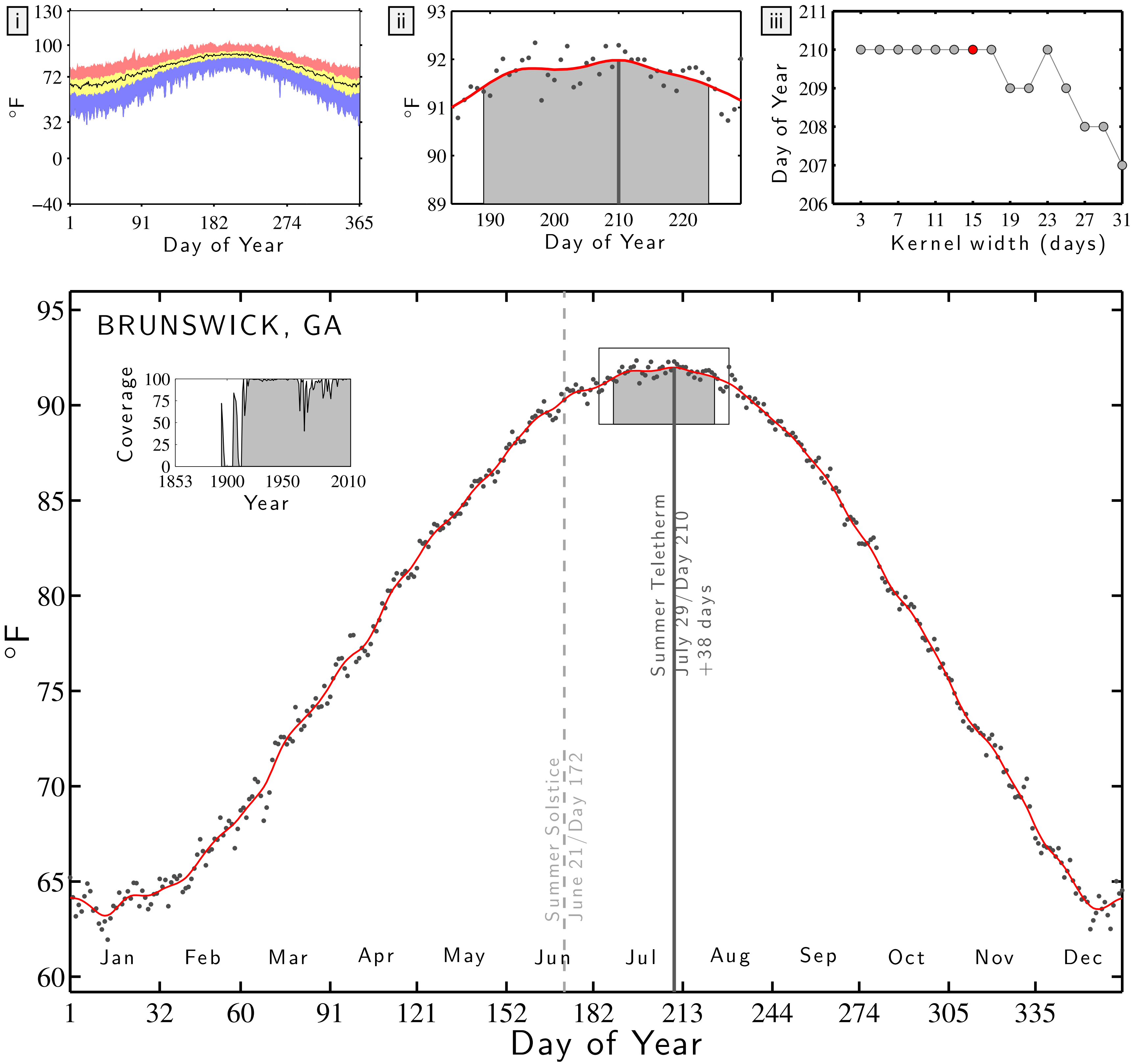

i
ii
iii
°F
Day of Year
Day of Year
Kernel width (days)
BRUNSWICK, GA
Coverage
Year
Summer Solstice
June 21/Day 172
Summer Teletherm
July 29/Day 210
+38 days
Jan
Feb
Mar
Apr
May
Jun
Jul
Aug
Sep
Oct
Nov
Dec

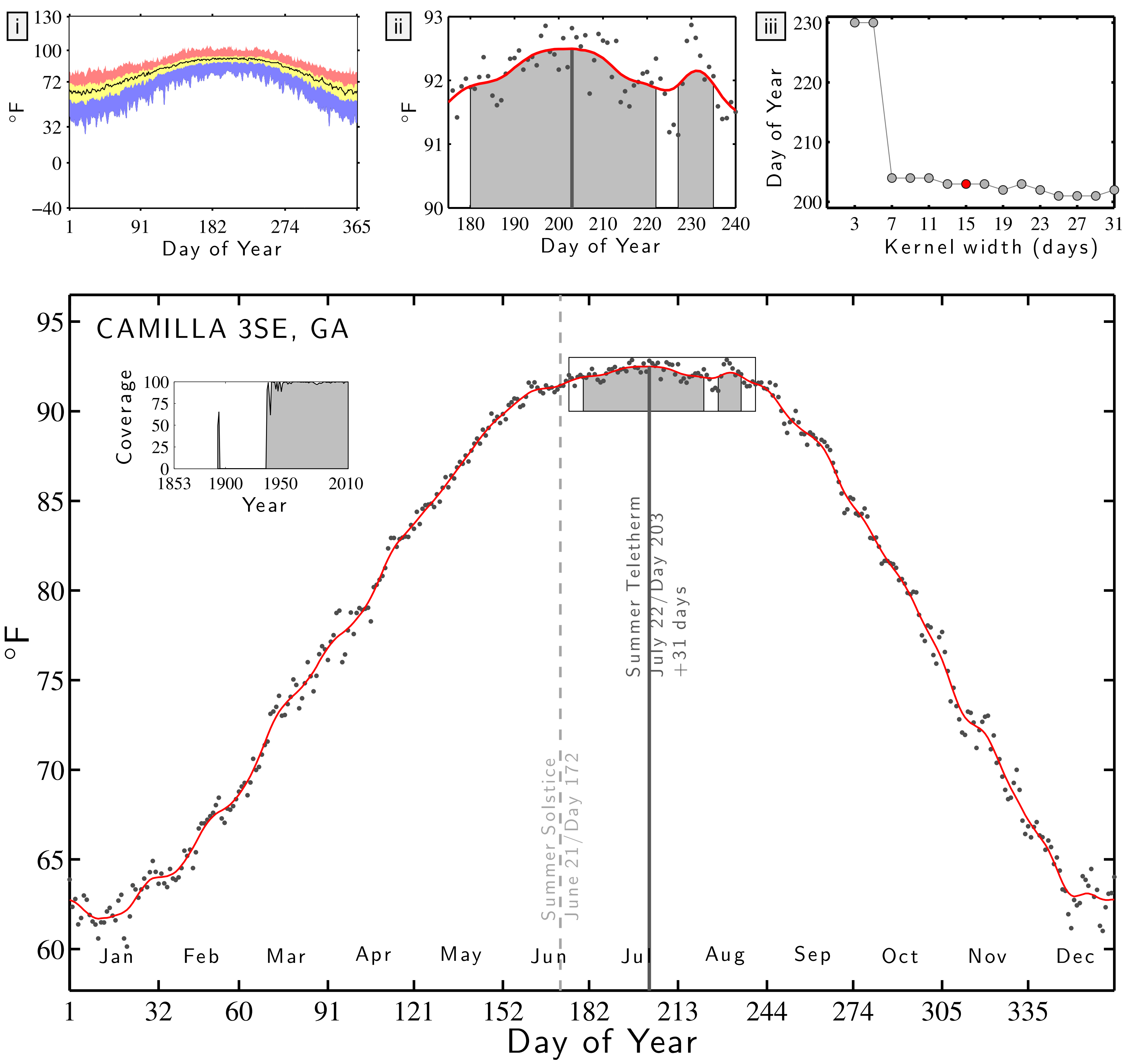

i
°F
Day of Year
ii
°F
Day of Year
iii
Day of Year
Kernel width (days)
CAMILLA 3SE, GA
Coverage
Year
Summer Solstice
June 21/Day 172
Summer Teletherm
July 22/Day 203
+31 days
Jan
Feb
Mar
Apr
May
Jun
Jul
Aug
Sep
Oct
Nov
Dec
°F
Day of Year

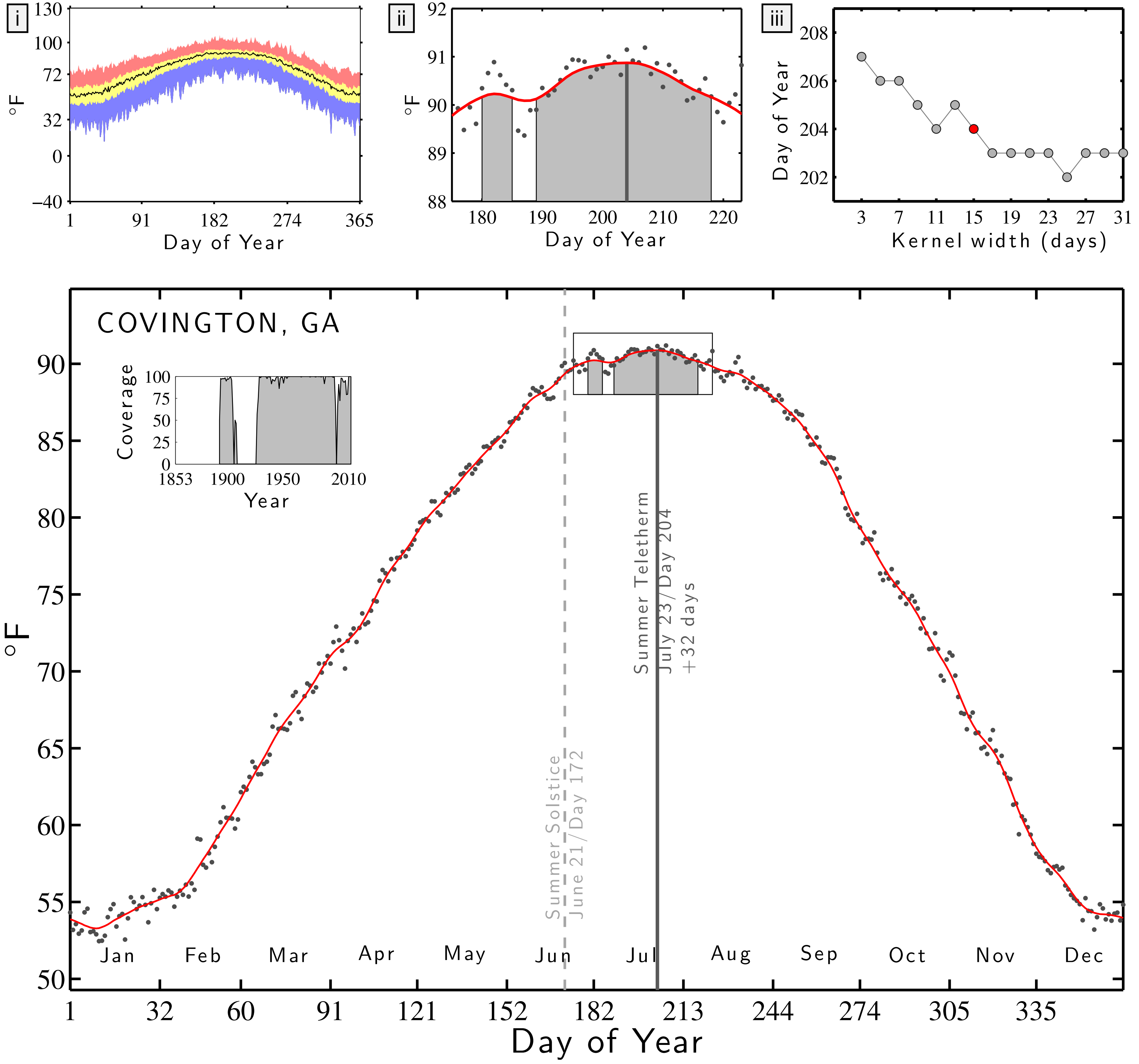

i
130
100
72
32
0
−40
°F
1
91
182
274
365
Day of Year
ii
92
91
90
89
88
°F
180
190
200
210
220
Day of Year
iii
208
206
204
202
Day of Year
3
7
11
15
19
23
27
31
Kernel width (days)
COVINGTON, GA
Coverage
100
75
50
25
0
1853
1900
1950
2010
Year
Summer Solstice
June 21/Day 172
Summer Teletherm
July 23/Day 204
+32 days
90
85
80
75
70
65
60
55
50
°F
Jan
Feb
Mar
Apr
May
Jun
Jul
Aug
Sep
Oct
Nov
Dec
1
32
60
91
121
152
182
213
244
274
305
335
Day of Year

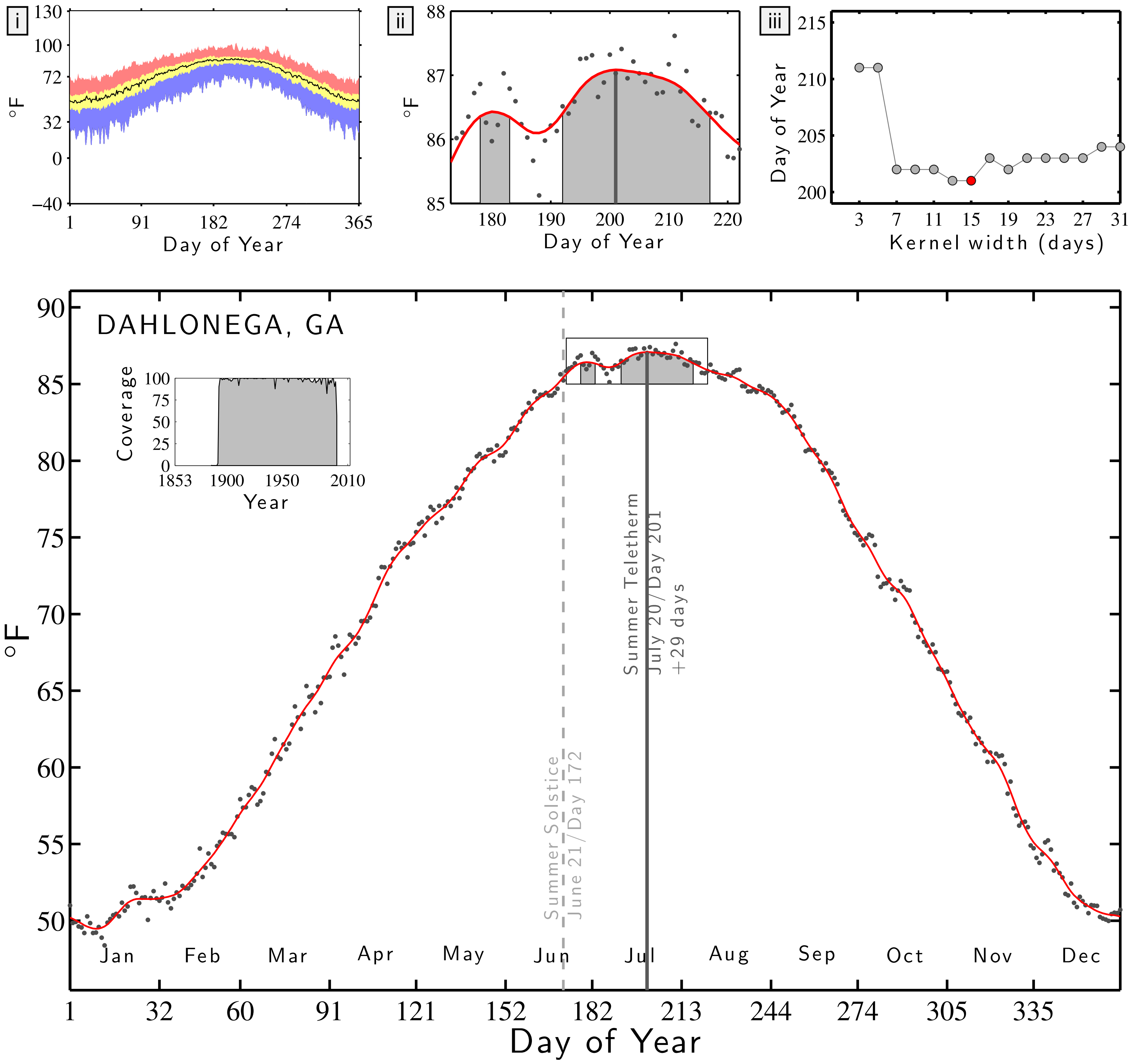

i
°F
Day of Year
ii
°F
Day of Year
iii
Day of Year
Kernel width (days)
DAHLONEGA, GA
Coverage
Year
Summer Solstice
June 21/Day 172
Summer Teletherm
July 20/Day 201
+29 days
Jan
Feb
Mar
Apr
May
Jun
Jul
Aug
Sep
Oct
Nov
Dec
°F
Day of Year

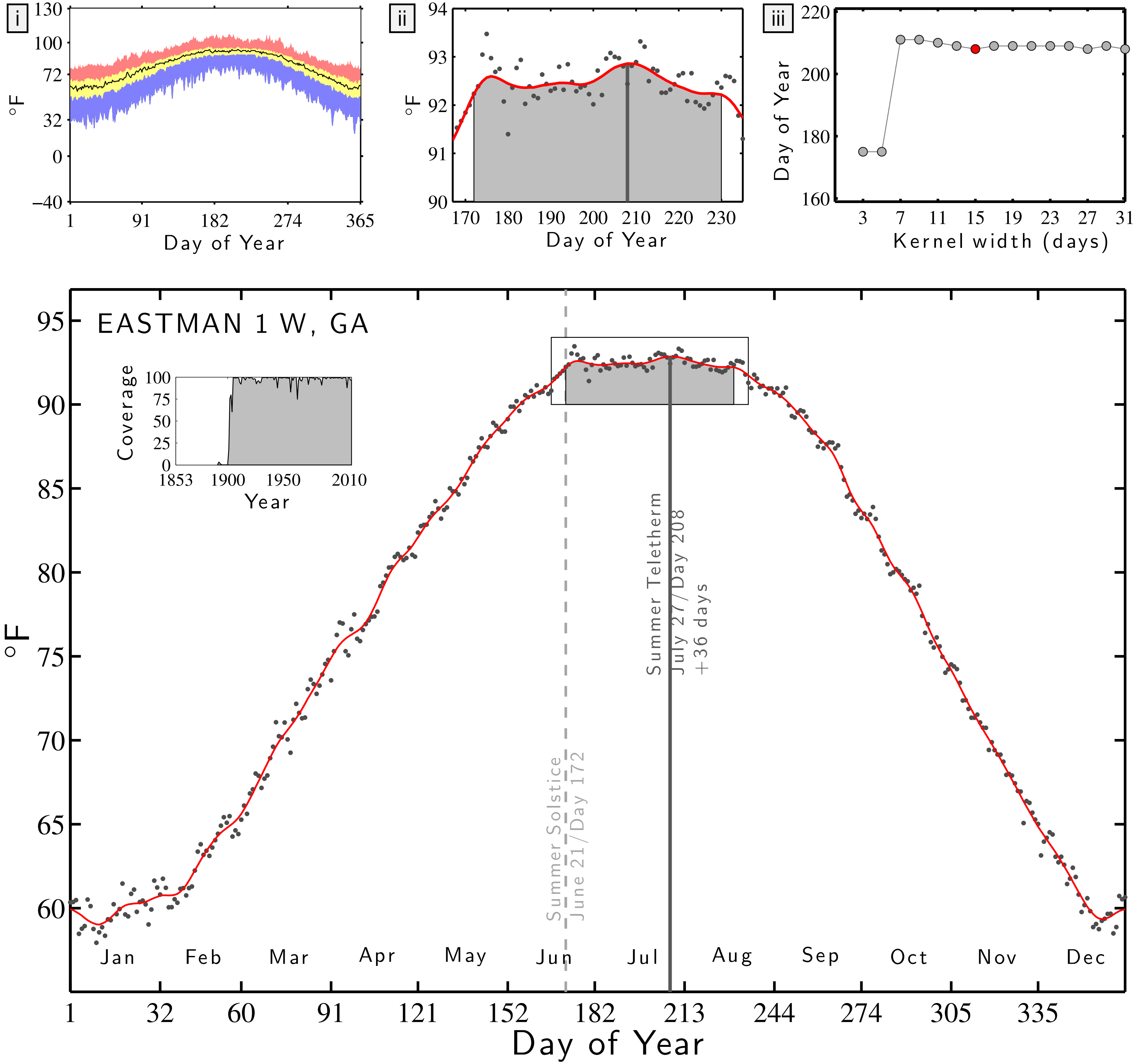
i
130
100
72
32
0
−40
°F
1
91
182
274
365
Day of Year
ii
94
93
92
91
90
°F
170
180
190
200
210
220
230
Day of Year
iii
220
200
180
160
Day of Year
3
7
11
15
19
23
27
31
Kernel width (days)
EASTMAN 1 W, GA
Coverage
100
75
50
25
0
1853
1900
1950
2010
Year
95
90
85
80
75
70
65
60
°F
Summer Solstice
June 21 / Day 172
Summer Teletherm
July 27 / Day 208
+36 days
Jan
Feb
Mar
Apr
May
Jun
Jul
Aug
Sep
Oct
Nov
Dec
1
32
60
91
121
152
182
213
244
274
305
335
Day of Year

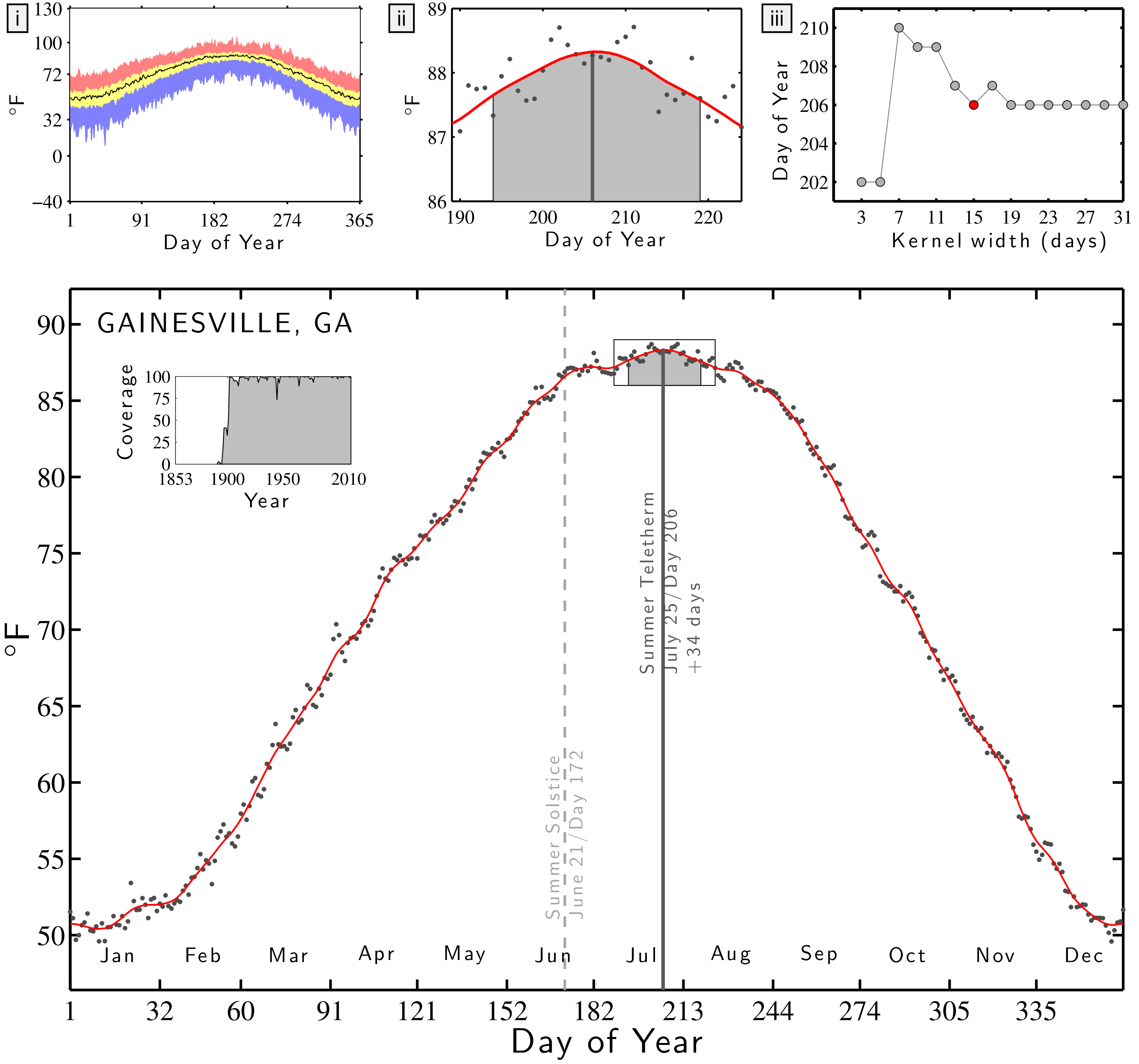

i
Day of Year
°F
ii
Day of Year
°F
iii
Day of Year
Kernel width (days)
GAINESVILLE, GA
Coverage
Year
Summer Solstice
June 21/Day 172
Summer Teletherm
July 25/Day 206
+34 days
Jan
Feb
Mar
Apr
May
Jun
Jul
Aug
Sep
Oct
Nov
Dec
Day of Year
°F

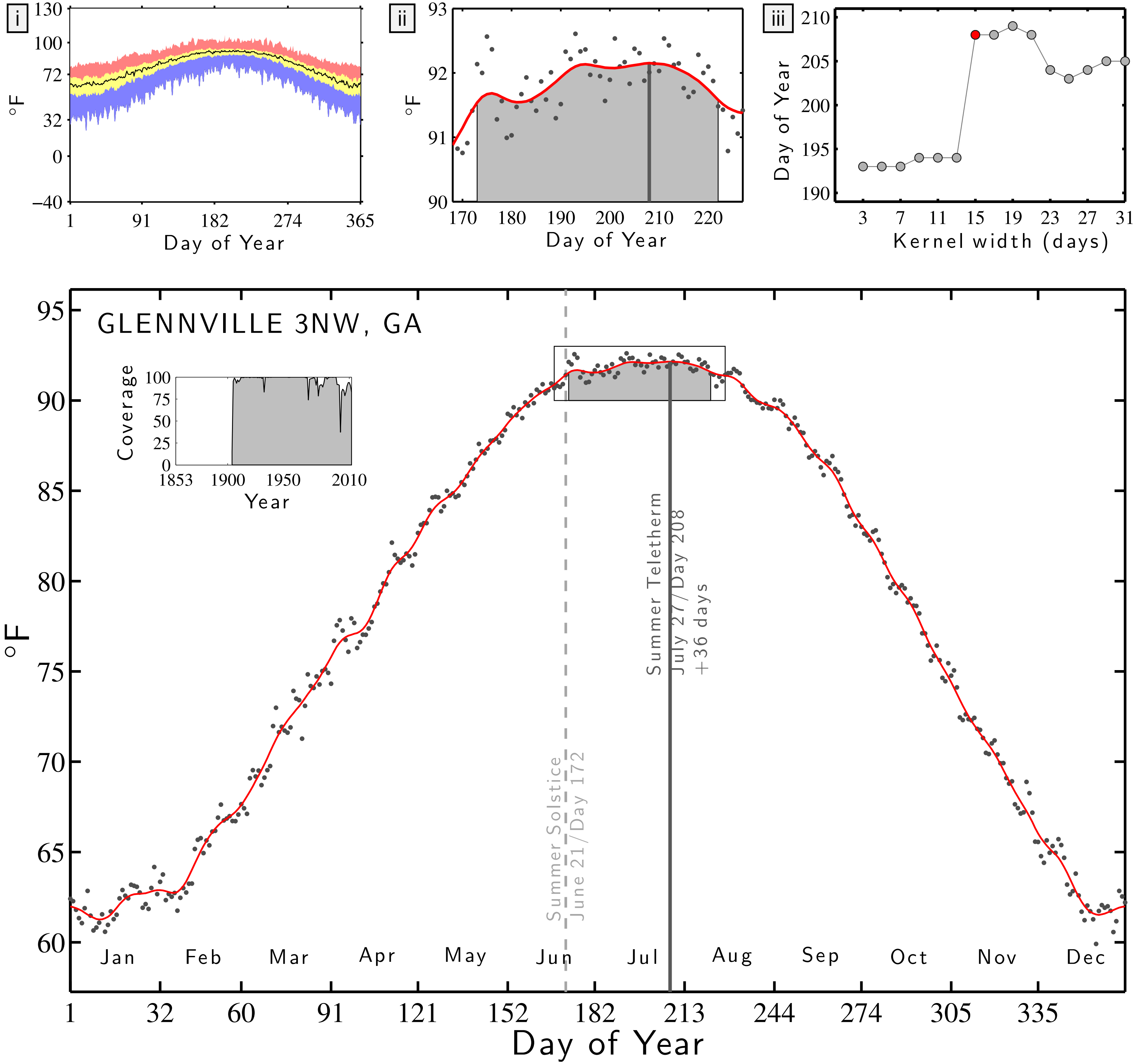

i
°F
130
100
72
32
0
−40
1
91
182
274
365
Day of Year
ii
93
92
91
90
170
180
190
200
210
220
Day of Year
iii
Day of Year
210
205
200
195
190
3
7
11
15
19
23
27
31
Kernel width (days)
GLENNVILLE 3NW, GA
Coverage
100
75
50
25
0
1853
1900
1950
2010
Year
Summer Teletherm
July 27/Day 208
+36 days
Summer Solstice
June 21/Day 172
Jan
Feb
Mar
Apr
May
Jun
Jul
Aug
Sep
Oct
Nov
Dec
95
90
85
80
75
70
65
60
1
32
60
91
121
152
182
213
244
274
305
335
Day of Year

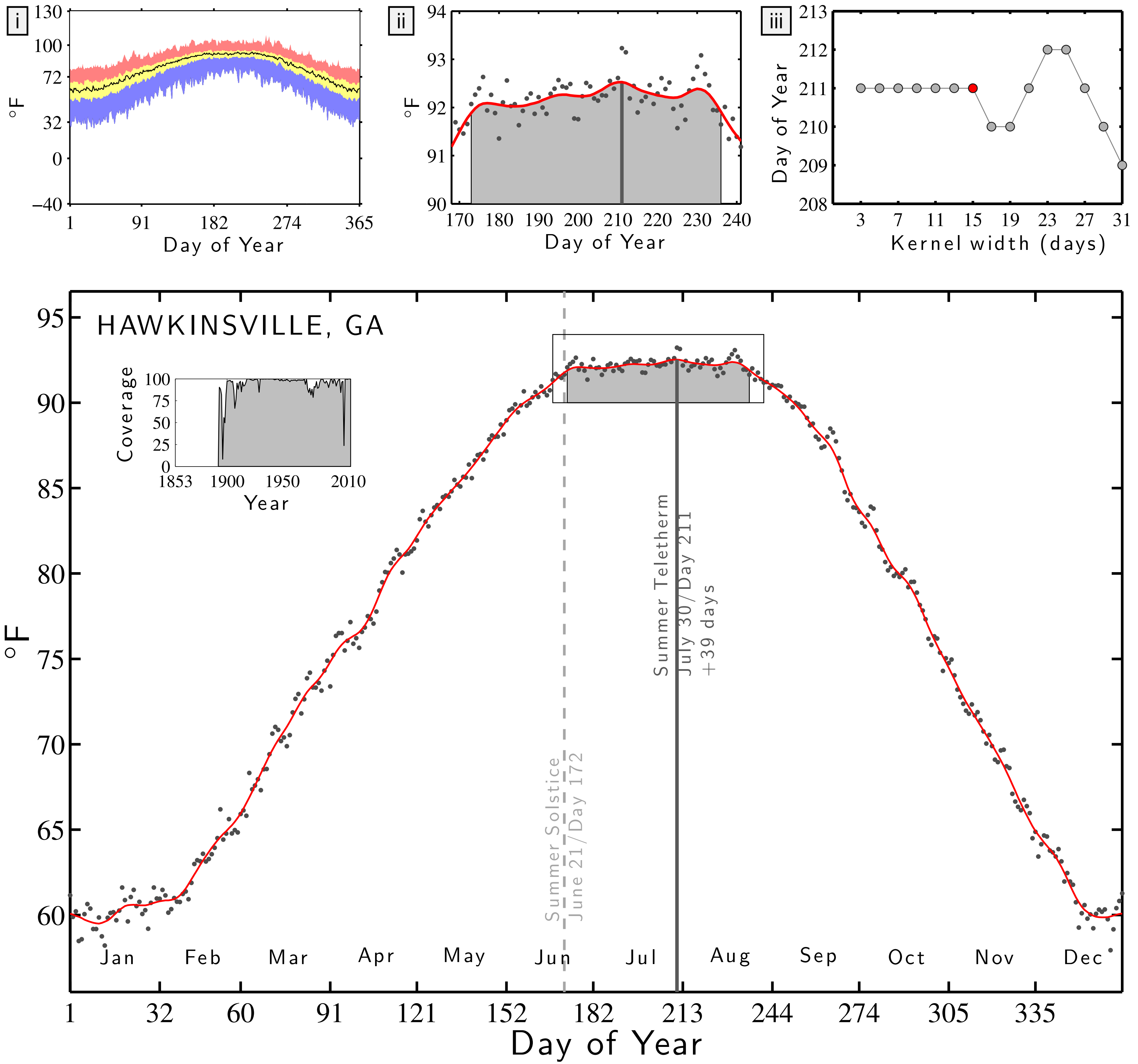

i
°F
Day of Year
ii
°F
Day of Year
iii
Day of Year
Kernel width (days)
HAWKINSVILLE, GA
Coverage
Year
Summer Solstice
June 21/Day 172
Summer Teletherm
July 30/Day 211
+39 days
Jan
Feb
Mar
Apr
May
Jun
Jul
Aug
Sep
Oct
Nov
Dec
°F
Day of Year

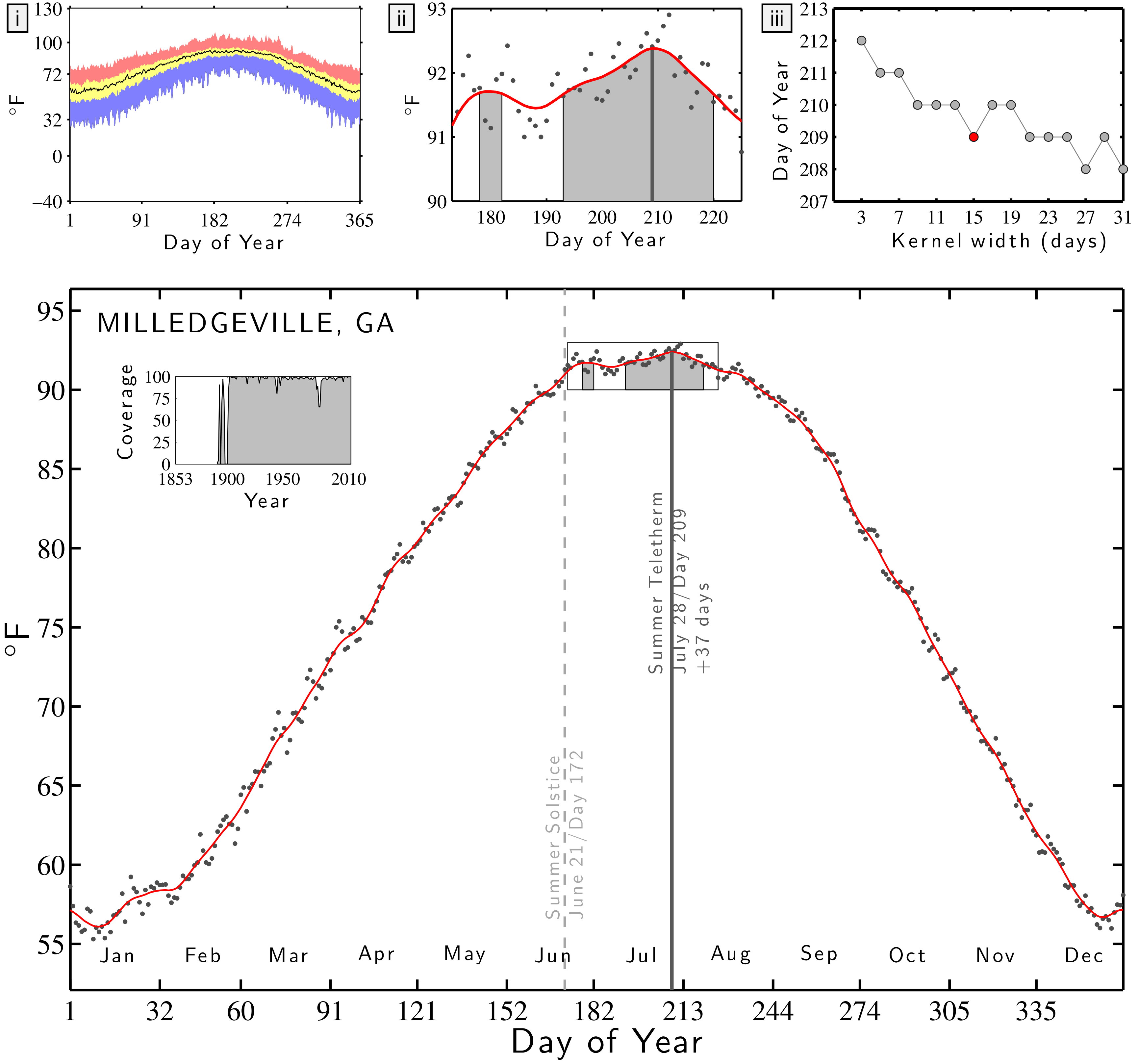

i
°F
Day of Year
ii
°F
Day of Year
iii
Day of Year
Kernel width (days)
MILLEDGEVILLE, GA
Coverage
Year
Summer Solstice
June 21/Day 172
Summer Teletherm
July 28/Day 209
+37 days
°F
Day of Year

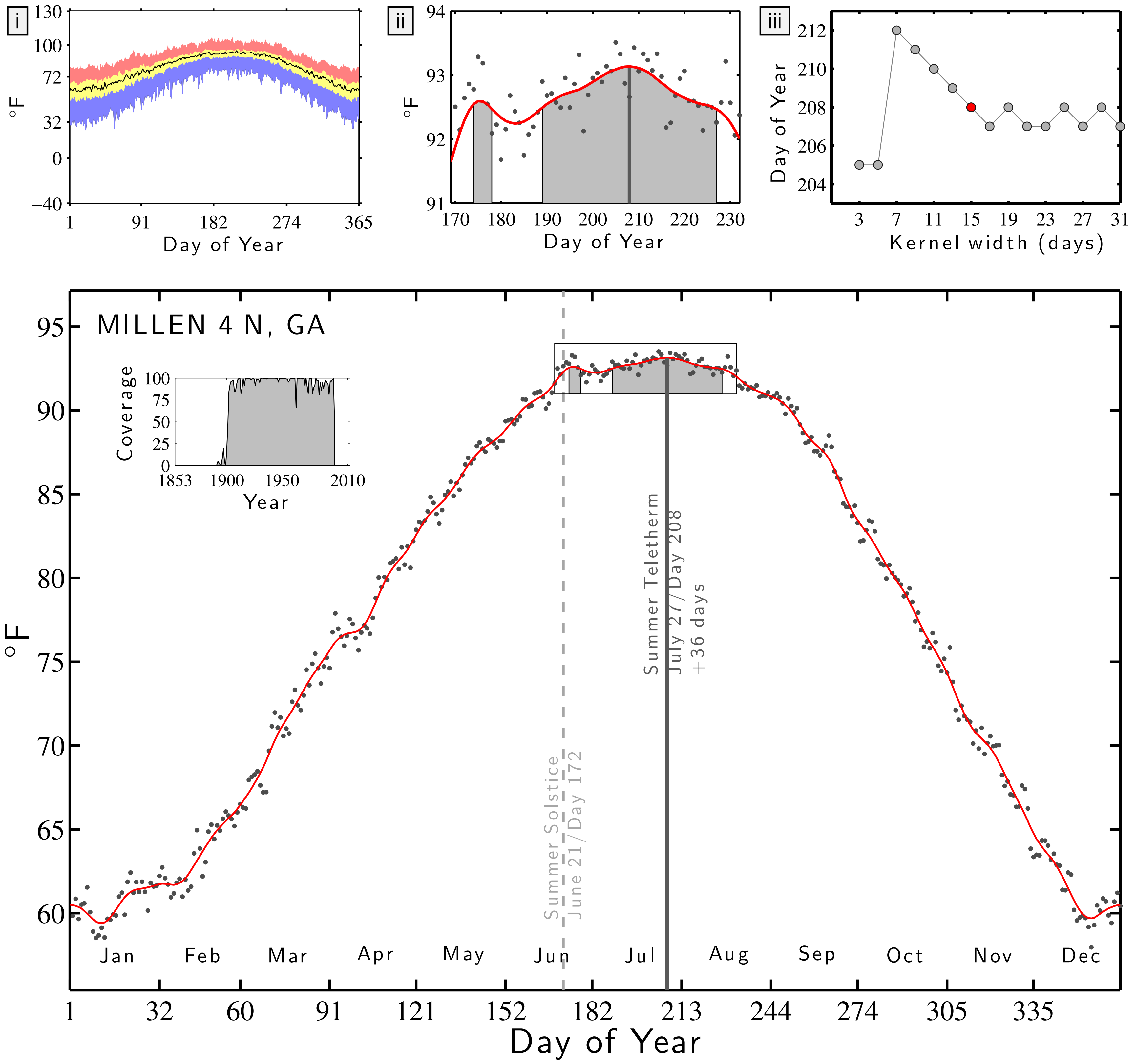

i
ii
iii
Day of Year
°F
Kernel width (days)
MILLEN 4 N, GA
Coverage
Year
Summer Solstice
June 21 / Day 172
Summer Teletherm
July 27 / Day 208
+36 days
Jan
Feb
Mar
Apr
May
Jun
Jul
Aug
Sep
Oct
Nov
Dec

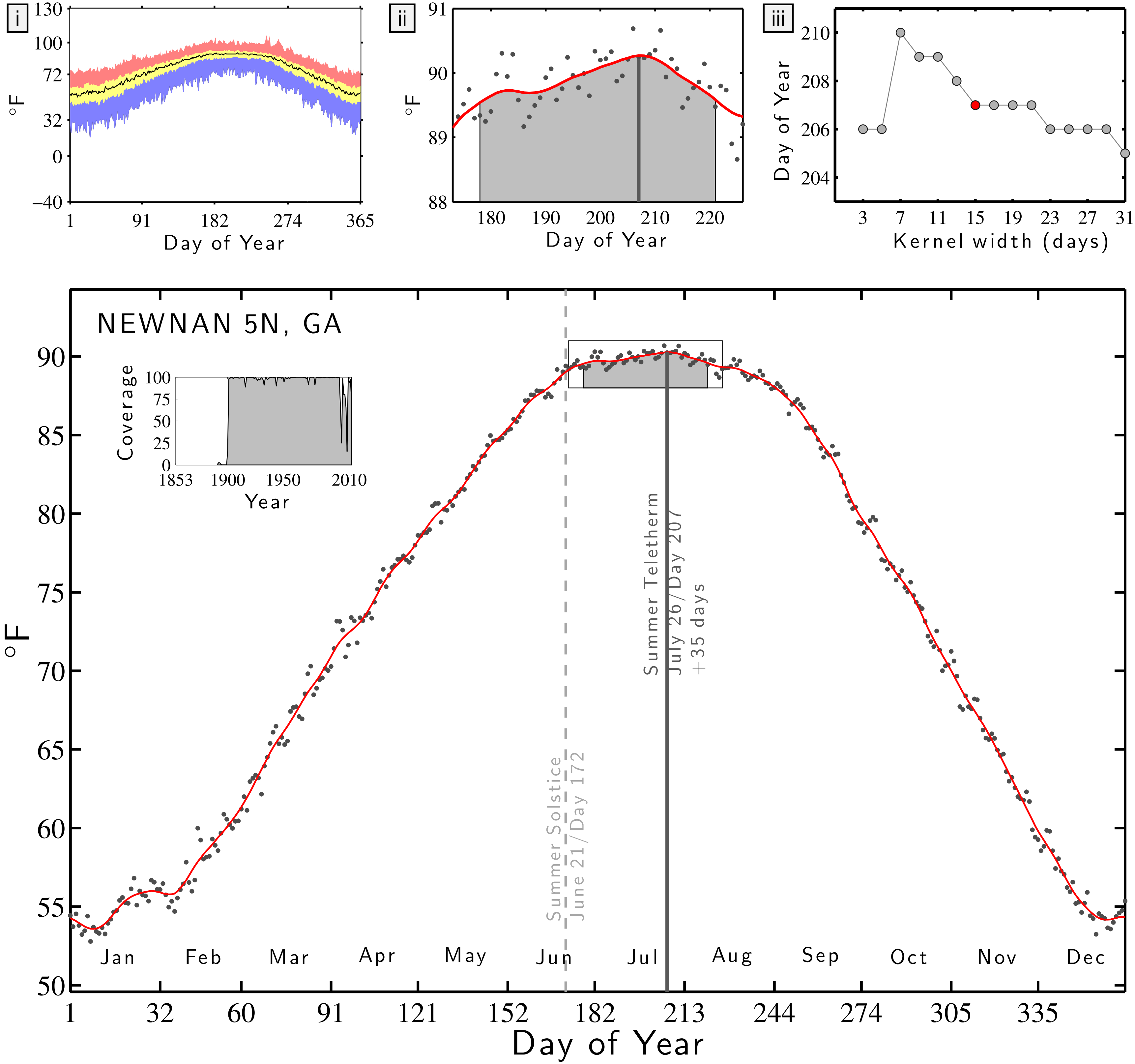

i
°F
Day of Year
ii
°F
Day of Year
iii
Day of Year
Kernel width (days)
NEWNAN 5N, GA
Coverage
Year
Summer Solstice
June 21 / Day 172
Summer Teletherm
July 26 / Day 207
+35 days
Jan
Feb
Mar
Apr
May
Jun
Jul
Aug
Sep
Oct
Nov
Dec
°F
Day of Year

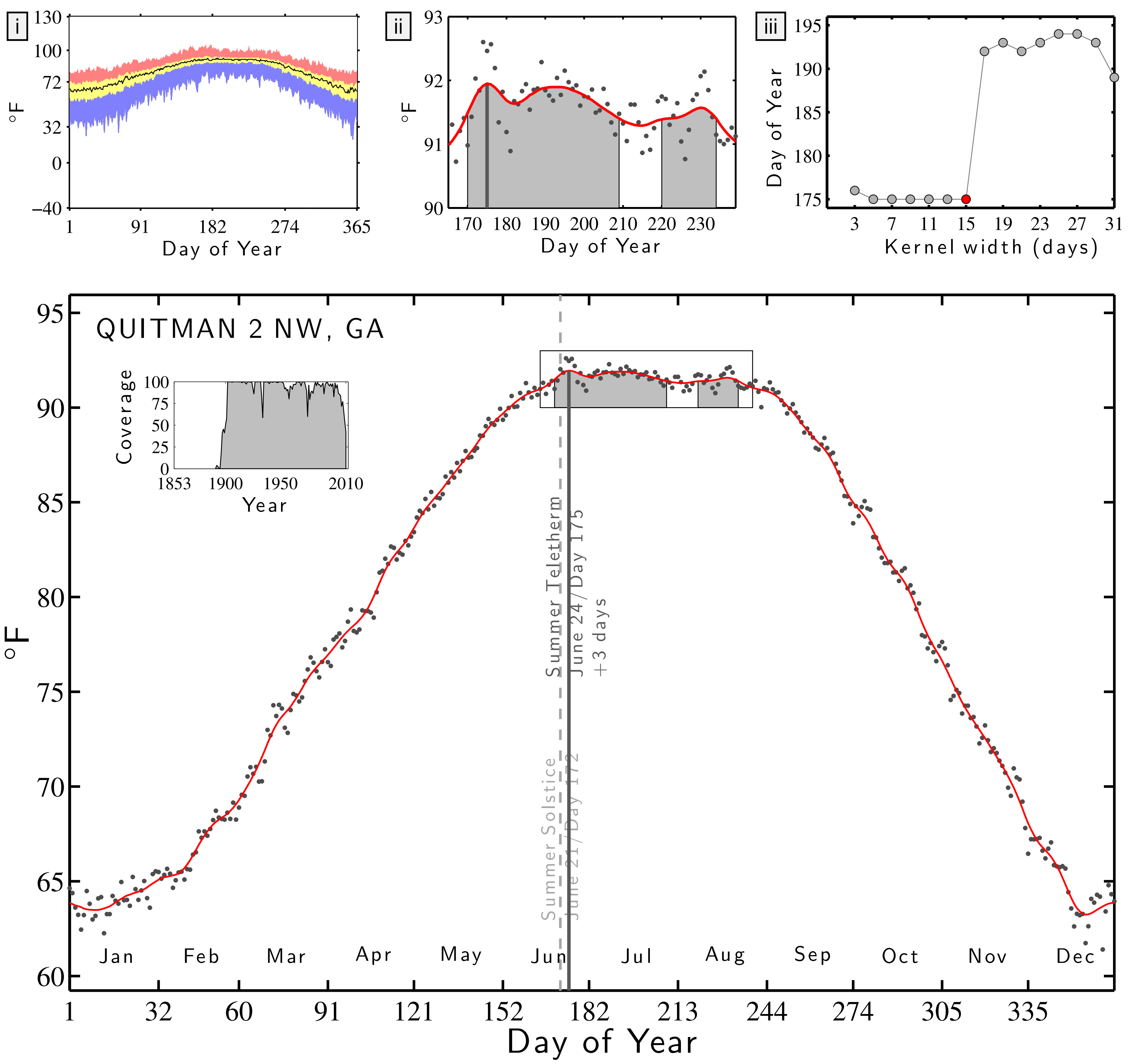

i
ii
iii
Day of Year
Kernel width (days)
QUITMAN 2 NW, GA
Coverage
Year
Summer Teletherm
June 24/Day 175
+3 days
Summer Solstice
June 21/Day 172
Jan
Feb
Mar
Apr
May
Jun
Jul
Aug
Sep
Oct
Nov
Dec
°F

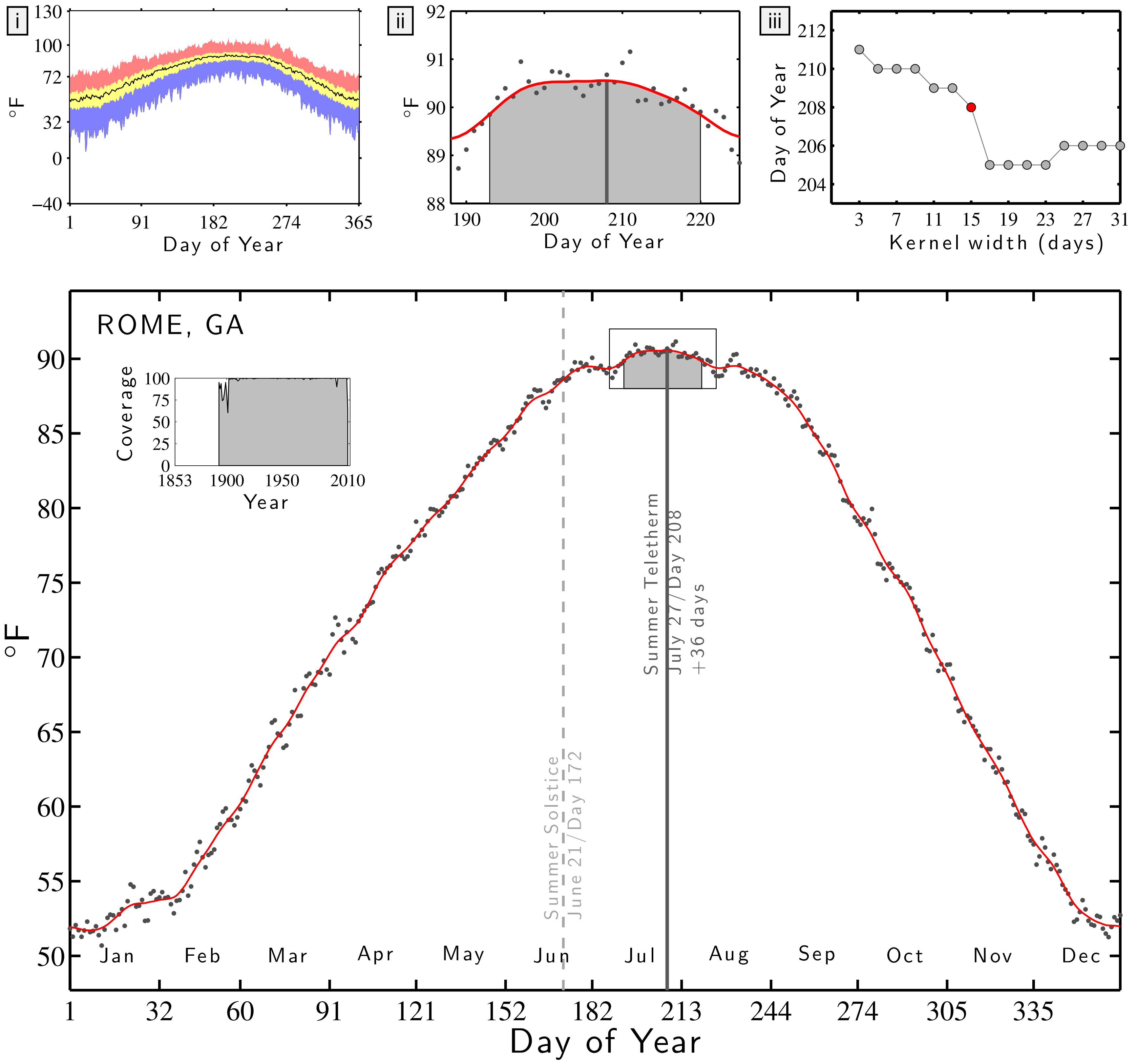

i
°F
Day of Year
ii
Day of Year
iii
Day of Year
Kernel width (days)
ROME, GA
Coverage
Year
Summer Solstice
June 21 / Day 172
Summer Teletherm
July 27 / Day 208
+36 days
Jan
Feb
Mar
Apr
May
Jun
Jul
Aug
Sep
Oct
Nov
Dec

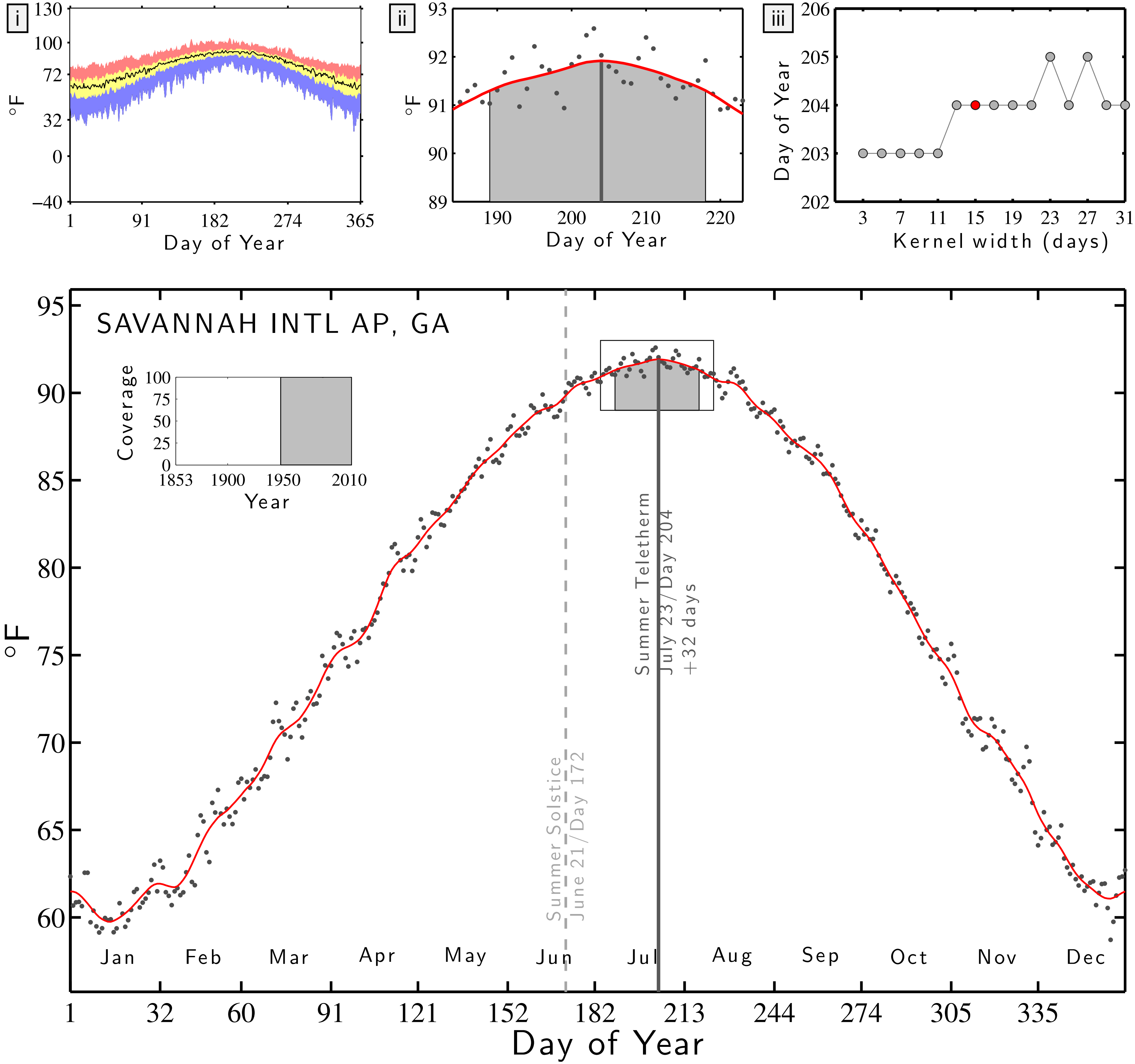

i
130
100
72
32
0
−40
°F
1
91
182
274
365
Day of Year
ii
93
92
91
90
89
°F
190
200
210
220
Day of Year
iii
206
205
204
203
202
Day of Year
3
7
11
15
19
23
27
31
Kernel width (days)
SAVANNAH INTL AP, GA
95
90
85
80
75
70
65
60
°F
Coverage
100
75
50
25
0
1853
1900
1950
2010
Year
Summer Solstice
June 21 / Day 172
Summer Teletherm
July 23 / Day 204
+32 days
Jan
Feb
Mar
Apr
May
Jun
Jul
Aug
Sep
Oct
Nov
Dec
1
32
60
91
121
152
182
213
244
274
305
335
Day of Year

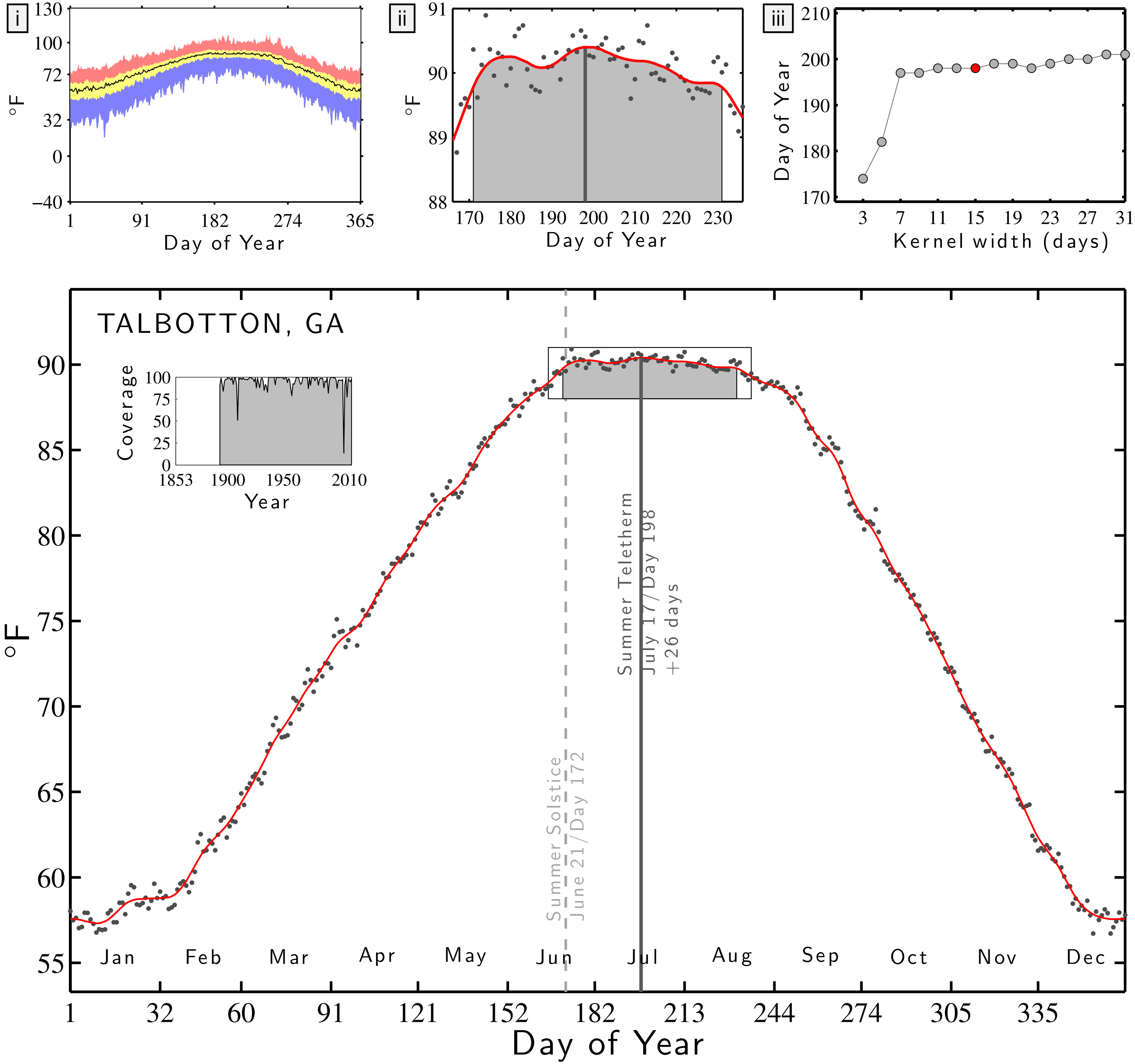

i
ii
iii
°F
Day of Year
Kernel width (days)
TALBOTTON, GA
Coverage
Year
Summer Solstice
June 21/Day 172
Summer Teletherm
July 17/Day 198
+26 days
Jan
Feb
Mar
Apr
May
Jun
Jul
Aug
Sep
Oct
Nov
Dec

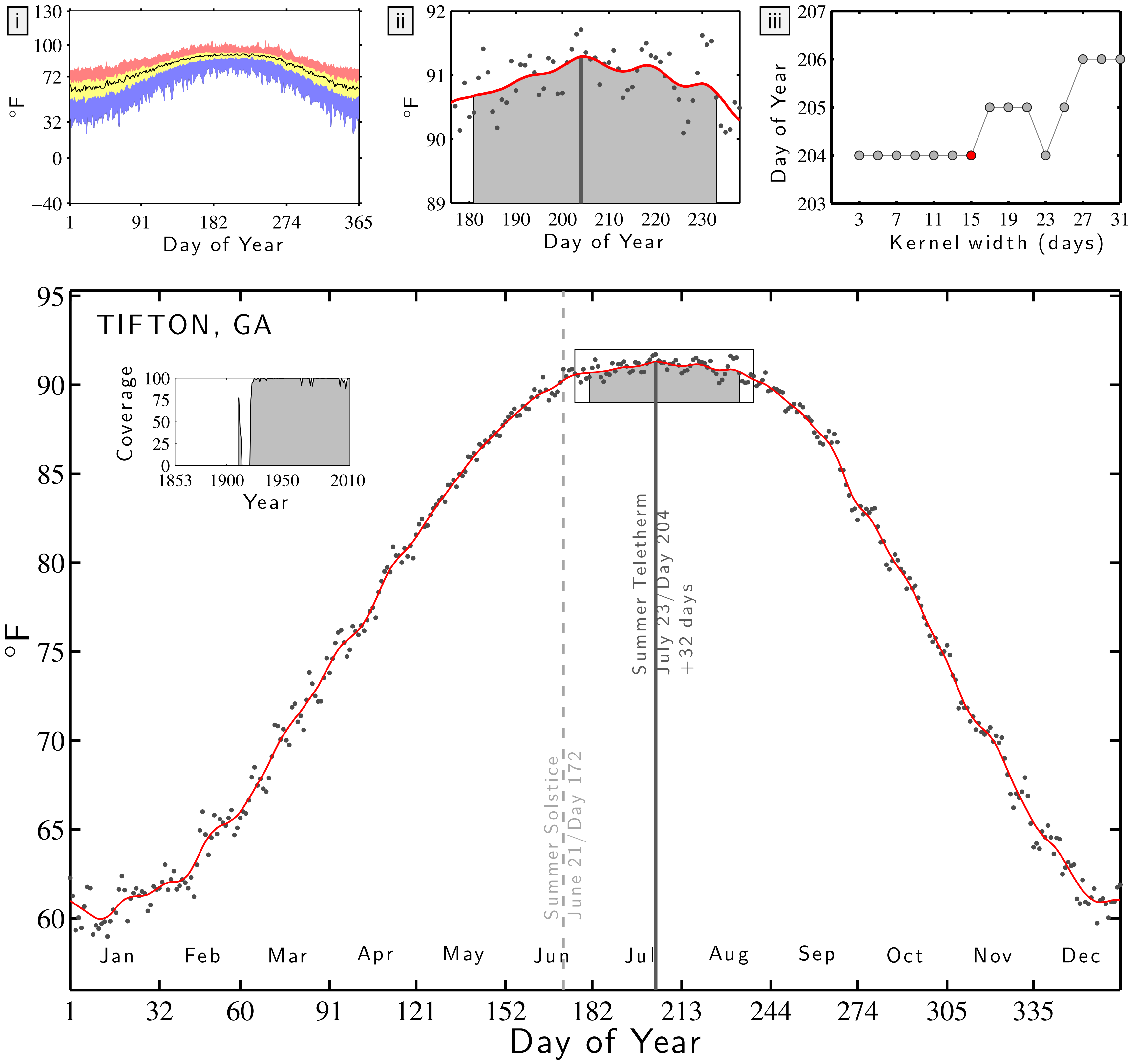

i
ii
iii
°F
Day of Year
Kernel width (days)
Day of Year
TIFTON, GA
Coverage
Year
Summer Solstice
June 21 / Day 172
Summer Teletherm
July 23 / Day 204
+32 days
Jan
Feb
Mar
Apr
May
Jun
Jul
Aug
Sep
Oct
Nov
Dec

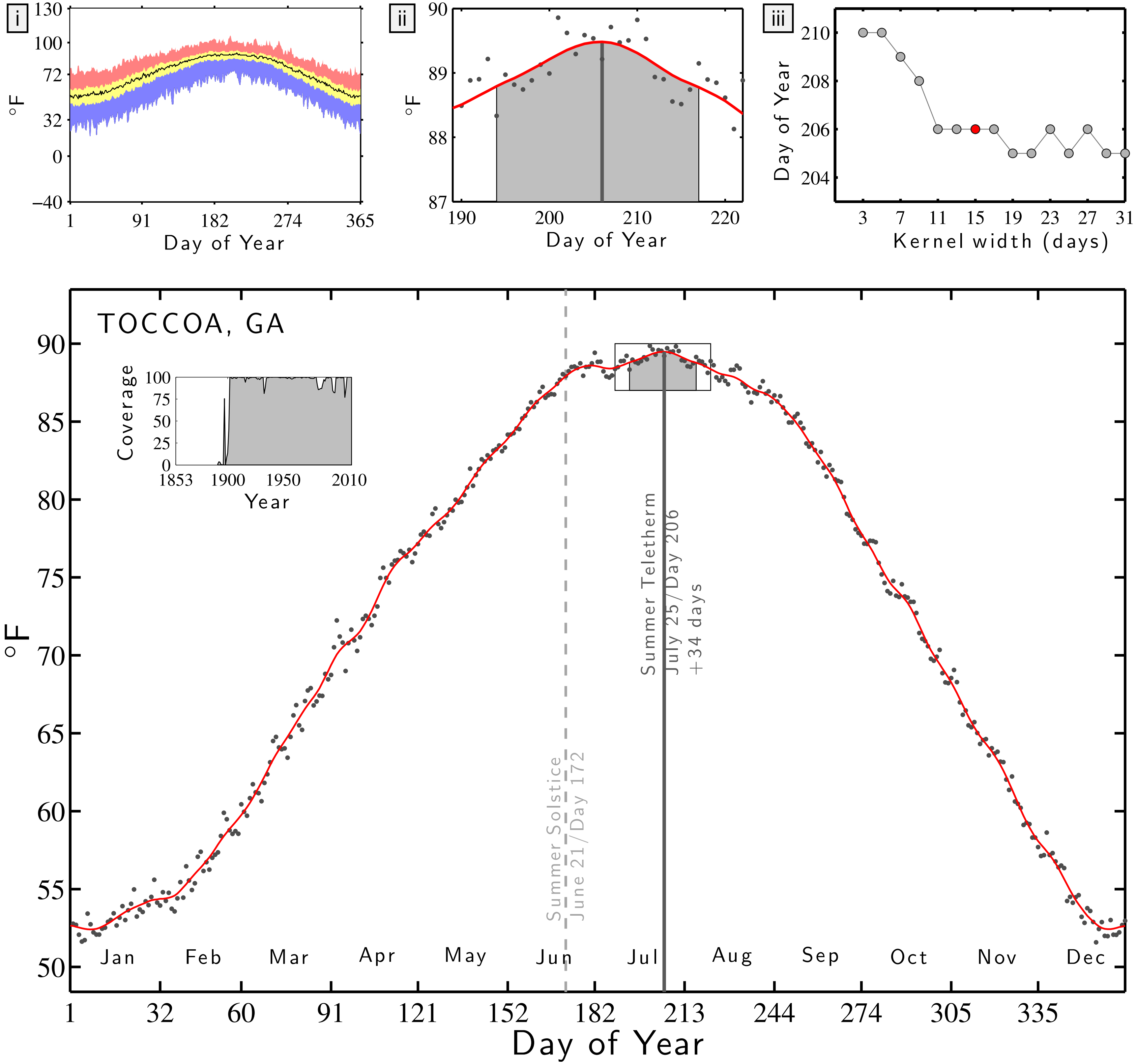

i
ii
iii
°F
Day of Year
Kernel width (days)
TOCCOA, GA
Coverage
Year
Summer Solstice
June 21/Day 172
Summer Teletherm
July 25/Day 206
+34 days
Jan
Feb
Mar
Apr
May
Jun
Jul
Aug
Sep
Oct
Nov
Dec

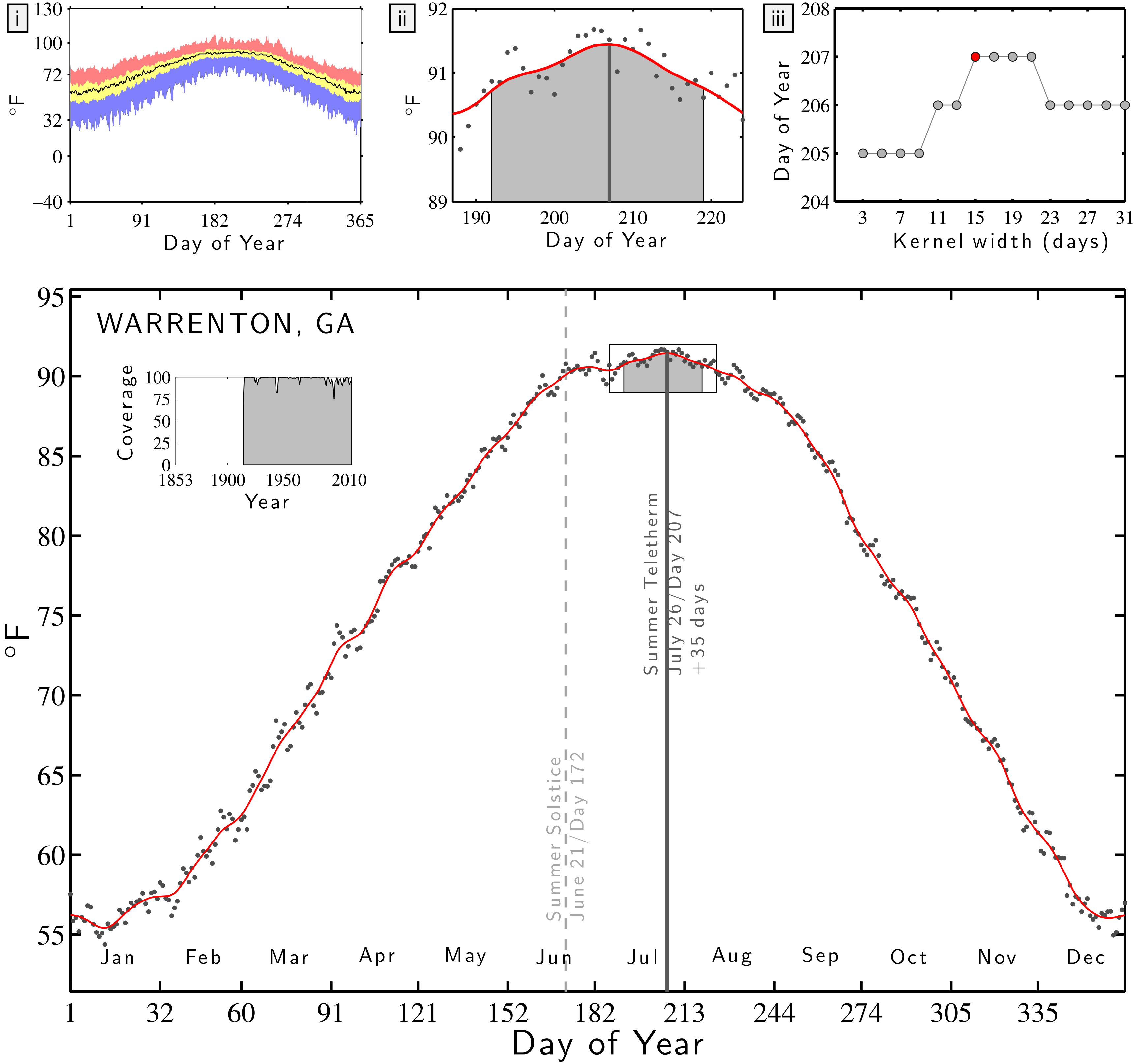

i
ii
iii
°F
Day of Year
Kernel width (days)
WARRENTON, GA
Coverage
Year
Summer Solstice
June 21 / Day 172
Summer Teletherm
July 26 / Day 207
+35 days
Jan
Feb
Mar
Apr
May
Jun
Jul
Aug
Sep
Oct
Nov
Dec

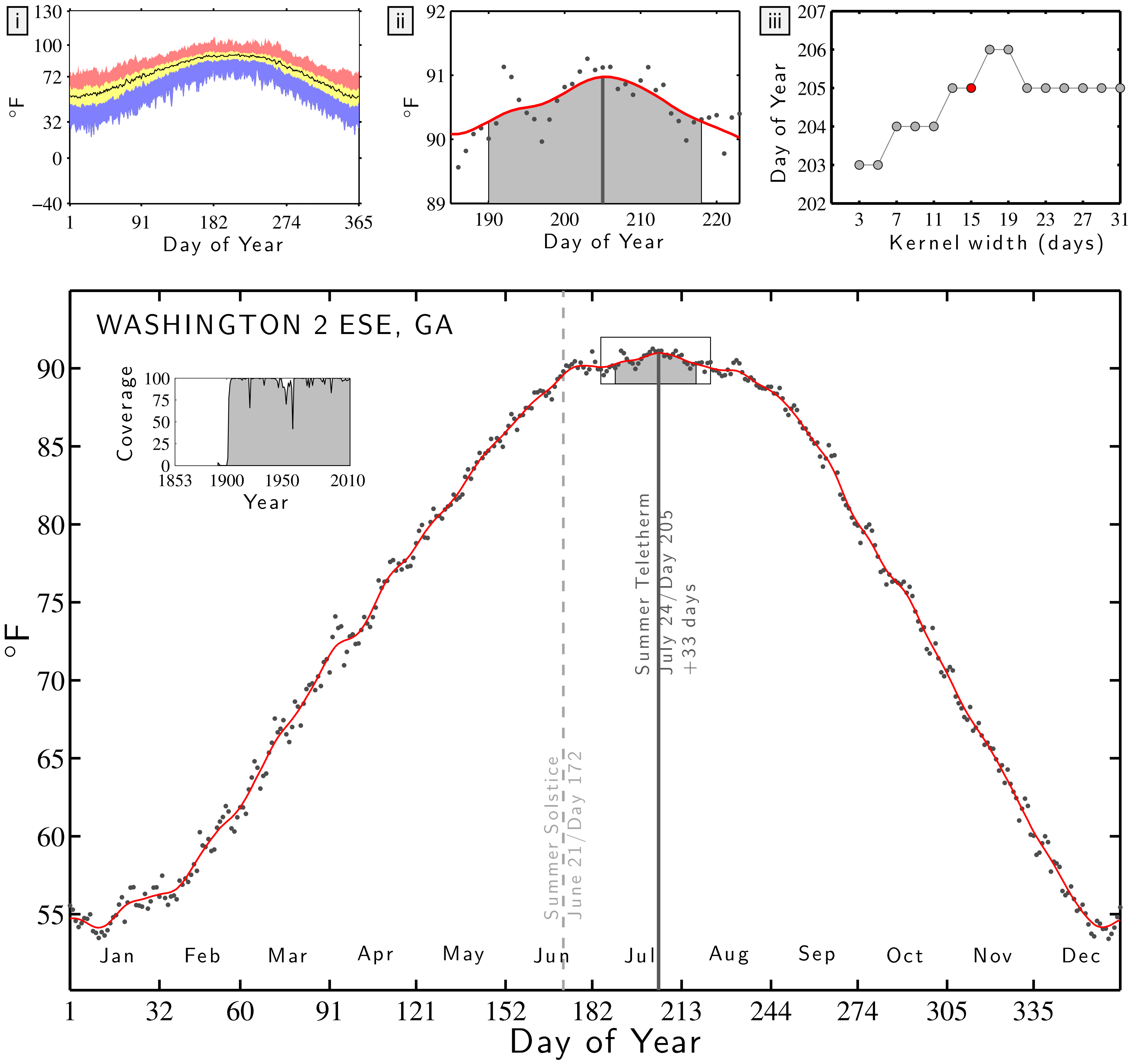

i
ii
iii
°F
Day of Year
Day of Year
Kernel width (days)
WASHINGTON 2 ESE, GA
Coverage
Year
Summer Solstice
June 21/Day 172
Summer Teletherm
July 24/Day 205
+33 days
Jan
Feb
Mar
Apr
May
Jun
Jul
Aug
Sep
Oct
Nov
Dec
Day of Year

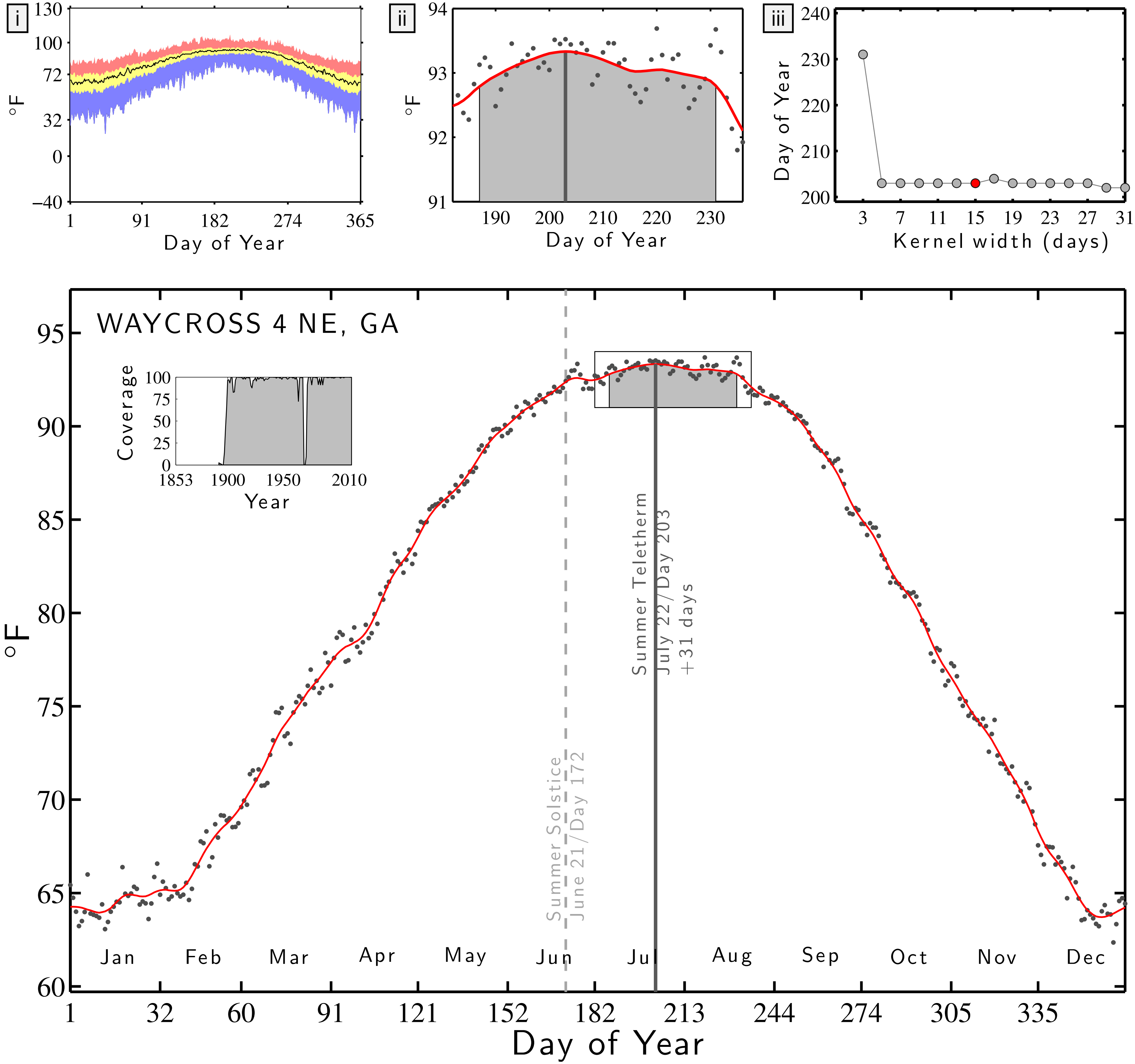

i
ii
iii
°F
Day of Year
Kernel width (days)
WAYCROSS 4 NE, GA
Coverage
Year
Summer Solstice
June 21/Day 172
Summer Teletherm
July 22/Day 203
+31 days
Jan
Feb
Mar
Apr
May
Jun
Jul
Aug
Sep
Oct
Nov
Dec

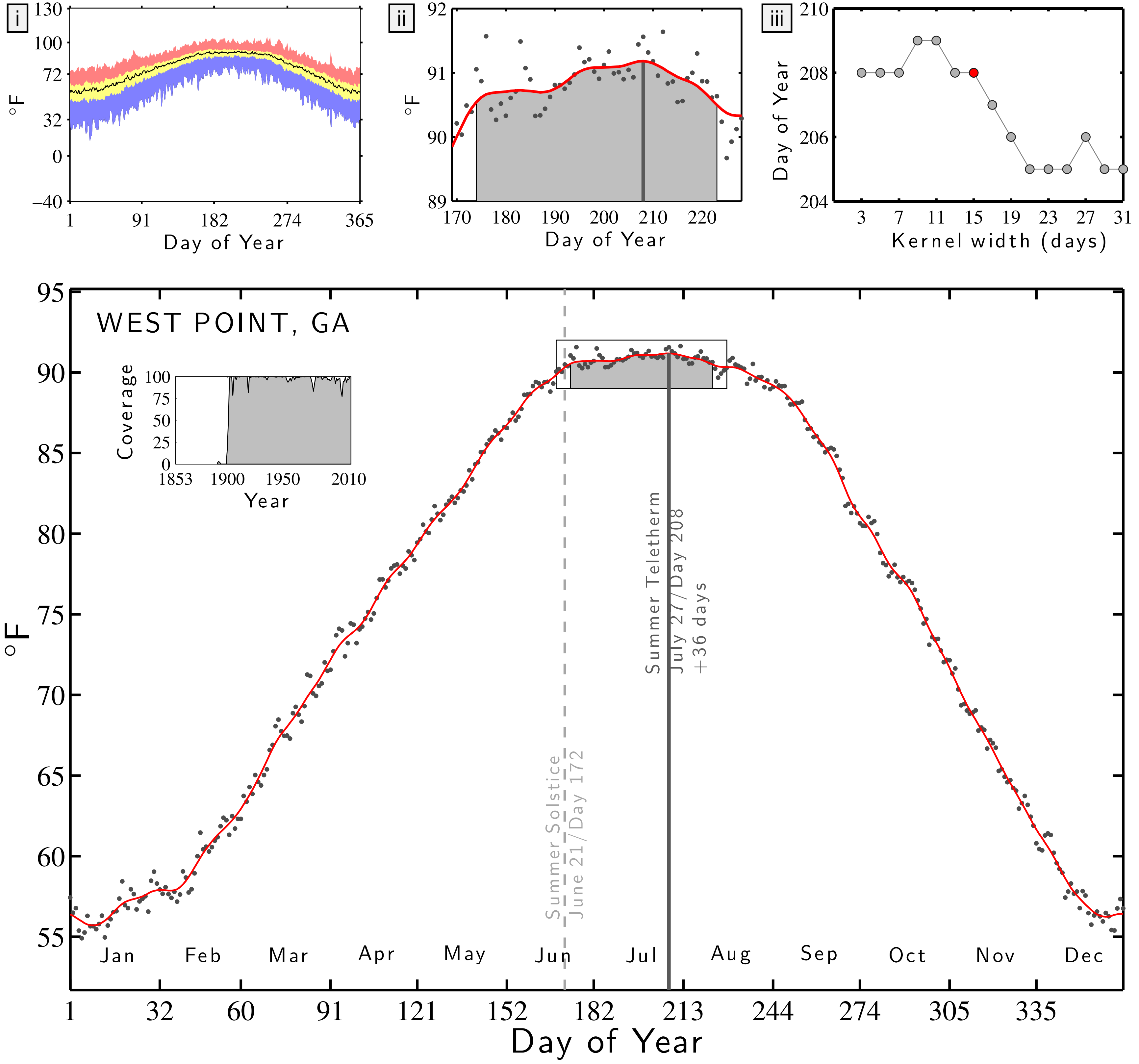

i
°F
Day of Year
ii
iii
Day of Year
Kernel width (days)
WEST POINT, GA
Coverage
Year
Summer Solstice
June 21 / Day 172
Summer Teletherm
July 27 / Day 208
+36 days
Jan
Feb
Mar
Apr
May
Jun
Jul
Aug
Sep
Oct
Nov
Dec

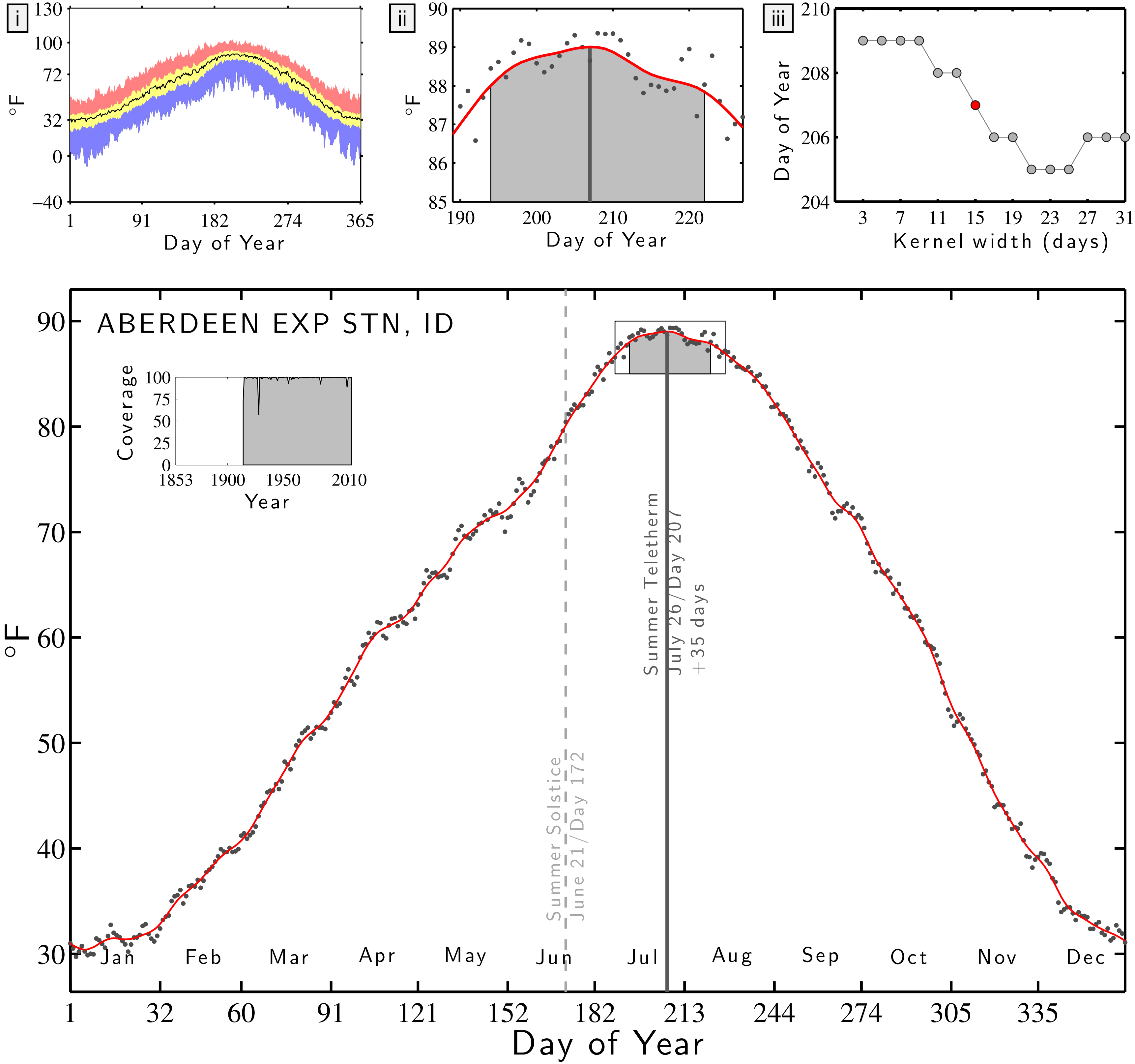
i
ii
iii
Day of Year
Kernel width (days)
ABERDEEN EXP STN, ID
Coverage
Year
Summer Solstice
June 21 / Day 172
Summer Teletherm
July 26 / Day 207
+35 days
Jan
Feb
Mar
Apr
May
Jun
Jul
Aug
Sep
Oct
Nov
Dec
°F
Day of Year

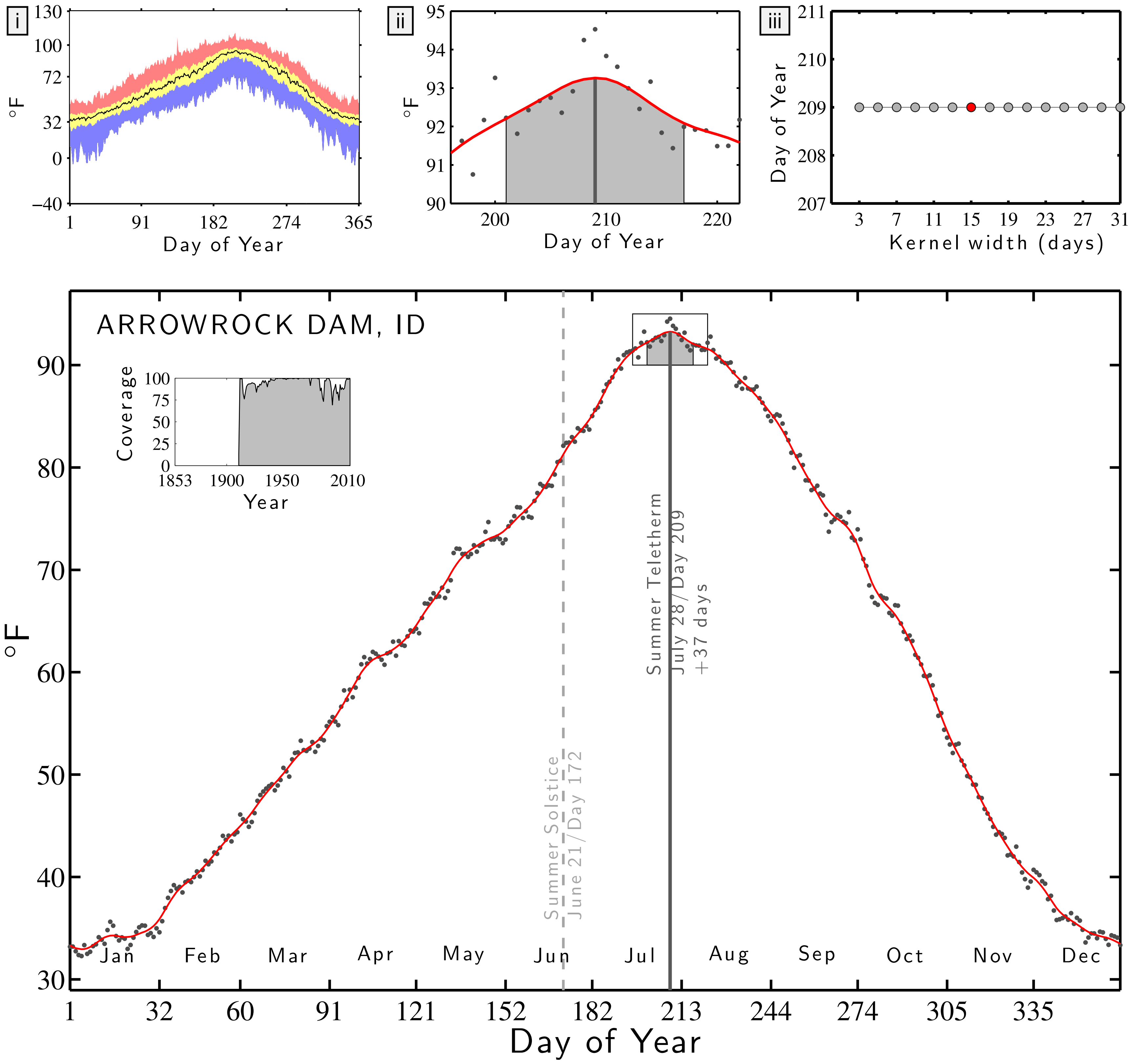

i
ii
iii
°F
Day of Year
Day of Year
Kernel width (days)
ARROWROCK DAM, ID
Coverage
Year
Summer Solstice
June 21/Day 172
Summer Teletherm
July 28/Day 209
+37 days
Jan
Feb
Mar
Apr
May
Jun
Jul
Aug
Sep
Oct
Nov
Dec
°F
Day of Year

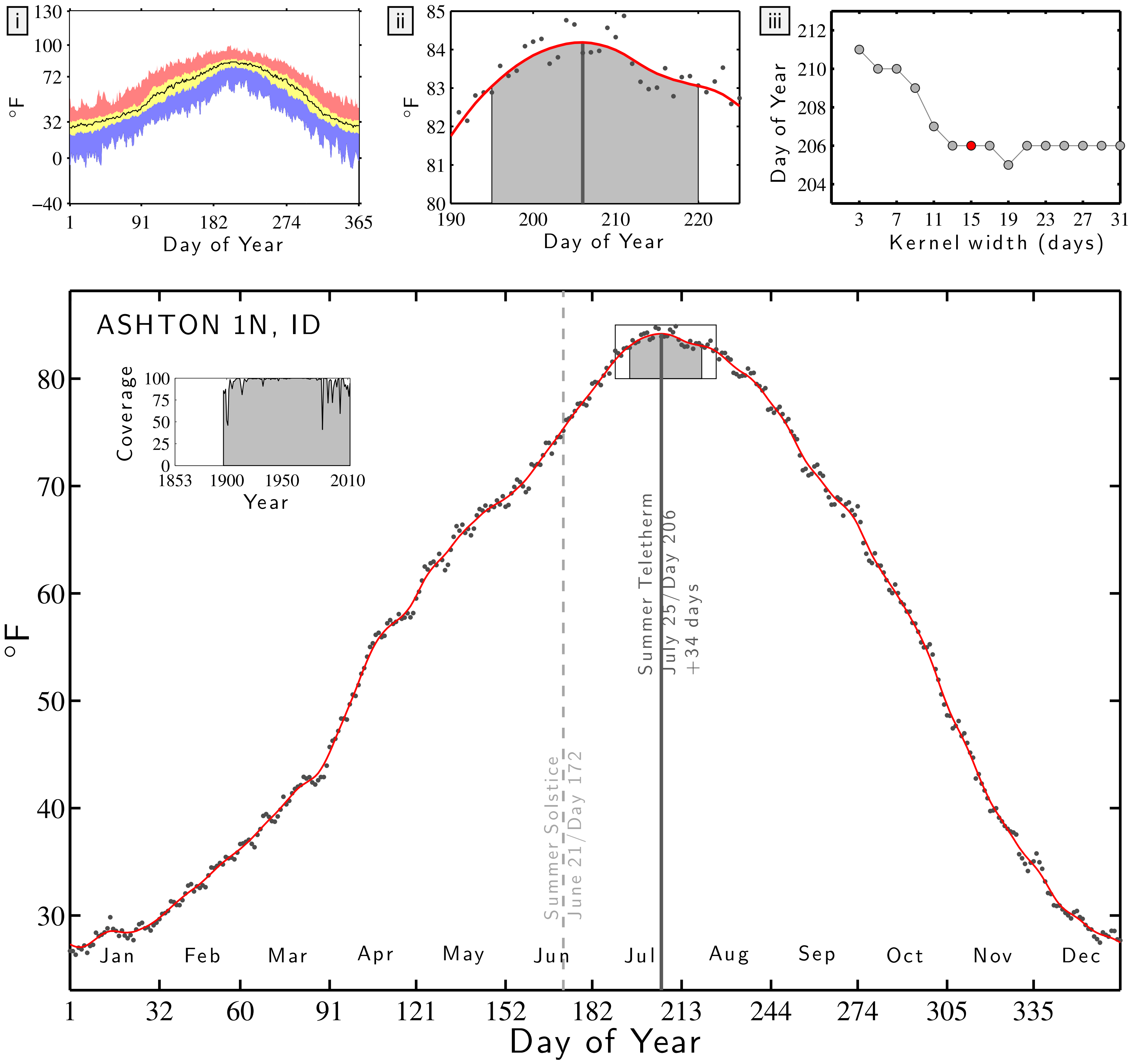

i
ii
iii
°F
Day of Year
Day of Year
Kernel width (days)
ASHTON 1N, ID
Coverage
Year
Summer Solstice
June 21/Day 172
Summer Teletherm
July 25/Day 206
+34 days
Jan
Feb
Mar
Apr
May
Jun
Jul
Aug
Sep
Oct
Nov
Dec

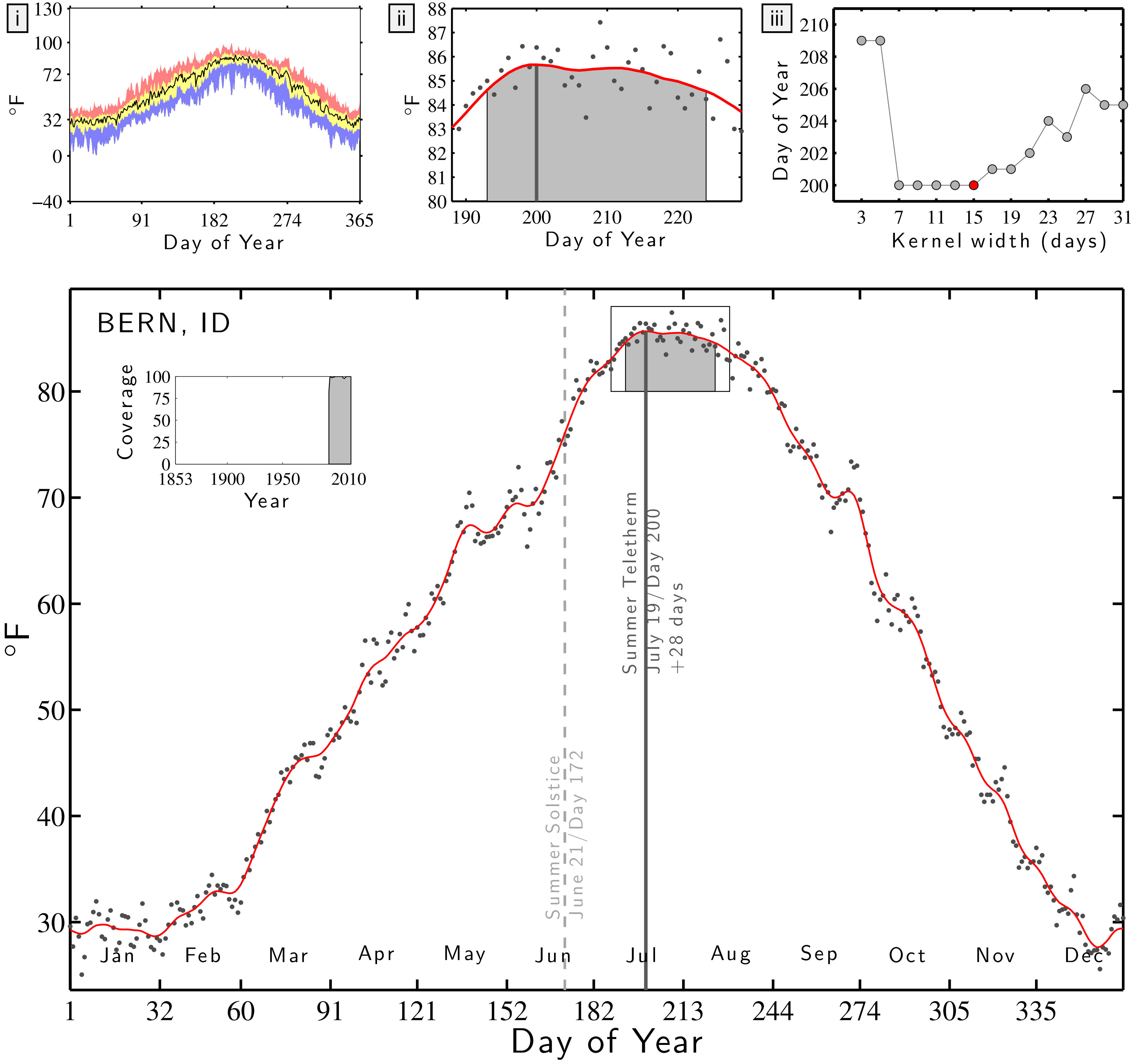
i
ii
iii
Day of Year
Kernel width (days)
BERN, ID
Coverage
Year
Summer Solstice
June 21/Day 172
Summer Teletherm
July 19/Day 200
+28 days
Jan
Feb
Mar
Apr
May
Jun
Jul
Aug
Sep
Oct
Nov
Dec
°F
Day of Year

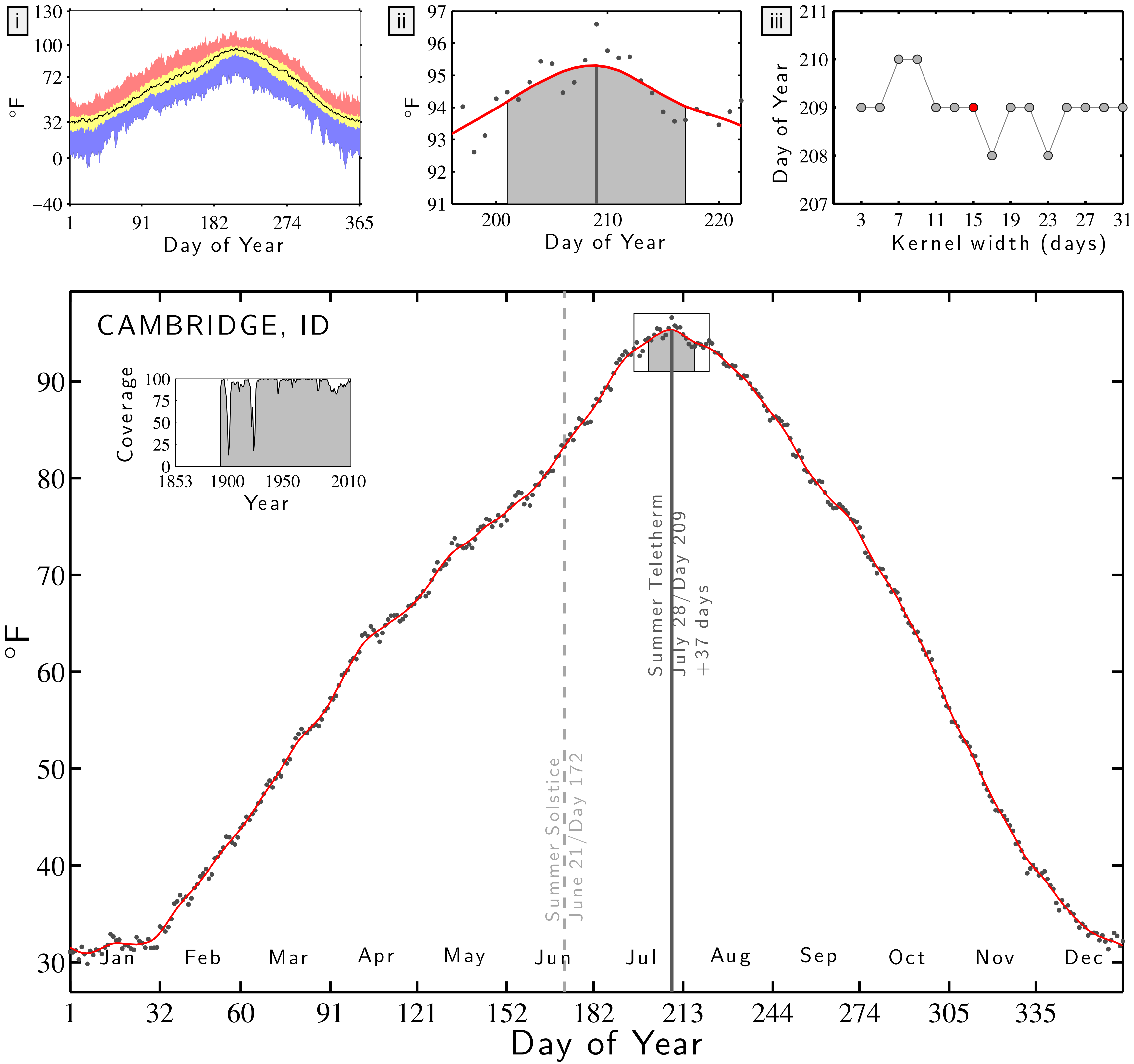

i
ii
iii
°F
Day of Year
Kernel width (days)
CAMBRIDGE, ID
Coverage
Year
Summer Solstice
June 21/Day 172
Summer Teletherm
July 28/Day 209
+37 days
Jan
Feb
Mar
Apr
May
Jun
Jul
Aug
Sep
Oct
Nov
Dec

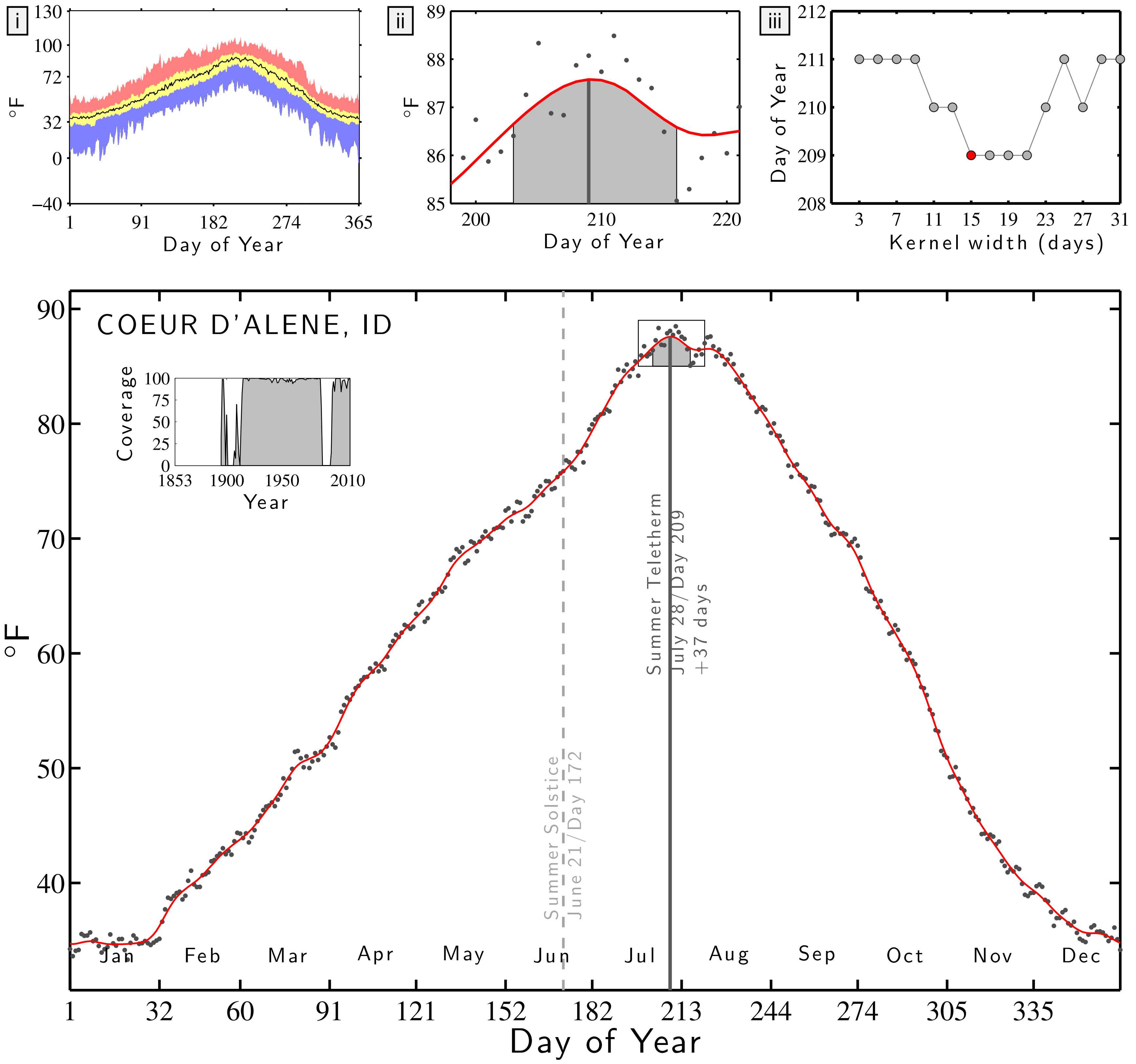

i
°F
Day of Year
ii
°F
Day of Year
iii
Day of Year
Kernel width (days)
COEUR D'ALENE, ID
Coverage
Year
Summer Solstice
June 21/Day 172
Summer Teletherm
July 28/Day 209
+37 days
Jan
Feb
Mar
Apr
May
Jun
Jul
Aug
Sep
Oct
Nov
Dec
°F
Day of Year

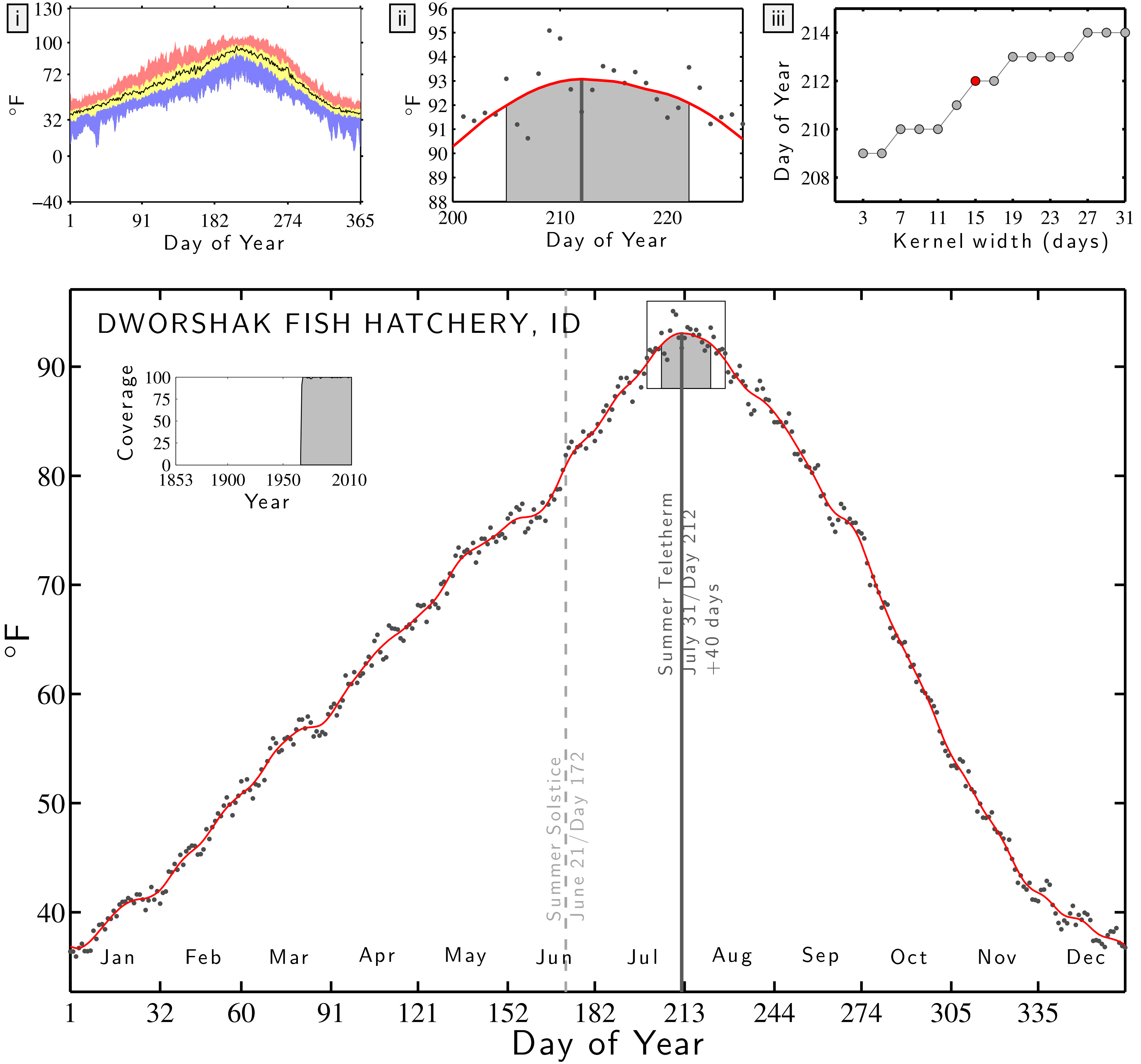

i
ii
iii
°F
Day of Year
Kernel width (days)
DWORSHAK FISH HATCHERY, ID
Coverage
Year
Summer Solstice
June 21/Day 172
Summer Teletherm
July 31/Day 212
+40 days
Jan
Feb
Mar
Apr
May
Jun
Jul
Aug
Sep
Oct
Nov
Dec

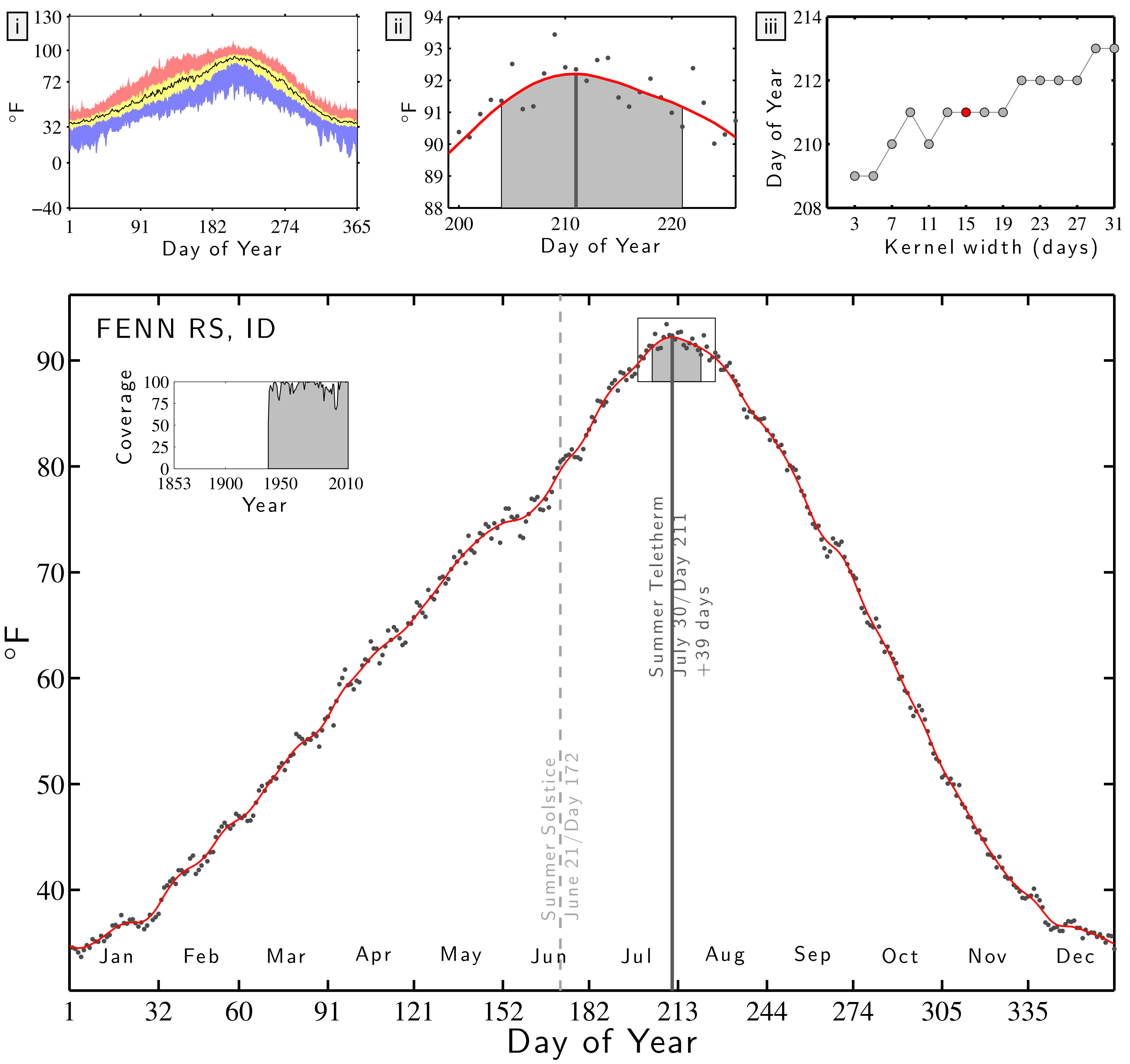

i
°F
Day of Year
ii
iii
Day of Year
Kernel width (days)
FENN RS, ID
Coverage
Year
Summer Solstice
June 21/Day 172
Summer Teletherm
July 30/Day 211
+39 days
Jan
Feb
Mar
Apr
May
Jun
Jul
Aug
Sep
Oct
Nov
Dec

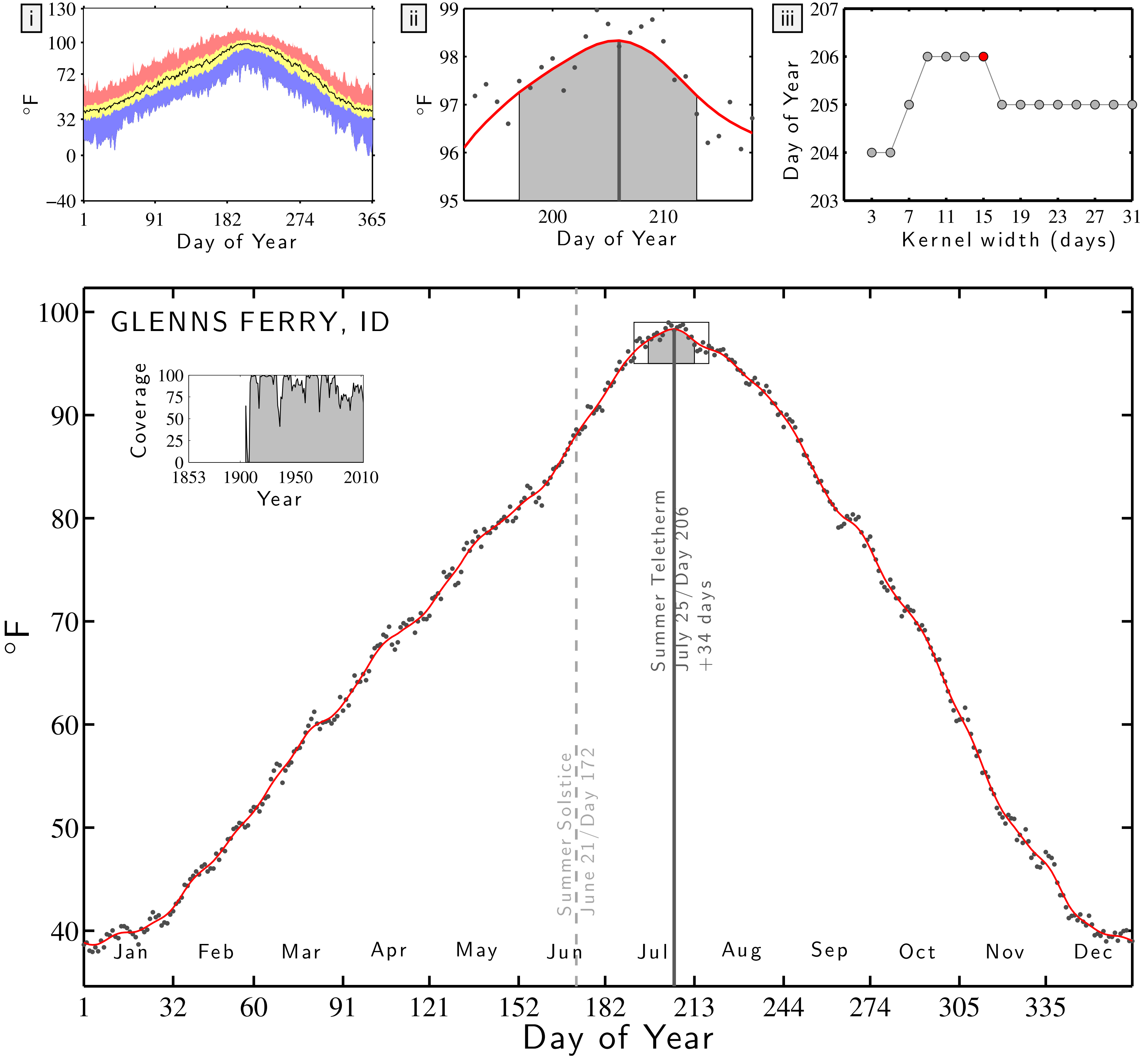

i
°F
Day of Year
ii
°F
Day of Year
iii
Day of Year
Kernel width (days)
GLENNS FERRY, ID
Coverage
Year
Summer Solstice
June 21/Day 172
Summer Teletherm
July 25/Day 206
+34 days
Jan
Feb
Mar
Apr
May
Jun
Jul
Aug
Sep
Oct
Nov
Dec
°F
Day of Year

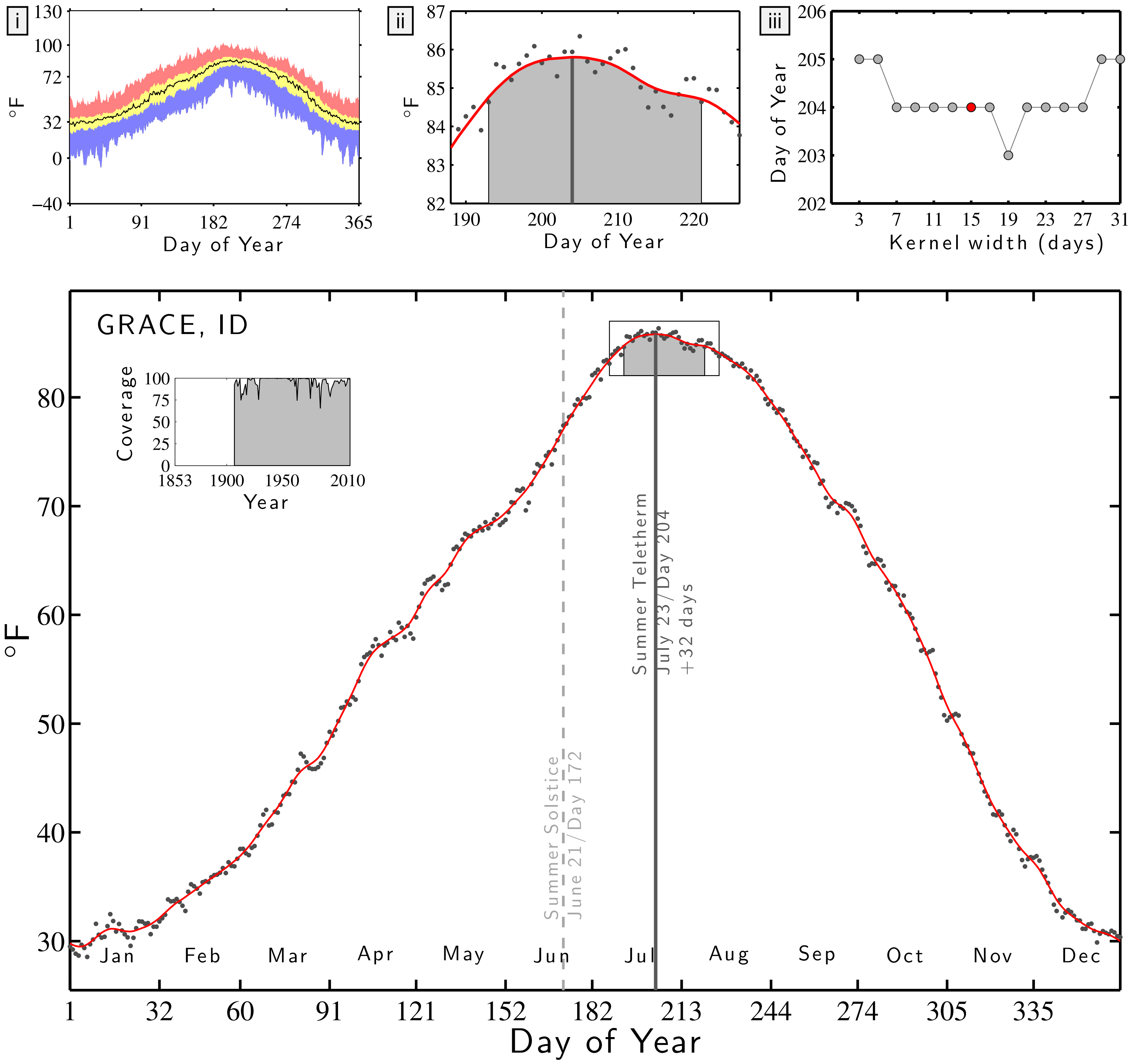

i
ii
iii
°F
Day of Year
Day of Year
Kernel width (days)
GRACE, ID
Coverage
Year
1853 1900 1950 2010
Summer Telethern
July 23/Day 204
+32 days
Summer Solstice
June 21/Day 172
Jan Feb Mar Apr May Jun Jul Aug Sep Oct Nov Dec
1 32 60 91 121 152 182 213 244 274 305 335
Day of Year

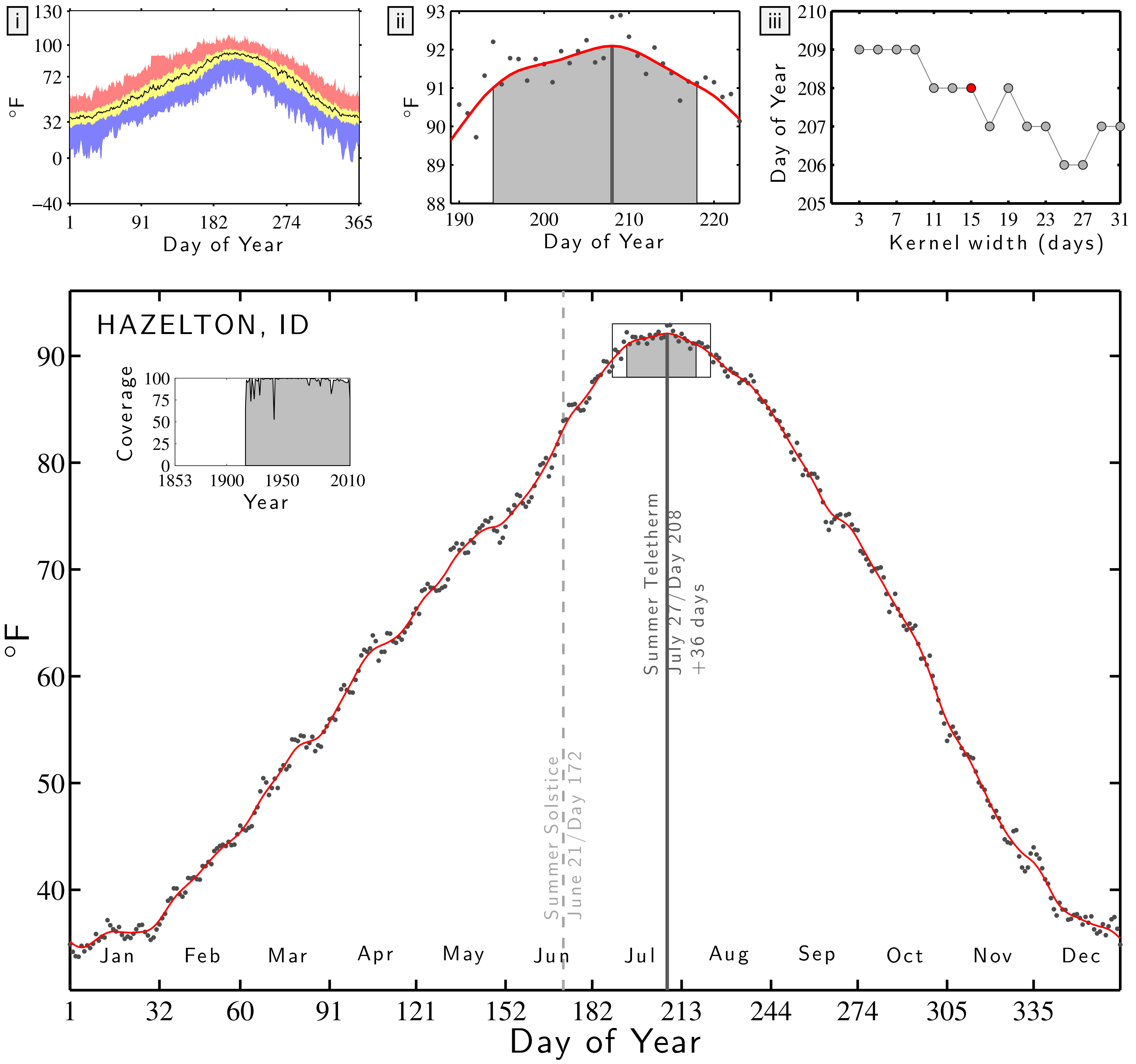

i
ii
iii
°F
Day of Year
Day of Year
Kernel width (days)
HAZELTON, ID
Coverage
Year
Summer Solstice
June 21 / Day 172
Summer Teletherm
July 27 / Day 208
+36 days
Jan
Feb
Mar
Apr
May
Jun
Jul
Aug
Sep
Oct
Nov
Dec

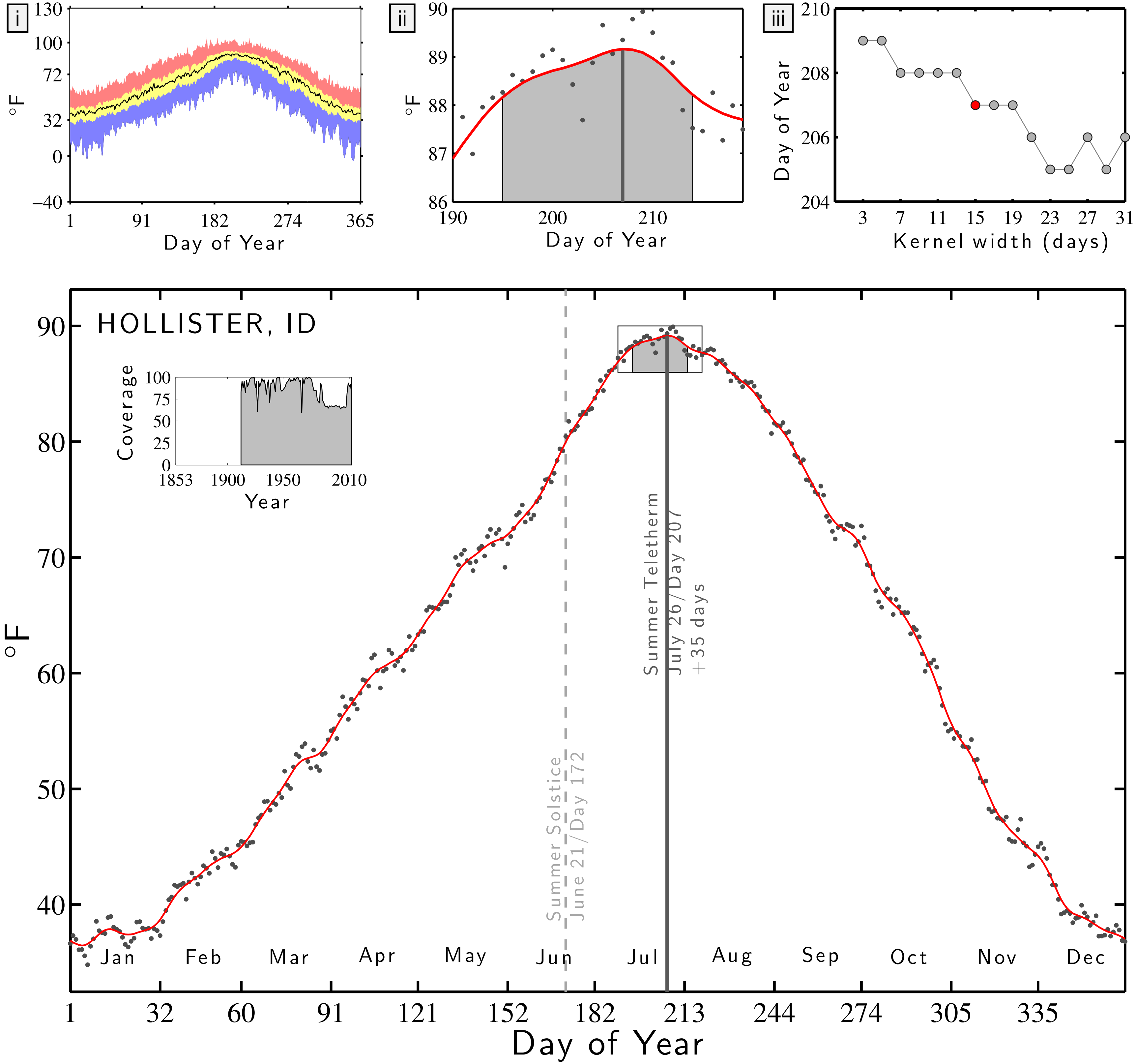

i
°F
Day of Year
ii
°F
Day of Year
iii
Day of Year
Kernel width (days)
HOLLISTER, ID
Coverage
Year
Summer Solstice
June 21 / Day 172
Summer Teletherm
July 26 / Day 207
+35 days
Jan
Feb
Mar
Apr
May
Jun
Jul
Aug
Sep
Oct
Nov
Dec
°F
Day of Year

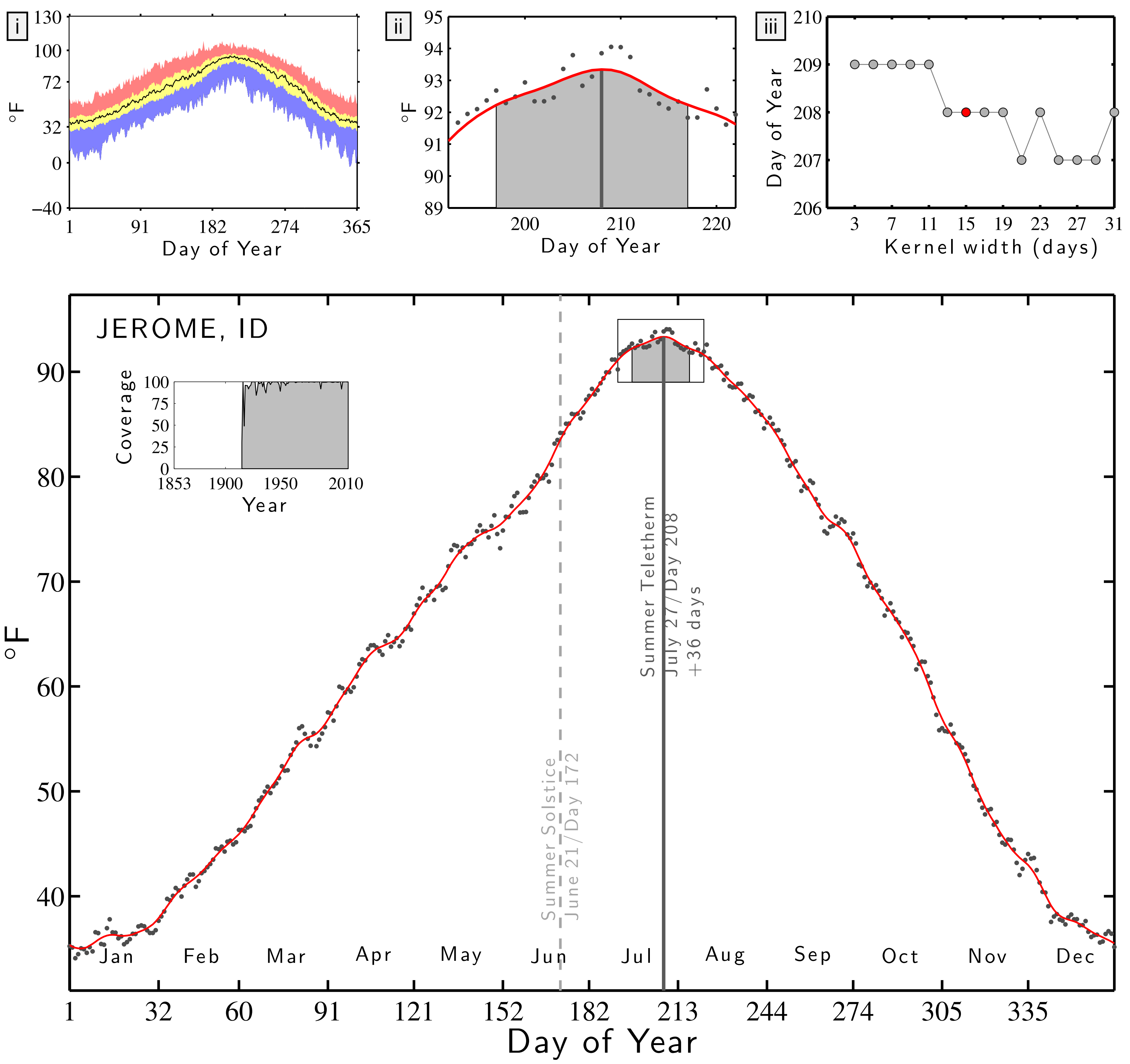
i
°F
Day of Year
ii
°F
Day of Year
iii
Day of Year
Kernel width (days)
JEROME, ID
Coverage
Year
Summer Solstice
June 21/Day 172
Summer Teletherm
July 27/Day 208
+36 days
Jan
Feb
Mar
Apr
May
Jun
Jul
Aug
Sep
Oct
Nov
Dec
°F
Day of Year

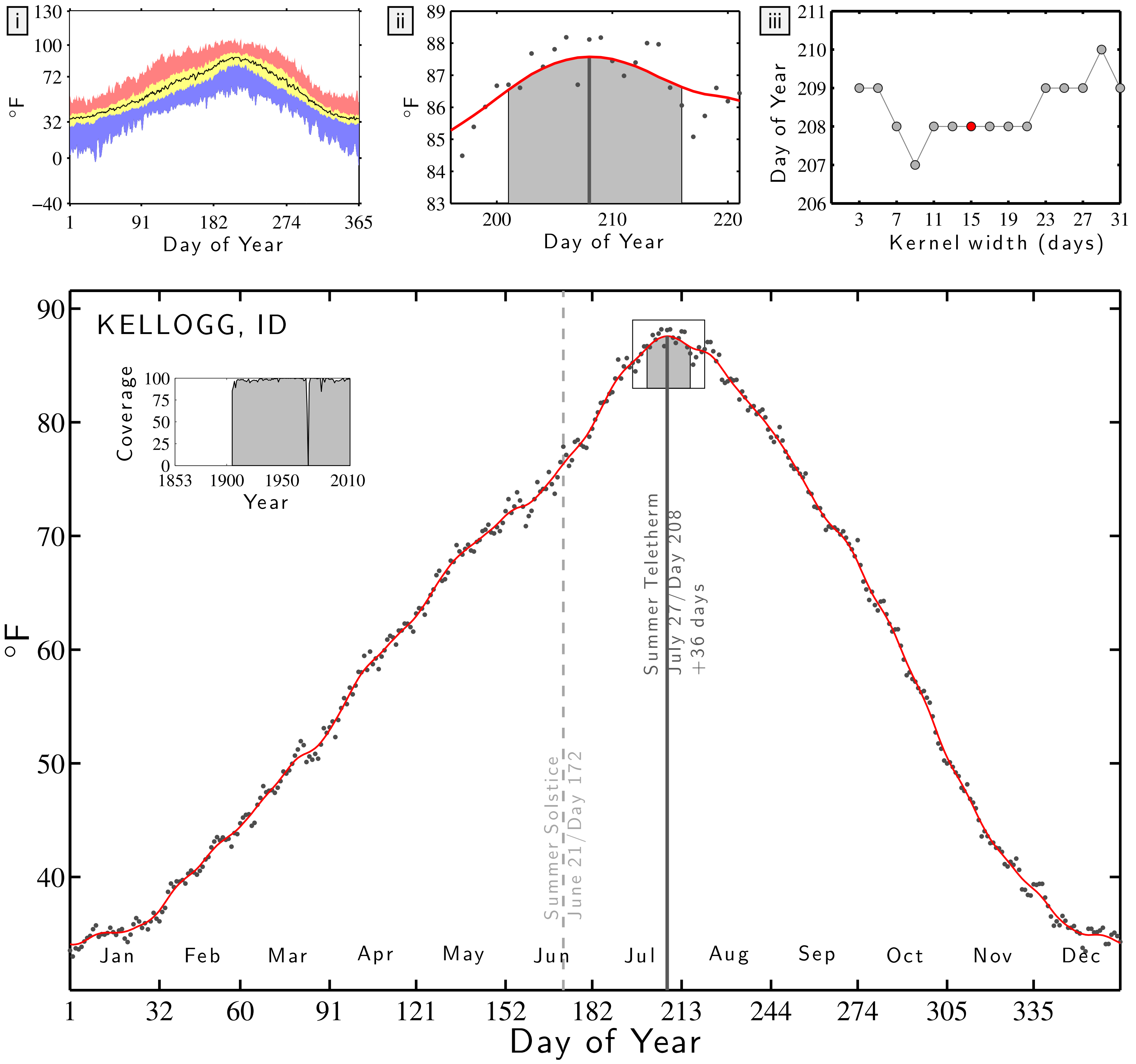

i
°F
Day of Year
ii
°F
Day of Year
iii
Day of Year
Kernel width (days)
KELLOGG, ID
Coverage
Year
Summer Solstice
June 21 / Day 172
Summer Teletherm
July 27 / Day 208
+36 days
Jan
Feb
Mar
Apr
May
Jun
Jul
Aug
Sep
Oct
Nov
Dec
°F
Day of Year

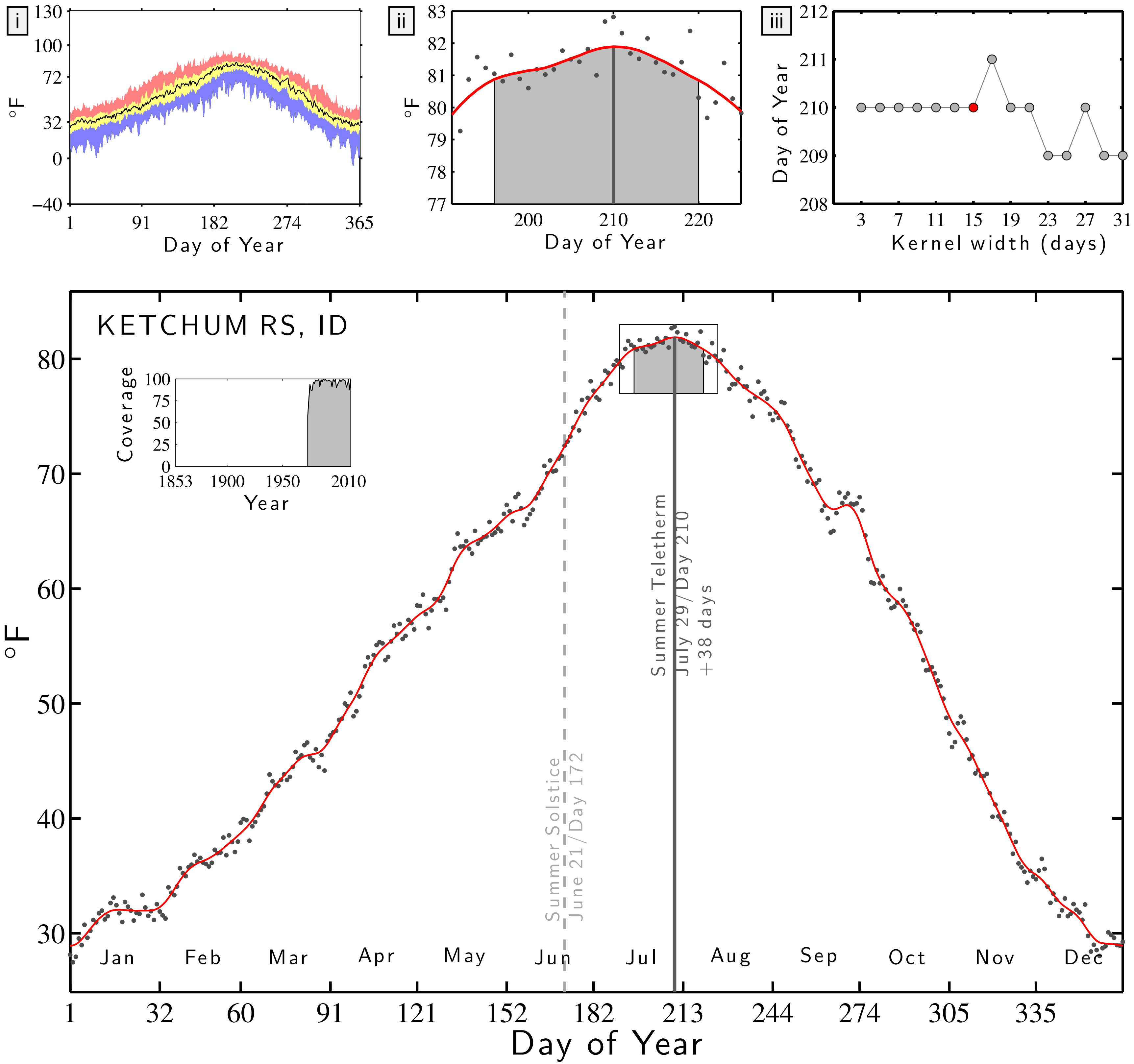

i
130
100
72
32
0
−40
°F
1
91
182
274
365
Day of Year
ii
83
82
81
80
79
78
77
°F
200
210
220
Day of Year
iii
212
211
210
209
208
Day of Year
3
7
11
15
19
23
27
31
Kernel width (days)
KETCHUM RS, ID
Coverage
100
75
50
25
0
1853
1900
1950
2010
Year
80
70
60
50
40
30
°F
Summer Solstice
June 21 / Day 172
Summer Teletherm
July 29 / Day 210
+38 days
Jan
Feb
Mar
Apr
May
Jun
Jul
Aug
Sep
Oct
Nov
Dec
1
32
60
91
121
152
182
213
244
274
305
335
Day of Year

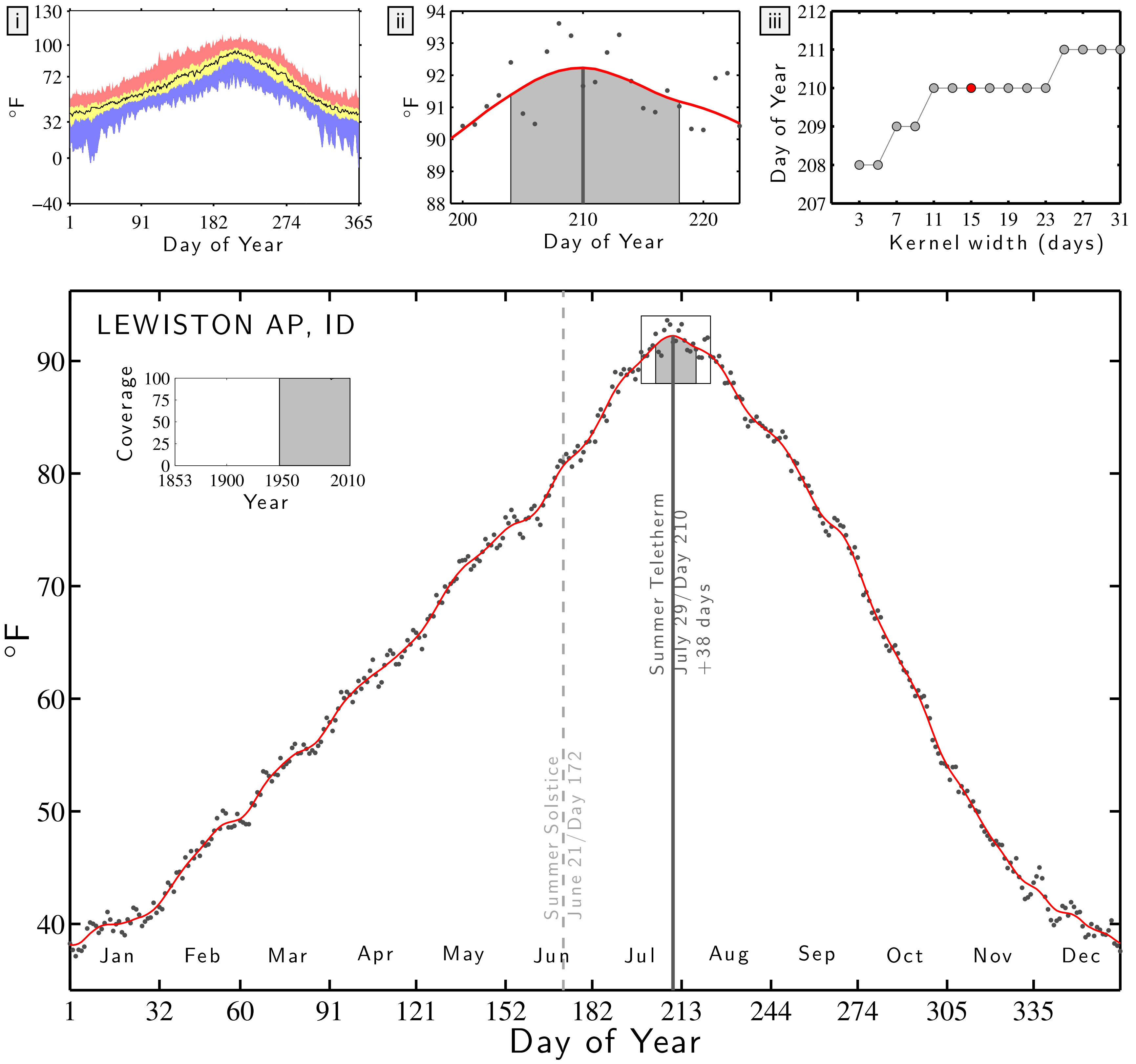

i
ii
iii
°F
Day of Year
Kernel width (days)
LEWISTON AP, ID
Coverage
Year
Summer Solstice
June 21 / Day 172
Summer Teletherm
July 29 / Day 210
+38 days
Jan
Feb
Mar
Apr
May
Jun
Jul
Aug
Sep
Oct
Nov
Dec

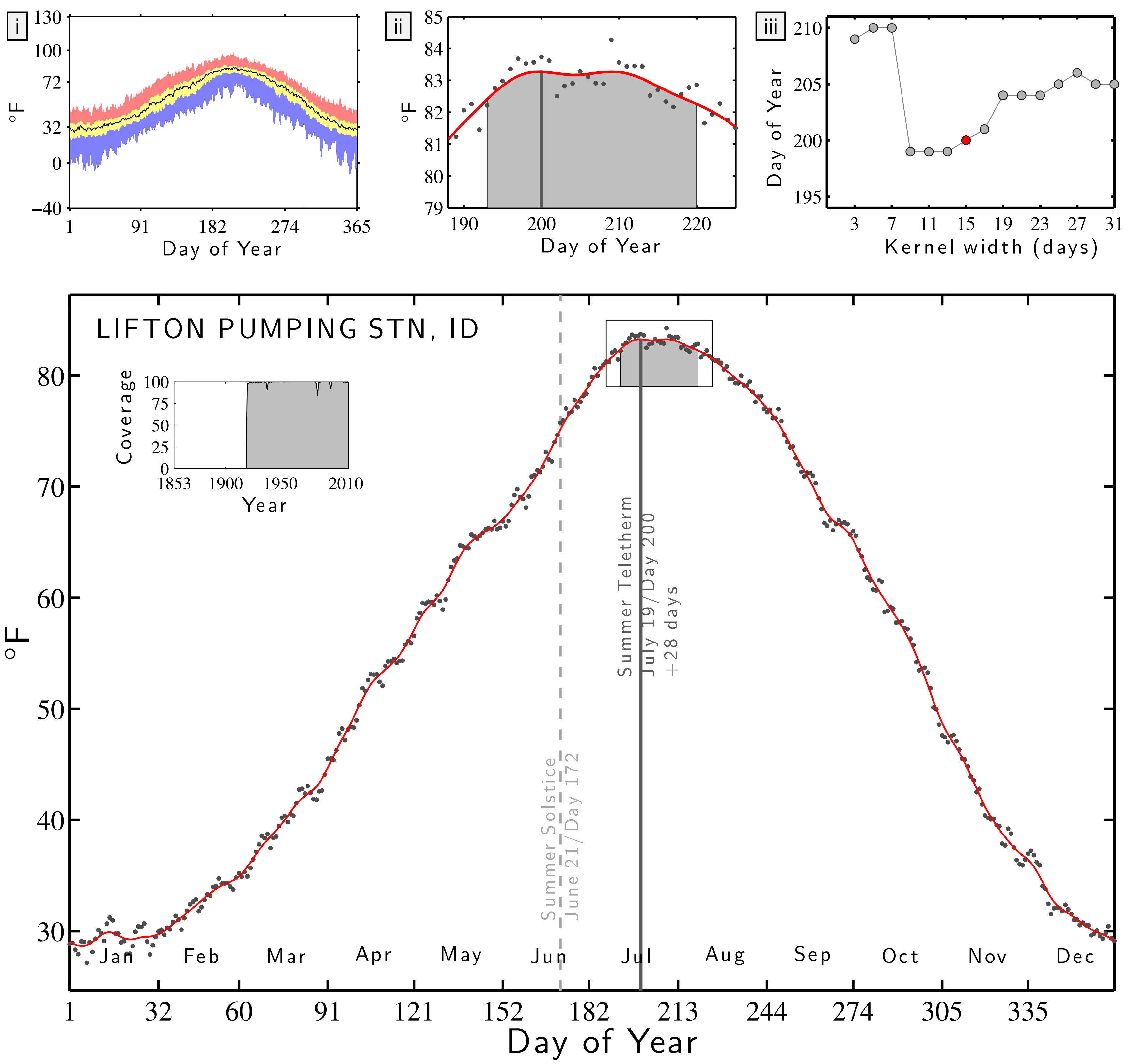

i
ii
iii
Day of Year
Kernel width (days)
°F
Day of Year
LIFTON PUMPING STN, ID
Coverage
Year
Summer Solstice
June 21/Day 172
Summer Teletherm
July 19/Day 200
+28 days
Jan
Feb
Mar
Apr
May
Jun
Jul
Aug
Sep
Oct
Nov
Dec

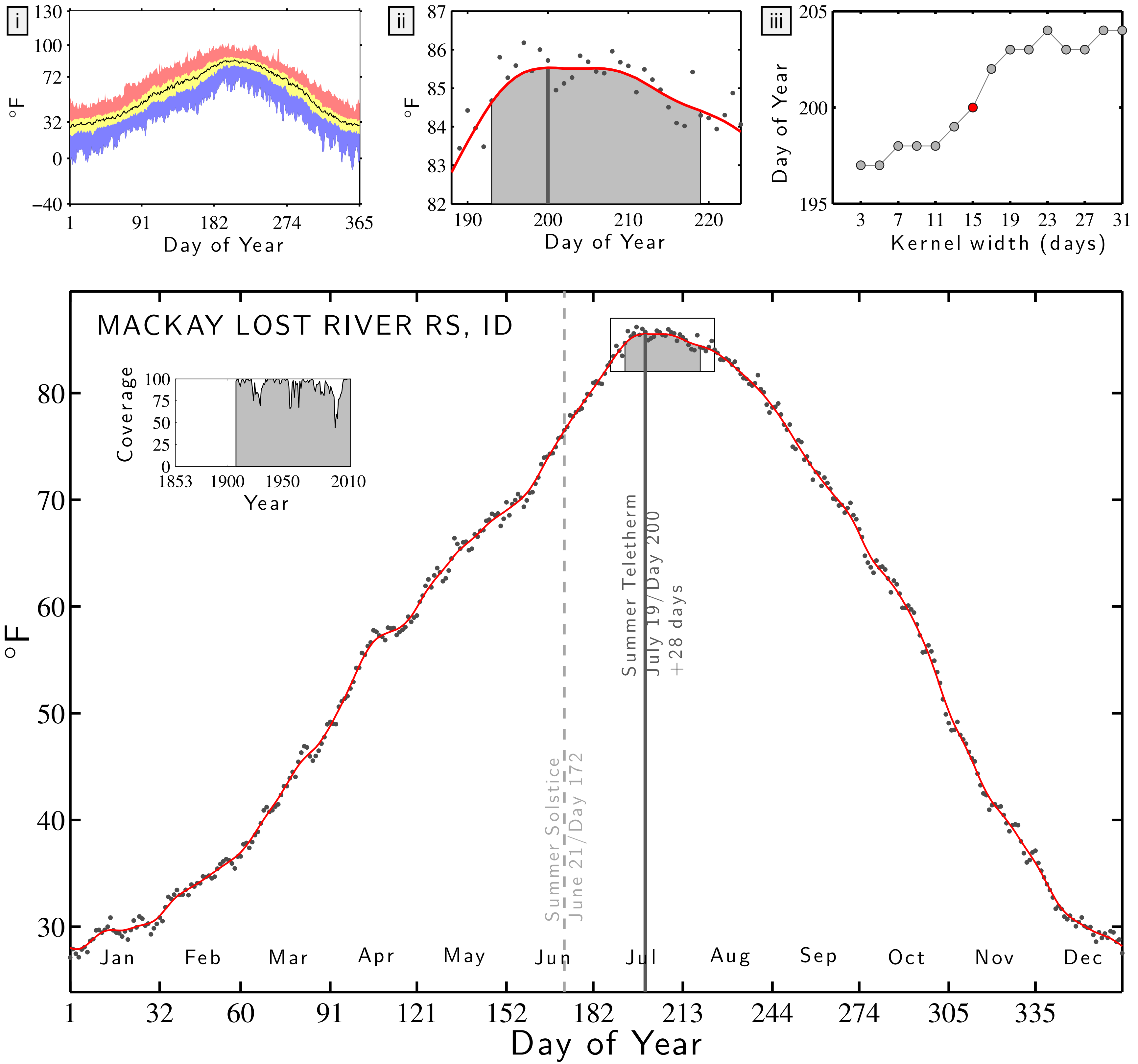

i
°F
130
100
72
32
0
−40
1
91
182
274
365
Day of Year
ii
87
86
85
84
83
82
190
200
210
220
Day of Year
iii
205
200
195
Day of Year
3
7
11
15
19
23
27
31
Kernel width (days)
MACKAY LOST RIVER RS, ID
Coverage
100
75
50
25
0
1853
1900
1950
2010
Year
80
70
60
50
40
30
Summer Solstice
June 21/Day 172
Summer Teletherm
July 19/Day 200
+28 days
Jan
Feb
Mar
Apr
May
Jun
Jul
Aug
Sep
Oct
Nov
Dec
1
32
60
91
121
152
182
213
244
274
305
335
Day of Year

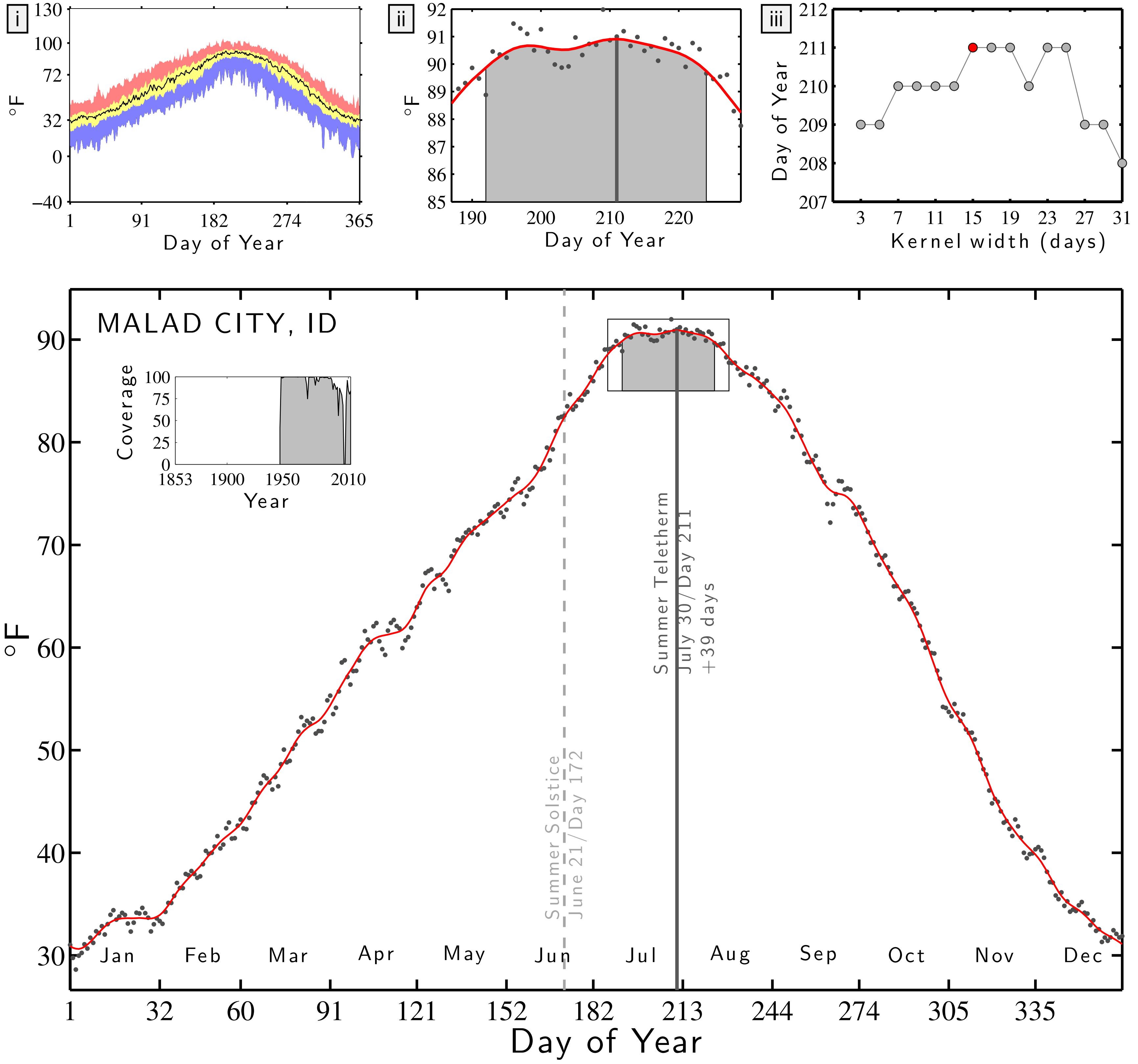

i
°F
Day of Year
ii
°F
Day of Year
iii
Day of Year
Kernel width (days)
MALAD CITY, ID
Coverage
Year
Summer Solstice
June 21 / Day 172
Summer Teletherm
July 30 / Day 211
+39 days
Jan
Feb
Mar
Apr
May
Jun
Jul
Aug
Sep
Oct
Nov
Dec
°F
Day of Year

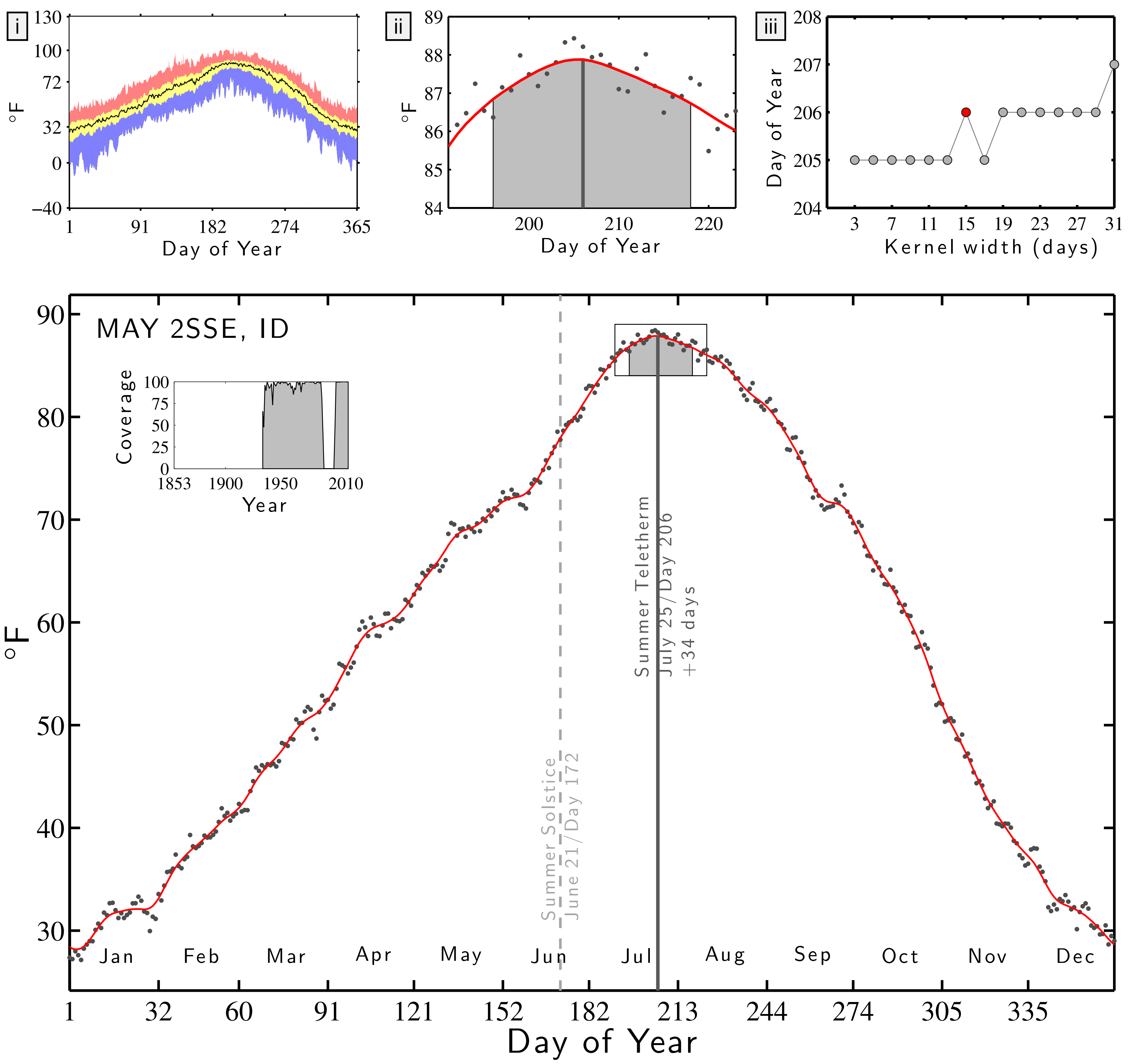

i
°F
Day of Year
ii
°F
Day of Year
iii
Day of Year
Kernel width (days)
MAY 2SSE, ID
Coverage
Year
Summer Solstice
June 21/Day 172
Summer Teletherm
July 25/Day 206
+34 days
Jan
Feb
Mar
Apr
May
Jun
Jul
Aug
Sep
Oct
Nov
Dec
°F
Day of Year

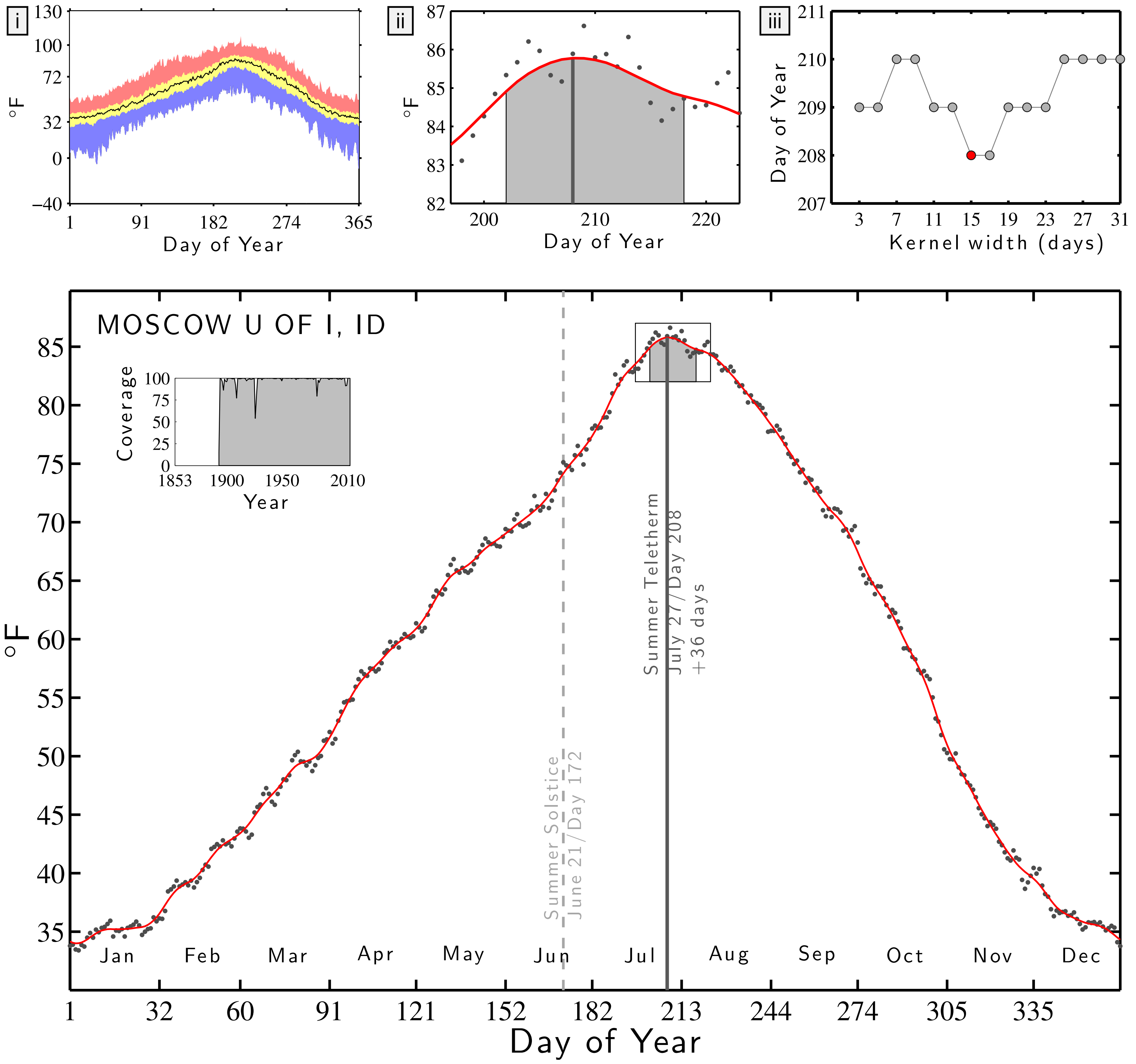

i
Day of Year
°F
ii
Day of Year
°F
iii
Day of Year
Kernel width (days)
MOSCOW U OF I, ID
Coverage
Year
Summer Solstice
June 21 / Day 172
Summer Teletherm
July 27 / Day 208
+36 days
Jan
Feb
Mar
Apr
May
Jun
Jul
Aug
Sep
Oct
Nov
Dec
Day of Year
°F

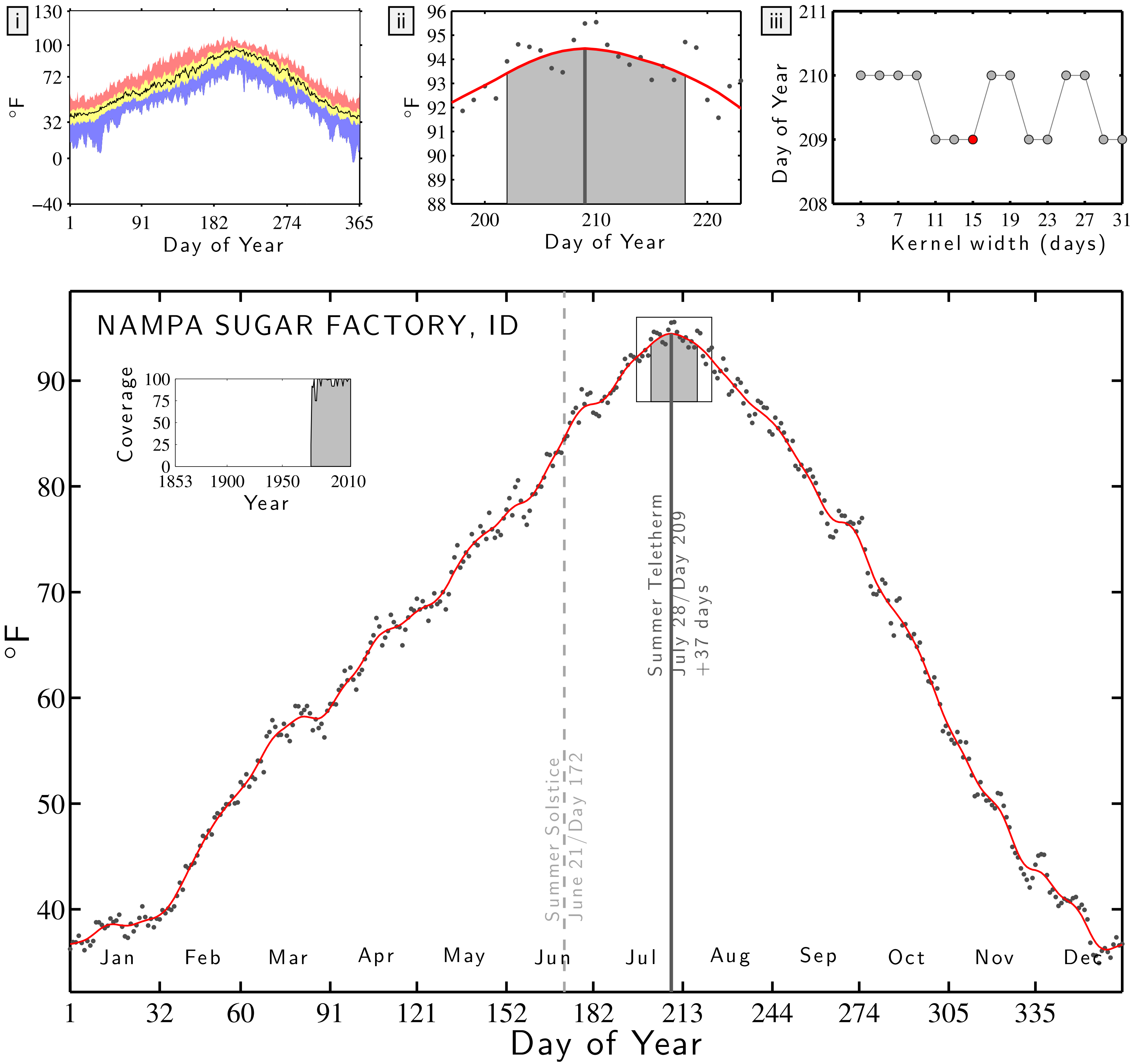

i
°F
Day of Year
ii
Day of Year
iii
Day of Year
Kernel width (days)
NAMPA SUGAR FACTORY, ID
Coverage
Year
Summer Solstice
June 21/Day 172
Summer Teletherm
July 28/Day 209
+37 days
Jan
Feb
Mar
Apr
May
Jun
Jul
Aug
Sep
Oct
Nov
Dec
°F
Day of Year

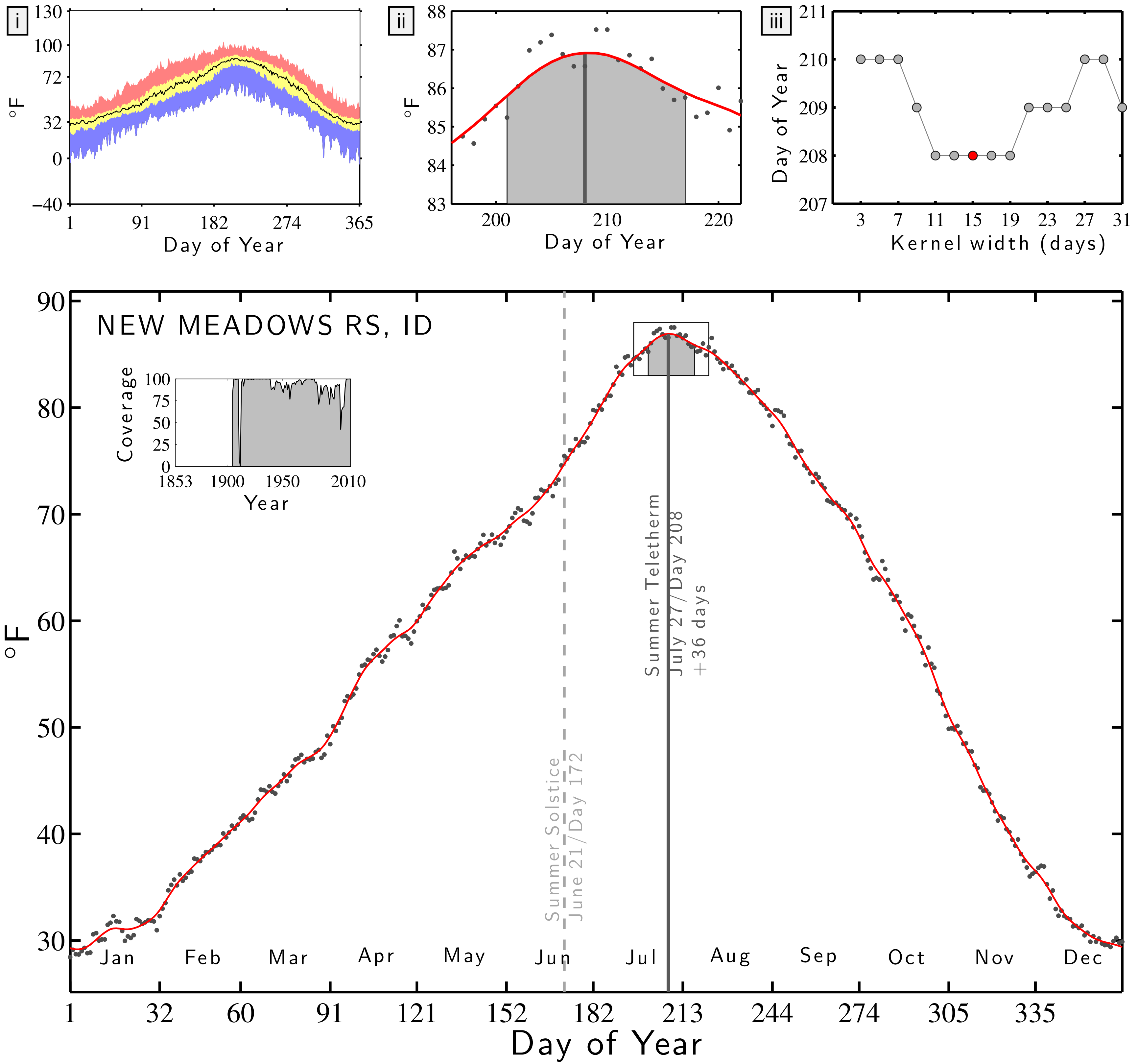

i
ii
iii
NEW MEADOWS RS, ID
Coverage
Year
Summer Solstice
June 21 / Day 172
Summer Teletherm
July 27 / Day 208
+36 days
Day of Year
Kernel width (days)
°F
Jan
Feb
Mar
Apr
May
Jun
Jul
Aug
Sep
Oct
Nov
Dec

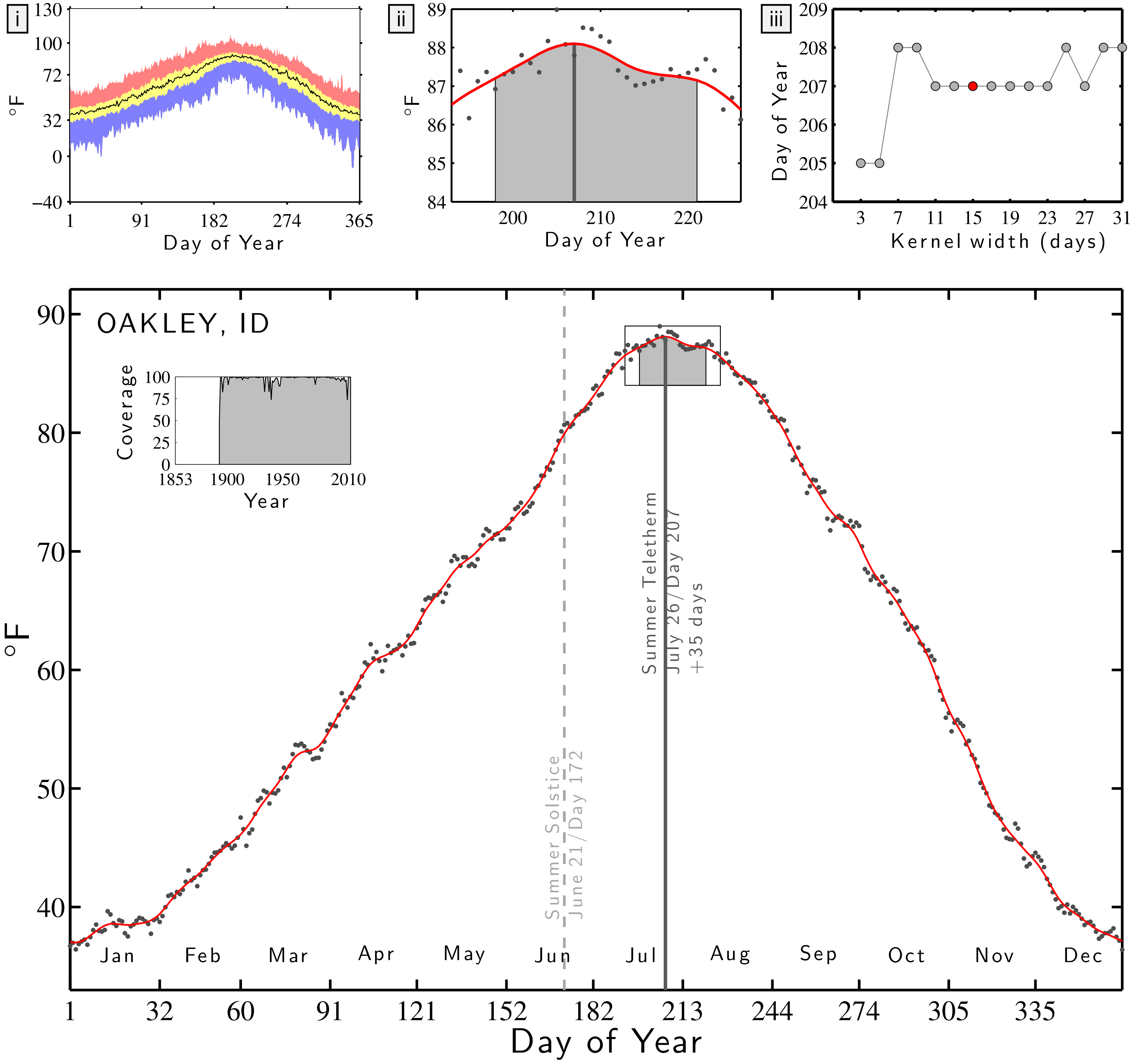

i
°F
Day of Year
ii
°F
Day of Year
iii
Day of Year
Kernel width (days)
OAKLEY, ID
Coverage
Year
1853
1900
1950
2010
Summer Solstice
June 21 / Day 172
Summer Teletherm
July 26 / Day 207
+35 days
Jan
Feb
Mar
Apr
May
Jun
Jul
Aug
Sep
Oct
Nov
Dec
°F
Day of Year

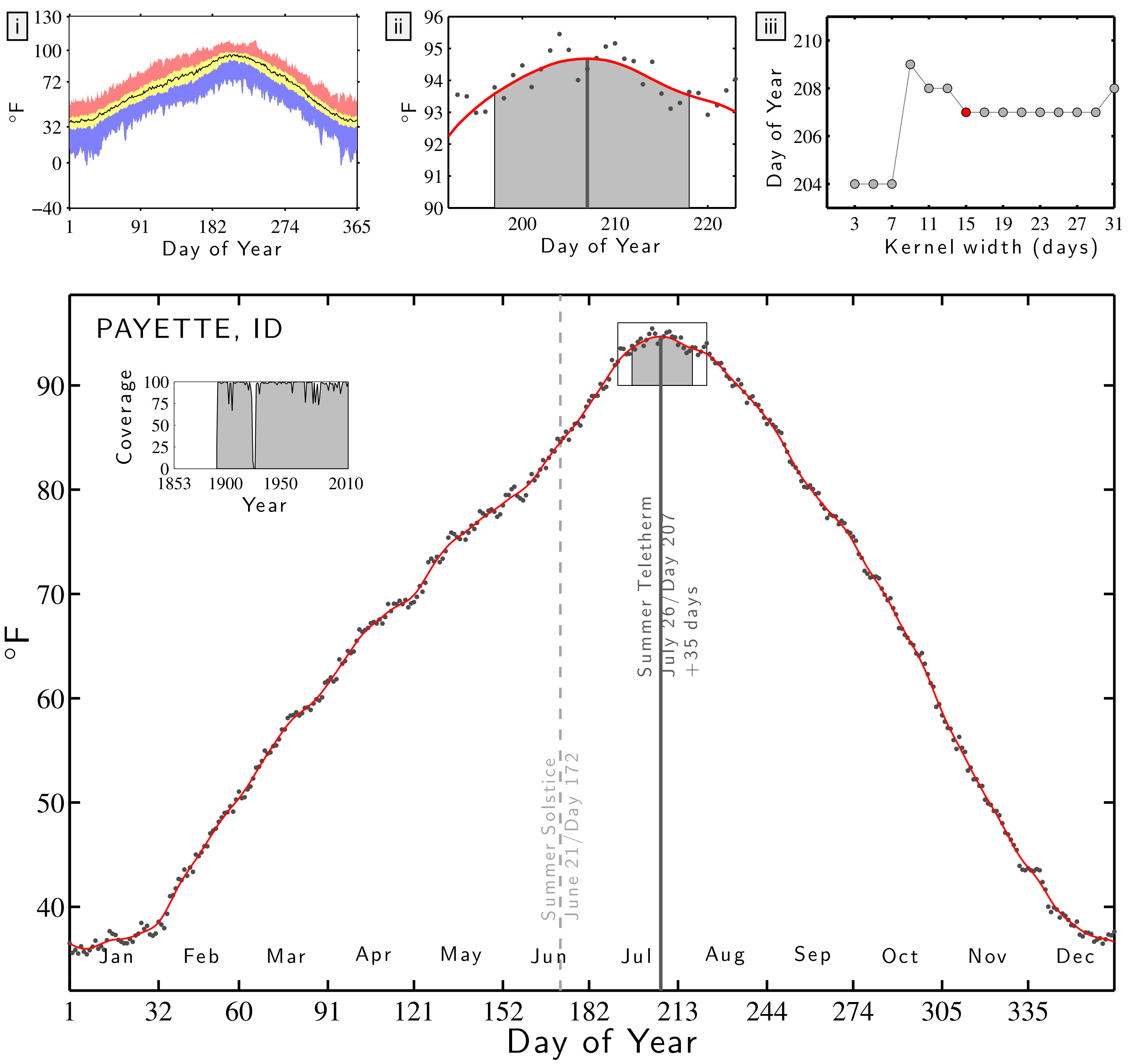

i
ii
iii
Day of Year
°F
Kernel width (days)
PAYETTE, ID
Coverage
Year
Summer Solstice
June 21 / Day 172
Summer Teletherm
July 26 / Day 207
+35 days
Jan
Feb
Mar
Apr
May
Jun
Jul
Aug
Sep
Oct
Nov
Dec

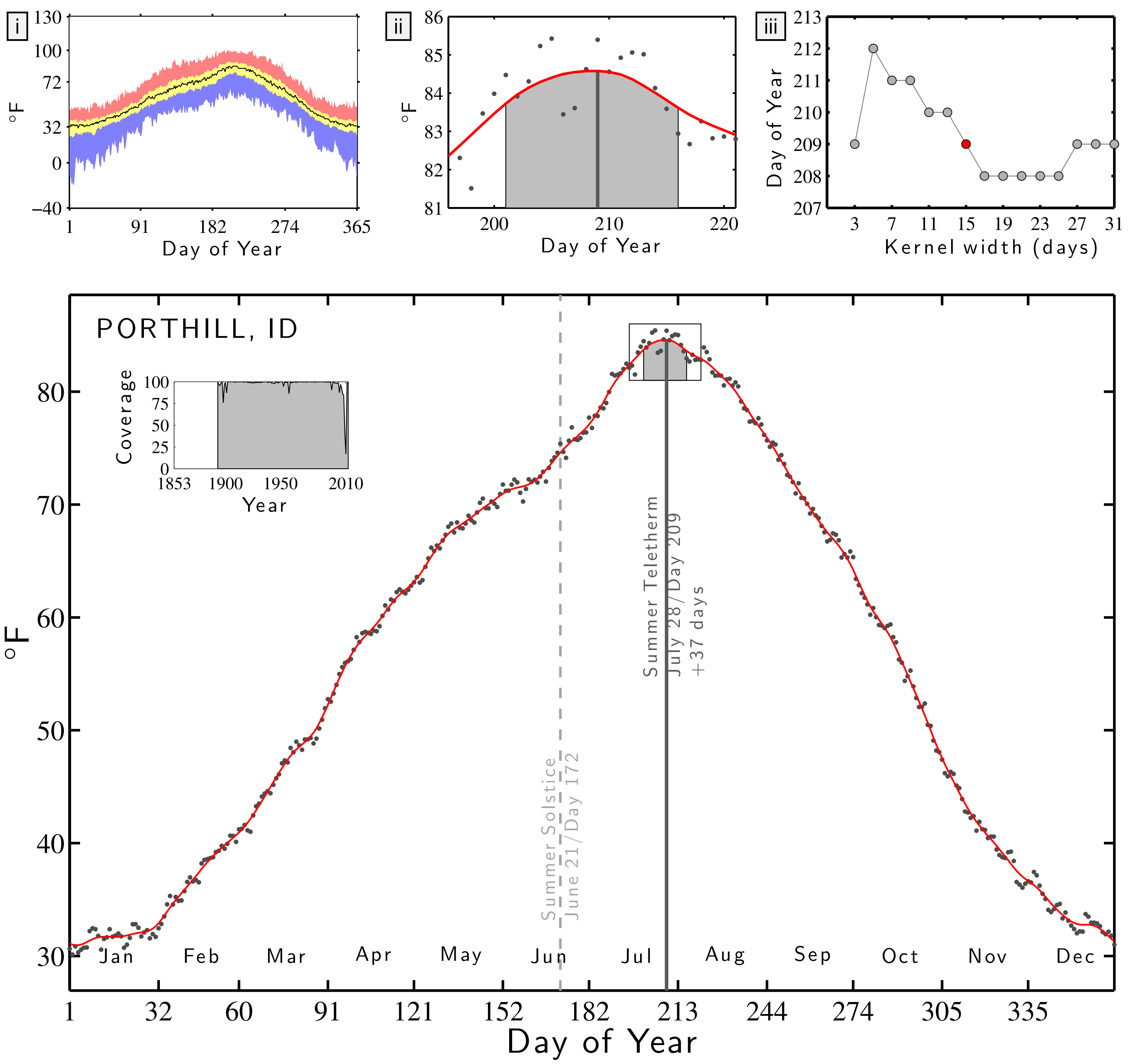
i
°F
130
100
72
32
0
−40
1
91
182
274
365
Day of Year
ii
86
85
84
83
82
81
200
210
220
iii
213
212
211
210
209
208
207
Day of Year
3
7
11
15
19
23
27
31
Kernel width (days)
PORTHILL, ID
Coverage
100
75
50
25
1853
1900
1950
2010
Year
80
70
60
50
40
30
Summer Solstice
June 21/Day 172
Summer Teletherm
July 28/Day 209
+37 days
Jan
Feb
Mar
Apr
May
Jun
Jul
Aug
Sep
Oct
Nov
Dec
1
32
60
91
121
152
182
213
244
274
305
335

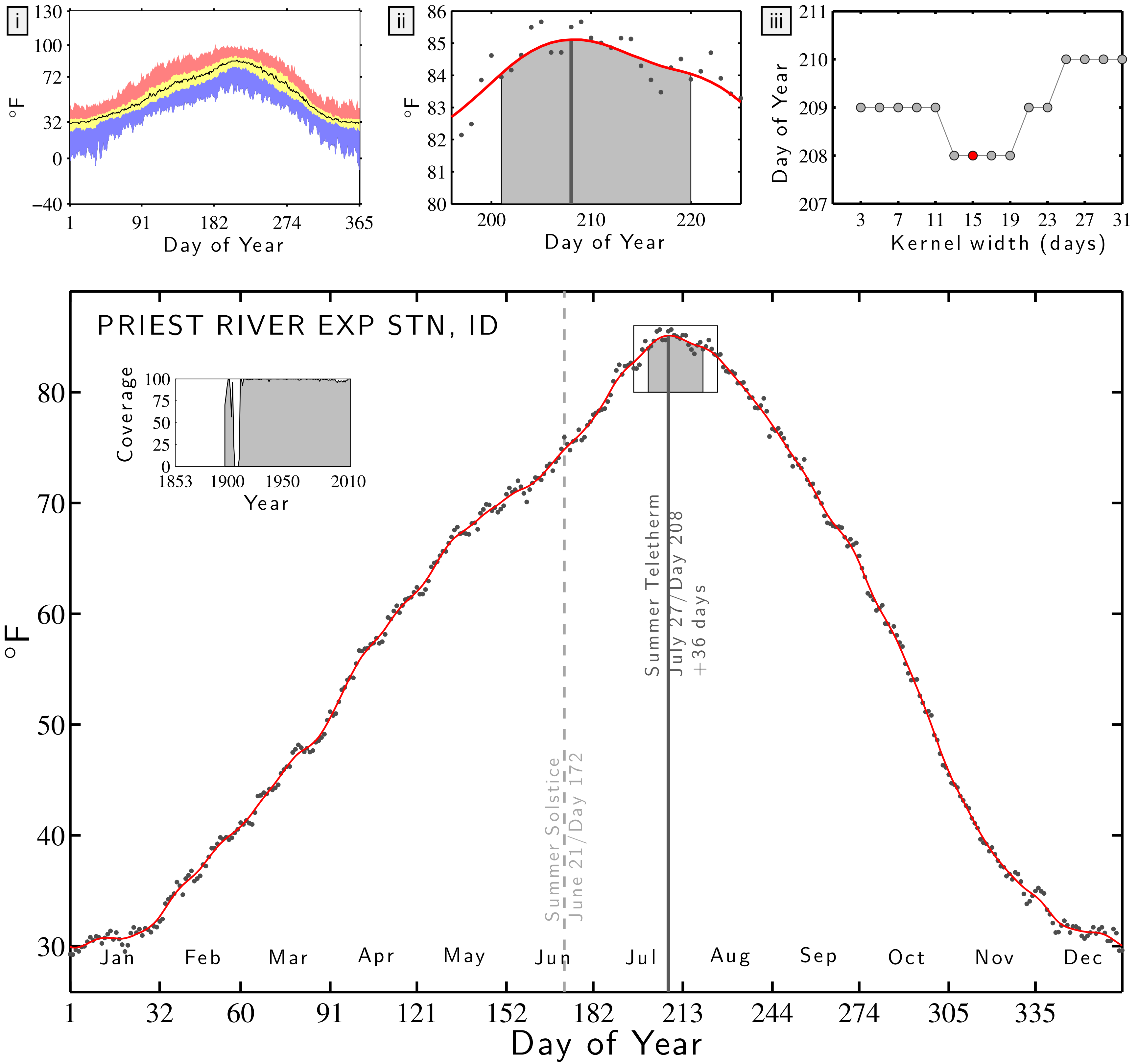

i
ii
iii
°F
Day of Year
Day of Year
Kernel width (days)
PRIEST RIVER EXP STN, ID
Coverage
Year
Summer Solstice
June 21 / Day 172
Summer Teletherm
July 27 / Day 208
+36 days
Jan
Feb
Mar
Apr
May
Jun
Jul
Aug
Sep
Oct
Nov
Dec
°F
Day of Year

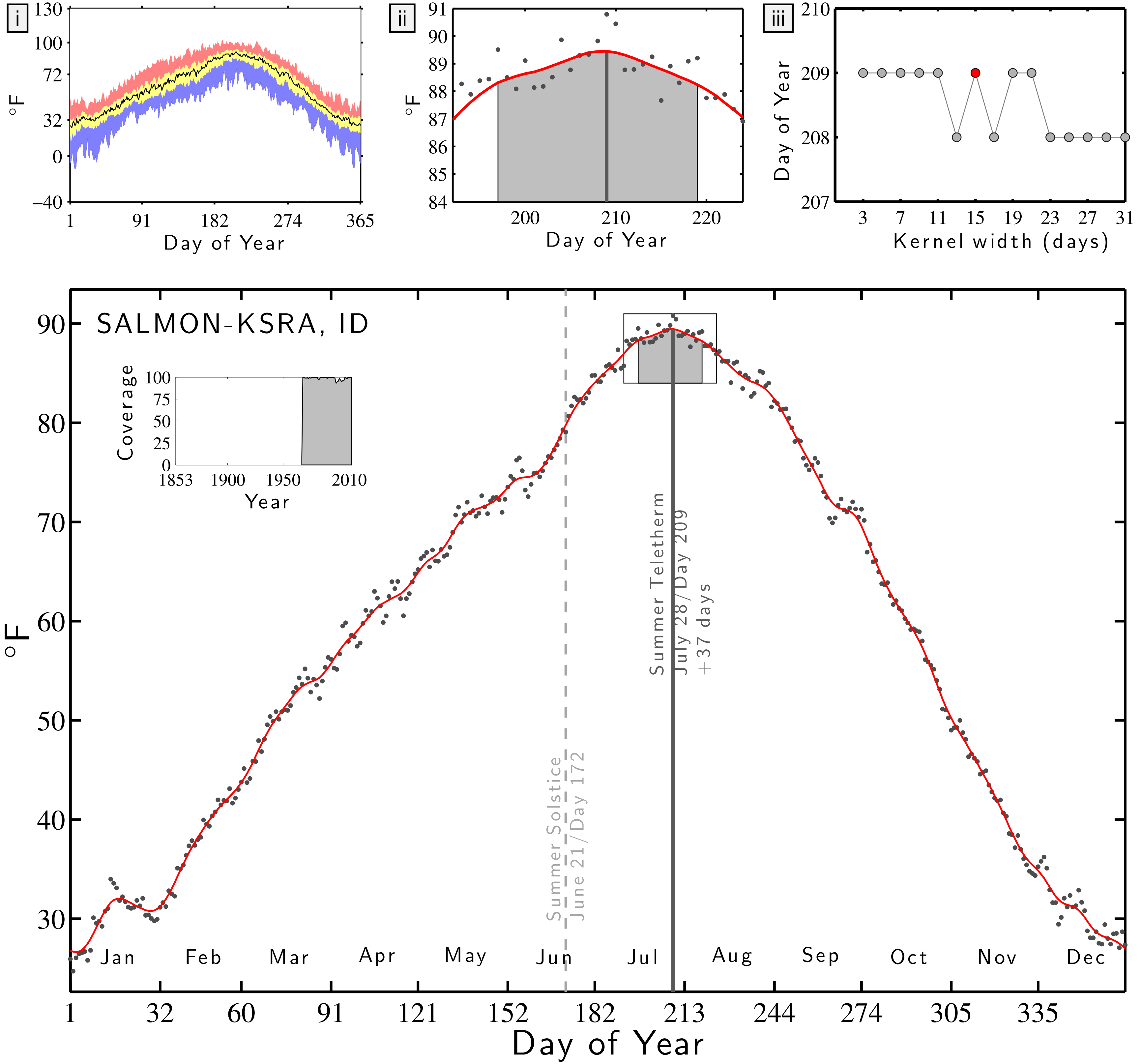

i
ii
iii
Day of Year
Kernel width (days)
SALMON-KSRA, ID
Coverage
Year
Summer Solstice
June 21/Day 172
Summer Teletherm
July 28/Day 209
+37 days
Jan
Feb
Mar
Apr
May
Jun
Jul
Aug
Sep
Oct
Nov
Dec
°F
Day of Year

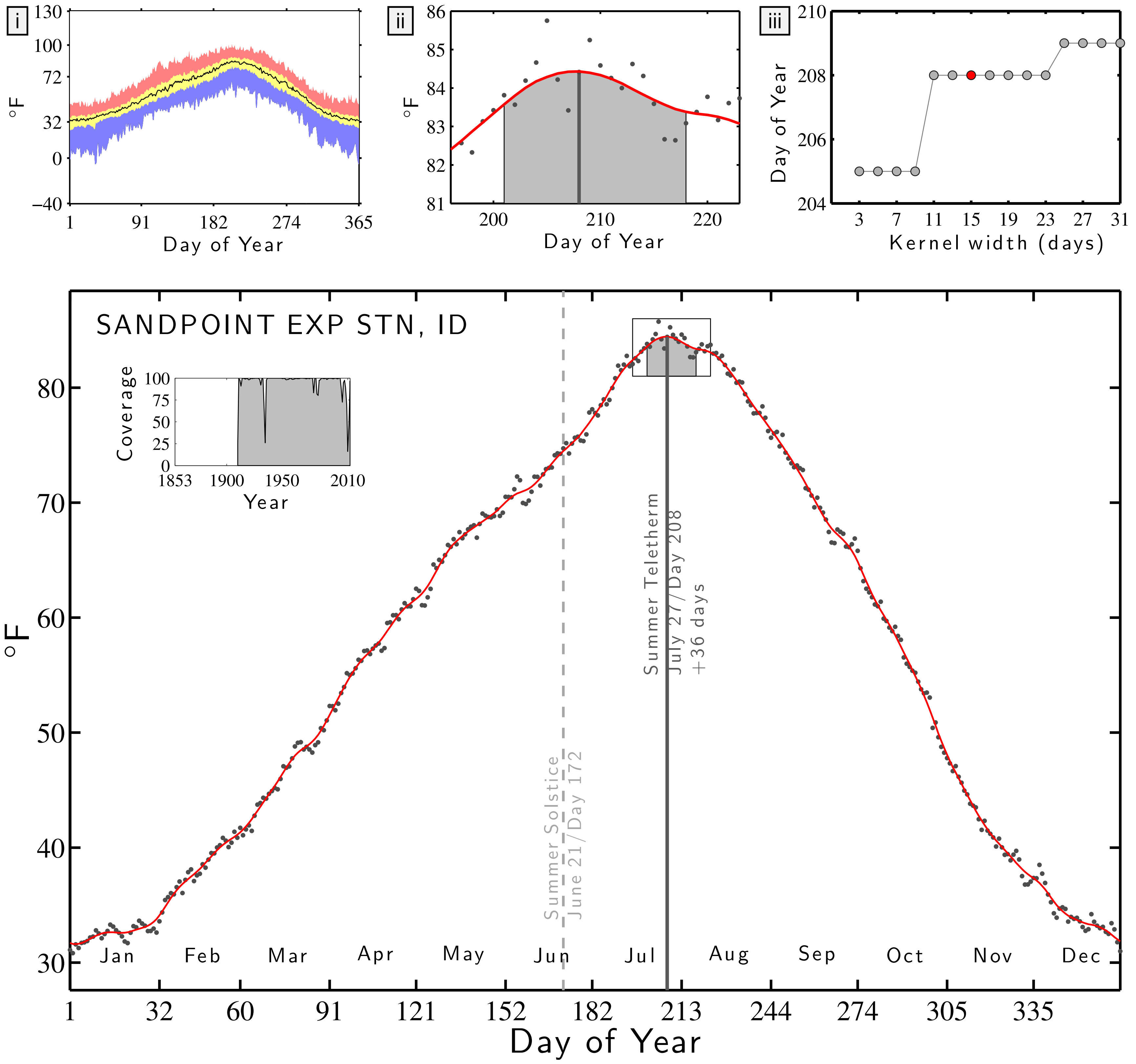

i
°F
Day of Year
ii
°F
Day of Year
iii
Day of Year
Kernel width (days)
SANDPOINT EXP STN, ID
Coverage
Year
Summer Solstice
June 21 / Day 172
Summer Teletherm
July 27 / Day 208
+36 days
Jan
Feb
Mar
Apr
May
Jun
Jul
Aug
Sep
Oct
Nov
Dec
°F
Day of Year

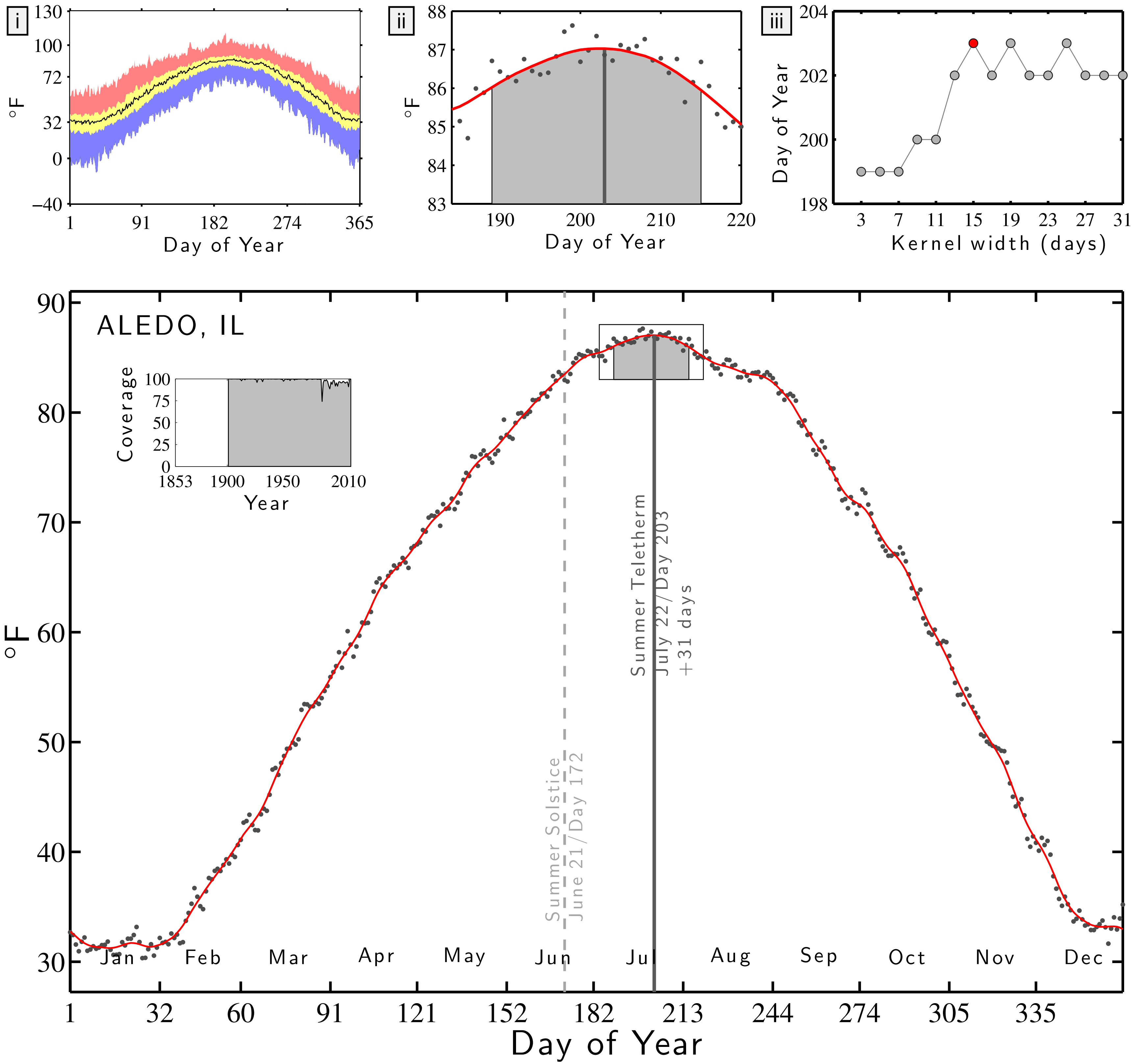

i
ii
iii
Day of Year
Kernel width (days)
ALEDO, IL
Coverage
Year
Summer Solstice
June 21/Day 172
Summer Teletherm
July 22/Day 203
+31 days
Jan
Feb
Mar
Apr
May
Jun
Jul
Aug
Sep
Oct
Nov
Dec
°F
Day of Year

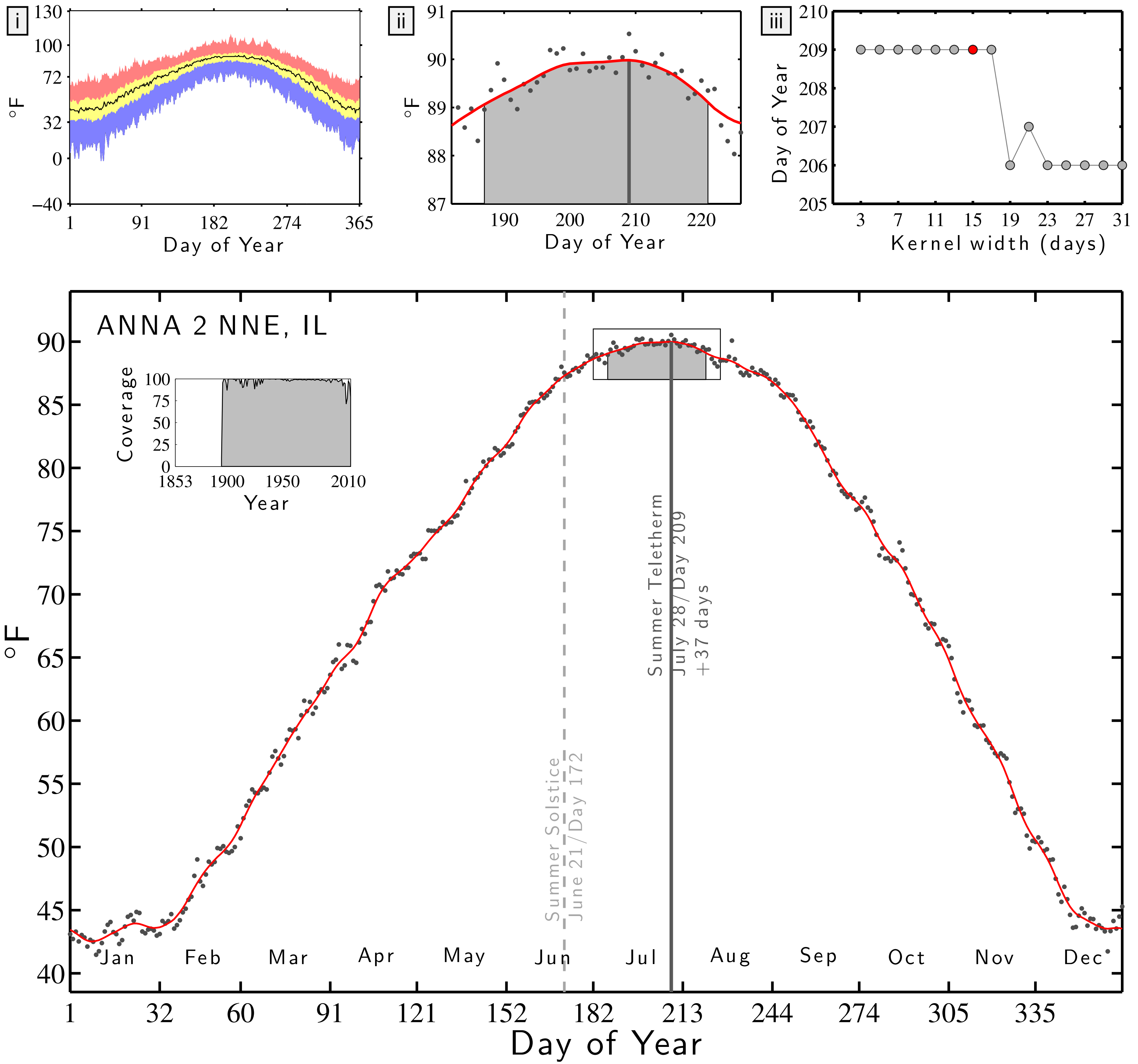

i
°F
Day of Year
ii
°F
Day of Year
iii
Day of Year
Kernel width (days)
ANNA 2 NNE, IL
Coverage
Year
Summer Solstice
June 21/Day 172
Summer Teletherm
July 28/Day 209
+37 days
Jan
Feb
Mar
Apr
May
Jun
Jul
Aug
Sep
Oct
Nov
Dec
°F
Day of Year

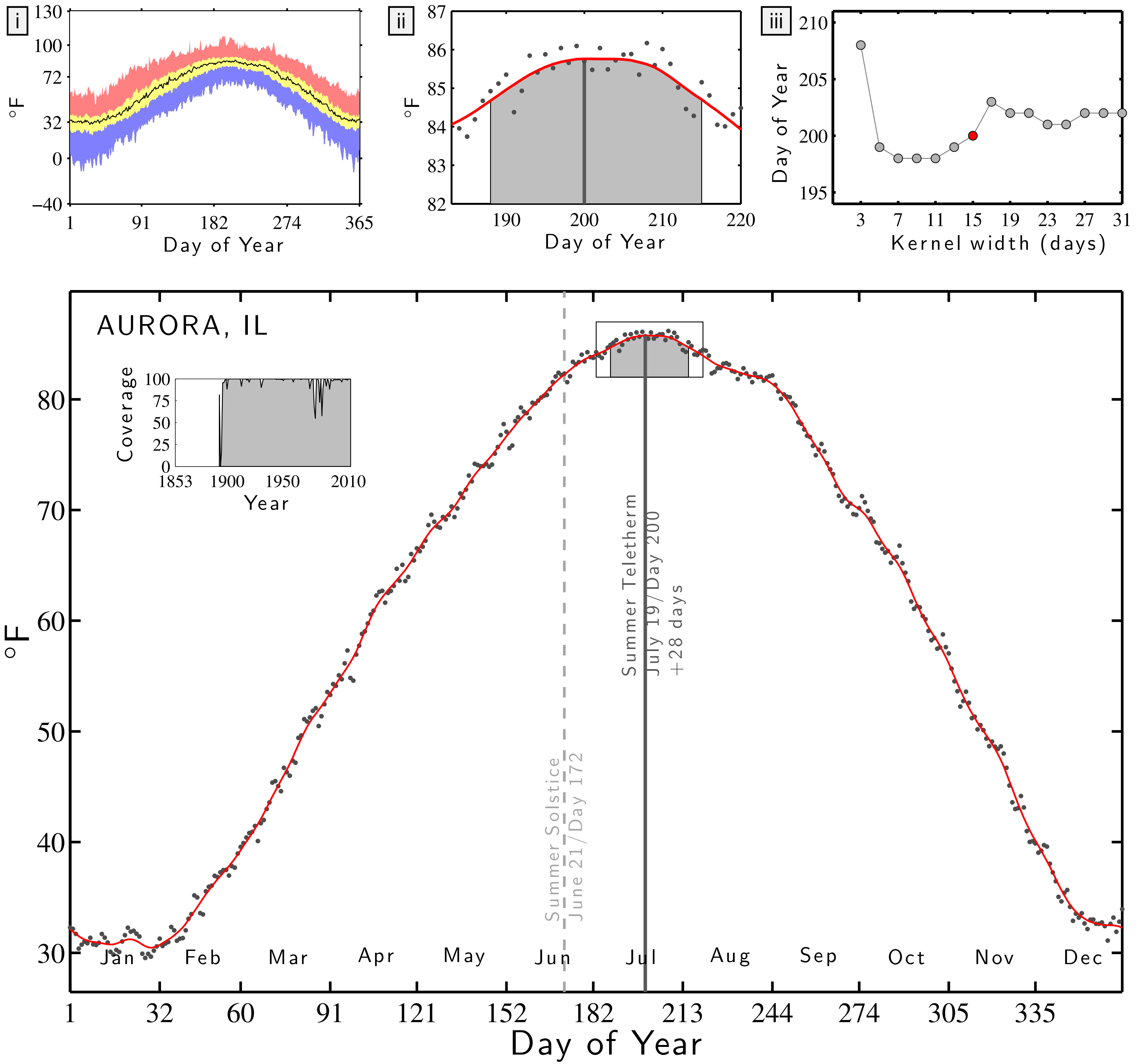

i
°F
Day of Year
ii
°F
Day of Year
iii
Day of Year
Kernel width (days)
AURORA, IL
Coverage
Year
Summer Solstice
June 21/Day 172
Summer Teletherm
July 19/Day 200
+28 days
Jan
Feb
Mar
Apr
May
Jun
Jul
Aug
Sep
Oct
Nov
Dec
°F
Day of Year

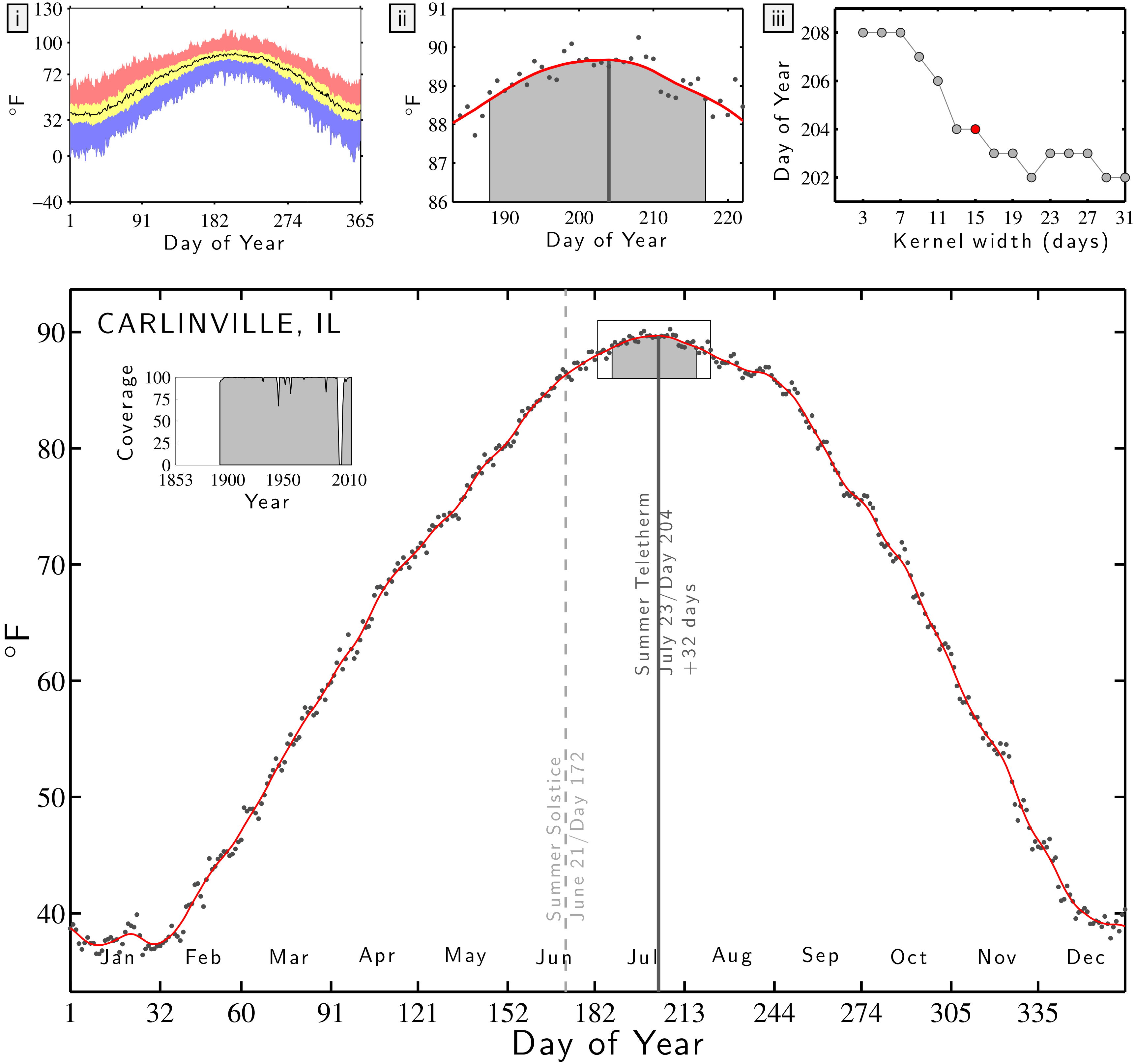

i
°F
Day of Year
ii
°F
Day of Year
iii
Day of Year
Kernel width (days)
CARLINVILLE, IL
Coverage
Year
Summer Solstice
June 21 / Day 172
Summer Teletherm
July 23 / Day 204
+32 days
Jan
Feb
Mar
Apr
May
Jun
Jul
Aug
Sep
Oct
Nov
Dec
°F
Day of Year

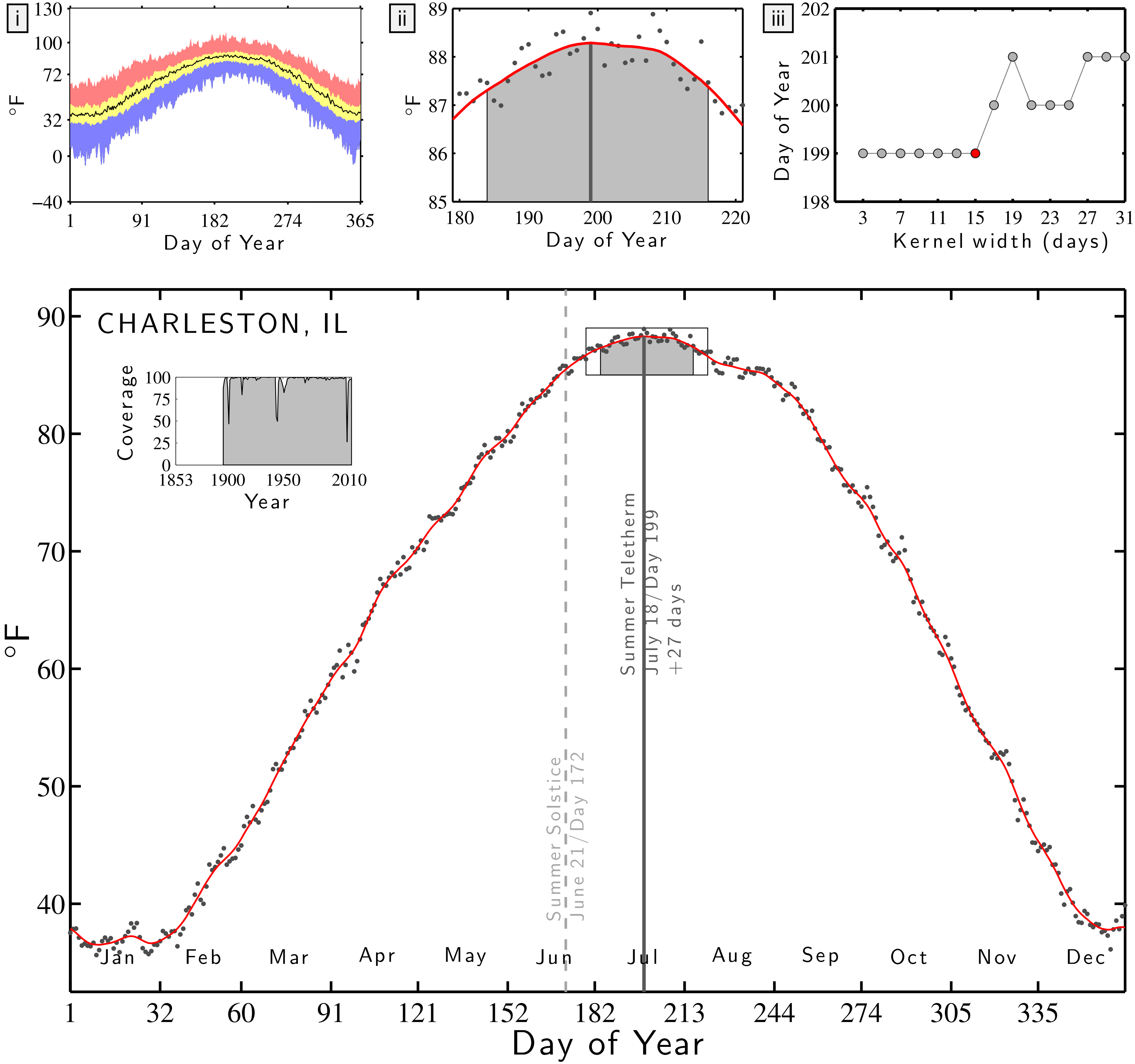

i
°F
130
100
72
32
0
−40
1
91
182
274
365
Day of Year
ii
°F
89
88
87
86
85
180
190
200
210
220
Day of Year
iii
Day of Year
202
201
200
199
198
3
7
11
15
19
23
27
31
Kernel width (days)
CHARLESTON, IL
Coverage
100
75
50
25
0
1853
1900
1950
2010
Year
Summer Solstice
June 21/Day 172
Summer Teletherm
July 18/Day 199
+27 days
°F
90
80
70
60
50
40
Jan
Feb
Mar
Apr
May
Jun
Jul
Aug
Sep
Oct
Nov
Dec
1
32
60
91
121
152
182
213
244
274
305
335
Day of Year

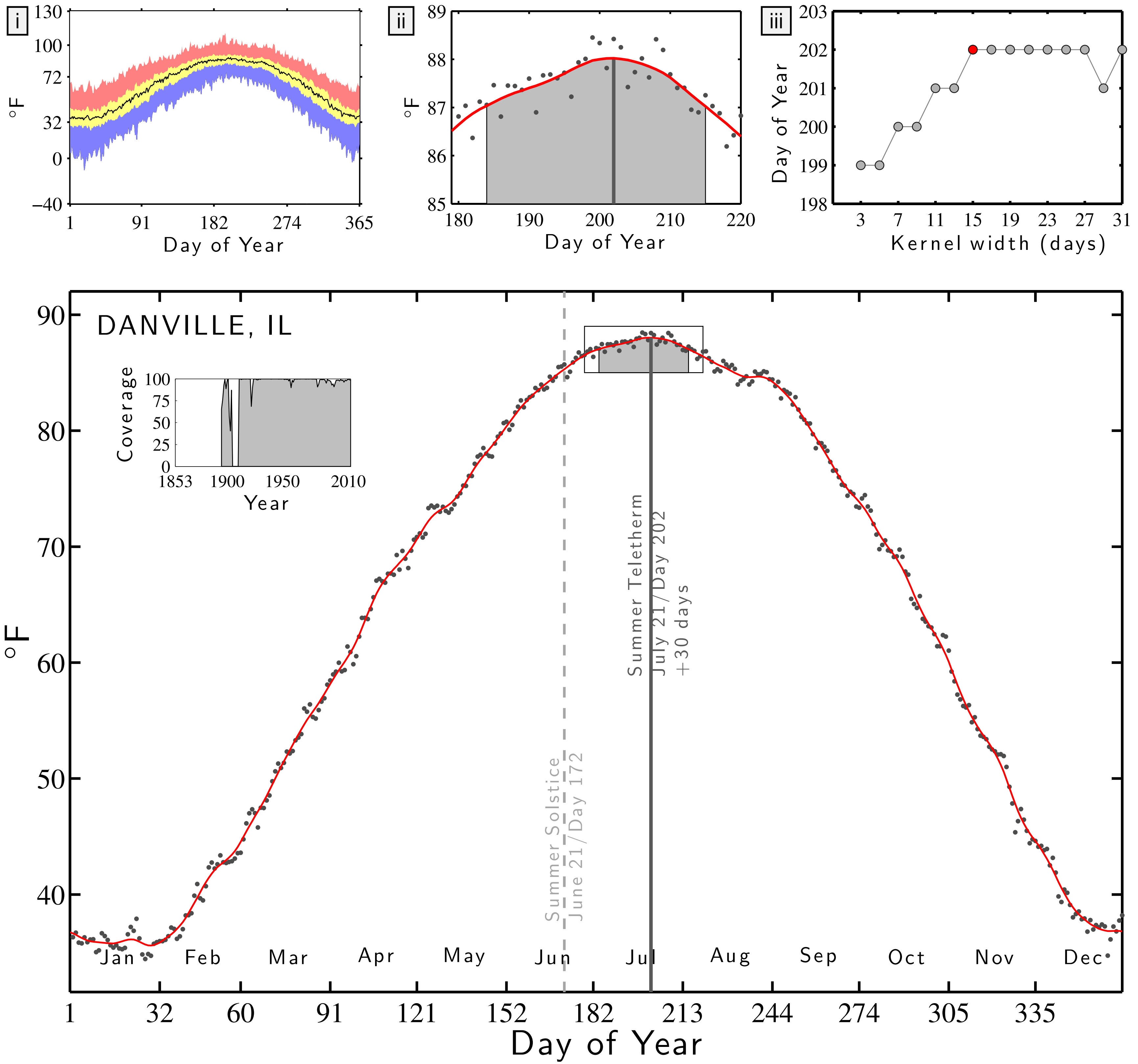

i
ii
iii
Day of Year
Kernel width (days)
DANVILLE, IL
Coverage
Year
Summer Solstice
June 21 / Day 172
Summer Teletherm
July 21 / Day 202
+30 days
Jan
Feb
Mar
Apr
May
Jun
Jul
Aug
Sep
Oct
Nov
Dec
°F

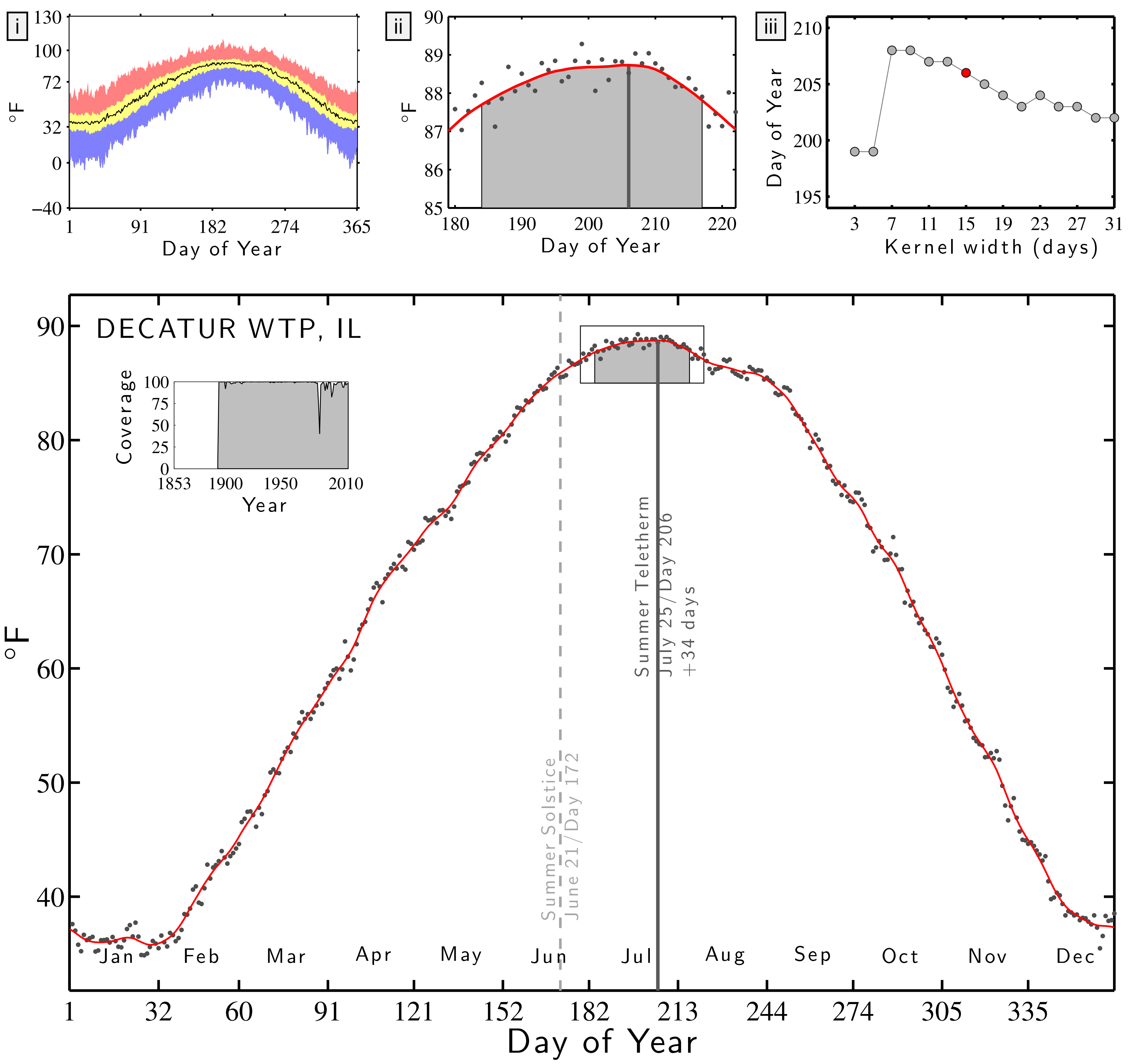

i
°F
Day of Year
ii
°F
Day of Year
iii
Day of Year
Kernel width (days)
DECATUR WTP, IL
Coverage
Year
Summer Solstice
June 21/Day 172
Summer Teletherm
July 25/Day 206
+34 days
Jan
Feb
Mar
Apr
May
Jun
Jul
Aug
Sep
Oct
Nov
Dec
°F
Day of Year

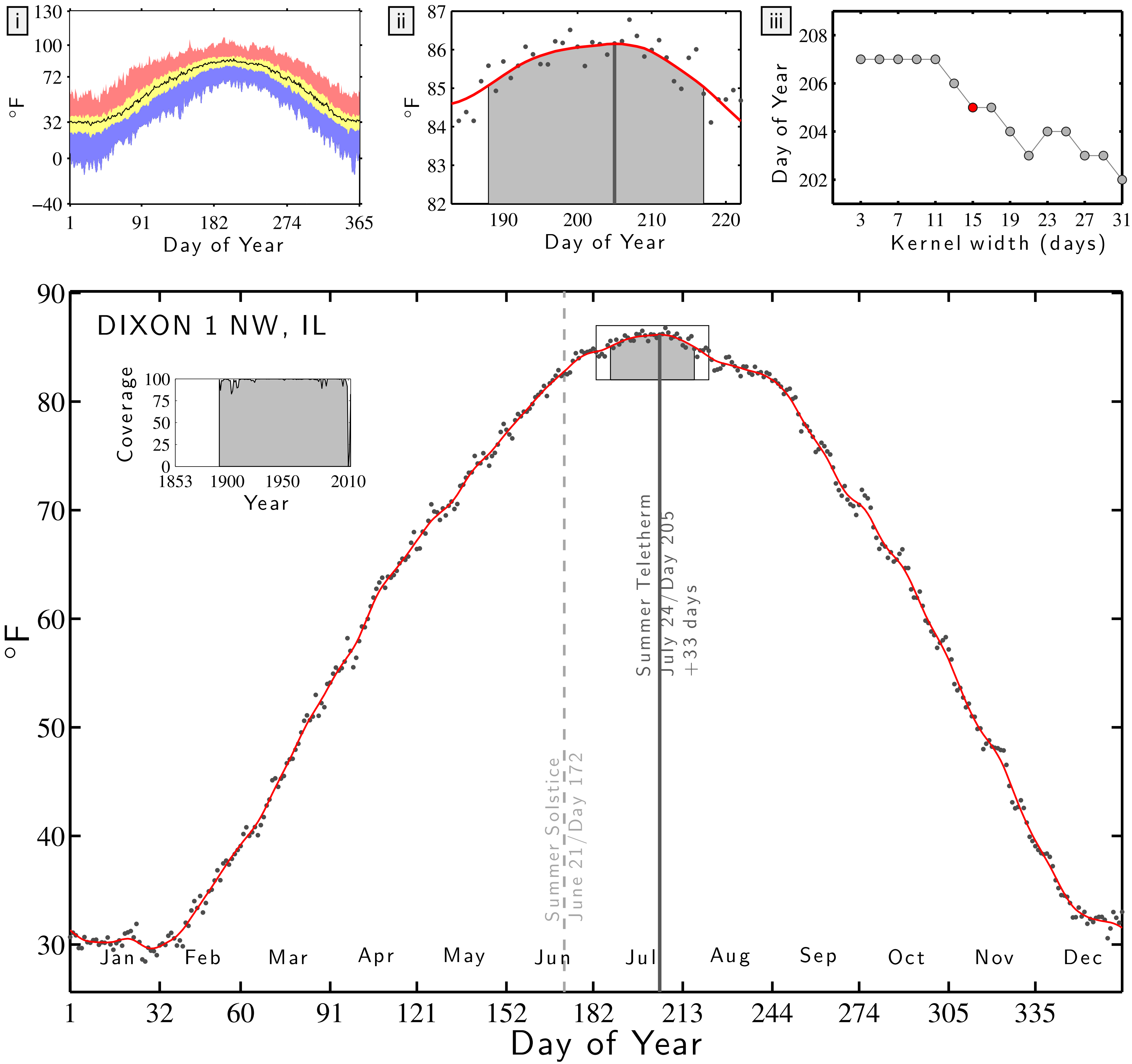

i
ii
iii
°F
Day of Year
Day of Year
Kernel width (days)
DIXON 1 NW, IL
Coverage
Year
Summer Solstice
June 21 / Day 172
Summer Teletherm
July 24 / Day 205
+33 days
Jan
Feb
Mar
Apr
May
Jun
Jul
Aug
Sep
Oct
Nov
Dec
Day of Year

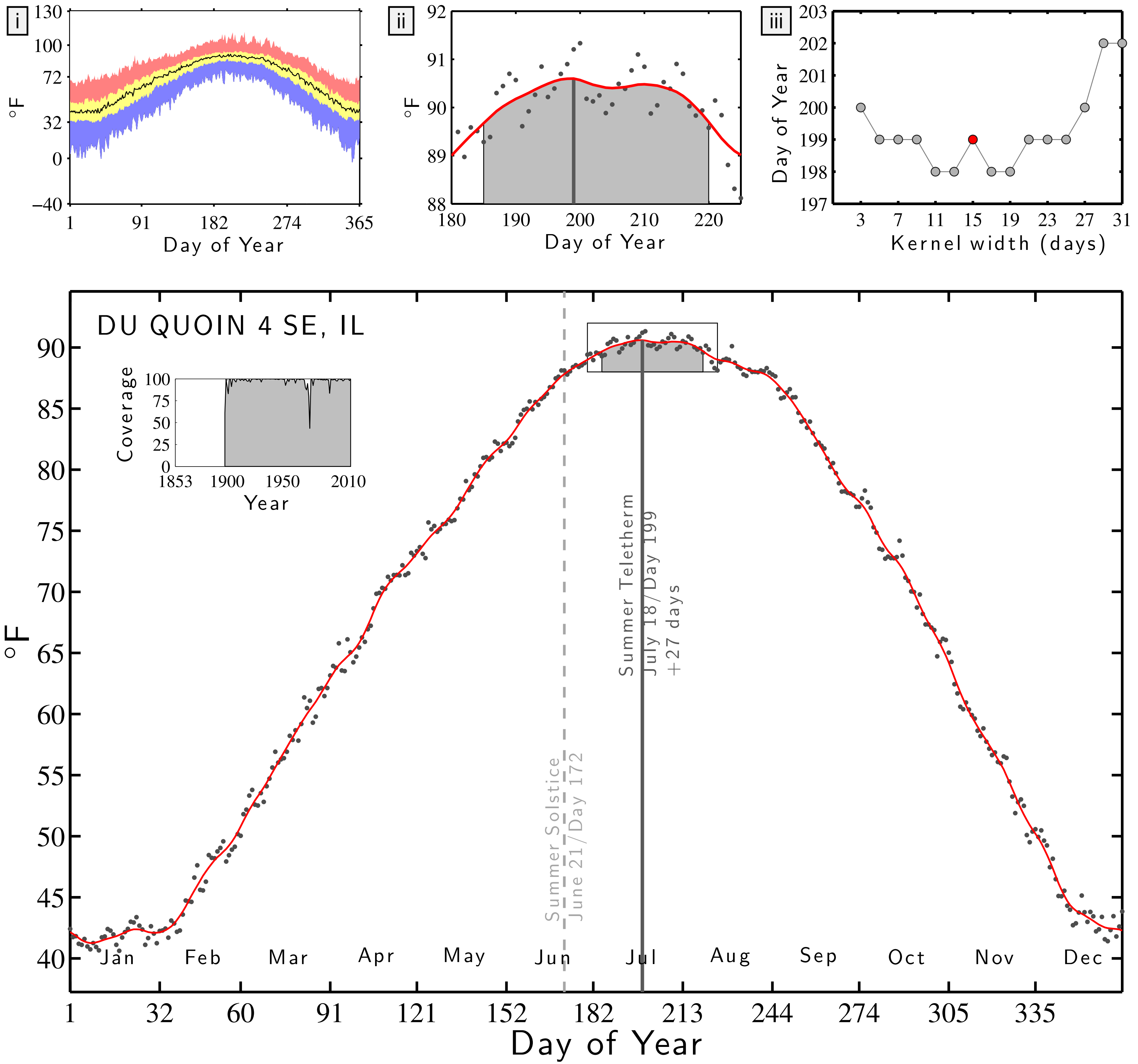

i
ii
iii
°F
Day of Year
Day of Year
Kernel width (days)
DU QUOIN 4 SE, IL
Coverage
Year
Summer Solstice
June 21/Day 172
Summer Teletherm
July 18/Day 199
+27 days
Jan
Feb
Mar
Apr
May
Jun
Jul
Aug
Sep
Oct
Nov
Dec
Day of Year

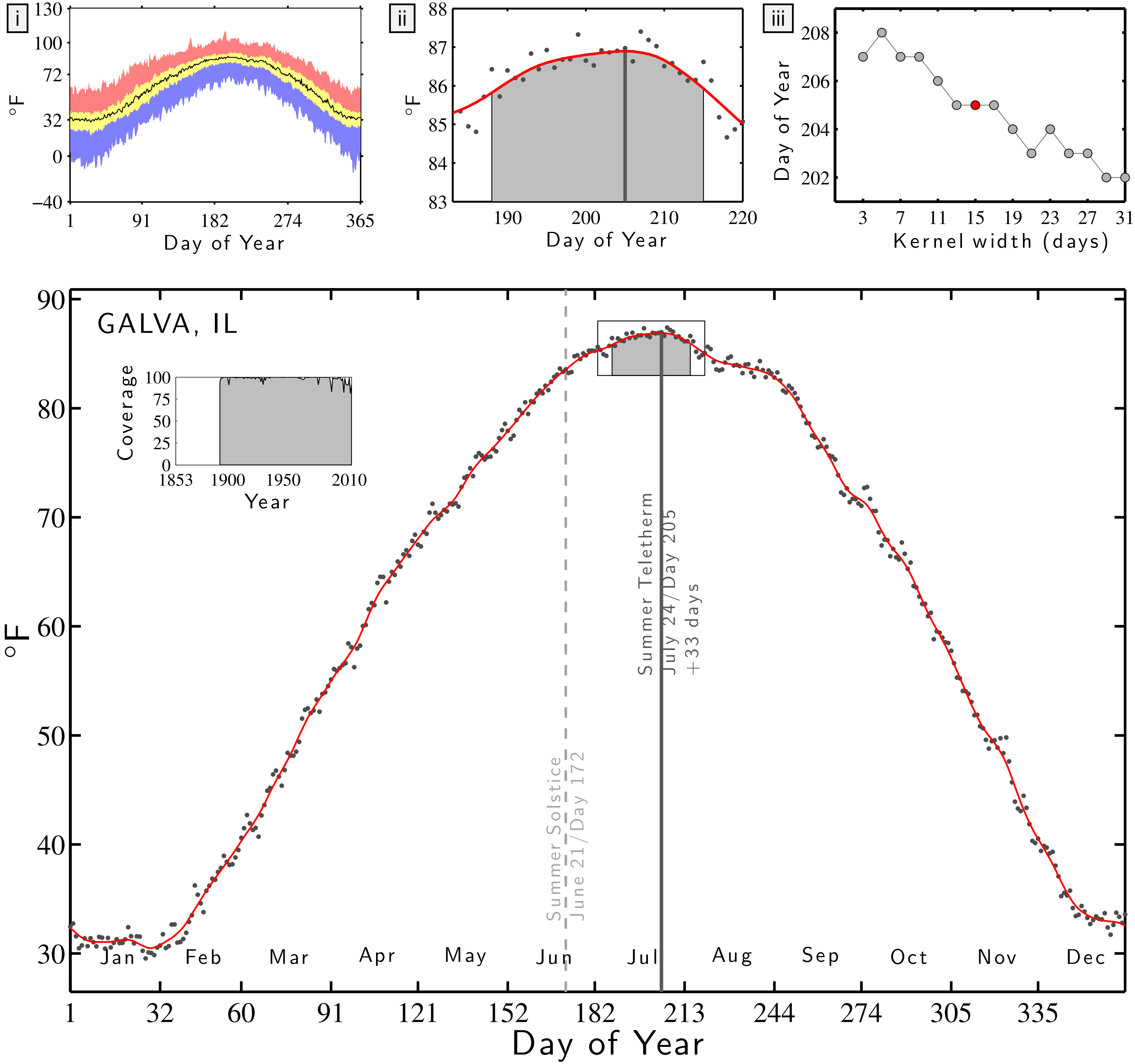

i
°F
Day of Year
ii
°F
Day of Year
iii
Day of Year
Kernel width (days)
GALVA, IL
Coverage
Year
Summer Solstice
June 21 / Day 172
Summer Teletherm
July 24 / Day 205
+33 days
Jan
Feb
Mar
Apr
May
Jun
Jul
Aug
Sep
Oct
Nov
Dec
°F
Day of Year

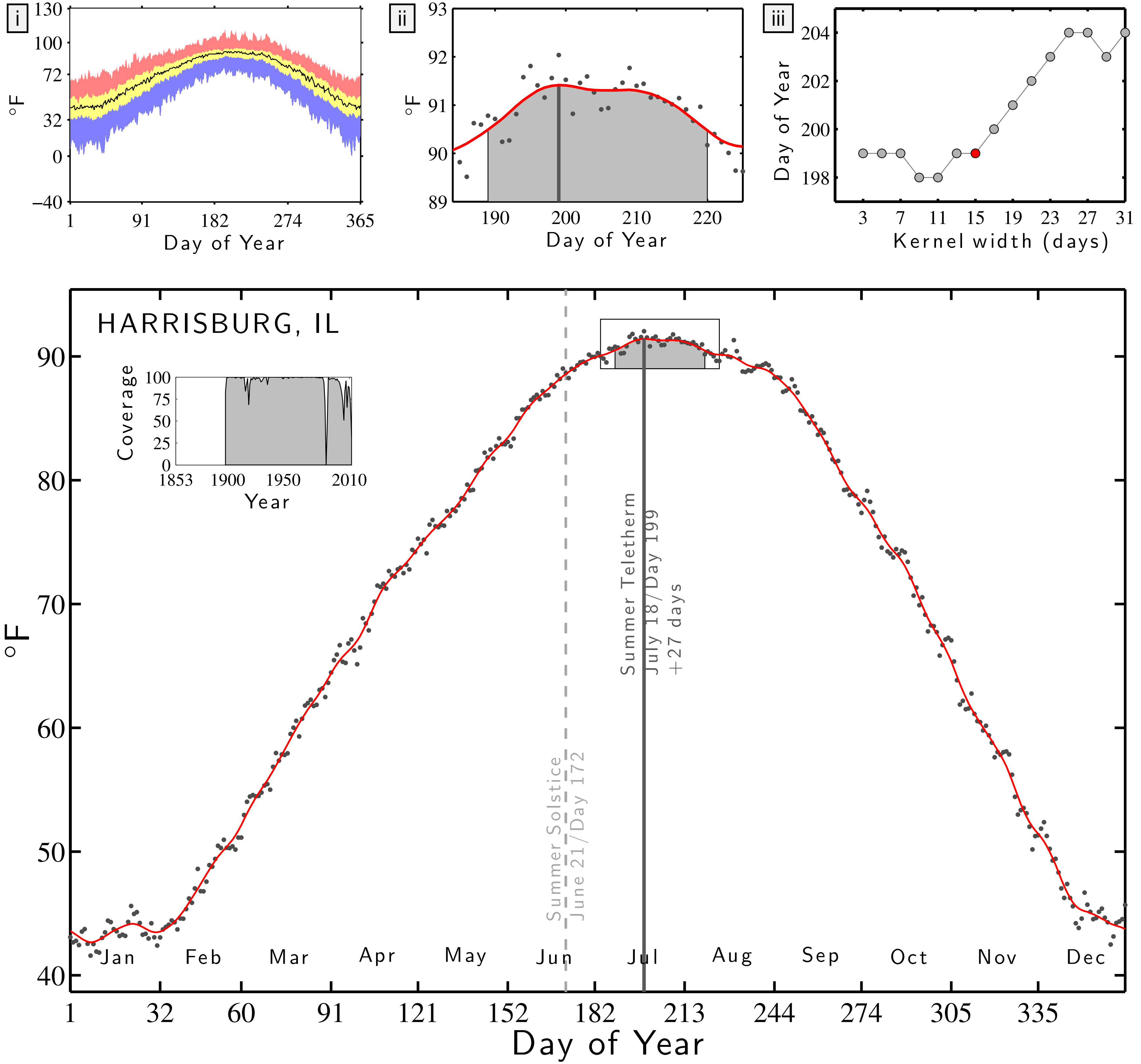

i
ii
iii
°F
Day of Year
Kernel width (days)
HARRISBURG, IL
Coverage
Year
Summer Teletherm
July 18/Day 199
+27 days
Summer Solstice
June 21/Day 172
Jan
Feb
Mar
Apr
May
Jun
Jul
Aug
Sep
Oct
Nov
Dec

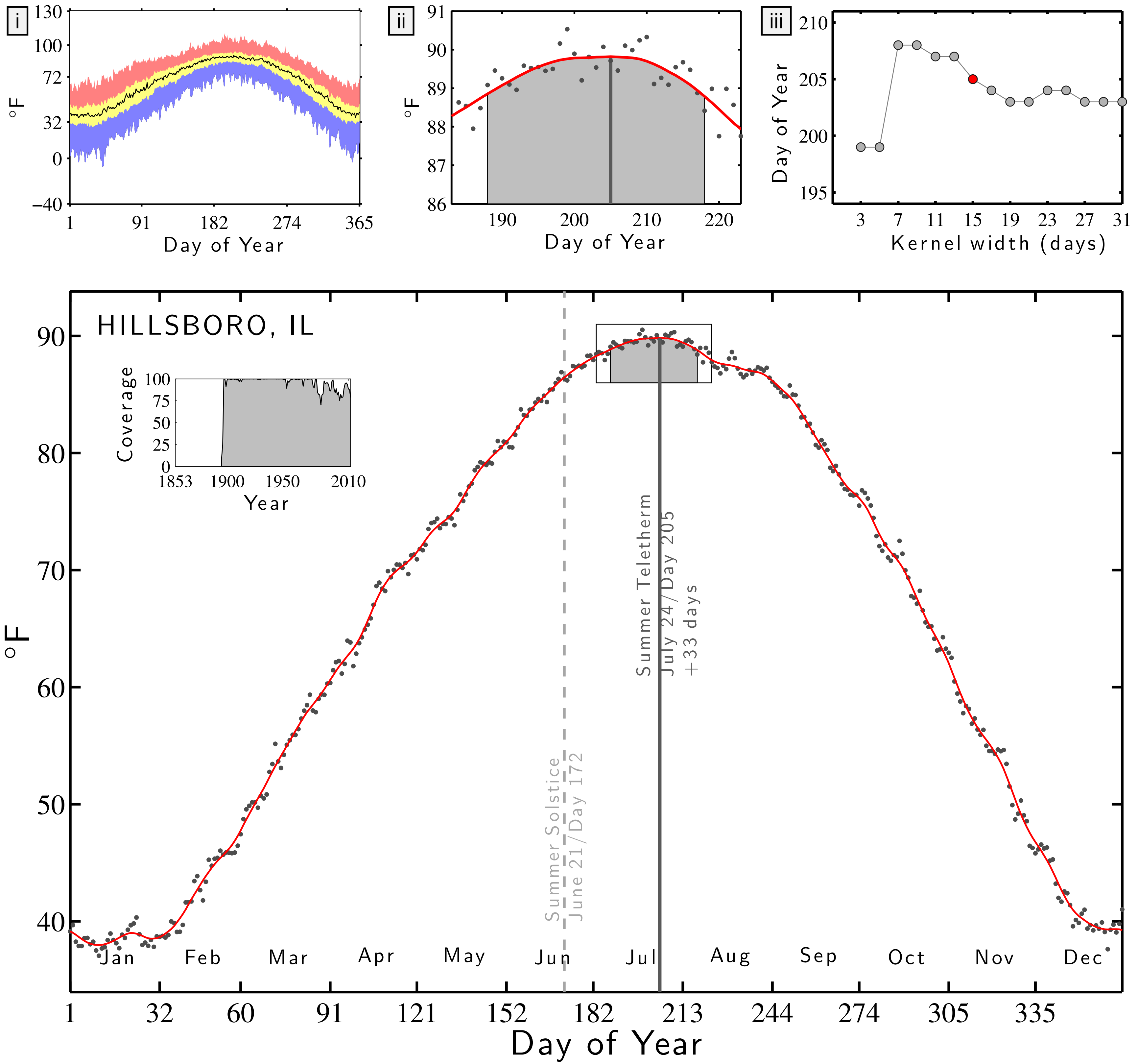

i
ii
iii
°F
Day of Year
Day of Year
Kernel width (days)
HILLSBORO, IL
Coverage
Year
Summer Solstice
June 21 / Day 172
Summer Teletherm
July 24 / Day 205
+33 days
Jan
Feb
Mar
Apr
May
Jun
Jul
Aug
Sep
Oct
Nov
Dec
Day of Year

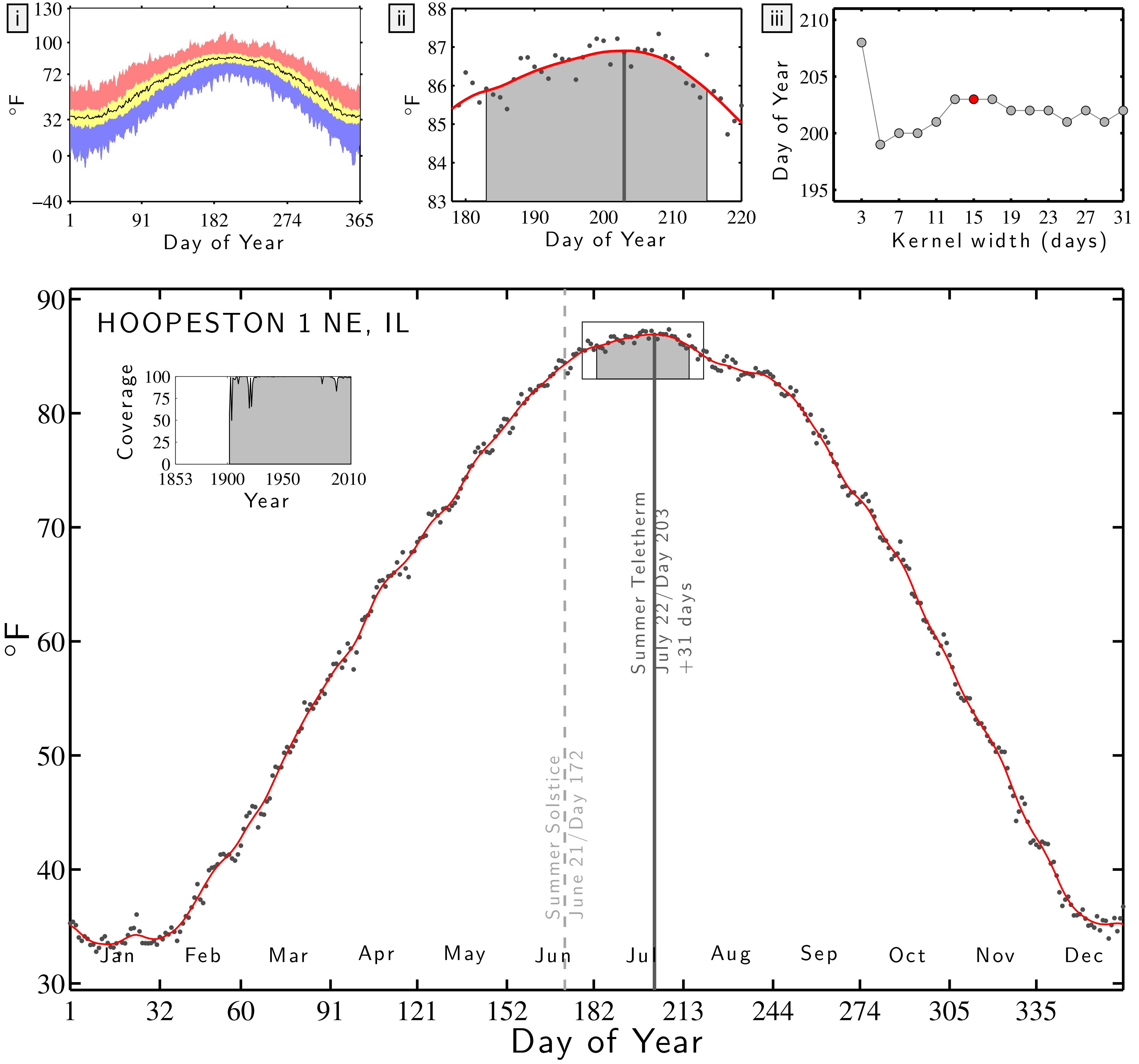

i
ii
iii
°F
Day of Year
Day of Year
Kernel width (days)
HOOPESTON 1 NE, IL
Coverage
Year
Summer Solstice
June 21/Day 172
Summer Teletherm
July 22/Day 203
+31 days
Jan
Feb
Mar
Apr
May
Jun
Jul
Aug
Sep
Oct
Nov
Dec

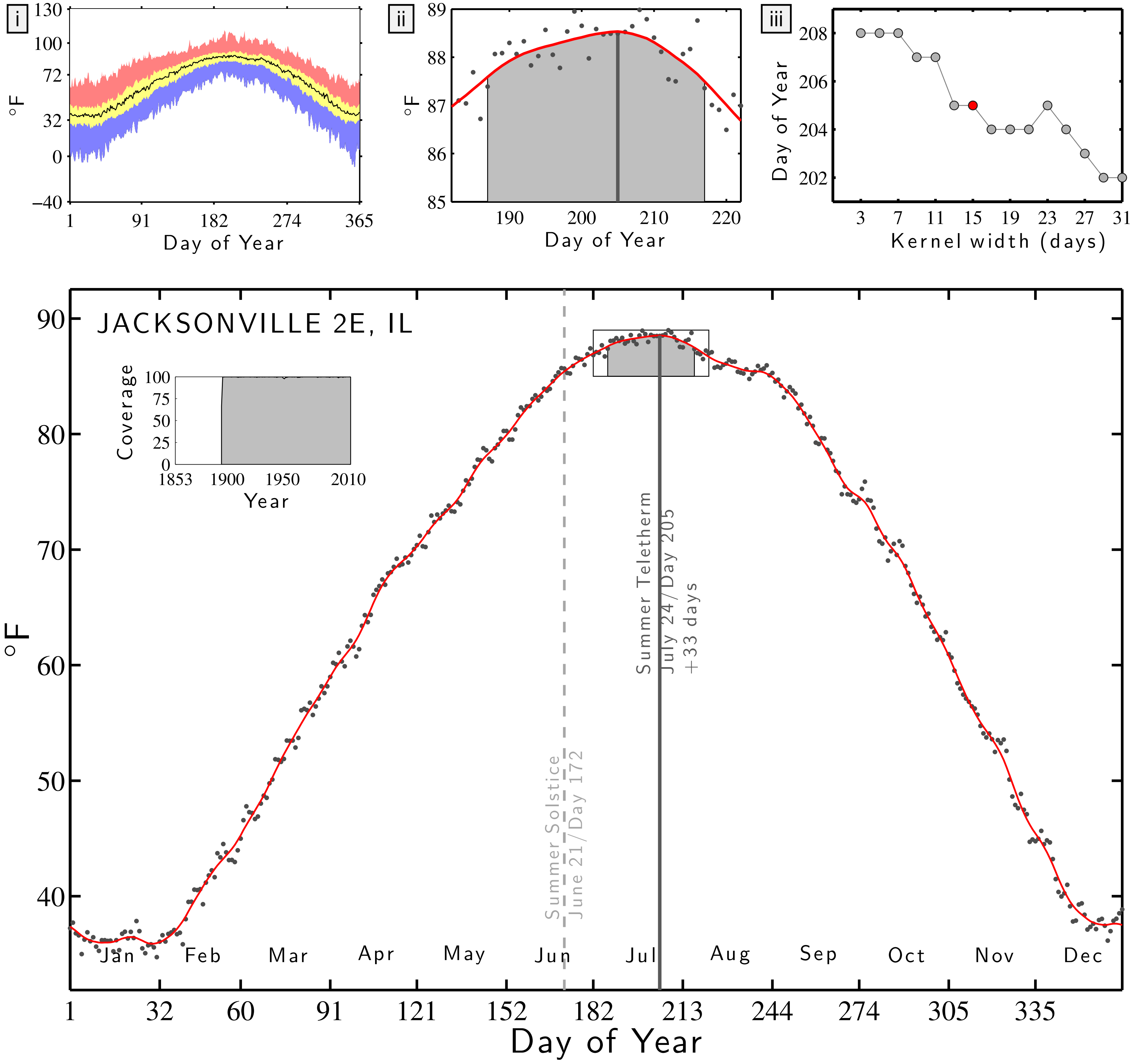
i
°F
Day of Year
ii
Day of Year
iii
Day of Year
Kernel width (days)
JACKSONVILLE 2E, IL
Coverage
Year
Summer Solstice
June 21 / Day 172
Summer Teletherm
July 24 / Day 205
+33 days
Jan
Feb
Mar
Apr
May
Jun
Jul
Aug
Sep
Oct
Nov
Dec
°F
Day of Year

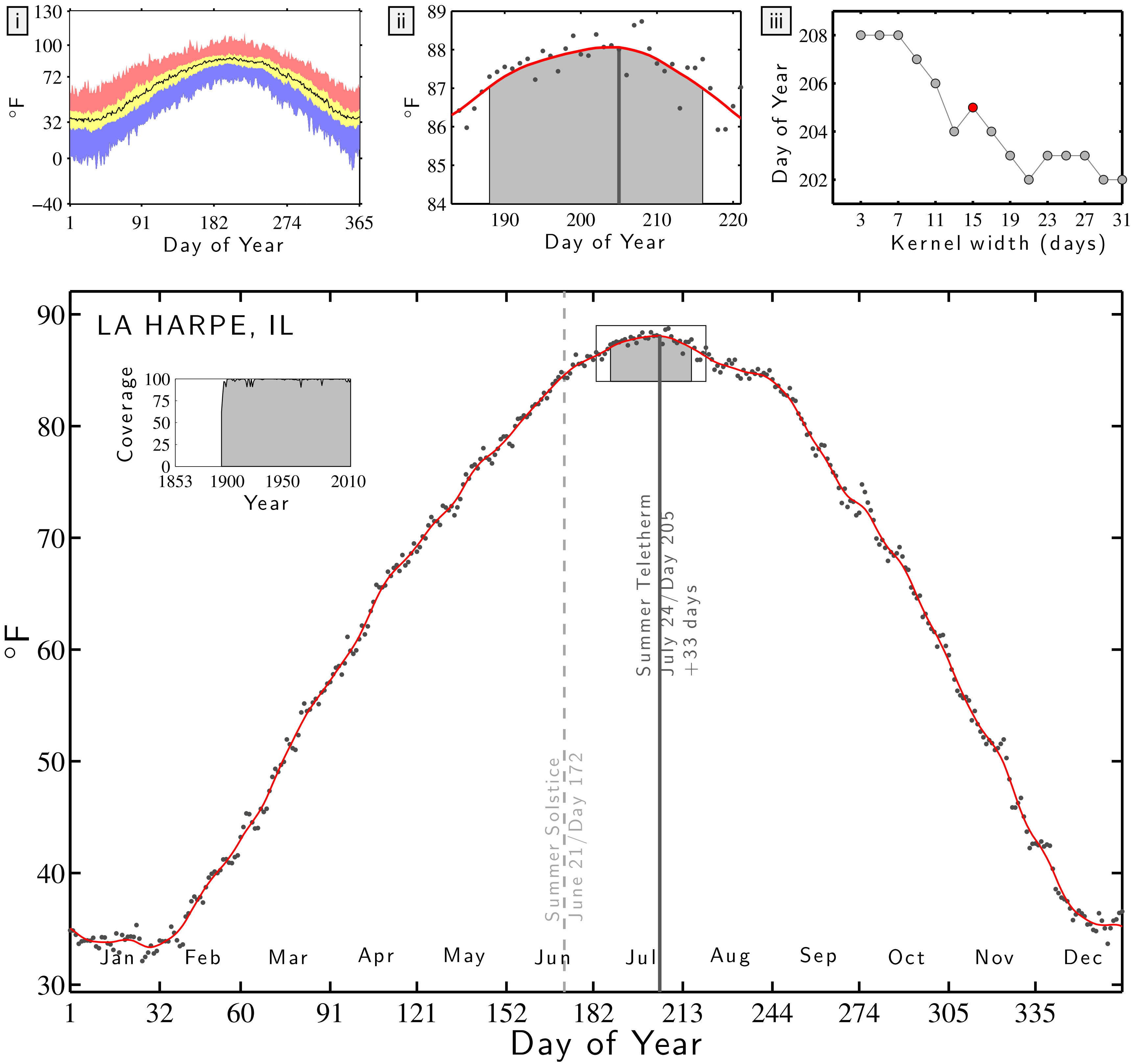

i
ii
iii
LA HARPE, IL
Coverage
Year
Summer Solstice
June 21 / Day 172
Summer Teletherm
July 24 / Day 205
+33 days
Jan
Feb
Mar
Apr
May
Jun
Jul
Aug
Sep
Oct
Nov
Dec
°F
Day of Year
Kernel width (days)

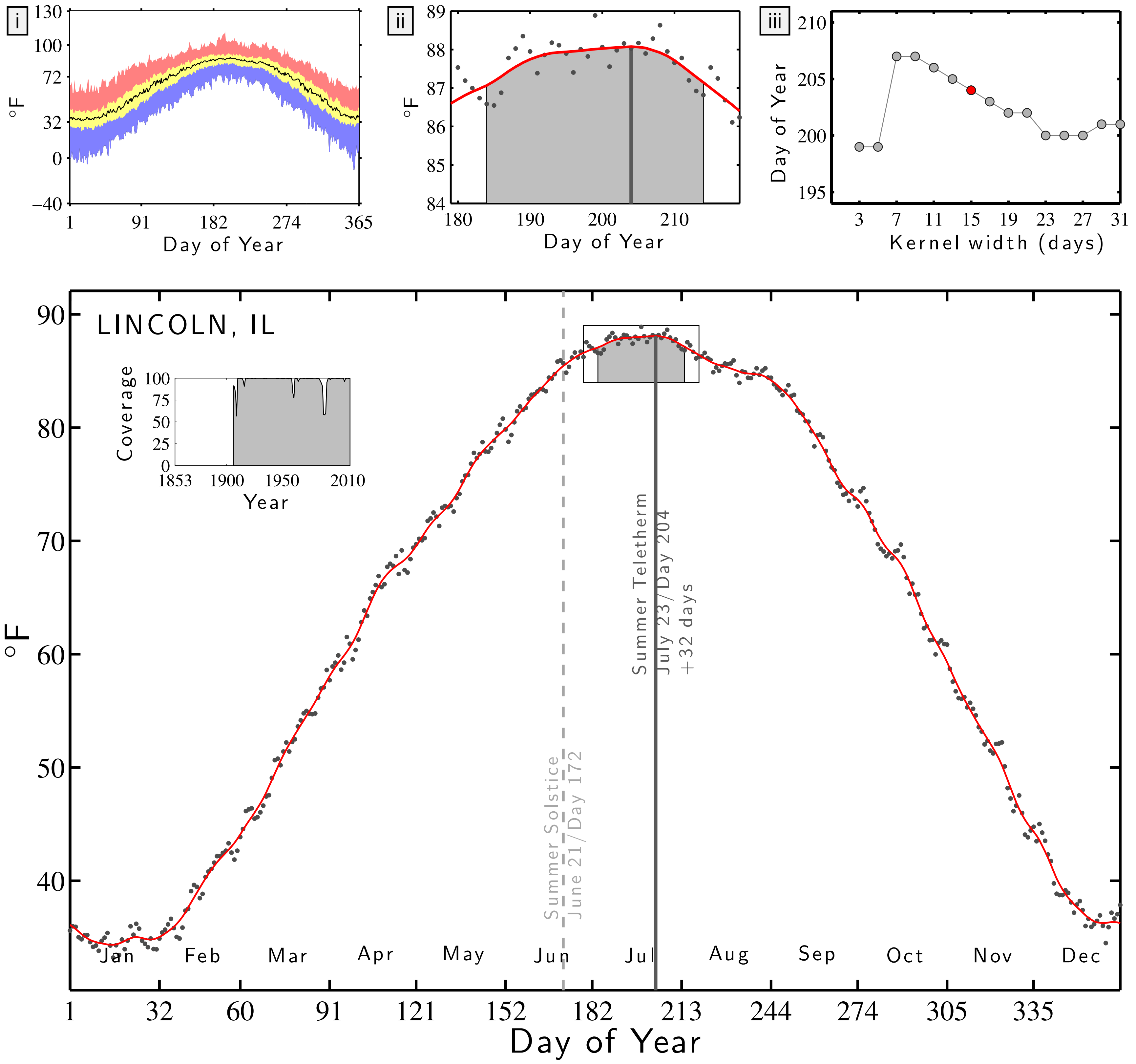

i
°F
Day of Year
ii
°F
Day of Year
iii
Day of Year
Kernel width (days)
LINCOLN, IL
Coverage
Year
Summer Solstice
June 21 / Day 172
Summer Teletherm
July 23 / Day 204
+32 days
Jan
Feb
Mar
Apr
May
Jun
Jul
Aug
Sep
Oct
Nov
Dec
°F
Day of Year

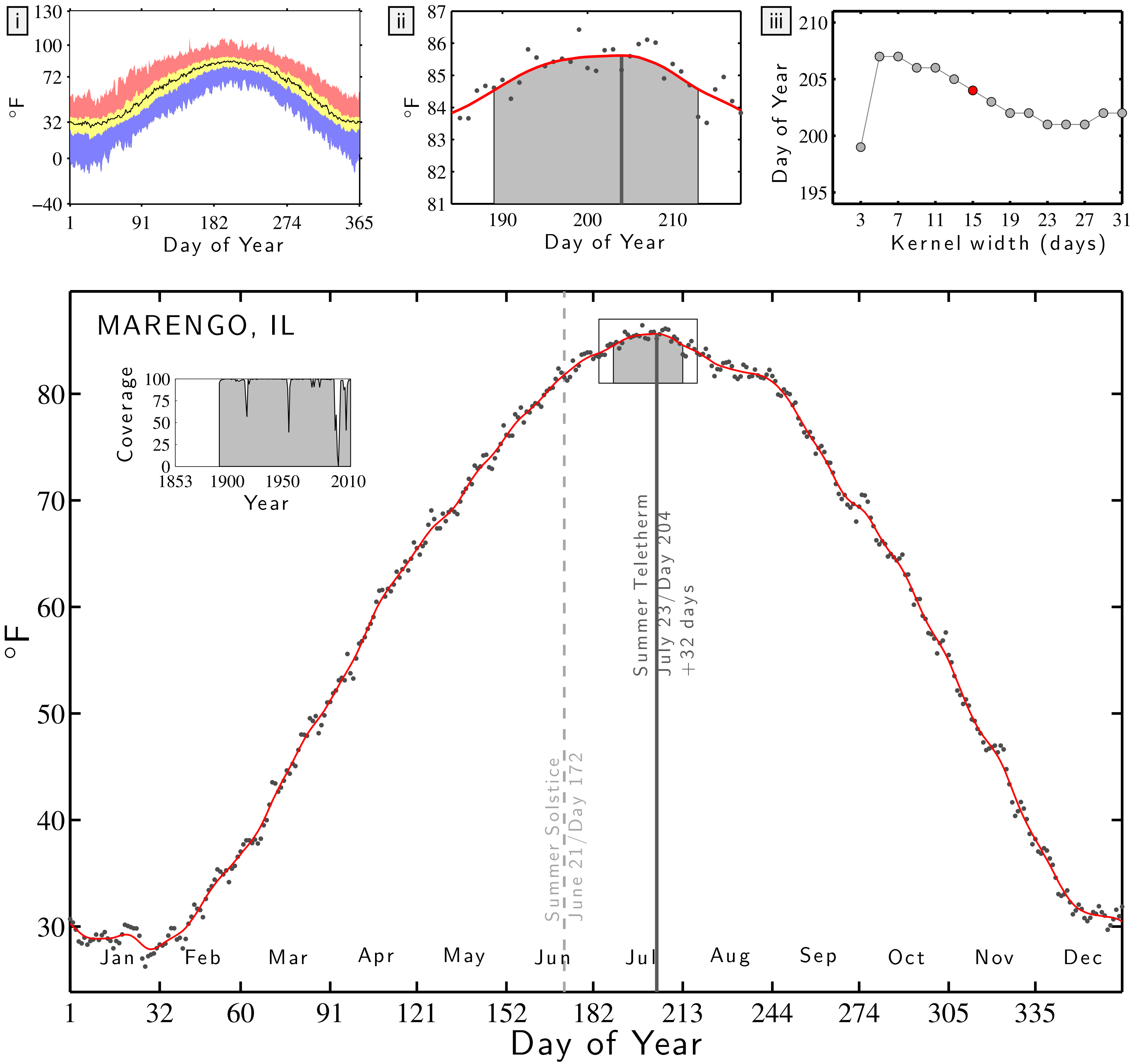

i
130
100
72
32
0
−40
°F
1
91
182
274
365
Day of Year
ii
87
86
85
84
83
82
81
°F
190
200
210
Day of Year
iii
210
205
200
195
Day of Year
3
7
11
15
19
23
27
31
Kernel width (days)
MARENGO, IL
Coverage
100
75
50
25
0
1853
1900
1950
2010
Year
80
70
60
50
40
30
°F
Summer Solstice
June 21 / Day 172
Summer Teletherm
July 23 / Day 204
+32 days
Jan
Feb
Mar
Apr
May
Jun
Jul
Aug
Sep
Oct
Nov
Dec
1
32
60
91
121
152
182
213
244
274
305
335
Day of Year

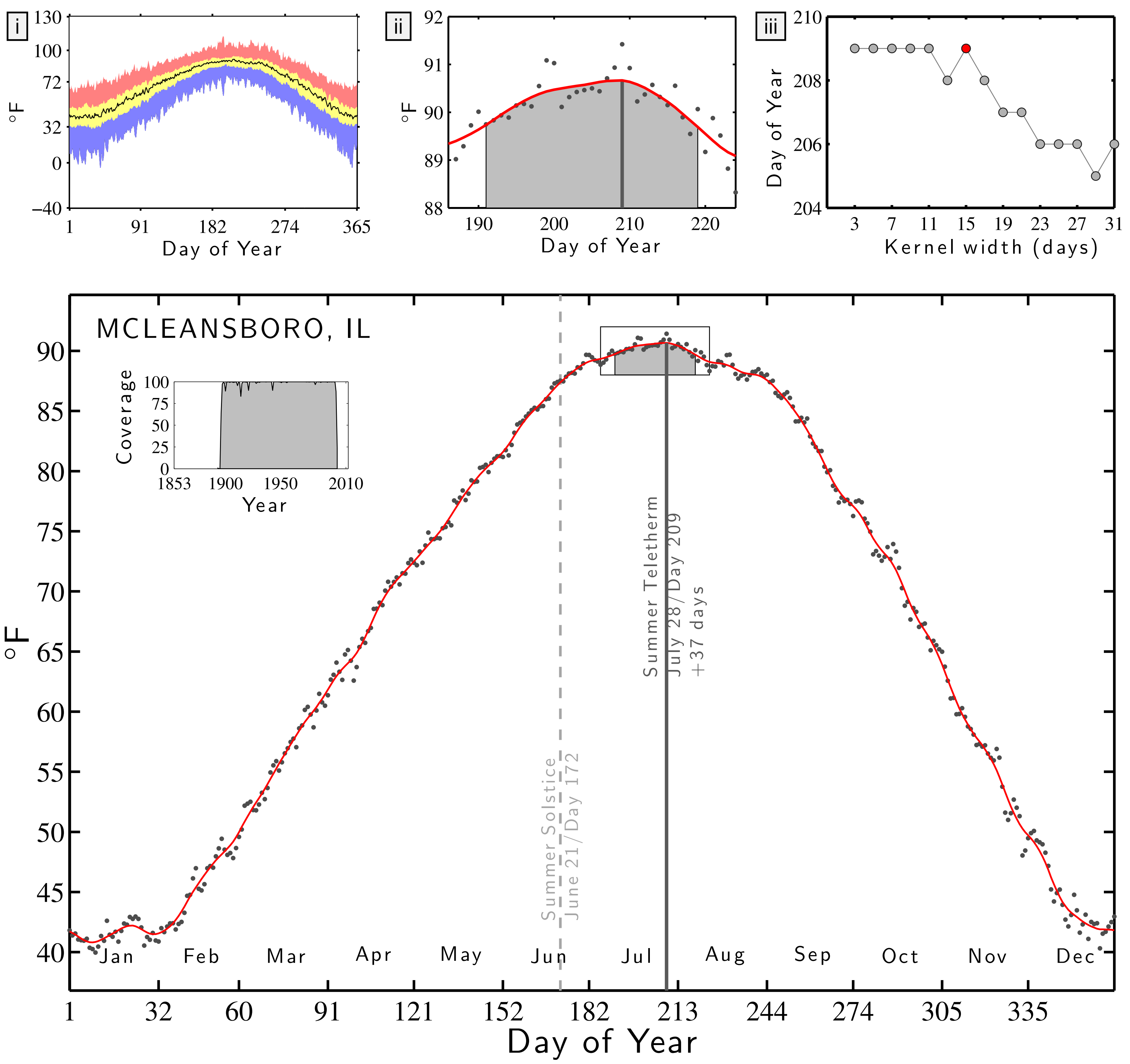

i
ii
iii
°F
Day of Year
Day of Year
Kernel width (days)
MCLEANSBORO, IL
Coverage
Year
Summer Solstice
June 21/Day 172
Summer Teletherm
July 28/Day 209
+37 days
Jan
Feb
Mar
Apr
May
Jun
Jul
Aug
Sep
Oct
Nov
Dec
Day of Year

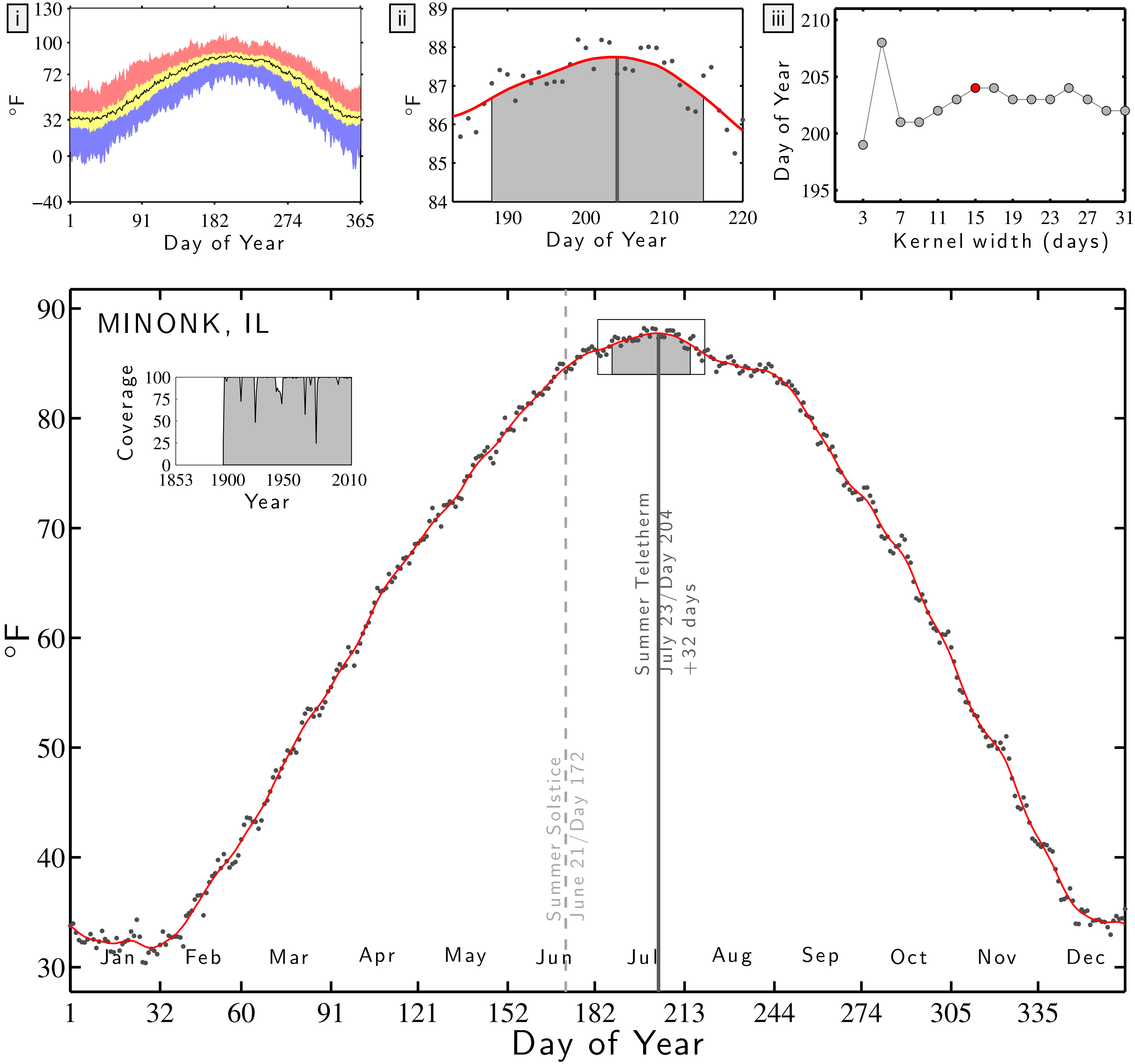

i
°F
Day of Year
ii
°F
Day of Year
iii
Day of Year
Kernel width (days)
MINONK, IL
Coverage
Year
Summer Solstice
June 21 / Day 172
Summer Teletherm
July 23 / Day 204
+32 days
Jan
Feb
Mar
Apr
May
Jun
Jul
Aug
Sep
Oct
Nov
Dec
°F
Day of Year

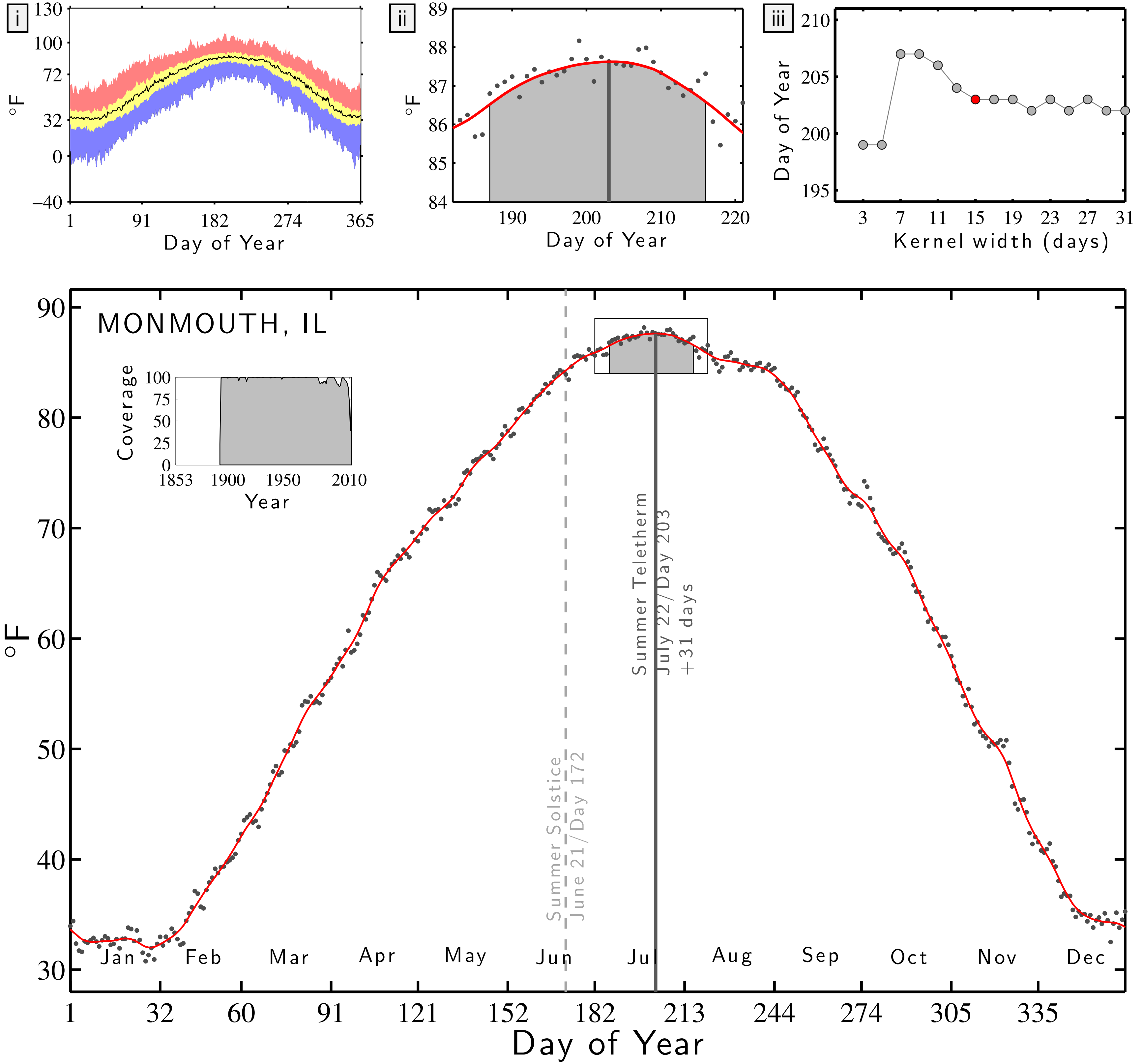

i
°F
Day of Year
ii
°F
Day of Year
iii
Day of Year
Kernel width (days)
MONMOUTH, IL
Coverage
Year
Summer Solstice
June 21/Day 172
Summer Teletherm
July 22/Day 203
+31 days
Jan
Feb
Mar
Apr
May
Jun
Jul
Aug
Sep
Oct
Nov
Dec
°F
Day of Year

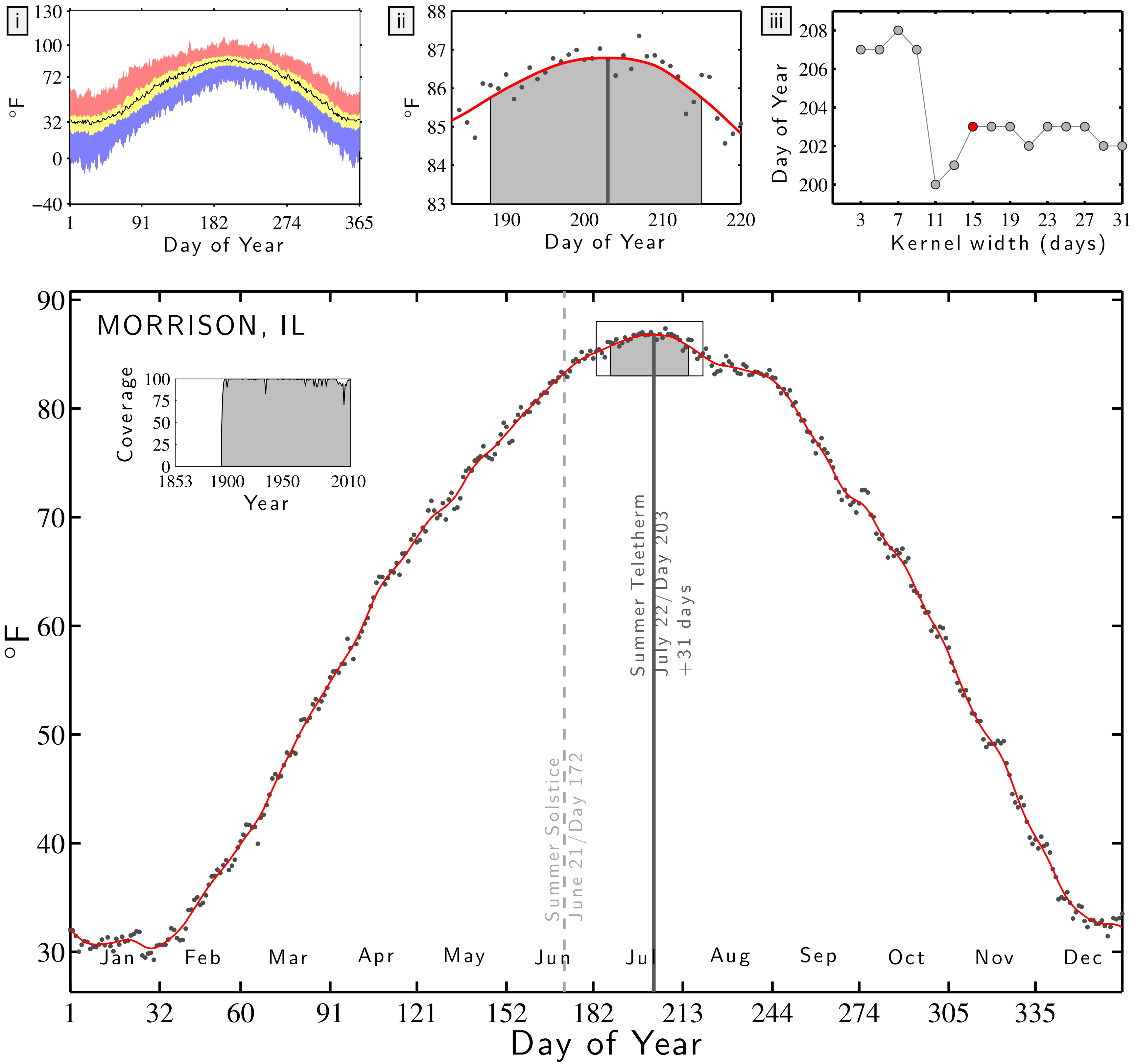

i
ii
iii
°F
Day of Year
Kernel width (days)
MORRISON, IL
Coverage
Year
Summer Solstice
June 21/Day 172
Summer Teletherm
July 22/Day 203
+31 days
Jan
Feb
Mar
Apr
May
Jun
Jul
Aug
Sep
Oct
Nov
Dec

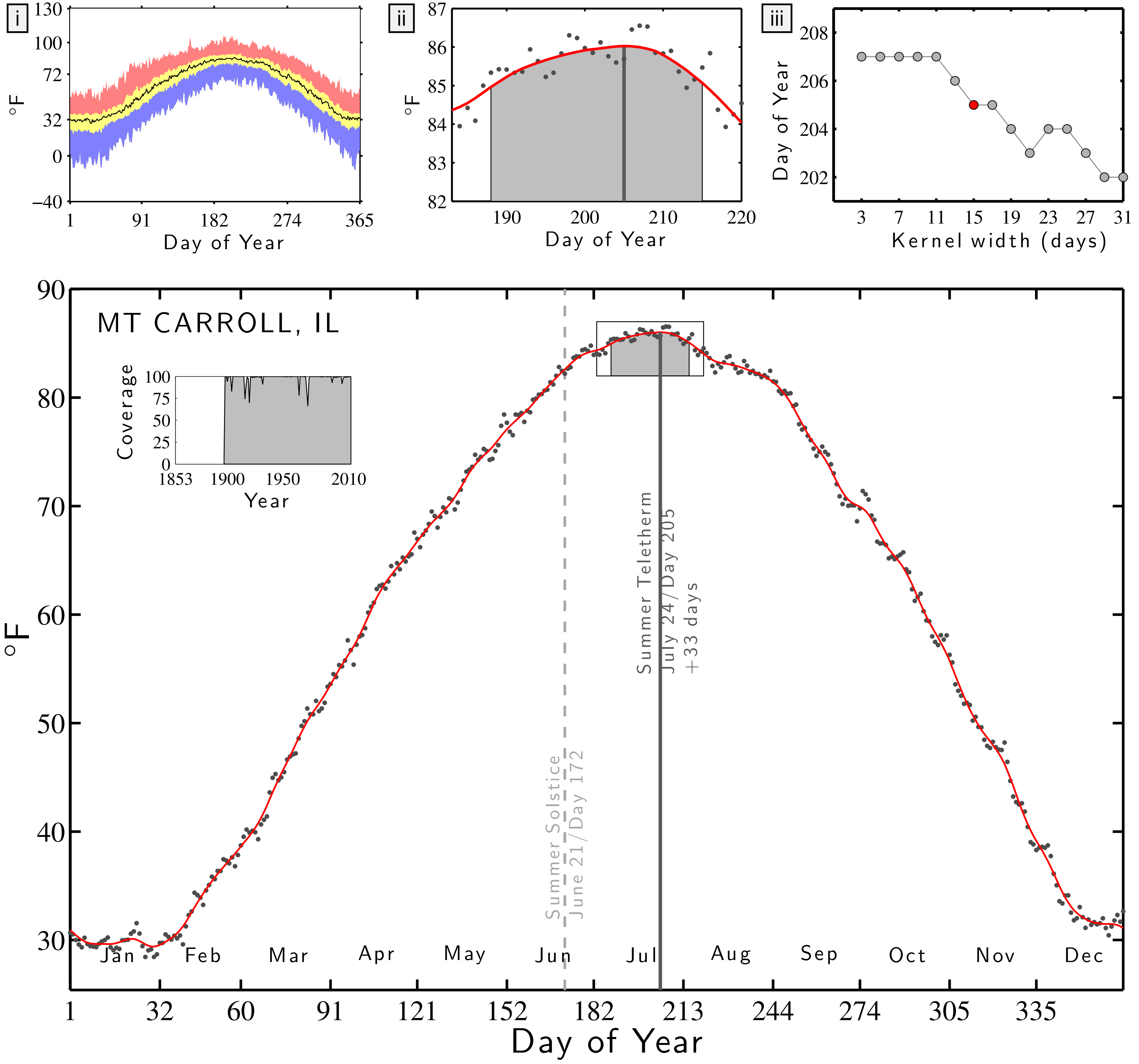

i
°F
Day of Year
ii
°F
Day of Year
iii
Day of Year
Kernel width (days)
MT CARROLL, IL
Coverage
Year
Summer Solstice
June 21 / Day 172
Summer Teletherm
July 24 / Day 205
+33 days
Jan
Feb
Mar
Apr
May
Jun
Jul
Aug
Sep
Oct
Nov
Dec
°F
Day of Year

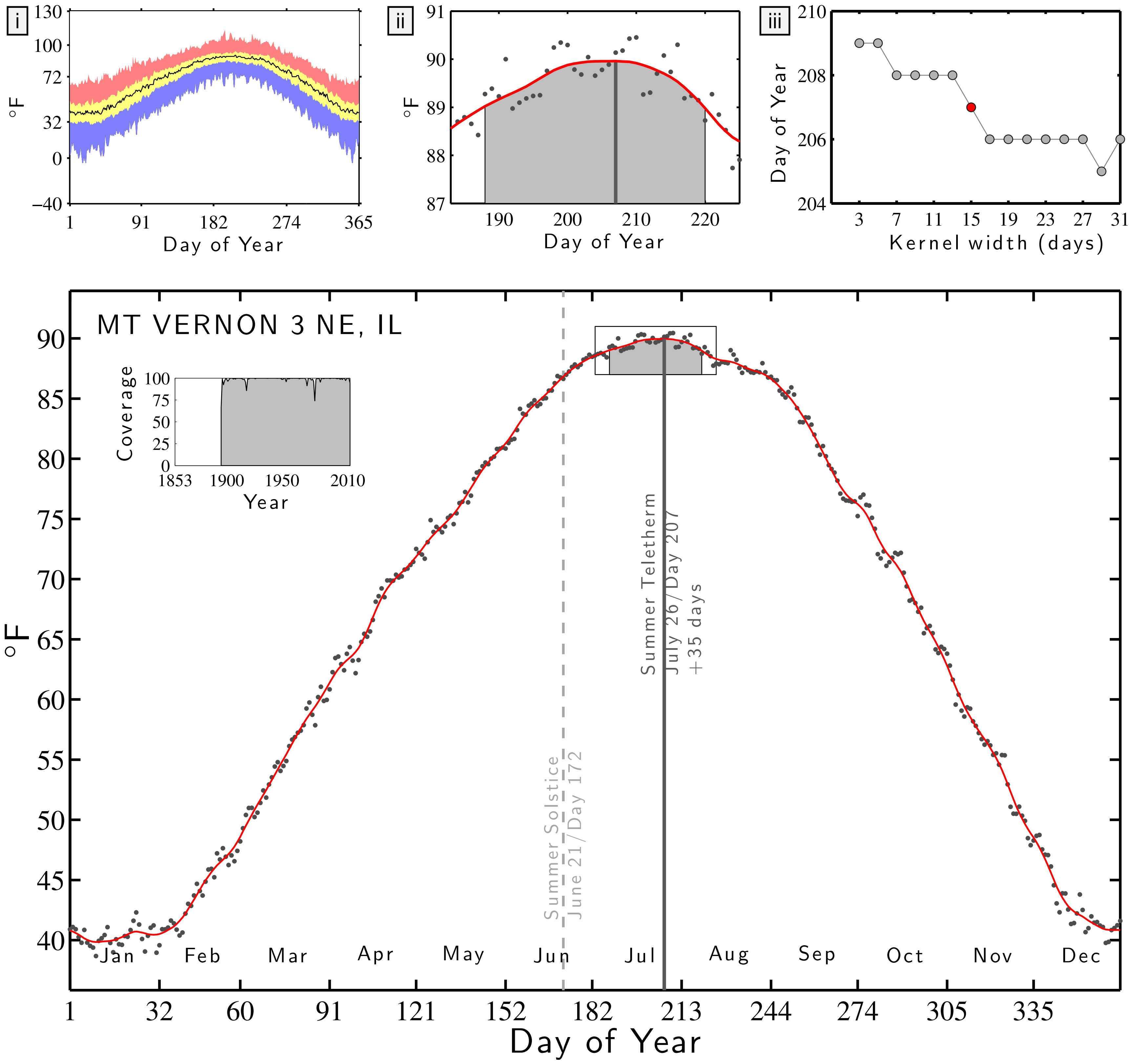

i
°F
130
100
72
32
0
−40
1
91
182
274
365
Day of Year
ii
°F
91
90
89
88
87
190
200
210
220
Day of Year
iii
Day of Year
210
208
206
204
3
7
11
15
19
23
27
31
Kernel width (days)
MT VERNON 3 NE, IL
Coverage
100
75
50
25
0
1853
1900
1950
2010
Year
Summer Solstice
June 21 / Day 172
Summer Teletherm
July 26 / Day 207
+35 days
Jan
Feb
Mar
Apr
May
Jun
Jul
Aug
Sep
Oct
Nov
Dec
°F
90
85
80
75
70
65
60
55
50
45
40
1
32
60
91
121
152
182
213
244
274
305
335
Day of Year

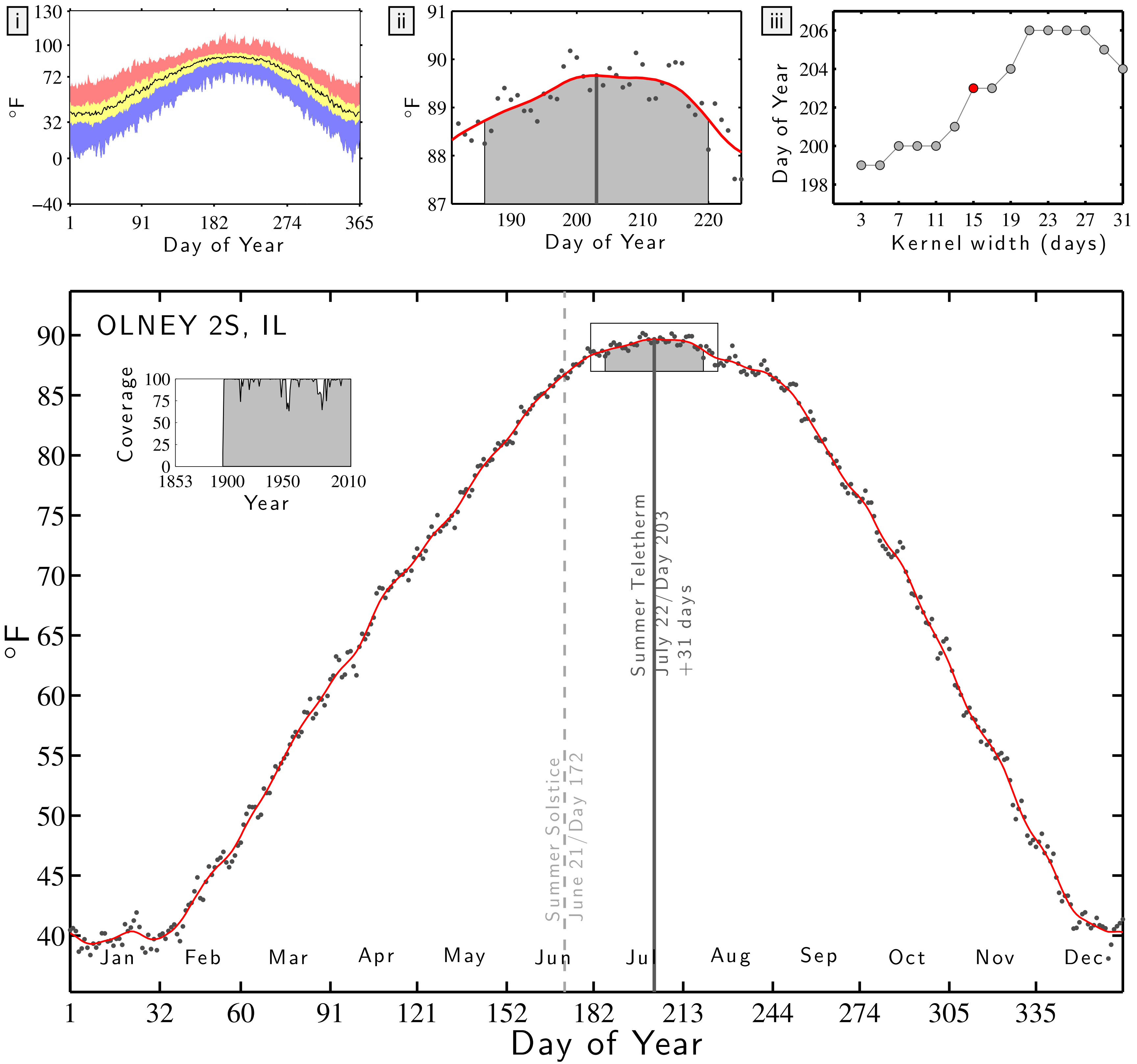

i
ii
iii
°F
Day of Year
Day of Year
Kernel width (days)
OLNEY 2S, IL
Coverage
Year
1853
1900
1950
2010
Summer Solstice
June 21/Day 172
Summer Teletherm
July 22/Day 203
+31 days
Jan
Feb
Mar
Apr
May
Jun
Jul
Aug
Sep
Oct
Nov
Dec

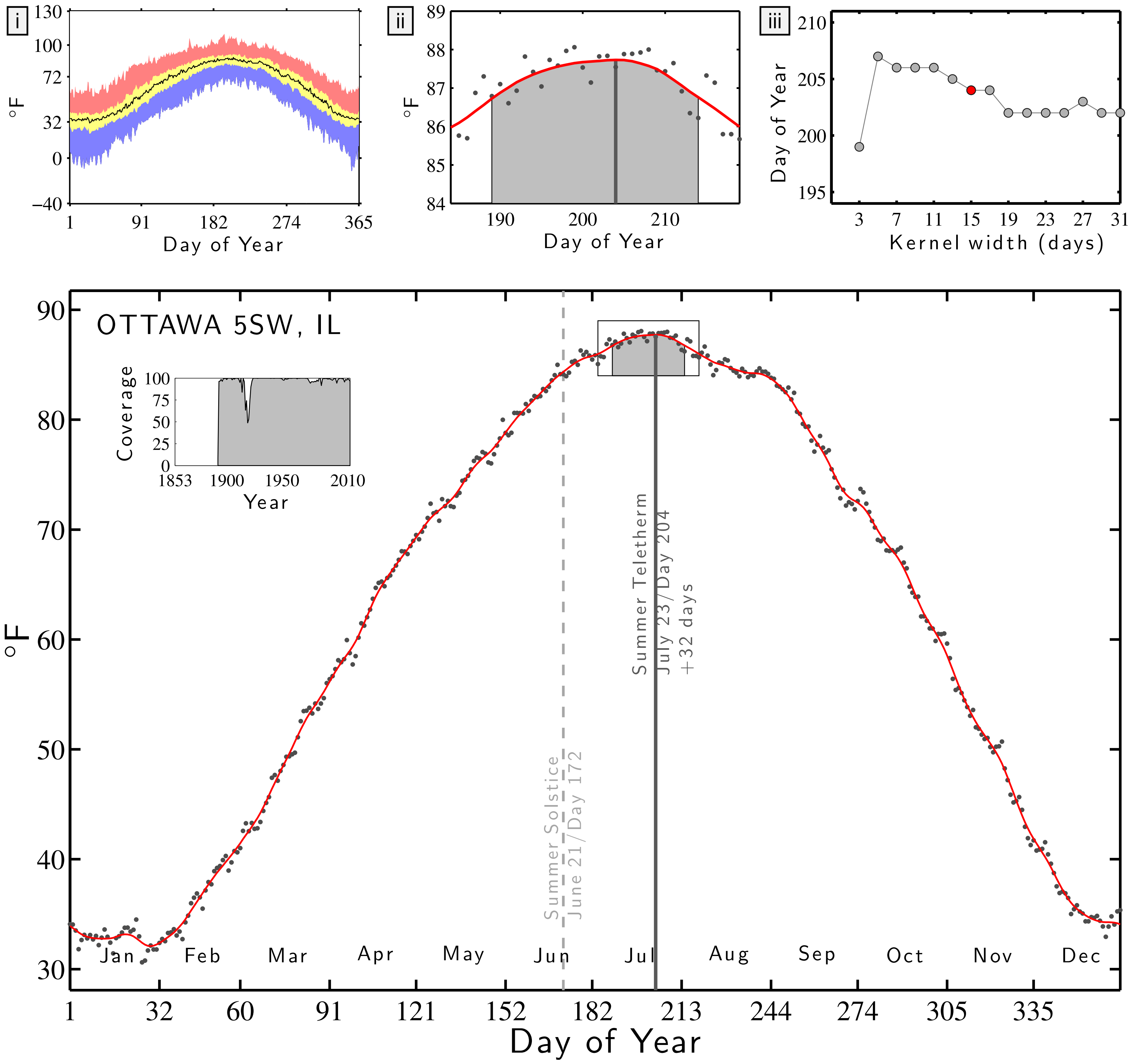

i
ii
iii
°F
Day of Year
Day of Year
Kernel width (days)
OTTAWA 5SW, IL
Coverage
Year
Summer Solstice
June 21/Day 172
Summer Teletherm
July 23/Day 204
+32 days
Jan
Feb
Mar
Apr
May
Jun
Jul
Aug
Sep
Oct
Nov
Dec
Day of Year

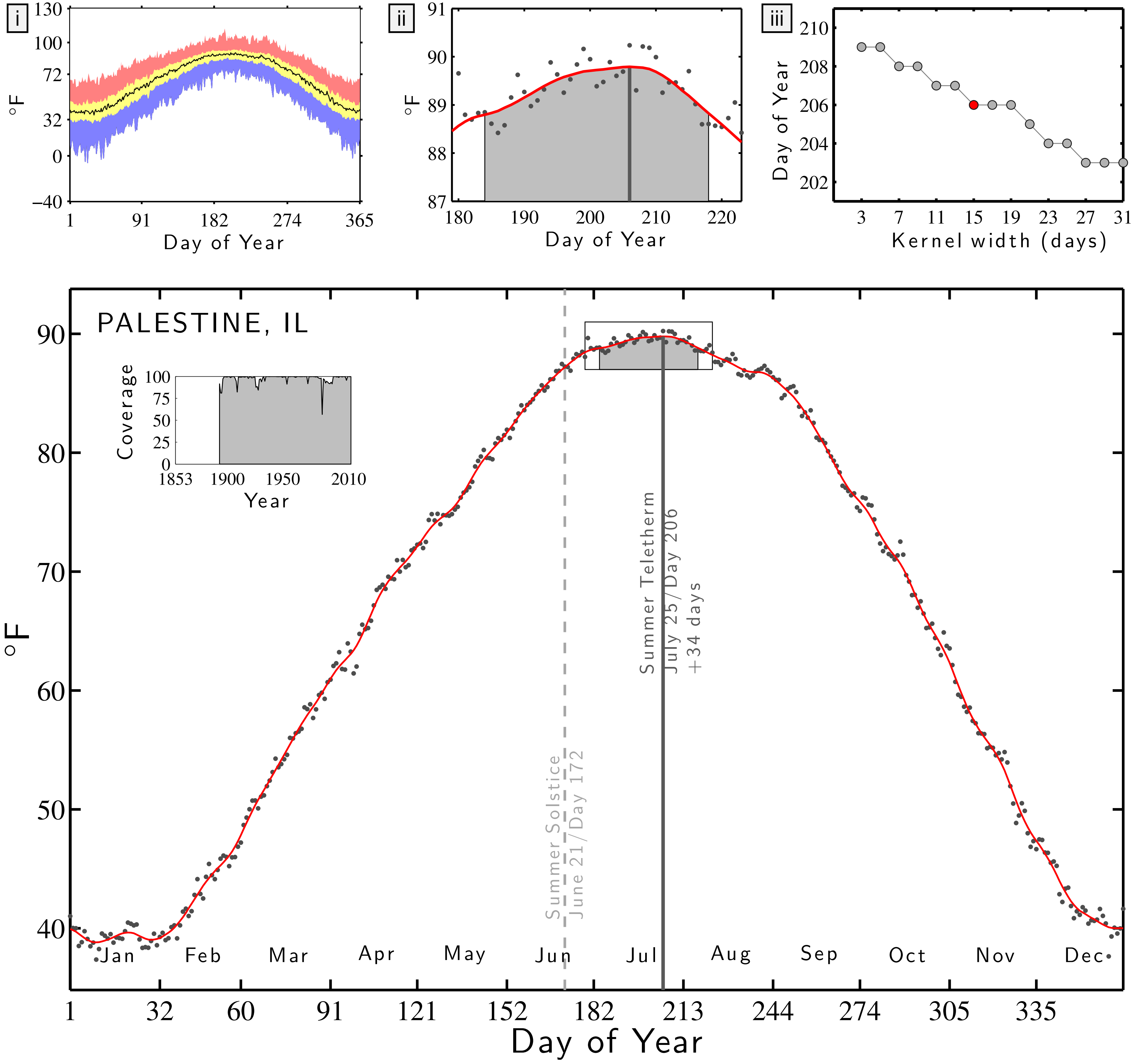
i
ii
iii
Day of Year
Kernel width (days)
°F
Day of Year
PALESTINE, IL
Coverage
Year
Summer Solstice
June 21/Day 172
Summer Teletherm
July 25/Day 206
+34 days
Jan
Feb
Mar
Apr
May
Jun
Jul
Aug
Sep
Oct
Nov
Dec

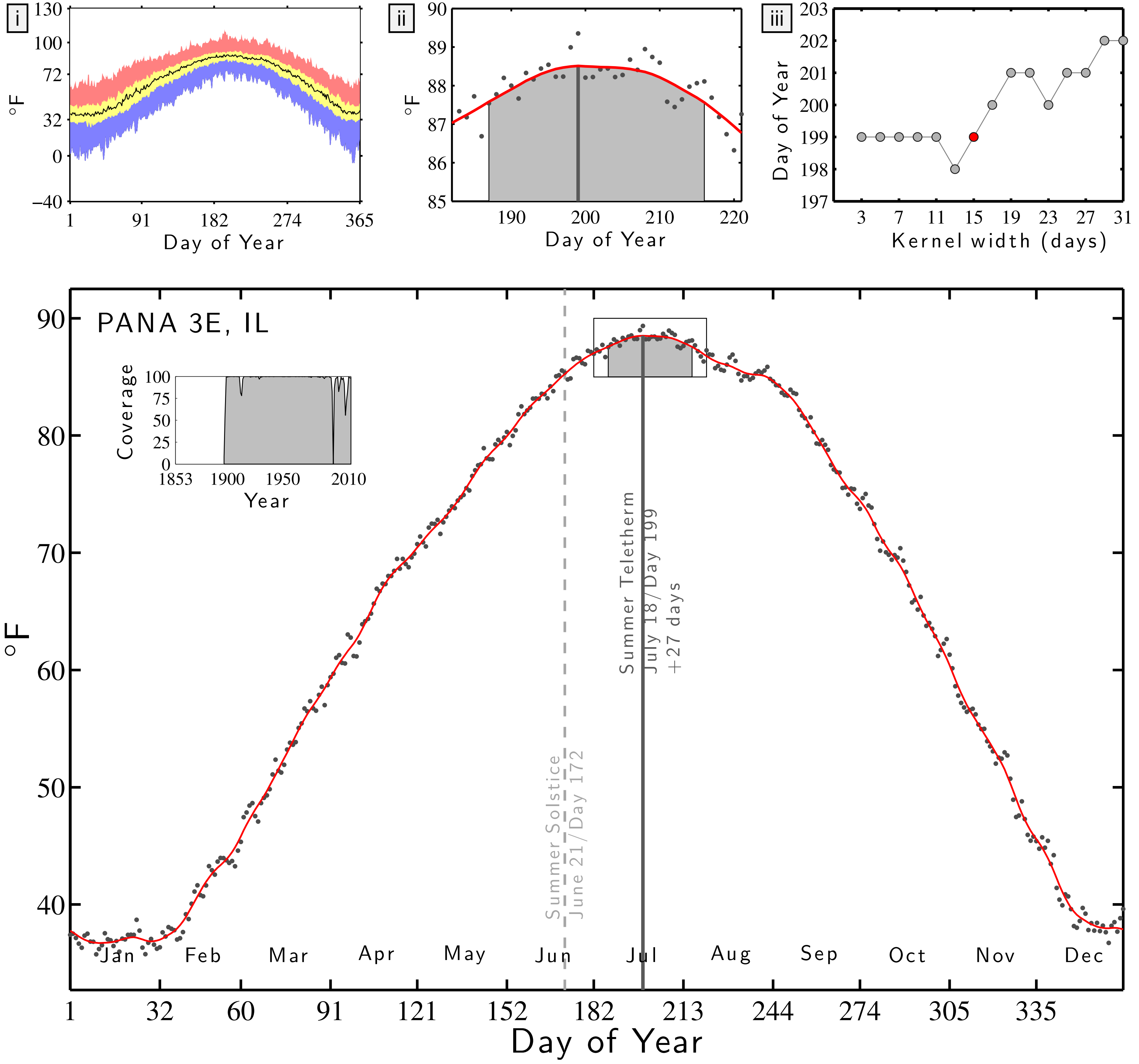

i
°F
Day of Year
ii
iii
Day of Year
Kernel width (days)
PANA 3E, IL
Coverage
Year
Summer Solstice
June 21/Day 172
Summer Teletherm
July 18/Day 199
+27 days
Jan
Feb
Mar
Apr
May
Jun
Jul
Aug
Sep
Oct
Nov
Dec

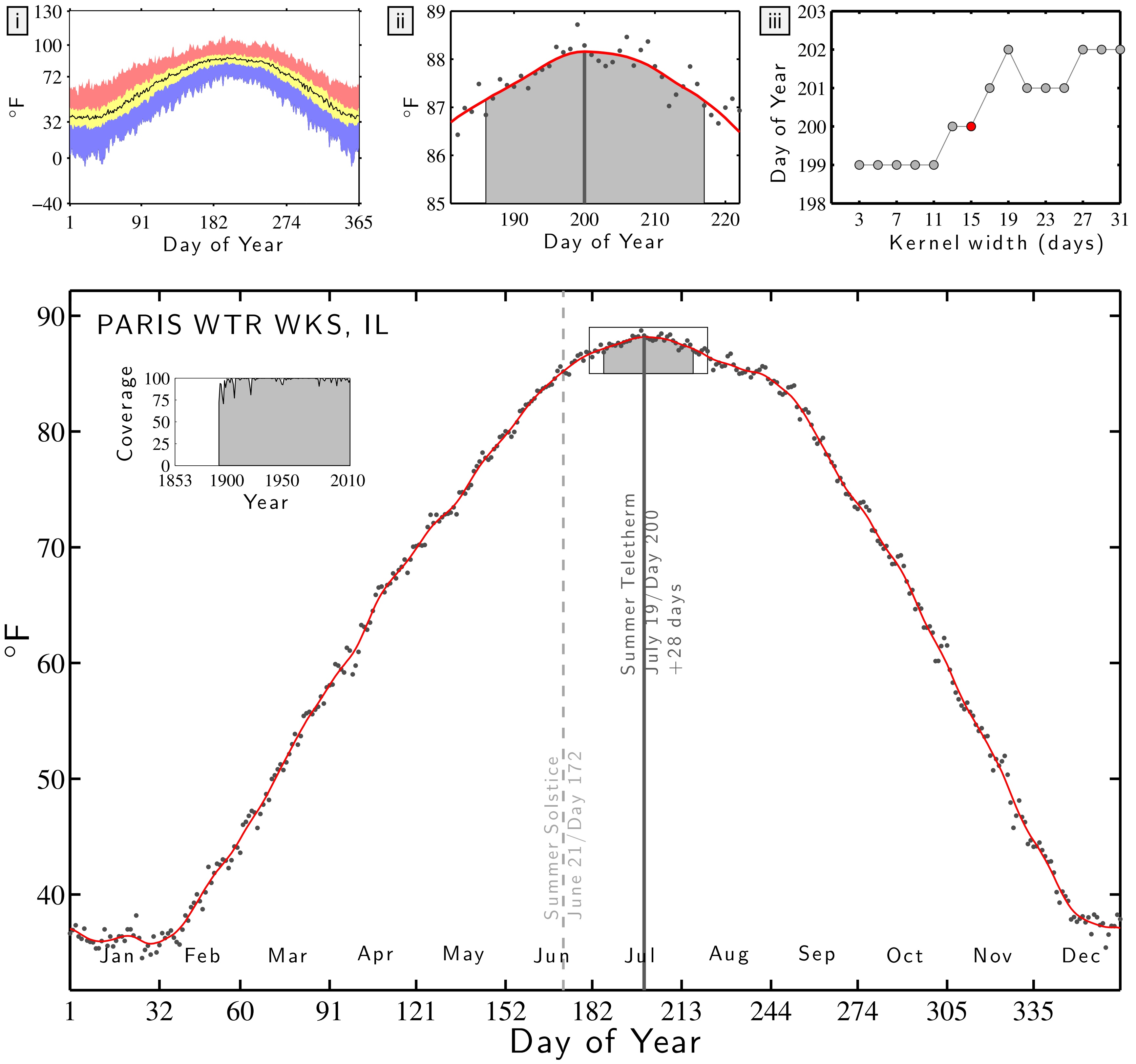

i
ii
iii
°F
Day of Year
Kernel width (days)
PARIS WTR WKS, IL
Coverage
Year
Summer Solstice
June 21/Day 172
Summer Teletherm
July 19/Day 200
+28 days
Jan
Feb
Mar
Apr
May
Jun
Jul
Aug
Sep
Oct
Nov
Dec

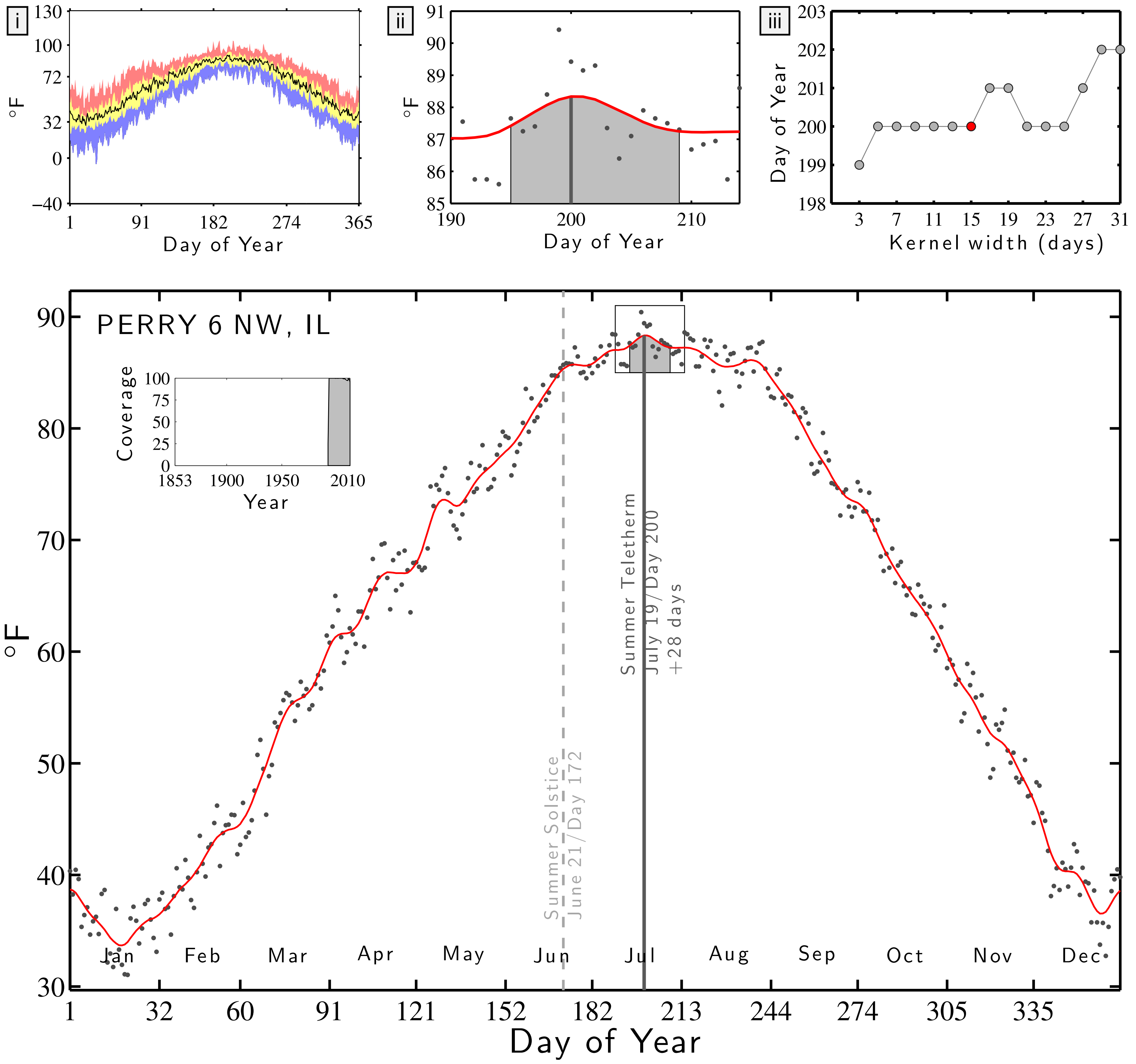

i
°F
Day of Year
ii
°F
Day of Year
iii
Day of Year
Kernel width (days)
PERRY 6 NW, IL
Coverage
Year
Summer Solstice
June 21/Day 172
Summer Teletherm
July 19/Day 200
+28 days
Jan
Feb
Mar
Apr
May
Jun
Jul
Aug
Sep
Oct
Nov
Dec
°F
Day of Year

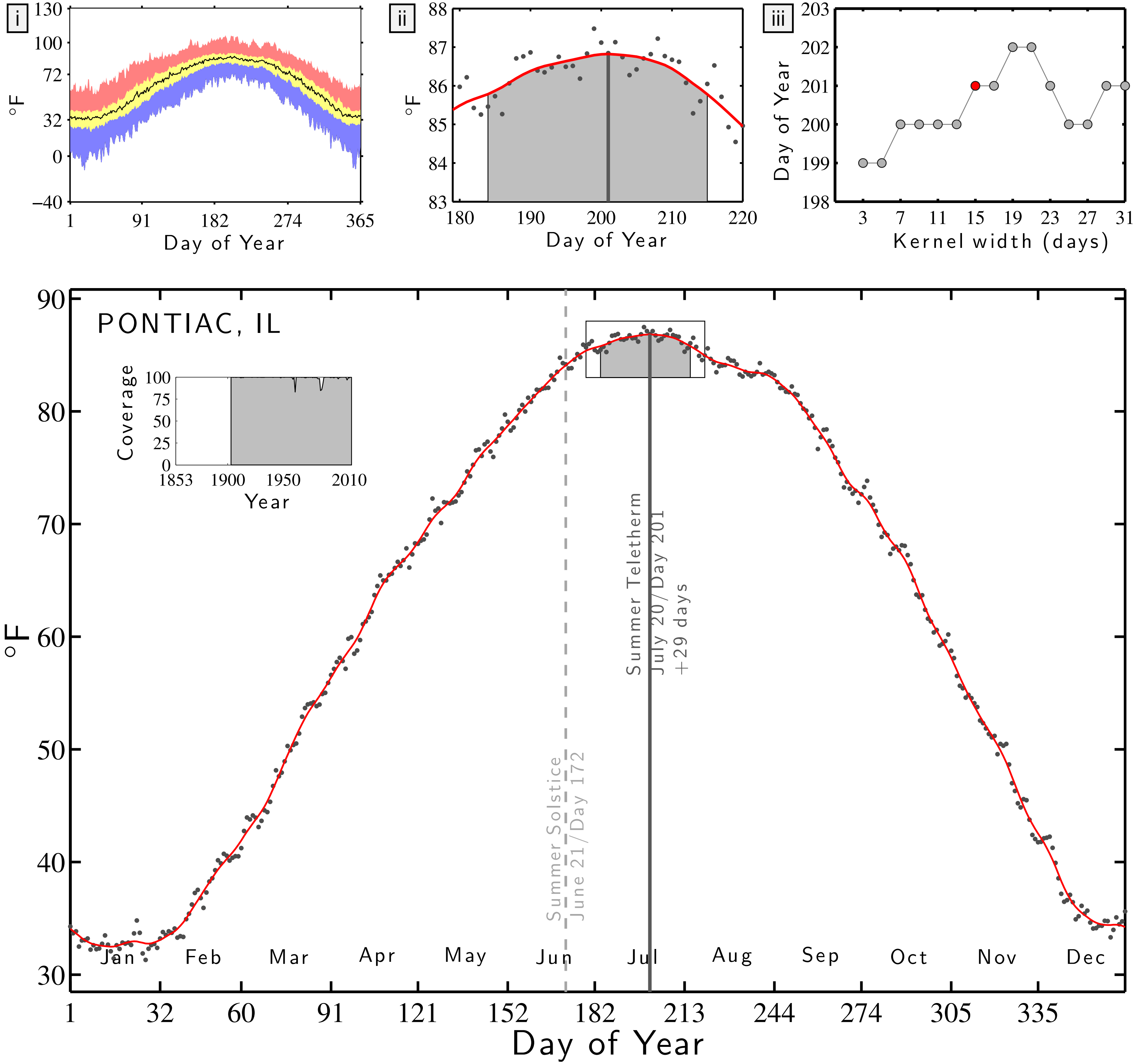

i
°F
Day of Year
ii
°F
Day of Year
iii
Day of Year
Kernel width (days)
PONTIAC, IL
Coverage
Year
Summer Solstice
June 21 / Day 172
Summer Teletherm
July 20 / Day 201
+29 days
Jan
Feb
Mar
Apr
May
Jun
Jul
Aug
Sep
Oct
Nov
Dec
°F
Day of Year

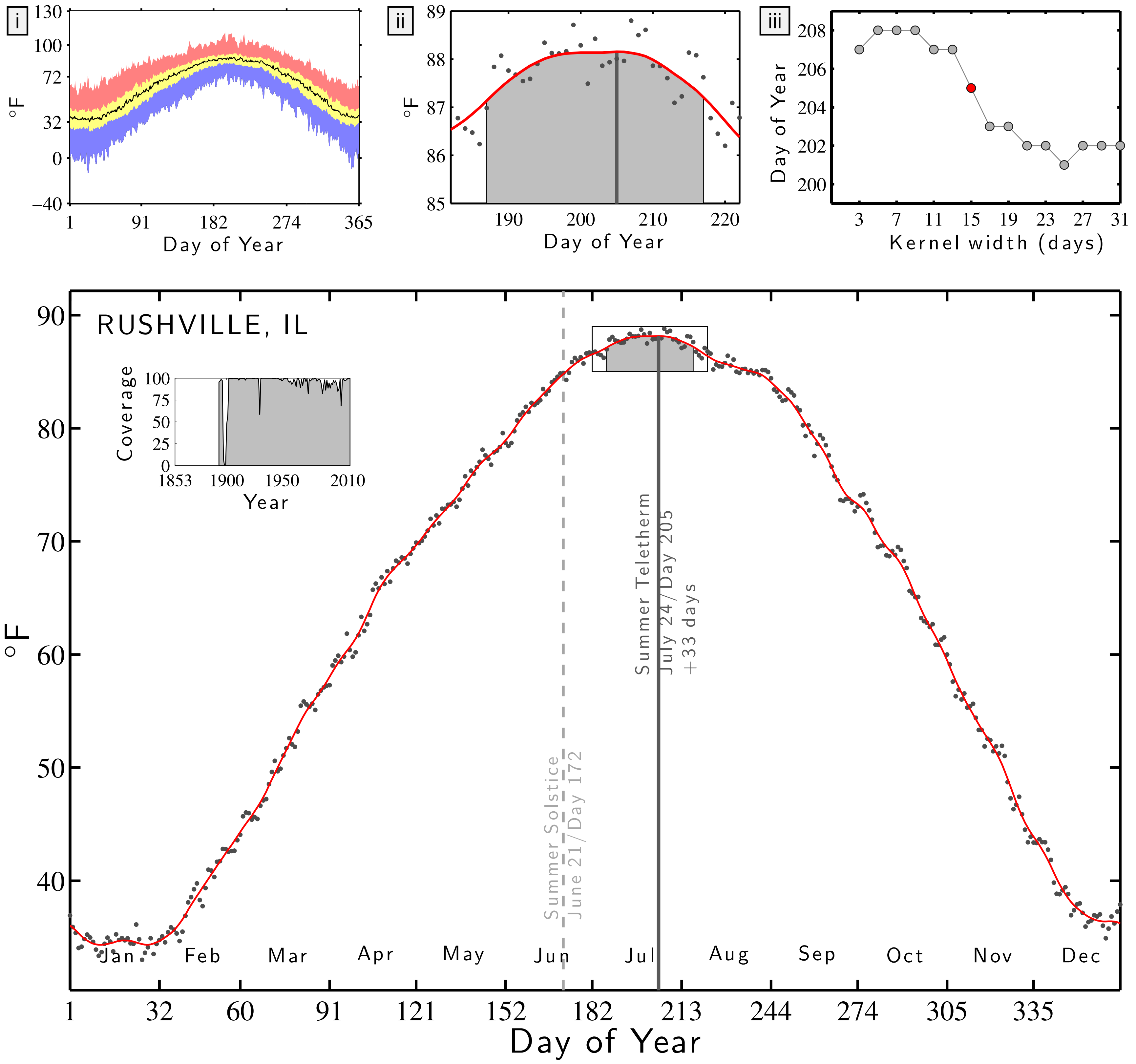

i
130
100
72
32
0
−40
°F
1
91
182
274
365
Day of Year
ii
89
88
87
86
85
°F
190
200
210
220
Day of Year
iii
208
206
204
202
200
Day of Year
3
7
11
15
19
23
27
31
Kernel width (days)
RUSHVILLE, IL
Coverage
100
75
50
25
0
1853
1900
1950
2010
Year
Summer Solstice
June 21 / Day 172
Summer Teletherm
July 24 / Day 205
+33 days
90
80
70
60
50
40
°F
Jan
Feb
Mar
Apr
May
Jun
Jul
Aug
Sep
Oct
Nov
Dec
1
32
60
91
121
152
182
213
244
274
305
335
Day of Year

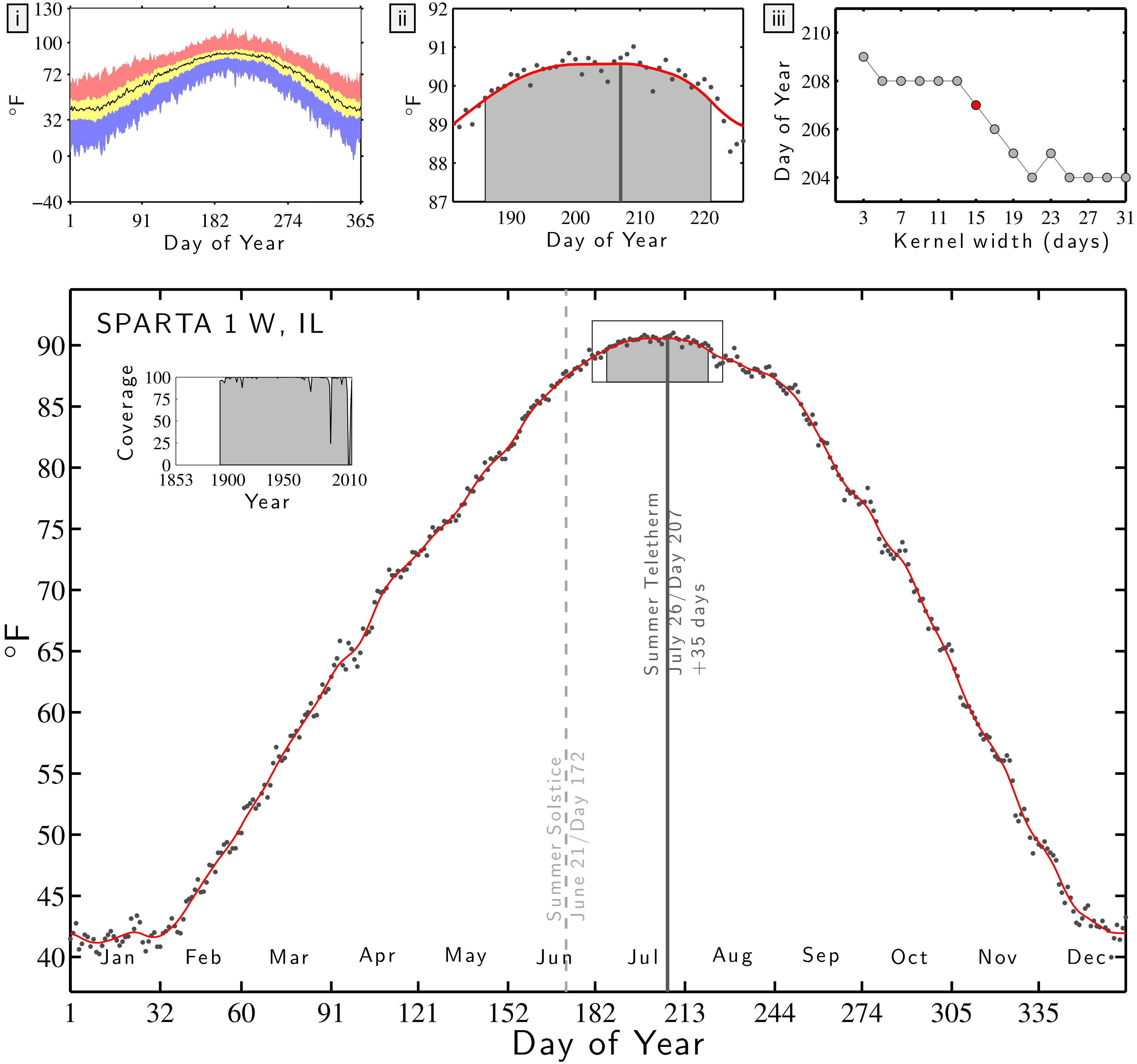

i
°F
Day of Year
ii
Day of Year
iii
Day of Year
Kernel width (days)
SPARTA 1 W, IL
Coverage
Year
Summer Solstice
June 21 / Day 172
Summer Teletherm
July 26 / Day 207
+35 days
Jan
Feb
Mar
Apr
May
Jun
Jul
Aug
Sep
Oct
Nov
Dec
Day of Year

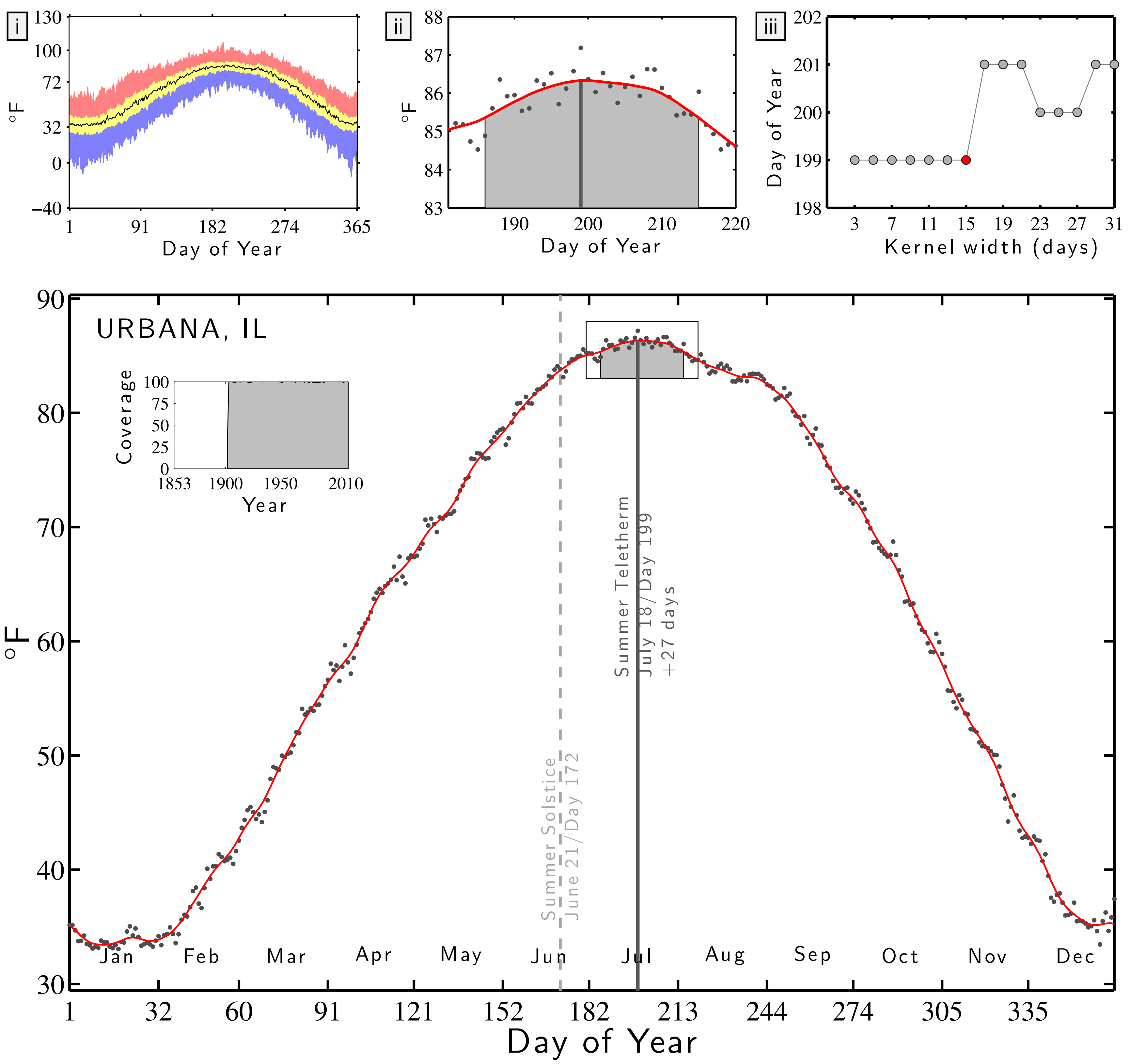

i
°F
130
100
72
32
0
−40
1
91
182
274
365
Day of Year
ii
°F
88
87
86
85
84
83
190
200
210
220
Day of Year
iii
Day of Year
202
201
200
199
198
3
7
11
15
19
23
27
31
Kernel width (days)
URBANA, IL
Coverage
100
75
50
25
0
1853
1900
1950
2010
Year
Summer Solstice
June 21/Day 172
Summer Teletherm
July 18/Day 199
+27 days
Jan
Feb
Mar
Apr
May
Jun
Jul
Aug
Sep
Oct
Nov
Dec
°F
90
80
70
60
50
40
30
1
32
60
91
121
152
182
213
244
274
305
335
Day of Year

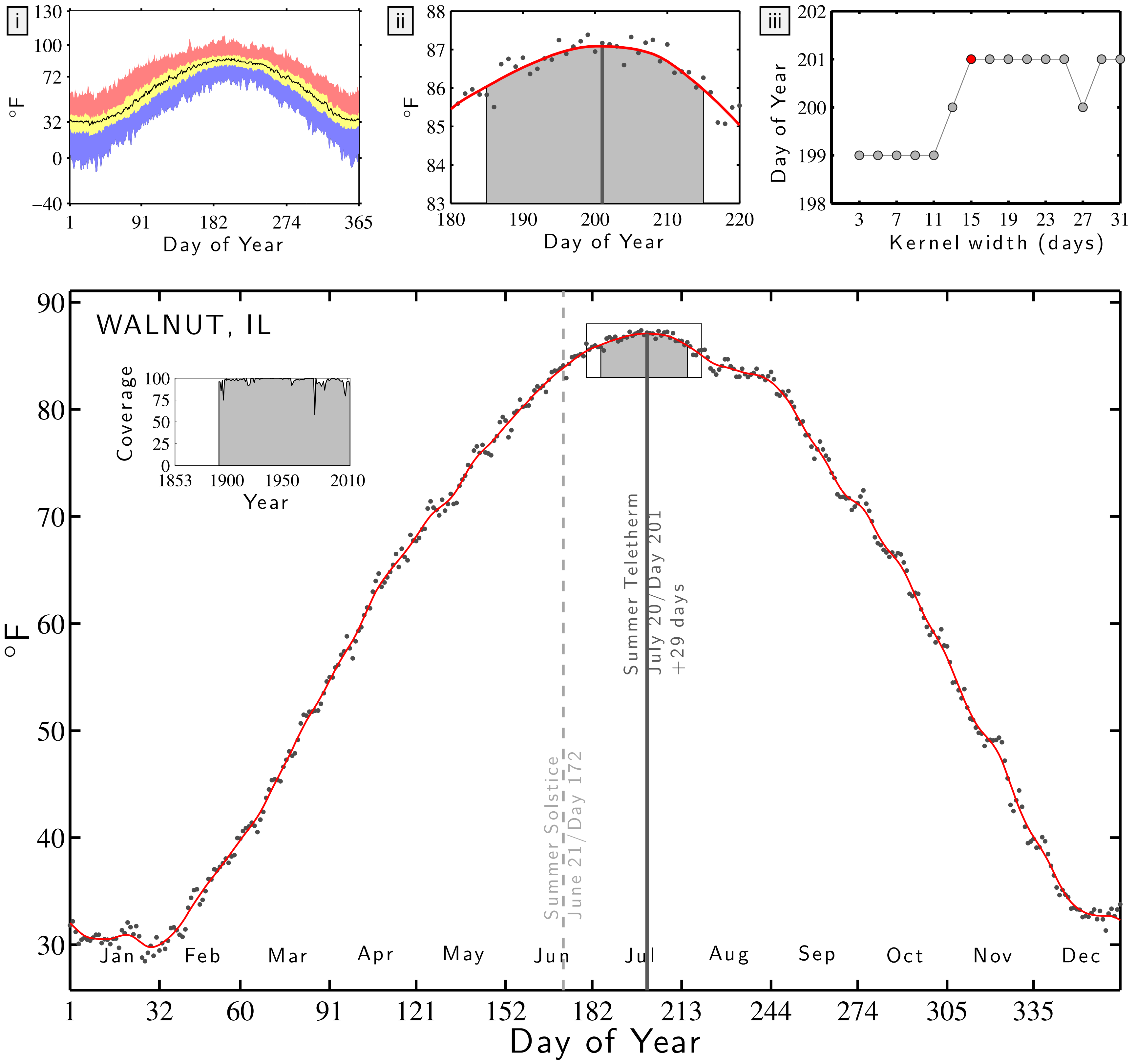

i
°F
Day of Year
ii
°F
Day of Year
iii
Day of Year
Kernel width (days)
WALNUT, IL
Coverage
Year
Summer Solstice
June 21 / Day 172
Summer Teletherm
July 20 / Day 201
+29 days
Jan
Feb
Mar
Apr
May
Jun
Jul
Aug
Sep
Oct
Nov
Dec
°F
Day of Year

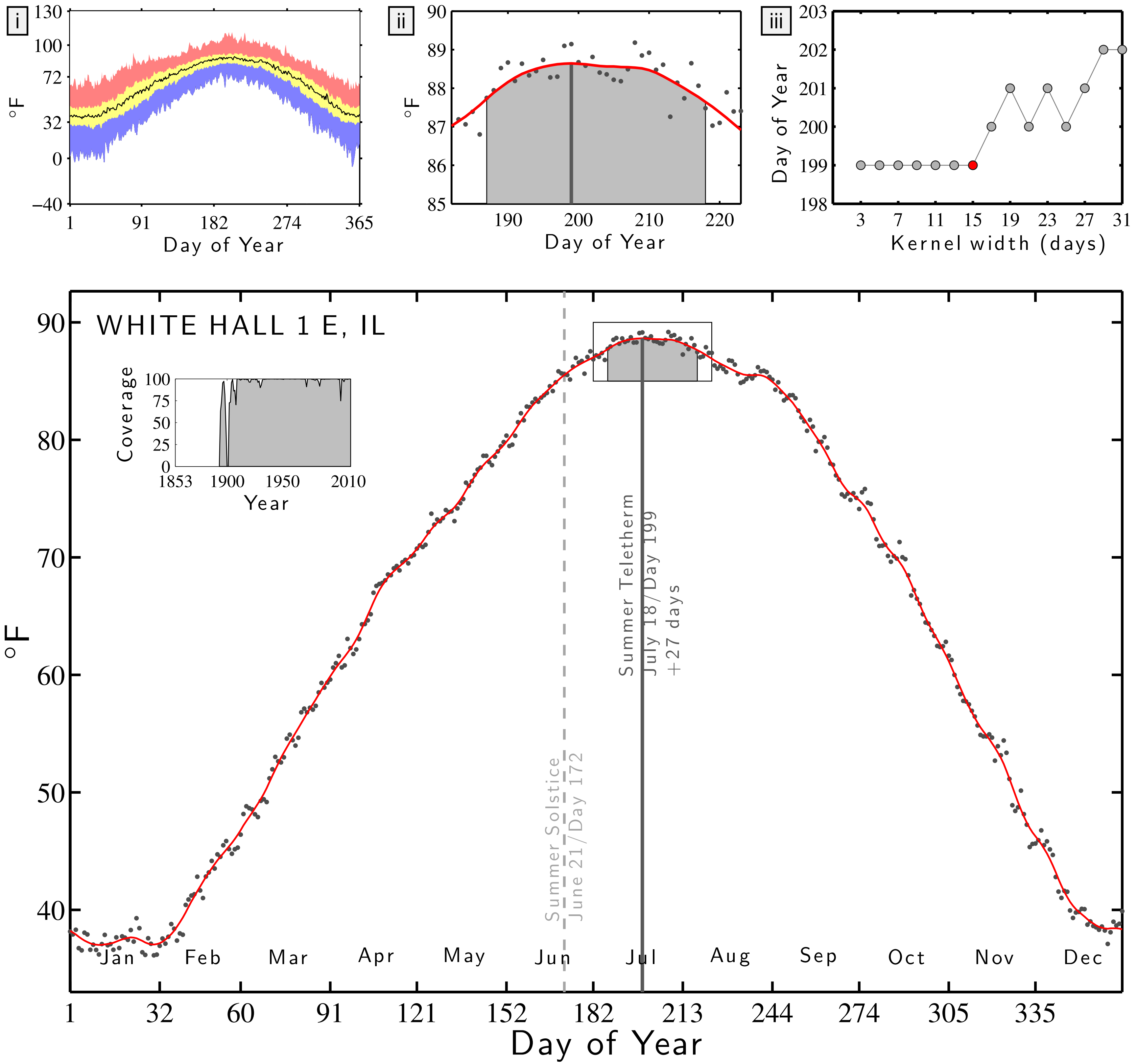

i
ii
iii
°F
Day of Year
Day of Year
Kernel width (days)
WHITE HALL 1 E, IL
Coverage
Year
Summer Solstice
June 21/Day 172
Summer Teletherm
July 18/Day 199
+27 days
Jan
Feb
Mar
Apr
May
Jun
Jul
Aug
Sep
Oct
Nov
Dec
Day of Year

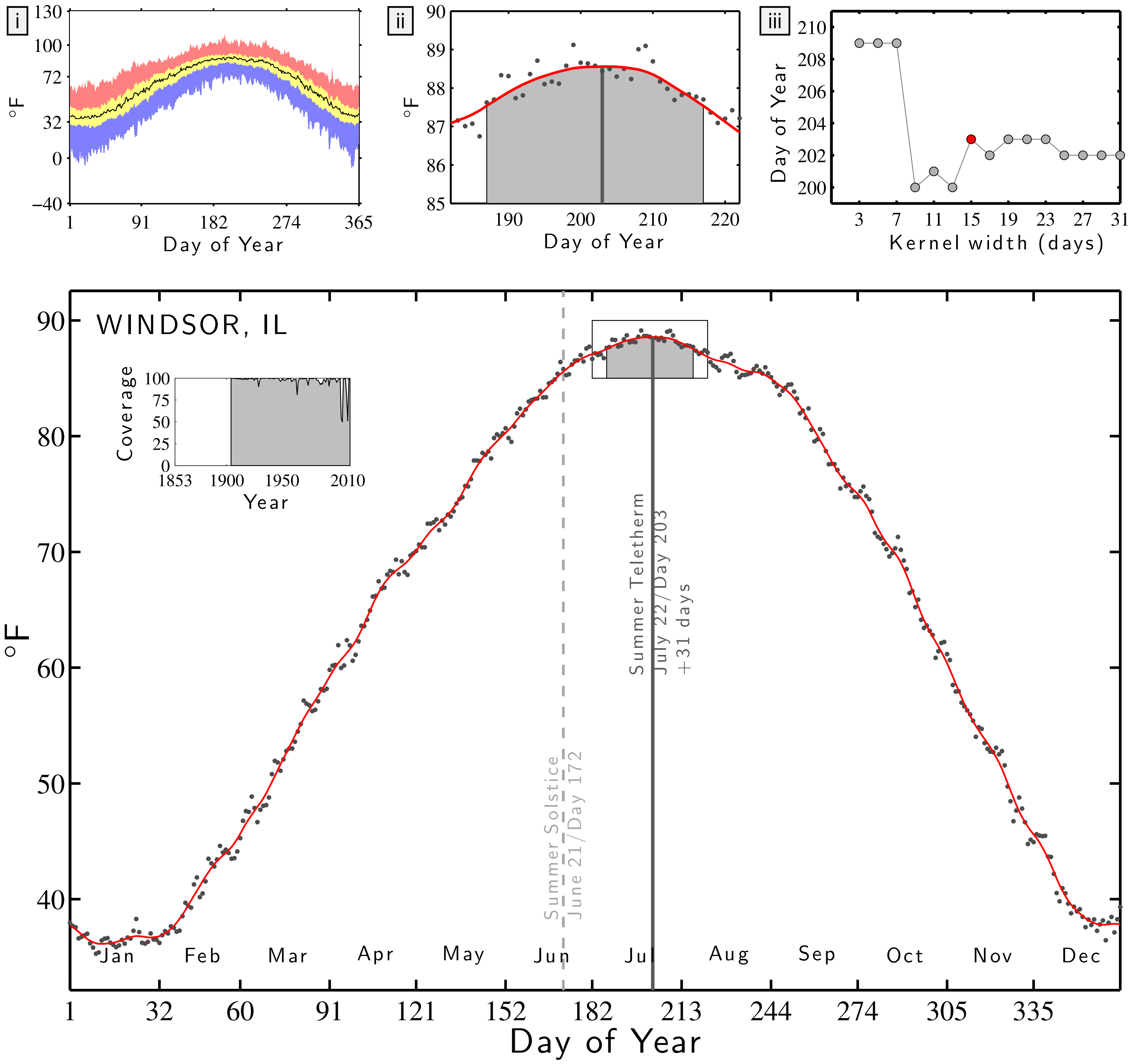

i
°F
Day of Year
ii
Day of Year
iii
Day of Year
Kernel width (days)
WINDSOR, IL
Coverage
Year
Summer Solstice
June 21/Day 172
Summer Teletherm
July 22/Day 203
+31 days
Jan
Feb
Mar
Apr
May
Jun
Jul
Aug
Sep
Oct
Nov
Dec
Day of Year

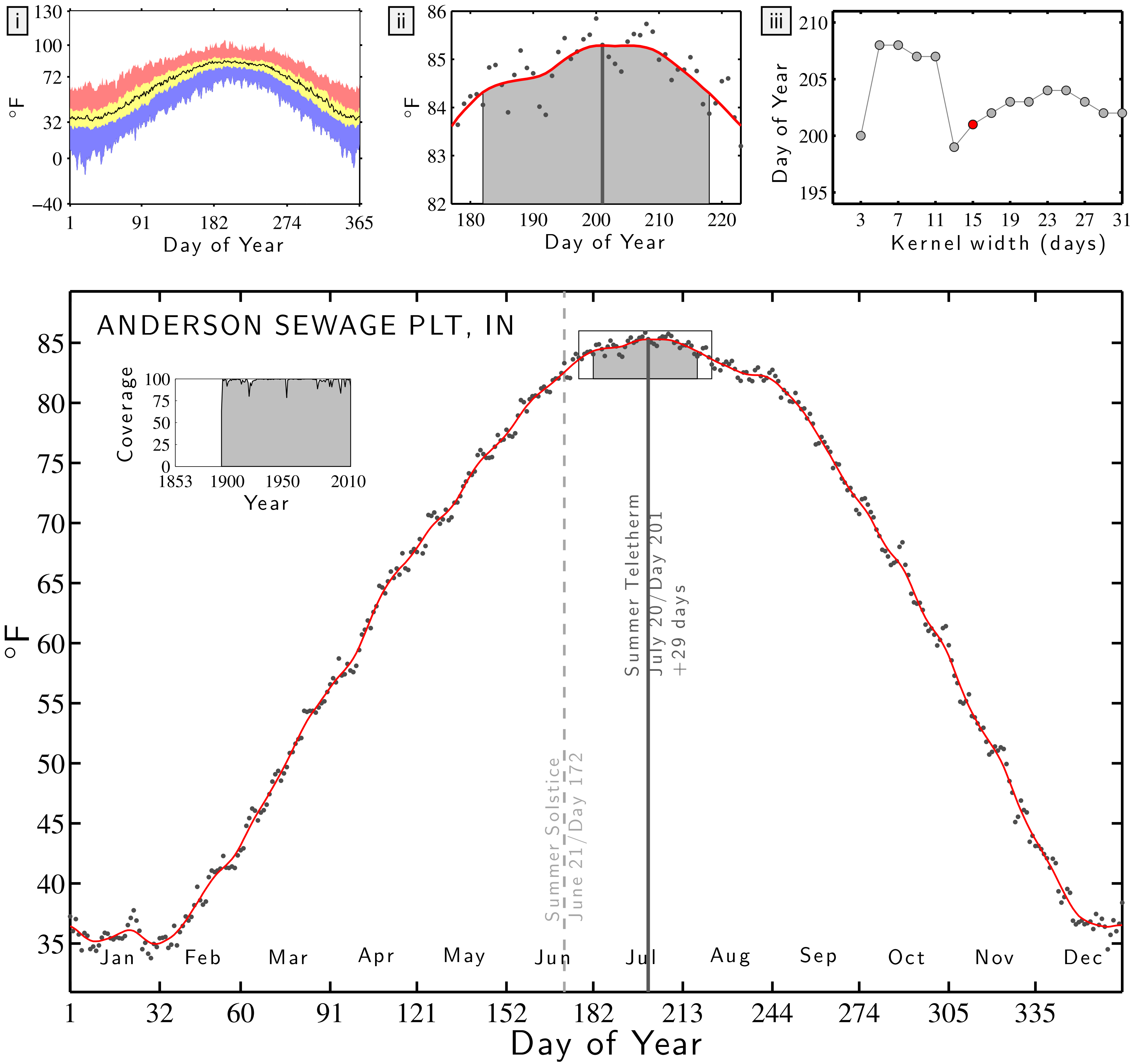

i
°F
Day of Year
ii
°F
Day of Year
iii
Day of Year
Kernel width (days)
ANDERSON SEWAGE PLT, IN
Coverage
Year
Summer Solstice
June 21 / Day 172
Summer Teletherm
July 20 / Day 201
+29 days
Jan
Feb
Mar
Apr
May
Jun
Jul
Aug
Sep
Oct
Nov
Dec
°F
Day of Year

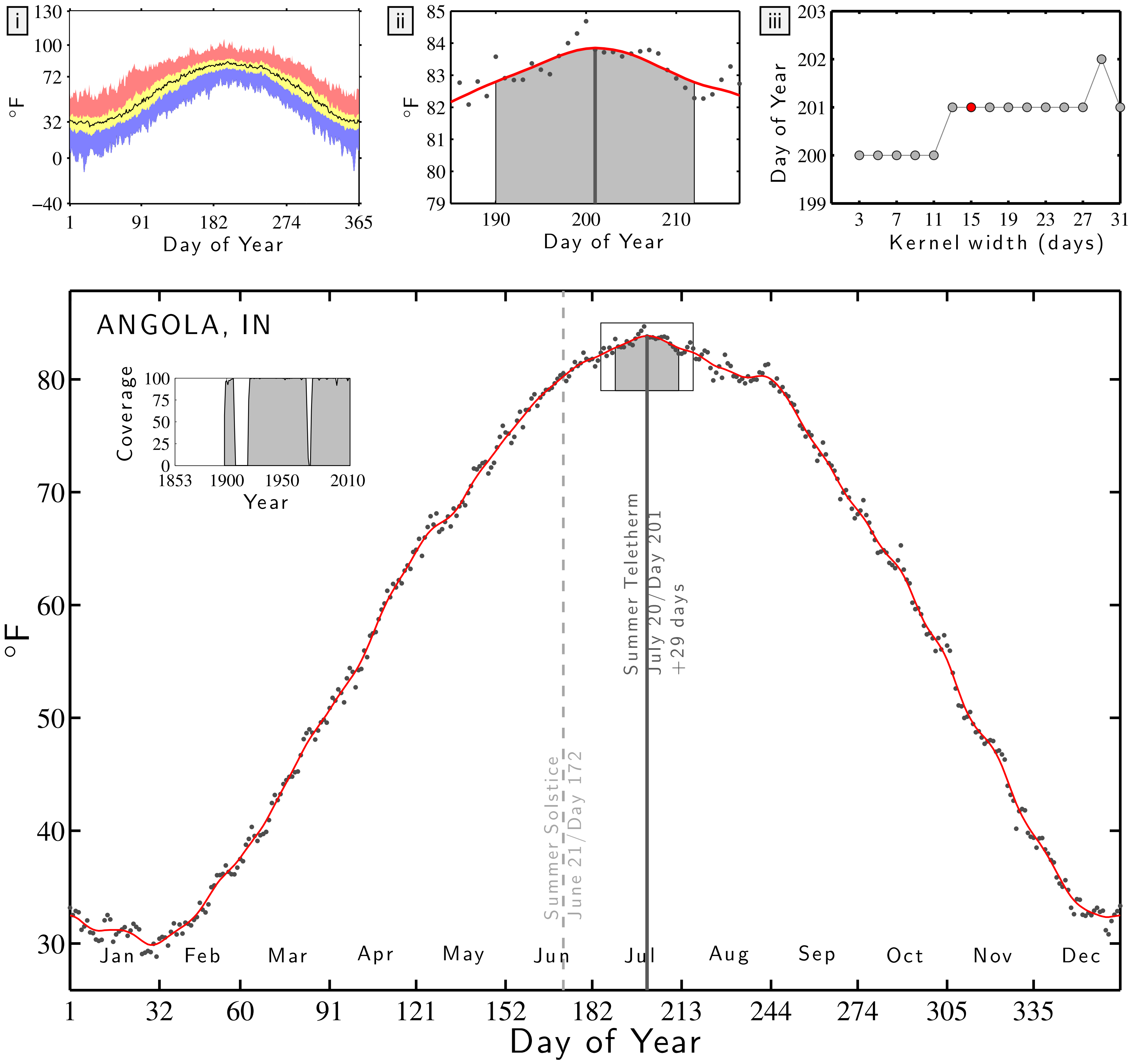

i
°F
130
100
72
32
0
−40
1
91
182
274
365
Day of Year
ii
85
84
83
82
81
80
79
190
200
210
iii
Day of Year
203
202
201
200
199
3
7
11
15
19
23
27
31
Kernel width (days)
ANGOLA, IN
Coverage
100
75
50
25
0
1853
1900
1950
2010
Year
Summer Solstice
June 21/Day 172
Summer Teletherm
July 20/Day 201
+29 days
80
70
60
50
40
30
Jan
Feb
Mar
Apr
May
Jun
Jul
Aug
Sep
Oct
Nov
Dec
1
32
60
91
121
152
182
213
244
274
305
335
Day of Year

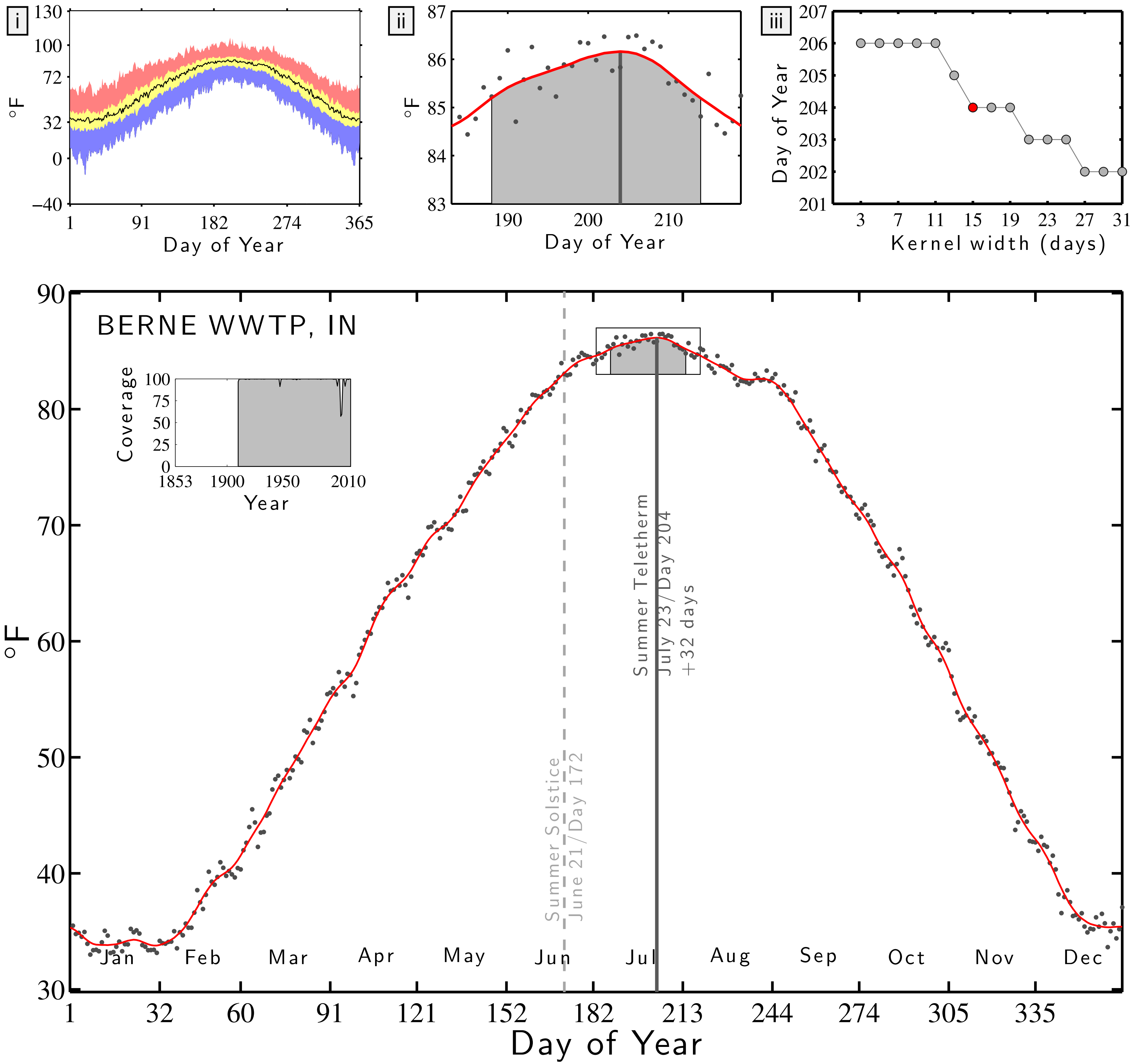

i
ii
iii
°F
Day of Year
Kernel width (days)
BERNE WWTP, IN
Coverage
Year
Summer Solstice
June 21 / Day 172
Summer Teletherm
July 23 / Day 204
+32 days
Jan
Feb
Mar
Apr
May
Jun
Jul
Aug
Sep
Oct
Nov
Dec

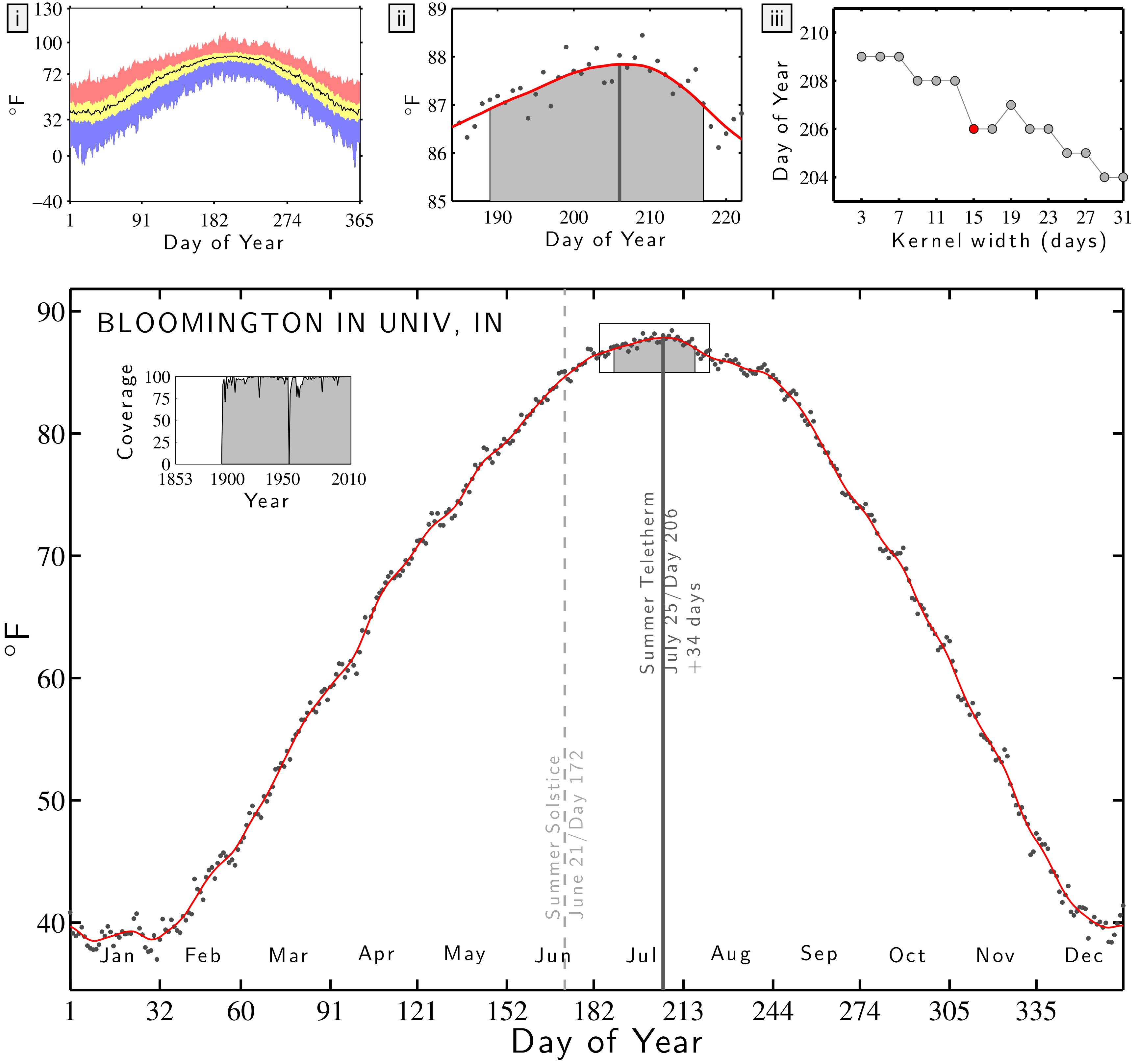

i
ii
iii
Day of Year
Kernel width (days)
°F
Day of Year
BLOOMINGTON IN UNIV, IN
Coverage
Year
Summer Solstice
June 21/Day 172
Summer Teletherm
July 25/Day 206
+34 days
Jan
Feb
Mar
Apr
May
Jun
Jul
Aug
Sep
Oct
Nov
Dec

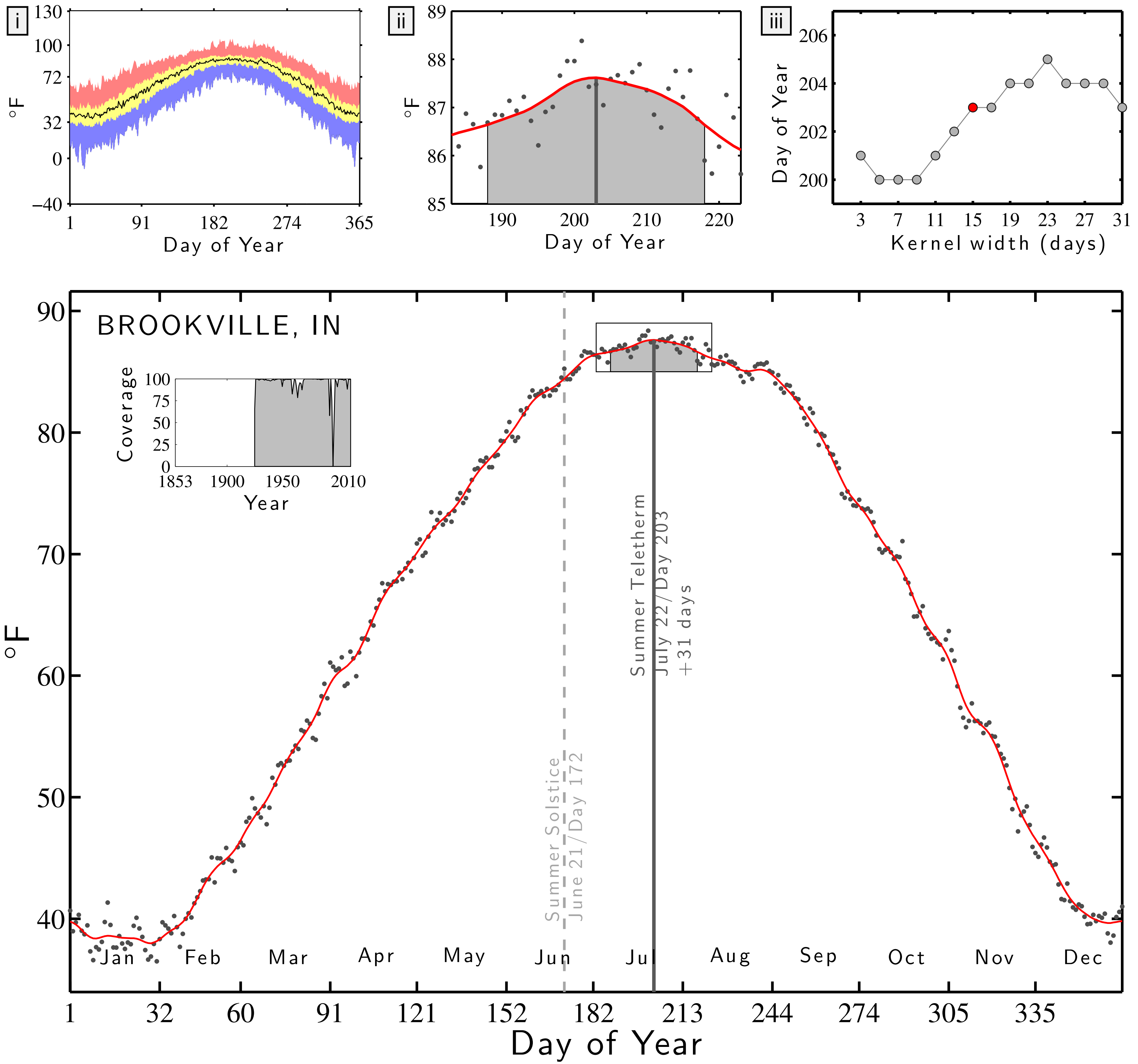

i
ii
iii
°F
Day of Year
Kernel width (days)
BROOKVILLE, IN
Coverage
Year
Summer Solstice
June 21/Day 172
Summer Teletherm
July 22/Day 203
+31 days
Jan
Feb
Mar
Apr
May
Jun
Jul
Aug
Sep
Oct
Nov
Dec

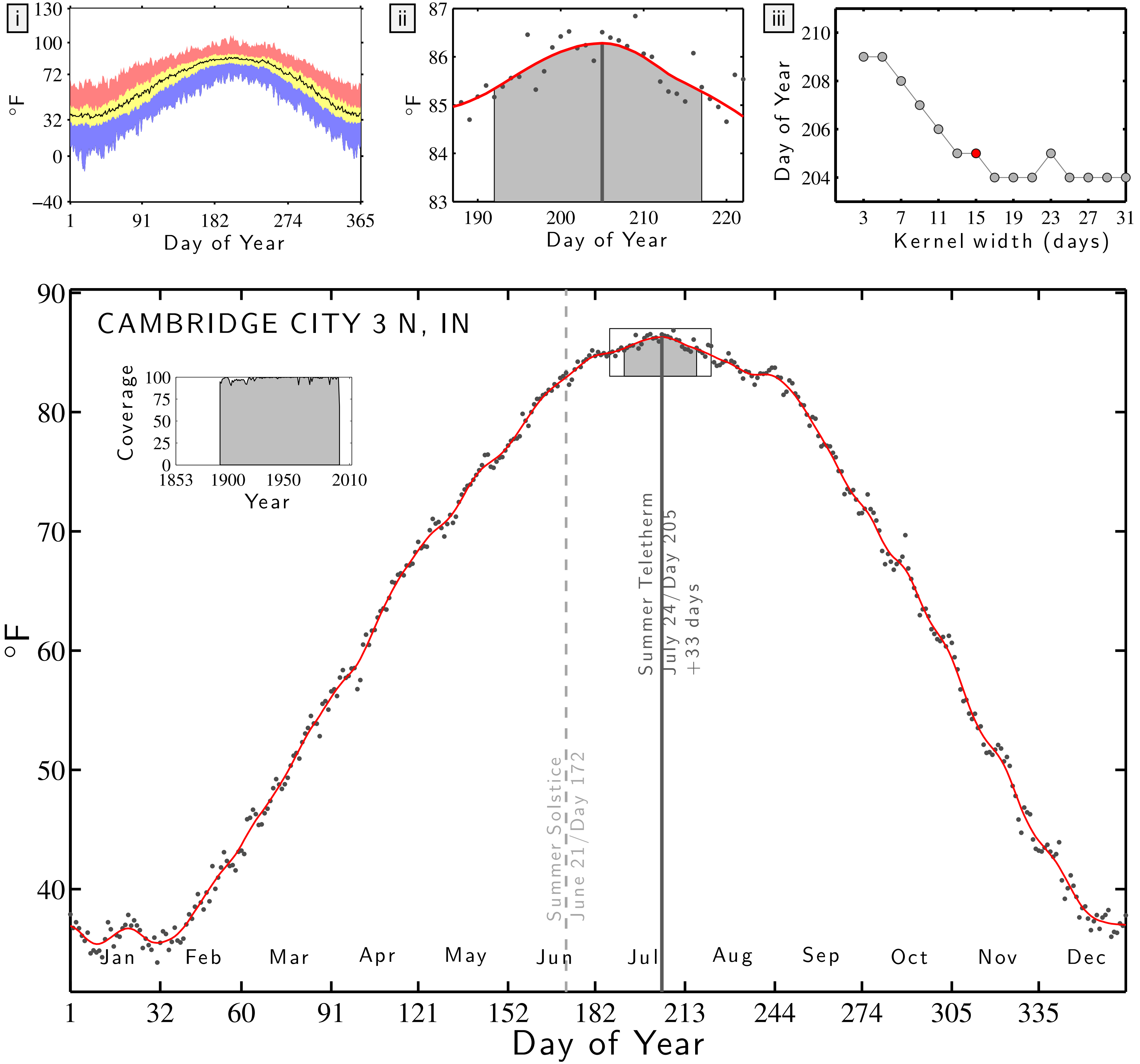

i
°F
Day of Year
ii
°F
Day of Year
iii
Day of Year
Kernel width (days)
CAMBRIDGE CITY 3 N, IN
Coverage
Year
Summer Solstice
June 21/Day 172
Summer Teletherm
July 24/Day 205
+33 days
Jan
Feb
Mar
Apr
May
Jun
Jul
Aug
Sep
Oct
Nov
Dec
°F
Day of Year

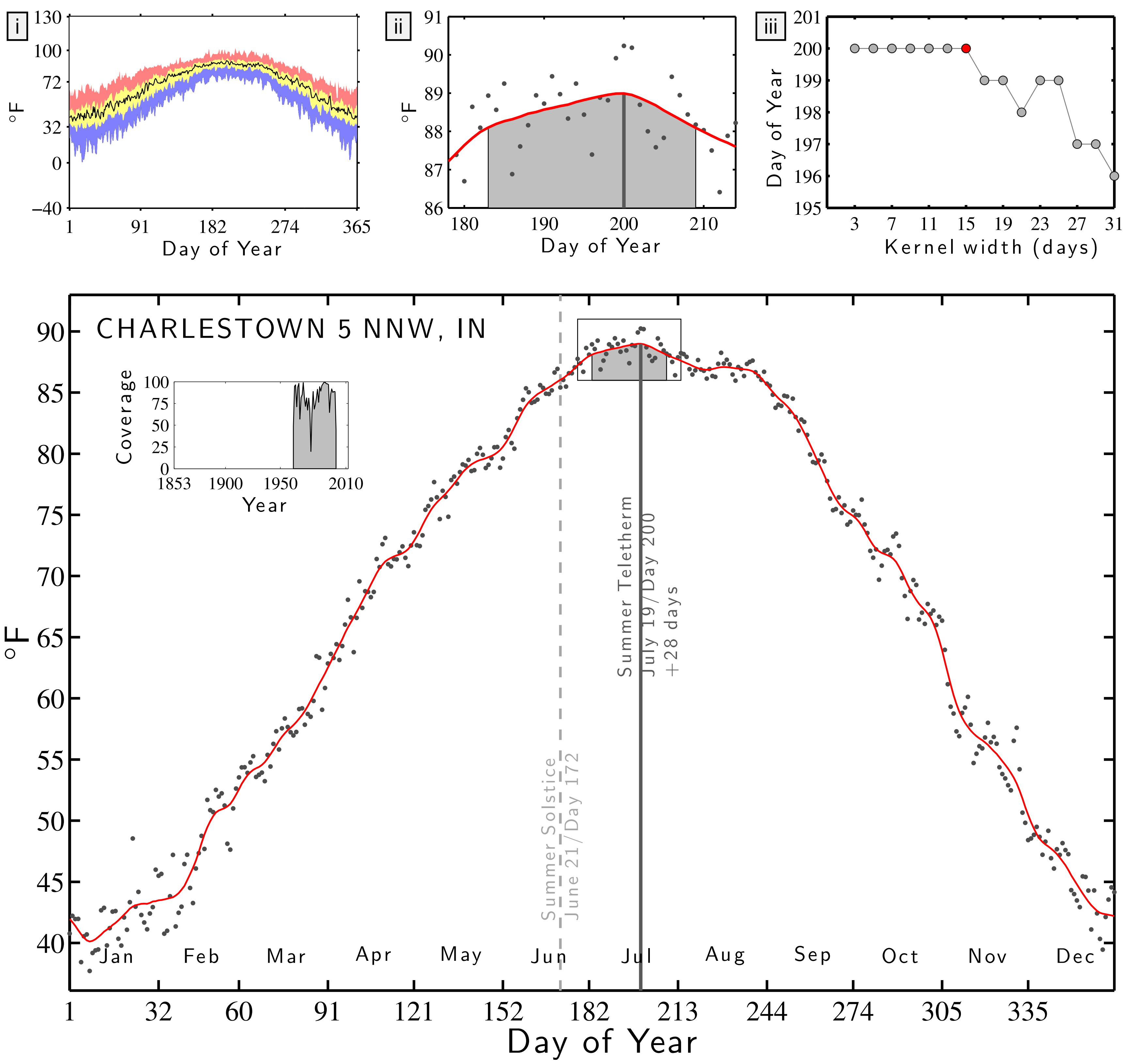

i
°F
Day of Year
ii
°F
Day of Year
iii
Day of Year
Kernel width (days)
CHARLESTOWN 5 NNW, IN
Coverage
Year
Summer Solstice
June 21/Day 172
Summer Teletherm
July 19/Day 200
+28 days
Jan
Feb
Mar
Apr
May
Jun
Jul
Aug
Sep
Oct
Nov
Dec
°F
Day of Year

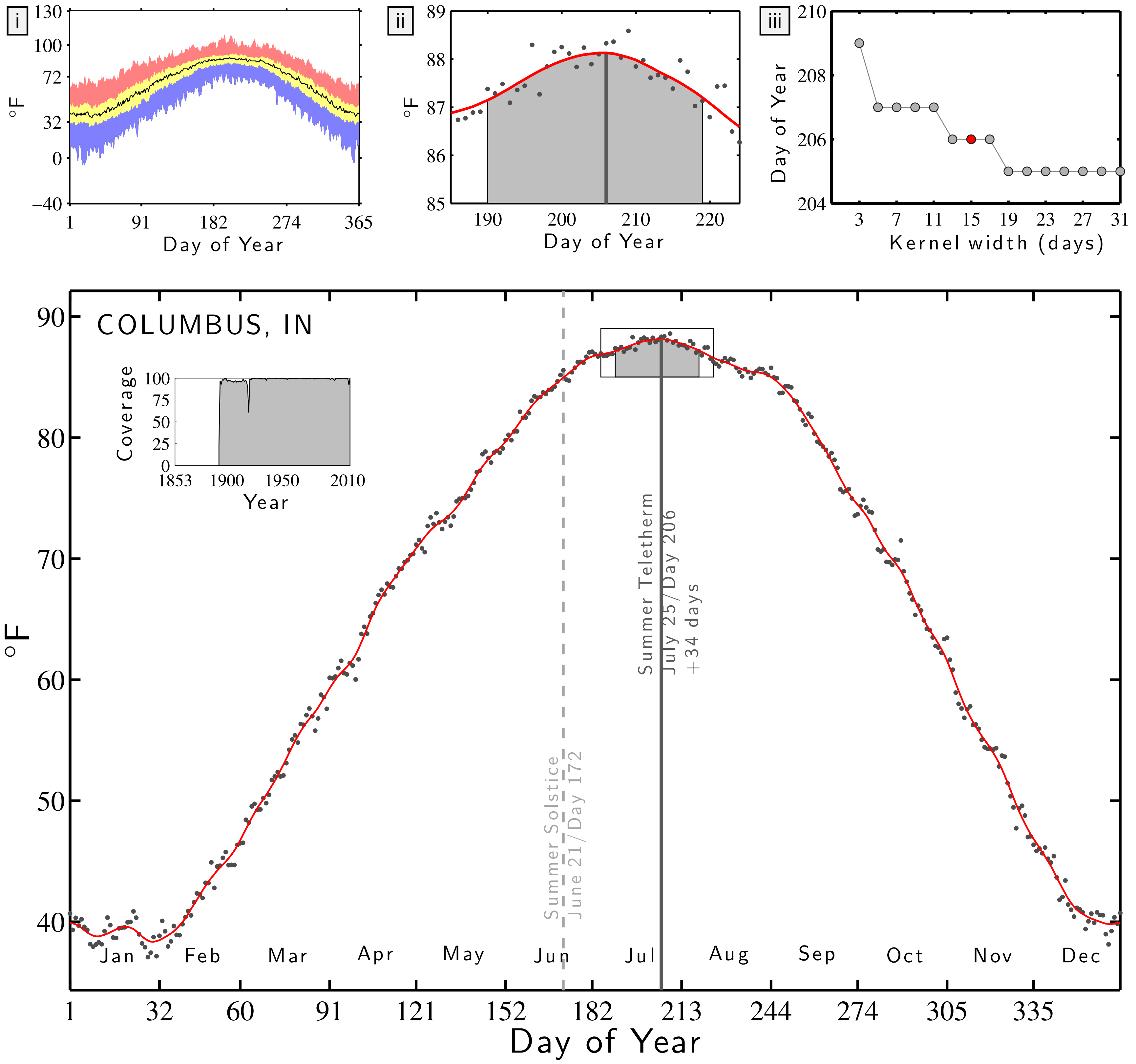

i
°F
Day of Year
ii
°F
Day of Year
iii
Day of Year
Kernel width (days)
COLUMBUS, IN
Coverage
Year
Summer Solstice
June 21/Day 172
Summer Teletherm
July 25/Day 206
+34 days
Jan
Feb
Mar
Apr
May
Jun
Jul
Aug
Sep
Oct
Nov
Dec
°F
Day of Year

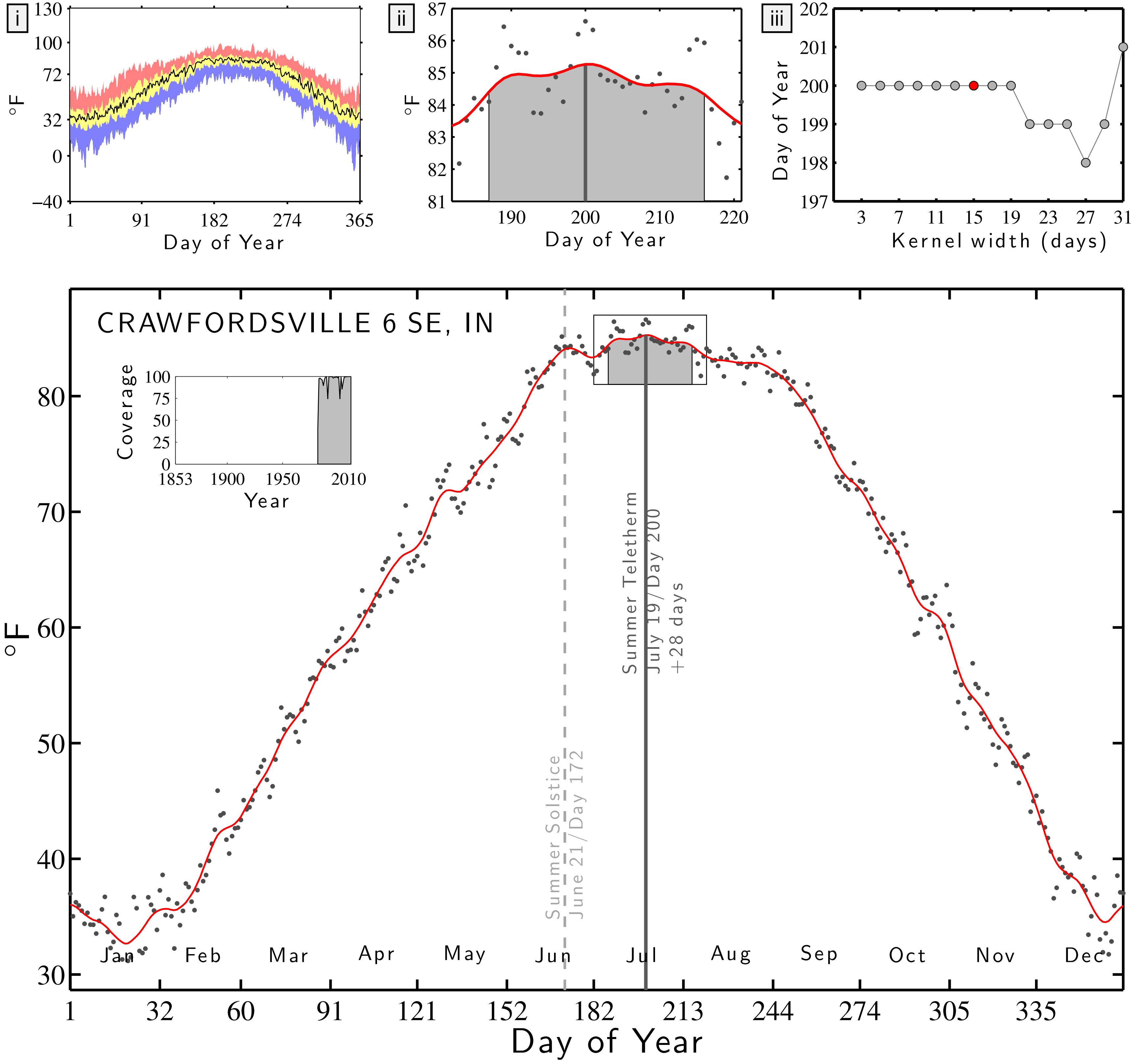

i
ii
iii
Day of Year
Day of Year
Kernel width (days)
CRAWFORDSVILLE 6 SE, IN
Coverage
Year
Summer Solstice
June 21/Day 172
Summer Teletherm
July 19/Day 200
+28 days
Jan
Feb
Mar
Apr
May
Jun
Jul
Aug
Sep
Oct
Nov
Dec
Day of Year

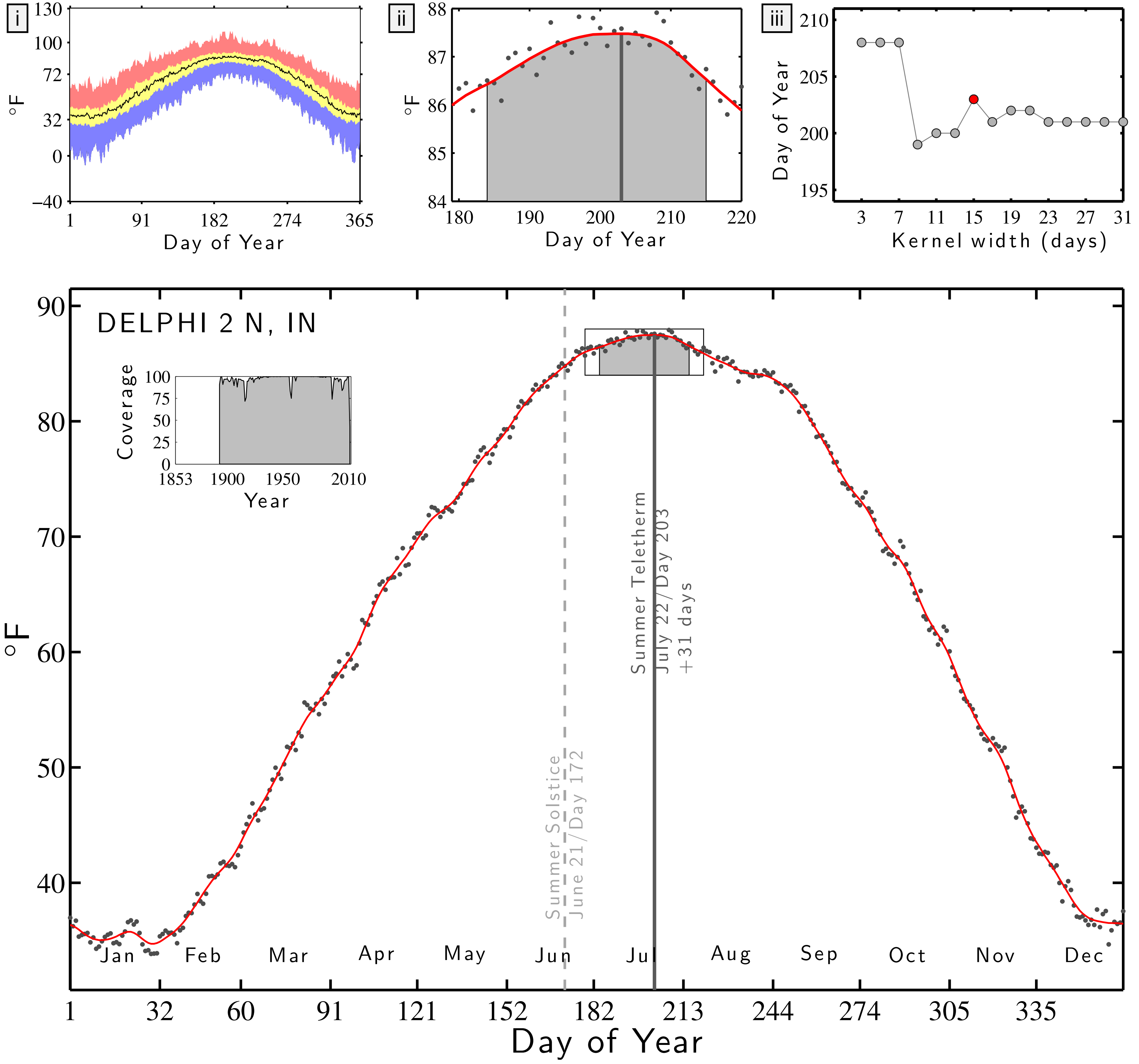

i
ii
iii
°F
Day of Year
Day of Year
Kernel width (days)
DELPHI 2 N, IN
Coverage
Year
Summer Solstice
June 21 / Day 172
Summer Teletherm
July 22 / Day 203
+31 days
Jan
Feb
Mar
Apr
May
Jun
Jul
Aug
Sep
Oct
Nov
Dec
Day of Year

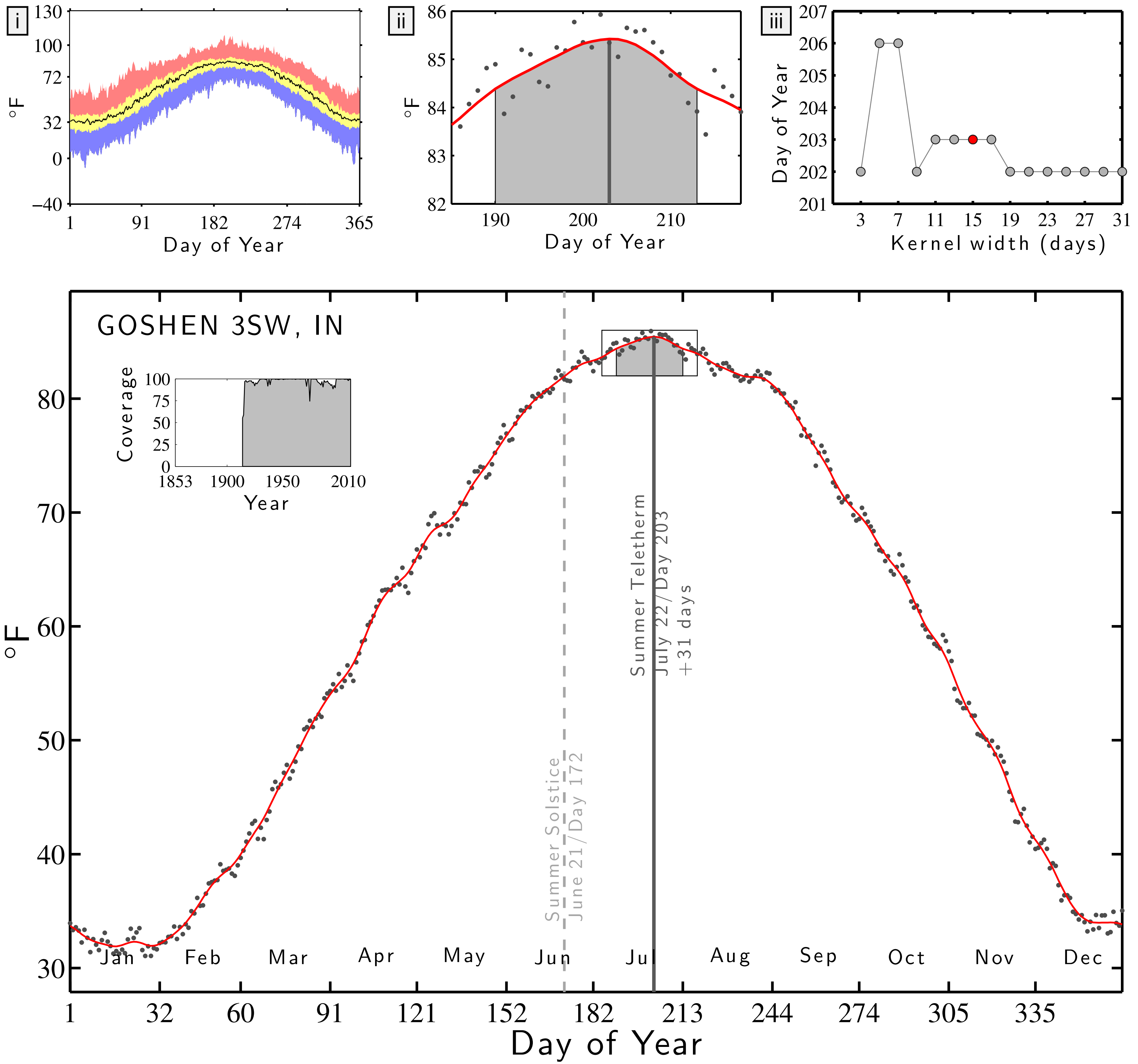

i
°F
Day of Year
ii
°F
Day of Year
iii
Day of Year
Kernel width (days)
GOSHEN 3SW, IN
Coverage
Year
Summer Solstice
June 21/Day 172
Summer Teletherm
July 22/Day 203
+31 days
Jan
Feb
Mar
Apr
May
Jun
Jul
Aug
Sep
Oct
Nov
Dec
°F
Day of Year

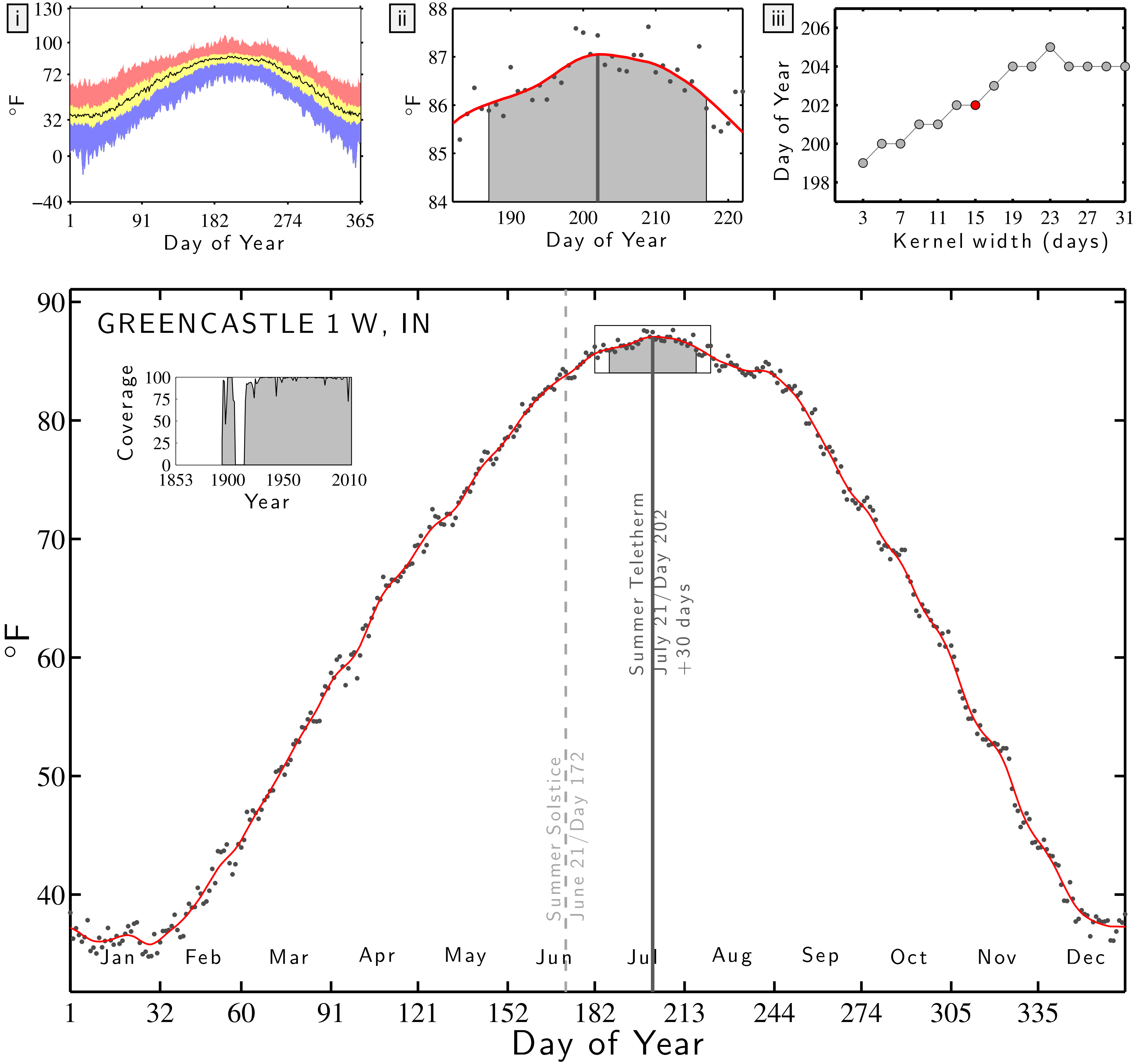

i
ii
iii
°F
Day of Year
Day of Year
Kernel width (days)
GREENCASTLE 1 W, IN
Coverage
Year
1853
1900
1950
2010
Summer Solstice
June 21/Day 172
Summer Teletherm
July 21/Day 202
+30 days
Jan
Feb
Mar
Apr
May
Jun
Jul
Aug
Sep
Oct
Nov
Dec
1
32
60
91
121
152
182
213
244
274
305
335

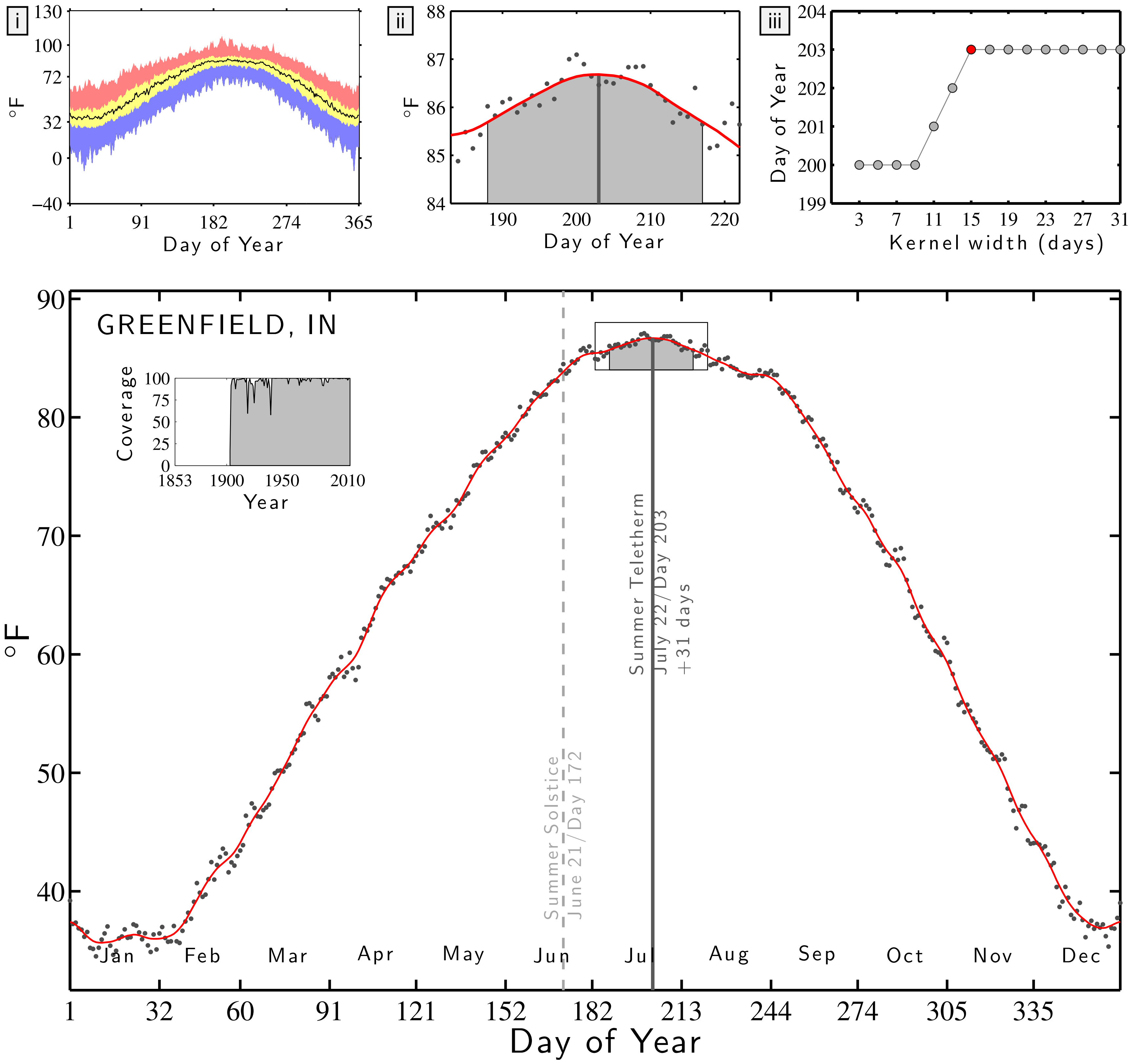

i
°F
Day of Year
ii
°F
Day of Year
iii
Day of Year
Kernel width (days)
GREENFIELD, IN
Coverage
Year
Summer Solstice
June 21/Day 172
Summer Teletherm
July 22/Day 203
+31 days
Jan
Feb
Mar
Apr
May
Jun
Jul
Aug
Sep
Oct
Nov
Dec
°F
Day of Year

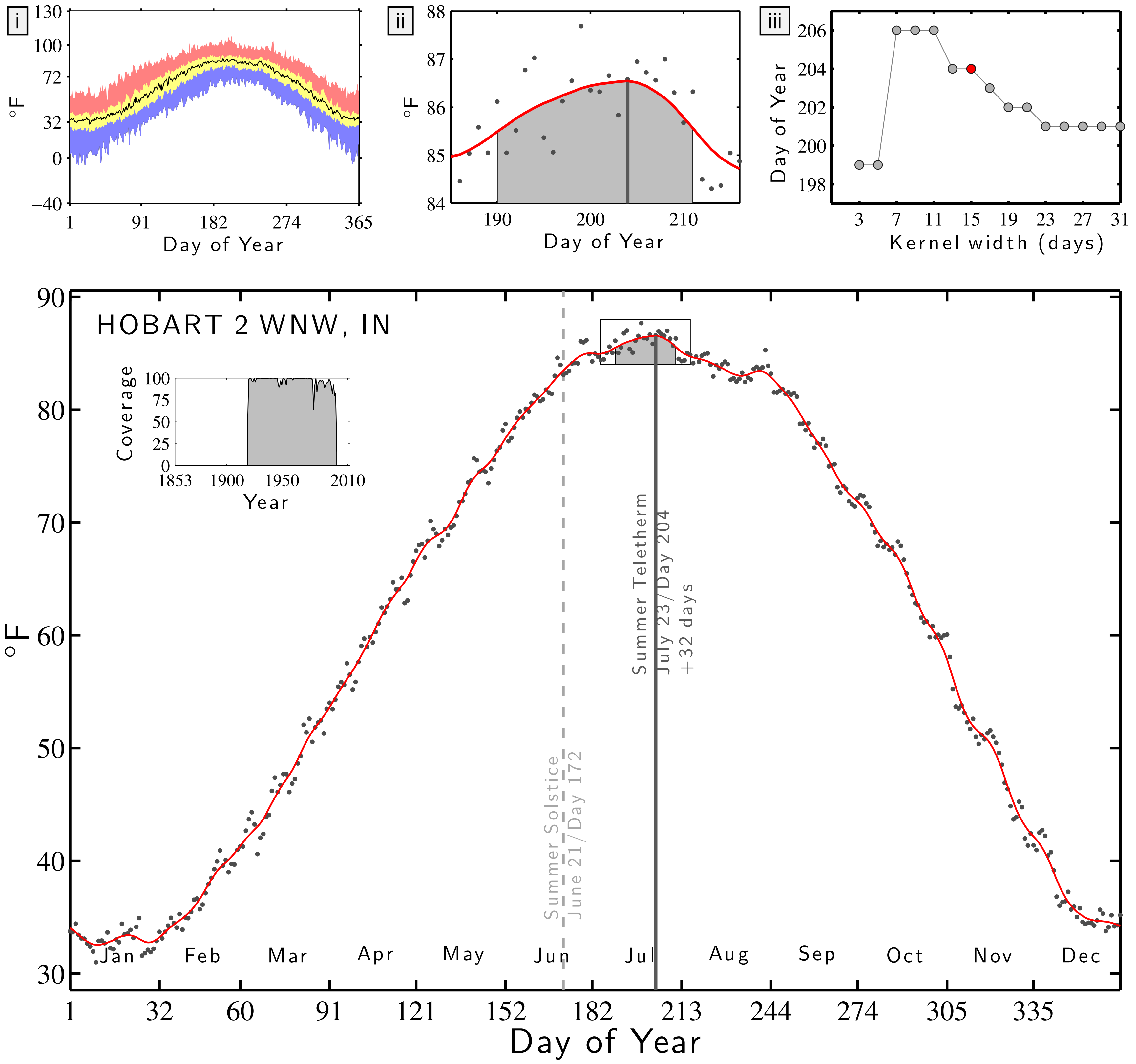

i
ii
iii
°F
Day of Year
Day of Year
Day of Year
Kernel width (days)
HOBART 2 WNW, IN
Coverage
Year
Summer Solstice
June 21 / Day 172
Summer Teletherm
July 23 / Day 204
+32 days
Jan
Feb
Mar
Apr
May
Jun
Jul
Aug
Sep
Oct
Nov
Dec
°F
Day of Year

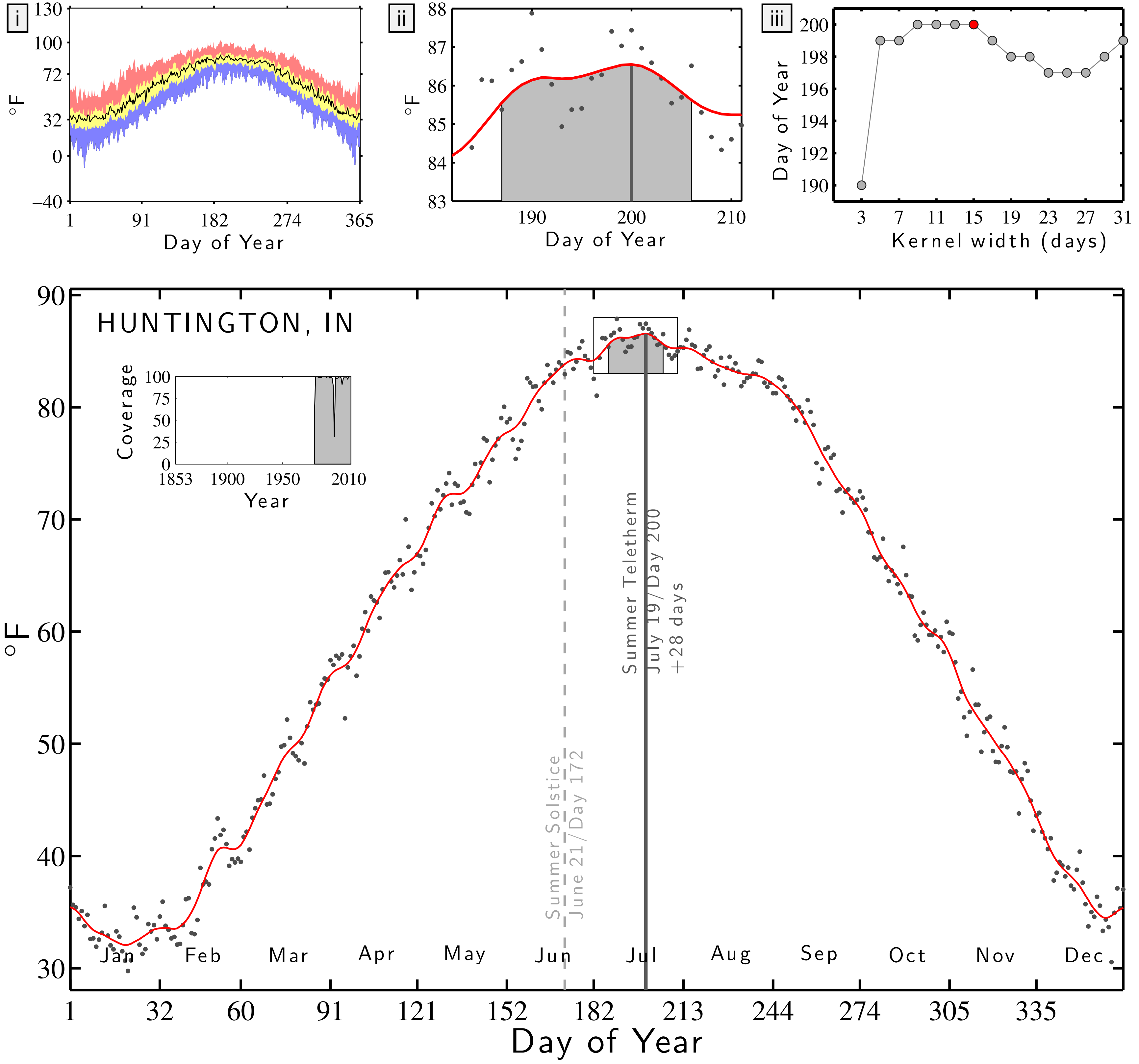

i
ii
iii
°F
Day of Year
Day of Year
Kernel width (days)
HUNTINGTON, IN
Coverage
Year
Summer Solstice
June 21 / Day 172
Summer Teletherm
July 19 / Day 200
+28 days
Jan
Feb
Mar
Apr
May
Jun
Jul
Aug
Sep
Oct
Nov
Dec

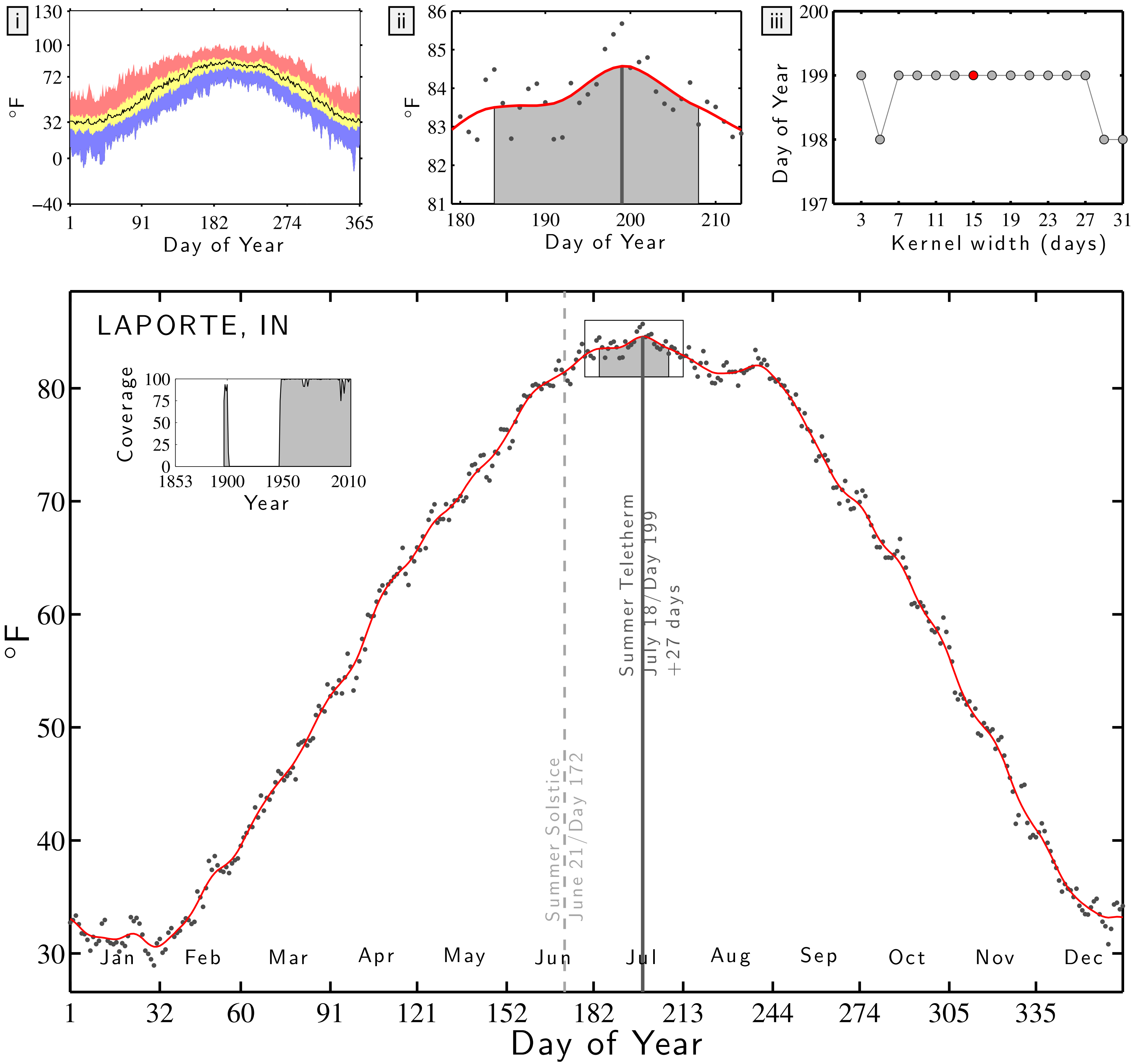

i
ii
iii
°F
Day of Year
Kernel width (days)
LAPORTE, IN
Coverage
Year
Summer Solstice
June 21/Day 172
Summer Teletherm
July 18/Day 199
+27 days
Jan
Feb
Mar
Apr
May
Jun
Jul
Aug
Sep
Oct
Nov
Dec

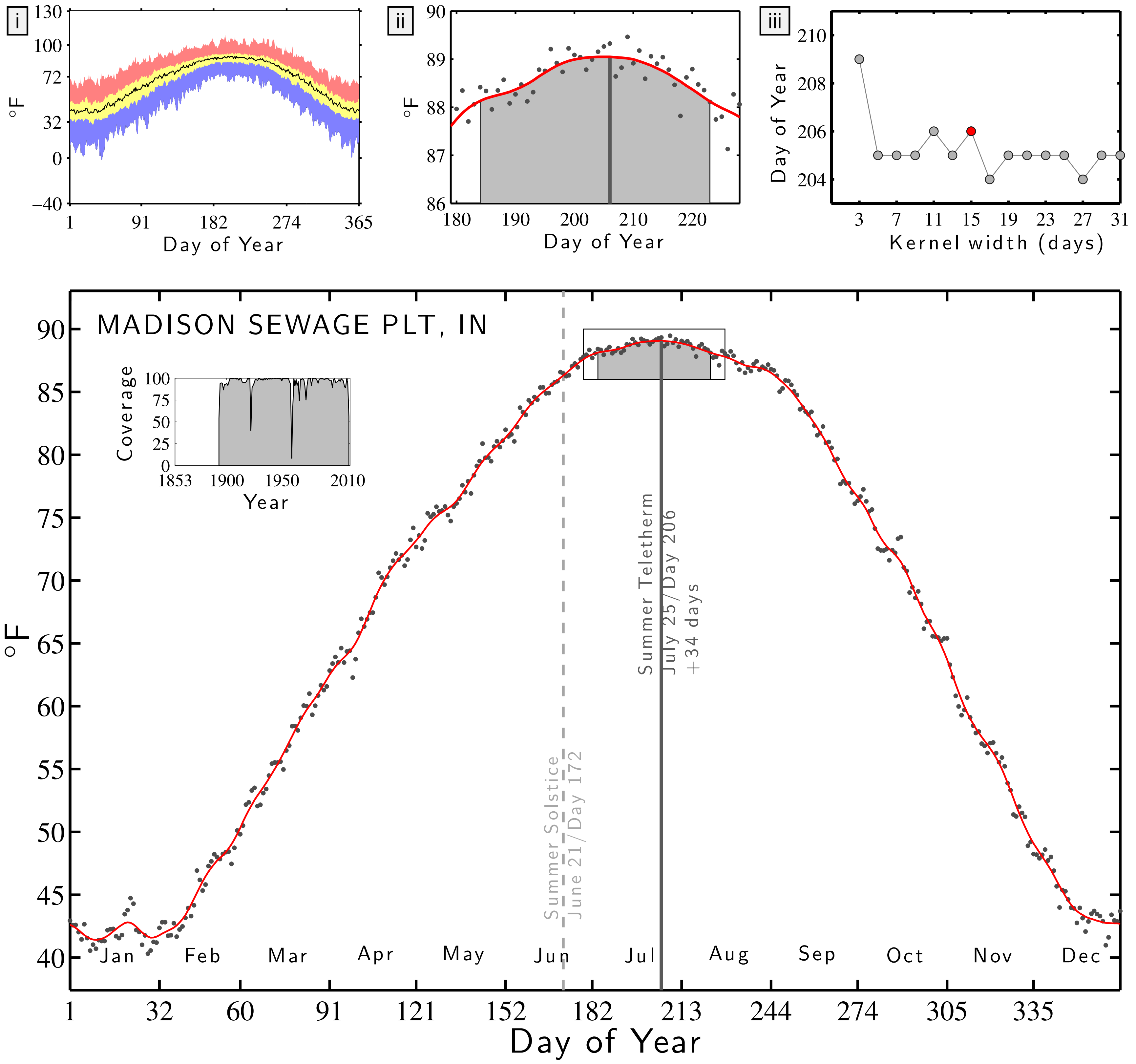

i
ii
iii
MADISON SEWAGE PLT, IN
Summer Solstice
June 21/Day 172
Summer Teletherm
July 25/Day 206
+34 days
Coverage
Year
Day of Year
Kernel width (days)
°F
Jan
Feb
Mar
Apr
May
Jun
Jul
Aug
Sep
Oct
Nov
Dec

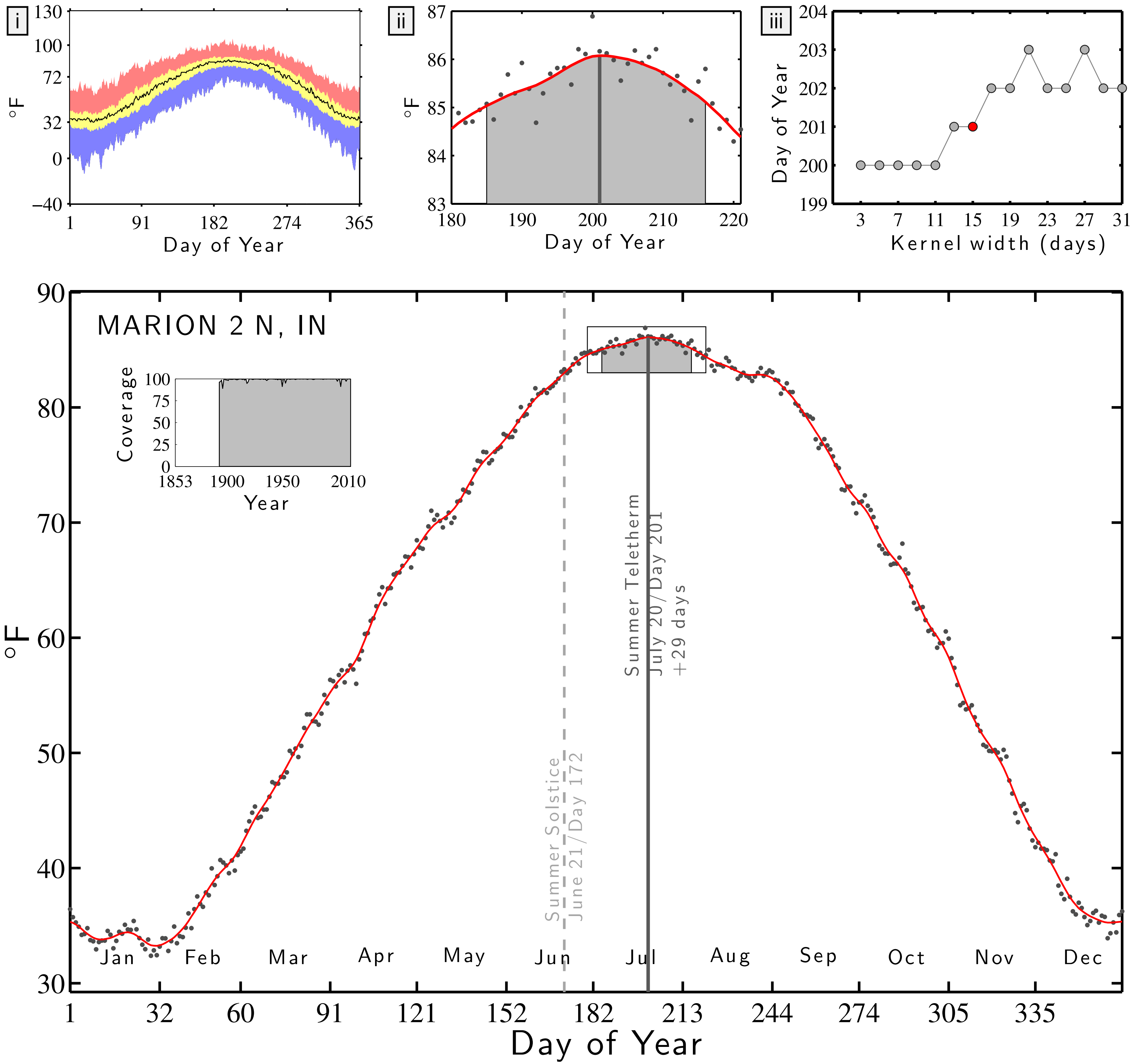

i
ii
iii
Day of Year
Kernel width (days)
MARION 2 N, IN
Coverage
Year
Summer Solstice
June 21/Day 172
Summer Teletherm
July 20/Day 201
+29 days
Jan
Feb
Mar
Apr
May
Jun
Jul
Aug
Sep
Oct
Nov
Dec
°F
Day of Year

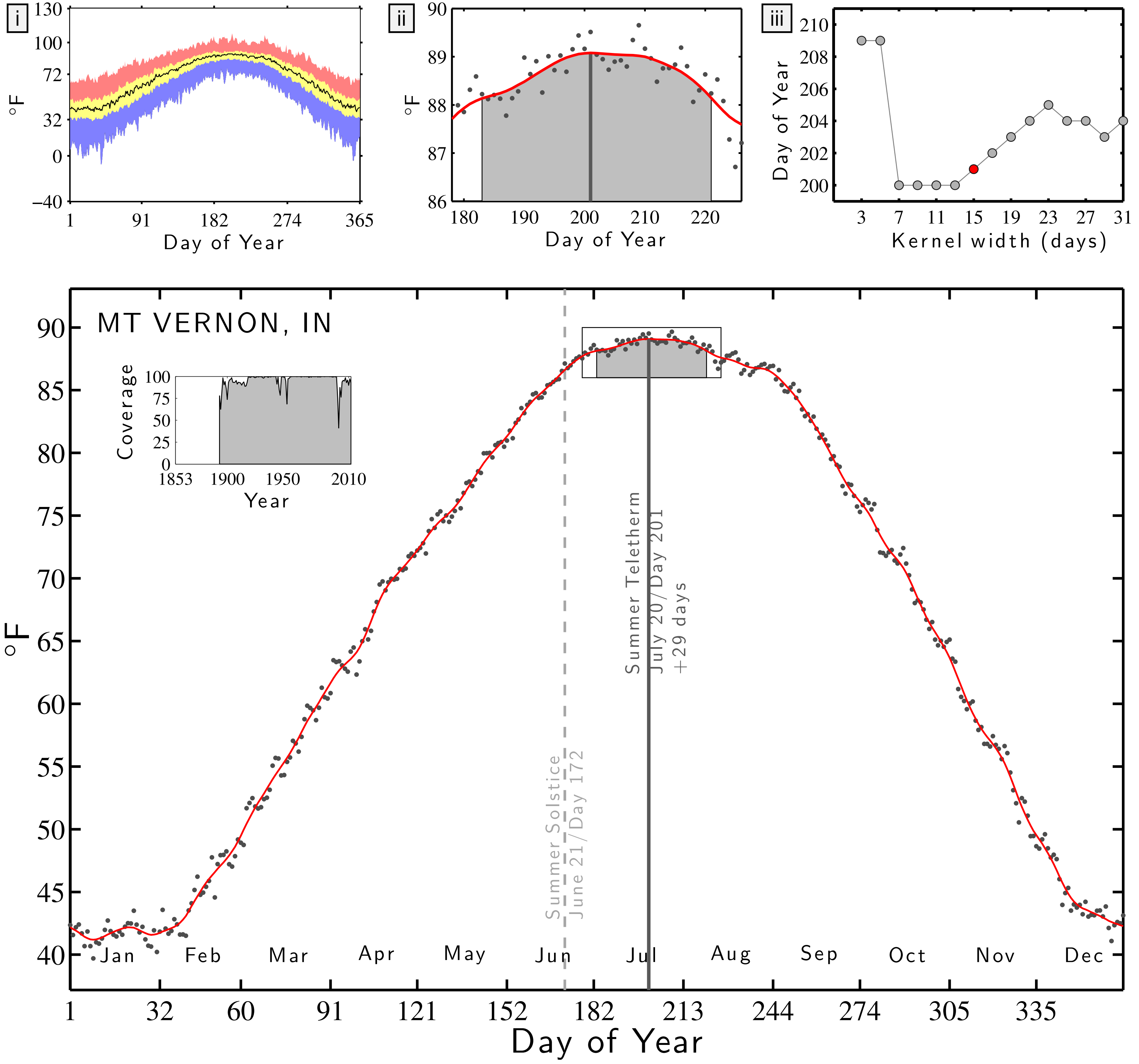

i
ii
iii
°F
Day of Year
Day of Year
Kernel width (days)
MT VERNON, IN
Coverage
Year
Summer Solstice
June 21/Day 172
Summer Telotherm
July 20/Day 201
+29 days
Jan
Feb
Mar
Apr
May
Jun
Jul
Aug
Sep
Oct
Nov
Dec

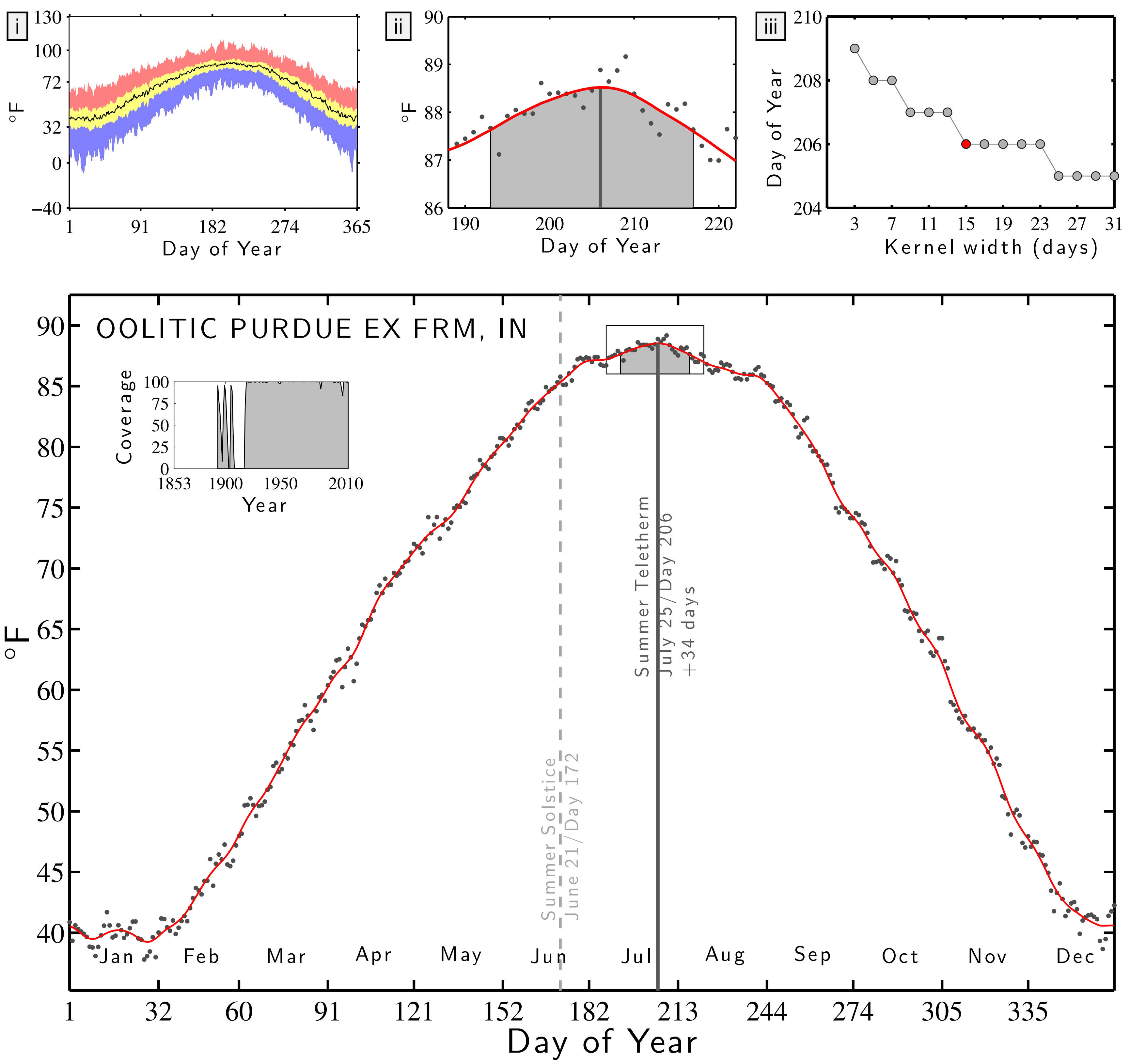

i
ii
iii
°F
Day of Year
Day of Year
Kernel width (days)
OOLITIC PURDUE EX FRM, IN
Coverage
Year
Summer Solstice
June 21/Day 172
Summer Teletherm
July 25/Day 206
+34 days
Jan
Feb
Mar
Apr
May
Jun
Jul
Aug
Sep
Oct
Nov
Dec
Day of Year

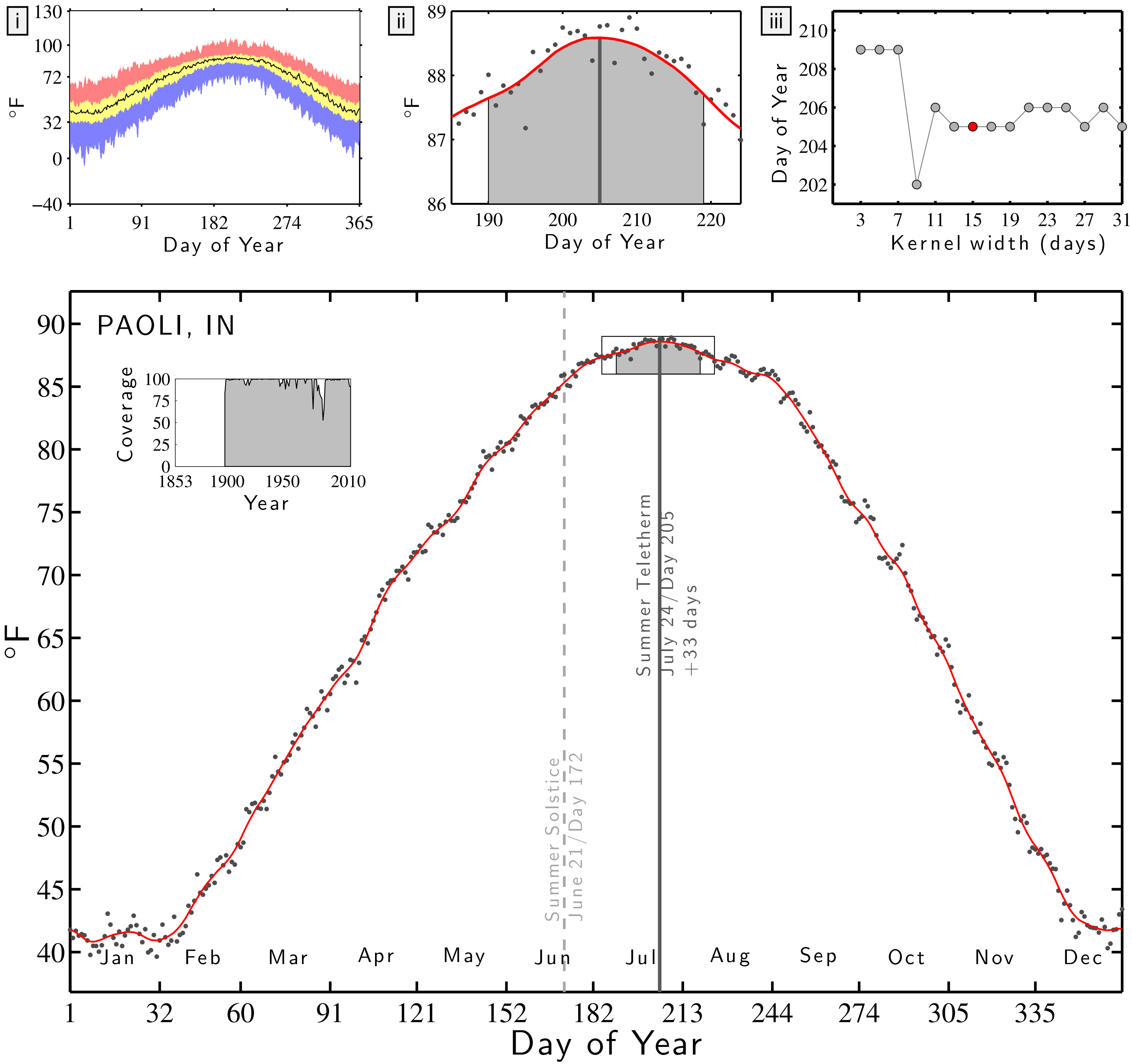

i
°F
Day of Year
ii
°F
Day of Year
iii
Day of Year
Kernel width (days)
PAOLI, IN
Coverage
Year
Summer Solstice
June 21 / Day 172
Summer Teletherm
July 24 / Day 205
+33 days
Jan
Feb
Mar
Apr
May
Jun
Jul
Aug
Sep
Oct
Nov
Dec
°F
Day of Year

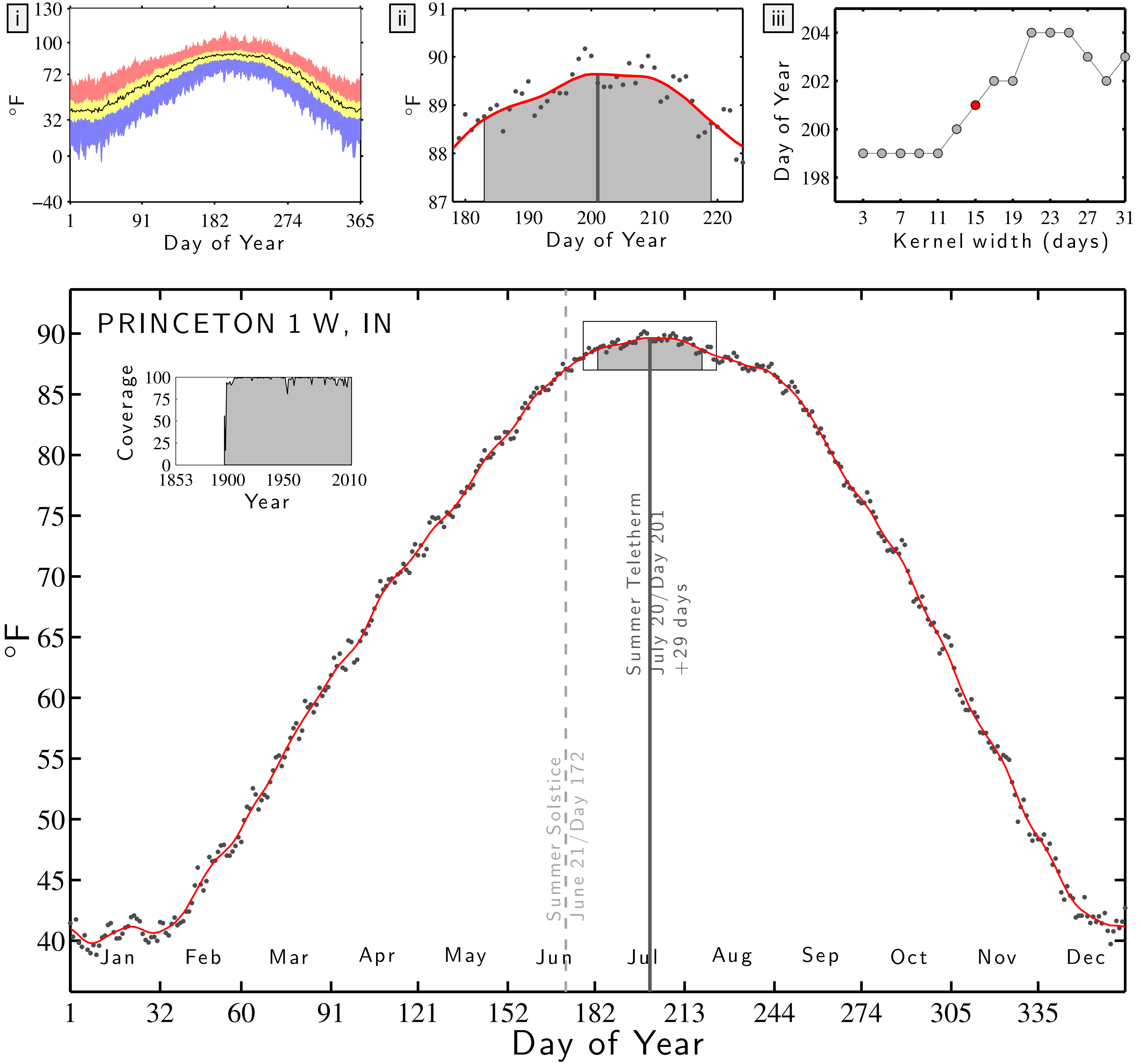

i
130
100
72
32
0
−40
°F
1
91
182
274
365
Day of Year
ii
91
90
89
88
87
°F
180
190
200
210
220
Day of Year
iii
204
202
200
198
Day of Year
3
7
11
15
19
23
27
31
Kernel width (days)
PRINCETON 1 W, IN
Coverage
100
75
50
25
0
1853
1900
1950
2010
Year
Summer Solstice
June 21/Day 172
Summer Teletherm
July 20/Day 201
+29 days
90
85
80
75
70
65
60
55
50
45
40
°F
Jan
Feb
Mar
Apr
May
Jun
Jul
Aug
Sep
Oct
Nov
Dec
1
32
60
91
121
152
182
213
244
274
305
335
Day of Year

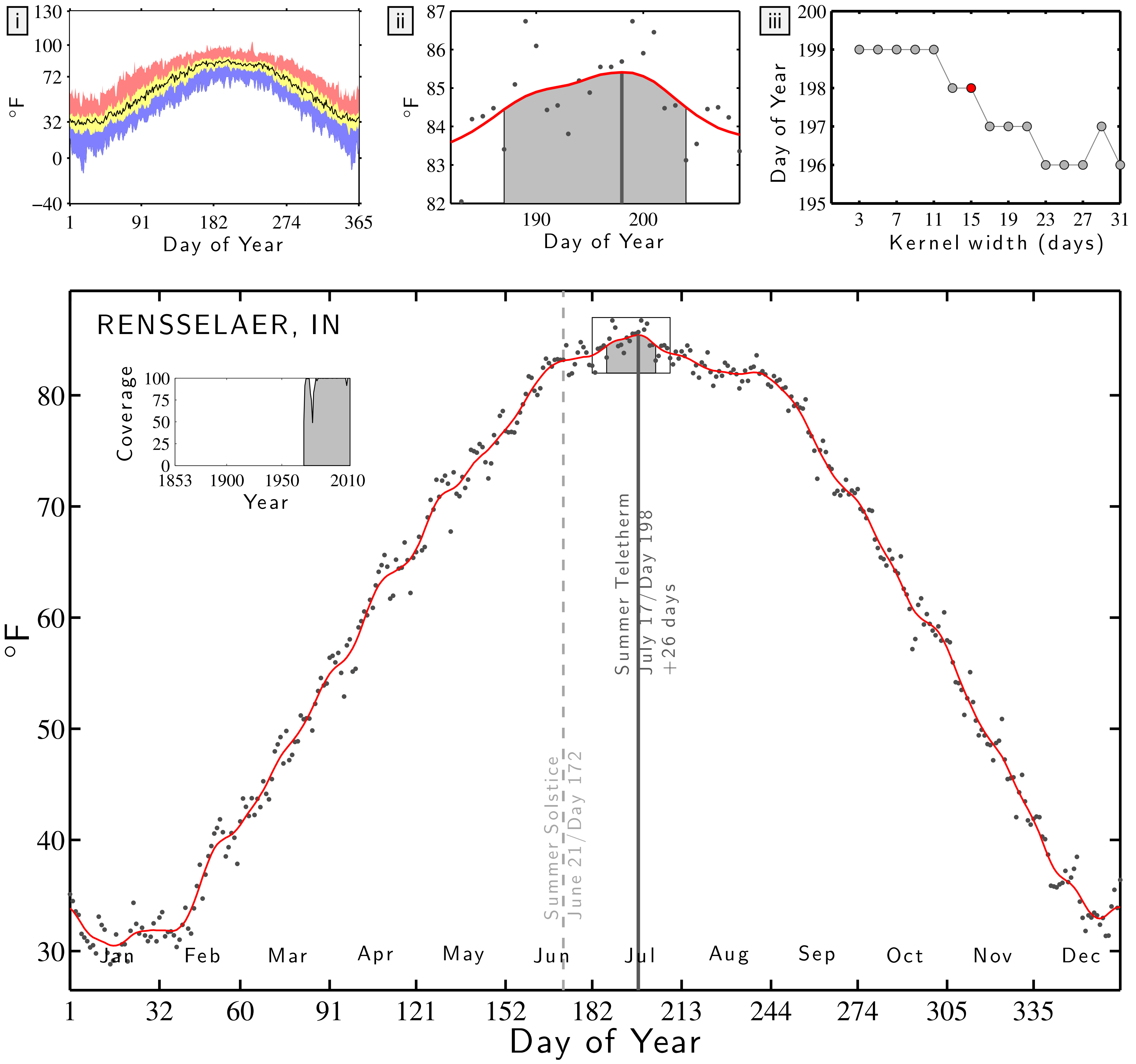

i
°F
130
100
72
32
0
−40
1
91
182
274
365
Day of Year
ii
°F
87
86
85
84
83
82
190
200
Day of Year
iii
Day of Year
200
199
198
197
196
195
3
7
11
15
19
23
27
31
Kernel width (days)
RENSSELAER, IN
Coverage
100
75
50
25
0
1853
1900
1950
2010
Year
Summer Solstice
June 21/Day 172
Summer Teletherm
July 17/Day 198
+26 days
°F
80
70
60
50
40
30
Jan
Feb
Mar
Apr
May
Jun
Jul
Aug
Sep
Oct
Nov
Dec
1
32
60
91
121
152
182
213
244
274
305
335
Day of Year

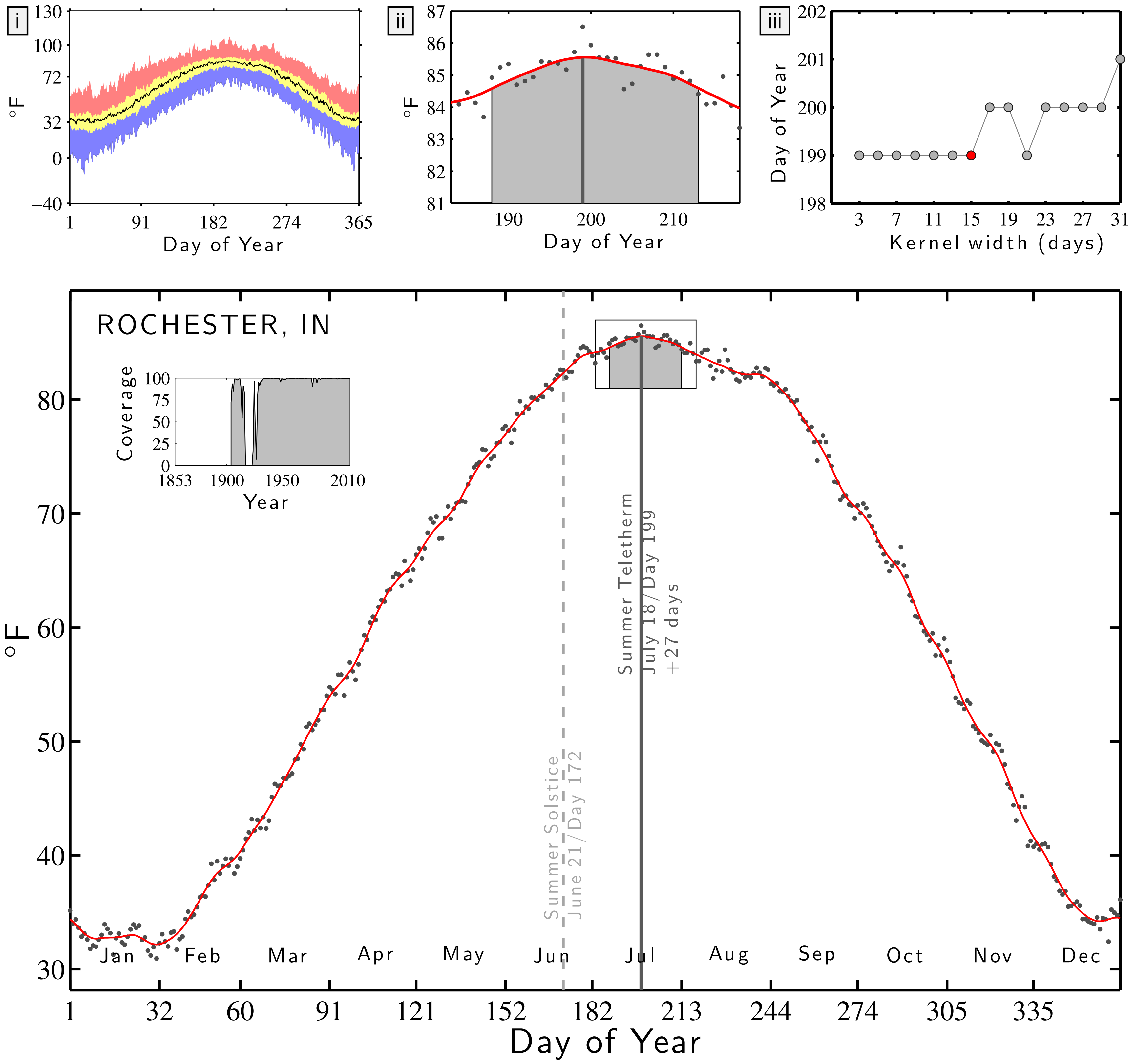

i
°F
Day of Year
ii
°F
Day of Year
iii
Day of Year
Kernel width (days)
ROCHESTER, IN
Coverage
Year
Summer Solstice
June 21/Day 172
Summer Teletherm
July 18/Day 199
+27 days
Jan
Feb
Mar
Apr
May
Jun
Jul
Aug
Sep
Oct
Nov
Dec
°F
Day of Year

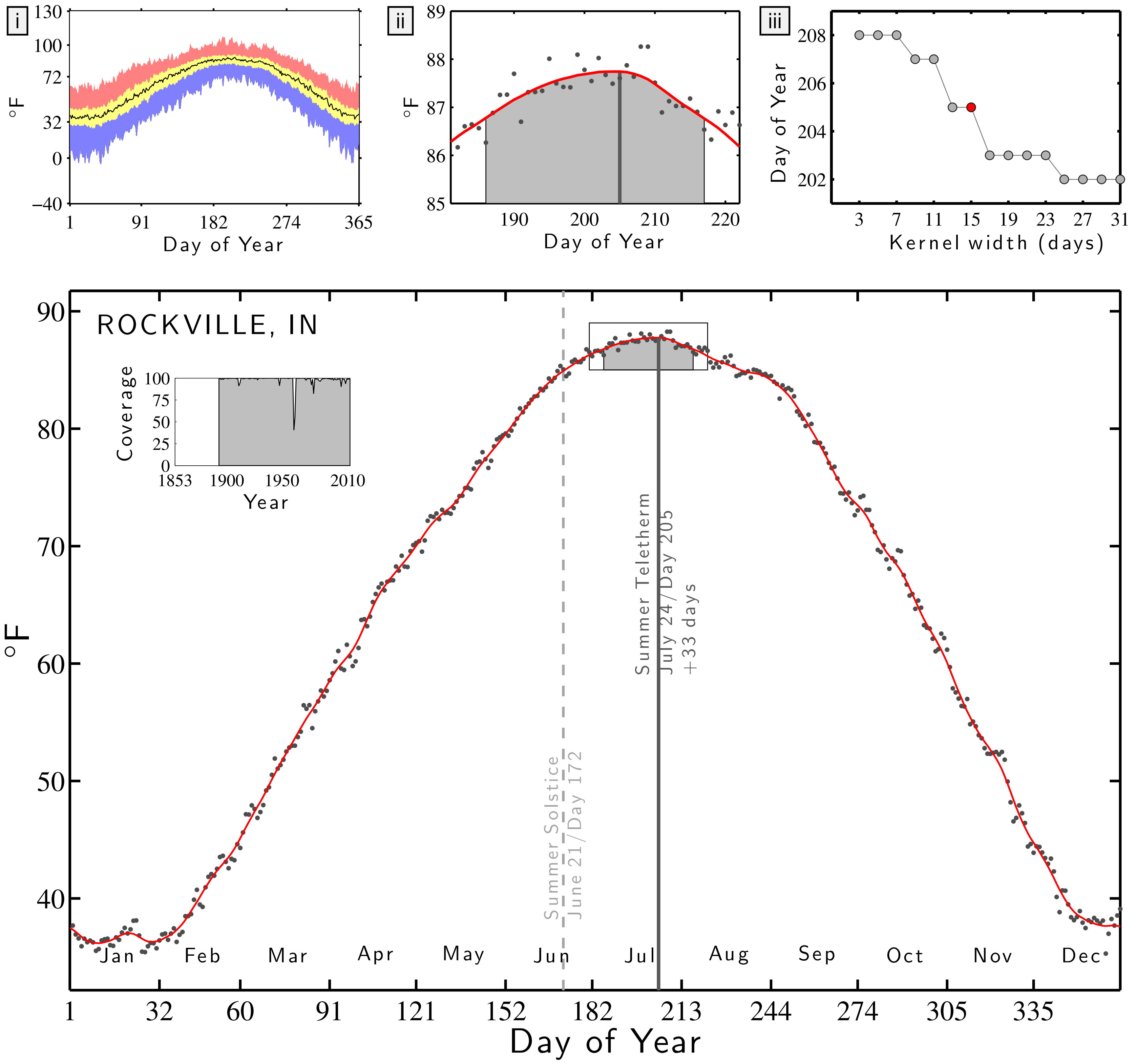

i
°F
Day of Year
ii
°F
Day of Year
iii
Day of Year
Kernel width (days)
ROCKVILLE, IN
Coverage
Year
Summer Solstice
June 21 / Day 172
Summer Teletherm
July 24 / Day 205
+33 days
Jan
Feb
Mar
Apr
May
Jun
Jul
Aug
Sep
Oct
Nov
Dec
°F
Day of Year

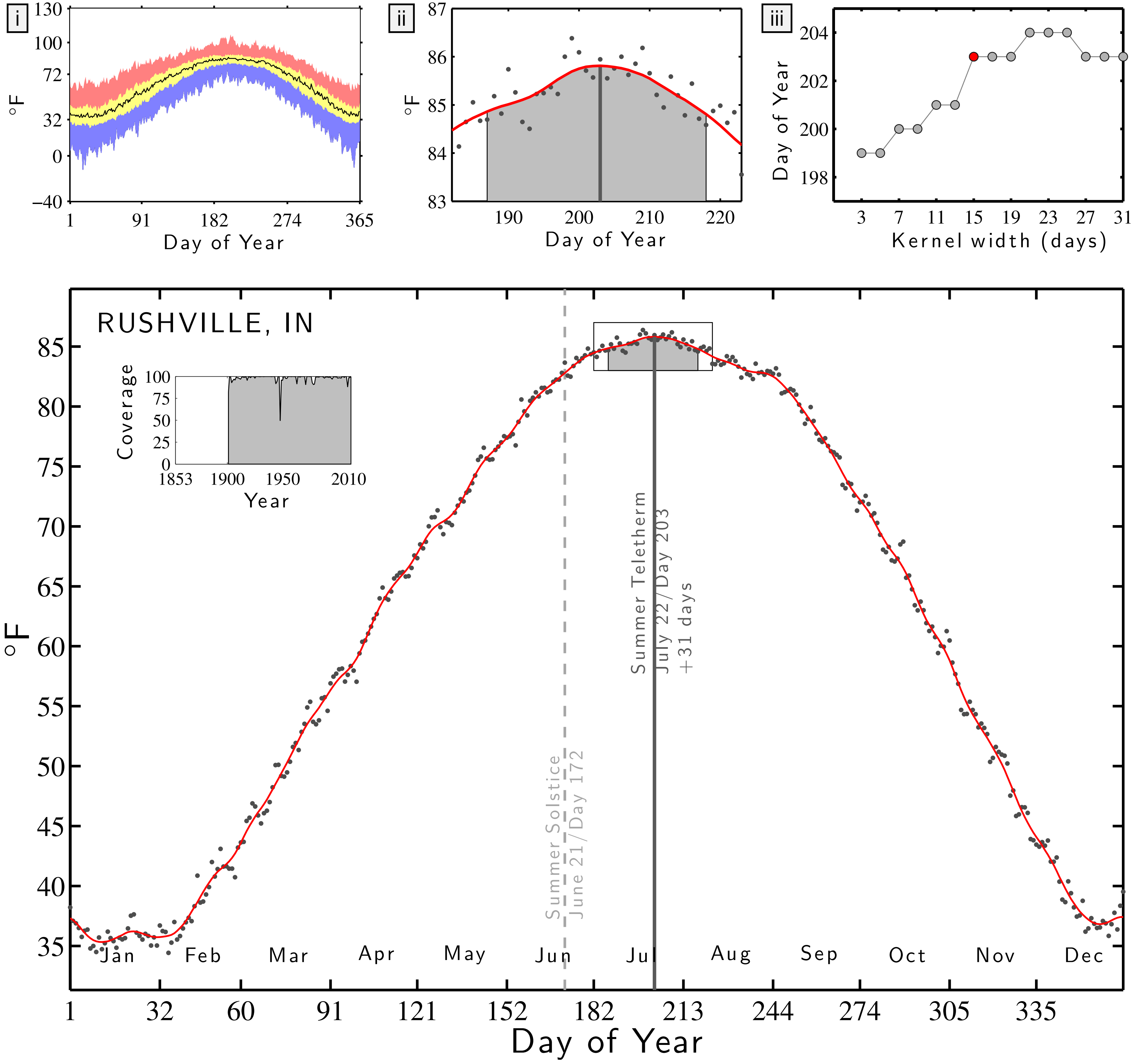

i
ii
iii
°F
Day of Year
Kernel width (days)
RUSHVILLE, IN
Coverage
Year
Summer Solstice
June 21/Day 172
Summer Teletherm
July 22/Day 203
+31 days
Jan
Feb
Mar
Apr
May
Jun
Jul
Aug
Sep
Oct
Nov
Dec

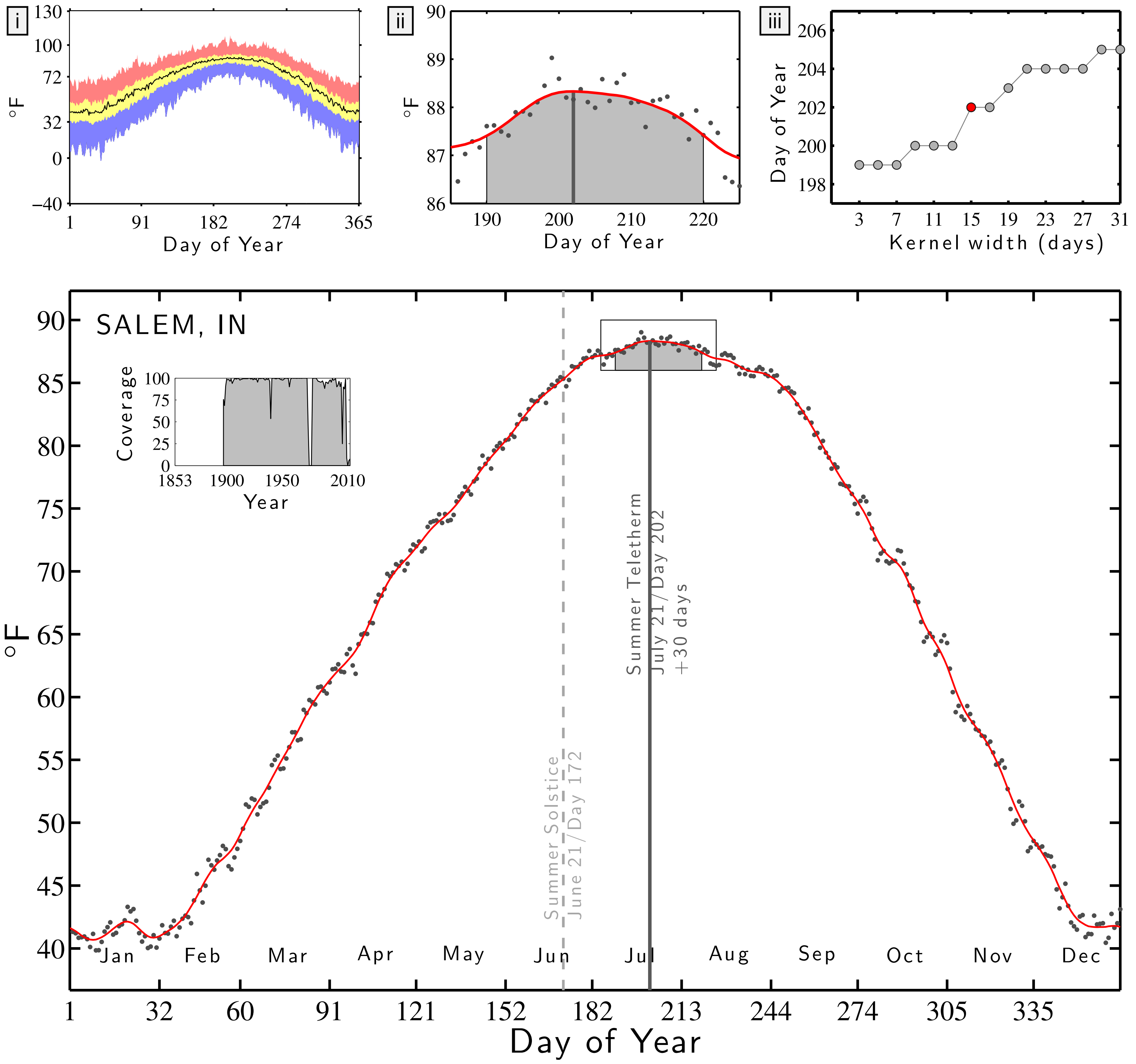

i
°F
Day of Year
ii
°F
Day of Year
iii
Day of Year
Kernel width (days)
SALEM, IN
Coverage
Year
Summer Solstice
June 21 / Day 172
Summer Teletherm
July 21 / Day 202
+30 days
Jan
Feb
Mar
Apr
May
Jun
Jul
Aug
Sep
Oct
Nov
Dec
°F
Day of Year

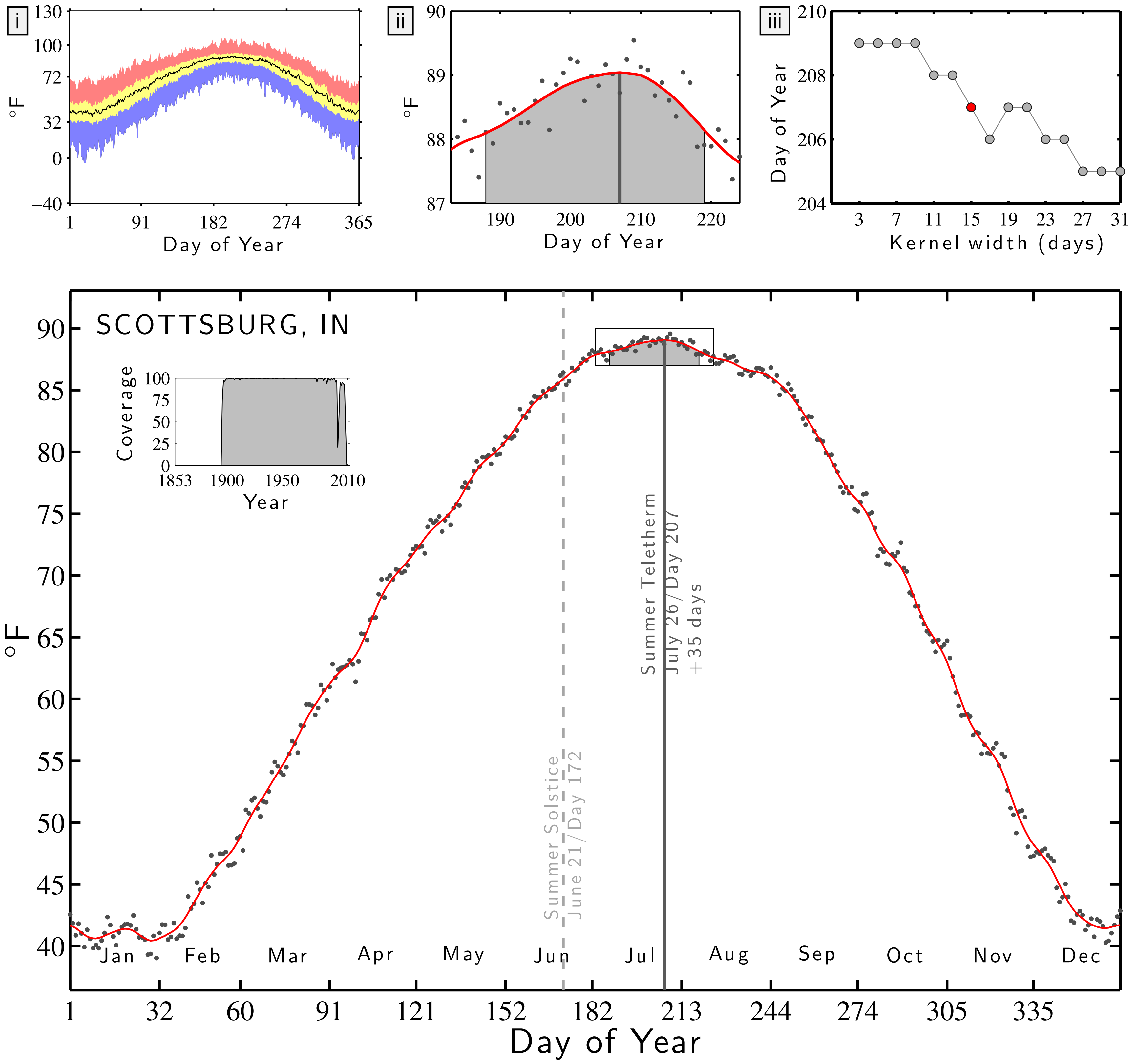

i
130
100
72
32
0
−40
°F
1
91
182
274
365
Day of Year
ii
90
89
88
87
°F
190
200
210
220
Day of Year
iii
210
208
206
204
Day of Year
3
7
11
15
19
23
27
31
Kernel width (days)
SCOTTSBURG, IN
Coverage
100
75
50
25
0
1853
1900
1950
2010
Year
Summer Solstice
June 21/Day 172
Summer Teletherm
July 26/Day 207
+35 days
Jan
Feb
Mar
Apr
May
Jun
Jul
Aug
Sep
Oct
Nov
Dec
°F
1
32
60
91
121
152
182
213
244
274
305
335
Day of Year

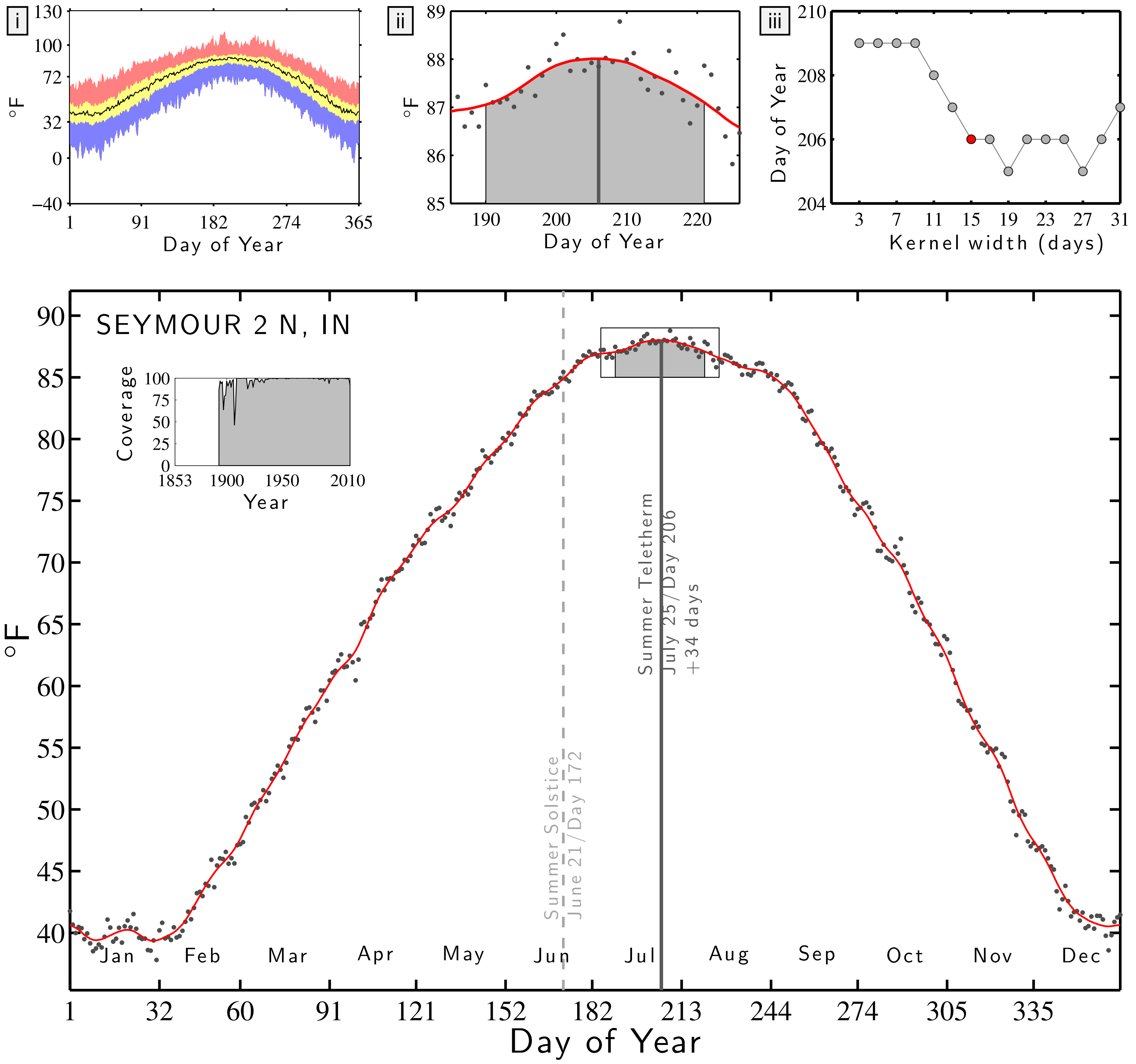

i
°F
Day of Year
ii
°F
Day of Year
iii
Day of Year
Kernel width (days)
SEYMOUR 2 N, IN
Coverage
Year
Summer Solstice
June 21/Day 172
Summer Teletherm
July 25/Day 206
+34 days
Jan
Feb
Mar
Apr
May
Jun
Jul
Aug
Sep
Oct
Nov
Dec
°F
Day of Year

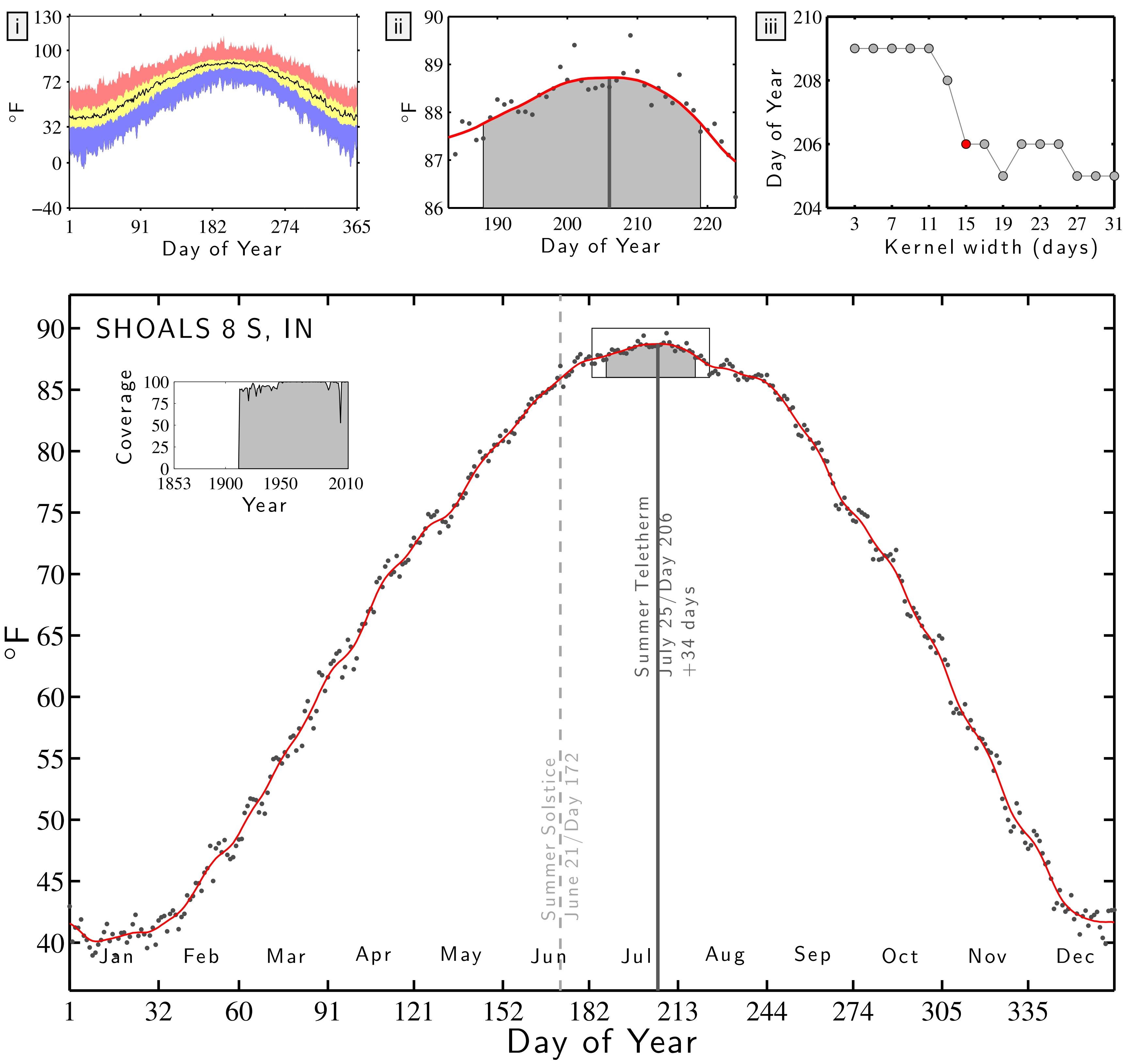

i
ii
iii
°F
Day of Year
Day of Year
Kernel width (days)
SHOALS 8 S, IN
Coverage
Year
Summer Solstice
June 21/Day 172
Summer Teletherm
July 25/Day 206
+34 days
Jan
Feb
Mar
Apr
May
Jun
Jul
Aug
Sep
Oct
Nov
Dec
°F
Day of Year

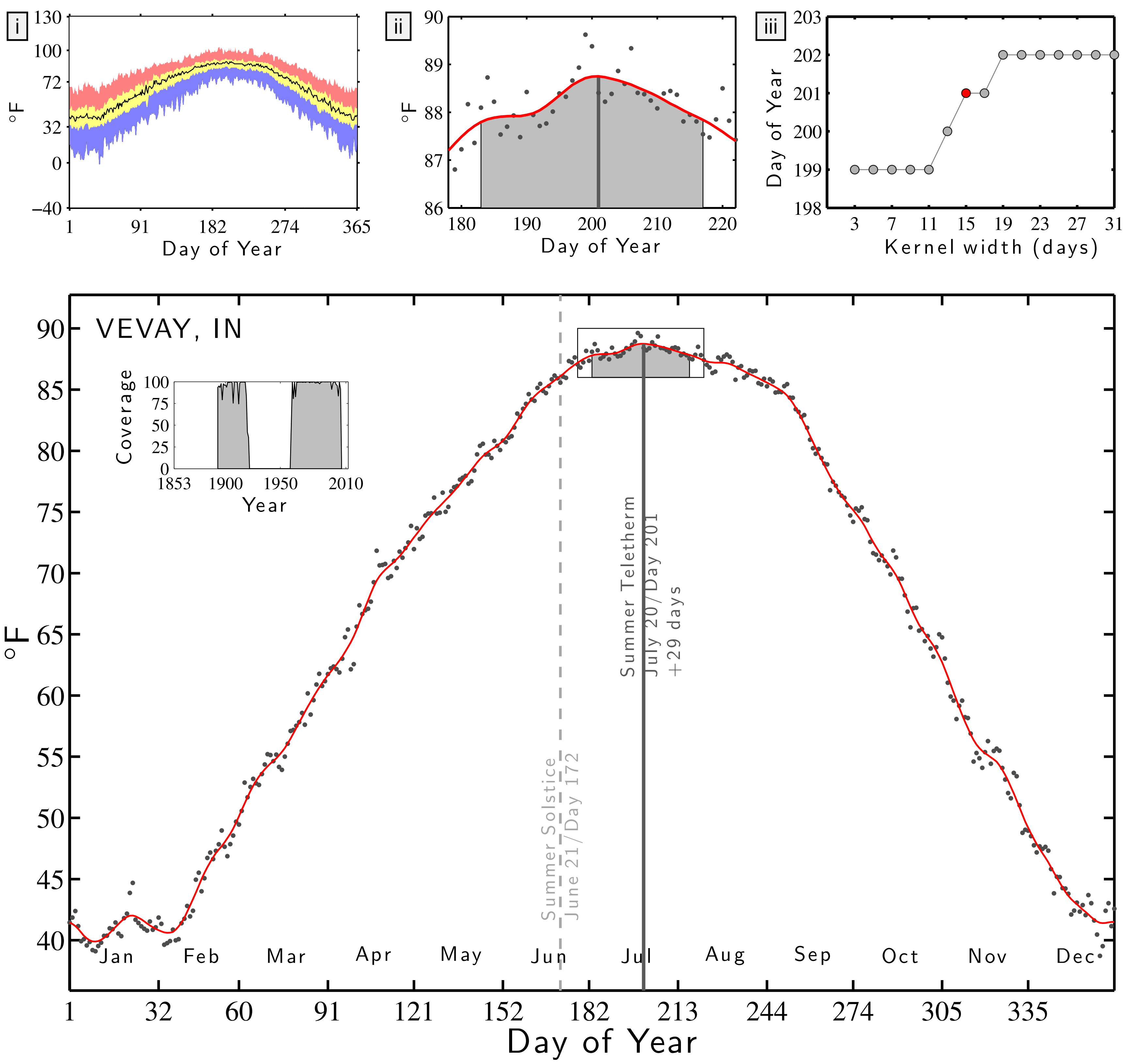

i
°F
Day of Year
ii
°F
Day of Year
iii
Day of Year
Kernel width (days)
VEVAY, IN
Coverage
Year
Summer Solstice
June 21 / Day 172
Summer Teletherm
July 20 / Day 201
+29 days
Jan
Feb
Mar
Apr
May
Jun
Jul
Aug
Sep
Oct
Nov
Dec
°F
Day of Year

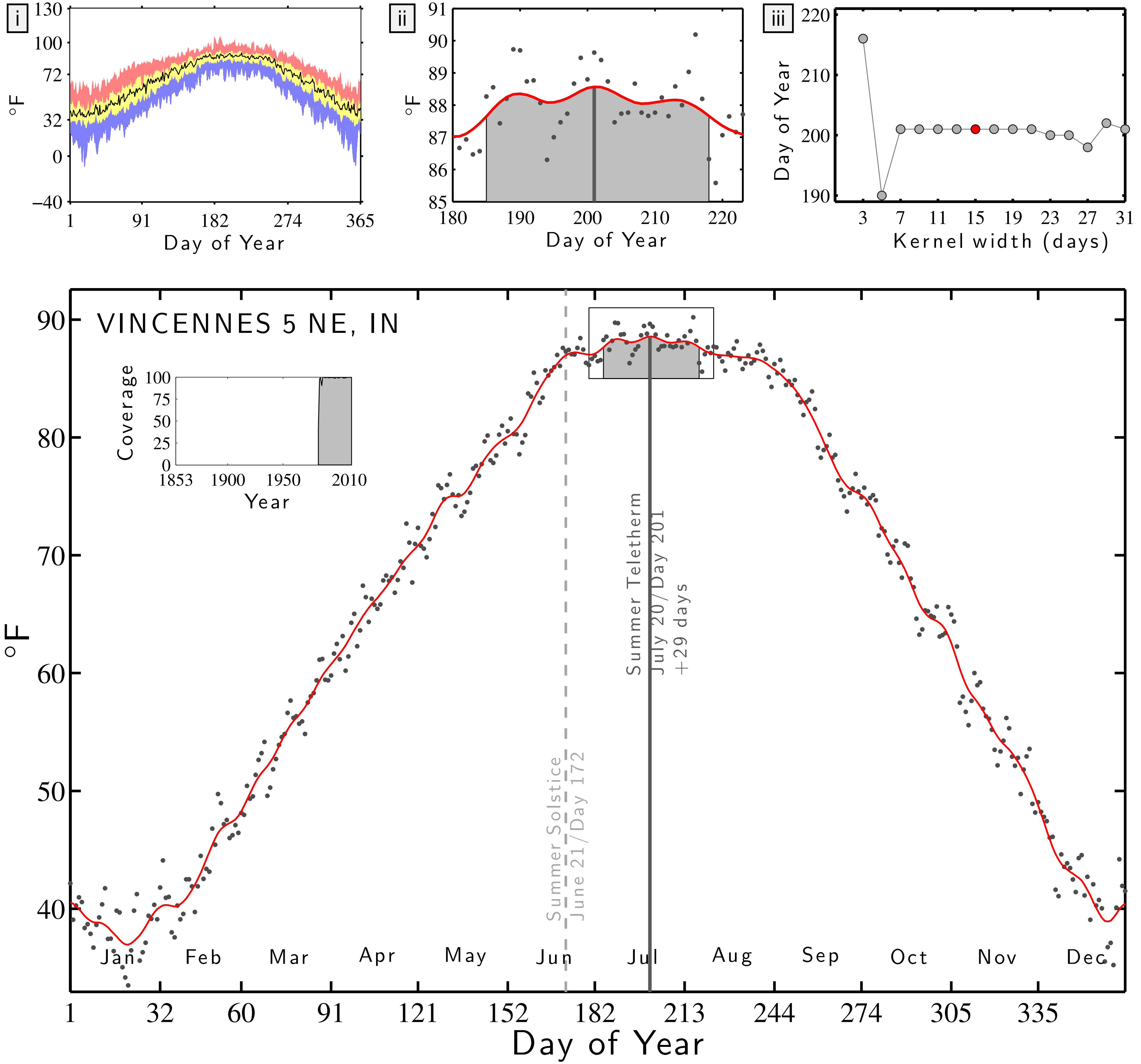

i
°F
Day of Year
ii
°F
Day of Year
iii
Day of Year
Kernel width (days)
VINCENNES 5 NE, IN
Coverage
Year
Summer Solstice
June 21/Day 172
Summer Teletherm
July 20/Day 201
+29 days
Jan
Feb
Mar
Apr
May
Jun
Jul
Aug
Sep
Oct
Nov
Dec
°F
Day of Year

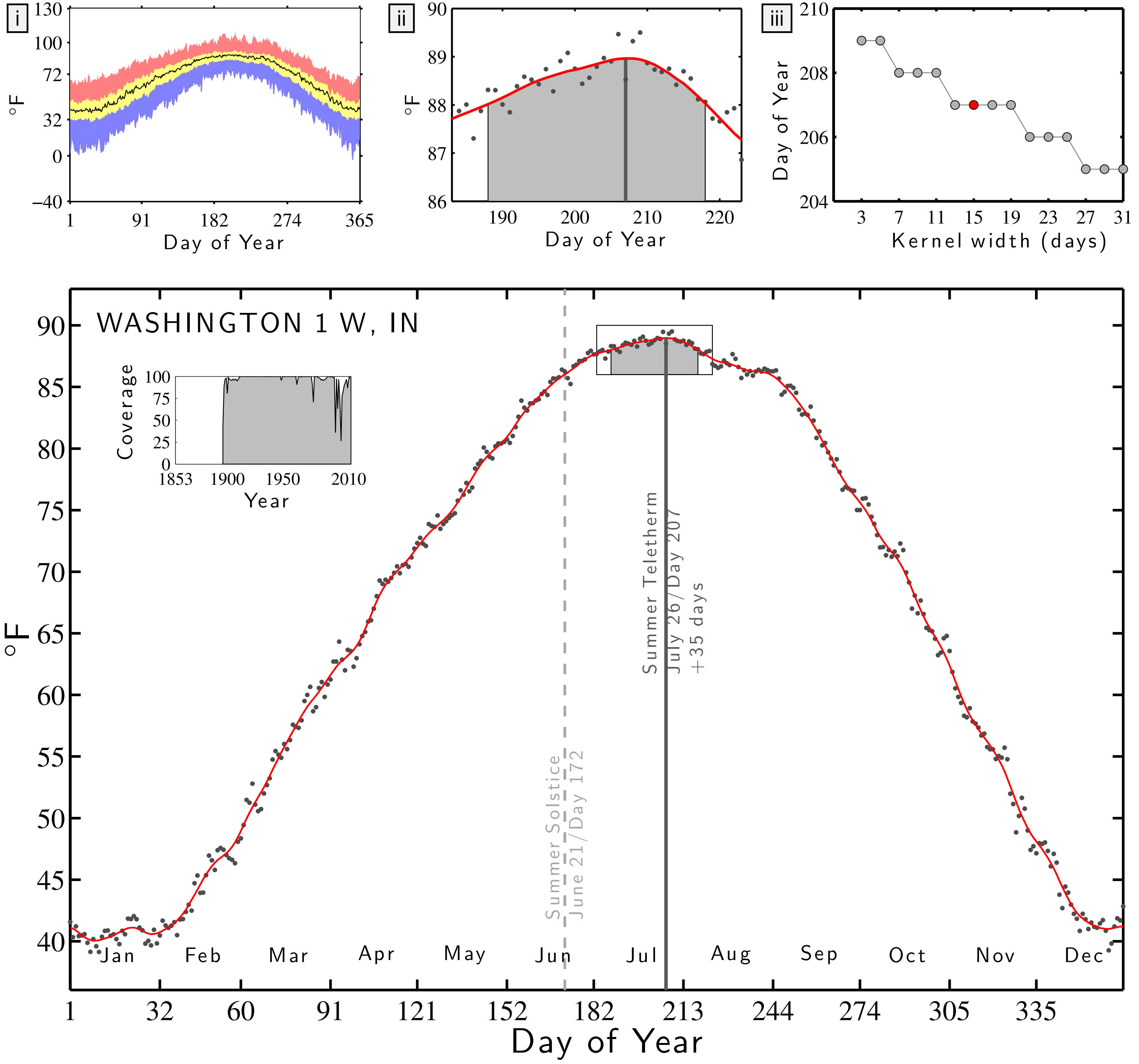

i
130
100
72
32
0
−40
°F
1
91
182
274
365
Day of Year
ii
90
89
88
87
86
190
200
210
220
Day of Year
iii
210
208
206
204
Day of Year
3
7
11
15
19
23
27
31
Kernel width (days)
WASHINGTON 1 W, IN
Coverage
100
75
50
25
0
1853
1900
1950
2010
Year
Summer Solstice
June 21 / Day 172
Summer Teletherm
July 26 / Day 207
+35 days
Jan
Feb
Mar
Apr
May
Jun
Jul
Aug
Sep
Oct
Nov
Dec
90
85
80
75
70
65
60
55
50
45
40
1
32
60
91
121
152
182
213
244
274
305
335
Day of Year

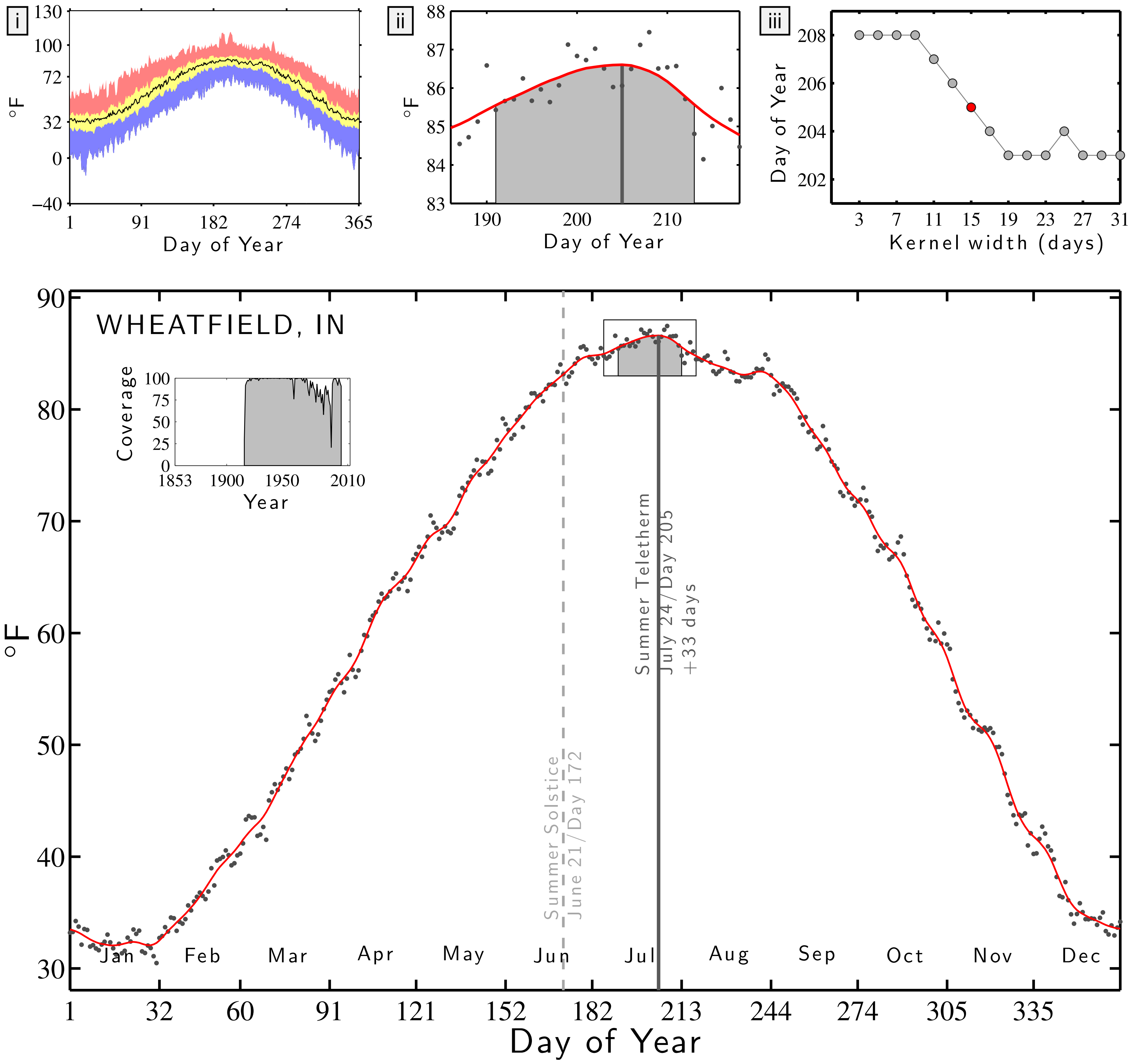

i
°F
Day of Year
ii
°F
Day of Year
iii
Day of Year
Kernel width (days)
WHEATFIELD, IN
Coverage
Year
Summer Solstice
June 21 / Day 172
Summer Teletherm
July 24 / Day 205
+33 days
Jan
Feb
Mar
Apr
May
Jun
Jul
Aug
Sep
Oct
Nov
Dec
°F
Day of Year

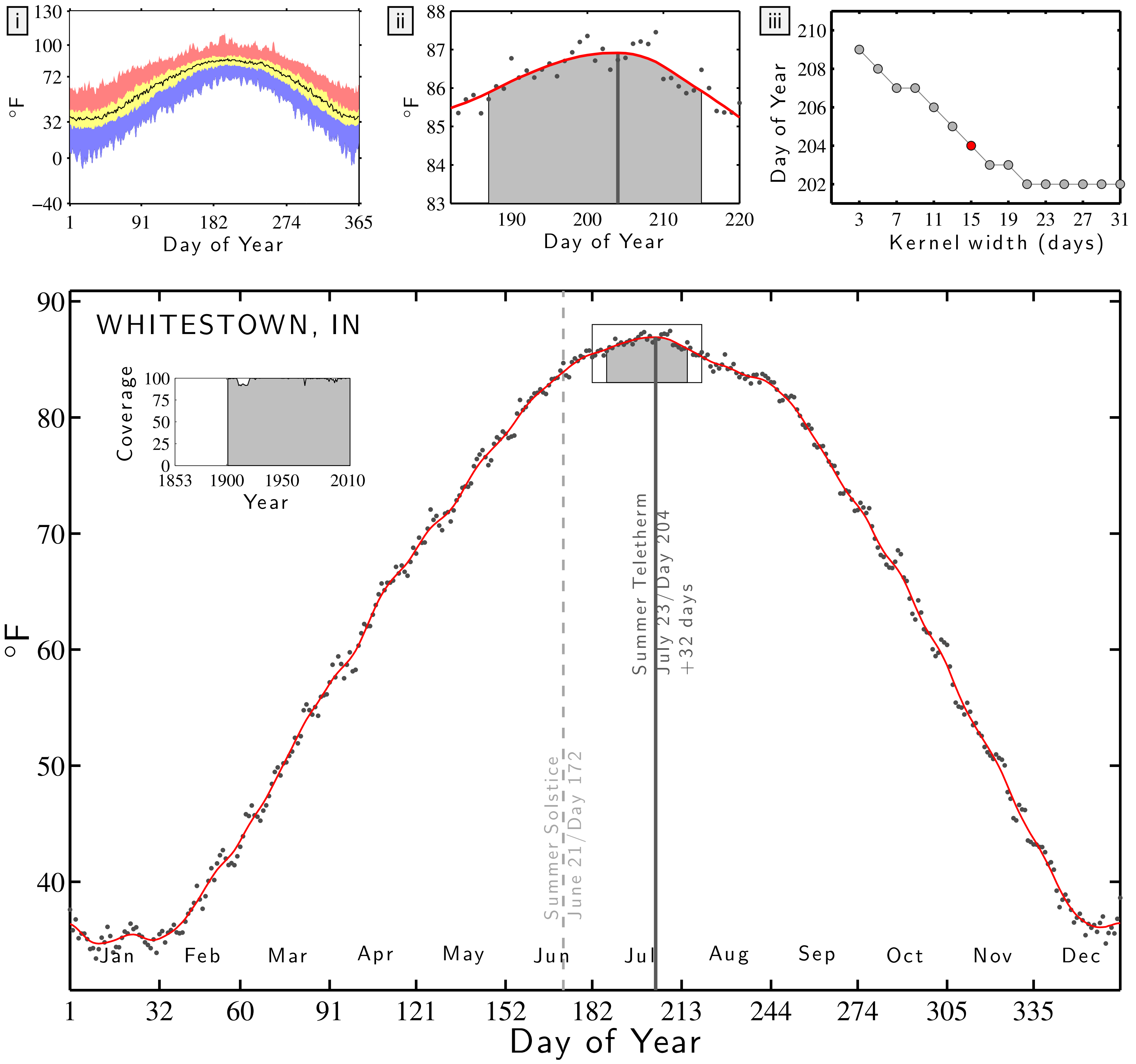

i
ii
iii
°F
Day of Year
Kernel width (days)
WHITESTOWN, IN
Coverage
Year
Summer Solstice
June 21 / Day 172
Summer Teletherm
July 23 / Day 204
+32 days
Jan
Feb
Mar
Apr
May
Jun
Jul
Aug
Sep
Oct
Nov
Dec

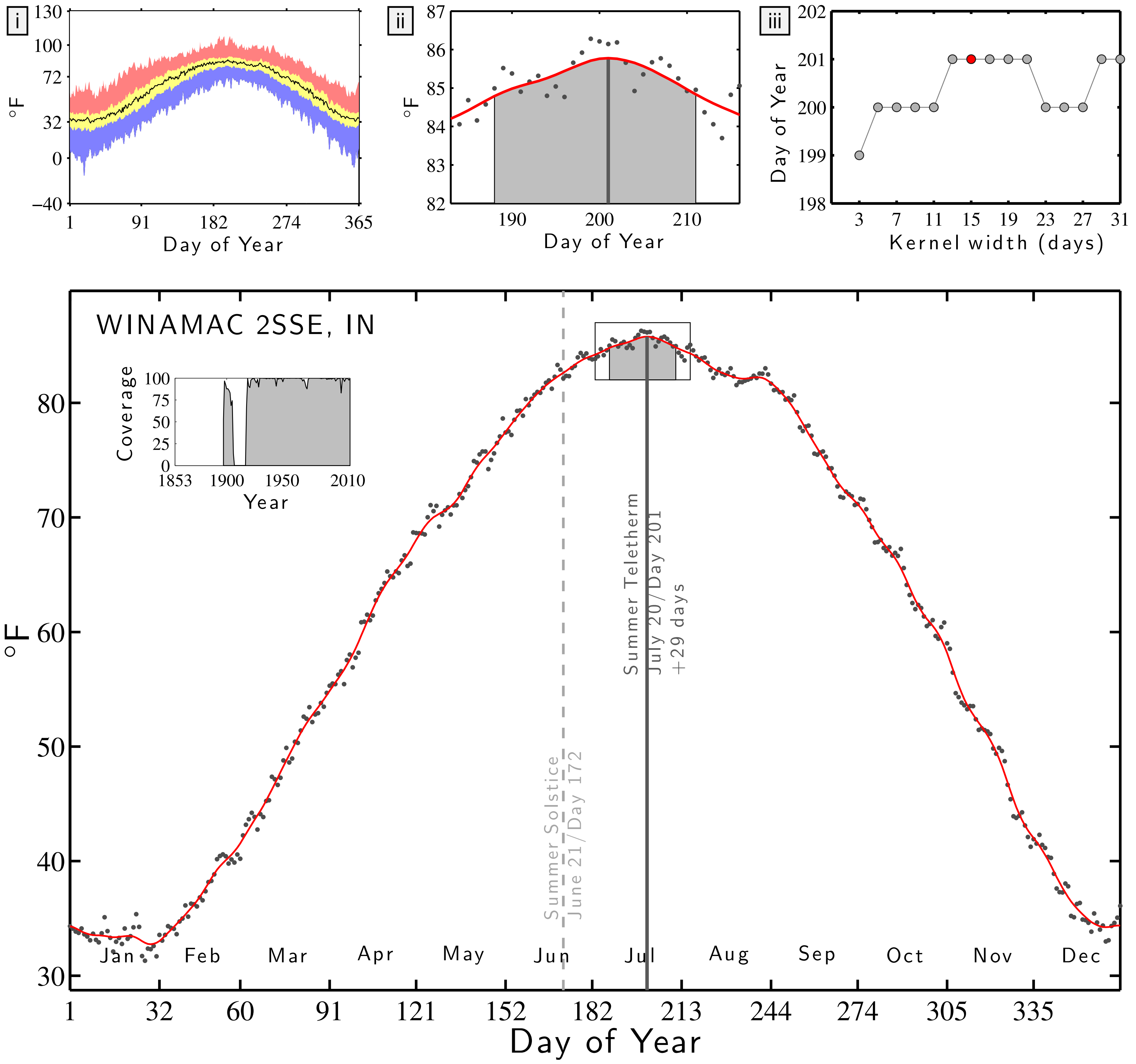

i
130
100
72
32
0
−40
°F
1
91
182
274
365
Day of Year
ii
87
86
85
84
83
82
°F
190
200
210
Day of Year
iii
202
201
200
199
198
Day of Year
3
7
11
15
19
23
27
31
Kernel width (days)
WINAMAC 2SSE, IN
Coverage
100
75
50
25
0
1853
1900
1950
2010
Year
°F
80
70
60
50
40
30
Summer Solstice
June 21/Day 172
Summer Teletherm
July 20/Day 201
+29 days
Jan
Feb
Mar
Apr
May
Jun
Jul
Aug
Sep
Oct
Nov
Dec
1
32
60
91
121
152
182
213
244
274
305
335
Day of Year

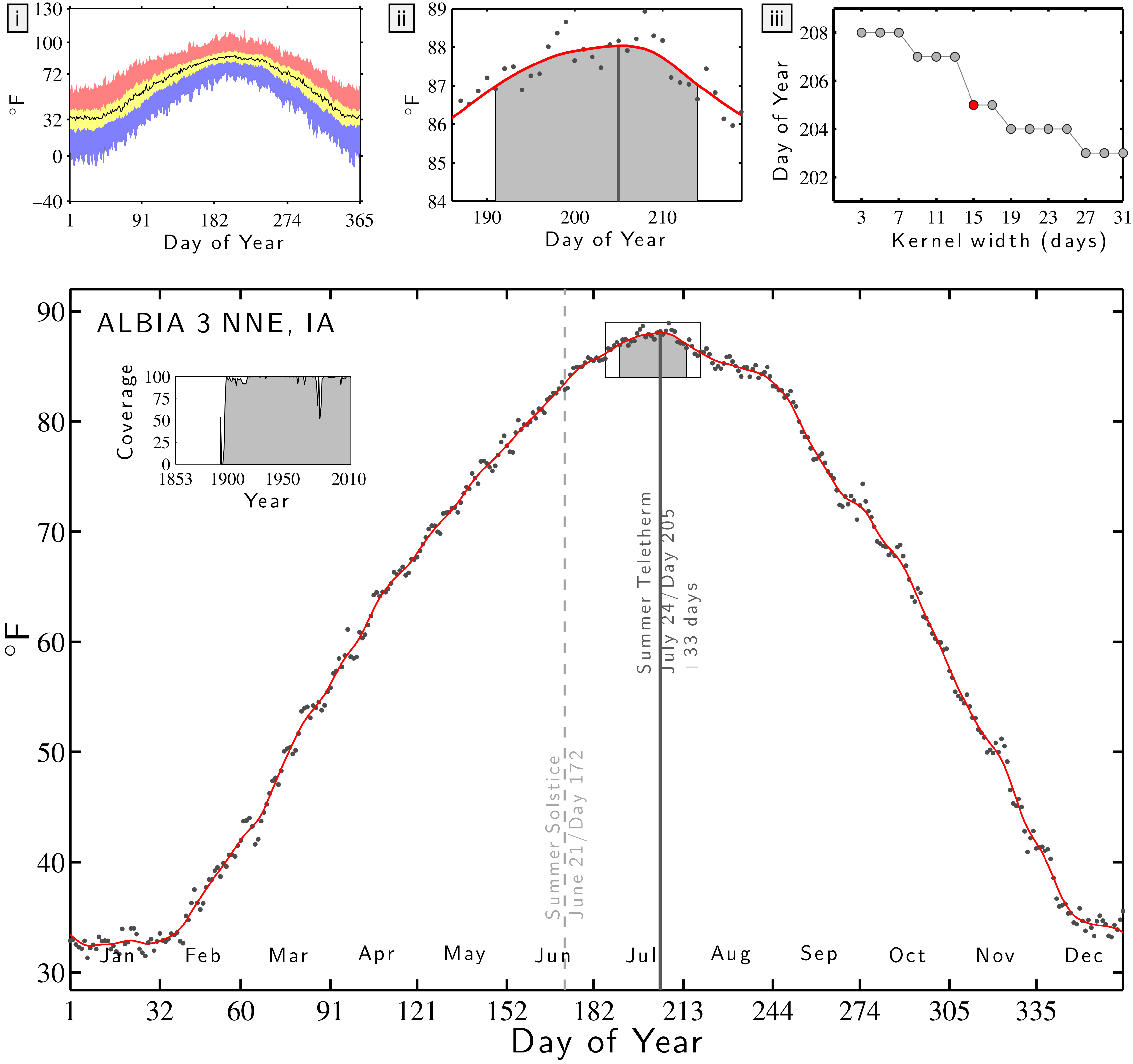
i
ii
iii
Day of Year
Kernel width (days)
°F
ALBIA 3 NNE, IA
Coverage
Year
Summer Solstice
June 21/Day 172
Summer Teletherm
July 24/Day 205
+33 days
Jan
Feb
Mar
Apr
May
Jun
Jul
Aug
Sep
Oct
Nov
Dec
Day of Year

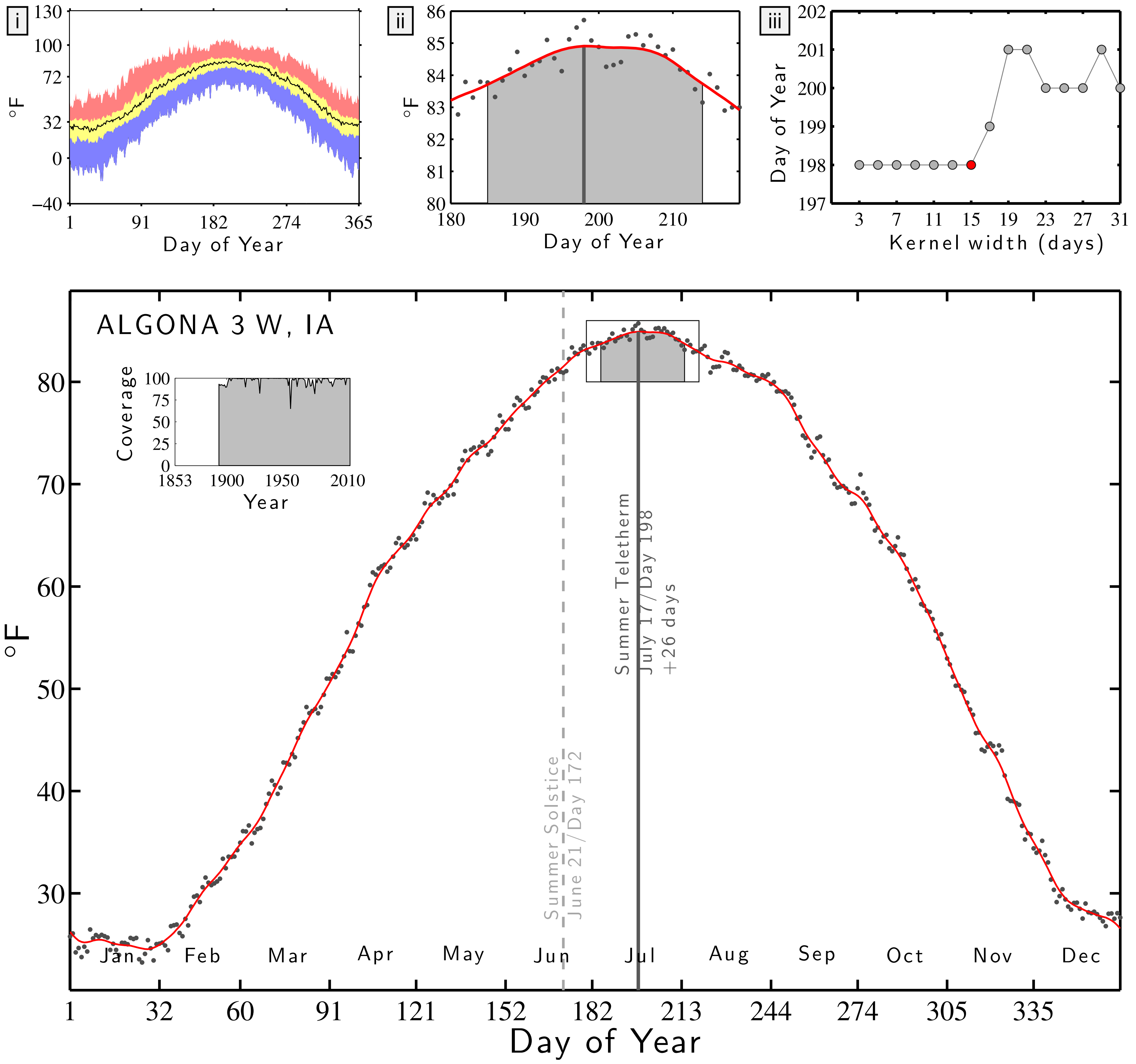

i
ii
iii
°F
Day of Year
Kernel width (days)
ALGONA 3 W, IA
Coverage
Year
Summer Solstice
June 21/Day 172
Summer Teletherm
July 17/Day 198
+26 days
Jan
Feb
Mar
Apr
May
Jun
Jul
Aug
Sep
Oct
Nov
Dec

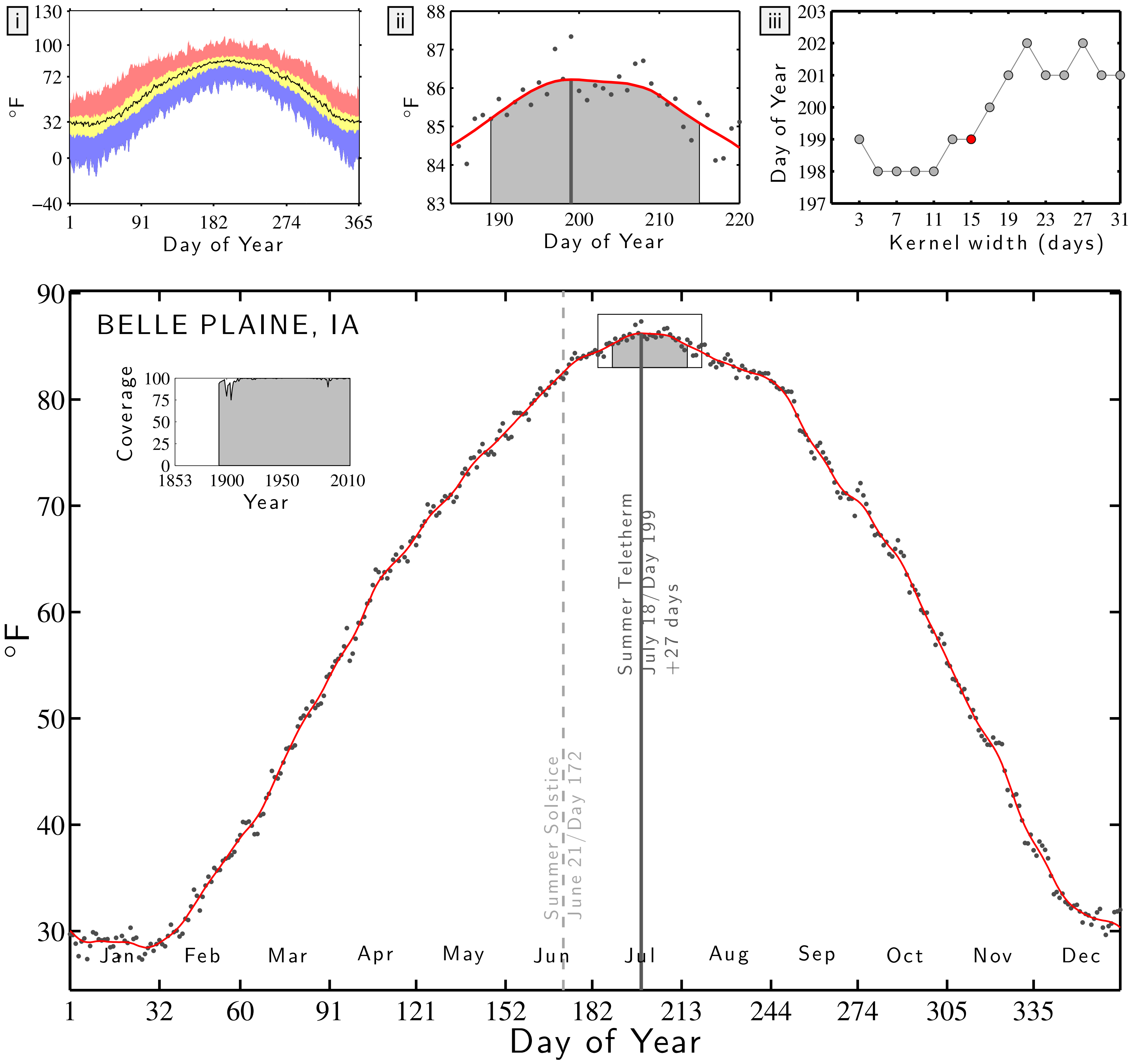

i
ii
iii
°F
Day of Year
Day of Year
Kernel width (days)
BELLE PLAINE, IA
Coverage
Year
Summer Solstice
June 21/Day 172
Summer Teletherm
July 18/Day 199
+27 days
Jan
Feb
Mar
Apr
May
Jun
Jul
Aug
Sep
Oct
Nov
Dec
°F
Day of Year

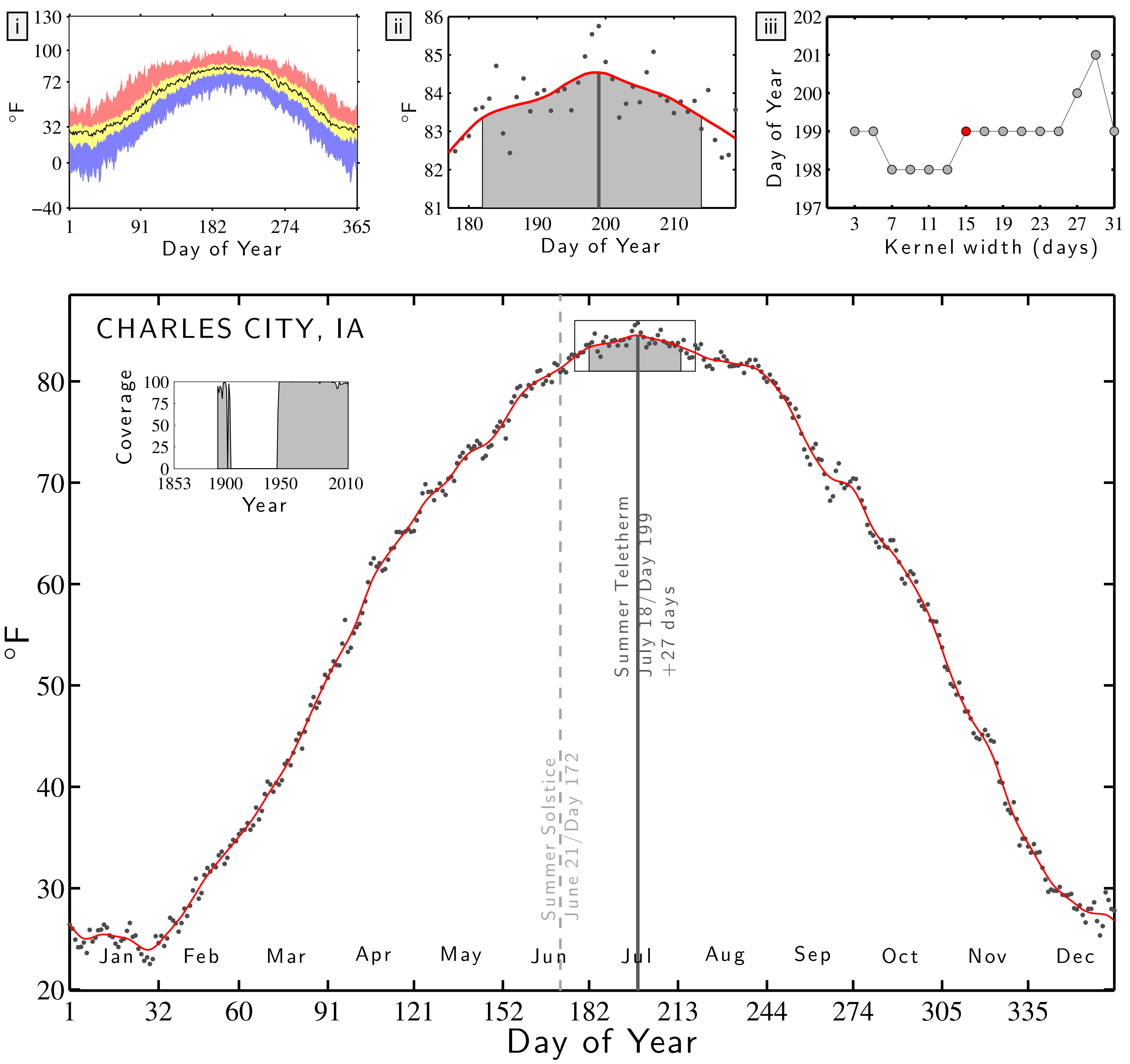
i
°F
Day of Year
ii
°F
Day of Year
iii
Day of Year
Kernel width (days)
CHARLES CITY, IA
Coverage
Year
Summer Solstice
June 21/Day 172
Summer Teletherm
July 18/Day 199
+27 days
Jan
Feb
Mar
Apr
May
Jun
Jul
Aug
Sep
Oct
Nov
Dec
°F
Day of Year

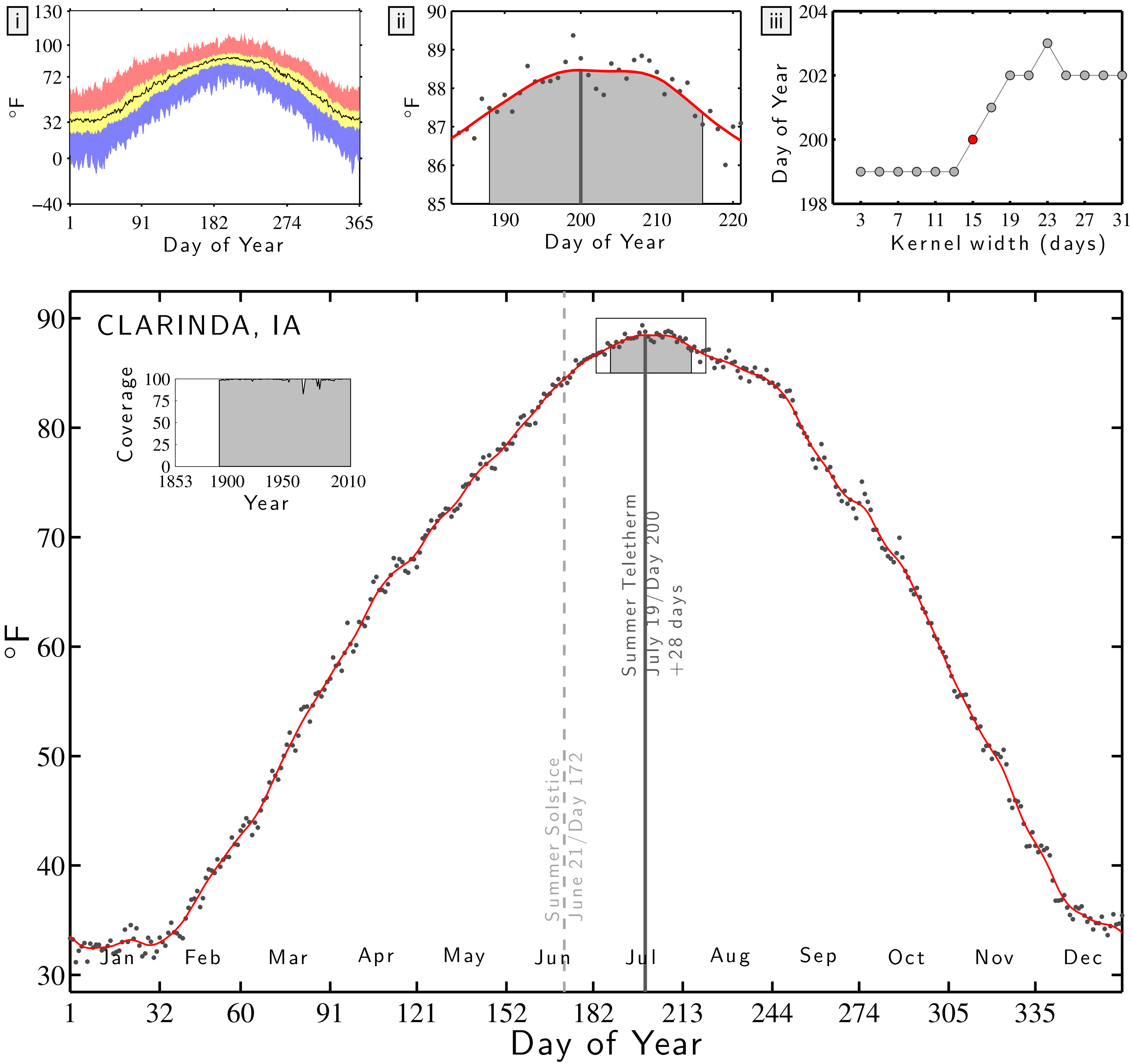

i
°F
130
100
72
32
0
−40
1
91
182
274
365
Day of Year
ii
90
89
88
87
86
85
190
200
210
220
Day of Year
iii
Day of Year
204
202
200
198
3
7
11
15
19
23
27
31
Kernel width (days)
CLARINDA, IA
Coverage
100
75
50
25
0
1853
1900
1950
2010
Year
Summer Solstice
June 21/Day 172
Summer Teletherm
July 19/Day 200
+28 days
Jan
Feb
Mar
Apr
May
Jun
Jul
Aug
Sep
Oct
Nov
Dec
90
80
70
60
50
40
30
1
32
60
91
121
152
182
213
244
274
305
335
Day of Year

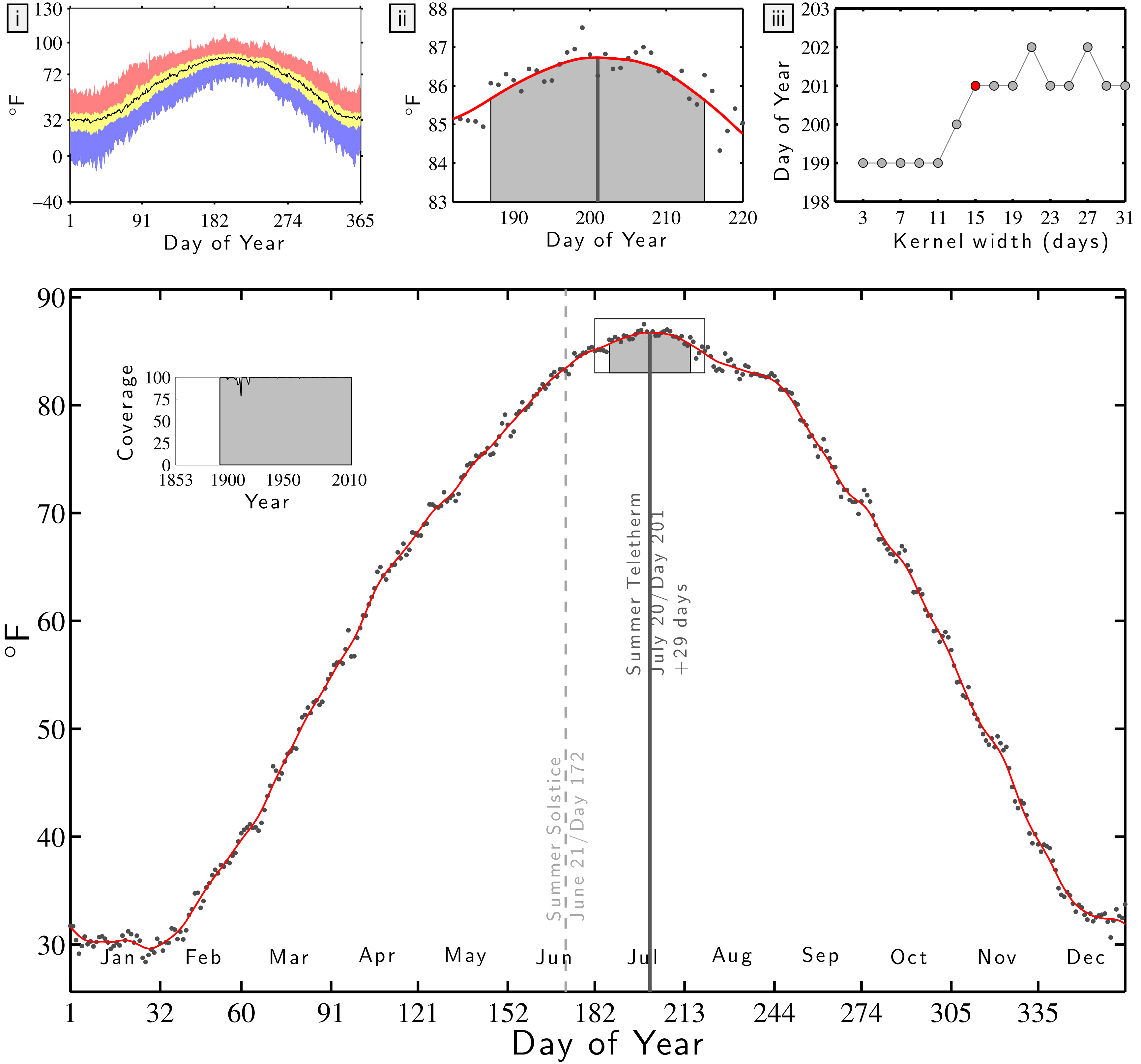

i
130
100
72
32
0
−40
°F
1
91
182
274
365
Day of Year
ii
88
87
86
85
84
83
°F
190
200
210
220
Day of Year
iii
203
202
201
200
199
198
Day of Year
3
7
11
15
19
23
27
31
Kernel width (days)
90
80
70
60
50
40
30
°F
Coverage
100
75
50
25
0
1853
1900
1950
2010
Year
Summer Solstice
June 21/Day 172
Summer Teletherm
July 20/Day 201
+29 days
Jan
Feb
Mar
Apr
May
Jun
Jul
Aug
Sep
Oct
Nov
Dec
1
32
60
91
121
152
182
213
244
274
305
335
Day of Year

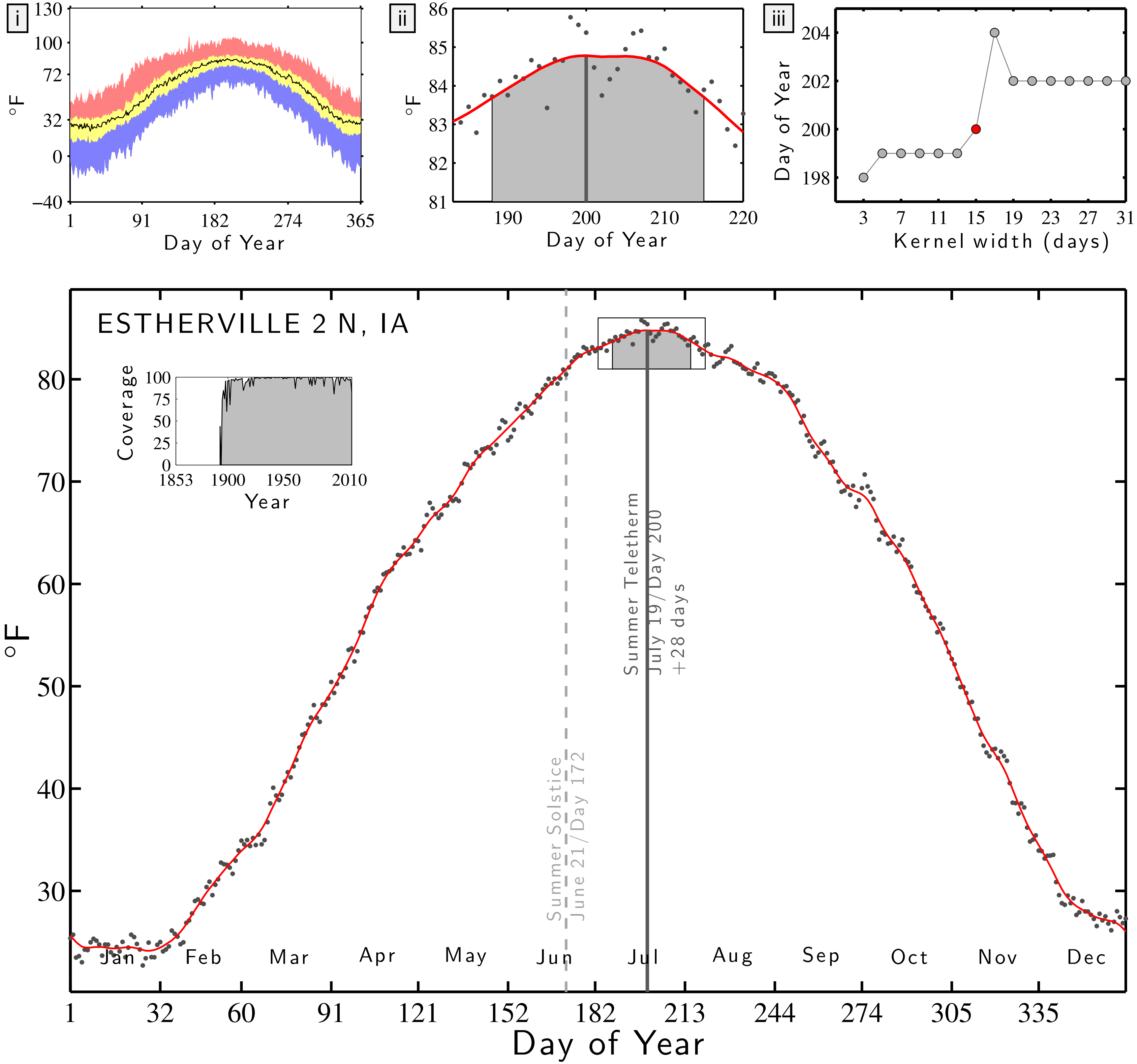

i
°F
Day of Year
ii
°F
Day of Year
iii
Day of Year
Kernel width (days)
ESTHERVILLE 2 N, IA
Coverage
Year
Summer Solstice
June 21/Day 172
Summer Teletherm
July 19/Day 200
+28 days
Jan
Feb
Mar
Apr
May
Jun
Jul
Aug
Sep
Oct
Nov
Dec
°F
Day of Year

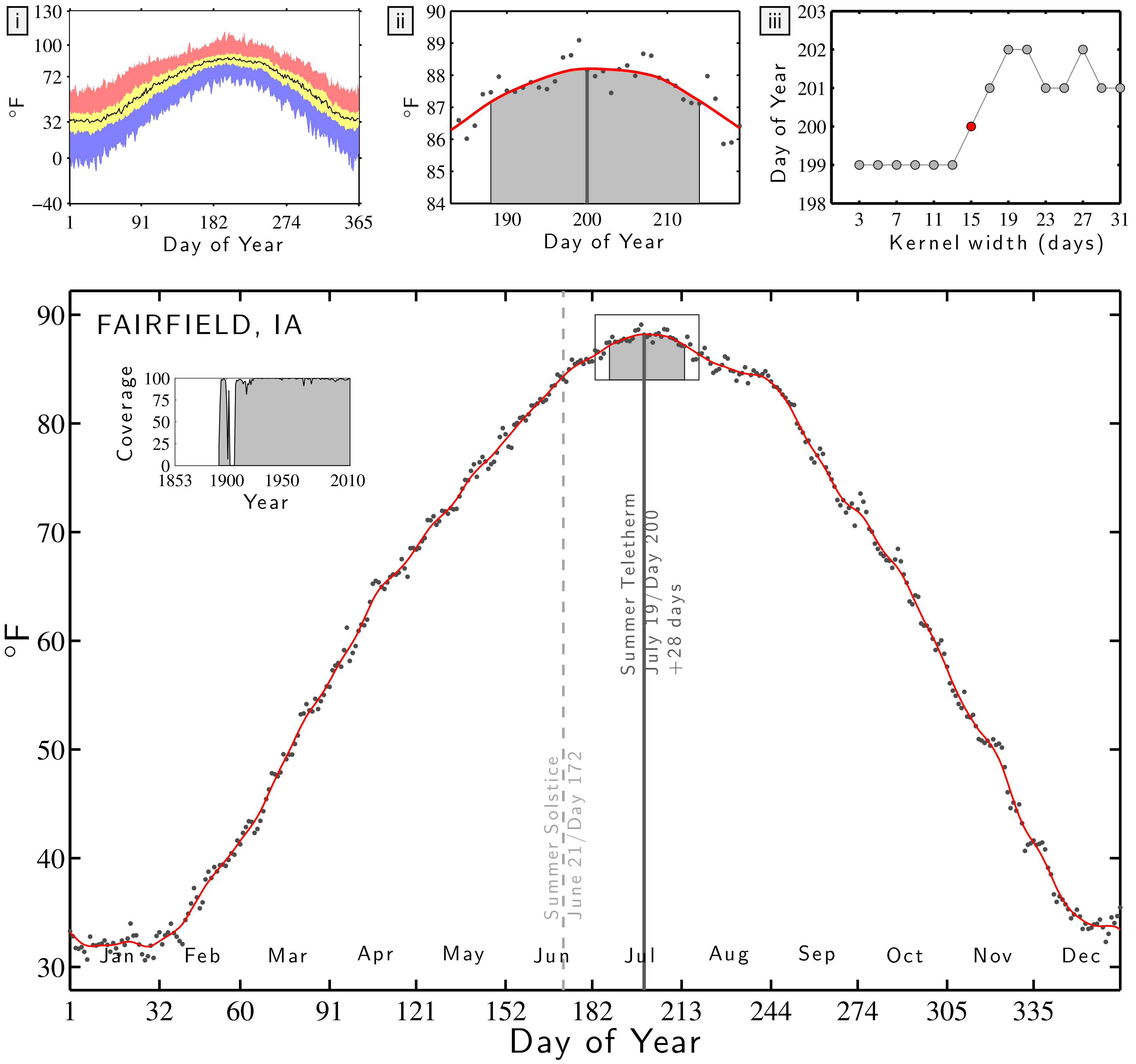

i
ii
iii
°F
Day of Year
Kernel width (days)
FAIRFIELD, IA
Coverage
Year
Summer Solstice
June 21/Day 172
Summer Teletherm
July 19/Day 200
+28 days
Jan
Feb
Mar
Apr
May
Jun
Jul
Aug
Sep
Oct
Nov
Dec

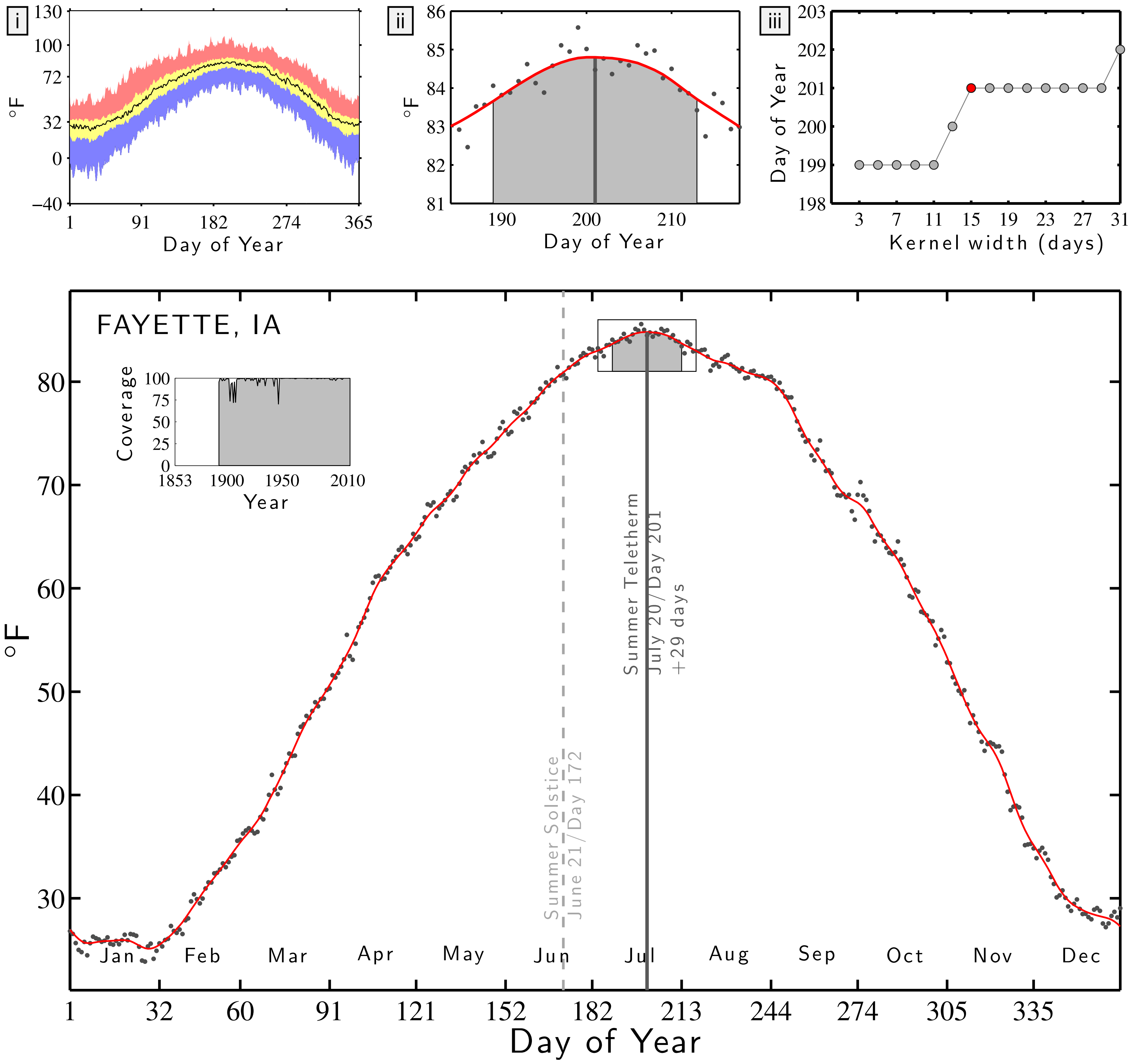
i
ii
iii
°F
Day of Year
Kernel width (days)
FAYETTE, IA
Coverage
Year
Summer Solstice
June 21/Day 172
Summer Teletherm
July 20/Day 201
+29 days
Jan
Feb
Mar
Apr
May
Jun
Jul
Aug
Sep
Oct
Nov
Dec

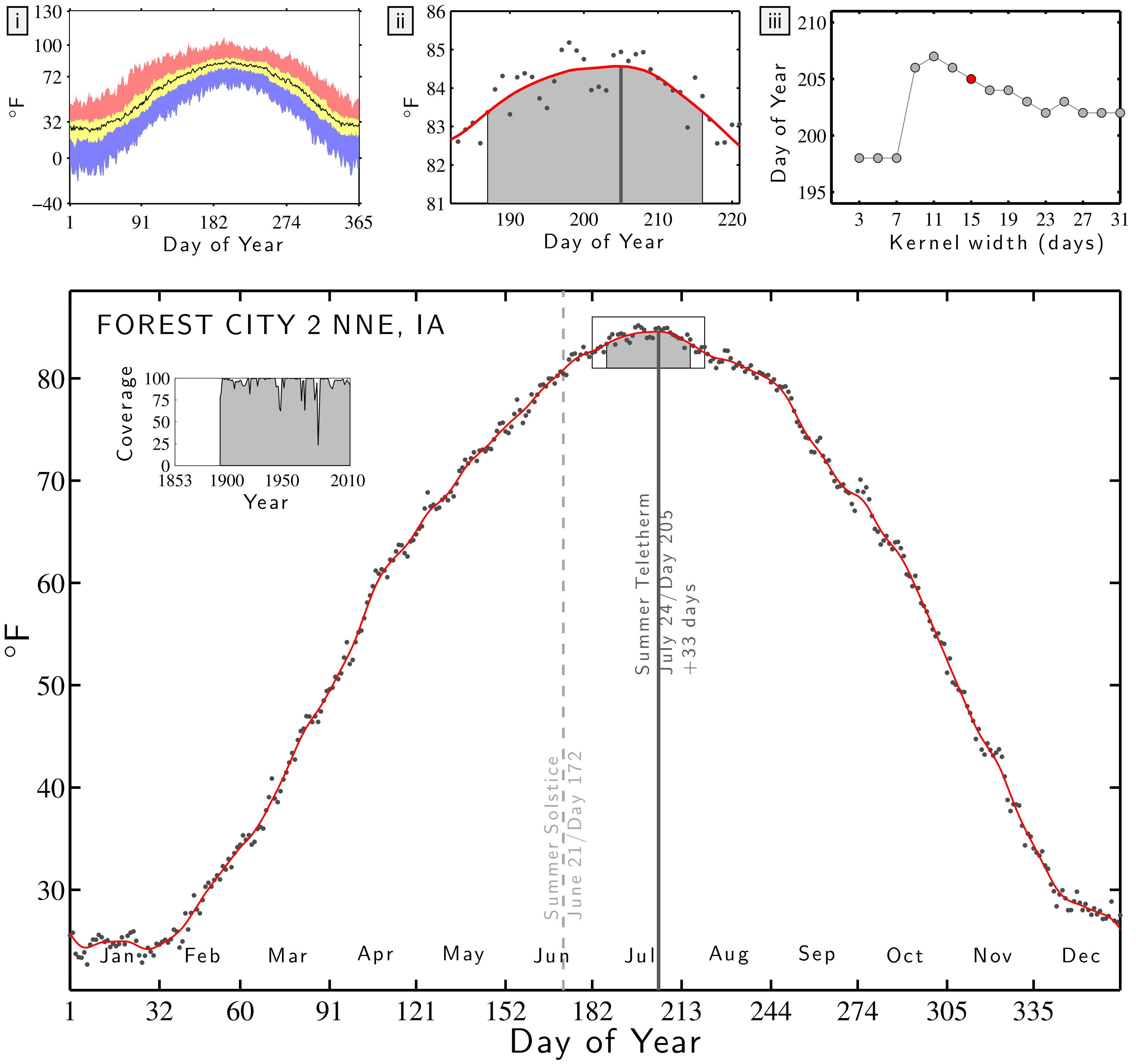
i
ii
iii
Day of Year
°F
Kernel width (days)
FOREST CITY 2 NNE, IA
Coverage
Year
Summer Solstice
June 21 / Day 172
Summer Teletherm
July 24 / Day 205
+33 days
Jan
Feb
Mar
Apr
May
Jun
Jul
Aug
Sep
Oct
Nov
Dec
Day of Year

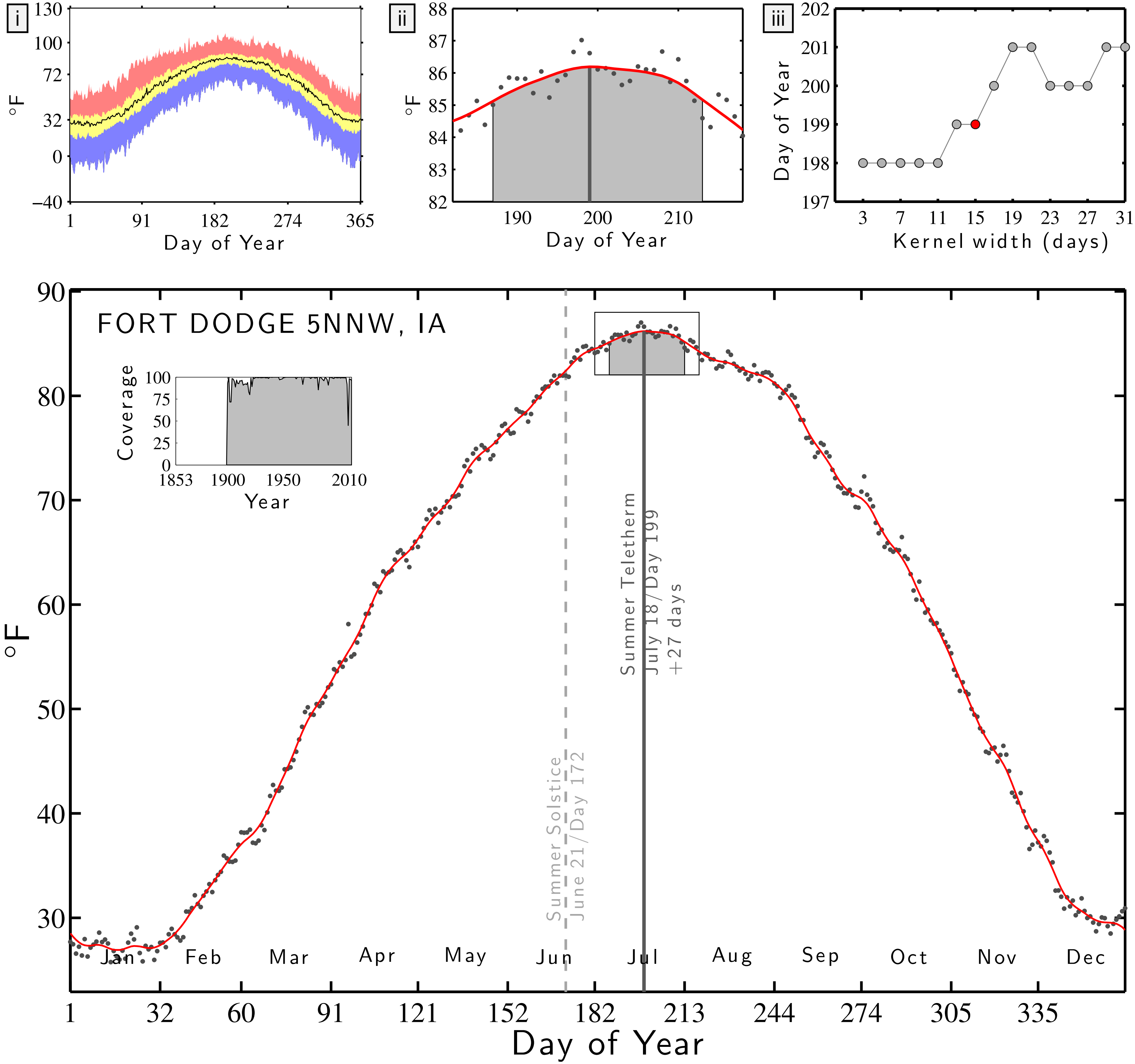

i
130
100
72
32
0
−40
°F
1
91
182
274
365
Day of Year
ii
88
87
86
85
84
83
82
°F
190
200
210
Day of Year
iii
202
201
200
199
198
197
Day of Year
3
7
11
15
19
23
27
31
Kernel width (days)
FORT DODGE 5NNW, IA
Coverage
100
75
50
25
0
1853
1900
1950
2010
Year
Summer Solstice
June 21/Day 172
Summer Teletherm
July 18/Day 199
+27 days
90
80
70
60
50
40
30
°F
Jan
Feb
Mar
Apr
May
Jun
Jul
Aug
Sep
Oct
Nov
Dec
1
32
60
91
121
152
182
213
244
274
305
335
Day of Year

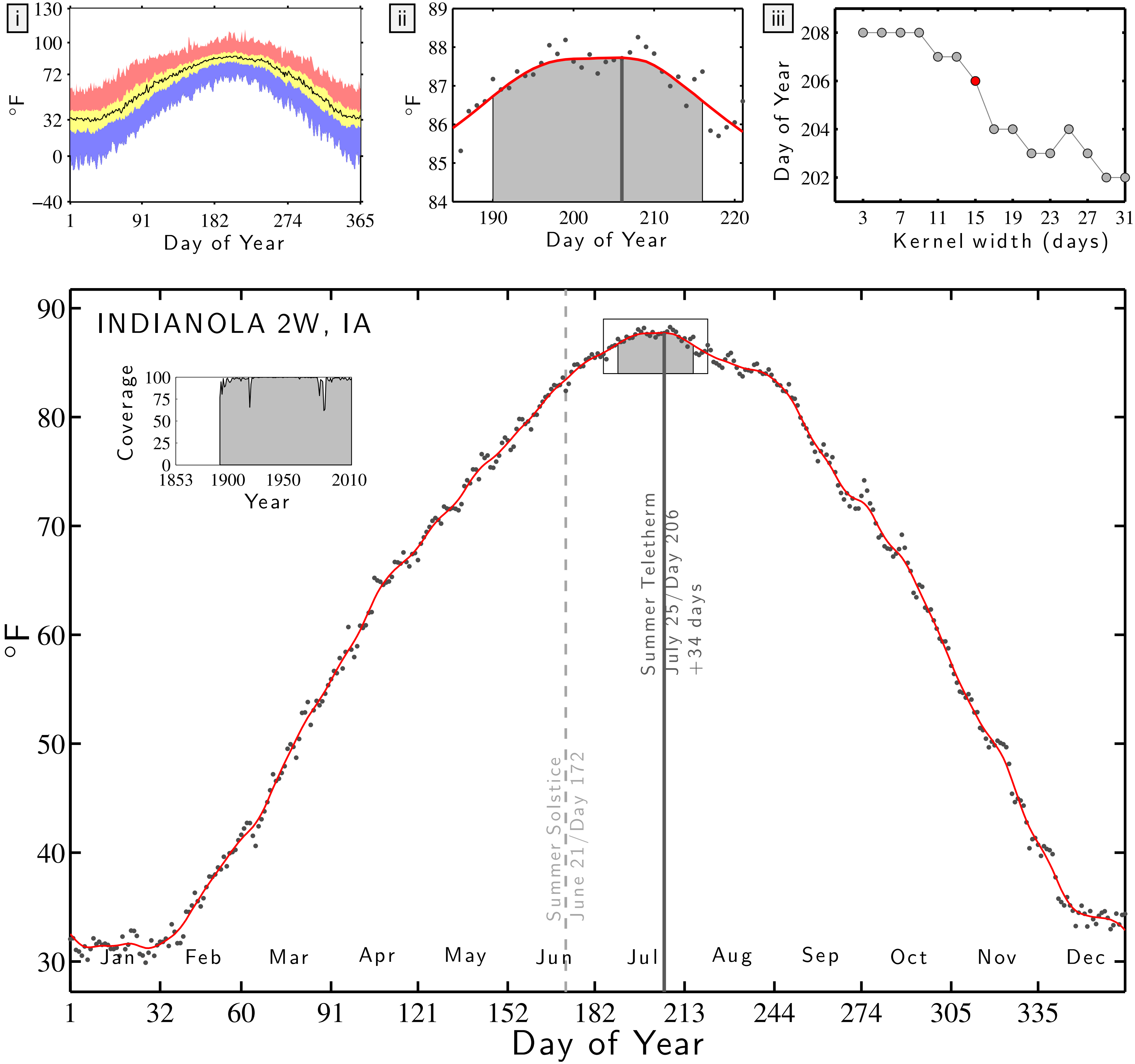

i
ii
iii
°F
Day of Year
Day of Year
Kernel width (days)
INDIANOLA 2W, IA
Coverage
Year
Summer Solstice
June 21/Day 172
Summer Teletherm
July 25/Day 206
+34 days
Jan
Feb
Mar
Apr
May
Jun
Jul
Aug
Sep
Oct
Nov
Dec

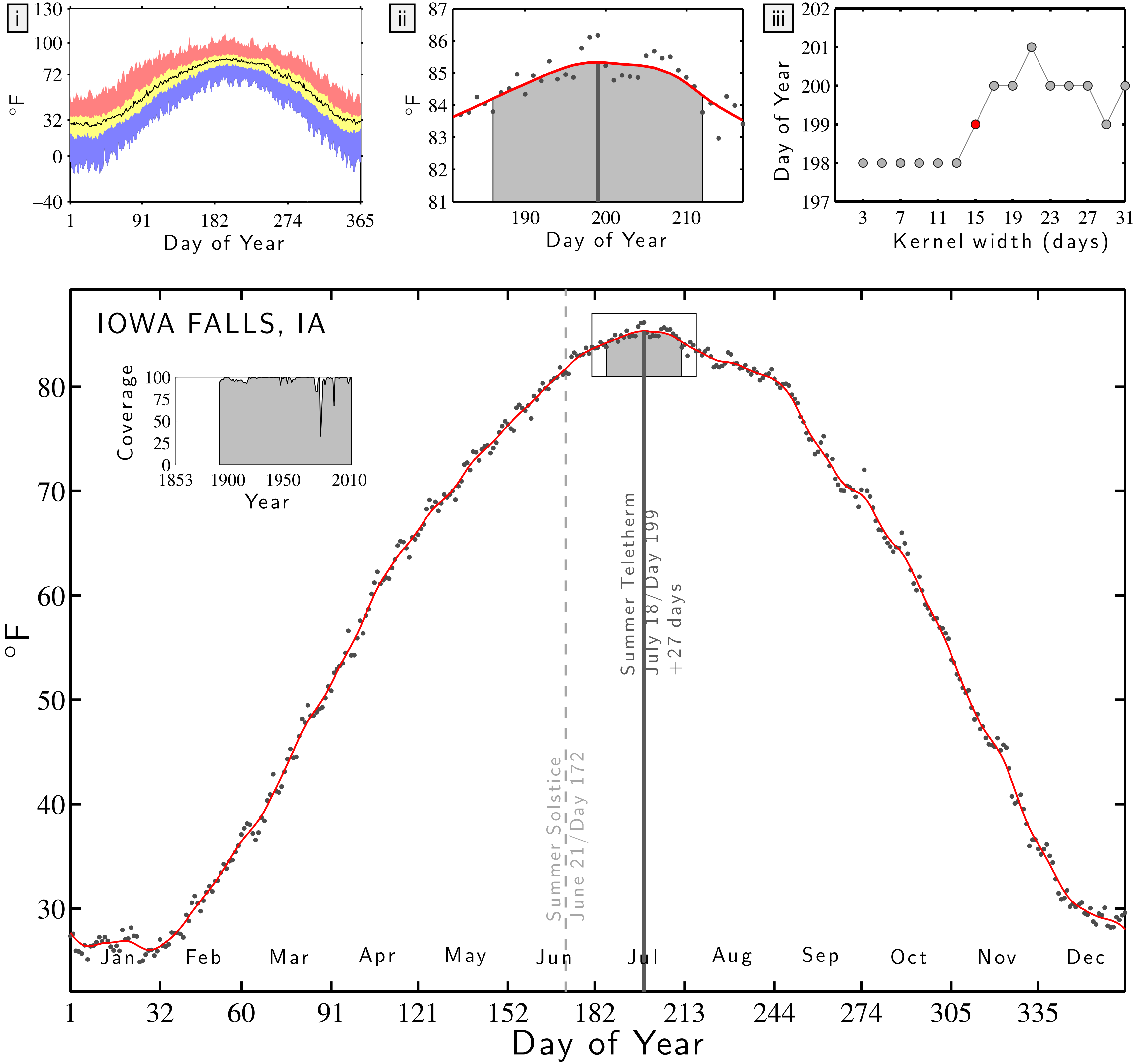

i
°F
130
100
72
32
0
−40
1
91
182
274
365
Day of Year
ii
°F
87
86
85
84
83
82
81
190
200
210
Day of Year
iii
Day of Year
202
201
200
199
198
197
3
7
11
15
19
23
27
31
Kernel width (days)
IOWA FALLS, IA
Coverage
100
75
50
25
0
1853
1900
1950
2010
Year
Summer Solstice
June 21 / Day 172
Summer Teletherm
July 18 / Day 199
+27 days
Jan
Feb
Mar
Apr
May
Jun
Jul
Aug
Sep
Oct
Nov
Dec
°F
80
70
60
50
40
30
1
32
60
91
121
152
182
213
244
274
305
335
Day of Year

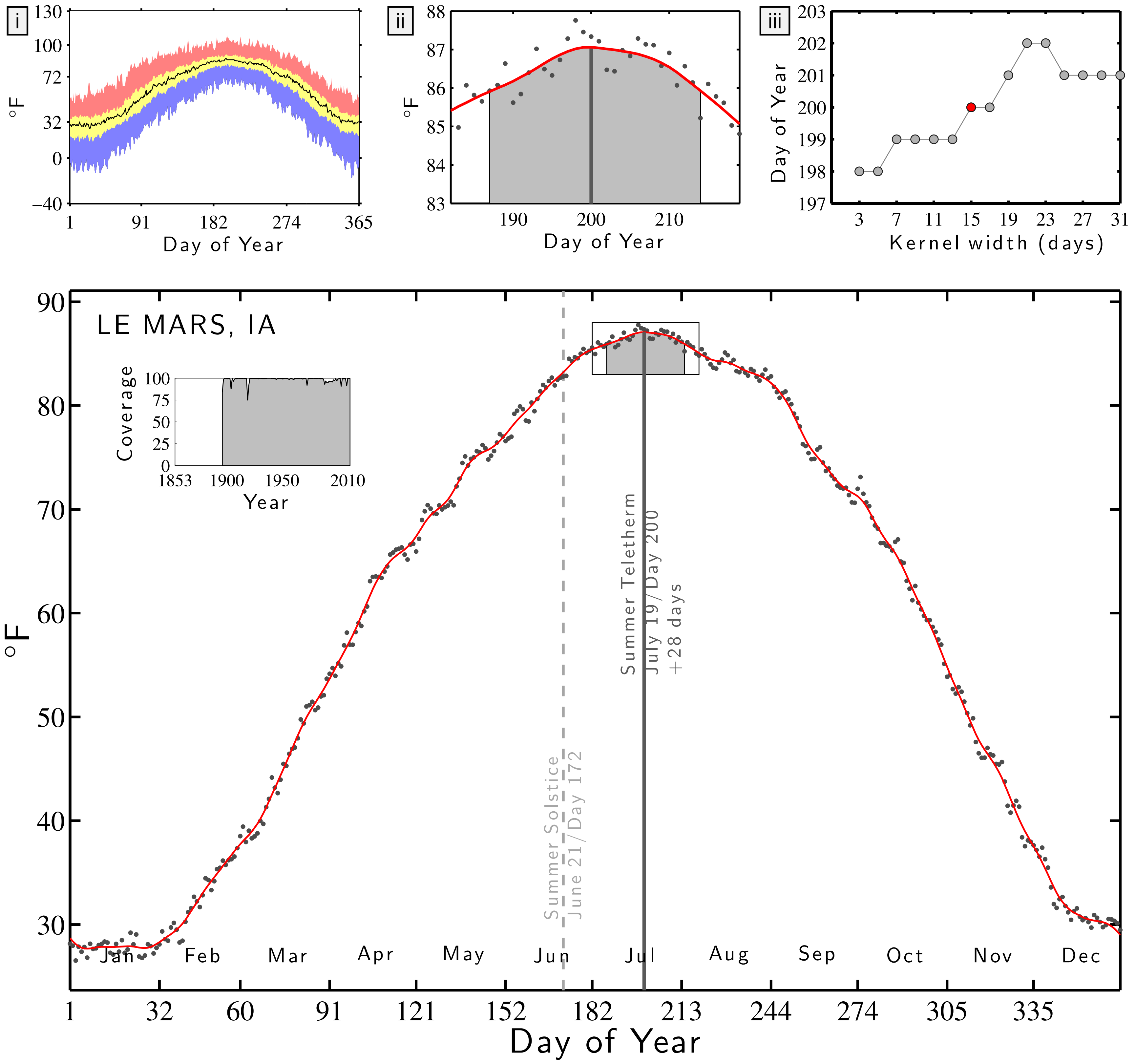

i
°F
Day of Year
ii
°F
Day of Year
iii
Day of Year
Kernel width (days)
LE MARS, IA
Coverage
Year
Summer Solstice
June 21/Day 172
Summer Teletherm
July 19/Day 200
+28 days
Jan
Feb
Mar
Apr
May
Jun
Jul
Aug
Sep
Oct
Nov
Dec
°F
Day of Year

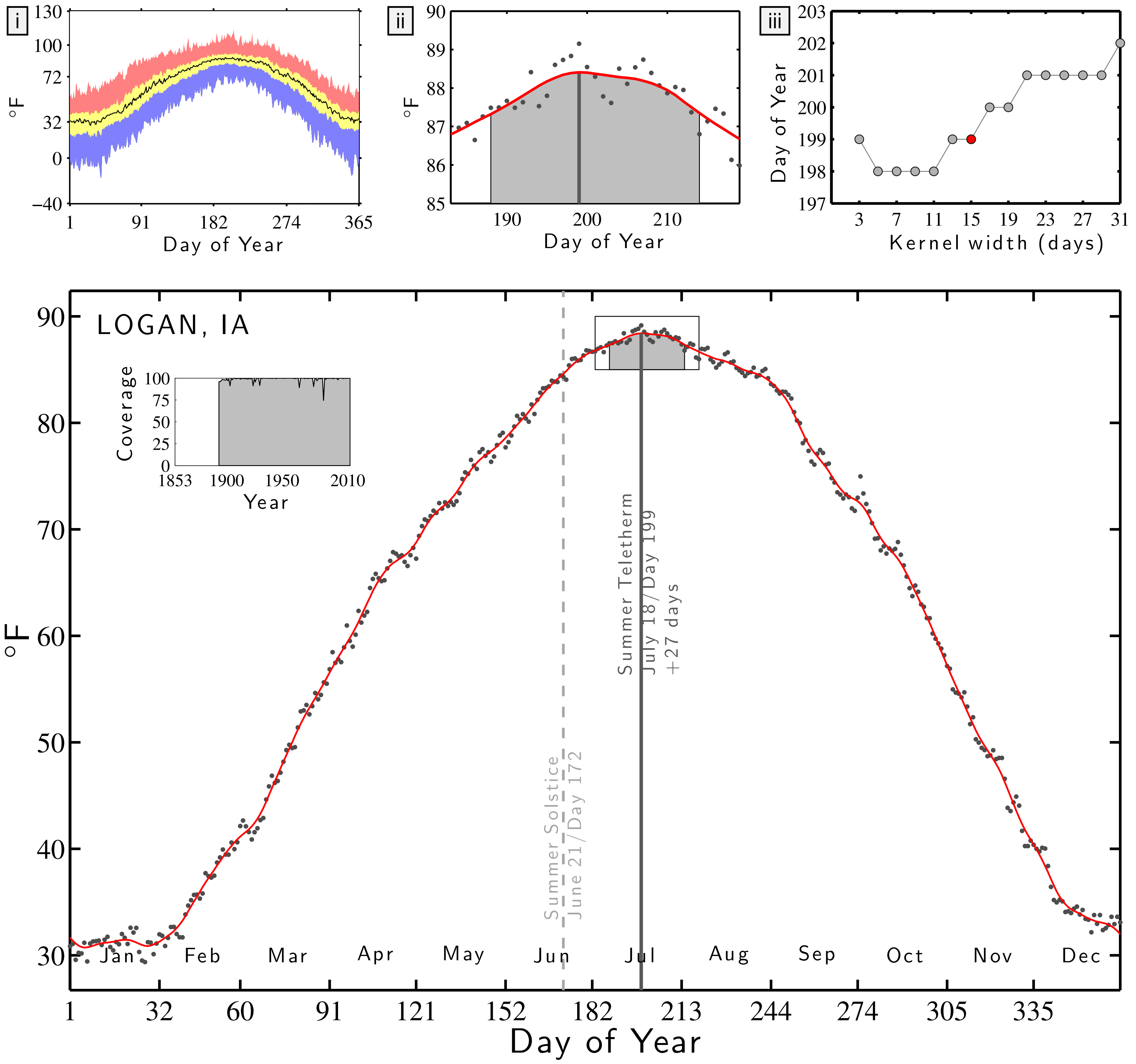

i
°F
130
100
72
32
0
−40
1
91
182
274
365
Day of Year
ii
90
89
88
87
86
85
190
200
210
Day of Year
iii
Day of Year
203
202
201
200
199
198
197
3
7
11
15
19
23
27
31
Kernel width (days)
LOGAN, IA
Coverage
100
75
50
25
0
1853
1900
1950
2010
Year
Summer Solstice
June 21/Day 172
Summer Teletherm
July 18/Day 199
+27 days
Jan
Feb
Mar
Apr
May
Jun
Jul
Aug
Sep
Oct
Nov
Dec
90
80
70
60
50
40
30
1
32
60
91
121
152
182
213
244
274
305
335
Day of Year

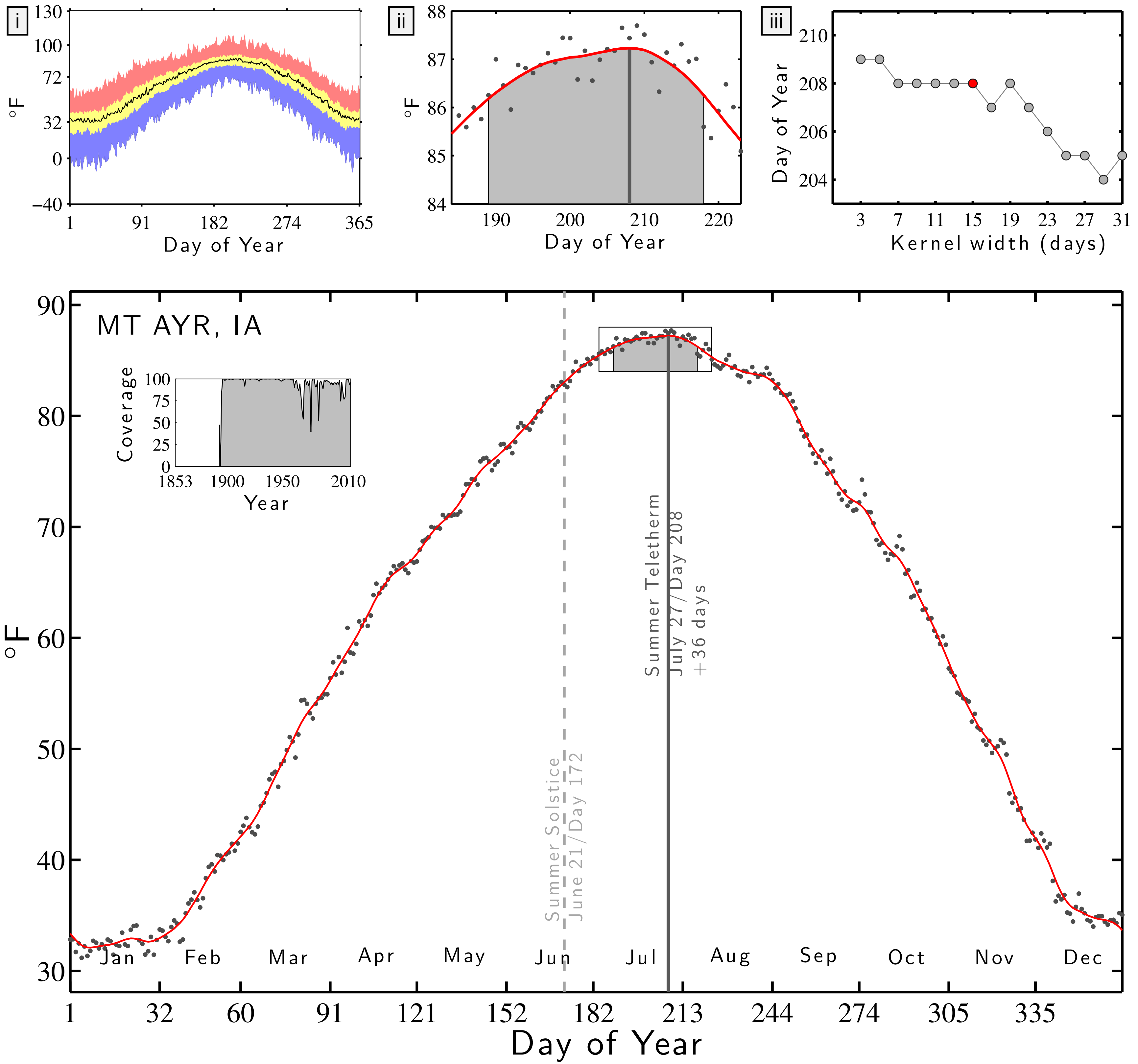

i
°F
Day of Year
ii
°F
Day of Year
iii
Day of Year
Kernel width (days)
MT AYR, IA
Coverage
Year
Summer Solstice
June 21/Day 172
Summer Teletherm
July 27/Day 208
+36 days
Jan
Feb
Mar
Apr
May
Jun
Jul
Aug
Sep
Oct
Nov
Dec
°F
Day of Year

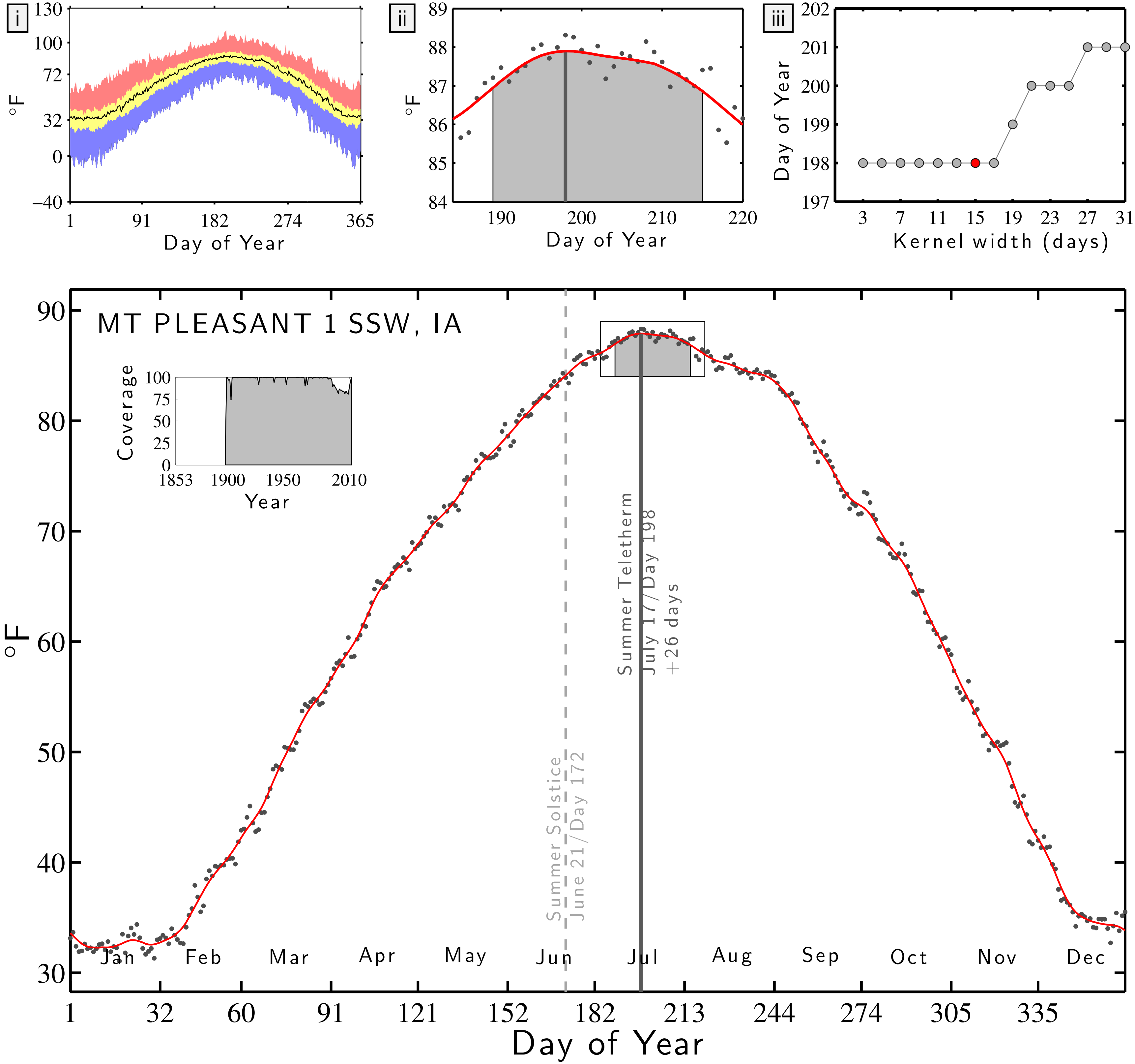

i
130
100
72
32
0
−40
°F
1
91
182
274
365
Day of Year
ii
89
88
87
86
85
84
°F
190
200
210
220
Day of Year
iii
202
201
200
199
198
197
Day of Year
3
7
11
15
19
23
27
31
Kernel width (days)
MT PLEASANT 1 SSW, IA
Coverage
100
75
50
25
0
1853
1900
1950
2010
Year
Summer Solstice
June 21/Day 172
Summer Teletherm
July 17/Day 198
+26 days
Jan
Feb
Mar
Apr
May
Jun
Jul
Aug
Sep
Oct
Nov
Dec
90
80
70
60
50
40
30
°F
1
32
60
91
121
152
182
213
244
274
305
335
Day of Year

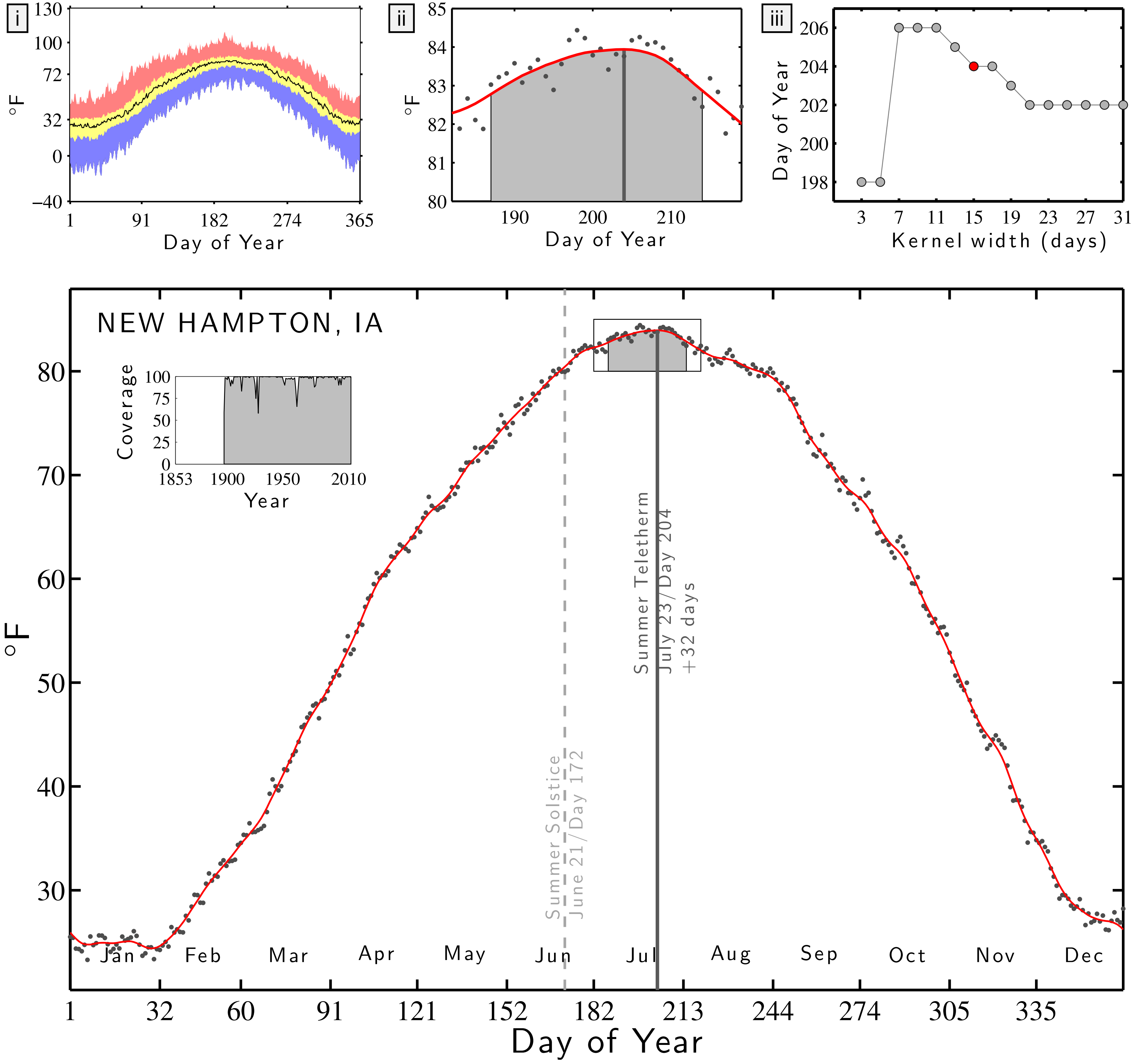

i
°F
Day of Year
ii
°F
Day of Year
iii
Day of Year
Kernel width (days)
NEW HAMPTON, IA
Coverage
Year
Summer Solstice
June 21 / Day 172
Summer Teletherm
July 23 / Day 204
+32 days
Jan
Feb
Mar
Apr
May
Jun
Jul
Aug
Sep
Oct
Nov
Dec
°F
Day of Year

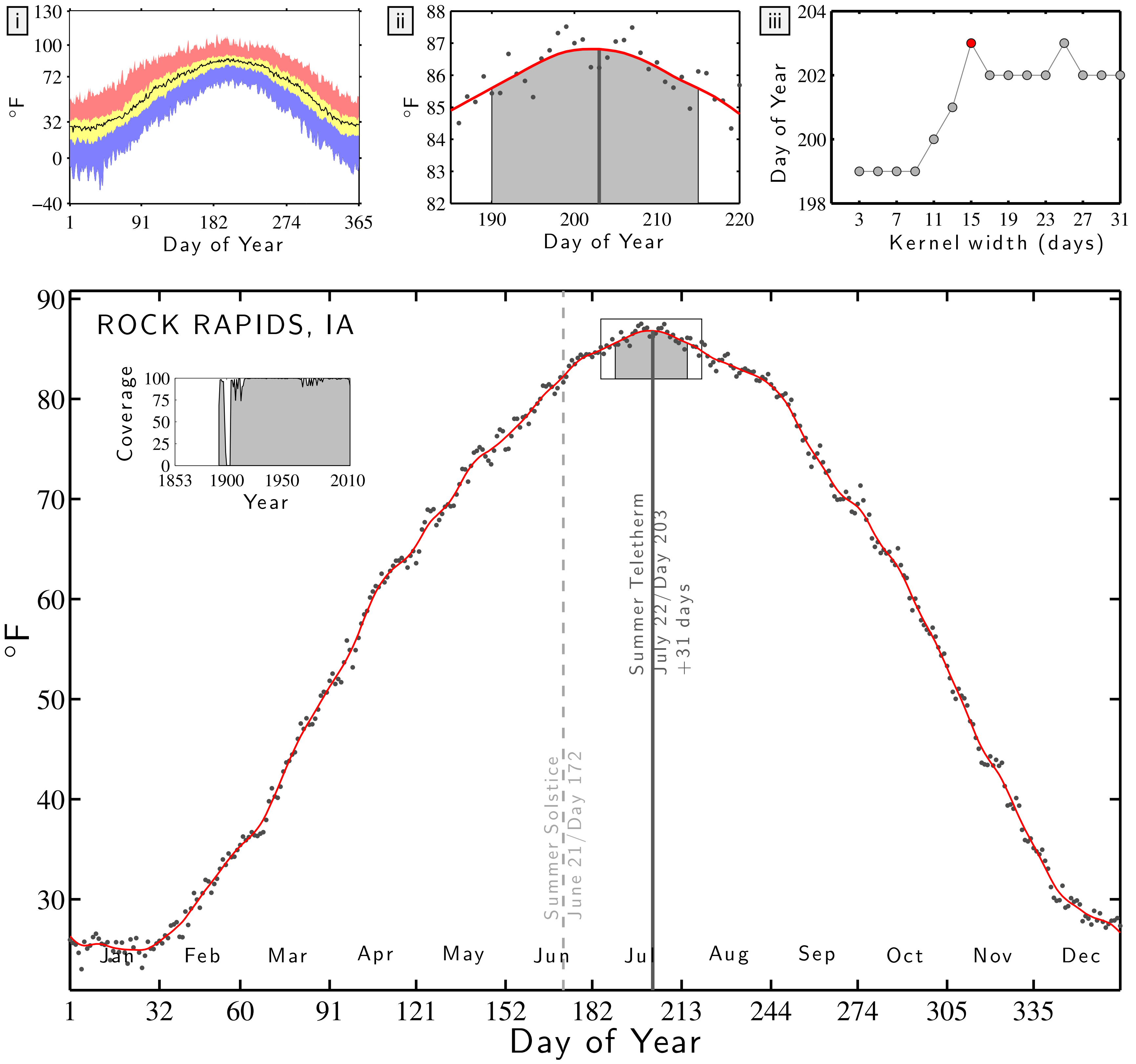

i
ii
iii
ROCK RAPIDS, IA
Coverage
Year
Summer Solstice
June 21/Day 172
Summer Teletherm
July 22/Day 203
+31 days
Day of Year
Kernel width (days)
°F
Jan Feb Mar Apr May Jun Jul Aug Sep Oct Nov Dec

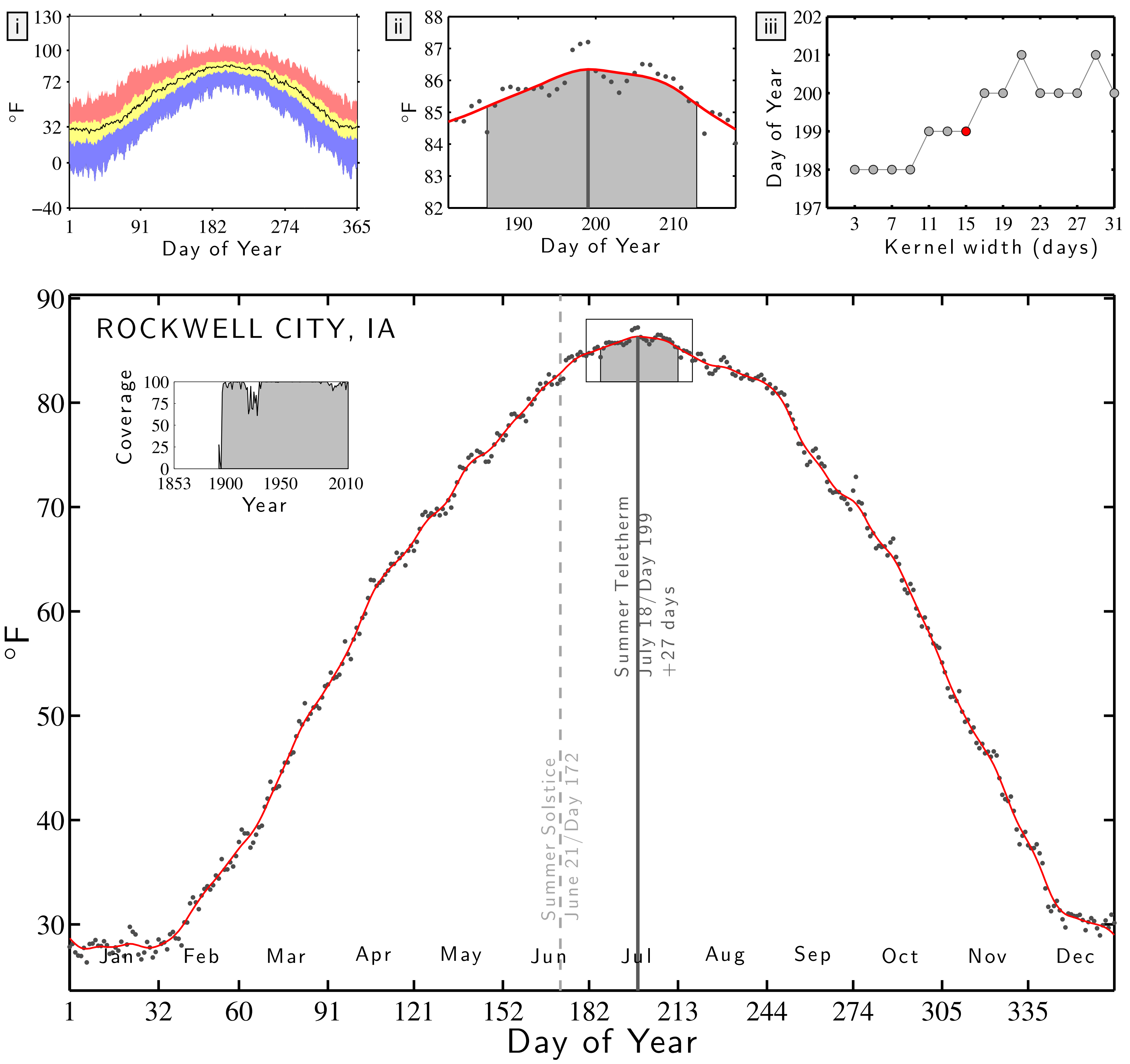

i
ii
iii
°F
Day of Year
Kernel width (days)
ROCKWELL CITY, IA
Coverage
Year
Summer Solstice
June 21/Day 172
Summer Teletherm
July 18/Day 199
+27 days
Jan
Feb
Mar
Apr
May
Jun
Jul
Aug
Sep
Oct
Nov
Dec

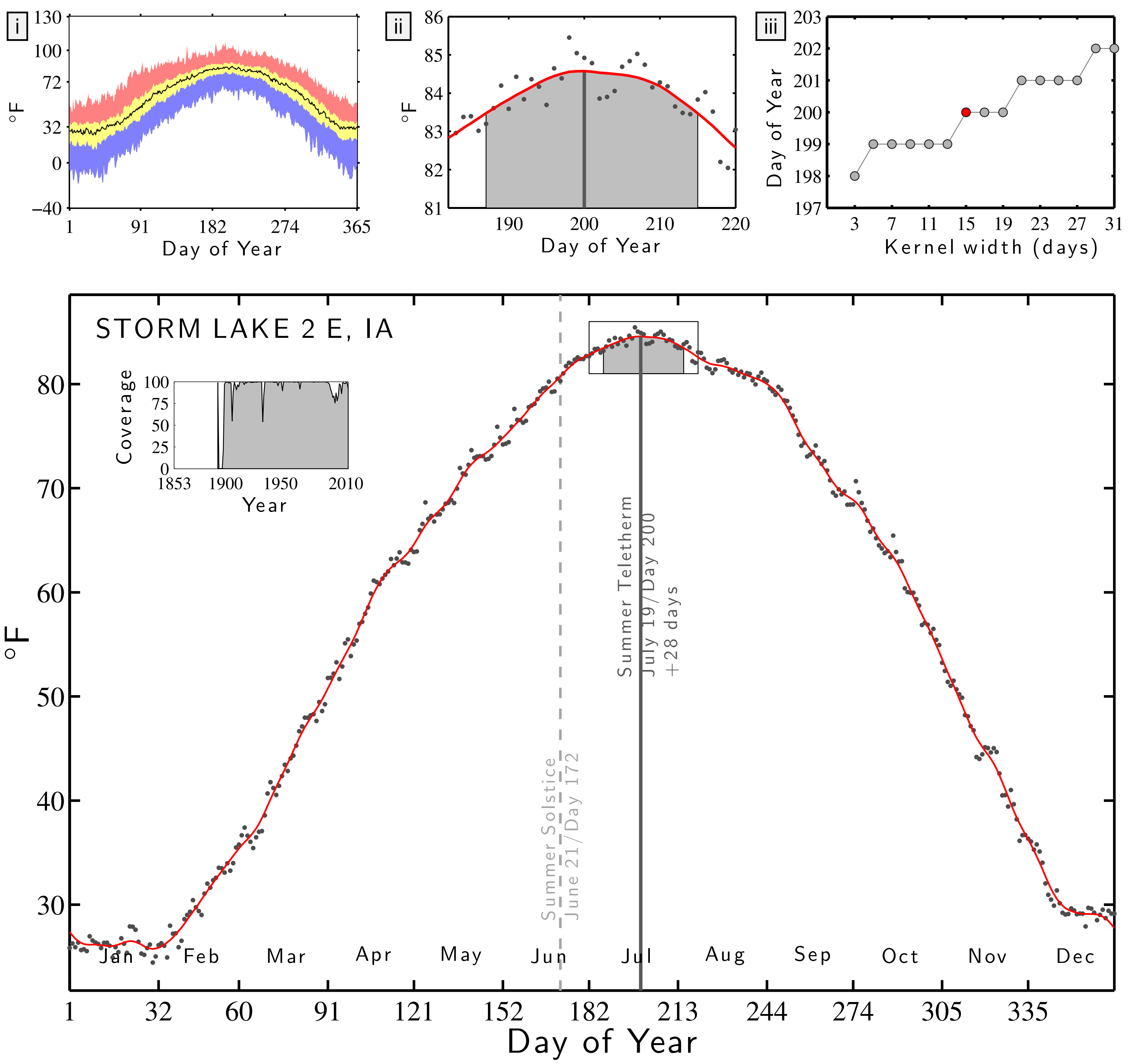

i
°F
Day of Year
ii
°F
Day of Year
iii
Day of Year
Kernel width (days)
STORM LAKE 2 E, IA
Coverage
Year
Summer Solstice
June 21/Day 172
Summer Teletherm
July 19/Day 200
+28 days
Jan
Feb
Mar
Apr
May
Jun
Jul
Aug
Sep
Oct
Nov
Dec
°F
Day of Year

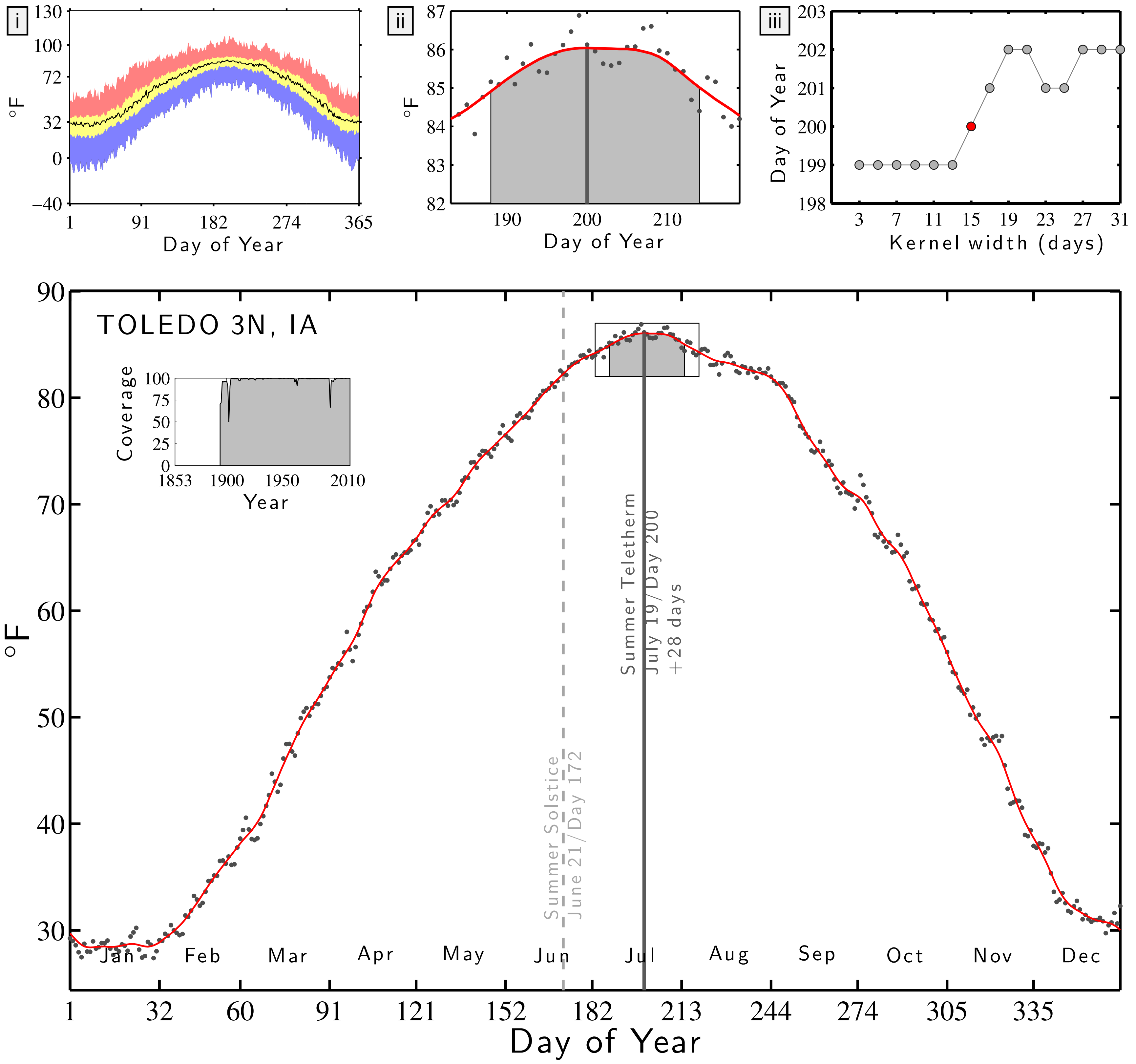

i
°F
Day of Year
ii
°F
Day of Year
iii
Day of Year
Kernel width (days)
TOLEDO 3N, IA
Coverage
Year
Summer Solstice
June 21/Day 172
Summer Teletherm
July 19/Day 200
+28 days
Jan
Feb
Mar
Apr
May
Jun
Jul
Aug
Sep
Oct
Nov
Dec
°F
Day of Year

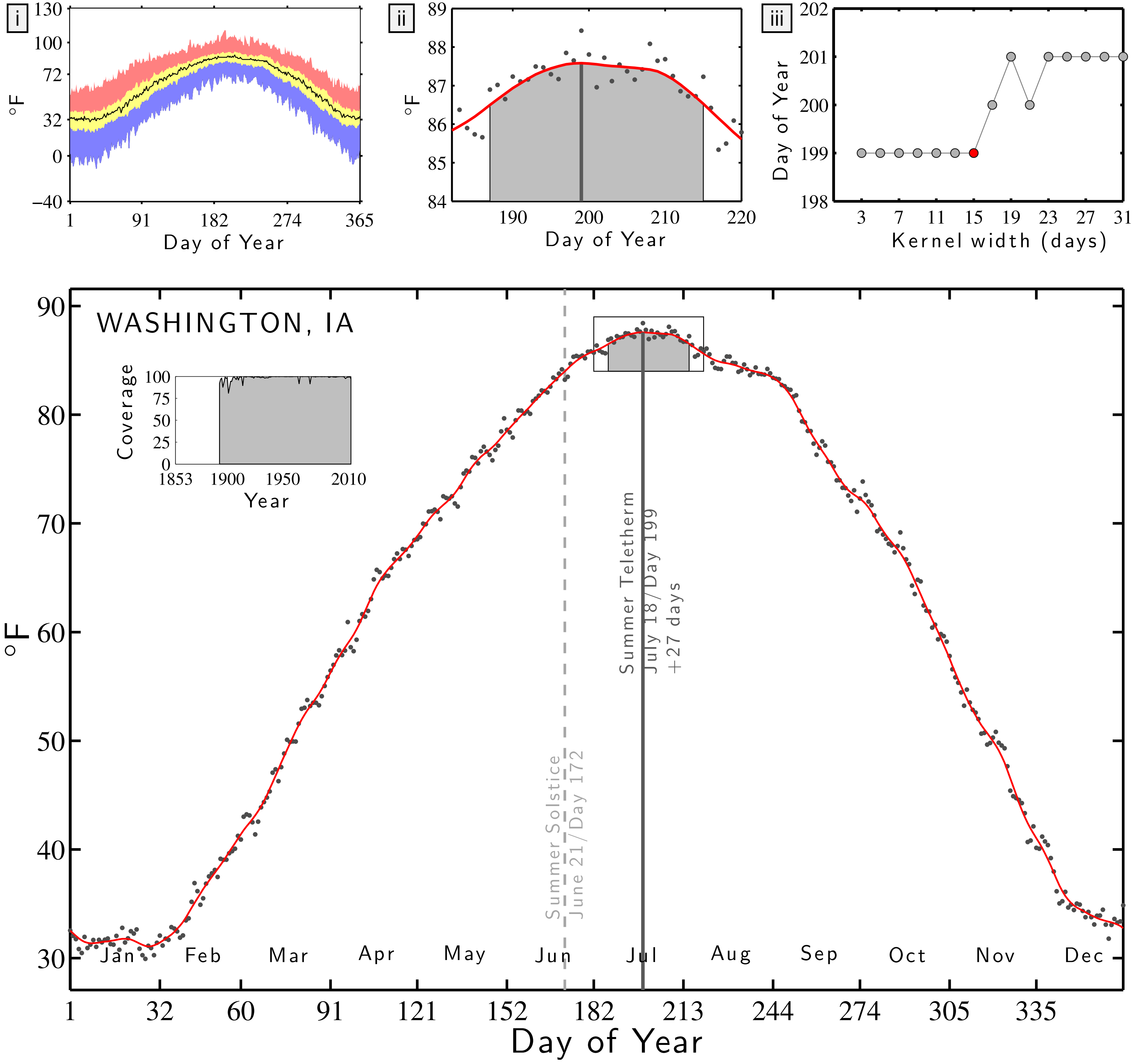

i
°F
Day of Year
ii
°F
Day of Year
iii
Day of Year
Kernel width (days)
WASHINGTON, IA
Coverage
Year
Summer Solstice
June 21/Day 172
Summer Teletherm
July 18/Day 199
+27 days
Jan
Feb
Mar
Apr
May
Jun
Jul
Aug
Sep
Oct
Nov
Dec
°F
Day of Year

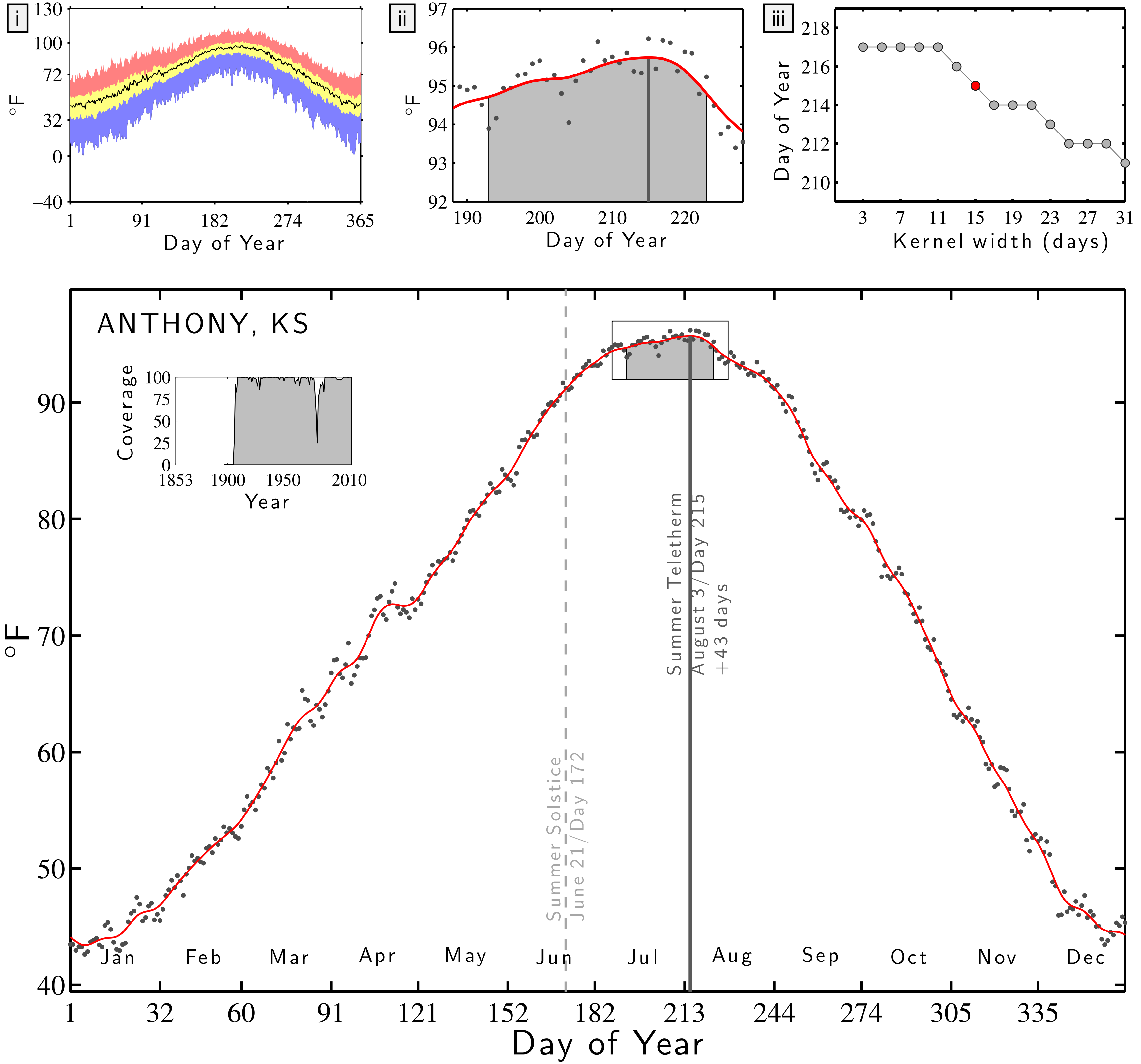

i
°F
Day of Year
ii
°F
Day of Year
iii
Day of Year
Kernel width (days)
ANTHONY, KS
Coverage
Year
Summer Solstice
June 21 / Day 172
Summer Teletherm
August 3 / Day 215
+43 days
Jan
Feb
Mar
Apr
May
Jun
Jul
Aug
Sep
Oct
Nov
Dec
°F
Day of Year

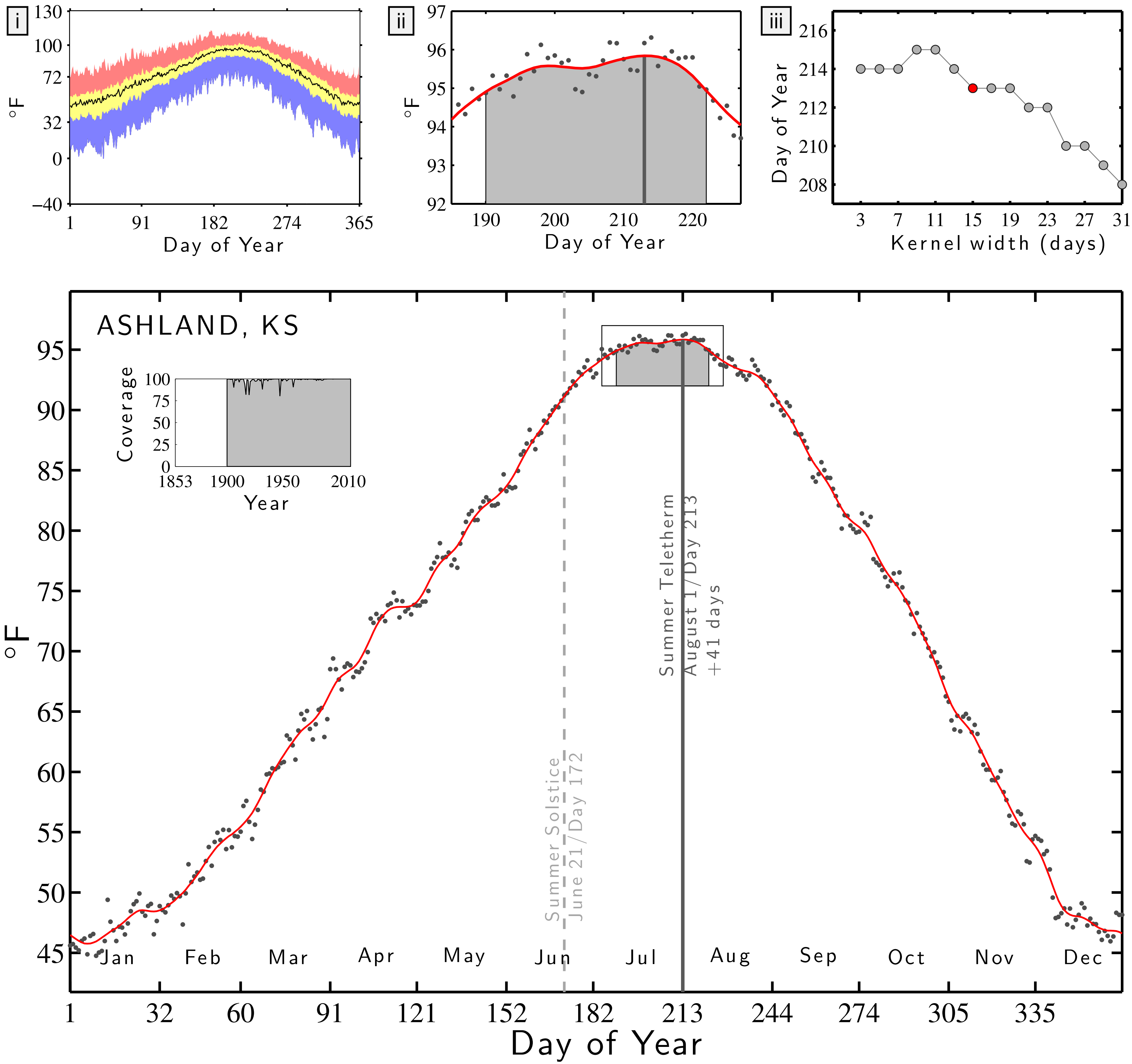

i
°F
Day of Year
ii
°F
Day of Year
iii
Day of Year
Kernel width (days)
ASHLAND, KS
Coverage
Year
Summer Solstice
June 21/Day 172
Summer Teletherm
August 1/Day 213
+41 days
Jan Feb Mar Apr May Jun Jul Aug Sep Oct Nov Dec
°F
Day of Year

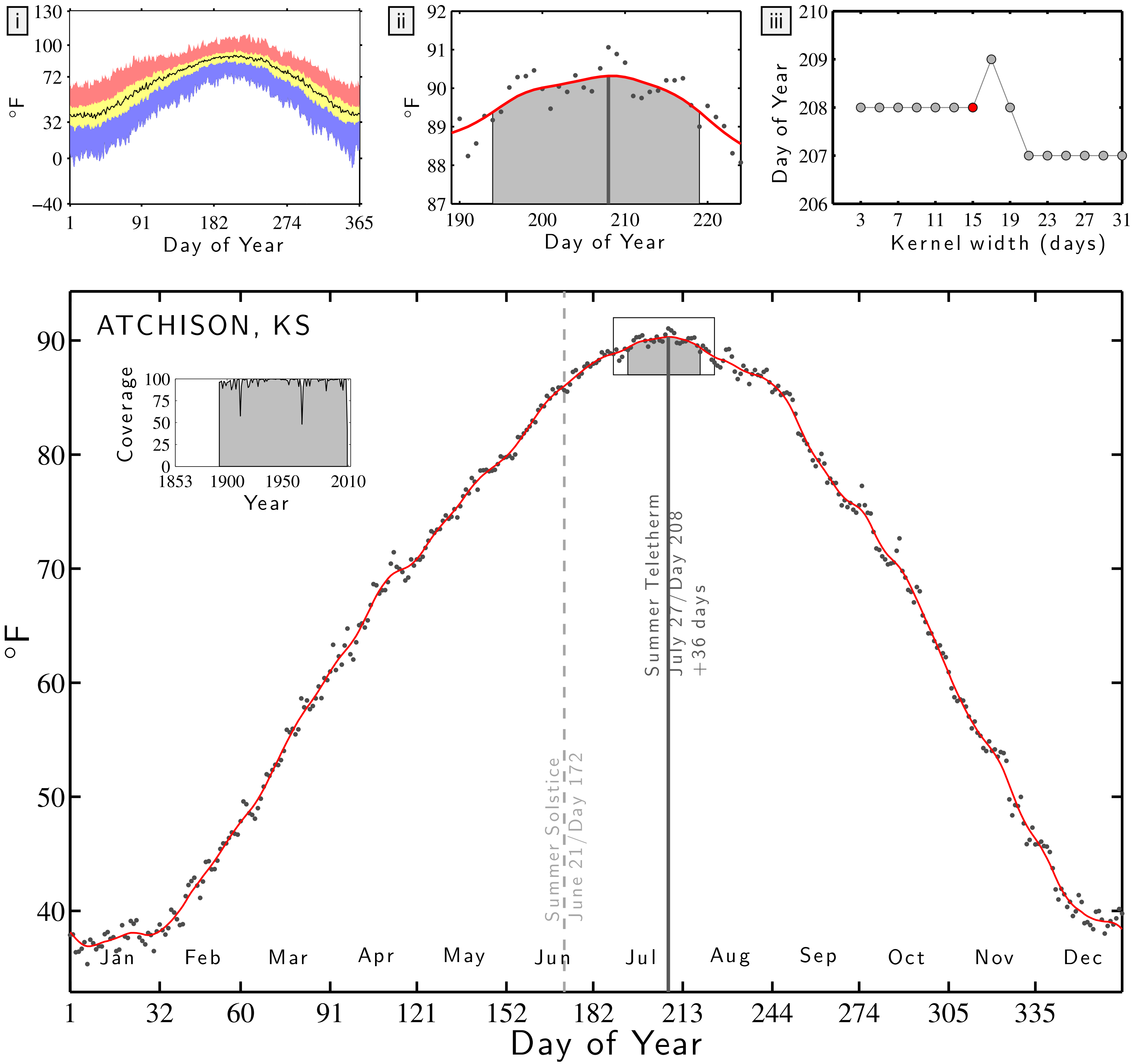

i
°F
Day of Year
ii
iii
Day of Year
Kernel width (days)
ATCHISON, KS
Coverage
Year
Summer Solstice
June 21 / Day 172
Summer Teletherm
July 27 / Day 208
+36 days
Jan
Feb
Mar
Apr
May
Jun
Jul
Aug
Sep
Oct
Nov
Dec
Day of Year
°F

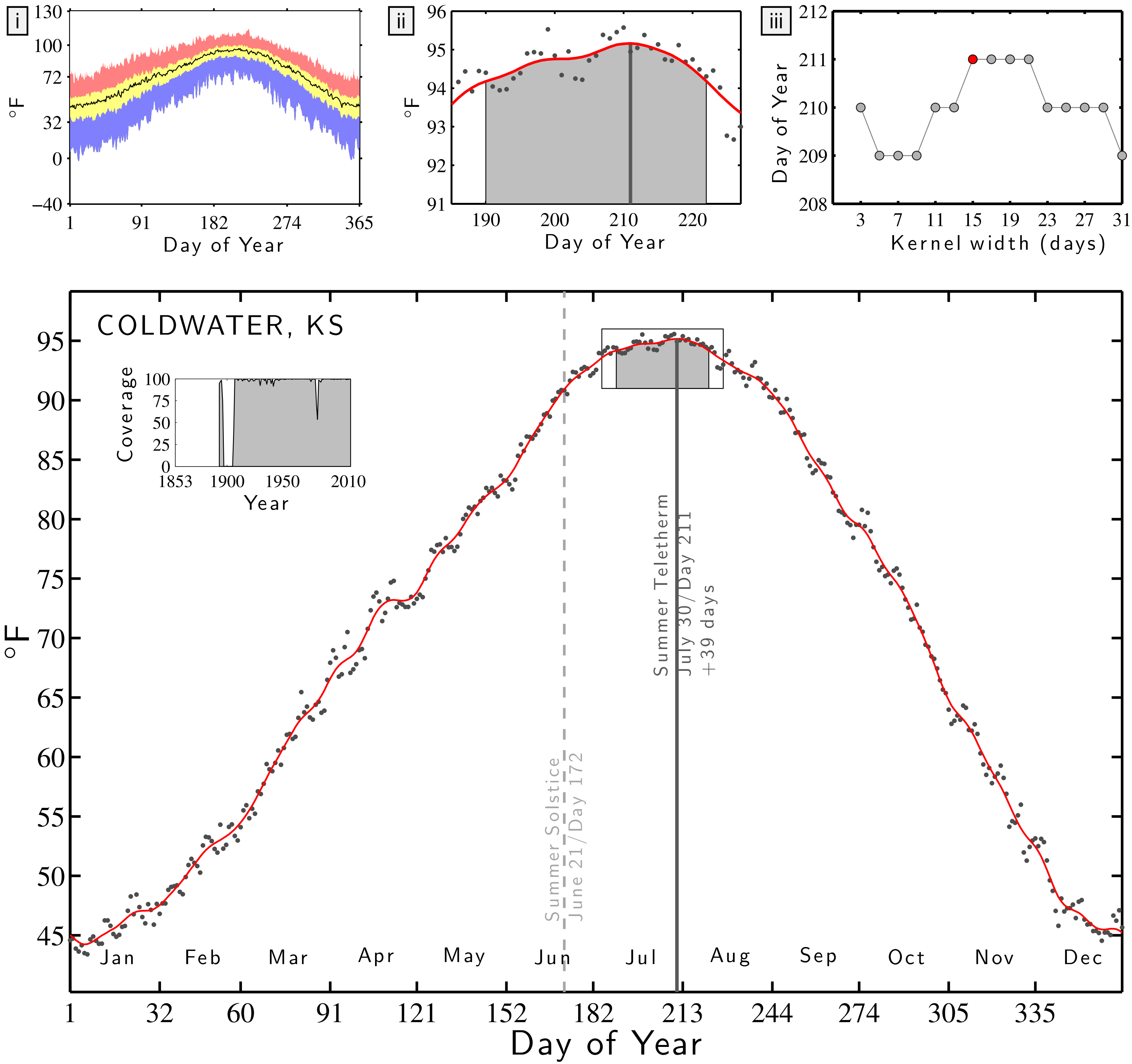

i
°F
Day of Year
ii
°F
Day of Year
iii
Day of Year
Kernel width (days)
COLDWATER, KS
Coverage
Year
1853
1900
1950
2010
Summer Solstice
June 21/Day 172
Summer Telетherm
July 30/Day 211
+39 days
Jan
Feb
Mar
Apr
May
Jun
Jul
Aug
Sep
Oct
Nov
Dec
°F
Day of Year

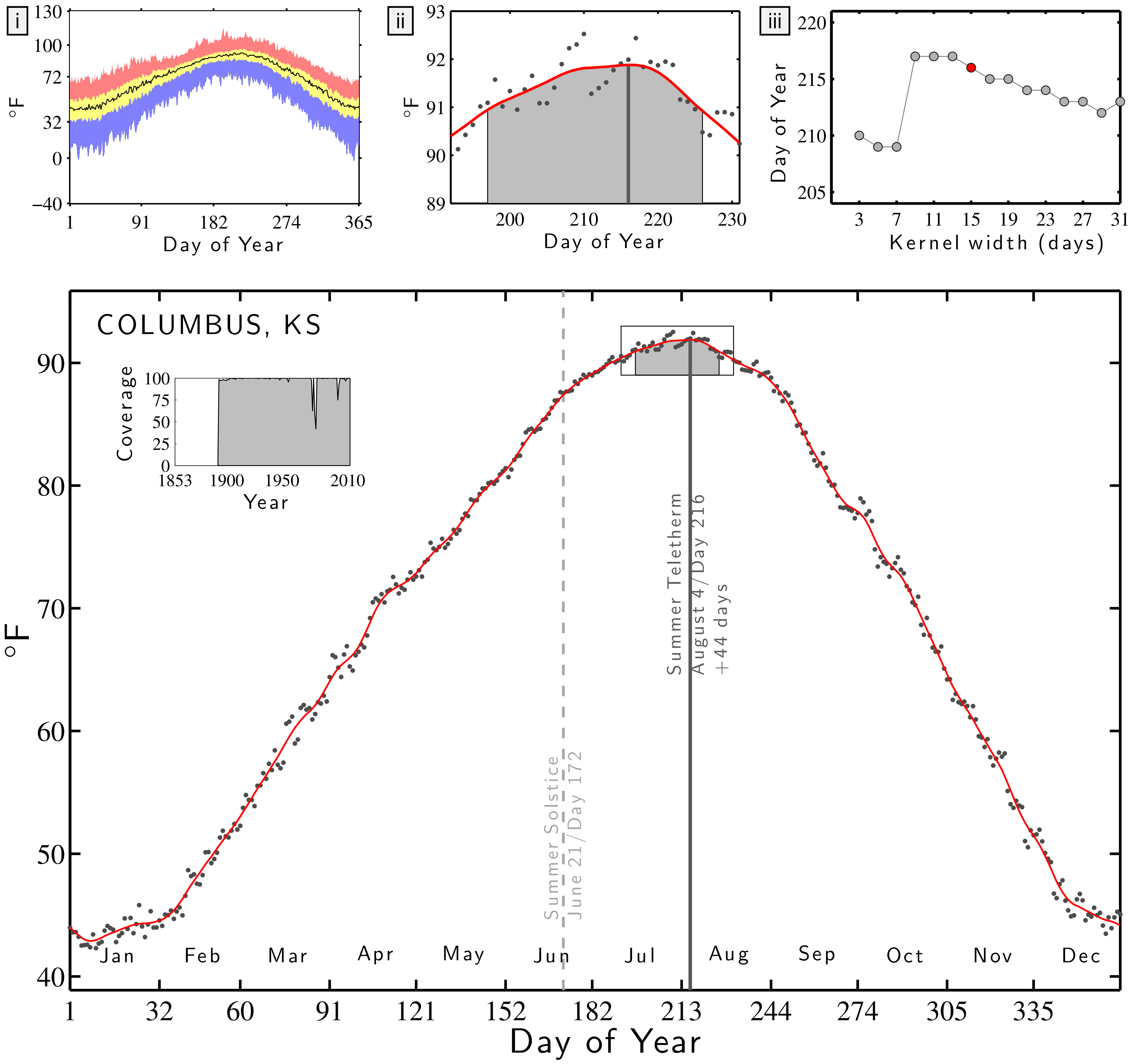

i
ii
iii
°F
Day of Year
Kernel width (days)
Day of Year
COLUMBUS, KS
Coverage
Year
Summer Solstice
June 21 / Day 172
Summer Teletherm
August 4 / Day 216
+44 days
Jan
Feb
Mar
Apr
May
Jun
Jul
Aug
Sep
Oct
Nov
Dec

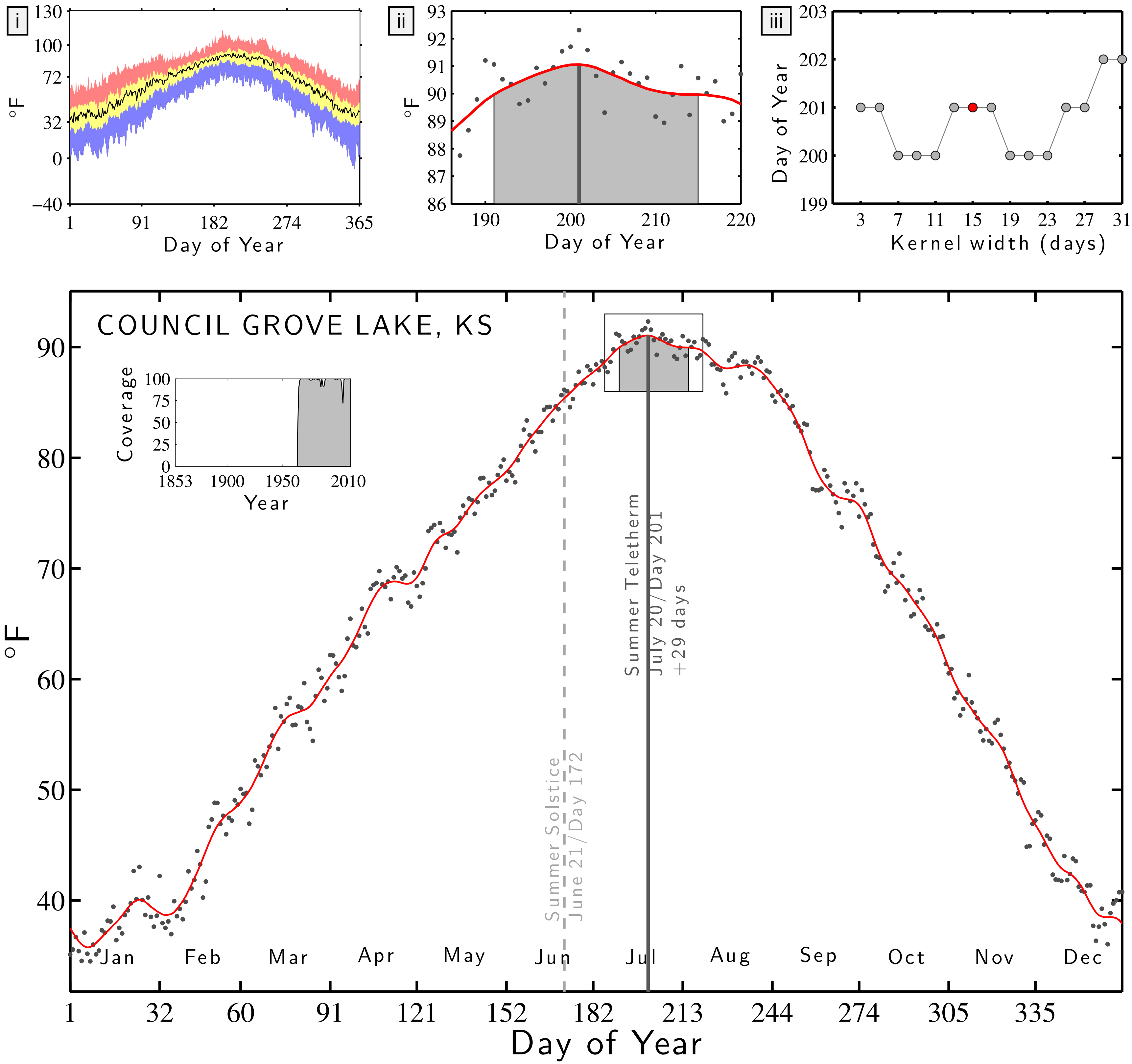

i
ii
iii
COUNCIL GROVE LAKE, KS
Coverage
Year
Summer Solstice
June 21 / Day 172
Summer Teletherm
July 20 / Day 201
+29 days
°F
Day of Year
Kernel width (days)
Jan
Feb
Mar
Apr
May
Jun
Jul
Aug
Sep
Oct
Nov
Dec

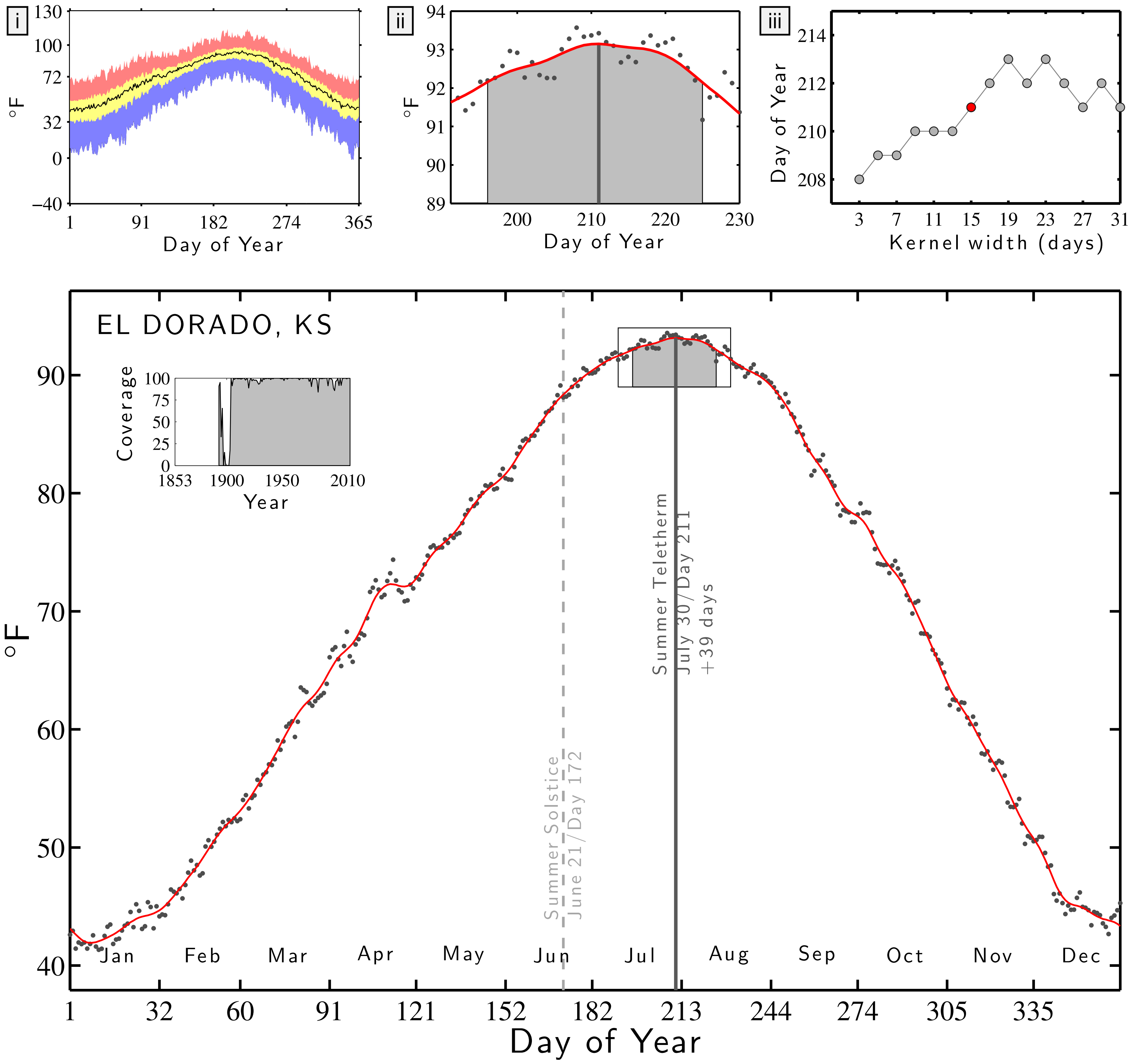

i
°F
Day of Year
ii
°F
Day of Year
iii
Day of Year
Kernel width (days)
EL DORADO, KS
Coverage
Year
Summer Solstice
June 21/Day 172
Summer Teletherm
July 30/Day 211
+39 days
Jan
Feb
Mar
Apr
May
Jun
Jul
Aug
Sep
Oct
Nov
Dec
°F
Day of Year

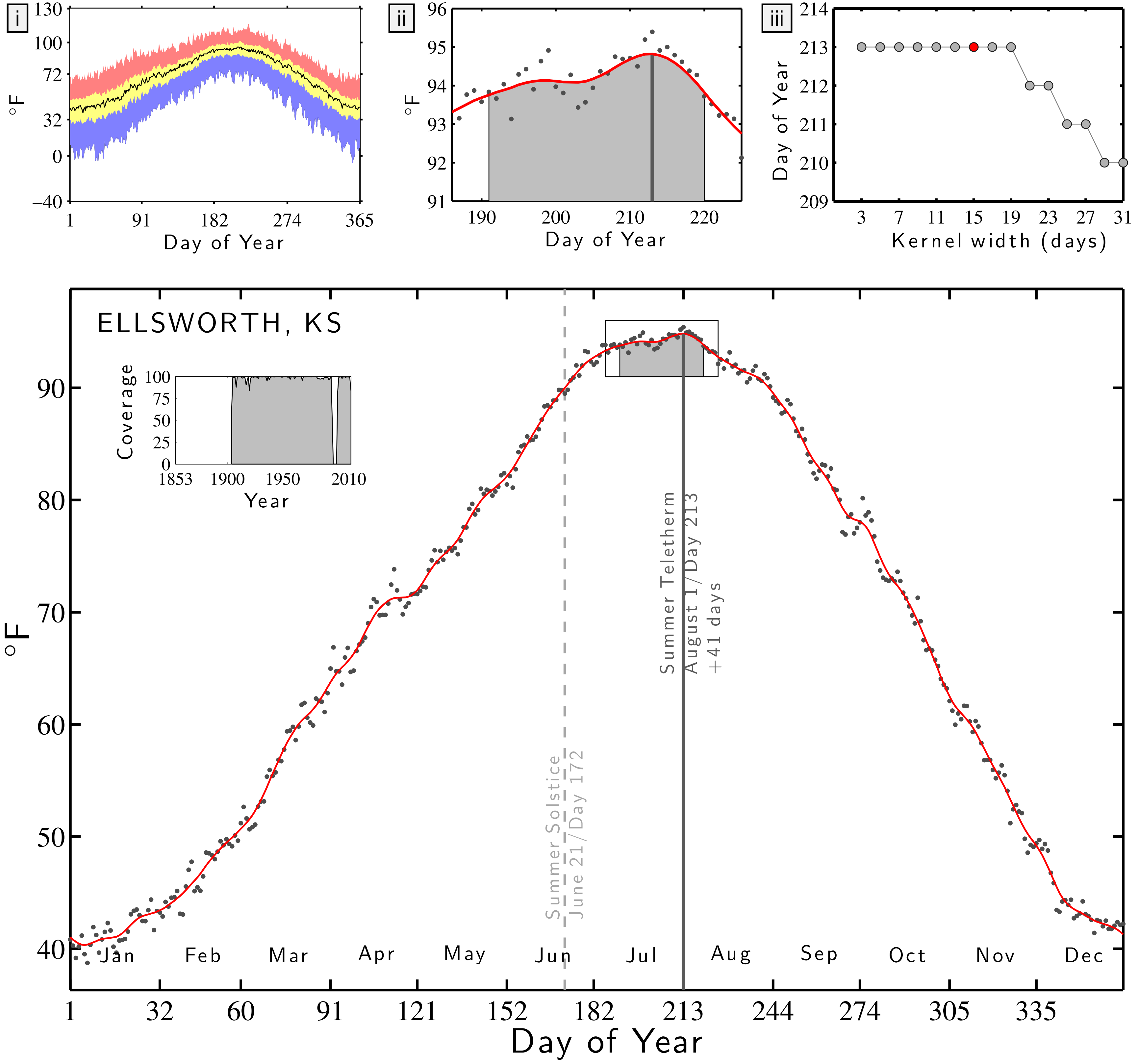

i
°F
Day of Year
ii
°F
Day of Year
iii
Day of Year
Kernel width (days)
ELLSWORTH, KS
Coverage
Year
Summer Solstice
June 21 / Day 172
Summer Teletherm
August 1 / Day 213
+41 days
Jan
Feb
Mar
Apr
May
Jun
Jul
Aug
Sep
Oct
Nov
Dec
°F
Day of Year

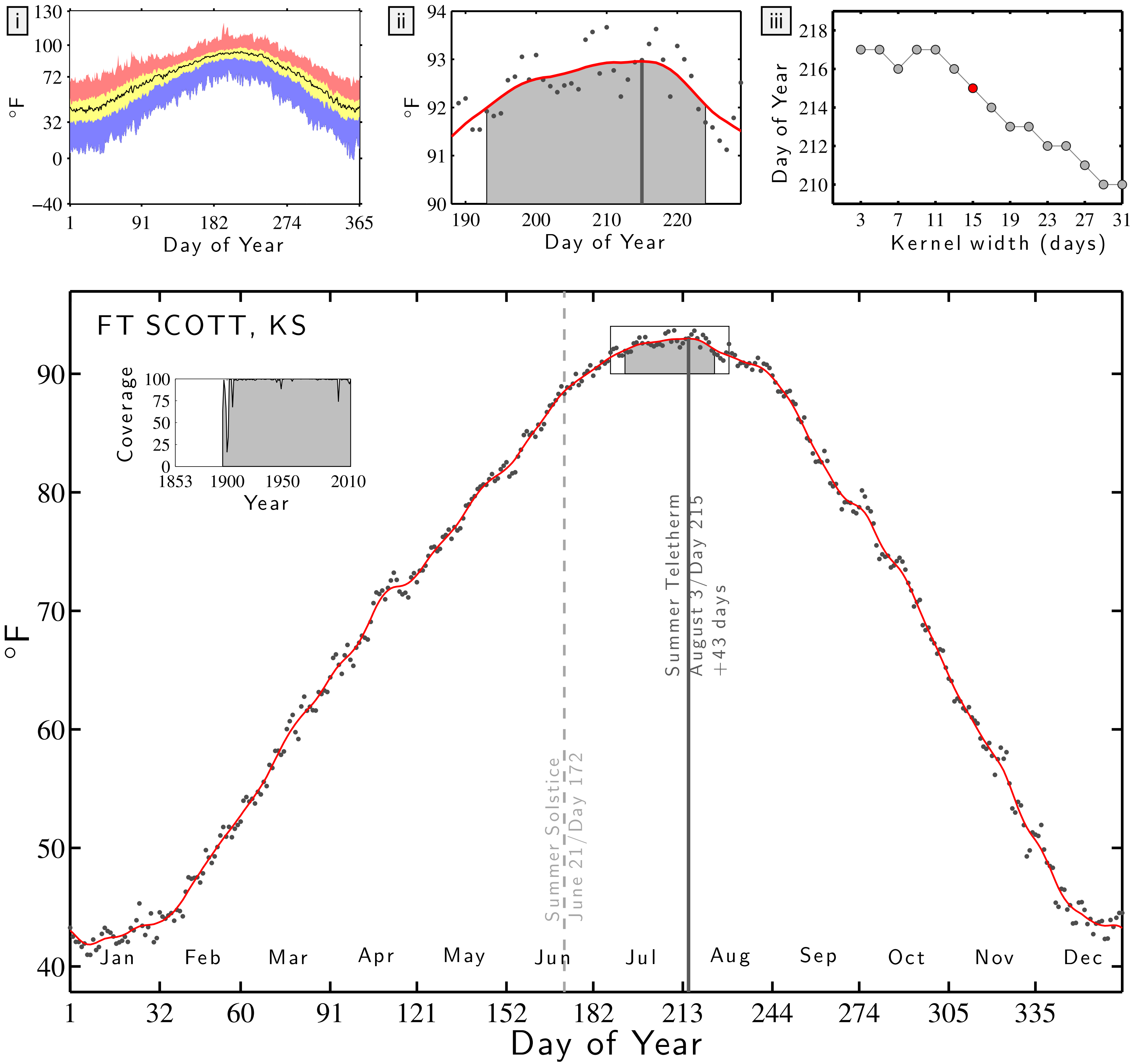

i
°F
Day of Year
ii
°F
Day of Year
iii
Day of Year
Kernel width (days)
FT SCOTT, KS
Coverage
Year
Summer Solstice
June 21 / Day 172
Summer Teletherm
August 3 / Day 215
+43 days
Jan
Feb
Mar
Apr
May
Jun
Jul
Aug
Sep
Oct
Nov
Dec
°F
Day of Year

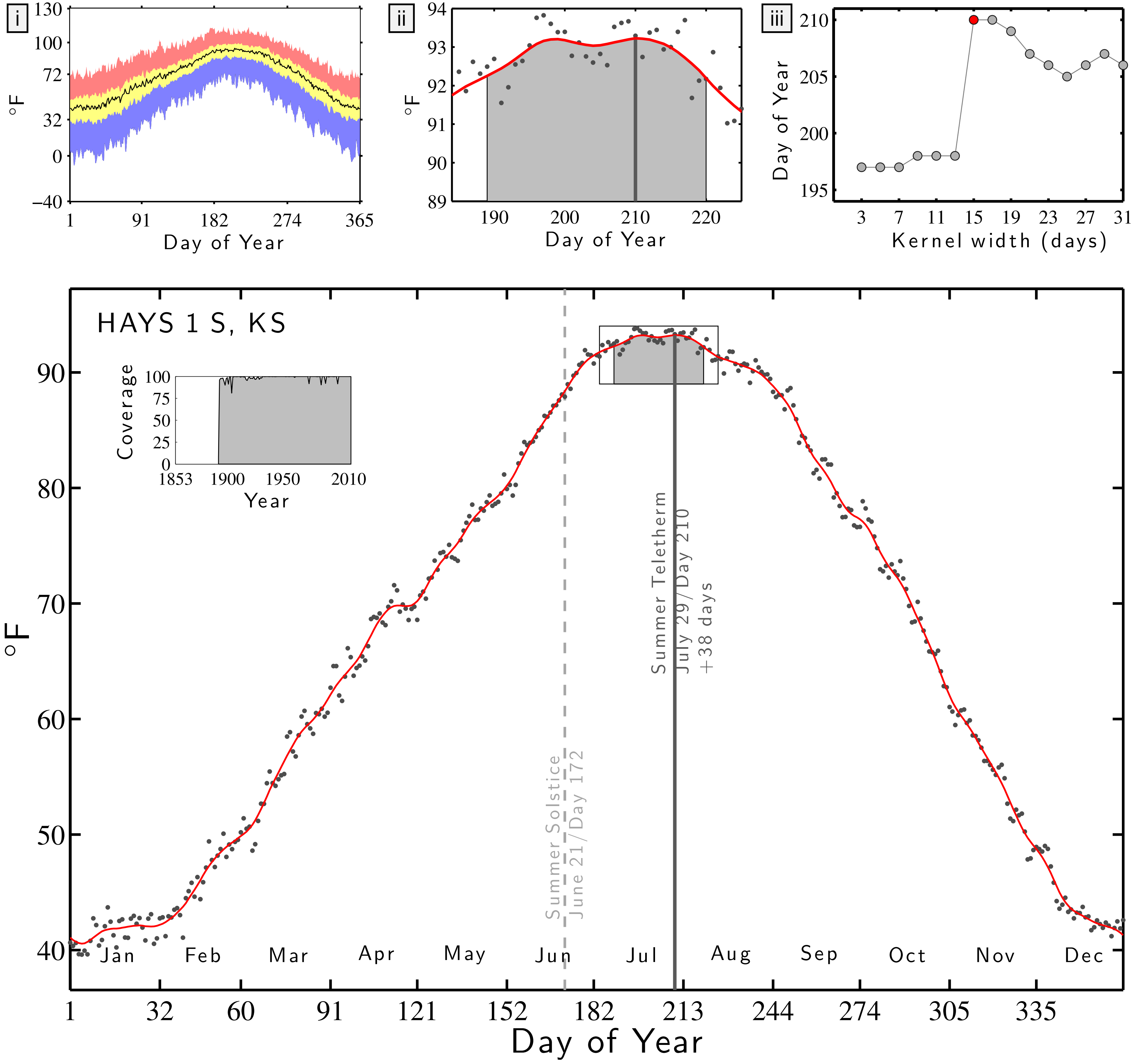

i
ii
iii
°F
Day of Year
Day of Year
Kernel width (days)
HAYS 1 S, KS
Coverage
Year
Summer Solstice
June 21 / Day 172
Summer Teletherm
July 29 / Day 210
+38 days
Jan
Feb
Mar
Apr
May
Jun
Jul
Aug
Sep
Oct
Nov
Dec

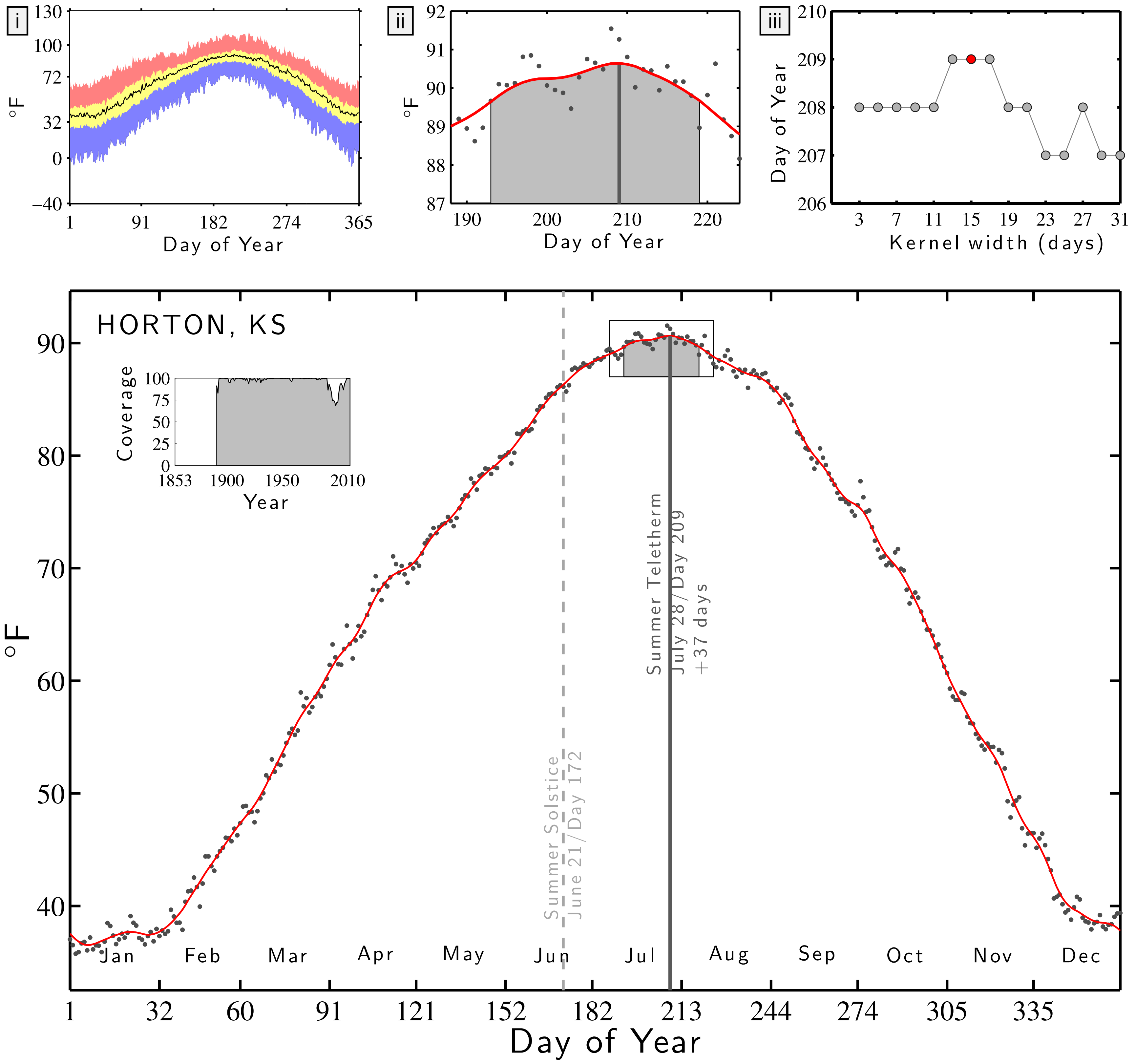

i
°F
Day of Year
ii
°F
Day of Year
iii
Day of Year
Kernel width (days)
HORTON, KS
Coverage
Year
Summer Solstice
June 21/Day 172
Summer Teletherm
July 28/Day 209
+37 days
Jan
Feb
Mar
Apr
May
Jun
Jul
Aug
Sep
Oct
Nov
Dec
°F
Day of Year

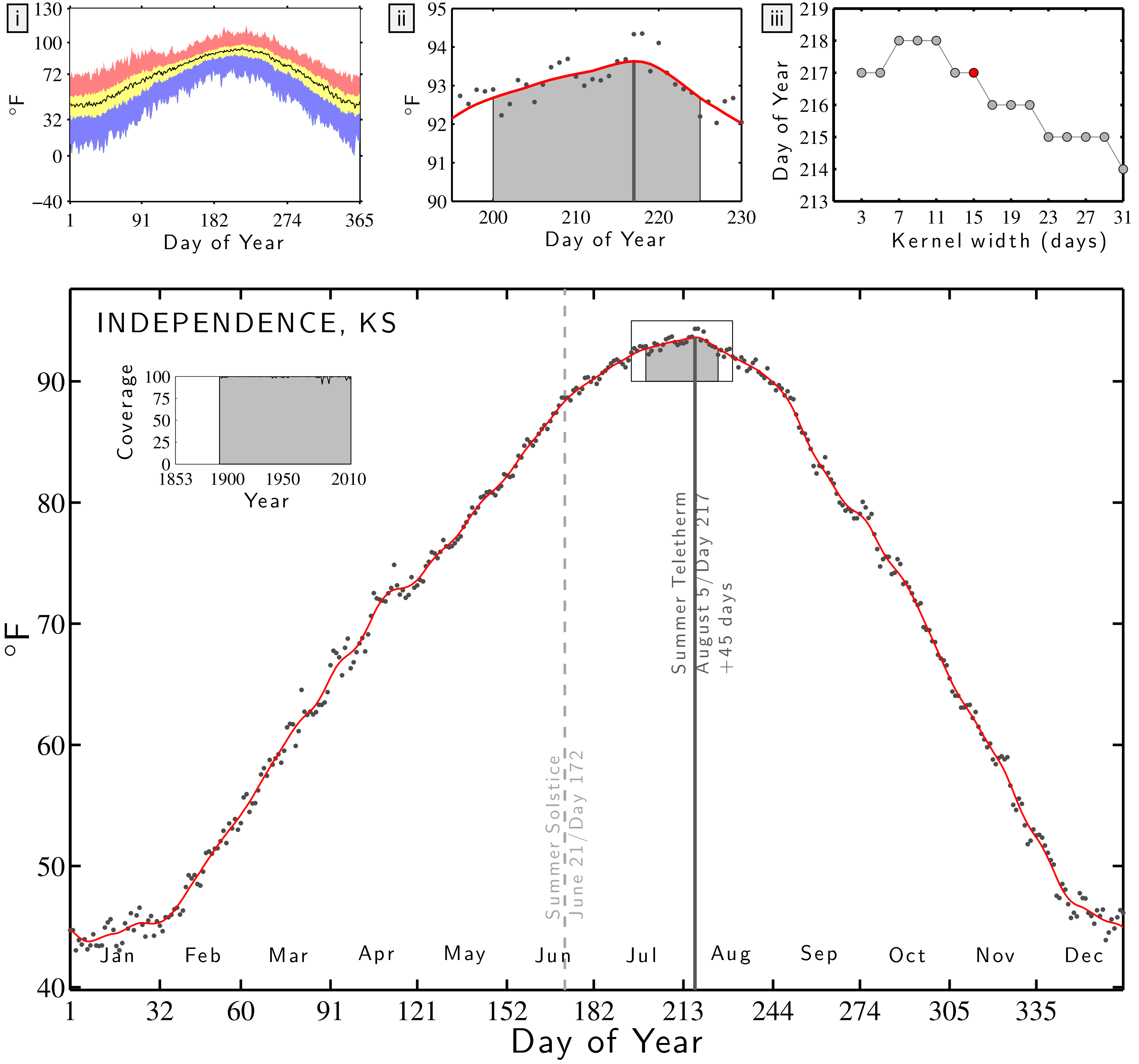

i
ii
iii
°F
Day of Year
Day of Year
Kernel width (days)
INDEPENDENCE, KS
Coverage
Year
1853
1900
1950
2010
Summer Solstice
June 21/Day 172
Summer Teletherm
August 5/Day 217
+45 days
Jan
Feb
Mar
Apr
May
Jun
Jul
Aug
Sep
Oct
Nov
Dec
Day of Year

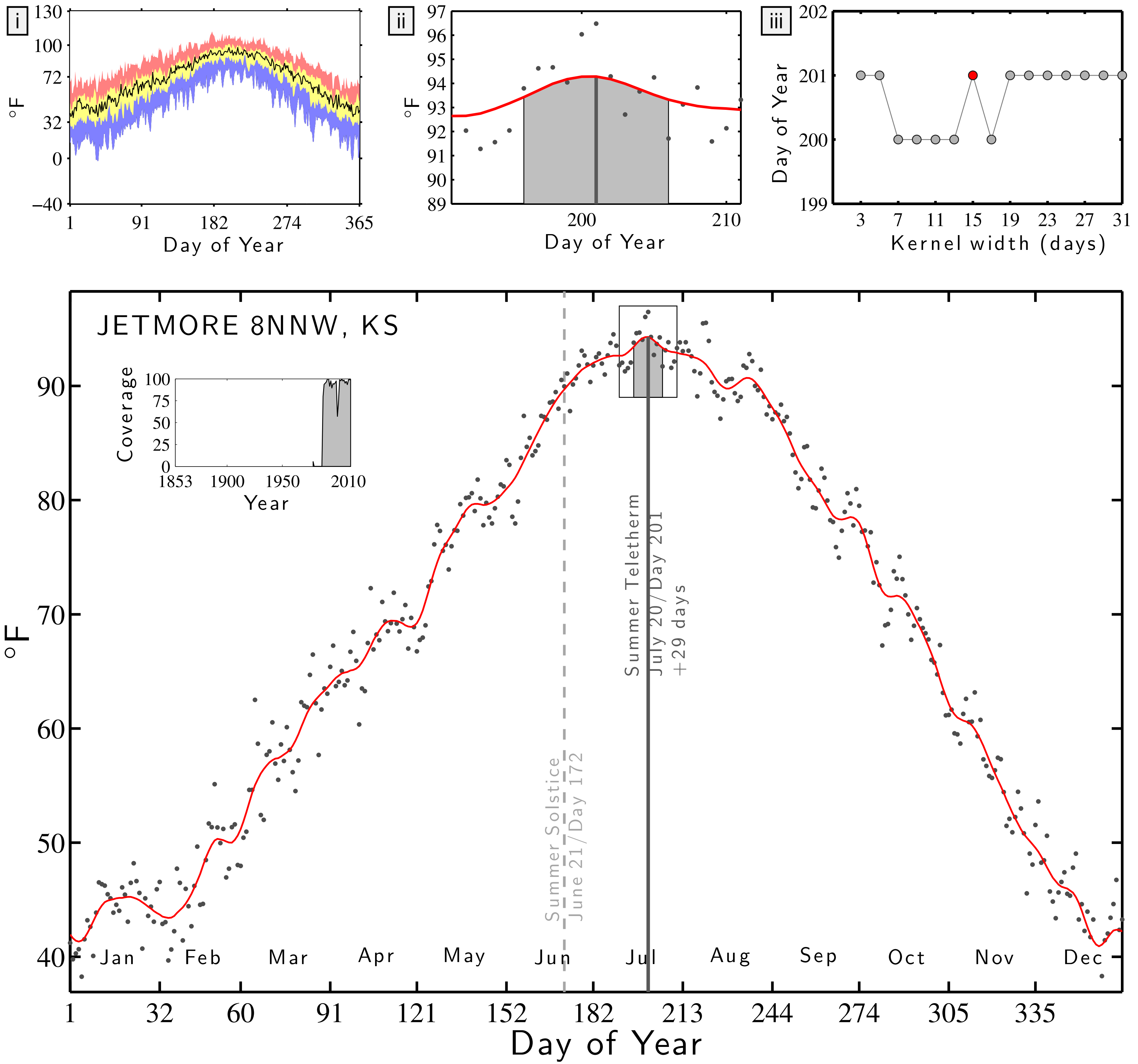

i
ii
iii
°F
Day of Year
Day of Year
Kernel width (days)
JETMORE 8NNW, KS
Coverage
Year
Summer Solstice
June 21/Day 172
Summer Teletherm
July 20/Day 201
+29 days
Jan
Feb
Mar
Apr
May
Jun
Jul
Aug
Sep
Oct
Nov
Dec
°F
Day of Year

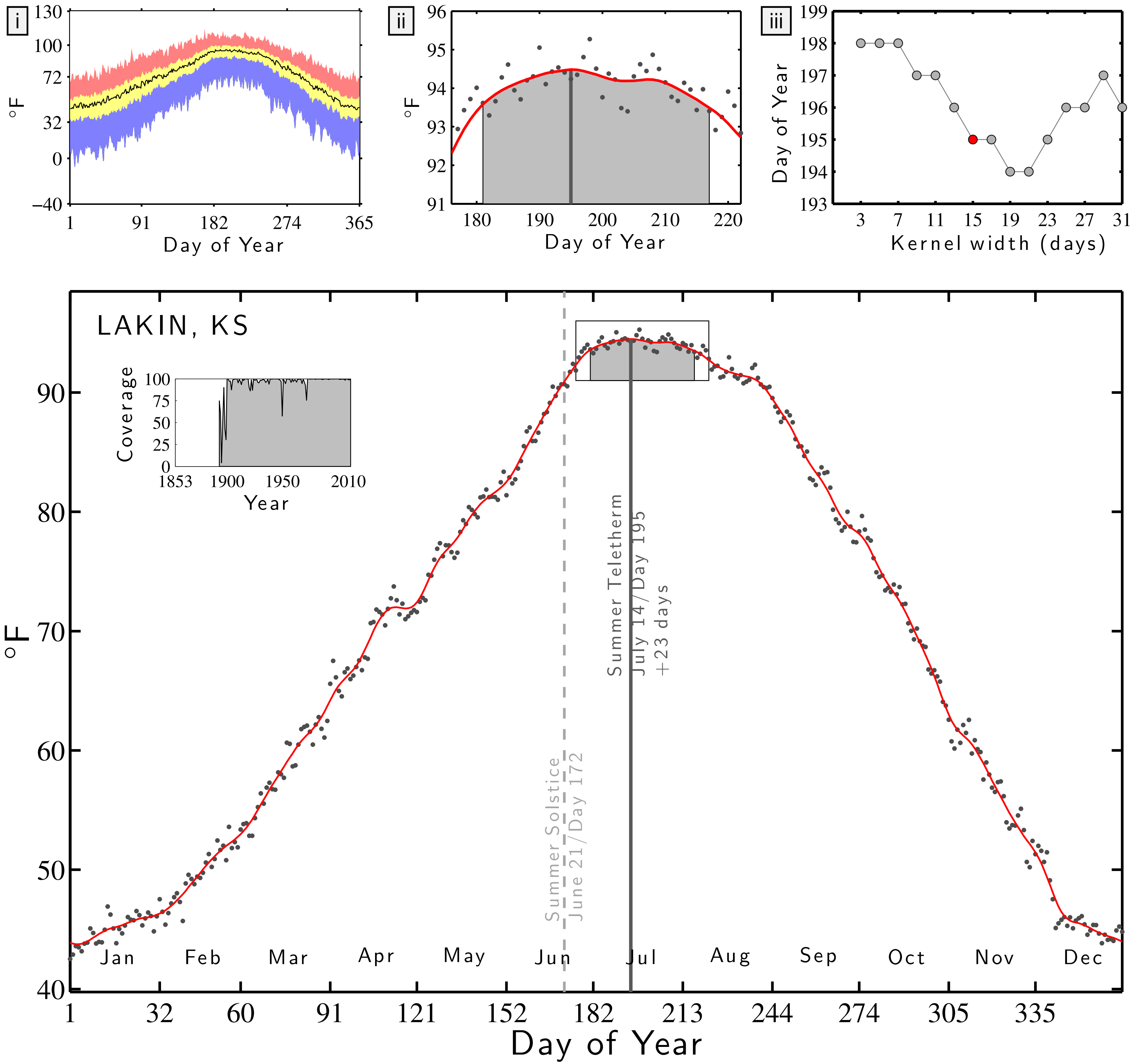

i
°F
Day of Year
ii
°F
Day of Year
iii
Day of Year
Kernel width (days)
LAKIN, KS
Coverage
Year
Summer Solstice
June 21/Day 172
Summer Teletherm
July 14/Day 195
+23 days
Jan
Feb
Mar
Apr
May
Jun
Jul
Aug
Sep
Oct
Nov
Dec
°F
Day of Year

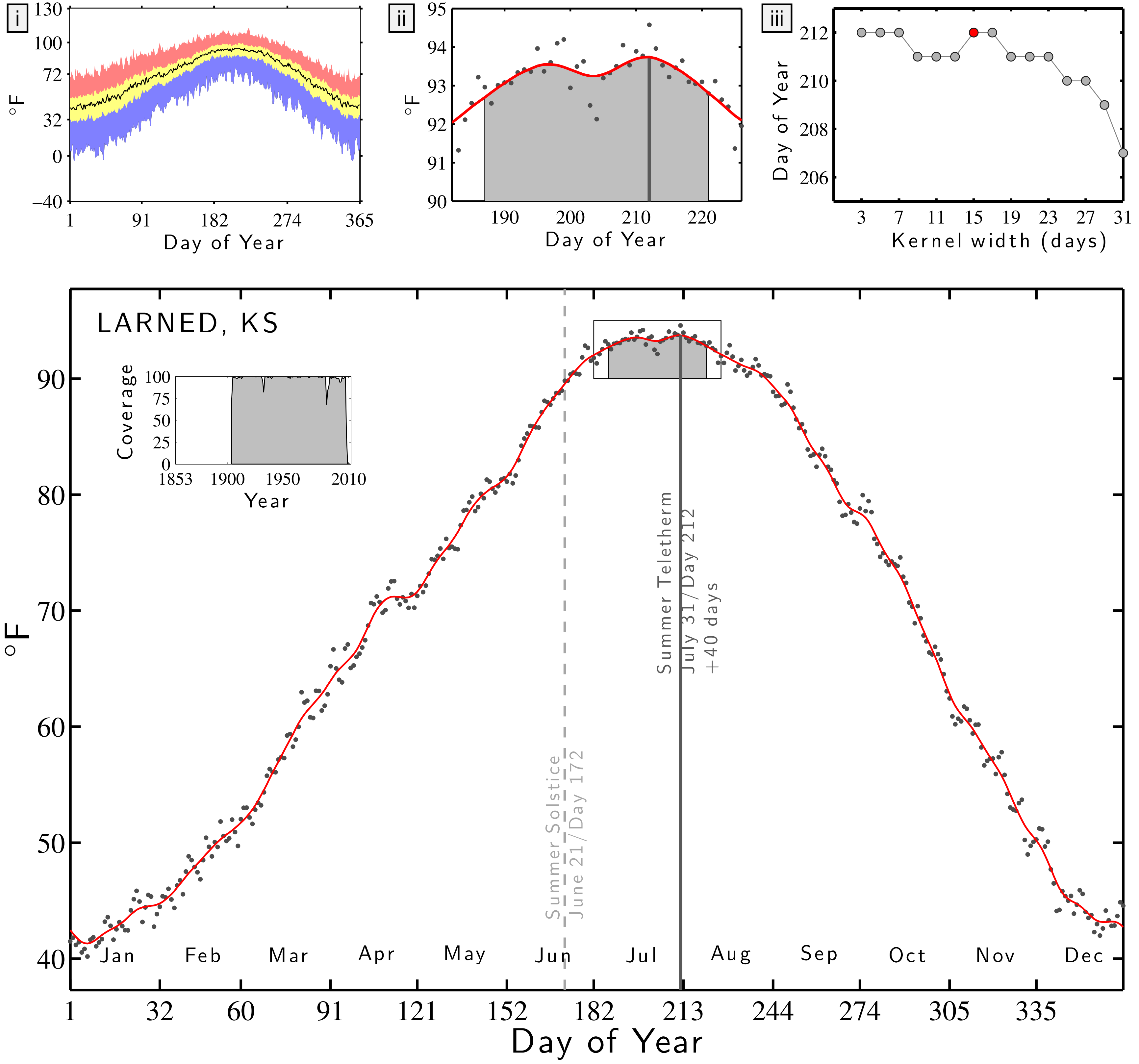

i
ii
iii
°F
Day of Year
Day of Year
Kernel width (days)
LARNED, KS
Coverage
Year
Summer Solstice
June 21 / Day 172
Summer Teletherm
July 31 / Day 212
+40 days
Jan
Feb
Mar
Apr
May
Jun
Jul
Aug
Sep
Oct
Nov
Dec

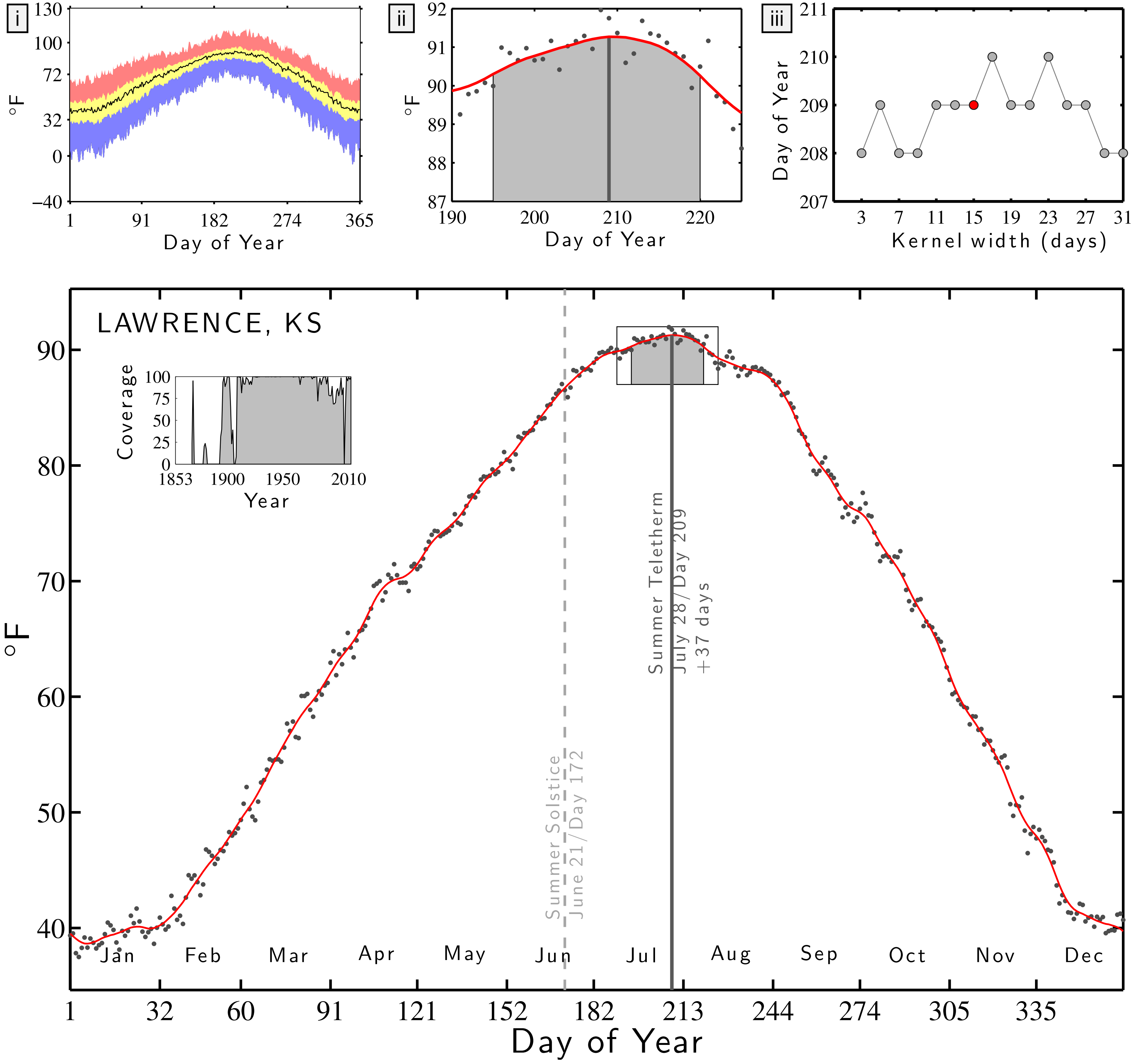

i
130
100
72
32
0
−40
°F
1
91
182
274
365
Day of Year
ii
92
91
90
89
88
87
°F
190
200
210
220
Day of Year
iii
211
210
209
208
207
Day of Year
3
7
11
15
19
23
27
31
Kernel width (days)
LAWRENCE, KS
Coverage
100
75
50
25
0
1853
1900
1950
2010
Year
Summer Solstice
June 21/Day 172
Summer Teletherm
July 28/Day 209
+37 days
°F
90
80
70
60
50
40
Jan
Feb
Mar
Apr
May
Jun
Jul
Aug
Sep
Oct
Nov
Dec
1
32
60
91
121
152
182
213
244
274
305
335
Day of Year

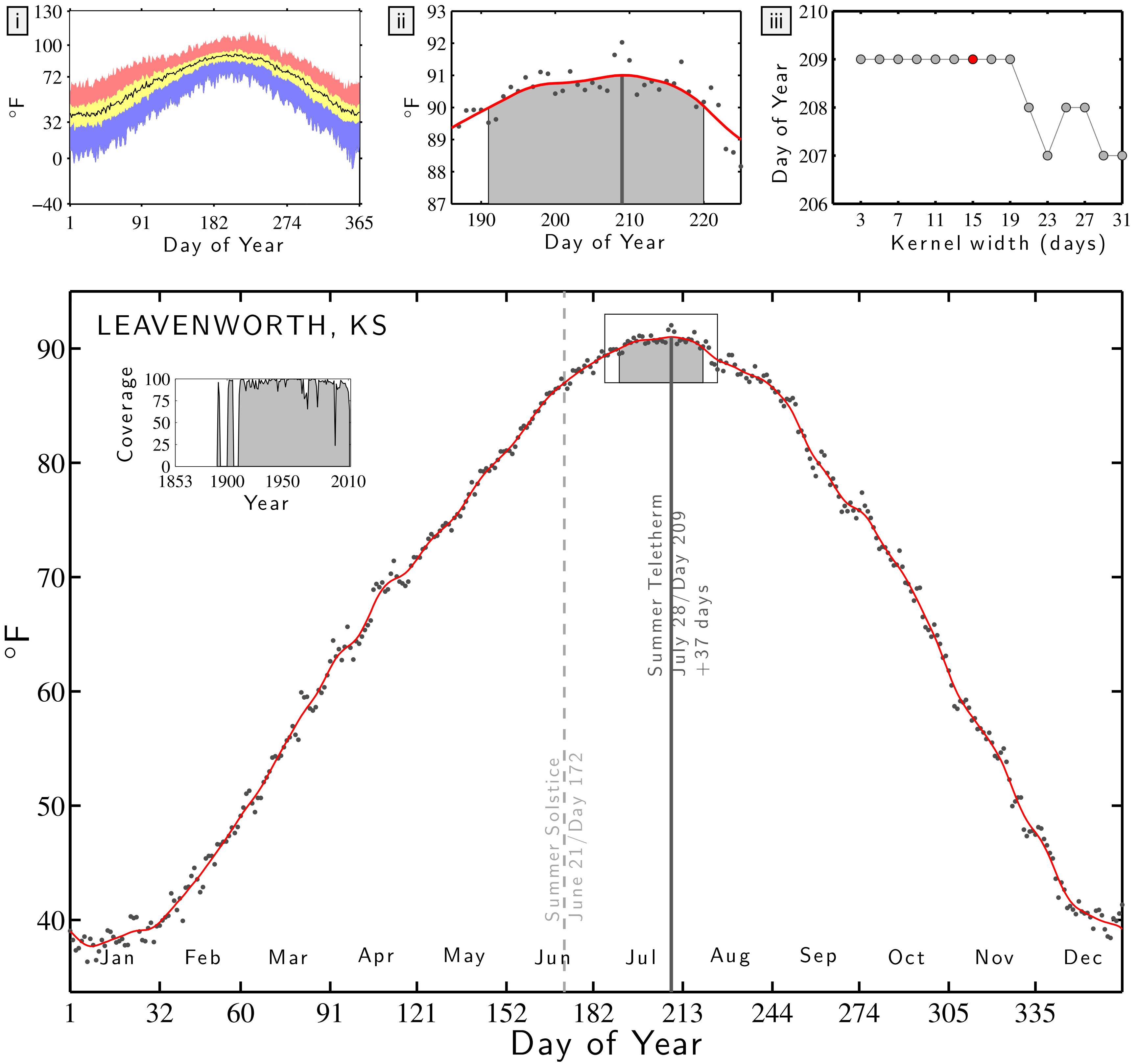
i
°F
Day of Year
ii
°F
Day of Year
iii
Day of Year
Kernel width (days)
LEAVENWORTH, KS
Coverage
Year
Summer Solstice
June 21/Day 172
Summer Teletherm
July 28/Day 209
+37 days
Jan
Feb
Mar
Apr
May
Jun
Jul
Aug
Sep
Oct
Nov
Dec
°F
Day of Year

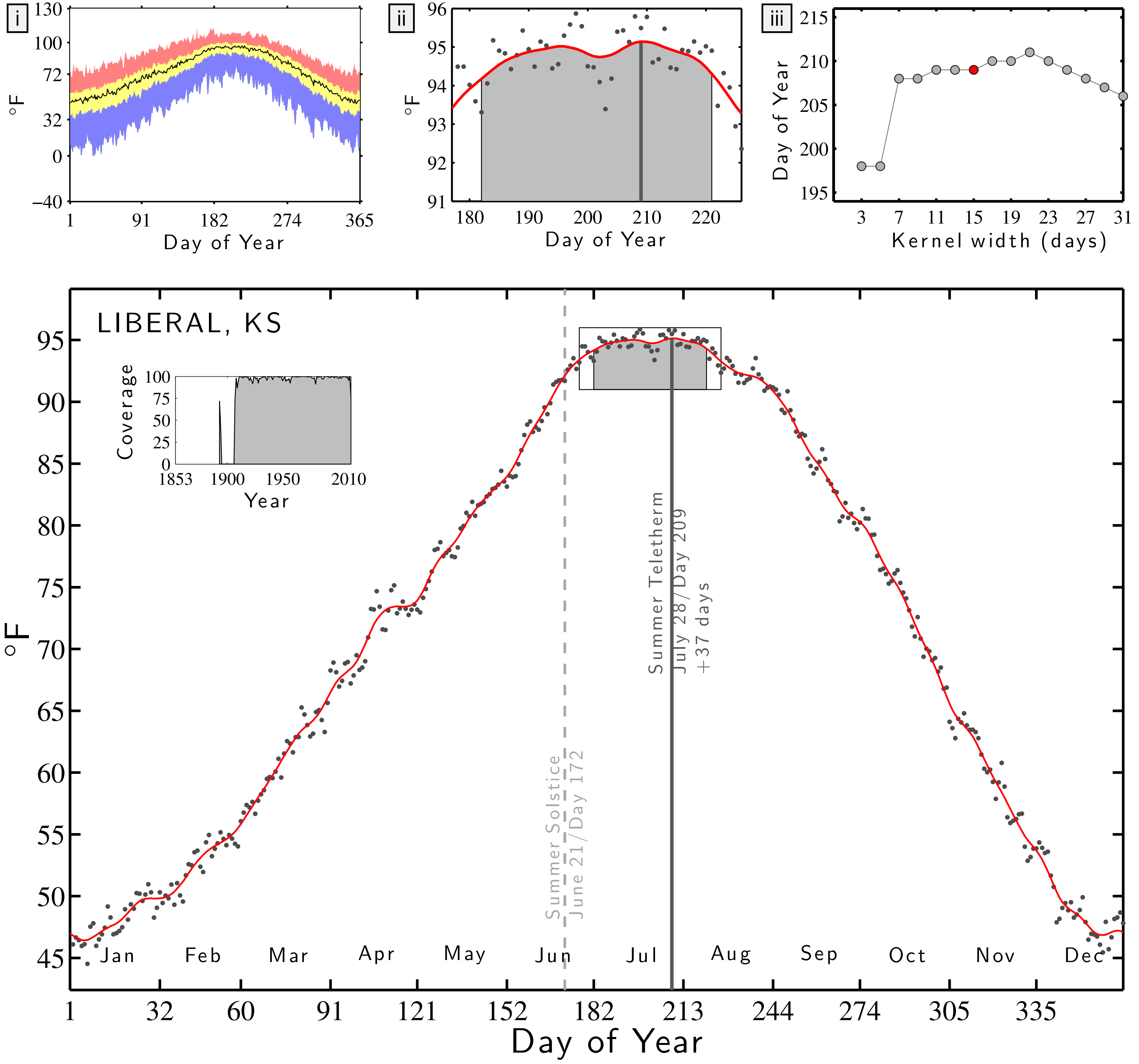

i
°F
130
100
72
32
0
−40
1
91
182
274
365
Day of Year
ii
°F
96
95
94
93
92
91
180
190
200
210
220
Day of Year
iii
Day of Year
215
210
205
200
195
3
7
11
15
19
23
27
31
Kernel width (days)
LIBERAL, KS
Coverage
100
75
50
25
0
1853
1900
1950
2010
Year
Summer Solstice
June 21/Day 172
Summer Teletherm
July 28/Day 209
+37 days
Jan
Feb
Mar
Apr
May
Jun
Jul
Aug
Sep
Oct
Nov
Dec
°F
95
90
85
80
75
70
65
60
55
50
45
1
32
60
91
121
152
182
213
244
274
305
335
Day of Year

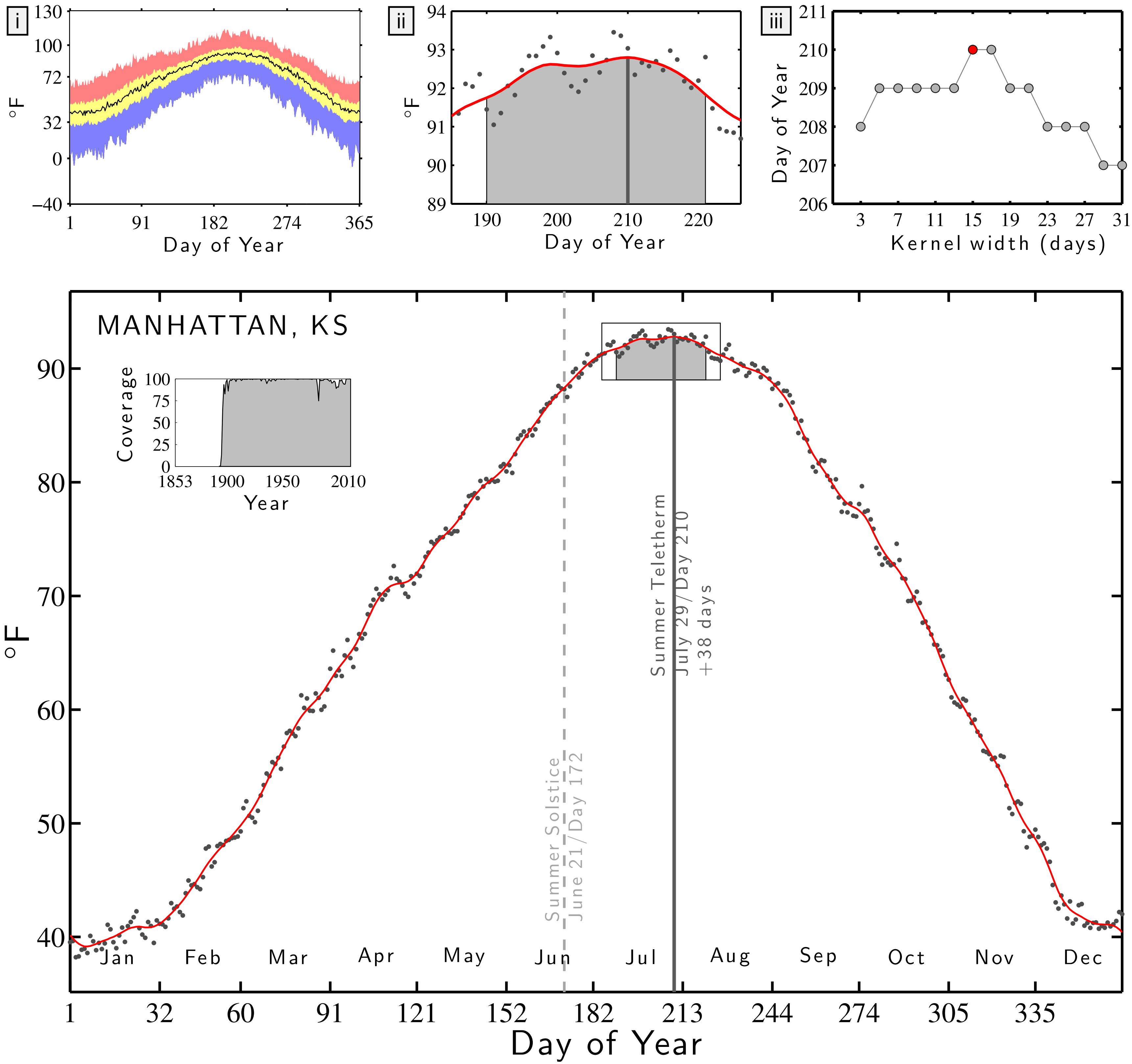

i
°F
Day of Year
ii
°F
Day of Year
iii
Day of Year
Kernel width (days)
MANHATTAN, KS
Coverage
Year
Summer Solstice
June 21/Day 172
Summer Teletherm
July 29/Day 210
+38 days
Jan
Feb
Mar
Apr
May
Jun
Jul
Aug
Sep
Oct
Nov
Dec
°F
Day of Year

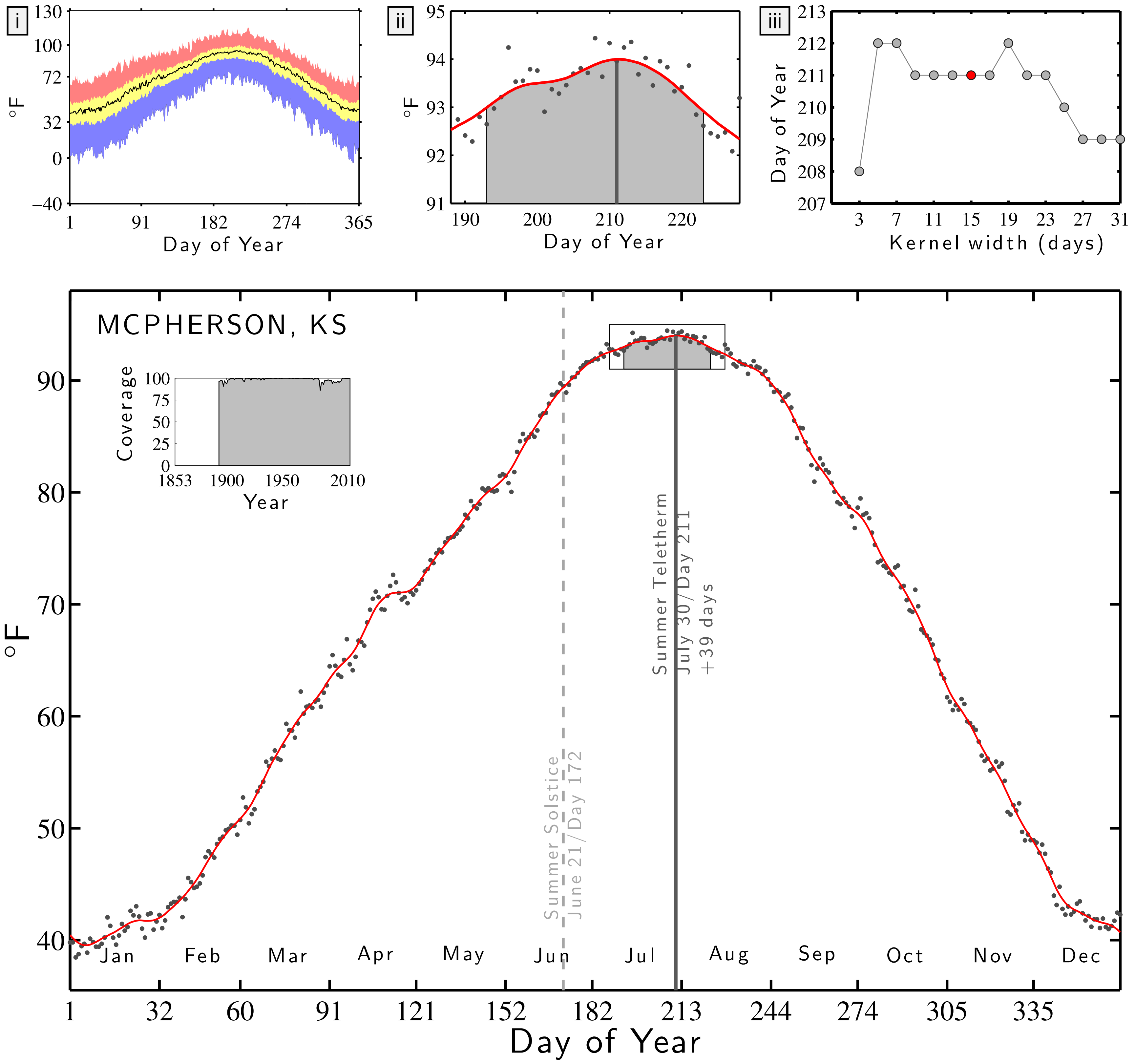

i
°F
Day of Year
ii
°F
Day of Year
iii
Day of Year
Kernel width (days)
MCPHERSON, KS
Coverage
Year
Summer Solstice
June 21/Day 172
Summer Teletherm
July 30/Day 211
+39 days
Jan
Feb
Mar
Apr
May
Jun
Jul
Aug
Sep
Oct
Nov
Dec
°F
Day of Year

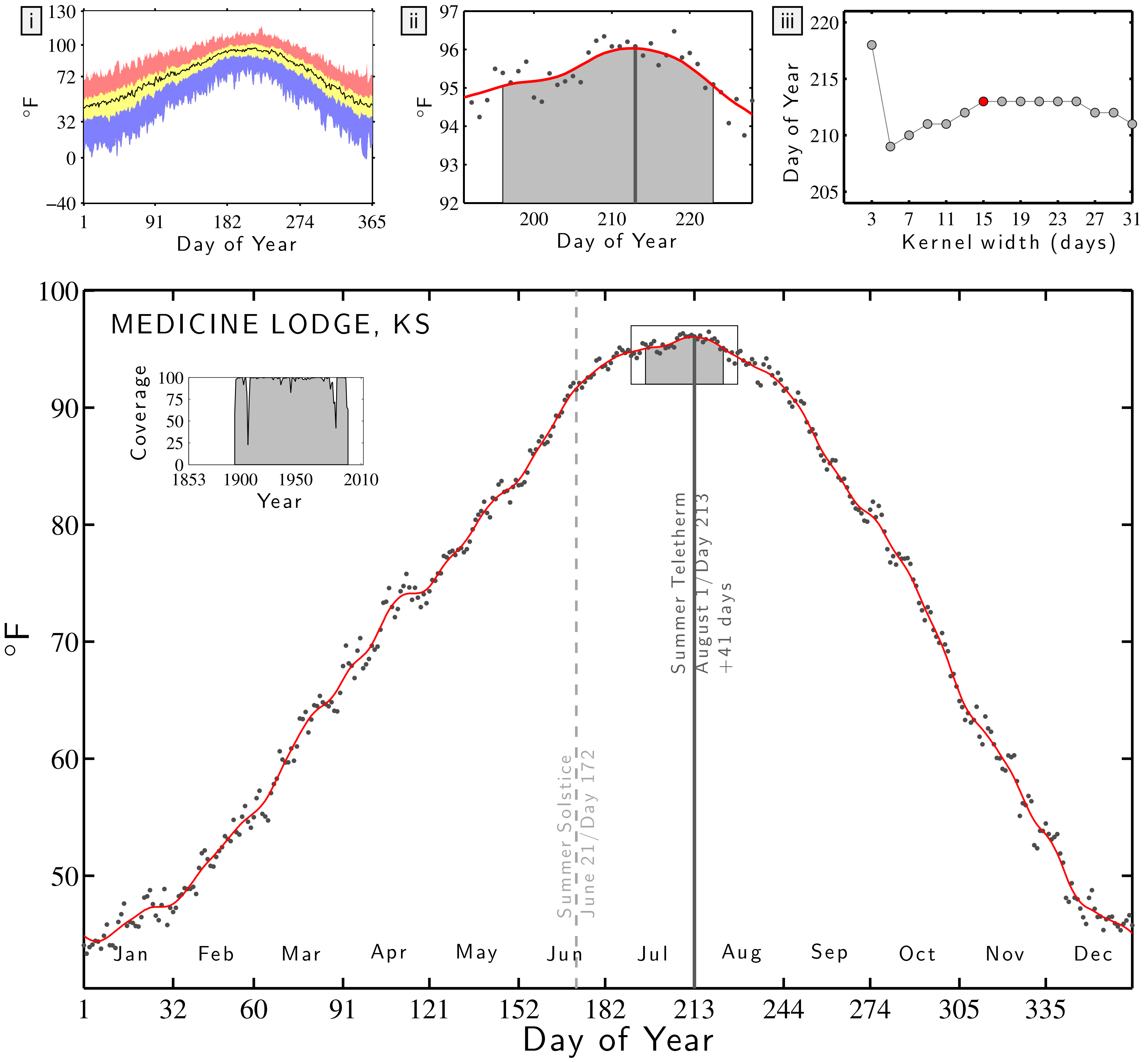

i
°F
Day of Year
ii
°F
Day of Year
iii
Day of Year
Kernel width (days)
MEDICINE LODGE, KS
Coverage
Year
Summer Solstice
June 21/Day 172
Summer Teletherm
August 1/Day 213
+41 days
Jan
Feb
Mar
Apr
May
Jun
Jul
Aug
Sep
Oct
Nov
Dec
°F
Day of Year

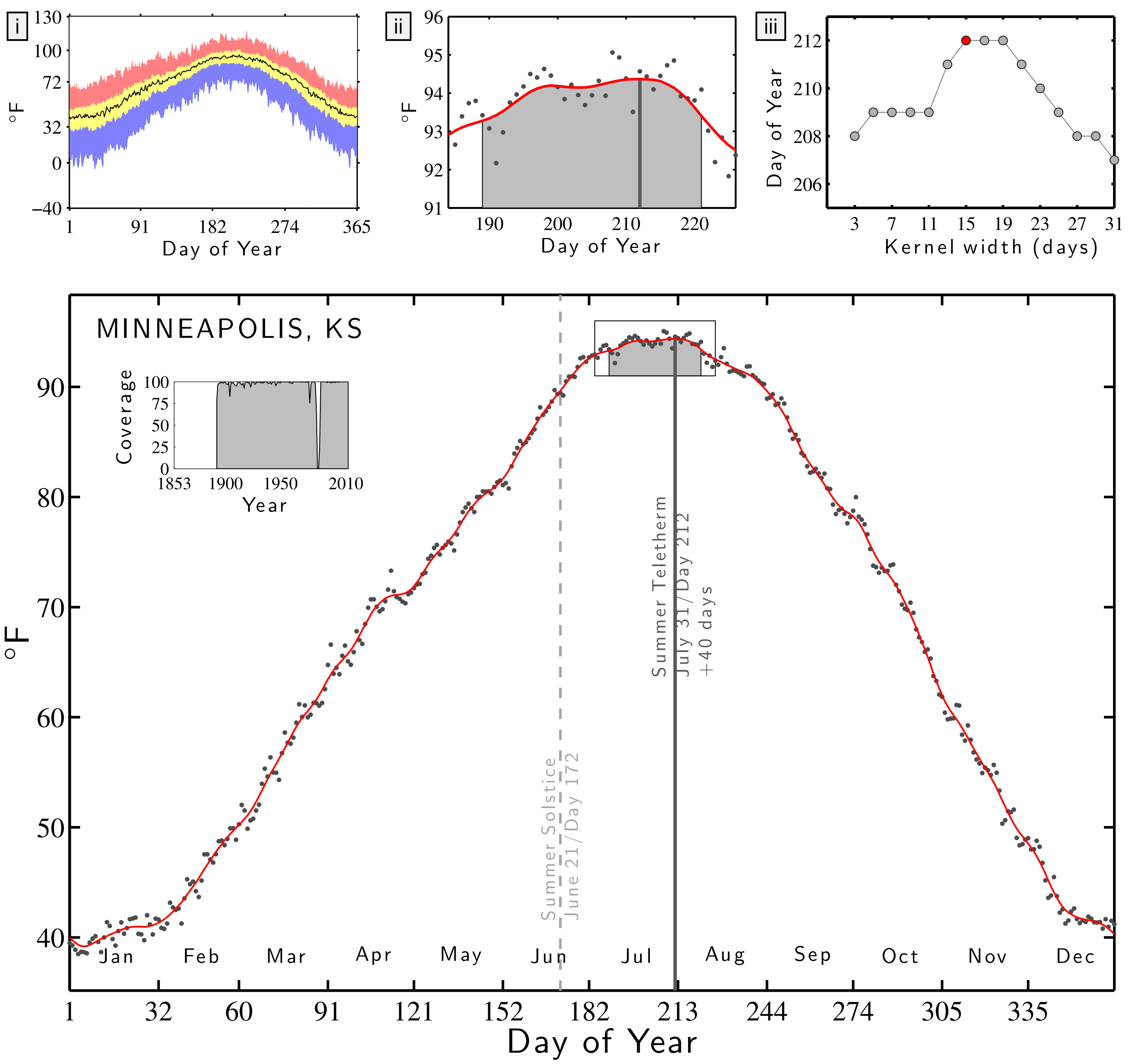

i
ii
iii
Day of Year
Kernel width (days)
MINNEAPOLIS, KS
Coverage
Year
Summer Solstice
June 21/Day 172
Summer Teletherm
July 31/Day 212
+40 days
Jan
Feb
Mar
Apr
May
Jun
Jul
Aug
Sep
Oct
Nov
Dec
°F
Day of Year

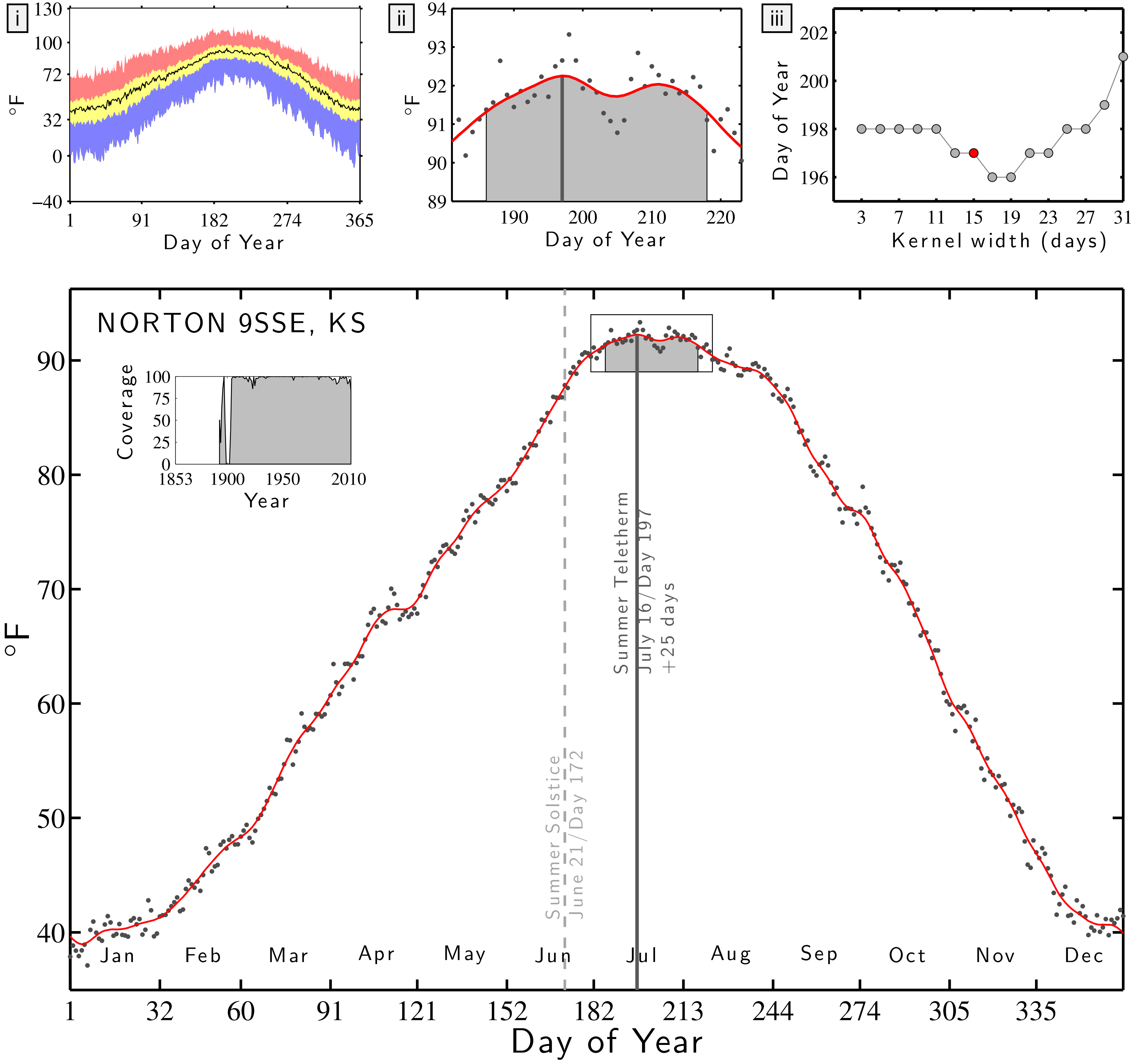

i
ii
iii
Day of Year
°F
Kernel width (days)
NORTON 9SSE, KS
Coverage
Year
Summer Solstice
June 21/Day 172
Summer Teletherm
July 16/Day 197
+25 days
Jan
Feb
Mar
Apr
May
Jun
Jul
Aug
Sep
Oct
Nov
Dec

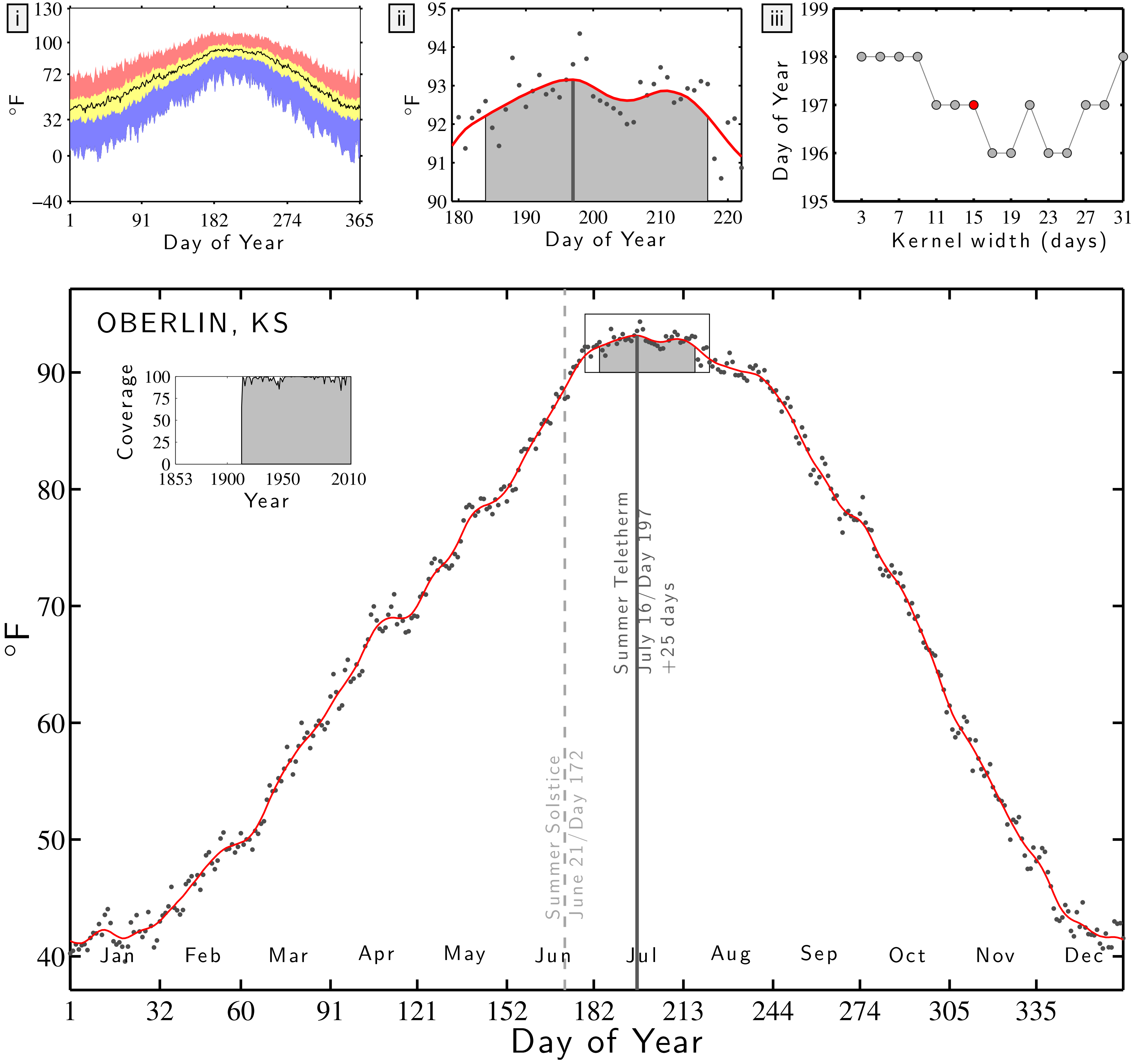

i
°F
Day of Year
ii
°F
Day of Year
iii
Day of Year
Kernel width (days)
OBERLIN, KS
Coverage
Year
Summer Solstice
June 21/Day 172
Summer Teletherm
July 16/Day 197
+25 days
Jan
Feb
Mar
Apr
May
Jun
Jul
Aug
Sep
Oct
Nov
Dec
°F
Day of Year

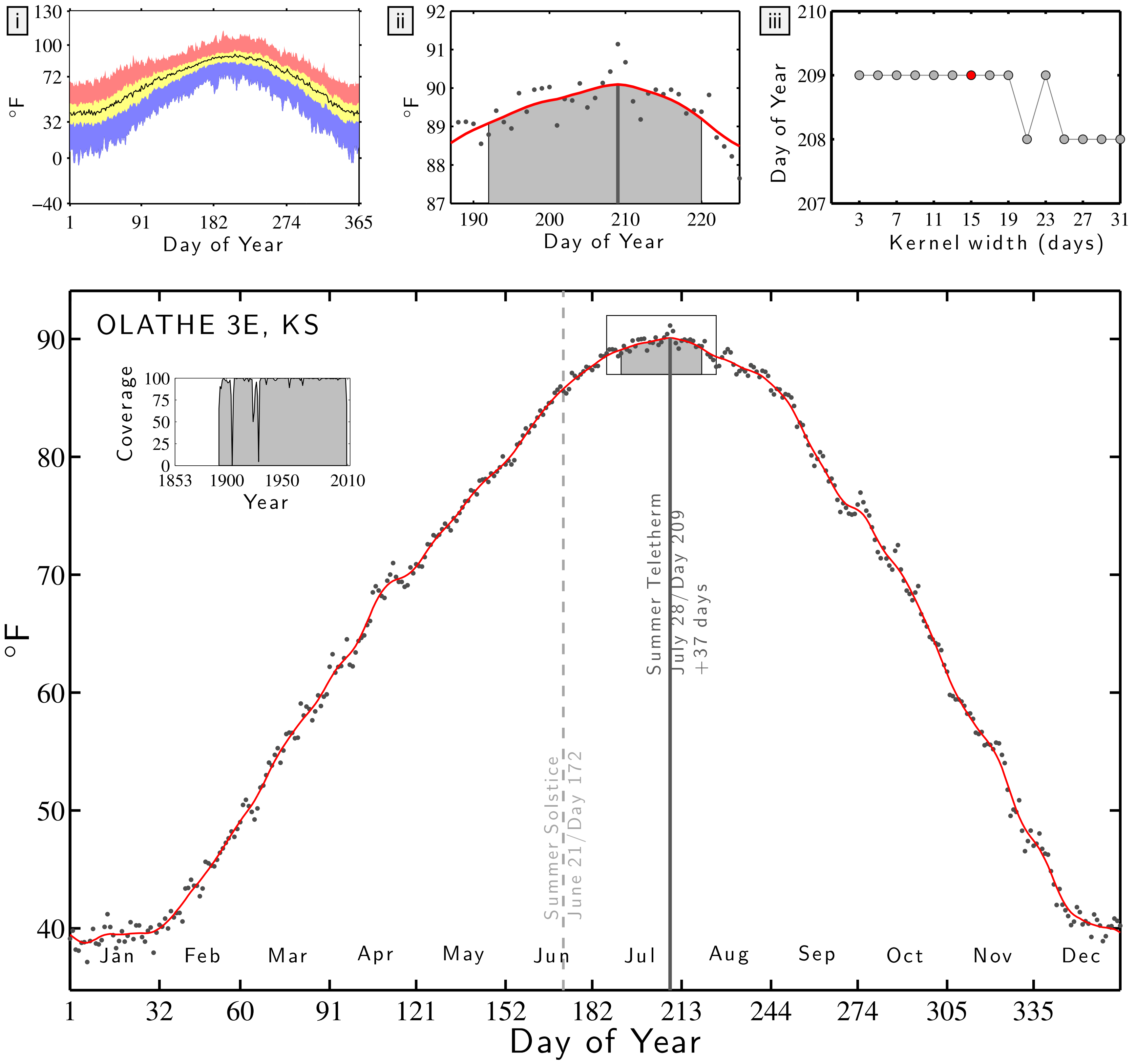

i
130
100
72
32
0
−40
°F
1
91
182
274
365
Day of Year
ii
92
91
90
89
88
87
°F
190
200
210
220
Day of Year
iii
210
209
208
207
Day of Year
3
7
11
15
19
23
27
31
Kernel width (days)
OLATHE 3E, KS
Coverage
100
75
50
25
0
1853
1900
1950
2010
Year
Summer Solstice
June 21/Day 172
Summer Teletherm
July 28/Day 209
+37 days
90
80
70
60
50
40
°F
Jan
Feb
Mar
Apr
May
Jun
Jul
Aug
Sep
Oct
Nov
Dec
1
32
60
91
121
152
182
213
244
274
305
335
Day of Year

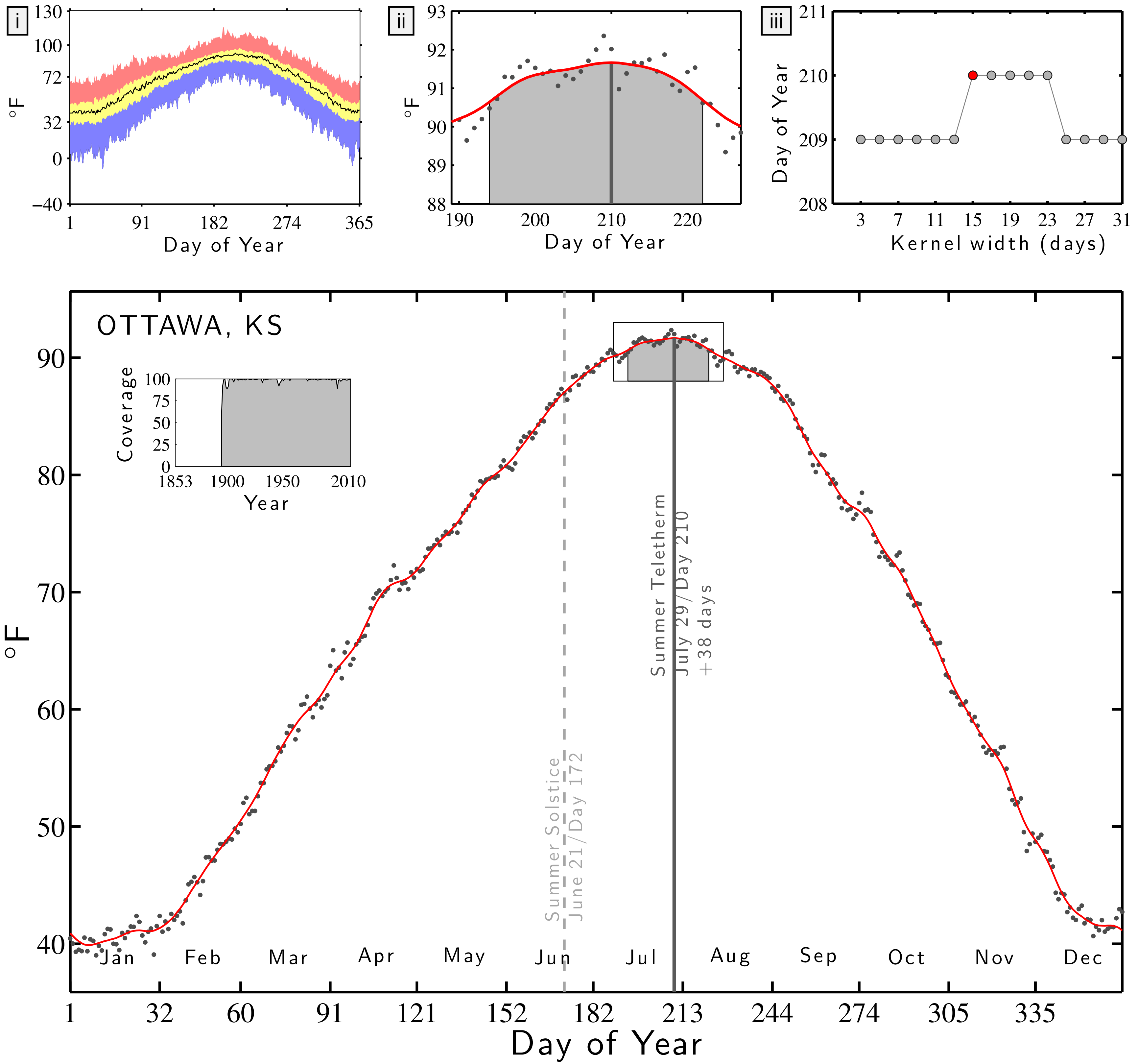

i
°F
Day of Year
ii
°F
Day of Year
iii
Day of Year
Kernel width (days)
OTTAWA, KS
Coverage
Year
Summer Solstice
June 21 / Day 172
Summer Teletherm
July 29 / Day 210
+38 days
Jan
Feb
Mar
Apr
May
Jun
Jul
Aug
Sep
Oct
Nov
Dec
°F
Day of Year

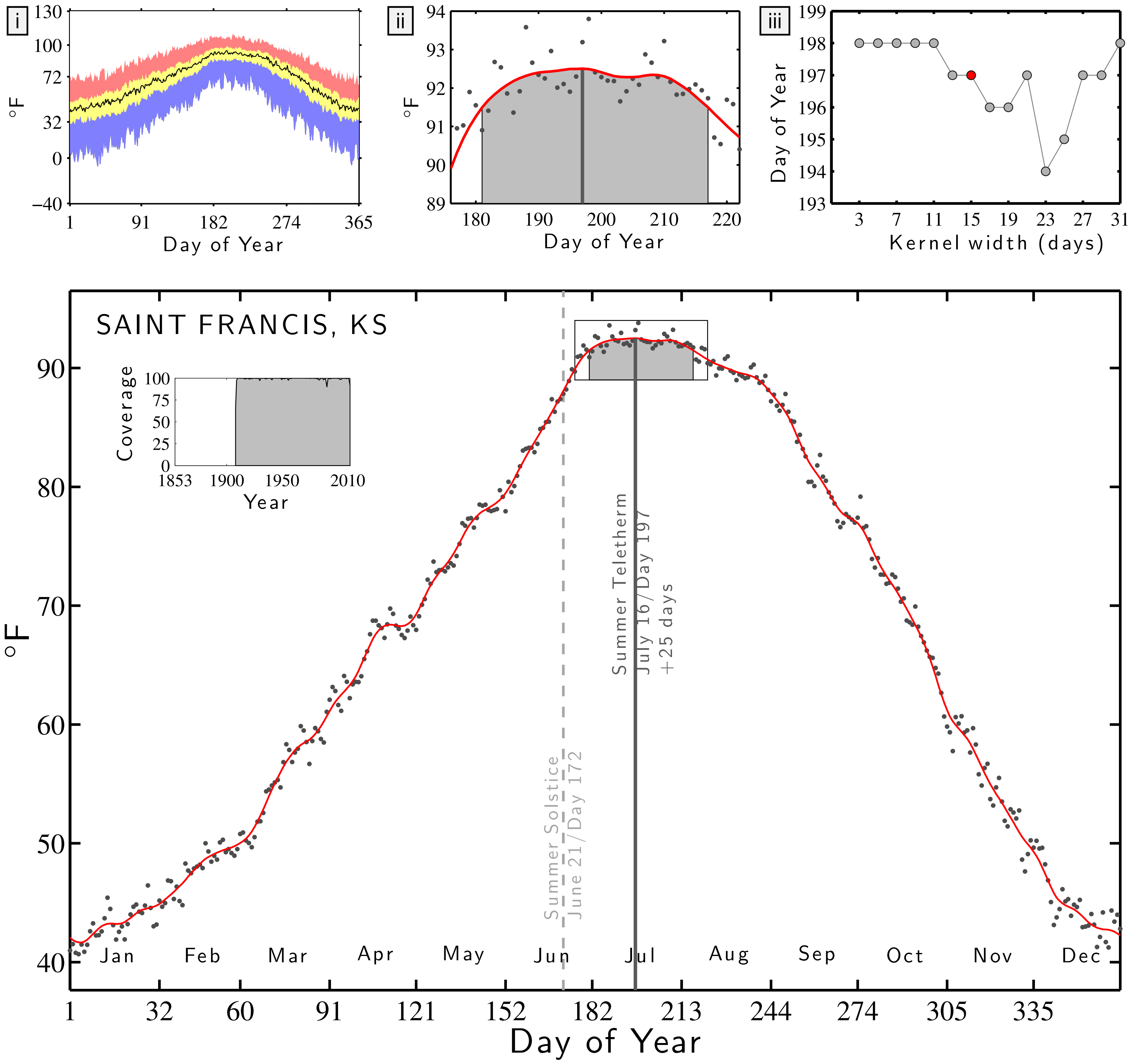

i
ii
iii
°F
Day of Year
Day of Year
Kernel width (days)
SAINT FRANCIS, KS
Coverage
Year
Summer Solstice
June 21 / Day 172
Summer Teletherm
July 16 / Day 197
+25 days
Jan
Feb
Mar
Apr
May
Jun
Jul
Aug
Sep
Oct
Nov
Dec

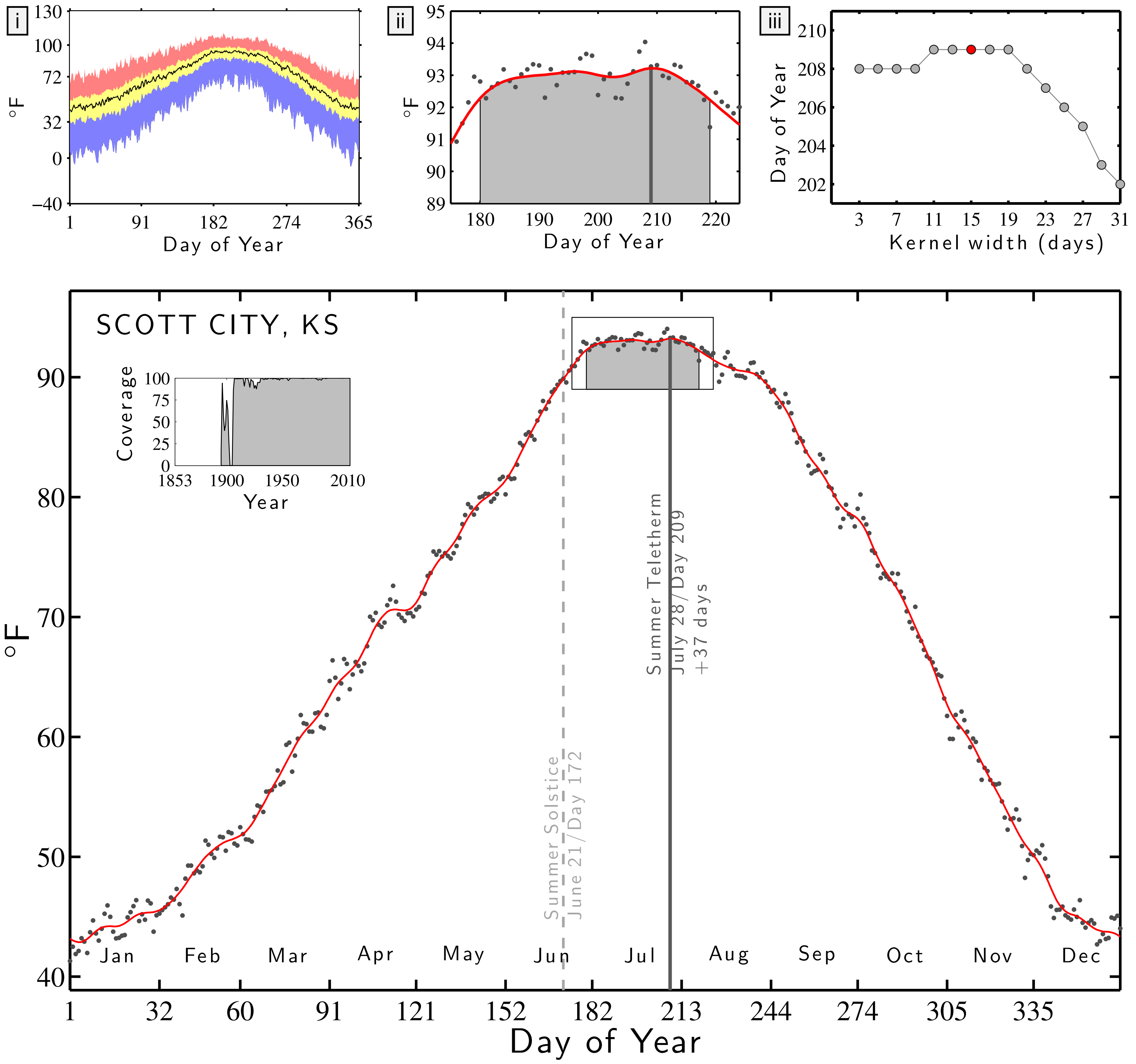

i
°F
Day of Year
ii
°F
Day of Year
iii
Day of Year
Kernel width (days)
SCOTT CITY, KS
Coverage
Year
Summer Solstice
June 21/Day 172
Summer Teletherm
July 28/Day 209
+37 days
Jan
Feb
Mar
Apr
May
Jun
Jul
Aug
Sep
Oct
Nov
Dec
°F
Day of Year

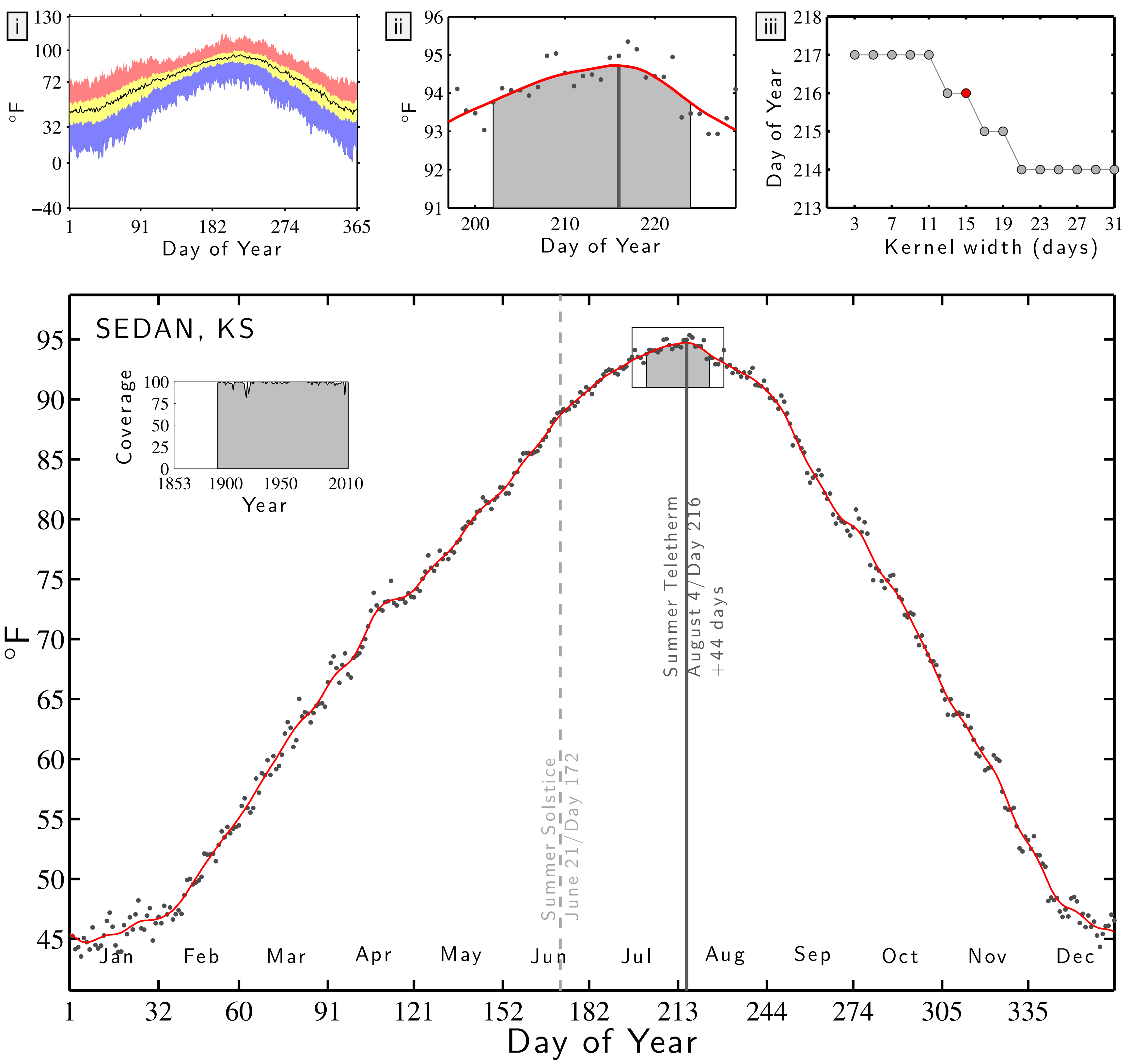

i
ii
iii
°F
Day of Year
Day of Year
Kernel width (days)
SEDAN, KS
Coverage
Year
Summer Solstice
June 21 / Day 172
Summer Teletherm
August 4 / Day 216
+44 days
Jan
Feb
Mar
Apr
May
Jun
Jul
Aug
Sep
Oct
Nov
Dec
Day of Year

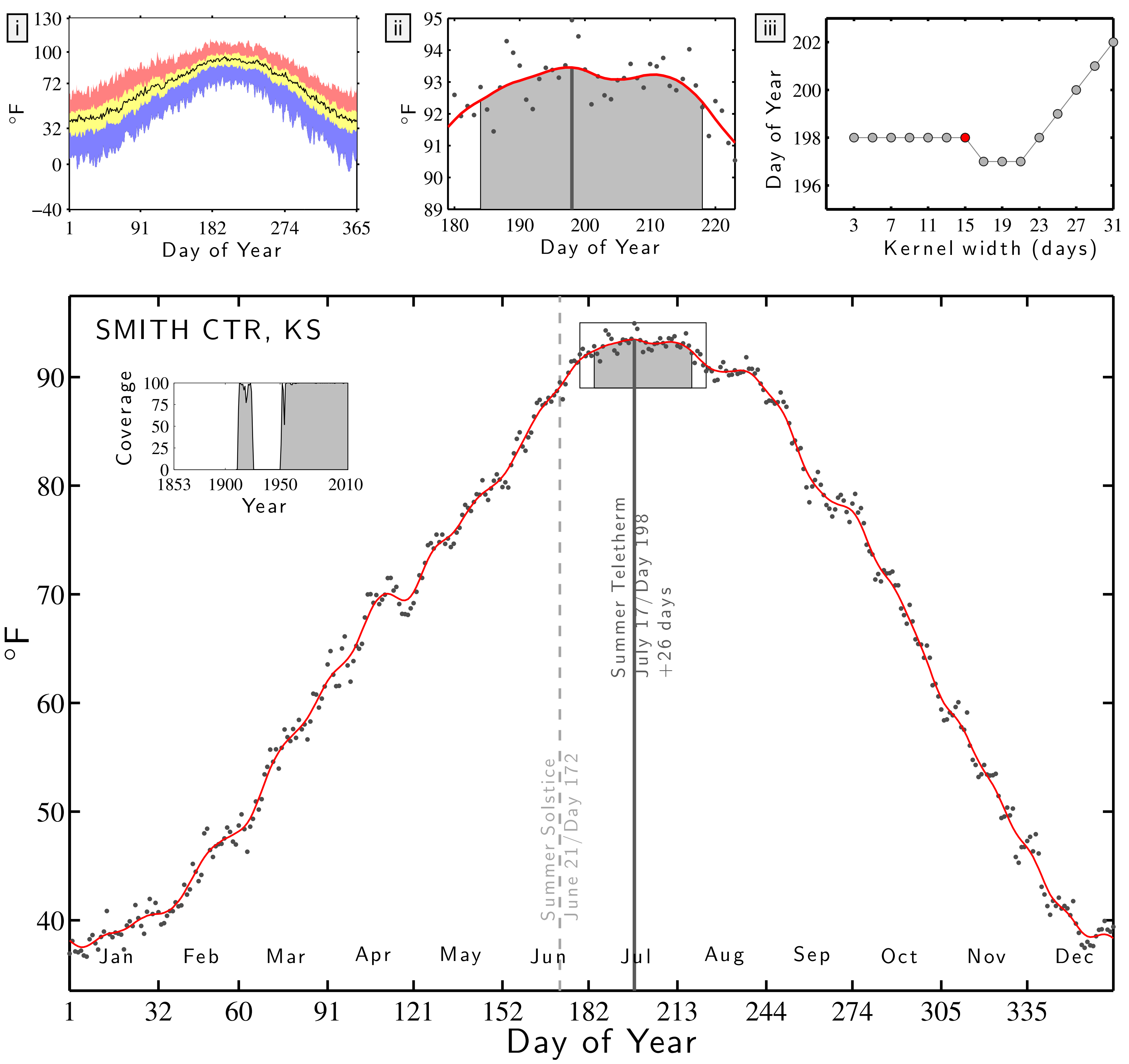

i
°F
Day of Year
ii
°F
Day of Year
iii
Day of Year
Kernel width (days)
SMITH CTR, KS
Coverage
Year
Summer Solstice
June 21 / Day 172
Summer Teletherm
July 17 / Day 198
+26 days
Jan
Feb
Mar
Apr
May
Jun
Jul
Aug
Sep
Oct
Nov
Dec
°F
Day of Year

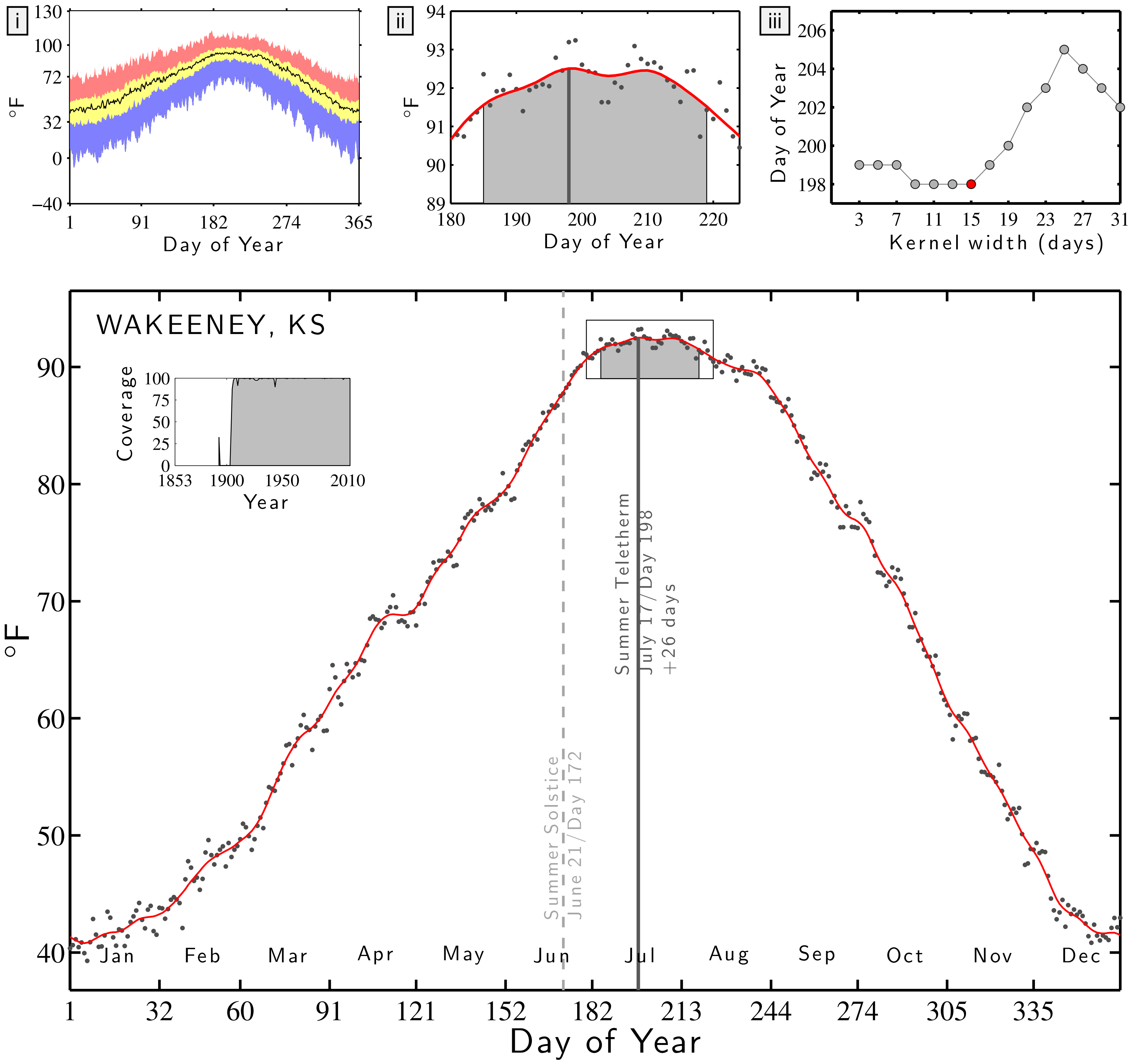

i
°F
Day of Year
ii
°F
Day of Year
iii
Day of Year
Kernel width (days)
WAKEENEY, KS
Coverage
Year
Summer Solstice
June 21/Day 172
Summer Teletherm
July 17/Day 198
+26 days
Jan
Feb
Mar
Apr
May
Jun
Jul
Aug
Sep
Oct
Nov
Dec
°F
Day of Year

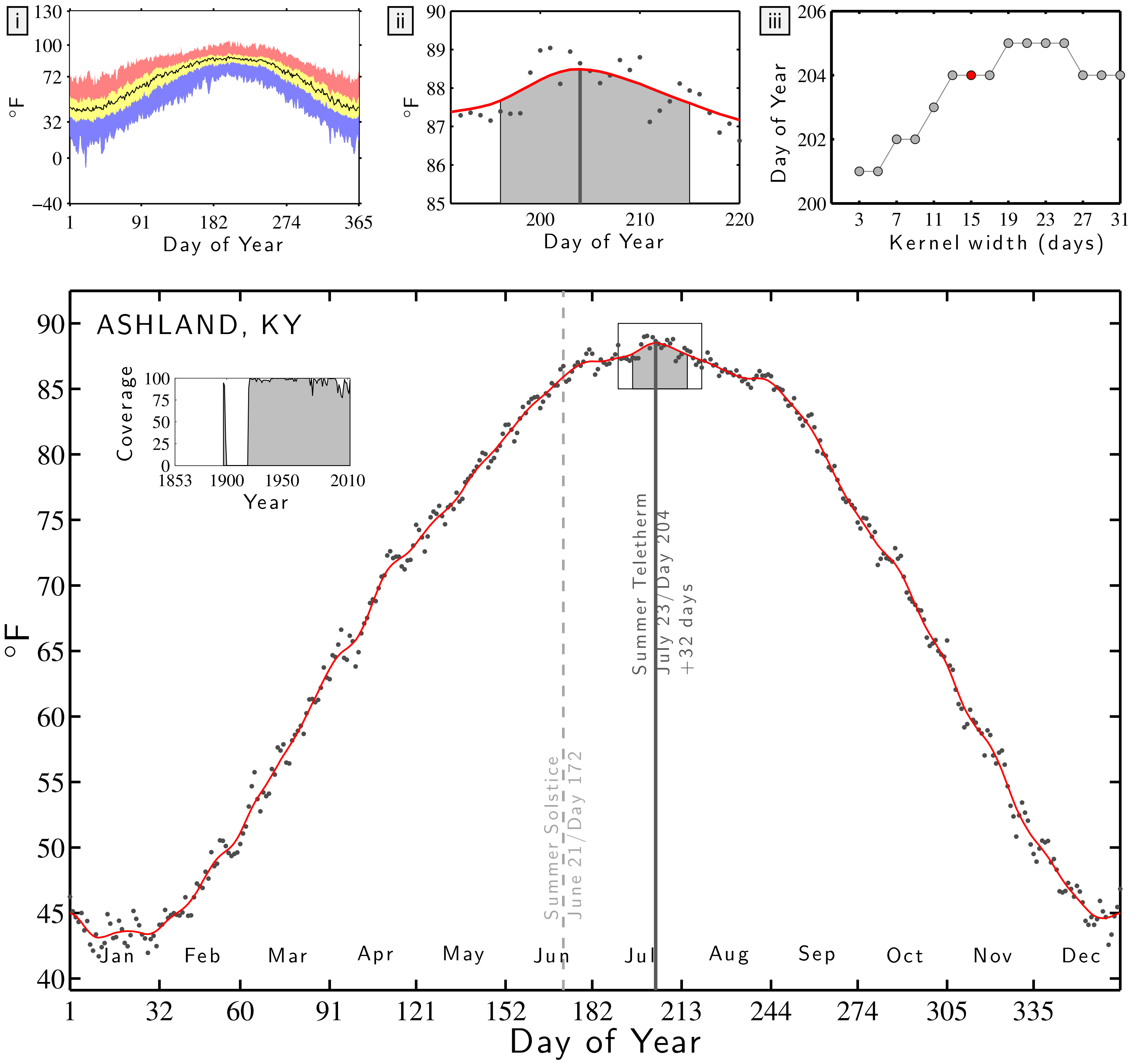

i
ii
iii
ASHLAND, KY
Coverage
Year
Summer Solstice
June 21 / Day 172
Summer Teletherm
July 23 / Day 204
+32 days
Jan
Feb
Mar
Apr
May
Jun
Jul
Aug
Sep
Oct
Nov
Dec
Day of Year
Kernel width (days)
°F

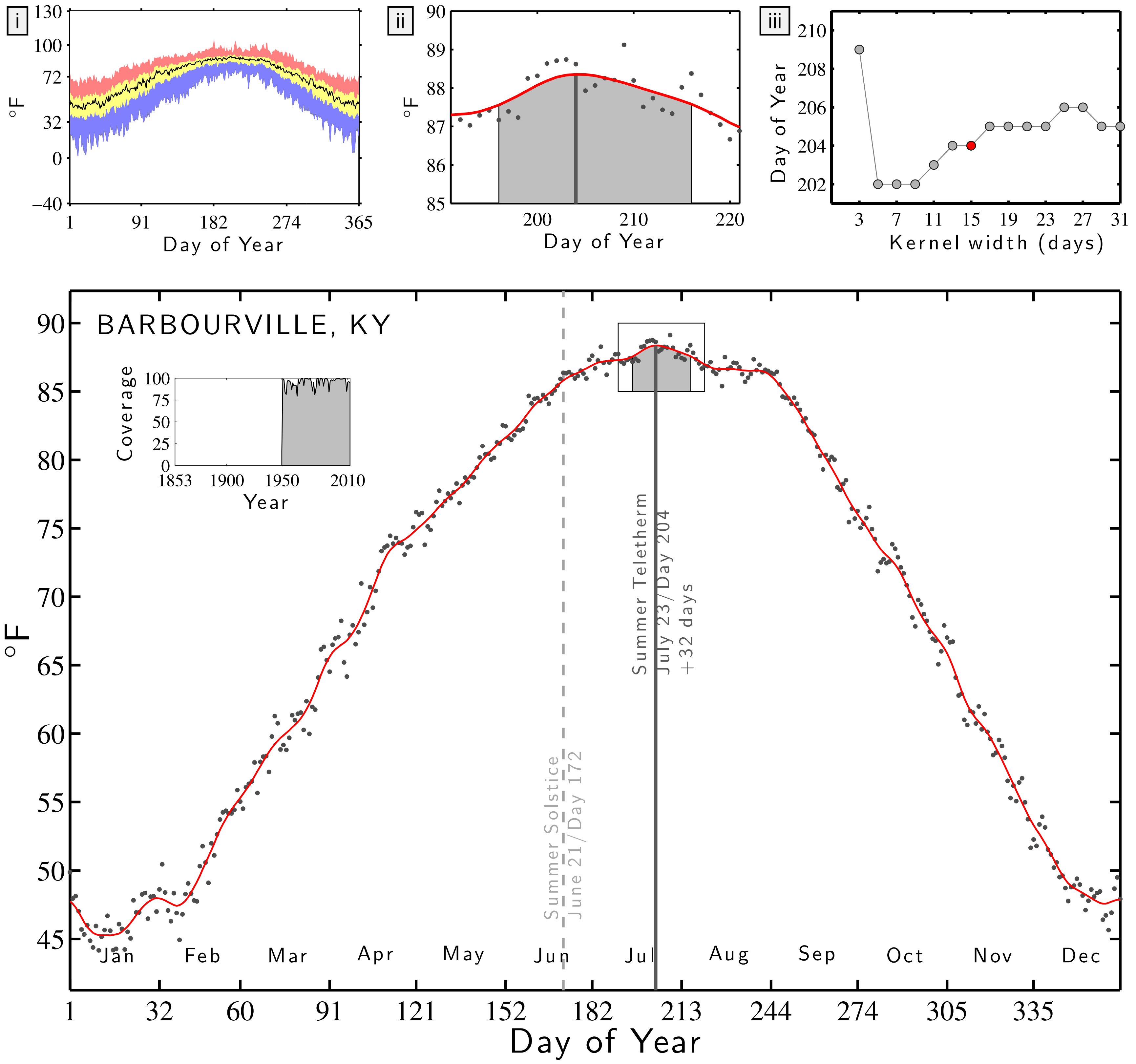

i
ii
iii
°F
Day of Year
Day of Year
Kernel width (days)
BARBOURVILLE, KY
Coverage
Year
Summer Solstice
June 21 / Day 172
Summer Teletherm
July 23 / Day 204
+32 days
Jan
Feb
Mar
Apr
May
Jun
Jul
Aug
Sep
Oct
Nov
Dec

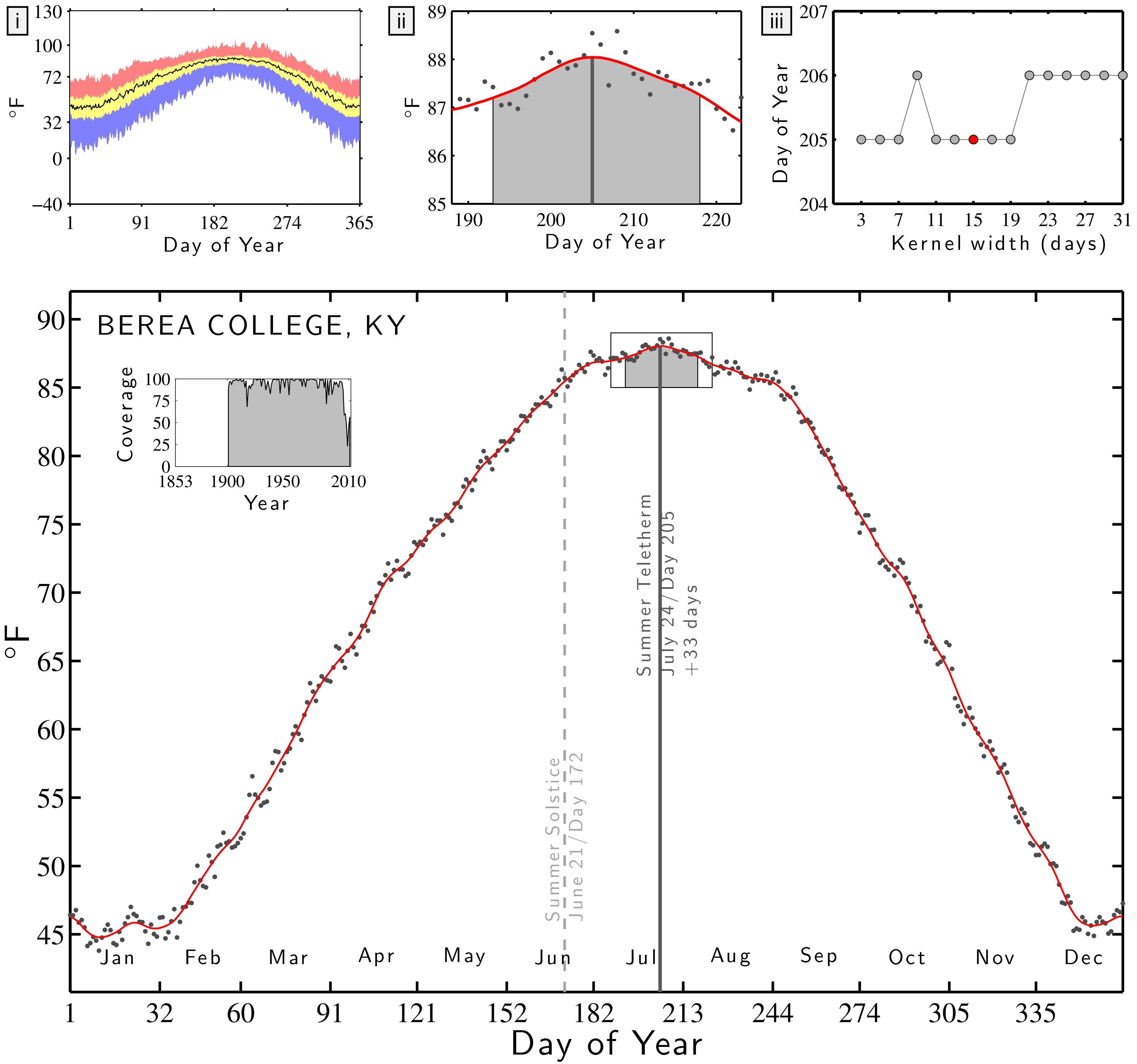

i
ii
iii
°F
Day of Year
Day of Year
Kernel width (days)
BEREA COLLEGE, KY
Coverage
Year
Summer Solstice
June 21 / Day 172
Summer Teletherm
July 24 / Day 205
+33 days
Jan
Feb
Mar
Apr
May
Jun
Jul
Aug
Sep
Oct
Nov
Dec
Day of Year

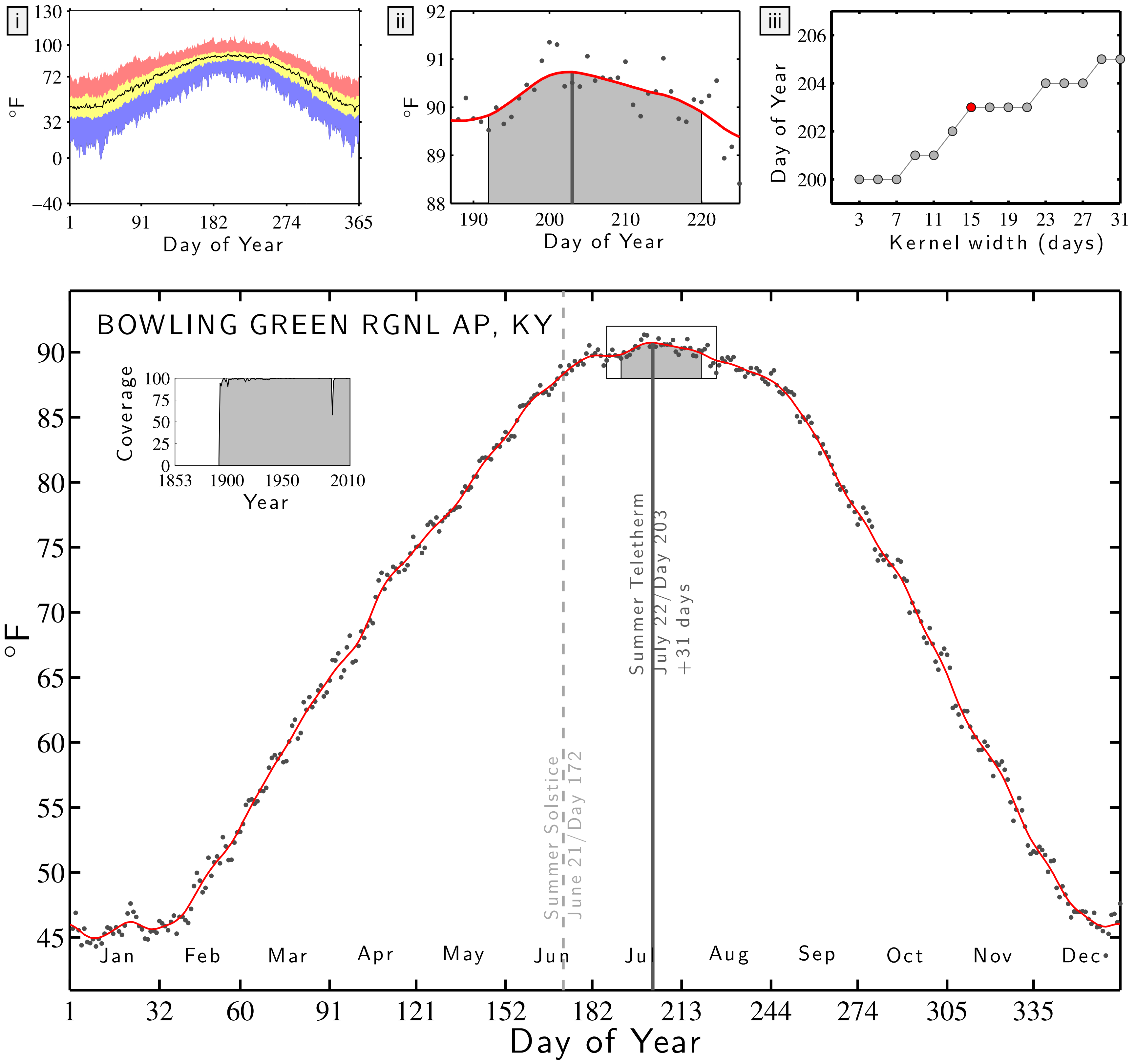

i
ii
iii
Day of Year
°F
Kernel width (days)
BOWLING GREEN RGNL AP, KY
Coverage
Year
Summer Solstice
June 21/Day 172
Summer Teletherm
July 22/Day 203
+31 days
Jan
Feb
Mar
Apr
May
Jun
Jul
Aug
Sep
Oct
Nov
Dec

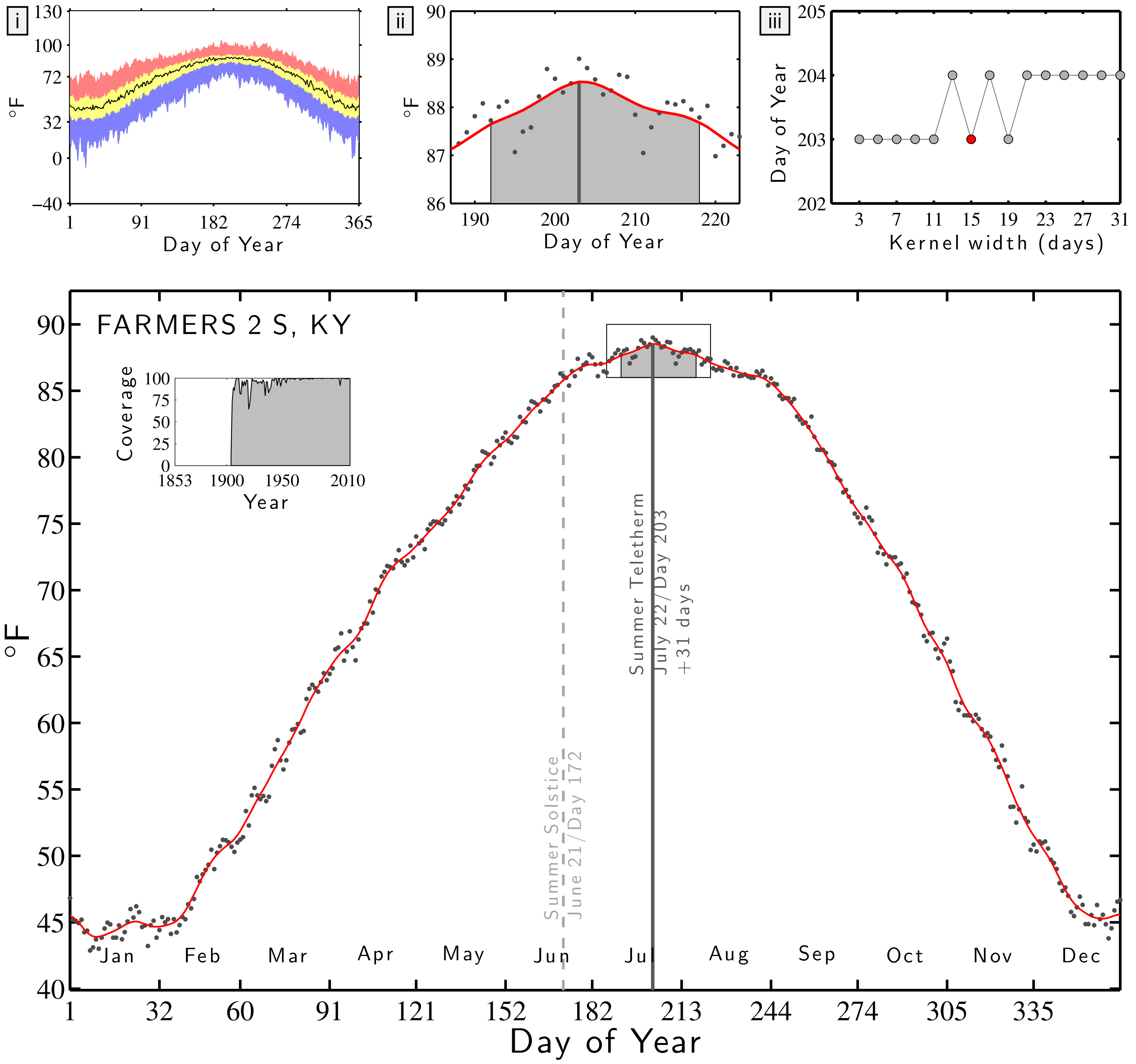

i
°F
Day of Year
ii
Day of Year
iii
Day of Year
Kernel width (days)
FARMERS 2 S, KY
Coverage
Year
Summer Solstice
June 21/Day 172
Summer Teletherm
July 22/Day 203
+31 days
Jan
Feb
Mar
Apr
May
Jun
Jul
Aug
Sep
Oct
Nov
Dec
Day of Year

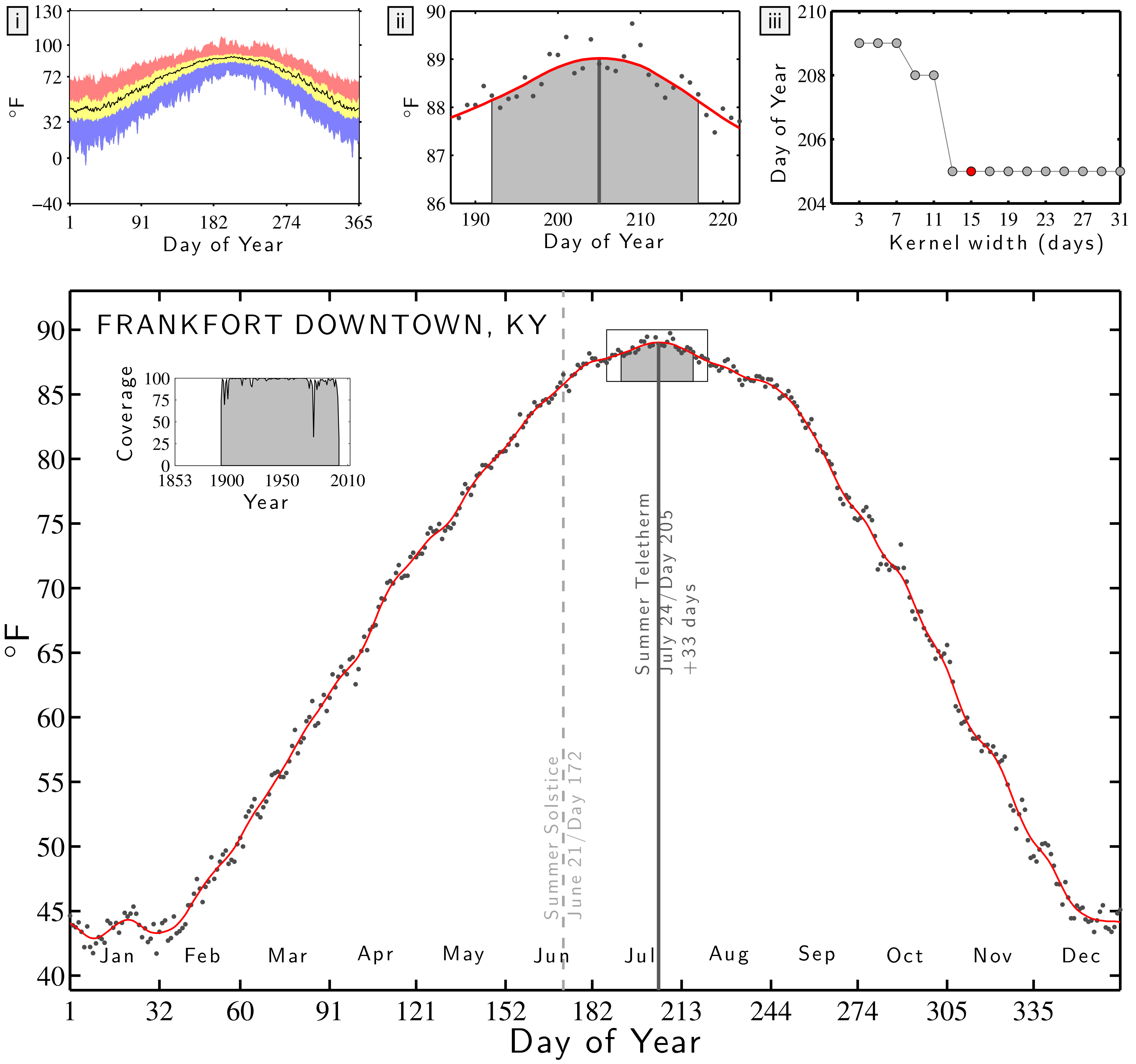

i
°F
130
100
72
32
0
−40
1
91
182
274
365
Day of Year
ii
°F
90
89
88
87
86
190
200
210
220
Day of Year
iii
Day of Year
210
208
206
204
3
7
11
15
19
23
27
31
Kernel width (days)
FRANKFORT DOWNTOWN, KY
Coverage
100
75
50
25
0
1853
1900
1950
2010
Year
Summer Solstice
June 21 / Day 172
Summer Teletherm
July 24 / Day 205
+33 days
°F
90
85
80
75
70
65
60
55
50
45
40
Jan
Feb
Mar
Apr
May
Jun
Jul
Aug
Sep
Oct
Nov
Dec
1
32
60
91
121
152
182
213
244
274
305
335
Day of Year

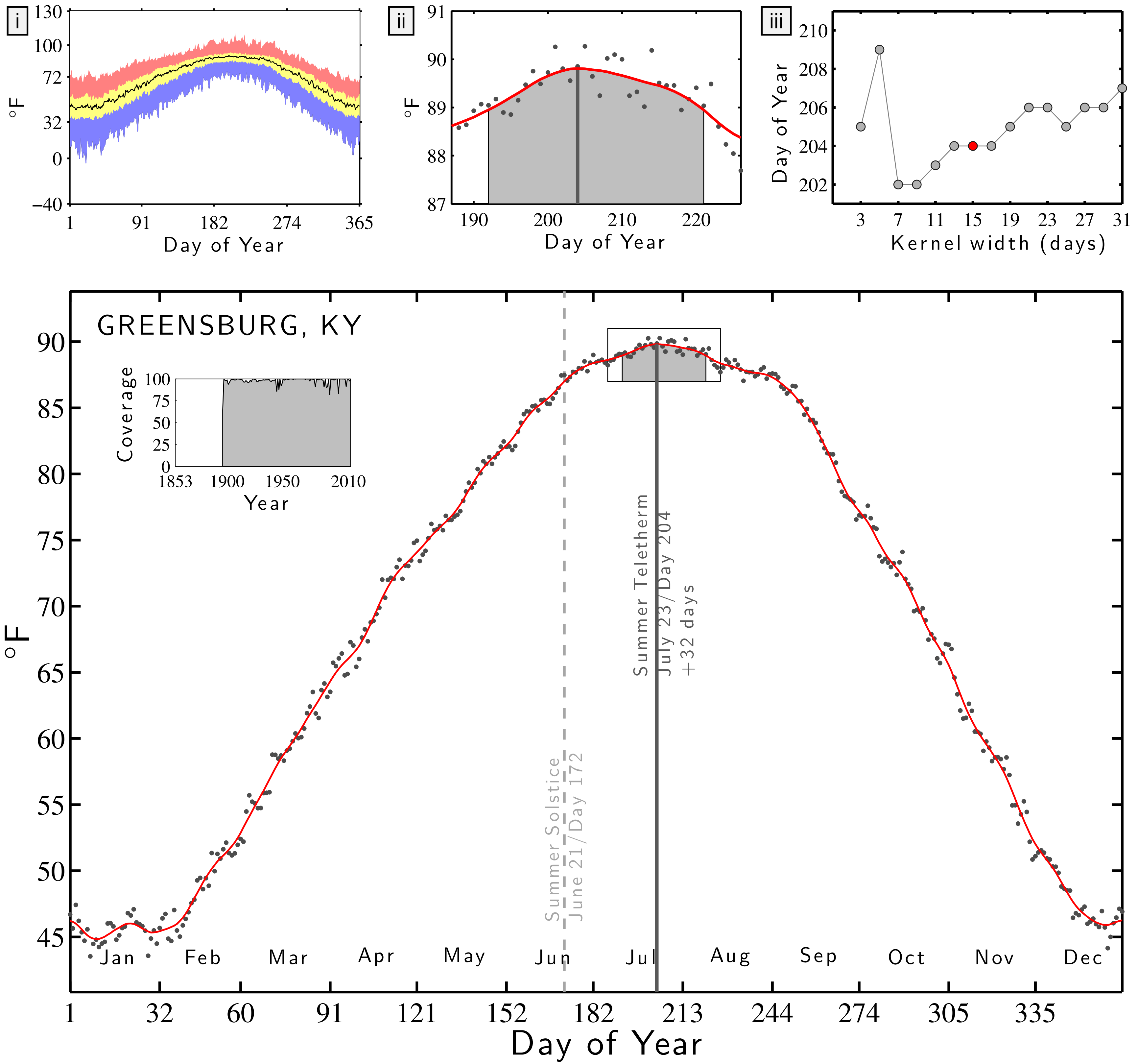

i
ii
iii
GREENSBURG, KY
Coverage
Year
Summer Solstice
June 21 / Day 172
Summer Teletherm
July 23 / Day 204
+32 days
Jan
Feb
Mar
Apr
May
Jun
Jul
Aug
Sep
Oct
Nov
Dec
Day of Year
°F
Kernel width (days)

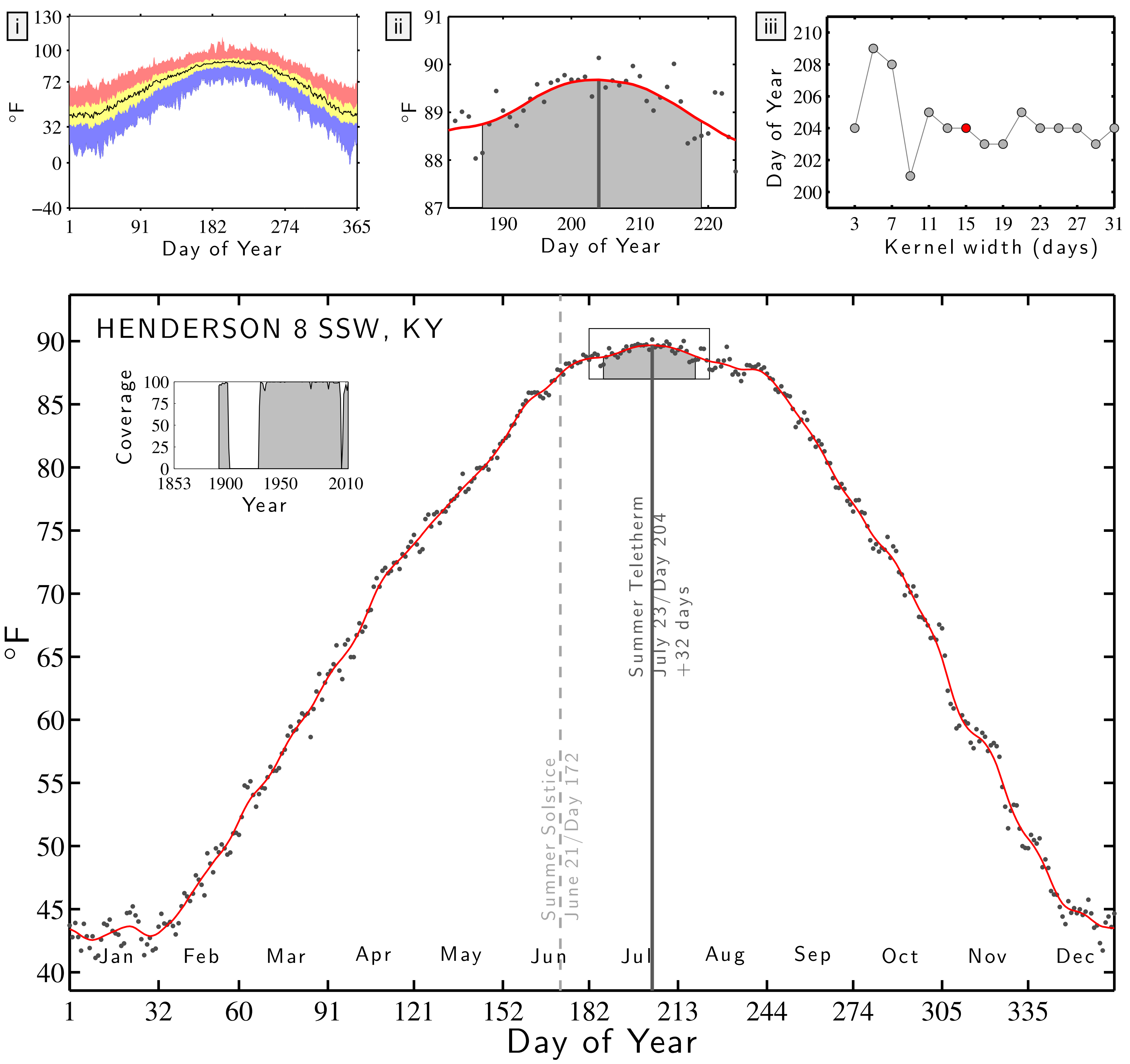

i
ii
iii
°F
Day of Year
Day of Year
Kernel width (days)
HENDERSON 8 SSW, KY
Coverage
Year
1853
1900
1950
2010
Summer Solstice
June 21 / Day 172
Summer Teletherm
July 23 / Day 204
+32 days
Jan
Feb
Mar
Apr
May
Jun
Jul
Aug
Sep
Oct
Nov
Dec

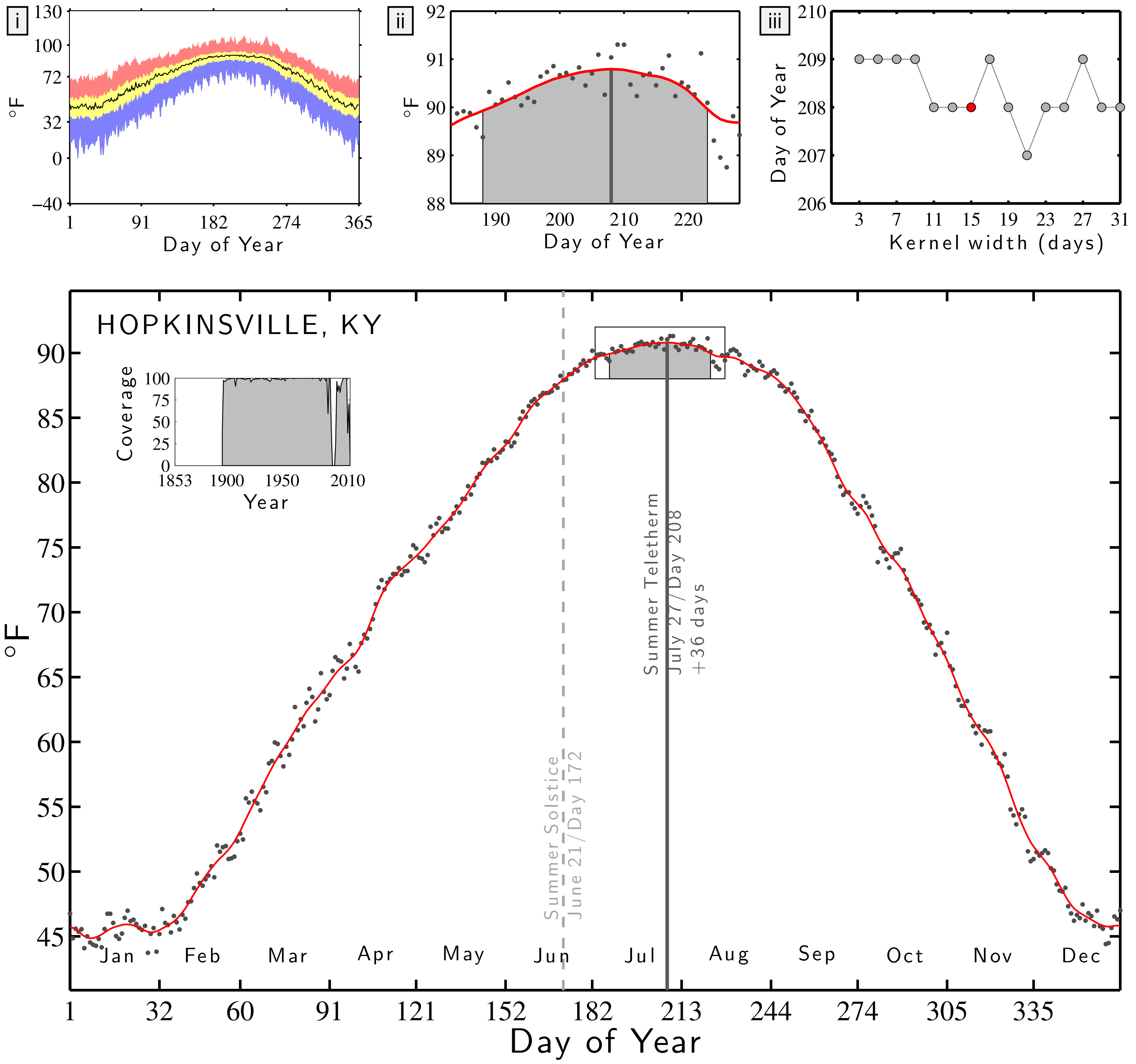

i
°F
Day of Year
ii
°F
Day of Year
iii
Day of Year
Kernel width (days)
HOPKINSVILLE, KY
Coverage
Year
Summer Solstice
June 21/Day 172
Summer Teletherm
July 27/Day 208
+36 days
Jan
Feb
Mar
Apr
May
Jun
Jul
Aug
Sep
Oct
Nov
Dec
°F
Day of Year

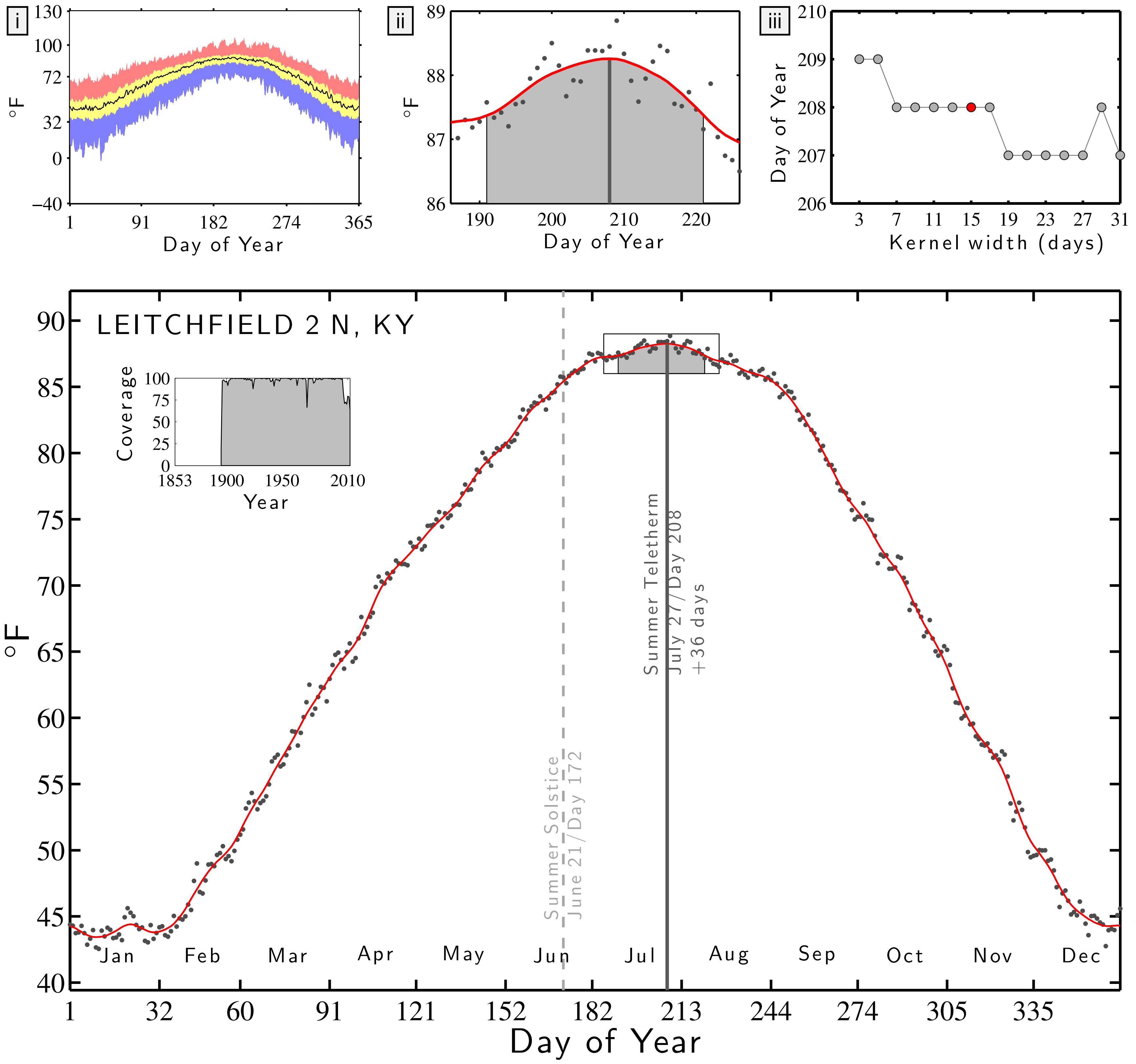

i
°F
Day of Year
ii
°F
Day of Year
iii
Day of Year
Kernel width (days)
LEITCHFIELD 2 N, KY
Coverage
Year
Summer Solstice
June 21 / Day 172
Summer Teletherm
July 27 / Day 208
+36 days
Jan
Feb
Mar
Apr
May
Jun
Jul
Aug
Sep
Oct
Nov
Dec
°F
Day of Year

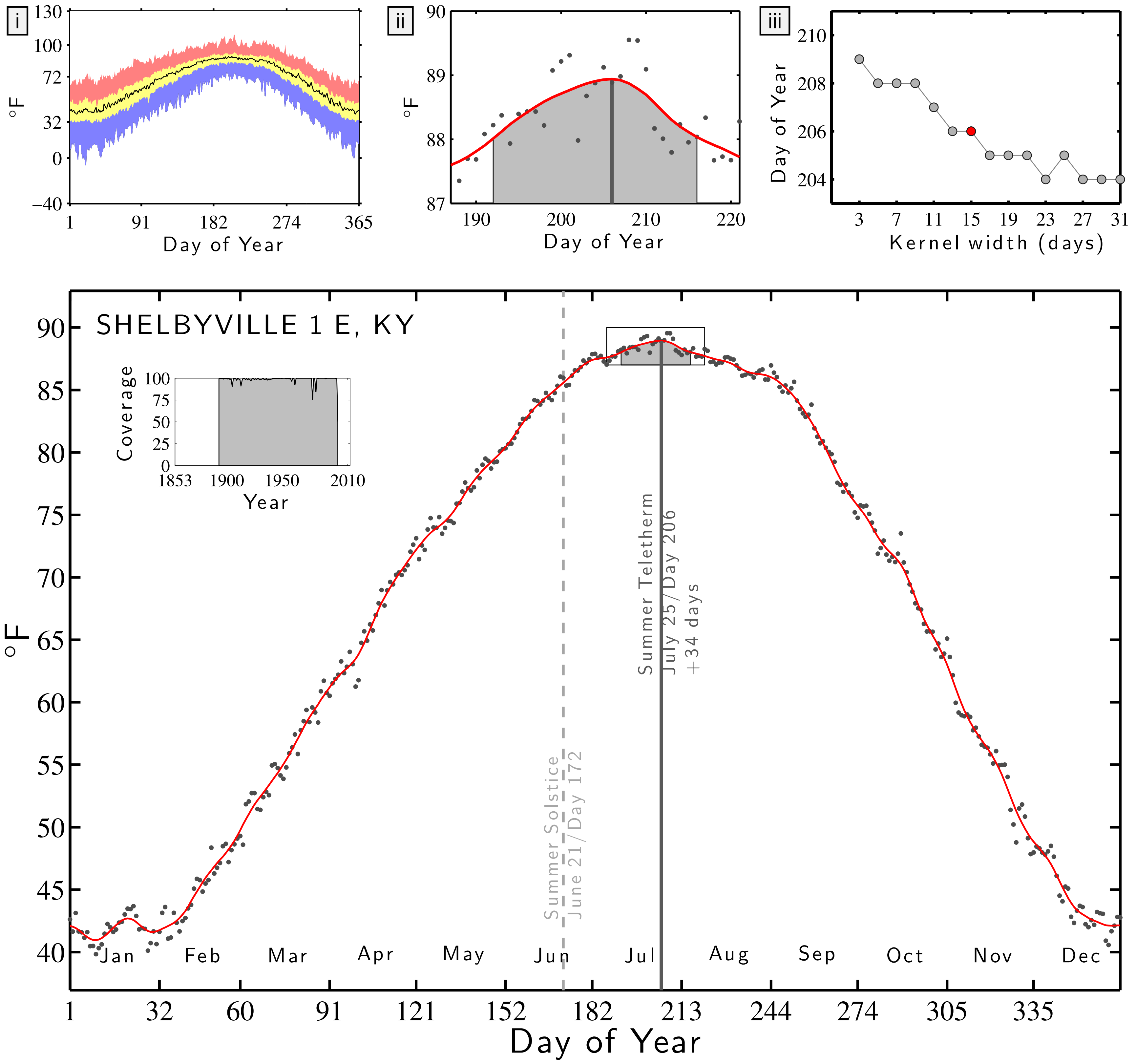

i
°F
Day of Year
ii
°F
Day of Year
iii
Day of Year
Kernel width (days)
SHELBYVILLE 1 E, KY
Coverage
Year
Summer Solstice
June 21/Day 172
Summer Teletherm
July 25/Day 206
+34 days
Jan
Feb
Mar
Apr
May
Jun
Jul
Aug
Sep
Oct
Nov
Dec
°F
Day of Year

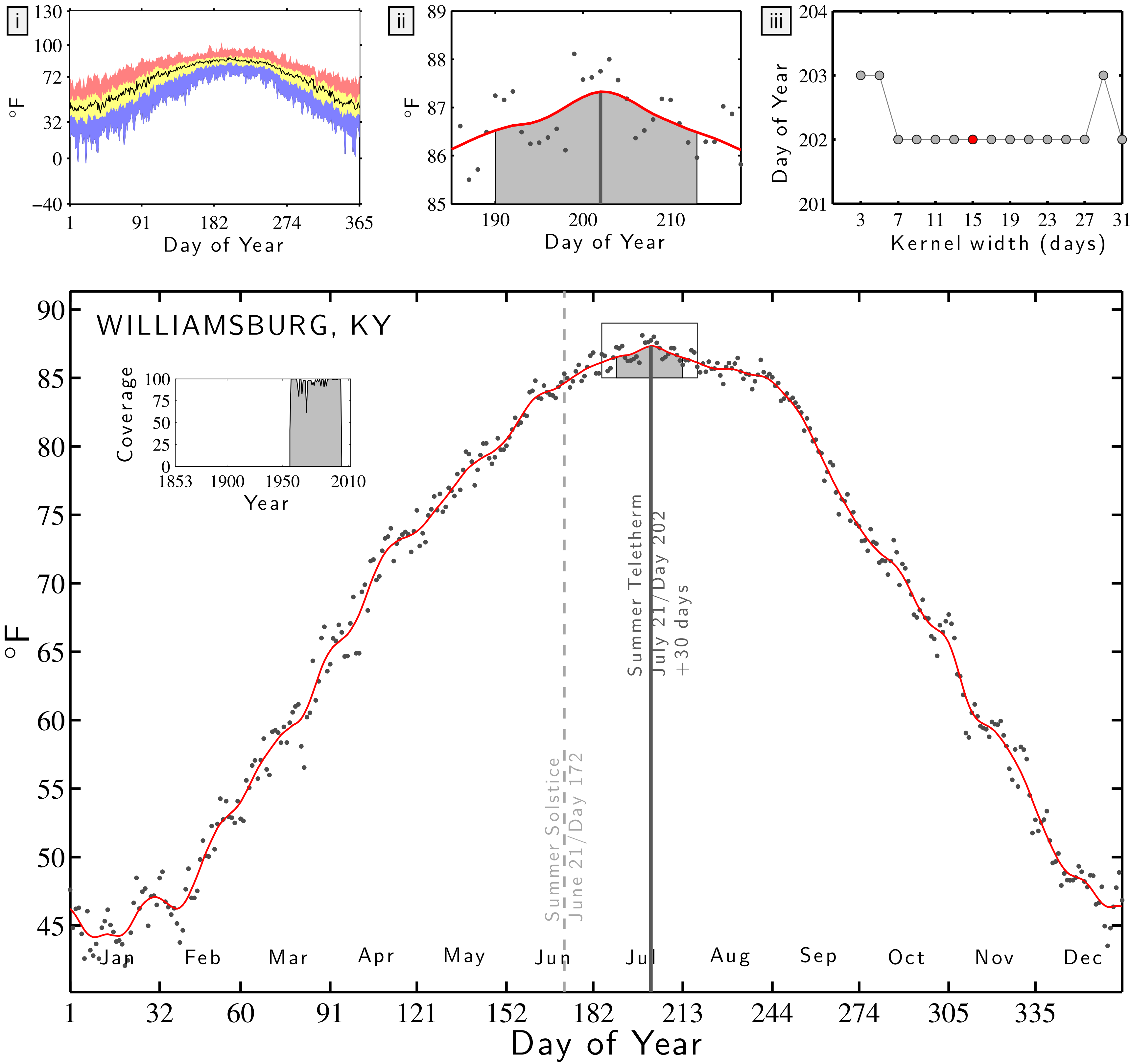

i
°F
Day of Year
ii
°F
Day of Year
iii
Day of Year
Kernel width (days)
WILLIAMSBURG, KY
Coverage
Year
Summer Solstice
June 21 / Day 172
Summer Teletherm
July 21 / Day 202
+30 days
Jan
Feb
Mar
Apr
May
Jun
Jul
Aug
Sep
Oct
Nov
Dec
°F
Day of Year

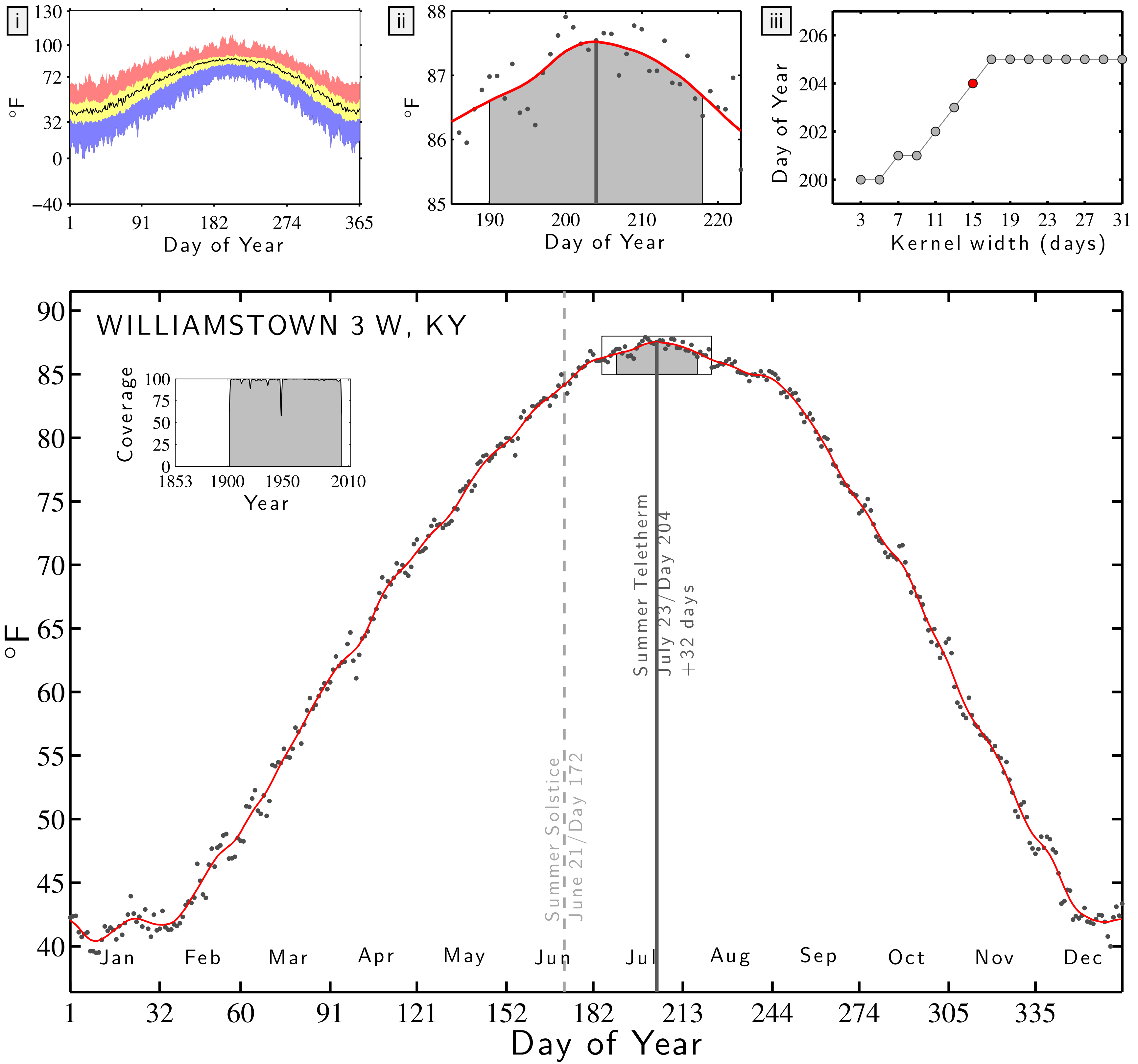

i
°F
Day of Year
ii
°F
Day of Year
iii
Day of Year
Kernel width (days)
WILLIAMSTOWN 3 W, KY
Coverage
Year
Summer Solstice
June 21/Day 172
Summer Teletherm
July 23/Day 204
+32 days
Jan
Feb
Mar
Apr
May
Jun
Jul
Aug
Sep
Oct
Nov
Dec
°F
Day of Year

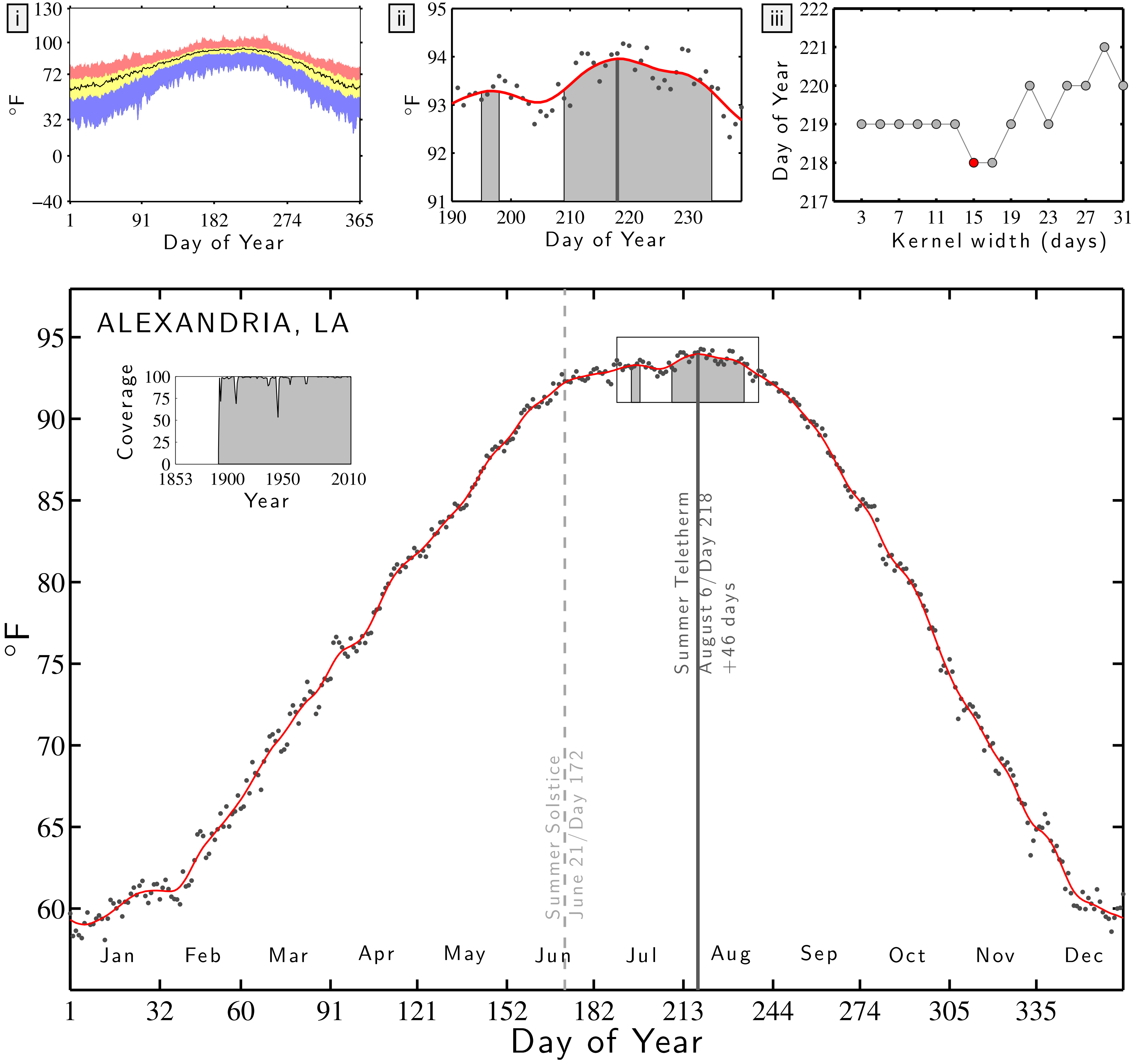

i
°F
Day of Year
ii
°F
Day of Year
iii
Day of Year
Kernel width (days)
ALEXANDRIA, LA
Coverage
Year
Summer Solstice
June 21 / Day 172
Summer Teletherm
August 6 / Day 218
+46 days
Jan
Feb
Mar
Apr
May
Jun
Jul
Aug
Sep
Oct
Nov
Dec
°F
Day of Year

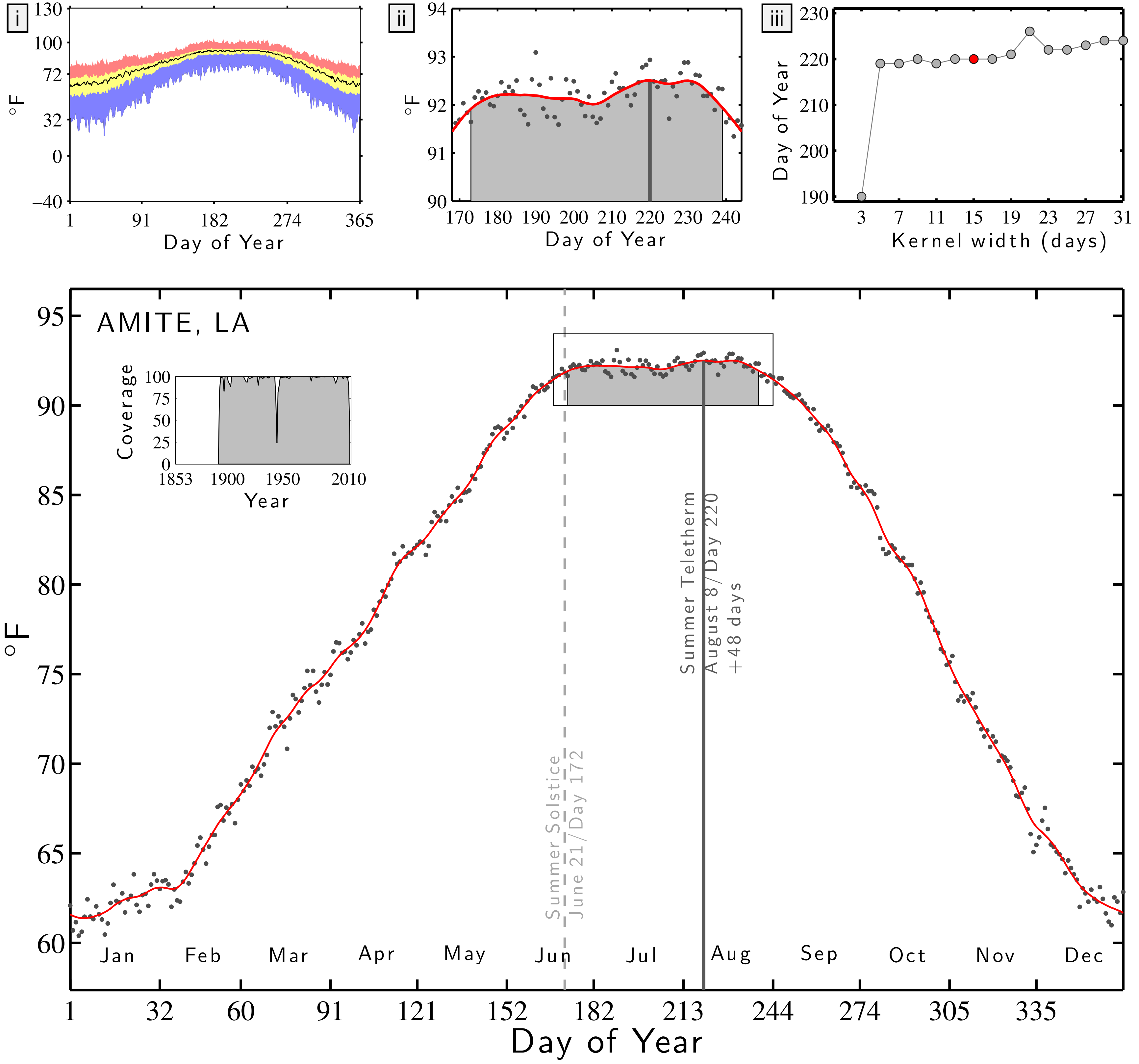

i
°F
Day of Year
ii
°F
Day of Year
iii
Day of Year
Kernel width (days)
AMITE, LA
Coverage
Year
Summer Solstice
June 21/Day 172
Summer Teletherm
August 8/Day 220
+48 days
Jan
Feb
Mar
Apr
May
Jun
Jul
Aug
Sep
Oct
Nov
Dec
°F
Day of Year

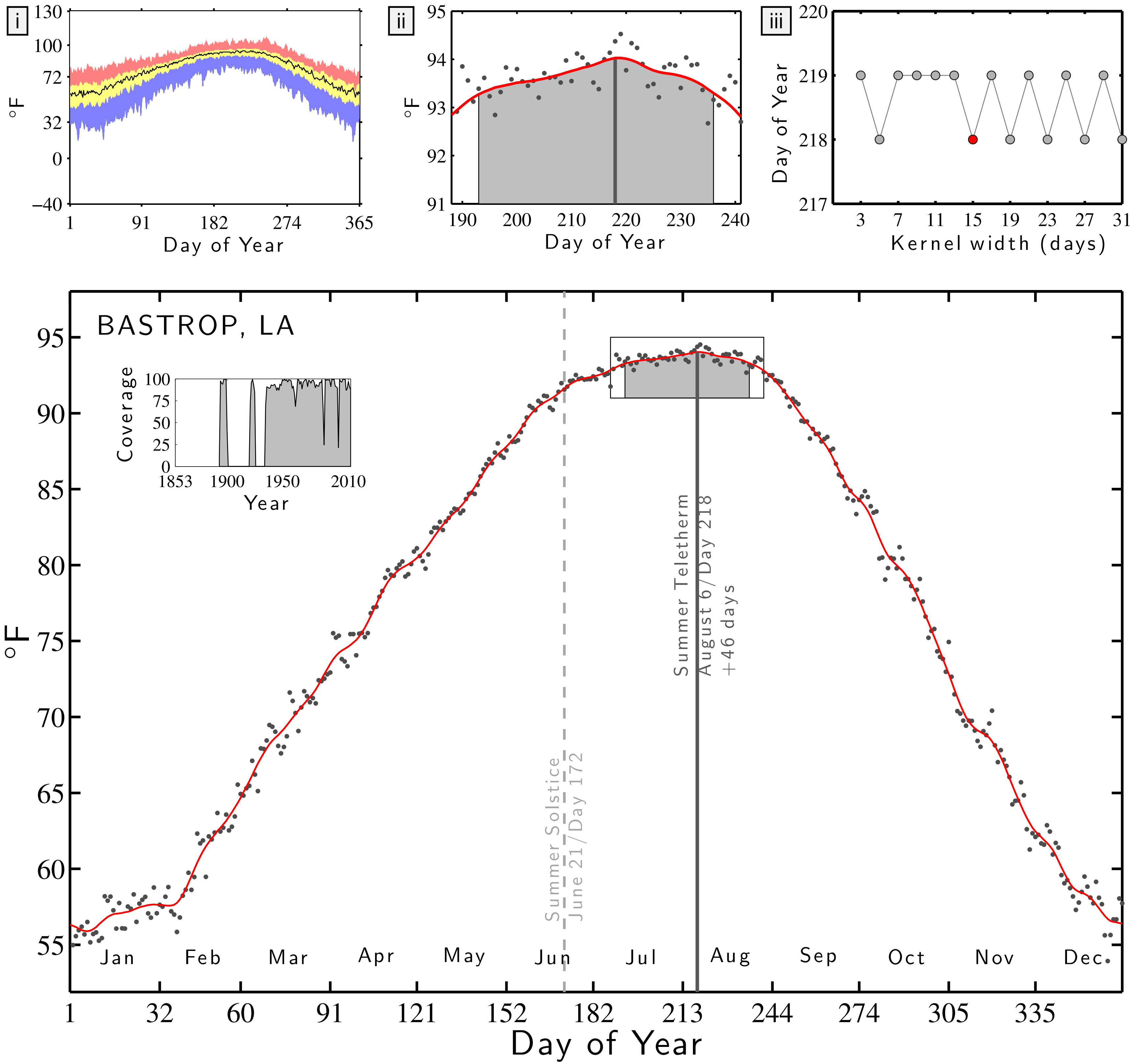

i
°F
Day of Year
ii
°F
Day of Year
iii
Day of Year
Kernel width (days)
BASTROP, LA
Coverage
Year
Summer Solstice
June 21 / Day 172
Summer Teletherm
August 6 / Day 218
+46 days
Jan
Feb
Mar
Apr
May
Jun
Jul
Aug
Sep
Oct
Nov
Dec
°F
Day of Year

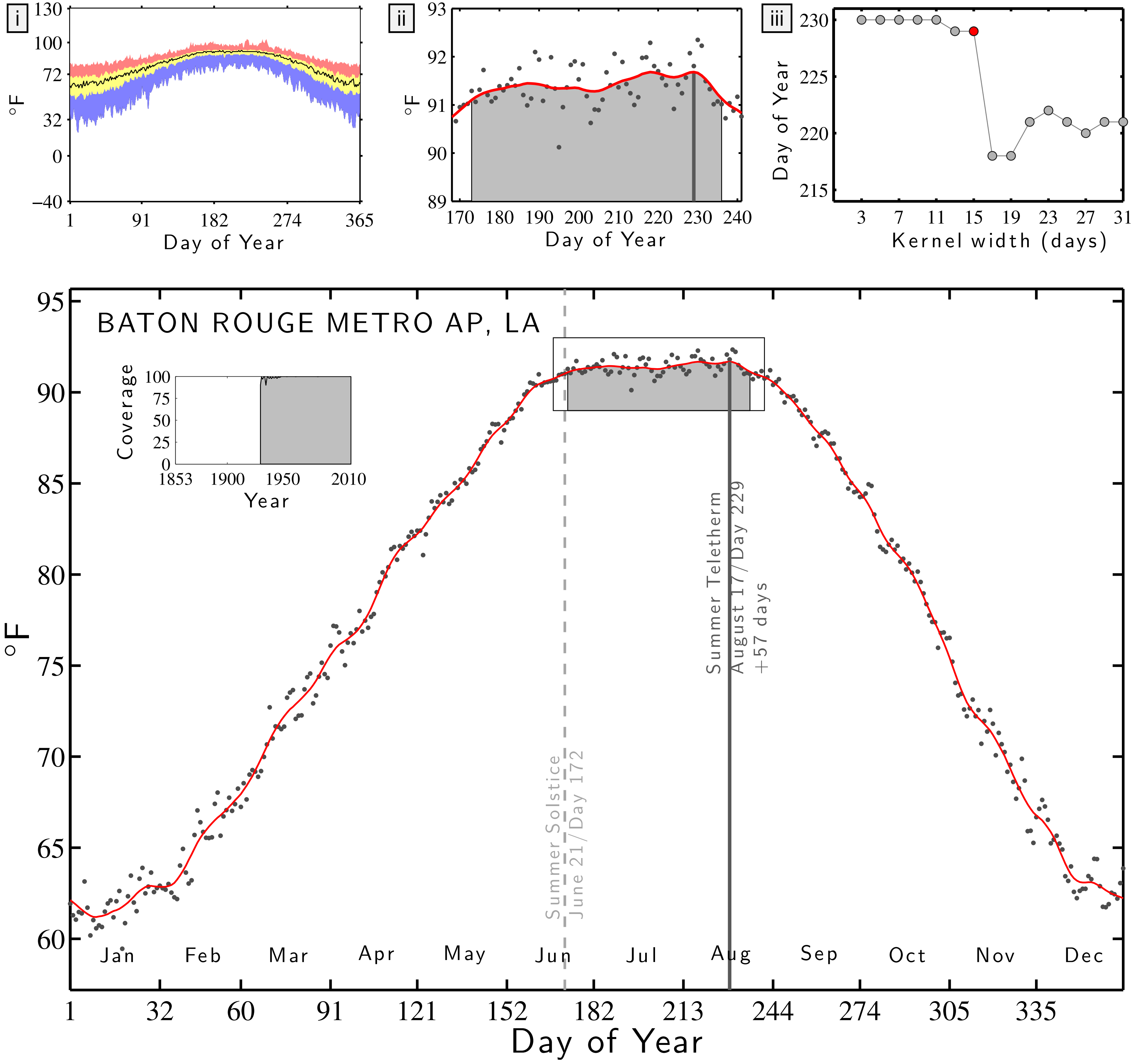

i
ii
iii
BATON ROUGE METRO AP, LA
Coverage
Year
Summer Solstice
June 21 / Day 172
Summer Teletherm
August 17 / Day 229
+57 days
Jan
Feb
Mar
Apr
May
Jun
Jul
Aug
Sep
Oct
Nov
Dec
°F
Day of Year
Kernel width (days)

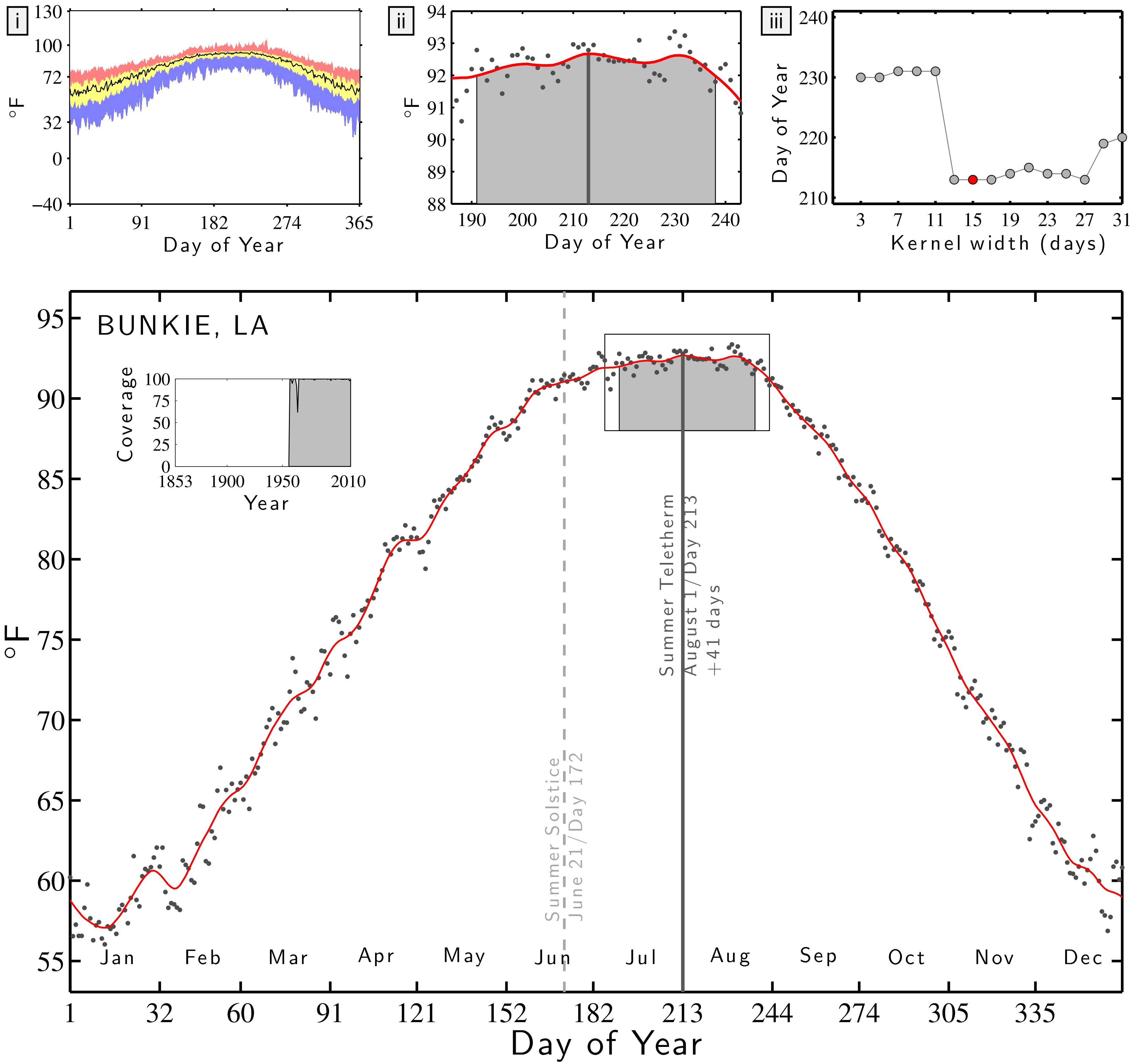

i
ii
iii
Day of Year
°F
Kernel width (days)
BUNKIE, LA
Coverage
Year
Summer Solstice
June 21 / Day 172
Summer Teletherm
August 1 / Day 213
+41 days
Jan
Feb
Mar
Apr
May
Jun
Jul
Aug
Sep
Oct
Nov
Dec

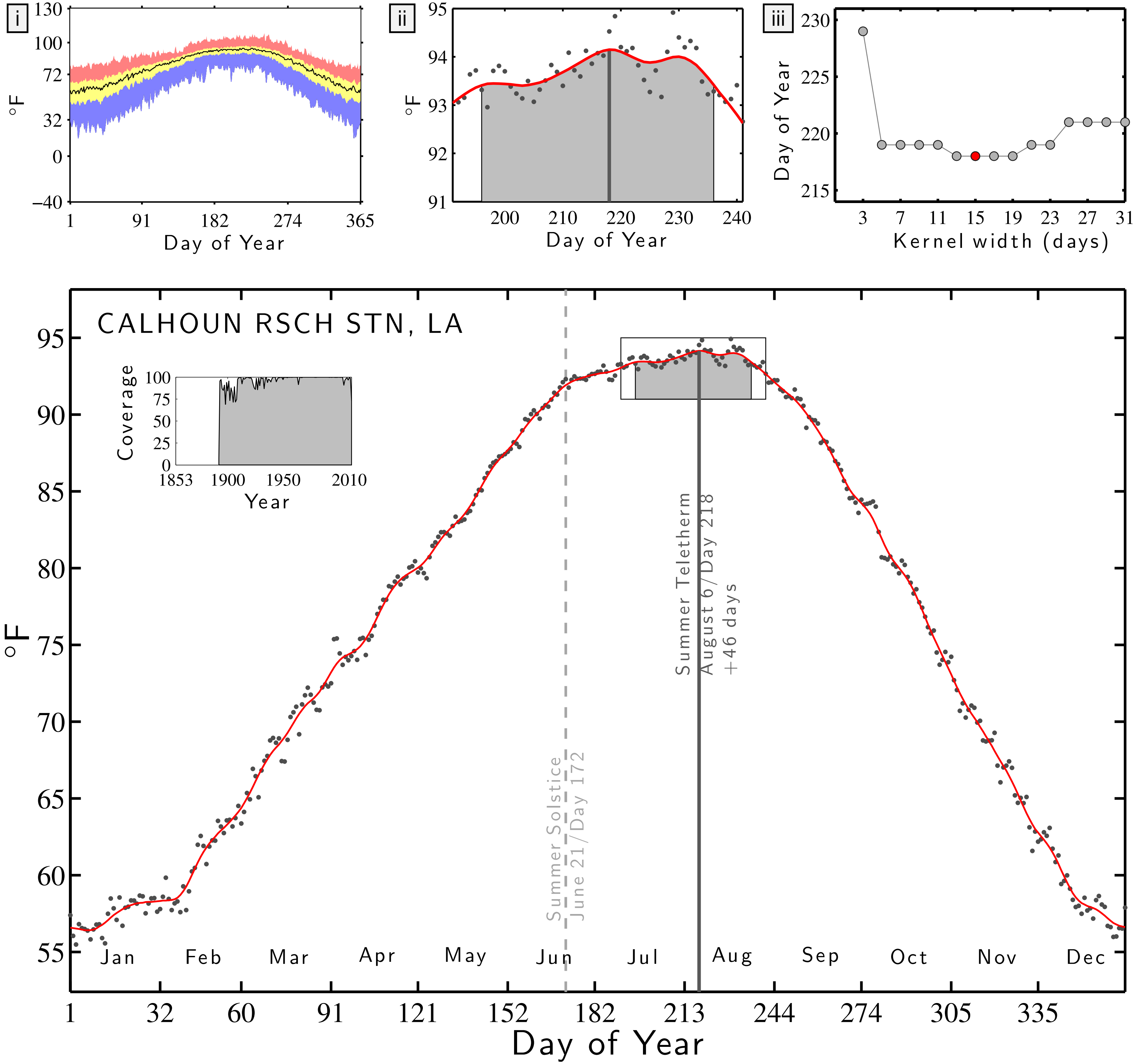

i
ii
iii
°F
Day of Year
Day of Year
Kernel width (days)
CALHOUN RSCH STN, LA
Coverage
Year
Summer Solstice
June 21/Day 172
Summer Teletherm
August 6/Day 218
+46 days
Jan
Feb
Mar
Apr
May
Jun
Jul
Aug
Sep
Oct
Nov
Dec

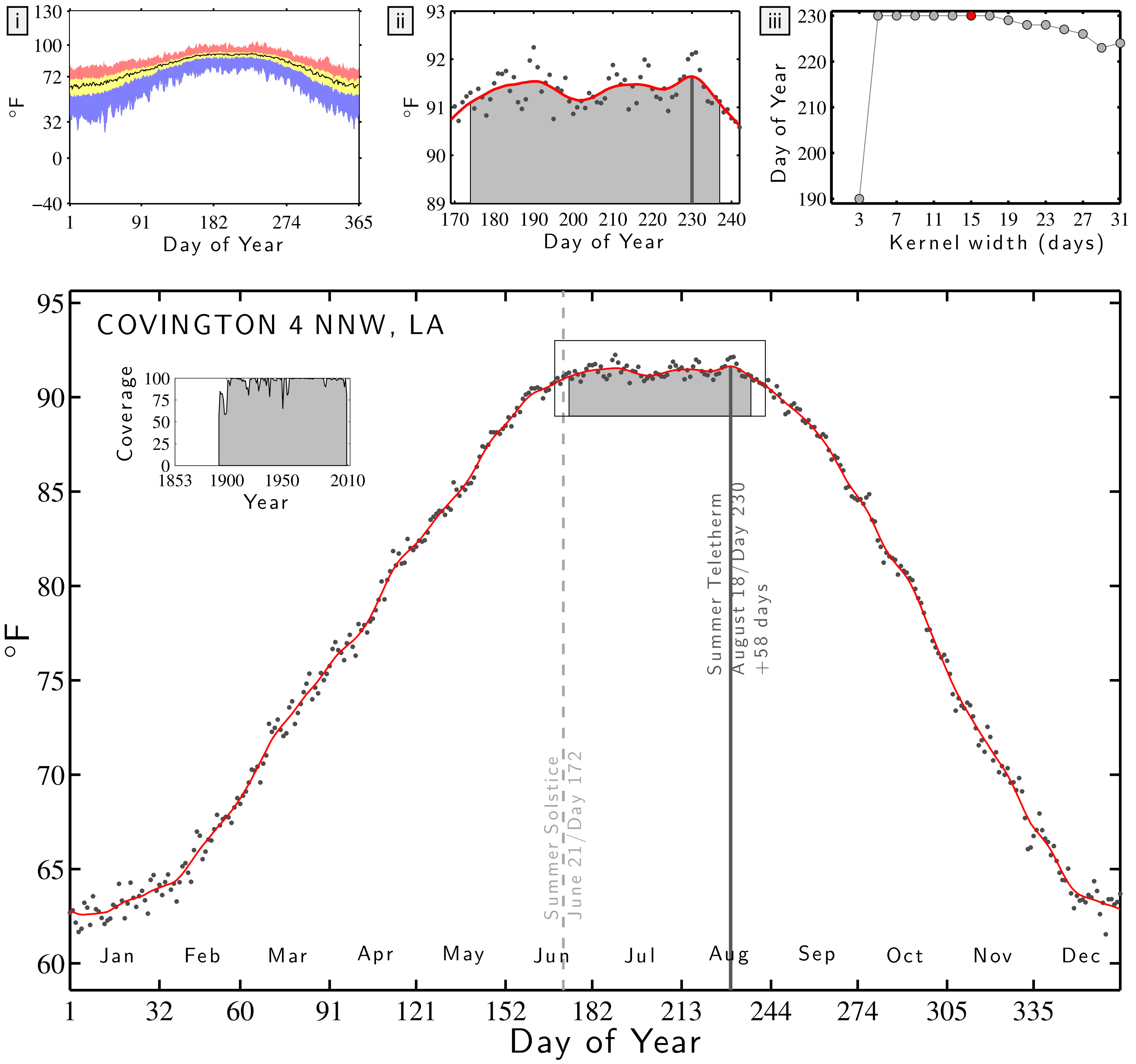

i
ii
iii
°F
Day of Year
Day of Year
Kernel width (days)
COVINGTON 4 NNW, LA
Coverage
Year
Summer Solstice
June 21/Day 172
Summer Teletherm
August 18/Day 230
+58 days
Jan
Feb
Mar
Apr
May
Jun
Jul
Aug
Sep
Oct
Nov
Dec
°F
Day of Year

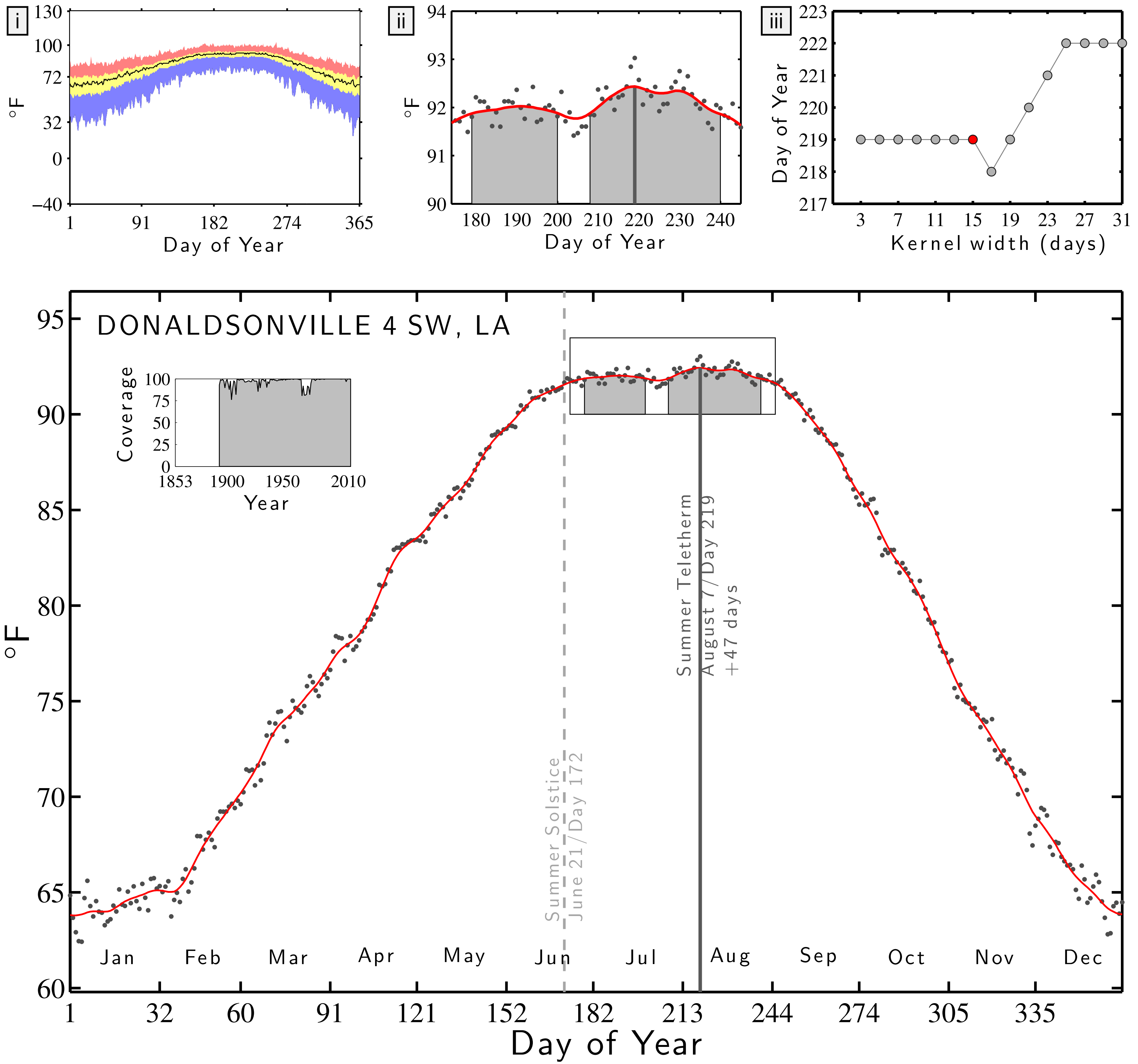

i
ii
iii
Day of Year
°F
Kernel width (days)
DONALDSONVILLE 4 SW, LA
Coverage
Year
Summer Solstice
June 21/Day 172
Summer Teletherm
August 7/Day 219
+47 days
Jan
Feb
Mar
Apr
May
Jun
Jul
Aug
Sep
Oct
Nov
Dec

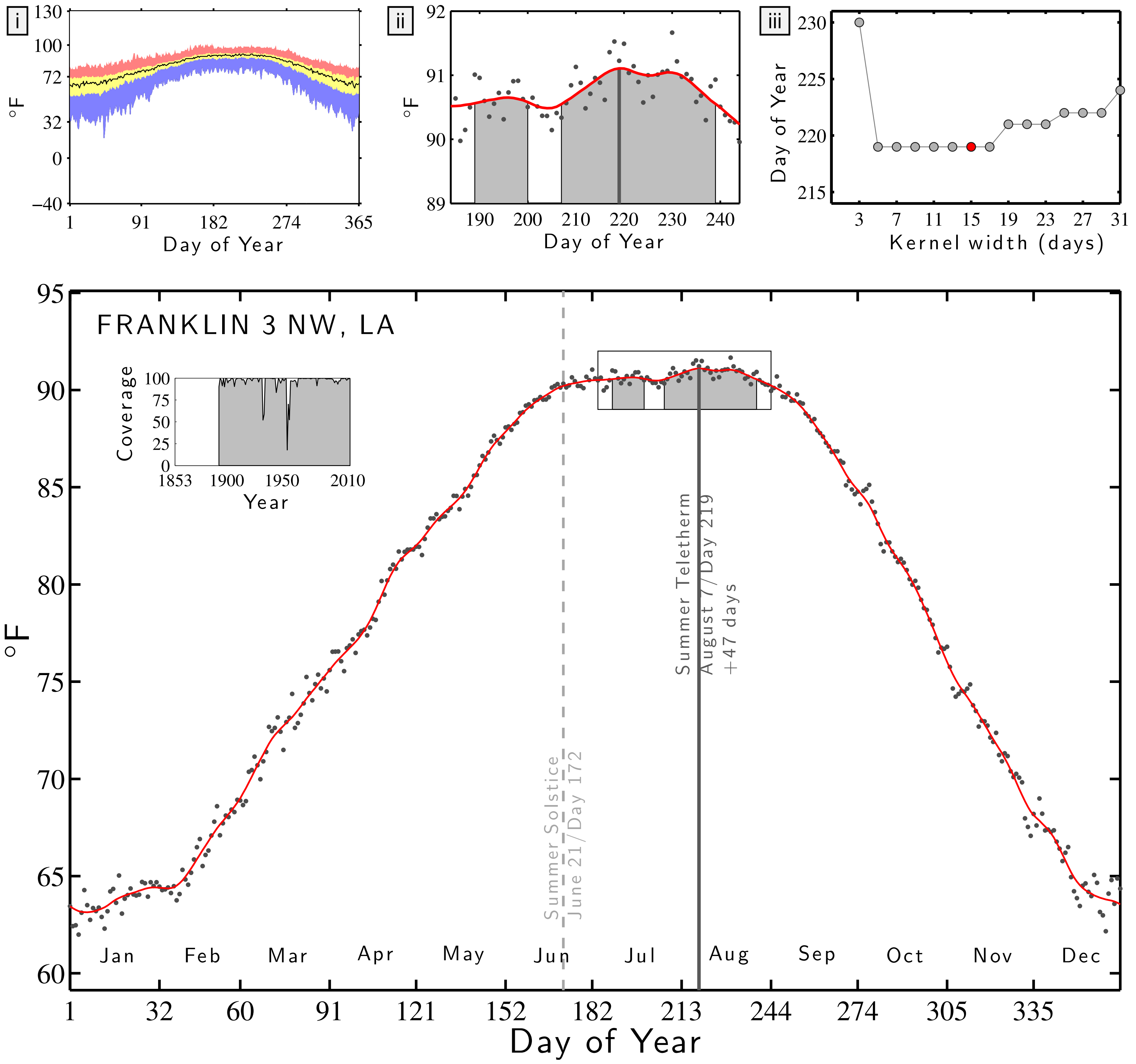

i
ii
iii
Day of Year
°F
Kernel width (days)
FRANKLIN 3 NW, LA
Coverage
Year
Summer Solstice
June 21 / Day 172
Summer Teletherm
August 7 / Day 219
+47 days
Jan
Feb
Mar
Apr
May
Jun
Jul
Aug
Sep
Oct
Nov
Dec

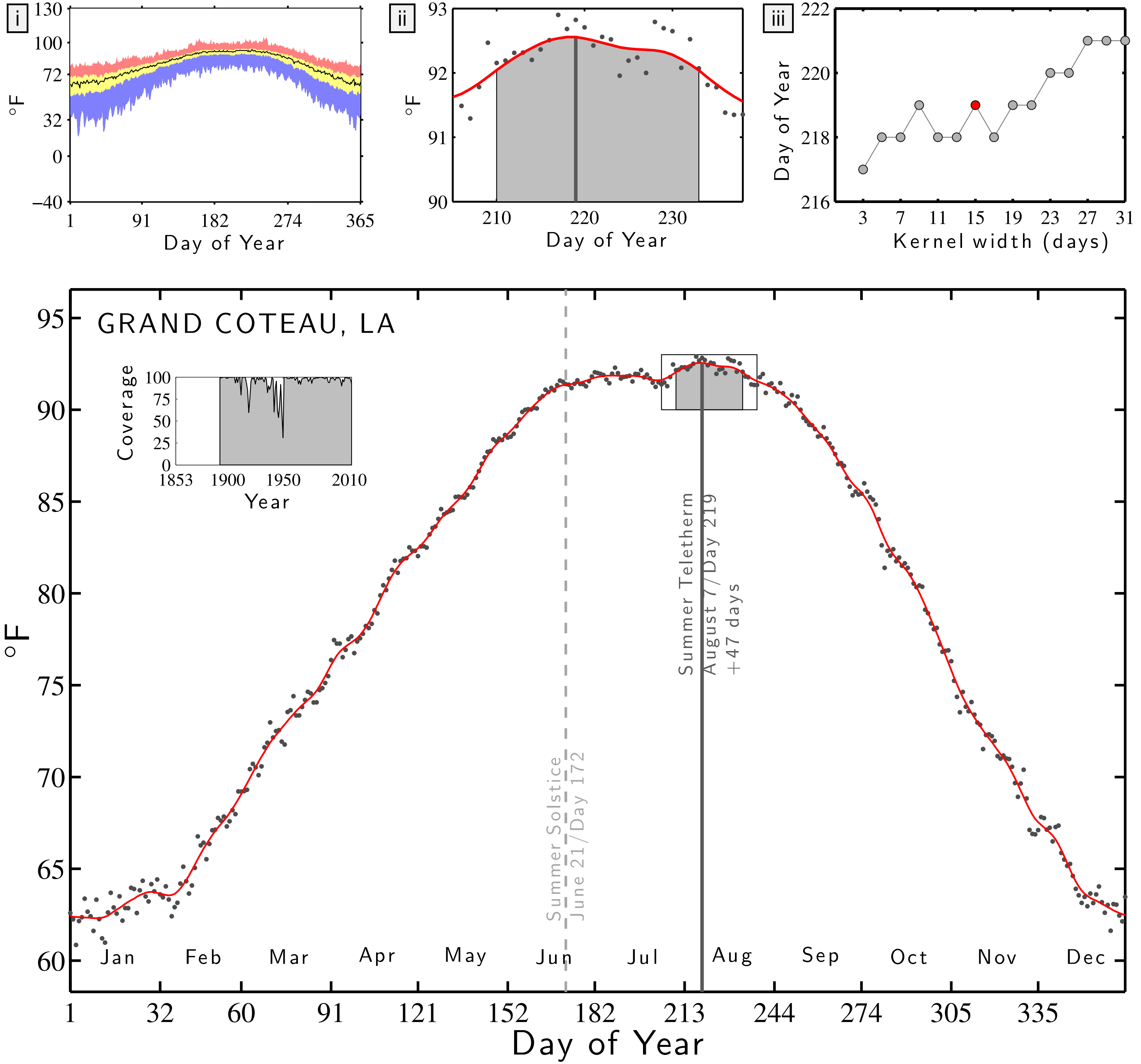

i
ii
iii
°F
Day of Year
Day of Year
Kernel width (days)
GRAND COTEAU, LA
Coverage
Year
Summer Solstice
June 21 / Day 172
Summer Teletherm
August 7 / Day 219
+47 days
Jan
Feb
Mar
Apr
May
Jun
Jul
Aug
Sep
Oct
Nov
Dec

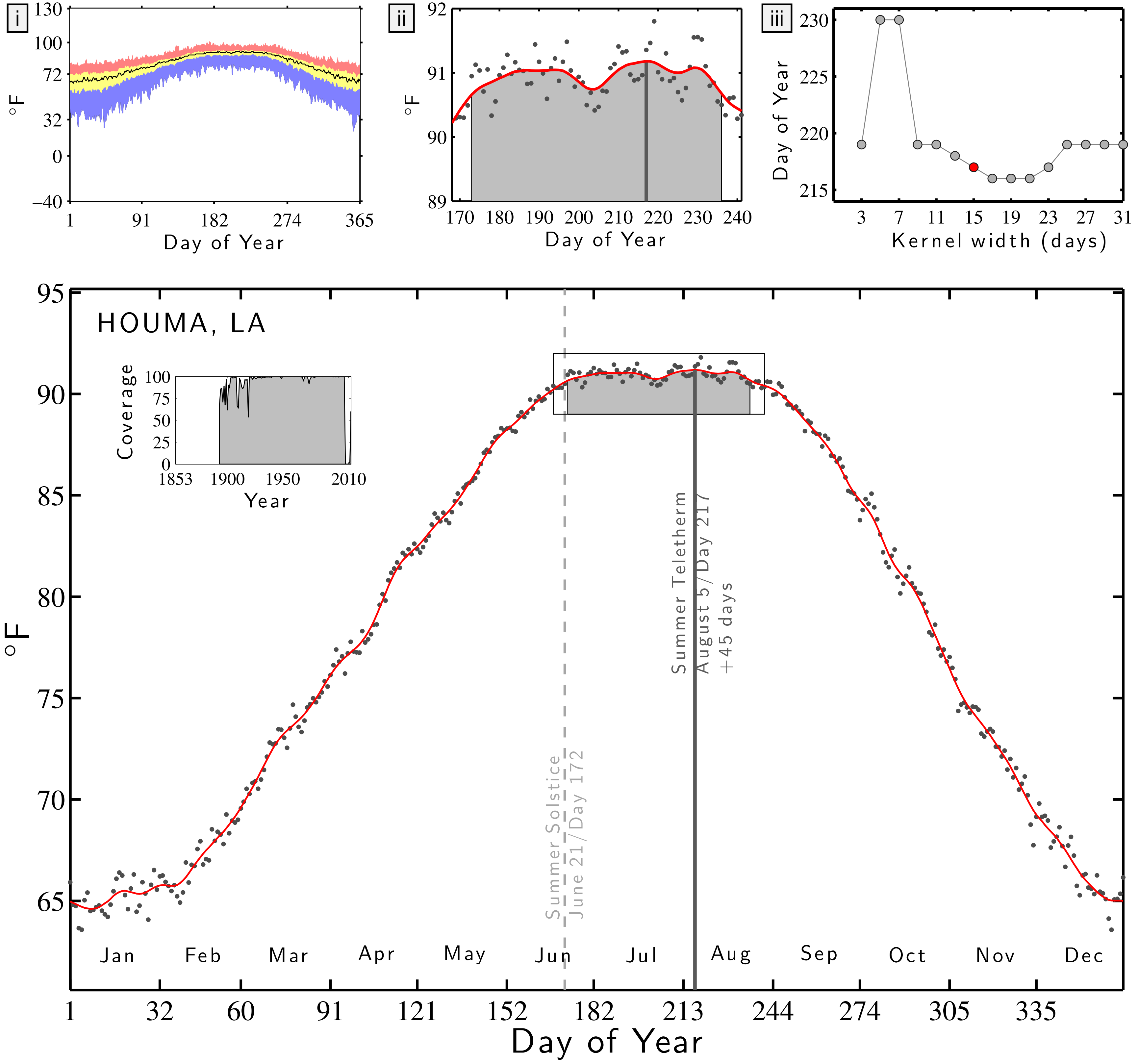

i
130
100
72
32
0
−40
°F
1
91
182
274
365
Day of Year
ii
92
91
90
89
°F
170
180
190
200
210
220
230
240
Day of Year
iii
230
225
220
215
Day of Year
3
7
11
15
19
23
27
31
Kernel width (days)
HOUMA, LA
Coverage
100
75
50
25
0
1853
1900
1950
2010
Year
95
90
85
80
75
70
65
°F
Summer Teletherm
August 5/Day 217
+45 days
Summer Solstice
June 21/Day 172
Jan
Feb
Mar
Apr
May
Jun
Jul
Aug
Sep
Oct
Nov
Dec
1
32
60
91
121
152
182
213
244
274
305
335
Day of Year

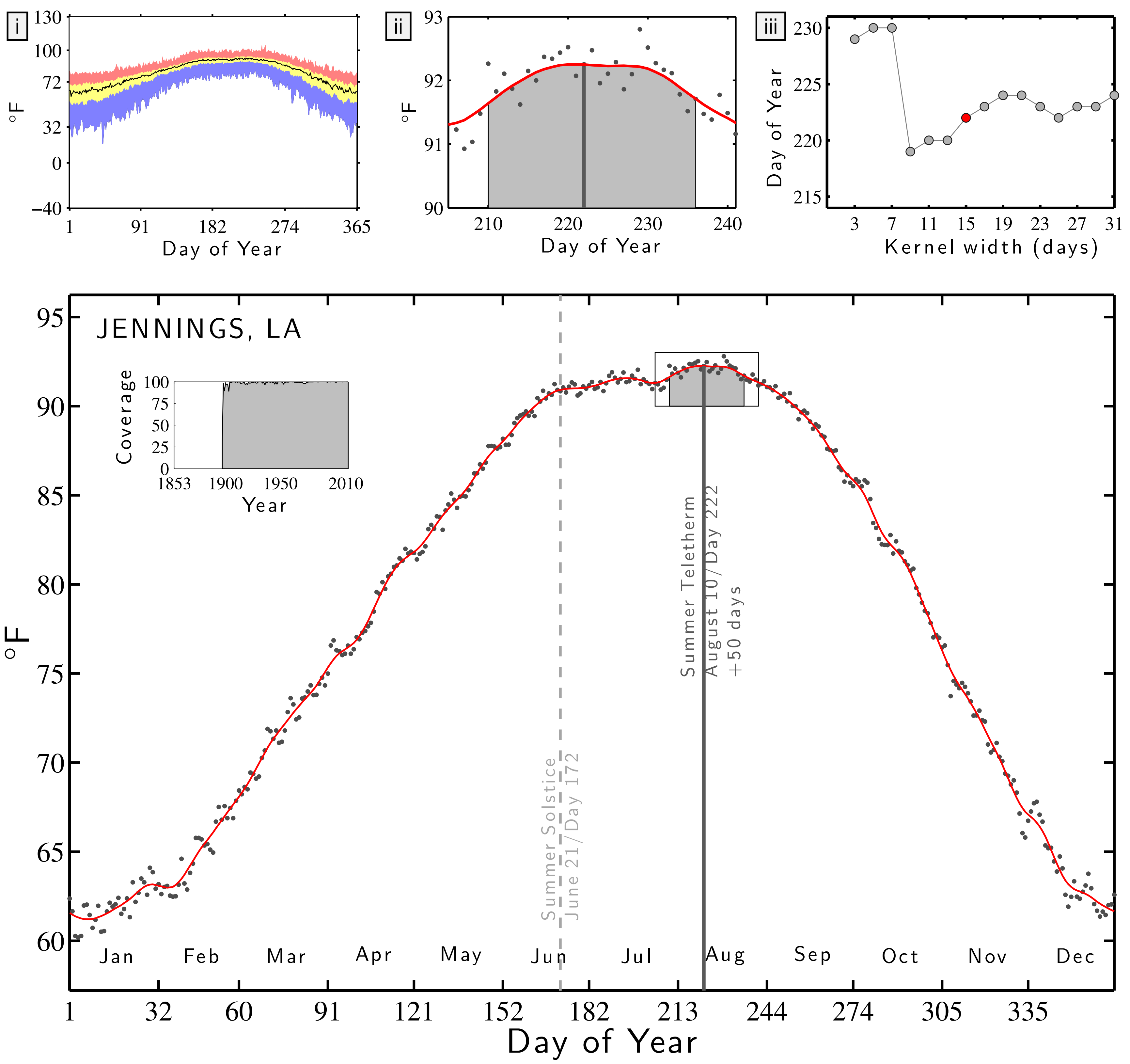
i
ii
iii
JENNINGS, LA
Coverage
Year
Summer Solstice
June 21/Day 172
Summer Teletherm
August 10/Day 222
+50 days
°F
Day of Year
Kernel width (days)
Jan
Feb
Mar
Apr
May
Jun
Jul
Aug
Sep
Oct
Nov
Dec

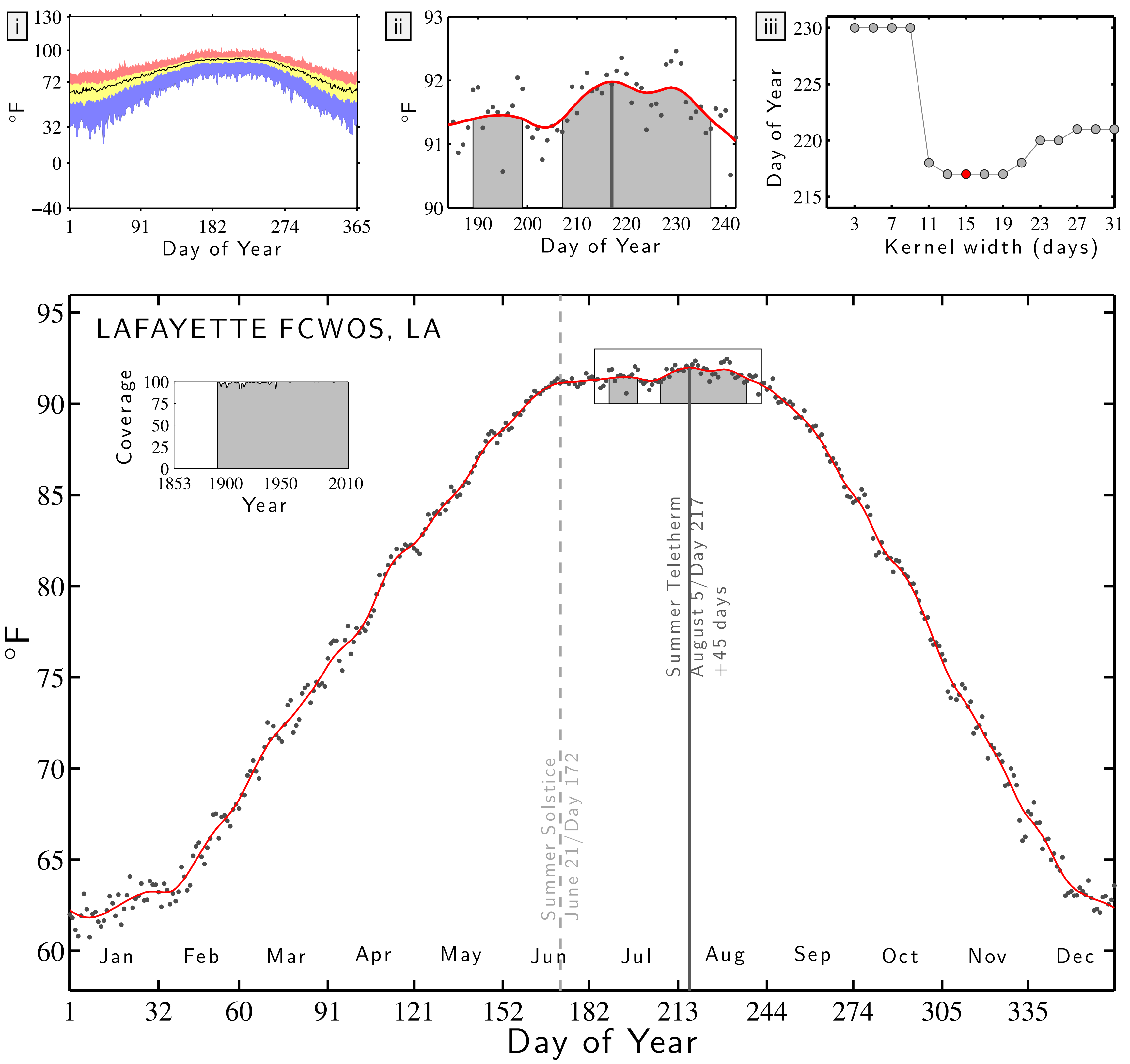

i
ii
iii
LAFAYETTE FCWOS, LA
Summer Solstice
June 21 / Day 172
Summer Teletherm
August 5 / Day 217
+45 days
Coverage
Year
Kernel width (days)
Day of Year
°F
Jan
Feb
Mar
Apr
May
Jun
Jul
Aug
Sep
Oct
Nov
Dec

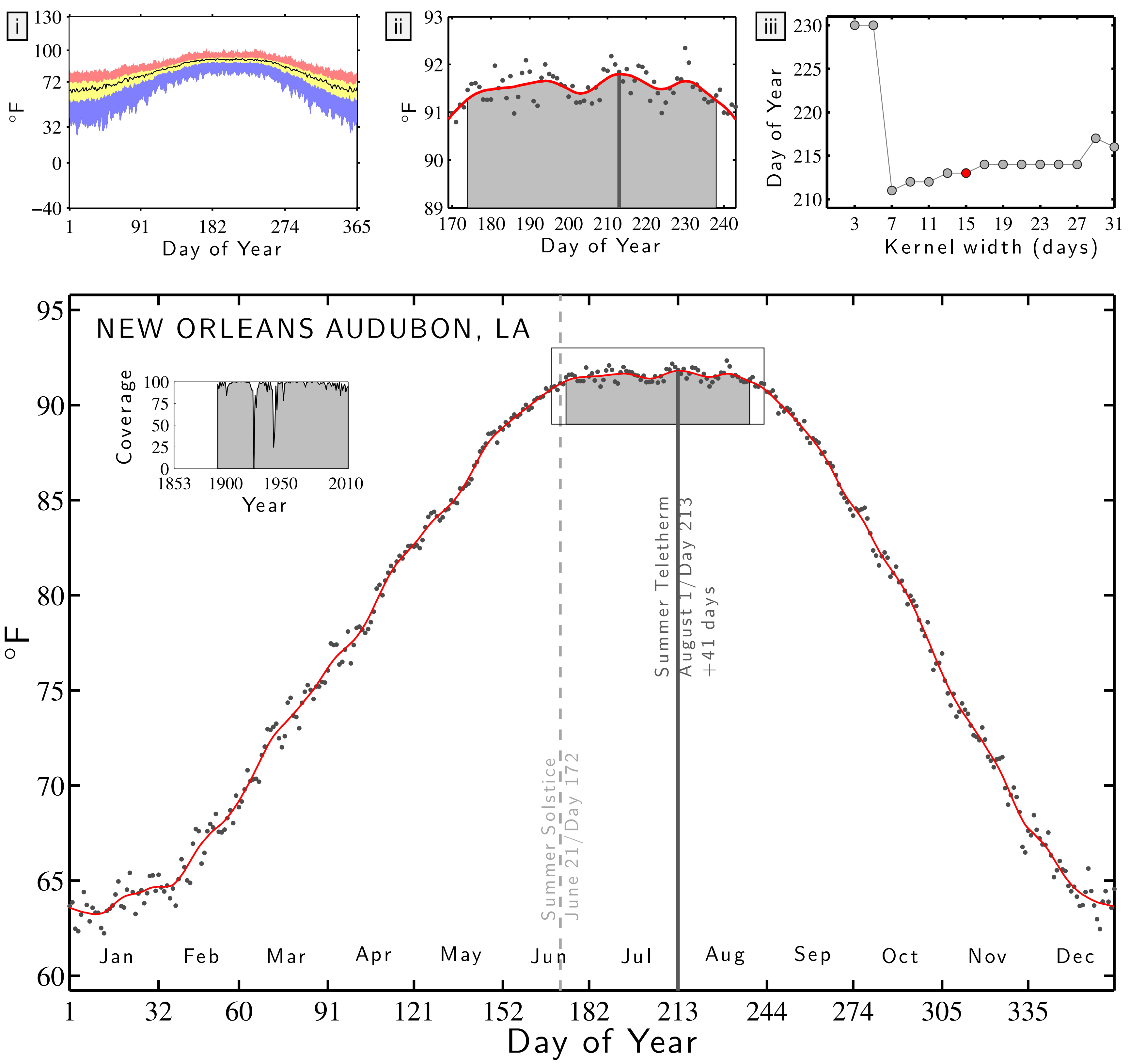

i
ii
iii
°F
Day of Year
Kernel width (days)
NEW ORLEANS AUDUBON, LA
Coverage
Year
Summer Solstice
June 21 / Day 172
Summer Teletherm
August 1 / Day 213
+41 days
Jan
Feb
Mar
Apr
May
Jun
Jul
Aug
Sep
Oct
Nov
Dec

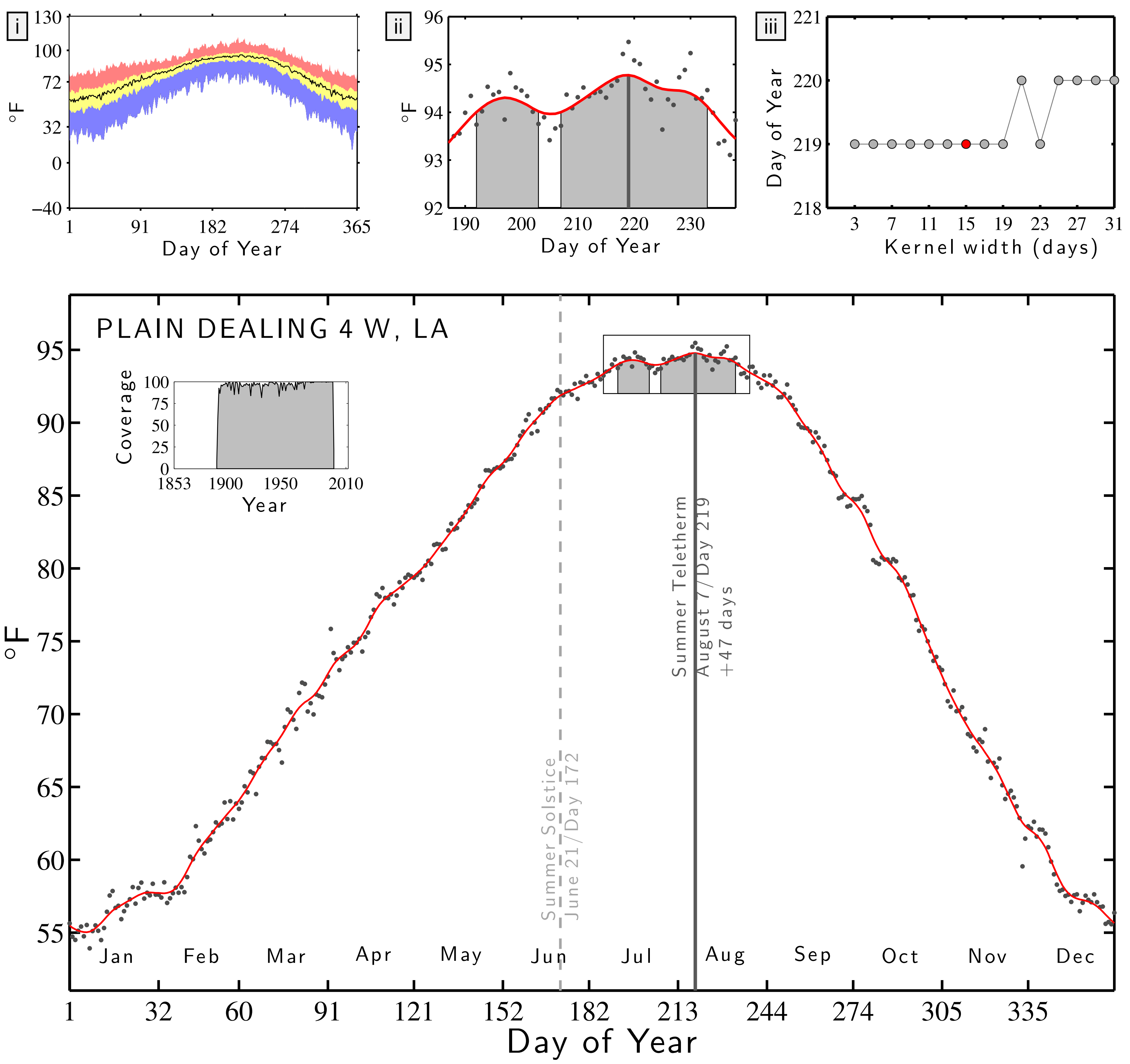

i
°F
Day of Year
ii
iii
Day of Year
Kernel width (days)
PLAIN DEALING 4 W, LA
Coverage
Year
Summer Solstice
June 21 / Day 172
Summer Teletherm
August 7 / Day 219
+47 days
Jan
Feb
Mar
Apr
May
Jun
Jul
Aug
Sep
Oct
Nov
Dec

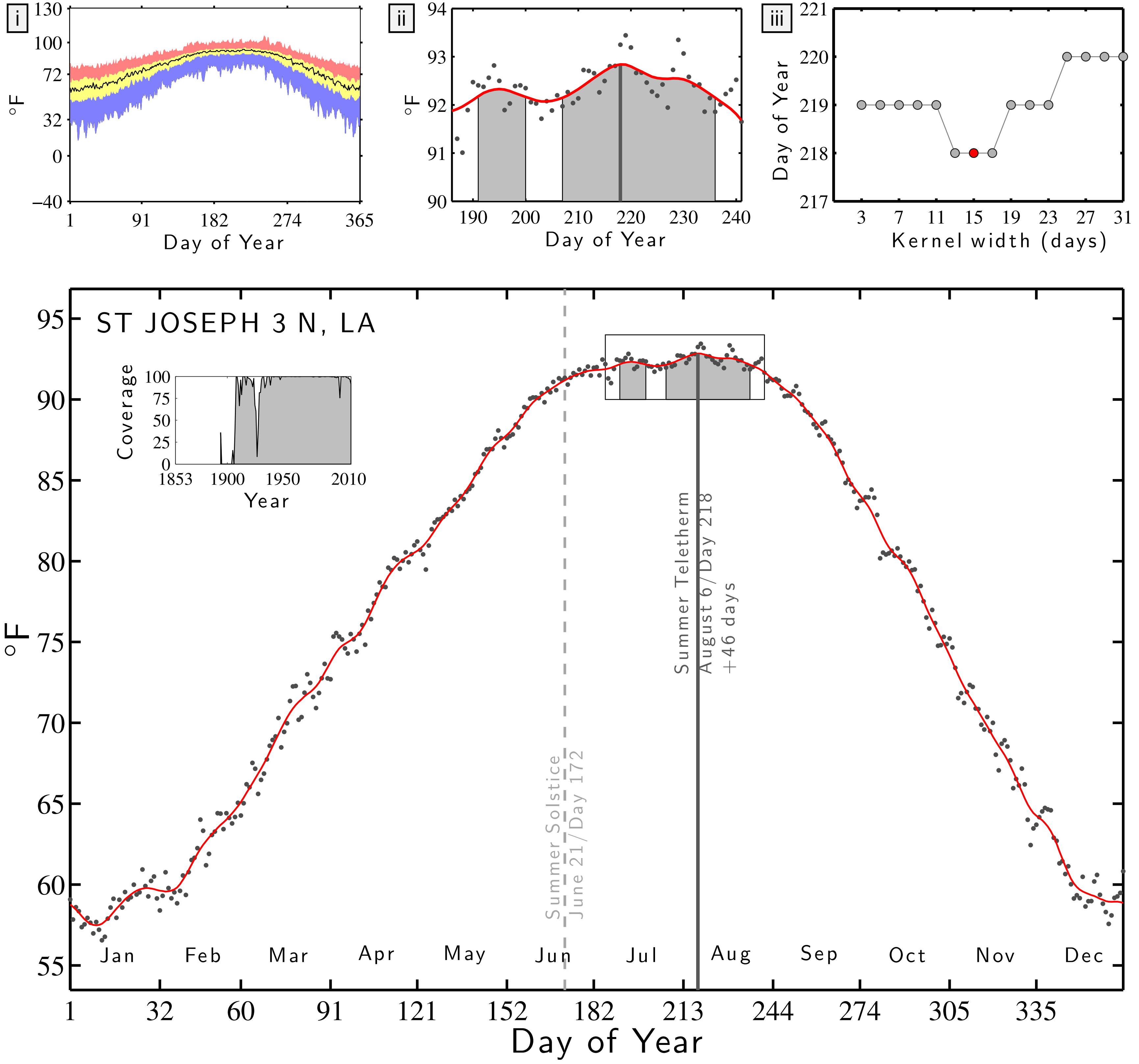

i
ii
iii
°F
Day of Year
Day of Year
Kernel width (days)
ST JOSEPH 3 N, LA
Coverage
Year
Summer Solstice
June 21/Day 172
Summer Teletherm
August 6/Day 218
+46 days
Jan
Feb
Mar
Apr
May
Jun
Jul
Aug
Sep
Oct
Nov
Dec

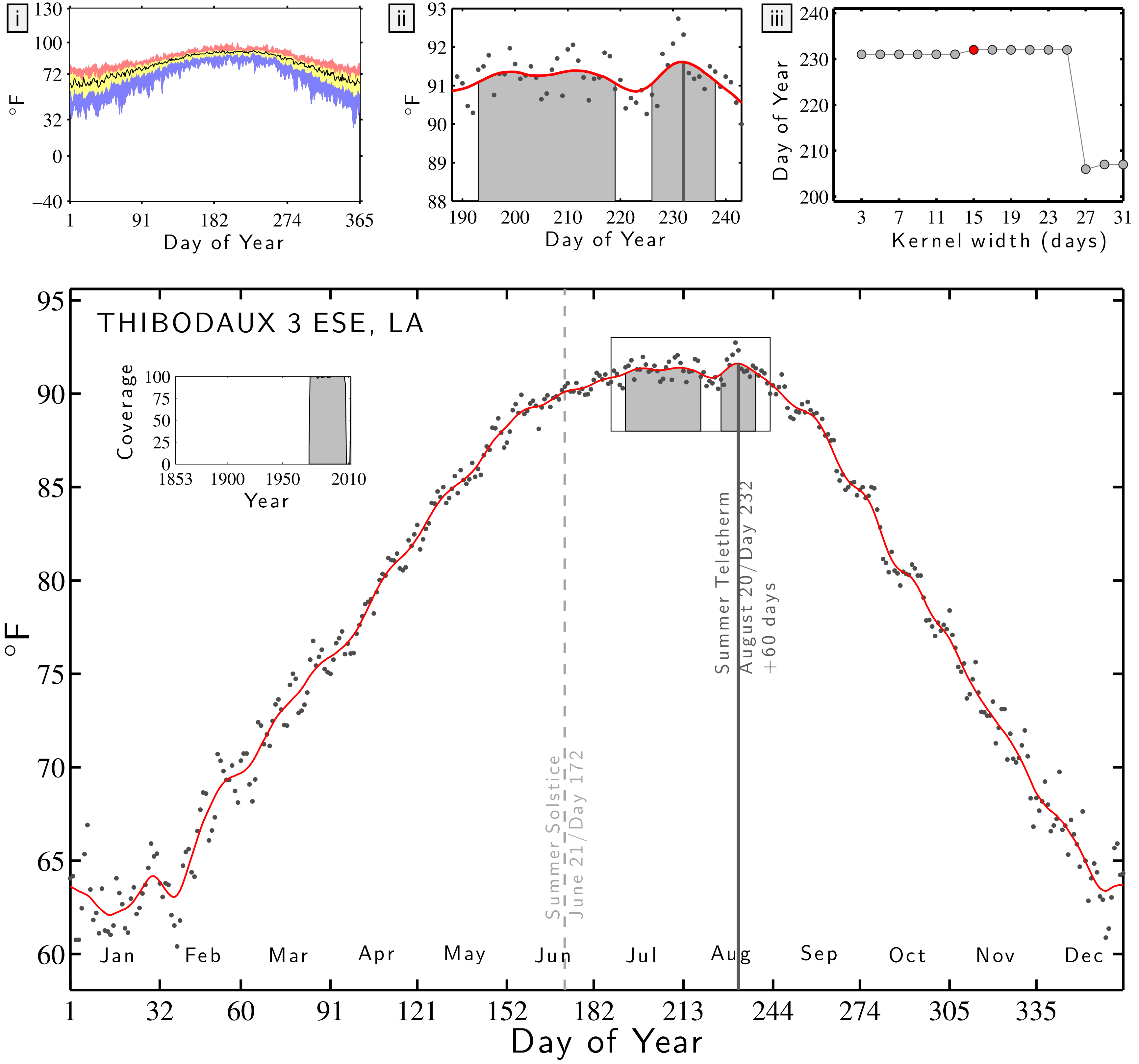

i
ii
iii
°F
Day of Year
Kernel width (days)
THIBODAUX 3 ESE, LA
Coverage
Year
Summer Solstice
June 21 / Day 172
Summer Teletherm
August 20 / Day 232
+60 days
Jan
Feb
Mar
Apr
May
Jun
Jul
Aug
Sep
Oct
Nov
Dec

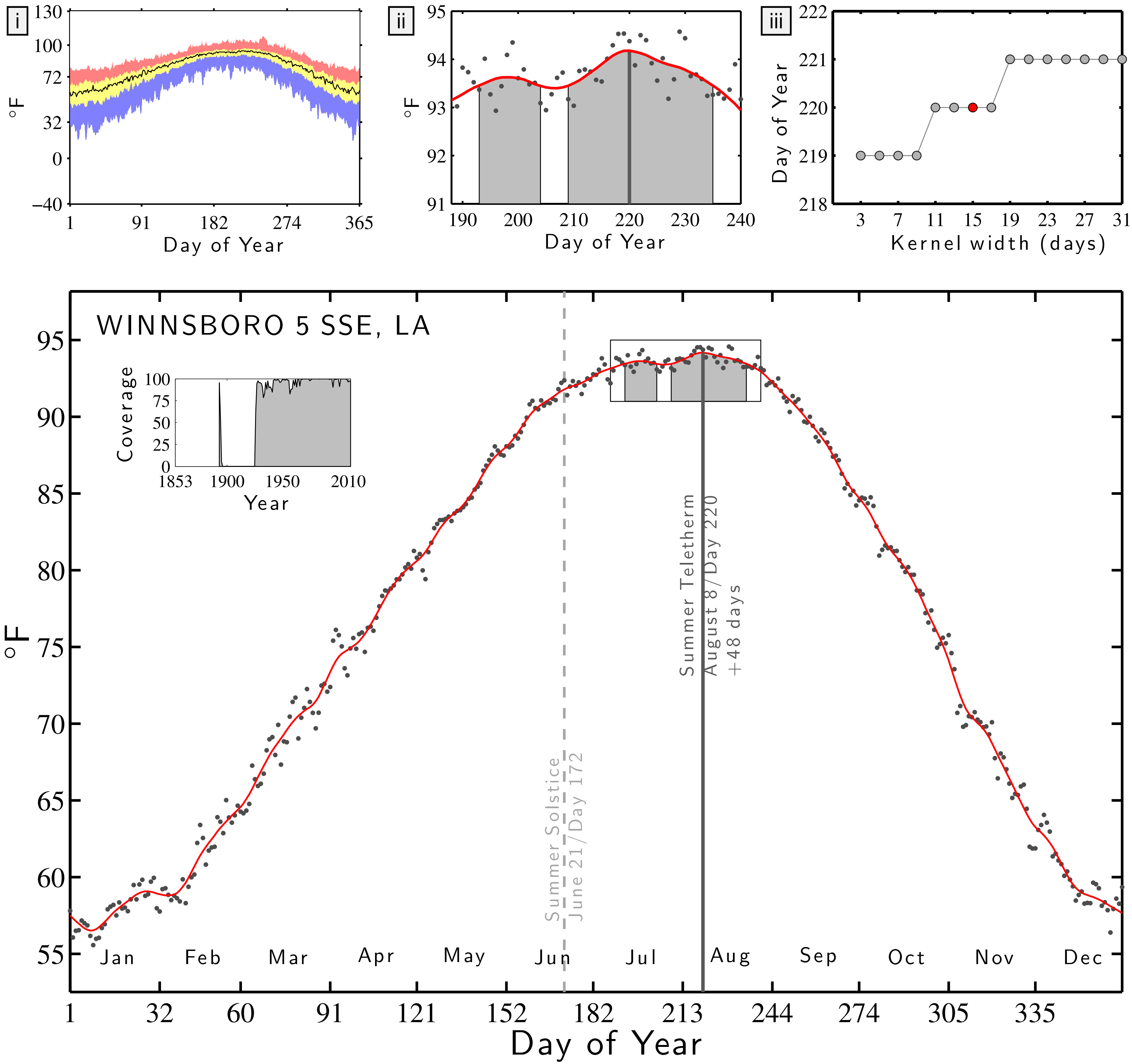

i
°F
Day of Year
ii
°F
Day of Year
iii
Day of Year
Kernel width (days)
WINNSBORO 5 SSE, LA
Coverage
Year
Summer Solstice
June 21/Day 172
Summer Teletherm
August 8/Day 220
+48 days
Jan
Feb
Mar
Apr
May
Jun
Jul
Aug
Sep
Oct
Nov
Dec
°F
Day of Year

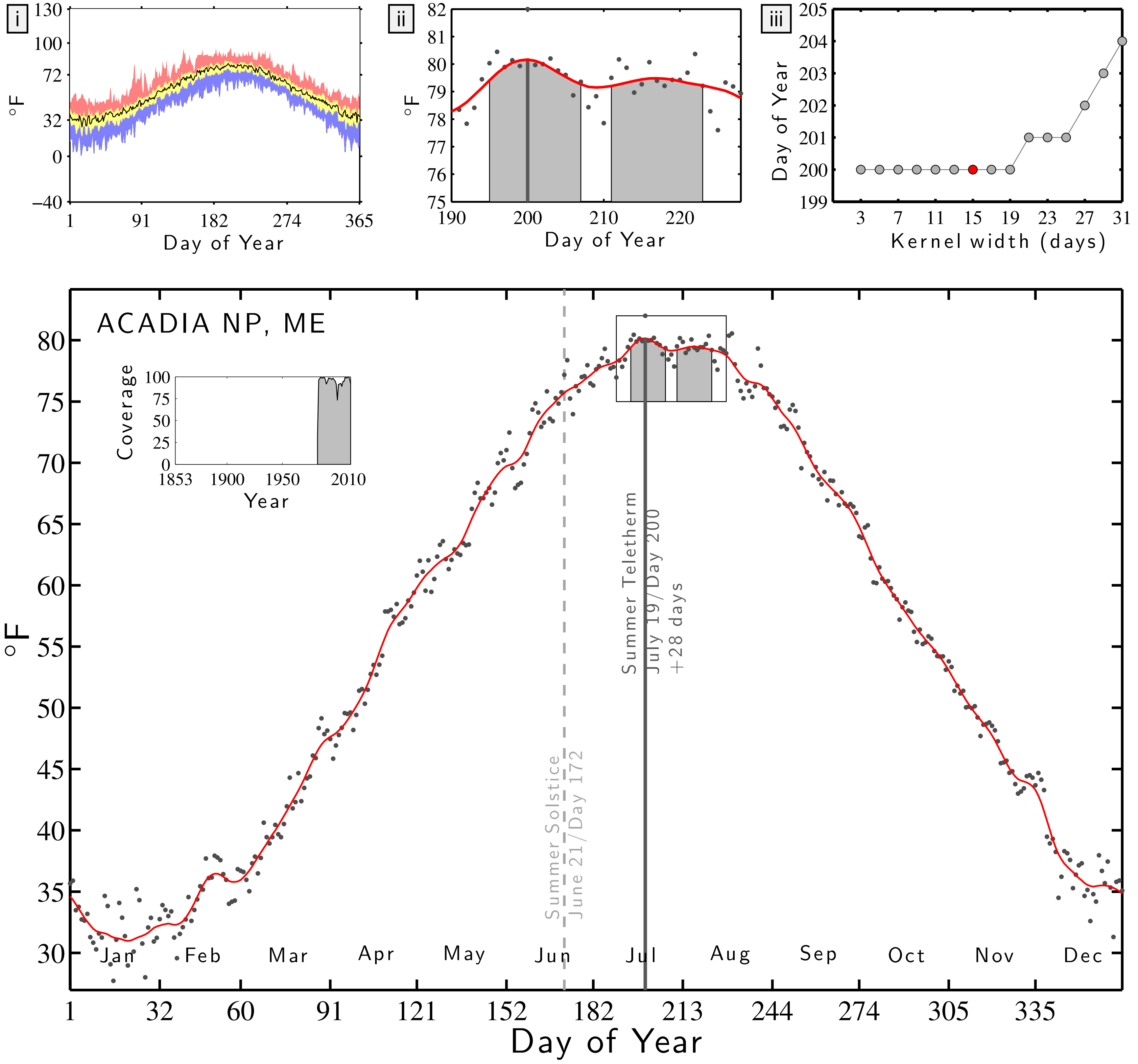

i
°F
Day of Year
ii
Day of Year
iii
Day of Year
Kernel width (days)
ACADIA NP, ME
Coverage
Year
Summer Solstice
June 21 / Day 172
Summer Teletherm
July 19 / Day 200
+28 days
Jan
Feb
Mar
Apr
May
Jun
Jul
Aug
Sep
Oct
Nov
Dec
Day of Year

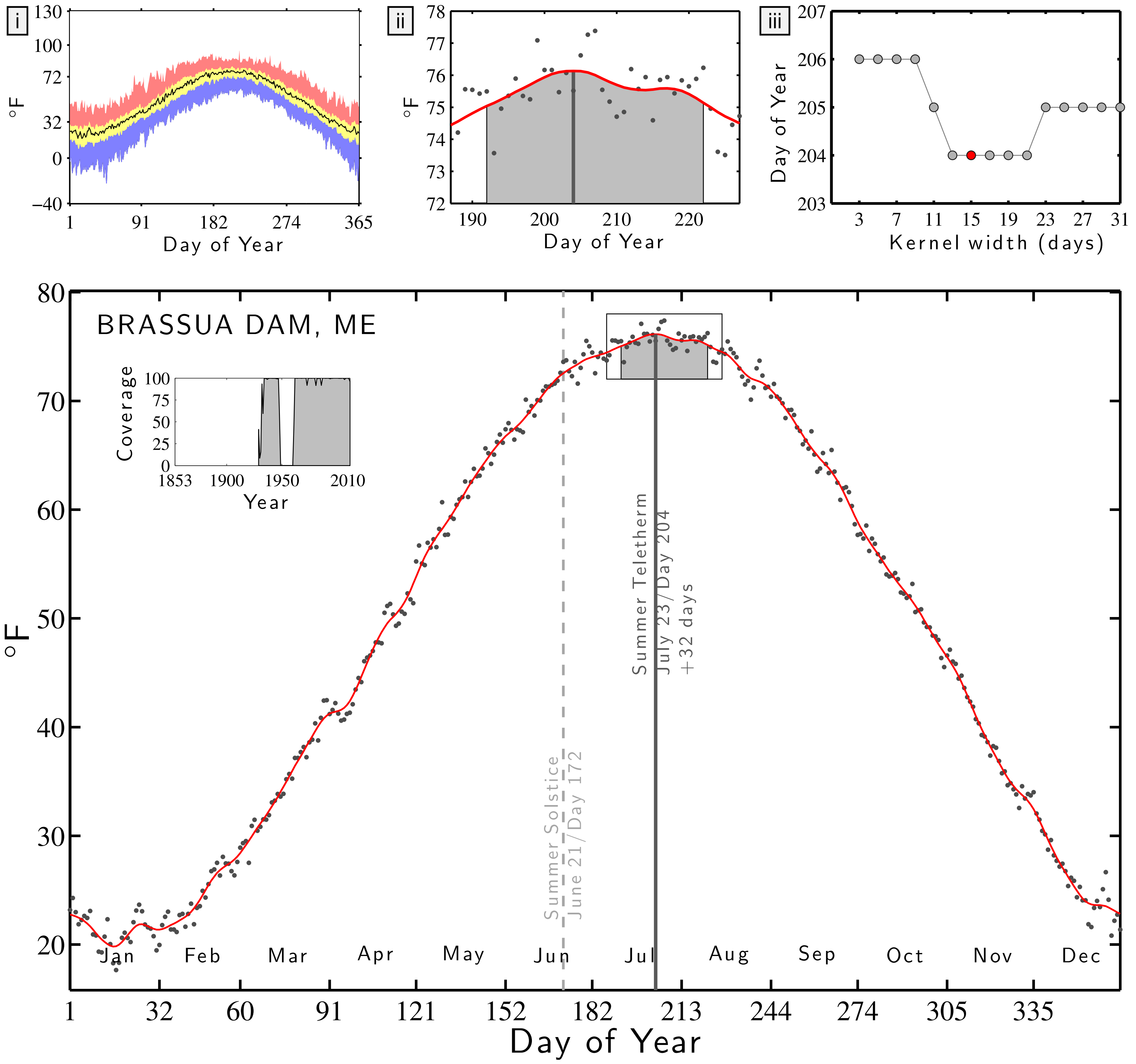

i
°F
Day of Year
ii
°F
Day of Year
iii
Day of Year
Kernel width (days)
BRASSUA DAM, ME
Coverage
Year
Summer Solstice
June 21 / Day 172
Summer Teletherm
July 23 / Day 204
+32 days
Jan
Feb
Mar
Apr
May
Jun
Jul
Aug
Sep
Oct
Nov
Dec
°F
Day of Year

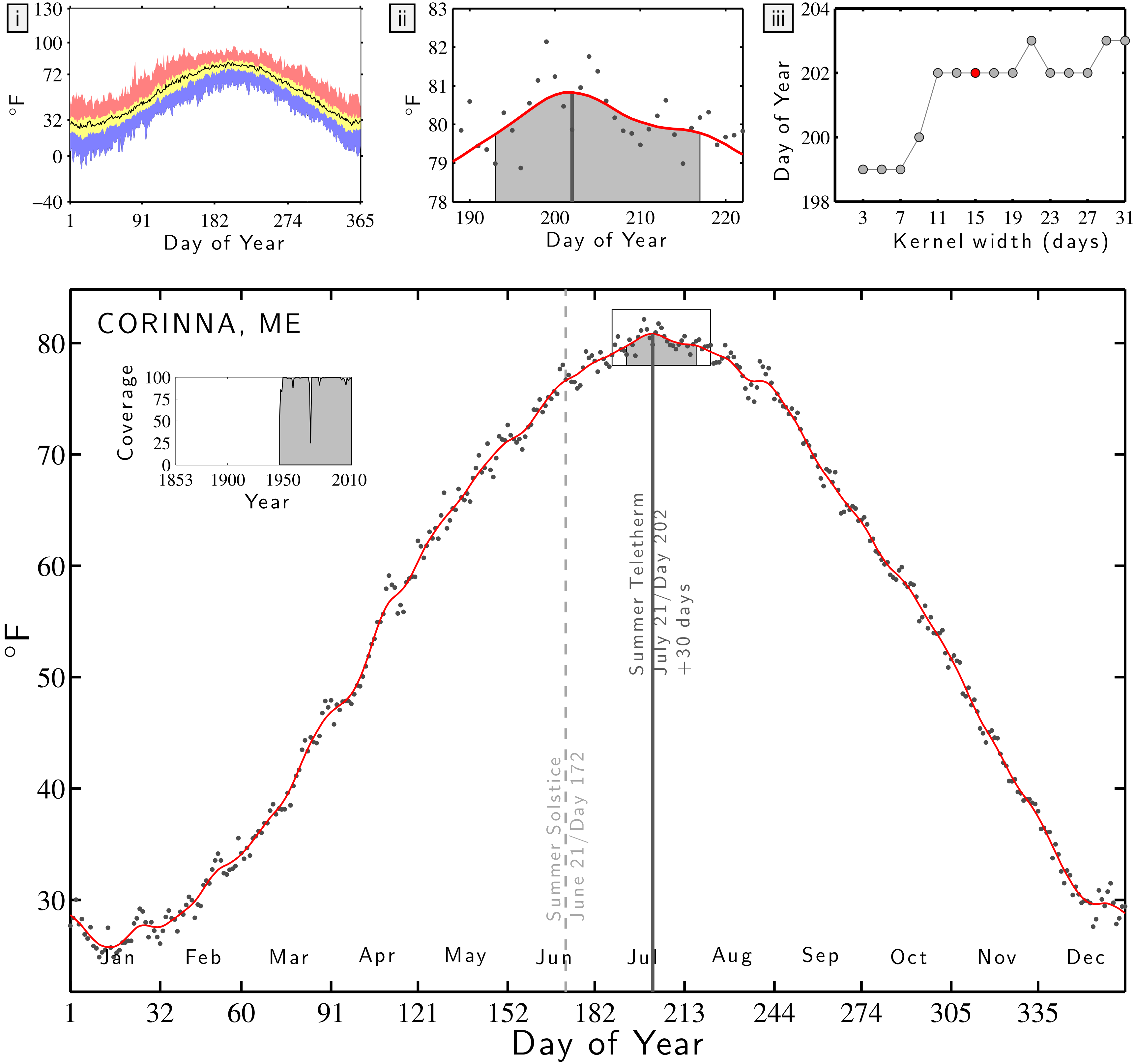

i
°F
Day of Year
ii
°F
Day of Year
iii
Day of Year
Kernel width (days)
CORINNA, ME
Coverage
Year
Summer Solstice
June 21 / Day 172
Summer Teletherm
July 21 / Day 202
+30 days
Jan
Feb
Mar
Apr
May
Jun
Jul
Aug
Sep
Oct
Nov
Dec
°F
Day of Year

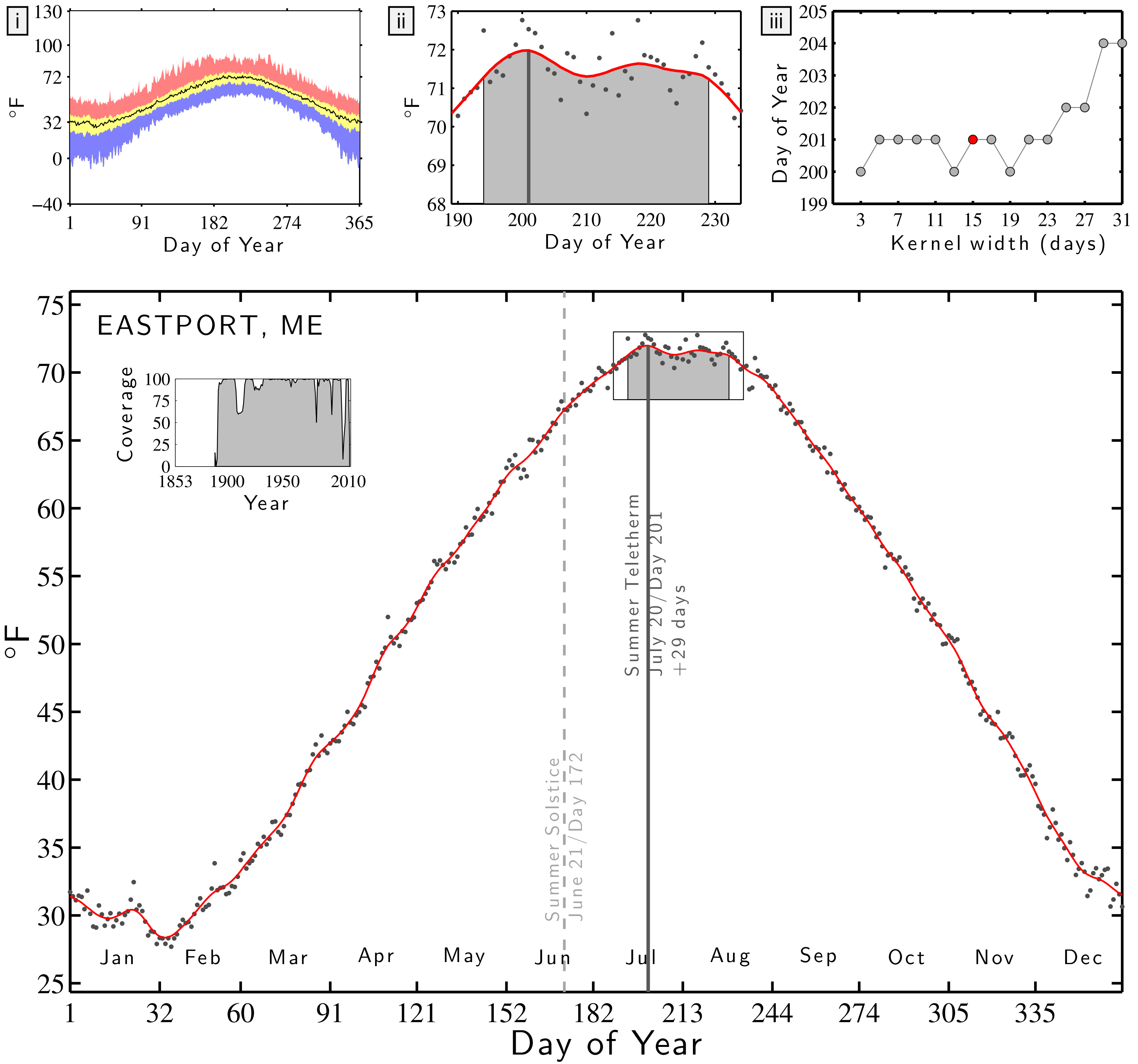

i
ii
iii
°F
Day of Year
Day of Year
Kernel width (days)
EASTPORT, ME
Coverage
Year
Summer Solstice
June 21 / Day 172
Summer Teletherm
July 20 / Day 201
+29 days
Jan
Feb
Mar
Apr
May
Jun
Jul
Aug
Sep
Oct
Nov
Dec
Day of Year

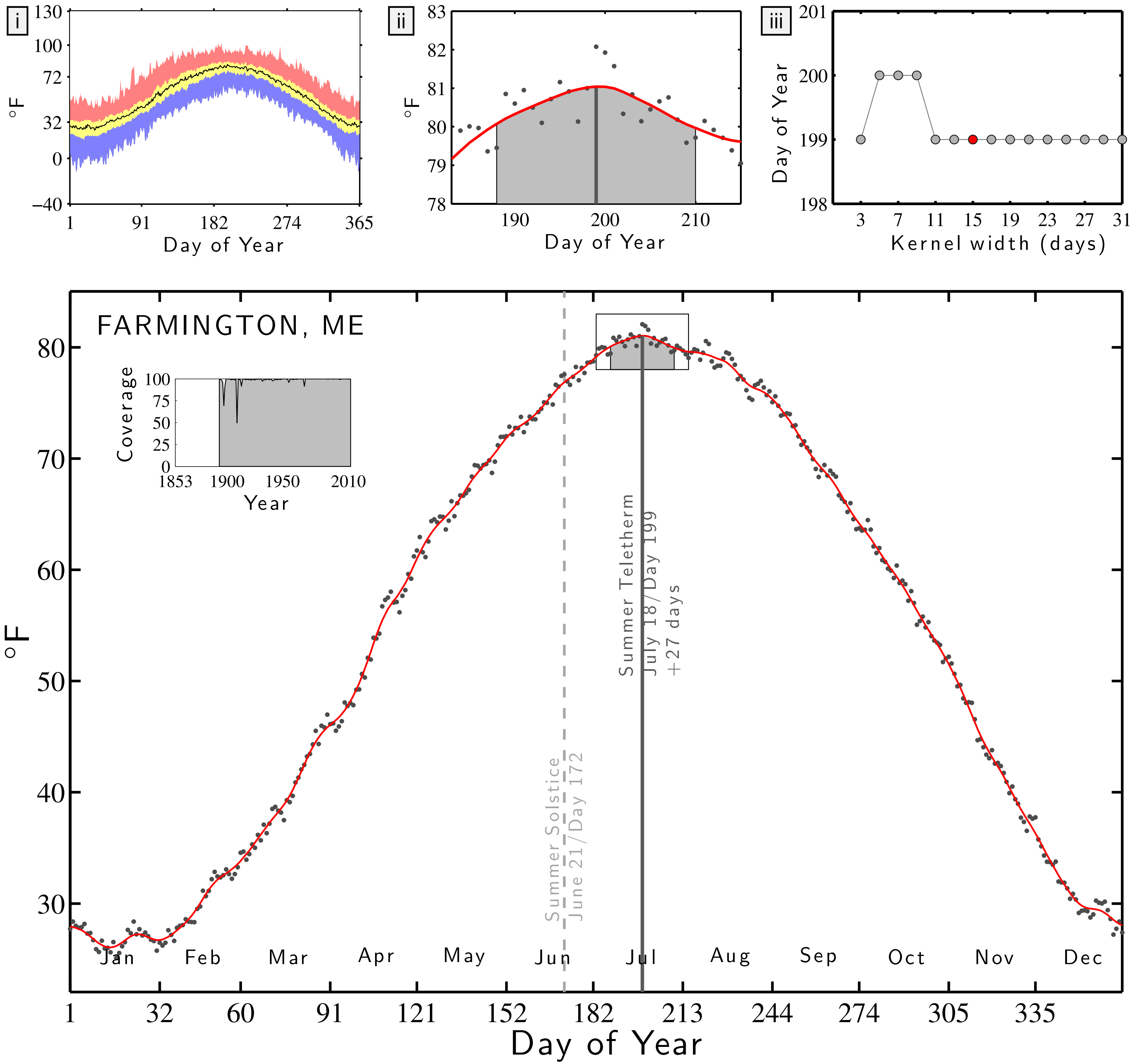

i
°F
Day of Year
ii
°F
Day of Year
iii
Day of Year
Kernel width (days)
FARMINGTON, ME
Coverage
Year
Summer Solstice
June 21/Day 172
Summer Teletherm
July 18/Day 199
+27 days
Jan
Feb
Mar
Apr
May
Jun
Jul
Aug
Sep
Oct
Nov
Dec
°F
Day of Year

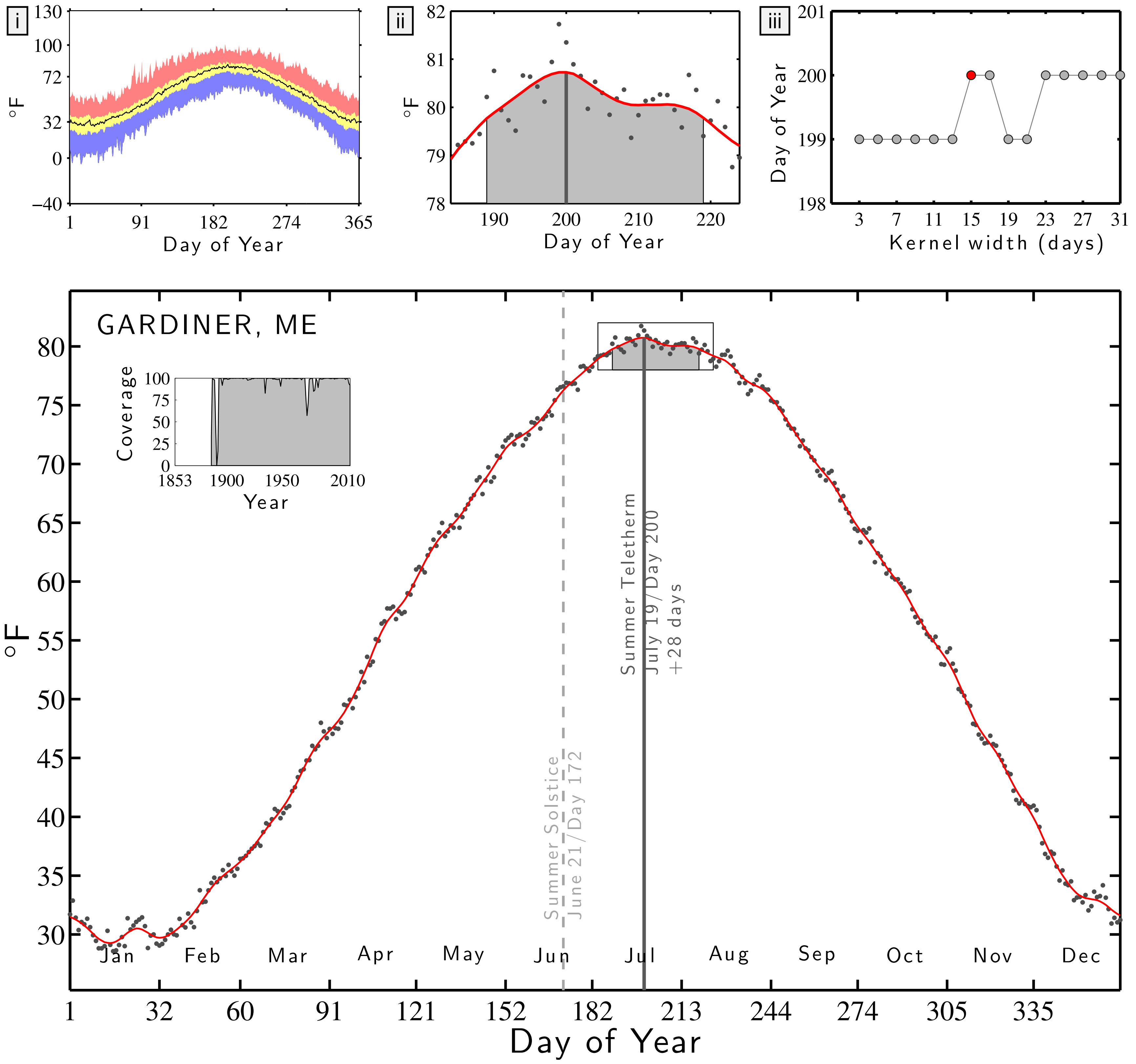

i
°F
Day of Year
ii
°F
Day of Year
iii
Day of Year
Kernel width (days)
GARDINER, ME
Coverage
Year
Summer Solstice
June 21/Day 172
Summer Teletherm
July 19/Day 200
+28 days
Jan
Feb
Mar
Apr
May
Jun
Jul
Aug
Sep
Oct
Nov
Dec
°F
Day of Year

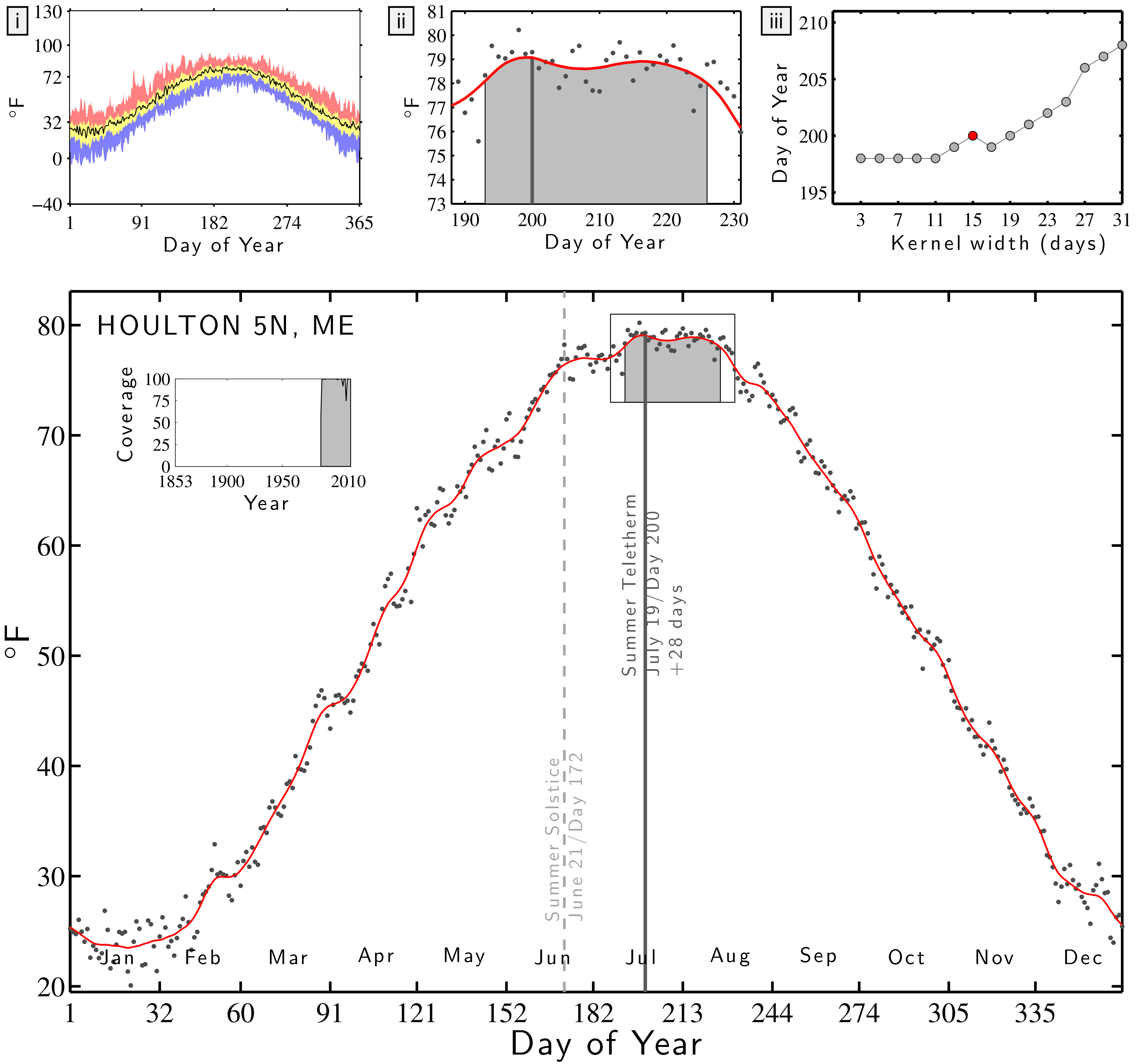

i
°F
Day of Year
ii
°F
Day of Year
iii
Day of Year
Kernel width (days)
HOULTON 5N, ME
Coverage
Year
Summer Solstice
June 21/Day 172
Summer Teletherm
July 19/Day 200
+28 days
Jan
Feb
Mar
Apr
May
Jun
Jul
Aug
Sep
Oct
Nov
Dec
°F
Day of Year

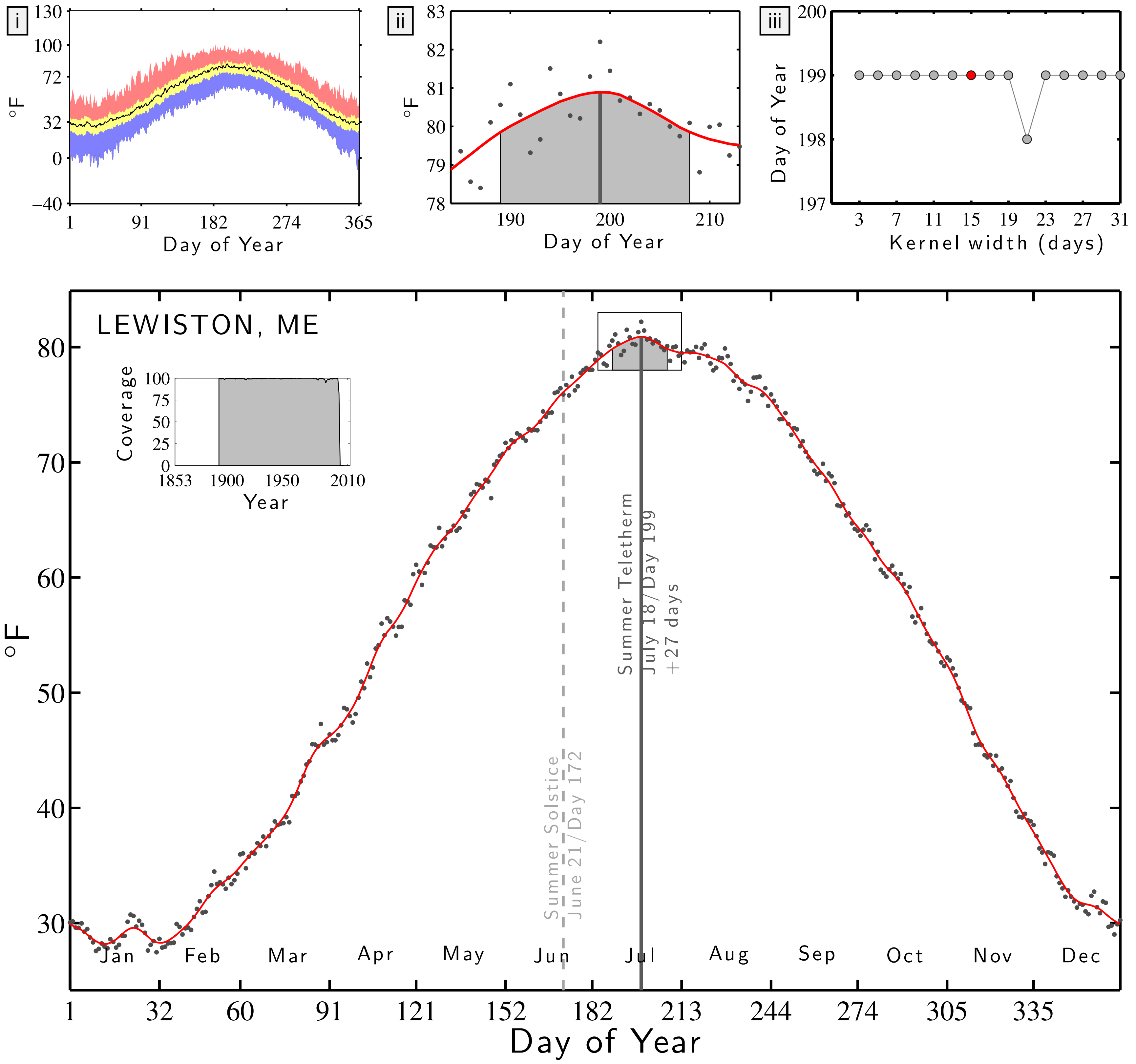

i
ii
iii
°F
Day of Year
Day of Year
Kernel width (days)
LEWISTON, ME
Coverage
Year
Summer Solstice
June 21/Day 172
Summer Teletherm
July 18/Day 199
+27 days
Jan
Feb
Mar
Apr
May
Jun
Jul
Aug
Sep
Oct
Nov
Dec
Day of Year

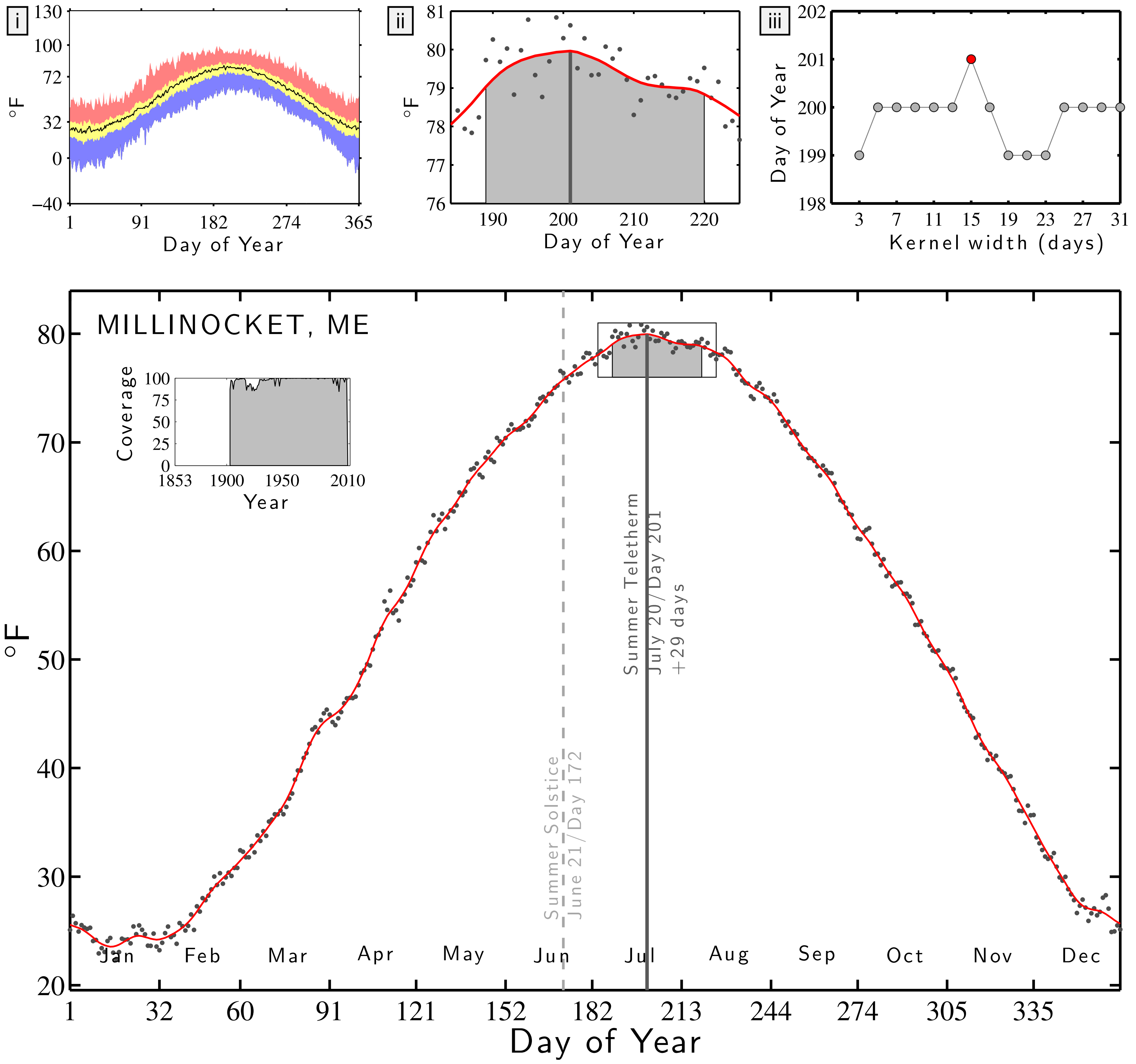

i
ii
iii
°F
Day of Year
Kernel width (days)
MILLINOCKET, ME
Coverage
Year
Summer Solstice
June 21 / Day 172
Summer Teletherm
July 20 / Day 201
+29 days
Jan
Feb
Mar
Apr
May
Jun
Jul
Aug
Sep
Oct
Nov
Dec

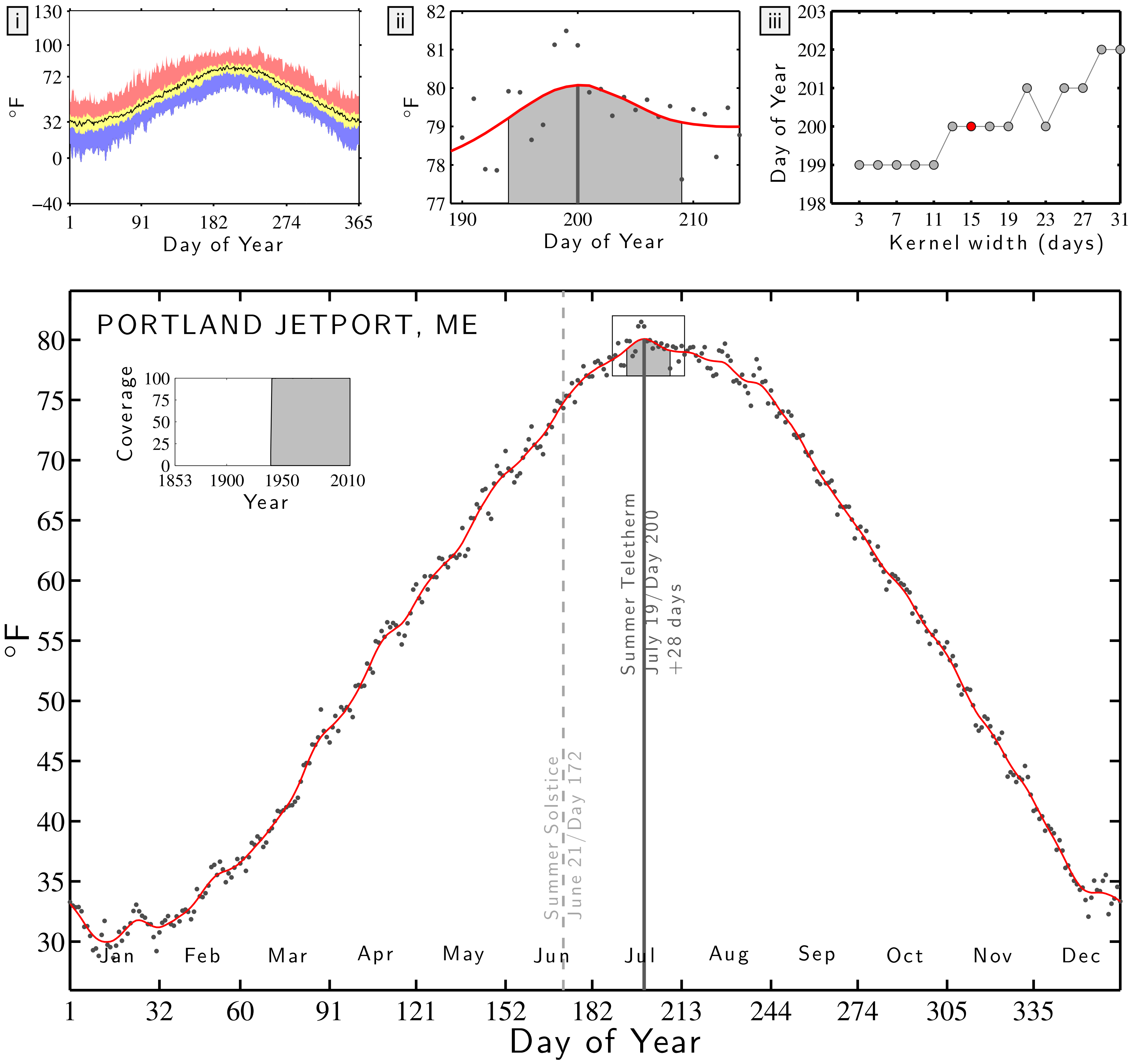

i
°F
Day of Year
ii
°F
Day of Year
iii
Day of Year
Kernel width (days)
PORTLAND JETPORT, ME
Coverage
Year
Summer Solstice
June 21/Day 172
Summer Teletherm
July 19/Day 200
+28 days
Jan
Feb
Mar
Apr
May
Jun
Jul
Aug
Sep
Oct
Nov
Dec
°F
Day of Year

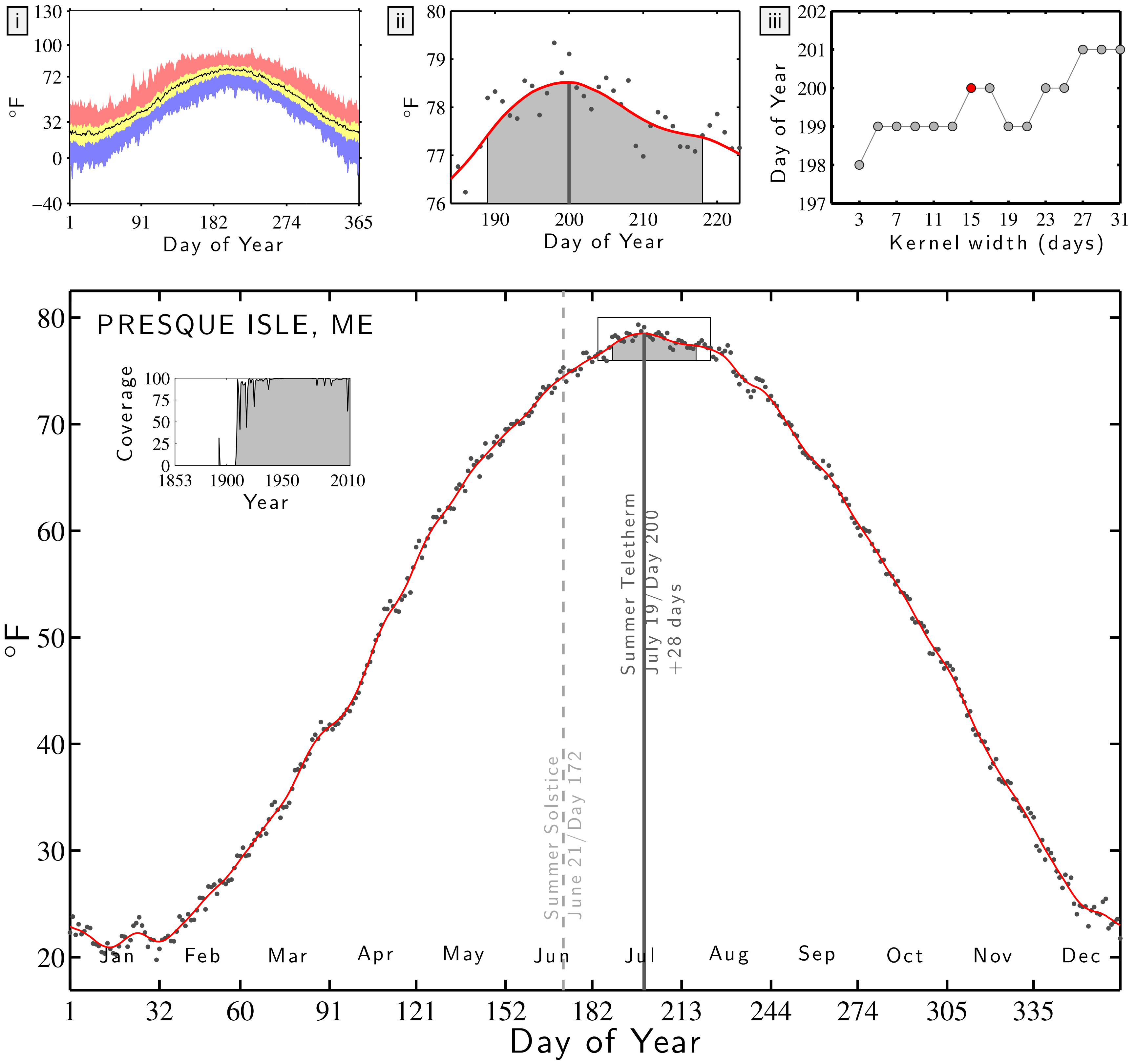

i
°F
Day of Year
ii
°F
Day of Year
iii
Day of Year
Kernel width (days)
PRESQUE ISLE, ME
Coverage
Year
Summer Solstice
June 21/Day 172
Summer Teletherm
July 19/Day 200
+28 days
Jan
Feb
Mar
Apr
May
Jun
Jul
Aug
Sep
Oct
Nov
Dec
°F
Day of Year

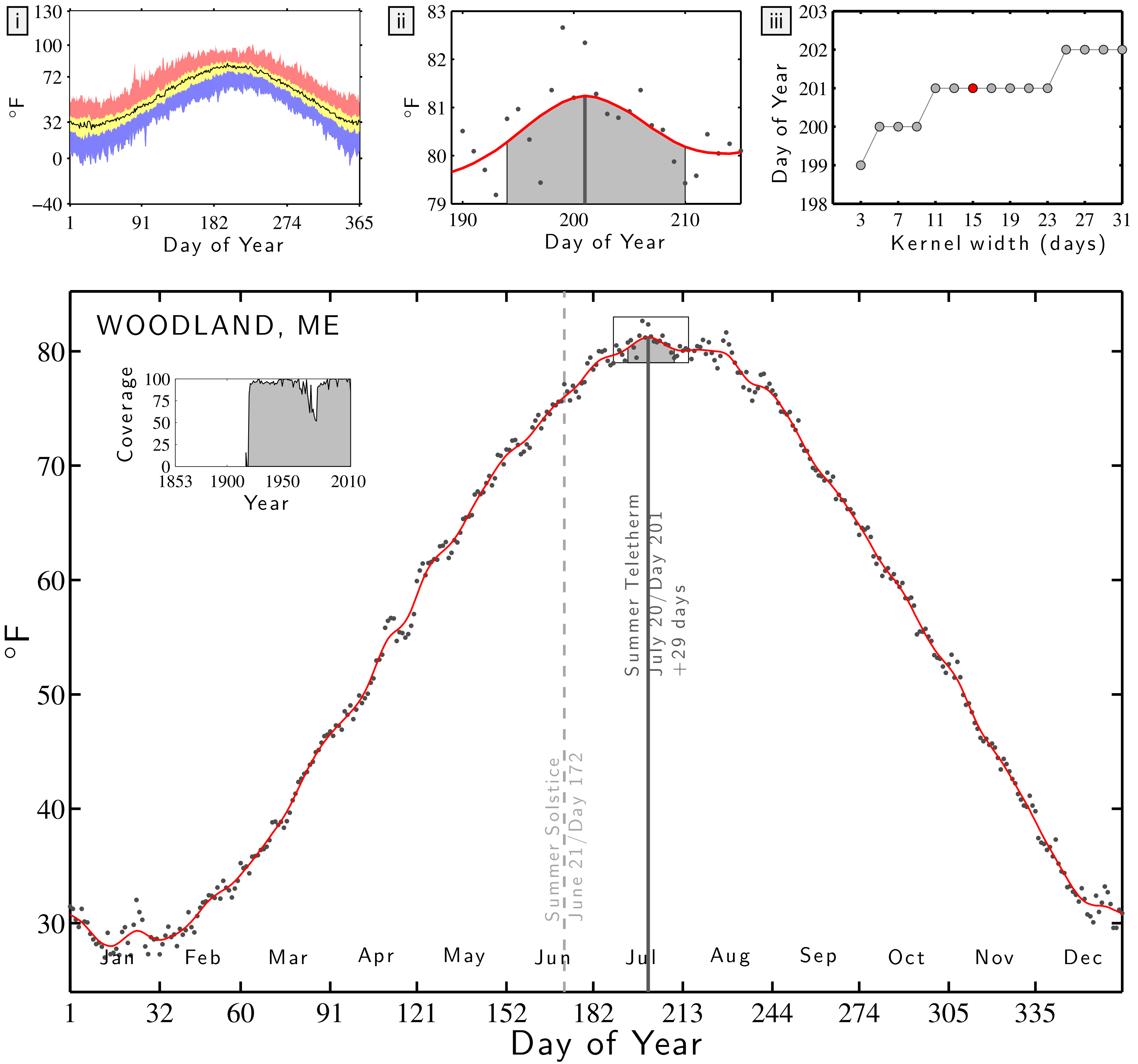

i
°F
Day of Year
ii
°F
Day of Year
iii
Day of Year
Kernel width (days)
WOODLAND, ME
Coverage
Year
Summer Solstice
June 21/Day 172
Summer Teletherm
July 20/Day 201
+29 days
Jan
Feb
Mar
Apr
May
Jun
Jul
Aug
Sep
Oct
Nov
Dec
°F
Day of Year

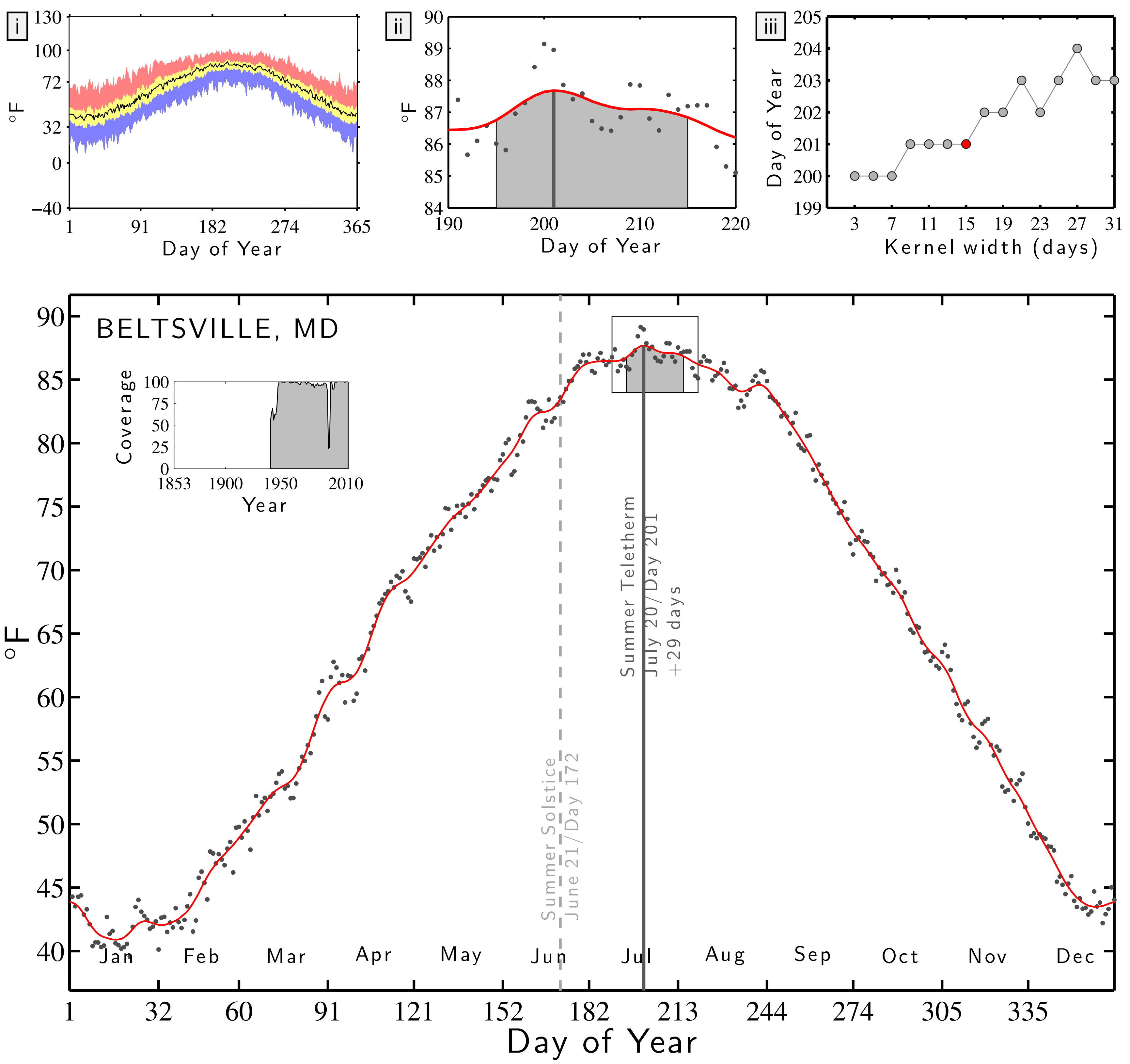

i
ii
iii
°F
Day of Year
Day of Year
Kernel width (days)
BELTSVILLE, MD
Coverage
Year
Summer Solstice
June 21/Day 172
Summer Teletherm
July 20/Day 201
+29 days
Jan
Feb
Mar
Apr
May
Jun
Jul
Aug
Sep
Oct
Nov
Dec

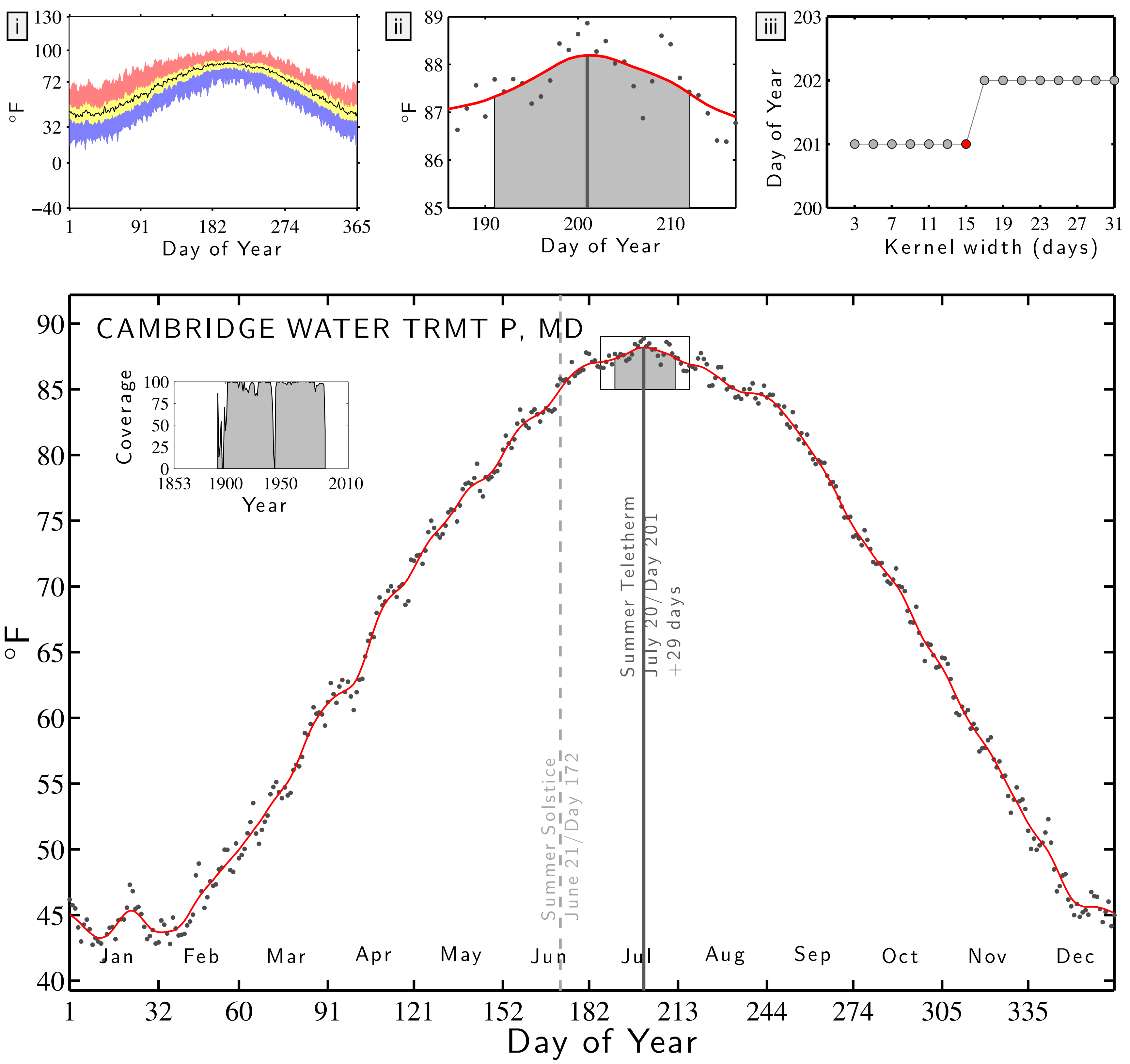

i
°F
130
100
72
32
0
−40
1
91
182
274
365
Day of Year
ii
89
88
87
86
85
190
200
210
Day of Year
iii
Day of Year
203
202
201
200
3
7
11
15
19
23
27
31
Kernel width (days)
CAMBRIDGE WATER TRMT P, MD
Coverage
100
75
50
25
0
1853
1900
1950
2010
Year
Summer Solstice
June 21/Day 172
Summer Teletherm
July 20/Day 201
+29 days
90
85
80
75
70
65
60
55
50
45
40
Jan
Feb
Mar
Apr
May
Jun
Jul
Aug
Sep
Oct
Nov
Dec
1
32
60
91
121
152
182
213
244
274
305
335
Day of Year

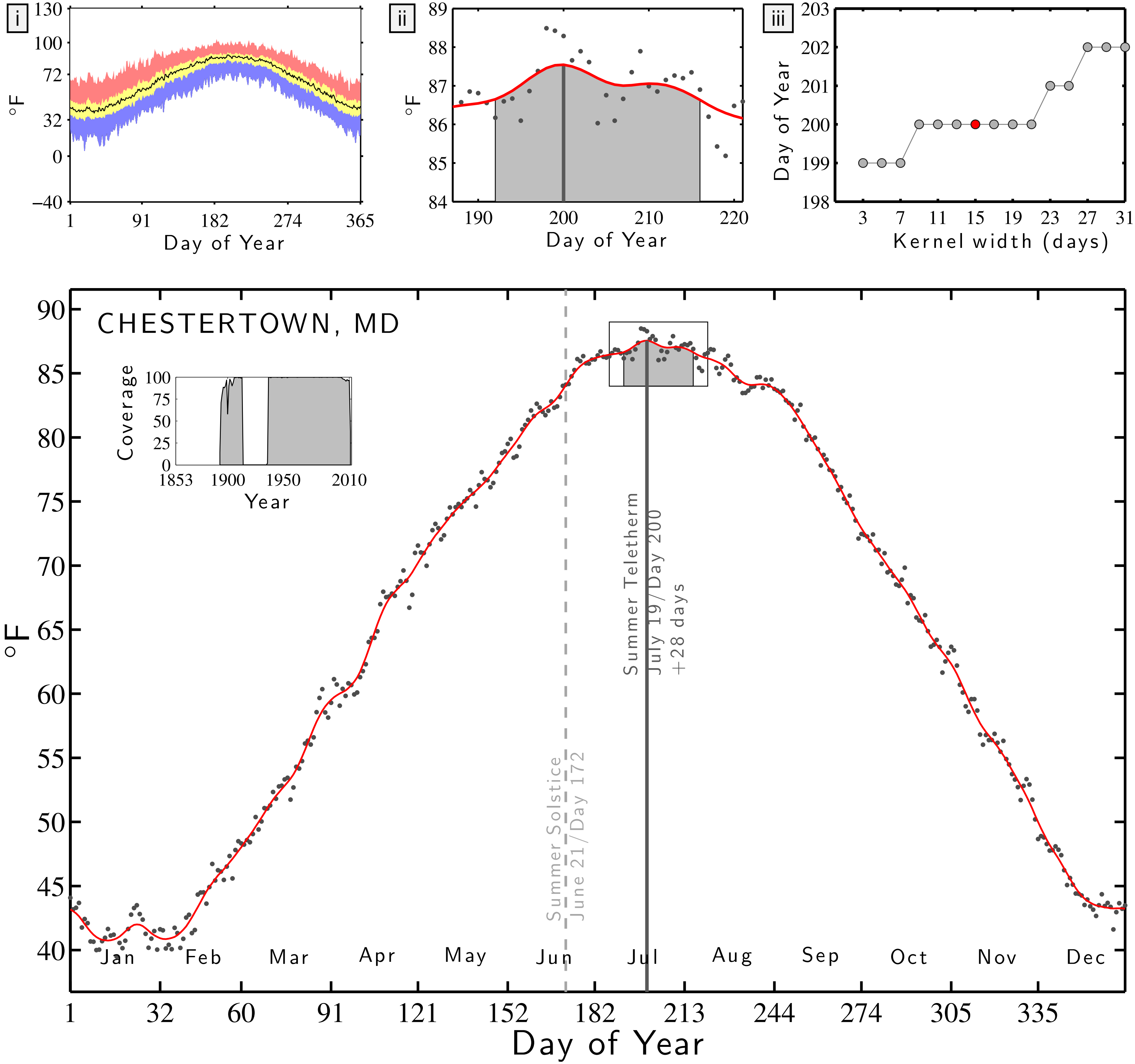

i
°F
Day of Year
ii
°F
Day of Year
iii
Day of Year
Kernel width (days)
CHESTERTOWN, MD
Coverage
Year
Summer Solstice
June 21/Day 172
Summer Teletherm
July 19/Day 200
+28 days
Jan
Feb
Mar
Apr
May
Jun
Jul
Aug
Sep
Oct
Nov
Dec
°F
Day of Year

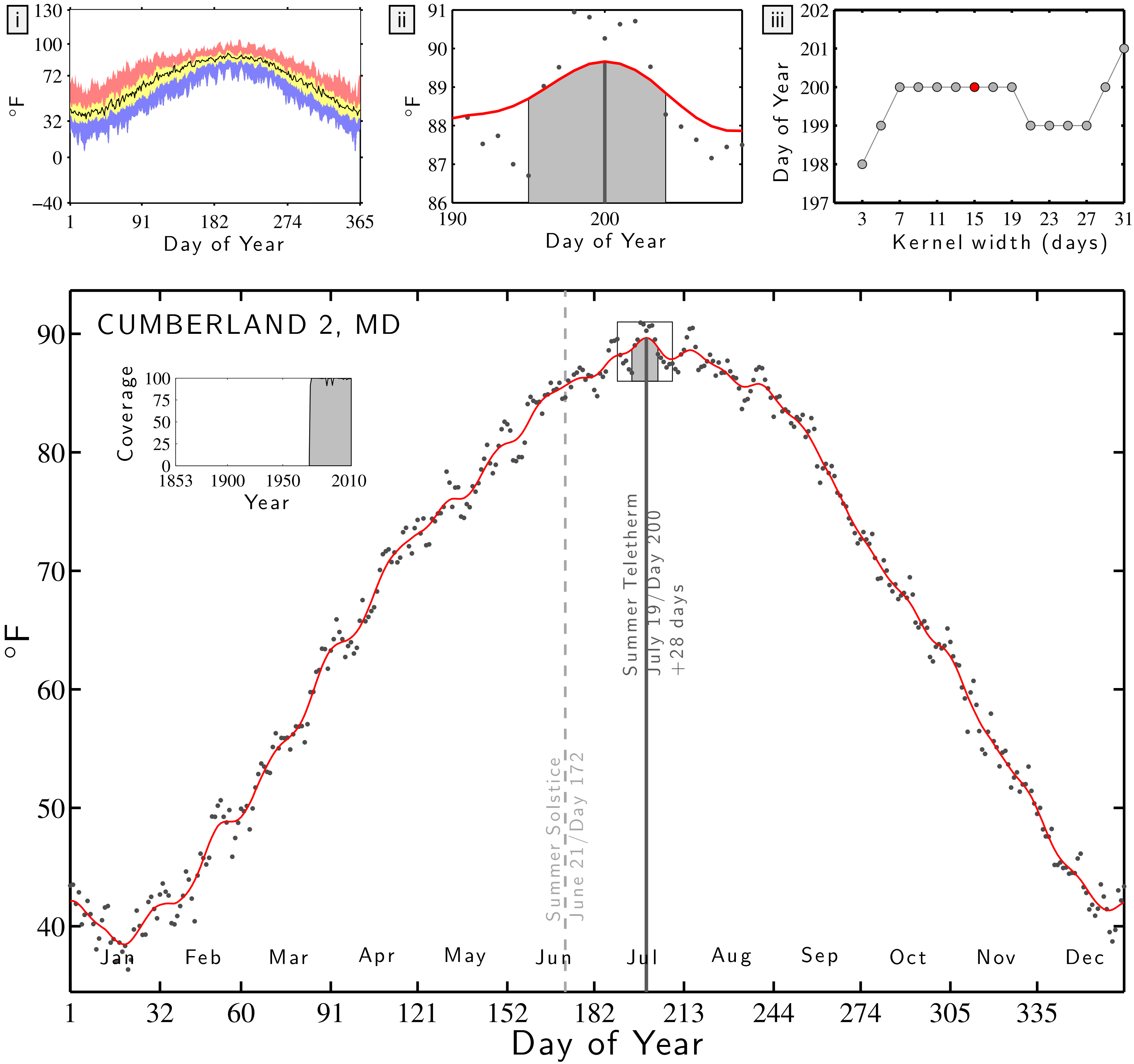

i
°F
Day of Year
ii
°F
Day of Year
iii
Day of Year
Kernel width (days)
CUMBERLAND 2, MD
Coverage
Year
Summer Solstice
June 21/Day 172
Summer Teletherm
July 19/Day 200
+28 days
Jan
Feb
Mar
Apr
May
Jun
Jul
Aug
Sep
Oct
Nov
Dec
°F
Day of Year

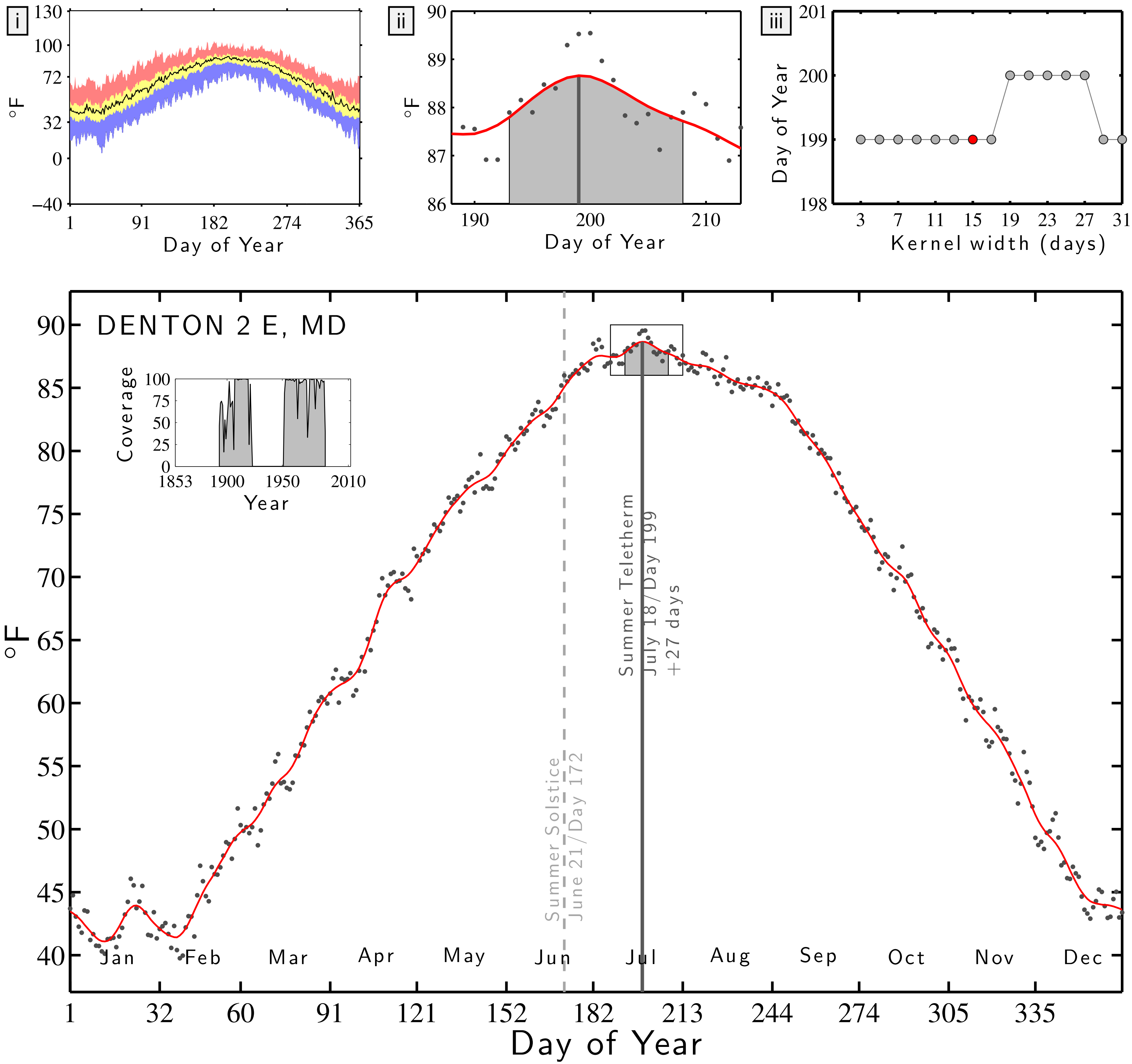
i
°F
Day of Year
ii
°F
Day of Year
iii
Day of Year
Kernel width (days)
DENTON 2 E, MD
Coverage
Year
Summer Solstice
June 21/Day 172
Summer Teletherm
July 18/Day 199
+27 days
Jan
Feb
Mar
Apr
May
Jun
Jul
Aug
Sep
Oct
Nov
Dec
°F
Day of Year

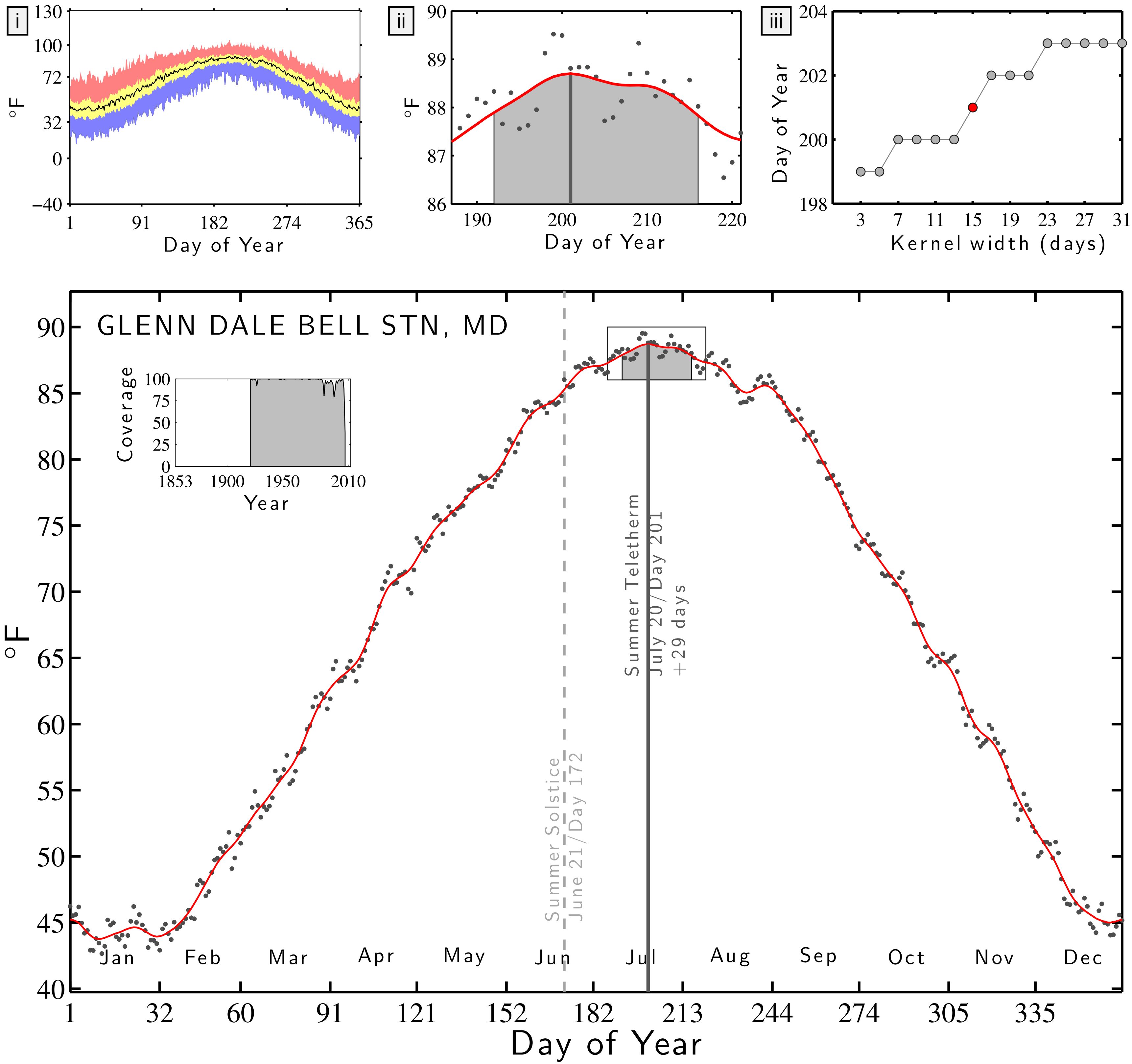

i
ii
iii
°F
Day of Year
Kernel width (days)
GLENN DALE BELL STN, MD
Coverage
Year
Summer Solstice
June 21/Day 172
Summer Teletherm
July 20/Day 201
+29 days
Jan
Feb
Mar
Apr
May
Jun
Jul
Aug
Sep
Oct
Nov
Dec

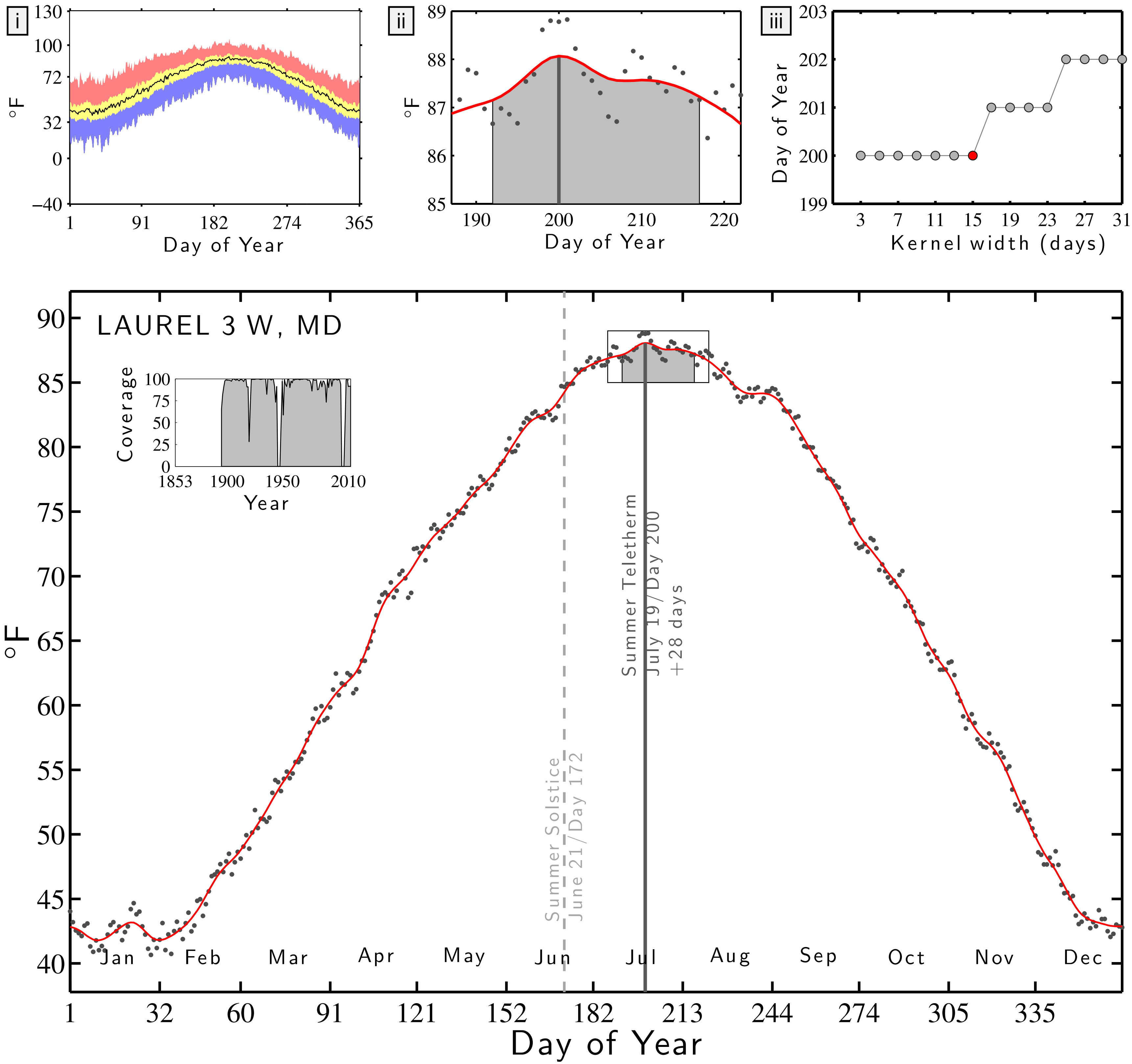

i
130
100
72
32
0
−40
°F
1
91
182
274
365
Day of Year
ii
89
88
87
86
85
°F
190
200
210
220
Day of Year
iii
203
202
201
200
199
Day of Year
3
7
11
15
19
23
27
31
Kernel width (days)
LAUREL 3 W, MD
Coverage
100
75
50
25
0
1853
1900
1950
2010
Year
Summer Solstice
June 21/Day 172
Summer Teletherm
July 19/Day 200
+28 days
90
85
80
75
70
65
60
55
50
45
40
°F
Jan
Feb
Mar
Apr
May
Jun
Jul
Aug
Sep
Oct
Nov
Dec
1
32
60
91
121
152
182
213
244
274
305
335
Day of Year

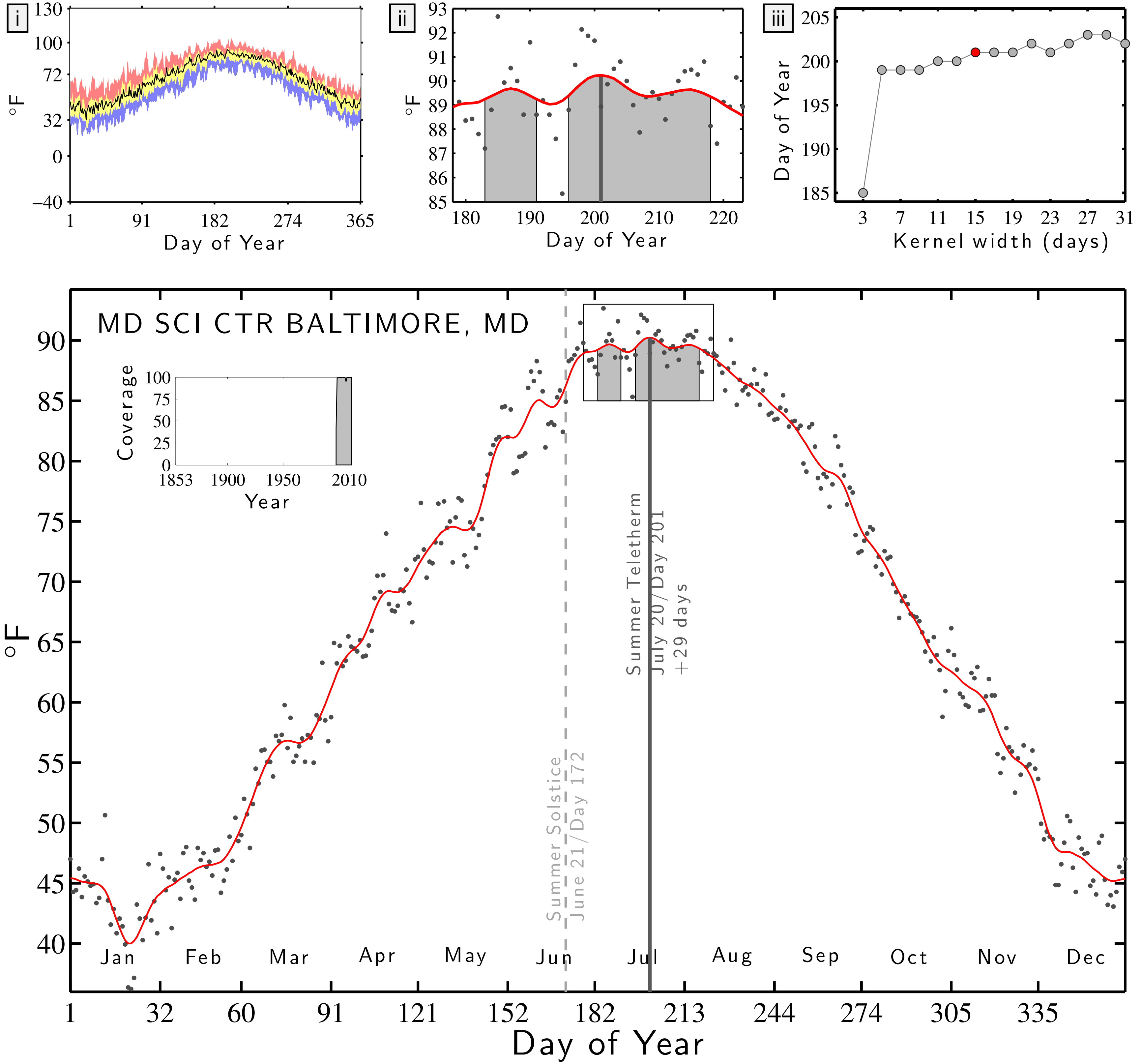

i
ii
iii
°F
Day of Year
Kernel width (days)
MD SCI CTR BALTIMORE, MD
Coverage
Year
Summer Solstice
June 21/Day 172
Summer Teletherm
July 20/Day 201
+29 days
Jan
Feb
Mar
Apr
May
Jun
Jul
Aug
Sep
Oct
Nov
Dec

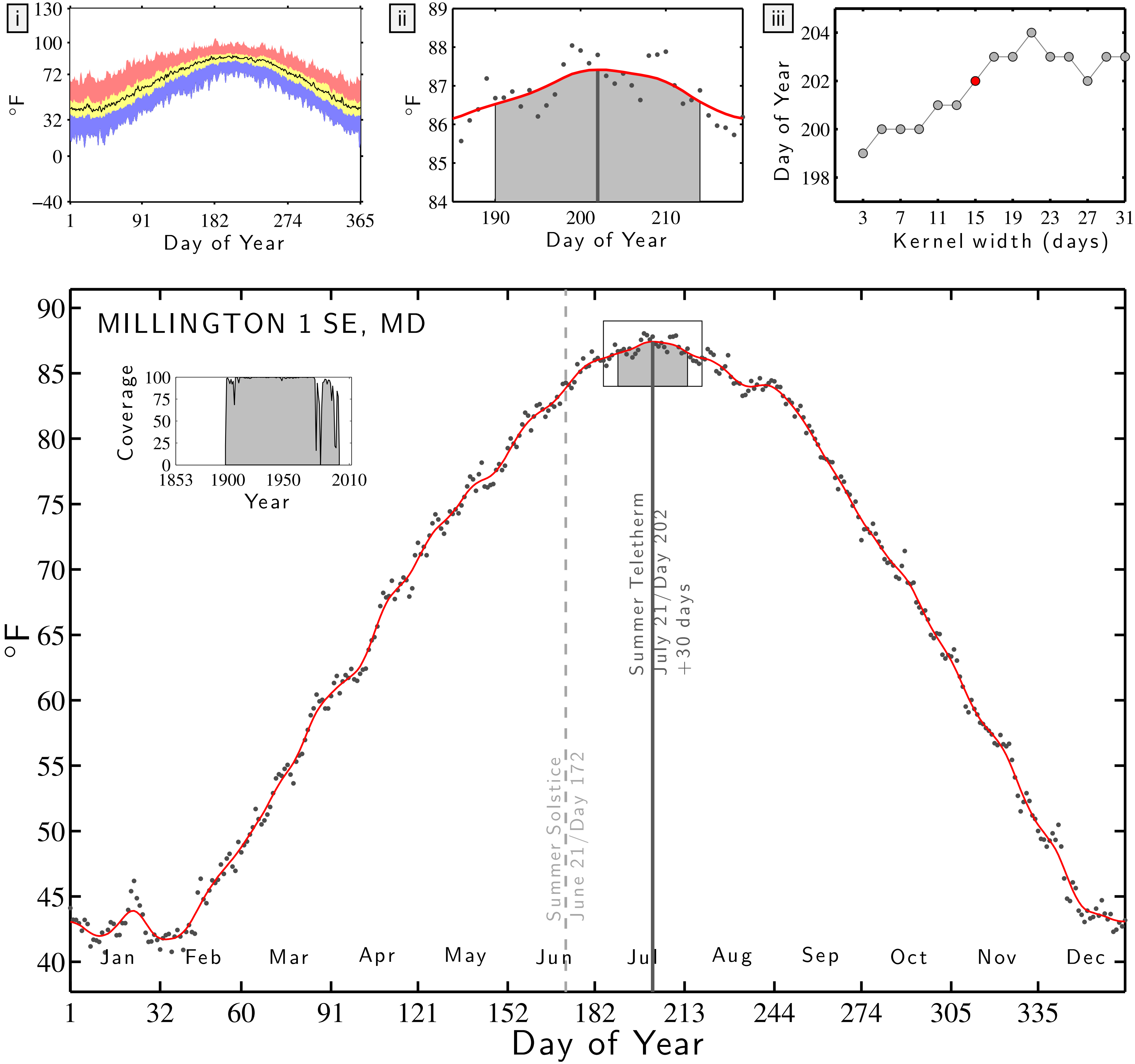
i
ii
iii
MILLINGTON 1 SE, MD
Coverage
Year
Summer Solstice
June 21 / Day 172
Summer Teletherm
July 21 / Day 202
+30 days
Kernel width (days)
Day of Year
°F
Jan
Feb
Mar
Apr
May
Jun
Jul
Aug
Sep
Oct
Nov
Dec

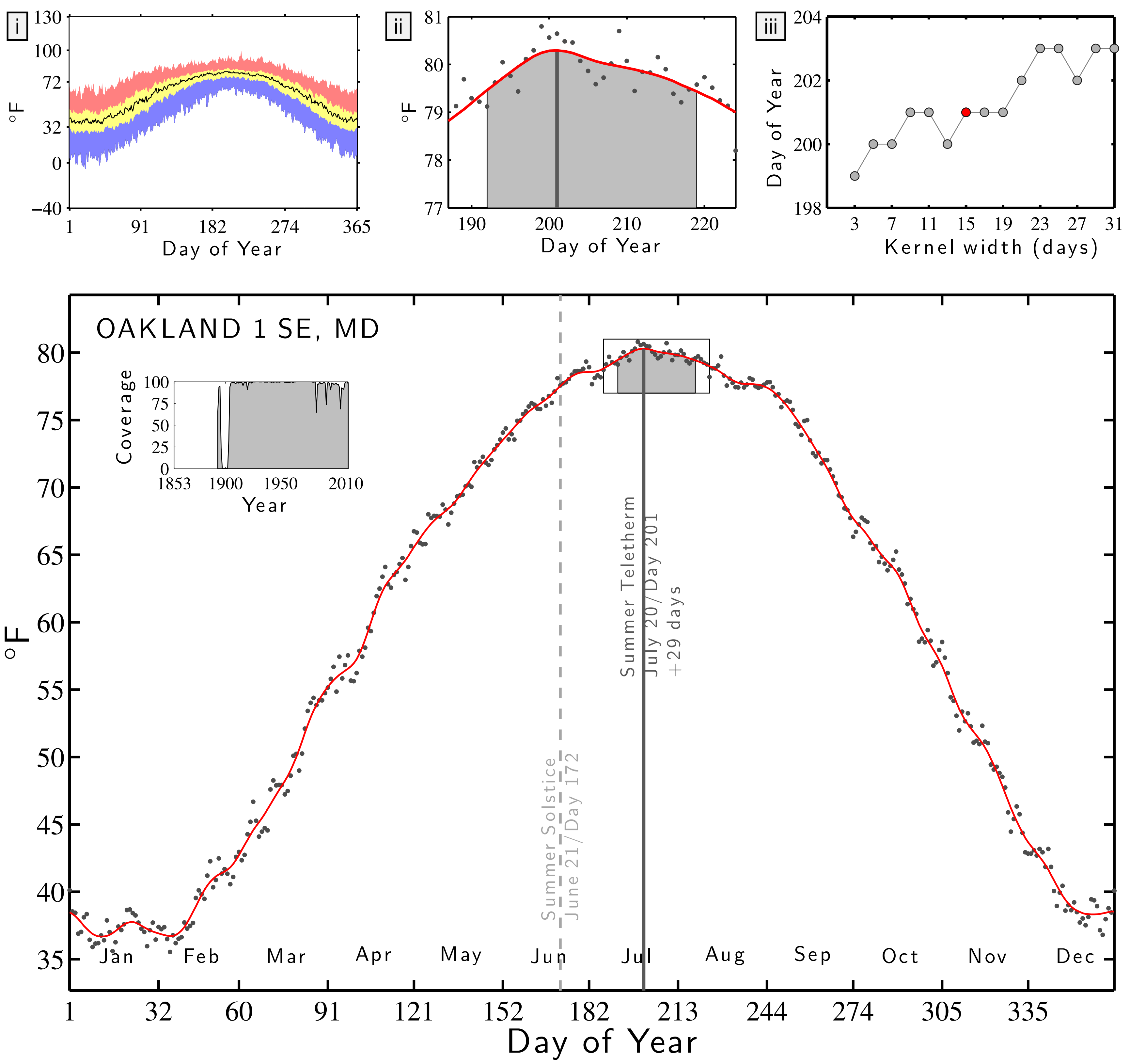
i
130
100
72
32
0
−40
°F
1
91
182
274
365
Day of Year
ii
81
80
79
78
77
°F
190
200
210
220
Day of Year
iii
204
202
200
198
Day of Year
3
7
11
15
19
23
27
31
Kernel width (days)
OAKLAND 1 SE, MD
Coverage
100
75
50
25
0
1853
1900
1950
2010
Year
80
75
70
65
60
55
50
45
40
35
°F
Summer Solstice
June 21 / Day 172
Summer Teletherm
July 20 / Day 201
+29 days
Jan
Feb
Mar
Apr
May
Jun
Jul
Aug
Sep
Oct
Nov
Dec
1
32
60
91
121
152
182
213
244
274
305
335
Day of Year

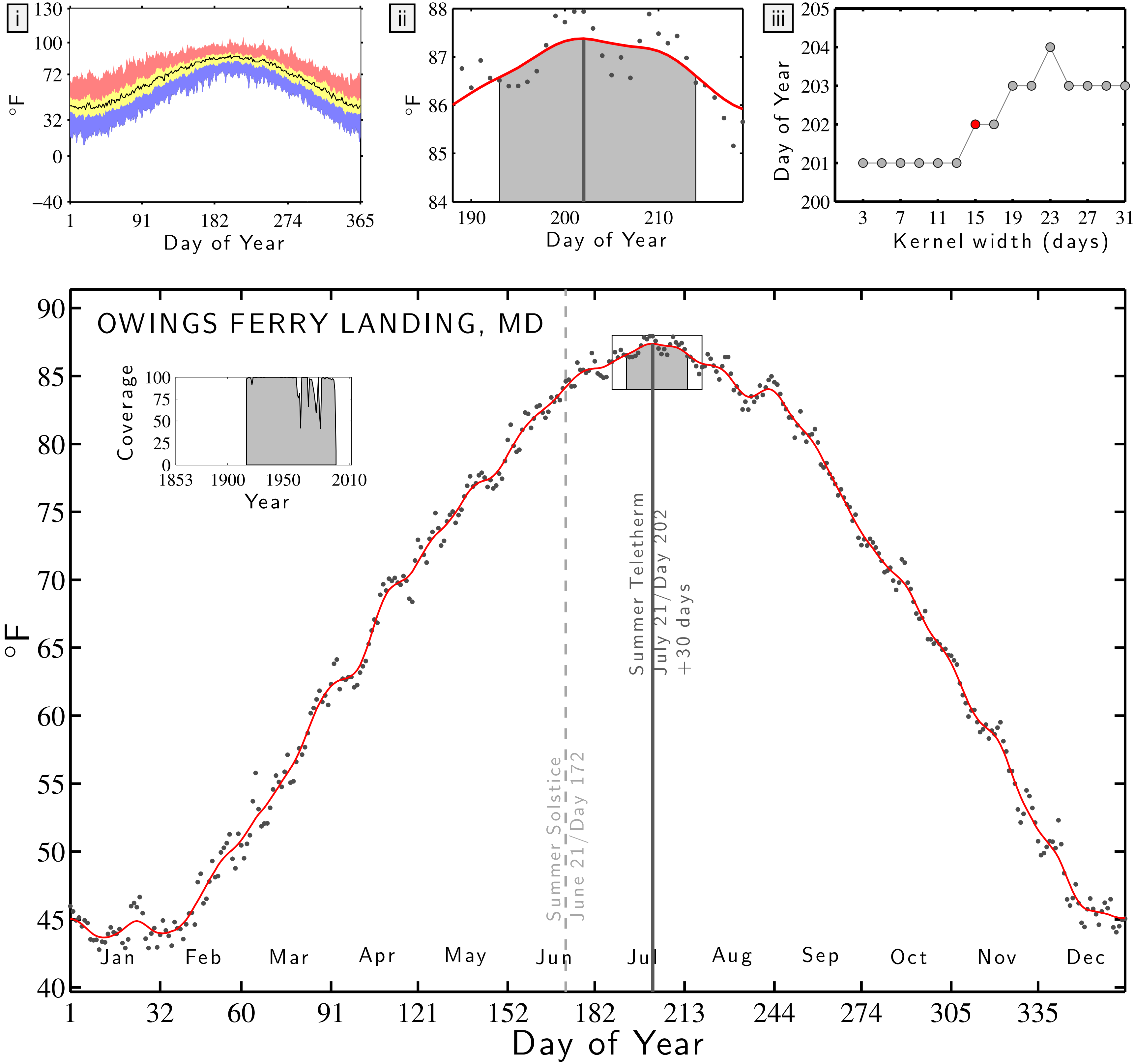
i
ii
iii
°F
Day of Year
Kernel width (days)
OWINGS FERRY LANDING, MD
Coverage
Year
Summer Solstice
June 21 / Day 172
Summer Teletherm
July 21 / Day 202
+30 days
Jan
Feb
Mar
Apr
May
Jun
Jul
Aug
Sep
Oct
Nov
Dec

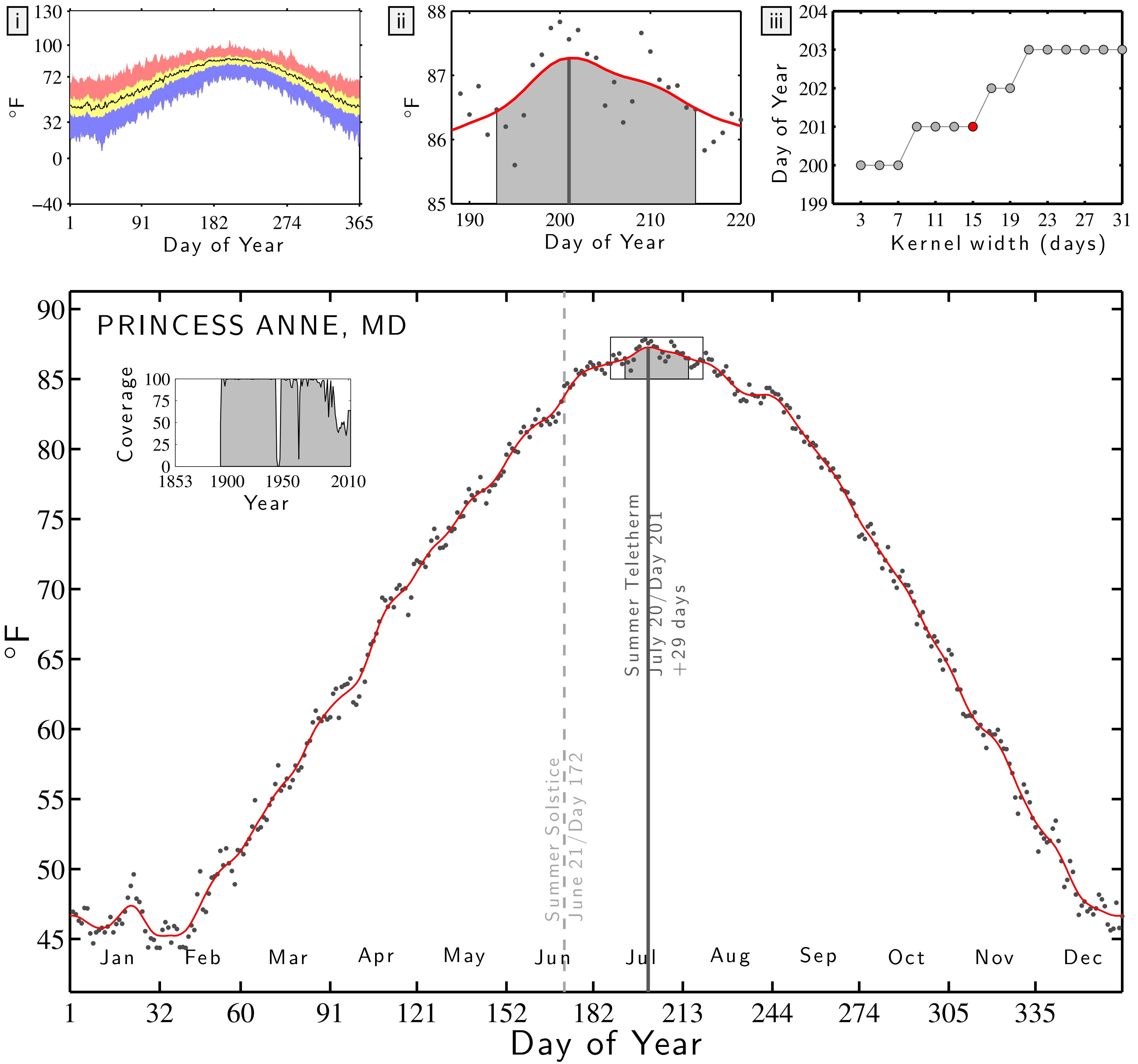

i
ii
iii
°F
Day of Year
Day of Year
Kernel width (days)
PRINCESS ANNE, MD
Coverage
Year
Summer Solstice
June 21 / Day 172
Summer Teletherm
July 20 / Day 201
+29 days
Jan
Feb
Mar
Apr
May
Jun
Jul
Aug
Sep
Oct
Nov
Dec
Day of Year

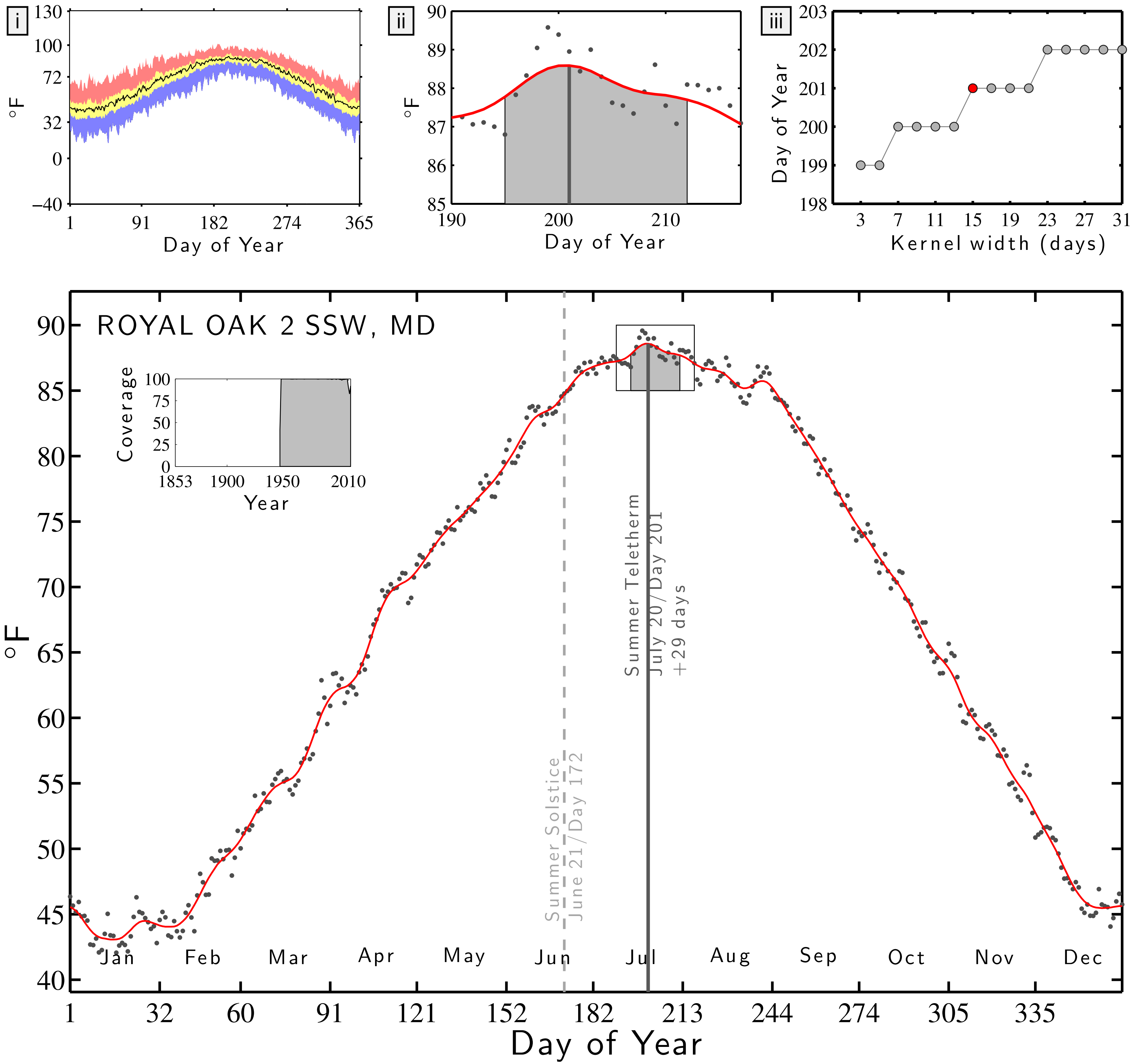

i
ii
iii
°F
Day of Year
Kernel width (days)
ROYAL OAK 2 SSW, MD
Coverage
Year
Summer Solstice
June 21 / Day 172
Summer Teletherm
July 20 / Day 201
+29 days
Jan
Feb
Mar
Apr
May
Jun
Jul
Aug
Sep
Oct
Nov
Dec

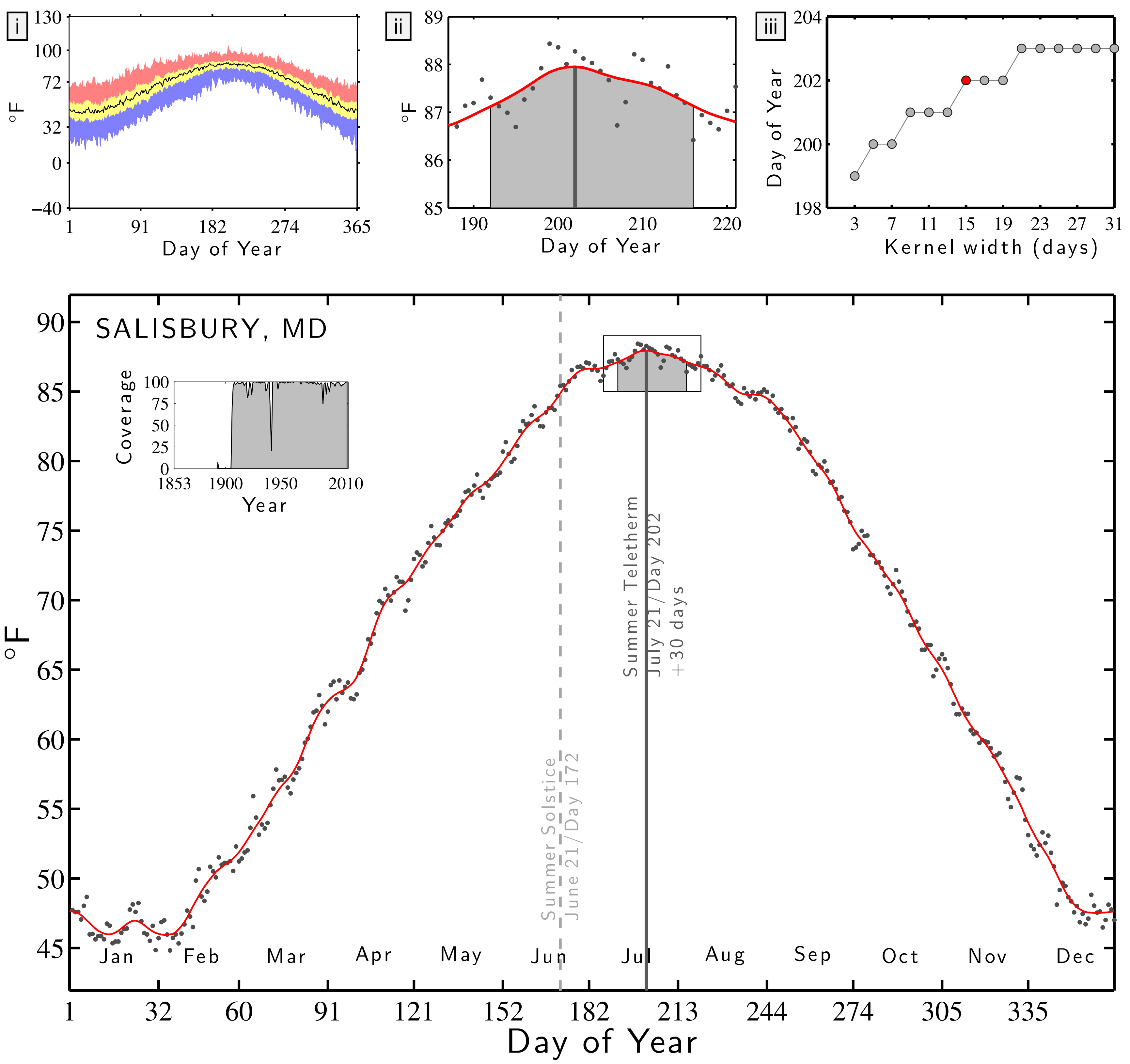

i
ii
iii
°F
Day of Year
Kernel width (days)
SALISBURY, MD
Coverage
Year
1853
1900
1950
2010
Summer Solstice
June 21 / Day 172
Summer Teletherm
July 21 / Day 202
+30 days
Jan
Feb
Mar
Apr
May
Jun
Jul
Aug
Sep
Oct
Nov
Dec

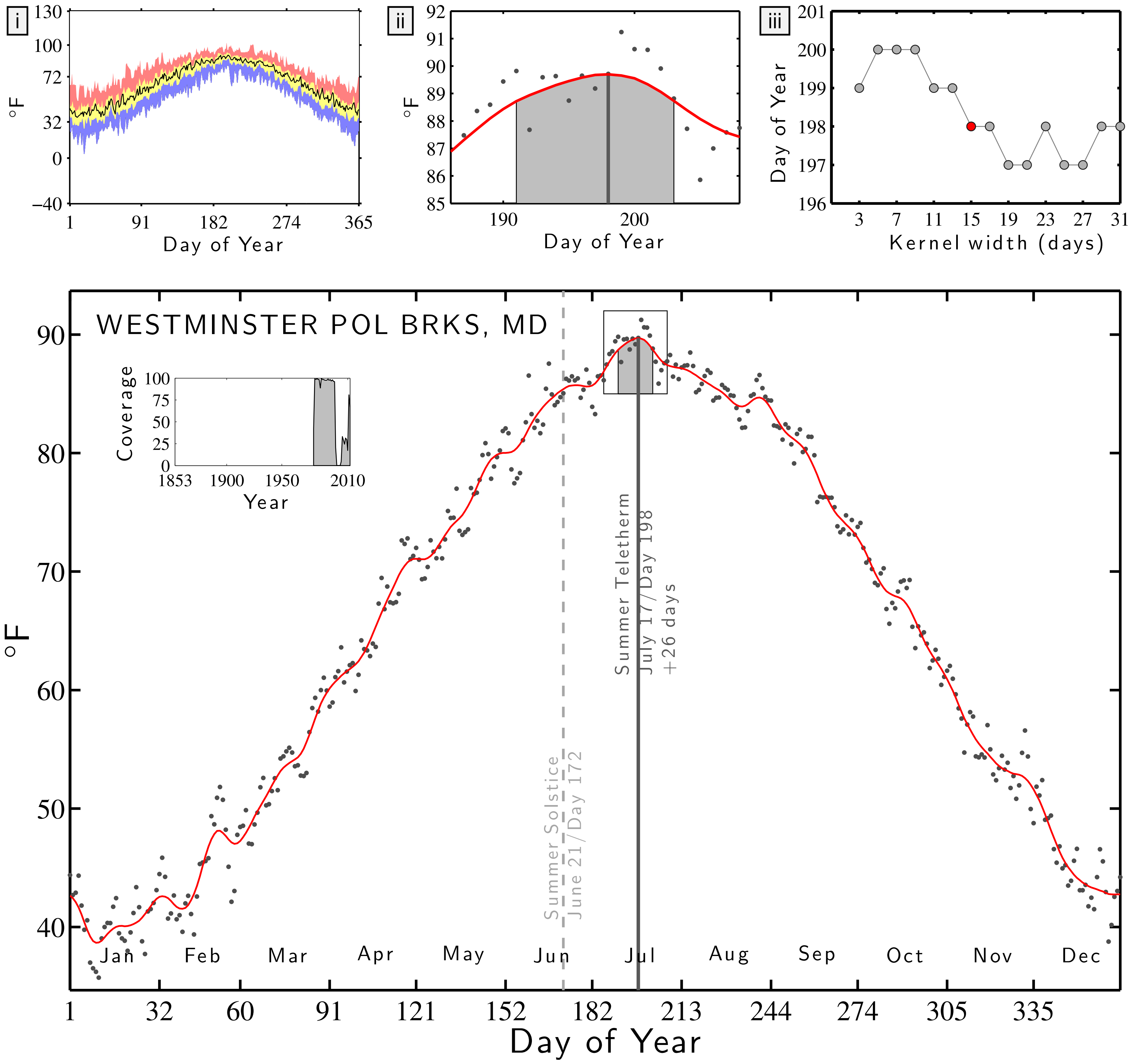

i
°F
Day of Year
ii
°F
Day of Year
iii
Day of Year
Kernel width (days)
WESTMINSTER POL BRKS, MD
Coverage
Year
Summer Solstice
June 21 / Day 172
Summer Teletherm
July 17 / Day 198
+26 days
Jan
Feb
Mar
Apr
May
Jun
Jul
Aug
Sep
Oct
Nov
Dec
°F
Day of Year

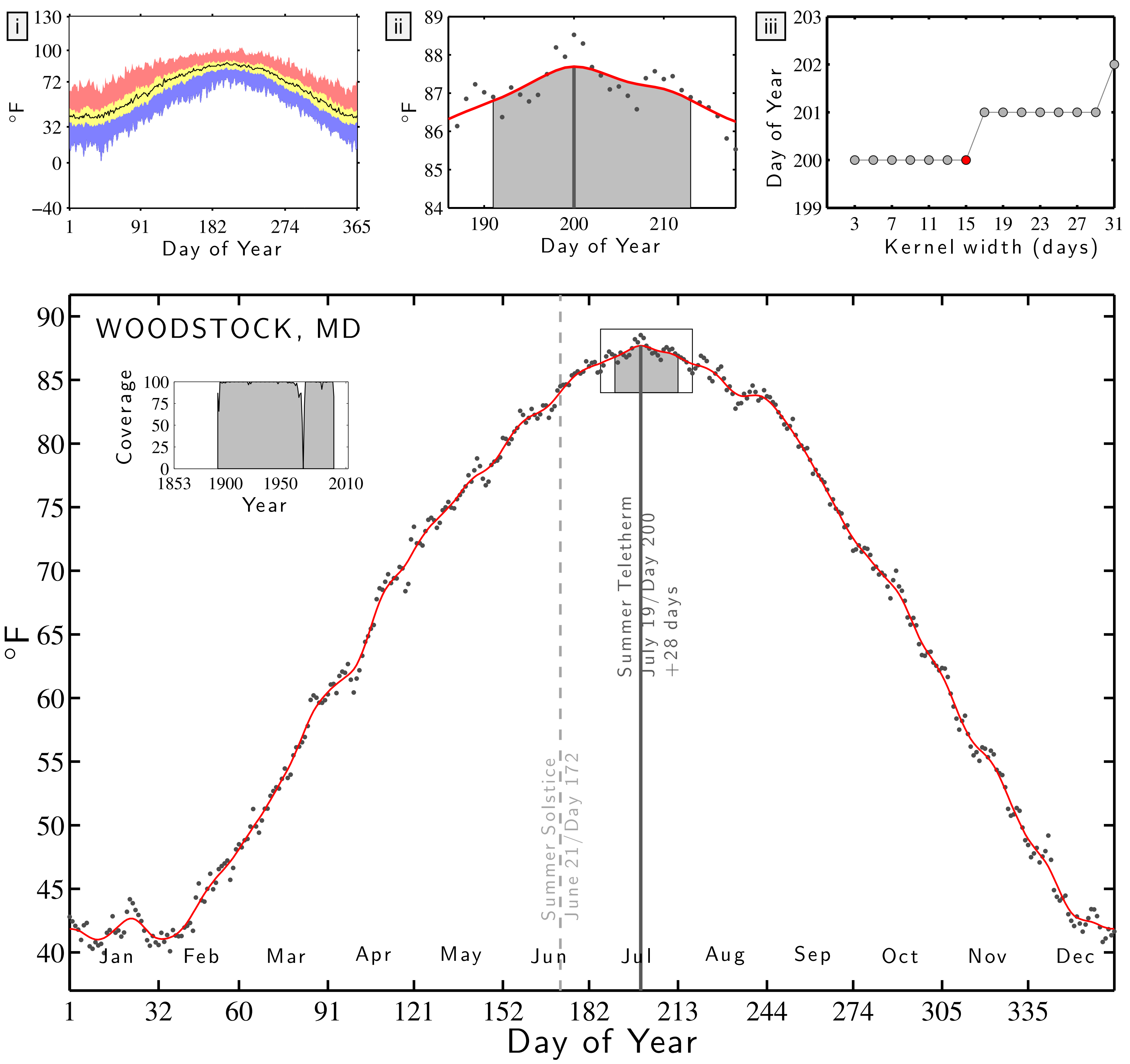

i
°F
Day of Year
ii
°F
Day of Year
iii
Day of Year
Kernel width (days)
WOODSTOCK, MD
Coverage
Year
Summer Solstice
June 21/Day 172
Summer Teletherm
July 19/Day 200
+28 days
Jan
Feb
Mar
Apr
May
Jun
Jul
Aug
Sep
Oct
Nov
Dec
°F
Day of Year

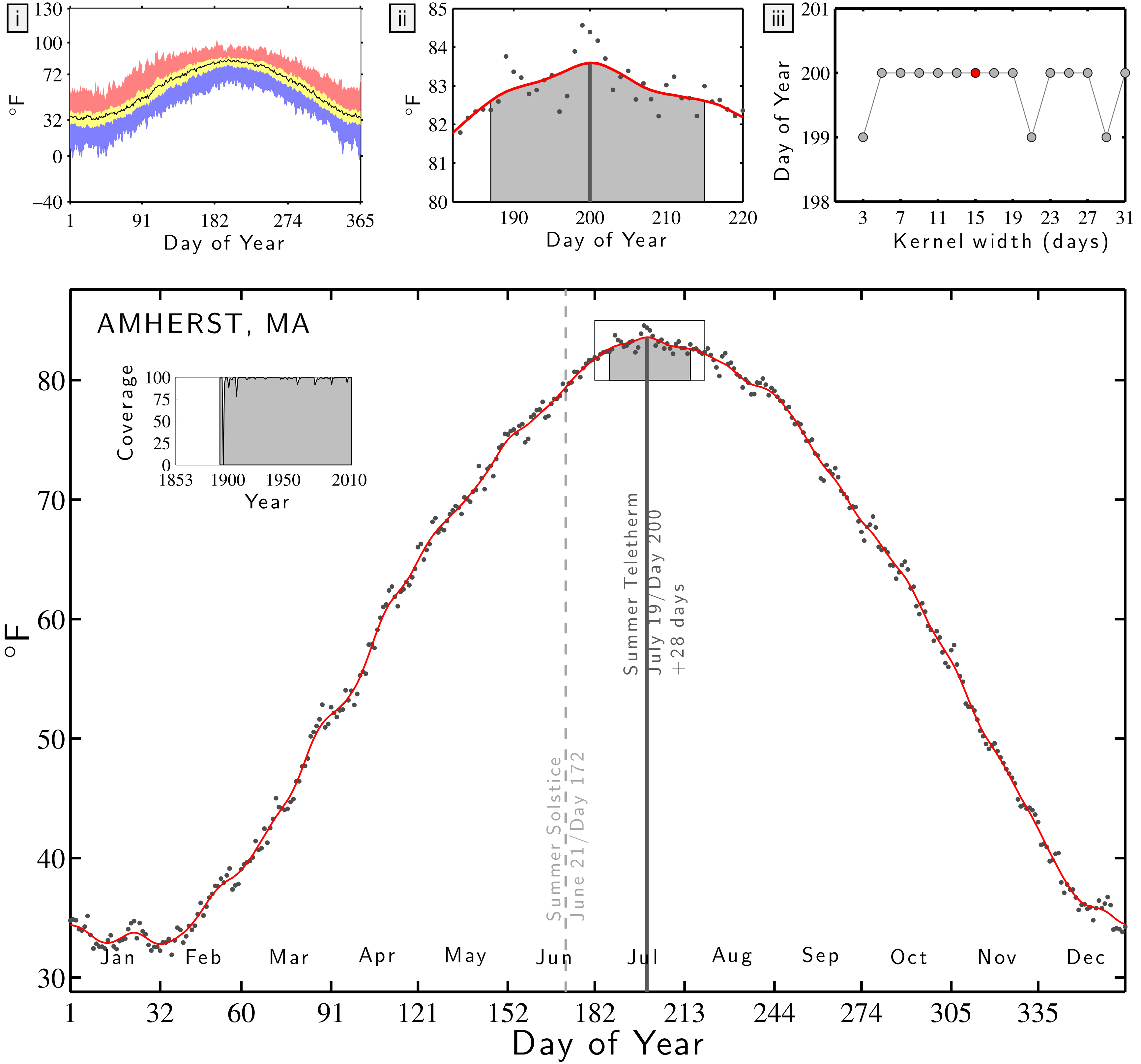

i
°F
Day of Year
ii
°F
Day of Year
iii
Day of Year
Kernel width (days)
AMHERST, MA
Coverage
Year
°F
Summer Solstice
June 21/Day 172
Summer Teletherm
July 19/Day 200
+28 days
Jan
Feb
Mar
Apr
May
Jun
Jul
Aug
Sep
Oct
Nov
Dec
Day of Year

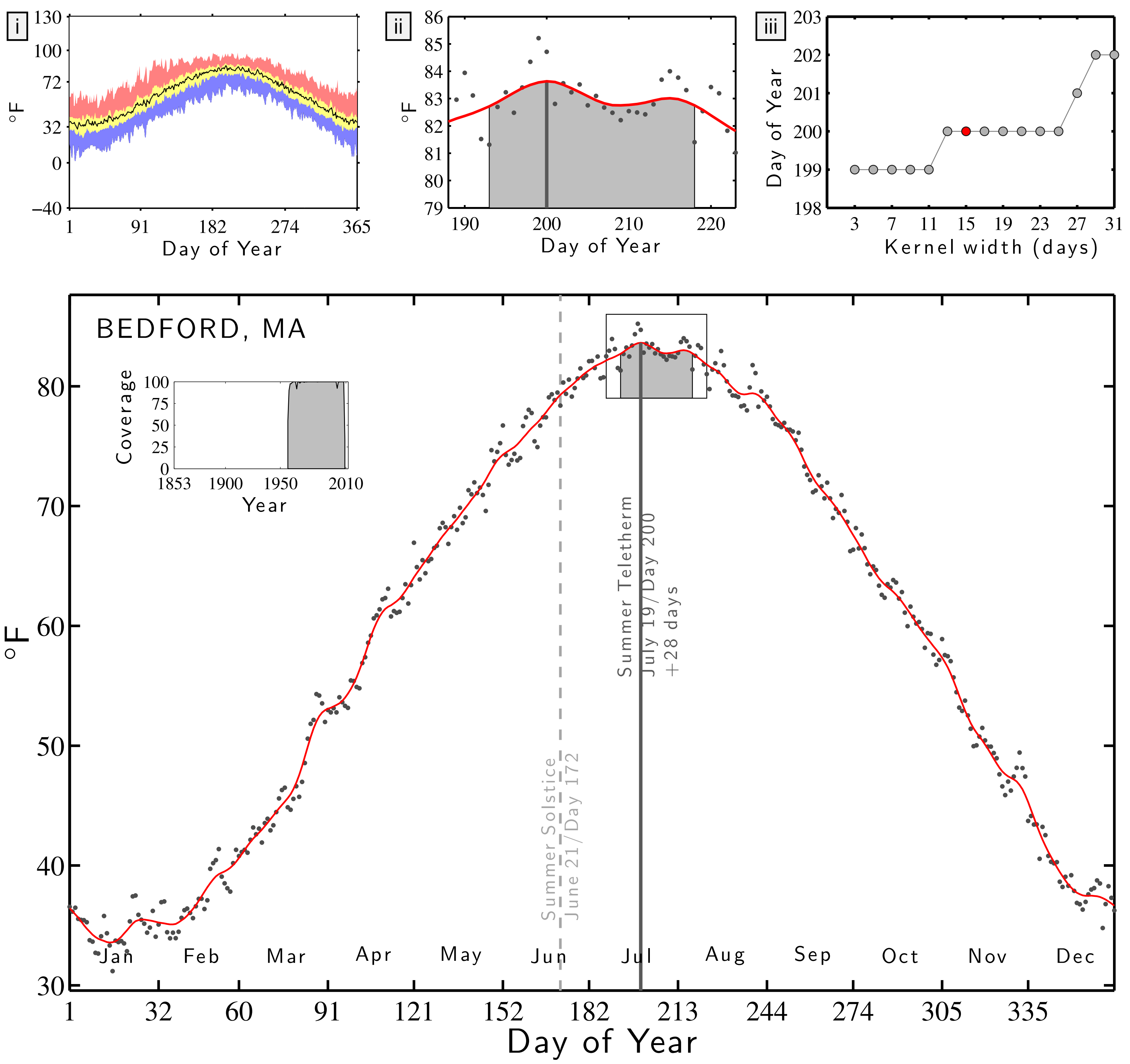

i
ii
iii
°F
Day of Year
Kernel width (days)
Day of Year
BEDFORD, MA
Coverage
Year
Summer Solstice
June 21/Day 172
Summer Teletherm
July 19/Day 200
+28 days
Jan
Feb
Mar
Apr
May
Jun
Jul
Aug
Sep
Oct
Nov
Dec

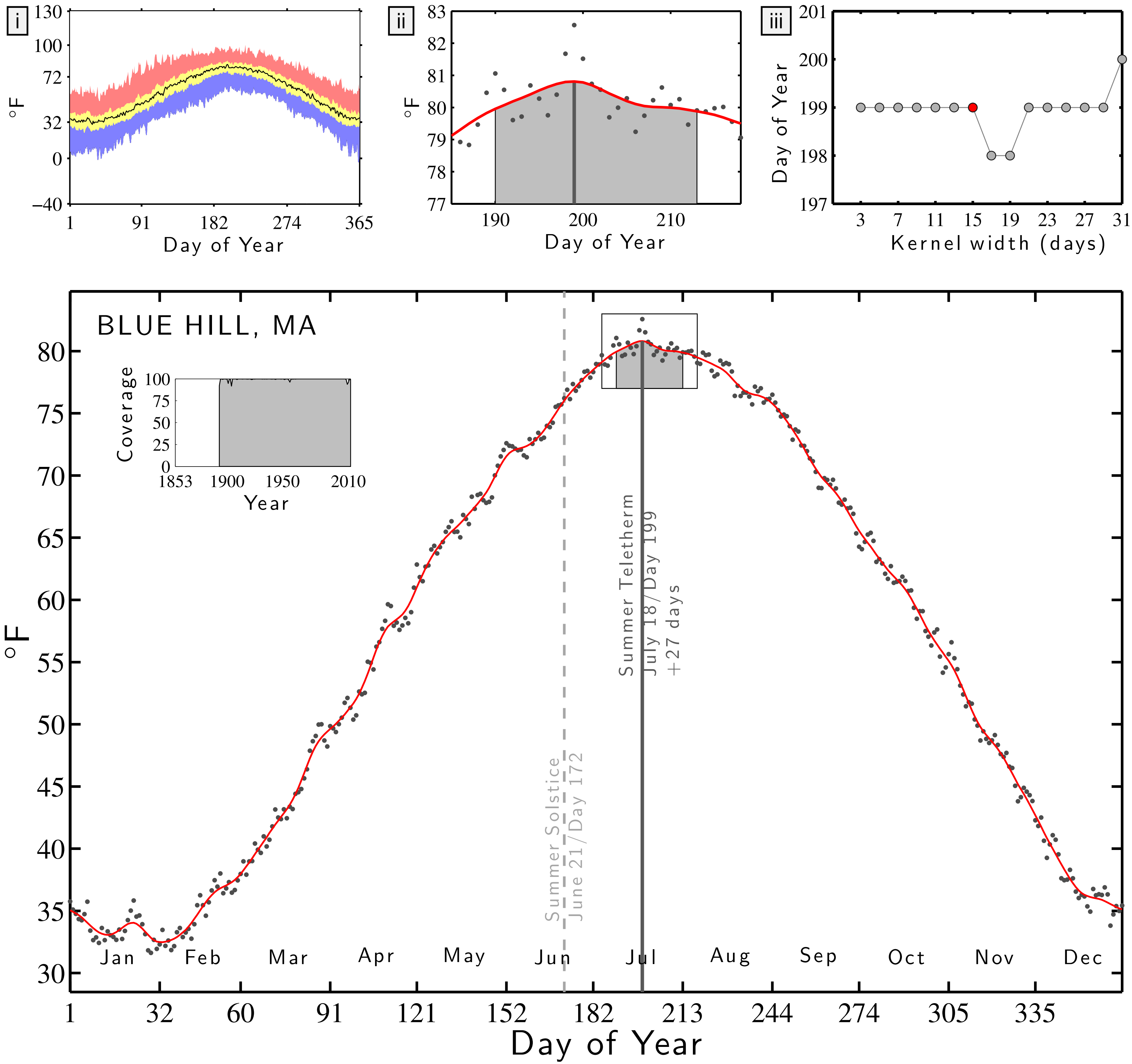

i
ii
iii
°F
Day of Year
Kernel width (days)
BLUE HILL, MA
Coverage
Year
1853
1900
1950
2010
Summer Solstice
June 21/Day 172
Summer Teletherm
July 18/Day 199
+27 days
Jan
Feb
Mar
Apr
May
Jun
Jul
Aug
Sep
Oct
Nov
Dec

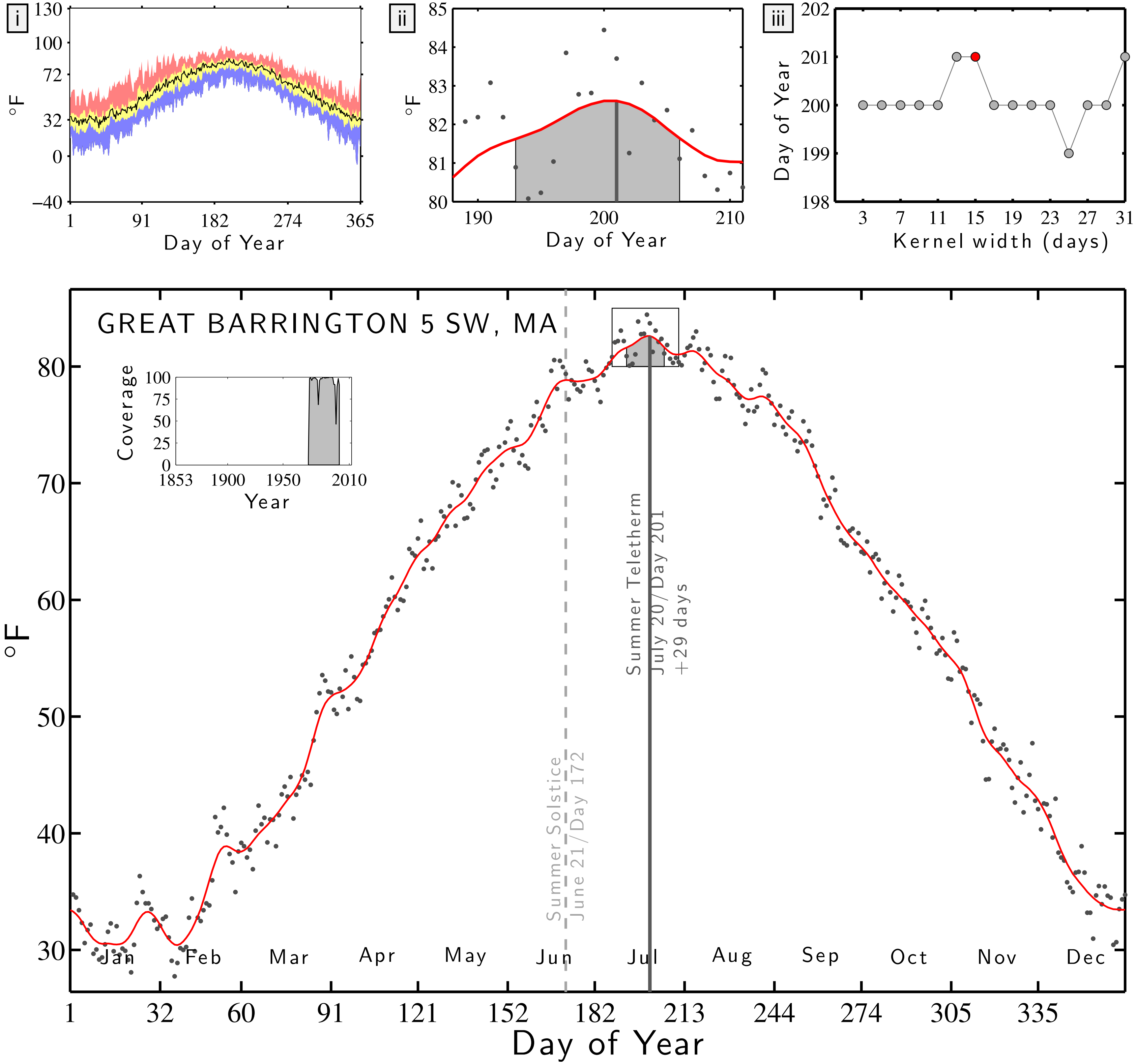

i
ii
iii
°F
Day of Year
Kernel width (days)
GREAT BARRINGTON 5 SW, MA
Coverage
Year
Summer Solstice
June 21 / Day 172
Summer Teletherm
July 20 / Day 201
+29 days
Jan
Feb
Mar
Apr
May
Jun
Jul
Aug
Sep
Oct
Nov
Dec

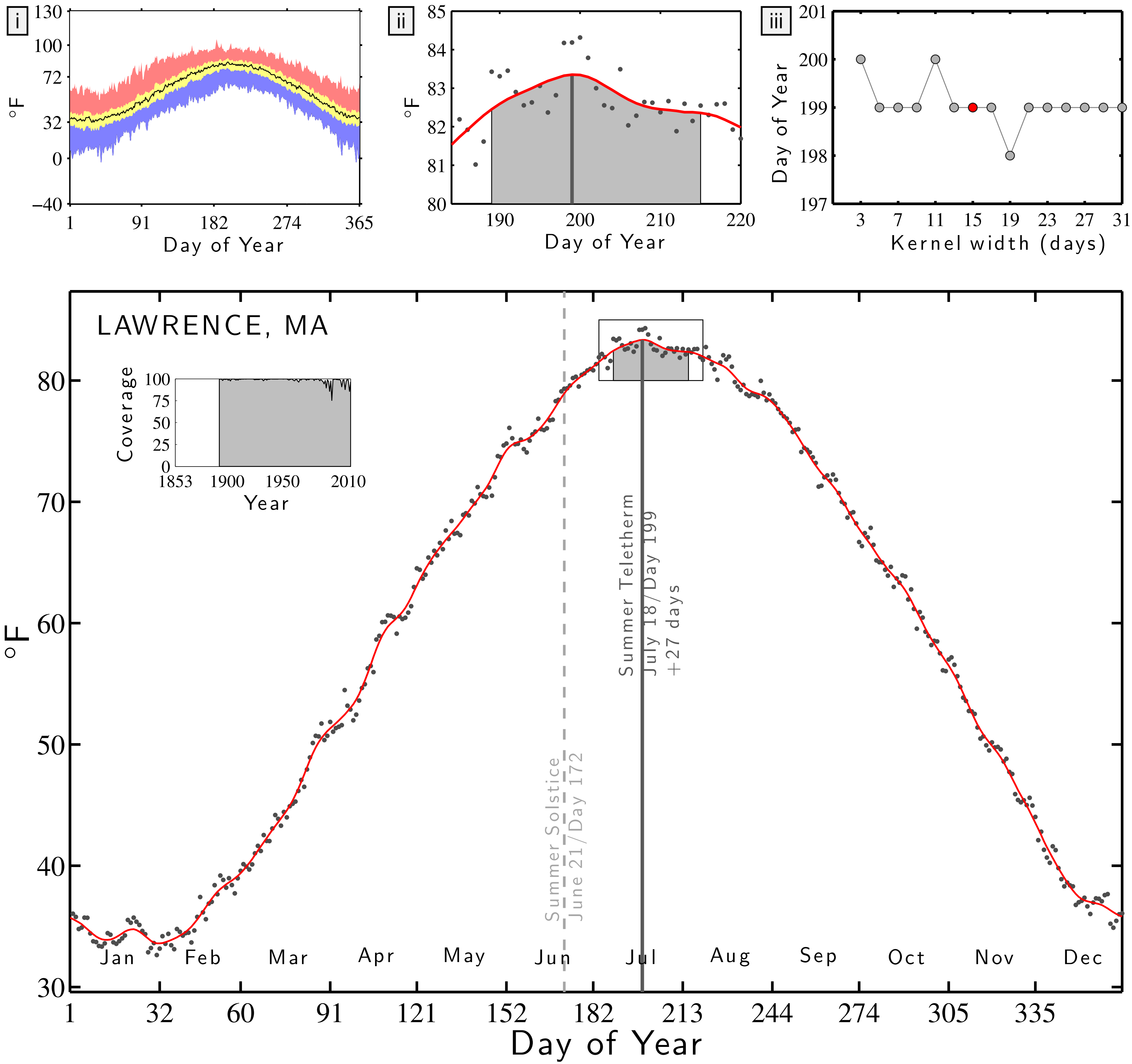

i
ii
iii
°F
Day of Year
Kernel width (days)
LAWRENCE, MA
Coverage
Year
Summer Solstice
June 21/Day 172
Summer Teletherm
July 18/Day 199
+27 days
Jan
Feb
Mar
Apr
May
Jun
Jul
Aug
Sep
Oct
Nov
Dec

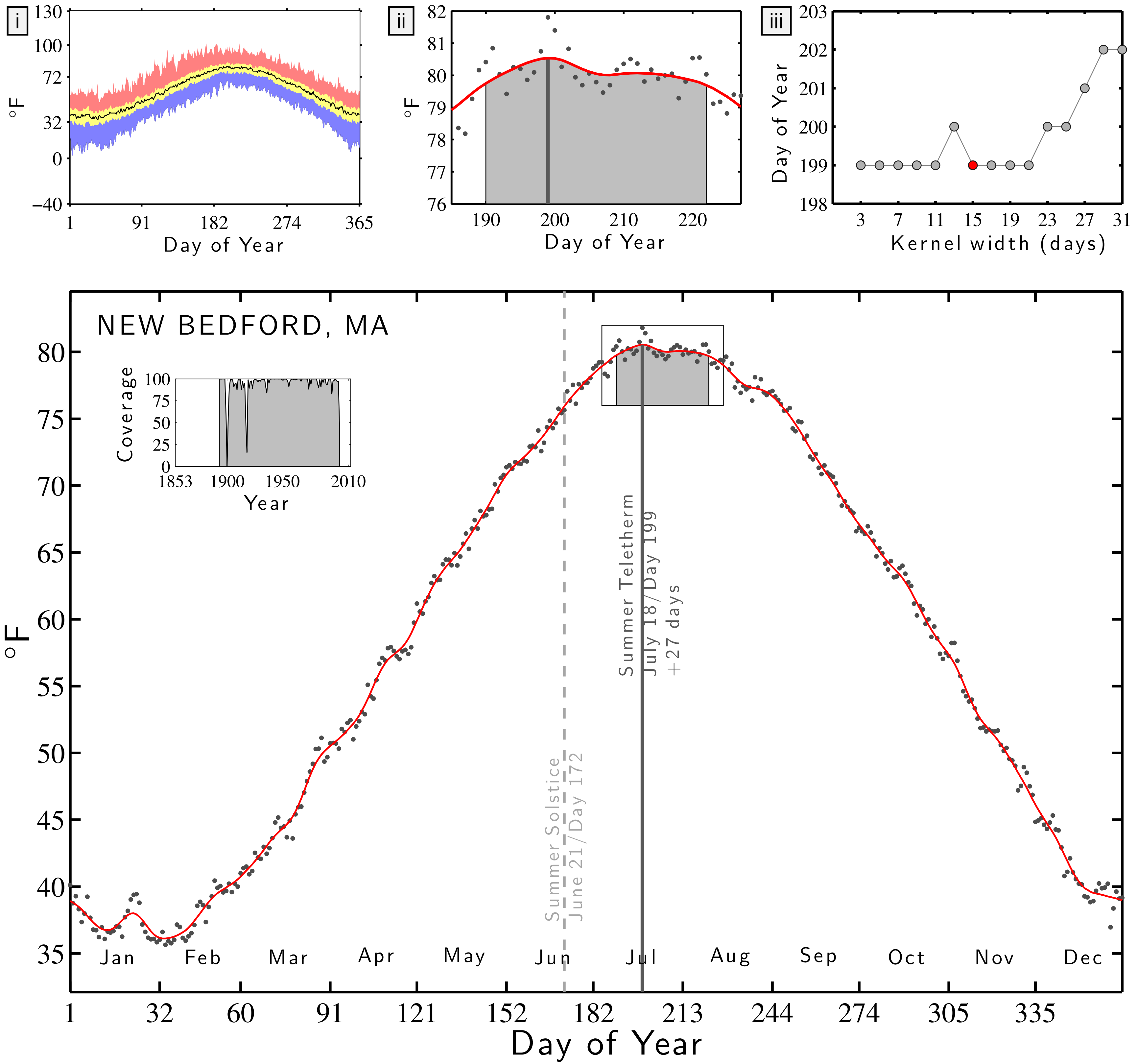

i
ii
iii
°F
Day of Year
Kernel width (days)
NEW BEDFORD, MA
Coverage
Year
Summer Solstice
June 21/Day 172
Summer Teletherm
July 18/Day 199
+27 days
Jan
Feb
Mar
Apr
May
Jun
Jul
Aug
Sep
Oct
Nov
Dec

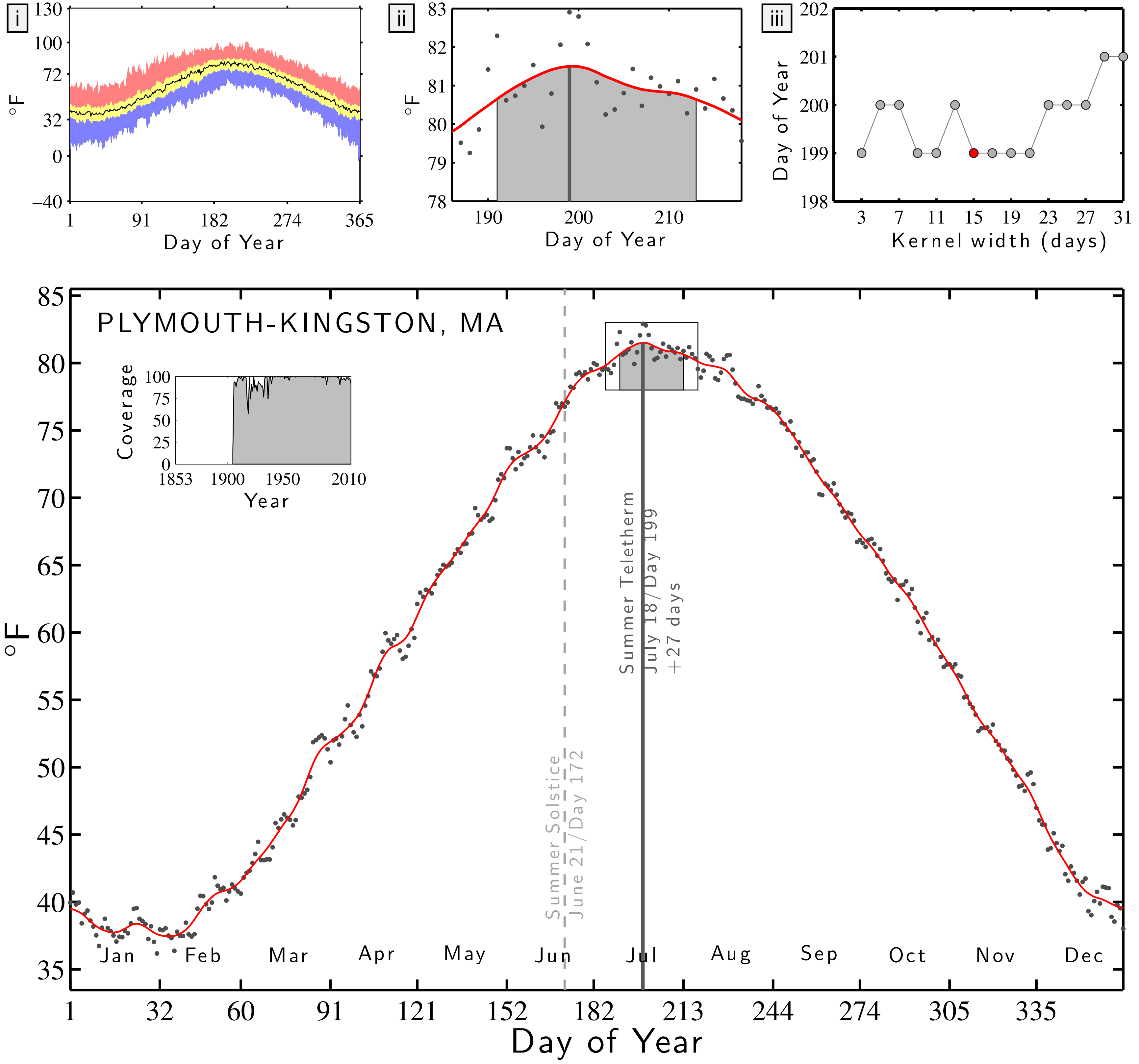

i
°F
Day of Year
ii
°F
Day of Year
iii
Day of Year
Kernel width (days)
PLYMOUTH-KINGSTON, MA
Coverage
Year
Summer Solstice
June 21/Day 172
Summer Teletherm
July 18/Day 199
+27 days
Jan
Feb
Mar
Apr
May
Jun
Jul
Aug
Sep
Oct
Nov
Dec
°F
Day of Year

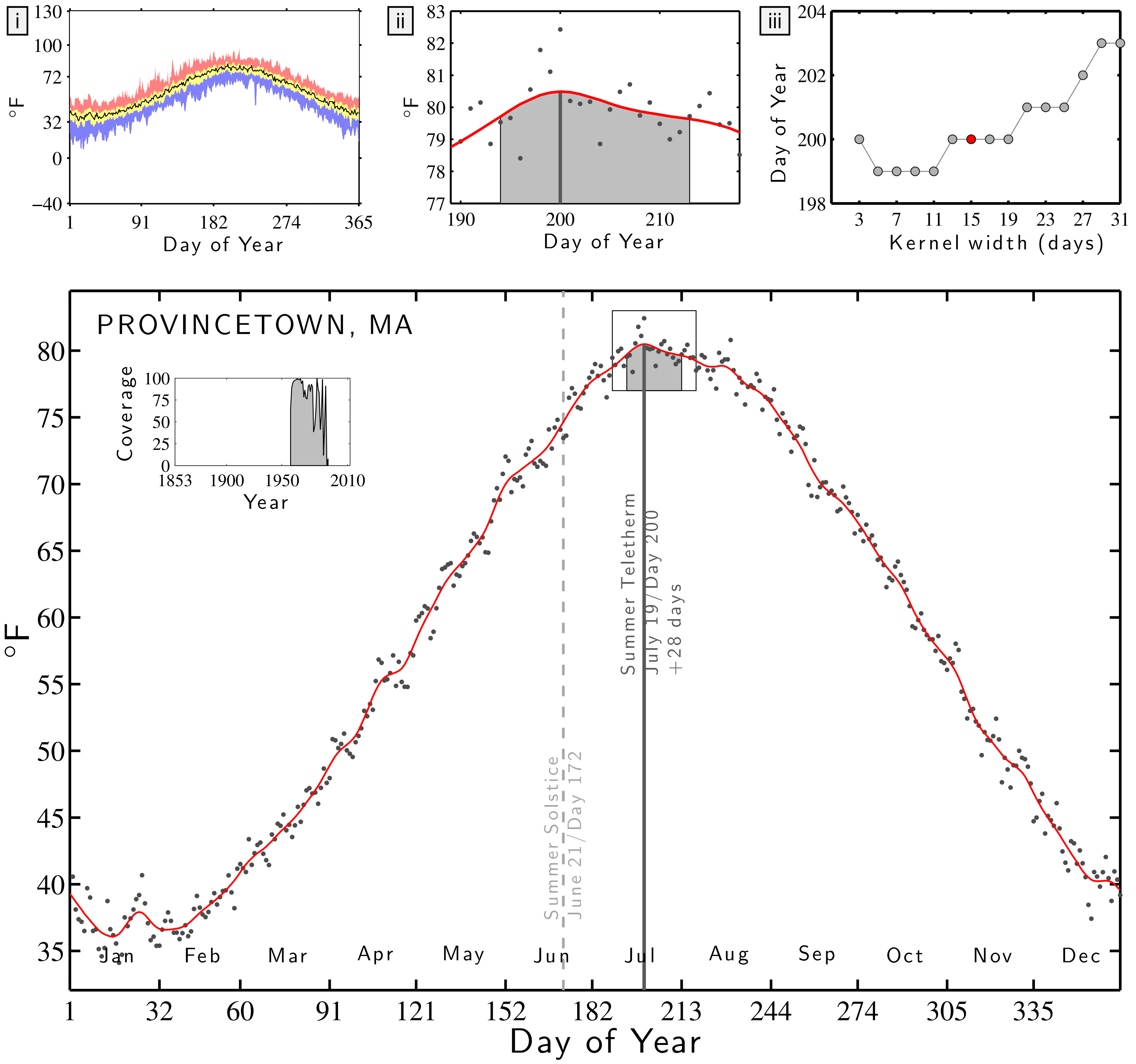

i
ii
iii
Day of Year
°F
Kernel width (days)
PROVINCETOWN, MA
Coverage
Year
Summer Solstice
June 21/Day 172
Summer Teletherm
July 19/Day 200
+28 days
Jan
Feb
Mar
Apr
May
Jun
Jul
Aug
Sep
Oct
Nov
Dec

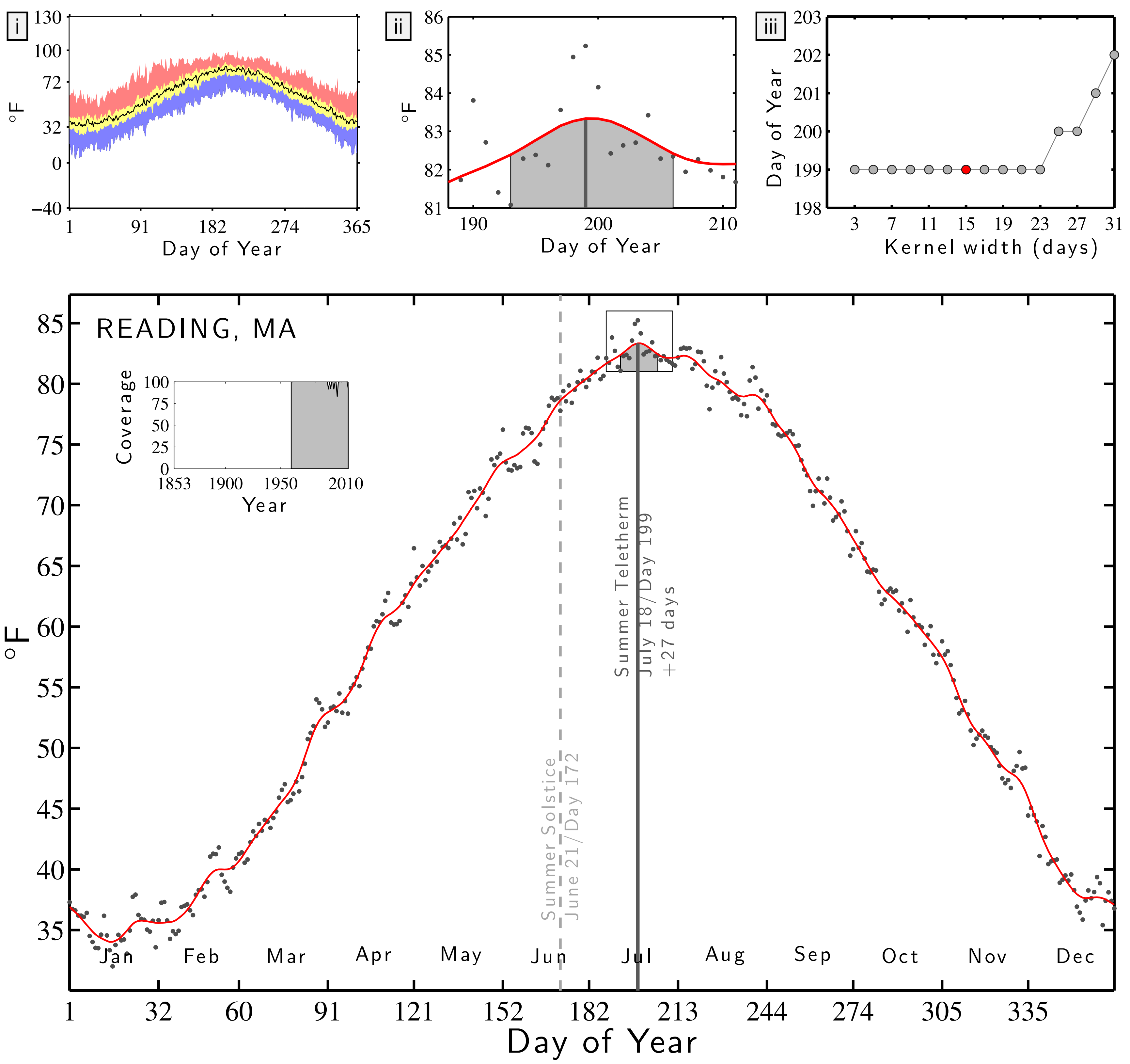
i
°F
Day of Year
ii
Day of Year
iii
Day of Year
Kernel width (days)
READING, MA
Coverage
Year
Summer Solstice
June 21/Day 172
Summer Teletherm
July 18/Day 199
+27 days
Jan
Feb
Mar
Apr
May
Jun
Jul
Aug
Sep
Oct
Nov
Dec
Day of Year

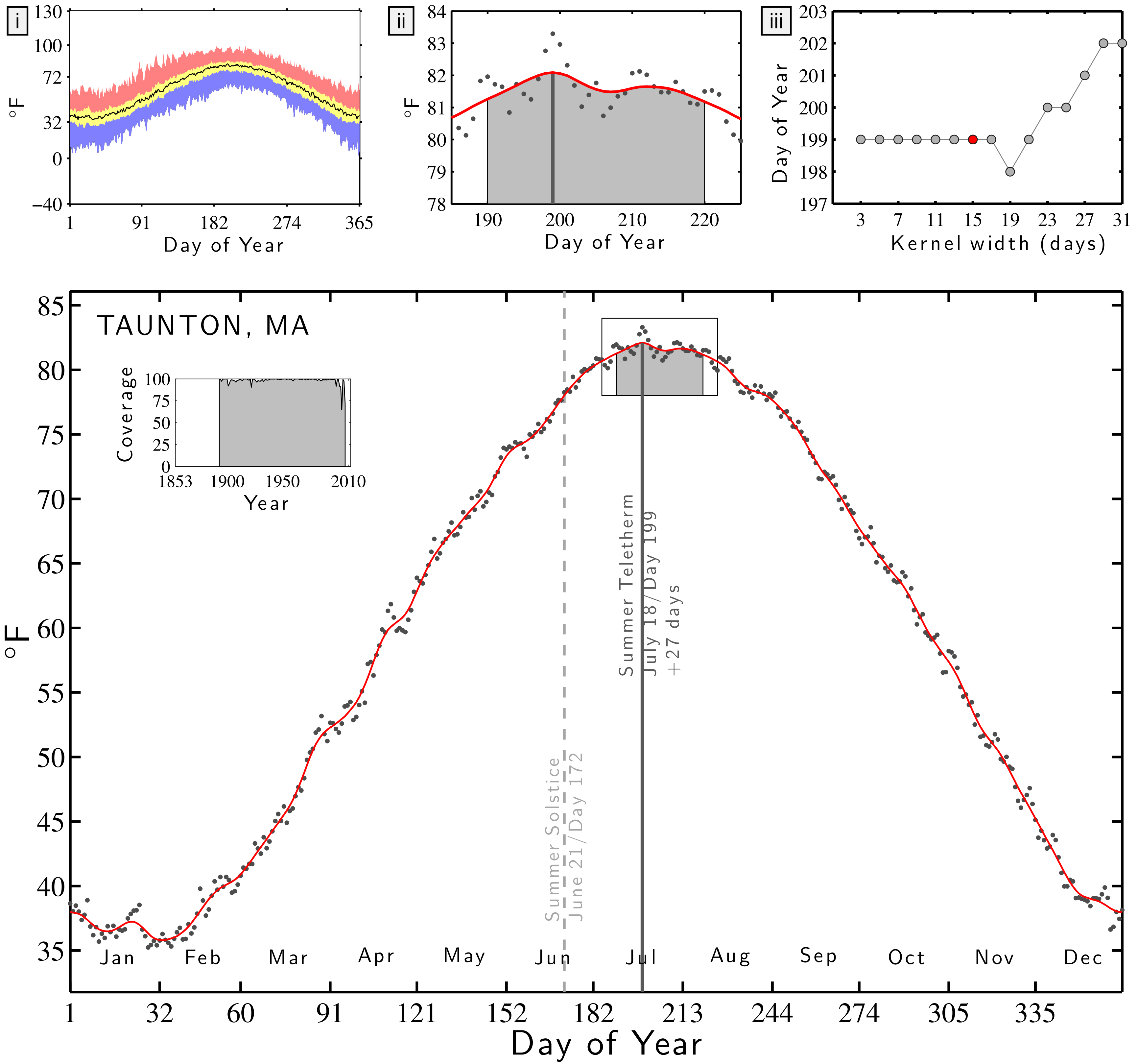

i
°F
Day of Year
ii
°F
Day of Year
iii
Day of Year
Kernel width (days)
TAUNTON, MA
Coverage
Year
Summer Solstice
June 21/Day 172
Summer Teletherm
July 18/Day 199
+27 days
Jan
Feb
Mar
Apr
May
Jun
Jul
Aug
Sep
Oct
Nov
Dec
°F
Day of Year

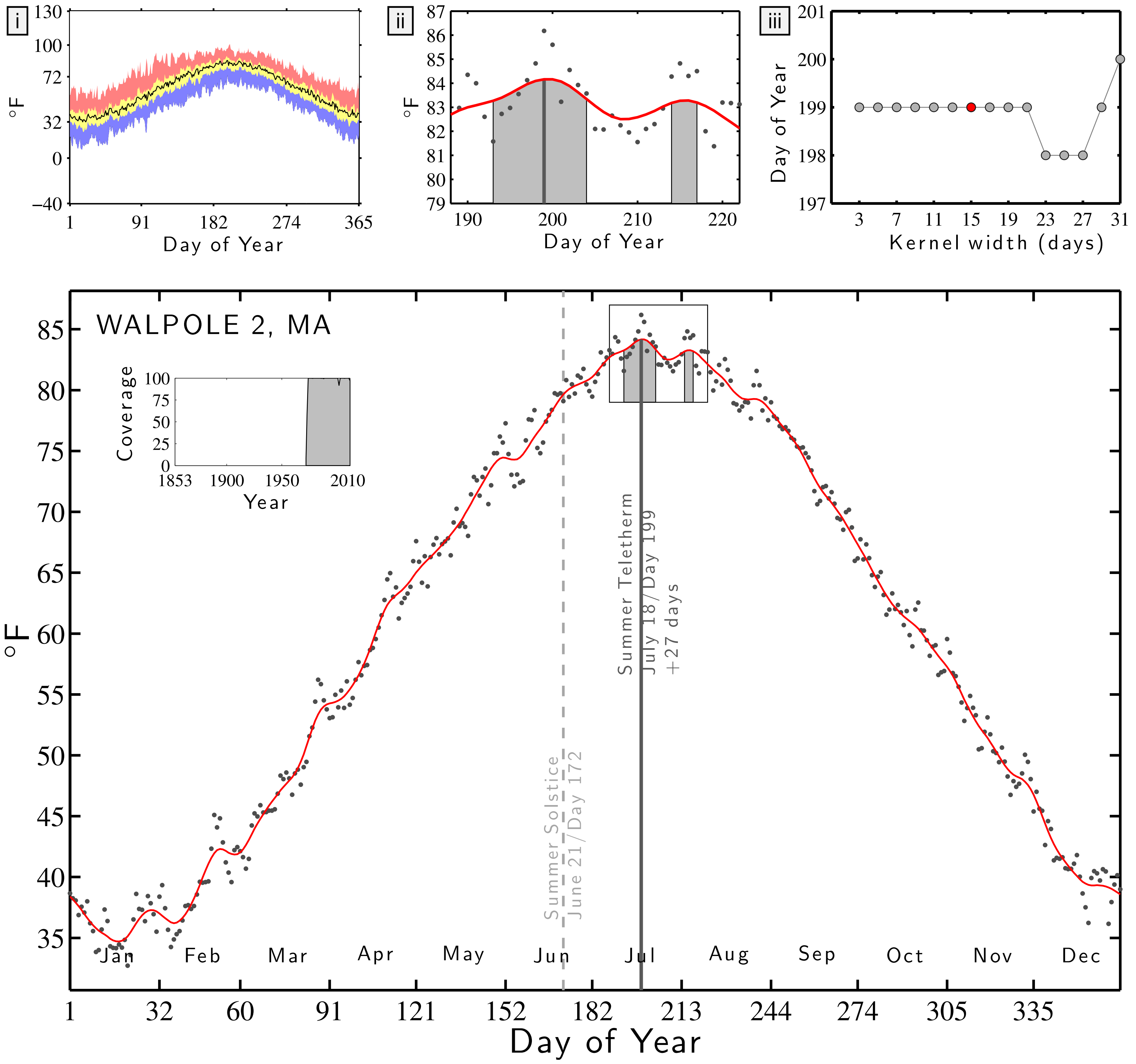

i
ii
iii
°F
Day of Year
Day of Year
Kernel width (days)
WALPOLE 2, MA
Coverage
Year
Summer Solstice
June 21/Day 172
Summer Teletherm
July 18/Day 199
+27 days
Jan
Feb
Mar
Apr
May
Jun
Jul
Aug
Sep
Oct
Nov
Dec

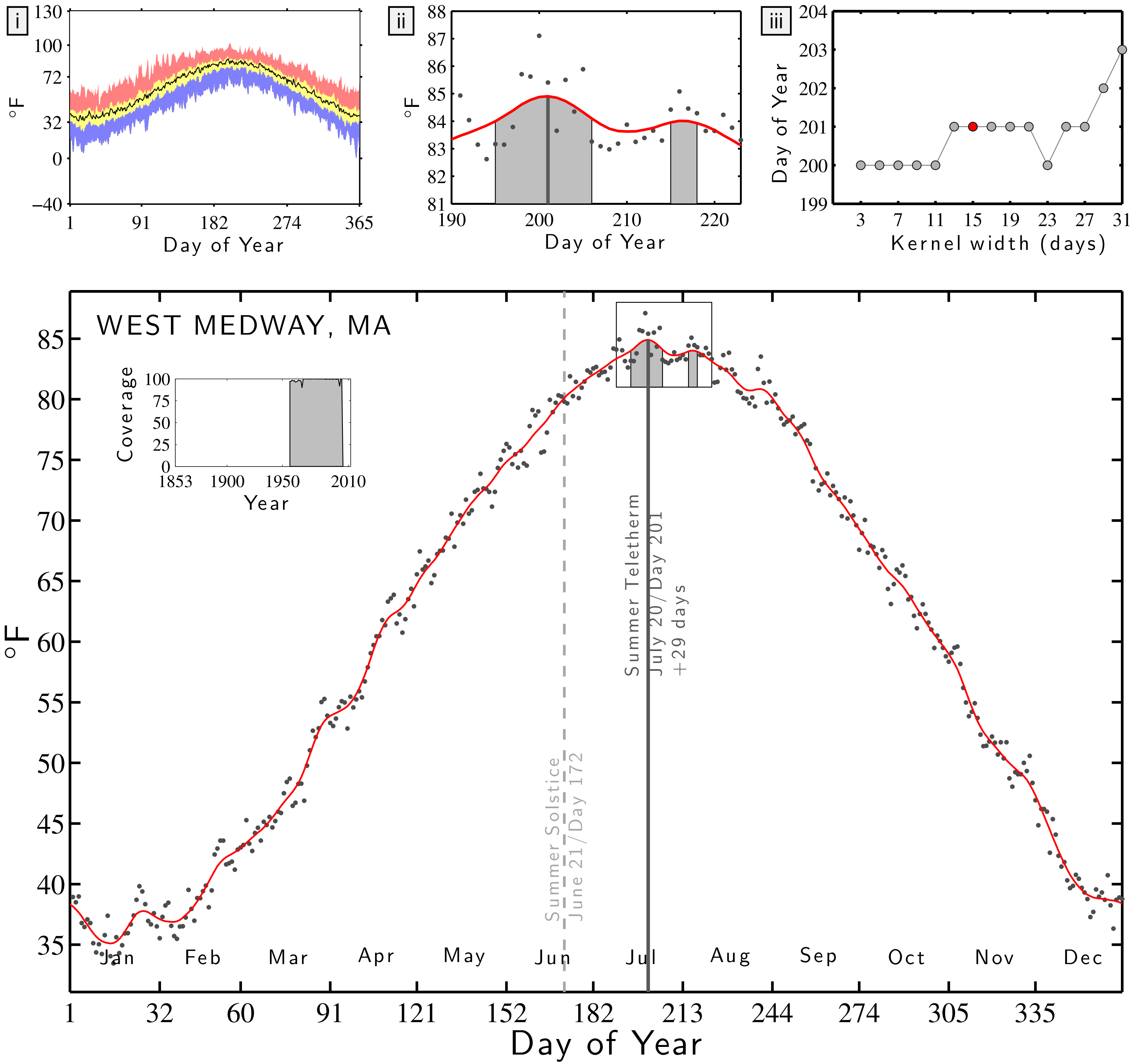

i
ii
iii
°F
Day of Year
Day of Year
Kernel width (days)
WEST MEDWAY, MA
Coverage
Year
Summer Solstice
June 21/Day 172
Summer Teletherm
July 20/Day 201
+29 days
Jan
Feb
Mar
Apr
May
Jun
Jul
Aug
Sep
Oct
Nov
Dec

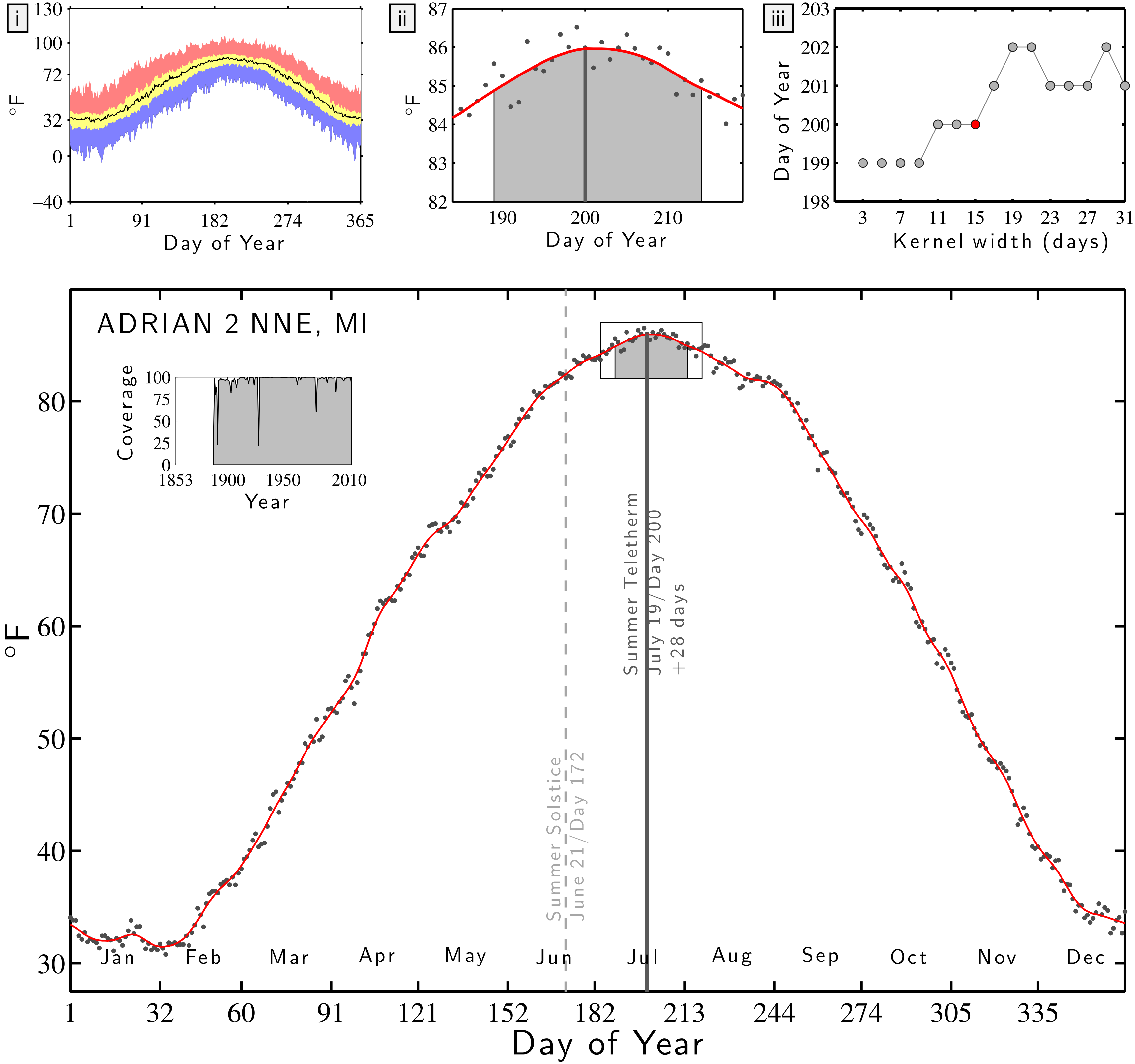

i
°F
Day of Year
ii
°F
Day of Year
iii
Day of Year
Kernel width (days)
ADRIAN 2 NNE, MI
Coverage
Year
Summer Solstice
June 21/Day 172
Summer Teletherm
July 19/Day 200
+28 days
Jan
Feb
Mar
Apr
May
Jun
Jul
Aug
Sep
Oct
Nov
Dec
°F
Day of Year

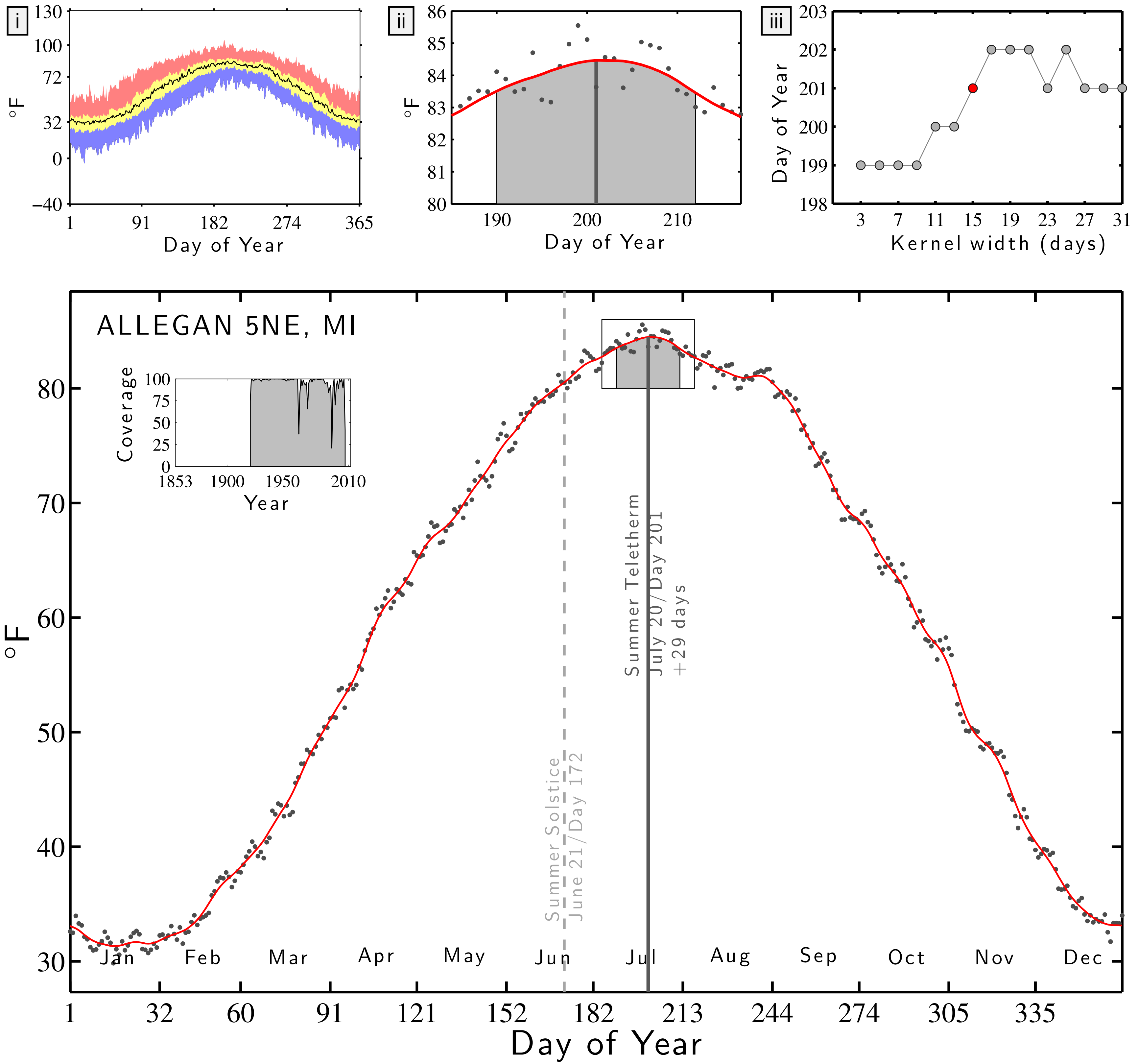
i
°F
Day of Year
ii
°F
Day of Year
iii
Day of Year
Kernel width (days)
ALLEGAN 5NE, MI
Coverage
Year
Summer Solstice
June 21/Day 172
Summer Teletherm
July 20/Day 201
+29 days
Jan
Feb
Mar
Apr
May
Jun
Jul
Aug
Sep
Oct
Nov
Dec
°F
Day of Year

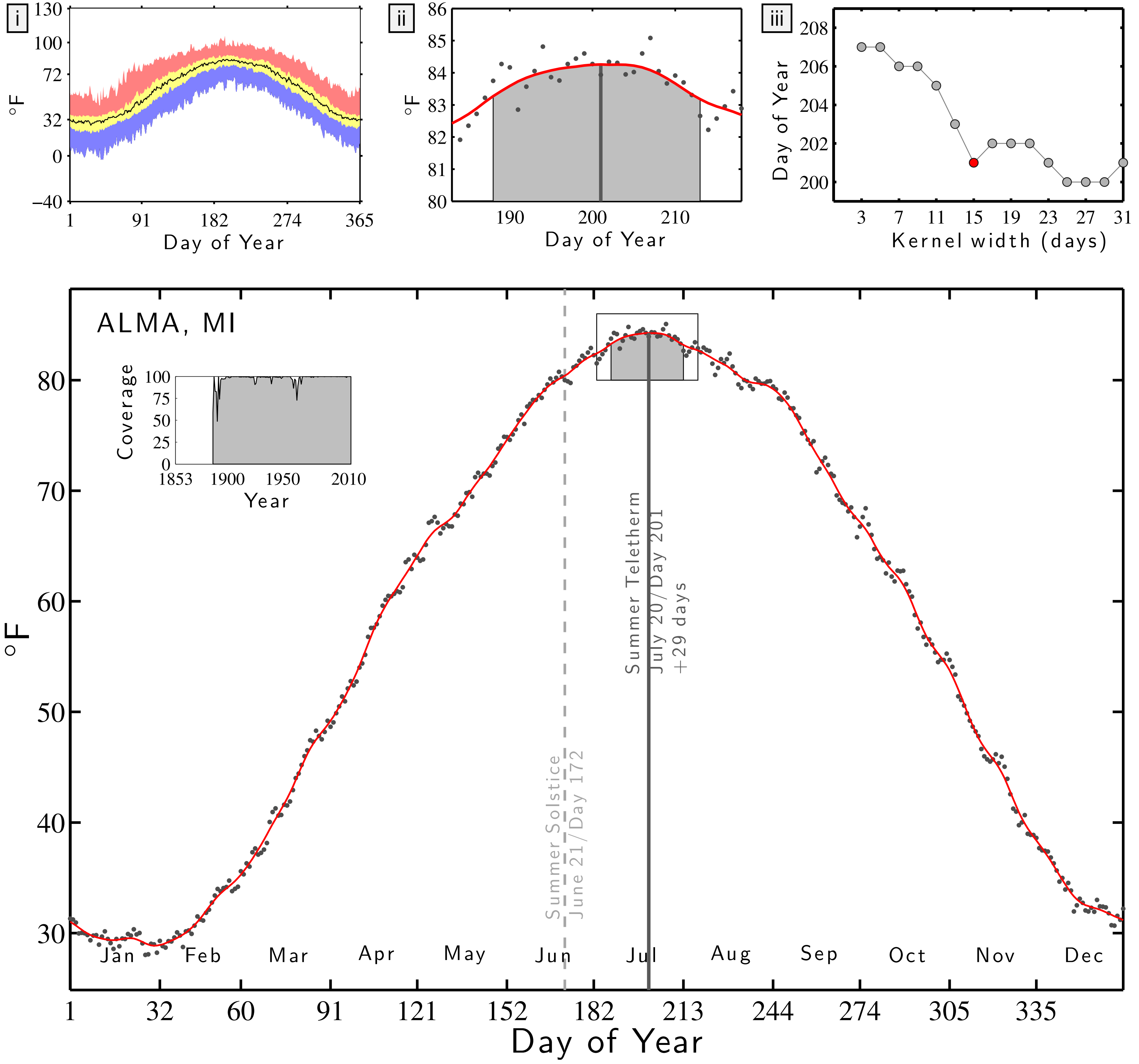

i
ii
iii
°F
Day of Year
Day of Year
Kernel width (days)
ALMA, MI
Coverage
Year
Summer Solstice
June 21/Day 172
Summer Teletherm
July 20/Day 201
+29 days
Jan
Feb
Mar
Apr
May
Jun
Jul
Aug
Sep
Oct
Nov
Dec
Day of Year

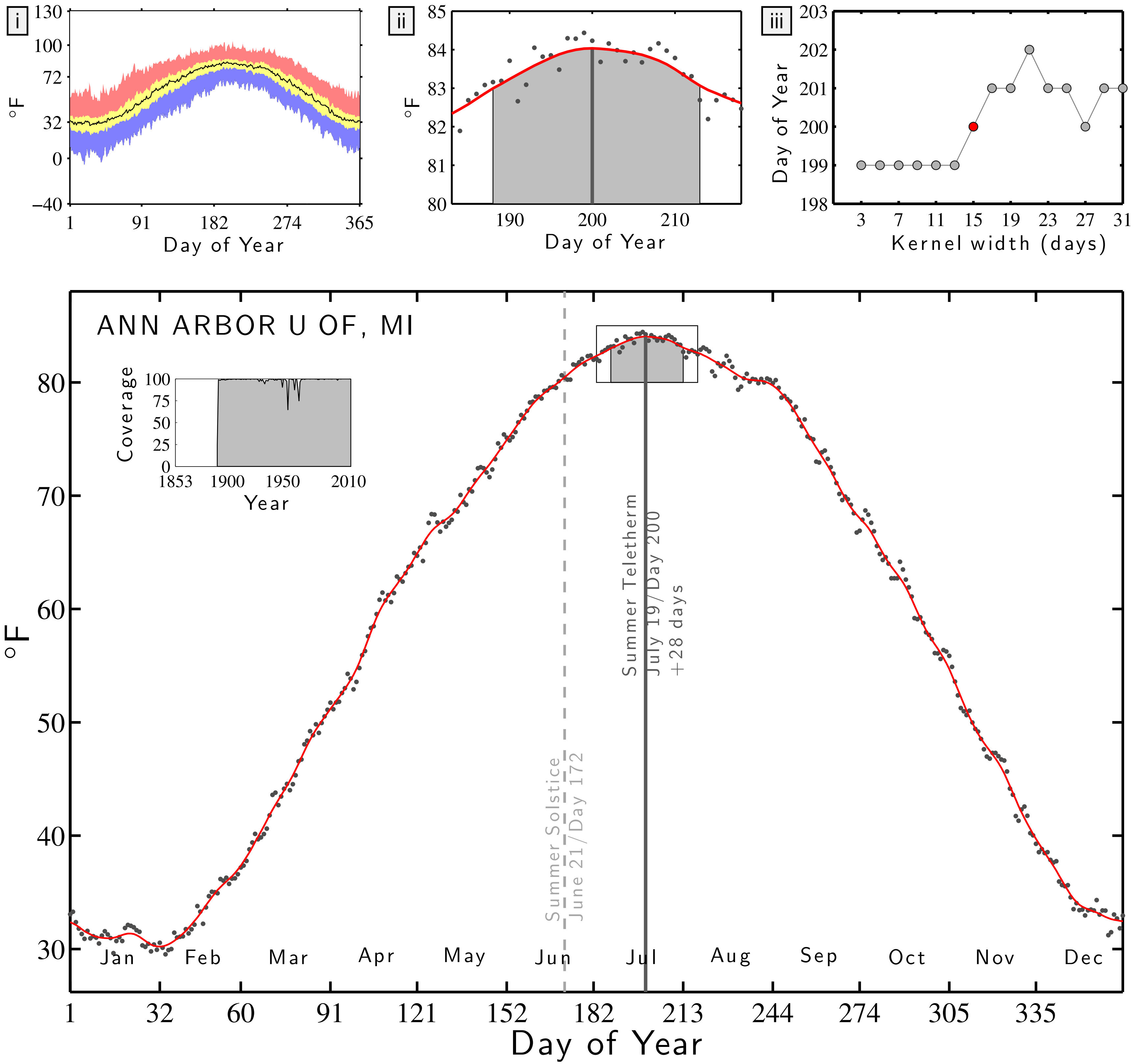

i
°F
Day of Year
ii
°F
Day of Year
iii
Day of Year
Kernel width (days)
ANN ARBOR U OF, MI
Coverage
Year
Summer Solstice
June 21/Day 172
Summer Teletherm
July 19/Day 200
+28 days
Jan
Feb
Mar
Apr
May
Jun
Jul
Aug
Sep
Oct
Nov
Dec
°F
Day of Year

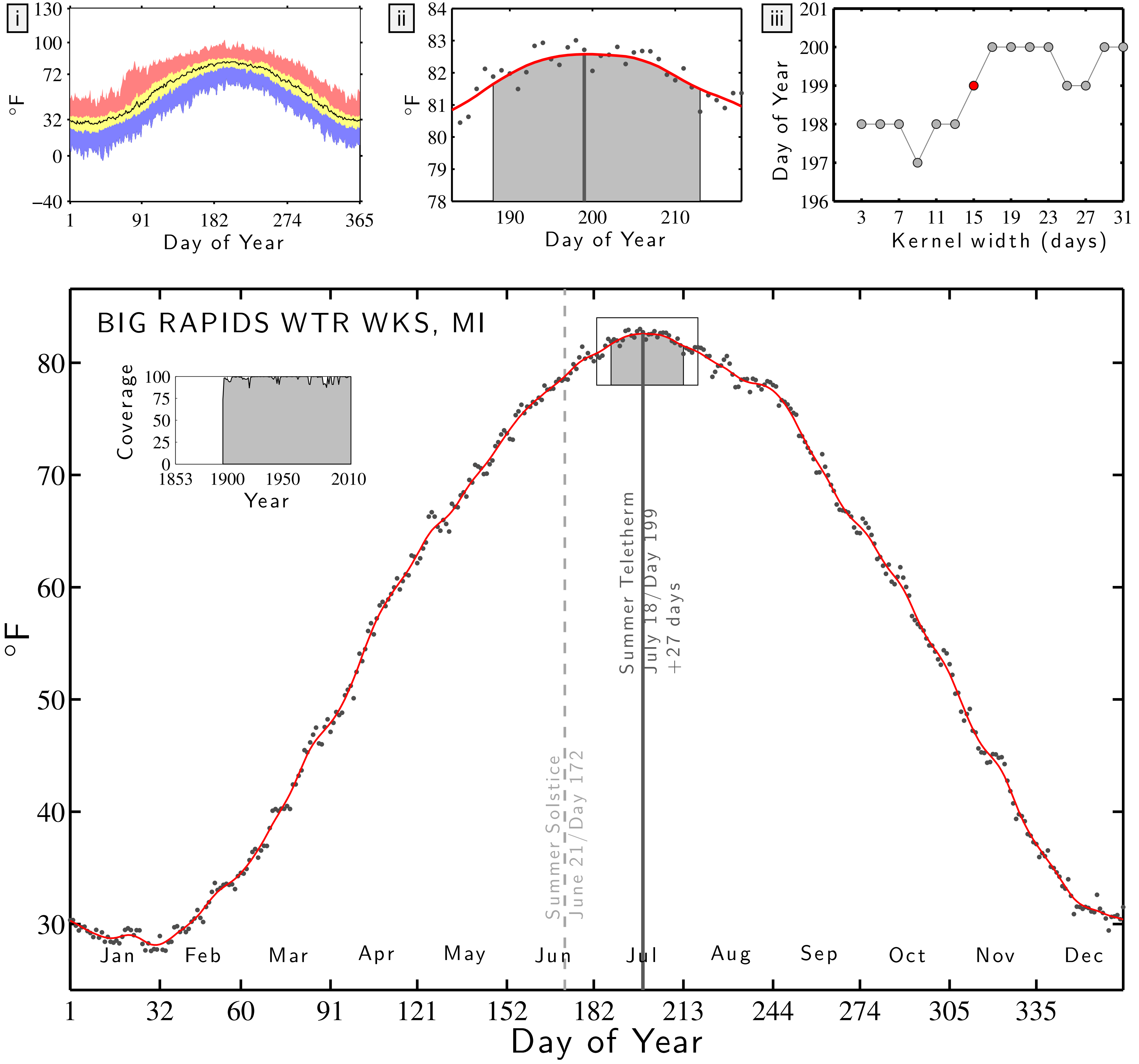

i
°F
130
100
72
32
0
−40
1
91
182
274
365
Day of Year
ii
°F
84
83
82
81
80
79
78
190
200
210
Day of Year
iii
Day of Year
201
200
199
198
197
196
3
7
11
15
19
23
27
31
Kernel width (days)
BIG RAPIDS WTR WKS, MI
Coverage
100
75
50
25
0
1853
1900
1950
2010
Year
°F
80
70
60
50
40
30
Summer Solstice
June 21/Day 172
Summer Teletherm
July 18/Day 199
+27 days
Jan
Feb
Mar
Apr
May
Jun
Jul
Aug
Sep
Oct
Nov
Dec
1
32
60
91
121
152
182
213
244
274
305
335
Day of Year

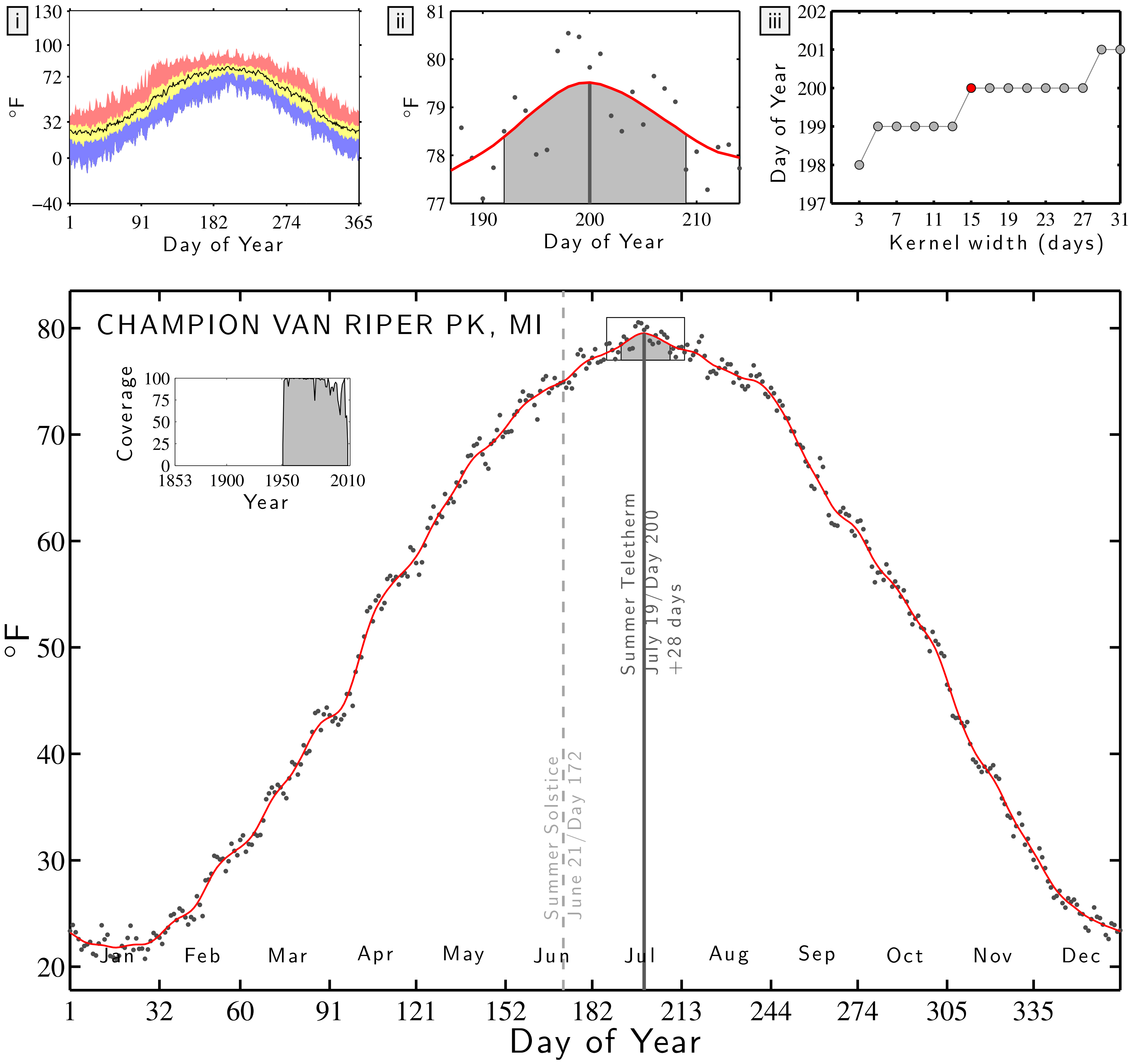

i
ii
iii
°F
Day of Year
Kernel width (days)
CHAMPION VAN RIPER PK, MI
Coverage
Year
Summer Solstice
June 21/Day 172
Summer Teletherm
July 19/Day 200
+28 days
Jan
Feb
Mar
Apr
May
Jun
Jul
Aug
Sep
Oct
Nov
Dec

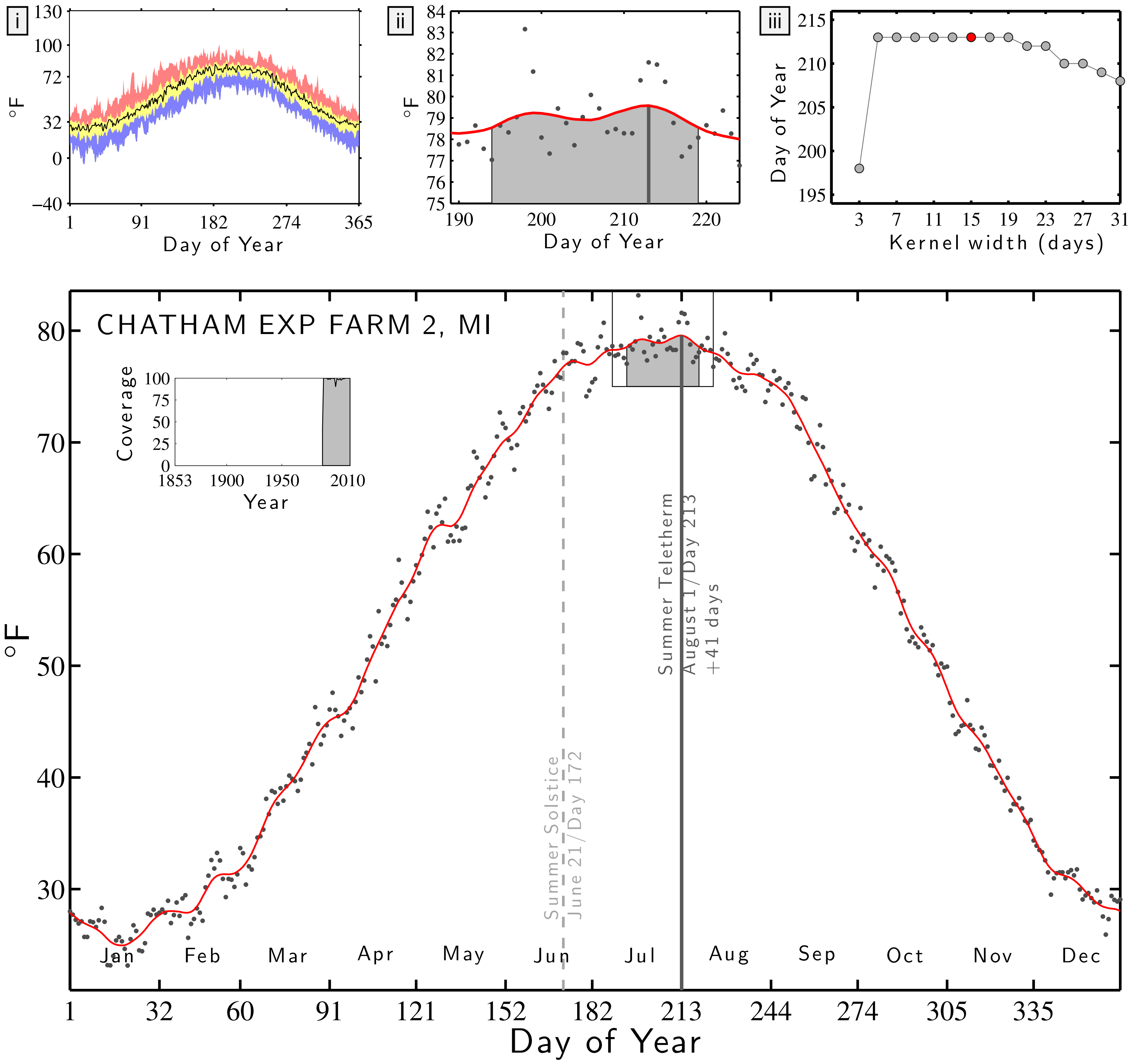

i
°F
Day of Year
ii
°F
Day of Year
iii
Day of Year
Kernel width (days)
CHATHAM EXP FARM 2, MI
Coverage
Year
Summer Solstice
June 21 / Day 172
Summer Teletherm
August 1 / Day 213
+41 days
Jan
Feb
Mar
Apr
May
Jun
Jul
Aug
Sep
Oct
Nov
Dec
°F
Day of Year

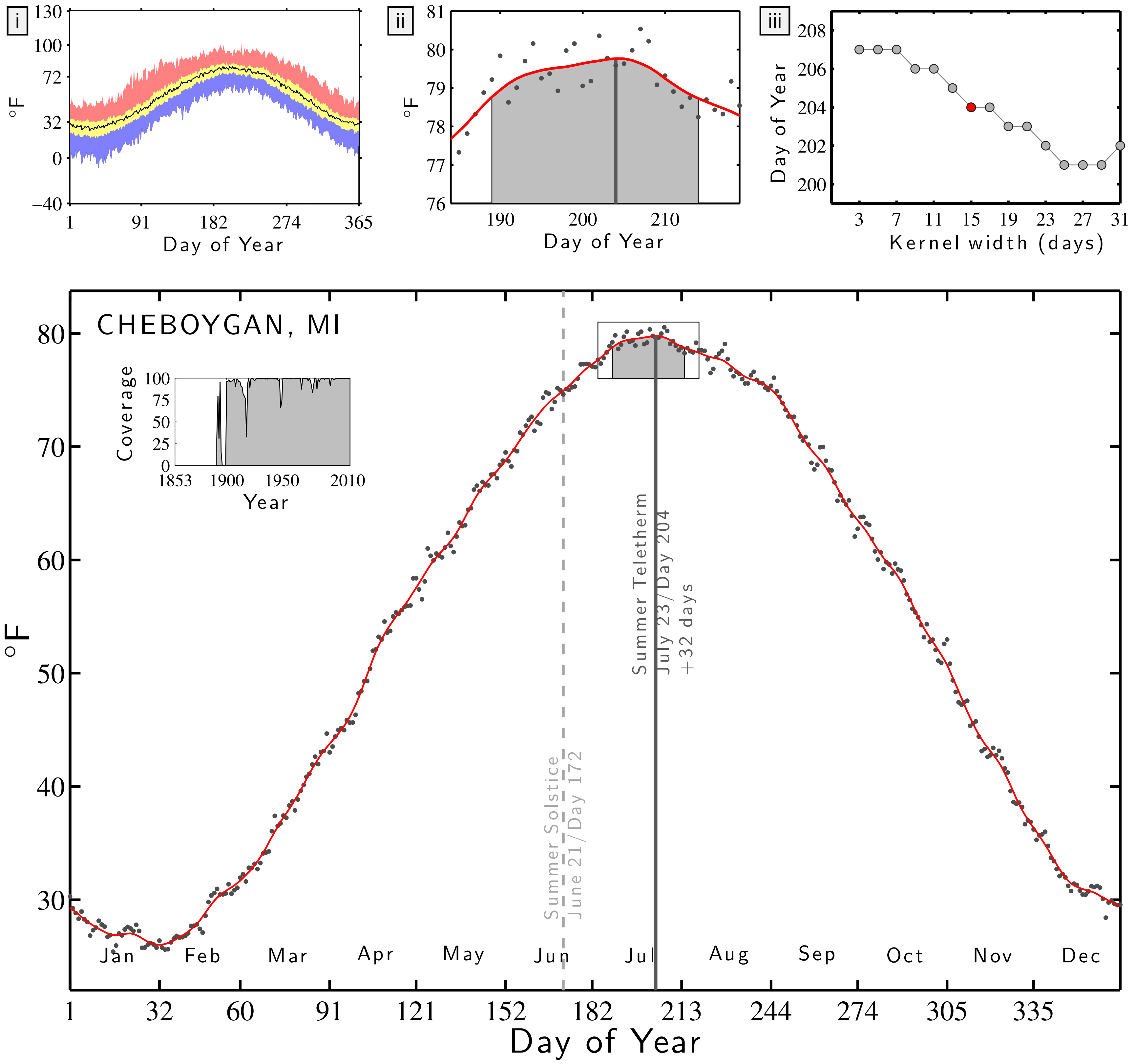

i
°F
Day of Year
ii
°F
Day of Year
iii
Day of Year
Kernel width (days)
CHEBOYGAN, MI
Coverage
Year
Summer Solstice
June 21 / Day 172
Summer Teletherm
July 23 / Day 204
+32 days
Jan Feb Mar Apr May Jun Jul Aug Sep Oct Nov Dec
°F
Day of Year

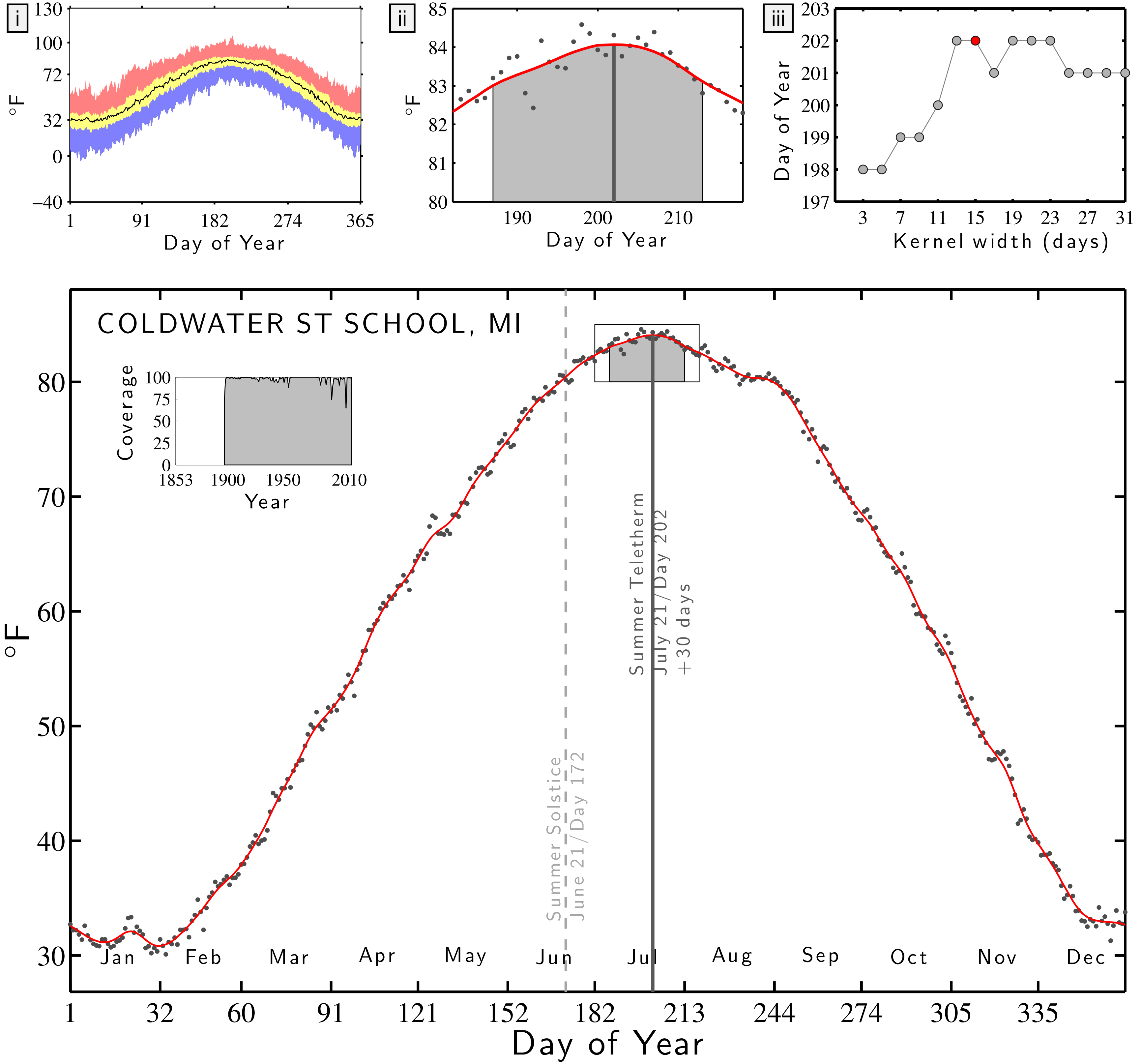

i
ii
iii
Day of Year
Kernel width (days)
°F
Day of Year
COLDWATER ST SCHOOL, MI
Coverage
Year
1853
1900
1950
2010
Summer Solstice
June 21/Day 172
Summer Teletherm
July 21/Day 202
+30 days
Jan
Feb
Mar
Apr
May
Jun
Jul
Aug
Sep
Oct
Nov
Dec

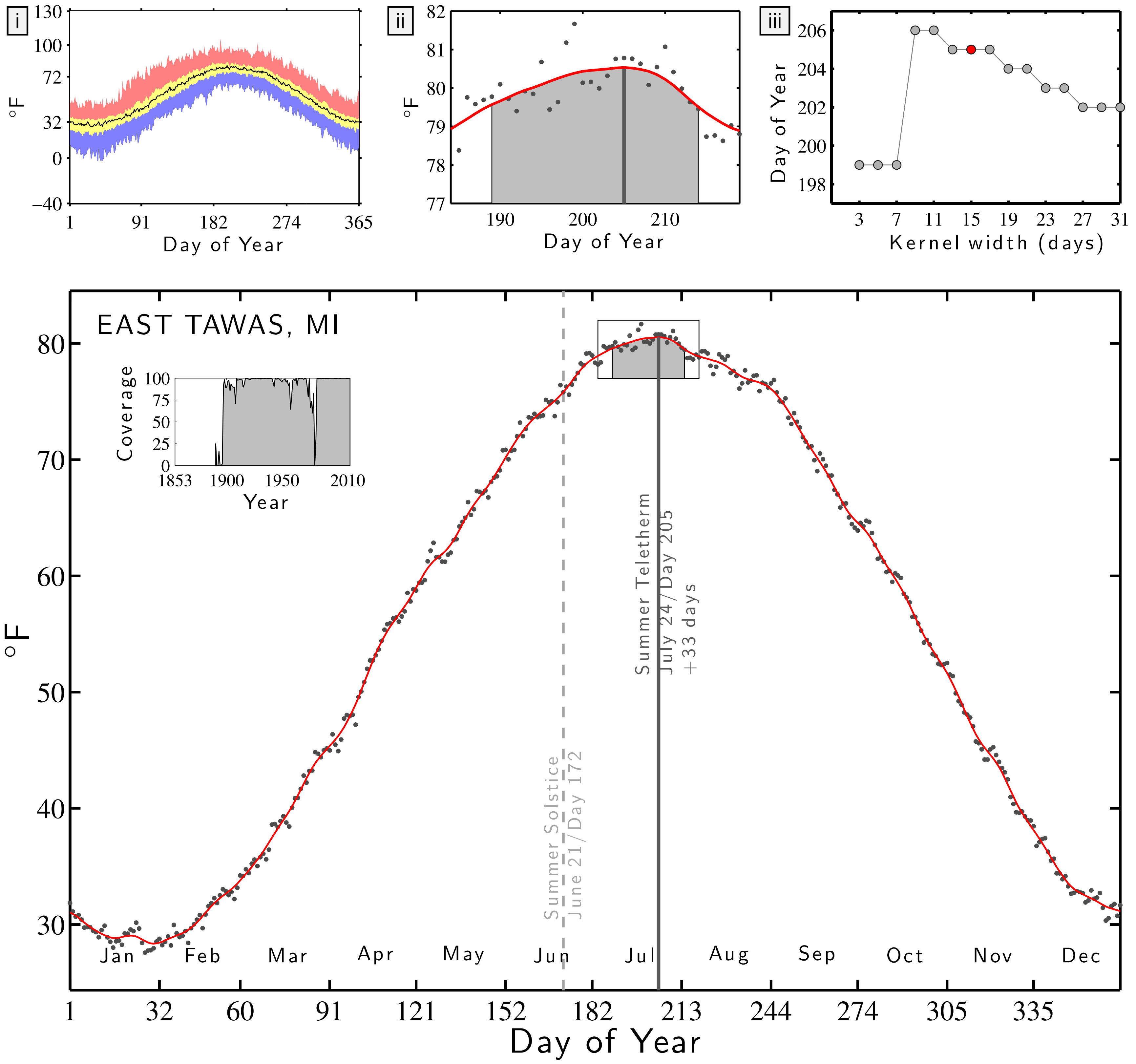

i
130
100
72
32
0
−40
°F
1
91
182
274
365
Day of Year
ii
82
81
80
79
78
77
°F
190
200
210
Day of Year
iii
206
204
202
200
198
Day of Year
3
7
11
15
19
23
27
31
Kernel width (days)
EAST TAWAS, MI
Coverage
100
75
50
25
0
1853
1900
1950
2010
Year
Summer Solstice
June 21/Day 172
Summer Teletherm
July 24/Day 205
+33 days
80
70
60
50
40
30
°F
Jan
Feb
Mar
Apr
May
Jun
Jul
Aug
Sep
Oct
Nov
Dec
1
32
60
91
121
152
182
213
244
274
305
335
Day of Year

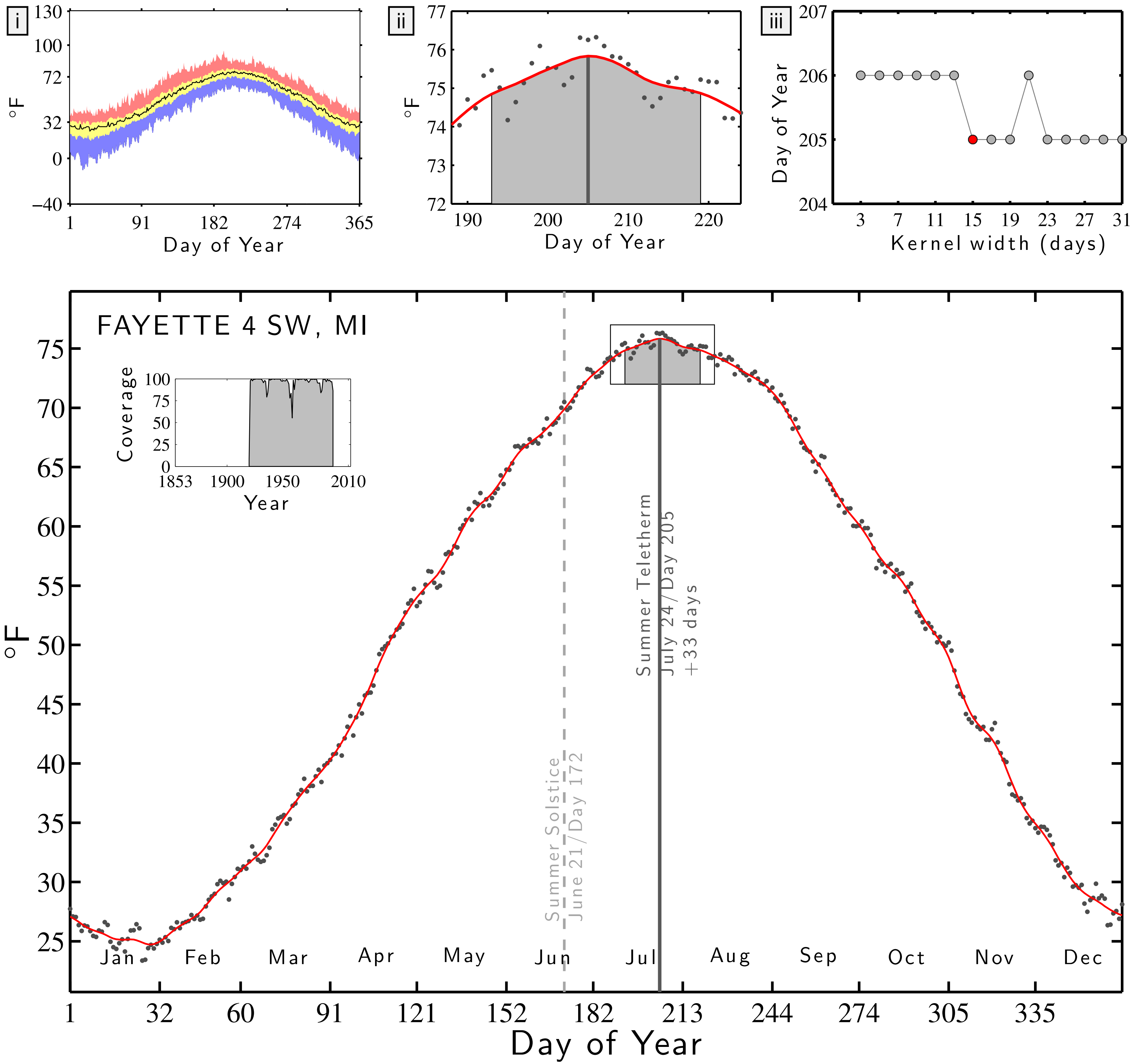
i
130
100
72
32
0
−40
°F
1
91
182
274
365
Day of Year
ii
77
76
75
74
73
72
°F
190
200
210
220
Day of Year
iii
207
206
205
204
Day of Year
3
7
11
15
19
23
27
31
Kernel width (days)
FAYETTE 4 SW, MI
Coverage
100
75
50
25
0
1853
1900
1950
2010
Year
Summer Solstice
June 21/Day 172
Summer Teletherm
July 24/Day 205
+33 days
75
70
65
60
55
50
45
40
35
30
25
°F
Jan
Feb
Mar
Apr
May
Jun
Jul
Aug
Sep
Oct
Nov
Dec
1
32
60
91
121
152
182
213
244
274
305
335
Day of Year

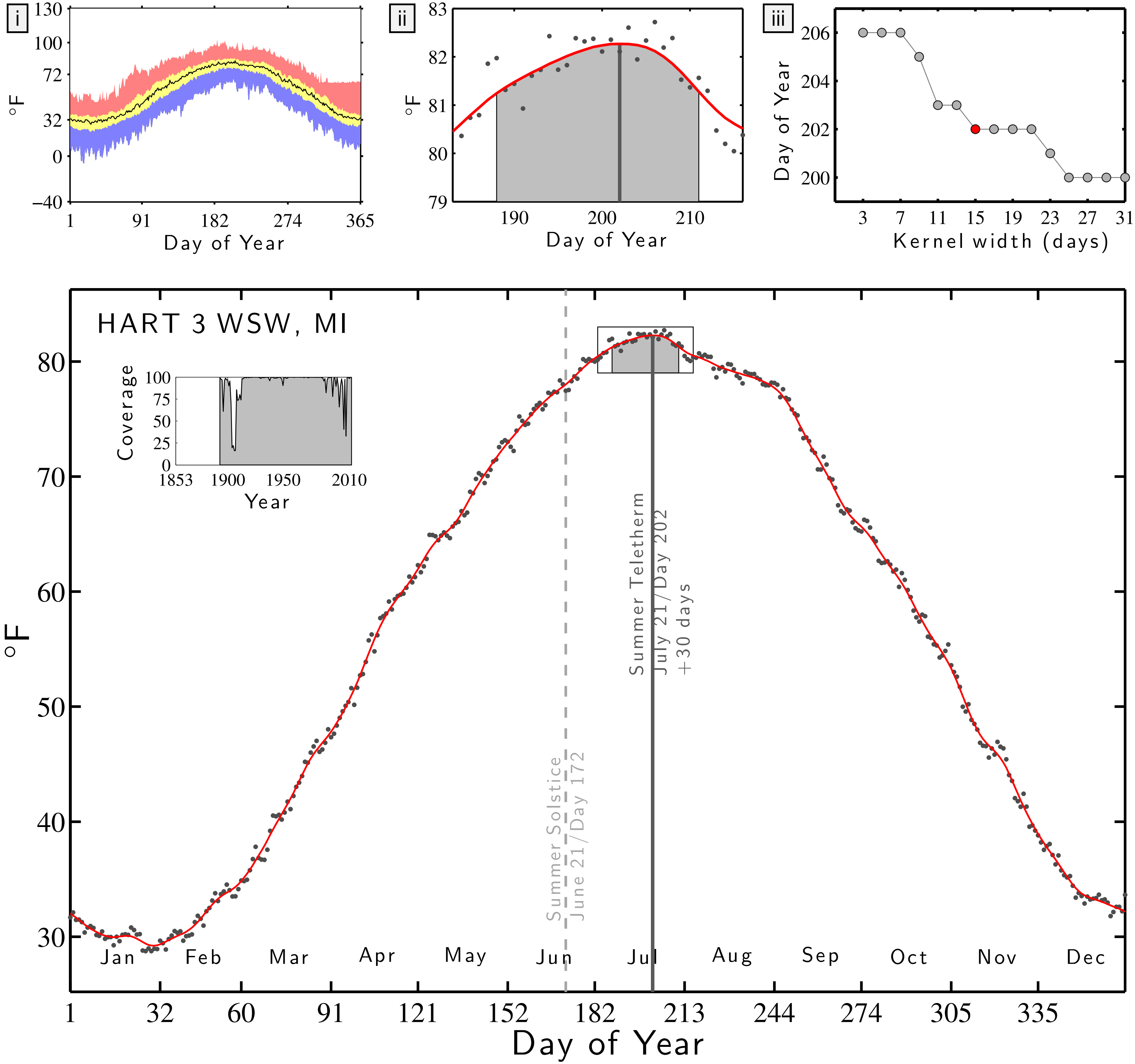

i
°F
130
100
72
32
0
−40
1
91
182
274
365
Day of Year
ii
°F
83
82
81
80
79
190
200
210
Day of Year
iii
Day of Year
206
204
202
200
3
7
11
15
19
23
27
31
Kernel width (days)
HART 3 WSW, MI
Coverage
100
75
50
25
0
1853
1900
1950
2010
Year
°F
80
70
60
50
40
30
Summer Solstice
June 21 / Day 172
Summer Teletherm
July 21 / Day 202
+30 days
Jan
Feb
Mar
Apr
May
Jun
Jul
Aug
Sep
Oct
Nov
Dec
1
32
60
91
121
152
182
213
244
274
305
335
Day of Year

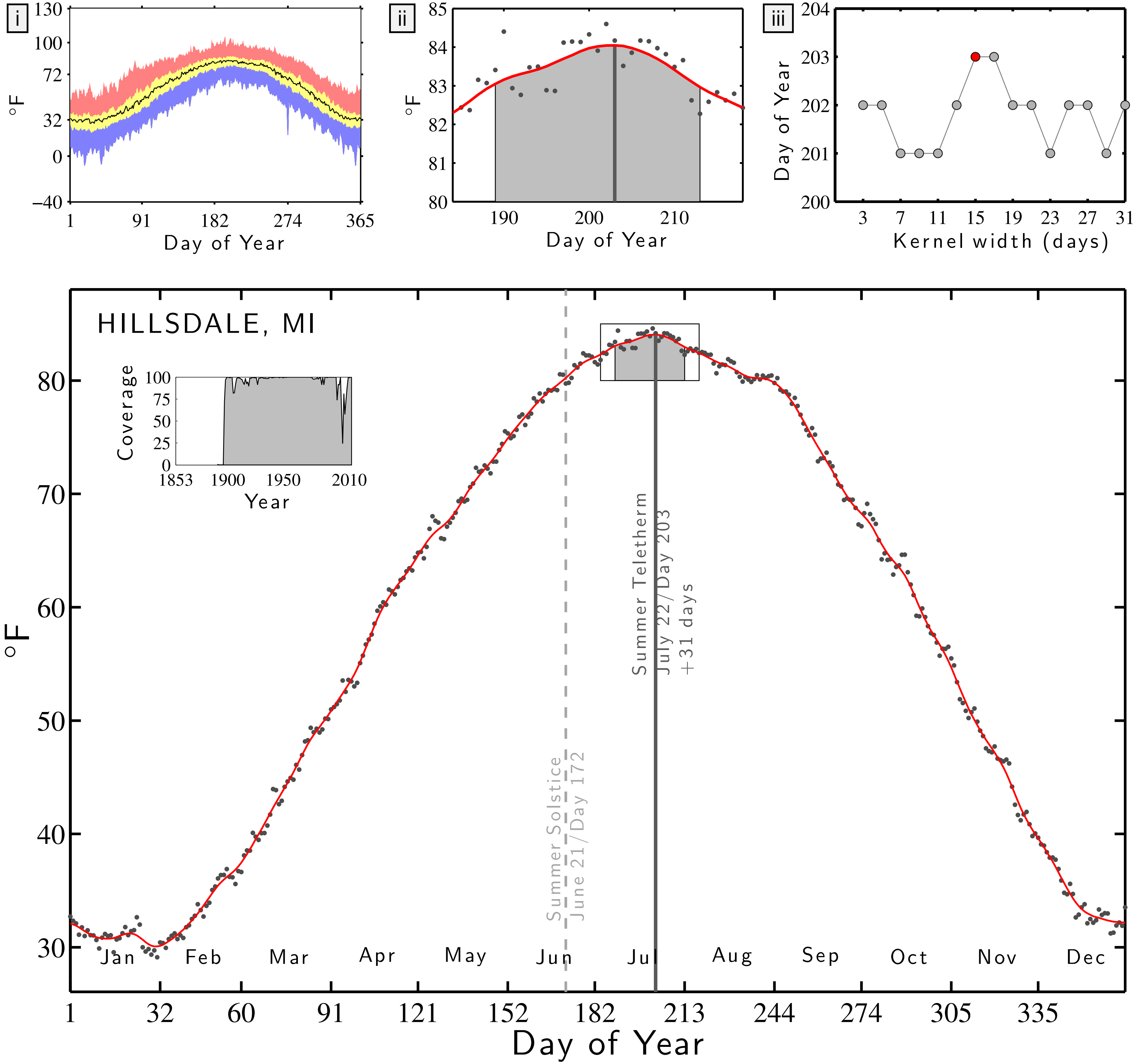

i
°F
Day of Year
ii
°F
Day of Year
iii
Day of Year
Kernel width (days)
HILLSDALE, MI
Coverage
Year
Summer Solstice
June 21/Day 172
Summer Teletherm
July 22/Day 203
+31 days
Jan
Feb
Mar
Apr
May
Jun
Jul
Aug
Sep
Oct
Nov
Dec
°F
Day of Year

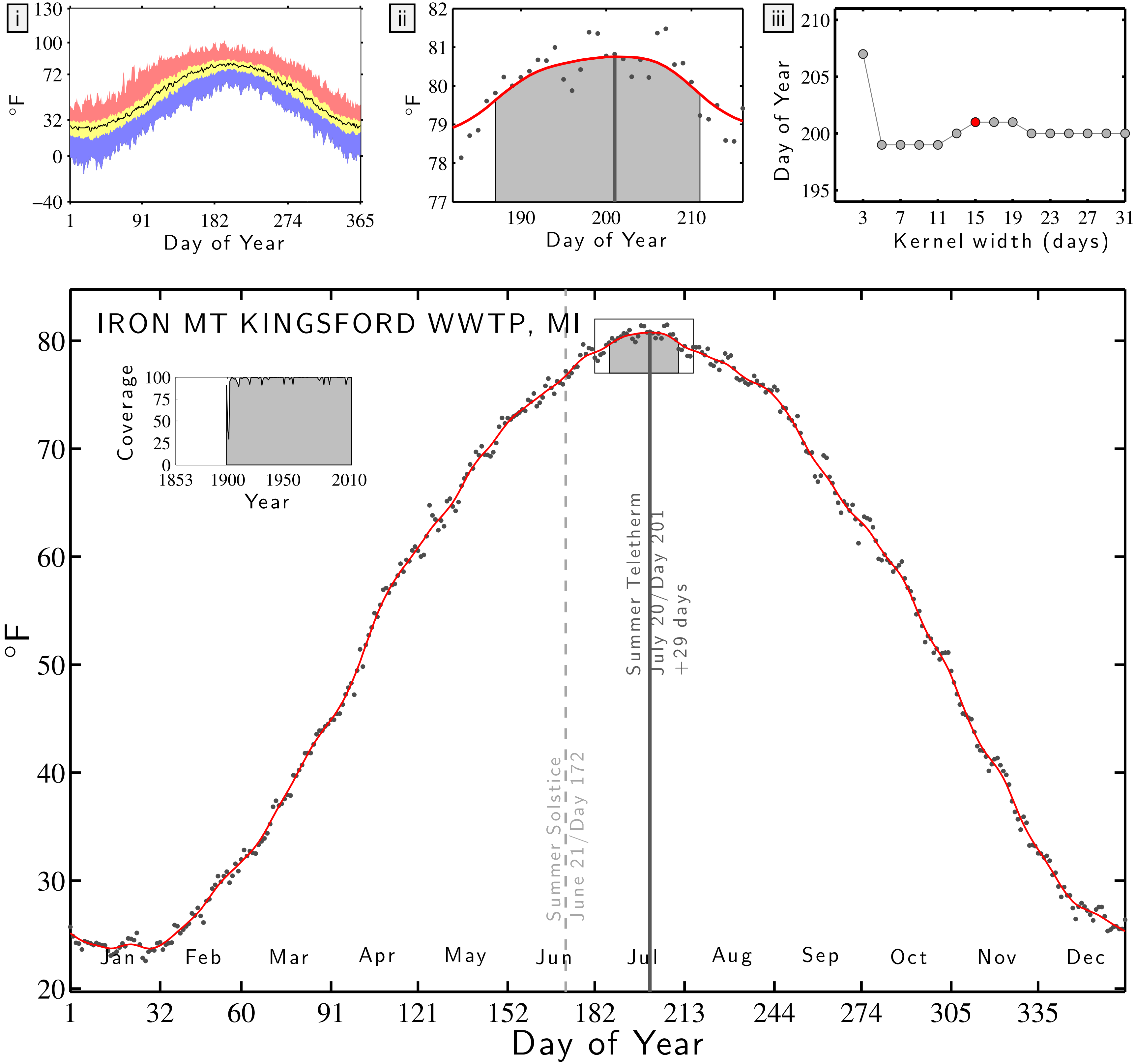

i
°F
Day of Year
ii
°F
Day of Year
iii
Day of Year
Kernel width (days)
IRON MT KINGSFORD WWTP, MI
Coverage
Year
Summer Solstice
June 21/Day 172
Summer Teletherm
July 20/Day 201
+29 days
Jan
Feb
Mar
Apr
May
Jun
Jul
Aug
Sep
Oct
Nov
Dec
°F
Day of Year

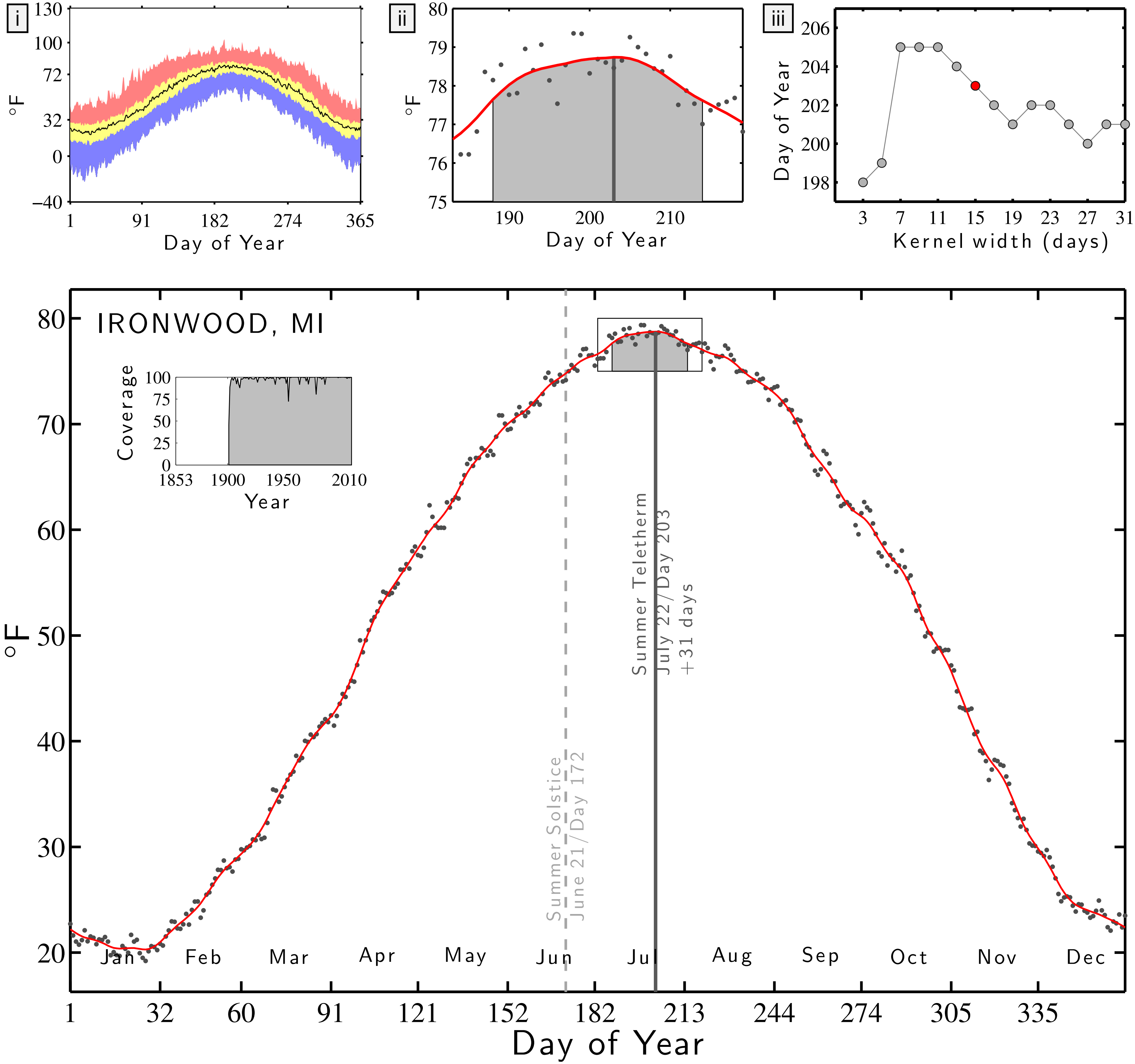

i
ii
iii
IRONWOOD, MI
Coverage
Year
Summer Solstice
June 21/Day 172
Summer Teletherm
July 22/Day 203
+31 days
Jan Feb Mar Apr May Jun Jul Aug Sep Oct Nov Dec
Day of Year
°F
Day of Year
Kernel width (days)

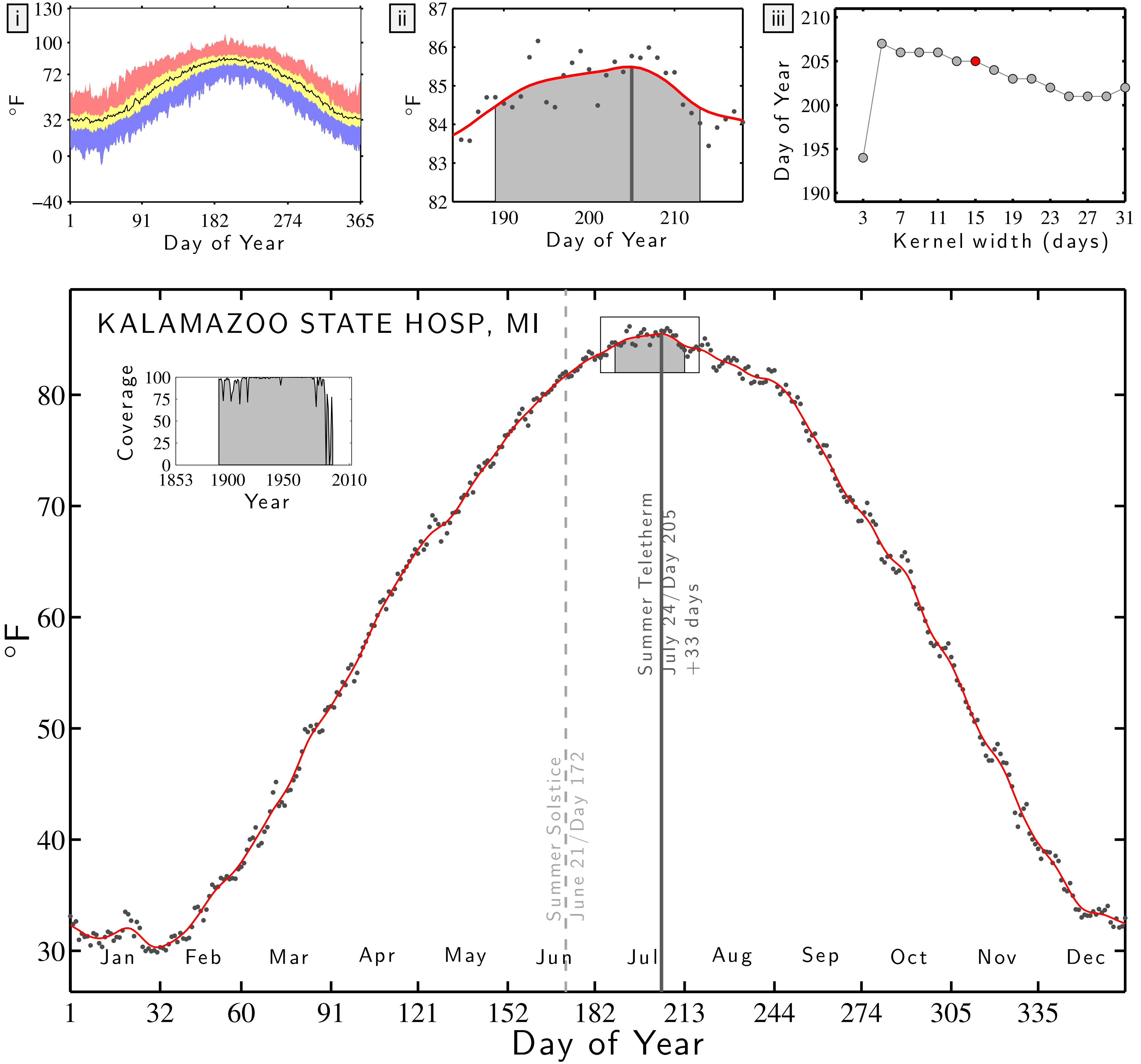

i
ii
iii
°F
Day of Year
Day of Year
Kernel width (days)
KALAMAZOO STATE HOSP, MI
Coverage
Year
Summer Solstice
June 21 / Day 172
Summer Teletherm
July 24 / Day 205
+33 days
Jan
Feb
Mar
Apr
May
Jun
Jul
Aug
Sep
Oct
Nov
Dec
°F
Day of Year

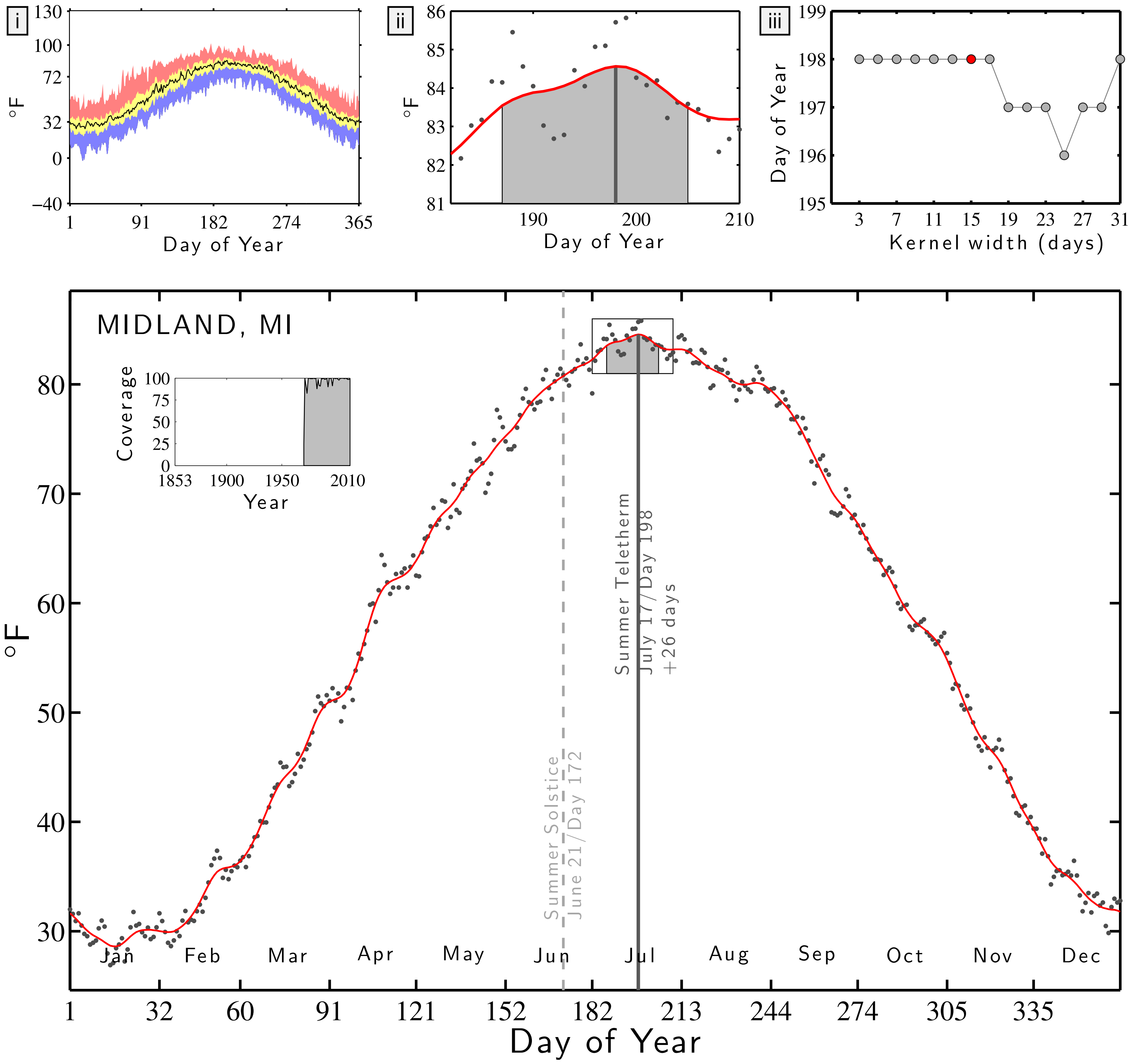

i
°F
Day of Year
ii
°F
Day of Year
iii
Day of Year
Kernel width (days)
MIDLAND, MI
Coverage
Year
Summer Solstice
June 21/Day 172
Summer Teletherm
July 17/Day 198
+26 days
Jan
Feb
Mar
Apr
May
Jun
Jul
Aug
Sep
Oct
Nov
Dec
°F
Day of Year

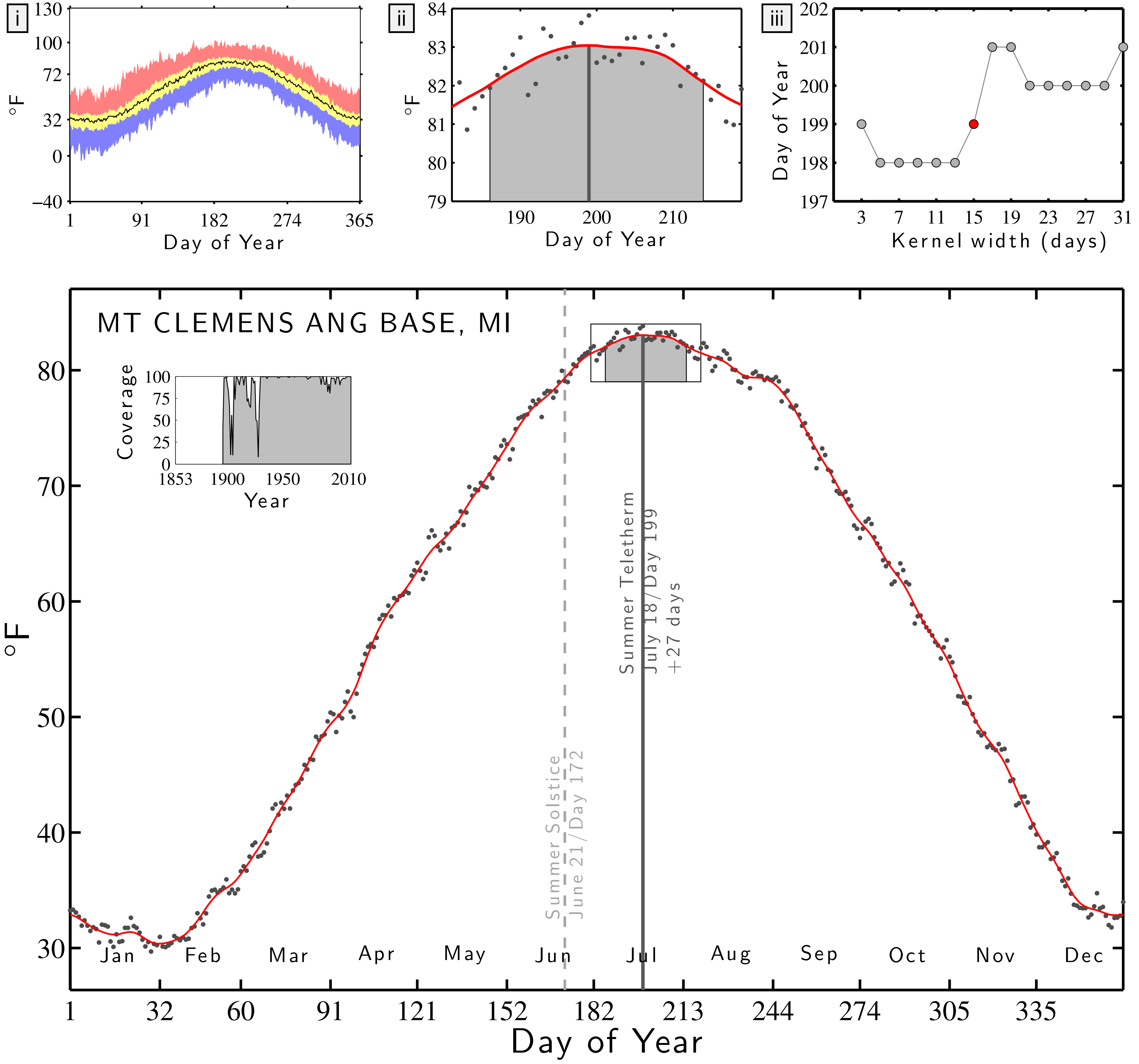

i
ii
iii
°F
Day of Year
Day of Year
Kernel width (days)
MT CLEMENS ANG BASE, MI
Coverage
Year
1853
1900
1950
2010
Summer Solstice
June 21/Day 172
Summer Teletherm
July 18/Day 199
+27 days
Jan
Feb
Mar
Apr
May
Jun
Jul
Aug
Sep
Oct
Nov
Dec

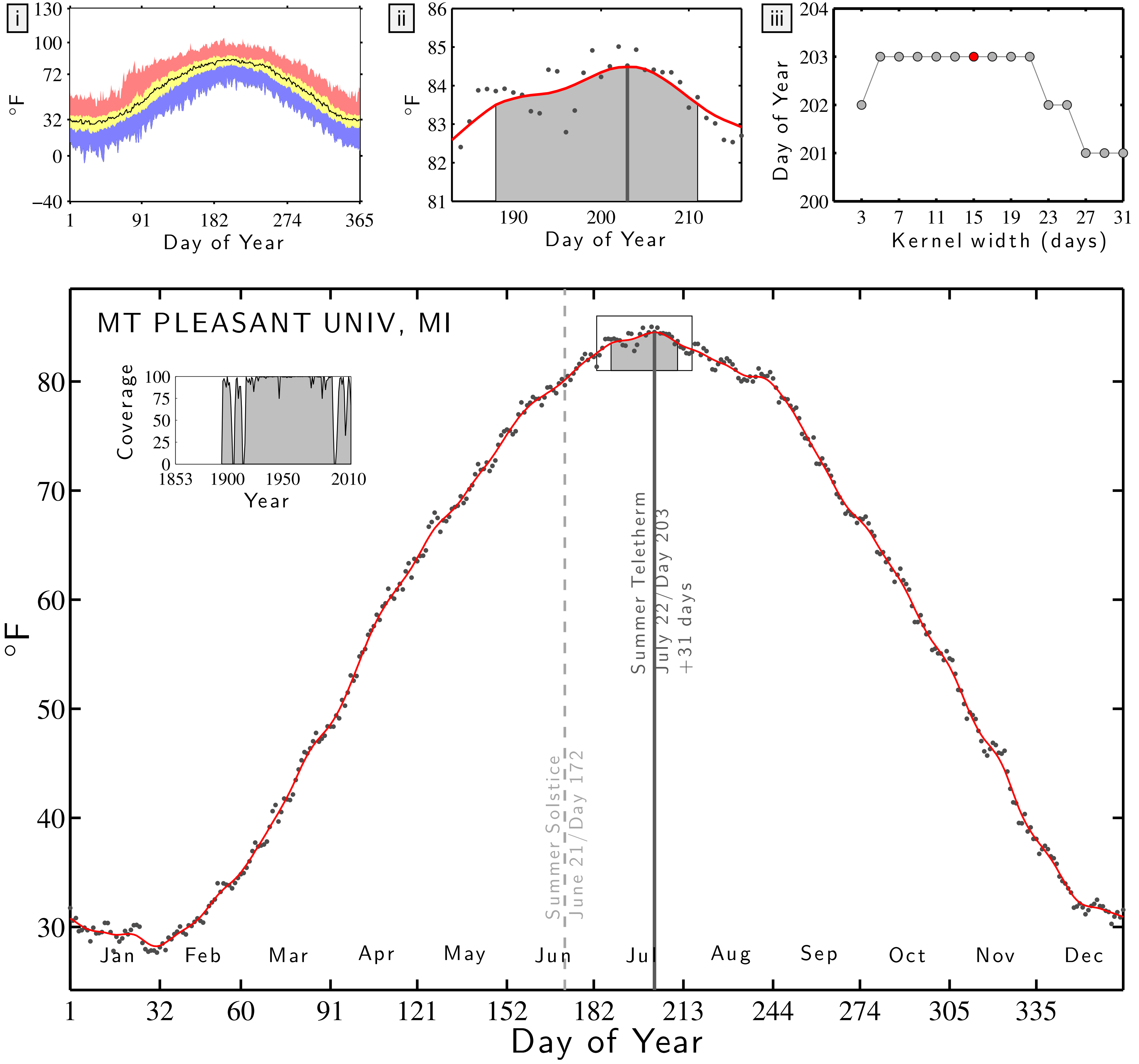

i
°F
Day of Year
ii
°F
Day of Year
iii
Day of Year
Kernel width (days)
MT PLEASANT UNIV, MI
Coverage
Year
Summer Solstice
June 21/Day 172
Summer Teletherm
July 22/Day 203
+31 days
Jan
Feb
Mar
Apr
May
Jun
Jul
Aug
Sep
Oct
Nov
Dec
°F
Day of Year

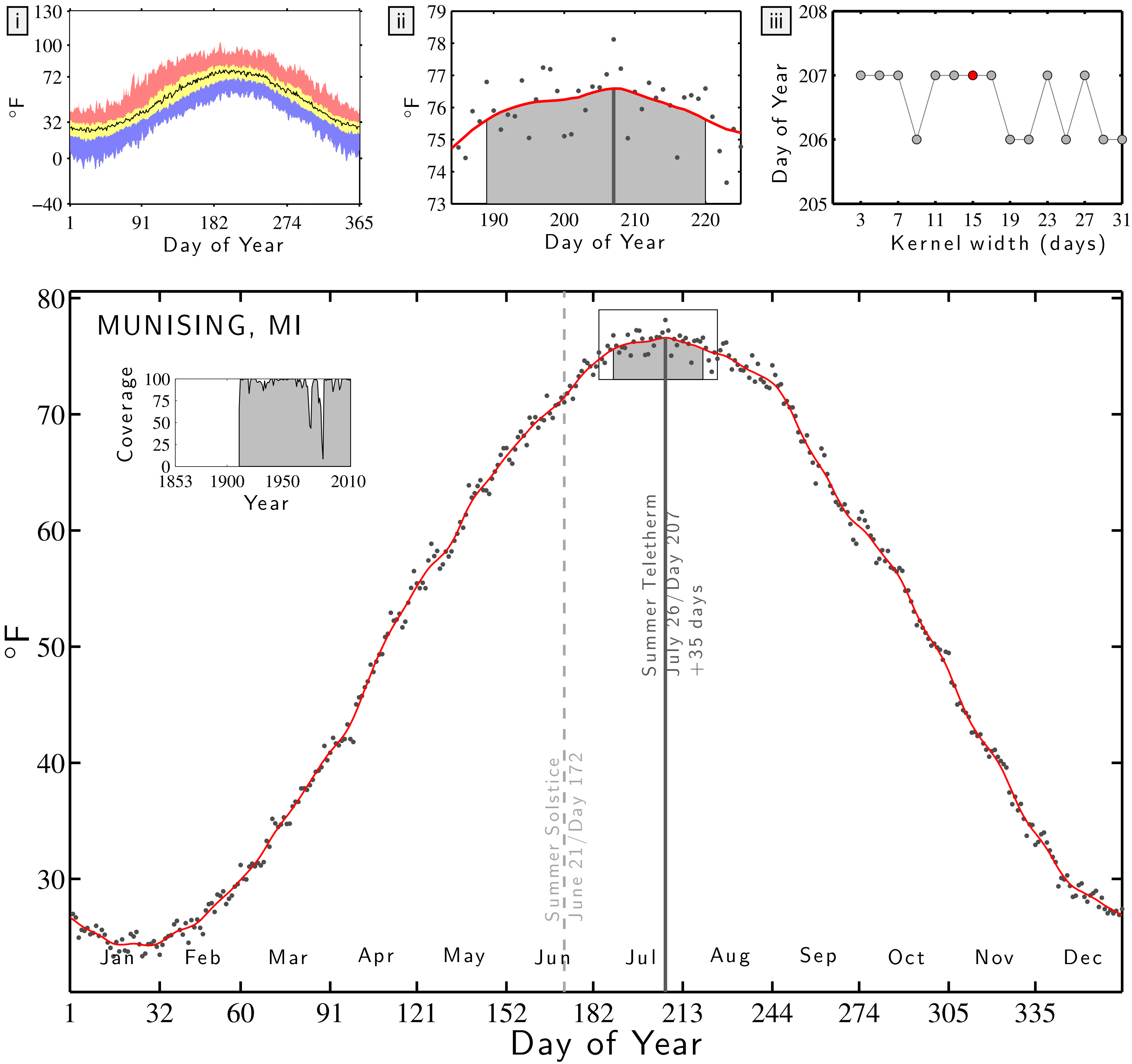

i
°F
Day of Year
ii
°F
Day of Year
iii
Day of Year
Kernel width (days)
MUNISING, MI
Coverage
Year
Summer Solstice
June 21 / Day 172
Summer Teletherm
July 26 / Day 207
+35 days
Jan
Feb
Mar
Apr
May
Jun
Jul
Aug
Sep
Oct
Nov
Dec
°F
Day of Year

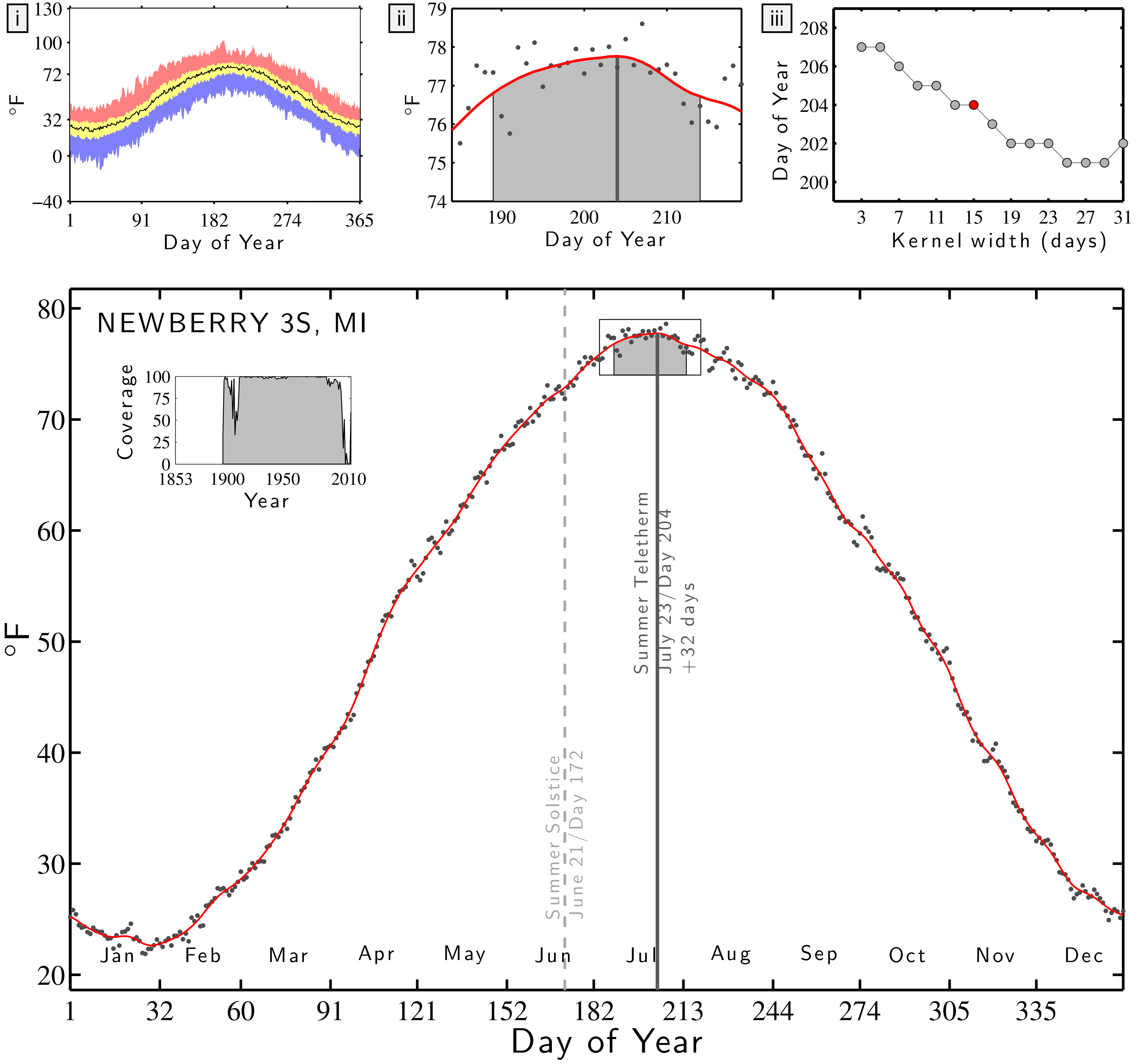

i
°F
Day of Year
ii
°F
Day of Year
iii
Day of Year
Kernel width (days)
NEWBERRY 3S, MI
Coverage
Year
Summer Solstice
June 21 / Day 172
Summer Teletherm
July 23 / Day 204
+32 days
Jan
Feb
Mar
Apr
May
Jun
Jul
Aug
Sep
Oct
Nov
Dec
°F
Day of Year

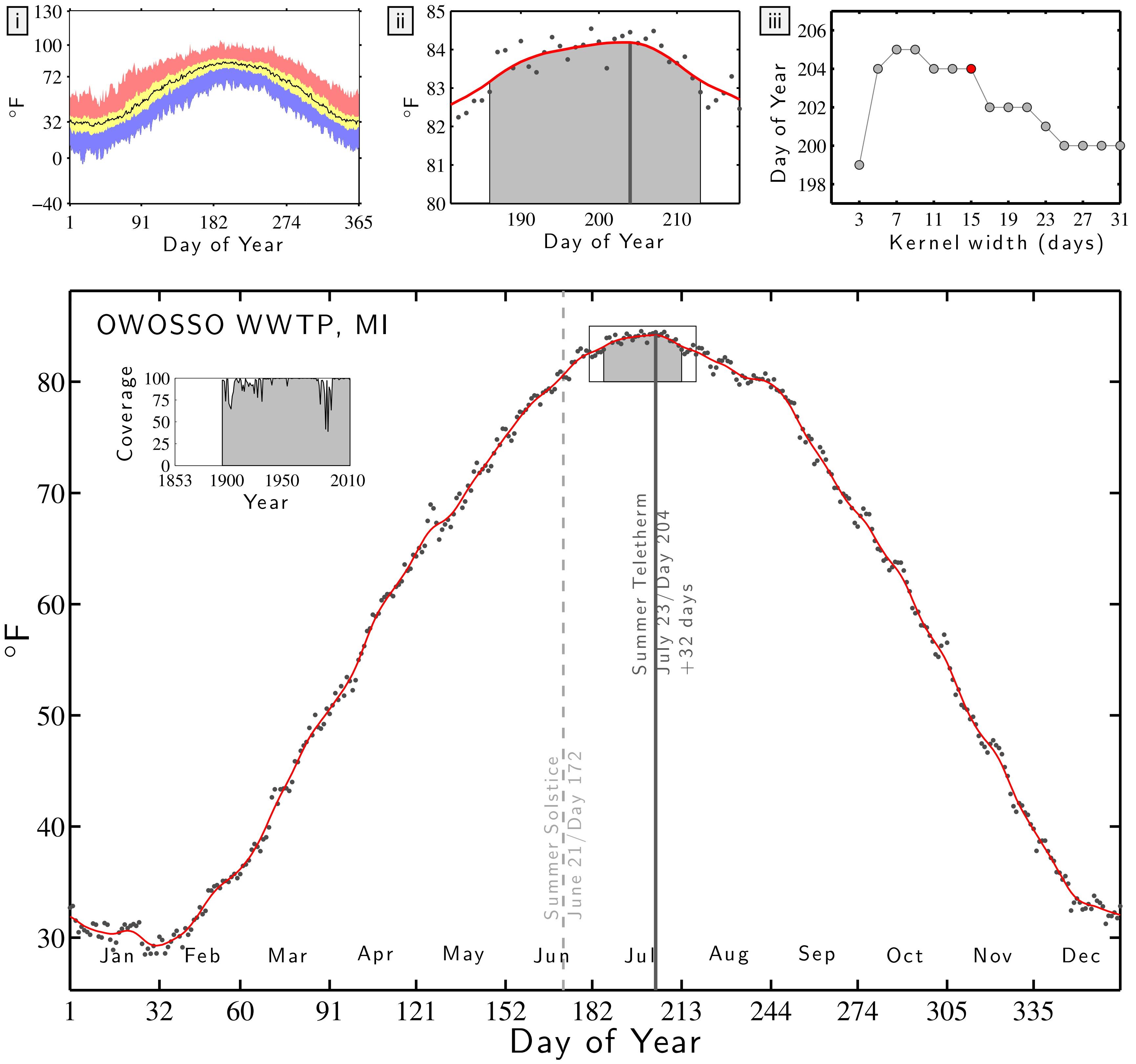
i
°F
Day of Year
ii
°F
Day of Year
iii
Day of Year
Kernel width (days)
OWOSSO WWTP, MI
Coverage
Year
Summer Solstice
June 21 / Day 172
Summer Teletherm
July 23 / Day 204
+32 days
Jan
Feb
Mar
Apr
May
Jun
Jul
Aug
Sep
Oct
Nov
Dec
°F
Day of Year

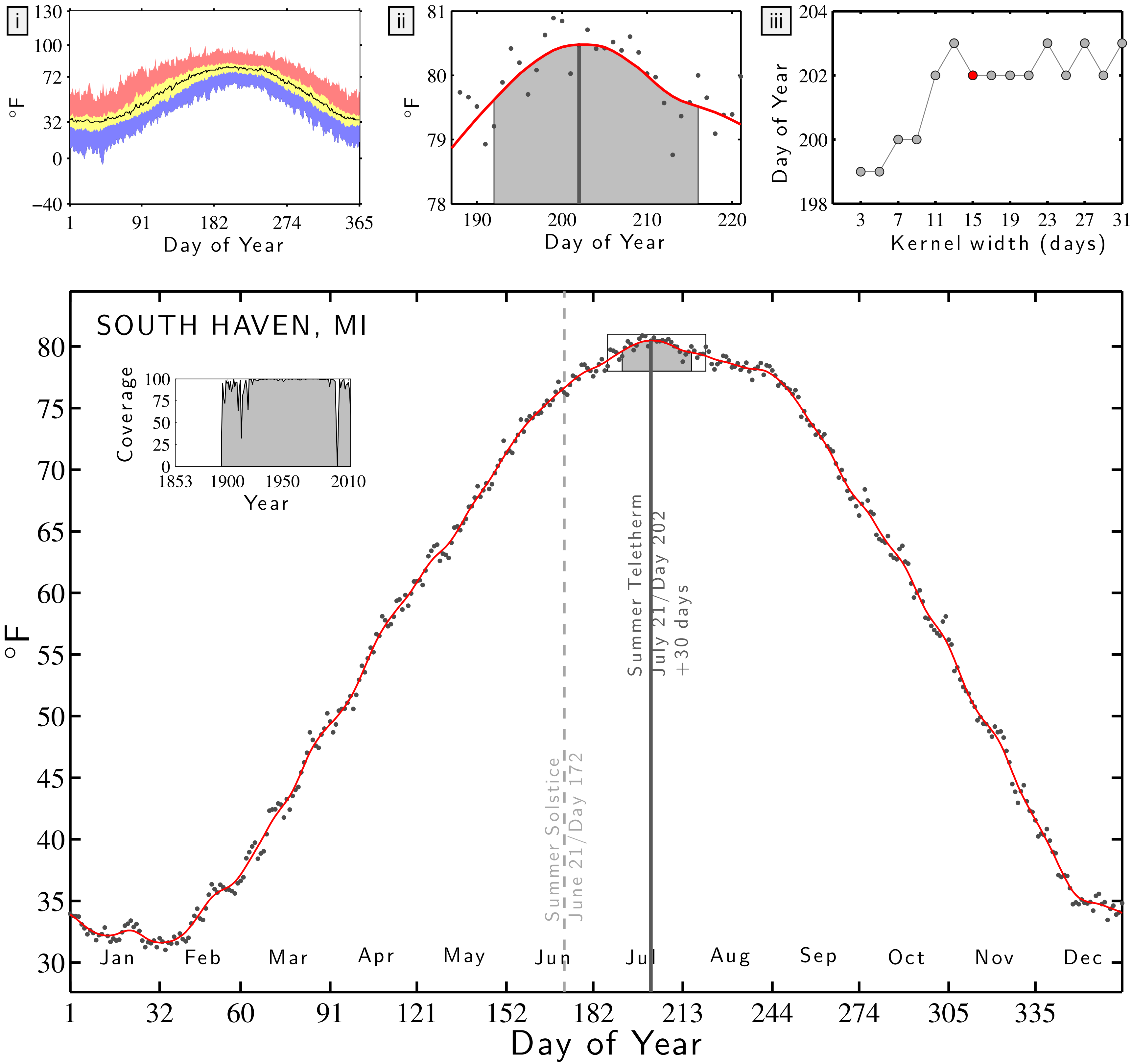
i
°F
Day of Year
ii
°F
Day of Year
iii
Day of Year
Kernel width (days)
SOUTH HAVEN, MI
Coverage
Year
Summer Solstice
June 21 / Day 172
Summer Teletherm
July 21 / Day 202
+30 days
Jan
Feb
Mar
Apr
May
Jun
Jul
Aug
Sep
Oct
Nov
Dec
°F
Day of Year

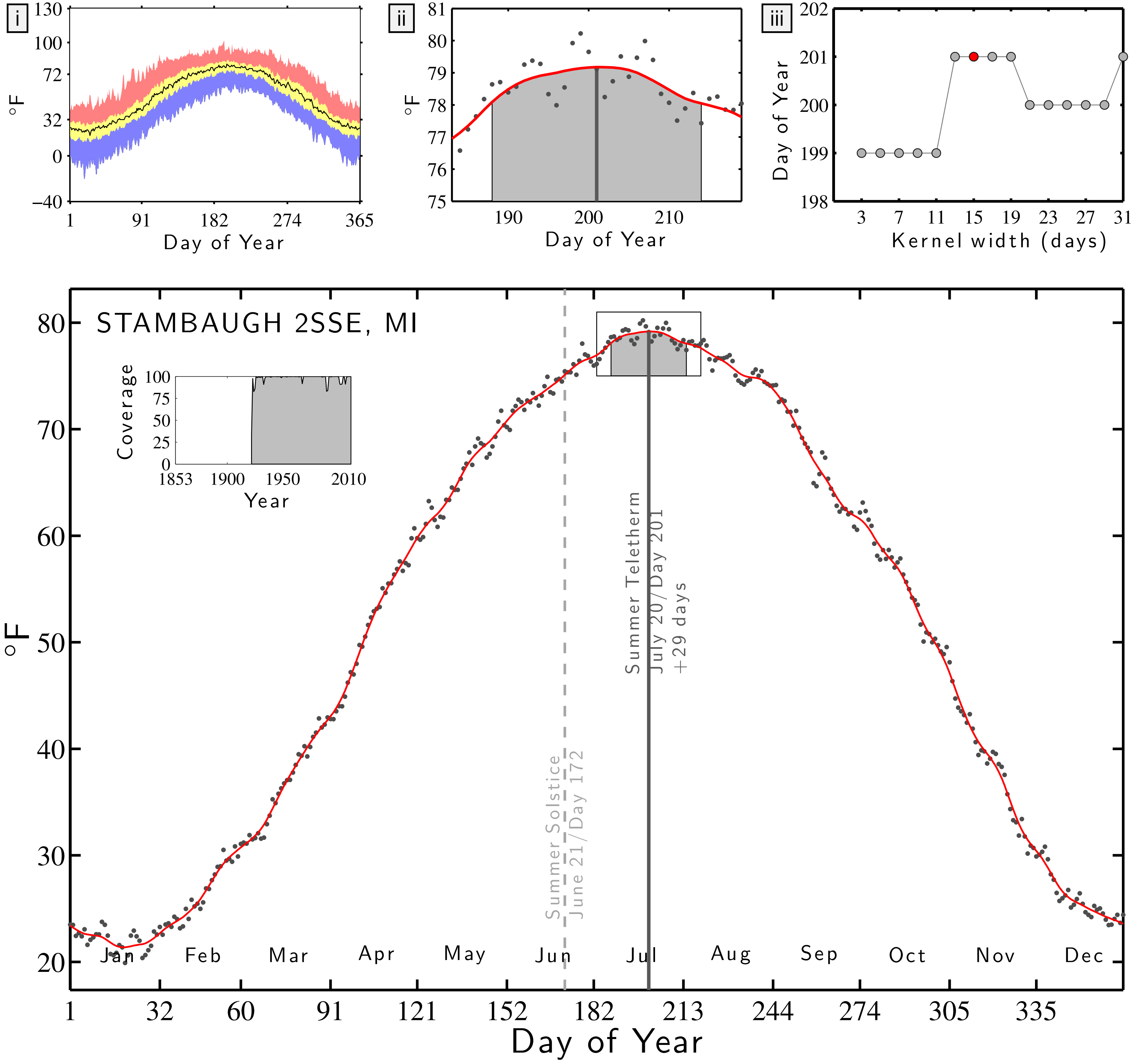

i
ii
iii
°F
Day of Year
Day of Year
Kernel width (days)
STAMBAUGH 2SSE, MI
Coverage
Year
Summer Solstice
June 21/Day 172
Summer Teletherm
July 20/Day 201
+29 days
Jan
Feb
Mar
Apr
May
Jun
Jul
Aug
Sep
Oct
Nov
Dec

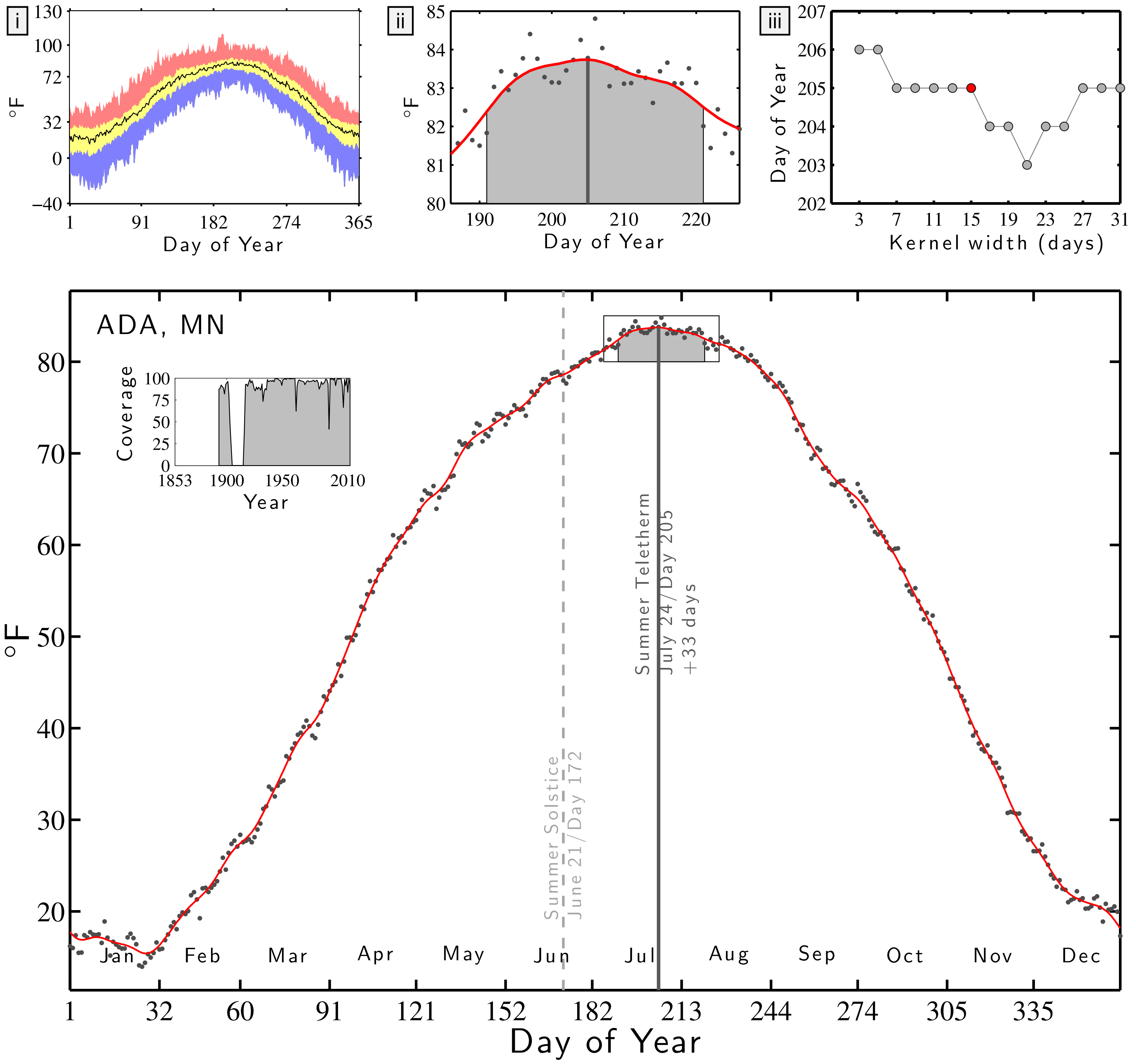

i
130
100
72
32
0
−40
°F
1
91
182
274
365
Day of Year
ii
85
84
83
82
81
80
°F
190
200
210
220
Day of Year
iii
207
206
205
204
203
202
Day of Year
3
7
11
15
19
23
27
31
Kernel width (days)
ADA, MN
Coverage
100
75
50
25
0
1853
1900
1950
2010
Year
80
70
60
50
40
30
20
°F
Summer Solstice
June 21/Day 172
Summer Teletherm
July 24/Day 205
+33 days
Jan
Feb
Mar
Apr
May
Jun
Jul
Aug
Sep
Oct
Nov
Dec
1
32
60
91
121
152
182
213
244
274
305
335
Day of Year

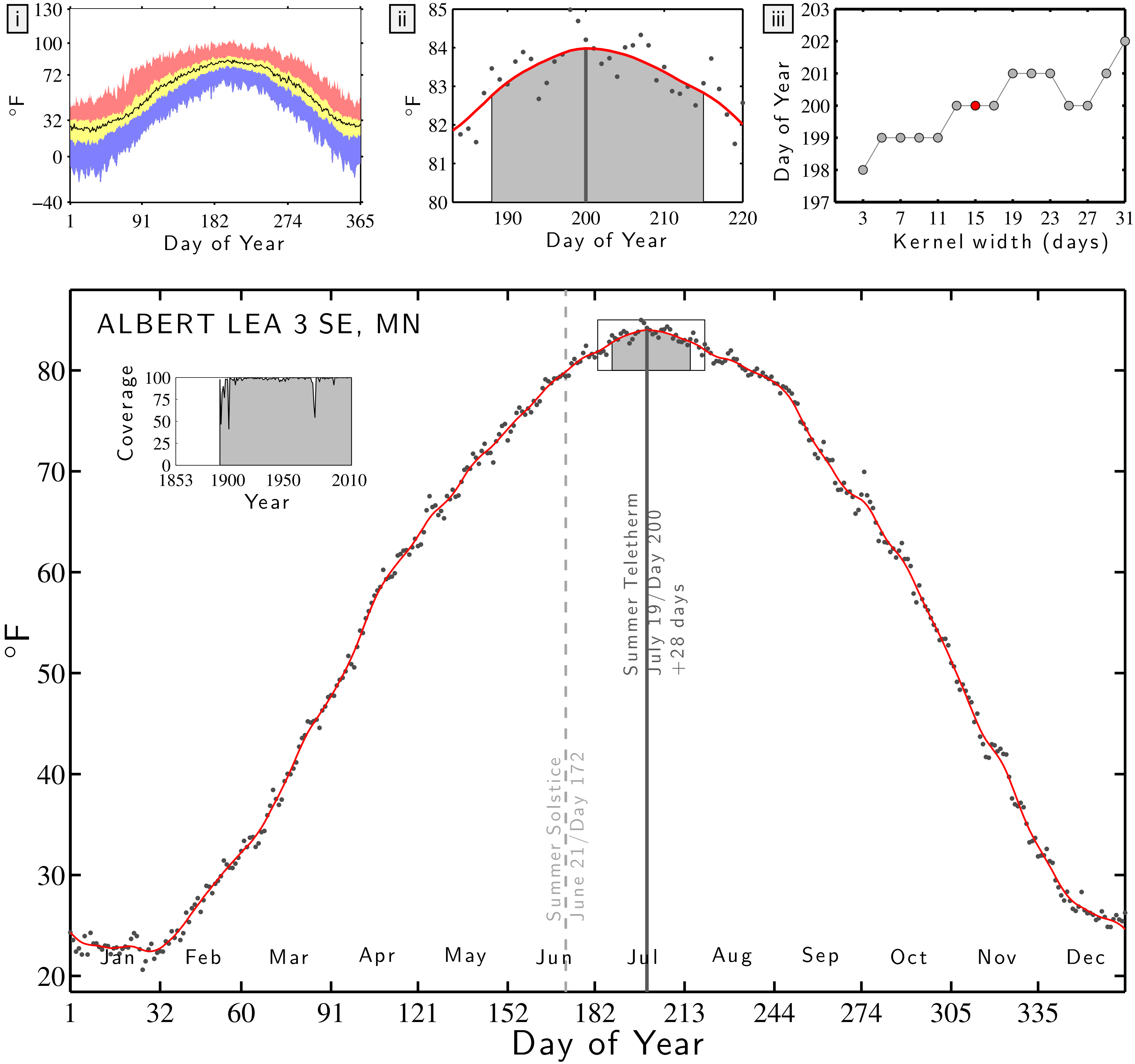

i
°F
Day of Year
ii
°F
Day of Year
iii
Day of Year
Kernel width (days)
ALBERT LEA 3 SE, MN
Coverage
Year
Summer Solstice
June 21/Day 172
Summer Teletherm
July 19/Day 200
+28 days
Jan
Feb
Mar
Apr
May
Jun
Jul
Aug
Sep
Oct
Nov
Dec
°F
Day of Year

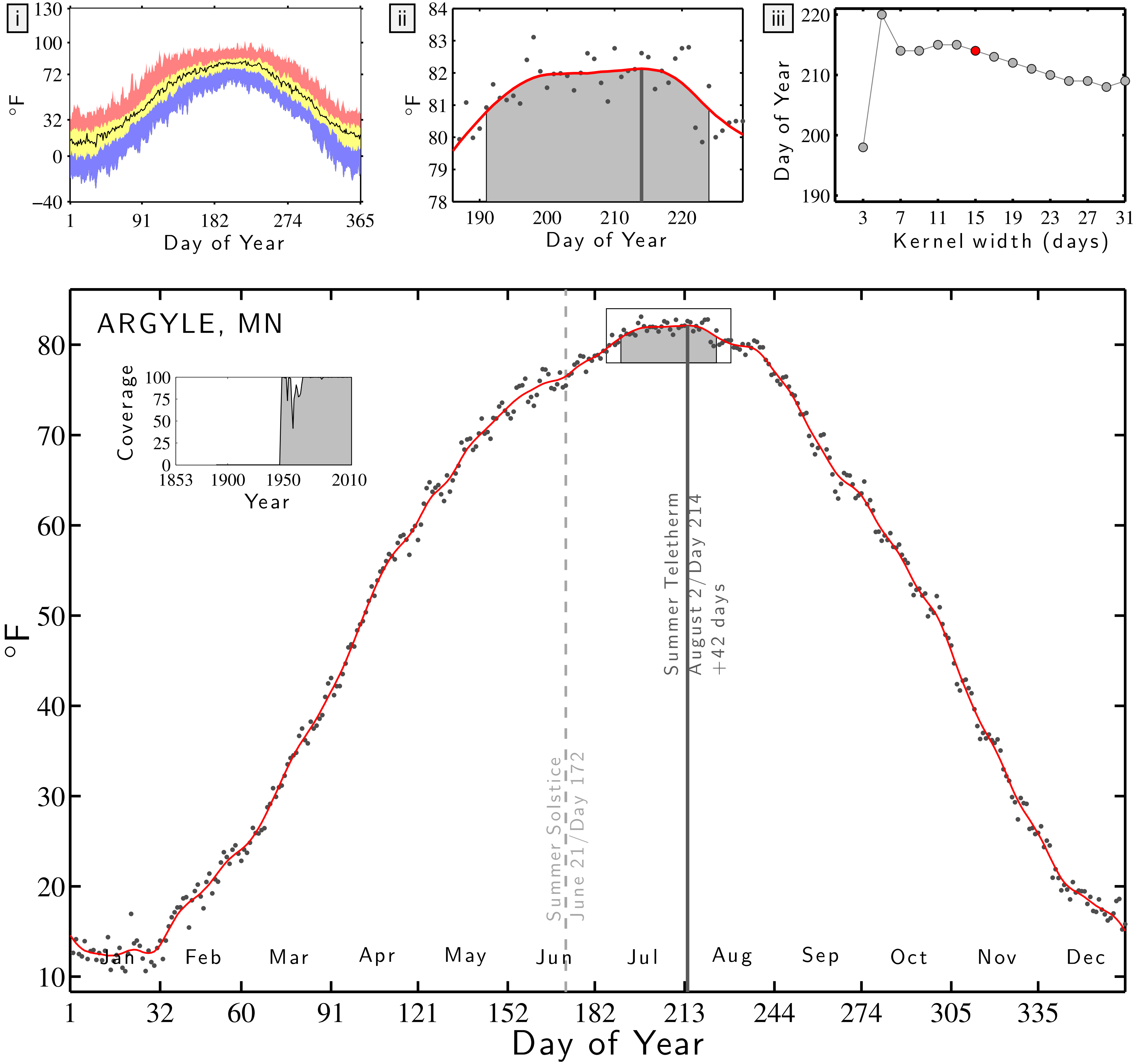

i
°F
Day of Year
ii
Day of Year
iii
Day of Year
Kernel width (days)
ARGYLE, MN
Coverage
Year
Summer Solstice
June 21/Day 172
Summer Teletherm
August 2/Day 214
+42 days
Jan
Feb
Mar
Apr
May
Jun
Jul
Aug
Sep
Oct
Nov
Dec
Day of Year

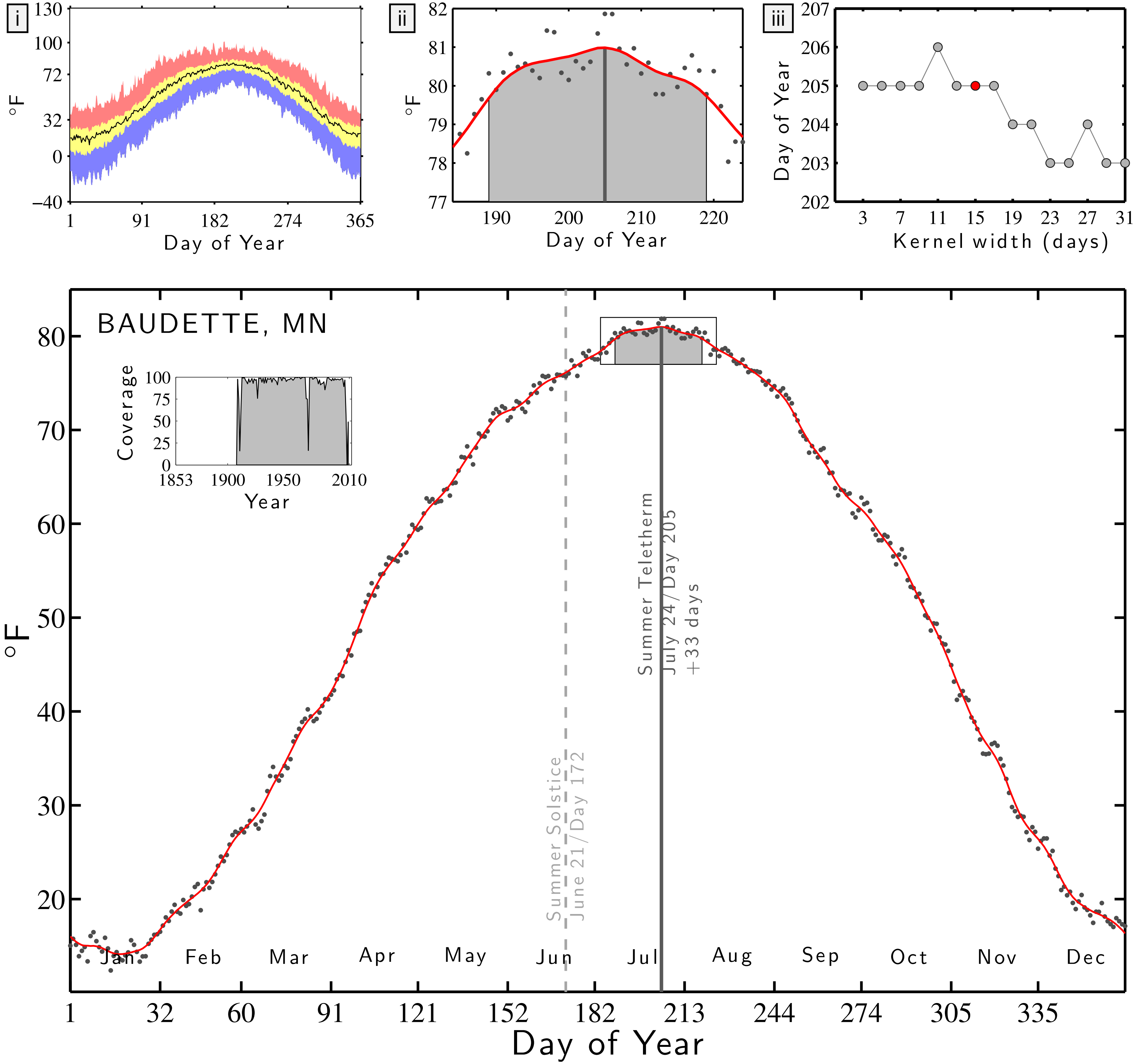

i
ii
iii
BAUDETTE, MN
Coverage
Year
Summer Solstice
June 21 / Day 172
Summer Teletherm
July 24 / Day 205
+33 days
Jan
Feb
Mar
Apr
May
Jun
Jul
Aug
Sep
Oct
Nov
Dec
°F
Day of Year
Day of Year
Kernel width (days)

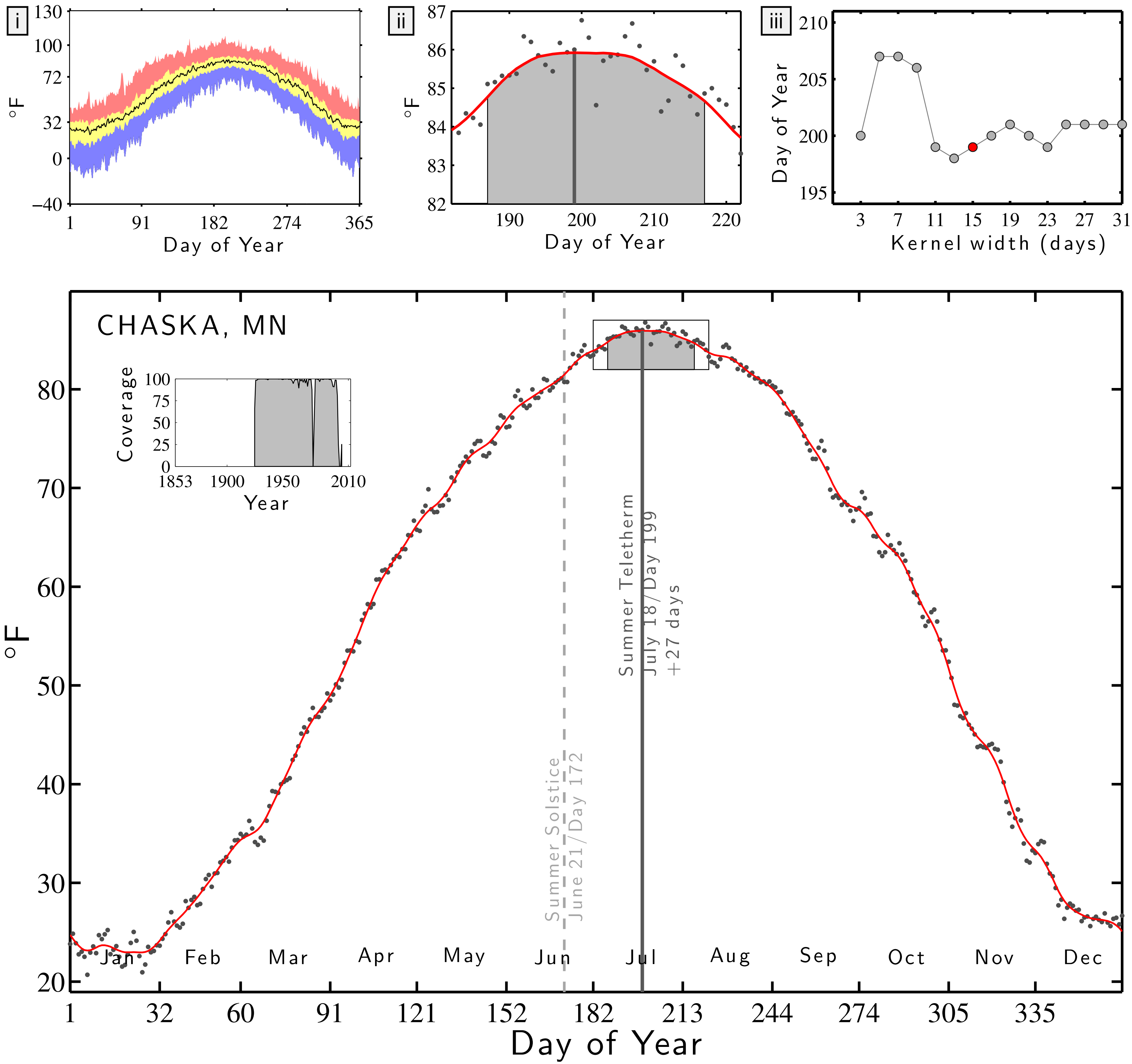

i
ii
iii
Day of Year
°F
Kernel width (days)
CHASKA, MN
Coverage
Year
Summer Solstice
June 21/Day 172
Summer Teletherm
July 18/Day 199
+27 days
Jan
Feb
Mar
Apr
May
Jun
Jul
Aug
Sep
Oct
Nov
Dec

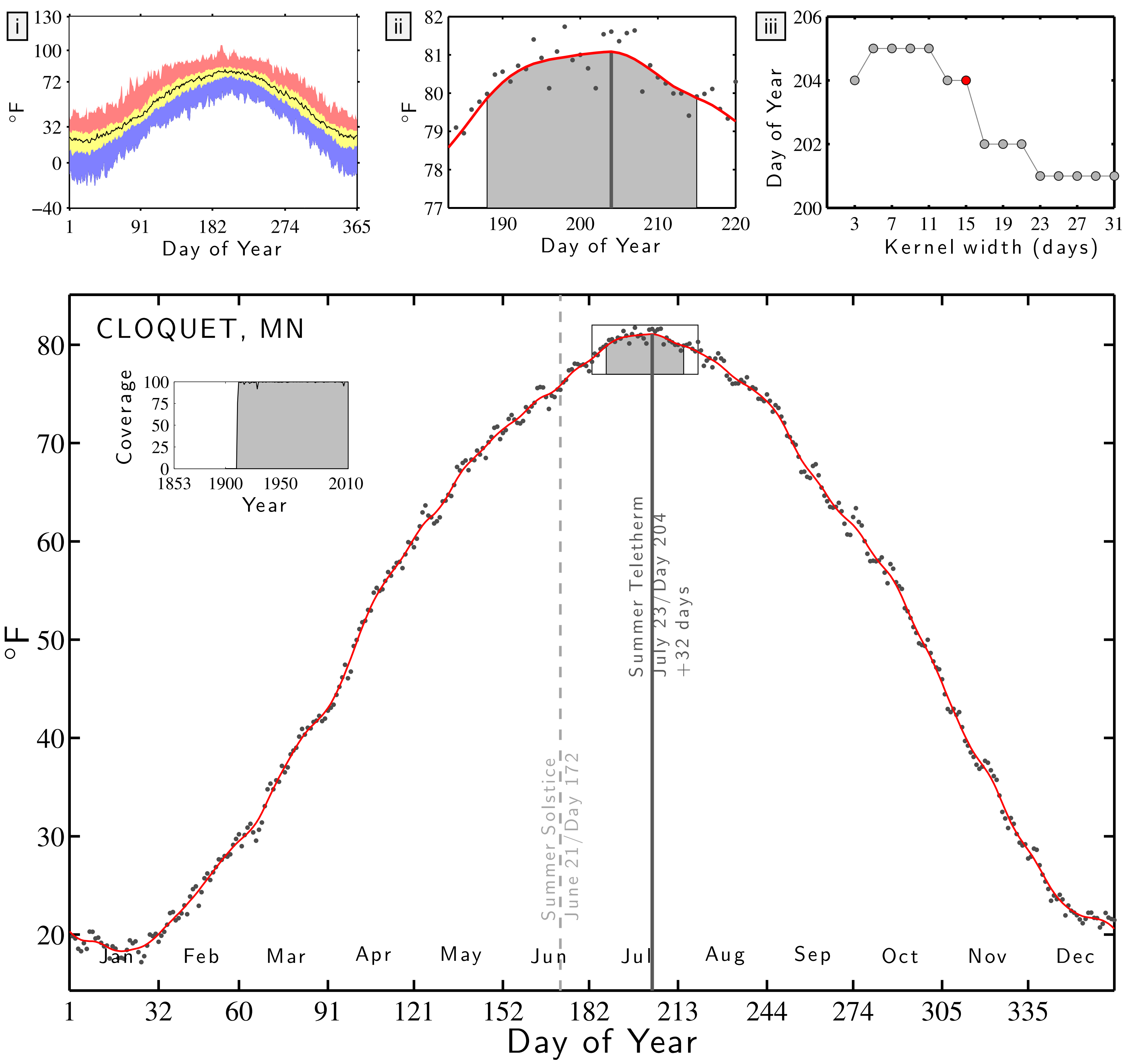

i
°F
Day of Year
ii
°F
Day of Year
iii
Day of Year
Kernel width (days)
CLOQUET, MN
Coverage
Year
Summer Solstice
June 21 / Day 172
Summer Teletherm
July 23 / Day 204
+32 days
Jan
Feb
Mar
Apr
May
Jun
Jul
Aug
Sep
Oct
Nov
Dec
°F
Day of Year

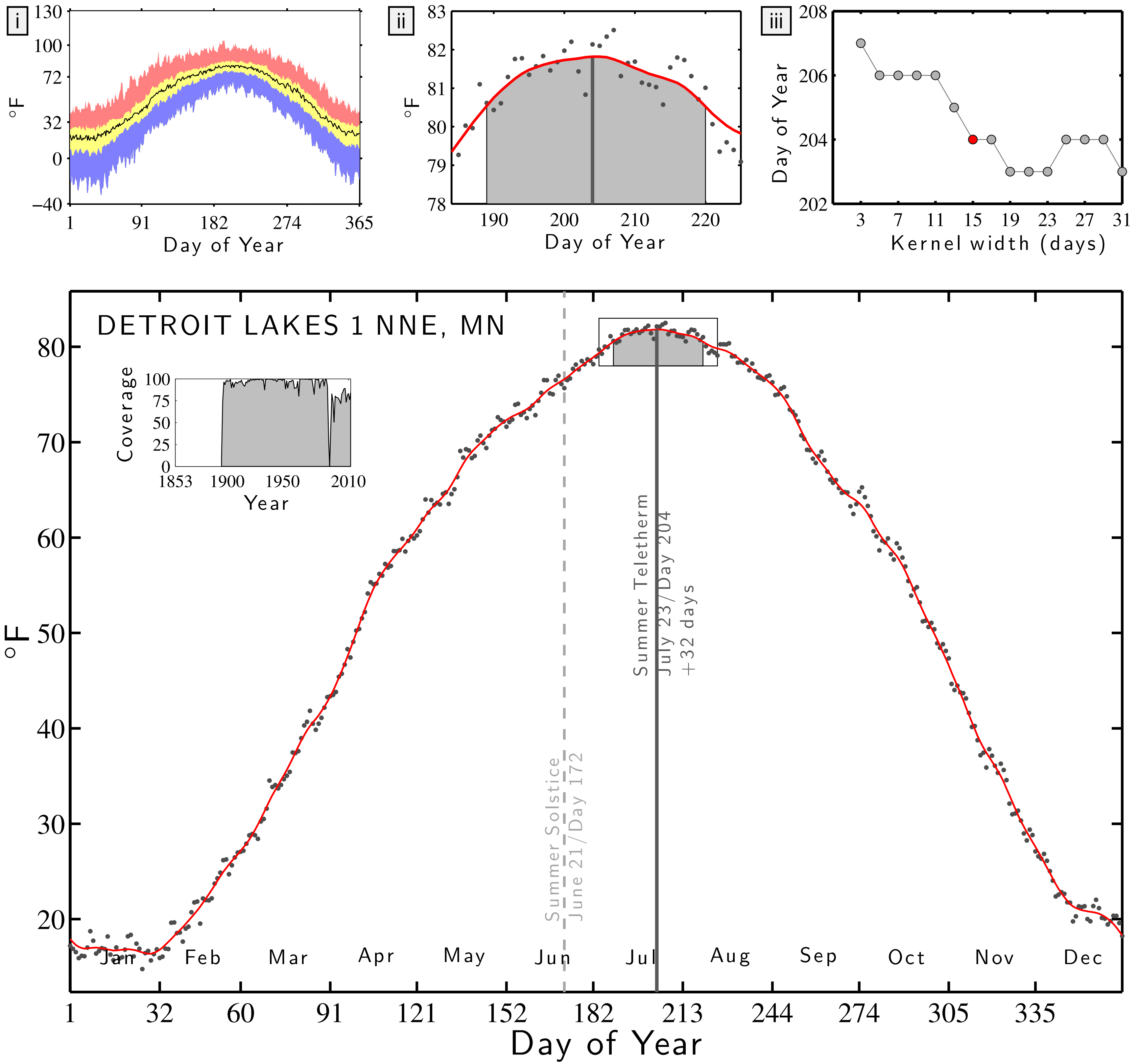

i
ii
iii
°F
Day of Year
Kernel width (days)
DETROIT LAKES 1 NNE, MN
Coverage
Year
Summer Solstice
June 21/Day 172
Summer Teletherm
July 23/Day 204
+32 days
Jan
Feb
Mar
Apr
May
Jun
Jul
Aug
Sep
Oct
Nov
Dec

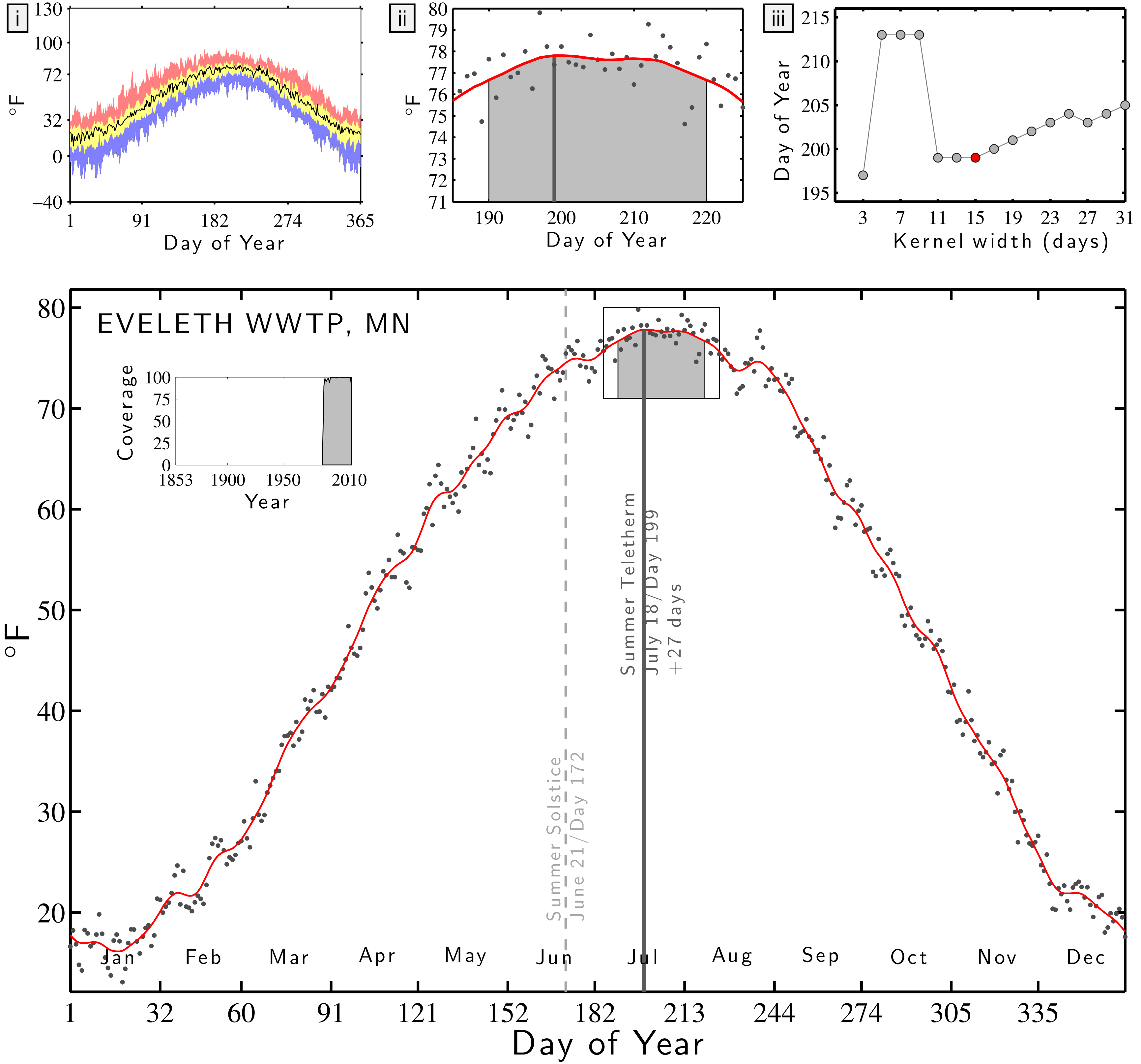
i
°F
Day of Year
ii
°F
Day of Year
iii
Day of Year
Kernel width (days)
EVELETH WWTP, MN
Coverage
Year
Summer Solstice
June 21/Day 172
Summer Teletherm
July 18/Day 199
+27 days
Jan
Feb
Mar
Apr
May
Jun
Jul
Aug
Sep
Oct
Nov
Dec
°F
Day of Year

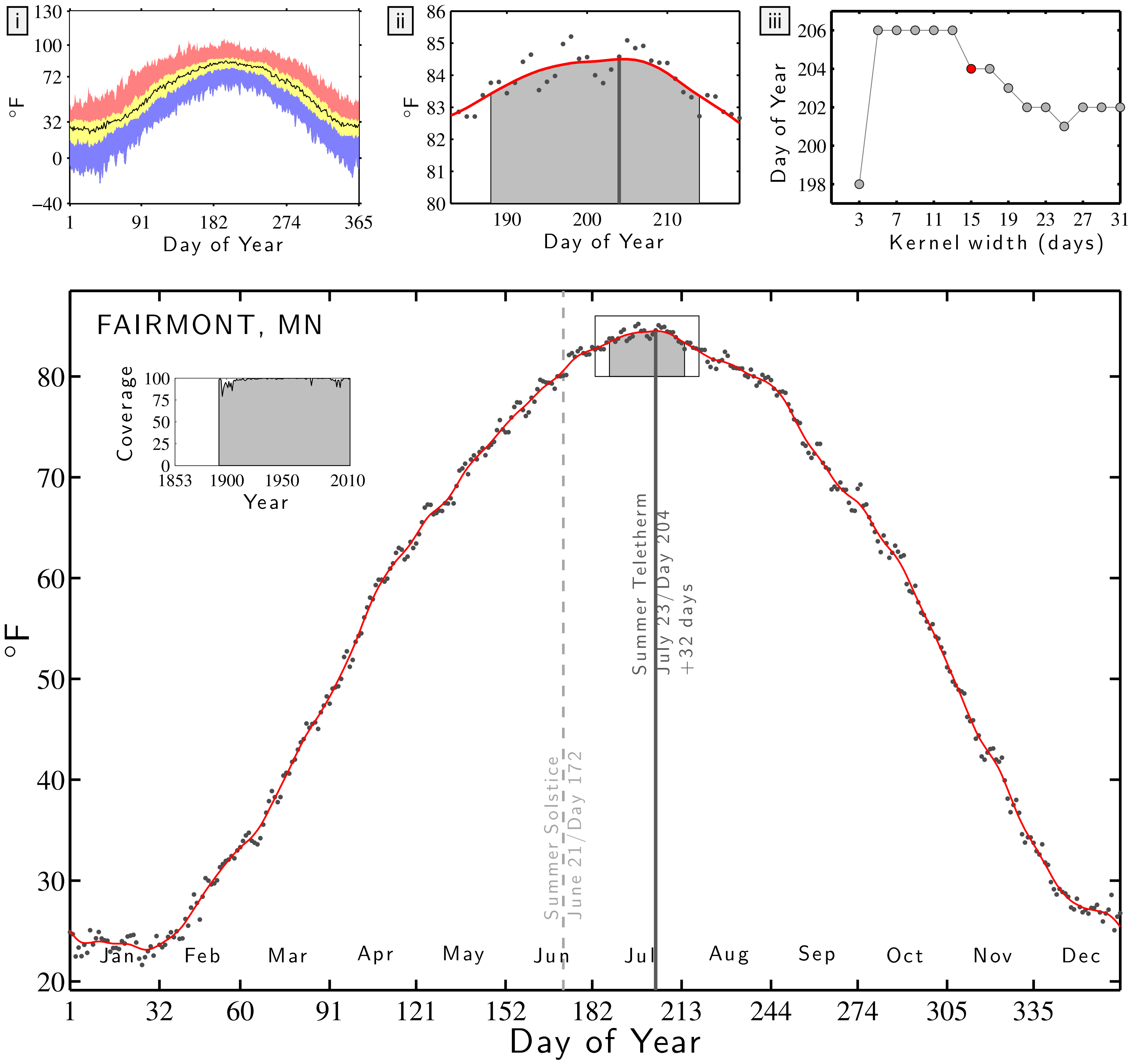

i
°F
Day of Year
ii
°F
Day of Year
iii
Day of Year
Kernel width (days)
FAIRMONT, MN
Coverage
Year
Summer Solstice
June 21 / Day 172
Summer Teletherm
July 23 / Day 204
+32 days
Jan
Feb
Mar
Apr
May
Jun
Jul
Aug
Sep
Oct
Nov
Dec
°F
Day of Year

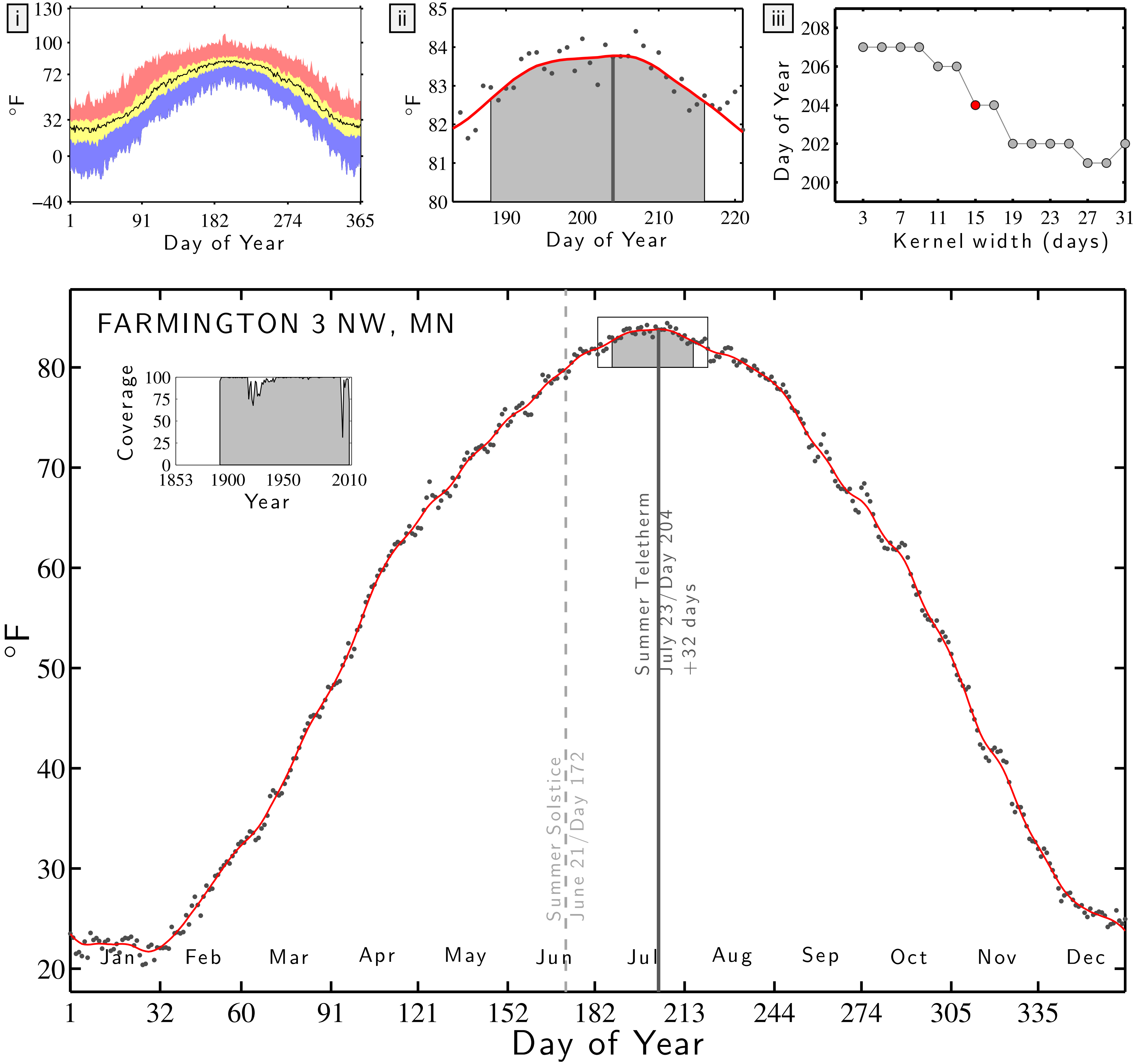

i
°F
Day of Year
ii
°F
Day of Year
iii
Day of Year
Kernel width (days)
FARMINGTON 3 NW, MN
Coverage
Year
Summer Solstice
June 21 / Day 172
Summer Teletherm
July 23 / Day 204
+32 days
Jan
Feb
Mar
Apr
May
Jun
Jul
Aug
Sep
Oct
Nov
Dec
°F
Day of Year

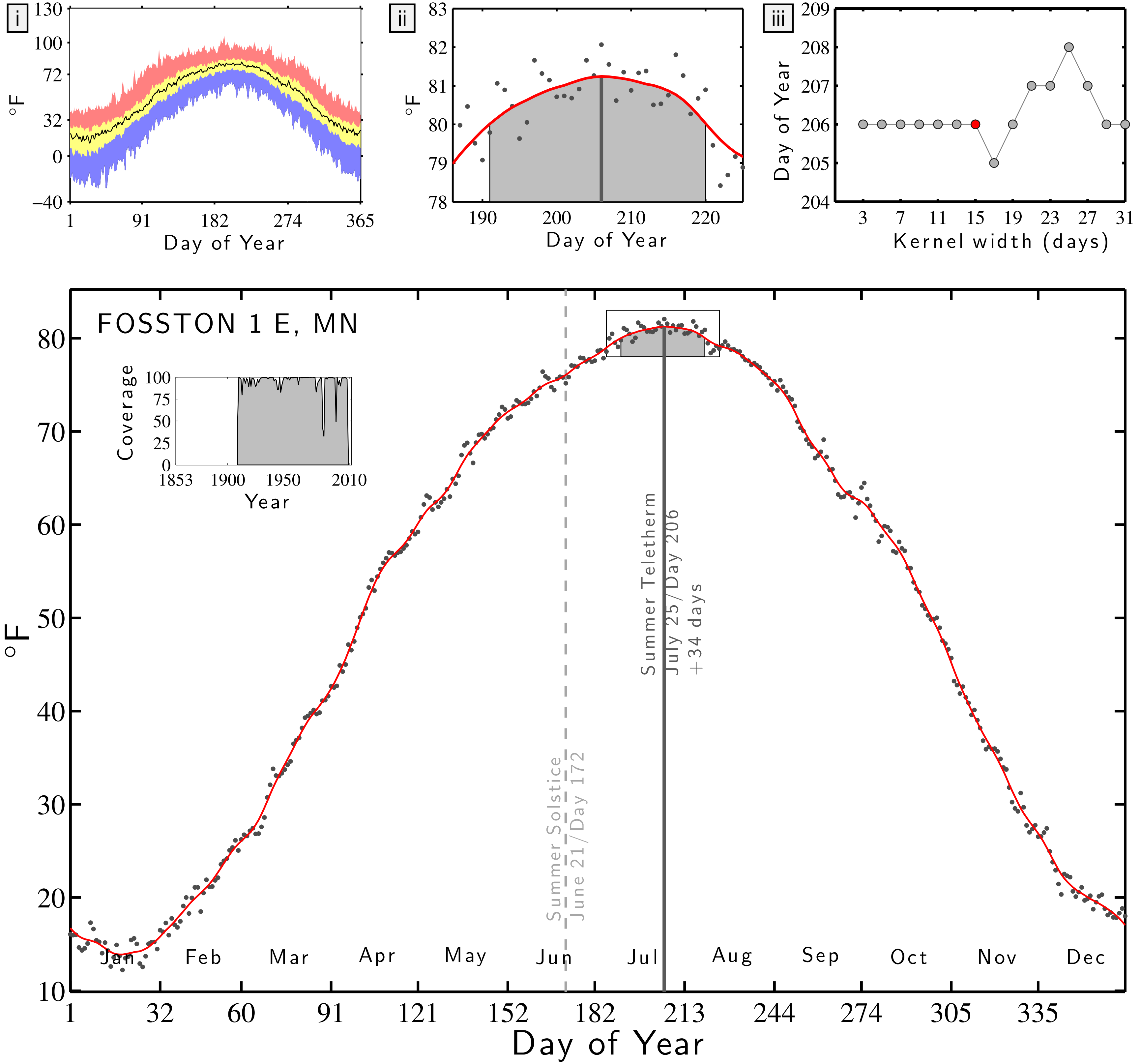

i
°F
Day of Year
ii
°F
Day of Year
iii
Day of Year
Kernel width (days)
FOSSTON 1 E, MN
Coverage
Year
Summer Solstice
June 21/Day 172
Summer Teletherm
July 25/Day 206
+34 days
Jan
Feb
Mar
Apr
May
Jun
Jul
Aug
Sep
Oct
Nov
Dec
°F
Day of Year

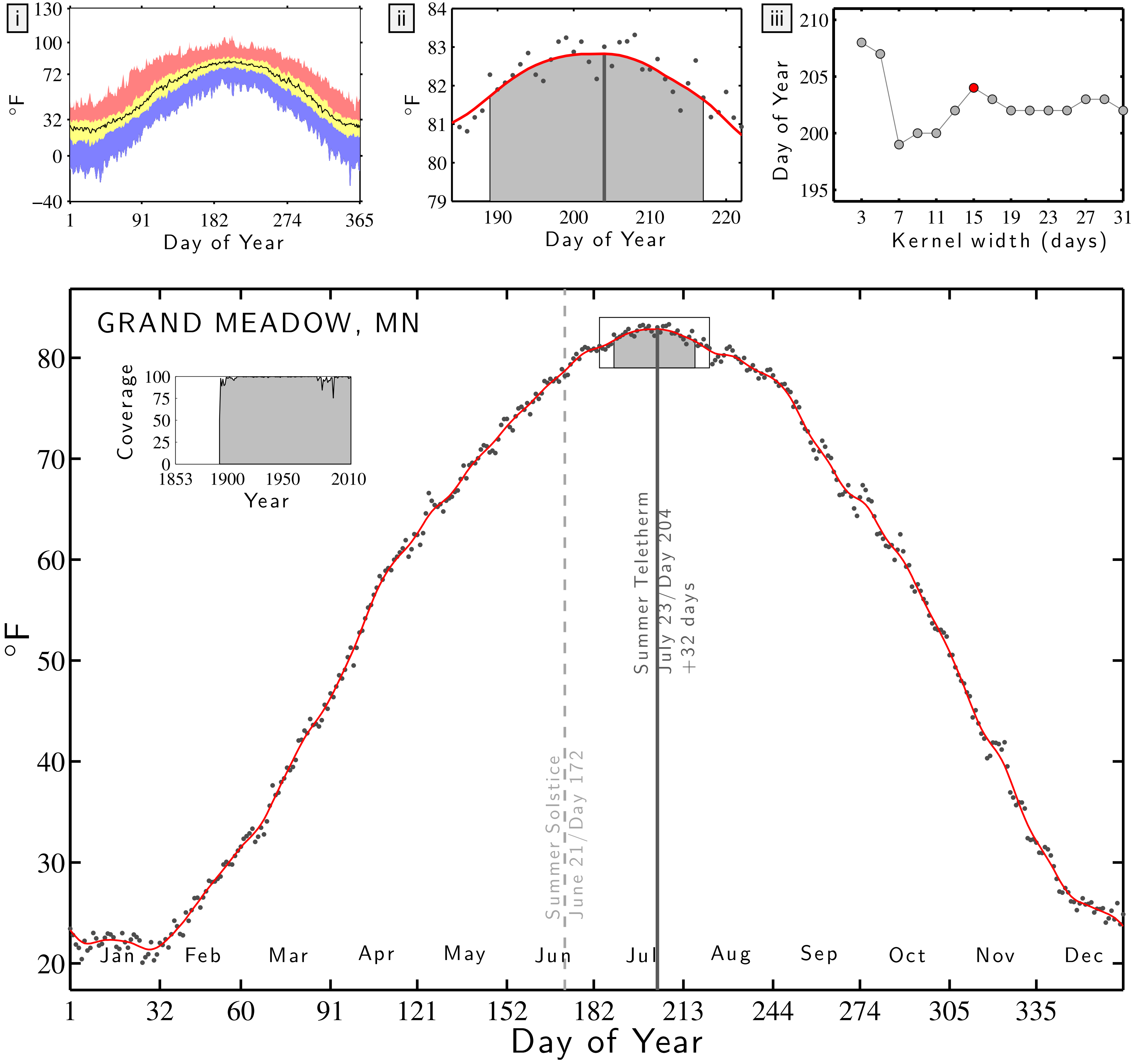

i
°F
130
100
72
32
0
−40
1
91
182
274
365
Day of Year
ii
84
83
82
81
80
79
190
200
210
220
Day of Year
iii
Day of Year
210
205
200
195
3
7
11
15
19
23
27
31
Kernel width (days)
GRAND MEADOW, MN
Coverage
100
75
50
25
0
1853
1900
1950
2010
Year
Summer Solstice
June 21 / Day 172
Summer Teletherm
July 23 / Day 204
+32 days
Jan
Feb
Mar
Apr
May
Jun
Jul
Aug
Sep
Oct
Nov
Dec
80
70
60
50
40
30
20
1
32
60
91
121
152
182
213
244
274
305
335
Day of Year

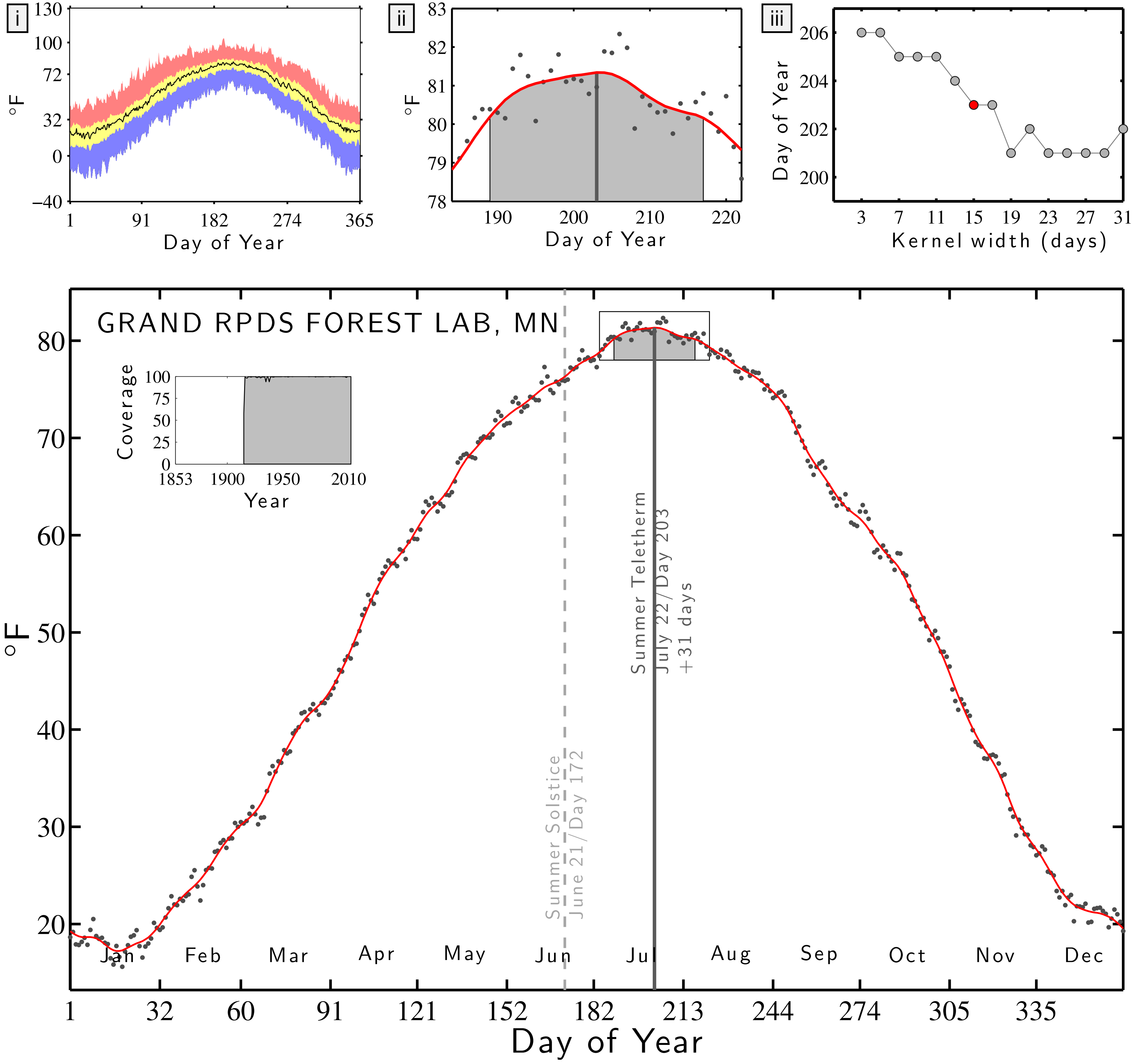

i
ii
iii
°F
Day of Year
Day of Year
Kernel width (days)
GRAND RPDS FOREST LAB, MN
Coverage
Year
Summer Solstice
June 21/Day 172
Summer Teletherm
July 22/Day 203
+31 days
Jan
Feb
Mar
Apr
May
Jun
Jul
Aug
Sep
Oct
Nov
Dec

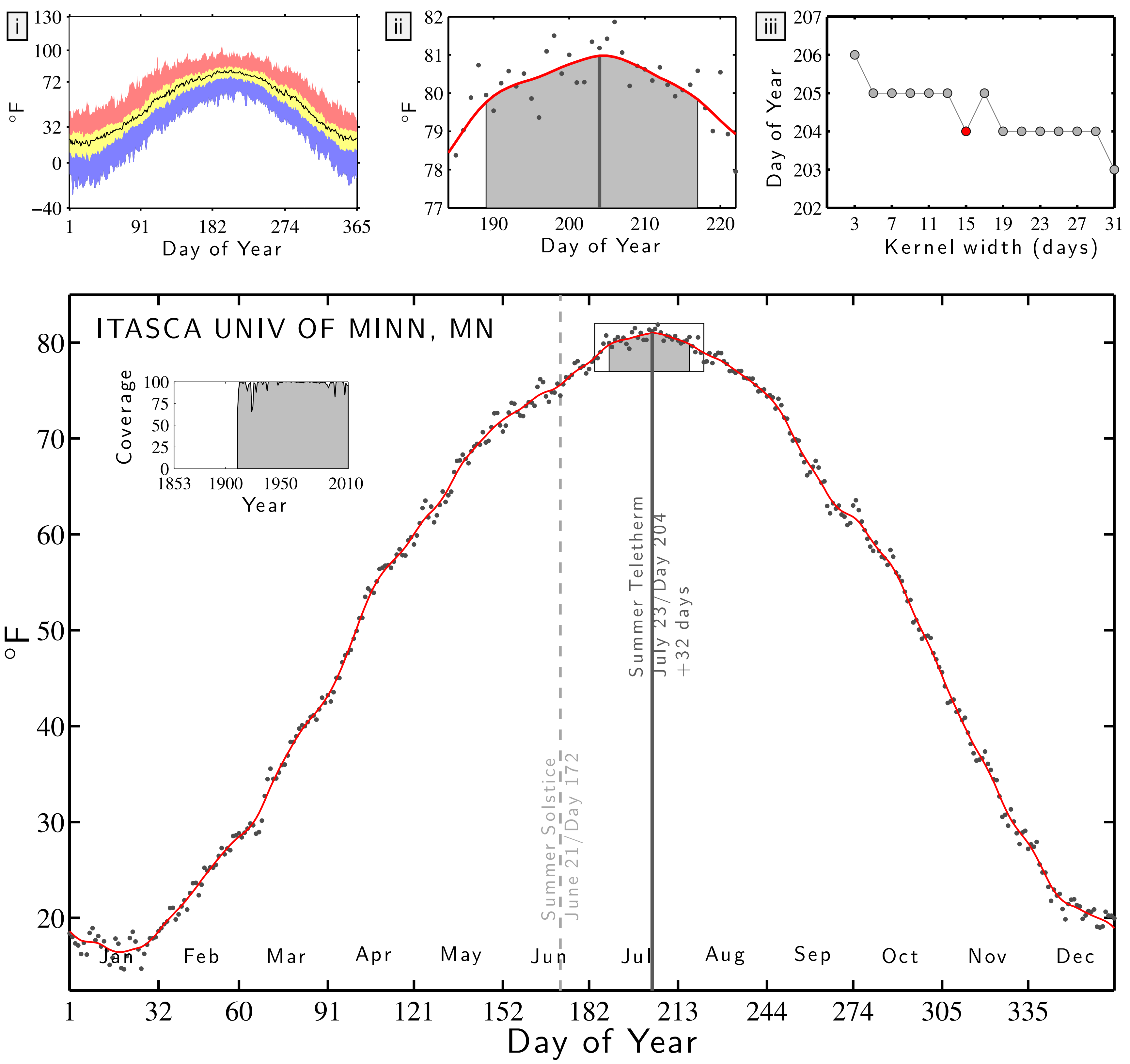

i
ii
iii
°F
Day of Year
Kernel width (days)
ITASCA UNIV OF MINN, MN
Coverage
Year
Summer Solstice
June 21 / Day 172
Summer Teletherm
July 23 / Day 204
+32 days
Jan
Feb
Mar
Apr
May
Jun
Jul
Aug
Sep
Oct
Nov
Dec

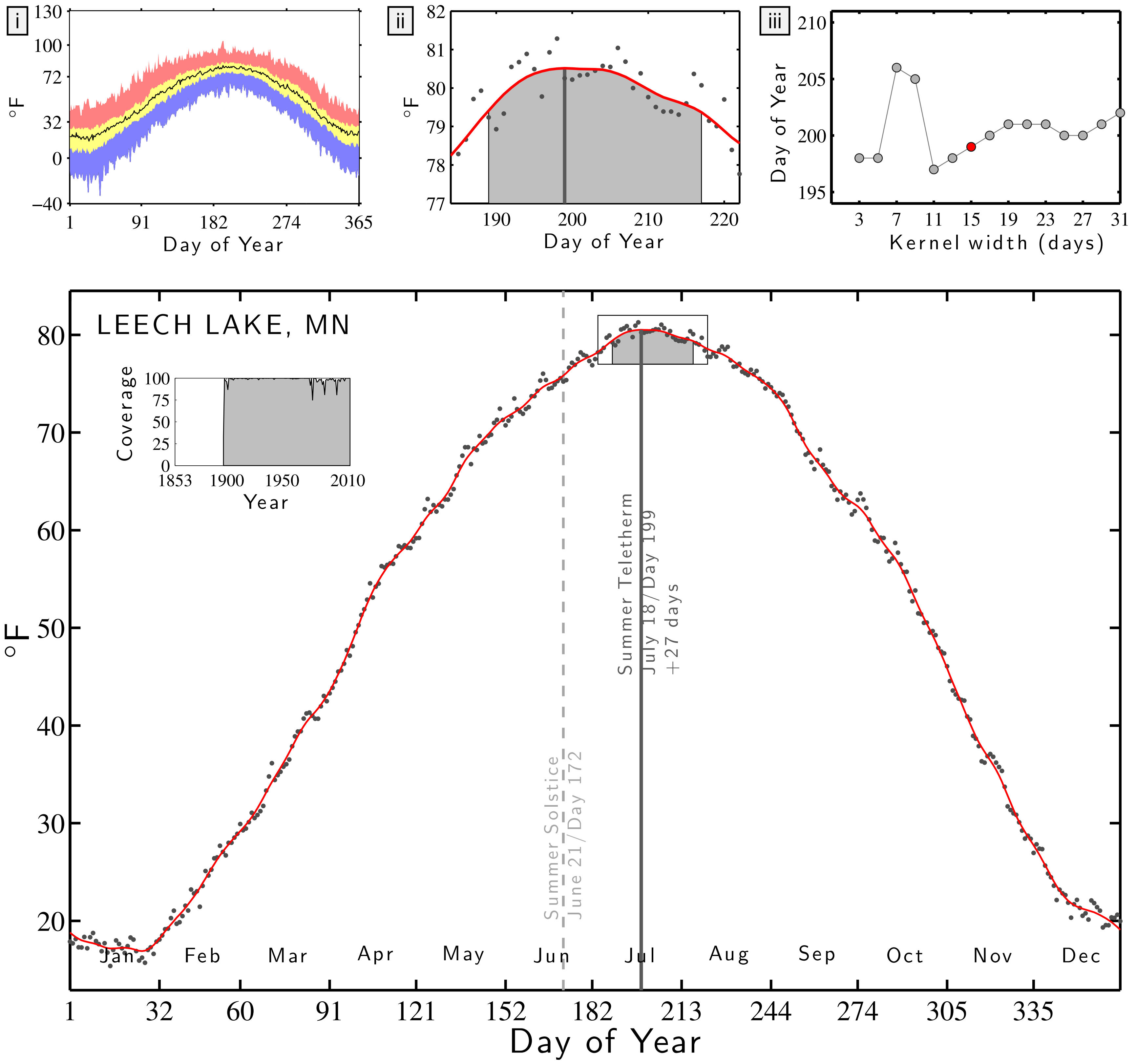

i
°F
Day of Year
ii
°F
Day of Year
iii
Day of Year
Kernel width (days)
LEECH LAKE, MN
Coverage
Year
Summer Solstice
June 21/Day 172
Summer Teletherm
July 18/Day 199
+27 days
Jan
Feb
Mar
Apr
May
Jun
Jul
Aug
Sep
Oct
Nov
Dec
°F
Day of Year

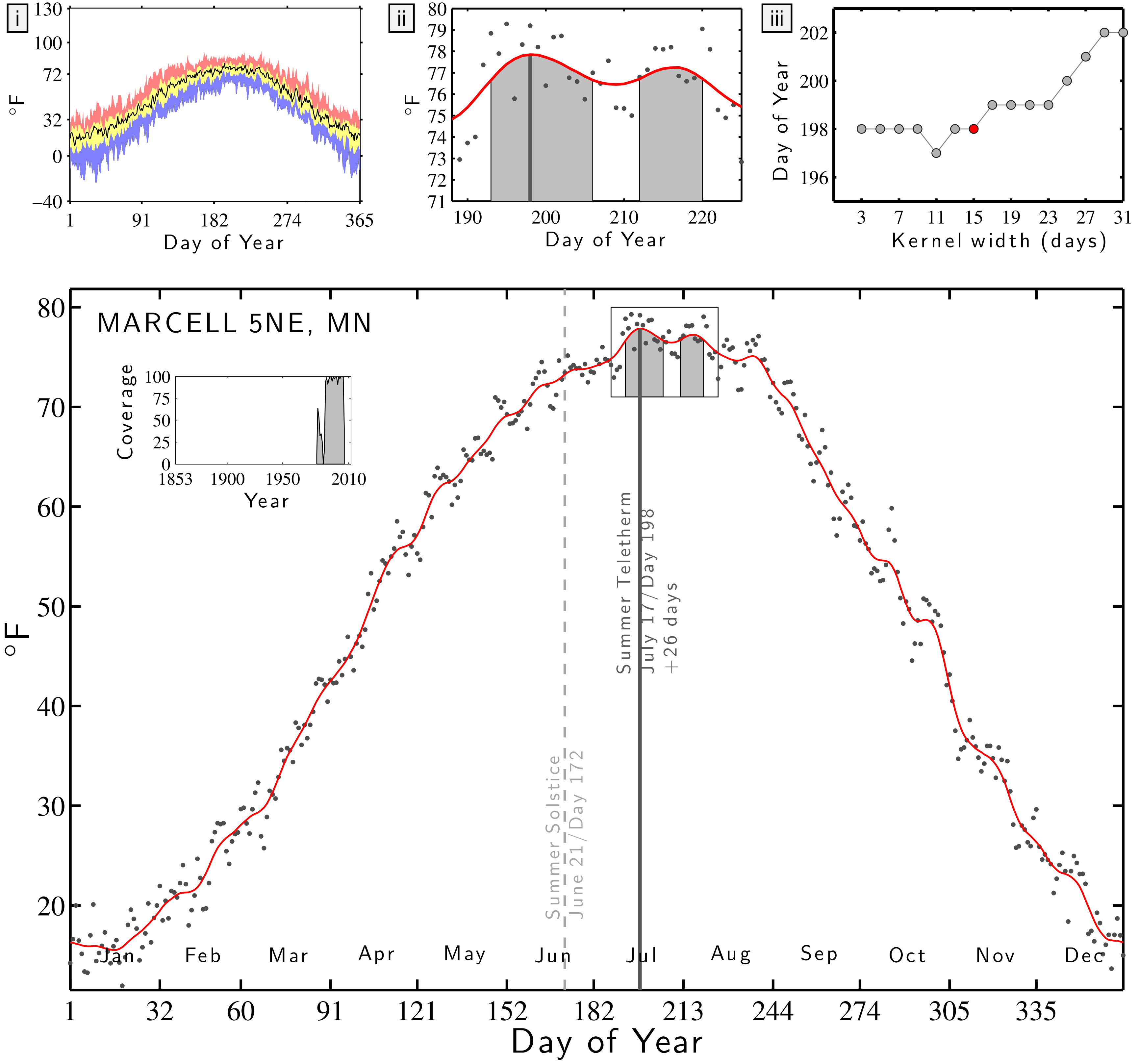

i
°F
130
100
72
32
0
−40
1
91
182
274
365
Day of Year
ii
°F
80
79
78
77
76
75
74
73
72
71
190
200
210
220
Day of Year
iii
Day of Year
202
200
198
196
3
7
11
15
19
23
27
31
Kernel width (days)
MARCELL 5NE, MN
Coverage
100
75
50
25
0
1853
1900
1950
2010
Year
°F
80
70
60
50
40
30
20
Summer Solstice
June 21 / Day 172
Summer Teletherm
July 17 / Day 198
+26 days
Jan
Feb
Mar
Apr
May
Jun
Jul
Aug
Sep
Oct
Nov
Dec
1
32
60
91
121
152
182
213
244
274
305
335
Day of Year

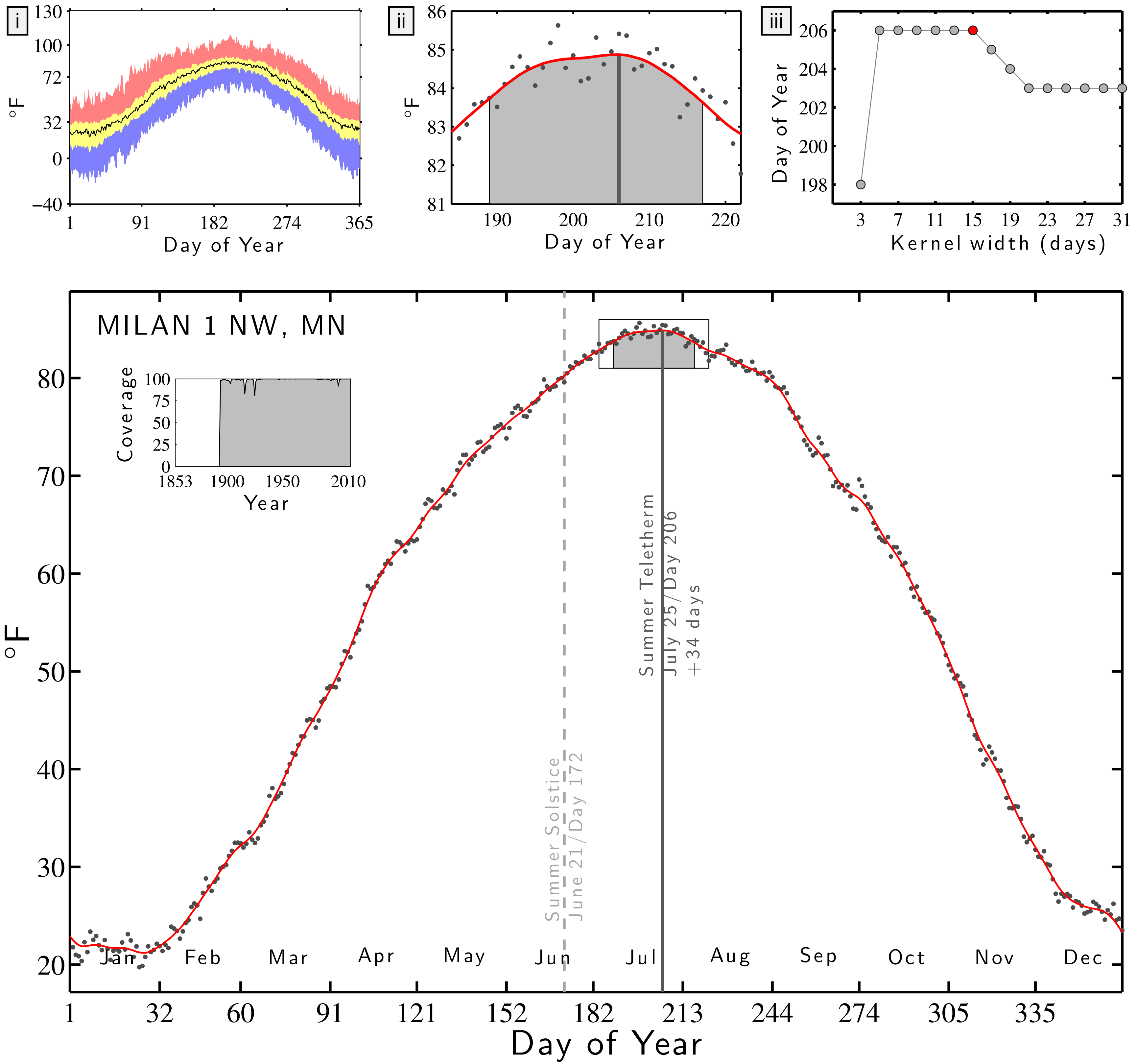

i
°F
Day of Year
ii
°F
Day of Year
iii
Day of Year
Kernel width (days)
MILAN 1 NW, MN
Coverage
Year
Summer Solstice
June 21/Day 172
Summer Teletherm
July 25/Day 206
+34 days
Jan
Feb
Mar
Apr
May
Jun
Jul
Aug
Sep
Oct
Nov
Dec
°F
Day of Year

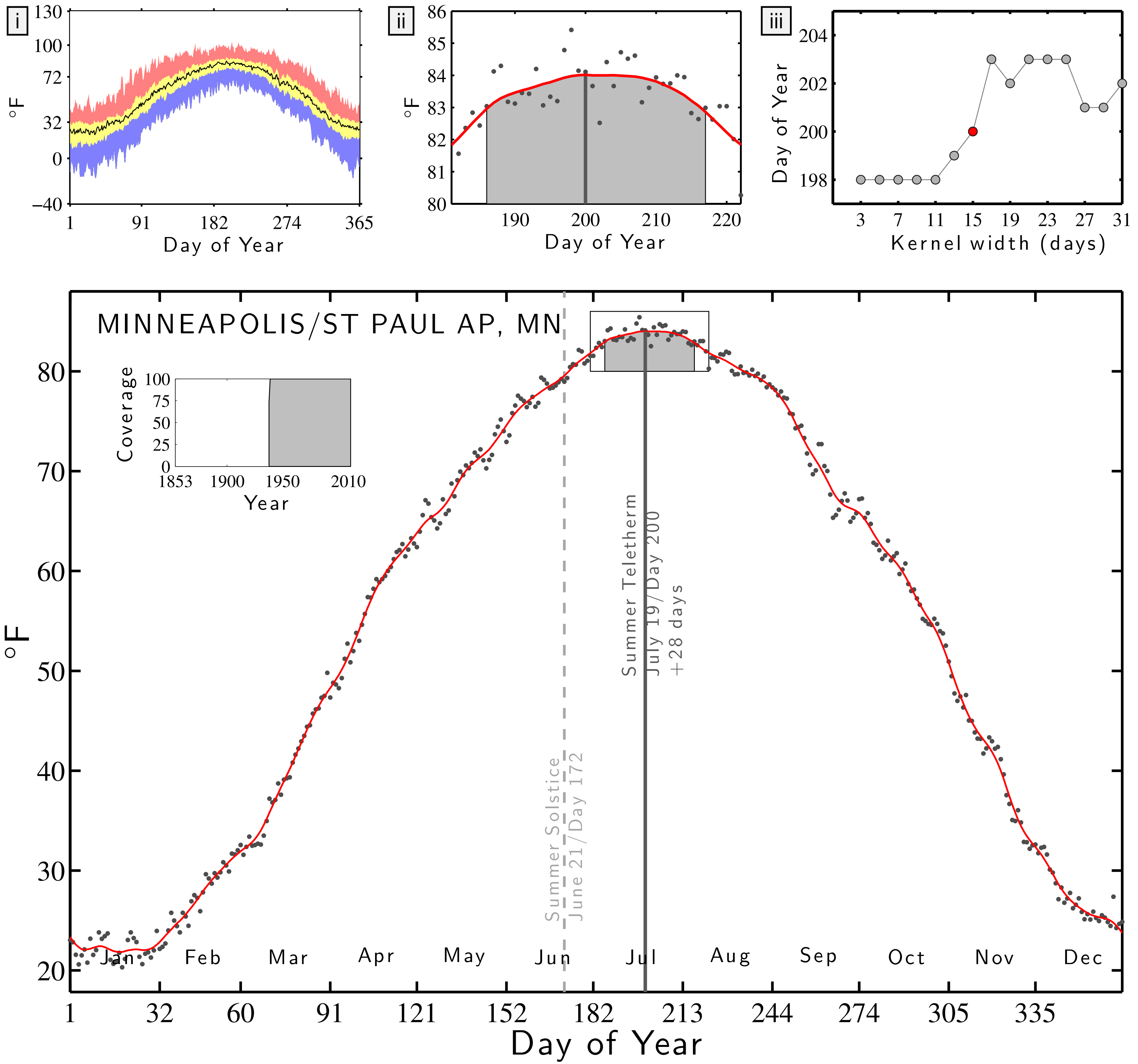

i
°F
Day of Year
ii
°F
Day of Year
iii
Day of Year
Kernel width (days)
MINNEAPOLIS/ST PAUL AP, MN
Coverage
Year
Summer Solstice
June 21/Day 172
Summer Teletherm
July 19/Day 200
+28 days
Jan
Feb
Mar
Apr
May
Jun
Jul
Aug
Sep
Oct
Nov
Dec
°F
Day of Year

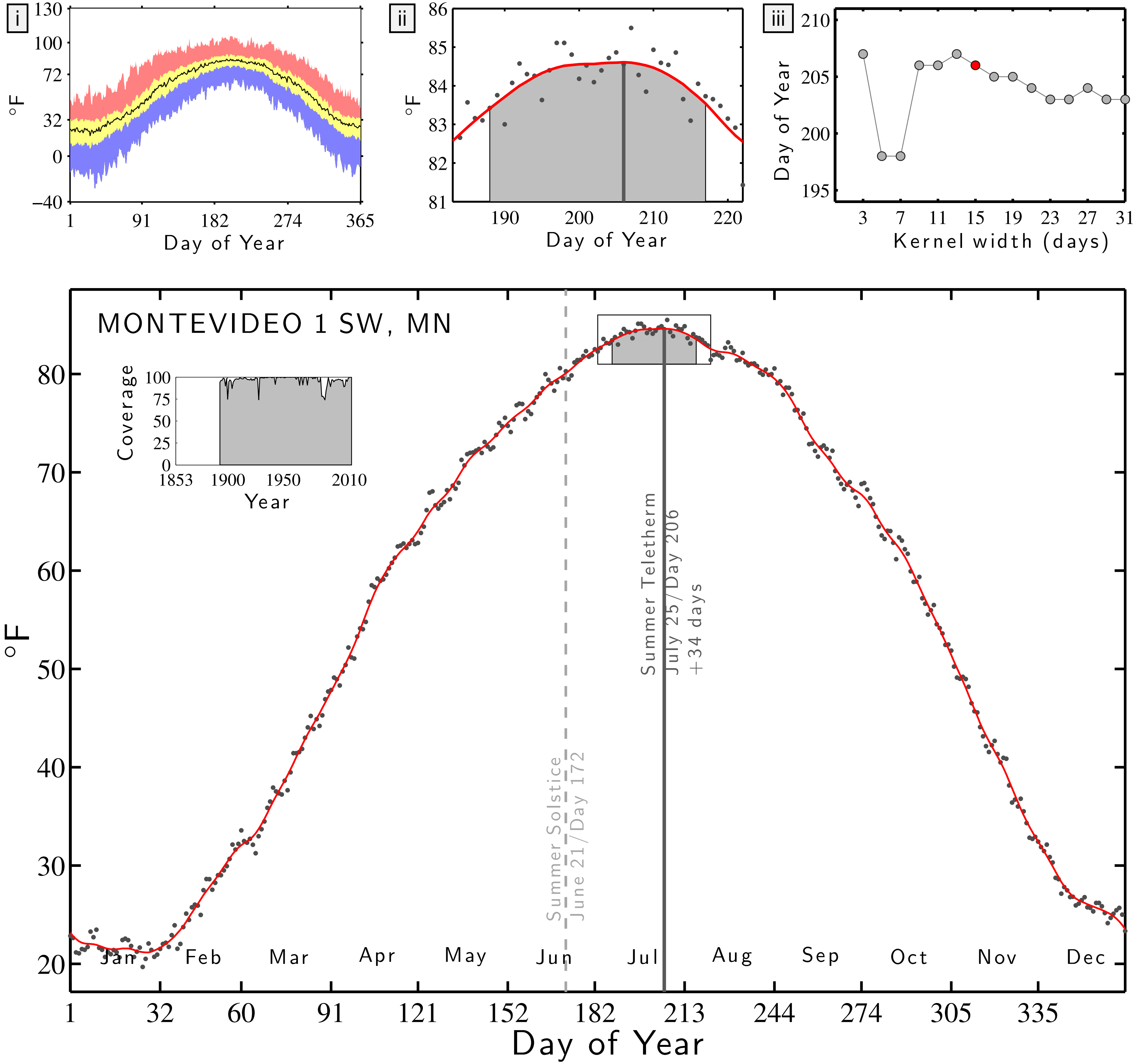

i
°F
Day of Year
ii
°F
Day of Year
iii
Day of Year
Kernel width (days)
MONTEVIDEO 1 SW, MN
Coverage
Year
Summer Solstice
June 21/Day 172
Summer Teletherm
July 25/Day 206
+34 days
Jan
Feb
Mar
Apr
May
Jun
Jul
Aug
Sep
Oct
Nov
Dec
°F
Day of Year

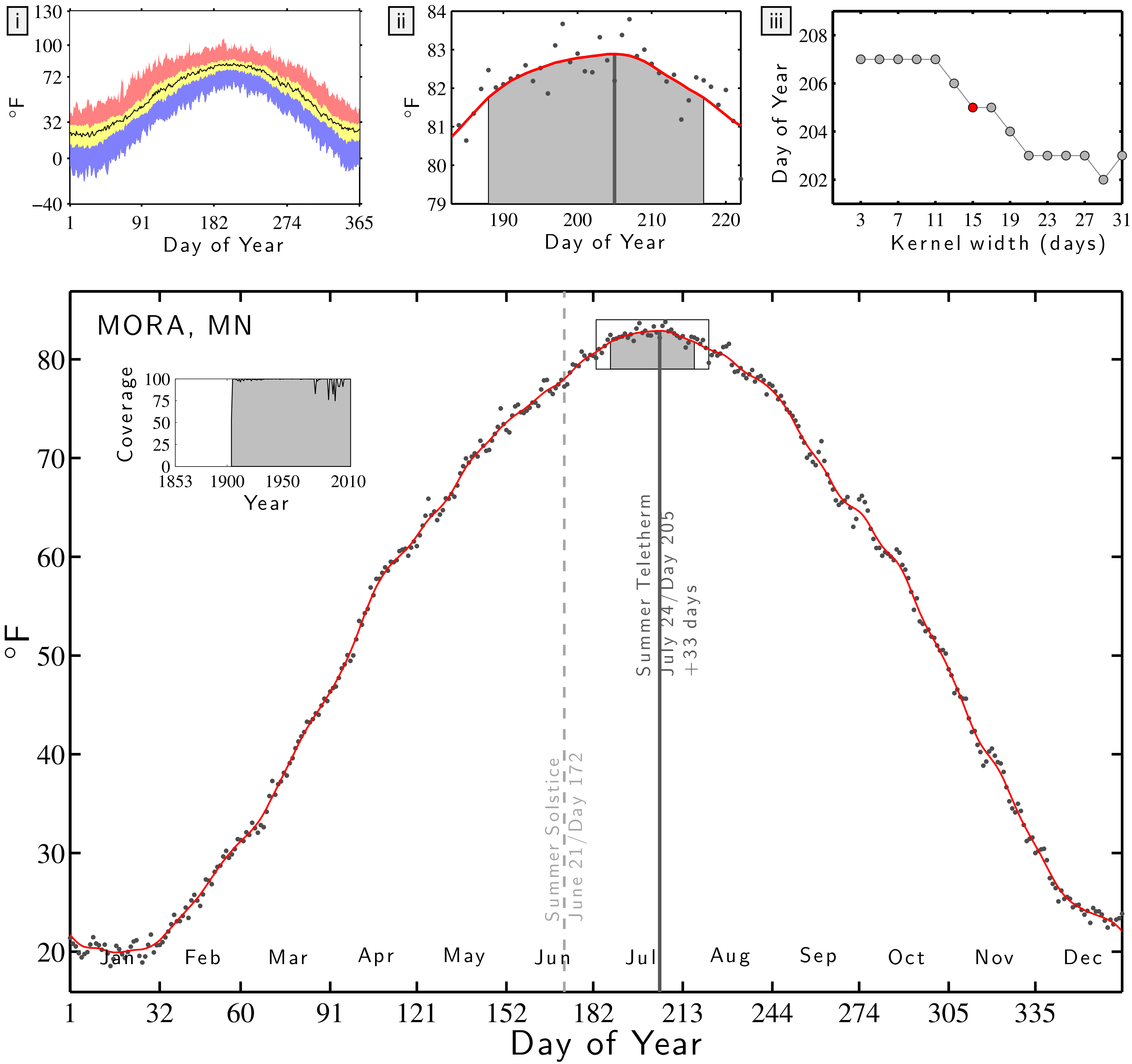

i
°F
Day of Year
ii
°F
Day of Year
iii
Day of Year
Kernel width (days)
MORA, MN
Coverage
Year
Summer Solstice
June 21 / Day 172
Summer Teletherm
July 24 / Day 205
+33 days
Jan
Feb
Mar
Apr
May
Jun
Jul
Aug
Sep
Oct
Nov
Dec
°F
Day of Year

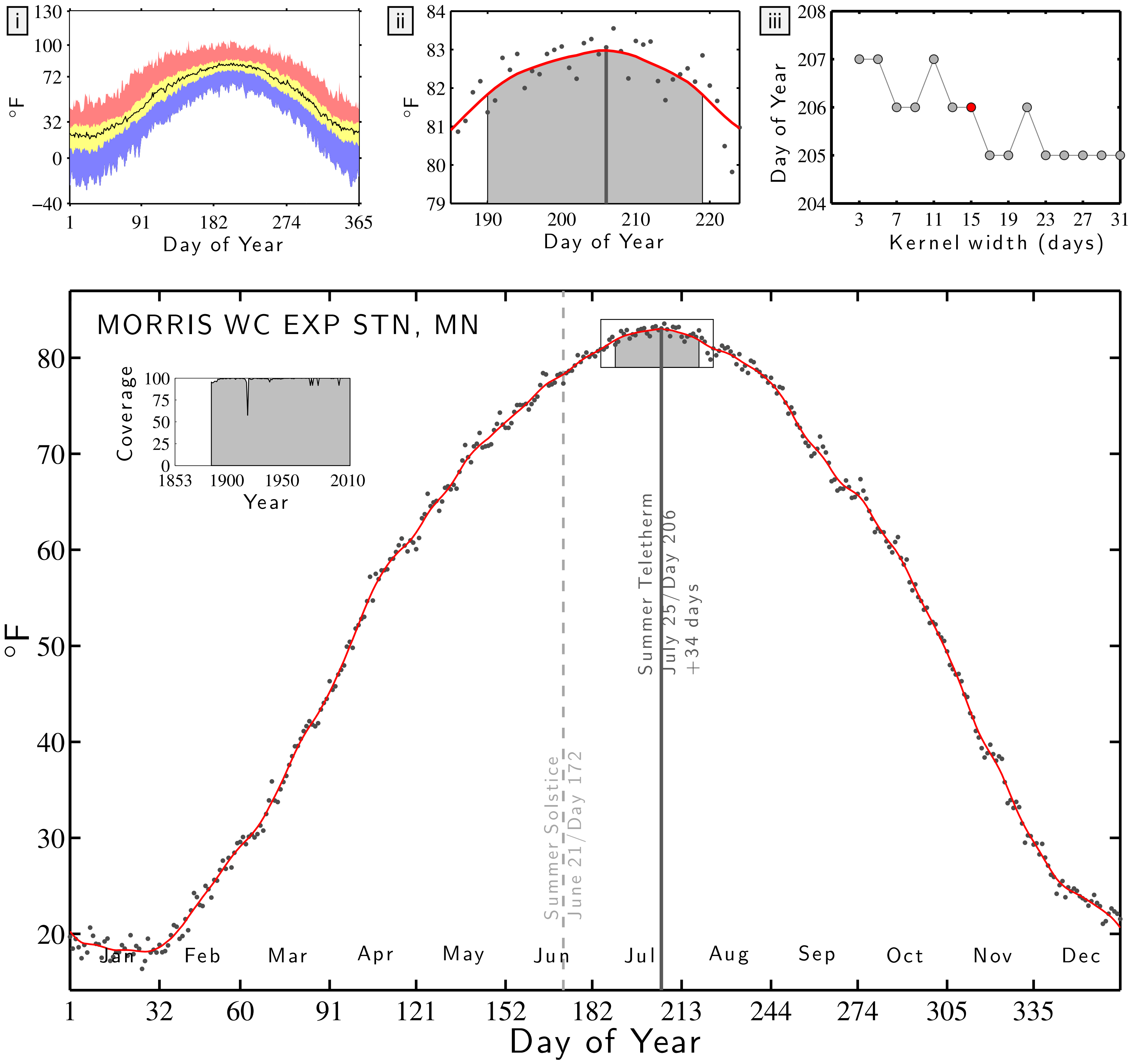
i
°F
Day of Year
ii
°F
Day of Year
iii
Day of Year
Kernel width (days)
MORRIS WC EXP STN, MN
Coverage
Year
Summer Solstice
June 21/Day 172
Summer Teletherm
July 25/Day 206
+34 days
Jan
Feb
Mar
Apr
May
Jun
Jul
Aug
Sep
Oct
Nov
Dec
°F
Day of Year

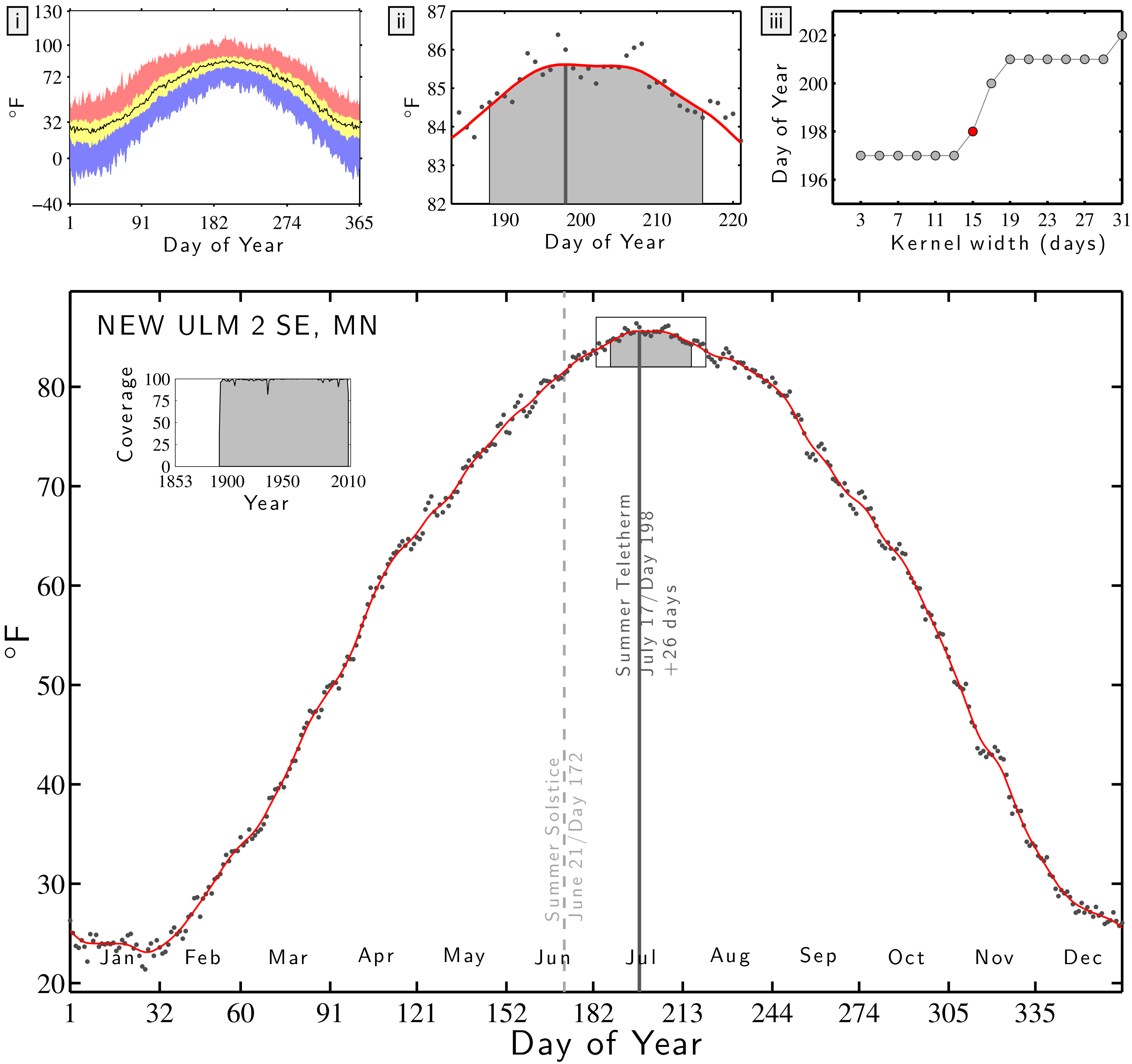

i
°F
Day of Year
ii
°F
Day of Year
iii
Day of Year
Kernel width (days)
NEW ULM 2 SE, MN
Coverage
Year
Summer Solstice
June 21/Day 172
Summer Teletherm
July 17/Day 198
+26 days
Jan
Feb
Mar
Apr
May
Jun
Jul
Aug
Sep
Oct
Nov
Dec
°F
Day of Year

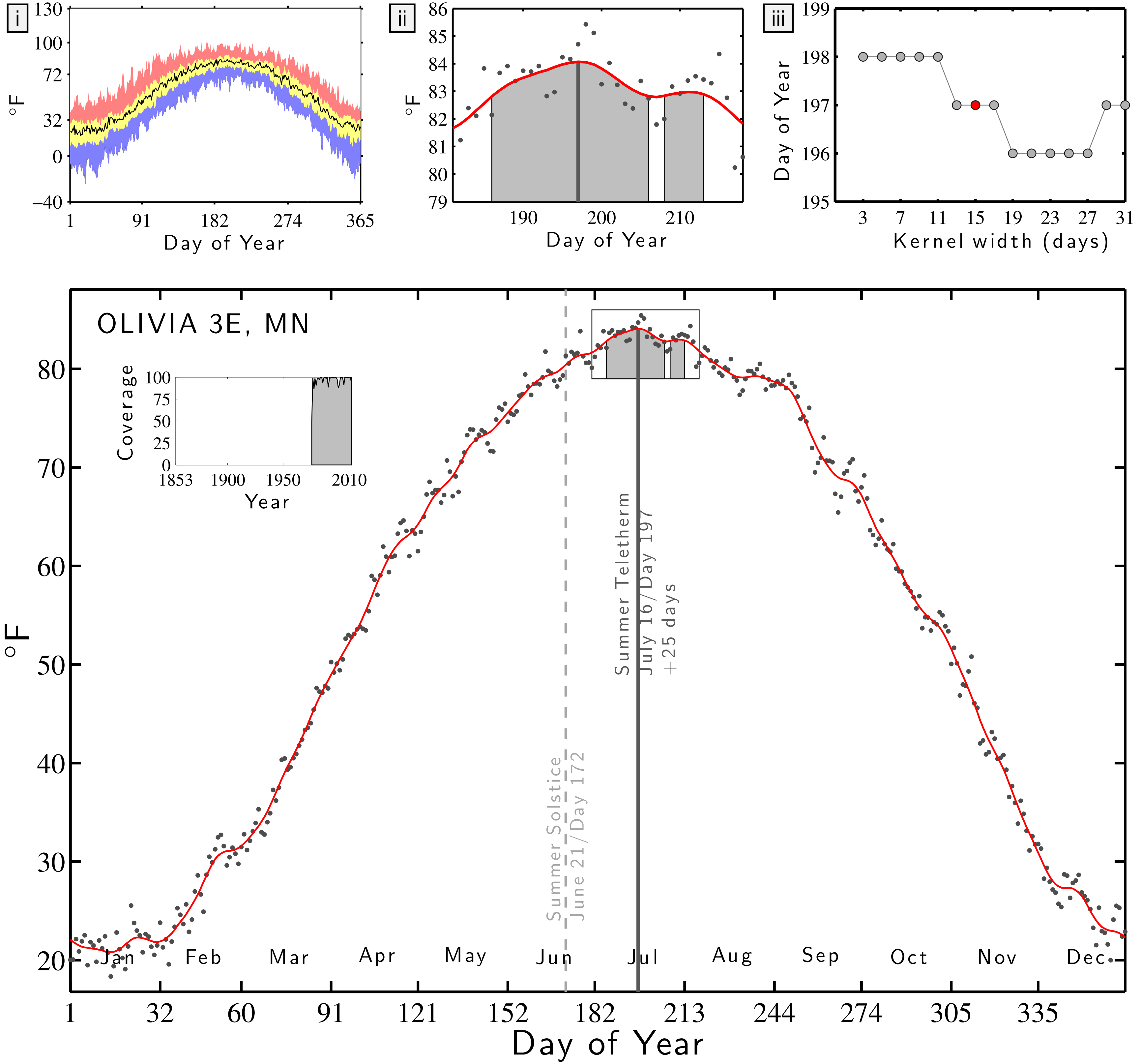

i
ii
iii
°F
Day of Year
Day of Year
Kernel width (days)
OLIVIA 3E, MN
Coverage
Year
Summer Solstice
June 21 / Day 172
Summer Teletherm
July 16 / Day 197
+25 days
Jan
Feb
Mar
Apr
May
Jun
Jul
Aug
Sep
Oct
Nov
Dec

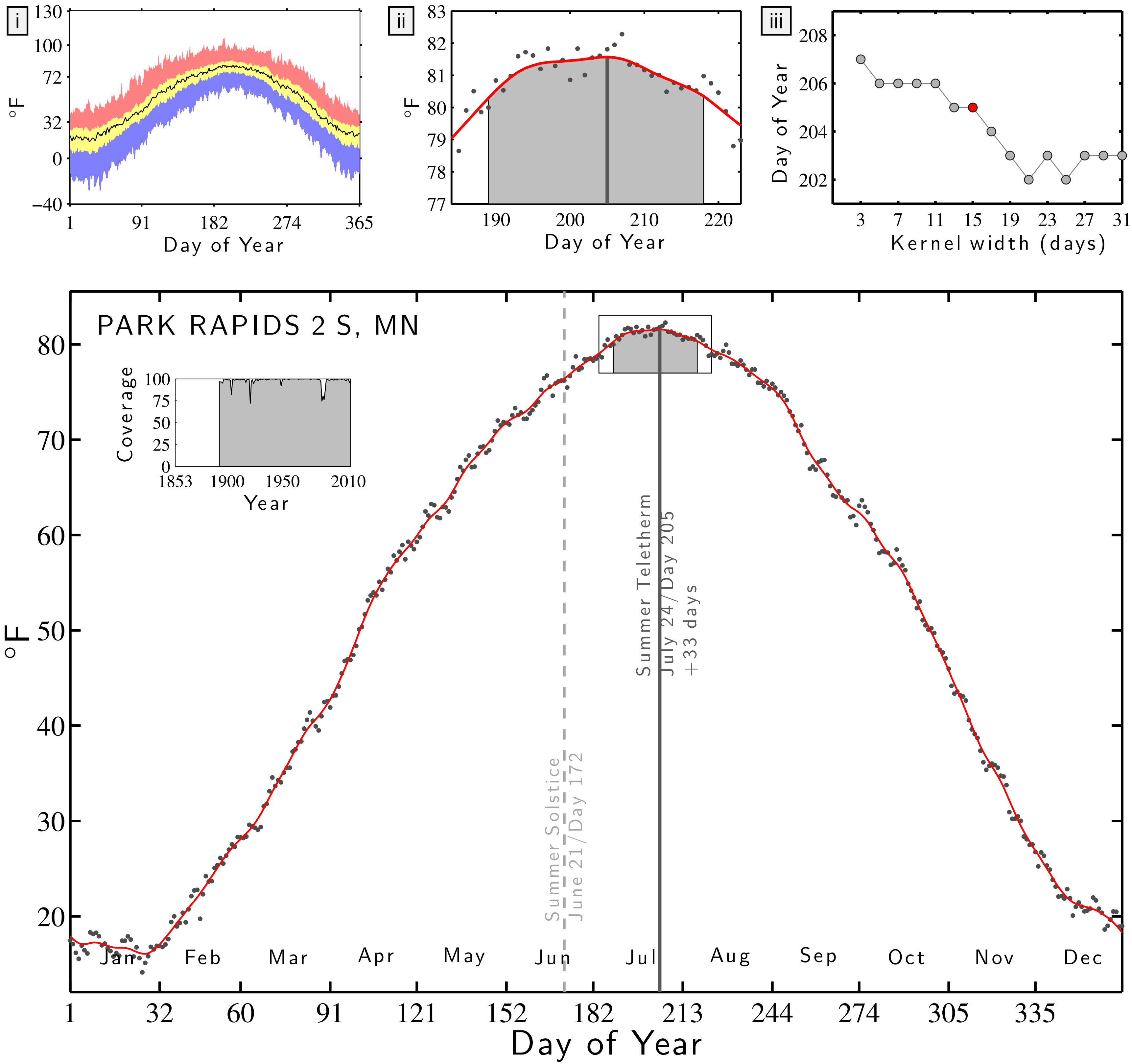

i
°F
130
100
72
32
0
−40
1
91
182
274
365
Day of Year
ii
°F
83
82
81
80
79
78
77
190
200
210
220
Day of Year
iii
Day of Year
208
206
204
202
3
7
11
15
19
23
27
31
Kernel width (days)
PARK RAPIDS 2 S, MN
Coverage
100
75
50
25
0
1853
1900
1950
2010
Year
Summer Solstice
June 21 / Day 172
Summer Teletherm
July 24 / Day 205
+33 days
°F
80
70
60
50
40
30
20
Jan
Feb
Mar
Apr
May
Jun
Jul
Aug
Sep
Oct
Nov
Dec
1
32
60
91
121
152
182
213
244
274
305
335
Day of Year

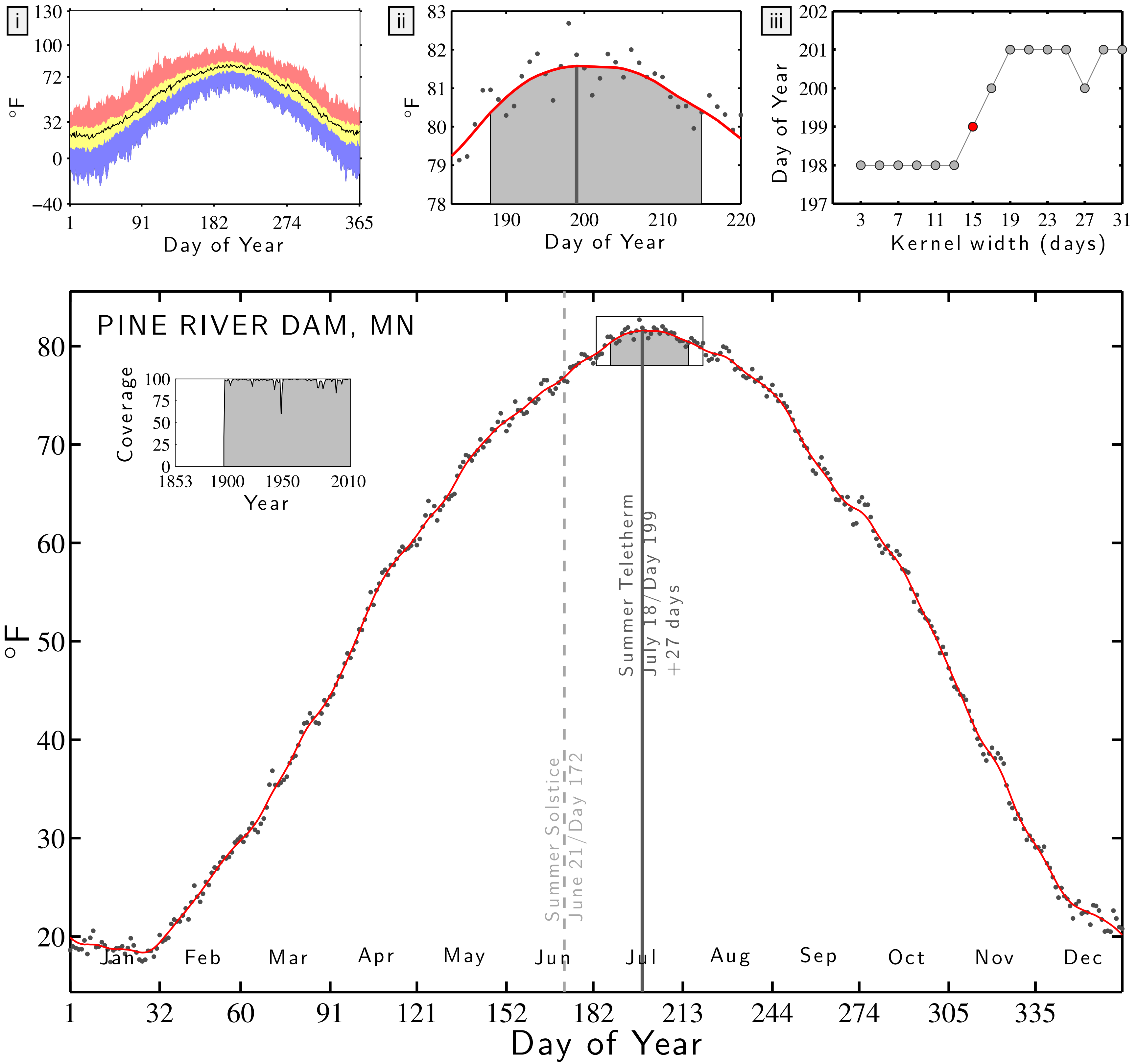

i
°F
Day of Year
ii
°F
Day of Year
iii
Day of Year
Kernel width (days)
PINE RIVER DAM, MN
Coverage
Year
Summer Solstice
June 21/Day 172
Summer Teletherm
July 18/Day 199
+27 days
Jan
Feb
Mar
Apr
May
Jun
Jul
Aug
Sep
Oct
Nov
Dec
°F
Day of Year

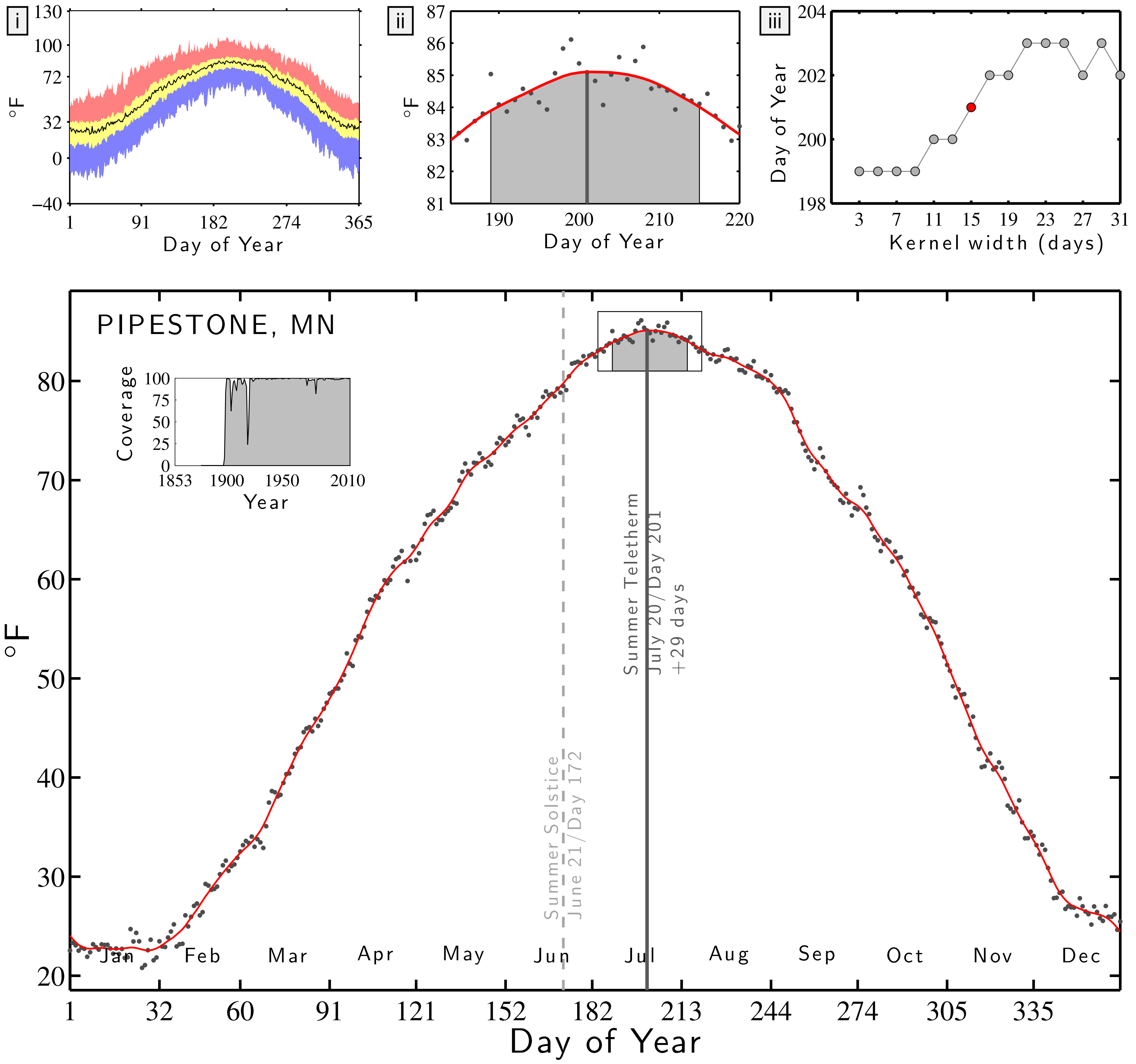

i
ii
iii
°F
Day of Year
Kernel width (days)
PIPESTONE, MN
Coverage
Year
Summer Solstice
June 21/Day 172
Summer Telotherm
July 20/Day 201
+29 days
Jan
Feb
Mar
Apr
May
Jun
Jul
Aug
Sep
Oct
Nov
Dec

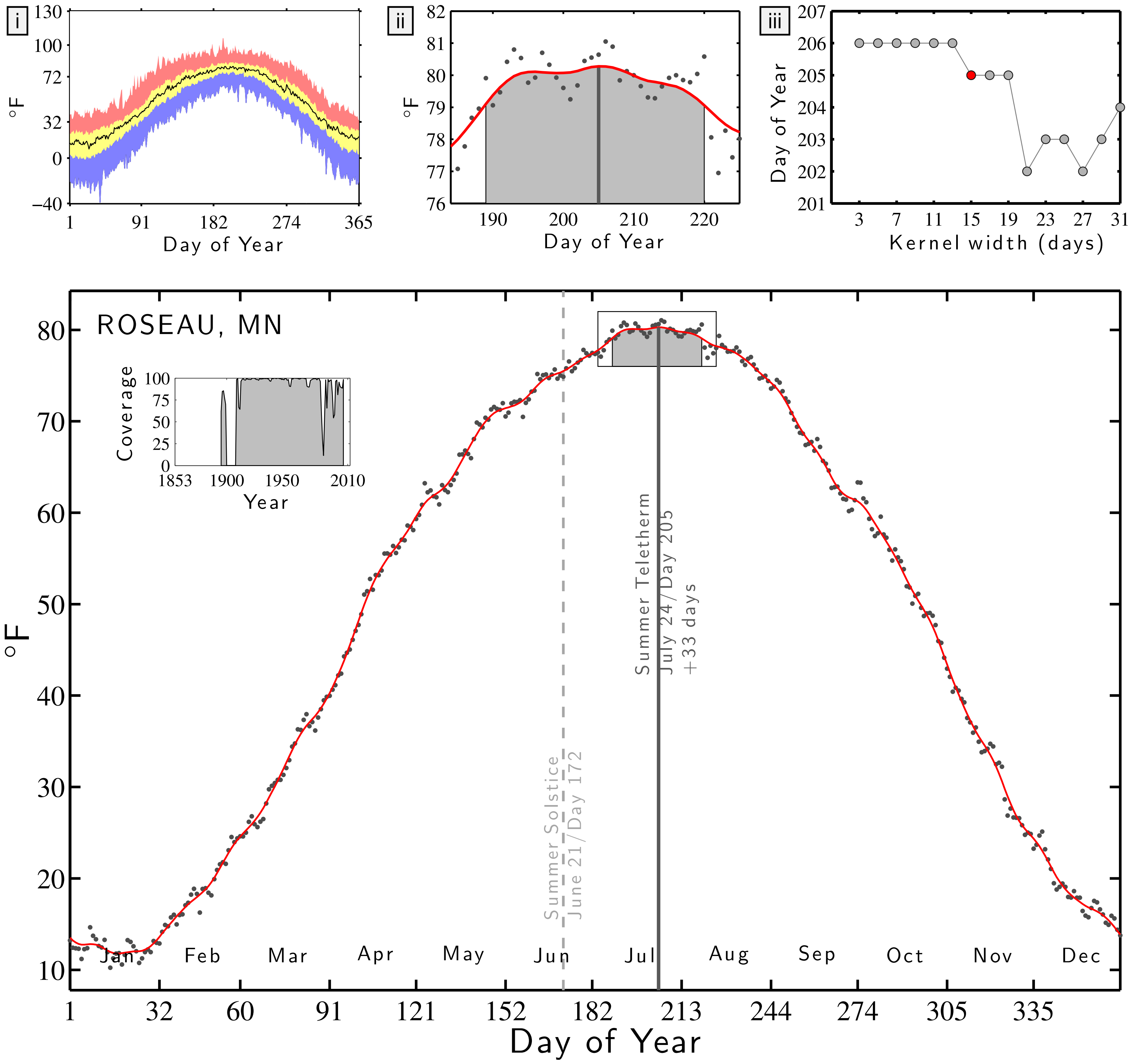

i
°F
Day of Year
ii
°F
Day of Year
iii
Day of Year
Kernel width (days)
ROSEAU, MN
Coverage
Year
Summer Solstice
June 21 / Day 172
Summer Teletherm
July 24 / Day 205
+33 days
Jan
Feb
Mar
Apr
May
Jun
Jul
Aug
Sep
Oct
Nov
Dec
°F
Day of Year

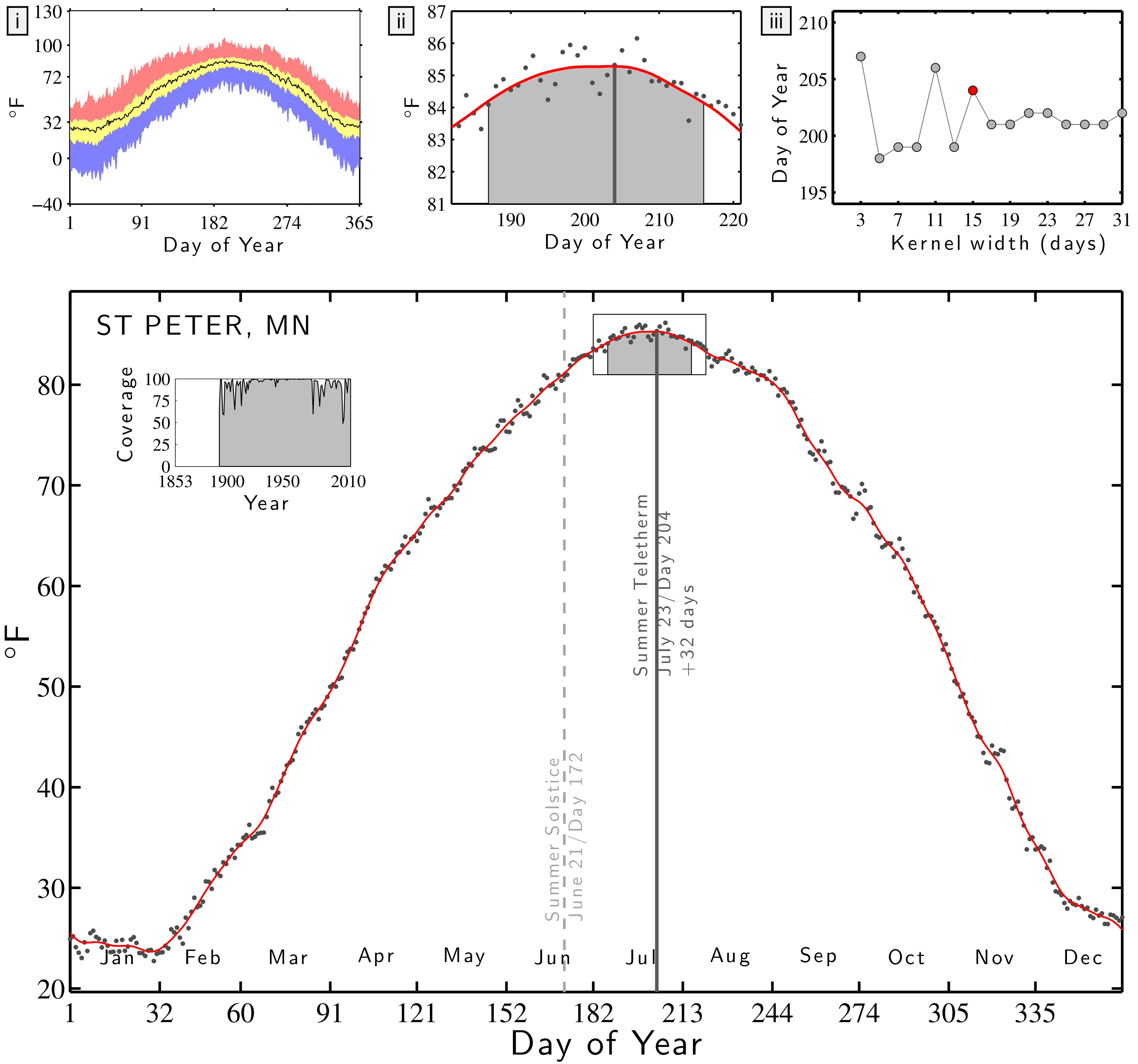

i
ii
iii
Day of Year
Kernel width (days)
ST PETER, MN
Coverage
Year
Summer Solstice
June 21 / Day 172
Summer Teletherm
July 23 / Day 204
+32 days
Jan
Feb
Mar
Apr
May
Jun
Jul
Aug
Sep
Oct
Nov
Dec
°F
Day of Year

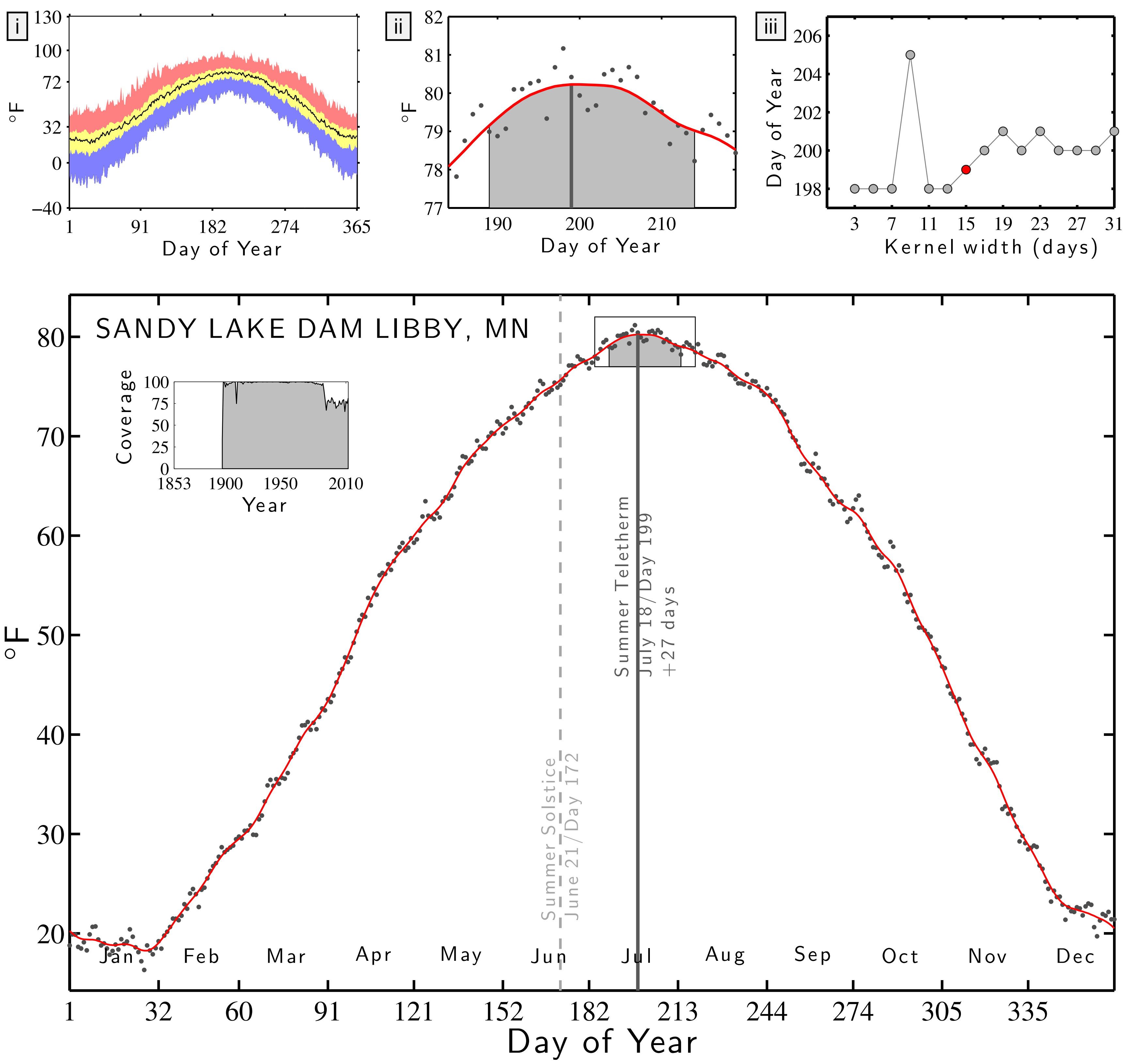

i
ii
iii
°F
Day of Year
Kernel width (days)
SANDY LAKE DAM LIBBY, MN
Coverage
Year
Summer Solstice
June 21/Day 172
Summer Teletherm
July 18/Day 199
+27 days
Jan
Feb
Mar
Apr
May
Jun
Jul
Aug
Sep
Oct
Nov
Dec

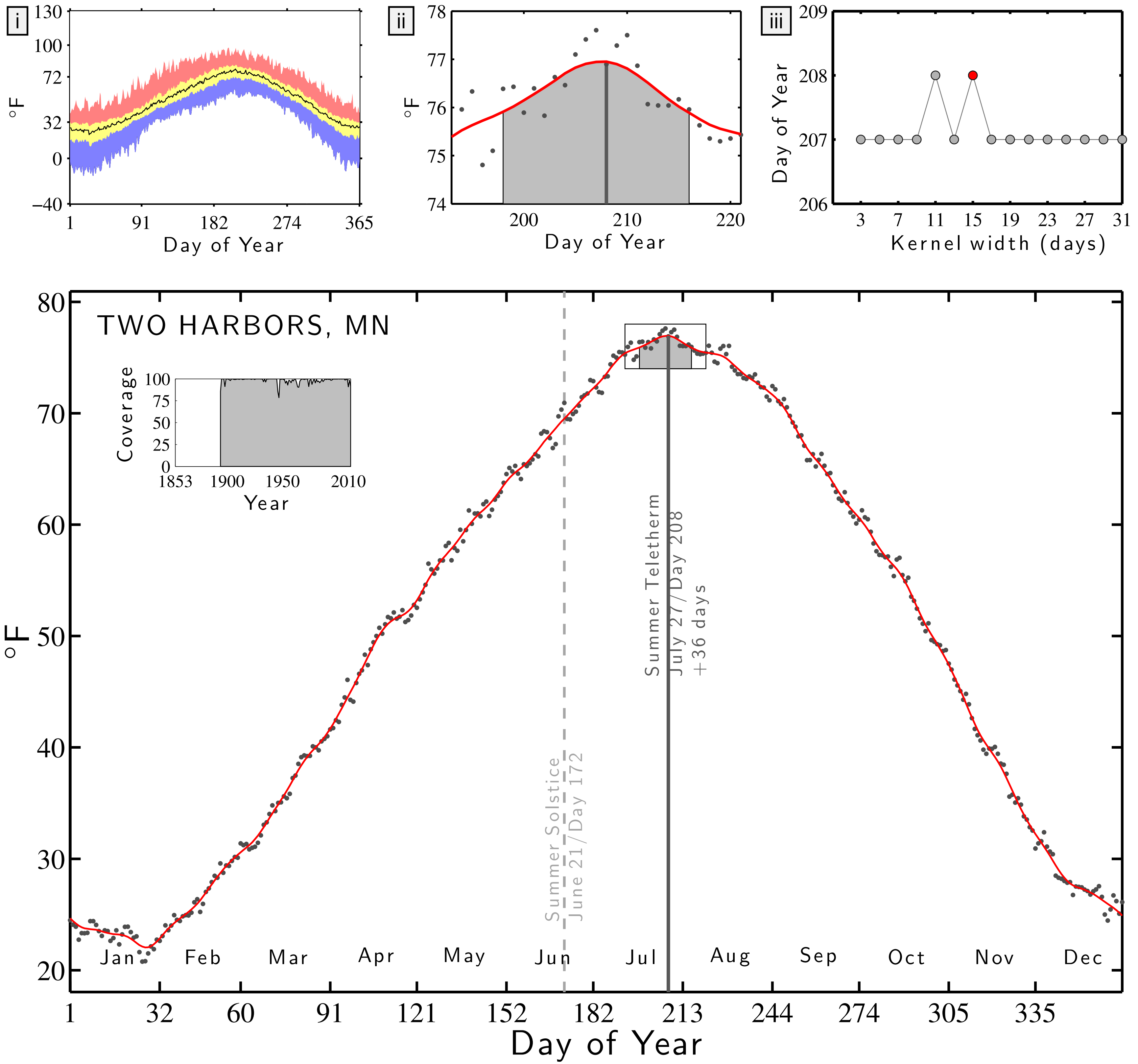

i
°F
Day of Year
ii
°F
Day of Year
iii
Day of Year
Kernel width (days)
TWO HARBORS, MN
Coverage
Year
Summer Solstice
June 21 / Day 172
Summer Teletherm
July 27 / Day 208
+36 days
Jan
Feb
Mar
Apr
May
Jun
Jul
Aug
Sep
Oct
Nov
Dec
°F
Day of Year

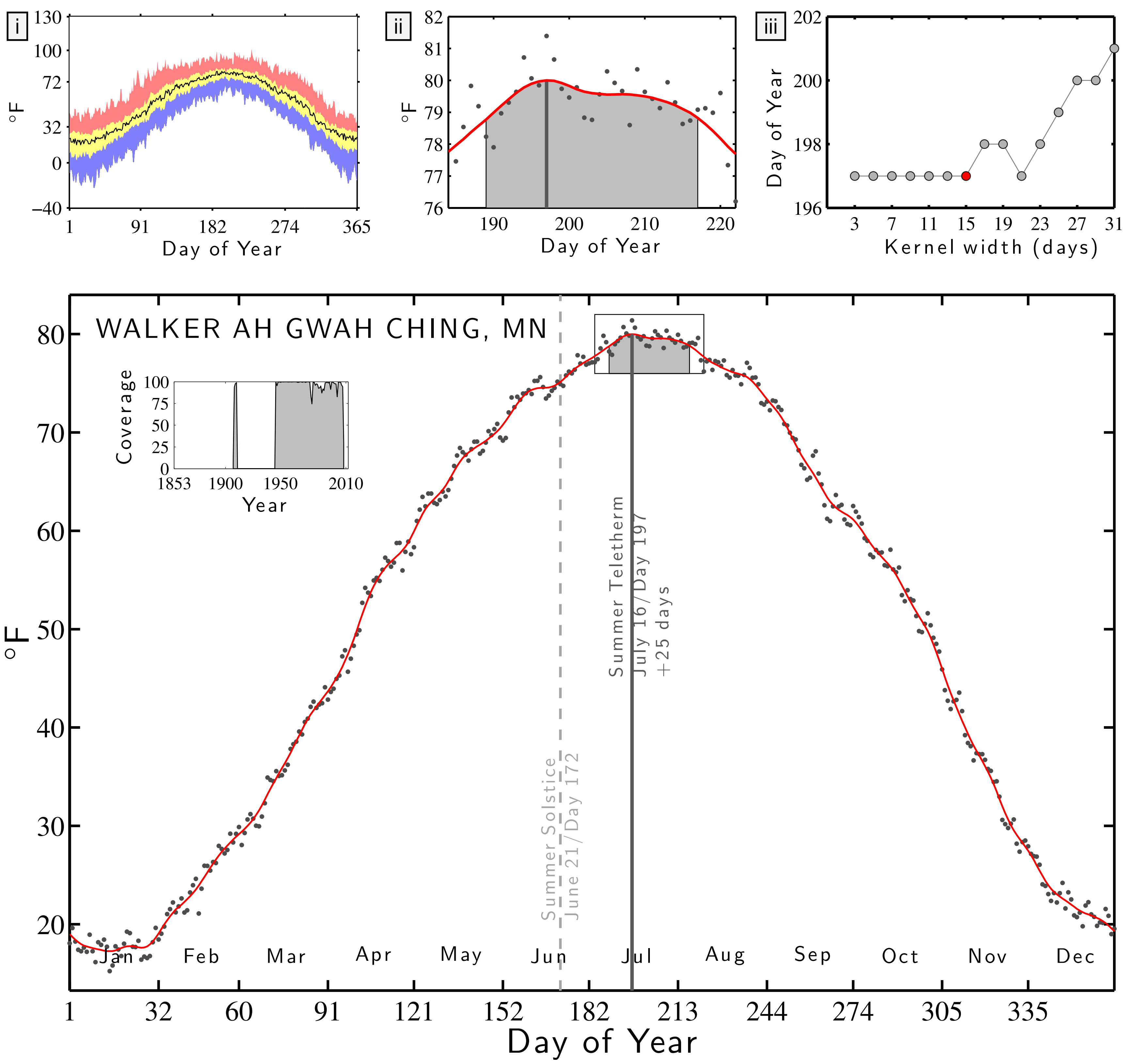

i
ii
iii
°F
Day of Year
Kernel width (days)
WALKER AH GWAH CHING, MN
Coverage
Year
Summer Solstice
June 21/Day 172
Summer Teletherm
July 16/Day 197
+25 days
Jan
Feb
Mar
Apr
May
Jun
Jul
Aug
Sep
Oct
Nov
Dec

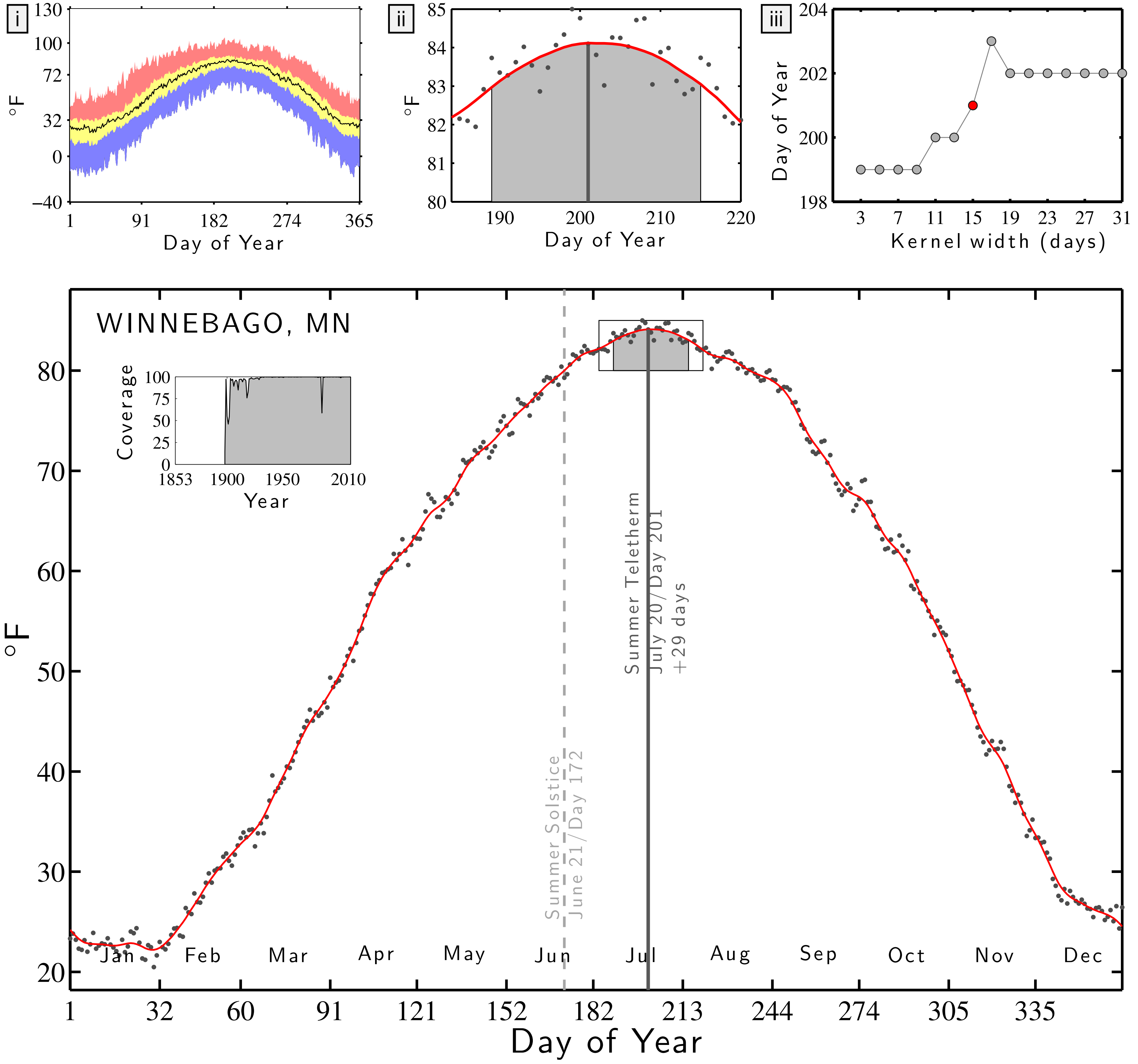

i
ii
iii
Day of Year
Kernel width (days)
WINNEBAGO, MN
Coverage
Year
Summer Solstice
June 21 / Day 172
Summer Teletherm
July 20 / Day 201
+29 days
Jan
Feb
Mar
Apr
May
Jun
Jul
Aug
Sep
Oct
Nov
Dec
°F
Day of Year

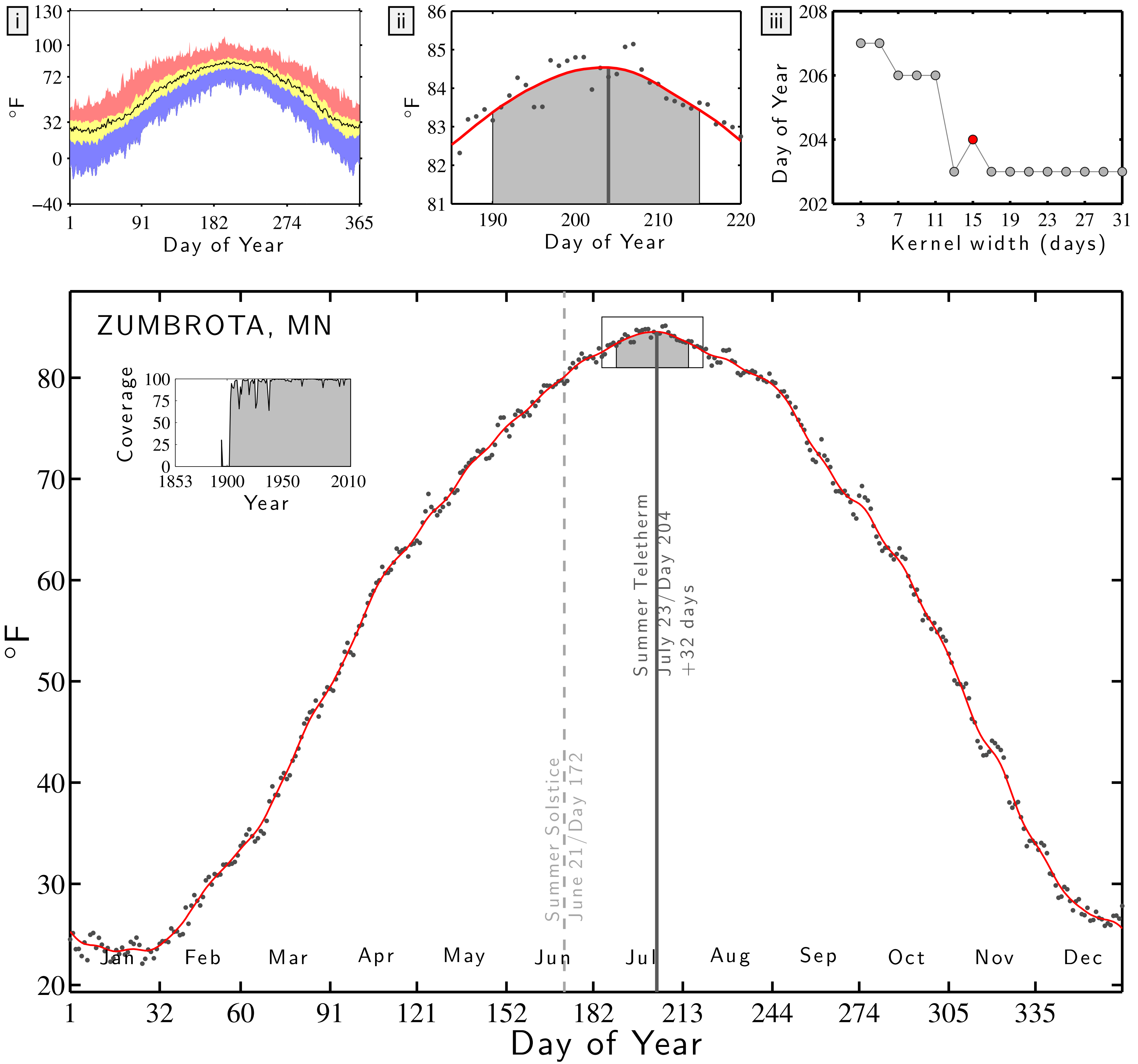

i
ii
iii
°F
Day of Year
Kernel width (days)
ZUMBROTA, MN
Coverage
Year
Summer Solstice
June 21/Day 172
Summer Teletherm
July 23/Day 204
+32 days
Jan
Feb
Mar
Apr
May
Jun
Jul
Aug
Sep
Oct
Nov
Dec

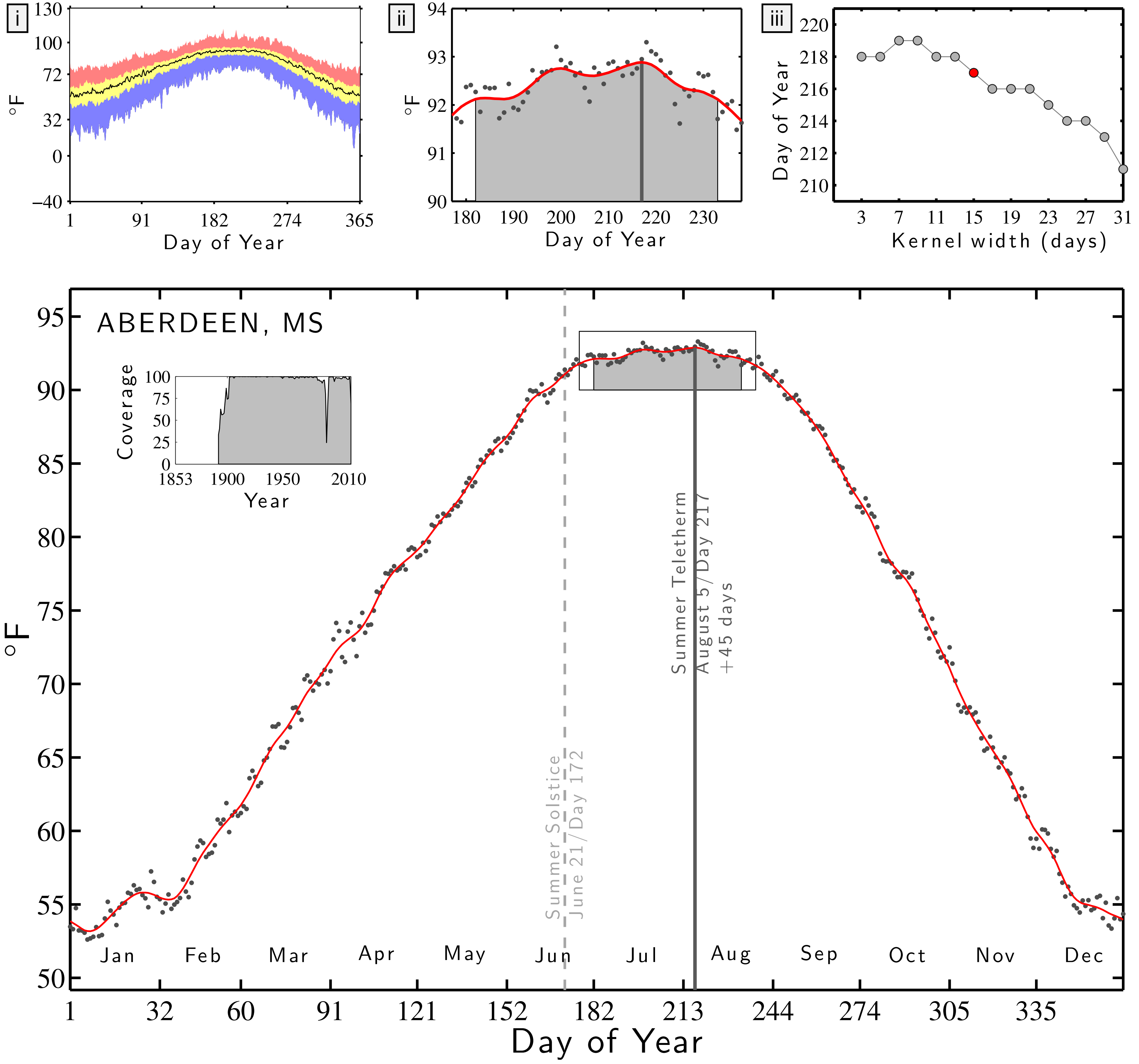

i
130
100
72
32
0
−40
°F
1
91
182
274
365
Day of Year
ii
94
93
92
91
90
°F
180
190
200
210
220
230
Day of Year
iii
220
218
216
214
212
210
Day of Year
3
7
11
15
19
23
27
31
Kernel width (days)
ABERDEEN, MS
Coverage
100
75
50
25
0
1853
1900
1950
2010
Year
95
90
85
80
75
70
65
60
55
50
°F
Summer Solstice
June 21/Day 172
Summer Teletherm
August 5/Day 217
+45 days
Jan
Feb
Mar
Apr
May
Jun
Jul
Aug
Sep
Oct
Nov
Dec
1
32
60
91
121
152
182
213
244
274
305
335
Day of Year

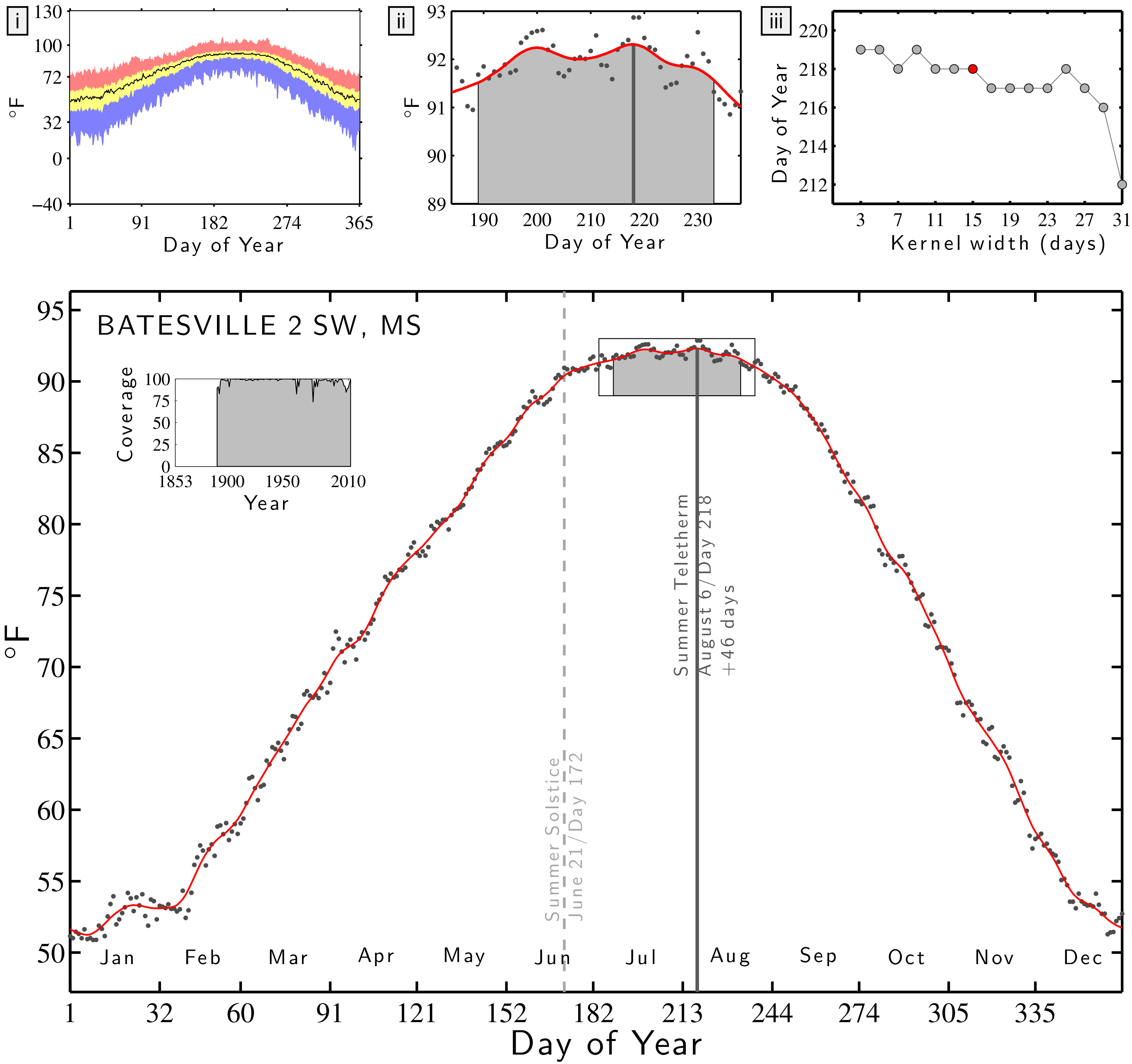

i
°F
Day of Year
ii
°F
Day of Year
iii
Day of Year
Kernel width (days)
BATESVILLE 2 SW, MS
Coverage
Year
Summer Solstice
June 21/Day 172
Summer Teletherm
August 6/Day 218
+46 days
Jan
Feb
Mar
Apr
May
Jun
Jul
Aug
Sep
Oct
Nov
Dec
°F
Day of Year

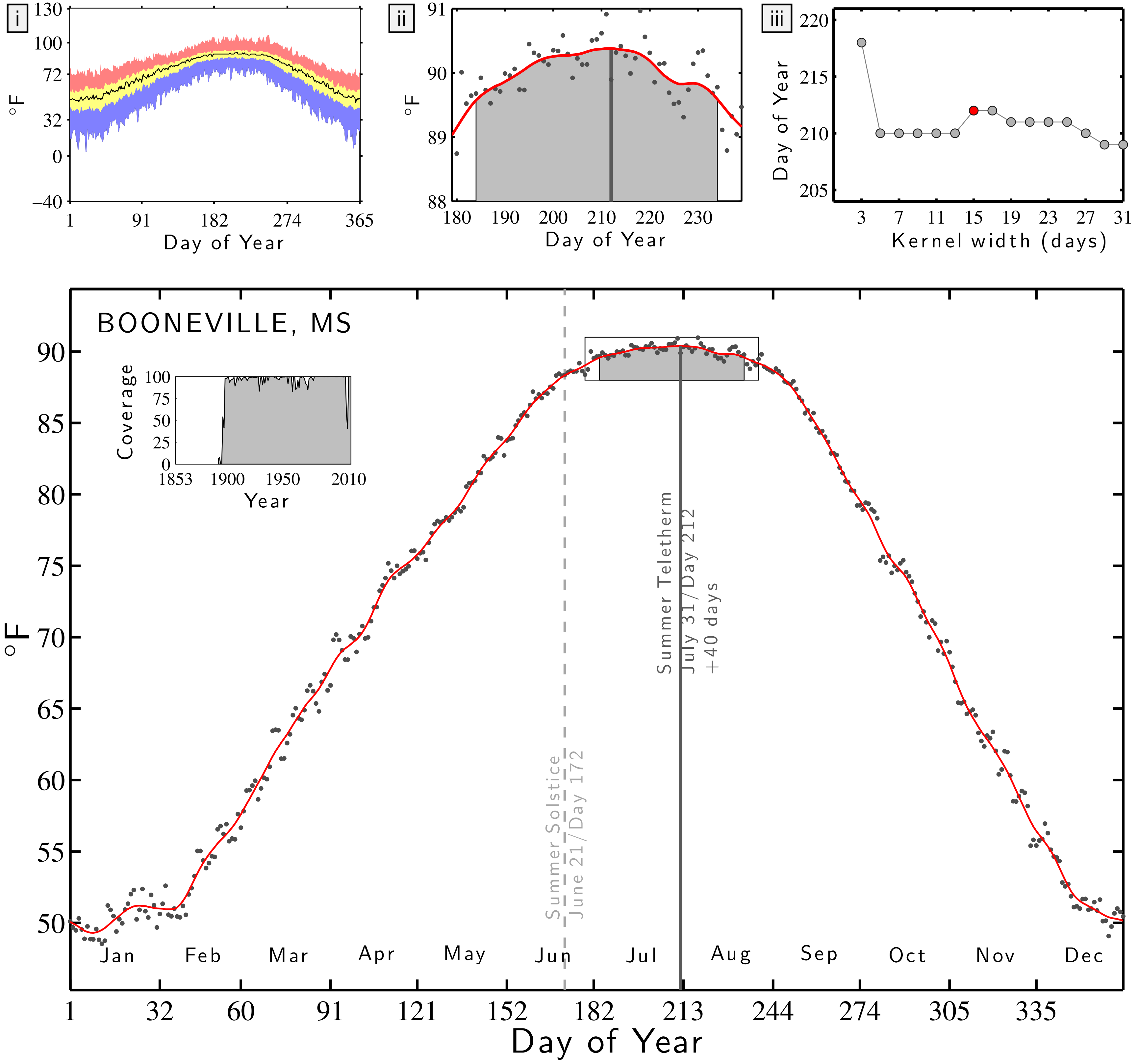

i
°F
130
100
72
32
0
−40
1
91
182
274
365
Day of Year
ii
91
90
89
88
180
190
200
210
220
230
Day of Year
iii
Day of Year
220
215
210
205
3
7
11
15
19
23
27
31
Kernel width (days)
BOONEVILLE, MS
Coverage
100
75
50
25
0
1853
1900
1950
2010
Year
Summer Solstice
June 21 / Day 172
Summer Teletherm
July 31 / Day 212
+40 days
°F
90
85
80
75
70
65
60
55
50
Jan
Feb
Mar
Apr
May
Jun
Jul
Aug
Sep
Oct
Nov
Dec
1
32
60
91
121
152
182
213
244
274
305
335
Day of Year

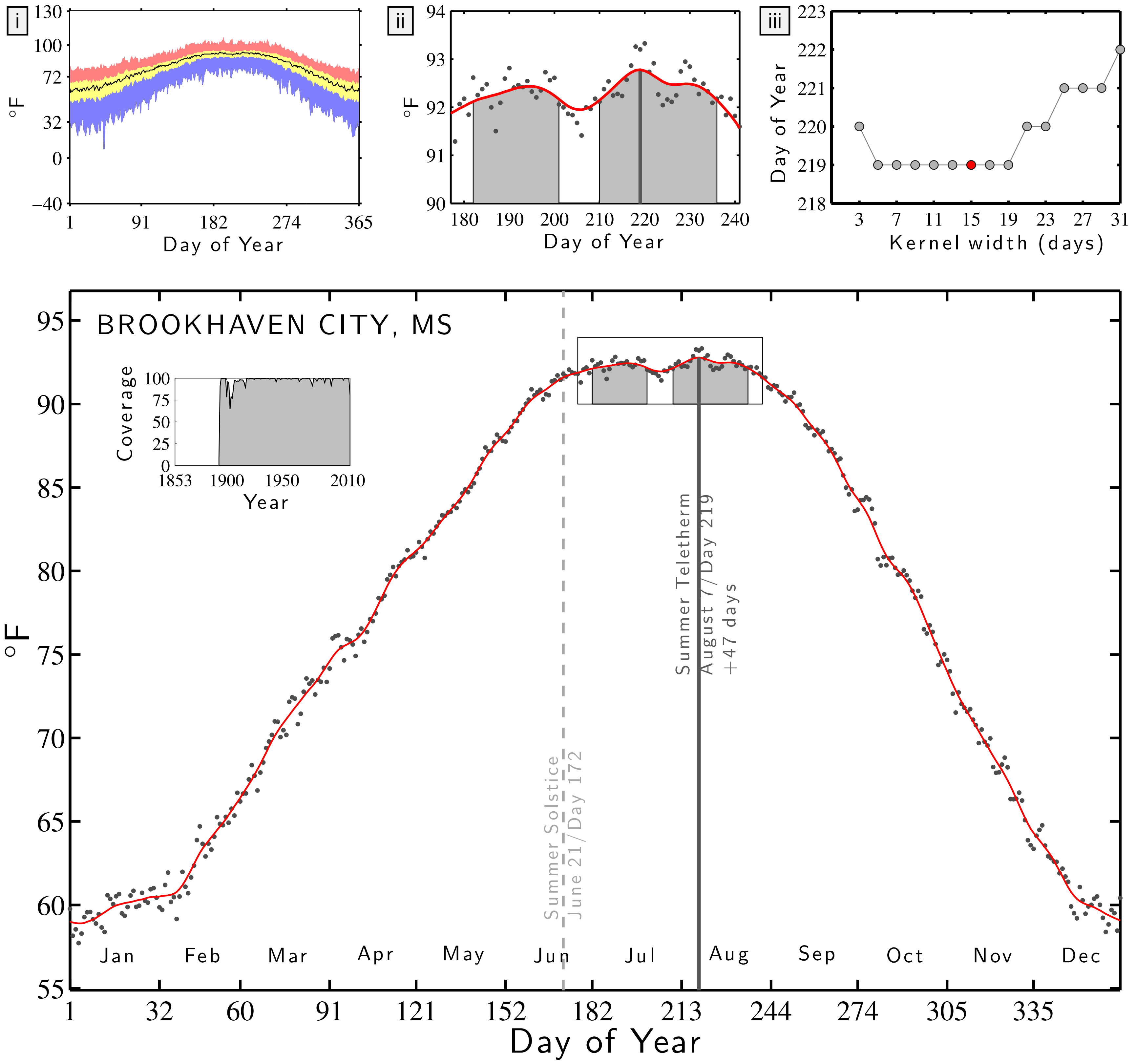
i
ii
iii
°F
Day of Year
Day of Year
Kernel width (days)
BROOKHAVEN CITY, MS
Coverage
Year
Summer Solstice
June 21 / Day 172
Summer Teletherm
August 7 / Day 219
+47 days
Jan
Feb
Mar
Apr
May
Jun
Jul
Aug
Sep
Oct
Nov
Dec
°F
Day of Year

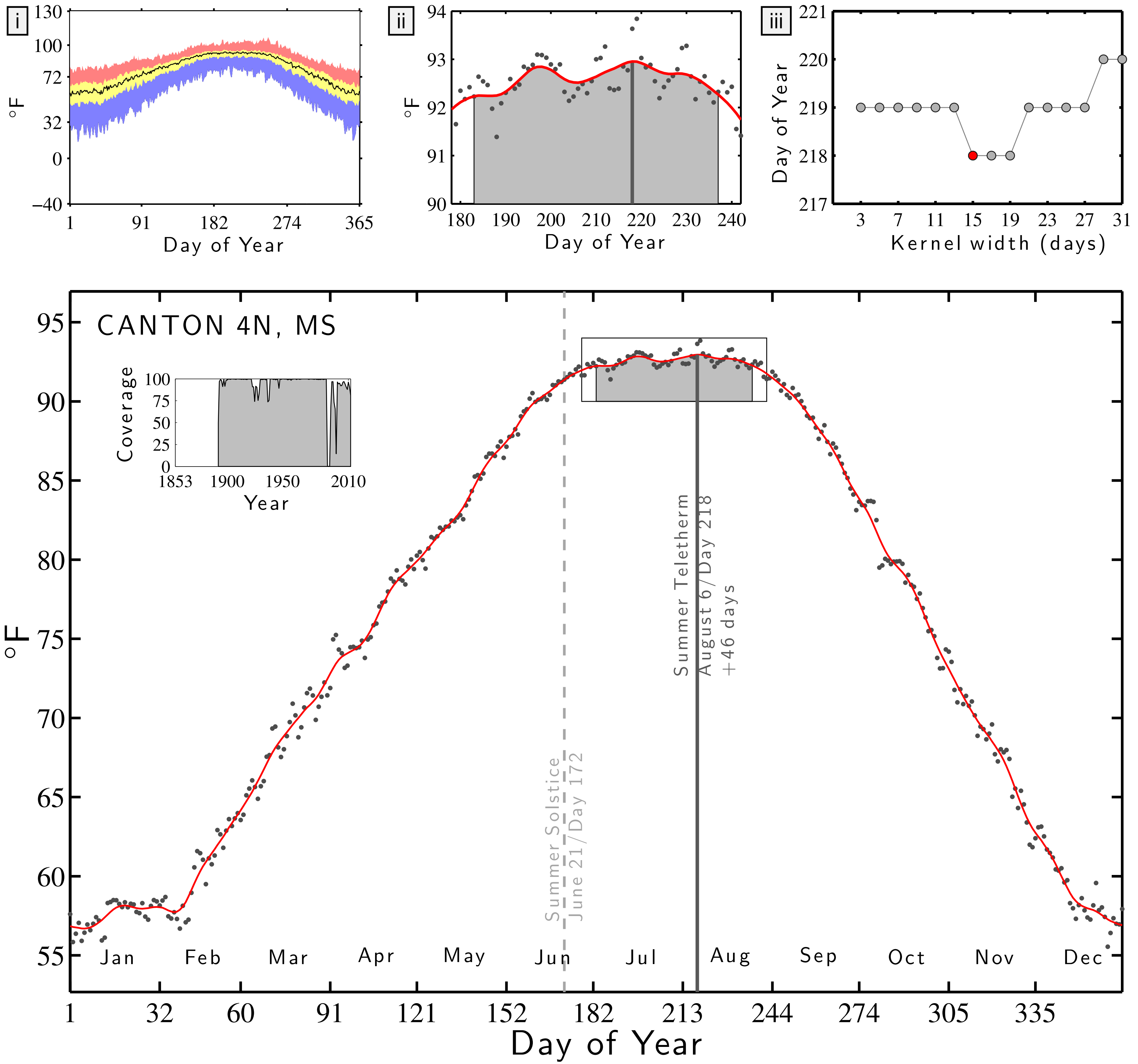

i
ii
iii
Day of Year
Kernel width (days)
CANTON 4N, MS
Coverage
Year
Summer Solstice
June 21 / Day 172
Summer Teletherm
August 6 / Day 218
+46 days
Jan
Feb
Mar
Apr
May
Jun
Jul
Aug
Sep
Oct
Nov
Dec
°F
Day of Year

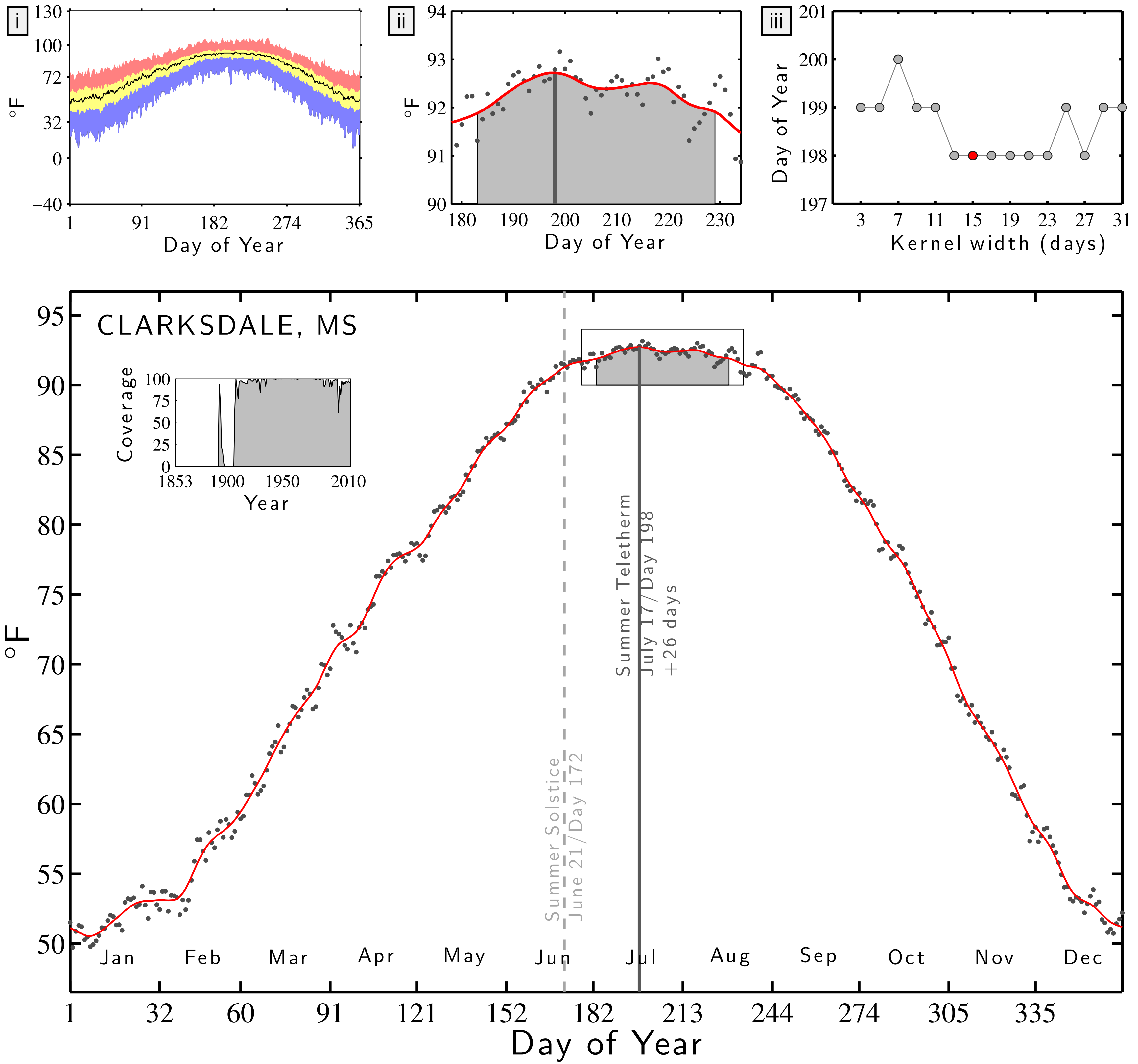

i
°F
130
100
72
32
0
−40
1
91
182
274
365
Day of Year
ii
94
93
92
91
90
180
190
200
210
220
230
Day of Year
iii
Day of Year
201
200
199
198
197
3
7
11
15
19
23
27
31
Kernel width (days)
CLARKSDALE, MS
Coverage
100
75
50
25
0
1853
1900
1950
2010
Year
Summer Solstice
June 21/Day 172
Summer Teletherm
July 17/Day 198
+26 days
95
90
85
80
75
70
65
60
55
50
Jan
Feb
Mar
Apr
May
Jun
Jul
Aug
Sep
Oct
Nov
Dec
1
32
60
91
121
152
182
213
244
274
305
335
Day of Year

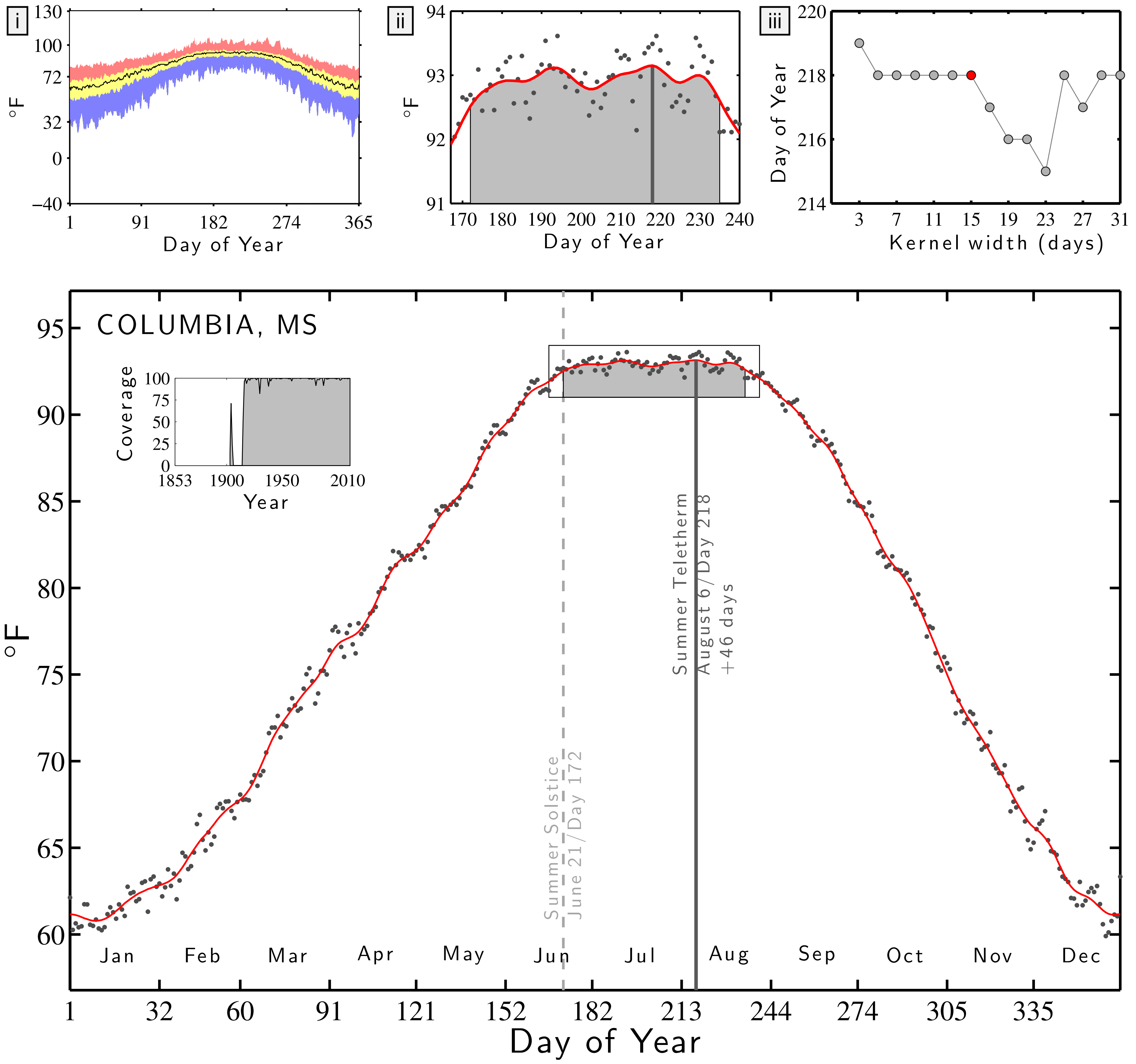

i
°F
Day of Year
ii
°F
Day of Year
iii
Day of Year
Kernel width (days)
COLUMBIA, MS
Coverage
Year
Summer Solstice
June 21/Day 172
Summer Teletherm
August 6/Day 218
+46 days
Jan
Feb
Mar
Apr
May
Jun
Jul
Aug
Sep
Oct
Nov
Dec
°F
Day of Year

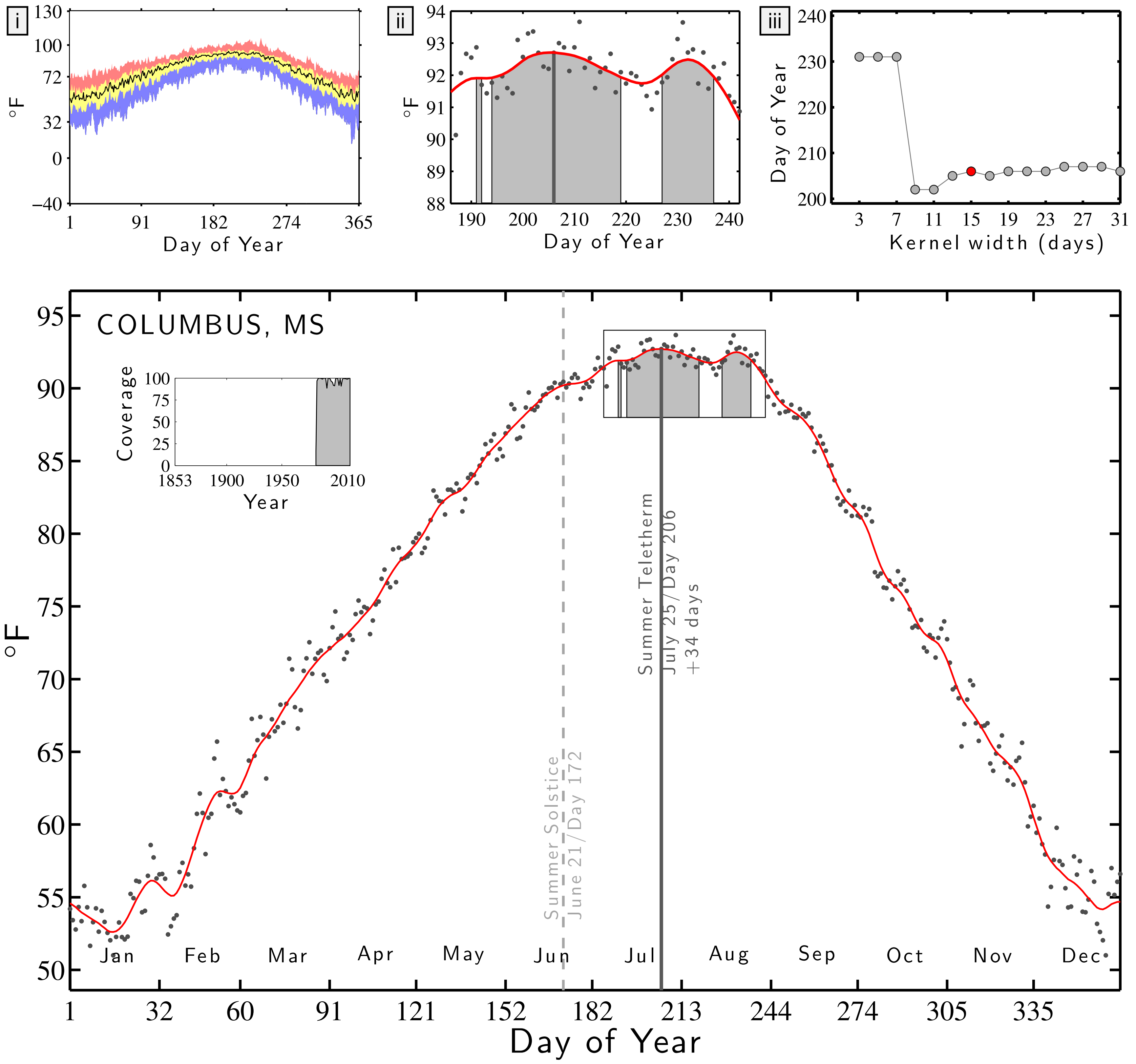

i
°F
Day of Year
ii
°F
Day of Year
iii
Day of Year
Kernel width (days)
COLUMBUS, MS
Coverage
Year
Summer Solstice
June 21/Day 172
Summer Teletherm
July 25/Day 206
+34 days
Jan
Feb
Mar
Apr
May
Jun
Jul
Aug
Sep
Oct
Nov
Dec
°F
Day of Year

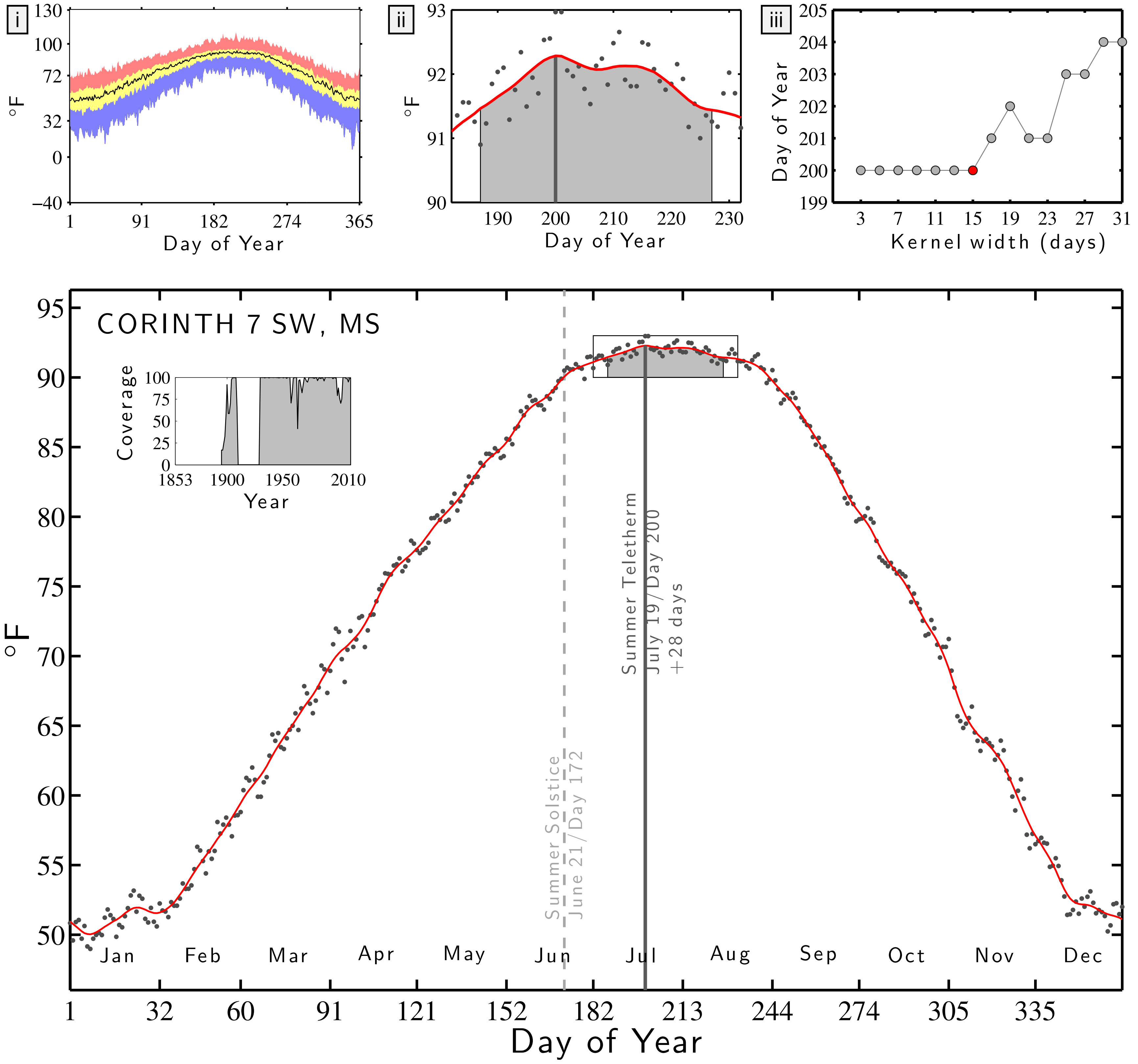
i
ii
iii
°F
Day of Year
Day of Year
Kernel width (days)
CORINTH 7 SW, MS
Coverage
Year
Summer Solstice
June 21/Day 172
Summer Teletherm
July 19/Day 200
+28 days
Jan
Feb
Mar
Apr
May
Jun
Jul
Aug
Sep
Oct
Nov
Dec
°F
Day of Year

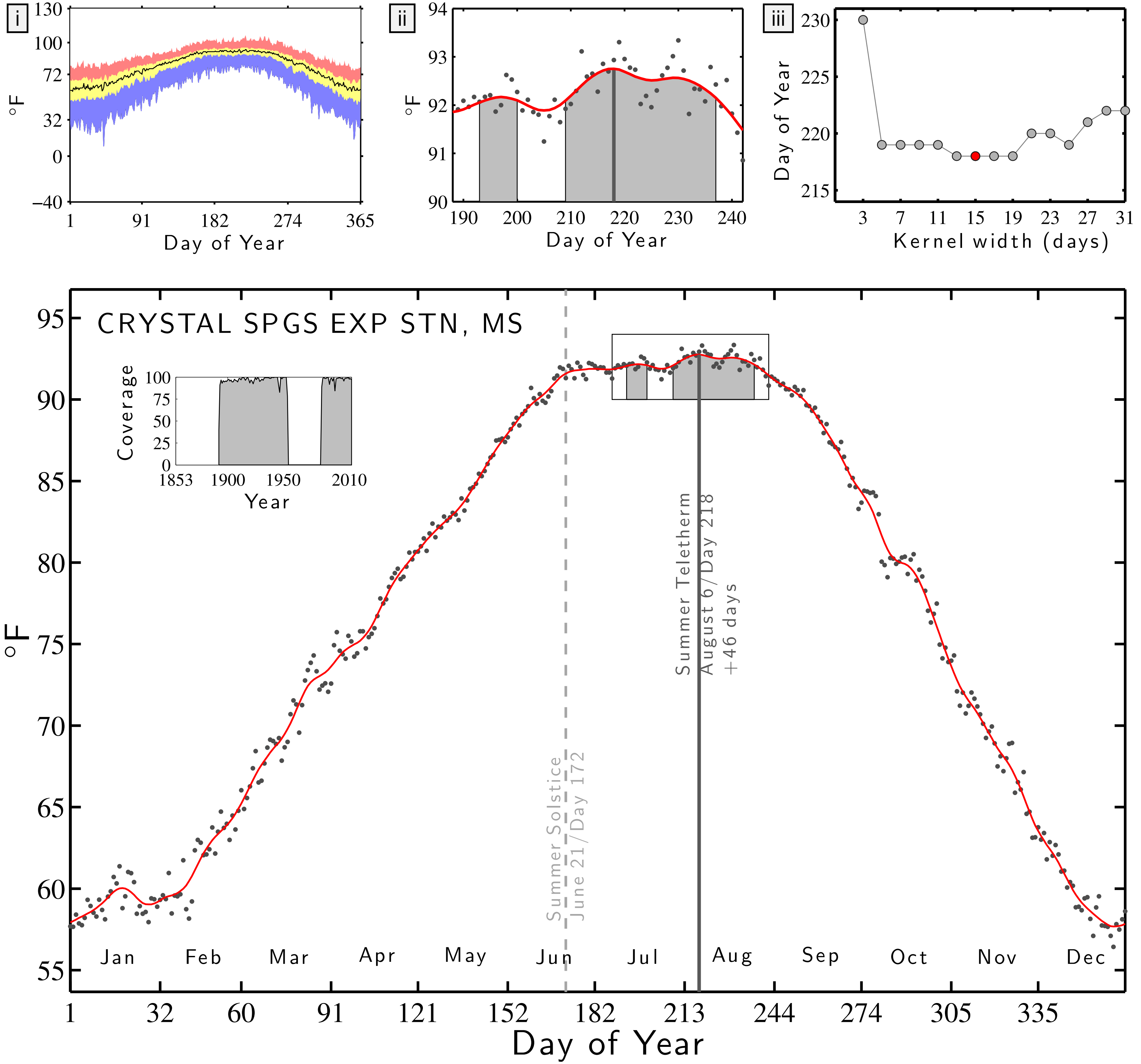

i
ii
iii
°F
Day of Year
Kernel width (days)
CRYSTAL SPGS EXP STN, MS
Coverage
Year
Summer Solstice
June 21 / Day 172
Summer Teletherm
August 6 / Day 218
+46 days
Jan
Feb
Mar
Apr
May
Jun
Jul
Aug
Sep
Oct
Nov
Dec

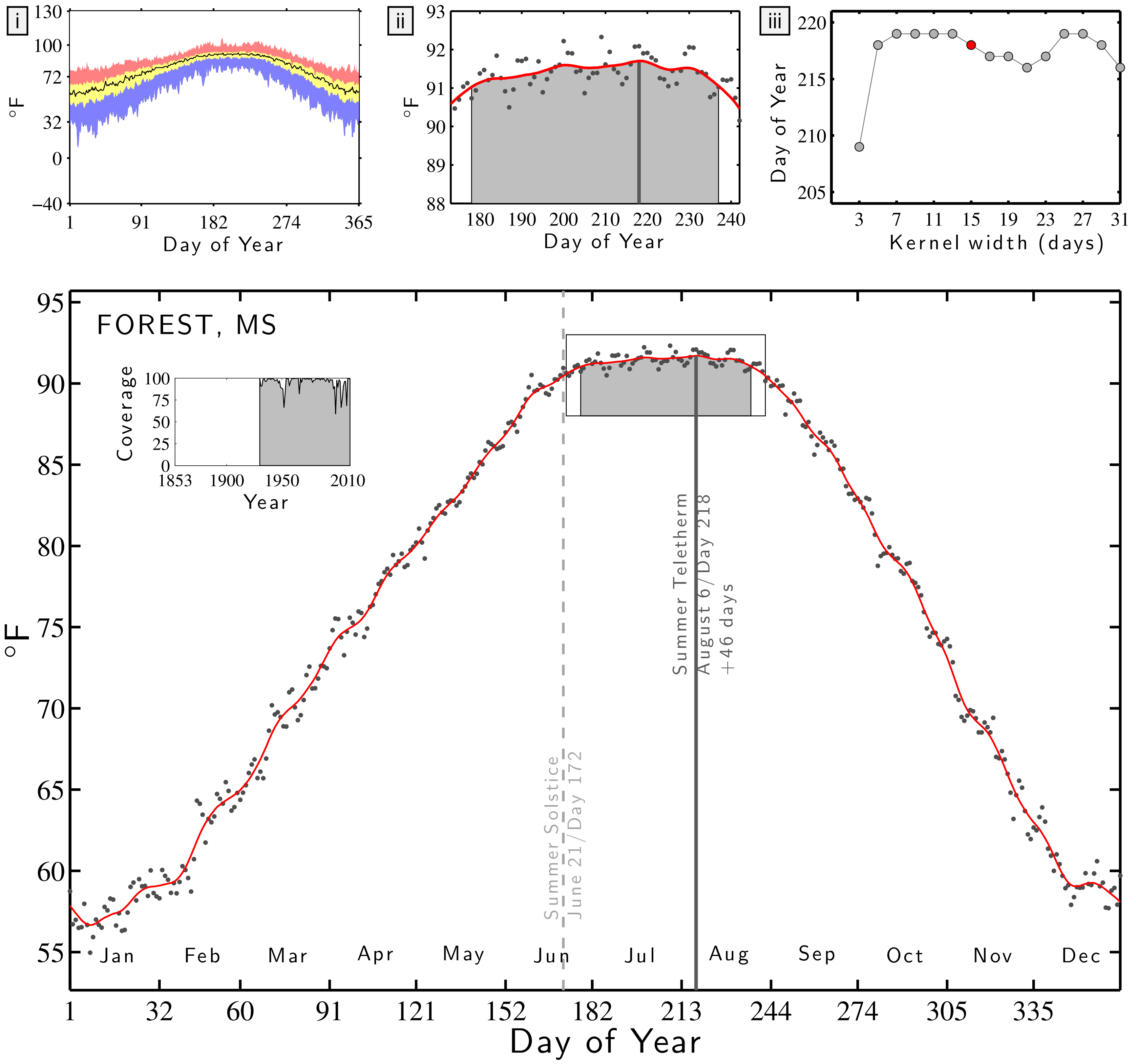

i
°F
Day of Year
ii
°F
Day of Year
iii
Day of Year
Kernel width (days)
FOREST, MS
Coverage
Year
1853
1900
1950
2010
Summer Solstice
June 21/Day 172
Summer Teletherm
August 6/Day 218
+46 days
Jan
Feb
Mar
Apr
May
Jun
Jul
Aug
Sep
Oct
Nov
Dec
°F
Day of Year

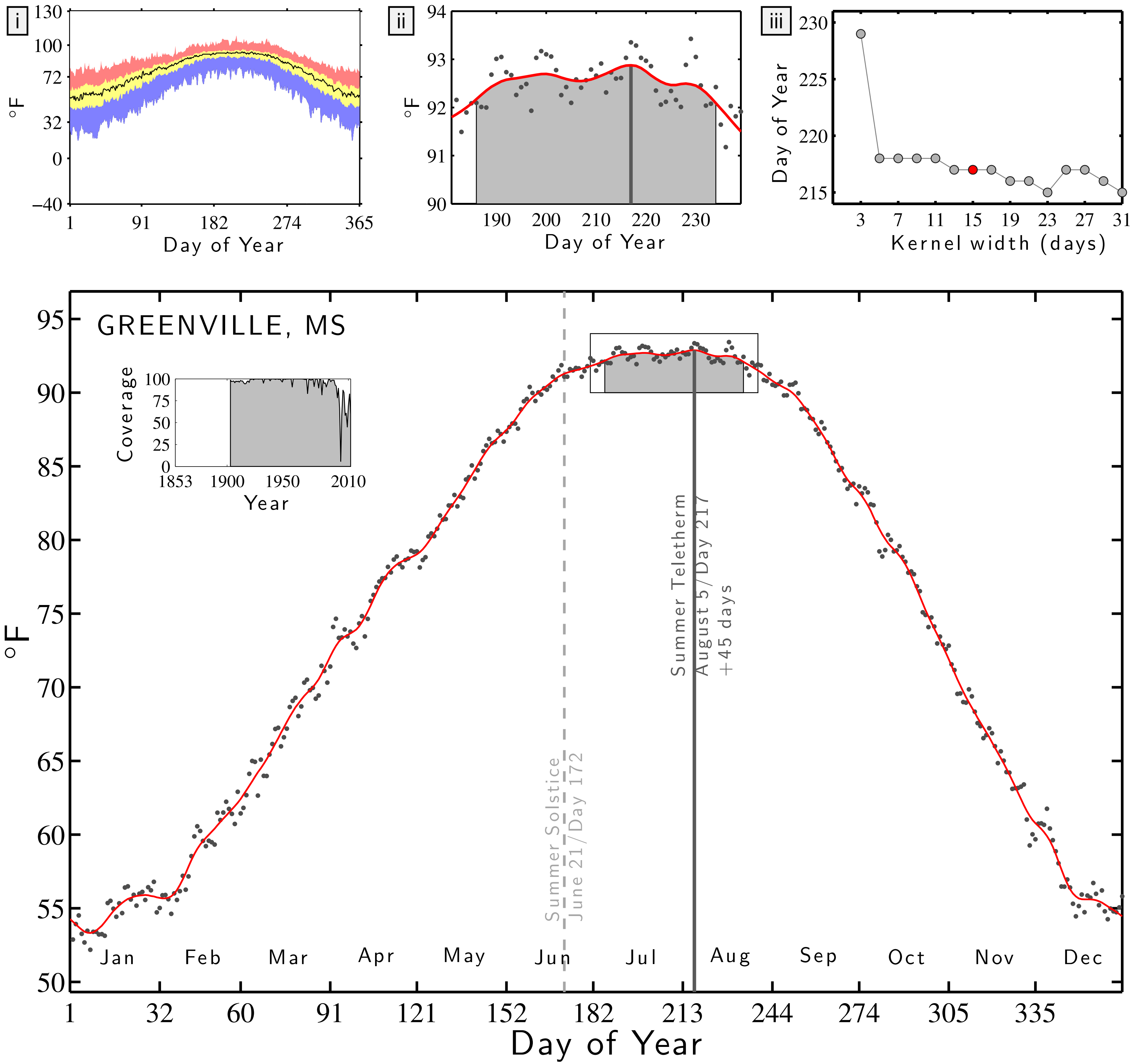
i
°F
Day of Year
ii
°F
Day of Year
iii
Day of Year
Kernel width (days)
GREENVILLE, MS
Coverage
Year
Summer Solstice
June 21/Day 172
Summer Teletherm
August 5/Day 217
+45 days
Jan
Feb
Mar
Apr
May
Jun
Jul
Aug
Sep
Oct
Nov
Dec
°F
Day of Year

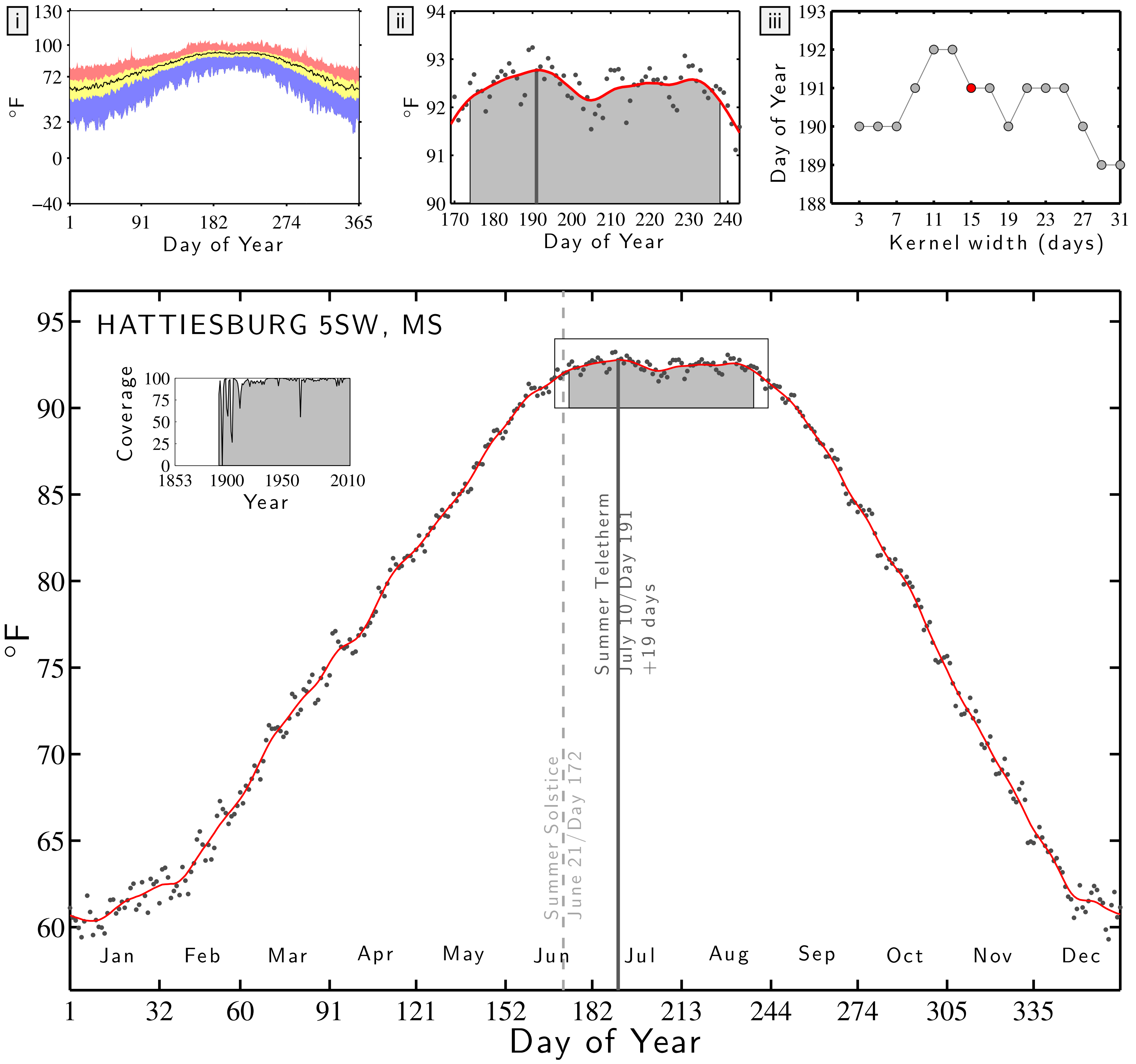

i
ii
iii
°F
Day of Year
Day of Year
Kernel width (days)
HATTIESBURG 5SW, MS
Coverage
Year
Summer Solstice
June 21/Day 172
Summer Teletherm
July 10/Day 191
+19 days
Jan
Feb
Mar
Apr
May
Jun
Jul
Aug
Sep
Oct
Nov
Dec

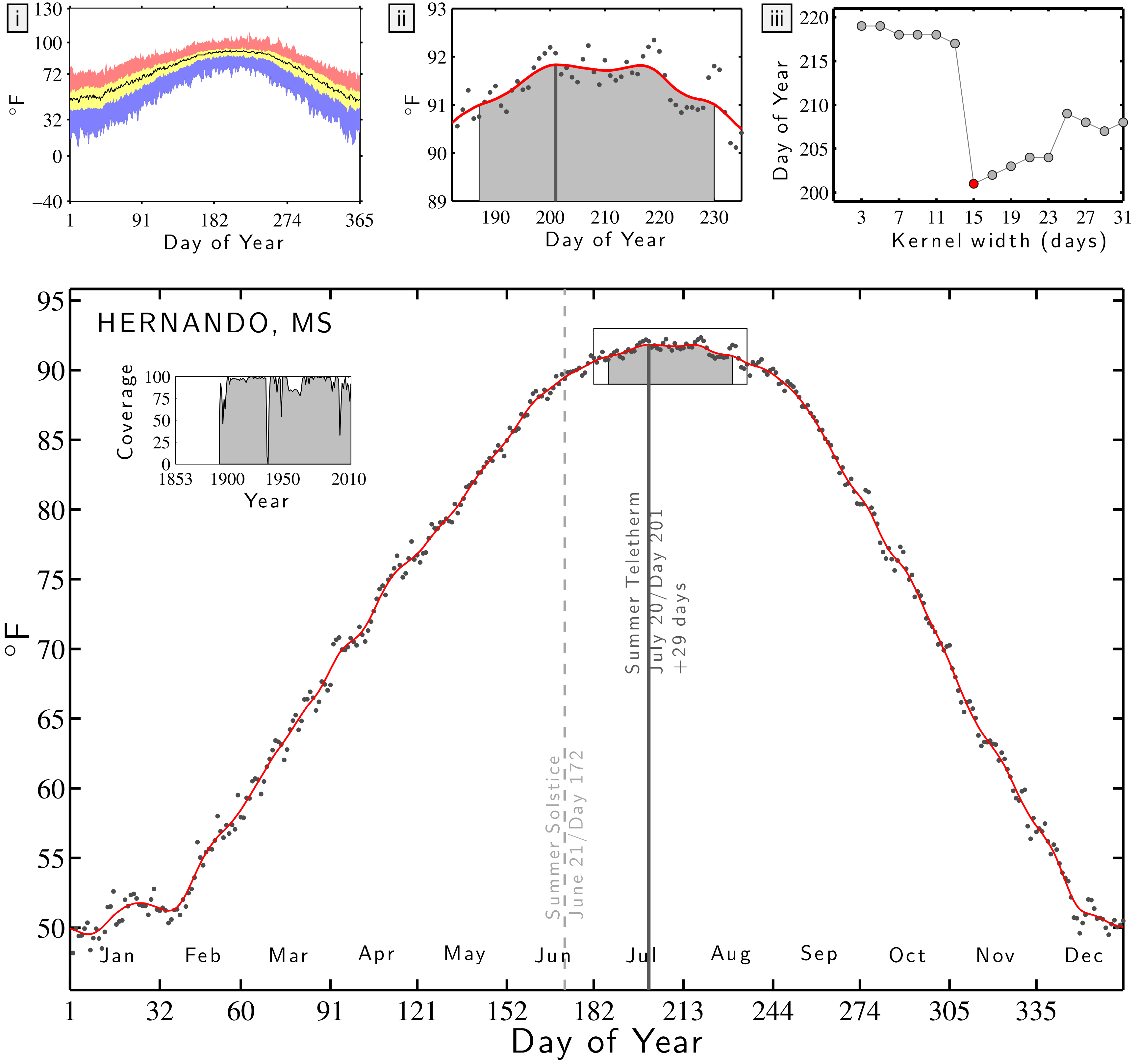

i
°F
Day of Year
ii
°F
Day of Year
iii
Day of Year
Kernel width (days)
HERNANDO, MS
Coverage
Year
Summer Solstice
June 21/Day 172
Summer Teletherm
July 20/Day 201
+29 days
Jan
Feb
Mar
Apr
May
Jun
Jul
Aug
Sep
Oct
Nov
Dec
°F
Day of Year

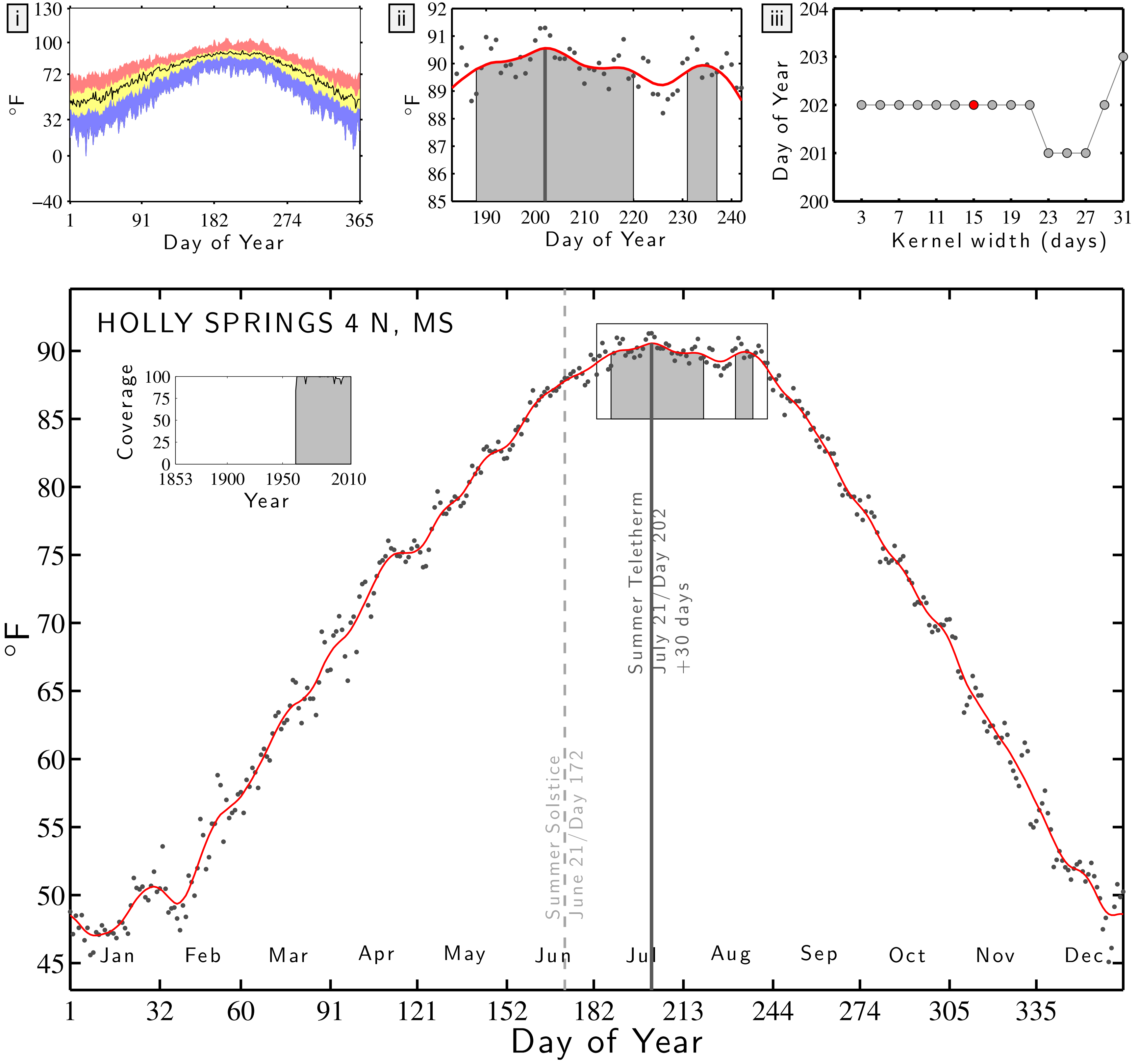

i
°F
Day of Year
ii
°F
Day of Year
iii
Day of Year
Kernel width (days)
HOLLY SPRINGS 4 N, MS
Coverage
Year
Summer Solstice
June 21 / Day 172
Summer Teletherm
July 21 / Day 202
+30 days
Jan
Feb
Mar
Apr
May
Jun
Jul
Aug
Sep
Oct
Nov
Dec
°F
Day of Year

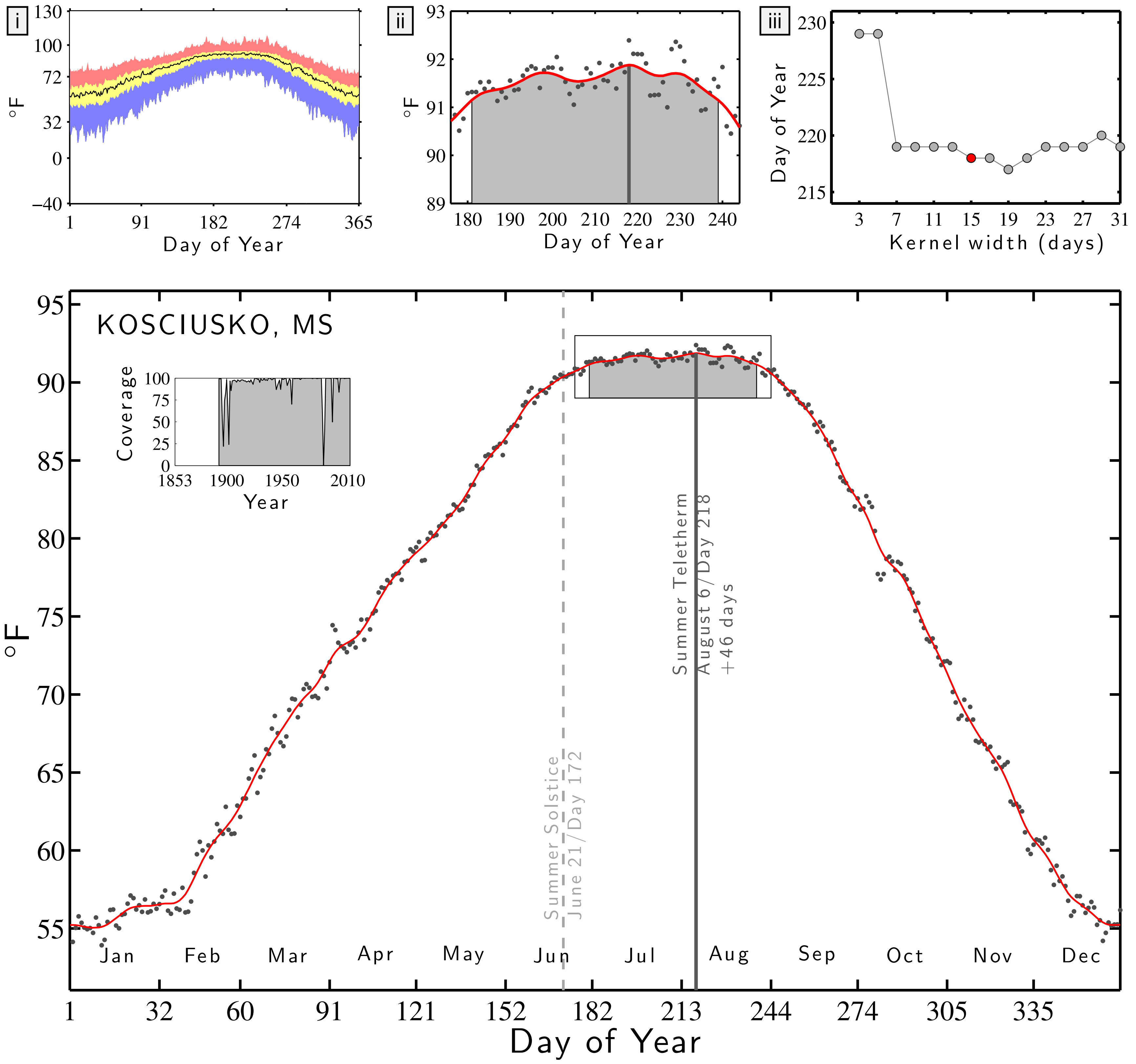

i
ii
iii
°F
Day of Year
Kernel width (days)
KOSCIUSKO, MS
Coverage
Year
Summer Solstice
June 21/Day 172
Summer Teletherm
August 6/Day 218
+46 days
Jan
Feb
Mar
Apr
May
Jun
Jul
Aug
Sep
Oct
Nov
Dec

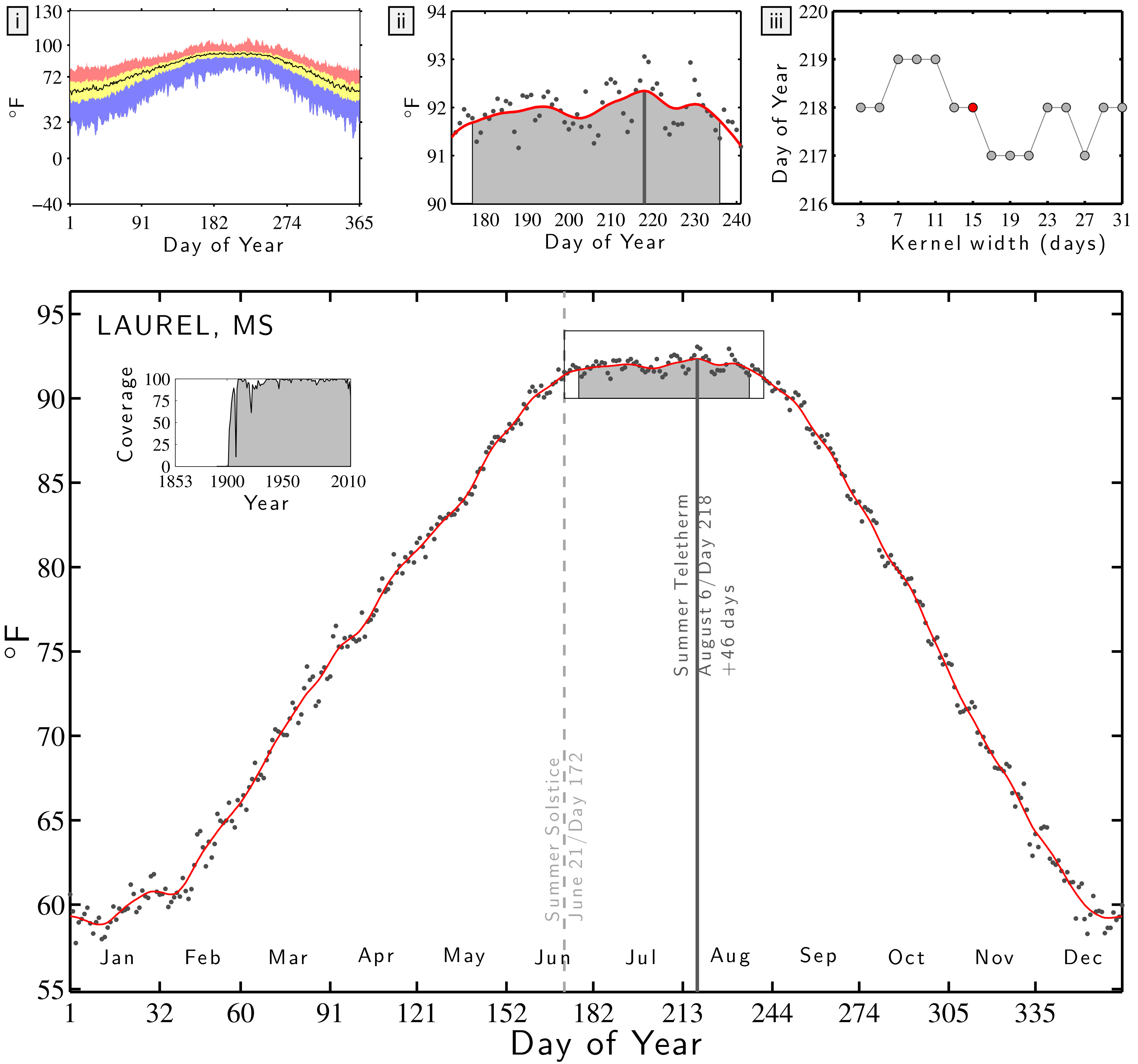

i
°F
Day of Year
ii
iii
Day of Year
Kernel width (days)
LAUREL, MS
Coverage
Year
Summer Solstice
June 21/Day 172
Summer Teletherm
August 6/Day 218
+46 days
Jan
Feb
Mar
Apr
May
Jun
Jul
Aug
Sep
Oct
Nov
Dec
Day of Year

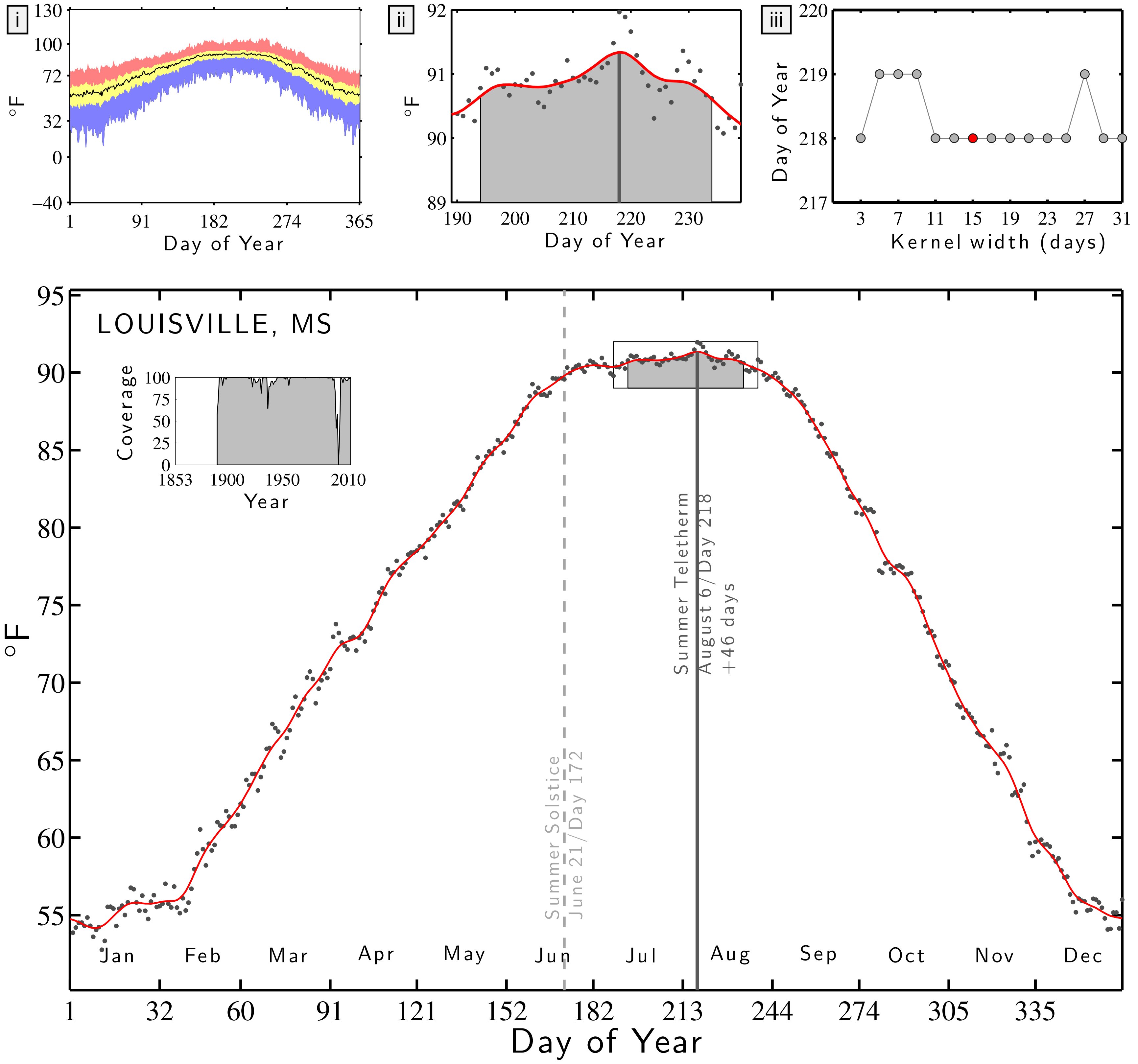

i
°F
Day of Year
ii
°F
Day of Year
iii
Day of Year
Kernel width (days)
LOUISVILLE, MS
Coverage
Year
1853 1900 1950 2010
Summer Solstice
June 21 / Day 172
Summer Teletherm
August 6 / Day 218
+46 days
Jan Feb Mar Apr May Jun Jul Aug Sep Oct Nov Dec
°F
Day of Year

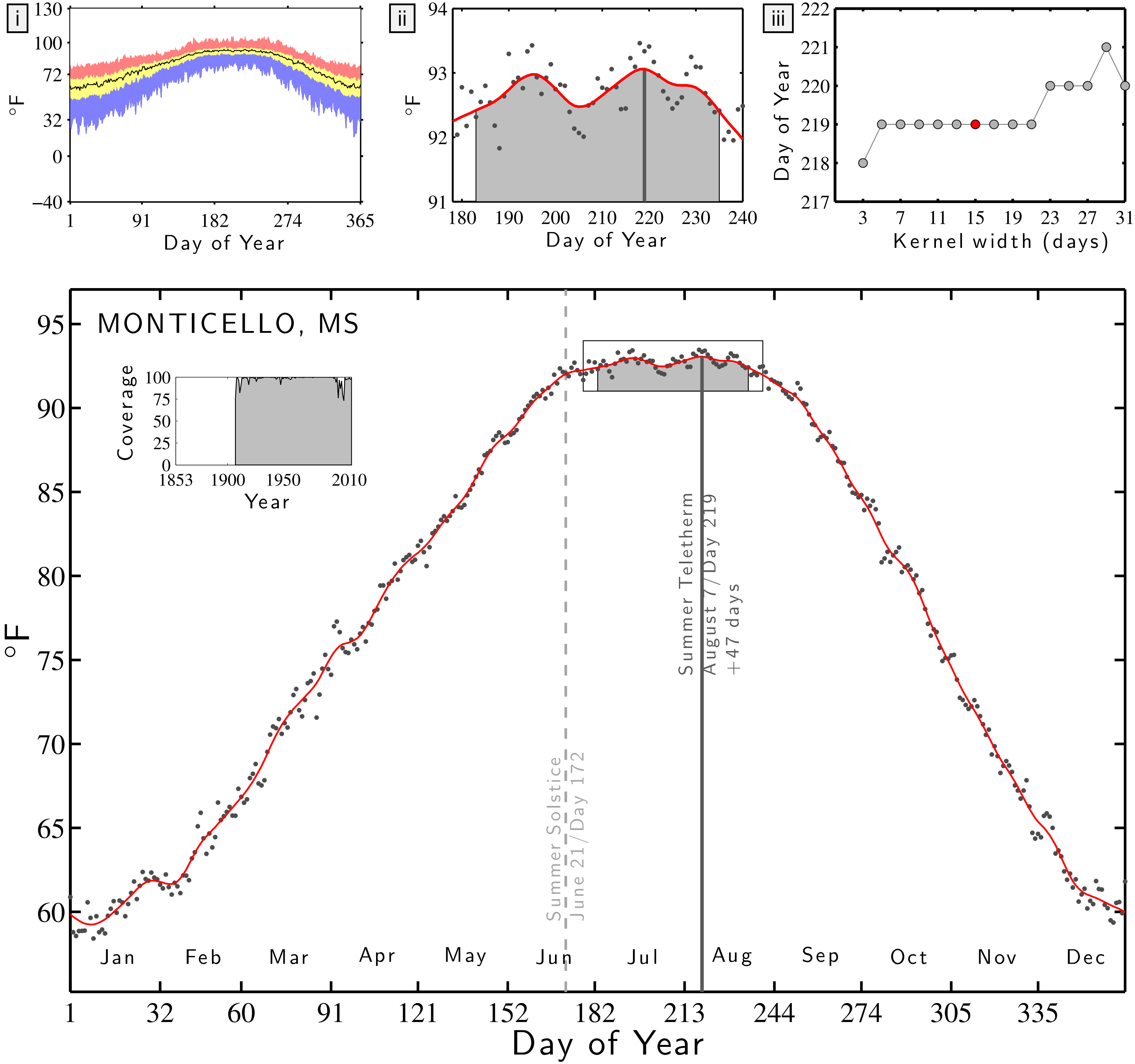

i
ii
iii
°F
Day of Year
Day of Year
Kernel width (days)
MONTICELLO, MS
Coverage
Year
Summer Solstice
June 21 / Day 172
Summer Teletherm
August 7 / Day 219
+47 days
Jan
Feb
Mar
Apr
May
Jun
Jul
Aug
Sep
Oct
Nov
Dec
Day of Year

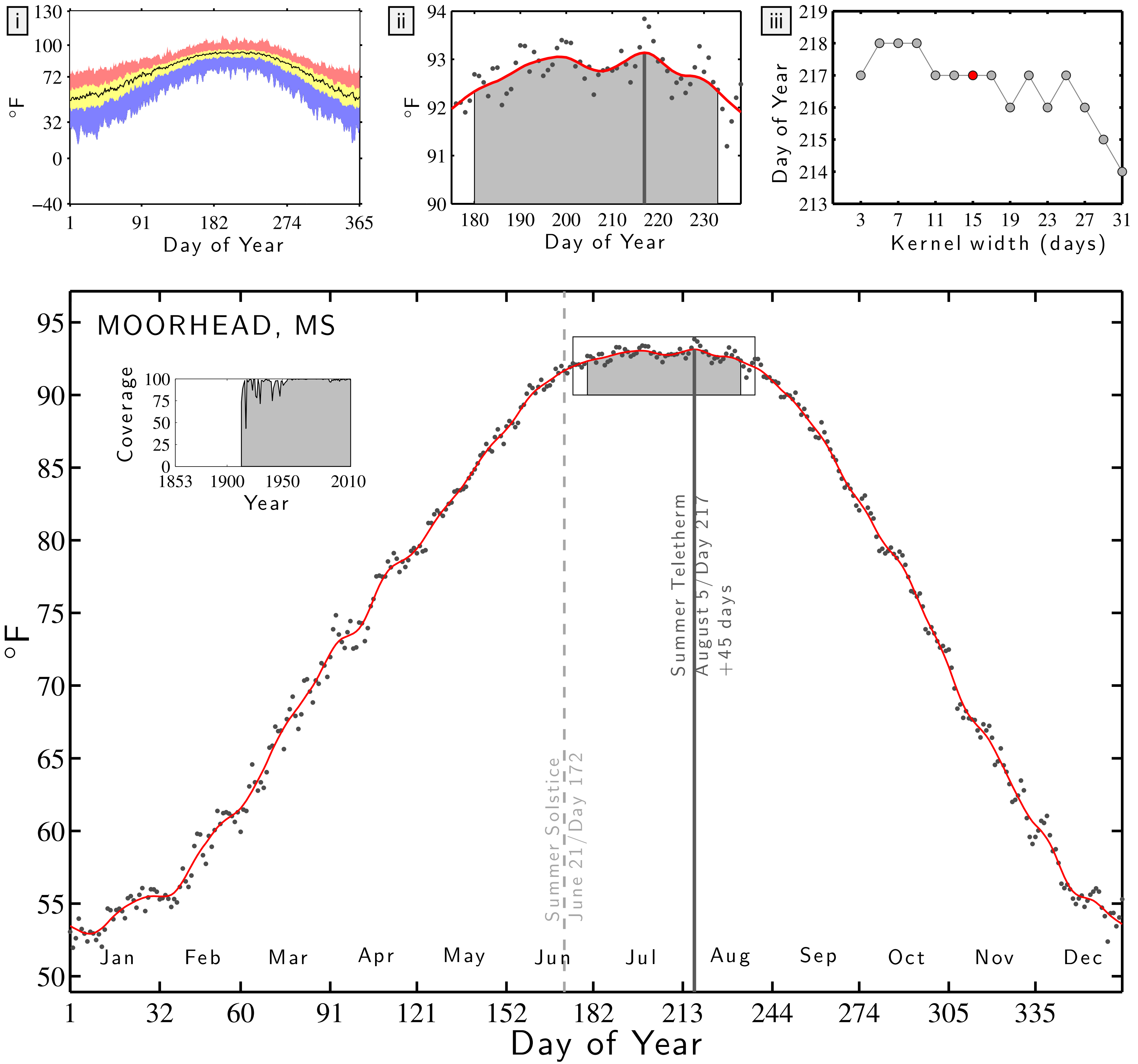

i
°F
Day of Year
ii
°F
Day of Year
iii
Day of Year
Kernel width (days)
MOORHEAD, MS
Coverage
Year
Summer Solstice
June 21/Day 172
Summer Teletherm
August 5/Day 217
+45 days
Jan
Feb
Mar
Apr
May
Jun
Jul
Aug
Sep
Oct
Nov
Dec
°F
Day of Year

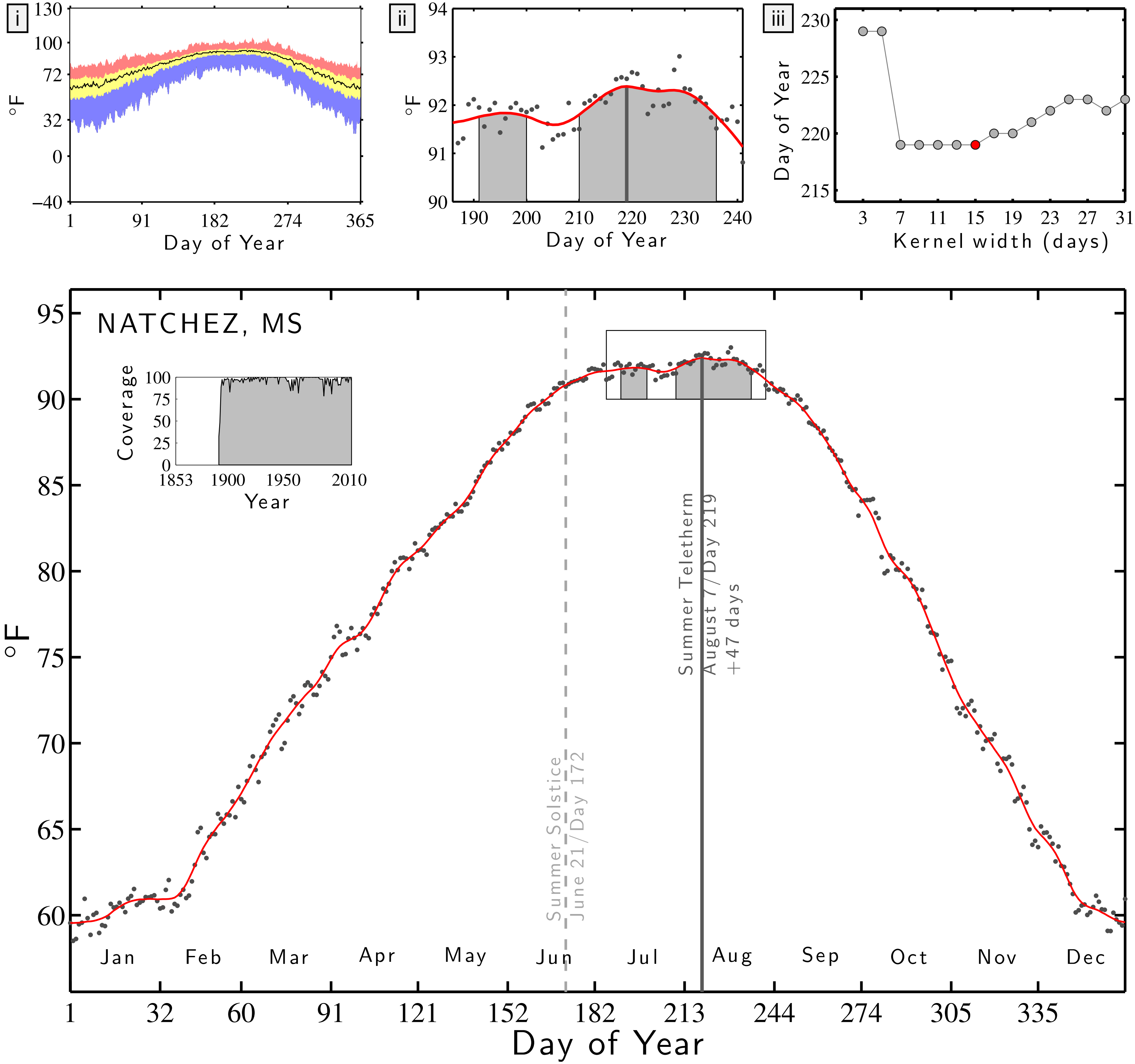
i
°F
130
100
72
32
0
−40
1
91
182
274
365
Day of Year
ii
°F
94
93
92
91
90
190
200
210
220
230
240
Day of Year
iii
Day of Year
230
225
220
215
3
7
11
15
19
23
27
31
Kernel width (days)
NATCHEZ, MS
Coverage
100
75
50
25
0
1853
1900
1950
2010
Year
°F
95
90
85
80
75
70
65
60
Summer Solstice
June 21 / Day 172
Summer Teletherm
August 7 / Day 219
+47 days
Jan
Feb
Mar
Apr
May
Jun
Jul
Aug
Sep
Oct
Nov
Dec
1
32
60
91
121
152
182
213
244
274
305
335
Day of Year

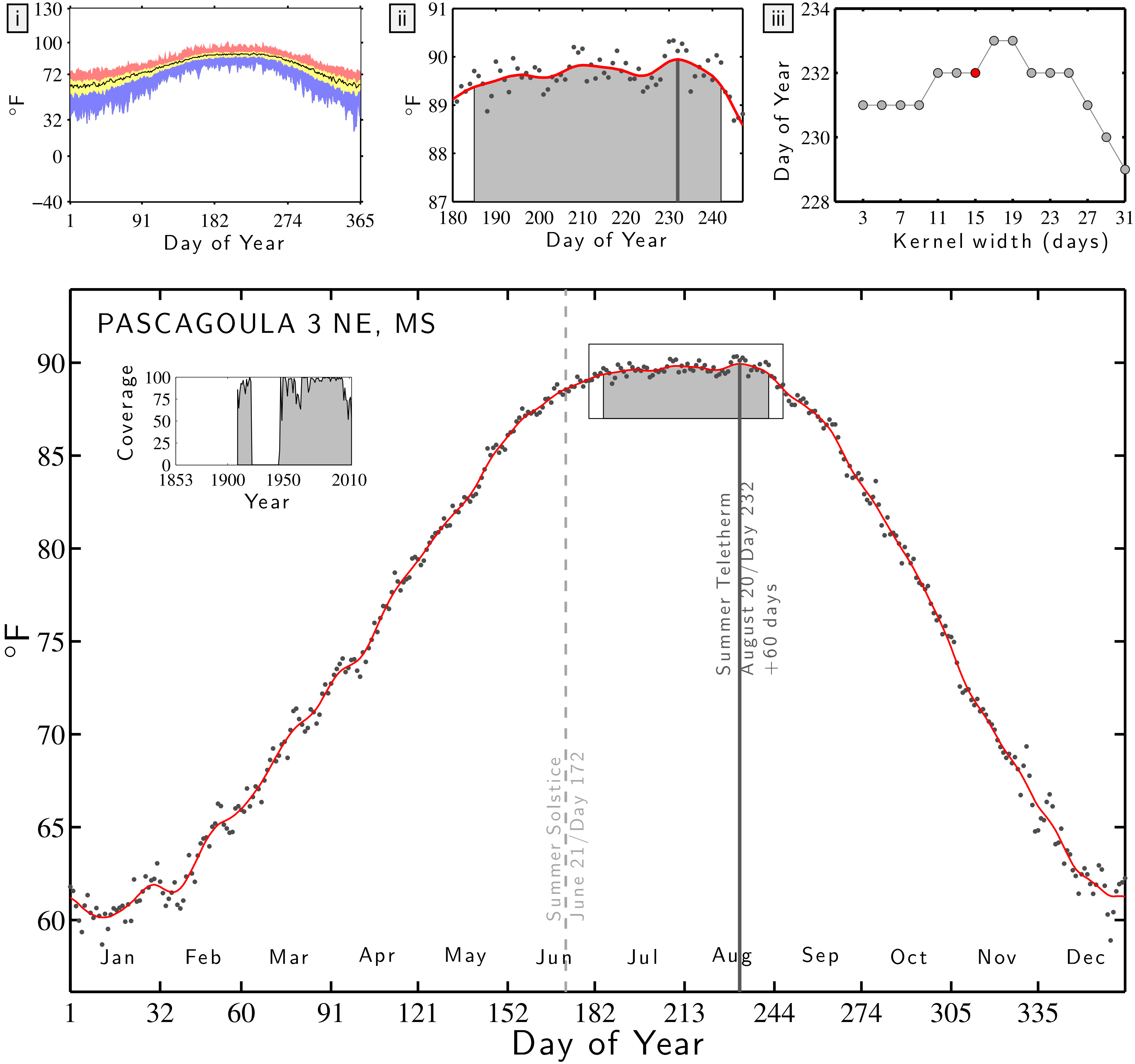

i
°F
Day of Year
ii
°F
Day of Year
iii
Day of Year
Kernel width (days)
PASCAGOULA 3 NE, MS
Coverage
Year
Summer Solstice
June 21 / Day 172
Summer Teletherm
August 20 / Day 232
+60 days
Jan
Feb
Mar
Apr
May
Jun
Jul
Aug
Sep
Oct
Nov
Dec
°F
Day of Year

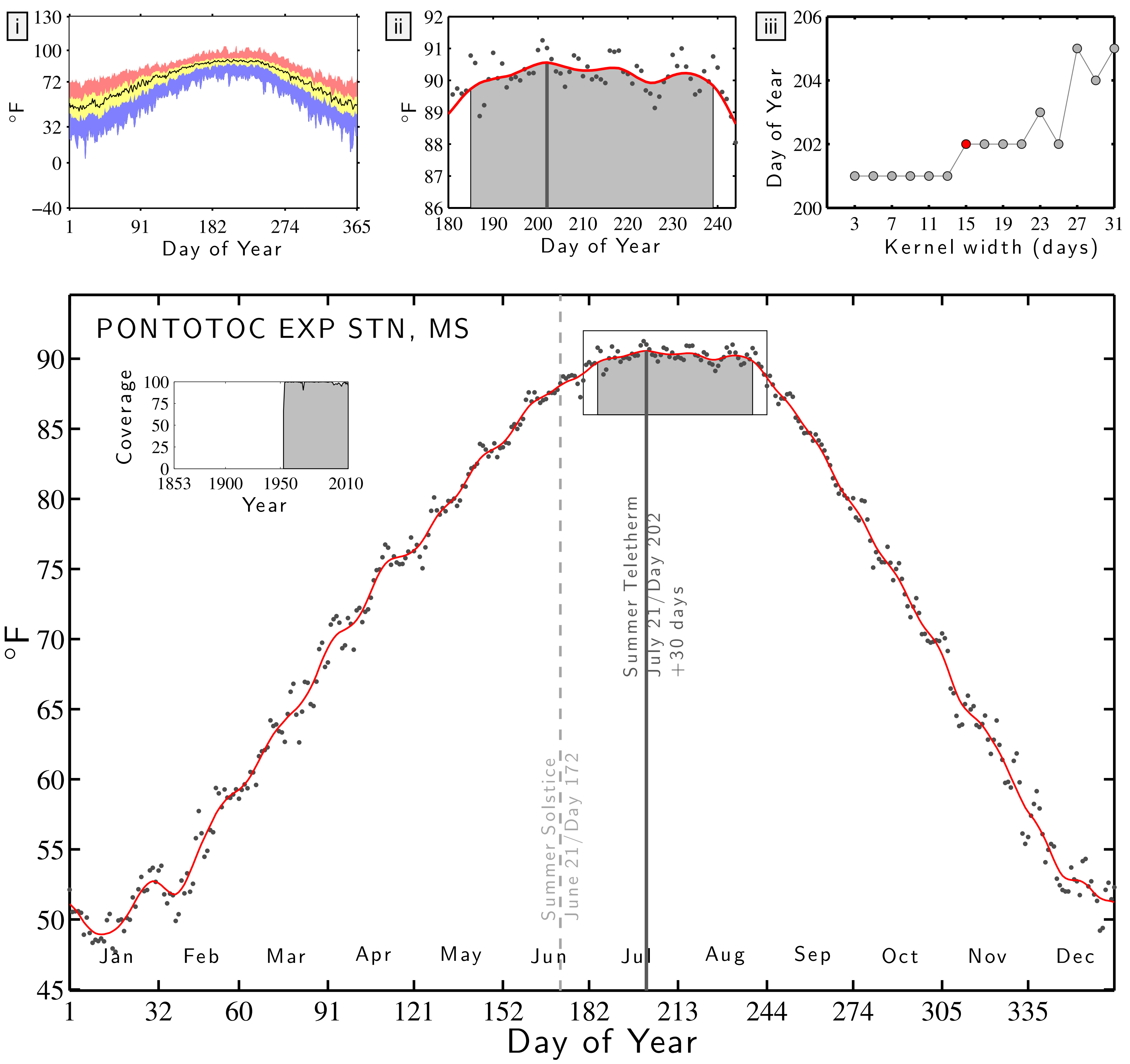

i
°F
Day of Year
ii
°F
Day of Year
iii
Day of Year
Kernel width (days)
PONTOTOC EXP STN, MS
Coverage
Year
Summer Solstice
June 21 / Day 172
Summer Teletherm
July 21 / Day 202
+30 days
Jan
Feb
Mar
Apr
May
Jun
Jul
Aug
Sep
Oct
Nov
Dec
°F
Day of Year

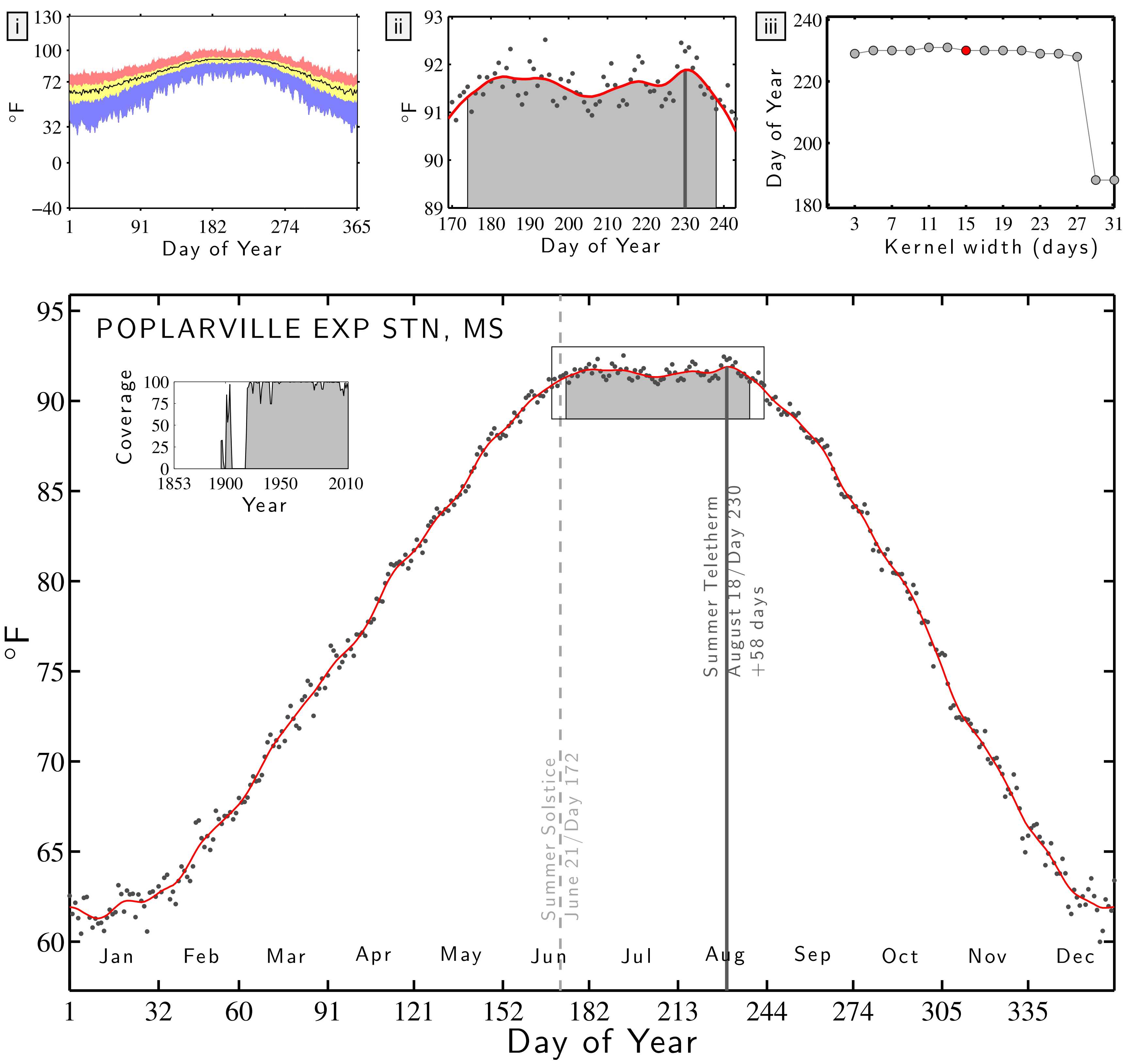
i
130
100
72
32
0
−40
°F
1
91
182
274
365
Day of Year
ii
93
92
91
90
89
°F
170
180
190
200
210
220
230
240
Day of Year
iii
240
220
200
180
Day of Year
3
7
11
15
19
23
27
31
Kernel width (days)
POPLARVILLE EXP STN, MS
Coverage
100
75
50
25
0
1853
1900
1950
2010
Year
95
90
85
80
75
70
65
60
°F
Summer Solstice
June 21/Day 172
Summer Teletherm
August 18/Day 230
+58 days
Jan
Feb
Mar
Apr
May
Jun
Jul
Aug
Sep
Oct
Nov
Dec
1
32
60
91
121
152
182
213
244
274
305
335
Day of Year

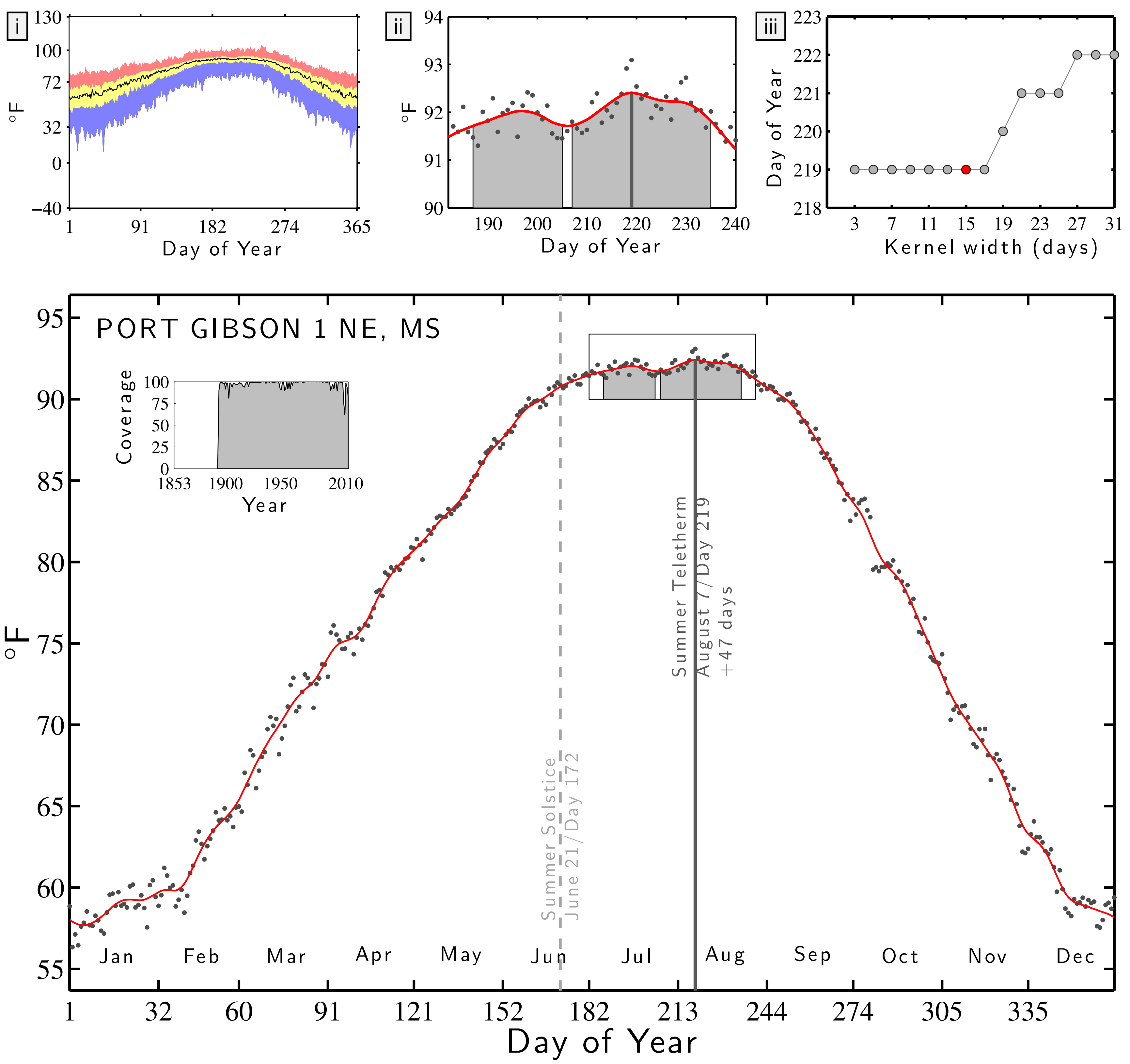

i
°F
Day of Year
ii
°F
Day of Year
iii
Day of Year
Kernel width (days)
PORT GIBSON 1 NE, MS
Coverage
Year
Summer Solstice
June 21 / Day 172
Summer Teletherm
August 7 / Day 219
+47 days
Jan
Feb
Mar
Apr
May
Jun
Jul
Aug
Sep
Oct
Nov
Dec
°F
Day of Year

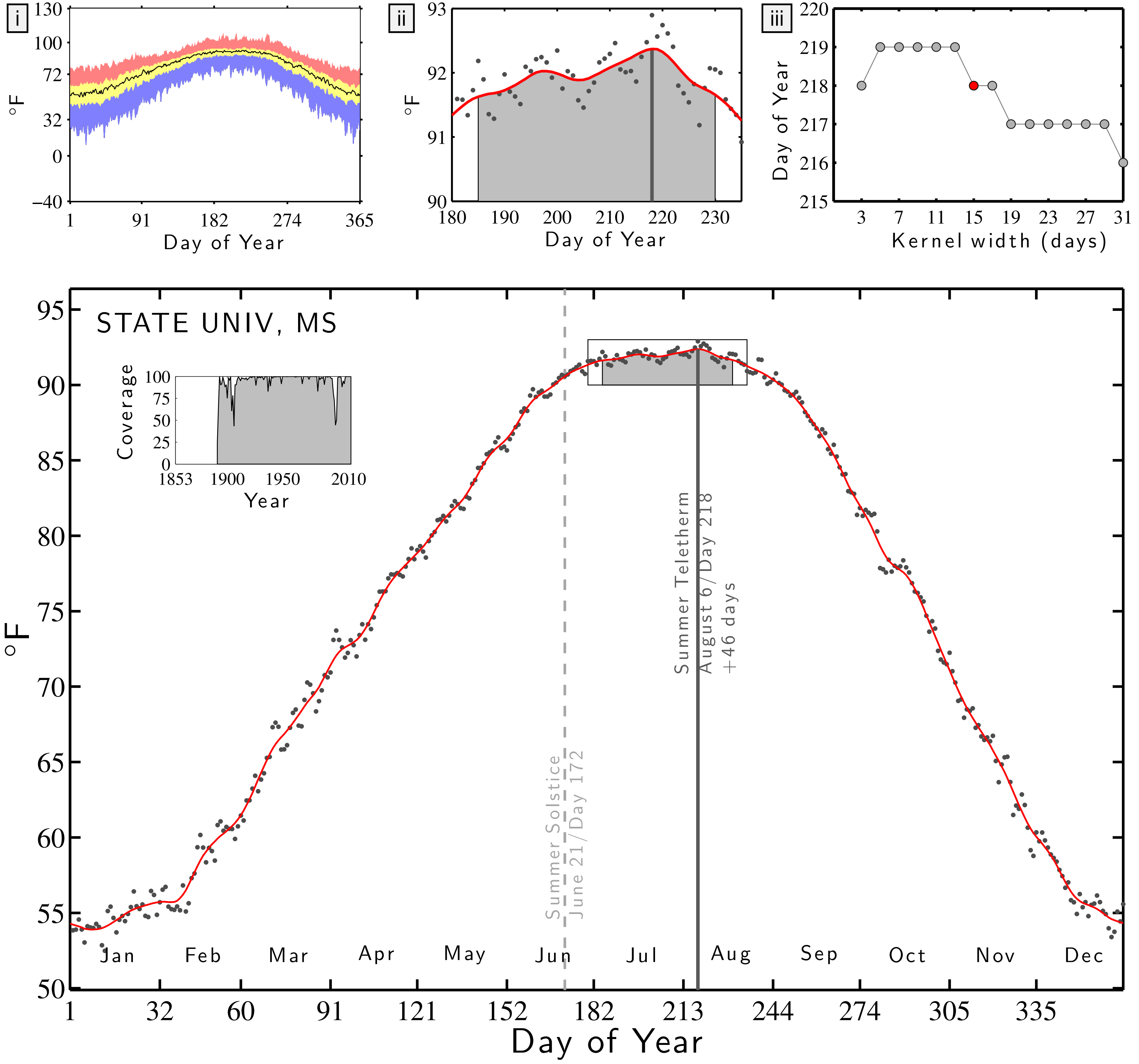
i
130
100
72
32
0
−40
°F
1
91
182
274
365
Day of Year
ii
93
92
91
90
°F
180
190
200
210
220
230
Day of Year
iii
220
219
218
217
216
215
Day of Year
3
7
11
15
19
23
27
31
Kernel width (days)
STATE UNIV, MS
Coverage
100
75
50
25
0
1853
1900
1950
2010
Year
Summer Solstice
June 21/Day 172
Summer Teletherm
August 6/Day 218
+46 days
95
90
85
80
75
70
65
60
55
50
°F
Jan
Feb
Mar
Apr
May
Jun
Jul
Aug
Sep
Oct
Nov
Dec
1
32
60
91
121
152
182
213
244
274
305
335
Day of Year

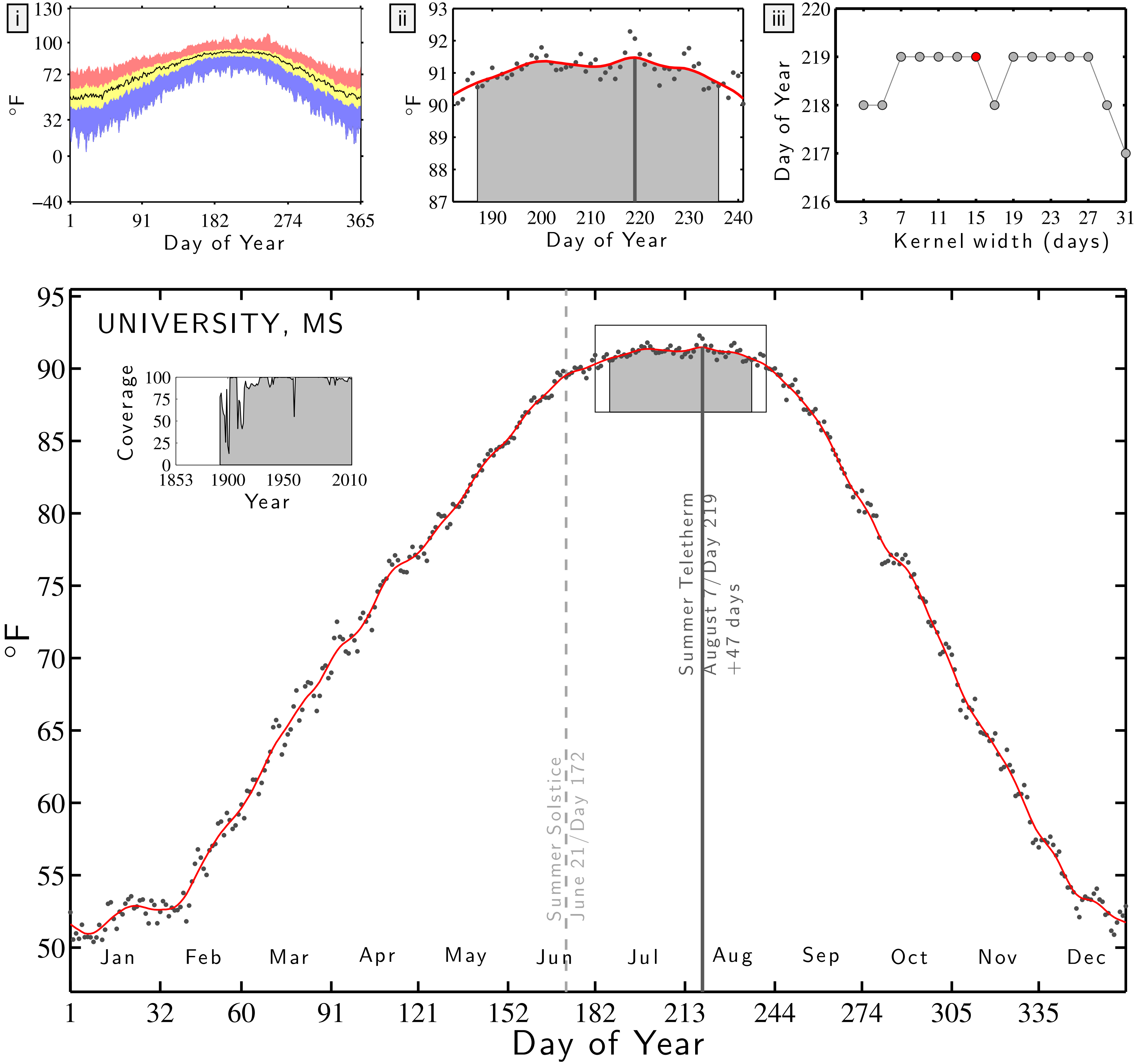

i
°F
Day of Year
ii
°F
Day of Year
iii
Day of Year
Kernel width (days)
UNIVERSITY, MS
Coverage
Year
Summer Solstice
June 21 / Day 172
Summer Teletherm
August 7 / Day 219
+47 days
Jan
Feb
Mar
Apr
May
Jun
Jul
Aug
Sep
Oct
Nov
Dec
°F
Day of Year

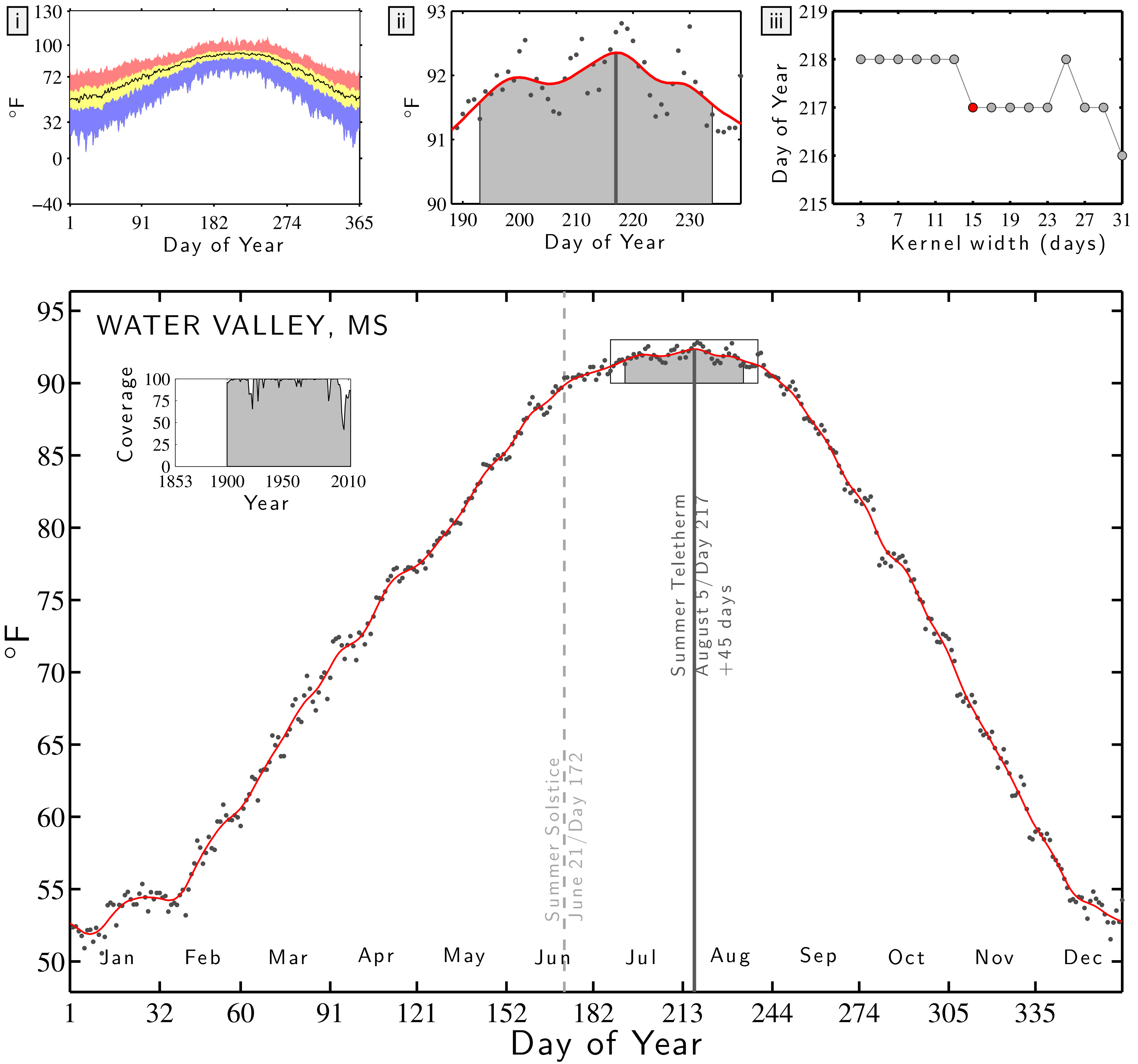

i
130
100
72
32
0
−40
°F
1
91
182
274
365
Day of Year
ii
93
92
91
90
°F
190
200
210
220
230
Day of Year
iii
219
218
217
216
215
Day of Year
3
7
11
15
19
23
27
31
Kernel width (days)
WATER VALLEY, MS
Coverage
100
75
50
25
0
1853
1900
1950
2010
Year
95
90
85
80
75
70
65
60
55
50
°F
Summer Solstice
June 21/Day 172
Summer Teletherm
August 5/Day 217
+45 days
Jan
Feb
Mar
Apr
May
Jun
Jul
Aug
Sep
Oct
Nov
Dec
1
32
60
91
121
152
182
213
244
274
305
335
Day of Year

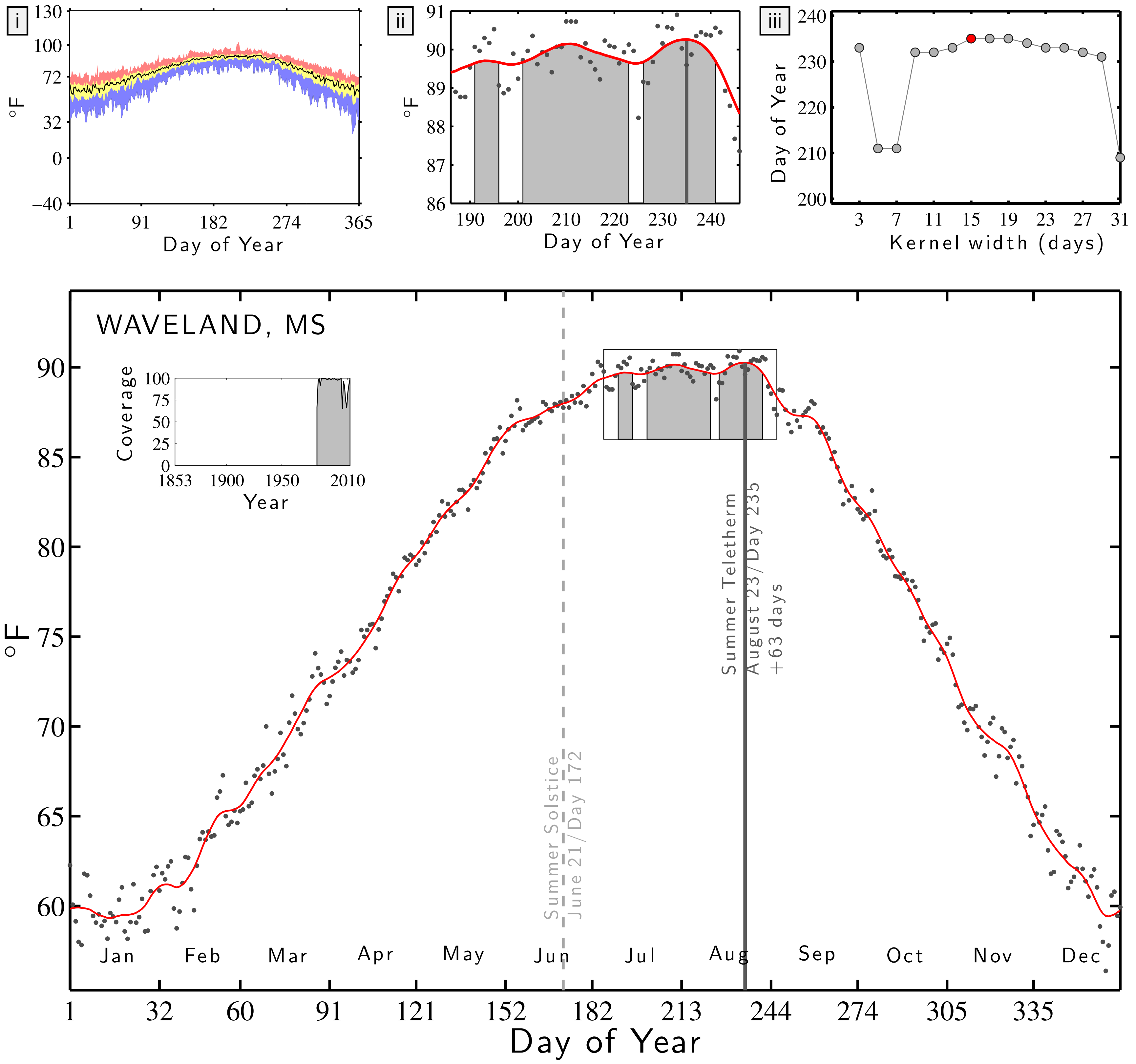

i
ii
iii
°F
Day of Year
Kernel width (days)
WAVELAND, MS
Coverage
Year
Summer Solstice
June 21 / Day 172
Summer Teletherm
August 23 / Day 235
+63 days
Jan
Feb
Mar
Apr
May
Jun
Jul
Aug
Sep
Oct
Nov
Dec

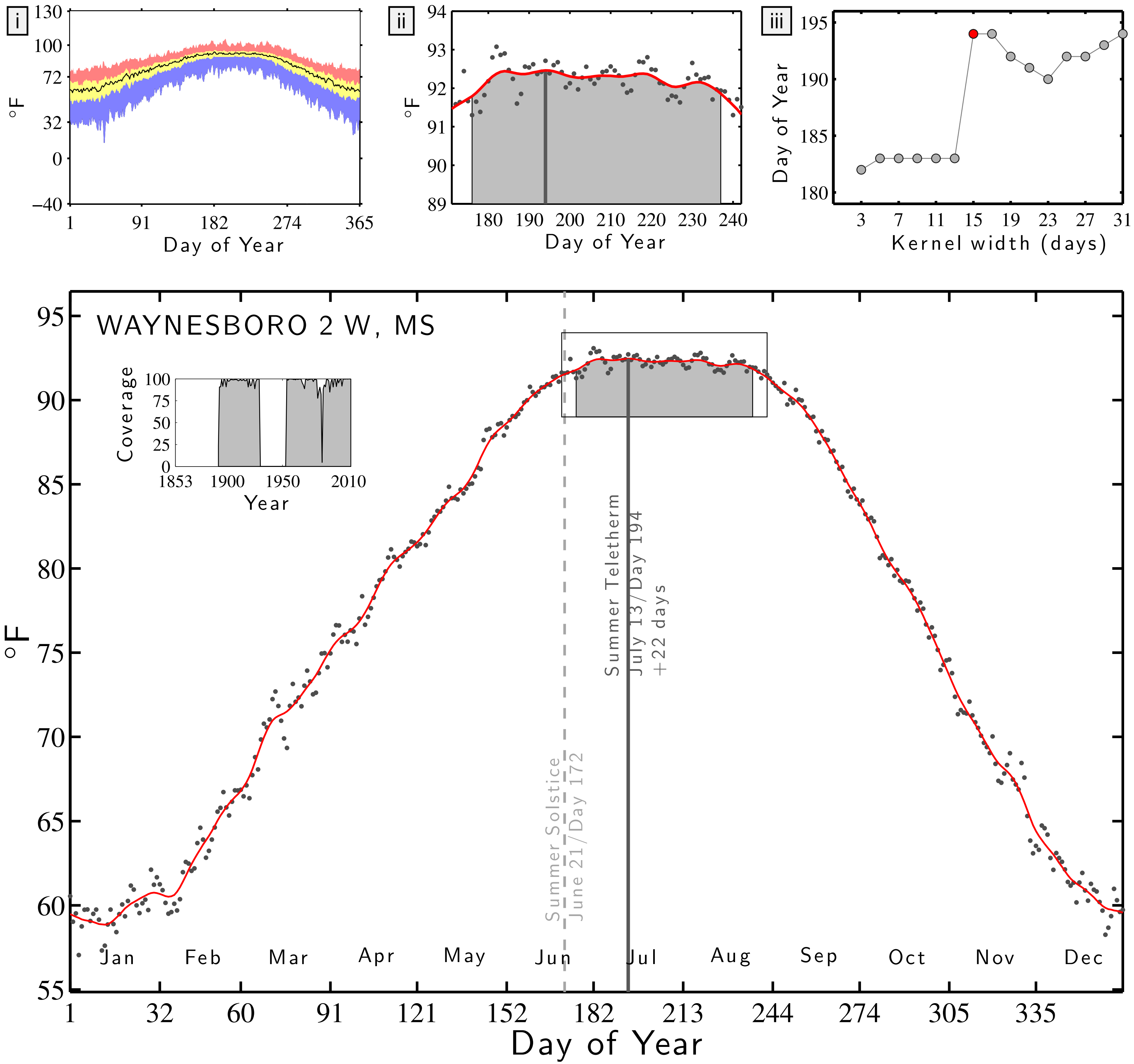

i
°F
Day of Year
ii
°F
Day of Year
iii
Day of Year
Kernel width (days)
WAYNESBORO 2 W, MS
Coverage
Year
Summer Solstice
June 21/Day 172
Summer Teletherm
July 13/Day 194
+22 days
Jan
Feb
Mar
Apr
May
Jun
Jul
Aug
Sep
Oct
Nov
Dec
°F
Day of Year

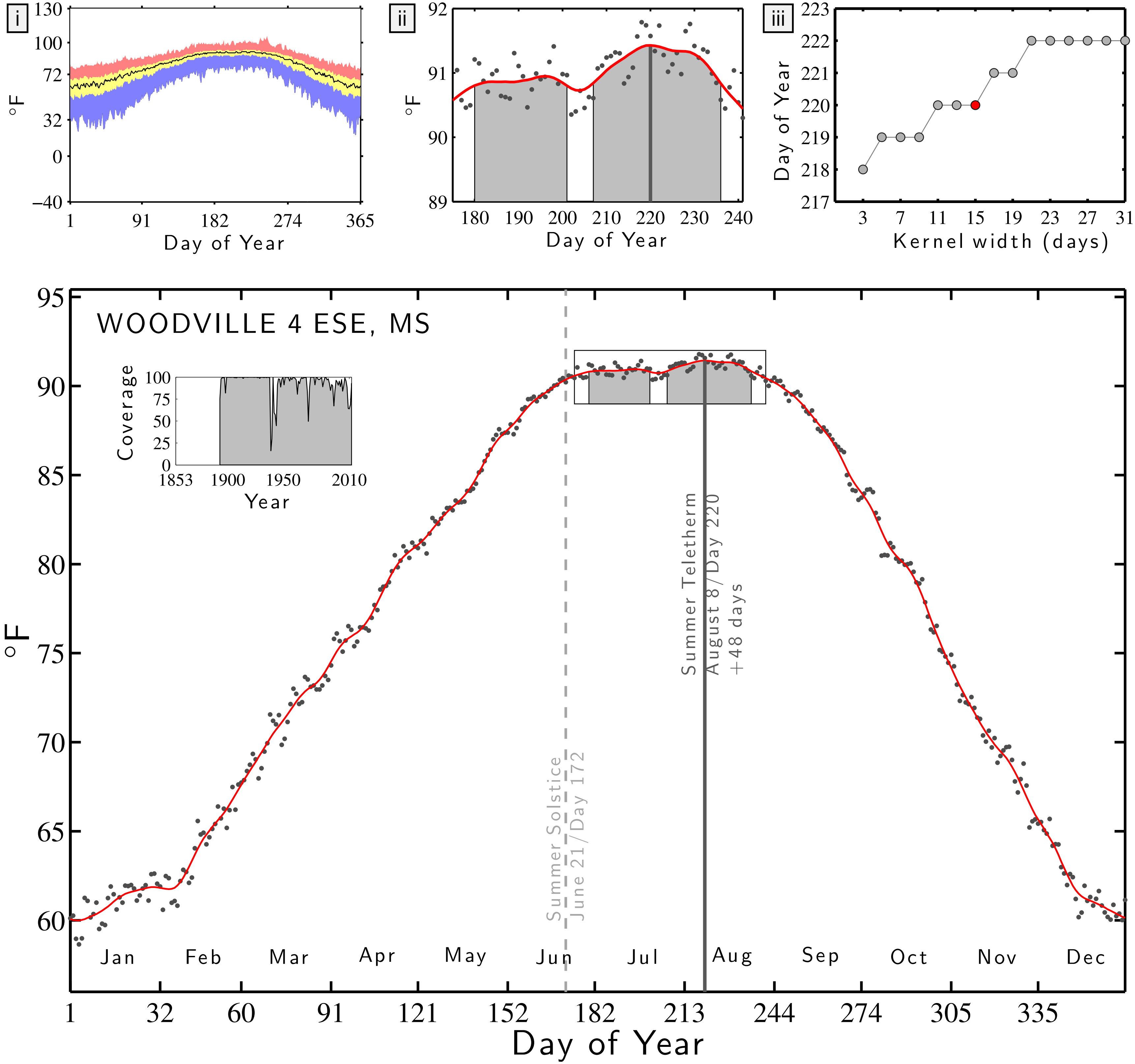

i
°F
Day of Year
ii
°F
Day of Year
iii
Day of Year
Kernel width (days)
WOODVILLE 4 ESE, MS
Coverage
Year
Summer Solstice
June 21/Day 172
Summer Teletherm
August 8/Day 220
+48 days
Jan
Feb
Mar
Apr
May
Jun
Jul
Aug
Sep
Oct
Nov
Dec
°F
Day of Year

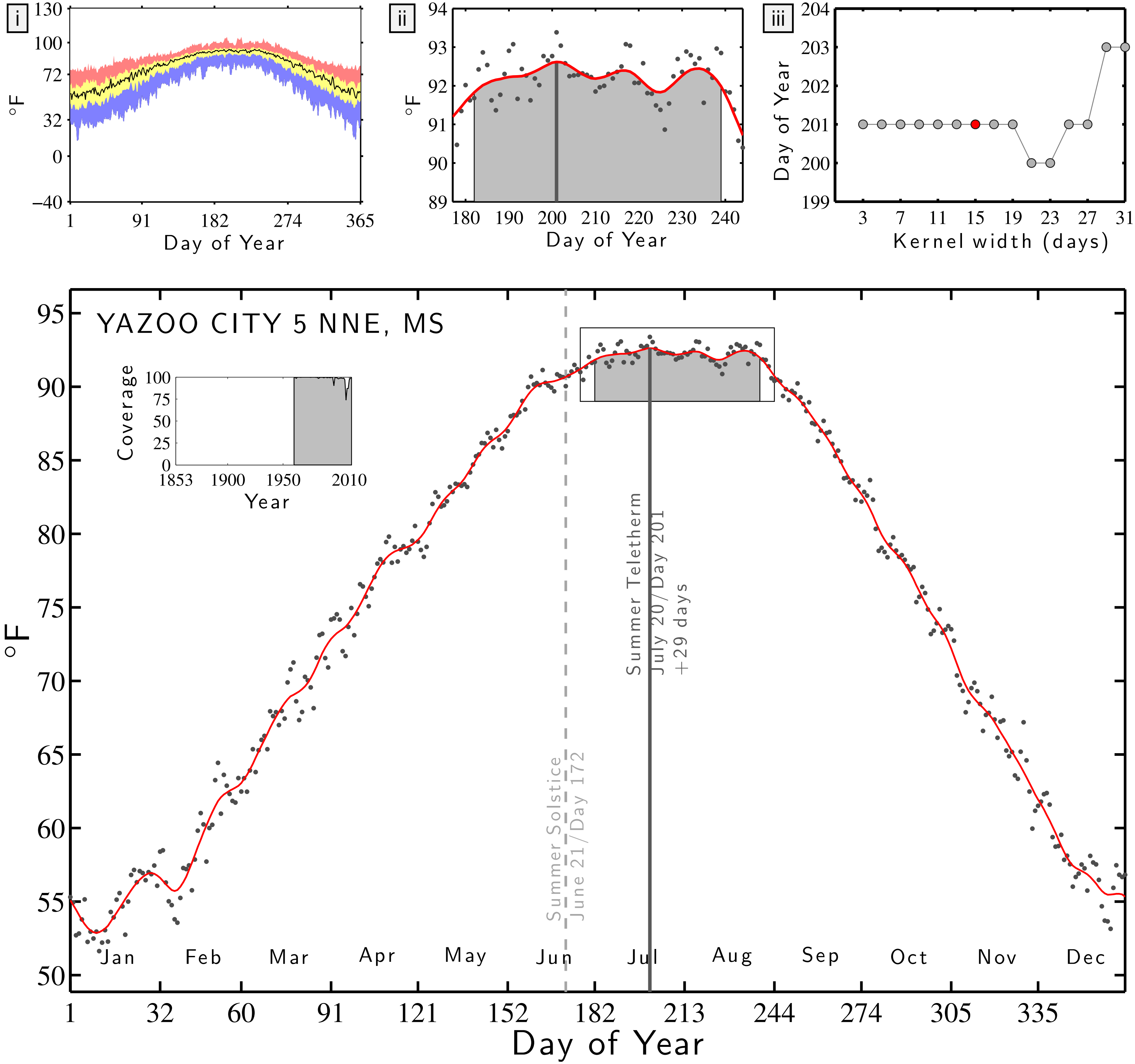

i
°F
Day of Year
ii
°F
Day of Year
iii
Day of Year
Kernel width (days)
YAZOO CITY 5 NNE, MS
Coverage
Year
Summer Solstice
June 21 / Day 172
Summer Teletherm
July 20 / Day 201
+29 days
Jan
Feb
Mar
Apr
May
Jun
Jul
Aug
Sep
Oct
Nov
Dec
°F
Day of Year

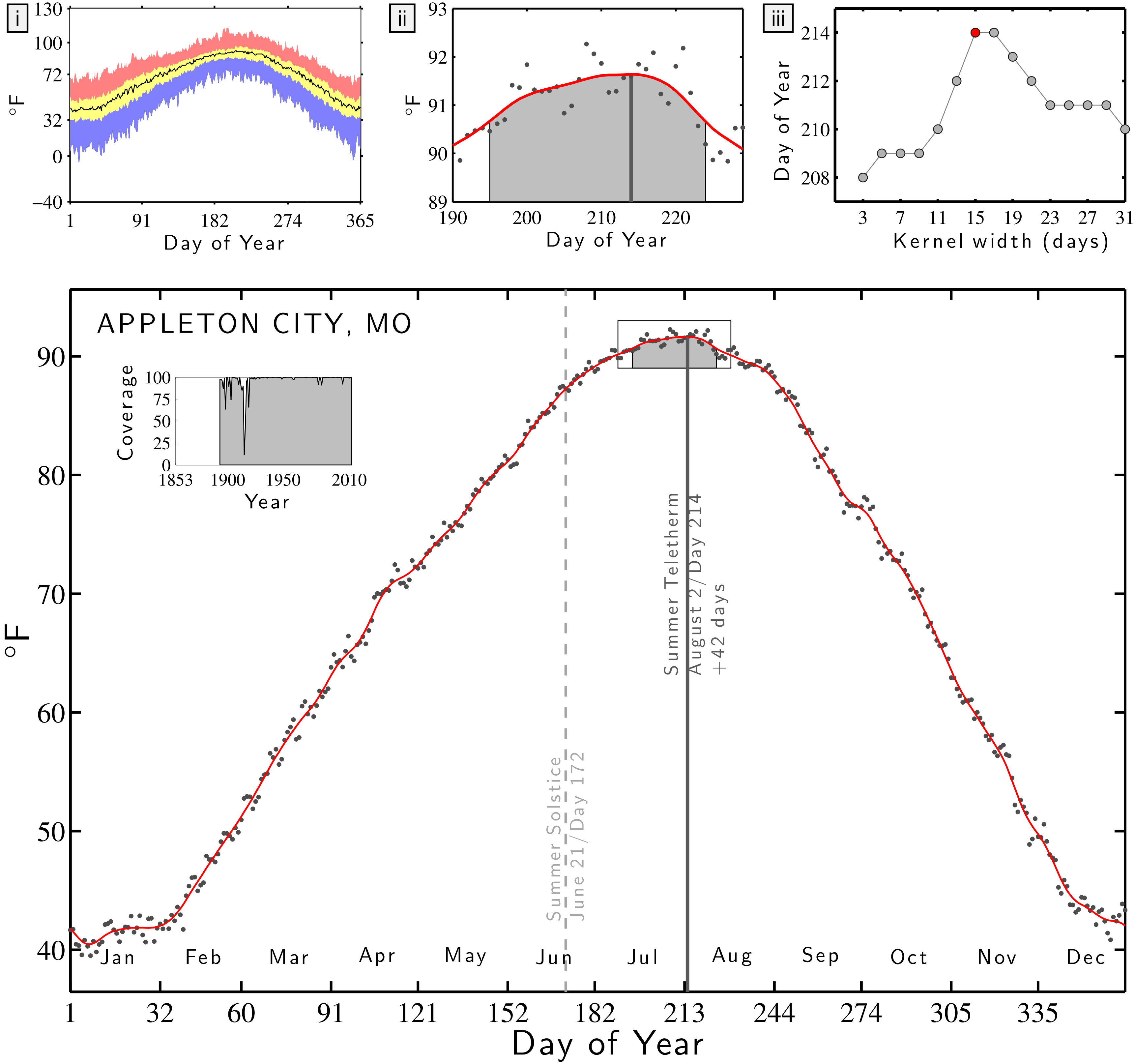

i
ii
iii
°F
Day of Year
Kernel width (days)
APPLETON CITY, MO
Coverage
Year
1853
1900
1950
2010
Summer Solstice
June 21 / Day 172
Summer Teletherm
August 2 / Day 214
+42 days
Jan
Feb
Mar
Apr
May
Jun
Jul
Aug
Sep
Oct
Nov
Dec

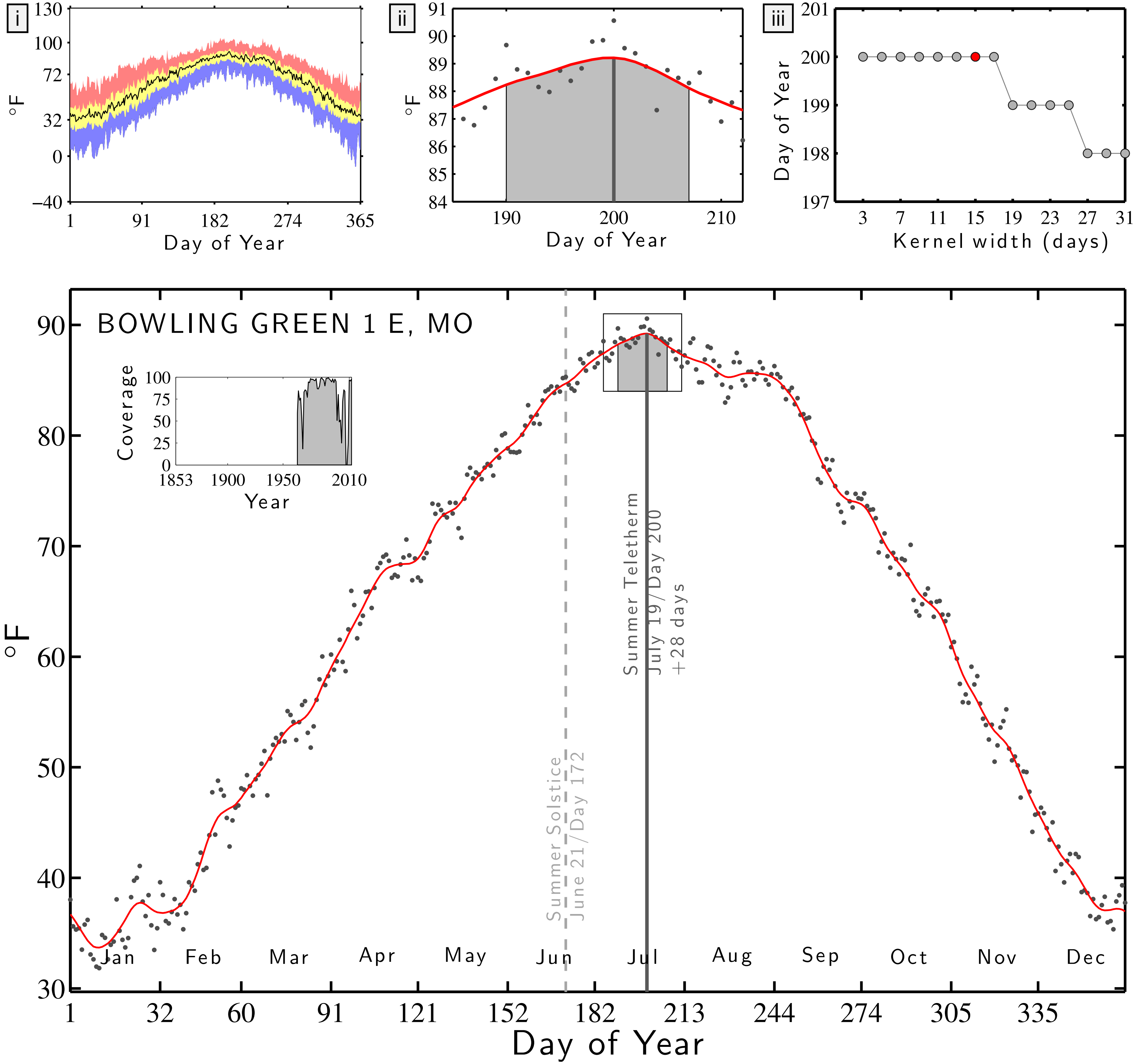

i
°F
Day of Year
ii
°F
Day of Year
iii
Day of Year
Kernel width (days)
BOWLING GREEN 1 E, MO
Coverage
Year
Summer Solstice
June 21/Day 172
Summer Teletherm
July 19/Day 200
+28 days
Jan
Feb
Mar
Apr
May
Jun
Jul
Aug
Sep
Oct
Nov
Dec
°F
Day of Year

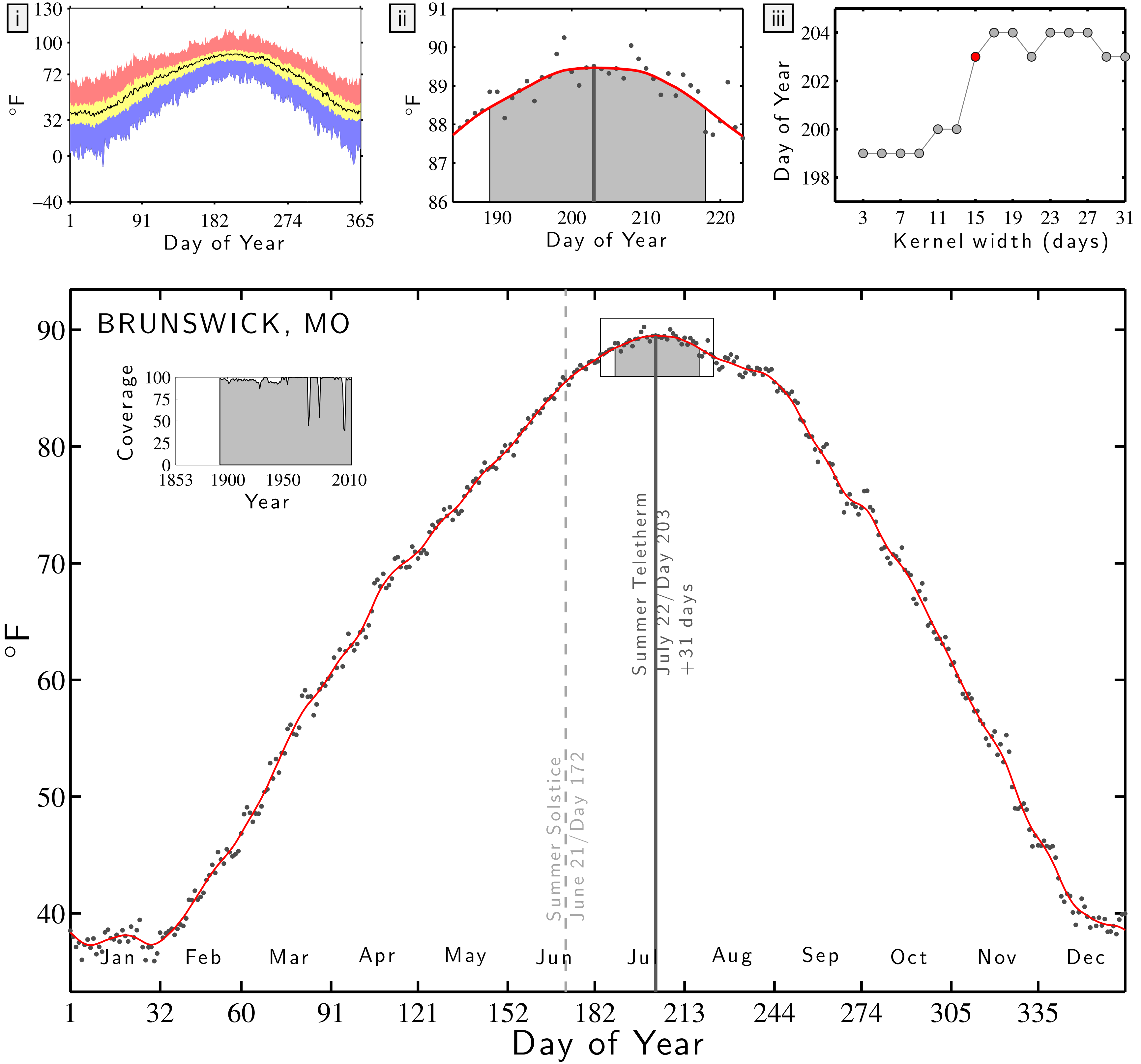

i
°F
Day of Year
ii
°F
Day of Year
iii
Day of Year
Kernel width (days)
BRUNSWICK, MO
Coverage
Year
Summer Solstice
June 21/Day 172
Summer Teletherm
July 22/Day 203
+31 days
Jan
Feb
Mar
Apr
May
Jun
Jul
Aug
Sep
Oct
Nov
Dec
°F
Day of Year

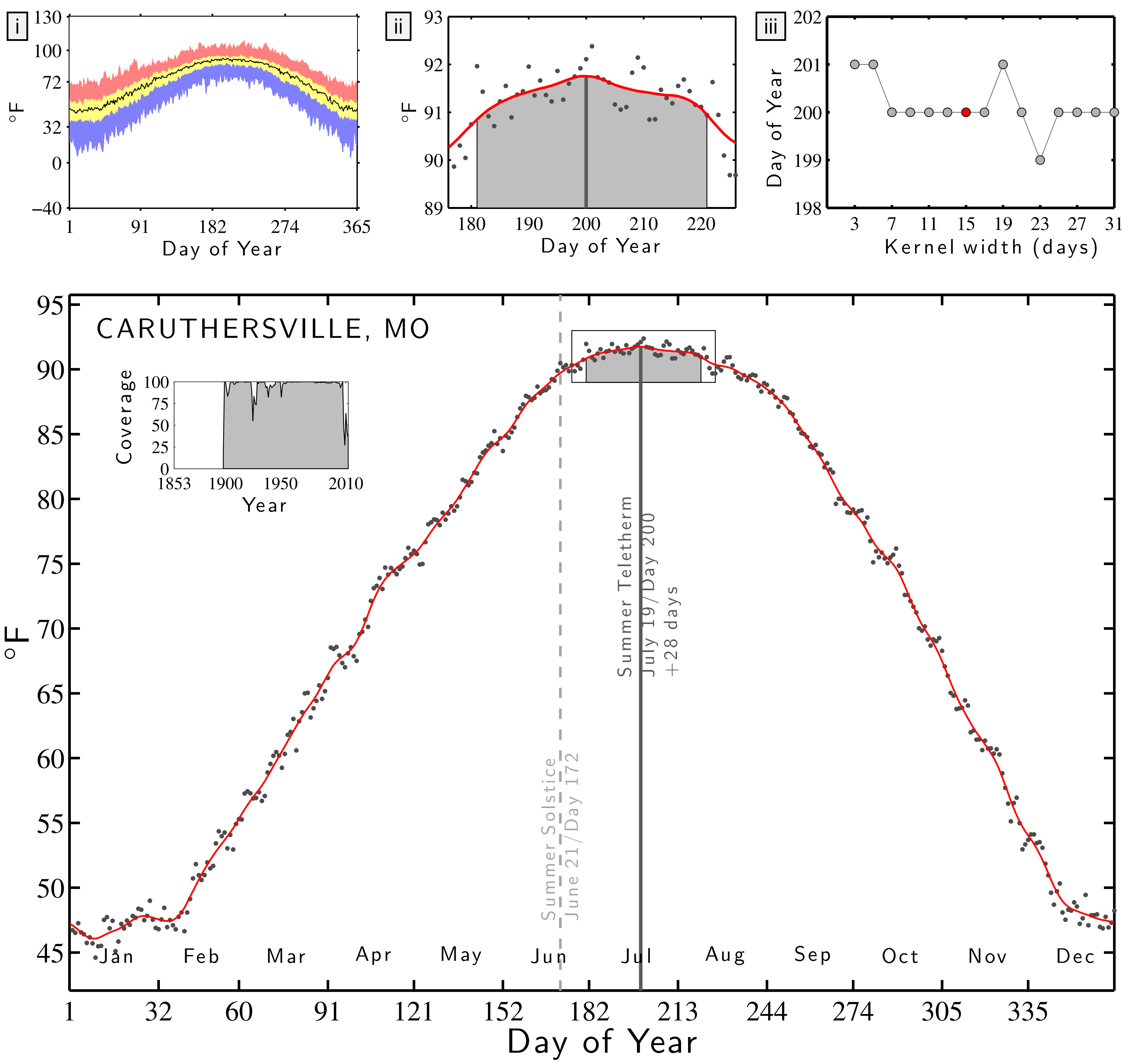
i
ii
iii
CARUTHERSVILLE, MO
Day of Year
°F
Kernel width (days)
Coverage
Year
Summer Solstice
June 21/Day 172
Summer Teletherm
July 19/Day 200
+28 days
Jan
Feb
Mar
Apr
May
Jun
Jul
Aug
Sep
Oct
Nov
Dec

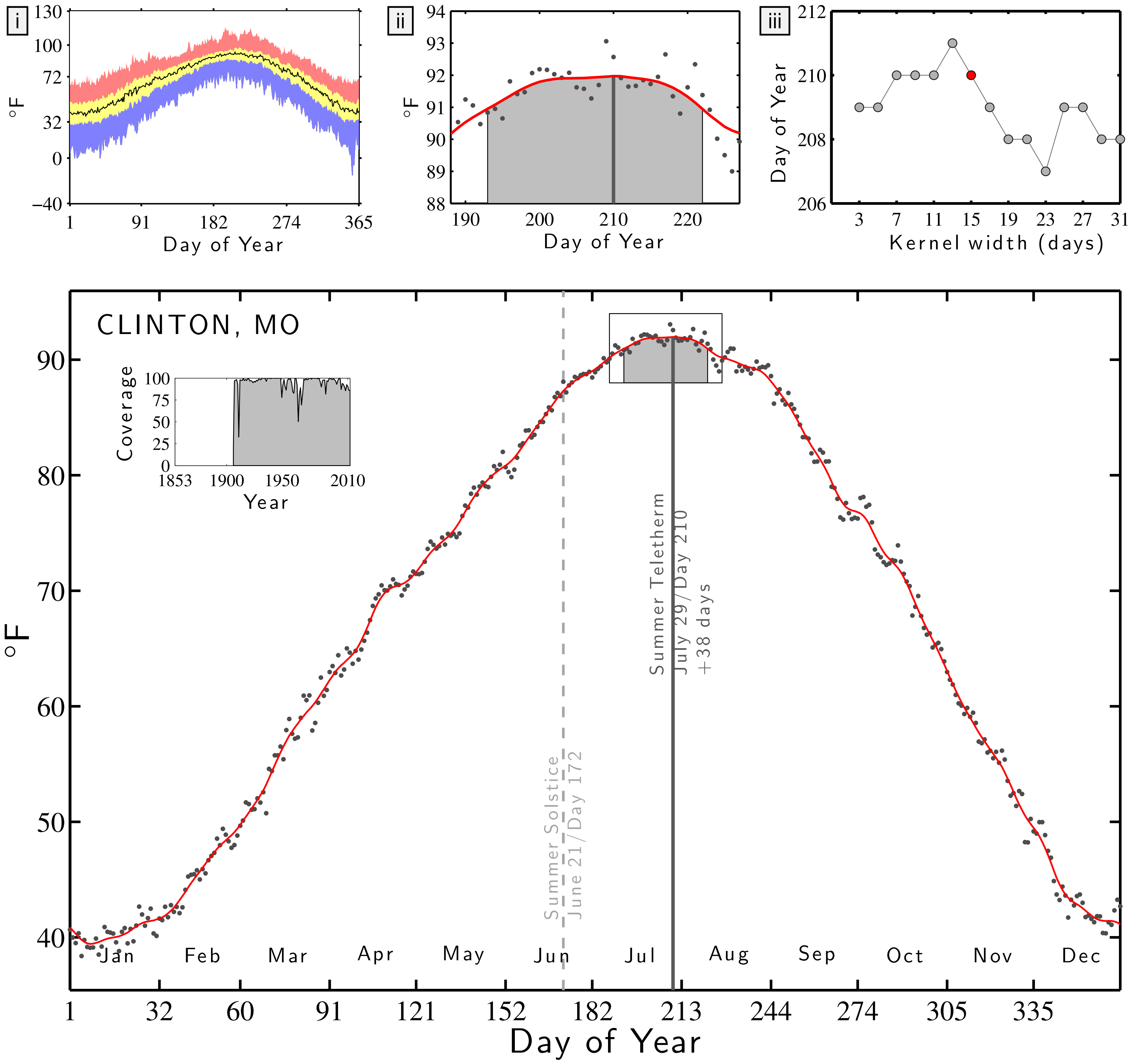

i
°F
130
100
72
32
0
−40
1
91
182
274
365
Day of Year
ii
94
93
92
91
90
89
88
190
200
210
220
iii
212
210
208
206
Day of Year
3
5
7
9
11
13
15
17
19
21
23
25
27
29
31
Kernel width (days)
CLINTON, MO
Coverage
100
75
50
25
0
1853
1900
1950
2010
Year
Summer Solstice
June 21/Day 172
Summer Teletherm
July 29/Day 210
+38 days
90
80
70
60
50
40
Jan
Feb
Mar
Apr
May
Jun
Jul
Aug
Sep
Oct
Nov
Dec
1
32
60
91
121
152
182
213
244
274
305
335

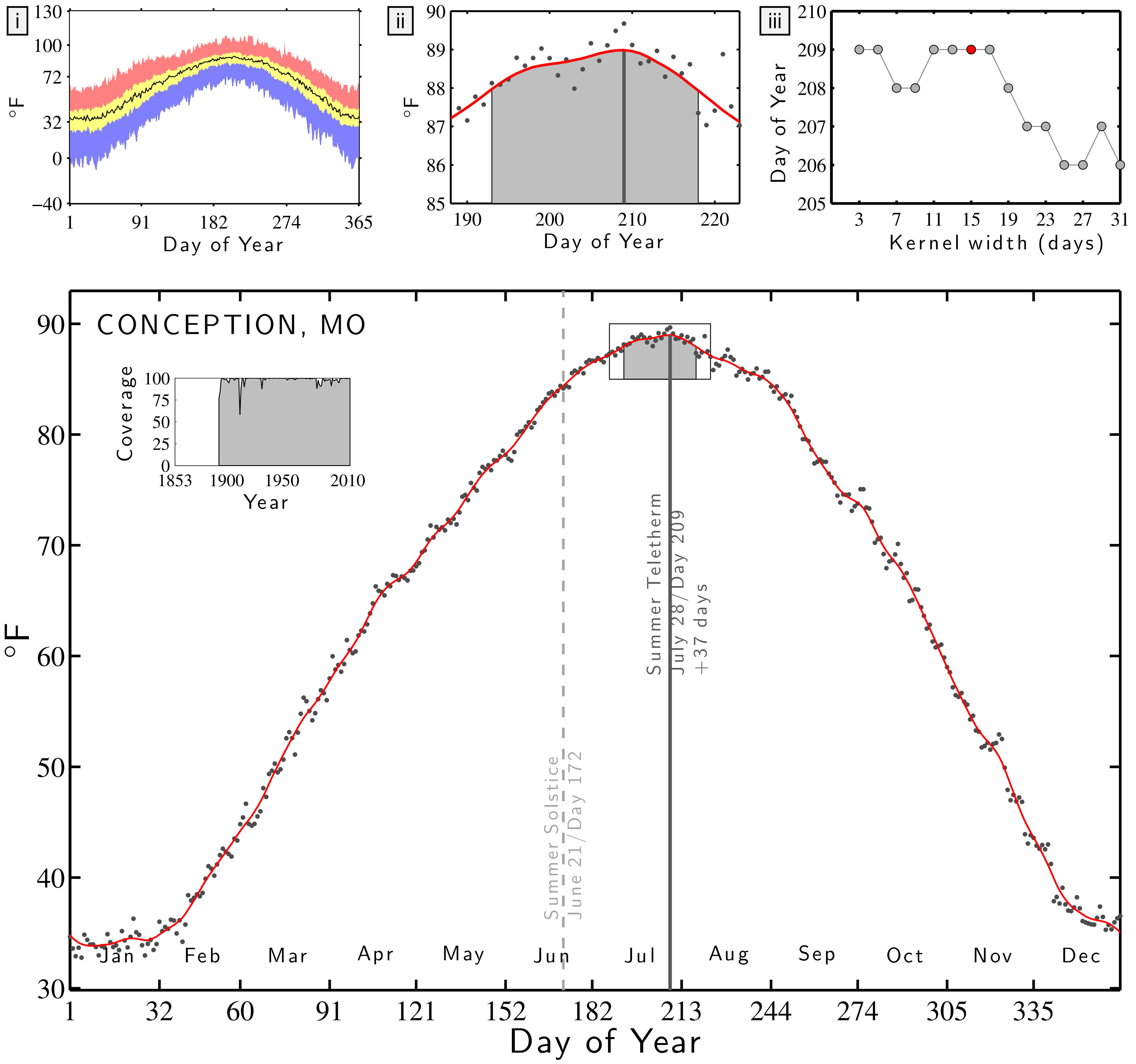

i
ii
iii
°F
Day of Year
Kernel width (days)
Day of Year
CONCEPTION, MO
Coverage
Year
Summer Solstice
June 21/Day 172
Summer Teletherm
July 28/Day 209
+37 days
Jan
Feb
Mar
Apr
May
Jun
Jul
Aug
Sep
Oct
Nov
Dec

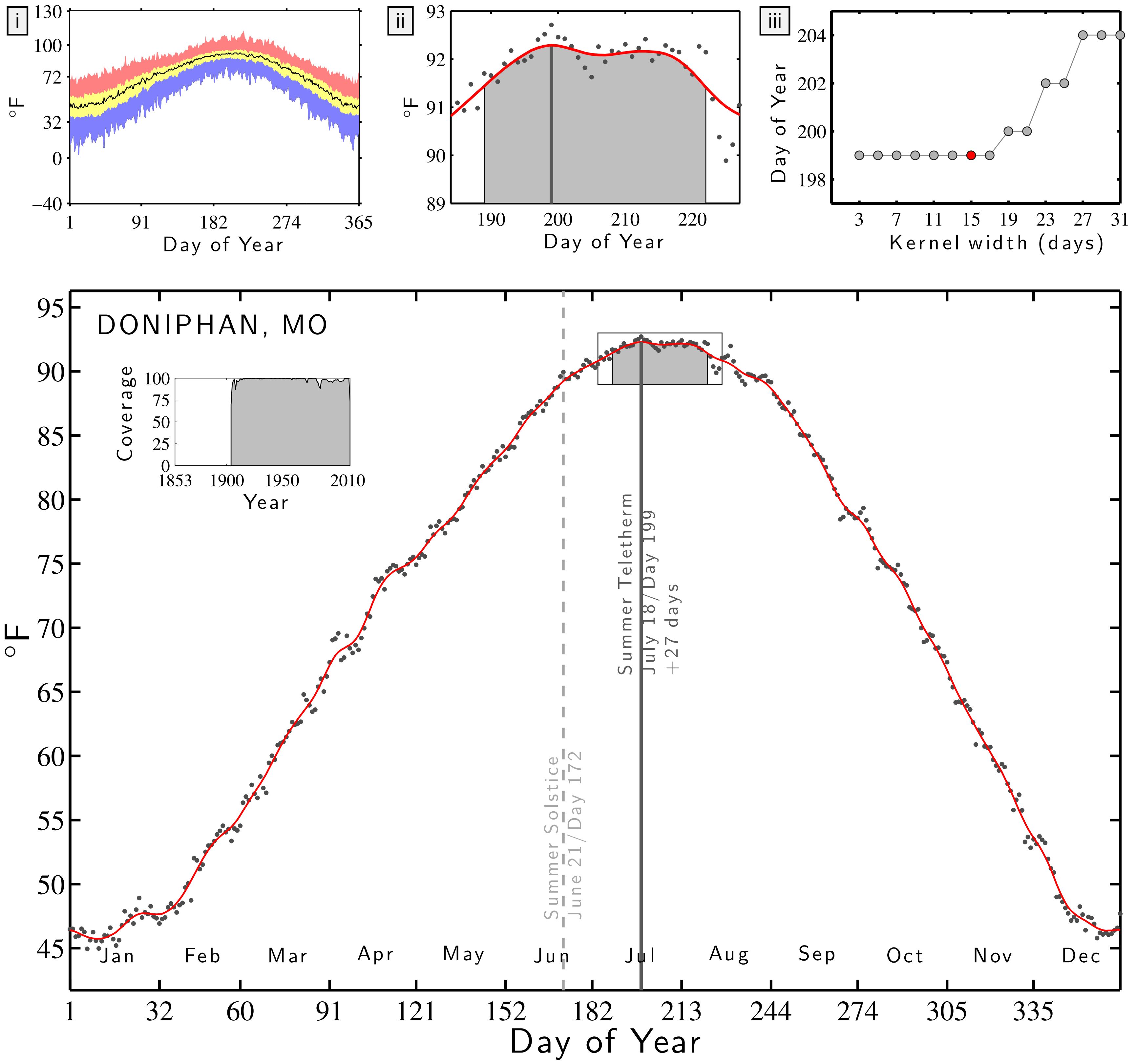

i
ii
iii
°F
Day of Year
Kernel width (days)
DONIPHAN, MO
Coverage
Year
Summer Solstice
June 21/Day 172
Summer Teletherm
July 18/Day 199
+27 days
Jan
Feb
Mar
Apr
May
Jun
Jul
Aug
Sep
Oct
Nov
Dec

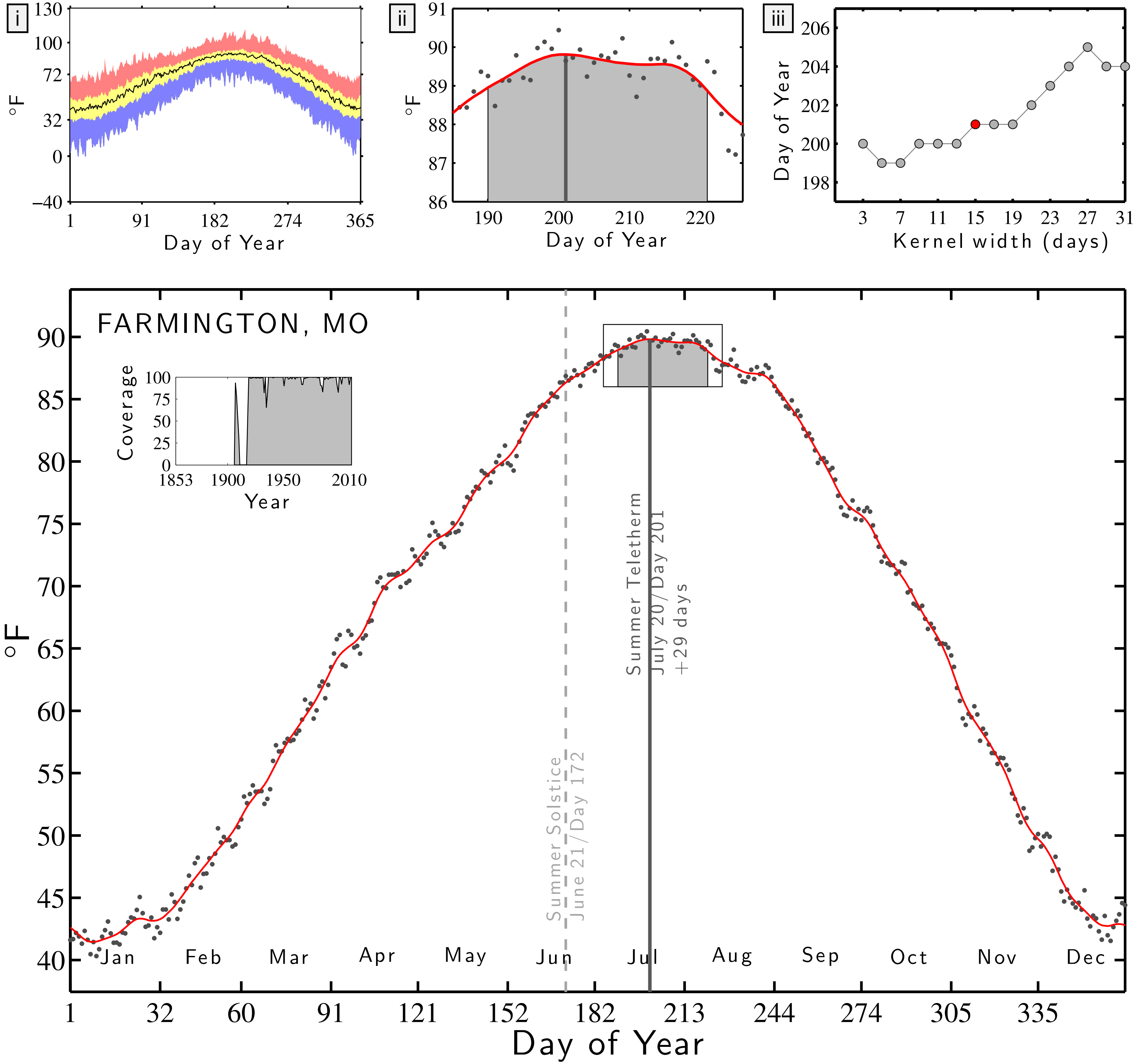

i
°F
Day of Year
ii
°F
Day of Year
iii
Day of Year
Kernel width (days)
FARMINGTON, MO
Coverage
Year
Summer Solstice
June 21/Day 172
Summer Teletherm
July 20/Day 201
+29 days
Jan
Feb
Mar
Apr
May
Jun
Jul
Aug
Sep
Oct
Nov
Dec
°F
Day of Year

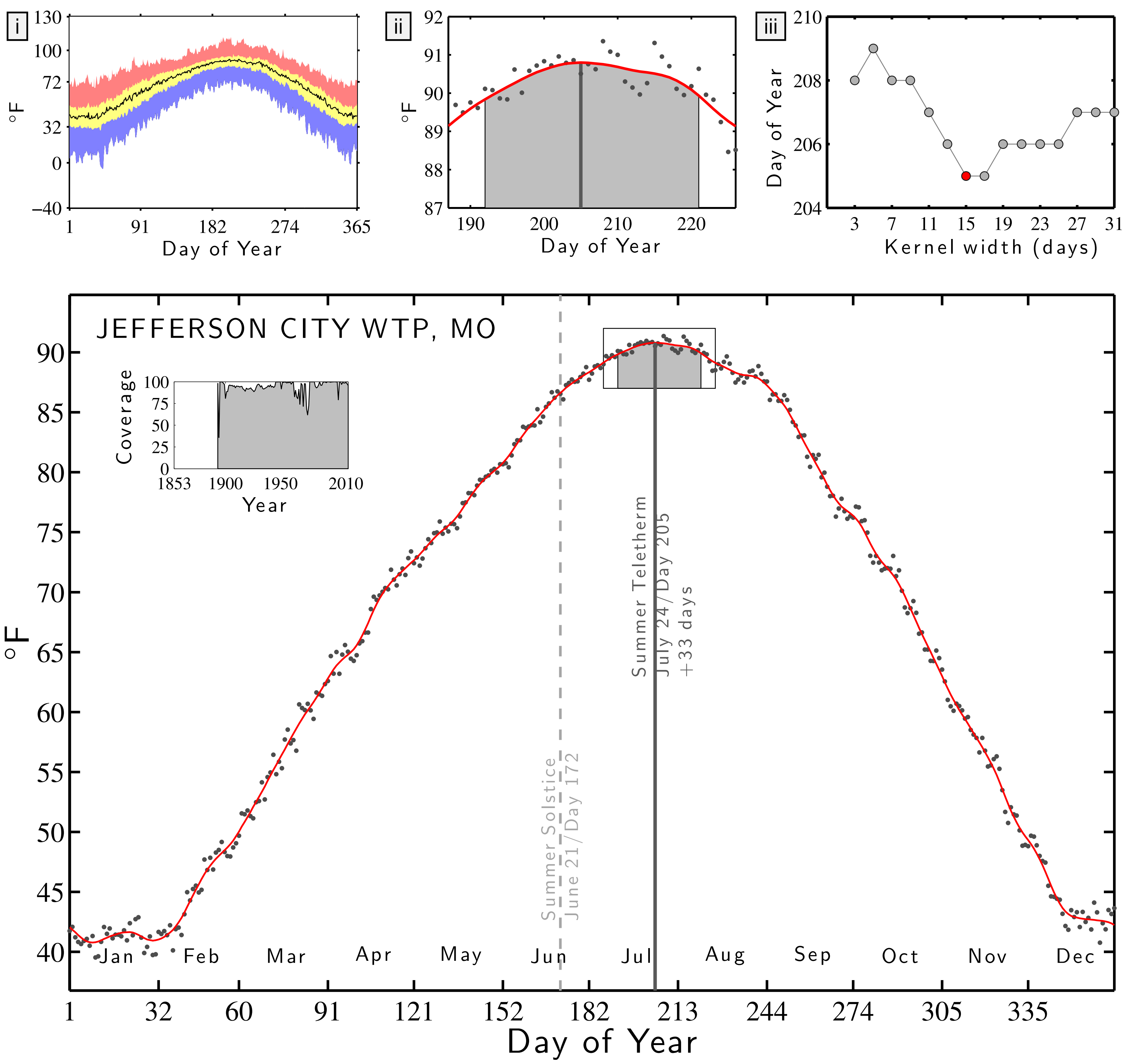

i
°F
Day of Year
ii
iii
Day of Year
Kernel width (days)
JEFFERSON CITY WTP, MO
Coverage
Year
Summer Solstice
June 21/Day 172
Summer Teletherm
July 24/Day 205
+33 days
Jan
Feb
Mar
Apr
May
Jun
Jul
Aug
Sep
Oct
Nov
Dec
°F
Day of Year

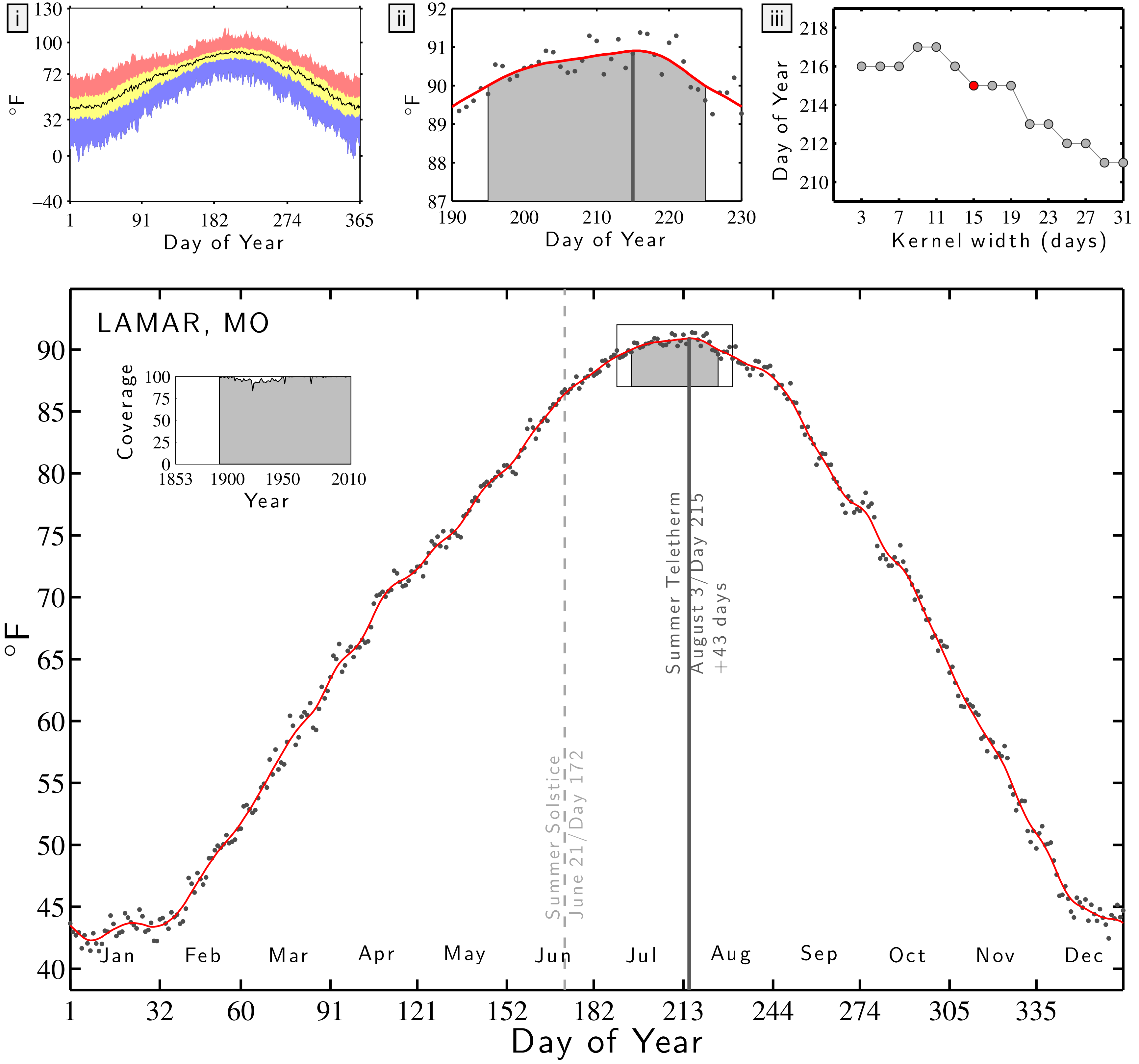

i
ii
iii
LAMAR, MO
Day of Year
°F
Kernel width (days)
Coverage
Year
Summer Solstice
June 21 / Day 172
Summer Teletherm
August 3 / Day 215
+43 days
Jan
Feb
Mar
Apr
May
Jun
Jul
Aug
Sep
Oct
Nov
Dec

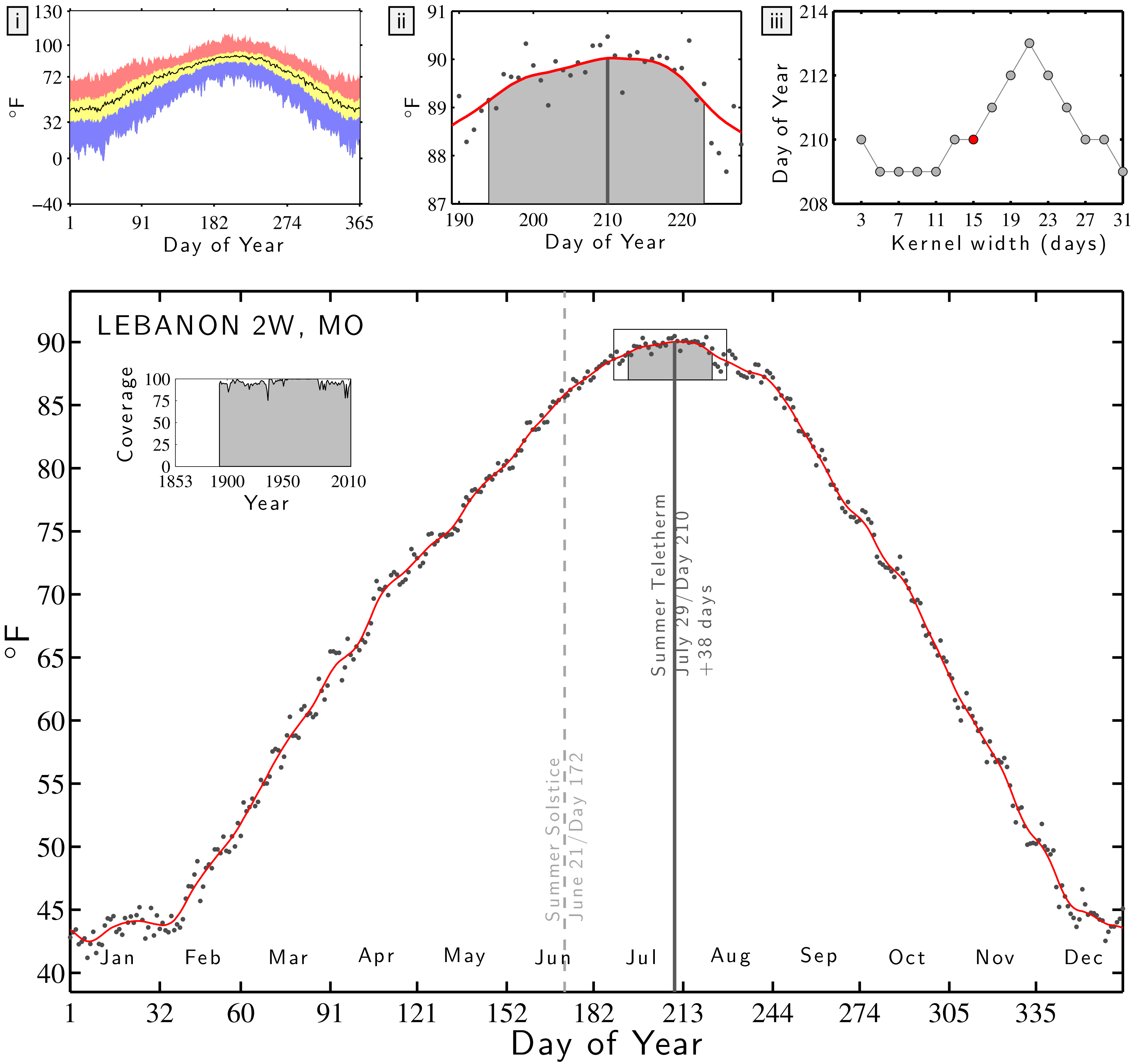

i
ii
iii
°F
Day of Year
Kernel width (days)
LEBANON 2W, MO
Coverage
Year
Summer Solstice
June 21/Day 172
Summer Teletherm
July 29/Day 210
+38 days
Jan
Feb
Mar
Apr
May
Jun
Jul
Aug
Sep
Oct
Nov
Dec

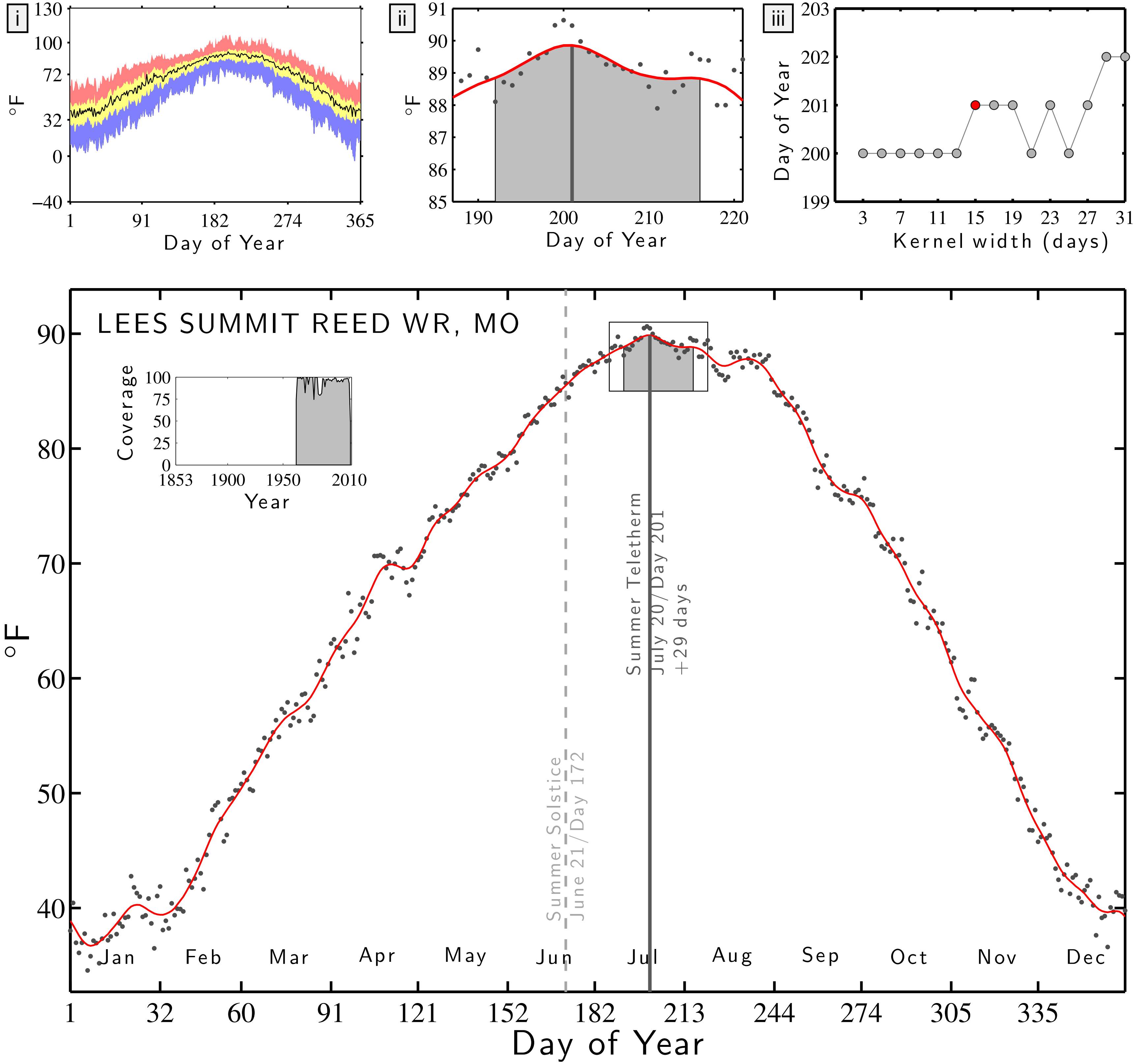

i
130
100
72
32
0
−40
°F
1
91
182
274
365
Day of Year
ii
91
90
89
88
87
86
85
°F
190
200
210
220
Day of Year
iii
203
202
201
200
199
Day of Year
3
7
11
15
19
23
27
31
Kernel width (days)
LEES SUMMIT REED WR, MO
Coverage
100
75
50
25
0
1853
1900
1950
2010
Year
Summer Solstice
June 21/Day 172
Summer Teletherm
July 20/Day 201
+29 days
90
80
70
60
50
40
°F
Jan
Feb
Mar
Apr
May
Jun
Jul
Aug
Sep
Oct
Nov
Dec
1
32
60
91
121
152
182
213
244
274
305
335
Day of Year

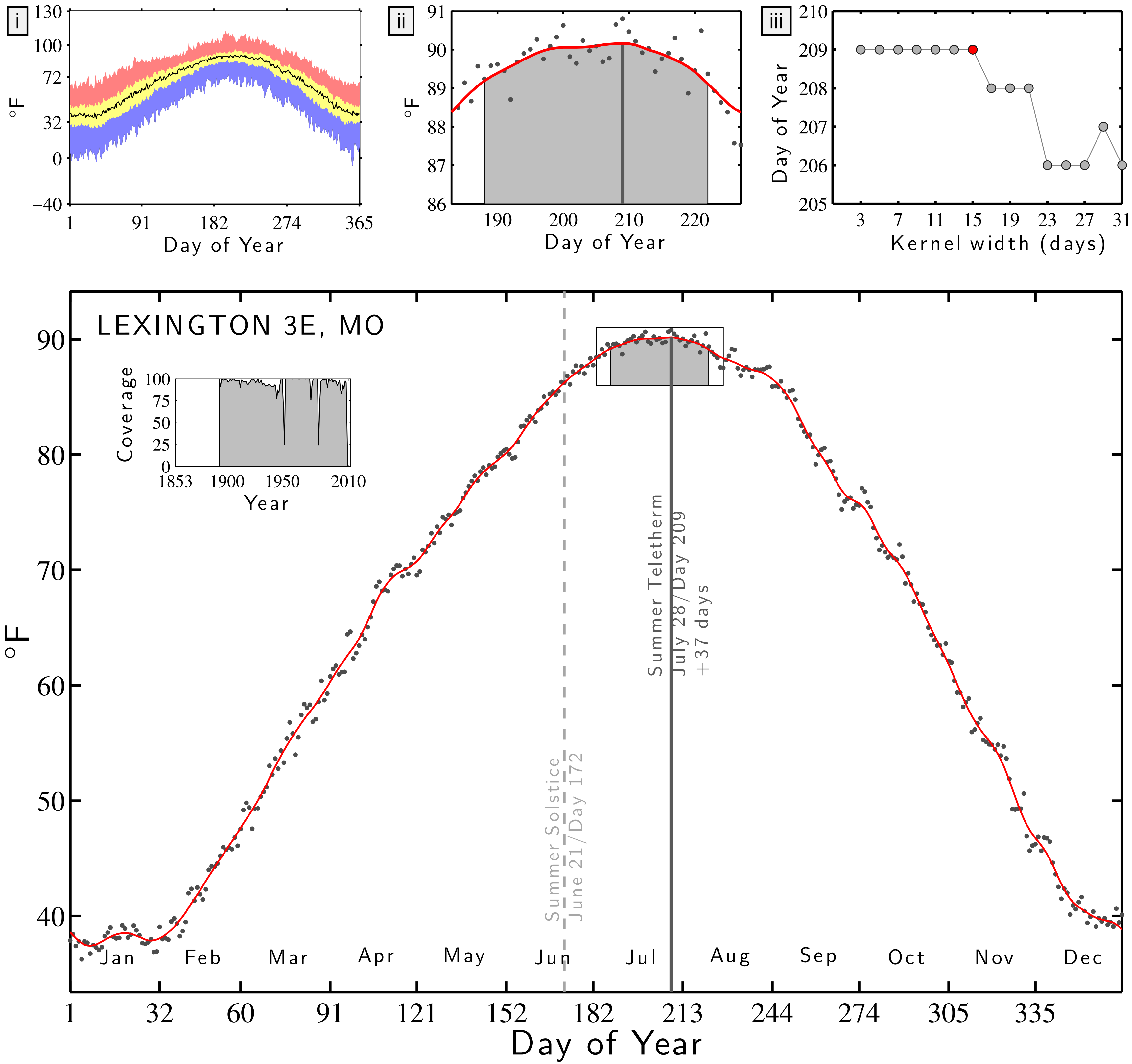

i
°F
Day of Year
ii
Day of Year
iii
Day of Year
Kernel width (days)
LEXINGTON 3E, MO
Coverage
Year
Summer Solstice
June 21/Day 172
Summer Teletherm
July 28/Day 209
+37 days
Jan
Feb
Mar
Apr
May
Jun
Jul
Aug
Sep
Oct
Nov
Dec
Day of Year

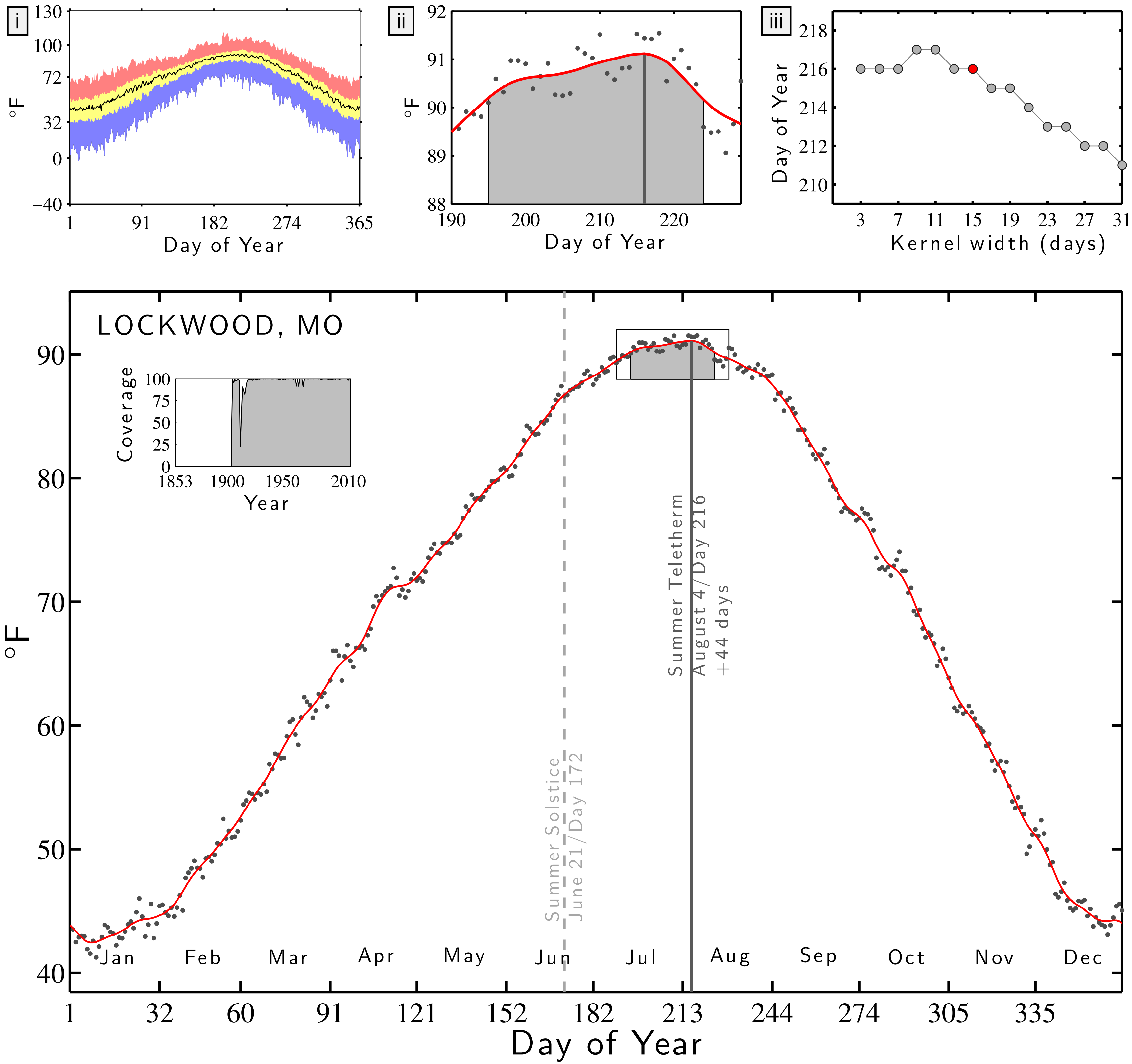

i
°F
Day of Year
ii
°F
Day of Year
iii
Day of Year
Kernel width (days)
LOCKWOOD, MO
Coverage
Year
Summer Solstice
June 21 / Day 172
Summer Teletherm
August 4 / Day 216
+44 days
Jan
Feb
Mar
Apr
May
Jun
Jul
Aug
Sep
Oct
Nov
Dec
°F
Day of Year

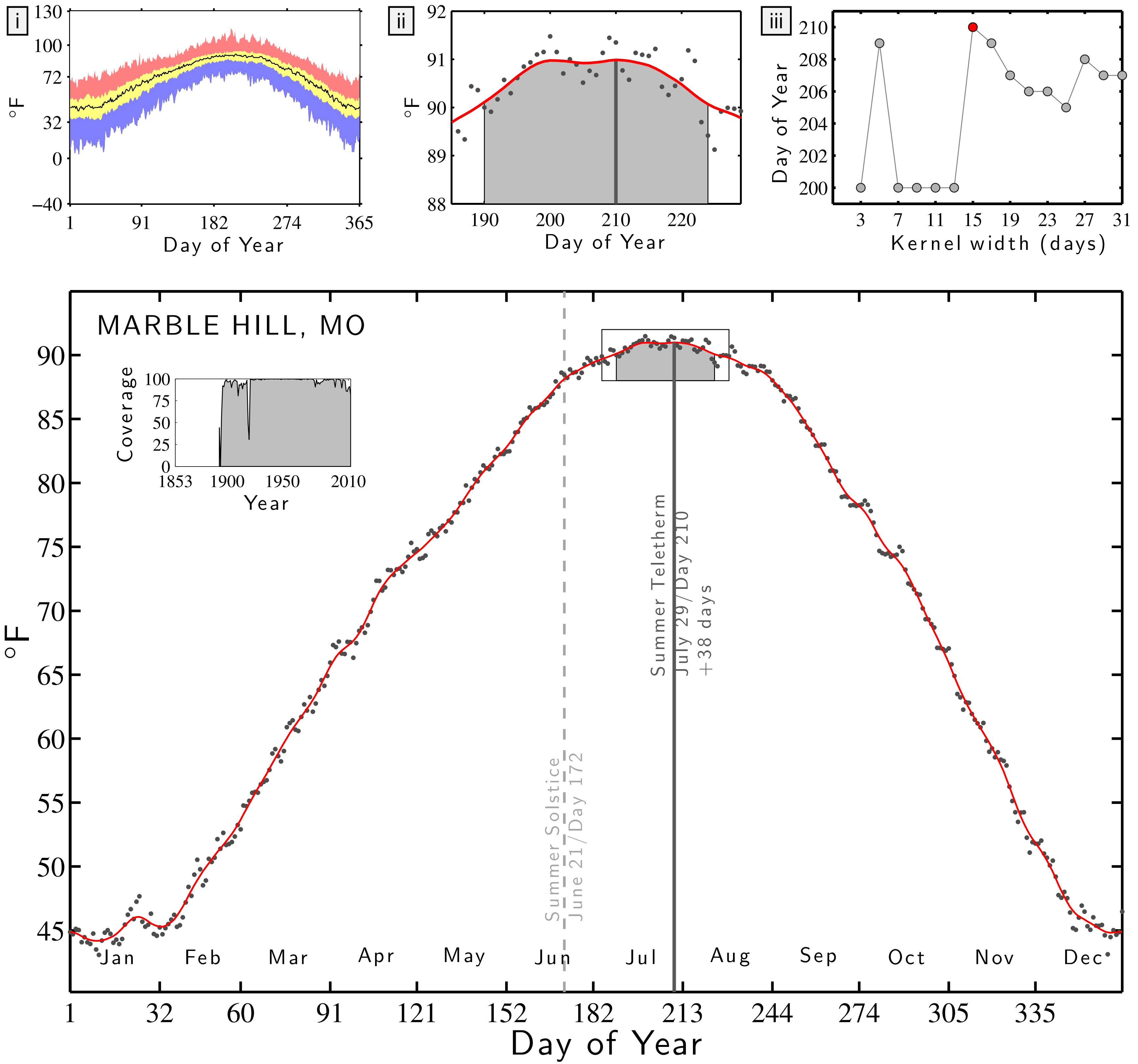

i
ii
iii
Day of Year
Kernel width (days)
°F
MARBLE HILL, MO
Coverage
Year
Summer Solstice
June 21 / Day 172
Summer Teletherm
July 29 / Day 210
+38 days
Jan
Feb
Mar
Apr
May
Jun
Jul
Aug
Sep
Oct
Nov
Dec

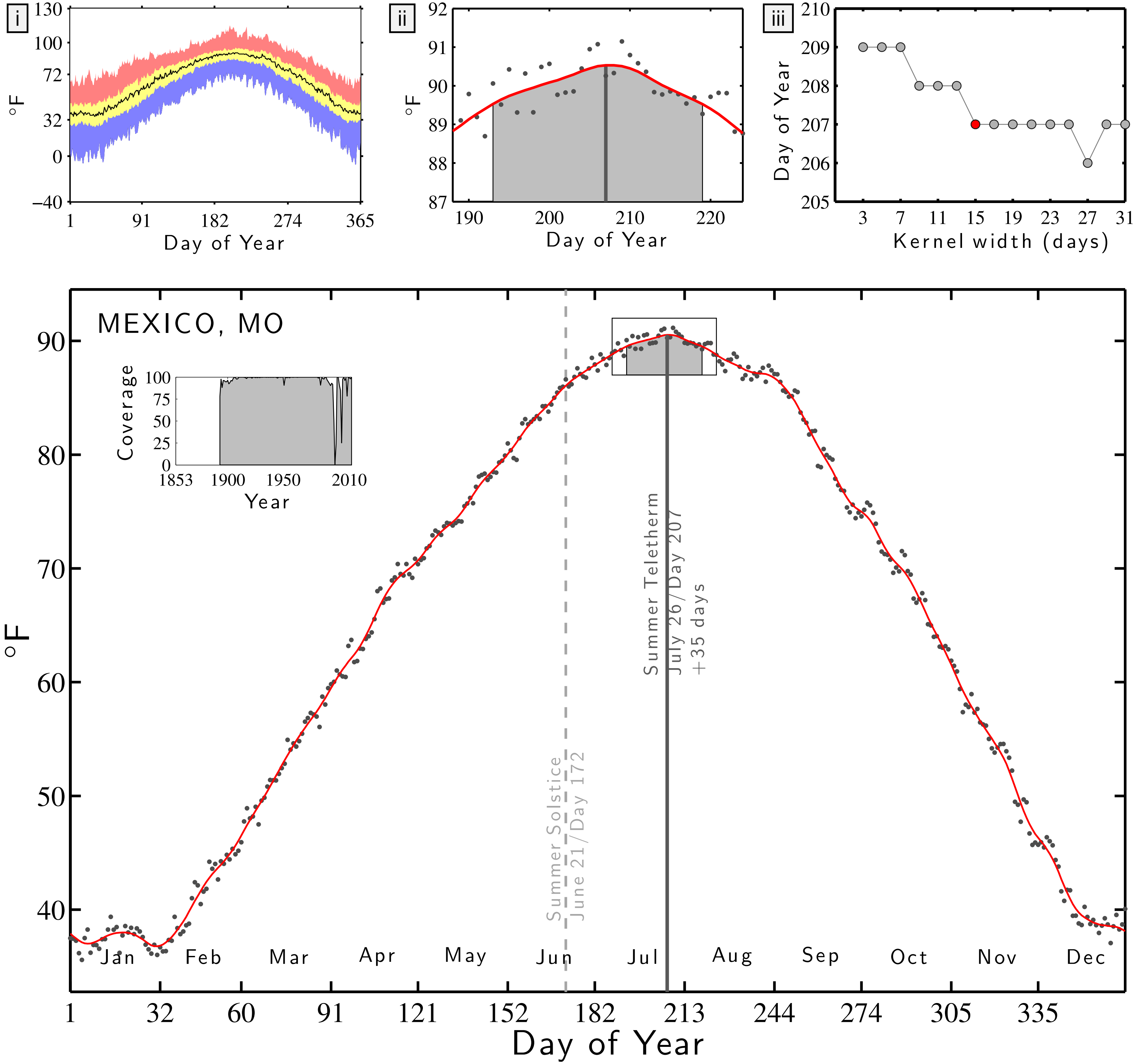
i
°F
Day of Year
ii
Day of Year
iii
Day of Year
Kernel width (days)
MEXICO, MO
Coverage
Year
Summer Solstice
June 21/Day 172
Summer Teletherm
July 26/Day 207
+35 days
Jan
Feb
Mar
Apr
May
Jun
Jul
Aug
Sep
Oct
Nov
Dec
Day of Year

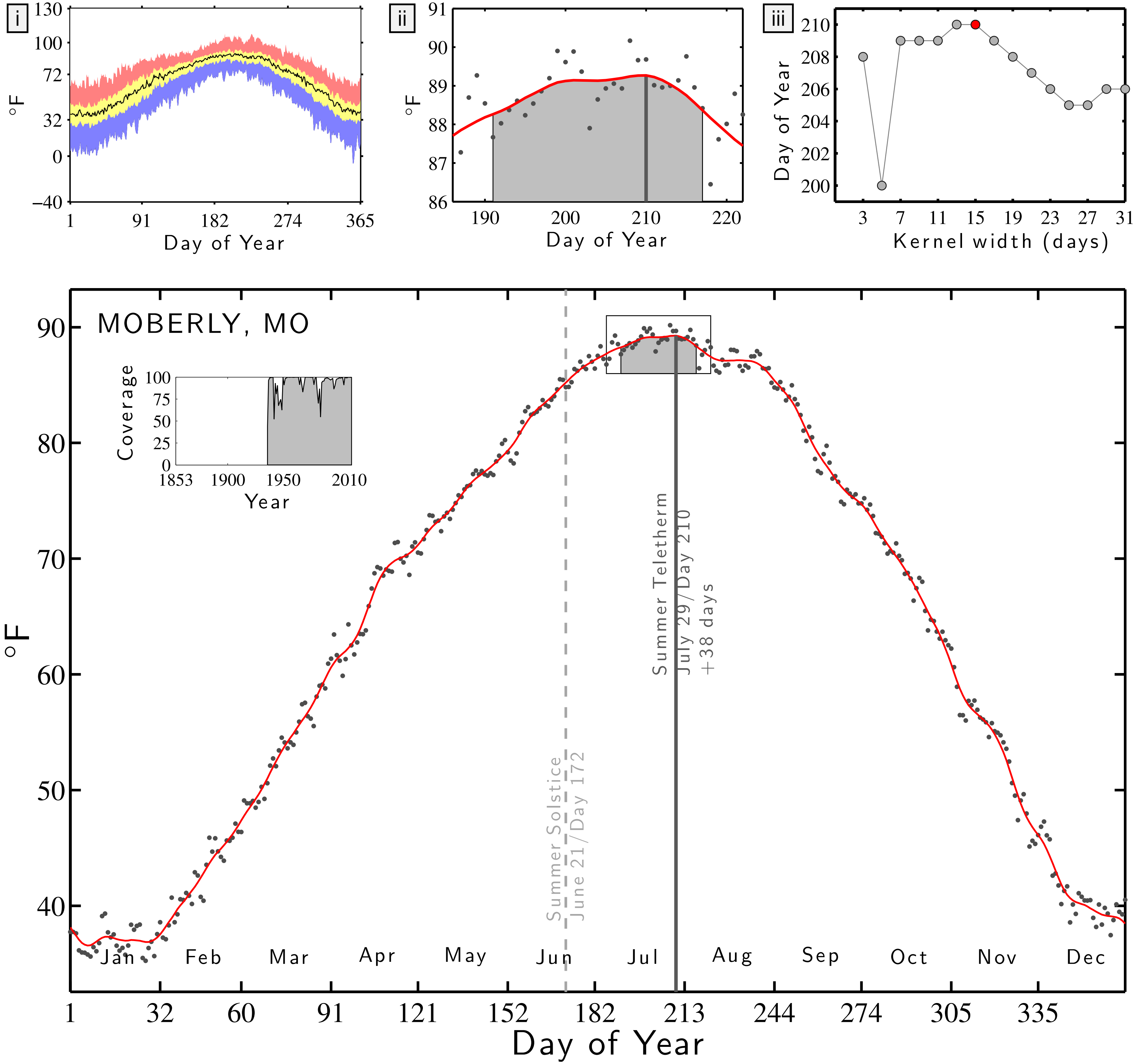

i
°F
Day of Year
ii
°F
Day of Year
iii
Day of Year
Kernel width (days)
MOBERLY, MO
Coverage
Year
Summer Solstice
June 21 / Day 172
Summer Teletherm
July 29 / Day 210
+38 days
Jan
Feb
Mar
Apr
May
Jun
Jul
Aug
Sep
Oct
Nov
Dec
°F
Day of Year

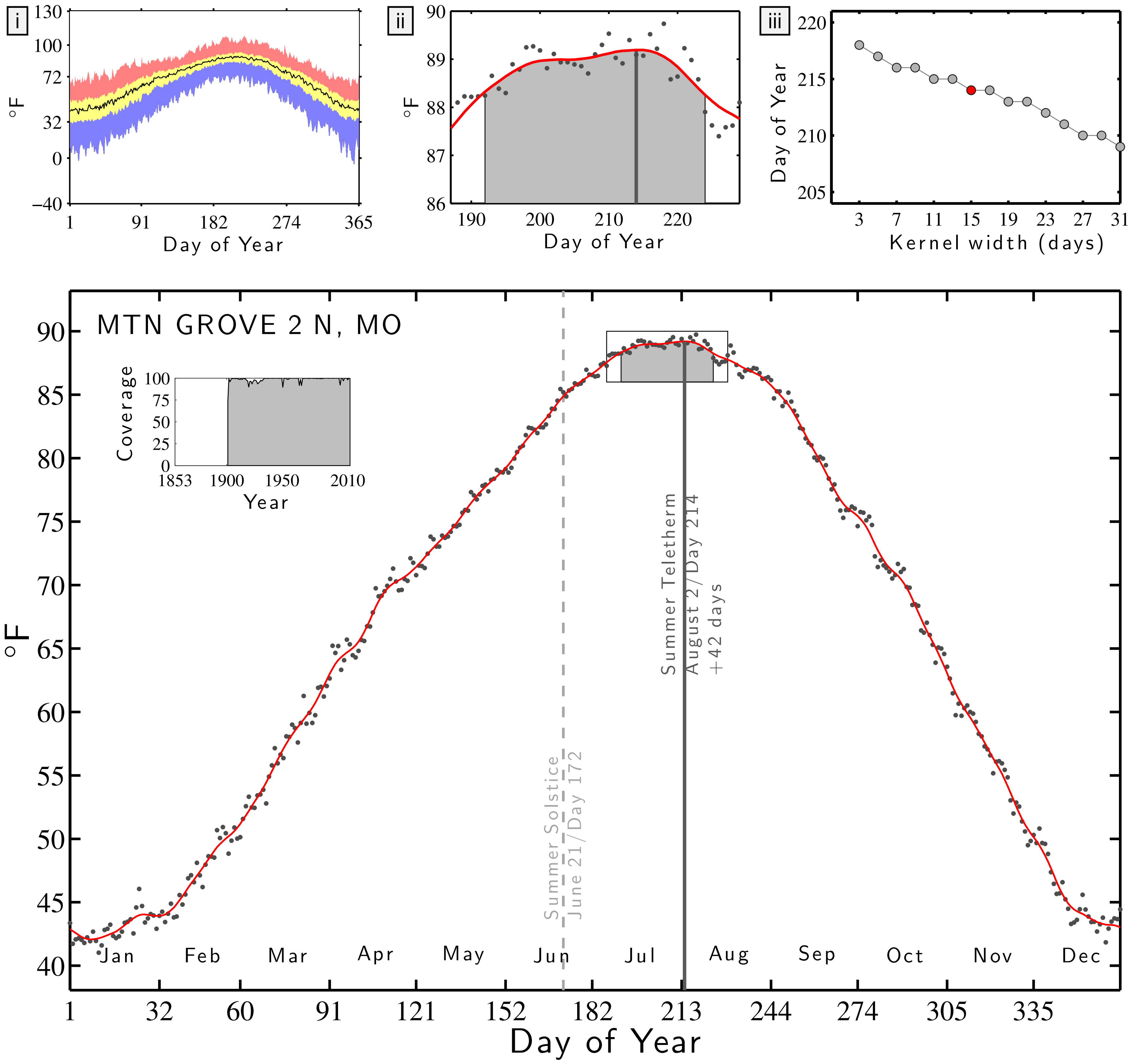

i
°F
Day of Year
ii
°F
Day of Year
iii
Day of Year
Kernel width (days)
MTN GROVE 2 N, MO
Coverage
Year
Summer Solstice
June 21 / Day 172
Summer Teletherm
August 2 / Day 214
+42 days
Jan
Feb
Mar
Apr
May
Jun
Jul
Aug
Sep
Oct
Nov
Dec
°F
Day of Year

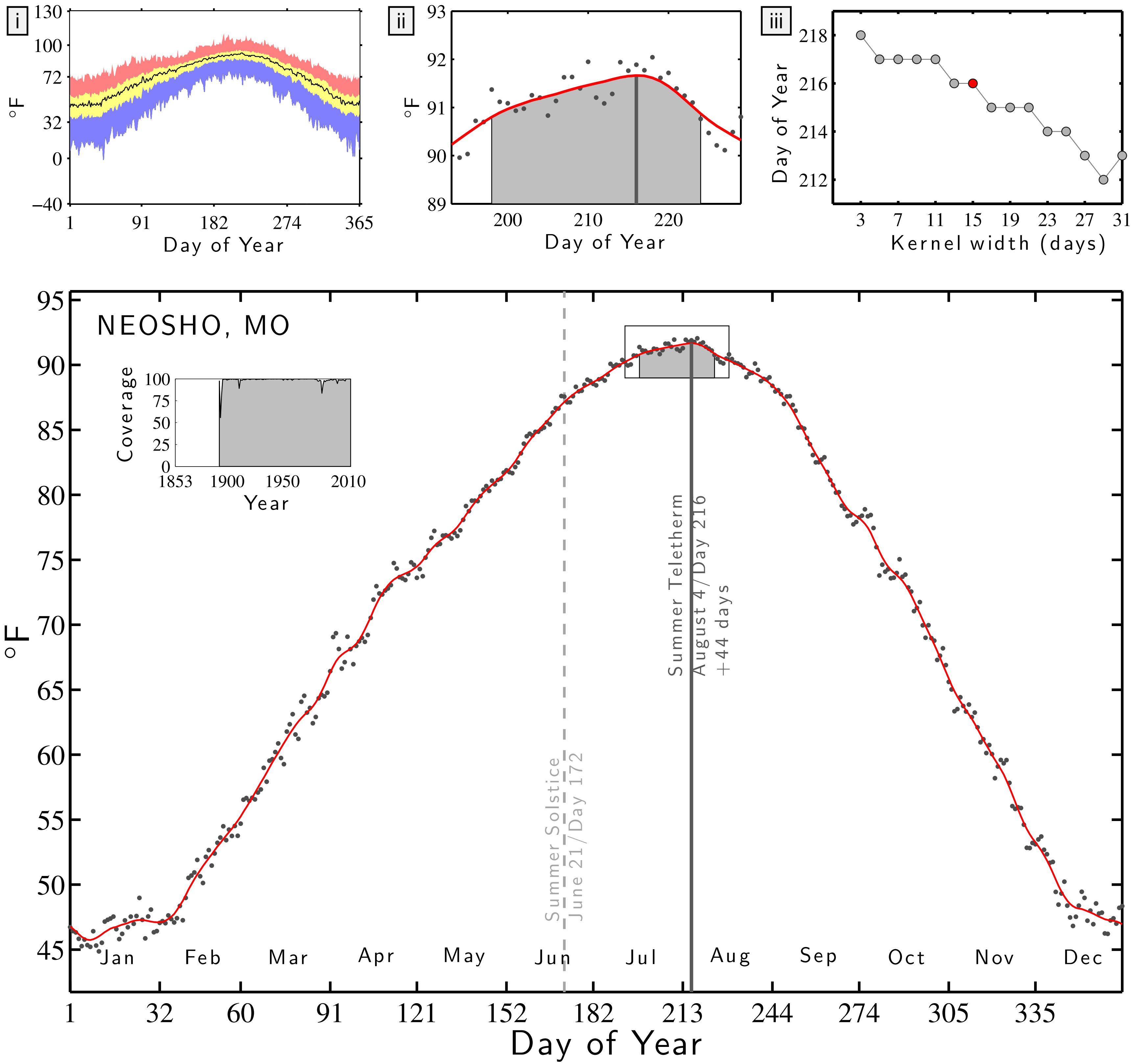

i
ii
iii
Day of Year
Kernel width (days)
NEOSHO, MO
Coverage
Year
Summer Solstice
June 21 / Day 172
Summer Teletherm
August 4 / Day 216
+44 days
°F
Jan
Feb
Mar
Apr
May
Jun
Jul
Aug
Sep
Oct
Nov
Dec
Day of Year

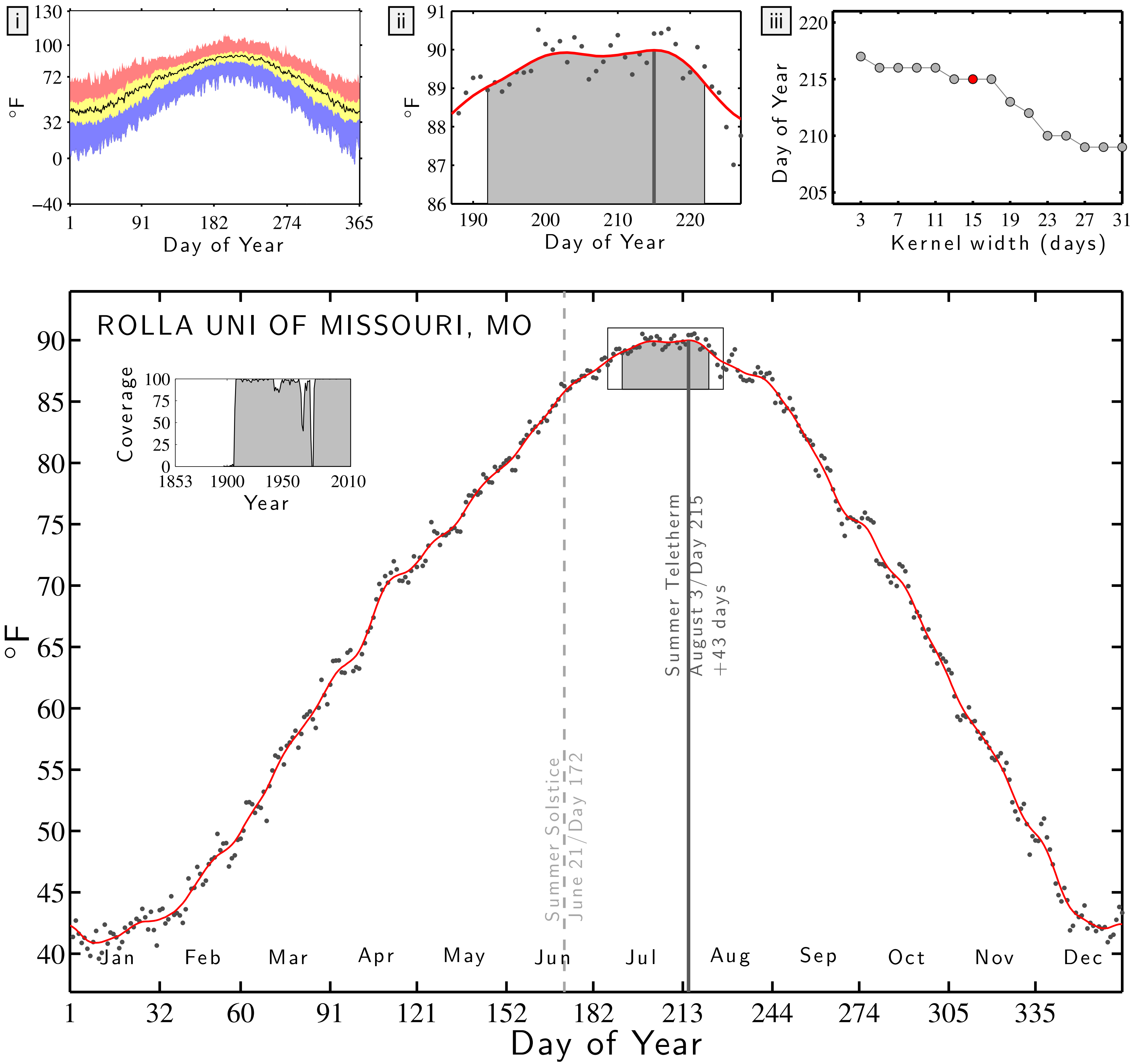

i
ii
iii
°F
Day of Year
Day of Year
Kernel width (days)
ROLLA UNI OF MISSOURI, MO
Coverage
Year
Summer Solstice
June 21 / Day 172
Summer Teletherm
August 3 / Day 215
+43 days
Jan
Feb
Mar
Apr
May
Jun
Jul
Aug
Sep
Oct
Nov
Dec

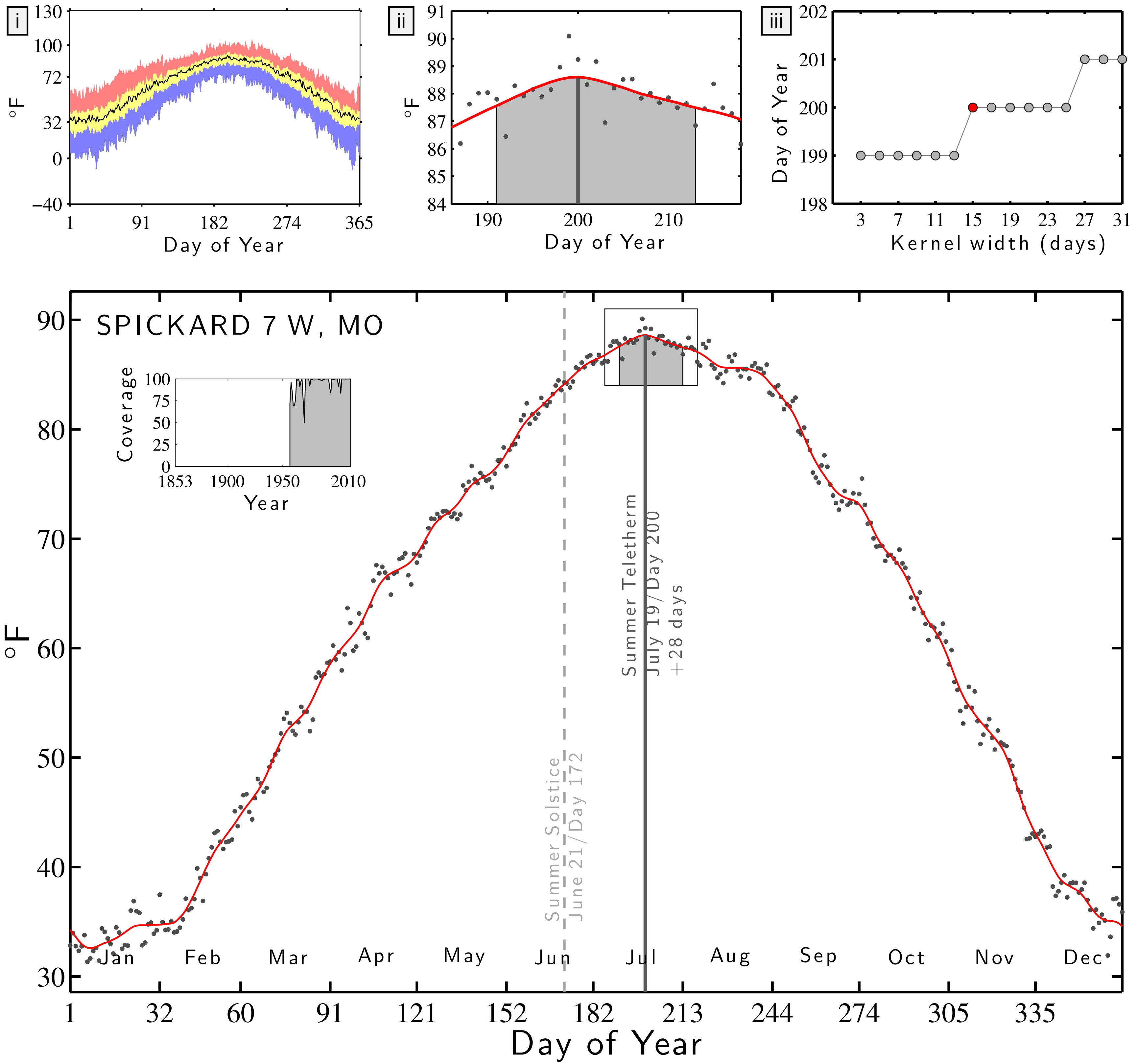

i
130
100
72
32
0
−40
°F
1
91
182
274
365
Day of Year
ii
91
90
89
88
87
86
85
84
°F
190
200
210
Day of Year
iii
202
201
200
199
198
Day of Year
3
7
11
15
19
23
27
31
Kernel width (days)
SPICKARD 7 W, MO
Coverage
100
75
50
25
0
1853
1900
1950
2010
Year
90
80
70
60
50
40
30
°F
Summer Solstice
June 21/Day 172
Summer Teletherm
July 19/Day 200
+28 days
Jan
Feb
Mar
Apr
May
Jun
Jul
Aug
Sep
Oct
Nov
Dec
1
32
60
91
121
152
182
213
244
274
305
335
Day of Year

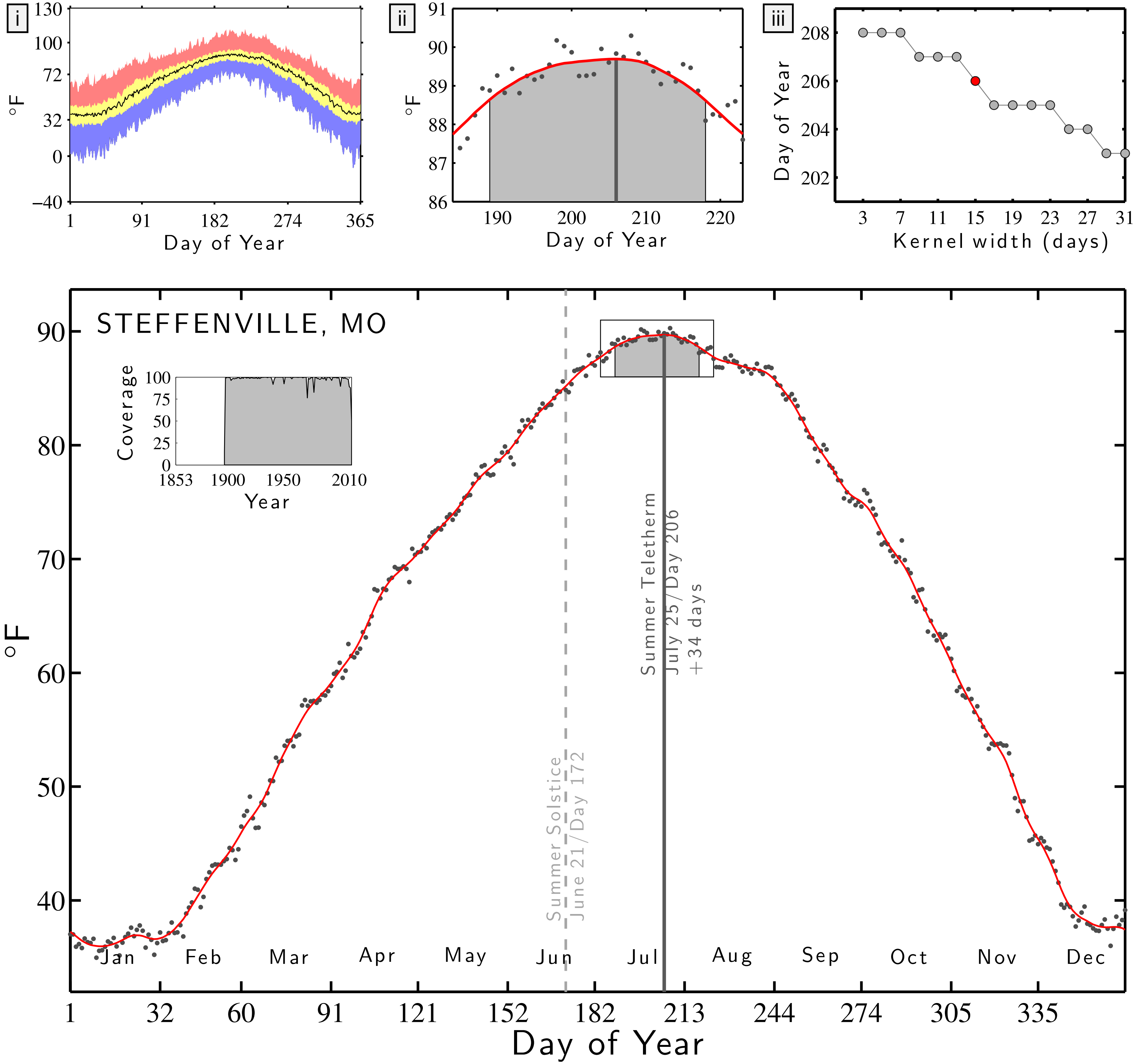

i
°F
Day of Year
ii
°F
Day of Year
iii
Day of Year
Kernel width (days)
STEFFENVILLE, MO
Coverage
Year
Summer Solstice
June 21/Day 172
Summer Teletherm
July 25/Day 206
+34 days
Jan
Feb
Mar
Apr
May
Jun
Jul
Aug
Sep
Oct
Nov
Dec
°F
Day of Year

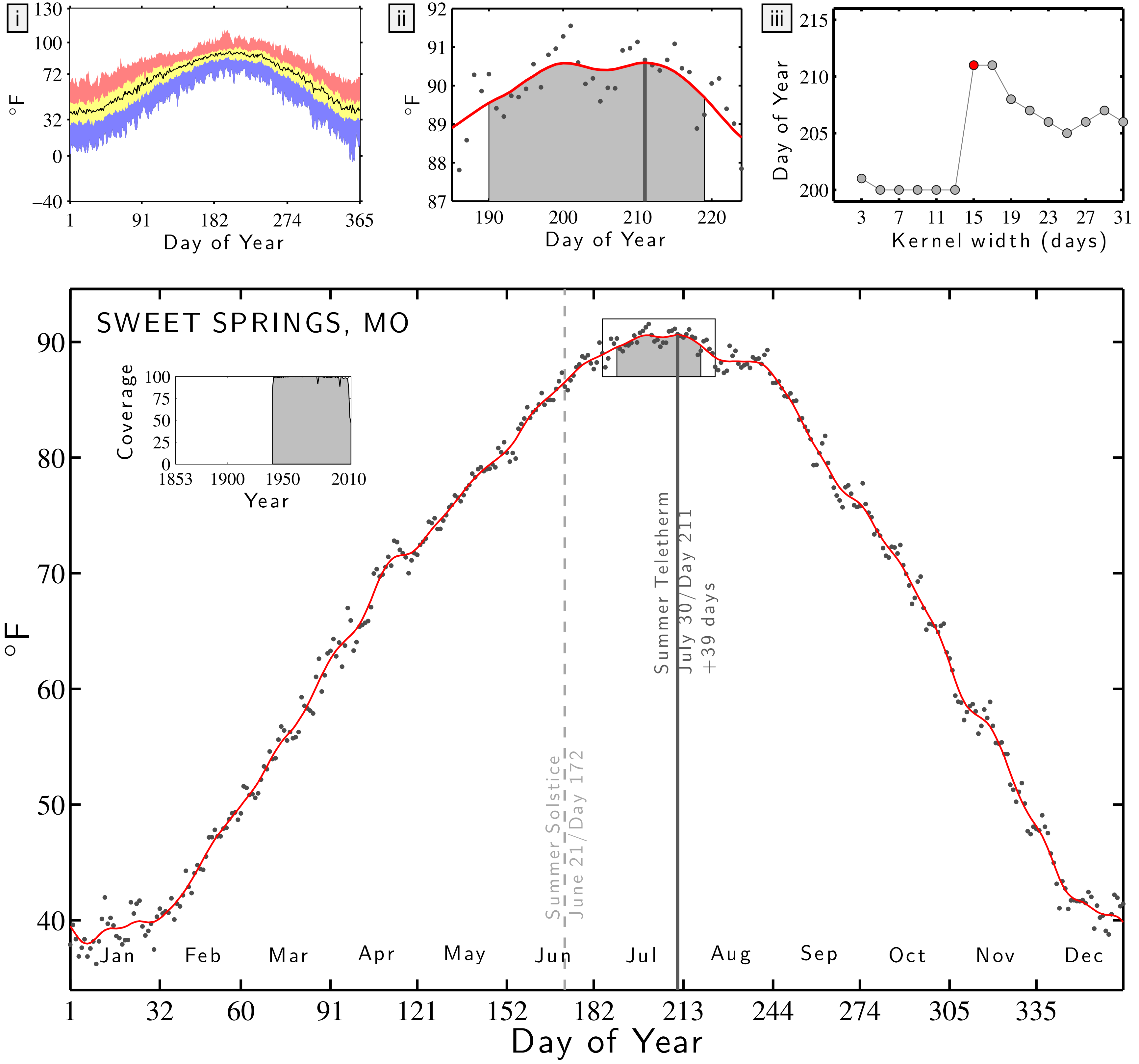

i
°F
Day of Year
ii
Day of Year
iii
Day of Year
Kernel width (days)
SWEET SPRINGS, MO
Coverage
Year
Summer Solstice
June 21/Day 172
Summer Teletherm
July 30/Day 211
+39 days
Jan
Feb
Mar
Apr
May
Jun
Jul
Aug
Sep
Oct
Nov
Dec
Day of Year

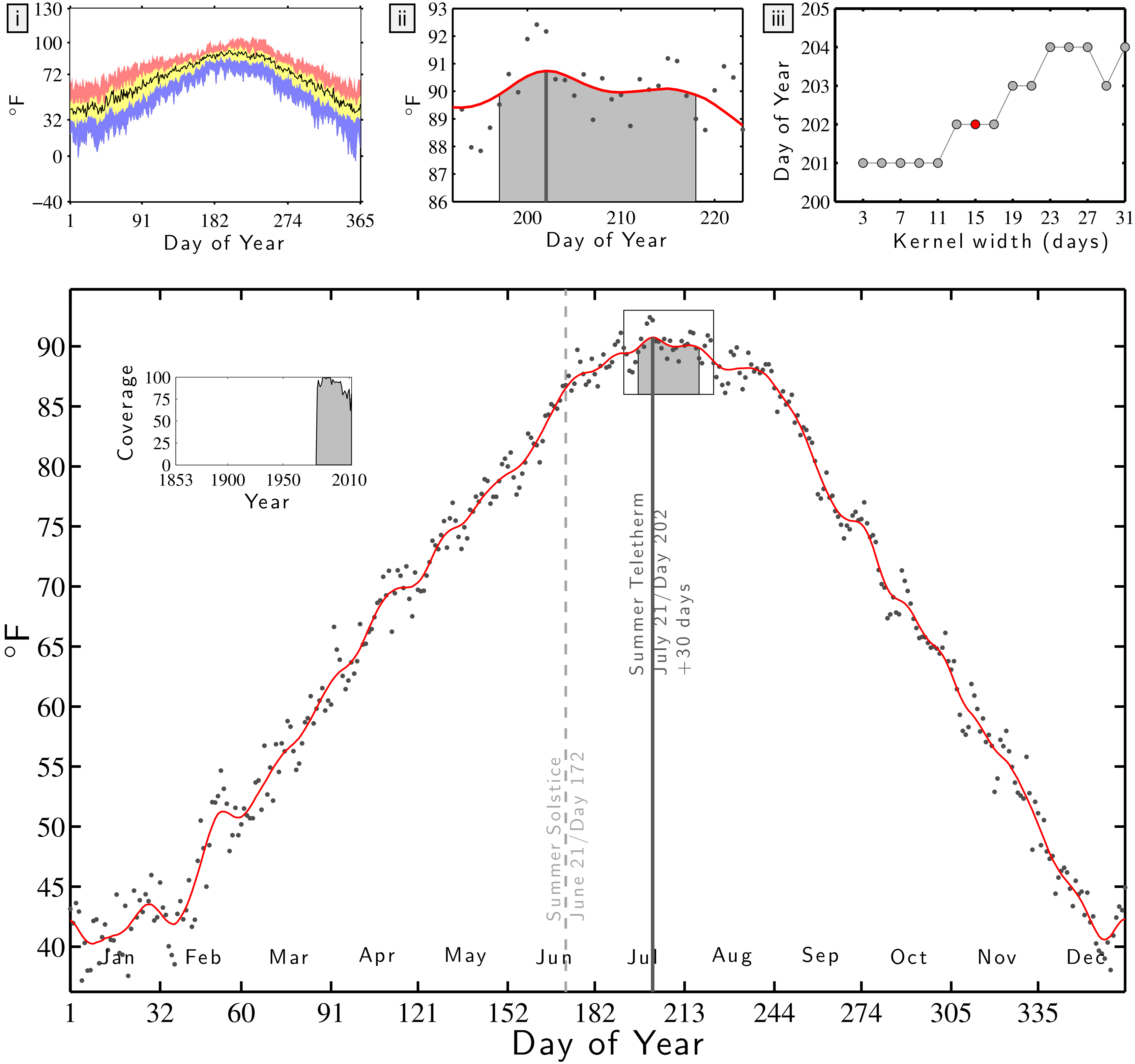

i
130
100
72
32
0
−40
°F
1
91
182
274
365
Day of Year
ii
93
92
91
90
89
88
87
86
°F
200
210
220
Day of Year
iii
205
204
203
202
201
200
Day of Year
3
7
11
15
19
23
27
31
Kernel width (days)
Coverage
100
75
50
25
0
1853
1900
1950
2010
Year
Summer Solstice
June 21 / Day 172
Summer Teletherm
July 21 / Day 202
+30 days
90
85
80
75
70
65
60
55
50
45
40
°F
Jan
Feb
Mar
Apr
May
Jun
Jul
Aug
Sep
Oct
Nov
Dec
1
32
60
91
121
152
182
213
244
274
305
335
Day of Year

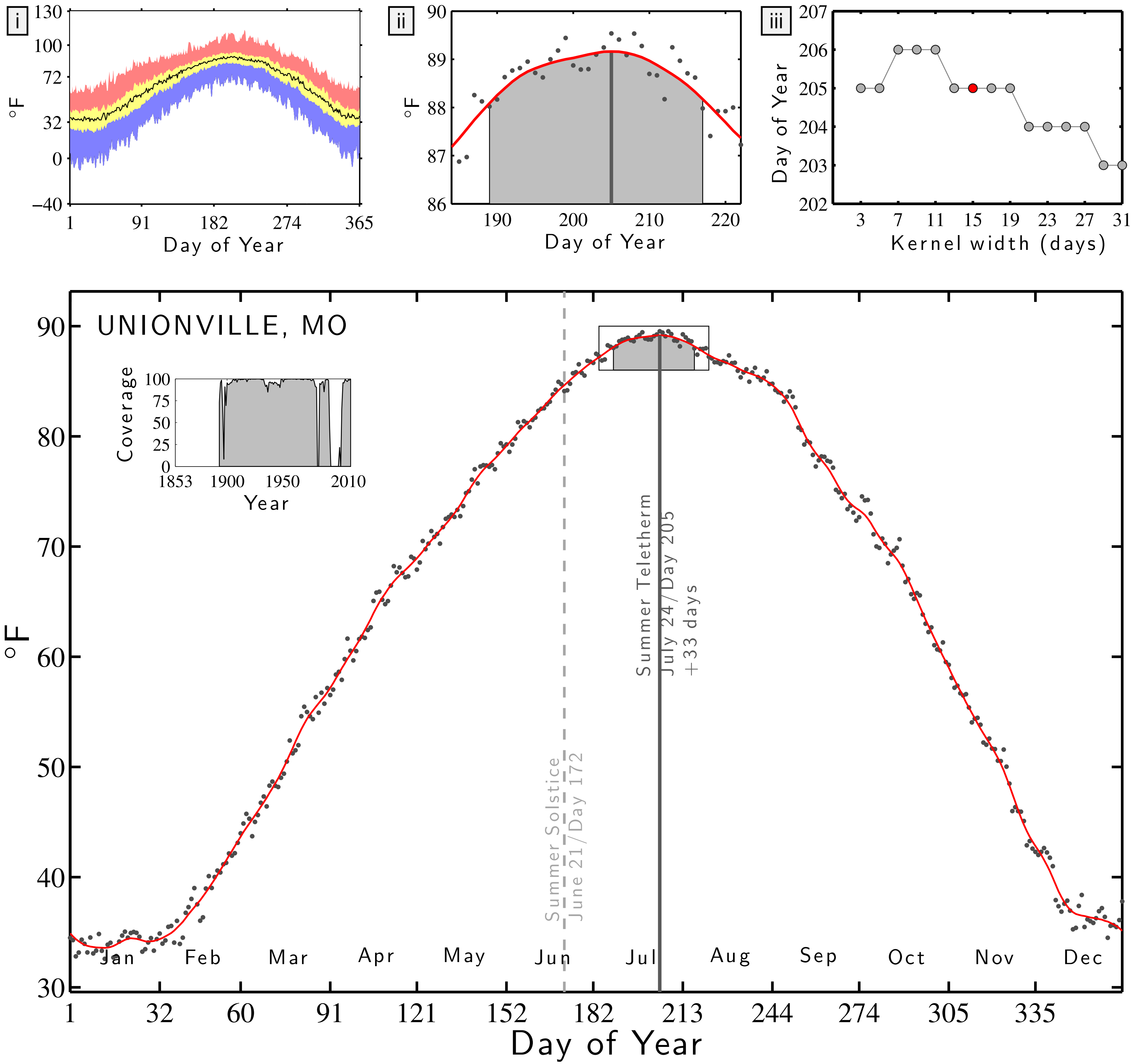

i
ii
iii
°F
Day of Year
Kernel width (days)
UNIONVILLE, MO
Coverage
Year
Summer Solstice
June 21 / Day 172
Summer Teletherm
July 24 / Day 205
+33 days
Jan
Feb
Mar
Apr
May
Jun
Jul
Aug
Sep
Oct
Nov
Dec

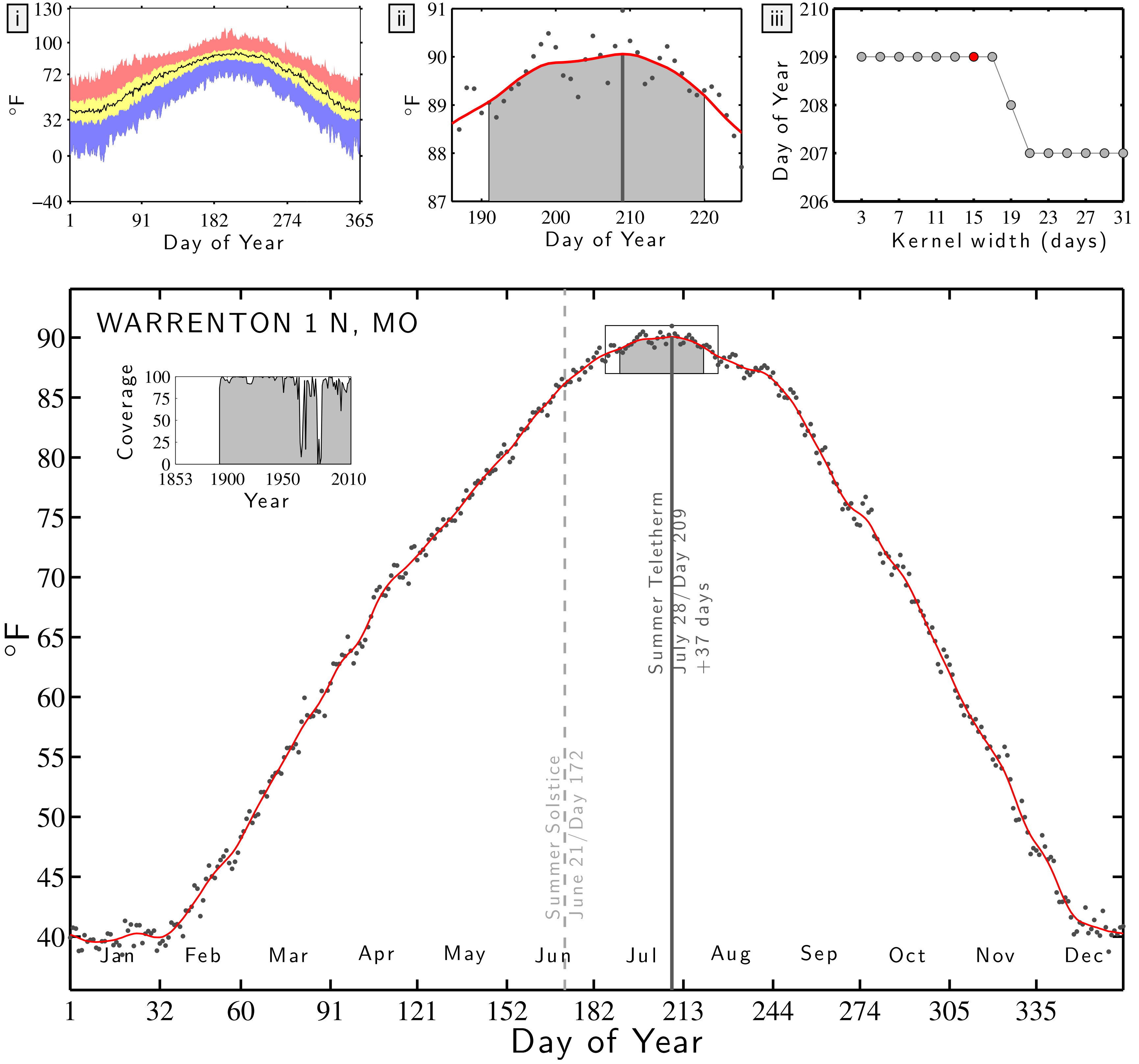

i
ii
iii
Day of Year
°F
Kernel width (days)
WARRENTON 1 N, MO
Coverage
Year
Summer Solstice
June 21/Day 172
Summer Teletherm
July 28/Day 209
+37 days
Jan
Feb
Mar
Apr
May
Jun
Jul
Aug
Sep
Oct
Nov
Dec

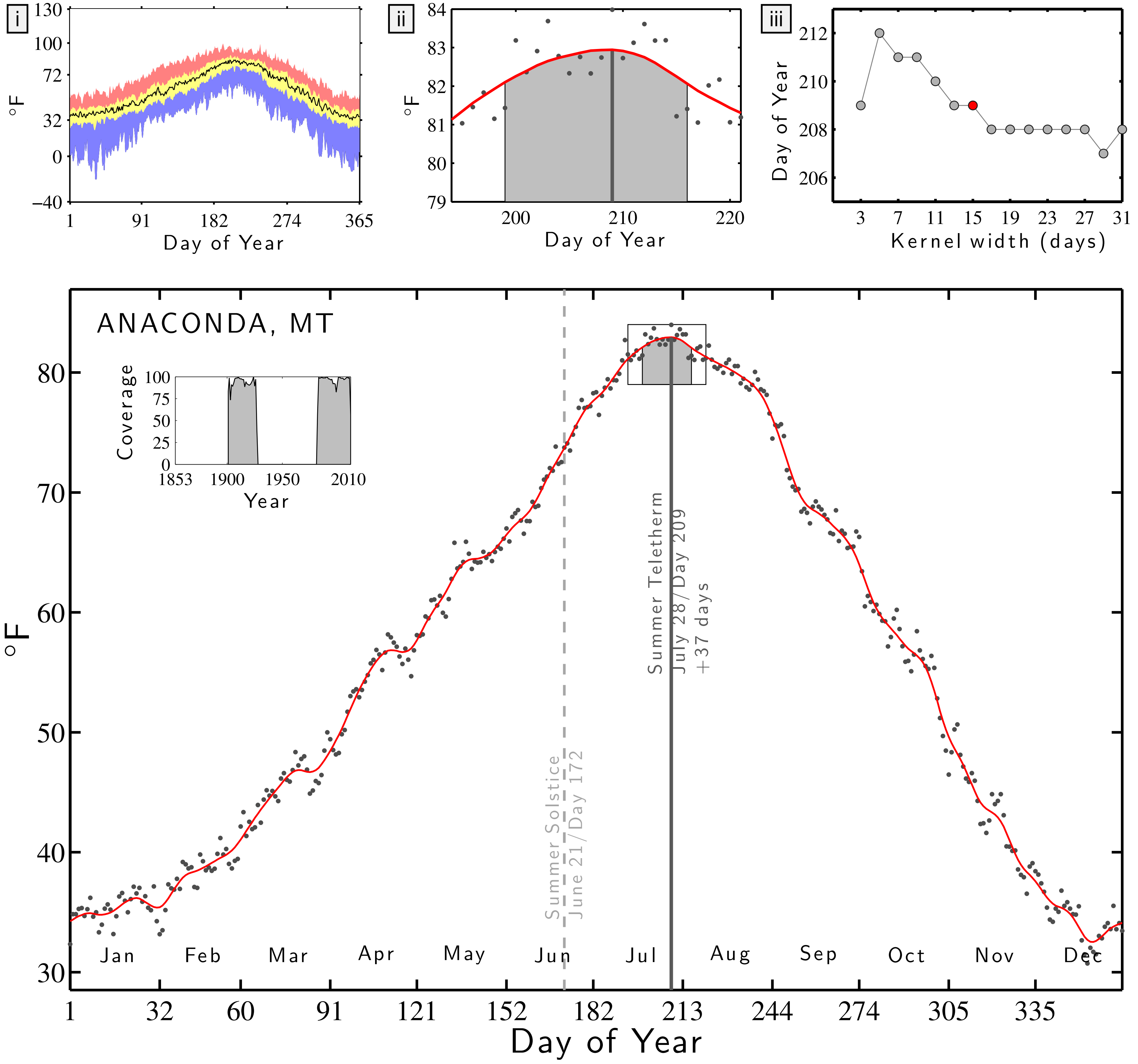

i
°F
Day of Year
ii
°F
Day of Year
iii
Day of Year
Kernel width (days)
ANACONDA, MT
Coverage
Year
Summer Solstice
June 21/Day 172
Summer Teletherm
July 28/Day 209
+37 days
Jan
Feb
Mar
Apr
May
Jun
Jul
Aug
Sep
Oct
Nov
Dec
°F
Day of Year

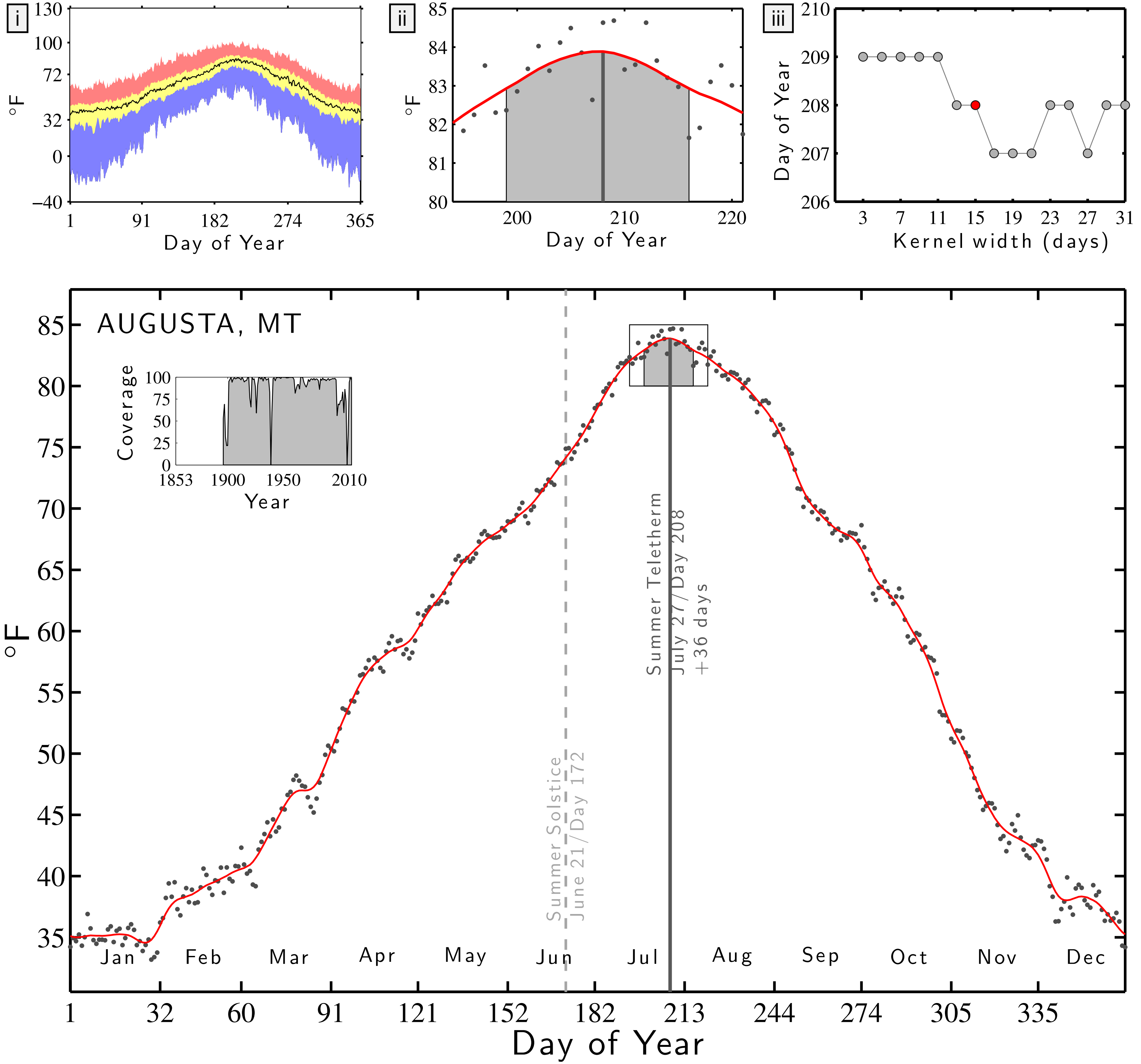

i
ii
iii
°F
Day of Year
Kernel width (days)
AUGUSTA, MT
Coverage
Year
Summer Solstice
June 21 / Day 172
Summer Teletherm
July 27 / Day 208
+36 days
Jan
Feb
Mar
Apr
May
Jun
Jul
Aug
Sep
Oct
Nov
Dec

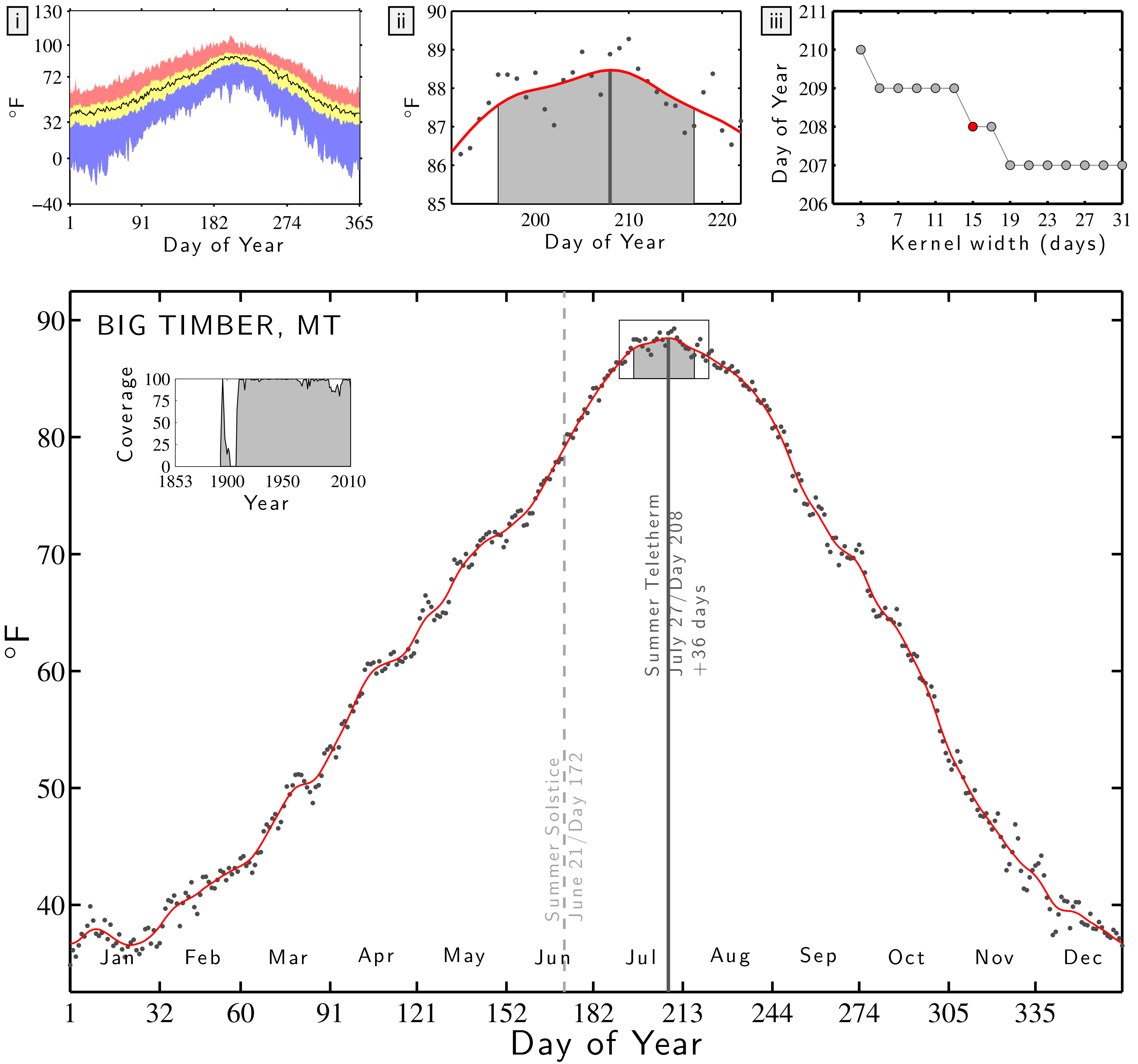

i
ii
iii
°F
Day of Year
Day of Year
Kernel width (days)
BIG TIMBER, MT
Coverage
Year
Summer Solstice
June 21 / Day 172
Summer Teletherm
July 27 / Day 208
+36 days
Jan
Feb
Mar
Apr
May
Jun
Jul
Aug
Sep
Oct
Nov
Dec

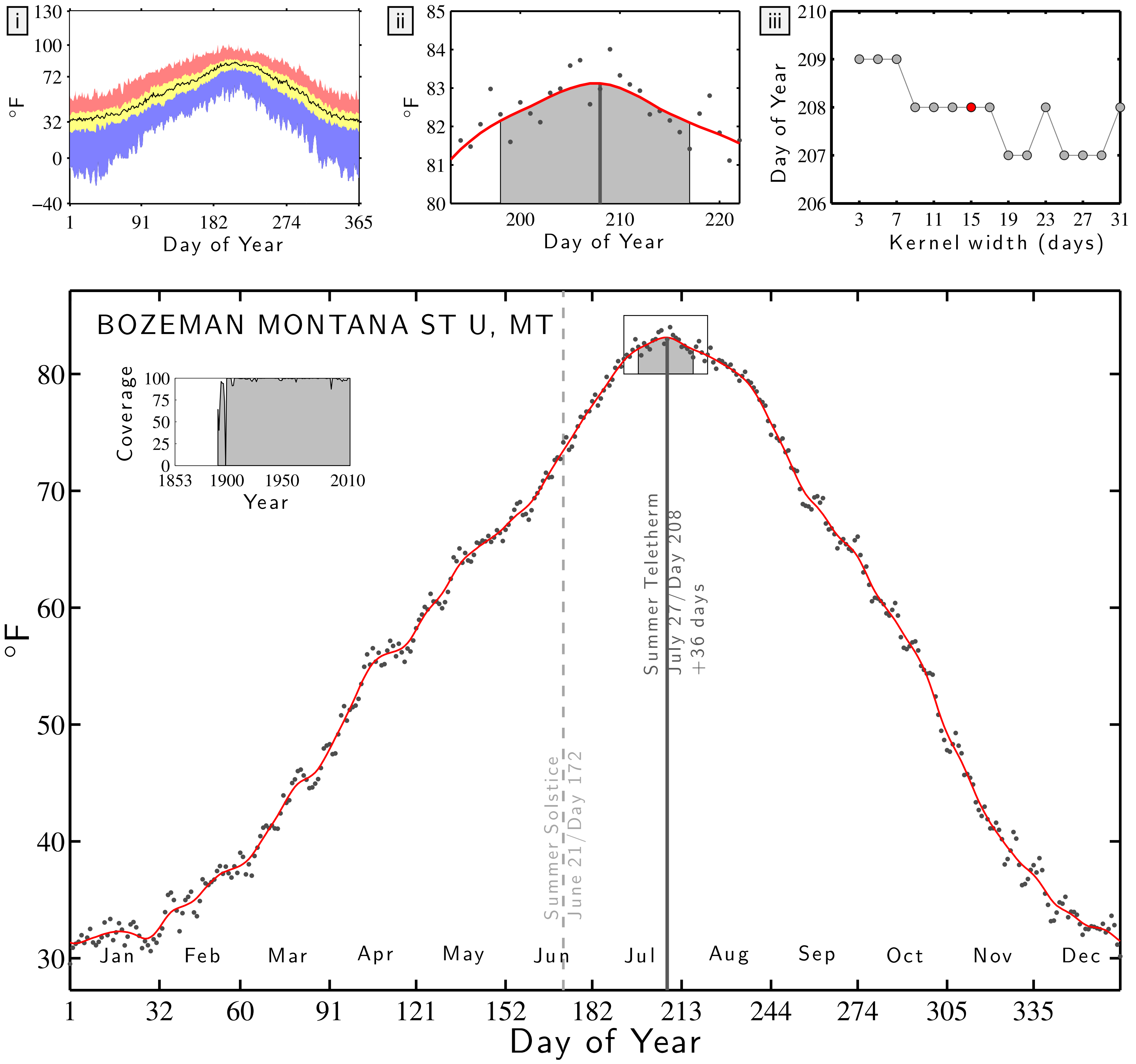
i
ii
iii
°F
Day of Year
Day of Year
Kernel width (days)
BOZEMAN MONTANA ST U, MT
Coverage
Year
Summer Solstice
June 21 / Day 172
Summer Teletherm
July 27 / Day 208
+36 days
Jan
Feb
Mar
Apr
May
Jun
Jul
Aug
Sep
Oct
Nov
Dec
°F
Day of Year

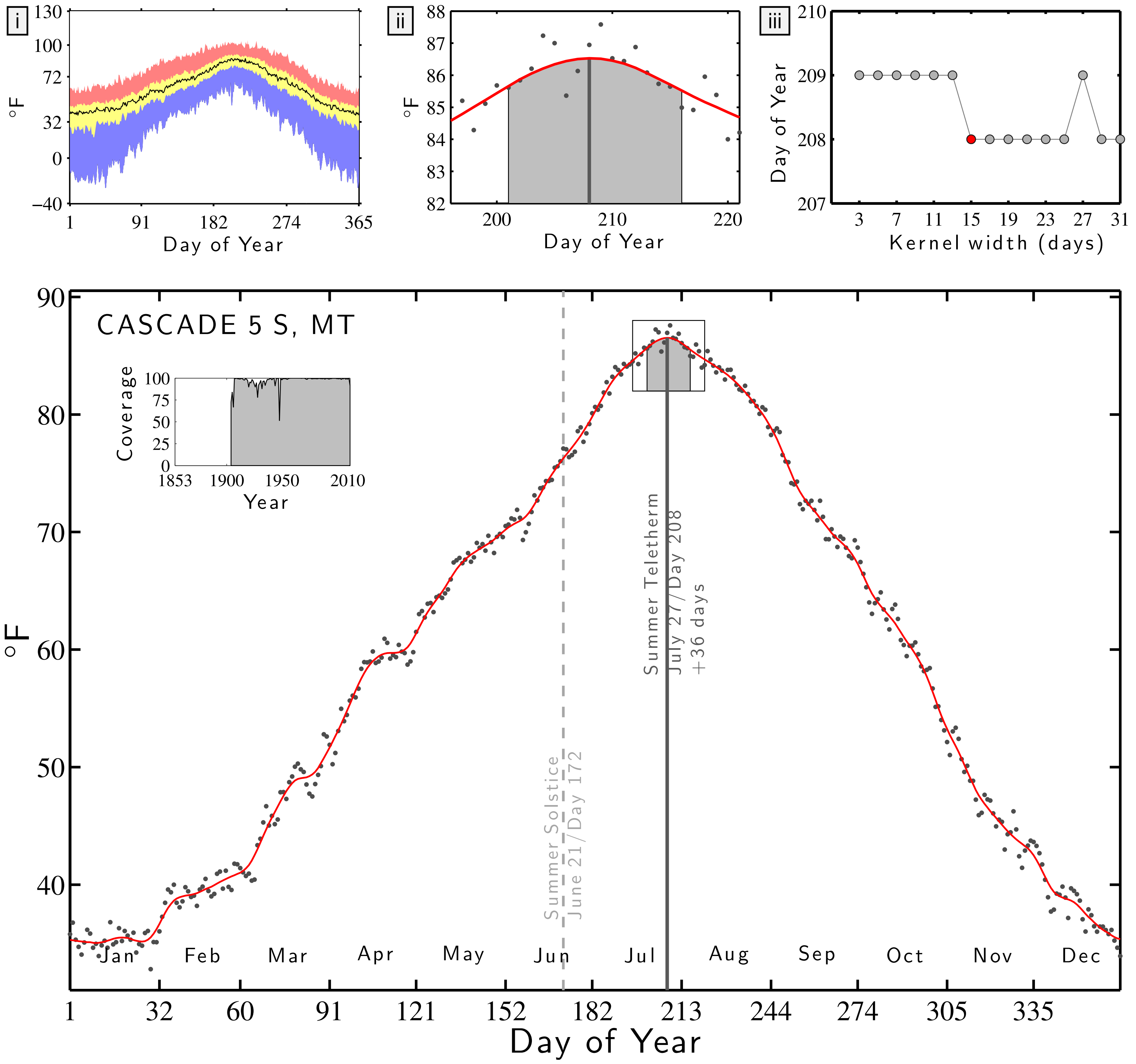

i
ii
iii
CASCADE 5 S, MT
Coverage
Year
Summer Solstice
June 21 / Day 172
Summer Teletherm
July 27 / Day 208
+36 days
Day of Year
Kernel width (days)
°F
Jan
Feb
Mar
Apr
May
Jun
Jul
Aug
Sep
Oct
Nov
Dec

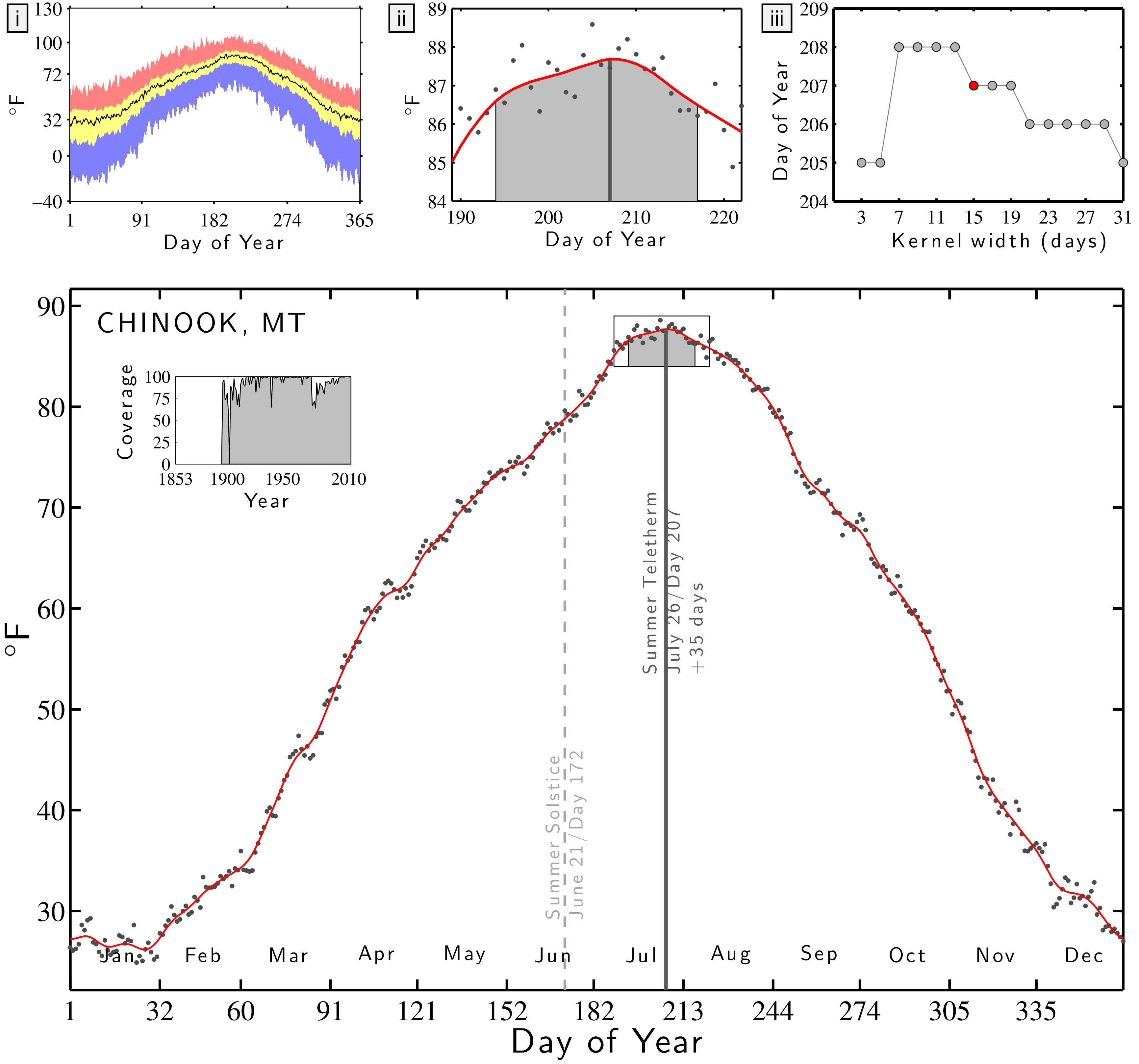

i
°F
Day of Year
ii
°F
Day of Year
iii
Day of Year
Kernel width (days)
CHINOOK, MT
Coverage
Year
Summer Solstice
June 21 / Day 172
Summer Teletherm
July 26 / Day 207
+35 days
Jan
Feb
Mar
Apr
May
Jun
Jul
Aug
Sep
Oct
Nov
Dec
°F
Day of Year

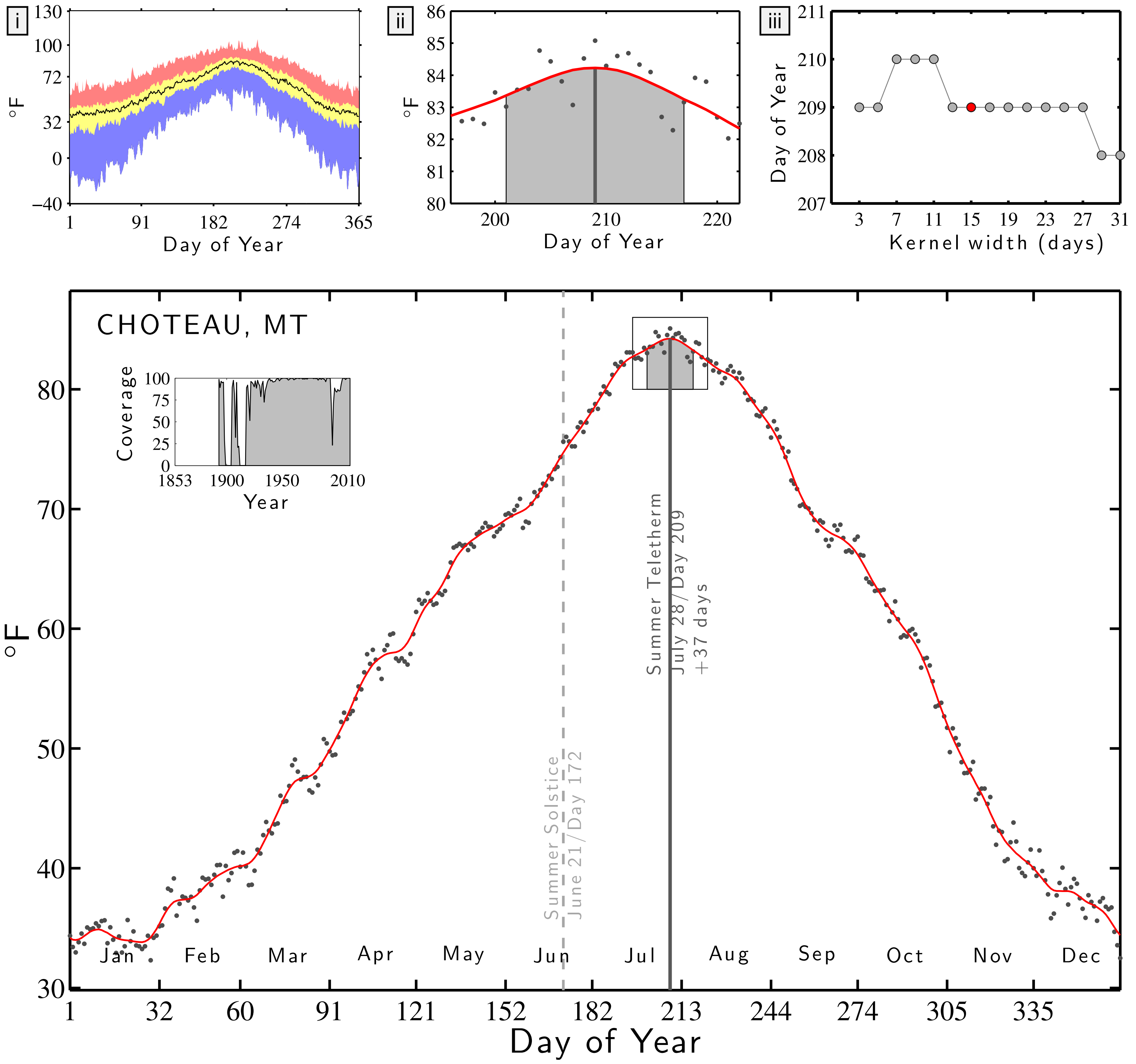

i
ii
iii
°F
Day of Year
Day of Year
Kernel width (days)
CHOTEAU, MT
Coverage
Year
Summer Solstice
June 21/Day 172
Summer Teletherm
July 28/Day 209
+37 days
Jan
Feb
Mar
Apr
May
Jun
Jul
Aug
Sep
Oct
Nov
Dec
°F
Day of Year

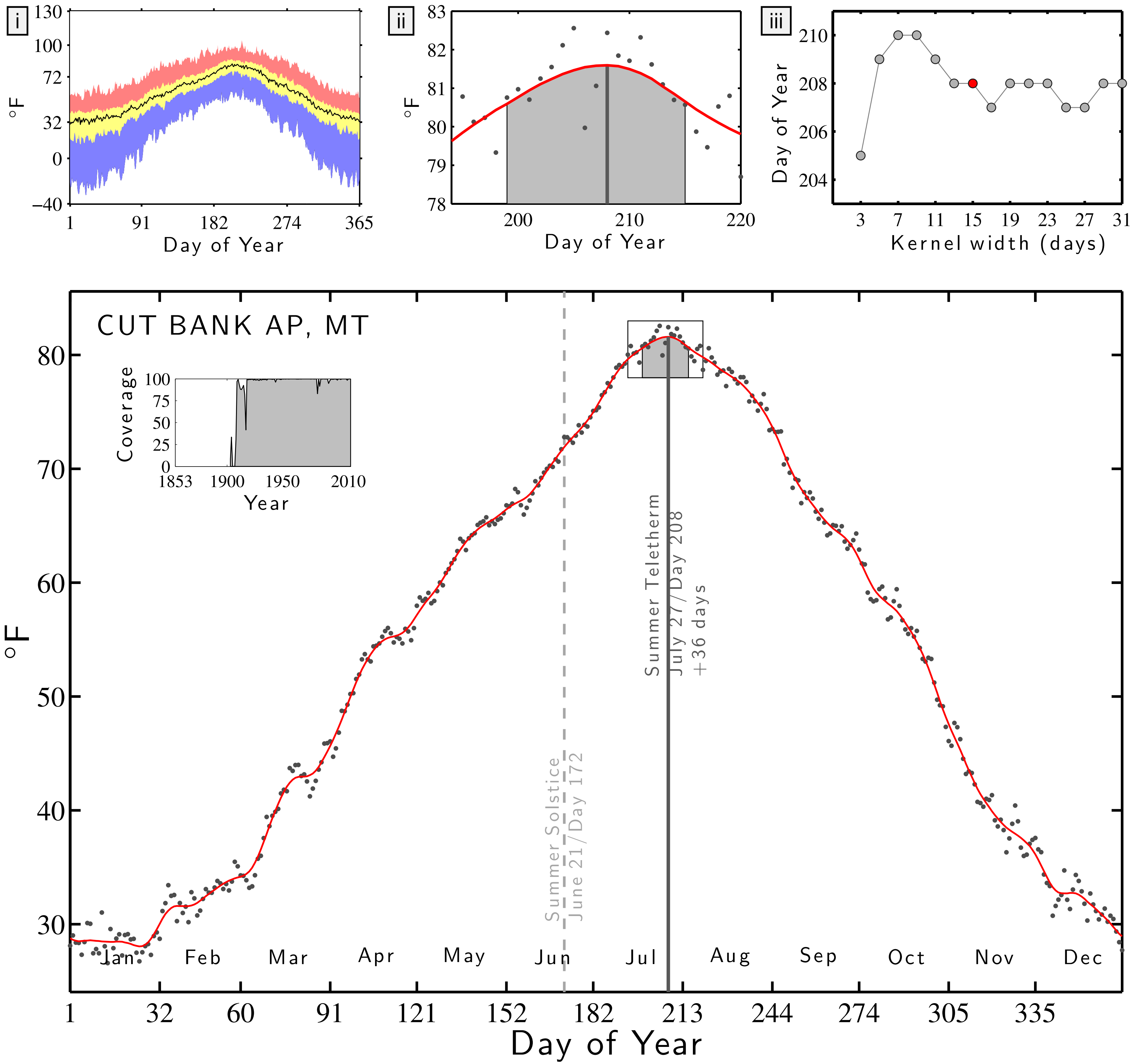
i
°F
Day of Year
ii
°F
Day of Year
iii
Day of Year
Kernel width (days)
CUT BANK AP, MT
Coverage
Year
Summer Solstice
June 21 / Day 172
Summer Teletherm
July 27 / Day 208
+36 days
Jan
Feb
Mar
Apr
May
Jun
Jul
Aug
Sep
Oct
Nov
Dec
°F
Day of Year

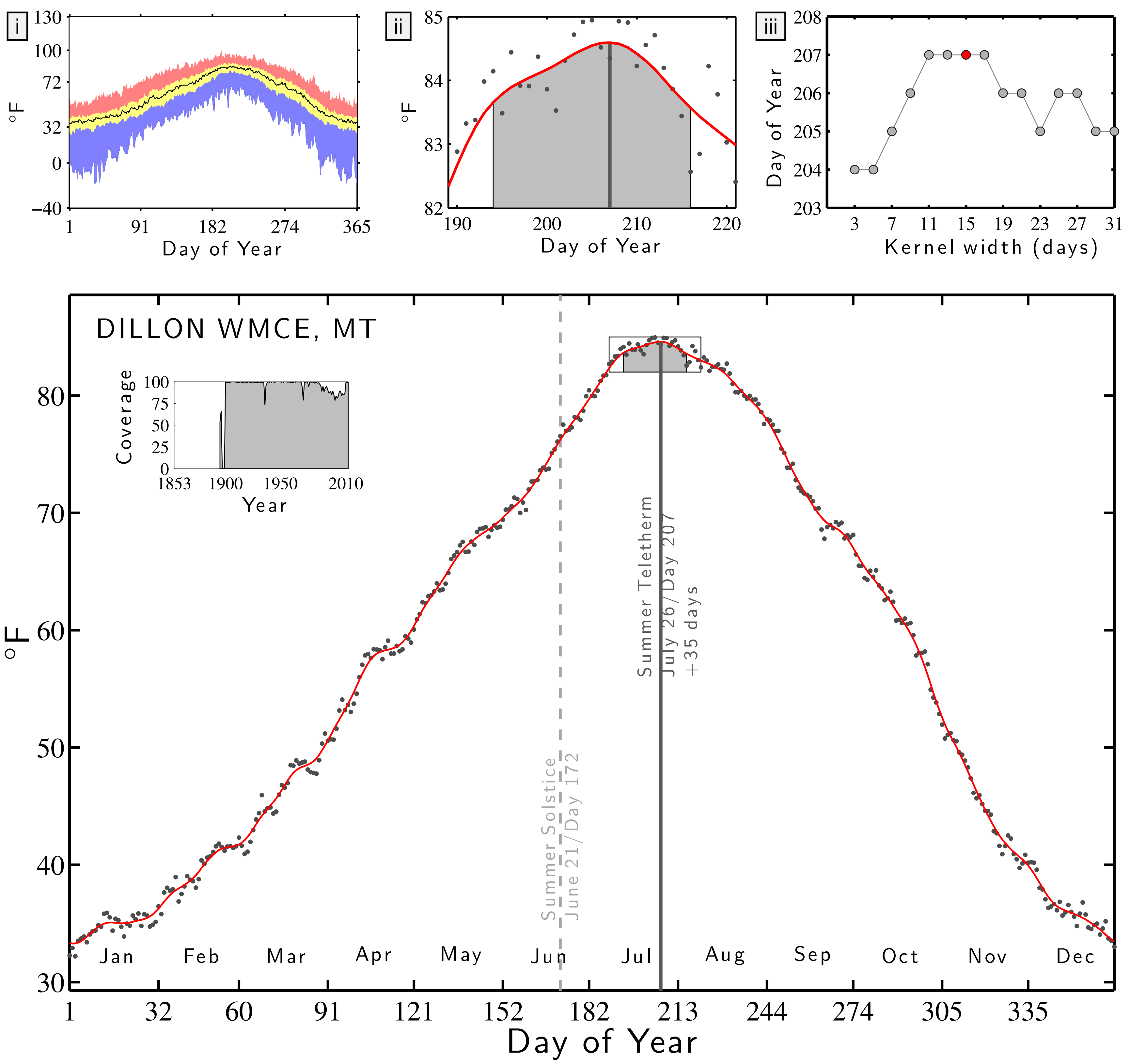

i
ii
iii
DILLON WMCE, MT
Coverage
Year
Summer Solstice
June 21 / Day 172
Summer Teletherm
July 26 / Day 207
+35 days
Day of Year
Kernel width (days)
°F
Jan
Feb
Mar
Apr
May
Jun
Jul
Aug
Sep
Oct
Nov
Dec

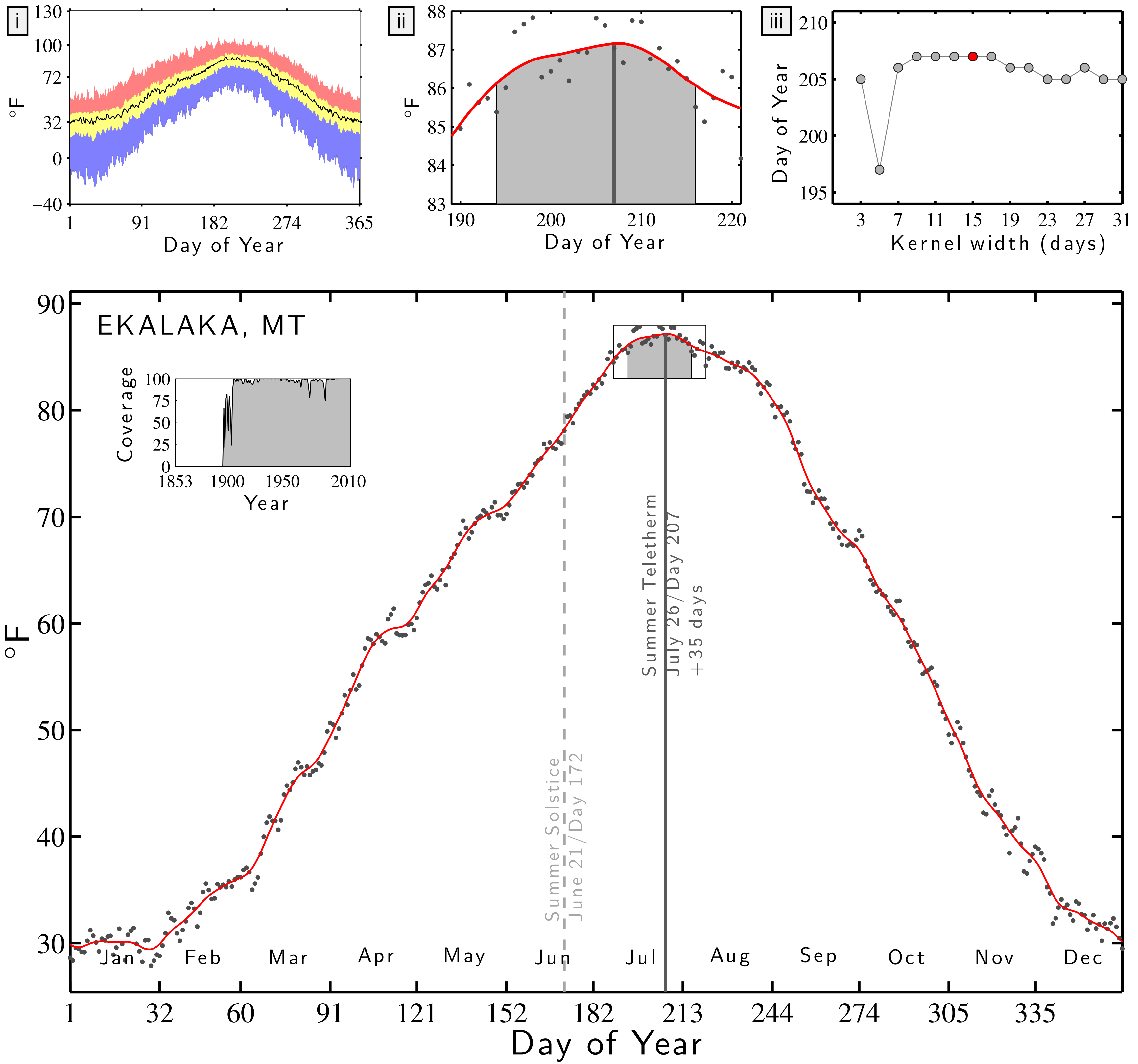

i
°F
Day of Year
ii
°F
Day of Year
iii
Day of Year
Kernel width (days)
EKALAKA, MT
Coverage
Year
Summer Solstice
June 21 / Day 172
Summer Teletherm
July 26 / Day 207
+35 days
Jan
Feb
Mar
Apr
May
Jun
Jul
Aug
Sep
Oct
Nov
Dec
°F
Day of Year

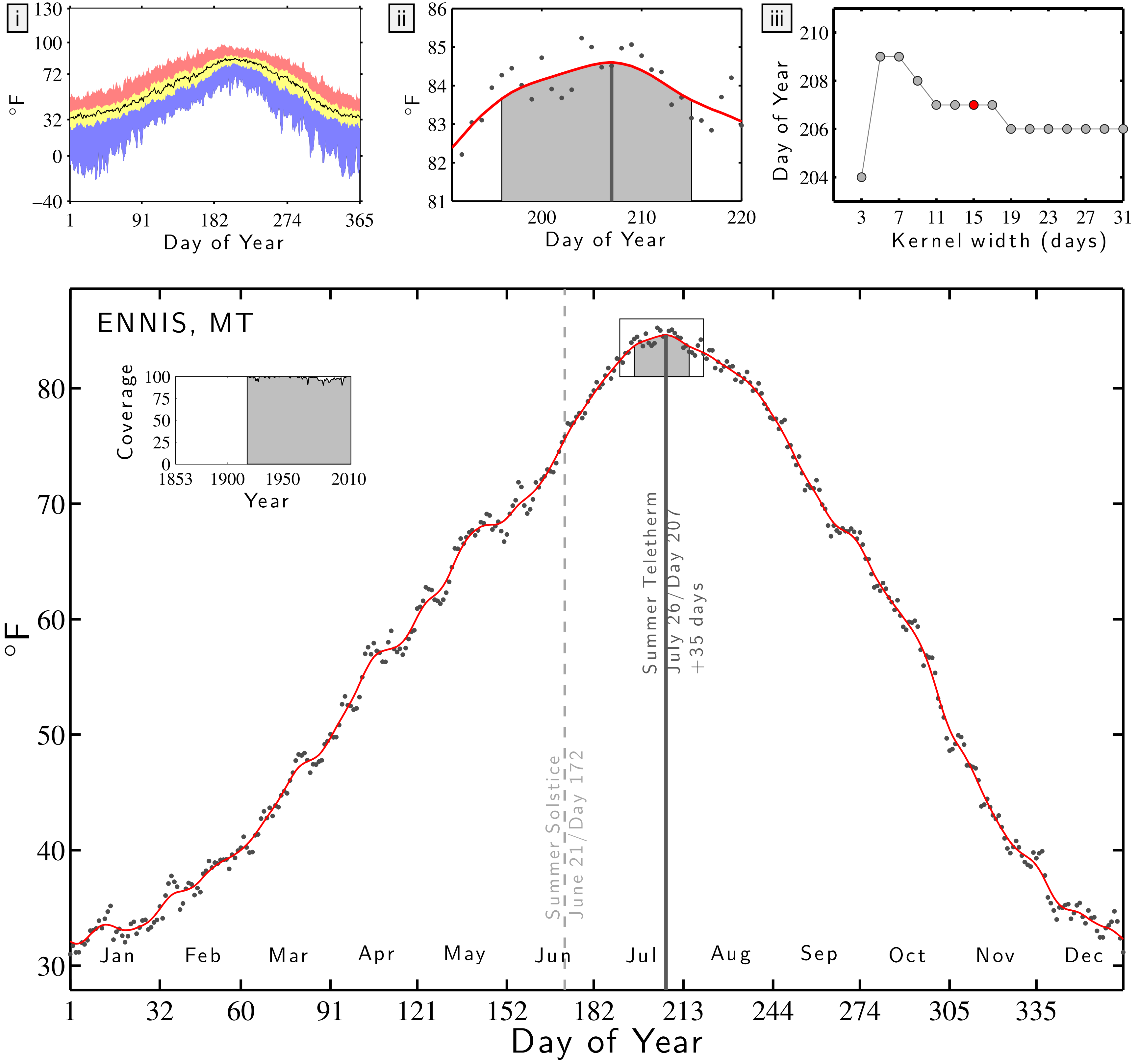

i
130
100
72
32
0
−40
°F
1
91
182
274
365
Day of Year
ii
86
85
84
83
82
81
°F
200
210
220
Day of Year
iii
210
208
206
204
Day of Year
3
7
11
15
19
23
27
31
Kernel width (days)
ENNIS, MT
Coverage
100
75
50
25
0
1853
1900
1950
2010
Year
°F
80
70
60
50
40
30
Summer Solstice
June 21 / Day 172
Summer Teletherm
July 26 / Day 207
+35 days
Jan
Feb
Mar
Apr
May
Jun
Jul
Aug
Sep
Oct
Nov
Dec
1
32
60
91
121
152
182
213
244
274
305
335
Day of Year

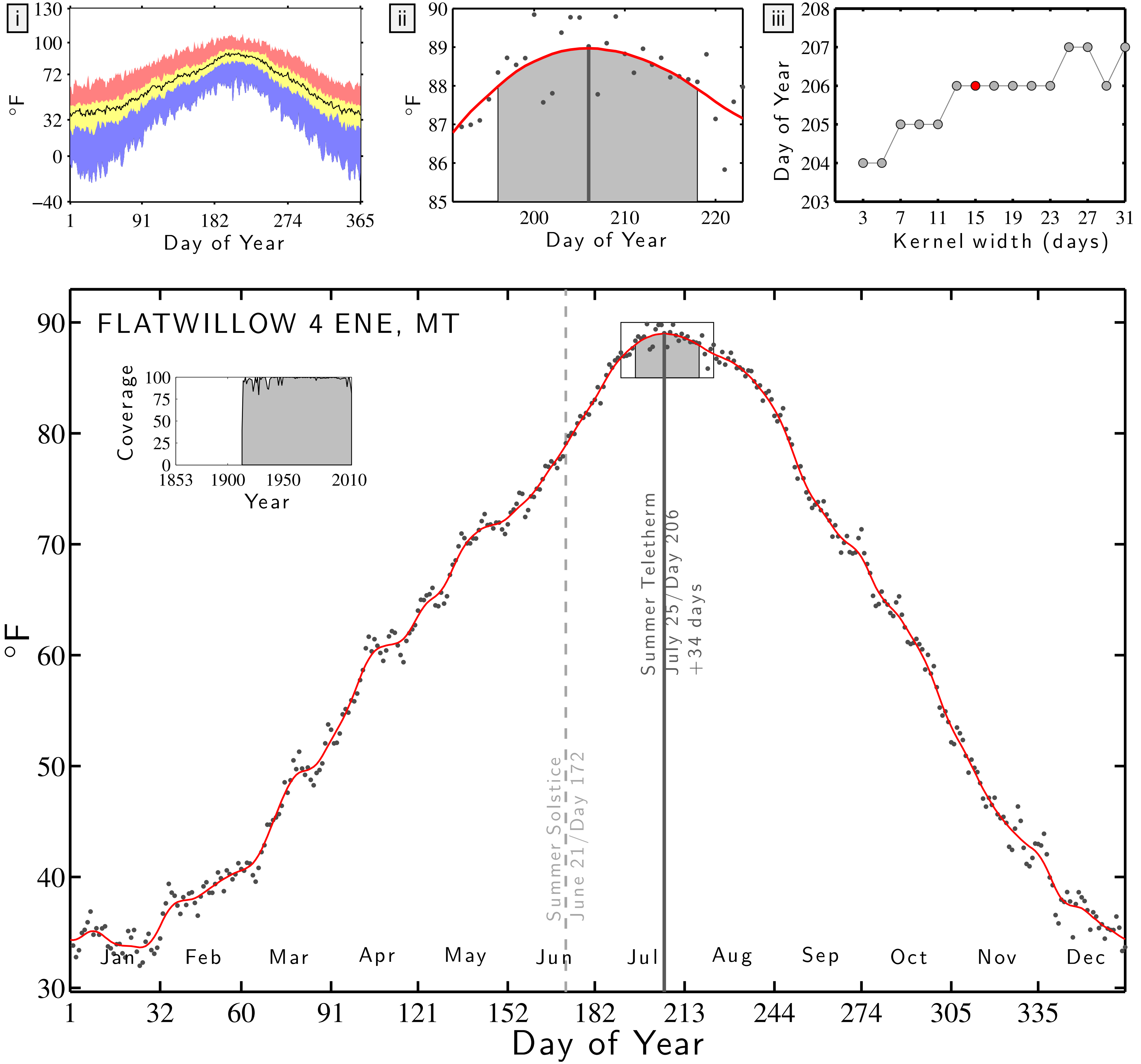

i
130
100
72
32
0
−40
°F
1
91
182
274
365
Day of Year
ii
90
89
88
87
86
85
°F
200
210
220
Day of Year
iii
208
207
206
205
204
203
Day of Year
3
7
11
15
19
23
27
31
Kernel width (days)
FLATWILLOW 4 ENE, MT
Coverage
100
75
50
25
0
1853
1900
1950
2010
Year
Summer Solstice
June 21/Day 172
Summer Teletherm
July 25/Day 206
+34 days
Jan
Feb
Mar
Apr
May
Jun
Jul
Aug
Sep
Oct
Nov
Dec
°F
90
80
70
60
50
40
30
1
32
60
91
121
152
182
213
244
274
305
335
Day of Year

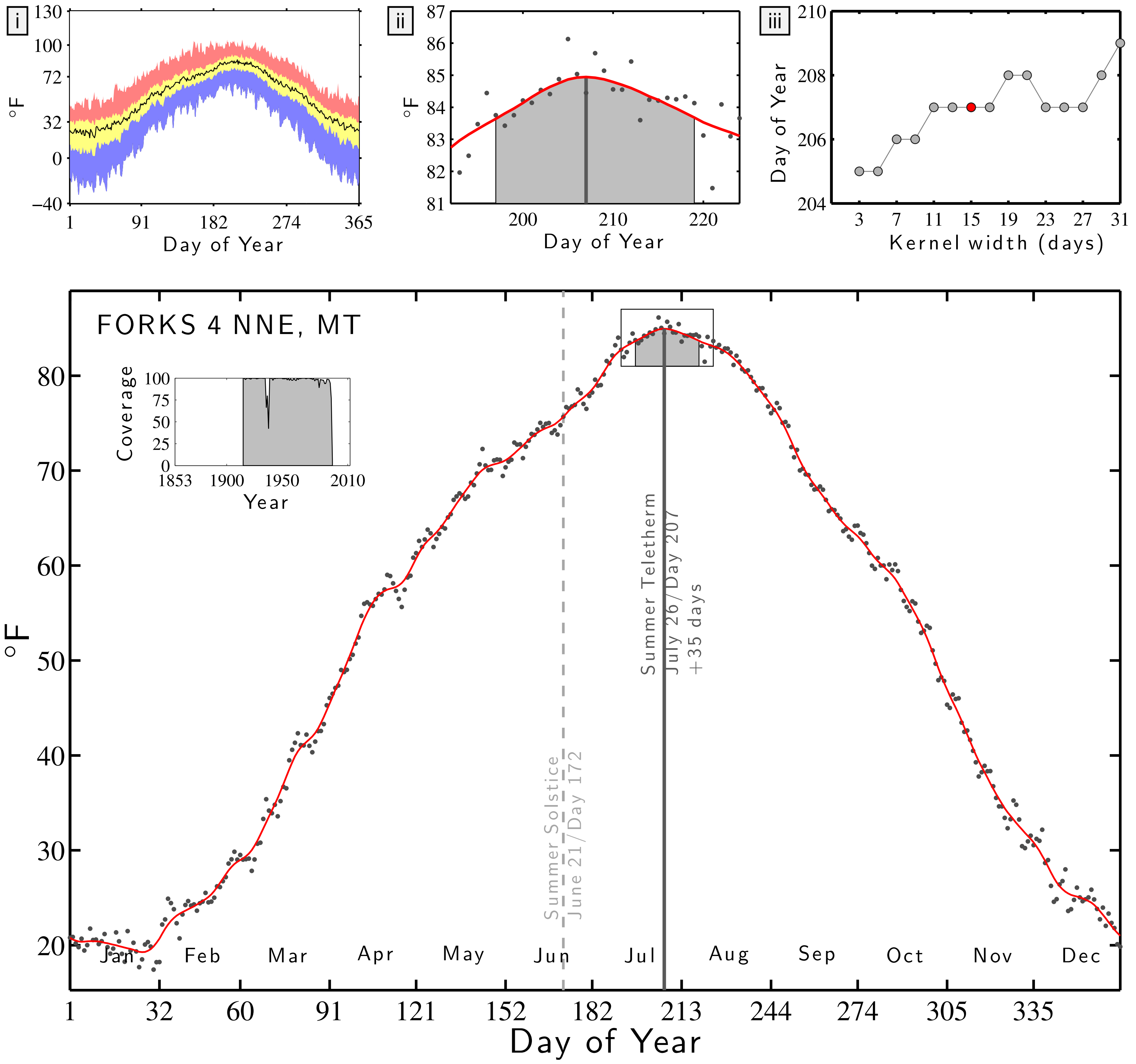
i
°F
Day of Year
ii
°F
Day of Year
iii
Day of Year
Kernel width (days)
FORKS 4 NNE, MT
Coverage
Year
Summer Solstice
June 21 / Day 172
Summer Teletherm
July 26 / Day 207
+35 days
Jan
Feb
Mar
Apr
May
Jun
Jul
Aug
Sep
Oct
Nov
Dec
°F
Day of Year

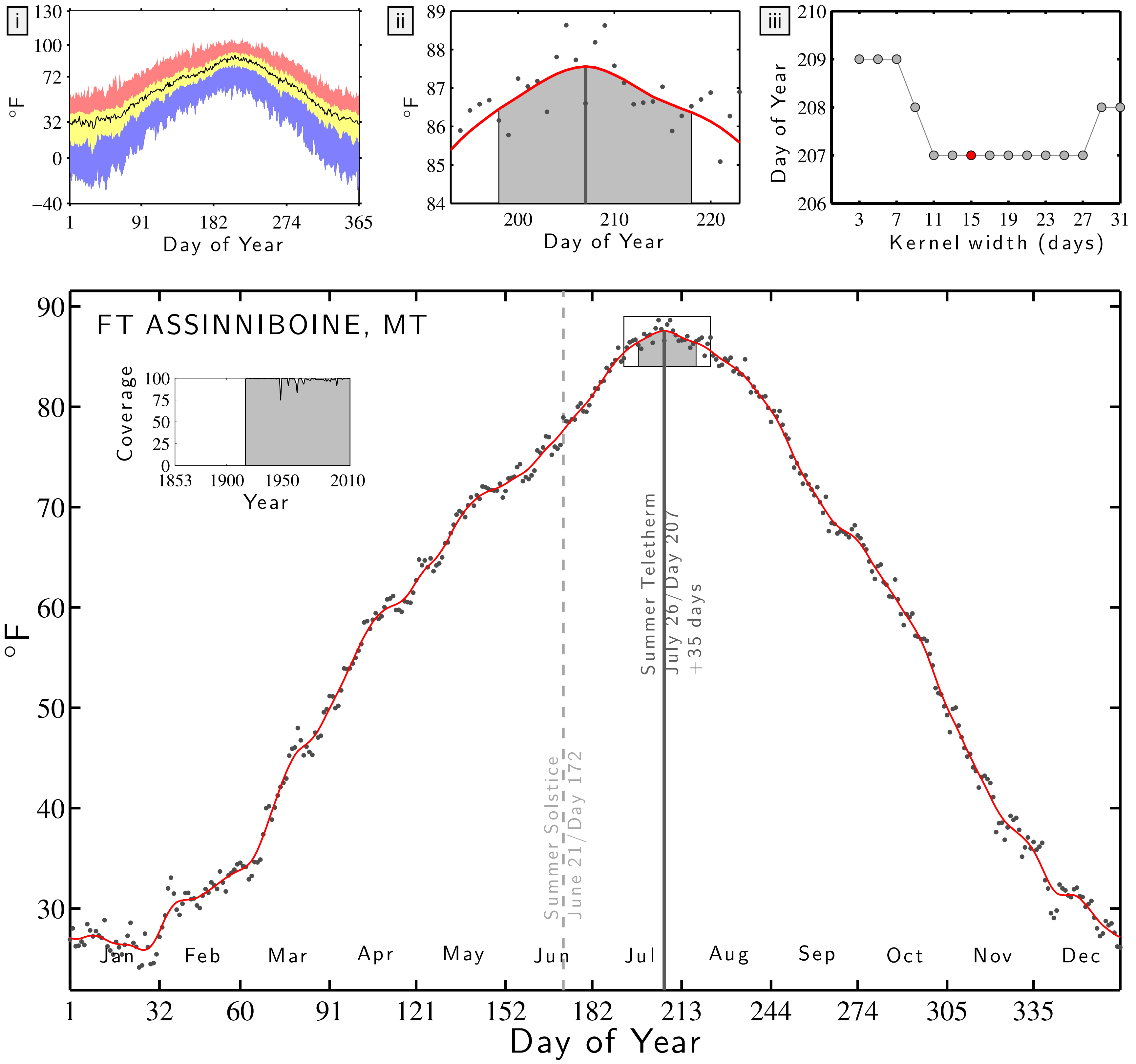

i
°F
Day of Year
ii
Day of Year
iii
Day of Year
Kernel width (days)
FT ASSINNIBOINE, MT
Coverage
Year
Summer Solstice
June 21 / Day 172
Summer Teletherm
July 26 / Day 207
+35 days
Jan
Feb
Mar
Apr
May
Jun
Jul
Aug
Sep
Oct
Nov
Dec
Day of Year

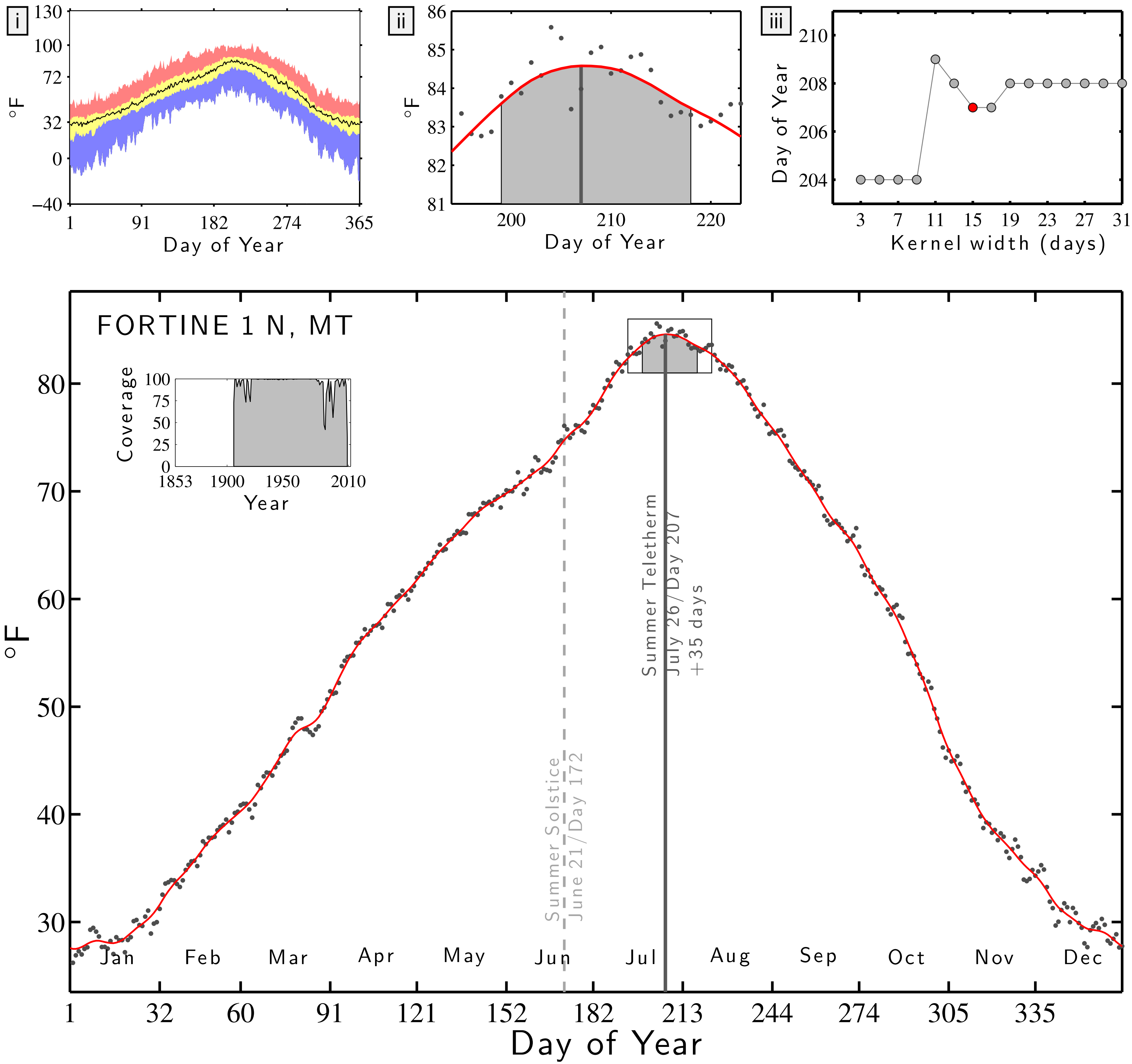

i
°F
Day of Year
ii
°F
Day of Year
iii
Day of Year
Kernel width (days)
FORTINE 1 N, MT
Coverage
Year
Summer Solstice
June 21 / Day 172
Summer Teletherm
July 26 / Day 207
+35 days
Jan
Feb
Mar
Apr
May
Jun
Jul
Aug
Sep
Oct
Nov
Dec
°F
Day of Year

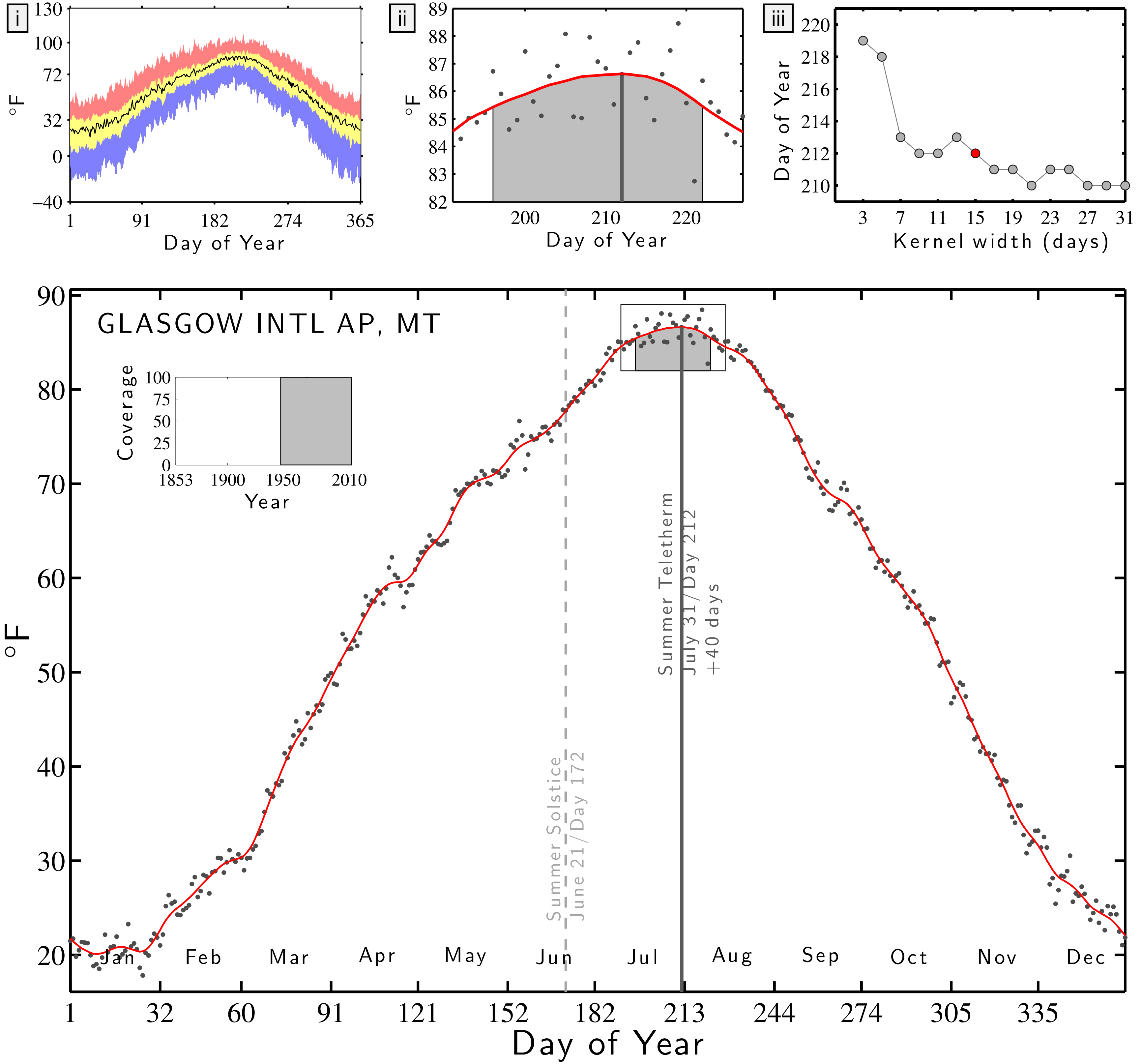
i
°F
Day of Year
ii
°F
Day of Year
iii
Day of Year
Kernel width (days)
GLASGOW INTL AP, MT
Coverage
Year
Summer Solstice
June 21 / Day 172
Summer Teletherm
July 31 / Day 212
+40 days
Jan
Feb
Mar
Apr
May
Jun
Jul
Aug
Sep
Oct
Nov
Dec
°F
Day of Year

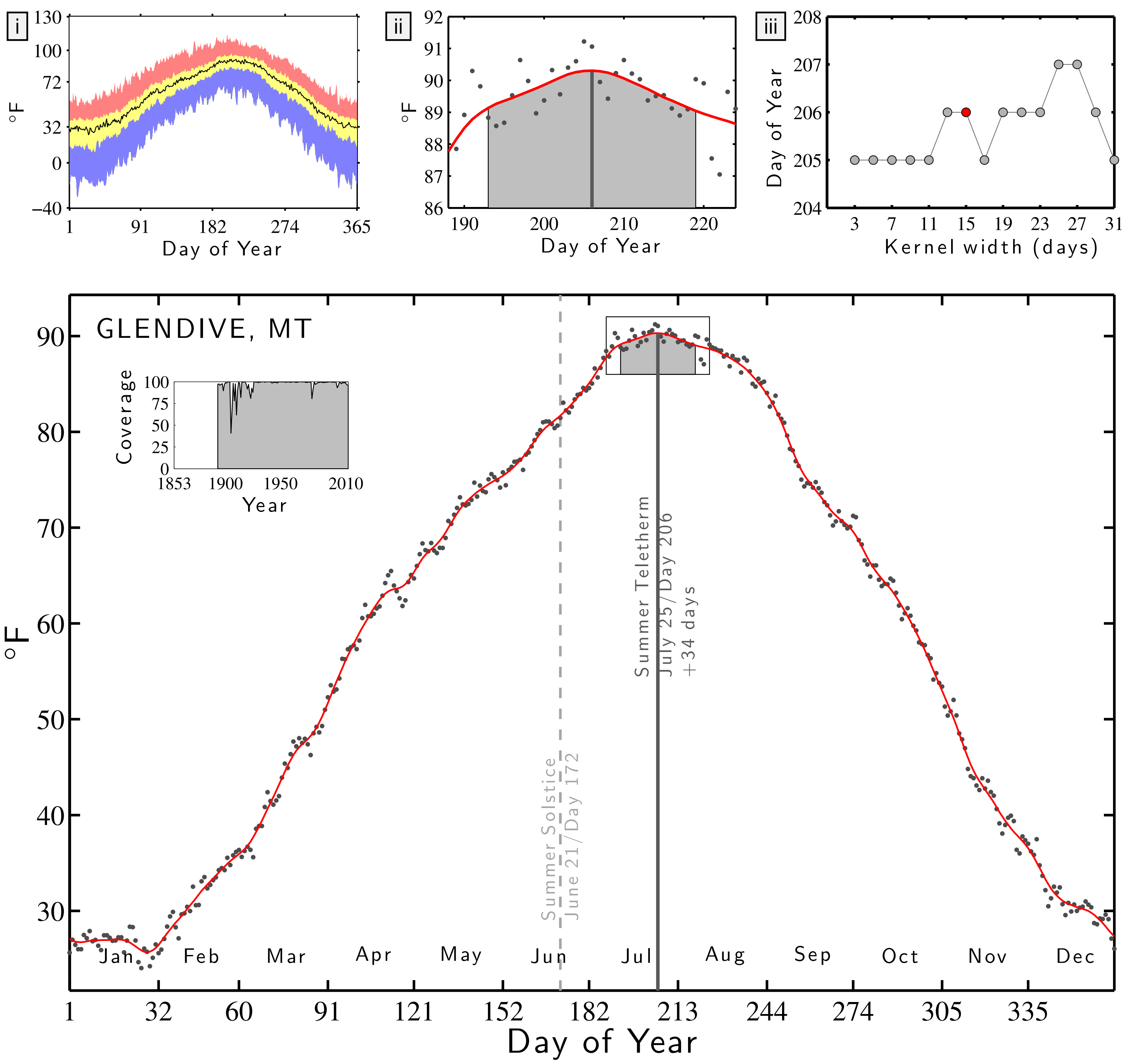

i
°F
Day of Year
ii
°F
Day of Year
iii
Day of Year
Kernel width (days)
GLENDIVE, MT
Coverage
Year
Summer Solstice
June 21/Day 172
Summer Teletherm
July 25/Day 206
+34 days
Jan
Feb
Mar
Apr
May
Jun
Jul
Aug
Sep
Oct
Nov
Dec
°F
Day of Year

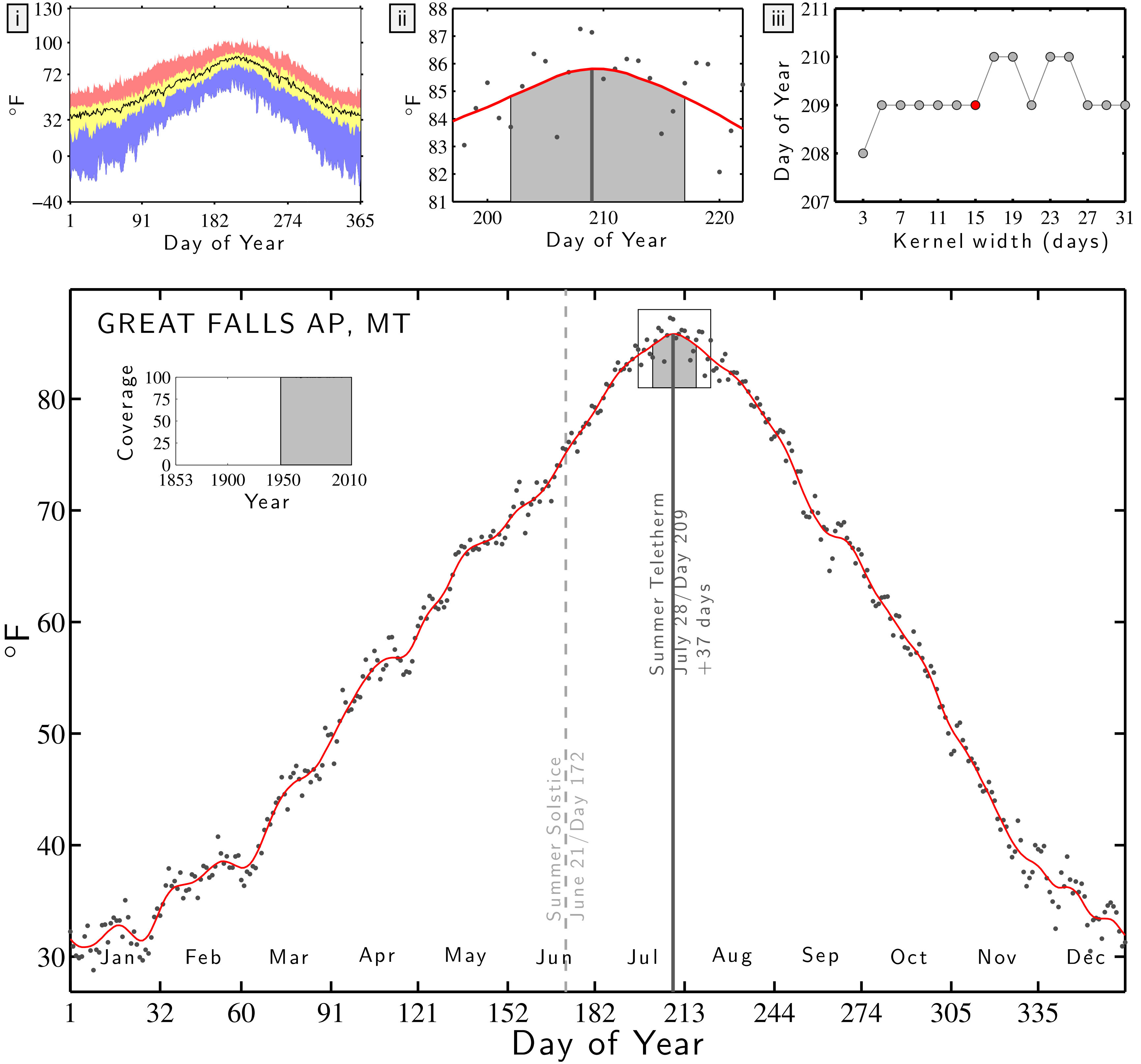

i
ii
iii
°F
Day of Year
Day of Year
Kernel width (days)
GREAT FALLS AP, MT
Coverage
Year
Summer Solstice
June 21/Day 172
Summer Teletherm
July 28/Day 209
+37 days
Jan
Feb
Mar
Apr
May
Jun
Jul
Aug
Sep
Oct
Nov
Dec

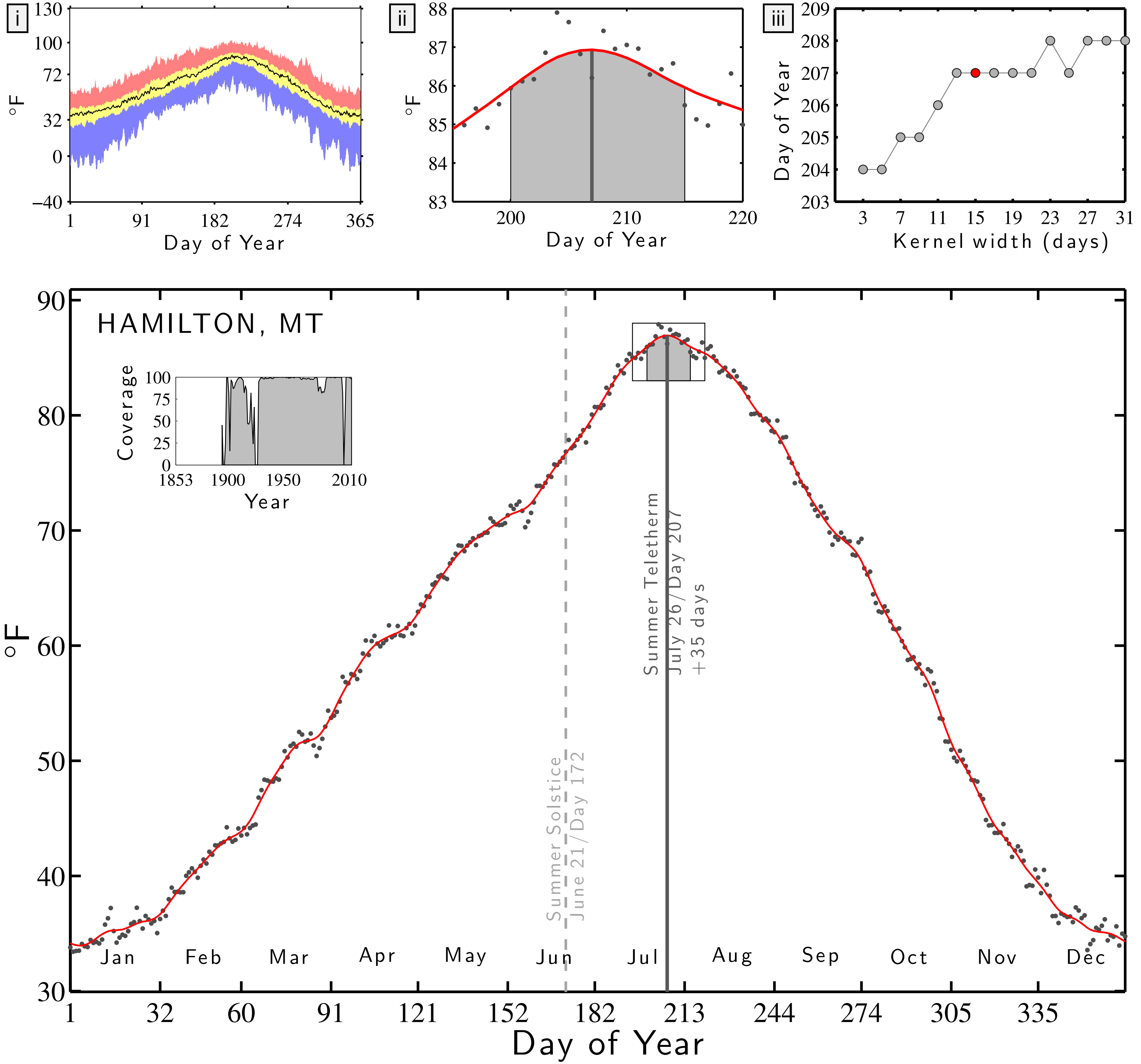

i
°F
Day of Year
ii
°F
Day of Year
iii
Day of Year
Kernel width (days)
HAMILTON, MT
Coverage
Year
Summer Solstice
June 21 / Day 172
Summer Teletherm
July 26 / Day 207
+35 days
Jan
Feb
Mar
Apr
May
Jun
Jul
Aug
Sep
Oct
Nov
Dec
°F
Day of Year

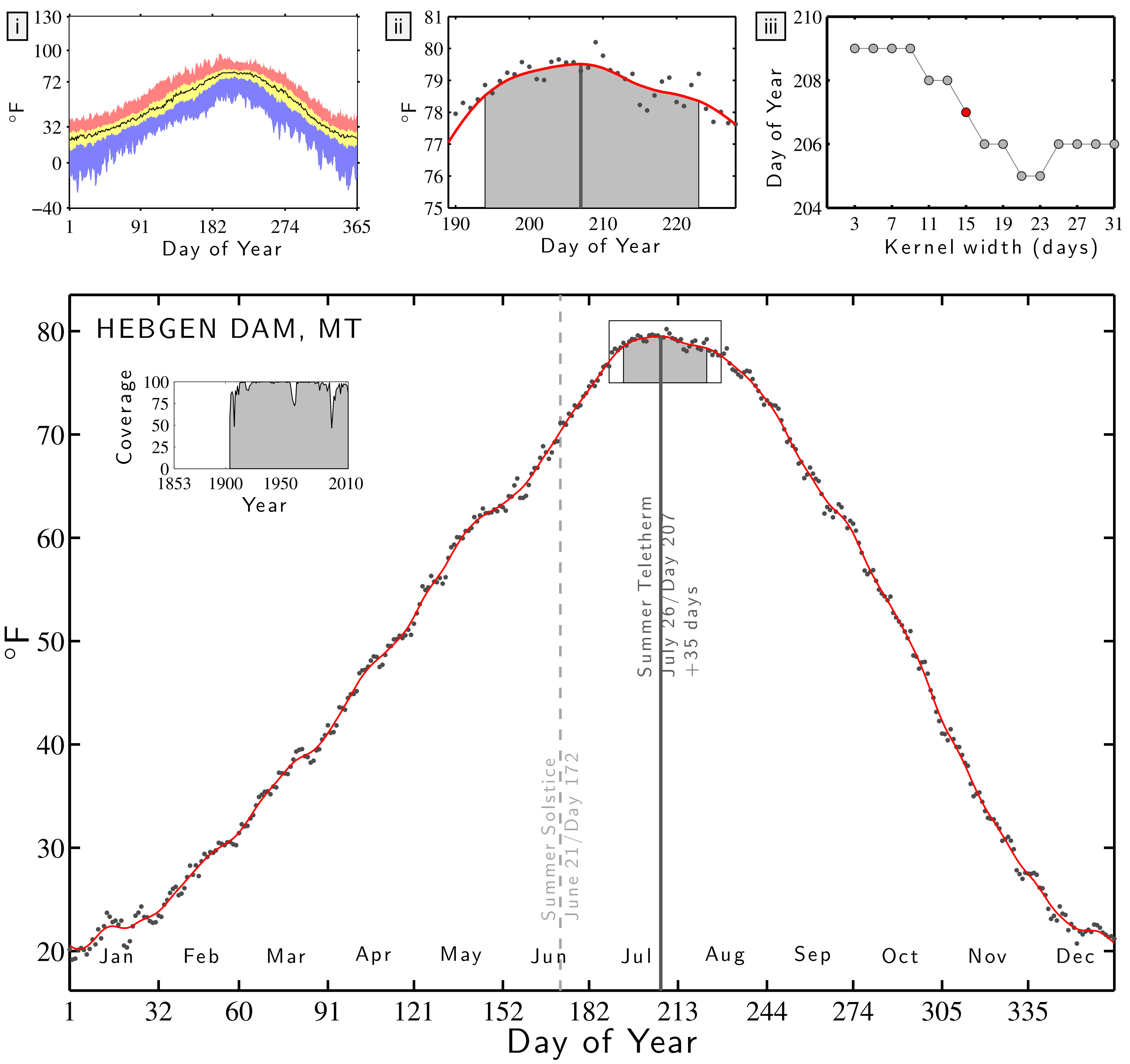

i
ii
iii
°F
Day of Year
Kernel width (days)
HEBGEN DAM, MT
Coverage
Year
Summer Solstice
June 21 / Day 172
Summer Teletherm
July 26 / Day 207
+35 days
Jan
Feb
Mar
Apr
May
Jun
Jul
Aug
Sep
Oct
Nov
Dec

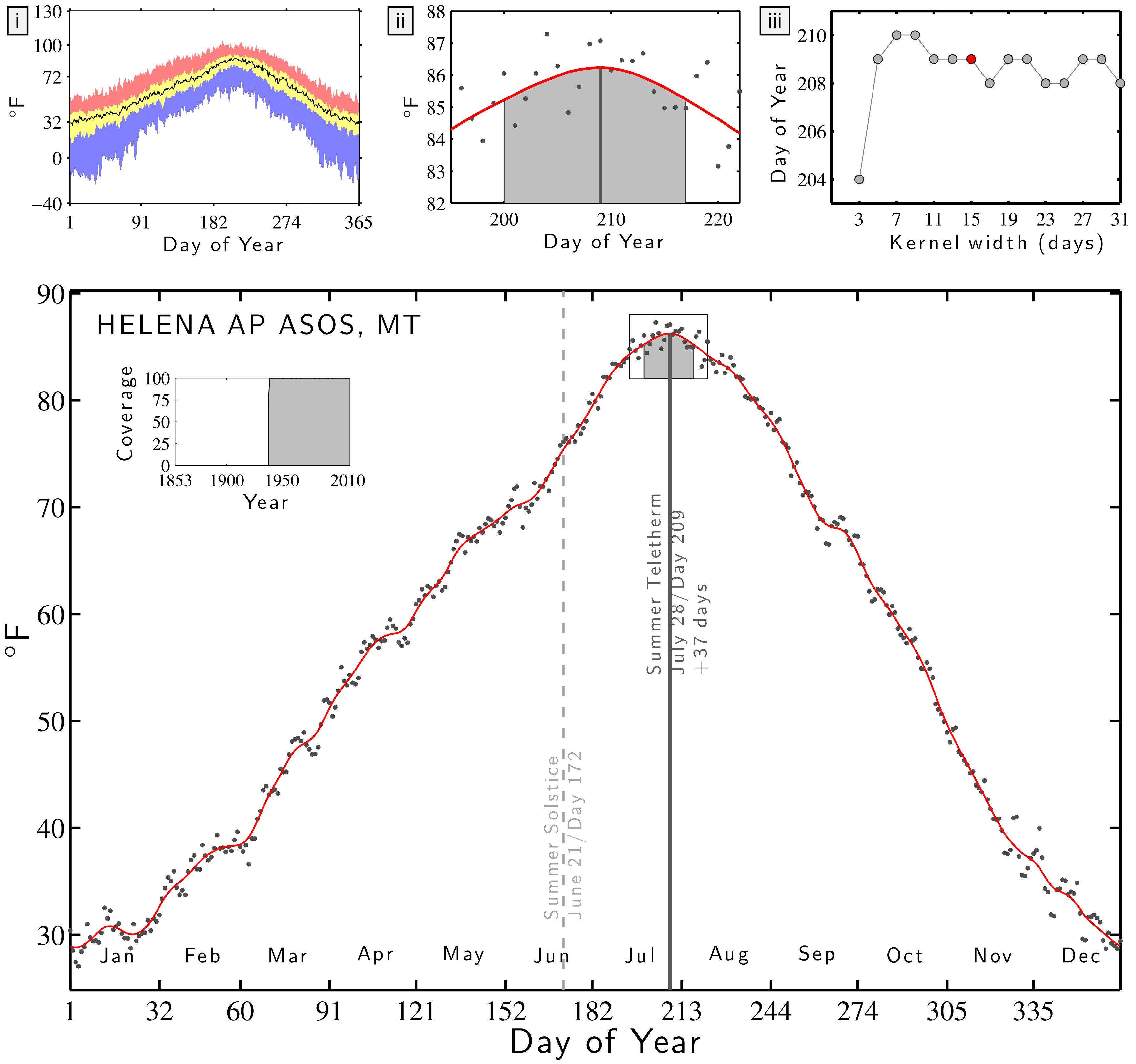

i
ii
iii
HELENA AP ASOS, MT
Coverage
Year
Summer Solstice
June 21/Day 172
Summer Teletherm
July 28/Day 209
+37 days
Jan
Feb
Mar
Apr
May
Jun
Jul
Aug
Sep
Oct
Nov
Dec
°F
Day of Year
Kernel width (days)

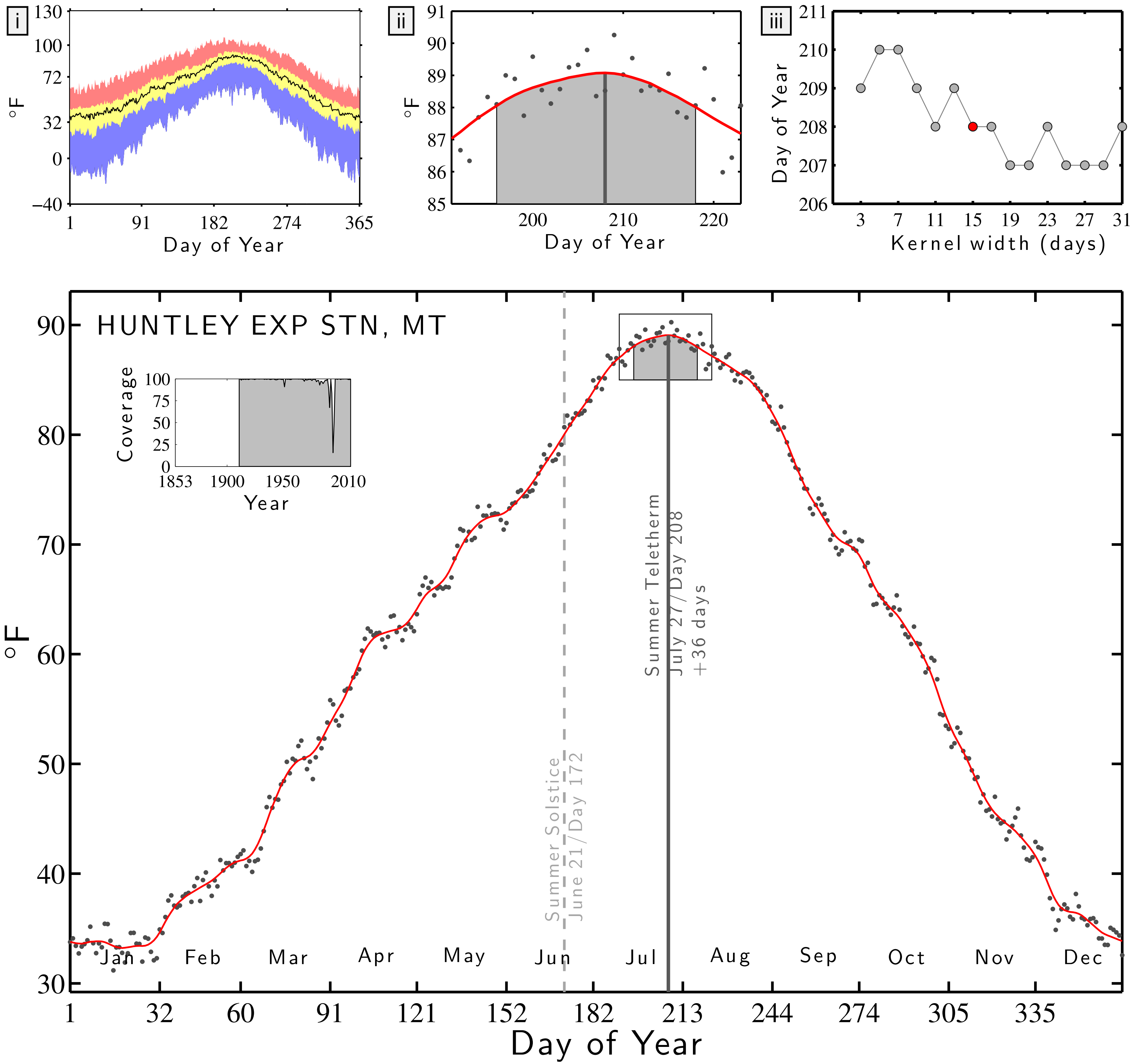

i
°F
Day of Year
ii
°F
Day of Year
iii
Day of Year
Kernel width (days)
HUNTLEY EXP STN, MT
Coverage
Year
Summer Solstice
June 21/Day 172
Summer Teletherm
July 27/Day 208
+36 days
Jan
Feb
Mar
Apr
May
Jun
Jul
Aug
Sep
Oct
Nov
Dec
°F
Day of Year

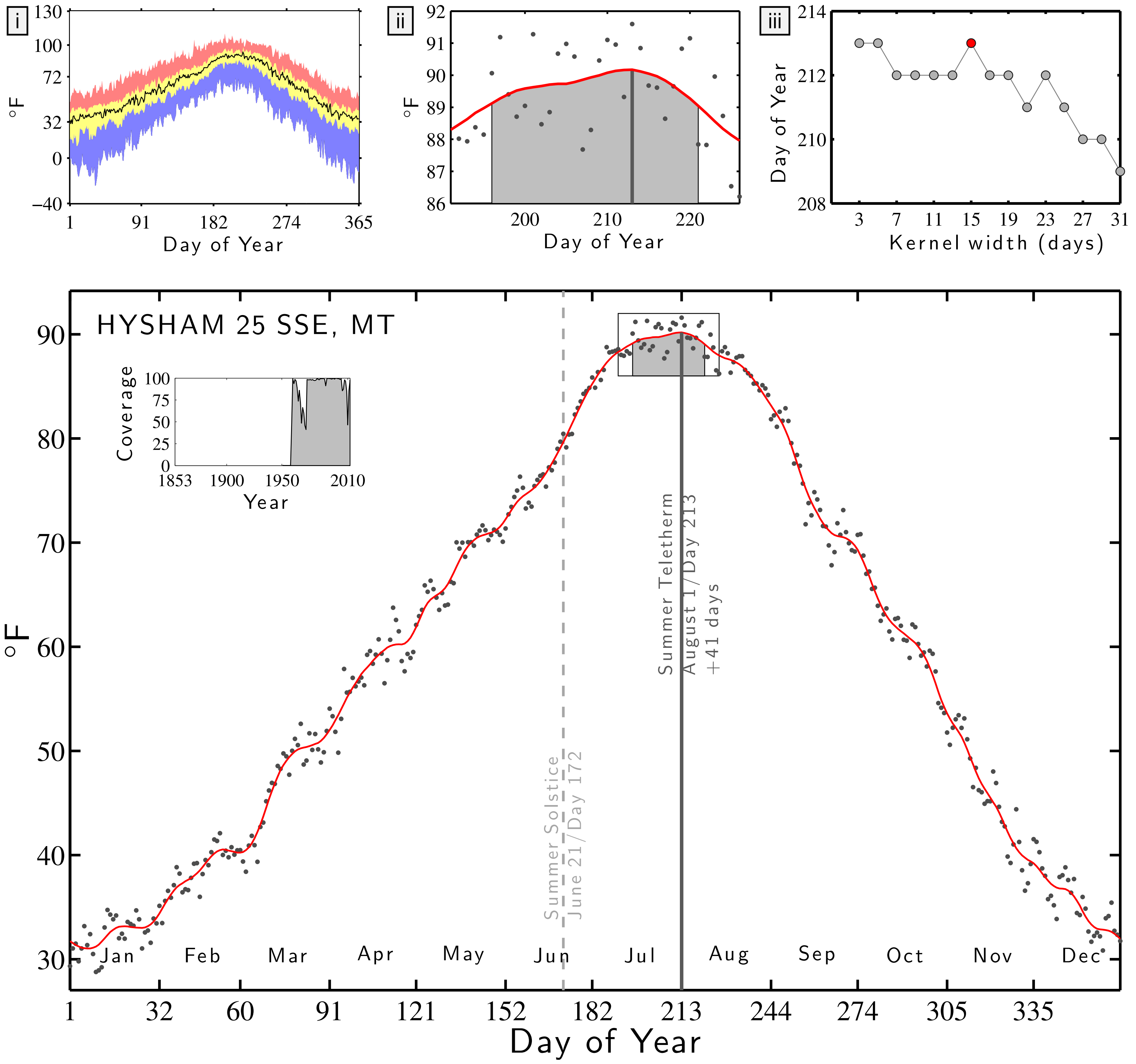

i
ii
iii
°F
Day of Year
Day of Year
Kernel width (days)
HYSHAM 25 SSE, MT
Coverage
Year
Summer Solstice
June 21 / Day 172
Summer Teletherm
August 1 / Day 213
+41 days
Jan
Feb
Mar
Apr
May
Jun
Jul
Aug
Sep
Oct
Nov
Dec

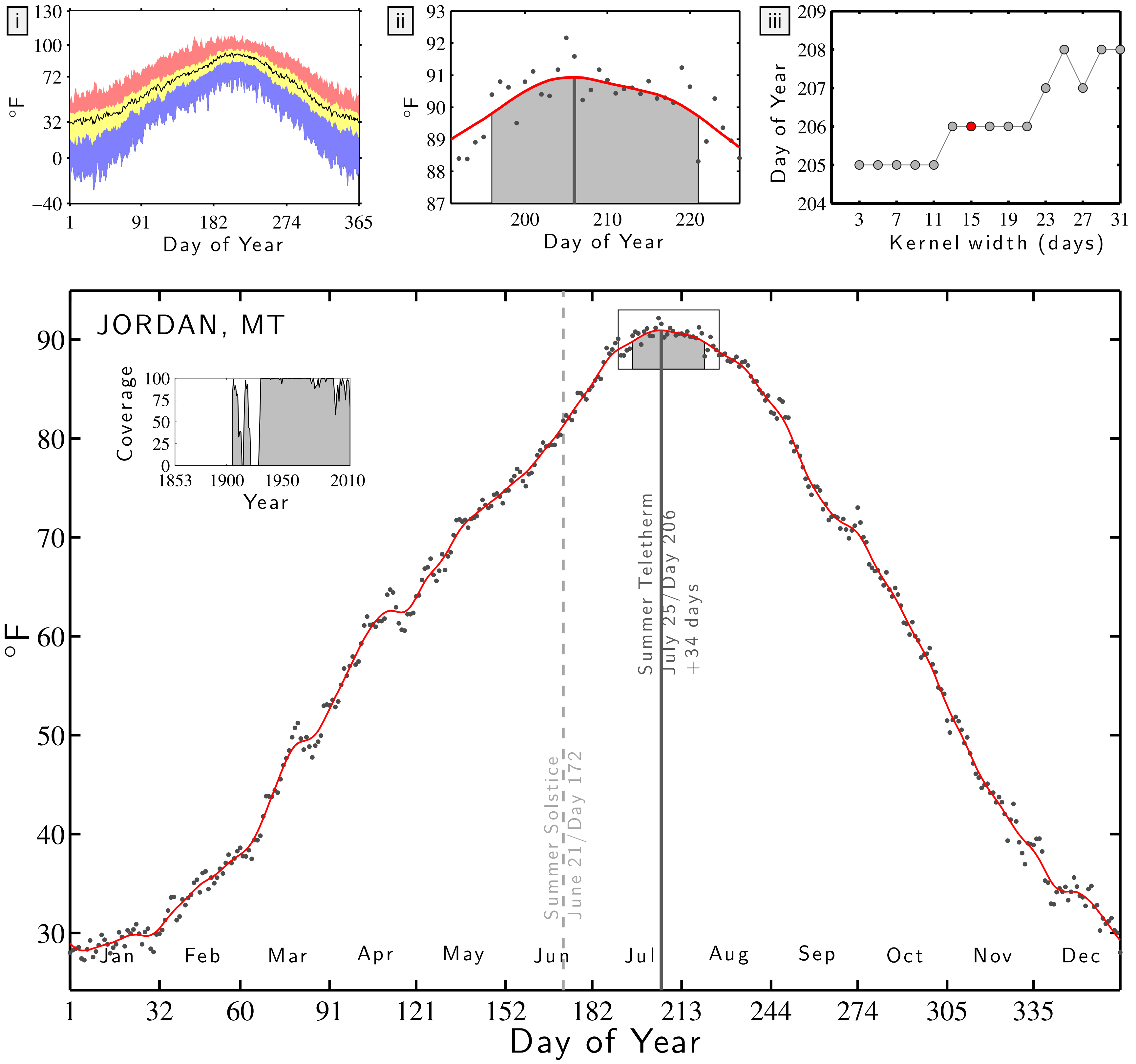

i
°F
Day of Year
ii
°F
Day of Year
iii
Day of Year
Kernel width (days)
JORDAN, MT
Coverage
Year
Summer Solstice
June 21/Day 172
Summer Teletherm
July 25/Day 206
+34 days
Jan
Feb
Mar
Apr
May
Jun
Jul
Aug
Sep
Oct
Nov
Dec
°F
Day of Year

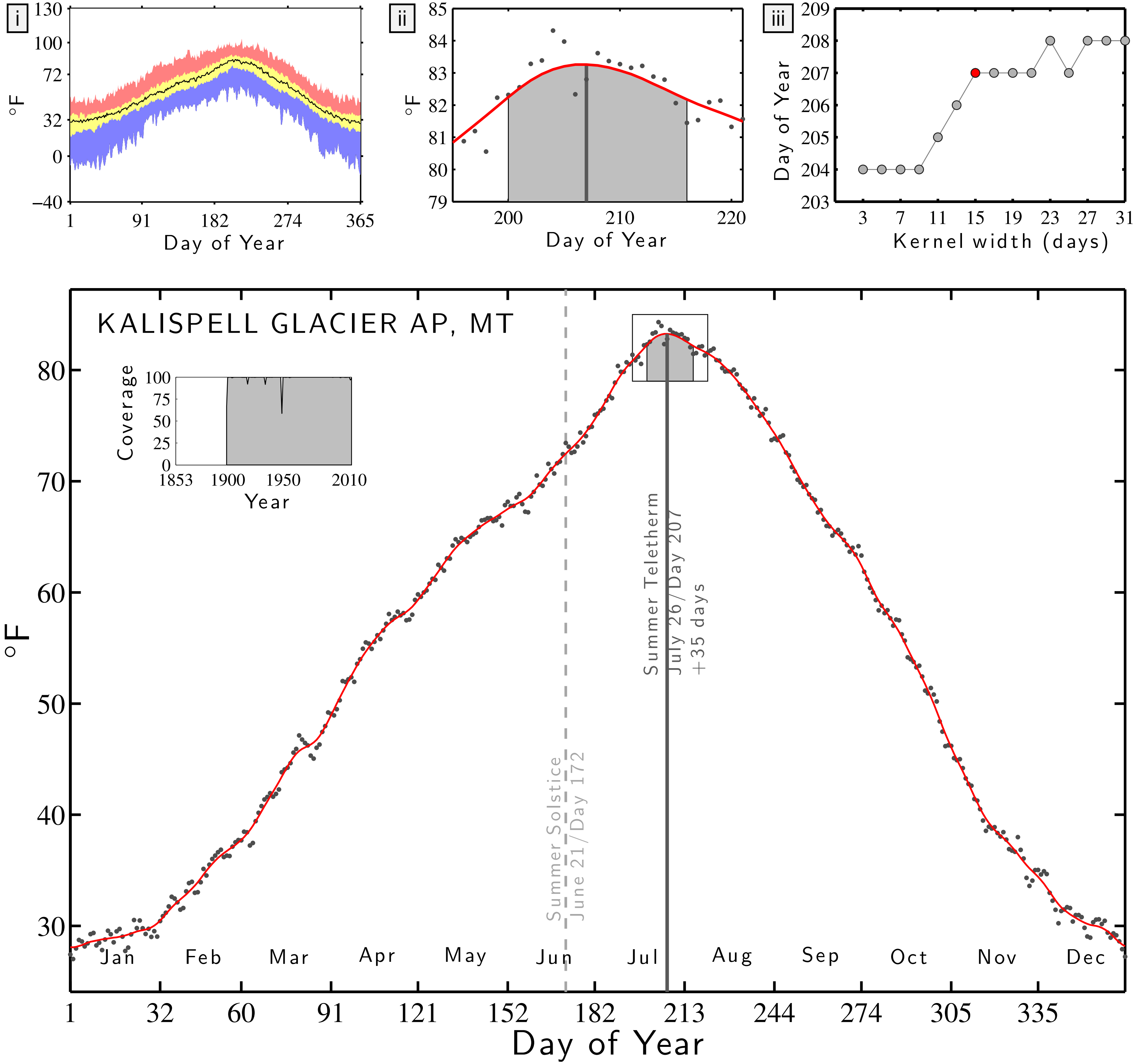

i
130
100
72
32
0
−40
°F
1
91
182
274
365
Day of Year
ii
85
84
83
82
81
80
79
°F
200
210
220
Day of Year
iii
209
208
207
206
205
204
203
Day of Year
3
7
11
15
19
23
27
31
Kernel width (days)
KALISPELL GLACIER AP, MT
Coverage
100
75
50
25
0
1853
1900
1950
2010
Year
Summer Solstice
June 21 / Day 172
Summer Teletherm
July 26 / Day 207
+35 days
80
70
60
50
40
30
°F
Jan
Feb
Mar
Apr
May
Jun
Jul
Aug
Sep
Oct
Nov
Dec
1
32
60
91
121
152
182
213
244
274
305
335
Day of Year

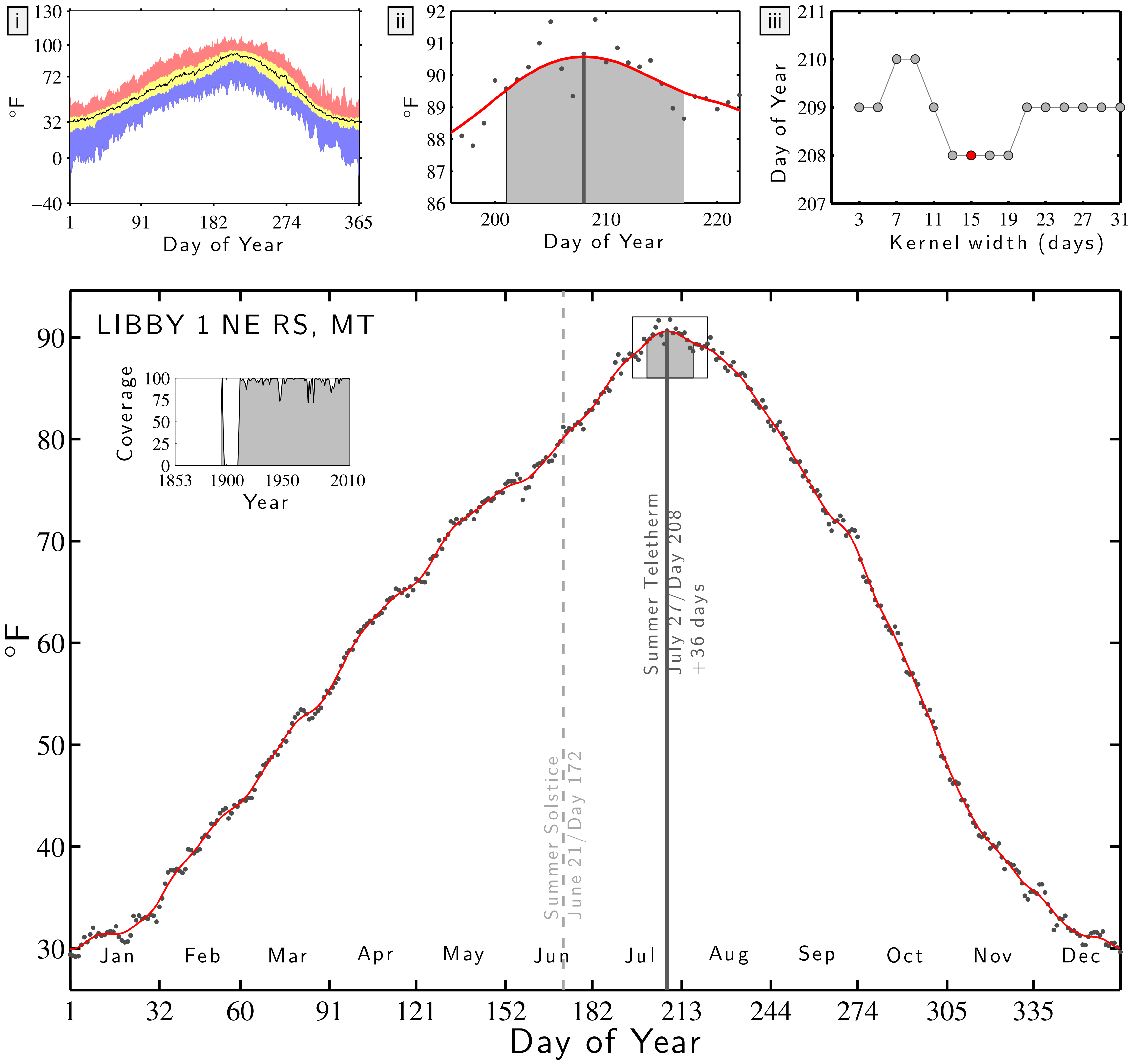
i
°F
Day of Year
ii
°F
Day of Year
iii
Day of Year
Kernel width (days)
LIBBY 1 NE RS, MT
Coverage
Year
Summer Solstice
June 21 / Day 172
Summer Teletherm
July 27 / Day 208
+36 days
Jan
Feb
Mar
Apr
May
Jun
Jul
Aug
Sep
Oct
Nov
Dec
°F
Day of Year

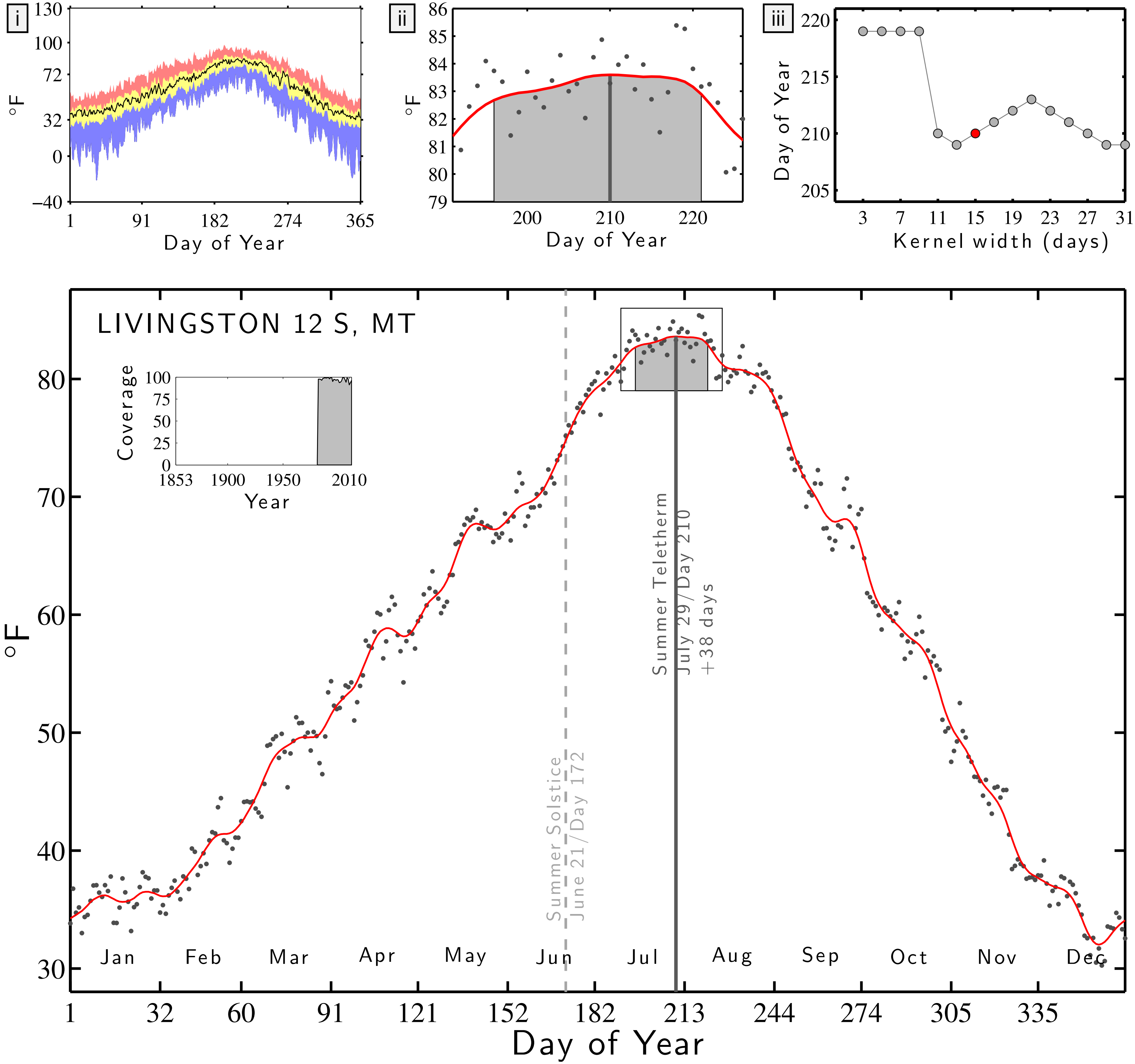

i
130
100
72
32
0
−40
°F
1
91
182
274
365
Day of Year
ii
86
85
84
83
82
81
80
79
°F
200
210
220
Day of Year
iii
220
215
210
205
Day of Year
3
7
11
15
19
23
27
31
Kernel width (days)
LIVINGSTON 12 S, MT
Coverage
100
75
50
25
0
1853
1900
1950
2010
Year
Summer Solstice
June 21 / Day 172
Summer Teletherm
July 29 / Day 210
+38 days
80
70
60
50
40
30
°F
Jan
Feb
Mar
Apr
May
Jun
Jul
Aug
Sep
Oct
Nov
Dec
1
32
60
91
121
152
182
213
244
274
305
335
Day of Year

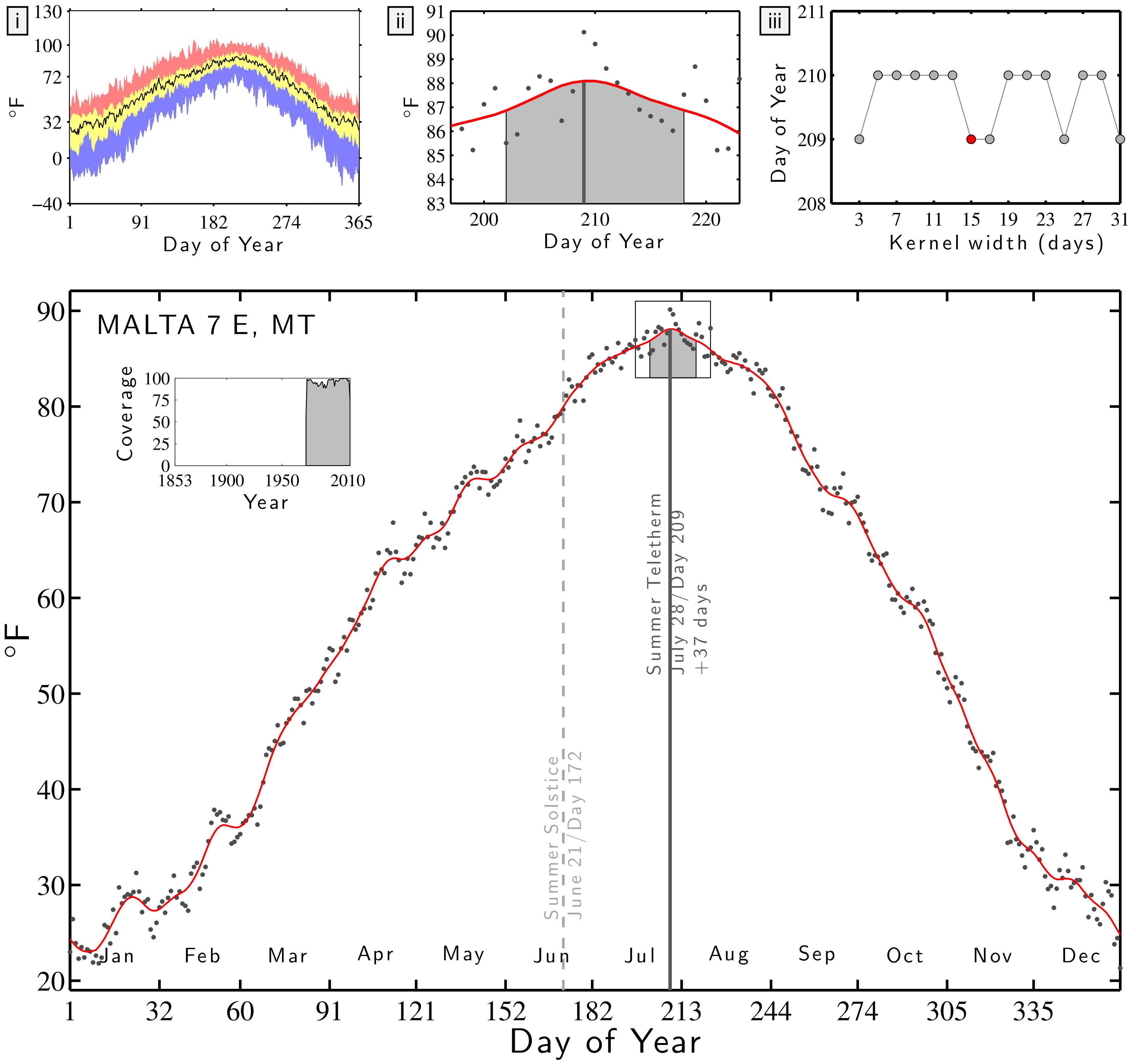

i
°F
Day of Year
ii
°F
Day of Year
iii
Day of Year
Kernel width (days)
MALTA 7 E, MT
Coverage
Year
Summer Solstice
June 21/Day 172
Summer Teletherm
July 28/Day 209
+37 days
Jan
Feb
Mar
Apr
May
Jun
Jul
Aug
Sep
Oct
Nov
Dec
°F
Day of Year

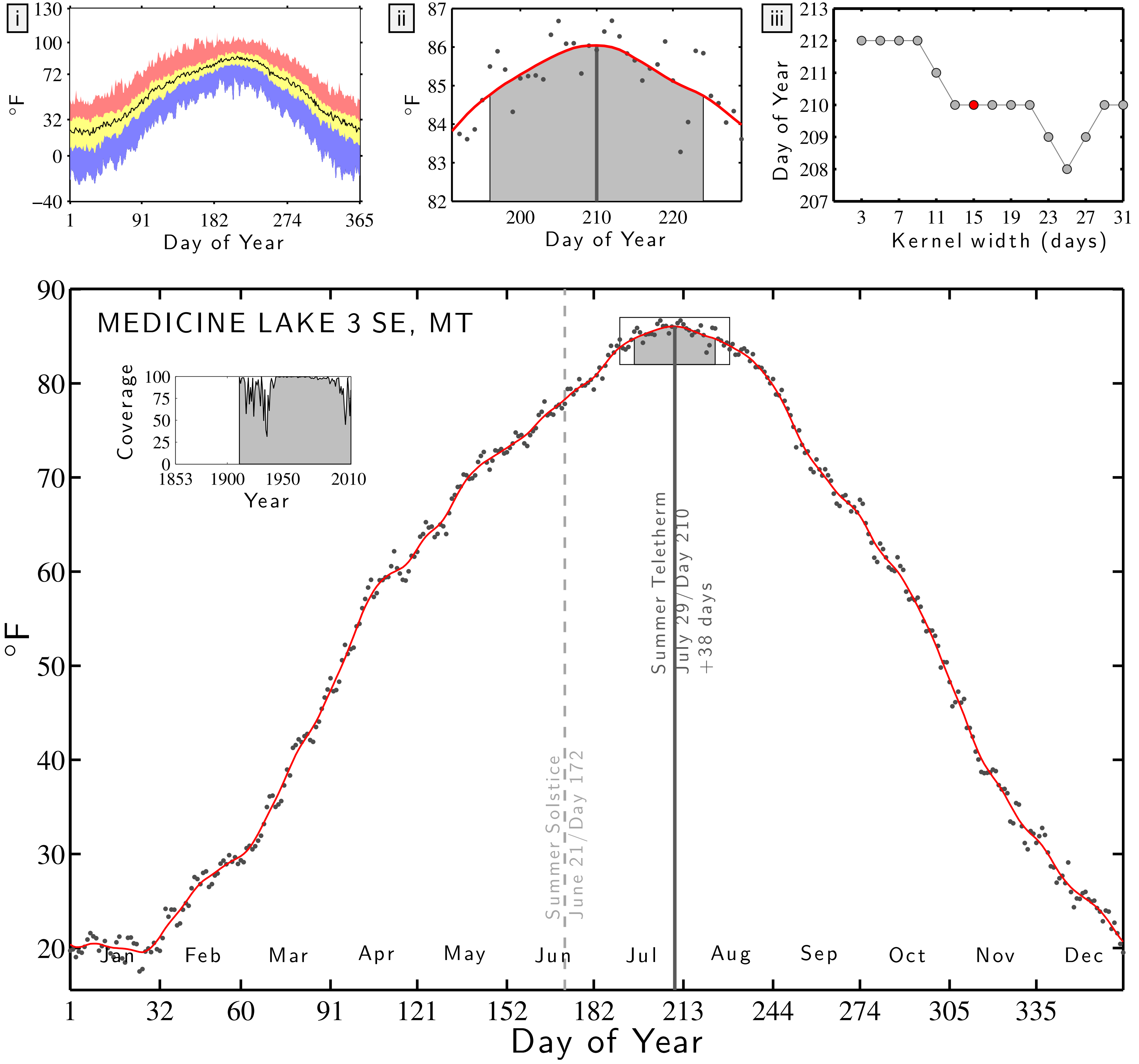

i
°F
Day of Year
ii
°F
Day of Year
iii
Day of Year
Kernel width (days)
MEDICINE LAKE 3 SE, MT
Coverage
Year
Summer Solstice
June 21/Day 172
Summer Teletherm
July 29/Day 210
+38 days
Jan
Feb
Mar
Apr
May
Jun
Jul
Aug
Sep
Oct
Nov
Dec
°F
Day of Year

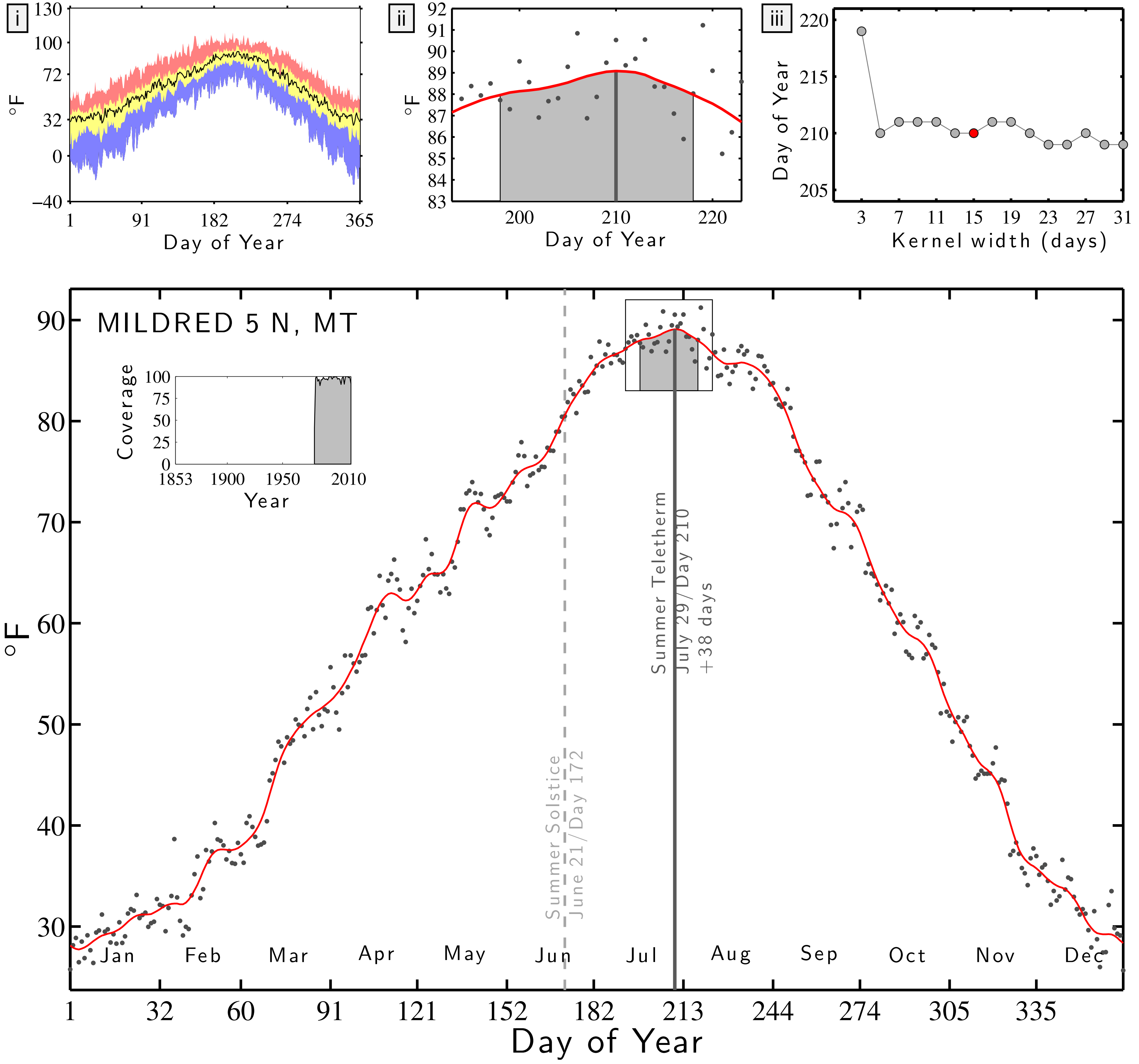

i
ii
iii
°F
Day of Year
Kernel width (days)
MILDRED 5 N, MT
Coverage
Year
Summer Solstice
June 21 / Day 172
Summer Teletherm
July 29 / Day 210
+38 days
Jan
Feb
Mar
Apr
May
Jun
Jul
Aug
Sep
Oct
Nov
Dec

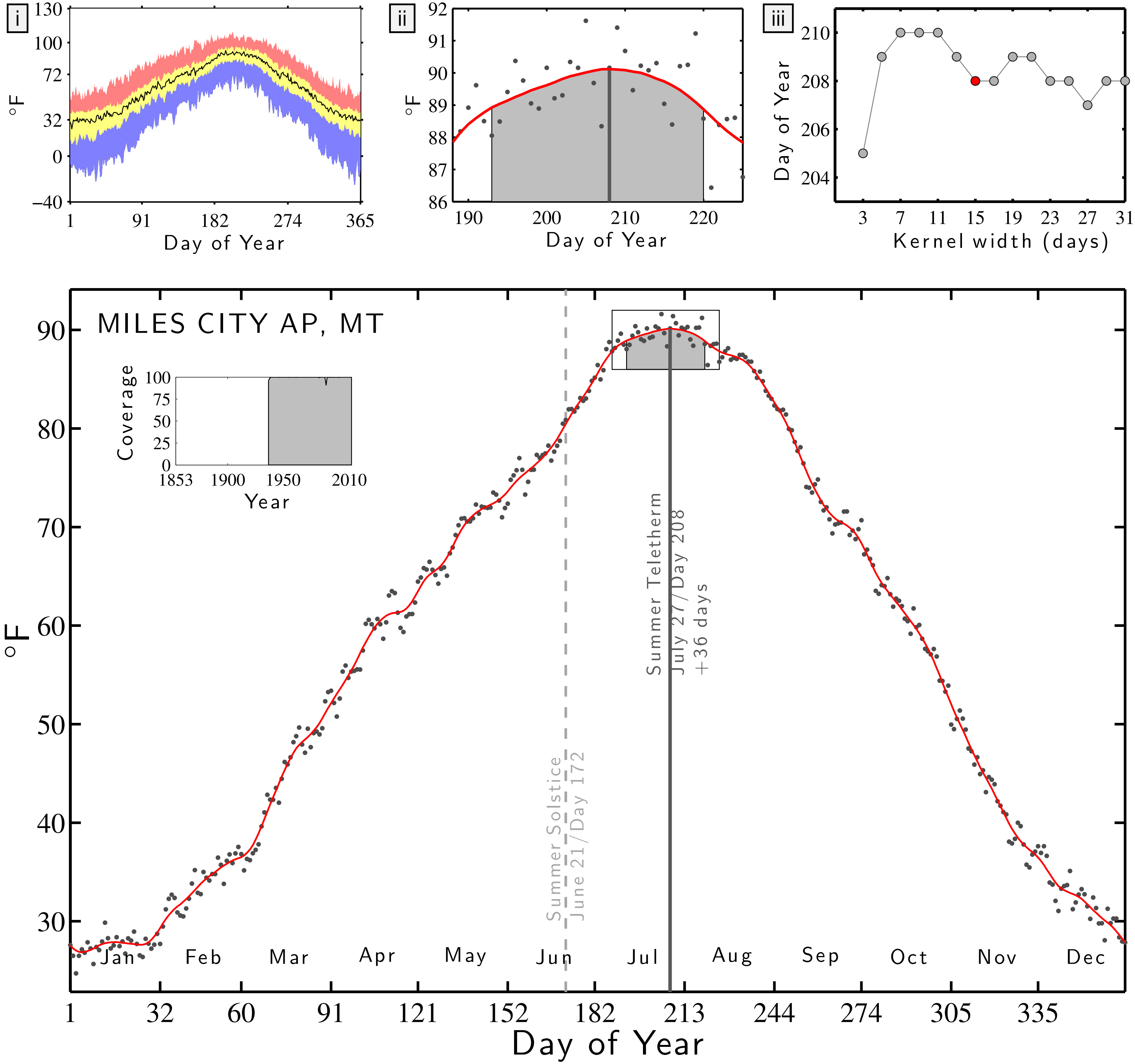

i
°F
Day of Year
ii
°F
Day of Year
iii
Day of Year
Kernel width (days)
MILES CITY AP, MT
Coverage
Year
Summer Solstice
June 21 / Day 172
Summer Teletherm
July 27 / Day 208
+36 days
Jan
Feb
Mar
Apr
May
Jun
Jul
Aug
Sep
Oct
Nov
Dec
°F
Day of Year

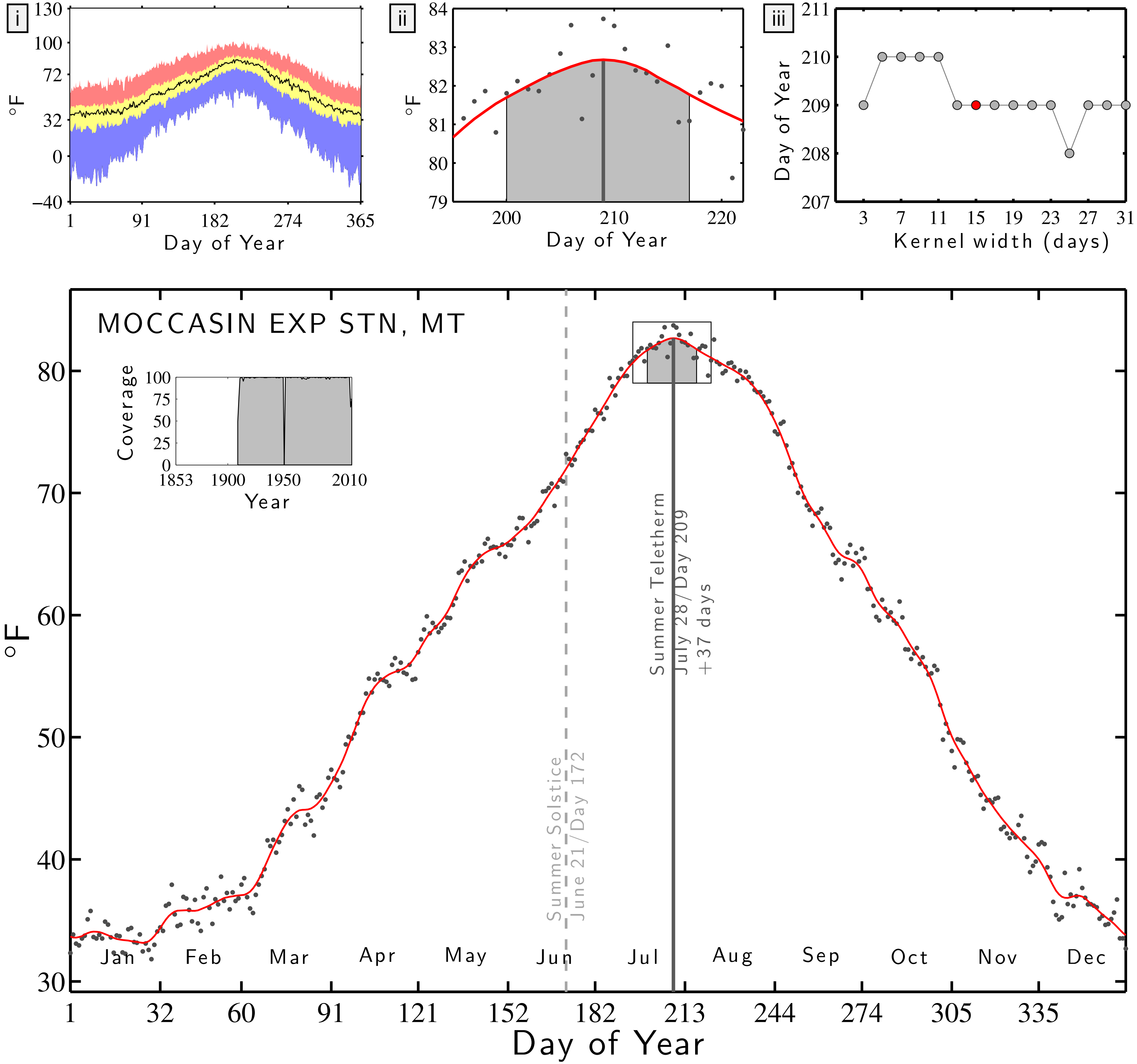

i
°F
Day of Year
ii
°F
Day of Year
iii
Day of Year
Kernel width (days)
MOCCASIN EXP STN, MT
Coverage
Year
Summer Solstice
June 21/Day 172
Summer Teletherm
July 28/Day 209
+37 days
Jan
Feb
Mar
Apr
May
Jun
Jul
Aug
Sep
Oct
Nov
Dec
°F
Day of Year

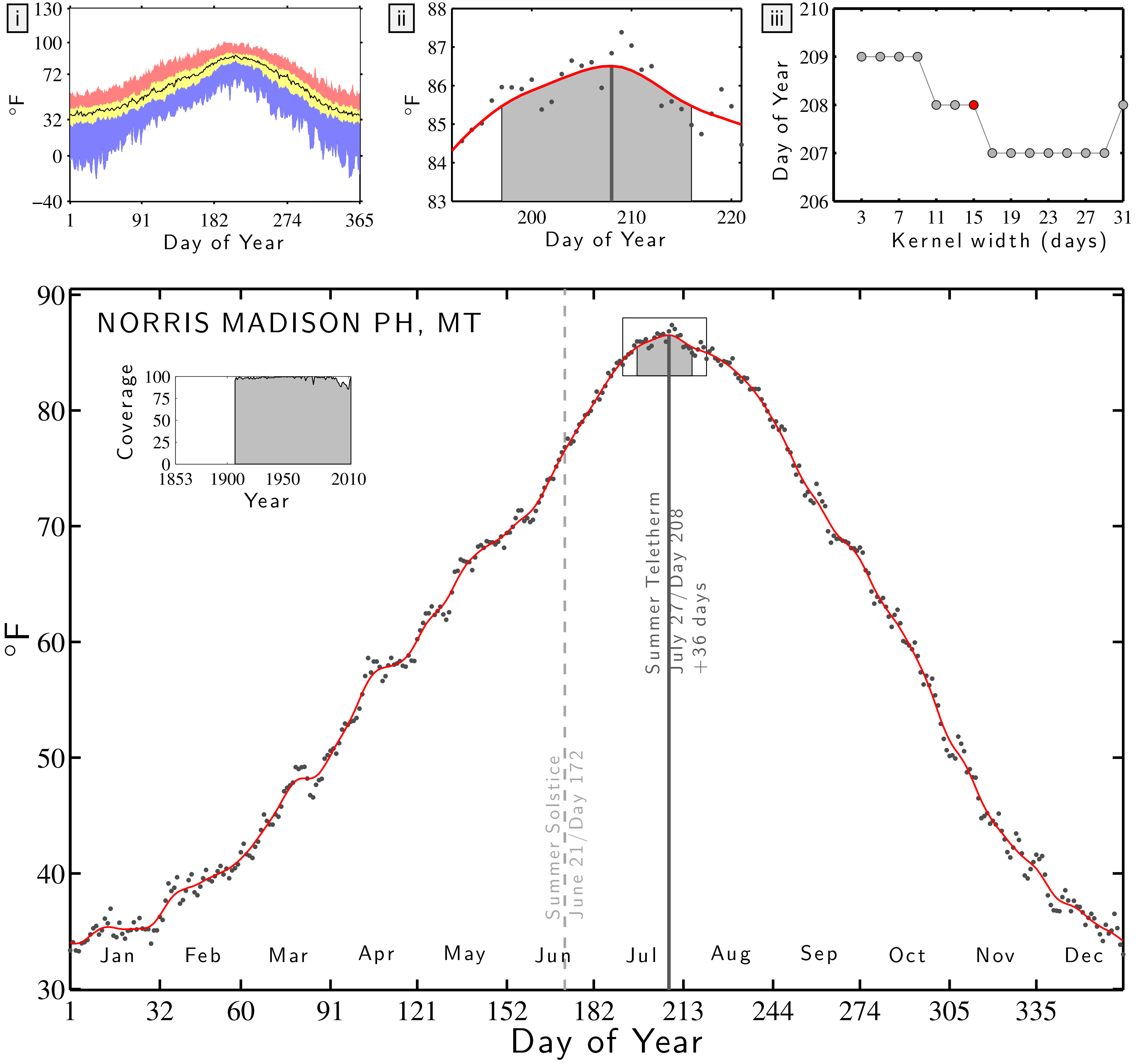

i
ii
iii
Day of Year
Kernel width (days)
°F
NORRIS MADISON PH, MT
Coverage
Year
Summer Solstice
June 21 / Day 172
Summer Teletherm
July 27 / Day 208
+36 days
Jan
Feb
Mar
Apr
May
Jun
Jul
Aug
Sep
Oct
Nov
Dec

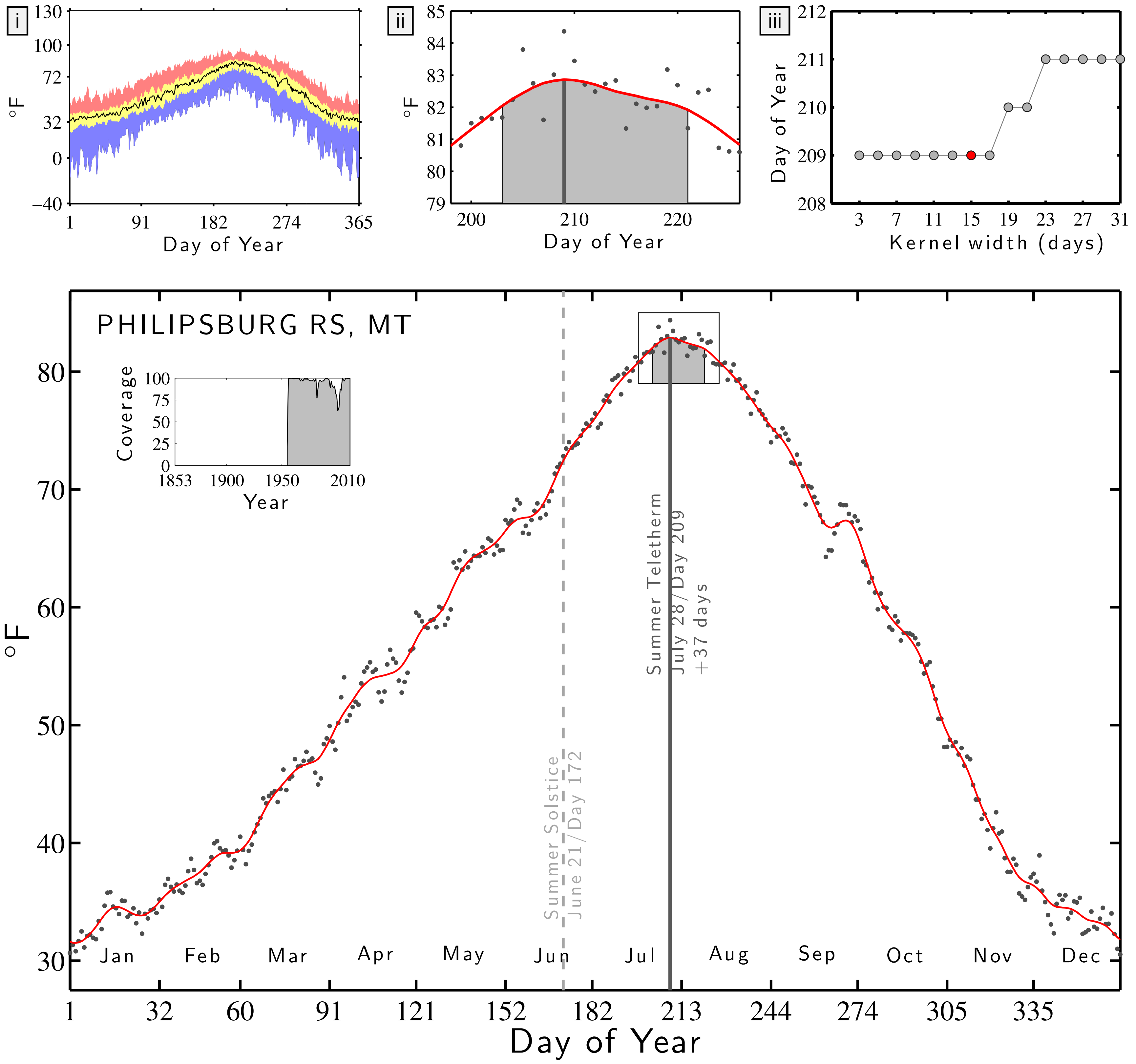
i
°F
Day of Year
ii
°F
Day of Year
iii
Day of Year
Kernel width (days)
PHILIPSBURG RS, MT
Coverage
Year
Summer Solstice
June 21/Day 172
Summer Teletherm
July 28/Day 209
+37 days
Jan
Feb
Mar
Apr
May
Jun
Jul
Aug
Sep
Oct
Nov
Dec
°F
Day of Year

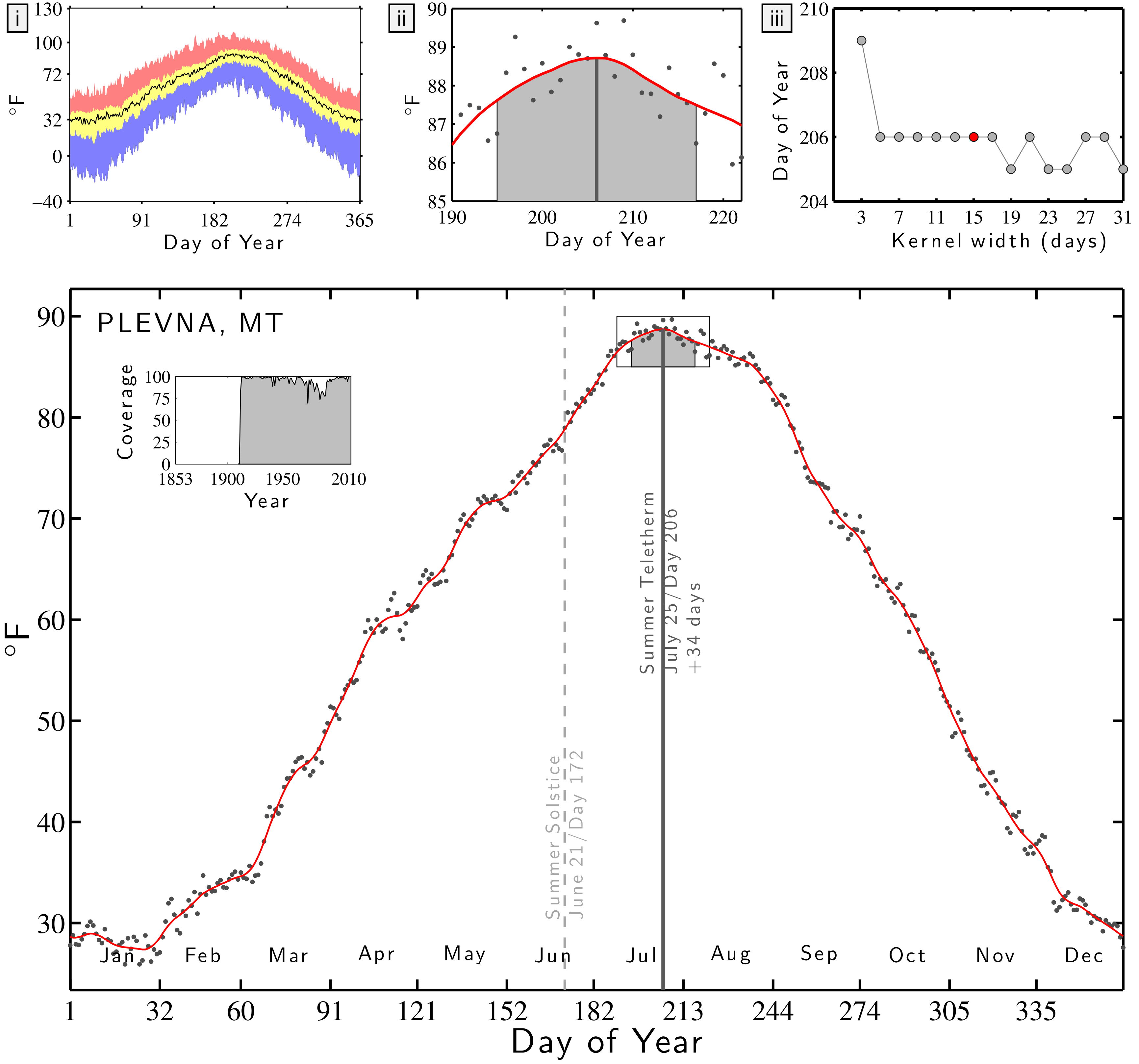

i
°F
130
100
72
32
0
−40
1
91
182
274
365
Day of Year
ii
°F
90
89
88
87
86
85
190
200
210
220
Day of Year
iii
Day of Year
210
208
206
204
3
7
11
15
19
23
27
31
Kernel width (days)
PLEVNA, MT
Coverage
100
75
50
25
0
1853
1900
1950
2010
Year
Summer Solstice
June 21/Day 172
Summer Teletherm
July 25/Day 206
+34 days
°F
90
80
70
60
50
40
30
Jan
Feb
Mar
Apr
May
Jun
Jul
Aug
Sep
Oct
Nov
Dec
1
32
60
91
121
152
182
213
244
274
305
335
Day of Year

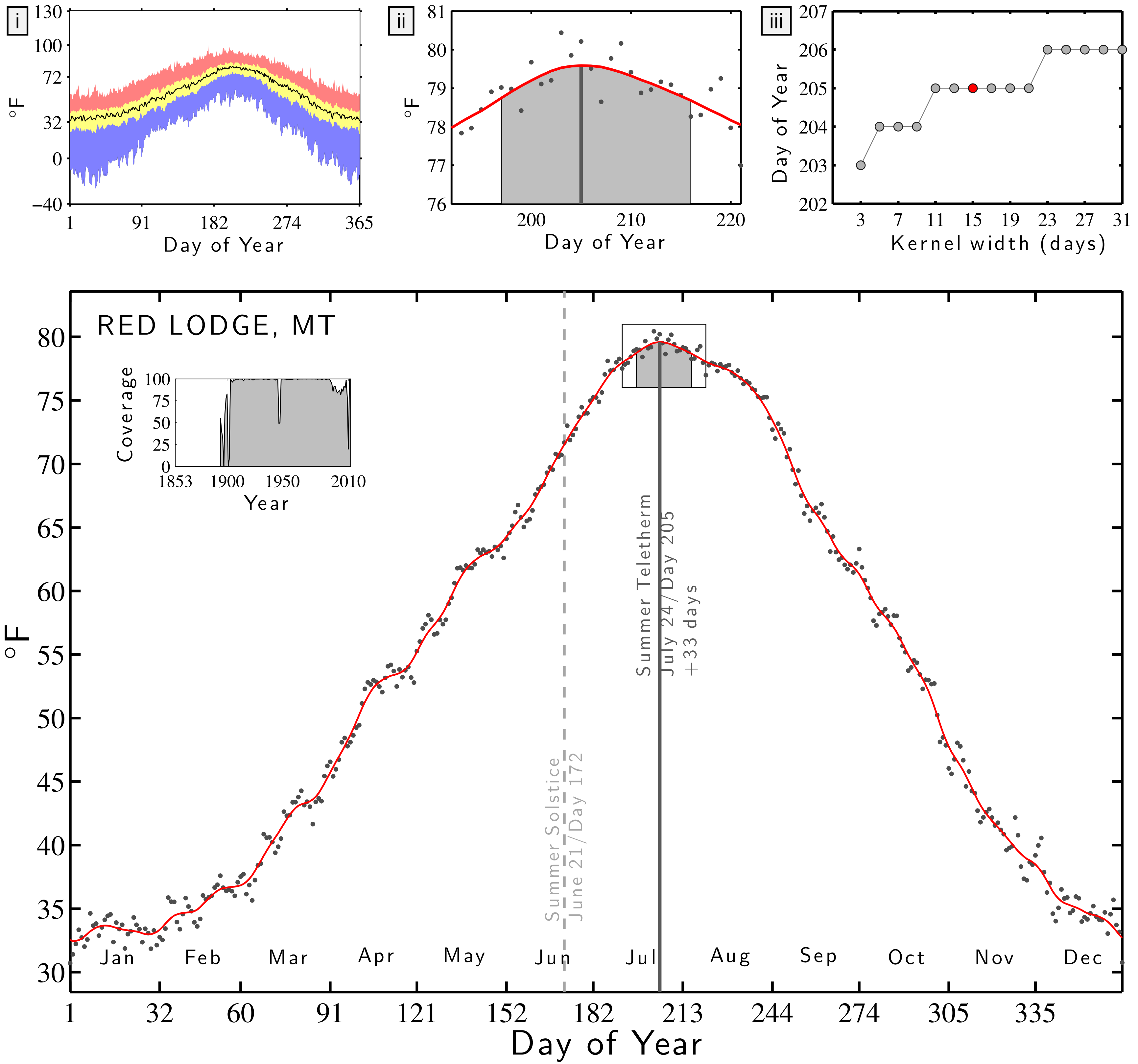

i
°F
Day of Year
ii
°F
Day of Year
iii
Day of Year
Kernel width (days)
RED LODGE, MT
Coverage
Year
Summer Solstice
June 21 / Day 172
Summer Teletherm
July 24 / Day 205
+33 days
Jan
Feb
Mar
Apr
May
Jun
Jul
Aug
Sep
Oct
Nov
Dec
°F
Day of Year

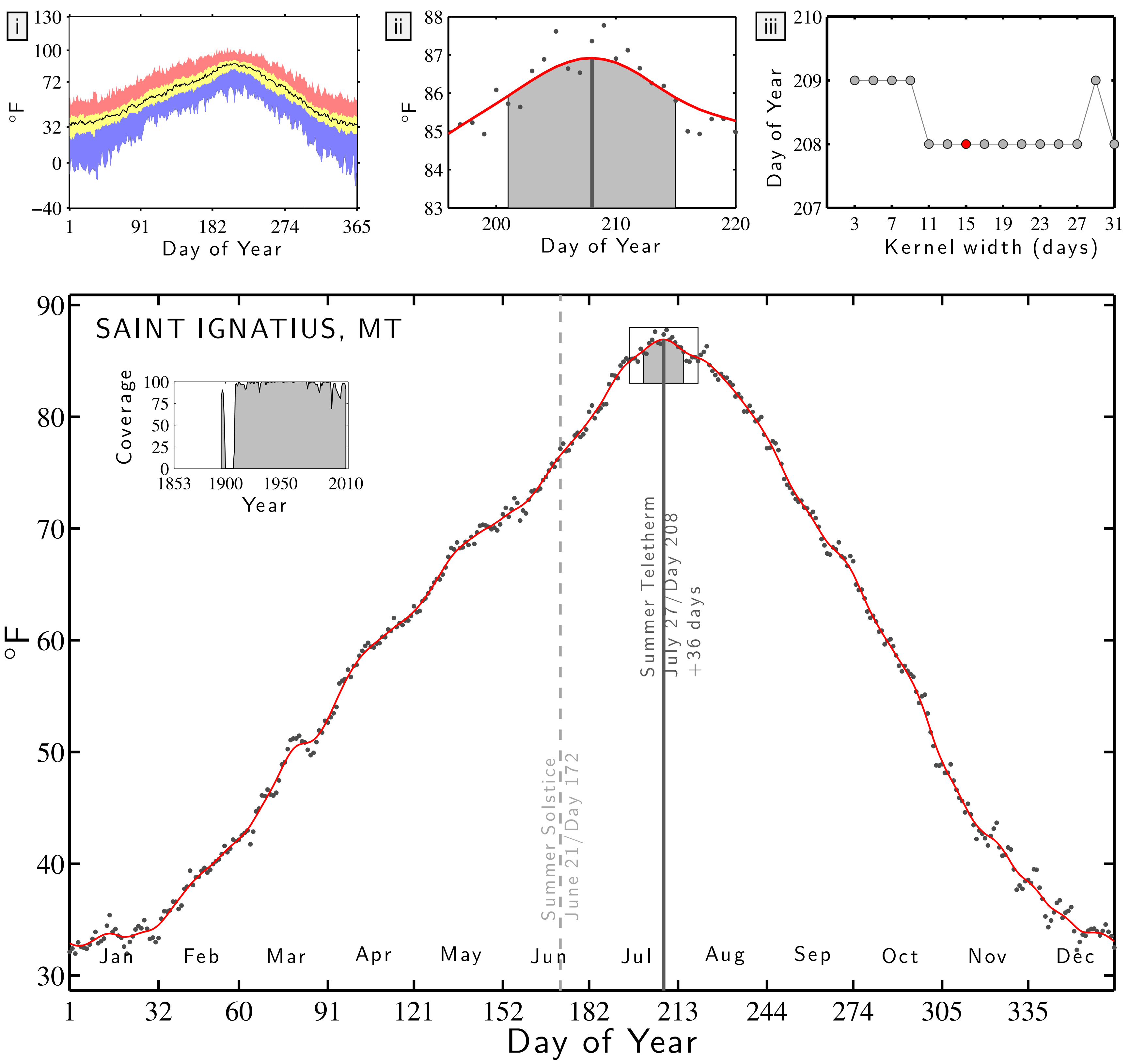

i
130
100
72
32
0
−40
°F
1
91
182
274
365
Day of Year
ii
88
87
86
85
84
83
°F
200
210
220
Day of Year
iii
210
209
208
207
Day of Year
3
7
11
15
19
23
27
31
Kernel width (days)
SAINT IGNATIUS, MT
Coverage
100
75
50
25
0
1853
1900
1950
2010
Year
Summer Solstice
June 21 / Day 172
Summer Teletherm
July 27 / Day 208
+36 days
Jan
Feb
Mar
Apr
May
Jun
Jul
Aug
Sep
Oct
Nov
Dec
90
80
70
60
50
40
30
°F
1
32
60
91
121
152
182
213
244
274
305
335
Day of Year

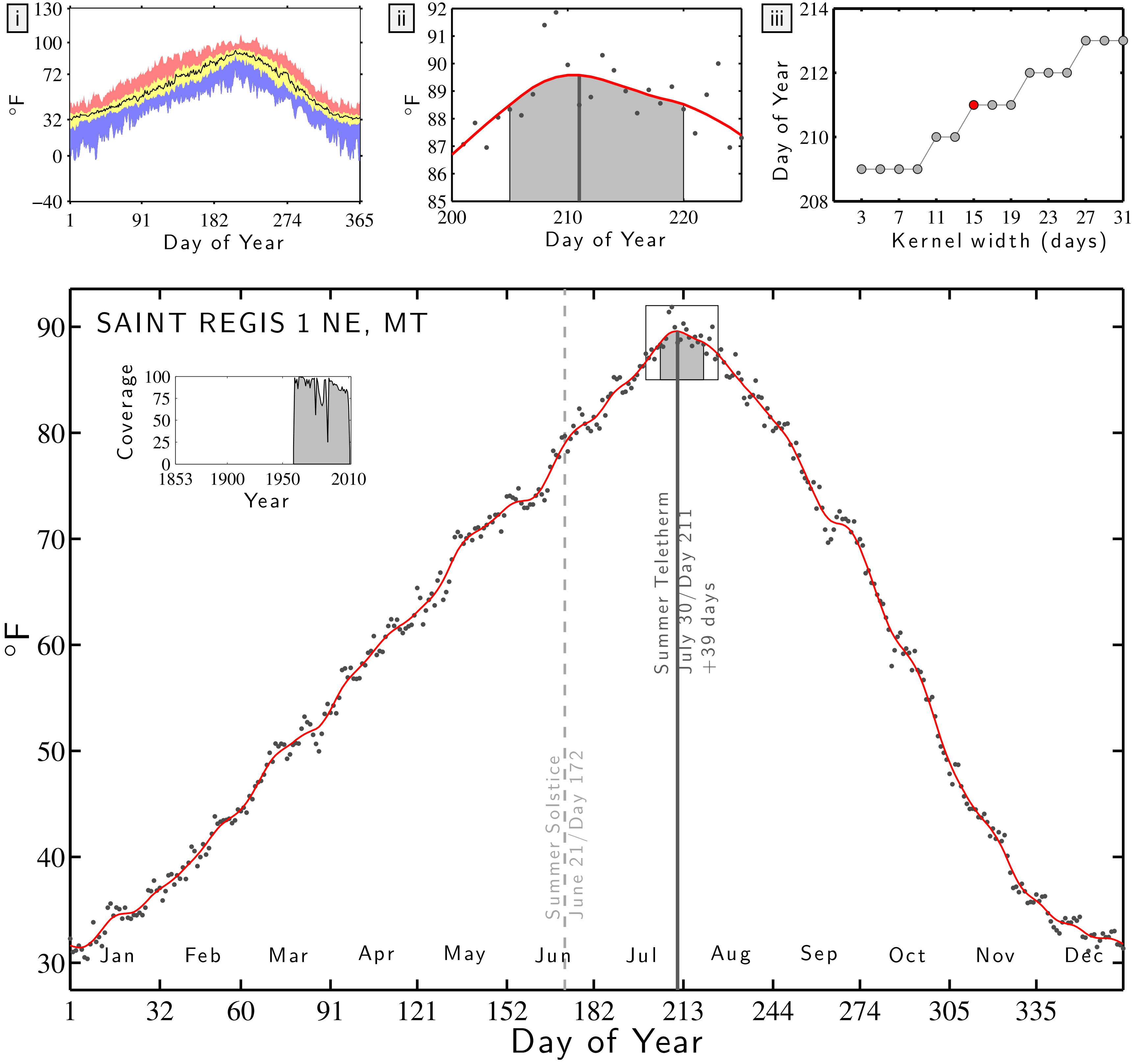

i
ii
iii
Day of Year
Kernel width (days)
°F
SAINT REGIS 1 NE, MT
Coverage
Year
Summer Solstice
June 21/Day 172
Summer Teletherm
July 30/Day 211
+39 days
Jan
Feb
Mar
Apr
May
Jun
Jul
Aug
Sep
Oct
Nov
Dec

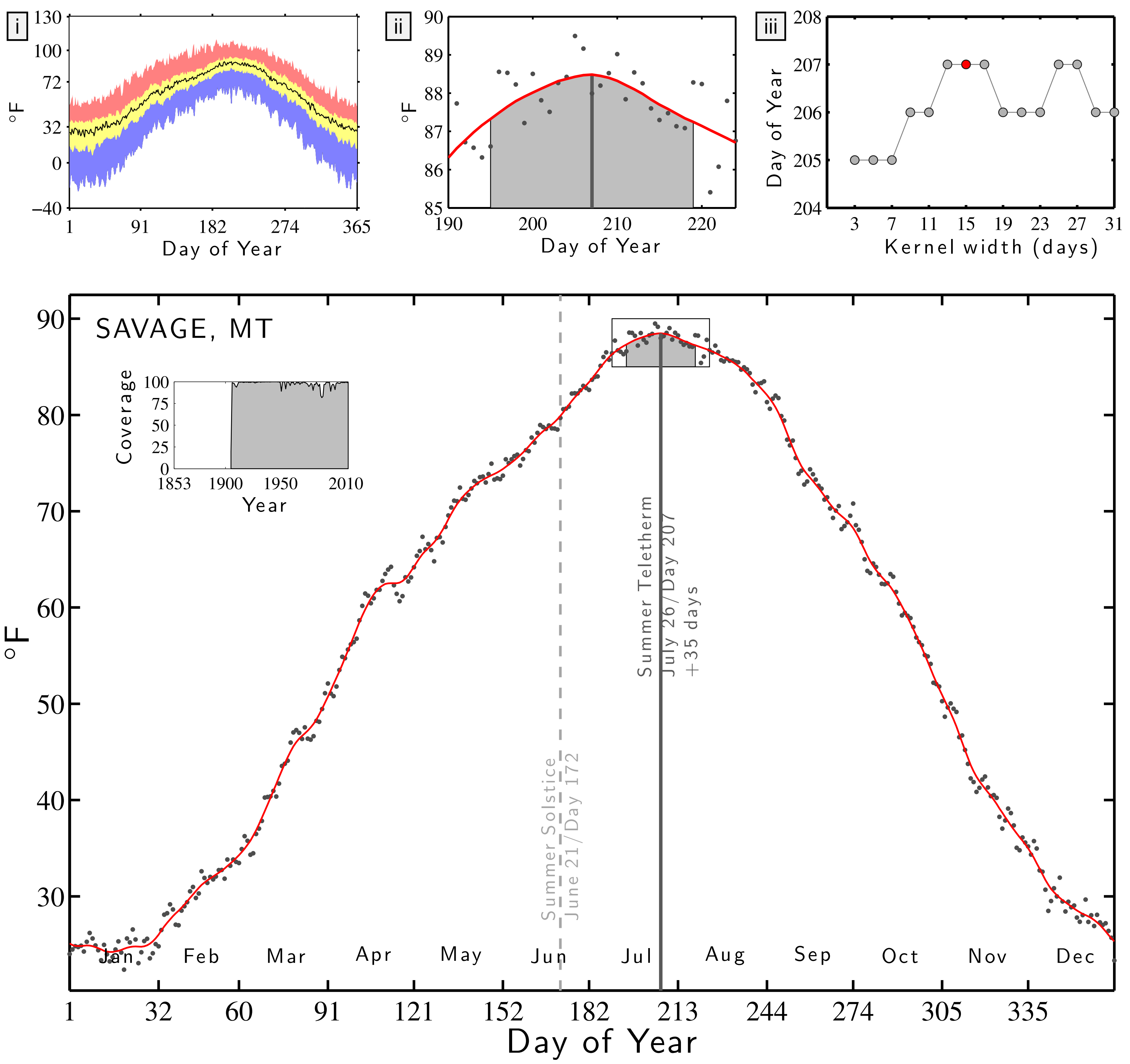

i
°F
Day of Year
ii
°F
Day of Year
iii
Day of Year
Kernel width (days)
SAVAGE, MT
Coverage
Year
Summer Solstice
June 21 / Day 172
Summer Teletherm
July 26 / Day 207
+35 days
Jan
Feb
Mar
Apr
May
Jun
Jul
Aug
Sep
Oct
Nov
Dec
°F
Day of Year

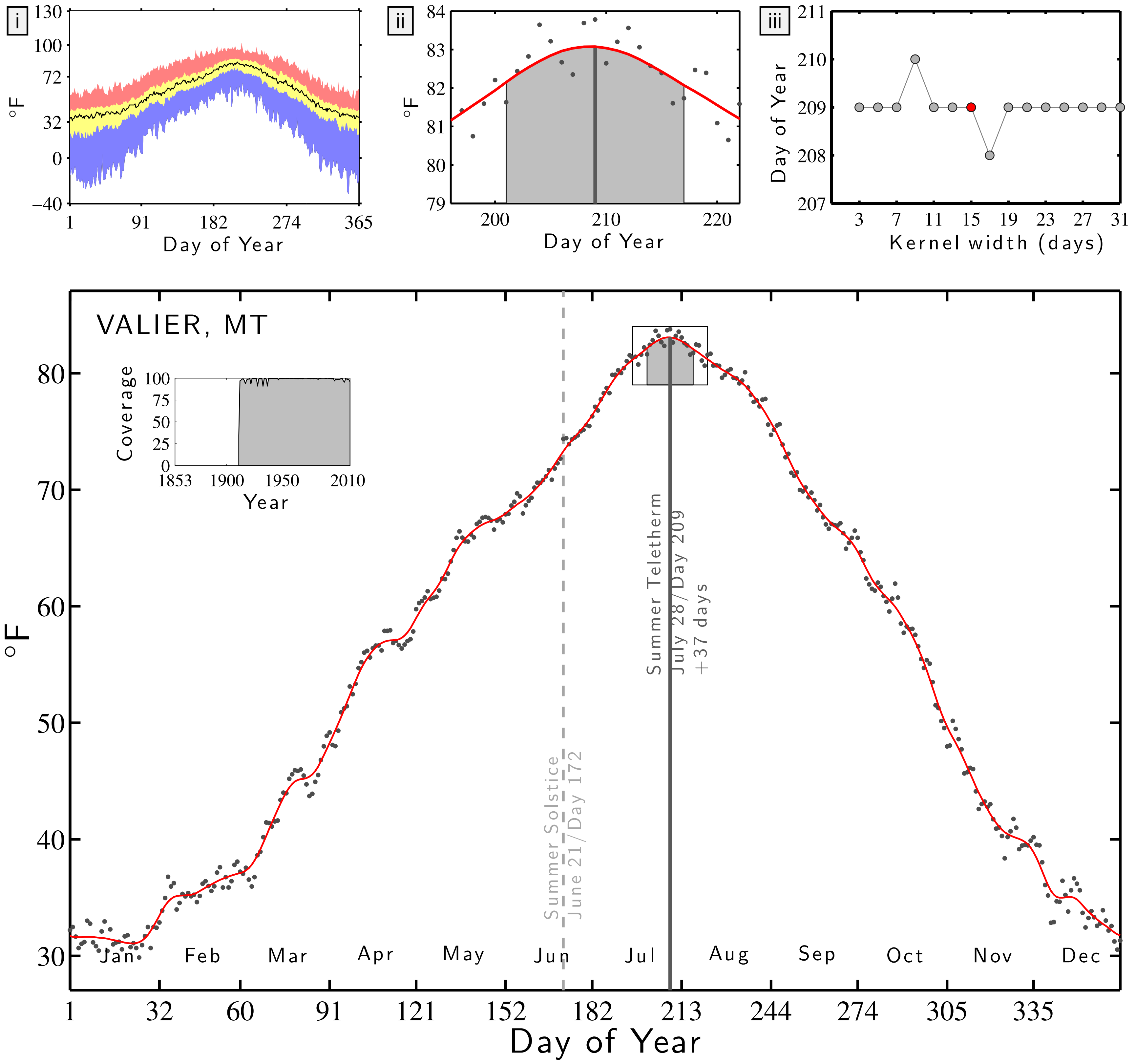

i
°F
Day of Year
ii
°F
Day of Year
iii
Day of Year
Kernel width (days)
VALIER, MT
Coverage
Year
Summer Solstice
June 21/Day 172
Summer Teletherm
July 28/Day 209
+37 days
Jan
Feb
Mar
Apr
May
Jun
Jul
Aug
Sep
Oct
Nov
Dec
°F
Day of Year

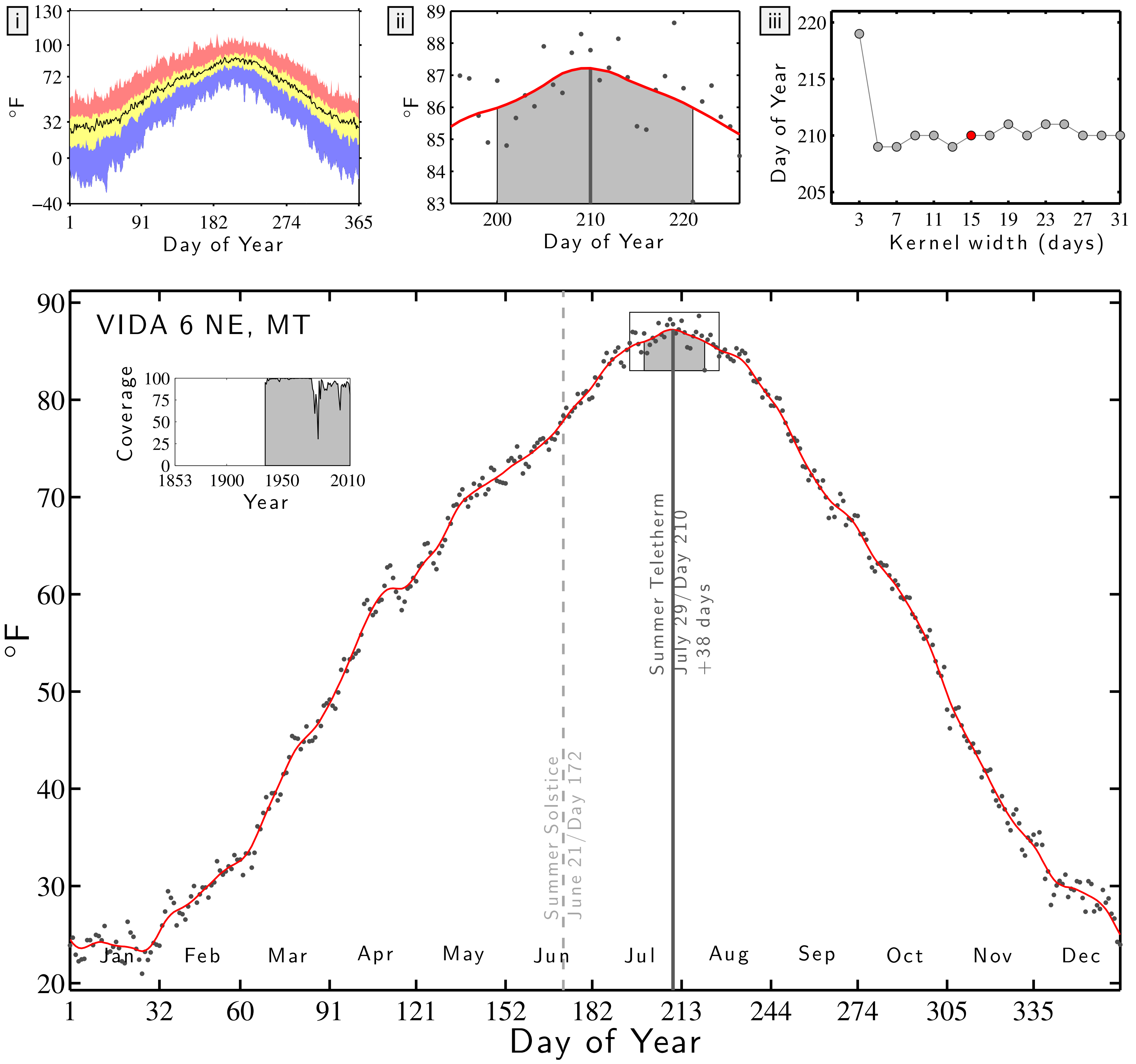

i
°F
Day of Year
ii
°F
Day of Year
iii
Day of Year
Kernel width (days)
VIDA 6 NE, MT
Coverage
Year
Summer Solstice
June 21 / Day 172
Summer Teletherm
July 29 / Day 210
+38 days
Jan
Feb
Mar
Apr
May
Jun
Jul
Aug
Sep
Oct
Nov
Dec
°F
Day of Year

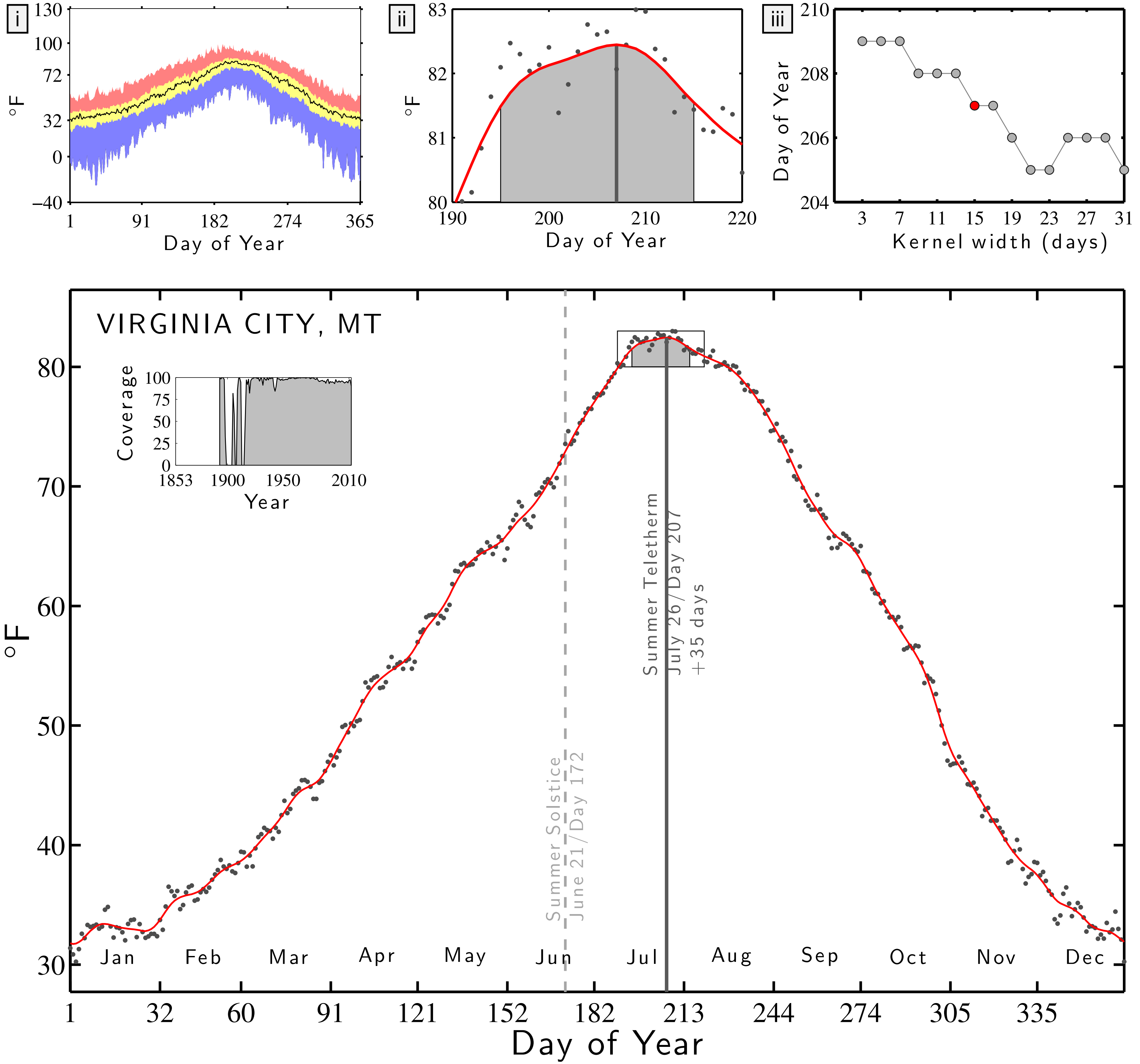

i
ii
iii
°F
Day of Year
Kernel width (days)
VIRGINIA CITY, MT
Coverage
Year
Summer Solstice
June 21 / Day 172
Summer Teletherm
July 26 / Day 207
+35 days
Jan
Feb
Mar
Apr
May
Jun
Jul
Aug
Sep
Oct
Nov
Dec

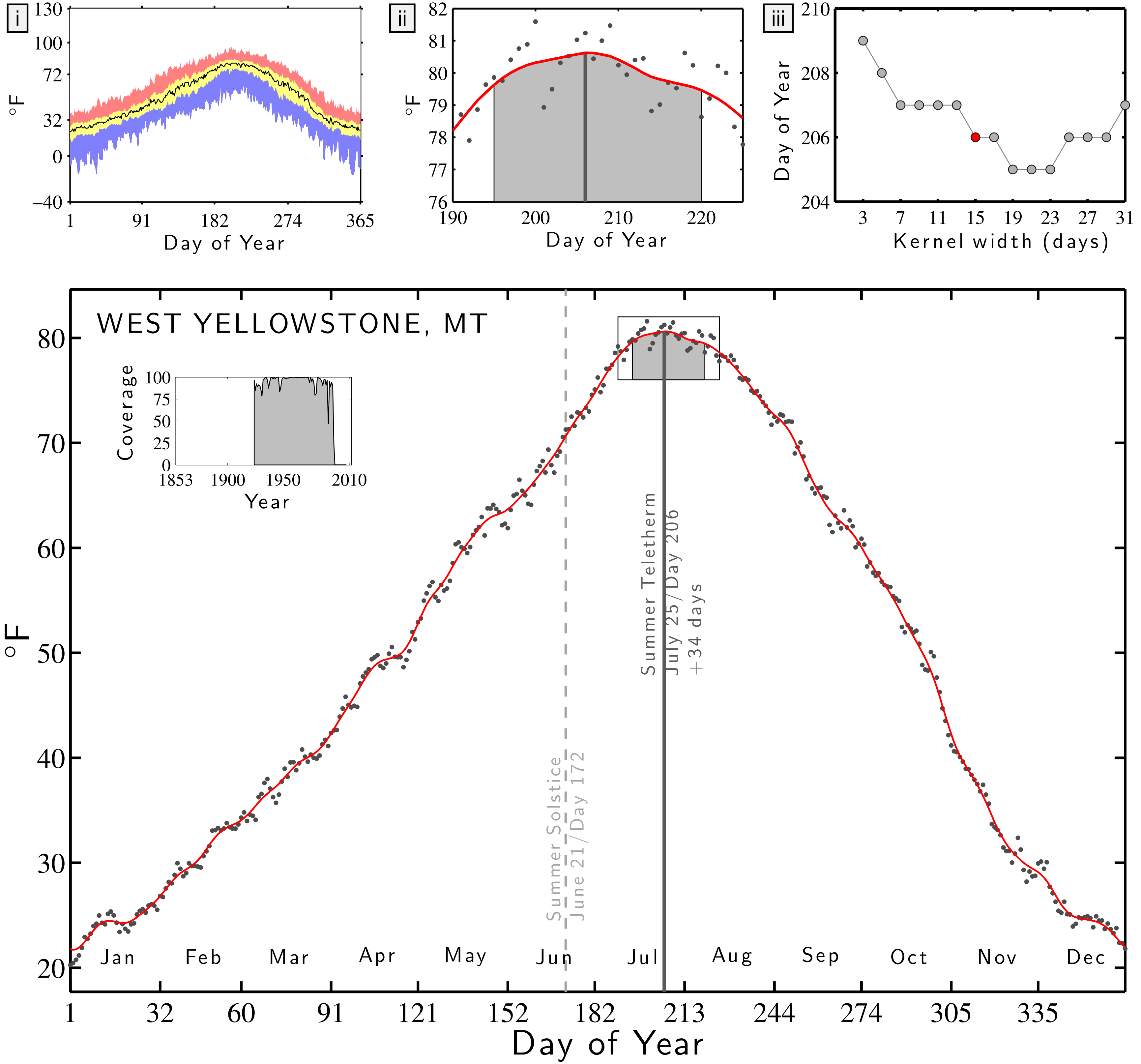

i
ii
iii
WEST YELLOWSTONE, MT
Coverage
Year
Summer Solstice
June 21/Day 172
Summer Teletherm
July 25/Day 206
+34 days
Day of Year
°F
Kernel width (days)
Jan
Feb
Mar
Apr
May
Jun
Jul
Aug
Sep
Oct
Nov
Dec

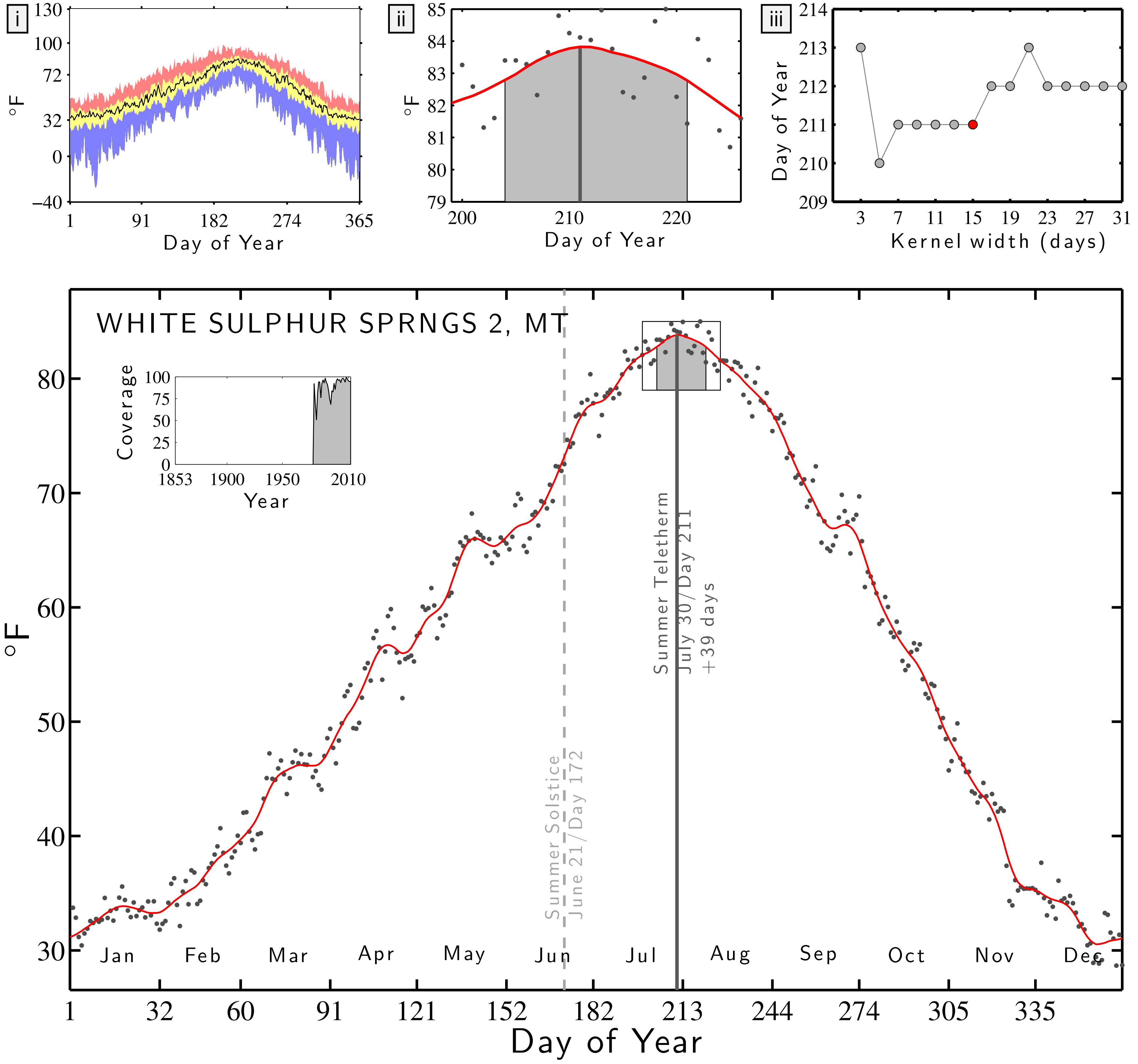

i
°F
Day of Year
ii
°F
Day of Year
iii
Day of Year
Kernel width (days)
WHITE SULPHUR SPRNGS 2, MT
Coverage
Year
Summer Solstice
June 21 / Day 172
Summer Teletherm
July 30 / Day 211
+39 days
Jan
Feb
Mar
Apr
May
Jun
Jul
Aug
Sep
Oct
Nov
Dec
°F
Day of Year

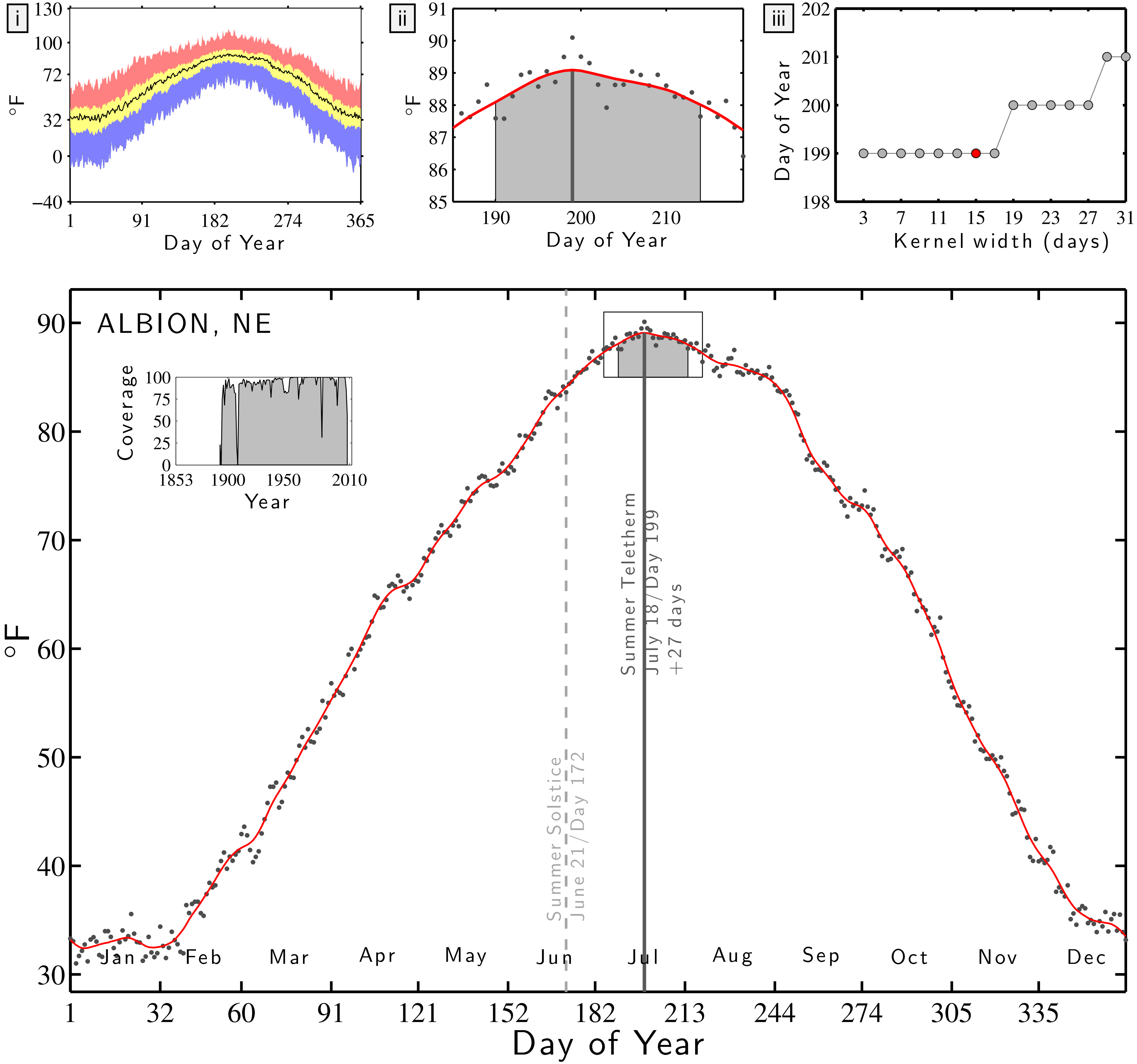

i
°F
Day of Year
ii
°F
Day of Year
iii
Day of Year
Kernel width (days)
ALBION, NE
Coverage
Year
Summer Solstice
June 21/Day 172
Summer Teletherm
July 18/Day 199
+27 days
Jan
Feb
Mar
Apr
May
Jun
Jul
Aug
Sep
Oct
Nov
Dec
°F
Day of Year

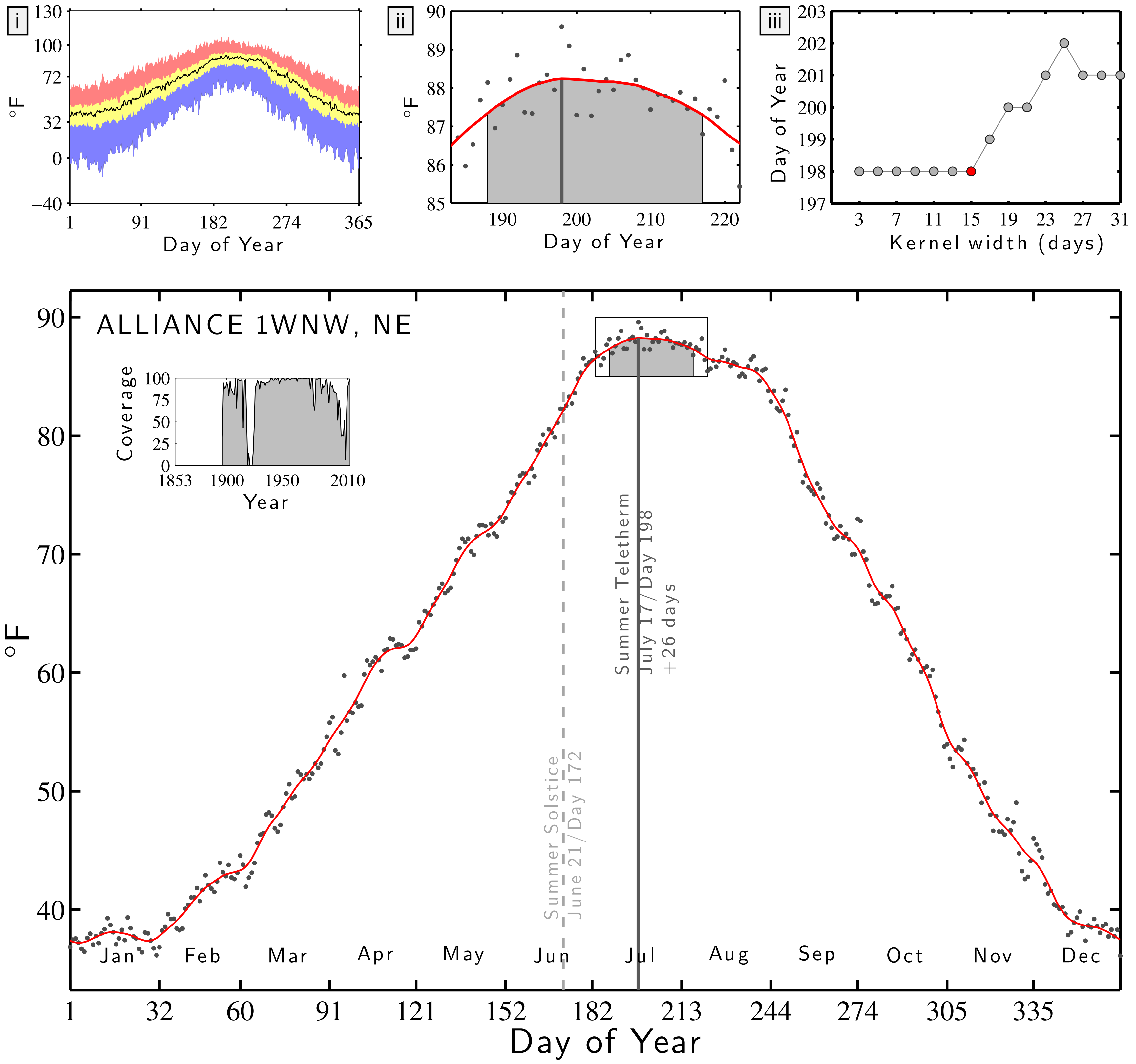

i
ii
iii
Day of Year
Kernel width (days)
°F
Day of Year
ALLIANCE 1WNW, NE
Coverage
Year
Summer Solstice
June 21/Day 172
Summer Teletherm
July 17/Day 198
+26 days
Jan
Feb
Mar
Apr
May
Jun
Jul
Aug
Sep
Oct
Nov
Dec

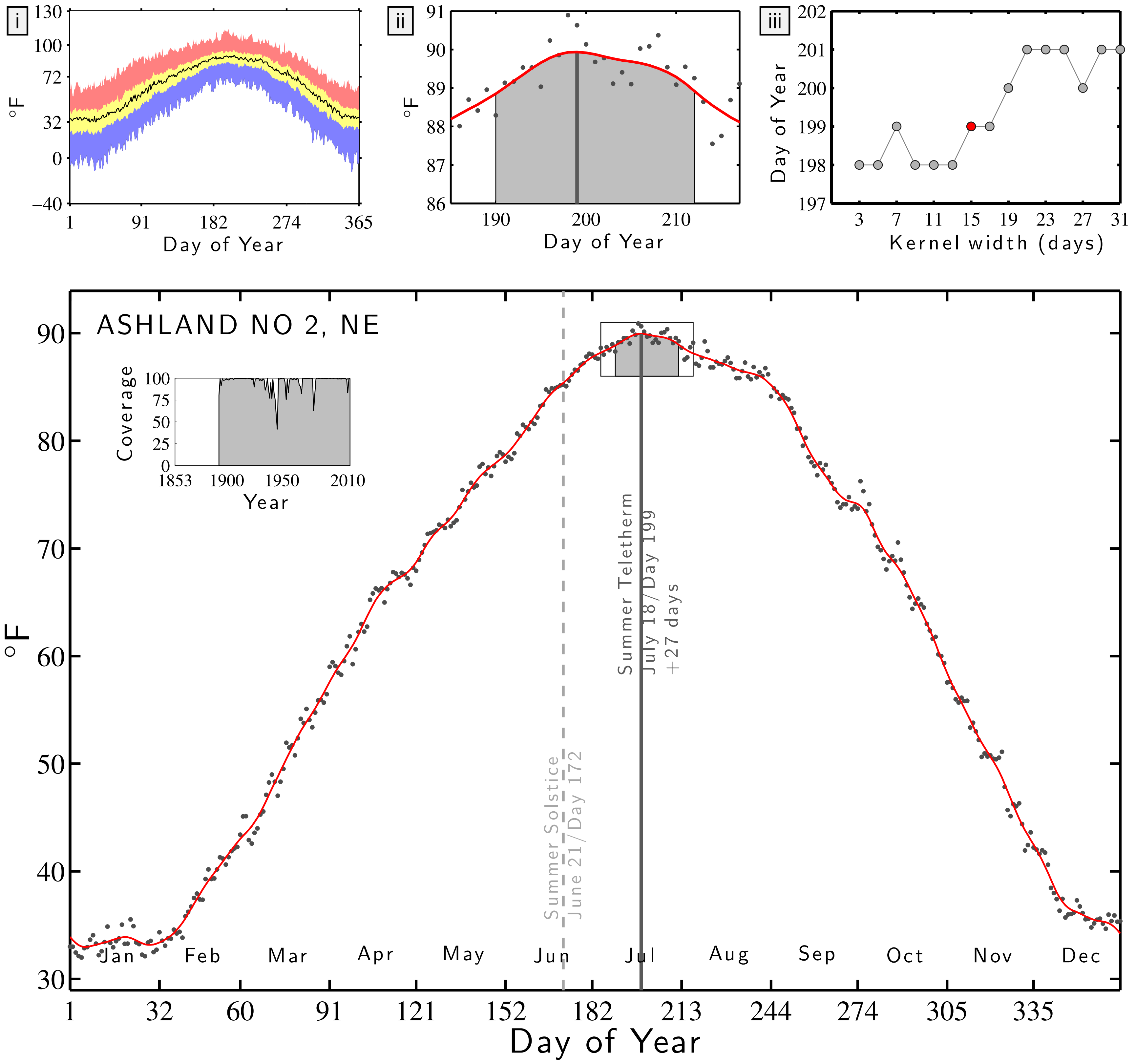

i
°F
Day of Year
ii
°F
Day of Year
iii
Day of Year
Kernel width (days)
ASHLAND NO 2, NE
Coverage
Year
Summer Solstice
June 21 / Day 172
Summer Teletherm
July 18 / Day 199
+27 days
Jan
Feb
Mar
Apr
May
Jun
Jul
Aug
Sep
Oct
Nov
Dec
°F
Day of Year

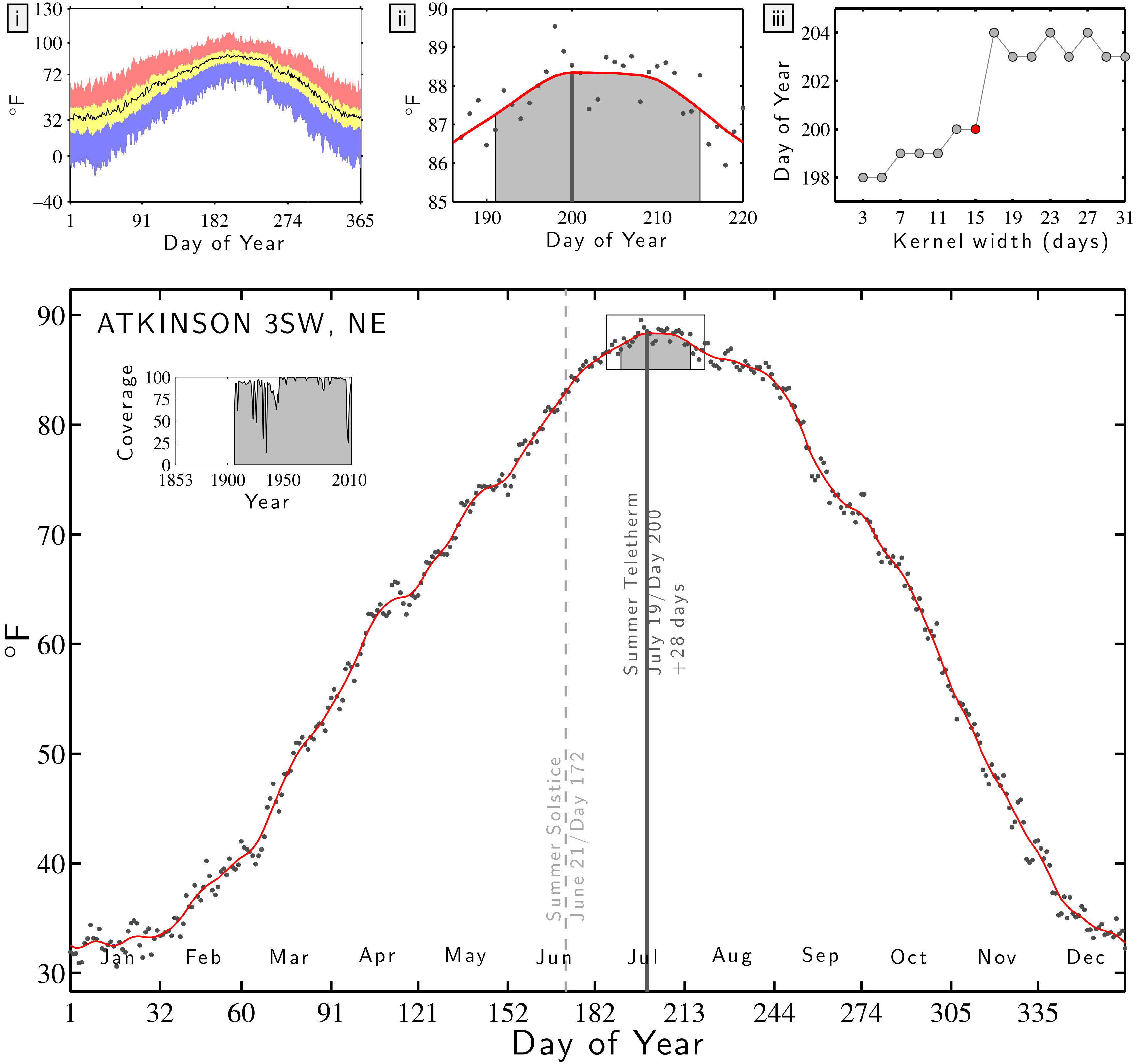

i
°F
Day of Year
ii
°F
Day of Year
iii
Day of Year
Kernel width (days)
ATKINSON 3SW, NE
Coverage
Year
Summer Solstice
June 21/Day 172
Summer Teletherm
July 19/Day 200
+28 days
Jan
Feb
Mar
Apr
May
Jun
Jul
Aug
Sep
Oct
Nov
Dec
°F
Day of Year

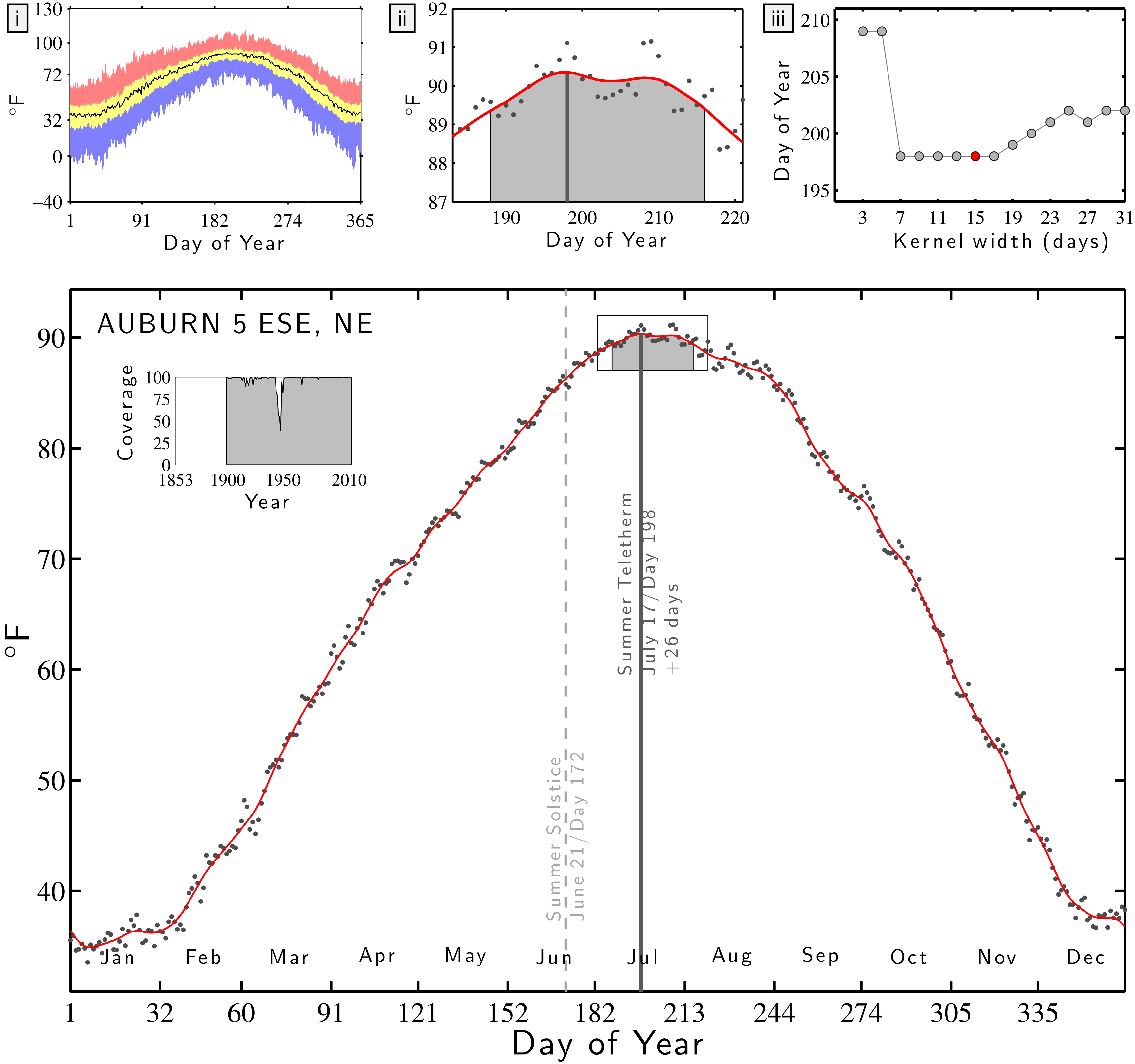

i
°F
Day of Year
ii
Day of Year
iii
Day of Year
Kernel width (days)
AUBURN 5 ESE, NE
Coverage
Year
Summer Solstice
June 21/Day 172
Summer Teletherm
July 17/Day 198
+26 days
Jan
Feb
Mar
Apr
May
Jun
Jul
Aug
Sep
Oct
Nov
Dec
Day of Year

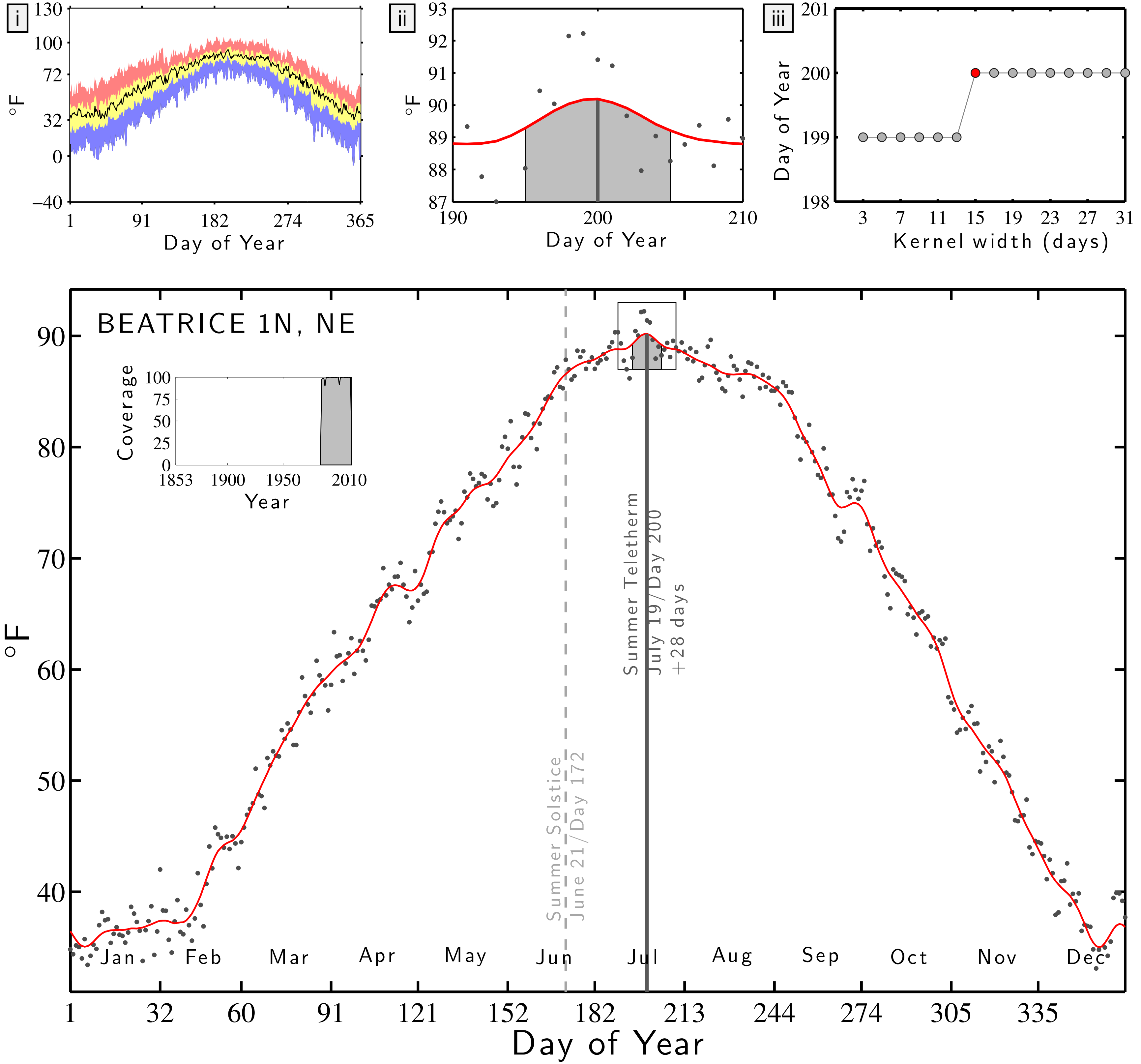

i
130
100
72
32
0
−40
°F
1
91
182
274
365
Day of Year
ii
93
92
91
90
89
88
87
°F
190
200
210
Day of Year
iii
201
200
199
198
Day of Year
3
7
11
15
19
23
27
31
Kernel width (days)
BEATRICE 1N, NE
Coverage
100
75
50
25
0
1853
1900
1950
2010
Year
Summer Solstice
June 21/Day 172
Summer Teletherm
July 19/Day 200
+28 days
90
80
70
60
50
40
°F
Jan
Feb
Mar
Apr
May
Jun
Jul
Aug
Sep
Oct
Nov
Dec
1
32
60
91
121
152
182
213
244
274
305
335
Day of Year

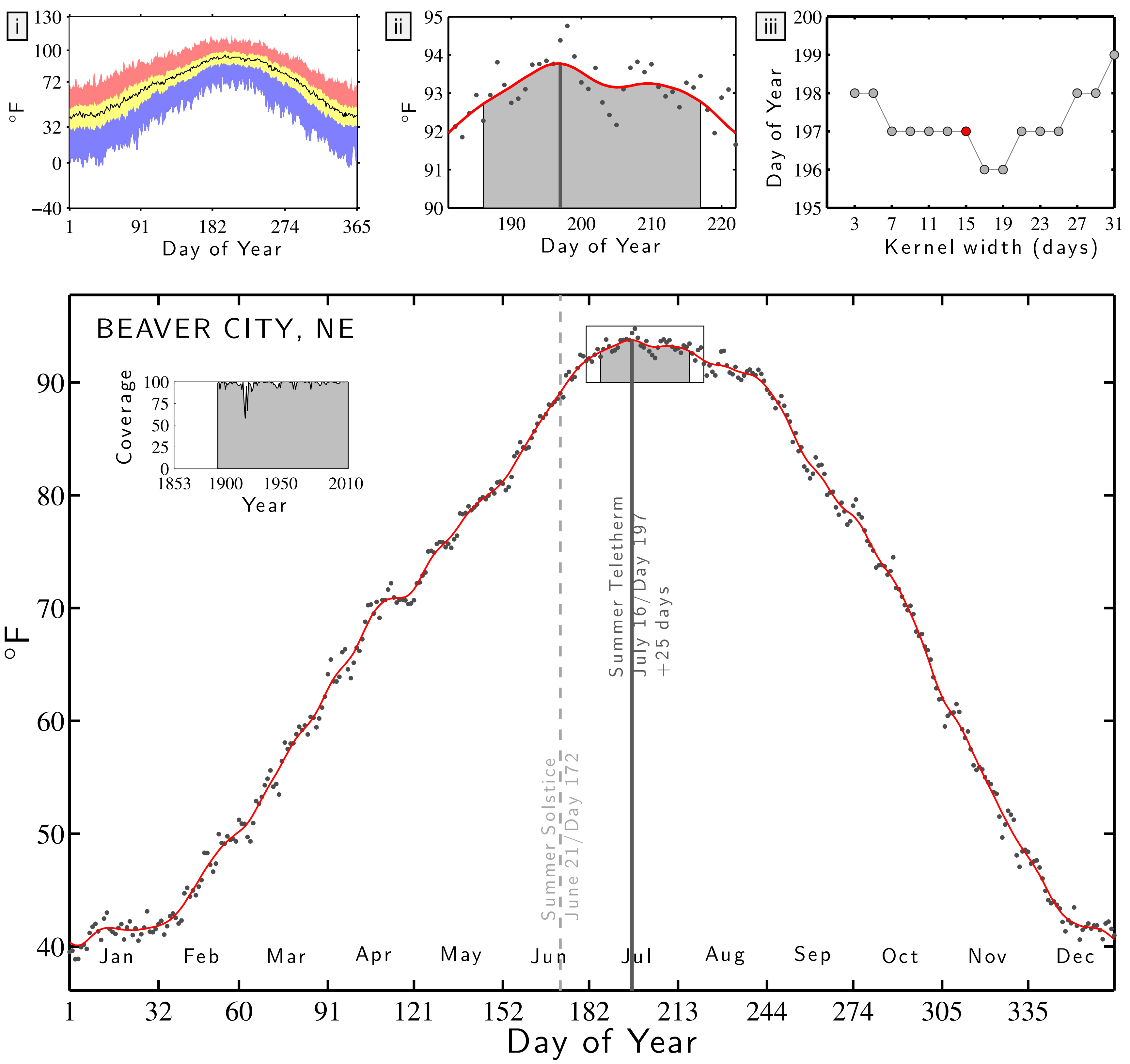

i
°F
Day of Year
ii
°F
Day of Year
iii
Day of Year
Kernel width (days)
BEAVER CITY, NE
Coverage
Year
Summer Solstice
June 21/Day 172
Summer Teletherm
July 16/Day 197
+25 days
Jan
Feb
Mar
Apr
May
Jun
Jul
Aug
Sep
Oct
Nov
Dec
°F
Day of Year

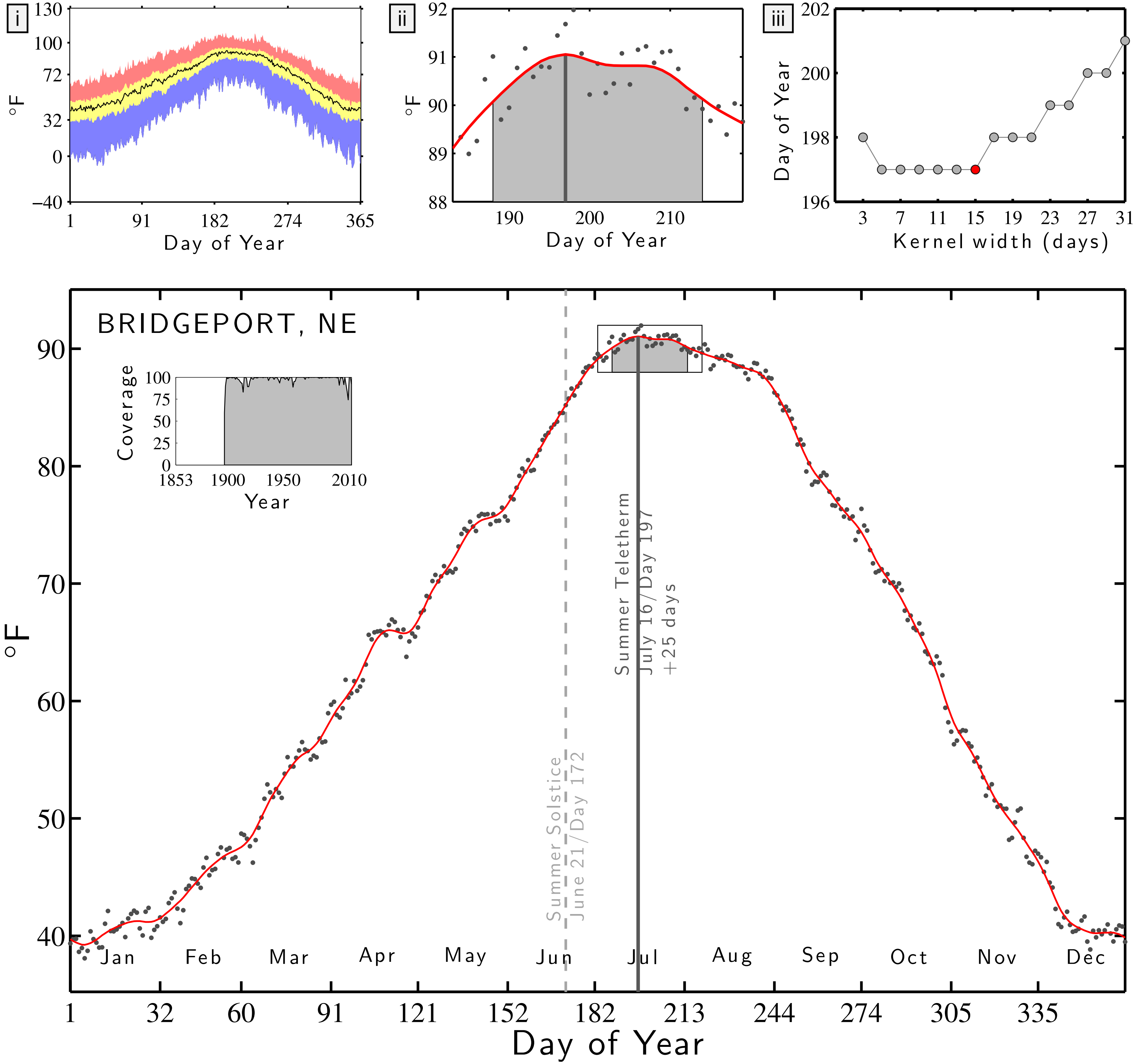

i
°F
130
100
72
32
0
−40
1
91
182
274
365
Day of Year
ii
92
91
90
89
88
190
200
210
iii
Day of Year
202
200
198
196
3
7
11
15
19
23
27
31
Kernel width (days)
BRIDGEPORT, NE
Coverage
100
75
50
25
0
1853
1900
1950
2010
Year
Summer Solstice
June 21/Day 172
Summer Teletherm
July 16/Day 197
+25 days
Jan
Feb
Mar
Apr
May
Jun
Jul
Aug
Sep
Oct
Nov
Dec
90
80
70
60
50
40
1
32
60
91
121
152
182
213
244
274
305
335

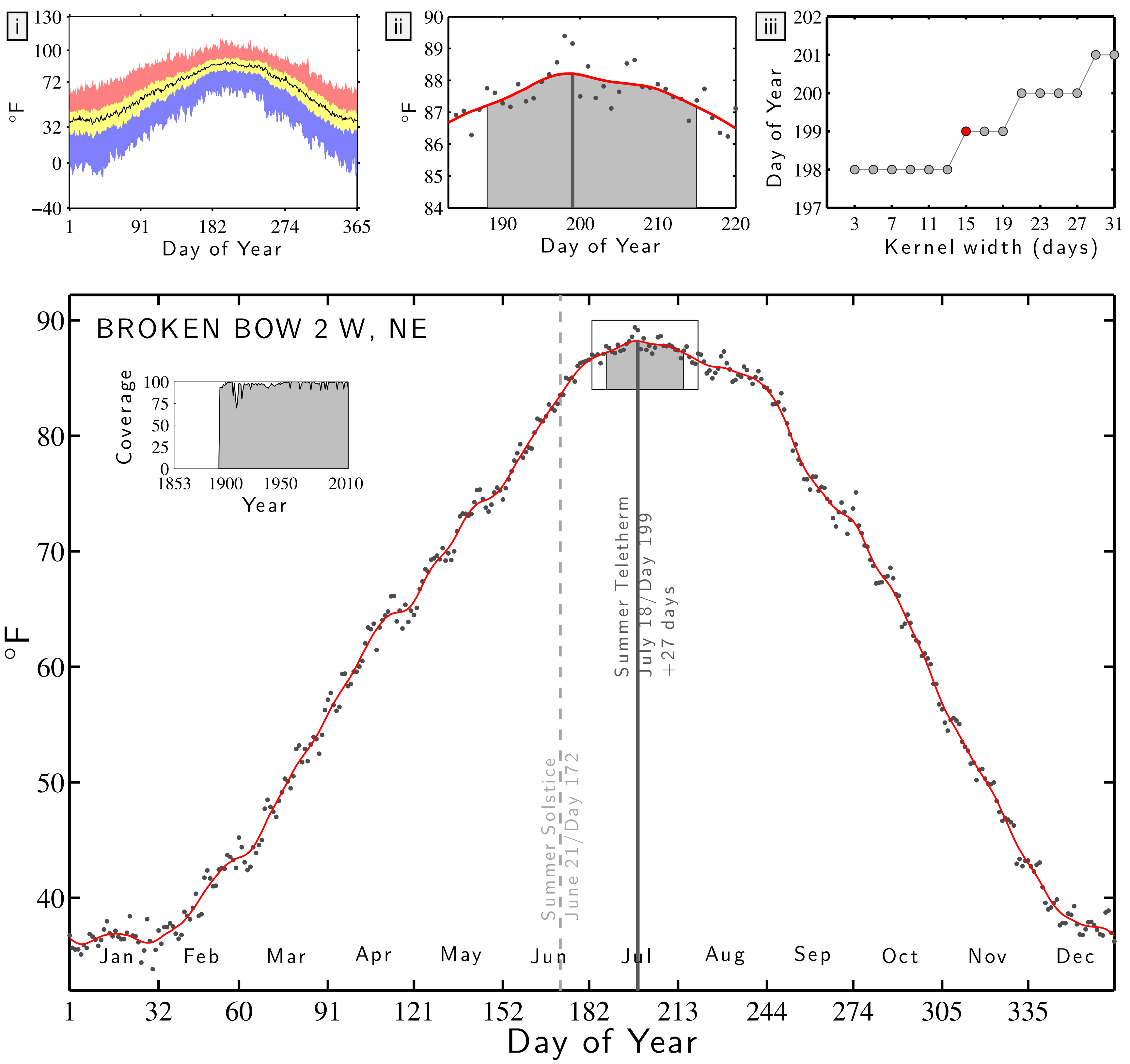

i
°F
Day of Year
ii
°F
Day of Year
iii
Day of Year
Kernel width (days)
BROKEN BOW 2 W, NE
Coverage
Year
Summer Solstice
June 21/Day 172
Summer Teletherm
July 18/Day 199
+27 days
Jan
Feb
Mar
Apr
May
Jun
Jul
Aug
Sep
Oct
Nov
Dec
°F
Day of Year

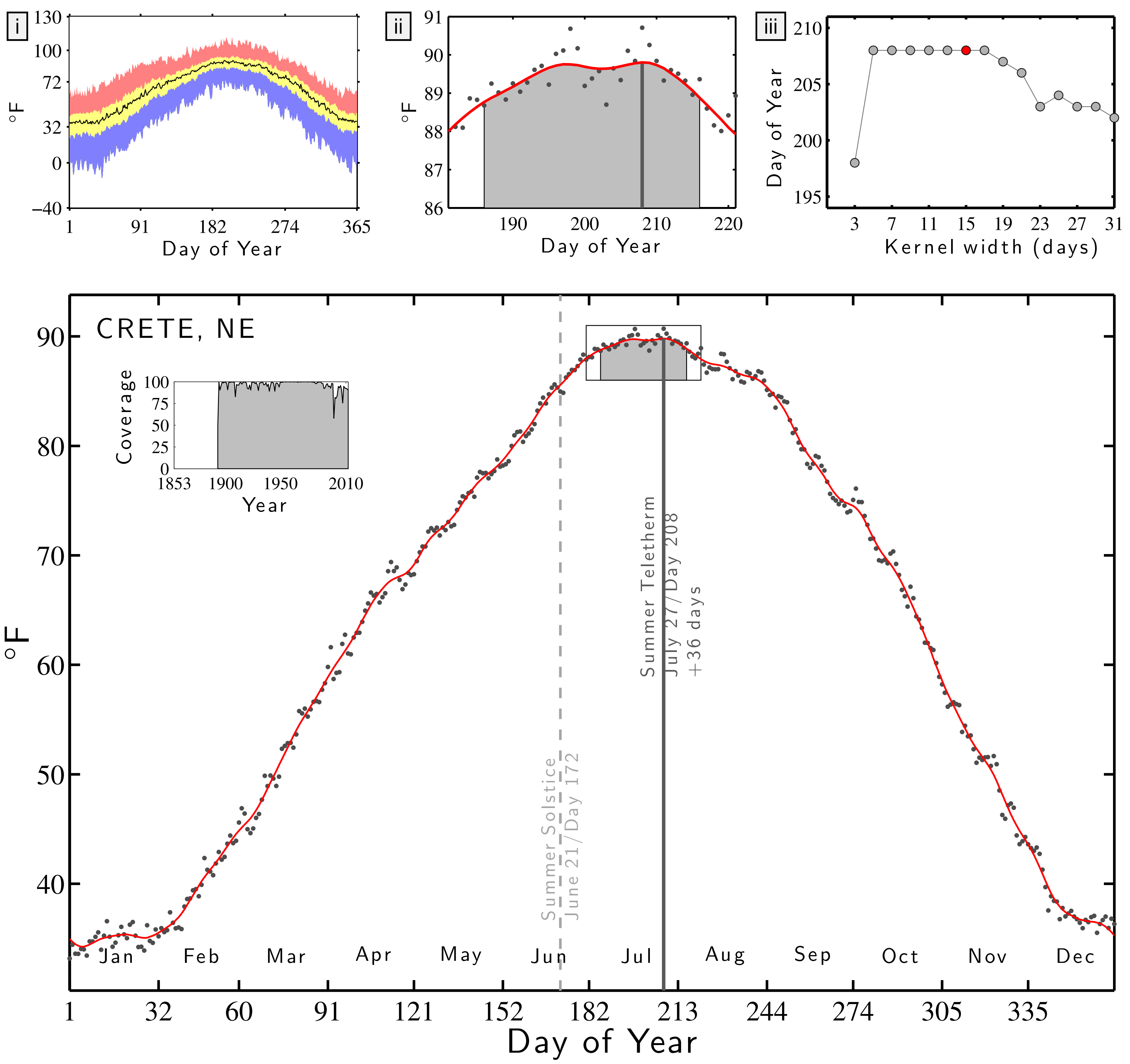
i
°F
Day of Year
ii
°F
Day of Year
iii
Day of Year
Kernel width (days)
CRETE, NE
Coverage
Year
Summer Solstice
June 21 / Day 172
Summer Teletherm
July 27 / Day 208
+36 days
Jan
Feb
Mar
Apr
May
Jun
Jul
Aug
Sep
Oct
Nov
Dec
°F
Day of Year

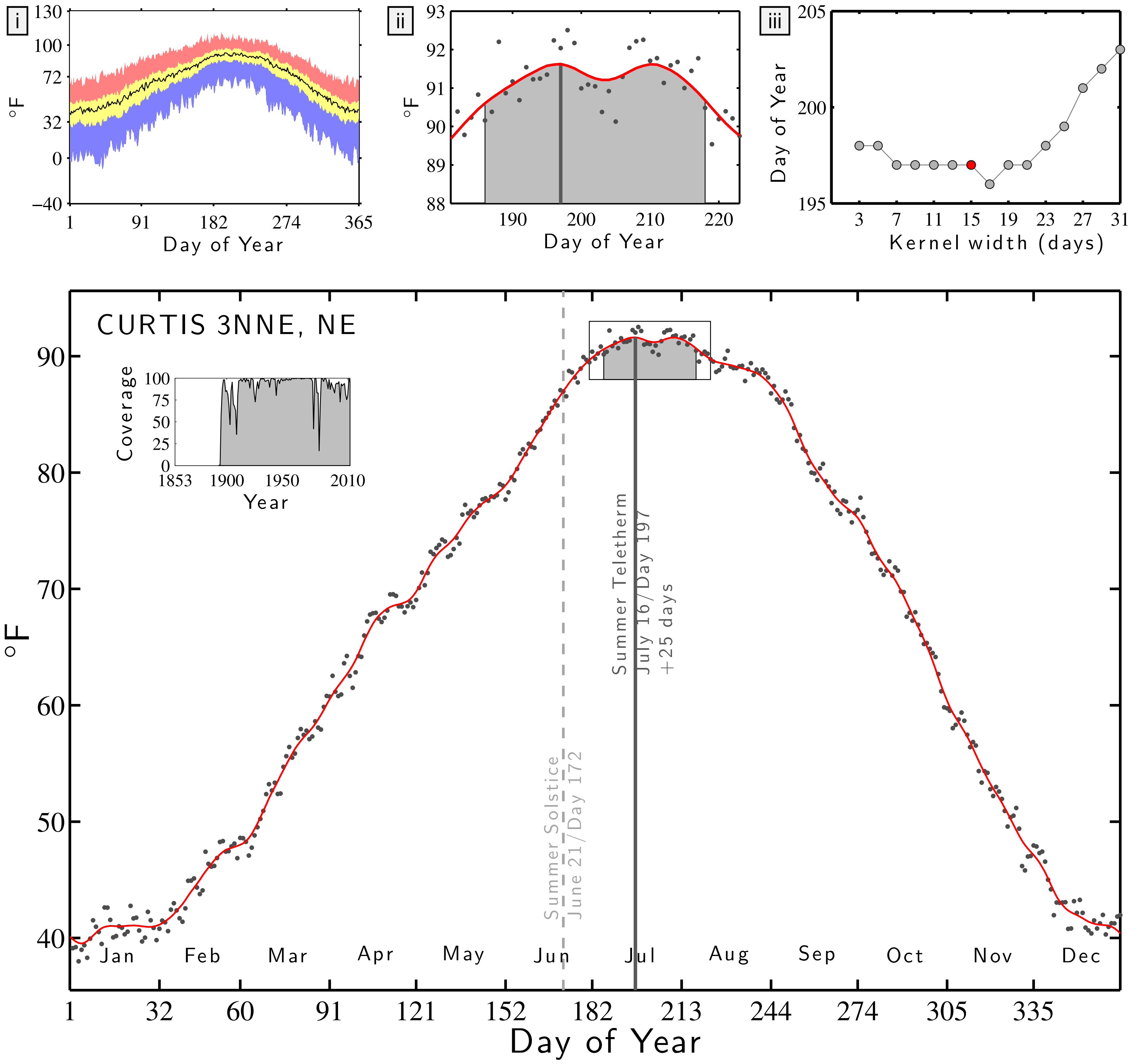

i
°F
Day of Year
ii
°F
Day of Year
iii
Day of Year
Kernel width (days)
CURTIS 3NNE, NE
Coverage
Year
Summer Solstice
June 21/Day 172
Summer Teletherm
July 16/Day 197
+25 days
Jan
Feb
Mar
Apr
May
Jun
Jul
Aug
Sep
Oct
Nov
Dec
°F
Day of Year

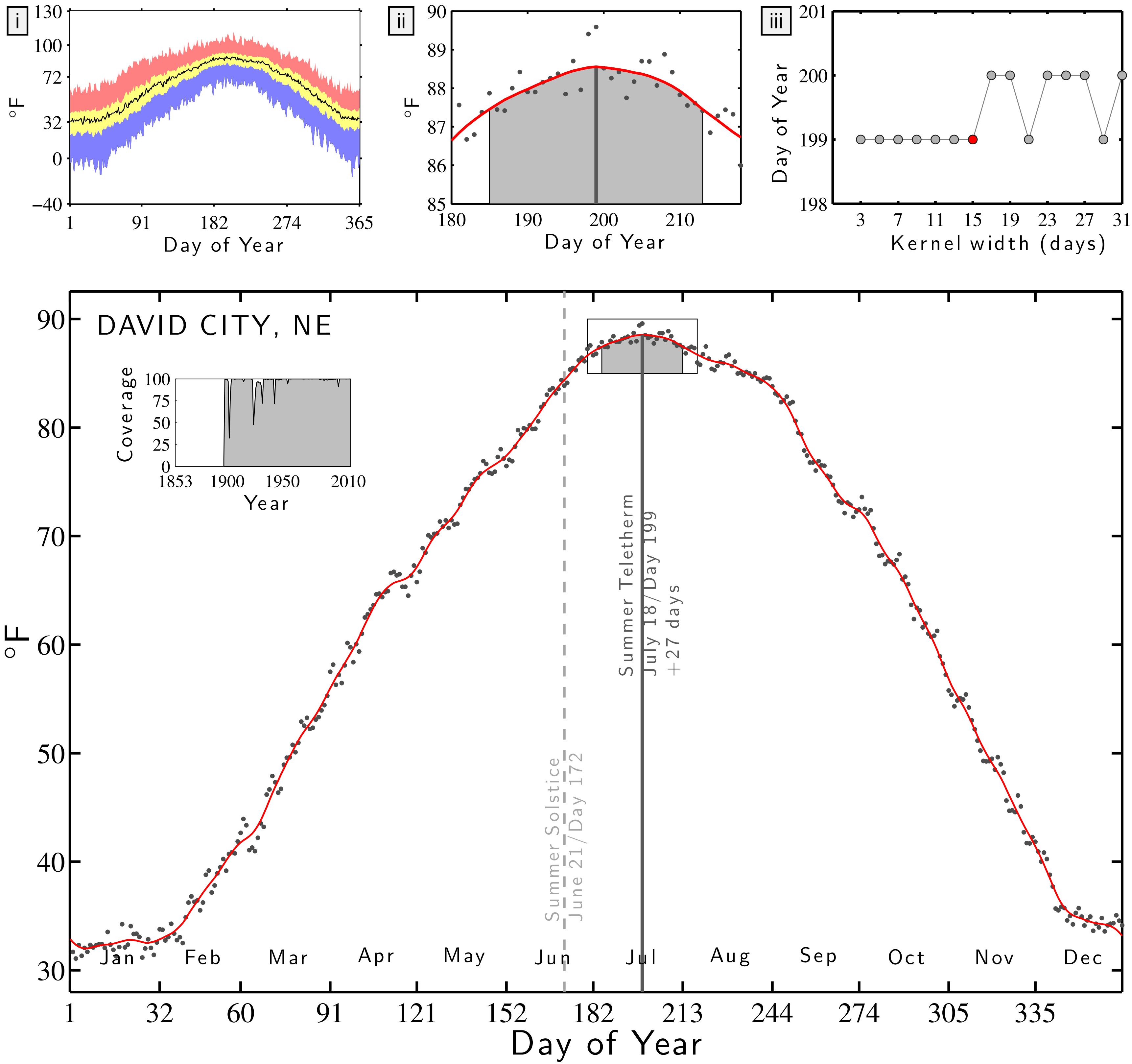

i
°F
Day of Year
ii
°F
Day of Year
iii
Day of Year
Kernel width (days)
DAVID CITY, NE
Coverage
Year
Summer Solstice
June 21/Day 172
Summer Teletherm
July 18/Day 199
+27 days
Jan
Feb
Mar
Apr
May
Jun
Jul
Aug
Sep
Oct
Nov
Dec
°F
Day of Year

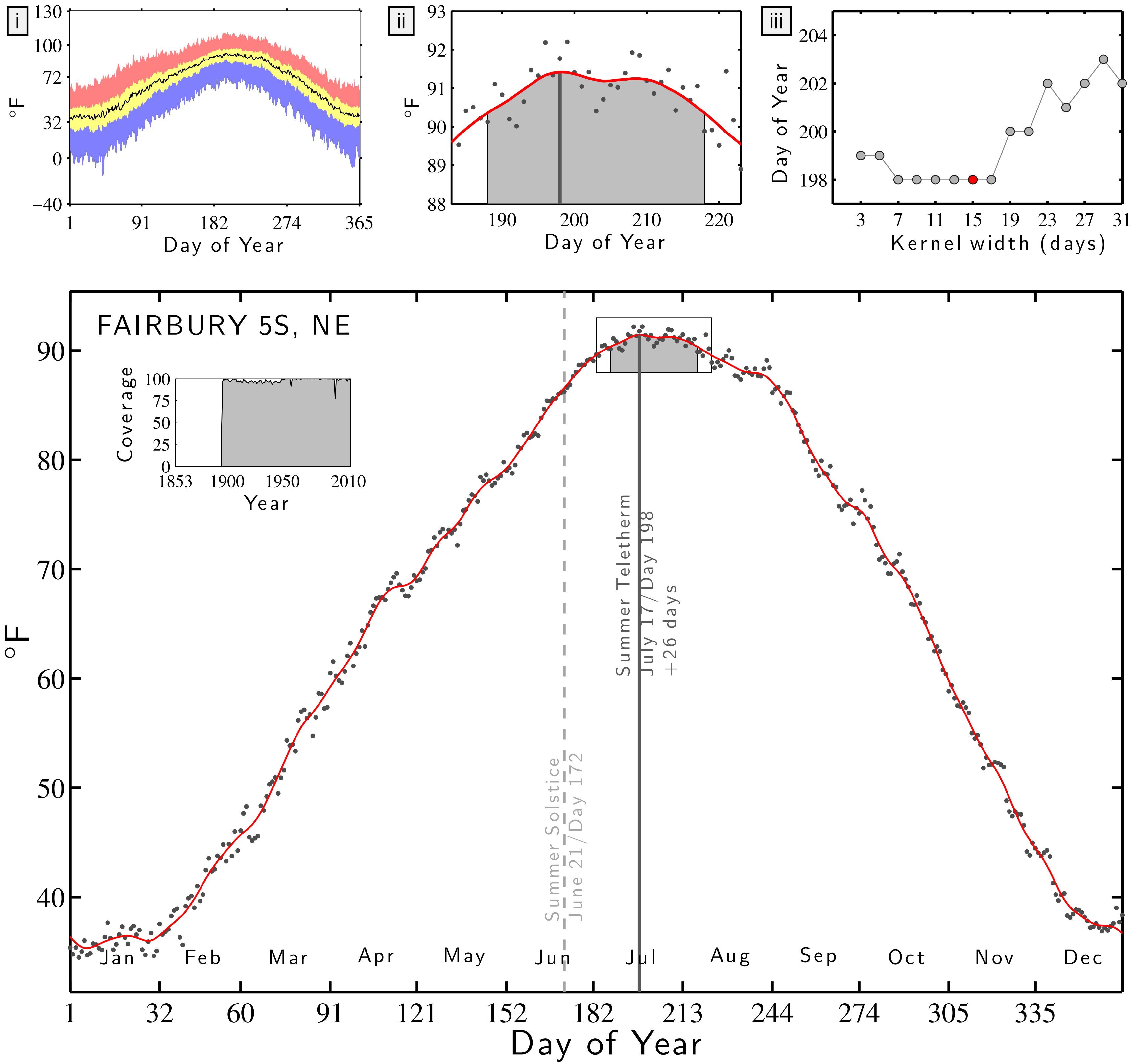

i
°F
Day of Year
ii
°F
Day of Year
iii
Day of Year
Kernel width (days)
FAIRBURY 5S, NE
Coverage
Year
Summer Solstice
June 21/Day 172
Summer Teletherm
July 17/Day 198
+26 days
Jan
Feb
Mar
Apr
May
Jun
Jul
Aug
Sep
Oct
Nov
Dec
°F
Day of Year

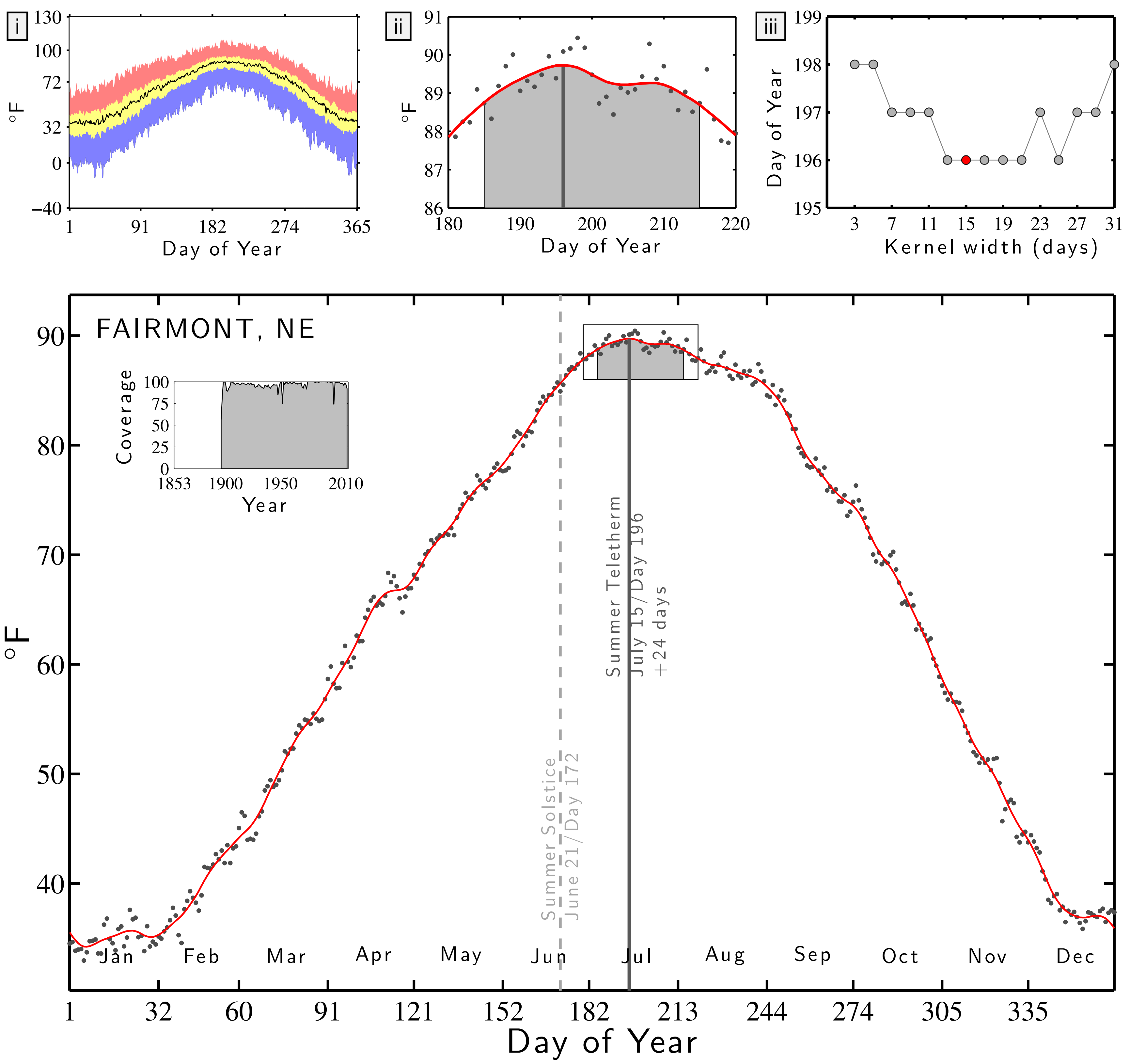

i
°F
Day of Year
ii
°F
Day of Year
iii
Day of Year
Kernel width (days)
FAIRMONT, NE
Coverage
Year
Summer Solstice
June 21/Day 172
Summer Teletherm
July 15/Day 196
+24 days
Jan
Feb
Mar
Apr
May
Jun
Jul
Aug
Sep
Oct
Nov
Dec
°F
Day of Year

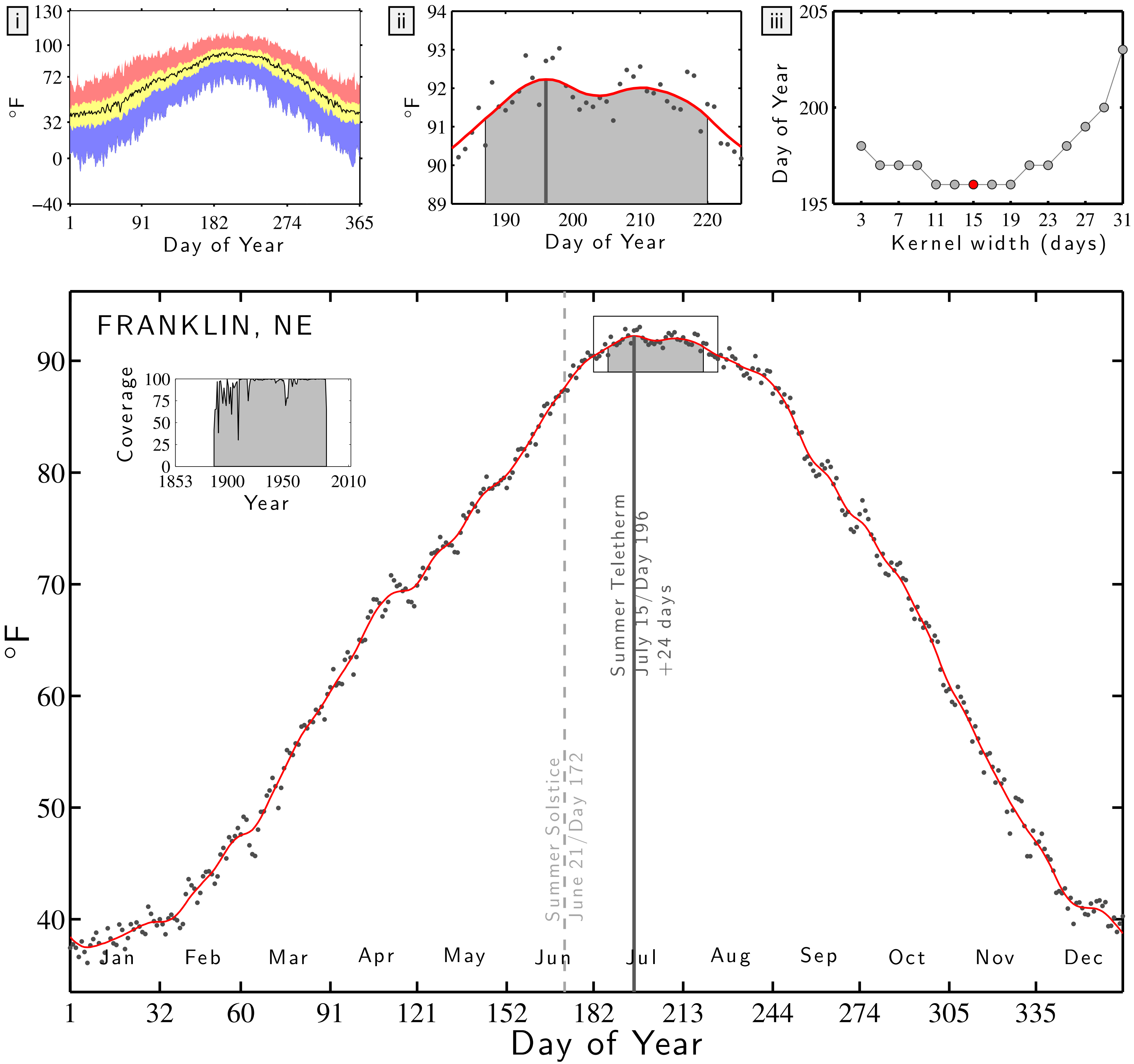
i
ii
iii
°F
Day of Year
Kernel width (days)
FRANKLIN, NE
Coverage
Year
Summer Solstice
June 21/Day 172
Summer Teletherm
July 15/Day 196
+24 days
Jan
Feb
Mar
Apr
May
Jun
Jul
Aug
Sep
Oct
Nov
Dec
Day of Year

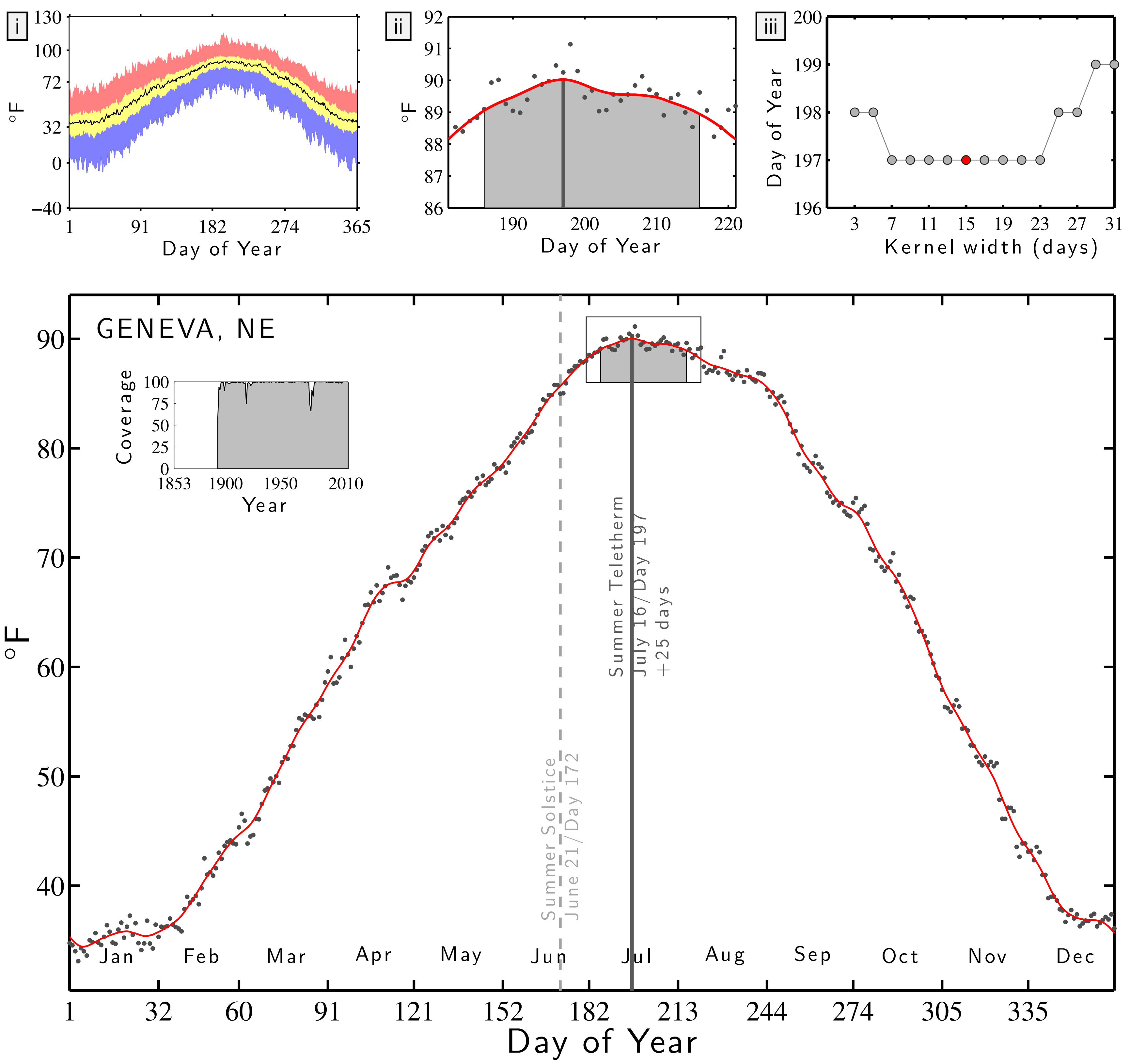

i
°F
130
100
72
32
0
−40
1
91
182
274
365
Day of Year
ii
°F
92
91
90
89
88
87
86
190
200
210
220
Day of Year
iii
Day of Year
200
199
198
197
196
3
7
11
15
19
23
27
31
Kernel width (days)
GENEVA, NE
Coverage
100
75
50
25
0
1853
1900
1950
2010
Year
Summer Solstice
June 21/Day 172
Summer Teletherm
July 16/Day 197
+25 days
°F
90
80
70
60
50
40
Jan
Feb
Mar
Apr
May
Jun
Jul
Aug
Sep
Oct
Nov
Dec
1
32
60
91
121
152
182
213
244
274
305
335
Day of Year

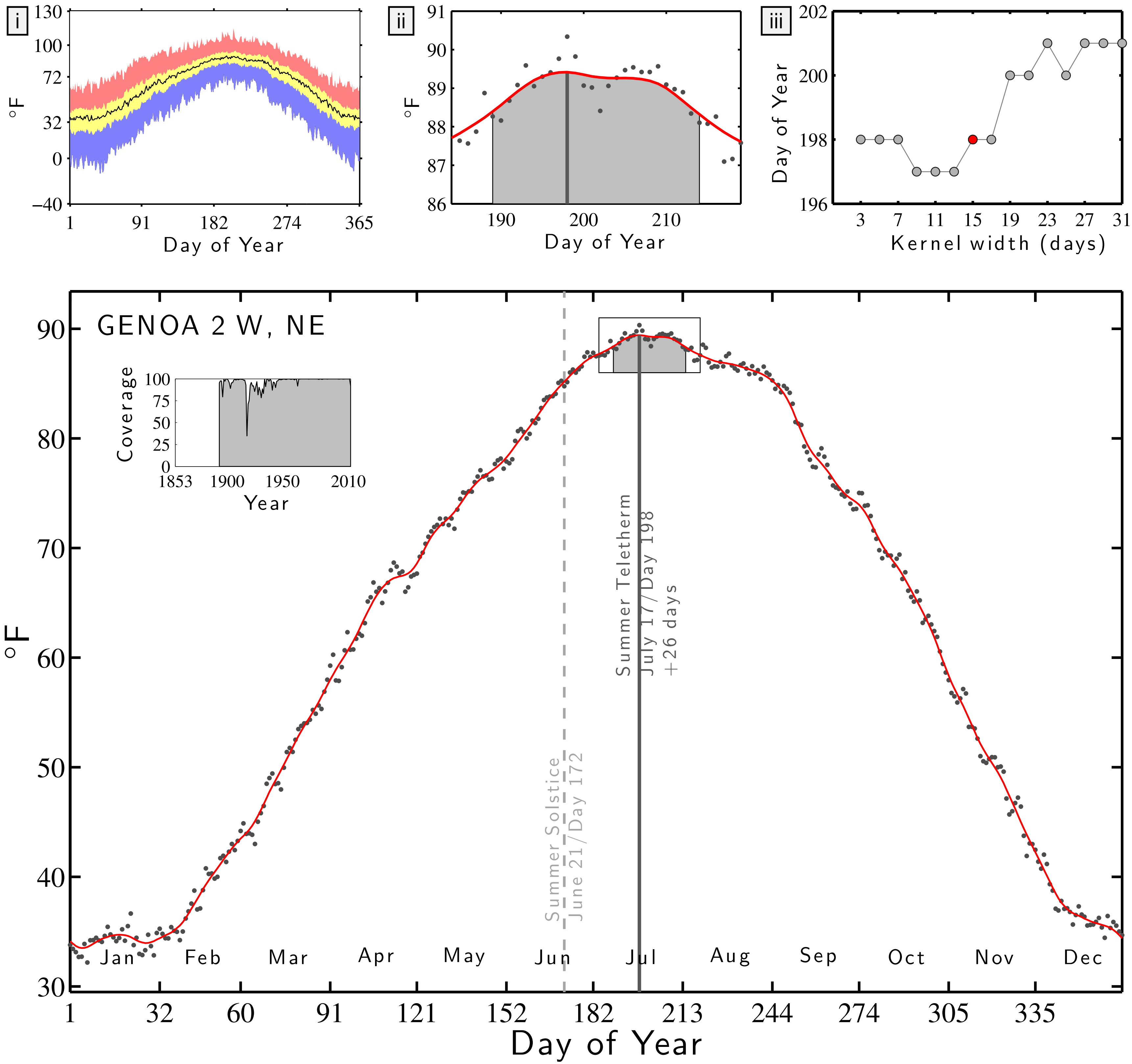

i
ii
iii
°F
Day of Year
Kernel width (days)
GENOA 2 W, NE
Coverage
Year
Summer Solstice
June 21/Day 172
Summer Teletherm
July 17/Day 198
+26 days
Jan
Feb
Mar
Apr
May
Jun
Jul
Aug
Sep
Oct
Nov
Dec

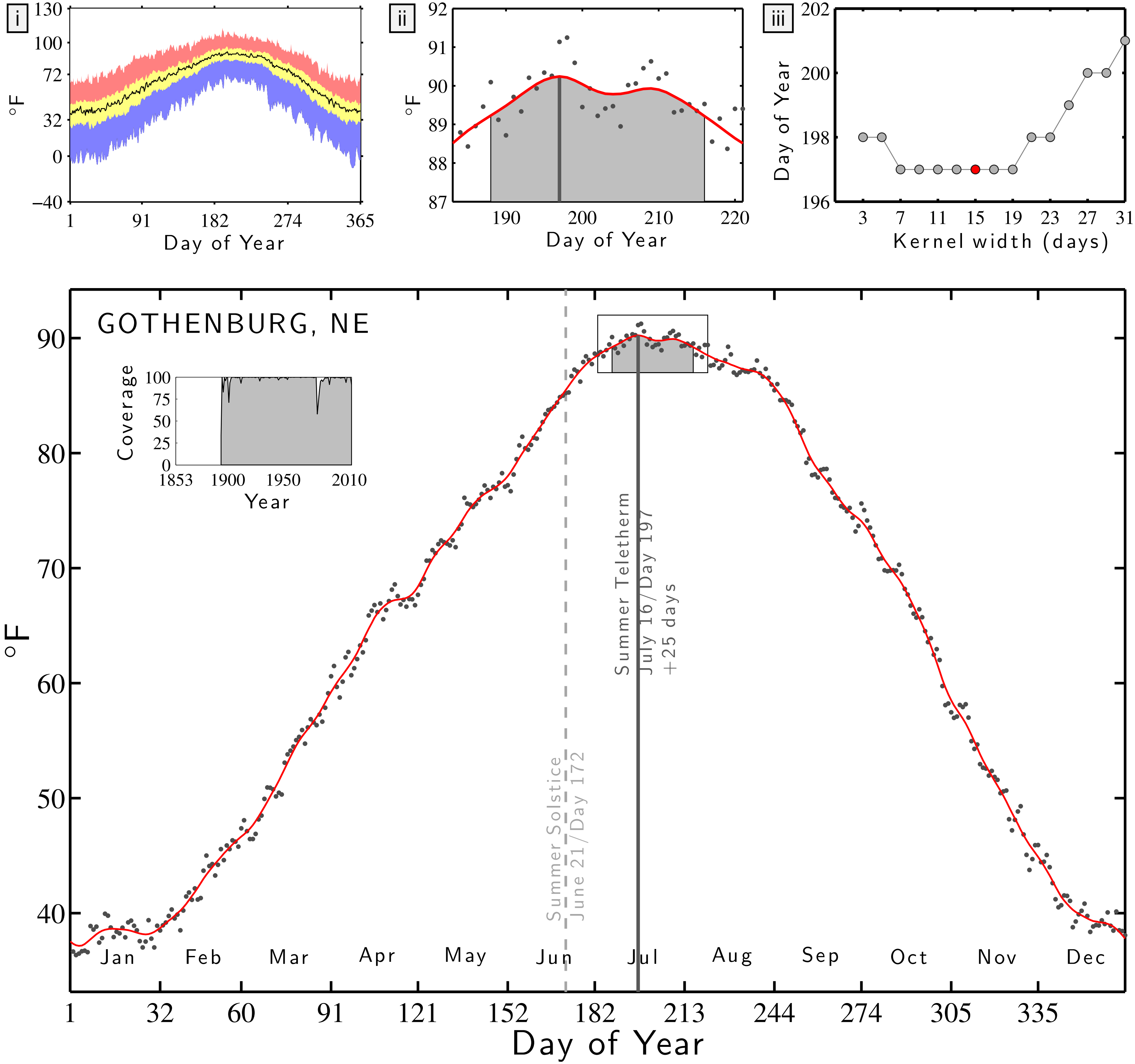

i
ii
iii
°F
Day of Year
Day of Year
Kernel width (days)
GOTHENBURG, NE
Coverage
Year
Summer Solstice
June 21/Day 172
Summer Teletherm
July 16/Day 197
+25 days
Jan
Feb
Mar
Apr
May
Jun
Jul
Aug
Sep
Oct
Nov
Dec
°F
Day of Year

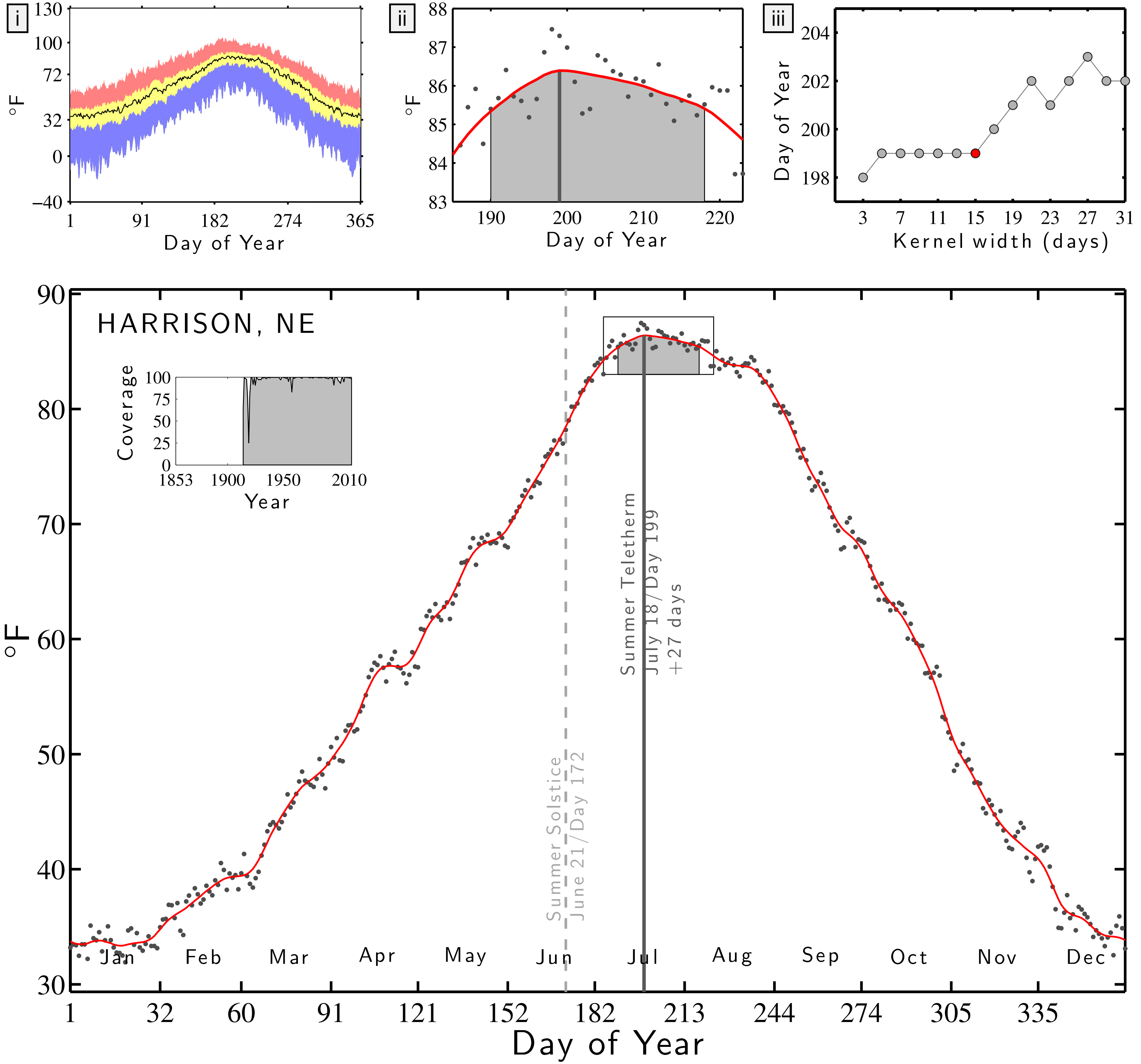

i
°F
Day of Year
ii
°F
Day of Year
iii
Day of Year
Kernel width (days)
HARRISON, NE
Coverage
Year
Summer Solstice
June 21/Day 172
Summer Teletherm
July 18/Day 199
+27 days
Jan
Feb
Mar
Apr
May
Jun
Jul
Aug
Sep
Oct
Nov
Dec
°F
Day of Year

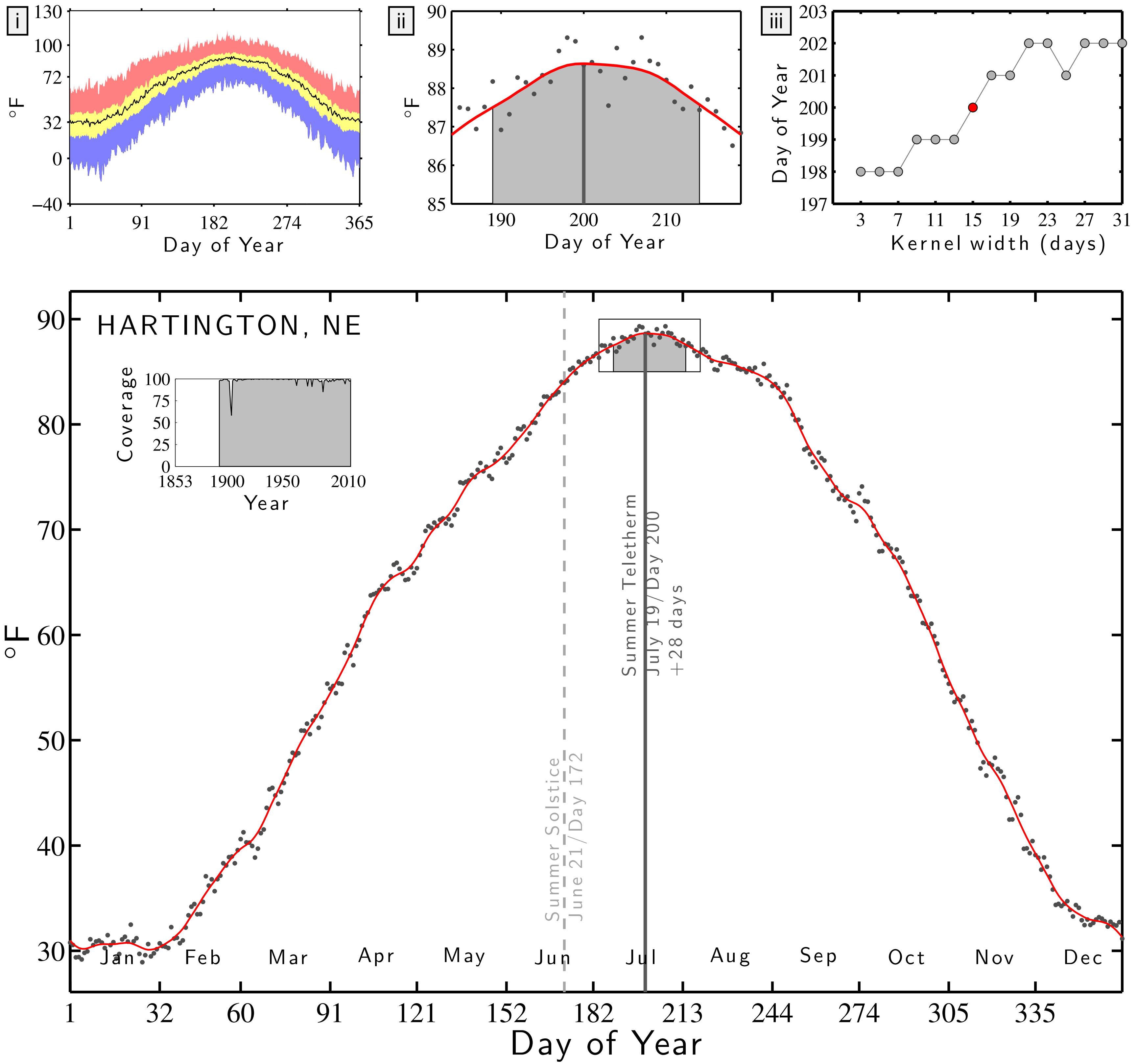

i
°F
Day of Year
ii
°F
Day of Year
iii
Day of Year
Kernel width (days)
HARTINGTON, NE
Coverage
Year
Summer Solstice
June 21/Day 172
Summer Teletherm
July 19/Day 200
+28 days
Jan
Feb
Mar
Apr
May
Jun
Jul
Aug
Sep
Oct
Nov
Dec
°F
Day of Year

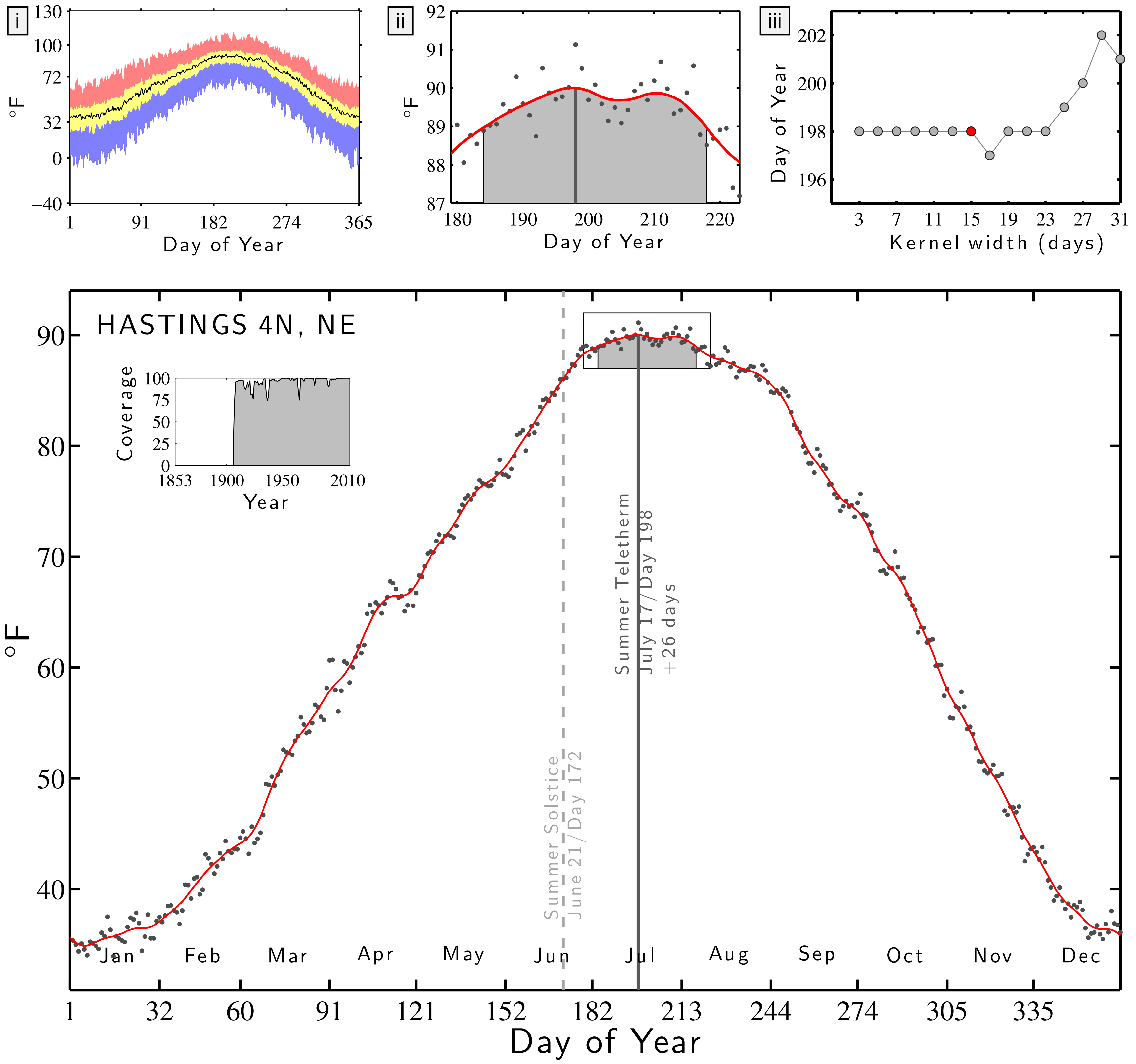

i
ii
iii
°F
Day of Year
Day of Year
Kernel width (days)
HASTINGS 4N, NE
Coverage
Year
Summer Solstice
June 21/Day 172
Summer Teletherm
July 17/Day 198
+26 days
Jan
Feb
Mar
Apr
May
Jun
Jul
Aug
Sep
Oct
Nov
Dec

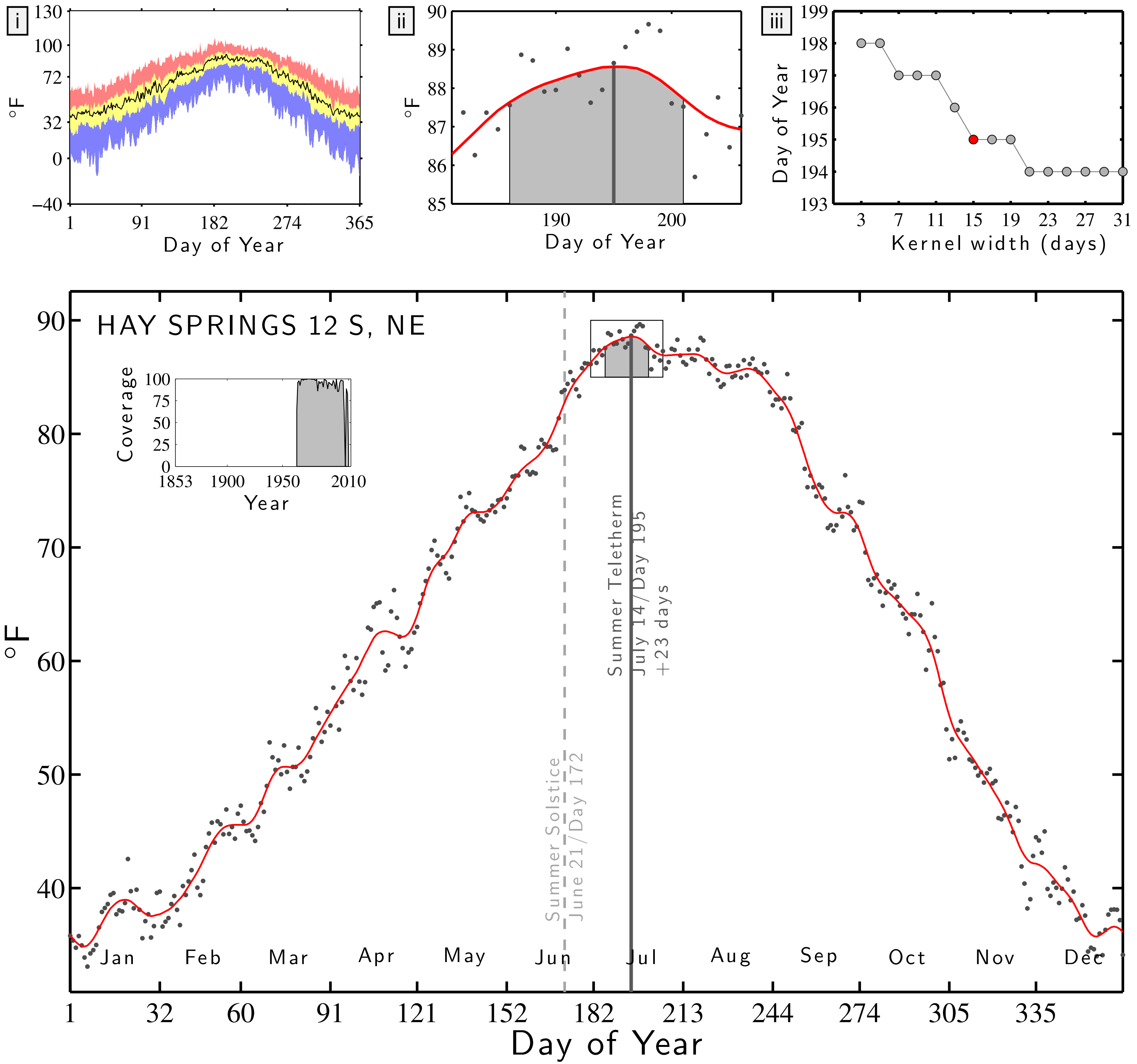

i
°F
Day of Year
ii
°F
Day of Year
iii
Day of Year
Kernel width (days)
HAY SPRINGS 12 S, NE
Coverage
Year
Summer Solstice
June 21/Day 172
Summer Teletherm
July 14/Day 195
+23 days
Jan
Feb
Mar
Apr
May
Jun
Jul
Aug
Sep
Oct
Nov
Dec
°F
Day of Year

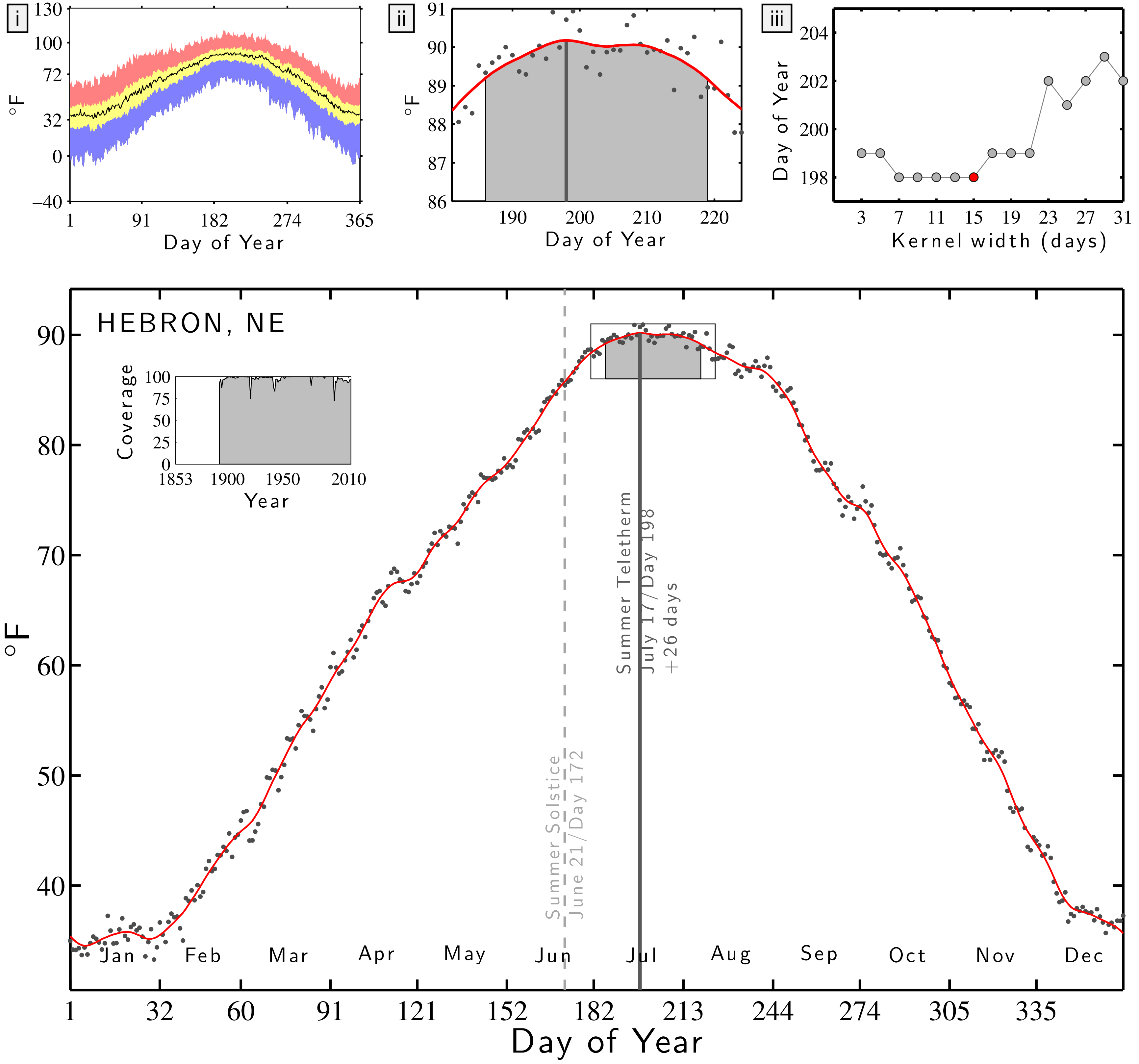

i
130
100
72
32
0
−40
°F
1
91
182
274
365
Day of Year
ii
91
90
89
88
87
86
°F
190
200
210
220
Day of Year
iii
204
202
200
198
Day of Year
3
7
11
15
19
23
27
31
Kernel width (days)
HEBRON, NE
Coverage
100
75
50
25
0
1853
1900
1950
2010
Year
Summer Solstice
June 21 / Day 172
Summer Teletherm
July 17 / Day 198
+26 days
90
80
70
60
50
40
°F
Jan
Feb
Mar
Apr
May
Jun
Jul
Aug
Sep
Oct
Nov
Dec
1
32
60
91
121
152
182
213
244
274
305
335
Day of Year

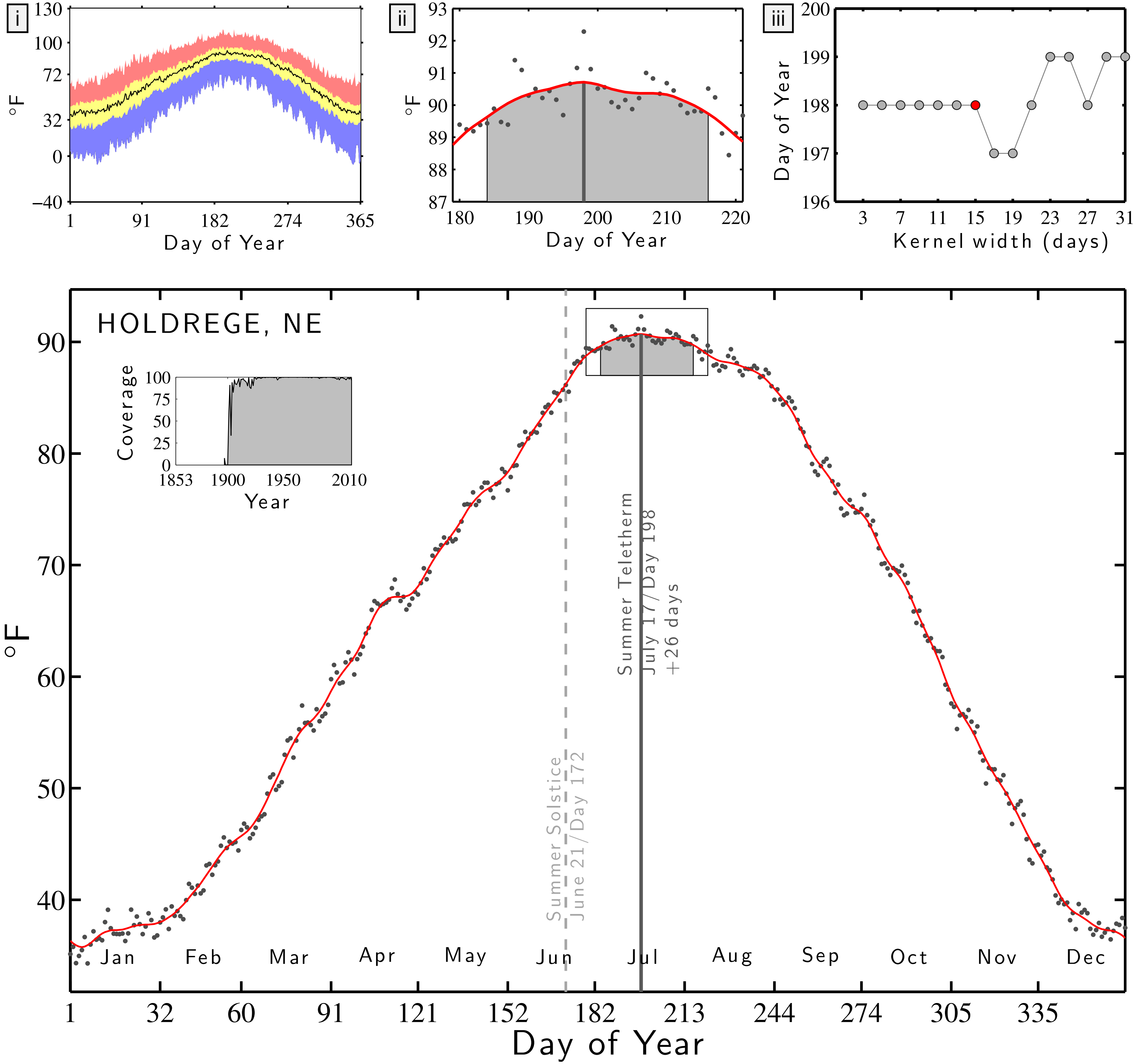

i
130
100
72
32
0
−40
°F
1
91
182
274
365
Day of Year
ii
93
92
91
90
89
88
87
°F
180
190
200
210
220
Day of Year
iii
200
199
198
197
196
Day of Year
3
7
11
15
19
23
27
31
Kernel width (days)
HOLDREGE, NE
Coverage
100
75
50
25
0
1853
1900
1950
2010
Year
Summer Solstice
June 21/Day 172
Summer Teletherm
July 17/Day 198
+26 days
Jan
Feb
Mar
Apr
May
Jun
Jul
Aug
Sep
Oct
Nov
Dec
90
80
70
60
50
40
°F
1
32
60
91
121
152
182
213
244
274
305
335
Day of Year

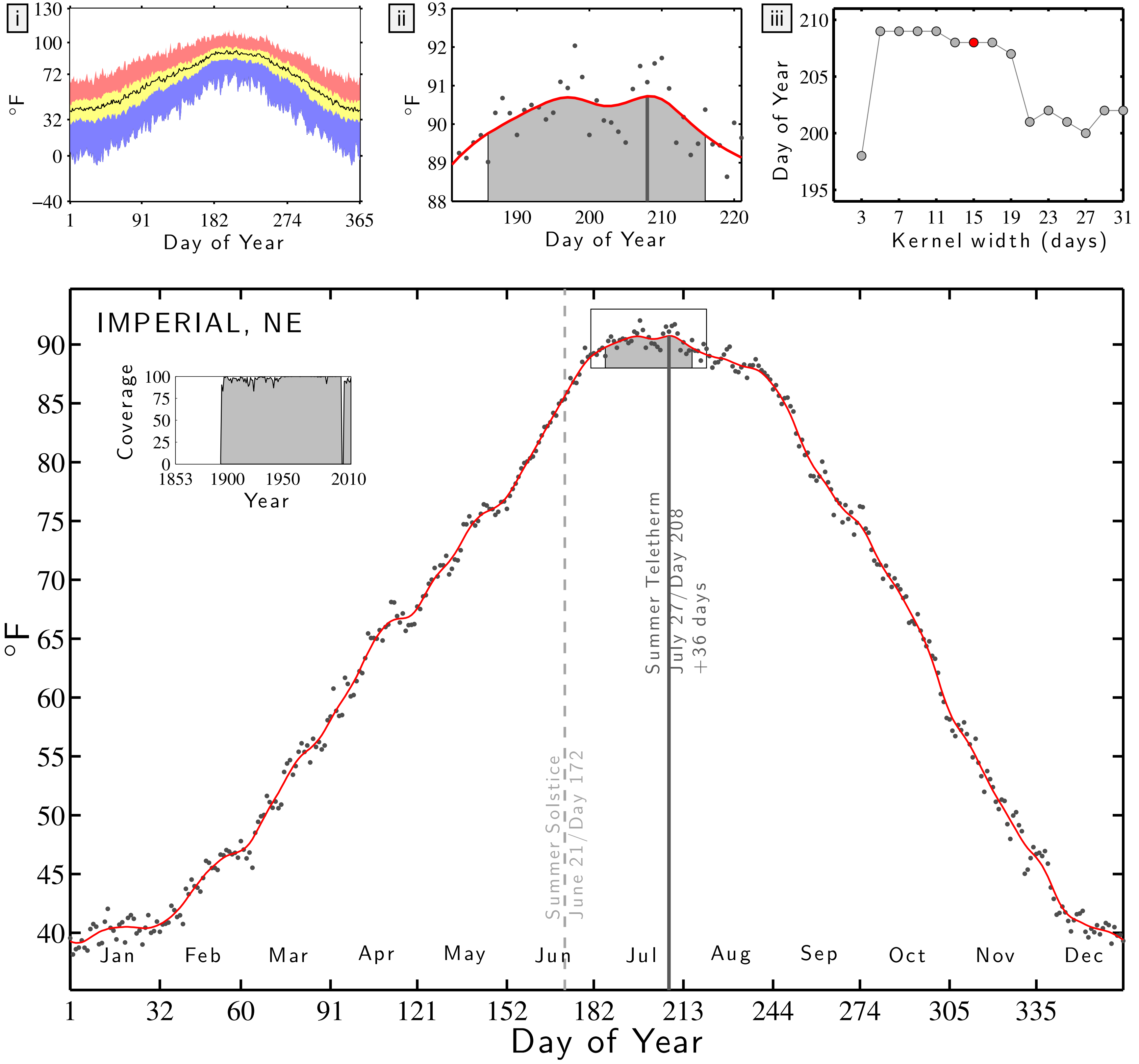

i
ii
iii
Day of Year
°F
Kernel width (days)
IMPERIAL, NE
Coverage
Year
Summer Solstice
June 21 / Day 172
Summer Teletherm
July 27 / Day 208
+36 days
Jan
Feb
Mar
Apr
May
Jun
Jul
Aug
Sep
Oct
Nov
Dec

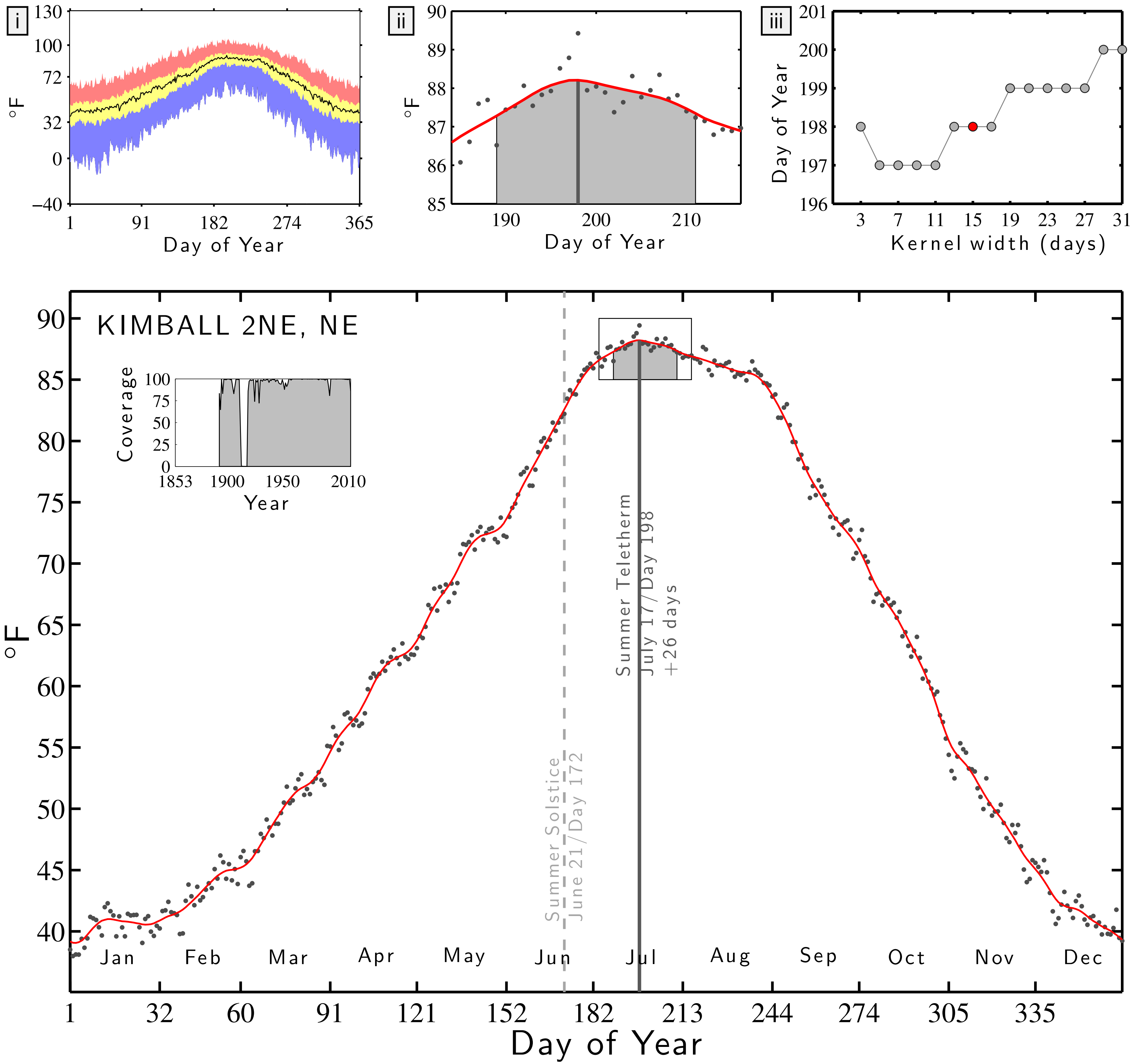

i
ii
iii
°F
Day of Year
Kernel width (days)
KIMBALL 2NE, NE
Coverage
Year
Summer Solstice
June 21/Day 172
Summer Teletherm
July 17/Day 198
+26 days
Jan
Feb
Mar
Apr
May
Jun
Jul
Aug
Sep
Oct
Nov
Dec

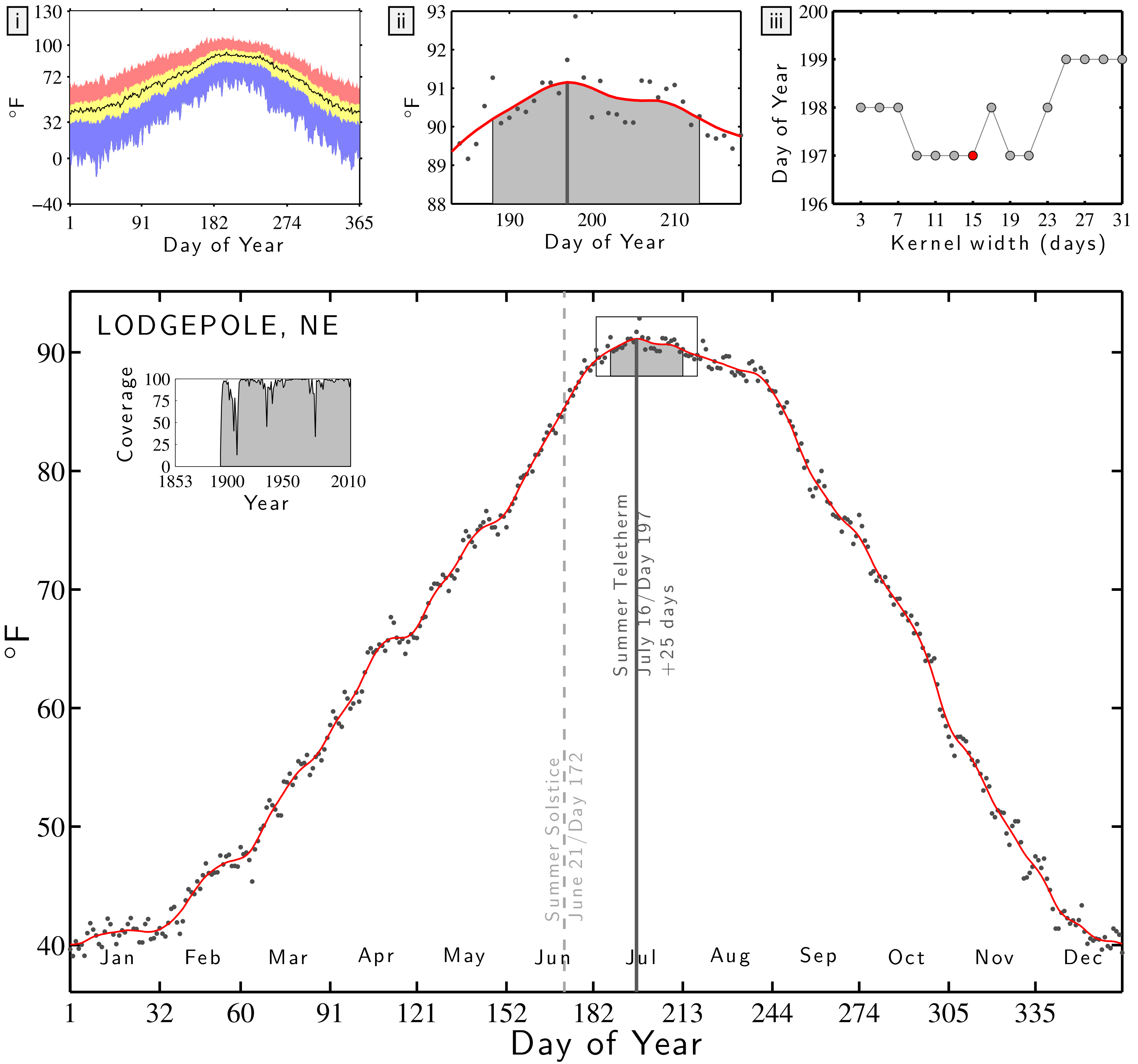

i
ii
iii
°F
Day of Year
Kernel width (days)
LODGEPOLE, NE
Coverage
Year
Summer Solstice
June 21/Day 172
Summer Teletherm
July 16/Day 197
+25 days
Jan
Feb
Mar
Apr
May
Jun
Jul
Aug
Sep
Oct
Nov
Dec

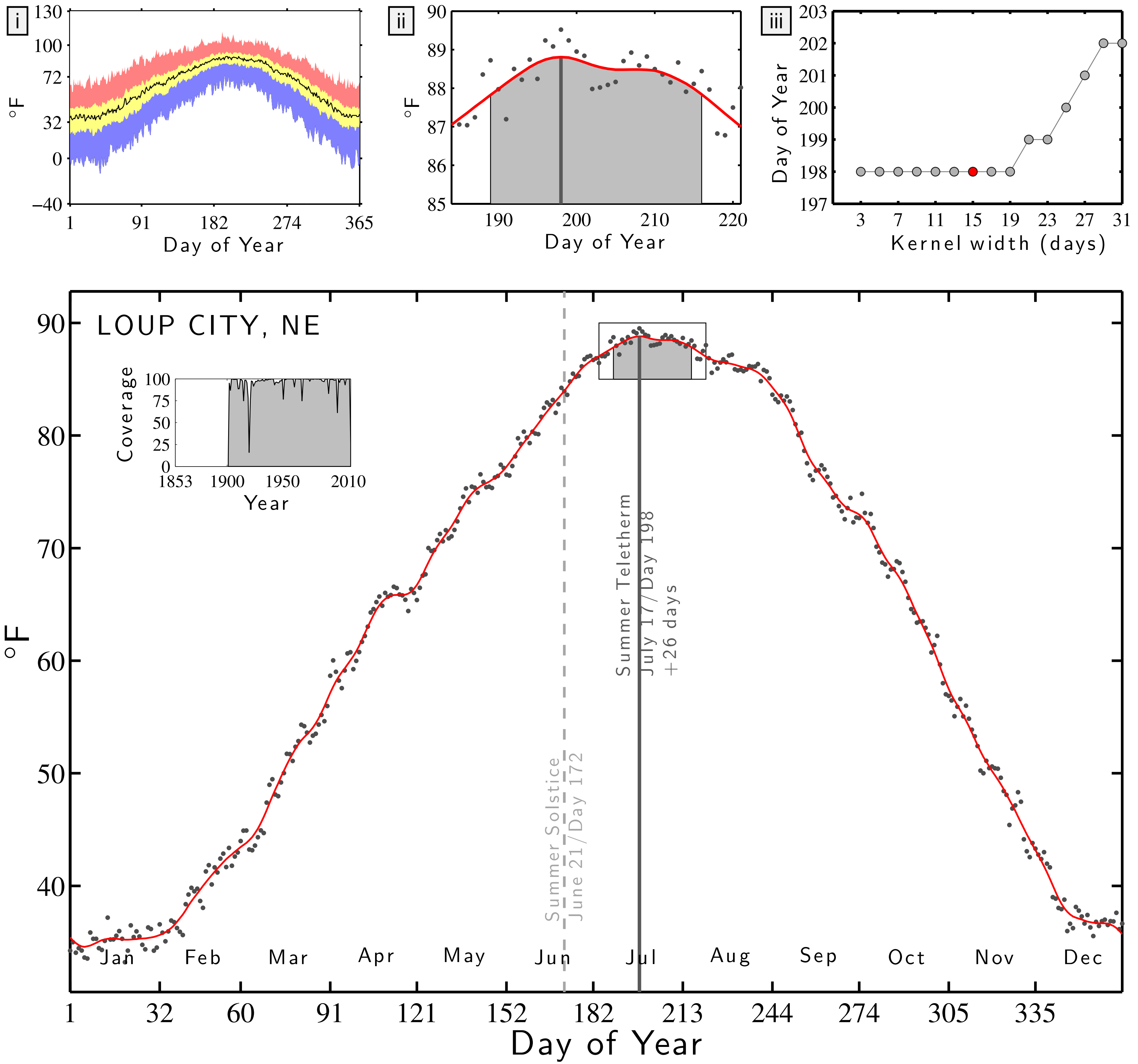
i
°F
Day of Year
ii
°F
Day of Year
iii
Day of Year
Kernel width (days)
LOUP CITY, NE
Coverage
Year
Summer Solstice
June 21 / Day 172
Summer Teletherm
July 17 / Day 198
+26 days
Jan
Feb
Mar
Apr
May
Jun
Jul
Aug
Sep
Oct
Nov
Dec
°F
Day of Year

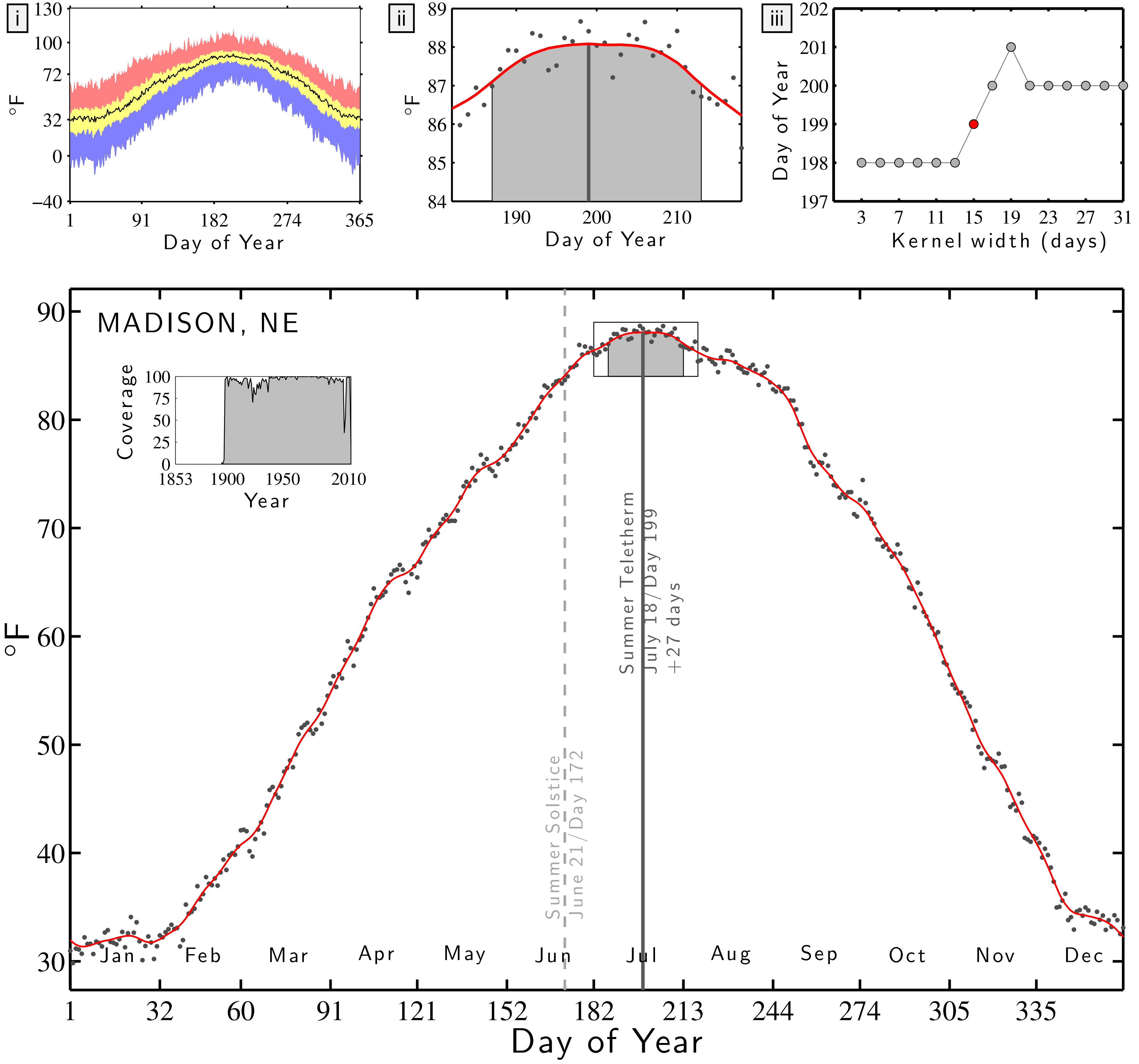

i
ii
iii
°F
Day of Year
Day of Year
Kernel width (days)
MADISON, NE
Coverage
Year
Summer Solstice
June 21/Day 172
Summer Teletherm
July 18/Day 199
+27 days
Jan
Feb
Mar
Apr
May
Jun
Jul
Aug
Sep
Oct
Nov
Dec

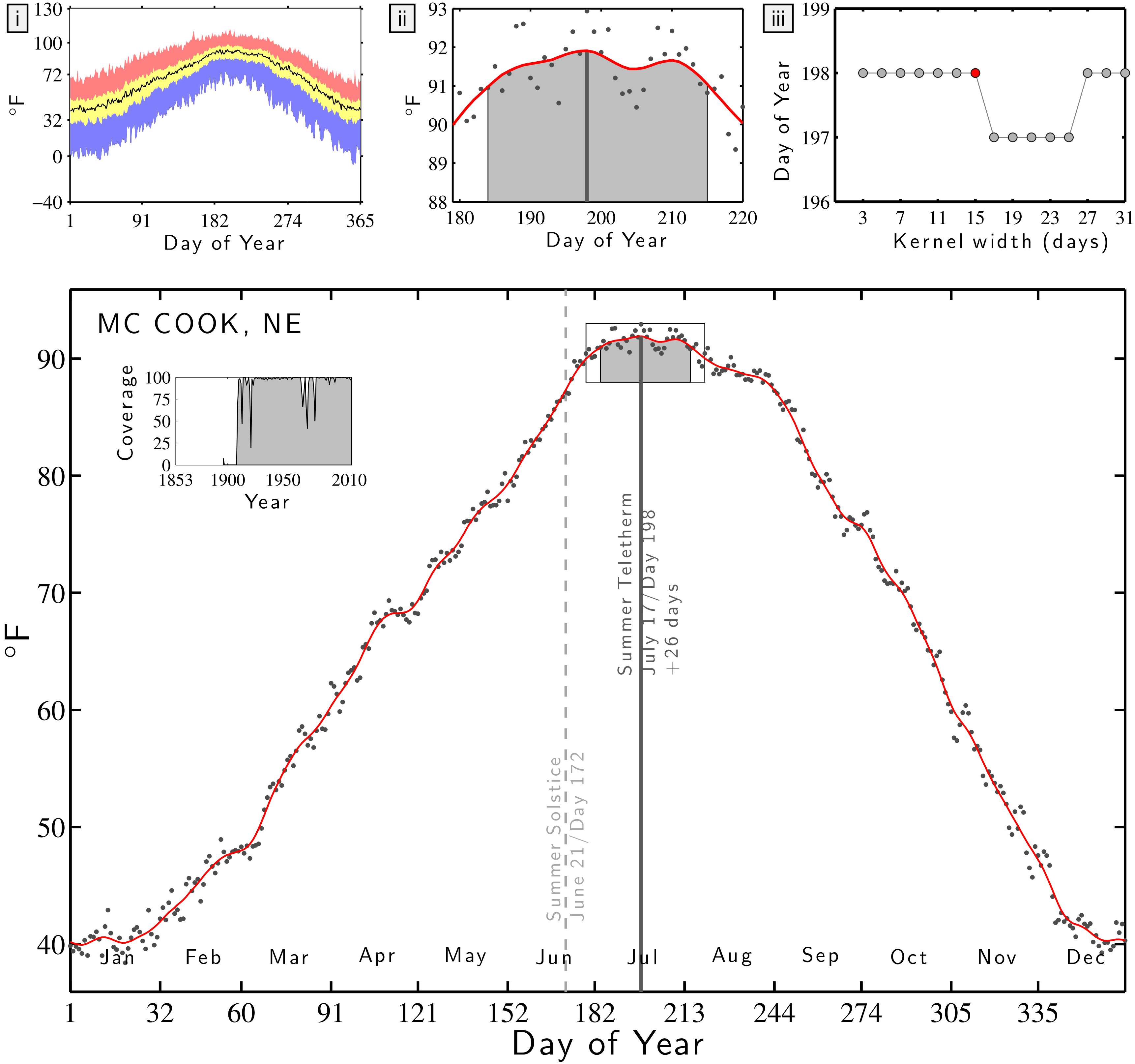

i
130
100
72
32
0
−40
°F
1
91
182
274
365
Day of Year
ii
93
92
91
90
89
88
°F
180
190
200
210
220
Day of Year
iii
199
198
197
196
Day of Year
3
7
11
15
19
23
27
31
Kernel width (days)
MC COOK, NE
Coverage
100
75
50
25
0
1853
1900
1950
2010
Year
Summer Solstice
June 21 / Day 172
Summer Teletherm
July 17 / Day 198
+26 days
Jan
Feb
Mar
Apr
May
Jun
Jul
Aug
Sep
Oct
Nov
Dec
90
80
70
60
50
40
°F
1
32
60
91
121
152
182
213
244
274
305
335
Day of Year

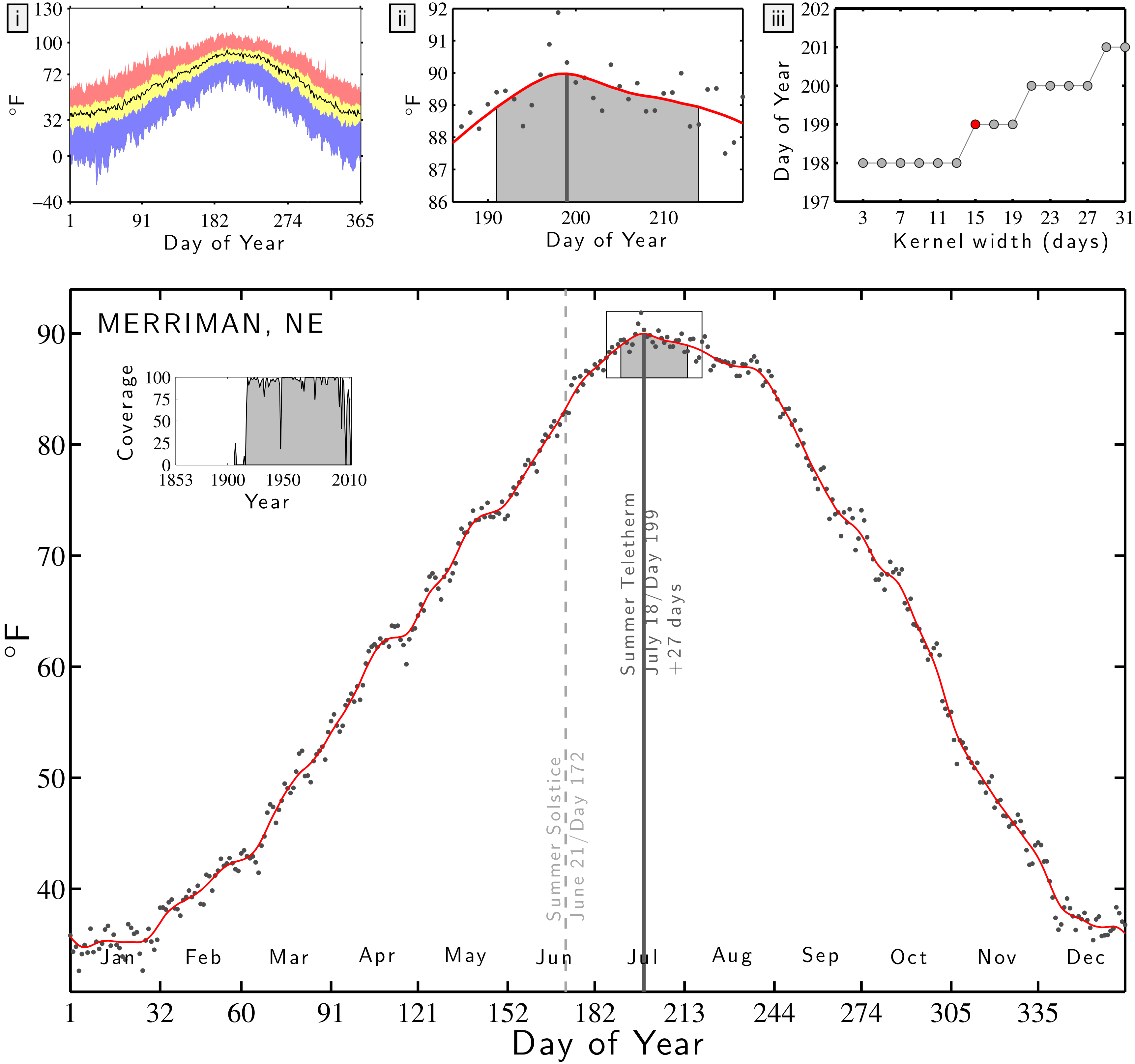

i
ii
iii
°F
Day of Year
Kernel width (days)
MERRIMAN, NE
Coverage
Year
Summer Solstice
June 21/Day 172
Summer Teletherm
July 18/Day 199
+27 days
Jan
Feb
Mar
Apr
May
Jun
Jul
Aug
Sep
Oct
Nov
Dec

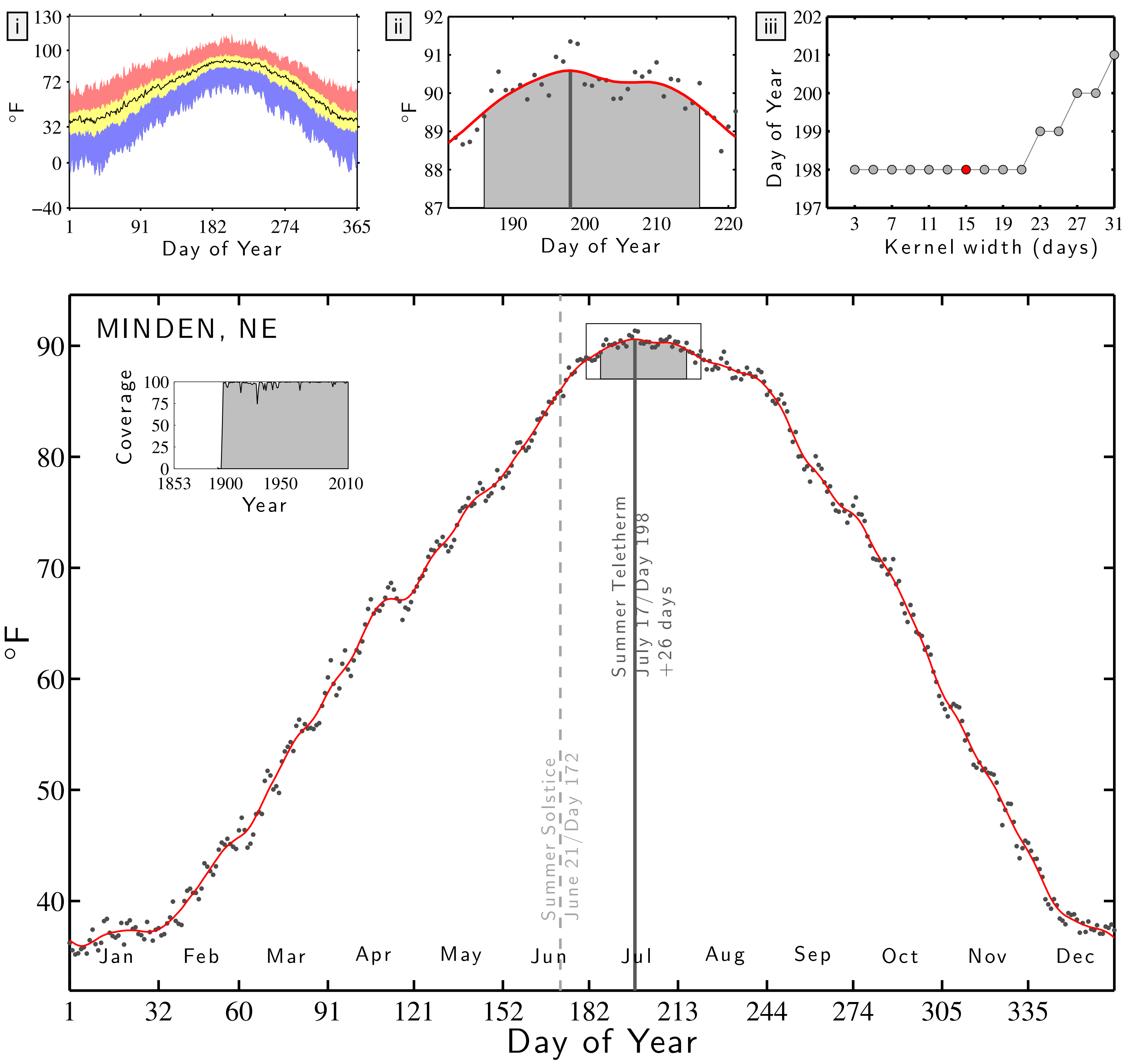

i
°F
Day of Year
ii
°F
Day of Year
iii
Day of Year
Kernel width (days)
MINDEN, NE
Coverage
Year
Summer Solstice
June 21/Day 172
Summer Teletherm
July 17/Day 198
+26 days
Jan
Feb
Mar
Apr
May
Jun
Jul
Aug
Sep
Oct
Nov
Dec
°F
Day of Year

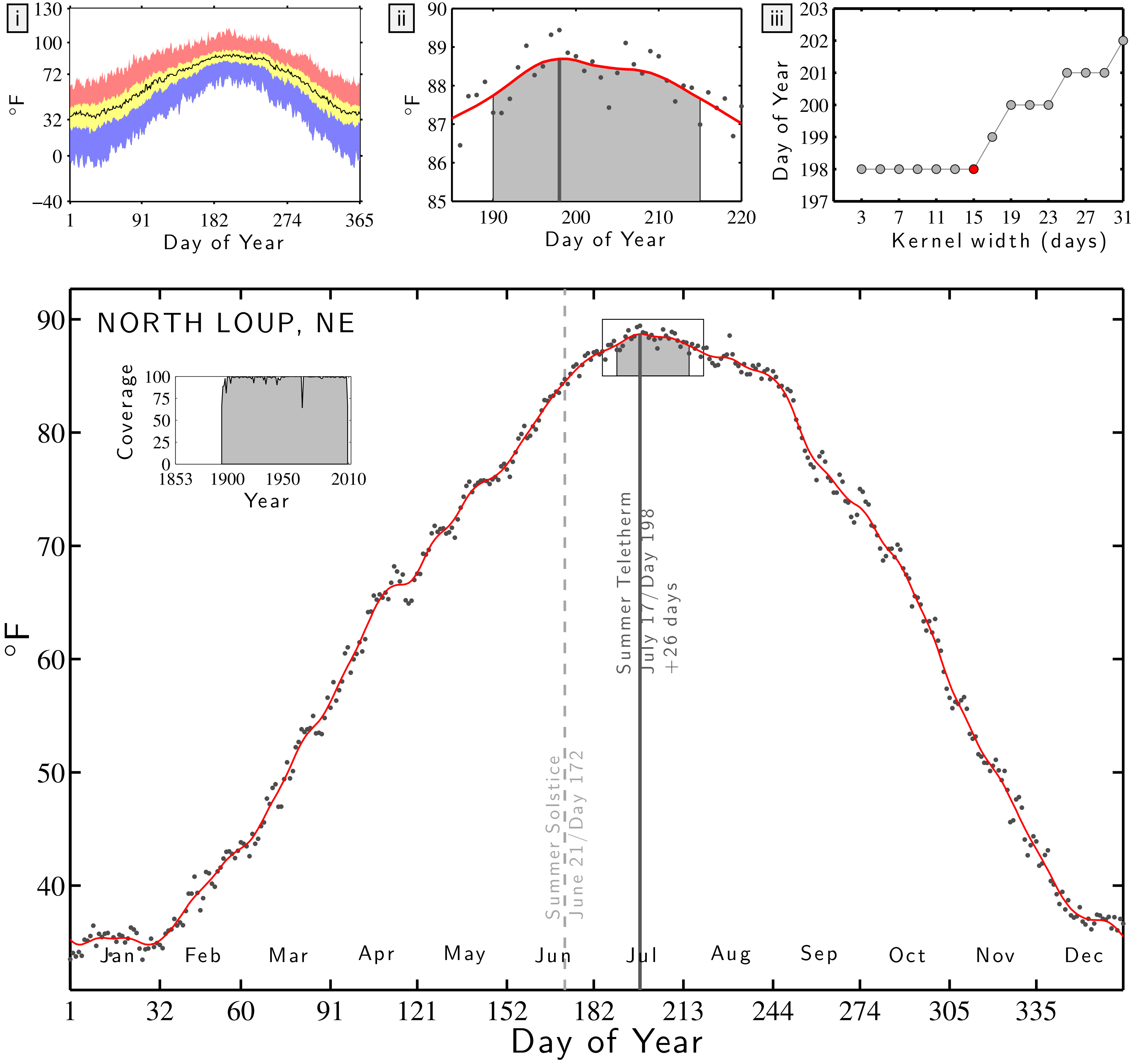

i
°F
Day of Year
ii
°F
Day of Year
iii
Day of Year
Kernel width (days)
NORTH LOUP, NE
Coverage
Year
Summer Solstice
June 21/Day 172
Summer Teletherm
July 17/Day 198
+26 days
Jan
Feb
Mar
Apr
May
Jun
Jul
Aug
Sep
Oct
Nov
Dec
°F
Day of Year

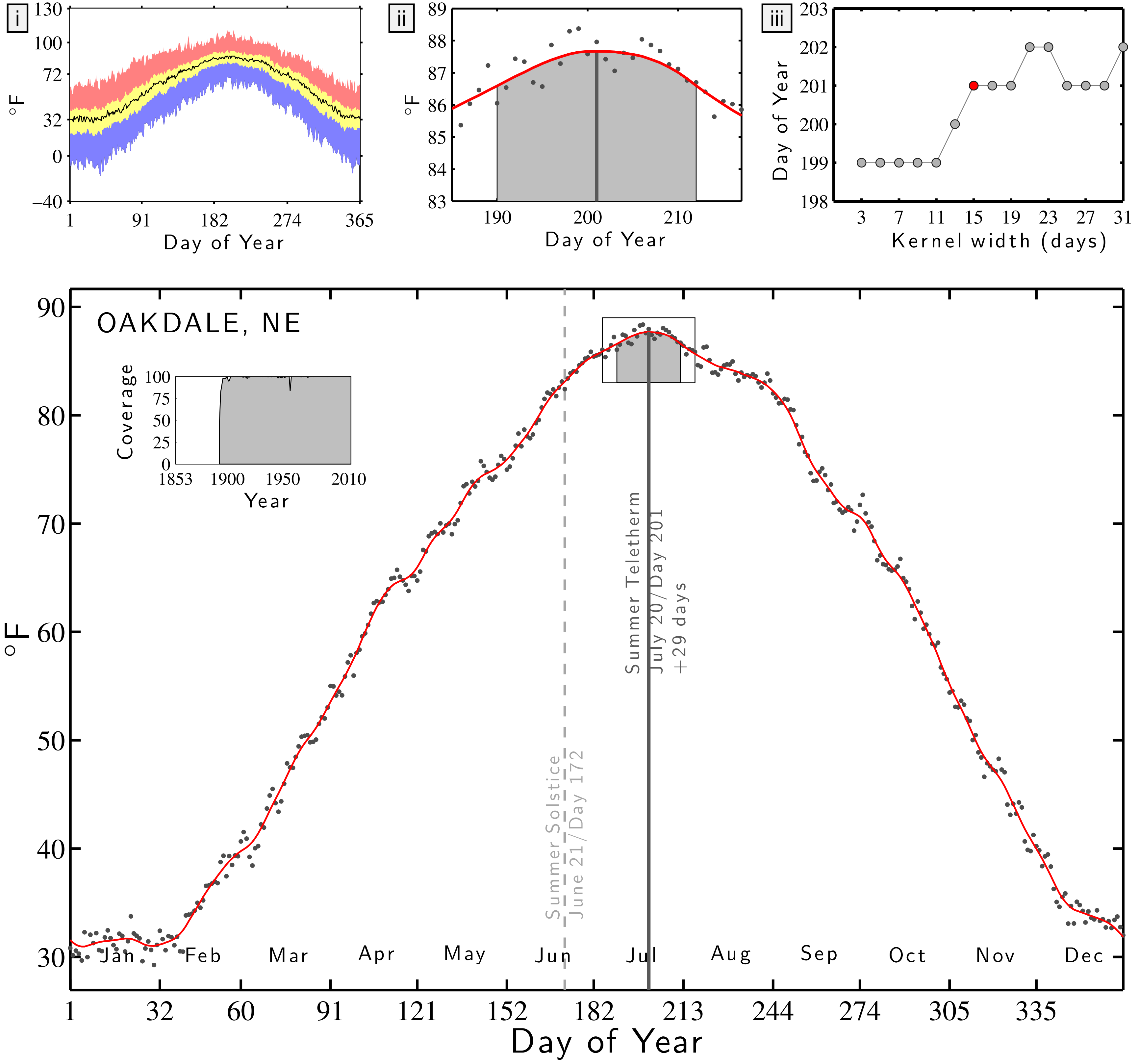

i
°F
Day of Year
ii
°F
Day of Year
iii
Day of Year
Kernel width (days)
OAKDALE, NE
Coverage
Year
Summer Solstice
June 21/Day 172
Summer Teletherm
July 20/Day 201
+29 days
Jan
Feb
Mar
Apr
May
Jun
Jul
Aug
Sep
Oct
Nov
Dec
°F
Day of Year

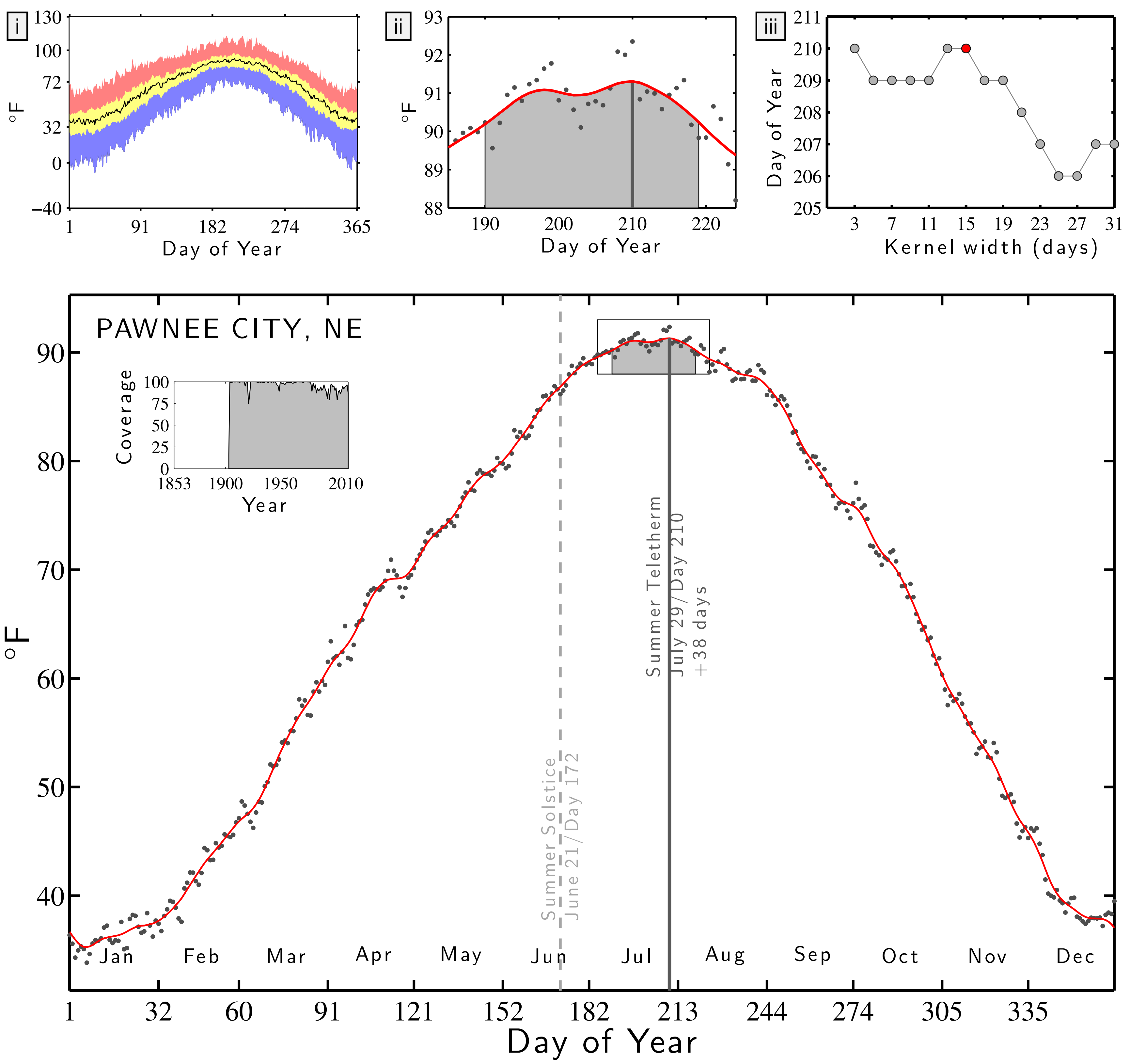

i
°F
130
100
72
32
0
−40
1
91
182
274
365
Day of Year
ii
°F
93
92
91
90
89
88
190
200
210
220
Day of Year
iii
Day of Year
211
210
209
208
207
206
205
3
7
11
15
19
23
27
31
Kernel width (days)
PAWNEE CITY, NE
Coverage
100
75
50
25
0
1853
1900
1950
2010
Year
°F
90
80
70
60
50
40
Summer Solstice
June 21 / Day 172
Summer Teletherm
July 29 / Day 210
+38 days
Jan
Feb
Mar
Apr
May
Jun
Jul
Aug
Sep
Oct
Nov
Dec
1
32
60
91
121
152
182
213
244
274
305
335
Day of Year

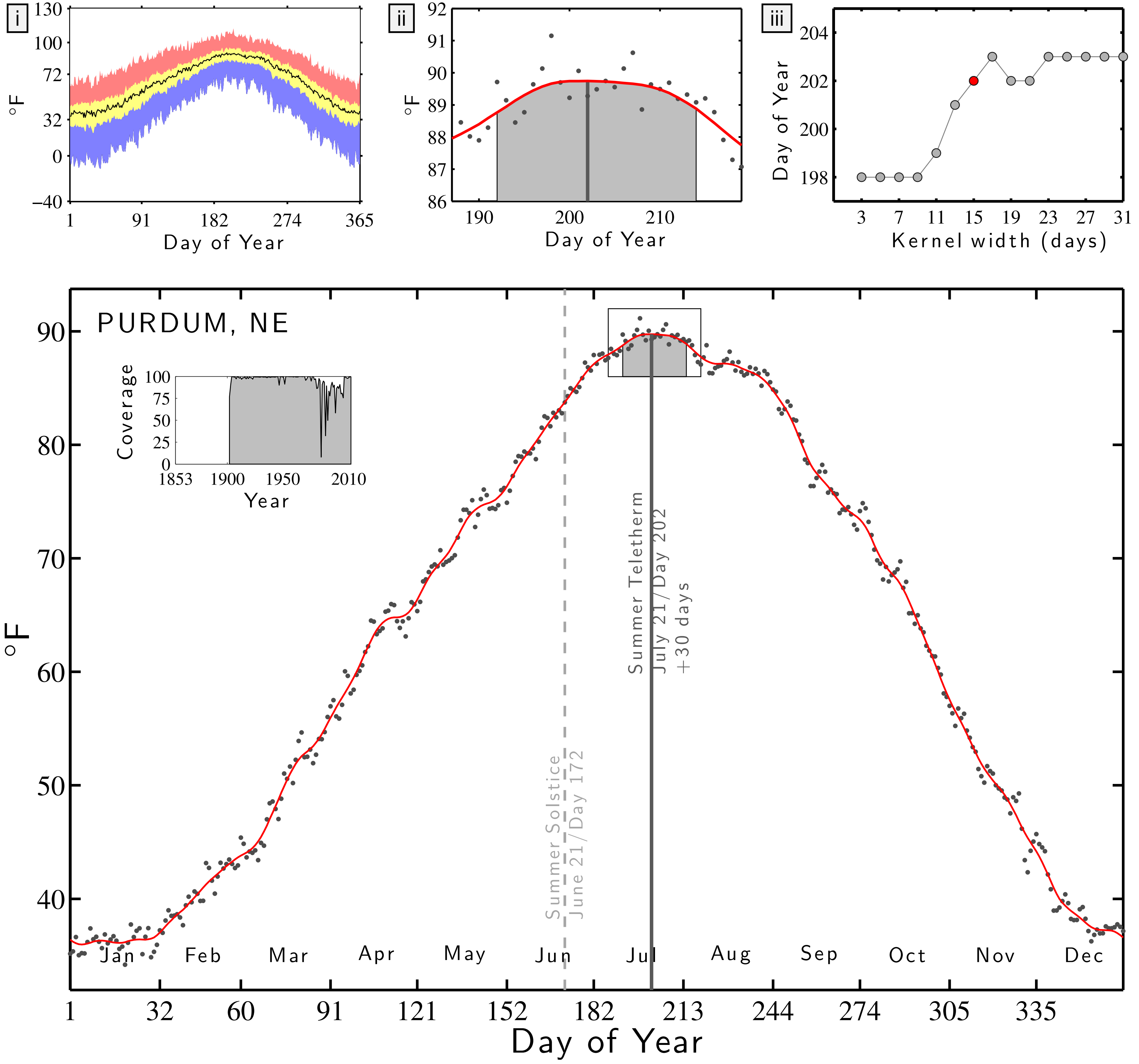

i
°F
130
100
72
32
0
−40
1
91
182
274
365
Day of Year
ii
92
91
90
89
88
87
86
190
200
210
Day of Year
iii
Day of Year
204
202
200
198
3
7
11
15
19
23
27
31
Kernel width (days)
PURDUM, NE
Coverage
100
75
50
25
0
1853
1900
1950
2010
Year
Summer Solstice
June 21 / Day 172
Summer Teletherm
July 21 / Day 202
+30 days
Jan
Feb
Mar
Apr
May
Jun
Jul
Aug
Sep
Oct
Nov
Dec
90
80
70
60
50
40
1
32
60
91
121
152
182
213
244
274
305
335
Day of Year

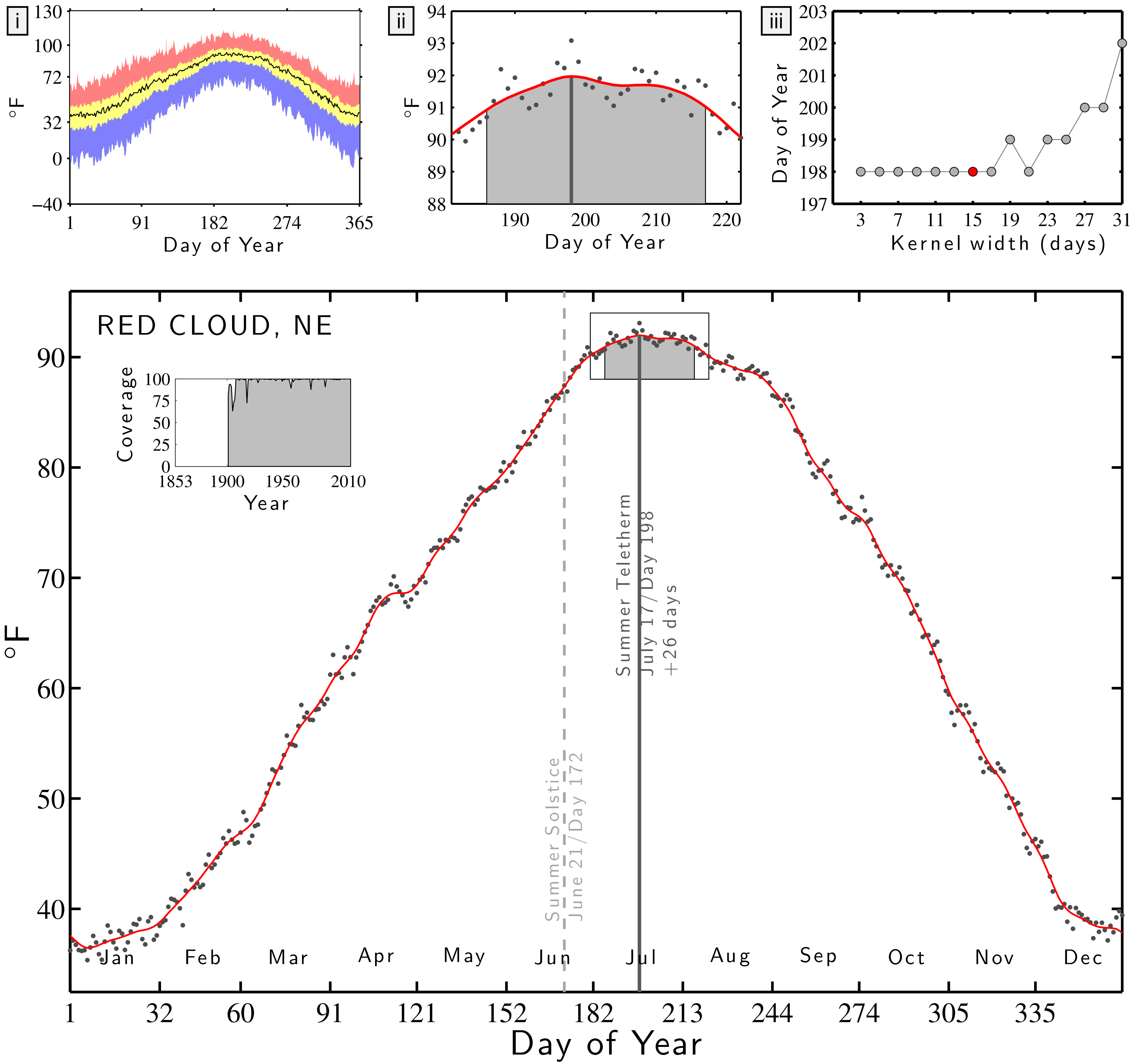

i
ii
iii
°F
Day of Year
Day of Year
Kernel width (days)
RED CLOUD, NE
Coverage
Year
Summer Solstice
June 21/Day 172
Summer Teletherm
July 17/Day 198
+26 days
Jan
Feb
Mar
Apr
May
Jun
Jul
Aug
Sep
Oct
Nov
Dec

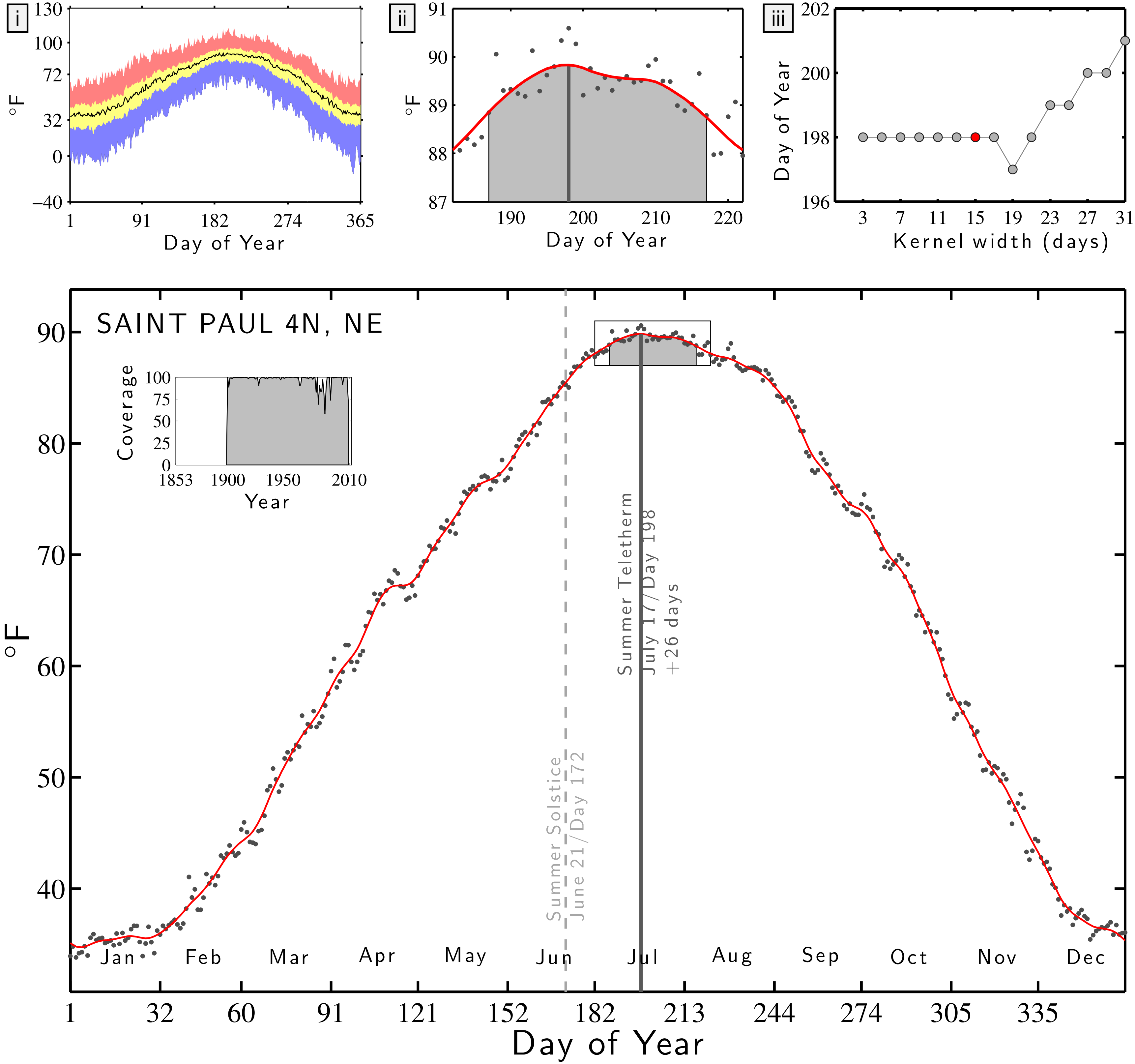

i
ii
iii
°F
Day of Year
Kernel width (days)
Day of Year
SAINT PAUL 4N, NE
Coverage
Year
Summer Solstice
June 21 / Day 172
Summer Teletherm
July 17 / Day 198
+26 days
Jan
Feb
Mar
Apr
May
Jun
Jul
Aug
Sep
Oct
Nov
Dec

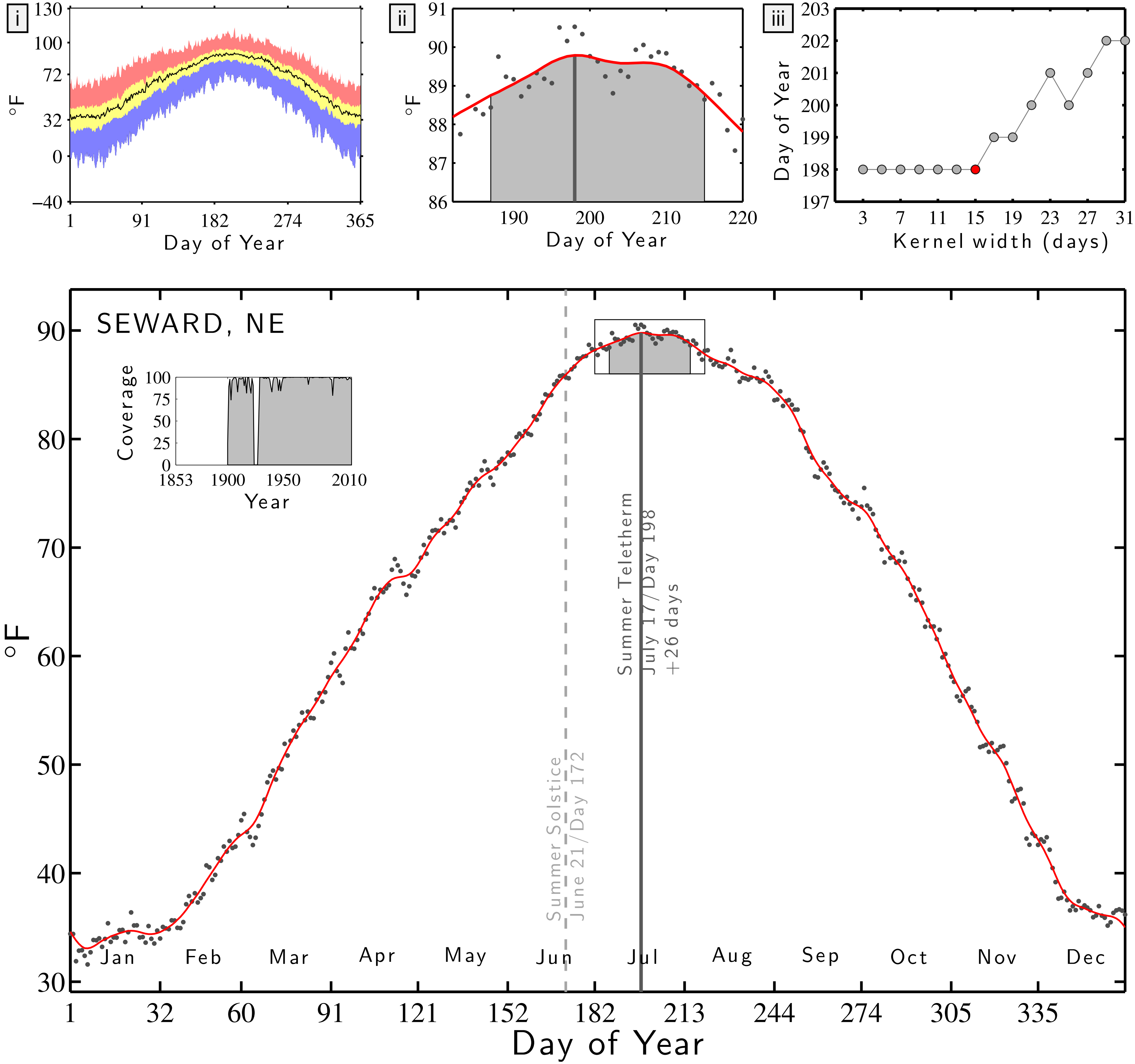

i
°F
Day of Year
ii
°F
Day of Year
iii
Day of Year
Kernel width (days)
SEWARD, NE
Coverage
Year
Summer Solstice
June 21 / Day 172
Summer Teletherm
July 17 / Day 198
+26 days
Jan
Feb
Mar
Apr
May
Jun
Jul
Aug
Sep
Oct
Nov
Dec
°F
Day of Year

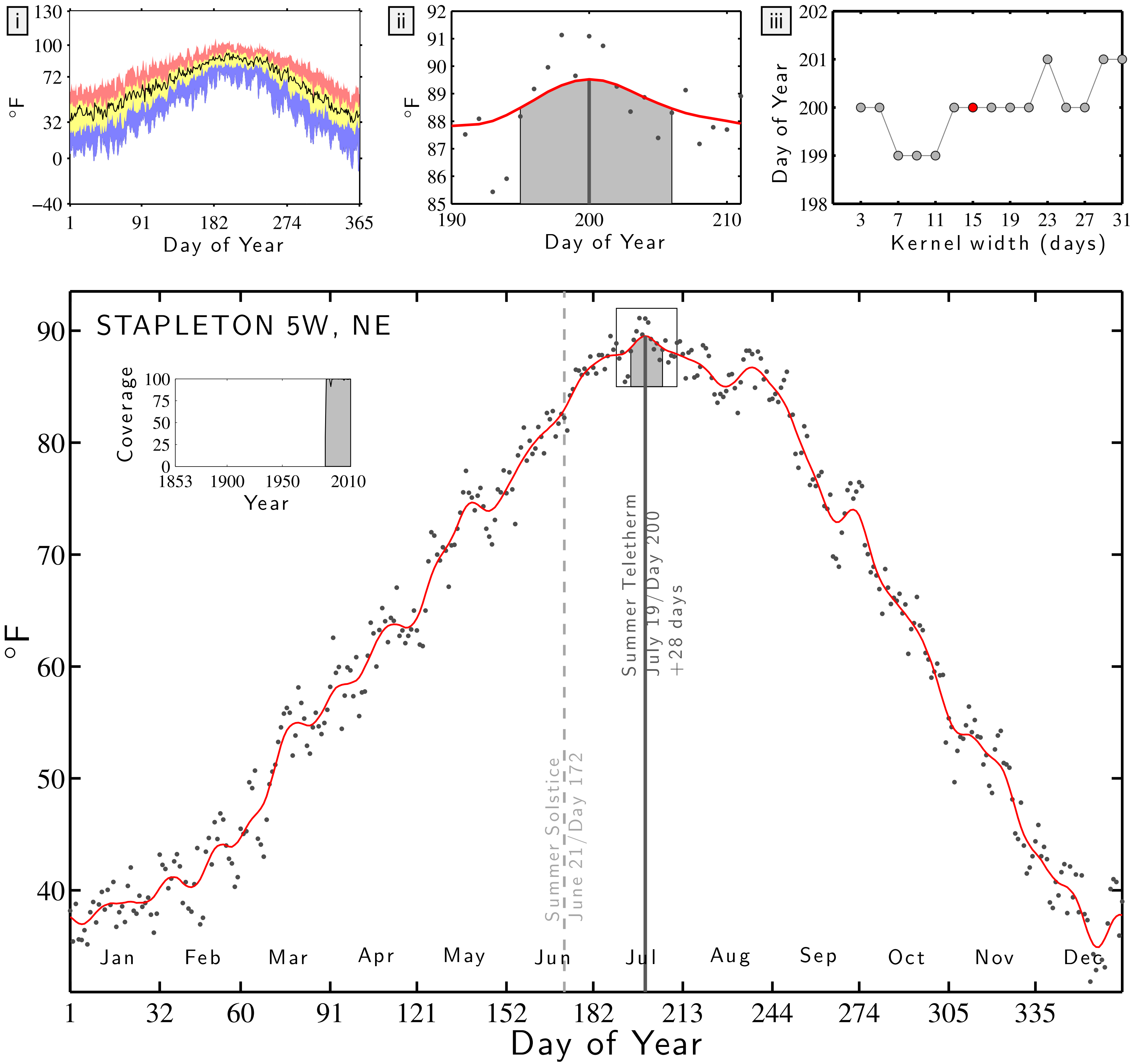

i
ii
iii
°F
Day of Year
Kernel width (days)
STAPLETON 5W, NE
Coverage
Year
Summer Solstice
June 21/Day 172
Summer Teletherm
July 19/Day 200
+28 days
Jan
Feb
Mar
Apr
May
Jun
Jul
Aug
Sep
Oct
Nov
Dec

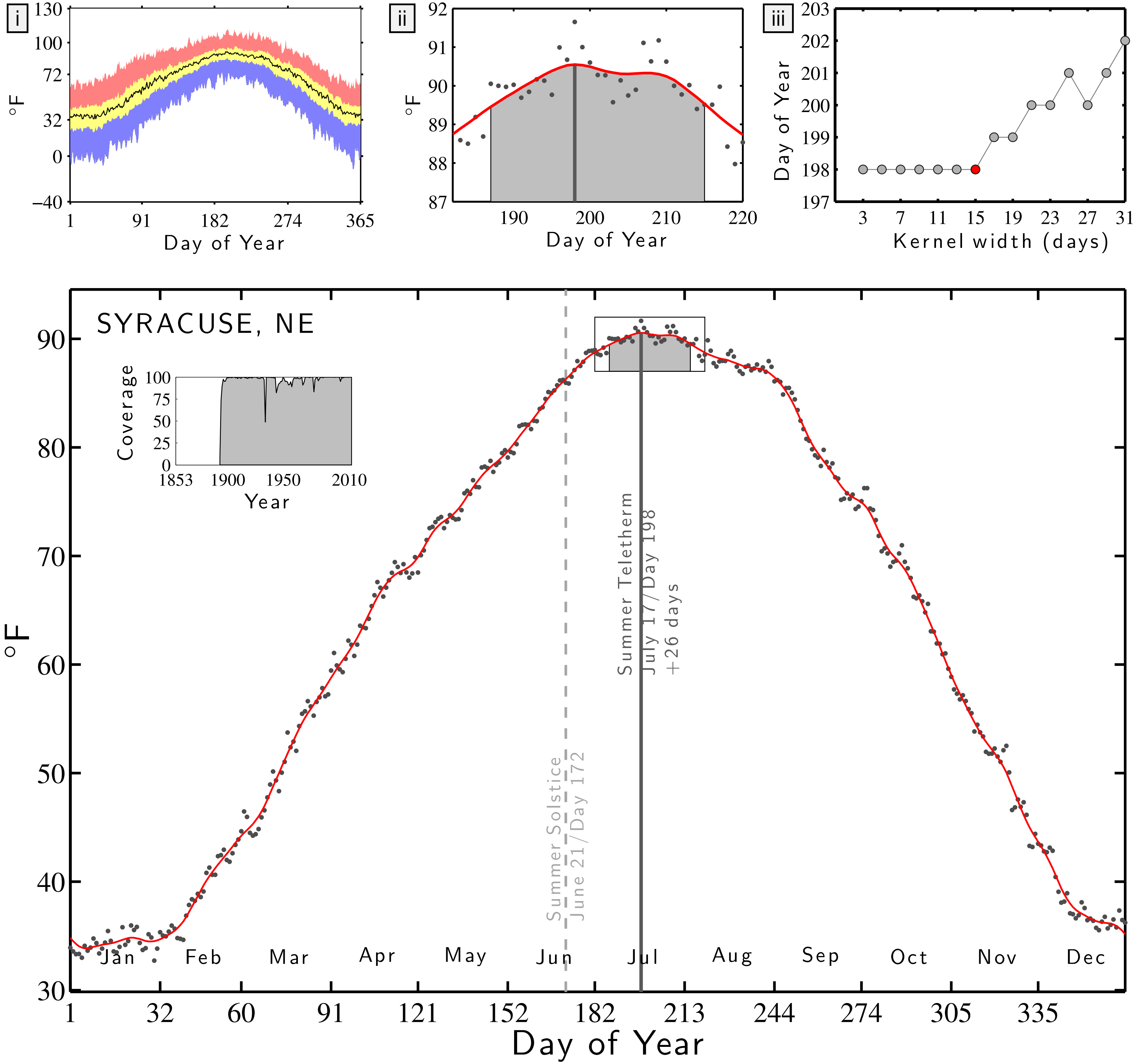

i
ii
iii
Day of Year
Kernel width (days)
SYRACUSE, NE
Coverage
Year
Summer Solstice
June 21/Day 172
Summer Teletherm
July 17/Day 198
+26 days
Jan
Feb
Mar
Apr
May
Jun
Jul
Aug
Sep
Oct
Nov
Dec
°F

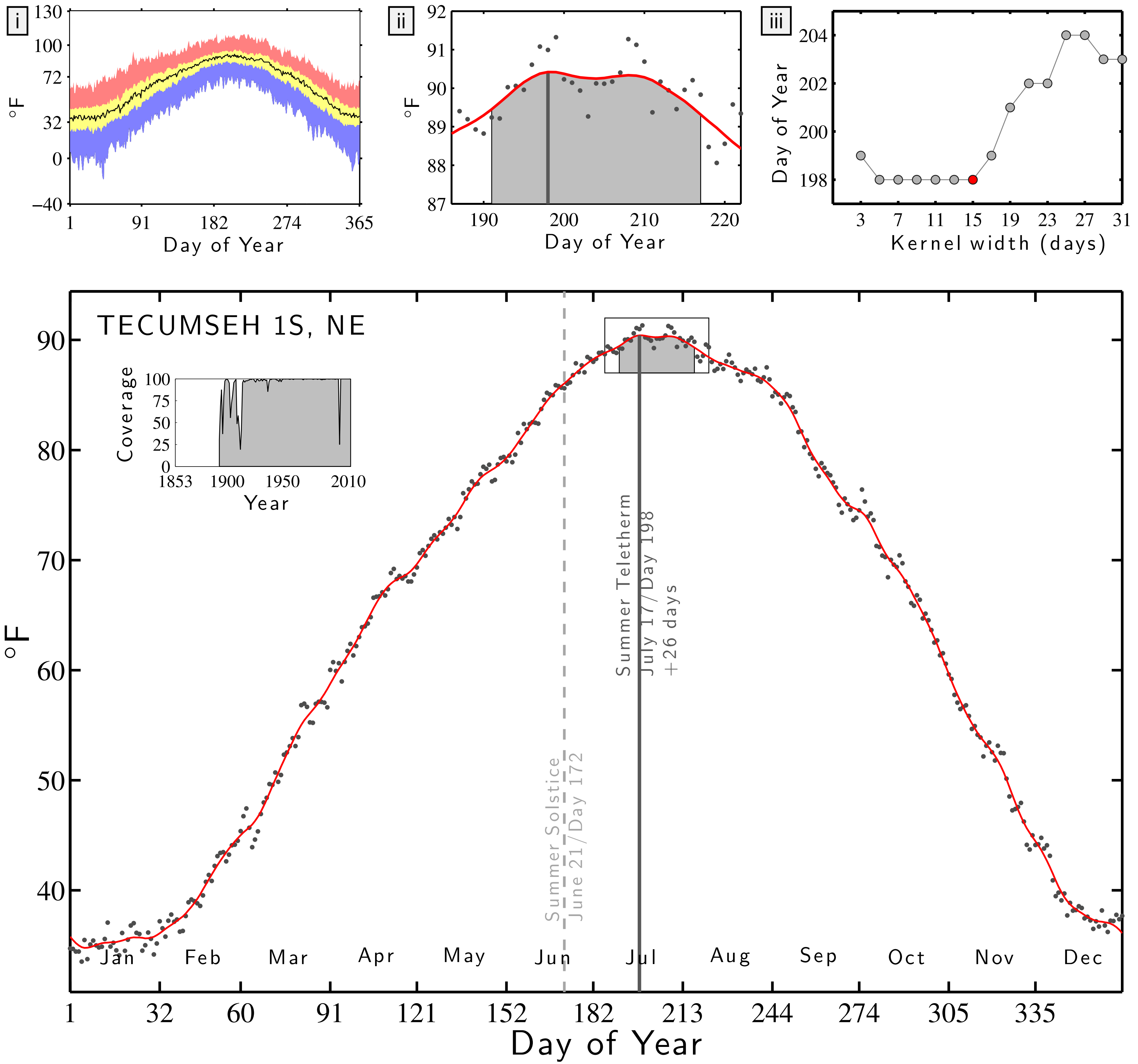

i
ii
iii
°F
Day of Year
Kernel width (days)
TECUMSEH 1S, NE
Coverage
Year
Summer Solstice
June 21 / Day 172
Summer Teletherm
July 17 / Day 198
+26 days
Jan
Feb
Mar
Apr
May
Jun
Jul
Aug
Sep
Oct
Nov
Dec

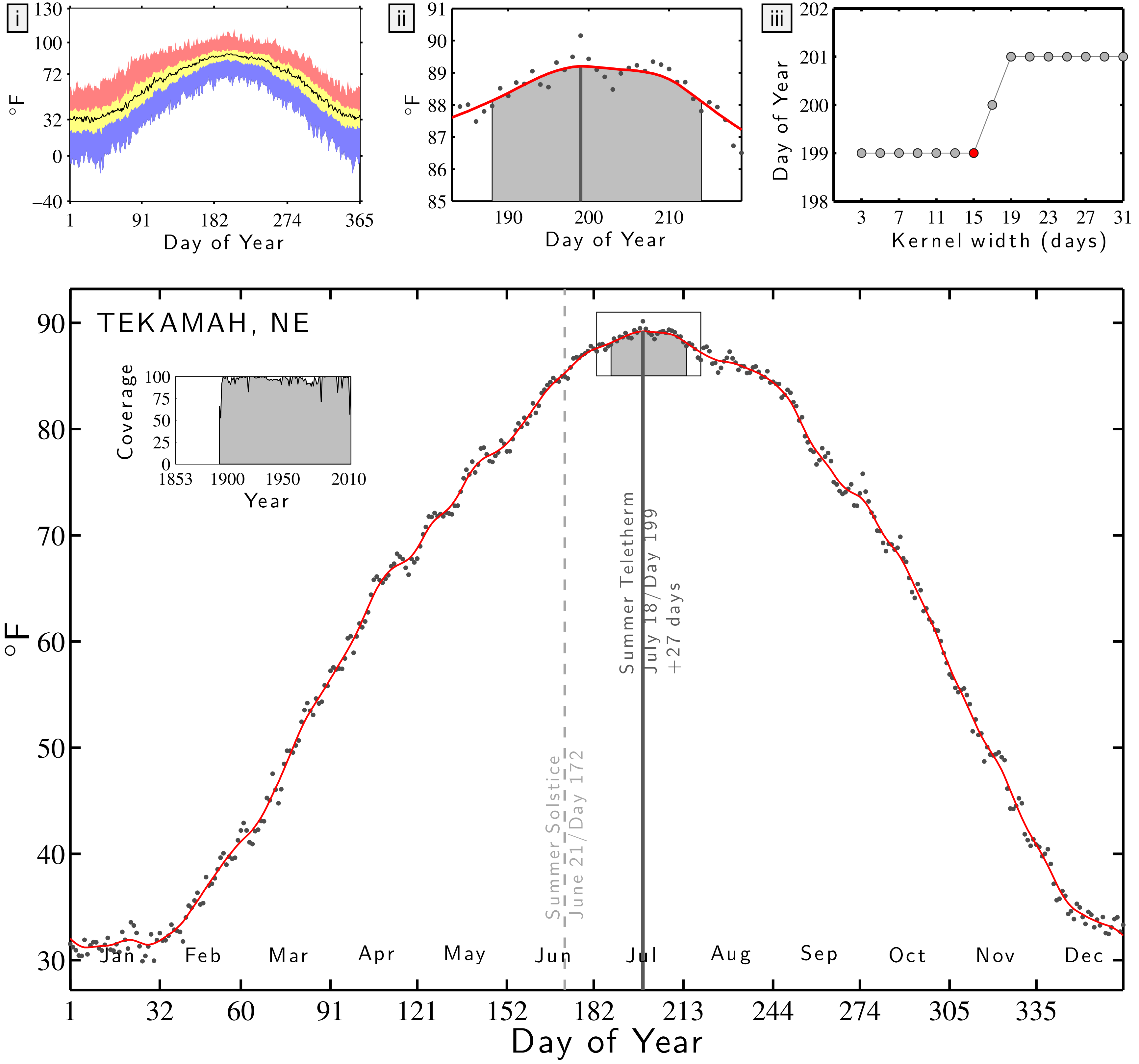
i
°F
Day of Year
ii
°F
Day of Year
iii
Day of Year
Kernel width (days)
TEKAMAH, NE
Coverage
Year
Summer Solstice
June 21/Day 172
Summer Teletherm
July 18/Day 199
+27 days
Jan
Feb
Mar
Apr
May
Jun
Jul
Aug
Sep
Oct
Nov
Dec
°F
Day of Year

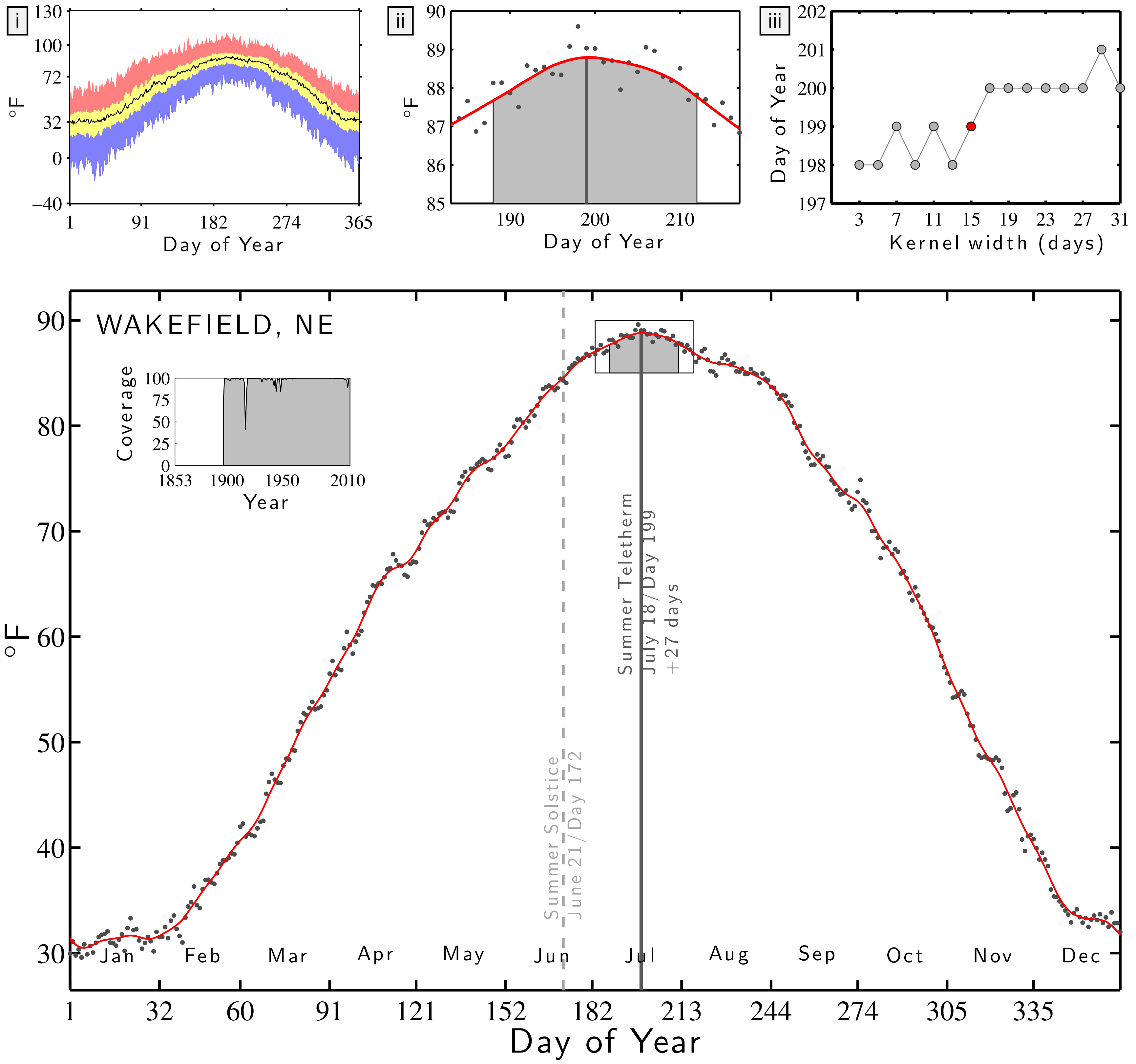

i
°F
Day of Year
ii
°F
Day of Year
iii
Day of Year
Kernel width (days)
WAKEFIELD, NE
Coverage
Year
Summer Solstice
June 21/Day 172
Summer Teletherm
July 18/Day 199
+27 days
Jan
Feb
Mar
Apr
May
Jun
Jul
Aug
Sep
Oct
Nov
Dec
°F
Day of Year

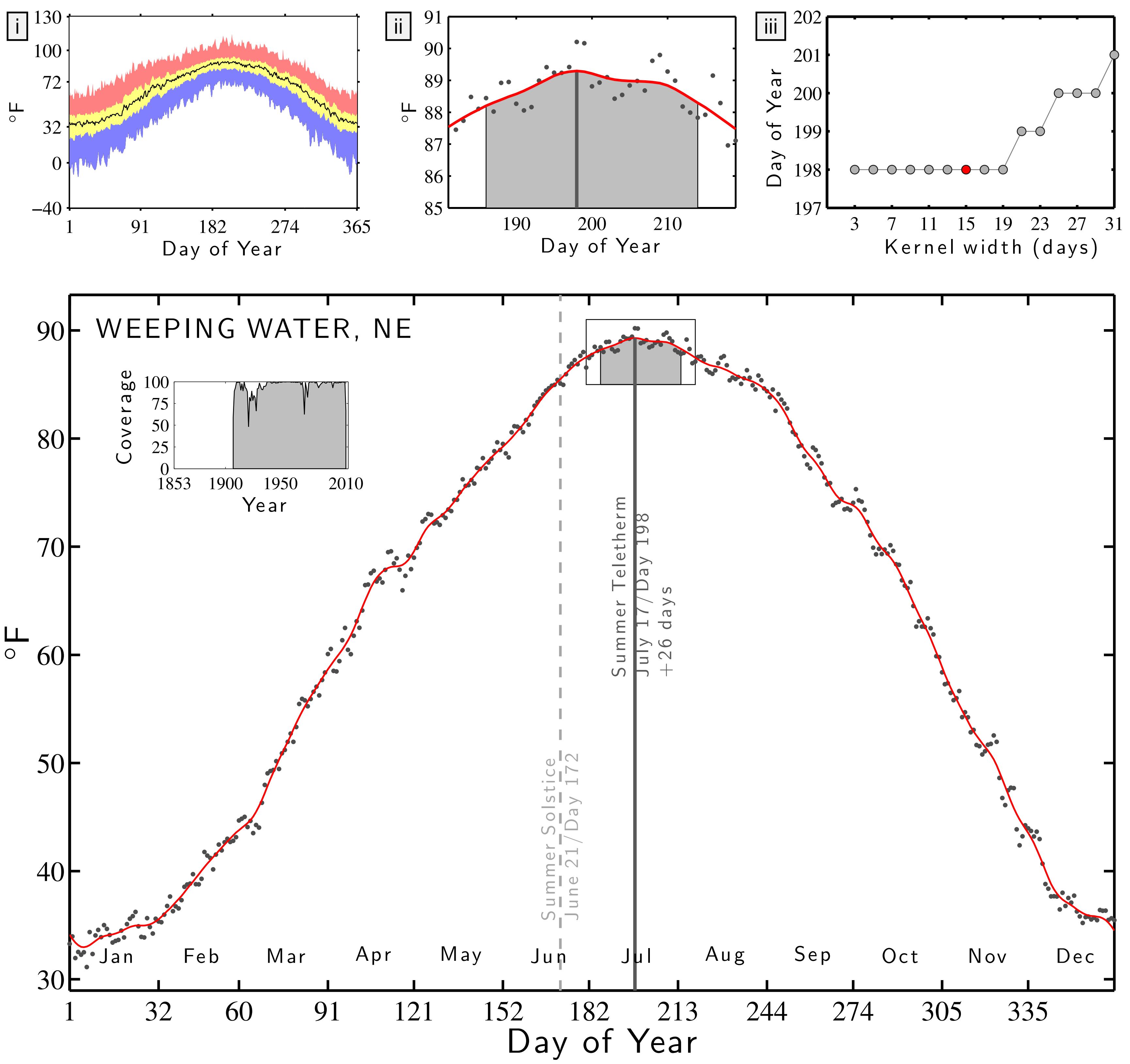

i
ii
iii
°F
Day of Year
Kernel width (days)
WEEPING WATER, NE
Coverage
Year
Summer Solstice
June 21 / Day 172
Summer Teletherm
July 17 / Day 198
+26 days
Jan
Feb
Mar
Apr
May
Jun
Jul
Aug
Sep
Oct
Nov
Dec

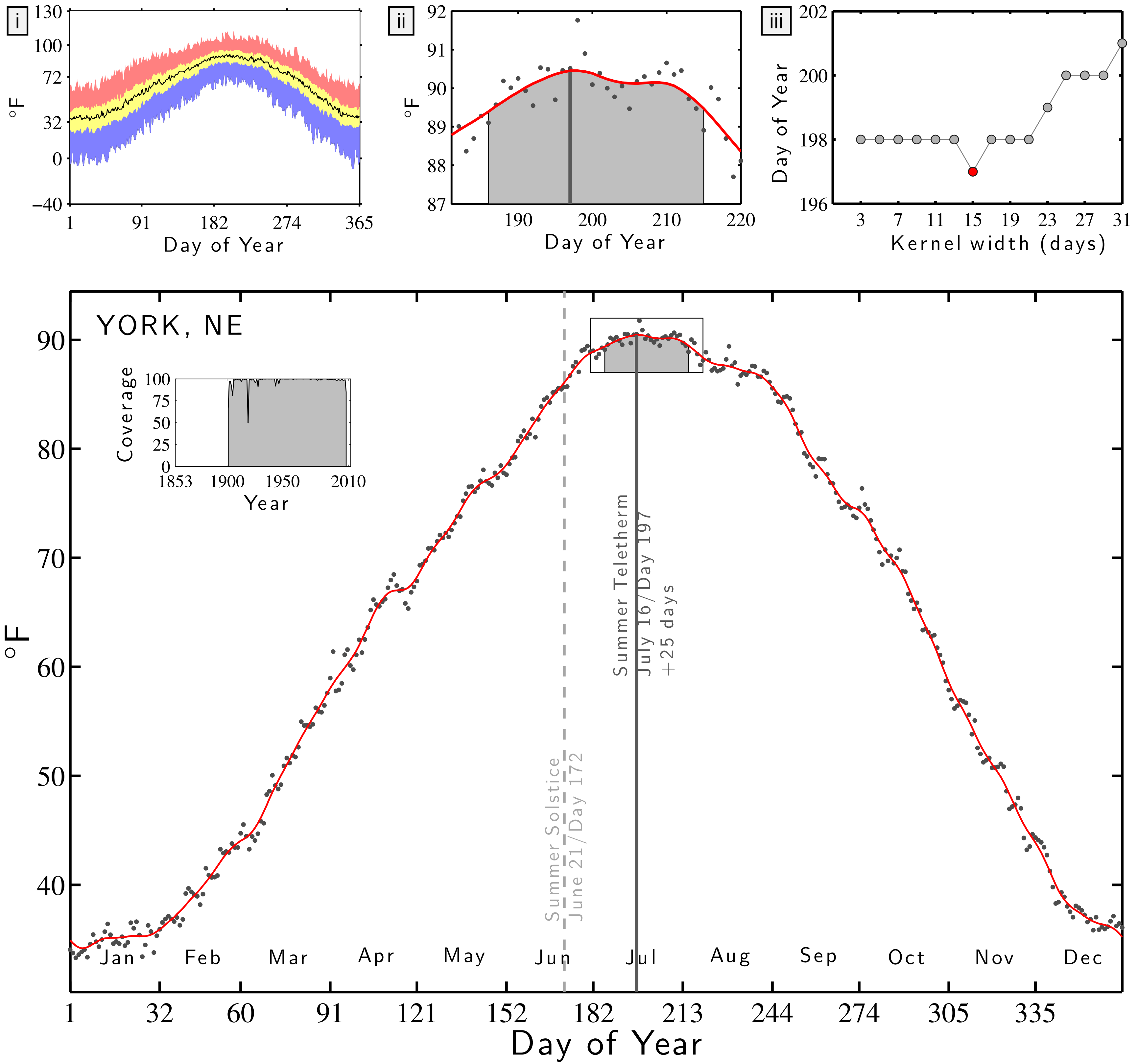

i
ii
iii
°F
Day of Year
Kernel width (days)
YORK, NE
Coverage
Year
Summer Solstice
June 21/Day 172
Summer Teletherm
July 16/Day 197
+25 days
Jan
Feb
Mar
Apr
May
Jun
Jul
Aug
Sep
Oct
Nov
Dec

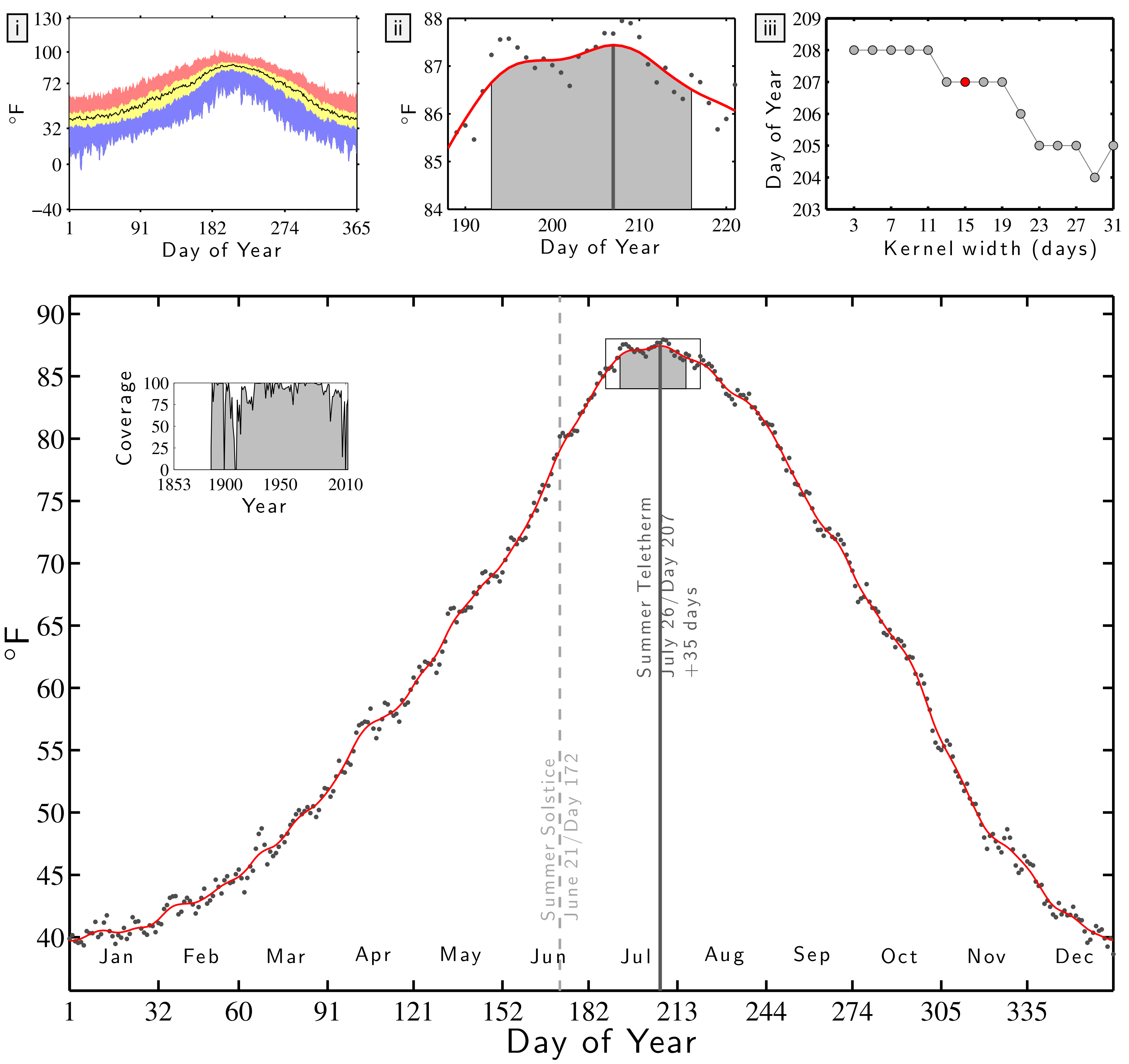

i
°F
Day of Year
ii
iii
Day of Year
Kernel width (days)
Coverage
Year
Summer Solstice
June 21 / Day 172
Summer Teletherm
July 26 / Day 207
+35 days
Jan
Feb
Mar
Apr
May
Jun
Jul
Aug
Sep
Oct
Nov
Dec
Day of Year

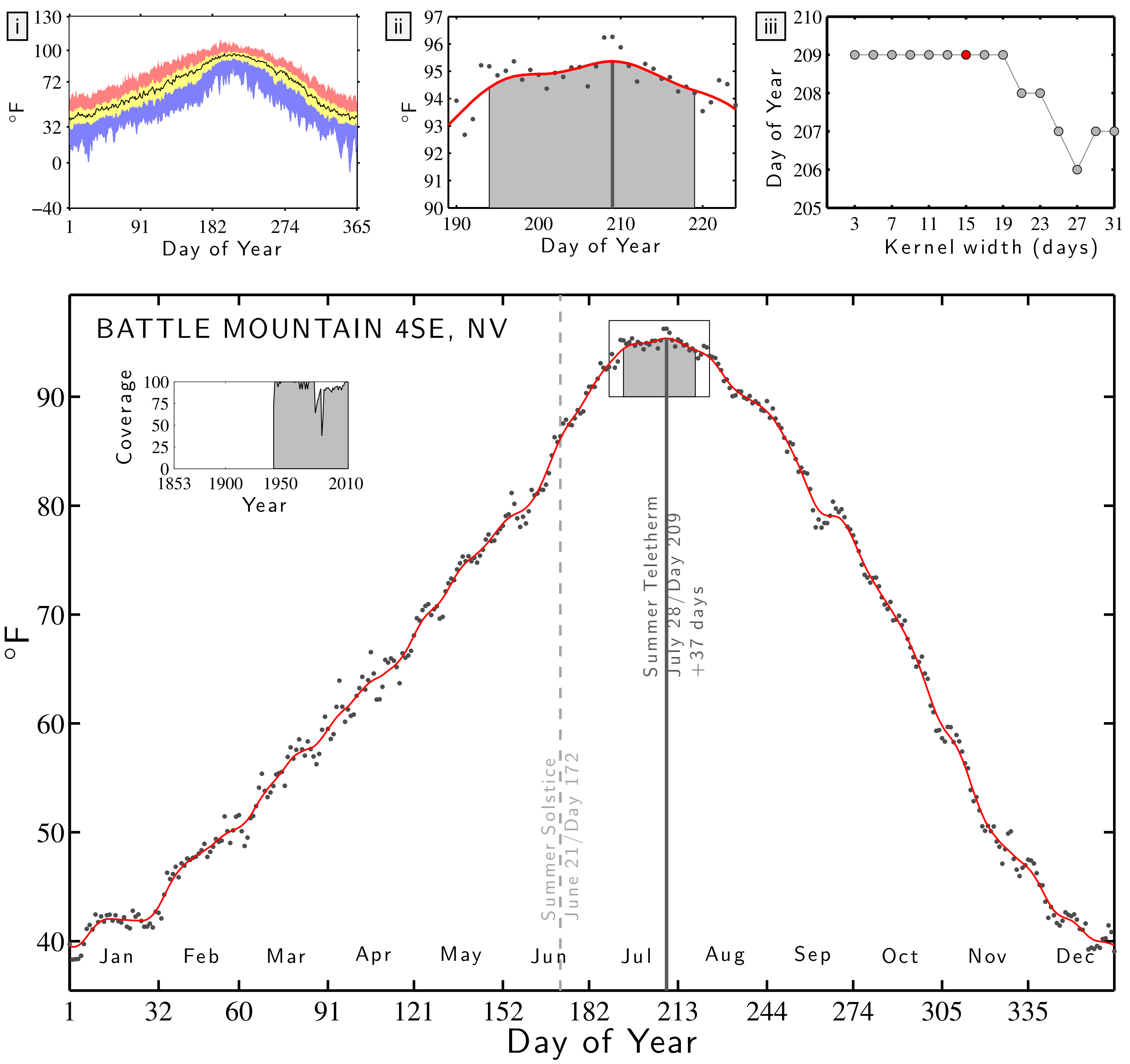

i
°F
Day of Year
ii
°F
Day of Year
iii
Day of Year
Kernel width (days)
BATTLE MOUNTAIN 4SE, NV
Coverage
Year
Summer Solstice
June 21/Day 172
Summer Teletherm
July 28/Day 209
+37 days
Jan
Feb
Mar
Apr
May
Jun
Jul
Aug
Sep
Oct
Nov
Dec
°F
Day of Year

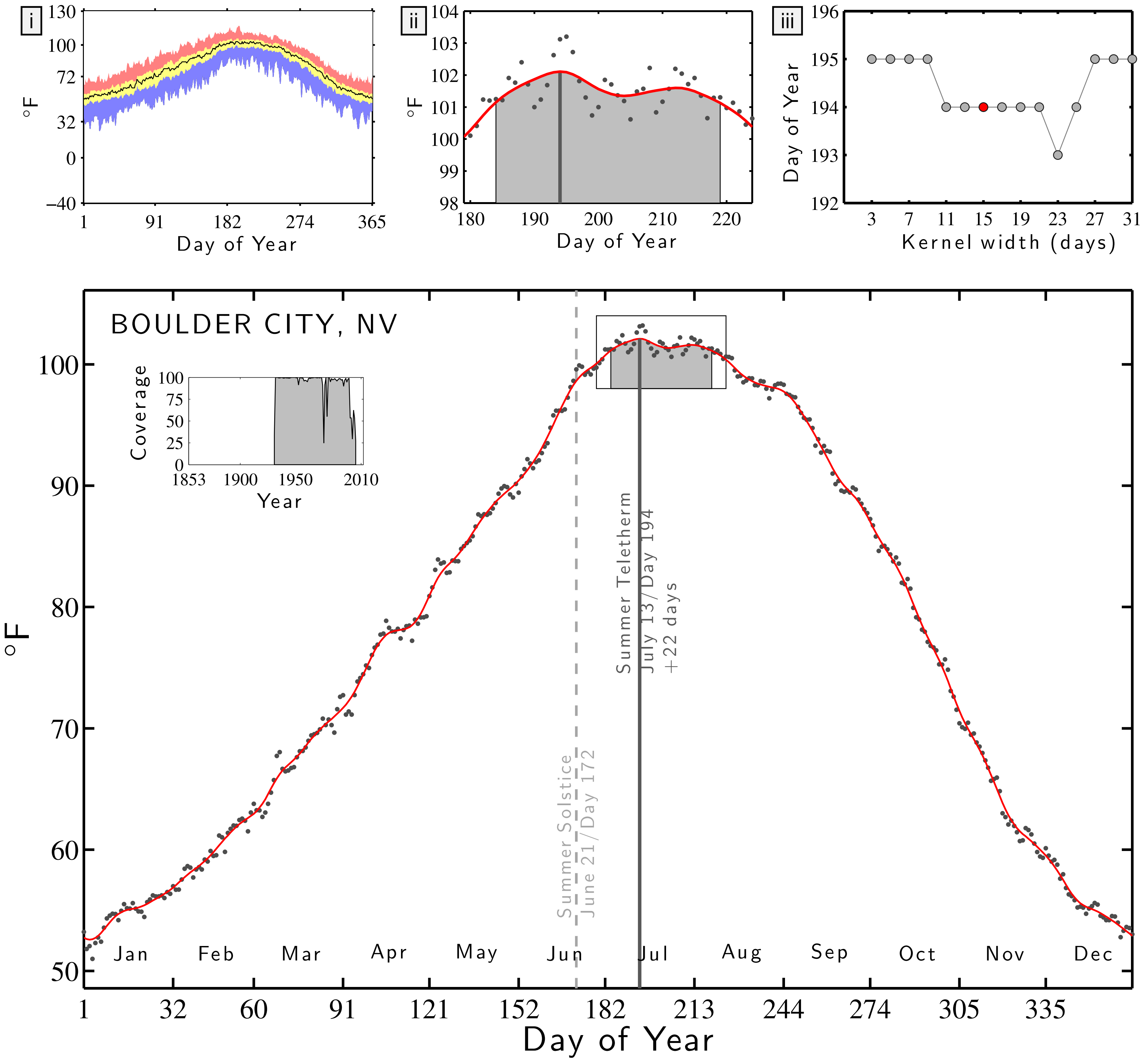
i
ii
iii
°F
Day of Year
Day of Year
Kernel width (days)
BOULDER CITY, NV
Coverage
Year
Summer Solstice
June 21/Day 172
Summer Teletherm
July 13/Day 194
+22 days
Jan
Feb
Mar
Apr
May
Jun
Jul
Aug
Sep
Oct
Nov
Dec

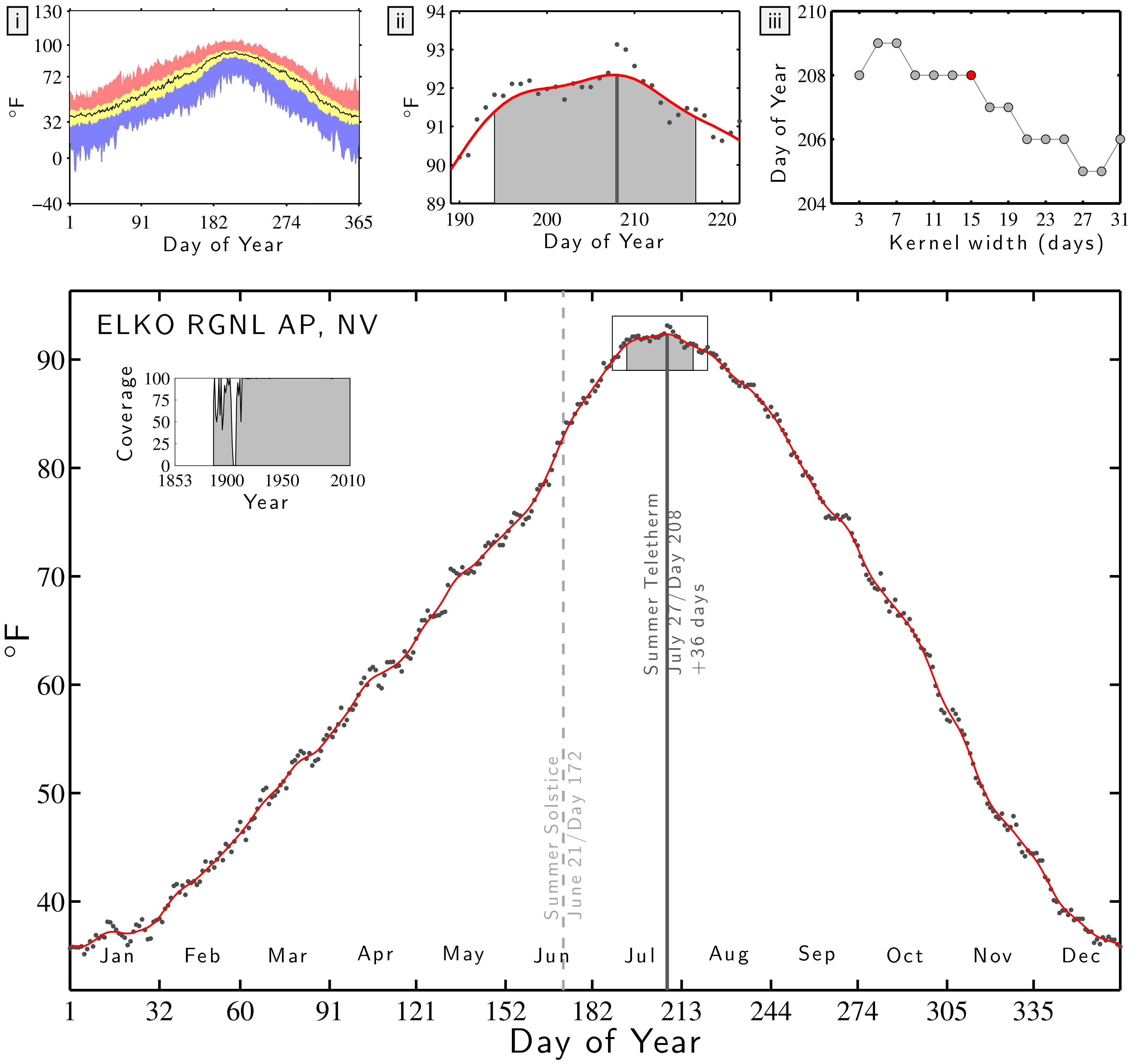

i
°F
Day of Year
ii
°F
Day of Year
iii
Day of Year
Kernel width (days)
ELKO RGNL AP, NV
Coverage
Year
Summer Solstice
June 21 / Day 172
Summer Teletherm
July 27 / Day 208
+36 days
Jan
Feb
Mar
Apr
May
Jun
Jul
Aug
Sep
Oct
Nov
Dec
°F
Day of Year

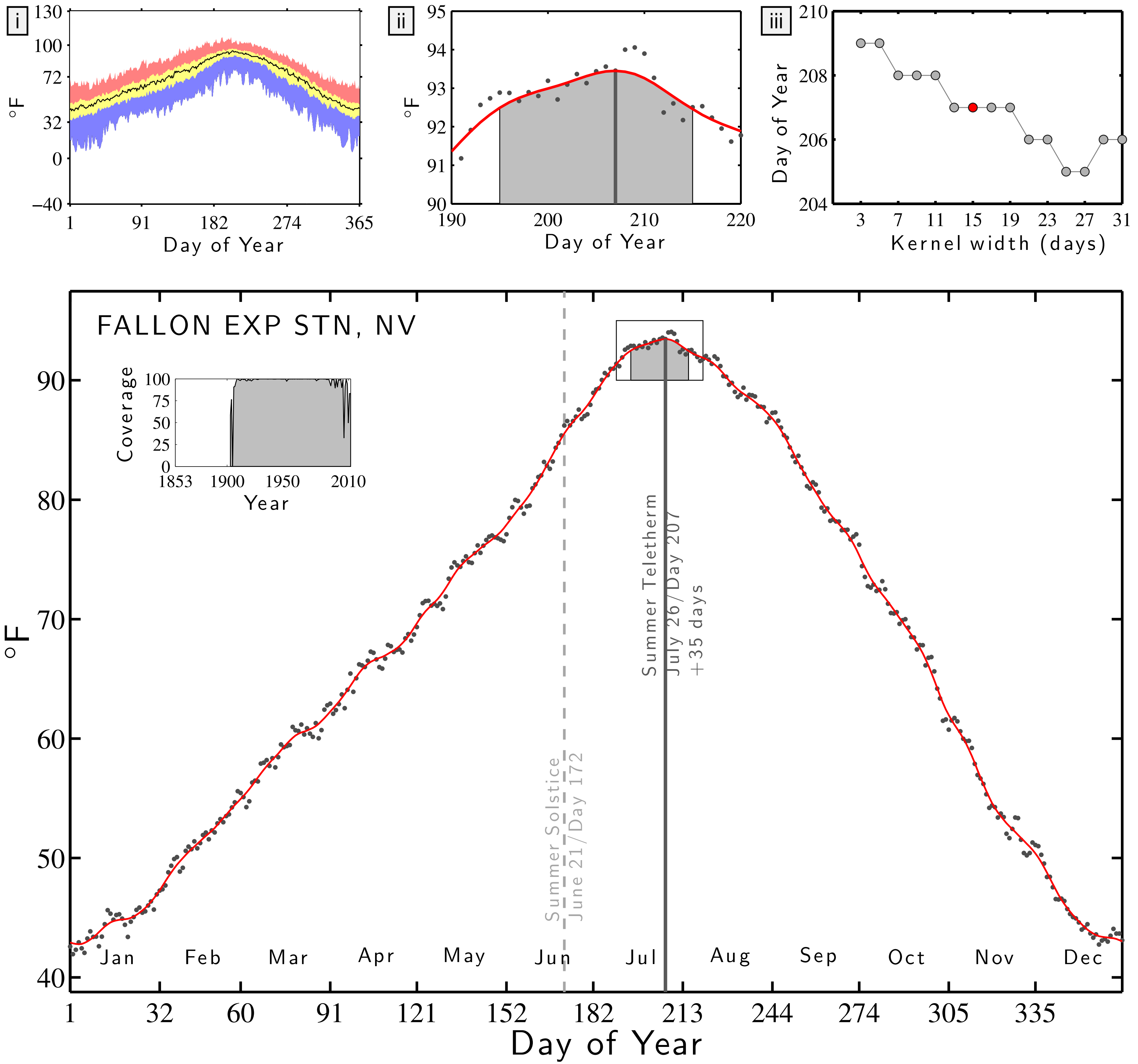

i
ii
iii
°F
Day of Year
Kernel width (days)
Day of Year
FALLON EXP STN, NV
Coverage
Year
Summer Solstice
June 21 / Day 172
Summer Teletherm
July 26 / Day 207
+35 days
Jan
Feb
Mar
Apr
May
Jun
Jul
Aug
Sep
Oct
Nov
Dec

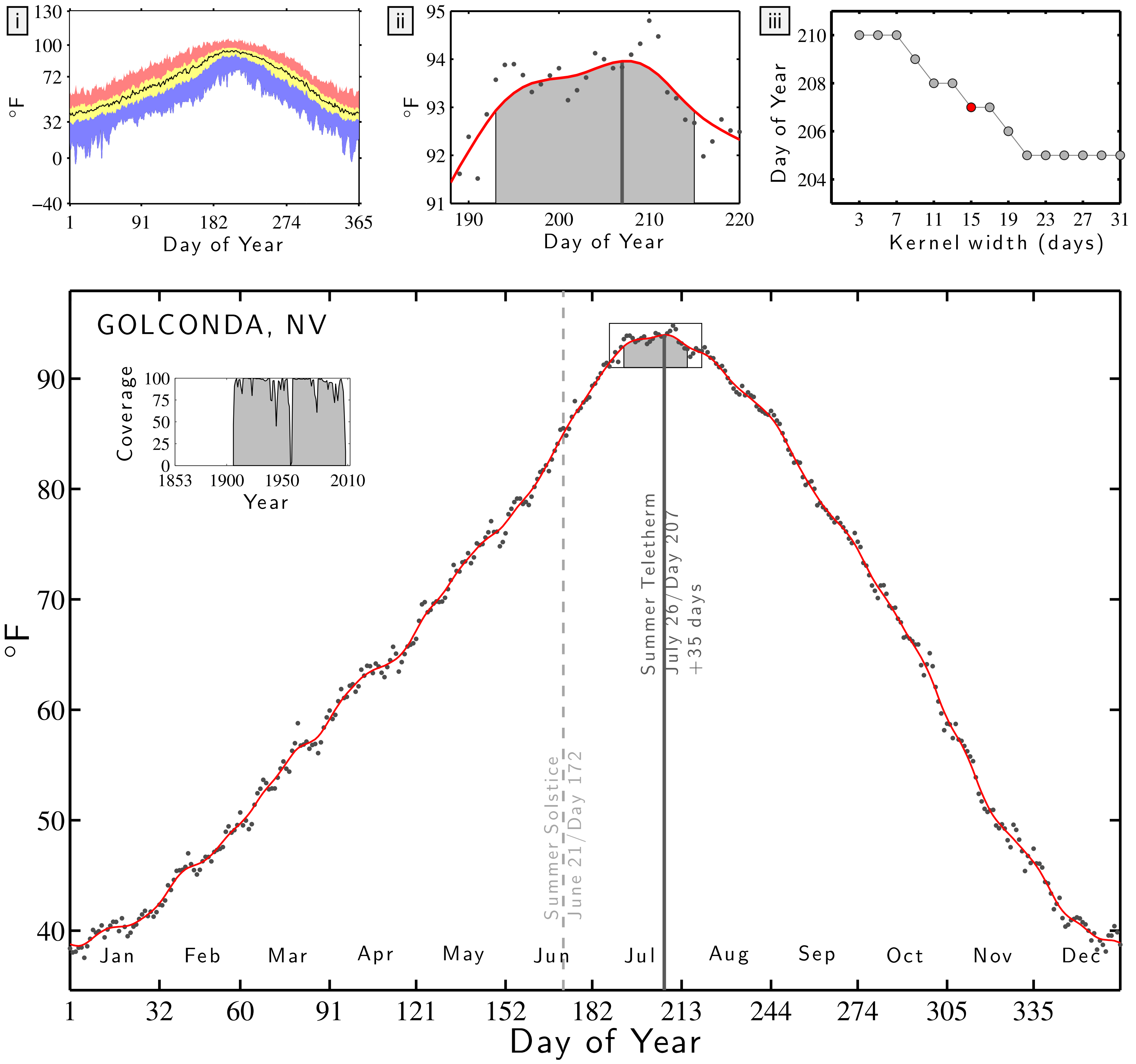

i
°F
Day of Year
ii
°F
Day of Year
iii
Day of Year
Kernel width (days)
GOLCONDA, NV
Coverage
Year
Summer Solstice
June 21 / Day 172
Summer Teletherm
July 26 / Day 207
+35 days
Jan
Feb
Mar
Apr
May
Jun
Jul
Aug
Sep
Oct
Nov
Dec
°F
Day of Year

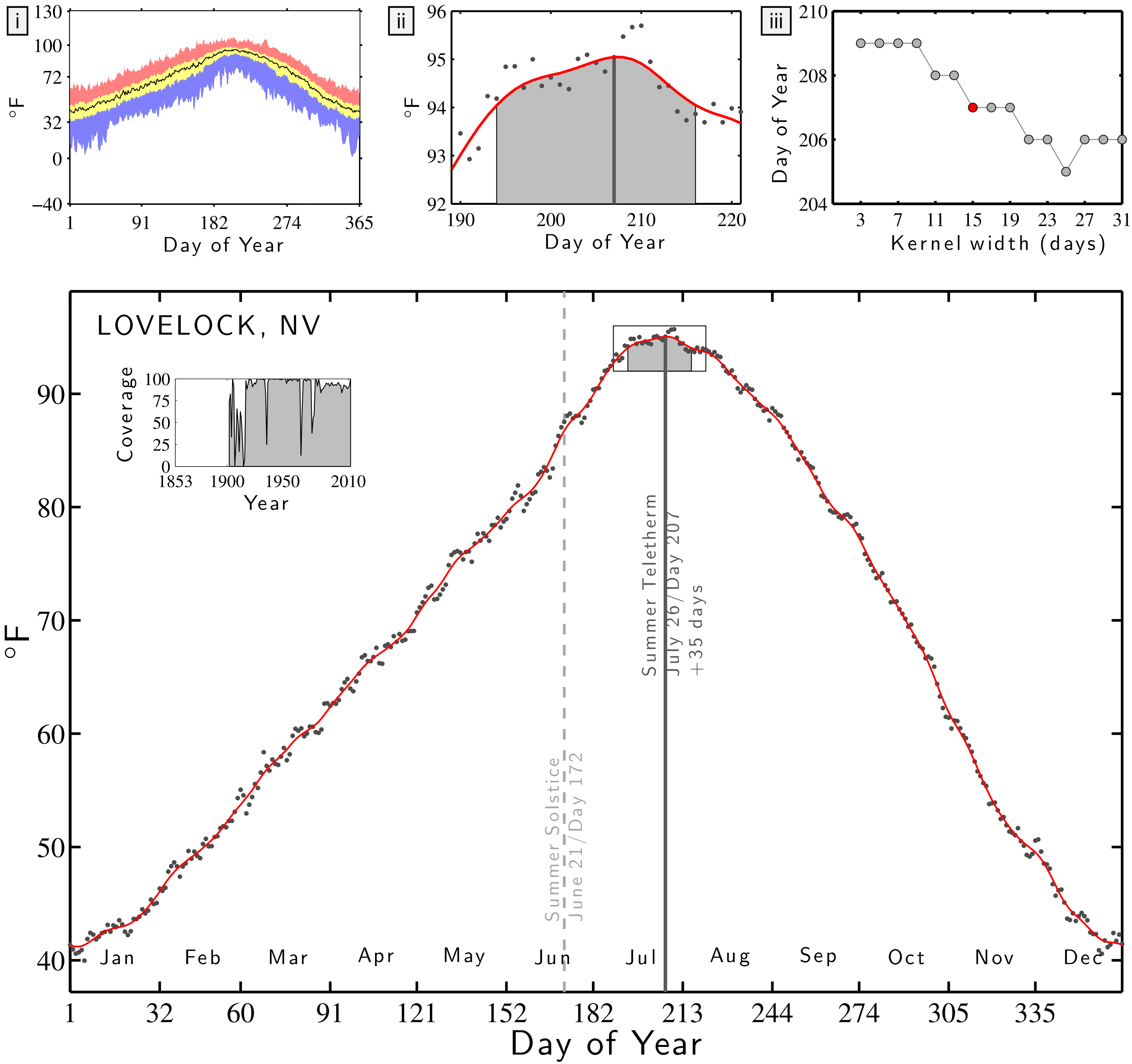

i
ii
iii
°F
Day of Year
Day of Year
Kernel width (days)
LOVELOCK, NV
Coverage
Year
1853
1900
1950
2010
Summer Solstice
June 21/Day 172
Summer Teletherm
July 26/Day 207
+35 days
Jan
Feb
Mar
Apr
May
Jun
Jul
Aug
Sep
Oct
Nov
Dec
°F
Day of Year

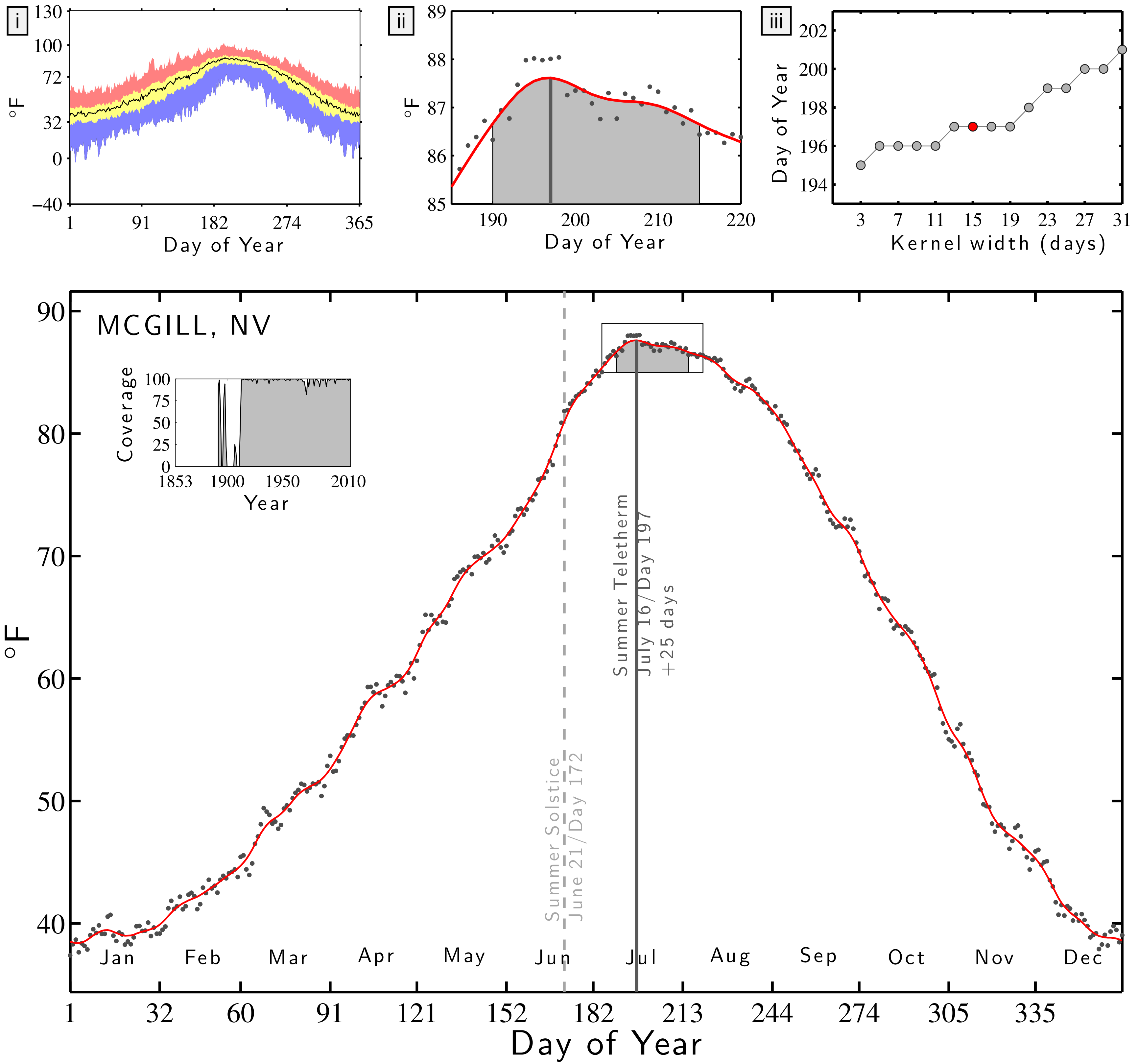

i
ii
iii
°F
Day of Year
Day of Year
Kernel width (days)
MCGILL, NV
Coverage
Year
Summer Solstice
June 21/Day 172
Summer Teletherm
July 16/Day 197
+25 days
Jan
Feb
Mar
Apr
May
Jun
Jul
Aug
Sep
Oct
Nov
Dec

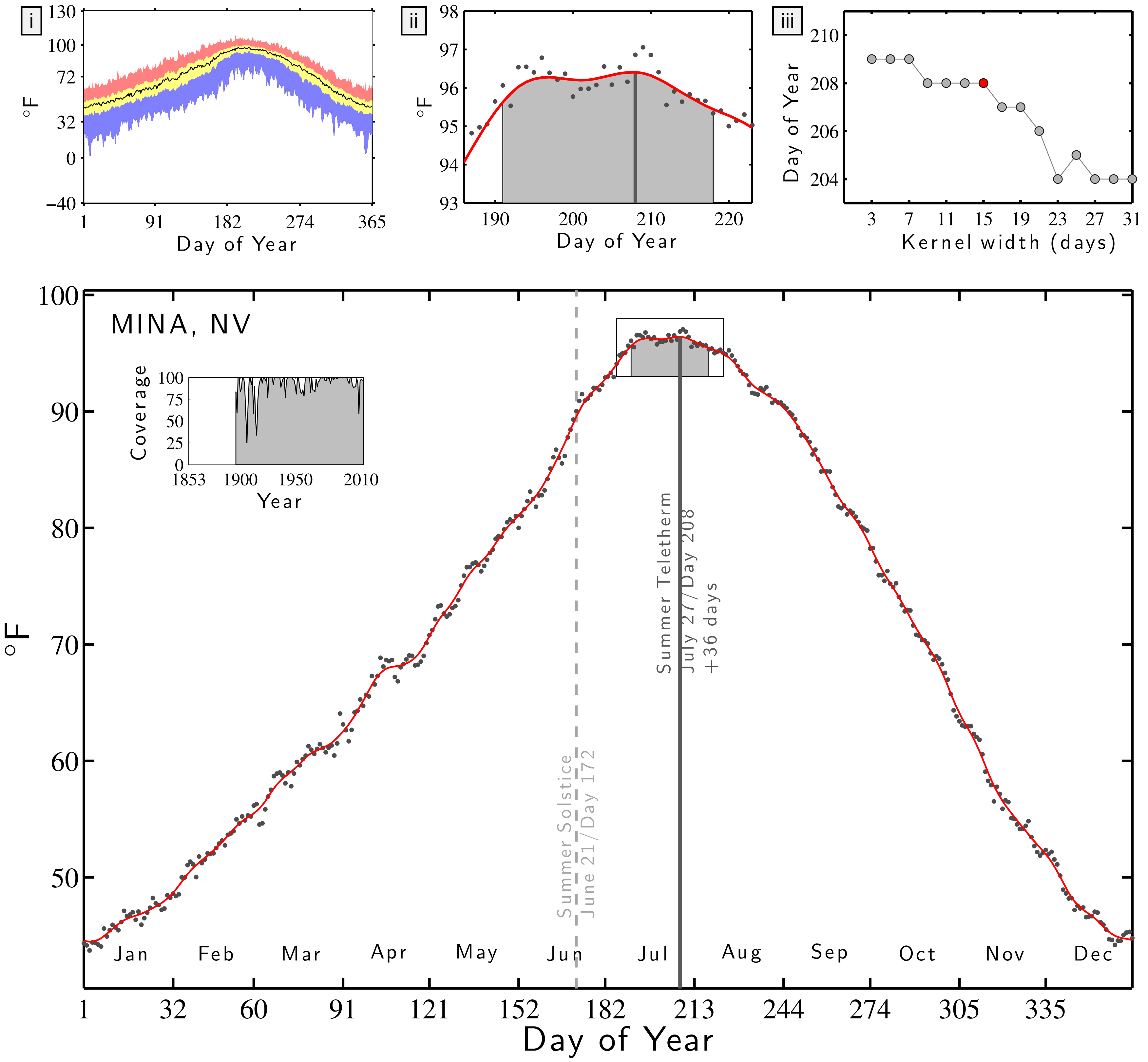

i
°F
Day of Year
ii
°F
Day of Year
iii
Day of Year
Kernel width (days)
MINA, NV
Coverage
Year
Summer Solstice
June 21/Day 172
Summer Teletherm
July 27/Day 208
+36 days
Jan
Feb
Mar
Apr
May
Jun
Jul
Aug
Sep
Oct
Nov
Dec
°F
Day of Year

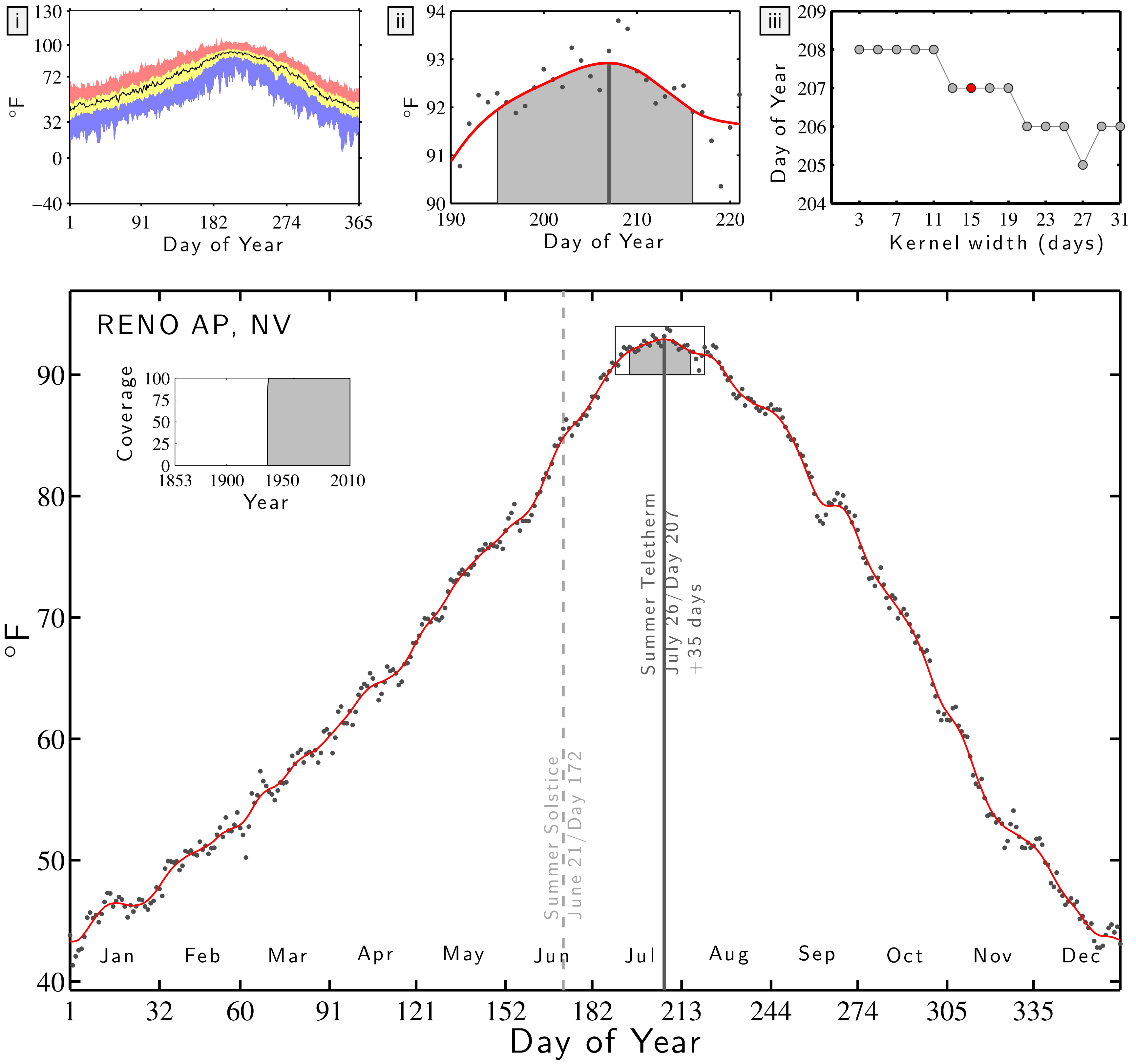

i
°F
130
100
72
32
0
−40
1
91
182
274
365
Day of Year
ii
°F
94
93
92
91
90
190
200
210
220
Day of Year
iii
Day of Year
209
208
207
206
205
204
3
7
11
15
19
23
27
31
Kernel width (days)
RENO AP, NV
Coverage
100
75
50
25
0
1853
1900
1950
2010
Year
°F
90
80
70
60
50
40
Summer Solstice
June 21 / Day 172
Summer Teletherm
July 26 / Day 207
+35 days
Jan
Feb
Mar
Apr
May
Jun
Jul
Aug
Sep
Oct
Nov
Dec
1
32
60
91
121
152
182
213
244
274
305
335
Day of Year

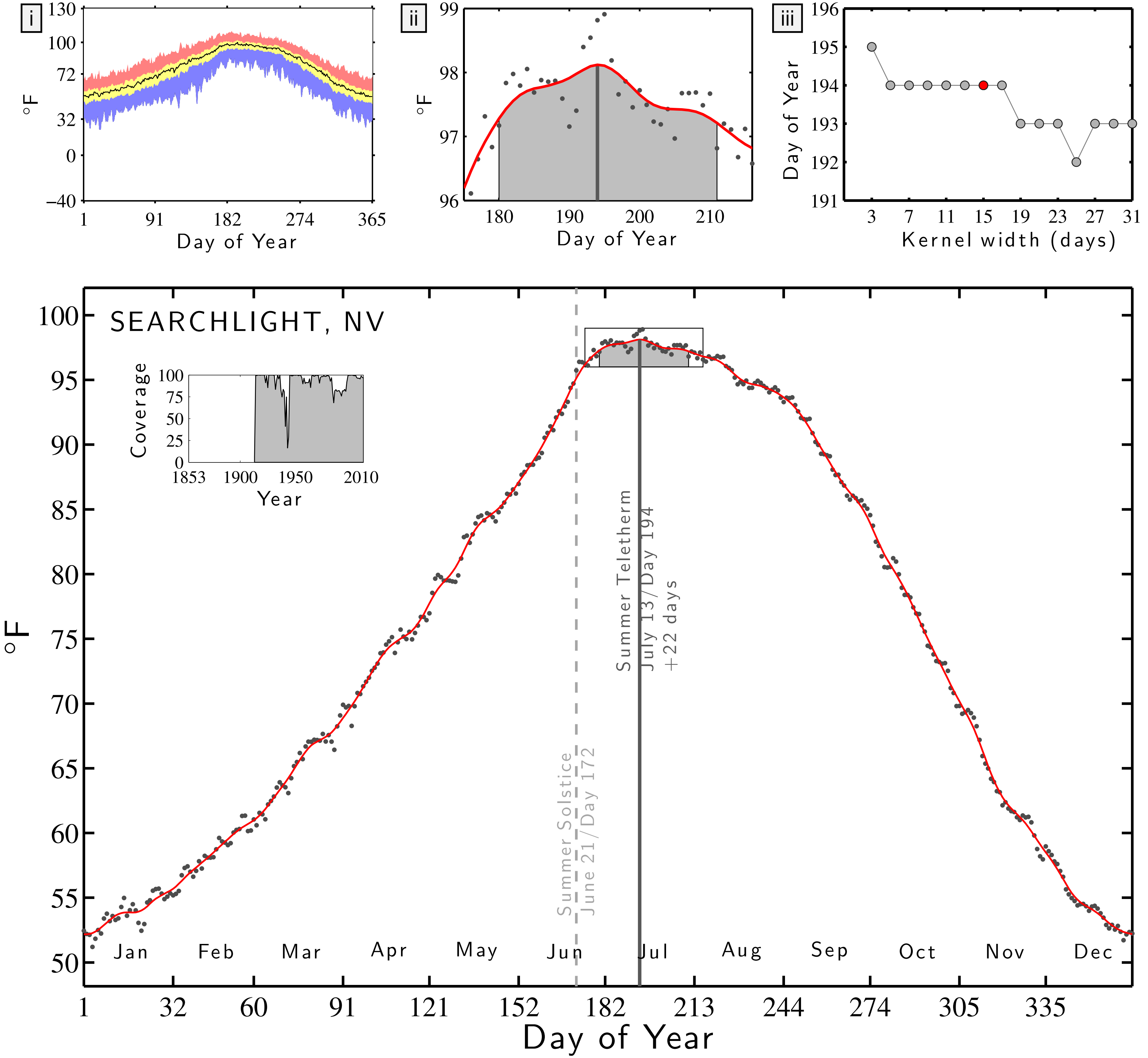

i
°F
Day of Year
ii
°F
Day of Year
iii
Day of Year
Kernel width (days)
SEARCHLIGHT, NV
Coverage
Year
Summer Solstice
June 21/Day 172
Summer Teletherm
July 13/Day 194
+22 days
Jan
Feb
Mar
Apr
May
Jun
Jul
Aug
Sep
Oct
Nov
Dec
°F
Day of Year

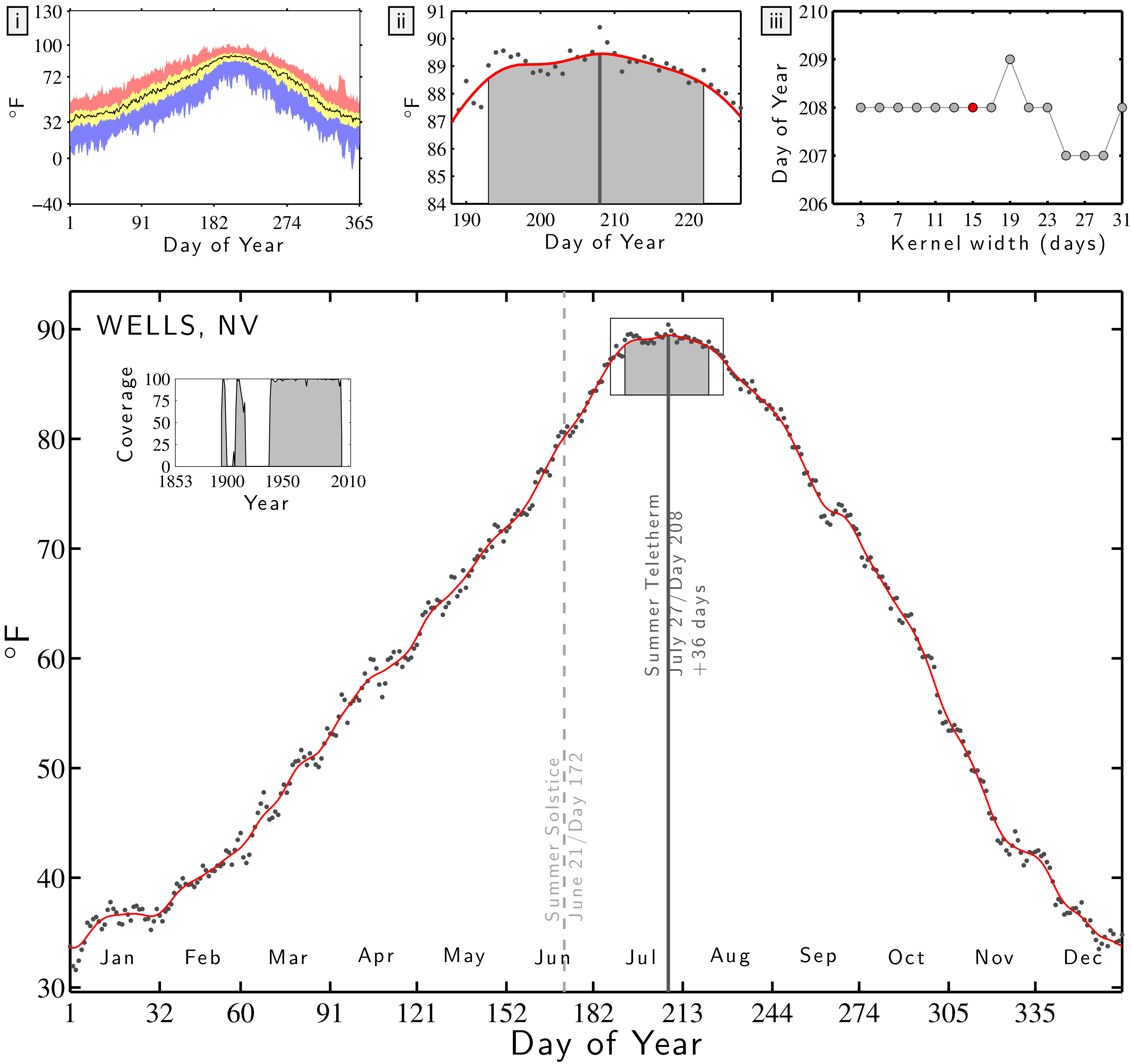

i
°F
Day of Year
ii
°F
Day of Year
iii
Day of Year
Kernel width (days)
WELLS, NV
Coverage
Year
Summer Solstice
June 21 / Day 172
Summer Teletherm
July 27 / Day 208
+36 days
Jan
Feb
Mar
Apr
May
Jun
Jul
Aug
Sep
Oct
Nov
Dec
°F
Day of Year

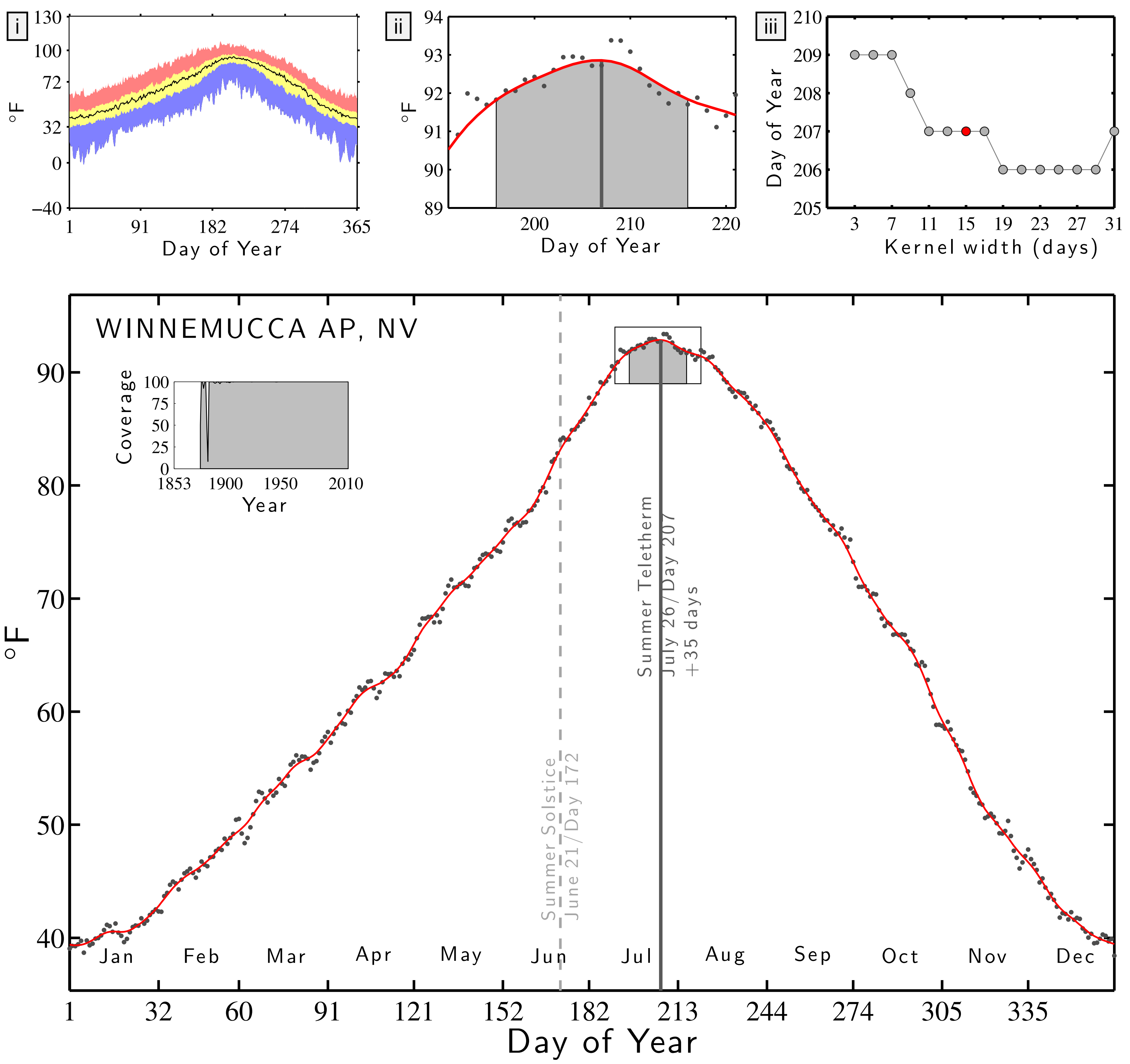
i
ii
iii
°F
Day of Year
Day of Year
Kernel width (days)
WINNEMUCCA AP, NV
Coverage
Year
Summer Solstice
June 21 / Day 172
Summer Teletherm
July 26 / Day 207
+35 days
Jan
Feb
Mar
Apr
May
Jun
Jul
Aug
Sep
Oct
Nov
Dec

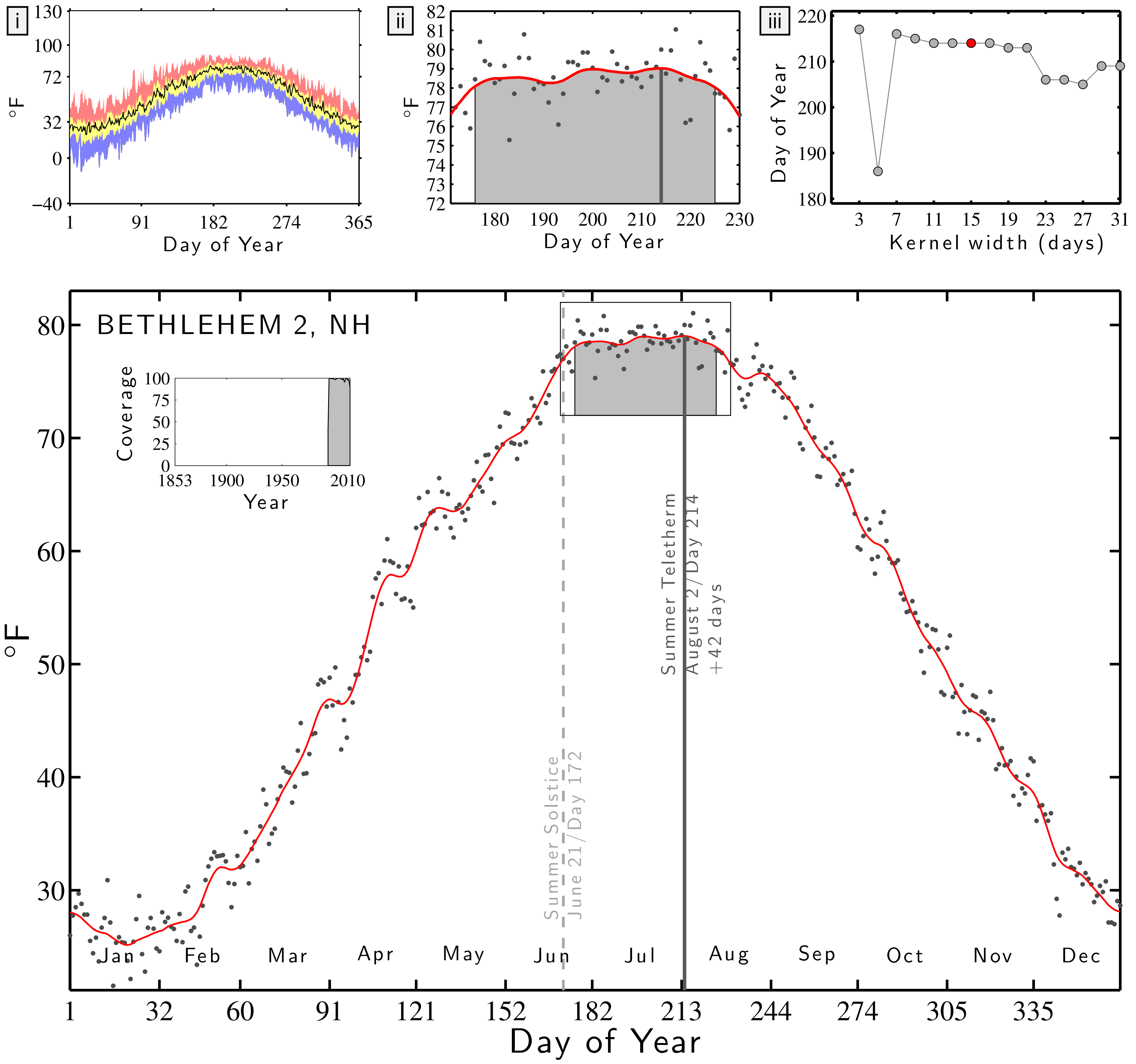

i
ii
iii
Day of Year
°F
Kernel width (days)
BETHLEHEM 2, NH
Coverage
Year
Summer Teletherm
August 2/Day 214
+42 days
Summer Solstice
June 21/Day 172
Jan
Feb
Mar
Apr
May
Jun
Jul
Aug
Sep
Oct
Nov
Dec

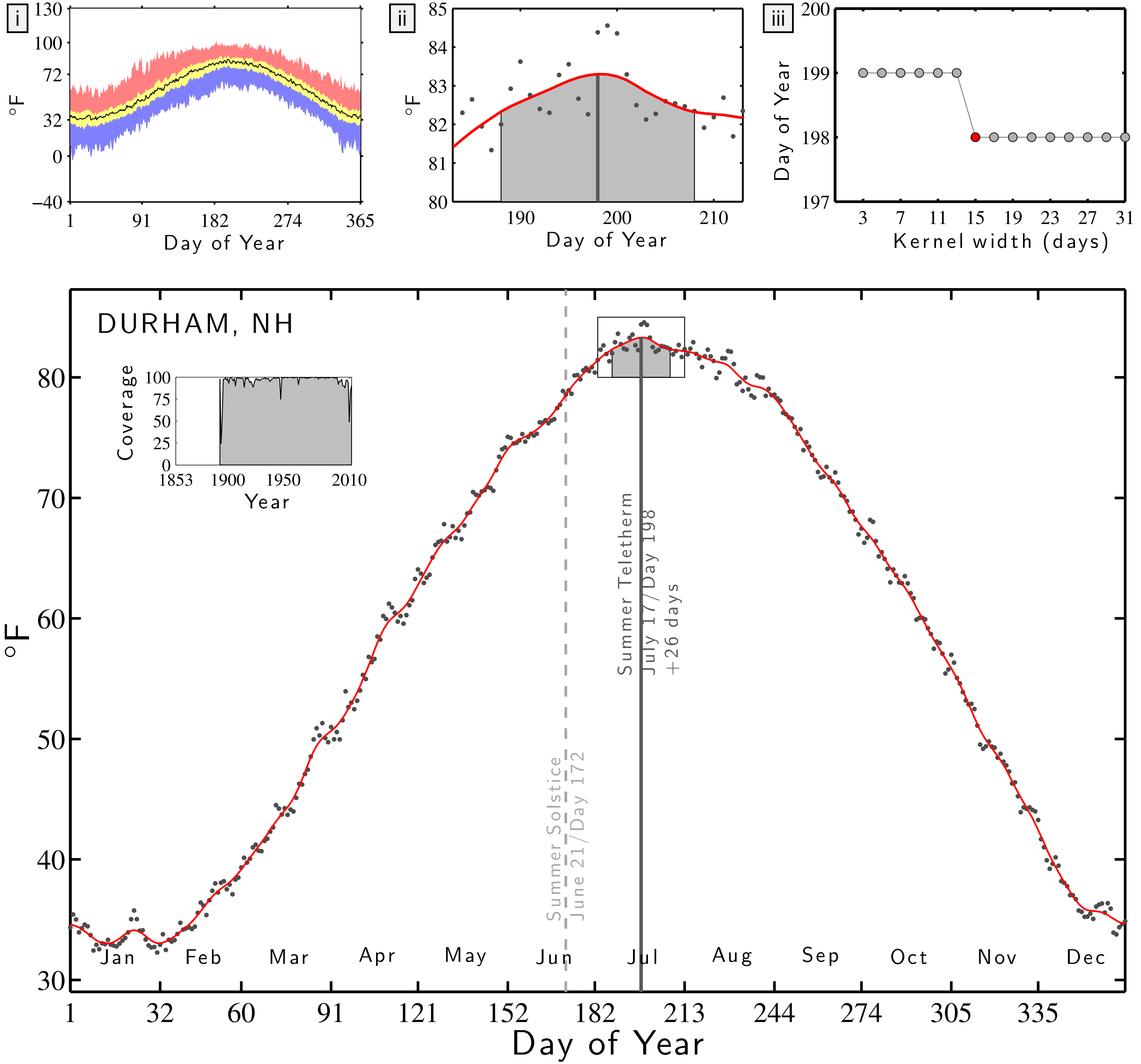

i
ii
iii
Day of Year
°F
Kernel width (days)
DURHAM, NH
Coverage
Year
Summer Solstice
June 21/Day 172
Summer Teletherm
July 17/Day 198
+26 days
Jan
Feb
Mar
Apr
May
Jun
Jul
Aug
Sep
Oct
Nov
Dec

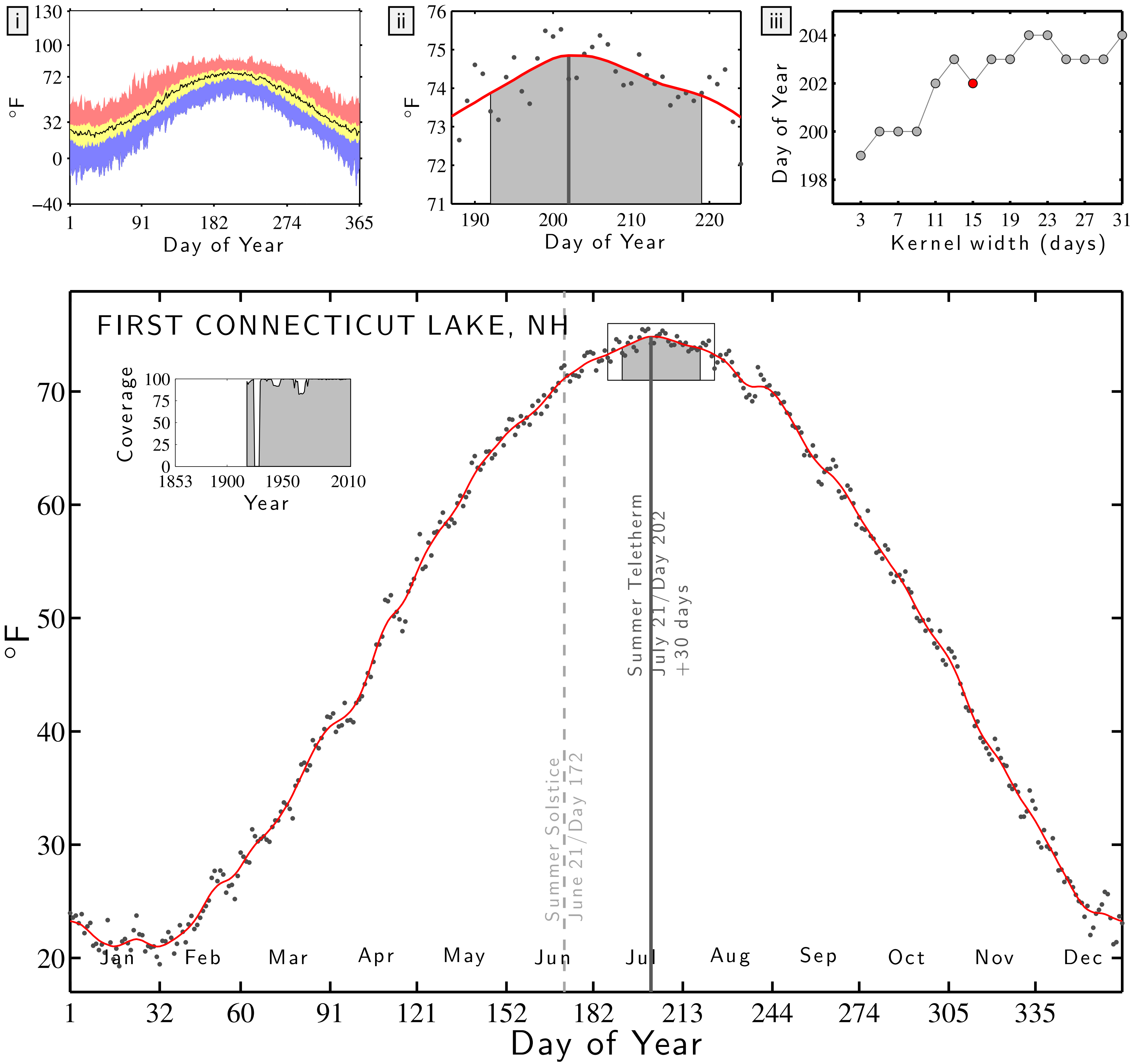

i
°F
Day of Year
ii
°F
Day of Year
iii
Day of Year
Kernel width (days)
FIRST CONNECTICUT LAKE, NH
Coverage
Year
Summer Solstice
June 21 / Day 172
Summer Teletherm
July 21 / Day 202
+30 days
Jan
Feb
Mar
Apr
May
Jun
Jul
Aug
Sep
Oct
Nov
Dec
°F
Day of Year

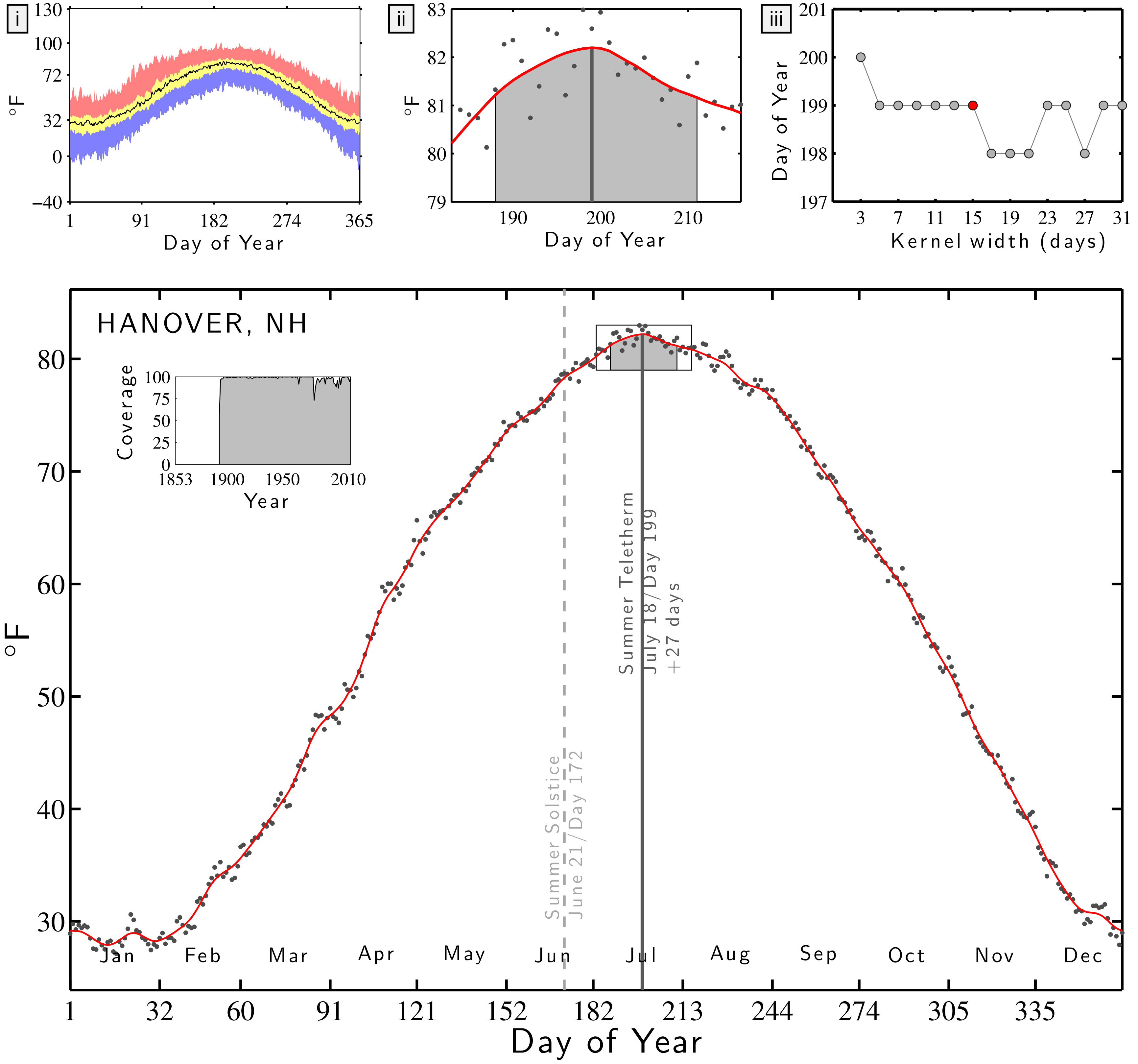

i
°F
Day of Year
ii
°F
Day of Year
iii
Day of Year
Kernel width (days)
HANOVER, NH
Coverage
Year
Summer Solstice
June 21 / Day 172
Summer Teletherm
July 18 / Day 199
+27 days
Jan
Feb
Mar
Apr
May
Jun
Jul
Aug
Sep
Oct
Nov
Dec
°F
Day of Year

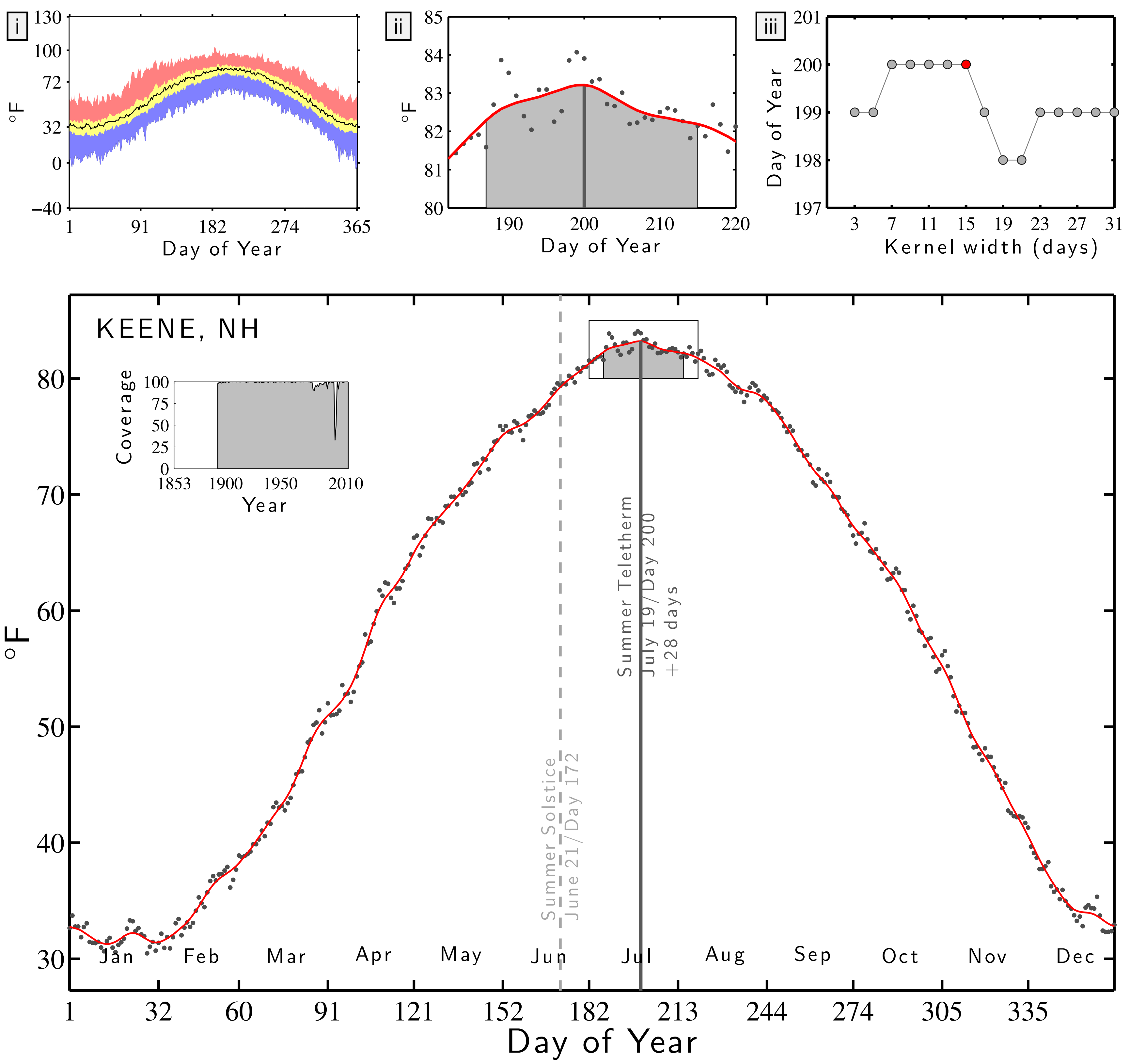
i
ii
iii
°F
Day of Year
Kernel width (days)
Day of Year
KEENE, NH
Coverage
Year
Summer Solstice
June 21/Day 172
Summer Teletherm
July 19/Day 200
+28 days
Jan
Feb
Mar
Apr
May
Jun
Jul
Aug
Sep
Oct
Nov
Dec

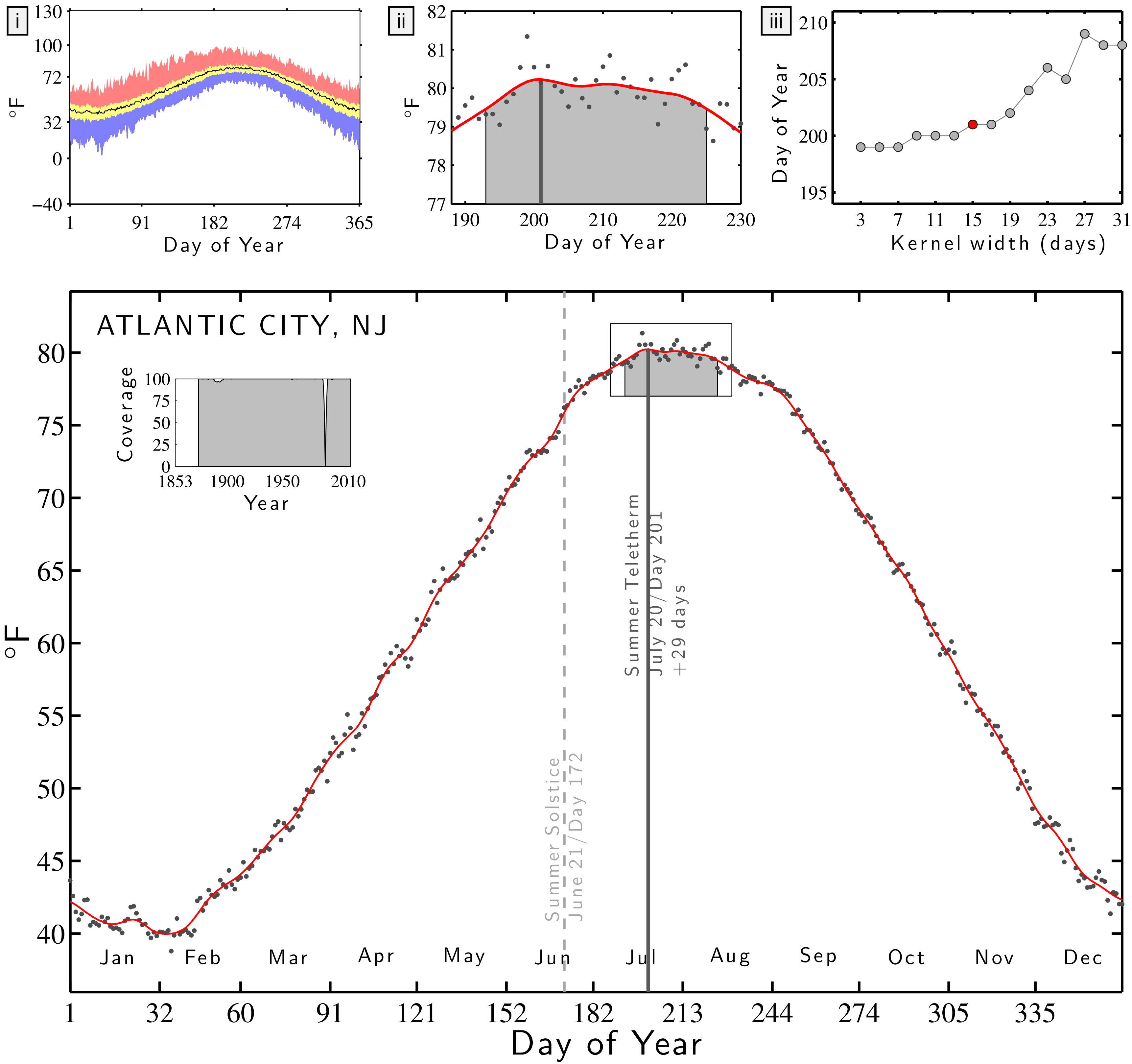

i
°F
Day of Year
ii
°F
Day of Year
iii
Day of Year
Kernel width (days)
ATLANTIC CITY, NJ
Coverage
Year
Summer Solstice
June 21/Day 172
Summer Teletherm
July 20/Day 201
+29 days
Jan
Feb
Mar
Apr
May
Jun
Jul
Aug
Sep
Oct
Nov
Dec
°F
Day of Year

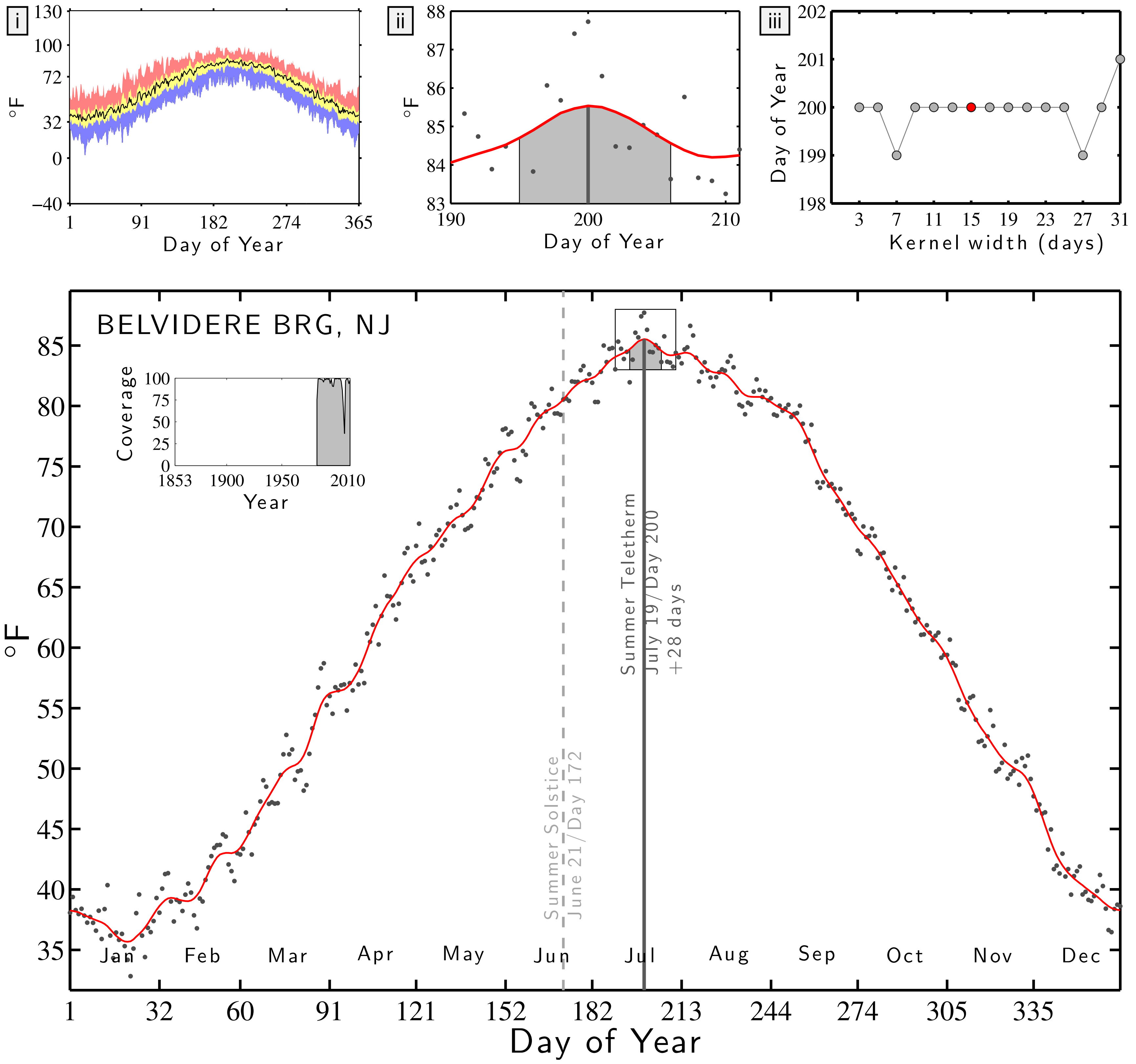

i
ii
iii
°F
Day of Year
Kernel width (days)
BELVIDERE BRG, NJ
Coverage
Year
Summer Solstice
June 21/Day 172
Summer Teletherm
July 19/Day 200
+28 days
Jan
Feb
Mar
Apr
May
Jun
Jul
Aug
Sep
Oct
Nov
Dec

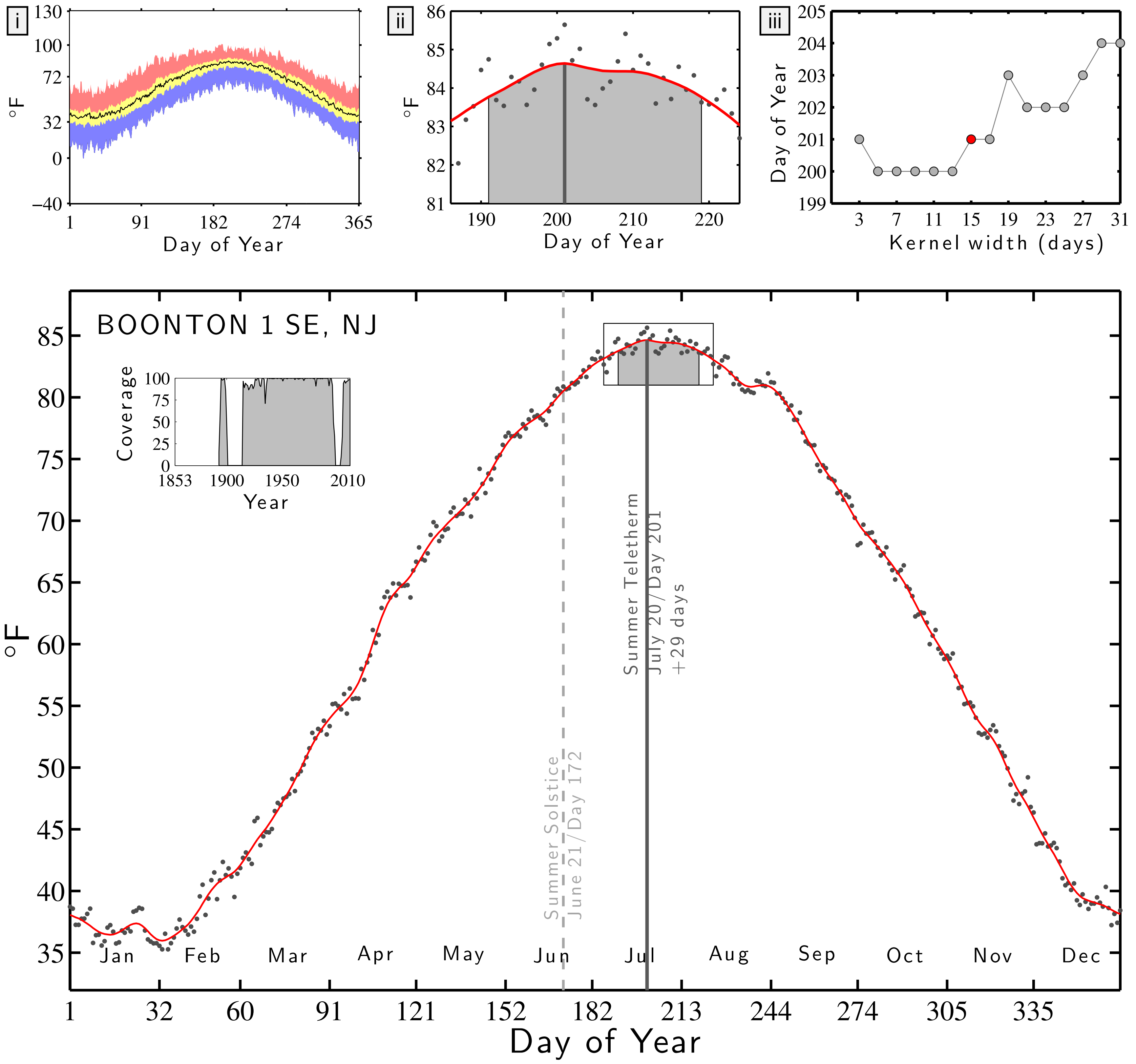

i
°F
Day of Year
ii
°F
Day of Year
iii
Day of Year
Kernel width (days)
BOONTON 1 SE, NJ
Coverage
Year
Summer Solstice
June 21/Day 172
Summer Teletherm
July 20/Day 201
+29 days
Jan
Feb
Mar
Apr
May
Jun
Jul
Aug
Sep
Oct
Nov
Dec
°F
Day of Year

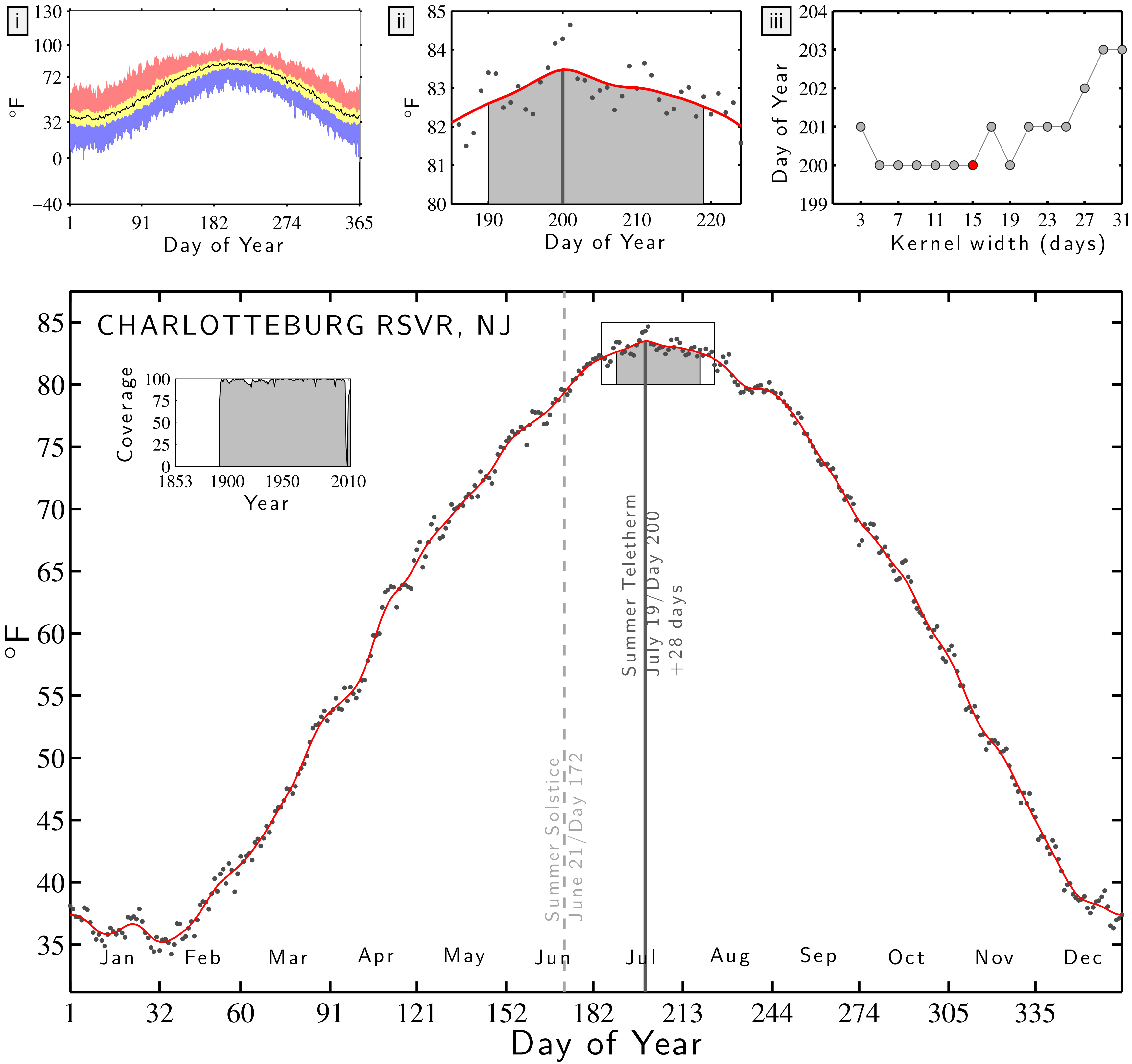

i
ii
iii
CHARLOTTEBURG RSVR, NJ
Coverage
Year
Summer Solstice
June 21/Day 172
Summer Teletherm
July 19/Day 200
+28 days
Day of Year
Kernel width (days)
°F
Jan
Feb
Mar
Apr
May
Jun
Jul
Aug
Sep
Oct
Nov
Dec

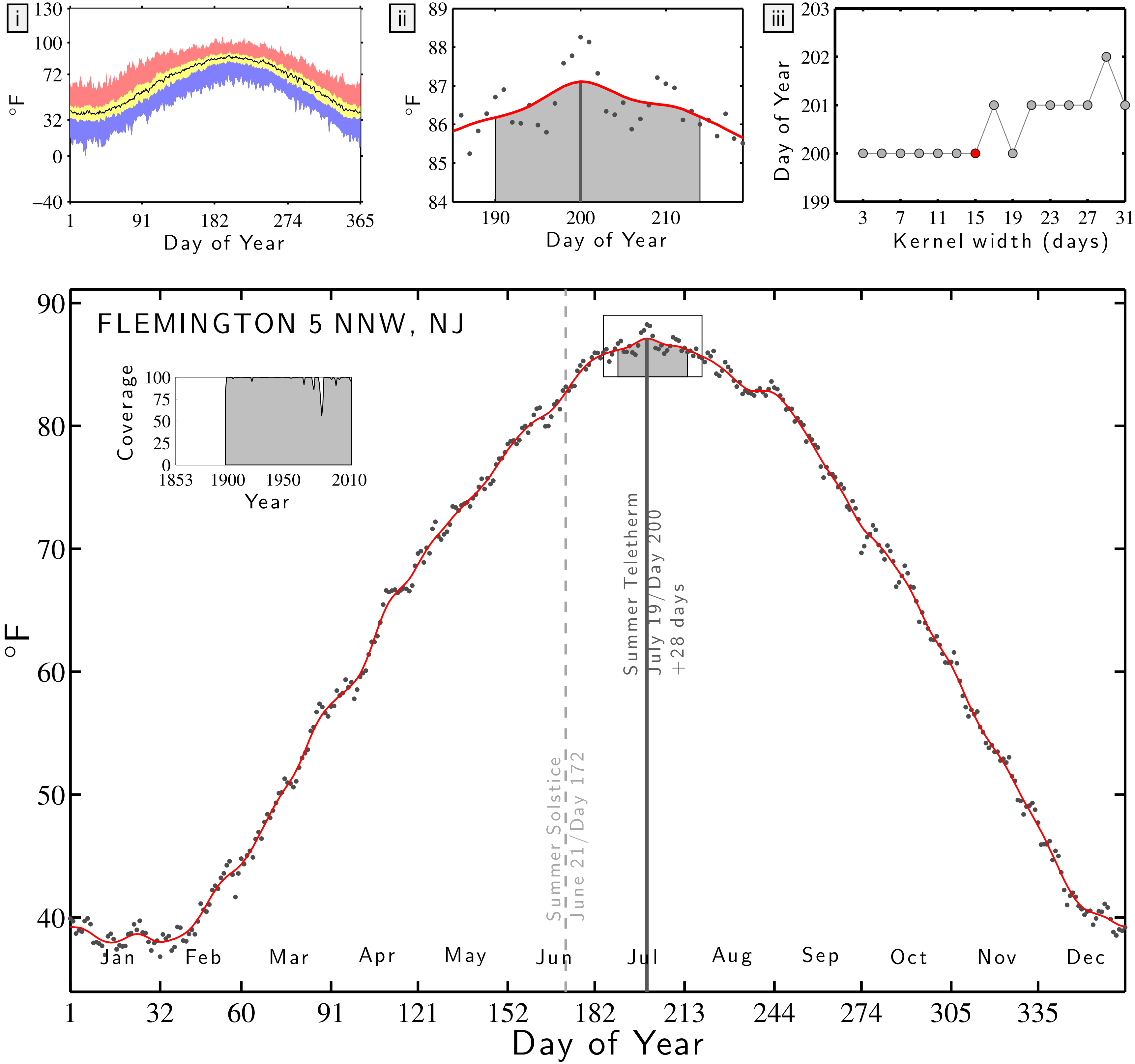
i
°F
Day of Year
ii
°F
Day of Year
iii
Day of Year
Kernel width (days)
FLEMINGTON 5 NNW, NJ
Coverage
Year
Summer Solstice
June 21/Day 172
Summer Teletherm
July 19/Day 200
+28 days
Jan
Feb
Mar
Apr
May
Jun
Jul
Aug
Sep
Oct
Nov
Dec
°F
Day of Year

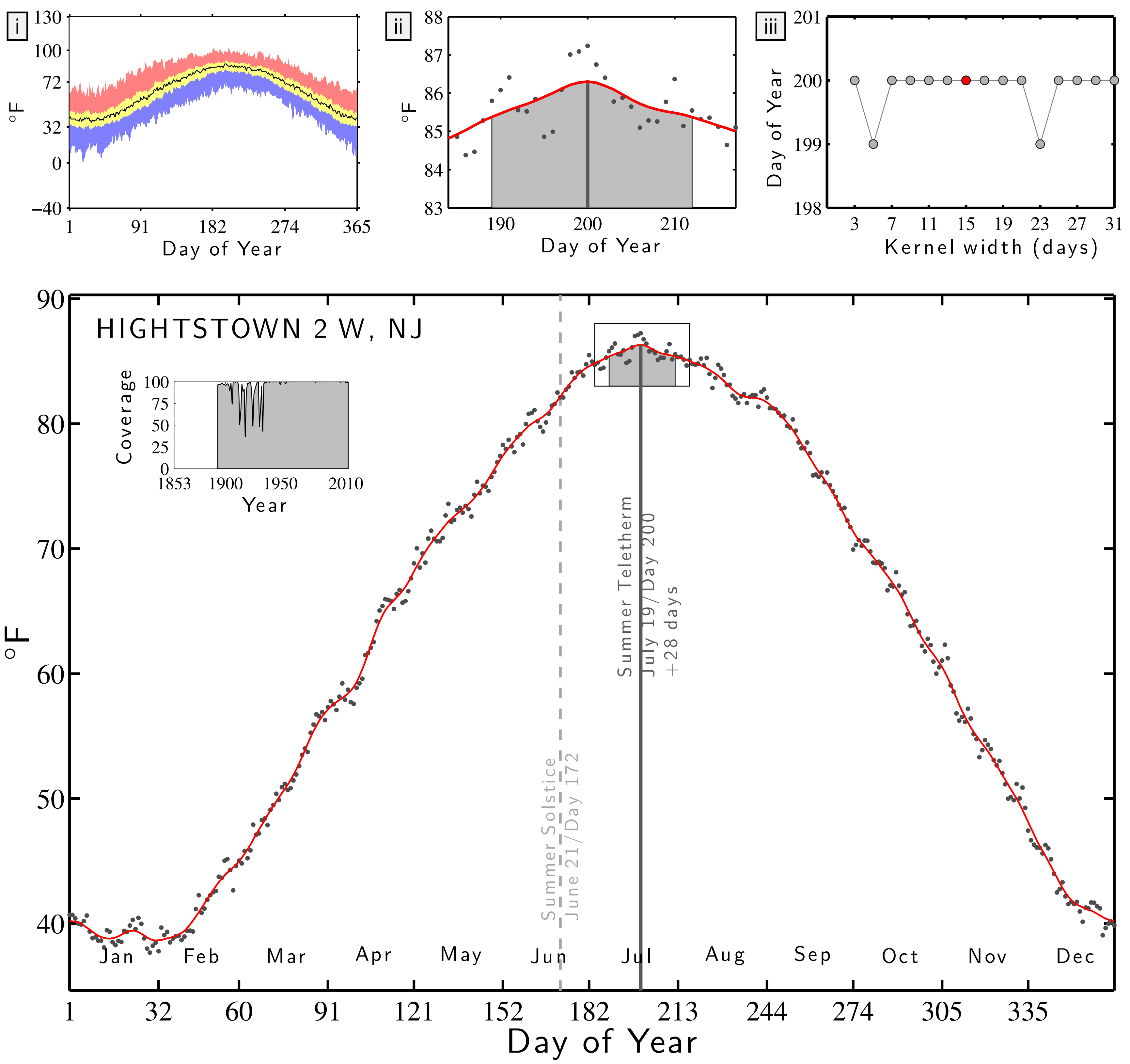

i
°F
Day of Year
ii
°F
Day of Year
iii
Day of Year
Kernel width (days)
HIGHTSTOWN 2 W, NJ
Coverage
Year
Summer Solstice
June 21/Day 172
Summer Teletherm
July 19/Day 200
+28 days
Jan
Feb
Mar
Apr
May
Jun
Jul
Aug
Sep
Oct
Nov
Dec
°F
Day of Year

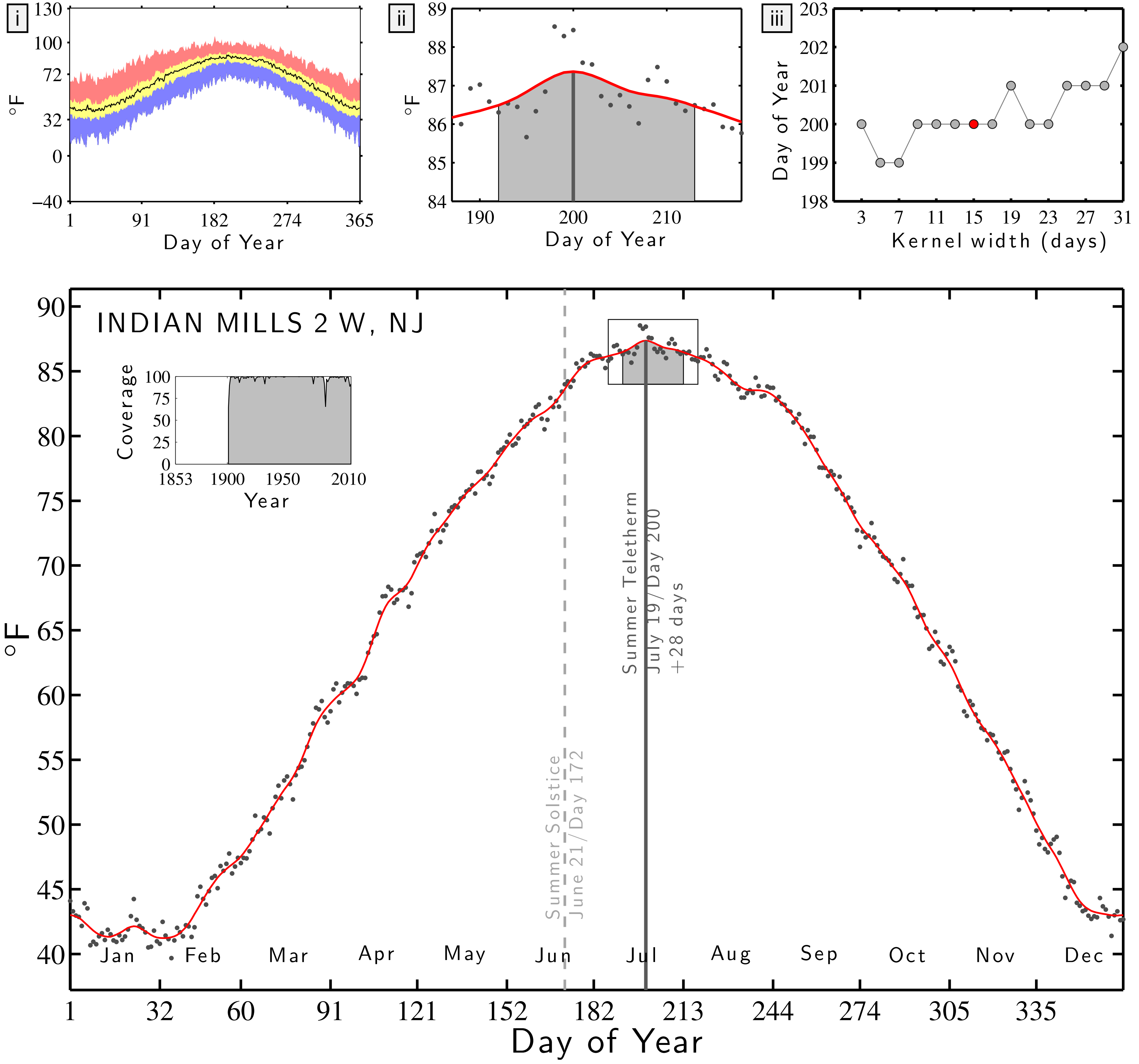

i
°F
Day of Year
ii
°F
Day of Year
iii
Day of Year
Kernel width (days)
INDIAN MILLS 2 W, NJ
Coverage
Year
Summer Solstice
June 21/Day 172
Summer Teletherm
July 19/Day 200
+28 days
Jan
Feb
Mar
Apr
May
Jun
Jul
Aug
Sep
Oct
Nov
Dec
°F
Day of Year

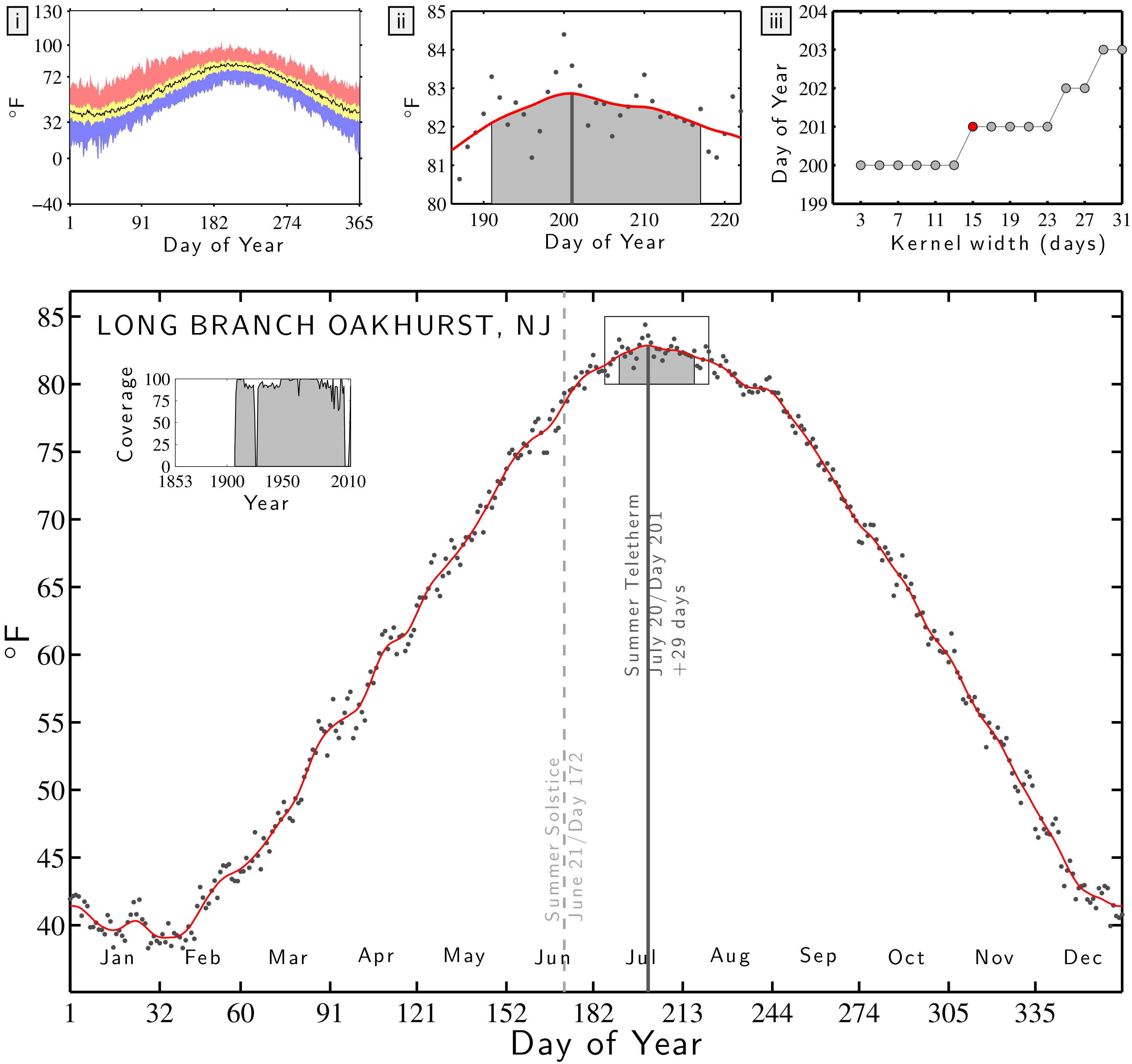

i
ii
iii
°F
Day of Year
Day of Year
Kernel width (days)
LONG BRANCH OAKHURST, NJ
Coverage
Year
Summer Solstice
June 21/Day 172
Summer Teletherm
July 20/Day 201
+29 days
Jan
Feb
Mar
Apr
May
Jun
Jul
Aug
Sep
Oct
Nov
Dec
Day of Year

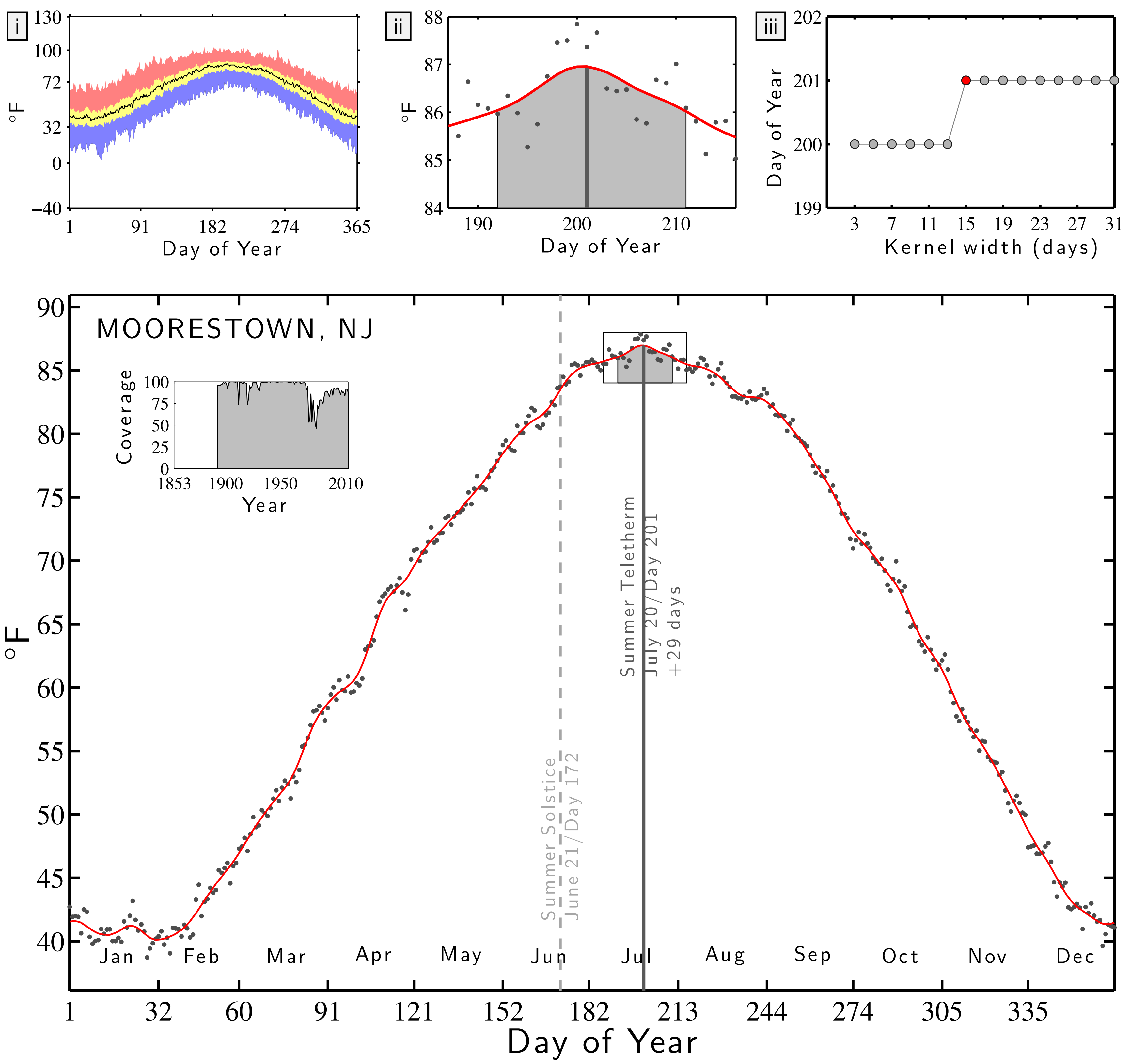

i
°F
Day of Year
ii
°F
Day of Year
iii
Day of Year
Kernel width (days)
MOORESTOWN, NJ
Coverage
Year
Summer Solstice
June 21/Day 172
Summer Teletherm
July 20/Day 201
+29 days
Jan
Feb
Mar
Apr
May
Jun
Jul
Aug
Sep
Oct
Nov
Dec
°F
Day of Year

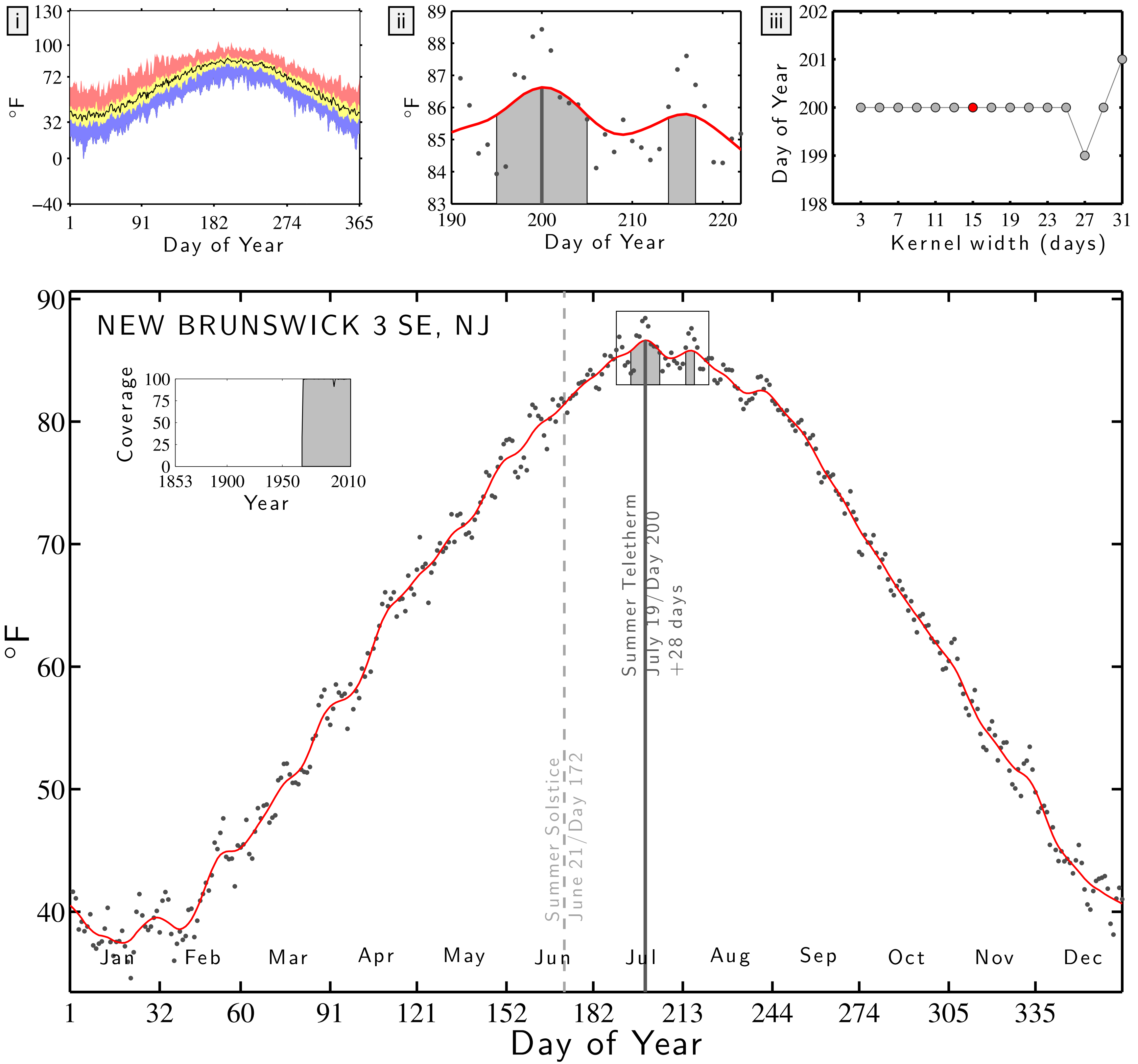

i
°F
Day of Year
ii
Day of Year
iii
Day of Year
Kernel width (days)
NEW BRUNSWICK 3 SE, NJ
Coverage
Year
Summer Solstice
June 21/Day 172
Summer Teletherm
July 19/Day 200
+28 days
Jan
Feb
Mar
Apr
May
Jun
Jul
Aug
Sep
Oct
Nov
Dec
Day of Year

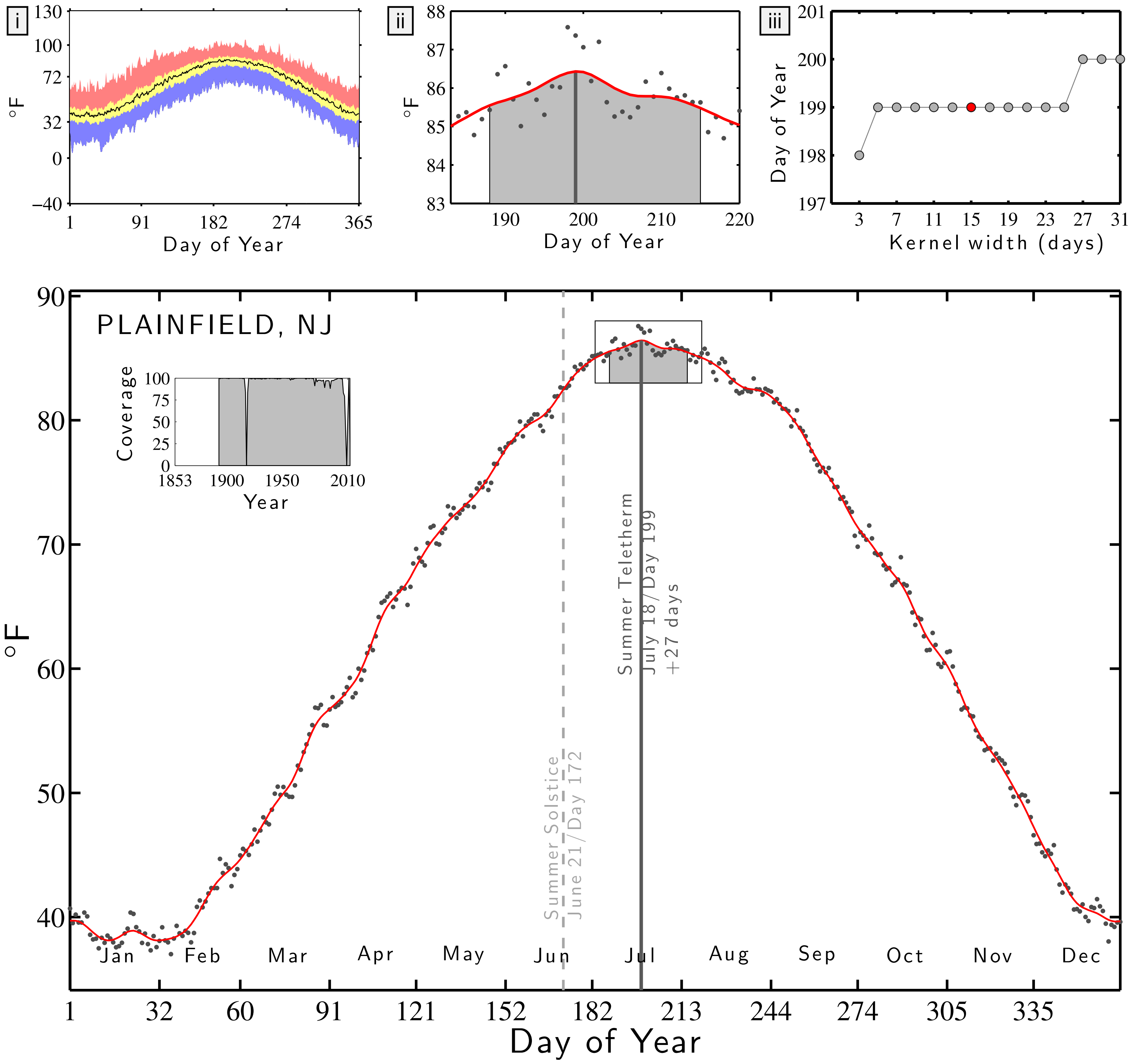

i
ii
iii
°F
Day of Year
Kernel width (days)
PLAINFIELD, NJ
Coverage
Year
Summer Solstice
June 21/Day 172
Summer Teletherm
July 18/Day 199
+27 days
Jan
Feb
Mar
Apr
May
Jun
Jul
Aug
Sep
Oct
Nov
Dec

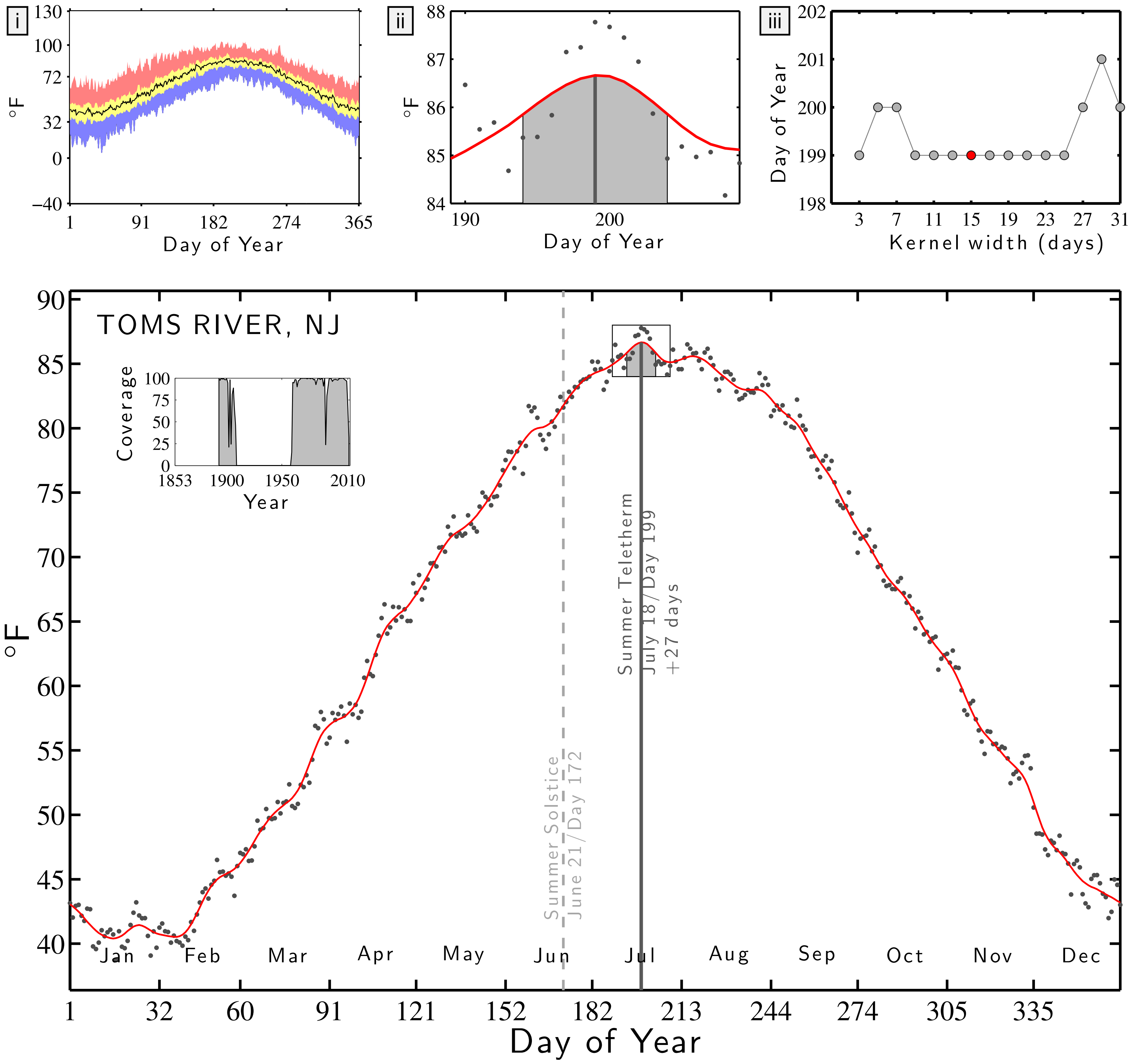

i
°F
Day of Year
ii
°F
Day of Year
iii
Day of Year
Kernel width (days)
TOMS RIVER, NJ
Coverage
Year
Summer Solstice
June 21/Day 172
Summer Teletherm
July 18/Day 199
+27 days
Jan
Feb
Mar
Apr
May
Jun
Jul
Aug
Sep
Oct
Nov
Dec
°F
Day of Year

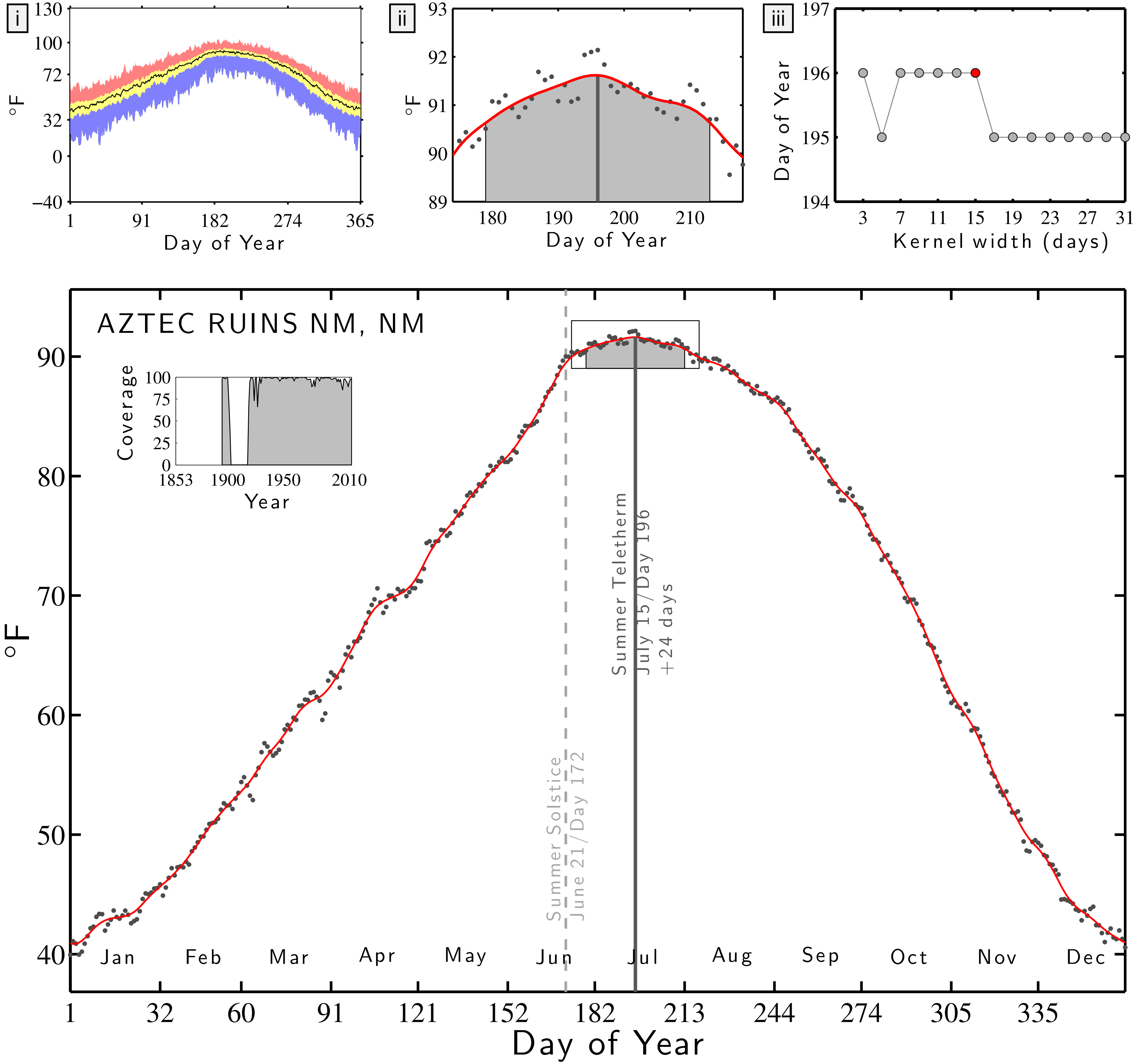

i
ii
iii
°F
Day of Year
Kernel width (days)
AZTEC RUINS NM, NM
Coverage
Year
Summer Solstice
June 21 / Day 172
Summer Teletherm
July 15 / Day 196
+24 days
Jan
Feb
Mar
Apr
May
Jun
Jul
Aug
Sep
Oct
Nov
Dec

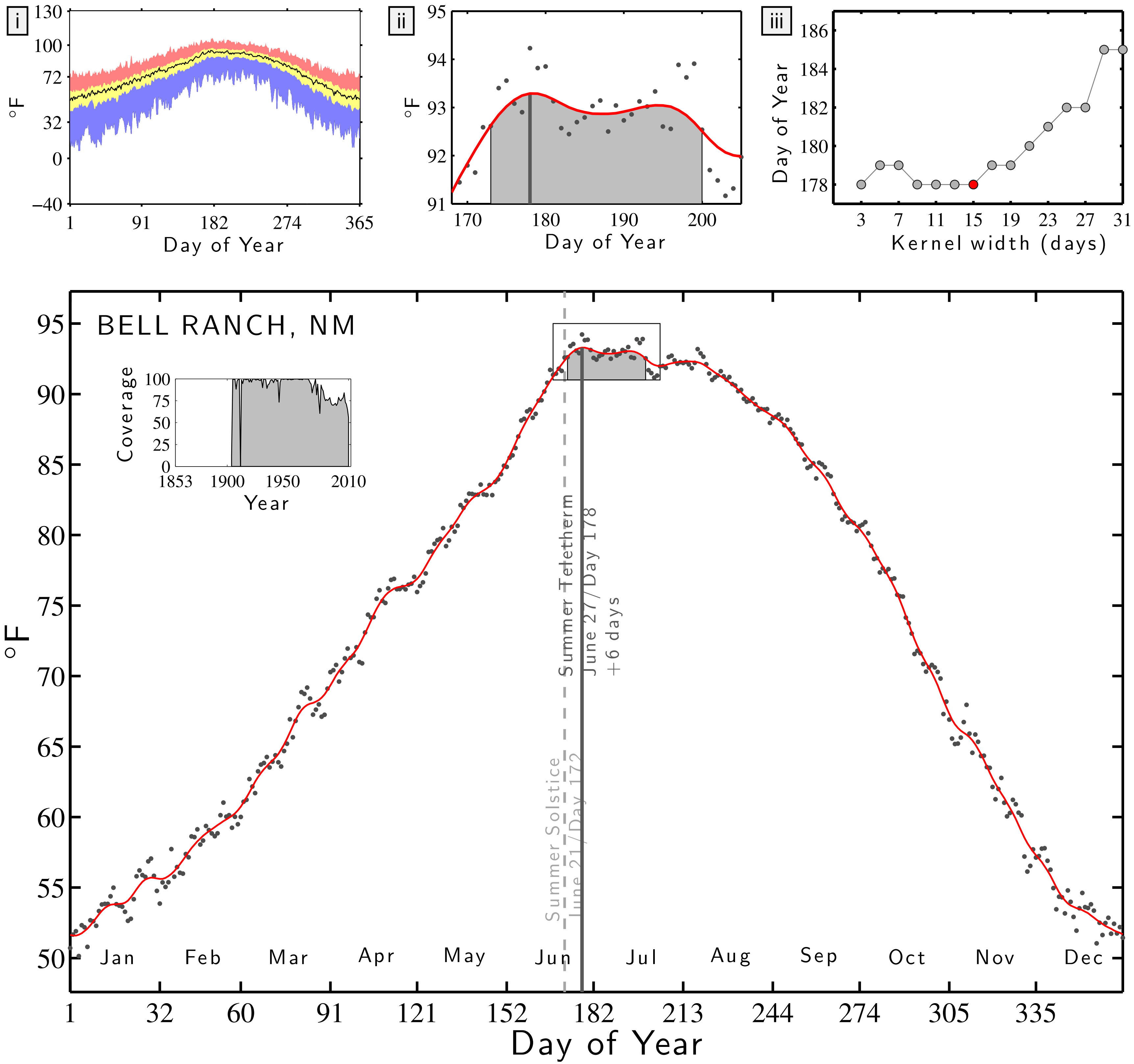

i
°F
130
100
72
32
0
−40
1
91
182
274
365
Day of Year
ii
°F
95
94
93
92
91
170
180
190
200
Day of Year
iii
Day of Year
186
184
182
180
178
3
7
11
15
19
23
27
31
Kernel width (days)
BELL RANCH, NM
Coverage
100
75
50
25
0
1853
1900
1950
2010
Year
Summer Teletherm
June 27 / Day 178
+6 days
Summer Solstice
June 21 / Day 172
Jan
Feb
Mar
Apr
May
Jun
Jul
Aug
Sep
Oct
Nov
Dec
95
90
85
80
75
70
65
60
55
50
°F
1
32
60
91
121
152
182
213
244
274
305
335
Day of Year

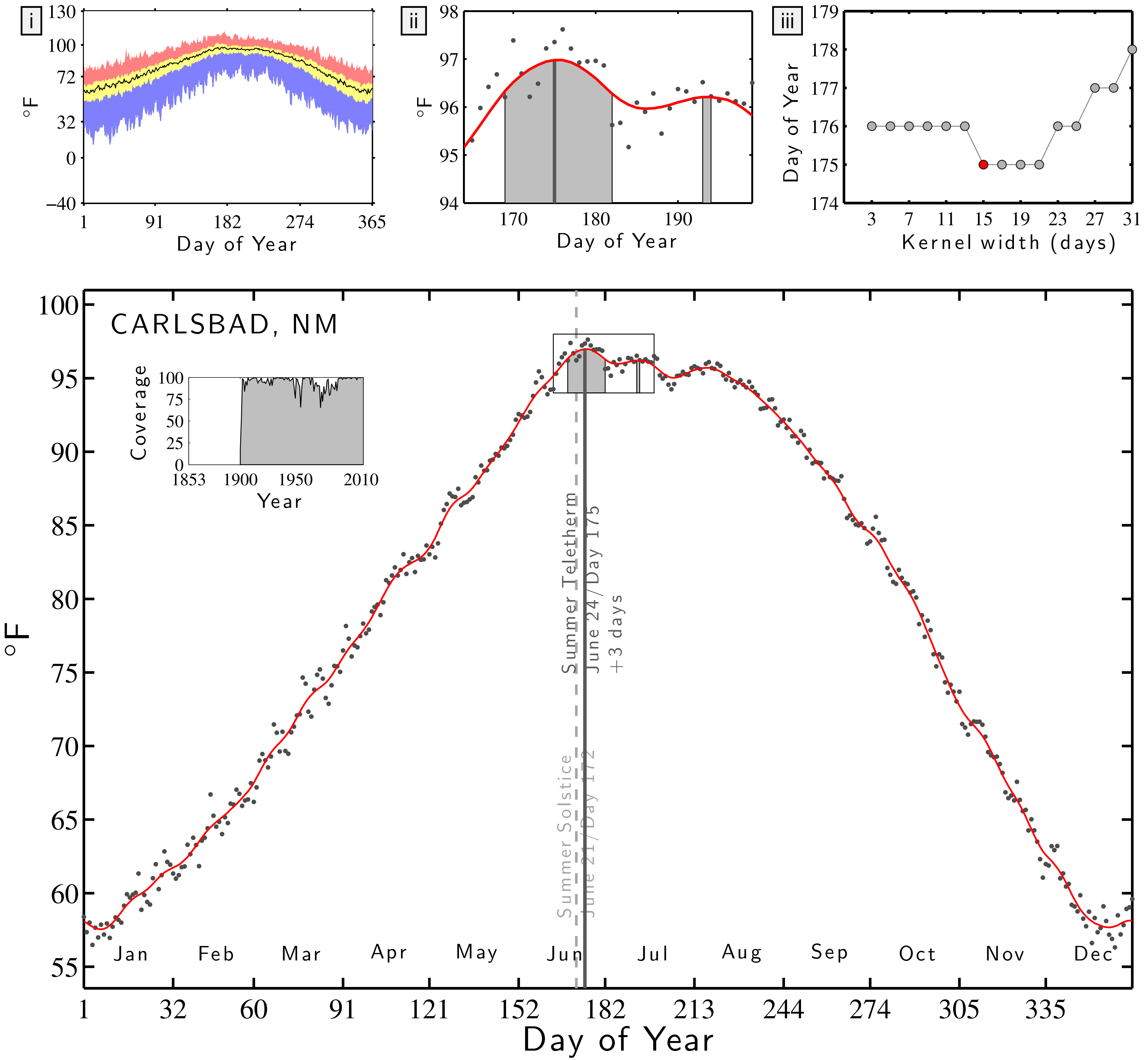

i
ii
iii
Day of Year
°F
Kernel width (days)
CARLSBAD, NM
Coverage
Year
Summer Teletherm
June 24/Day 175
+3 days
Summer Solstice
June 21/Day 172
Jan
Feb
Mar
Apr
May
Jun
Jul
Aug
Sep
Oct
Nov
Dec

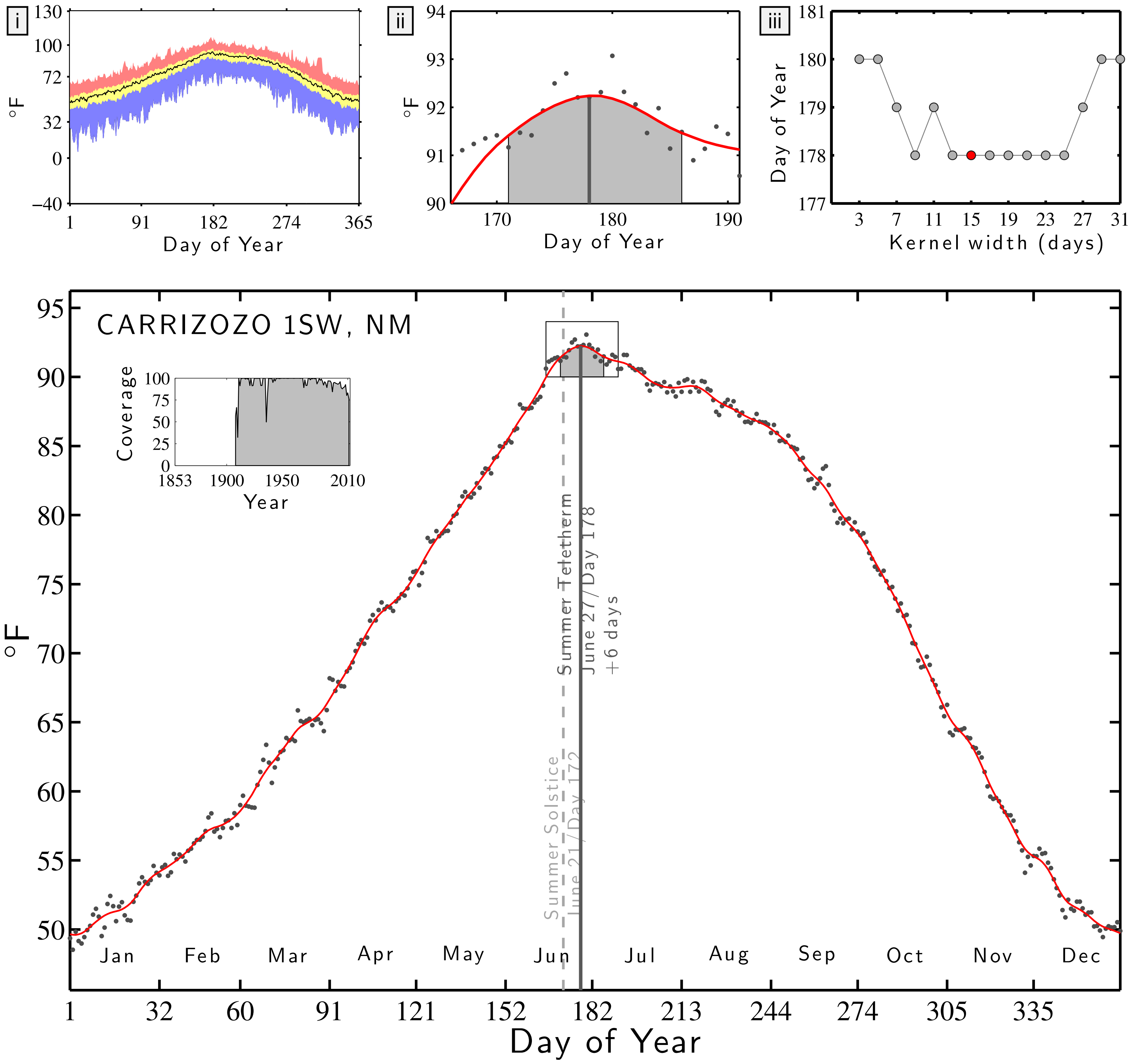

i
°F
Day of Year
ii
°F
Day of Year
iii
Day of Year
Kernel width (days)
CARRIZOZO 1SW, NM
Coverage
Year
Summer Solstice
June 21 / Day 172
Summer Teletherm
June 27 / Day 178
+6 days
Jan
Feb
Mar
Apr
May
Jun
Jul
Aug
Sep
Oct
Nov
Dec
°F
Day of Year

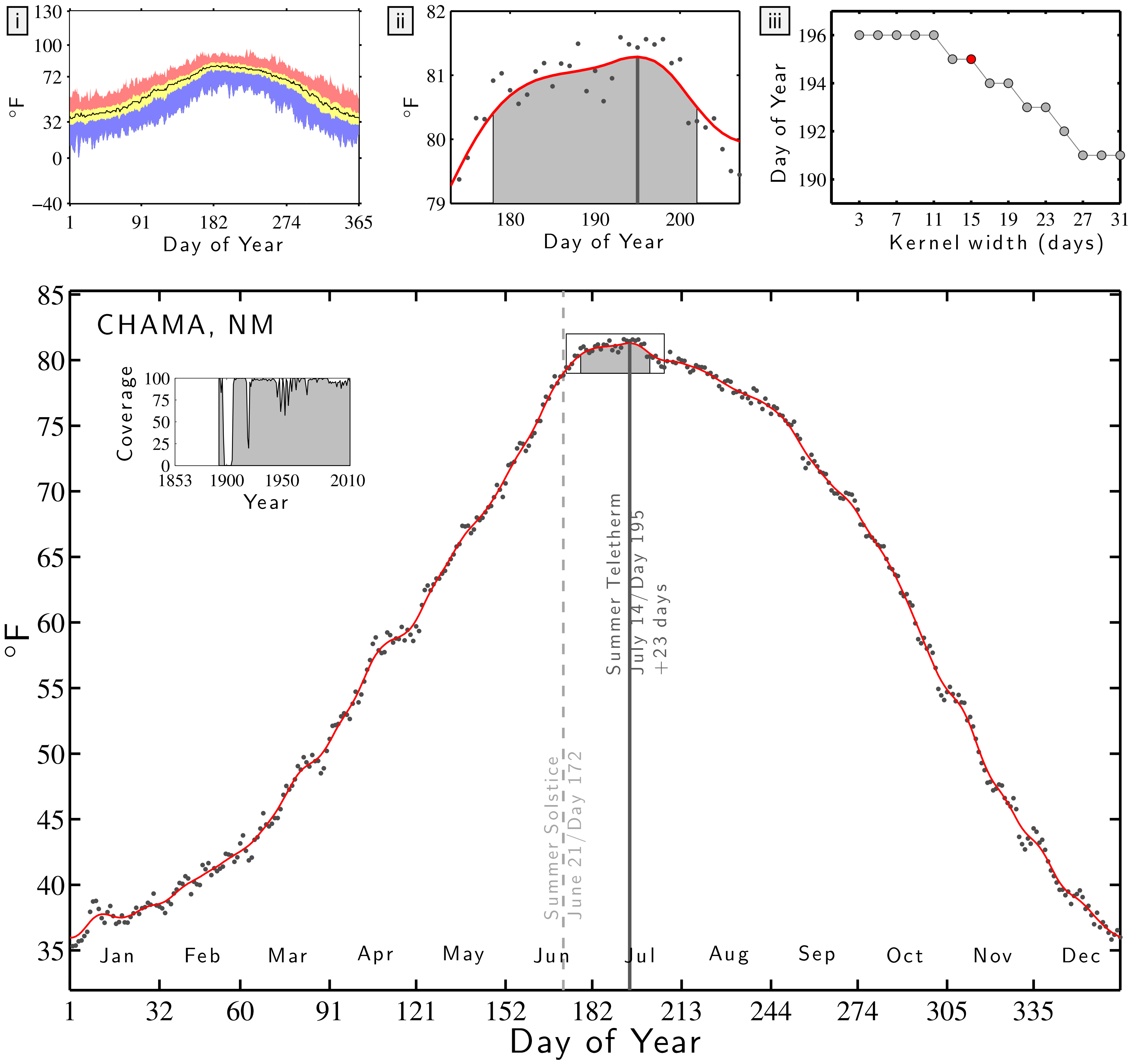

i
°F
Day of Year
ii
°F
Day of Year
iii
Day of Year
Kernel width (days)
CHAMA, NM
Coverage
Year
Summer Solstice
June 21/Day 172
Summer Teletherm
July 14/Day 195
+23 days
Jan
Feb
Mar
Apr
May
Jun
Jul
Aug
Sep
Oct
Nov
Dec
°F
Day of Year

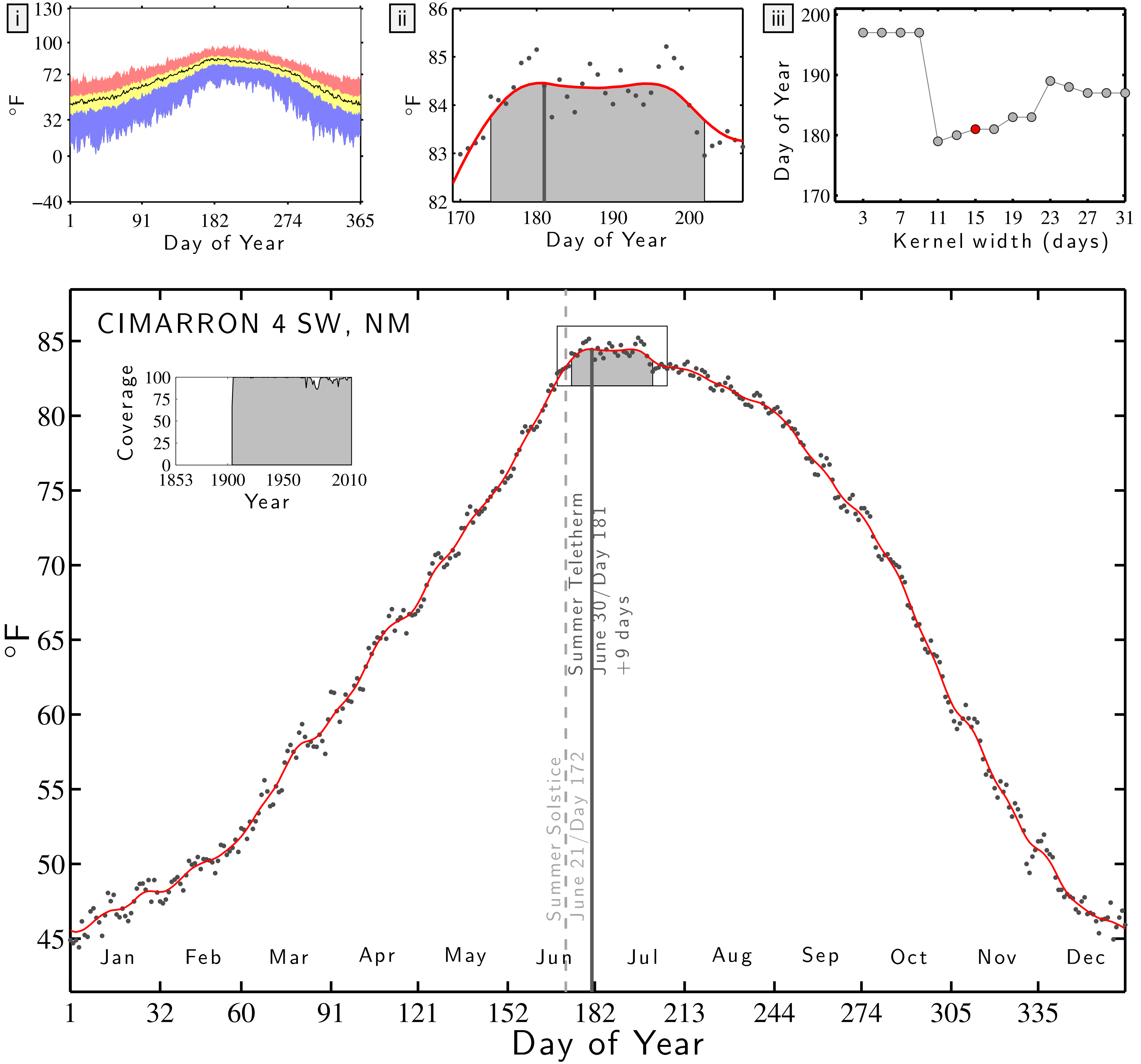

i
ii
iii
CIMARRON 4 SW, NM
Coverage
Year
Summer Solstice
June 21/Day 172
Summer Teletherm
June 30/Day 181
+9 days
Day of Year
Kernel width (days)
°F
Jan
Feb
Mar
Apr
May
Jun
Jul
Aug
Sep
Oct
Nov
Dec

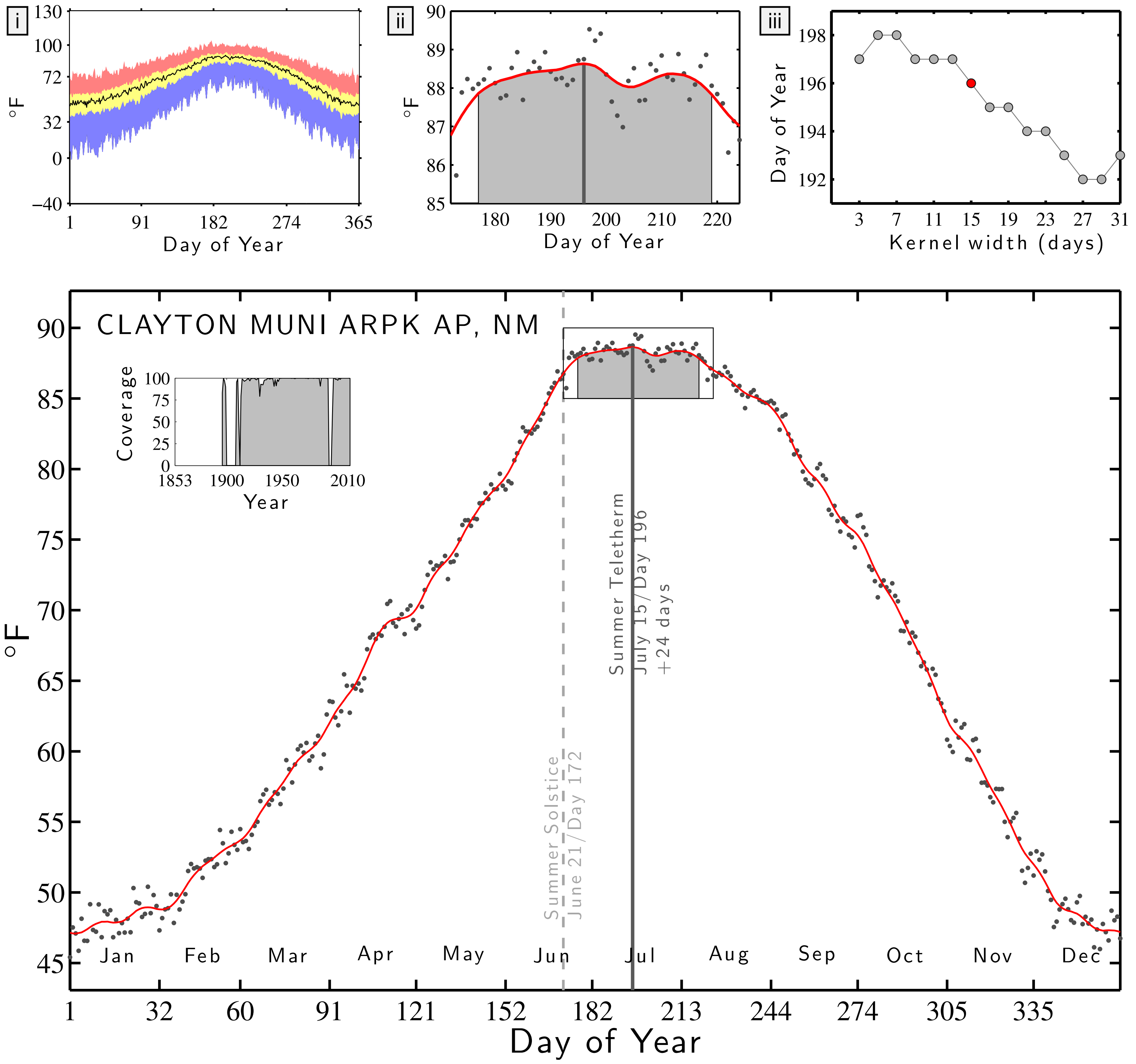

i
ii
iii
°F
Day of Year
Kernel width (days)
CLAYTON MUNI ARPK AP, NM
Coverage
Year
Summer Solstice
June 21/Day 172
Summer Teletherm
July 15/Day 196
+24 days
Jan
Feb
Mar
Apr
May
Jun
Jul
Aug
Sep
Oct
Nov
Dec

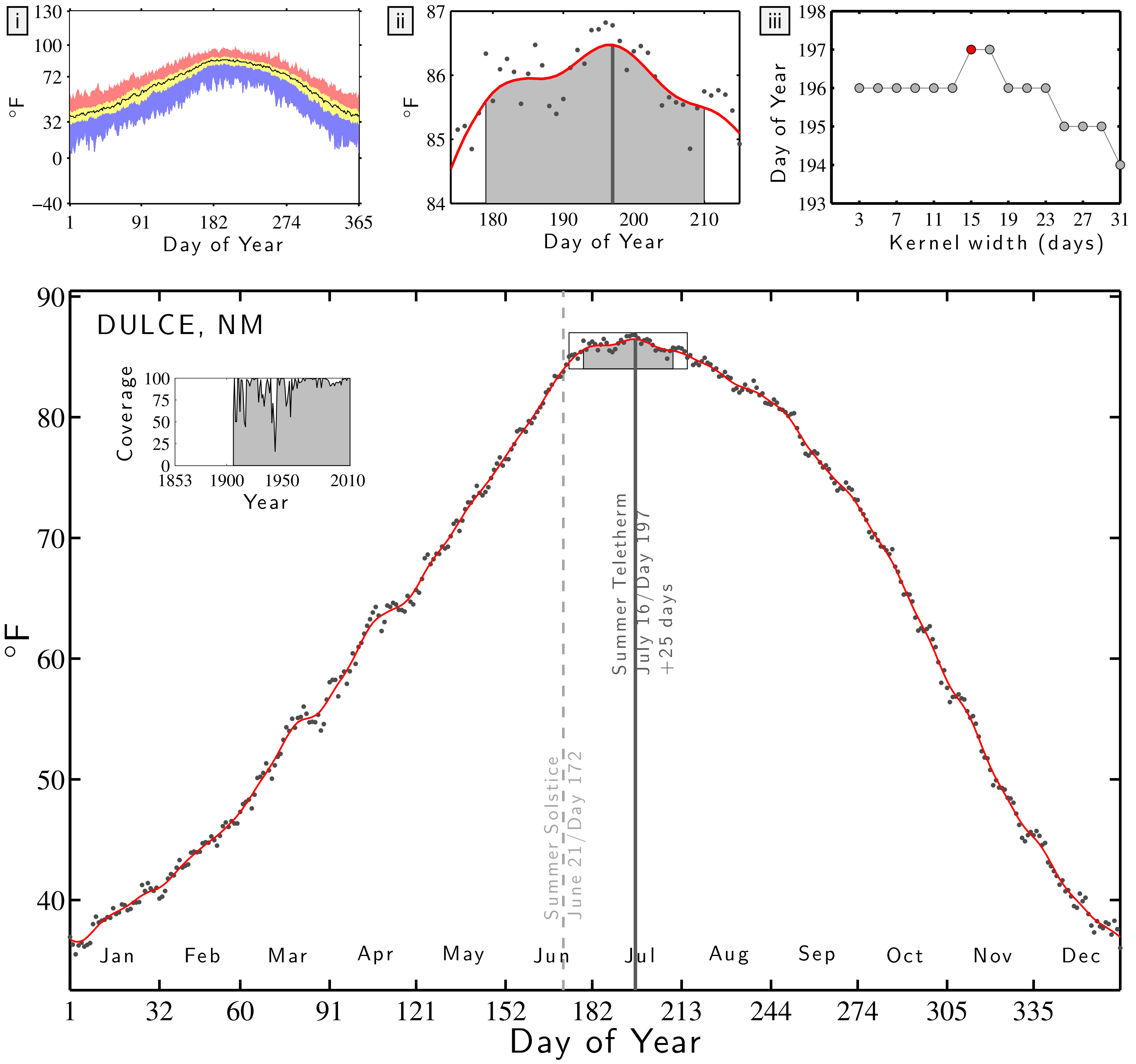

i
°F
Day of Year
ii
°F
Day of Year
iii
Day of Year
Kernel width (days)
DULCE, NM
Coverage
Year
Summer Solstice
June 21/Day 172
Summer Teletherm
July 16/Day 197
+25 days
Jan
Feb
Mar
Apr
May
Jun
Jul
Aug
Sep
Oct
Nov
Dec
°F
Day of Year

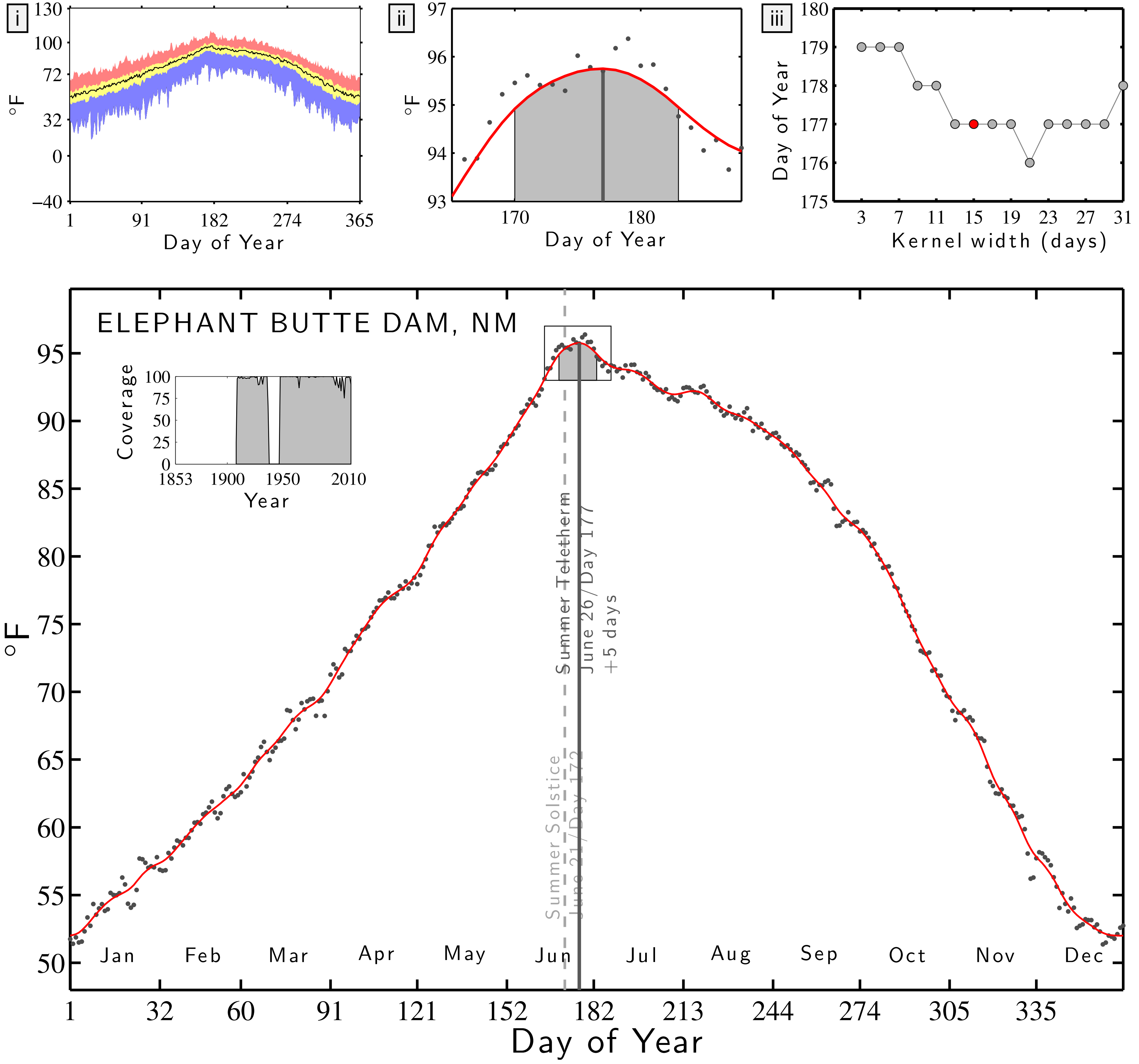

i
°F
Day of Year
ii
°F
Day of Year
iii
Day of Year
Kernel width (days)
ELEPHANT BUTTE DAM, NM
Coverage
Year
Summer Teletherm
June 26/Day 177
+5 days
Summer Solstice
June 21/Day 172
Jan
Feb
Mar
Apr
May
Jun
Jul
Aug
Sep
Oct
Nov
Dec
°F
Day of Year

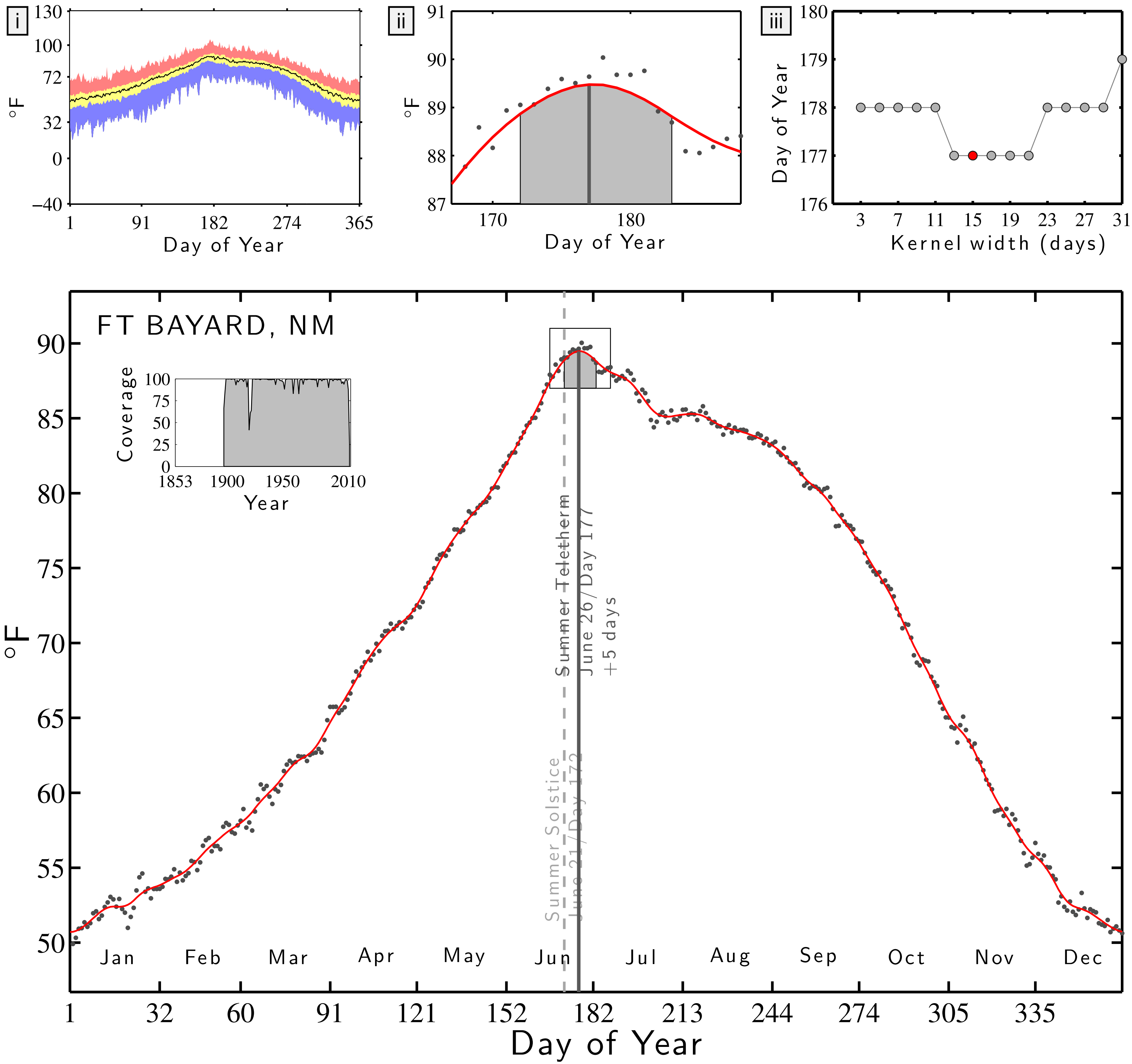

i
°F
Day of Year
ii
°F
Day of Year
iii
Day of Year
Kernel width (days)
FT BAYARD, NM
Coverage
Year
Summer Teletherm
June 26/Day 177
+5 days
Summer Solstice
June 21/Day 172
Jan
Feb
Mar
Apr
May
Jun
Jul
Aug
Sep
Oct
Nov
Dec
°F
Day of Year

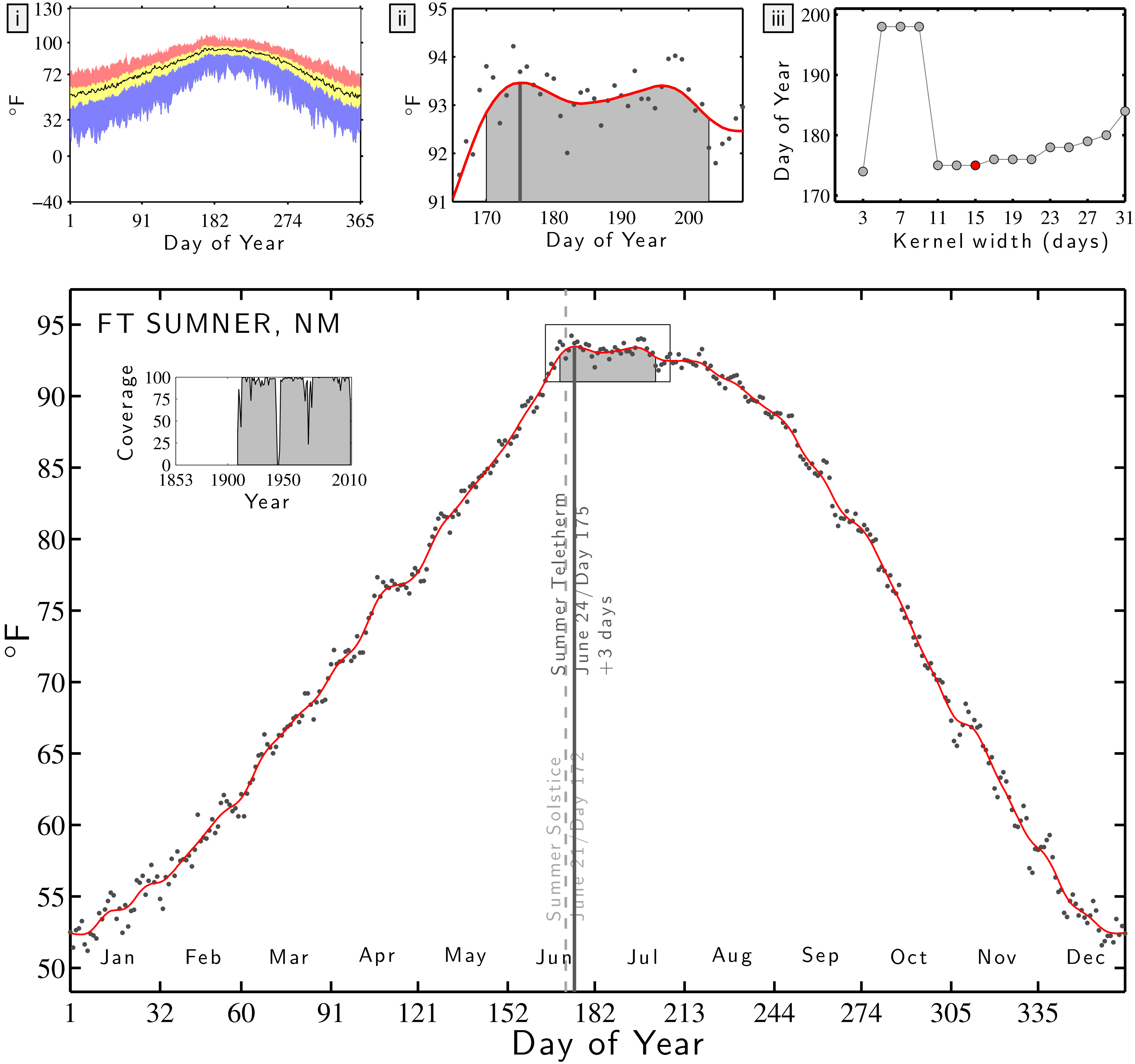

i
ii
iii
°F
Day of Year
Day of Year
Kernel width (days)
FT SUMNER, NM
Coverage
Year
Summer Teletherm
June 24/Day 175
+3 days
Summer Solstice
June 21/Day 172
Jan
Feb
Mar
Apr
May
Jun
Jul
Aug
Sep
Oct
Nov
Dec
Day of Year

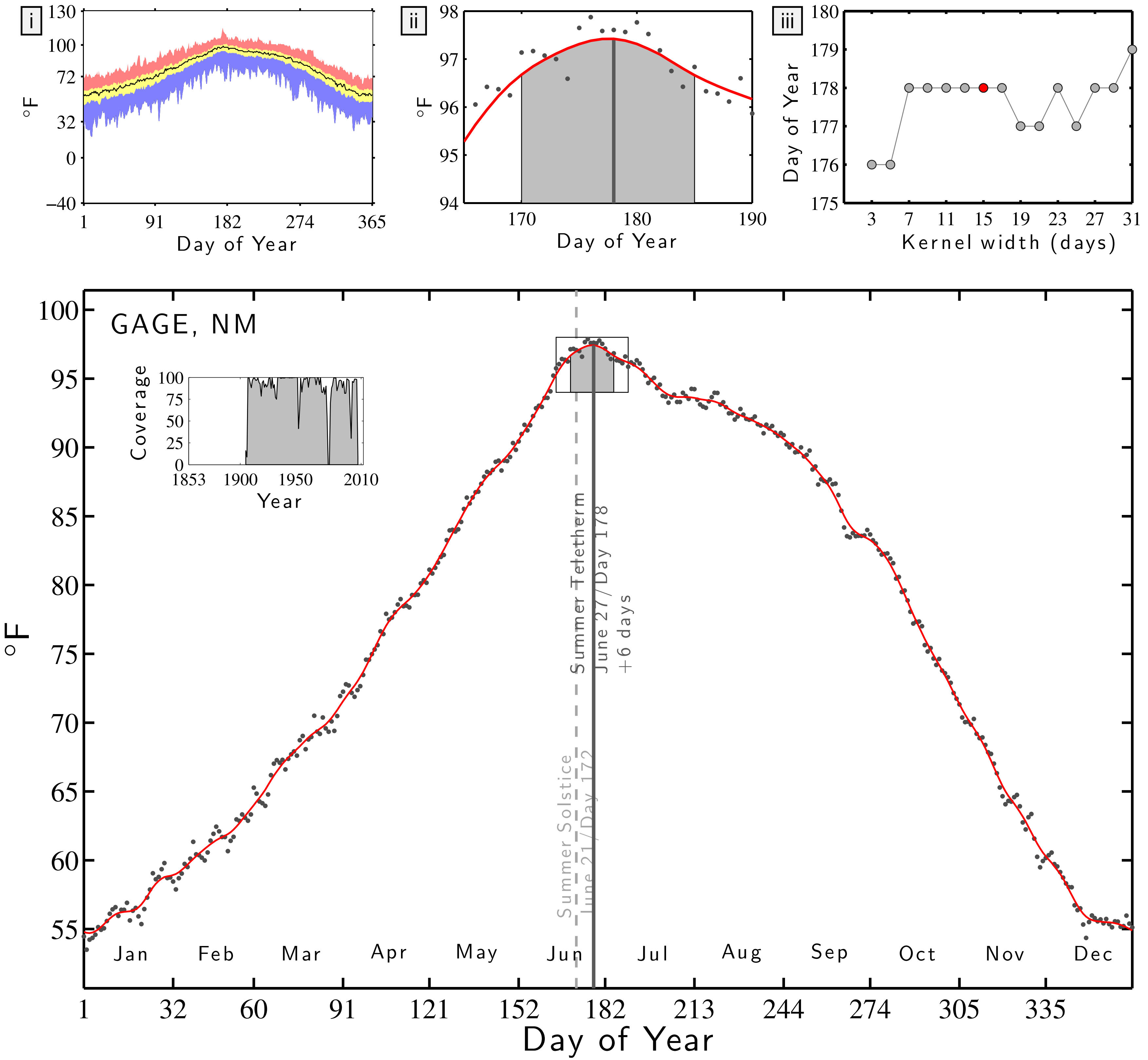

i
ii
iii
GAGE, NM
Coverage
Year
Summer Teletherm
June 27/Day 178
+6 days
Summer Solstice
June 21/Day 172
Day of Year
Kernel width (days)
°F
Jan
Feb
Mar
Apr
May
Jun
Jul
Aug
Sep
Oct
Nov
Dec

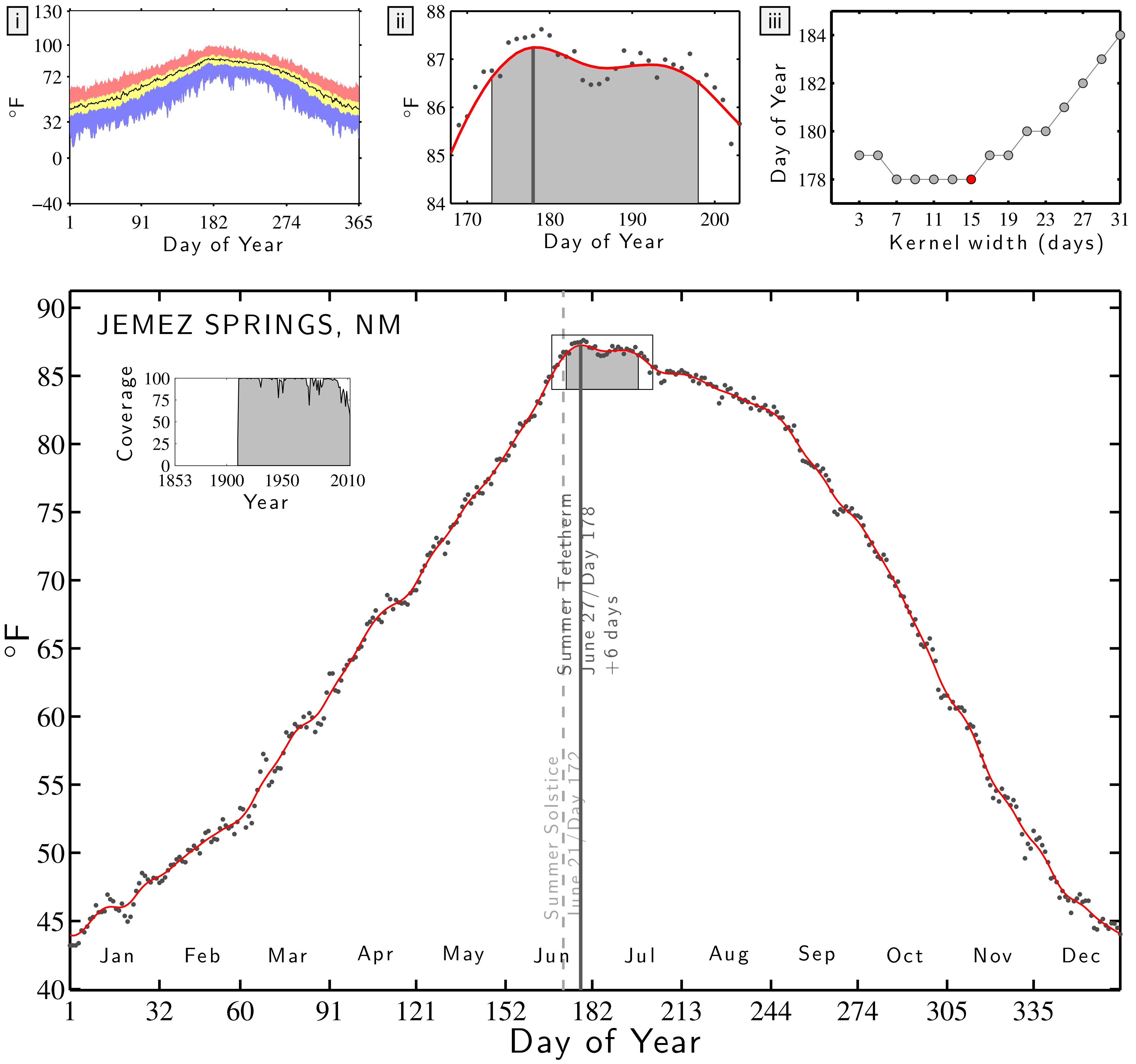

i
ii
iii
°F
Day of Year
Kernel width (days)
JEMEZ SPRINGS, NM
Coverage
Year
Summer Teletherm
June 27 / Day 178
+6 days
Summer Solstice
June 21 / Day 172
Jan
Feb
Mar
Apr
May
Jun
Jul
Aug
Sep
Oct
Nov
Dec

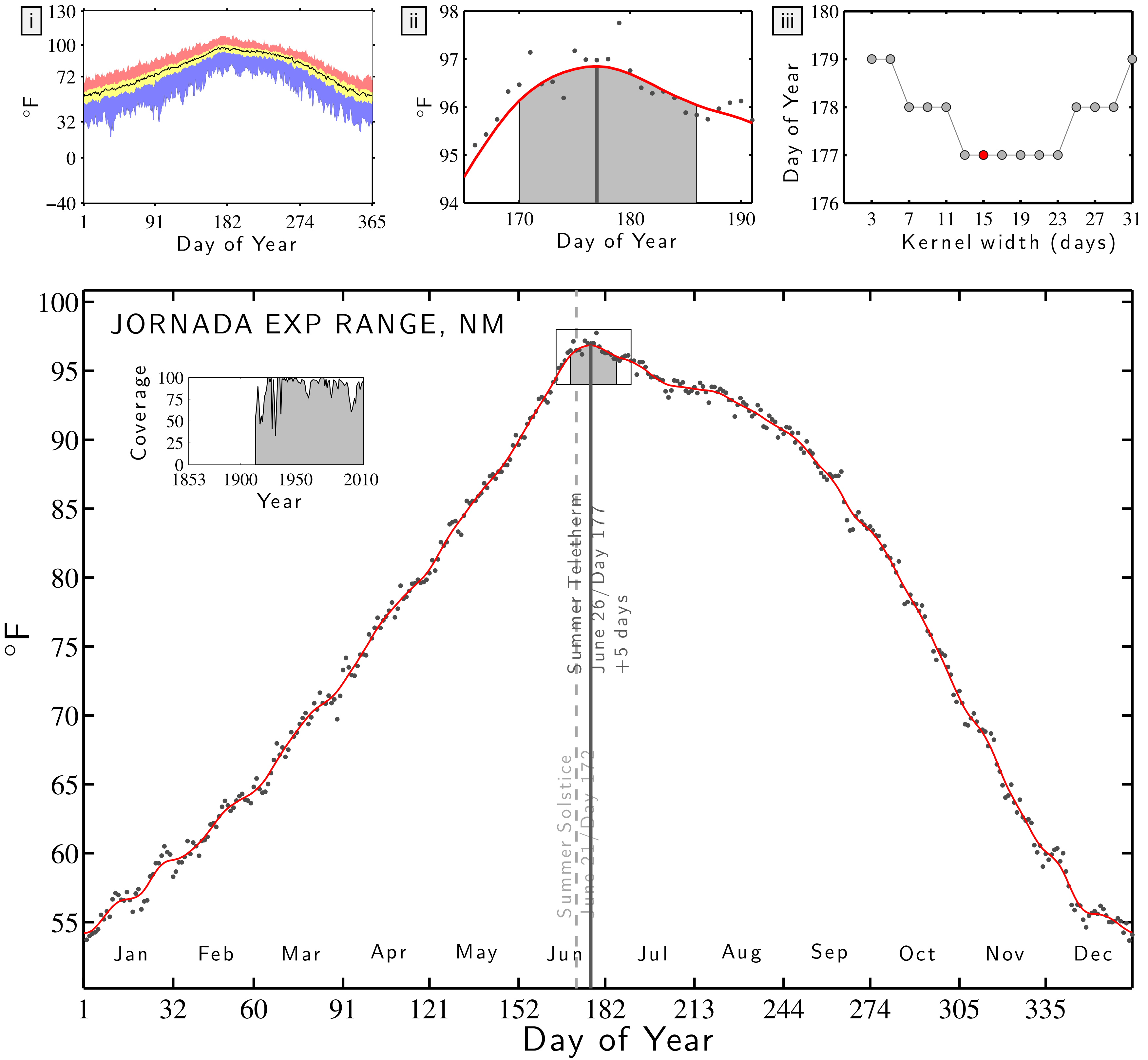

i
°F
Day of Year
ii
°F
Day of Year
iii
Day of Year
Kernel width (days)
JORNADA EXP RANGE, NM
Coverage
Year
Summer Teletherm
June 26/Day 177
+5 days
Summer Solstice
June 21/Day 172
Jan
Feb
Mar
Apr
May
Jun
Jul
Aug
Sep
Oct
Nov
Dec
°F
Day of Year

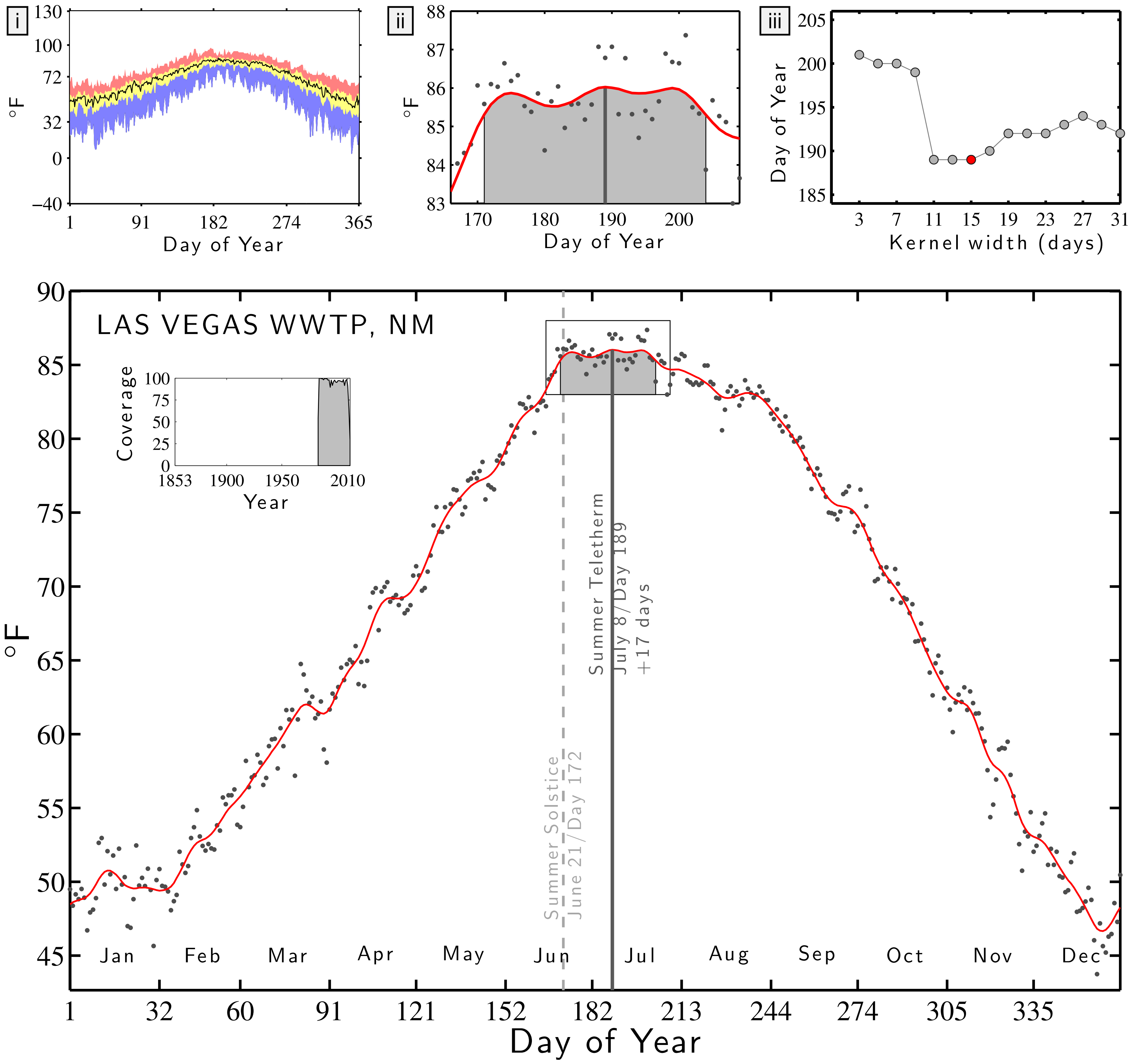

i
°F
Day of Year
ii
°F
Day of Year
iii
Day of Year
Kernel width (days)
LAS VEGAS WWTP, NM
Coverage
Year
Summer Solstice
June 21/Day 172
Summer Teletherm
July 8/Day 189
+17 days
Jan
Feb
Mar
Apr
May
Jun
Jul
Aug
Sep
Oct
Nov
Dec
°F
Day of Year

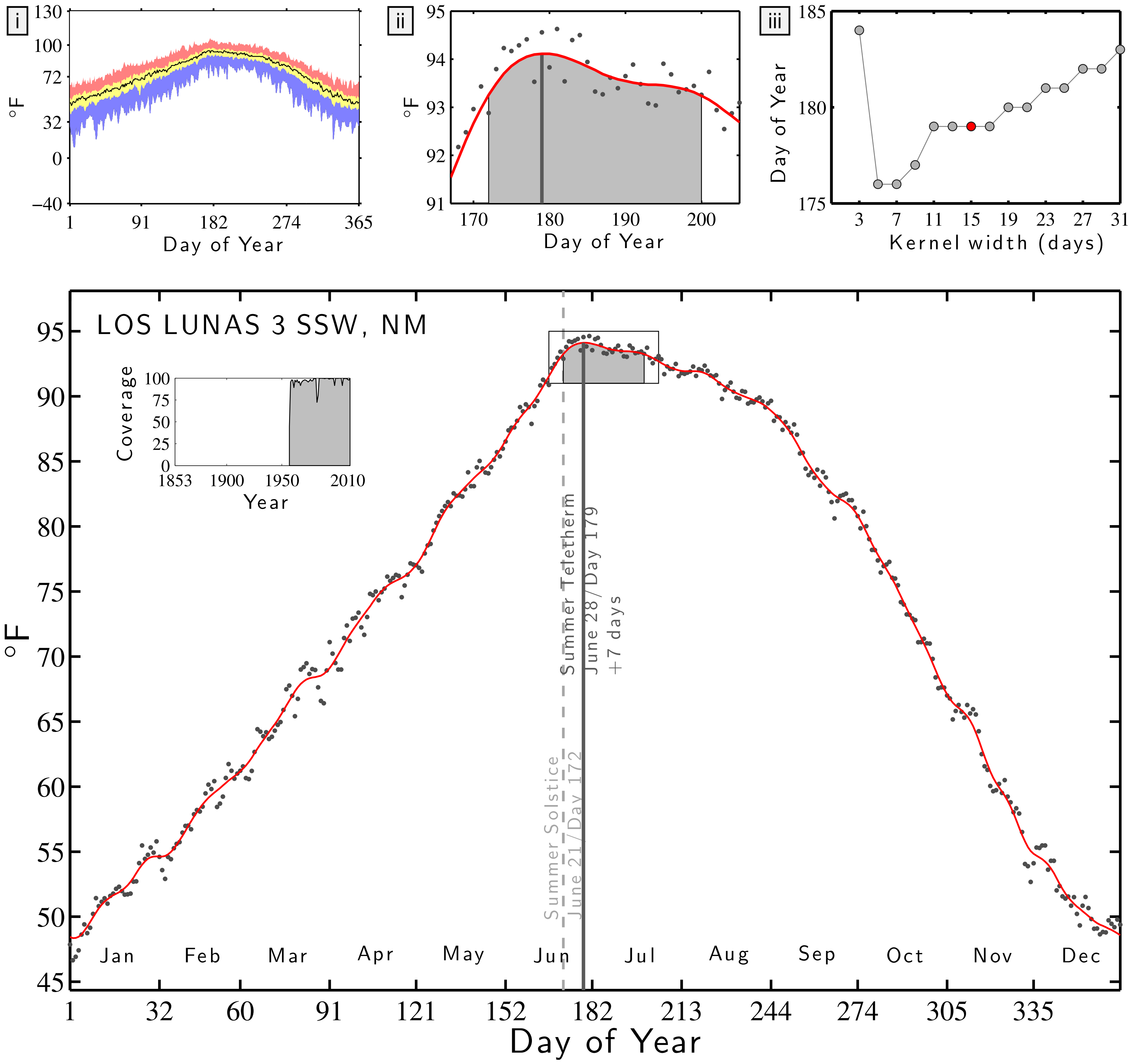

i
°F
Day of Year
ii
°F
Day of Year
iii
Day of Year
Kernel width (days)
LOS LUNAS 3 SSW, NM
Coverage
Year
Summer Solstice
June 21/Day 172
Summer Teletherm
June 28/Day 179
+7 days
Jan
Feb
Mar
Apr
May
Jun
Jul
Aug
Sep
Oct
Nov
Dec
°F
Day of Year

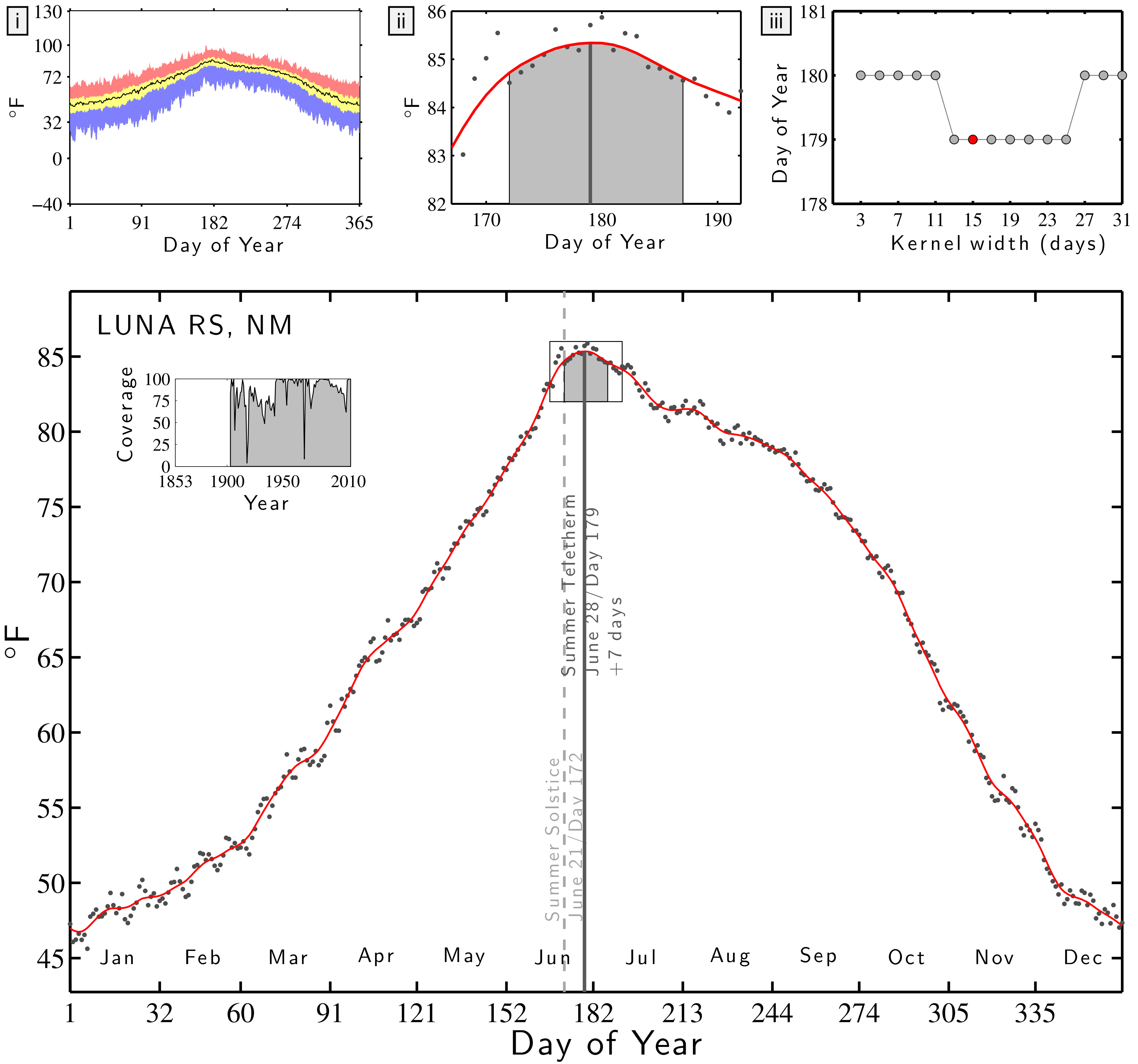

i
ii
iii
°F
Day of Year
Kernel width (days)
LUNA RS, NM
Coverage
Year
Summer Teletherm
June 28/Day 179
+7 days
Summer Solstice
June 21/Day 172
Jan
Feb
Mar
Apr
May
Jun
Jul
Aug
Sep
Oct
Nov
Dec

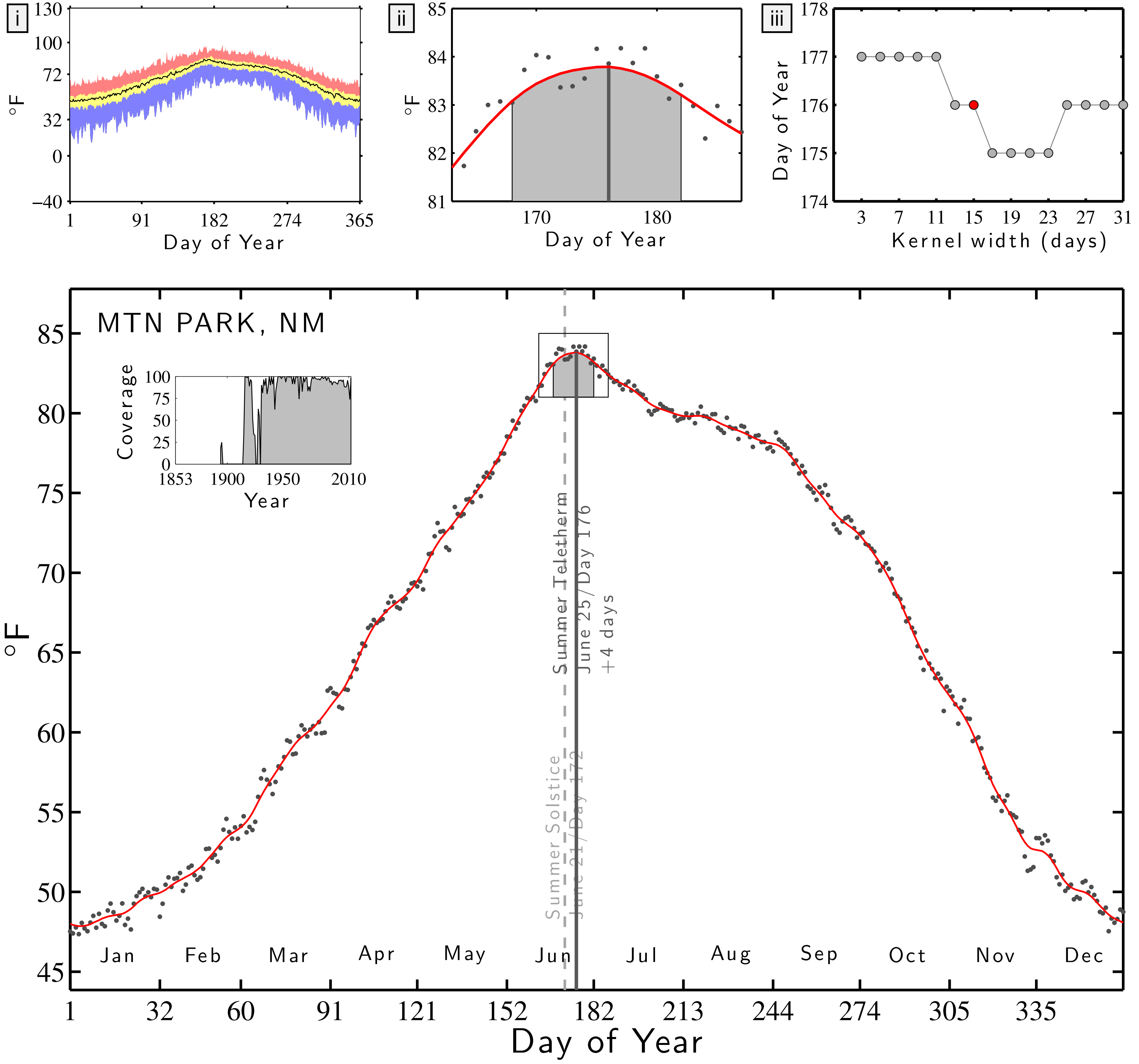

i
ii
iii
Day of Year
Kernel width (days)
MTN PARK, NM
Coverage
Year
Summer Teletherm
June 25/Day 176
+4 days
Summer Solstice
June 21/Day 172
Jan
Feb
Mar
Apr
May
Jun
Jul
Aug
Sep
Oct
Nov
Dec
°F
Day of Year

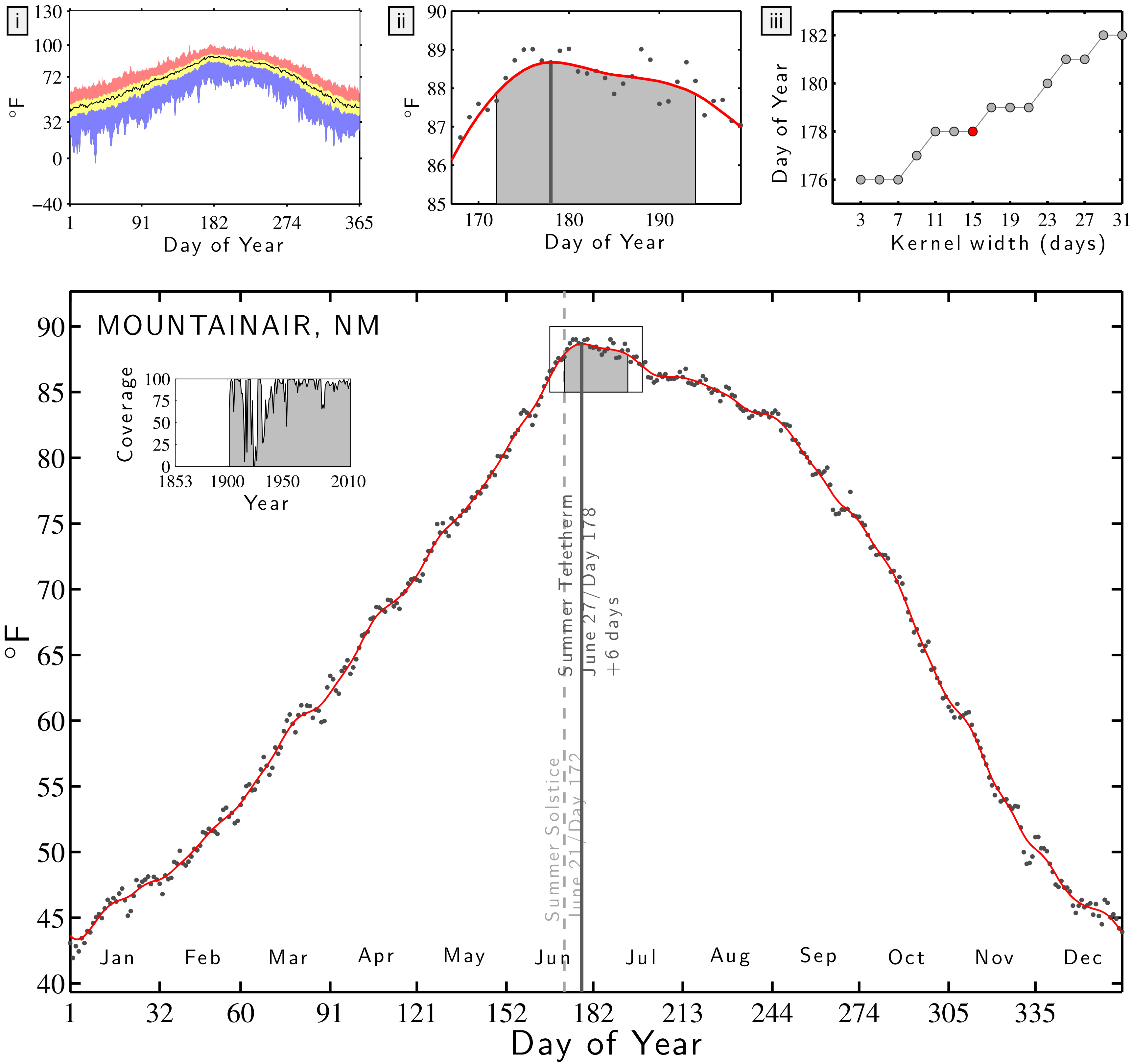

i
ii
iii
Day of Year
Kernel width (days)
MOUNTAINAIR, NM
Coverage
Year
Summer Solstice
June 21 / Day 172
Summer Teletherm
June 27 / Day 178
+6 days
Jan
Feb
Mar
Apr
May
Jun
Jul
Aug
Sep
Oct
Nov
Dec
°F
Day of Year

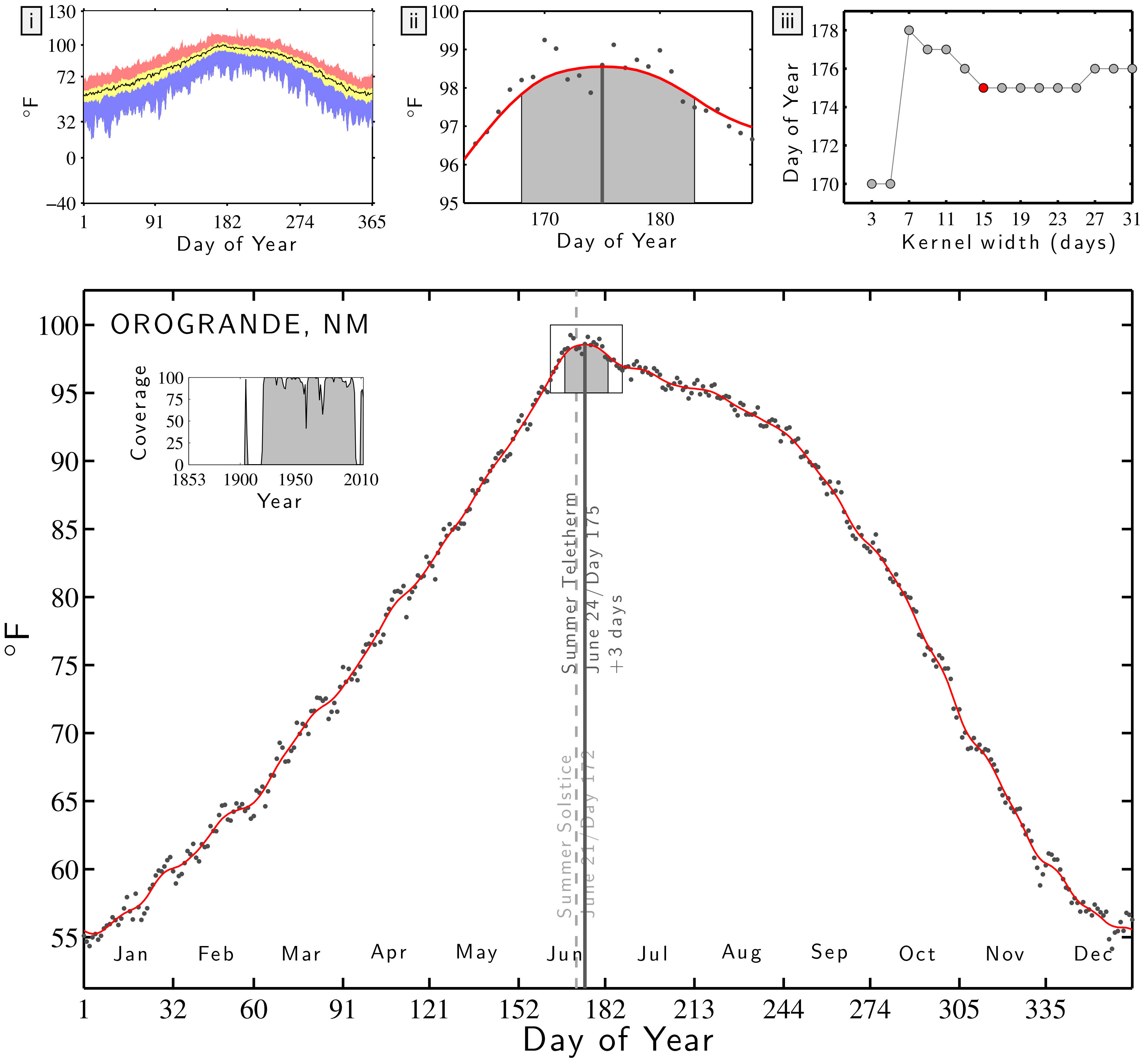
i
°F
Day of Year
ii
°F
Day of Year
iii
Day of Year
Kernel width (days)
OROGRANDE, NM
Coverage
Year
Summer Teletherm
June 24/Day 175
+3 days
Summer Solstice
June 21/Day 172
Jan
Feb
Mar
Apr
May
Jun
Jul
Aug
Sep
Oct
Nov
Dec
°F
Day of Year

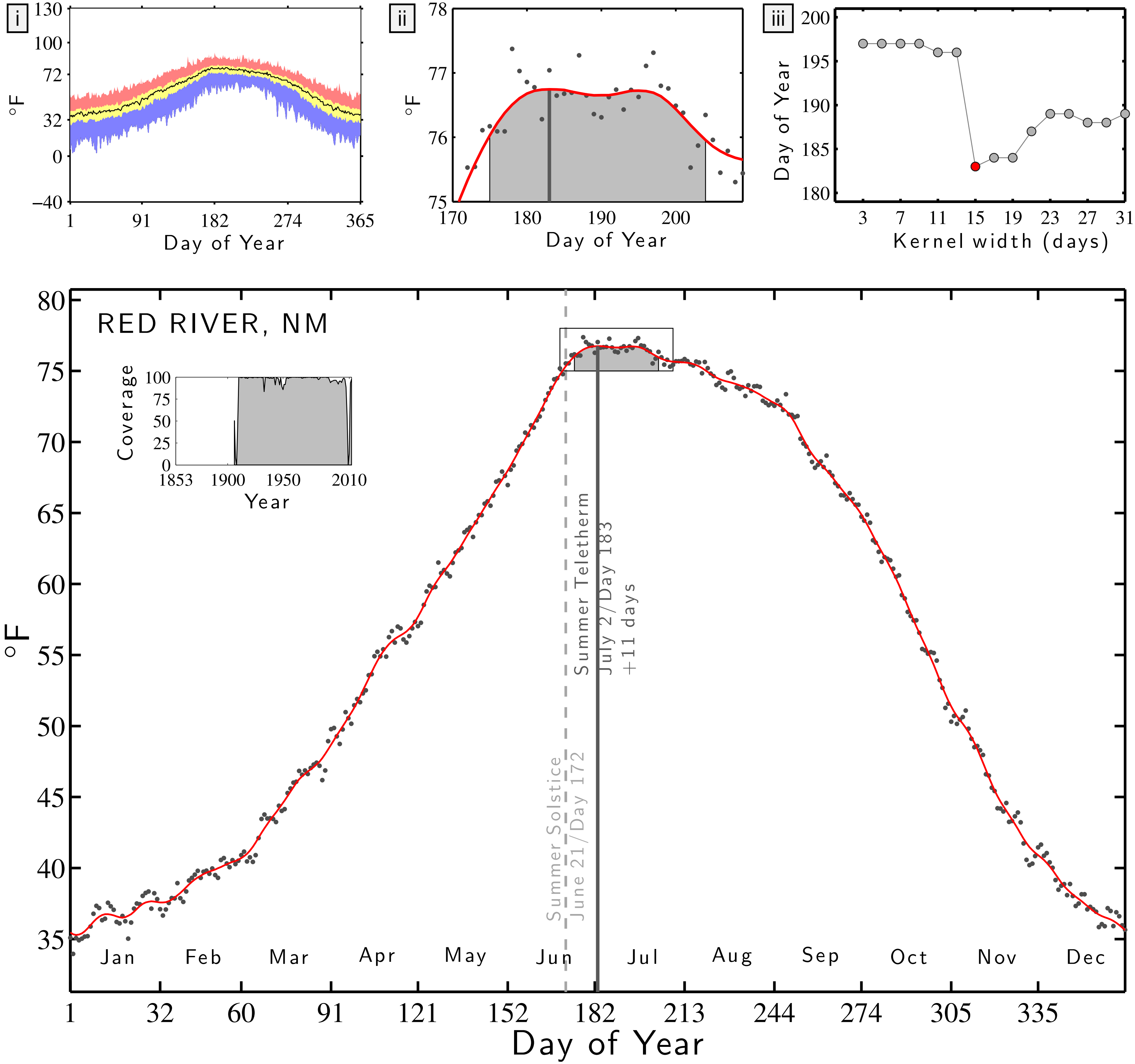

i
°F
Day of Year
ii
°F
Day of Year
iii
Day of Year
Kernel width (days)
RED RIVER, NM
Coverage
Year
Summer Solstice
June 21/Day 172
Summer Teletherm
July 2/Day 183
+11 days
Jan
Feb
Mar
Apr
May
Jun
Jul
Aug
Sep
Oct
Nov
Dec
°F
Day of Year

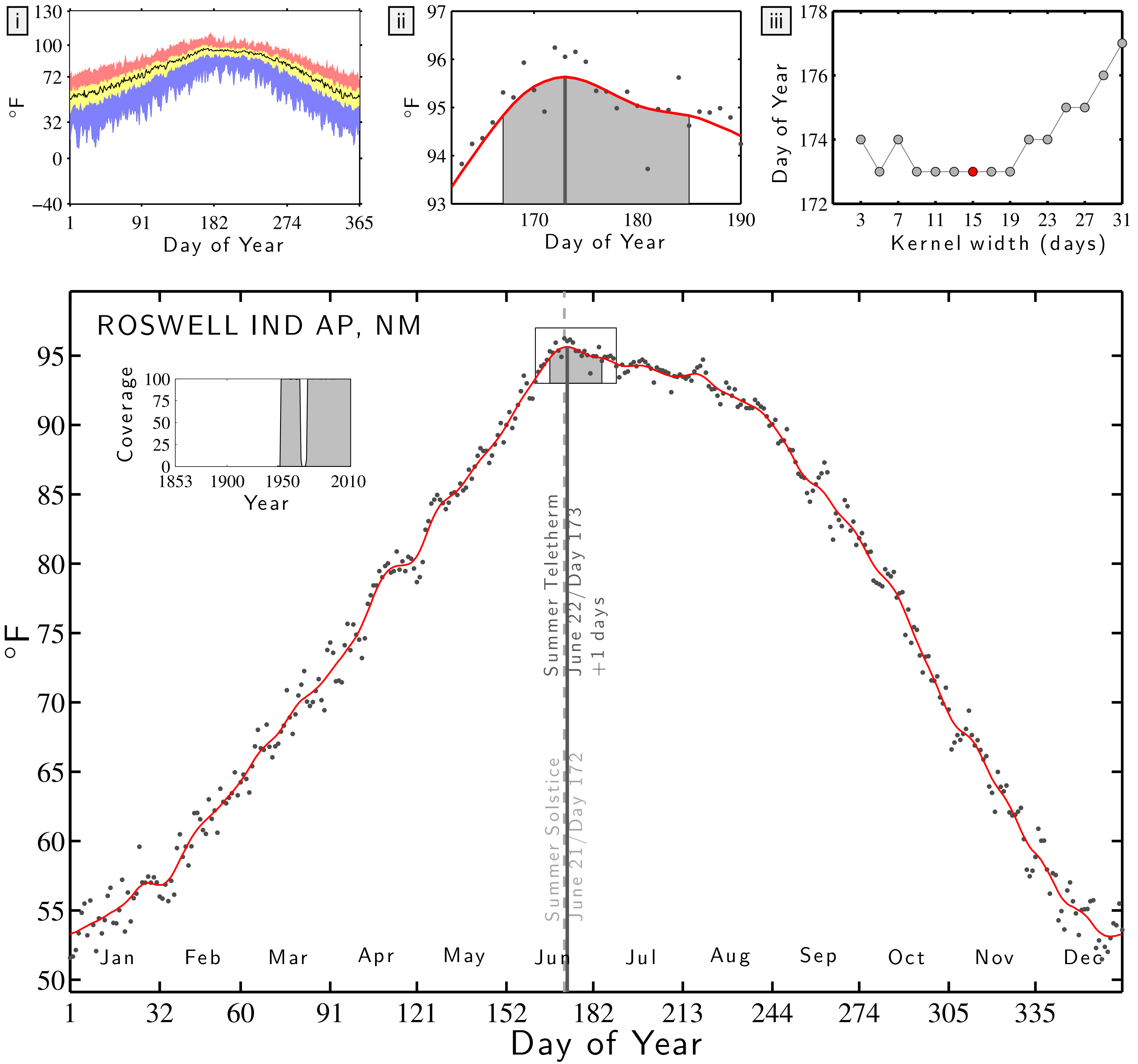

i
°F
130
100
72
32
0
−40
1
91
182
274
365
Day of Year
ii
97
96
95
94
93
170
180
190
Day of Year
iii
Day of Year
178
176
174
172
3
7
11
15
19
23
27
31
Kernel width (days)
ROSWELL IND AP, NM
Coverage
100
75
50
25
0
1853
1900
1950
2010
Year
Summer Telethern
June 22/Day 173
+1 days
Summer Solstice
June 21/Day 172
Jan
Feb
Mar
Apr
May
Jun
Jul
Aug
Sep
Oct
Nov
Dec
95
90
85
80
75
70
65
60
55
50
1
32
60
91
121
152
182
213
244
274
305
335
Day of Year

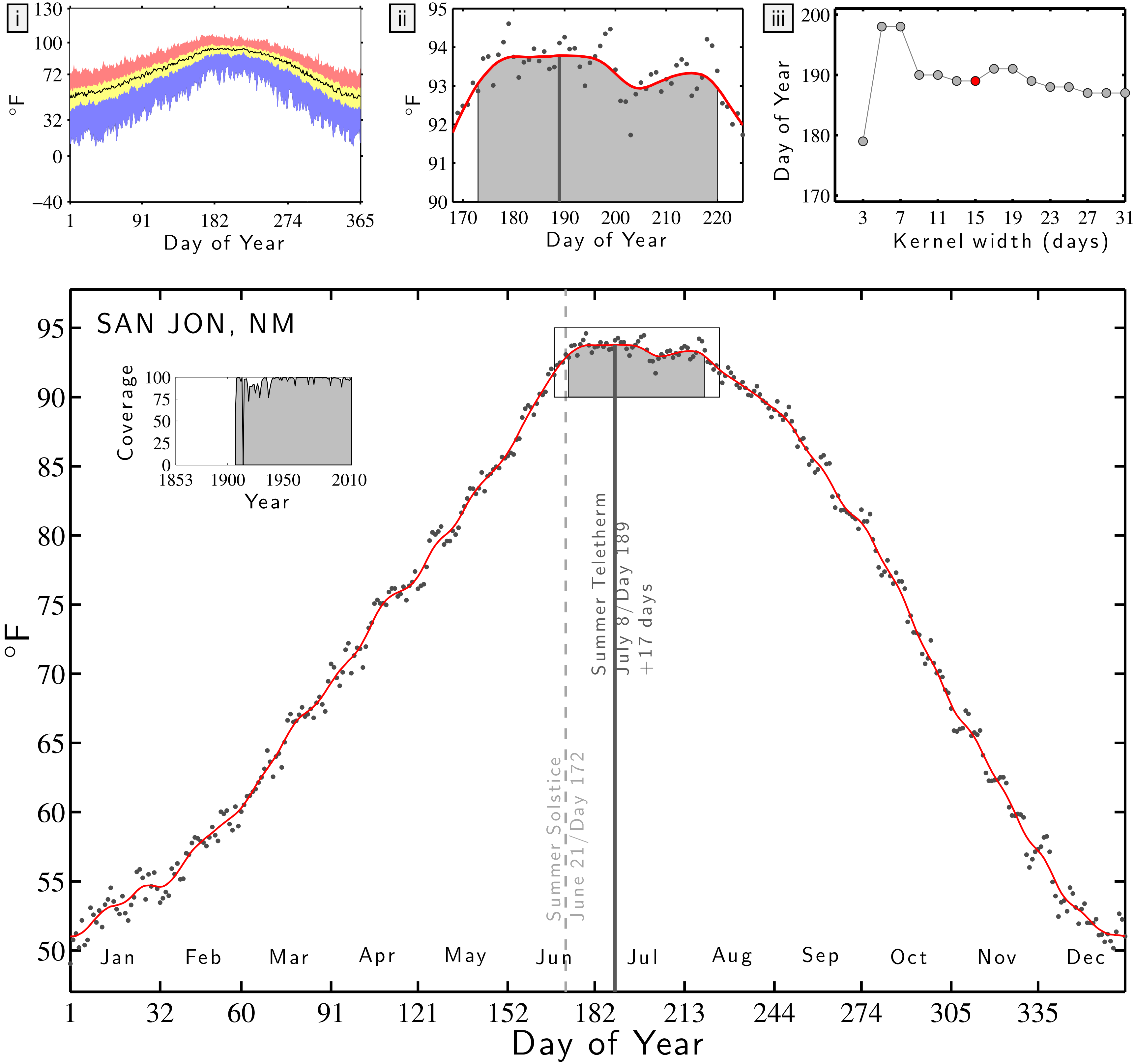

i
°F
Day of Year
ii
°F
Day of Year
iii
Day of Year
Kernel width (days)
SAN JON, NM
Coverage
Year
Summer Solstice
June 21/Day 172
Summer Teletherm
July 8/Day 189
+17 days
Jan
Feb
Mar
Apr
May
Jun
Jul
Aug
Sep
Oct
Nov
Dec
°F
Day of Year

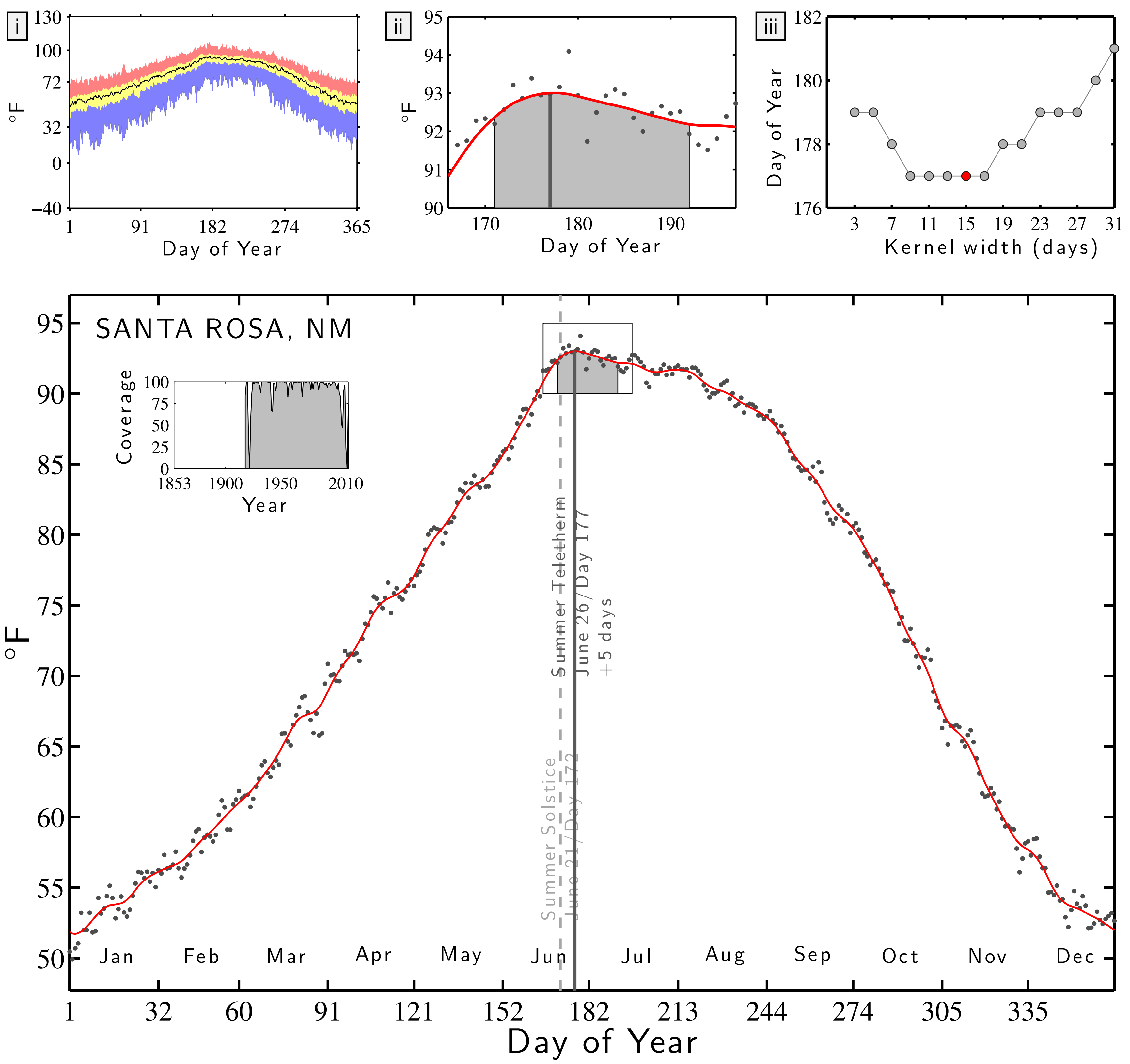

i
ii
iii
°F
Day of Year
Day of Year
Kernel width (days)
SANTA ROSA, NM
Coverage
Year
Summer Teletherm
June 26/Day 177
+5 days
Summer Solstice
June 21/Day 172
Jan
Feb
Mar
Apr
May
Jun
Jul
Aug
Sep
Oct
Nov
Dec
°F
Day of Year

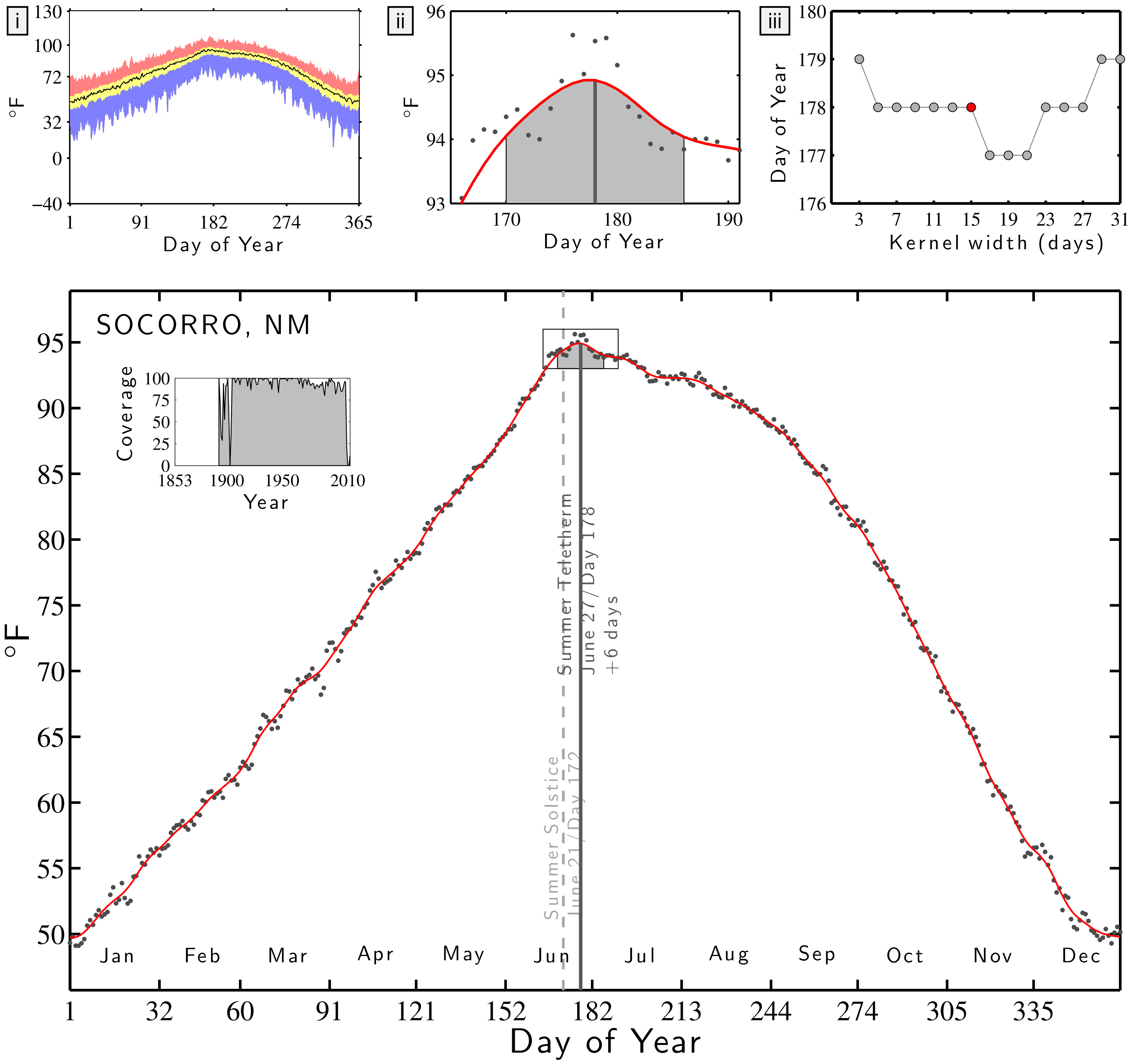

i
ii
iii
Day of Year
Kernel width (days)
SOCORRO, NM
Coverage
Year
Summer Teletherm
June 27 / Day 178
+6 days
Summer Solstice
June 21 / Day 172
Jan
Feb
Mar
Apr
May
Jun
Jul
Aug
Sep
Oct
Nov
Dec
°F
Day of Year

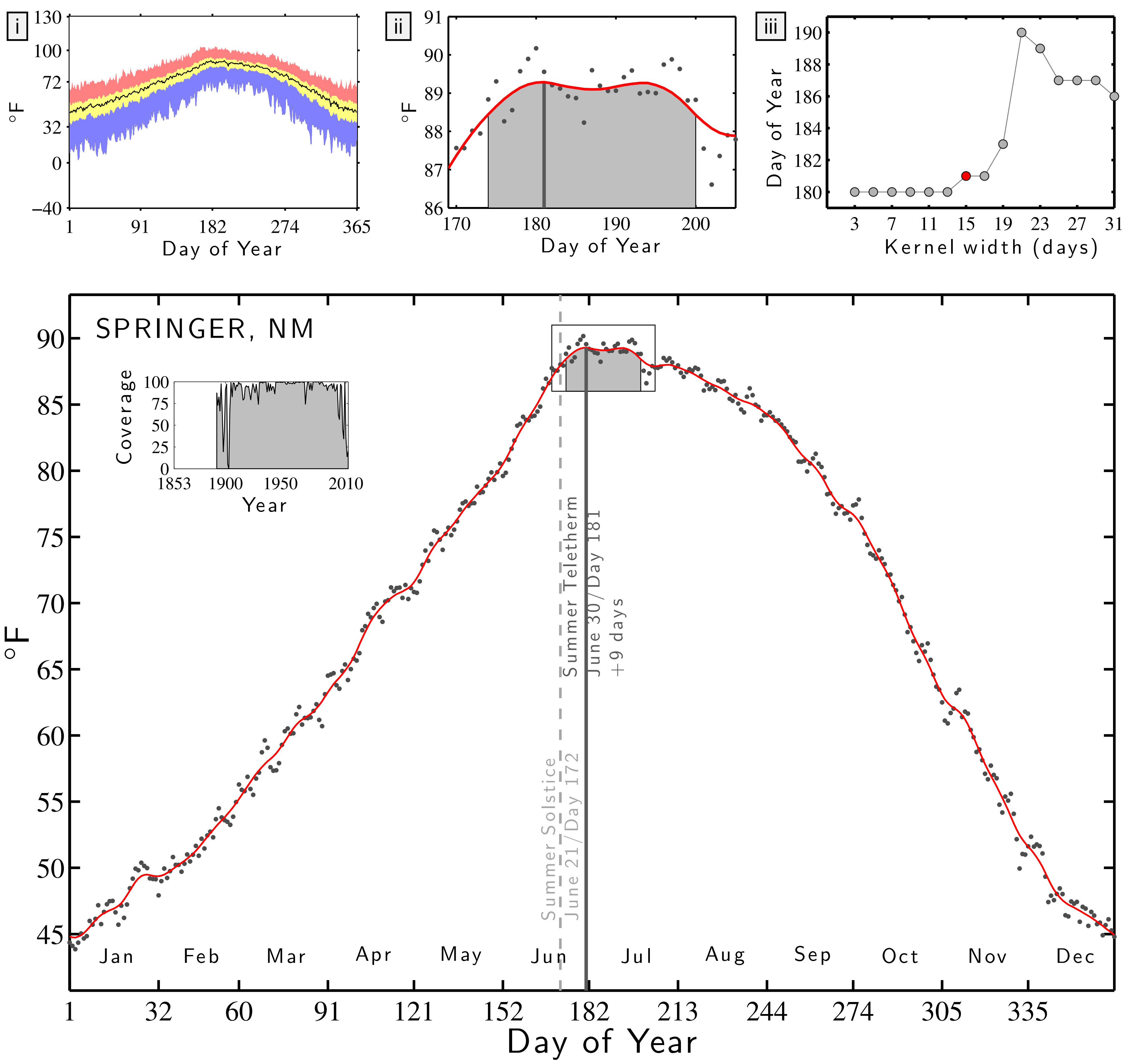

i
°F
Day of Year
ii
°F
Day of Year
iii
Day of Year
Kernel width (days)
SPRINGER, NM
Coverage
Year
Summer Solstice
June 21/Day 172
Summer Teletherm
June 30/Day 181
+9 days
Jan
Feb
Mar
Apr
May
Jun
Jul
Aug
Sep
Oct
Nov
Dec
°F
Day of Year

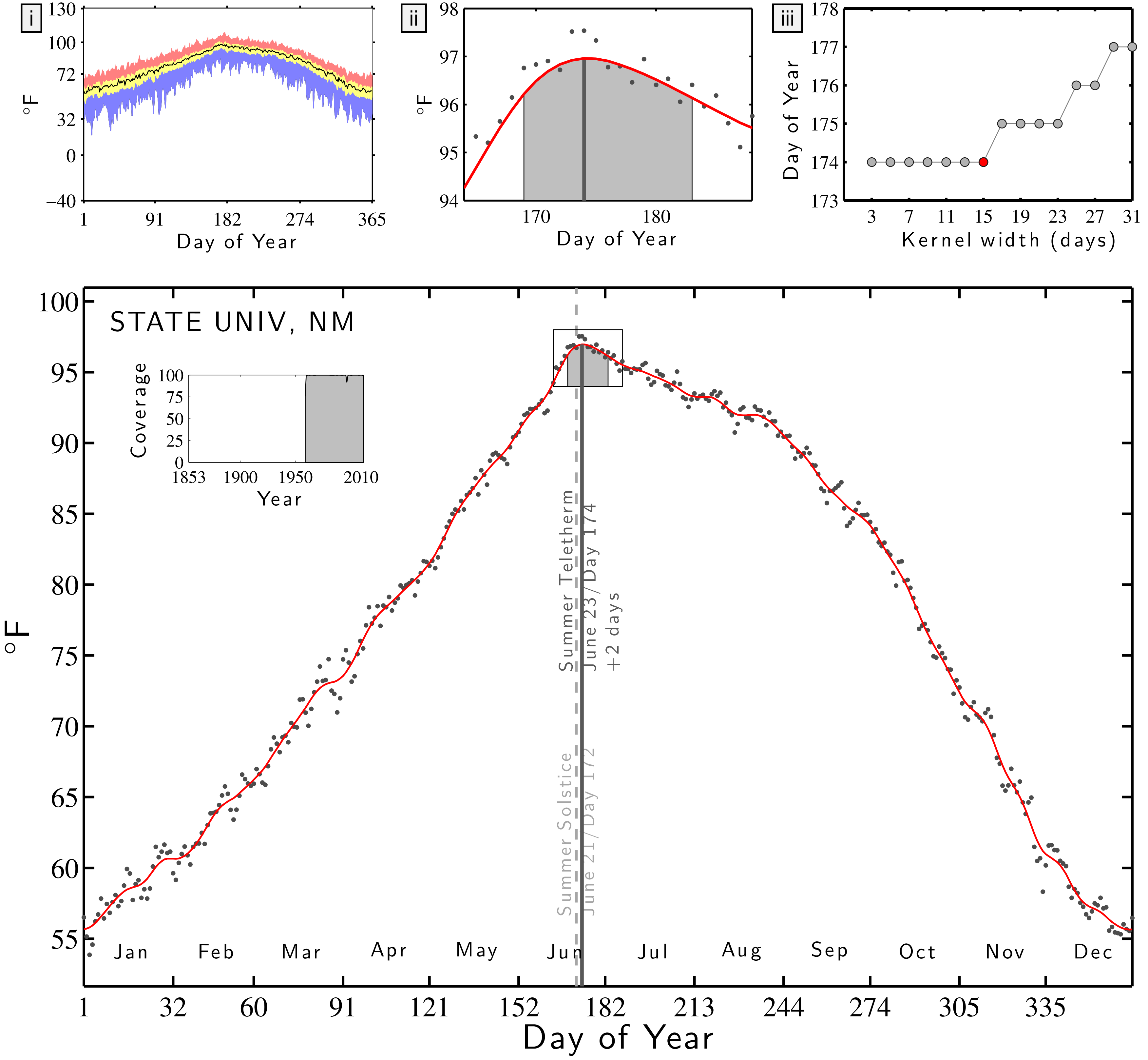

i
ii
iii
Day of Year
Kernel width (days)
STATE UNIV, NM
Coverage
Year
Summer Teletherm
June 23 / Day 174
+2 days
Summer Solstice
June 21 / Day 172
Jan
Feb
Mar
Apr
May
Jun
Jul
Aug
Sep
Oct
Nov
Dec
°F
Day of Year

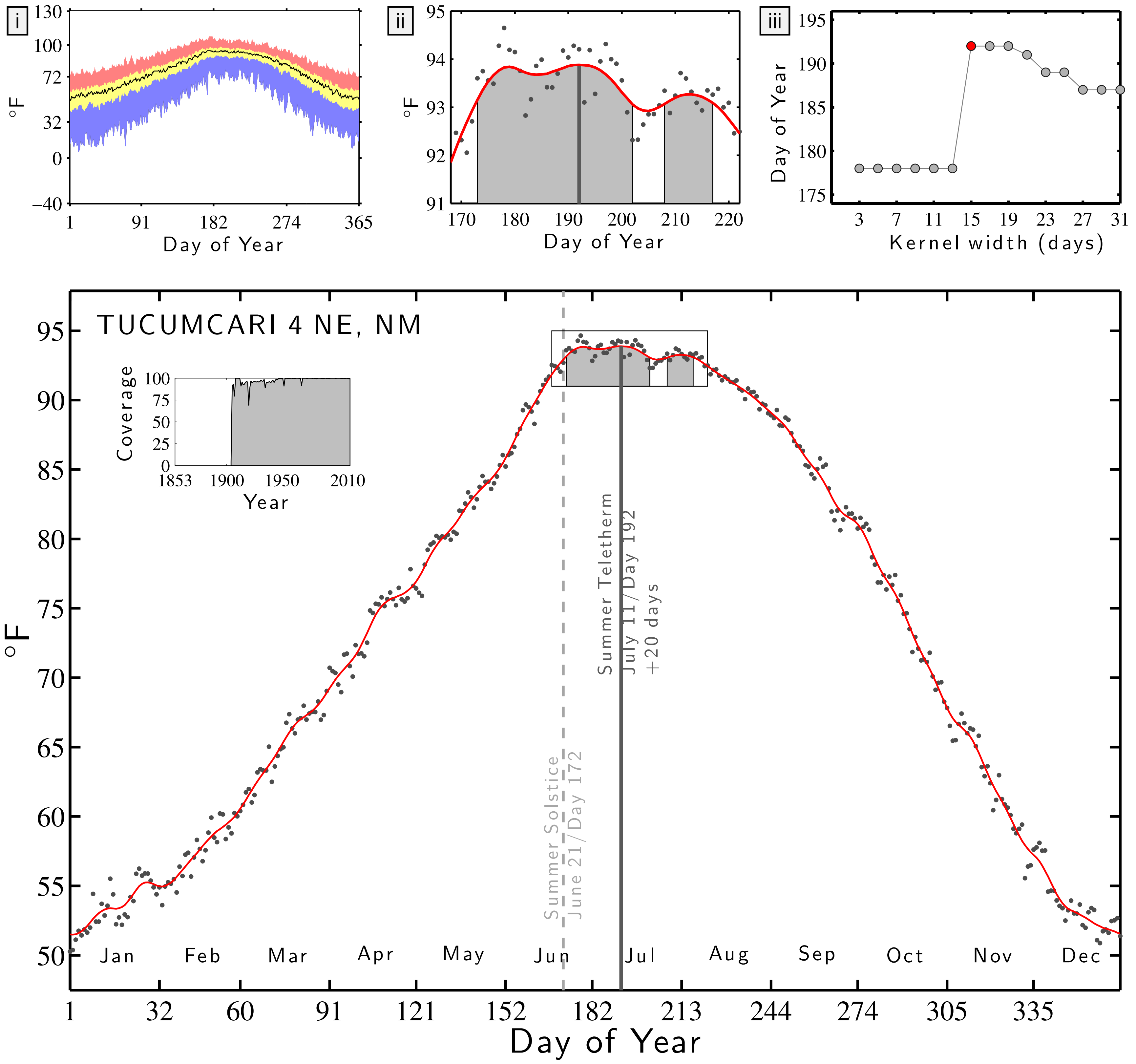

i
ii
iii
°F
Day of Year
Day of Year
Kernel width (days)
TUCUMCARI 4 NE, NM
Coverage
Year
Summer Solstice
June 21/Day 172
Summer Teletherm
July 11/Day 192
+20 days
Jan
Feb
Mar
Apr
May
Jun
Jul
Aug
Sep
Oct
Nov
Dec
Day of Year

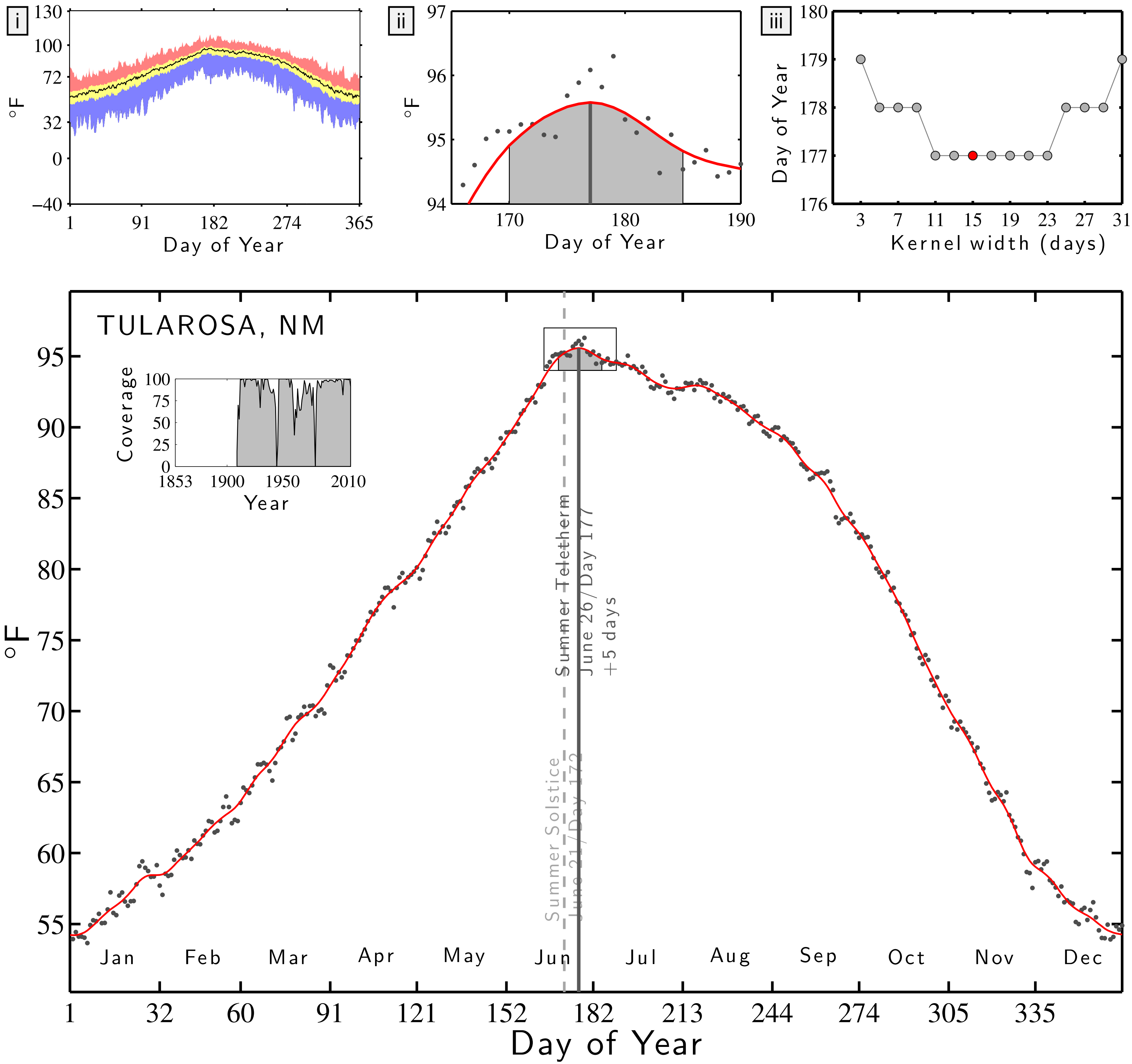

i
°F
Day of Year
ii
Day of Year
iii
Day of Year
Kernel width (days)
TULAROSA, NM
Coverage
Year
Summer Teletherm
June 26/Day 177
+5 days
Summer Solstice
June 21/Day 172
Jan
Feb
Mar
Apr
May
Jun
Jul
Aug
Sep
Oct
Nov
Dec
Day of Year

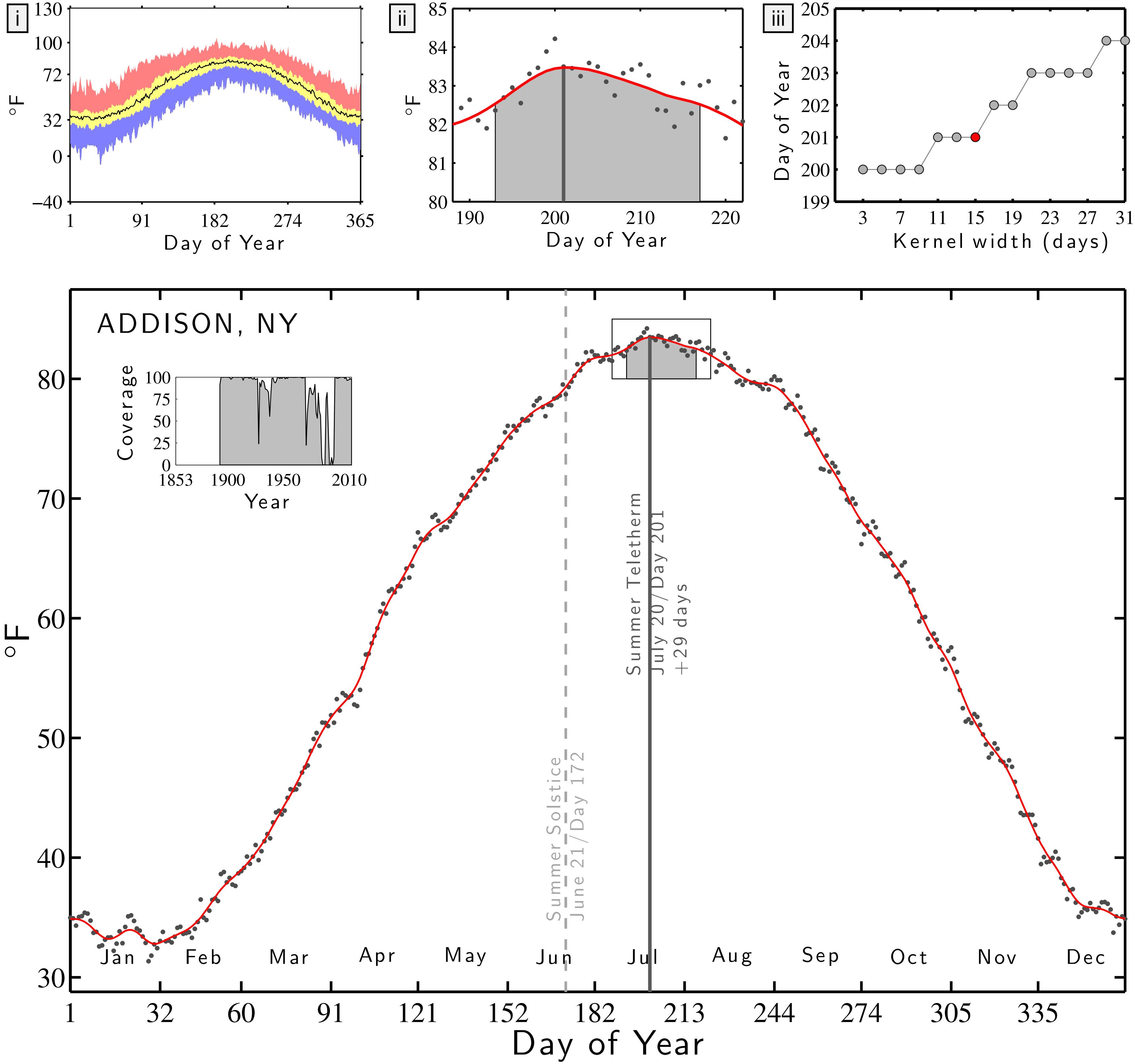

i
°F
Day of Year
ii
°F
Day of Year
iii
Day of Year
Kernel width (days)
ADDISON, NY
Coverage
Year
Summer Solstice
June 21/Day 172
Summer Teletherm
July 20/Day 201
+29 days
Jan
Feb
Mar
Apr
May
Jun
Jul
Aug
Sep
Oct
Nov
Dec
°F
Day of Year

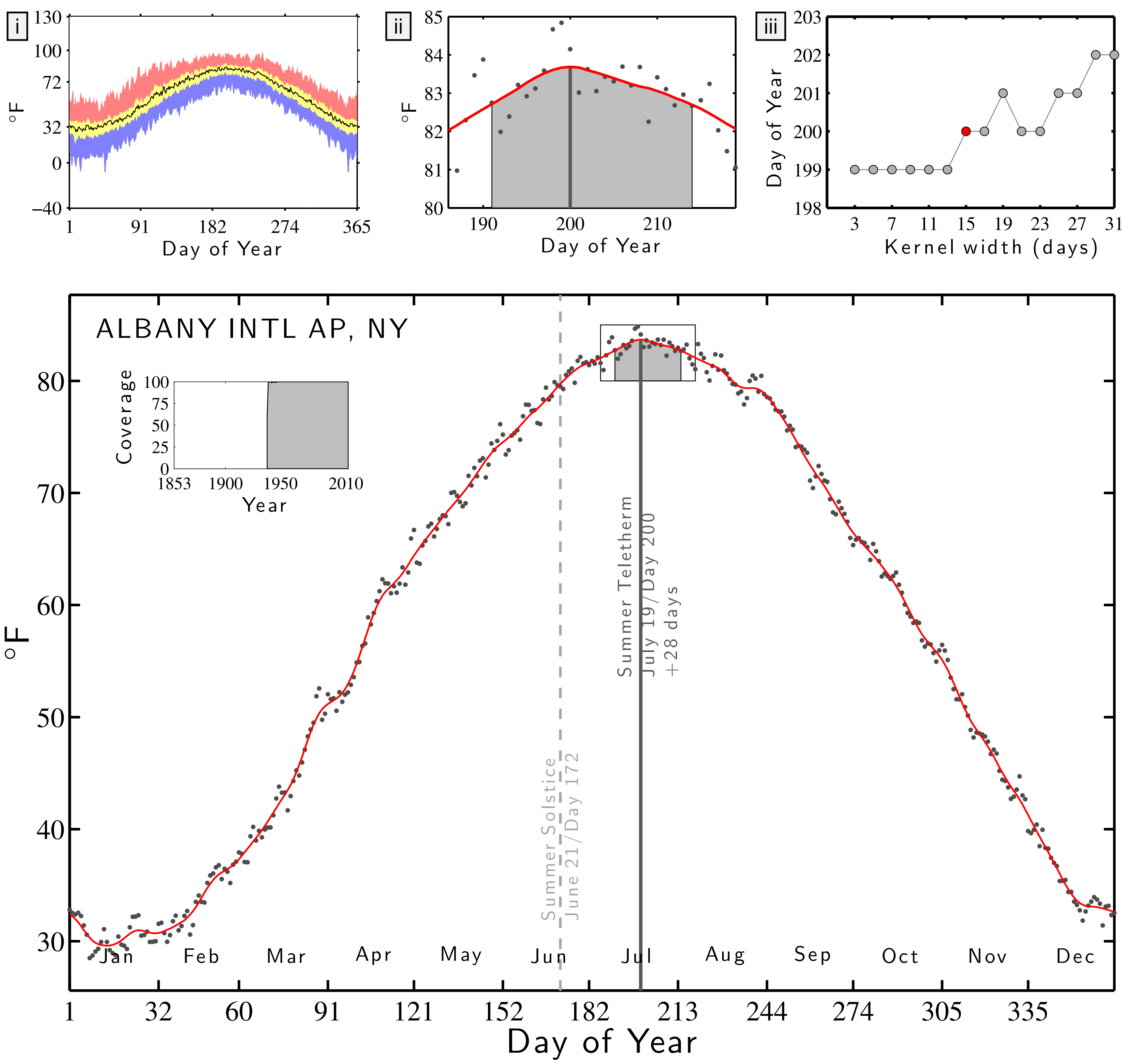

i
°F
130
100
72
32
0
−40
1
91
182
274
365
Day of Year
ii
85
84
83
82
81
80
190
200
210
Day of Year
iii
Day of Year
203
202
201
200
199
198
3
7
11
15
19
23
27
31
Kernel width (days)
ALBANY INTL AP, NY
Coverage
100
75
50
25
0
1853
1900
1950
2010
Year
80
70
60
50
40
30
Summer Solstice
June 21/Day 172
Summer Teletherm
July 19/Day 200
+28 days
Jan
Feb
Mar
Apr
May
Jun
Jul
Aug
Sep
Oct
Nov
Dec
1
32
60
91
121
152
182
213
244
274
305
335
Day of Year

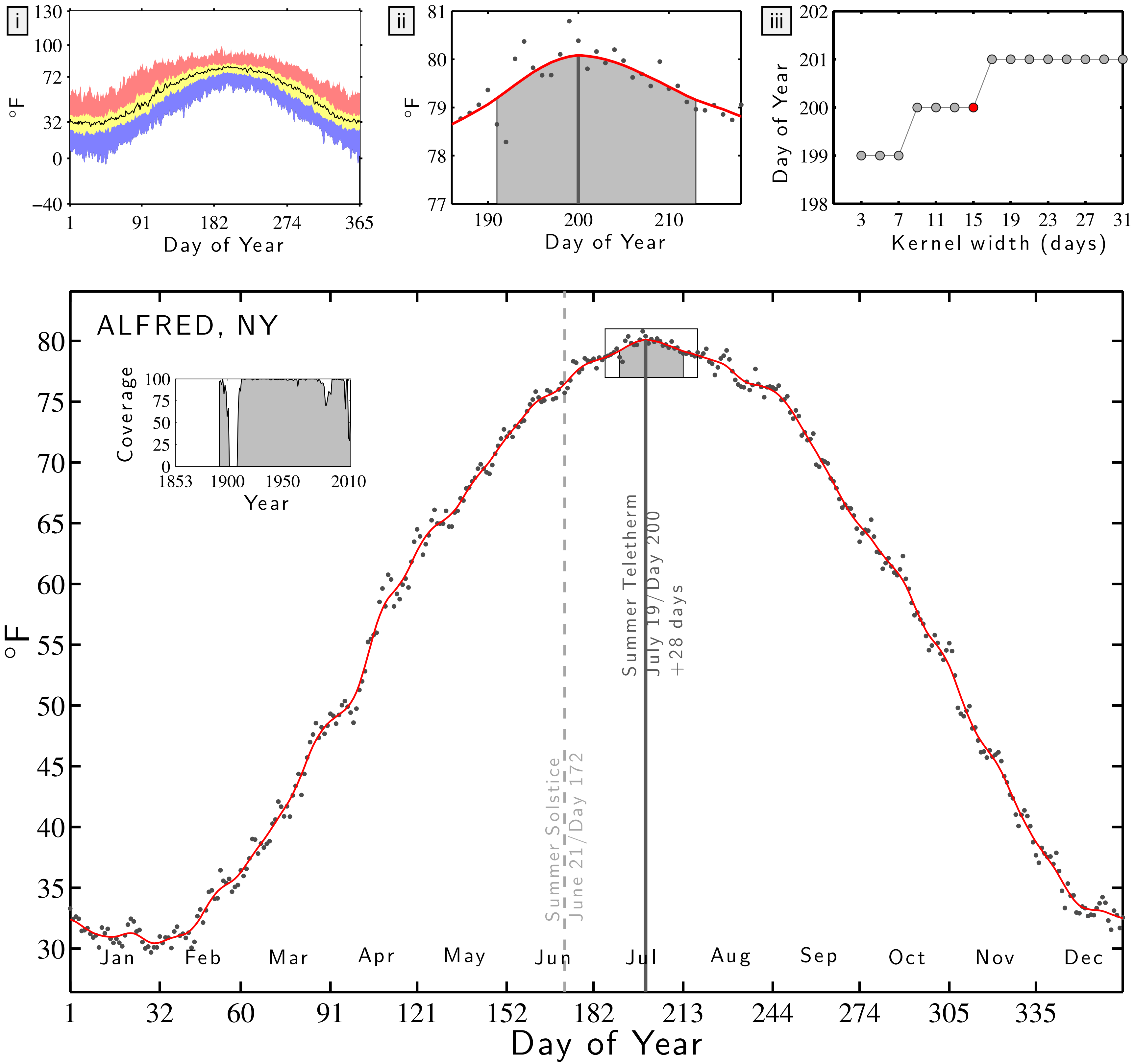
i
°F
Day of Year
ii
°F
Day of Year
iii
Day of Year
Kernel width (days)
ALFRED, NY
Coverage
Year
Summer Solstice
June 21/Day 172
Summer Teletherm
July 19/Day 200
+28 days
Jan
Feb
Mar
Apr
May
Jun
Jul
Aug
Sep
Oct
Nov
Dec
°F
Day of Year

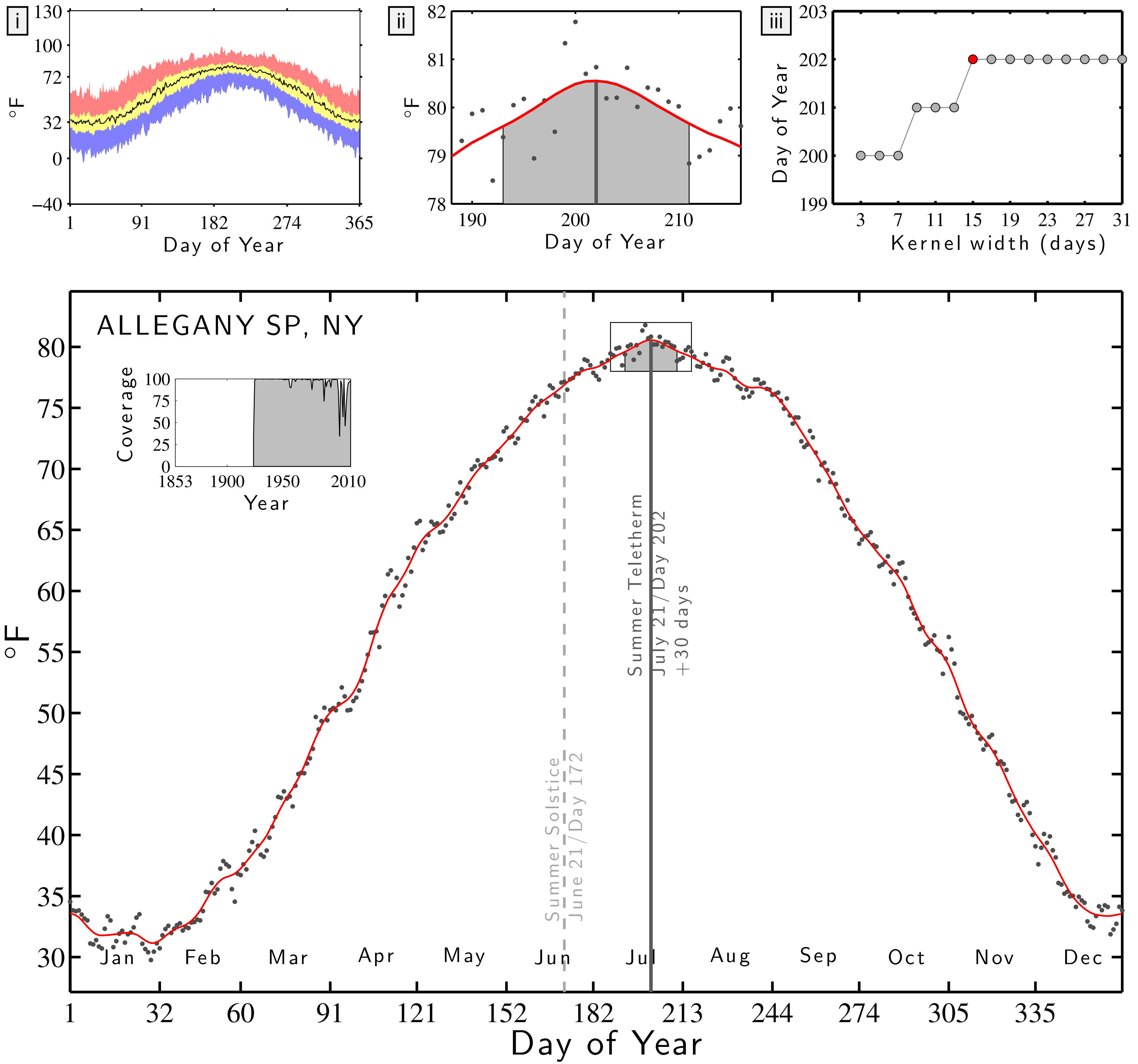

i
°F
Day of Year
ii
°F
Day of Year
iii
Day of Year
Kernel width (days)
ALLEGANY SP, NY
Coverage
Year
Summer Solstice
June 21 / Day 172
Summer Teletherm
July 21 / Day 202
+30 days
Jan
Feb
Mar
Apr
May
Jun
Jul
Aug
Sep
Oct
Nov
Dec
°F
Day of Year

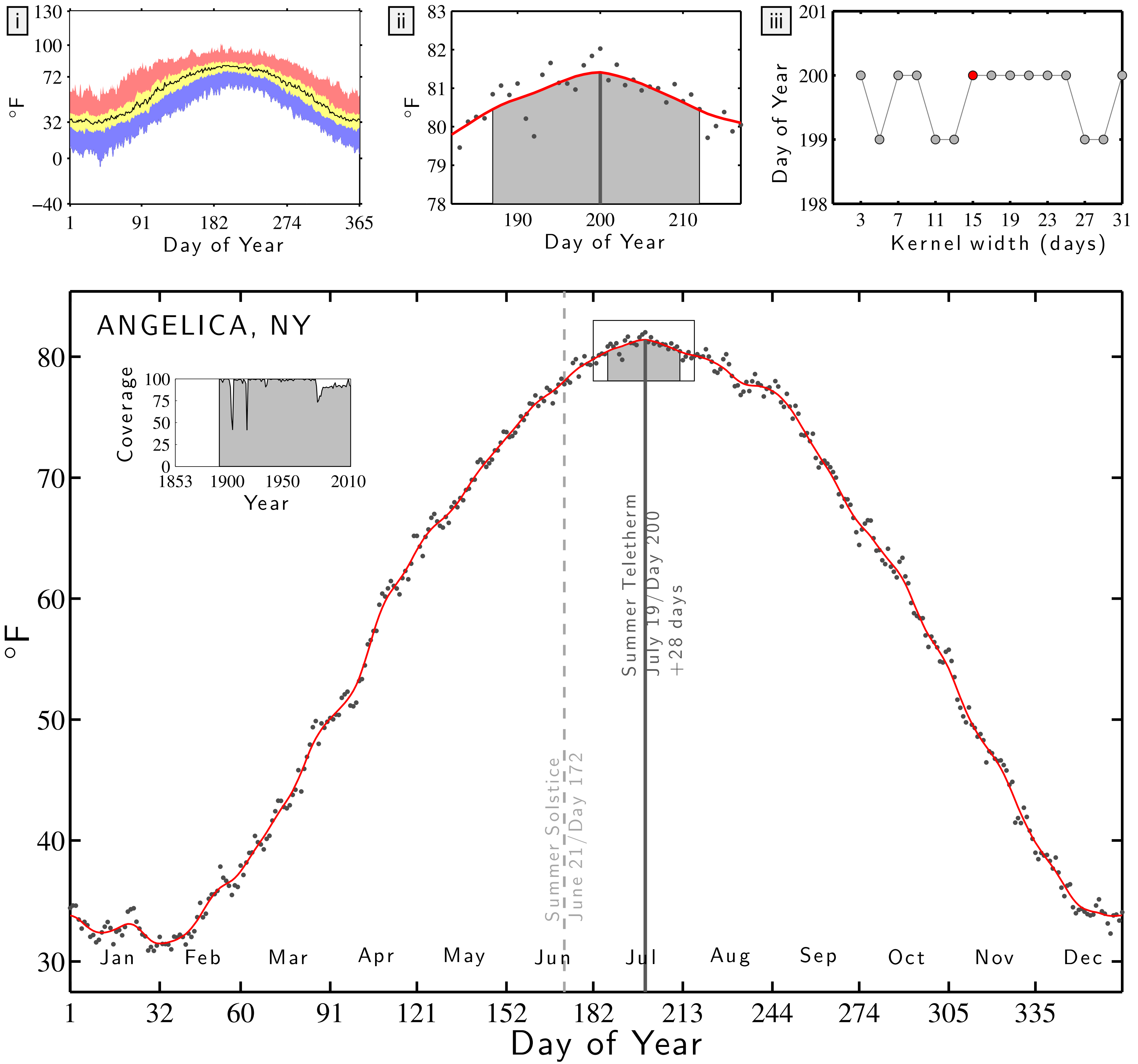

i
ii
iii
°F
Day of Year
Day of Year
Kernel width (days)
ANGELICA, NY
Coverage
Year
Summer Solstice
June 21/Day 172
Summer Teletherm
July 19/Day 200
+28 days
Jan
Feb
Mar
Apr
May
Jun
Jul
Aug
Sep
Oct
Nov
Dec

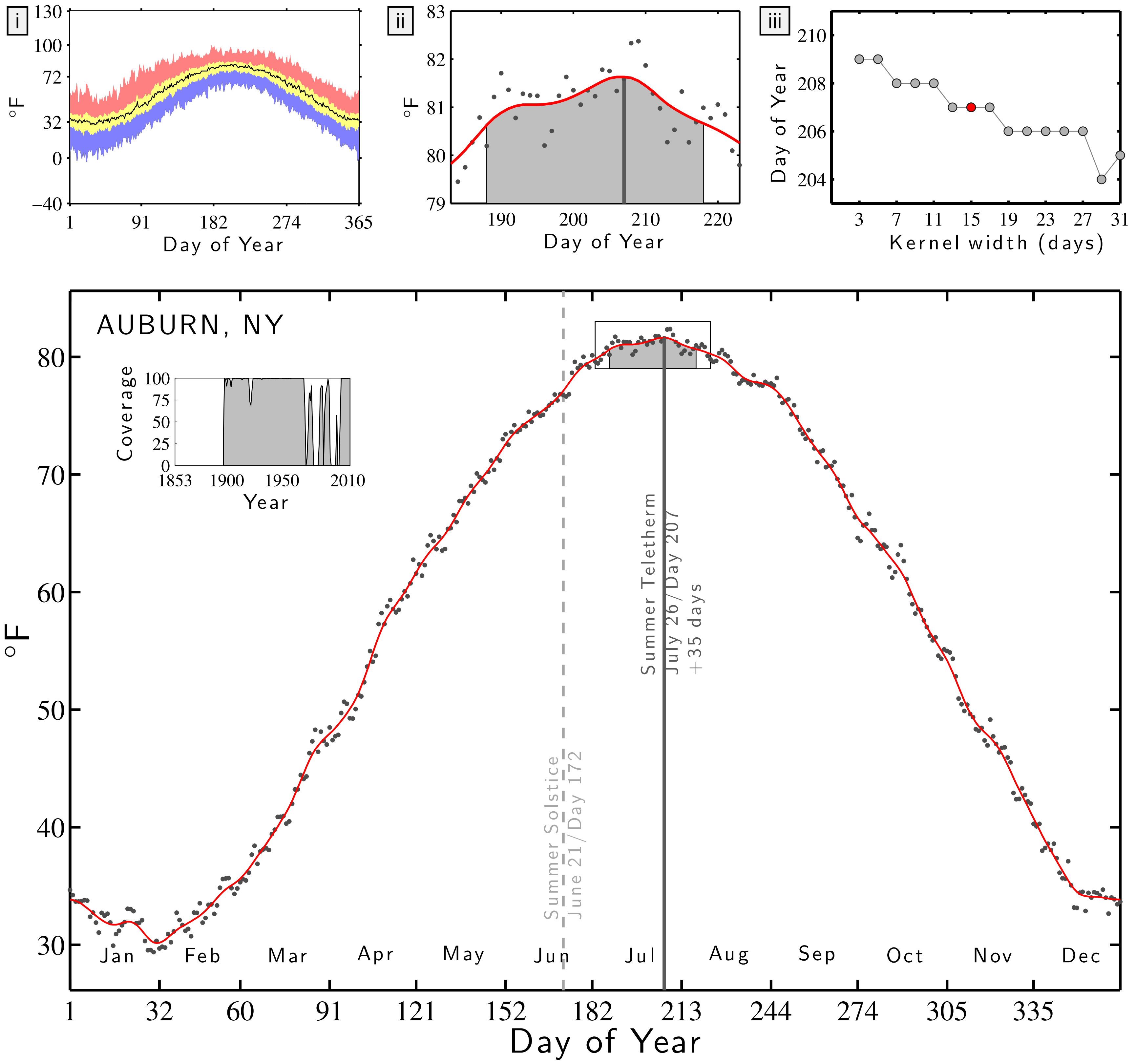

i
°F
Day of Year
ii
°F
Day of Year
iii
Day of Year
Kernel width (days)
AUBURN, NY
Coverage
Year
Summer Solstice
June 21 / Day 172
Summer Teletherm
July 26 / Day 207
+35 days
Jan
Feb
Mar
Apr
May
Jun
Jul
Aug
Sep
Oct
Nov
Dec
°F
Day of Year

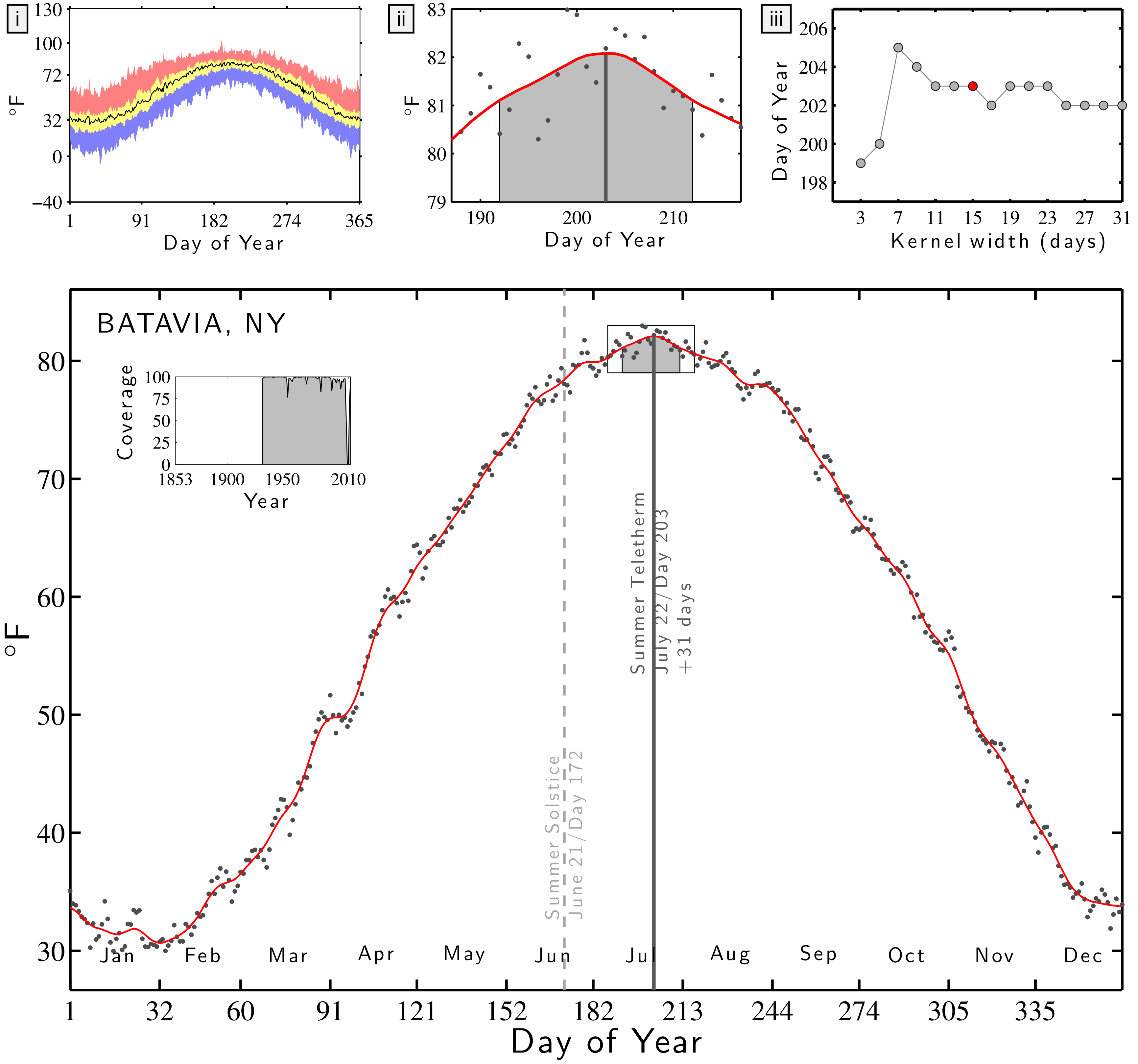

i
ii
iii
Day of Year
°F
Kernel width (days)
BATAVIA, NY
Coverage
Year
Summer Solstice
June 21 / Day 172
Summer Teletherm
July 22 / Day 203
+31 days
Jan
Feb
Mar
Apr
May
Jun
Jul
Aug
Sep
Oct
Nov
Dec

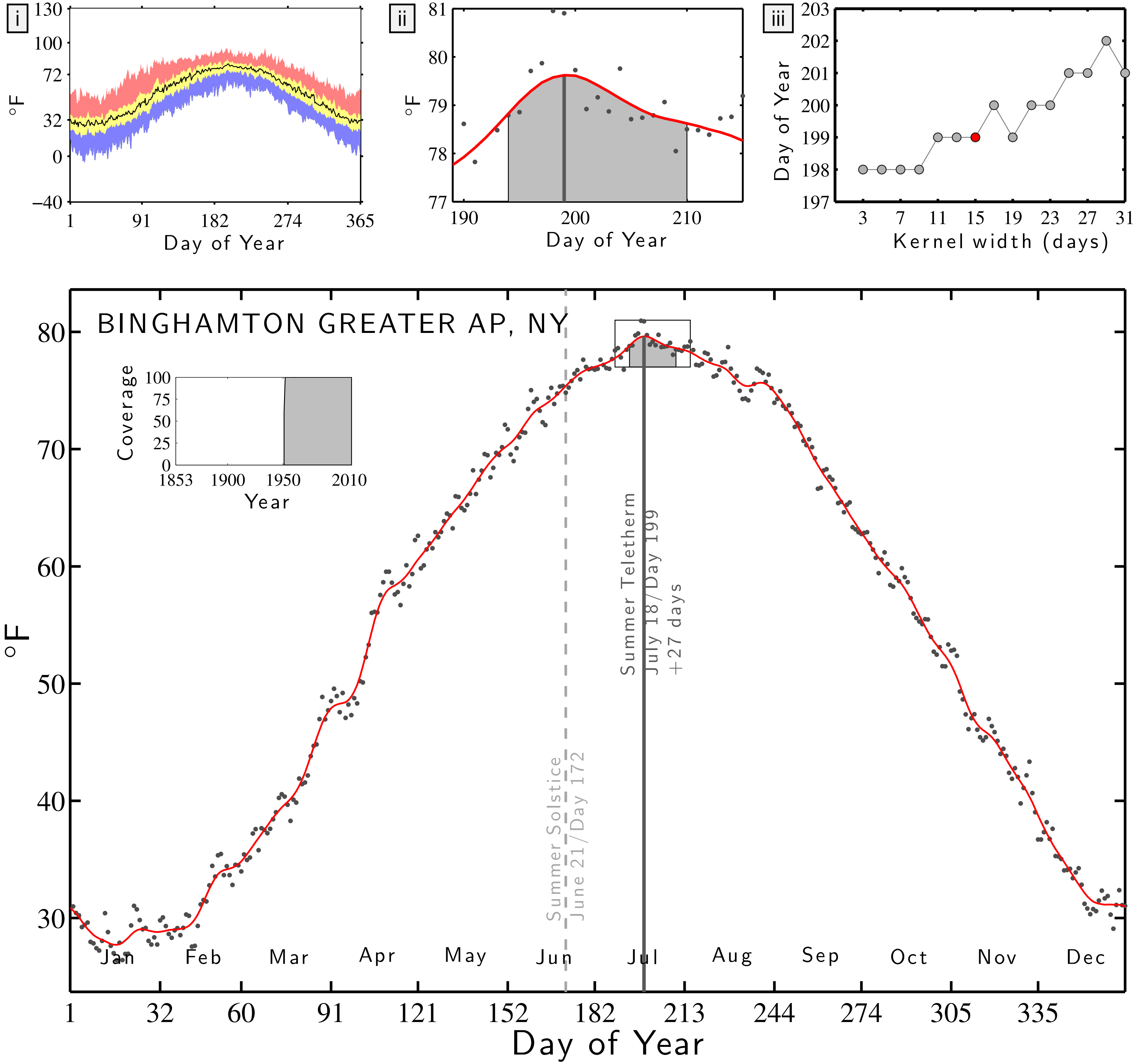

i
ii
iii
Day of Year
Kernel width (days)
BINGHAMTON GREATER AP, NY
Coverage
Year
Summer Solstice
June 21/Day 172
Summer Teletherm
July 18/Day 199
+27 days
Jan
Feb
Mar
Apr
May
Jun
Jul
Aug
Sep
Oct
Nov
Dec
°F

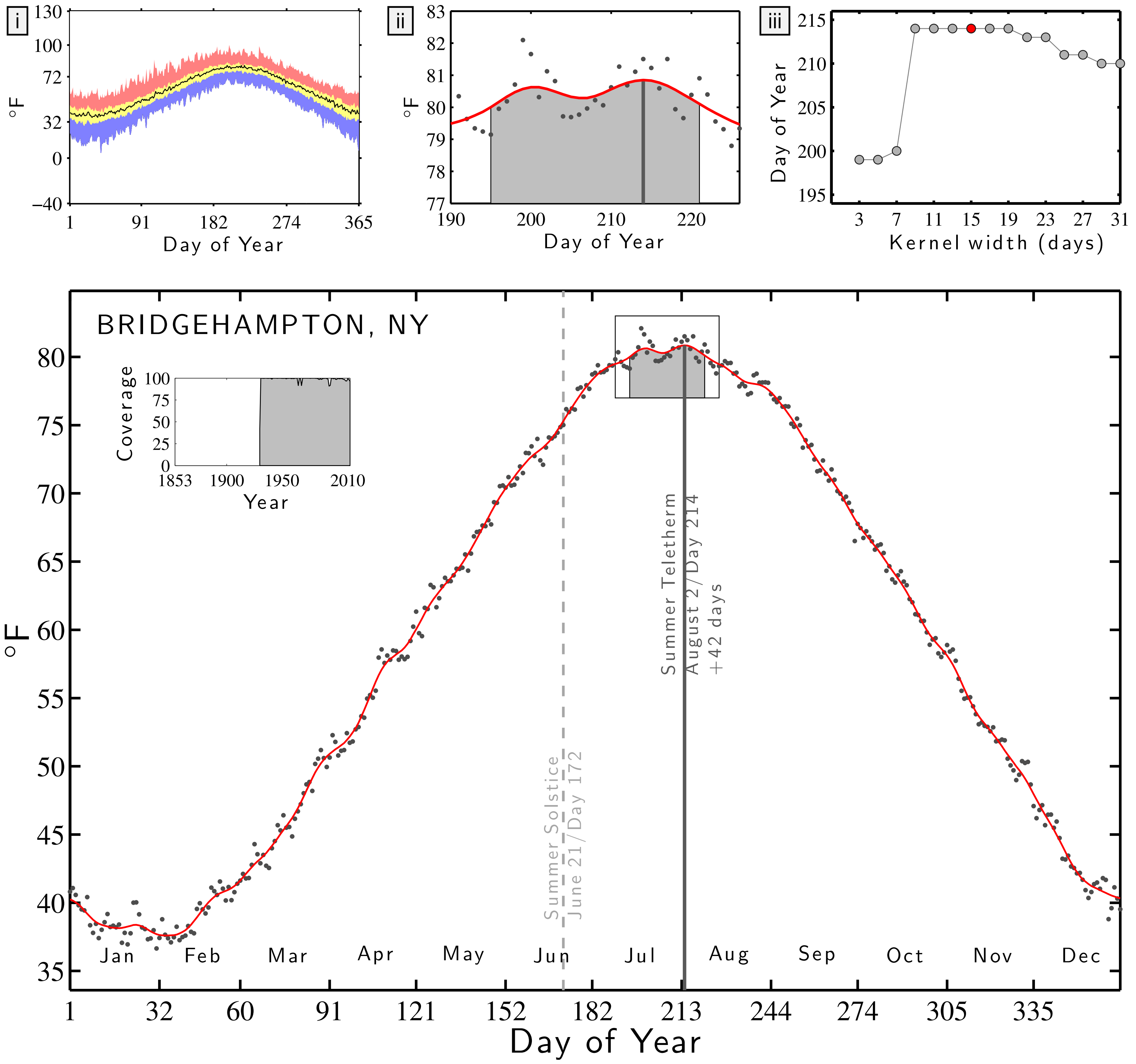

i
°F
Day of Year
ii
°F
Day of Year
iii
Day of Year
Kernel width (days)
BRIDGEHAMPTON, NY
Coverage
Year
Summer Solstice
June 21/Day 172
Summer Teletherm
August 2/Day 214
+42 days
Jan
Feb
Mar
Apr
May
Jun
Jul
Aug
Sep
Oct
Nov
Dec
°F
Day of Year

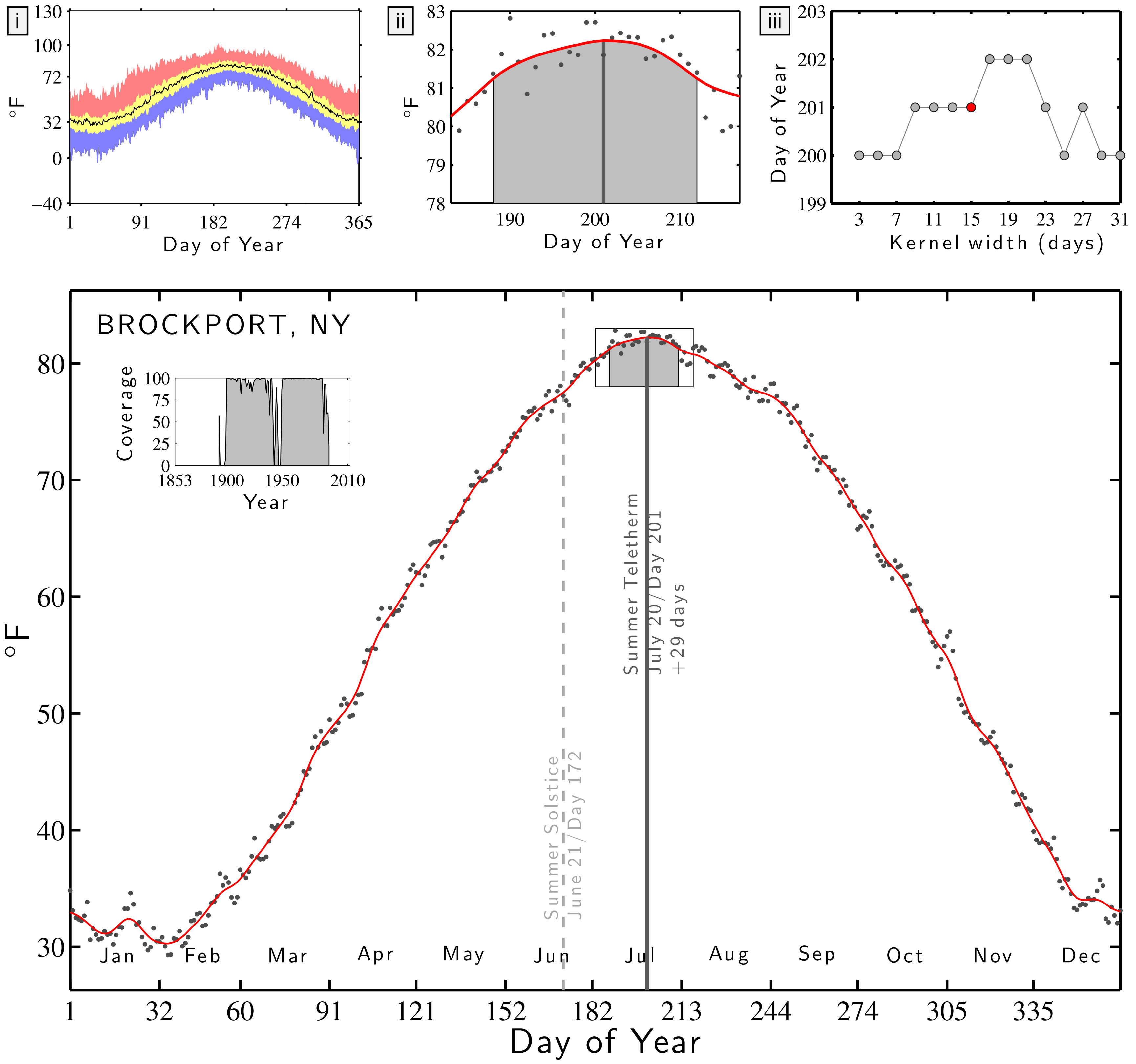

i
ii
iii
°F
Day of Year
Day of Year
Kernel width (days)
BROCKPORT, NY
Coverage
Year
Summer Solstice
June 21/Day 172
Summer Teletherm
July 20/Day 201
+29 days
Jan
Feb
Mar
Apr
May
Jun
Jul
Aug
Sep
Oct
Nov
Dec

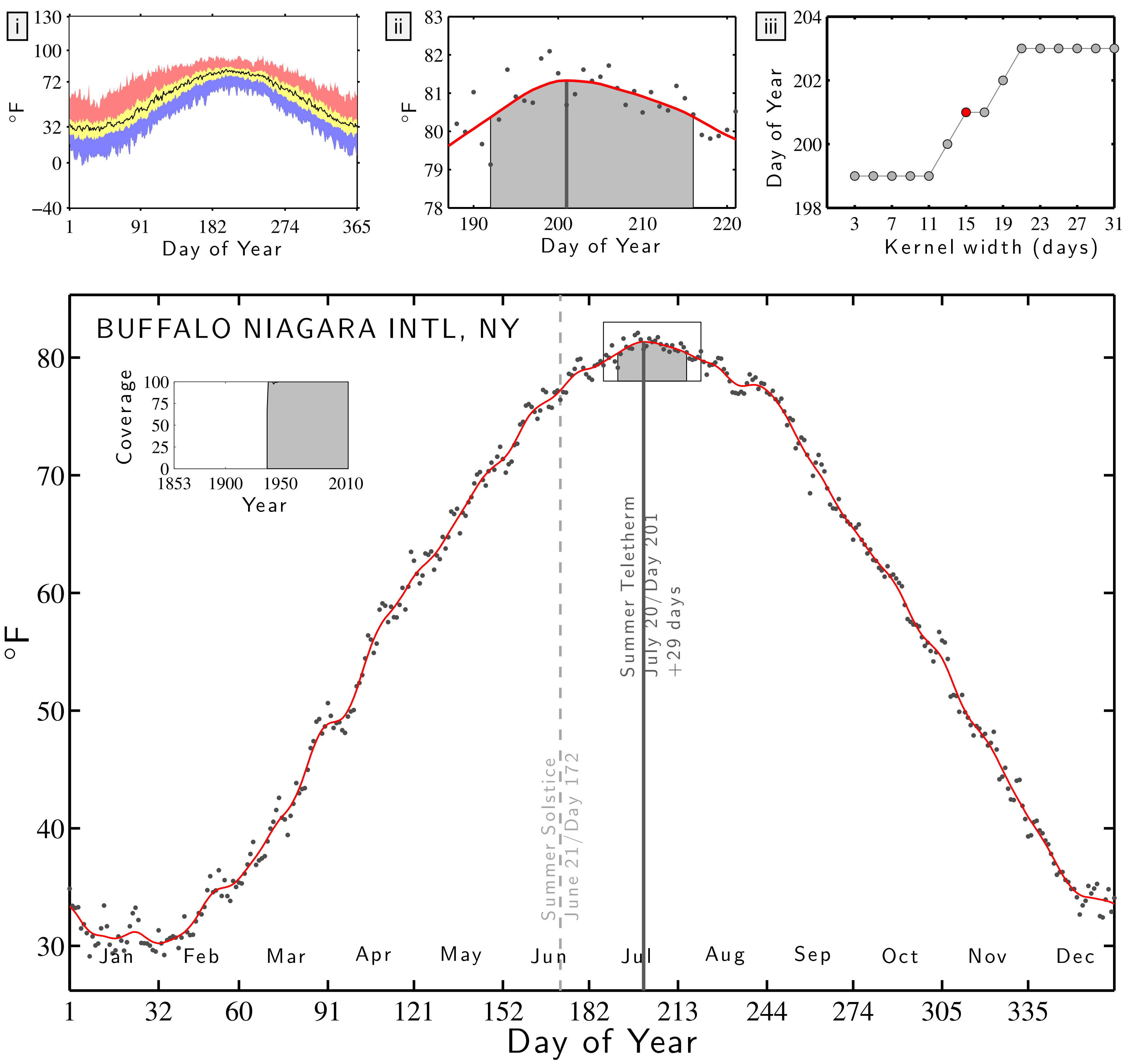
i
°F
Day of Year
ii
°F
Day of Year
iii
Day of Year
Kernel width (days)
BUFFALO NIAGARA INTL, NY
Coverage
Year
°F
Summer Solstice
June 21/Day 172
Summer Teletherm
July 20/Day 201
+29 days
Jan
Feb
Mar
Apr
May
Jun
Jul
Aug
Sep
Oct
Nov
Dec
Day of Year

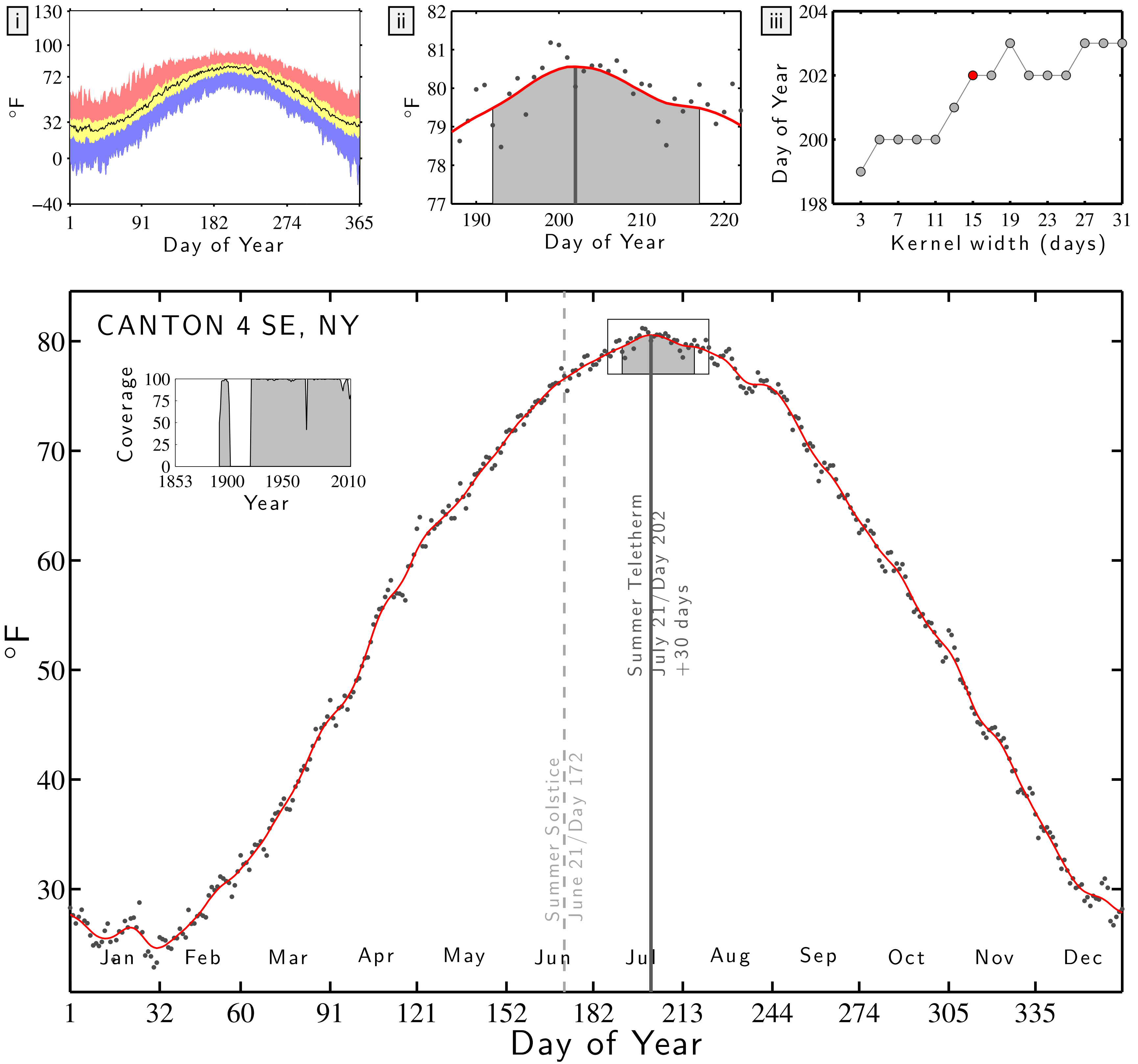

i
°F
Day of Year
ii
°F
Day of Year
iii
Day of Year
Kernel width (days)
CANTON 4 SE, NY
Coverage
Year
Summer Solstice
June 21 / Day 172
Summer Teletherm
July 21 / Day 202
+30 days
Jan
Feb
Mar
Apr
May
Jun
Jul
Aug
Sep
Oct
Nov
Dec
°F
Day of Year

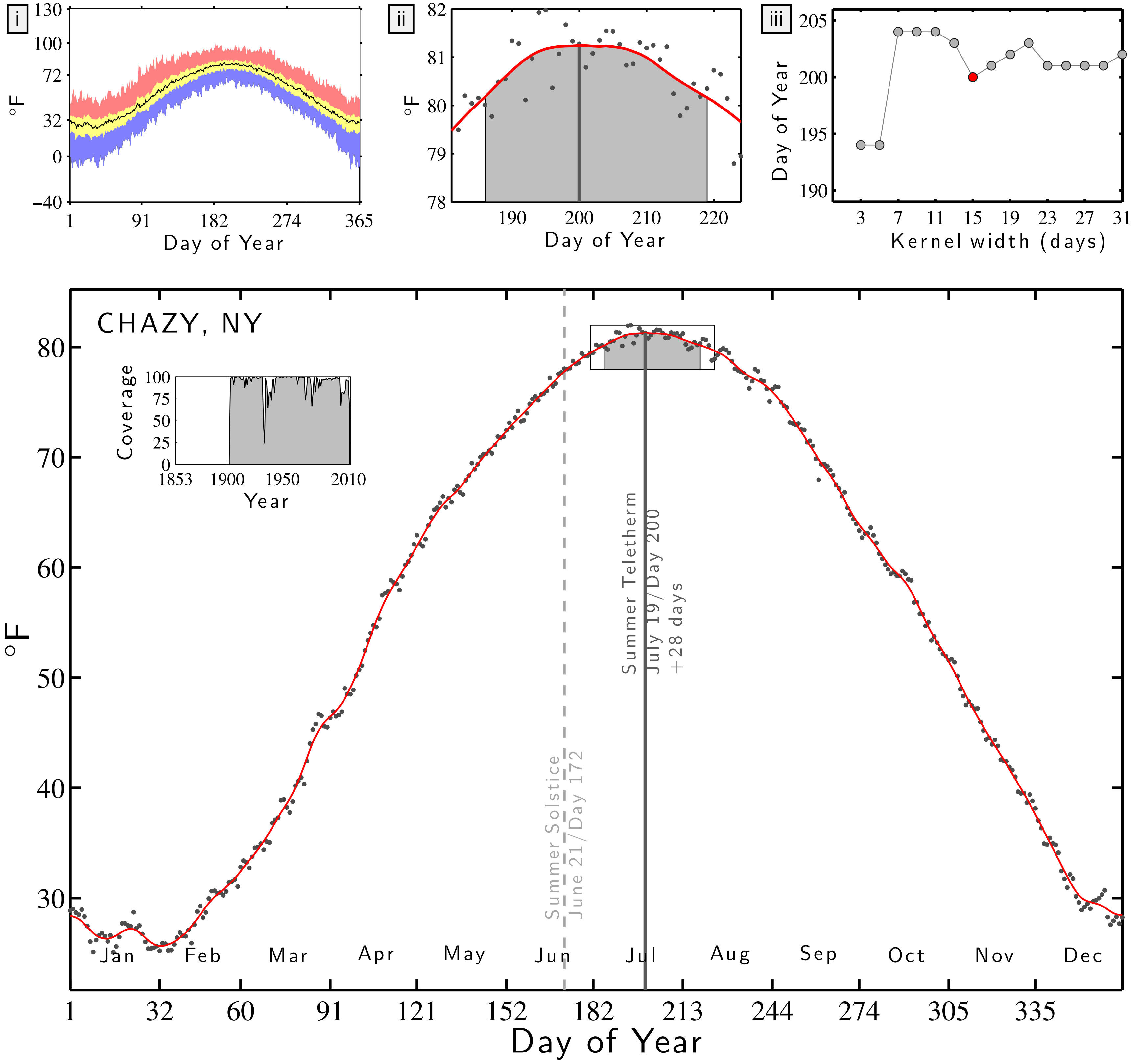
i
°F
Day of Year
ii
iii
Day of Year
Kernel width (days)
CHAZY, NY
Coverage
Year
Summer Solstice
June 21/Day 172
Summer Teletherm
July 19/Day 200
+28 days
Jan
Feb
Mar
Apr
May
Jun
Jul
Aug
Sep
Oct
Nov
Dec
Day of Year

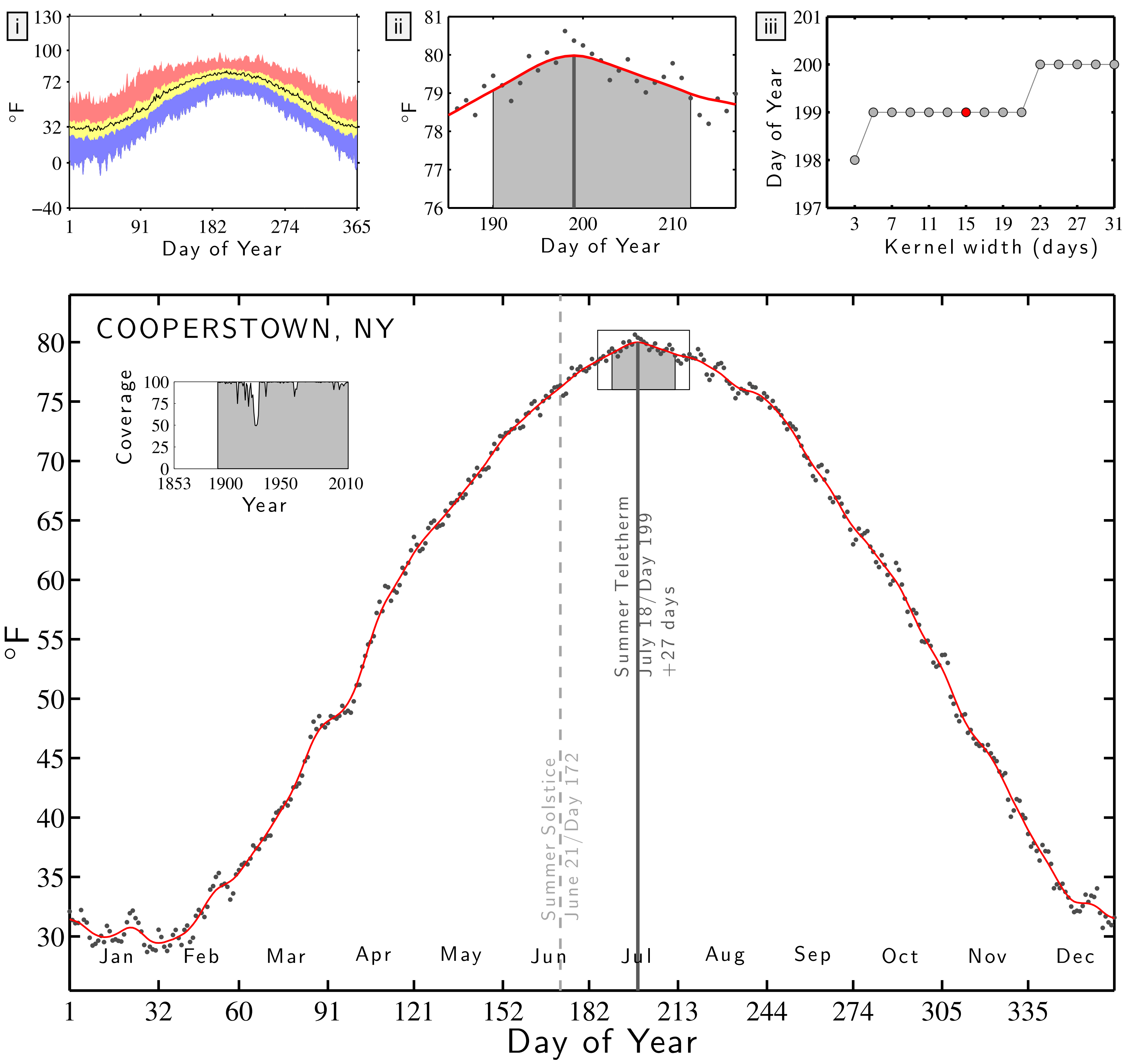

i
ii
iii
°F
Day of Year
Day of Year
Kernel width (days)
COOPERSTOWN, NY
Coverage
Year
Summer Solstice
June 21/Day 172
Summer Teletherm
July 18/Day 199
+27 days
Jan
Feb
Mar
Apr
May
Jun
Jul
Aug
Sep
Oct
Nov
Dec
Day of Year

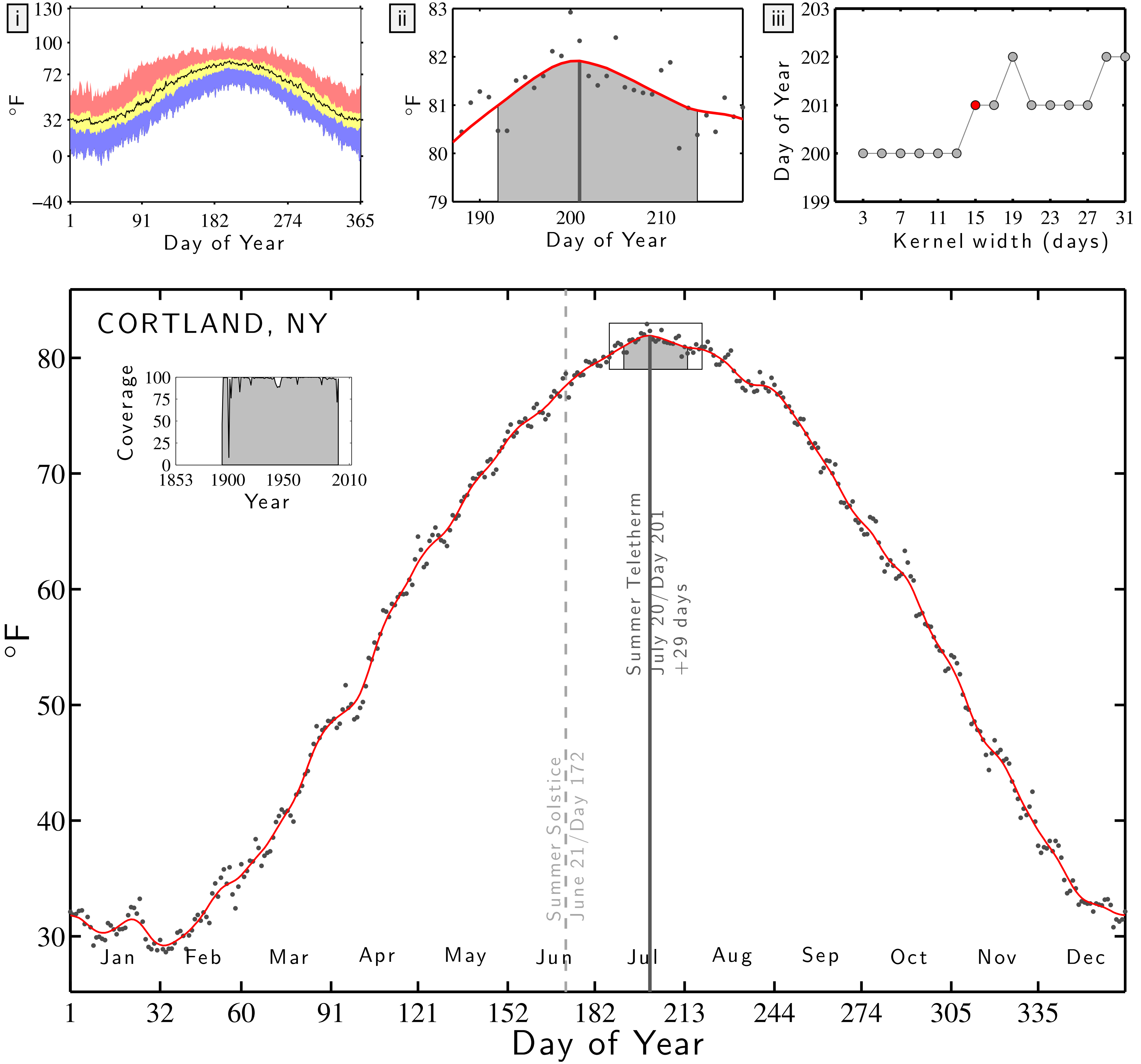

i
ii
iii
°F
Day of Year
Day of Year
Kernel width (days)
CORTLAND, NY
Coverage
Year
Summer Solstice
June 21 / Day 172
Summer Teletherm
July 20 / Day 201
+29 days
Jan
Feb
Mar
Apr
May
Jun
Jul
Aug
Sep
Oct
Nov
Dec

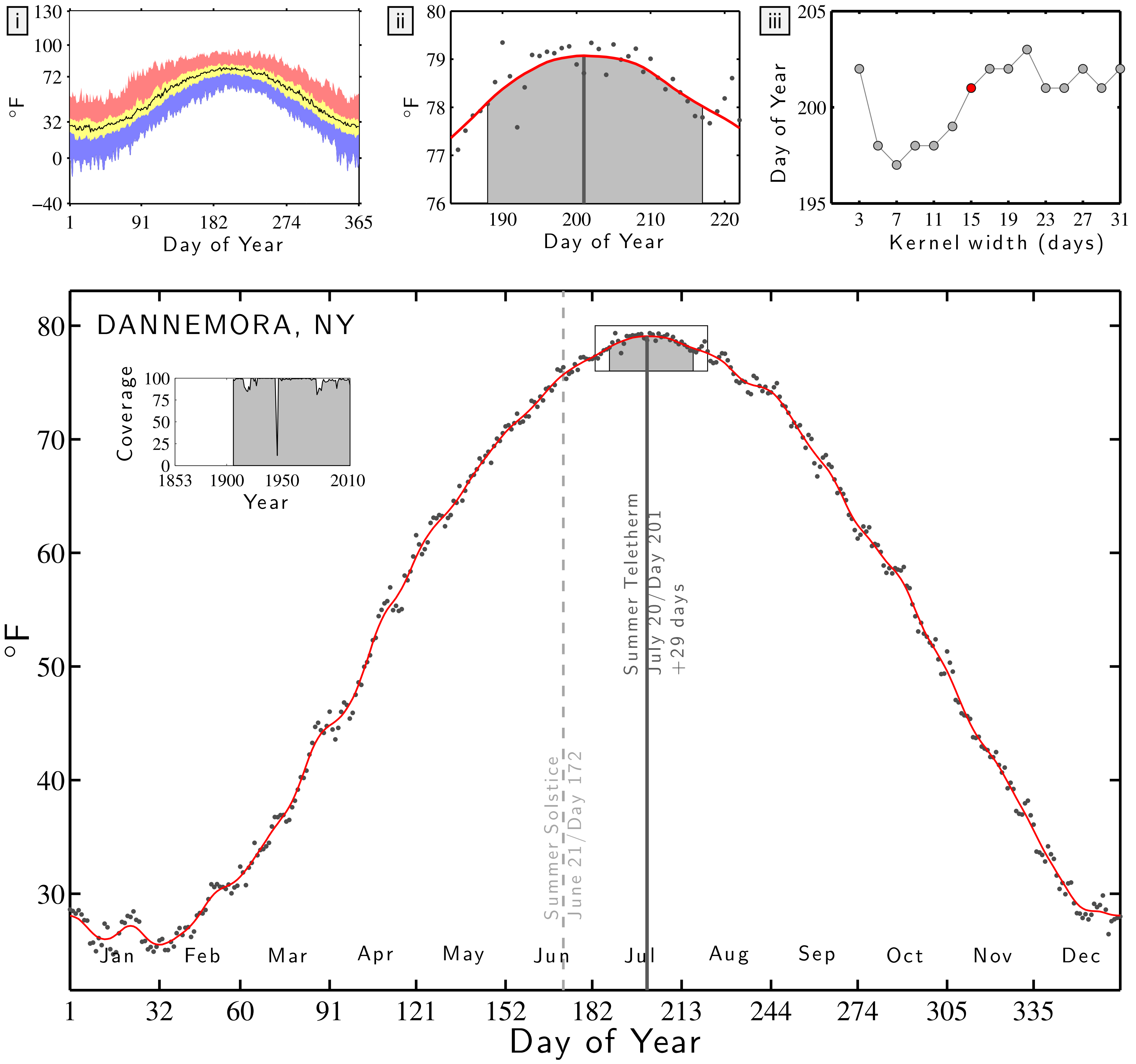

i
ii
iii
°F
Day of Year
Day of Year
Kernel width (days)
DANNEMORA, NY
Coverage
Year
Summer Solstice
June 21/Day 172
Summer Teletherm
July 20/Day 201
+29 days
Jan
Feb
Mar
Apr
May
Jun
Jul
Aug
Sep
Oct
Nov
Dec

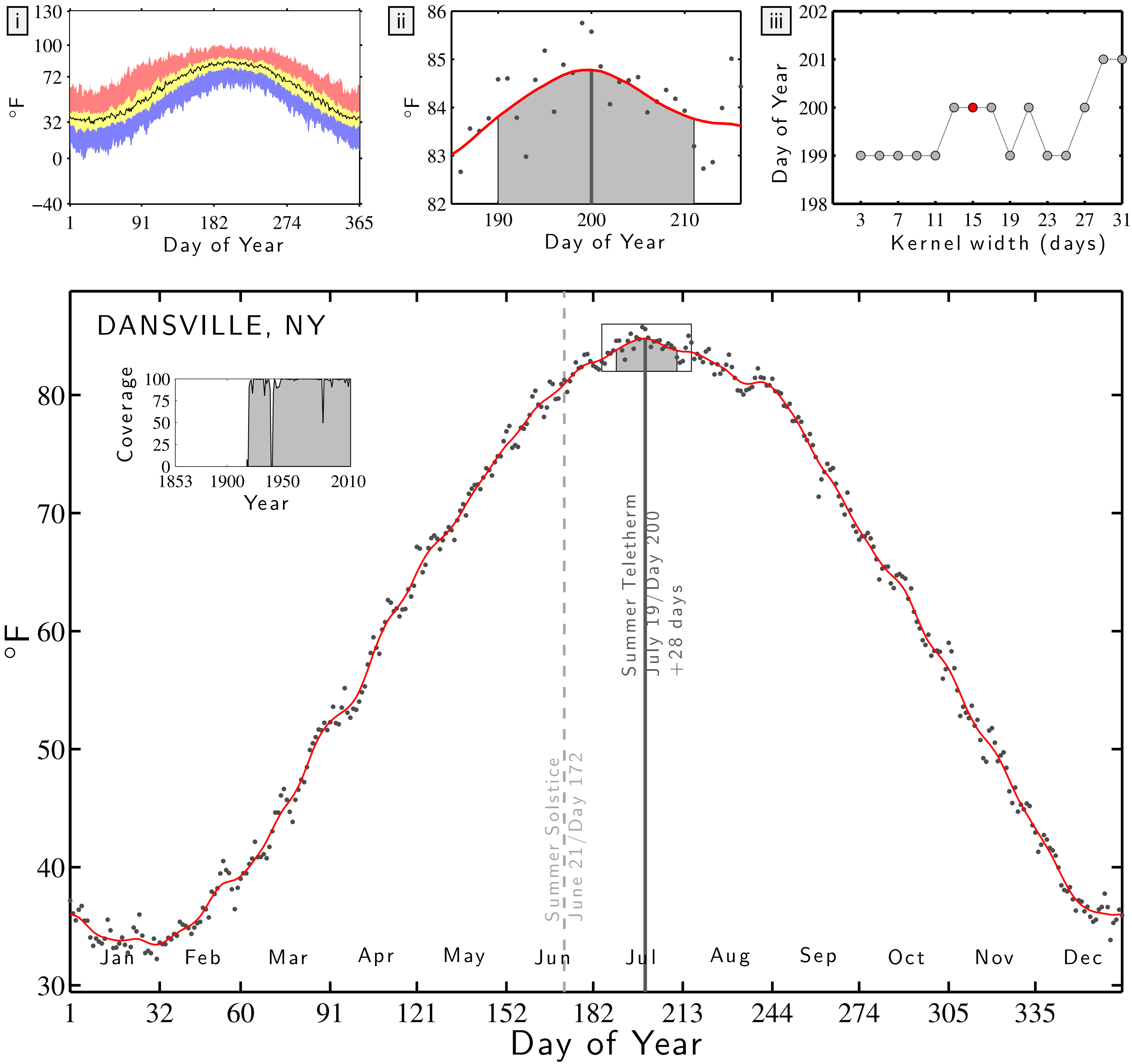

i
°F
Day of Year
ii
°F
Day of Year
iii
Day of Year
Kernel width (days)
DANSVILLE, NY
Coverage
Year
Summer Solstice
June 21/Day 172
Summer Teletherm
July 19/Day 200
+28 days
Jan
Feb
Mar
Apr
May
Jun
Jul
Aug
Sep
Oct
Nov
Dec
°F
Day of Year

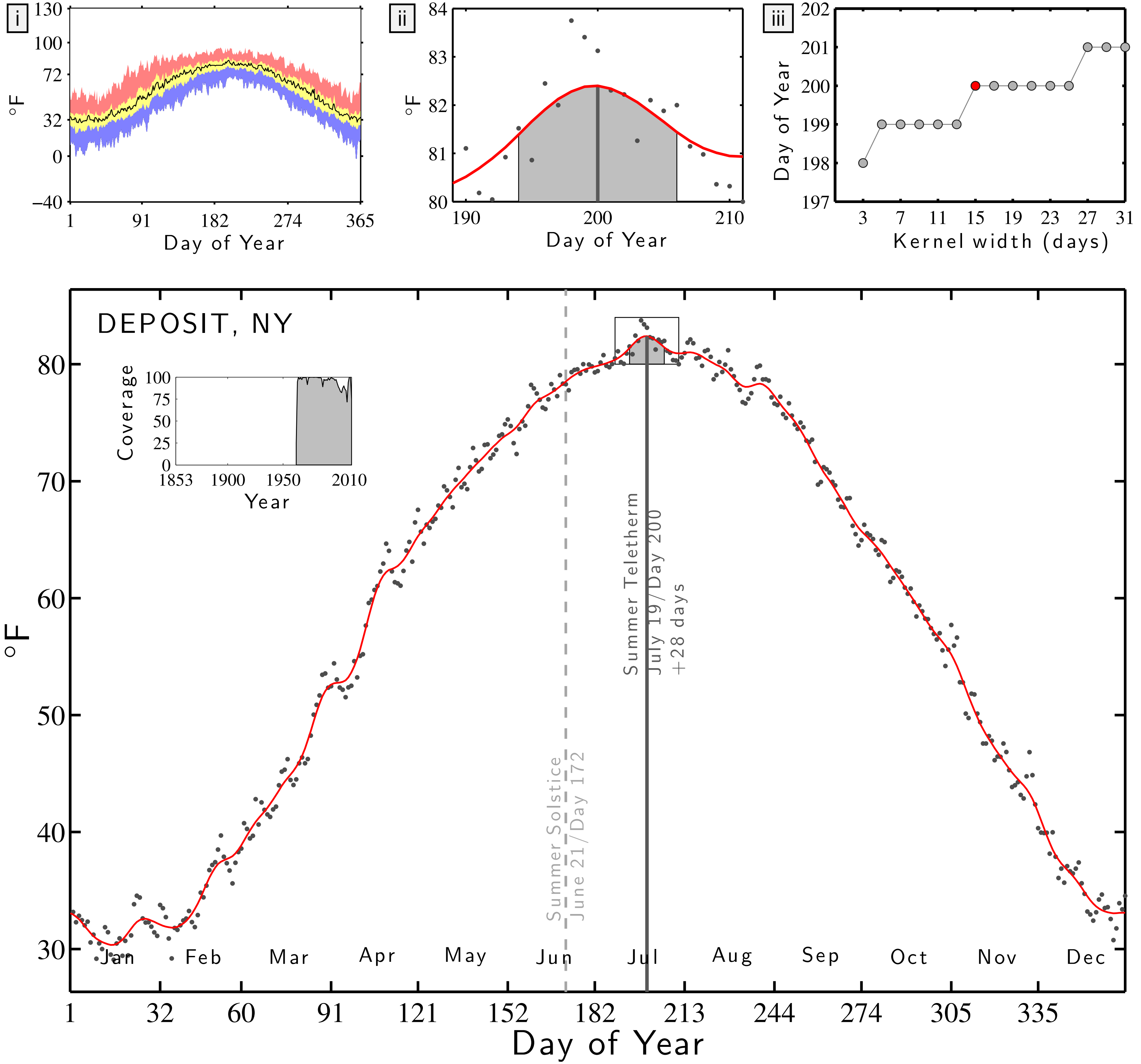

i
°F
Day of Year
ii
°F
Day of Year
iii
Day of Year
Kernel width (days)
DEPOSIT, NY
Coverage
Year
Summer Solstice
June 21/Day 172
Summer Teletherm
July 19/Day 200
+28 days
Jan
Feb
Mar
Apr
May
Jun
Jul
Aug
Sep
Oct
Nov
Dec
°F
Day of Year

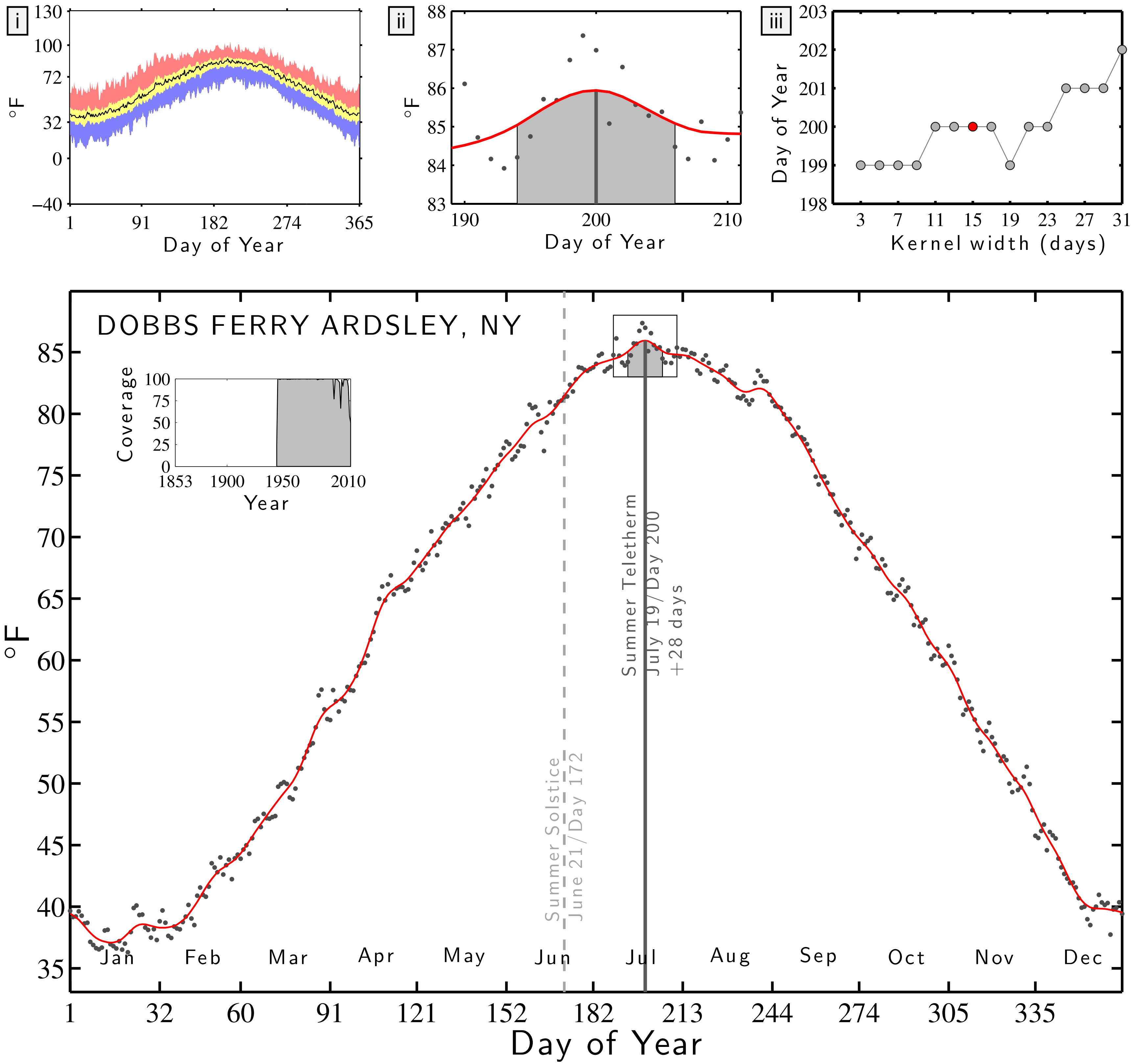

i
°F
Day of Year
ii
°F
Day of Year
iii
Day of Year
Kernel width (days)
DOBBS FERRY ARDSLEY, NY
Coverage
Year
Summer Solstice
June 21 / Day 172
Summer Teletherm
July 19 / Day 200
+28 days
Jan
Feb
Mar
Apr
May
Jun
Jul
Aug
Sep
Oct
Nov
Dec
°F
Day of Year

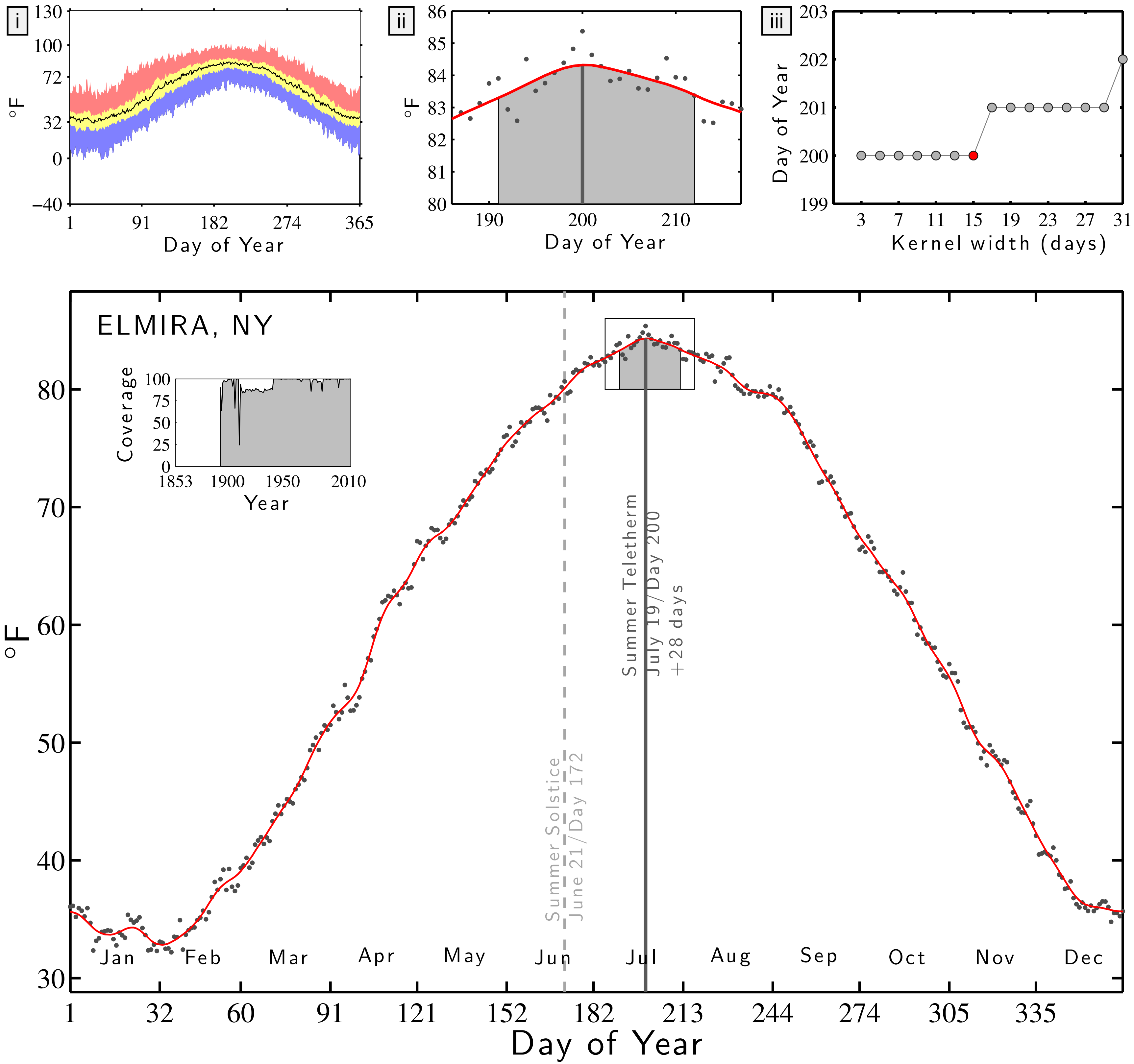

i
ii
iii
°F
Day of Year
Day of Year
Kernel width (days)
ELMIRA, NY
Coverage
Year
Summer Solstice
June 21/Day 172
Summer Teletherm
July 19/Day 200
+28 days
Jan
Feb
Mar
Apr
May
Jun
Jul
Aug
Sep
Oct
Nov
Dec
°F
Day of Year

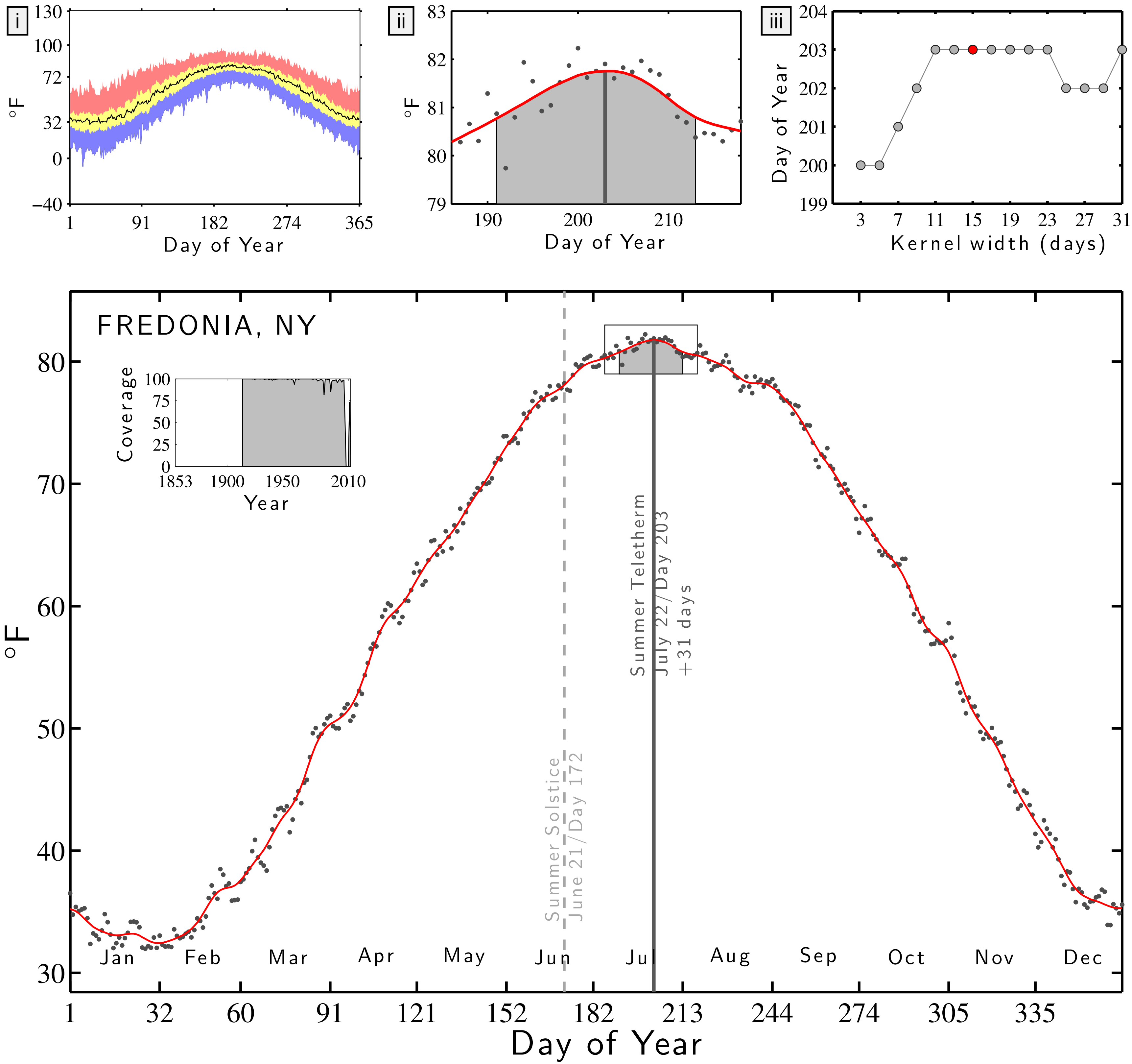
i
°F
Day of Year
ii
°F
Day of Year
iii
Day of Year
Kernel width (days)
FREDONIA, NY
Coverage
Year
Summer Solstice
June 21/Day 172
Summer Teletherm
July 22/Day 203
+31 days
Jan
Feb
Mar
Apr
May
Jun
Jul
Aug
Sep
Oct
Nov
Dec
°F
Day of Year

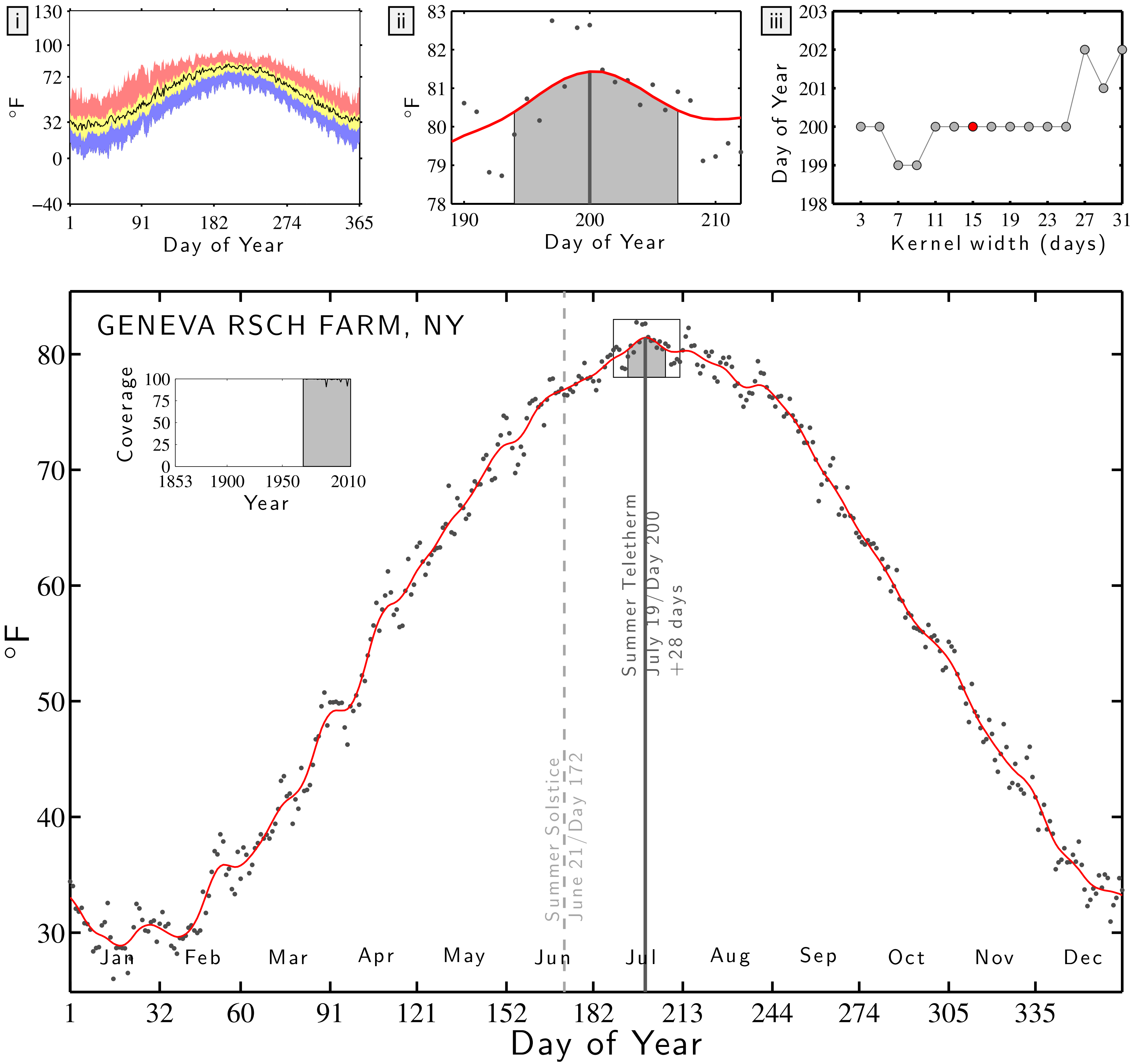

i
ii
iii
Day of Year
Kernel width (days)
GENEVA RSCH FARM, NY
Coverage
Year
Summer Solstice
June 21/Day 172
Summer Teletherm
July 19/Day 200
+28 days
Jan
Feb
Mar
Apr
May
Jun
Jul
Aug
Sep
Oct
Nov
Dec
°F
Day of Year

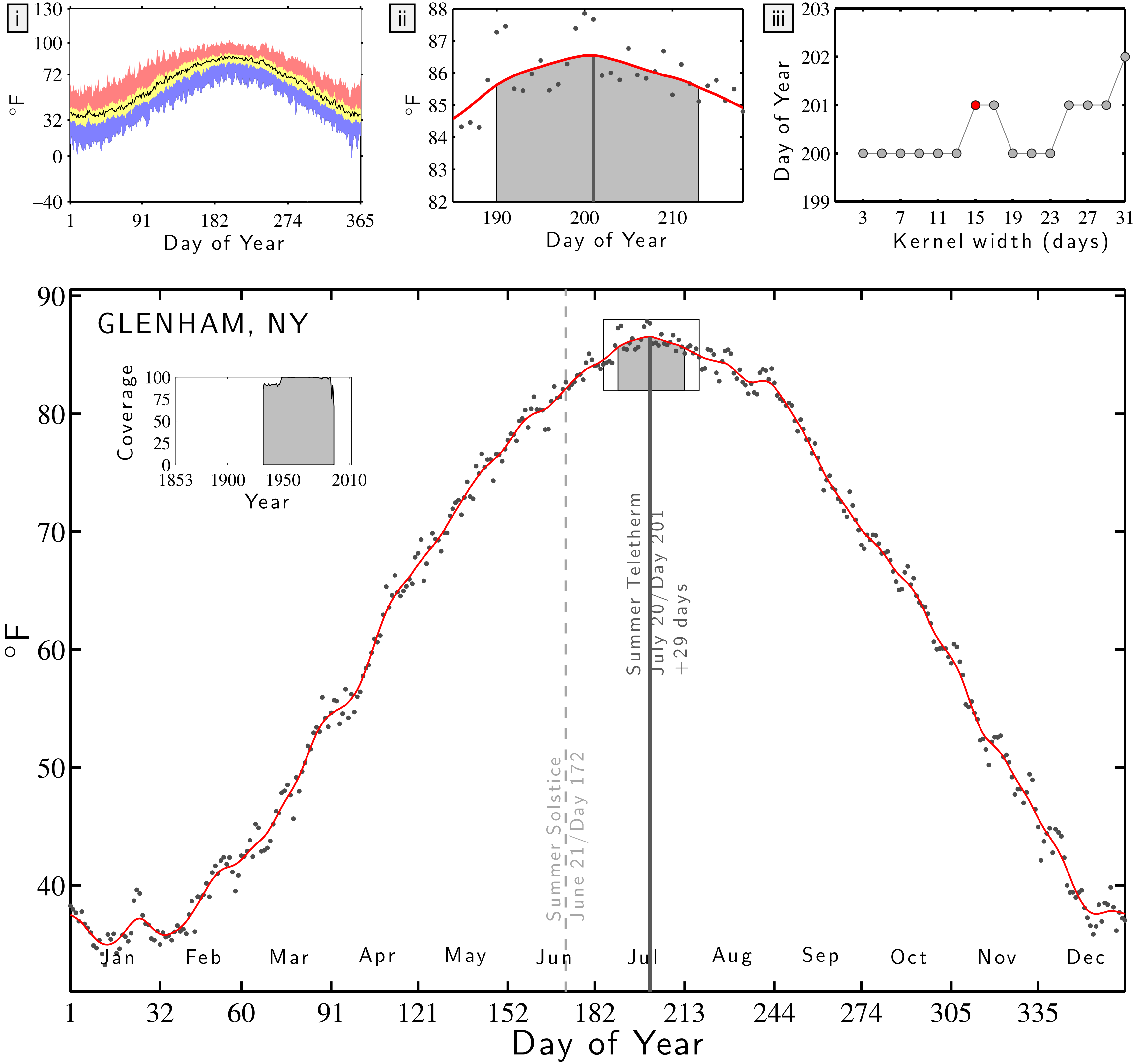

i
°F
130
100
72
32
0
−40
1
91
182
274
365
Day of Year
ii
88
87
86
85
84
83
82
190
200
210
Day of Year
iii
Day of Year
203
202
201
200
199
3
7
11
15
19
23
27
31
Kernel width (days)
GLENHAM, NY
Coverage
100
75
50
25
0
1853
1900
1950
2010
Year
90
80
70
60
50
40
Summer Solstice
June 21/Day 172
Summer Teletherm
July 20/Day 201
+29 days
Jan
Feb
Mar
Apr
May
Jun
Jul
Aug
Sep
Oct
Nov
Dec
1
32
60
91
121
152
182
213
244
274
305
335
Day of Year

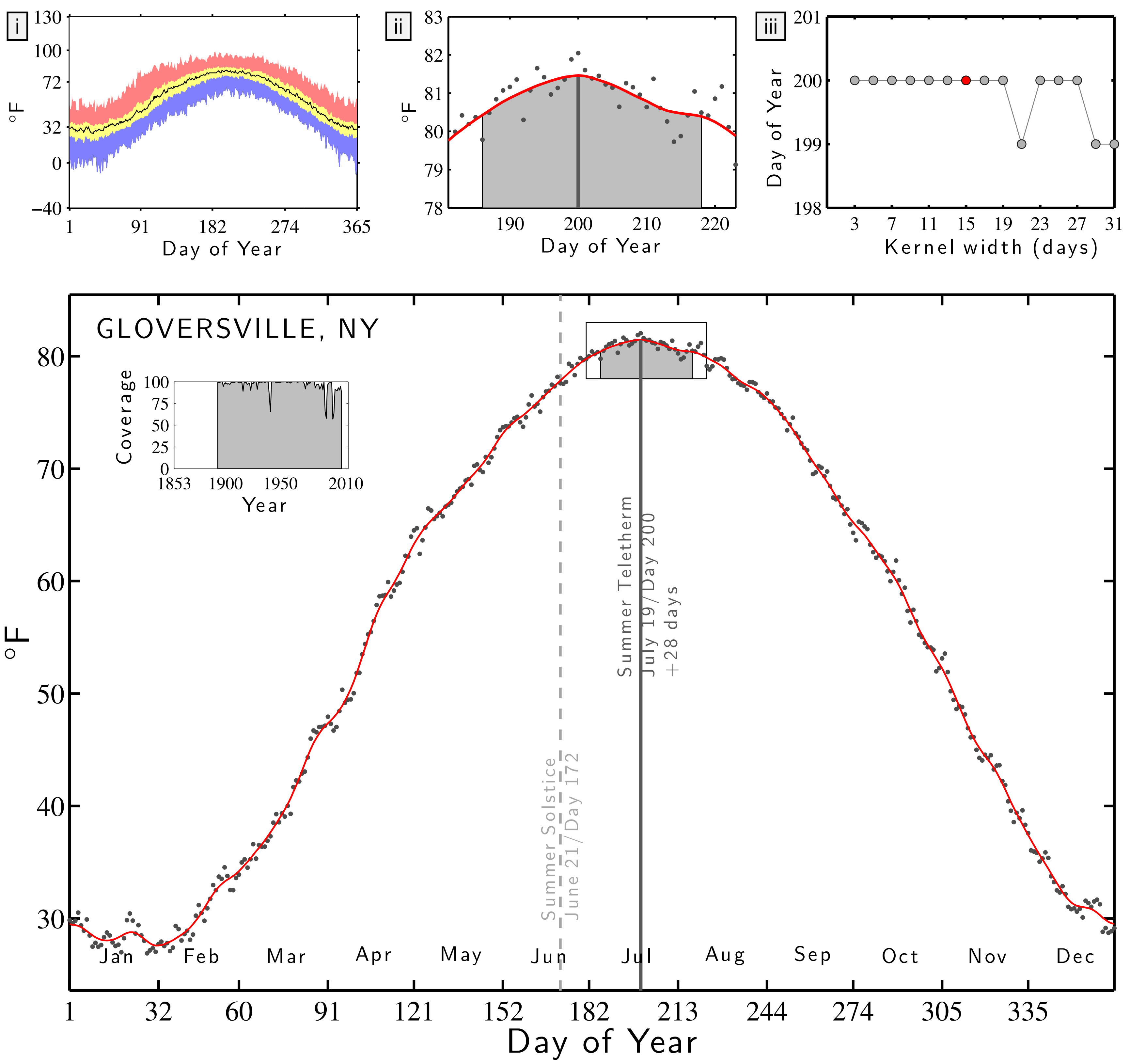

i
°F
130
100
72
32
0
−40
1
91
182
274
365
Day of Year
ii
°F
83
82
81
80
79
78
190
200
210
220
Day of Year
iii
Day of Year
201
200
199
198
3
7
11
15
19
23
27
31
Kernel width (days)
GLOVERSVILLE, NY
Coverage
100
75
50
25
0
1853
1900
1950
2010
Year
Summer Solstice
June 21/Day 172
Summer Teletherm
July 19/Day 200
+28 days
°F
80
70
60
50
40
30
Jan
Feb
Mar
Apr
May
Jun
Jul
Aug
Sep
Oct
Nov
Dec
1
32
60
91
121
152
182
213
244
274
305
335
Day of Year

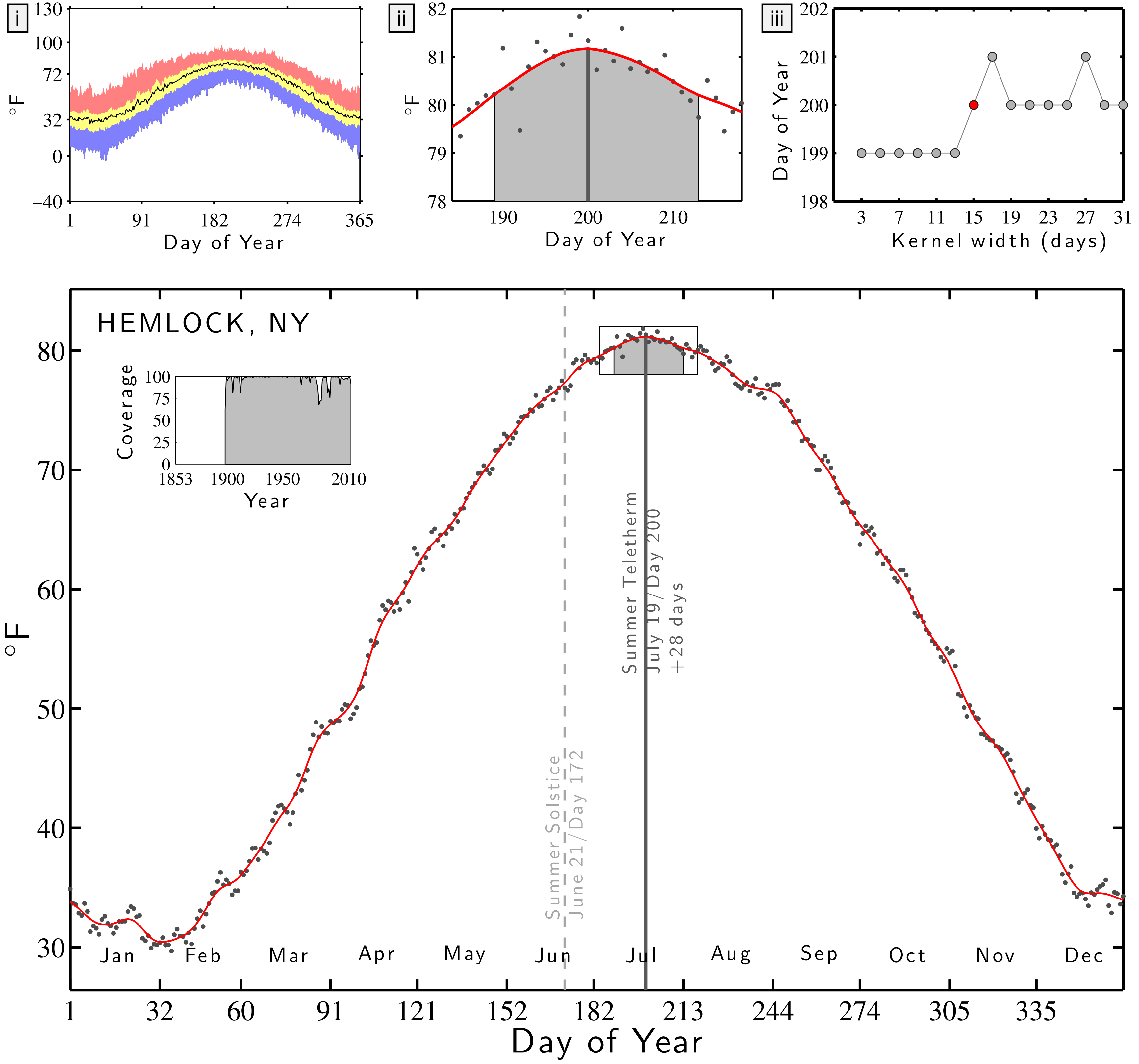

i
°F
Day of Year
ii
°F
Day of Year
iii
Day of Year
Kernel width (days)
HEMLOCK, NY
Coverage
Year
Summer Solstice
June 21/Day 172
Summer Teletherm
July 19/Day 200
+28 days
Jan
Feb
Mar
Apr
May
Jun
Jul
Aug
Sep
Oct
Nov
Dec
°F
Day of Year

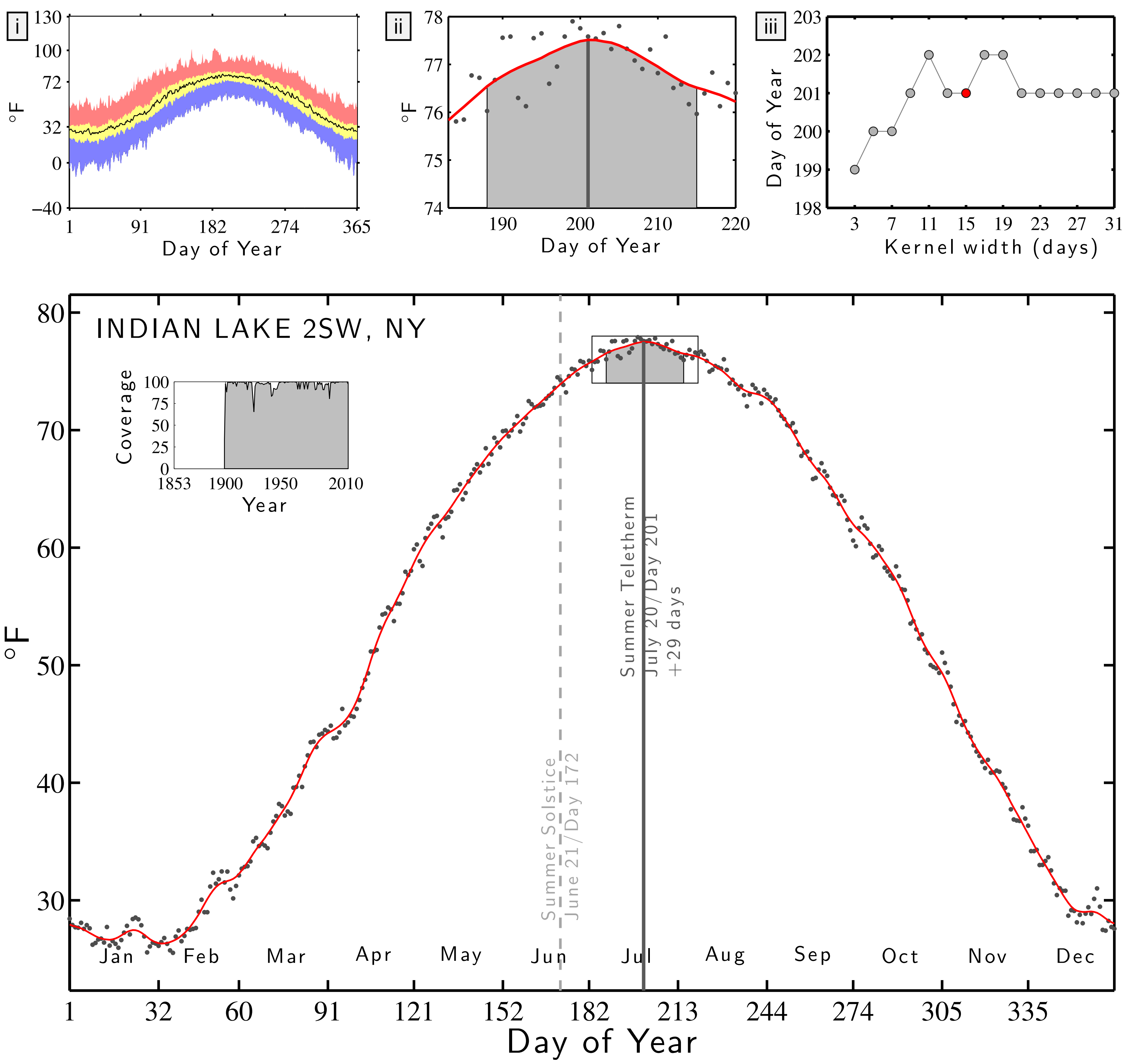

i
ii
iii
°F
Day of Year
Day of Year
Kernel width (days)
INDIAN LAKE 2SW, NY
Coverage
Year
Summer Solstice
June 21/Day 172
Summer Teletherm
July 20/Day 201
+29 days
Jan
Feb
Mar
Apr
May
Jun
Jul
Aug
Sep
Oct
Nov
Dec
Day of Year

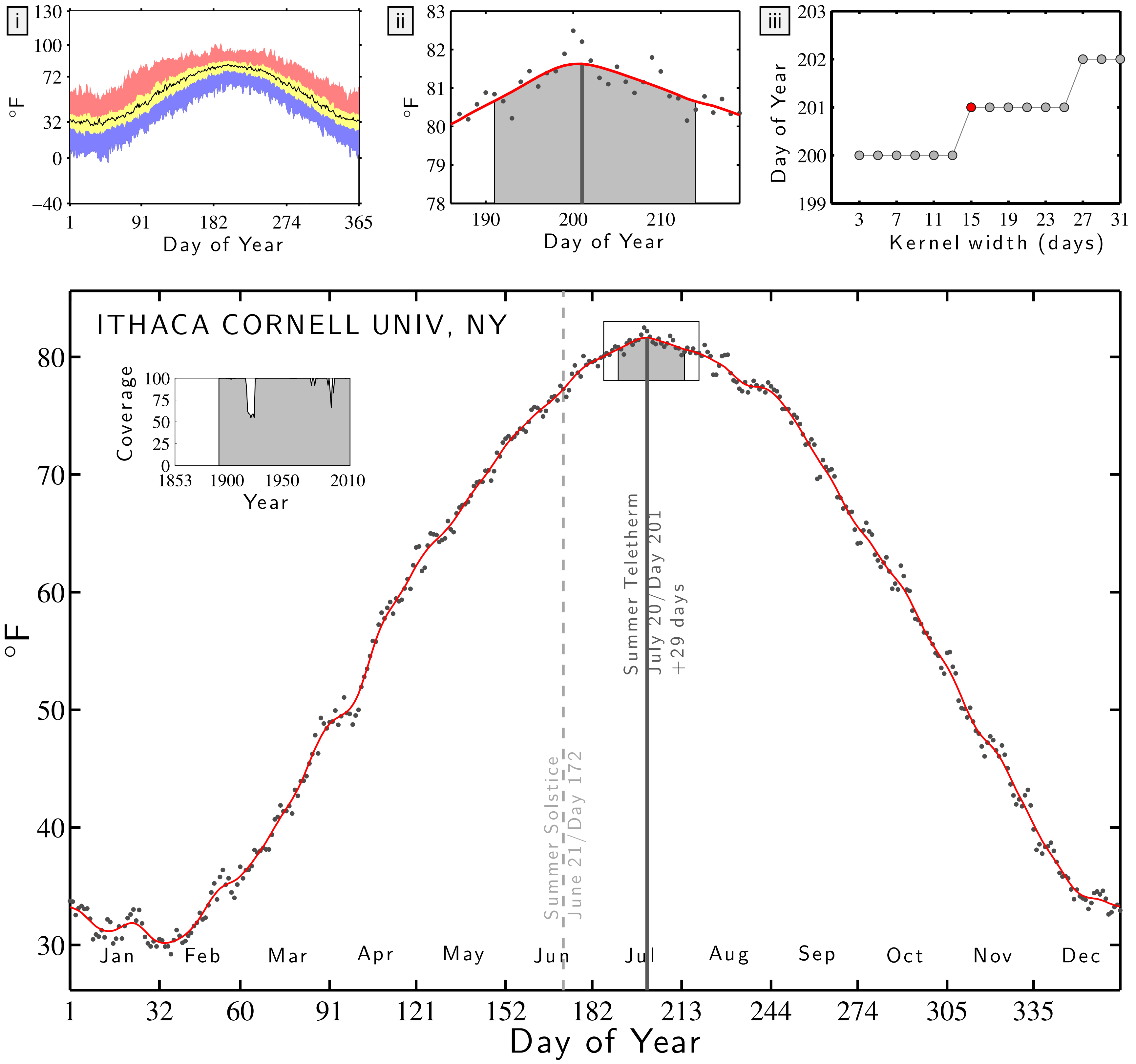

i
ii
iii
°F
Day of Year
Day of Year
Kernel width (days)
ITHACA CORNELL UNIV, NY
Coverage
Year
Summer Solstice
June 21/Day 172
Summer Teletherm
July 20/Day 201
+29 days
Jan
Feb
Mar
Apr
May
Jun
Jul
Aug
Sep
Oct
Nov
Dec

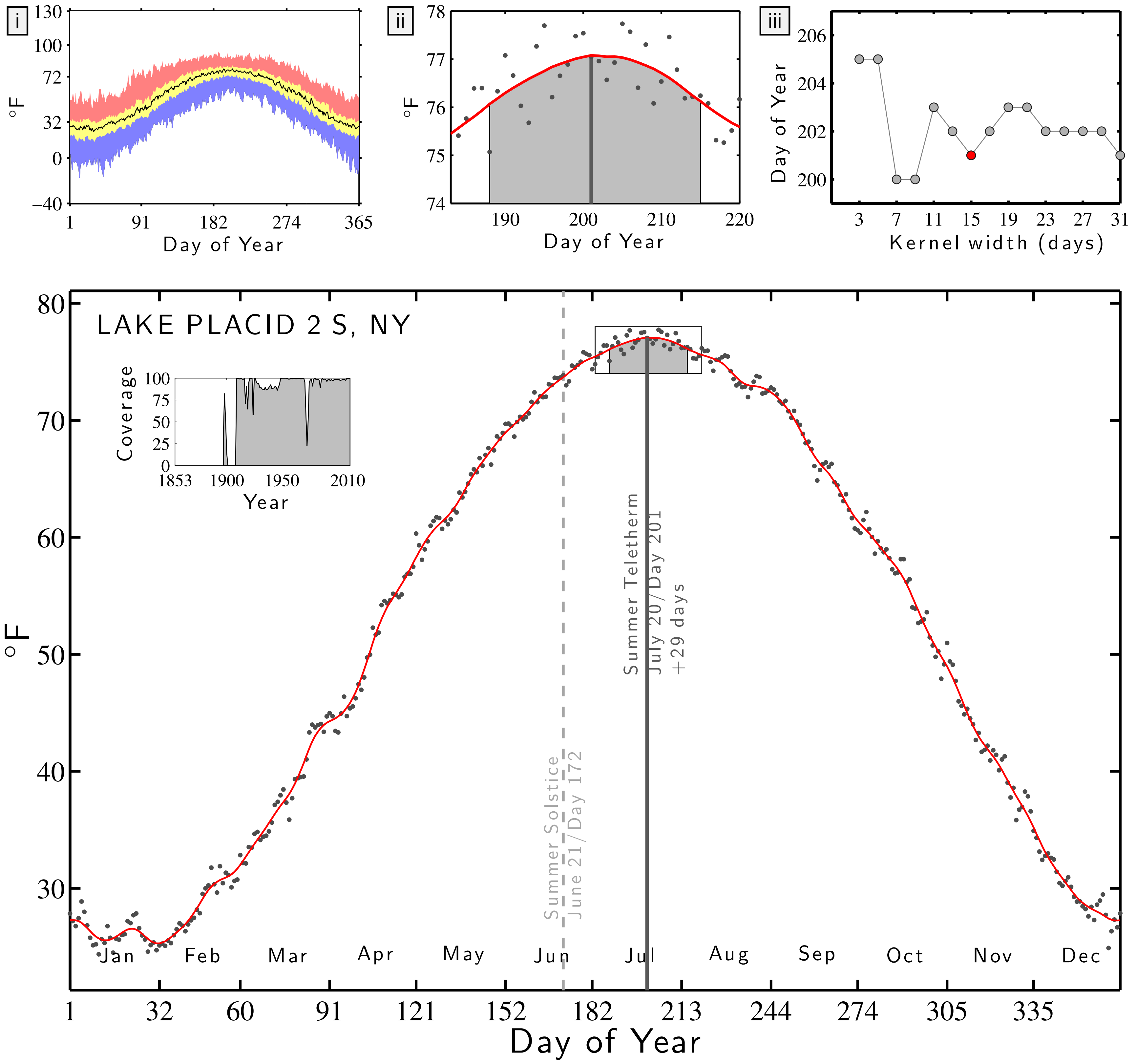
i
ii
iii
LAKE PLACID 2 S, NY
Coverage
Year
Summer Solstice
June 21/Day 172
Summer Teletherm
July 20/Day 201
+29 days
Jan
Feb
Mar
Apr
May
Jun
Jul
Aug
Sep
Oct
Nov
Dec
°F
Day of Year
Kernel width (days)

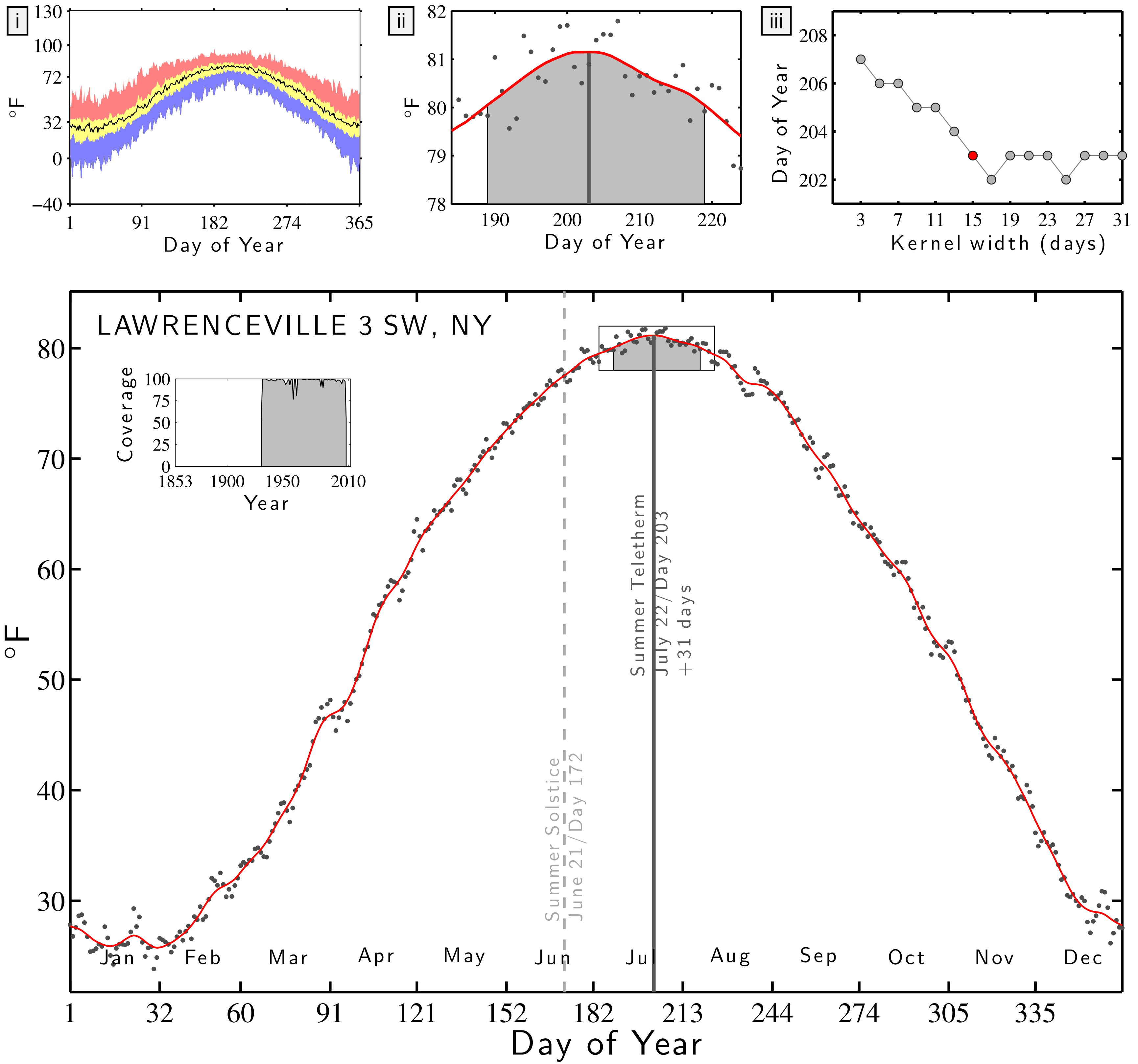

i
ii
iii
°F
Day of Year
Kernel width (days)
LAWRENCEVILLE 3 SW, NY
Coverage
Year
Summer Solstice
June 21 / Day 172
Summer Teletherm
July 22 / Day 203
+31 days
Jan
Feb
Mar
Apr
May
Jun
Jul
Aug
Sep
Oct
Nov
Dec

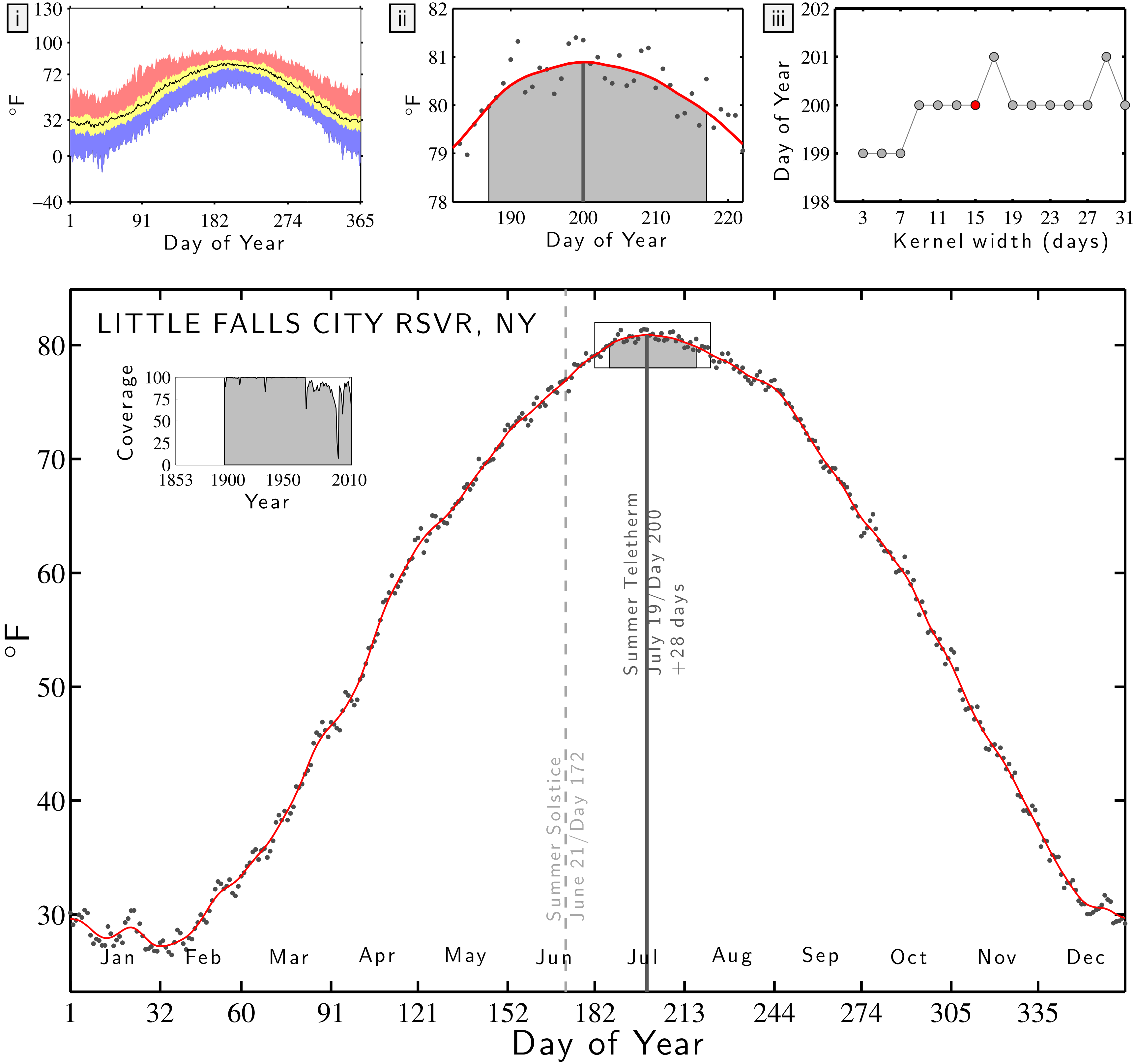

i
ii
iii
°F
Day of Year
Day of Year
Kernel width (days)
LITTLE FALLS CITY RSVR, NY
Coverage
Year
Summer Solstice
June 21/Day 172
Summer Teletherm
July 19/Day 200
+28 days
Jan
Feb
Mar
Apr
May
Jun
Jul
Aug
Sep
Oct
Nov
Dec

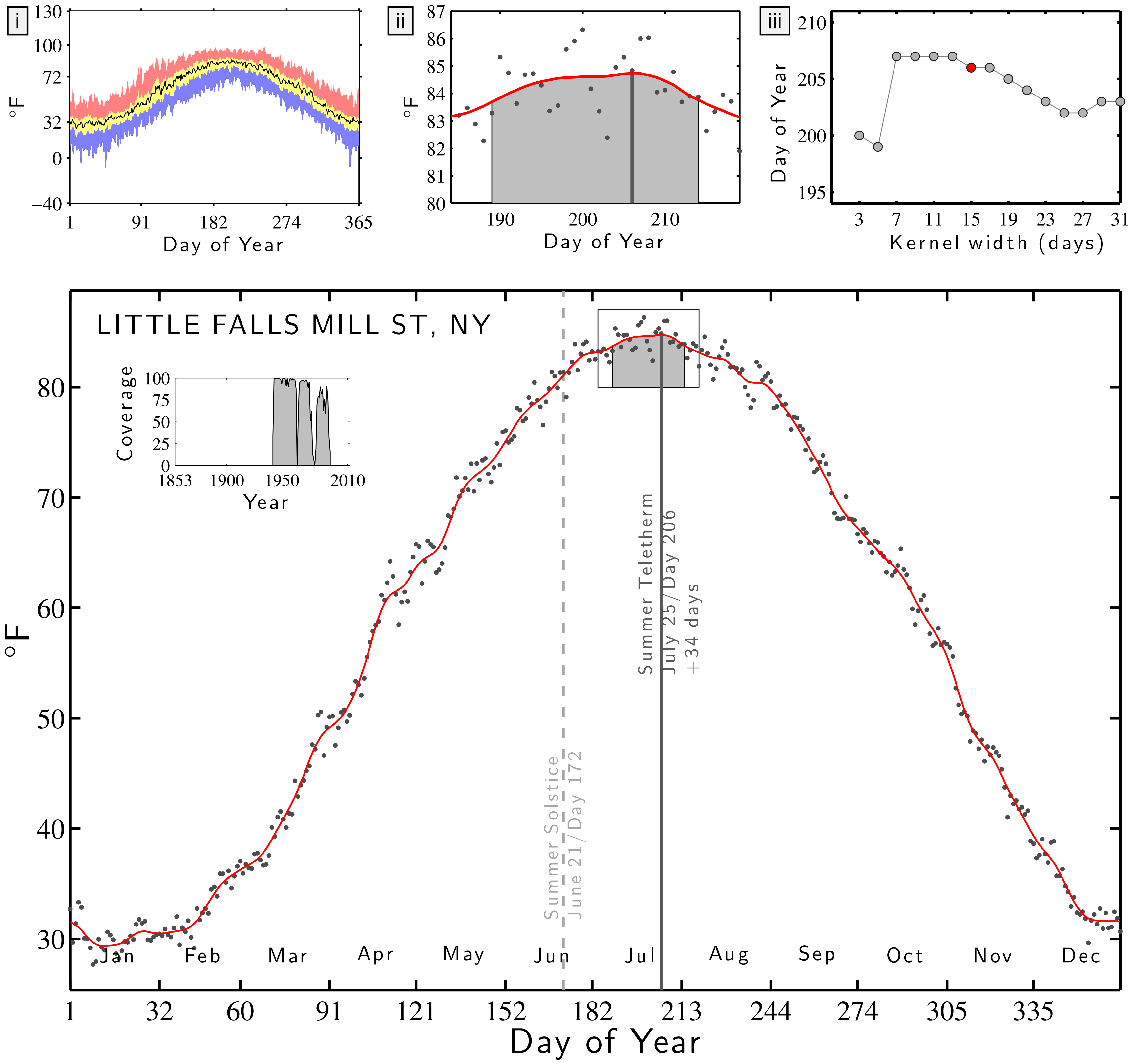

i
ii
iii
Day of Year
°F
Kernel width (days)
LITTLE FALLS MILL ST, NY
Coverage
Year
Summer Solstice
June 21/Day 172
Summer Teletherm
July 25/Day 206
+34 days
Jan
Feb
Mar
Apr
May
Jun
Jul
Aug
Sep
Oct
Nov
Dec
Day of Year

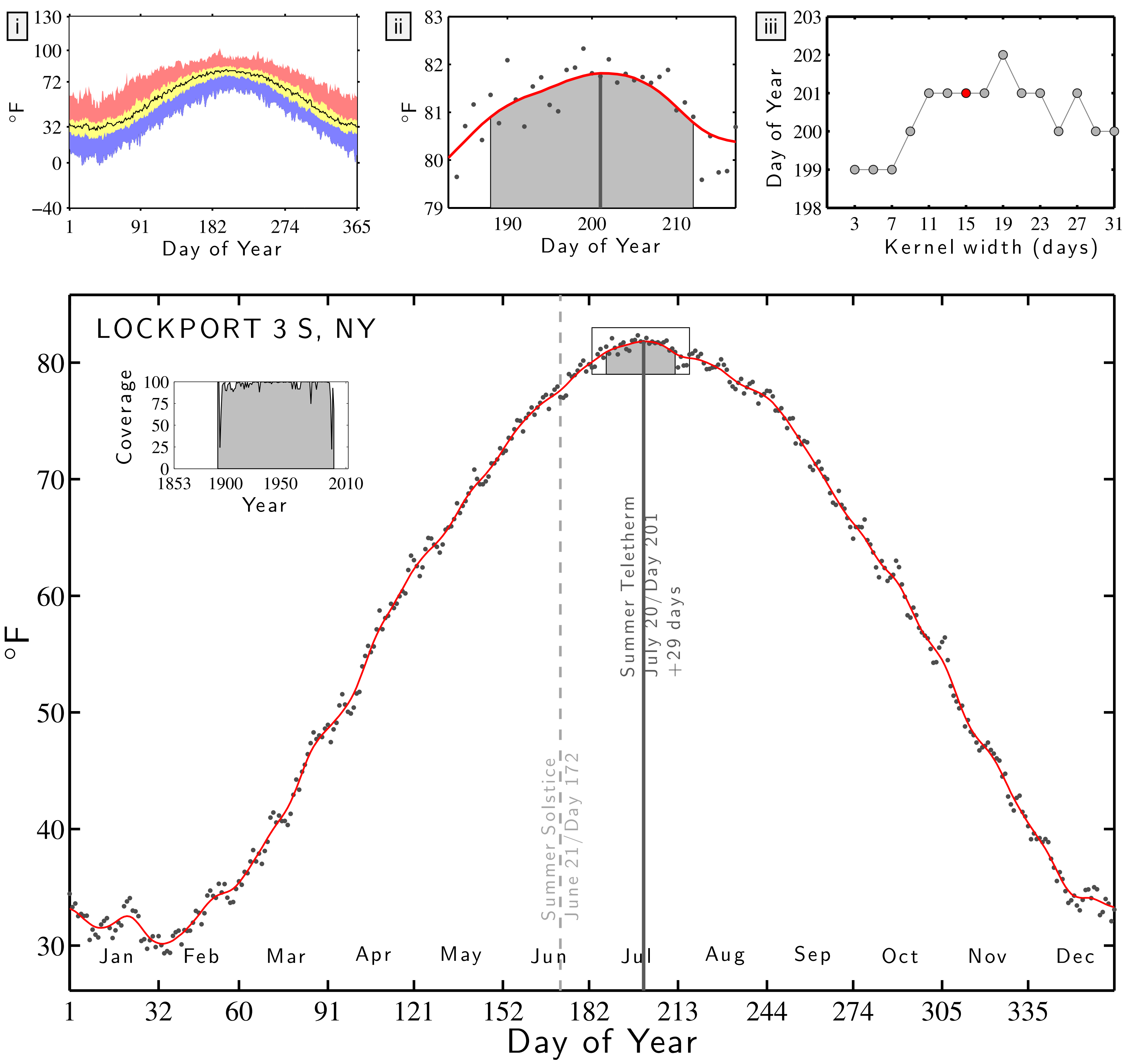

i
ii
iii
Day of Year
°F
Kernel width (days)
LOCKPORT 3 S, NY
Coverage
Year
Summer Solstice
June 21/Day 172
Summer Teletherm
July 20/Day 201
+29 days
Jan
Feb
Mar
Apr
May
Jun
Jul
Aug
Sep
Oct
Nov
Dec

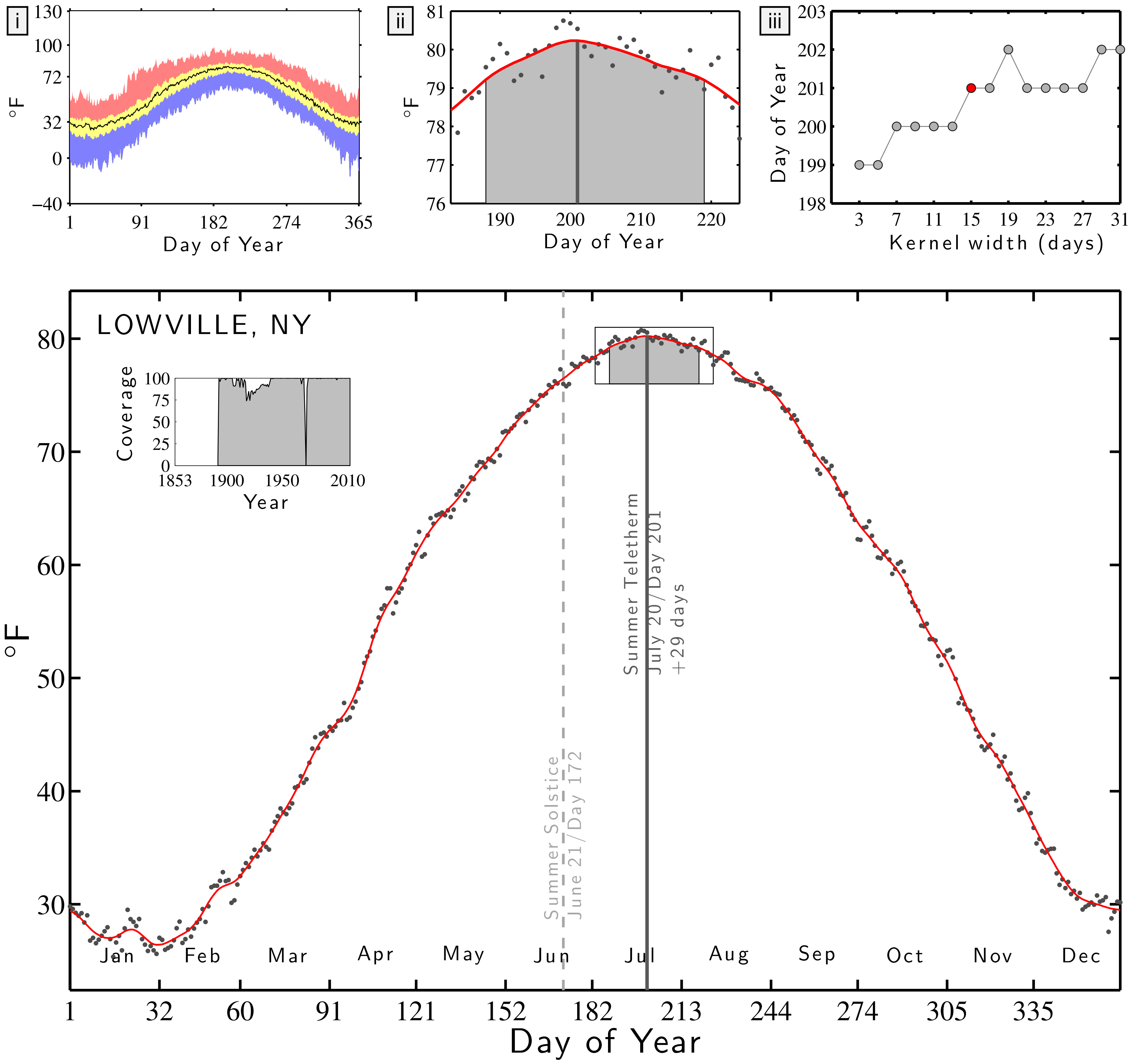

i
°F
Day of Year
ii
°F
Day of Year
iii
Day of Year
Kernel width (days)
LOWVILLE, NY
Coverage
Year
Summer Solstice
June 21/Day 172
Summer Teletherm
July 20/Day 201
+29 days
Jan
Feb
Mar
Apr
May
Jun
Jul
Aug
Sep
Oct
Nov
Dec
°F
Day of Year

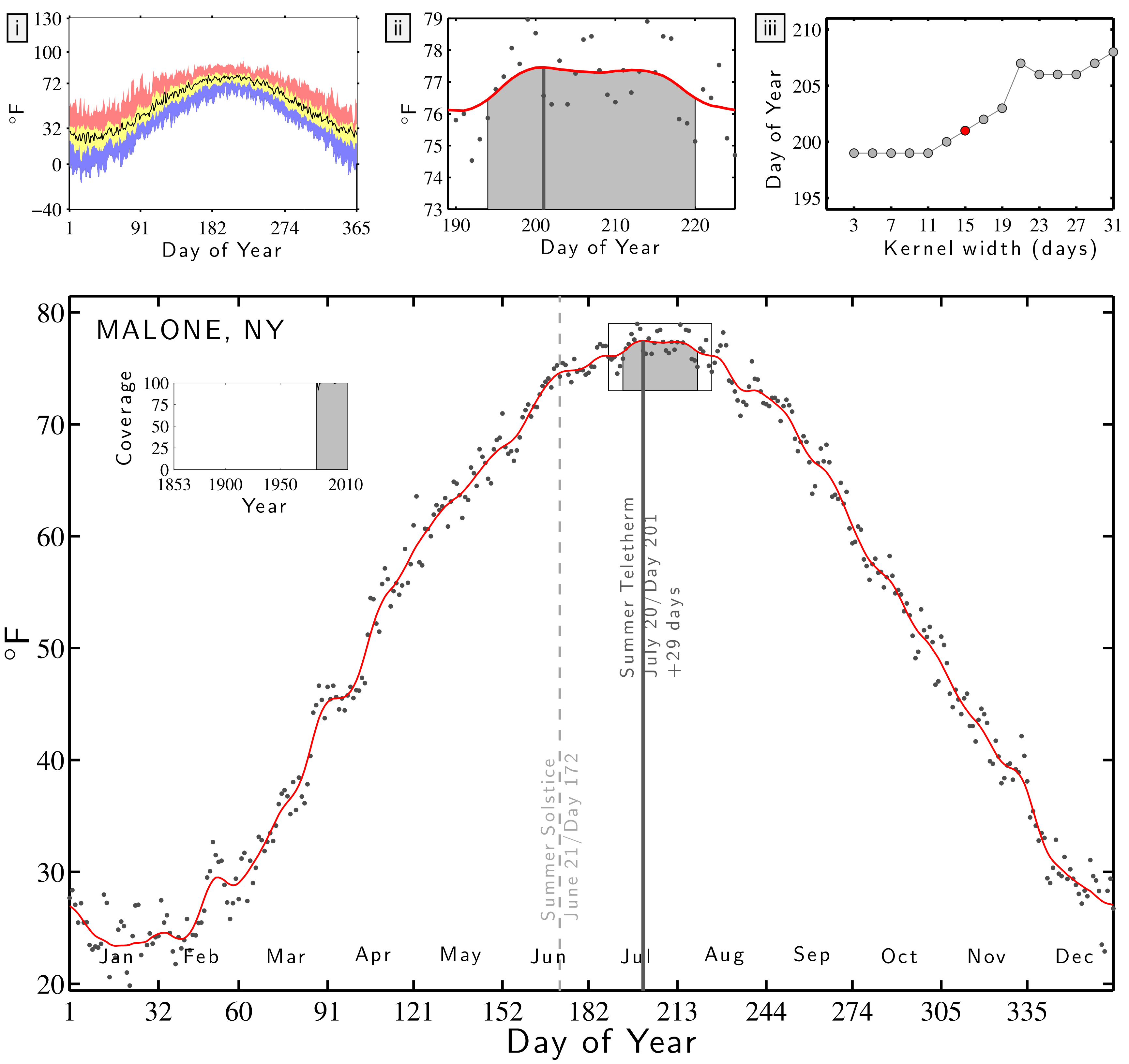

i
°F
130
100
72
32
0
−40
1
91
182
274
365
Day of Year
ii
79
78
77
76
75
74
73
190
200
210
220
Day of Year
iii
Day of Year
210
205
200
195
3
7
11
15
19
23
27
31
Kernel width (days)
MALONE, NY
Coverage
100
75
50
25
0
1853
1900
1950
2010
Year
Summer Solstice
June 21 / Day 172
Summer Teletherm
July 20 / Day 201
+29 days
80
70
60
50
40
30
20
Jan
Feb
Mar
Apr
May
Jun
Jul
Aug
Sep
Oct
Nov
Dec
1
32
60
91
121
152
182
213
244
274
305
335
Day of Year

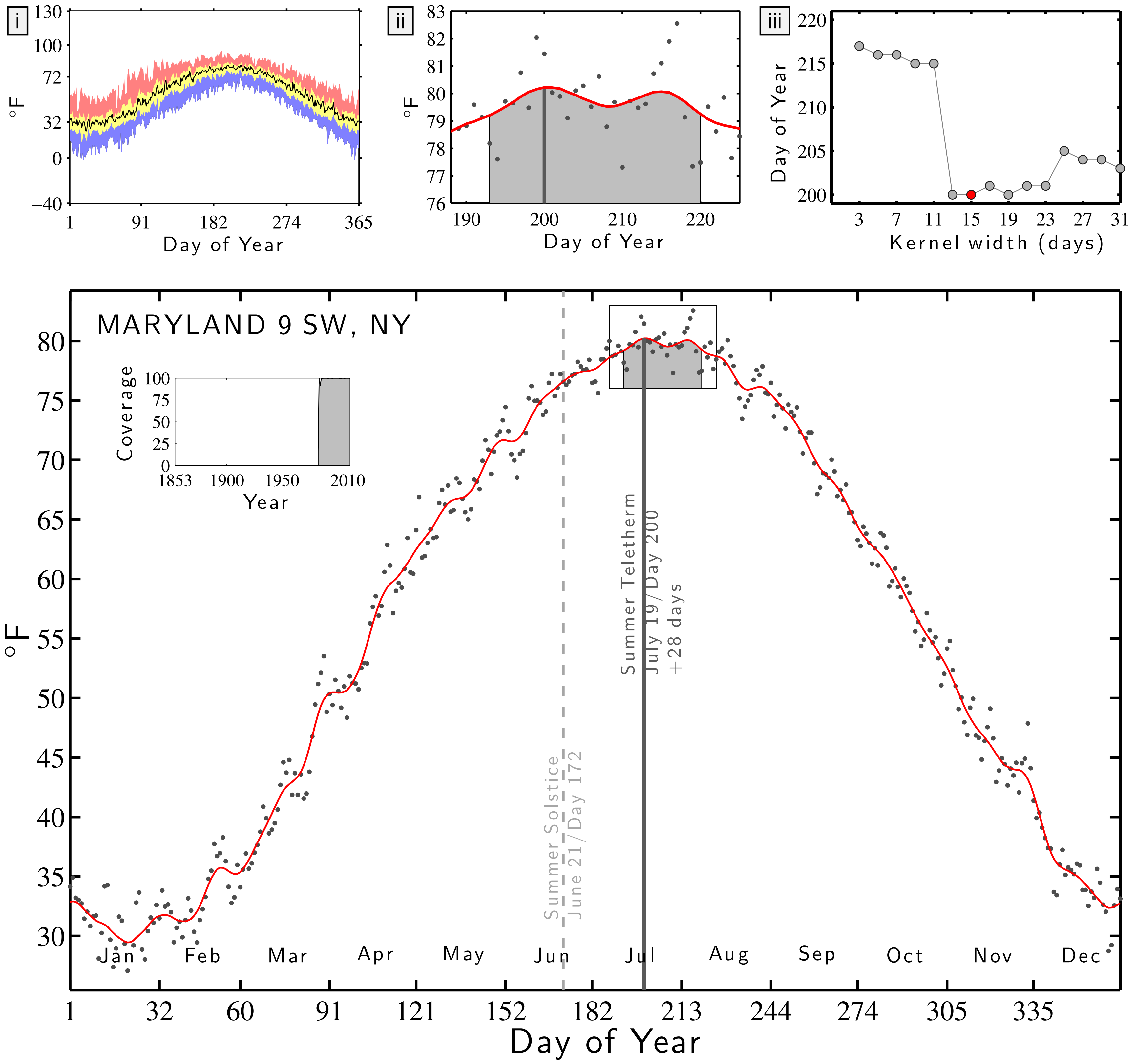

i
°F
Day of Year
ii
°F
Day of Year
iii
Day of Year
Kernel width (days)
MARYLAND 9 SW, NY
Coverage
Year
Summer Solstice
June 21/Day 172
Summer Teletherm
July 19/Day 200
+28 days
Jan
Feb
Mar
Apr
May
Jun
Jul
Aug
Sep
Oct
Nov
Dec
°F
Day of Year

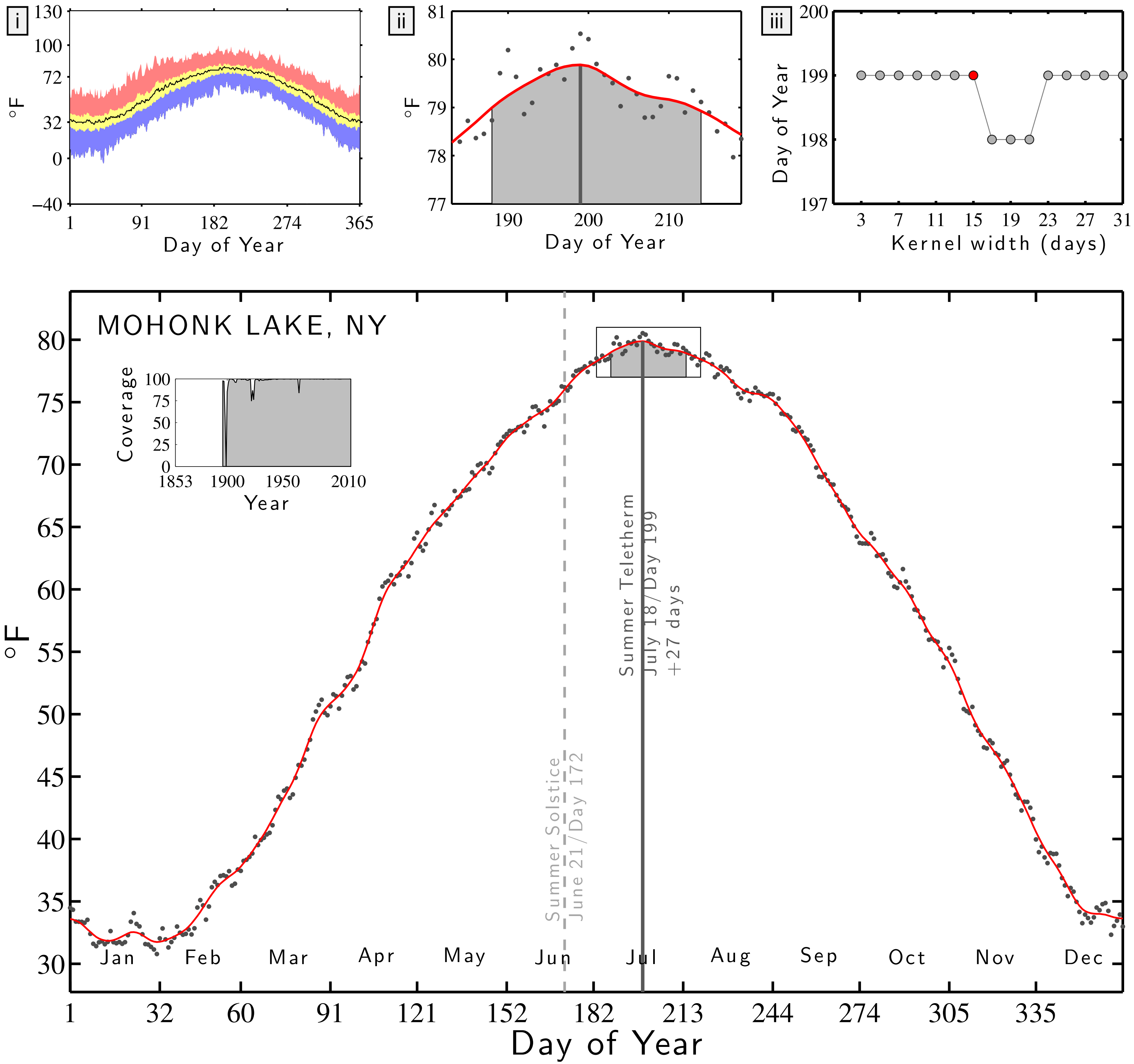

i
°F
Day of Year
ii
°F
Day of Year
iii
Day of Year
Kernel width (days)
MOHONK LAKE, NY
Coverage
Year
Summer Solstice
June 21/Day 172
Summer Teletherm
July 18/Day 199
+27 days
Jan
Feb
Mar
Apr
May
Jun
Jul
Aug
Sep
Oct
Nov
Dec
°F
Day of Year

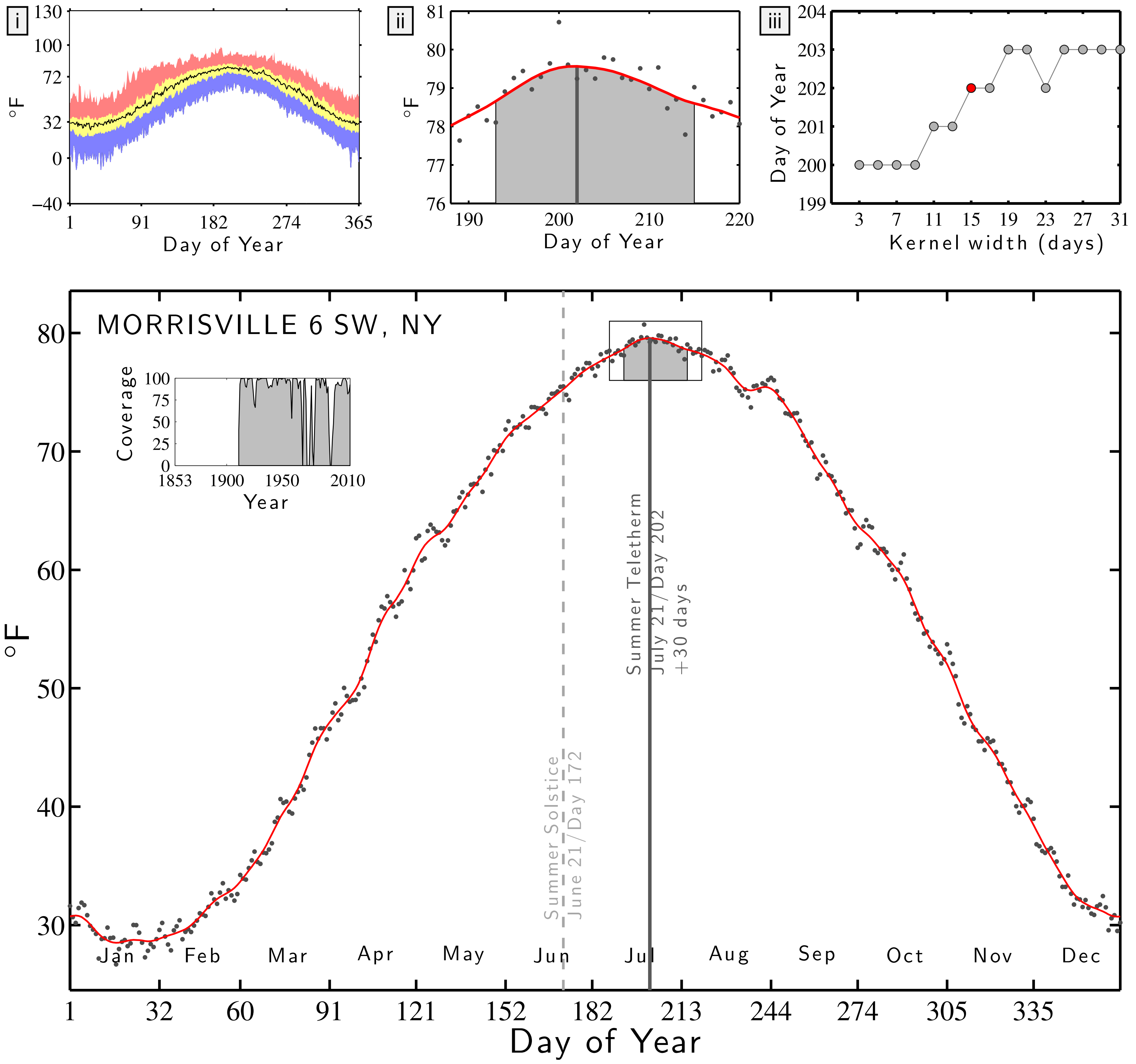

i
ii
iii
°F
Day of Year
Day of Year
Kernel width (days)
MORRISVILLE 6 SW, NY
Coverage
Year
Summer Solstice
June 21 / Day 172
Summer Teletherm
July 21 / Day 202
+30 days
Jan
Feb
Mar
Apr
May
Jun
Jul
Aug
Sep
Oct
Nov
Dec
Day of Year

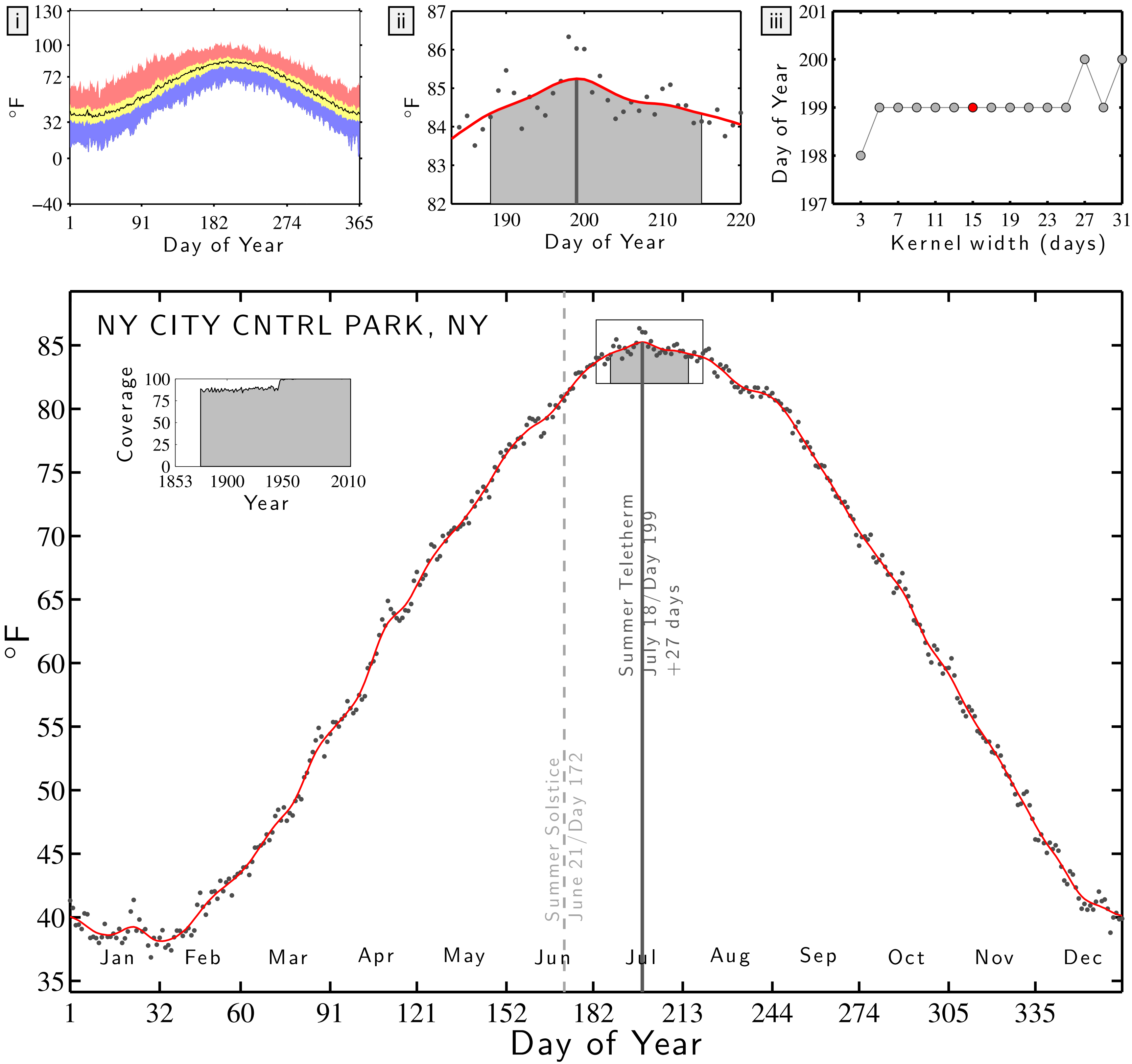

i
ii
iii
°F
Day of Year
Day of Year
Kernel width (days)
NY CITY CNTRL PARK, NY
Coverage
Year
Summer Solstice
June 21/Day 172
Summer Teletherm
July 18/Day 199
+27 days
Jan
Feb
Mar
Apr
May
Jun
Jul
Aug
Sep
Oct
Nov
Dec
°F
Day of Year

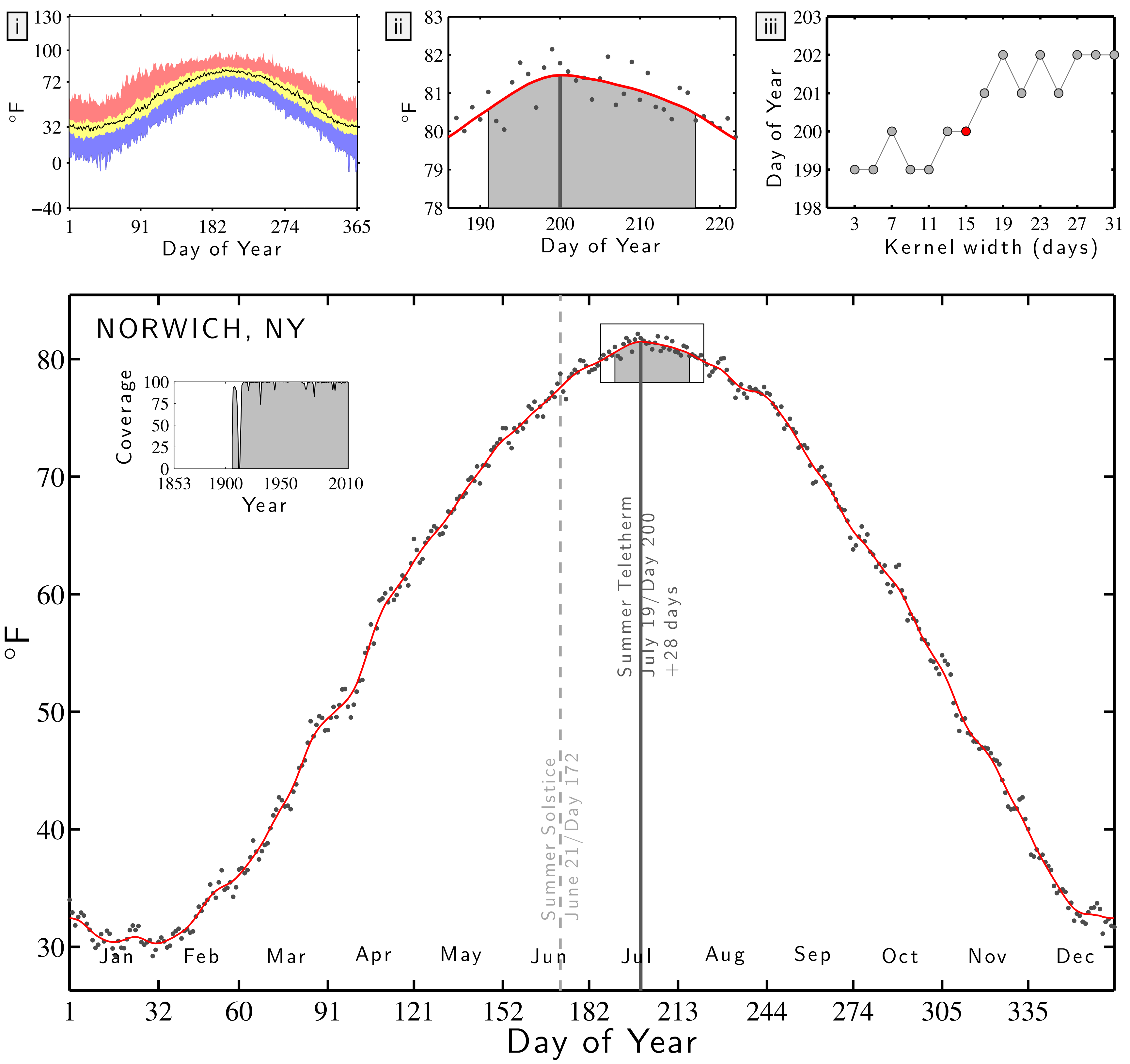

i
ii
iii
NORWICH, NY
Coverage
Year
Summer Solstice
June 21/Day 172
Summer Teletherm
July 19/Day 200
+28 days
Jan
Feb
Mar
Apr
May
Jun
Jul
Aug
Sep
Oct
Nov
Dec
Day of Year
°F
Kernel width (days)

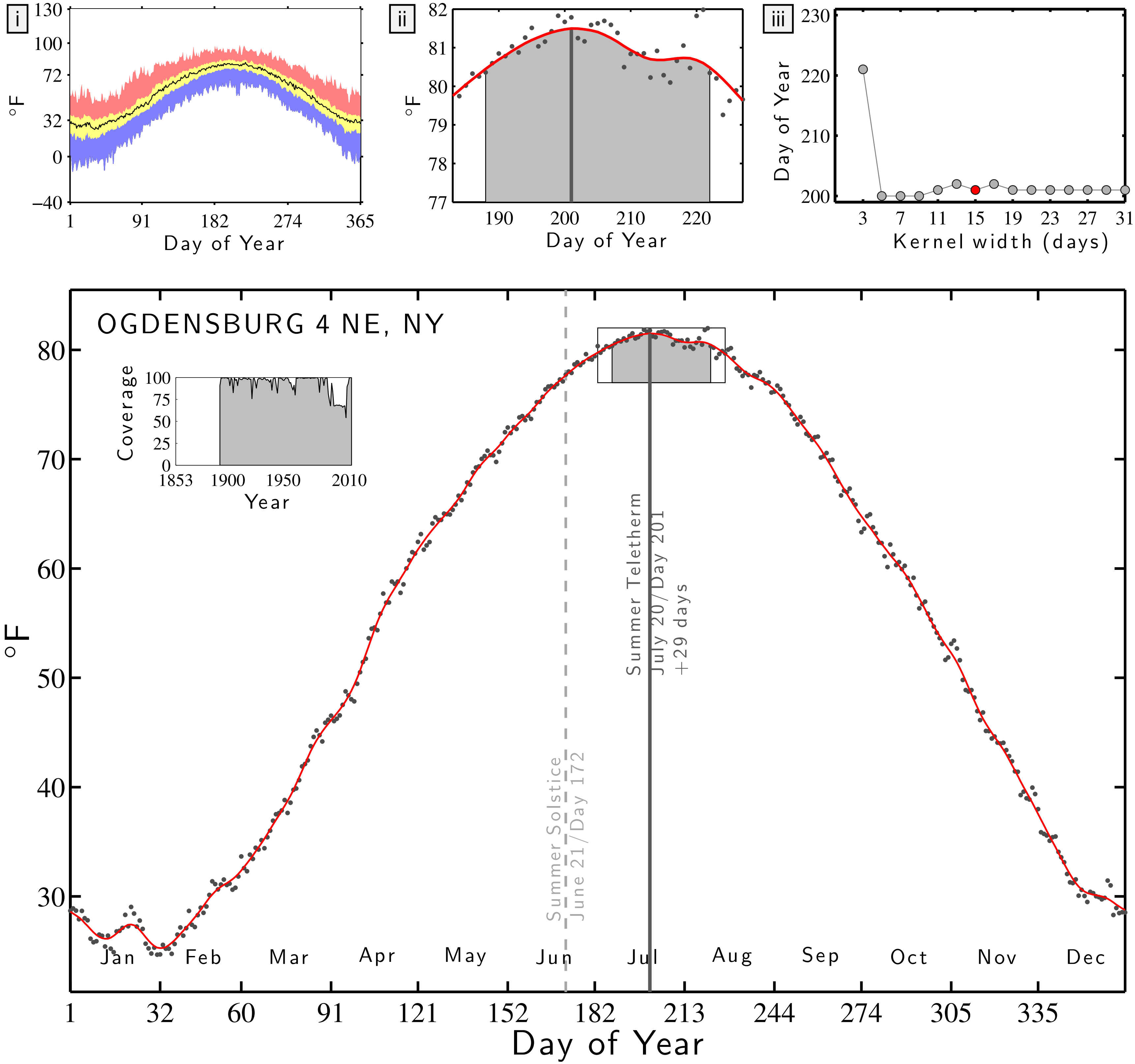

i
130
100
72
32
0
−40
°F
1
91
182
274
365
Day of Year
ii
82
81
80
79
78
77
°F
190
200
210
220
Day of Year
iii
230
220
210
200
Day of Year
3
7
11
15
19
23
27
31
Kernel width (days)
OGDENSBURG 4 NE, NY
Coverage
100
75
50
25
0
1853
1900
1950
2010
Year
Summer Solstice
June 21 / Day 172
Summer Teletherm
July 20 / Day 201
+29 days
80
70
60
50
40
30
°F
Jan
Feb
Mar
Apr
May
Jun
Jul
Aug
Sep
Oct
Nov
Dec
1
32
60
91
121
152
182
213
244
274
305
335
Day of Year

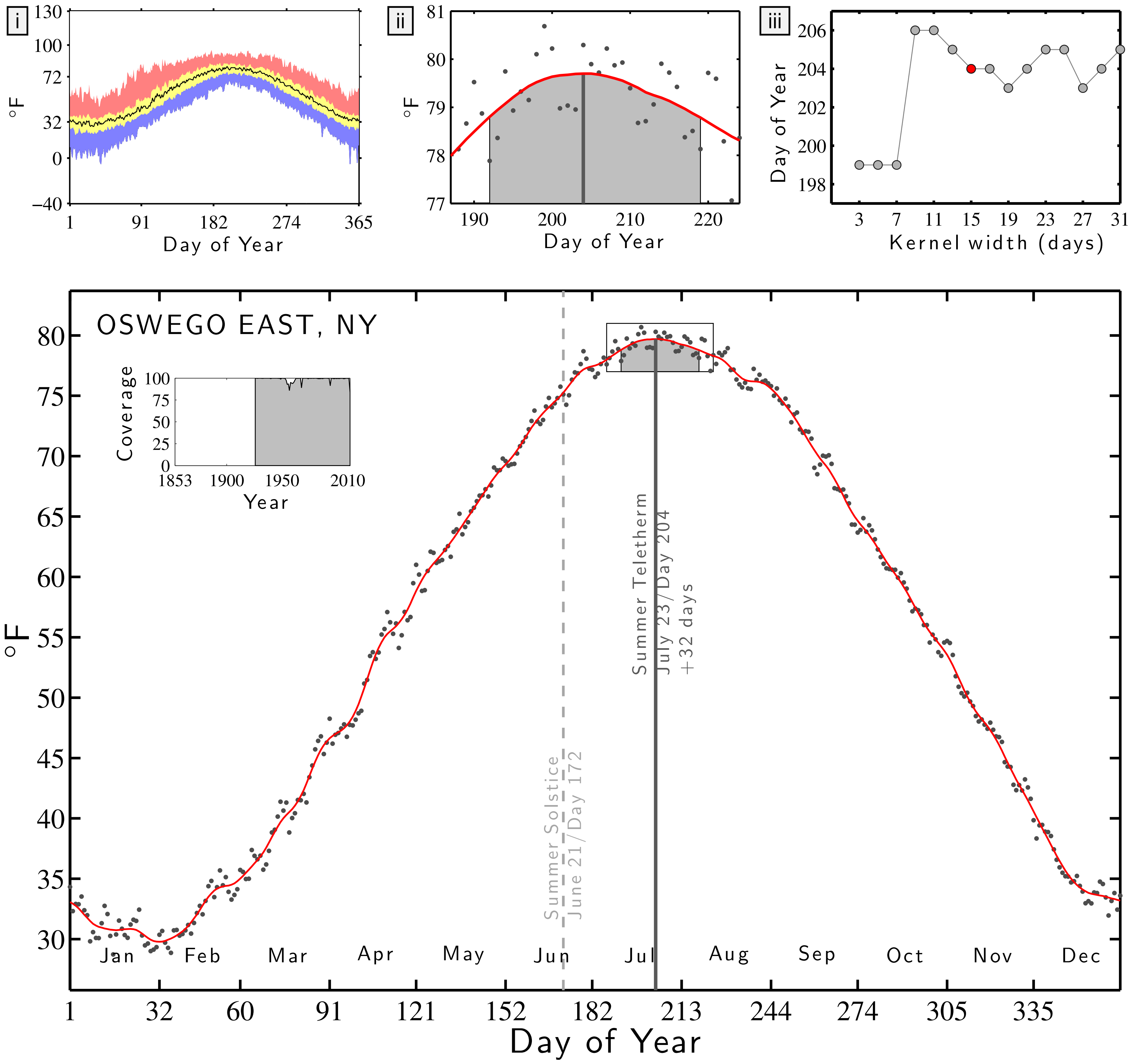

i
°F
Day of Year
ii
°F
Day of Year
iii
Day of Year
Kernel width (days)
OSWEGO EAST, NY
Coverage
Year
Summer Solstice
June 21 / Day 172
Summer Teletherm
July 23 / Day 204
+32 days
Jan
Feb
Mar
Apr
May
Jun
Jul
Aug
Sep
Oct
Nov
Dec
°F
Day of Year

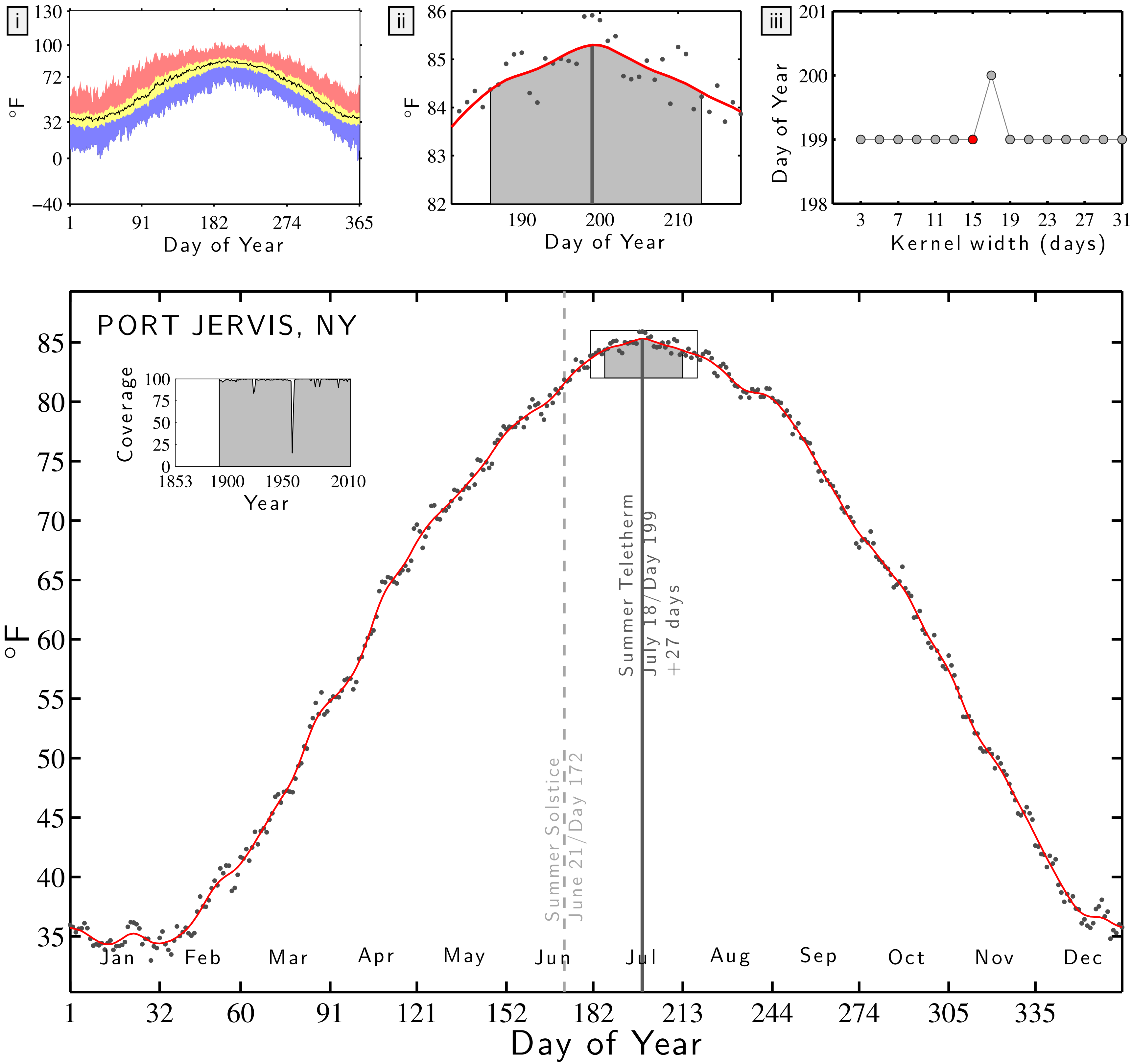
i
ii
iii
°F
Day of Year
Day of Year
Kernel width (days)
PORT JERVIS, NY
Coverage
Year
Summer Solstice
June 21/Day 172
Summer Teletherm
July 18/Day 199
+27 days
Jan
Feb
Mar
Apr
May
Jun
Jul
Aug
Sep
Oct
Nov
Dec
Day of Year

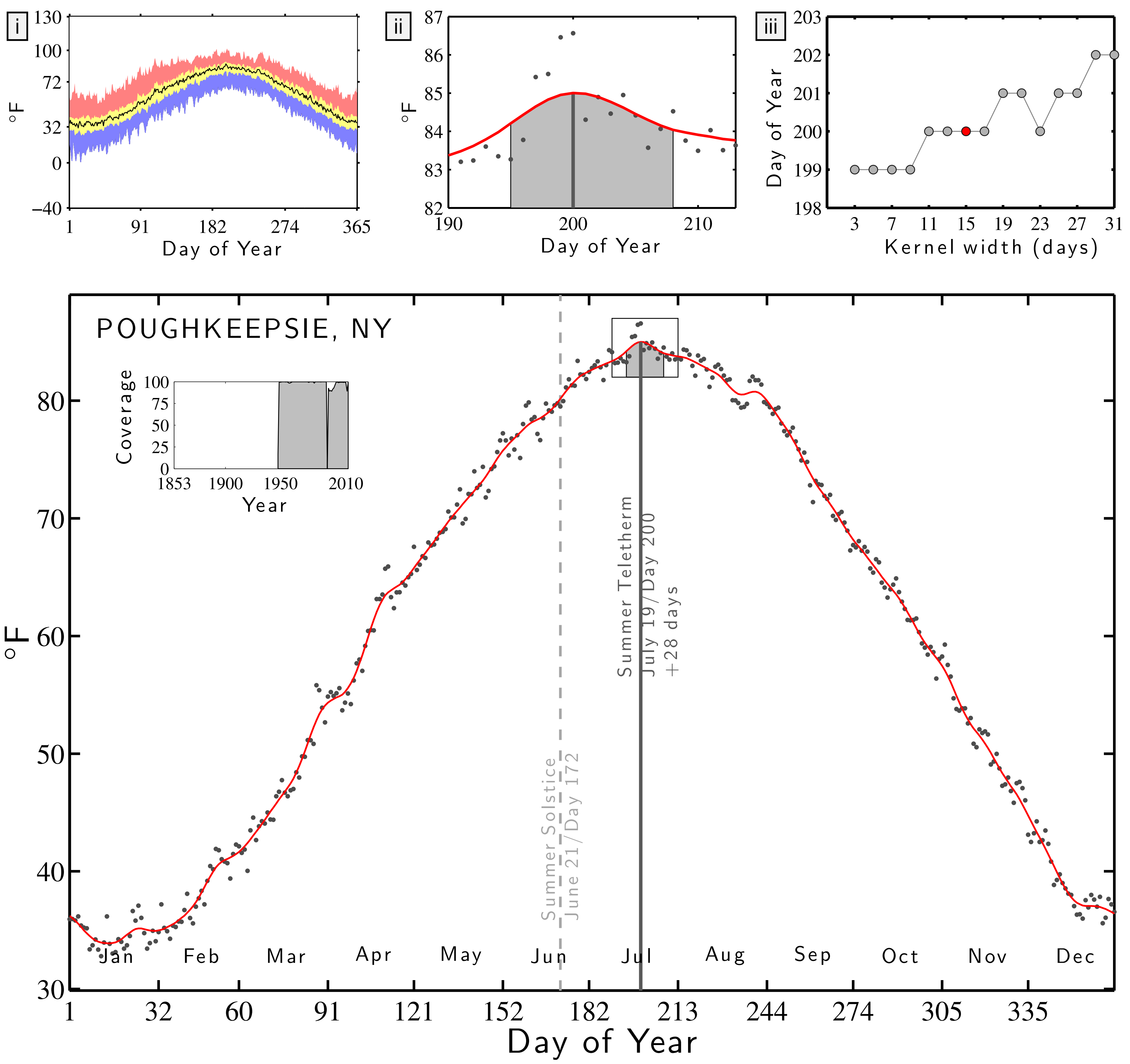

i
ii
iii
Day of Year
Kernel width (days)
POUGHKEEPSIE, NY
Coverage
Year
Summer Solstice
June 21/Day 172
Summer Teletherm
July 19/Day 200
+28 days
°F
Jan
Feb
Mar
Apr
May
Jun
Jul
Aug
Sep
Oct
Nov
Dec

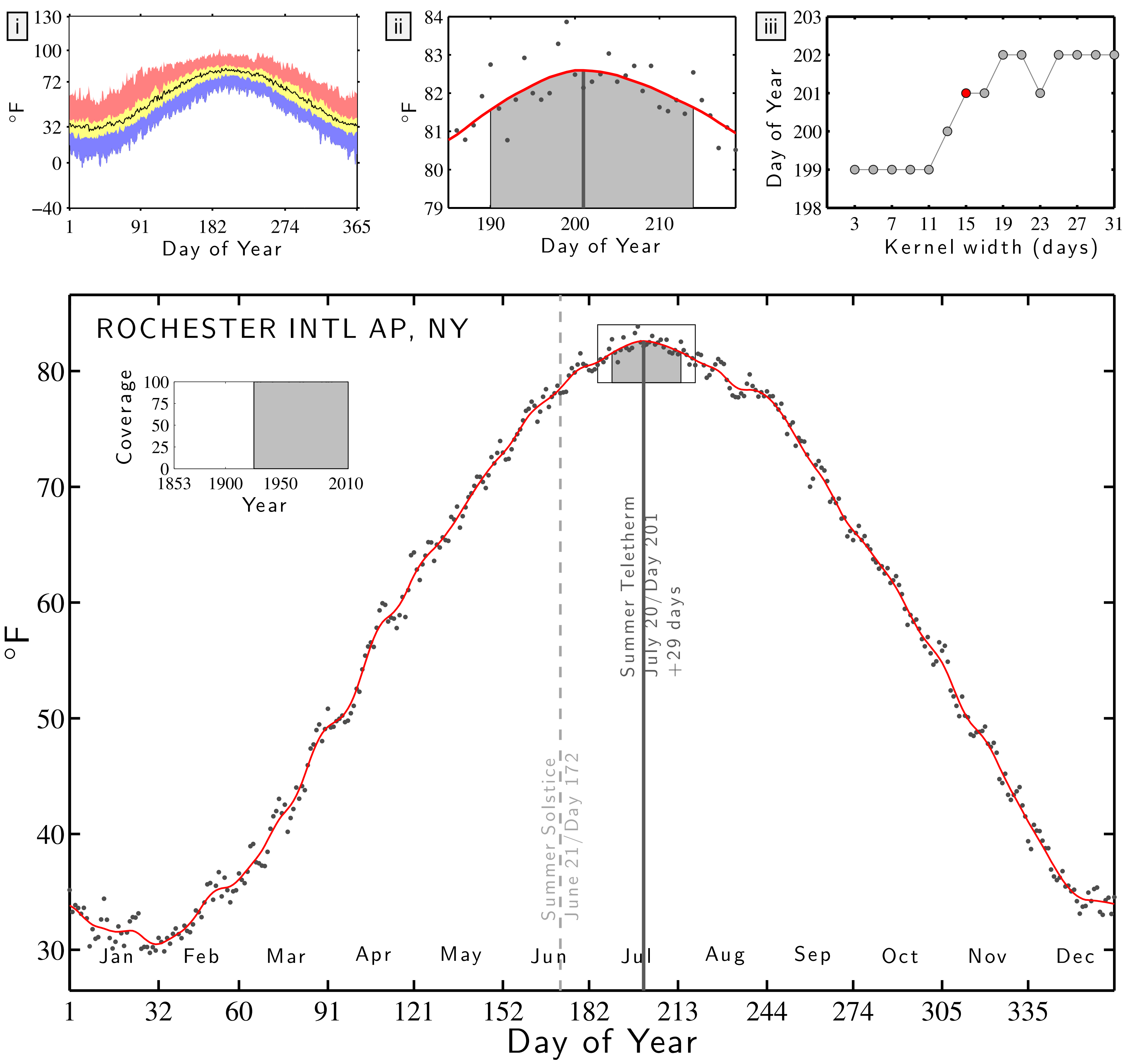

i
°F
Day of Year
ii
°F
Day of Year
iii
Day of Year
Kernel width (days)
ROCHESTER INTL AP, NY
Coverage
Year
Summer Solstice
June 21/Day 172
Summer Teletherm
July 20/Day 201
+29 days
Jan
Feb
Mar
Apr
May
Jun
Jul
Aug
Sep
Oct
Nov
Dec
°F
Day of Year

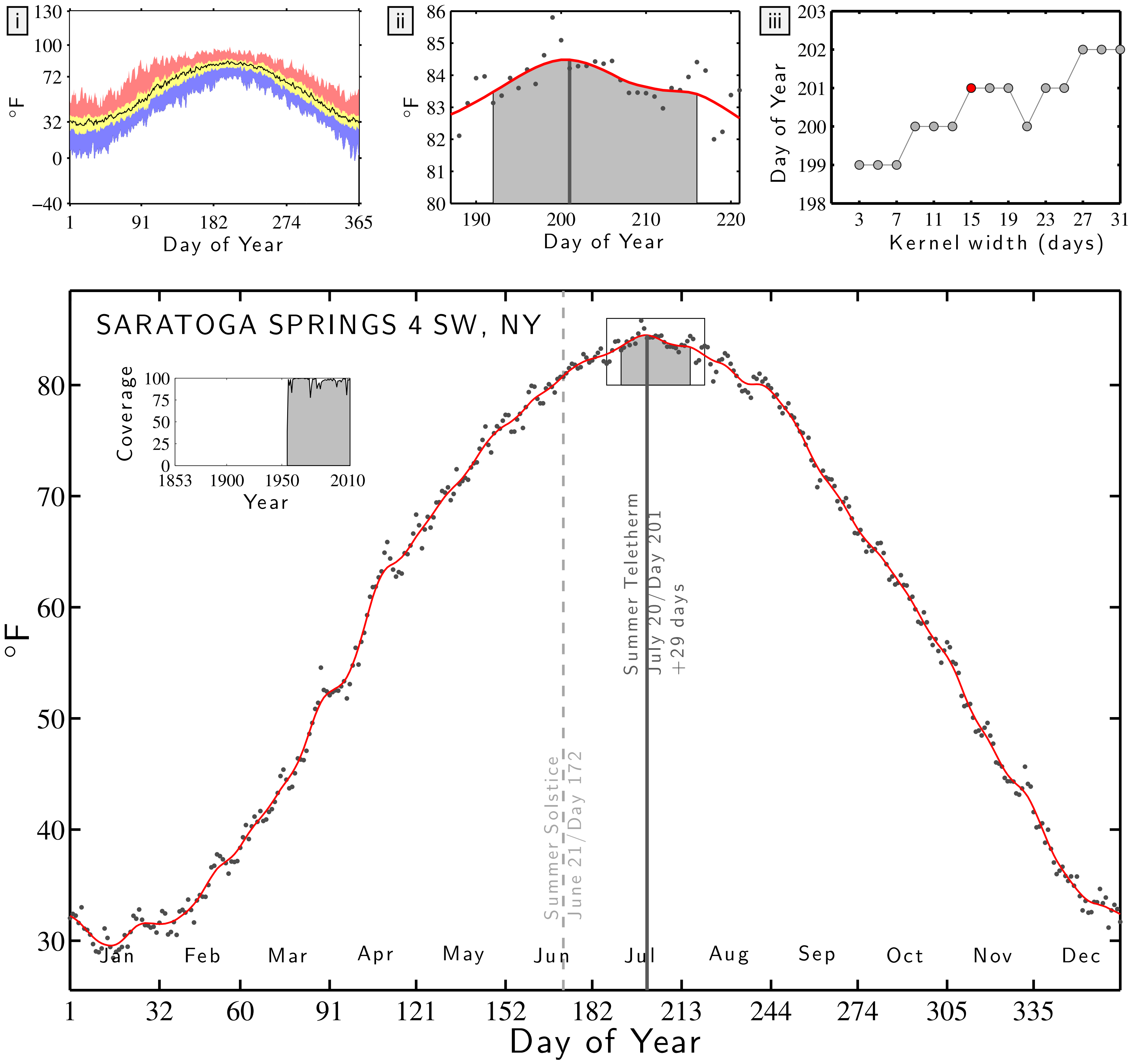

i
°F
Day of Year
ii
°F
Day of Year
iii
Day of Year
Kernel width (days)
SARATOGA SPRINGS 4 SW, NY
Coverage
Year
Summer Solstice
June 21 / Day 172
Summer Teletherm
July 20 / Day 201
+29 days
Jan
Feb
Mar
Apr
May
Jun
Jul
Aug
Sep
Oct
Nov
Dec
°F
Day of Year

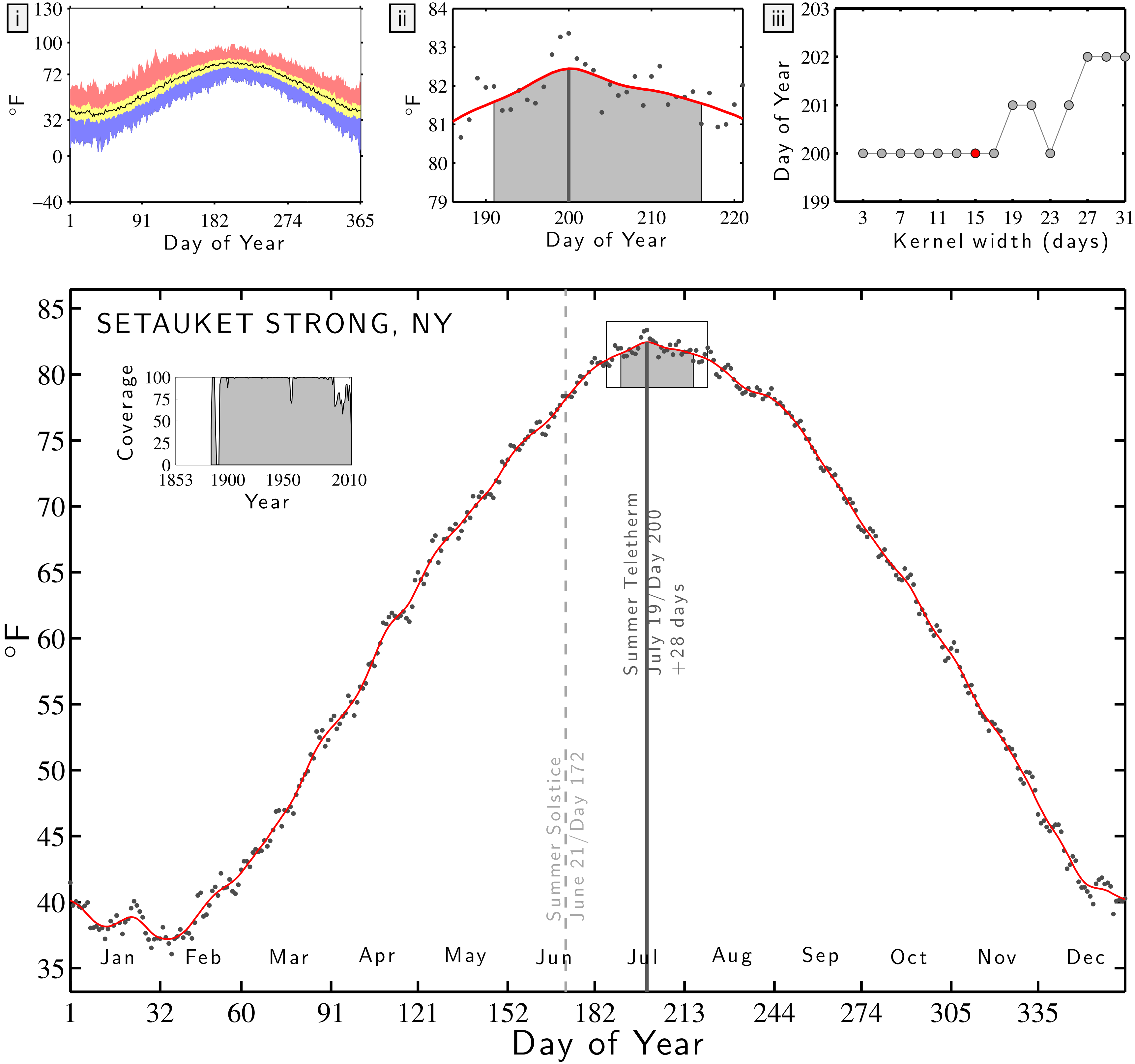

i
ii
iii
°F
Day of Year
Day of Year
Kernel width (days)
SETAUKET STRONG, NY
Coverage
Year
Summer Solstice
June 21/Day 172
Summer Teletherm
July 19/Day 200
+28 days
Jan
Feb
Mar
Apr
May
Jun
Jul
Aug
Sep
Oct
Nov
Dec
°F
Day of Year

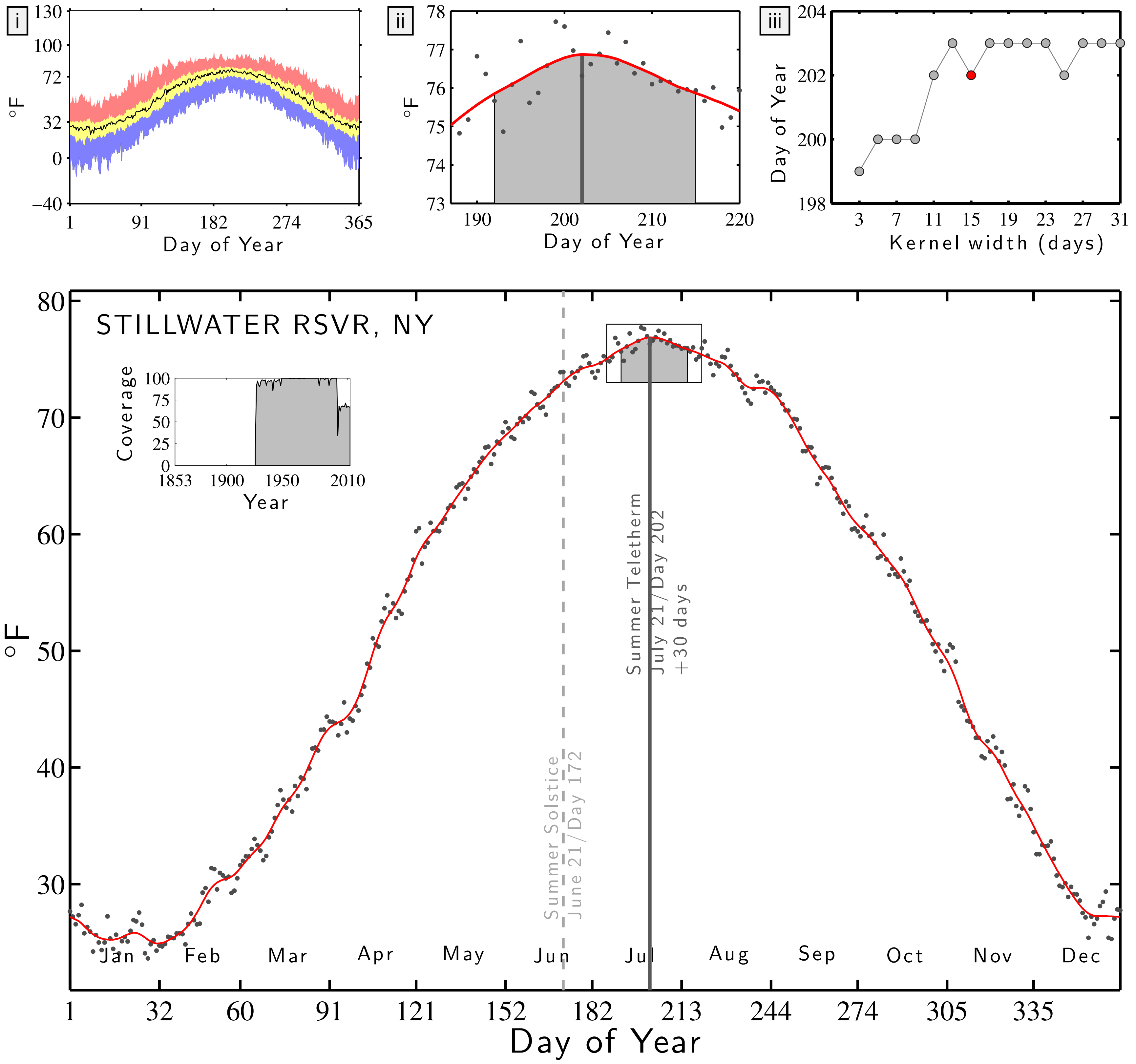

i
ii
iii
°F
Day of Year
Kernel width (days)
STILLWATER RSVR, NY
Coverage
Year
Summer Solstice
June 21/Day 172
Summer Teletherm
July 21/Day 202
+30 days
Jan
Feb
Mar
Apr
May
Jun
Jul
Aug
Sep
Oct
Nov
Dec

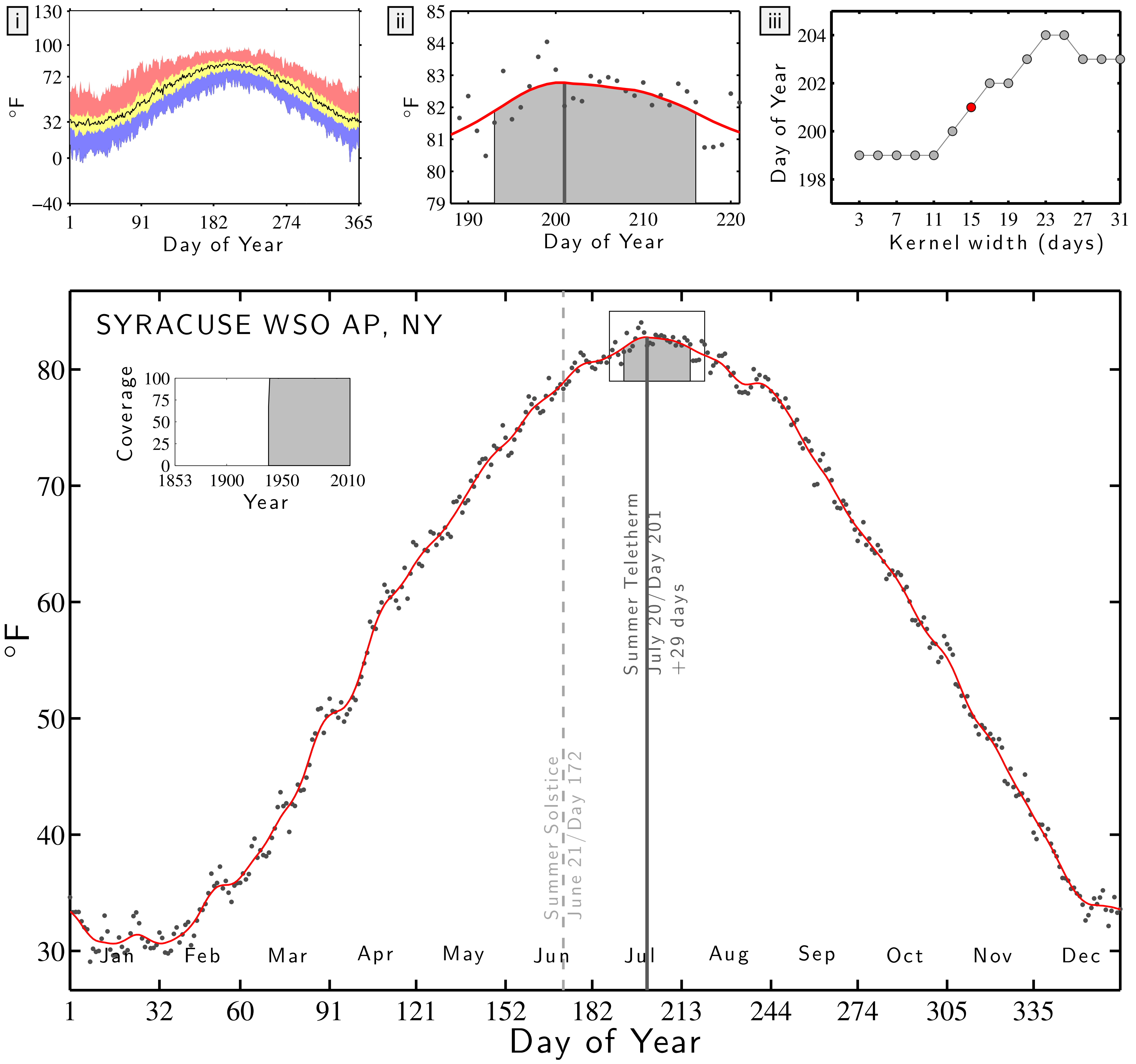

i
°F
Day of Year
ii
°F
Day of Year
iii
Day of Year
Kernel width (days)
SYRACUSE WSO AP, NY
Coverage
Year
Summer Solstice
June 21/Day 172
Summer Teletherm
July 20/Day 201
+29 days
Jan
Feb
Mar
Apr
May
Jun
Jul
Aug
Sep
Oct
Nov
Dec
°F
Day of Year

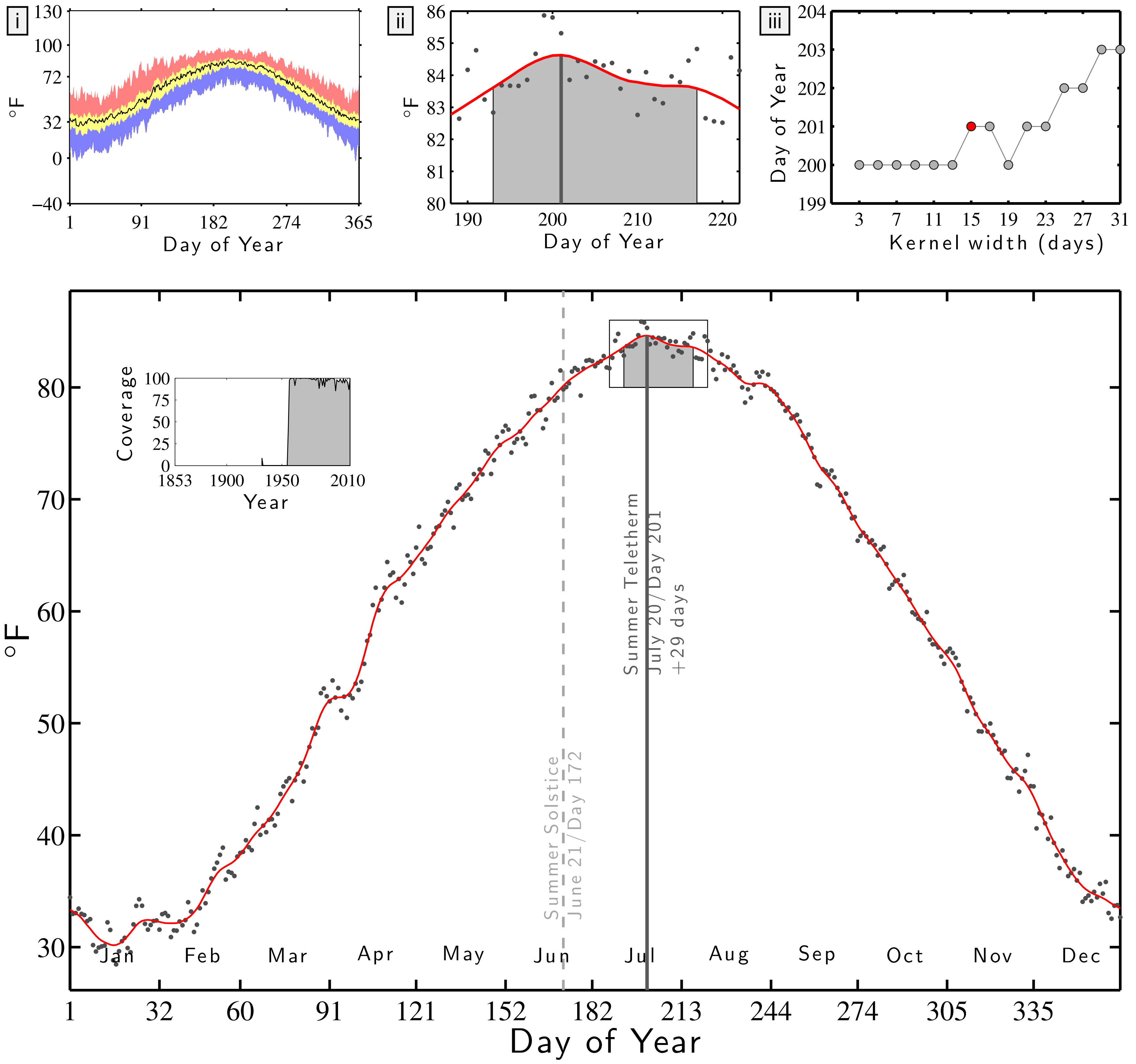
i
130
100
72
32
0
−40
°F
1
91
182
274
365
Day of Year
ii
86
85
84
83
82
81
80
°F
190
200
210
220
Day of Year
iii
204
203
202
201
200
199
Day of Year
3
7
11
15
19
23
27
31
Kernel width (days)
Coverage
100
75
50
25
0
1853
1900
1950
2010
Year
80
70
60
50
40
30
°F
Summer Solstice
June 21/Day 172
Summer Teletherm
July 20/Day 201
+29 days
Jan
Feb
Mar
Apr
May
Jun
Jul
Aug
Sep
Oct
Nov
Dec
1
32
60
91
121
152
182
213
244
274
305
335
Day of Year

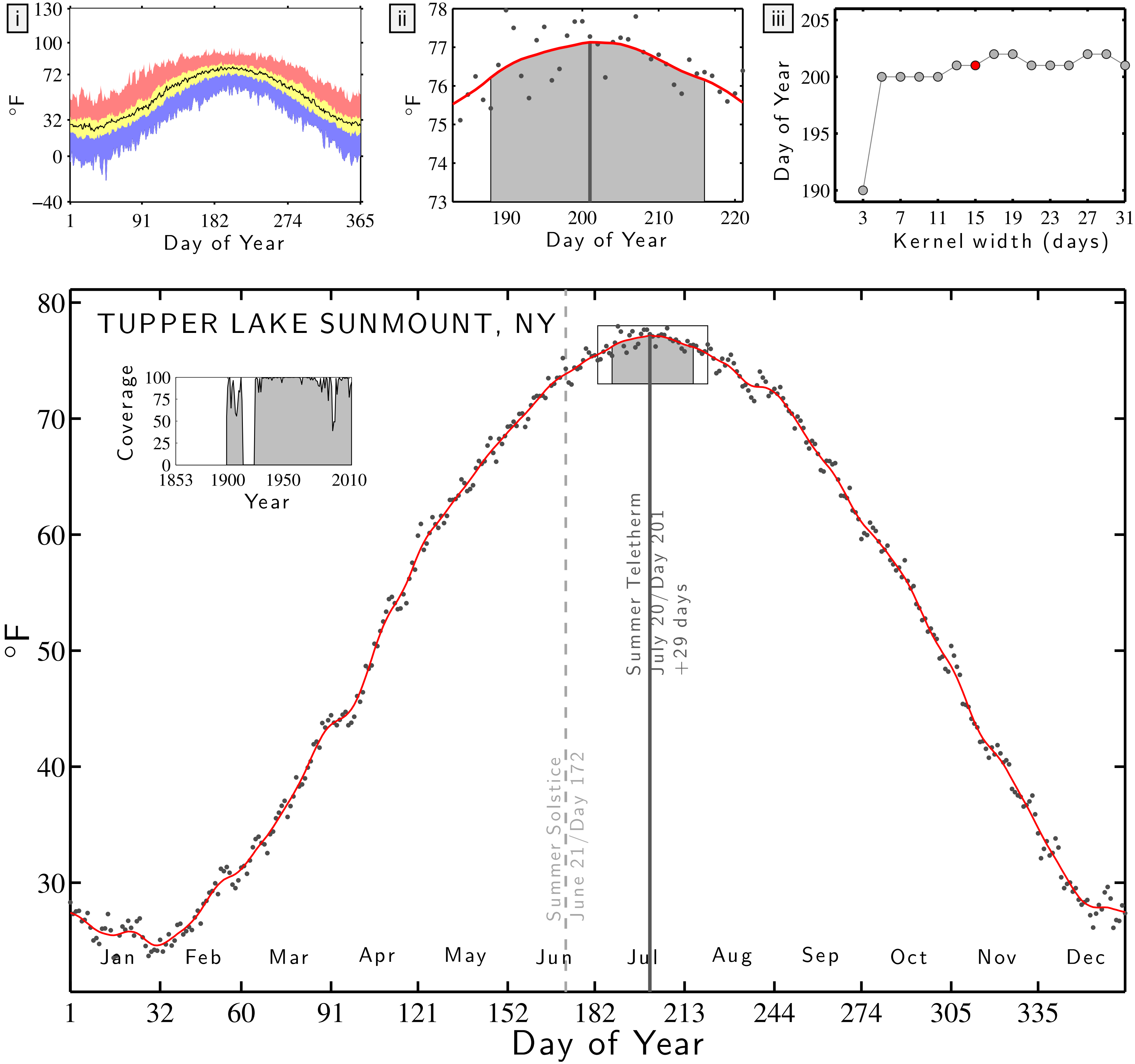

i
ii
iii
Day of Year
Kernel width (days)
TUPPER LAKE SUNMOUNT, NY
Coverage
Year
Summer Solstice
June 21/Day 172
Summer Teletherm
July 20/Day 201
+29 days
Jan
Feb
Mar
Apr
May
Jun
Jul
Aug
Sep
Oct
Nov
Dec
°F
Day of Year

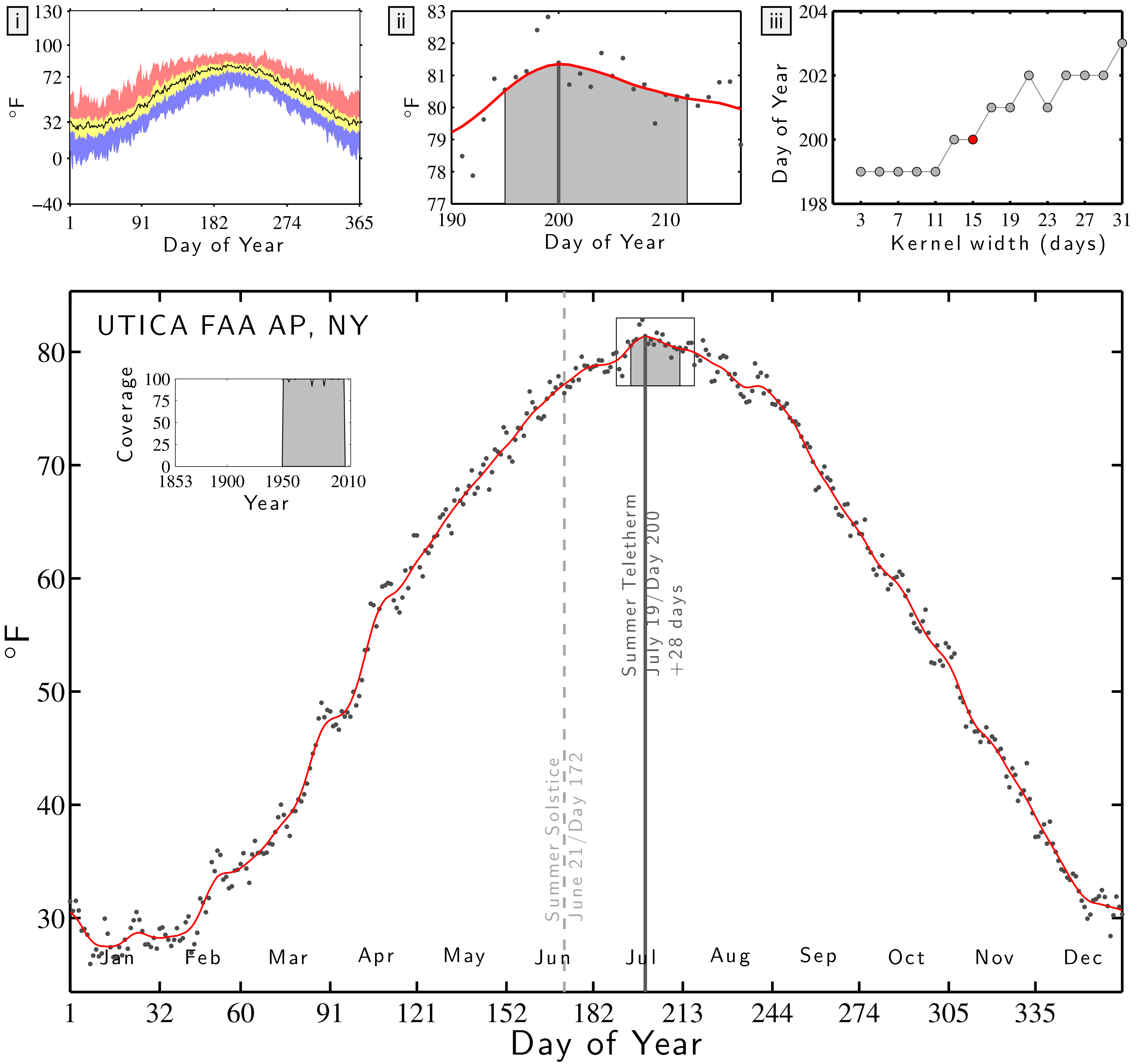

i
ii
iii
°F
Day of Year
Kernel width (days)
UTICA FAA AP, NY
Coverage
Year
Summer Solstice
June 21/Day 172
Summer Teletherm
July 19/Day 200
+28 days
Jan
Feb
Mar
Apr
May
Jun
Jul
Aug
Sep
Oct
Nov
Dec

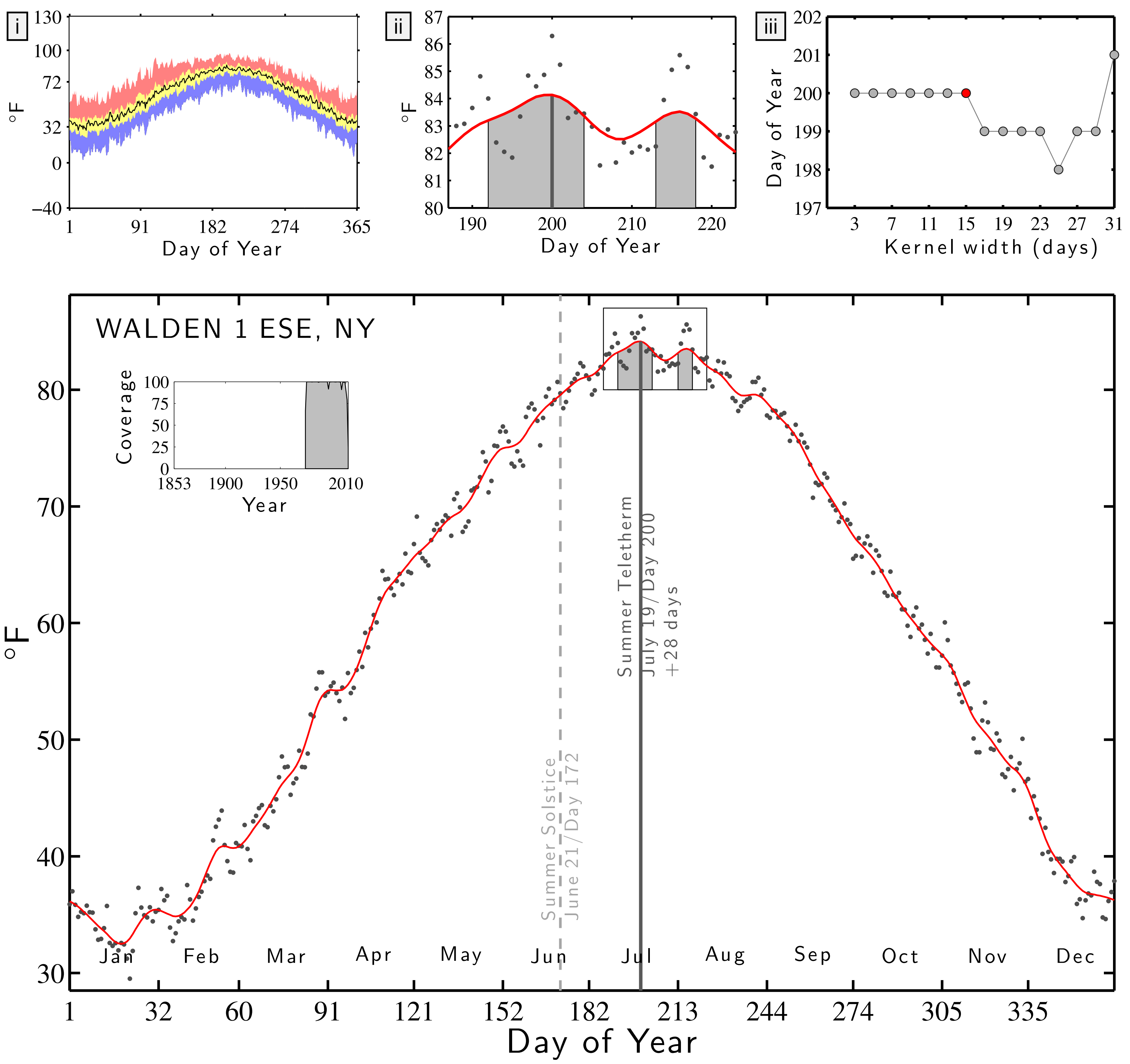

i
°F
Day of Year
ii
°F
Day of Year
iii
Day of Year
Kernel width (days)
WALDEN 1 ESE, NY
Coverage
Year
Summer Solstice
June 21/Day 172
Summer Teletherm
July 19/Day 200
+28 days
Jan
Feb
Mar
Apr
May
Jun
Jul
Aug
Sep
Oct
Nov
Dec
°F
Day of Year

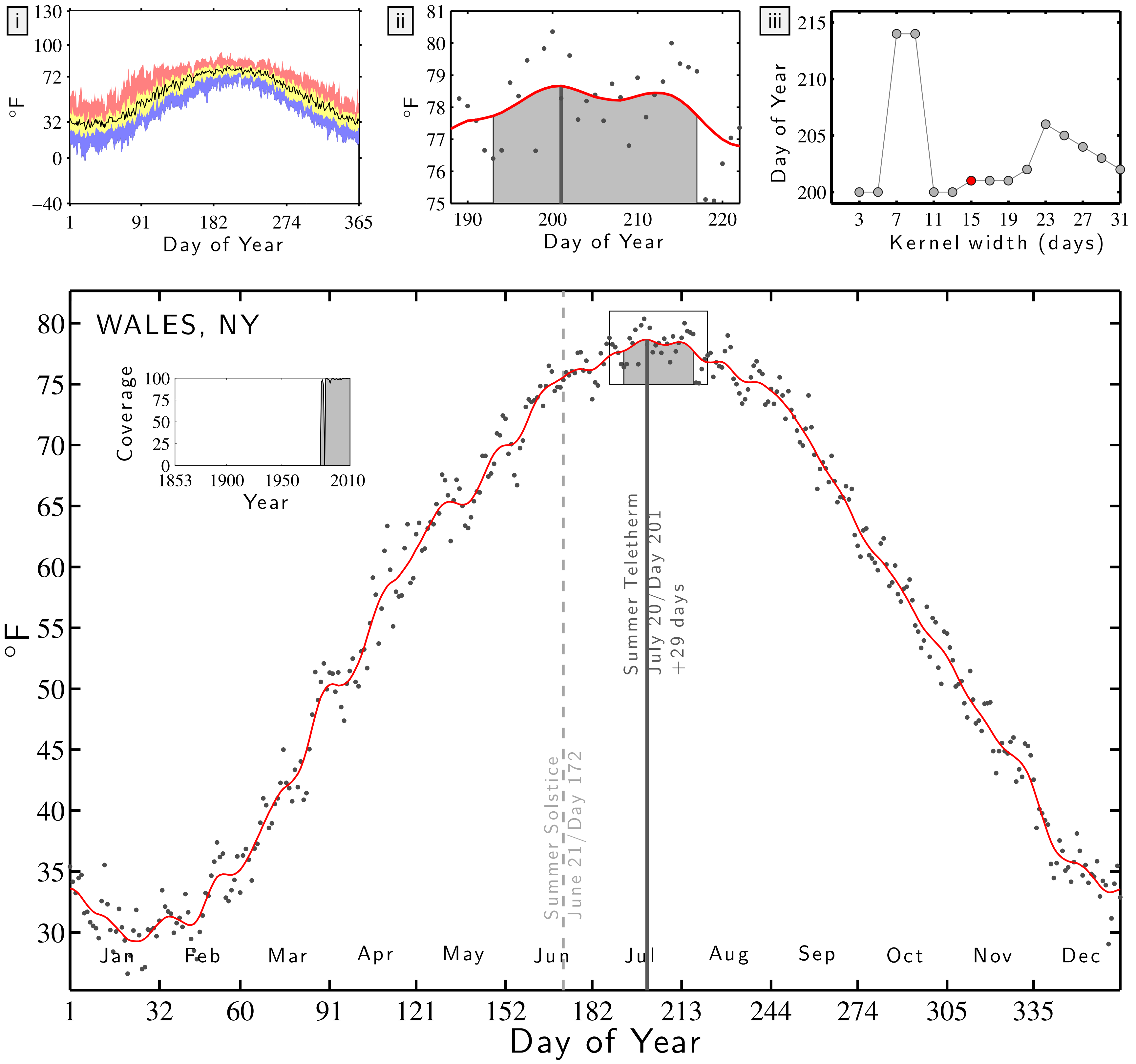

i
ii
iii
°F
Day of Year
Day of Year
Kernel width (days)
WALES, NY
Coverage
Year
Summer Solstice
June 21 / Day 172
Summer Teletherm
July 20 / Day 201
+29 days
Jan
Feb
Mar
Apr
May
Jun
Jul
Aug
Sep
Oct
Nov
Dec

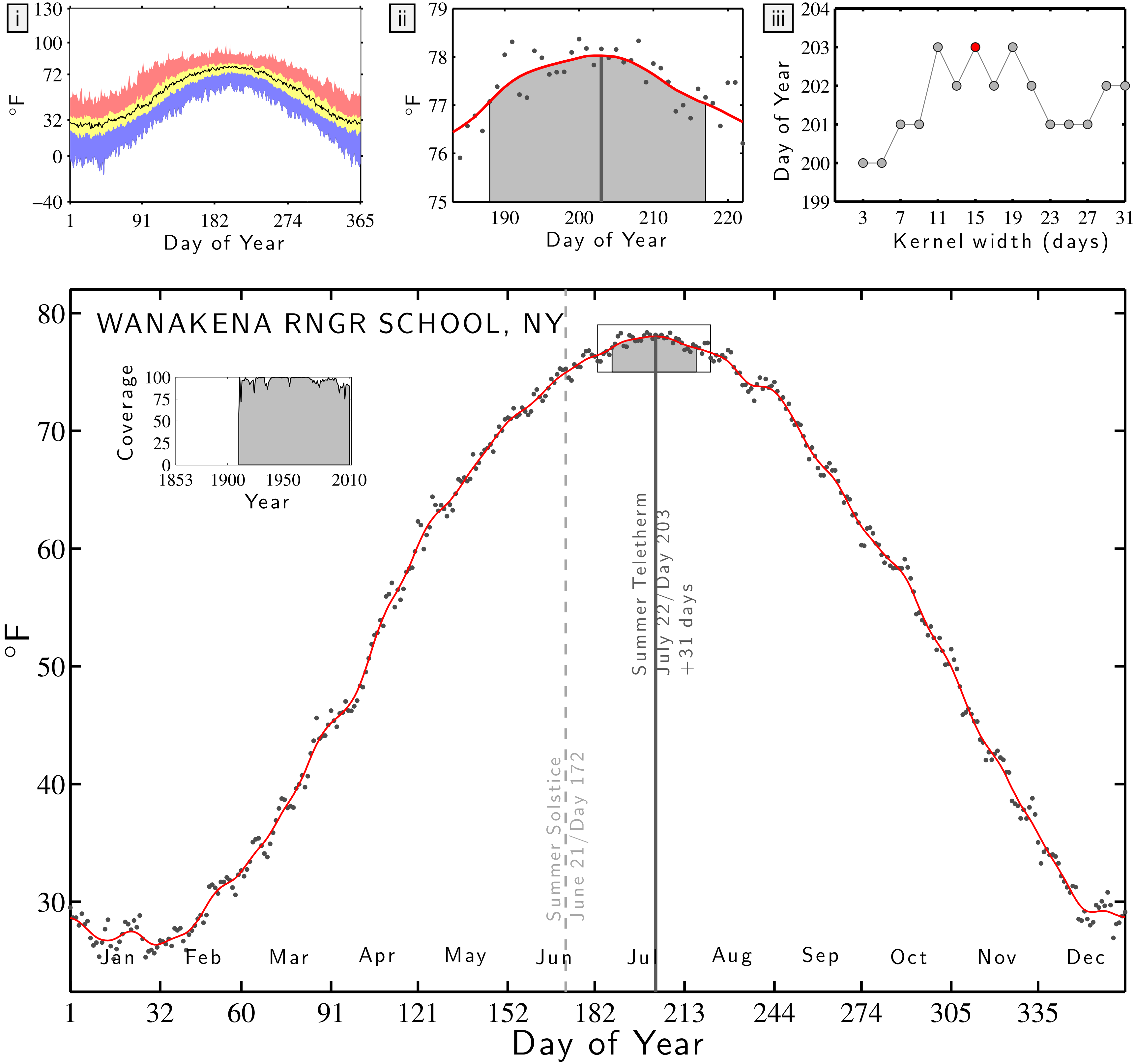

i
°F
Day of Year
ii
°F
Day of Year
iii
Day of Year
Kernel width (days)
WANAKENA RNGR SCHOOL, NY
Coverage
Year
Summer Solstice
June 21/Day 172
Summer Teletherm
July 22/Day 203
+31 days
Jan
Feb
Mar
Apr
May
Jun
Jul
Aug
Sep
Oct
Nov
Dec
°F
Day of Year

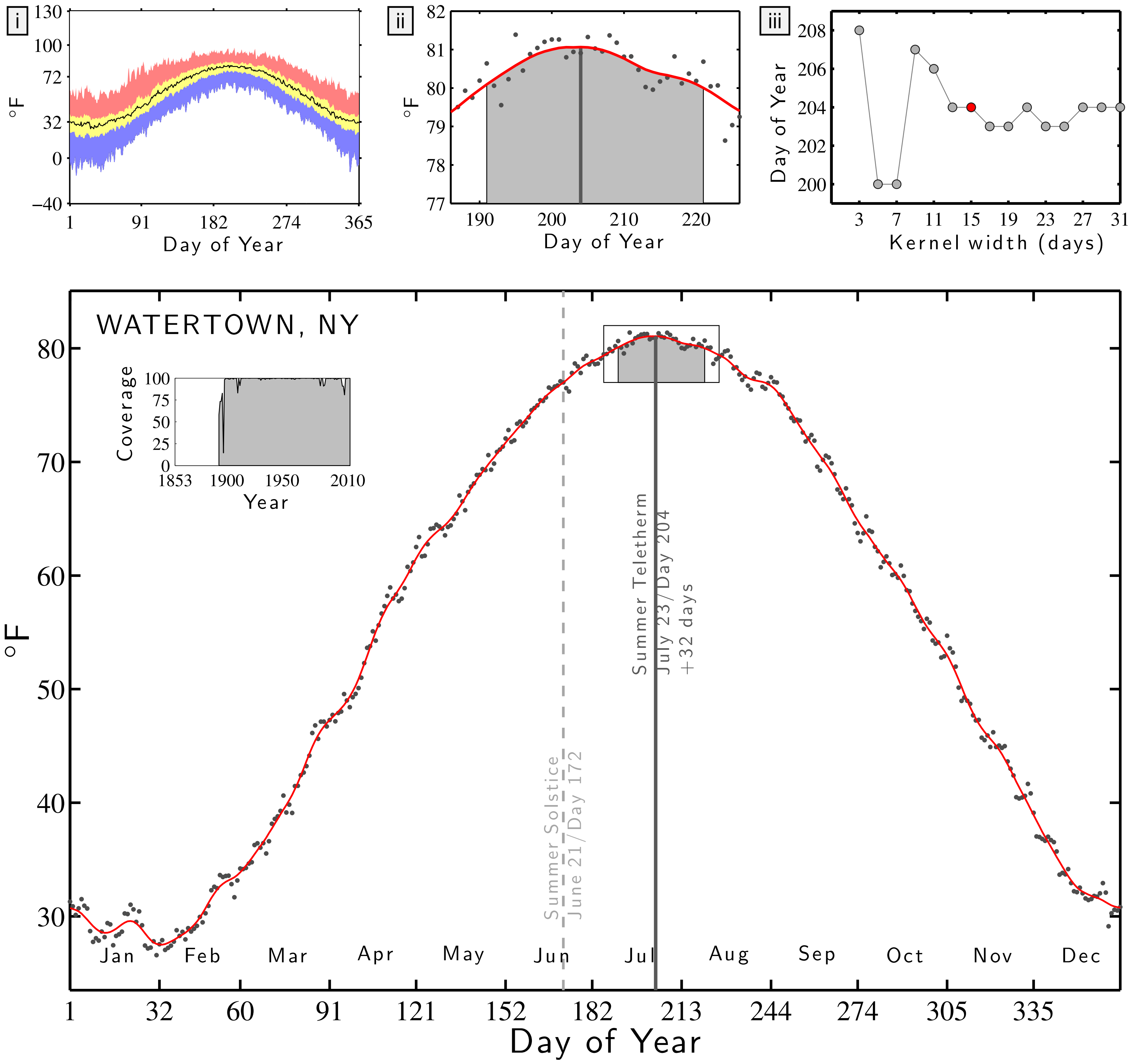

i
130
100
72
32
0
−40
°F
1
91
182
274
365
Day of Year
ii
82
81
80
79
78
77
°F
190
200
210
220
Day of Year
iii
208
206
204
202
200
Day of Year
3
7
11
15
19
23
27
31
Kernel width (days)
WATERTOWN, NY
Coverage
100
75
50
25
0
1853
1900
1950
2010
Year
Summer Solstice
June 21 / Day 172
Summer Teletherm
July 23 / Day 204
+32 days
80
70
60
50
40
30
°F
Jan
Feb
Mar
Apr
May
Jun
Jul
Aug
Sep
Oct
Nov
Dec
1
32
60
91
121
152
182
213
244
274
305
335
Day of Year

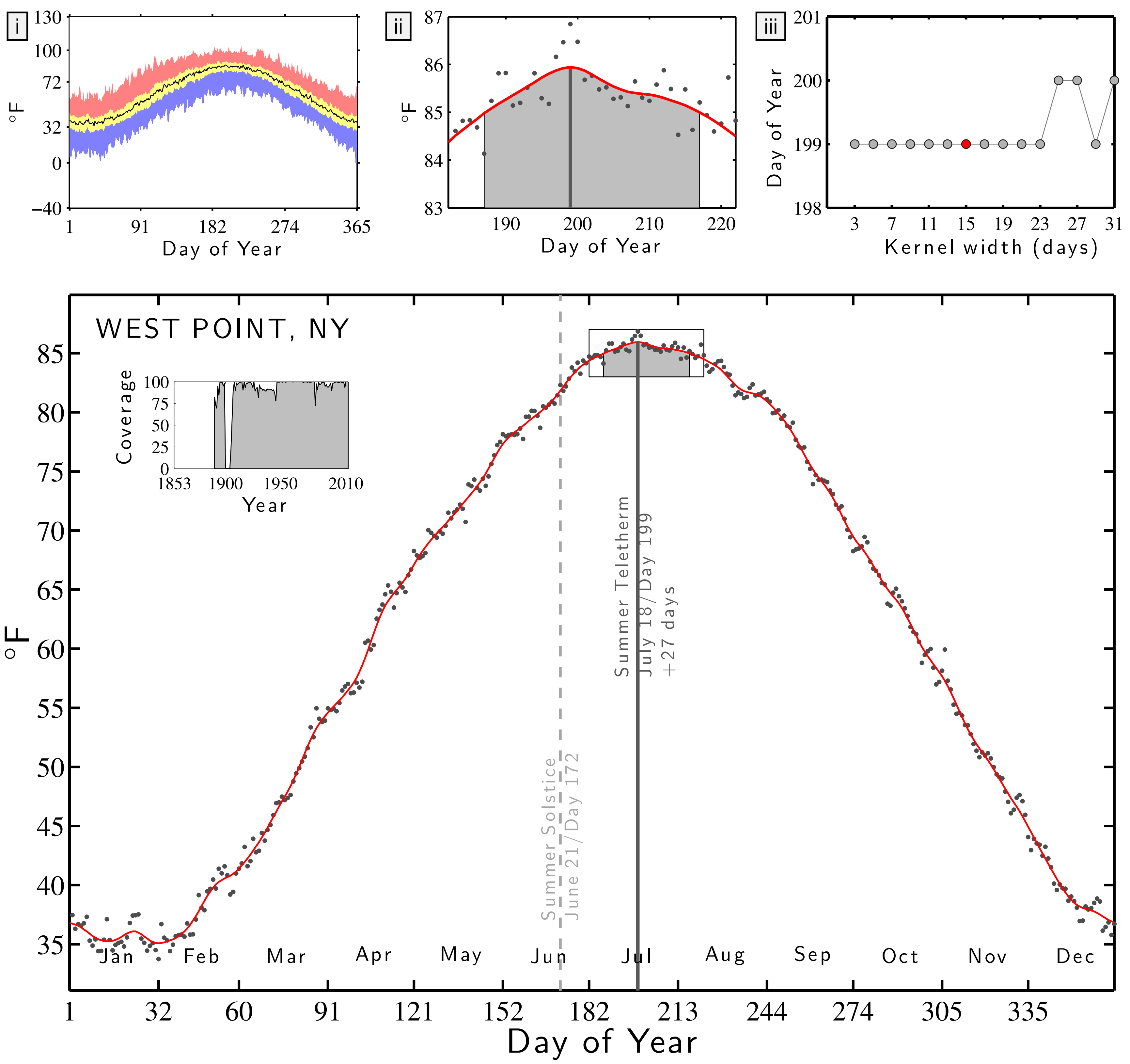

i
130
100
72
32
0
−40
°F
1
91
182
274
365
Day of Year
ii
87
86
85
84
83
°F
190
200
210
220
Day of Year
iii
201
200
199
198
Day of Year
3
7
11
15
19
23
27
31
Kernel width (days)
WEST POINT, NY
Coverage
100
75
50
25
0
1853
1900
1950
2010
Year
Summer Solstice
June 21/Day 172
Summer Teletherm
July 18/Day 199
+27 days
°F
85
80
75
70
65
60
55
50
45
40
35
Jan
Feb
Mar
Apr
May
Jun
Jul
Aug
Sep
Oct
Nov
Dec
1
32
60
91
121
152
182
213
244
274
305
335
Day of Year

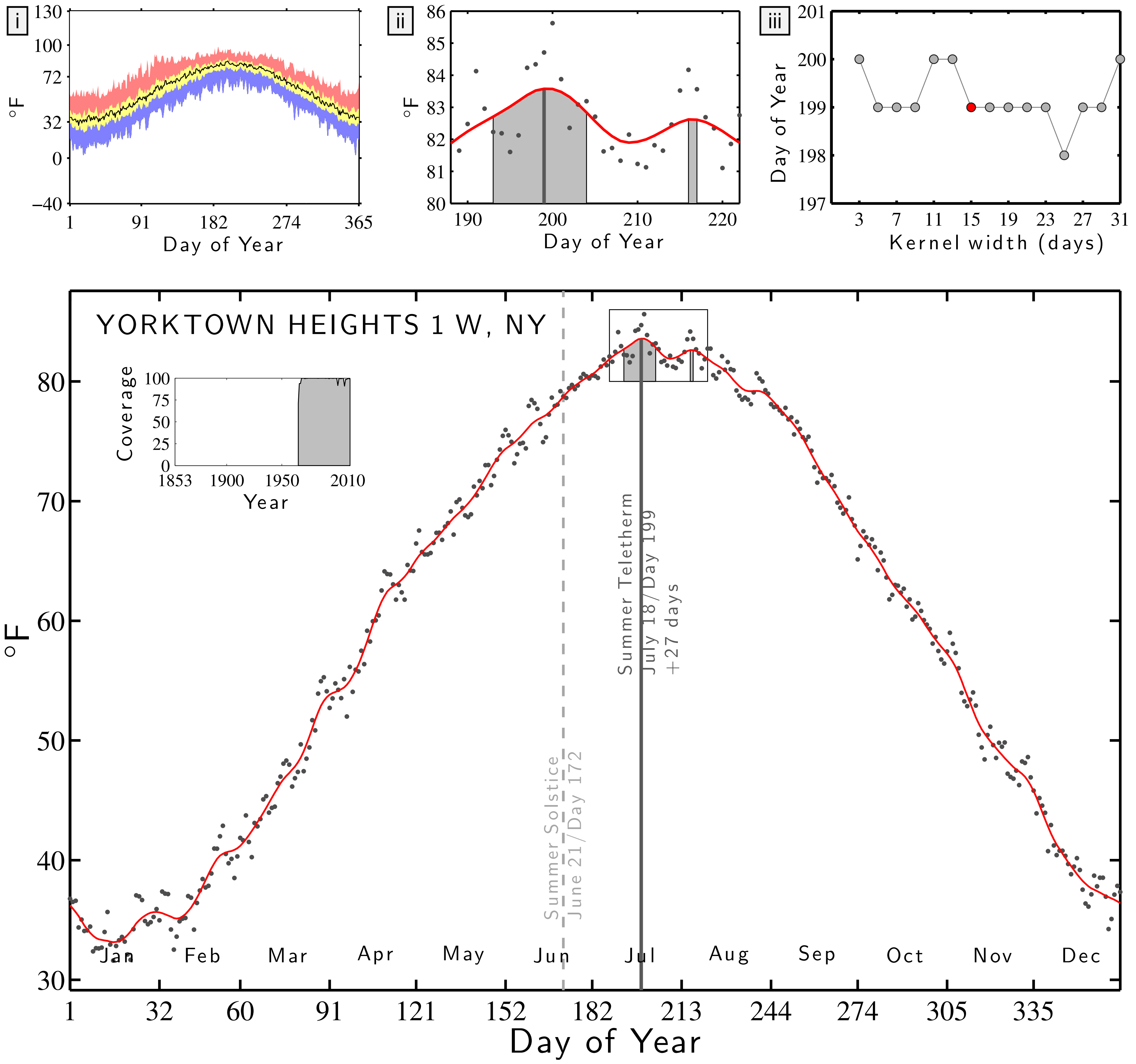

i
ii
iii
°F
Day of Year
Day of Year
Kernel width (days)
YORKTOWN HEIGHTS 1 W, NY
Coverage
Year
Summer Solstice
June 21/Day 172
Summer Teletherm
July 18/Day 199
+27 days
Jan
Feb
Mar
Apr
May
Jun
Jul
Aug
Sep
Oct
Nov
Dec
Day of Year

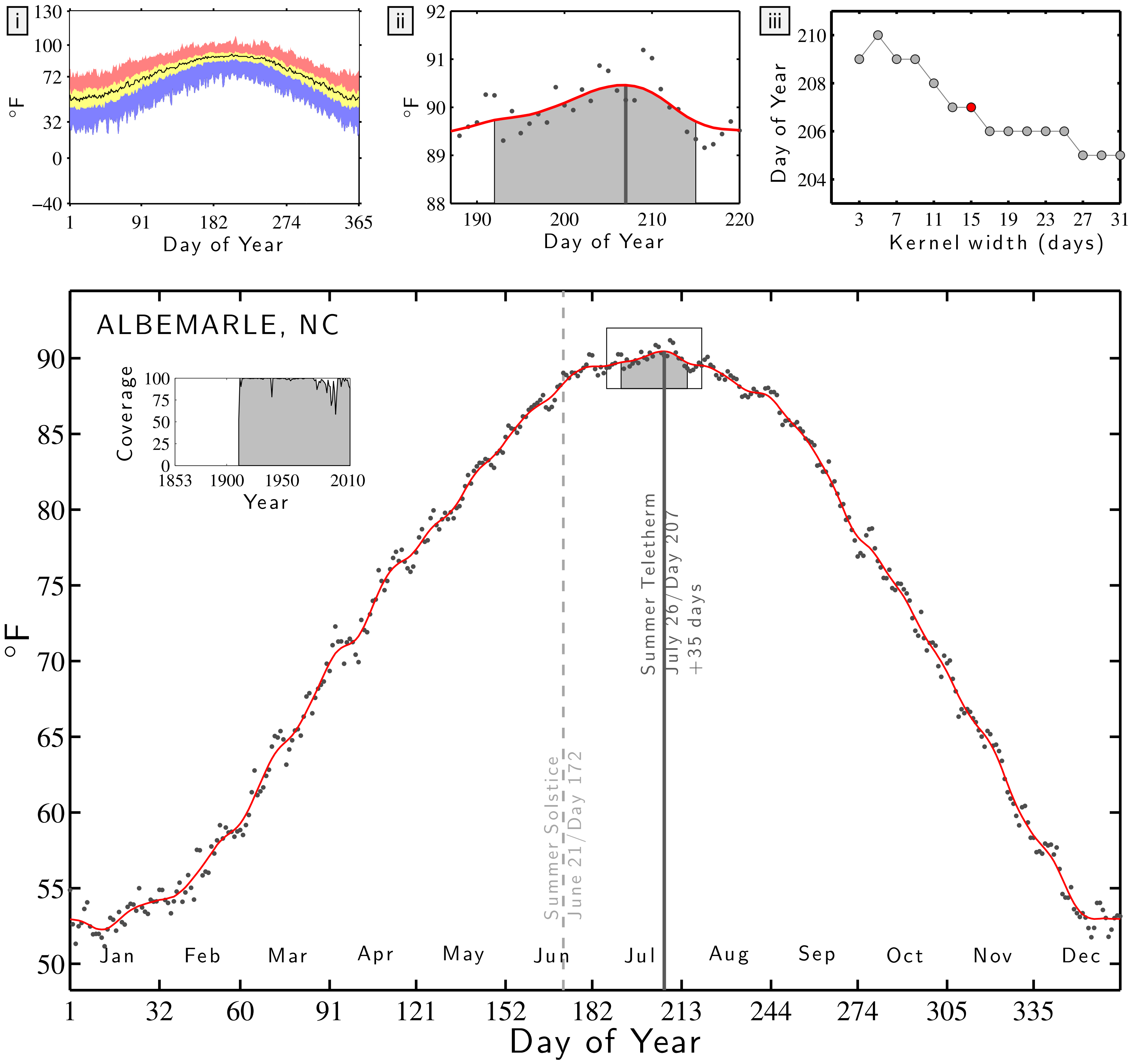
i
ii
iii
°F
Day of Year
Kernel width (days)
Day of Year
ALBEMARLE, NC
Coverage
Year
Summer Solstice
June 21/Day 172
Summer Teletherm
July 26/Day 207
+35 days
Jan
Feb
Mar
Apr
May
Jun
Jul
Aug
Sep
Oct
Nov
Dec

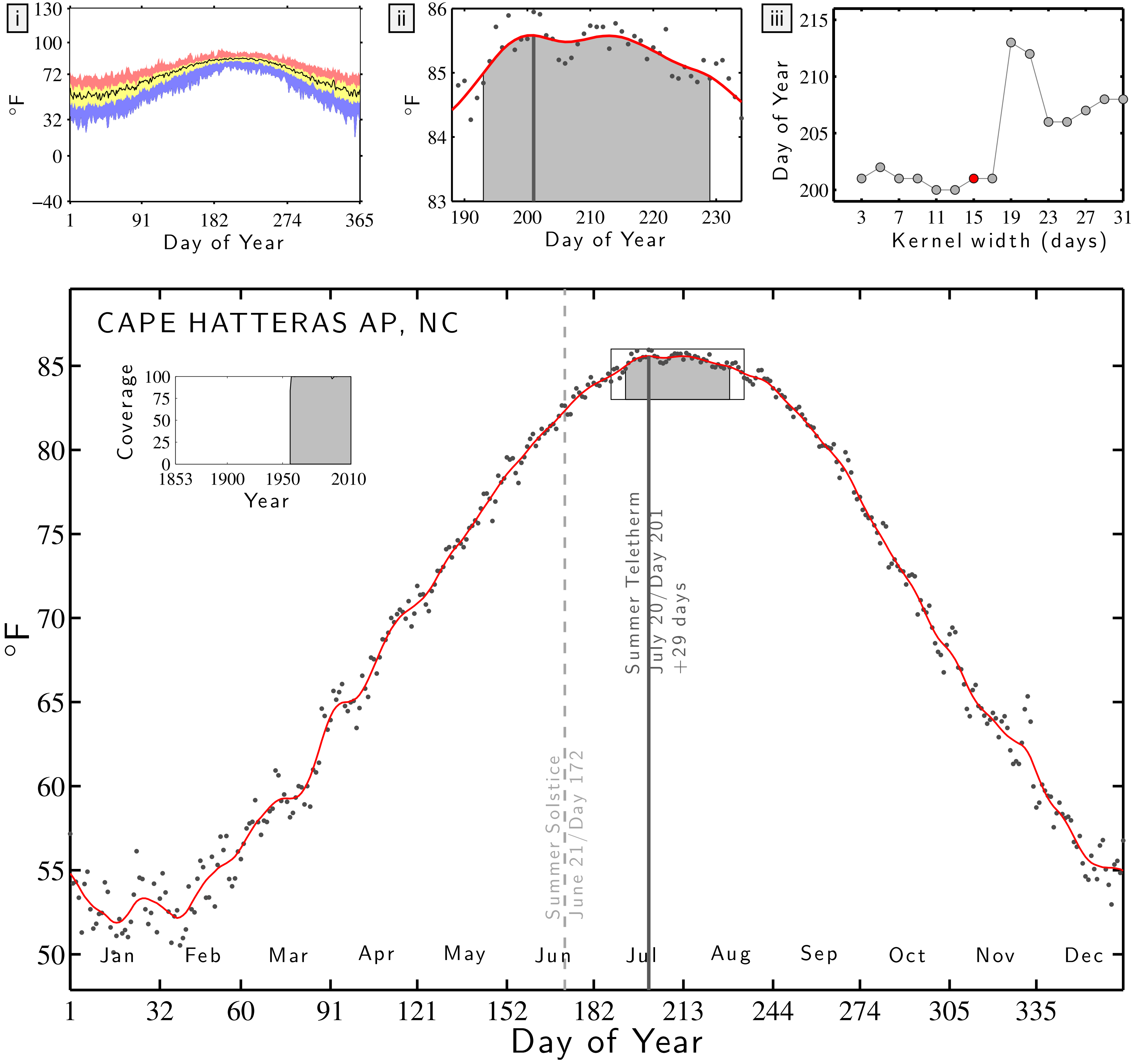

i
°F
Day of Year
ii
°F
Day of Year
iii
Day of Year
Kernel width (days)
CAPE HATTERAS AP, NC
Coverage
Year
Summer Solstice
June 21 / Day 172
Summer Teletherm
July 20 / Day 201
+29 days
Jan
Feb
Mar
Apr
May
Jun
Jul
Aug
Sep
Oct
Nov
Dec
°F
Day of Year

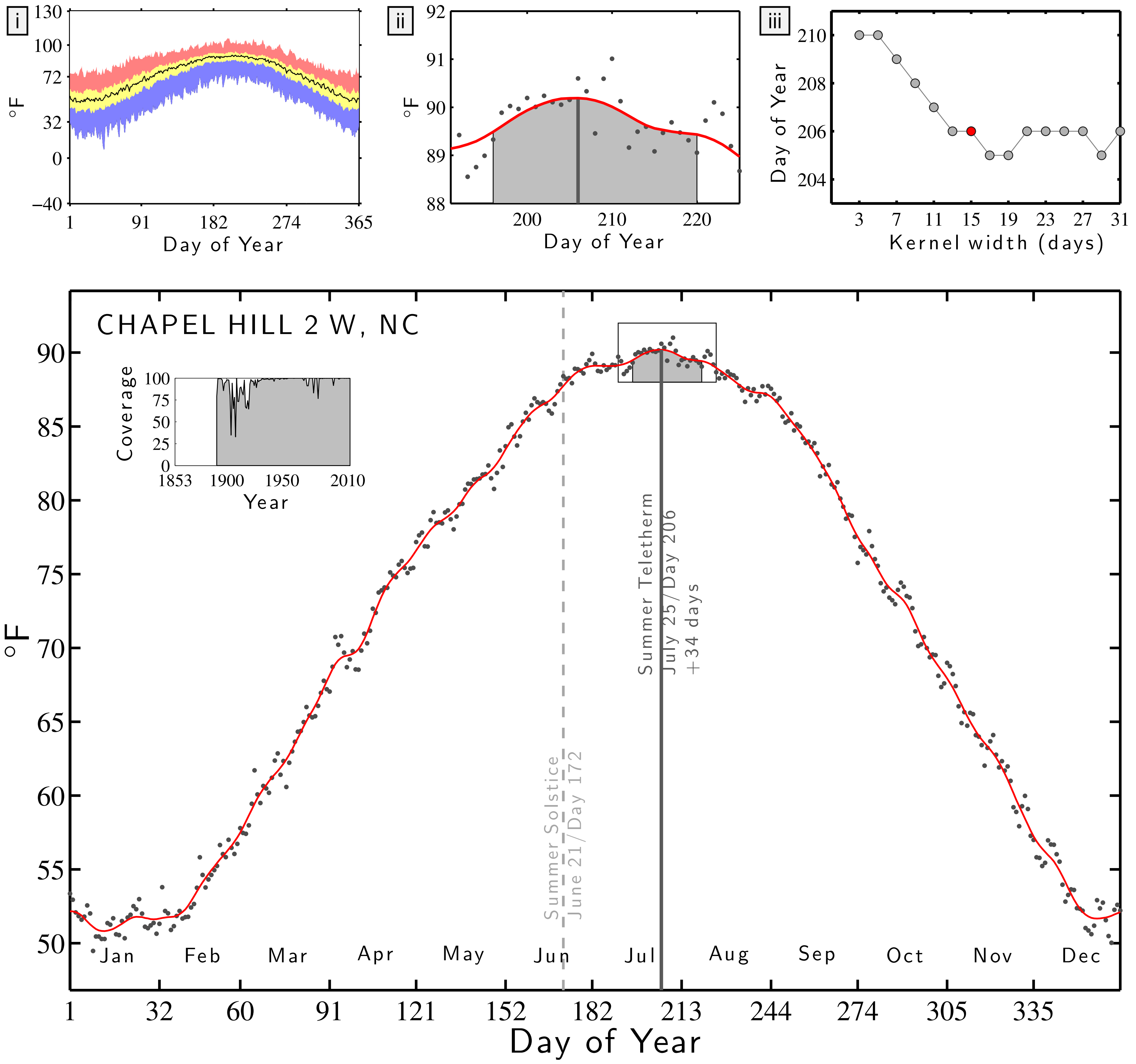

i
°F
Day of Year
ii
°F
Day of Year
iii
Day of Year
Kernel width (days)
CHAPEL HILL 2 W, NC
Coverage
Year
Summer Solstice
June 21/Day 172
Summer Teletherm
July 25/Day 206
+34 days
Jan
Feb
Mar
Apr
May
Jun
Jul
Aug
Sep
Oct
Nov
Dec
°F
Day of Year

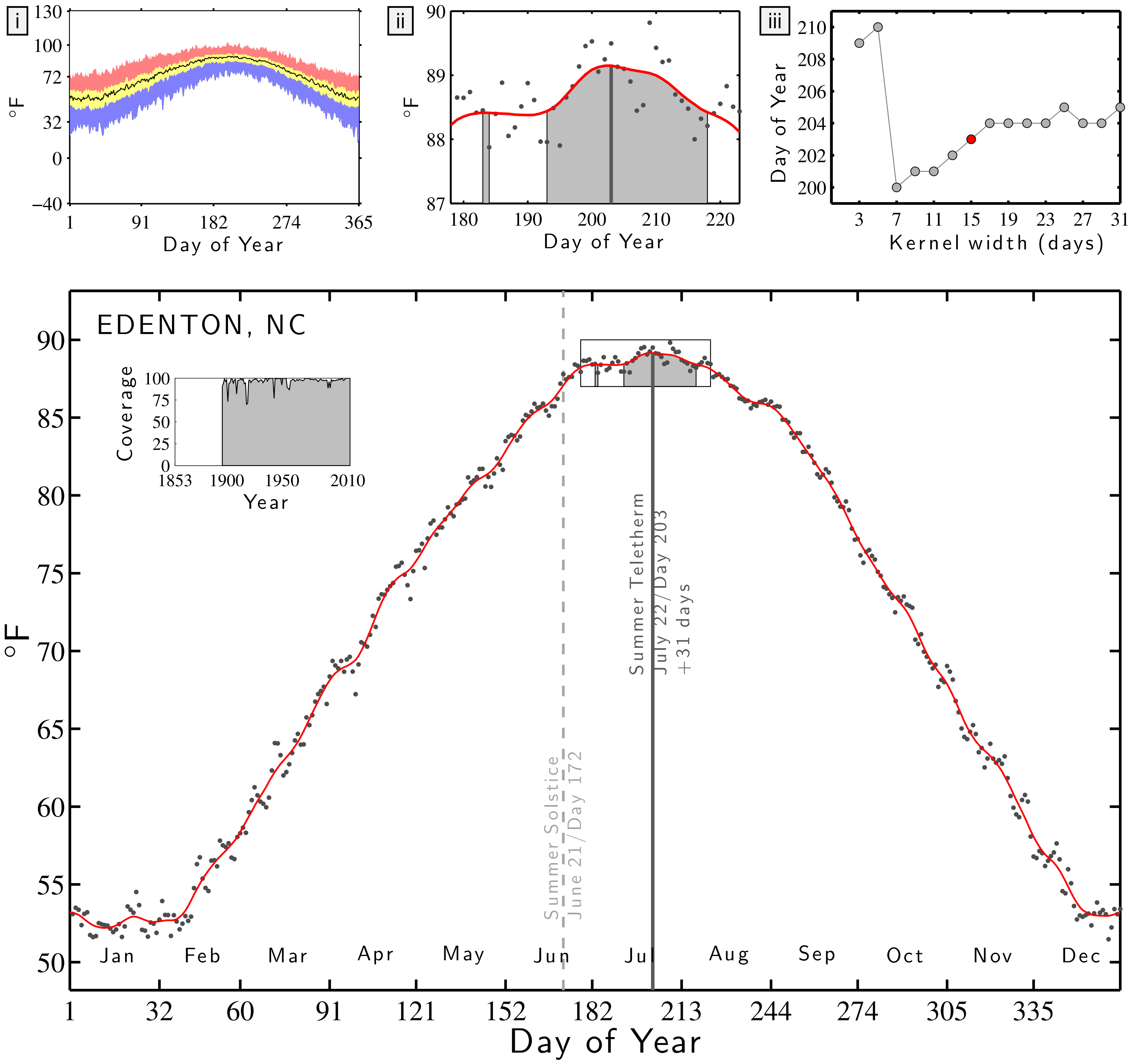

i
°F
Day of Year
ii
°F
Day of Year
iii
Day of Year
Kernel width (days)
EDENTON, NC
Coverage
Year
Summer Solstice
June 21/Day 172
Summer Teletherm
July 22/Day 203
+31 days
Jan
Feb
Mar
Apr
May
Jun
Jul
Aug
Sep
Oct
Nov
Dec
°F
Day of Year

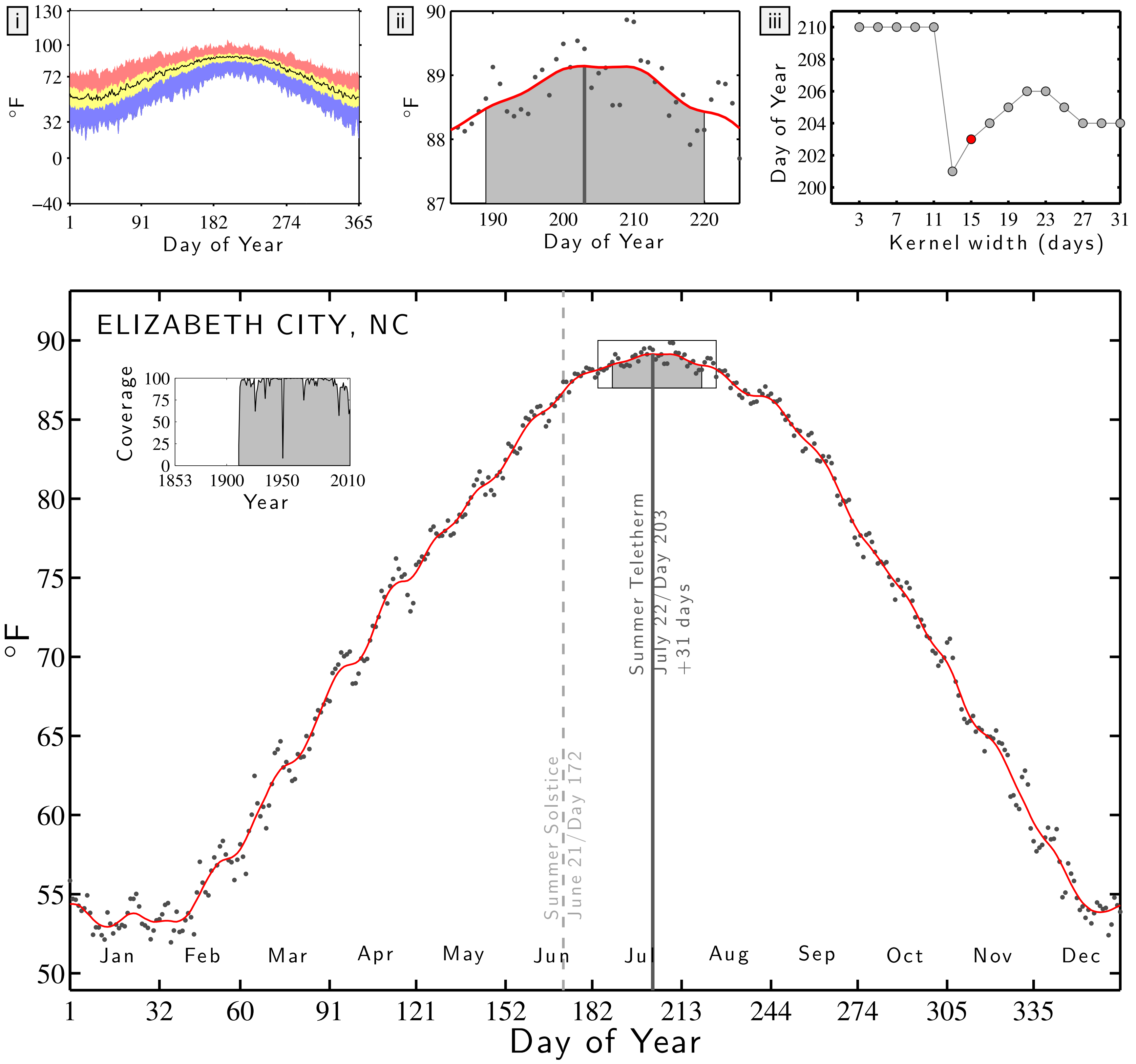

i
ii
iii
°F
Day of Year
Kernel width (days)
ELIZABETH CITY, NC
Coverage
Year
Summer Solstice
June 21/Day 172
Summer Teletherm
July 22/Day 203
+31 days
Jan
Feb
Mar
Apr
May
Jun
Jul
Aug
Sep
Oct
Nov
Dec

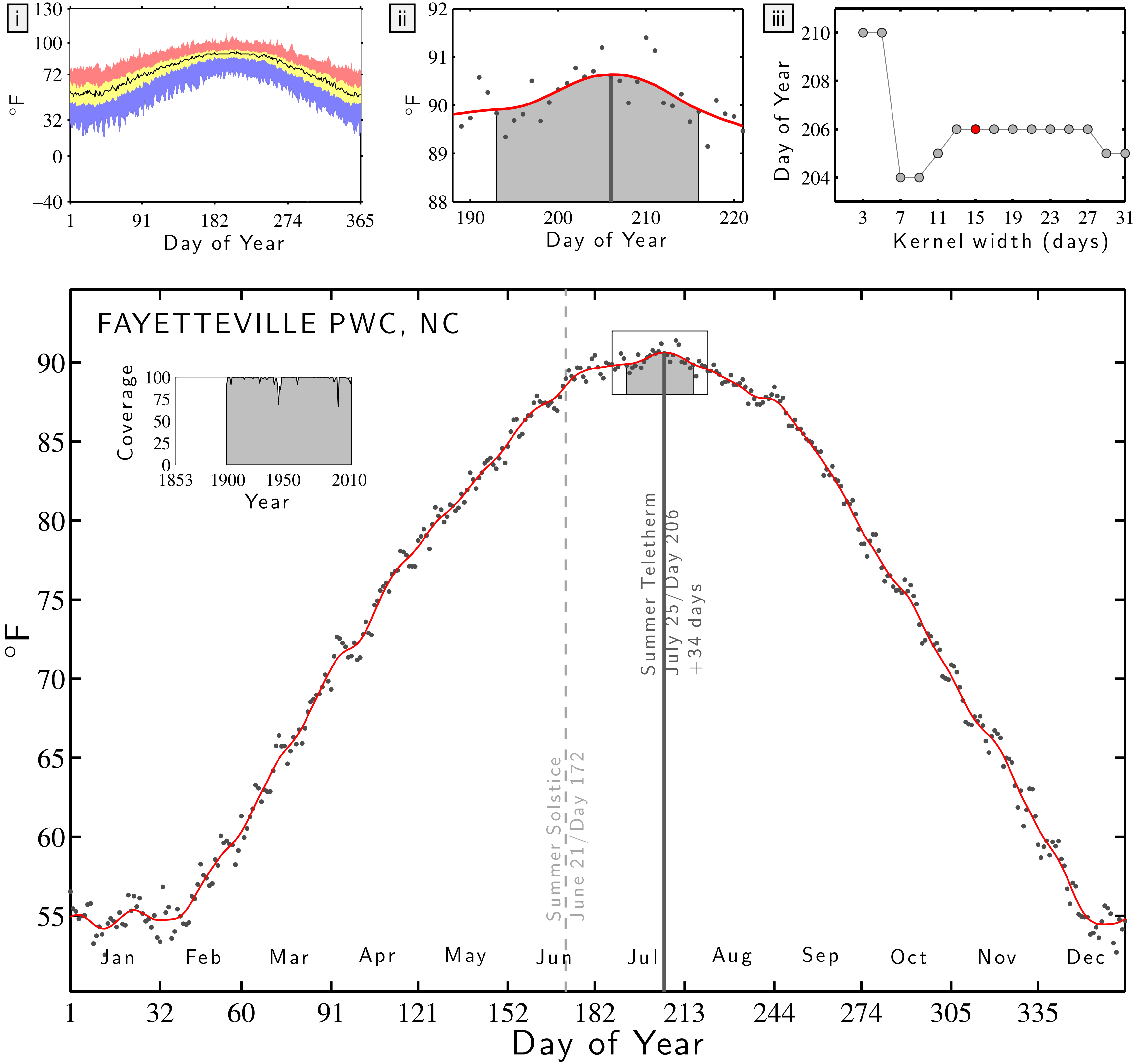

i
°F
Day of Year
ii
°F
Day of Year
iii
Day of Year
Kernel width (days)
FAYETTEVILLE PWC, NC
Coverage
Year
Summer Solstice
June 21/Day 172
Summer Teletherm
July 25/Day 206
+34 days
Jan
Feb
Mar
Apr
May
Jun
Jul
Aug
Sep
Oct
Nov
Dec
°F
Day of Year

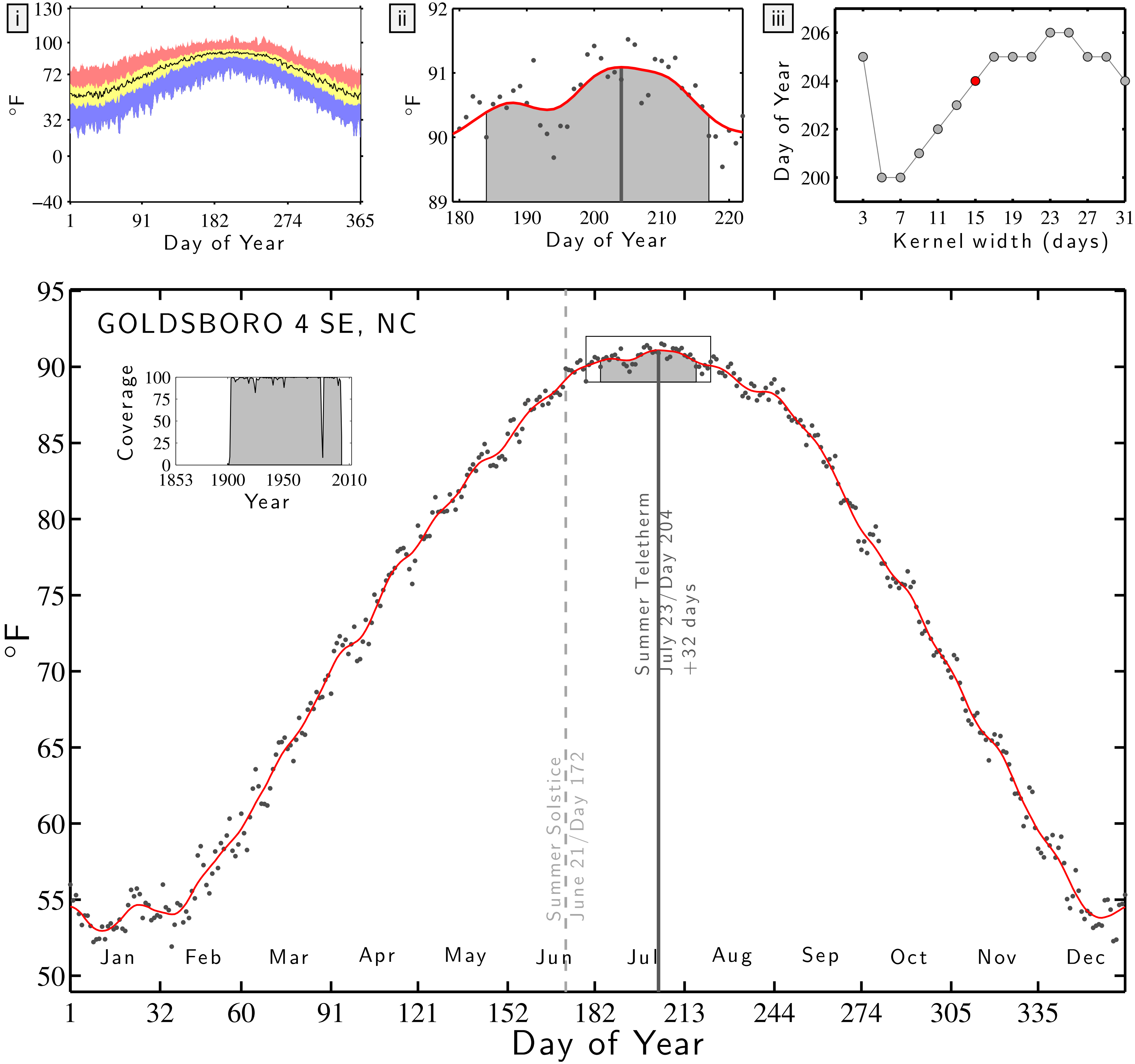

i
°F
Day of Year
ii
°F
Day of Year
iii
Day of Year
Kernel width (days)
GOLDSBORO 4 SE, NC
Coverage
Year
Summer Solstice
June 21 / Day 172
Summer Teletherm
July 23 / Day 204
+32 days
Jan Feb Mar Apr May Jun Jul Aug Sep Oct Nov Dec
°F
Day of Year

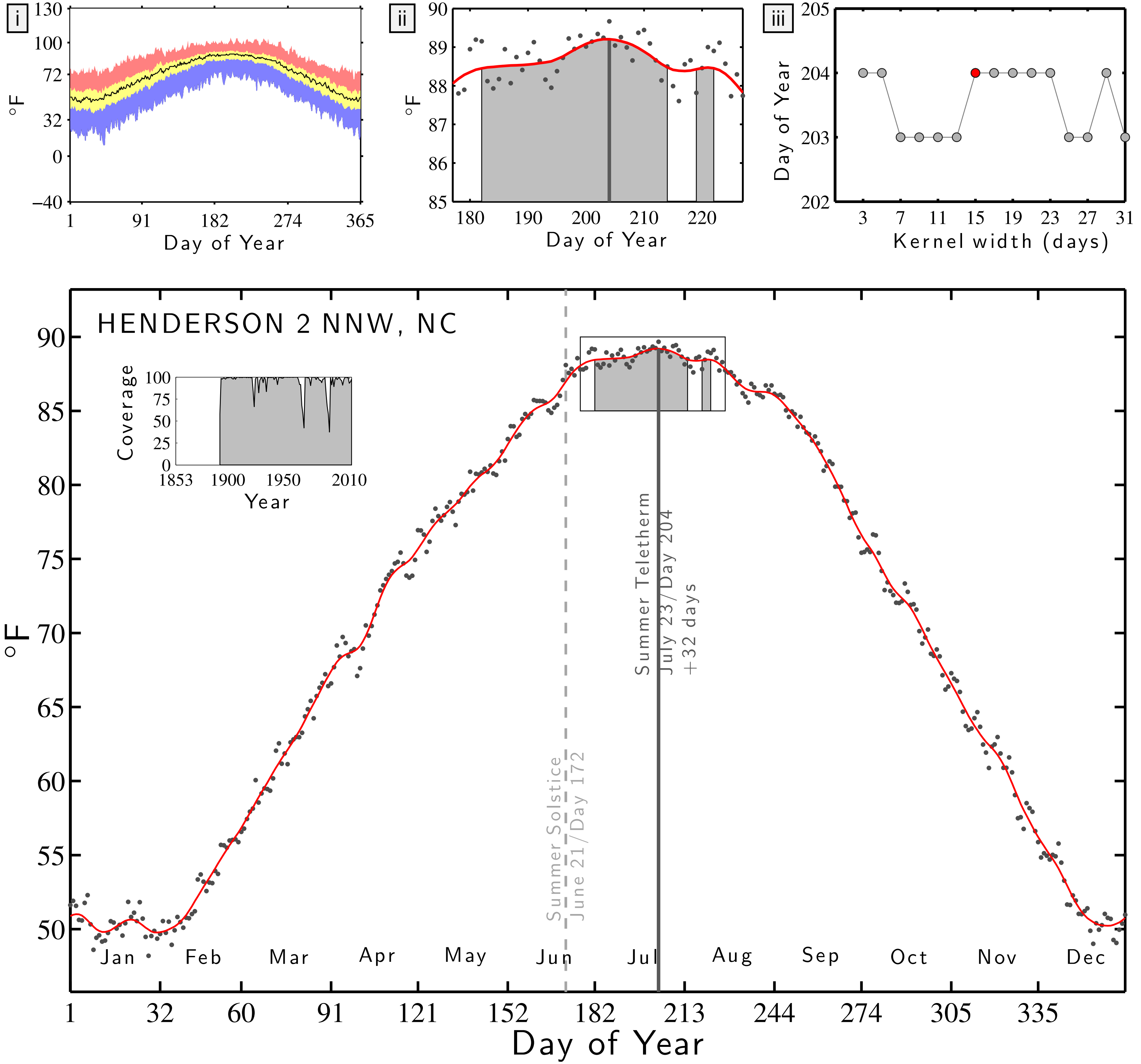

i
°F
Day of Year
ii
°F
Day of Year
iii
Day of Year
Kernel width (days)
HENDERSON 2 NNW, NC
Coverage
Year
Summer Solstice
June 21 / Day 172
Summer Teletherm
July 23 / Day 204
+32 days
Jan
Feb
Mar
Apr
May
Jun
Jul
Aug
Sep
Oct
Nov
Dec
°F
Day of Year

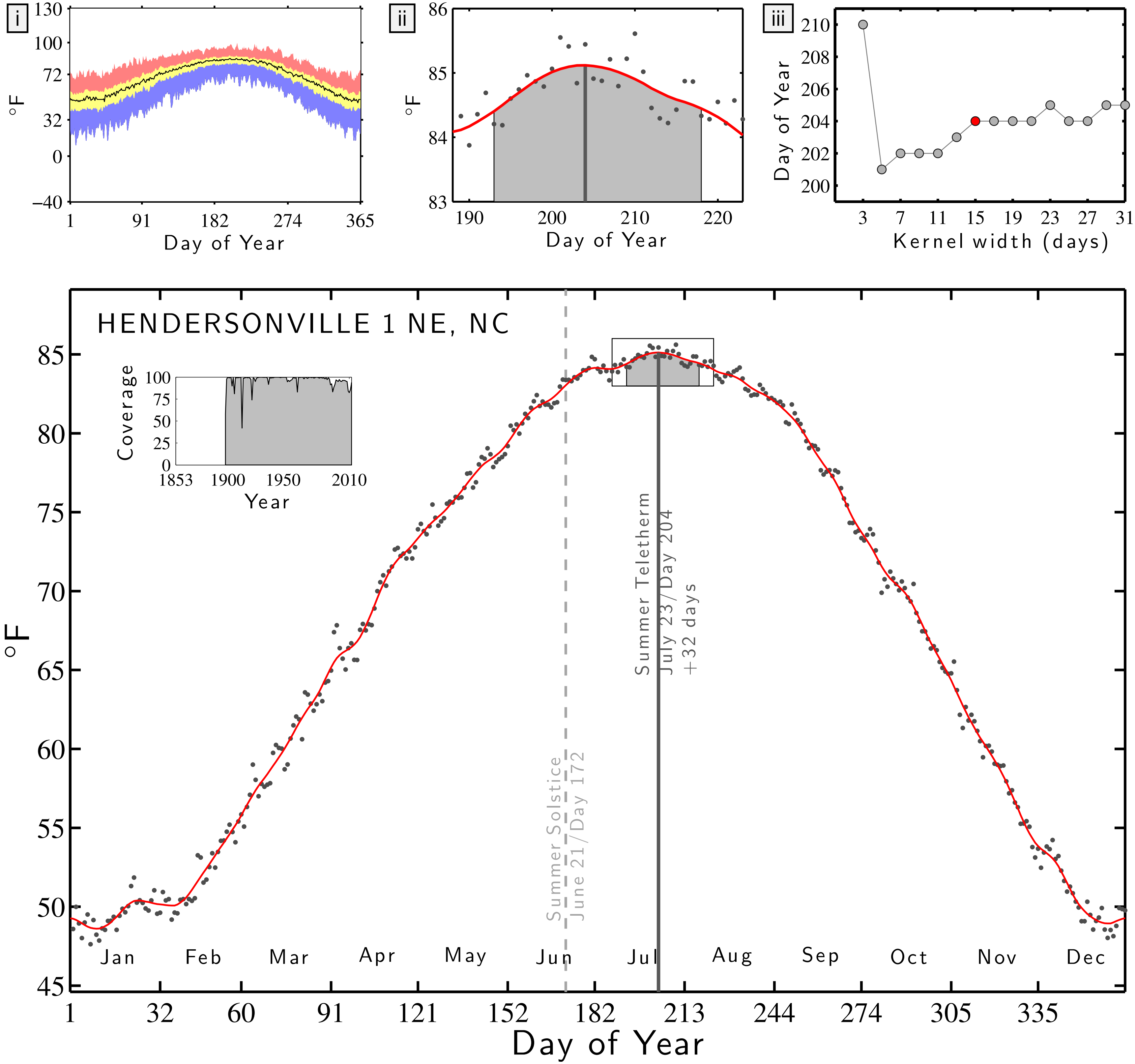

i
°F
Day of Year
ii
°F
Day of Year
iii
Day of Year
Kernel width (days)
HENDERSONVILLE 1 NE, NC
Coverage
Year
Summer Solstice
June 21 / Day 172
Summer Teletherm
July 23 / Day 204
+32 days
Jan
Feb
Mar
Apr
May
Jun
Jul
Aug
Sep
Oct
Nov
Dec
°F
Day of Year

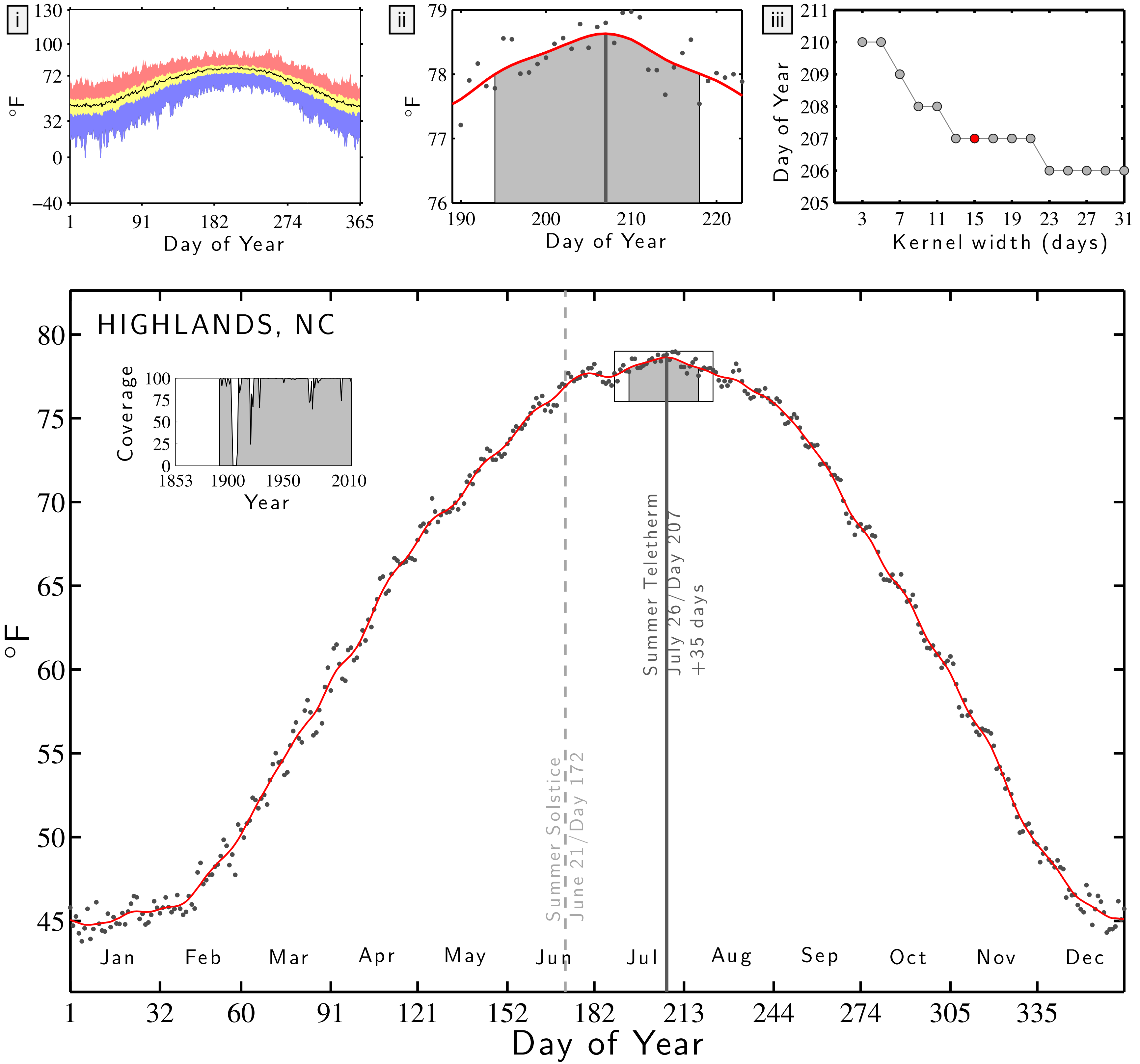

i
ii
iii
°F
Day of Year
Day of Year
Kernel width (days)
HIGHLANDS, NC
Coverage
Year
Summer Solstice
June 21 / Day 172
Summer Teletherm
July 26 / Day 207
+35 days
Jan
Feb
Mar
Apr
May
Jun
Jul
Aug
Sep
Oct
Nov
Dec
Day of Year

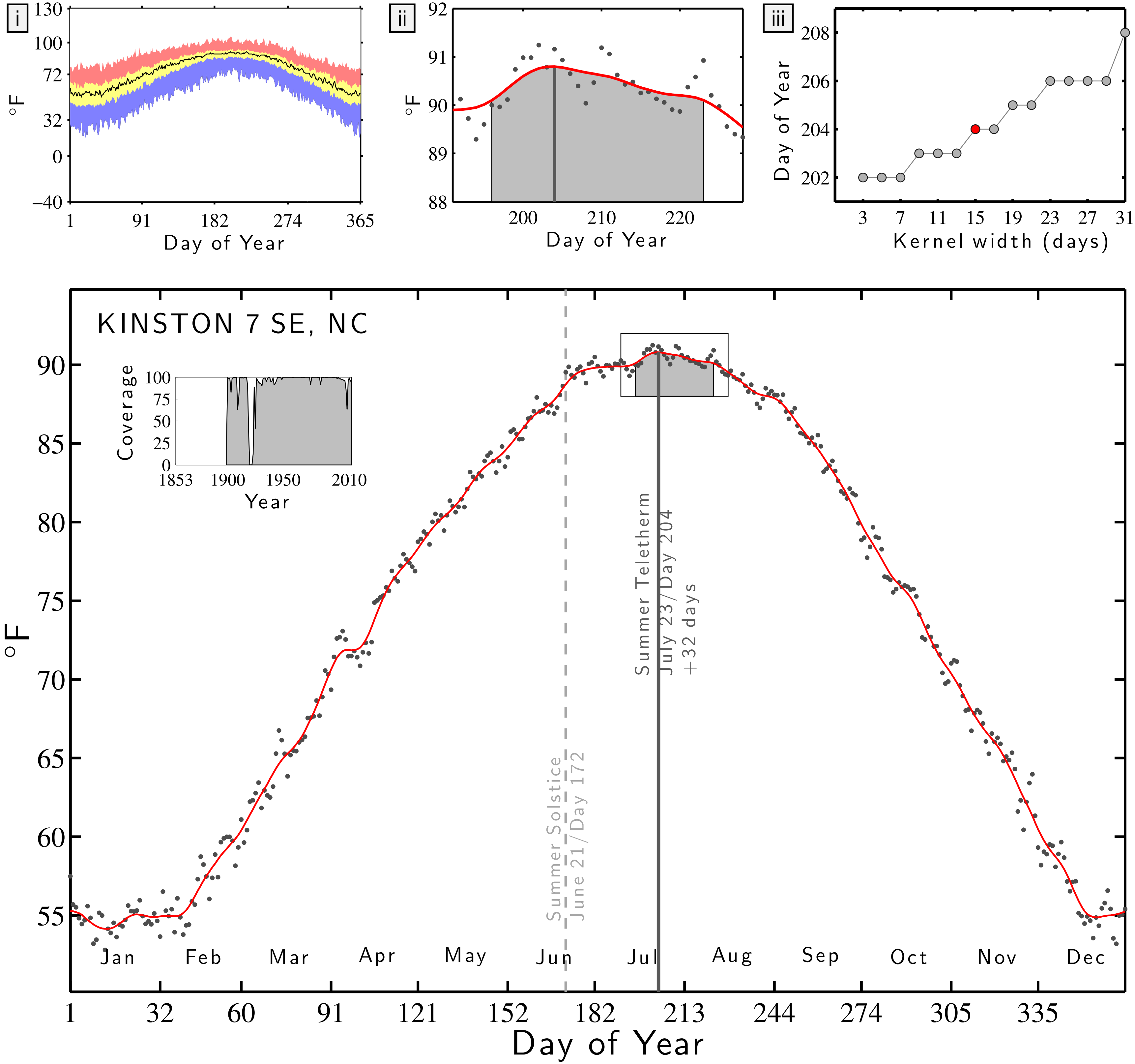

i
°F
130
100
72
32
0
−40
1
91
182
274
365
Day of Year
ii
°F
92
91
90
89
88
200
210
220
Day of Year
iii
Day of Year
208
206
204
202
3
7
11
15
19
23
27
31
Kernel width (days)
KINSTON 7 SE, NC
Coverage
100
75
50
25
0
1853
1900
1950
2010
Year
Summer Solstice
June 21 / Day 172
Summer Teletherm
July 23 / Day 204
+32 days
°F
90
85
80
75
70
65
60
55
Jan
Feb
Mar
Apr
May
Jun
Jul
Aug
Sep
Oct
Nov
Dec
1
32
60
91
121
152
182
213
244
274
305
335
Day of Year

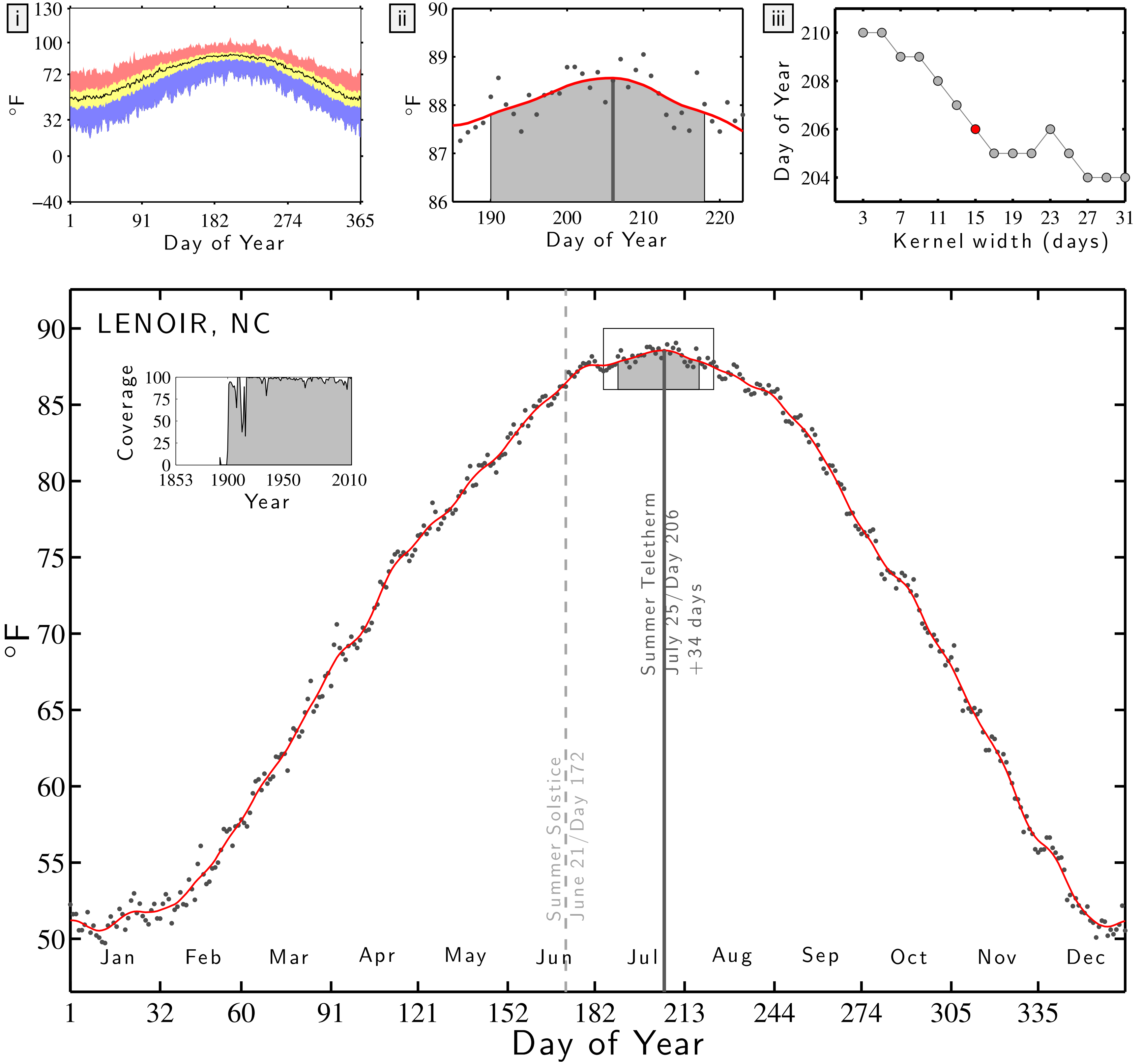

i
°F
130
100
72
32
0
−40
1
91
182
274
365
Day of Year
ii
°F
90
89
88
87
86
190
200
210
220
Day of Year
iii
Day of Year
210
208
206
204
3
7
11
15
19
23
27
31
Kernel width (days)
LENOIR, NC
Coverage
100
75
50
25
0
1853
1900
1950
2010
Year
Summer Solstice
June 21/Day 172
Summer Teletherm
July 25/Day 206
+34 days
°F
90
85
80
75
70
65
60
55
50
Jan
Feb
Mar
Apr
May
Jun
Jul
Aug
Sep
Oct
Nov
Dec
1
32
60
91
121
152
182
213
244
274
305
335
Day of Year

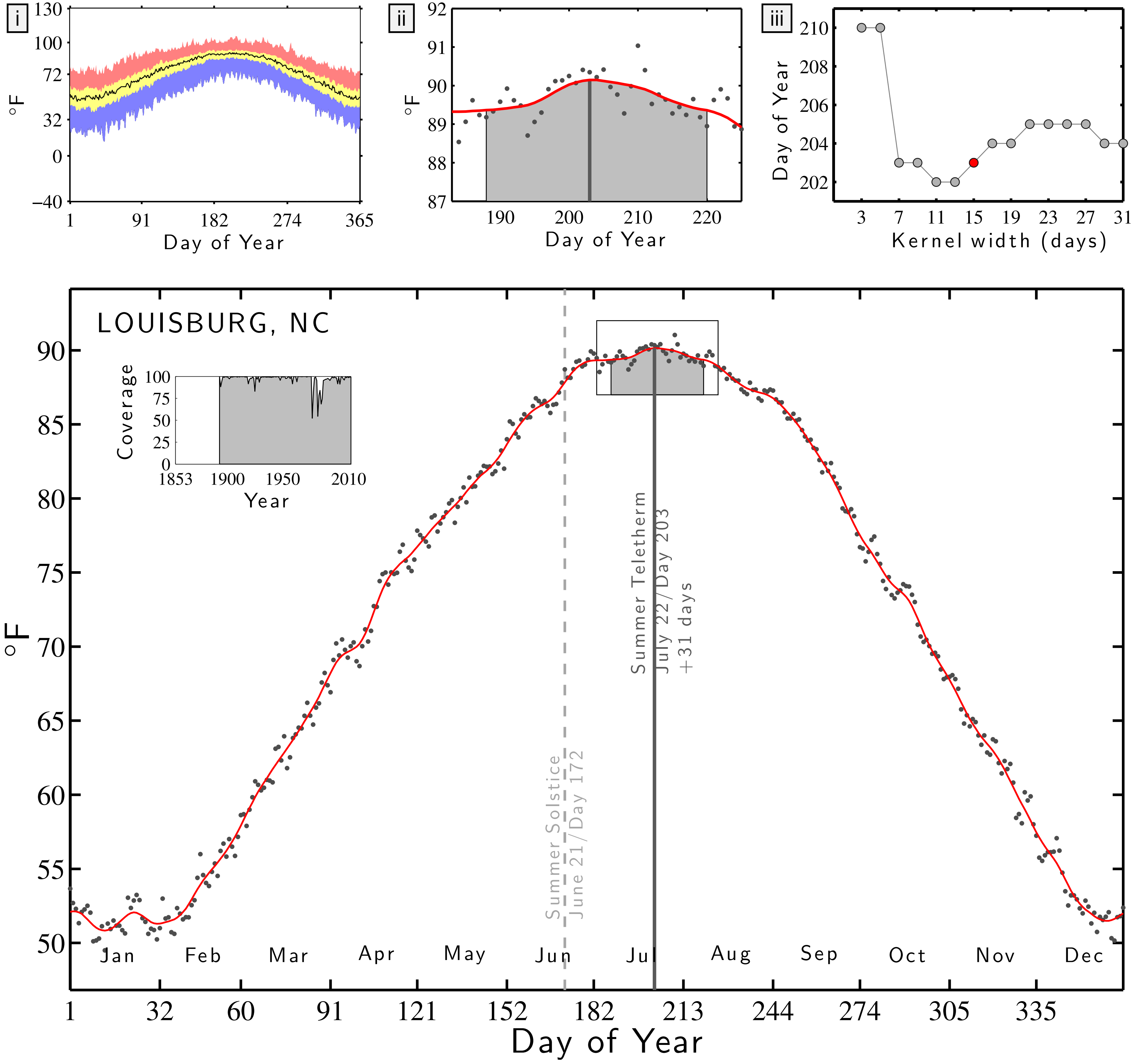

i
Day of Year
°F
ii
Day of Year
°F
iii
Day of Year
Kernel width (days)
LOUISBURG, NC
Coverage
Year
Summer Solstice
June 21/Day 172
Summer Teletherm
July 22/Day 203
+31 days
Jan
Feb
Mar
Apr
May
Jun
Jul
Aug
Sep
Oct
Nov
Dec
Day of Year
°F

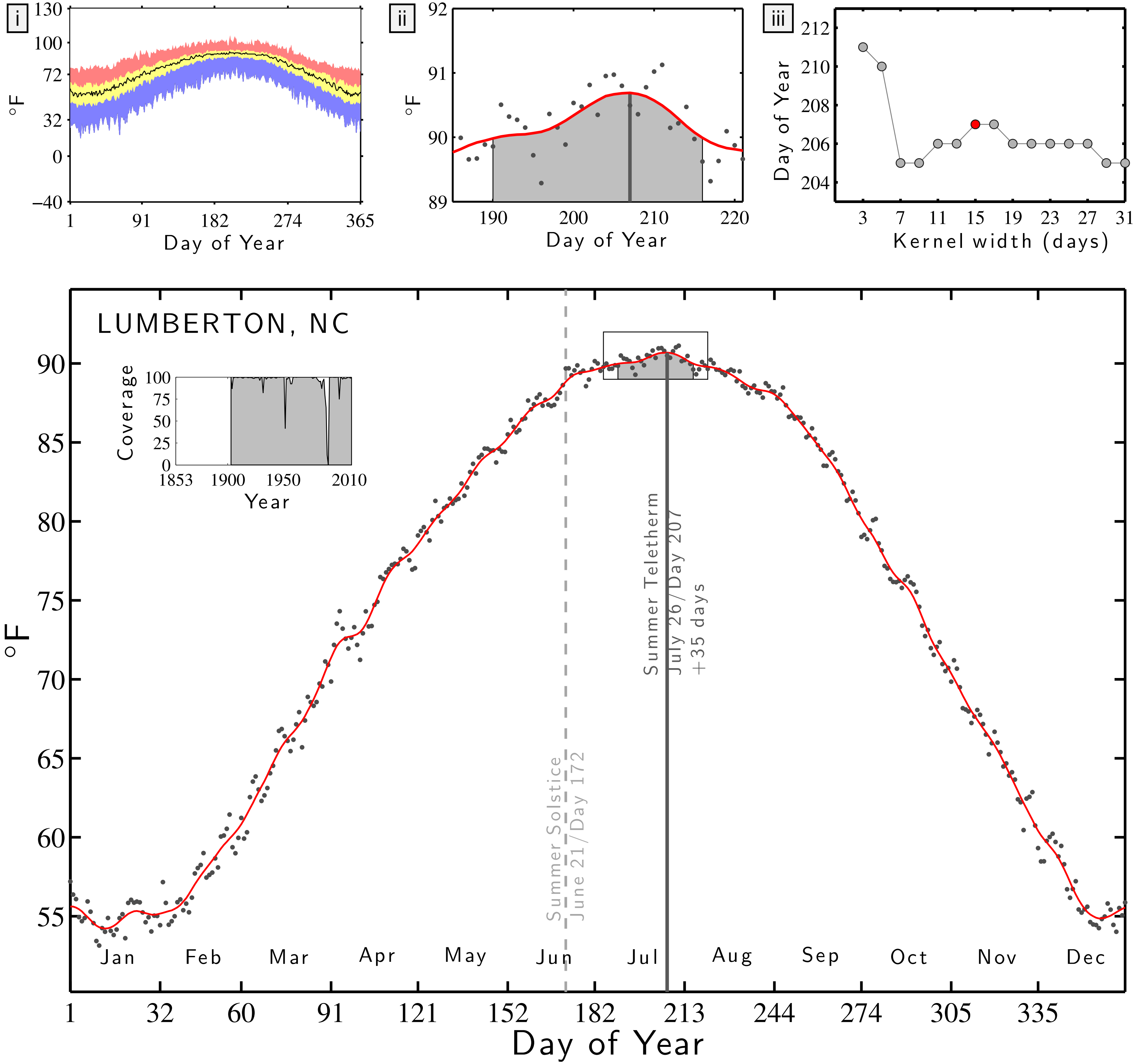

i
ii
iii
°F
Day of Year
Kernel width (days)
LUMBERTON, NC
Coverage
Year
Summer Solstice
June 21 / Day 172
Summer Teletherm
July 26 / Day 207
+35 days
Jan
Feb
Mar
Apr
May
Jun
Jul
Aug
Sep
Oct
Nov
Dec

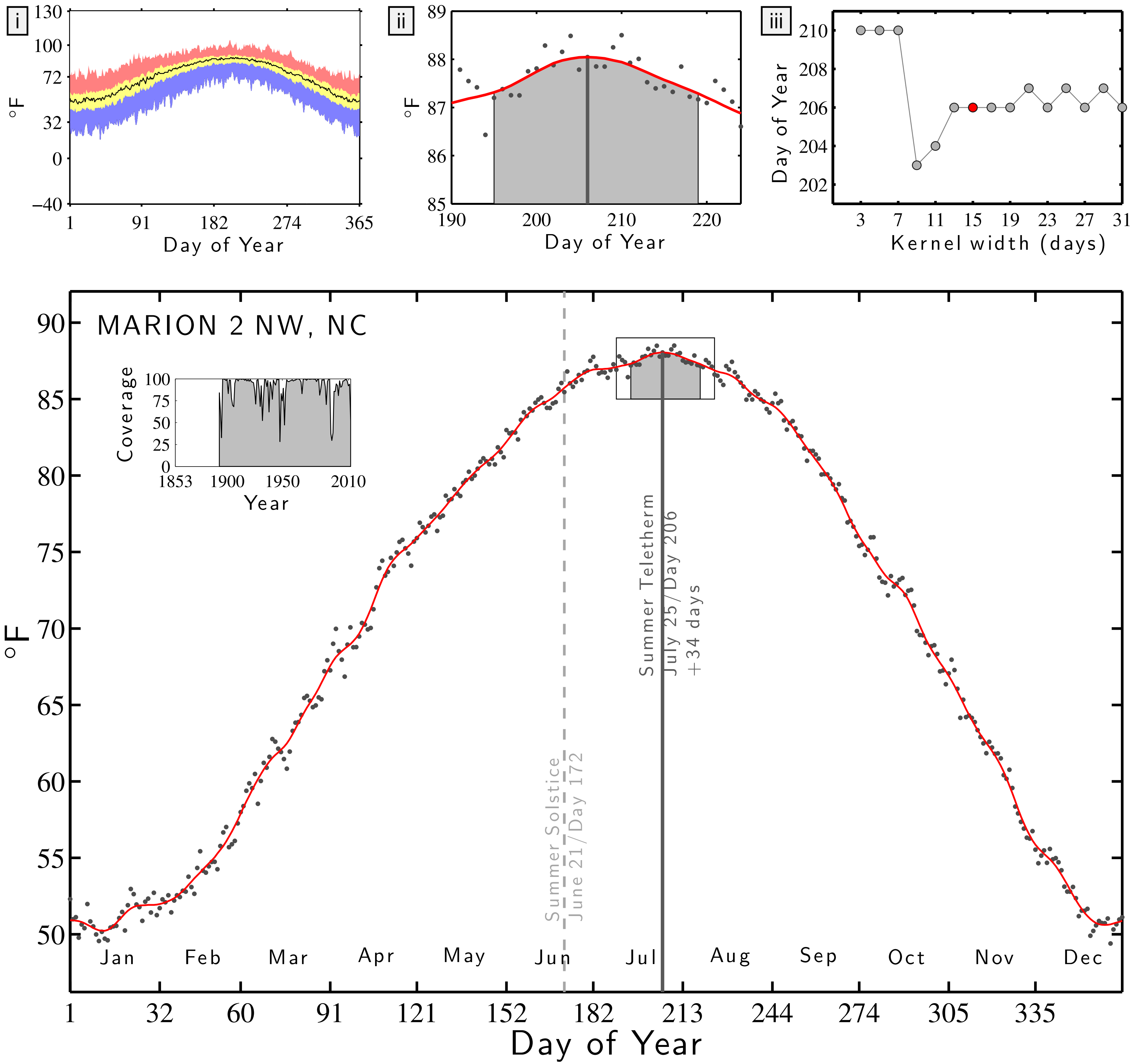

i
ii
iii
°F
Day of Year
Day of Year
Kernel width (days)
MARION 2 NW, NC
Coverage
Year
Summer Solstice
June 21/Day 172
Summer Teletherm
July 25/Day 206
+34 days
Jan
Feb
Mar
Apr
May
Jun
Jul
Aug
Sep
Oct
Nov
Dec
°F
Day of Year

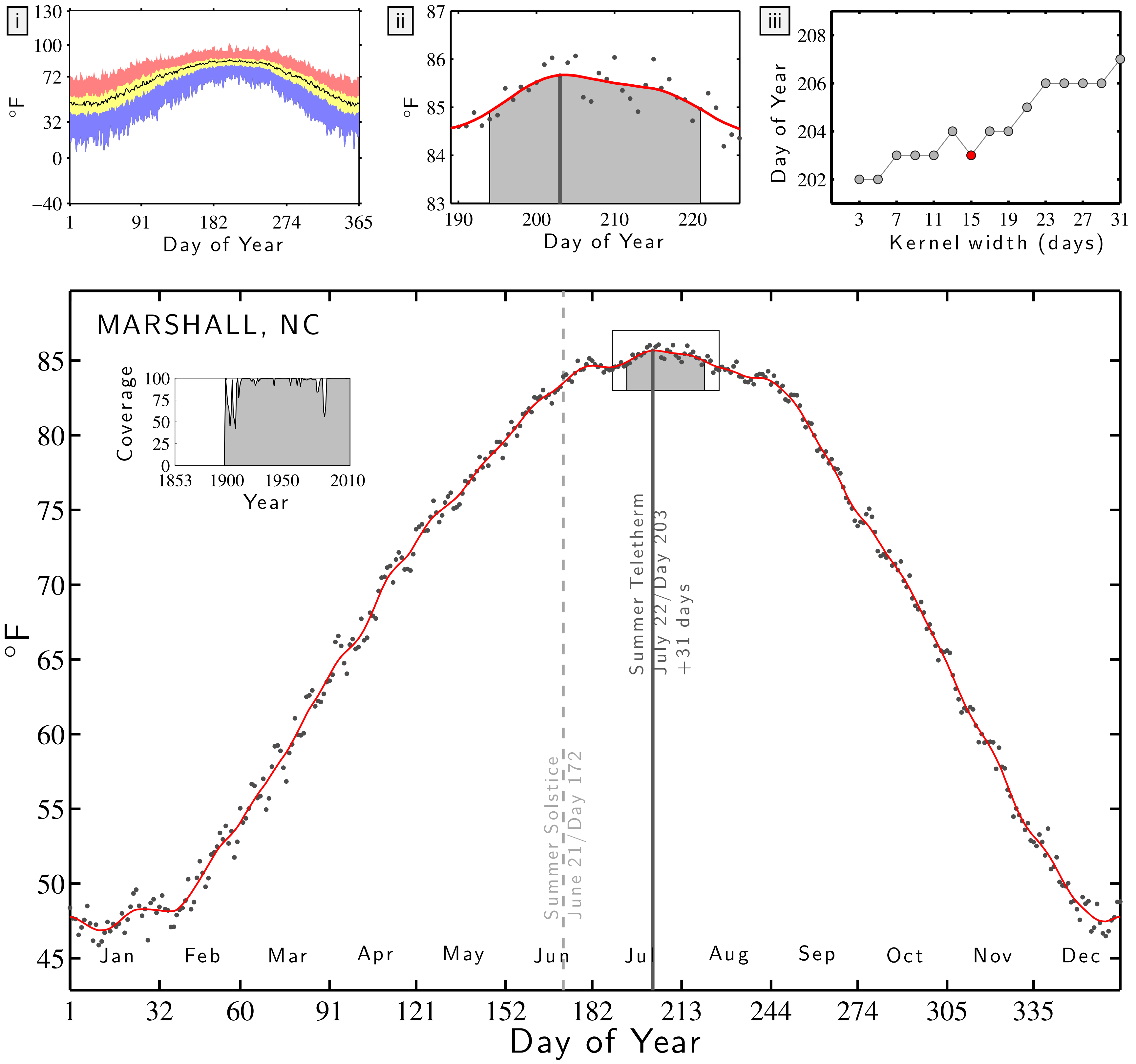

i
ii
iii
°F
Day of Year
Day of Year
Kernel width (days)
MARSHALL, NC
Coverage
Year
Summer Solstice
June 21 / Day 172
Summer Teletherm
July 22 / Day 203
+31 days
Jan
Feb
Mar
Apr
May
Jun
Jul
Aug
Sep
Oct
Nov
Dec
°F
Day of Year

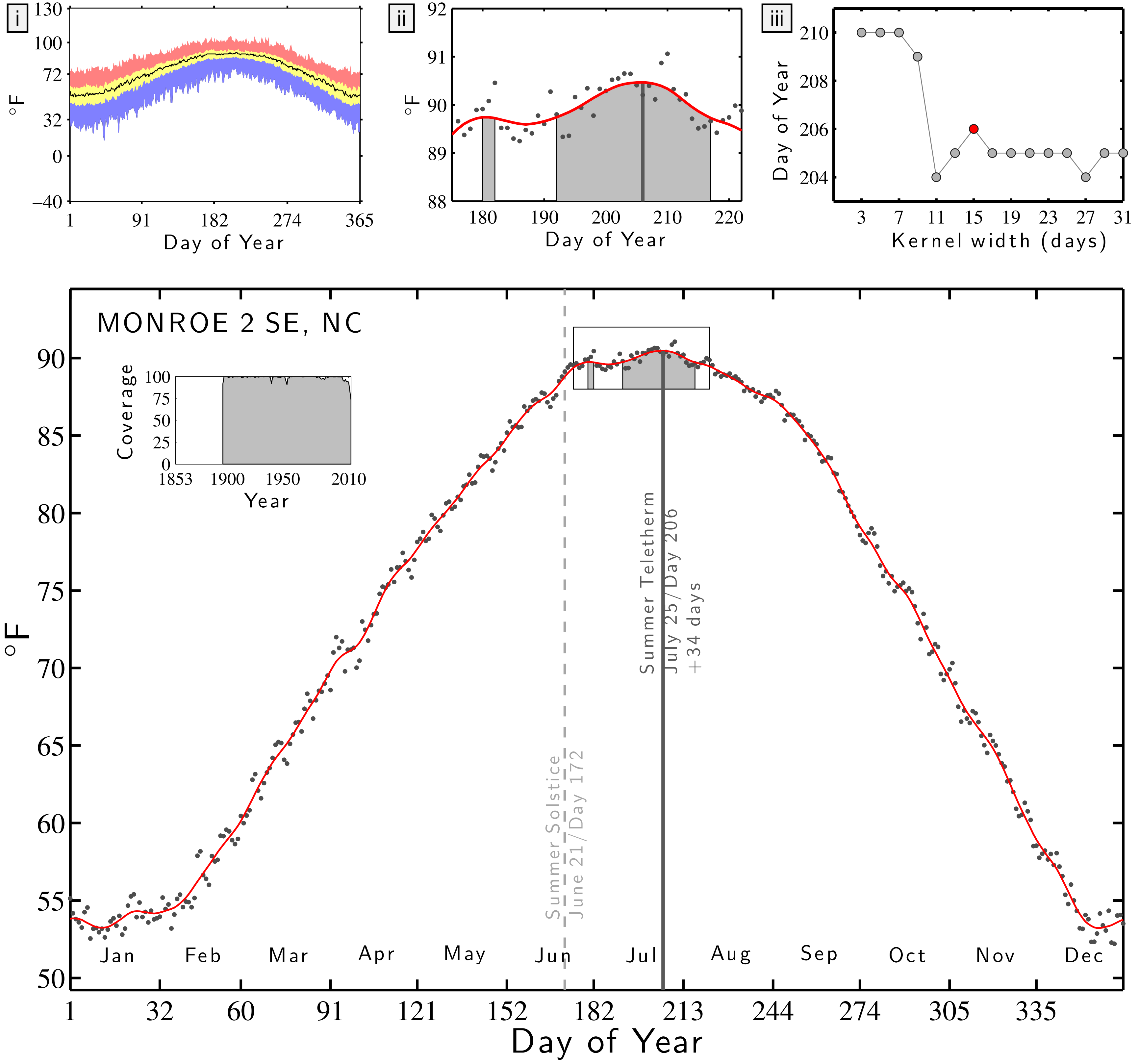

i
°F
130
100
72
32
0
−40
1
91
182
274
365
Day of Year
ii
92
91
90
89
88
180
190
200
210
220
Day of Year
iii
Day of Year
210
208
206
204
3
7
11
15
19
23
27
31
Kernel width (days)
MONROE 2 SE, NC
Coverage
100
75
50
25
0
1853
1900
1950
2010
Year
Summer Solstice
June 21/Day 172
Summer Teletherm
July 25/Day 206
+34 days
Jan
Feb
Mar
Apr
May
Jun
Jul
Aug
Sep
Oct
Nov
Dec
90
85
80
75
70
65
60
55
50
1
32
60
91
121
152
182
213
244
274
305
335
Day of Year

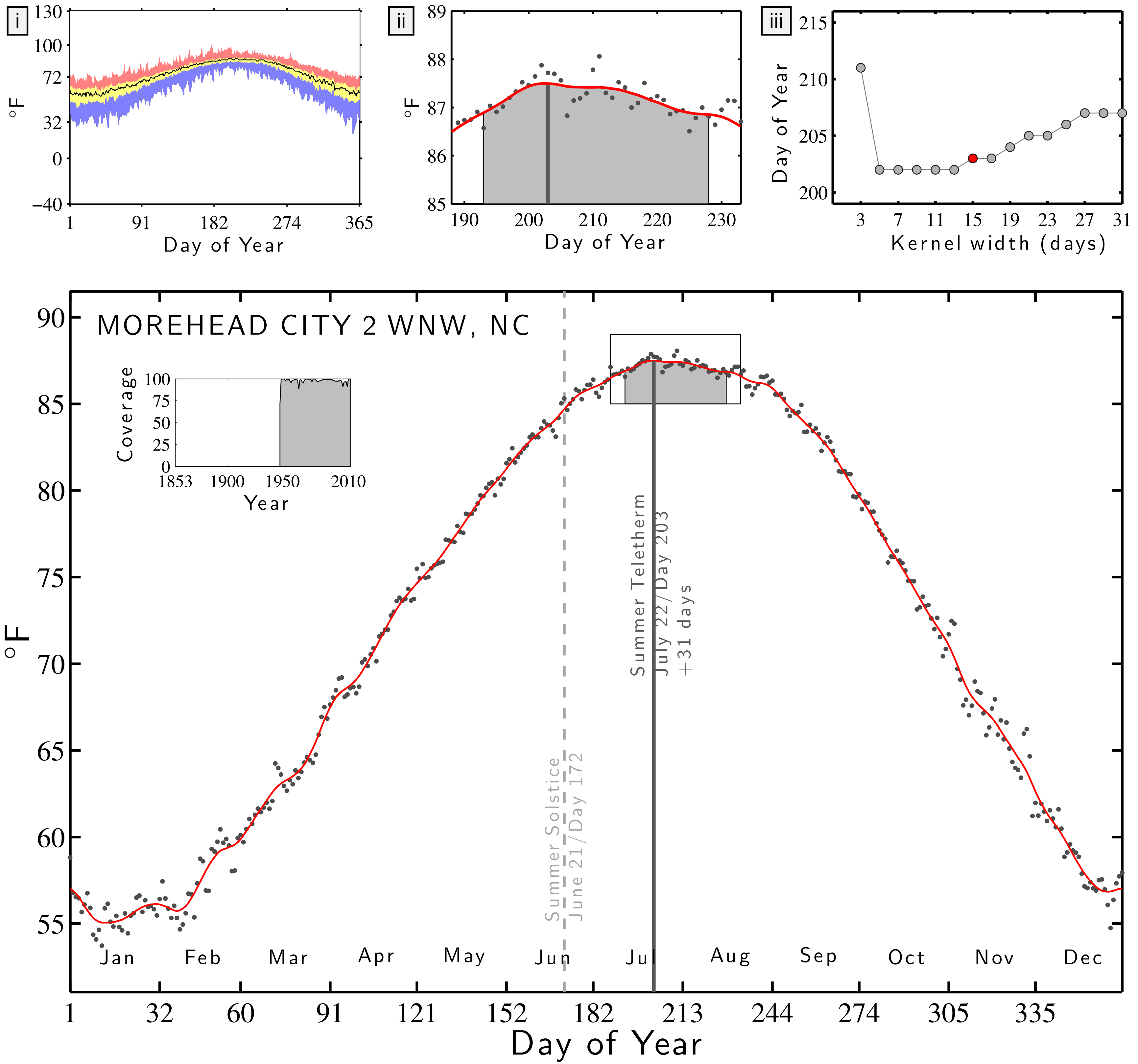

i
°F
130
100
72
32
0
−40
1
91
182
274
365
Day of Year
ii
°F
89
88
87
86
85
190
200
210
220
230
Day of Year
iii
Day of Year
215
210
205
200
3
7
11
15
19
23
27
31
Kernel width (days)
MOREHEAD CITY 2 WNW, NC
Coverage
100
75
50
25
0
1853
1900
1950
2010
Year
Summer Solstice
June 21/Day 172
Summer Teletherm
July 22/Day 203
+31 days
°F
90
85
80
75
70
65
60
55
Jan
Feb
Mar
Apr
May
Jun
Jul
Aug
Sep
Oct
Nov
Dec
1
32
60
91
121
152
182
213
244
274
305
335
Day of Year

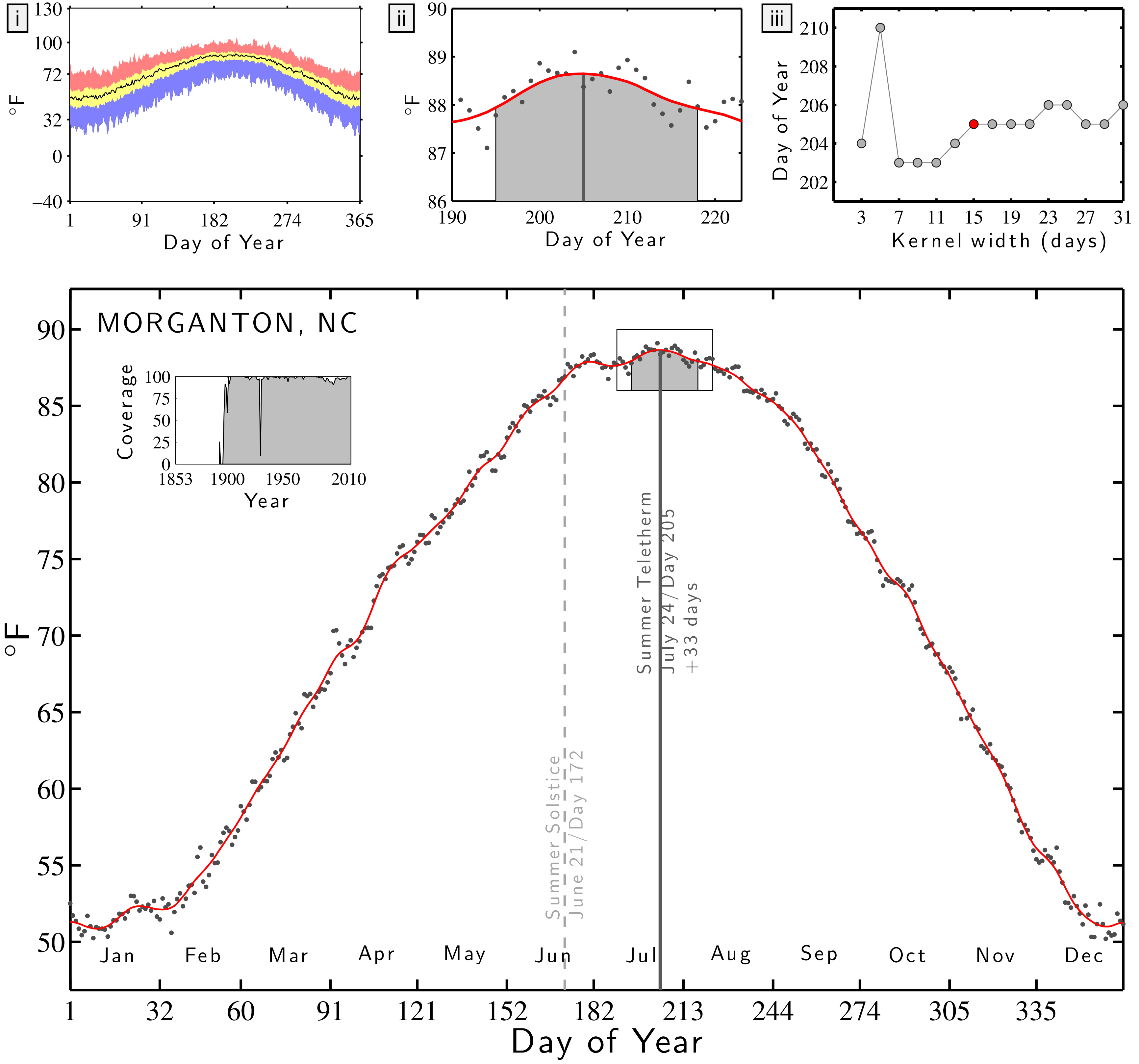

i
°F
Day of Year
ii
°F
Day of Year
iii
Day of Year
Kernel width (days)
MORGANTON, NC
Coverage
Year
Summer Solstice
June 21/Day 172
Summer Teletherm
July 24/Day 205
+33 days
Jan
Feb
Mar
Apr
May
Jun
Jul
Aug
Sep
Oct
Nov
Dec
°F
Day of Year

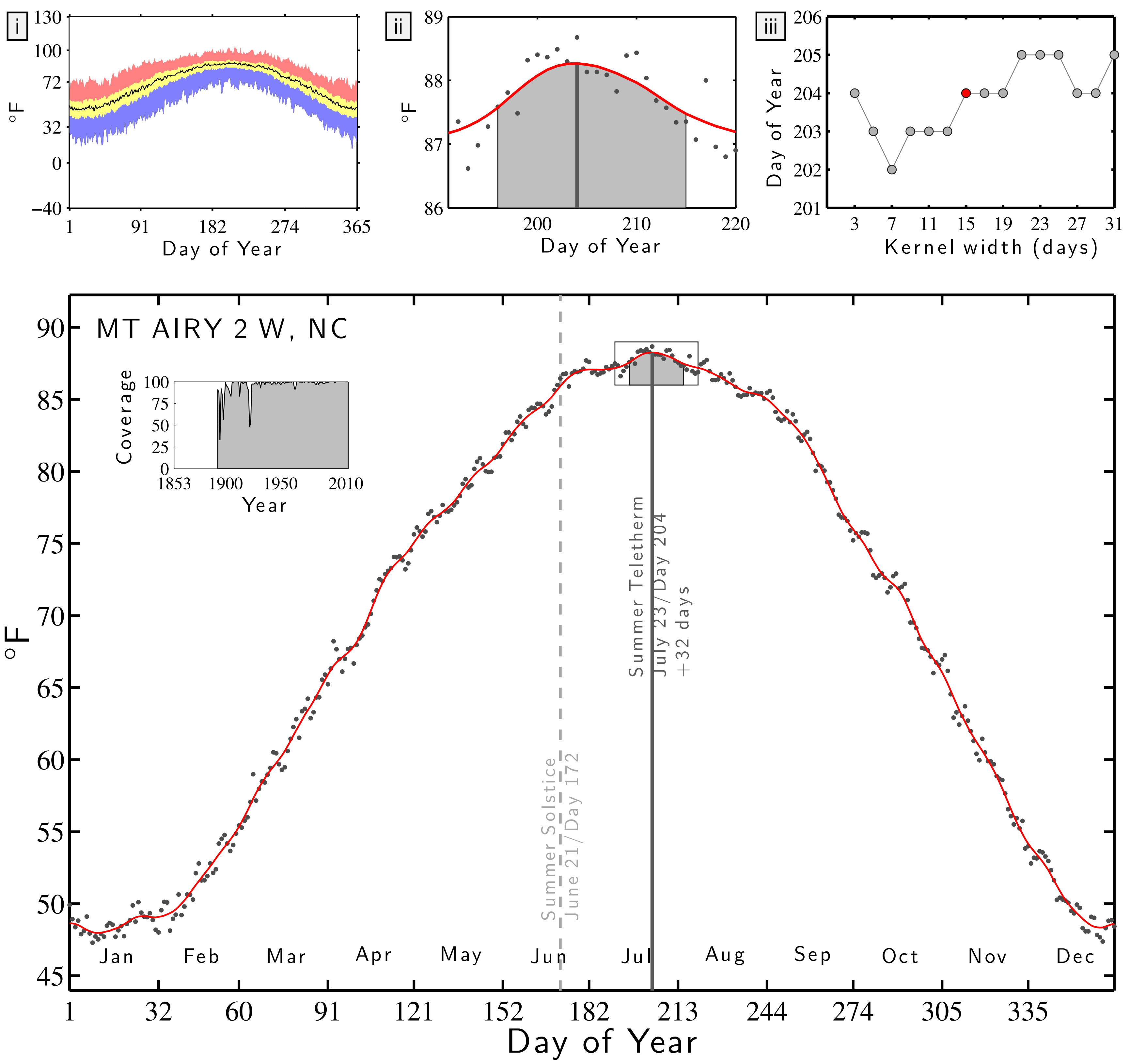

i
ii
iii
Day of Year
Kernel width (days)
MT AIRY 2 W, NC
Coverage
Year
Summer Solstice
June 21 / Day 172
Summer Teletherm
July 23 / Day 204
+32 days
Jan
Feb
Mar
Apr
May
Jun
Jul
Aug
Sep
Oct
Nov
Dec
°F
Day of Year

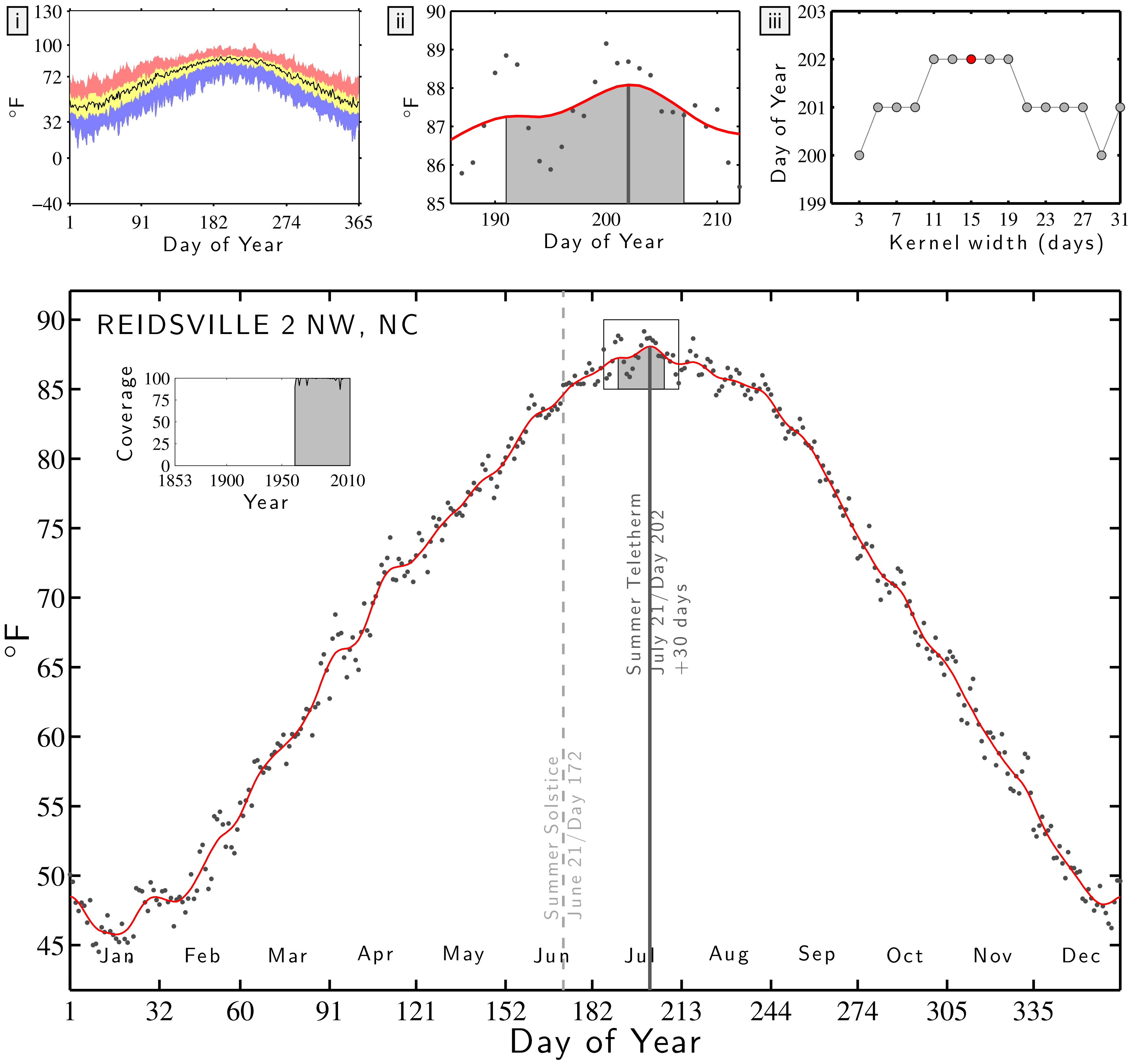

i
°F
Day of Year
ii
°F
Day of Year
iii
Day of Year
Kernel width (days)
REIDSVILLE 2 NW, NC
Coverage
Year
Summer Solstice
June 21 / Day 172
Summer Teletherm
July 21 / Day 202
+30 days
Jan
Feb
Mar
Apr
May
Jun
Jul
Aug
Sep
Oct
Nov
Dec
°F
Day of Year

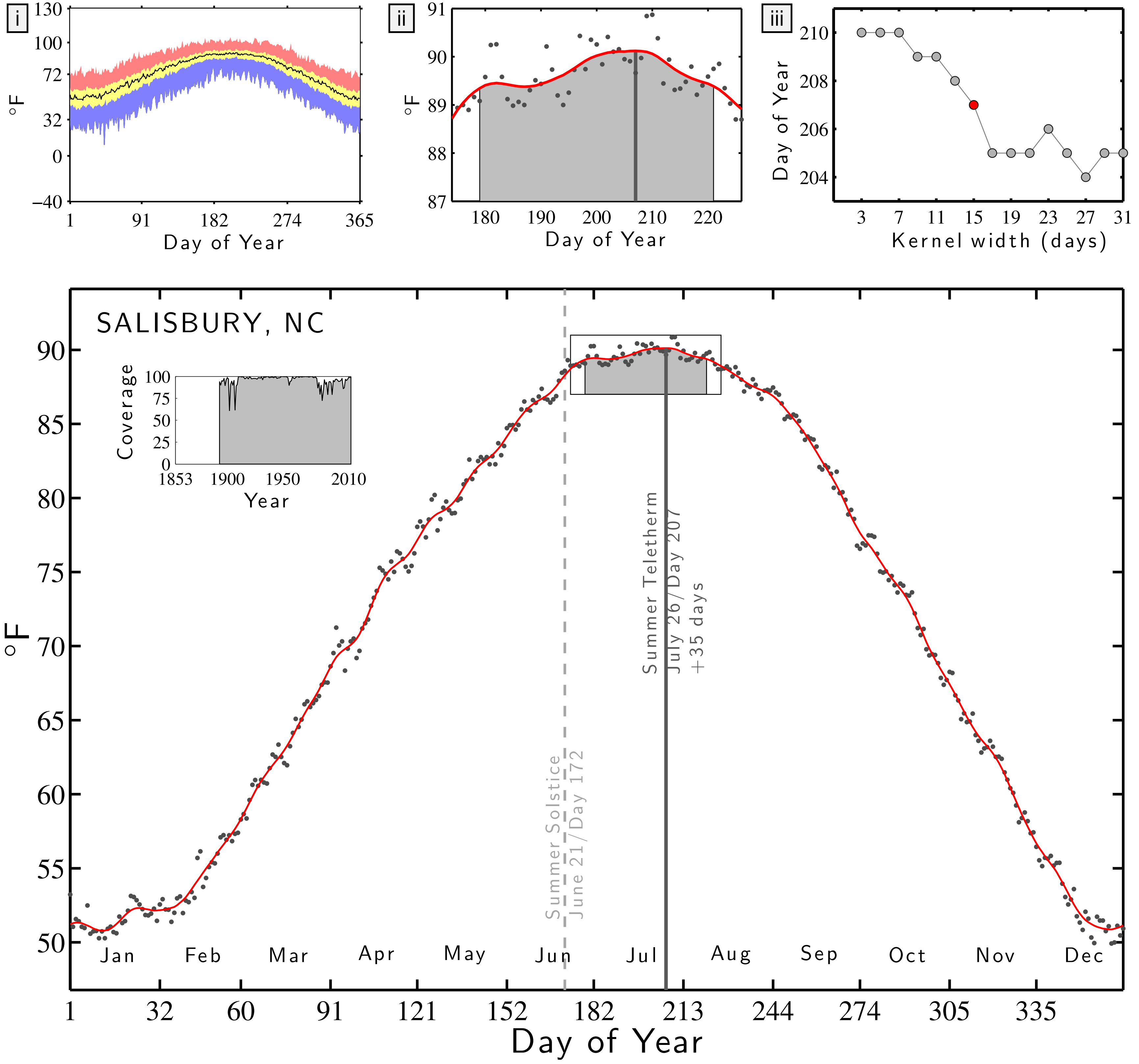

i
°F
Day of Year
ii
°F
Day of Year
iii
Day of Year
Kernel width (days)
SALISBURY, NC
Coverage
Year
Summer Solstice
June 21 / Day 172
Summer Teletherm
July 26 / Day 207
+35 days
Jan
Feb
Mar
Apr
May
Jun
Jul
Aug
Sep
Oct
Nov
Dec
°F
Day of Year

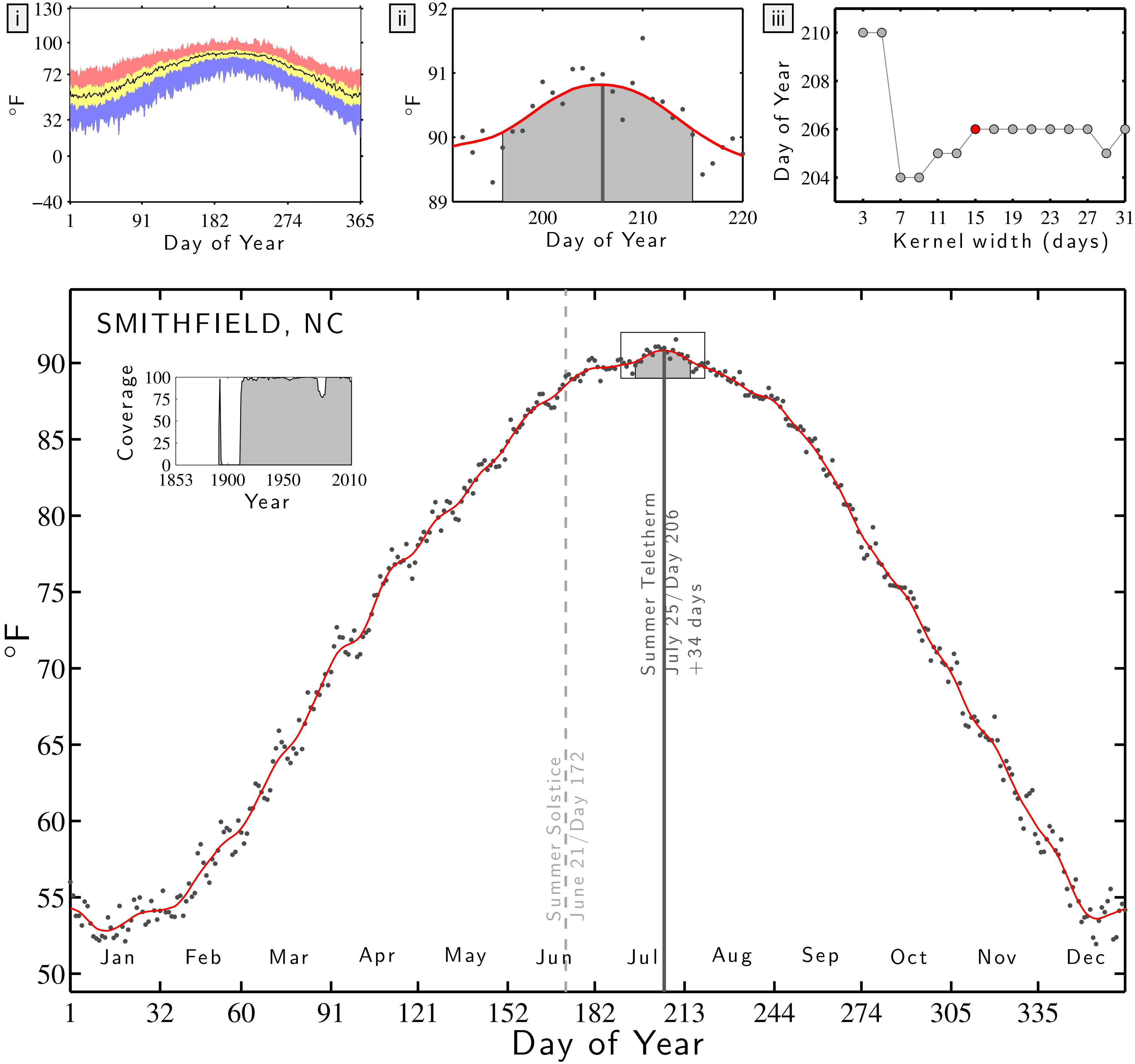
i
°F
130
100
72
32
0
−40
1
91
182
274
365
Day of Year
ii
°F
92
91
90
89
200
210
220
Day of Year
iii
Day of Year
210
208
206
204
3
7
11
15
19
23
27
31
Kernel width (days)
SMITHFIELD, NC
Coverage
100
75
50
25
0
1853
1900
1950
2010
Year
°F
90
85
80
75
70
65
60
55
50
Summer Solstice
June 21/Day 172
Summer Teletherm
July 25/Day 206
+34 days
Jan
Feb
Mar
Apr
May
Jun
Jul
Aug
Sep
Oct
Nov
Dec
1
32
60
91
121
152
182
213
244
274
305
335
Day of Year

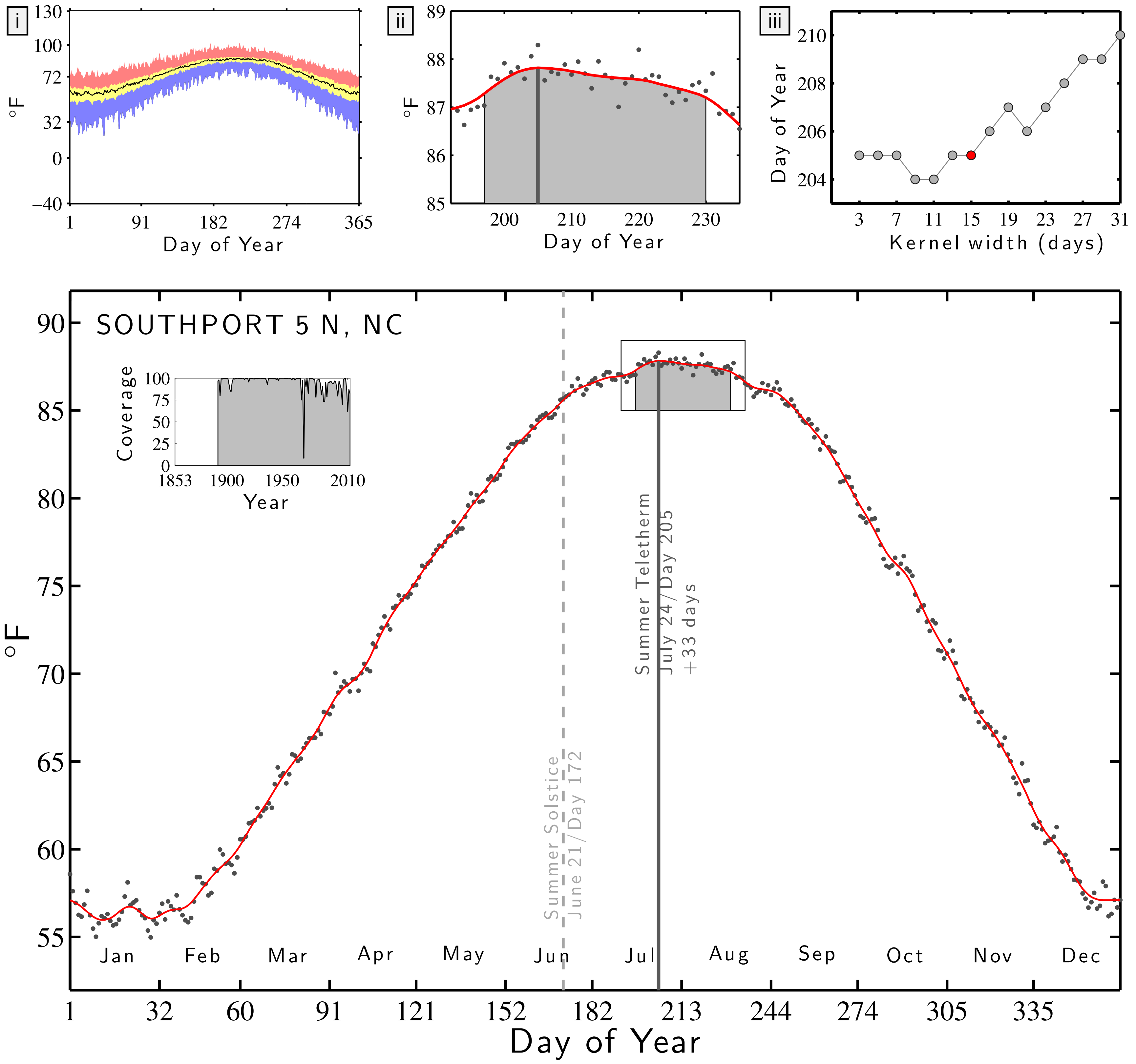

i
ii
iii
°F
Day of Year
Kernel width (days)
SOUTHPORT 5 N, NC
Coverage
Year
1853
1900
1950
2010
Summer Solstice
June 21 / Day 172
Summer Teletherm
July 24 / Day 205
+33 days
Jan
Feb
Mar
Apr
May
Jun
Jul
Aug
Sep
Oct
Nov
Dec

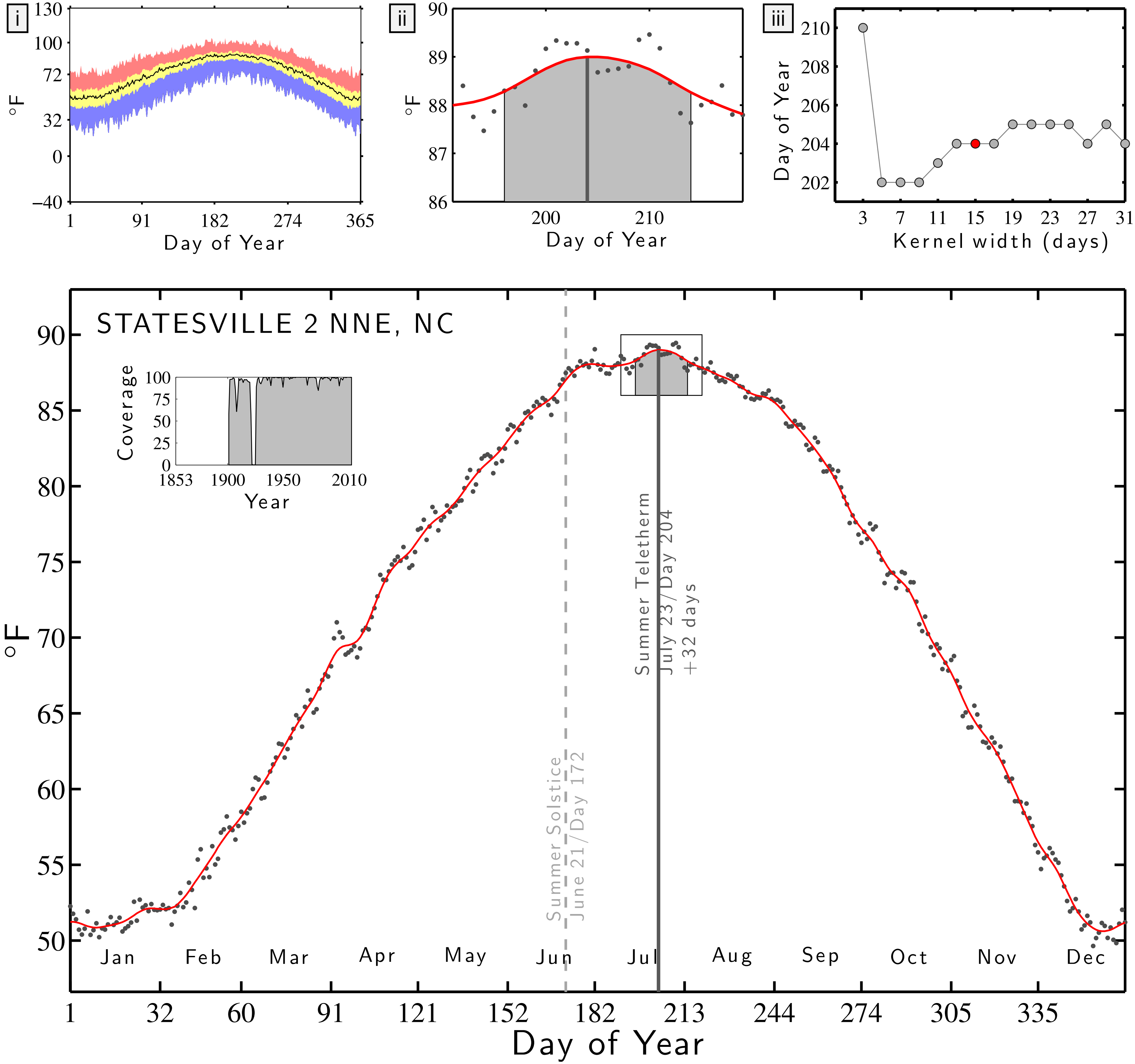

i
°F
Day of Year
ii
°F
Day of Year
iii
Day of Year
Kernel width (days)
STATESVILLE 2 NNE, NC
Coverage
Year
Summer Solstice
June 21 / Day 172
Summer Teletherm
July 23 / Day 204
+32 days
Jan
Feb
Mar
Apr
May
Jun
Jul
Aug
Sep
Oct
Nov
Dec
°F
Day of Year

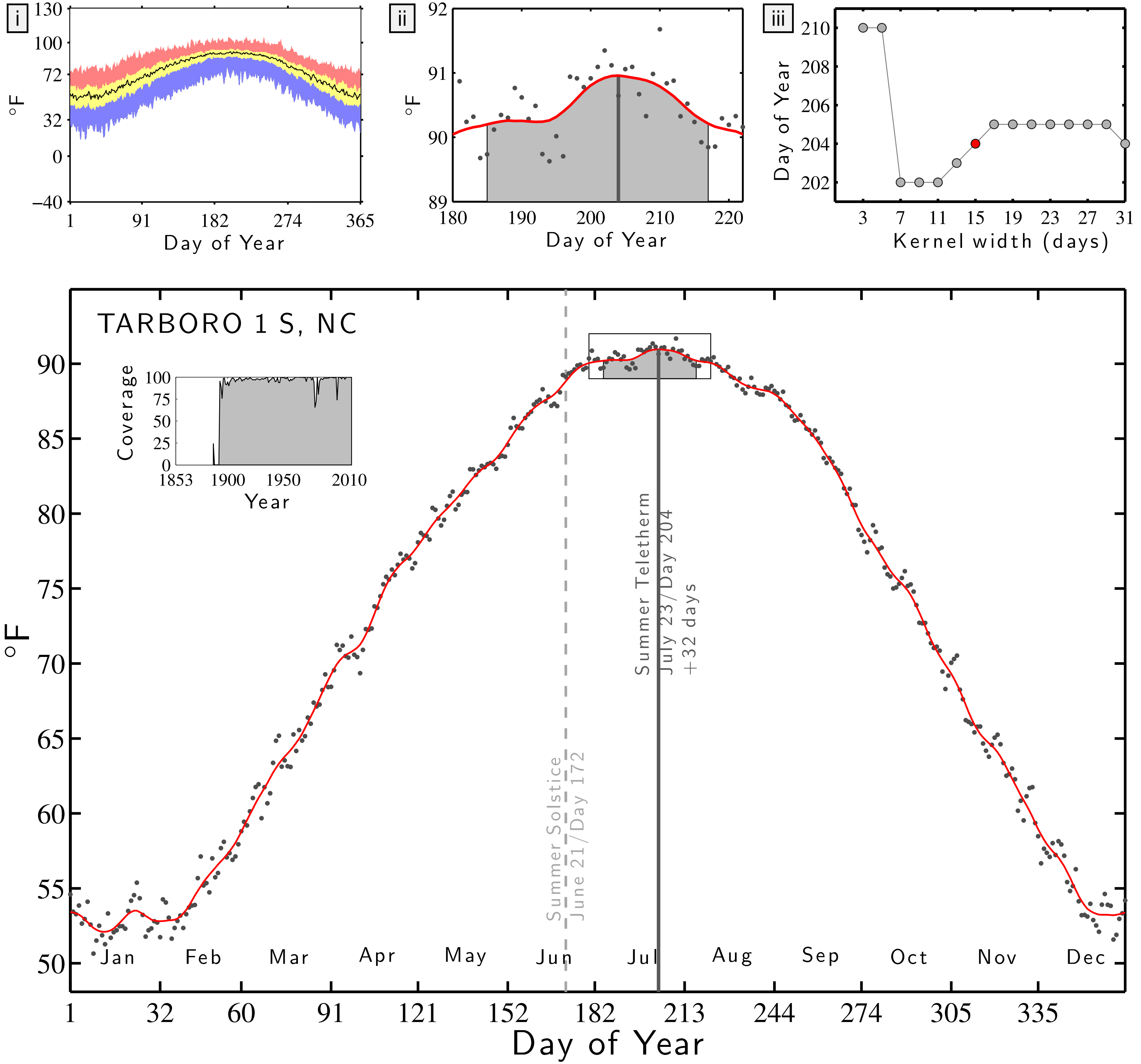
i
ii
iii
°F
Day of Year
Day of Year
Kernel width (days)
TARBORO 1 S, NC
Coverage
Year
Summer Solstice
June 21 / Day 172
Summer Teletherm
July 23 / Day 204
+32 days
Jan
Feb
Mar
Apr
May
Jun
Jul
Aug
Sep
Oct
Nov
Dec

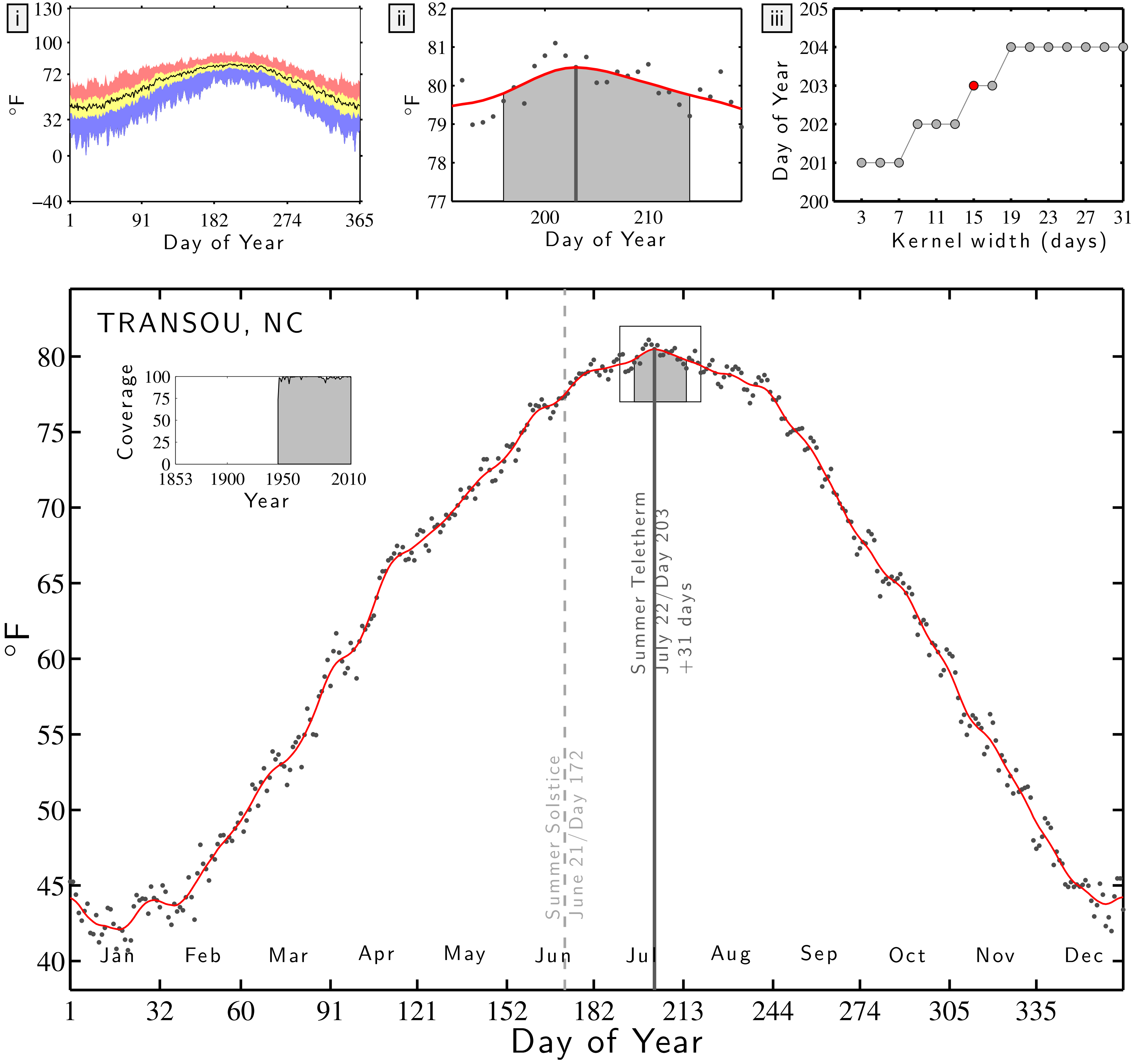

i
°F
Day of Year
ii
°F
Day of Year
iii
Day of Year
Kernel width (days)
TRANSOU, NC
Coverage
Year
Summer Solstice
June 21/Day 172
Summer Teletherm
July 22/Day 203
+31 days
Jan
Feb
Mar
Apr
May
Jun
Jul
Aug
Sep
Oct
Nov
Dec
°F
Day of Year

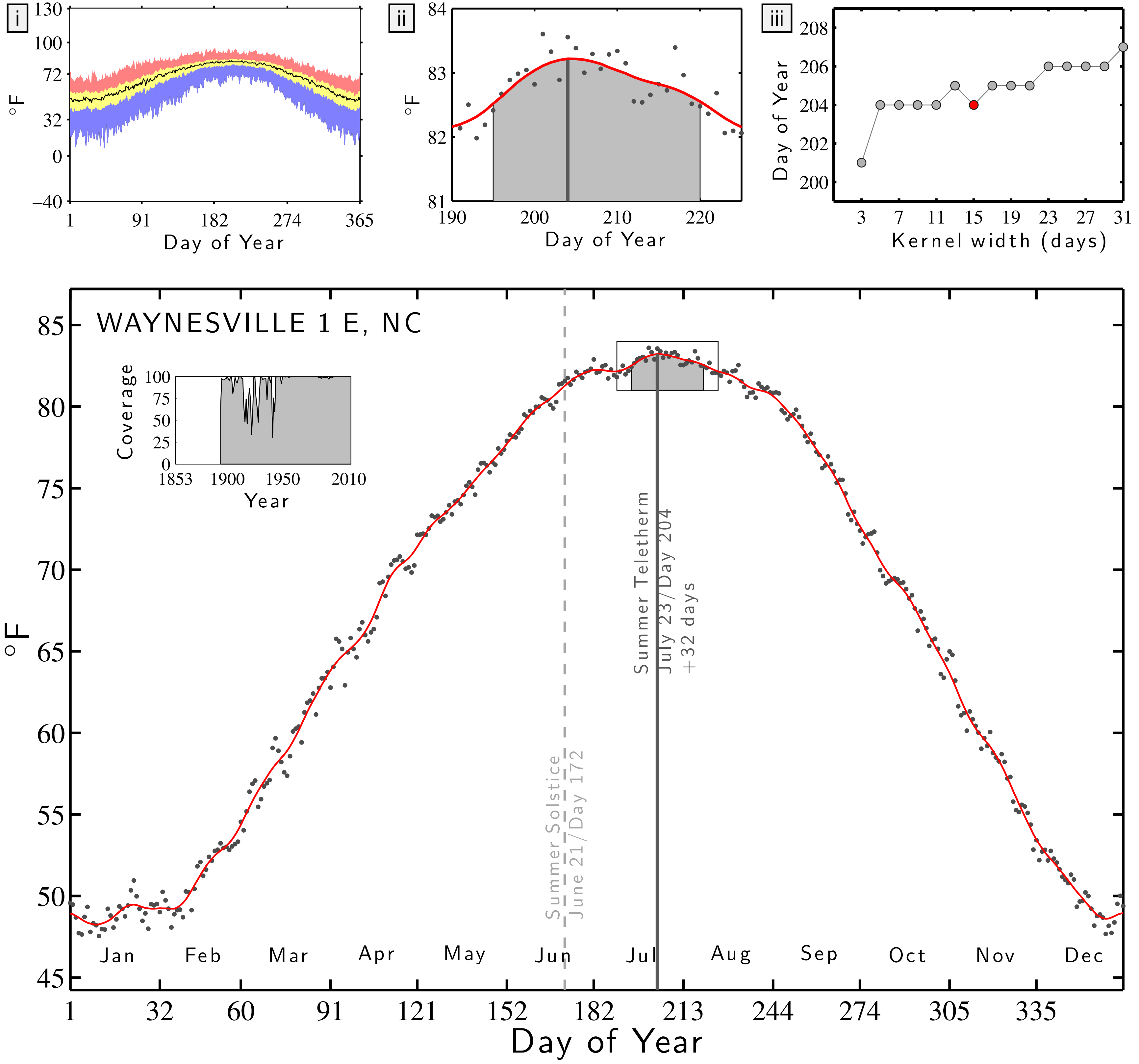

i
°F
Day of Year
ii
°F
Day of Year
iii
Day of Year
Kernel width (days)
WAYNESVILLE 1 E, NC
Coverage
Year
Summer Solstice
June 21 / Day 172
Summer Teletherm
July 23 / Day 204
+32 days
Jan
Feb
Mar
Apr
May
Jun
Jul
Aug
Sep
Oct
Nov
Dec
°F
Day of Year

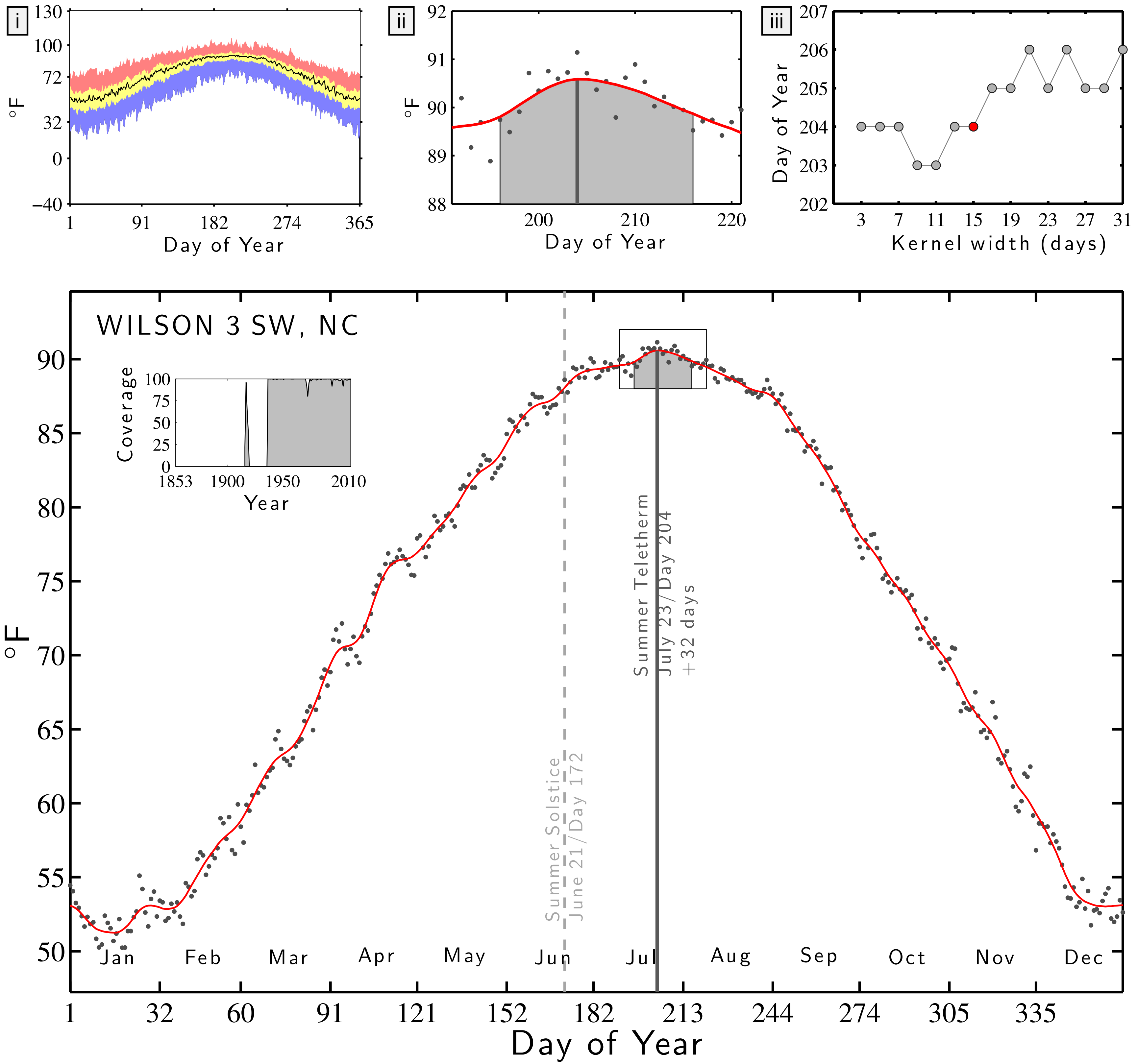

i
ii
iii
°F
Day of Year
Day of Year
Kernel width (days)
WILSON 3 SW, NC
Coverage
Year
Summer Solstice
June 21 / Day 172
Summer Teletherm
July 23 / Day 204
+32 days
Jan
Feb
Mar
Apr
May
Jun
Jul
Aug
Sep
Oct
Nov
Dec
Day of Year

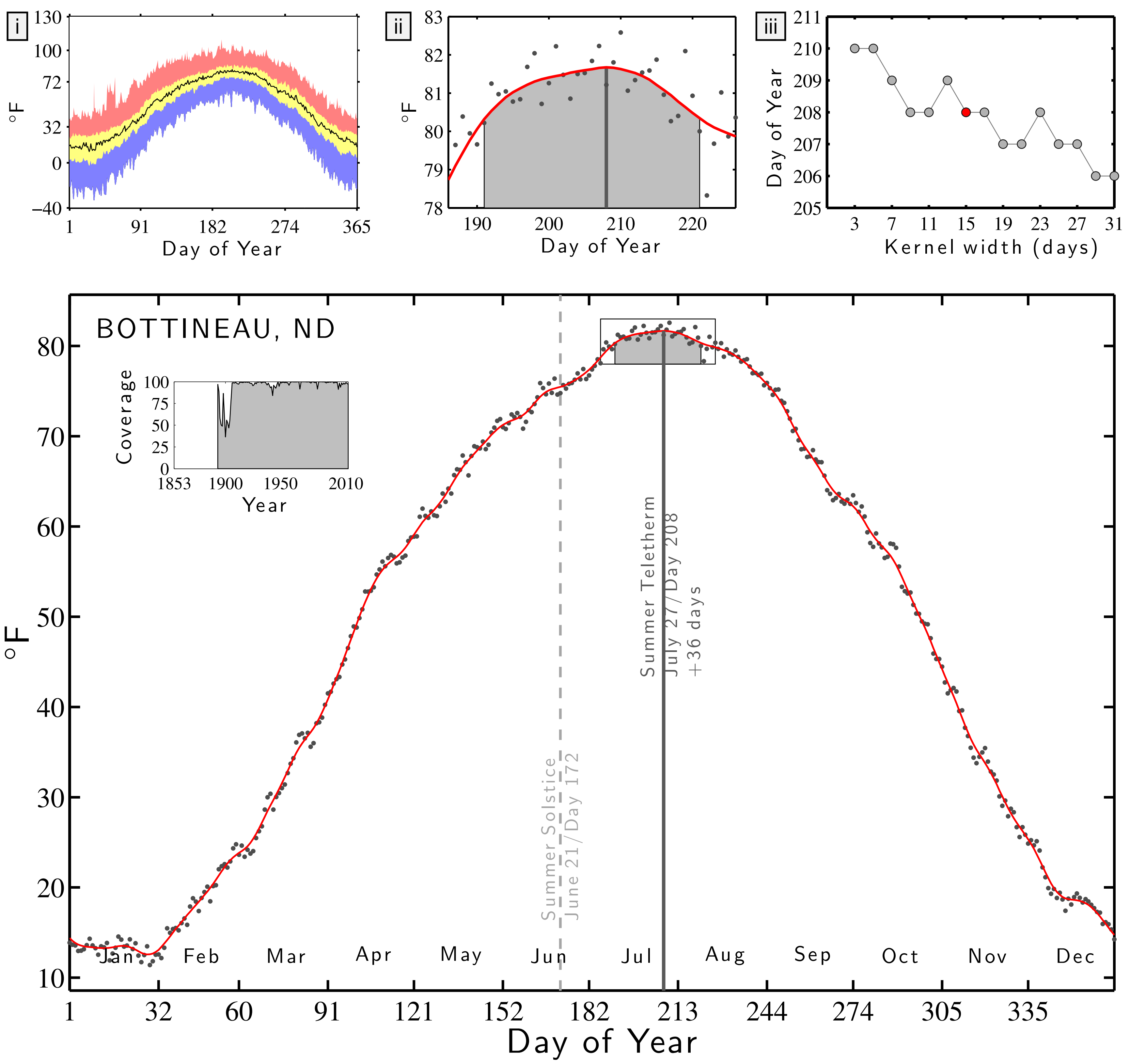

i
°F
Day of Year
ii
°F
Day of Year
iii
Day of Year
Kernel width (days)
BOTTINEAU, ND
Coverage
Year
Summer Solstice
June 21 / Day 172
Summer Teletherm
July 27 / Day 208
+36 days
Jan
Feb
Mar
Apr
May
Jun
Jul
Aug
Sep
Oct
Nov
Dec
°F
Day of Year

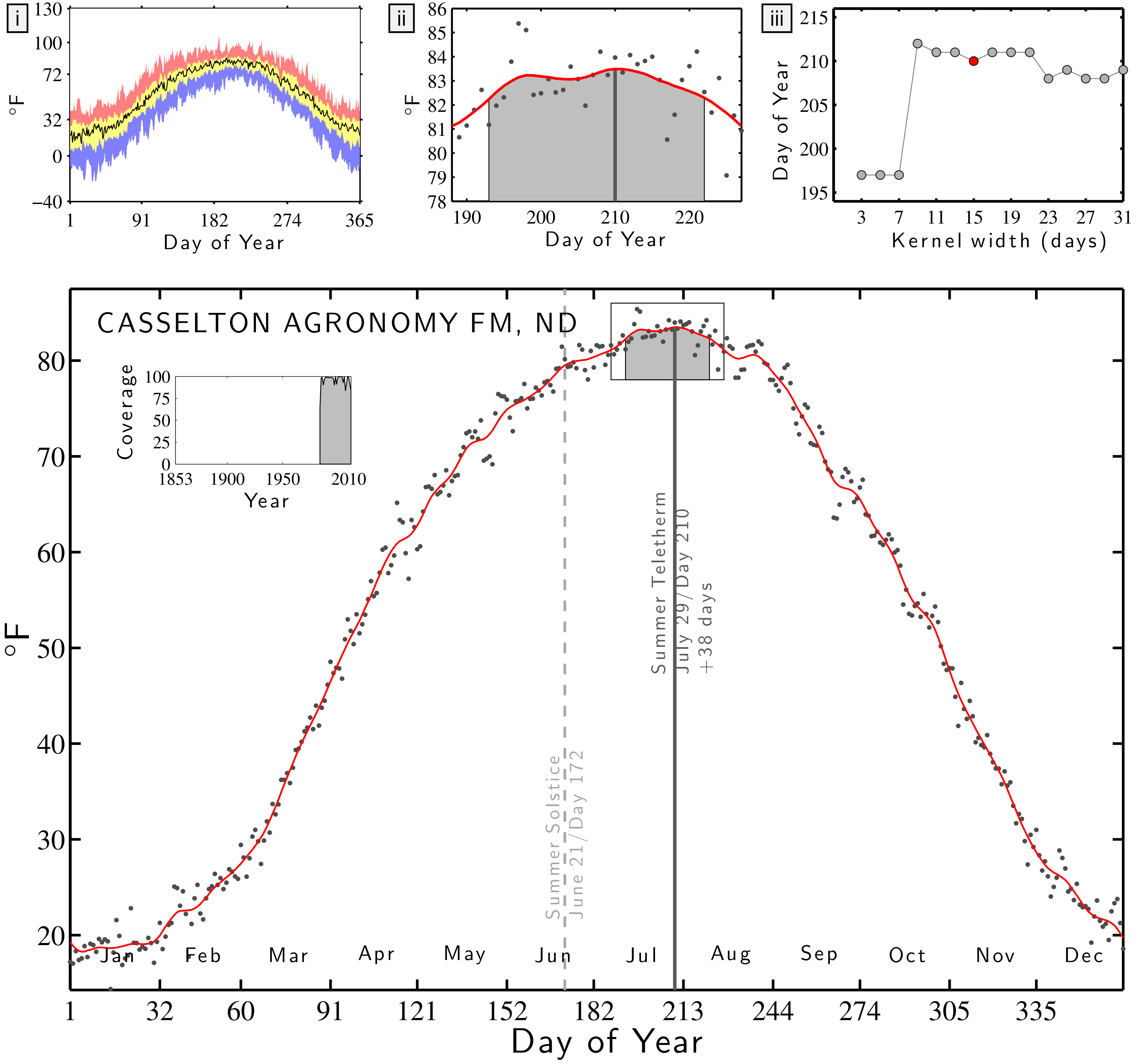

i
°F
Day of Year
ii
°F
Day of Year
iii
Day of Year
Kernel width (days)
CASSELTON AGRONOMY FM, ND
Coverage
Year
Summer Solstice
June 21 / Day 172
Summer Teletherm
July 29 / Day 210
+38 days
Jan
Feb
Mar
Apr
May
Jun
Jul
Aug
Sep
Oct
Nov
Dec
°F
Day of Year

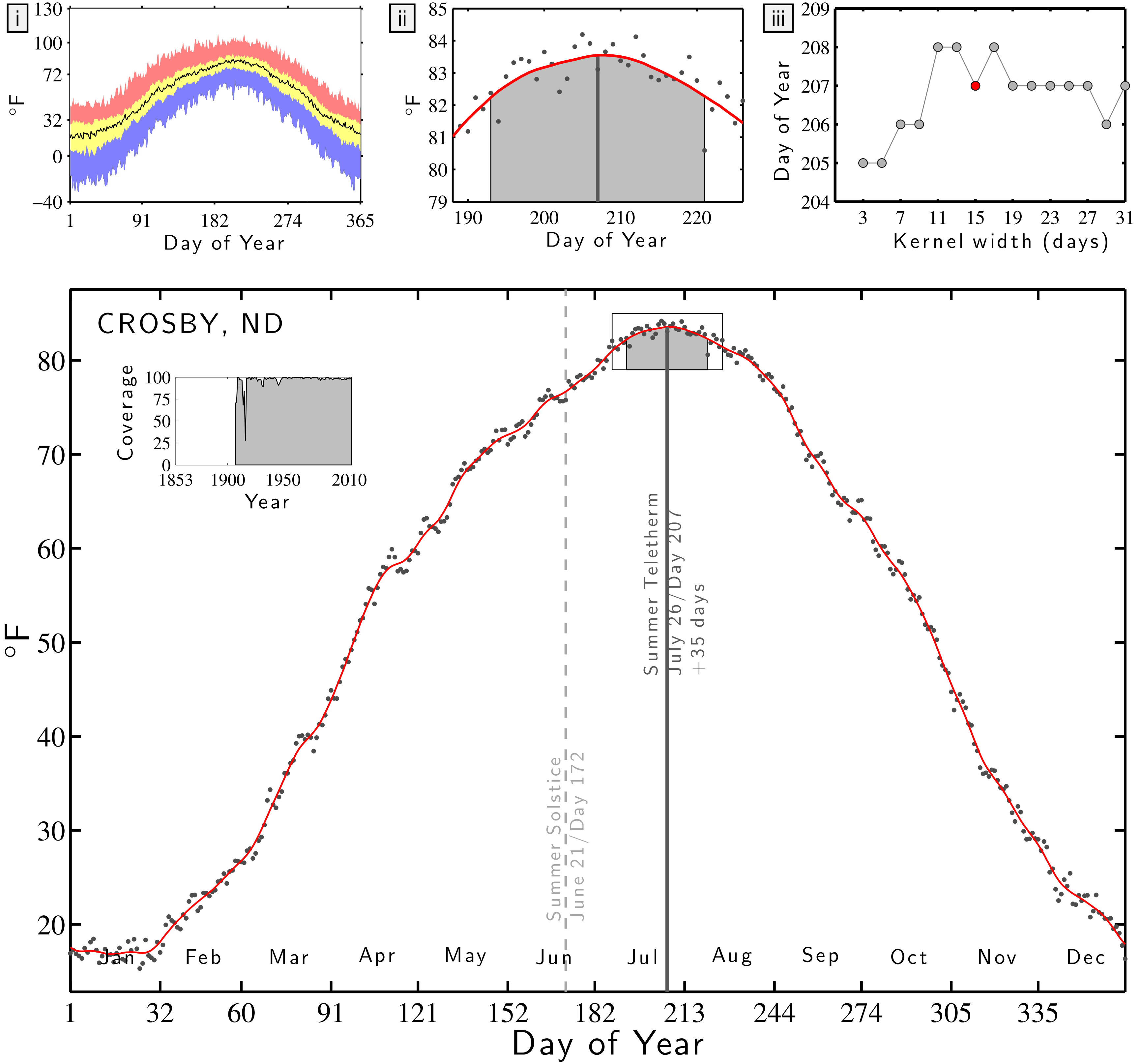

i
ii
iii
°F
Day of Year
Kernel width (days)
CROSBY, ND
Coverage
Year
Summer Solstice
June 21 / Day 172
Summer Teletherm
July 26 / Day 207
+35 days
Jan
Feb
Mar
Apr
May
Jun
Jul
Aug
Sep
Oct
Nov
Dec

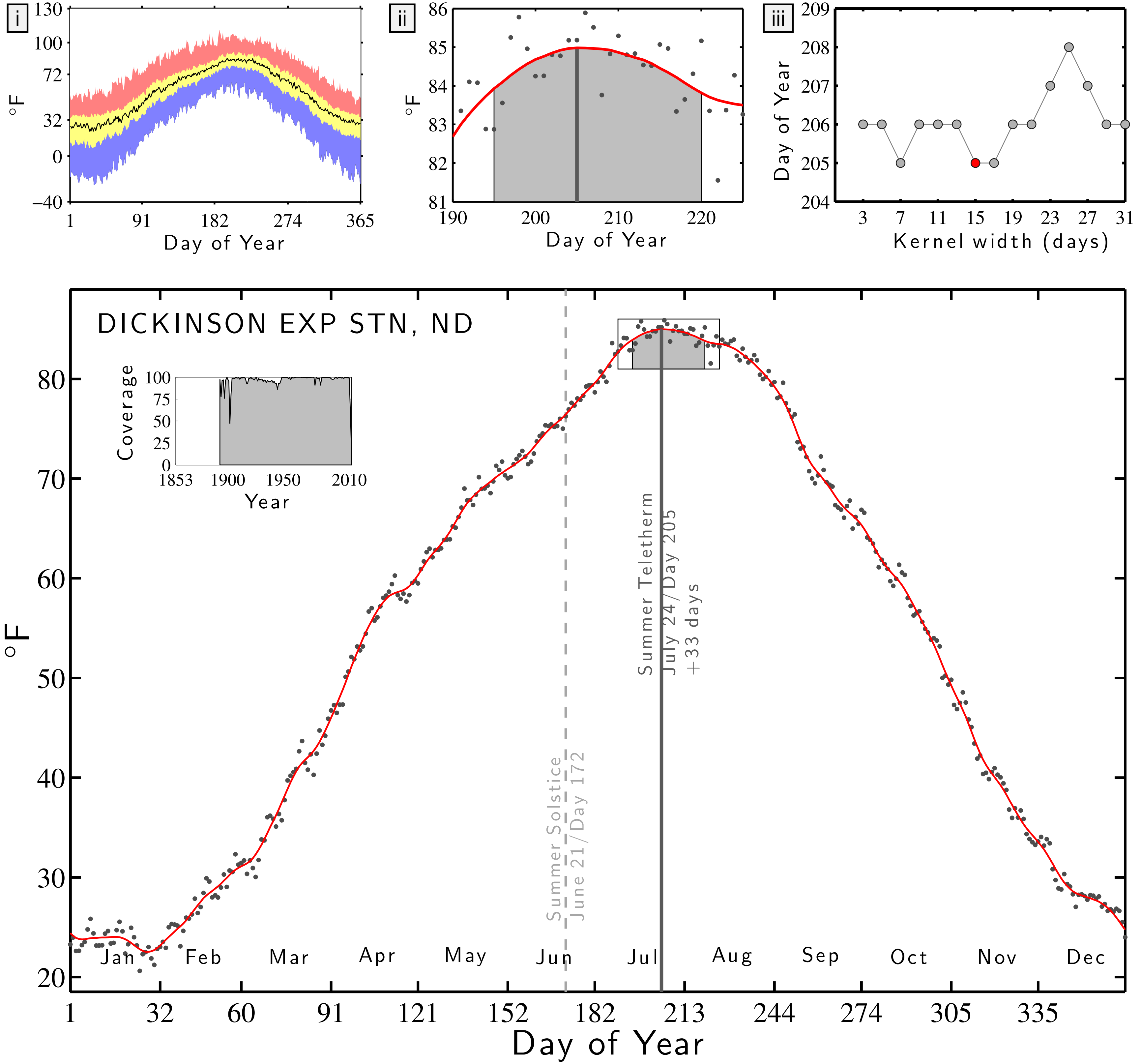

i
°F
Day of Year
ii
°F
Day of Year
iii
Day of Year
Kernel width (days)
DICKINSON EXP STN, ND
Coverage
Year
Summer Solstice
June 21 / Day 172
Summer Teletherm
July 24 / Day 205
+33 days
Jan
Feb
Mar
Apr
May
Jun
Jul
Aug
Sep
Oct
Nov
Dec
°F
Day of Year

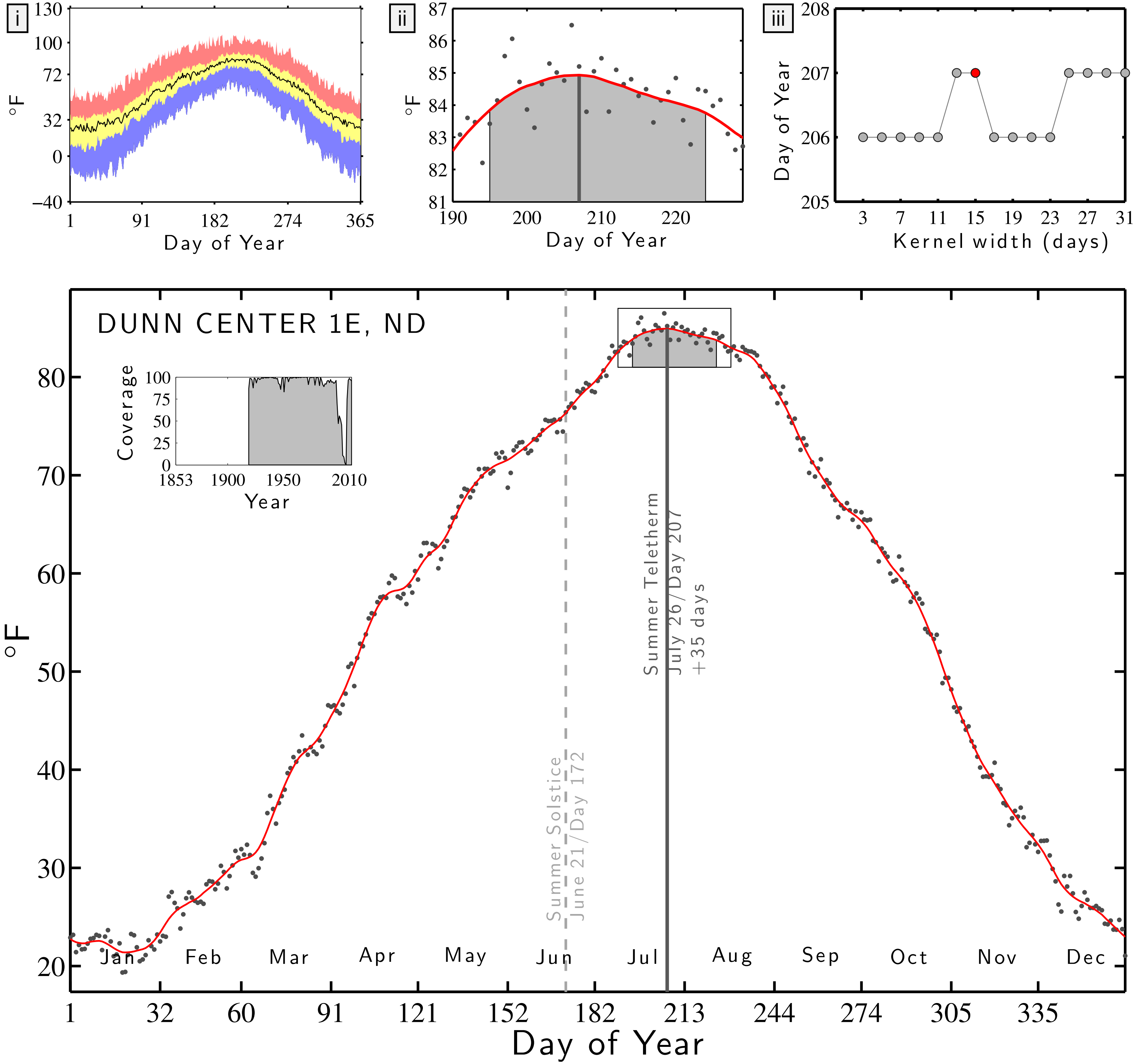

i
ii
iii
°F
Day of Year
Kernel width (days)
DUNN CENTER 1E, ND
Coverage
Year
Summer Solstice
June 21 / Day 172
Summer Teletherm
July 26 / Day 207
+35 days
Jan
Feb
Mar
Apr
May
Jun
Jul
Aug
Sep
Oct
Nov
Dec

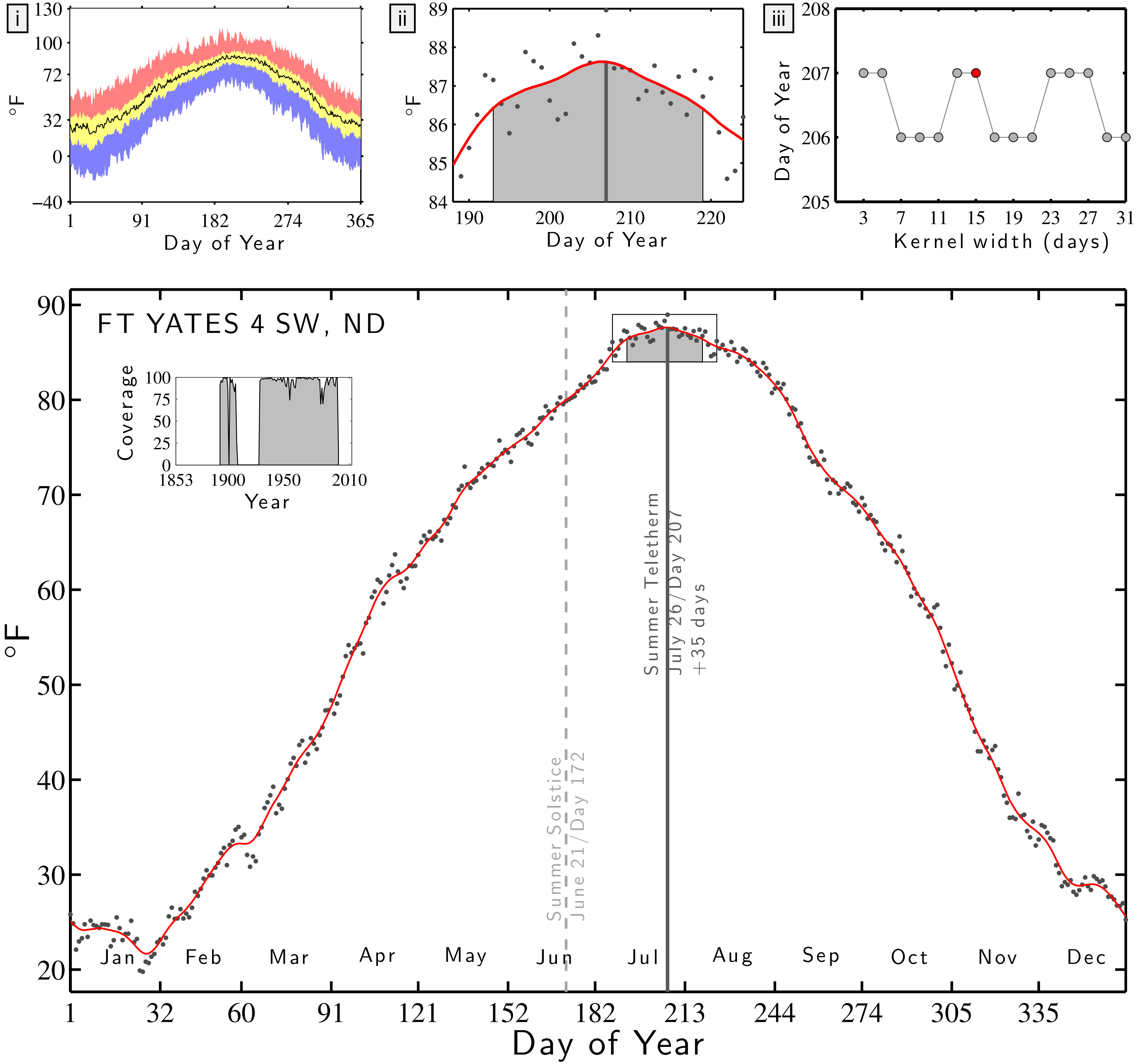

i
°F
Day of Year
ii
°F
Day of Year
iii
Day of Year
Kernel width (days)
FT YATES 4 SW, ND
Coverage
Year
Summer Solstice
June 21 / Day 172
Summer Teletherm
July 26 / Day 207
+35 days
Jan
Feb
Mar
Apr
May
Jun
Jul
Aug
Sep
Oct
Nov
Dec
°F
Day of Year

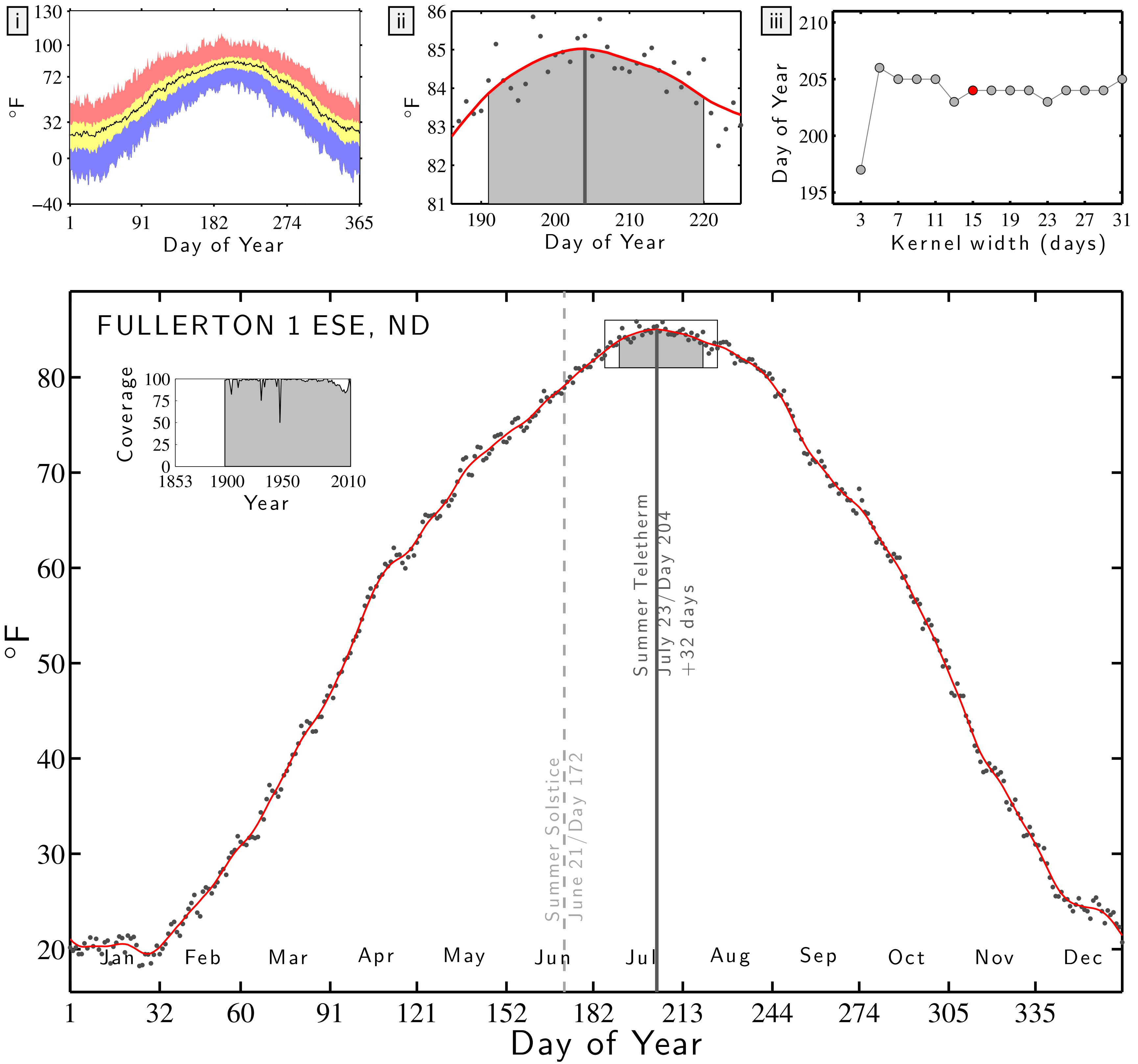

i
ii
iii
Day of Year
Kernel width (days)
FULLERTON 1 ESE, ND
Coverage
Year
Summer Solstice
June 21 / Day 172
Summer Teletherm
July 23 / Day 204
+32 days
Jan
Feb
Mar
Apr
May
Jun
Jul
Aug
Sep
Oct
Nov
Dec
°F
Day of Year

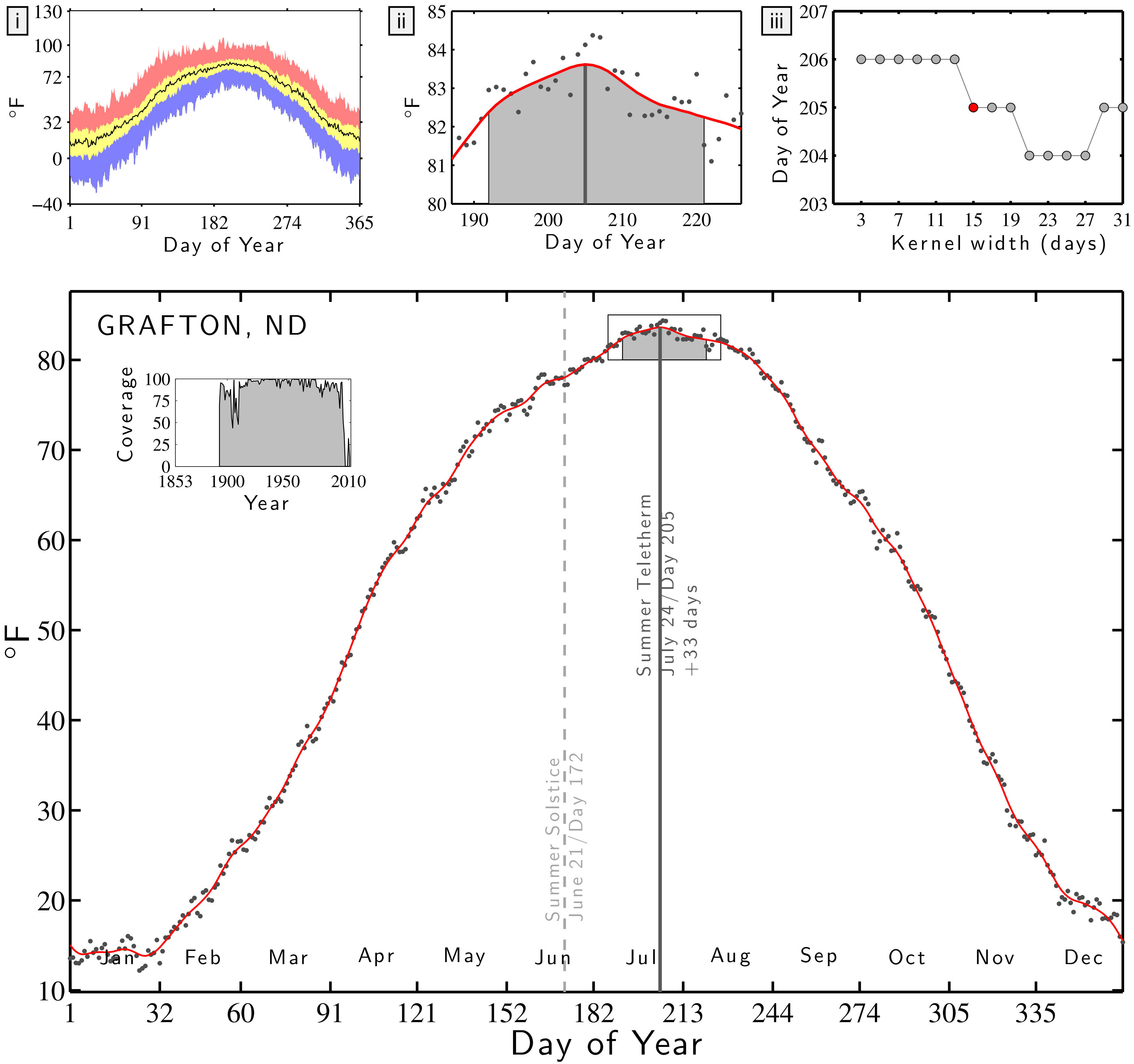

i
°F
Day of Year
ii
°F
Day of Year
iii
Day of Year
Kernel width (days)
GRAFTON, ND
Coverage
Year
Summer Solstice
June 21 / Day 172
Summer Teletherm
July 24 / Day 205
+33 days
Jan
Feb
Mar
Apr
May
Jun
Jul
Aug
Sep
Oct
Nov
Dec
°F
Day of Year

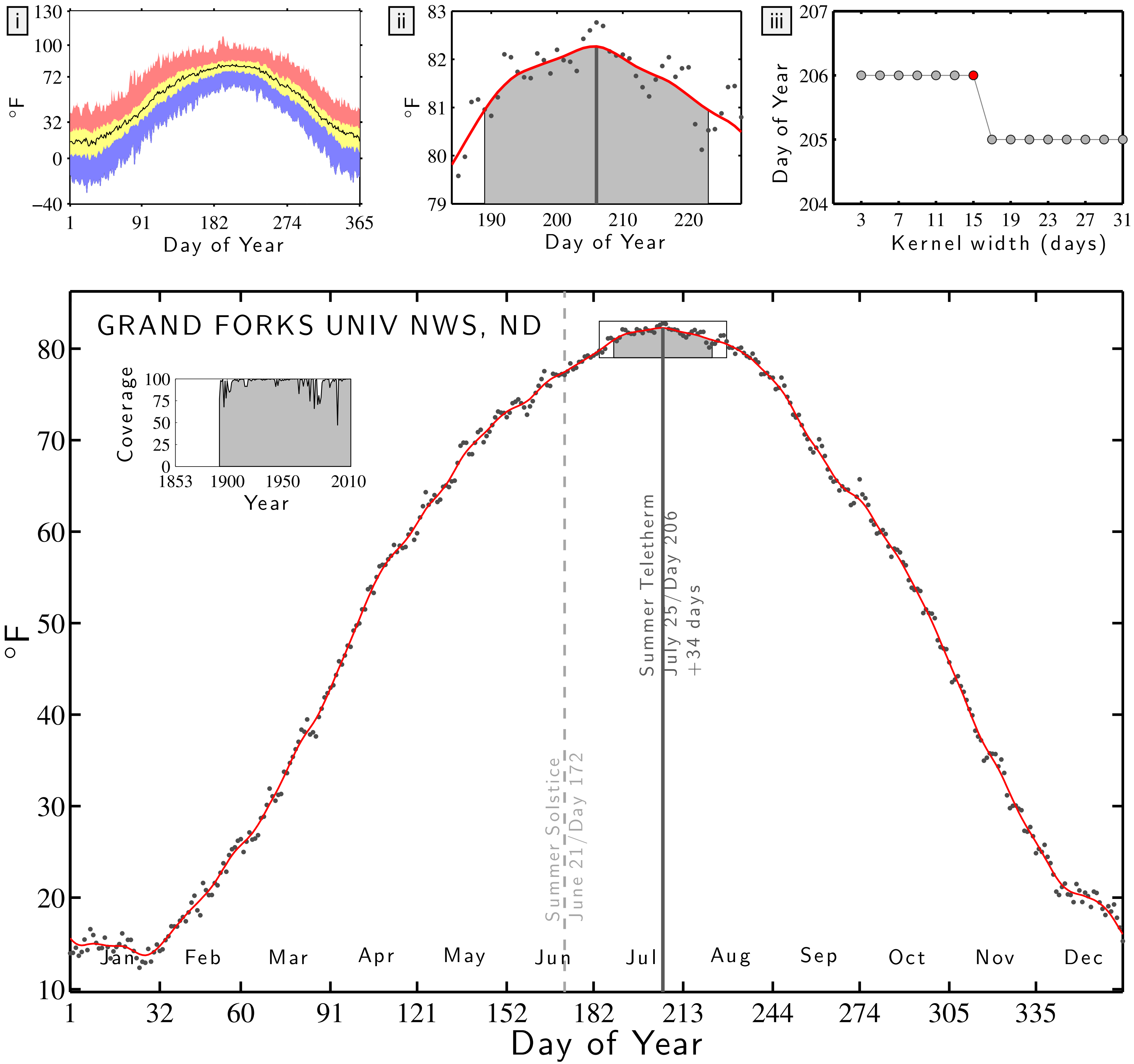

i
ii
iii
°F
Day of Year
Day of Year
Kernel width (days)
GRAND FORKS UNIV NWS, ND
Coverage
Year
1853
1900
1950
2010
Summer Solstice
June 21/Day 172
Summer Teletherm
July 25/Day 206
+34 days
Jan
Feb
Mar
Apr
May
Jun
Jul
Aug
Sep
Oct
Nov
Dec

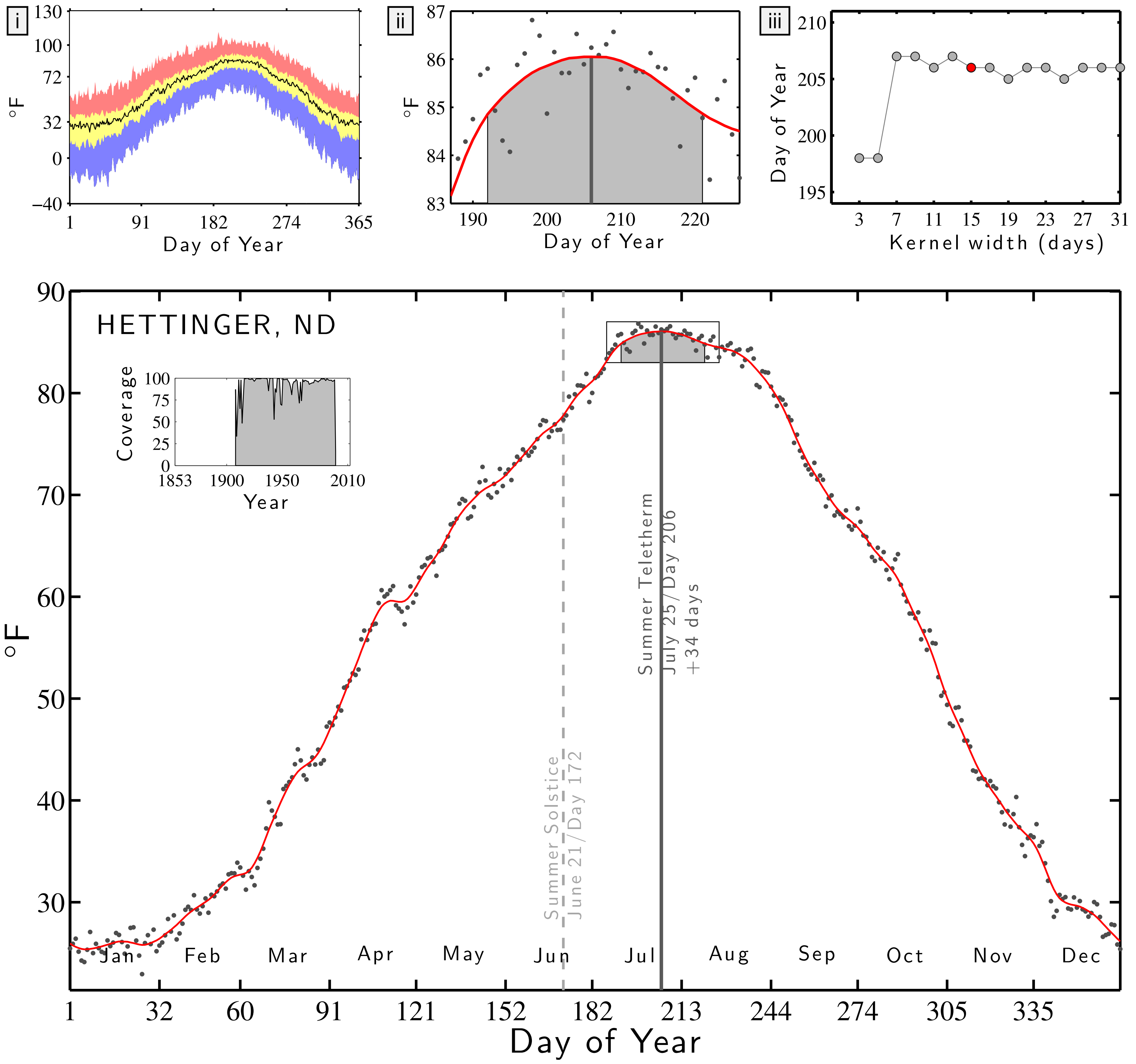

i
°F
130
100
72
32
0
−40
1
91
182
274
365
Day of Year
ii
°F
87
86
85
84
83
190
200
210
220
Day of Year
iii
Day of Year
210
205
200
195
3
7
11
15
19
23
27
31
Kernel width (days)
HETTINGER, ND
Coverage
100
75
50
25
0
1853
1900
1950
2010
Year
Summer Solstice
June 21/Day 172
Summer Teletherm
July 25/Day 206
+34 days
°F
90
80
70
60
50
40
30
Jan
Feb
Mar
Apr
May
Jun
Jul
Aug
Sep
Oct
Nov
Dec
1
32
60
91
121
152
182
213
244
274
305
335
Day of Year

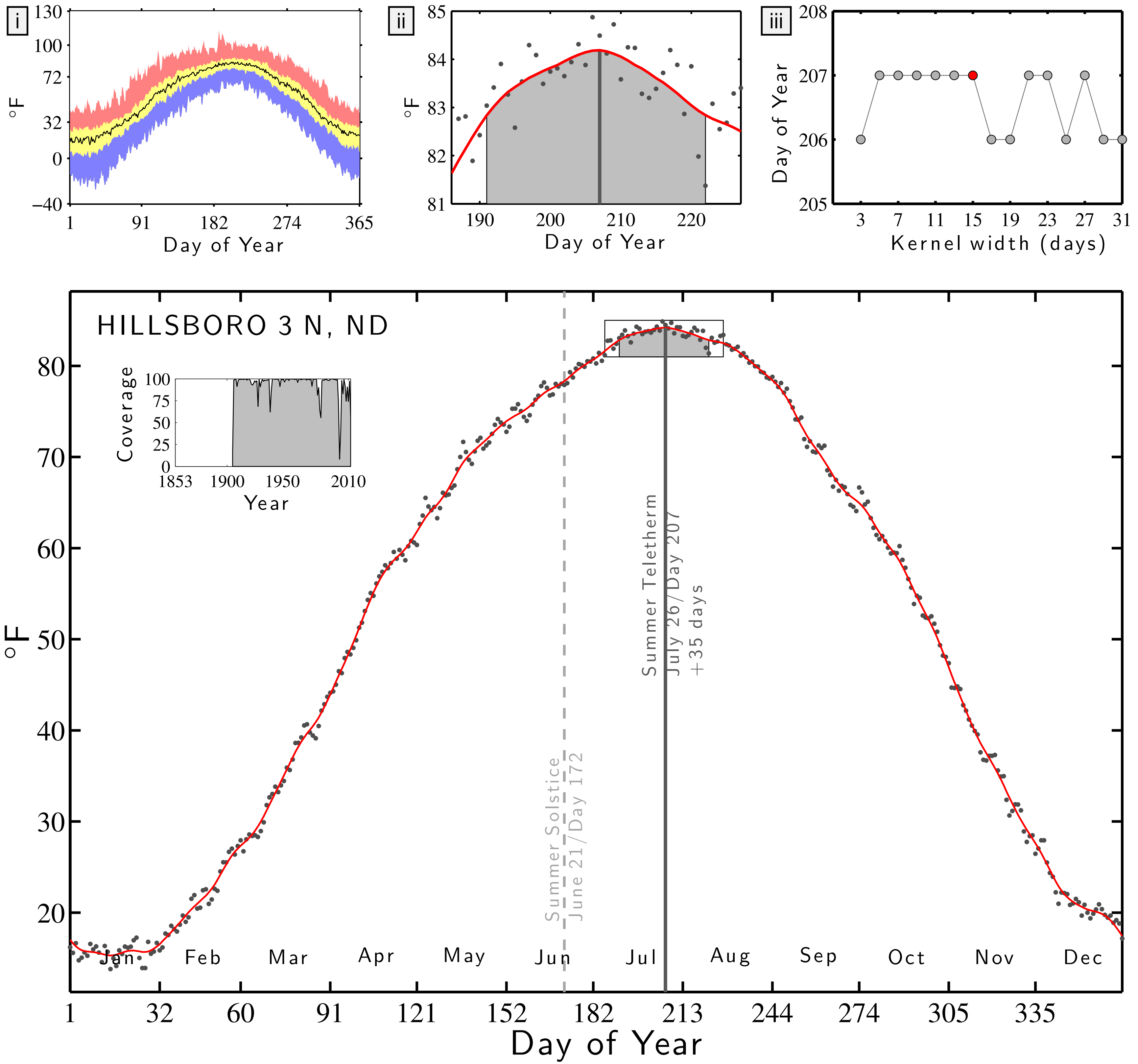

i
°F
Day of Year
ii
°F
Day of Year
iii
Day of Year
Kernel width (days)
HILLSBORO 3 N, ND
Coverage
Year
Summer Solstice
June 21 / Day 172
Summer Teletherm
July 26 / Day 207
+35 days
Jan
Feb
Mar
Apr
May
Jun
Jul
Aug
Sep
Oct
Nov
Dec
°F
Day of Year

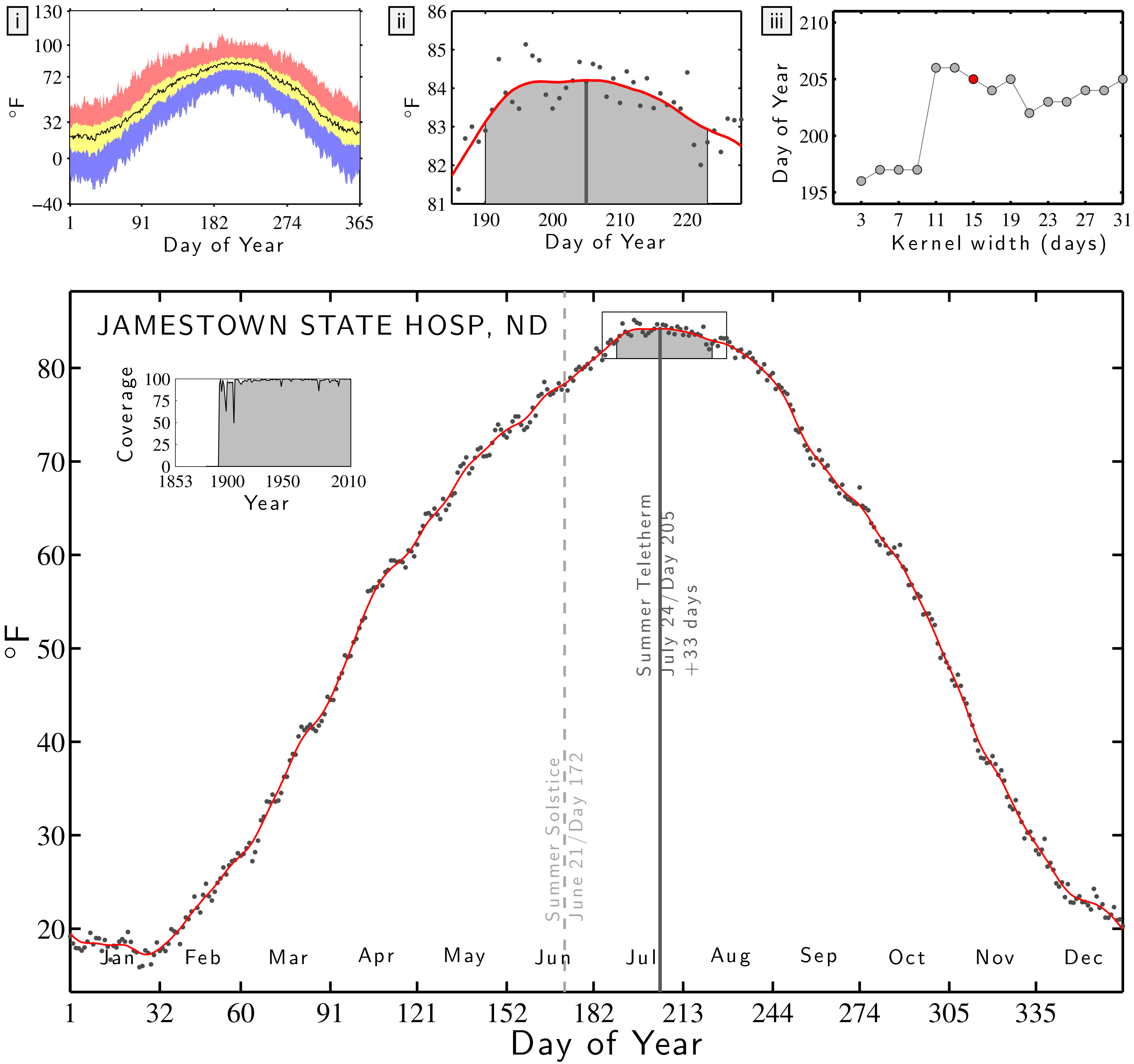

i
ii
iii
°F
Day of Year
Day of Year
Kernel width (days)
JAMESTOWN STATE HOSP, ND
Coverage
Year
Summer Solstice
June 21 / Day 172
Summer Teletherm
July 24 / Day 205
+33 days
Jan
Feb
Mar
Apr
May
Jun
Jul
Aug
Sep
Oct
Nov
Dec
°F
Day of Year

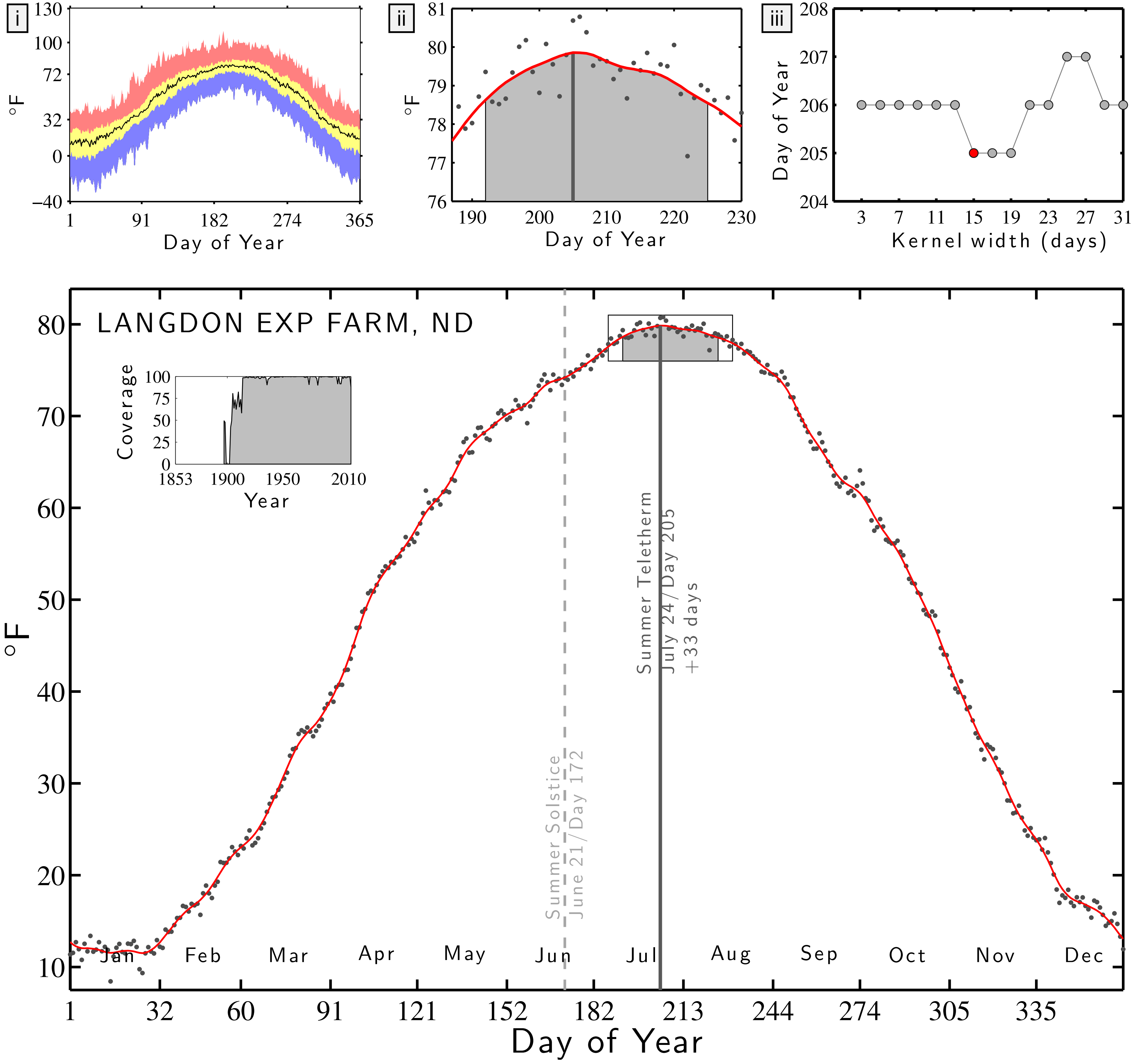

i
°F
Day of Year
ii
°F
Day of Year
iii
Day of Year
Kernel width (days)
LANGDON EXP FARM, ND
Coverage
Year
Summer Solstice
June 21 / Day 172
Summer Teletherm
July 24 / Day 205
+33 days
Jan
Feb
Mar
Apr
May
Jun
Jul
Aug
Sep
Oct
Nov
Dec
°F
Day of Year

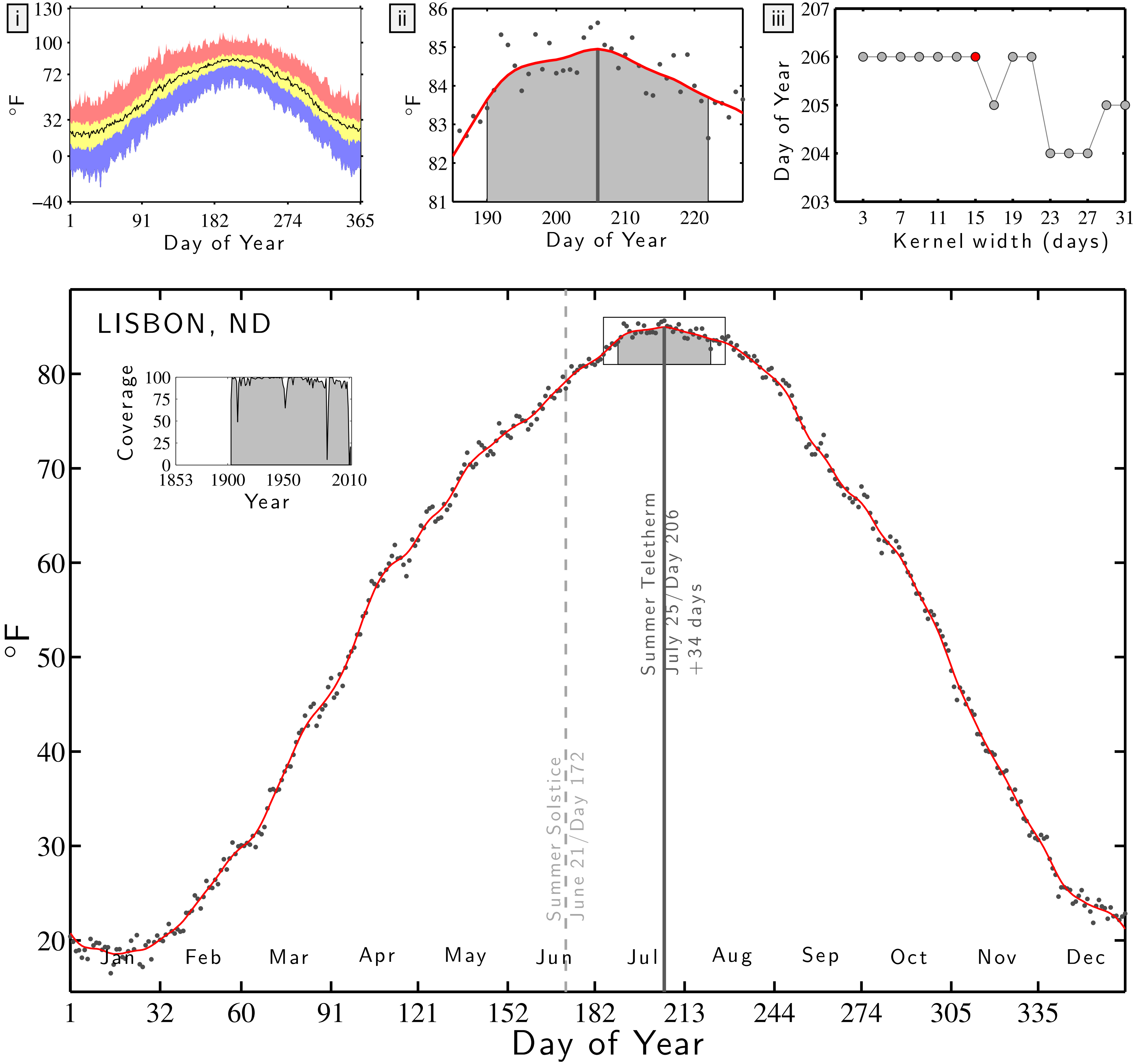

i
°F
Day of Year
ii
Day of Year
iii
Day of Year
Kernel width (days)
LISBON, ND
Coverage
Year
Summer Solstice
June 21/Day 172
Summer Teletherm
July 25/Day 206
+34 days
Jan
Feb
Mar
Apr
May
Jun
Jul
Aug
Sep
Oct
Nov
Dec
Day of Year

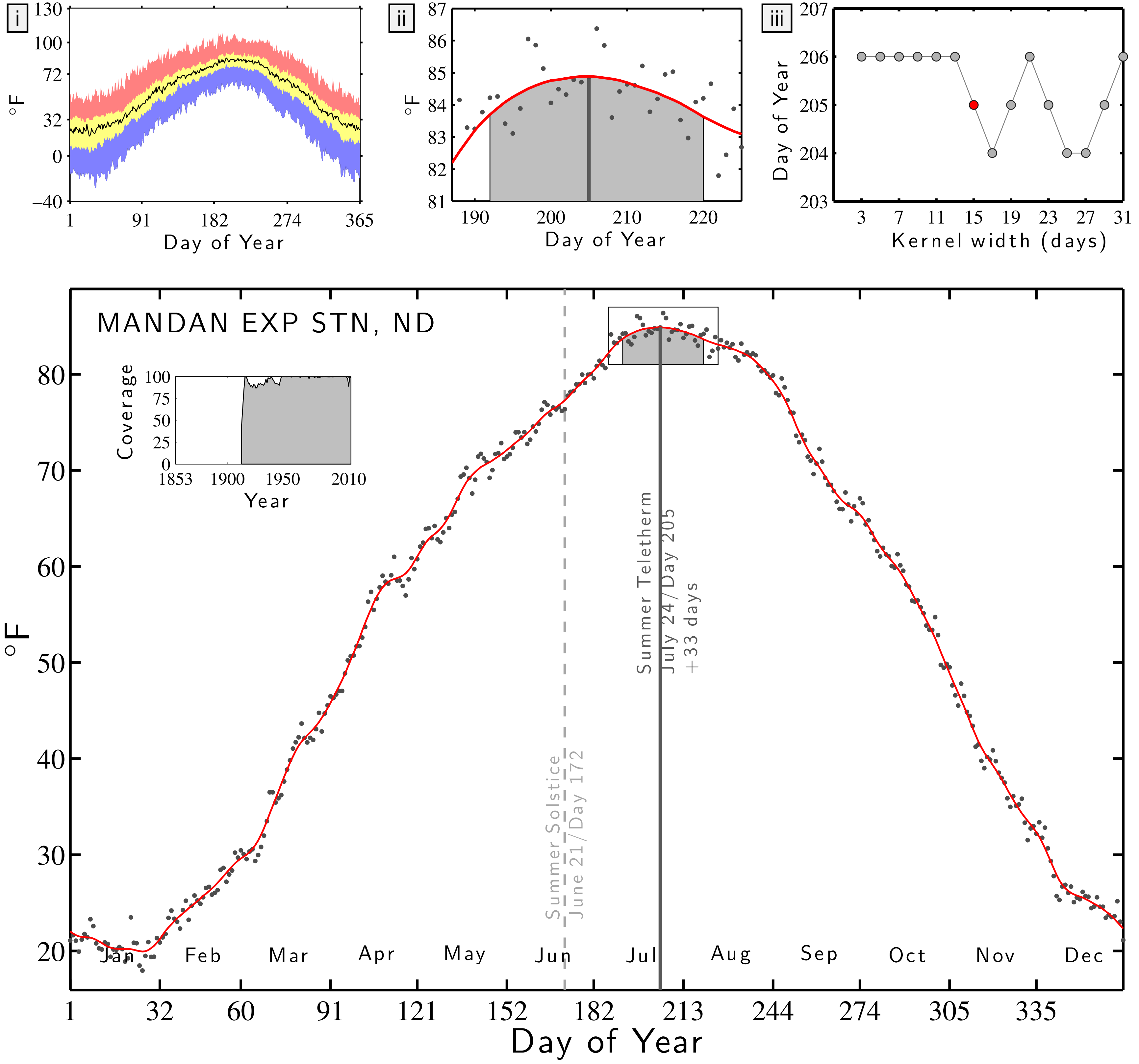

i
130
100
72
32
0
−40
°F
1
91
182
274
365
Day of Year
ii
87
86
85
84
83
82
81
°F
190
200
210
220
Day of Year
iii
207
206
205
204
203
Day of Year
3
7
11
15
19
23
27
31
Kernel width (days)
MANDAN EXP STN, ND
Coverage
100
75
50
25
0
1853
1900
1950
2010
Year
Summer Solstice
June 21 / Day 172
Summer Teletherm
July 24 / Day 205
+33 days
80
70
60
50
40
30
20
°F
Jan
Feb
Mar
Apr
May
Jun
Jul
Aug
Sep
Oct
Nov
Dec
1
32
60
91
121
152
182
213
244
274
305
335
Day of Year

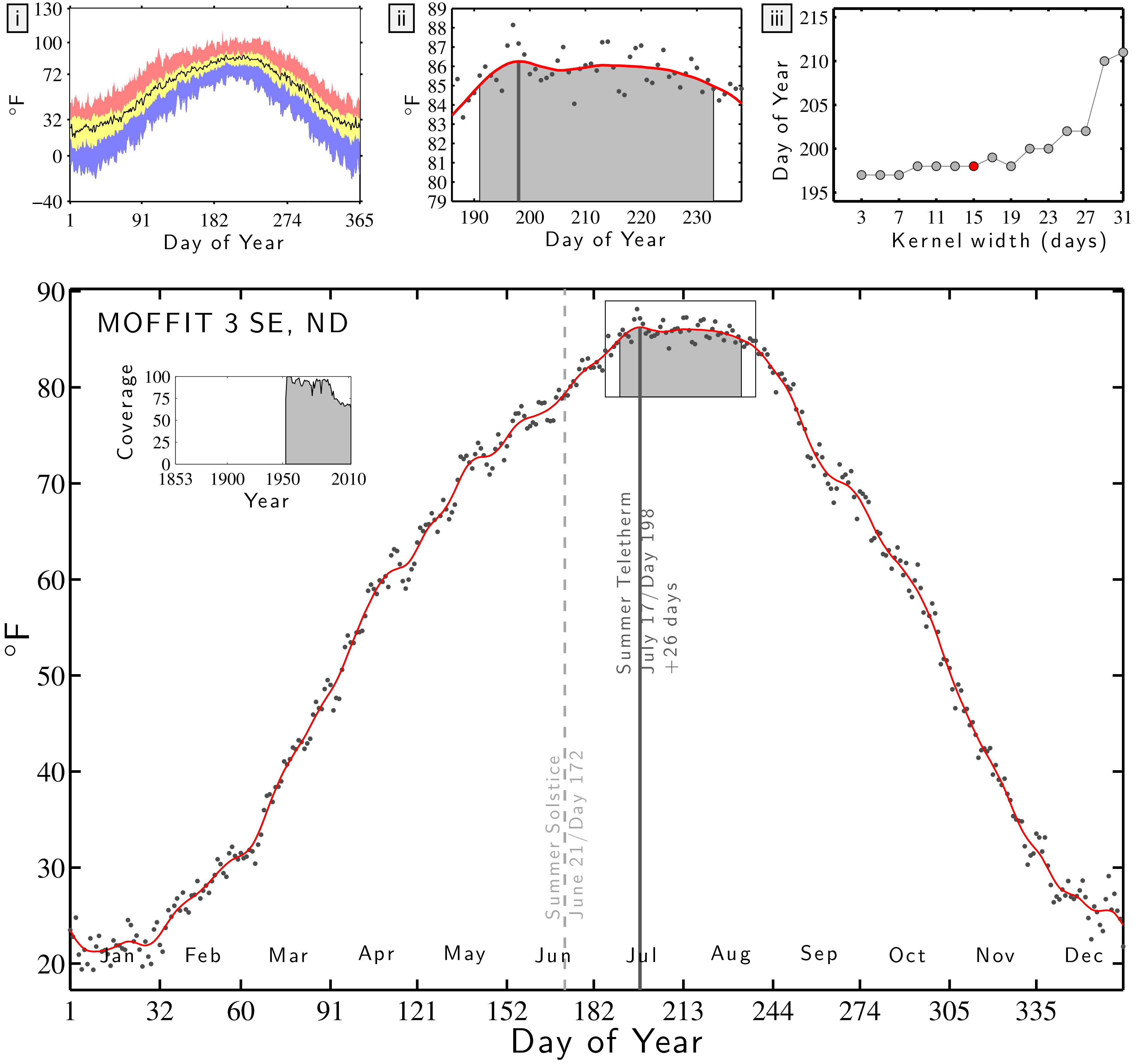

i
°F
Day of Year
ii
°F
Day of Year
iii
Day of Year
Kernel width (days)
MOFFIT 3 SE, ND
Coverage
Year
Summer Solstice
June 21 / Day 172
Summer Teletherm
July 17 / Day 198
+26 days
Jan
Feb
Mar
Apr
May
Jun
Jul
Aug
Sep
Oct
Nov
Dec
°F
Day of Year

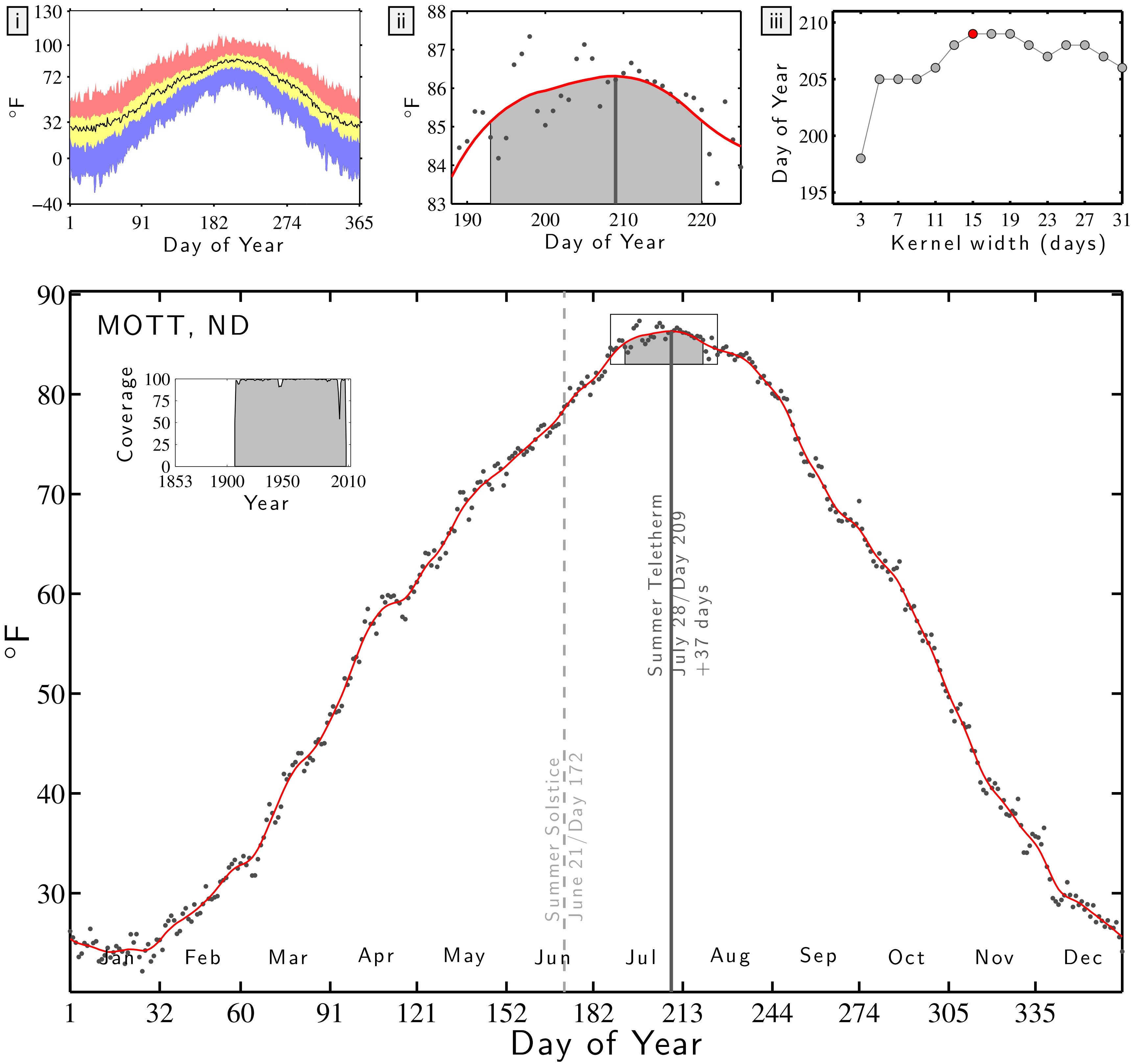

i
°F
Day of Year
ii
°F
Day of Year
iii
Day of Year
Kernel width (days)
MOTT, ND
Coverage
Year
Summer Solstice
June 21/Day 172
Summer Teletherm
July 28/Day 209
+37 days
Jan
Feb
Mar
Apr
May
Jun
Jul
Aug
Sep
Oct
Nov
Dec
°F
Day of Year

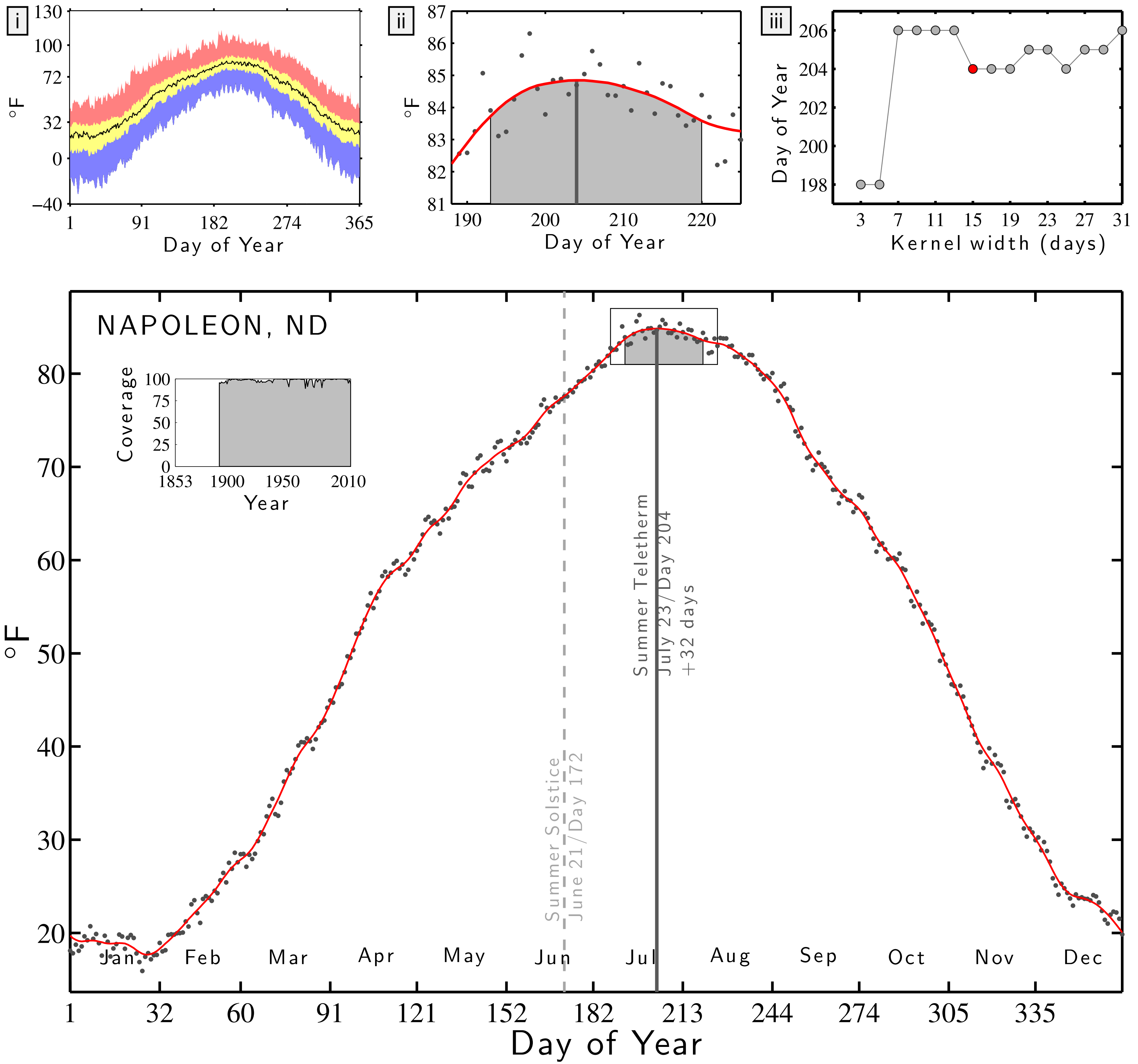

i
ii
iii
°F
Day of Year
Kernel width (days)
NAPOLEON, ND
Coverage
Year
Summer Solstice
June 21 / Day 172
Summer Teletherm
July 23 / Day 204
+32 days
Jan
Feb
Mar
Apr
May
Jun
Jul
Aug
Sep
Oct
Nov
Dec

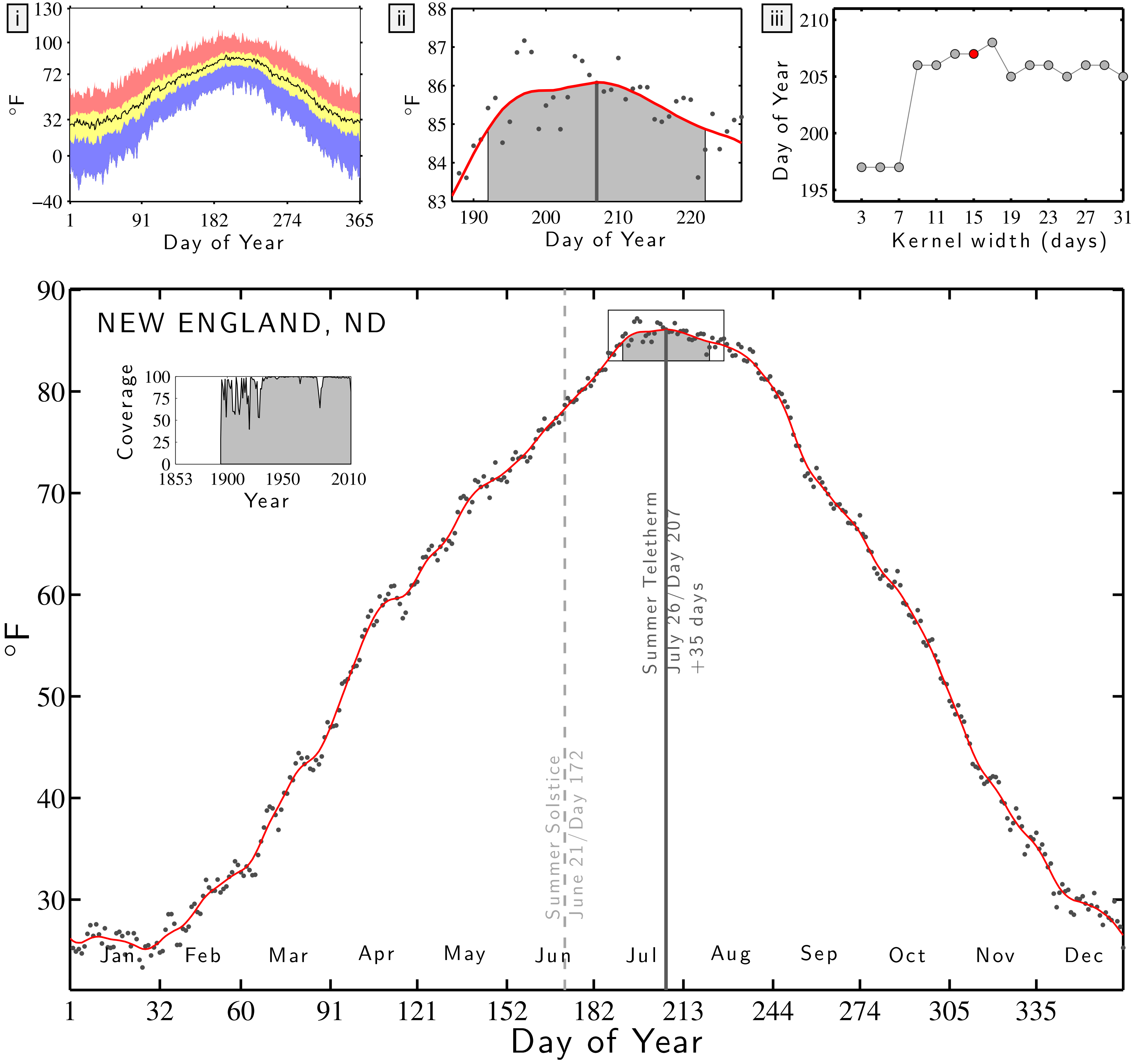

i
130
100
72
32
0
−40
°F
1
91
182
274
365
Day of Year
ii
88
87
86
85
84
83
°F
190
200
210
220
Day of Year
iii
210
205
200
195
Day of Year
3
7
11
15
19
23
27
31
Kernel width (days)
NEW ENGLAND, ND
Coverage
100
75
50
25
0
1853
1900
1950
2010
Year
90
80
70
60
50
40
30
°F
Summer Solstice
June 21 / Day 172
Summer Teletherm
July 26 / Day 207
+35 days
Jan
Feb
Mar
Apr
May
Jun
Jul
Aug
Sep
Oct
Nov
Dec
1
32
60
91
121
152
182
213
244
274
305
335
Day of Year

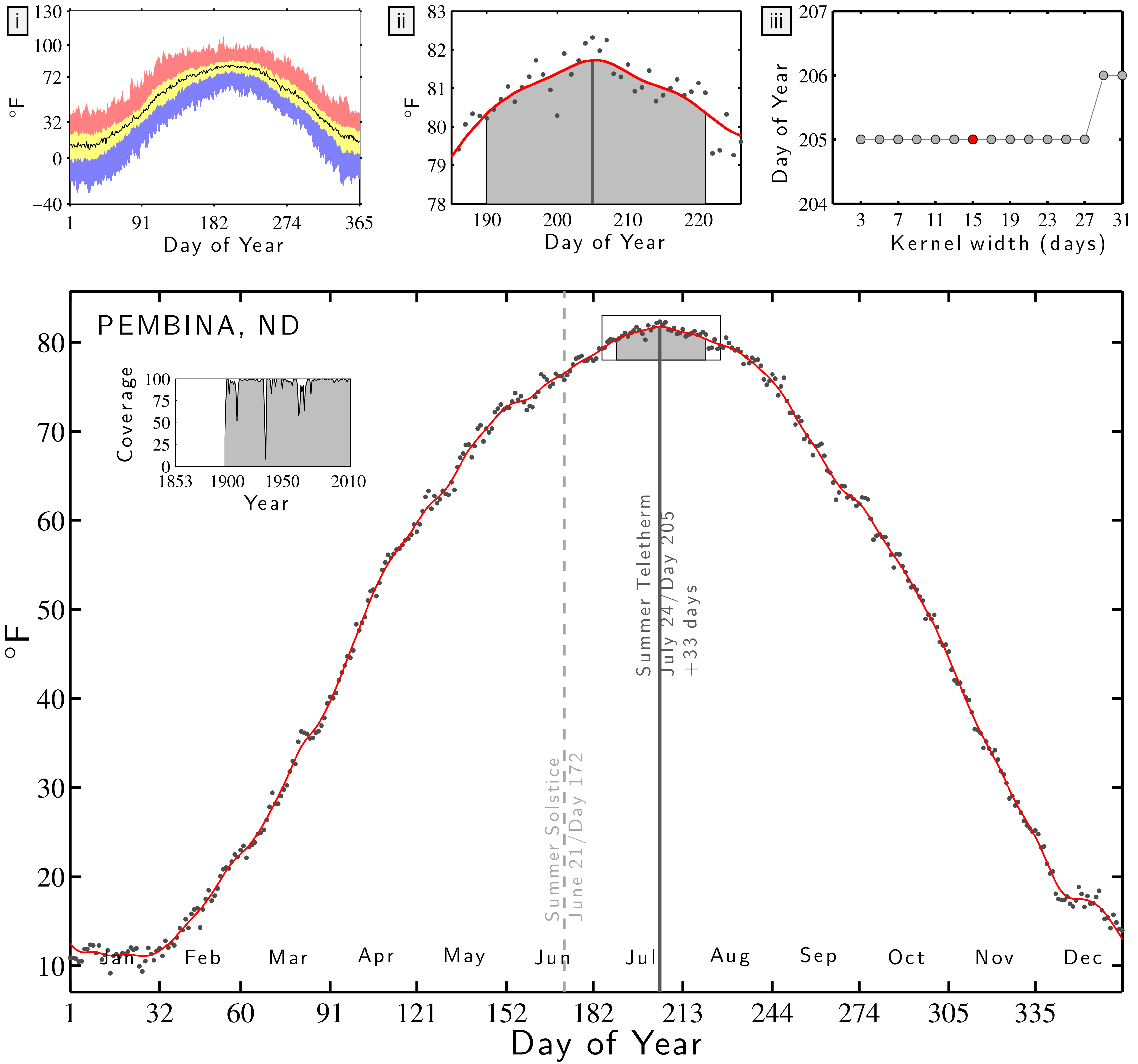

i
ii
iii
°F
Day of Year
Day of Year
Kernel width (days)
PEMBINA, ND
Coverage
Year
Summer Solstice
June 21/Day 172
Summer Teletherm
July 24/Day 205
+33 days
Jan
Feb
Mar
Apr
May
Jun
Jul
Aug
Sep
Oct
Nov
Dec

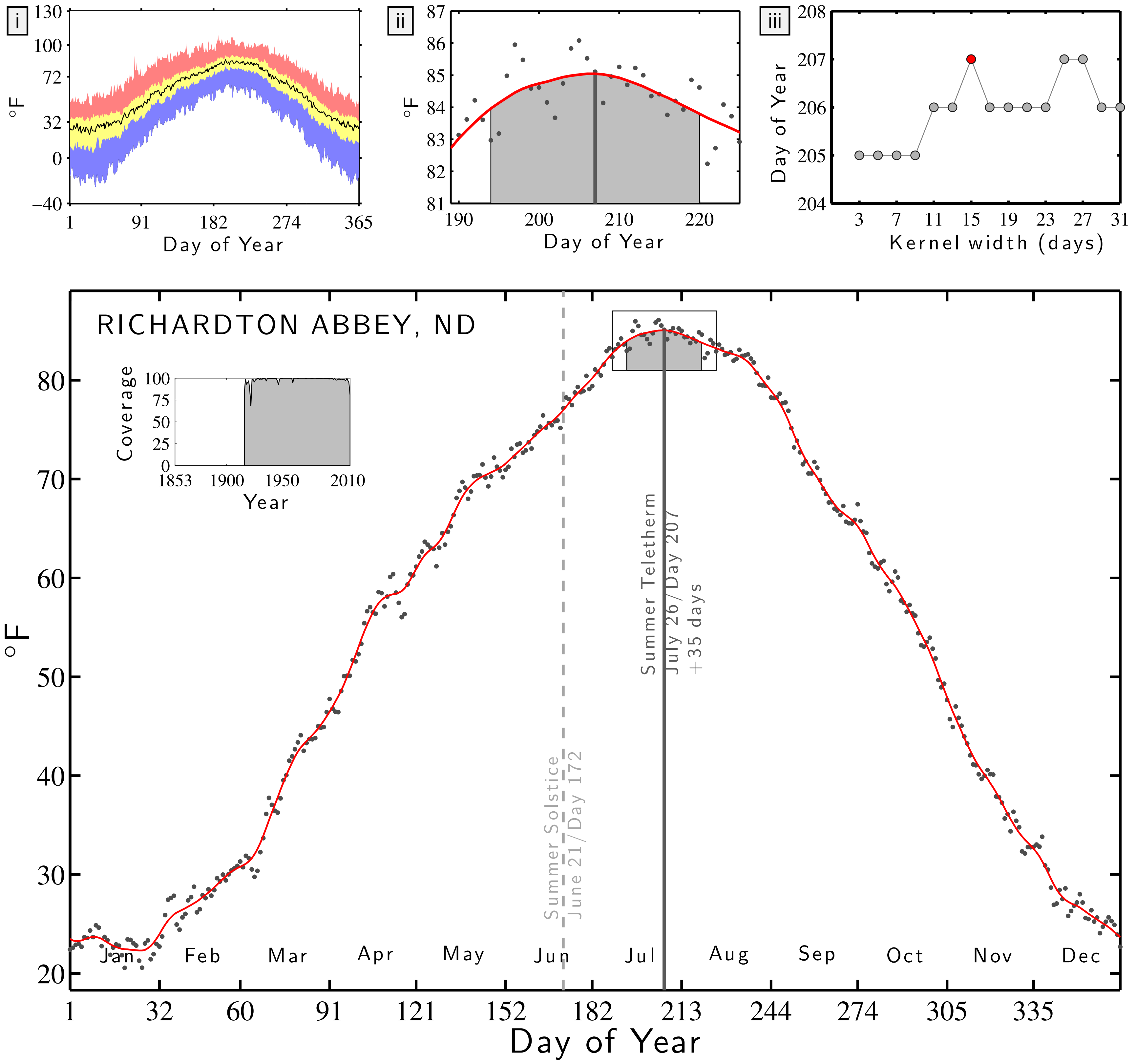

i
ii
iii
°F
Day of Year
Kernel width (days)
RICHARDTON ABBEY, ND
Coverage
Year
Summer Solstice
June 21 / Day 172
Summer Teletherm
July 26 / Day 207
+35 days
Jan
Feb
Mar
Apr
May
Jun
Jul
Aug
Sep
Oct
Nov
Dec

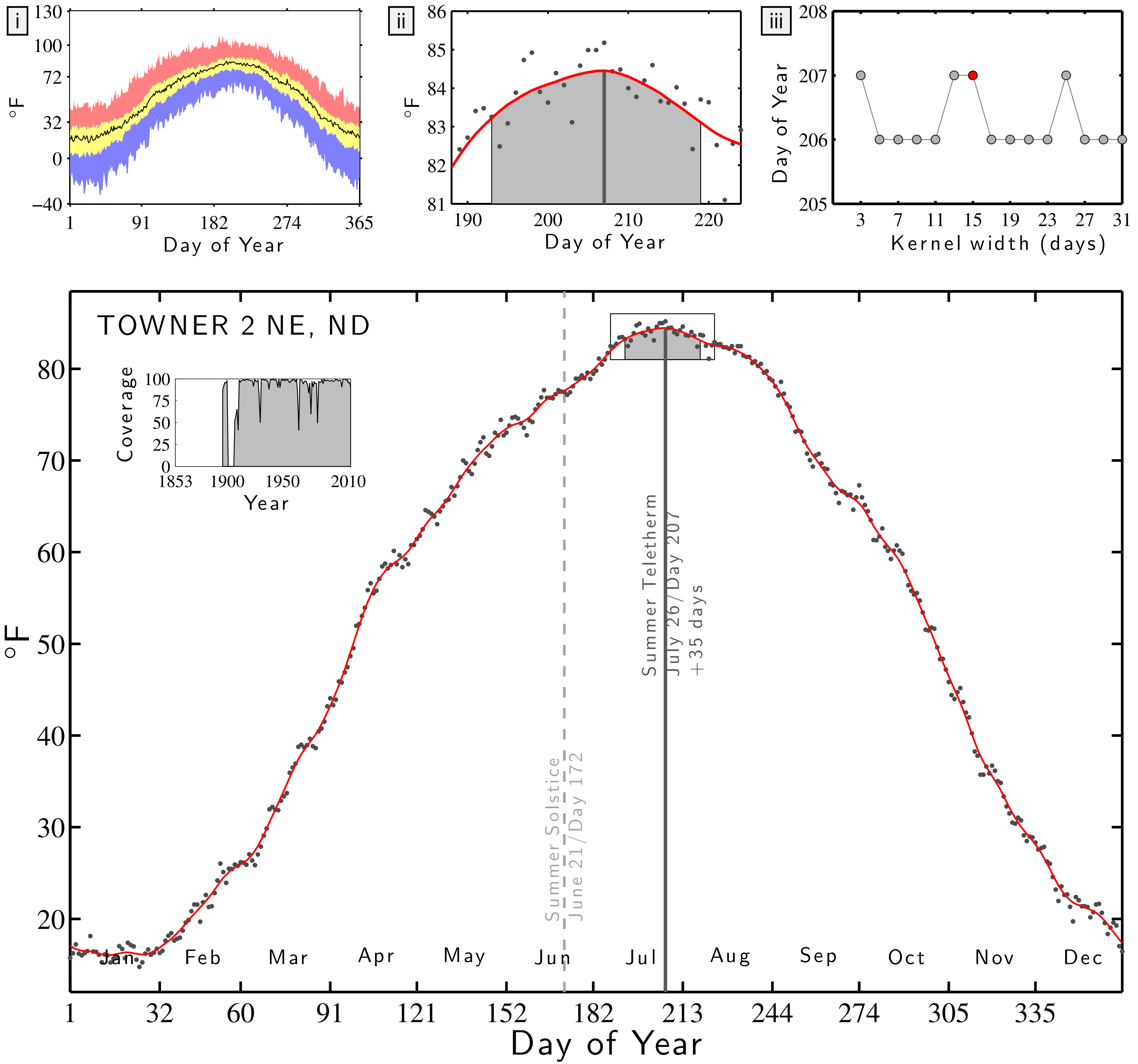

i
ii
iii
°F
Day of Year
Day of Year
Kernel width (days)
TOWNER 2 NE, ND
Coverage
Year
Summer Solstice
June 21 / Day 172
Summer Teletherm
July 26 / Day 207
+35 days
Jan
Feb
Mar
Apr
May
Jun
Jul
Aug
Sep
Oct
Nov
Dec
°F
Day of Year

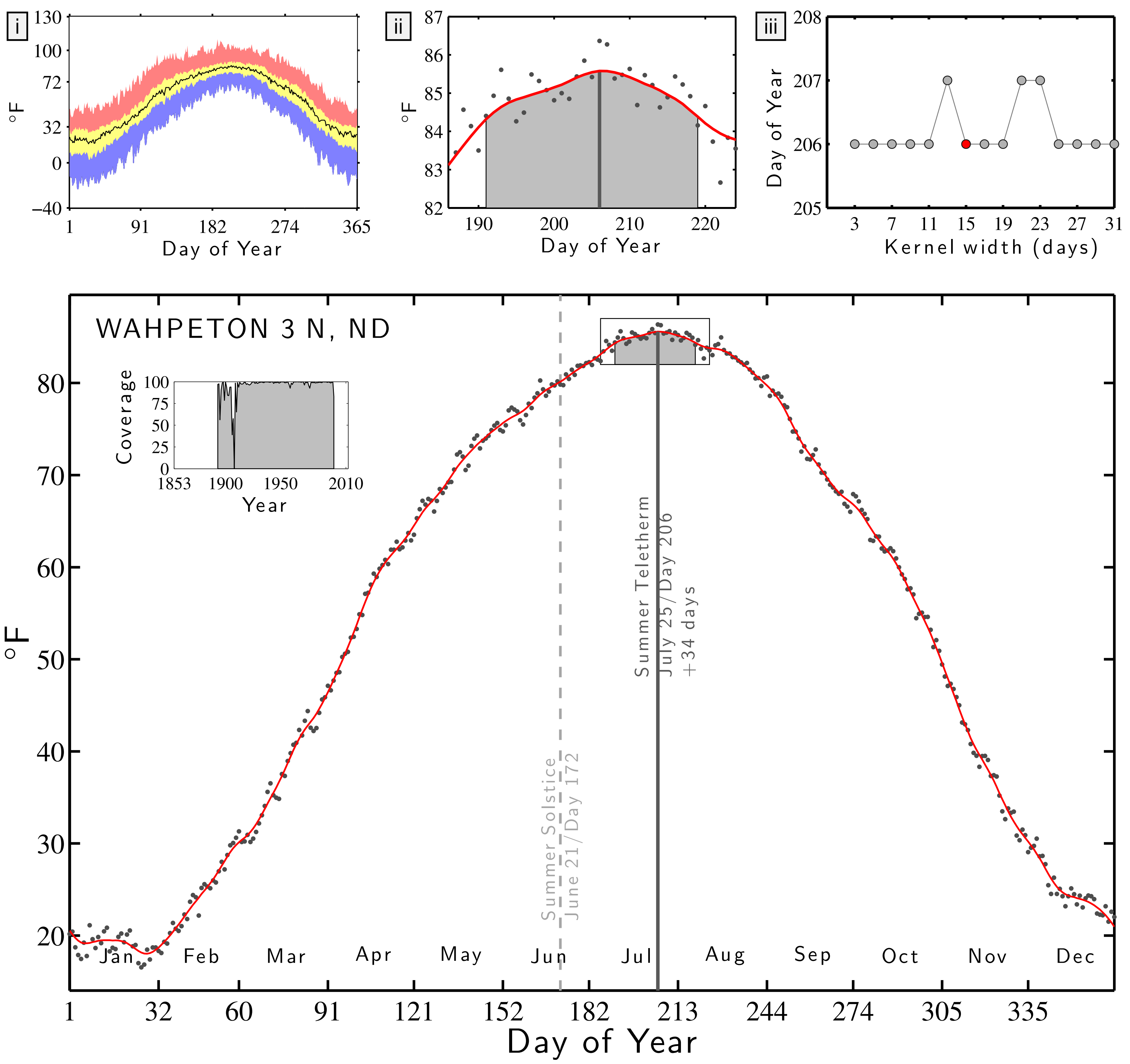

i
ii
iii
WAHPETON 3 N, ND
Coverage
Year
Summer Solstice
June 21/Day 172
Summer Teletherm
July 25/Day 206
+34 days
Kernel width (days)
Day of Year
°F
Jan
Feb
Mar
Apr
May
Jun
Jul
Aug
Sep
Oct
Nov
Dec

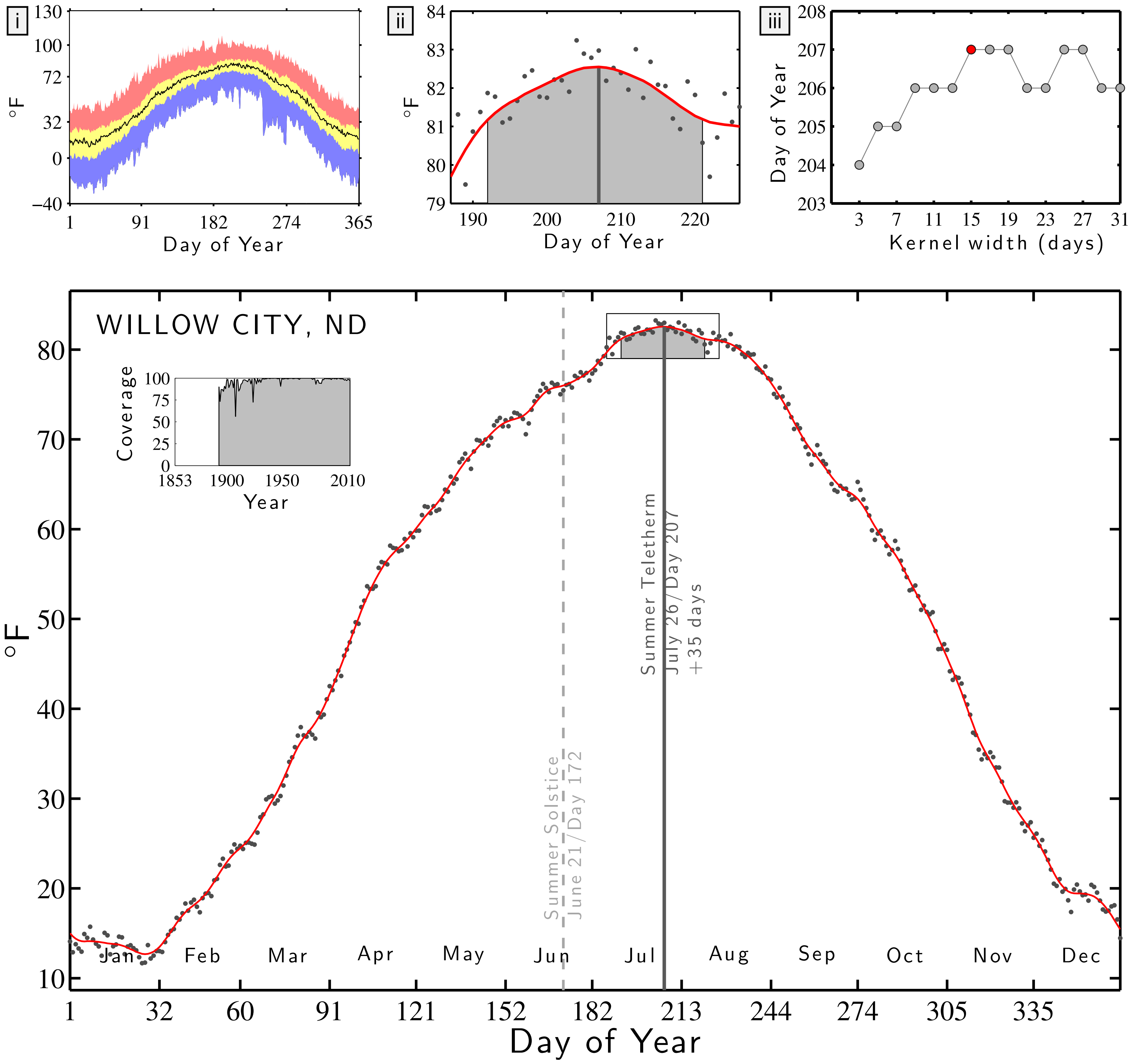

i
ii
iii
°F
Day of Year
Day of Year
Kernel width (days)
WILLOW CITY, ND
Coverage
Year
Summer Solstice
June 21 / Day 172
Summer Teletherm
July 26 / Day 207
+35 days
Jan
Feb
Mar
Apr
May
Jun
Jul
Aug
Sep
Oct
Nov
Dec

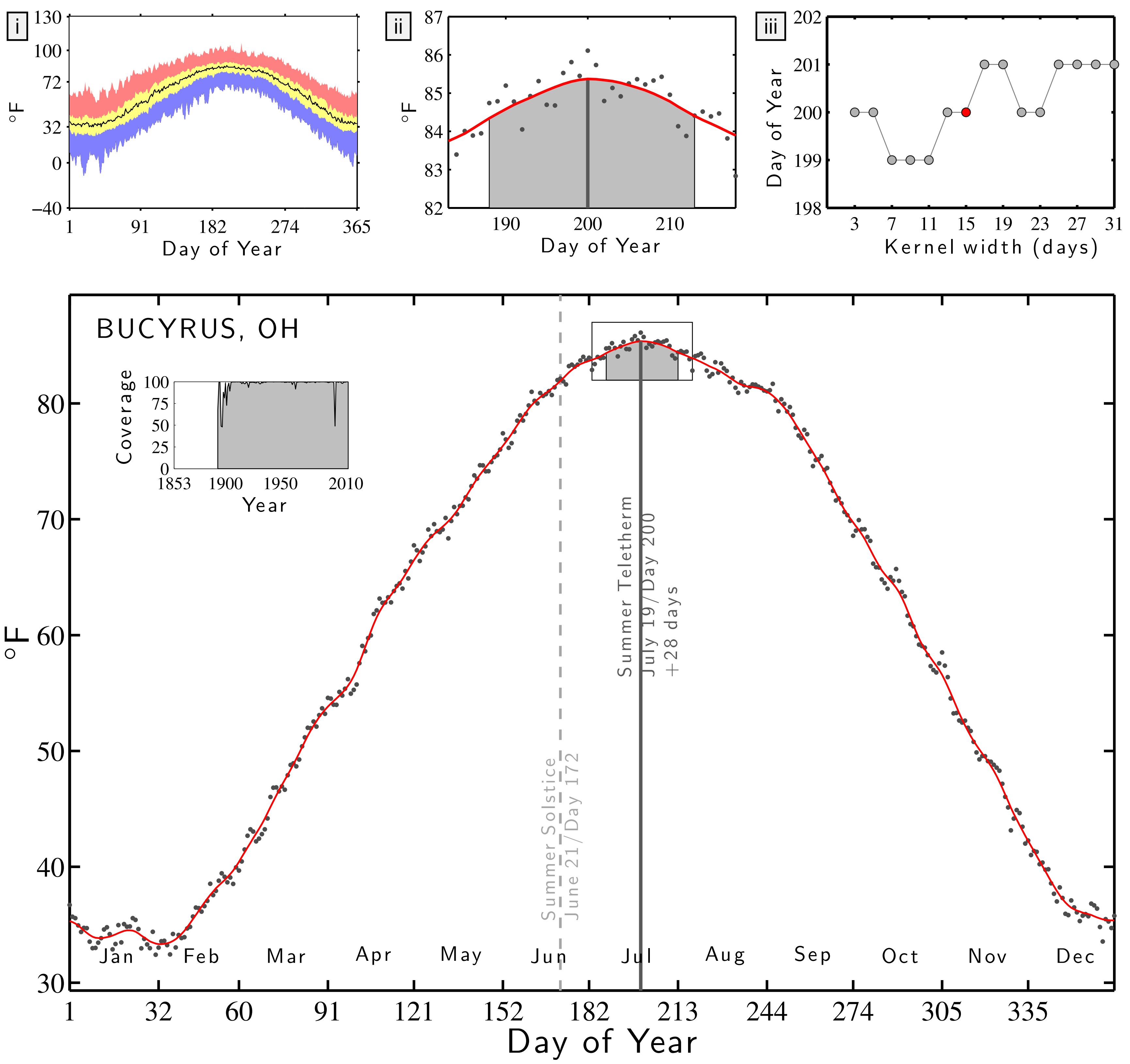

i
ii
iii
°F
Day of Year
Day of Year
Kernel width (days)
BUCYRUS, OH
Coverage
Year
Summer Solstice
June 21/Day 172
Summer Teletherm
July 19/Day 200
+28 days
Jan
Feb
Mar
Apr
May
Jun
Jul
Aug
Sep
Oct
Nov
Dec
Day of Year

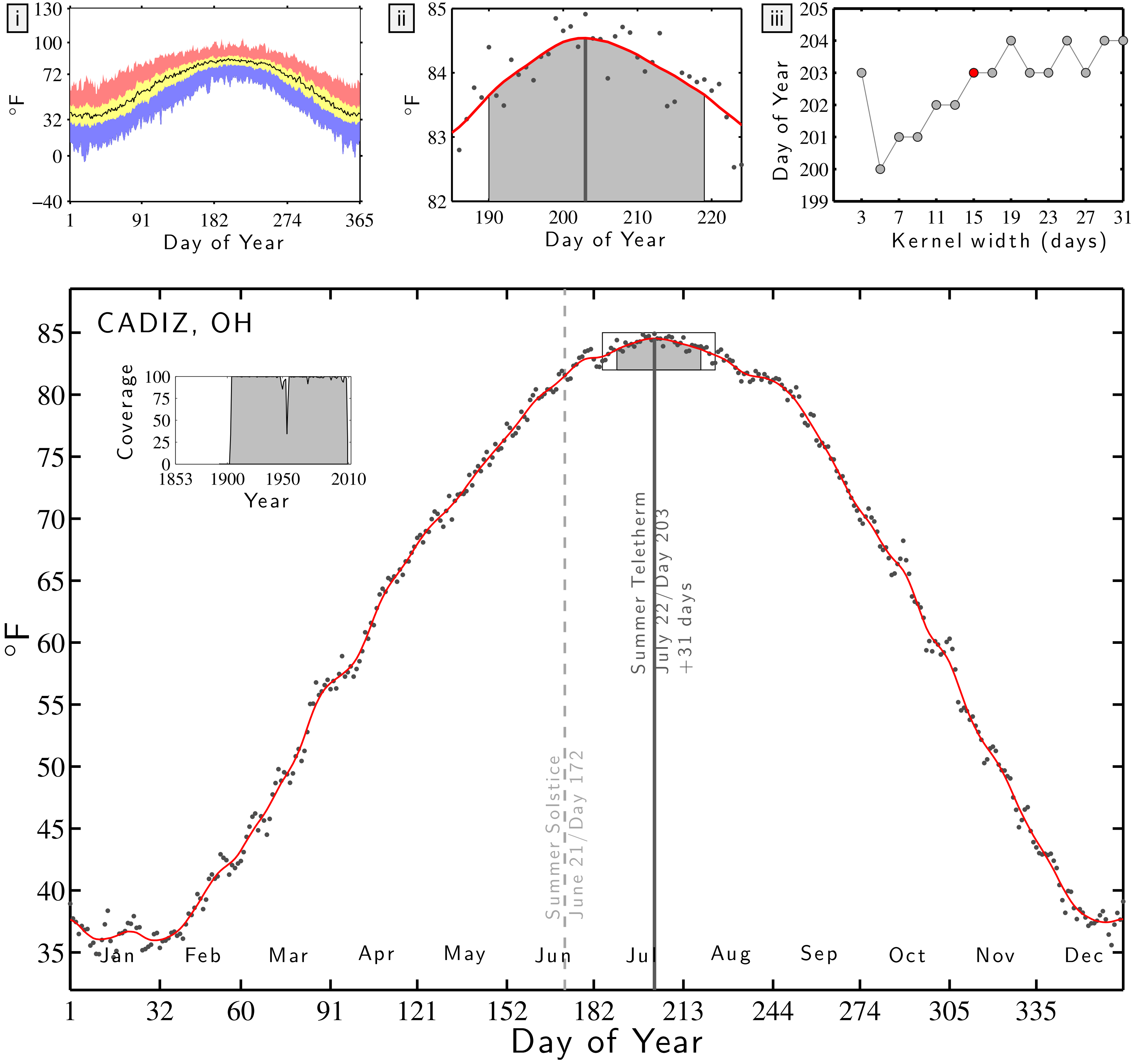
i
°F
Day of Year
ii
°F
Day of Year
iii
Day of Year
Kernel width (days)
CADIZ, OH
Coverage
Year
Summer Solstice
June 21/Day 172
Summer Teletherm
July 22/Day 203
+31 days
Jan
Feb
Mar
Apr
May
Jun
Jul
Aug
Sep
Oct
Nov
Dec
°F
Day of Year

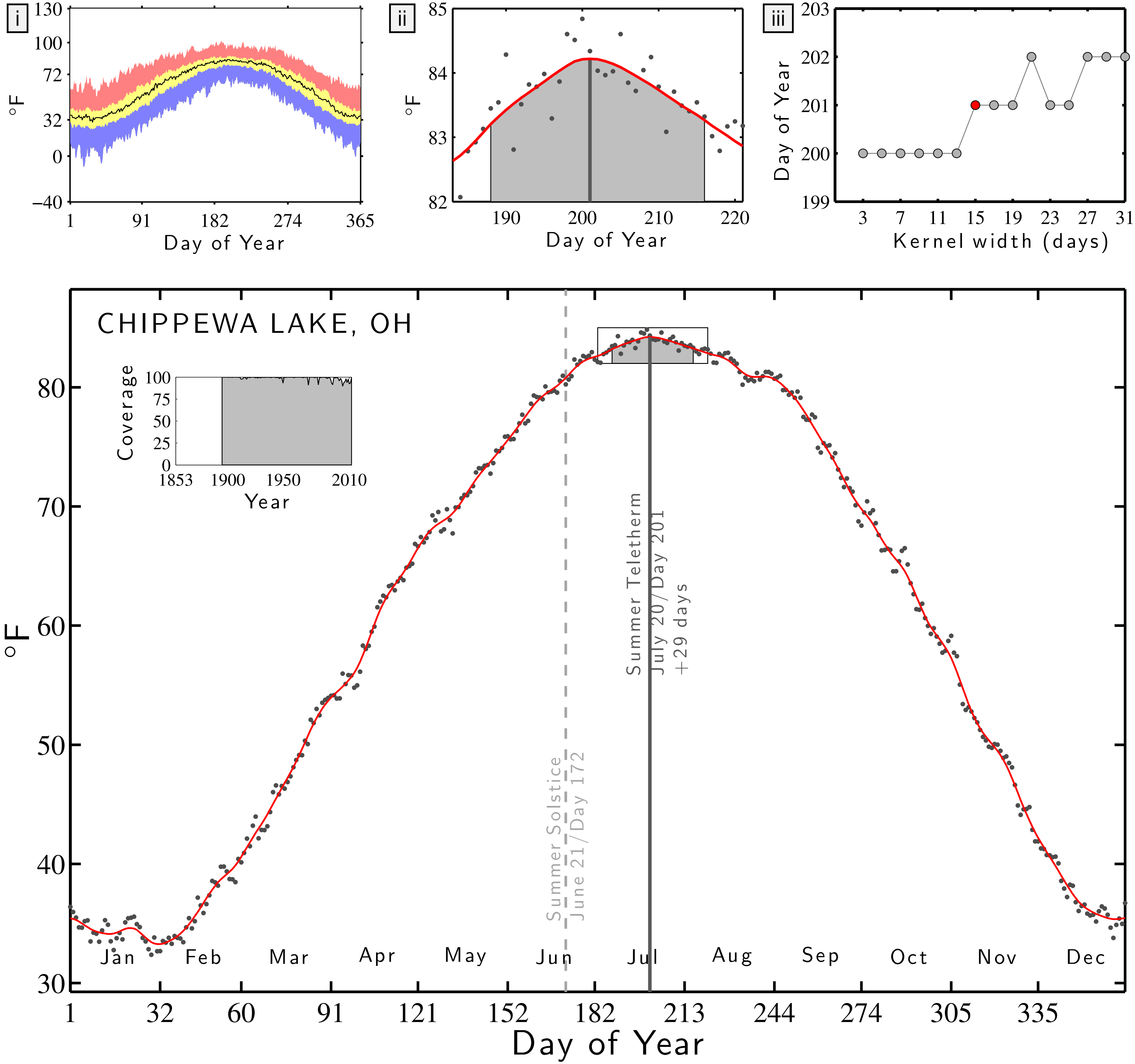

i
130
100
72
32
0
−40
°F
1
91
182
274
365
Day of Year
ii
85
84
83
82
°F
190
200
210
220
Day of Year
iii
203
202
201
200
199
Day of Year
3
7
11
15
19
23
27
31
Kernel width (days)
CHIPPEWA LAKE, OH
Coverage
100
75
50
25
0
1853
1900
1950
2010
Year
Summer Solstice
June 21/Day 172
Summer Teletherm
July 20/Day 201
+29 days
80
70
60
50
40
30
°F
Jan
Feb
Mar
Apr
May
Jun
Jul
Aug
Sep
Oct
Nov
Dec
1
32
60
91
121
152
182
213
244
274
305
335
Day of Year

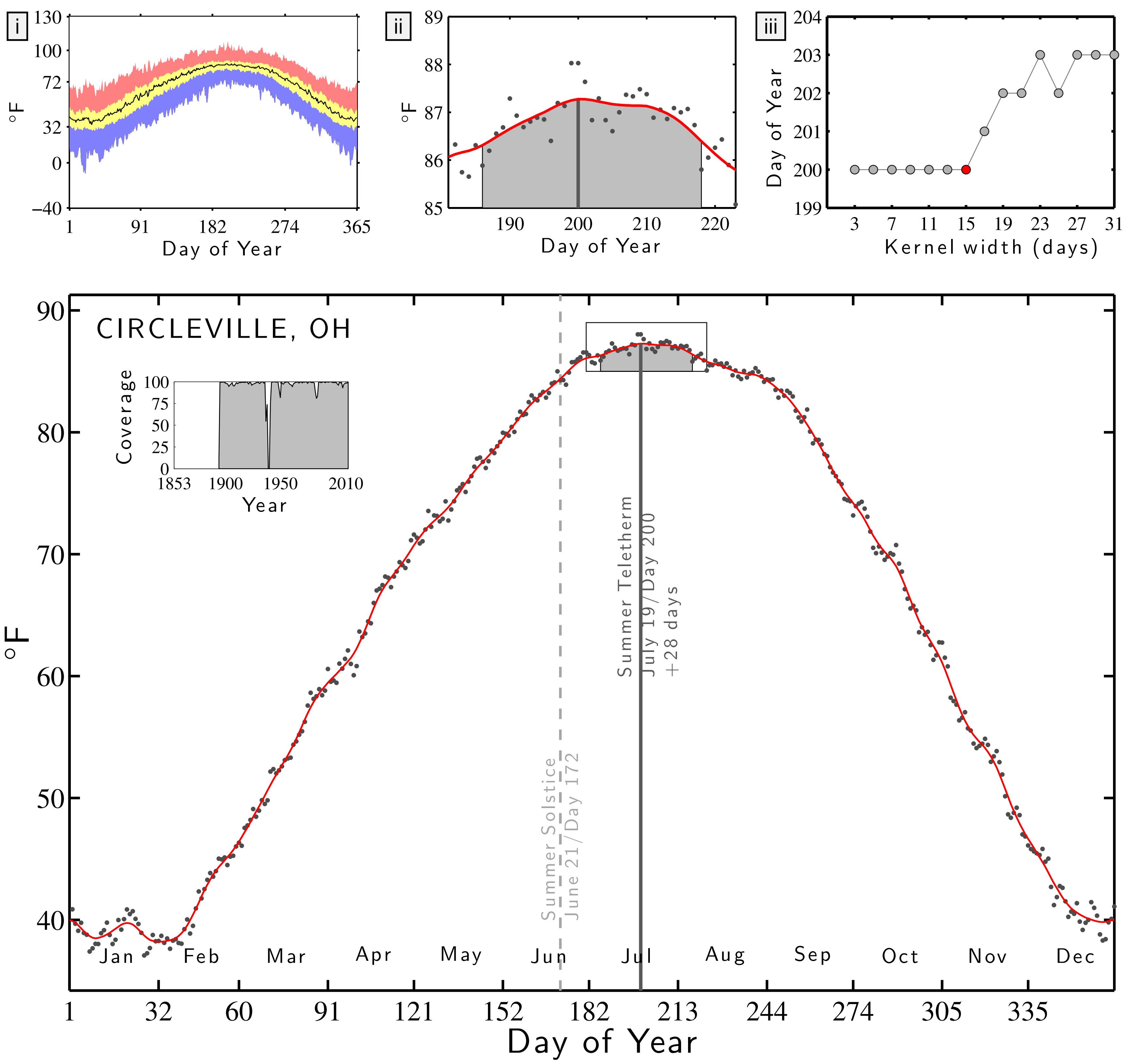

i
°F
Day of Year
ii
°F
Day of Year
iii
Day of Year
Kernel width (days)
CIRCLEVILLE, OH
Coverage
Year
Summer Solstice
June 21/Day 172
Summer Teletherm
July 19/Day 200
+28 days
Jan
Feb
Mar
Apr
May
Jun
Jul
Aug
Sep
Oct
Nov
Dec
°F
Day of Year

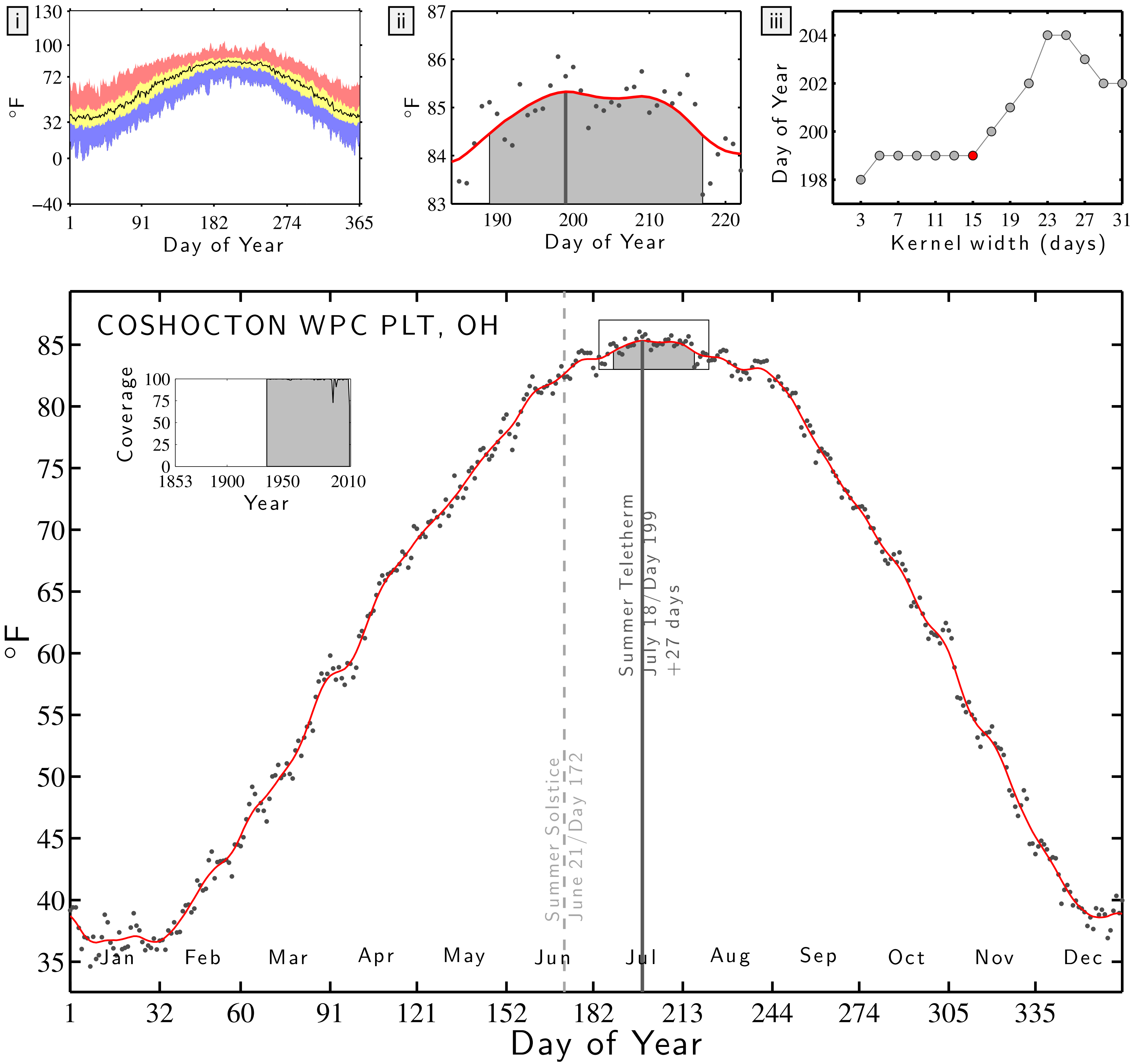

i
ii
iii
°F
Day of Year
Day of Year
Kernel width (days)
COSHOCTON WPC PLT, OH
Coverage
Year
Summer Solstice
June 21/Day 172
Summer Teletherm
July 18/Day 199
+27 days
Jan
Feb
Mar
Apr
May
Jun
Jul
Aug
Sep
Oct
Nov
Dec

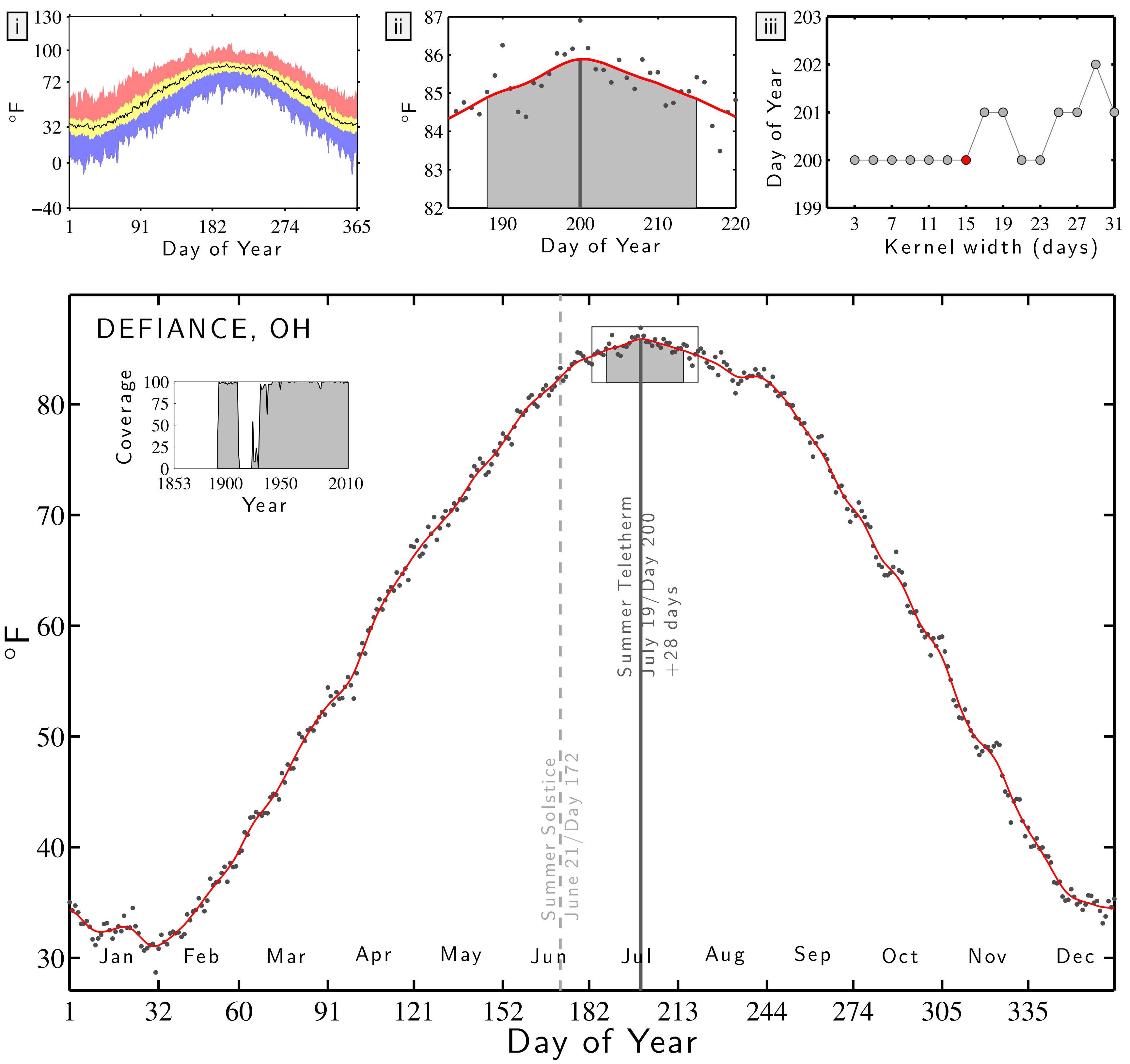

i
°F
Day of Year
ii
°F
Day of Year
iii
Day of Year
Kernel width (days)
DEFIANCE, OH
Coverage
Year
Summer Solstice
June 21/Day 172
Summer Teletherm
July 19/Day 200
+28 days
Jan
Feb
Mar
Apr
May
Jun
Jul
Aug
Sep
Oct
Nov
Dec
°F
Day of Year

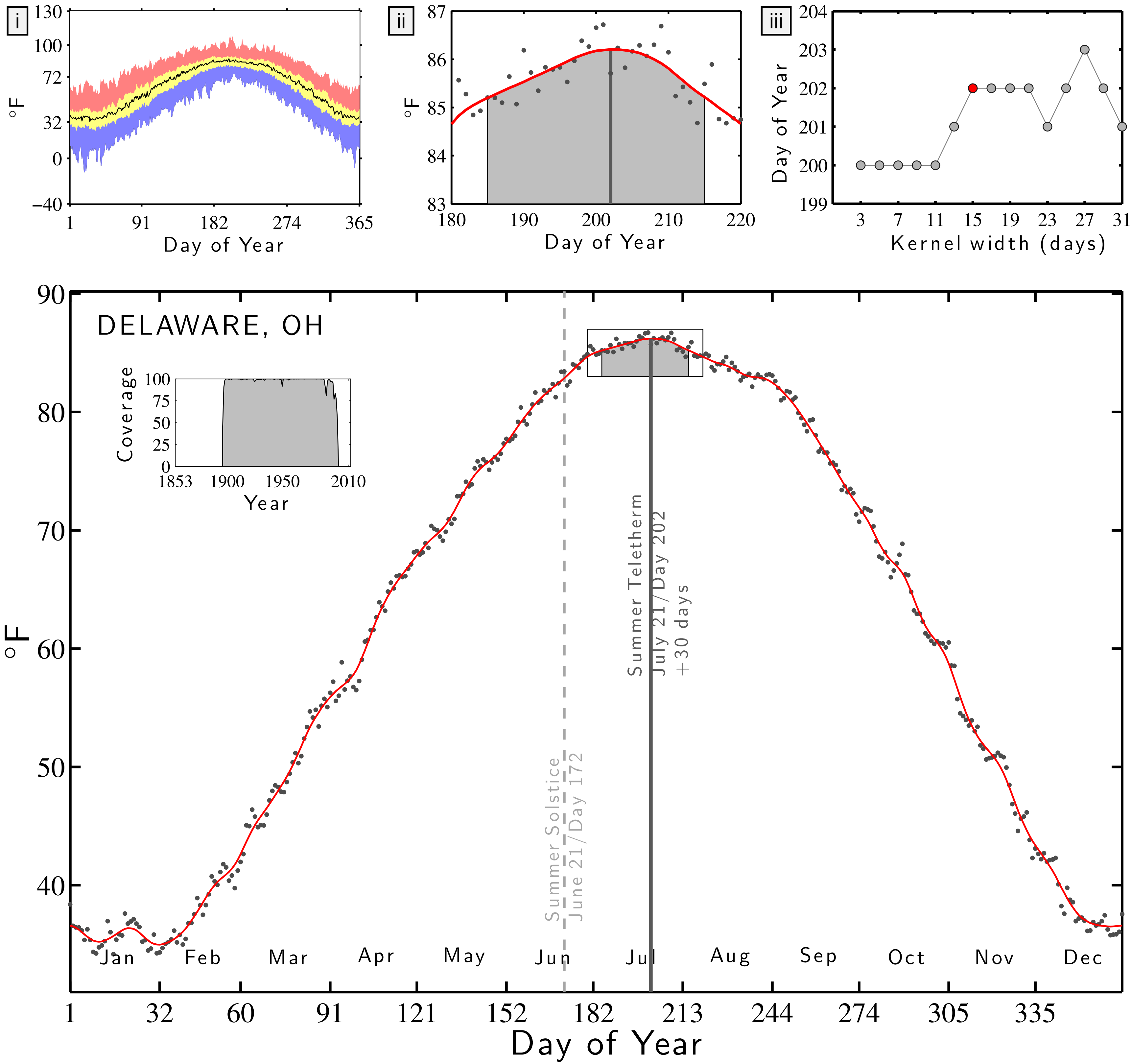

i
°F
Day of Year
ii
°F
Day of Year
iii
Day of Year
Kernel width (days)
DELAWARE, OH
Coverage
Year
1853
1900
1950
2010
Summer Solstice
June 21/Day 172
Summer Teletherm
July 21/Day 202
+30 days
Jan
Feb
Mar
Apr
May
Jun
Jul
Aug
Sep
Oct
Nov
Dec
°F
Day of Year

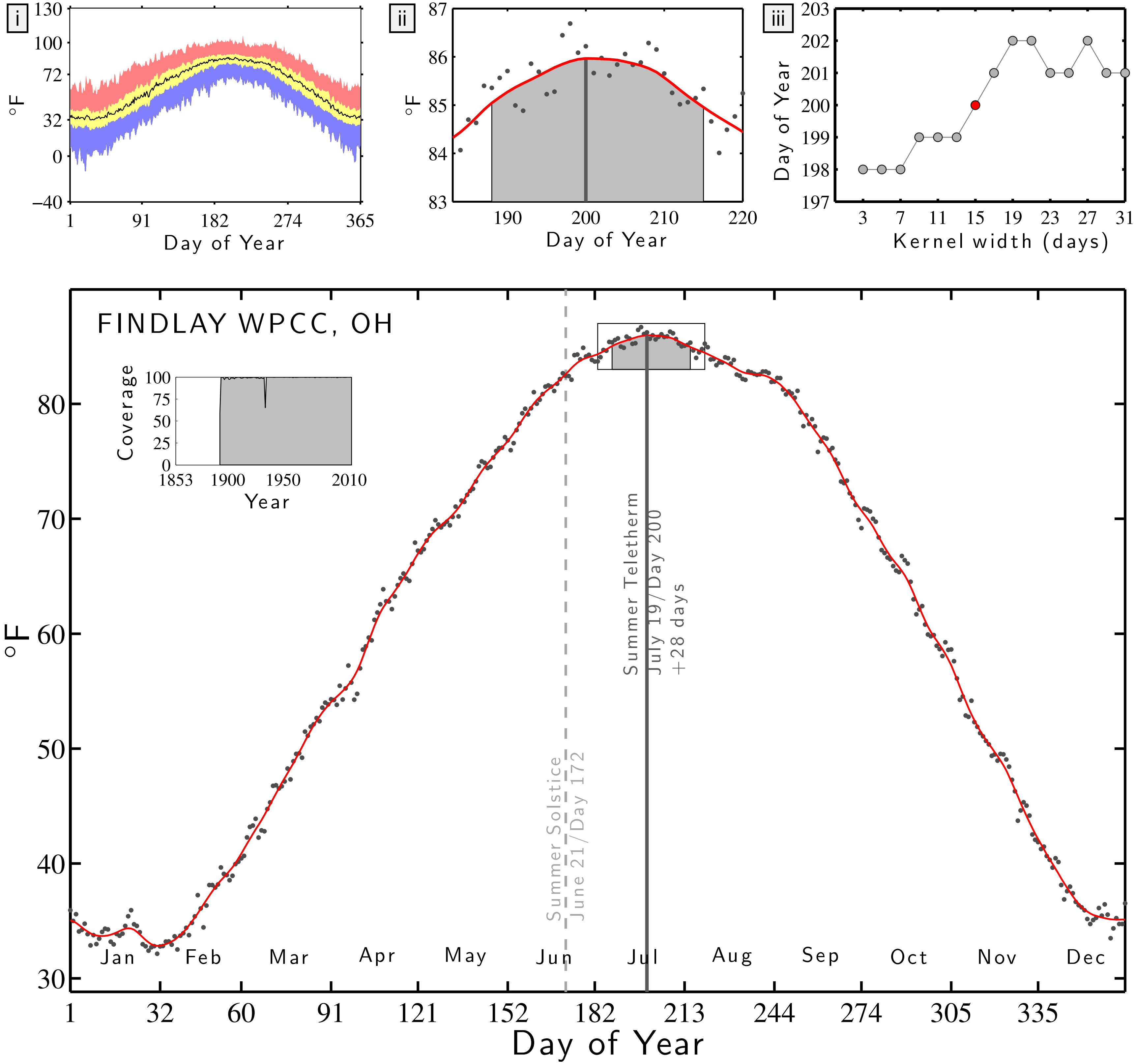

i
°F
130
100
72
32
0
−40
1
91
182
274
365
Day of Year
ii
87
86
85
84
83
190
200
210
220
Day of Year
iii
Day of Year
203
202
201
200
199
198
197
3
7
11
15
19
23
27
31
Kernel width (days)
FINDLAY WPCC, OH
Coverage
100
75
50
25
0
1853
1900
1950
2010
Year
Summer Solstice
June 21/Day 172
Summer Teletherm
July 19/Day 200
+28 days
Jan
Feb
Mar
Apr
May
Jun
Jul
Aug
Sep
Oct
Nov
Dec
80
70
60
50
40
30
1
32
60
91
121
152
182
213
244
274
305
335
Day of Year

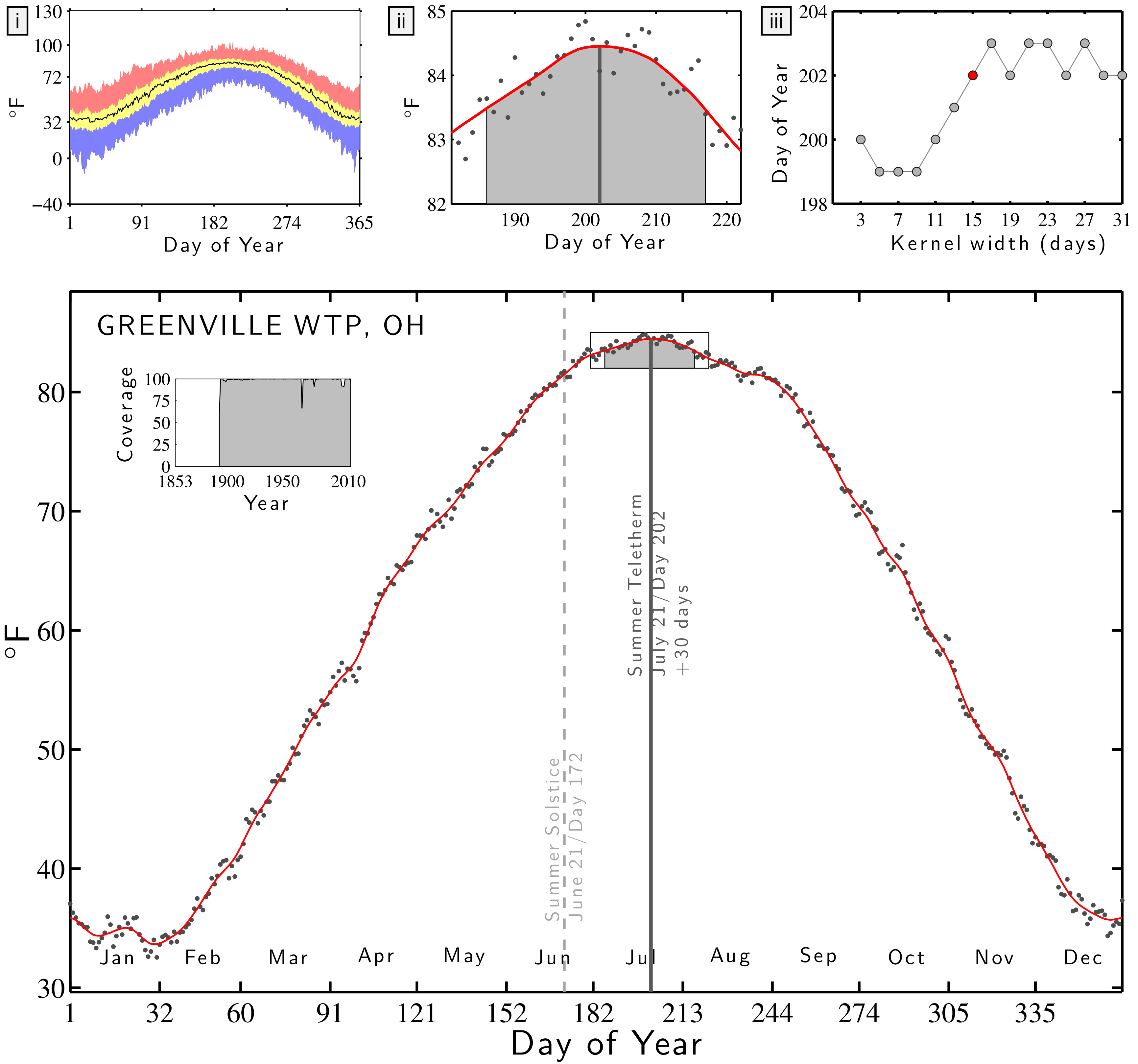

i
°F
Day of Year
ii
°F
Day of Year
iii
Day of Year
Kernel width (days)
GREENVILLE WTP, OH
Coverage
Year
Summer Solstice
June 21 / Day 172
Summer Teletherm
July 21 / Day 202
+30 days
Jan
Feb
Mar
Apr
May
Jun
Jul
Aug
Sep
Oct
Nov
Dec
°F
Day of Year

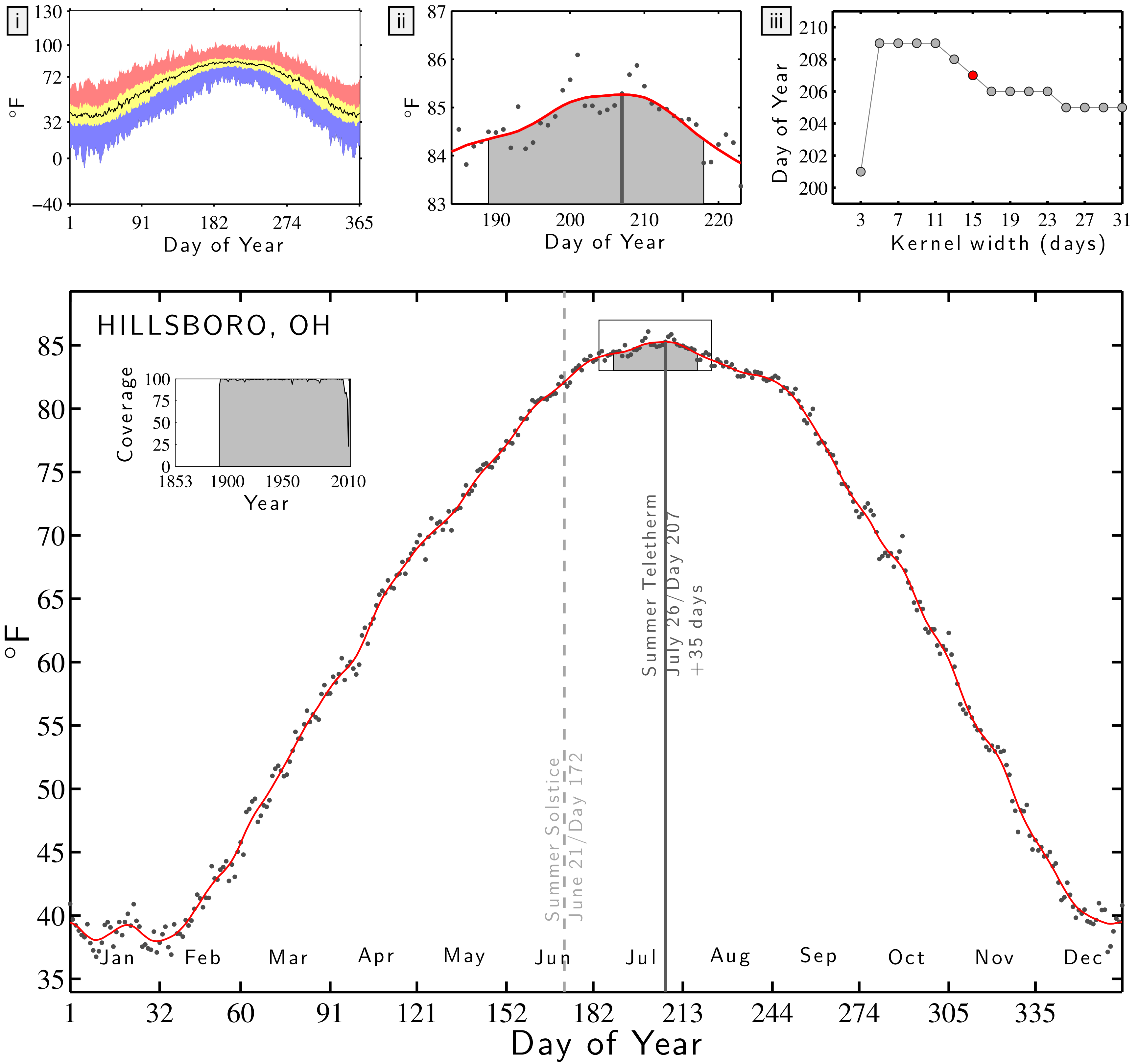

i
ii
iii
°F
Day of Year
Kernel width (days)
HILLSBORO, OH
Coverage
Year
Summer Solstice
June 21 / Day 172
Summer Teletherm
July 26 / Day 207
+35 days
Jan
Feb
Mar
Apr
May
Jun
Jul
Aug
Sep
Oct
Nov
Dec

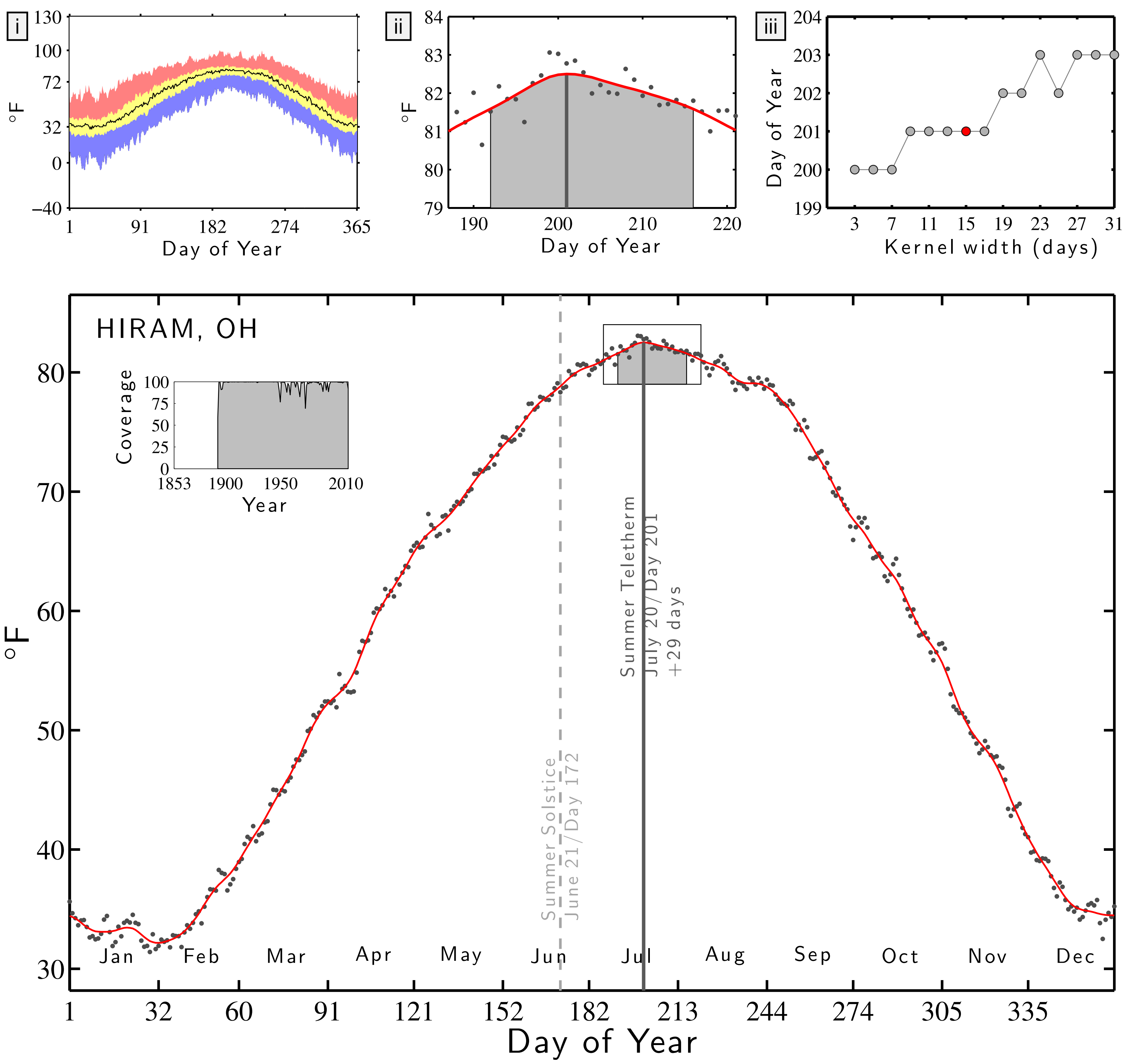

i
ii
iii
°F
Day of Year
Day of Year
Kernel width (days)
HIRAM, OH
Coverage
Year
Summer Solstice
June 21/Day 172
Summer Teletherm
July 20/Day 201
+29 days
Jan
Feb
Mar
Apr
May
Jun
Jul
Aug
Sep
Oct
Nov
Dec

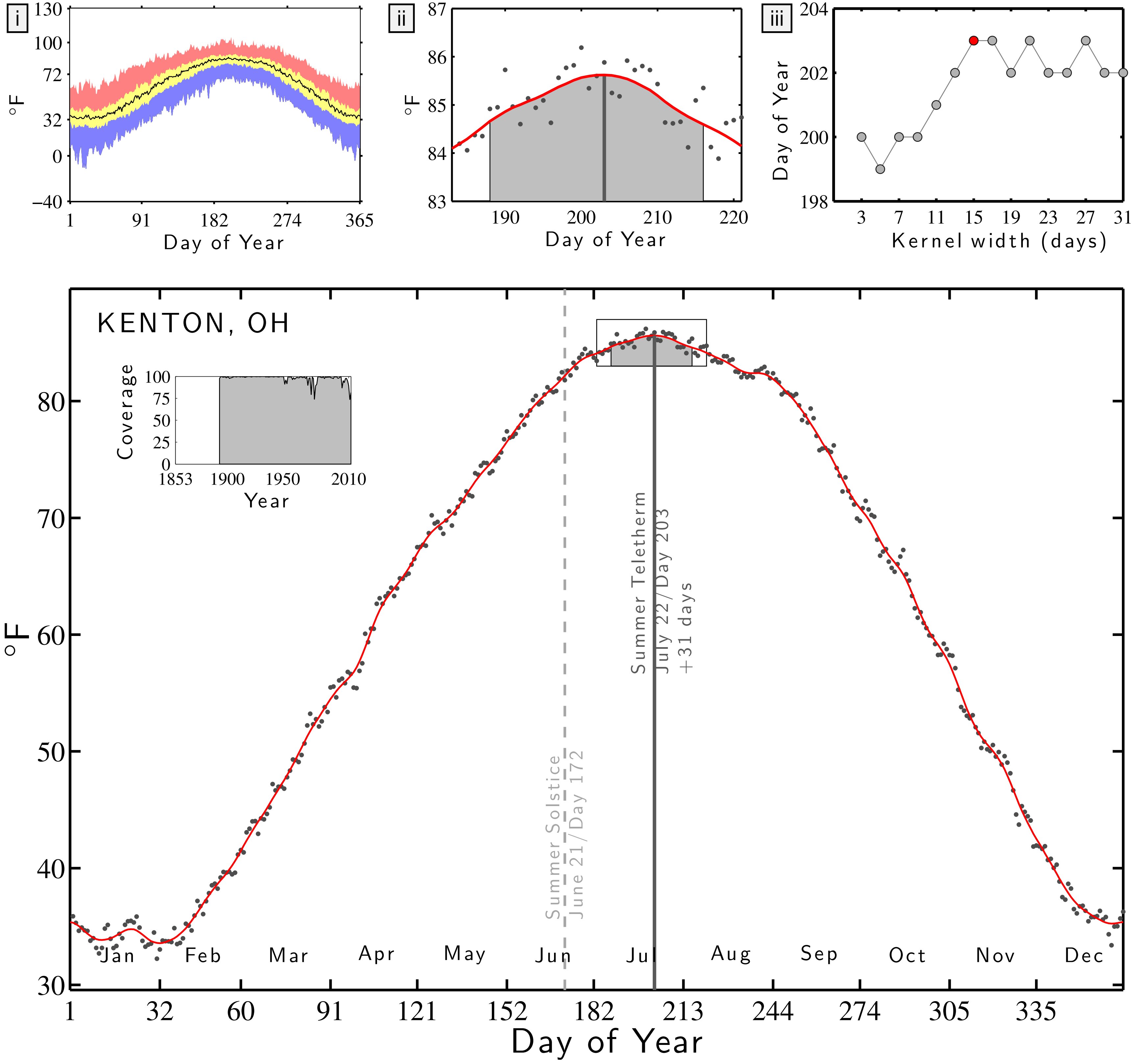

i
°F
Day of Year
ii
Day of Year
iii
Day of Year
Kernel width (days)
KENTON, OH
Coverage
Year
Summer Solstice
June 21/Day 172
Summer Teletherm
July 22/Day 203
+31 days
Jan
Feb
Mar
Apr
May
Jun
Jul
Aug
Sep
Oct
Nov
Dec
Day of Year

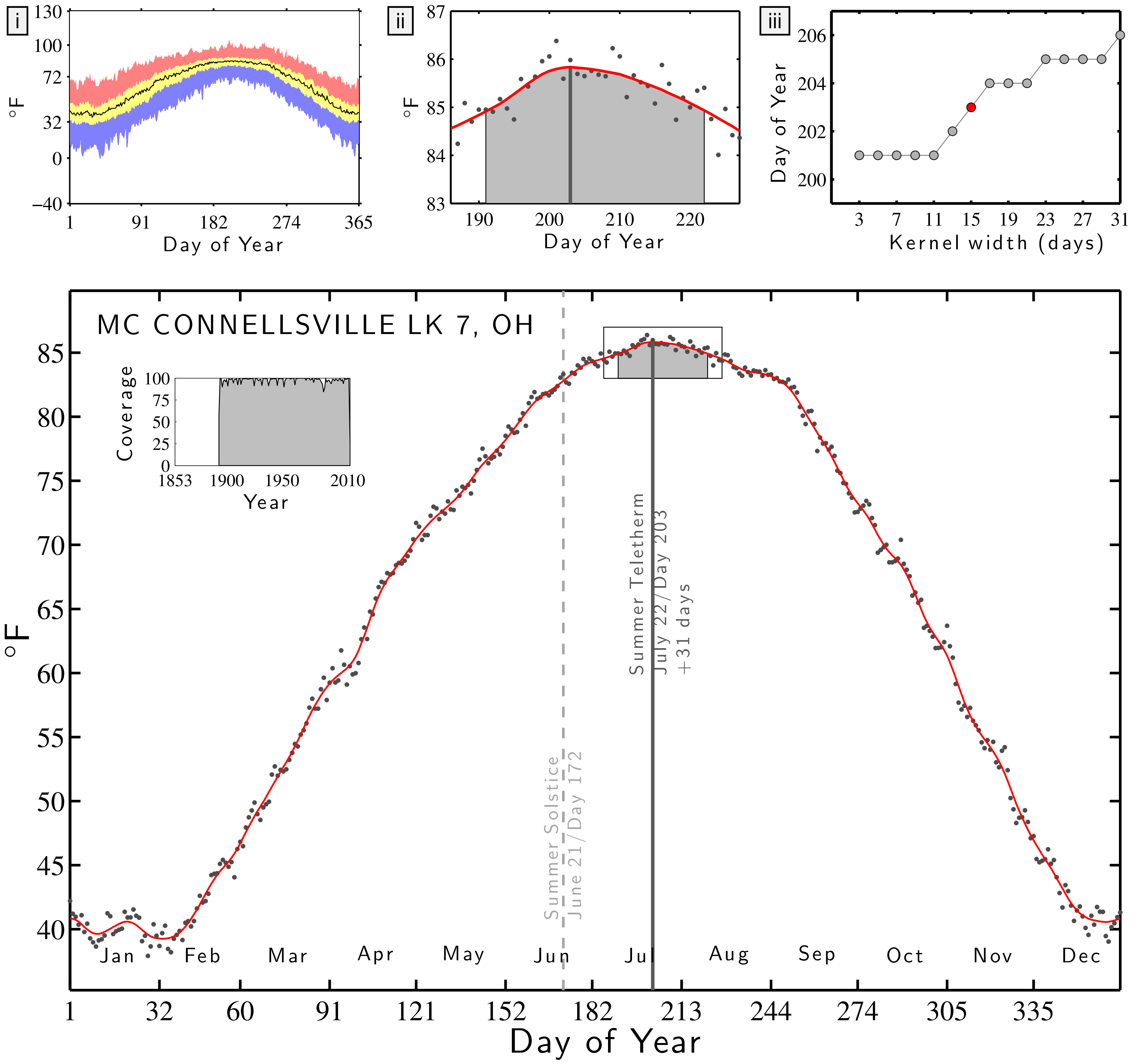

i
°F
Day of Year
ii
iii
Day of Year
Kernel width (days)
MC CONNELLSVILLE LK 7, OH
Coverage
Year
Summer Solstice
June 21/Day 172
Summer Teletherm
July 22/Day 203
+31 days
Jan
Feb
Mar
Apr
May
Jun
Jul
Aug
Sep
Oct
Nov
Dec

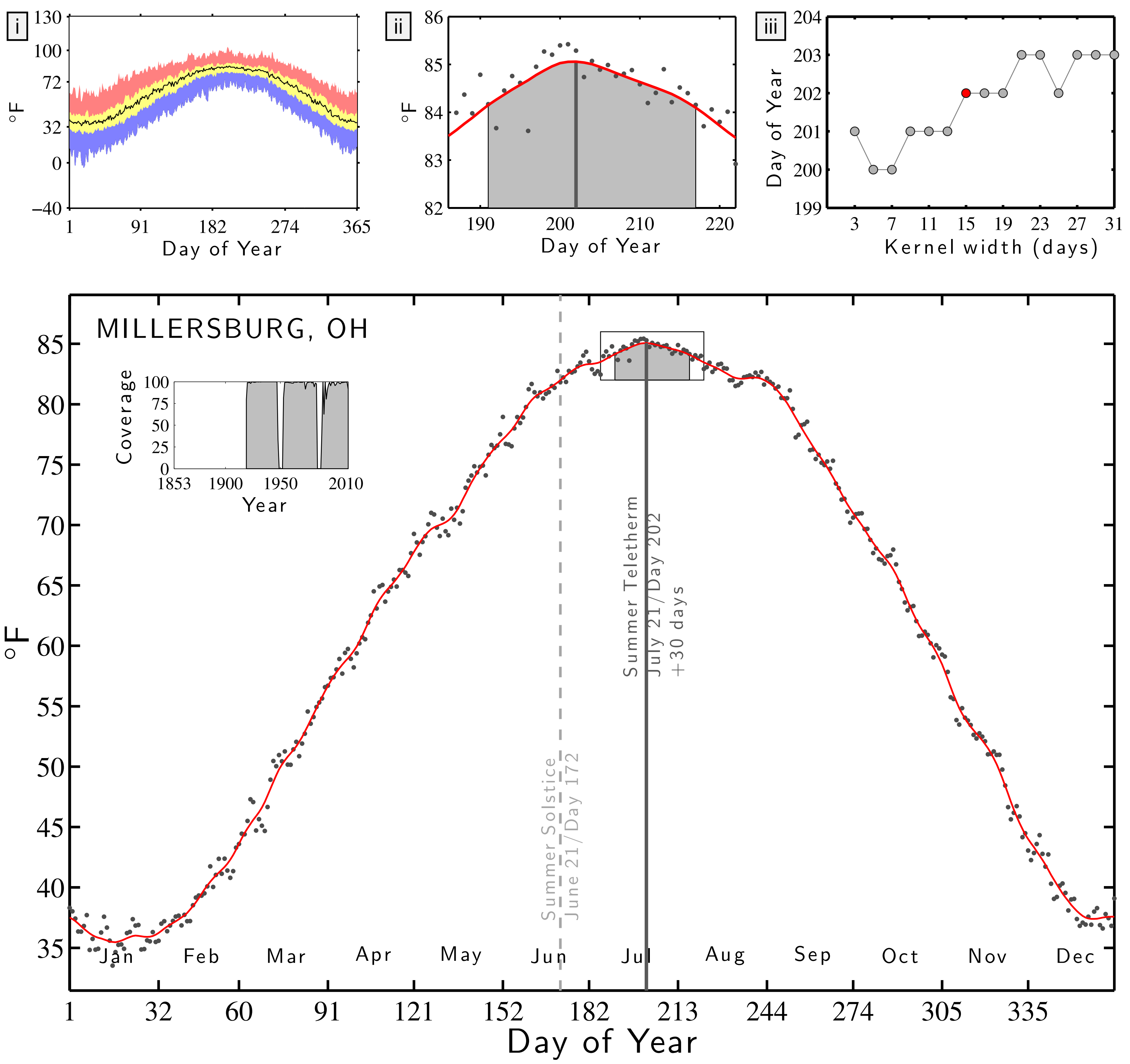

i
°F
Day of Year
ii
°F
Day of Year
iii
Day of Year
Kernel width (days)
MILLERSBURG, OH
Coverage
Year
Summer Solstice
June 21/Day 172
Summer Teletherm
July 21/Day 202
+30 days
Jan
Feb
Mar
Apr
May
Jun
Jul
Aug
Sep
Oct
Nov
Dec
°F
Day of Year

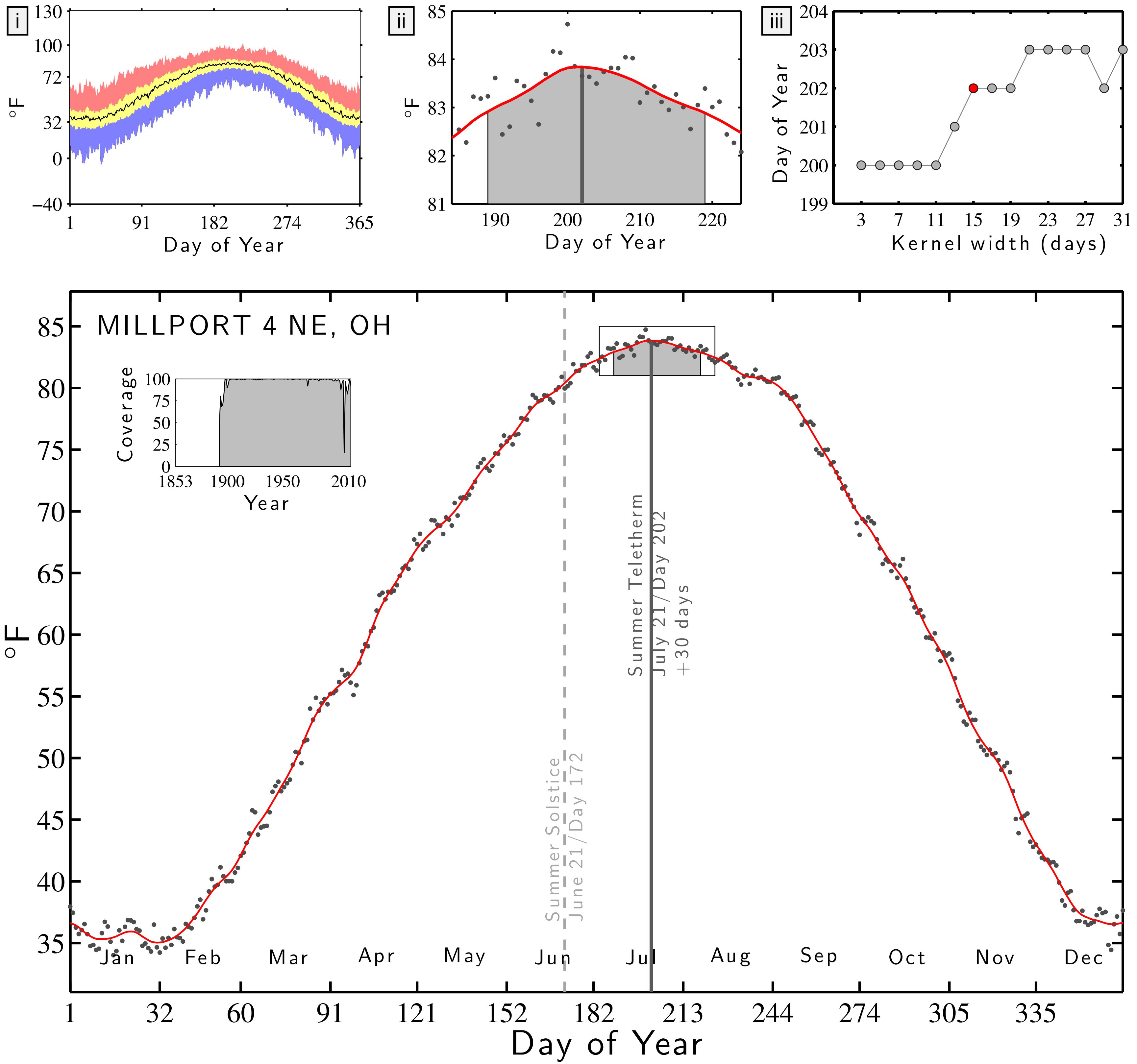

i
ii
iii
°F
Day of Year
Day of Year
Kernel width (days)
MILLPORT 4 NE, OH
Coverage
Year
Summer Solstice
June 21 / Day 172
Summer Teletherm
July 21 / Day 202
+30 days
Jan
Feb
Mar
Apr
May
Jun
Jul
Aug
Sep
Oct
Nov
Dec
Day of Year

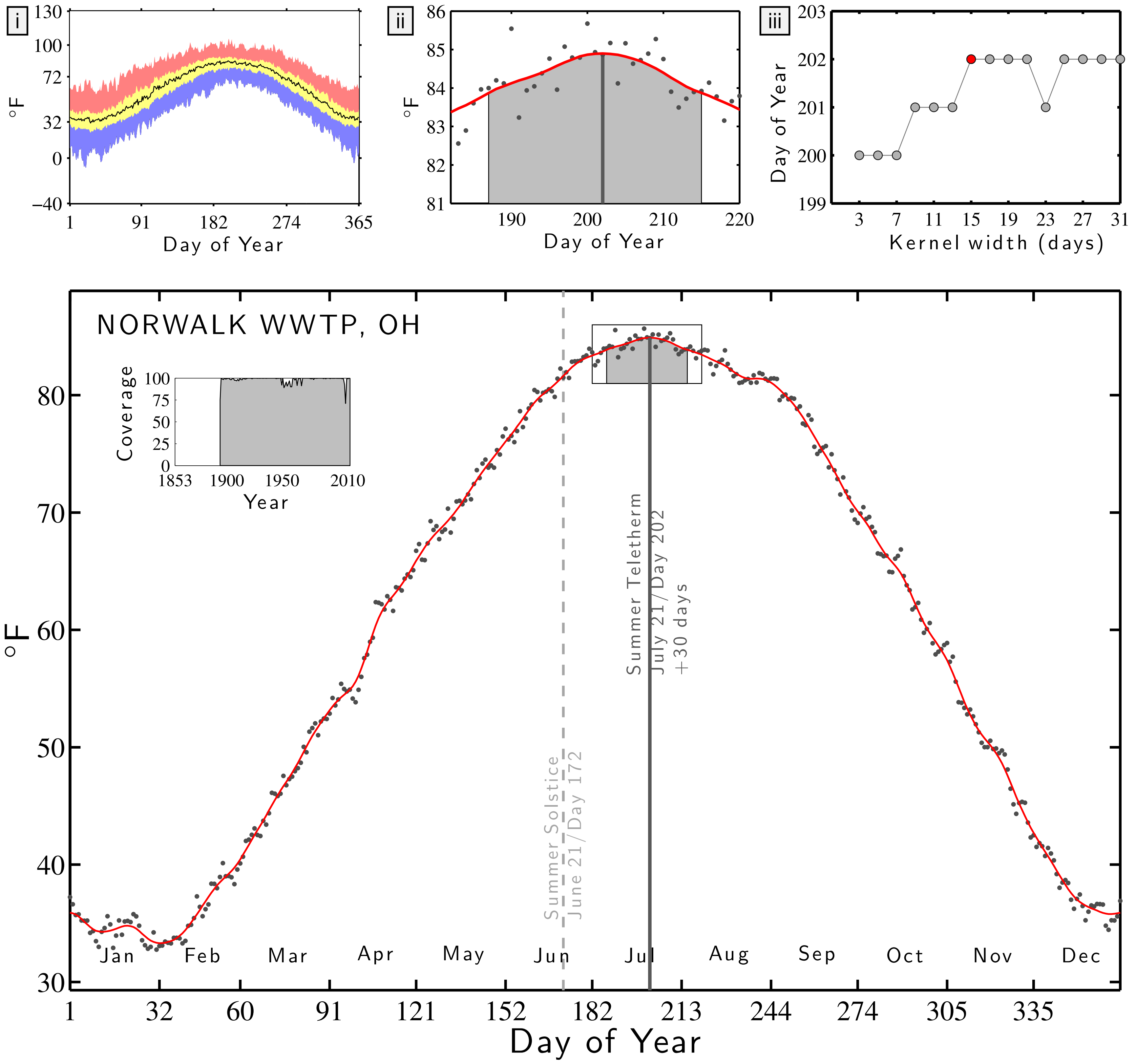

i
130
100
72
32
0
−40
°F
1
91
182
274
365
Day of Year
ii
86
85
84
83
82
81
°F
190
200
210
220
Day of Year
iii
203
202
201
200
199
Day of Year
3
7
11
15
19
23
27
31
Kernel width (days)
NORWALK WWTP, OH
Coverage
100
75
50
25
0
1853
1900
1950
2010
Year
Summer Solstice
June 21 / Day 172
Summer Teletherm
July 21 / Day 202
+30 days
80
70
60
50
40
30
°F
Jan
Feb
Mar
Apr
May
Jun
Jul
Aug
Sep
Oct
Nov
Dec
1
32
60
91
121
152
182
213
244
274
305
335
Day of Year

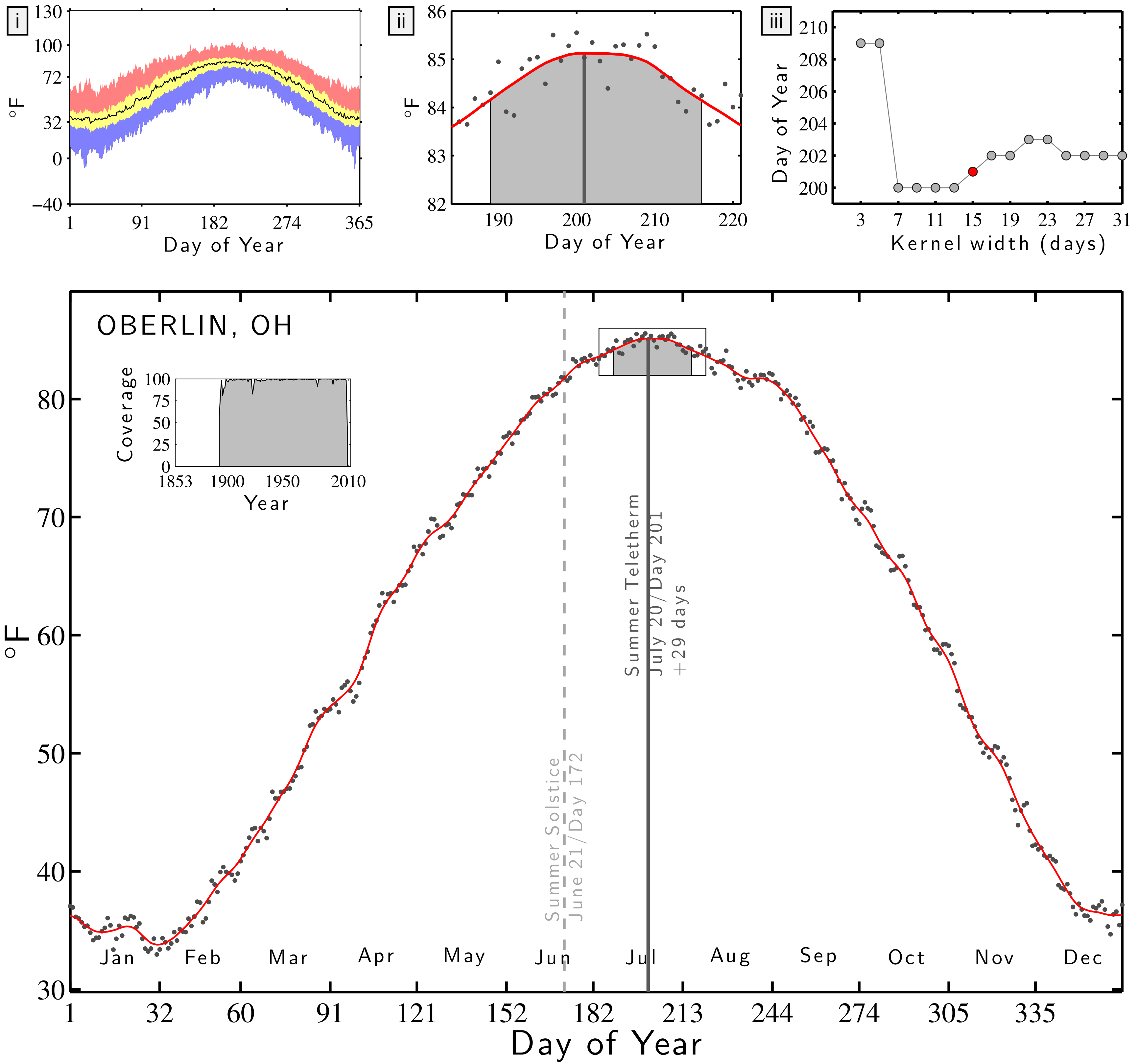

i
ii
iii
°F
Day of Year
Kernel width (days)
OBERLIN, OH
Coverage
Year
Summer Solstice
June 21/Day 172
Summer Teletherm
July 20/Day 201
+29 days
Jan
Feb
Mar
Apr
May
Jun
Jul
Aug
Sep
Oct
Nov
Dec

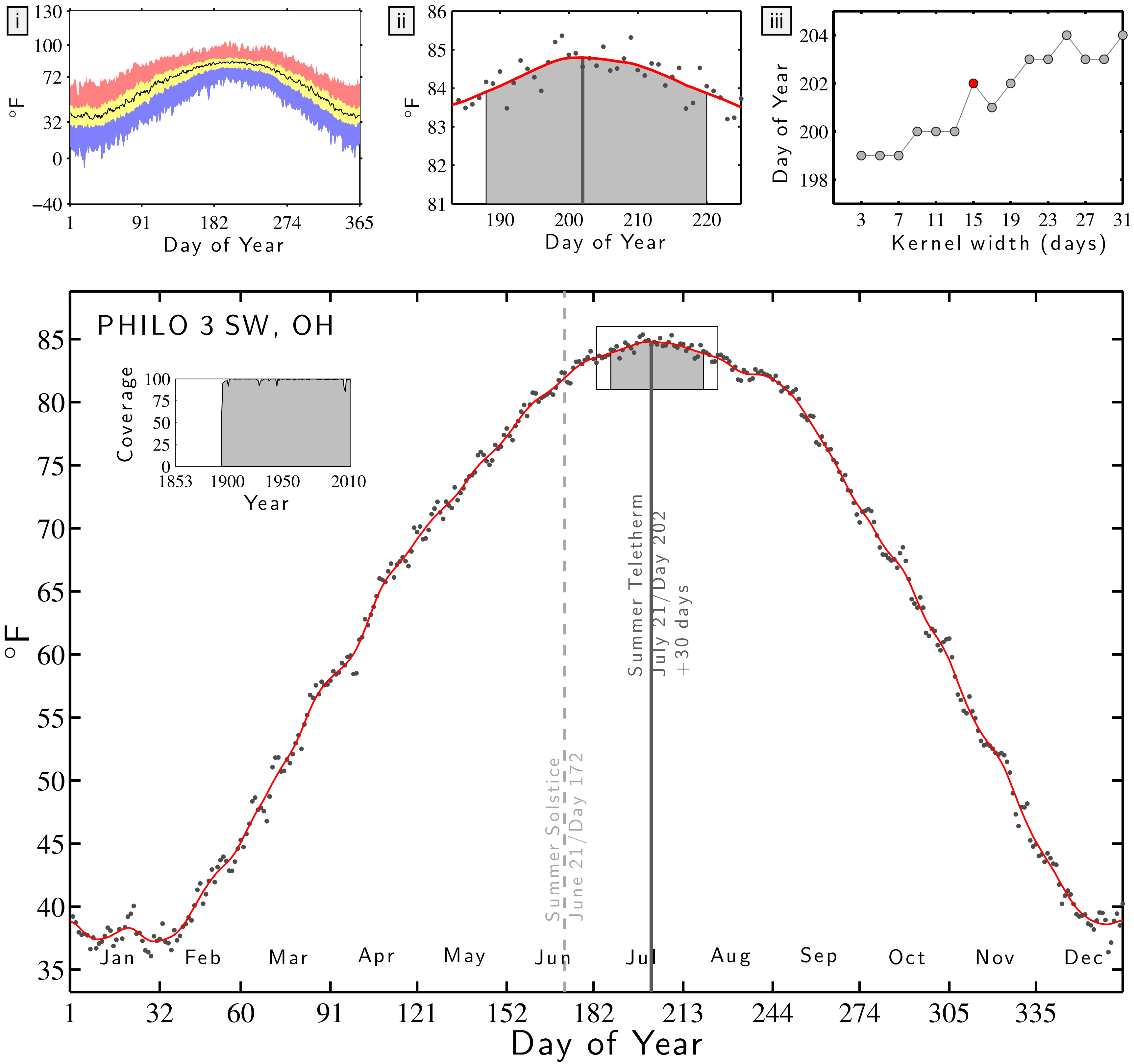

i
°F
Day of Year
ii
°F
Day of Year
iii
Day of Year
Kernel width (days)
PHILO 3 SW, OH
Coverage
Year
Summer Solstice
June 21 / Day 172
Summer Teletherm
July 21 / Day 202
+30 days
Jan
Feb
Mar
Apr
May
Jun
Jul
Aug
Sep
Oct
Nov
Dec
°F
Day of Year

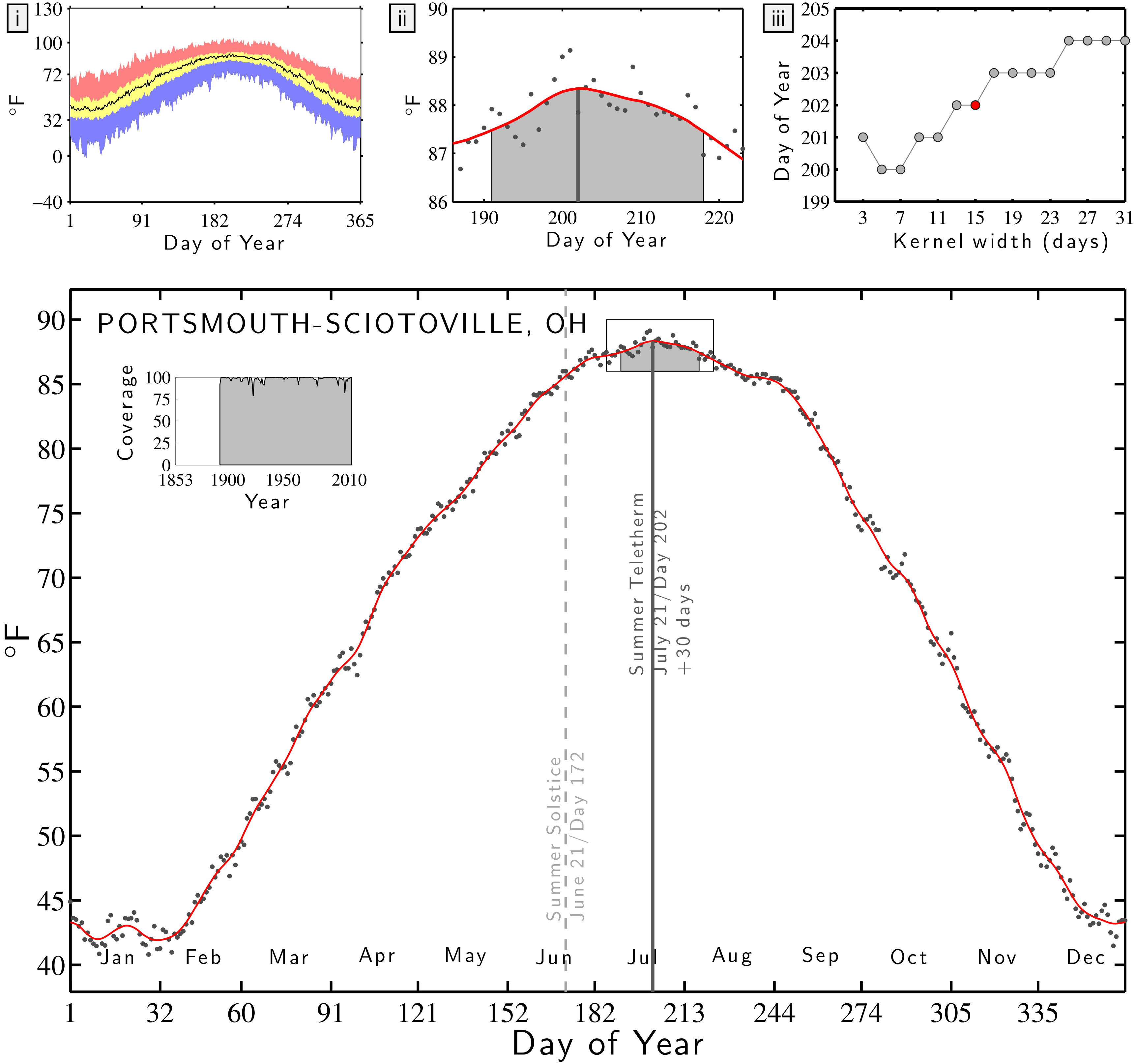
i
ii
iii
PORTSMOUTH-SCIOTOVILLE, OH
Coverage
Year
Summer Solstice
June 21/Day 172
Summer Teletherm
July 21/Day 202
+30 days
Kernel width (days)
Day of Year
°F
Jan
Feb
Mar
Apr
May
Jun
Jul
Aug
Sep
Oct
Nov
Dec

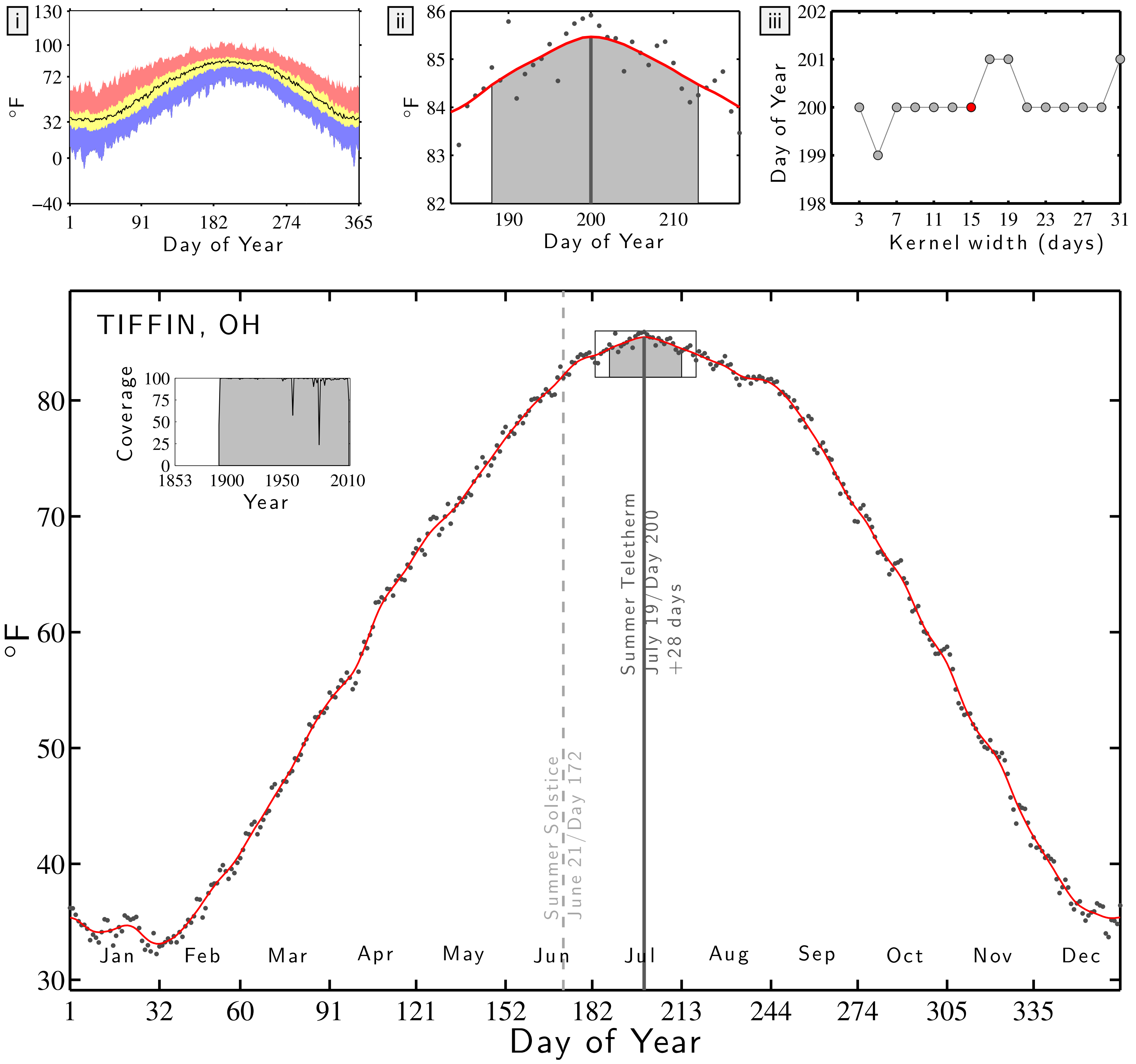

i
°F
Day of Year
ii
°F
Day of Year
iii
Day of Year
Kernel width (days)
TIFFIN, OH
Coverage
Year
Summer Solstice
June 21/Day 172
Summer Teletherm
July 19/Day 200
+28 days
Jan
Feb
Mar
Apr
May
Jun
Jul
Aug
Sep
Oct
Nov
Dec
°F
Day of Year

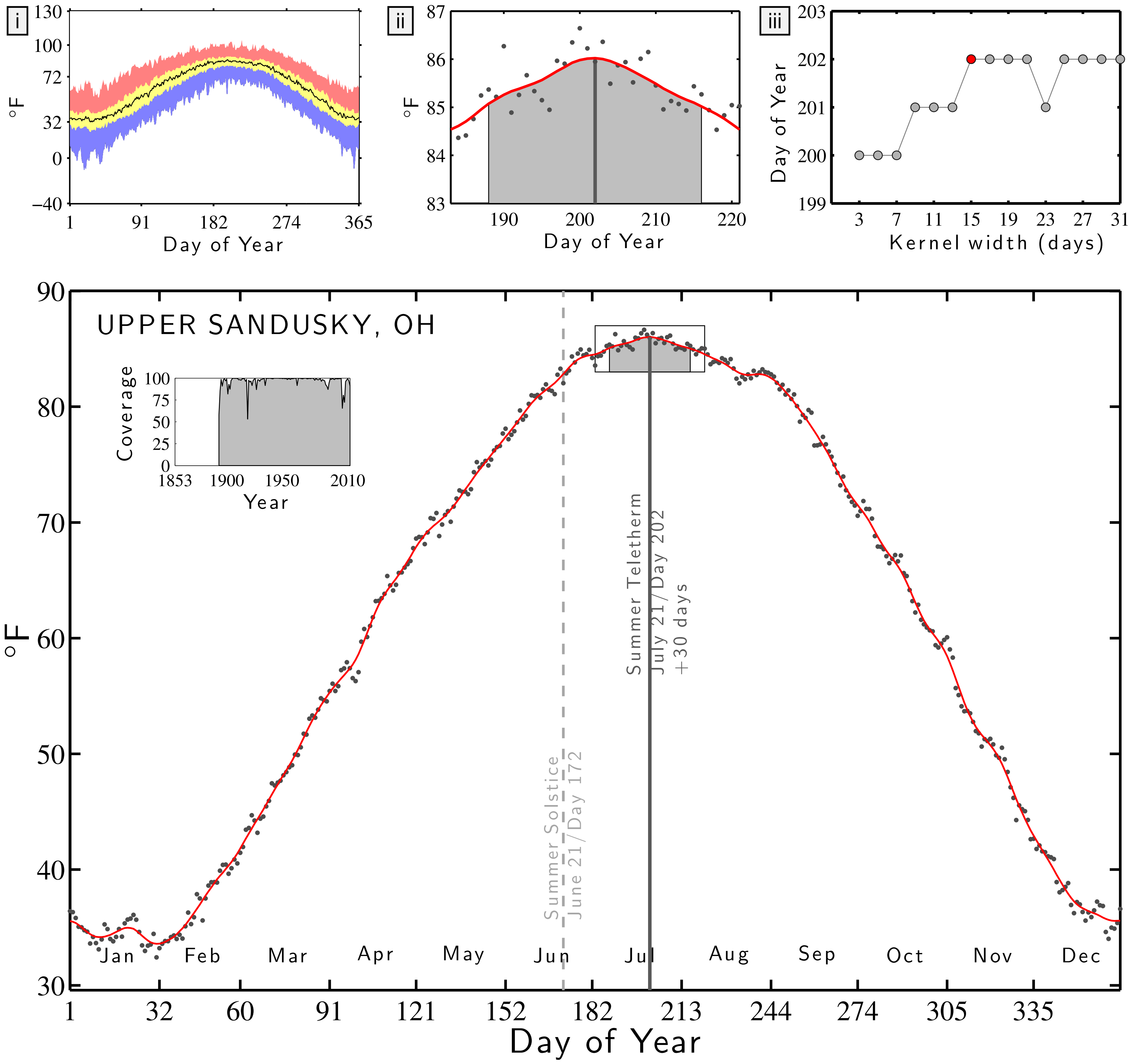

i
°F
Day of Year
ii
°F
Day of Year
iii
Day of Year
Kernel width (days)
UPPER SANDUSKY, OH
Coverage
Year
Summer Solstice
June 21 / Day 172
Summer Teletherm
July 21 / Day 202
+30 days
Jan
Feb
Mar
Apr
May
Jun
Jul
Aug
Sep
Oct
Nov
Dec
°F
Day of Year

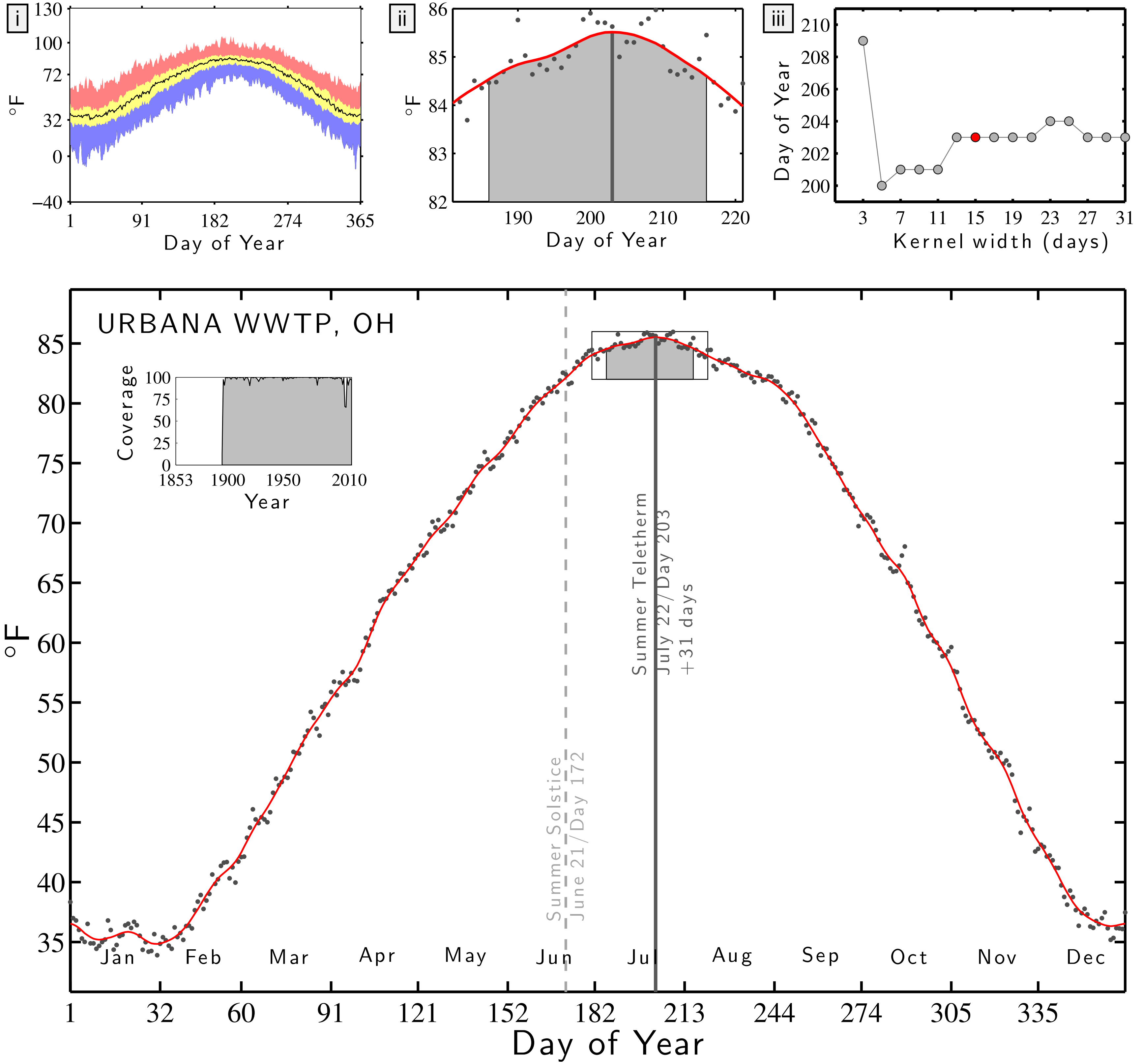

i
°F
Day of Year
ii
iii
Day of Year
Kernel width (days)
URBANA WWTP, OH
Coverage
Year
Summer Solstice
June 21/Day 172
Summer Teletherm
July 22/Day 203
+31 days
Jan
Feb
Mar
Apr
May
Jun
Jul
Aug
Sep
Oct
Nov
Dec

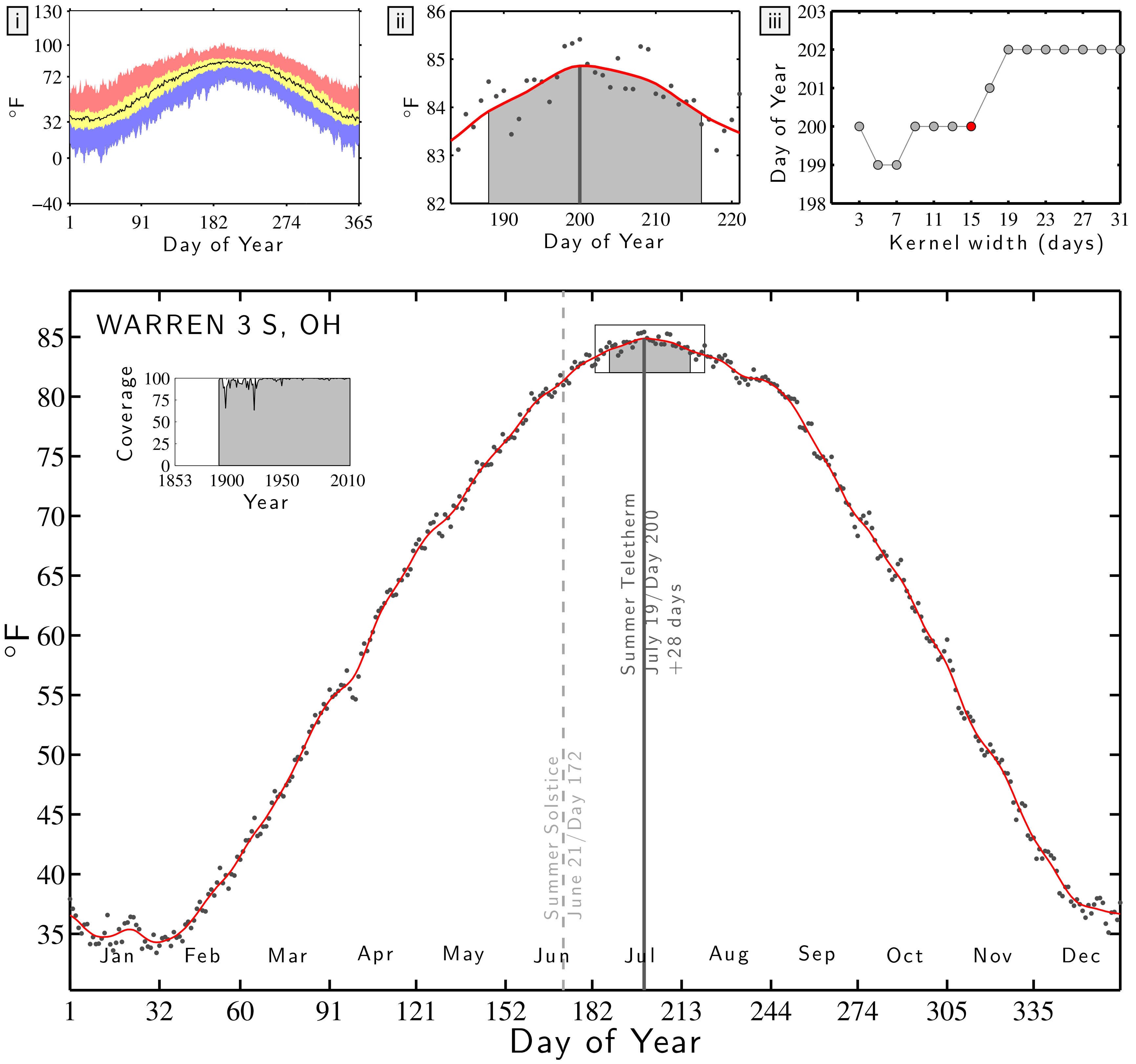

i
°F
130
100
72
32
0
−40
1
91
182
274
365
Day of Year
ii
86
85
84
83
82
190
200
210
220
Day of Year
iii
203
202
201
200
199
198
3
7
11
15
19
23
27
31
Kernel width (days)
Day of Year
WARREN 3 S, OH
Coverage
100
75
50
25
0
1853
1900
1950
2010
Year
Summer Solstice
June 21/Day 172
Summer Teletherm
July 19/Day 200
+28 days
85
80
75
70
65
60
55
50
45
40
35
Jan
Feb
Mar
Apr
May
Jun
Jul
Aug
Sep
Oct
Nov
Dec
1
32
60
91
121
152
182
213
244
274
305
335
Day of Year

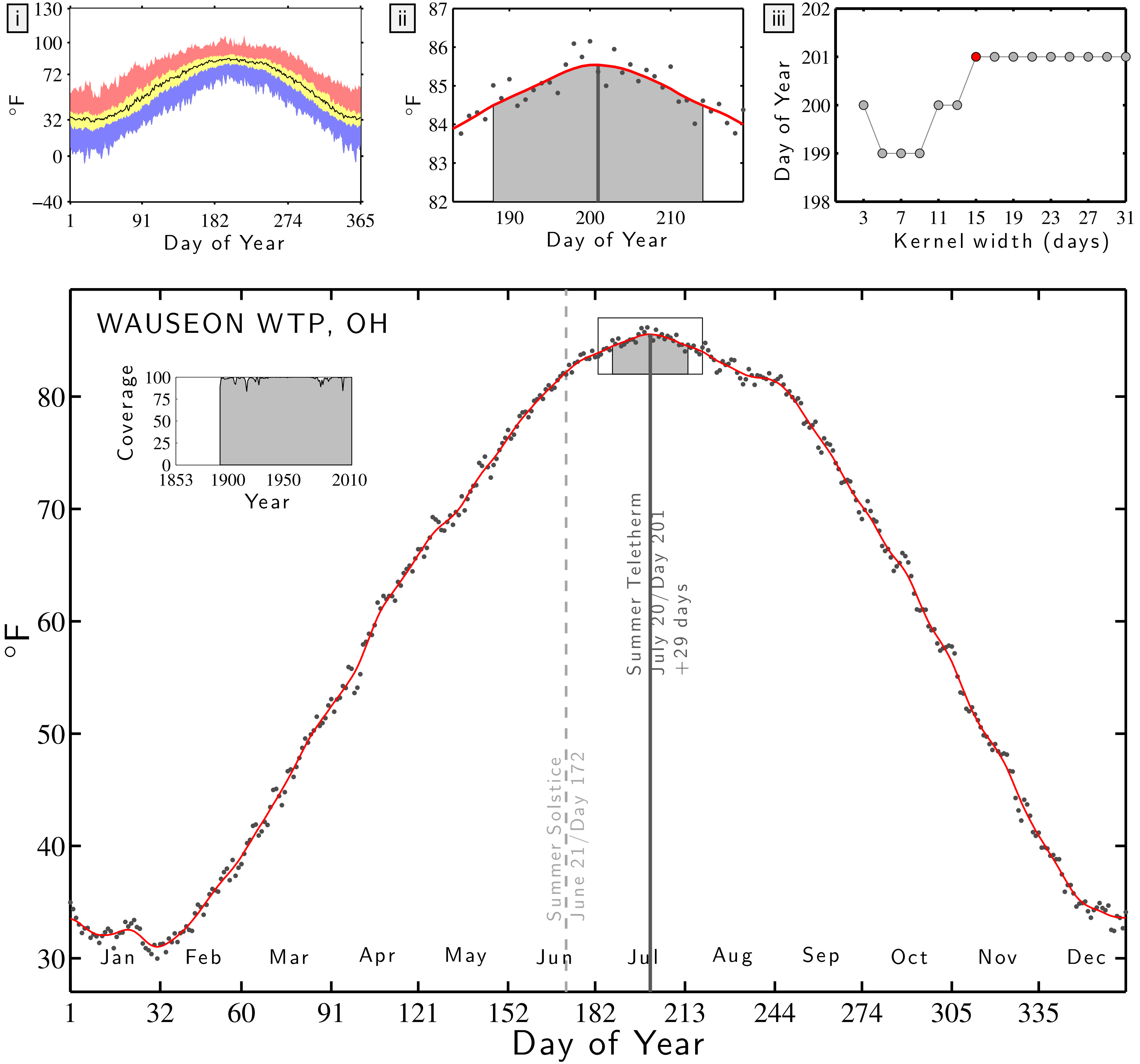

i
°F
Day of Year
ii
°F
Day of Year
iii
Day of Year
Kernel width (days)
WAUSEON WTP, OH
Coverage
Year
Summer Solstice
June 21/Day 172
Summer Teletherm
July 20/Day 201
+29 days
Jan
Feb
Mar
Apr
May
Jun
Jul
Aug
Sep
Oct
Nov
Dec
°F
Day of Year

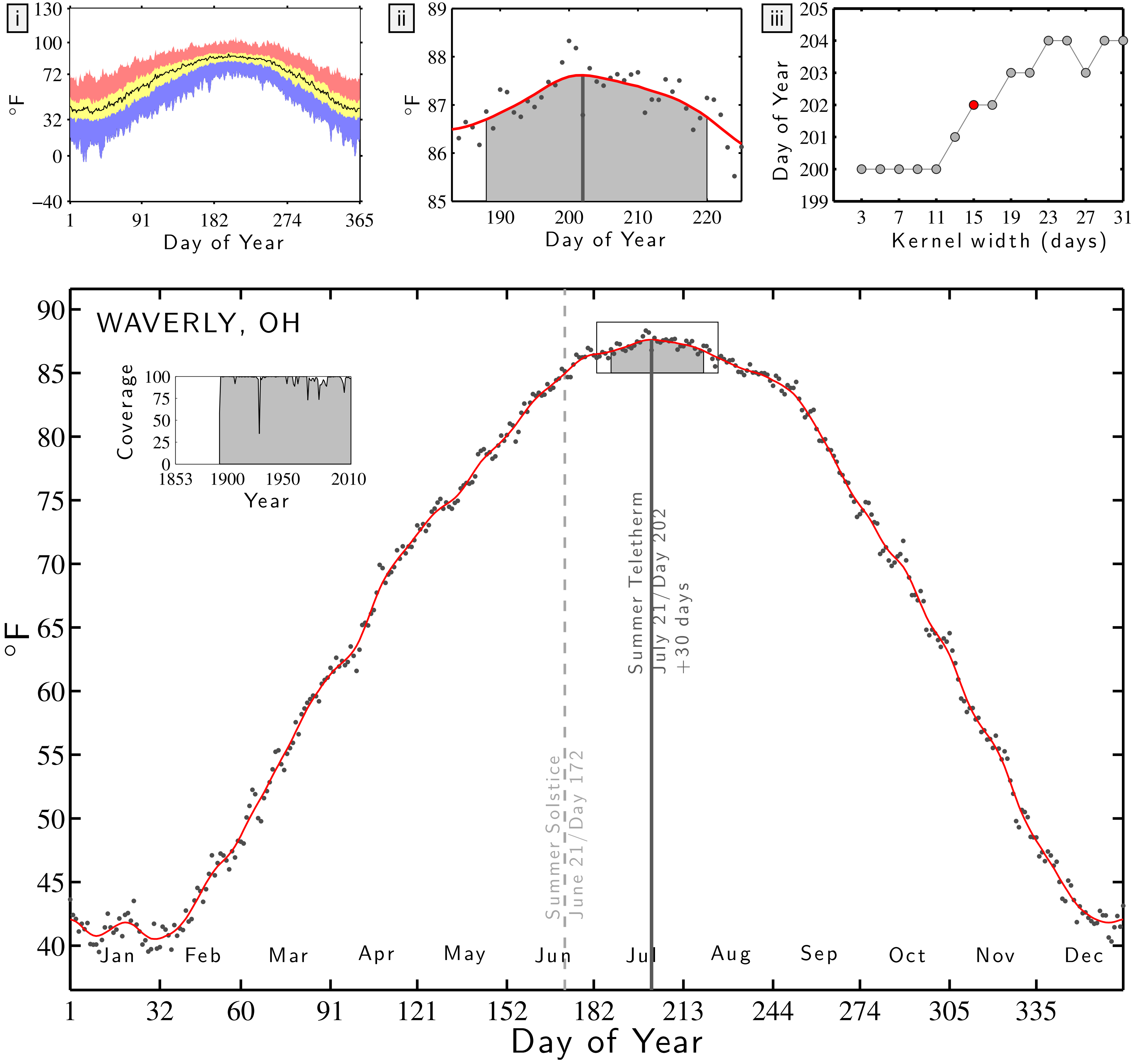

i
°F
Day of Year
ii
°F
Day of Year
iii
Day of Year
Kernel width (days)
WAVERLY, OH
Coverage
Year
Summer Solstice
June 21 / Day 172
Summer Teletherm
July 21 / Day 202
+30 days
Jan
Feb
Mar
Apr
May
Jun
Jul
Aug
Sep
Oct
Nov
Dec
°F
Day of Year

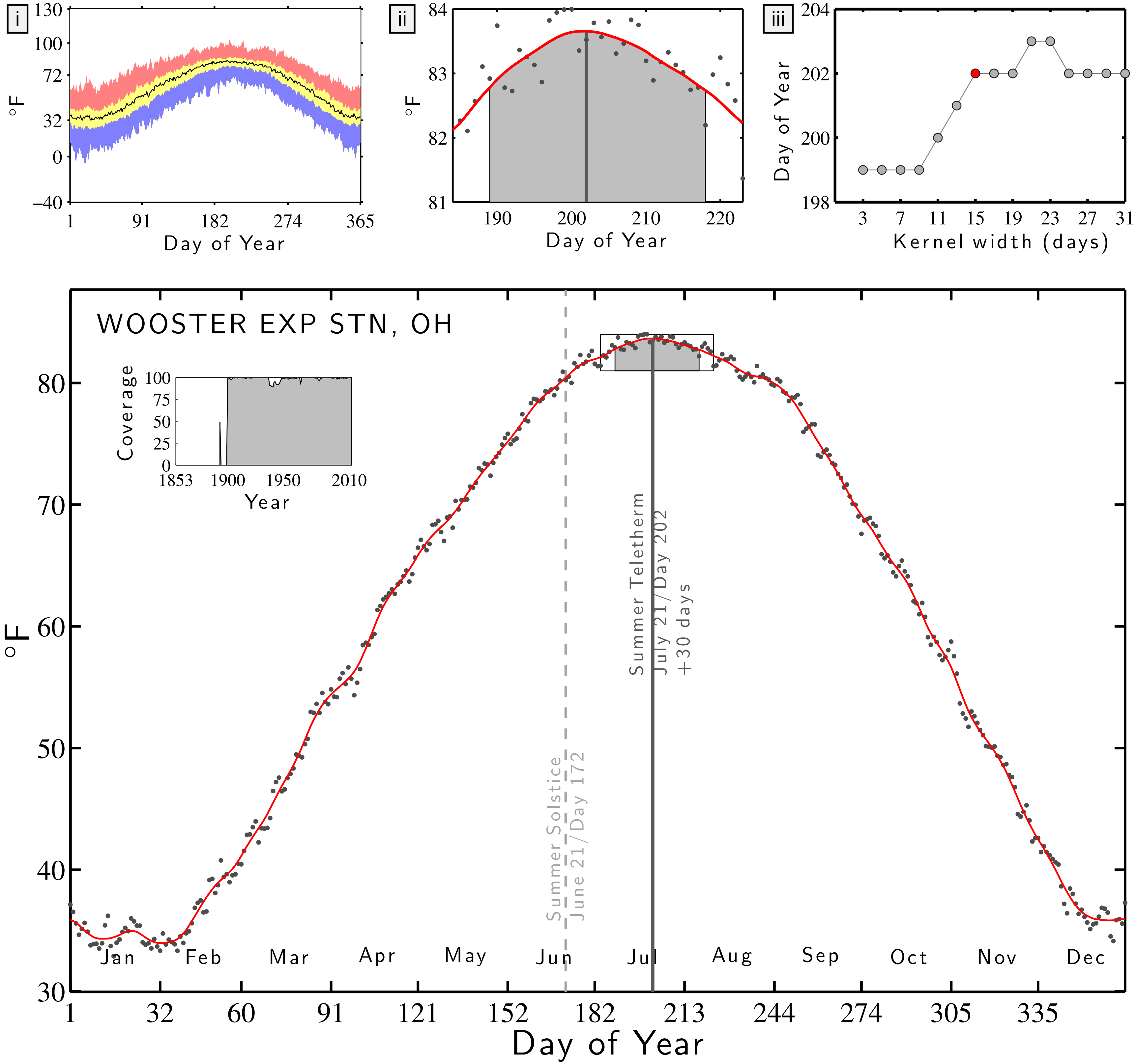

i
130
100
72
32
0
−40
°F
1
91
182
274
365
Day of Year
ii
84
83
82
81
°F
190
200
210
220
Day of Year
iii
204
202
200
198
Day of Year
3
7
11
15
19
23
27
31
Kernel width (days)
WOOSTER EXP STN, OH
Coverage
100
75
50
25
0
1853
1900
1950
2010
Year
Summer Solstice
June 21 / Day 172
Summer Teletherm
July 21 / Day 202
+30 days
Jan
Feb
Mar
Apr
May
Jun
Jul
Aug
Sep
Oct
Nov
Dec
80
70
60
50
40
30
°F
1
32
60
91
121
152
182
213
244
274
305
335
Day of Year

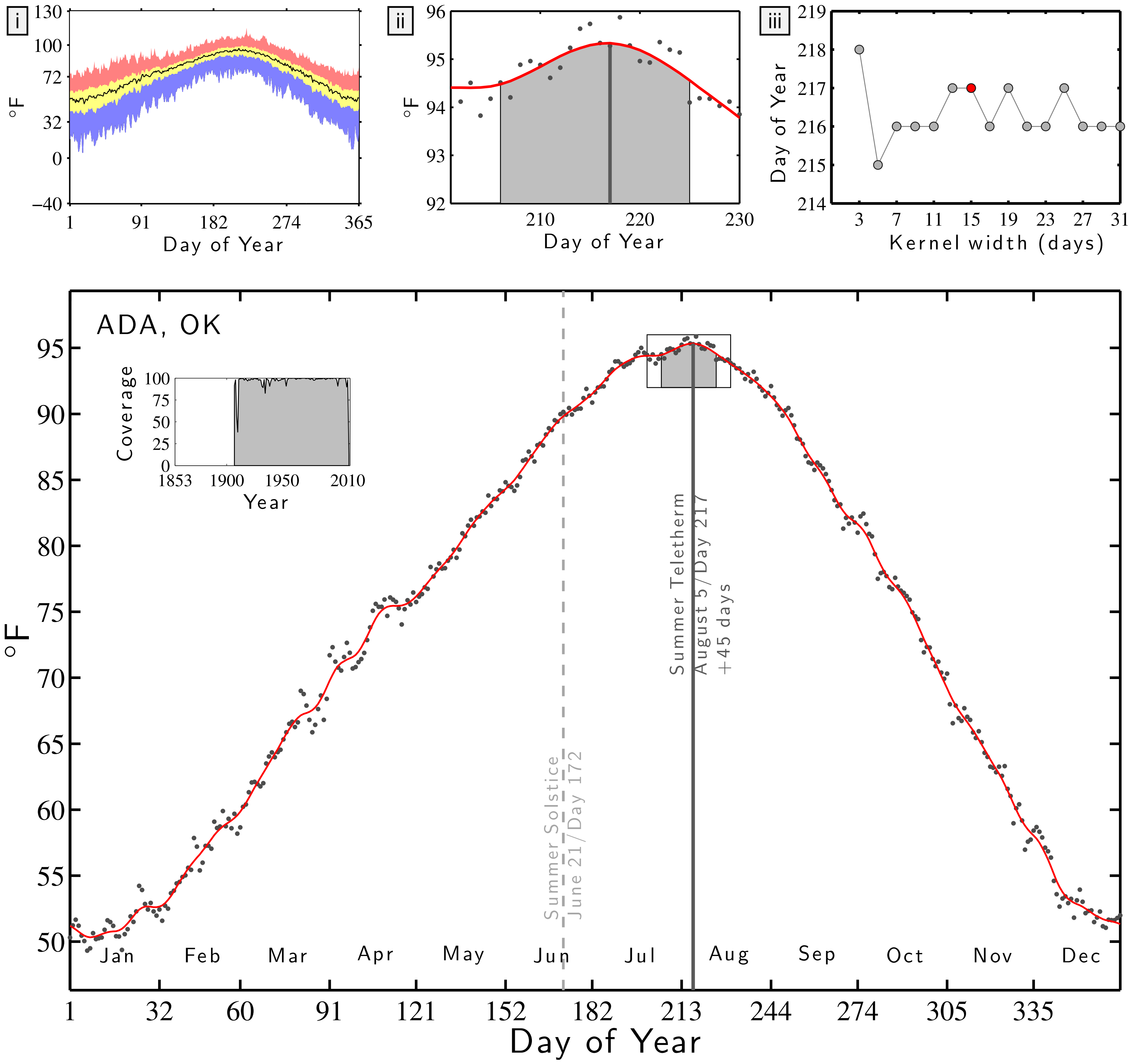

i
°F
Day of Year
ii
°F
Day of Year
iii
Day of Year
Kernel width (days)
ADA, OK
Coverage
Year
°F
Summer Solstice
June 21/Day 172
Summer Teletherm
August 5/Day 217
+45 days
Jan
Feb
Mar
Apr
May
Jun
Jul
Aug
Sep
Oct
Nov
Dec
Day of Year

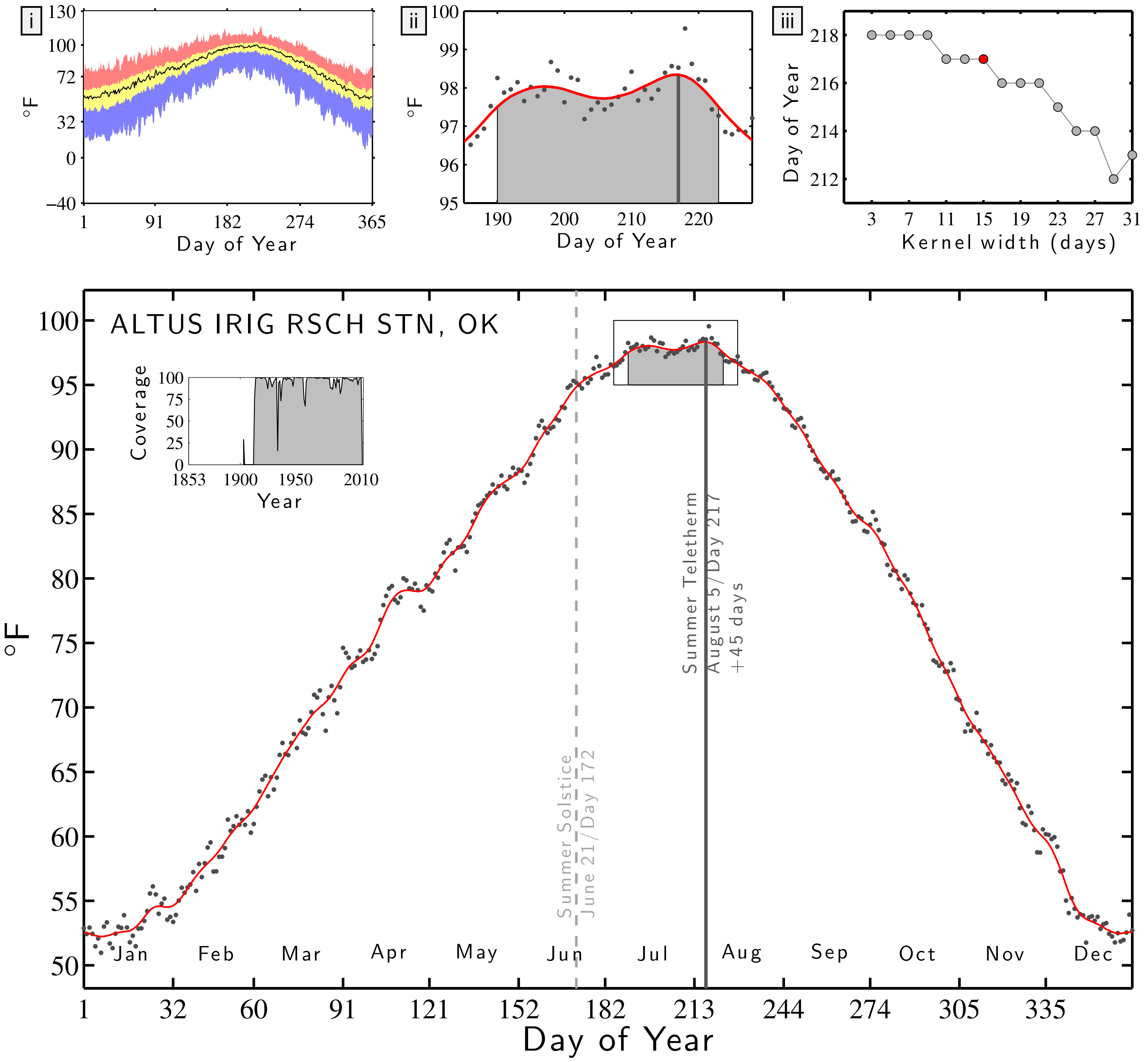

i
ii
iii
Day of Year
Kernel width (days)
ALTUS IRIG RSCH STN, OK
Coverage
Year
Summer Solstice
June 21/Day 172
Summer Teletherm
August 5/Day 217
+45 days
Jan
Feb
Mar
Apr
May
Jun
Jul
Aug
Sep
Oct
Nov
Dec
°F

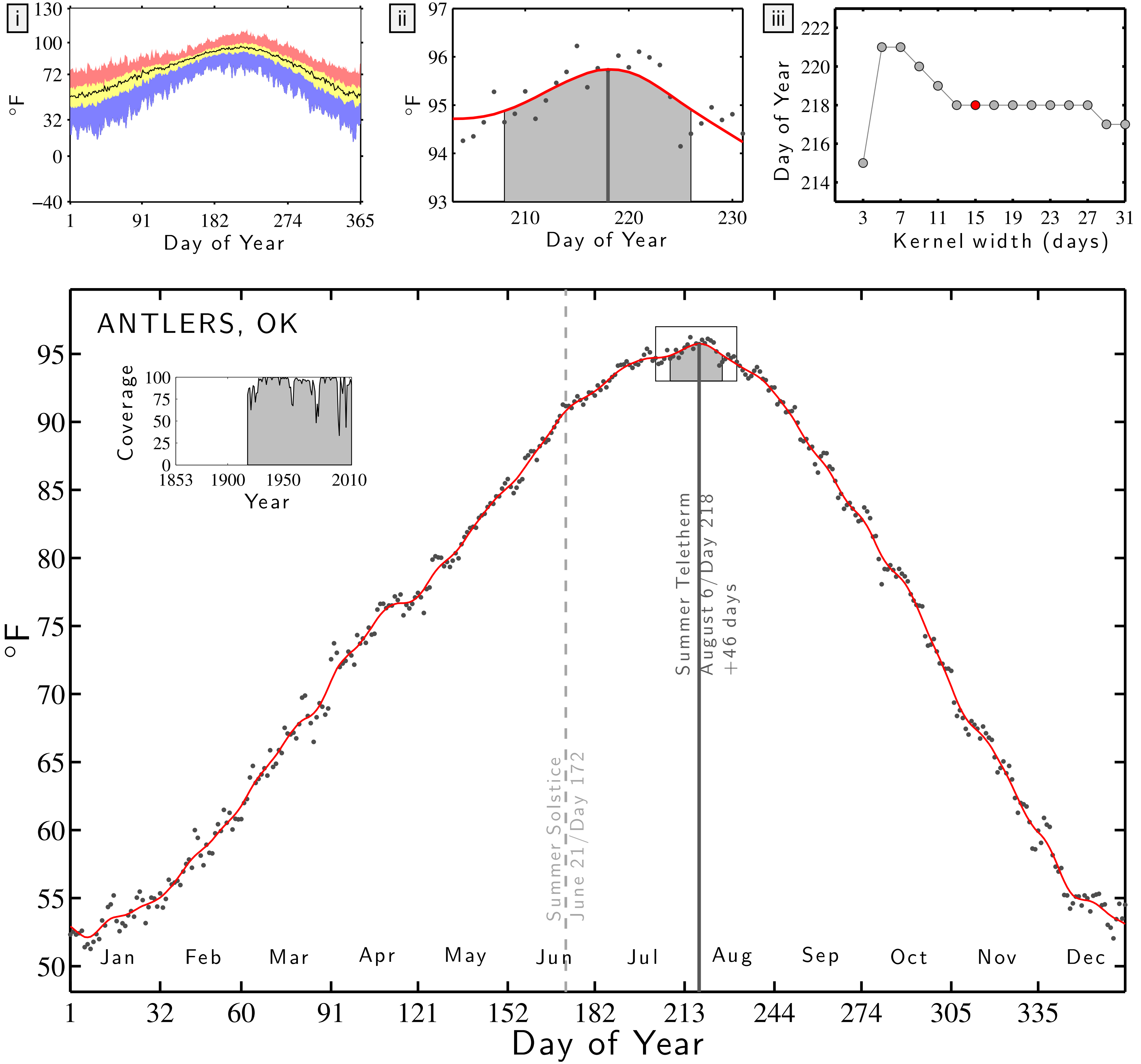

i
°F
Day of Year
ii
°F
Day of Year
iii
Day of Year
Kernel width (days)
ANTLERS, OK
Coverage
Year
Summer Solstice
June 21 / Day 172
Summer Teletherm
August 6 / Day 218
+46 days
Jan
Feb
Mar
Apr
May
Jun
Jul
Aug
Sep
Oct
Nov
Dec
°F
Day of Year

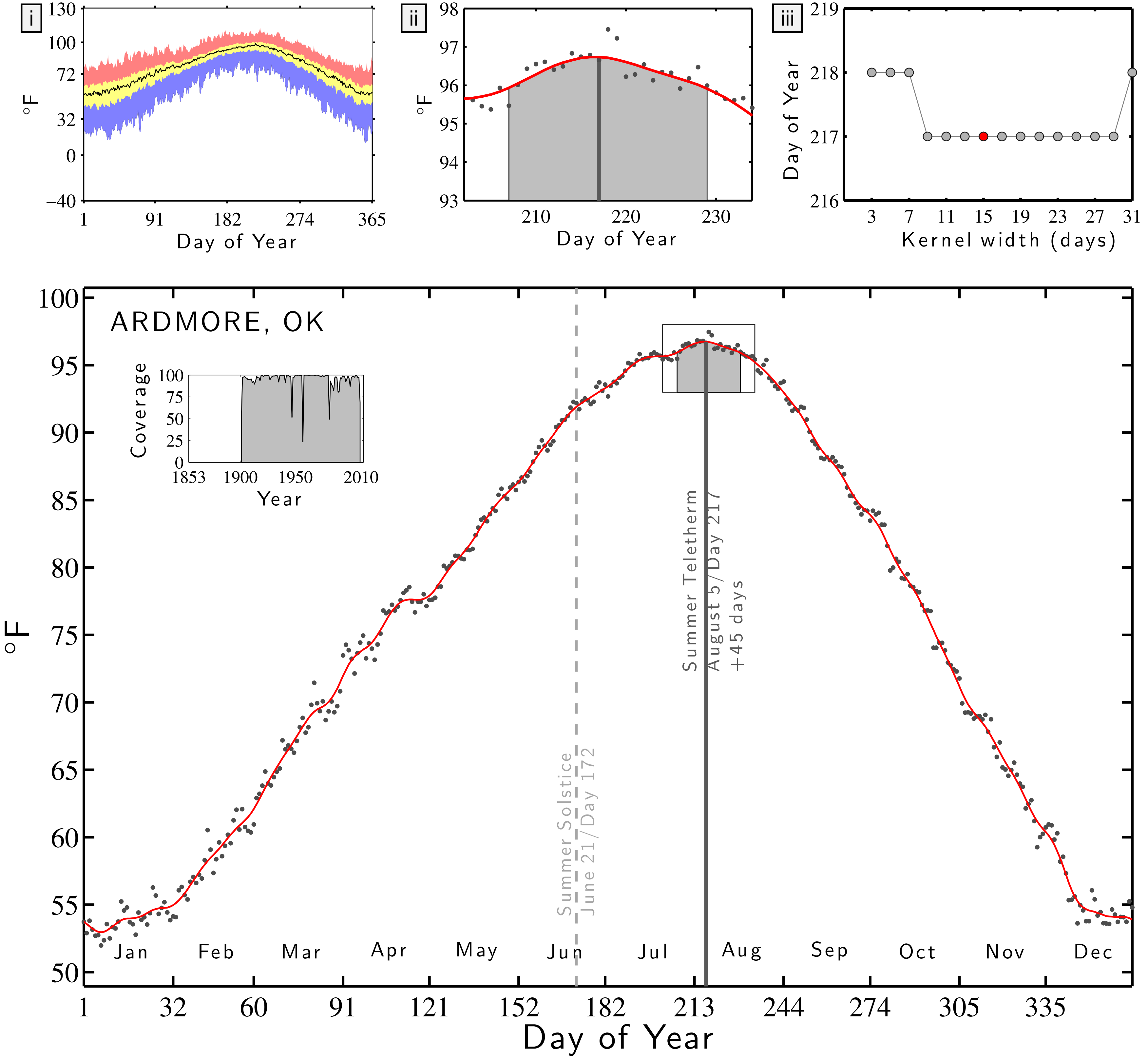

i
°F
Day of Year
ii
°F
Day of Year
iii
Day of Year
Kernel width (days)
ARDMORE, OK
Coverage
Year
Summer Solstice
June 21/Day 172
Summer Teletherm
August 5/Day 217
+45 days
Jan
Feb
Mar
Apr
May
Jun
Jul
Aug
Sep
Oct
Nov
Dec
°F
Day of Year

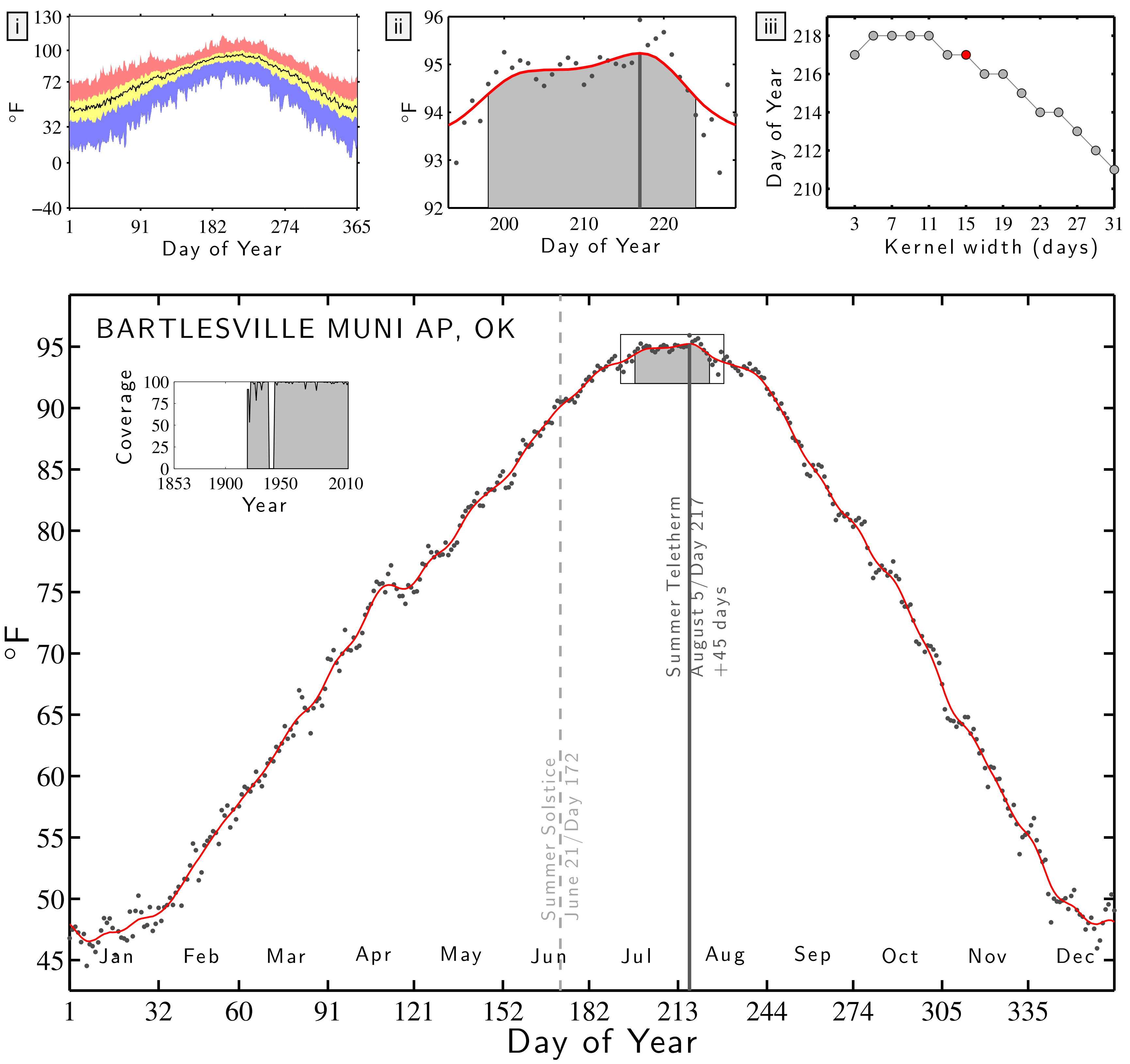

i
°F
Day of Year
ii
°F
Day of Year
iii
Day of Year
Kernel width (days)
BARTLESVILLE MUNI AP, OK
Coverage
Year
Summer Solstice
June 21/Day 172
Summer Teletherm
August 5/Day 217
+45 days
Jan
Feb
Mar
Apr
May
Jun
Jul
Aug
Sep
Oct
Nov
Dec
°F
Day of Year

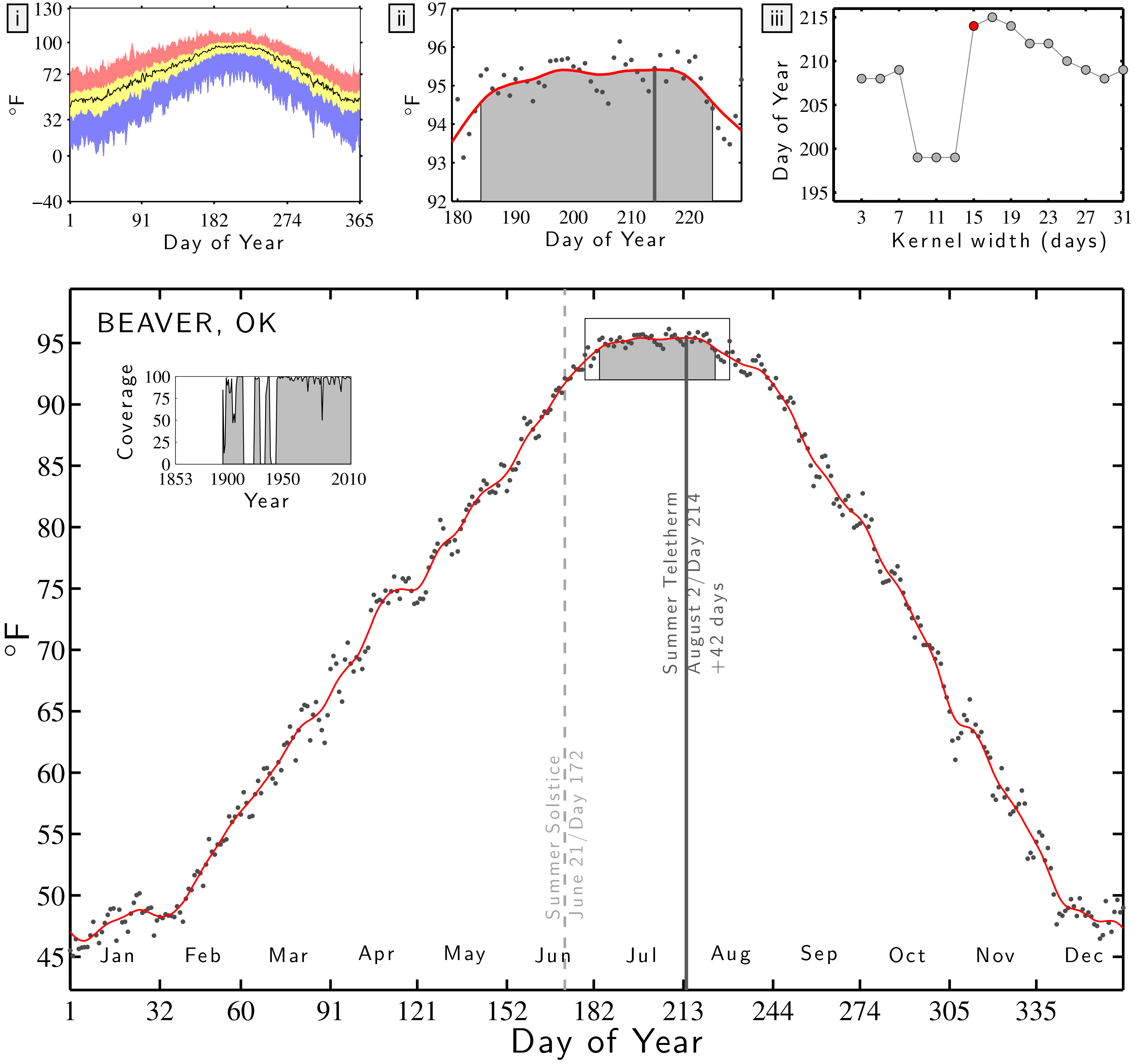

i
ii
iii
°F
Day of Year
Kernel width (days)
BEAVER, OK
Coverage
Year
Summer Solstice
June 21 / Day 172
Summer Teletherm
August 2 / Day 214
+42 days
Jan
Feb
Mar
Apr
May
Jun
Jul
Aug
Sep
Oct
Nov
Dec

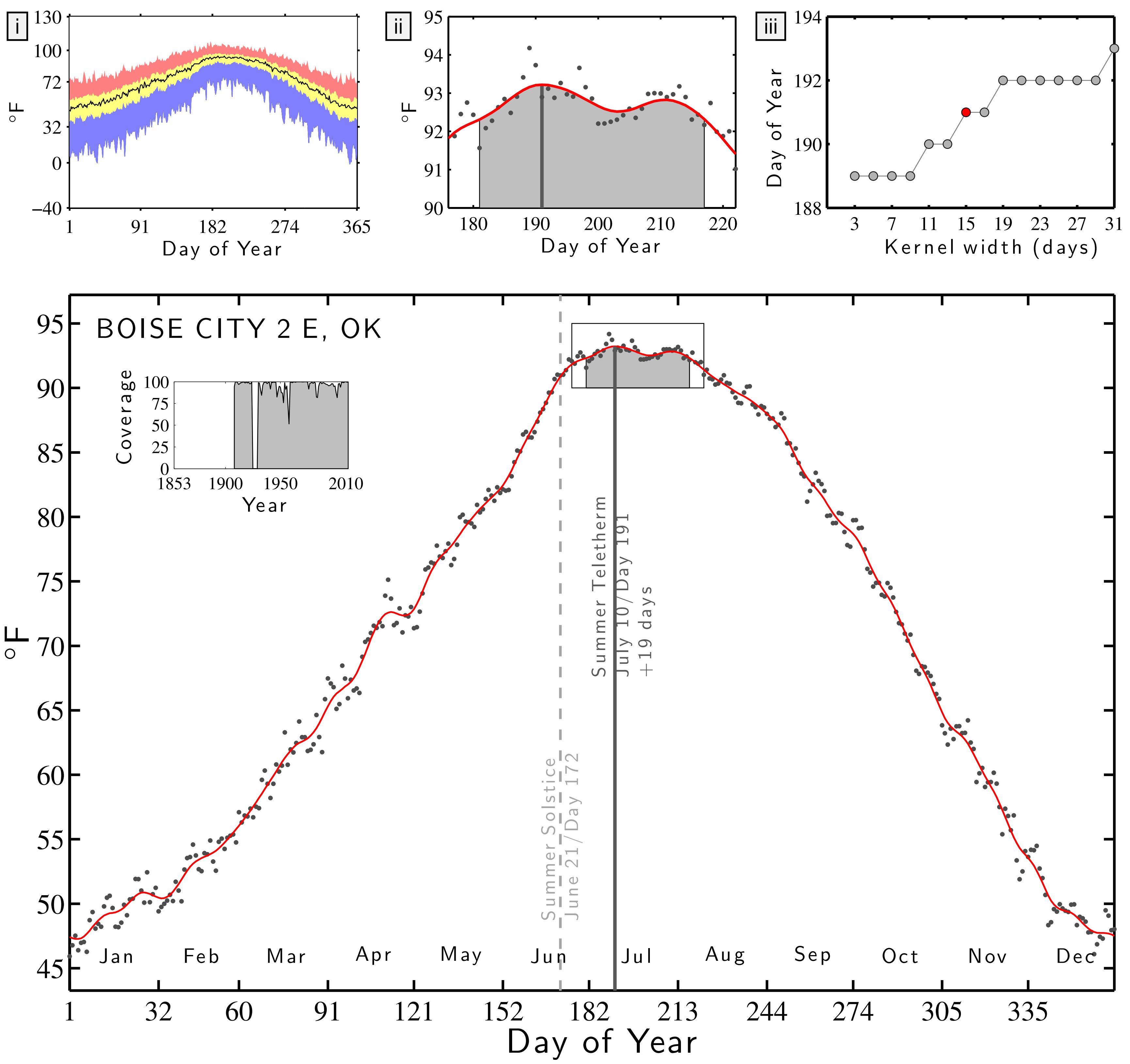

i
130
100
72
32
0
−40
°F
1
91
182
274
365
Day of Year
ii
95
94
93
92
91
90
°F
180
190
200
210
220
Day of Year
iii
194
192
190
188
Day of Year
3
7
11
15
19
23
27
31
Kernel width (days)
BOISE CITY 2 E, OK
Coverage
100
75
50
25
0
1853
1900
1950
2010
Year
Summer Solstice
June 21/Day 172
Summer Teletherm
July 10/Day 191
+19 days
95
90
85
80
75
70
65
60
55
50
45
°F
Jan
Feb
Mar
Apr
May
Jun
Jul
Aug
Sep
Oct
Nov
Dec
1
32
60
91
121
152
182
213
244
274
305
335
Day of Year

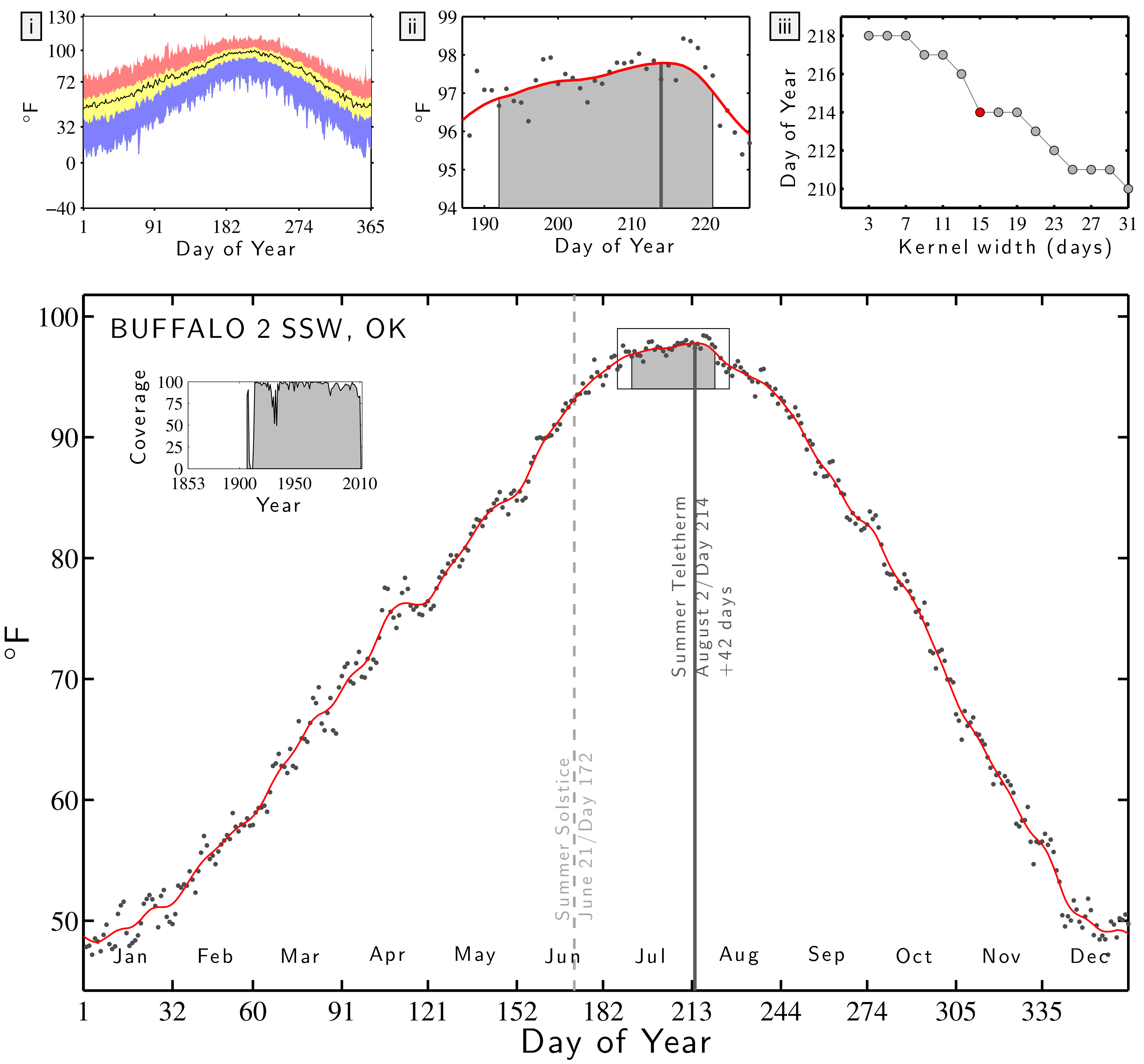

i
°F
130
100
72
32
0
−40
1
91
182
274
365
Day of Year
ii
99
98
97
96
95
94
190
200
210
220
iii
218
216
214
212
210
Day of Year
3
7
11
15
19
23
27
31
Kernel width (days)
BUFFALO 2 SSW, OK
Coverage
100
75
50
25
0
1853
1900
1950
2010
Year
Summer Solstice
June 21/Day 172
Summer Teletherm
August 2/Day 214
+42 days
Jan
Feb
Mar
Apr
May
Jun
Jul
Aug
Sep
Oct
Nov
Dec
90
80
70
60
50
32
60
121
152
213
244
305
335

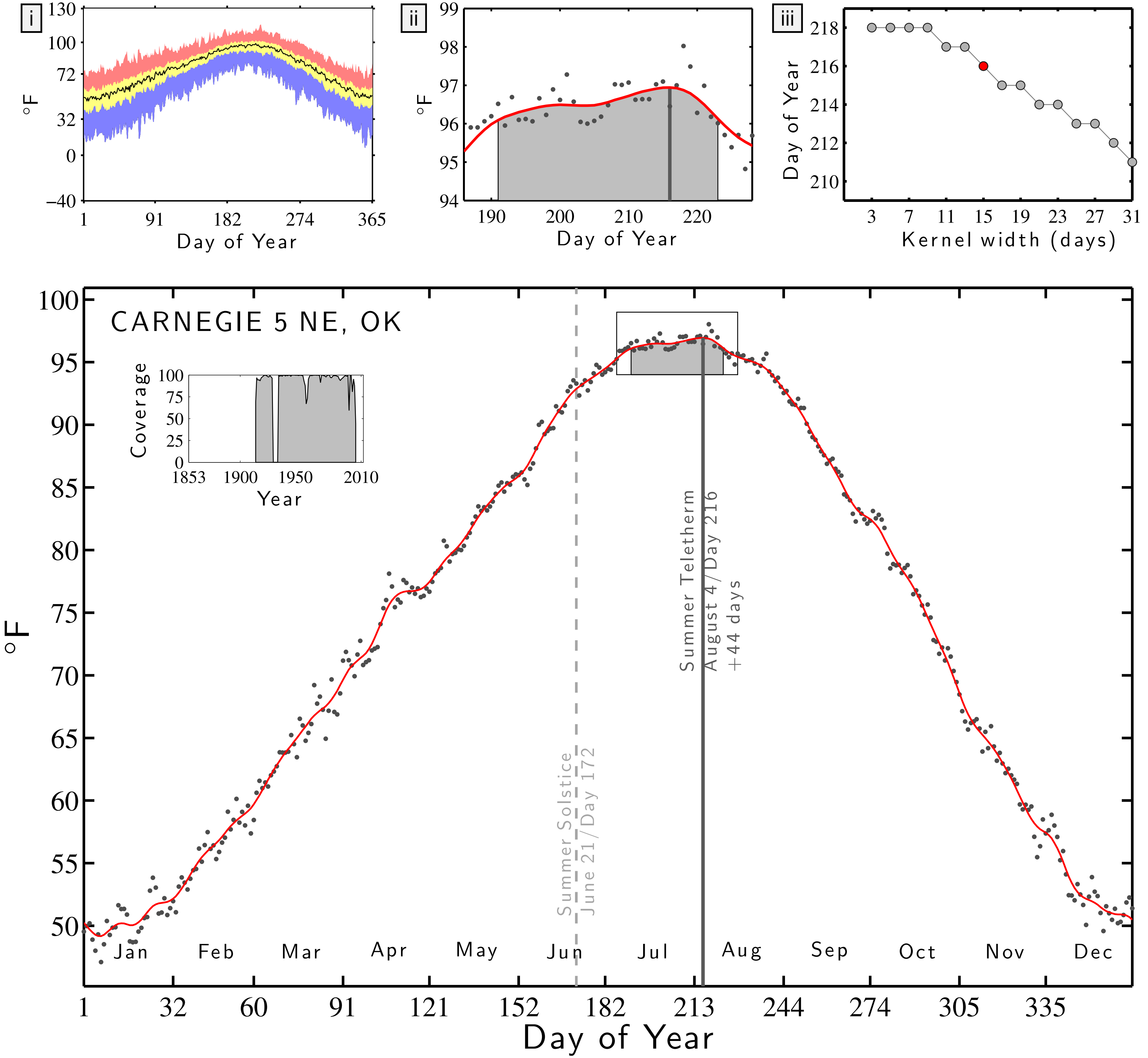

i
ii
iii
Day of Year
Kernel width (days)
°F
CARNEGIE 5 NE, OK
Coverage
Year
Summer Solstice
June 21/Day 172
Summer Teletherm
August 4/Day 216
+44 days
Jan
Feb
Mar
Apr
May
Jun
Jul
Aug
Sep
Oct
Nov
Dec
Day of Year

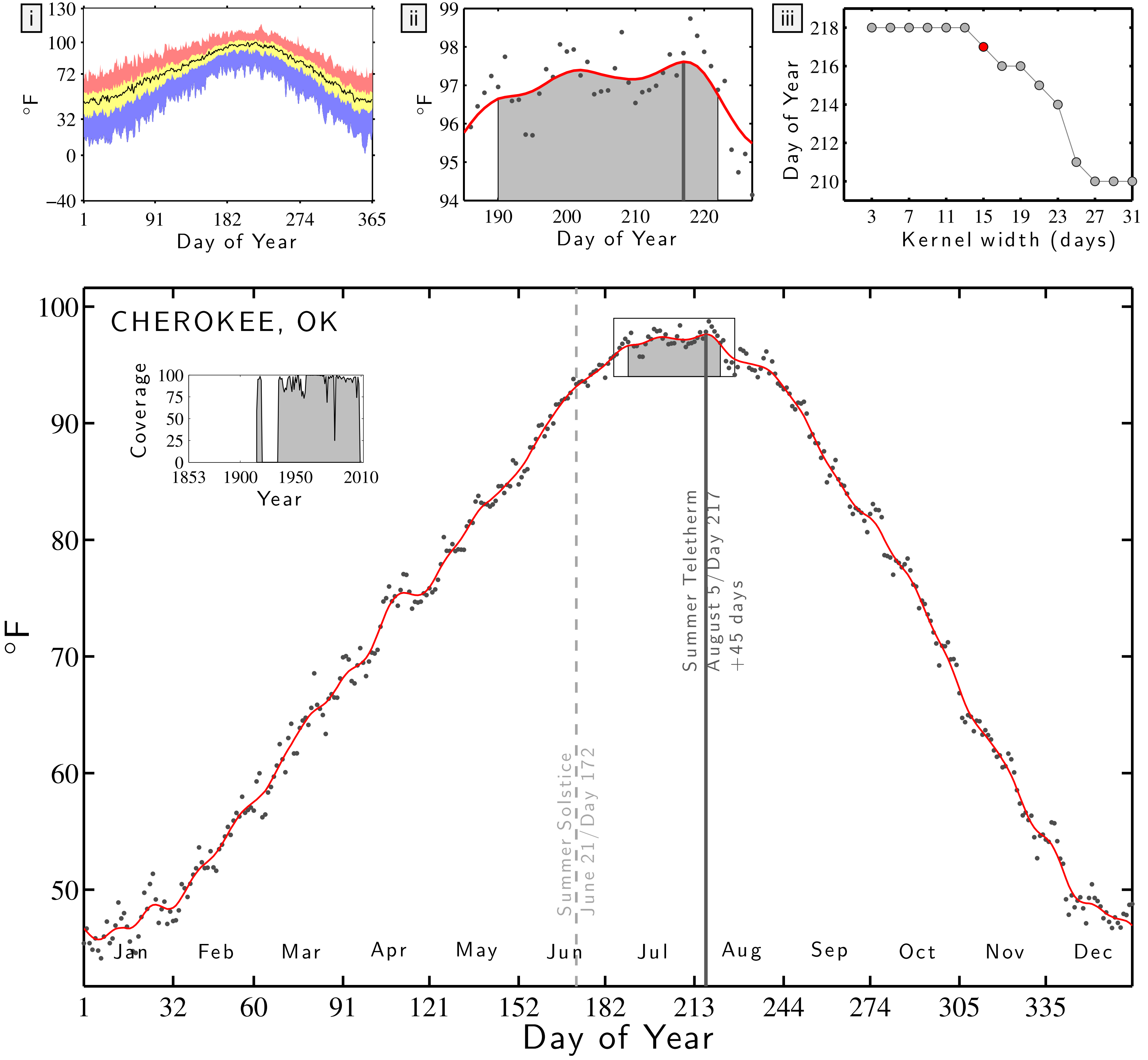

i
Day of Year
°F
ii
Day of Year
°F
iii
Kernel width (days)
Day of Year
CHEROKEE, OK
Coverage
Year
Summer Solstice
June 21/Day 172
Summer Teletherm
August 5/Day 217
+45 days
Jan
Feb
Mar
Apr
May
Jun
Jul
Aug
Sep
Oct
Nov
Dec
Day of Year
°F

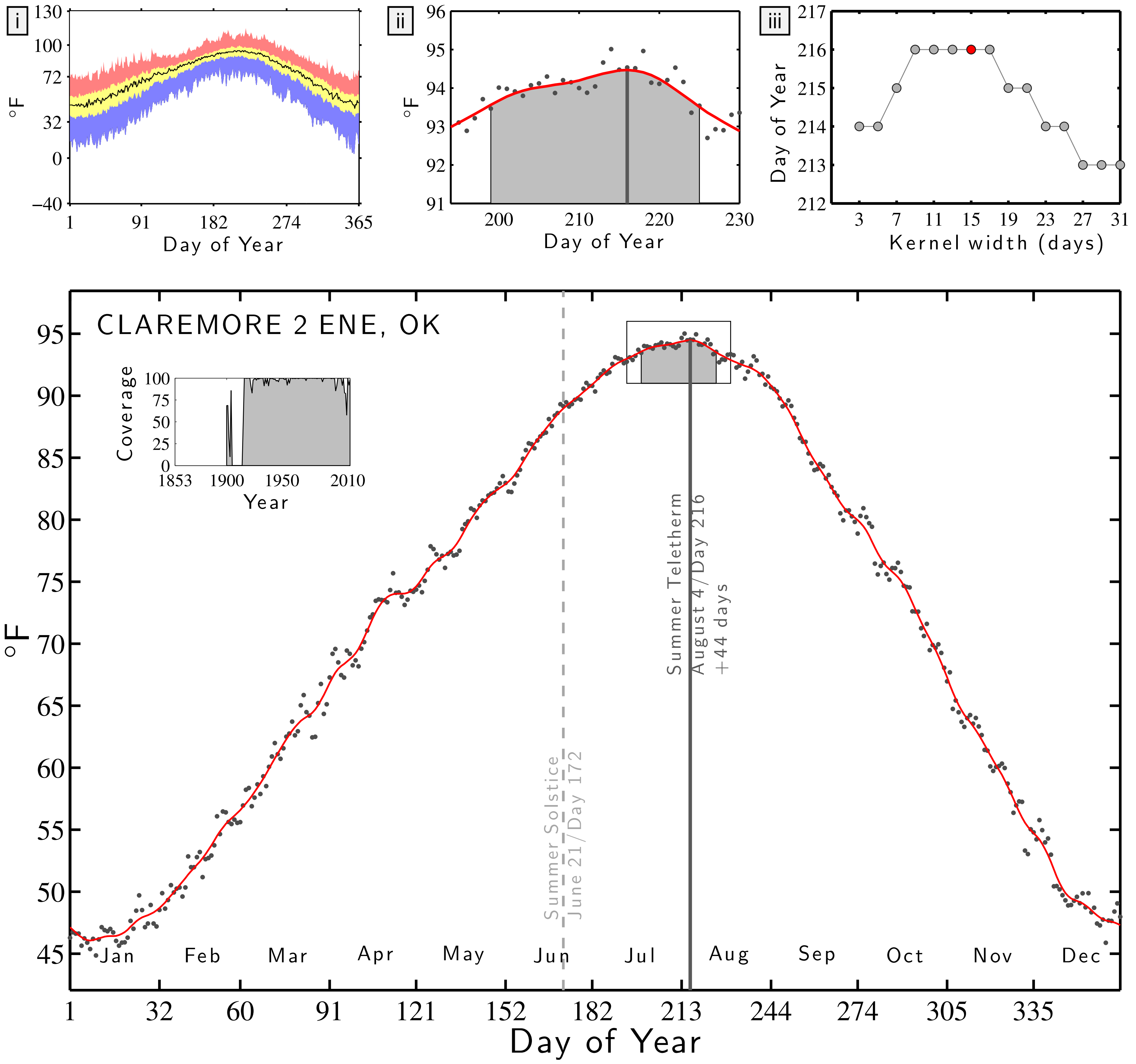
i
°F
Day of Year
ii
°F
Day of Year
iii
Day of Year
Kernel width (days)
CLAREMORE 2 ENE, OK
Coverage
Year
Summer Solstice
June 21 / Day 172
Summer Teletherm
August 4 / Day 216
+44 days
Jan
Feb
Mar
Apr
May
Jun
Jul
Aug
Sep
Oct
Nov
Dec
°F
Day of Year

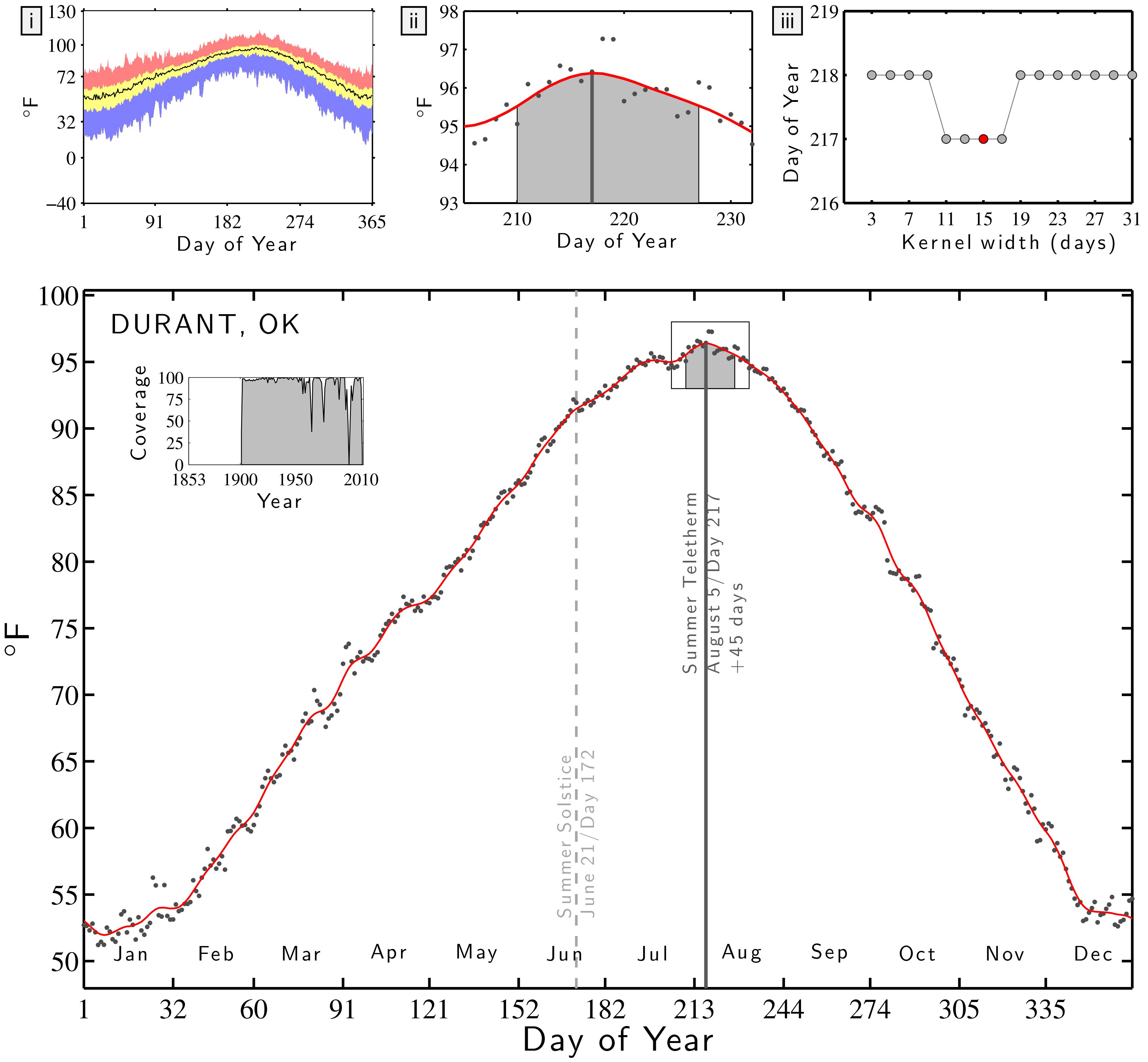

i
ii
iii
°F
Day of Year
Day of Year
Kernel width (days)
DURANT, OK
Coverage
Year
Summer Solstice
June 21/Day 172
Summer Teletherm
August 5/Day 217
+45 days
Jan
Feb
Mar
Apr
May
Jun
Jul
Aug
Sep
Oct
Nov
Dec

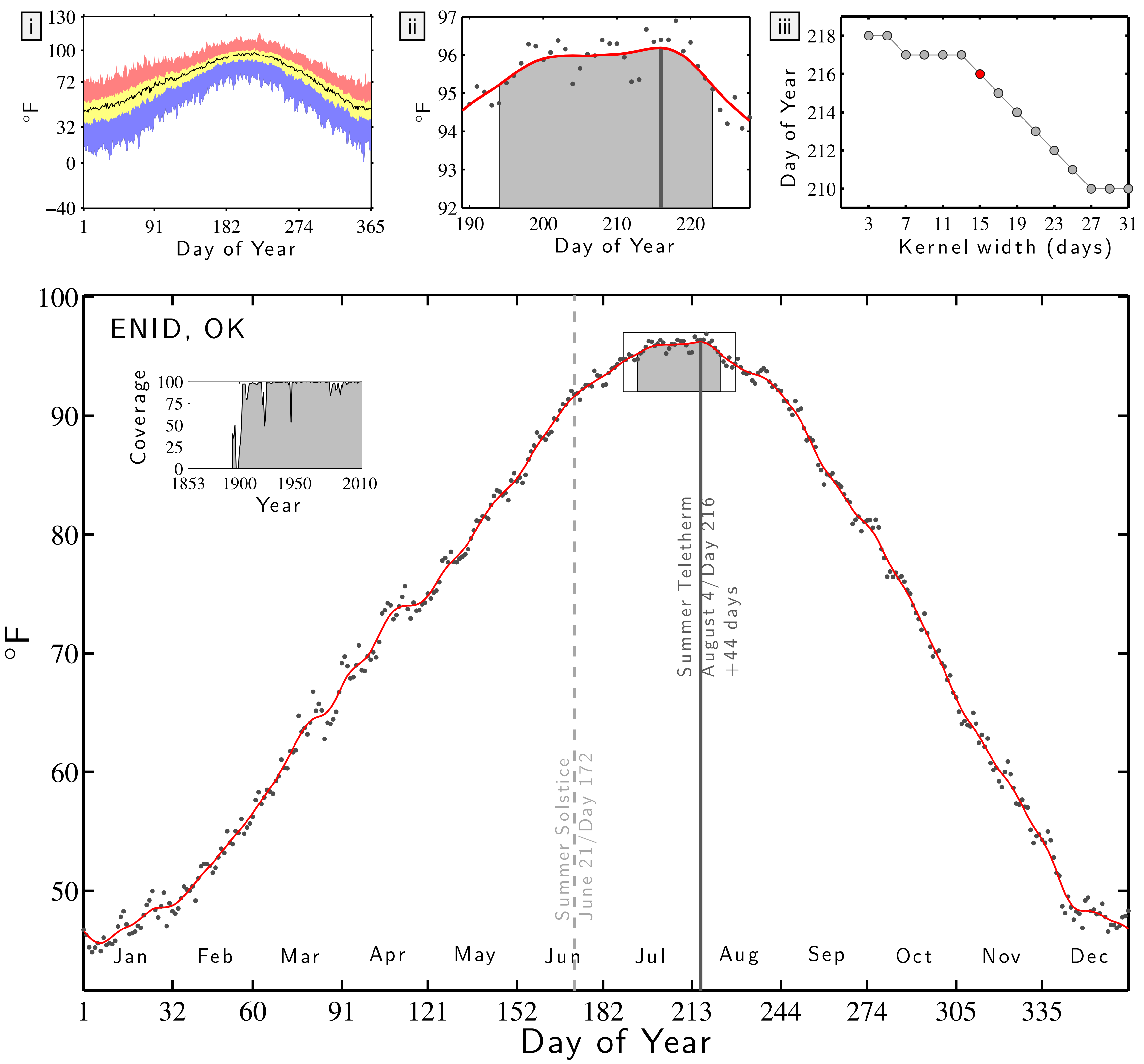

i
°F
Day of Year
ii
°F
Day of Year
iii
Day of Year
Kernel width (days)
ENID, OK
Coverage
Year
Summer Solstice
June 21/Day 172
Summer Teletherm
August 4/Day 216
+44 days
Jan
Feb
Mar
Apr
May
Jun
Jul
Aug
Sep
Oct
Nov
Dec
°F
Day of Year

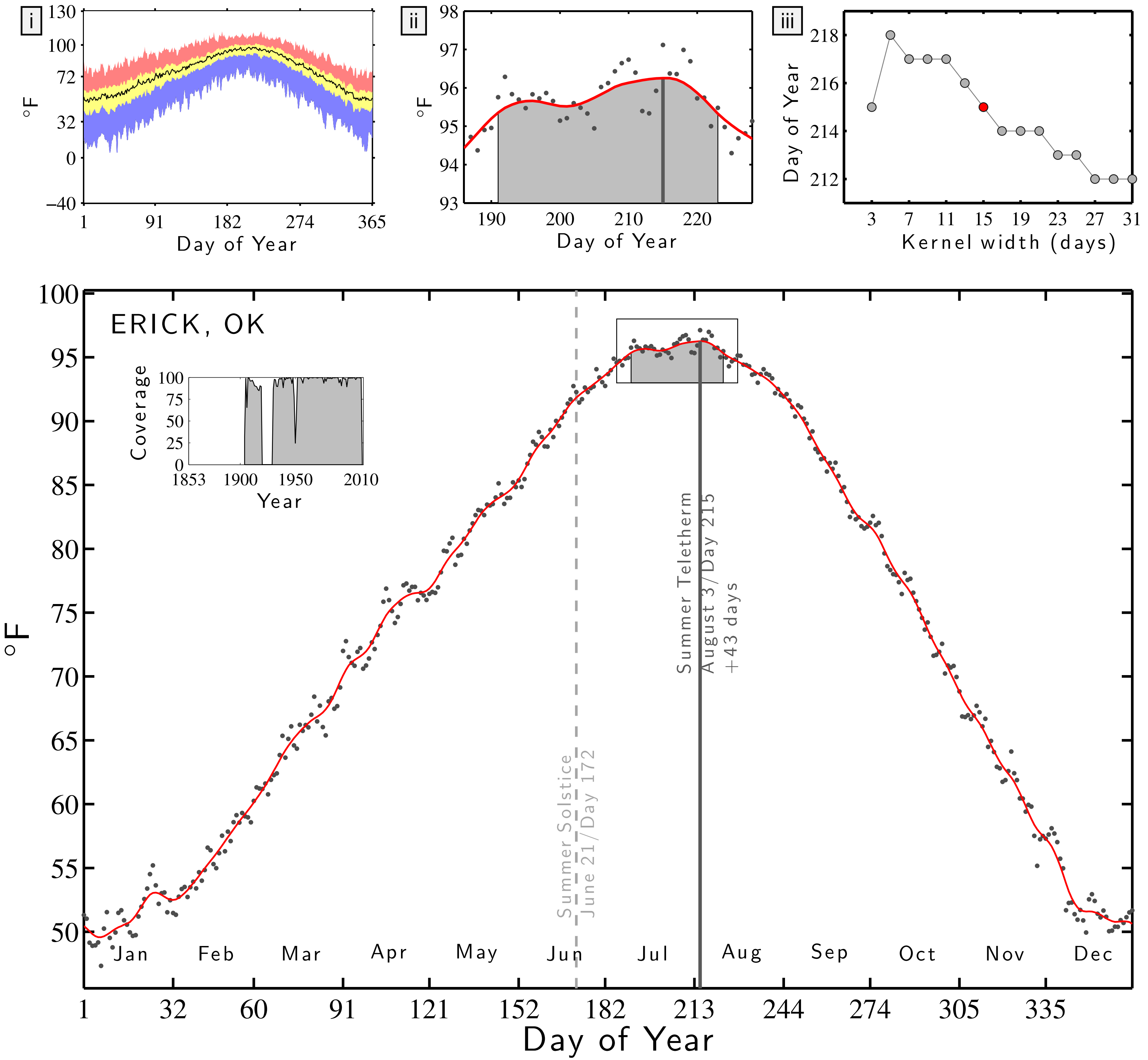

i
Day of Year
°F
ii
Day of Year
°F
iii
Kernel width (days)
Day of Year
ERICK, OK
Coverage
Year
Summer Solstice
June 21/Day 172
Summer Teletherm
August 3/Day 215
+43 days
Jan
Feb
Mar
Apr
May
Jun
Jul
Aug
Sep
Oct
Nov
Dec
Day of Year
°F

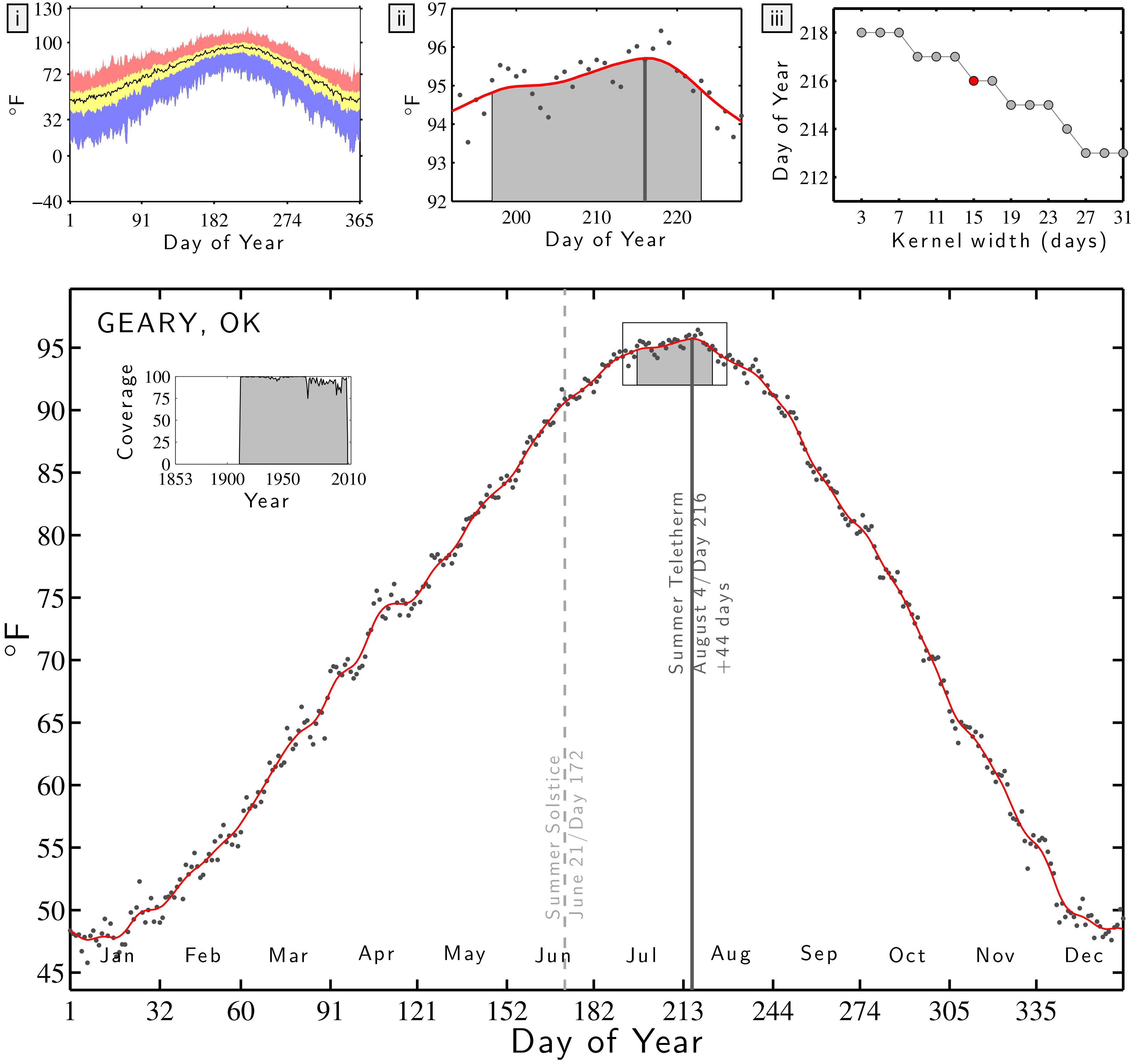

i
°F
Day of Year
ii
°F
Day of Year
iii
Day of Year
Kernel width (days)
GEARY, OK
Coverage
Year
Summer Solstice
June 21 / Day 172
Summer Teletherm
August 4 / Day 216
+44 days
Jan
Feb
Mar
Apr
May
Jun
Jul
Aug
Sep
Oct
Nov
Dec
°F
Day of Year

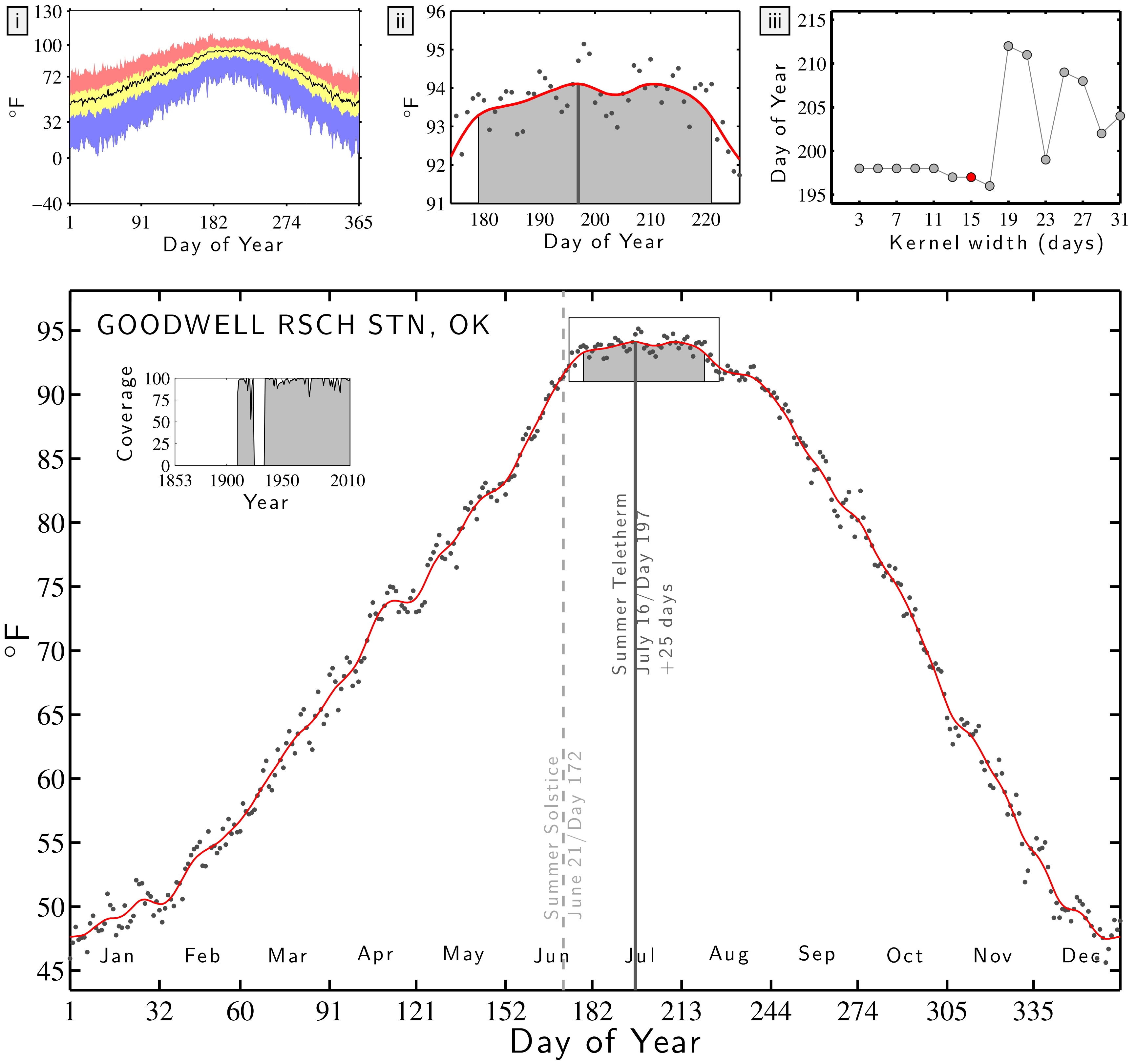

i
ii
iii
°F
Day of Year
Day of Year
Kernel width (days)
GOODWELL RSCH STN, OK
Coverage
Year
Summer Solstice
June 21/Day 172
Summer Teletherm
July 16/Day 197
+25 days
Jan
Feb
Mar
Apr
May
Jun
Jul
Aug
Sep
Oct
Nov
Dec

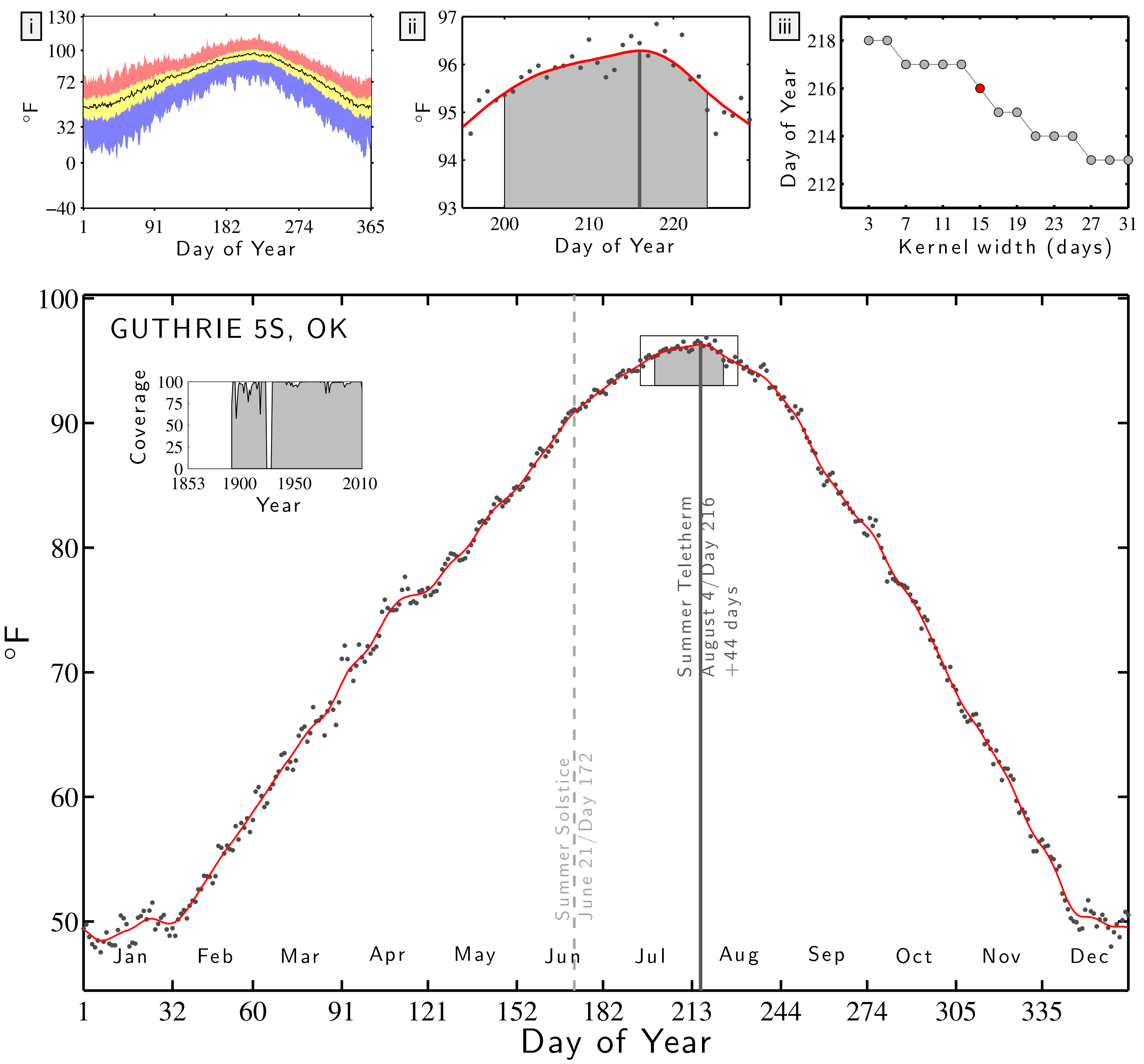

i
°F
Day of Year
ii
°F
Day of Year
iii
Day of Year
Kernel width (days)
GUTHRIE 5S, OK
Coverage
Year
Summer Solstice
June 21/Day 172
Summer Teletherm
August 4/Day 216
+44 days
Jan
Feb
Mar
Apr
May
Jun
Jul
Aug
Sep
Oct
Nov
Dec
°F
Day of Year

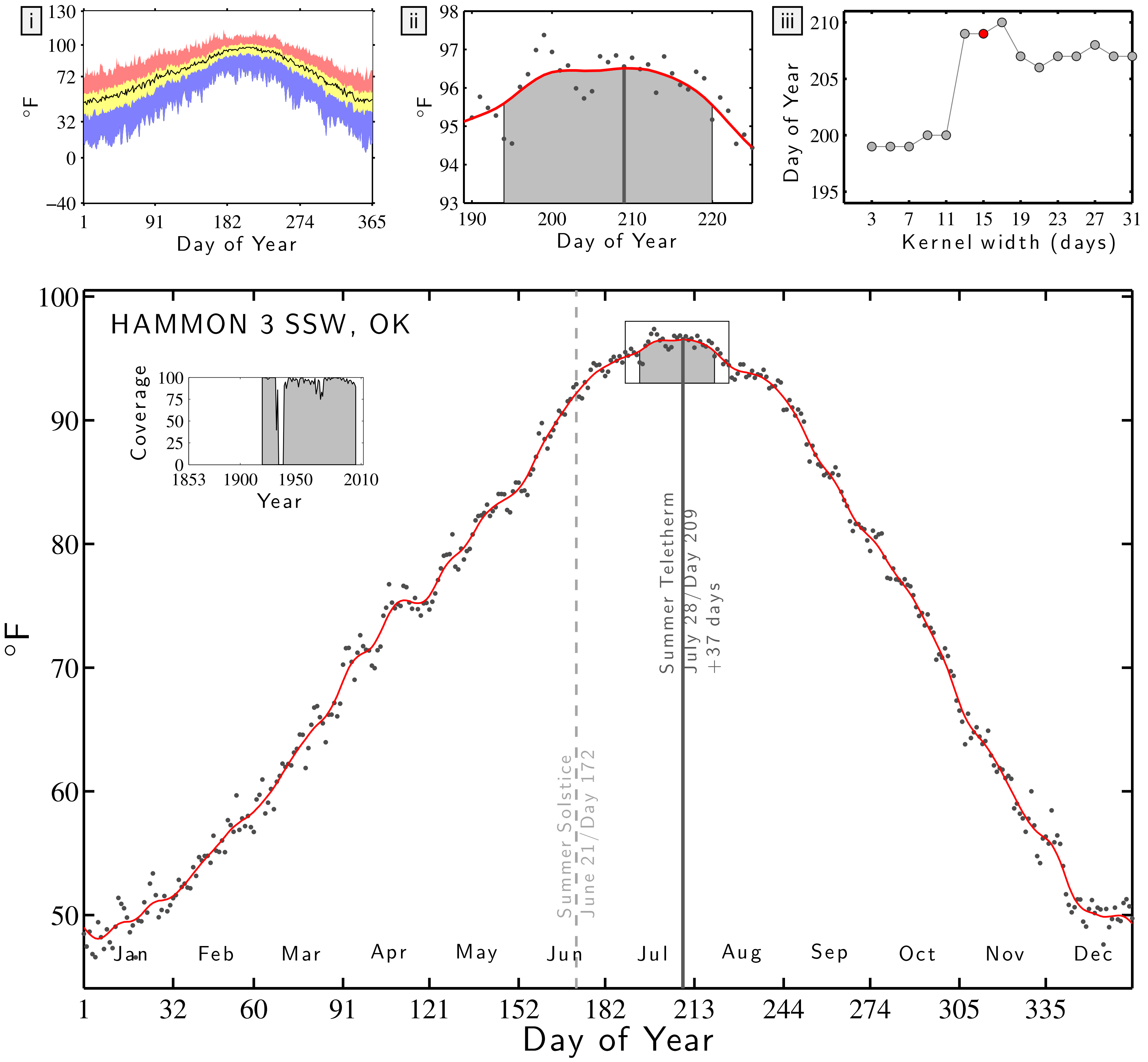

i
°F
Day of Year
ii
°F
Day of Year
iii
Day of Year
Kernel width (days)
HAMMON 3 SSW, OK
Coverage
Year
Summer Solstice
June 21/Day 172
Summer Teletherm
July 28/Day 209
+37 days
Jan
Feb
Mar
Apr
May
Jun
Jul
Aug
Sep
Oct
Nov
Dec
°F
Day of Year

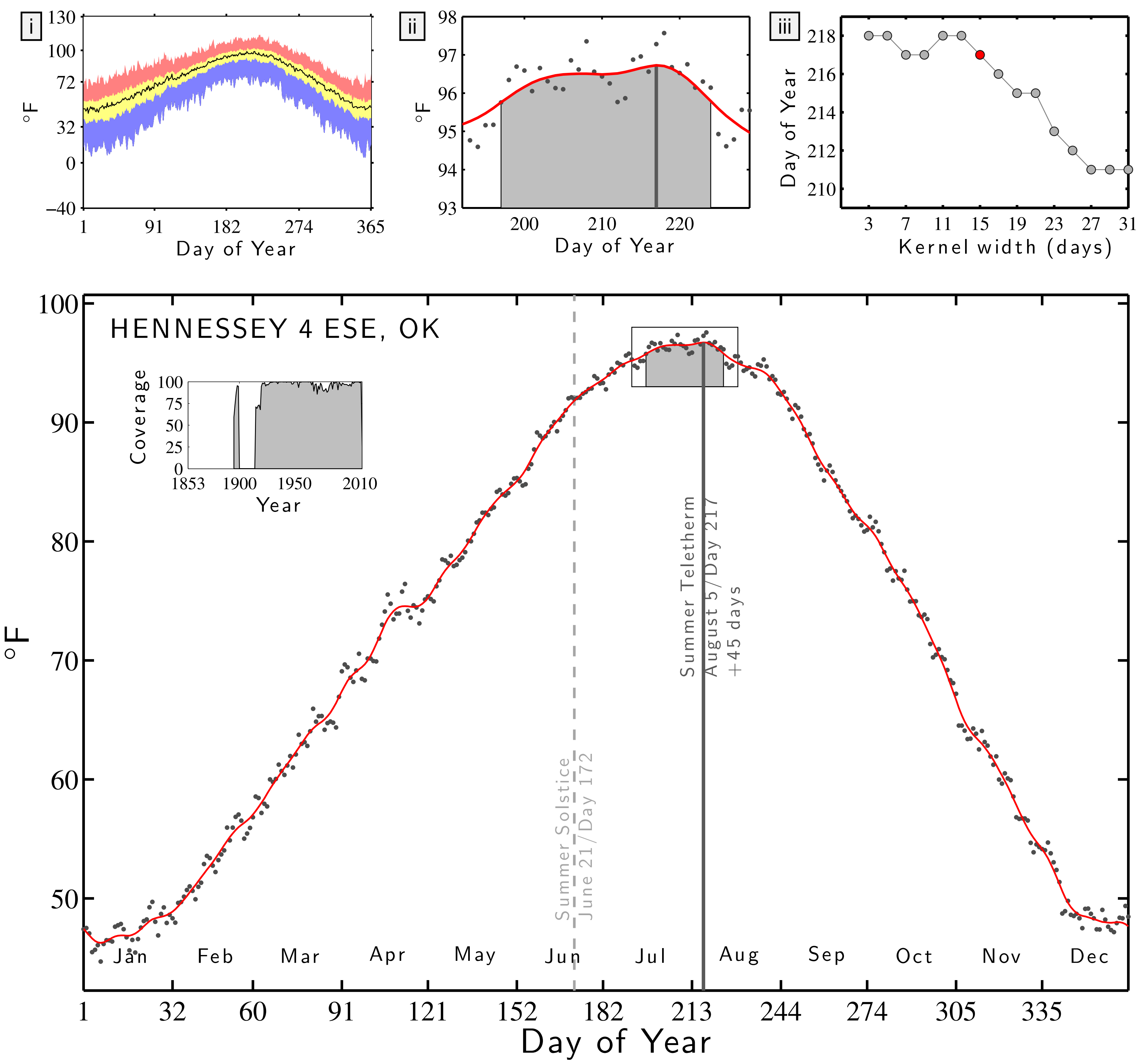

i
ii
iii
Day of Year
Kernel width (days)
HENNESSEY 4 ESE, OK
Coverage
Year
Summer Solstice
June 21/Day 172
Summer Teletherm
August 5/Day 217
+45 days
Jan
Feb
Mar
Apr
May
Jun
Jul
Aug
Sep
Oct
Nov
Dec
°F
Day of Year

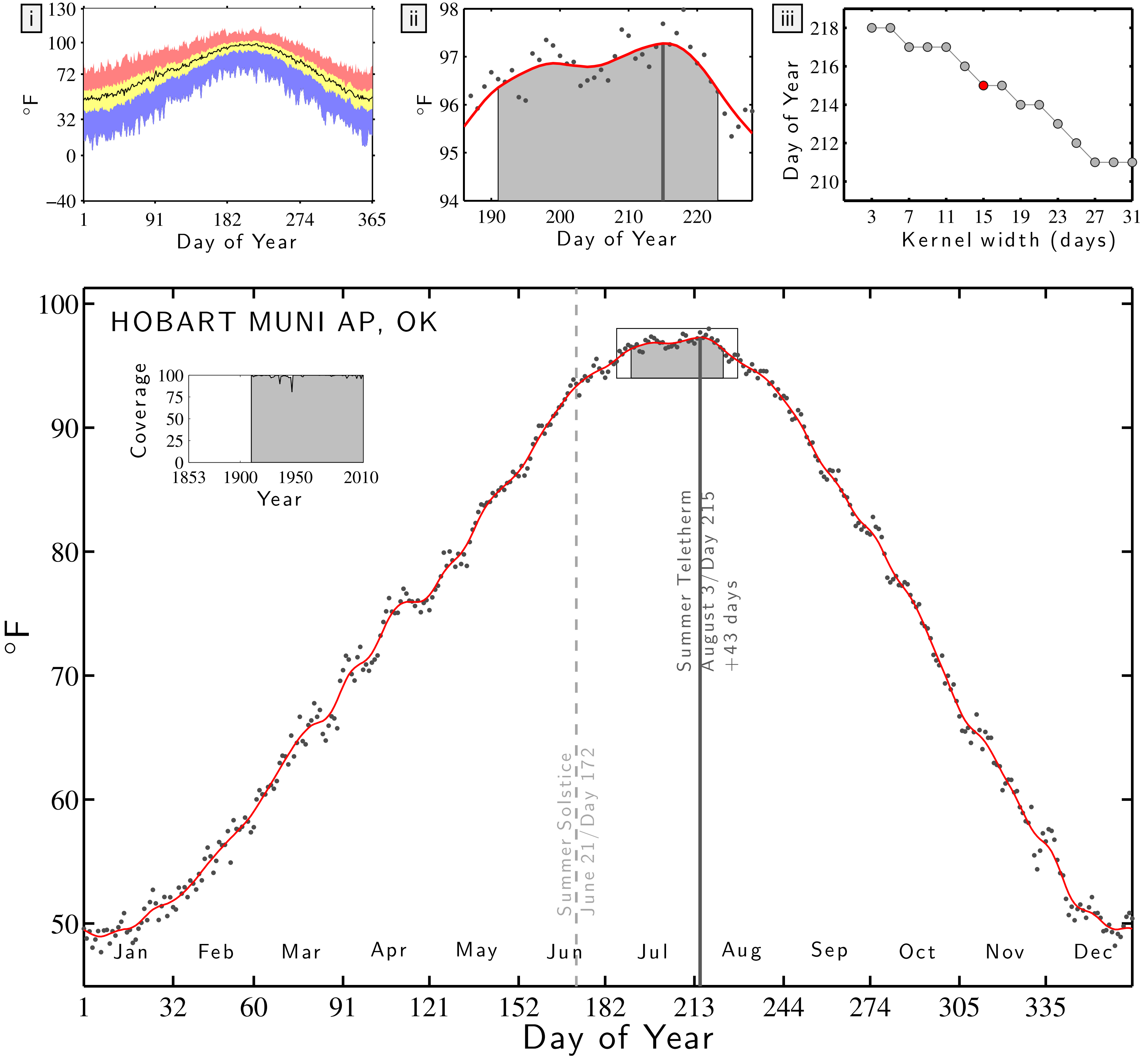

i
ii
iii
HOBART MUNI AP, OK
Coverage
Year
Summer Solstice
June 21/Day 172
Summer Teletherm
August 3/Day 215
+43 days
Jan
Feb
Mar
Apr
May
Jun
Jul
Aug
Sep
Oct
Nov
Dec
Day of Year
°F
Kernel width (days)

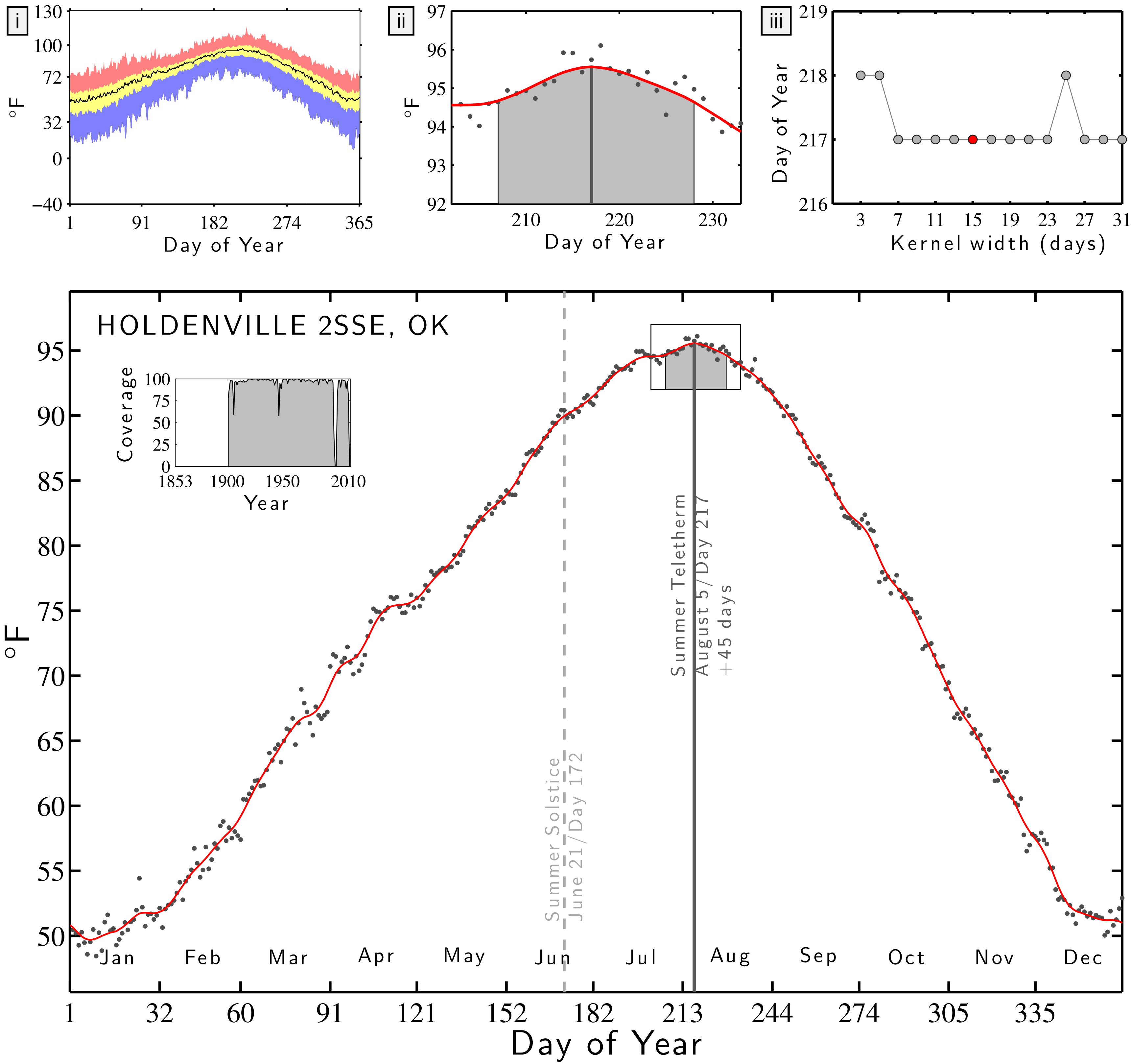

i
ii
iii
°F
Day of Year
Day of Year
Kernel width (days)
HOLDENVILLE 2SSE, OK
Coverage
Year
Summer Solstice
June 21 / Day 172
Summer Teletherm
August 5 / Day 217
+45 days
Jan
Feb
Mar
Apr
May
Jun
Jul
Aug
Sep
Oct
Nov
Dec
Day of Year

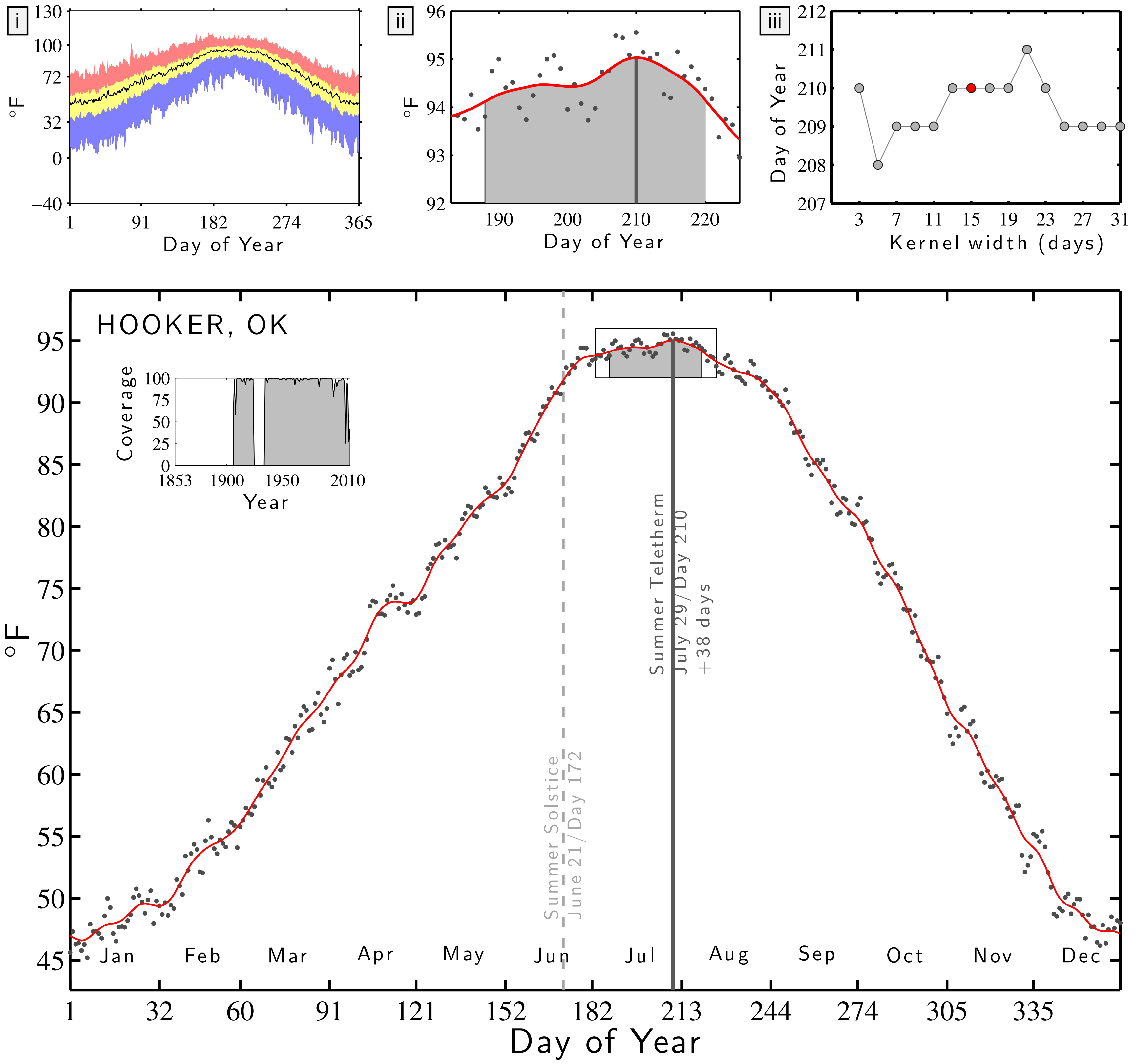

i
ii
iii
°F
Day of Year
Kernel width (days)
HOOKER, OK
Coverage
Year
Summer Solstice
June 21 / Day 172
Summer Teletherm
July 29 / Day 210
+38 days
Jan
Feb
Mar
Apr
May
Jun
Jul
Aug
Sep
Oct
Nov
Dec

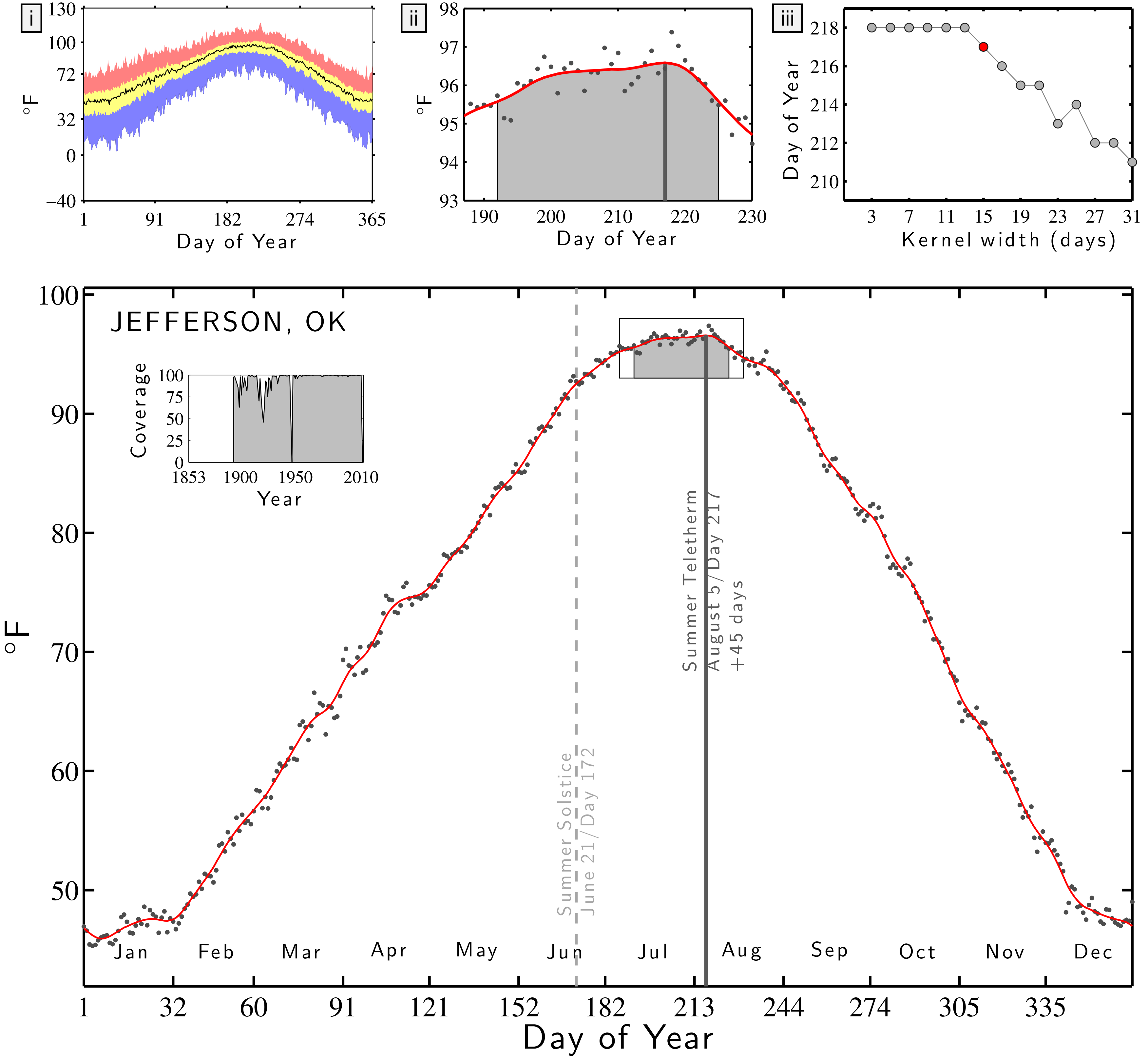

i
°F
Day of Year
ii
°F
Day of Year
iii
Day of Year
Kernel width (days)
JEFFERSON, OK
Coverage
Year
Summer Solstice
June 21/Day 172
Summer Teletherm
August 5/Day 217
+45 days
Jan
Feb
Mar
Apr
May
Jun
Jul
Aug
Sep
Oct
Nov
Dec
°F
Day of Year

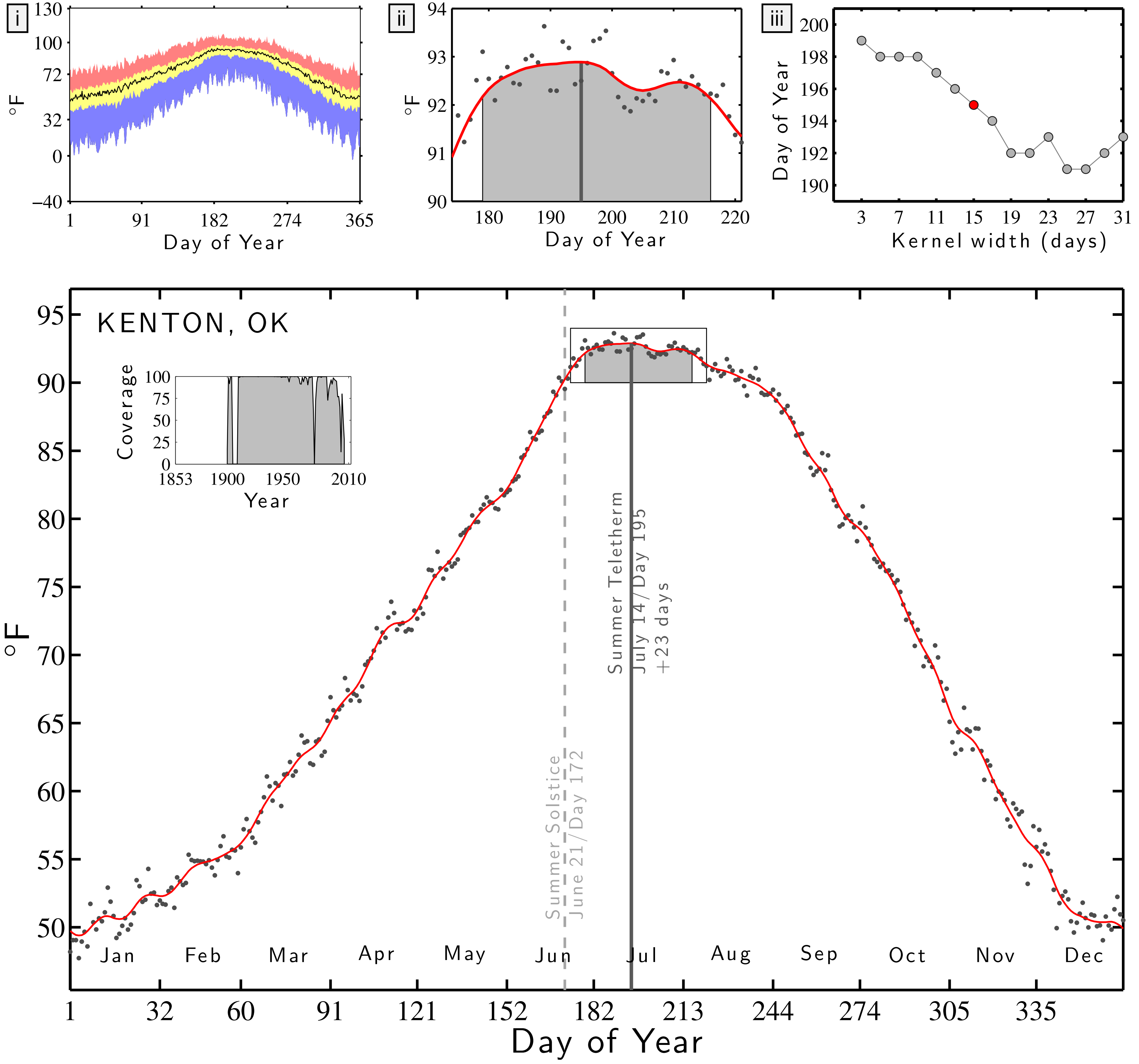

i
130
100
72
32
0
−40
°F
1
91
182
274
365
Day of Year
ii
94
93
92
91
90
°F
180
190
200
210
220
Day of Year
iii
200
198
196
194
192
190
Day of Year
3
7
11
15
19
23
27
31
Kernel width (days)
KENTON, OK
Coverage
100
75
50
25
0
1853
1900
1950
2010
Year
Summer Solstice
June 21/Day 172
Summer Teletherm
July 14/Day 195
+23 days
95
90
85
80
75
70
65
60
55
50
°F
Jan
Feb
Mar
Apr
May
Jun
Jul
Aug
Sep
Oct
Nov
Dec
1
32
60
91
121
152
182
213
244
274
305
335
Day of Year

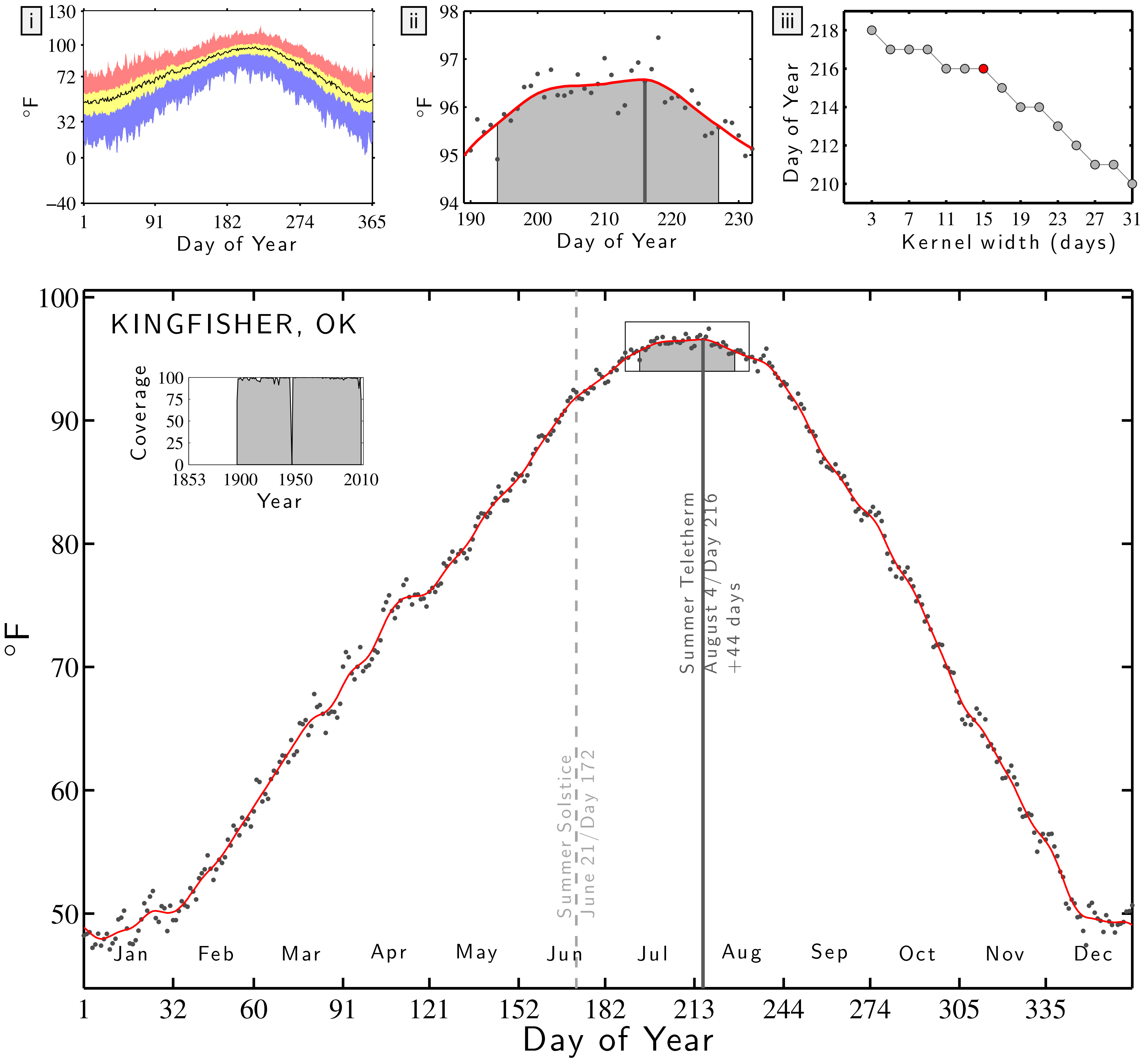

i
°F
Day of Year
ii
°F
Day of Year
iii
Day of Year
Kernel width (days)
KINGFISHER, OK
Coverage
Year
Summer Solstice
June 21/Day 172
Summer Teletherm
August 4/Day 216
+44 days
Jan
Feb
Mar
Apr
May
Jun
Jul
Aug
Sep
Oct
Nov
Dec
°F
Day of Year

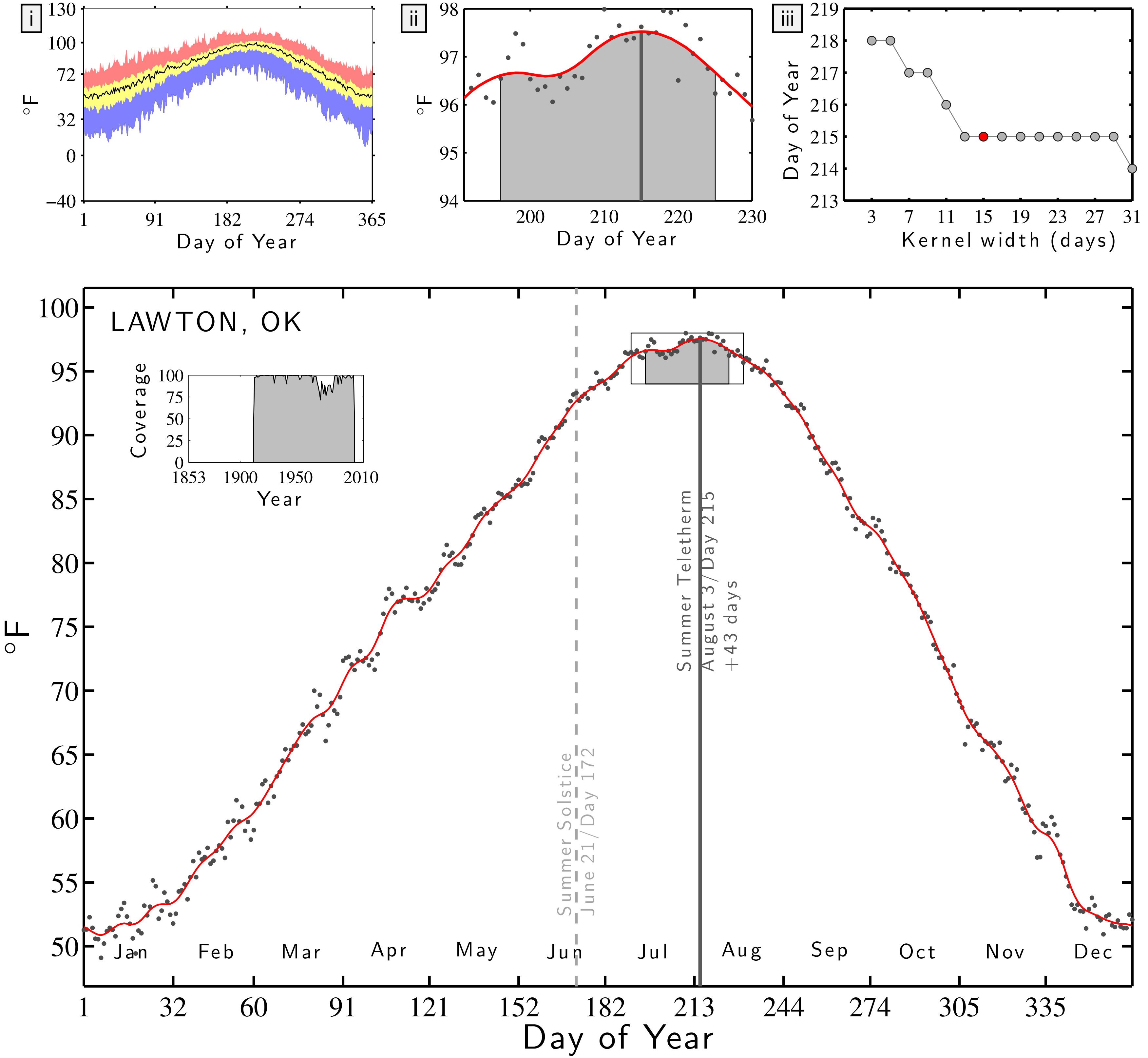

i
130
100
72
32
0
−40
°F
1
91
182
274
365
Day of Year
ii
98
97
96
95
94
°F
200
210
220
230
Day of Year
iii
219
218
217
216
215
214
213
Day of Year
3
7
11
15
19
23
27
31
Kernel width (days)
LAWTON, OK
Coverage
100
75
50
25
0
1853
1900
1950
2010
Year
Summer Solstice
June 21/Day 172
Summer Teletherm
August 3/Day 215
+43 days
°F
100
95
90
85
80
75
70
65
60
55
50
Jan
Feb
Mar
Apr
May
Jun
Jul
Aug
Sep
Oct
Nov
Dec
1
32
60
91
121
152
182
213
244
274
305
335
Day of Year

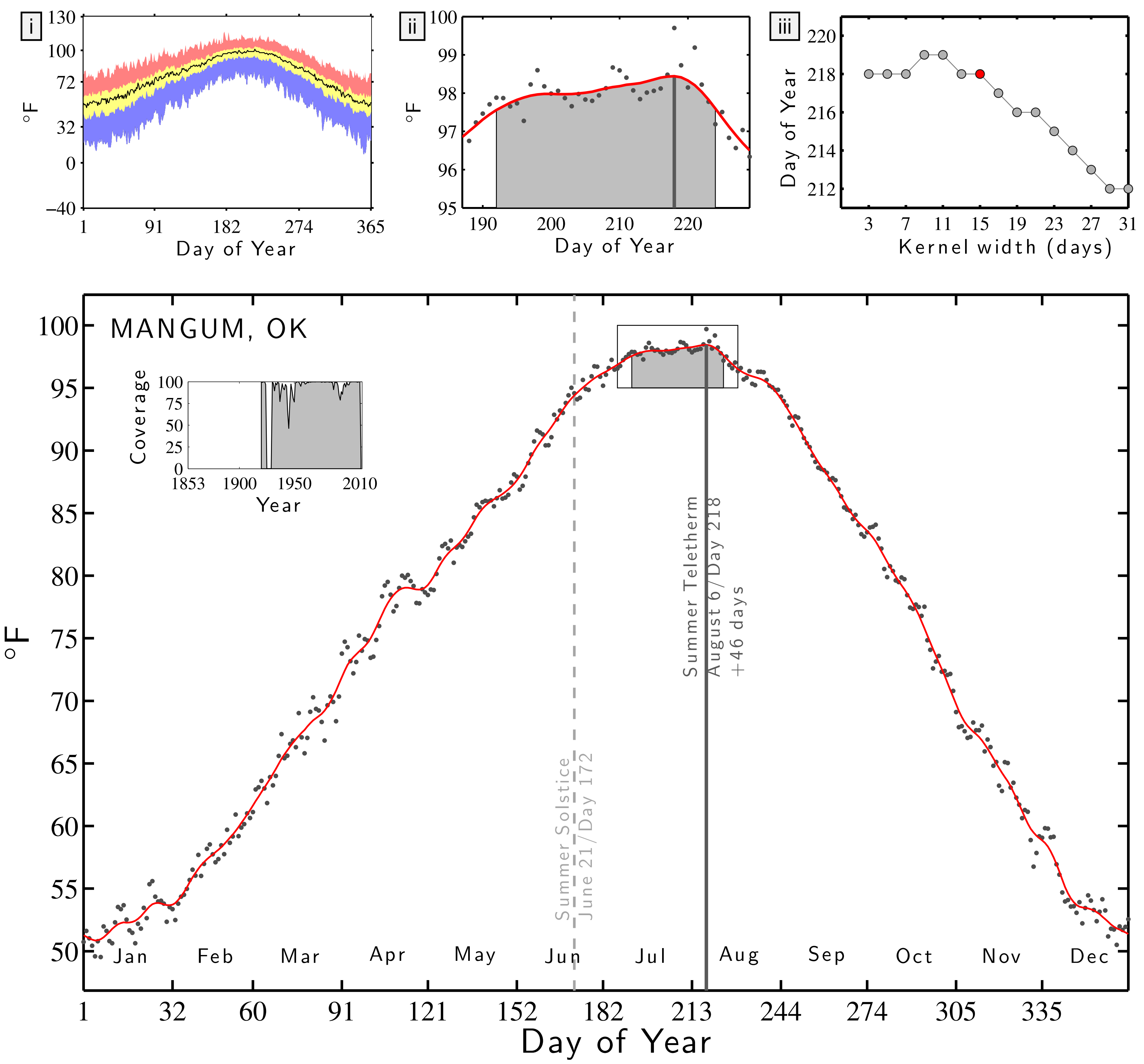

i
°F
Day of Year
ii
°F
Day of Year
iii
Day of Year
Kernel width (days)
MANGUM, OK
Coverage
Year
°F
Summer Solstice
June 21/Day 172
Summer Teletherm
August 6/Day 218
+46 days
Jan
Feb
Mar
Apr
May
Jun
Jul
Aug
Sep
Oct
Nov
Dec
Day of Year

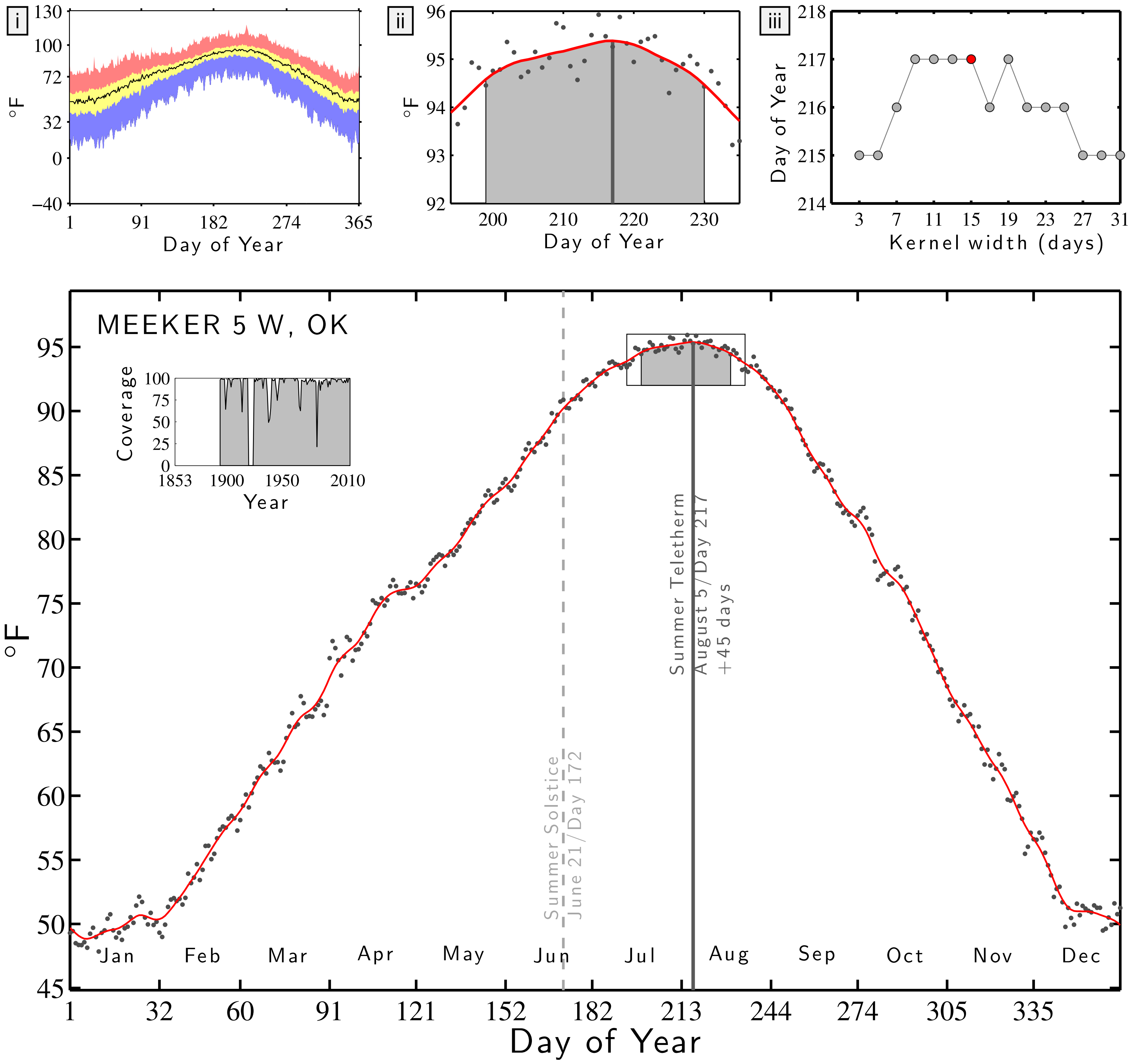

i
°F
130
100
72
32
0
−40
1
91
182
274
365
Day of Year
ii
96
95
94
93
92
200
210
220
230
iii
Day of Year
218
217
216
215
214
3
7
11
15
19
23
27
31
Kernel width (days)
MEEKER 5 W, OK
Coverage
100
75
50
25
0
1853
1900
1950
2010
Year
Summer Solstice
June 21/Day 172
Summer Teletherm
August 5/Day 217
+45 days
Jan
Feb
Mar
Apr
May
Jun
Jul
Aug
Sep
Oct
Nov
Dec
90
85
80
75
70
65
60
55
50
45
32
60
121
152
213
244
305
335

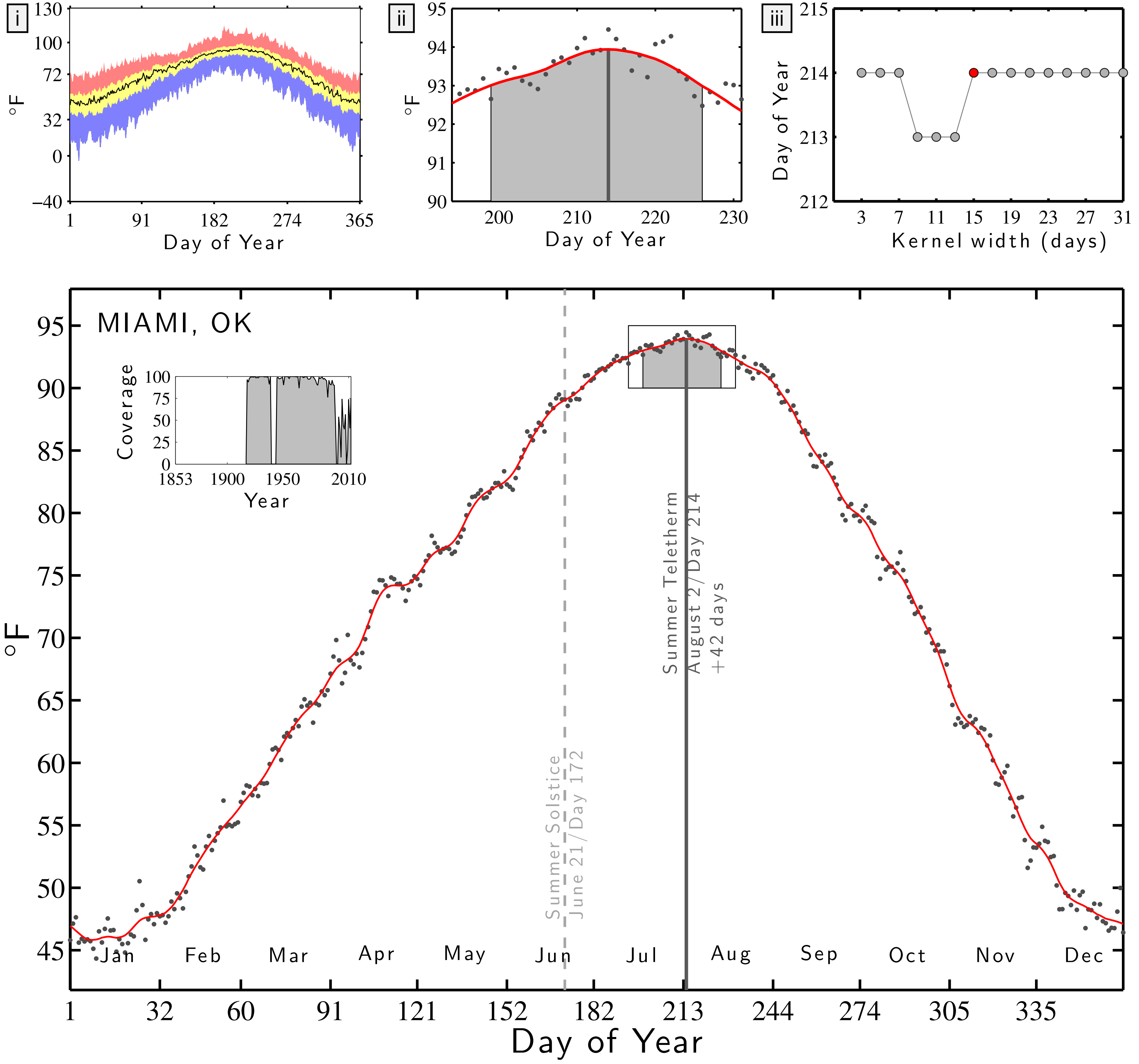

i
ii
iii
°F
Day of Year
Kernel width (days)
MIAMI, OK
Coverage
Year
Summer Solstice
June 21 / Day 172
Summer Teletherm
August 2 / Day 214
+42 days
Jan
Feb
Mar
Apr
May
Jun
Jul
Aug
Sep
Oct
Nov
Dec

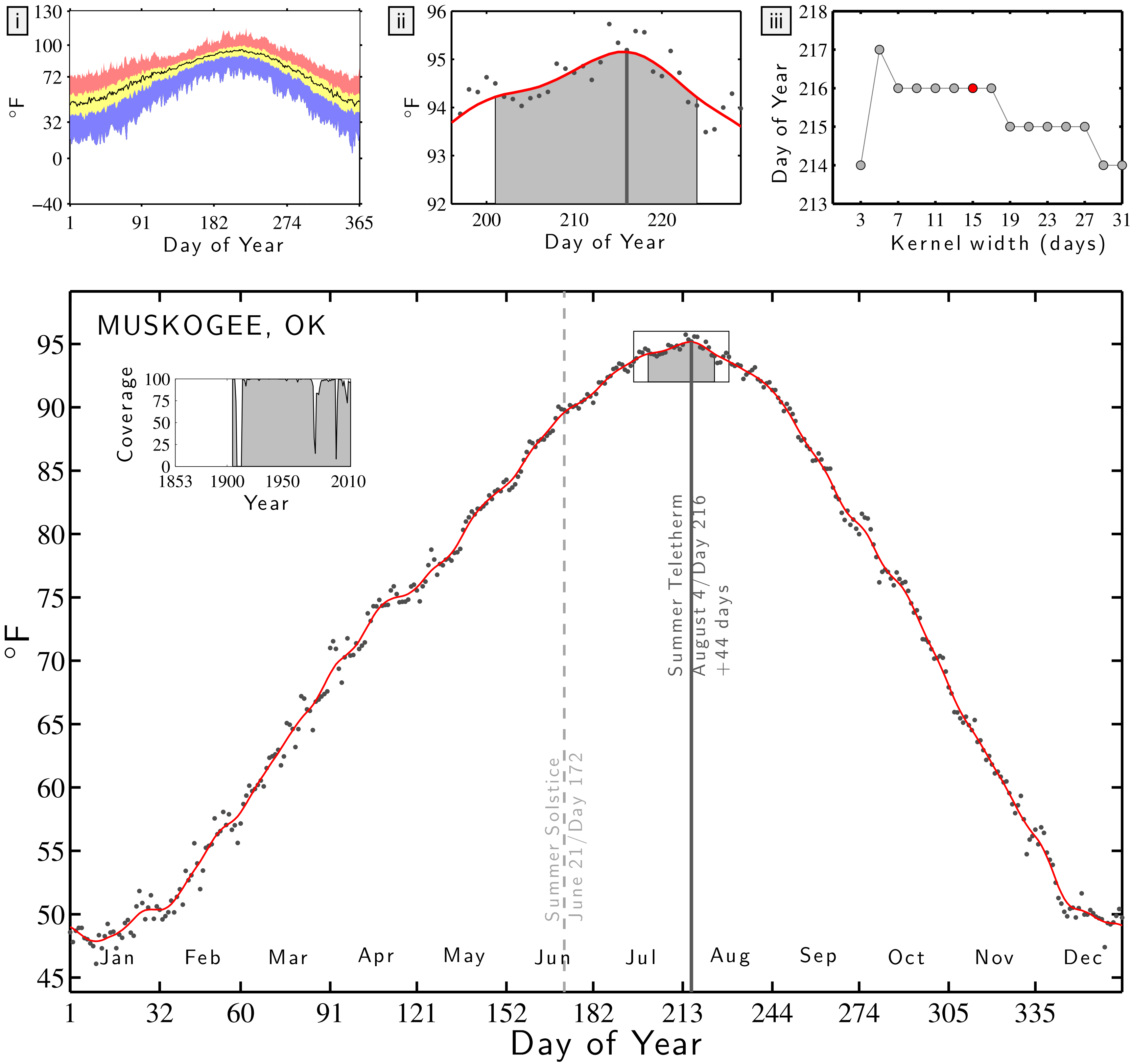

i
°F
Day of Year
ii
°F
Day of Year
iii
Day of Year
Kernel width (days)
MUSKOGEE, OK
Coverage
Year
Summer Solstice
June 21 / Day 172
Summer Teletherm
August 4 / Day 216
+44 days
Jan
Feb
Mar
Apr
May
Jun
Jul
Aug
Sep
Oct
Nov
Dec
°F
Day of Year

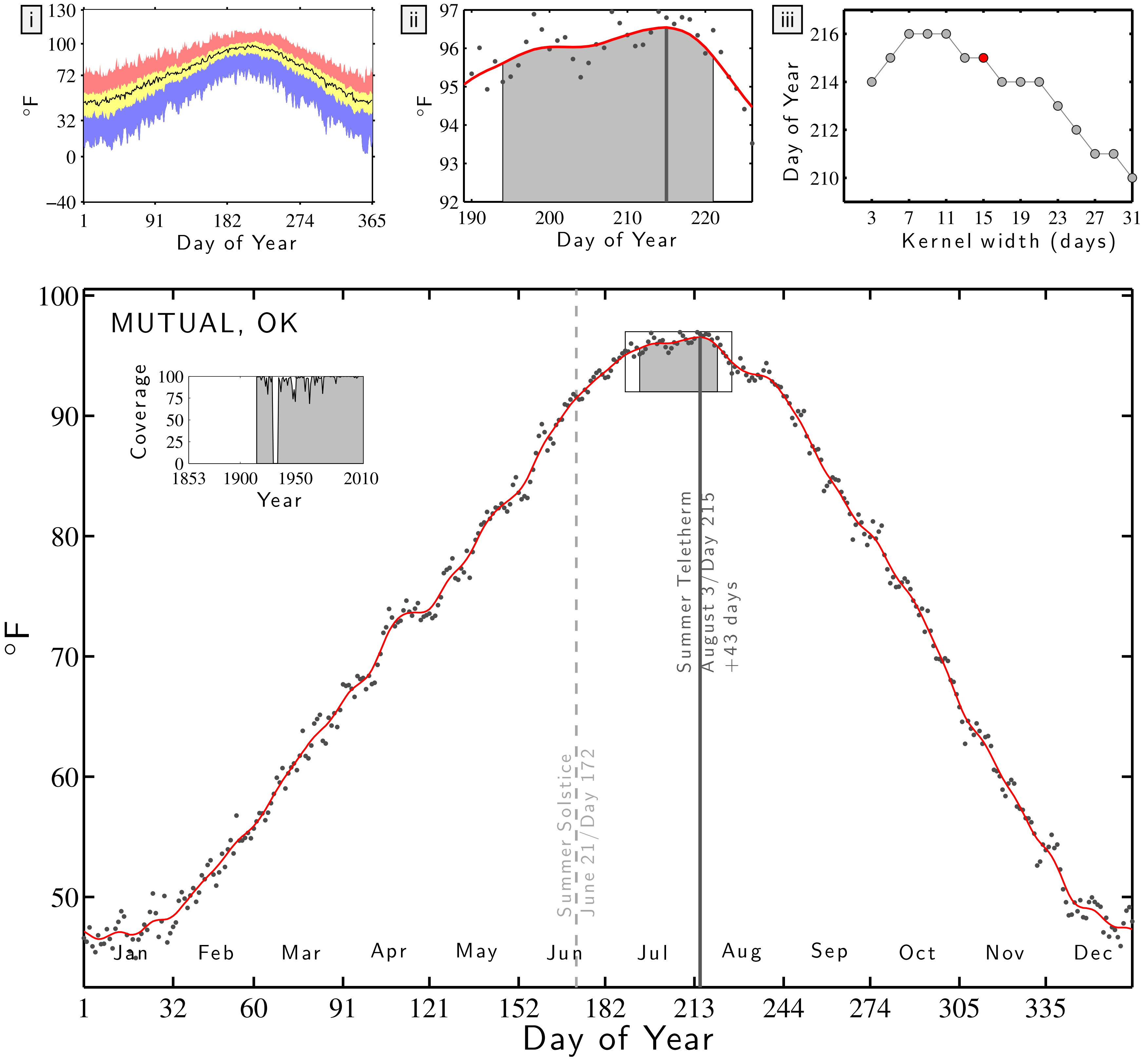

i
°F
Day of Year
ii
°F
Day of Year
iii
Day of Year
Kernel width (days)
MUTUAL, OK
Coverage
Year
Summer Solstice
June 21/Day 172
Summer Teletherm
August 3/Day 215
+43 days
Jan
Feb
Mar
Apr
May
Jun
Jul
Aug
Sep
Oct
Nov
Dec
°F
Day of Year

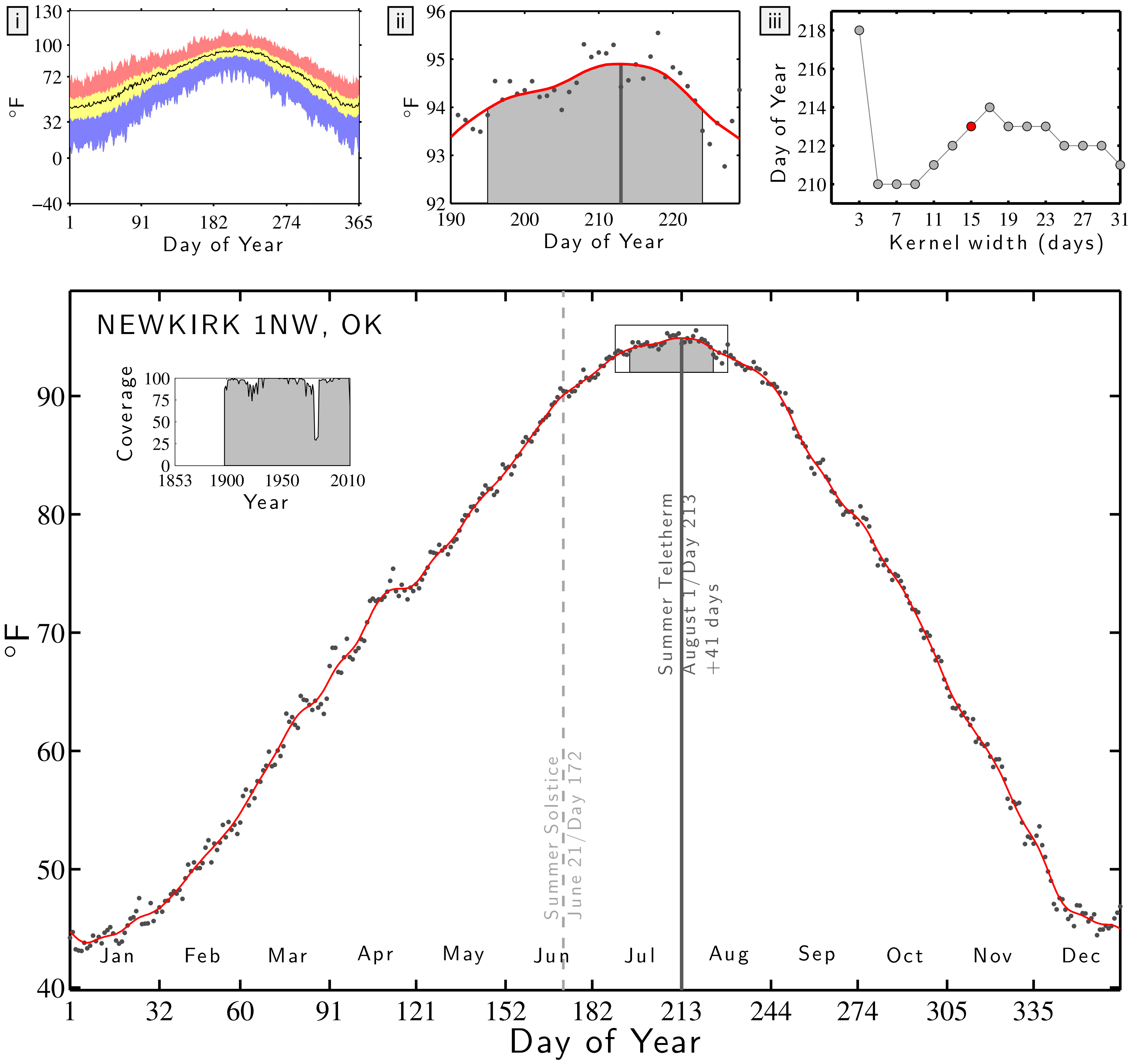

i
°F
Day of Year
ii
°F
Day of Year
iii
Day of Year
Kernel width (days)
NEWKIRK 1NW, OK
Coverage
Year
°F
Summer Solstice
June 21/Day 172
Summer Teletherm
August 1/Day 213
+41 days
Jan
Feb
Mar
Apr
May
Jun
Jul
Aug
Sep
Oct
Nov
Dec
Day of Year

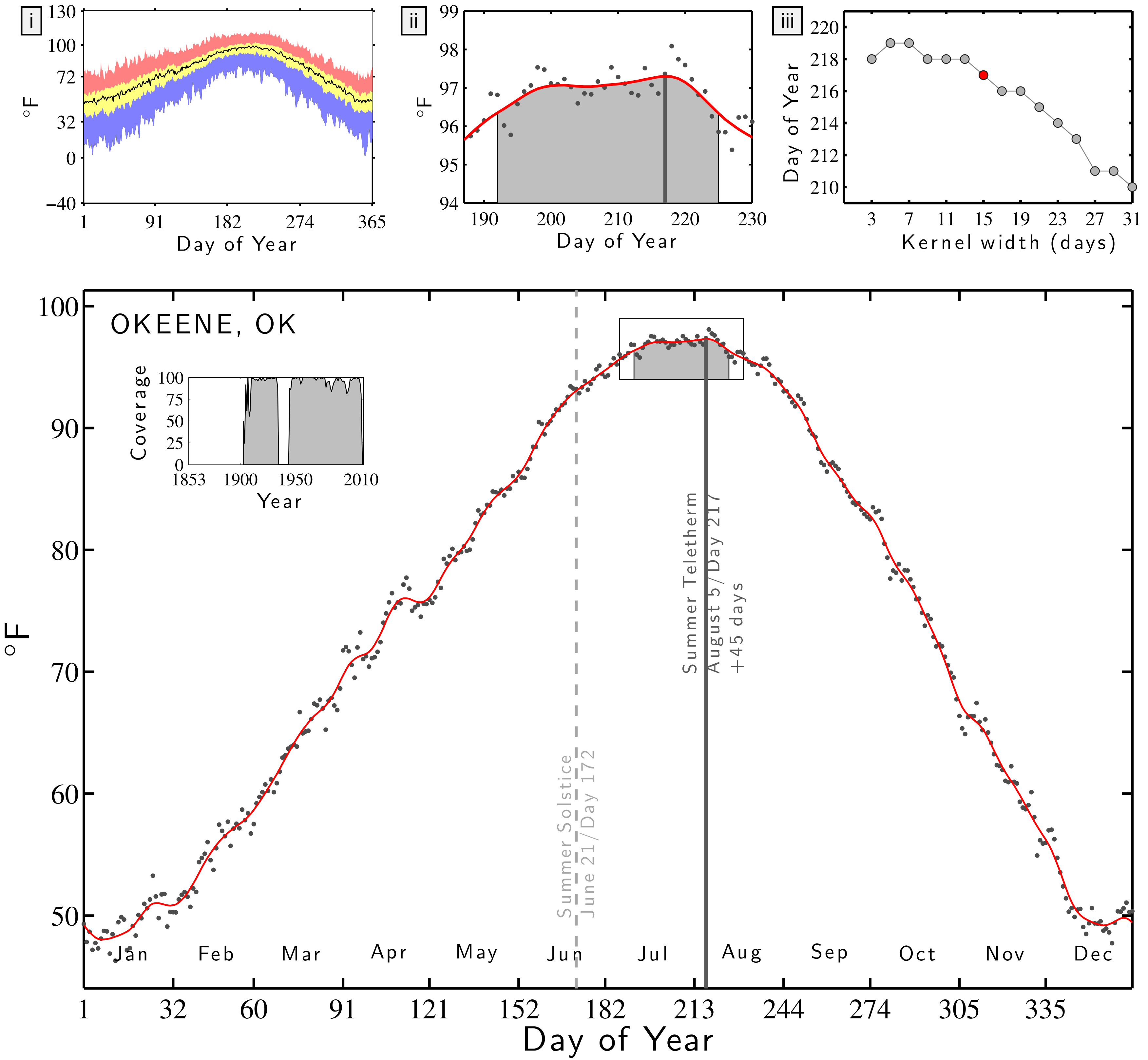

i
°F
Day of Year
ii
°F
Day of Year
iii
Day of Year
Kernel width (days)
OKEENE, OK
Coverage
Year
Summer Solstice
June 21/Day 172
Summer Teletherm
August 5/Day 217
+45 days
Jan
Feb
Mar
Apr
May
Jun
Jul
Aug
Sep
Oct
Nov
Dec
°F
Day of Year

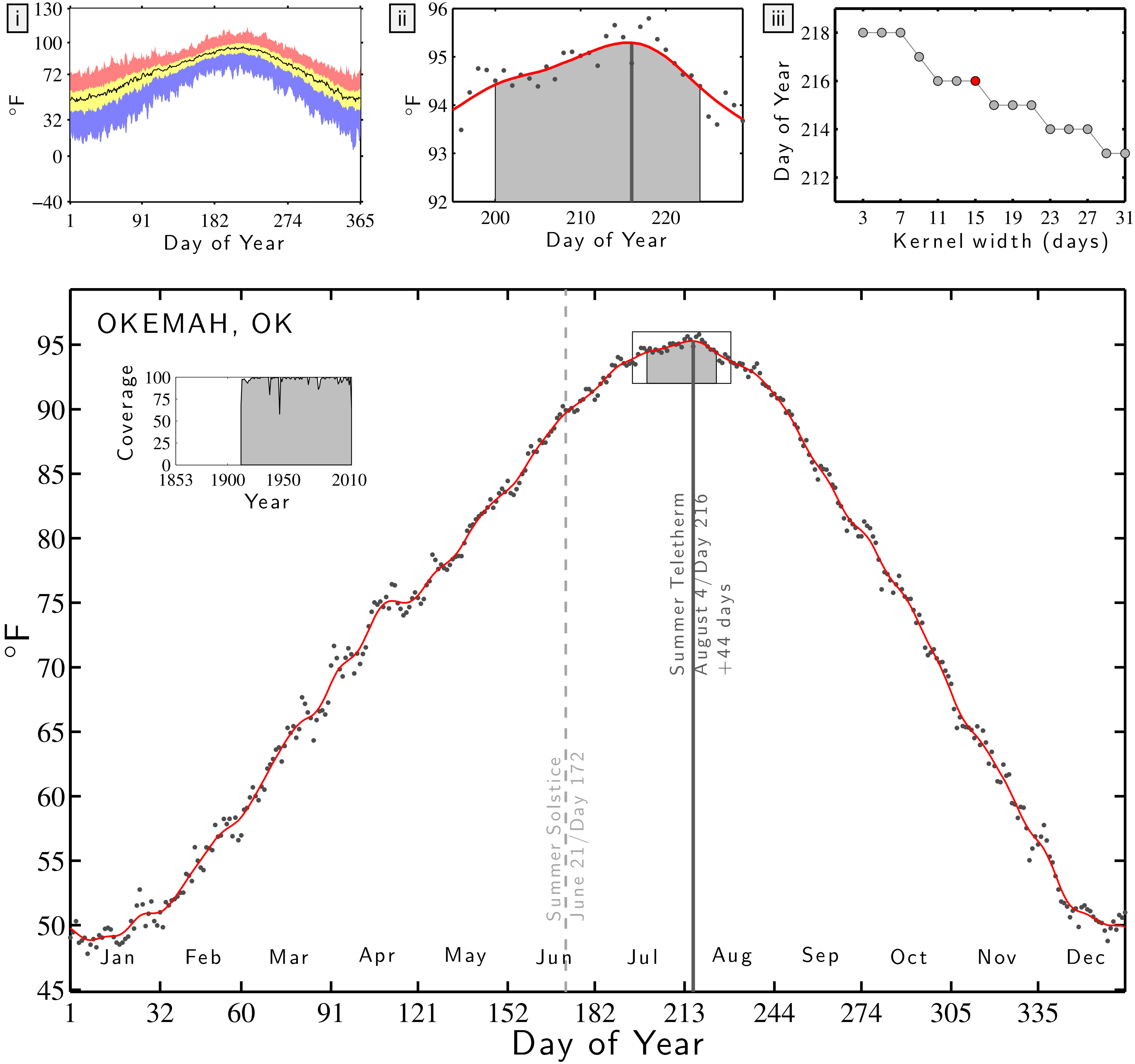

i
130
100
72
32
0
−40
°F
1
91
182
274
365
Day of Year
ii
96
95
94
93
92
°F
200
210
220
Day of Year
iii
218
216
214
212
Day of Year
3
7
11
15
19
23
27
31
Kernel width (days)
OKEMAH, OK
Coverage
100
75
50
25
0
1853
1900
1950
2010
Year
Summer Solstice
June 21/Day 172
Summer Teletherm
August 4/Day 216
+44 days
95
90
85
80
75
70
65
60
55
50
45
°F
Jan
Feb
Mar
Apr
May
Jun
Jul
Aug
Sep
Oct
Nov
Dec
1
32
60
91
121
152
182
213
244
274
305
335
Day of Year

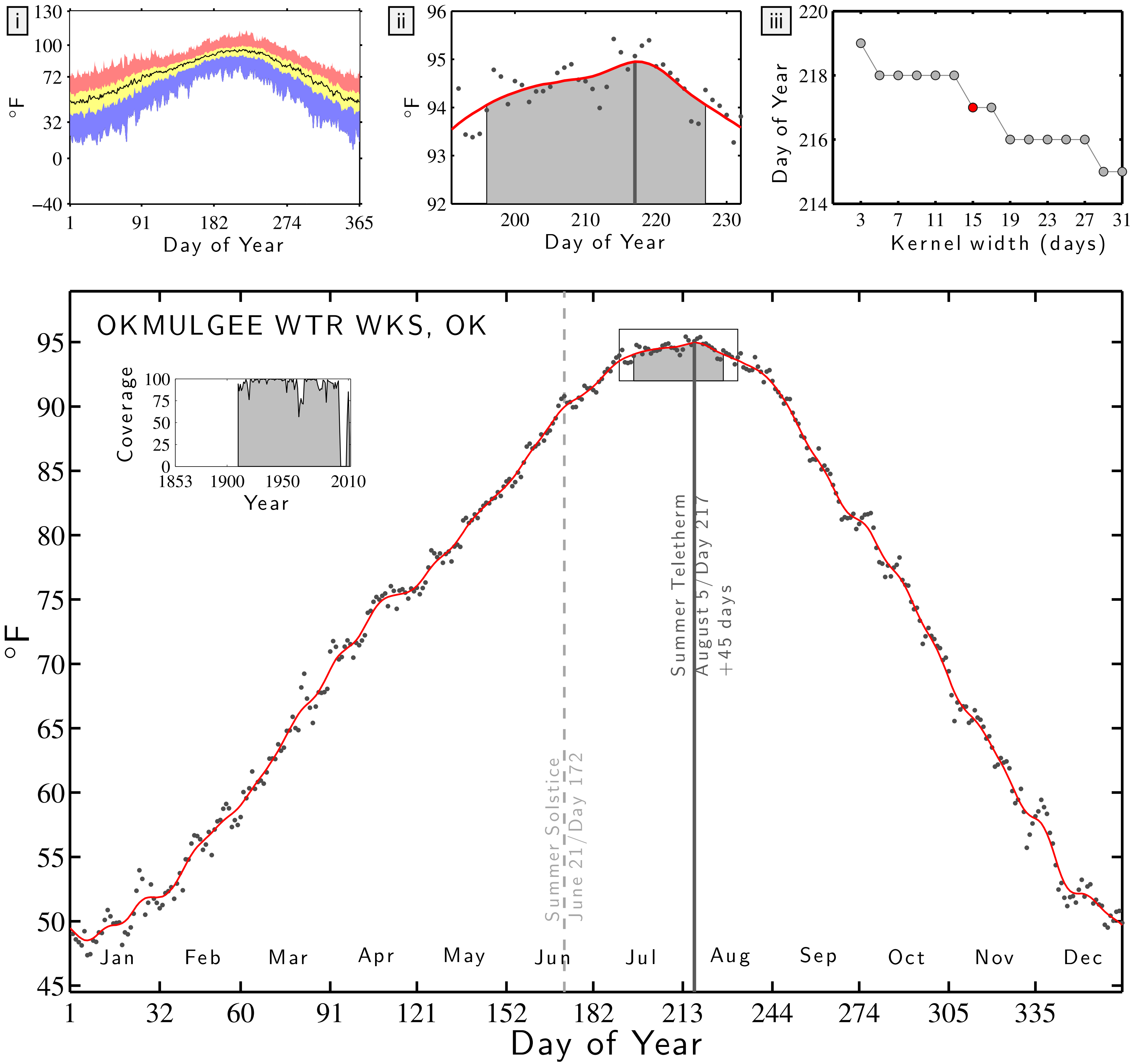

i
°F
Day of Year
ii
°F
Day of Year
iii
Day of Year
Kernel width (days)
OKMULGEE WTR WKS, OK
Coverage
Year
Summer Solstice
June 21/Day 172
Summer Teletherm
August 5/Day 217
+45 days
Jan
Feb
Mar
Apr
May
Jun
Jul
Aug
Sep
Oct
Nov
Dec
°F
Day of Year

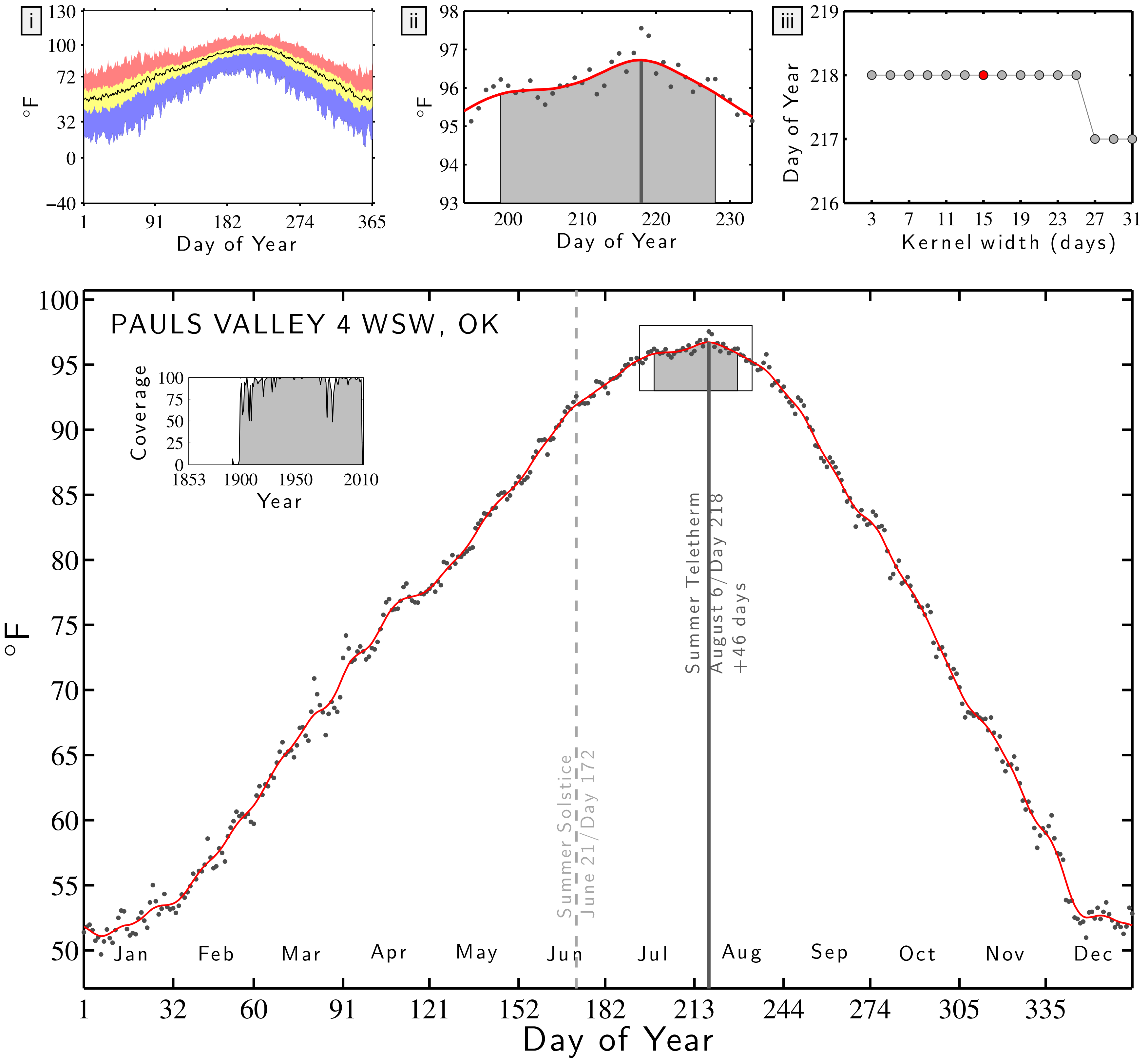

i
°F
Day of Year
ii
°F
Day of Year
iii
Day of Year
Kernel width (days)
PAULS VALLEY 4 WSW, OK
Coverage
Year
Summer Solstice
June 21/Day 172
Summer Teletherm
August 6/Day 218
+46 days
Jan
Feb
Mar
Apr
May
Jun
Jul
Aug
Sep
Oct
Nov
Dec
°F
Day of Year

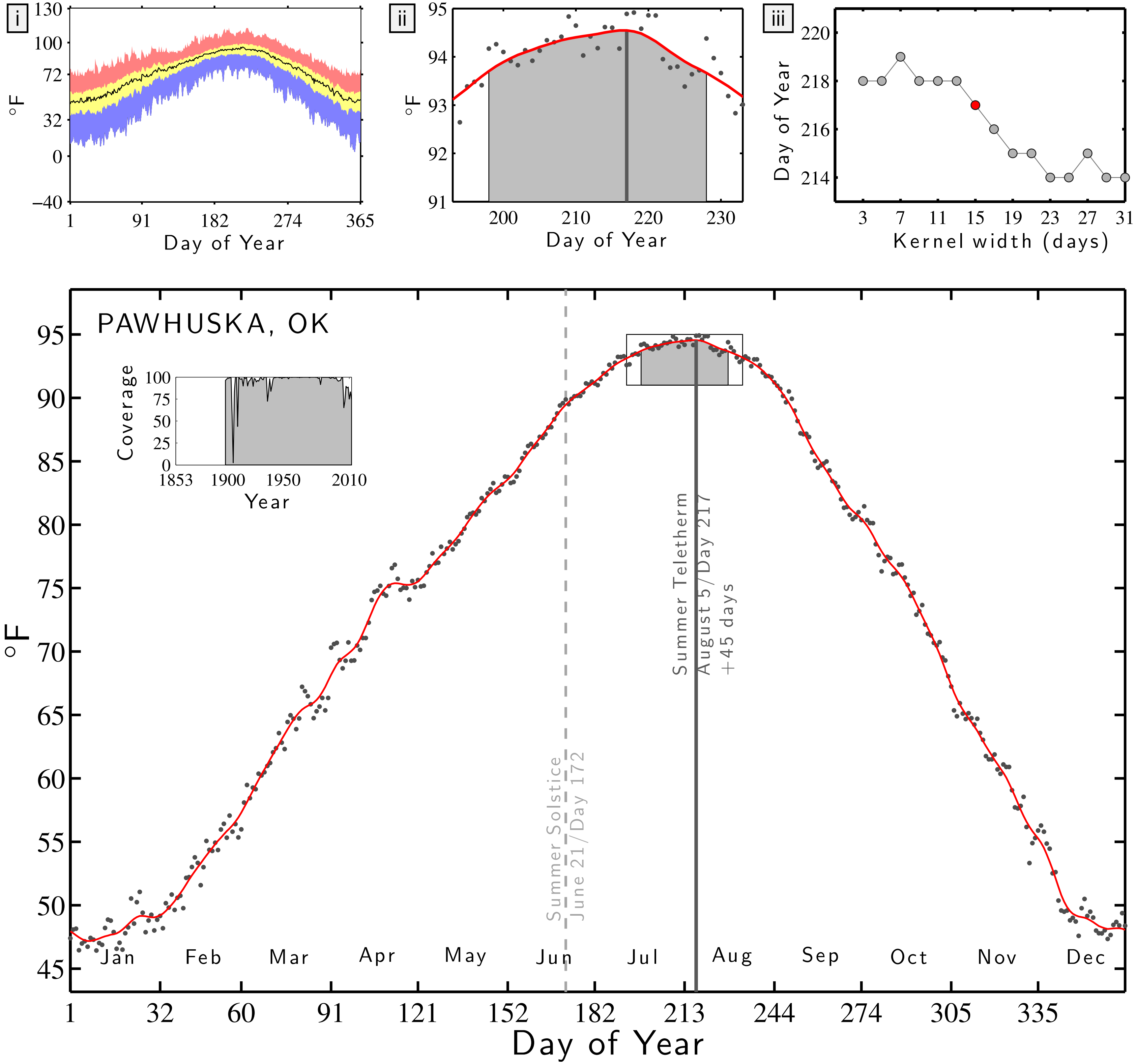

i
°F
Day of Year
ii
°F
Day of Year
iii
Day of Year
Kernel width (days)
PAWHUSKA, OK
Coverage
Year
Summer Solstice
June 21 / Day 172
Summer Teletherm
August 5 / Day 217
+45 days
Jan
Feb
Mar
Apr
May
Jun
Jul
Aug
Sep
Oct
Nov
Dec
°F
Day of Year

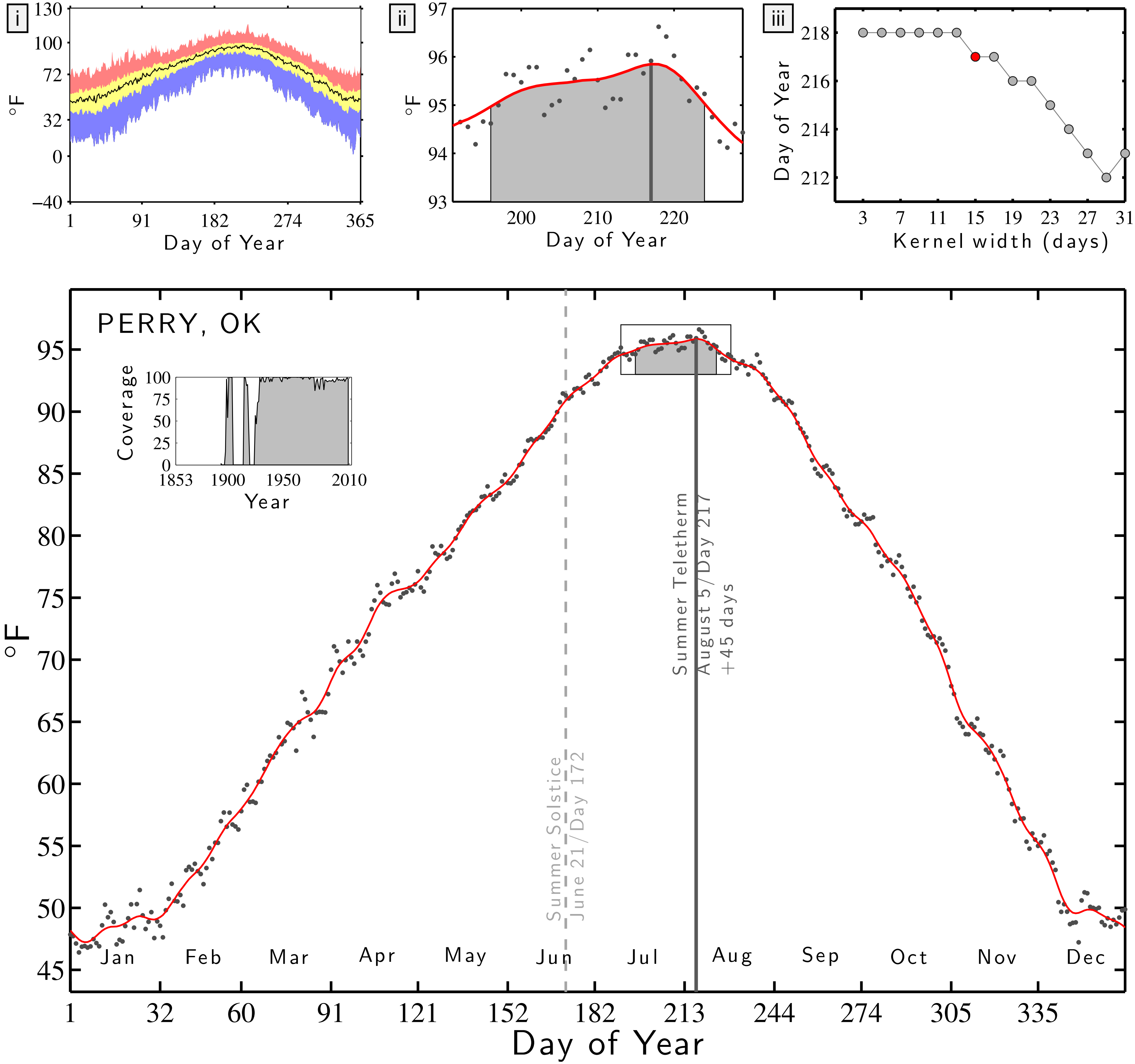

i
°F
Day of Year
ii
Day of Year
iii
Day of Year
Kernel width (days)
PERRY, OK
Coverage
Year
Summer Solstice
June 21/Day 172
Summer Teletherm
August 5/Day 217
+45 days
Jan
Feb
Mar
Apr
May
Jun
Jul
Aug
Sep
Oct
Nov
Dec
Day of Year

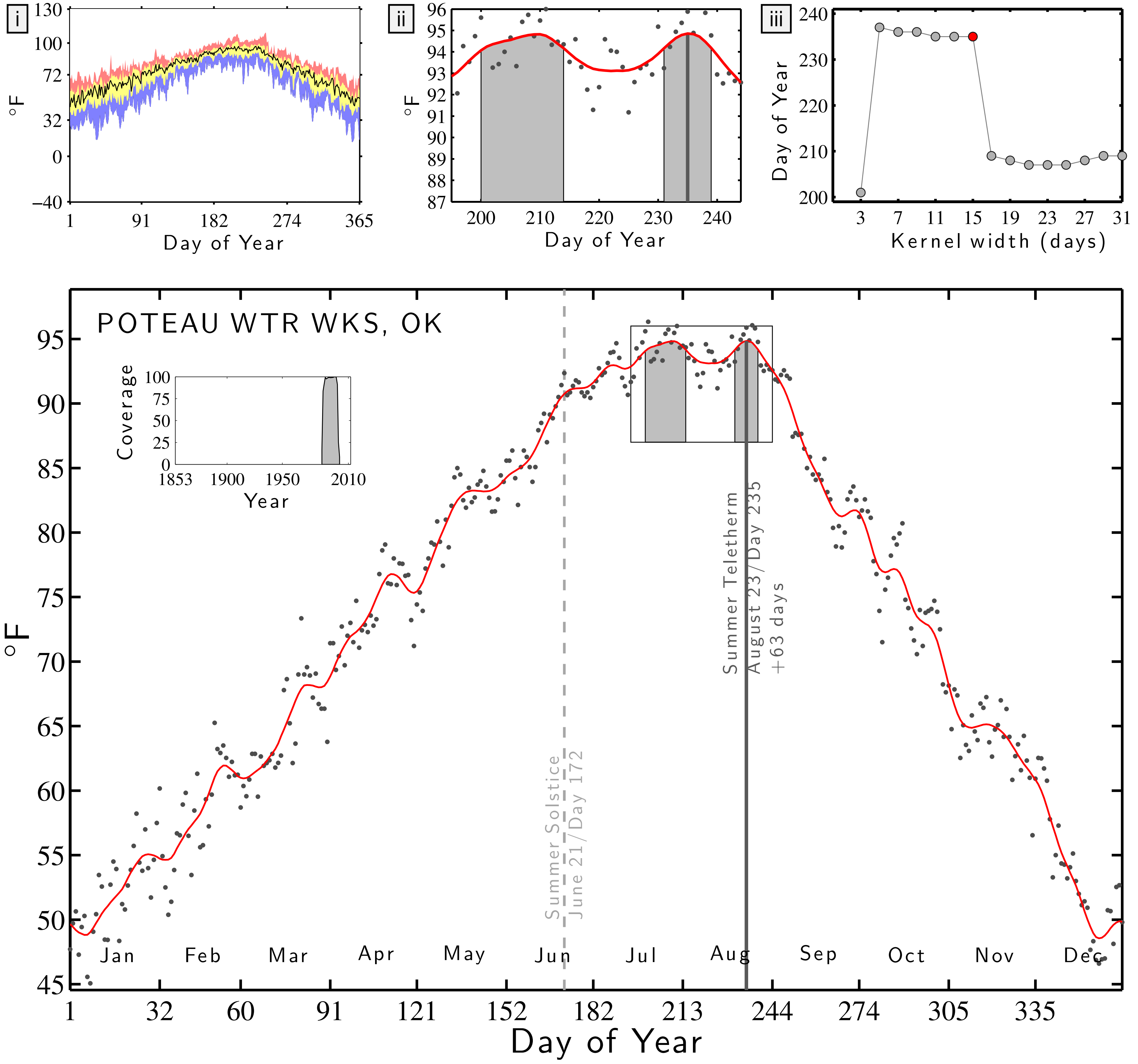

i
°F
130
100
72
32
0
–40
1
91
182
274
365
Day of Year
ii
°F
96
95
94
93
92
91
90
89
88
87
200
210
220
230
240
Day of Year
iii
Day of Year
240
230
220
210
200
3
7
11
15
19
23
27
31
Kernel width (days)
POTEAU WTR WKS, OK
Coverage
100
75
50
25
0
1853
1900
1950
2010
Year
Summer Solstice
June 21 / Day 172
Summer Teletherm
August 23 / Day 235
+63 days
°F
95
90
85
80
75
70
65
60
55
50
45
Jan
Feb
Mar
Apr
May
Jun
Jul
Aug
Sep
Oct
Nov
Dec
1
32
60
91
121
152
182
213
244
274
305
335
Day of Year

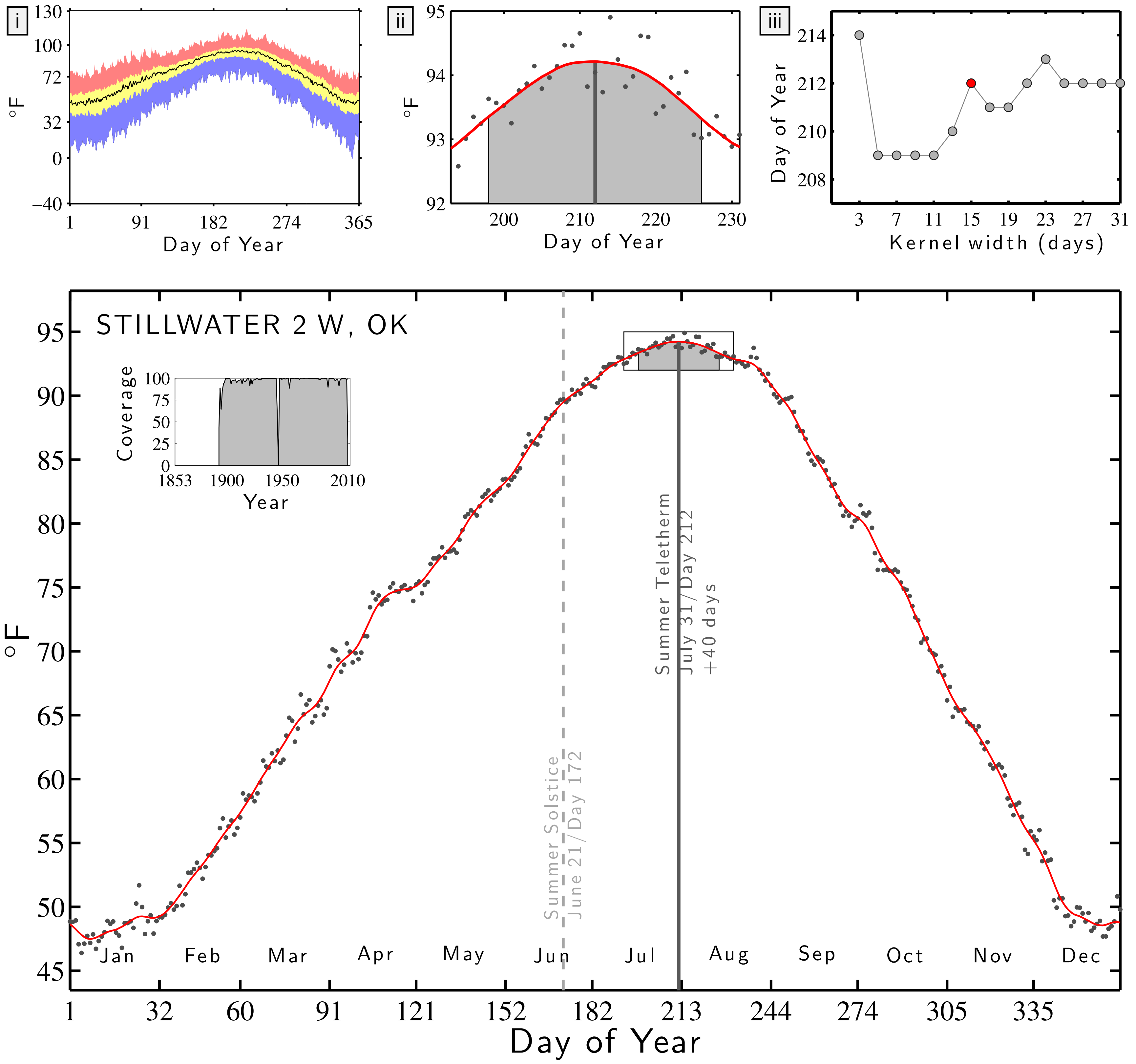

i
ii
iii
°F
Day of Year
Day of Year
Kernel width (days)
STILLWATER 2 W, OK
Coverage
Year
Summer Solstice
June 21 / Day 172
Summer Teletherm
July 31 / Day 212
+40 days
Jan
Feb
Mar
Apr
May
Jun
Jul
Aug
Sep
Oct
Nov
Dec
°F
Day of Year

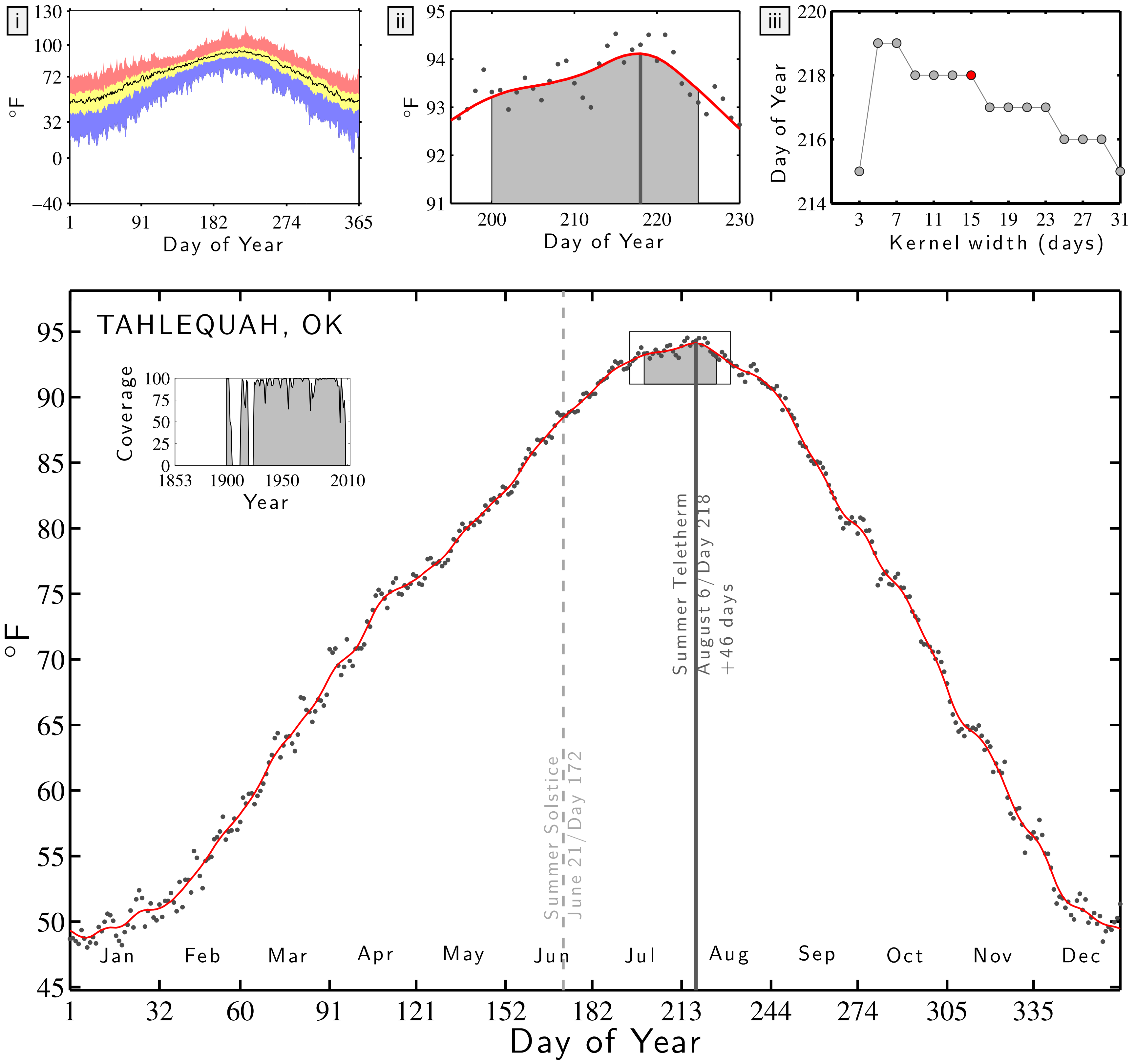

i
ii
iii
°F
Day of Year
Day of Year
Kernel width (days)
TAHLEQUAH, OK
Coverage
Year
Summer Solstice
June 21 / Day 172
Summer Teletherm
August 6 / Day 218
+46 days
Jan
Feb
Mar
Apr
May
Jun
Jul
Aug
Sep
Oct
Nov
Dec

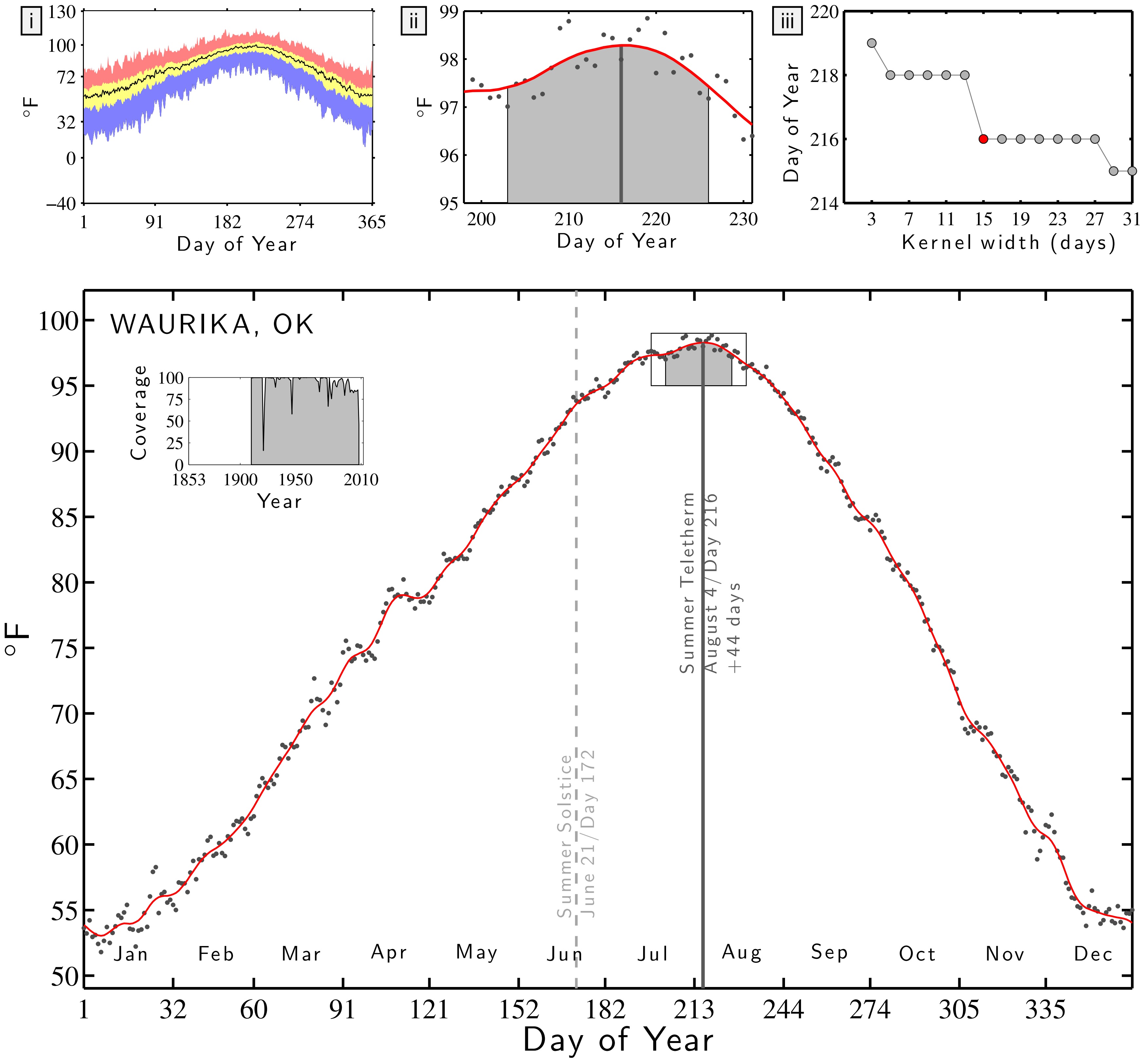

i
ii
iii
°F
Day of Year
Day of Year
Kernel width (days)
WAURIKA, OK
Coverage
Year
Summer Solstice
June 21/Day 172
Summer Teletherm
August 4/Day 216
+44 days
Jan
Feb
Mar
Apr
May
Jun
Jul
Aug
Sep
Oct
Nov
Dec

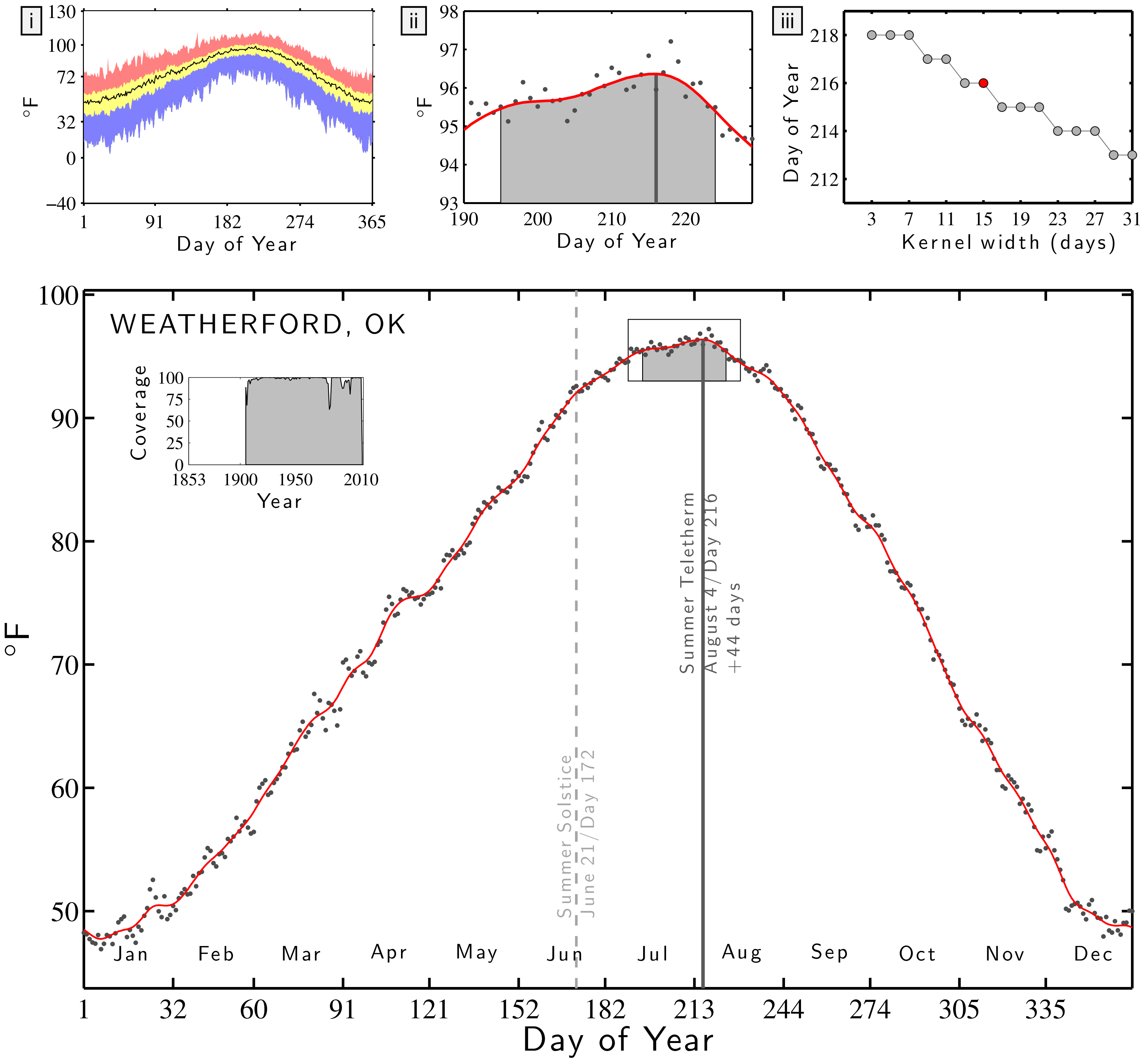

i
ii
iii
°F
Day of Year
Day of Year
Kernel width (days)
WEATHERFORD, OK
Coverage
Year
Summer Solstice
June 21/Day 172
Summer Teletherm
August 4/Day 216
+44 days
Jan
Feb
Mar
Apr
May
Jun
Jul
Aug
Sep
Oct
Nov
Dec

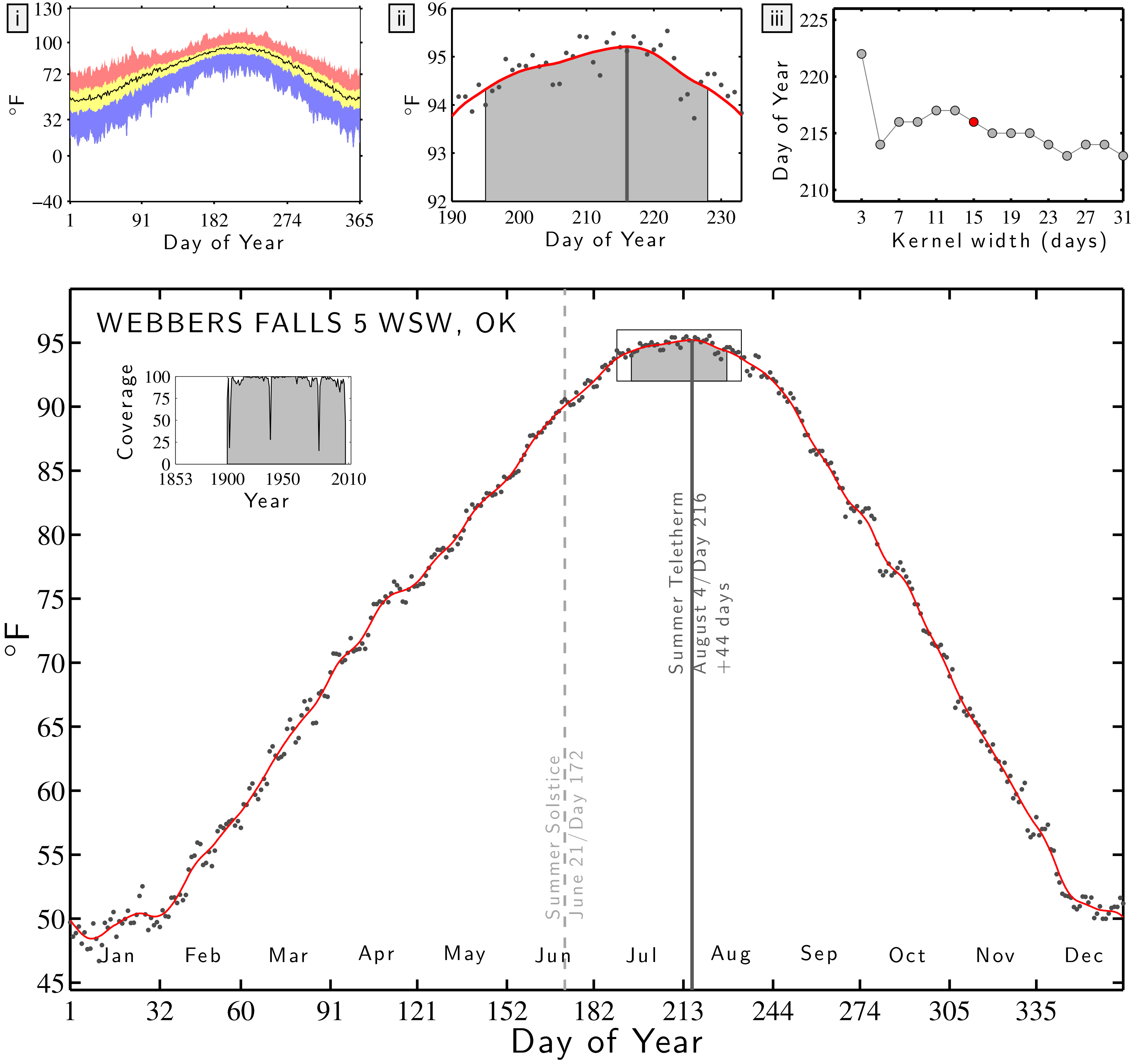

i
ii
iii
WEBBERS FALLS 5 WSW, OK
Summer Solstice
June 21 / Day 172
Summer Teletherm
August 4 / Day 216
+44 days
Coverage
Year
Day of Year
Kernel width (days)
°F
Jan
Feb
Mar
Apr
May
Jun
Jul
Aug
Sep
Oct
Nov
Dec

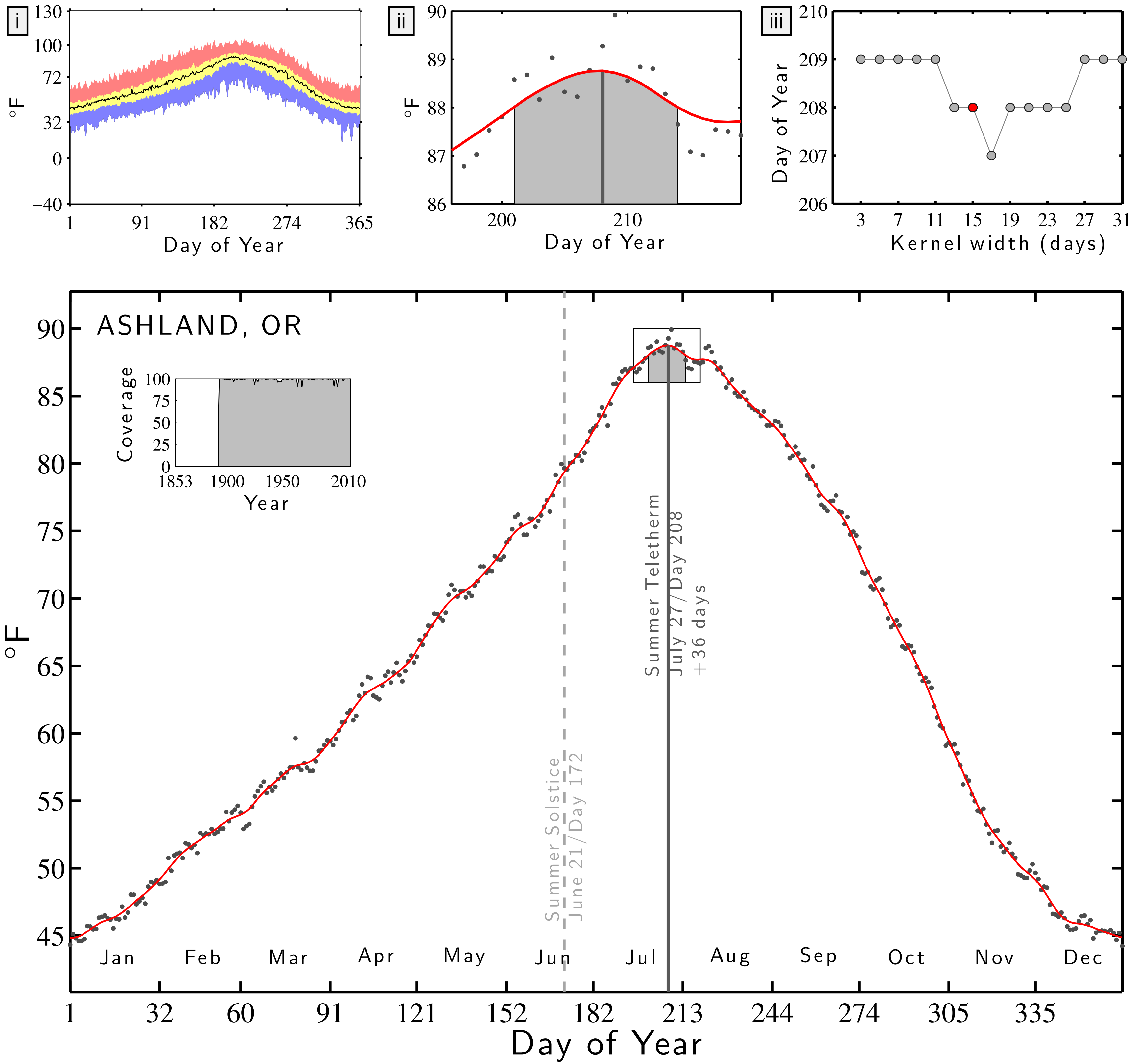
i
°F
Day of Year
ii
°F
Day of Year
iii
Day of Year
Kernel width (days)
ASHLAND, OR
Coverage
Year
Summer Solstice
June 21 / Day 172
Summer Teletherm
July 27 / Day 208
+36 days
Jan
Feb
Mar
Apr
May
Jun
Jul
Aug
Sep
Oct
Nov
Dec
°F
Day of Year

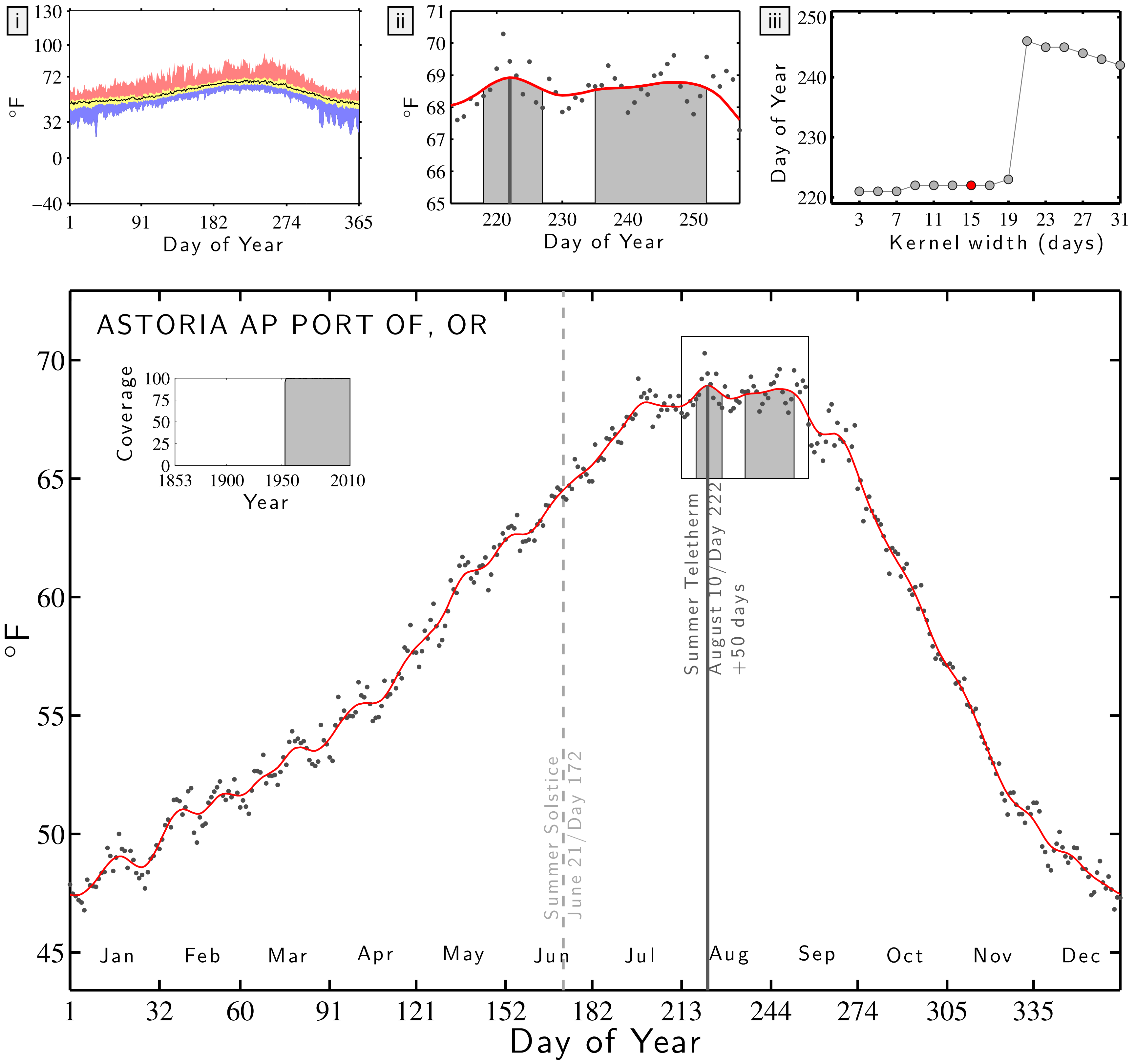

i
ii
iii
°F
Day of Year
Kernel width (days)
ASTORIA AP PORT OF, OR
Coverage
Year
Summer Solstice
June 21 / Day 172
Summer Teletherm
August 10 / Day 222
+50 days
Jan
Feb
Mar
Apr
May
Jun
Jul
Aug
Sep
Oct
Nov
Dec

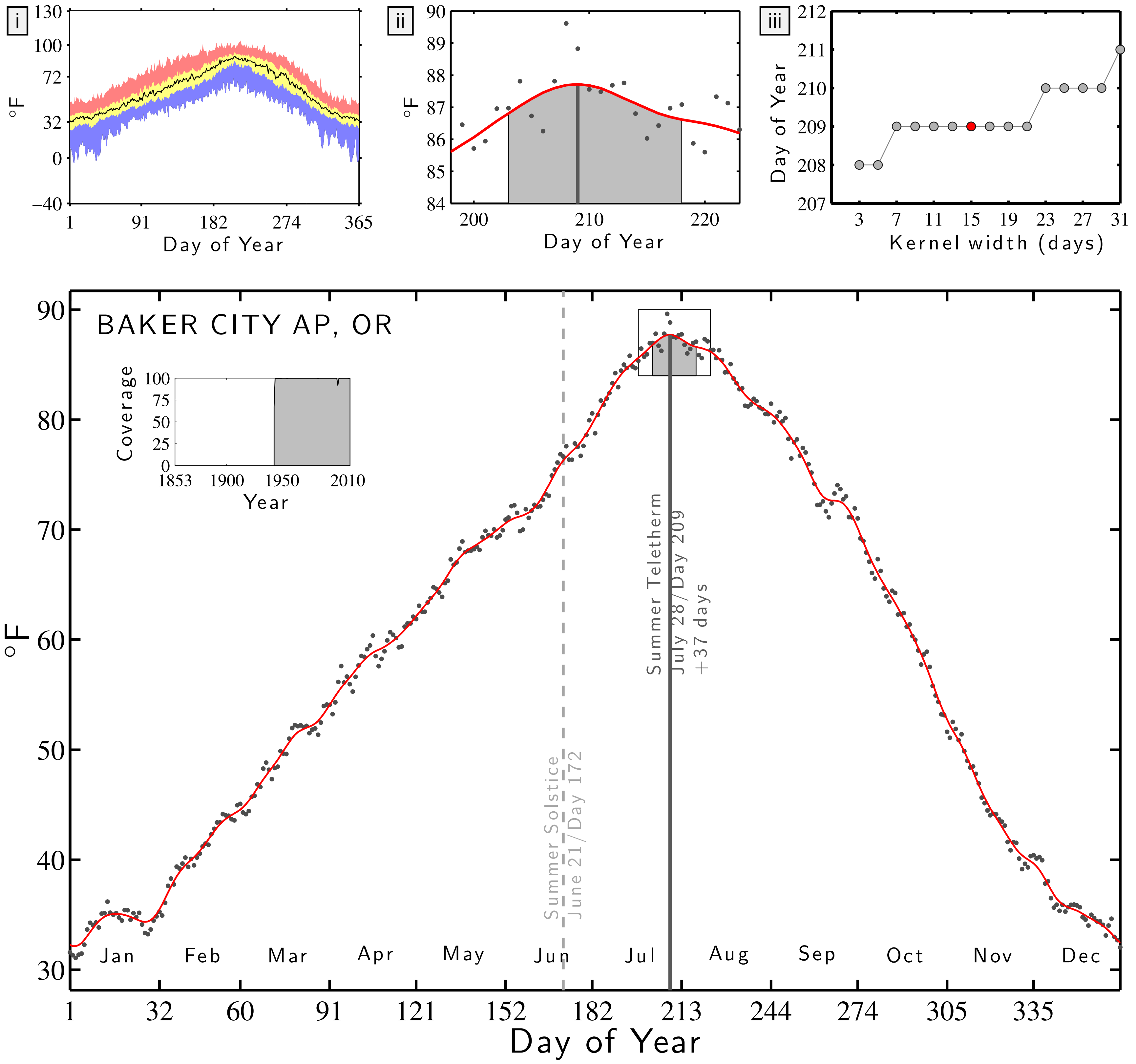

i
°F
Day of Year
ii
°F
Day of Year
iii
Day of Year
Kernel width (days)
BAKER CITY AP, OR
Coverage
Year
Summer Solstice
June 21/Day 172
Summer Teletherm
July 28/Day 209
+37 days
Jan
Feb
Mar
Apr
May
Jun
Jul
Aug
Sep
Oct
Nov
Dec
°F
Day of Year

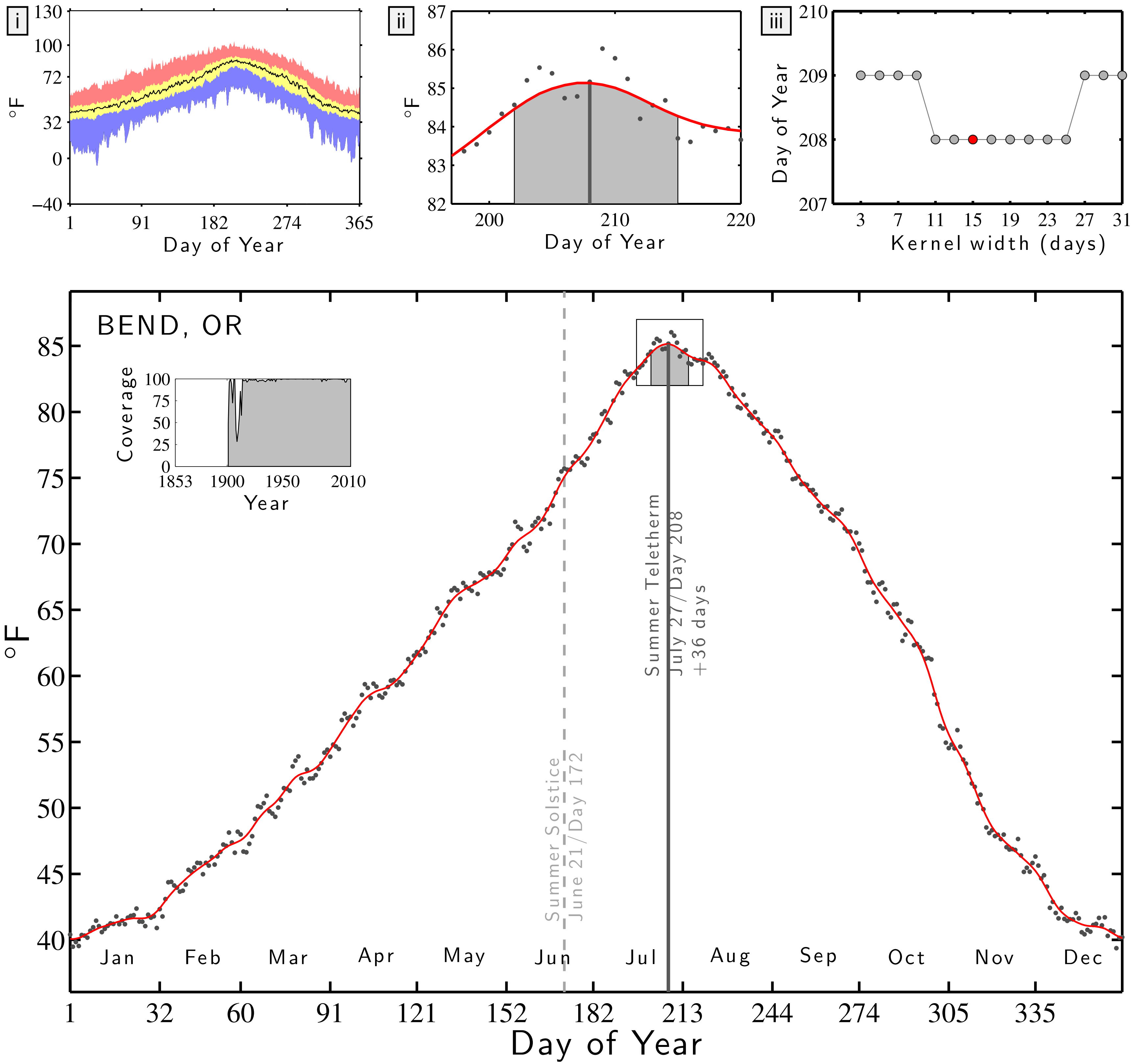
i
ii
iii
°F
Day of Year
Day of Year
Kernel width (days)
BEND, OR
Coverage
Year
Summer Solstice
June 21 / Day 172
Summer Telethern
July 27 / Day 208
+36 days
Jan
Feb
Mar
Apr
May
Jun
Jul
Aug
Sep
Oct
Nov
Dec

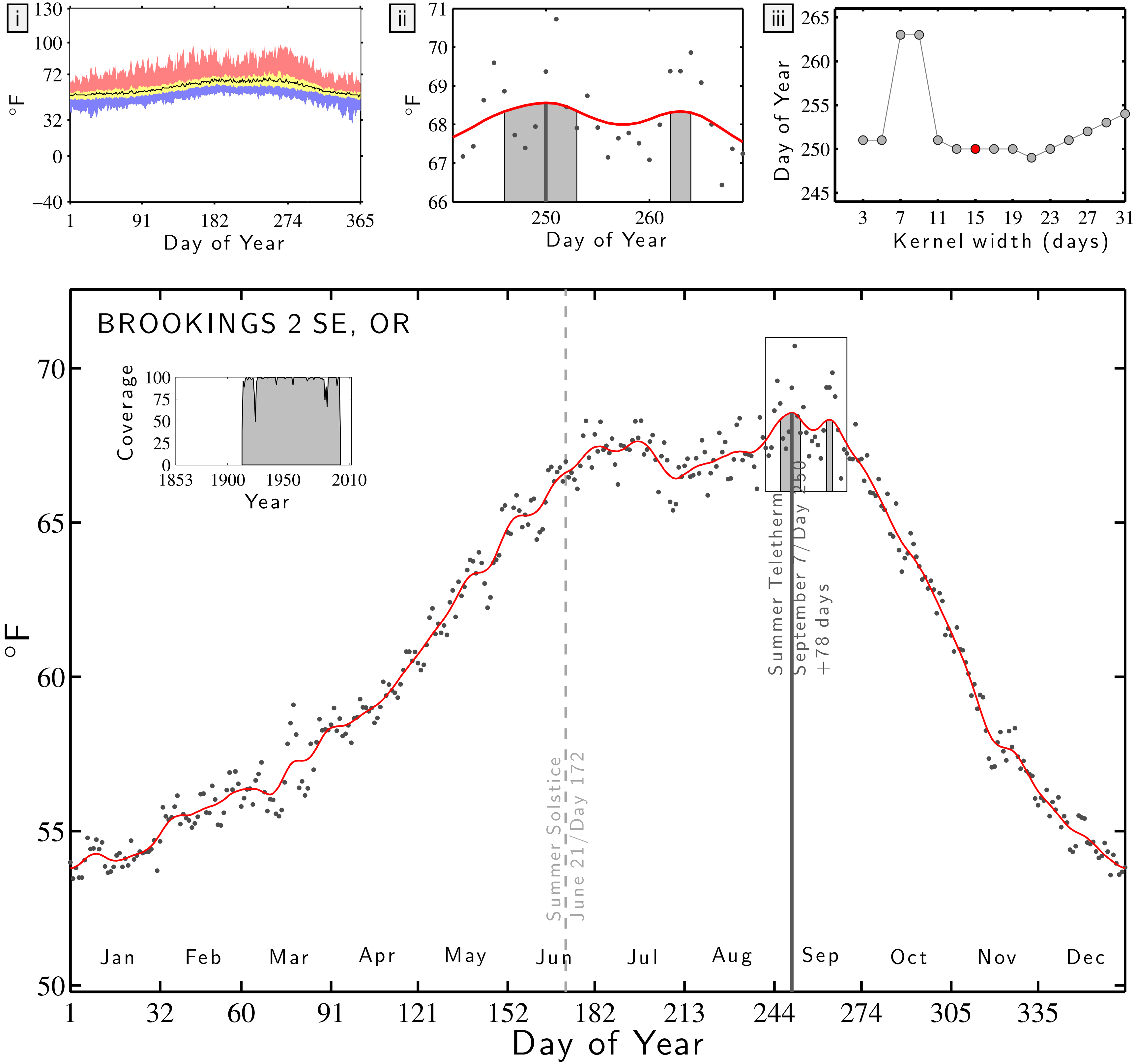

i
130
100
72
32
0
−40
°F
1
91
182
274
365
Day of Year
ii
71
70
69
68
67
66
°F
250
260
Day of Year
iii
265
260
255
250
245
Day of Year
3
7
11
15
19
23
27
31
Kernel width (days)
BROOKINGS 2 SE, OR
Coverage
100
75
50
25
0
1853
1900
1950
2010
Year
Summer Solstice
June 21/Day 172
Summer Teletherm
September 7/Day 250
+78 days
70
65
60
55
50
°F
Jan
Feb
Mar
Apr
May
Jun
Jul
Aug
Sep
Oct
Nov
Dec
1
32
60
91
121
152
182
213
244
274
305
335
Day of Year

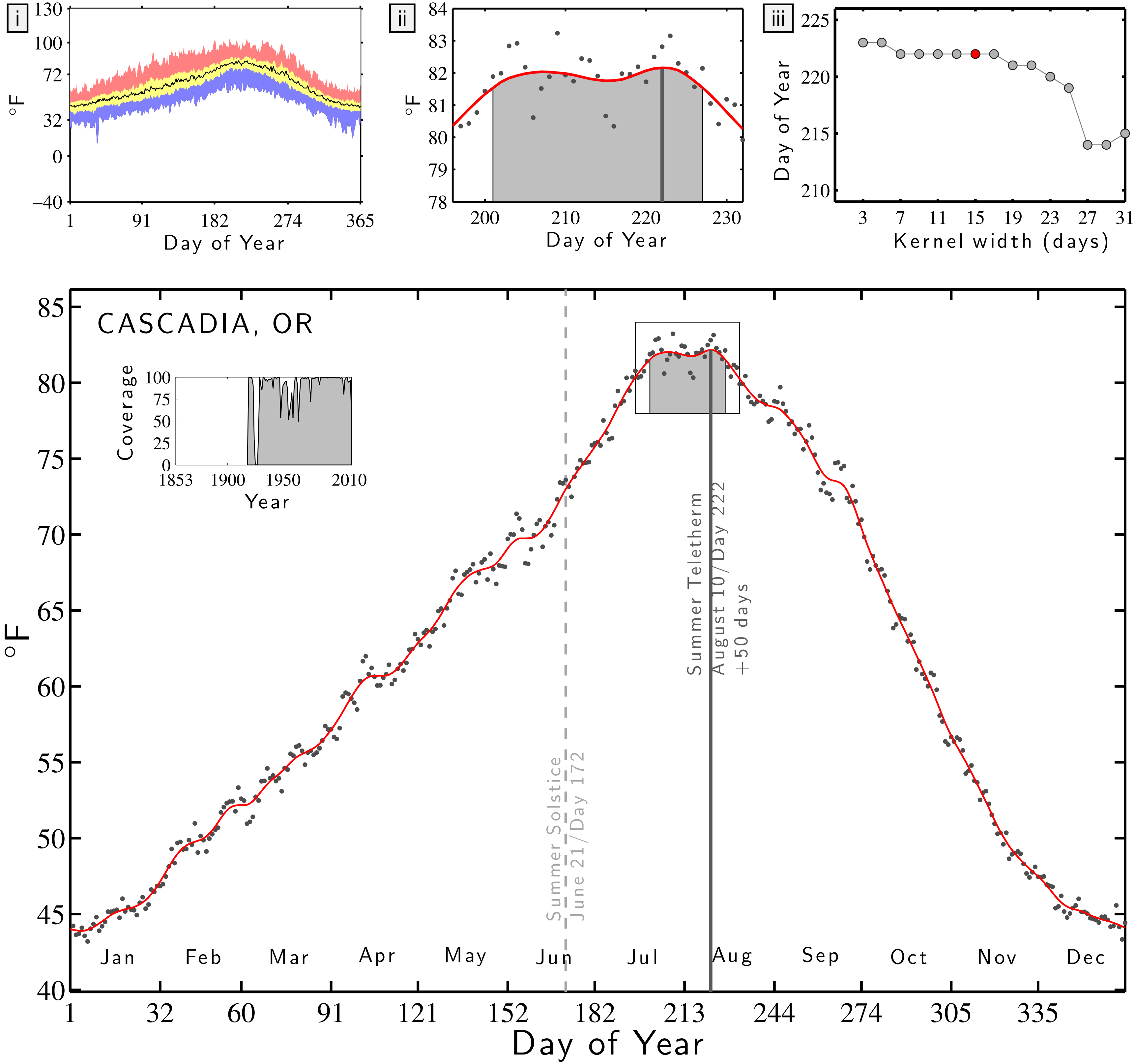

i
°F
Day of Year
ii
°F
Day of Year
iii
Day of Year
Kernel width (days)
CASCADIA, OR
Coverage
Year
Summer Solstice
June 21/Day 172
Summer Teletherm
August 10/Day 222
+50 days
Jan
Feb
Mar
Apr
May
Jun
Jul
Aug
Sep
Oct
Nov
Dec
°F
Day of Year

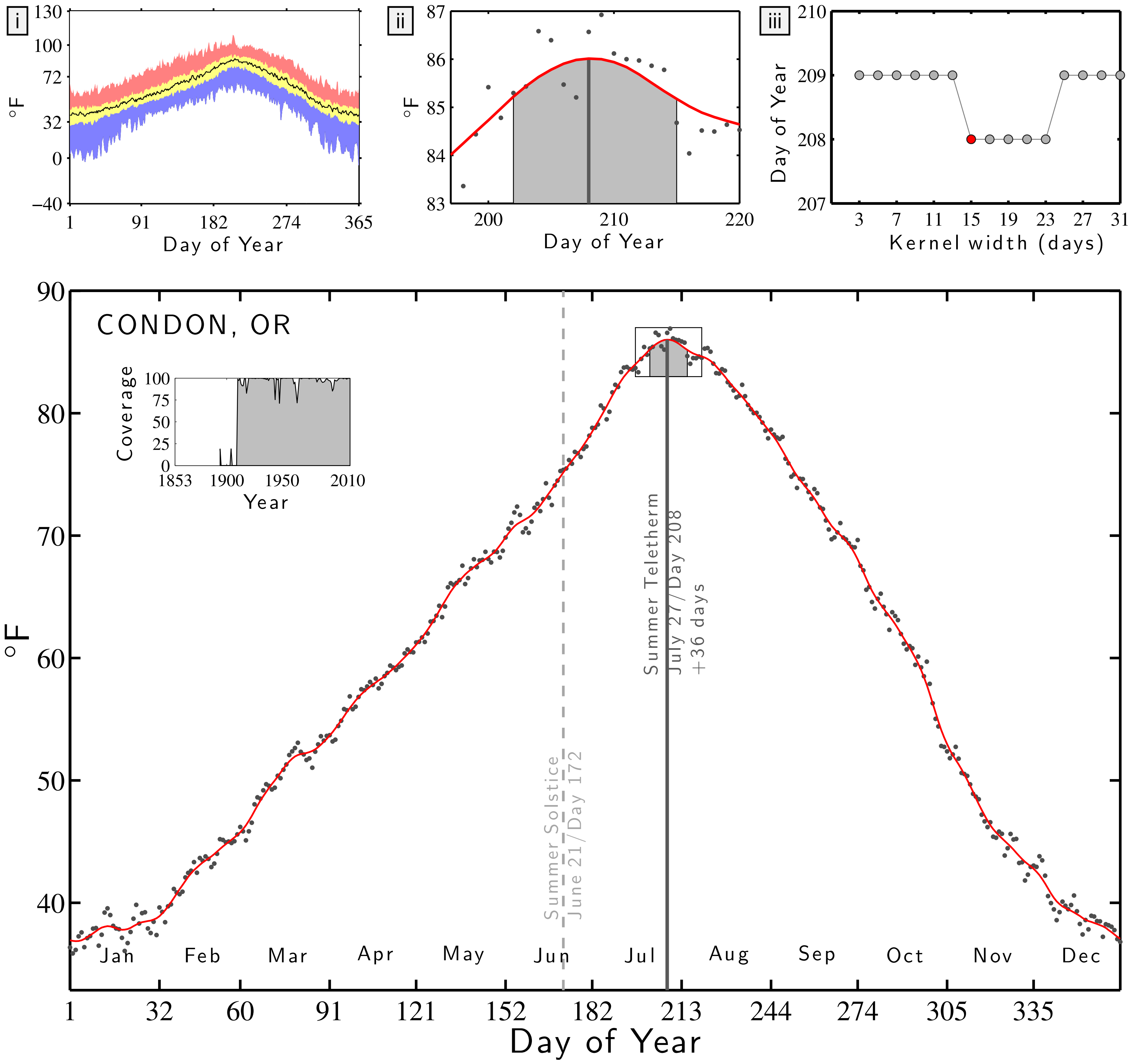

i
°F
Day of Year
ii
°F
Day of Year
iii
Day of Year
Kernel width (days)
CONDON, OR
Coverage
Year
Summer Solstice
June 21 / Day 172
Summer Teletherm
July 27 / Day 208
+36 days
Jan
Feb
Mar
Apr
May
Jun
Jul
Aug
Sep
Oct
Nov
Dec
°F
Day of Year

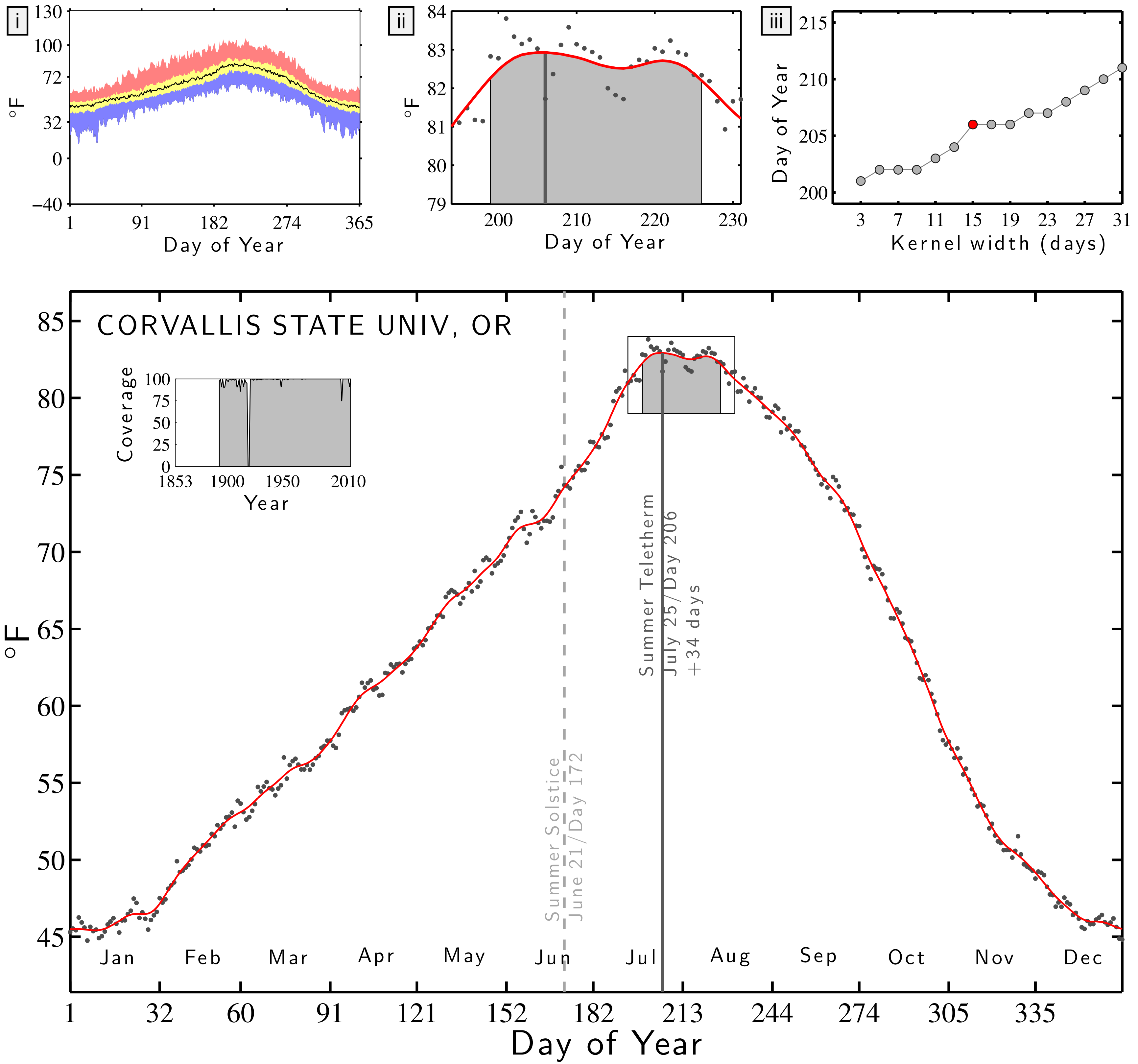

i
°F
Day of Year
ii
°F
Day of Year
iii
Day of Year
Kernel width (days)
CORVALLIS STATE UNIV, OR
Coverage
Year
Summer Solstice
June 21/Day 172
Summer Teletherm
July 25/Day 206
+34 days
Jan
Feb
Mar
Apr
May
Jun
Jul
Aug
Sep
Oct
Nov
Dec
°F
Day of Year

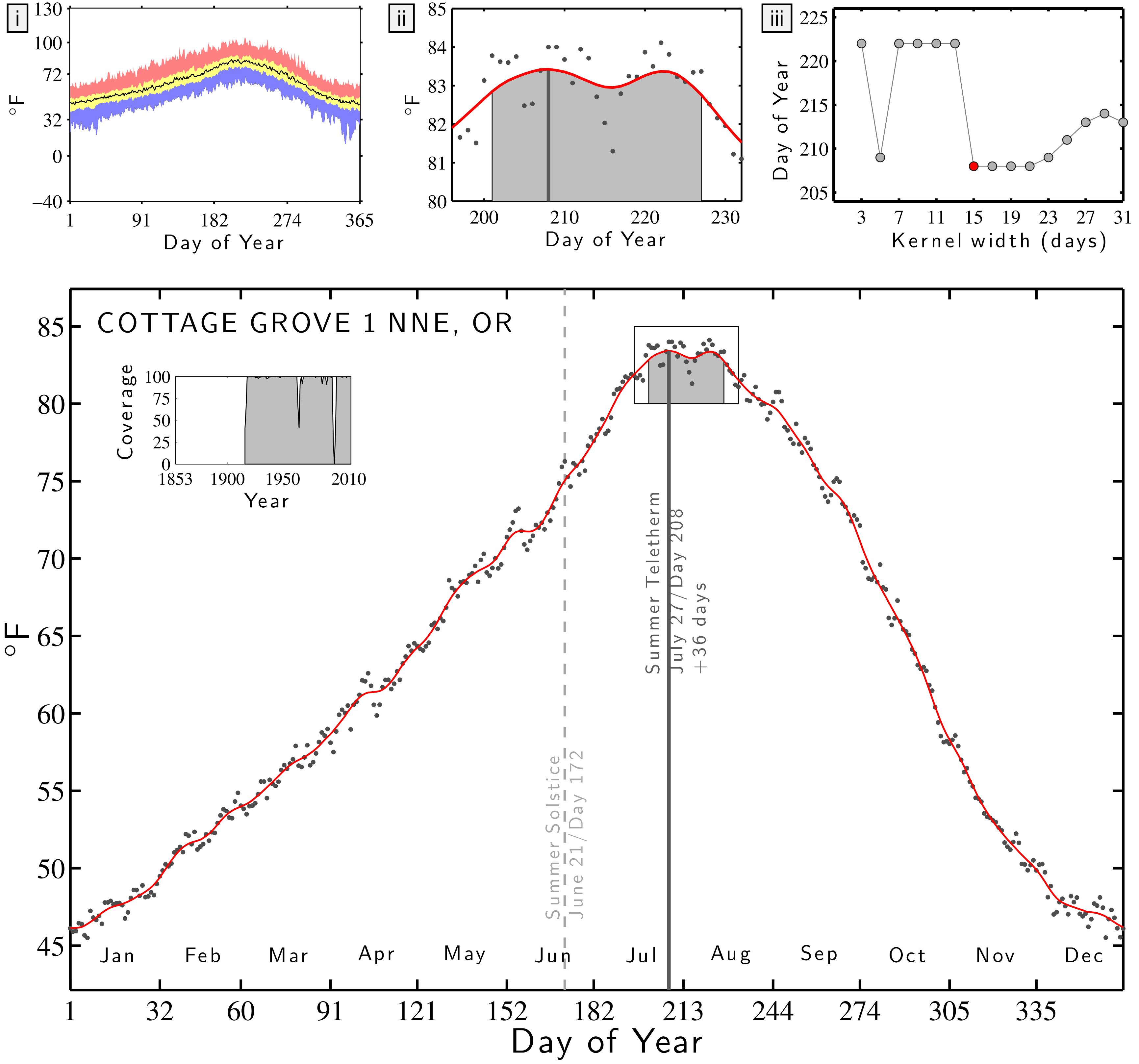

i
°F
130
100
72
32
0
−40
1
91
182
274
365
Day of Year
ii
85
84
83
82
81
80
200
210
220
230
Day of Year
iii
Day of Year
225
220
215
210
205
3
7
11
15
19
23
27
31
Kernel width (days)
COTTAGE GROVE 1 NNE, OR
Coverage
100
75
50
25
0
1853
1900
1950
2010
Year
Summer Solstice
June 21/Day 172
Summer Teletherm
July 27/Day 208
+36 days
85
80
75
70
65
60
55
50
45
Jan
Feb
Mar
Apr
May
Jun
Jul
Aug
Sep
Oct
Nov
Dec
1
32
60
91
121
152
182
213
244
274
305
335
Day of Year

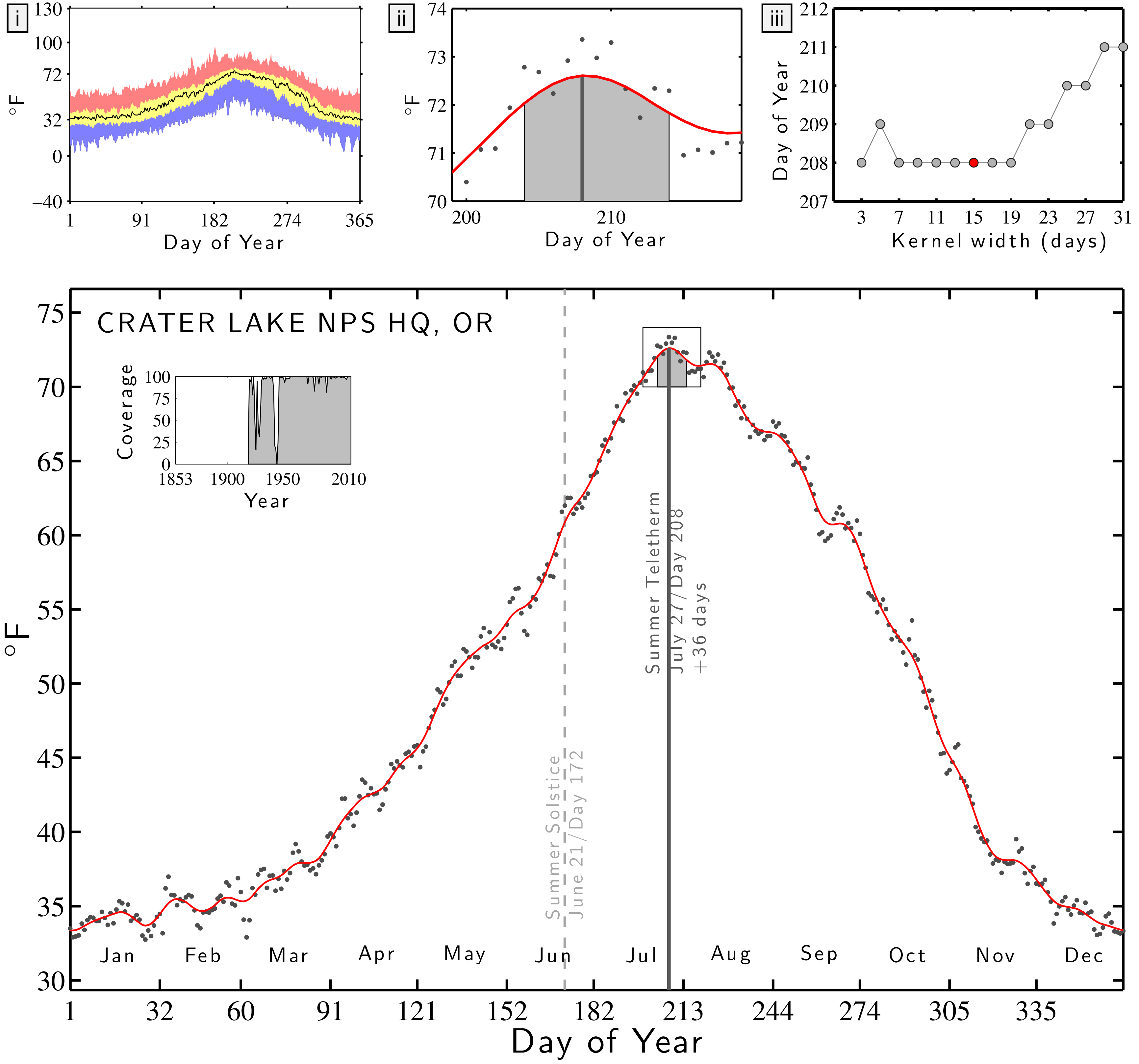
i
°F
Day of Year
ii
°F
Day of Year
iii
Day of Year
Kernel width (days)
CRATER LAKE NPS HQ, OR
Coverage
Year
Summer Solstice
June 21 / Day 172
Summer Teletherm
July 27 / Day 208
+36 days
Jan
Feb
Mar
Apr
May
Jun
Jul
Aug
Sep
Oct
Nov
Dec
°F
Day of Year

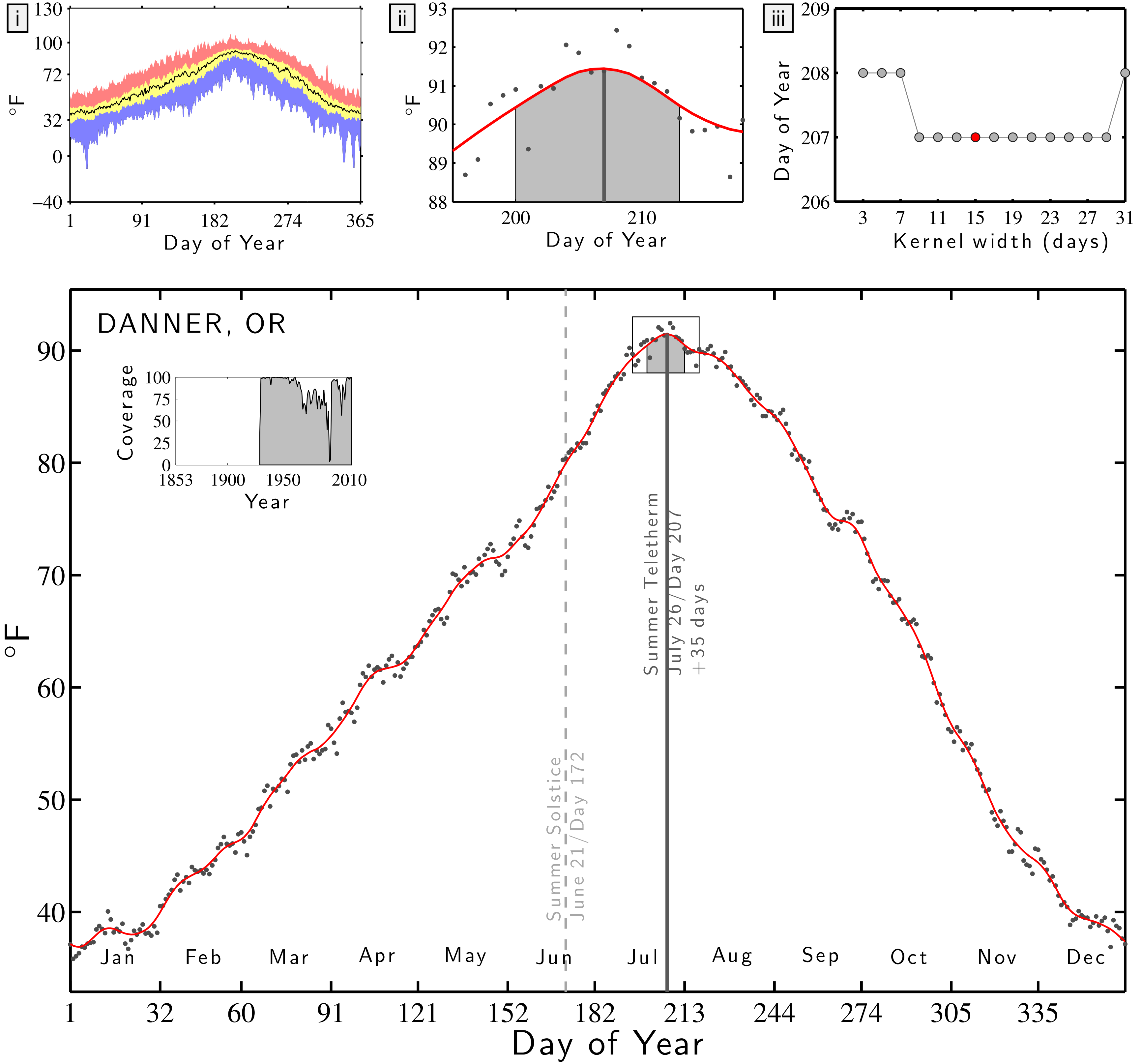

i
ii
iii
°F
Day of Year
Day of Year
Kernel width (days)
DANNER, OR
Coverage
Year
Summer Solstice
June 21 / Day 172
Summer Teletherm
July 26 / Day 207
+35 days
Jan
Feb
Mar
Apr
May
Jun
Jul
Aug
Sep
Oct
Nov
Dec
Day of Year
°F

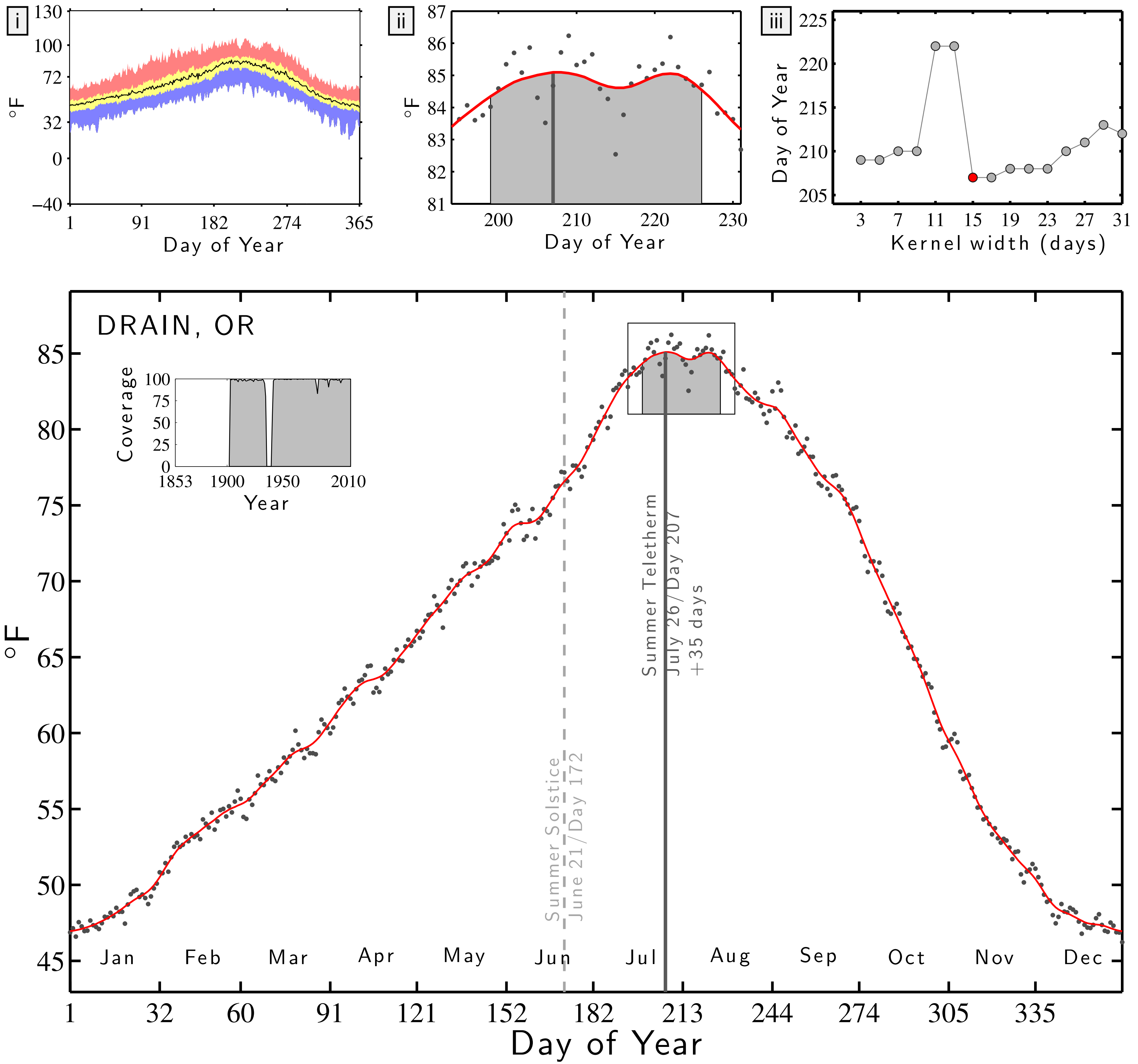

i
ii
iii
°F
Day of Year
Day of Year
Kernel width (days)
DRAIN, OR
Coverage
Year
Summer Solstice
June 21 / Day 172
Summer Teletherm
July 26 / Day 207
+35 days
Jan
Feb
Mar
Apr
May
Jun
Jul
Aug
Sep
Oct
Nov
Dec

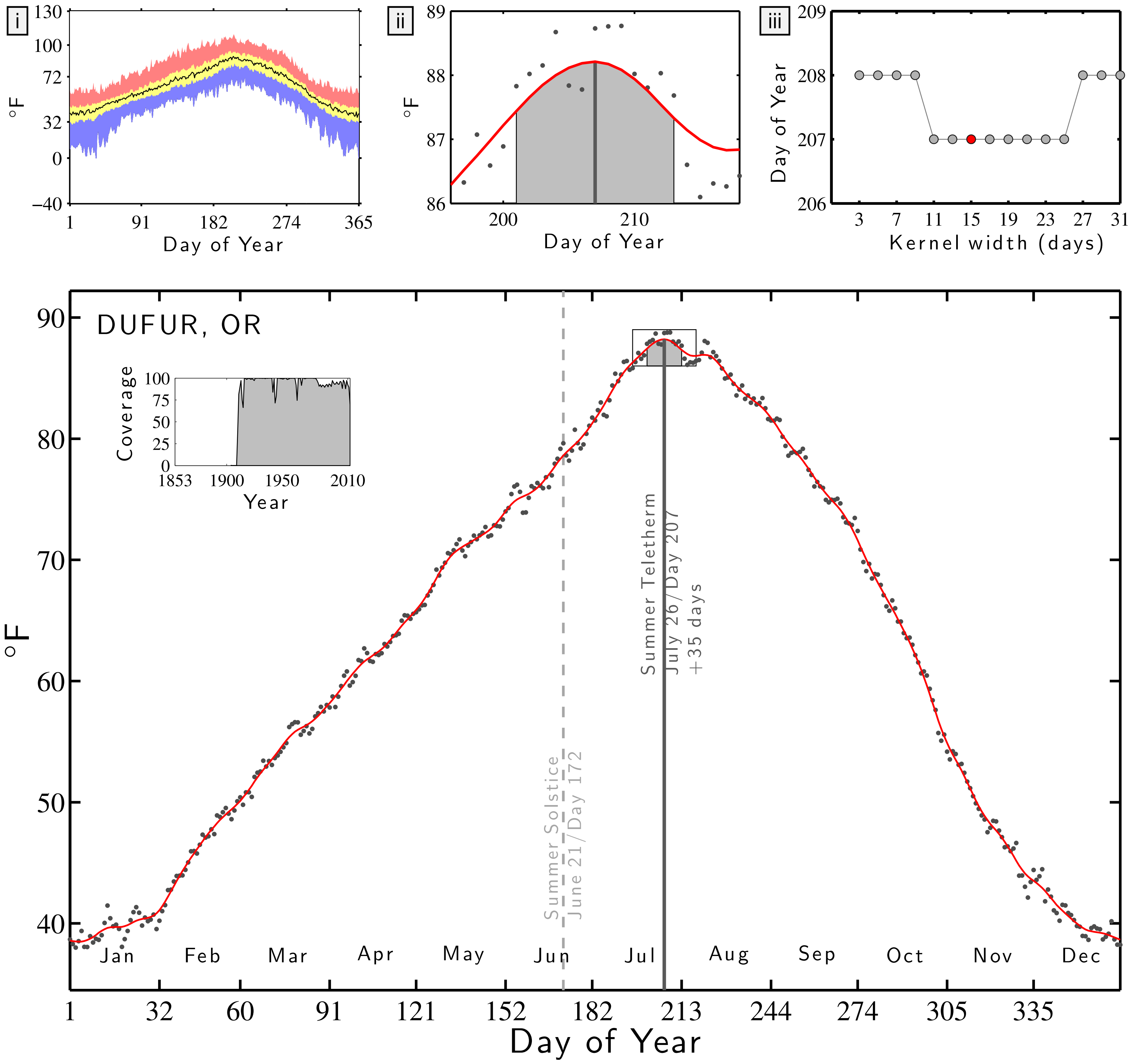

i
ii
iii
°F
Day of Year
Day of Year
Kernel width (days)
DUFUR, OR
Coverage
Year
Summer Solstice
June 21 / Day 172
Summer Teletherm
July 26 / Day 207
+35 days
Jan
Feb
Mar
Apr
May
Jun
Jul
Aug
Sep
Oct
Nov
Dec
Day of Year

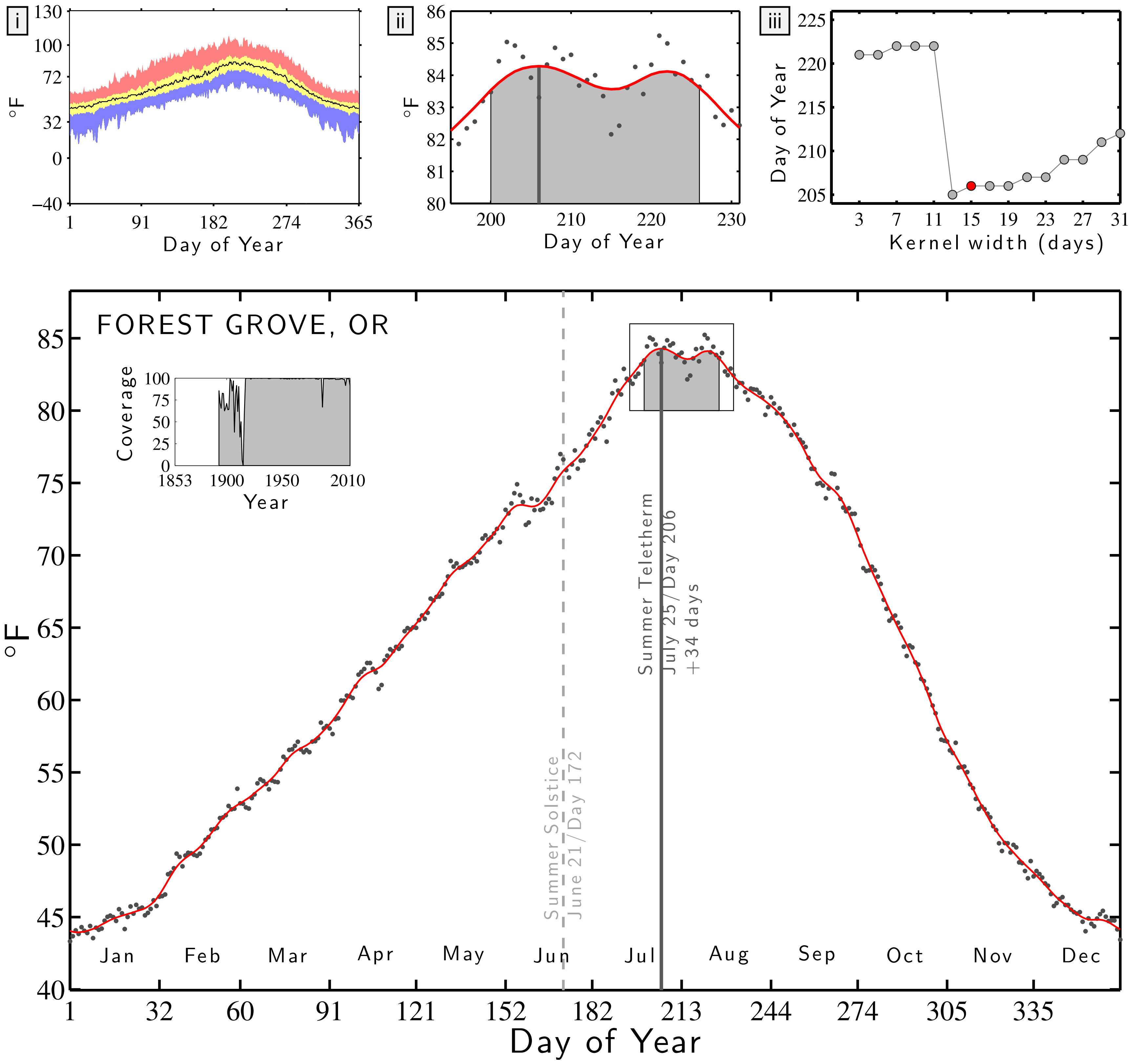

i
ii
iii
°F
Day of Year
Day of Year
Kernel width (days)
FOREST GROVE, OR
Coverage
Year
Summer Solstice
June 21/Day 172
Summer Teletherm
July 25/Day 206
+34 days
Jan
Feb
Mar
Apr
May
Jun
Jul
Aug
Sep
Oct
Nov
Dec
Day of Year

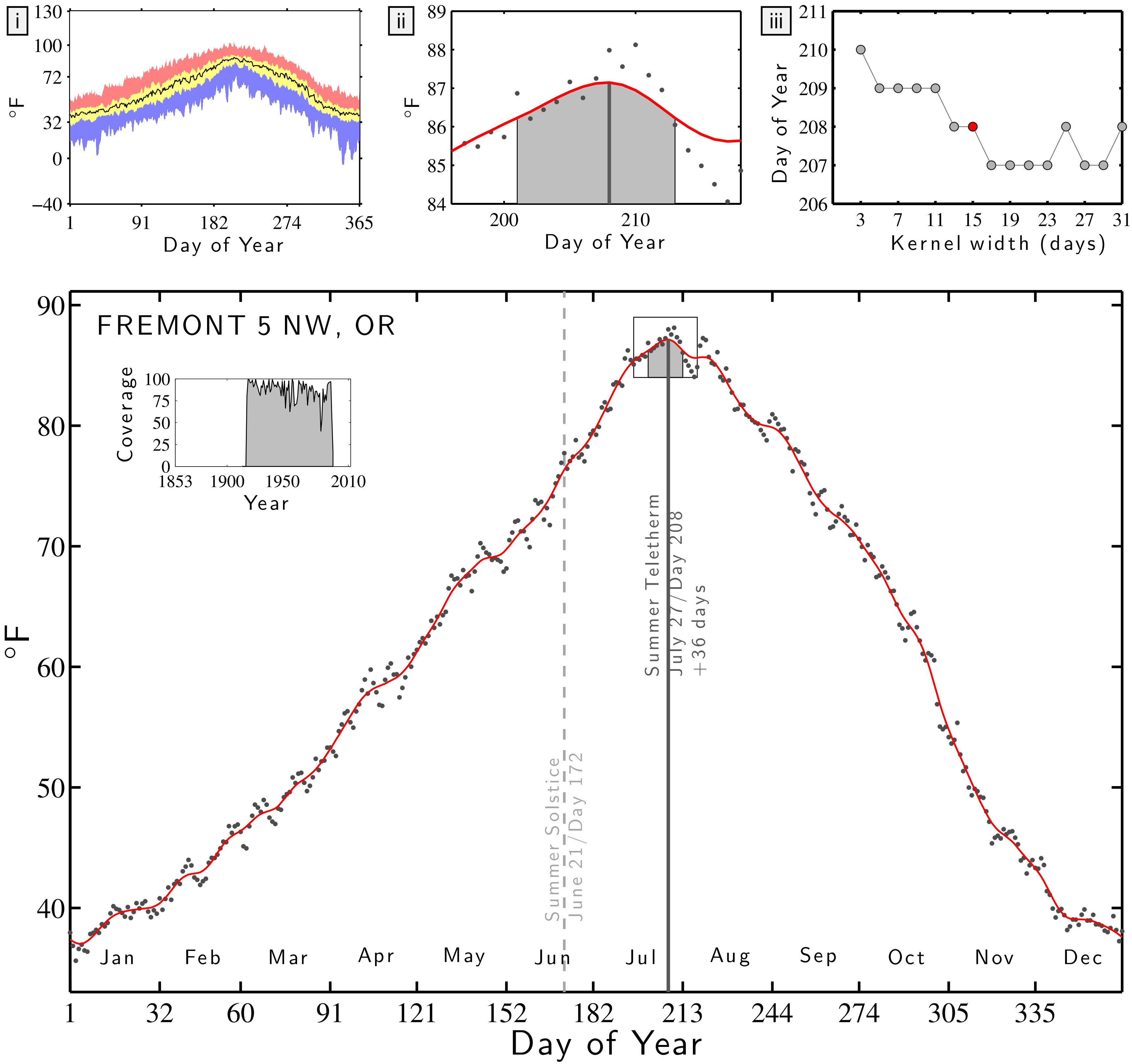

i
°F
Day of Year
ii
°F
Day of Year
iii
Day of Year
Kernel width (days)
FREMONT 5 NW, OR
Coverage
Year
Summer Solstice
June 21 / Day 172
Summer Teletherm
July 27 / Day 208
+36 days
Jan
Feb
Mar
Apr
May
Jun
Jul
Aug
Sep
Oct
Nov
Dec
°F
Day of Year

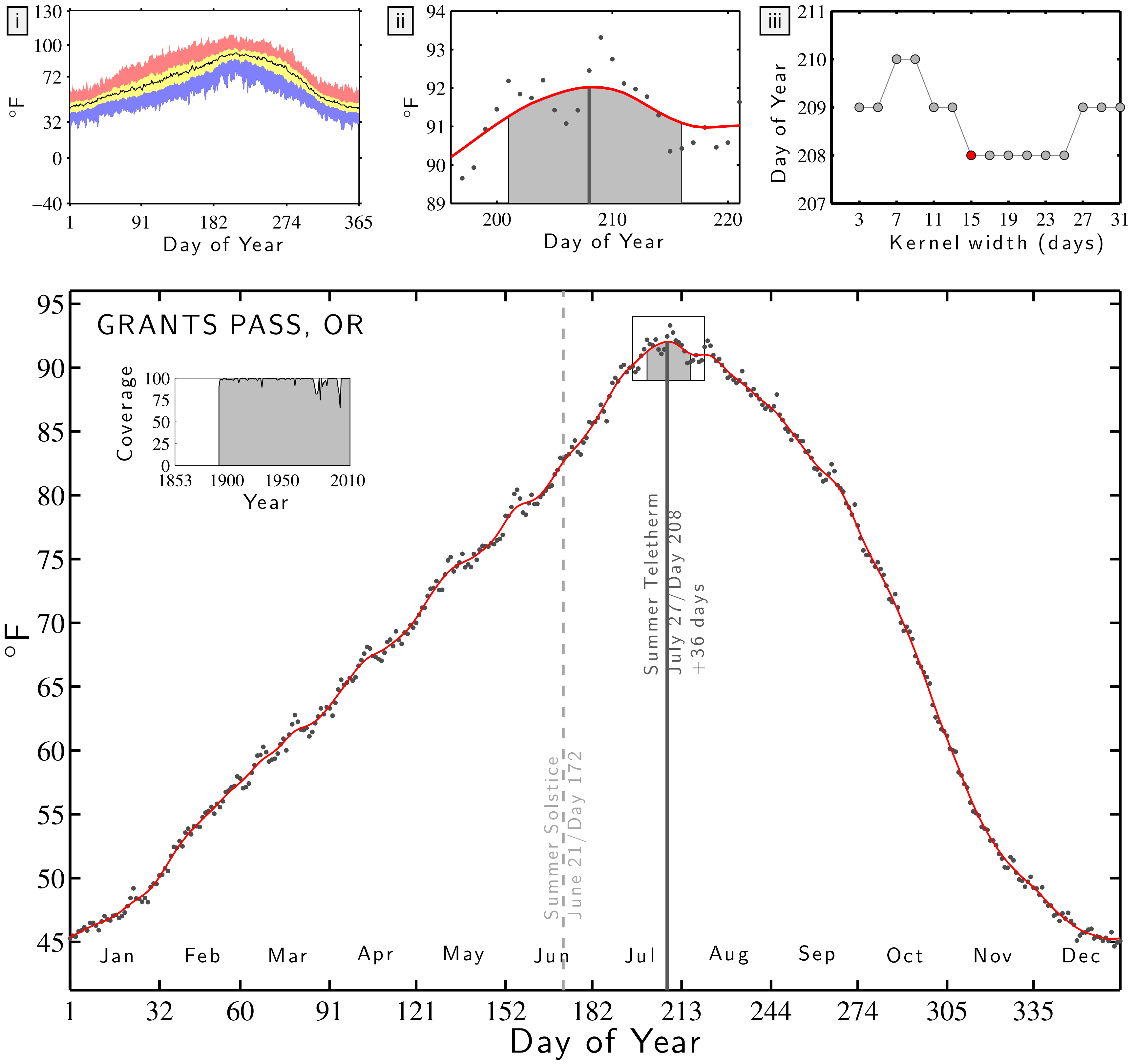

i
°F
Day of Year
ii
°F
Day of Year
iii
Day of Year
Kernel width (days)
GRANTS PASS, OR
Coverage
Year
Summer Solstice
June 21 / Day 172
Summer Teletherm
July 27 / Day 208
+36 days
Jan
Feb
Mar
Apr
May
Jun
Jul
Aug
Sep
Oct
Nov
Dec
°F
Day of Year

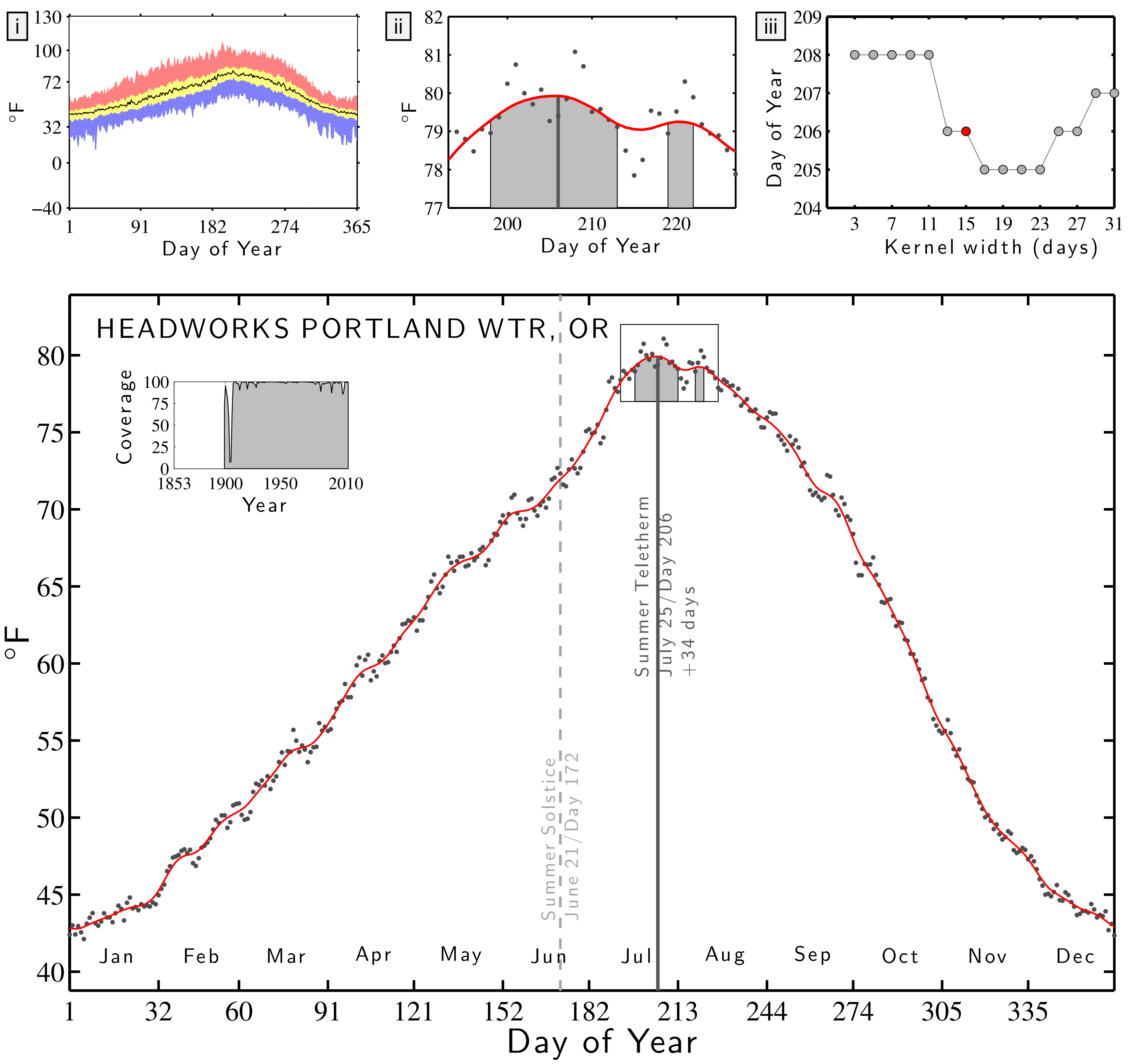

i
°F
Day of Year
ii
°F
Day of Year
iii
Day of Year
Kernel width (days)
HEADWORKS PORTLAND WTR, OR
Coverage
Year
Summer Solstice
June 21/Day 172
Summer Teletherm
July 25/Day 206
+34 days
Jan
Feb
Mar
Apr
May
Jun
Jul
Aug
Sep
Oct
Nov
Dec
°F
Day of Year

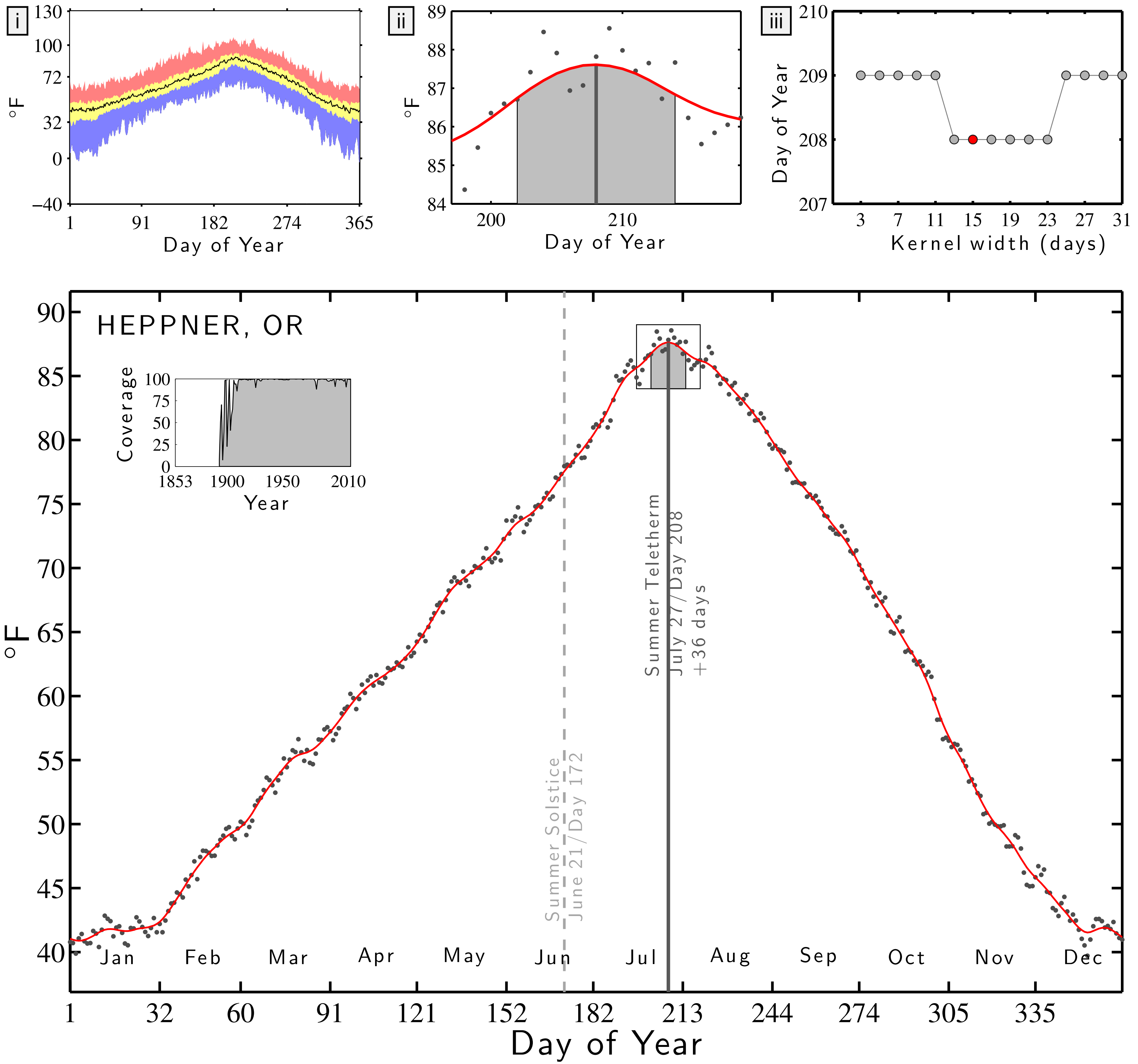
i
ii
iii
°F
Day of Year
Day of Year
Kernel width (days)
HEPPNER, OR
Coverage
Year
Summer Solstice
June 21 / Day 172
Summer Teletherm
July 27 / Day 208
+36 days
Jan
Feb
Mar
Apr
May
Jun
Jul
Aug
Sep
Oct
Nov
Dec

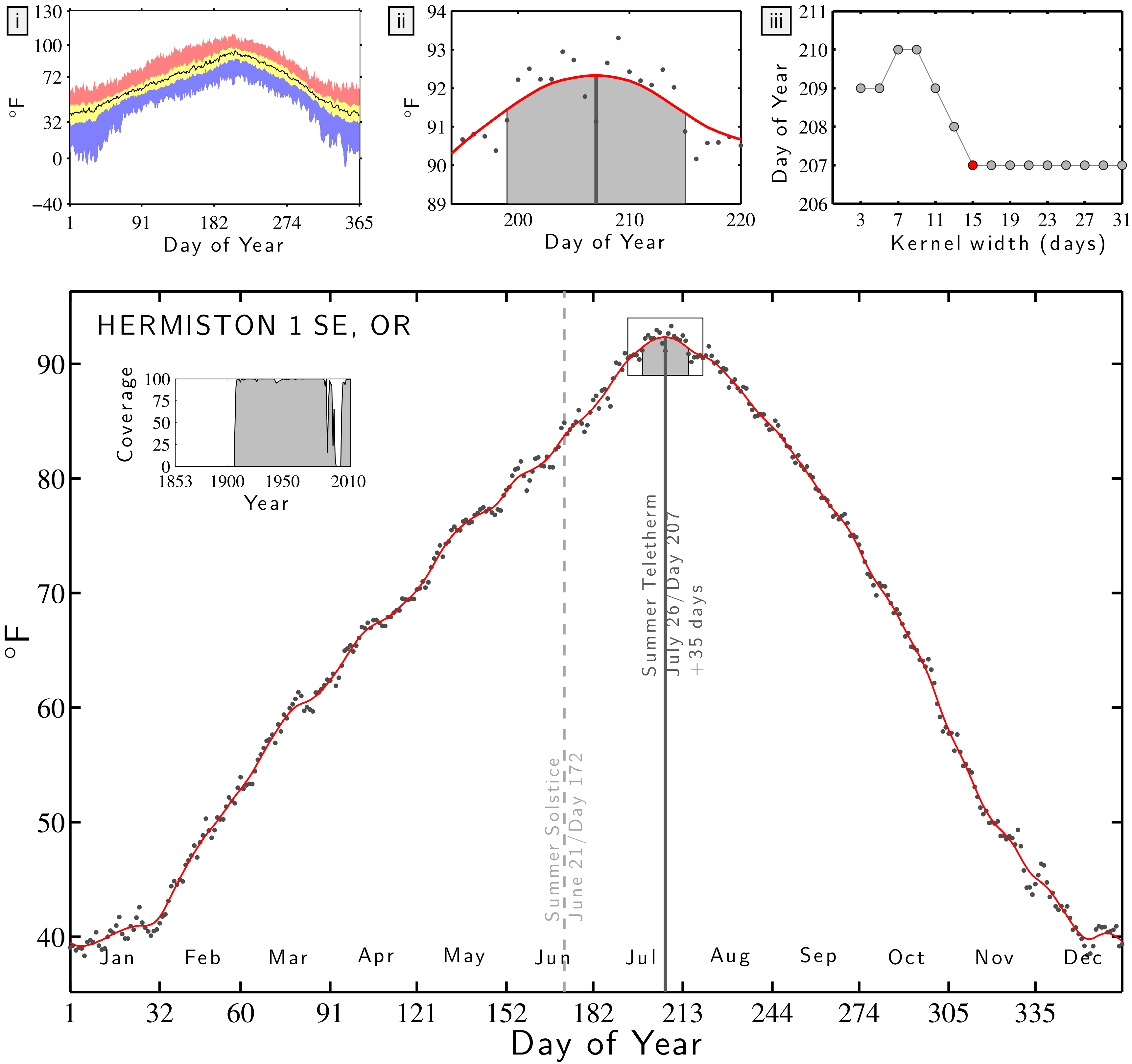
i
°F
Day of Year
ii
°F
Day of Year
iii
Day of Year
Kernel width (days)
HERMISTON 1 SE, OR
Coverage
Year
Summer Solstice
June 21 / Day 172
Summer Teletherm
July 26 / Day 207
+35 days
Jan
Feb
Mar
Apr
May
Jun
Jul
Aug
Sep
Oct
Nov
Dec
°F
Day of Year

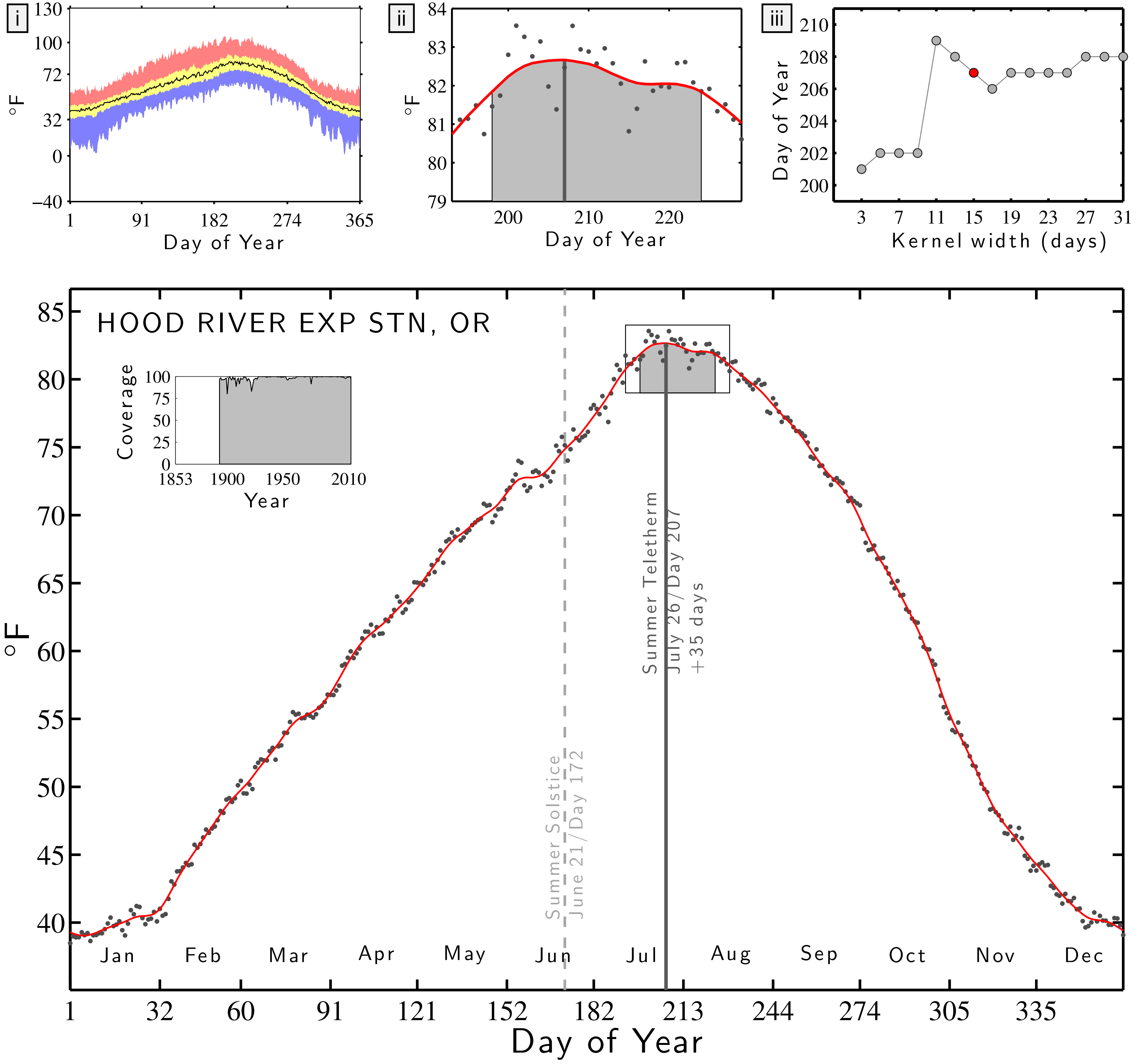

i
ii
iii
Day of Year
Kernel width (days)
°F
HOOD RIVER EXP STN, OR
Coverage
Year
Summer Solstice
June 21/Day 172
Summer Teletherm
July 26/Day 207
+35 days
Jan
Feb
Mar
Apr
May
Jun
Jul
Aug
Sep
Oct
Nov
Dec

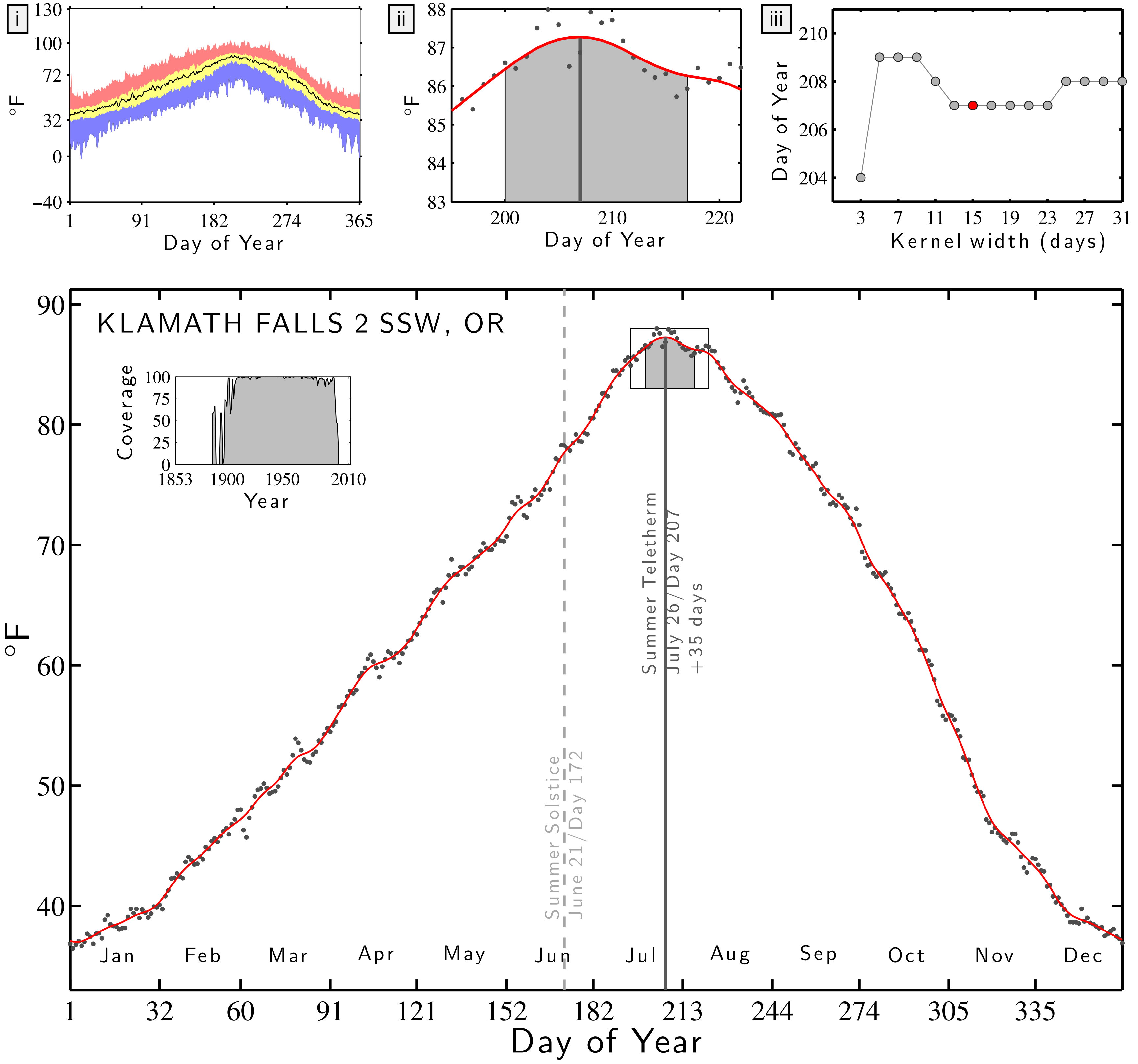

i
130
100
72
32
0
−40
°F
1
91
182
274
365
Day of Year
ii
88
87
86
85
84
83
°F
200
210
220
Day of Year
iii
210
208
206
204
Day of Year
3
7
11
15
19
23
27
31
Kernel width (days)
KLAMATH FALLS 2 SSW, OR
Coverage
100
75
50
25
0
1853
1900
1950
2010
Year
90
80
70
60
50
40
°F
Summer Solstice
June 21 / Day 172
Summer Teletherm
July 26 / Day 207
+35 days
Jan
Feb
Mar
Apr
May
Jun
Jul
Aug
Sep
Oct
Nov
Dec
1
32
60
91
121
152
182
213
244
274
305
335
Day of Year

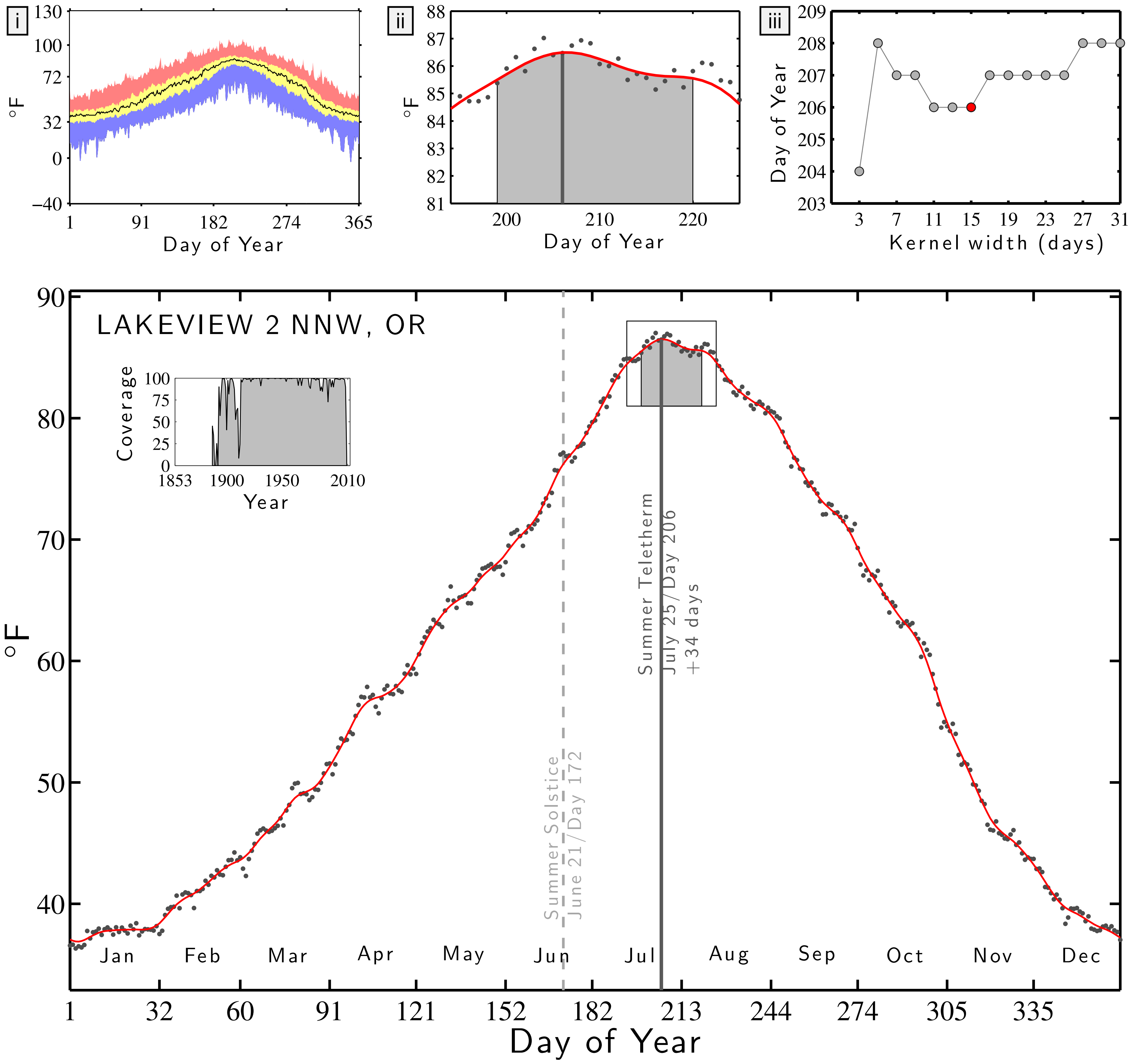

i
°F
Day of Year
ii
°F
Day of Year
iii
Day of Year
Kernel width (days)
LAKEVIEW 2 NNW, OR
Coverage
Year
Summer Solstice
June 21/Day 172
Summer Teletherm
July 25/Day 206
+34 days
Jan
Feb
Mar
Apr
May
Jun
Jul
Aug
Sep
Oct
Nov
Dec
°F
Day of Year

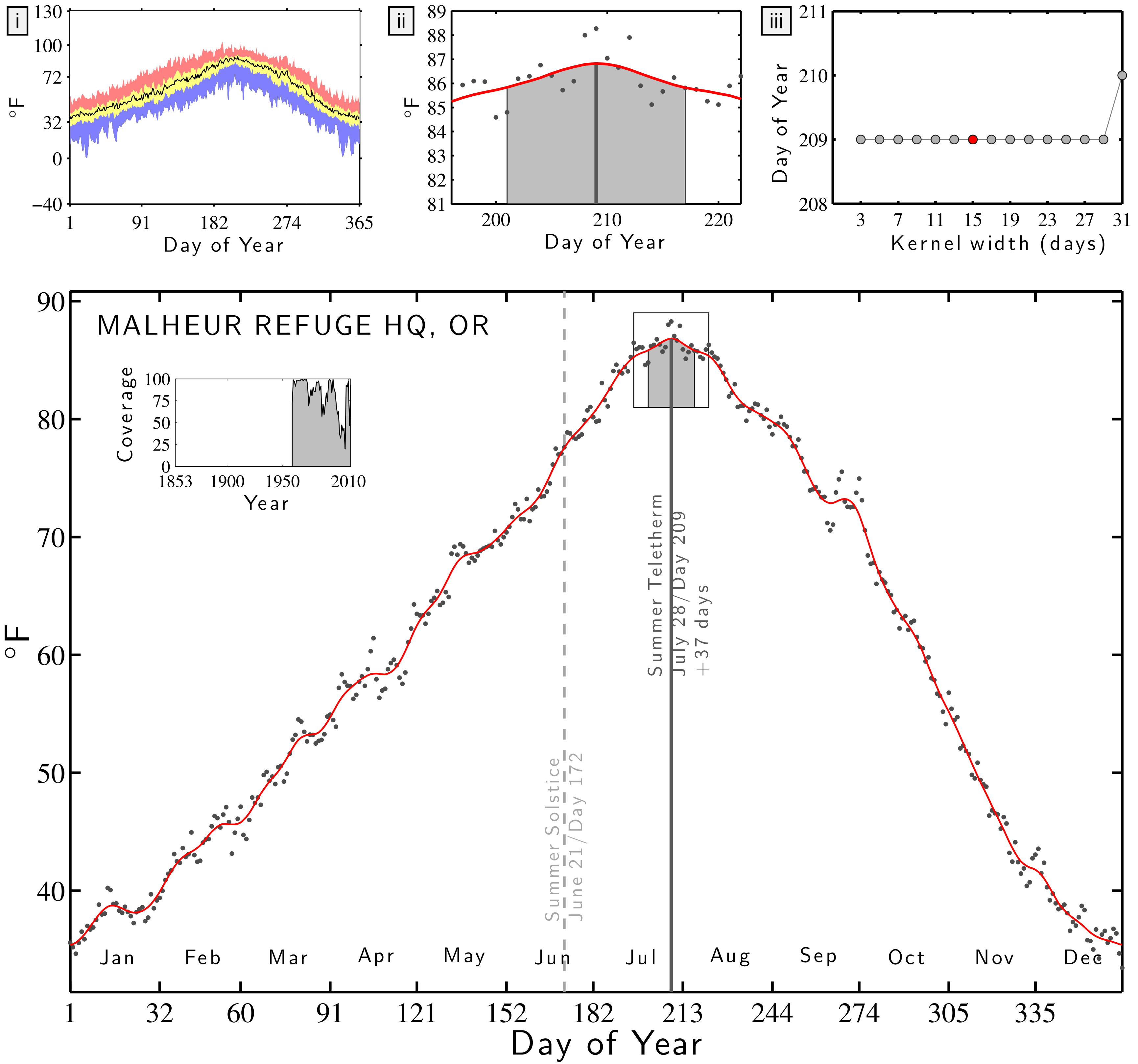

i
ii
iii
°F
Day of Year
Day of Year
Kernel width (days)
MALHEUR REFUGE HQ, OR
Coverage
Year
Summer Solstice
June 21/Day 172
Summer Teletherm
July 28/Day 209
+37 days
Jan
Feb
Mar
Apr
May
Jun
Jul
Aug
Sep
Oct
Nov
Dec

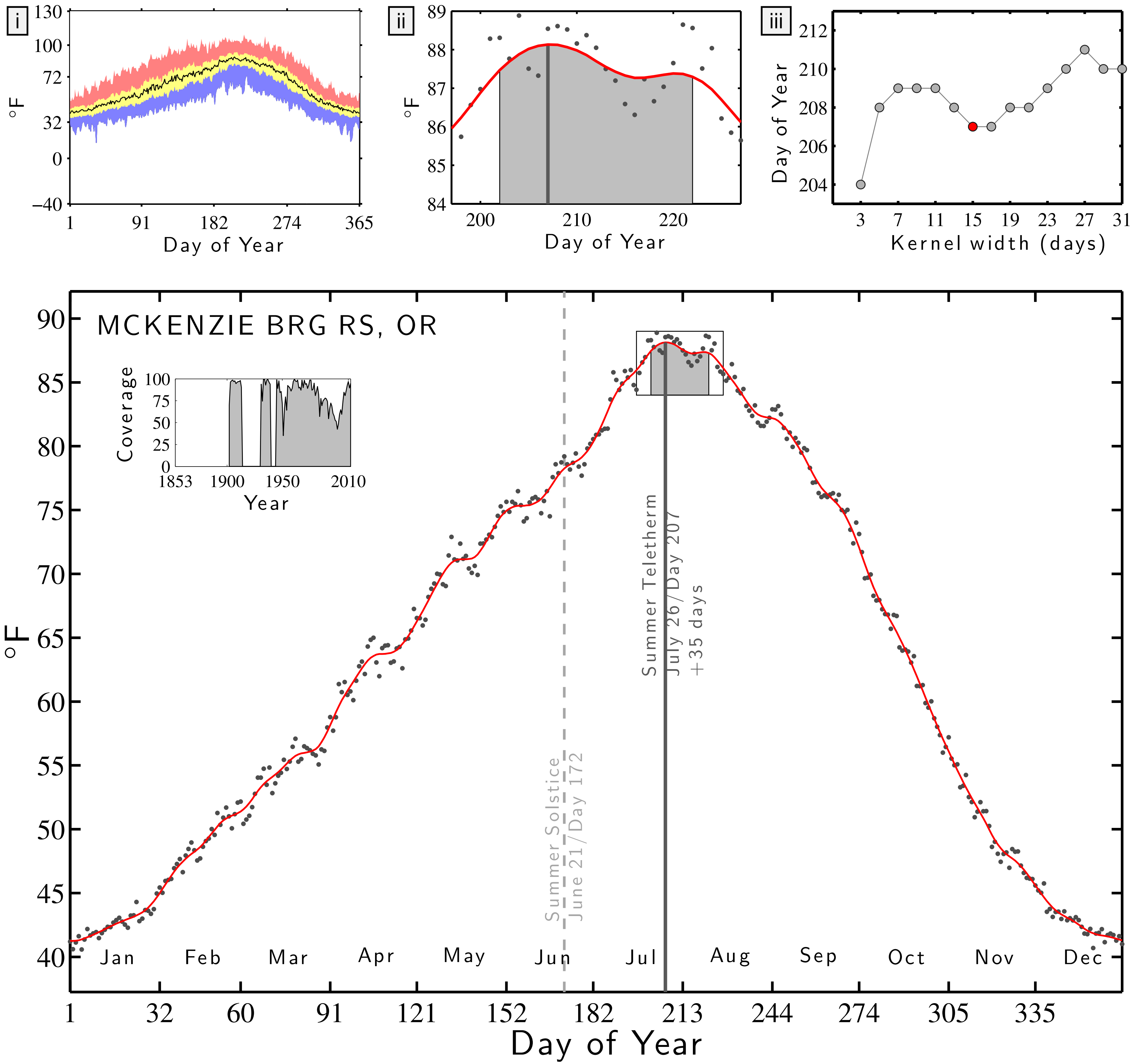

i
°F
Day of Year
ii
°F
Day of Year
iii
Day of Year
Kernel width (days)
MCKENZIE BRG RS, OR
Coverage
Year
Summer Solstice
June 21 / Day 172
Summer Teletherm
July 26 / Day 207
+35 days
Jan
Feb
Mar
Apr
May
Jun
Jul
Aug
Sep
Oct
Nov
Dec
°F
Day of Year

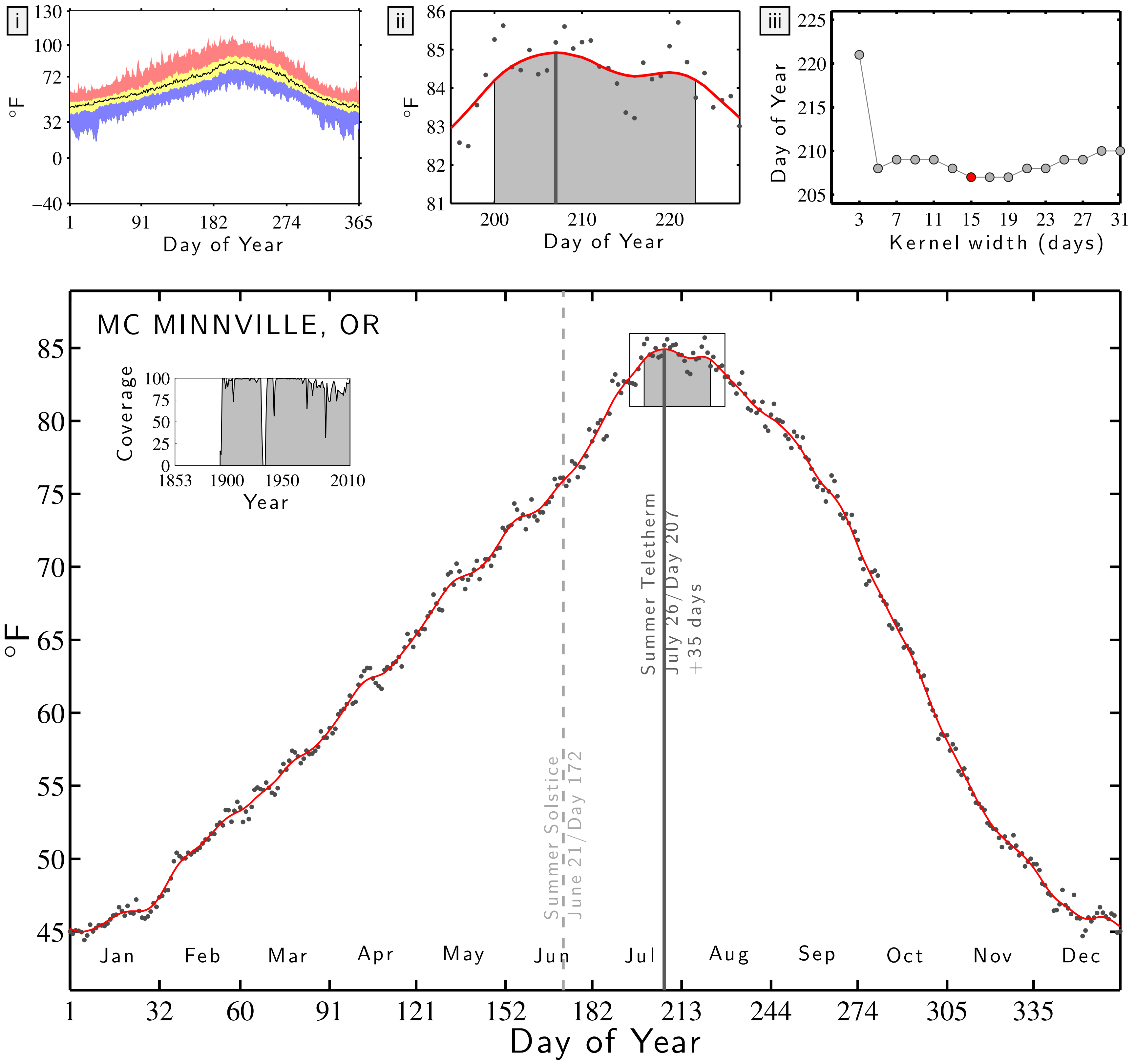

i
°F
Day of Year
ii
°F
Day of Year
iii
Day of Year
Kernel width (days)
MC MINNVILLE, OR
Coverage
Year
Summer Solstice
June 21/Day 172
Summer Teletherm
July 26/Day 207
+35 days
Jan
Feb
Mar
Apr
May
Jun
Jul
Aug
Sep
Oct
Nov
Dec
°F
Day of Year

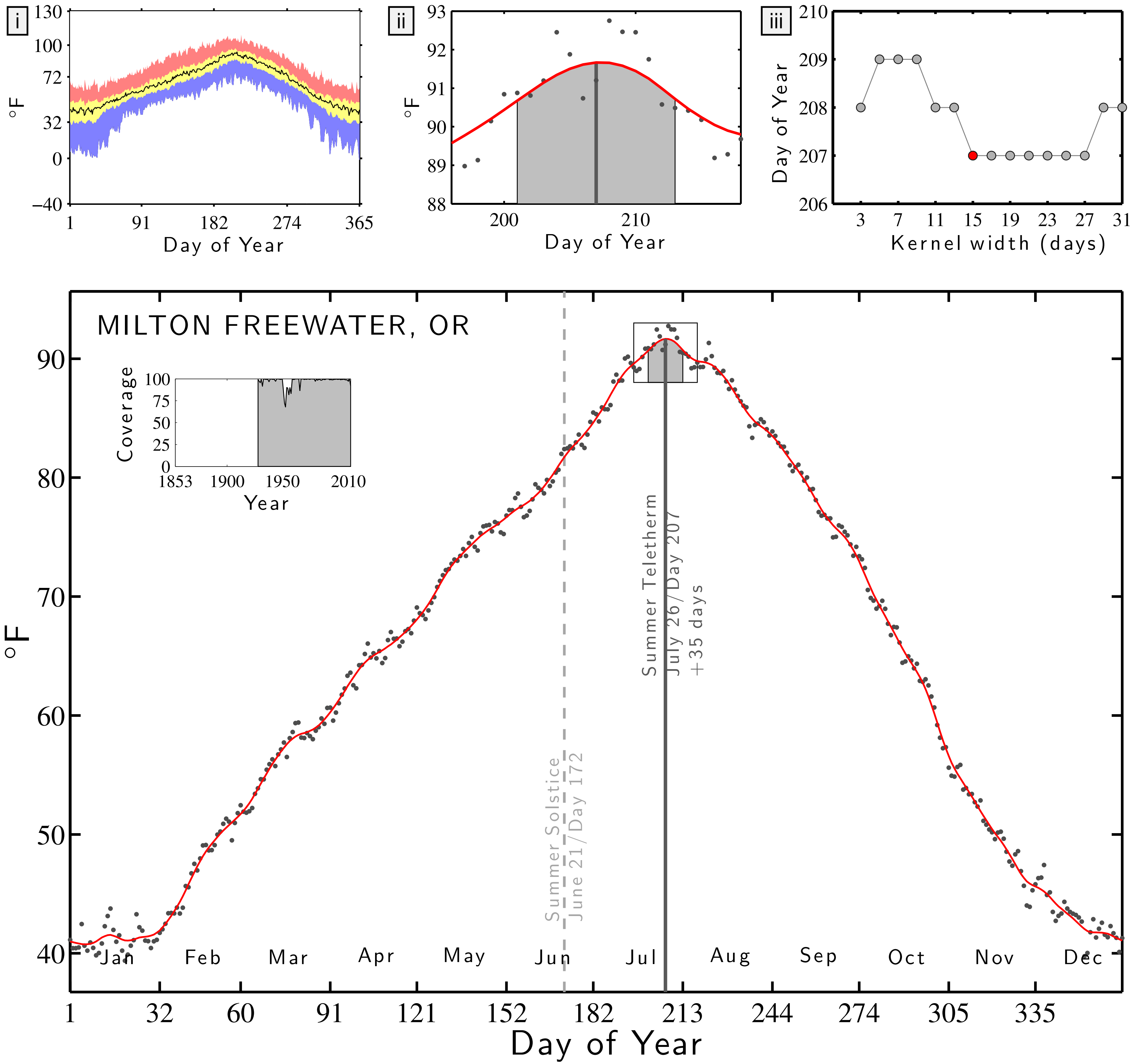

i
ii
iii
°F
Day of Year
Day of Year
Kernel width (days)
MILTON FREEWATER, OR
Coverage
Year
Summer Solstice
June 21 / Day 172
Summer Teletherm
July 26 / Day 207
+35 days
Jan
Feb
Mar
Apr
May
Jun
Jul
Aug
Sep
Oct
Nov
Dec

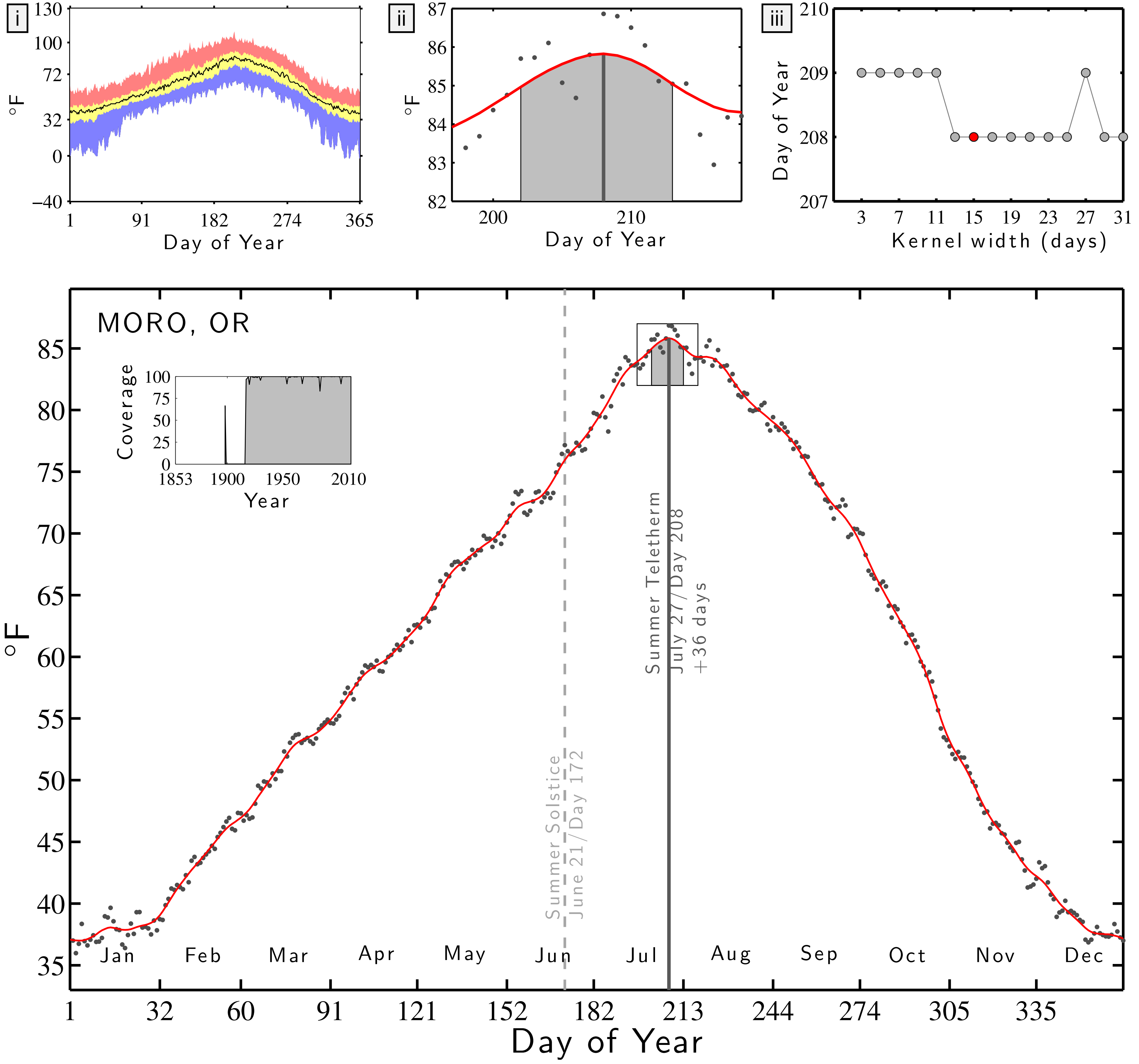

i
ii
iii
°F
Day of Year
Kernel width (days)
MORO, OR
Coverage
Year
Summer Solstice
June 21 / Day 172
Summer Teletherm
July 27 / Day 208
+36 days
Jan
Feb
Mar
Apr
May
Jun
Jul
Aug
Sep
Oct
Nov
Dec

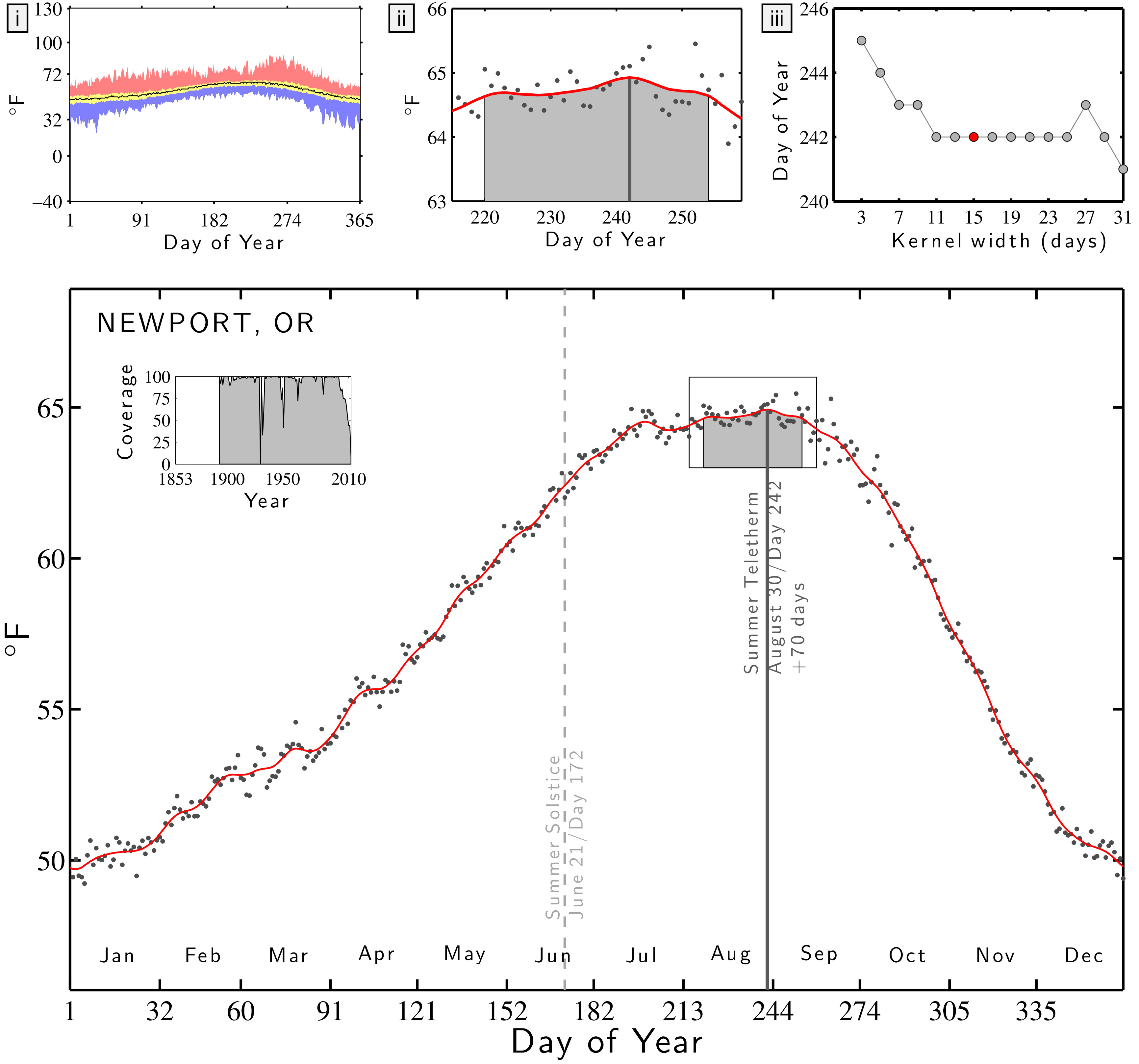

i
°F
Day of Year
ii
°F
Day of Year
iii
Day of Year
Kernel width (days)
NEWPORT, OR
Coverage
Year
Summer Solstice
June 21/Day 172
Summer Teletherm
August 30/Day 242
+70 days
Jan
Feb
Mar
Apr
May
Jun
Jul
Aug
Sep
Oct
Nov
Dec
°F
Day of Year

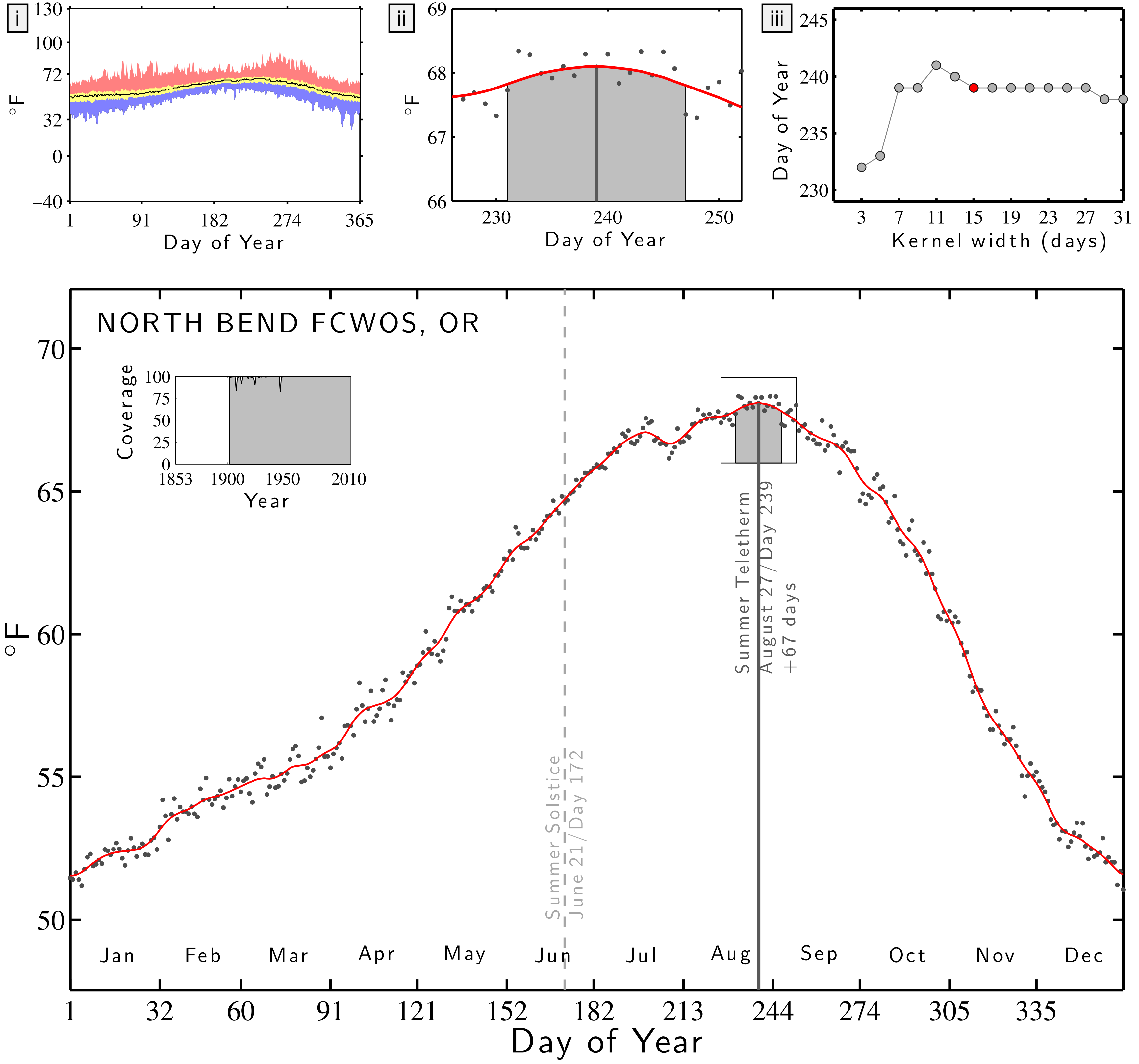

i
ii
iii
°F
Day of Year
Day of Year
Kernel width (days)
NORTH BEND FCWOS, OR
Coverage
Year
Summer Solstice
June 21 / Day 172
Summer Teletherm
August 27 / Day 239
+67 days
Jan
Feb
Mar
Apr
May
Jun
Jul
Aug
Sep
Oct
Nov
Dec

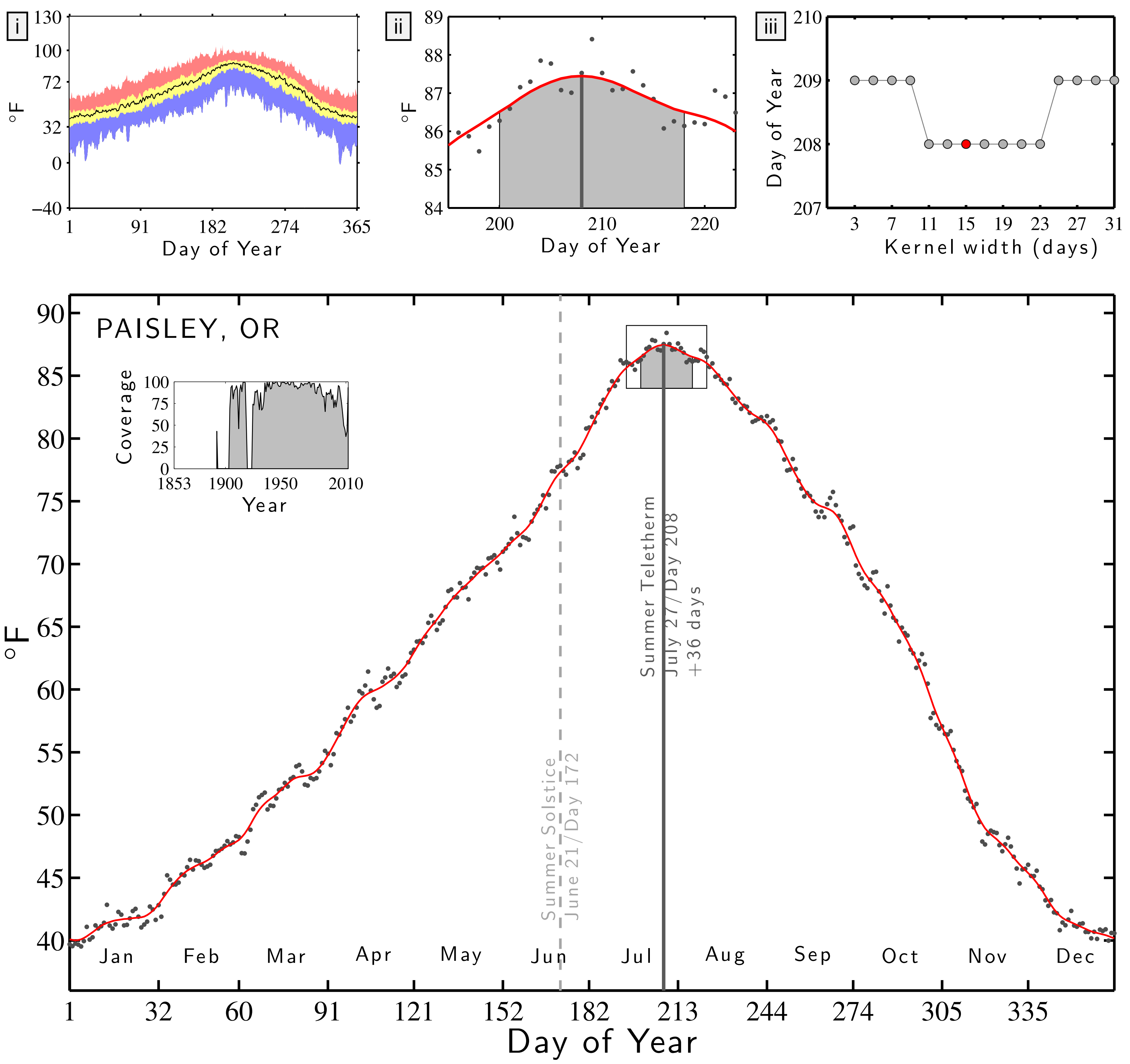

i
°F
Day of Year
ii
°F
Day of Year
iii
Day of Year
Kernel width (days)
PAISLEY, OR
Coverage
Year
Summer Solstice
June 21 / Day 172
Summer Teletherm
July 27 / Day 208
+36 days
Jan
Feb
Mar
Apr
May
Jun
Jul
Aug
Sep
Oct
Nov
Dec
°F
Day of Year

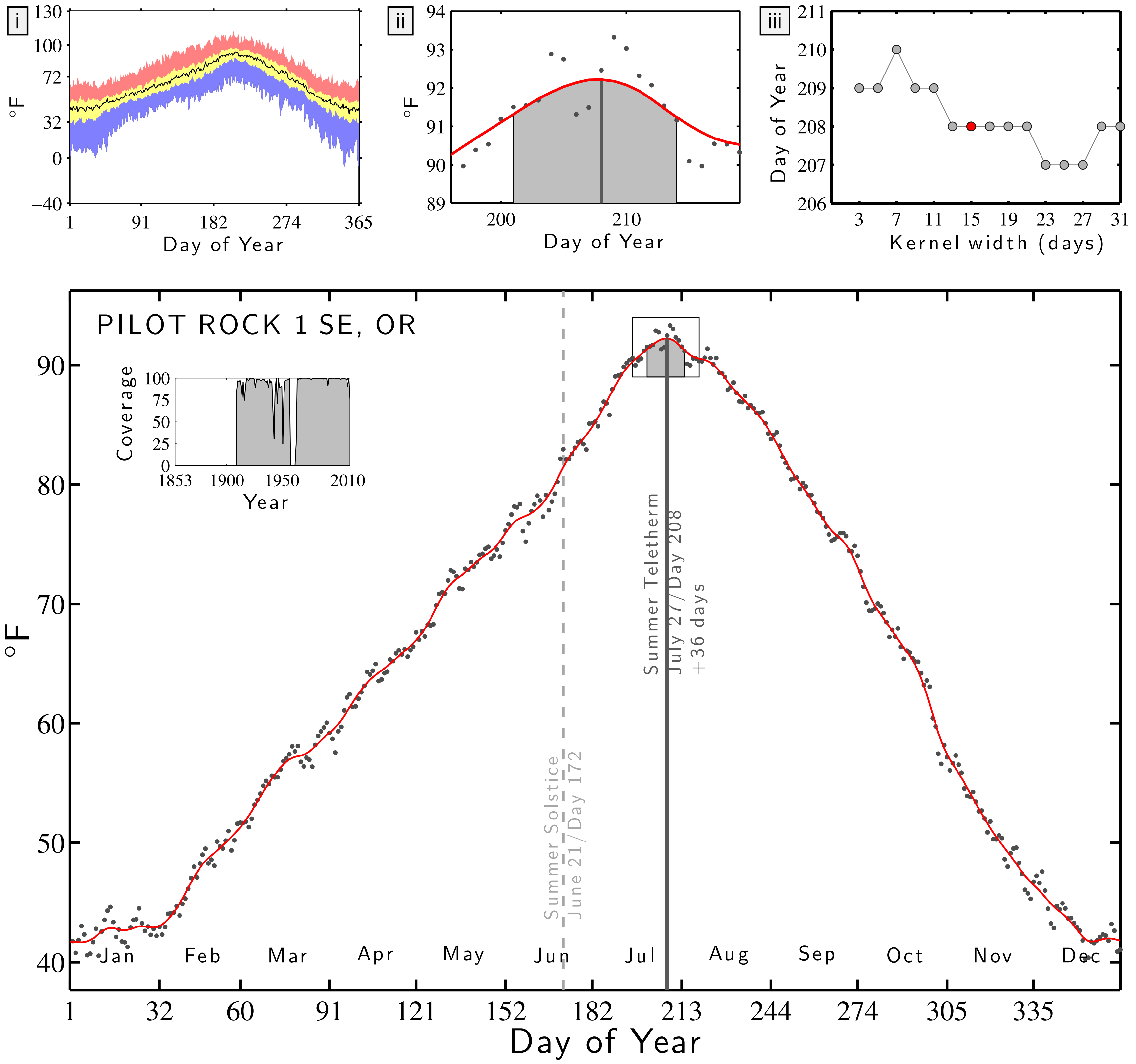

i
ii
iii
°F
Day of Year
Day of Year
Kernel width (days)
PILOT ROCK 1 SE, OR
Coverage
Year
Summer Solstice
June 21 / Day 172
Summer Teletherm
July 27 / Day 208
+36 days
Jan
Feb
Mar
Apr
May
Jun
Jul
Aug
Sep
Oct
Nov
Dec
Day of Year

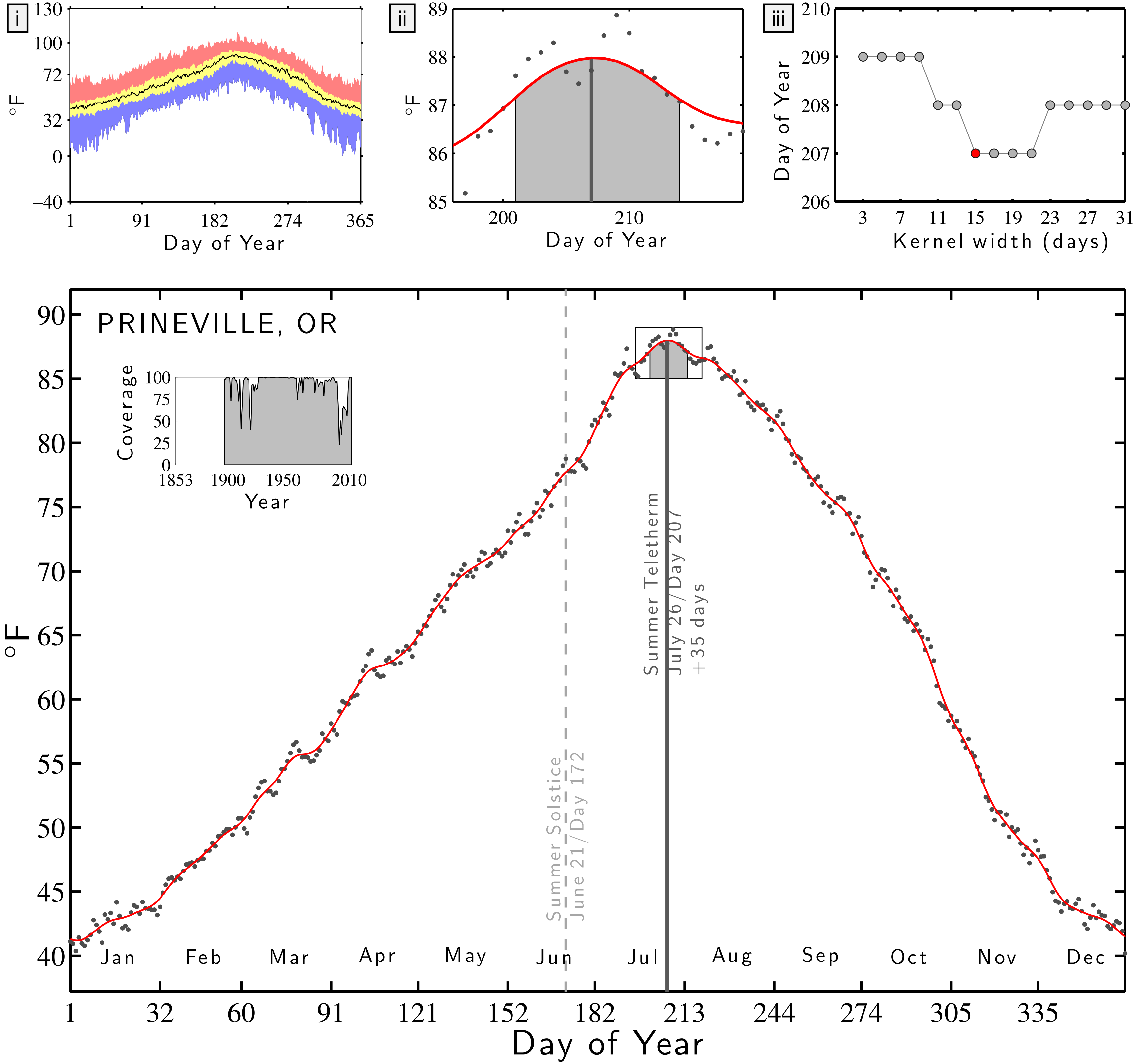

i
ii
iii
°F
Day of Year
Day of Year
Kernel width (days)
PRINEVILLE, OR
Coverage
Year
Summer Solstice
June 21 / Day 172
Summer Teletherm
July 26 / Day 207
+35 days
Jan
Feb
Mar
Apr
May
Jun
Jul
Aug
Sep
Oct
Nov
Dec
Day of Year

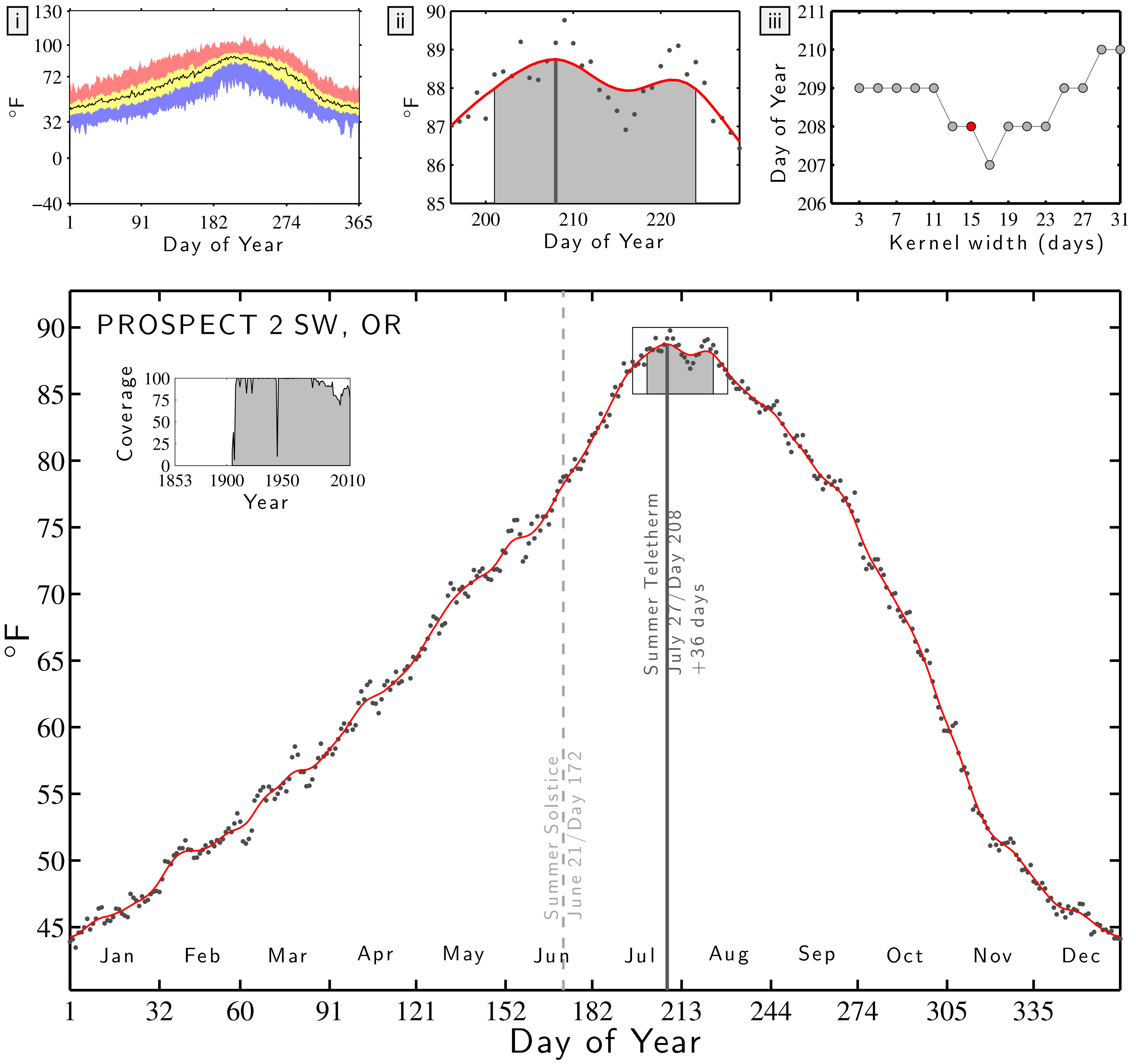
i
°F
Day of Year
ii
°F
Day of Year
iii
Day of Year
Kernel width (days)
PROSPECT 2 SW, OR
Coverage
Year
Summer Solstice
June 21 / Day 172
Summer Teletherm
July 27 / Day 208
+36 days
Jan Feb Mar Apr May Jun Jul Aug Sep Oct Nov Dec
°F
Day of Year

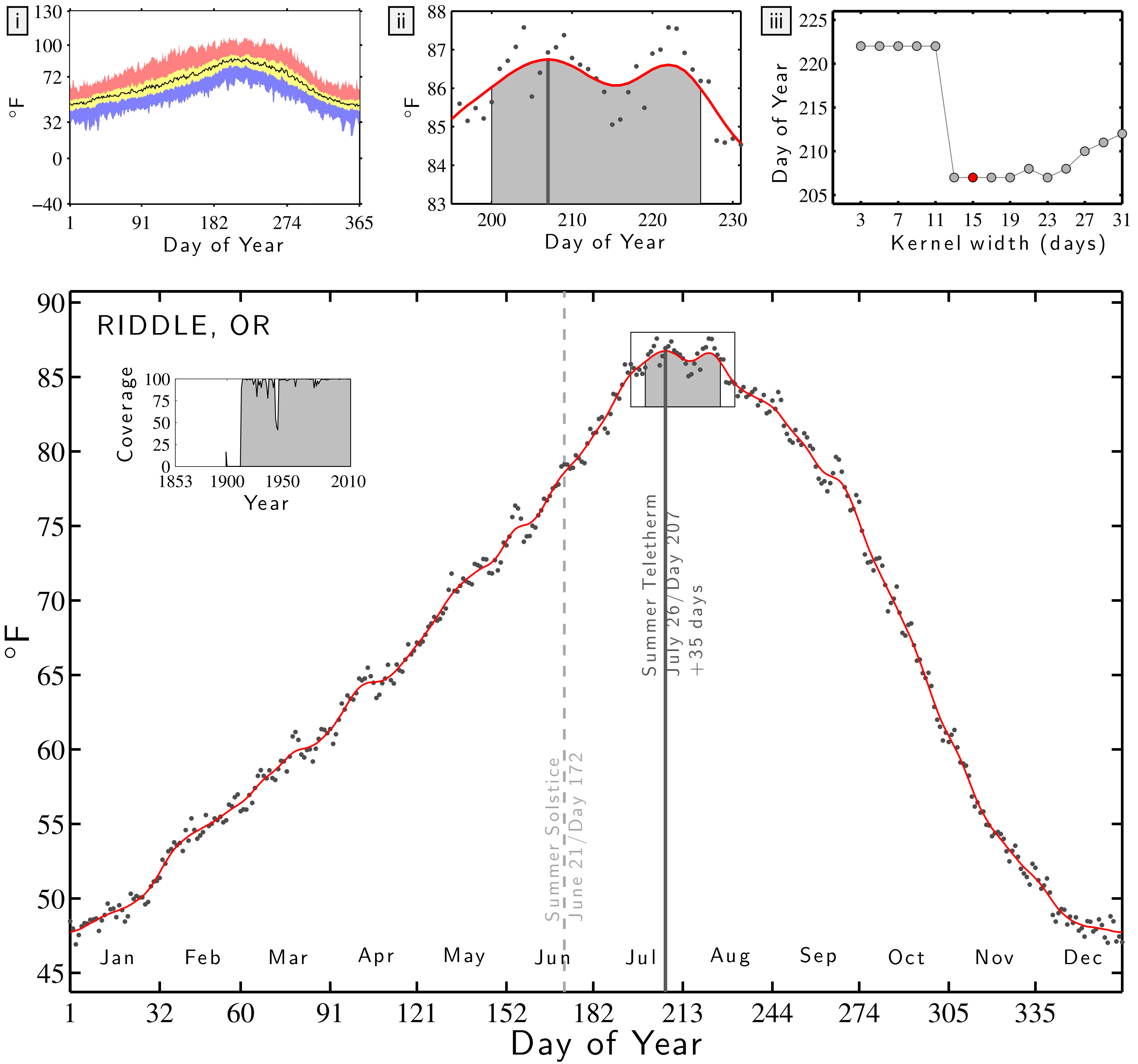

i
°F
Day of Year
ii
°F
Day of Year
iii
Day of Year
Kernel width (days)
RIDDLE, OR
Coverage
Year
Summer Solstice
June 21 / Day 172
Summer Teletherm
July 26 / Day 207
+35 days
Jan Feb Mar Apr May Jun Jul Aug Sep Oct Nov Dec
°F
Day of Year

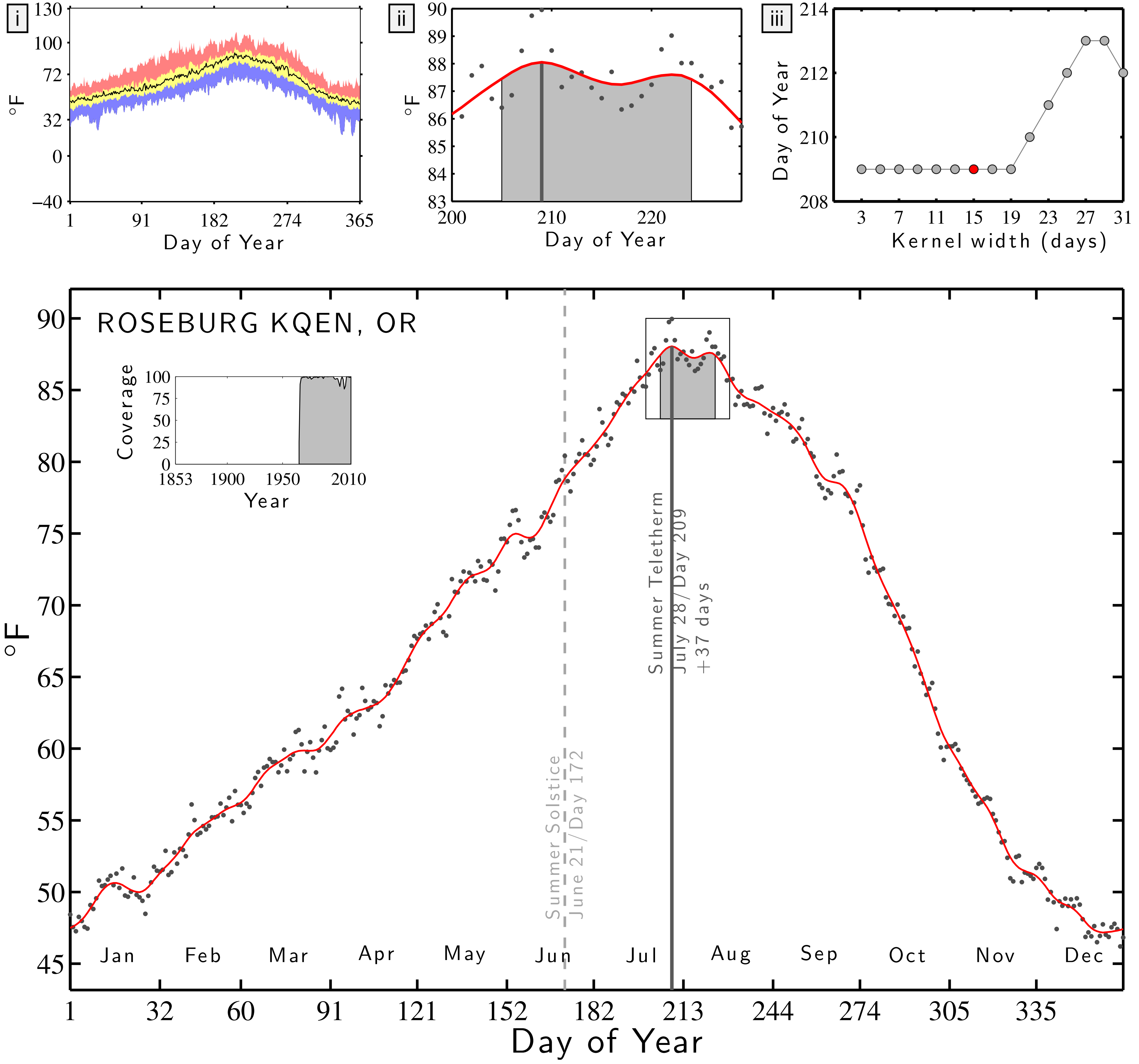

i
°F
Day of Year
ii
°F
Day of Year
iii
Day of Year
Kernel width (days)
ROSEBURG KQEN, OR
Coverage
Year
Summer Solstice
June 21 / Day 172
Summer Teletherm
July 28 / Day 209
+37 days
Jan
Feb
Mar
Apr
May
Jun
Jul
Aug
Sep
Oct
Nov
Dec
°F
Day of Year

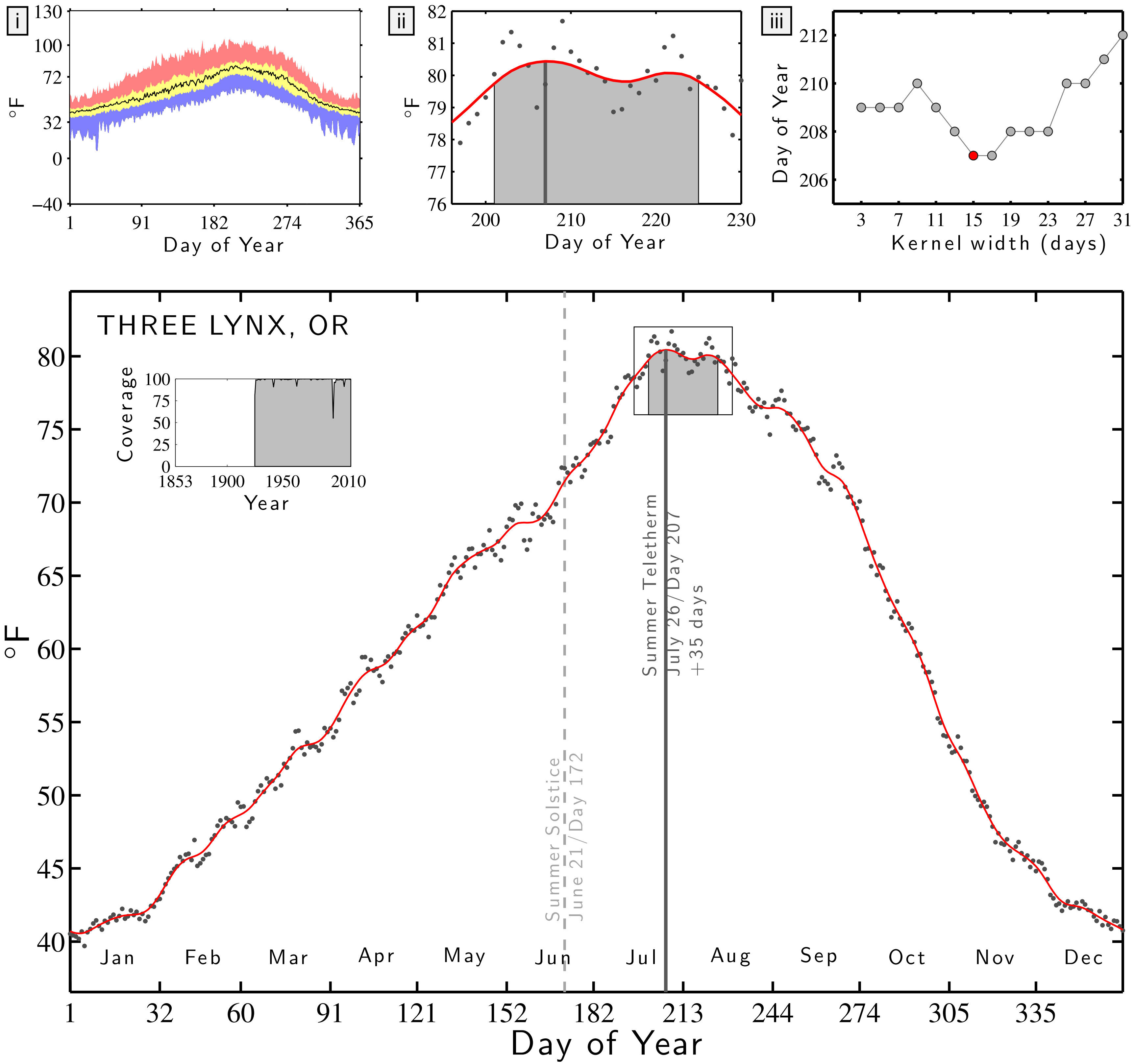

i
°F
Day of Year
ii
°F
Day of Year
iii
Day of Year
Kernel width (days)
THREE LYNX, OR
Coverage
Year
Summer Solstice
June 21 / Day 172
Summer Teletherm
July 26 / Day 207
+35 days
Jan
Feb
Mar
Apr
May
Jun
Jul
Aug
Sep
Oct
Nov
Dec
°F
Day of Year

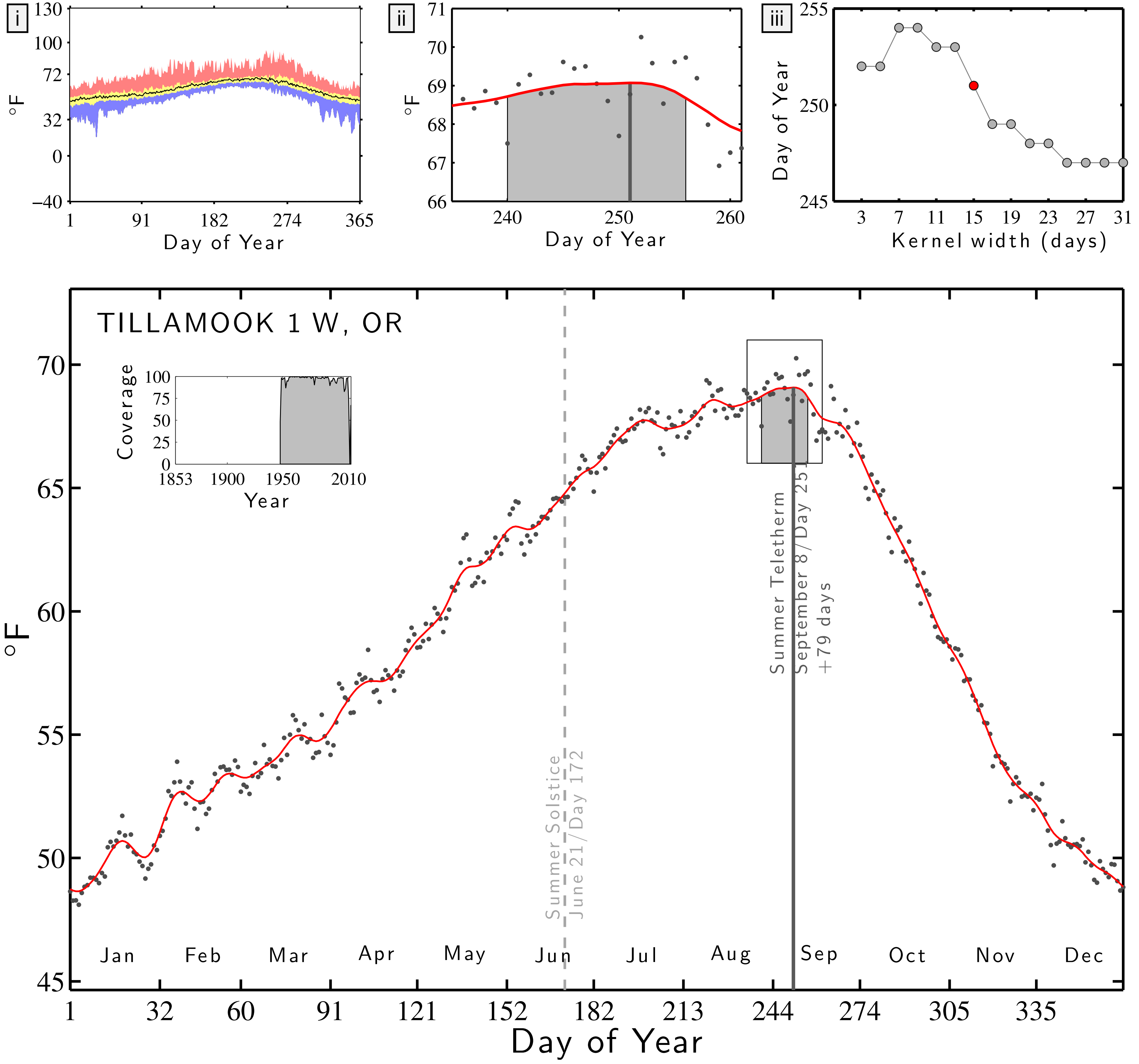

i
ii
iii
°F
Day of Year
Kernel width (days)
TILLAMOOK 1 W, OR
Coverage
Year
Summer Solstice
June 21/Day 172
Summer Teletherm
September 8/Day 251
+79 days
Jan
Feb
Mar
Apr
May
Jun
Jul
Aug
Sep
Oct
Nov
Dec

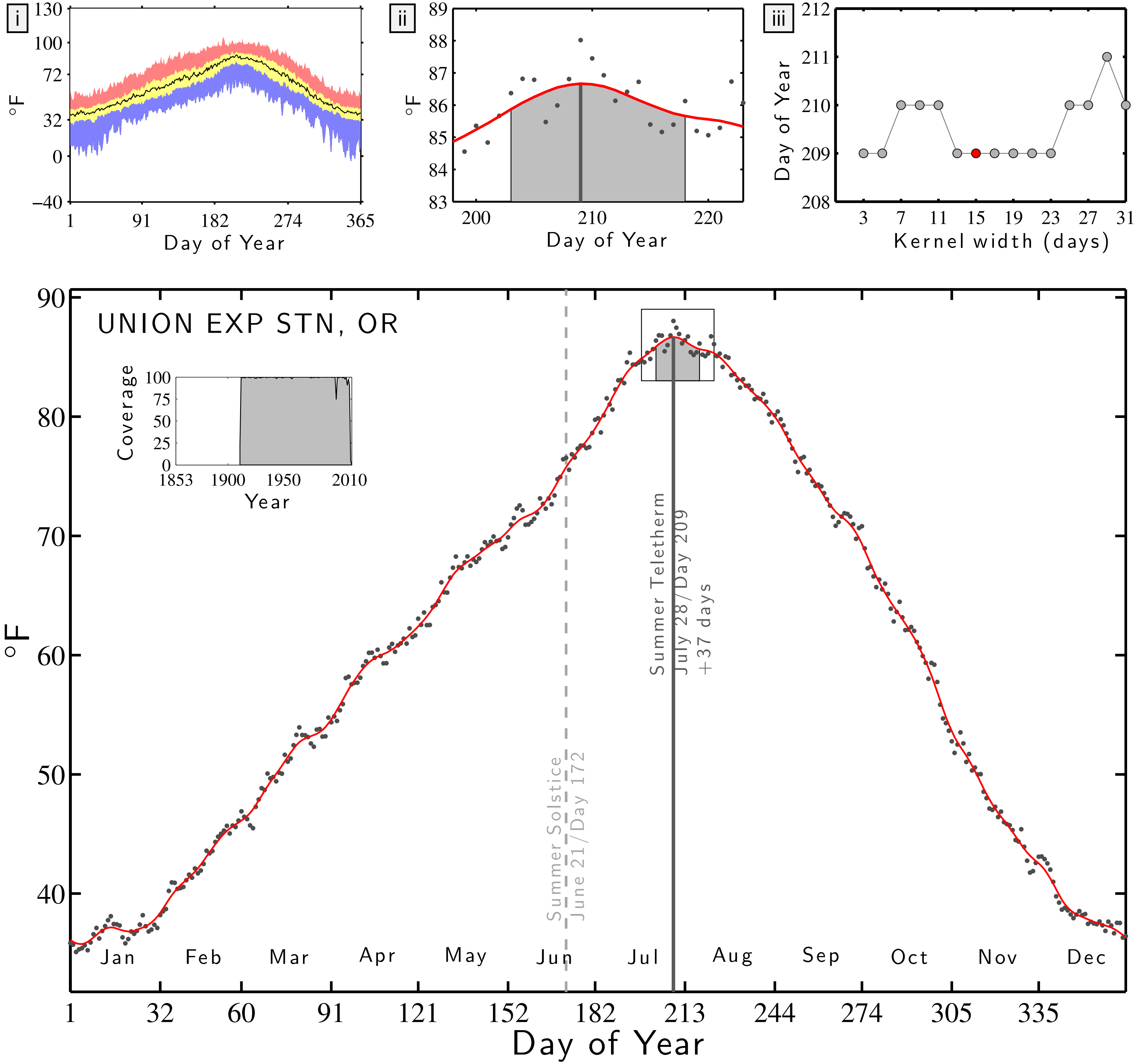

i
°F
130
100
72
32
0
−40
1
91
182
274
365
Day of Year
ii
89
88
87
86
85
84
83
200
210
220
Day of Year
iii
Day of Year
212
211
210
209
208
3
7
11
15
19
23
27
31
Kernel width (days)
UNION EXP STN, OR
Coverage
100
75
50
25
0
1853
1900
1950
2010
Year
Summer Solstice
June 21/Day 172
Summer Teletherm
July 28/Day 209
+37 days
90
80
70
60
50
40
Jan
Feb
Mar
Apr
May
Jun
Jul
Aug
Sep
Oct
Nov
Dec
1
32
60
91
121
152
182
213
244
274
305
335
Day of Year

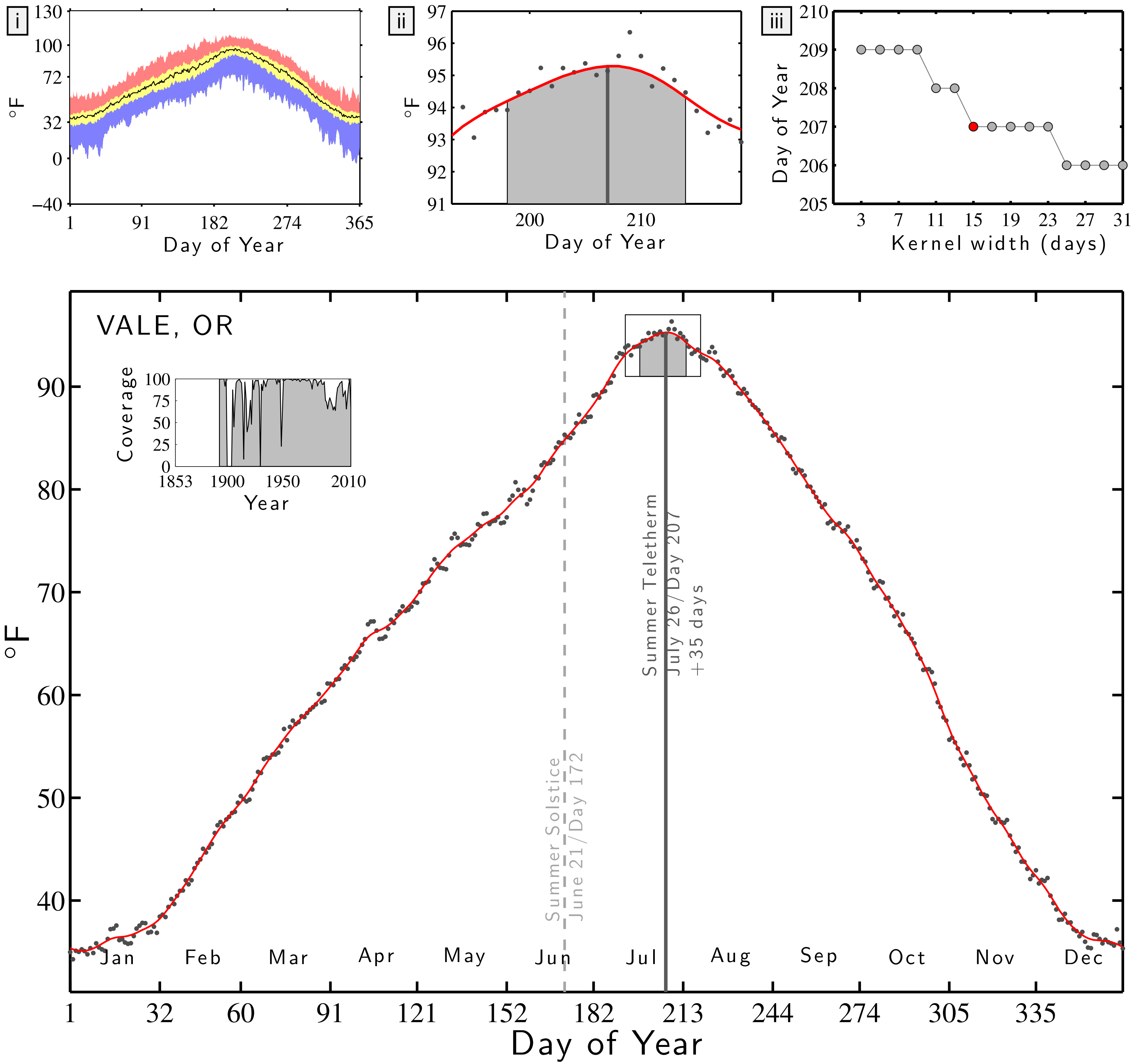

i
°F
Day of Year
ii
°F
Day of Year
iii
Day of Year
Kernel width (days)
VALE, OR
Coverage
Year
Summer Solstice
June 21 / Day 172
Summer Teletherm
July 26 / Day 207
+35 days
Jan
Feb
Mar
Apr
May
Jun
Jul
Aug
Sep
Oct
Nov
Dec
°F
Day of Year

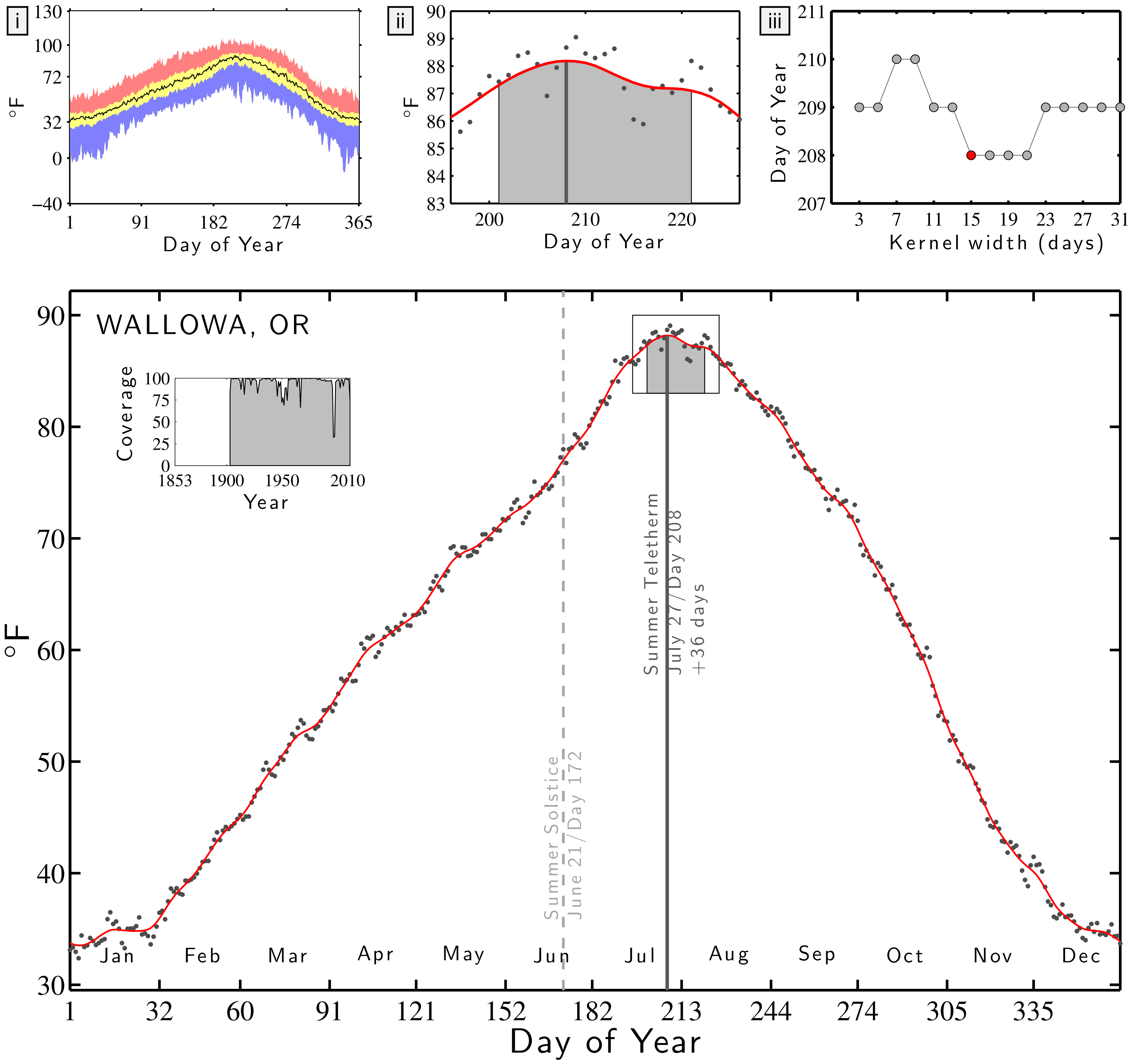

i
ii
iii
°F
Day of Year
Kernel width (days)
WALLOWA, OR
Coverage
Year
Summer Solstice
June 21 / Day 172
Summer Teletherm
July 27 / Day 208
+36 days
Jan
Feb
Mar
Apr
May
Jun
Jul
Aug
Sep
Oct
Nov
Dec

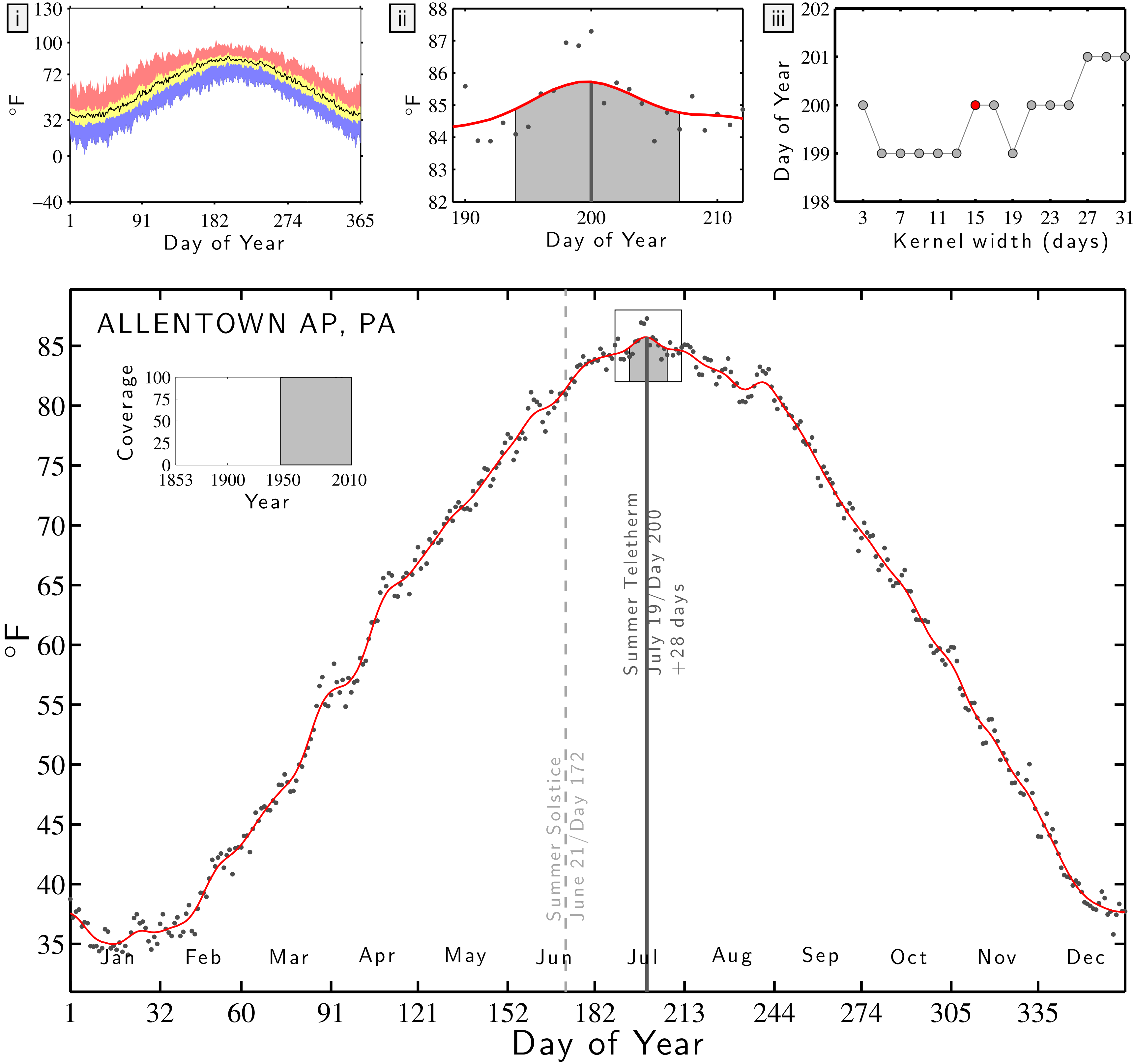

i
ii
iii
°F
Day of Year
Day of Year
Kernel width (days)
ALLENTOWN AP, PA
Coverage
Year
Summer Solstice
June 21/Day 172
Summer Teletherm
July 19/Day 200
+28 days
Jan
Feb
Mar
Apr
May
Jun
Jul
Aug
Sep
Oct
Nov
Dec

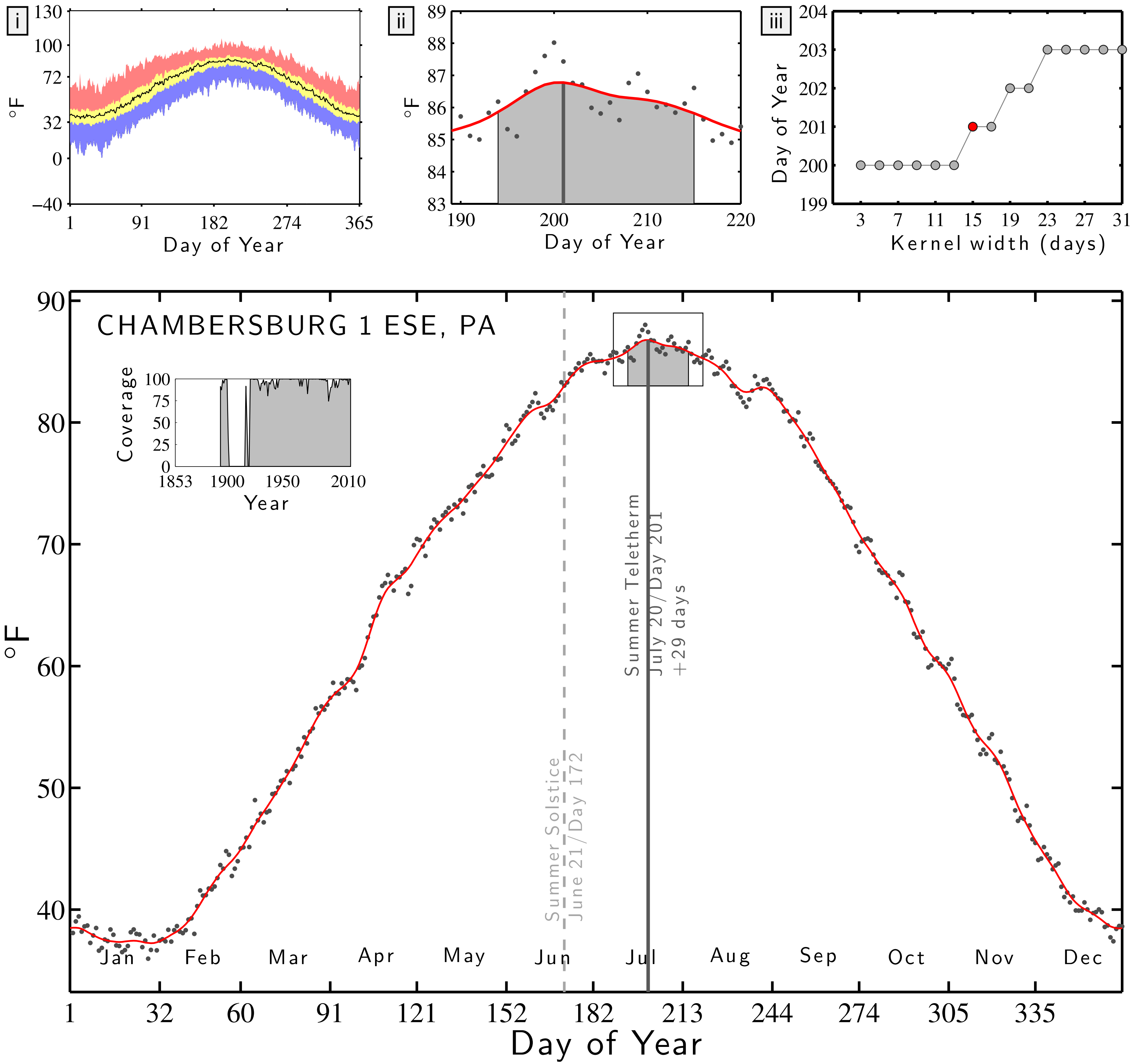

i
°F
Day of Year
ii
Day of Year
iii
Day of Year
Kernel width (days)
CHAMBERSBURG 1 ESE, PA
Coverage
Year
Summer Solstice
June 21 / Day 172
Summer Teletherm
July 20 / Day 201
+29 days
Jan
Feb
Mar
Apr
May
Jun
Jul
Aug
Sep
Oct
Nov
Dec
Day of Year

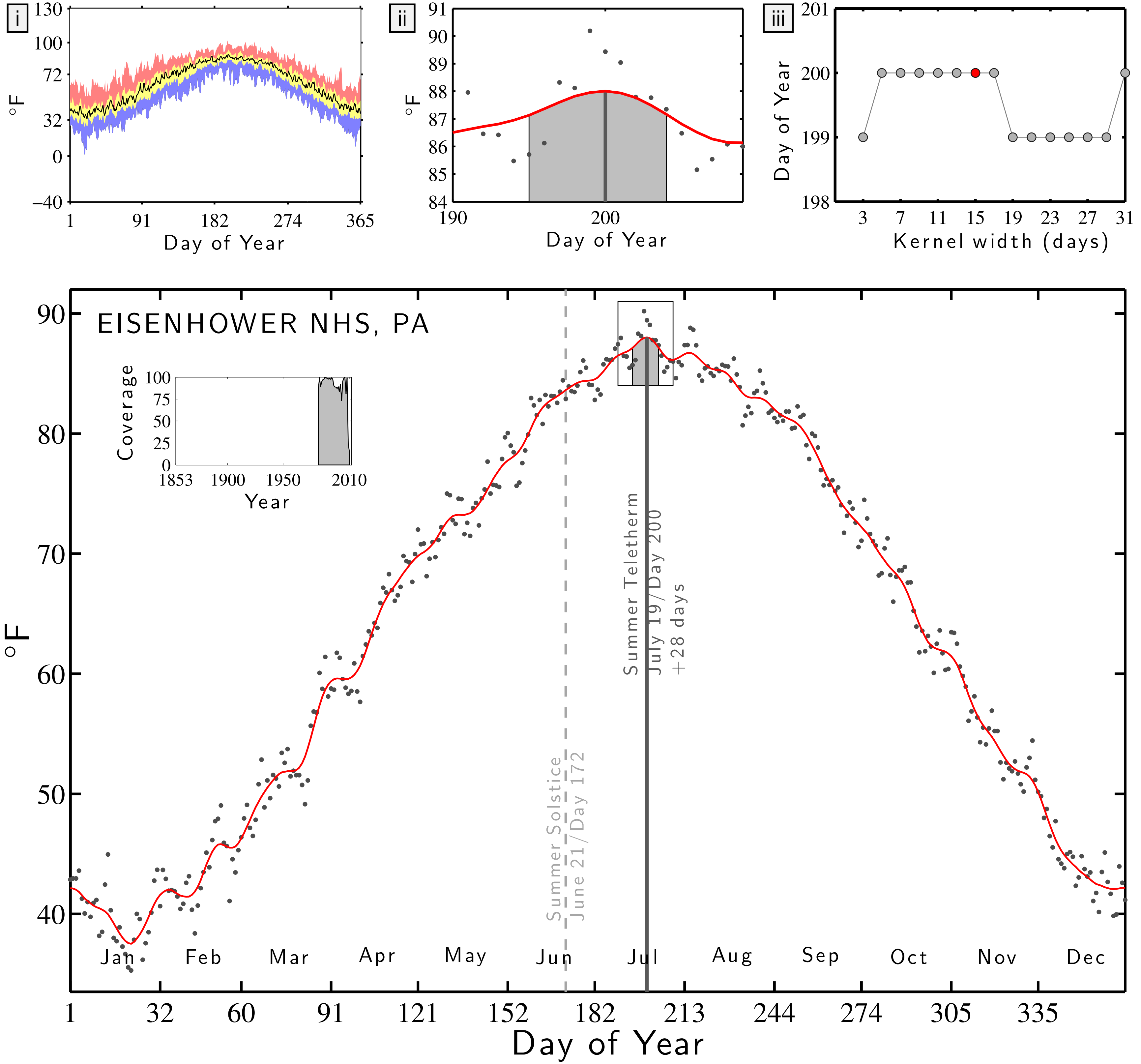

i
°F
Day of Year
ii
°F
Day of Year
iii
Day of Year
Kernel width (days)
EISENHOWER NHS, PA
Coverage
Year
Summer Solstice
June 21/Day 172
Summer Teletherm
July 19/Day 200
+28 days
Jan
Feb
Mar
Apr
May
Jun
Jul
Aug
Sep
Oct
Nov
Dec
°F
Day of Year

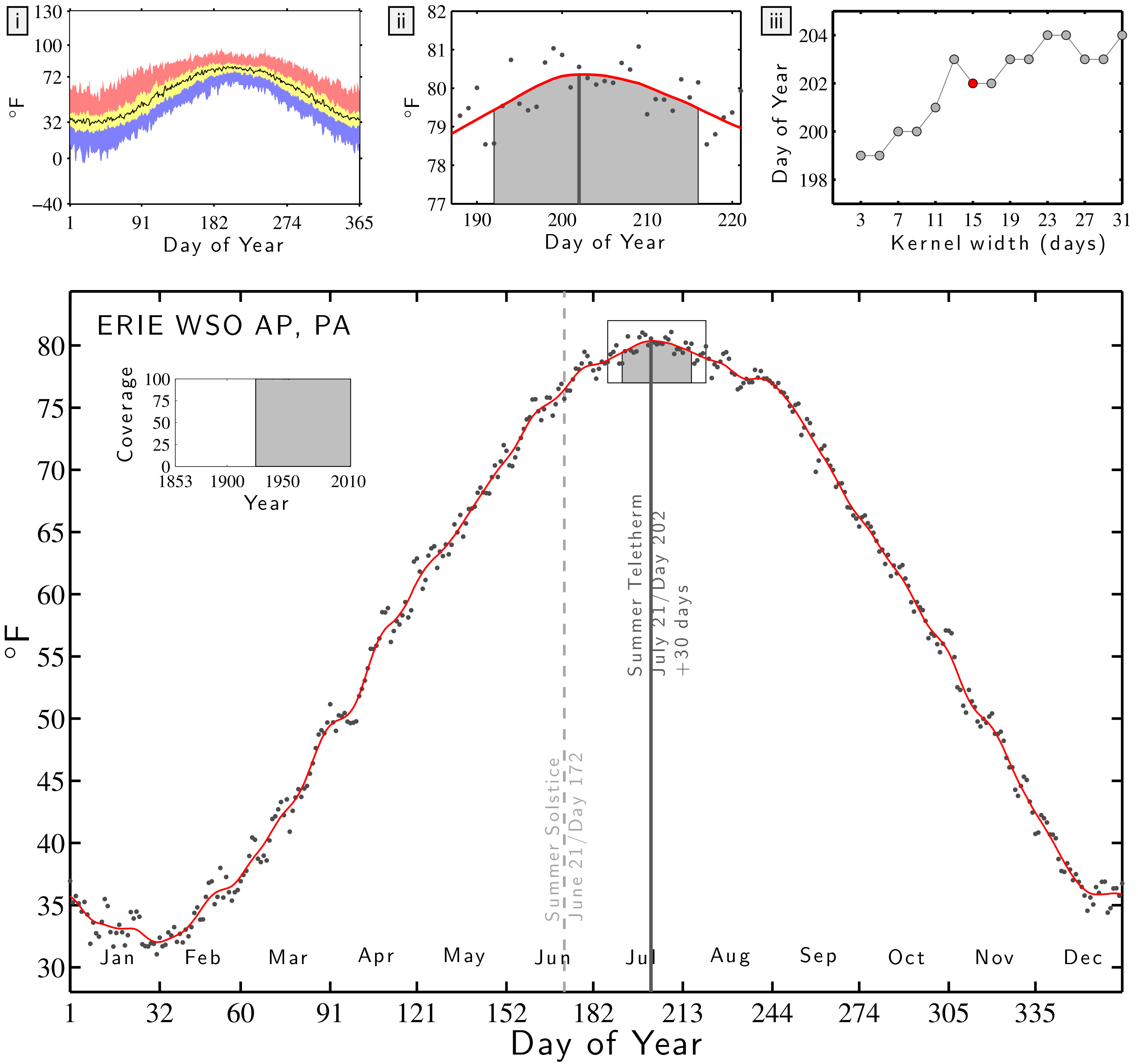

i
Day of Year
°F
ii
Day of Year
°F
iii
Kernel width (days)
Day of Year
ERIE WSO AP, PA
Coverage
Year
Summer Solstice
June 21 / Day 172
Summer Teletherm
July 21 / Day 202
+30 days
Jan
Feb
Mar
Apr
May
Jun
Jul
Aug
Sep
Oct
Nov
Dec
Day of Year
°F

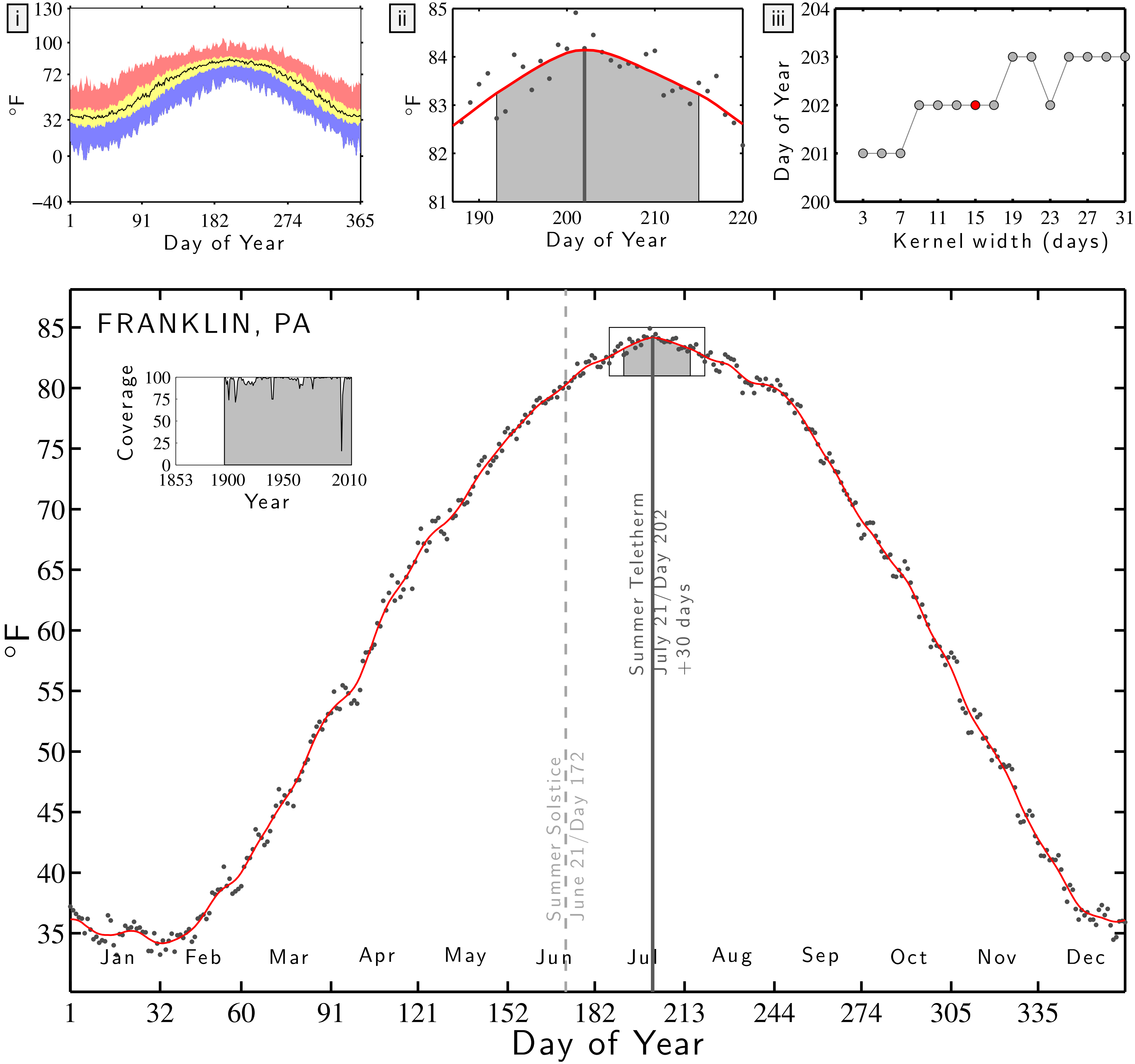

i
°F
130
100
72
32
0
−40
1
91
182
274
365
Day of Year
ii
85
84
83
82
81
190
200
210
220
Day of Year
iii
204
203
202
201
200
Day of Year
3
7
11
15
19
23
27
31
Kernel width (days)
FRANKLIN, PA
Coverage
100
75
50
25
0
1853
1900
1950
2010
Year
Summer Solstice
June 21/Day 172
Summer Teletherm
July 21/Day 202
+30 days
Jan
Feb
Mar
Apr
May
Jun
Jul
Aug
Sep
Oct
Nov
Dec
1
32
60
91
121
152
182
213
244
274
305
335
Day of Year

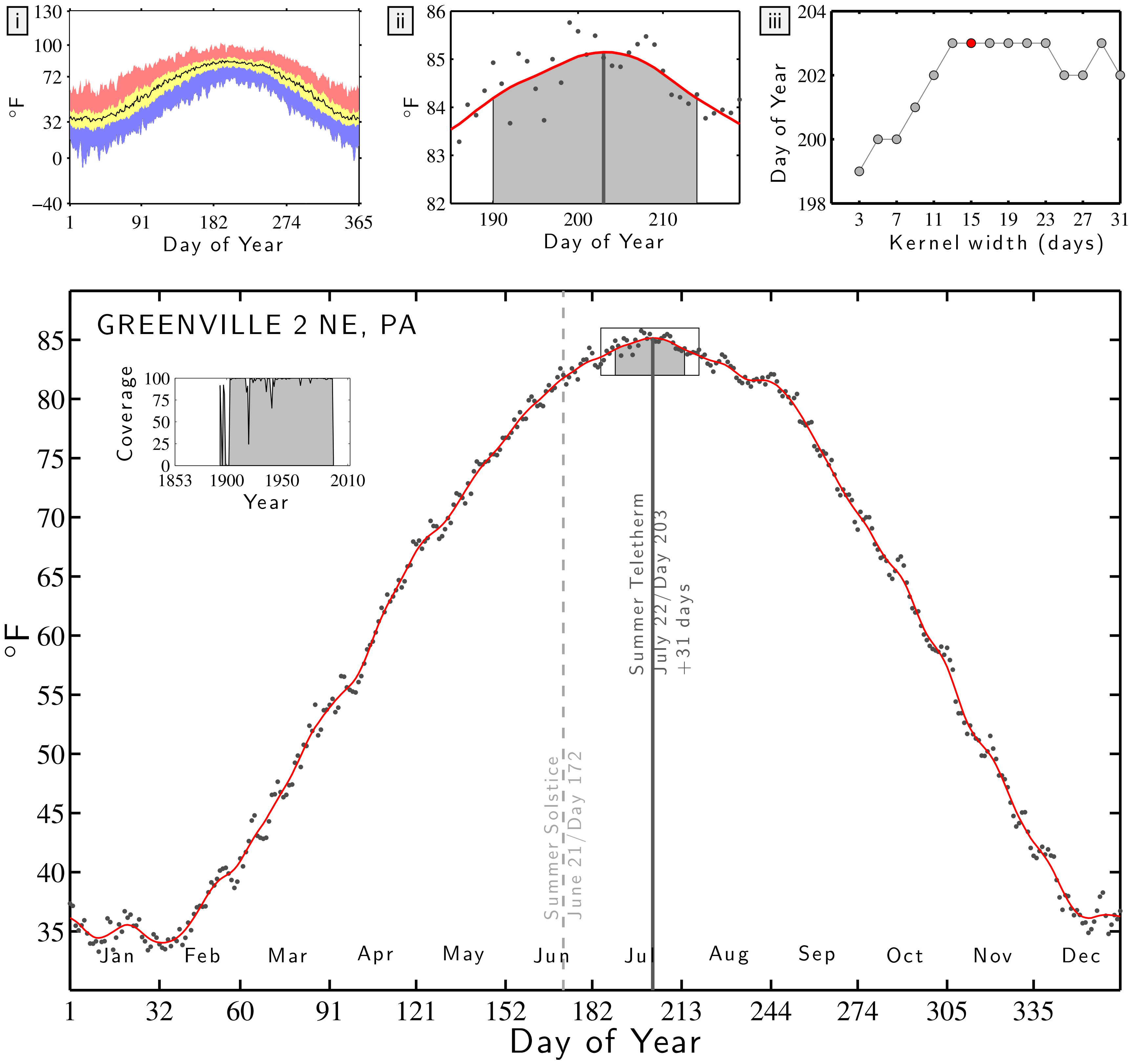

i
°F
Day of Year
ii
°F
Day of Year
iii
Day of Year
Kernel width (days)
GREENVILLE 2 NE, PA
Coverage
Year
Summer Solstice
June 21/Day 172
Summer Teletherm
July 22/Day 203
+31 days
Jan
Feb
Mar
Apr
May
Jun
Jul
Aug
Sep
Oct
Nov
Dec
°F
Day of Year

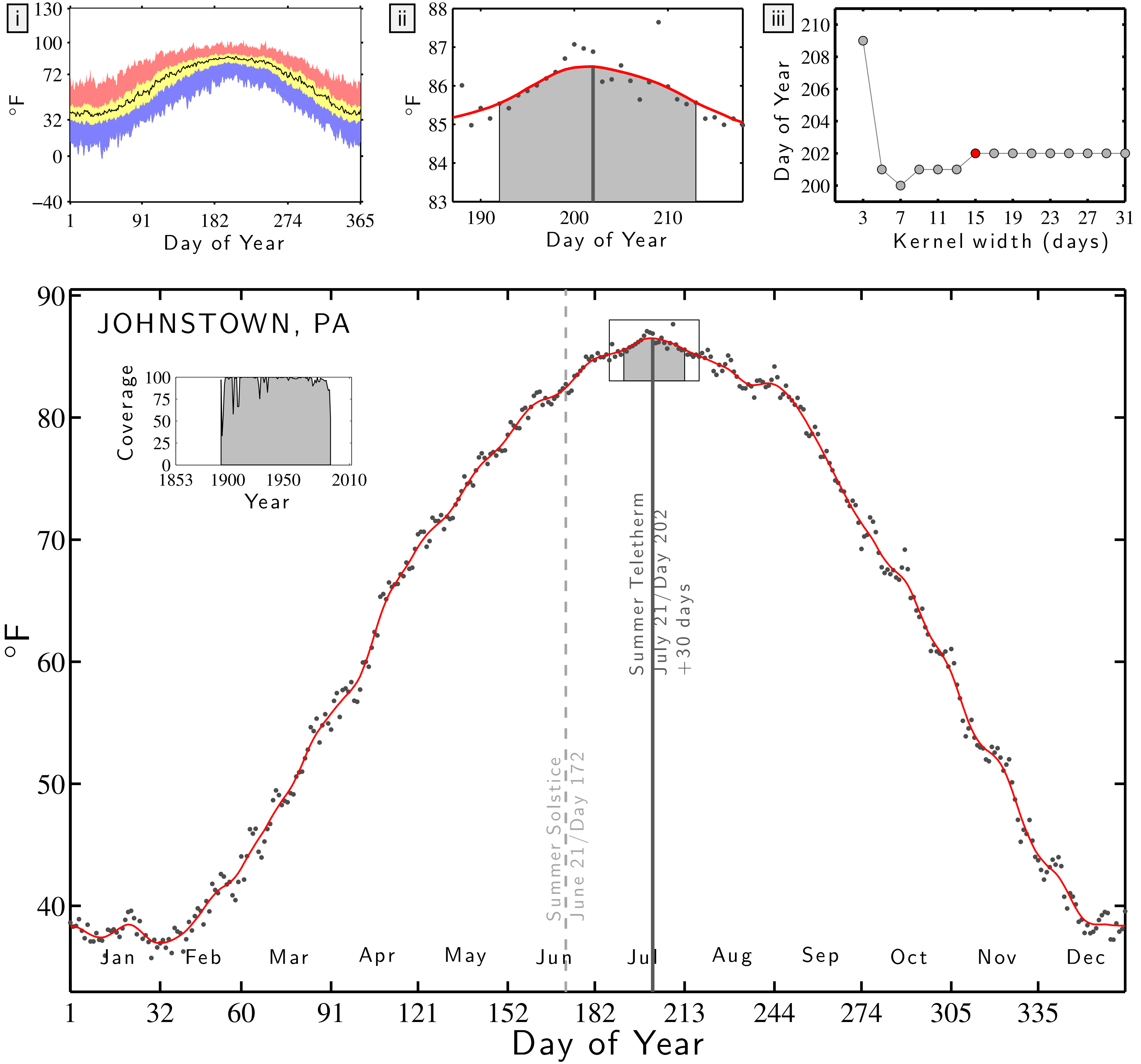

i
ii
iii
JOHNSTOWN, PA
Coverage
Year
Summer Solstice
June 21 / Day 172
Summer Teletherm
July 21 / Day 202
+30 days
Jan
Feb
Mar
Apr
May
Jun
Jul
Aug
Sep
Oct
Nov
Dec
Day of Year
°F
Kernel width (days)

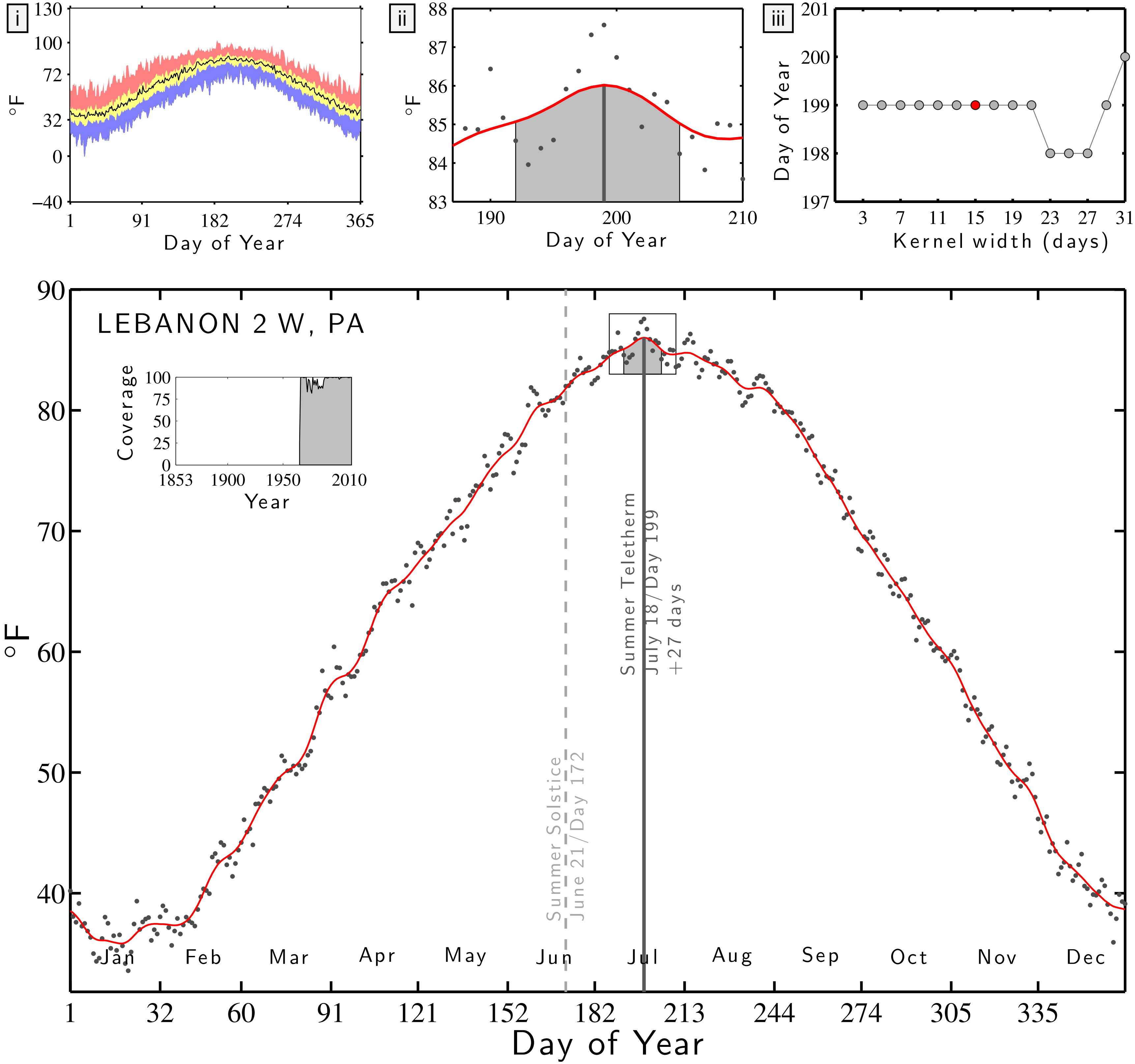

i
°F
Day of Year
ii
°F
Day of Year
iii
Day of Year
Kernel width (days)
LEBANON 2 W, PA
Coverage
Year
Summer Solstice
June 21 / Day 172
Summer Teletherm
July 18 / Day 199
+27 days
Jan
Feb
Mar
Apr
May
Jun
Jul
Aug
Sep
Oct
Nov
Dec
°F
Day of Year

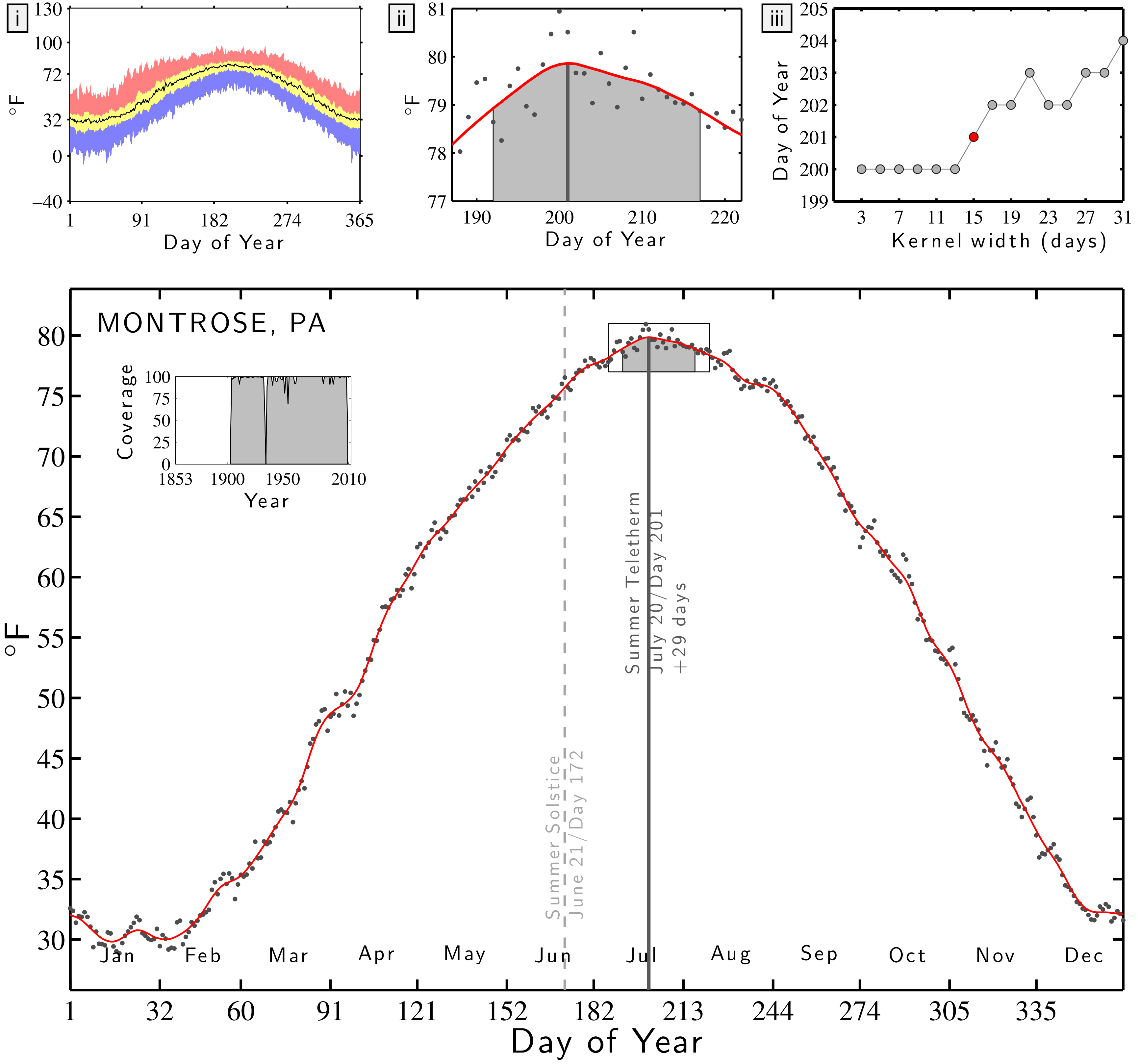

i
°F
Day of Year
ii
°F
Day of Year
iii
Day of Year
Kernel width (days)
MONTROSE, PA
Coverage
Year
Summer Solstice
June 21 / Day 172
Summer Teletherm
July 20 / Day 201
+29 days
Jan
Feb
Mar
Apr
May
Jun
Jul
Aug
Sep
Oct
Nov
Dec
°F
Day of Year

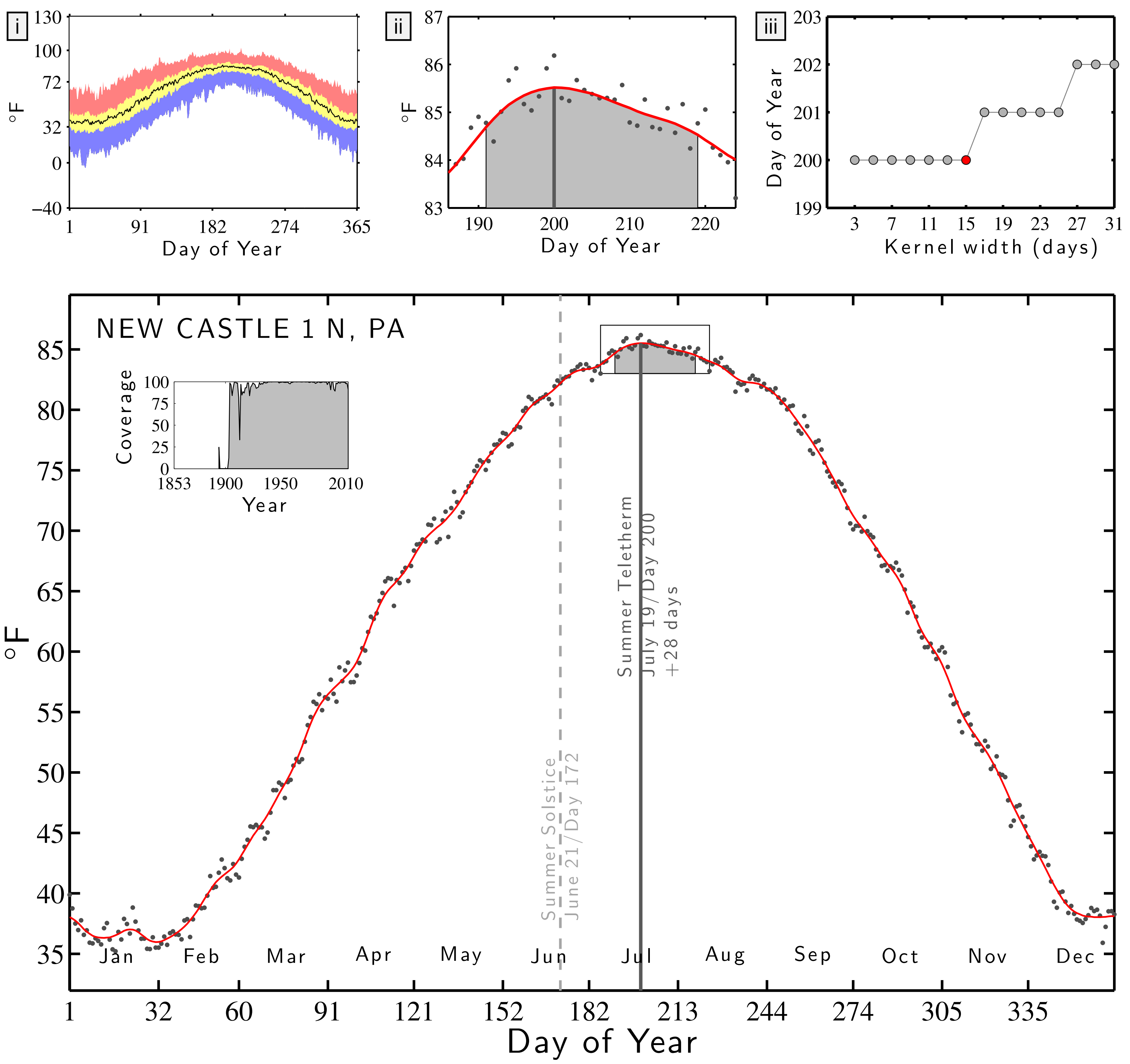

i
ii
iii
°F
Day of Year
Day of Year
Kernel width (days)
NEW CASTLE 1 N, PA
Coverage
Year
Summer Solstice
June 21/Day 172
Summer Teletherm
July 19/Day 200
+28 days
Jan
Feb
Mar
Apr
May
Jun
Jul
Aug
Sep
Oct
Nov
Dec
Day of Year

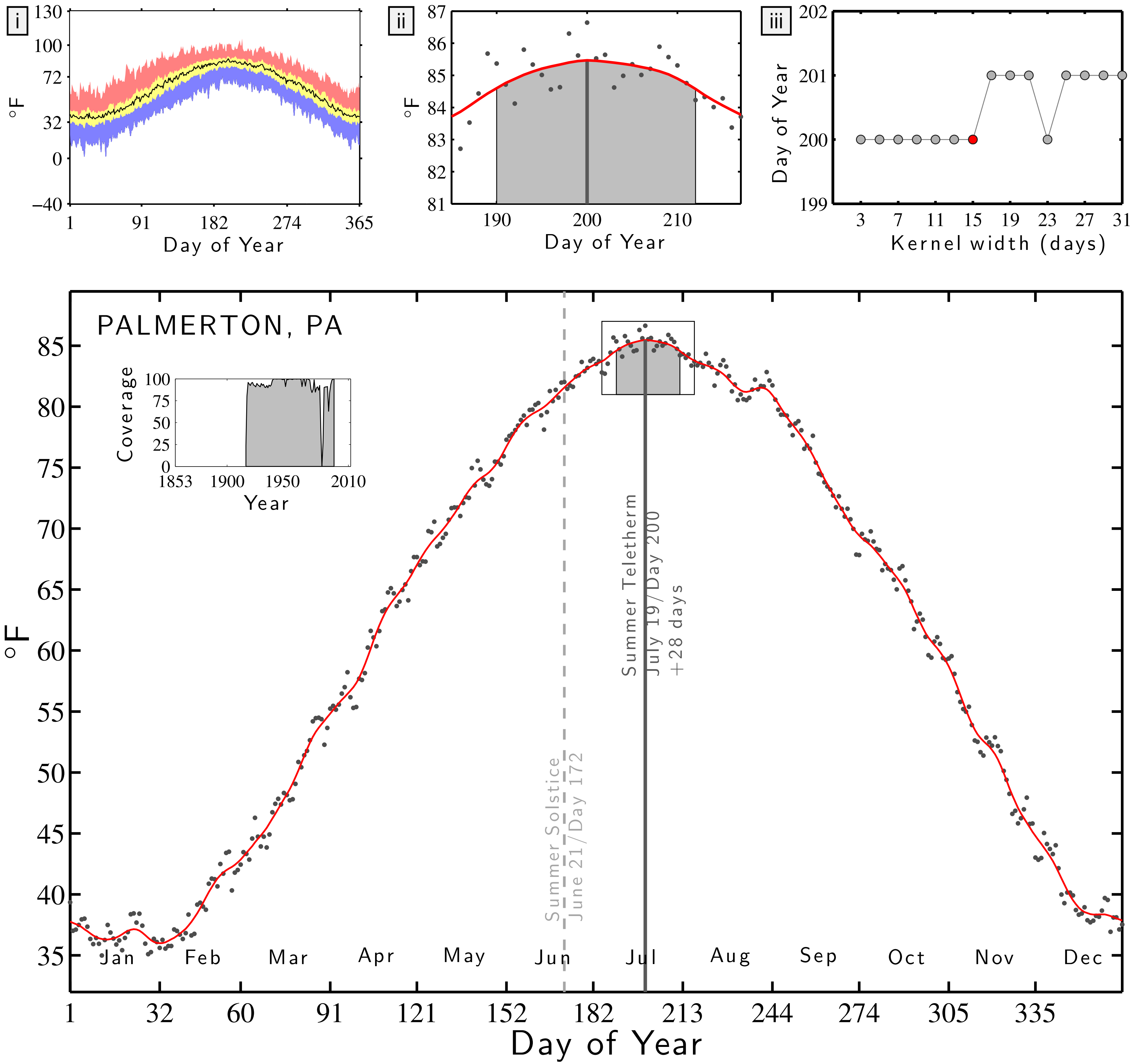

i
°F
Day of Year
ii
°F
Day of Year
iii
Day of Year
Kernel width (days)
PALMERTON, PA
Coverage
Year
Summer Solstice
June 21/Day 172
Summer Teletherm
July 19/Day 200
+28 days
Jan
Feb
Mar
Apr
May
Jun
Jul
Aug
Sep
Oct
Nov
Dec
°F
Day of Year

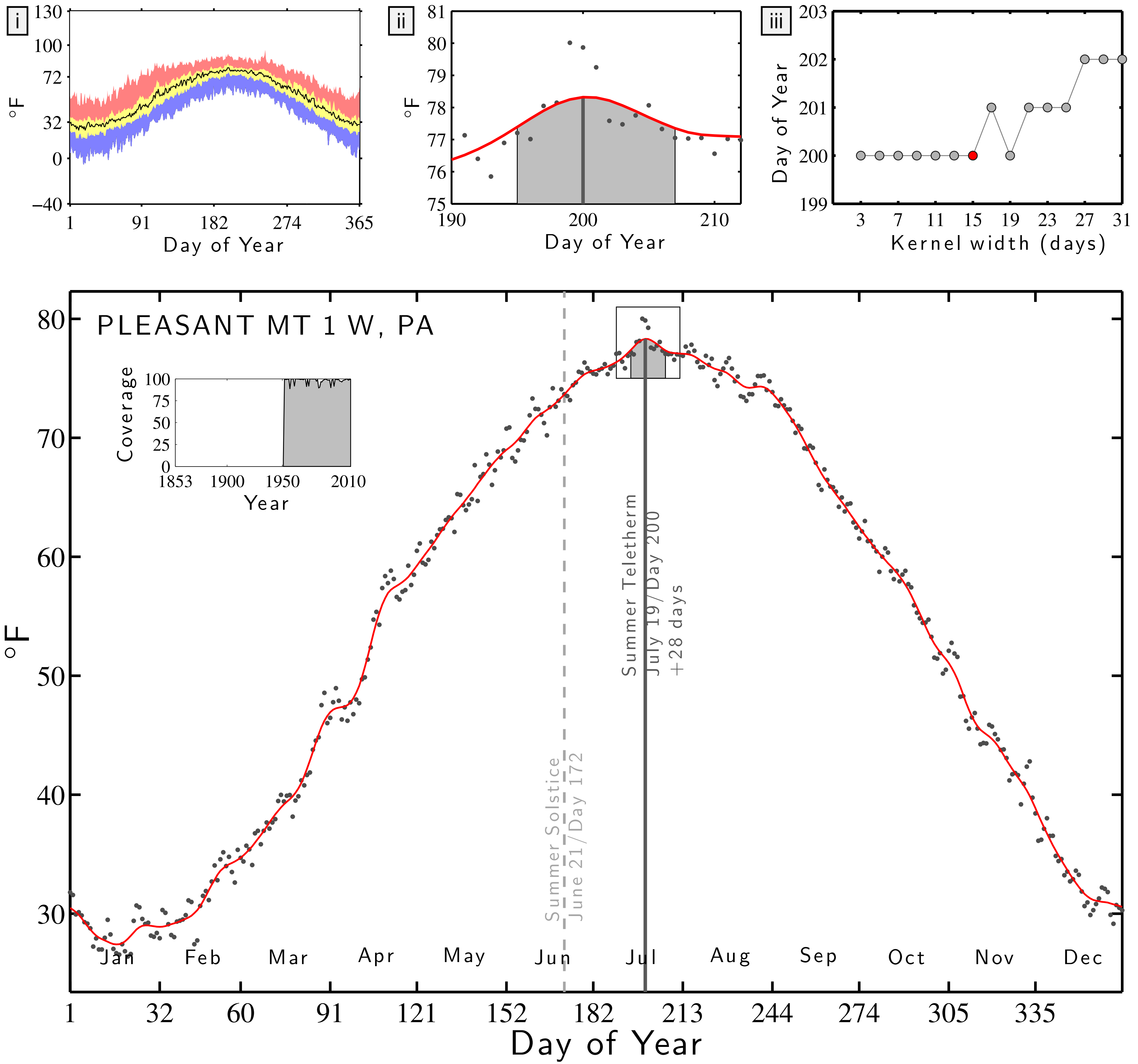

i
ii
iii
Day of Year
Kernel width (days)
°F
Day of Year
PLEASANT MT 1 W, PA
Coverage
Year
Summer Solstice
June 21/Day 172
Summer Teletherm
July 19/Day 200
+28 days
Jan
Feb
Mar
Apr
May
Jun
Jul
Aug
Sep
Oct
Nov
Dec

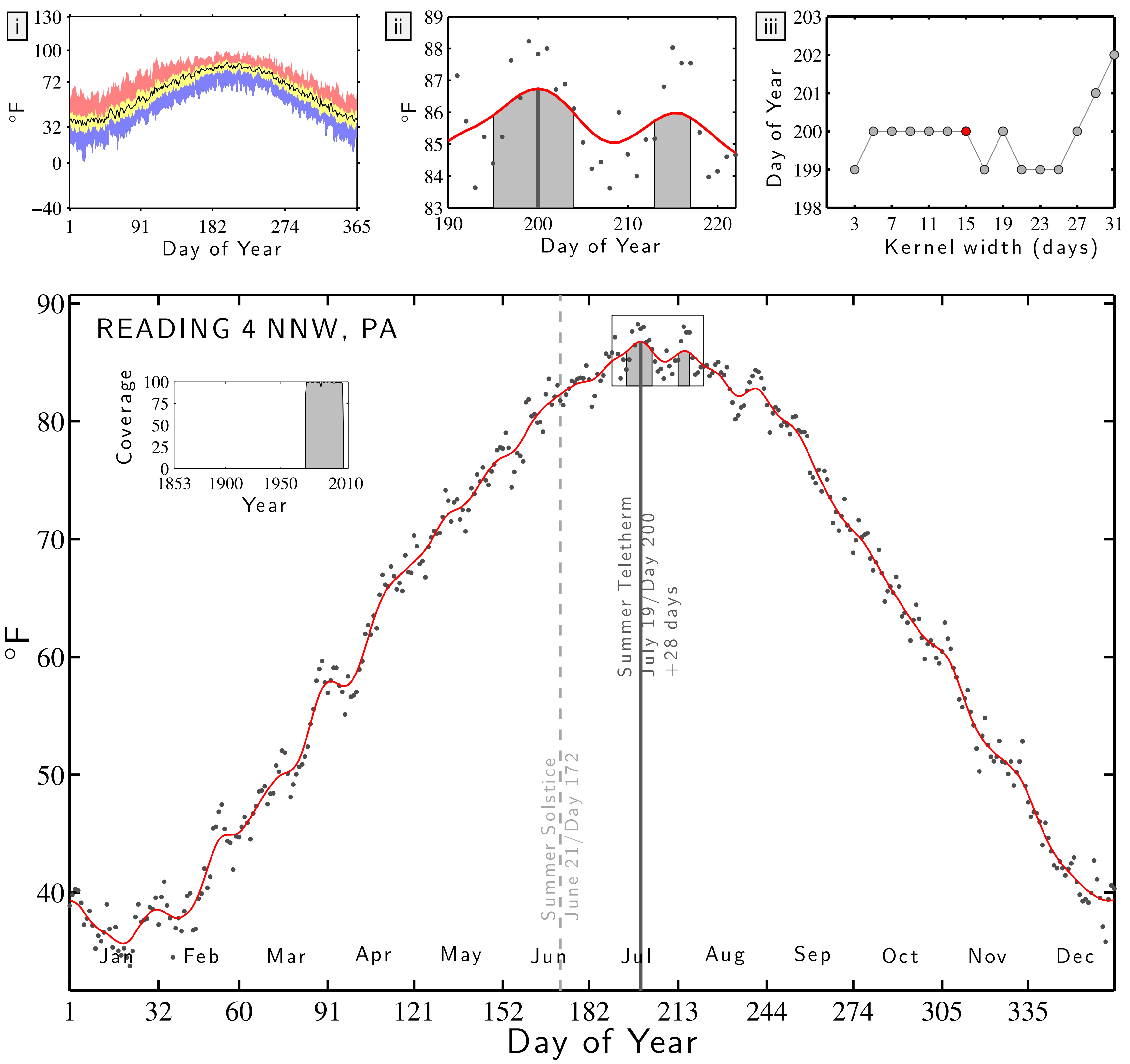

i
ii
iii
°F
Day of Year
Kernel width (days)
READING 4 NNW, PA
Coverage
Year
Summer Solstice
June 21/Day 172
Summer Teletherm
July 19/Day 200
+28 days
Jan
Feb
Mar
Apr
May
Jun
Jul
Aug
Sep
Oct
Nov
Dec

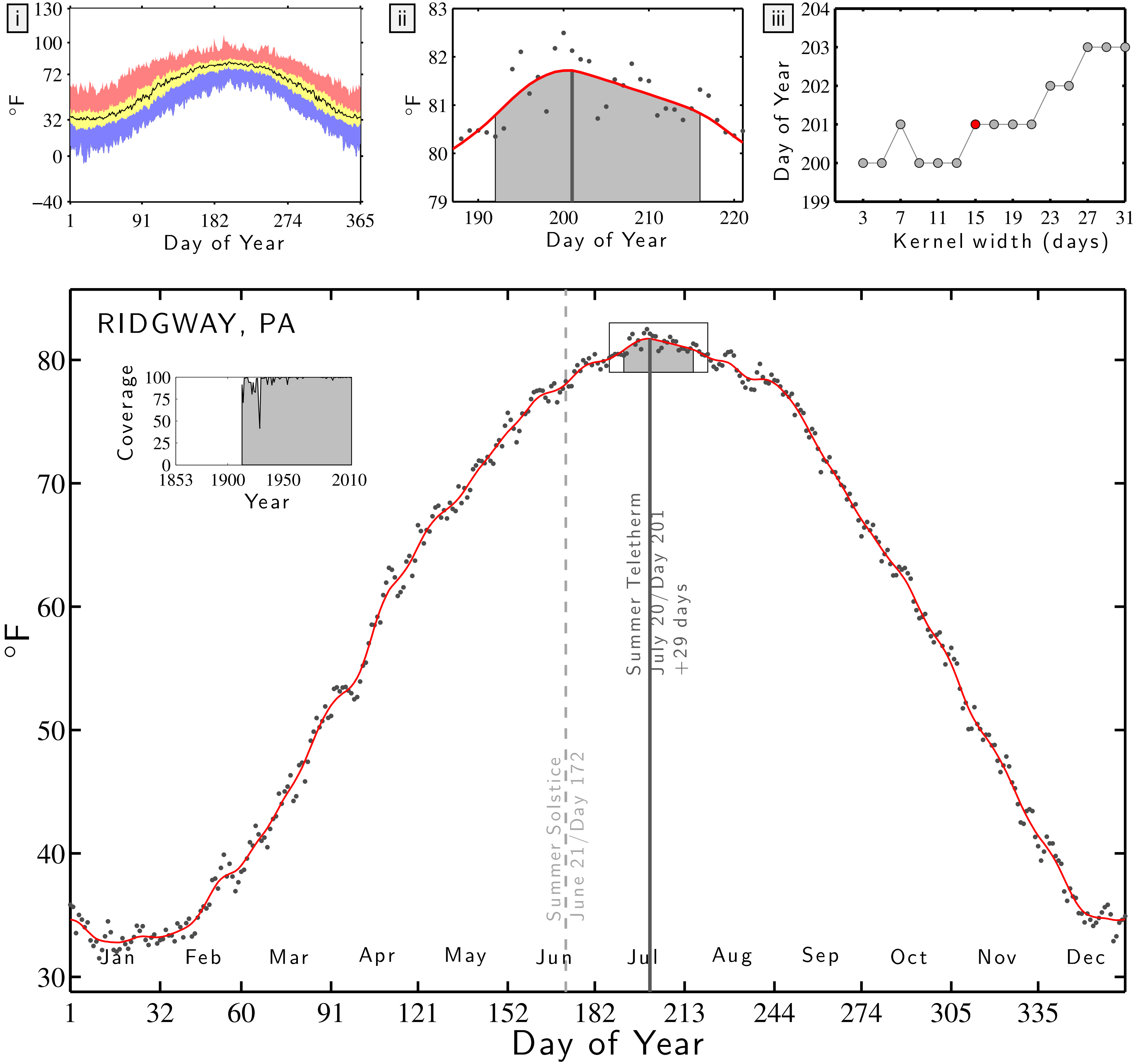

i
°F
Day of Year
ii
°F
Day of Year
iii
Day of Year
Kernel width (days)
RIDGWAY, PA
Coverage
Year
Summer Solstice
June 21/Day 172
Summer Teletherm
July 20/Day 201
+29 days
Jan
Feb
Mar
Apr
May
Jun
Jul
Aug
Sep
Oct
Nov
Dec
°F
Day of Year

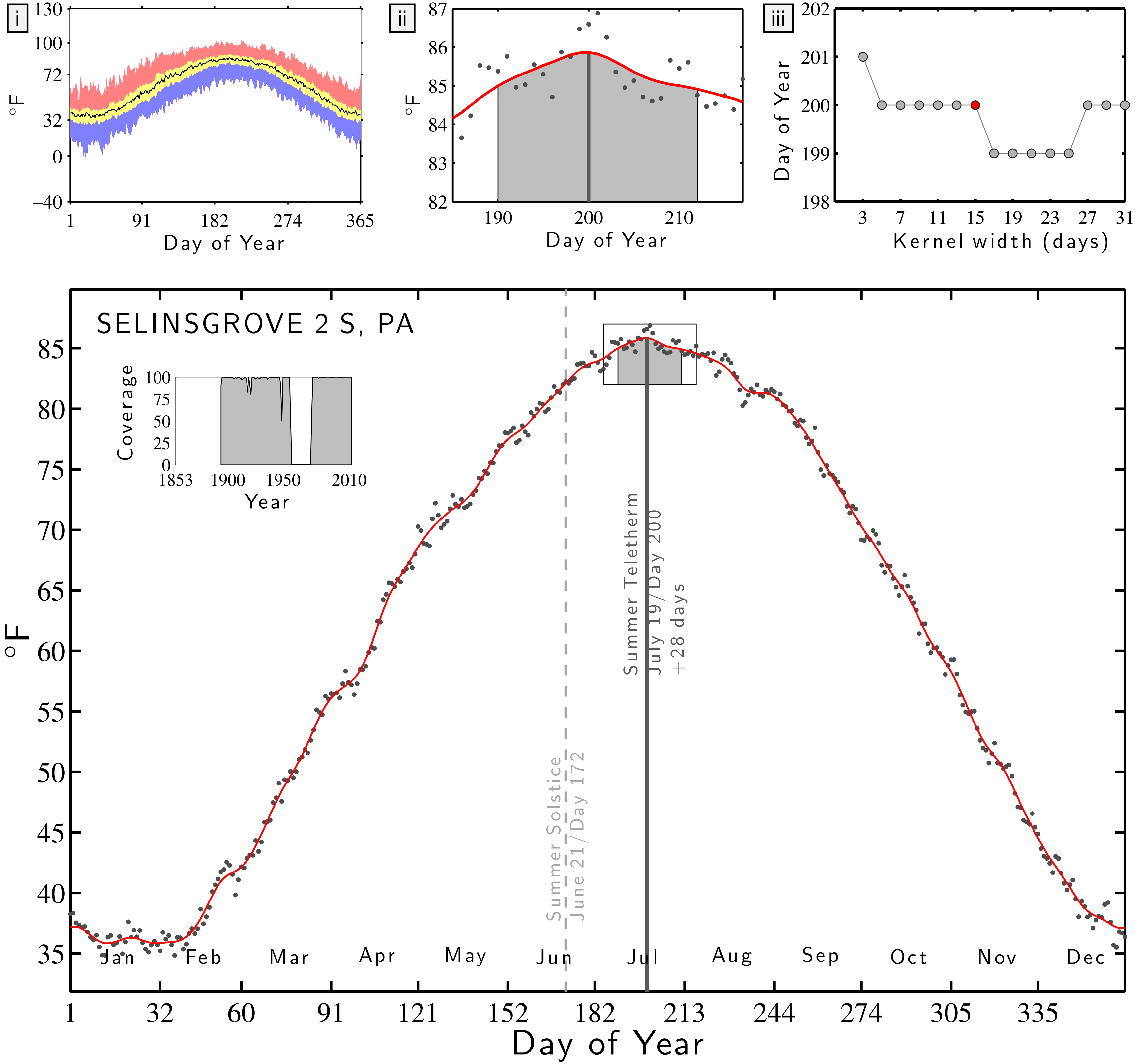

i
ii
iii
Day of Year
°F
Kernel width (days)
SELINSGROVE 2 S, PA
Coverage
Year
Summer Solstice
June 21/Day 172
Summer Teletherm
July 19/Day 200
+28 days
Jan
Feb
Mar
Apr
May
Jun
Jul
Aug
Sep
Oct
Nov
Dec

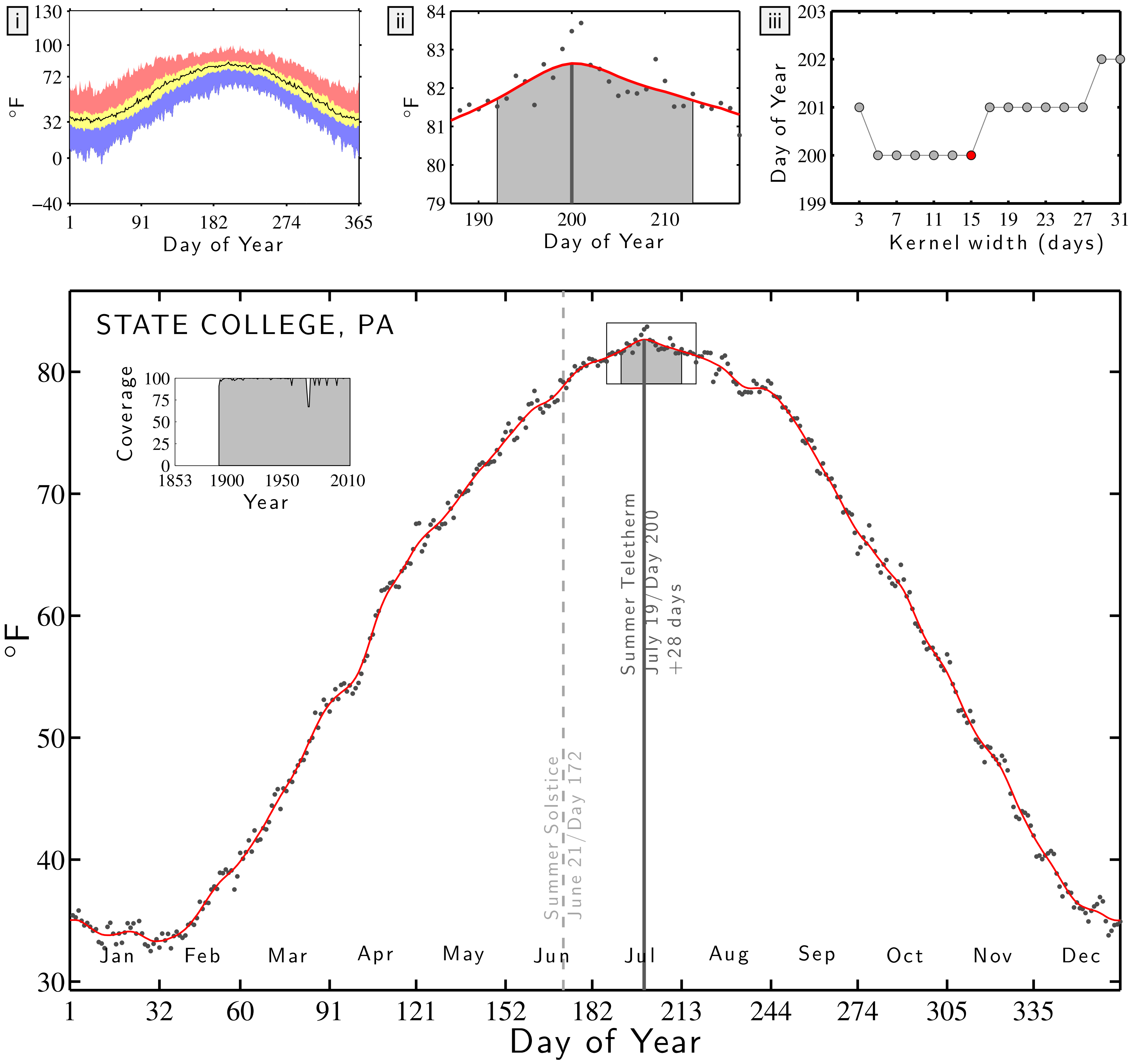

i
°F
Day of Year
ii
°F
Day of Year
iii
Day of Year
Kernel width (days)
STATE COLLEGE, PA
Coverage
Year
Summer Solstice
June 21/Day 172
Summer Teletherm
July 19/Day 200
+28 days
Jan
Feb
Mar
Apr
May
Jun
Jul
Aug
Sep
Oct
Nov
Dec
°F
Day of Year

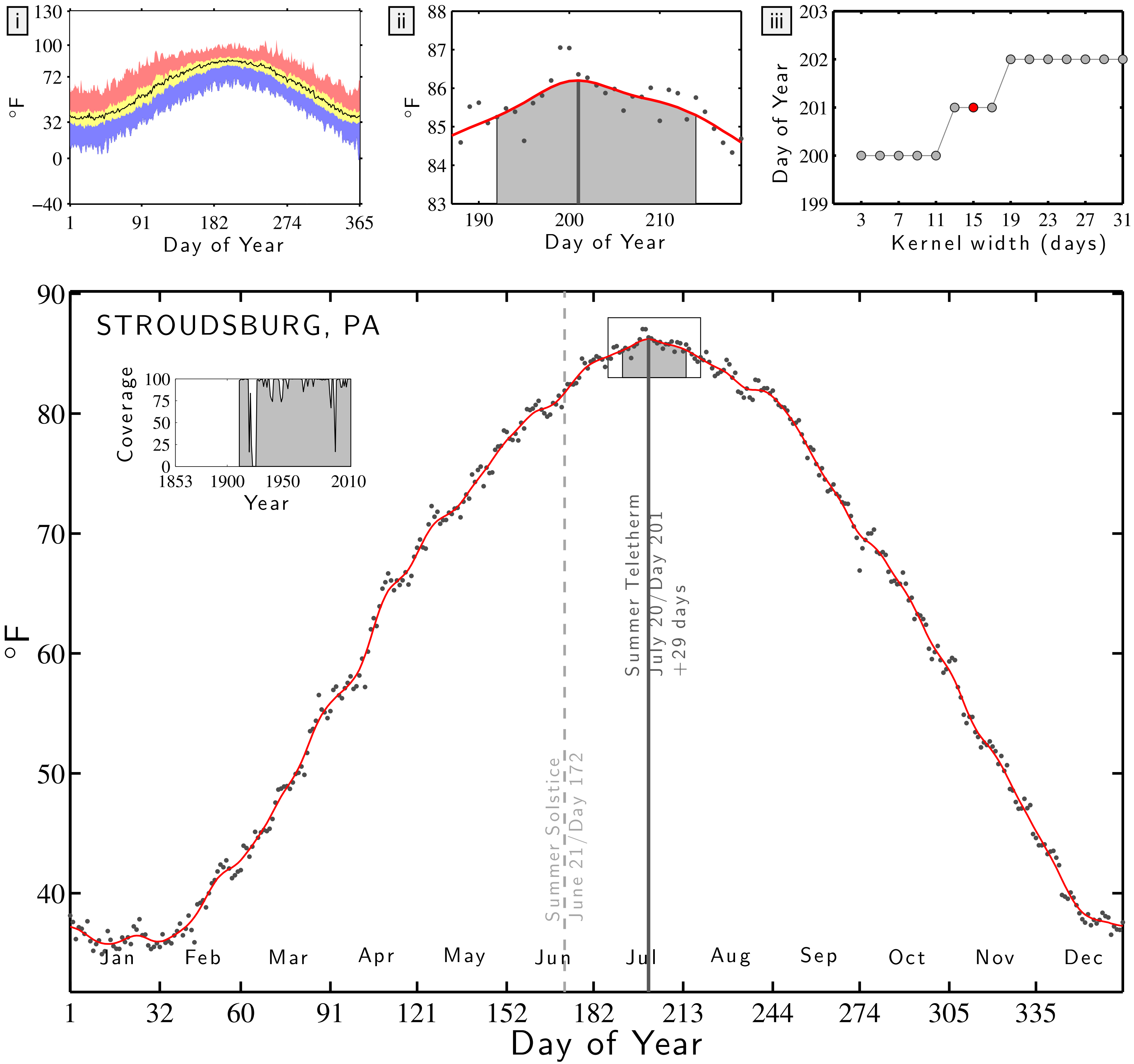

i
ii
iii
°F
Day of Year
Kernel width (days)
STROUDSBURG, PA
Coverage
Year
Summer Solstice
June 21/Day 172
Summer Teletherm
July 20/Day 201
+29 days
Jan
Feb
Mar
Apr
May
Jun
Jul
Aug
Sep
Oct
Nov
Dec

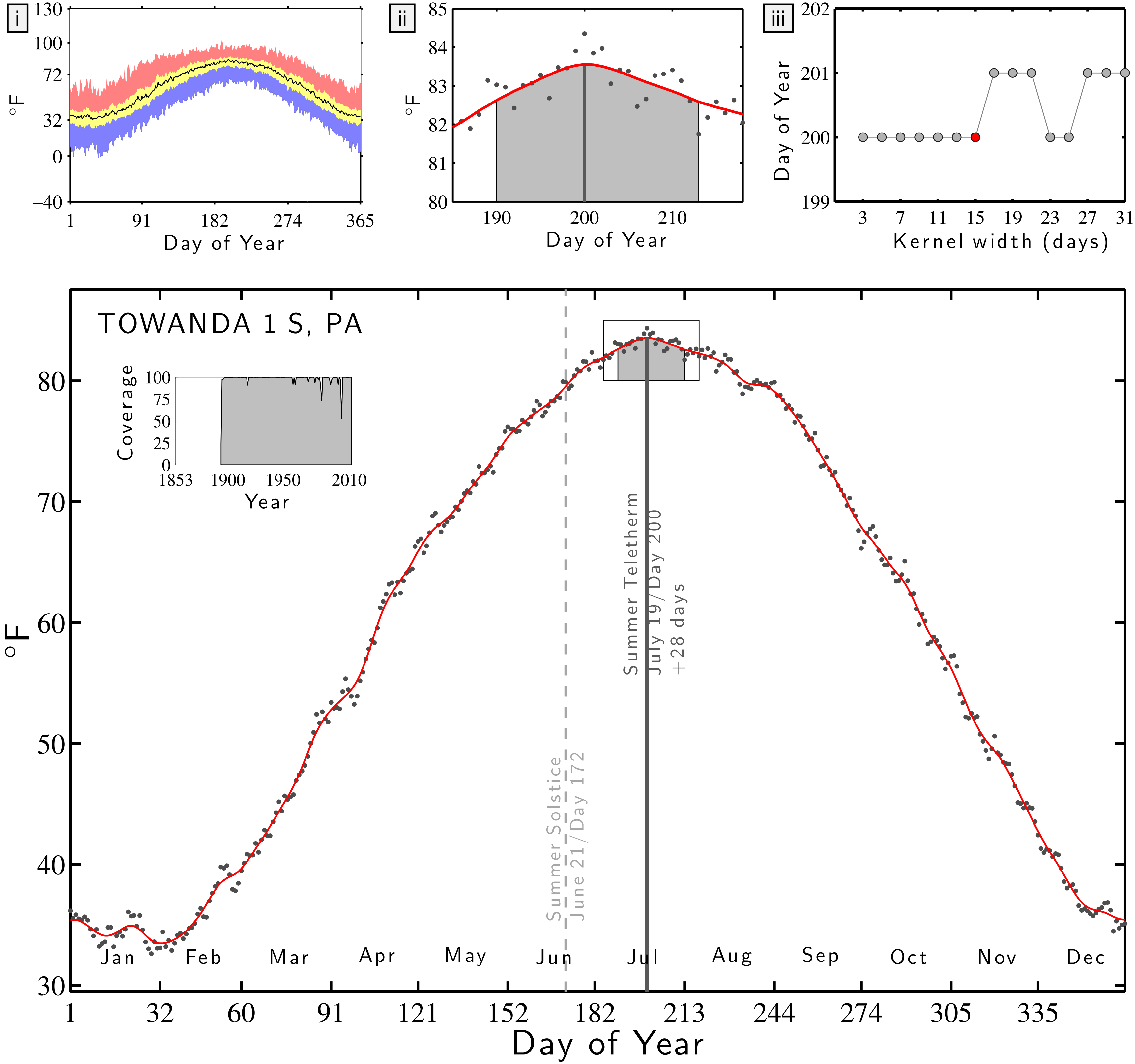

i
ii
iii
°F
Day of Year
Kernel width (days)
TOWANDA 1 S, PA
Coverage
Year
Summer Solstice
June 21/Day 172
Summer Teletherm
July 19/Day 200
+28 days
Jan
Feb
Mar
Apr
May
Jun
Jul
Aug
Sep
Oct
Nov
Dec

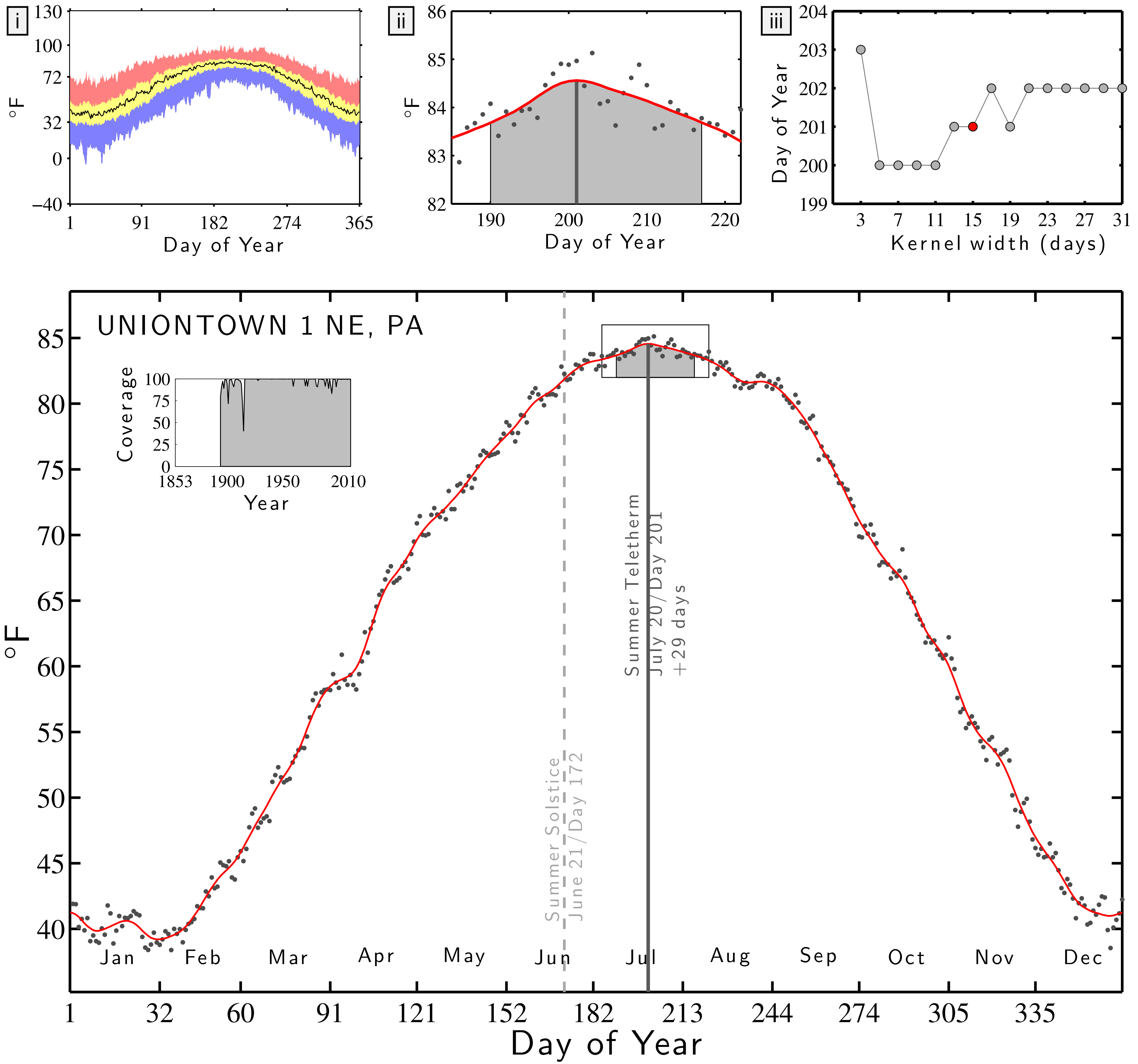

i
°F
Day of Year
ii
°F
Day of Year
iii
Day of Year
Kernel width (days)
UNIONTOWN 1 NE, PA
Coverage
Year
Summer Solstice
June 21/Day 172
Summer Teletherm
July 20/Day 201
+29 days
Jan
Feb
Mar
Apr
May
Jun
Jul
Aug
Sep
Oct
Nov
Dec
°F
Day of Year

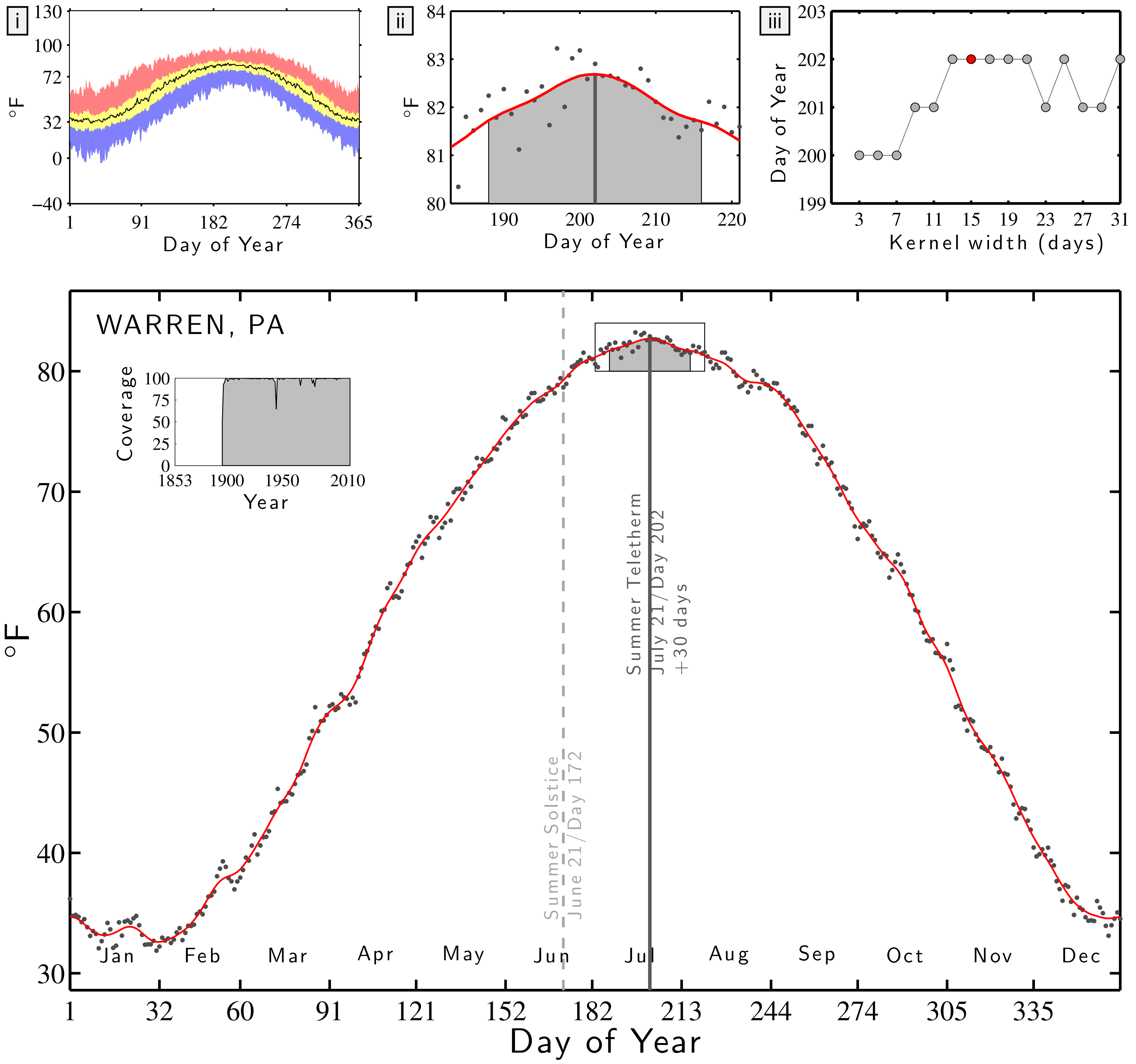
i
ii
iii
°F
Day of Year
Day of Year
Kernel width (days)
WARREN, PA
Coverage
Year
Summer Solstice
June 21 / Day 172
Summer Teletherm
July 21 / Day 202
+30 days
Jan
Feb
Mar
Apr
May
Jun
Jul
Aug
Sep
Oct
Nov
Dec

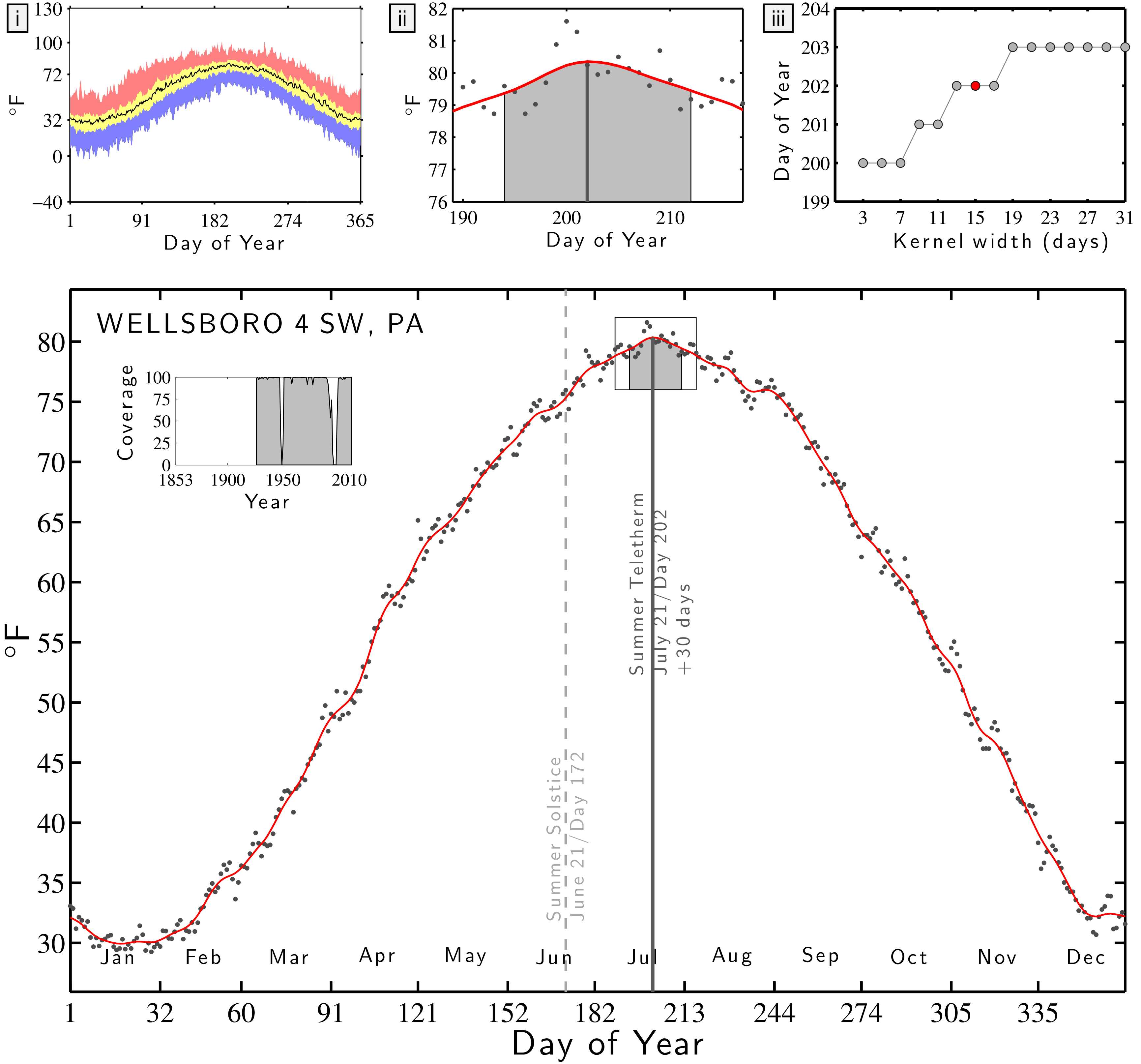

i
°F
Day of Year
ii
°F
Day of Year
iii
Day of Year
Kernel width (days)
WELLSBORO 4 SW, PA
Coverage
Year
Summer Solstice
June 21/Day 172
Summer Teletherm
July 21/Day 202
+30 days
Jan
Feb
Mar
Apr
May
Jun
Jul
Aug
Sep
Oct
Nov
Dec
°F
Day of Year

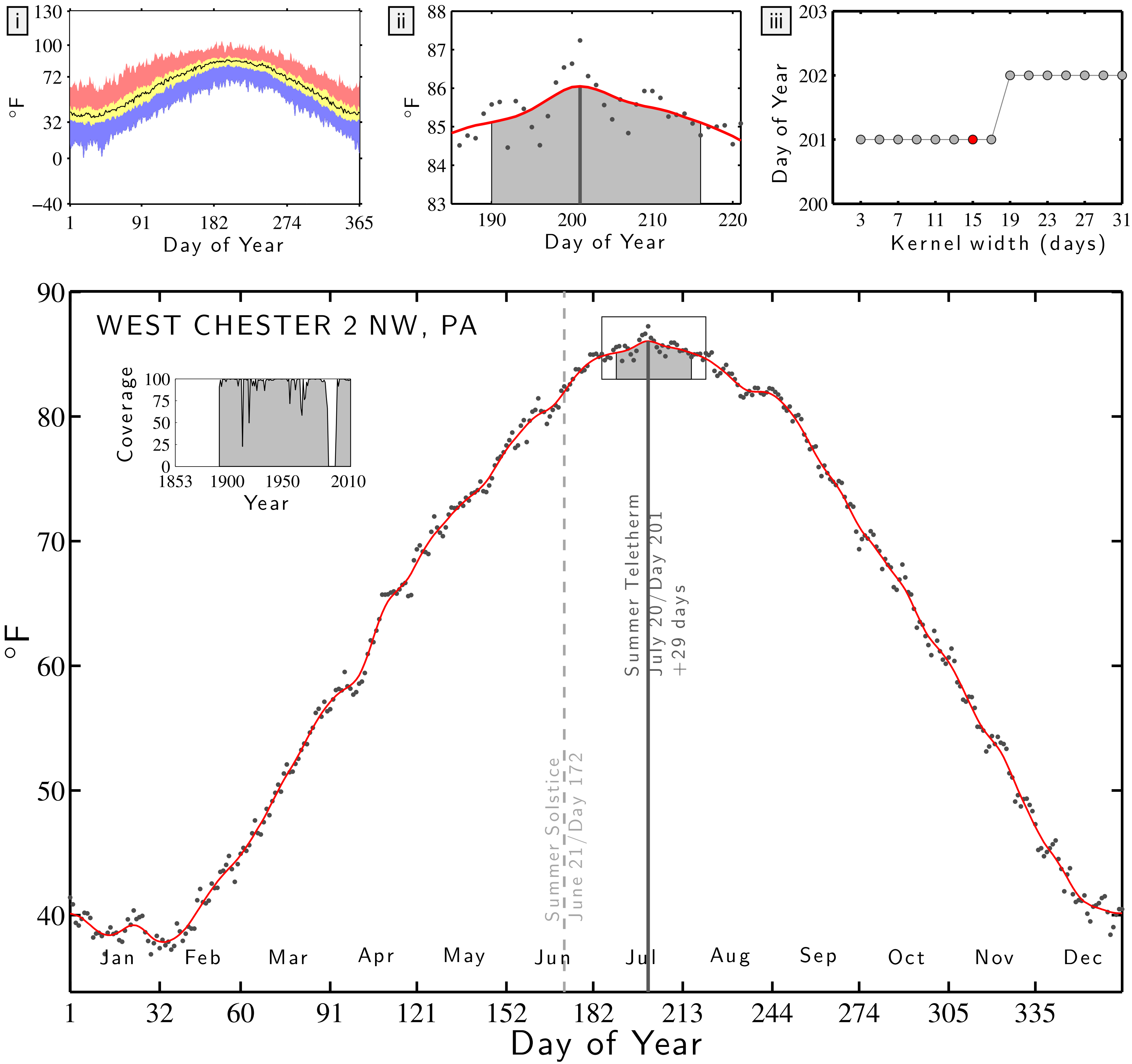

i
°F
Day of Year
ii
°F
Day of Year
iii
Day of Year
Kernel width (days)
WEST CHESTER 2 NW, PA
Coverage
Year
Summer Solstice
June 21 / Day 172
Summer Teletherm
July 20 / Day 201
+29 days
Jan
Feb
Mar
Apr
May
Jun
Jul
Aug
Sep
Oct
Nov
Dec
°F
Day of Year

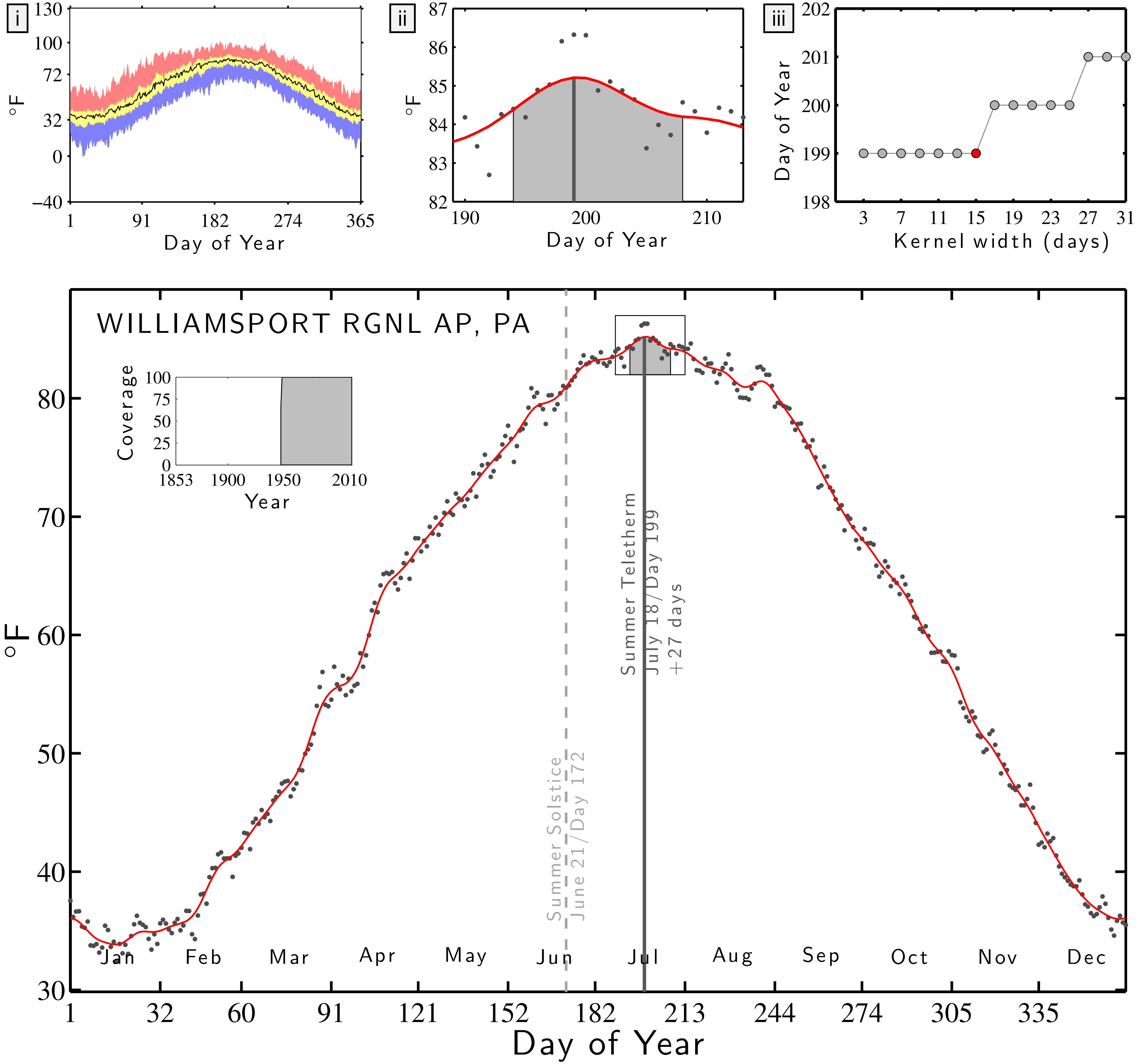

i
°F
Day of Year
ii
°F
Day of Year
iii
Day of Year
Kernel width (days)
WILLIAMSPORT RGNL AP, PA
Coverage
Year
Summer Solstice
June 21/Day 172
Summer Teletherm
July 18/Day 199
+27 days
Jan
Feb
Mar
Apr
May
Jun
Jul
Aug
Sep
Oct
Nov
Dec
°F
Day of Year

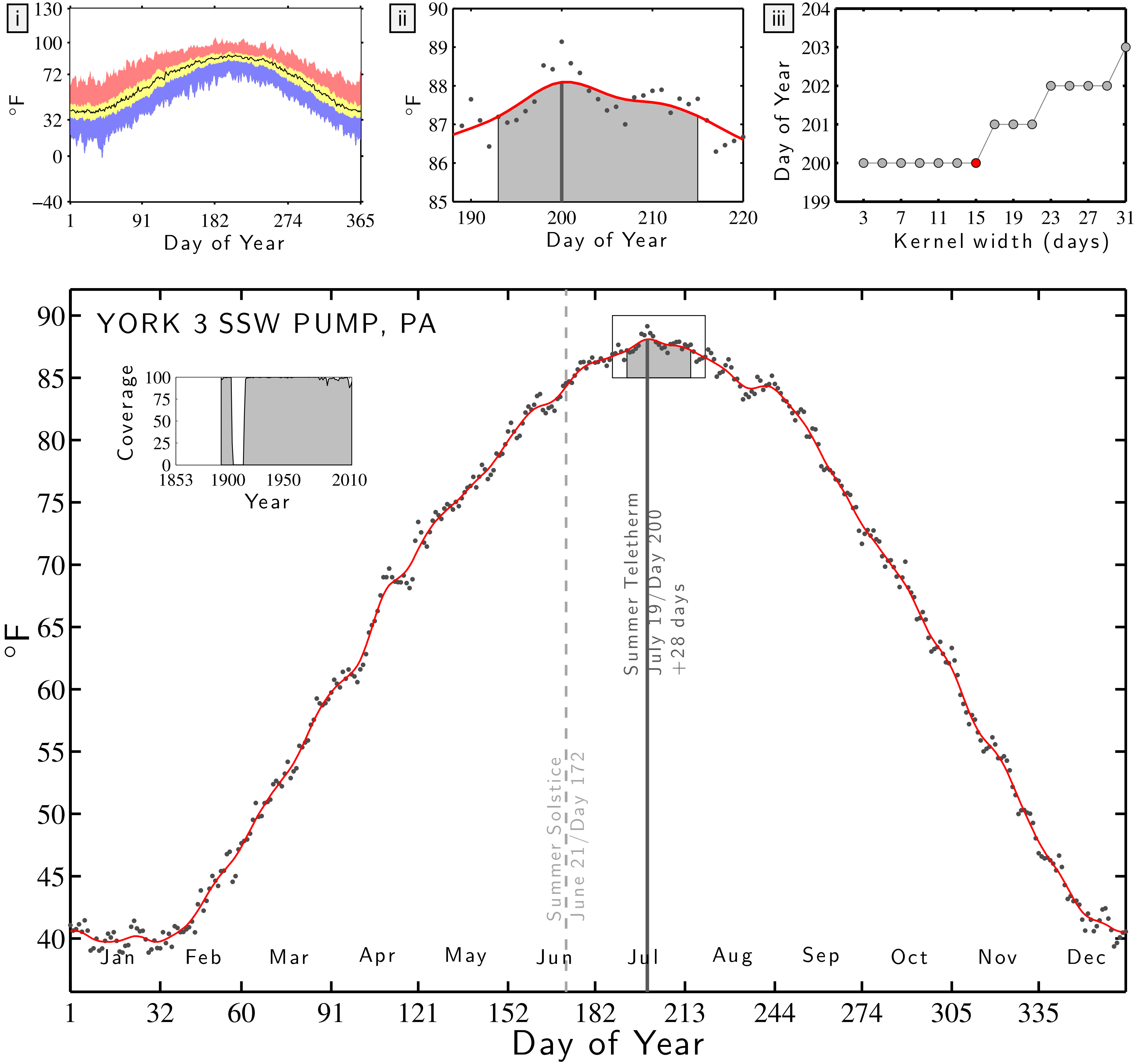

i
130
100
72
32
0
−40
°F
1
91
182
274
365
Day of Year
ii
90
89
88
87
86
85
°F
190
200
210
220
Day of Year
iii
204
203
202
201
200
199
Day of Year
3
7
11
15
19
23
27
31
Kernel width (days)
YORK 3 SSW PUMP, PA
Coverage
100
75
50
25
0
1853
1900
1950
2010
Year
Summer Solstice
June 21/Day 172
Summer Teletherm
July 19/Day 200
+28 days
Jan
Feb
Mar
Apr
May
Jun
Jul
Aug
Sep
Oct
Nov
Dec
°F
90
85
80
75
70
65
60
55
50
45
40
1
32
60
91
121
152
182
213
244
274
305
335
Day of Year

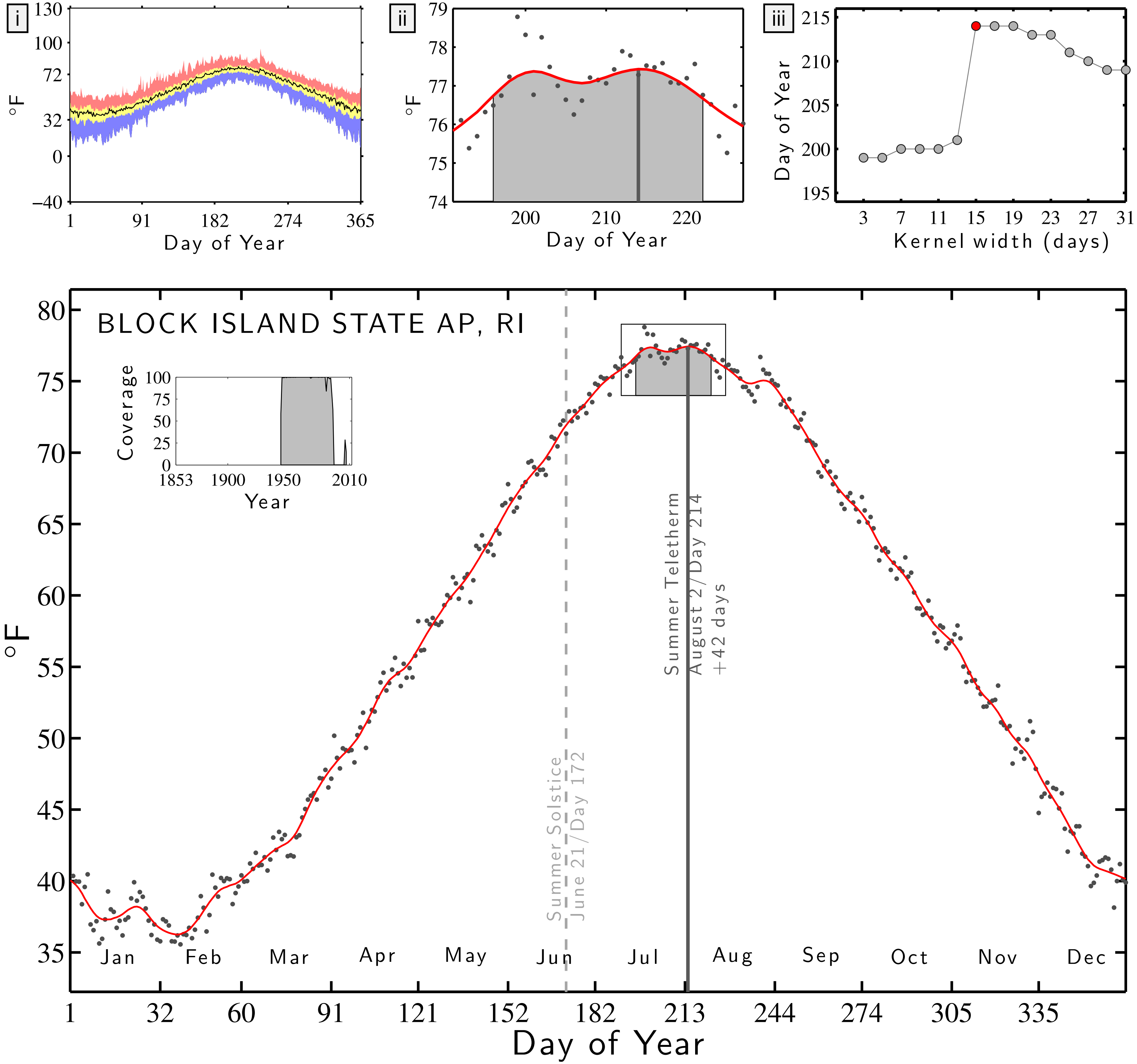

i
°F
130
100
72
32
0
−40
1
91
182
274
365
Day of Year
ii
79
78
77
76
75
74
200
210
220
Day of Year
iii
Day of Year
215
210
205
200
195
3
7
11
15
19
23
27
31
Kernel width (days)
BLOCK ISLAND STATE AP, RI
Coverage
100
75
50
25
0
1853
1900
1950
2010
Year
Summer Solstice
June 21/Day 172
Summer Teletherm
August 2/Day 214
+42 days
80
75
70
65
60
55
50
45
40
35
Jan
Feb
Mar
Apr
May
Jun
Jul
Aug
Sep
Oct
Nov
Dec
1
32
60
91
121
152
182
213
244
274
305
335
Day of Year

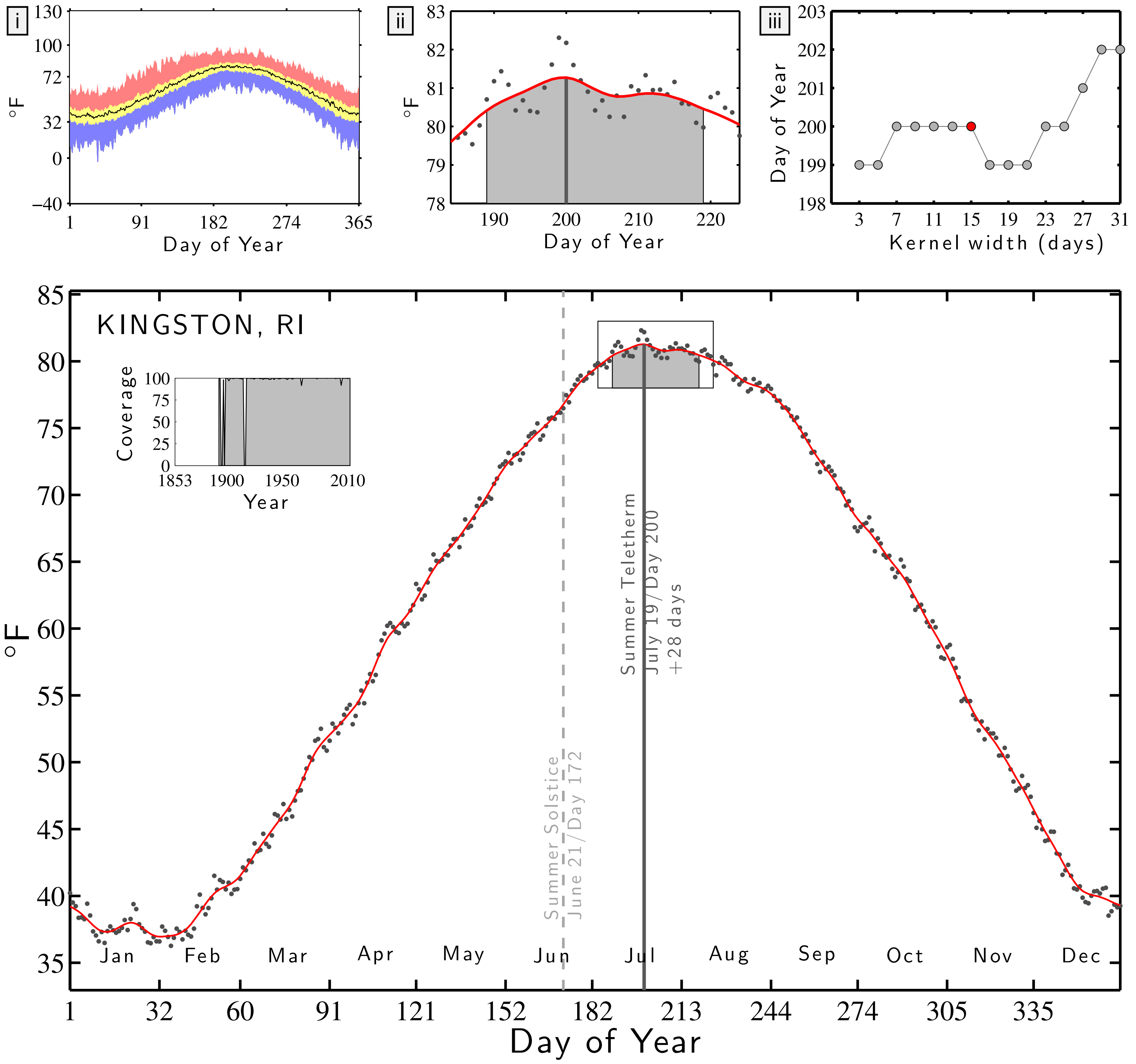

i
130
100
72
32
0
−40
°F
1
91
182
274
365
Day of Year
ii
83
82
81
80
79
78
°F
190
200
210
220
Day of Year
iii
203
202
201
200
199
198
Day of Year
3
7
11
15
19
23
27
31
Kernel width (days)
KINGSTON, RI
Coverage
100
75
50
25
0
1853
1900
1950
2010
Year
Summer Solstice
June 21/Day 172
Summer Teletherm
July 19/Day 200
+28 days
85
80
75
70
65
60
55
50
45
40
35
°F
Jan
Feb
Mar
Apr
May
Jun
Jul
Aug
Sep
Oct
Nov
Dec
1
32
60
91
121
152
182
213
244
274
305
335
Day of Year

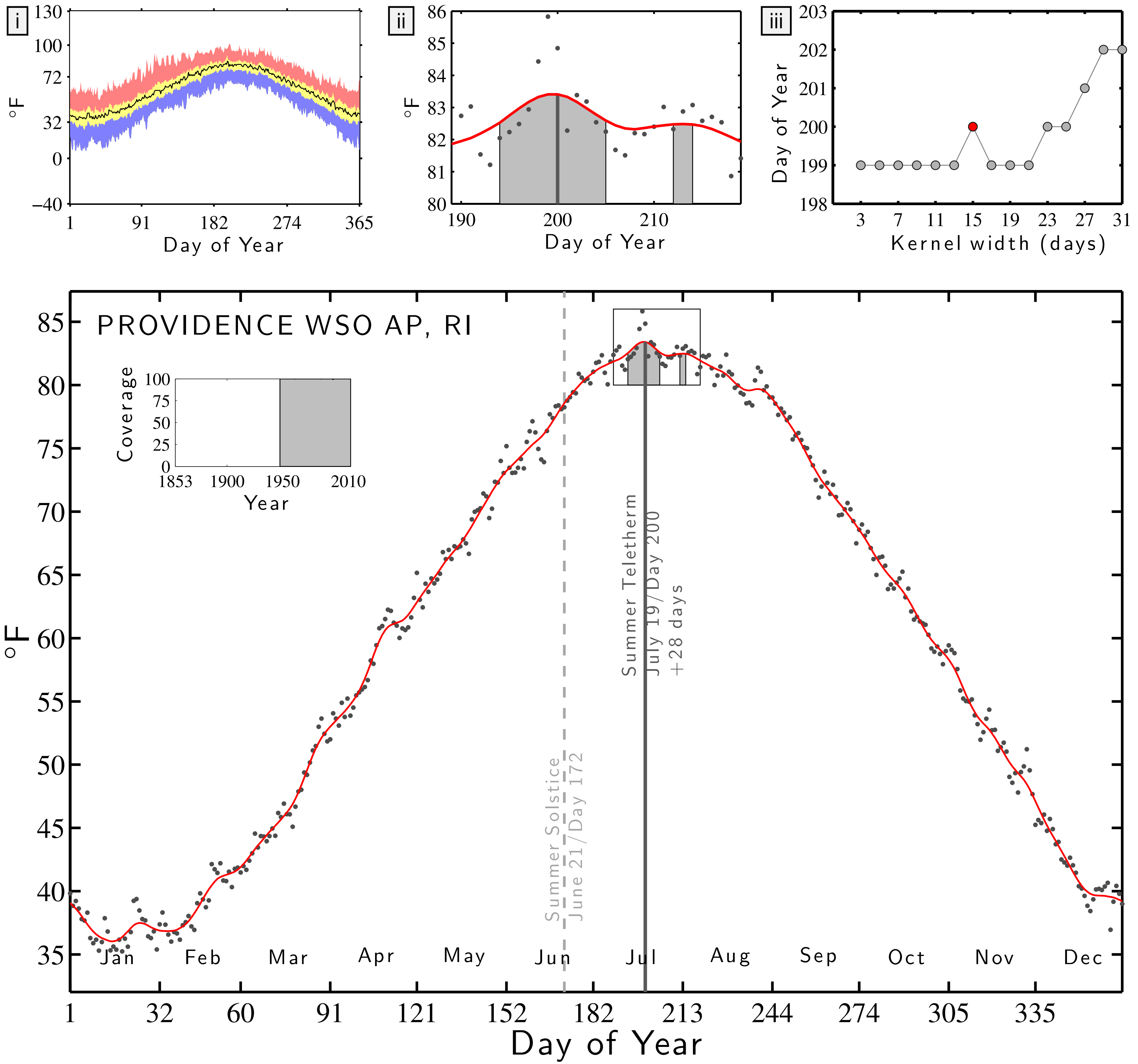

i
°F
Day of Year
ii
°F
Day of Year
iii
Day of Year
Kernel width (days)
PROVIDENCE WSO AP, RI
Coverage
Year
Summer Solstice
June 21/Day 172
Summer Teletherm
July 19/Day 200
+28 days
Jan
Feb
Mar
Apr
May
Jun
Jul
Aug
Sep
Oct
Nov
Dec
°F
Day of Year

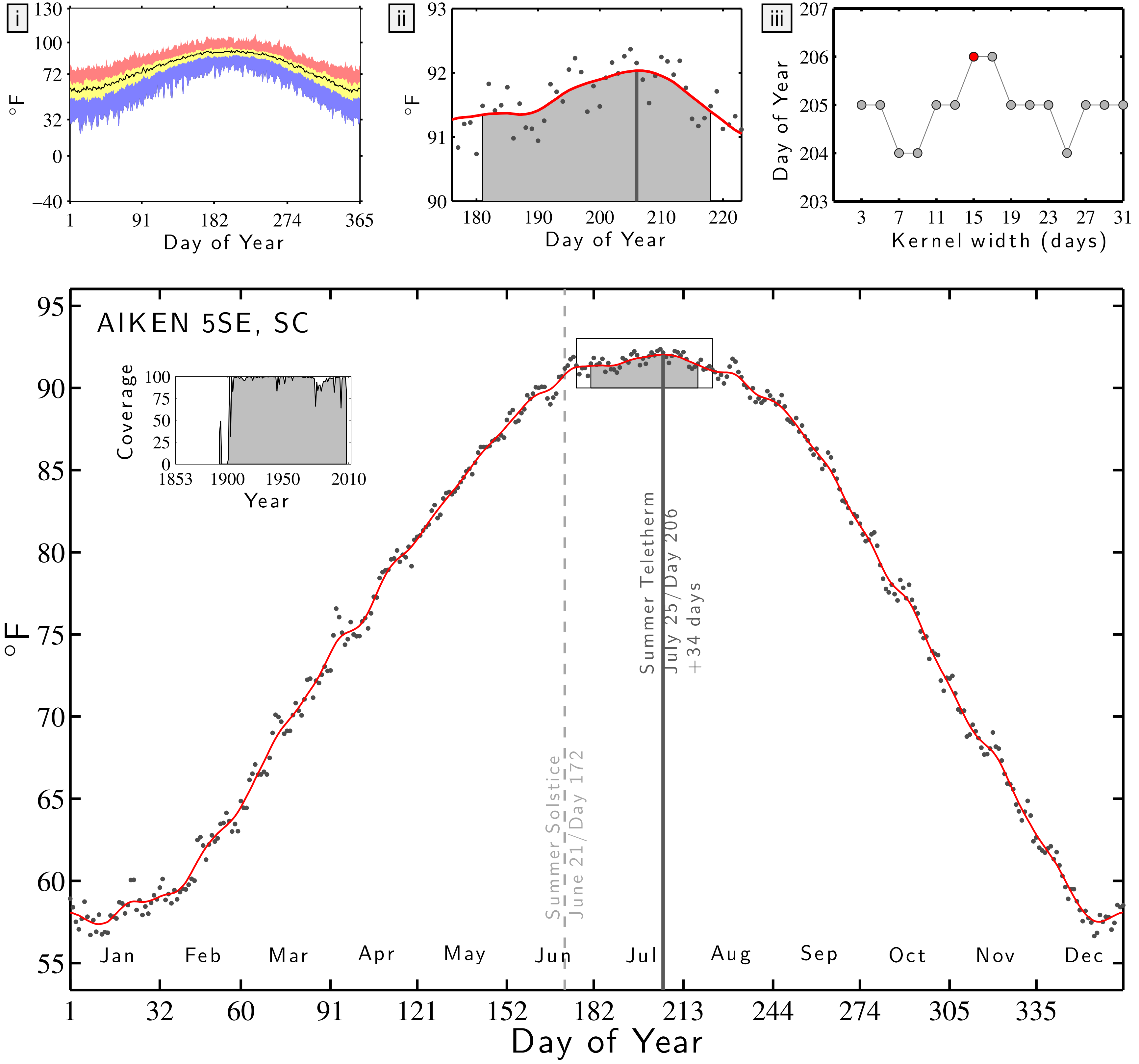

i
°F
Day of Year
ii
°F
Day of Year
iii
Day of Year
Kernel width (days)
AIKEN 5SE, SC
Coverage
Year
Summer Solstice
June 21/Day 172
Summer Teletherm
July 25/Day 206
+34 days
Jan
Feb
Mar
Apr
May
Jun
Jul
Aug
Sep
Oct
Nov
Dec
°F
Day of Year

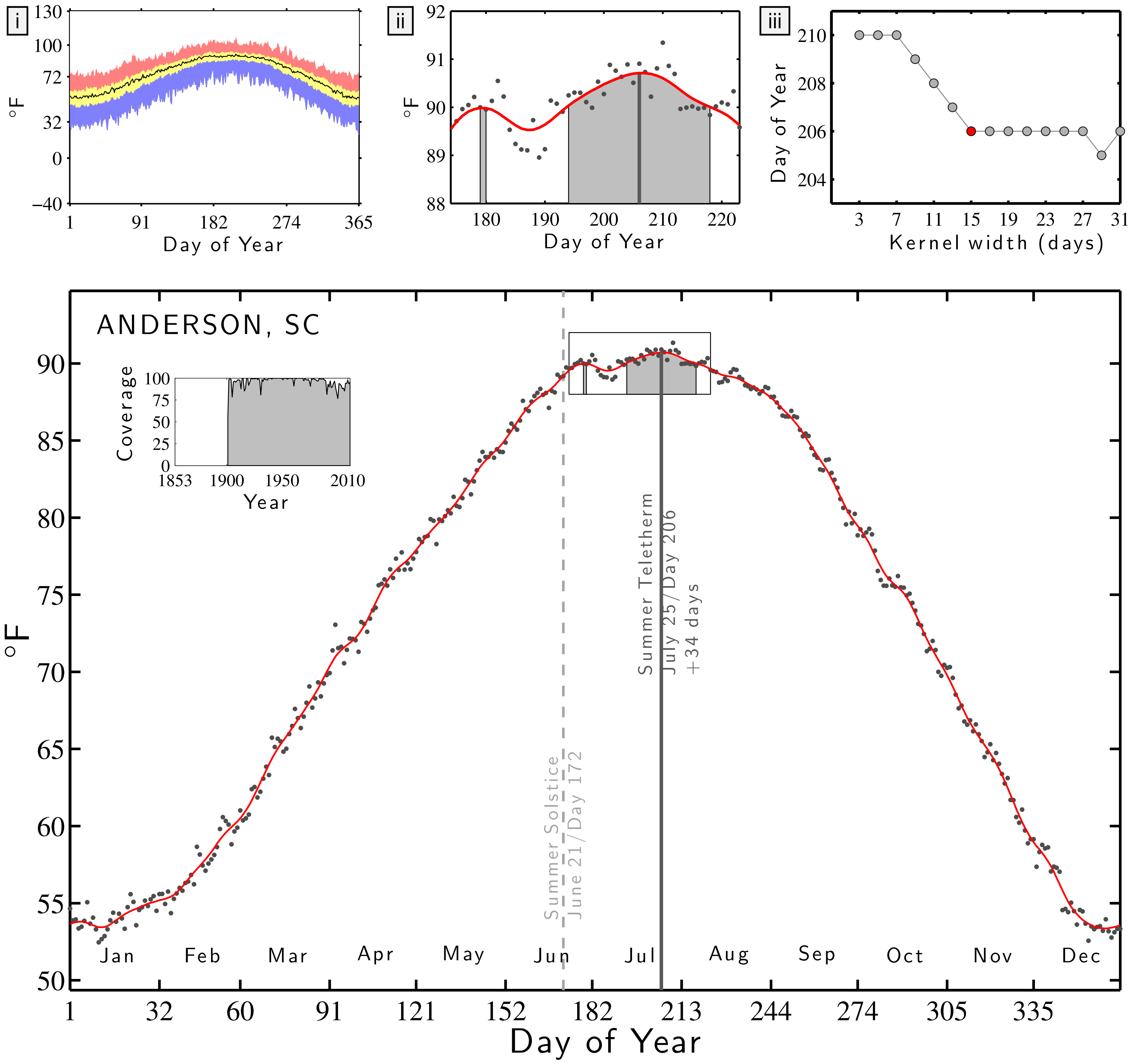

i
°F
Day of Year
ii
°F
Day of Year
iii
Day of Year
Kernel width (days)
ANDERSON, SC
Coverage
Year
Summer Solstice
June 21/Day 172
Summer Teletherm
July 25/Day 206
+34 days
Jan
Feb
Mar
Apr
May
Jun
Jul
Aug
Sep
Oct
Nov
Dec
°F
Day of Year

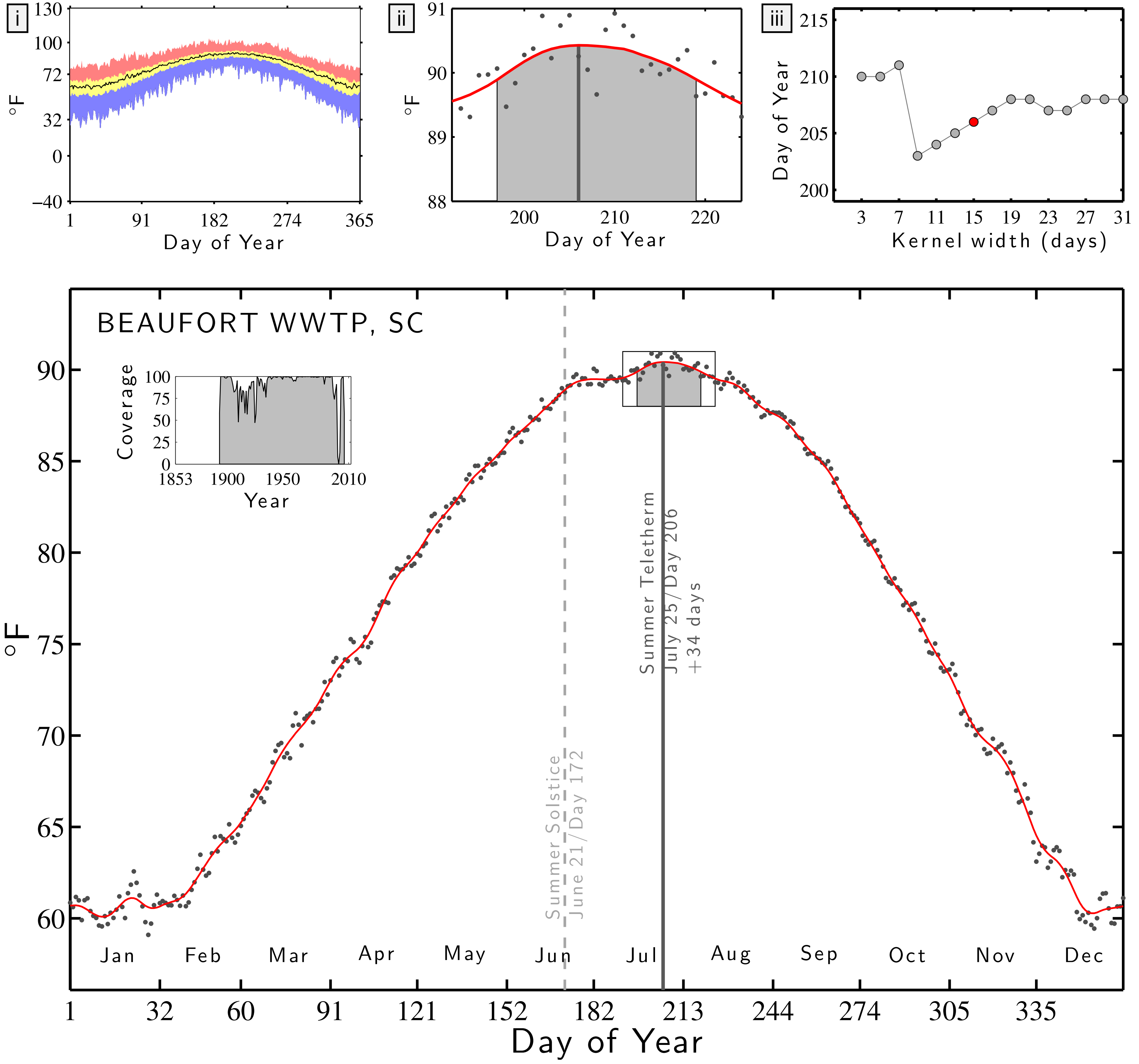

i
ii
iii
°F
Day of Year
Kernel width (days)
BEAUFORT WWTP, SC
Coverage
Year
Summer Solstice
June 21/Day 172
Summer Teletherm
July 25/Day 206
+34 days
Jan
Feb
Mar
Apr
May
Jun
Jul
Aug
Sep
Oct
Nov
Dec

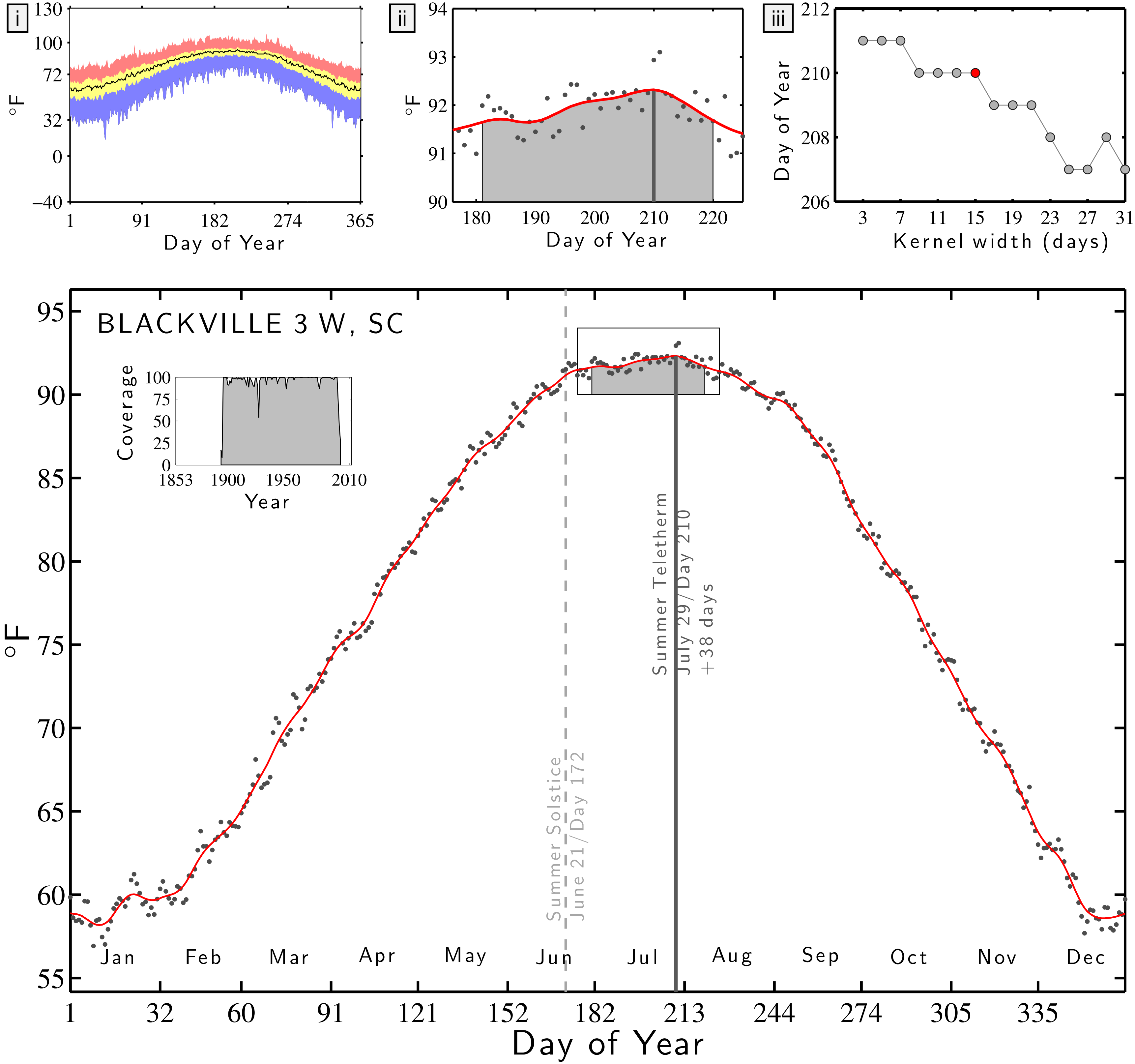

i
ii
iii
°F
Day of Year
Day of Year
Kernel width (days)
BLACKVILLE 3 W, SC
Coverage
Year
Summer Solstice
June 21 / Day 172
Summer Teletherm
July 29 / Day 210
+38 days
Jan
Feb
Mar
Apr
May
Jun
Jul
Aug
Sep
Oct
Nov
Dec

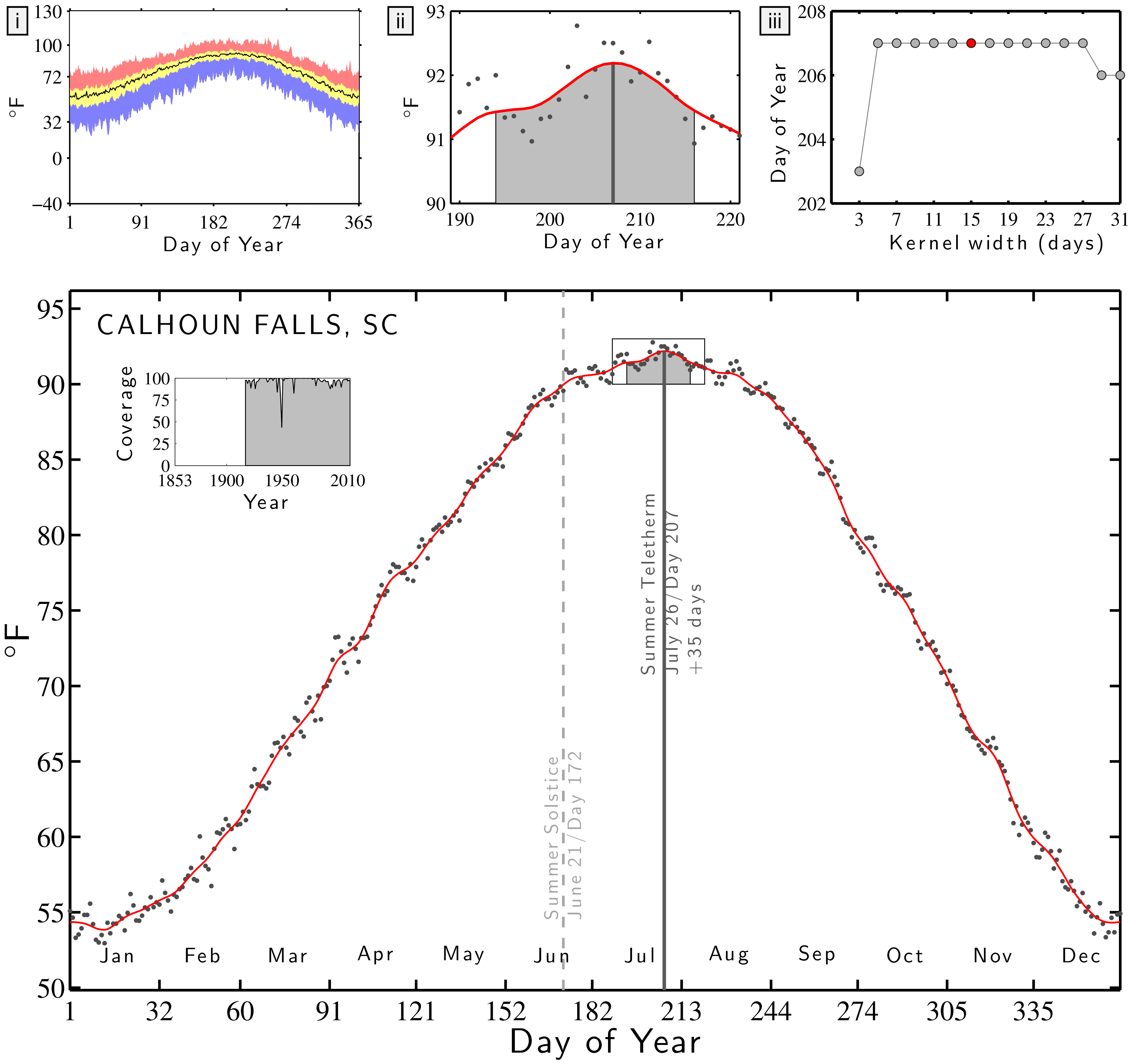

i
°F
Day of Year
ii
°F
Day of Year
iii
Day of Year
Kernel width (days)
CALHOUN FALLS, SC
Coverage
Year
Summer Solstice
June 21 / Day 172
Summer Teletherm
July 26 / Day 207
+35 days
Jan
Feb
Mar
Apr
May
Jun
Jul
Aug
Sep
Oct
Nov
Dec
°F
Day of Year

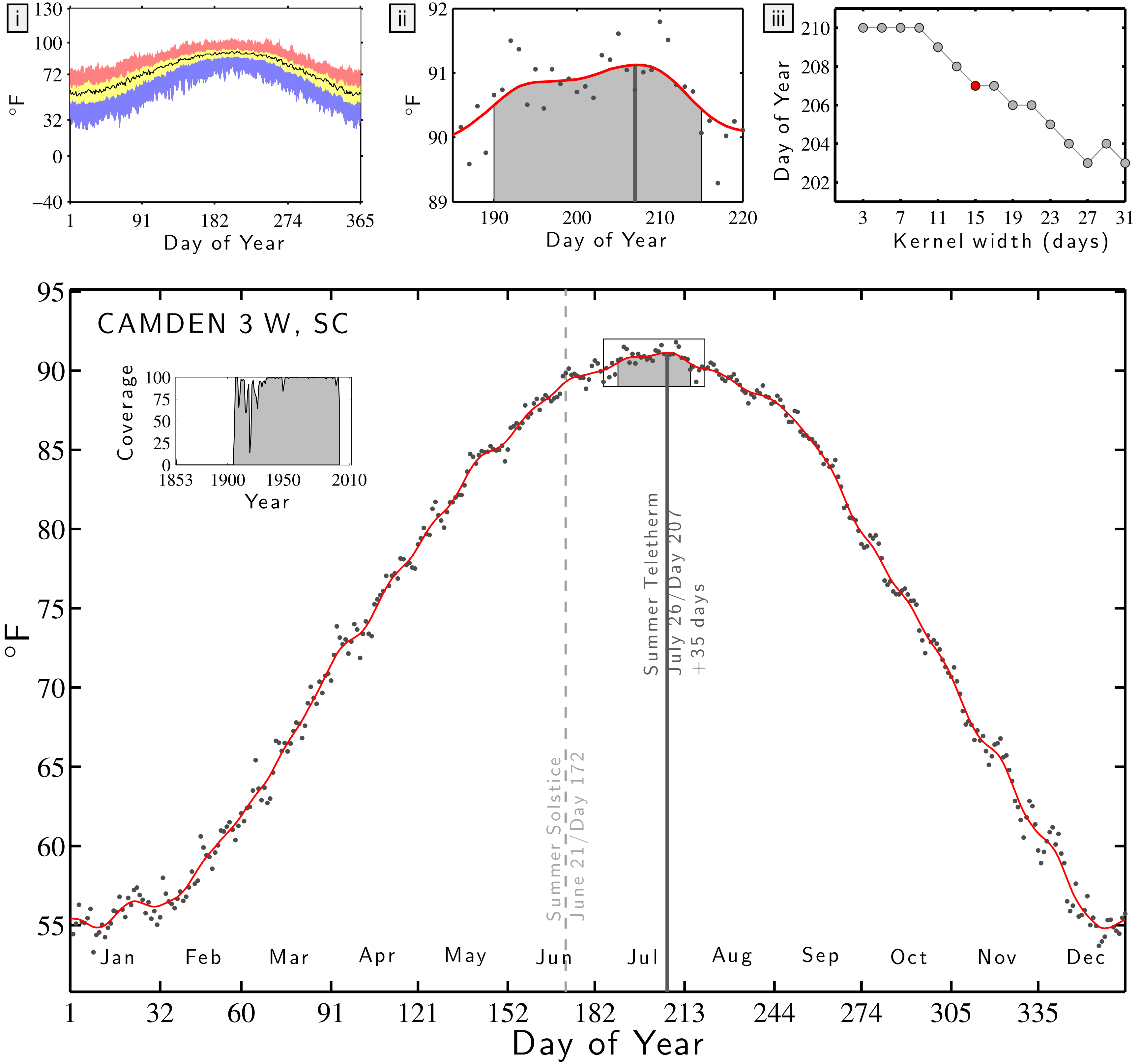

i
ii
iii
°F
Day of Year
Kernel width (days)
CAMDEN 3 W, SC
Coverage
Year
Summer Solstice
June 21 / Day 172
Summer Teletherm
July 26 / Day 207
+35 days
Jan
Feb
Mar
Apr
May
Jun
Jul
Aug
Sep
Oct
Nov
Dec

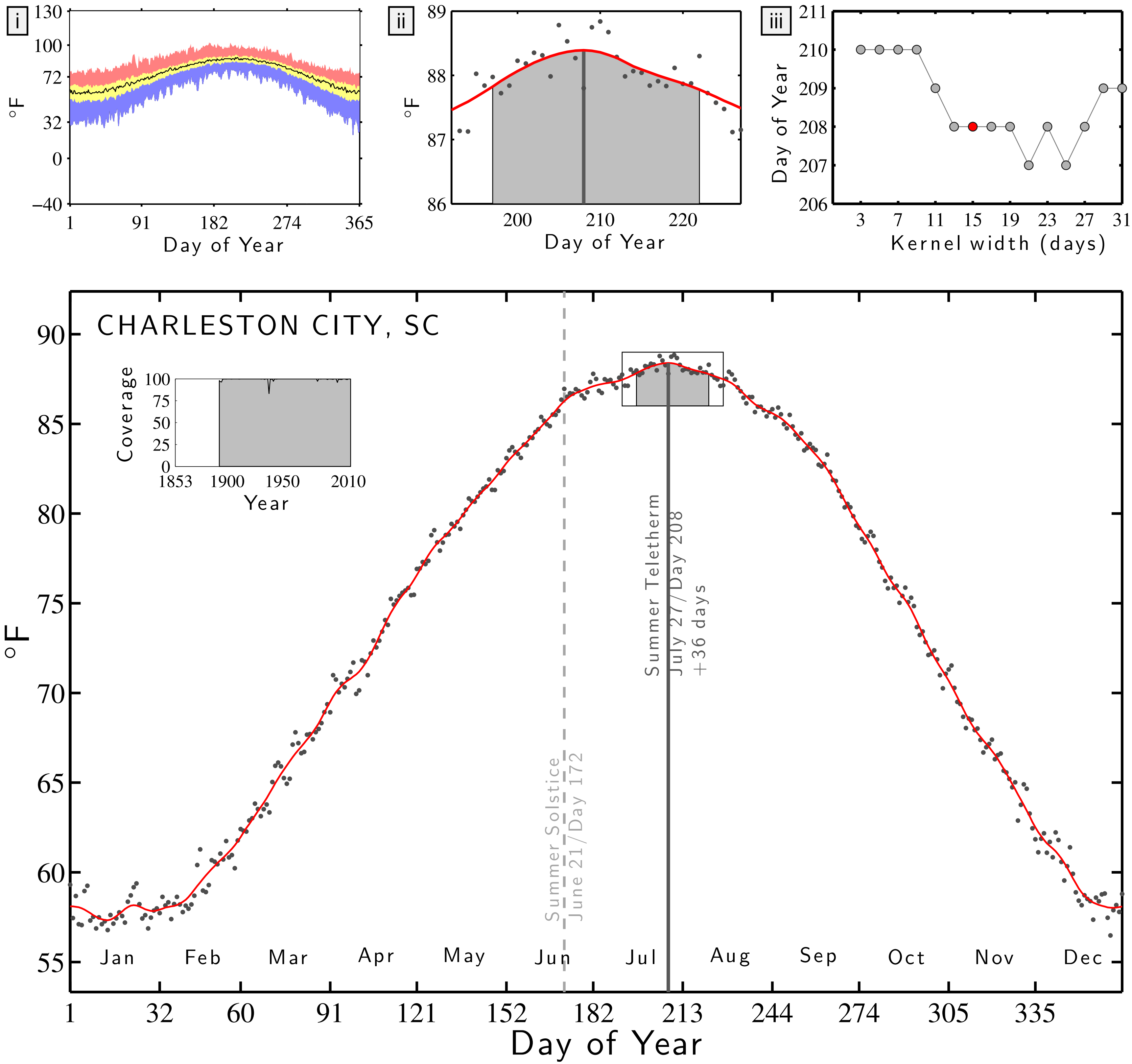

i
°F
Day of Year
ii
°F
Day of Year
iii
Day of Year
Kernel width (days)
CHARLESTON CITY, SC
Coverage
Year
Summer Solstice
June 21 / Day 172
Summer Teletherm
July 27 / Day 208
+36 days
Jan
Feb
Mar
Apr
May
Jun
Jul
Aug
Sep
Oct
Nov
Dec
°F
Day of Year

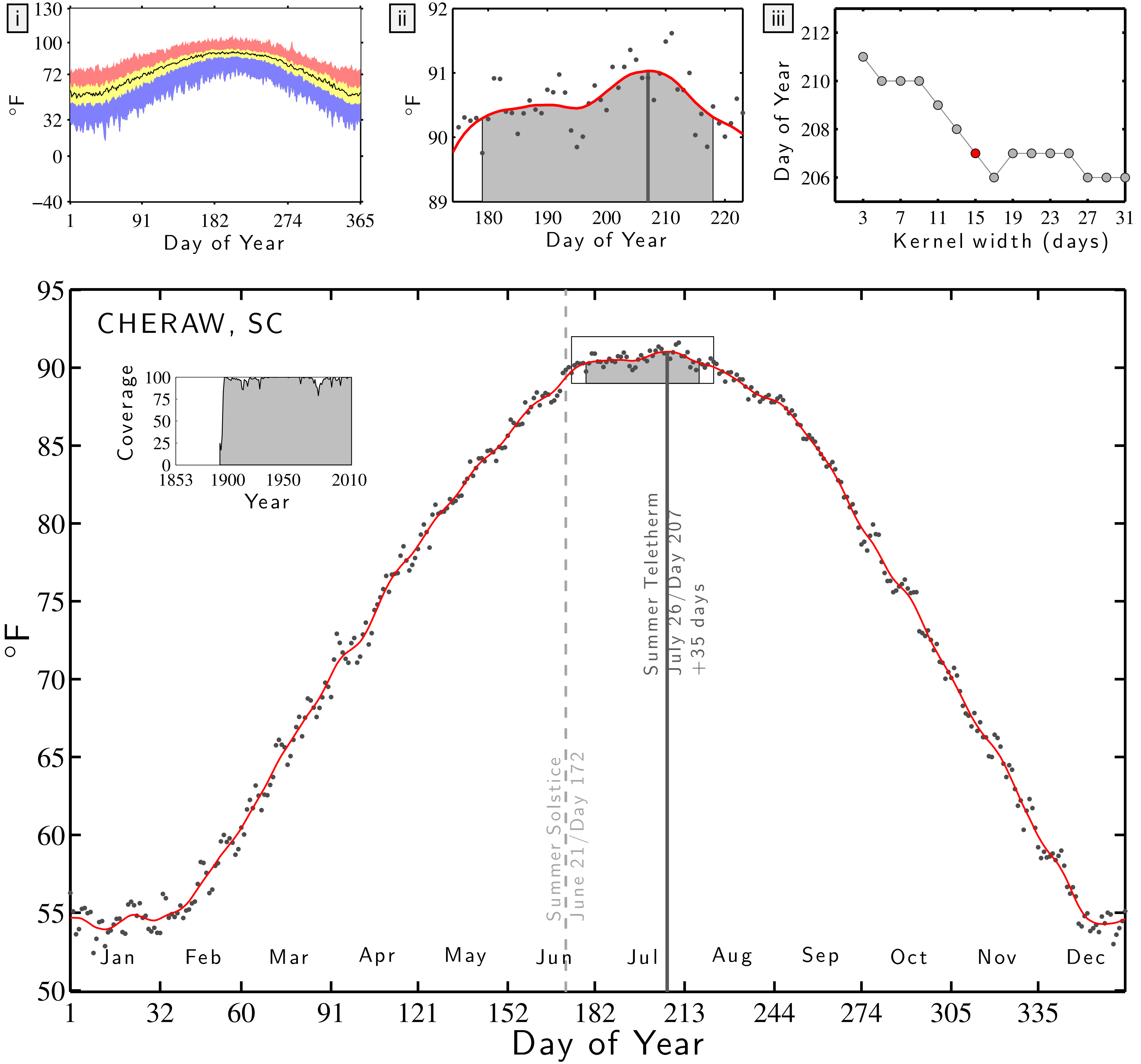

i
°F
Day of Year
ii
°F
Day of Year
iii
Day of Year
Kernel width (days)
CHERAW, SC
Coverage
Year
Summer Solstice
June 21 / Day 172
Summer Teletherm
July 26 / Day 207
+35 days
Jan
Feb
Mar
Apr
May
Jun
Jul
Aug
Sep
Oct
Nov
Dec
°F
Day of Year

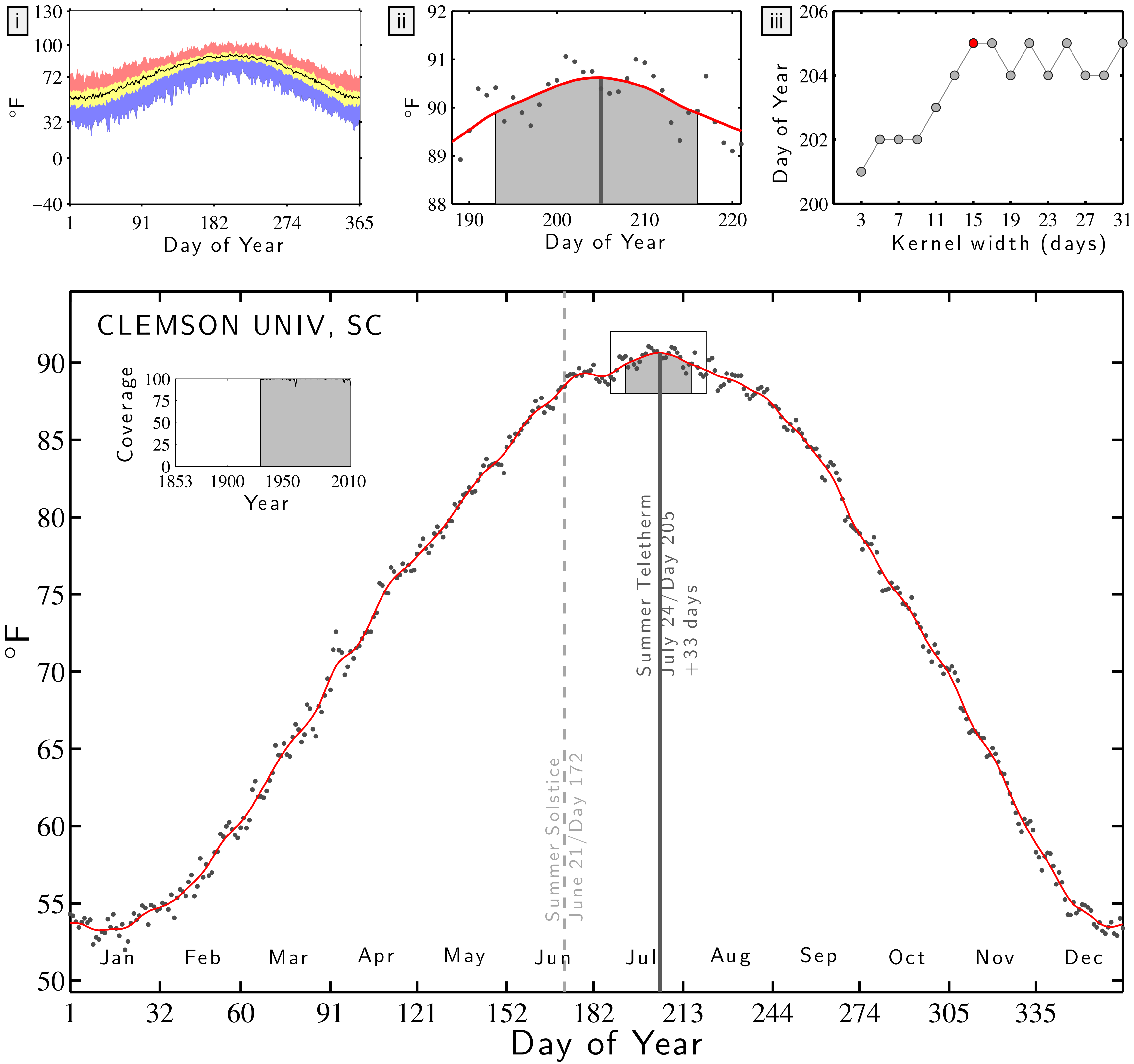

i
°F
Day of Year
ii
°F
Day of Year
iii
Day of Year
Kernel width (days)
CLEMSON UNIV, SC
Coverage
Year
Summer Solstice
June 21/Day 172
Summer Teletherm
July 24/Day 205
+33 days
Jan
Feb
Mar
Apr
May
Jun
Jul
Aug
Sep
Oct
Nov
Dec
°F
Day of Year

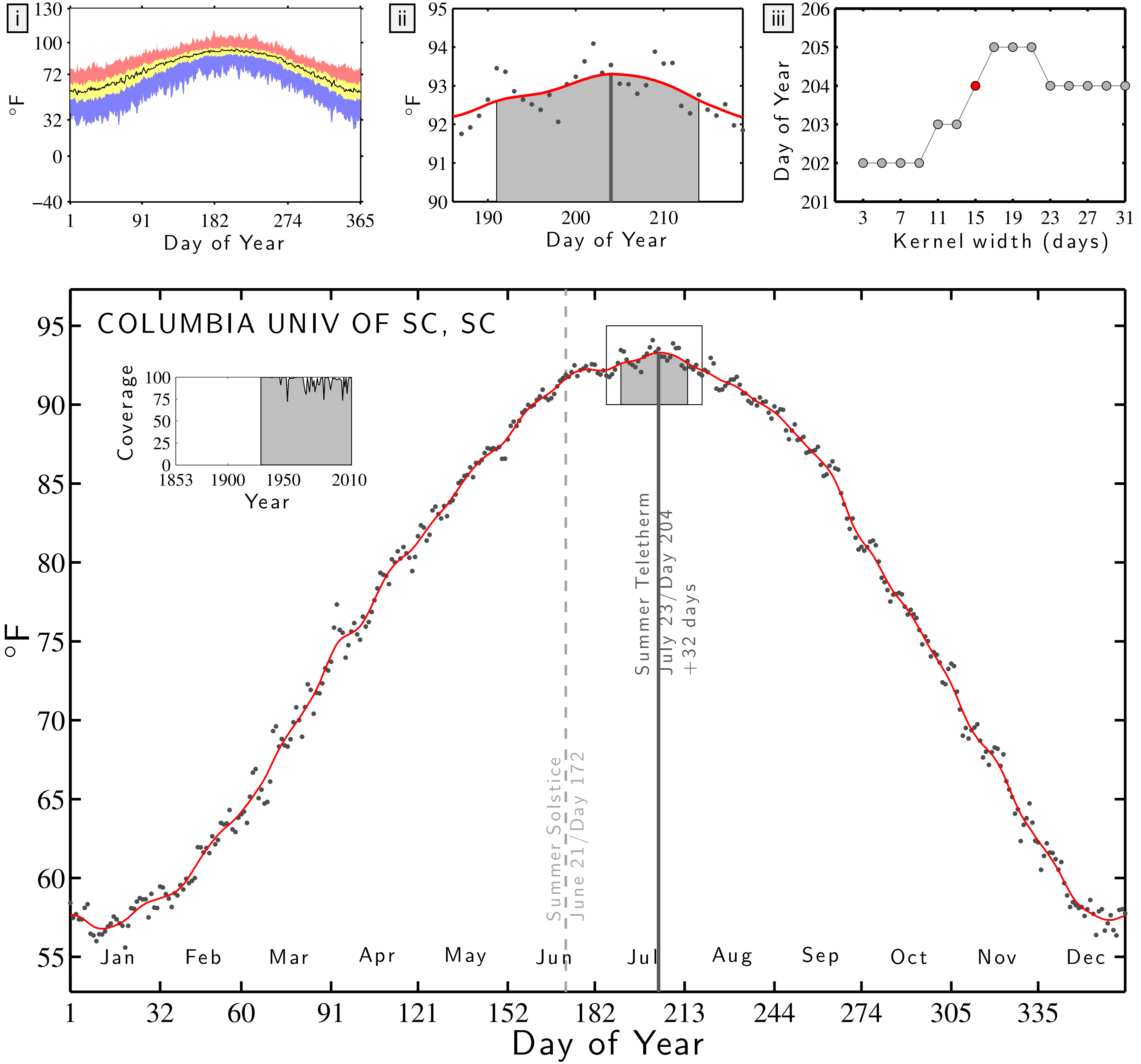

i
ii
iii
°F
Day of Year
Kernel width (days)
Day of Year
COLUMBIA UNIV OF SC, SC
Coverage
Year
Summer Solstice
June 21 / Day 172
Summer Teletherm
July 23 / Day 204
+32 days
Jan
Feb
Mar
Apr
May
Jun
Jul
Aug
Sep
Oct
Nov
Dec

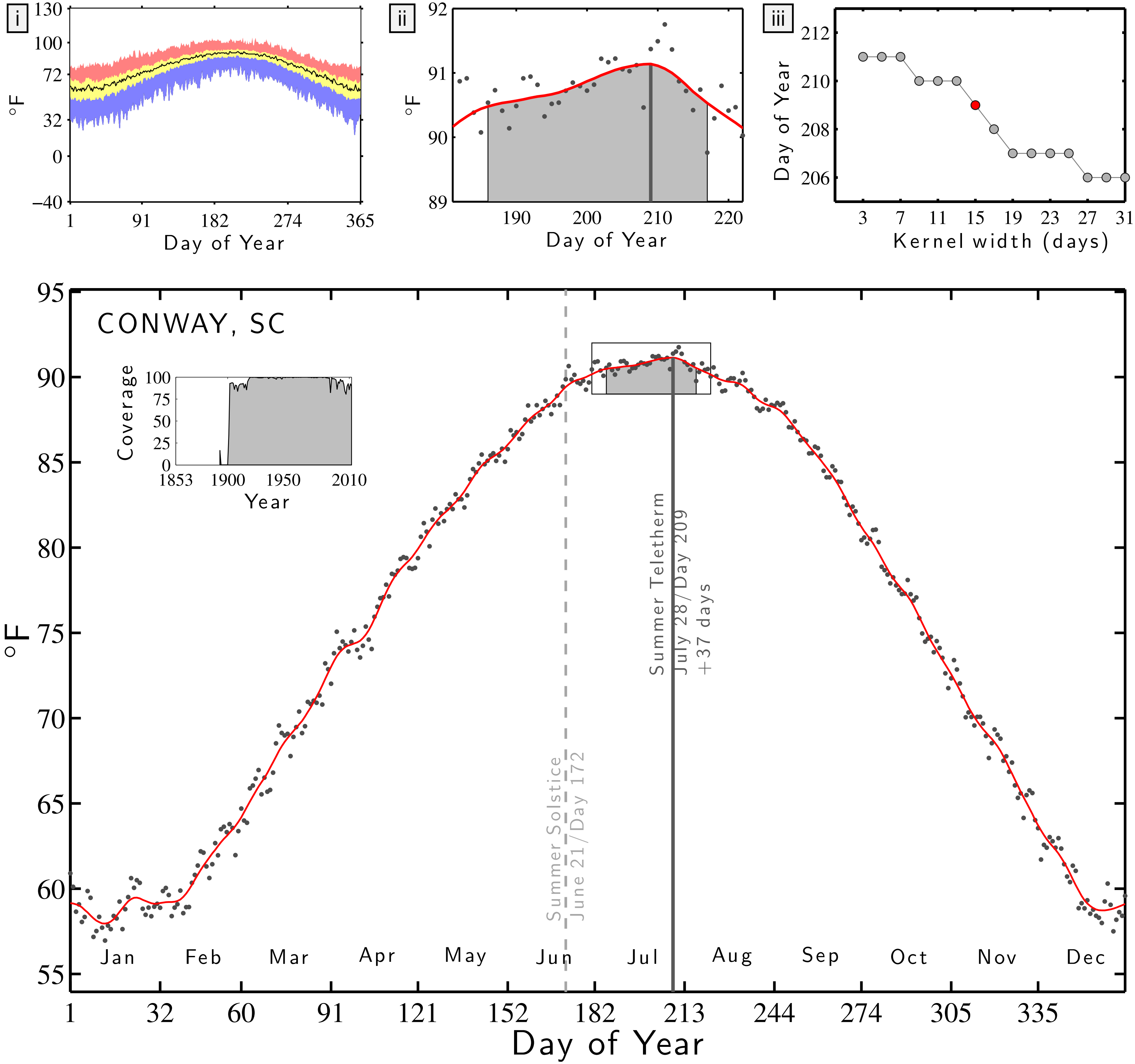

i
°F
Day of Year
ii
°F
Day of Year
iii
Day of Year
Kernel width (days)
CONWAY, SC
Coverage
Year
Summer Solstice
June 21/Day 172
Summer Teletherm
July 28/Day 209
+37 days
Jan
Feb
Mar
Apr
May
Jun
Jul
Aug
Sep
Oct
Nov
Dec
°F
Day of Year

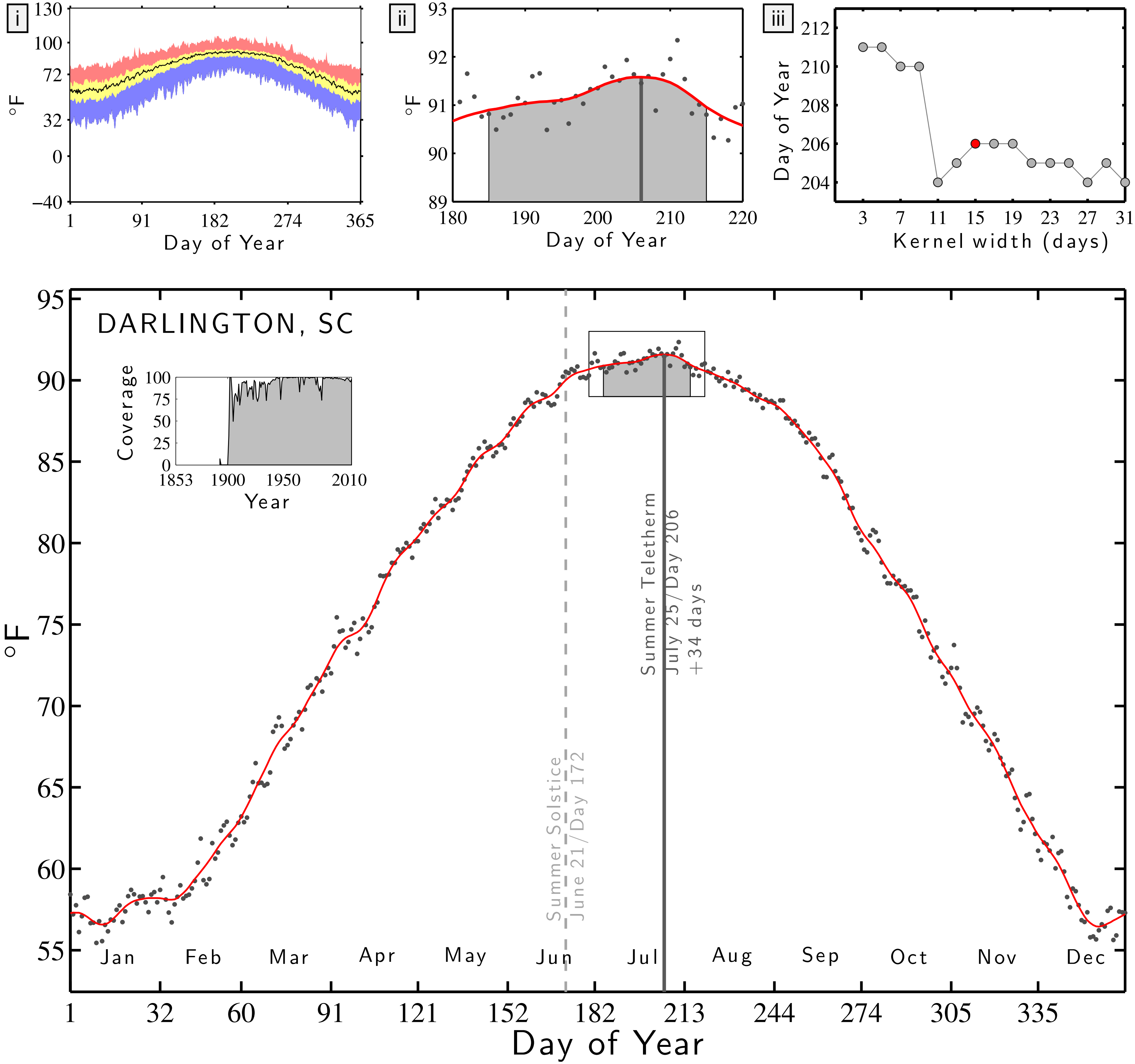

i
°F
Day of Year
ii
°F
Day of Year
iii
Day of Year
Kernel width (days)
DARLINGTON, SC
Coverage
Year
Summer Solstice
June 21/Day 172
Summer Teletherm
July 25/Day 206
+34 days
Jan
Feb
Mar
Apr
May
Jun
Jul
Aug
Sep
Oct
Nov
Dec
°F
Day of Year

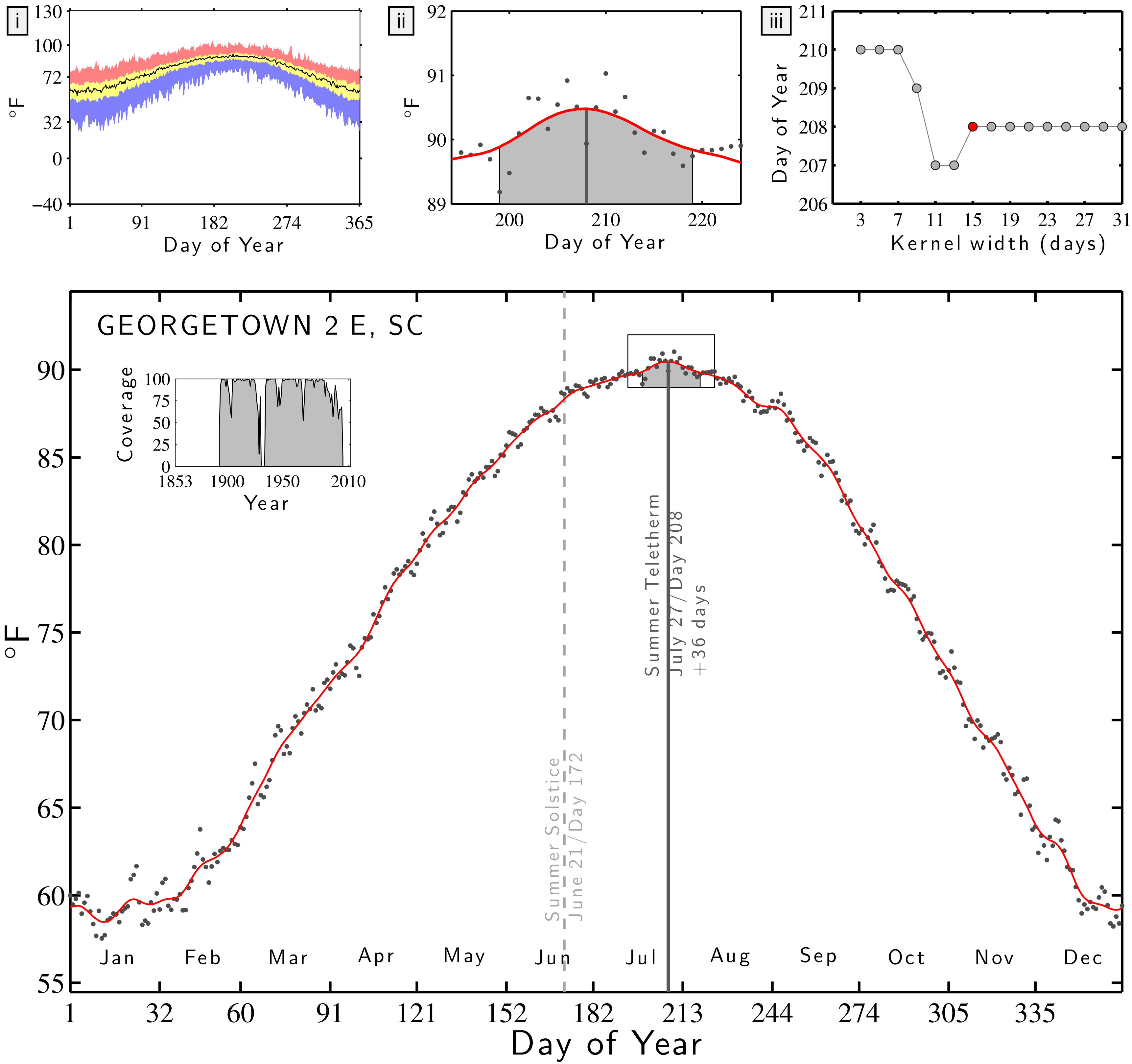

i
ii
iii
°F
Day of Year
Day of Year
Kernel width (days)
GEORGETOWN 2 E, SC
Coverage
Year
Summer Solstice
June 21/Day 172
Summer Teletherm
July 27/Day 208
+36 days
Jan
Feb
Mar
Apr
May
Jun
Jul
Aug
Sep
Oct
Nov
Dec
°F
Day of Year

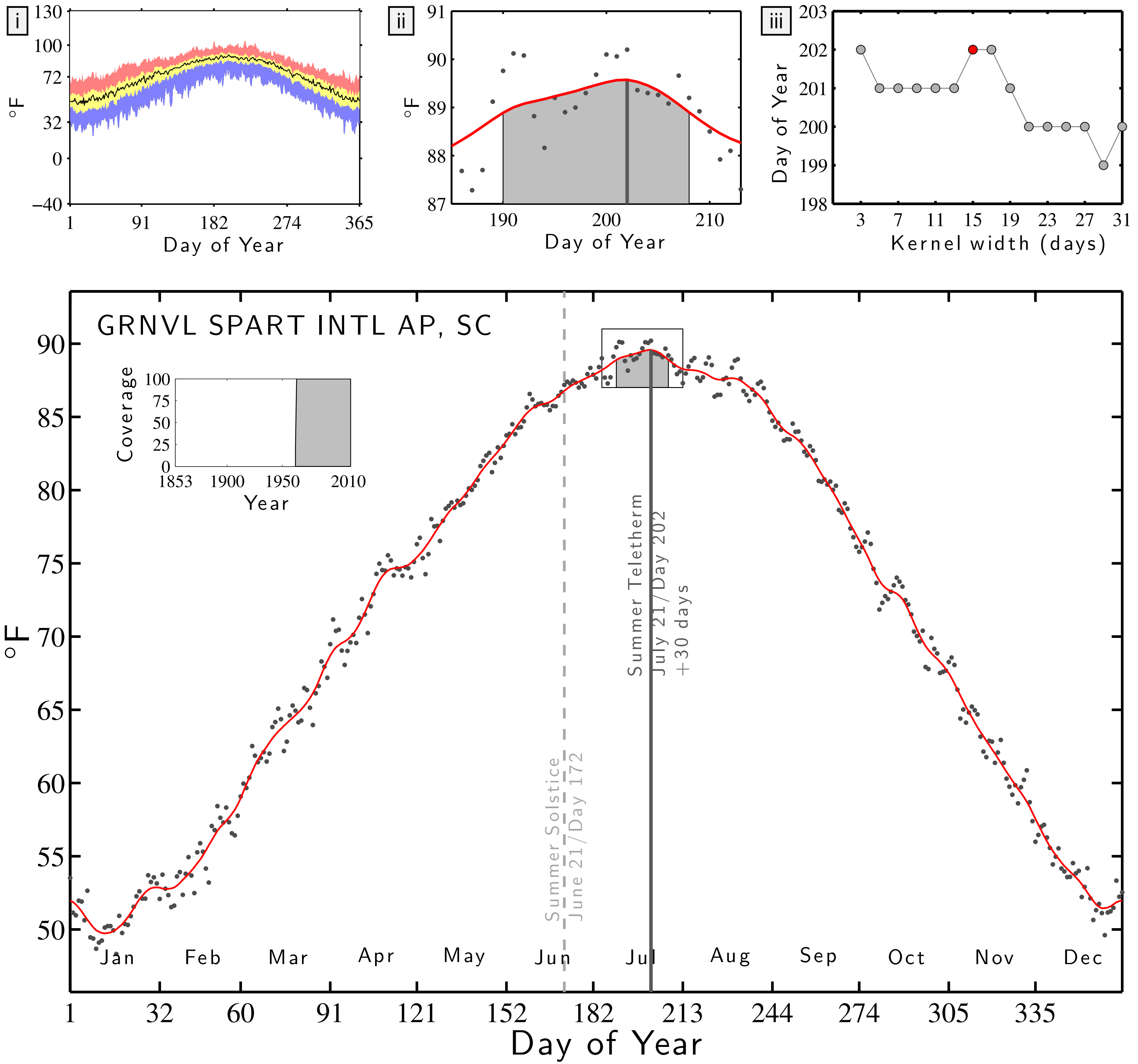

i
ii
iii
°F
Day of Year
Kernel width (days)
GRNVL SPART INTL AP, SC
Coverage
Year
Summer Solstice
June 21 / Day 172
Summer Teletherm
July 21 / Day 202
+30 days
Jan
Feb
Mar
Apr
May
Jun
Jul
Aug
Sep
Oct
Nov
Dec

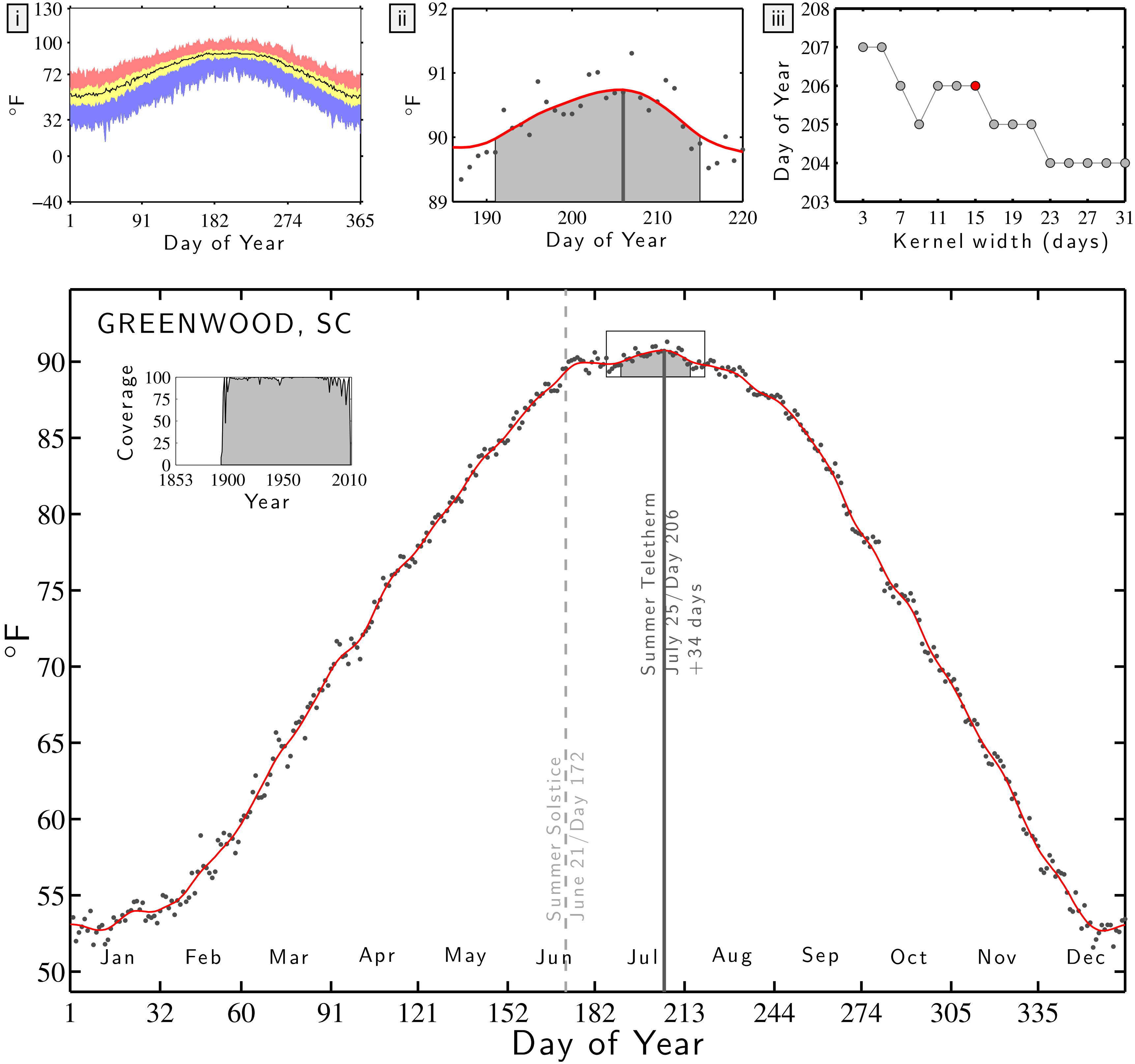

i
ii
iii
Day of Year
°F
Day of Year
Kernel width (days)
GREENWOOD, SC
Coverage
Year
Summer Solstice
June 21/Day 172
Summer Teletherm
July 25/Day 206
+34 days
Jan
Feb
Mar
Apr
May
Jun
Jul
Aug
Sep
Oct
Nov
Dec

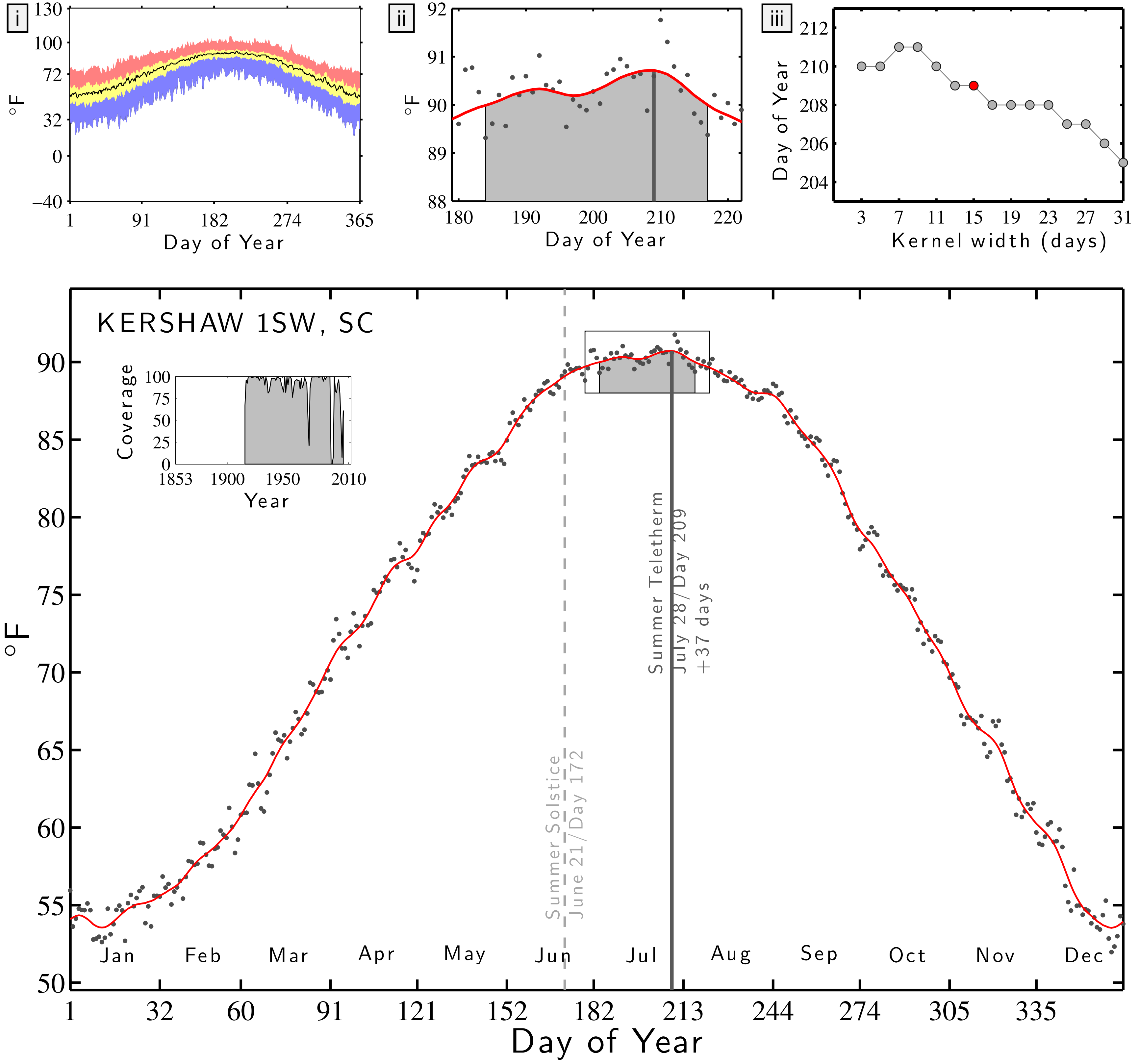

i
ii
iii
°F
Day of Year
Kernel width (days)
KERSHAW 1SW, SC
Coverage
Year
Summer Solstice
June 21/Day 172
Summer Teletherm
July 28/Day 209
+37 days
Jan
Feb
Mar
Apr
May
Jun
Jul
Aug
Sep
Oct
Nov
Dec

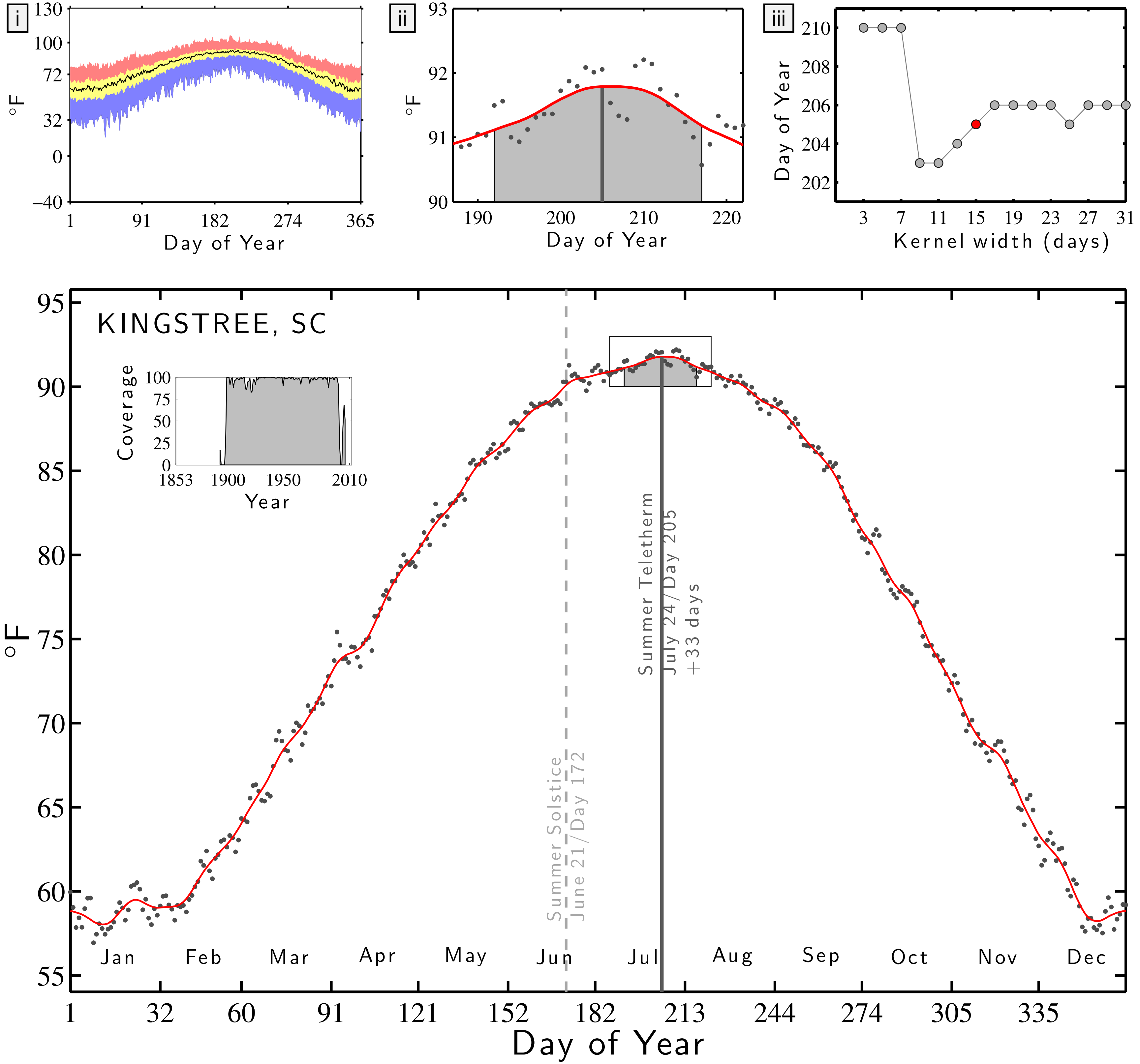

i
°F
Day of Year
ii
°F
Day of Year
iii
Day of Year
Kernel width (days)
KINGSTREE, SC
Coverage
Year
Summer Solstice
June 21/Day 172
Summer Teletherm
July 24/Day 205
+33 days
Jan
Feb
Mar
Apr
May
Jun
Jul
Aug
Sep
Oct
Nov
Dec
°F
Day of Year

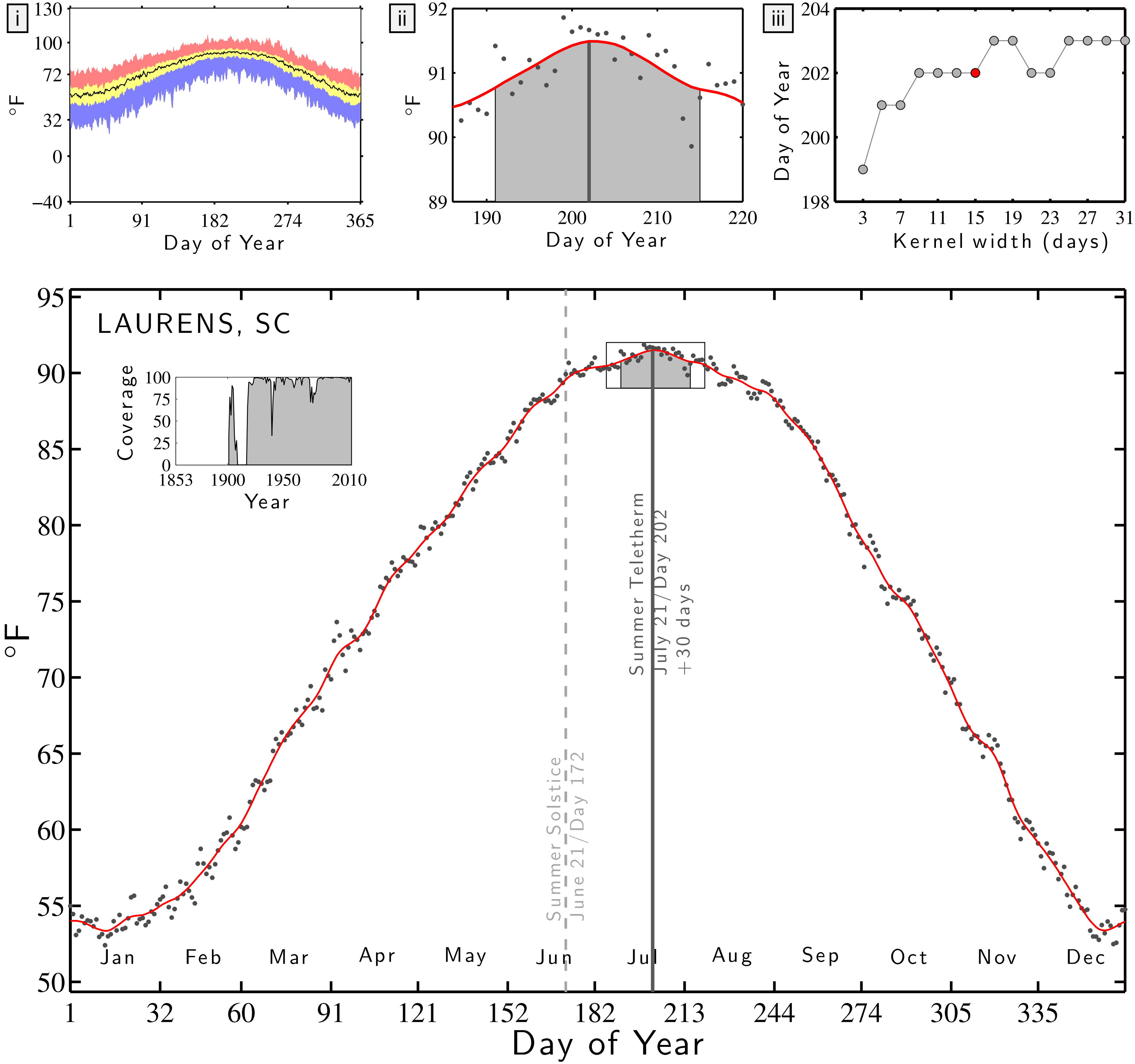

i
ii
iii
°F
Day of Year
Kernel width (days)
LAURENS, SC
Coverage
Year
Summer Solstice
June 21 / Day 172
Summer Teletherm
July 21 / Day 202
+30 days
Jan
Feb
Mar
Apr
May
Jun
Jul
Aug
Sep
Oct
Nov
Dec

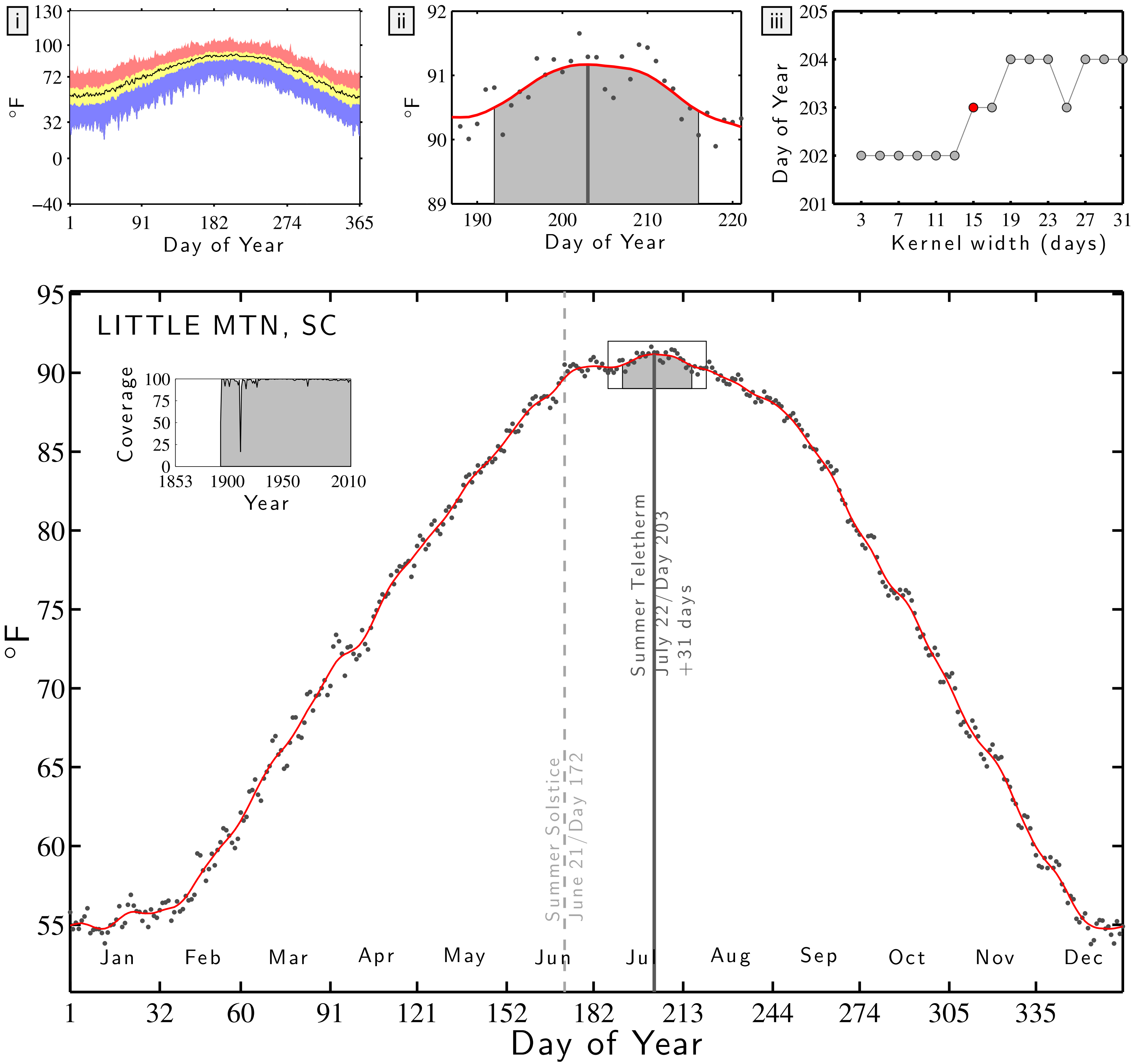

i
°F
Day of Year
ii
°F
Day of Year
iii
Day of Year
Kernel width (days)
LITTLE MTN, SC
Coverage
Year
Summer Solstice
June 21/Day 172
Summer Teletherm
July 22/Day 203
+31 days
Jan
Feb
Mar
Apr
May
Jun
Jul
Aug
Sep
Oct
Nov
Dec
°F
Day of Year

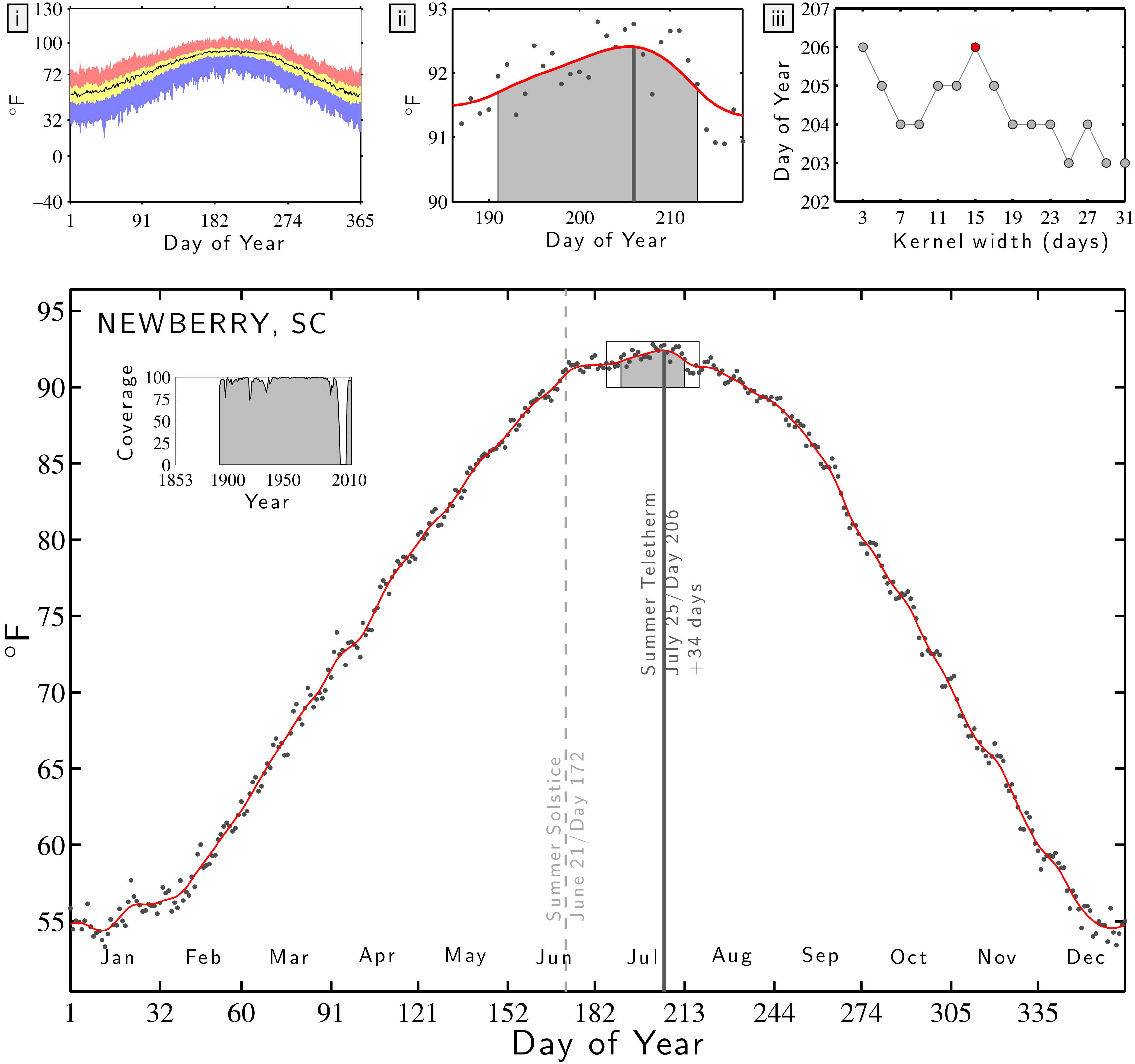
i
°F
Day of Year
ii
°F
Day of Year
iii
Day of Year
Kernel width (days)
NEWBERRY, SC
Coverage
Year
Summer Solstice
June 21/Day 172
Summer Teletherm
July 25/Day 206
+34 days
Jan
Feb
Mar
Apr
May
Jun
Jul
Aug
Sep
Oct
Nov
Dec
°F
Day of Year

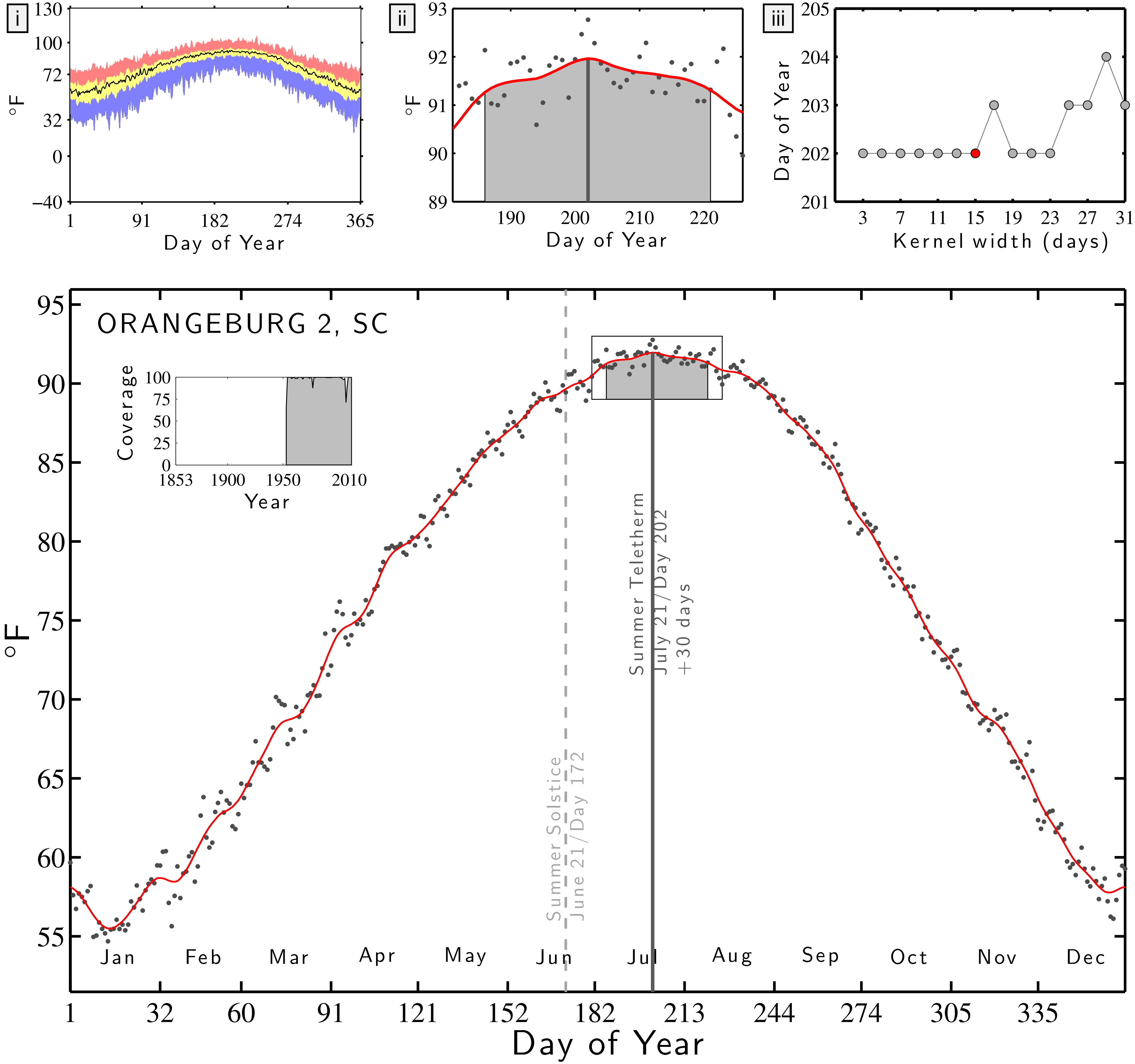

i
°F
130
100
72
32
0
−40
1
91
182
274
365
Day of Year
ii
93
92
91
90
89
190
200
210
220
Day of Year
iii
205
204
203
202
201
Day of Year
3
7
11
15
19
23
27
31
Kernel width (days)
ORANGEBURG 2, SC
Coverage
100
75
50
25
0
1853
1900
1950
2010
Year
95
90
85
80
75
70
65
60
55
Summer Solstice
June 21 / Day 172
Summer Teletherm
July 21 / Day 202
+30 days
Jan
Feb
Mar
Apr
May
Jun
Jul
Aug
Sep
Oct
Nov
Dec
1
32
60
91
121
152
182
213
244
274
305
335
Day of Year

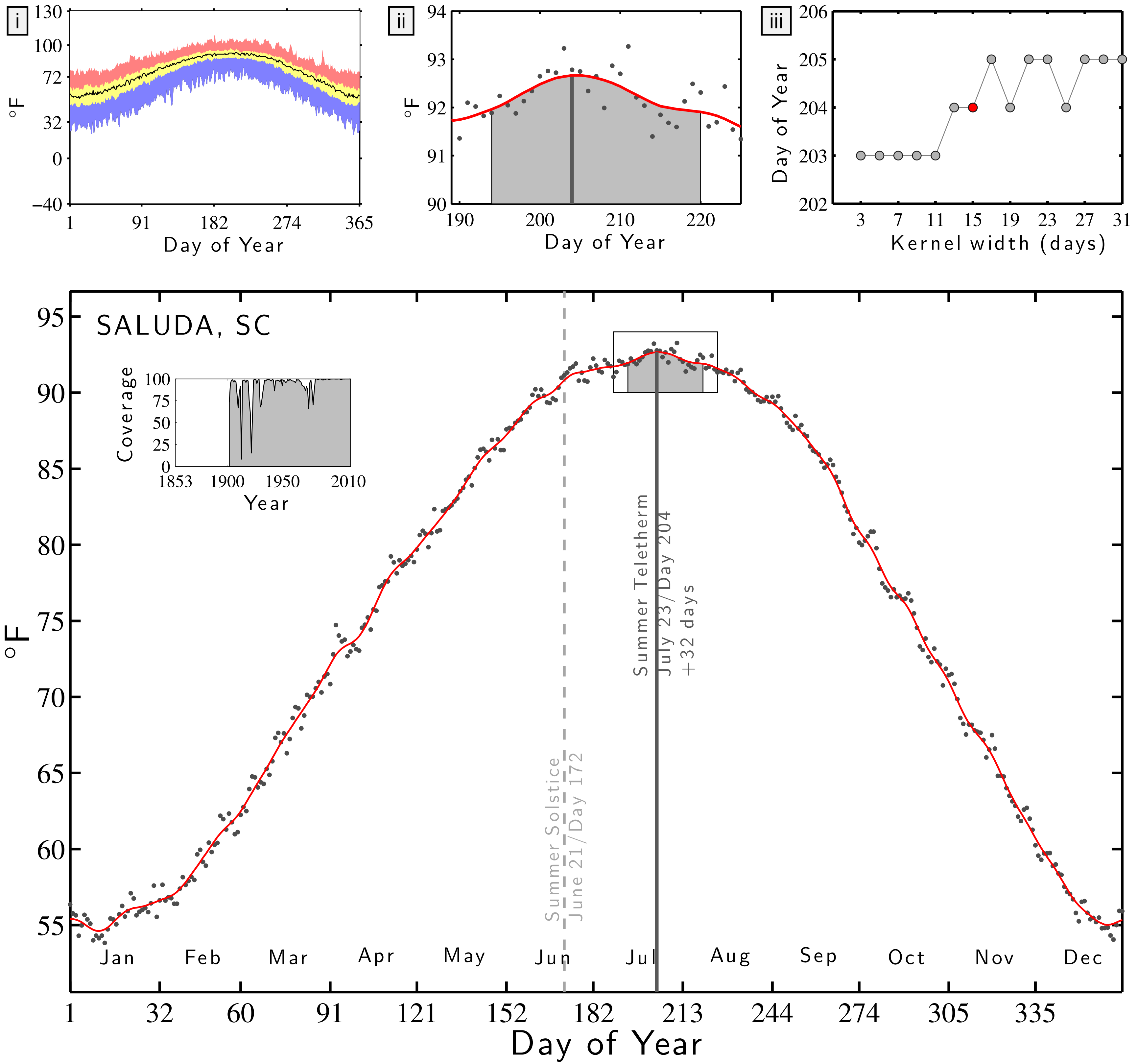

i
°F
Day of Year
ii
°F
Day of Year
iii
Day of Year
Kernel width (days)
SALUDA, SC
Coverage
Year
Summer Solstice
June 21/Day 172
Summer Teletherm
July 23/Day 204
+32 days
Jan
Feb
Mar
Apr
May
Jun
Jul
Aug
Sep
Oct
Nov
Dec
°F
Day of Year

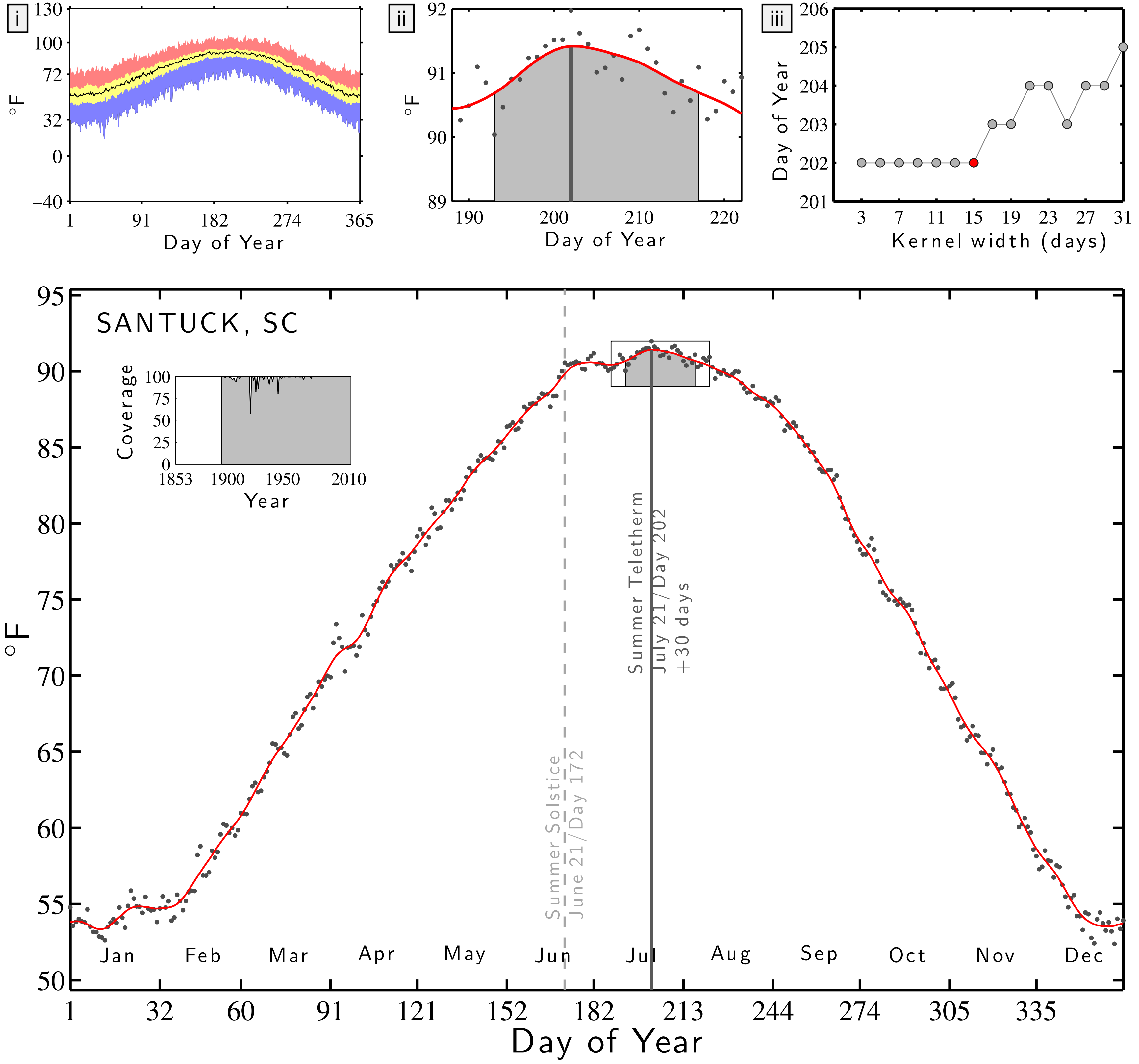

i
°F
Day of Year
ii
°F
Day of Year
iii
Day of Year
Kernel width (days)
SANTUCK, SC
Coverage
Year
Summer Solstice
June 21 / Day 172
Summer Teletherm
July 21 / Day 202
+30 days
Jan
Feb
Mar
Apr
May
Jun
Jul
Aug
Sep
Oct
Nov
Dec
°F
Day of Year

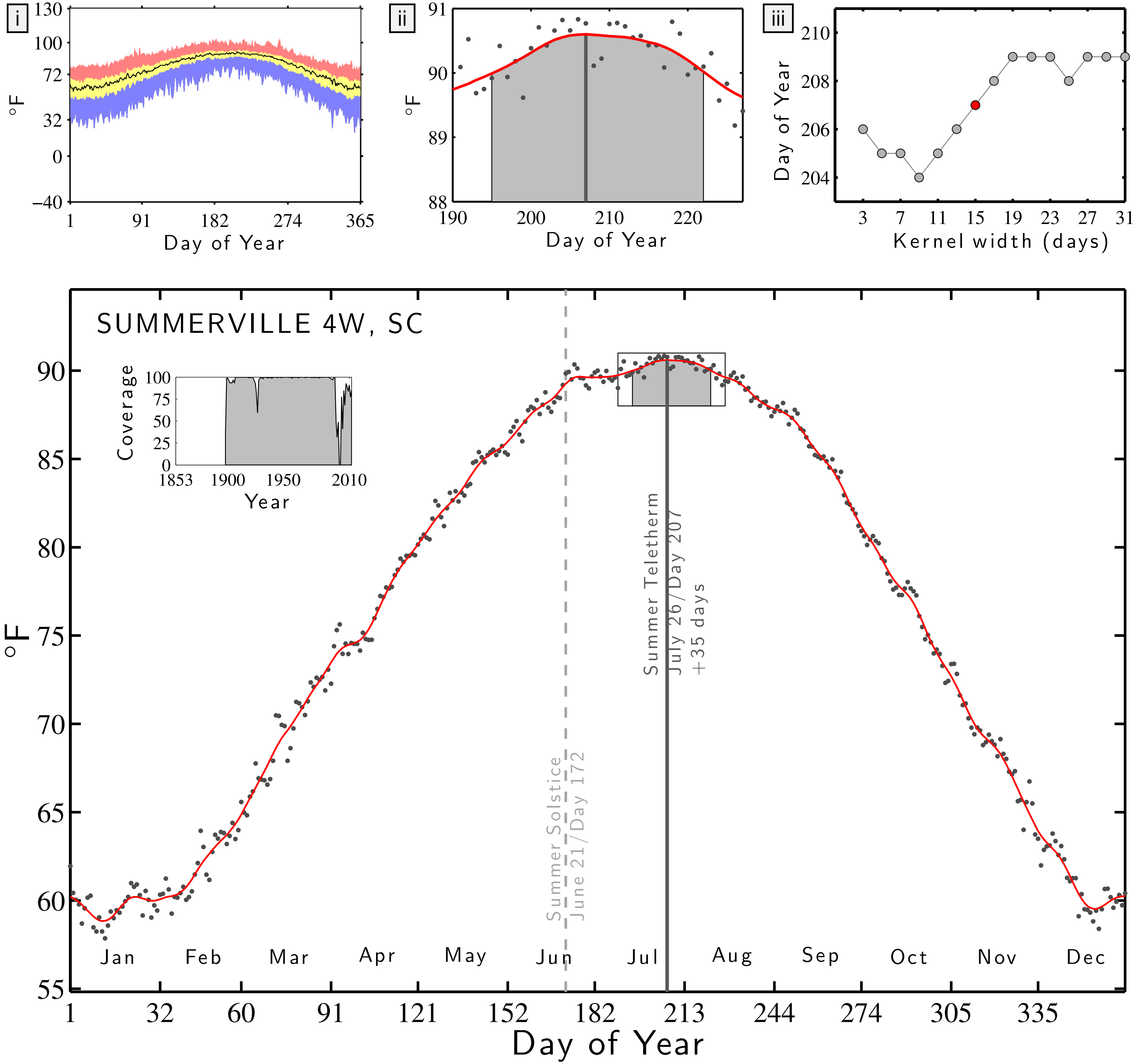

i
°F
Day of Year
ii
°F
Day of Year
iii
Day of Year
Kernel width (days)
SUMMERVILLE 4W, SC
Coverage
Year
Summer Solstice
June 21 / Day 172
Summer Teletherm
July 26 / Day 207
+35 days
Jan
Feb
Mar
Apr
May
Jun
Jul
Aug
Sep
Oct
Nov
Dec
°F
Day of Year

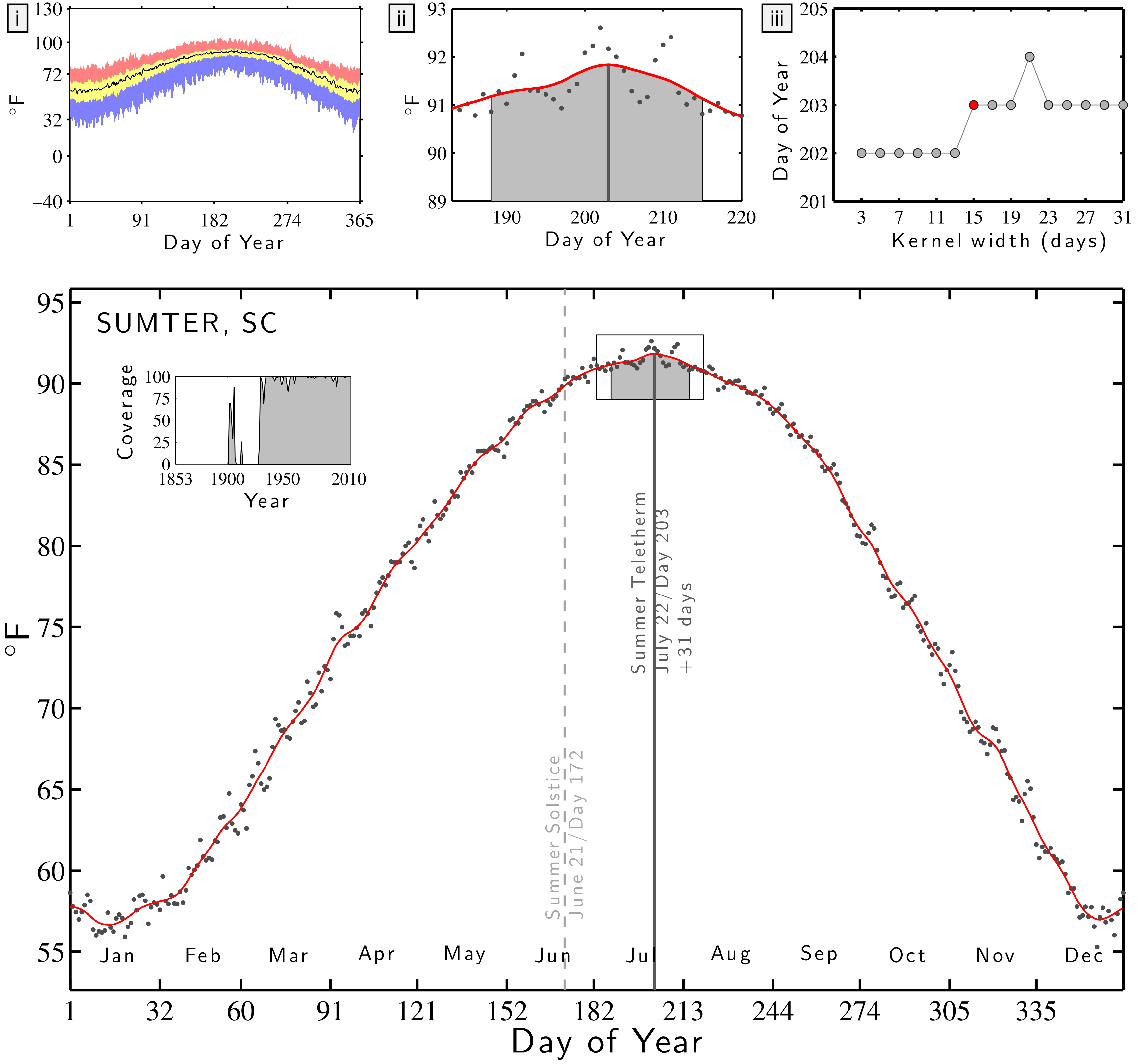

i
ii
iii
°F
Day of Year
Day of Year
Kernel width (days)
SUMTER, SC
Coverage
Year
Summer Solstice
June 21/Day 172
Summer Teletherm
July 22/Day 203
+31 days
Jan
Feb
Mar
Apr
May
Jun
Jul
Aug
Sep
Oct
Nov
Dec
Day of Year

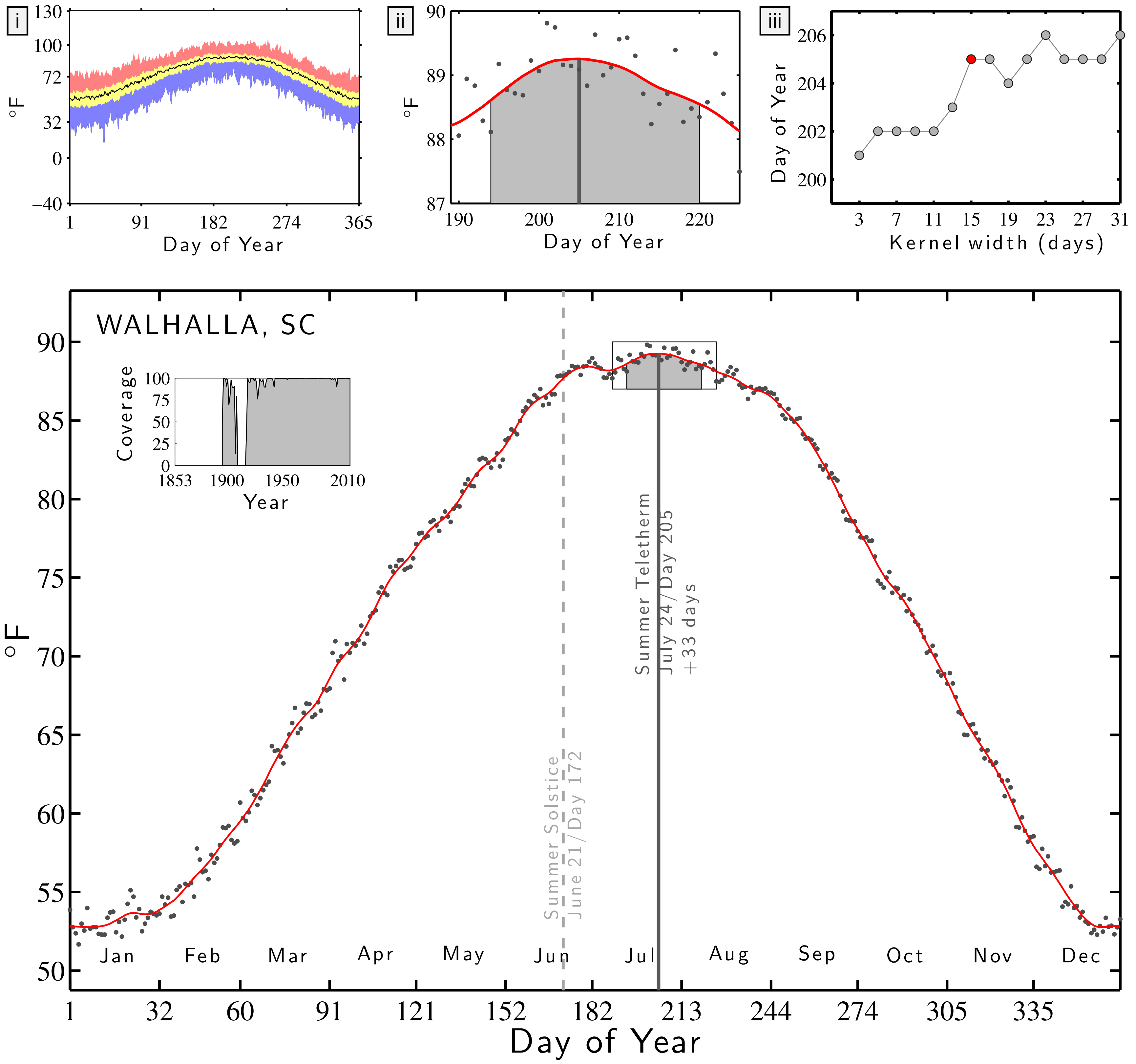

i
°F
Day of Year
ii
°F
Day of Year
iii
Day of Year
Kernel width (days)
WALHALLA, SC
Coverage
Year
Summer Solstice
June 21/Day 172
Summer Teletherm
July 24/Day 205
+33 days
Jan
Feb
Mar
Apr
May
Jun
Jul
Aug
Sep
Oct
Nov
Dec
°F
Day of Year

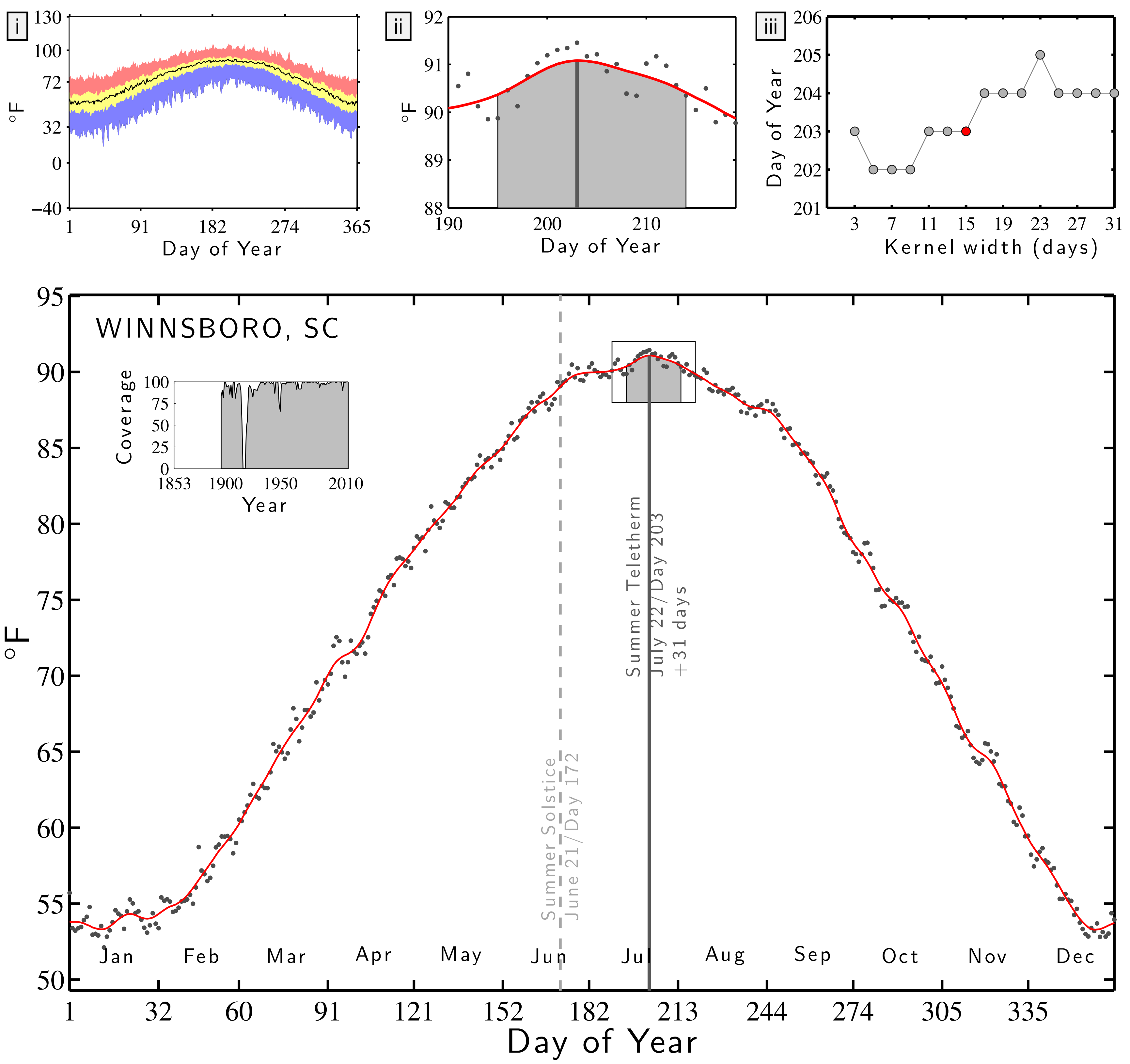

i
ii
iii
Day of Year
°F
Kernel width (days)
Day of Year
WINNSBORO, SC
Coverage
Year
1853
1900
1950
2010
Summer Solstice
June 21/Day 172
Summer Teletherm
July 22/Day 203
+31 days
Jan
Feb
Mar
Apr
May
Jun
Jul
Aug
Sep
Oct
Nov
Dec

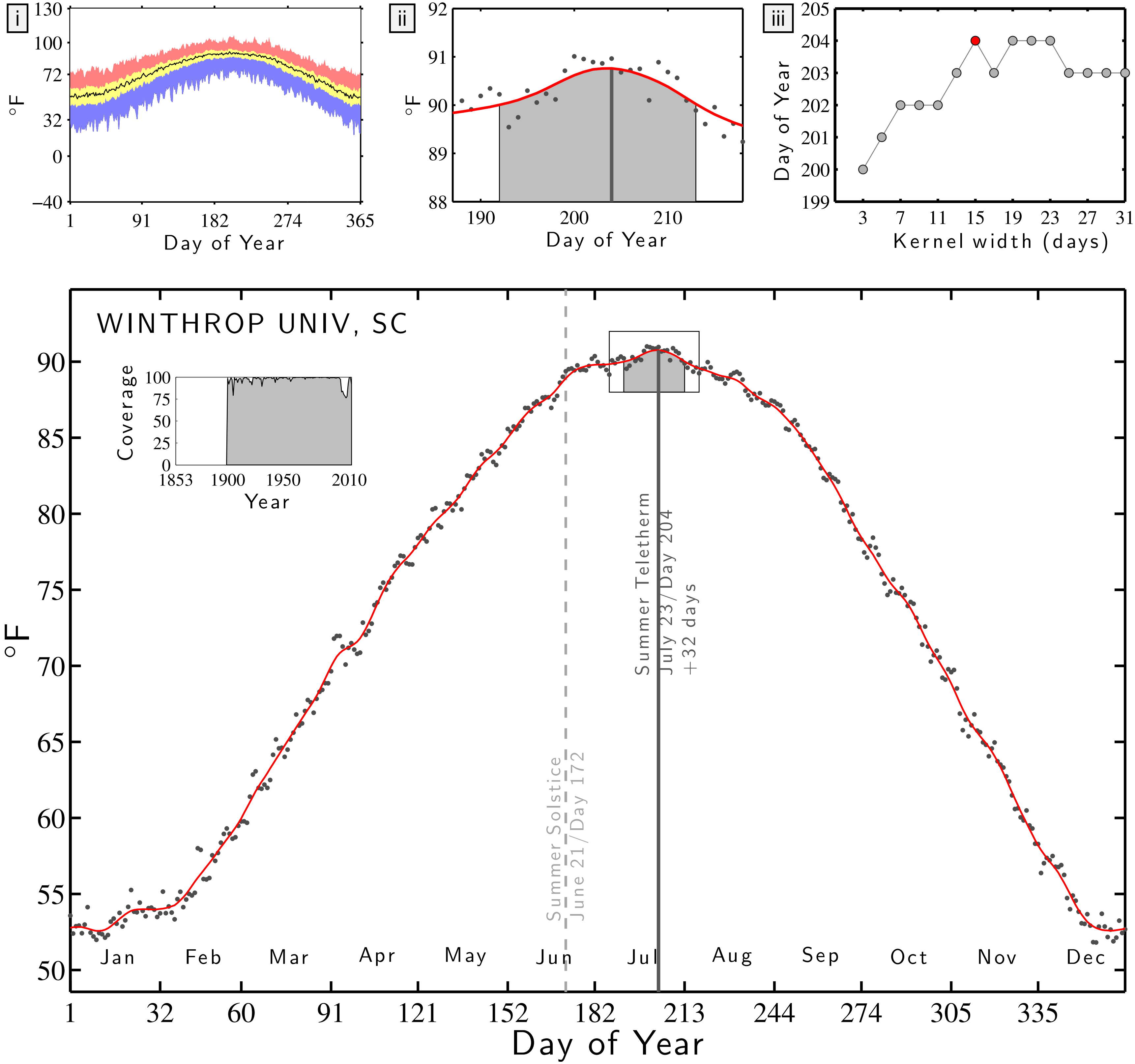

i
°F
Day of Year
ii
°F
Day of Year
iii
Day of Year
Kernel width (days)
WINTHROP UNIV, SC
Coverage
Year
Summer Solstice
June 21 / Day 172
Summer Teletherm
July 23 / Day 204
+32 days
Jan
Feb
Mar
Apr
May
Jun
Jul
Aug
Sep
Oct
Nov
Dec
°F
Day of Year

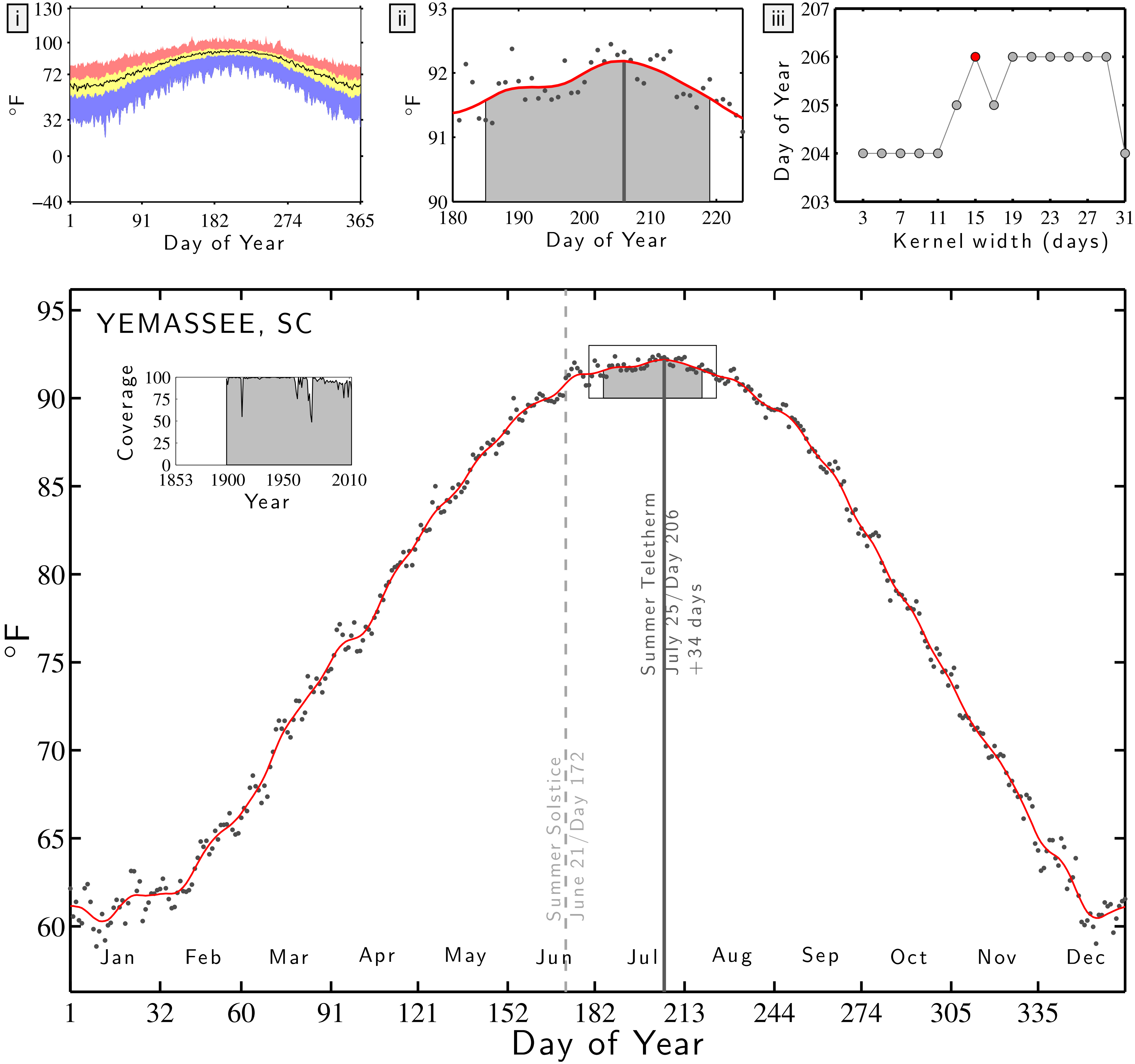

i
ii
iii
°F
Day of Year
Day of Year
Kernel width (days)
YEMASSEE, SC
Coverage
Year
Summer Solstice
June 21/Day 172
Summer Teletherm
July 25/Day 206
+34 days
Jan
Feb
Mar
Apr
May
Jun
Jul
Aug
Sep
Oct
Nov
Dec
Day of Year

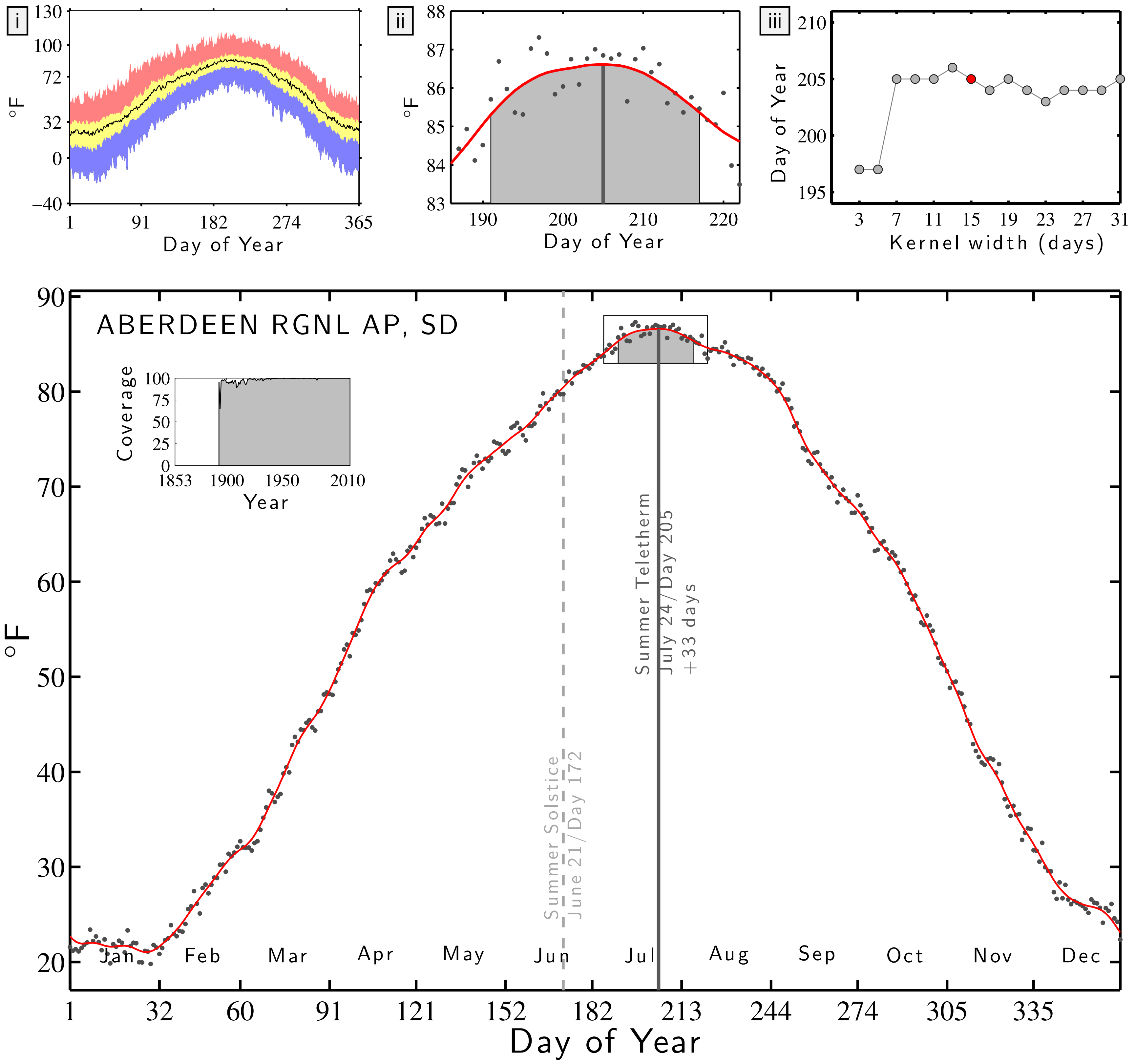
i
ii
iii
ABERDEEN RGNL AP, SD
Coverage
Year
Summer Solstice
June 21 / Day 172
Summer Teletherm
July 24 / Day 205
+33 days
Day of Year
Kernel width (days)
°F
Jan
Feb
Mar
Apr
May
Jun
Jul
Aug
Sep
Oct
Nov
Dec

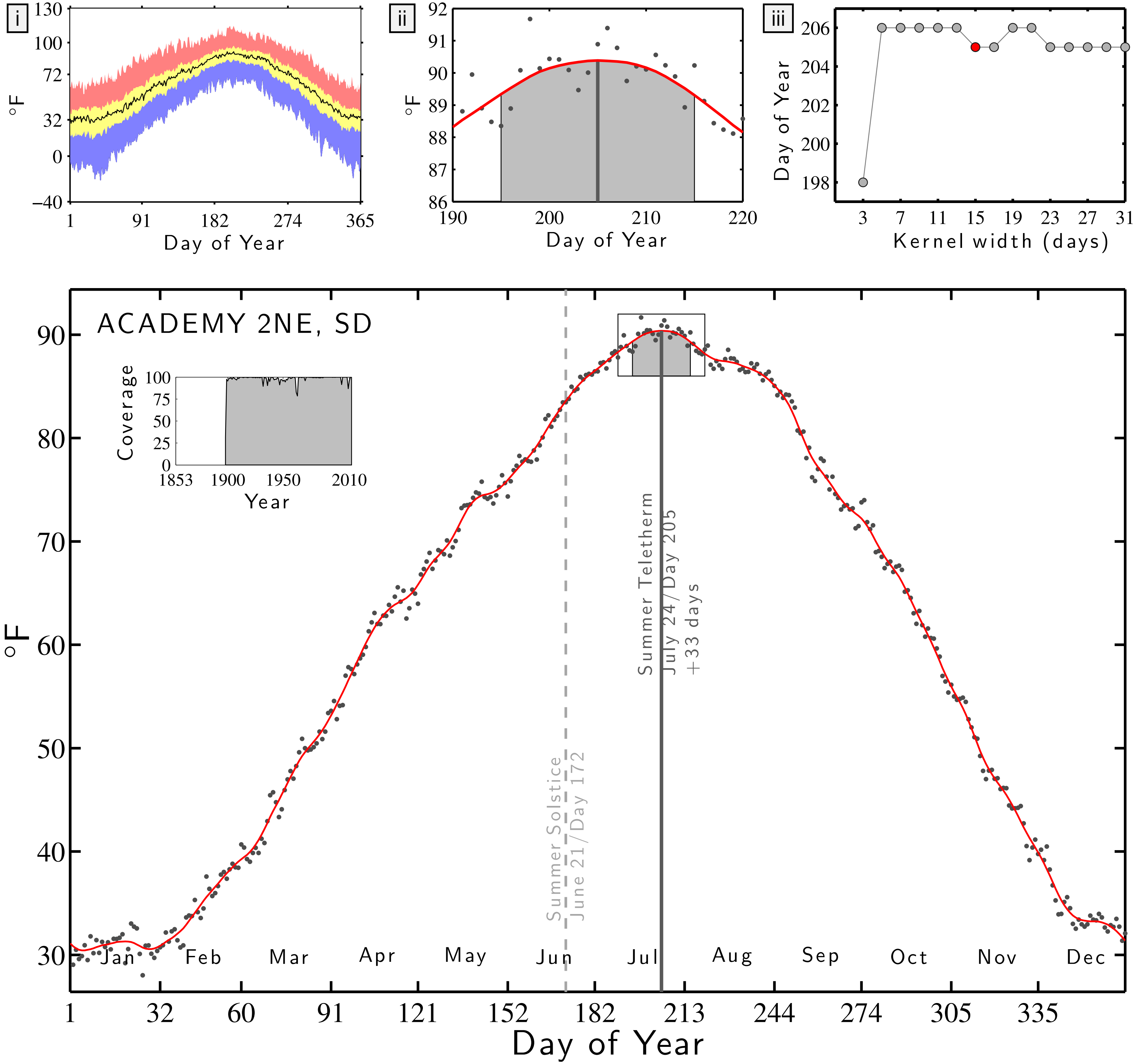

i
°F
Day of Year
ii
°F
Day of Year
iii
Day of Year
Kernel width (days)
ACADEMY 2NE, SD
Coverage
Year
Summer Solstice
June 21 / Day 172
Summer Teletherm
July 24 / Day 205
+33 days
Jan
Feb
Mar
Apr
May
Jun
Jul
Aug
Sep
Oct
Nov
Dec
°F
Day of Year

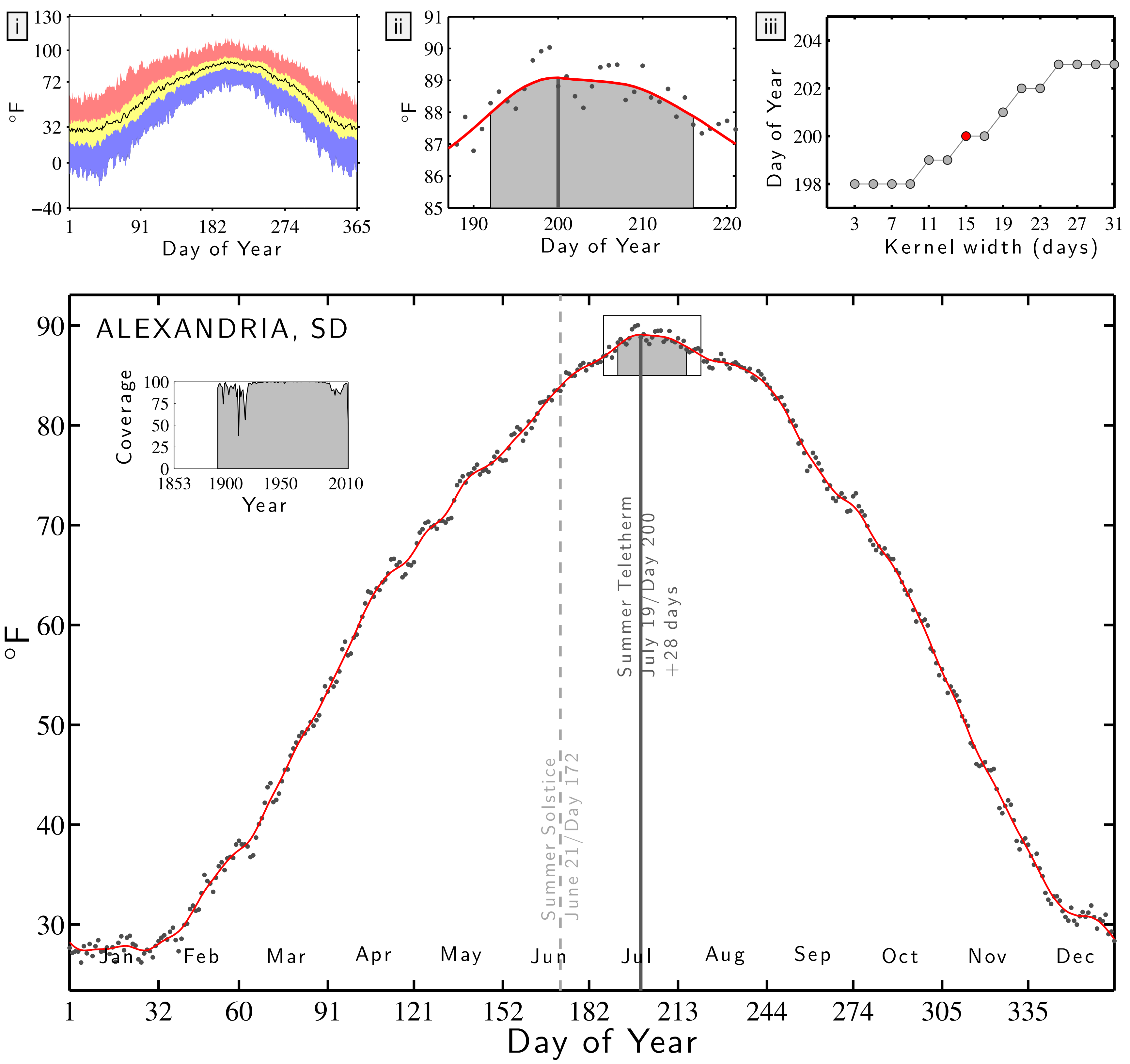

i
°F
Day of Year
ii
°F
Day of Year
iii
Day of Year
Kernel width (days)
ALEXANDRIA, SD
Coverage
Year
Summer Solstice
June 21/Day 172
Summer Teletherm
July 19/Day 200
+28 days
Jan
Feb
Mar
Apr
May
Jun
Jul
Aug
Sep
Oct
Nov
Dec
°F
Day of Year

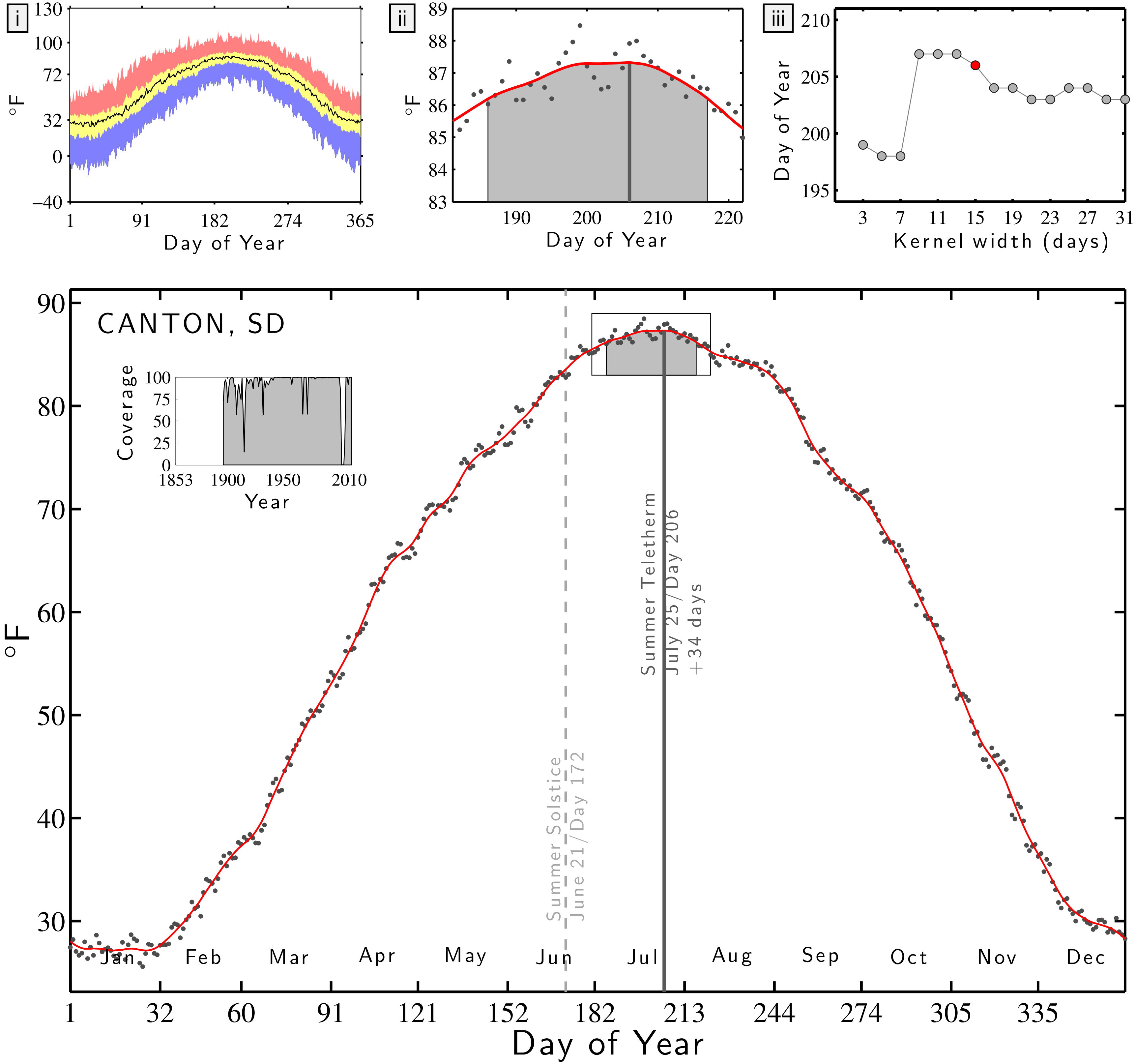

i
°F
Day of Year
ii
°F
Day of Year
iii
Day of Year
Kernel width (days)
CANTON, SD
Coverage
Year
Summer Solstice
June 21/Day 172
Summer Teletherm
July 25/Day 206
+34 days
Jan
Feb
Mar
Apr
May
Jun
Jul
Aug
Sep
Oct
Nov
Dec
°F
Day of Year

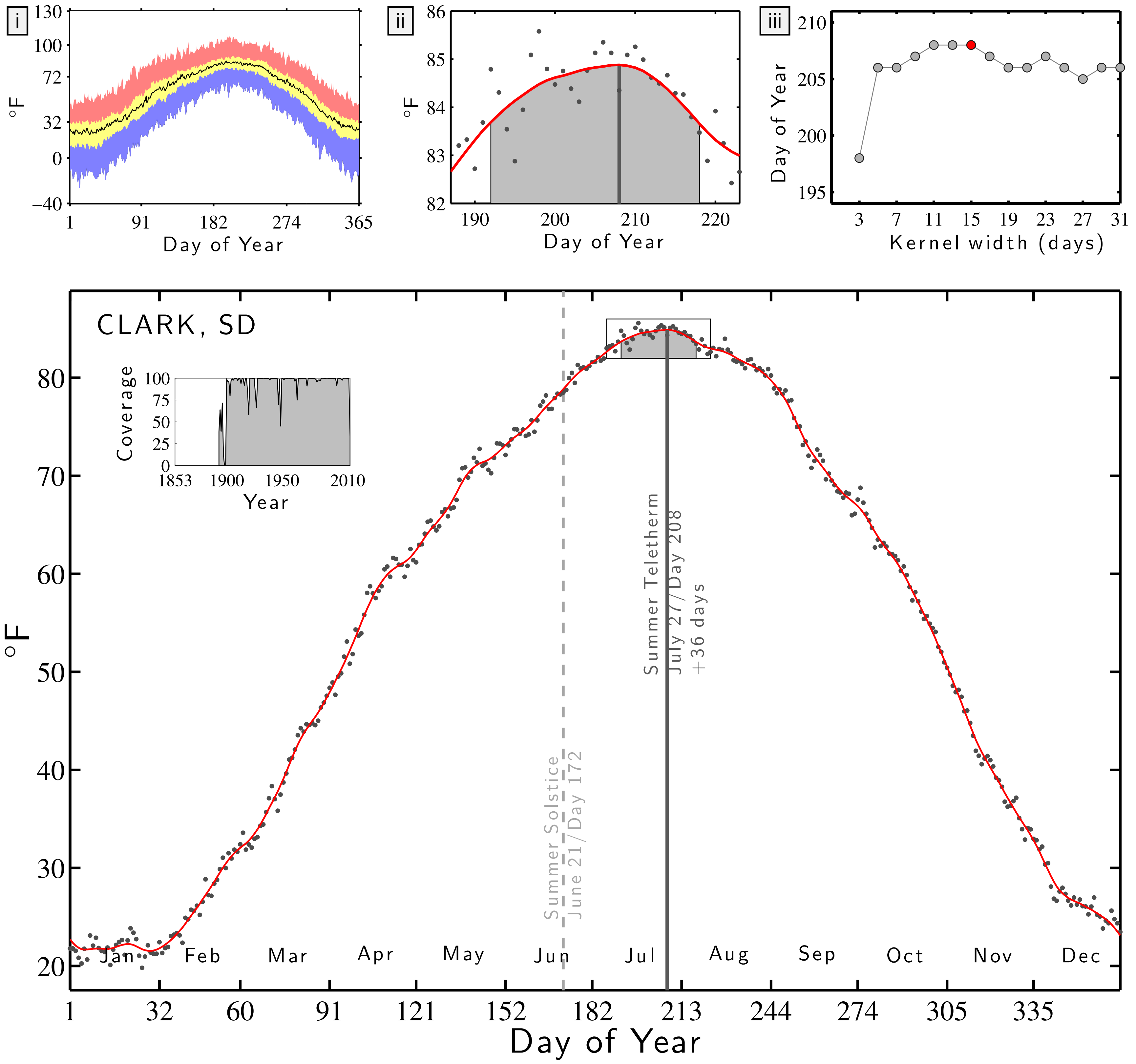

i
130
100
72
32
0
−40
°F
1
91
182
274
365
Day of Year
ii
86
85
84
83
82
°F
190
200
210
220
Day of Year
iii
210
205
200
195
Day of Year
3
7
11
15
19
23
27
31
Kernel width (days)
CLARK, SD
Coverage
100
75
50
25
0
1853
1900
1950
2010
Year
80
70
60
50
40
30
20
°F
Summer Solstice
June 21/Day 172
Summer Teletherm
July 27/Day 208
+36 days
Jan
Feb
Mar
Apr
May
Jun
Jul
Aug
Sep
Oct
Nov
Dec
1
32
60
91
121
152
182
213
244
274
305
335
Day of Year

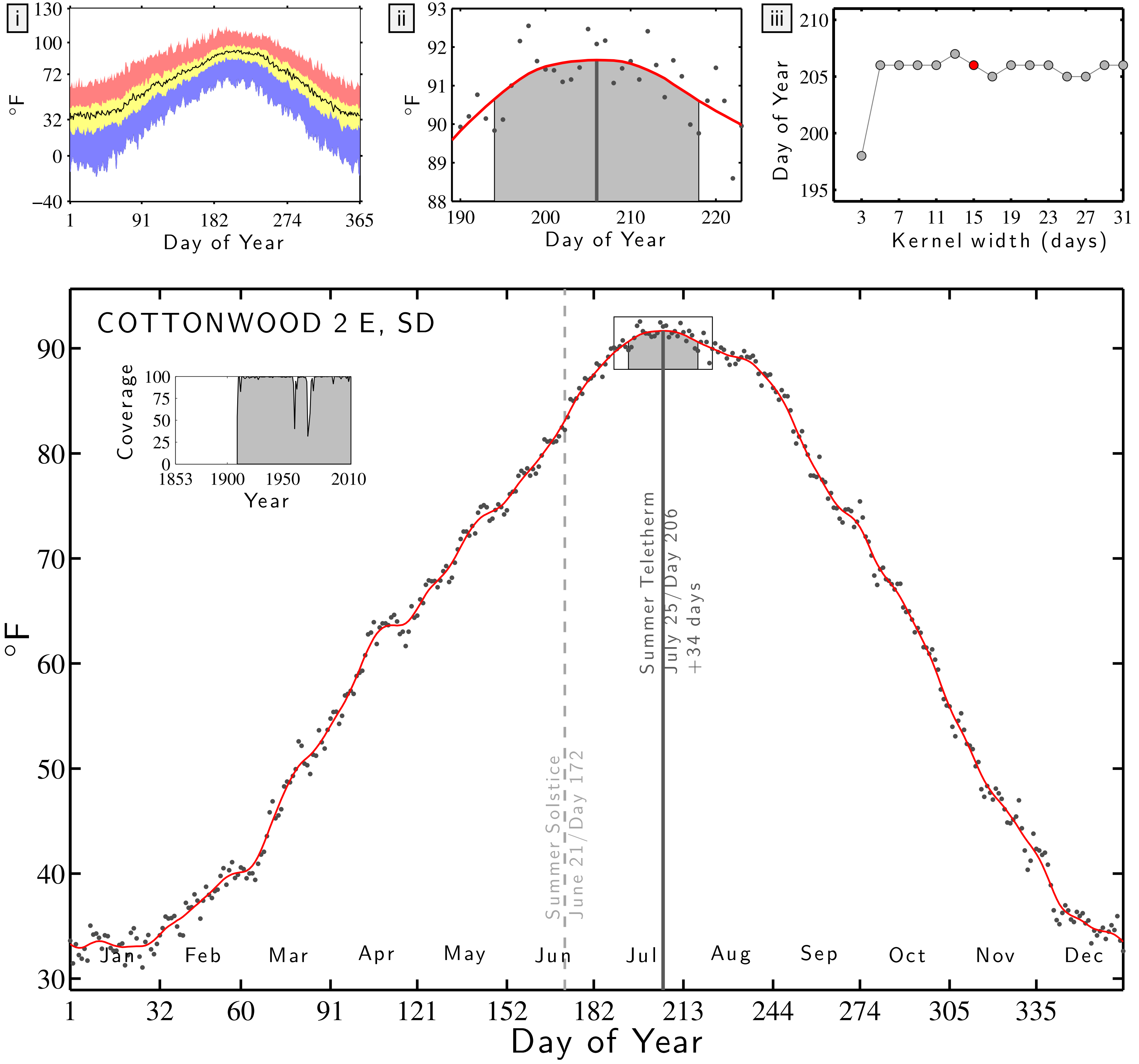
i
°F
Day of Year
ii
°F
Day of Year
iii
Day of Year
Kernel width (days)
COTTONWOOD 2 E, SD
Coverage
Year
Summer Solstice
June 21/Day 172
Summer Teletherm
July 25/Day 206
+34 days
Jan
Feb
Mar
Apr
May
Jun
Jul
Aug
Sep
Oct
Nov
Dec
°F
Day of Year

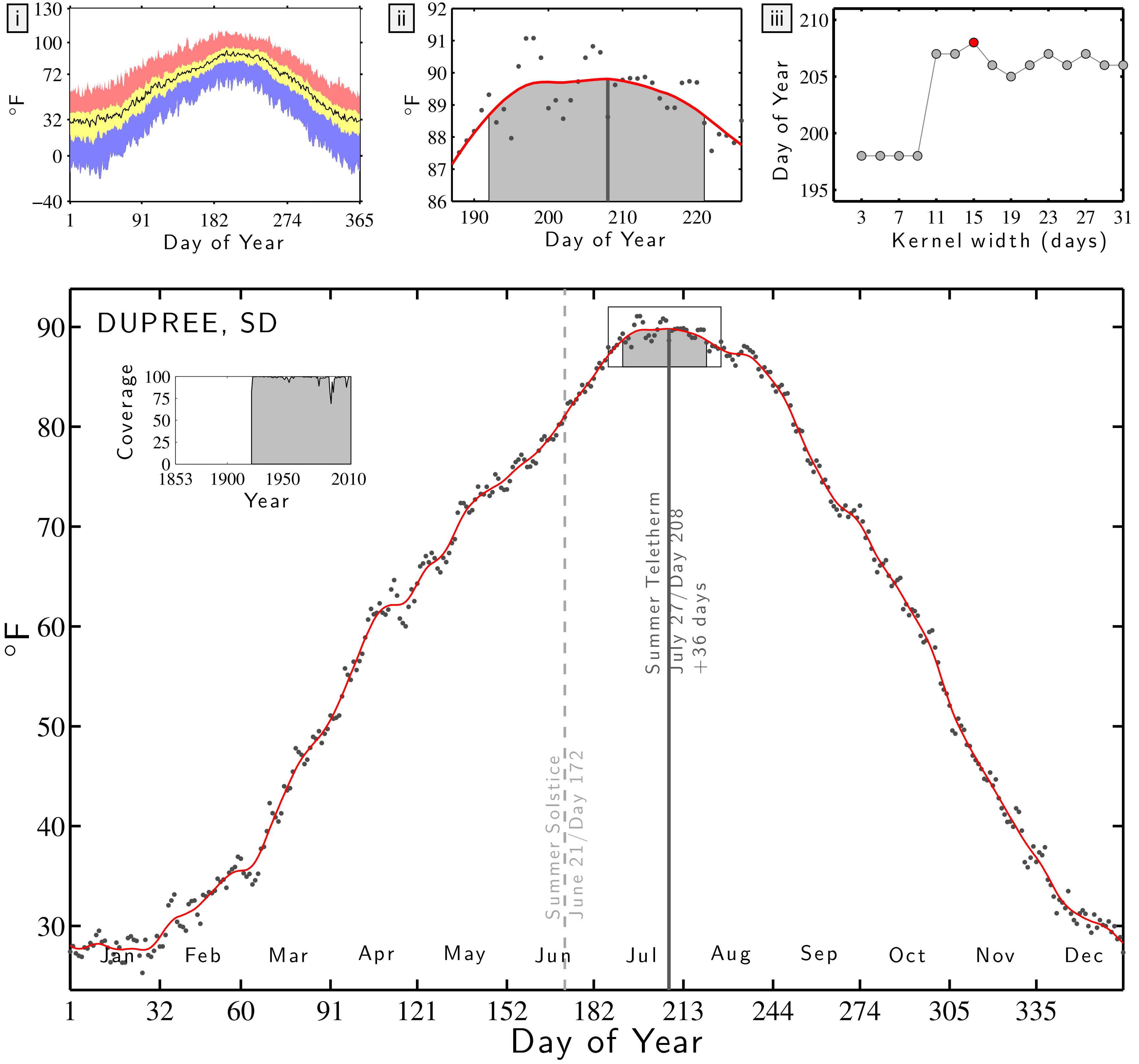

i
°F
Day of Year
ii
°F
Day of Year
iii
Day of Year
Kernel width (days)
DUPREE, SD
Coverage
Year
Summer Solstice
June 21 / Day 172
Summer Teletherm
July 27 / Day 208
+36 days
Jan
Feb
Mar
Apr
May
Jun
Jul
Aug
Sep
Oct
Nov
Dec
°F
Day of Year

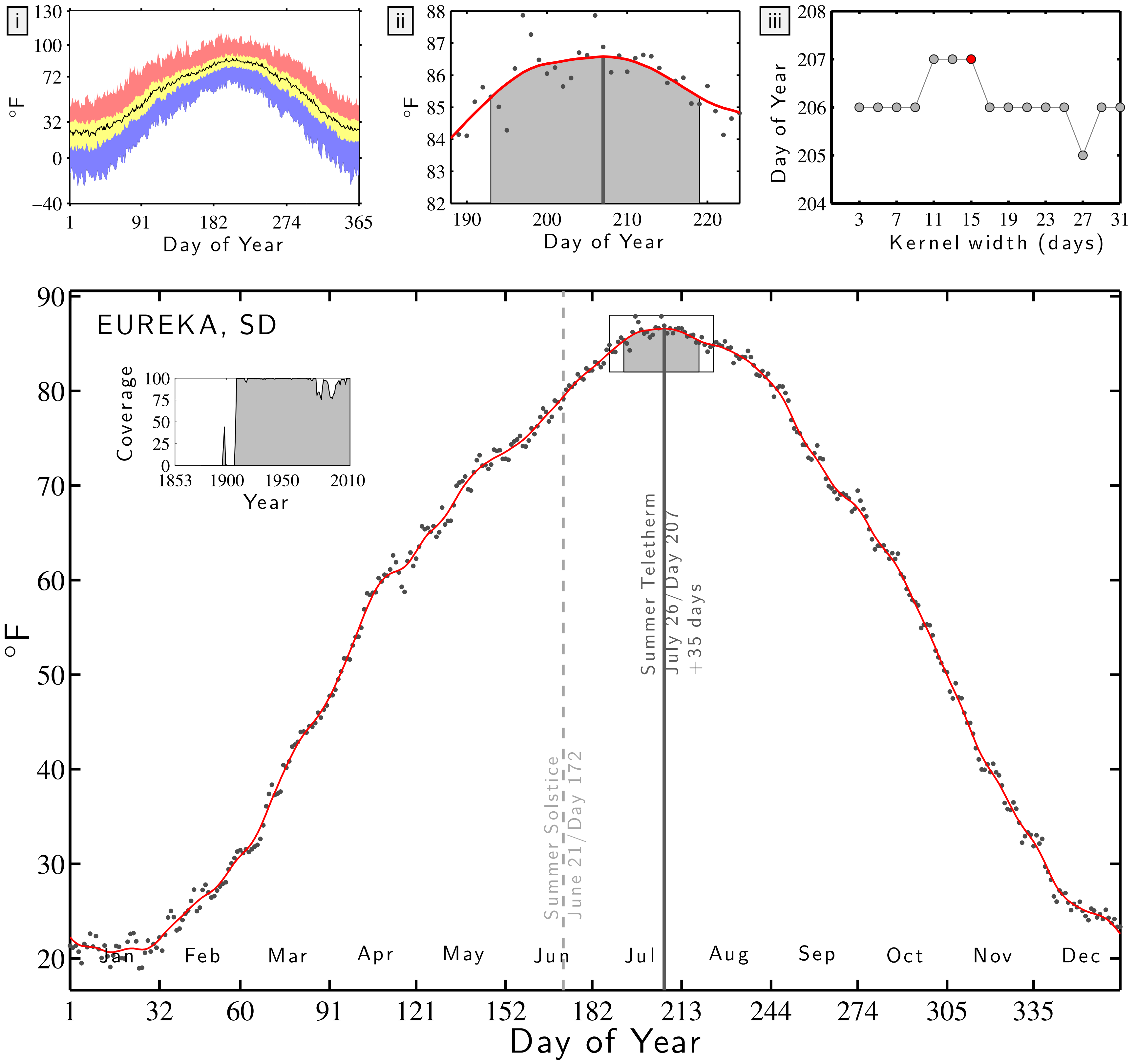

i
ii
iii
°F
Day of Year
Day of Year
Kernel width (days)
EUREKA, SD
Coverage
Year
1853
1900
1950
2010
Summer Solstice
June 21 / Day 172
Summer Teletherm
July 26 / Day 207
+35 days
Jan
Feb
Mar
Apr
May
Jun
Jul
Aug
Sep
Oct
Nov
Dec

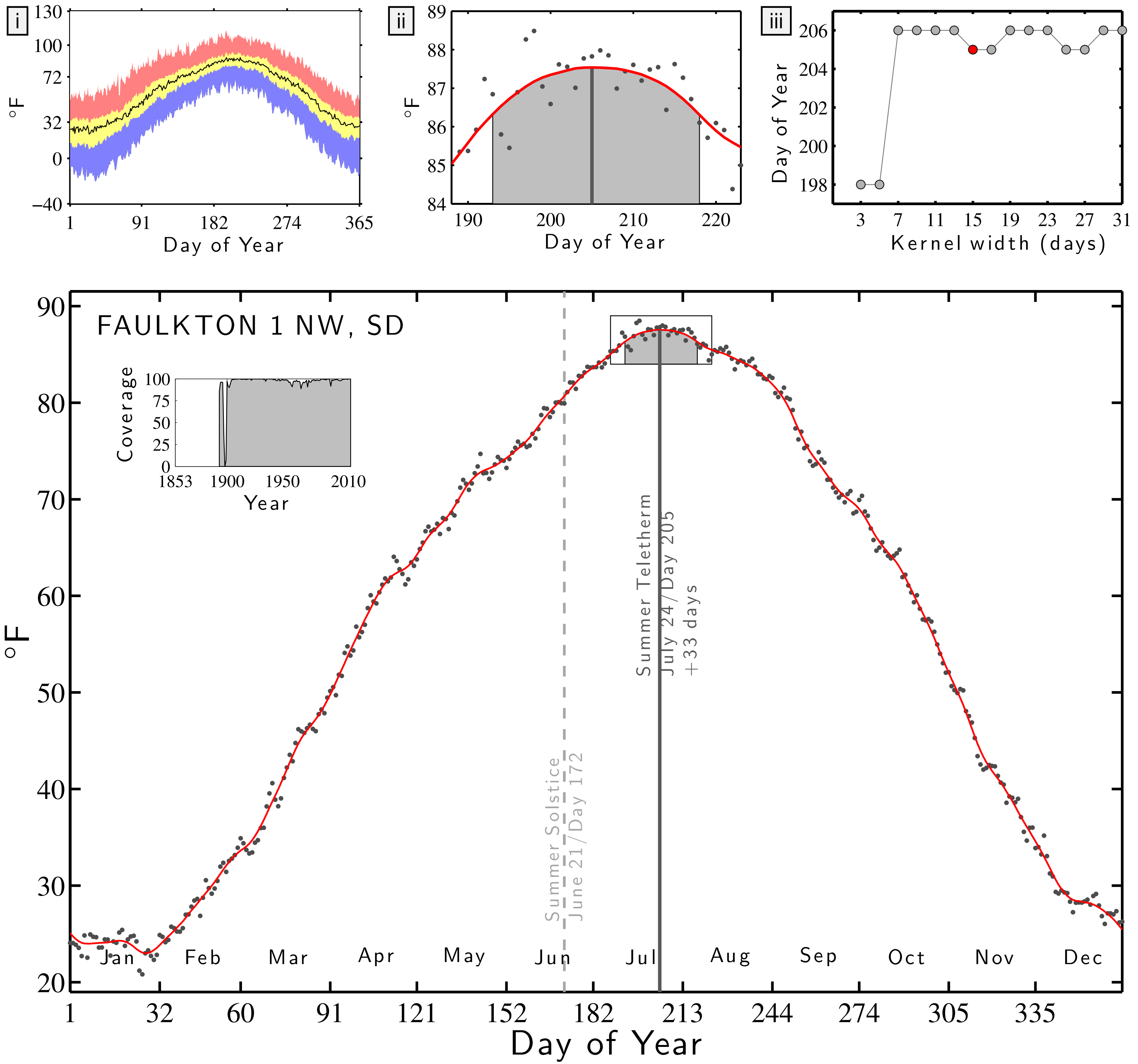

i
°F
Day of Year
ii
°F
Day of Year
iii
Day of Year
Kernel width (days)
FAULKTON 1 NW, SD
Coverage
Year
Summer Solstice
June 21 / Day 172
Summer Teletherm
July 24 / Day 205
+33 days
Jan
Feb
Mar
Apr
May
Jun
Jul
Aug
Sep
Oct
Nov
Dec
°F
Day of Year

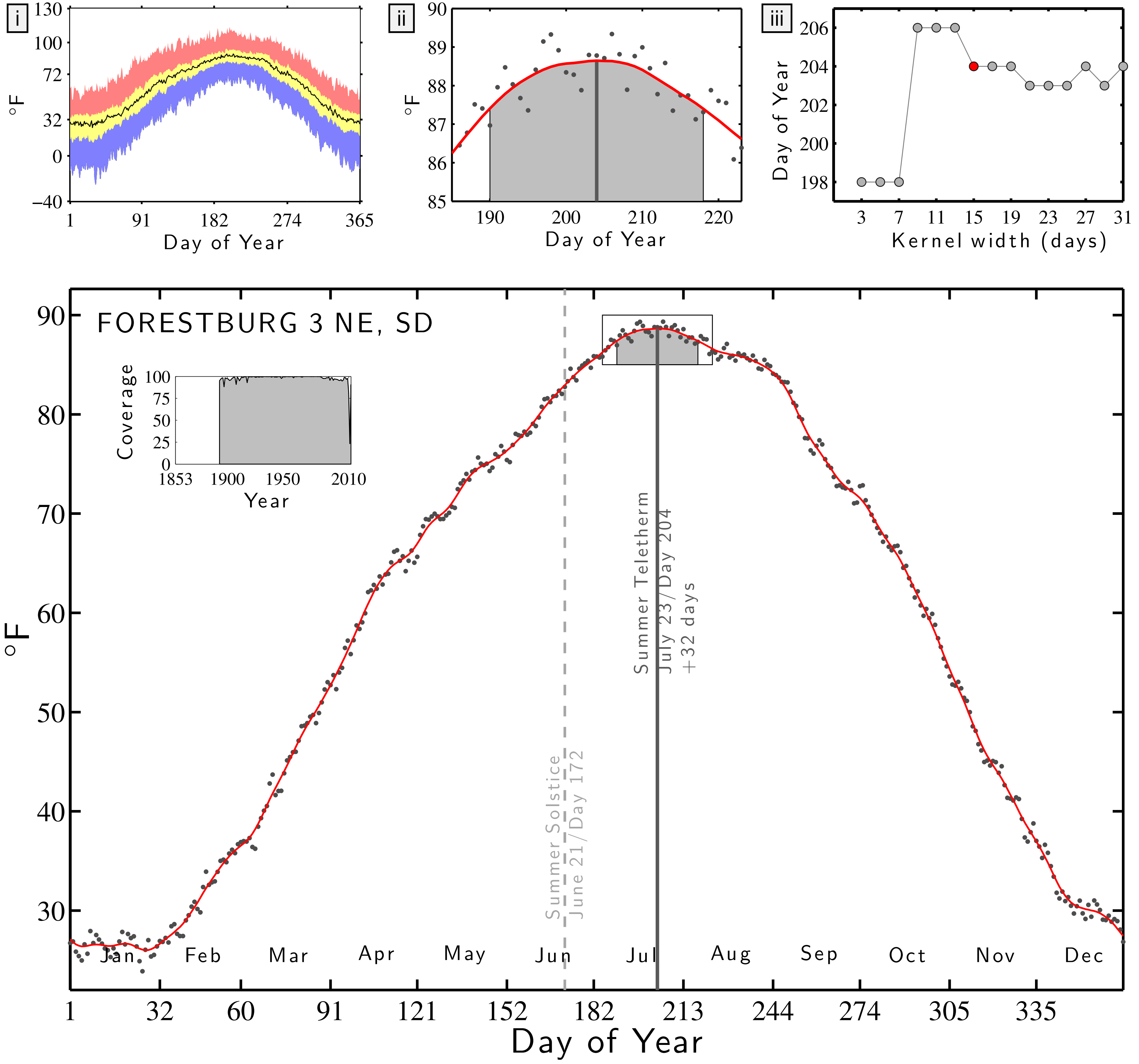

i
°F
Day of Year
ii
°F
Day of Year
iii
Day of Year
Kernel width (days)
FORESTBURG 3 NE, SD
Coverage
Year
Summer Solstice
June 21 / Day 172
Summer Teletherm
July 23 / Day 204
+32 days
Jan
Feb
Mar
Apr
May
Jun
Jul
Aug
Sep
Oct
Nov
Dec
°F
Day of Year

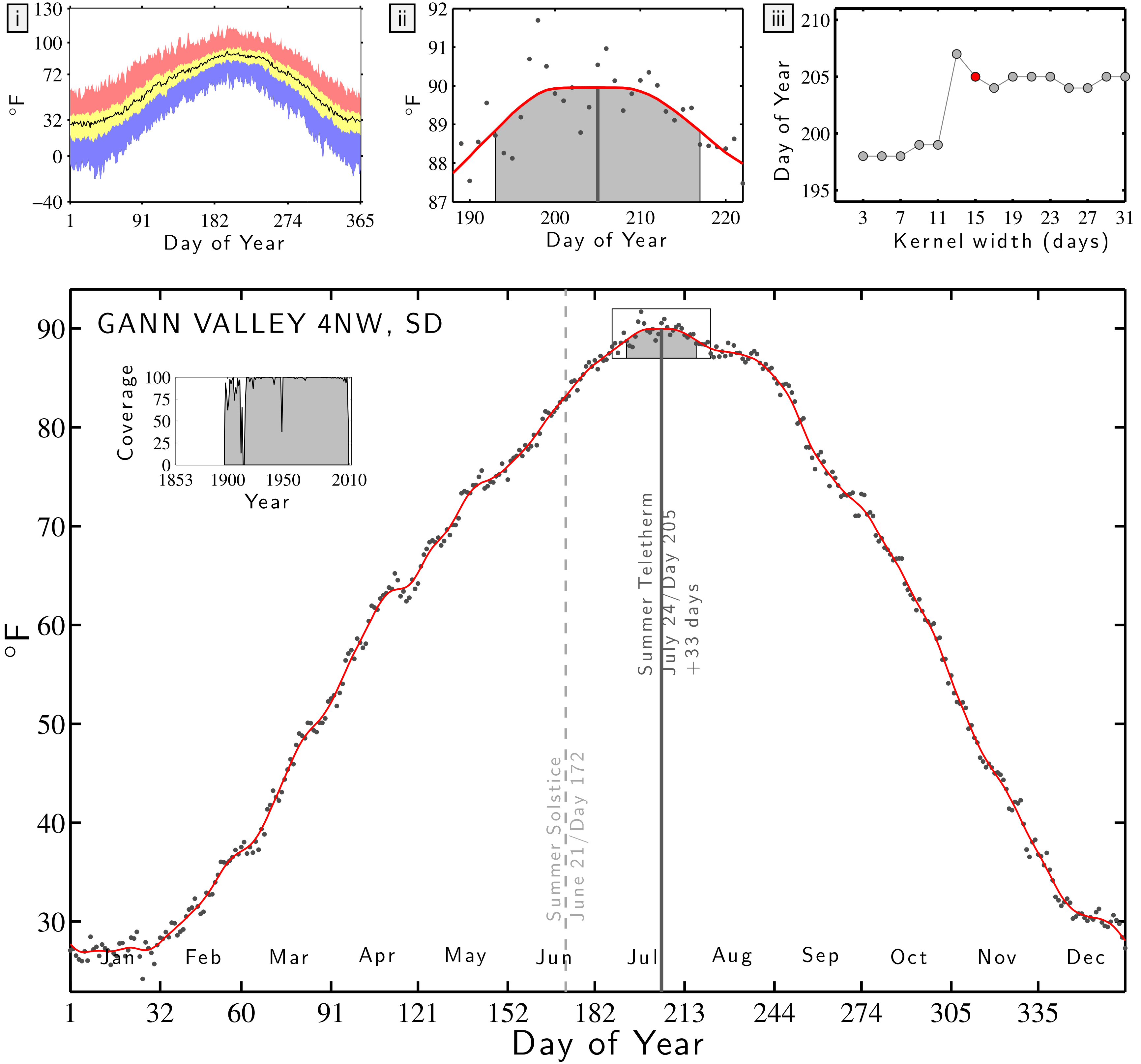

i
°F
Day of Year
ii
°F
Day of Year
iii
Day of Year
Kernel width (days)
GANN VALLEY 4NW, SD
Coverage
Year
Summer Solstice
June 21 / Day 172
Summer Teletherm
July 24 / Day 205
+33 days
Jan
Feb
Mar
Apr
May
Jun
Jul
Aug
Sep
Oct
Nov
Dec
°F
Day of Year

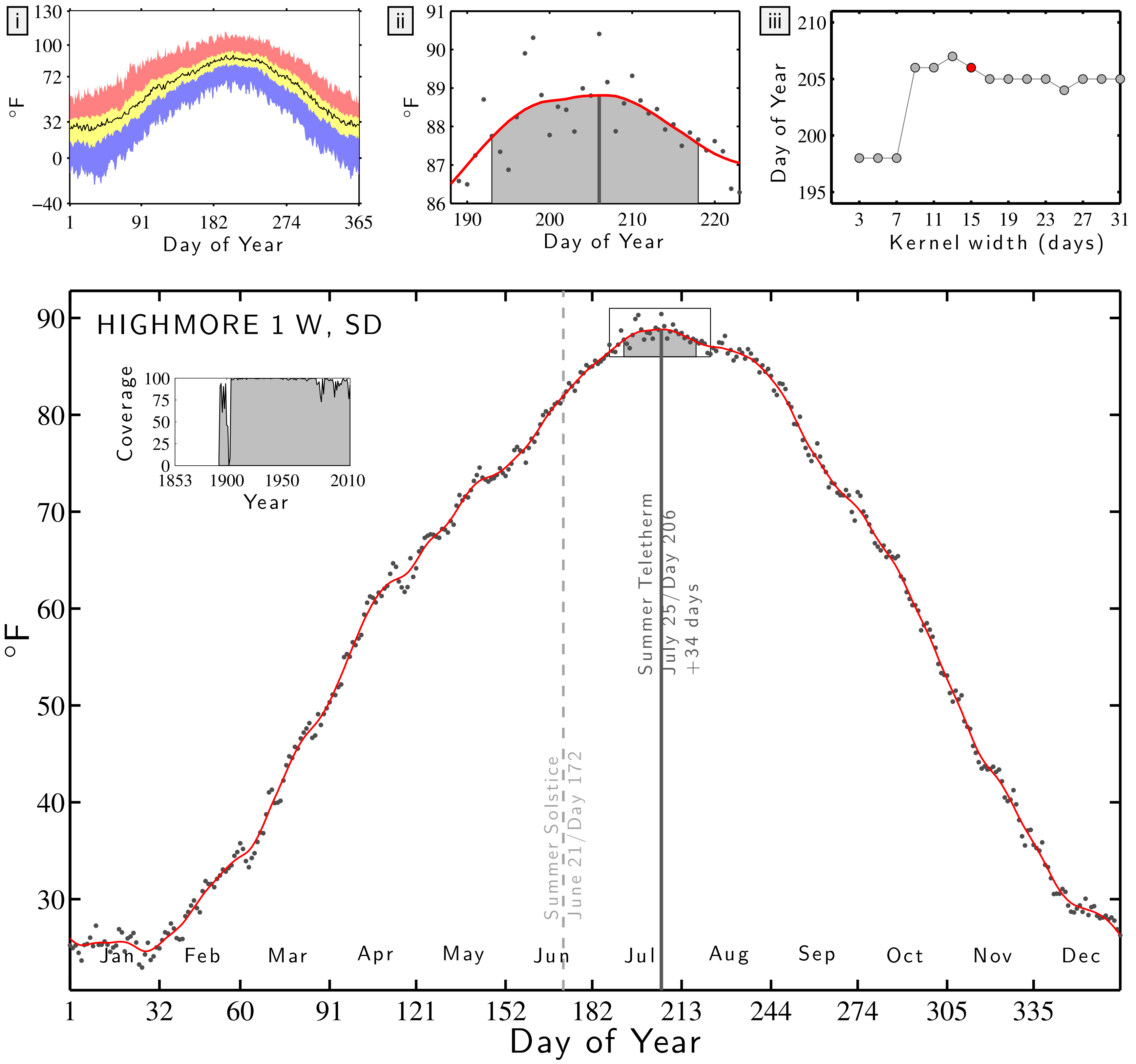

i
ii
iii
°F
Day of Year
Day of Year
Kernel width (days)
HIGHMORE 1 W, SD
Coverage
Year
Summer Solstice
June 21/Day 172
Summer Teletherm
July 25/Day 206
+34 days
Jan
Feb
Mar
Apr
May
Jun
Jul
Aug
Sep
Oct
Nov
Dec

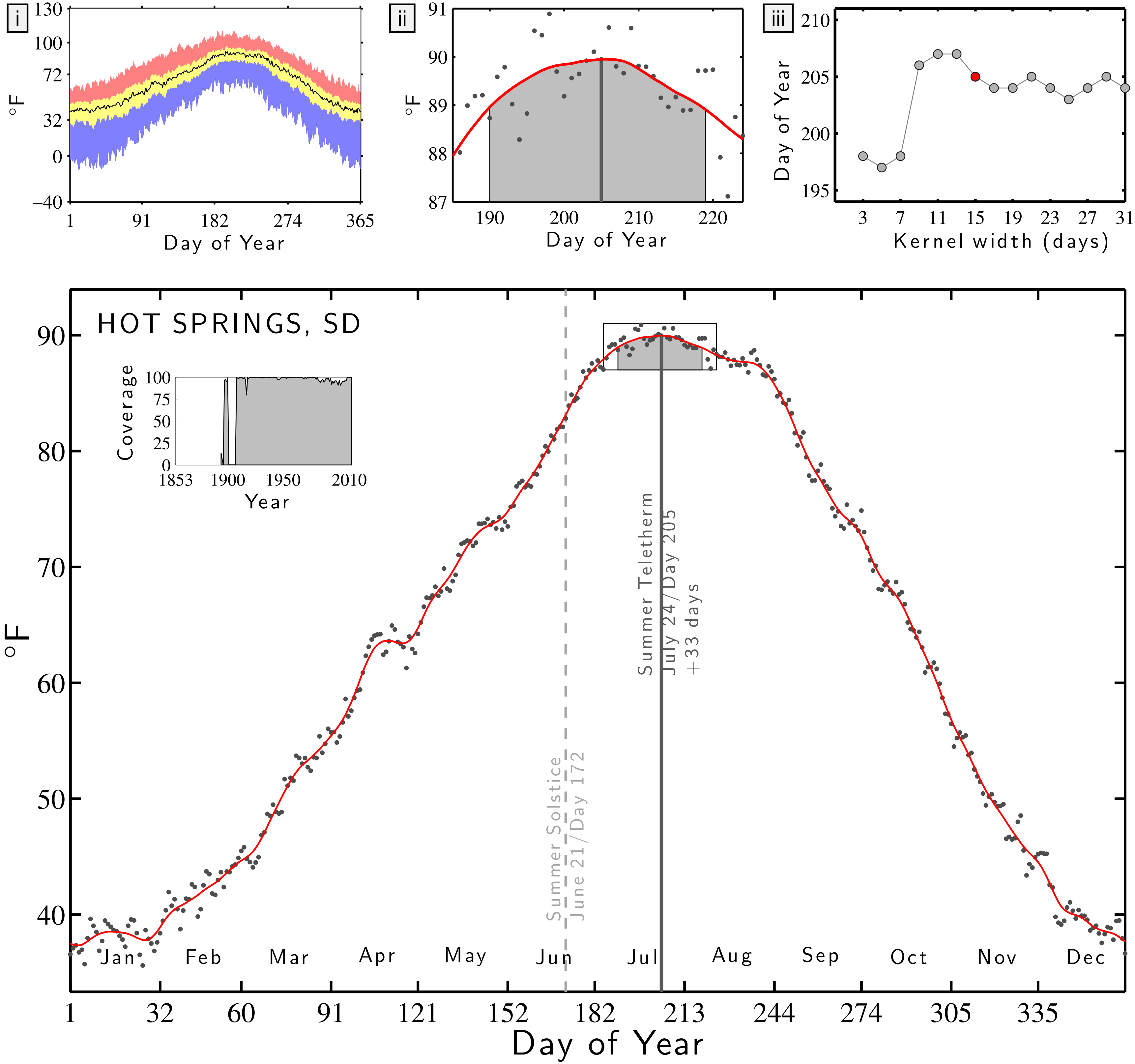

i
°F
Day of Year
ii
°F
Day of Year
iii
Day of Year
Kernel width (days)
HOT SPRINGS, SD
Coverage
Year
Summer Solstice
June 21 / Day 172
Summer Teletherm
July 24 / Day 205
+33 days
Jan
Feb
Mar
Apr
May
Jun
Jul
Aug
Sep
Oct
Nov
Dec
°F
Day of Year

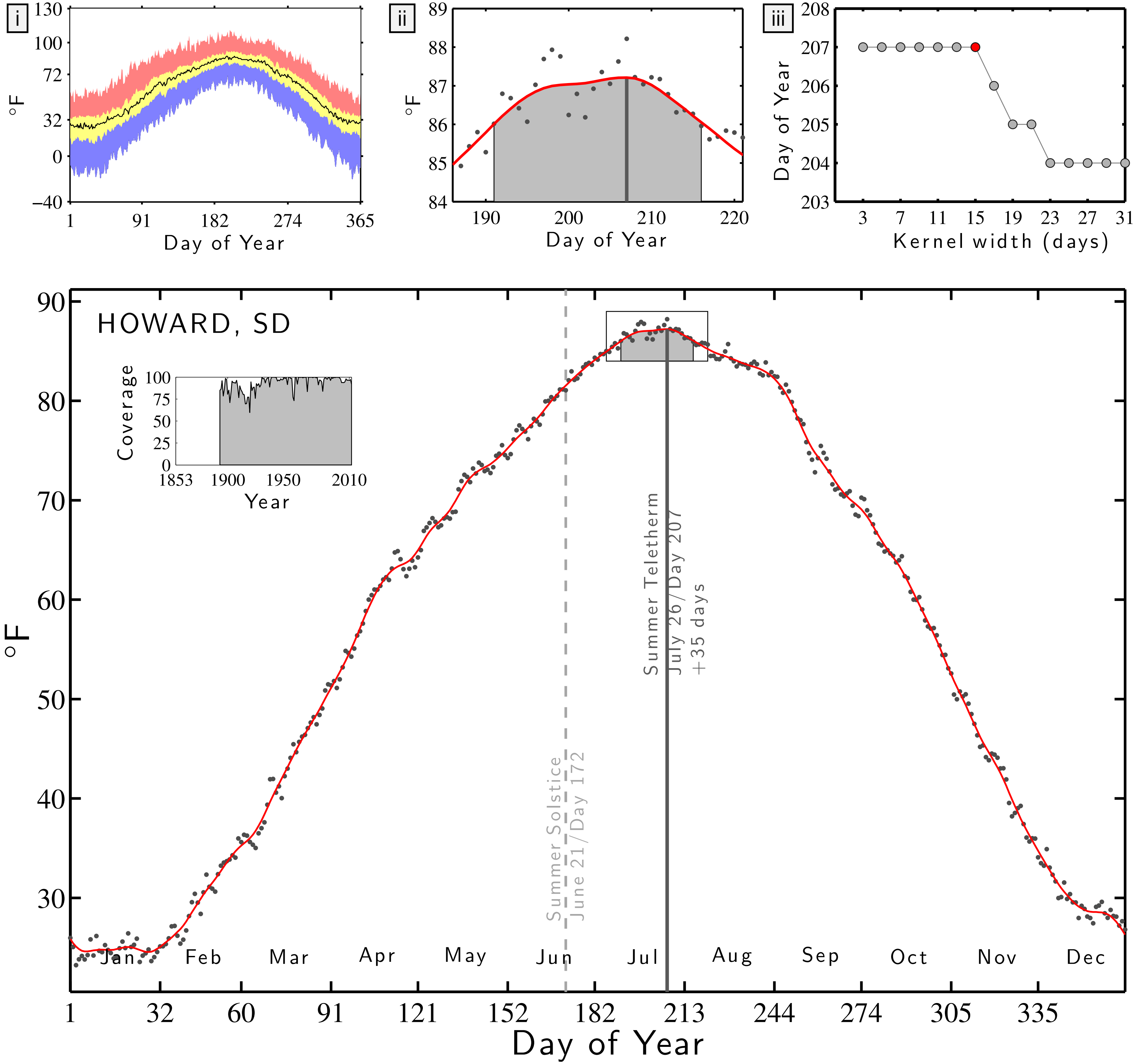

i
ii
iii
°F
Day of Year
Day of Year
Kernel width (days)
HOWARD, SD
Coverage
Year
Summer Solstice
June 21 / Day 172
Summer Teletherm
July 26 / Day 207
+35 days
Jan
Feb
Mar
Apr
May
Jun
Jul
Aug
Sep
Oct
Nov
Dec
Day of Year

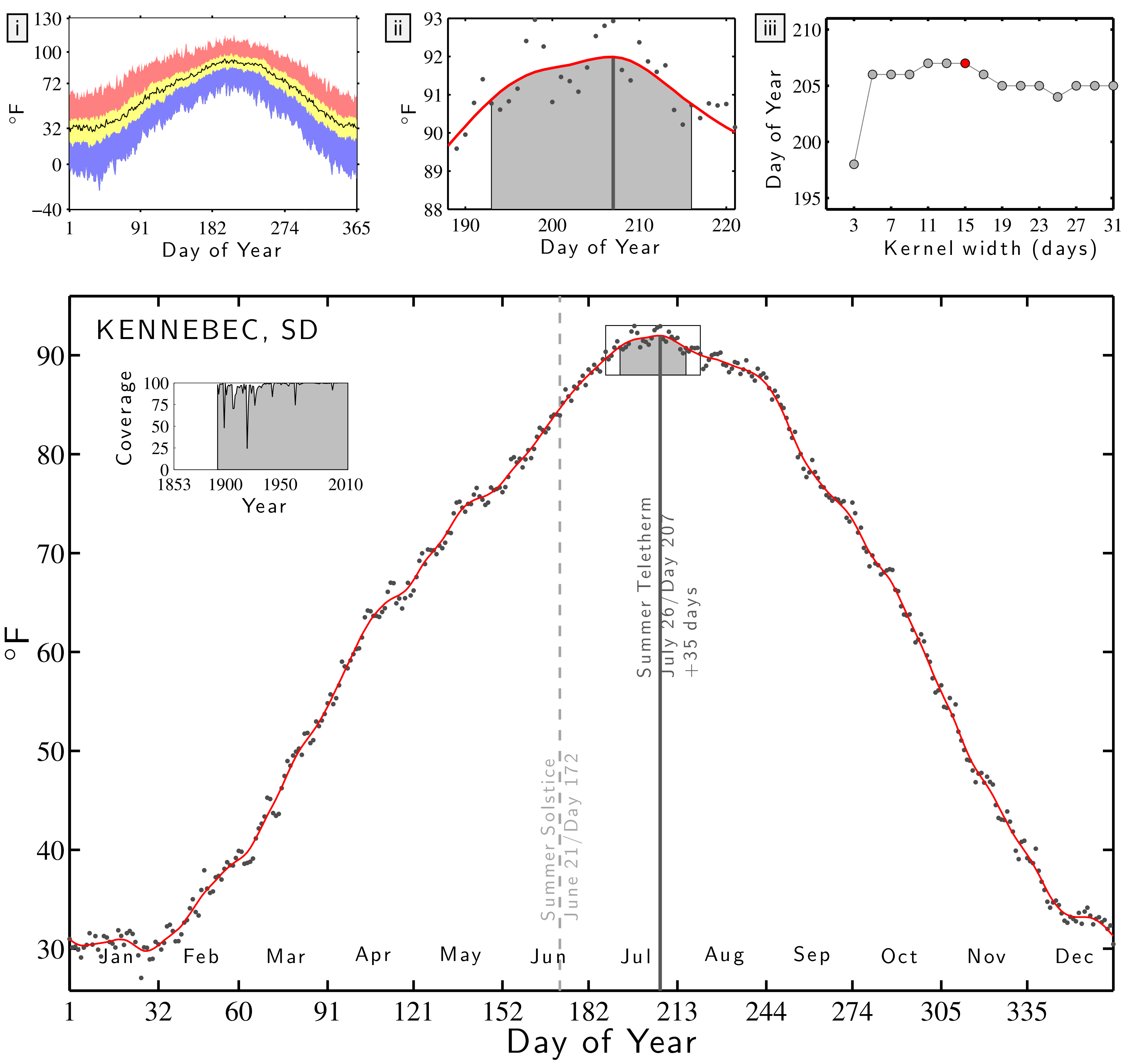

i
ii
iii
°F
Day of Year
Day of Year
Kernel width (days)
KENNEBEC, SD
Coverage
Year
Summer Solstice
June 21 / Day 172
Summer Teletherm
July 26 / Day 207
+35 days
Jan
Feb
Mar
Apr
May
Jun
Jul
Aug
Sep
Oct
Nov
Dec
Day of Year

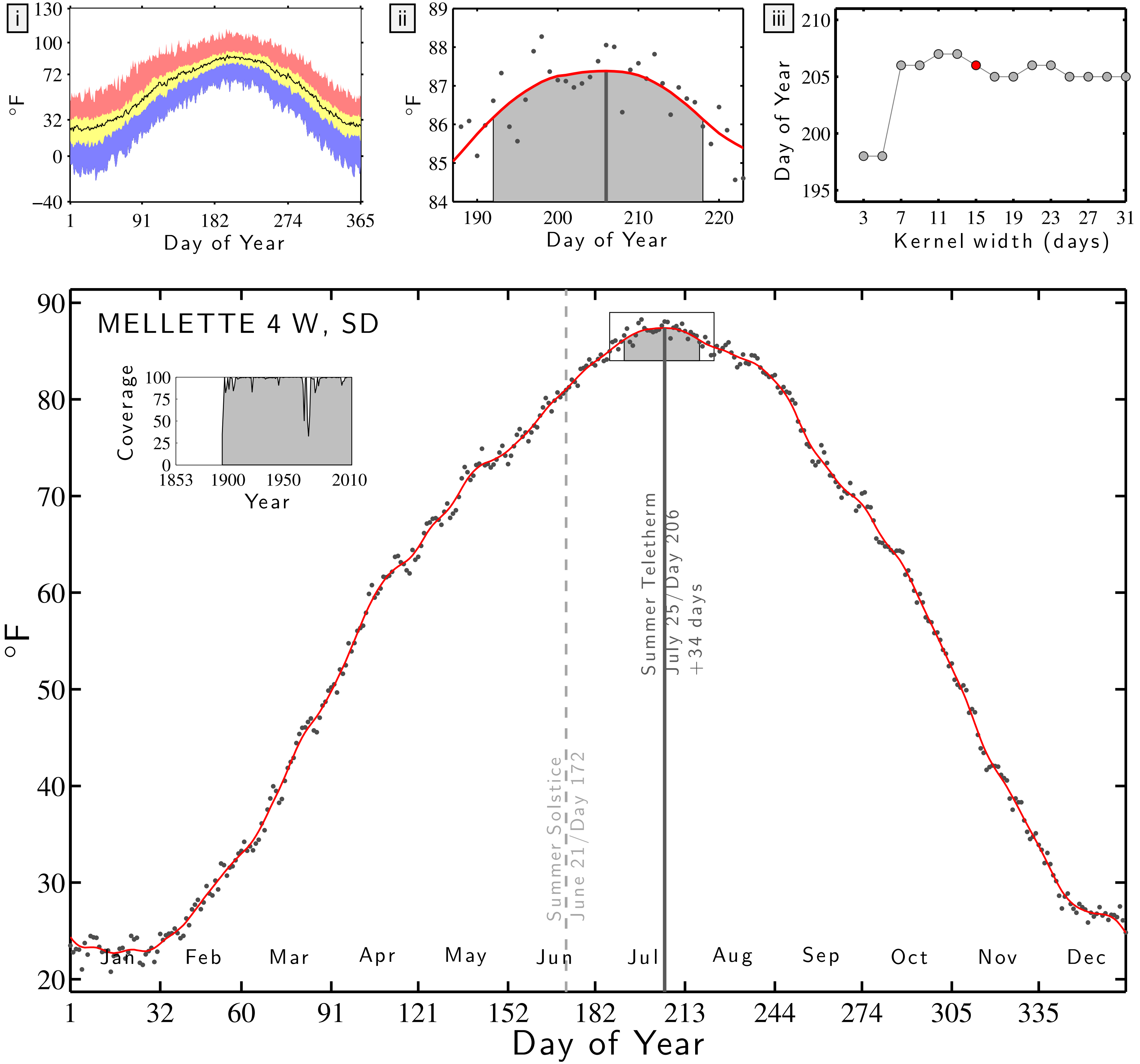
i
ii
iii
MELLETTE 4 W, SD
Coverage
Year
Summer Solstice
June 21/Day 172
Summer Teletherm
July 25/Day 206
+34 days
Day of Year
°F
Kernel width (days)
Jan
Feb
Mar
Apr
May
Jun
Jul
Aug
Sep
Oct
Nov
Dec

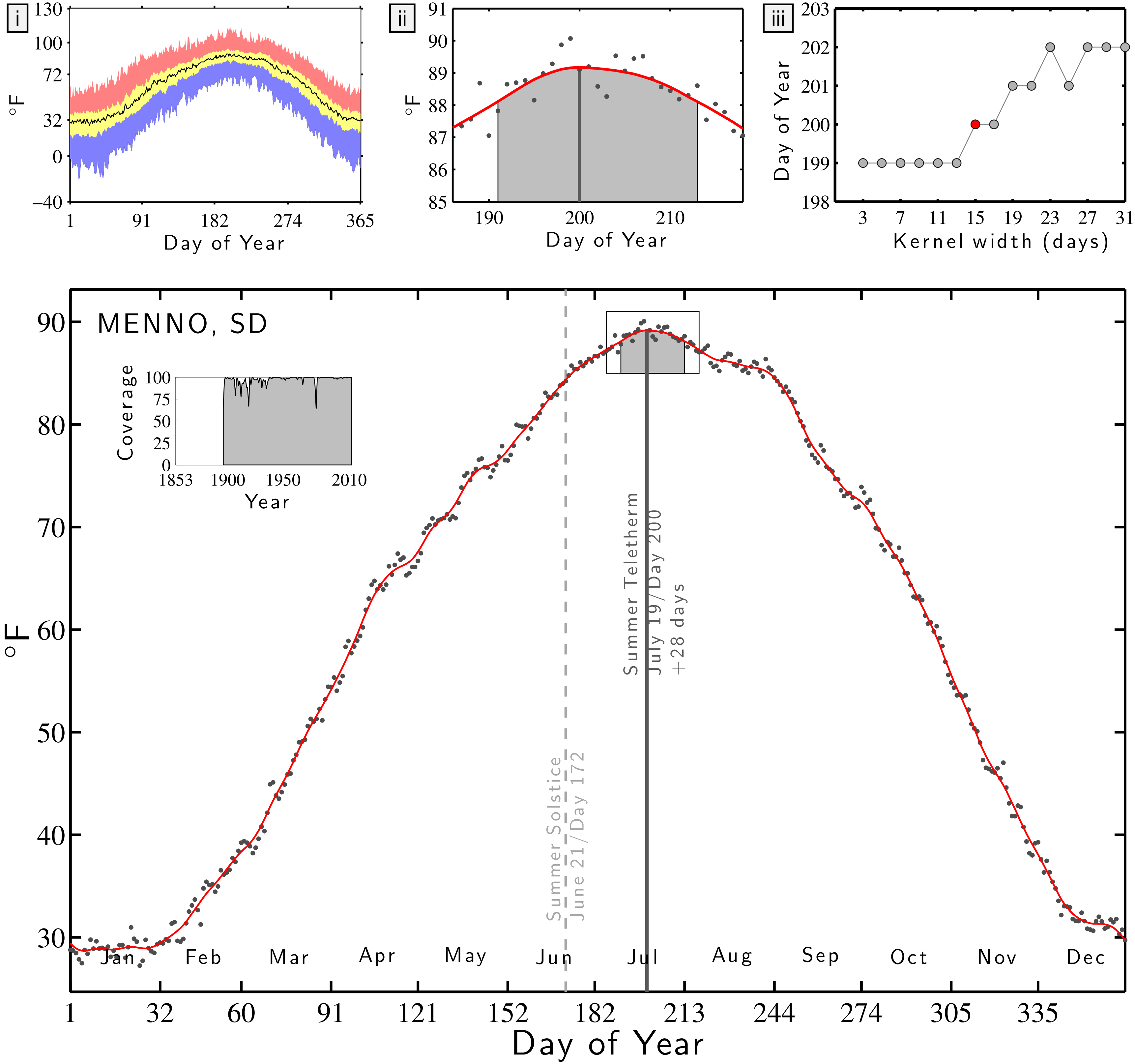

i
°F
130
100
72
32
0
−40
1
91
182
274
365
Day of Year
ii
91
90
89
88
87
86
85
190
200
210
Day of Year
iii
Day of Year
203
202
201
200
199
198
3
7
11
15
19
23
27
31
Kernel width (days)
MENNO, SD
Coverage
100
75
50
25
0
1853
1900
1950
2010
Year
Summer Solstice
June 21/Day 172
Summer Teletherm
July 19/Day 200
+28 days
90
80
70
60
50
40
30
Jan
Feb
Mar
Apr
May
Jun
Jul
Aug
Sep
Oct
Nov
Dec
1
32
60
91
121
152
182
213
244
274
305
335
Day of Year

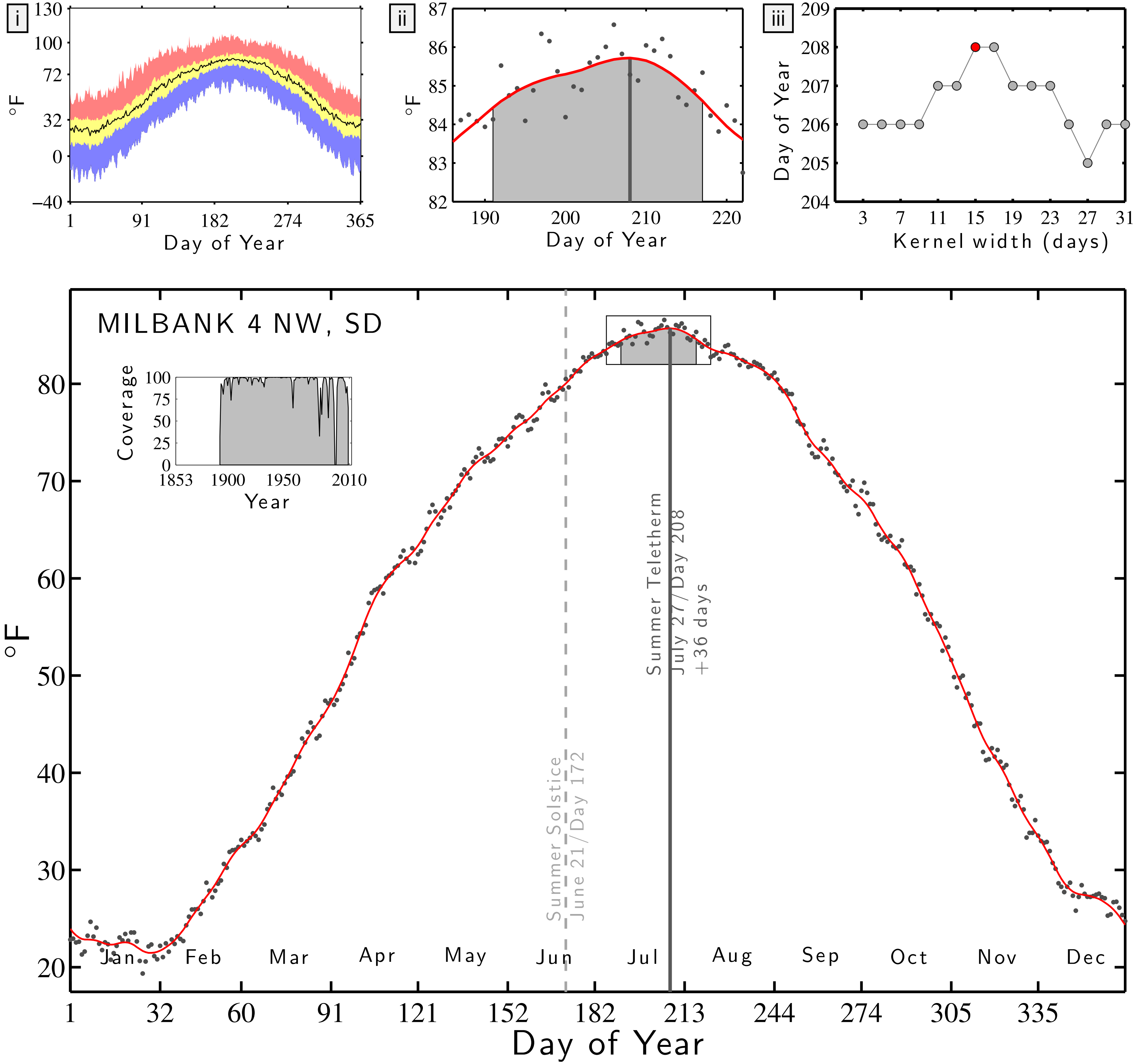

i
ii
iii
°F
Day of Year
Kernel width (days)
MILBANK 4 NW, SD
Coverage
Year
Summer Solstice
June 21 / Day 172
Summer Teletherm
July 27 / Day 208
+36 days
Jan
Feb
Mar
Apr
May
Jun
Jul
Aug
Sep
Oct
Nov
Dec

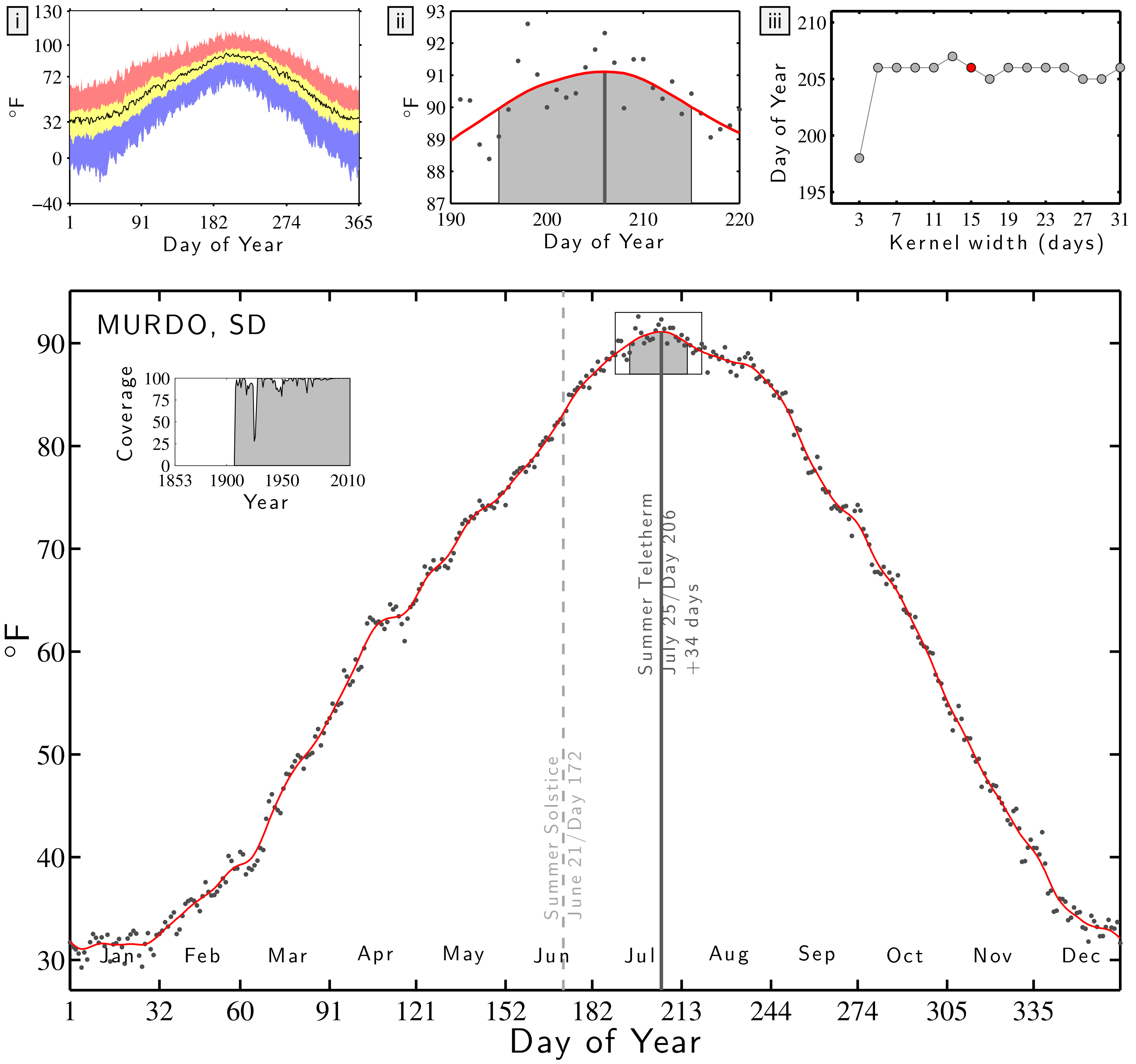

i
°F
Day of Year
ii
Day of Year
iii
Day of Year
Kernel width (days)
MURDO, SD
Coverage
Year
Summer Solstice
June 21/Day 172
Summer Teletherm
July 25/Day 206
+34 days
Jan
Feb
Mar
Apr
May
Jun
Jul
Aug
Sep
Oct
Nov
Dec
°F
Day of Year

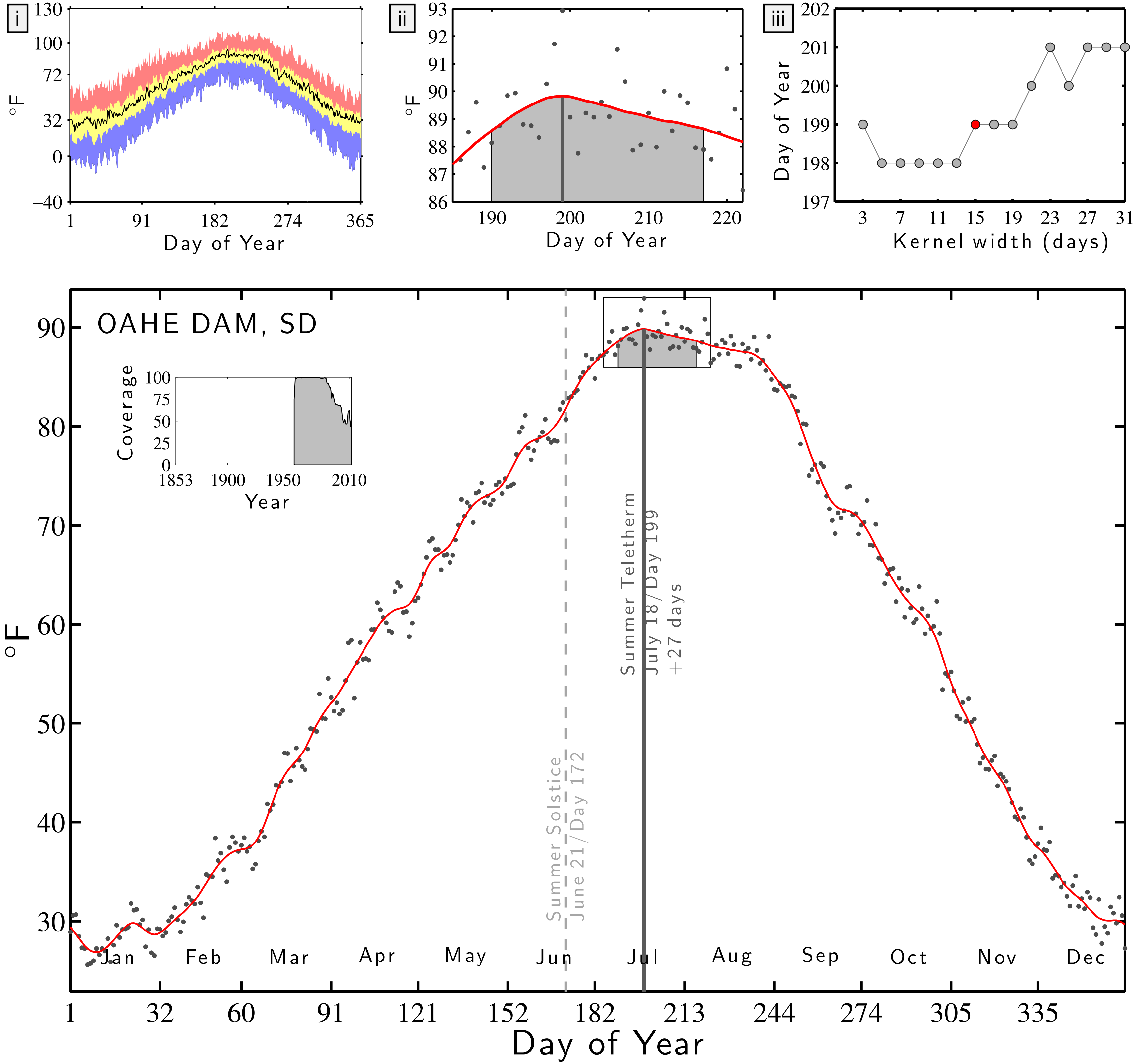

i
°F
Day of Year
ii
°F
Day of Year
iii
Day of Year
Kernel width (days)
OAHE DAM, SD
Coverage
Year
Summer Solstice
June 21 / Day 172
Summer Teletherm
July 18 / Day 199
+27 days
Jan
Feb
Mar
Apr
May
Jun
Jul
Aug
Sep
Oct
Nov
Dec
°F
Day of Year

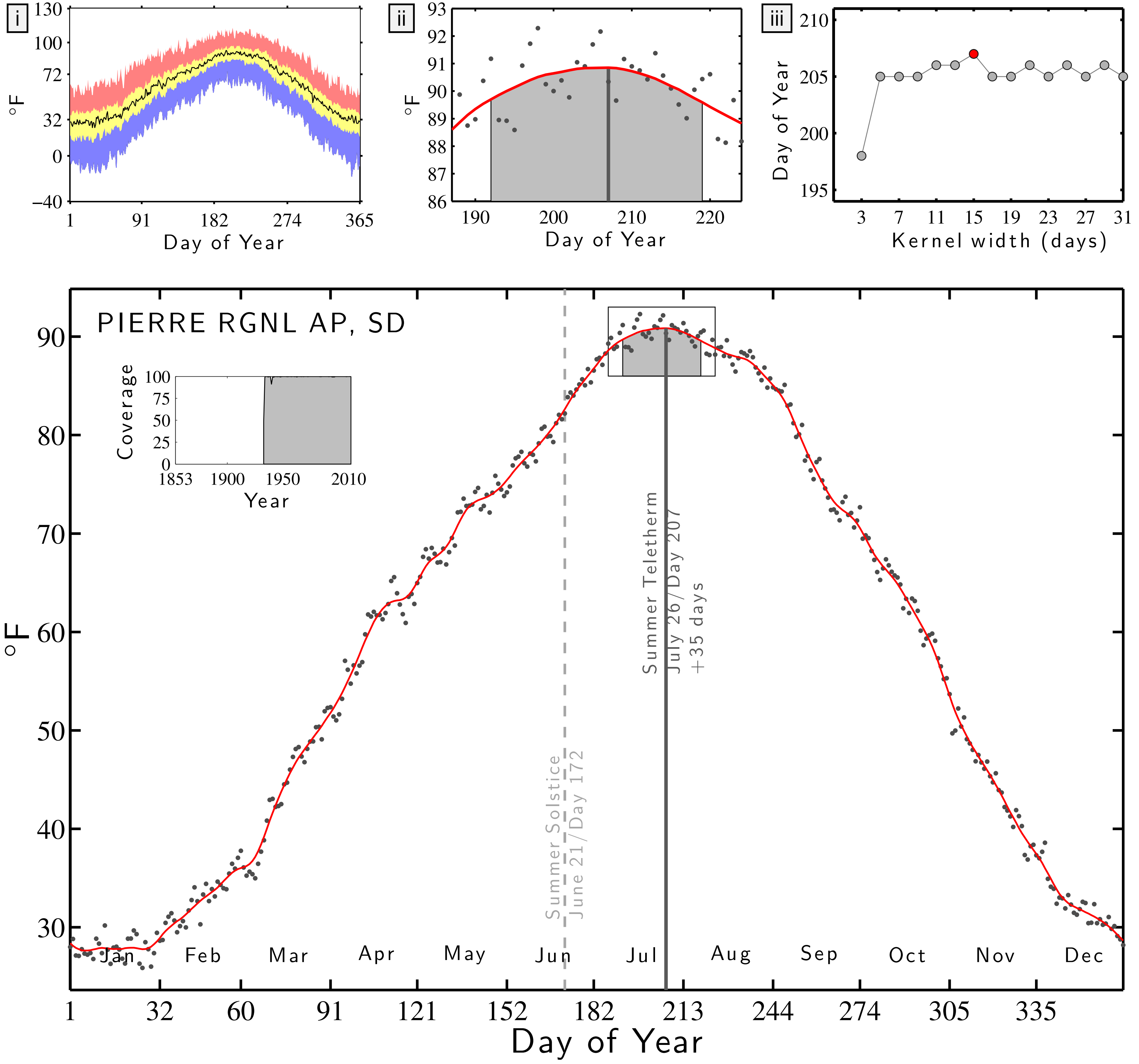

i
°F
Day of Year
ii
°F
Day of Year
iii
Day of Year
Kernel width (days)
PIERRE RGNL AP, SD
Coverage
Year
Summer Solstice
June 21 / Day 172
Summer Teletherm
July 26 / Day 207
+35 days
Jan
Feb
Mar
Apr
May
Jun
Jul
Aug
Sep
Oct
Nov
Dec
°F
Day of Year

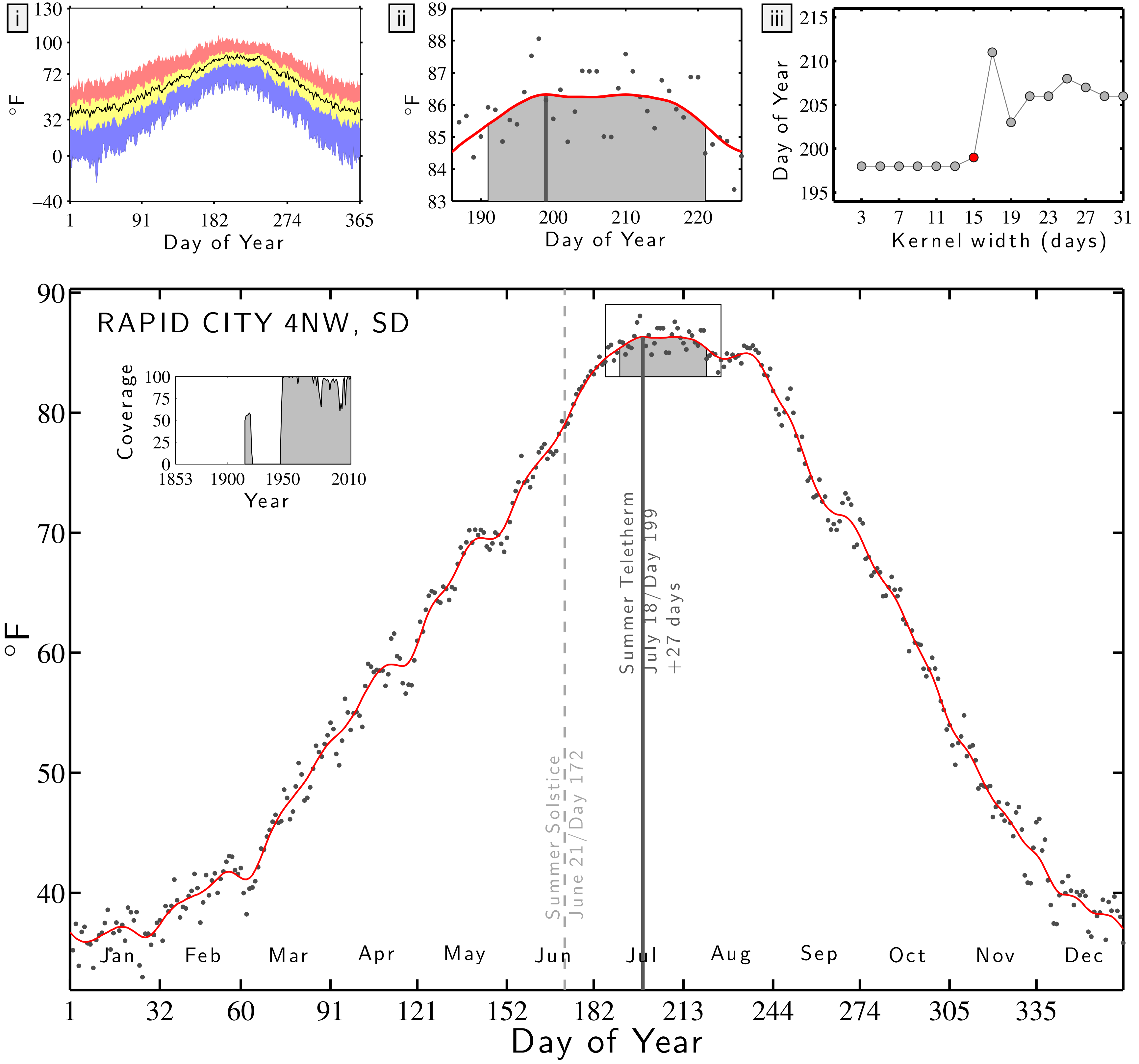

i
°F
Day of Year
ii
°F
Day of Year
iii
Day of Year
Kernel width (days)
RAPID CITY 4NW, SD
Coverage
Year
Summer Solstice
June 21/Day 172
Summer Teletherm
July 18/Day 199
+27 days
Jan
Feb
Mar
Apr
May
Jun
Jul
Aug
Sep
Oct
Nov
Dec
°F
Day of Year

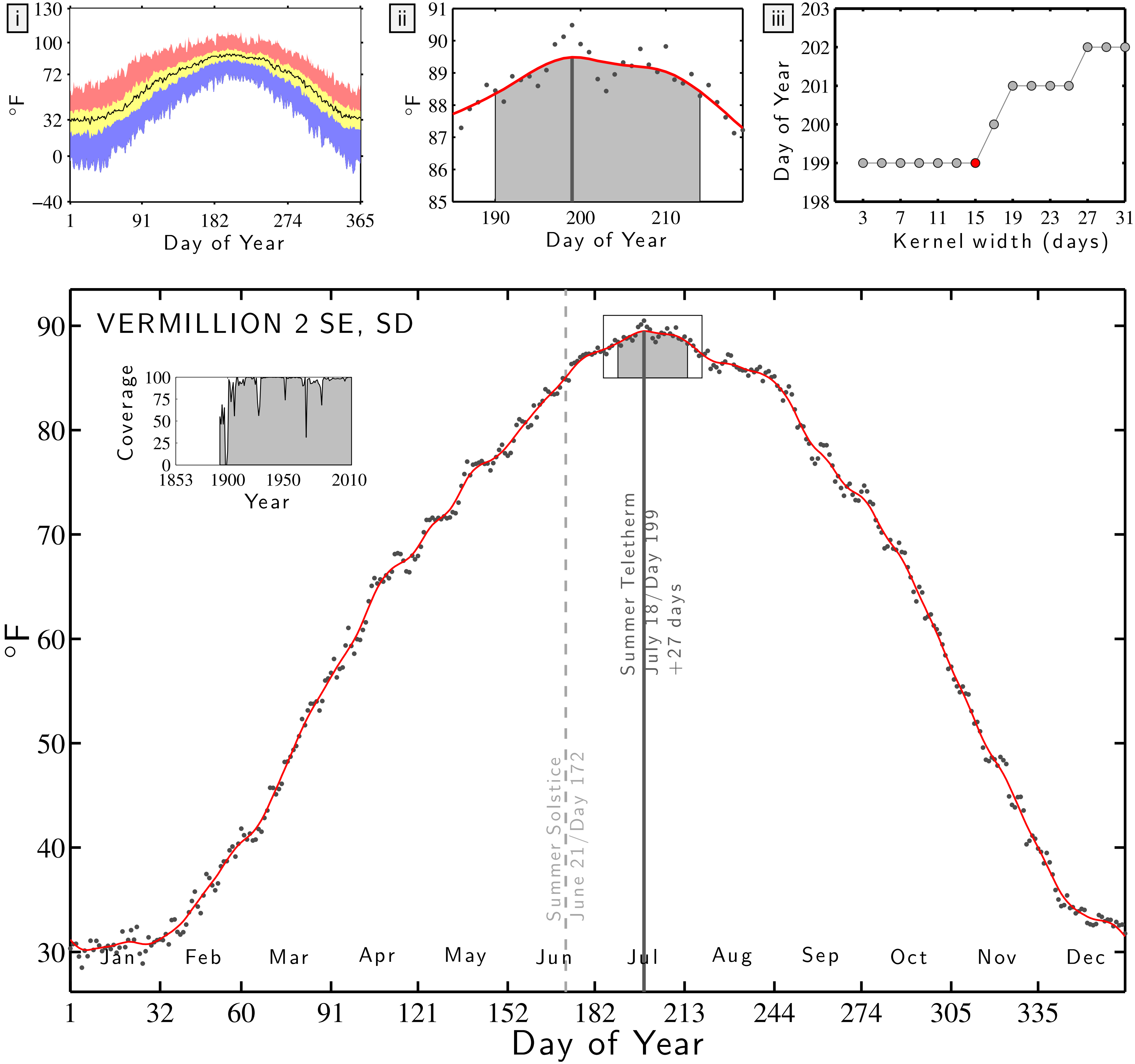

i
ii
iii
°F
Day of Year
Day of Year
Kernel width (days)
VERMILLION 2 SE, SD
Coverage
Year
Summer Solstice
June 21/Day 172
Summer Teletherm
July 18/Day 199
+27 days
Jan
Feb
Mar
Apr
May
Jun
Jul
Aug
Sep
Oct
Nov
Dec
Day of Year

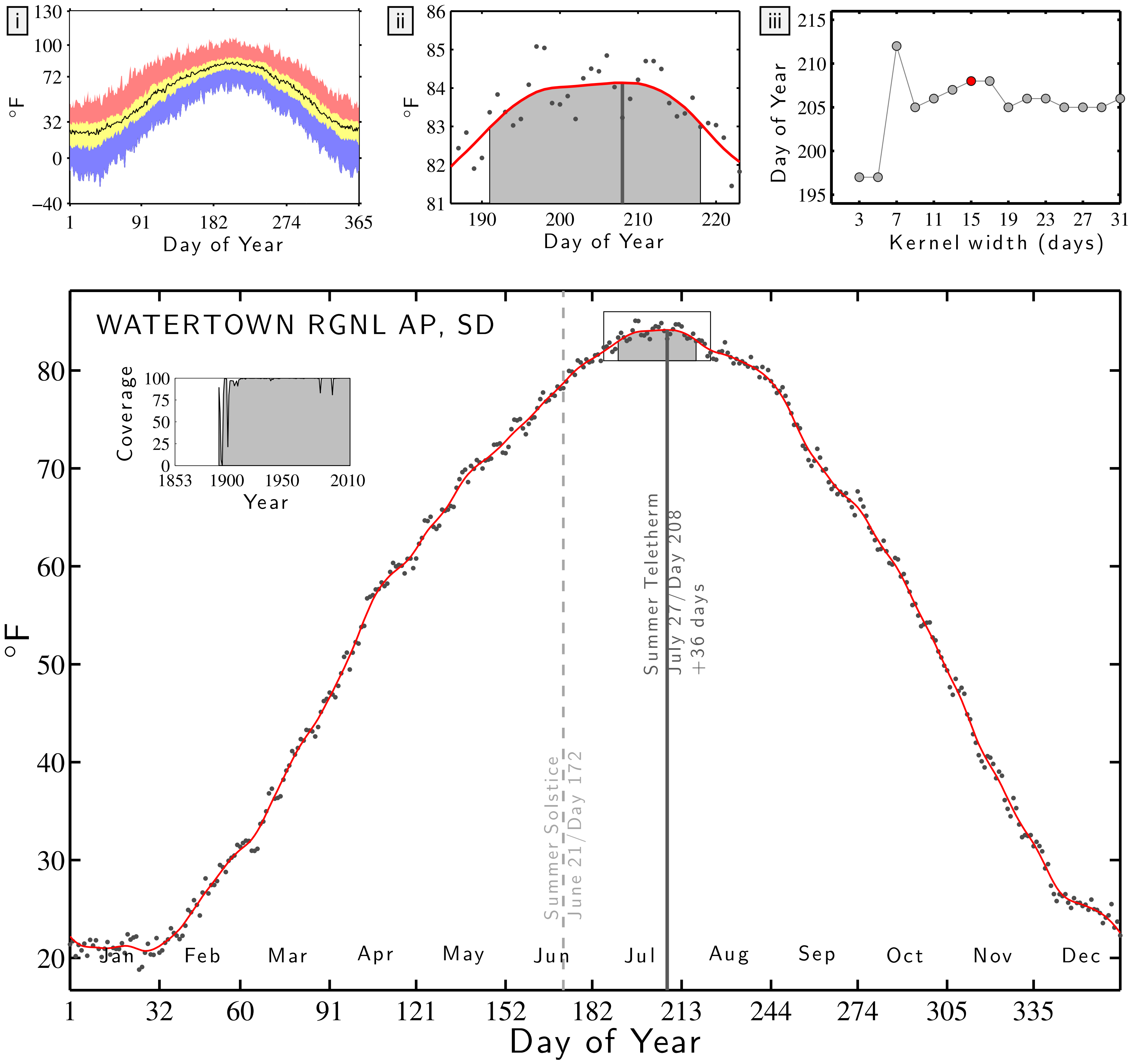

i
°F
Day of Year
ii
°F
Day of Year
iii
Day of Year
Kernel width (days)
WATERTOWN RGNL AP, SD
Coverage
Year
Summer Solstice
June 21 / Day 172
Summer Teletherm
July 27 / Day 208
+36 days
Jan
Feb
Mar
Apr
May
Jun
Jul
Aug
Sep
Oct
Nov
Dec
°F
Day of Year

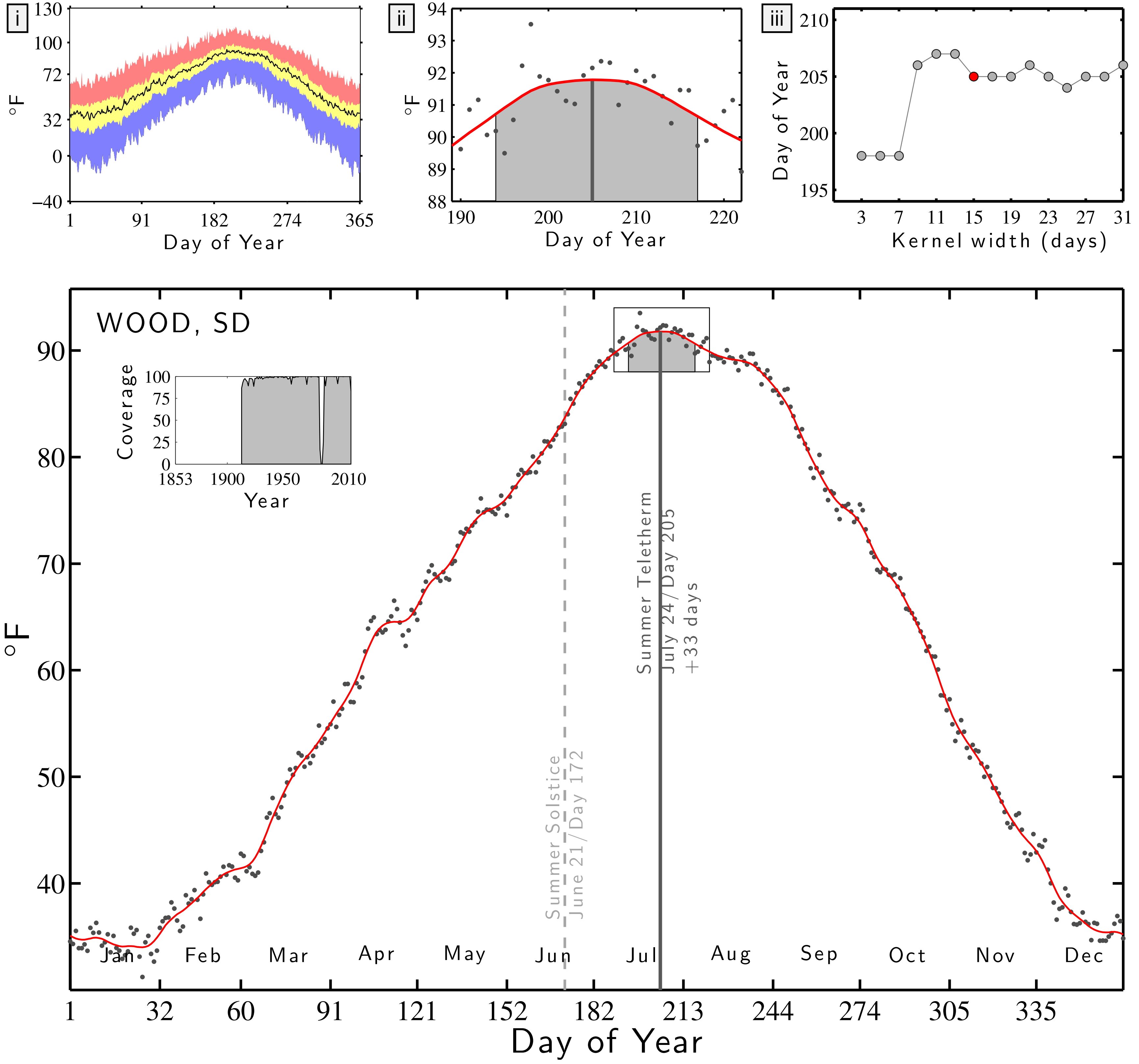

i
°F
Day of Year
ii
°F
Day of Year
iii
Day of Year
Kernel width (days)
WOOD, SD
Coverage
Year
Summer Solstice
June 21/Day 172
Summer Teletherm
July 24/Day 205
+33 days
Jan
Feb
Mar
Apr
May
Jun
Jul
Aug
Sep
Oct
Nov
Dec
°F
Day of Year

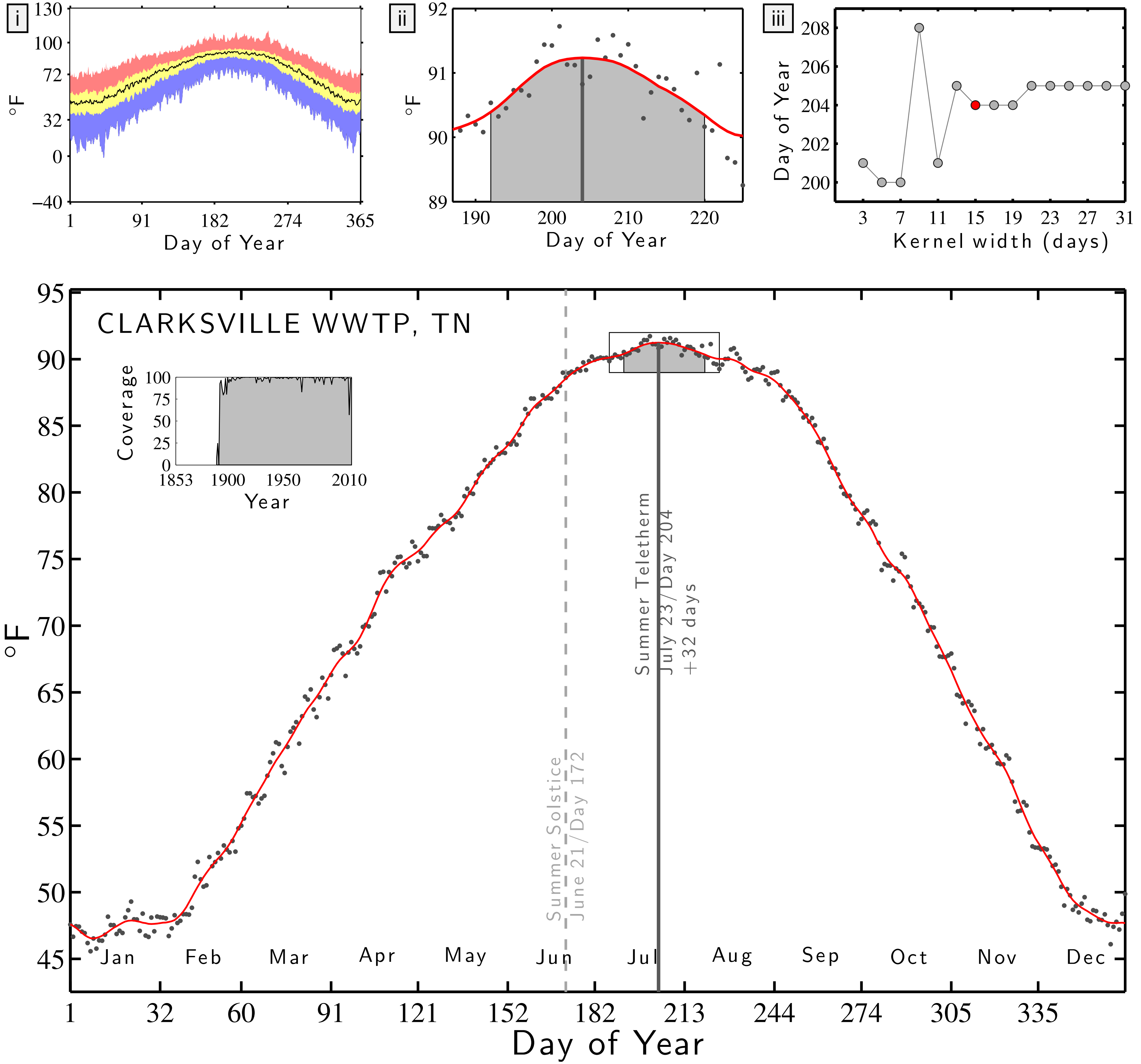
i
°F
130
100
72
32
0
−40
1
91
182
274
365
Day of Year
ii
°F
92
91
90
89
190
200
210
220
Day of Year
iii
Day of Year
208
206
204
202
200
3
7
11
15
19
23
27
31
Kernel width (days)
CLARKSVILLE WWTP, TN
Coverage
100
75
50
25
0
1853
1900
1950
2010
Year
Summer Solstice
June 21 / Day 172
Summer Teletherm
July 23 / Day 204
+32 days
°F
95
90
85
80
75
70
65
60
55
50
45
Jan
Feb
Mar
Apr
May
Jun
Jul
Aug
Sep
Oct
Nov
Dec
1
32
60
91
121
152
182
213
244
274
305
335
Day of Year

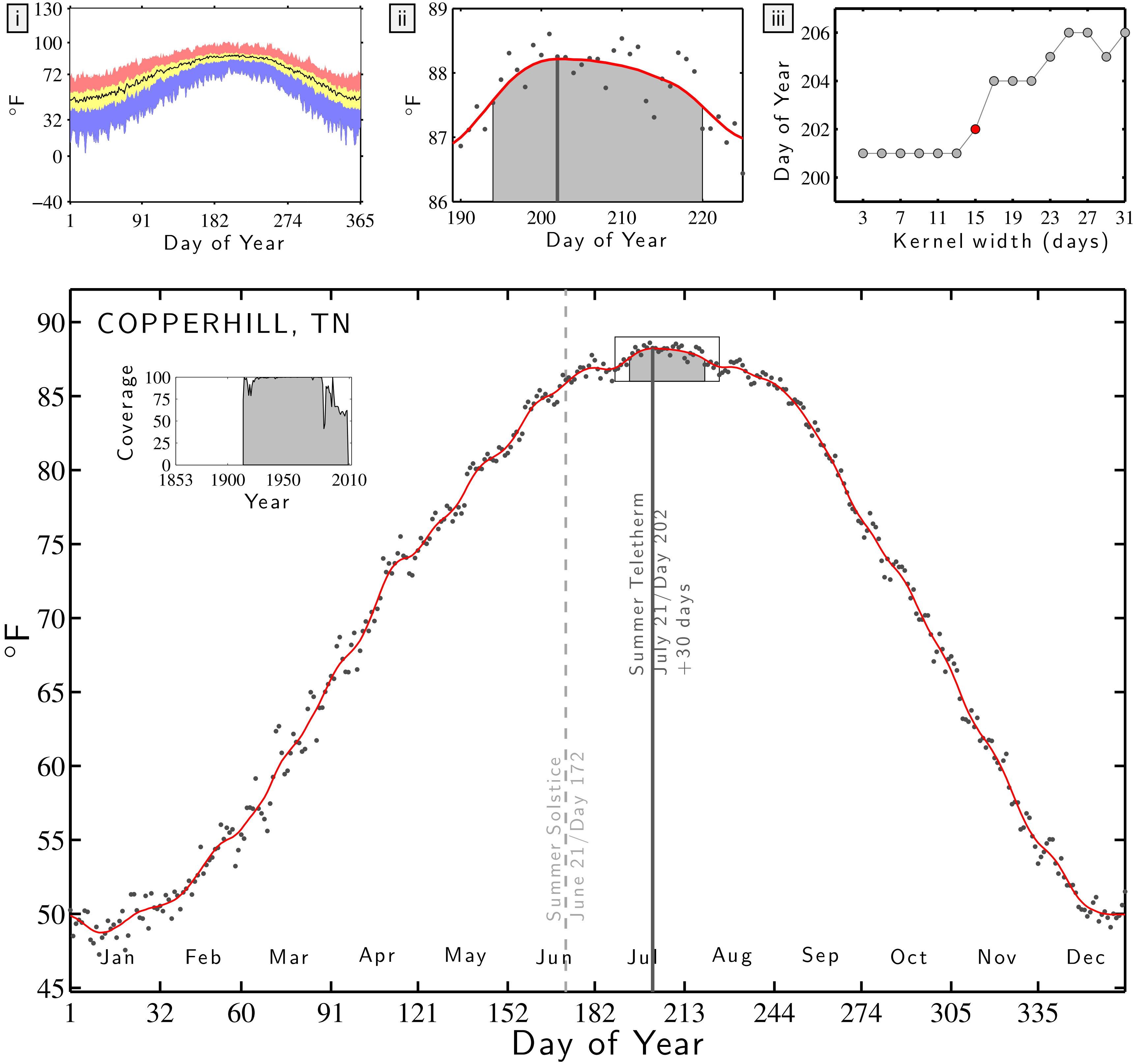
i
ii
iii
Day of Year
°F
Kernel width (days)
COPPERHILL, TN
Coverage
Year
Summer Solstice
June 21 / Day 172
Summer Teletherm
July 21 / Day 202
+30 days
Jan
Feb
Mar
Apr
May
Jun
Jul
Aug
Sep
Oct
Nov
Dec

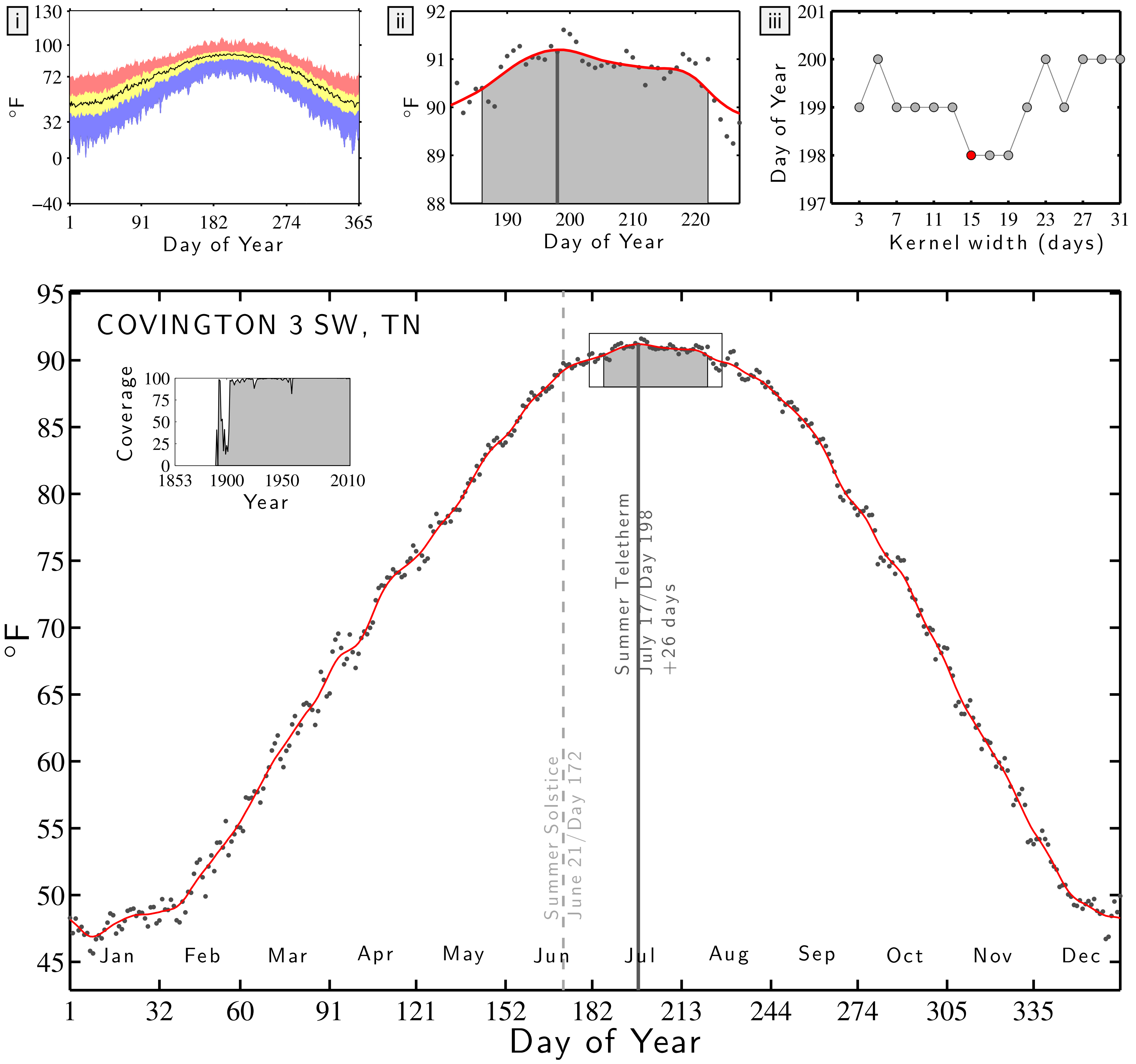

i
ii
iii
°F
Day of Year
Day of Year
Day of Year
Kernel width (days)
COVINGTON 3 SW, TN
Coverage
Year
Summer Solstice
June 21/Day 172
Summer Teletherm
July 17/Day 198
+26 days
Jan
Feb
Mar
Apr
May
Jun
Jul
Aug
Sep
Oct
Nov
Dec
Day of Year
°F

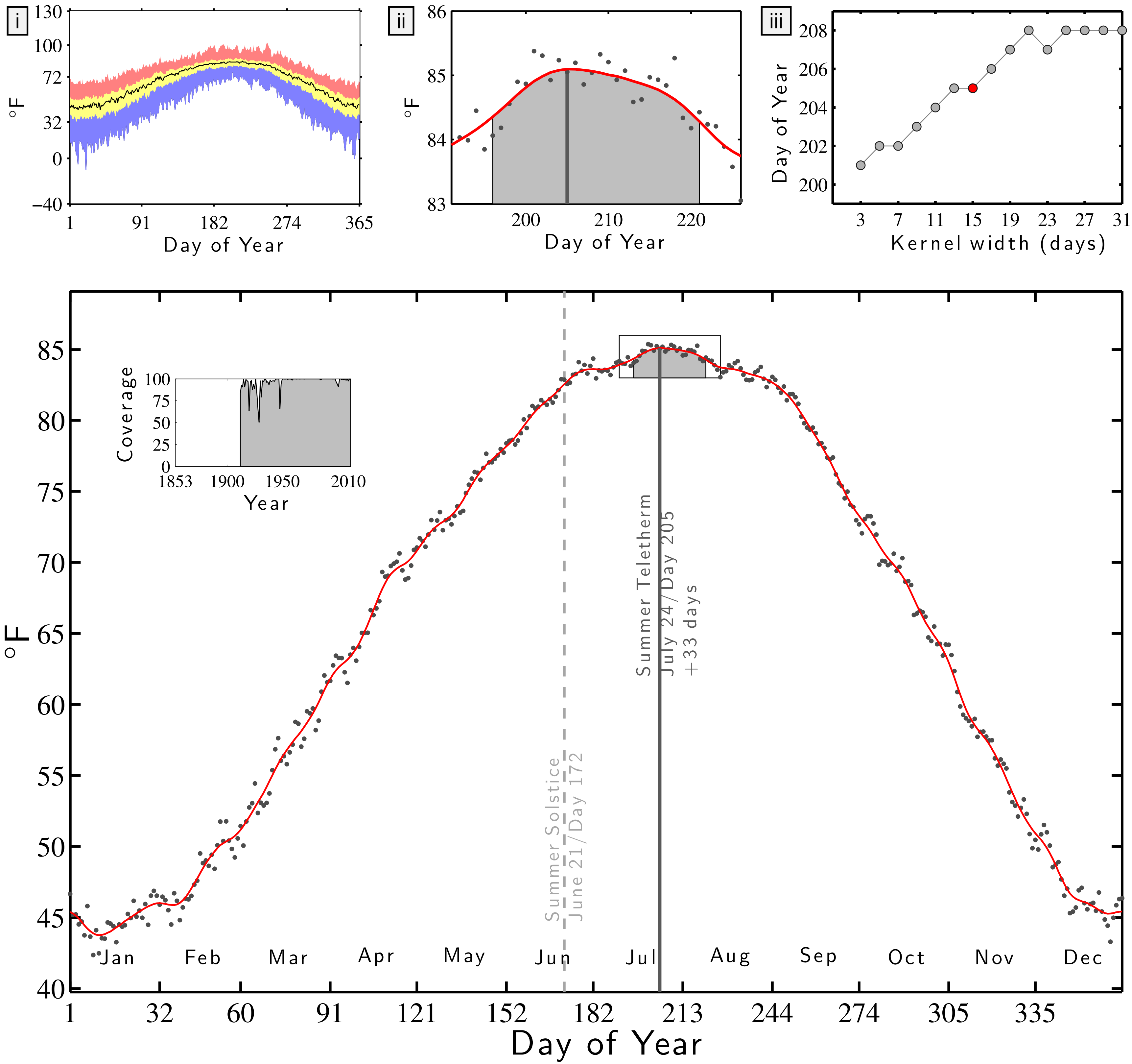

i
°F
Day of Year
ii
°F
Day of Year
iii
Day of Year
Kernel width (days)
Coverage
Year
°F
Summer Solstice
June 21 / Day 172
Summer Teletherm
July 24 / Day 205
+33 days
Jan
Feb
Mar
Apr
May
Jun
Jul
Aug
Sep
Oct
Nov
Dec
Day of Year

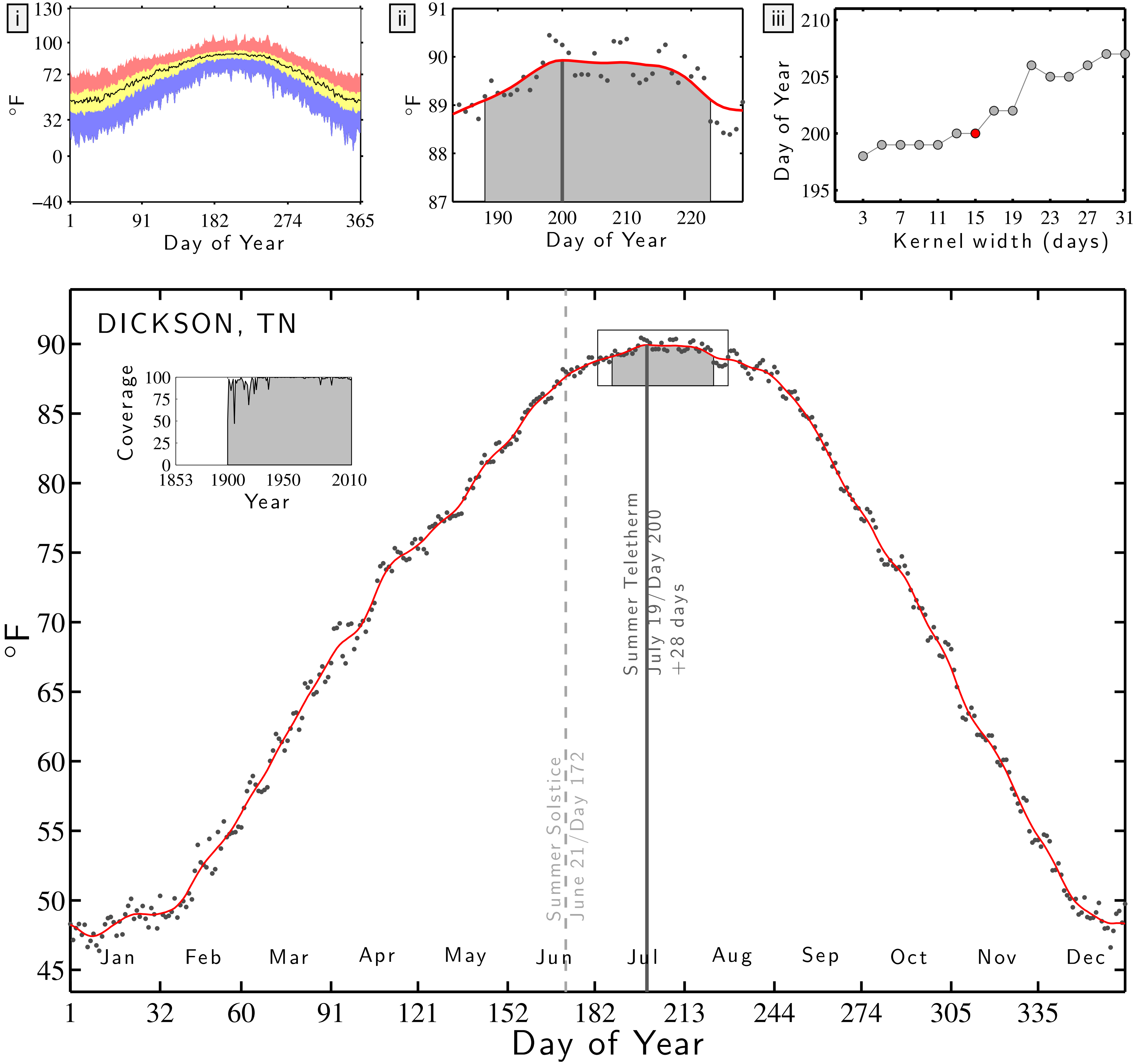

i
ii
iii
°F
Day of Year
Kernel width (days)
DICKSON, TN
Coverage
Year
Summer Solstice
June 21/Day 172
Summer Teletherm
July 19/Day 200
+28 days
Jan
Feb
Mar
Apr
May
Jun
Jul
Aug
Sep
Oct
Nov
Dec

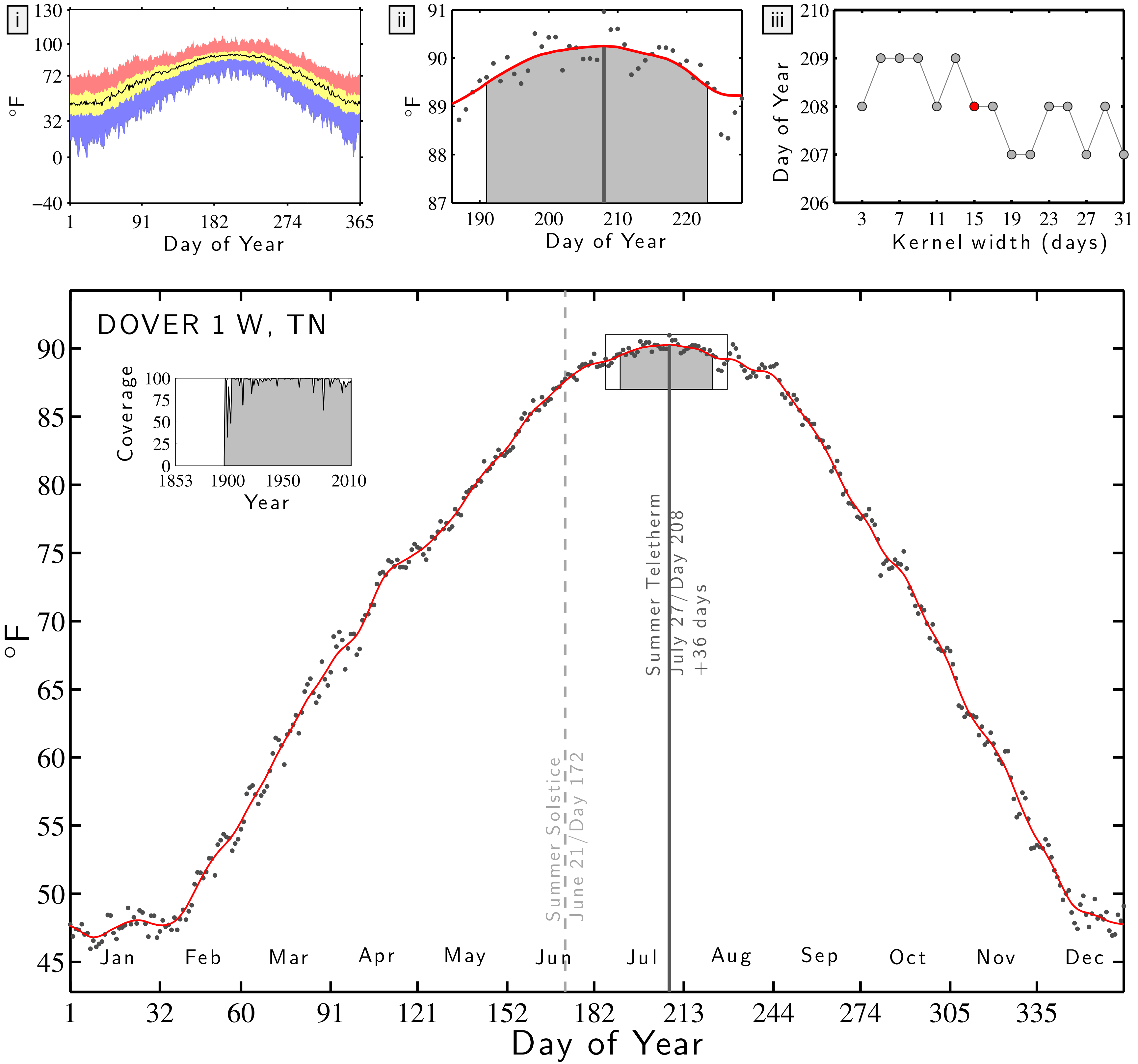

i
°F
Day of Year
ii
°F
Day of Year
iii
Day of Year
Kernel width (days)
DOVER 1 W, TN
Coverage
Year
Summer Solstice
June 21 / Day 172
Summer Teletherm
July 27 / Day 208
+36 days
Jan
Feb
Mar
Apr
May
Jun
Jul
Aug
Sep
Oct
Nov
Dec
°F
Day of Year

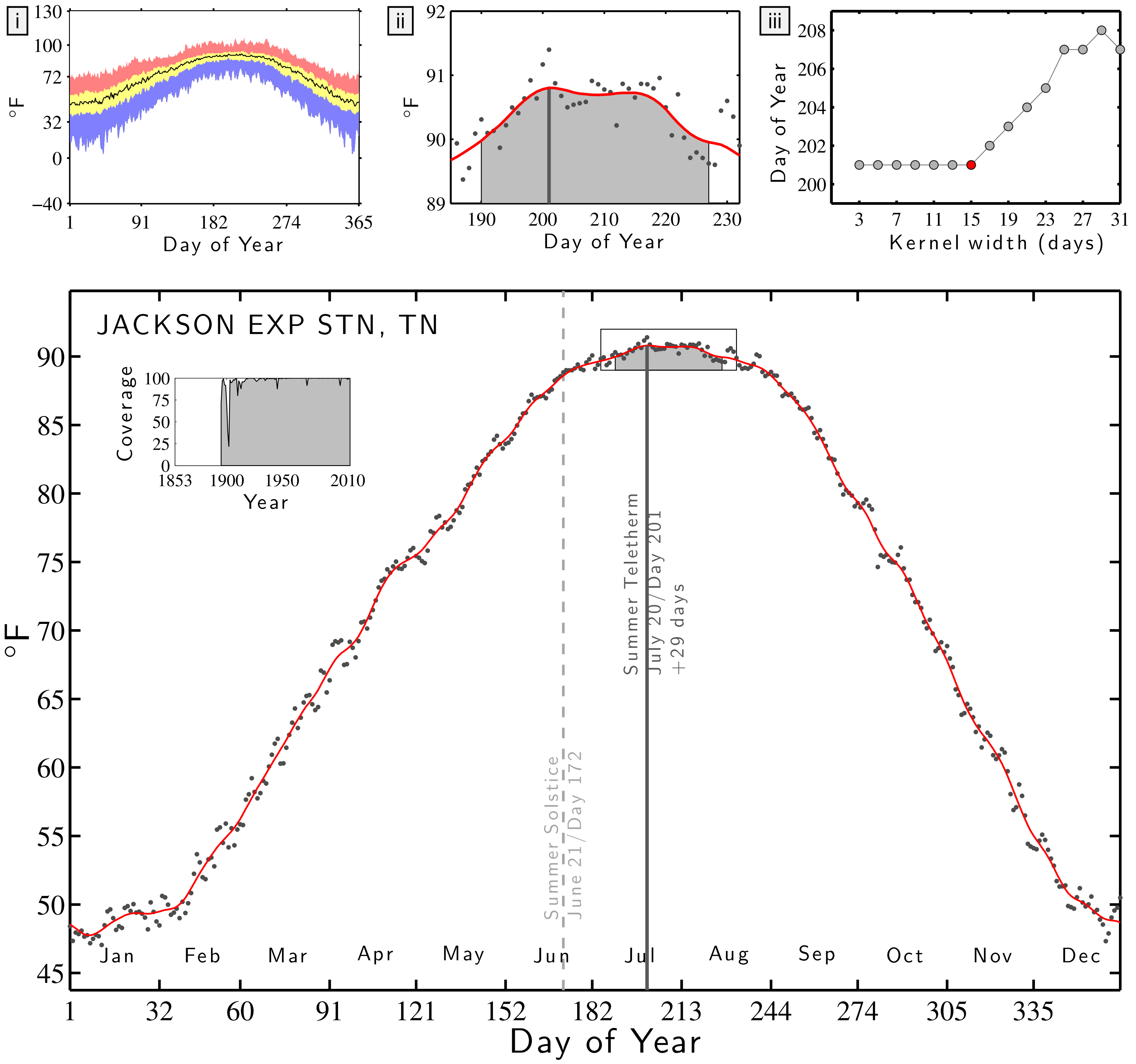

i
ii
iii
°F
Day of Year
Kernel width (days)
JACKSON EXP STN, TN
Coverage
Year
Summer Solstice
June 21/Day 172
Summer Teletherm
July 20/Day 201
+29 days
Jan
Feb
Mar
Apr
May
Jun
Jul
Aug
Sep
Oct
Nov
Dec

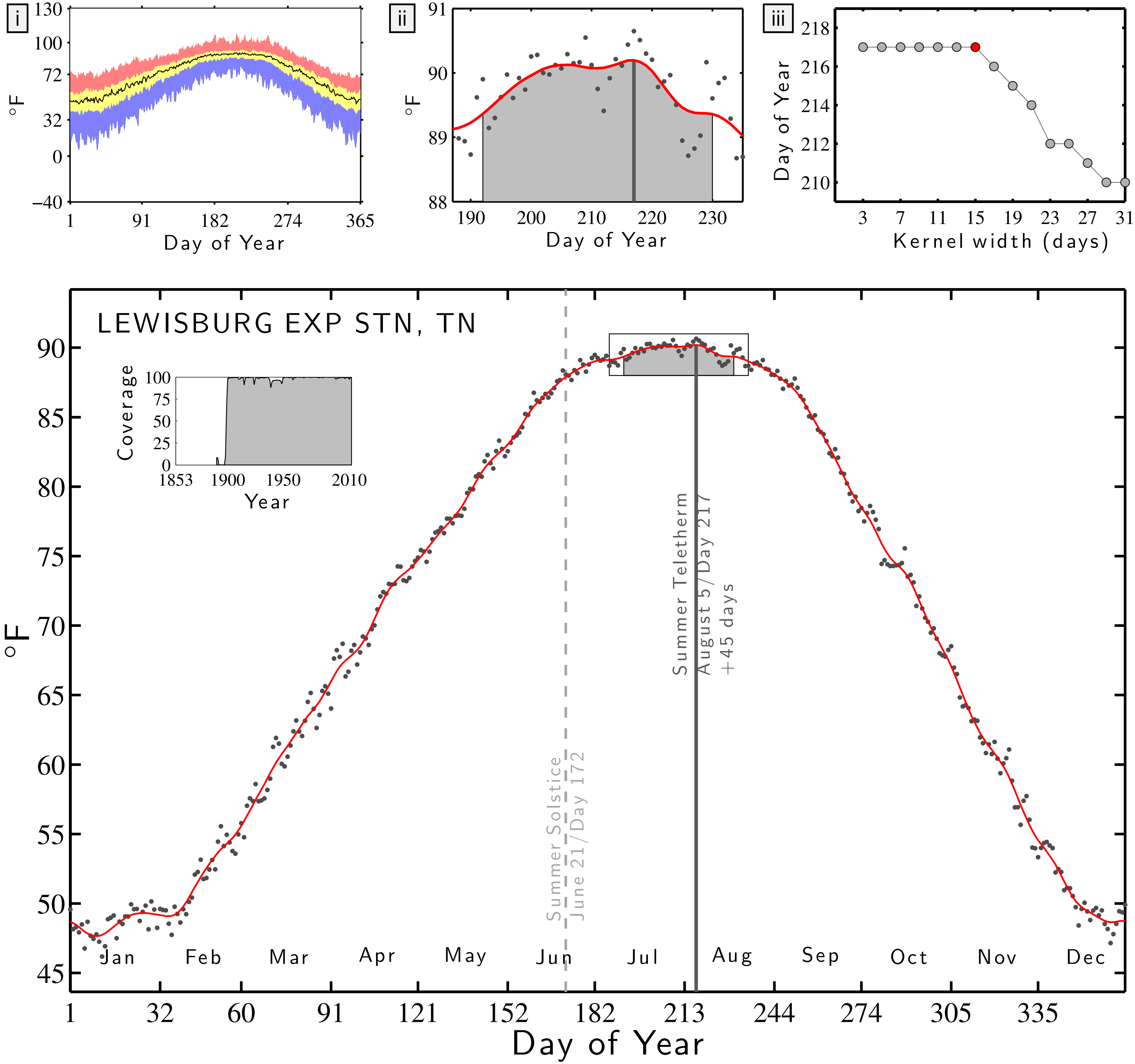

i
°F
Day of Year
ii
Day of Year
iii
Day of Year
Kernel width (days)
LEWISBURG EXP STN, TN
Coverage
Year
Summer Solstice
June 21/Day 172
Summer Teletherm
August 5/Day 217
+45 days
Jan
Feb
Mar
Apr
May
Jun
Jul
Aug
Sep
Oct
Nov
Dec
Day of Year

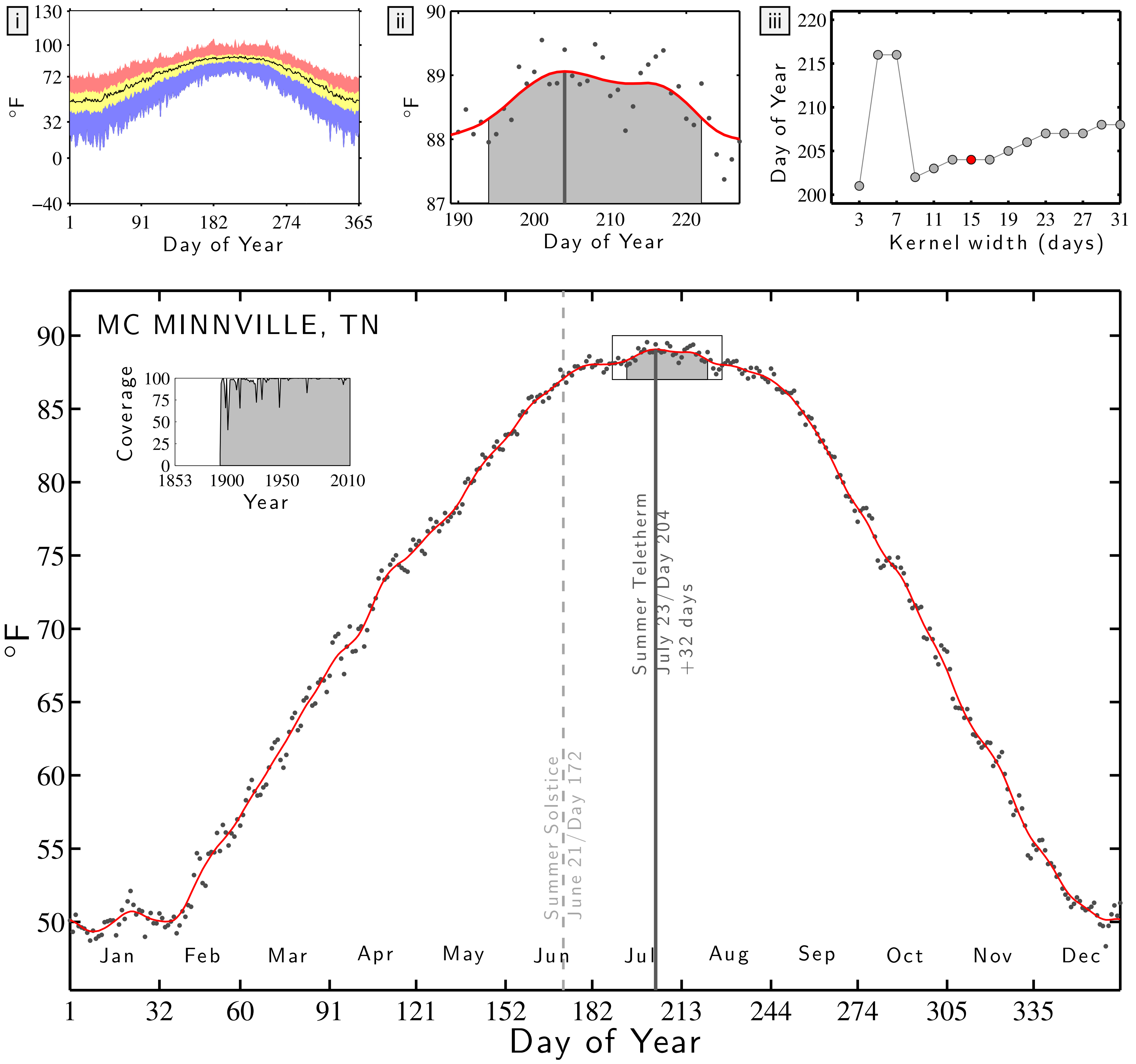

i
ii
iii
°F
Day of Year
Day of Year
Kernel width (days)
MC MINNVILLE, TN
Coverage
Year
Summer Solstice
June 21 / Day 172
Summer Teletherm
July 23 / Day 204
+32 days
Jan
Feb
Mar
Apr
May
Jun
Jul
Aug
Sep
Oct
Nov
Dec

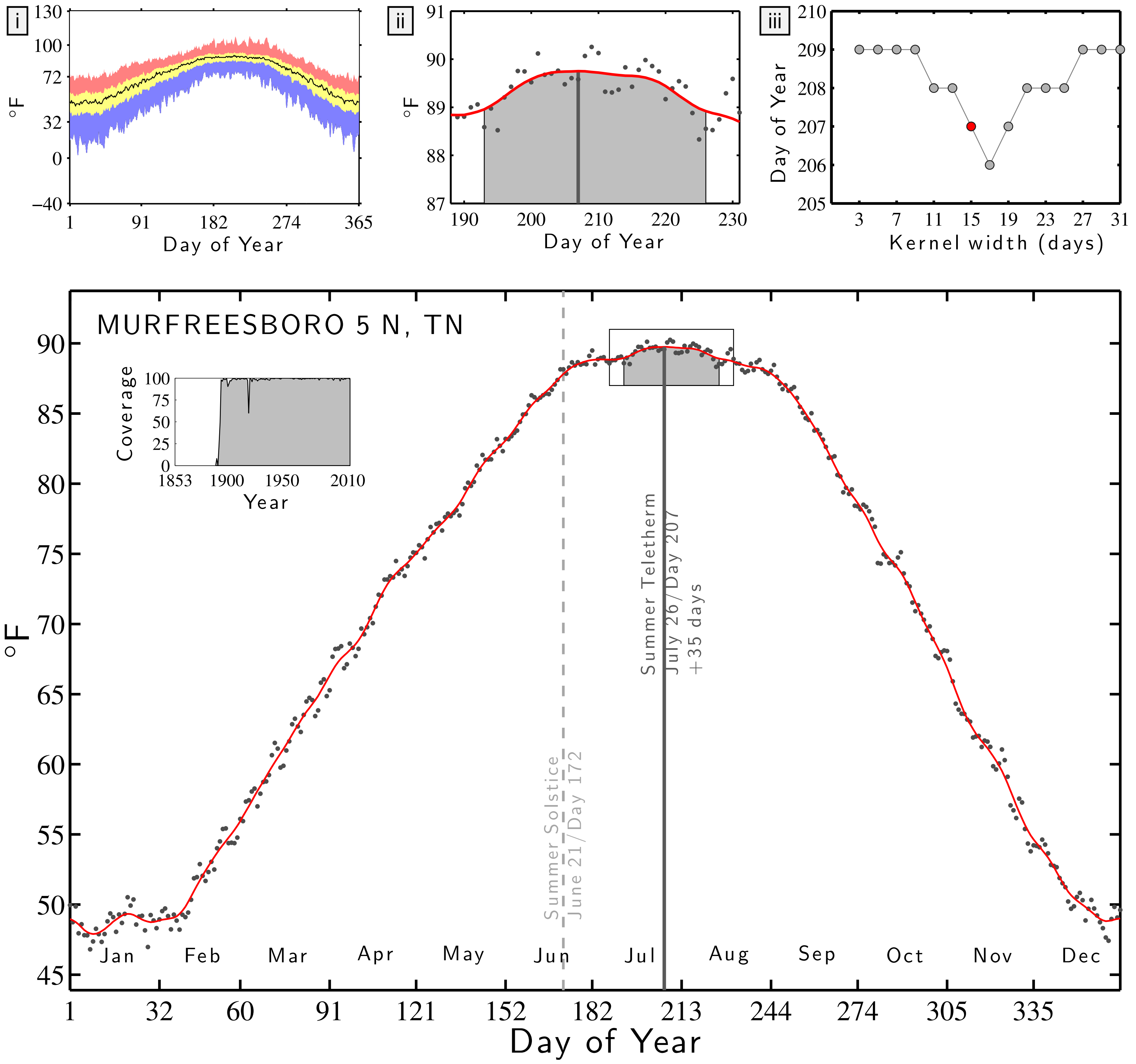

i
ii
iii
°F
Day of Year
Kernel width (days)
MURFREESBORO 5 N, TN
Coverage
Year
Summer Solstice
June 21 / Day 172
Summer Teletherm
July 26 / Day 207
+35 days
Jan
Feb
Mar
Apr
May
Jun
Jul
Aug
Sep
Oct
Nov
Dec

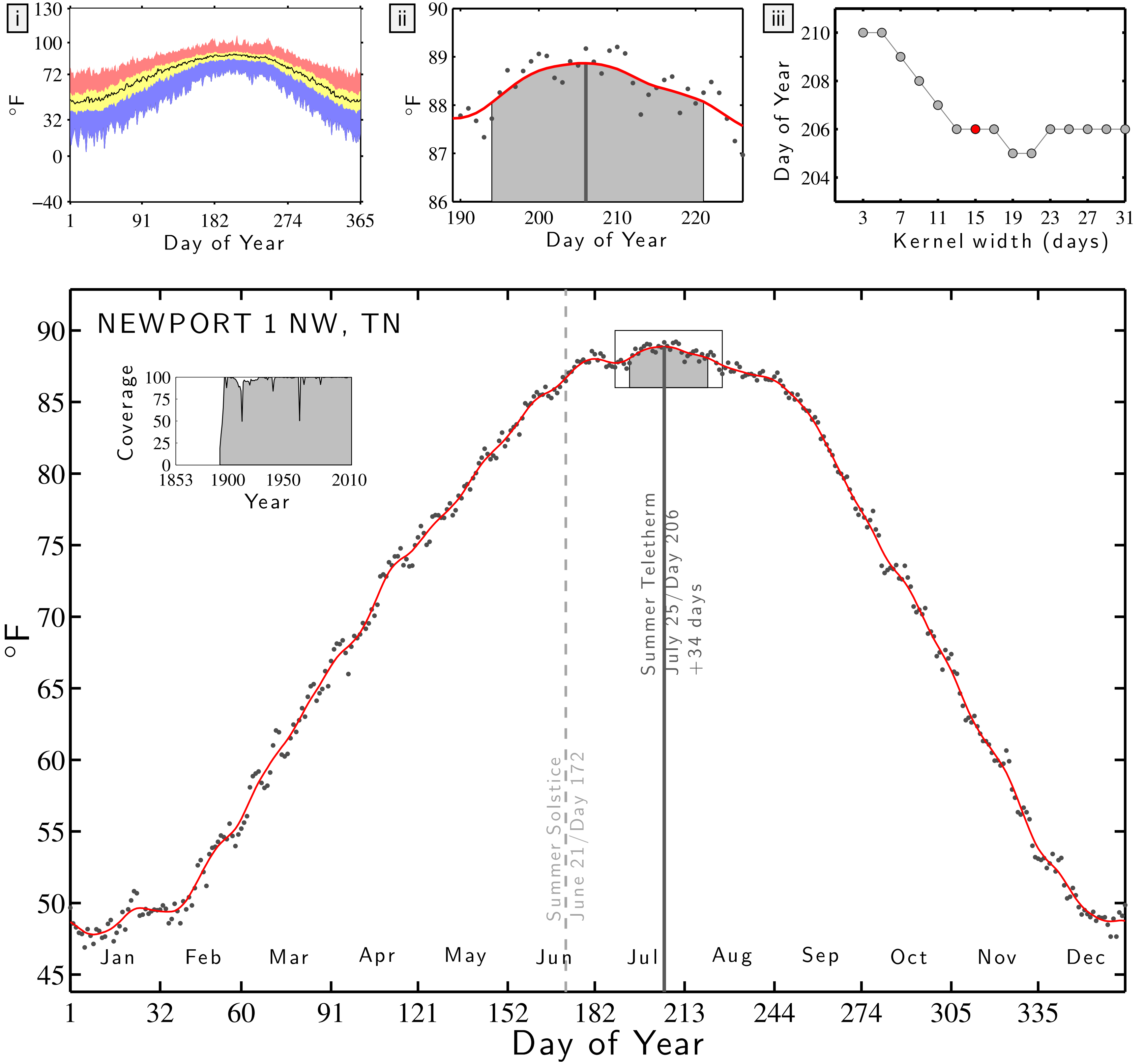

i
°F
Day of Year
ii
°F
Day of Year
iii
Day of Year
Kernel width (days)
NEWPORT 1 NW, TN
Coverage
Year
Summer Solstice
June 21/Day 172
Summer Teletherm
July 25/Day 206
+34 days
Jan
Feb
Mar
Apr
May
Jun
Jul
Aug
Sep
Oct
Nov
Dec
°F
Day of Year

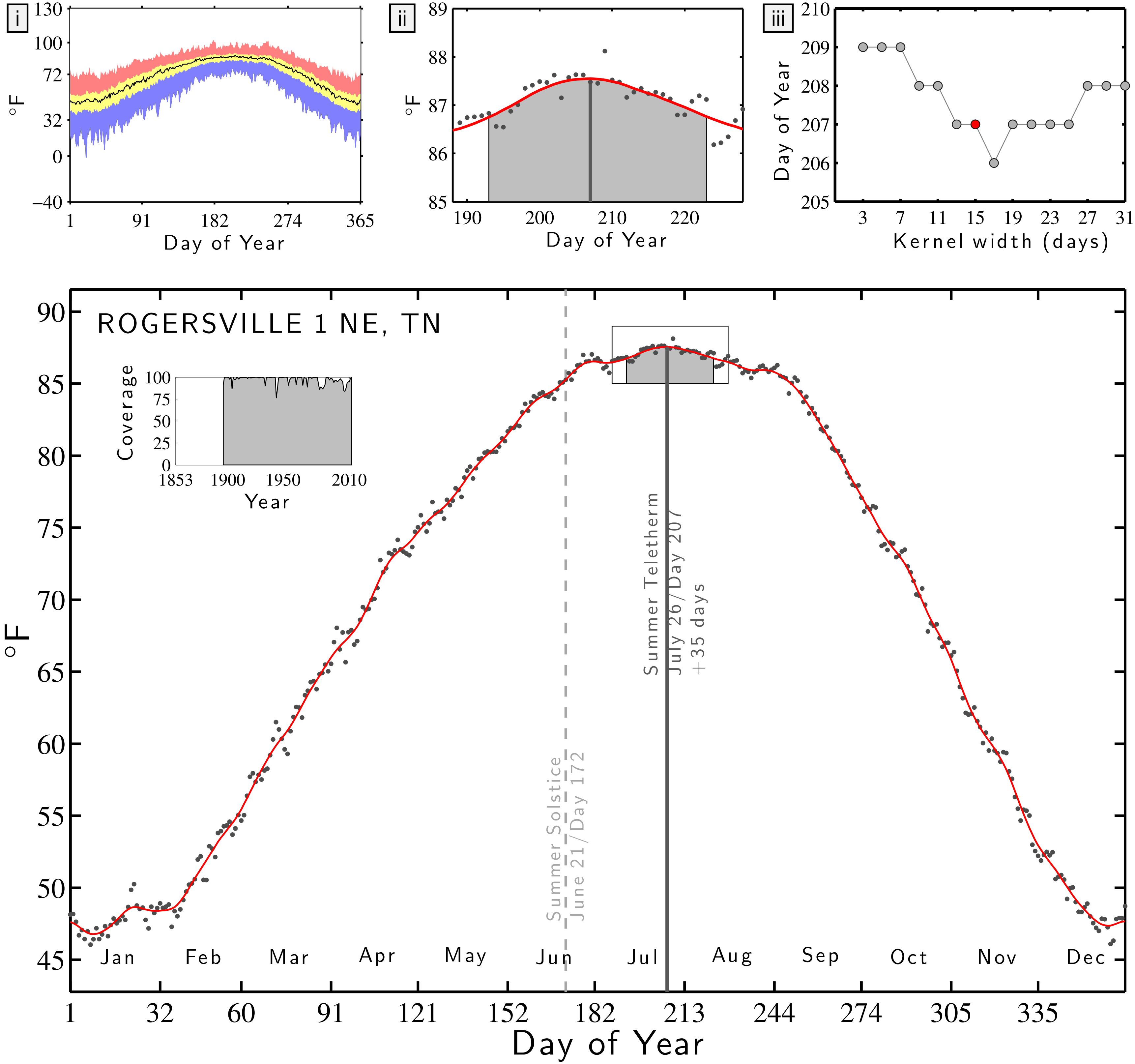

i
°F
Day of Year
ii
°F
Day of Year
iii
Day of Year
Kernel width (days)
ROGERSVILLE 1 NE, TN
Coverage
Year
Summer Solstice
June 21 / Day 172
Summer Teletherm
July 26 / Day 207
+35 days
Jan
Feb
Mar
Apr
May
Jun
Jul
Aug
Sep
Oct
Nov
Dec
°F
Day of Year

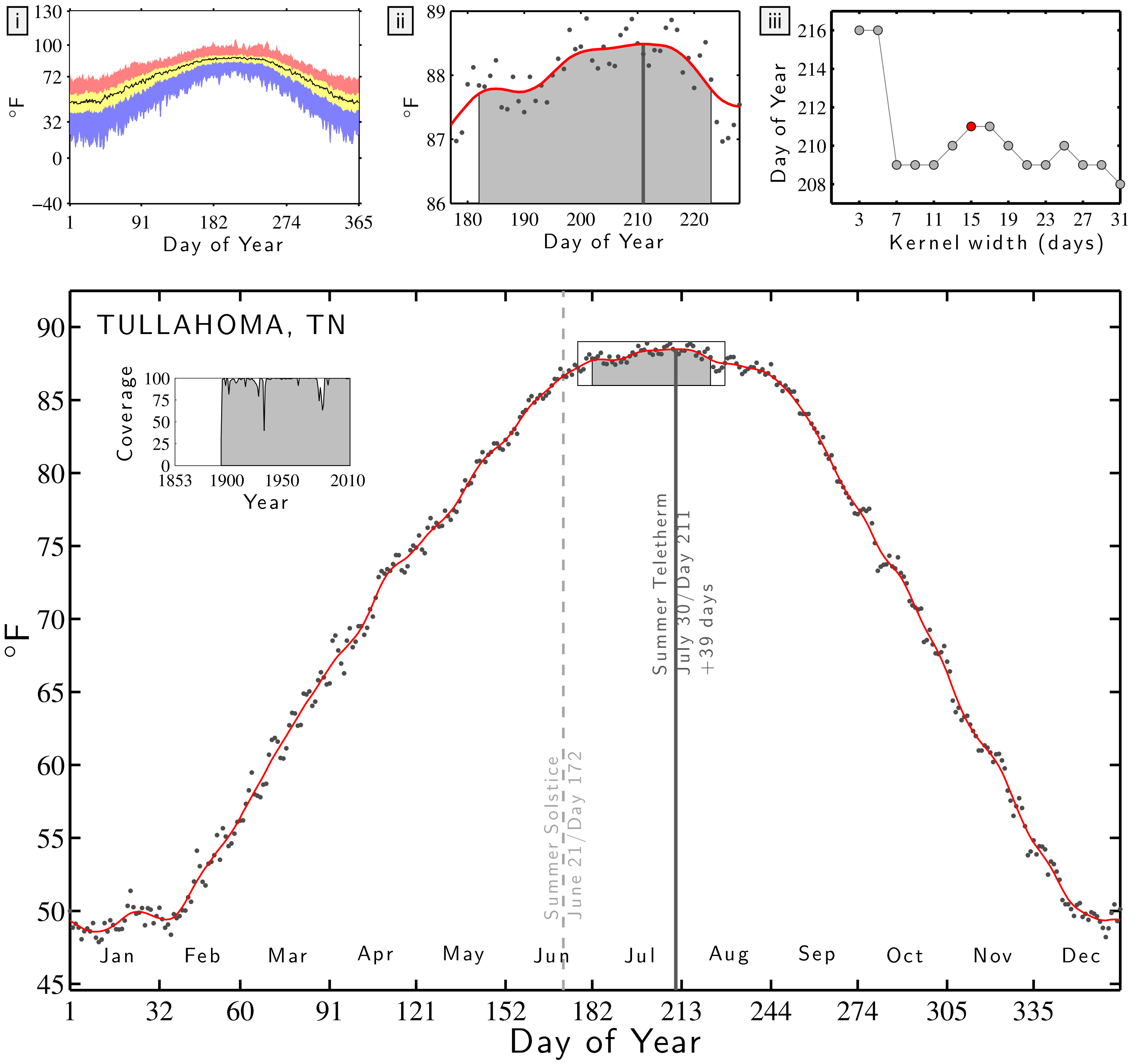

i
°F
Day of Year
ii
°F
Day of Year
iii
Day of Year
Kernel width (days)
TULLAHOMA, TN
Coverage
Year
Summer Solstice
June 21/Day 172
Summer Teletherm
July 30/Day 211
+39 days
Jan
Feb
Mar
Apr
May
Jun
Jul
Aug
Sep
Oct
Nov
Dec
°F
Day of Year

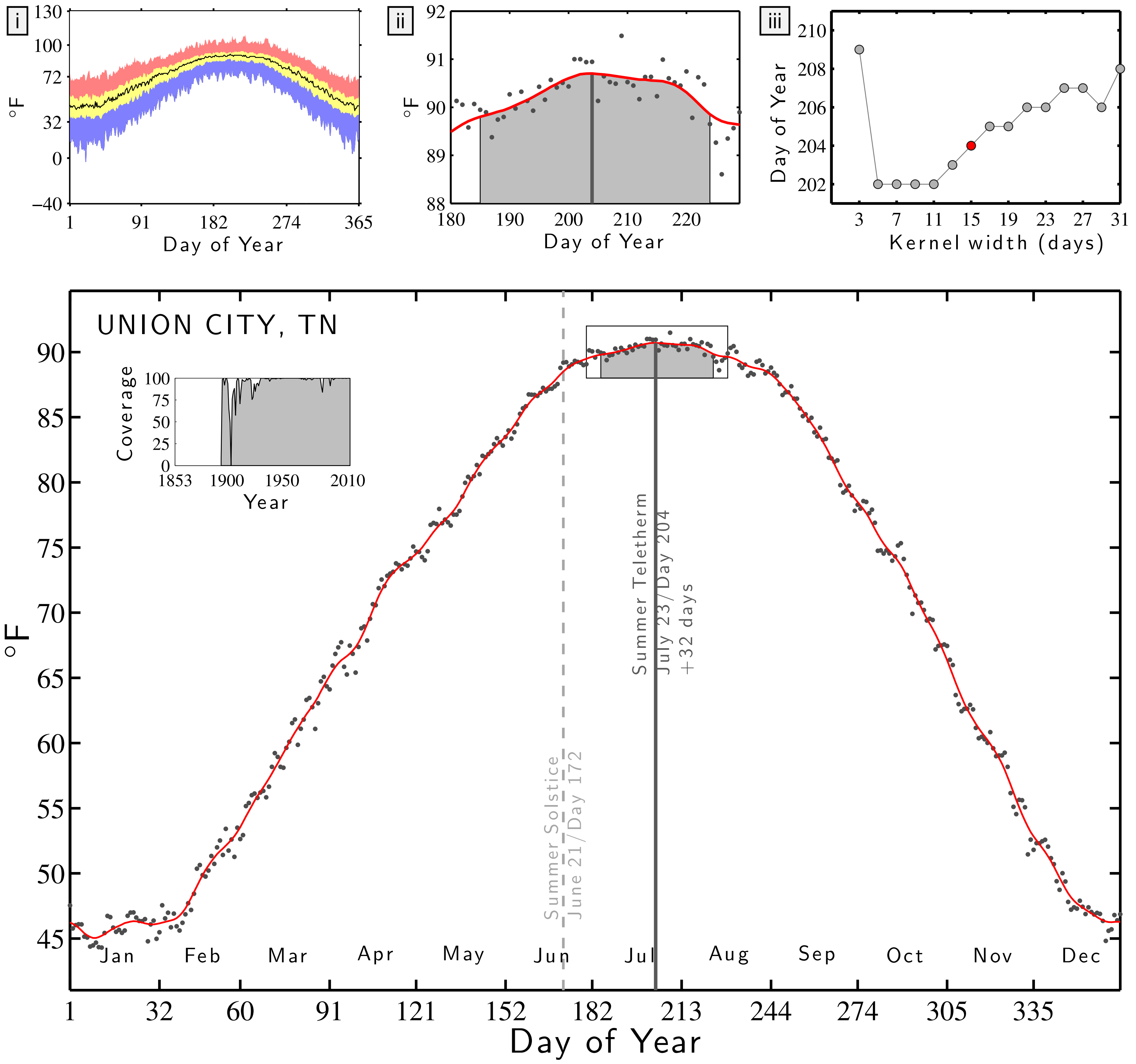

i
ii
iii
°F
Day of Year
Kernel width (days)
Day of Year
UNION CITY, TN
Coverage
Year
Summer Solstice
June 21 / Day 172
Summer Teletherm
July 23 / Day 204
+32 days
Jan
Feb
Mar
Apr
May
Jun
Jul
Aug
Sep
Oct
Nov
Dec

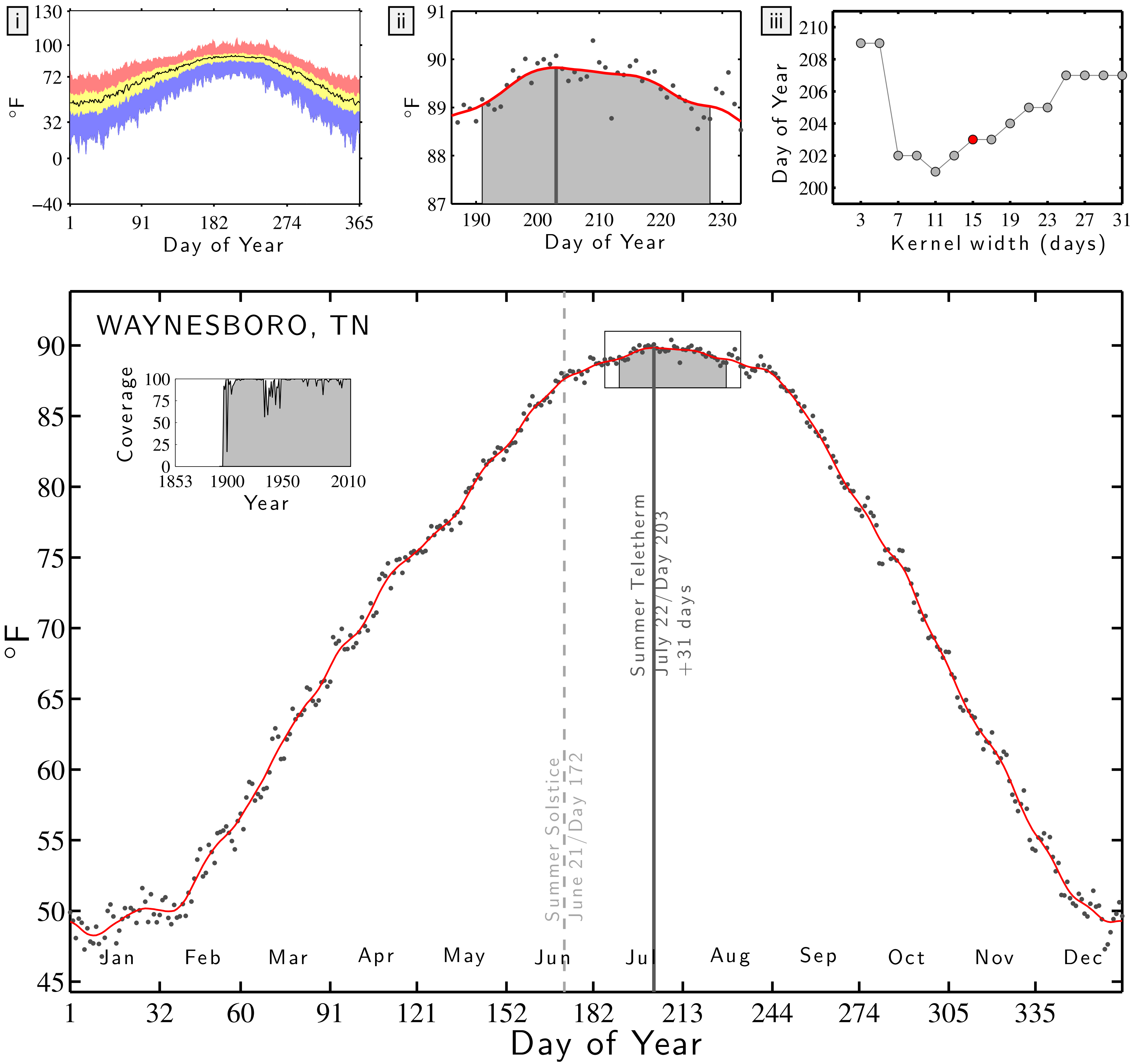

i
°F
130
100
72
32
0
−40
1
91
182
274
365
Day of Year
ii
91
90
89
88
87
190
200
210
220
230
Day of Year
iii
Day of Year
210
208
206
204
202
200
3
7
11
15
19
23
27
31
Kernel width (days)
WAYNESBORO, TN
Coverage
100
75
50
25
0
1853
1900
1950
2010
Year
Summer Solstice
June 21/Day 172
Summer Teletherm
July 22/Day 203
+31 days
90
85
80
75
70
65
60
55
50
45
Jan
Feb
Mar
Apr
May
Jun
Jul
Aug
Sep
Oct
Nov
Dec
1
32
60
91
121
152
182
213
244
274
305
335
Day of Year

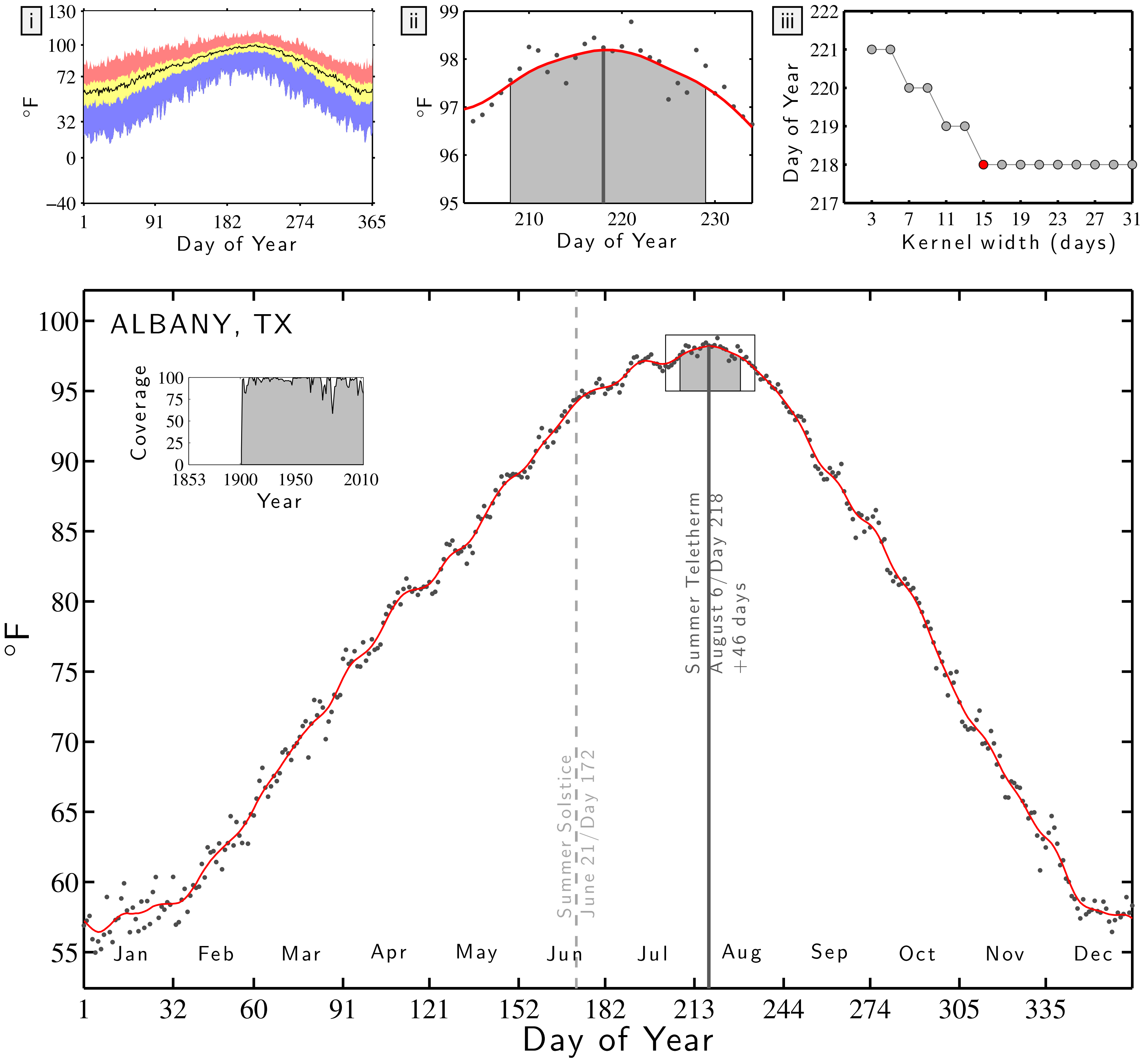

i
°F
Day of Year
ii
Day of Year
iii
Day of Year
Kernel width (days)
ALBANY, TX
Coverage
Year
Summer Solstice
June 21/Day 172
Summer Teletherm
August 6/Day 218
+46 days
Jan
Feb
Mar
Apr
May
Jun
Jul
Aug
Sep
Oct
Nov
Dec
°F
Day of Year

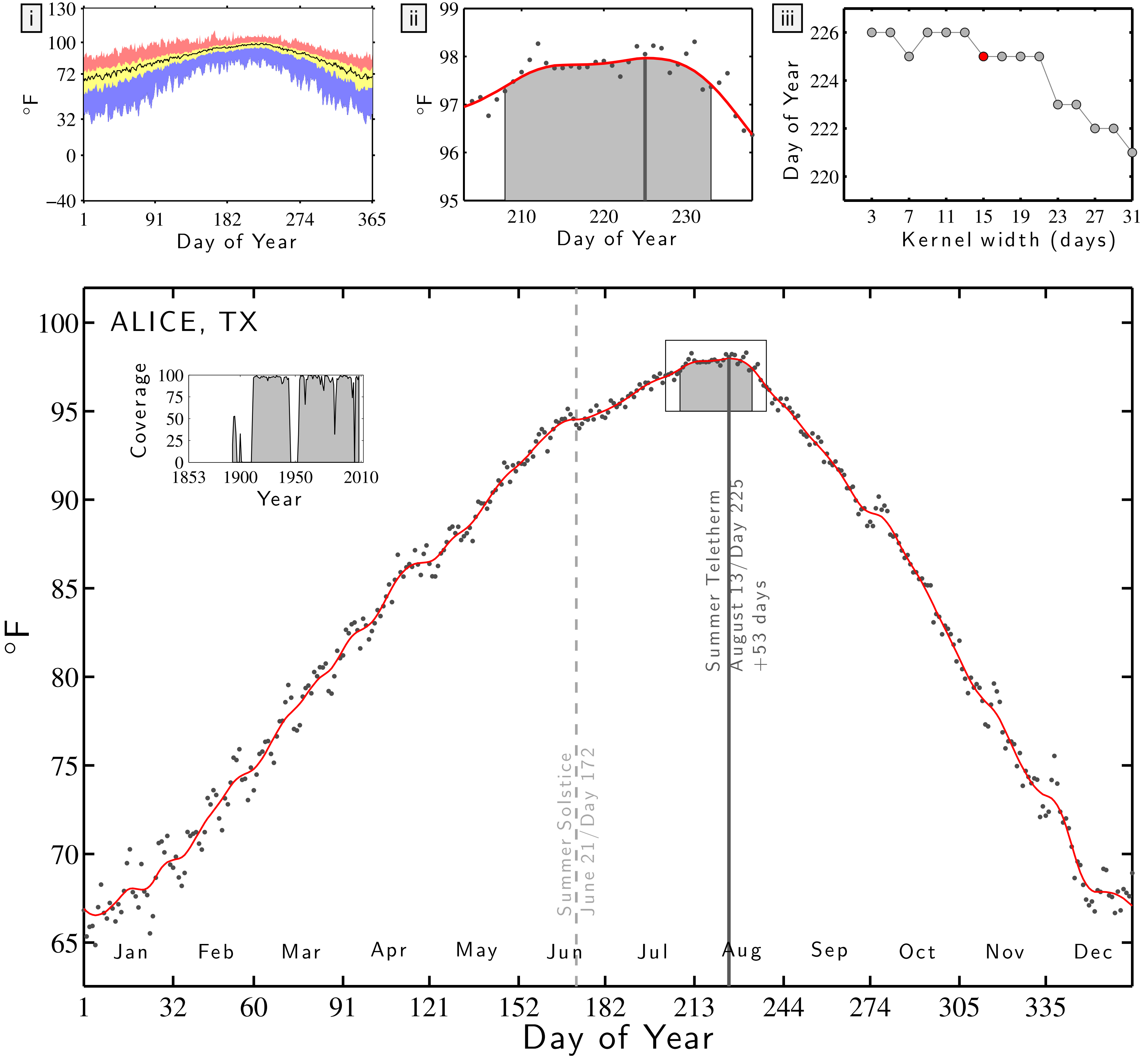

i
ii
iii
Day of Year
°F
Day of Year
Kernel width (days)
ALICE, TX
Coverage
Year
Summer Solstice
June 21/Day 172
Summer Teletherm
August 13/Day 225
+53 days
Jan
Feb
Mar
Apr
May
Jun
Jul
Aug
Sep
Oct
Nov
Dec
Day of Year
°F

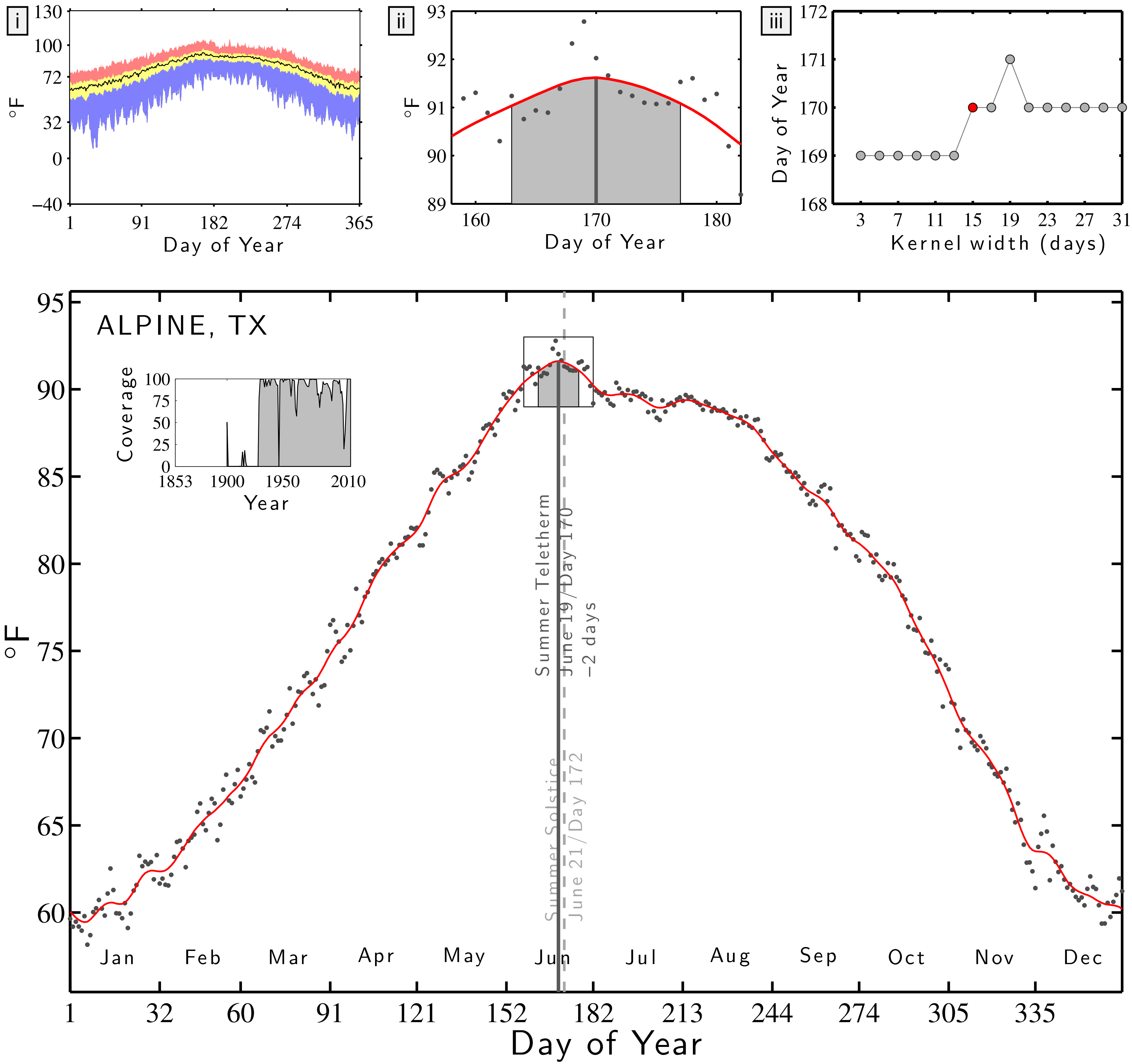

i
°F
Day of Year
ii
°F
Day of Year
iii
Day of Year
Kernel width (days)
ALPINE, TX
Coverage
Year
Summer Teletherm
June 19 / Day 170
−2 days
Summer Solstice
June 21 / Day 172
Jan
Feb
Mar
Apr
May
Jun
Jul
Aug
Sep
Oct
Nov
Dec
°F
Day of Year

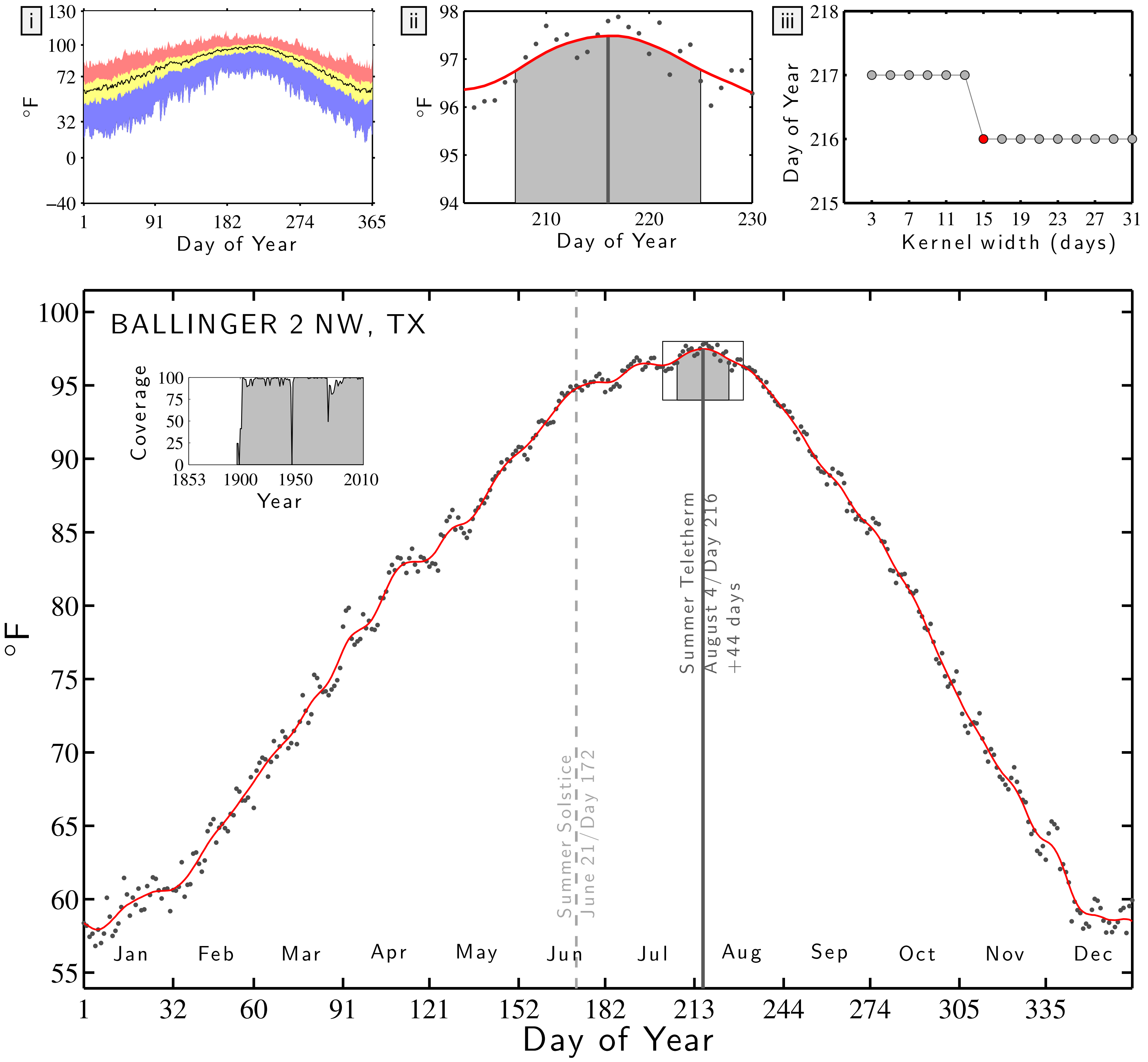

i
°F
Day of Year
ii
iii
Day of Year
Kernel width (days)
BALLINGER 2 NW, TX
Coverage
Year
Summer Solstice
June 21/Day 172
Summer Teletherm
August 4/Day 216
+44 days
Jan
Feb
Mar
Apr
May
Jun
Jul
Aug
Sep
Oct
Nov
Dec
Day of Year

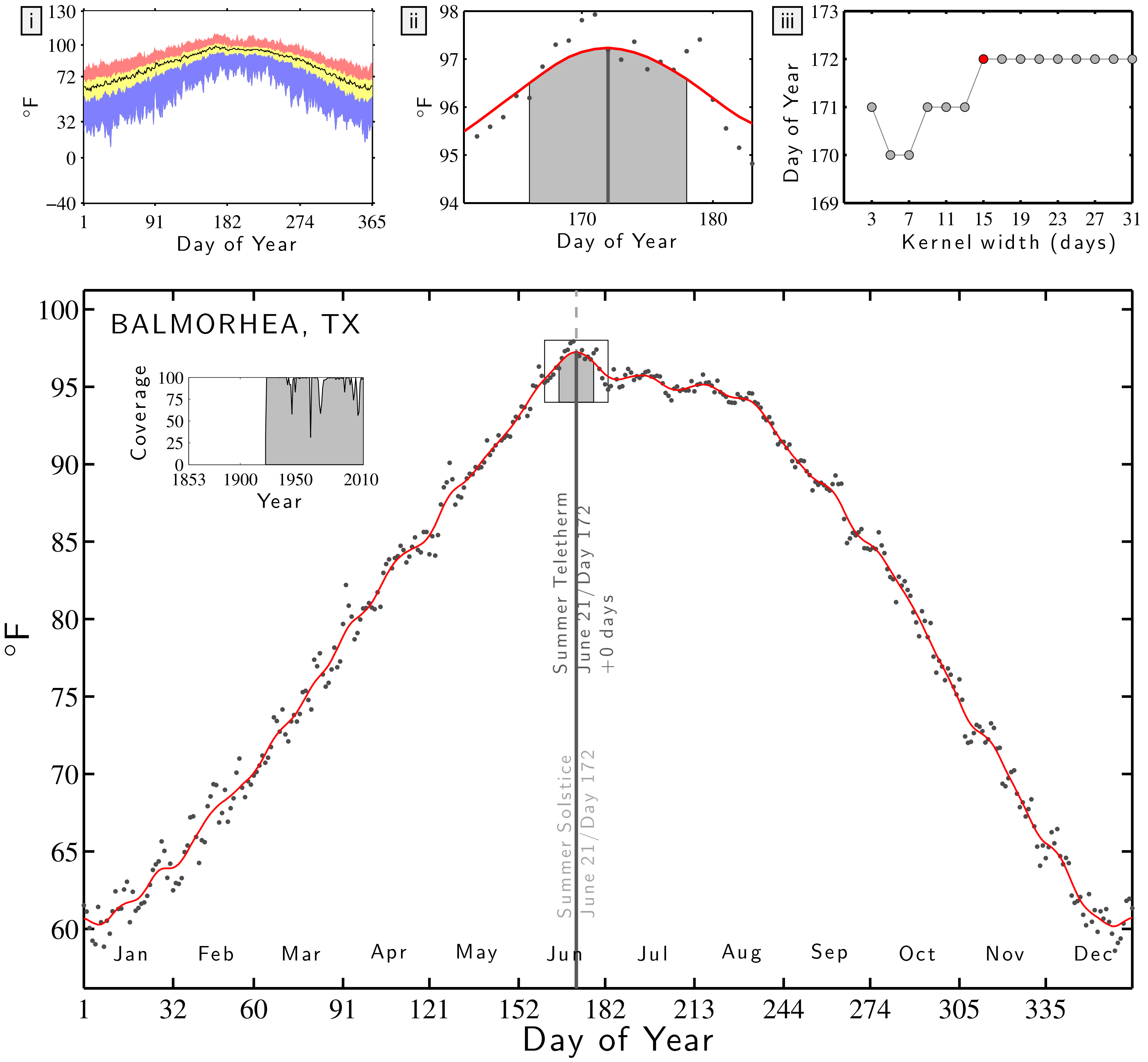

i
ii
iii
°F
Day of Year
Day of Year
Kernel width (days)
BALMORHEA, TX
Coverage
Year
Summer Teletherm
June 21/Day 172
+0 days
Summer Solstice
June 21/Day 172
Jan
Feb
Mar
Apr
May
Jun
Jul
Aug
Sep
Oct
Nov
Dec
°F
Day of Year

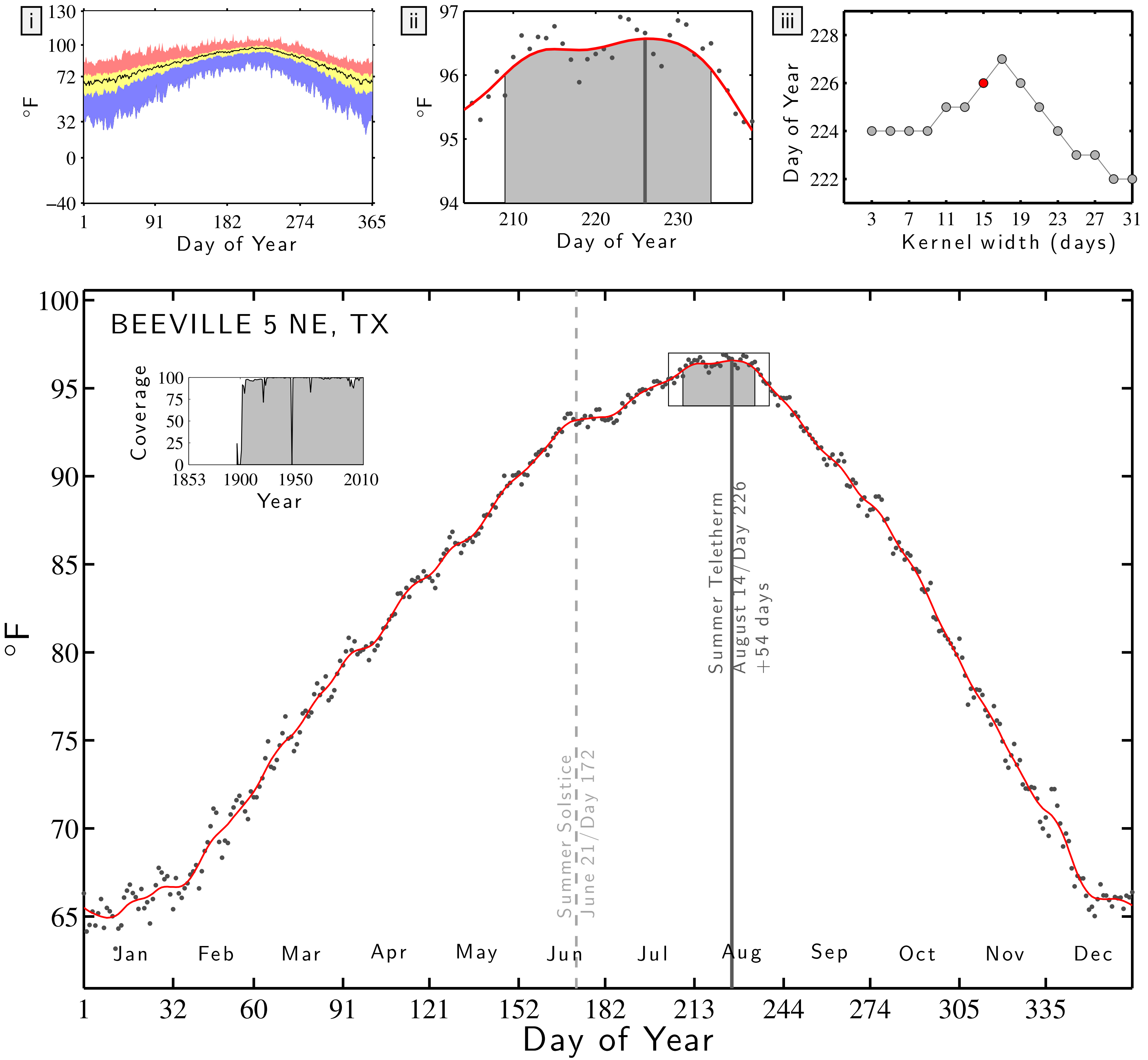

i
130
100
72
32
0
−40
°F
1
91
182
274
365
Day of Year
ii
97
96
95
94
°F
210
220
230
Day of Year
iii
228
226
224
222
Day of Year
3
7
11
15
19
23
27
31
Kernel width (days)
BEEVILLE 5 NE, TX
Coverage
100
75
50
25
0
1853
1900
1950
2010
Year
100
95
90
85
80
75
70
65
°F
Summer Solstice
June 21/Day 172
Summer Teletherm
August 14/Day 226
+54 days
Jan
Feb
Mar
Apr
May
Jun
Jul
Aug
Sep
Oct
Nov
Dec
1
32
60
91
121
152
182
213
244
274
305
335
Day of Year

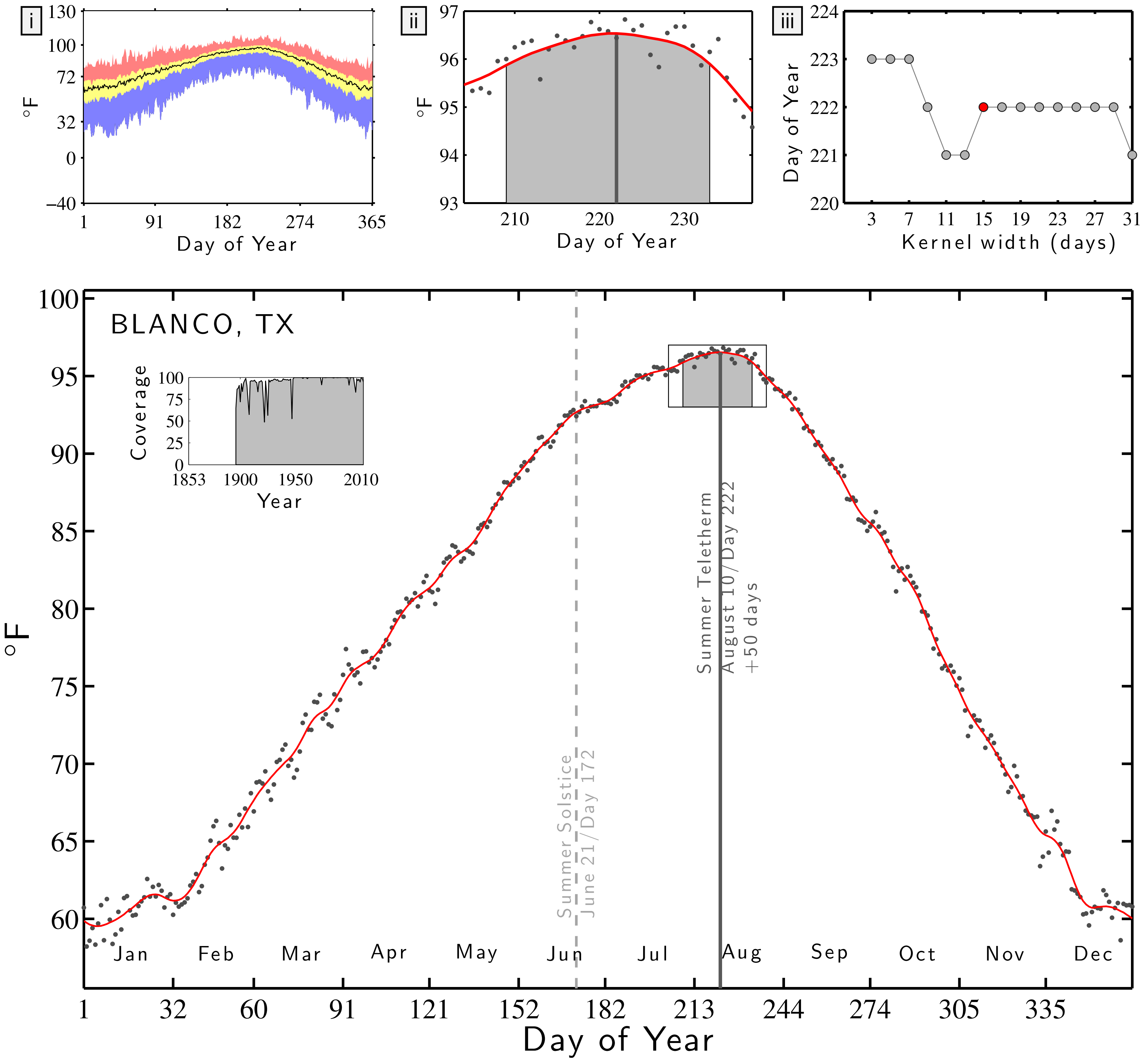

i
ii
iii
°F
Day of Year
Day of Year
Kernel width (days)
BLANCO, TX
Coverage
Year
Summer Solstice
June 21/Day 172
Summer Teletherm
August 10/Day 222
+50 days
Jan
Feb
Mar
Apr
May
Jun
Jul
Aug
Sep
Oct
Nov
Dec

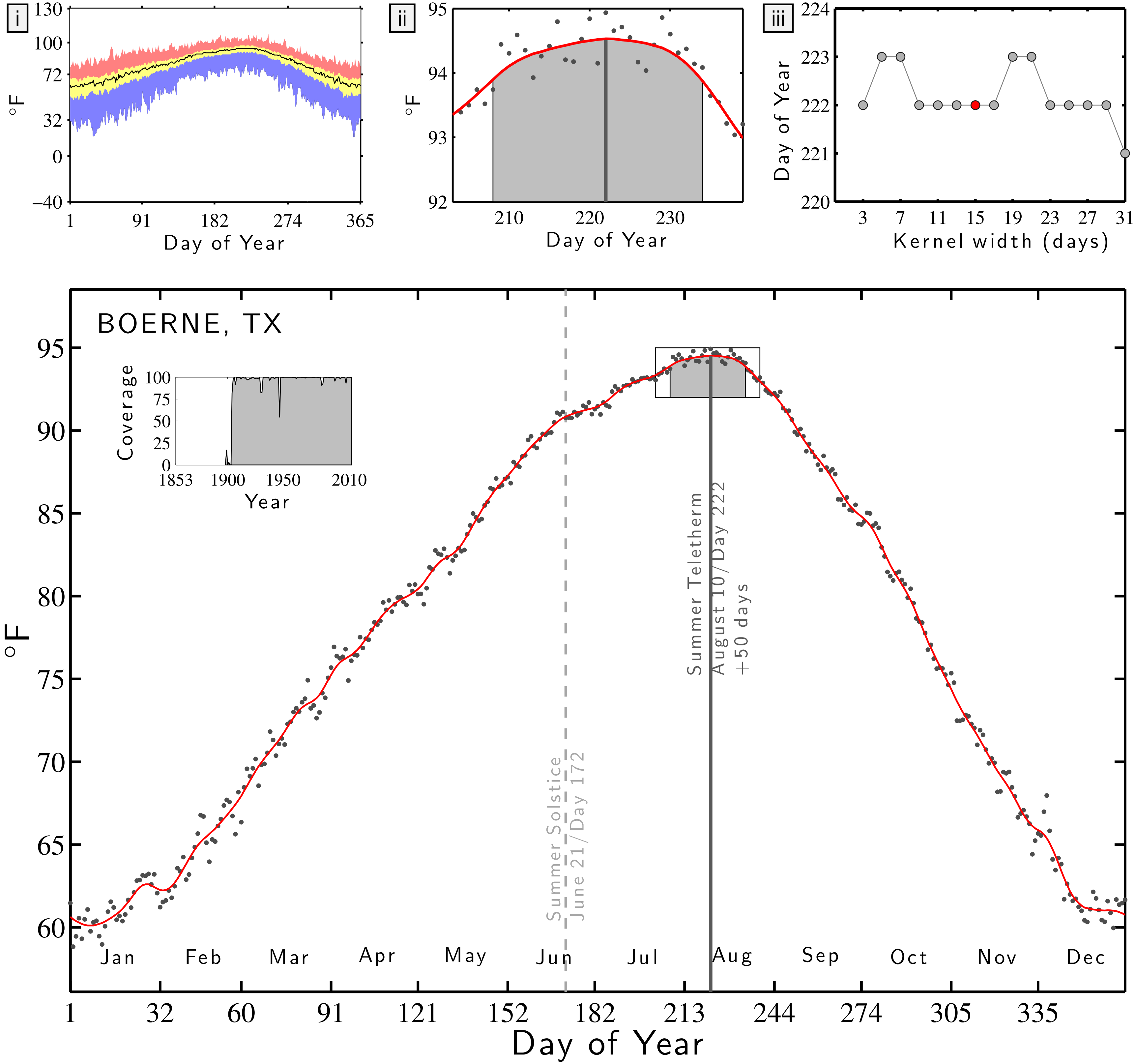

i
130
100
72
32
0
−40
°F
1
91
182
274
365
Day of Year
ii
95
94
93
92
°F
210
220
230
Day of Year
iii
224
223
222
221
220
Day of Year
3
7
11
15
19
23
27
31
Kernel width (days)
BOERNE, TX
Coverage
100
75
50
25
0
1853
1900
1950
2010
Year
Summer Solstice
June 21/Day 172
Summer Teletherm
August 10/Day 222
+50 days
°F
95
90
85
80
75
70
65
60
Jan
Feb
Mar
Apr
May
Jun
Jul
Aug
Sep
Oct
Nov
Dec
1
32
60
91
121
152
182
213
244
274
305
335
Day of Year

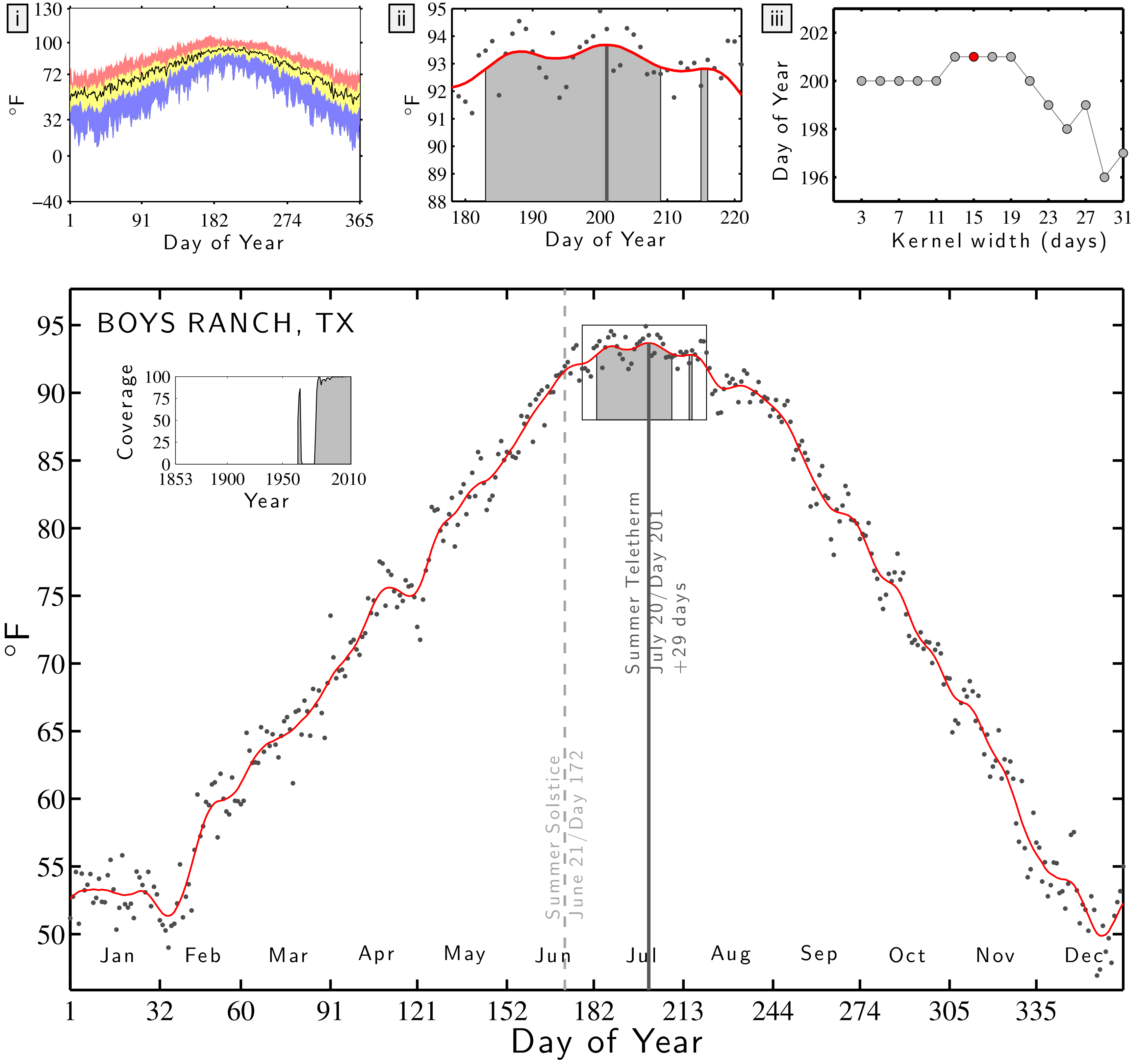

i
°F
Day of Year
ii
°F
Day of Year
iii
Day of Year
Kernel width (days)
BOYS RANCH, TX
Coverage
Year
Summer Solstice
June 21 / Day 172
Summer Teletherm
July 20 / Day 201
+29 days
Jan
Feb
Mar
Apr
May
Jun
Jul
Aug
Sep
Oct
Nov
Dec
°F
Day of Year

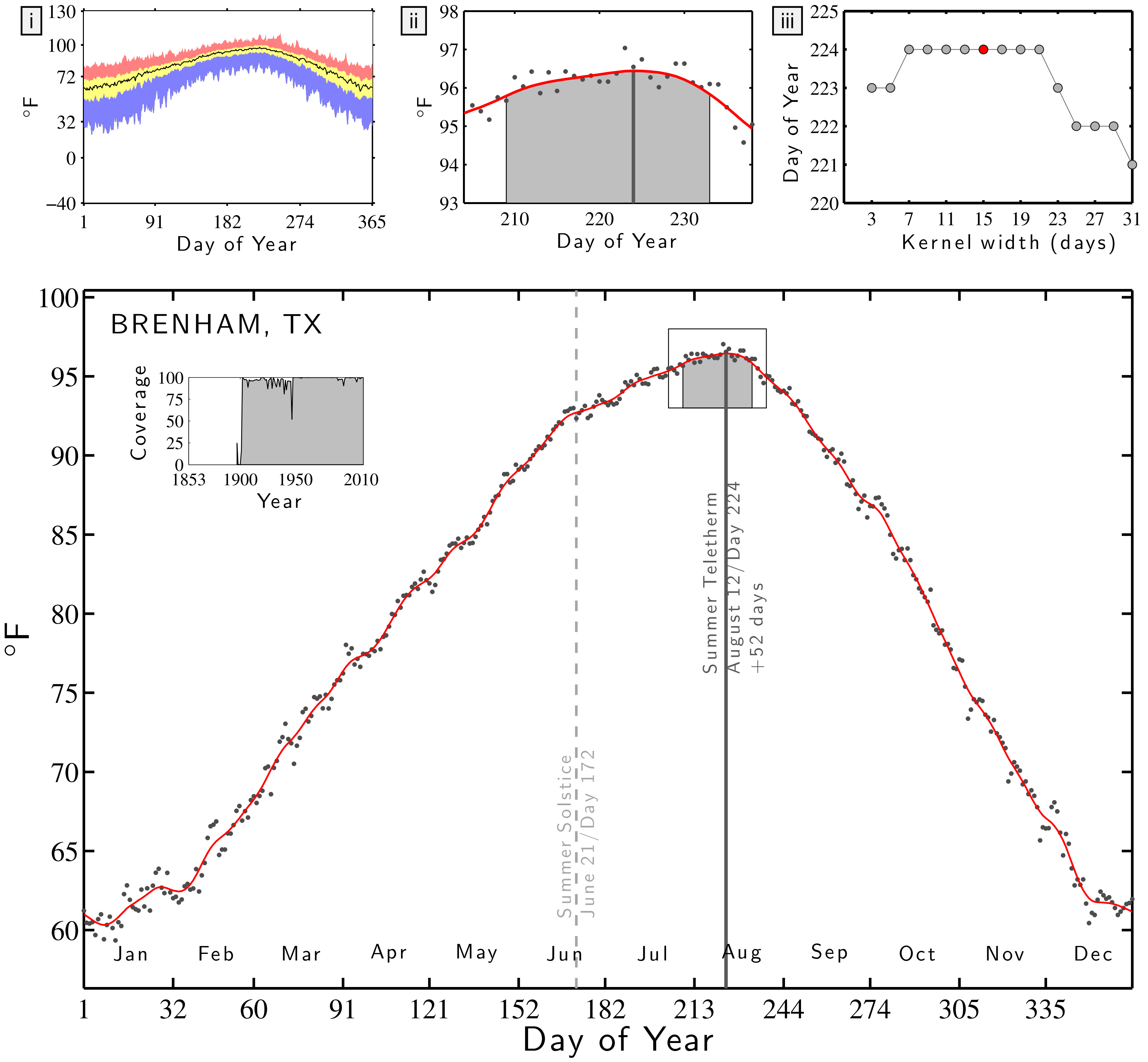

i
ii
iii
°F
Day of Year
Day of Year
Kernel width (days)
BRENHAM, TX
Coverage
Year
Summer Solstice
June 21/Day 172
Summer Teletherm
August 12/Day 224
+52 days
Jan
Feb
Mar
Apr
May
Jun
Jul
Aug
Sep
Oct
Nov
Dec

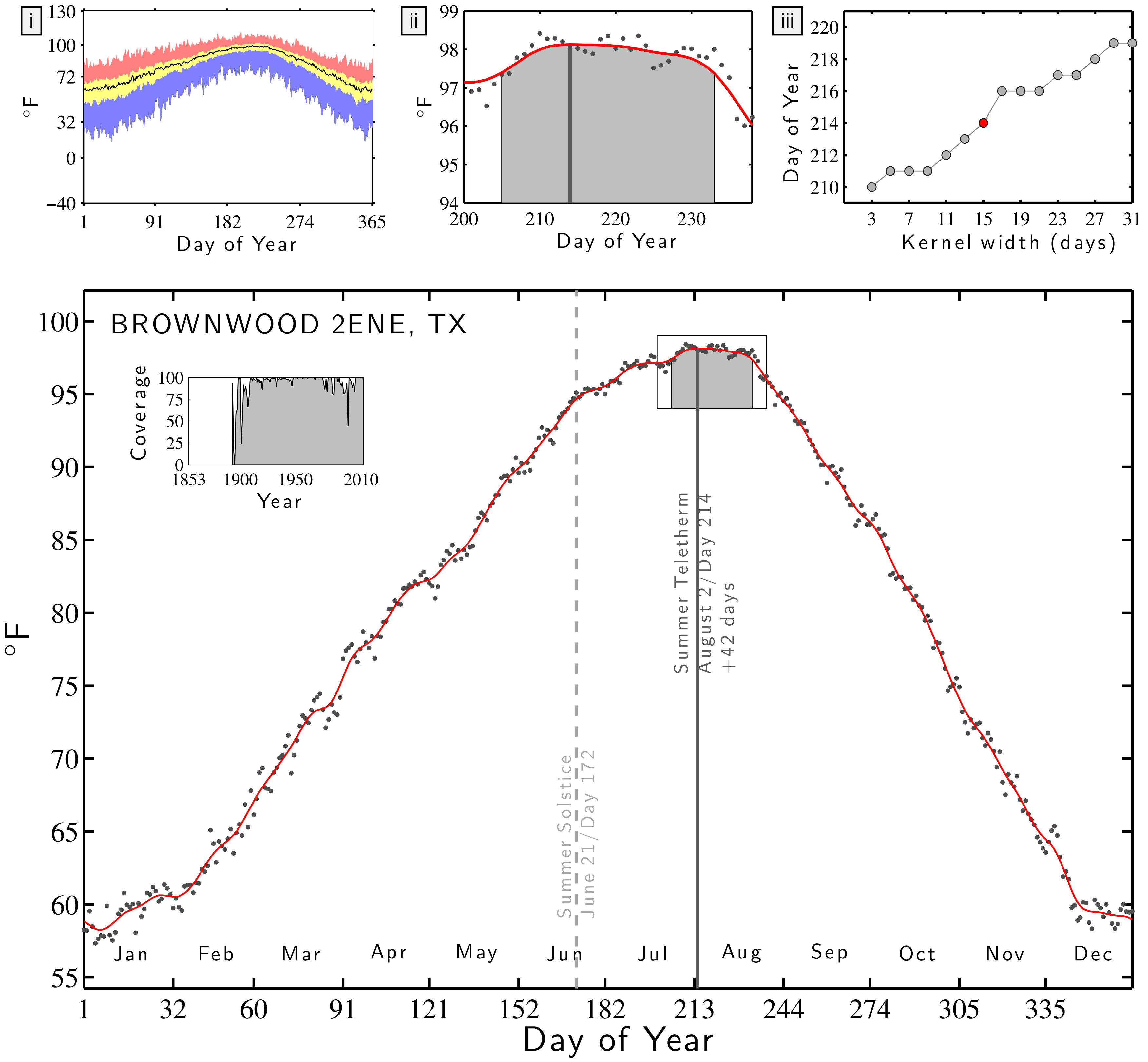

i
ii
iii
°F
Day of Year
Day of Year
Kernel width (days)
BROWNWOOD 2ENE, TX
Coverage
Year
Summer Solstice
June 21/Day 172
Summer Teletherm
August 2/Day 214
+42 days
Jan
Feb
Mar
Apr
May
Jun
Jul
Aug
Sep
Oct
Nov
Dec

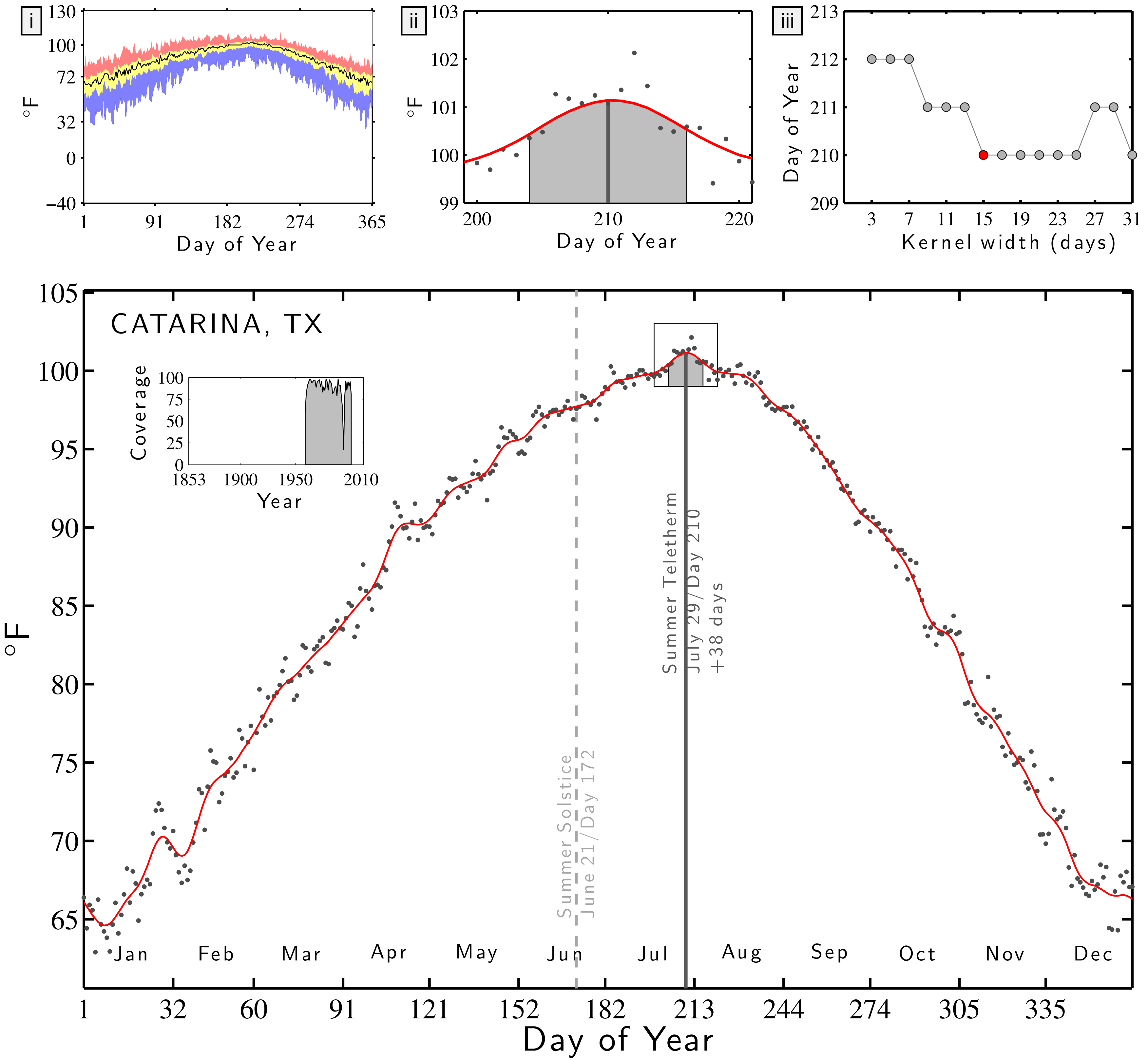

i
°F
Day of Year
ii
°F
Day of Year
iii
Day of Year
Kernel width (days)
CATARINA, TX
Coverage
Year
Summer Solstice
June 21/Day 172
Summer Teletherm
July 29/Day 210
+38 days
Jan
Feb
Mar
Apr
May
Jun
Jul
Aug
Sep
Oct
Nov
Dec
°F
Day of Year

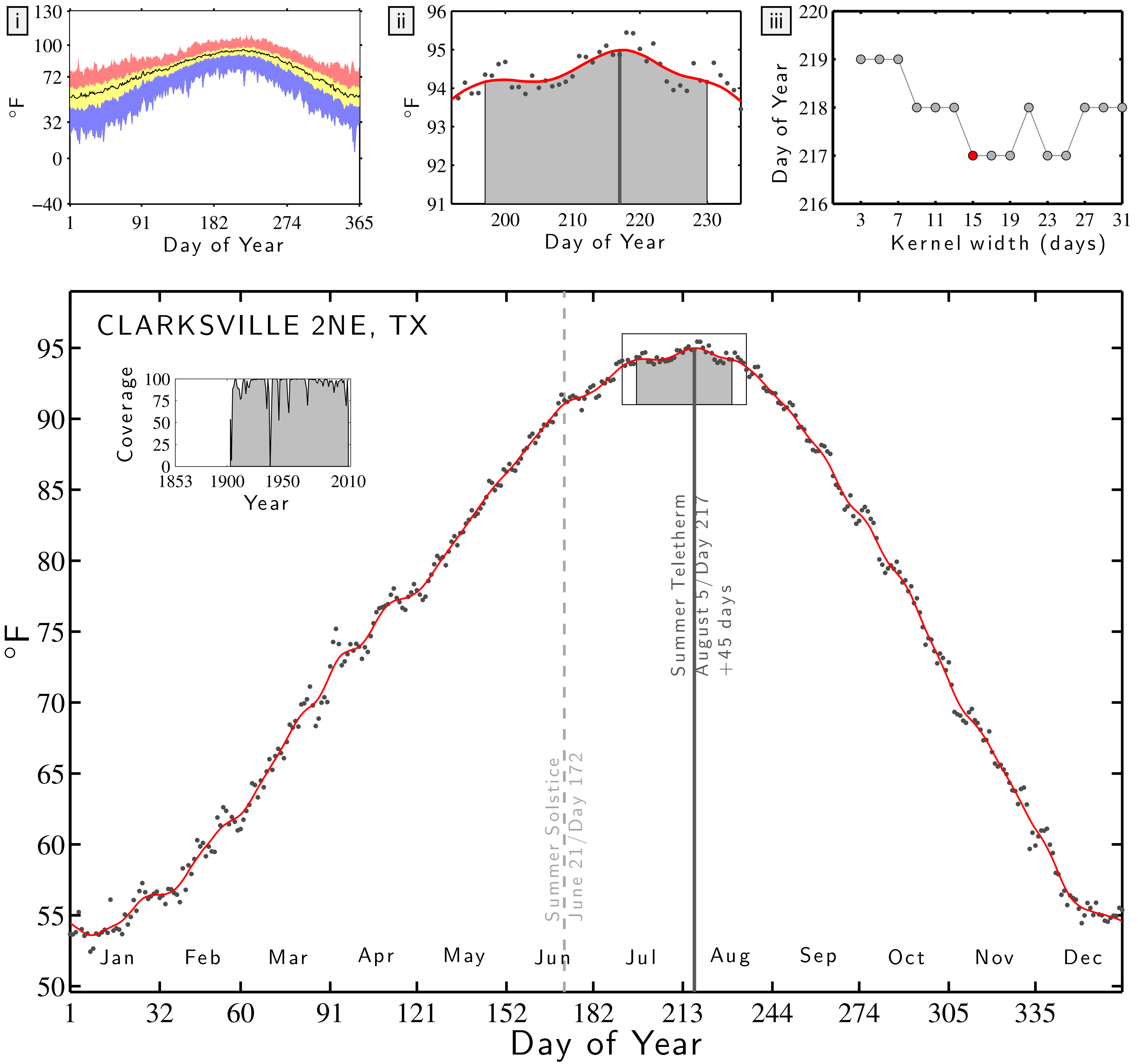

i
ii
iii
°F
Day of Year
Kernel width (days)
CLARKSVILLE 2NE, TX
Coverage
Year
Summer Solstice
June 21/Day 172
Summer Teletherm
August 5/Day 217
+45 days
Jan
Feb
Mar
Apr
May
Jun
Jul
Aug
Sep
Oct
Nov
Dec

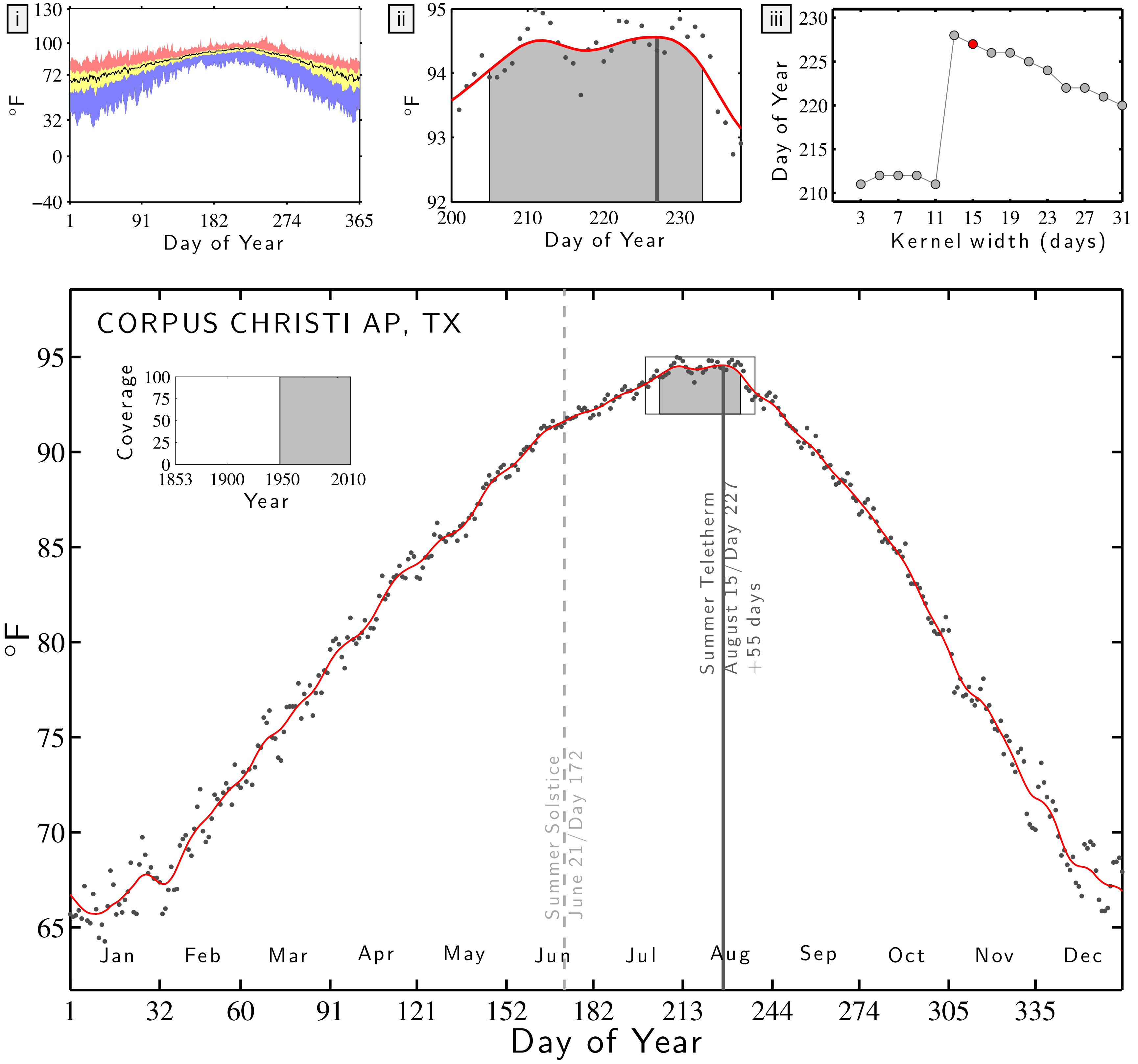

i
°F
130
100
72
32
0
−40
1
91
182
274
365
Day of Year
ii
95
94
93
92
200
210
220
230
Day of Year
iii
Day of Year
230
225
220
215
210
3
7
11
15
19
23
27
31
Kernel width (days)
CORPUS CHRISTI AP, TX
Coverage
100
75
50
25
0
1853
1900
1950
2010
Year
Summer Solstice
June 21 / Day 172
Summer Teletherm
August 15 / Day 227
+55 days
Jan
Feb
Mar
Apr
May
Jun
Jul
Aug
Sep
Oct
Nov
Dec
65
70
75
80
85
90
95
1
32
60
91
121
152
182
213
244
274
305
335
Day of Year

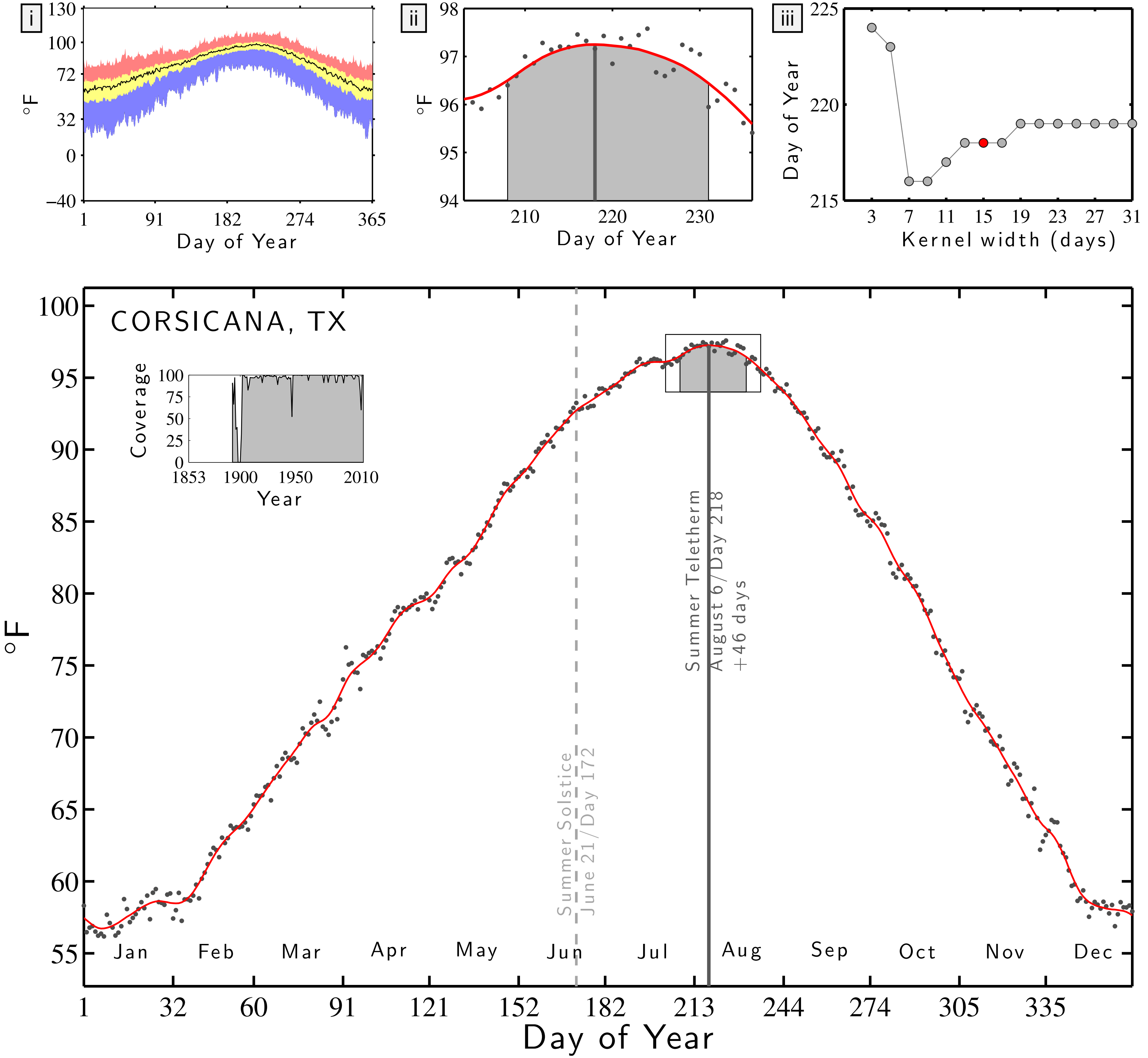

i
°F
Day of Year
ii
°F
Day of Year
iii
Day of Year
Kernel width (days)
CORSICANA, TX
Coverage
Year
Summer Solstice
June 21/Day 172
Summer Teletherm
August 6/Day 218
+46 days
Jan
Feb
Mar
Apr
May
Jun
Jul
Aug
Sep
Oct
Nov
Dec
°F
Day of Year

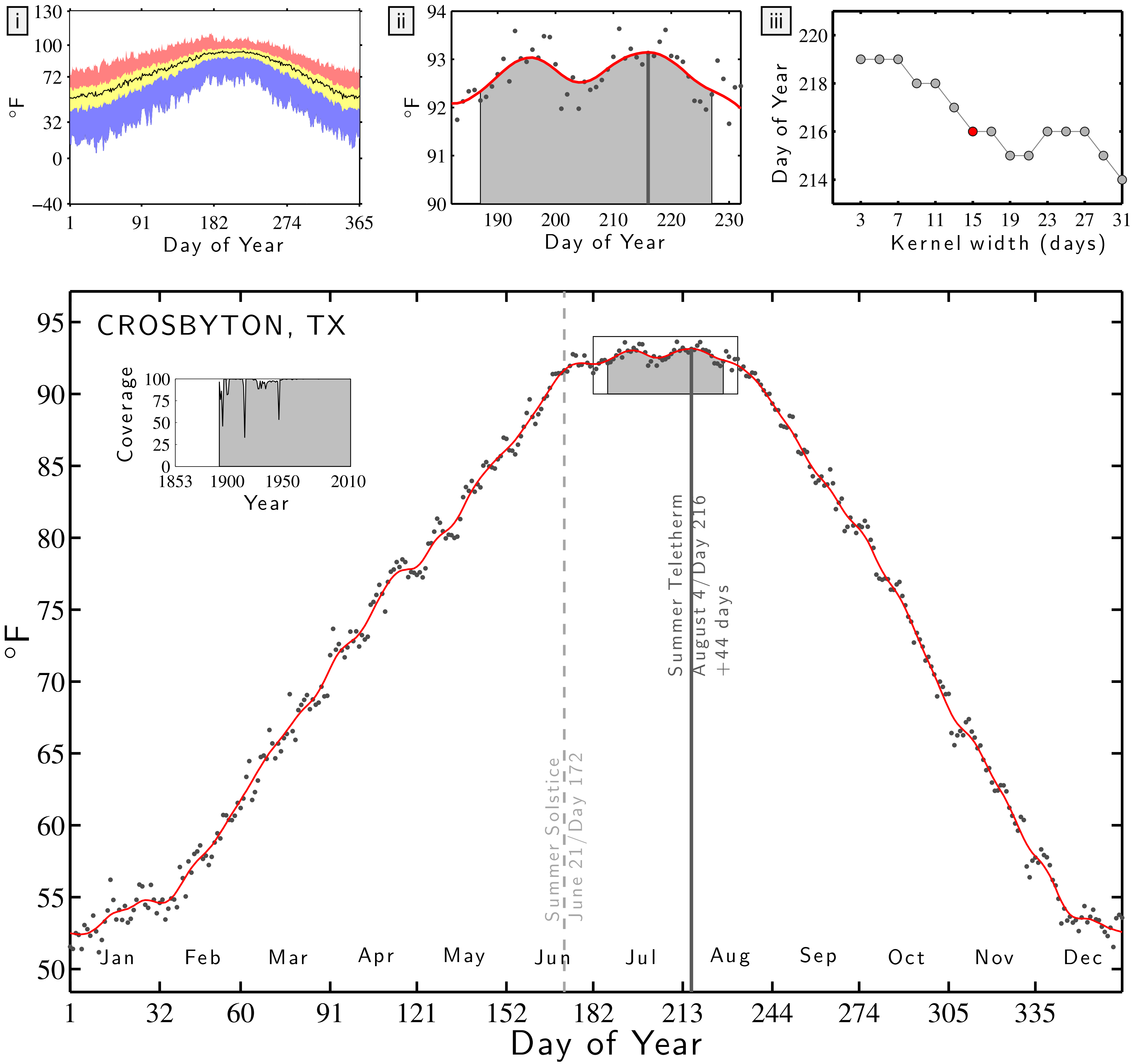

i
130
100
72
32
0
−40
°F
1
91
182
274
365
Day of Year
ii
94
93
92
91
90
°F
190
200
210
220
230
Day of Year
iii
220
218
216
214
Day of Year
3
7
11
15
19
23
27
31
Kernel width (days)
CROSBYTON, TX
95
90
85
80
75
70
65
60
55
50
°F
100
75
50
25
0
Coverage
1853
1900
1950
2010
Year
Summer Solstice
June 21 / Day 172
Summer Teletherm
August 4 / Day 216
+44 days
Jan
Feb
Mar
Apr
May
Jun
Jul
Aug
Sep
Oct
Nov
Dec
1
32
60
91
121
152
182
213
244
274
305
335
Day of Year

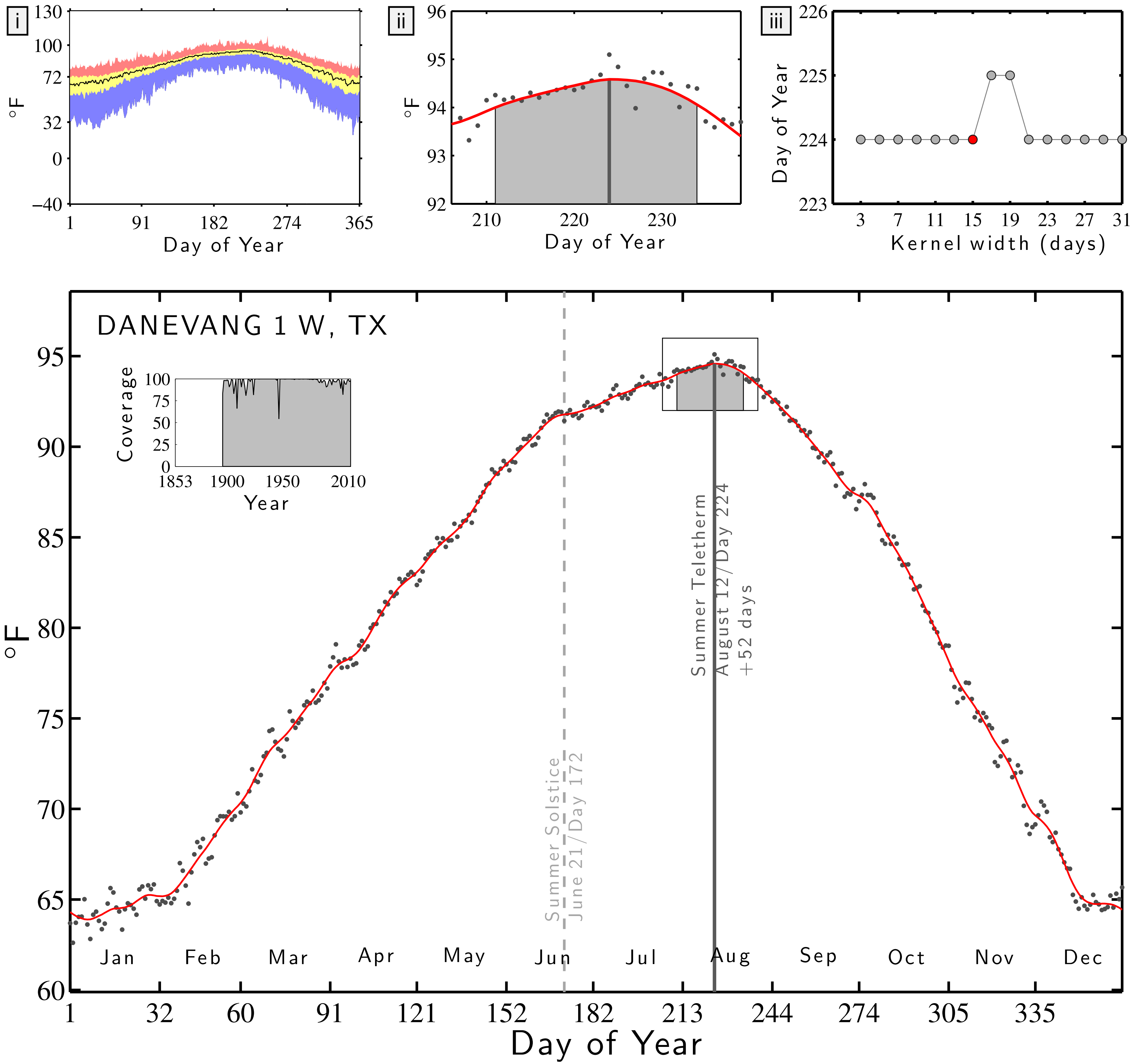

i
ii
iii
°F
Day of Year
Kernel width (days)
DANEVANG 1 W, TX
Coverage
Year
Summer Solstice
June 21 / Day 172
Summer Teletherm
August 12 / Day 224
+52 days
Jan
Feb
Mar
Apr
May
Jun
Jul
Aug
Sep
Oct
Nov
Dec

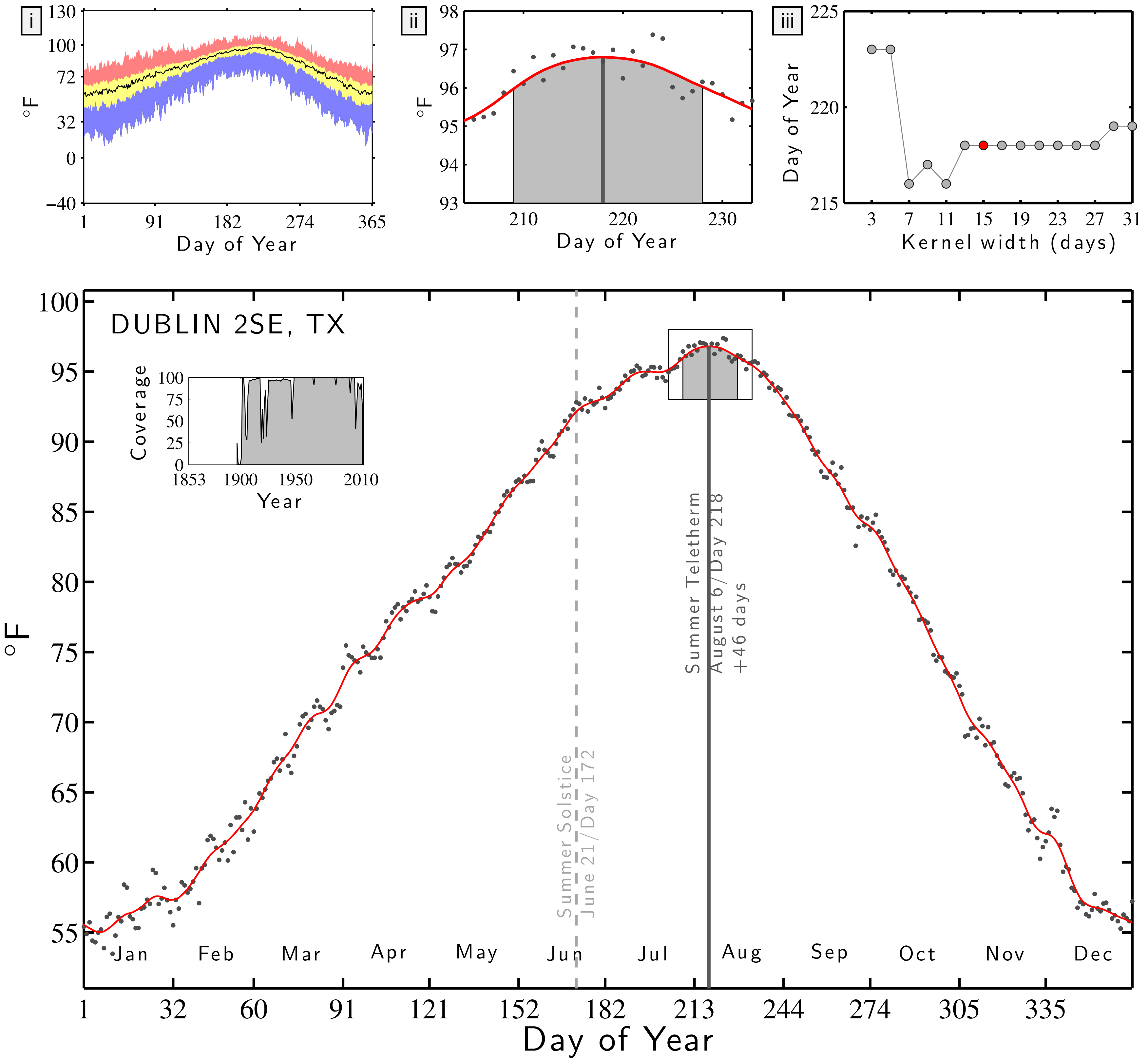

i
ii
iii
°F
Day of Year
Day of Year
Kernel width (days)
DUBLIN 2SE, TX
Coverage
Year
Summer Solstice
June 21/Day 172
Summer Teletherm
August 6/Day 218
+46 days
Jan
Feb
Mar
Apr
May
Jun
Jul
Aug
Sep
Oct
Nov
Dec
°F
Day of Year

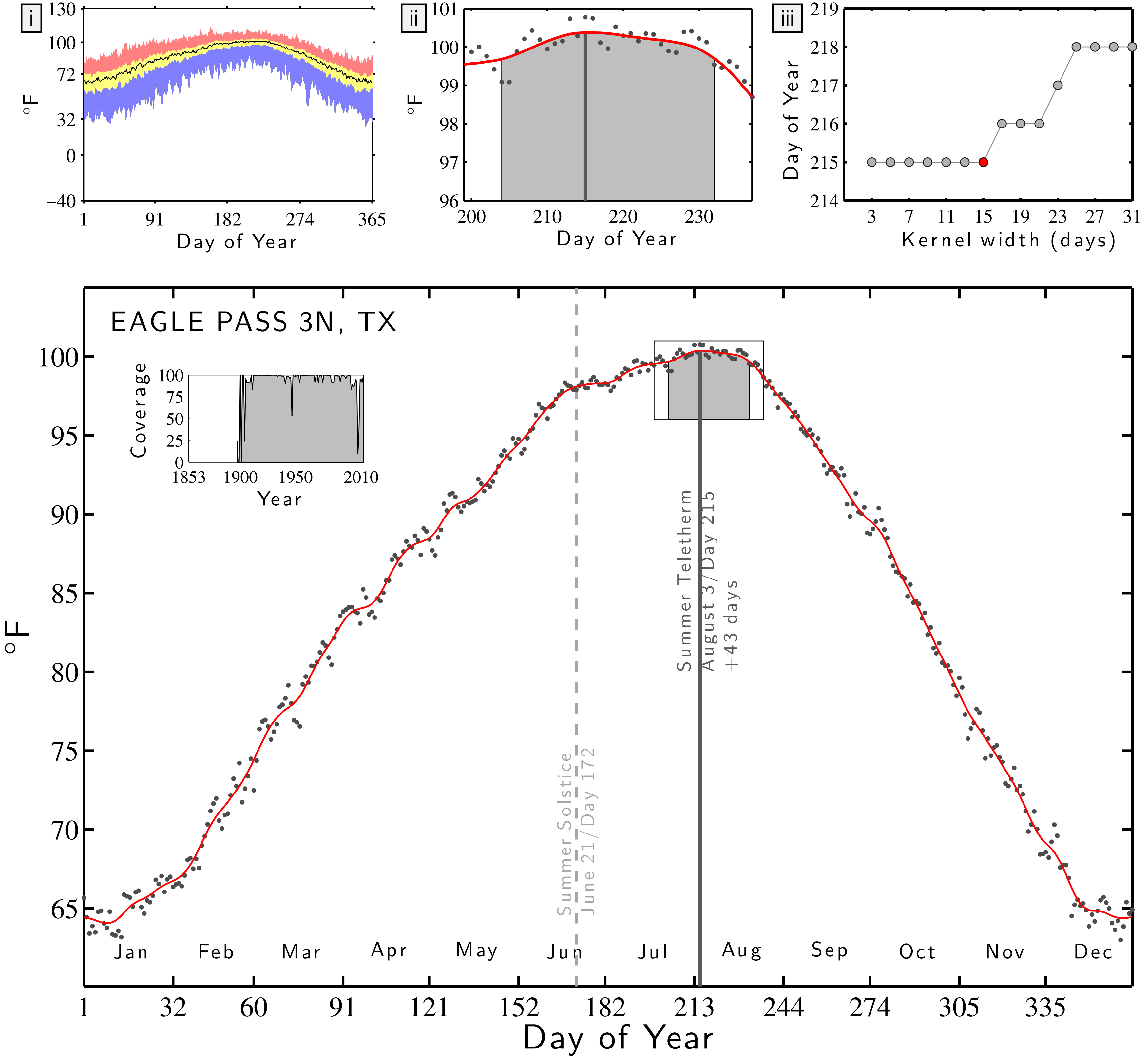

i
°F
Day of Year
ii
iii
Day of Year
Kernel width (days)
EAGLE PASS 3N, TX
Coverage
Year
Summer Solstice
June 21/Day 172
Summer Teletherm
August 3/Day 215
+43 days
Jan
Feb
Mar
Apr
May
Jun
Jul
Aug
Sep
Oct
Nov
Dec

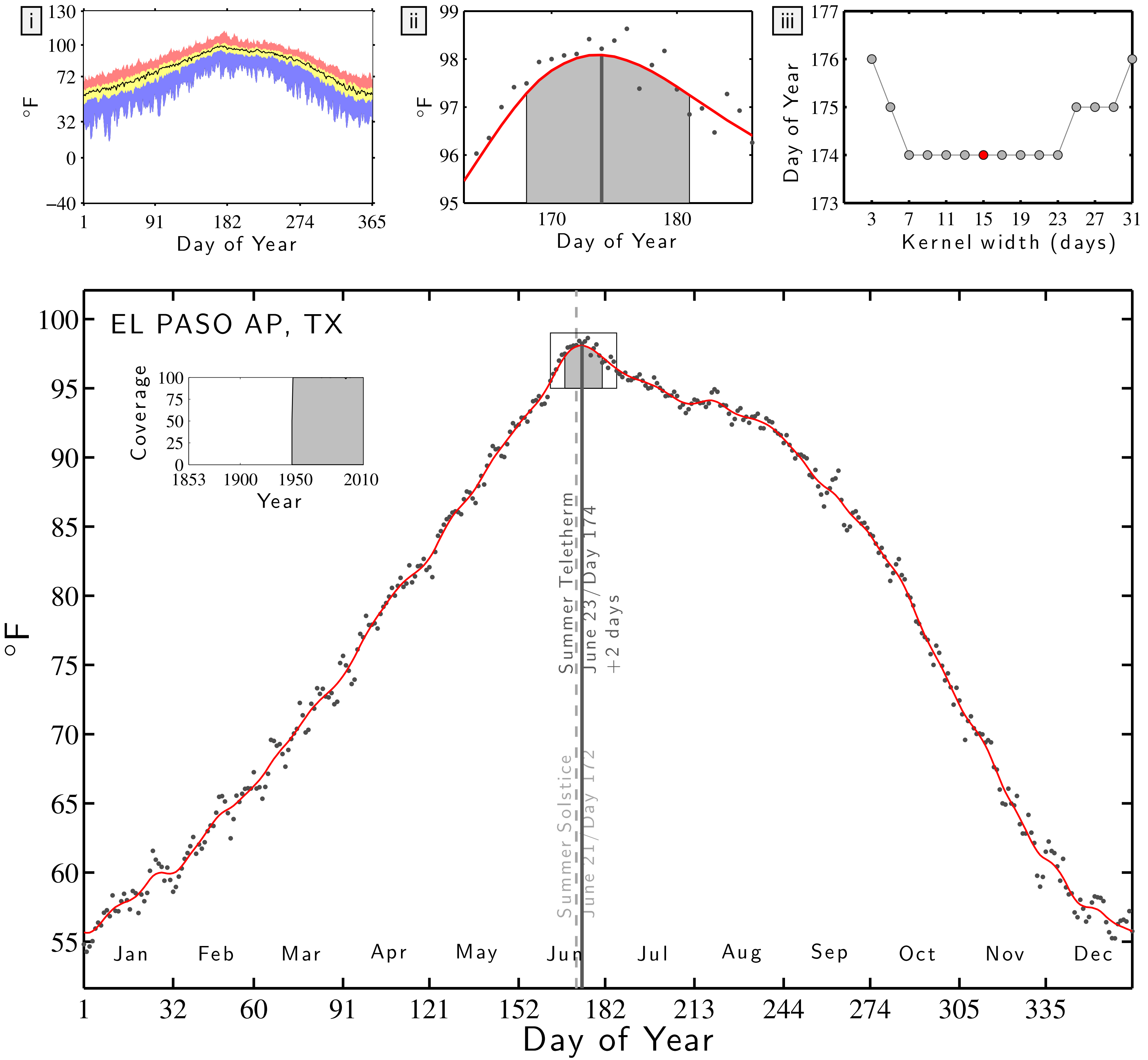

i
°F
Day of Year
ii
°F
Day of Year
iii
Day of Year
Kernel width (days)
EL PASO AP, TX
Coverage
Year
Summer Teletherm
June 23/Day 174
+2 days
Summer Solstice
June 21/Day 172
Jan
Feb
Mar
Apr
May
Jun
Jul
Aug
Sep
Oct
Nov
Dec
°F
Day of Year

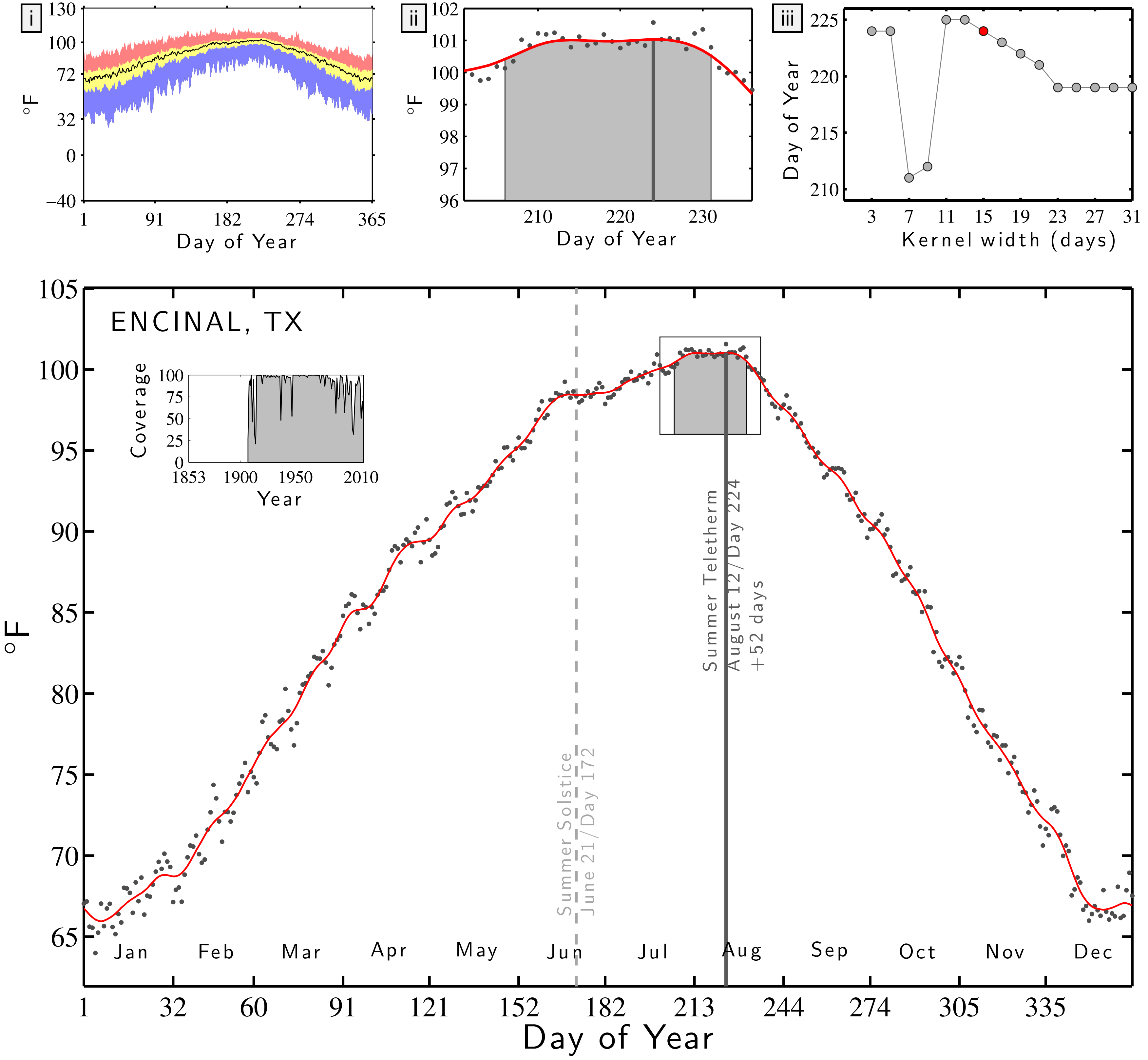

i
ii
iii
°F
Day of Year
Day of Year
Kernel width (days)
ENCINAL, TX
Coverage
Year
Summer Solstice
June 21/Day 172
Summer Teletherm
August 12/Day 224
+52 days
Jan
Feb
Mar
Apr
May
Jun
Jul
Aug
Sep
Oct
Nov
Dec

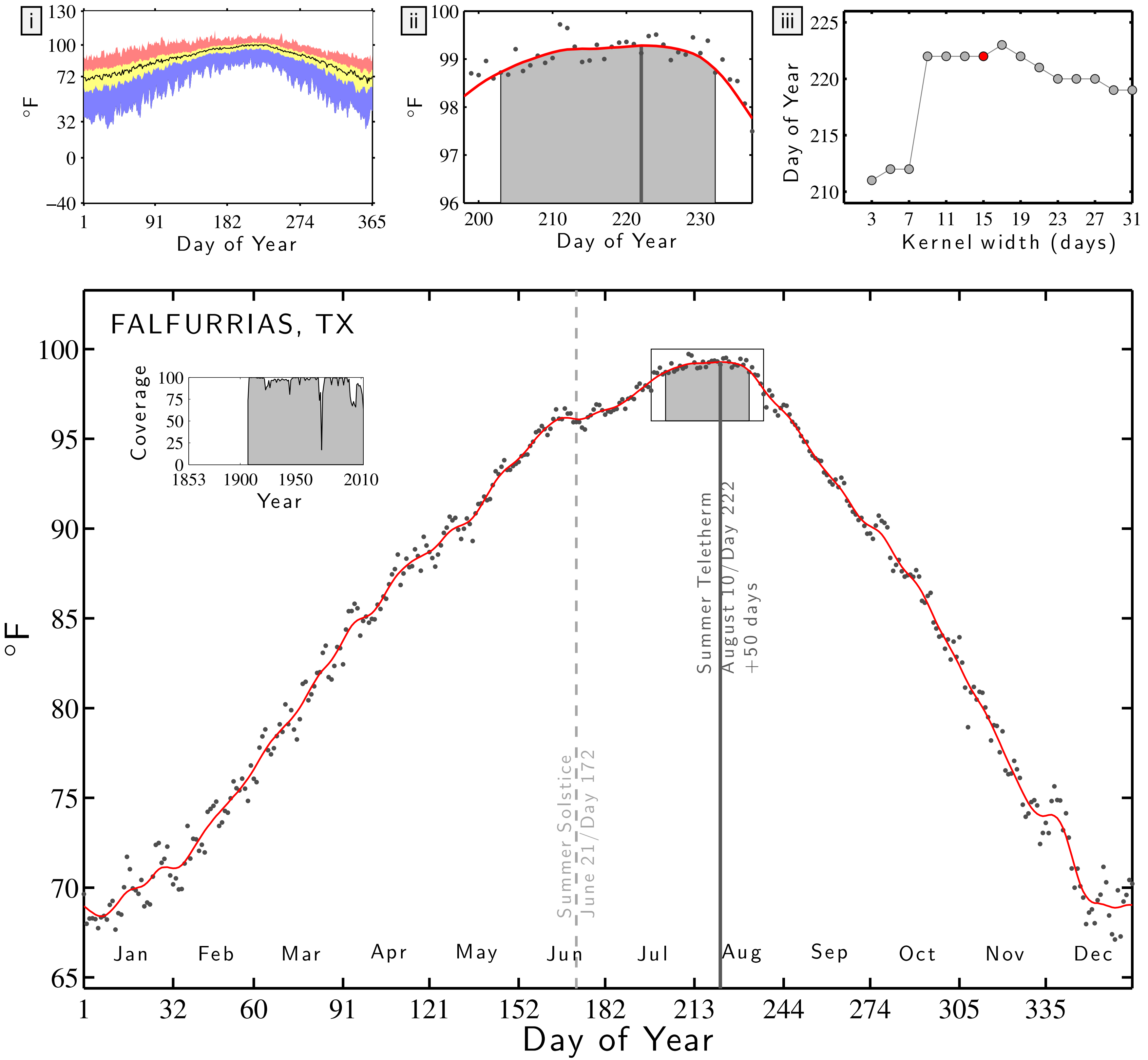

i
°F
Day of Year
ii
iii
Day of Year
Kernel width (days)
FALFURRIAS, TX
Coverage
Year
Summer Solstice
June 21/Day 172
Summer Teletherm
August 10/Day 222
+50 days
Jan
Feb
Mar
Apr
May
Jun
Jul
Aug
Sep
Oct
Nov
Dec

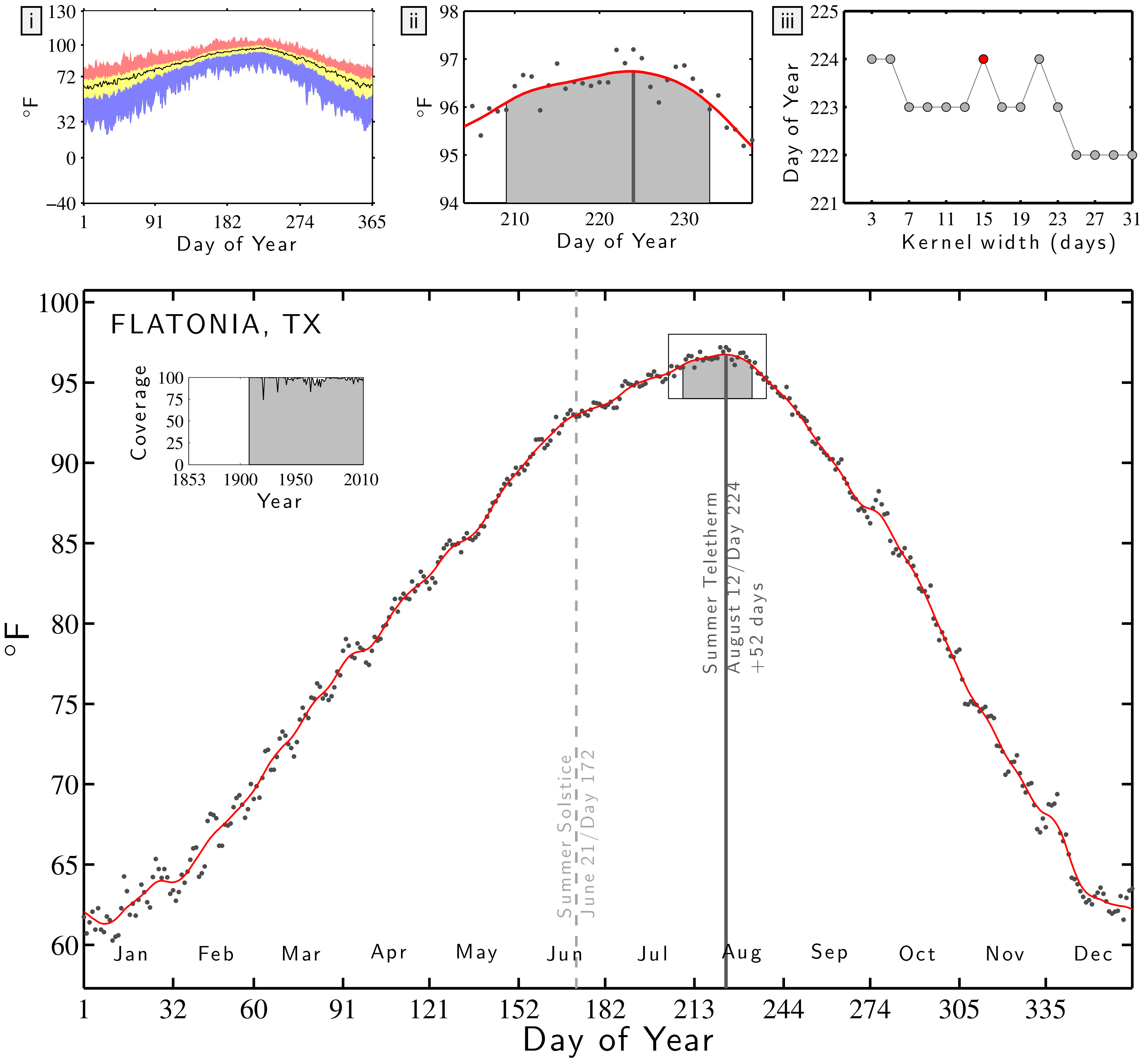

i
°F
130
100
72
32
0
−40
1
91
182
274
365
Day of Year
ii
98
97
96
95
94
210
220
230
Day of Year
iii
225
224
223
222
221
Day of Year
3
7
11
15
19
23
27
31
Kernel width (days)
FLATONIA, TX
Coverage
100
75
50
25
0
1853
1900
1950
2010
Year
Summer Solstice
June 21/Day 172
Summer Teletherm
August 12/Day 224
+52 days
Jan
Feb
Mar
Apr
May
Jun
Jul
Aug
Sep
Oct
Nov
Dec
°F
100
95
90
85
80
75
70
65
60
1
32
60
91
121
152
182
213
244
274
305
335
Day of Year

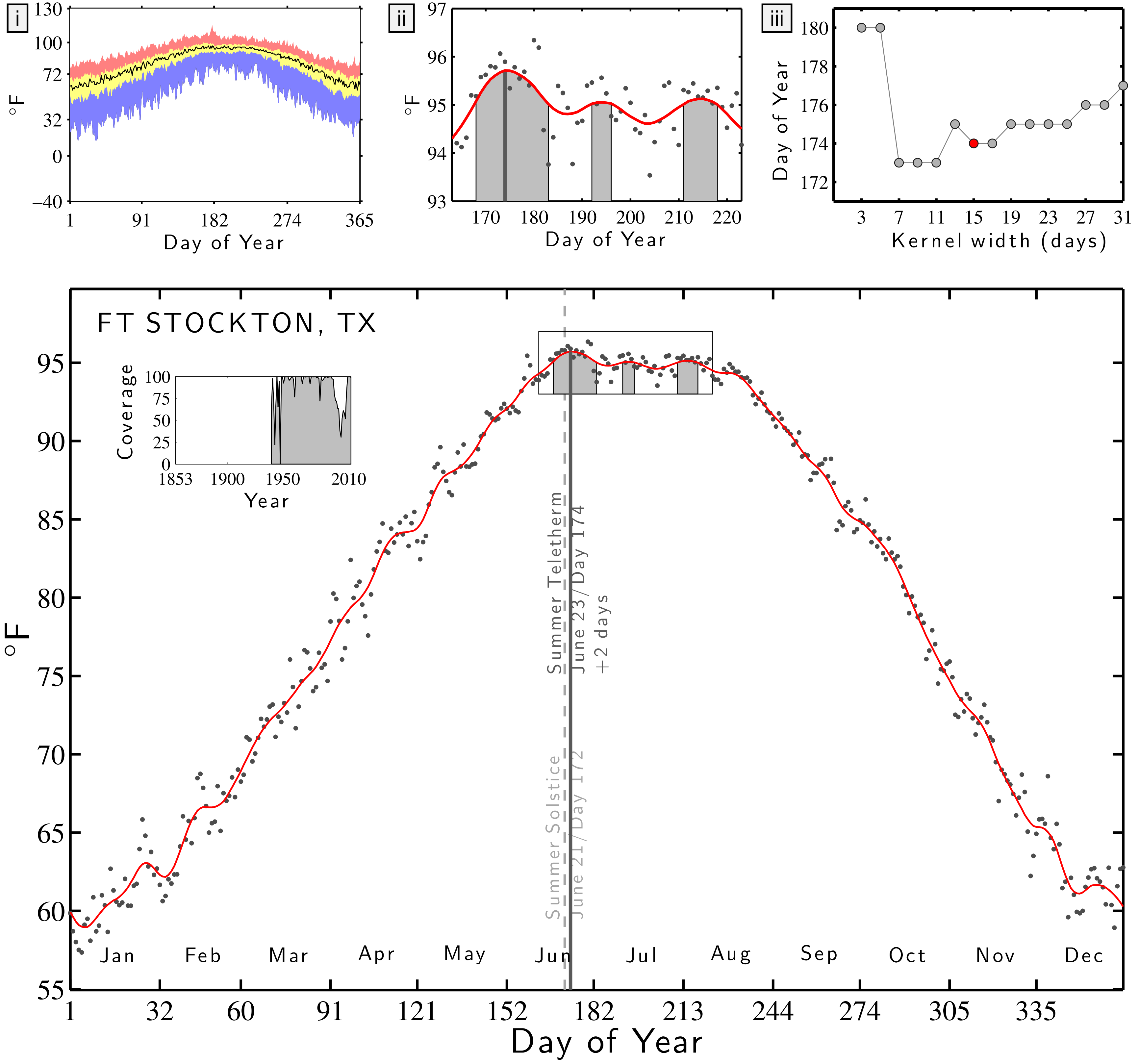

i
°F
Day of Year
ii
°F
Day of Year
iii
Day of Year
Kernel width (days)
FT STOCKTON, TX
Coverage
Year
Summer Teletherm
June 23 / Day 174
+2 days
Summer Solstice
June 21 / Day 172
Jan
Feb
Mar
Apr
May
Jun
Jul
Aug
Sep
Oct
Nov
Dec
°F
Day of Year

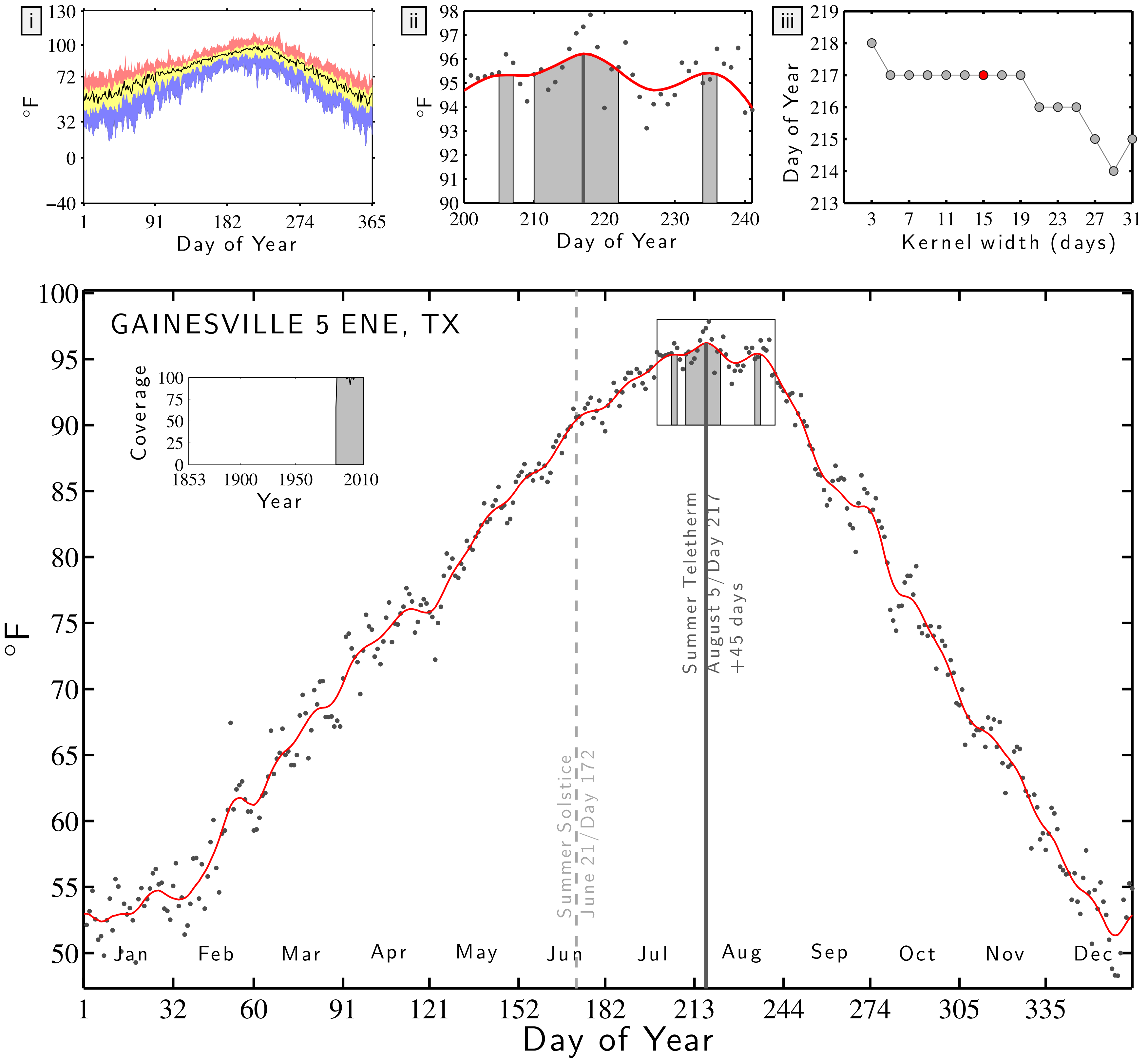

i
°F
Day of Year
ii
°F
Day of Year
iii
Day of Year
Kernel width (days)
GAINESVILLE 5 ENE, TX
Coverage
Year
Summer Solstice
June 21/Day 172
Summer Teletherm
August 5/Day 217
+45 days
Jan
Feb
Mar
Apr
May
Jun
Jul
Aug
Sep
Oct
Nov
Dec
°F
Day of Year

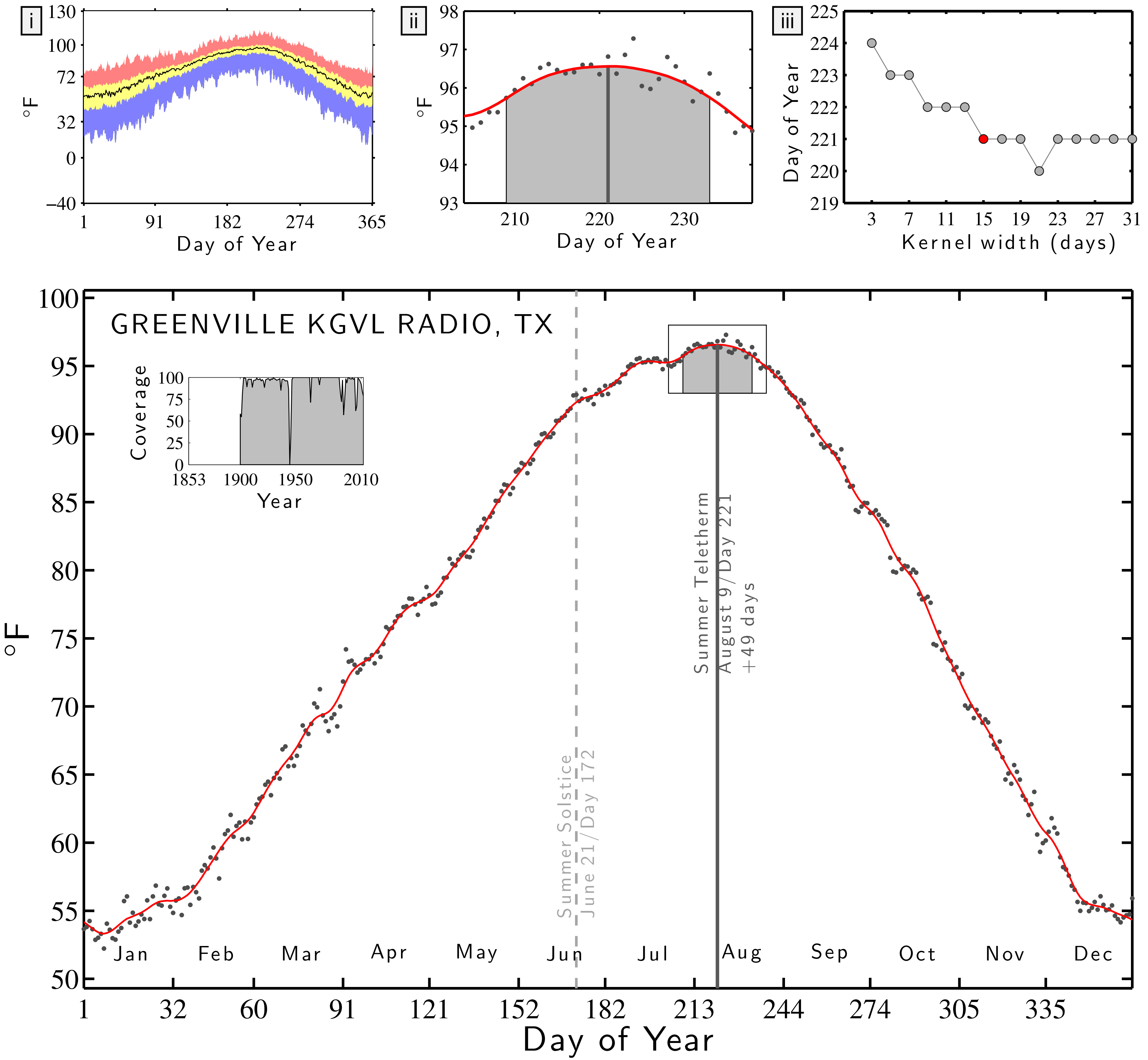

i
°F
Day of Year
ii
°F
Day of Year
iii
Day of Year
Kernel width (days)
GREENVILLE KGVL RADIO, TX
Coverage
Year
Summer Solstice
June 21/Day 172
Summer Teletherm
August 9/Day 221
+49 days
Jan
Feb
Mar
Apr
May
Jun
Jul
Aug
Sep
Oct
Nov
Dec
°F
Day of Year

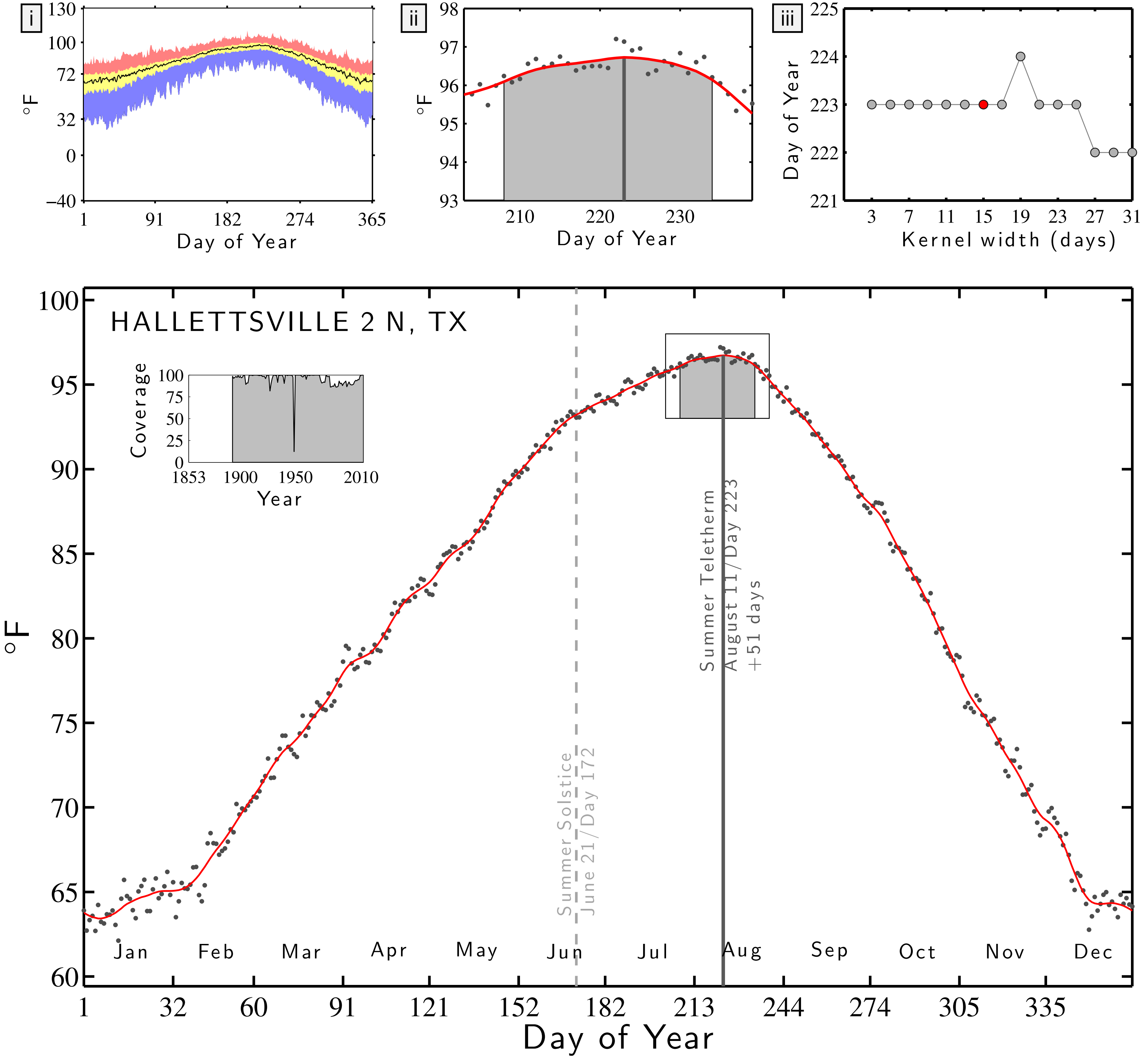

i
°F
130
100
72
32
0
−40
1
91
182
274
365
Day of Year
ii
98
97
96
95
94
93
210
220
230
iii
Day of Year
225
224
223
222
221
3
7
11
15
19
23
27
31
Kernel width (days)
HALLETTSVILLE 2 N, TX
Coverage
100
75
50
25
0
1853
1900
1950
2010
Year
Summer Solstice
June 21/Day 172
Summer Teletherm
August 11/Day 223
+51 days
Jan
Feb
Mar
Apr
May
Jun
Jul
Aug
Sep
Oct
Nov
Dec
100
95
90
85
80
75
70
65
60
1
32
60
91
121
152
182
213
244
274
305
335

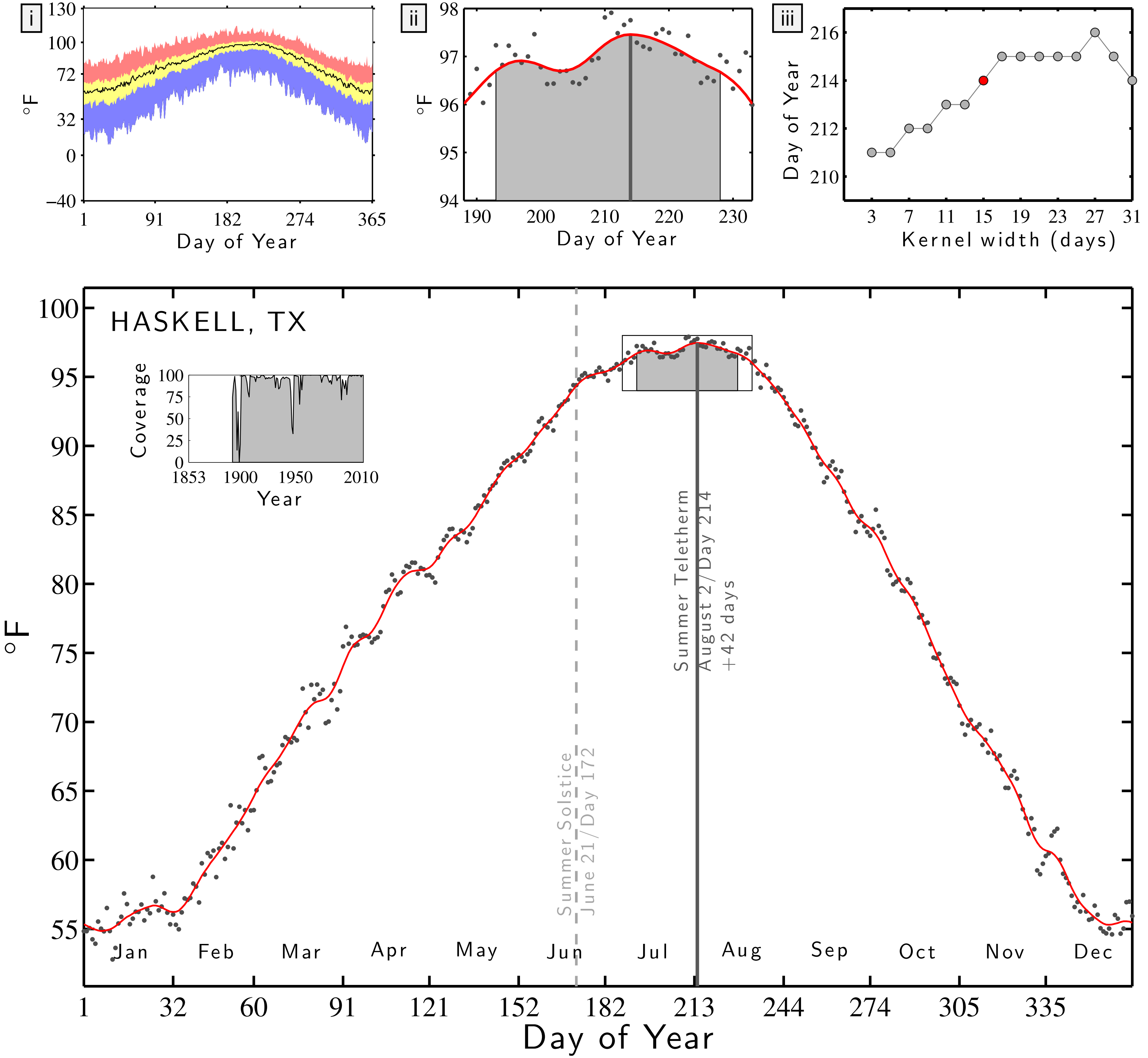

i
ii
iii
°F
Day of Year
Day of Year
Kernel width (days)
HASKELL, TX
Coverage
Year
Summer Solstice
June 21/Day 172
Summer Teletherm
August 2/Day 214
+42 days
Jan
Feb
Mar
Apr
May
Jun
Jul
Aug
Sep
Oct
Nov
Dec

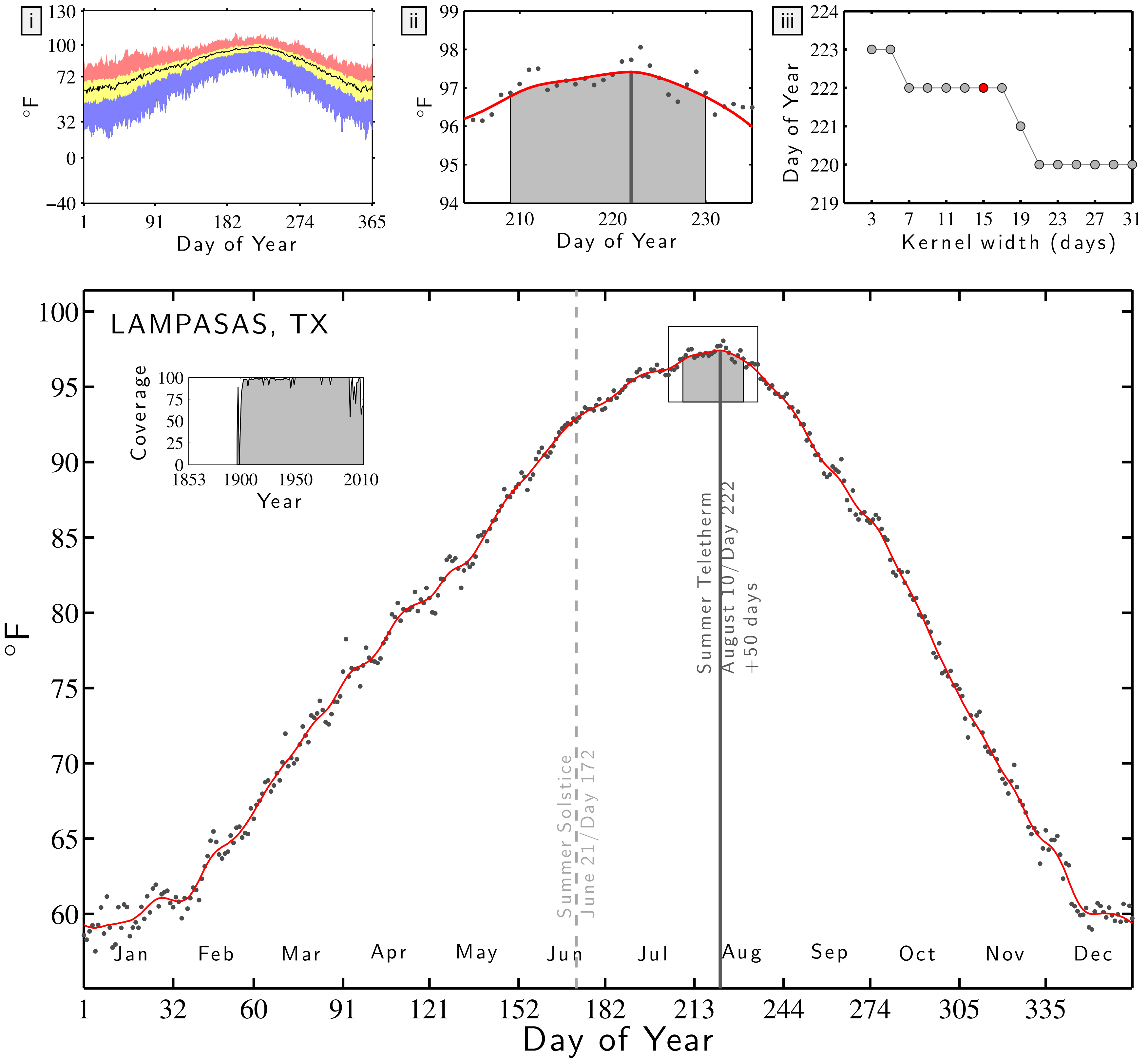

i
°F
Day of Year
ii
iii
Day of Year
Kernel width (days)
LAMPASAS, TX
Coverage
Year
Summer Solstice
June 21/Day 172
Summer Teletherm
August 10/Day 222
+50 days
Jan
Feb
Mar
Apr
May
Jun
Jul
Aug
Sep
Oct
Nov
Dec

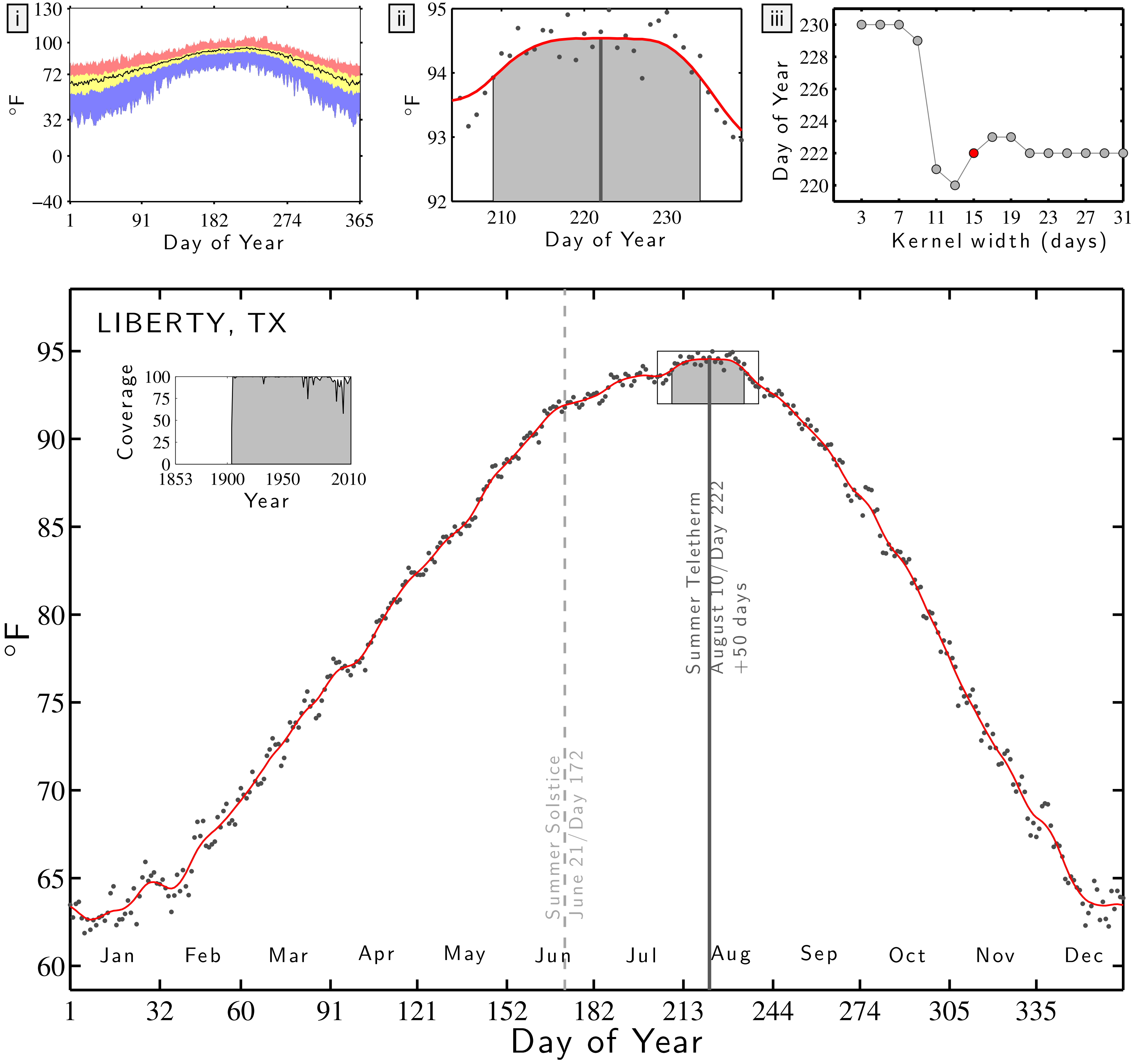

i
130
100
72
32
0
−40
°F
1
91
182
274
365
Day of Year
ii
95
94
93
92
°F
210
220
230
Day of Year
iii
230
228
226
224
222
220
Day of Year
3
7
11
15
19
23
27
31
Kernel width (days)
LIBERTY, TX
Coverage
100
75
50
25
0
1853
1900
1950
2010
Year
Summer Solstice
June 21/Day 172
Summer Teletherm
August 10/Day 222
+50 days
Jan
Feb
Mar
Apr
May
Jun
Jul
Aug
Sep
Oct
Nov
Dec
95
90
85
80
75
70
65
60
°F
1
32
60
91
121
152
182
213
244
274
305
335
Day of Year

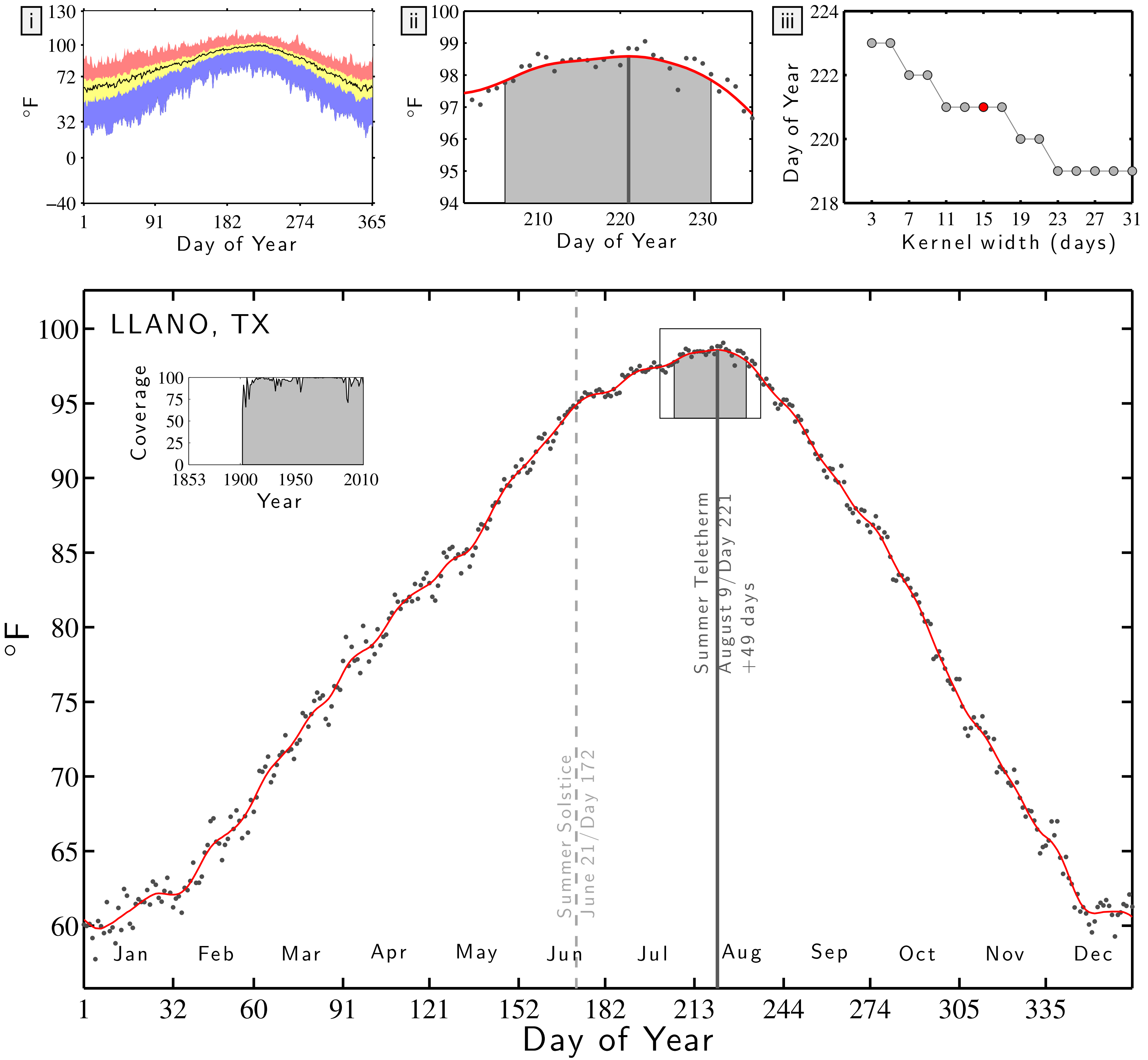
i
ii
iii
°F
Day of Year
Day of Year
Kernel width (days)
LLANO, TX
Coverage
Year
Summer Solstice
June 21/Day 172
Summer Teletherm
August 9/Day 221
+49 days
Jan
Feb
Mar
Apr
May
Jun
Jul
Aug
Sep
Oct
Nov
Dec

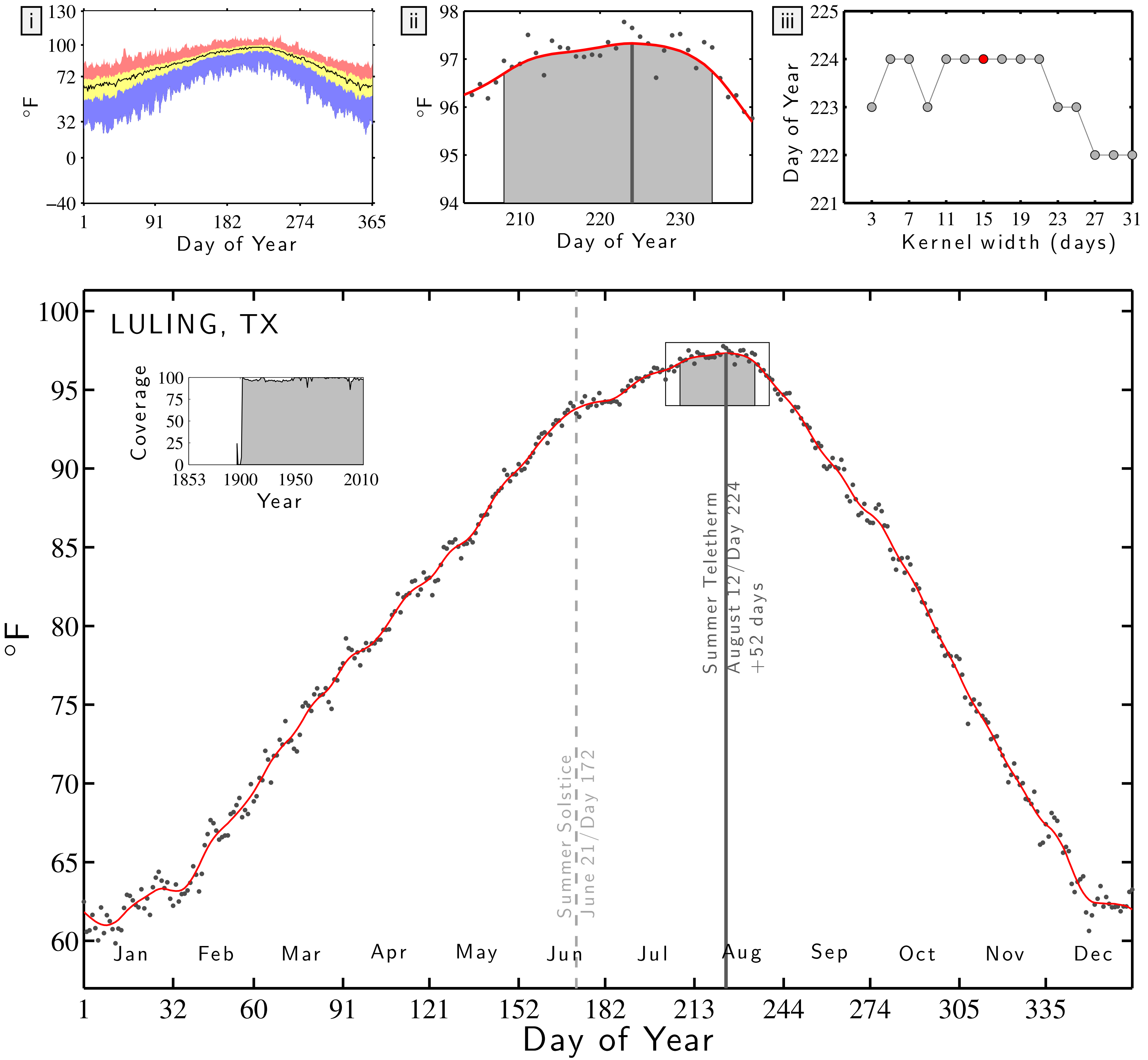

i
130
100
72
32
0
−40
°F
1
91
182
274
365
Day of Year
ii
98
97
96
95
94
°F
210
220
230
Day of Year
iii
225
224
223
222
221
Day of Year
3
7
11
15
19
23
27
31
Kernel width (days)
LULING, TX
Coverage
100
75
50
25
0
1853
1900
1950
2010
Year
Summer Solstice
June 21/Day 172
Summer Teletherm
August 12/Day 224
+52 days
Jan
Feb
Mar
Apr
May
Jun
Jul
Aug
Sep
Oct
Nov
Dec
°F
100
95
90
85
80
75
70
65
60
1
32
60
91
121
152
182
213
244
274
305
335
Day of Year

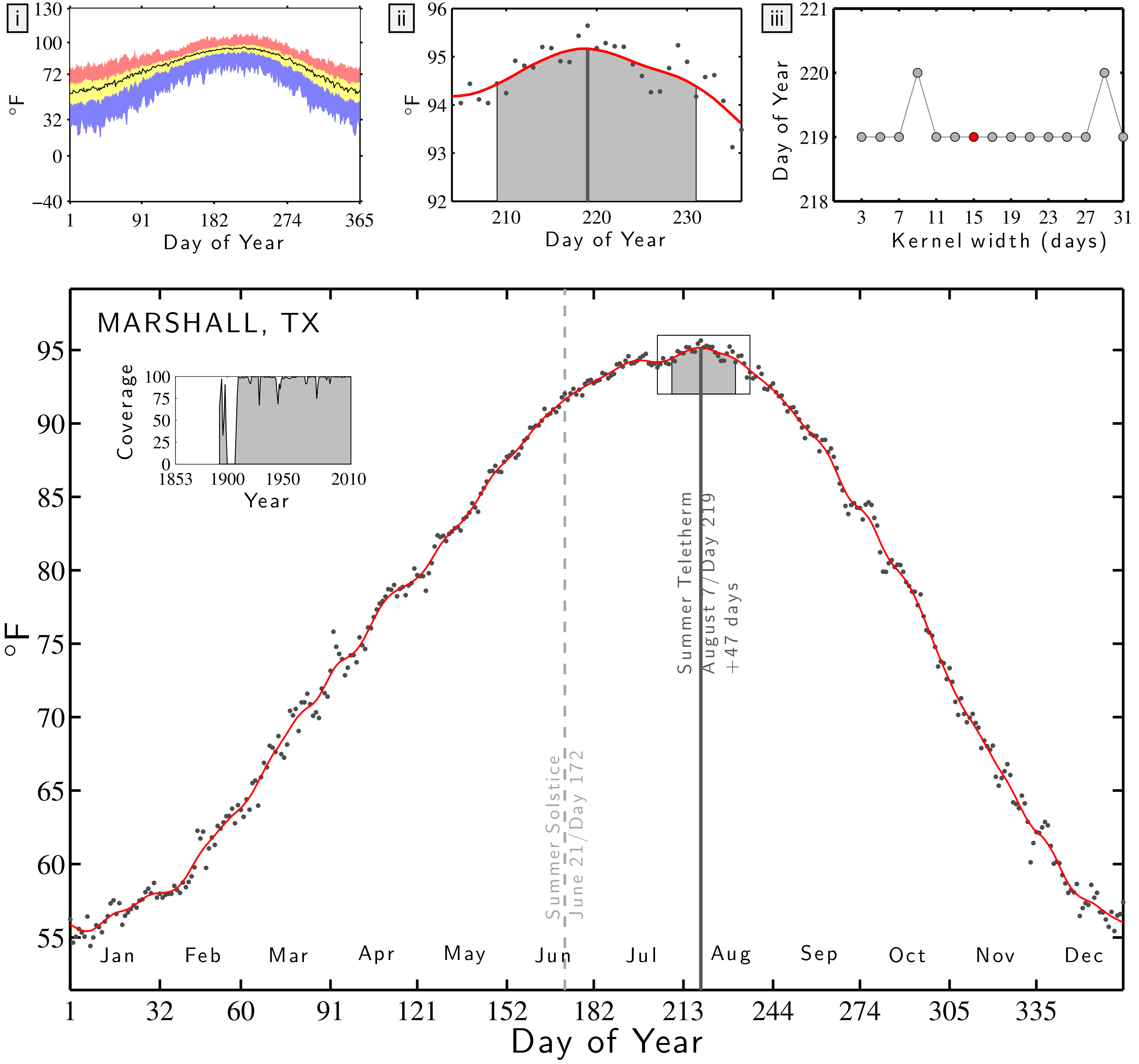
i
ii
iii
°F
Day of Year
Day of Year
Kernel width (days)
MARSHALL, TX
Coverage
Year
Summer Solstice
June 21 / Day 172
Summer Teletherm
August 7 / Day 219
+47 days
Jan
Feb
Mar
Apr
May
Jun
Jul
Aug
Sep
Oct
Nov
Dec

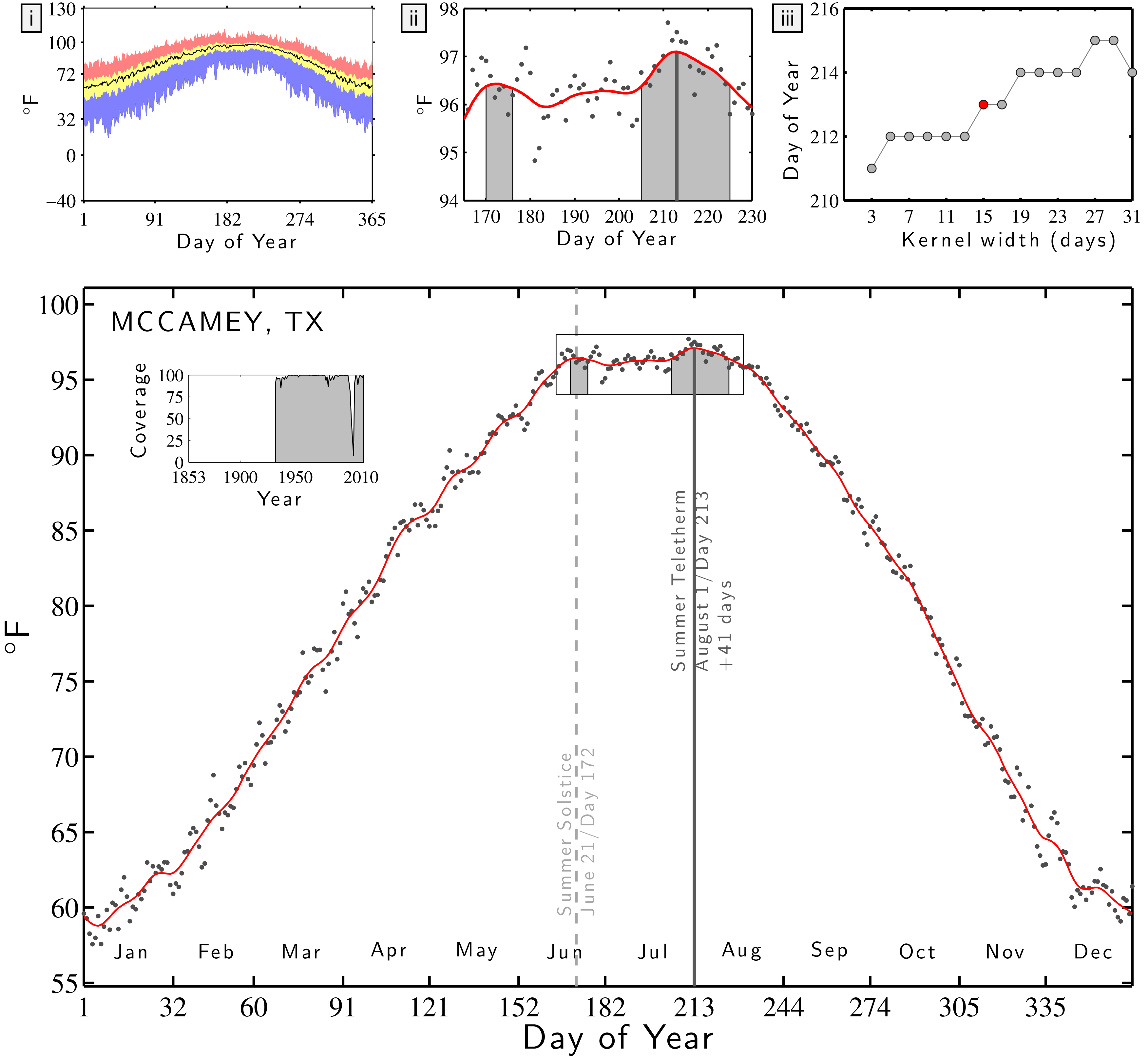

i
°F
Day of Year
ii
°F
Day of Year
iii
Day of Year
Kernel width (days)
MCCAMEY, TX
Coverage
Year
Summer Solstice
June 21/Day 172
Summer Teletherm
August 1/Day 213
+41 days
Jan
Feb
Mar
Apr
May
Jun
Jul
Aug
Sep
Oct
Nov
Dec
°F
Day of Year

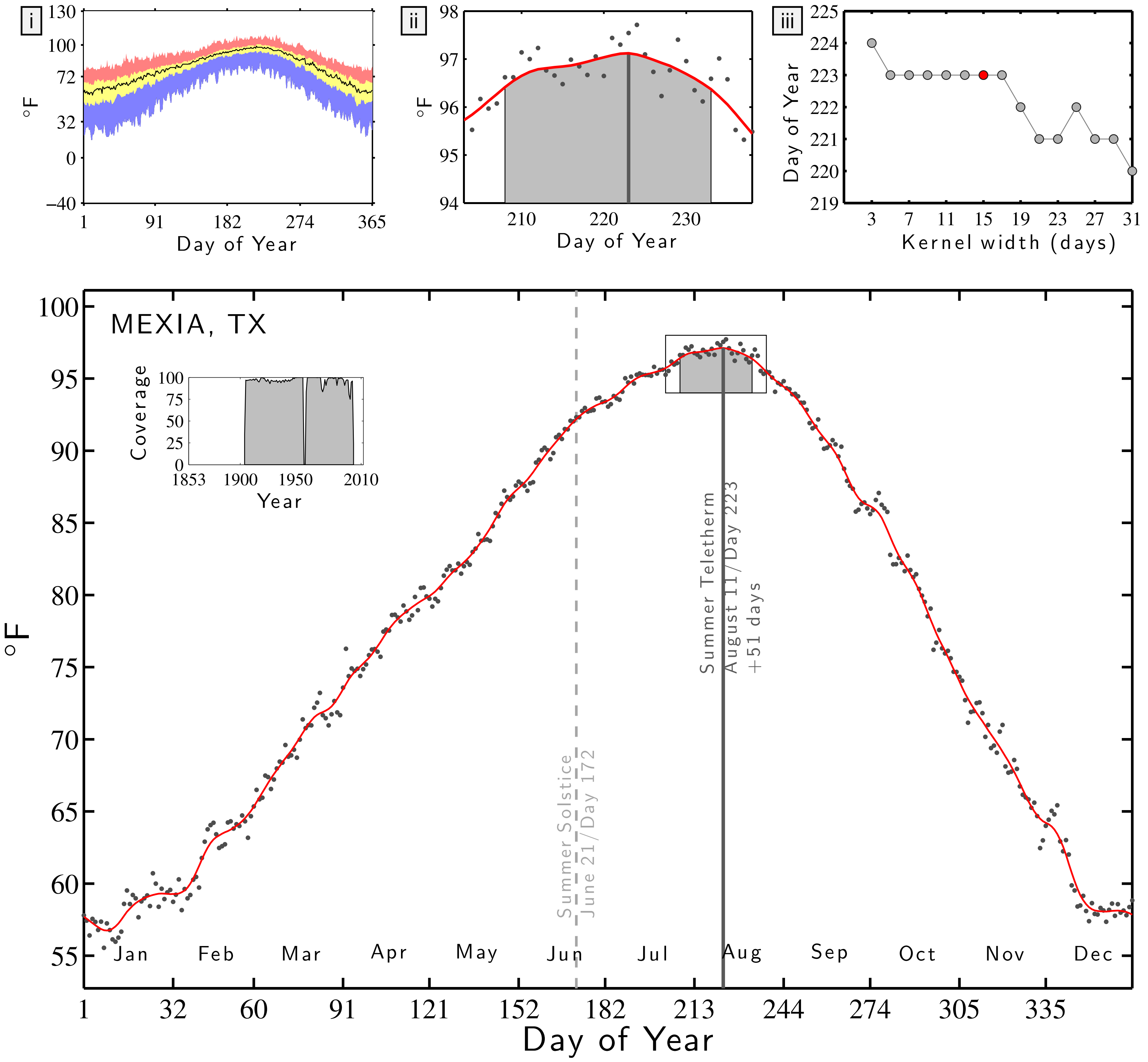

i
ii
iii
Day of Year
Kernel width (days)
MEXIA, TX
Coverage
Year
Summer Solstice
June 21/Day 172
Summer Teletherm
August 11/Day 223
+51 days
Jan
Feb
Mar
Apr
May
Jun
Jul
Aug
Sep
Oct
Nov
Dec
°F
Day of Year

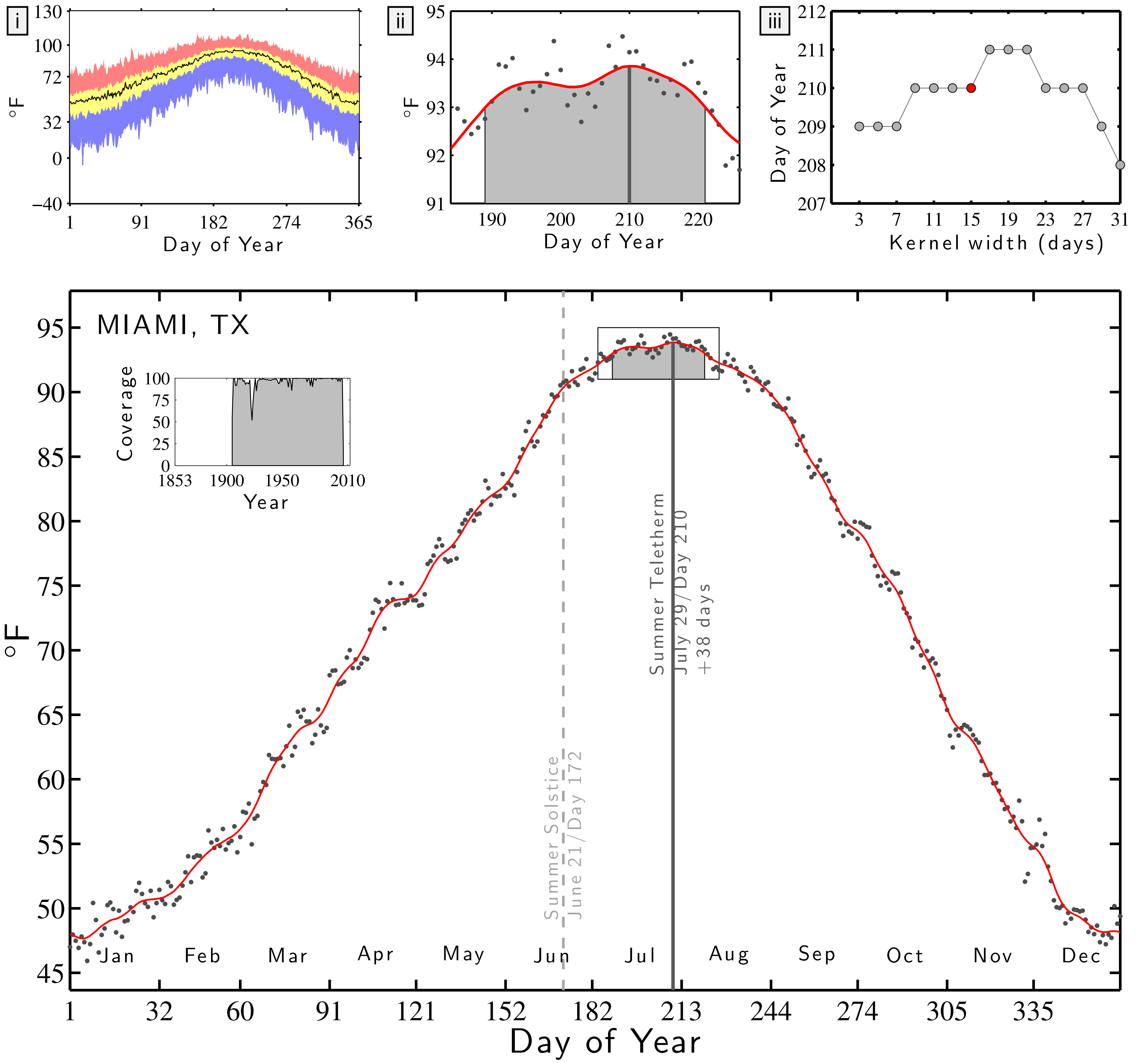

i
ii
iii
°F
Day of Year
Day of Year
Kernel width (days)
MIAMI, TX
Coverage
Year
Summer Solstice
June 21 / Day 172
Summer Teletherm
July 29 / Day 210
+38 days
Jan
Feb
Mar
Apr
May
Jun
Jul
Aug
Sep
Oct
Nov
Dec
Day of Year

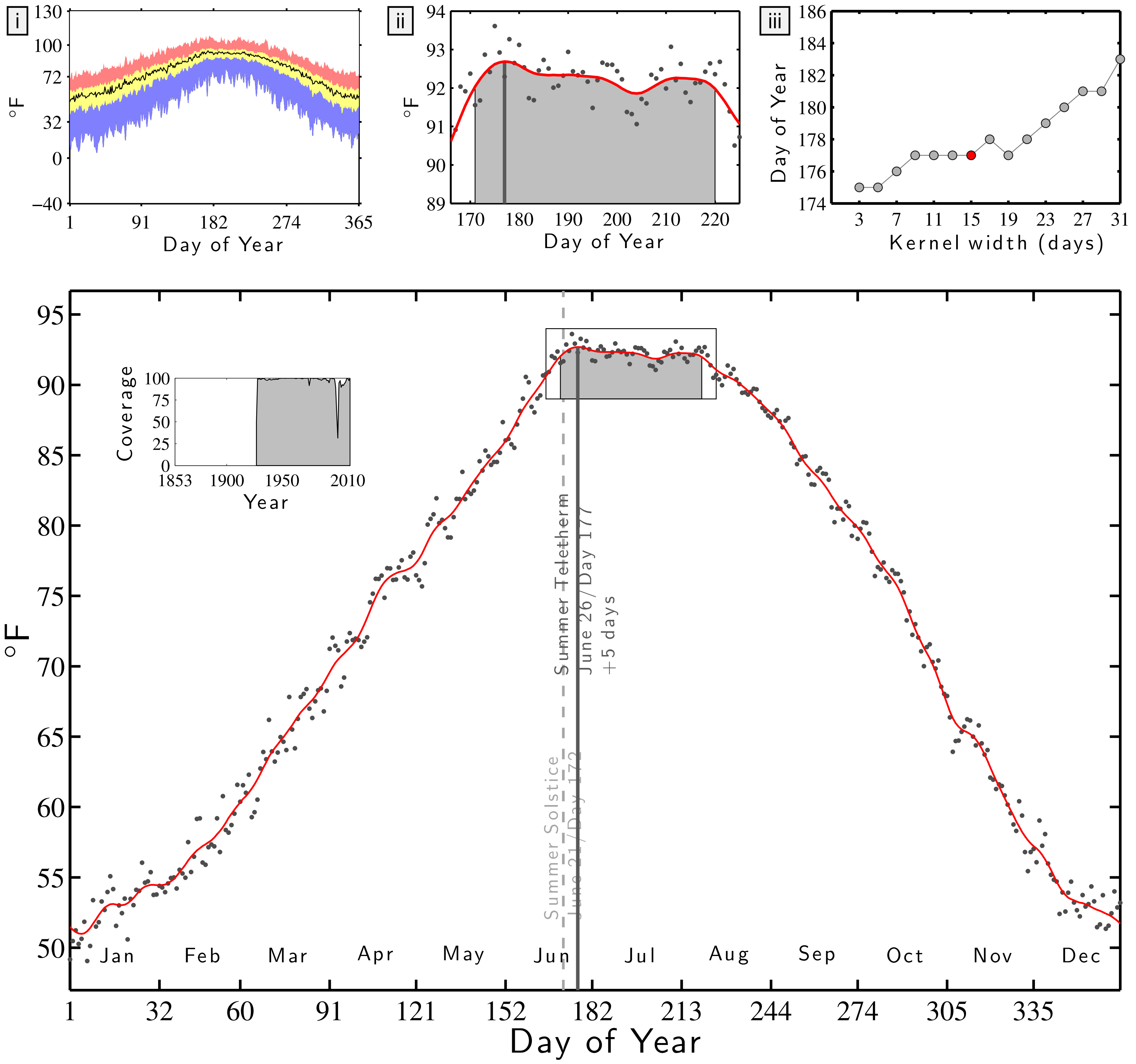

i
ii
iii
°F
Day of Year
Kernel width (days)
Coverage
Year
Summer Teletherm
June 26 / Day 177
+5 days
Summer Solstice
June 21 / Day 172
Jan
Feb
Mar
Apr
May
Jun
Jul
Aug
Sep
Oct
Nov
Dec

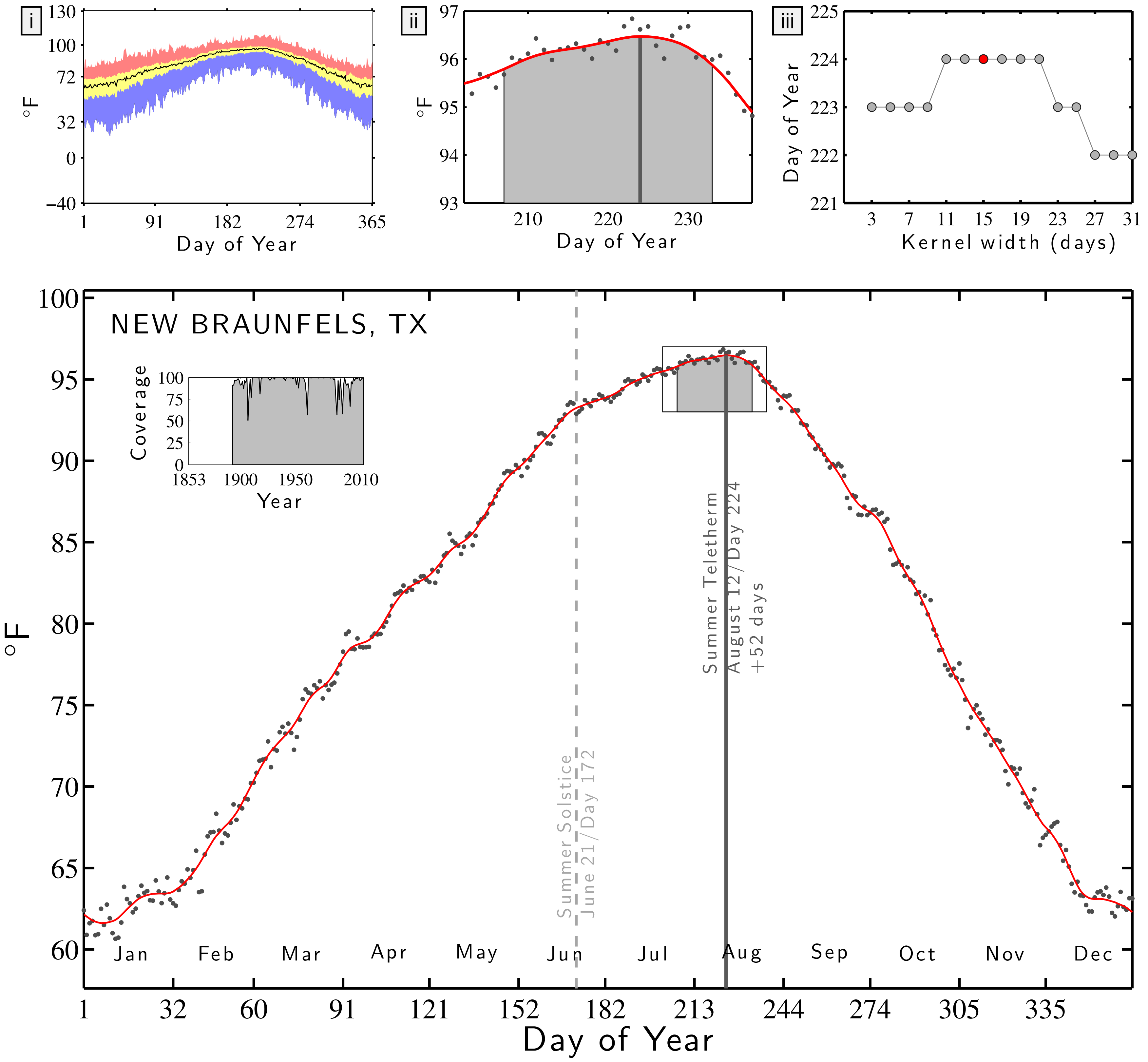

i
ii
iii
°F
Day of Year
Kernel width (days)
NEW BRAUNFELS, TX
Coverage
Year
Summer Solstice
June 21/Day 172
Summer Teletherm
August 12/Day 224
+52 days
Jan
Feb
Mar
Apr
May
Jun
Jul
Aug
Sep
Oct
Nov
Dec

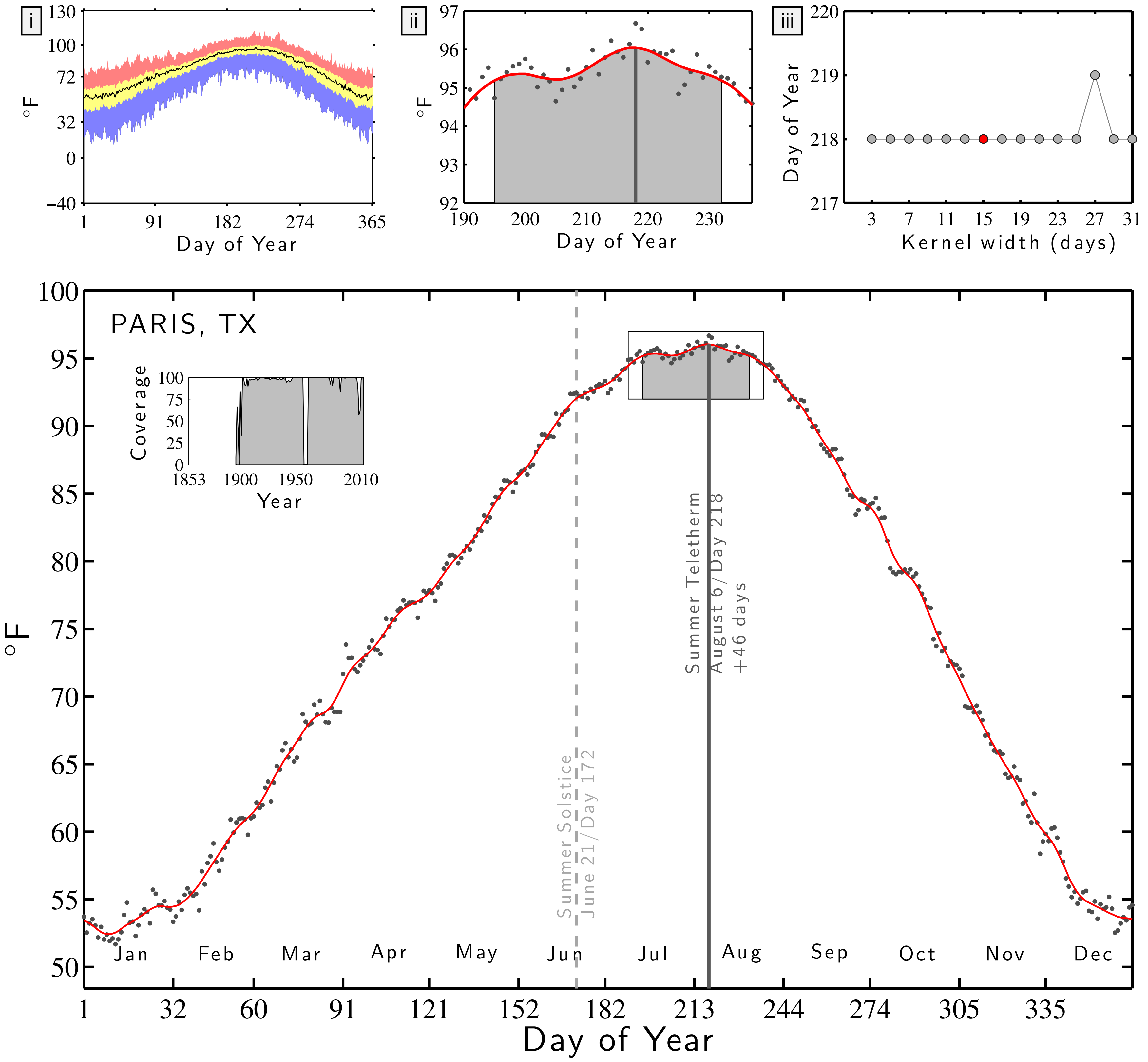

i
°F
Day of Year
ii
°F
Day of Year
iii
Day of Year
Kernel width (days)
PARIS, TX
Coverage
Year
Summer Solstice
June 21/Day 172
Summer Teletherm
August 6/Day 218
+46 days
Jan
Feb
Mar
Apr
May
Jun
Jul
Aug
Sep
Oct
Nov
Dec
°F
Day of Year

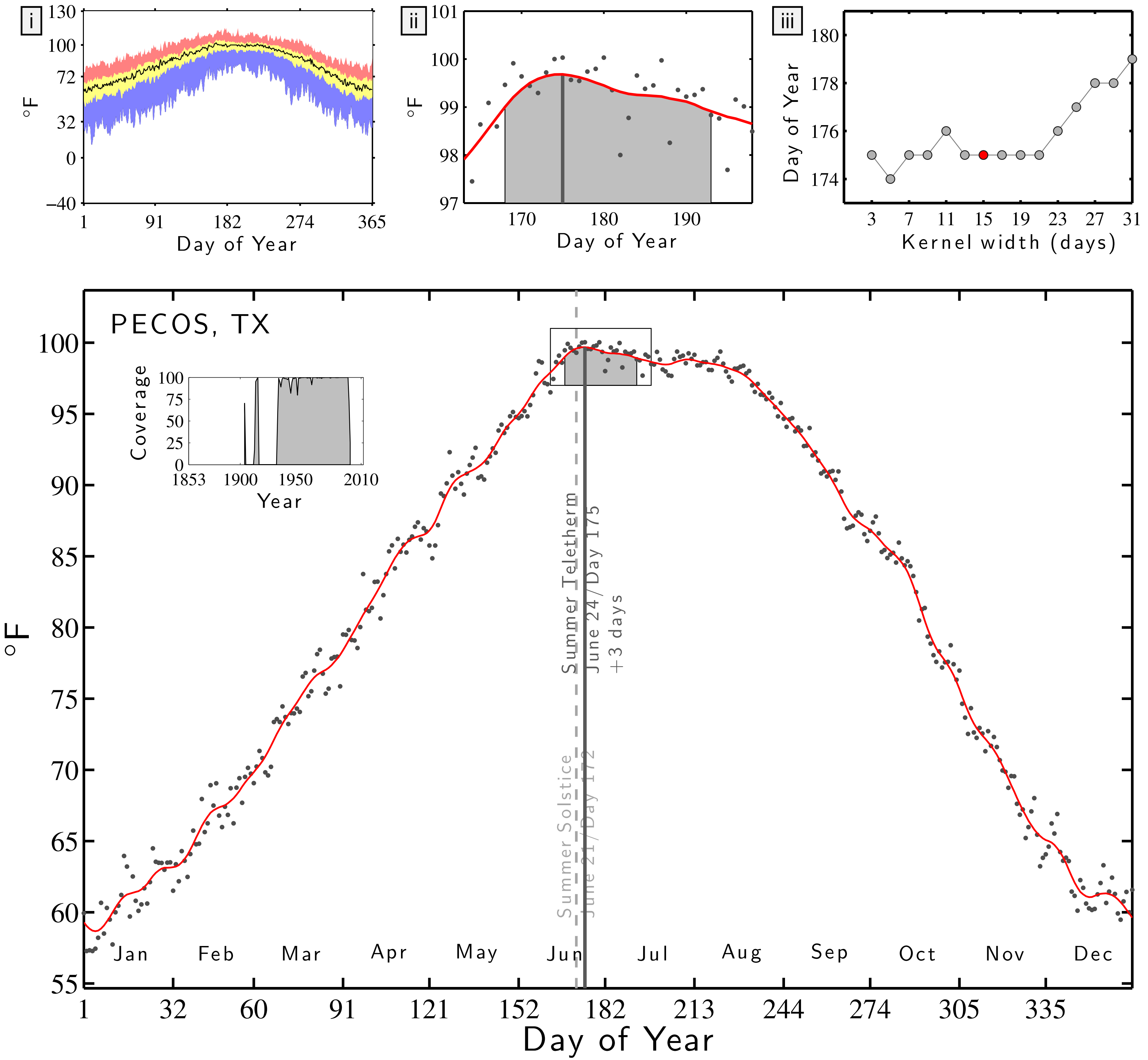

i
°F
Day of Year
ii
°F
Day of Year
iii
Day of Year
Kernel width (days)
PECOS, TX
Coverage
Year
Summer Teletherm
June 24/Day 175
+3 days
Summer Solstice
June 21/Day 172
Jan
Feb
Mar
Apr
May
Jun
Jul
Aug
Sep
Oct
Nov
Dec
°F
Day of Year

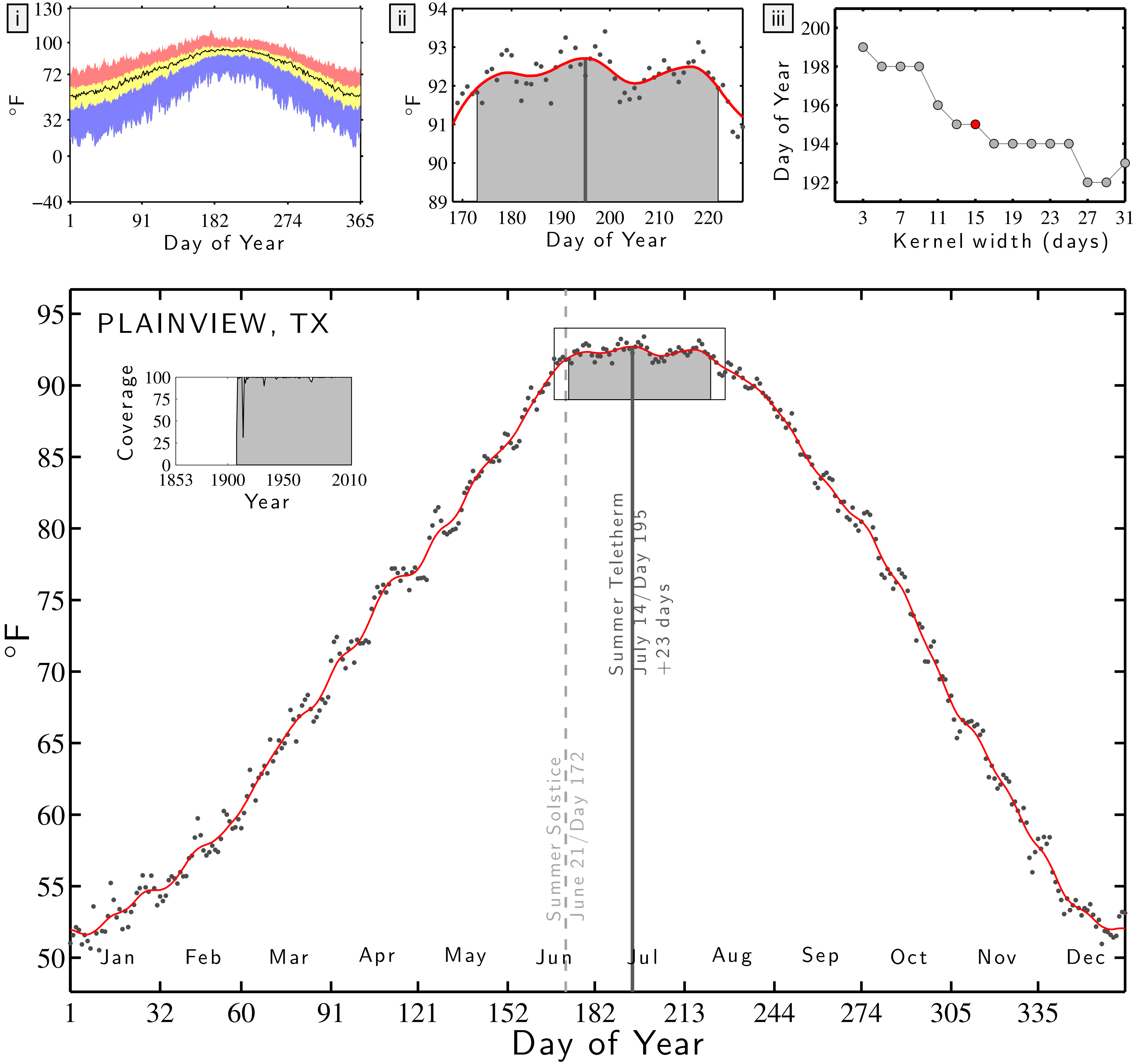

i
ii
iii
Day of Year
°F
Kernel width (days)
PLAINVIEW, TX
Coverage
Year
Summer Solstice
June 21/Day 172
Summer Teletherm
July 14/Day 195
+23 days
Jan
Feb
Mar
Apr
May
Jun
Jul
Aug
Sep
Oct
Nov
Dec

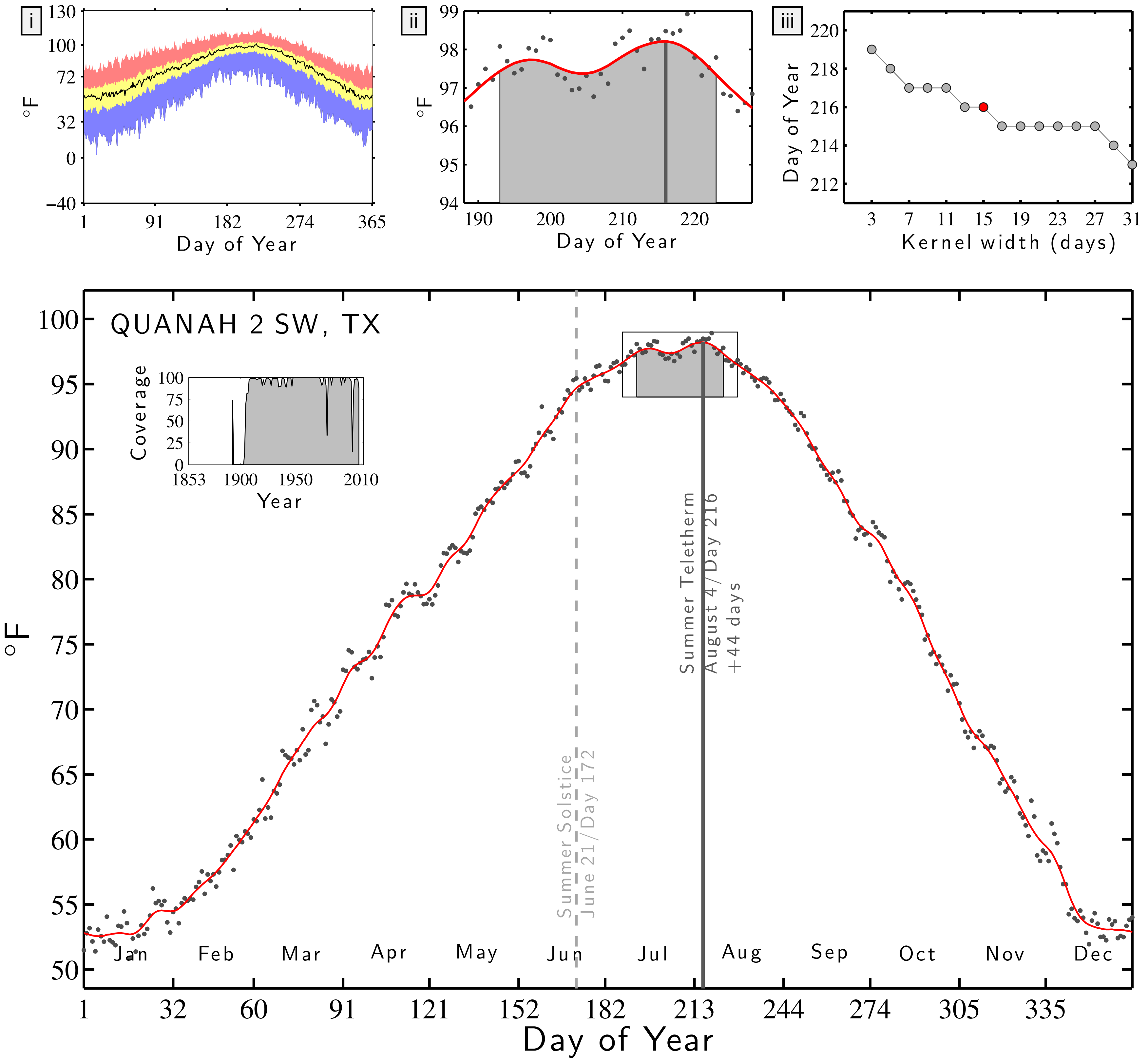

i
°F
Day of Year
ii
°F
Day of Year
iii
Day of Year
Kernel width (days)
QUANAH 2 SW, TX
Coverage
Year
Summer Solstice
June 21/Day 172
Summer Teletherm
August 4/Day 216
+44 days
Jan
Feb
Mar
Apr
May
Jun
Jul
Aug
Sep
Oct
Nov
Dec
°F
Day of Year

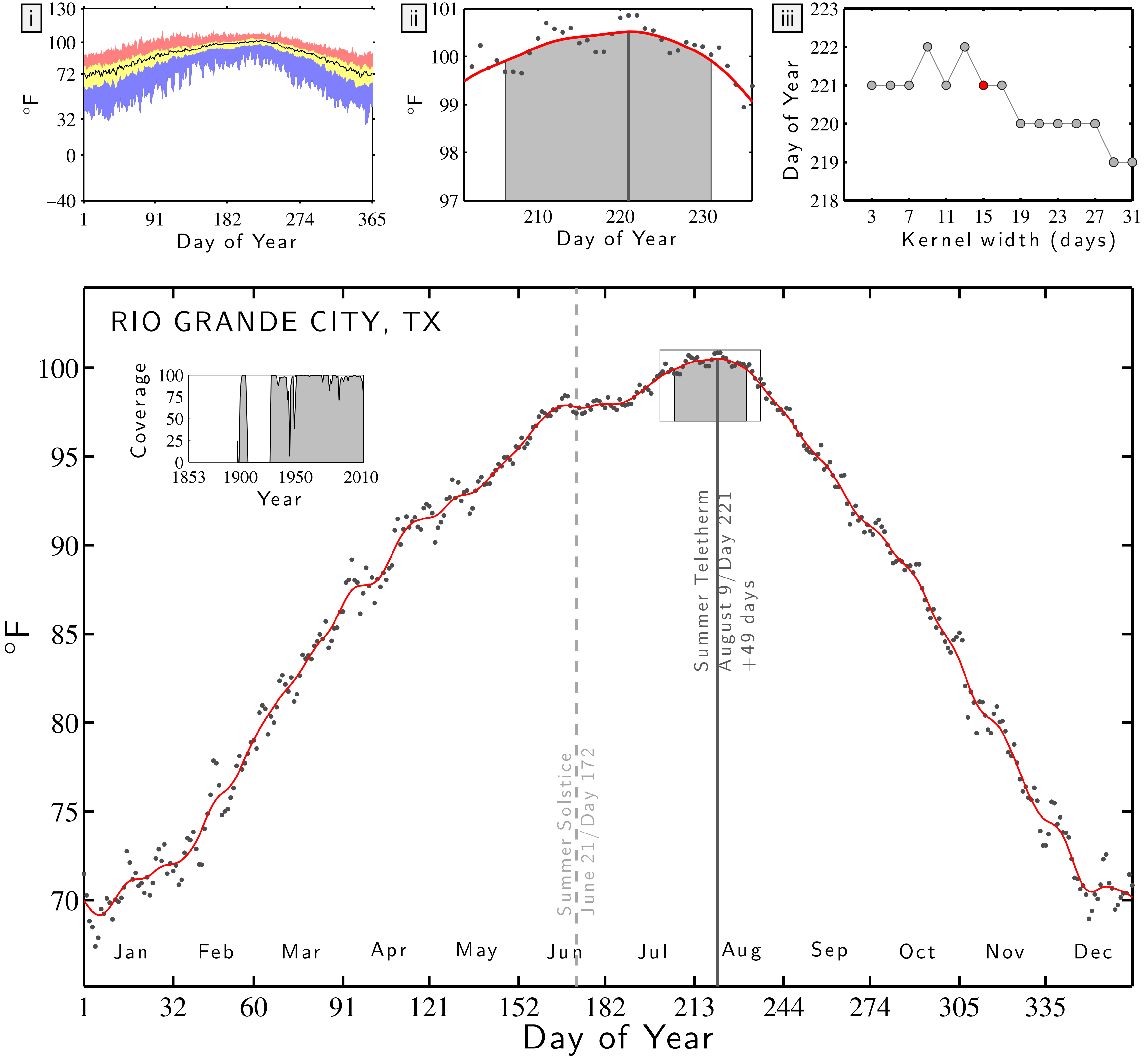

i
130
100
72
32
0
−40
°F
1
91
182
274
365
Day of Year
ii
101
100
99
98
97
°F
210
220
230
Day of Year
iii
223
222
221
220
219
218
Day of Year
3
7
11
15
19
23
27
31
Kernel width (days)
RIO GRANDE CITY, TX
Coverage
100
75
50
25
0
1853
1900
1950
2010
Year
100
95
90
85
80
75
70
°F
Summer Solstice
June 21/Day 172
Summer Teletherm
August 9/Day 221
+49 days
Jan
Feb
Mar
Apr
May
Jun
Jul
Aug
Sep
Oct
Nov
Dec
1
32
60
91
121
152
182
213
244
274
305
335
Day of Year

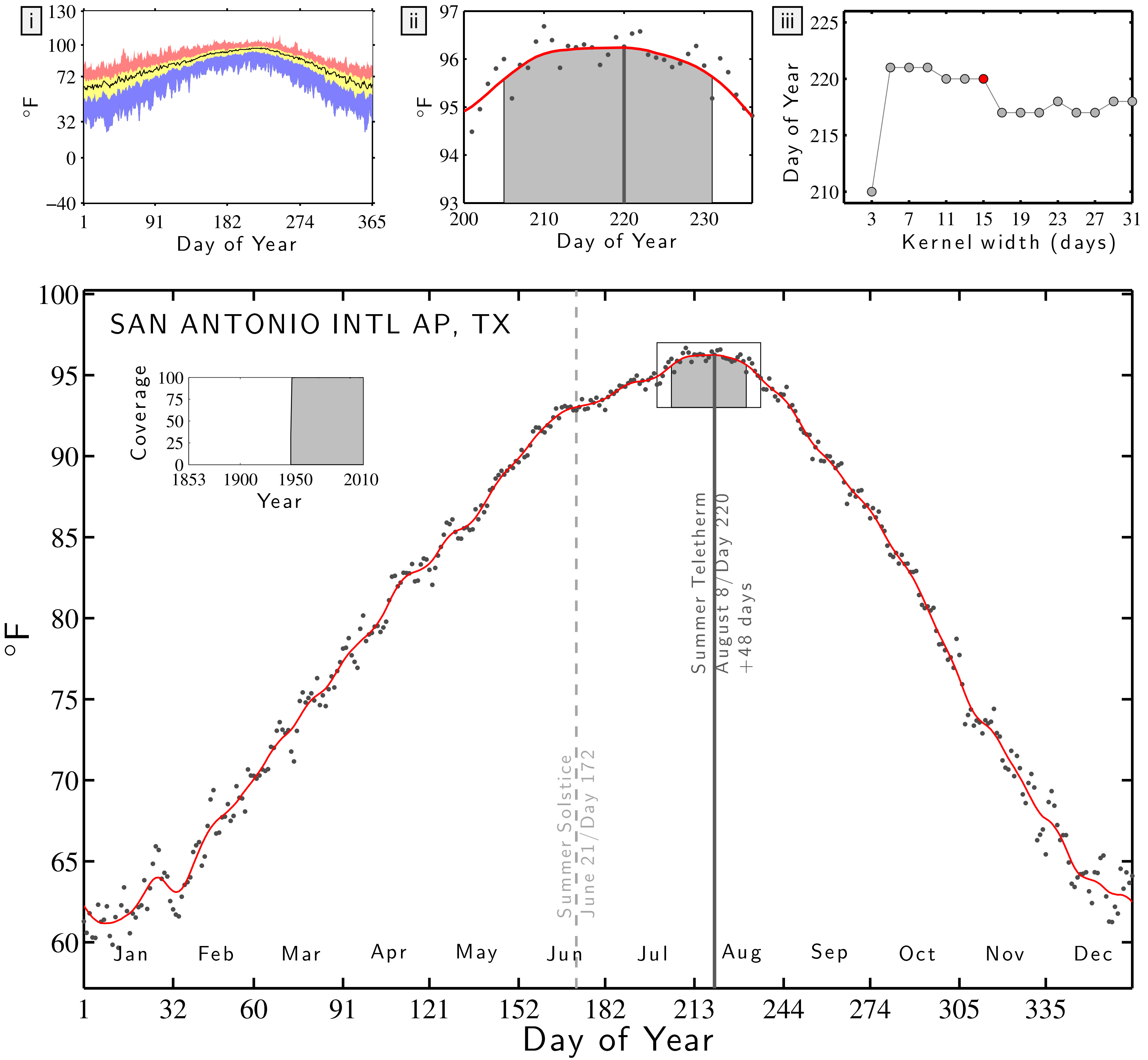

i
ii
iii
°F
Day of Year
Day of Year
Kernel width (days)
SAN ANTONIO INTL AP, TX
Coverage
Year
Summer Solstice
June 21/Day 172
Summer Teletherm
August 8/Day 220
+48 days
Jan
Feb
Mar
Apr
May
Jun
Jul
Aug
Sep
Oct
Nov
Dec

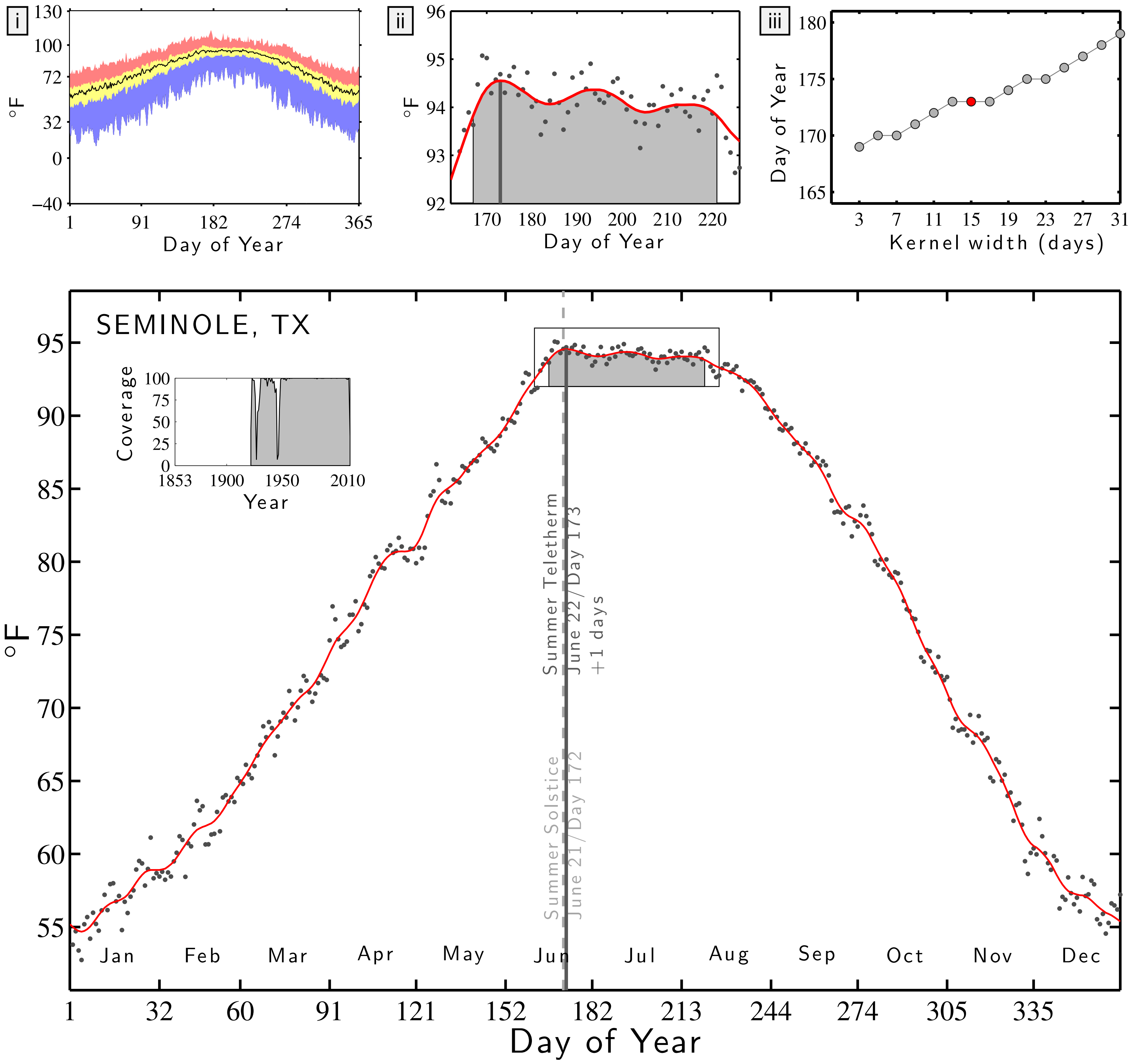

i
°F
Day of Year
ii
°F
Day of Year
iii
Day of Year
Kernel width (days)
SEMINOLE, TX
Coverage
Year
Summer Teletherm
June 22/Day 173
+1 days
Summer Solstice
June 21/Day 172
Jan
Feb
Mar
Apr
May
Jun
Jul
Aug
Sep
Oct
Nov
Dec
°F
Day of Year

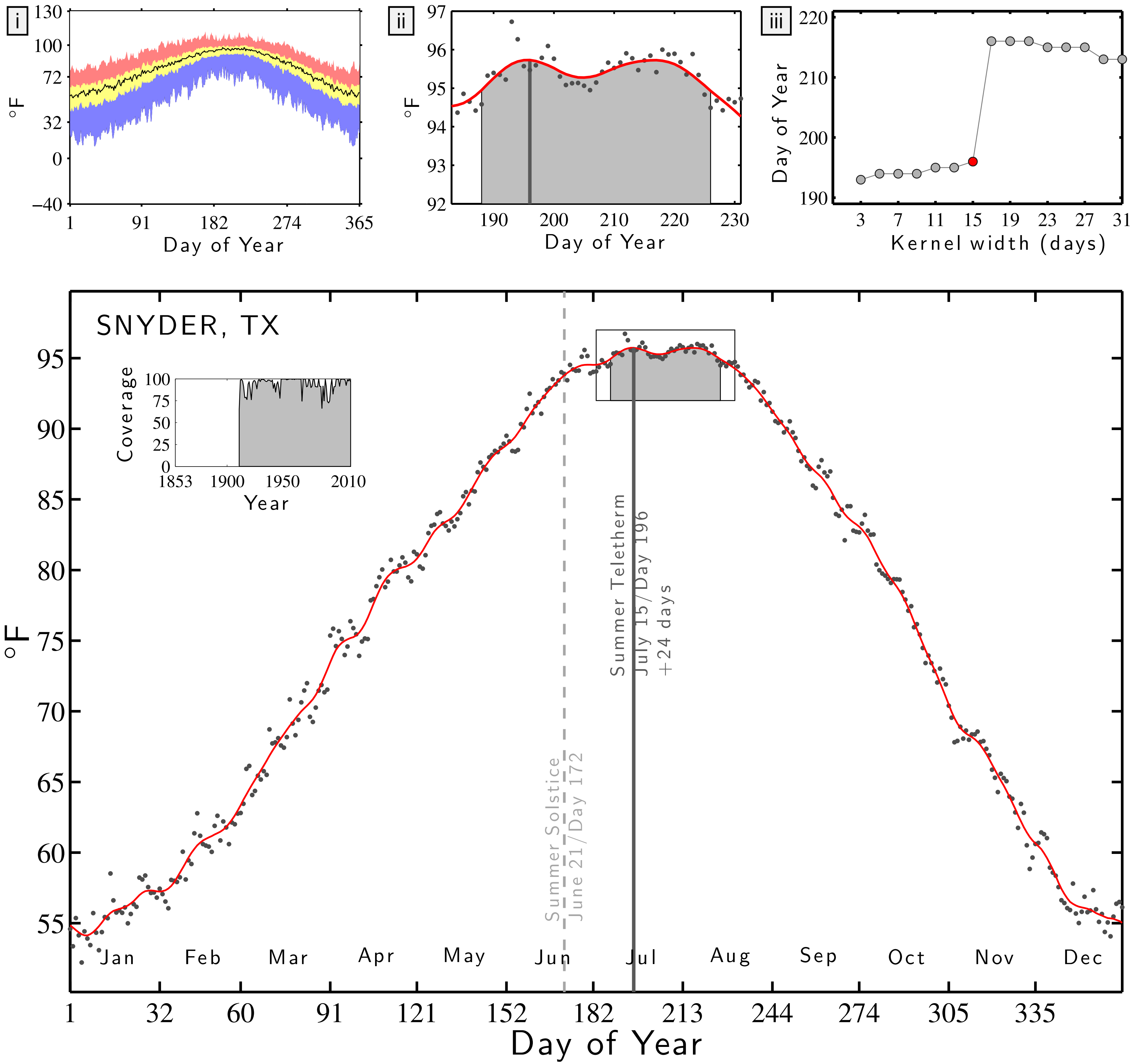

i
°F
Day of Year
ii
°F
Day of Year
iii
Day of Year
Kernel width (days)
SNYDER, TX
Coverage
Year
Summer Solstice
June 21 / Day 172
Summer Teletherm
July 15 / Day 196
+24 days
Jan
Feb
Mar
Apr
May
Jun
Jul
Aug
Sep
Oct
Nov
Dec
°F
Day of Year

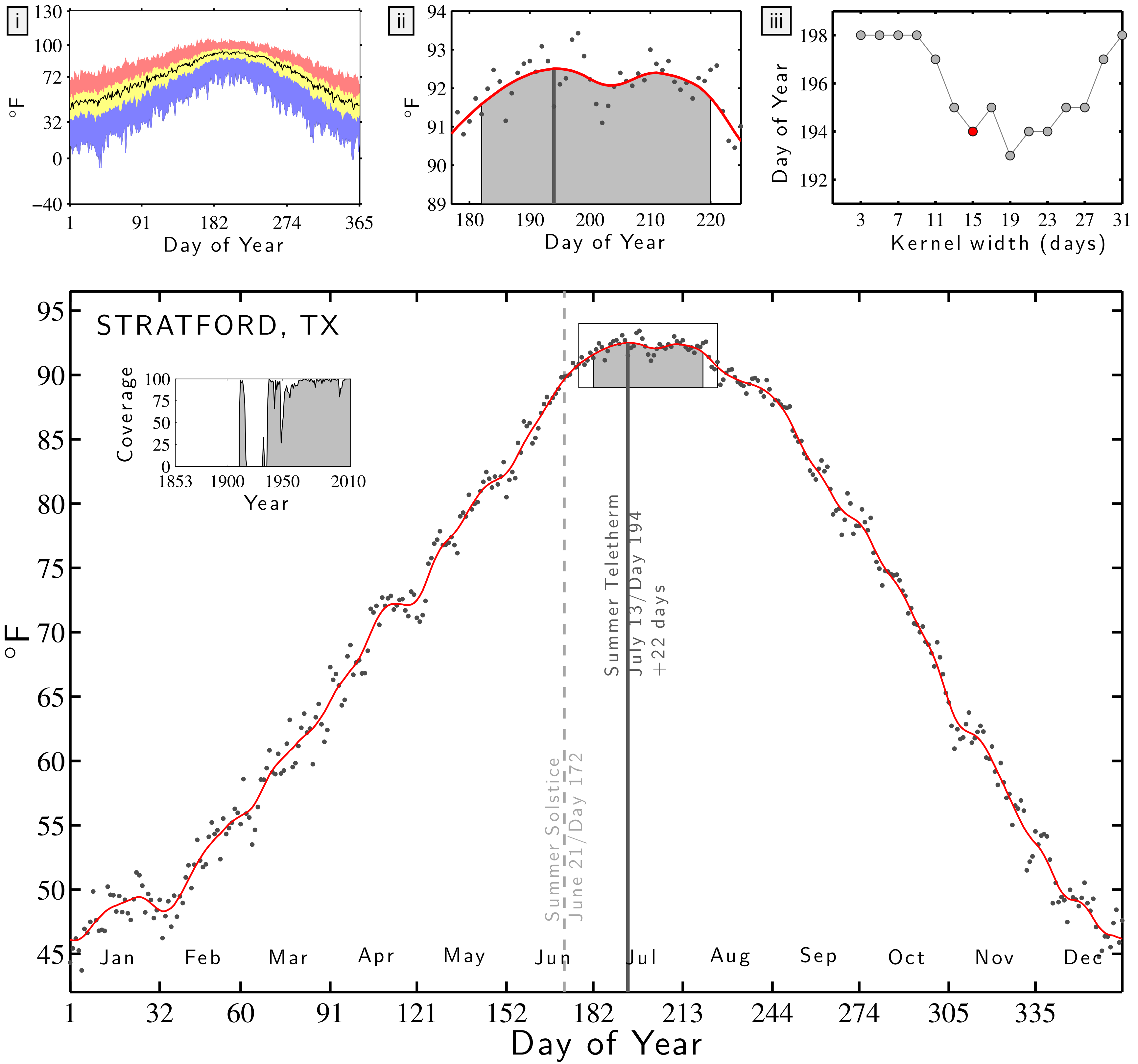

i
ii
iii
°F
Day of Year
Day of Year
Kernel width (days)
STRATFORD, TX
Coverage
Year
Summer Solstice
June 21/Day 172
Summer Teletherm
July 13/Day 194
+22 days
Jan
Feb
Mar
Apr
May
Jun
Jul
Aug
Sep
Oct
Nov
Dec

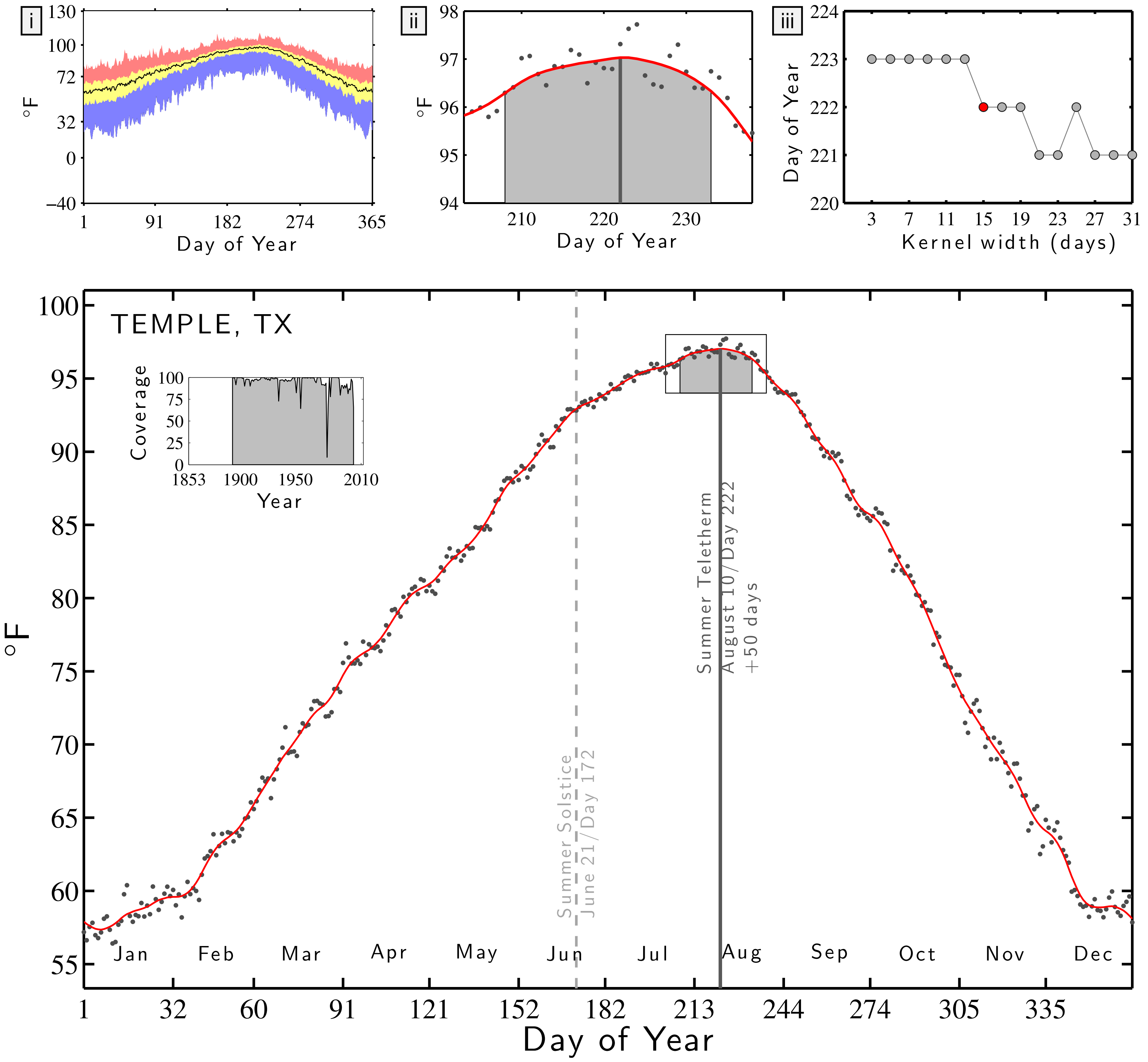

i
ii
iii
°F
Day of Year
Day of Year
Kernel width (days)
TEMPLE, TX
Coverage
Year
Summer Solstice
June 21/Day 172
Summer Teletherm
August 10/Day 222
+50 days
Jan
Feb
Mar
Apr
May
Jun
Jul
Aug
Sep
Oct
Nov
Dec

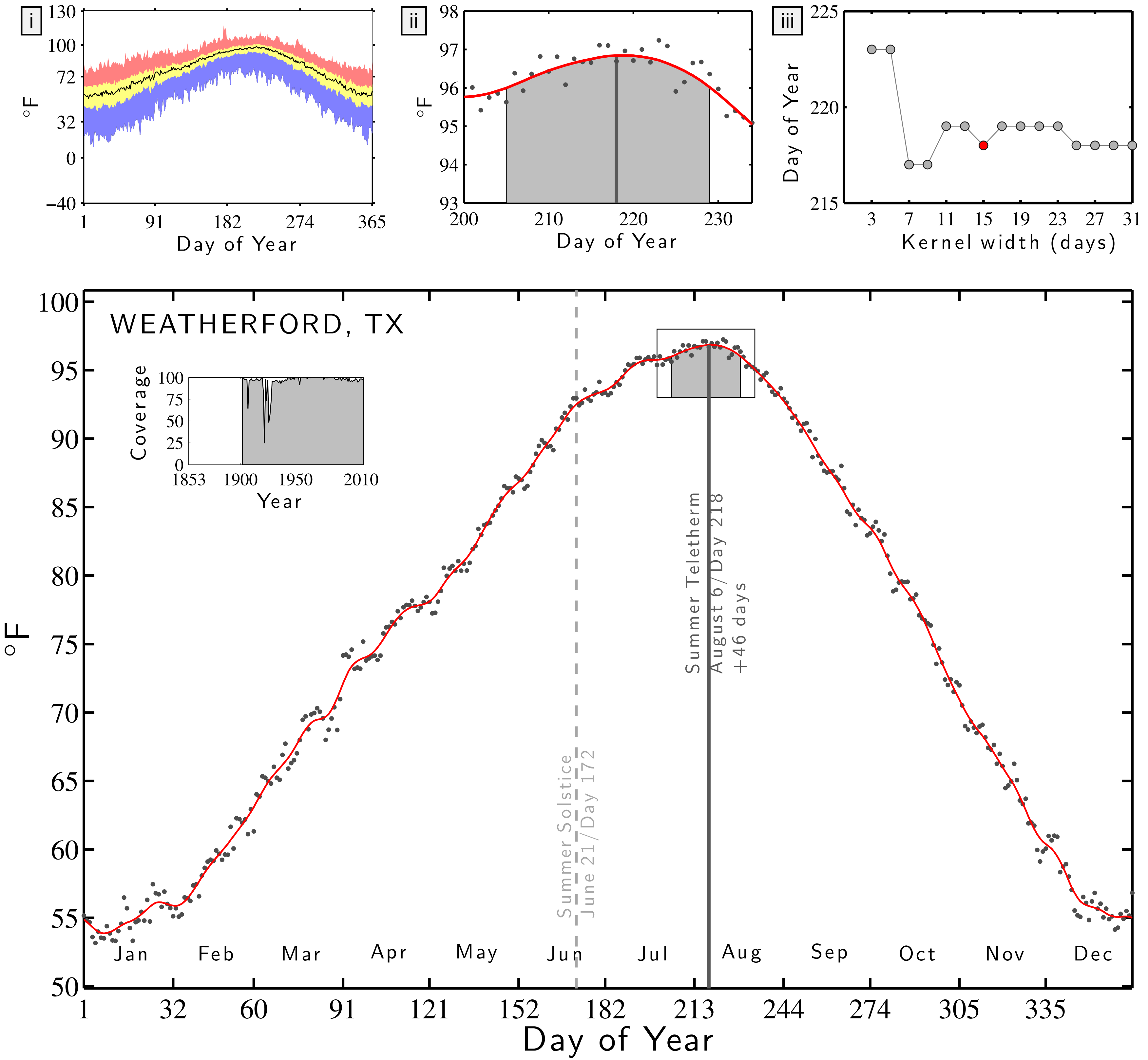

i
°F
Day of Year
ii
Day of Year
iii
Day of Year
Kernel width (days)
WEATHERFORD, TX
Coverage
Year
Summer Solstice
June 21/Day 172
Summer Teletherm
August 6/Day 218
+46 days
Jan
Feb
Mar
Apr
May
Jun
Jul
Aug
Sep
Oct
Nov
Dec
Day of Year

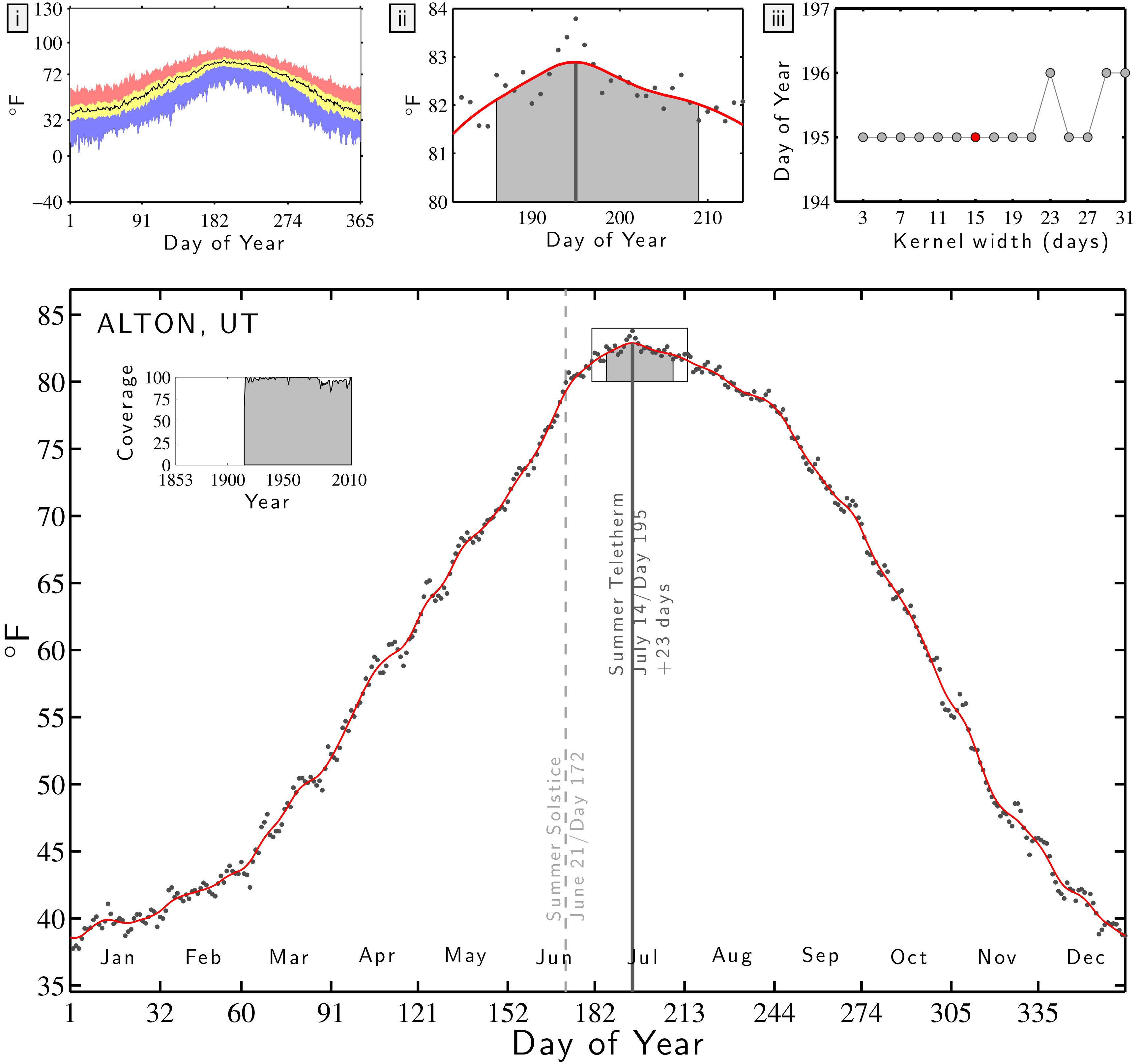

i
°F
Day of Year
ii
°F
Day of Year
iii
Day of Year
Kernel width (days)
ALTON, UT
Coverage
Year
Summer Solstice
June 21/Day 172
Summer Teletherm
July 14/Day 195
+23 days
Jan
Feb
Mar
Apr
May
Jun
Jul
Aug
Sep
Oct
Nov
Dec
°F
Day of Year

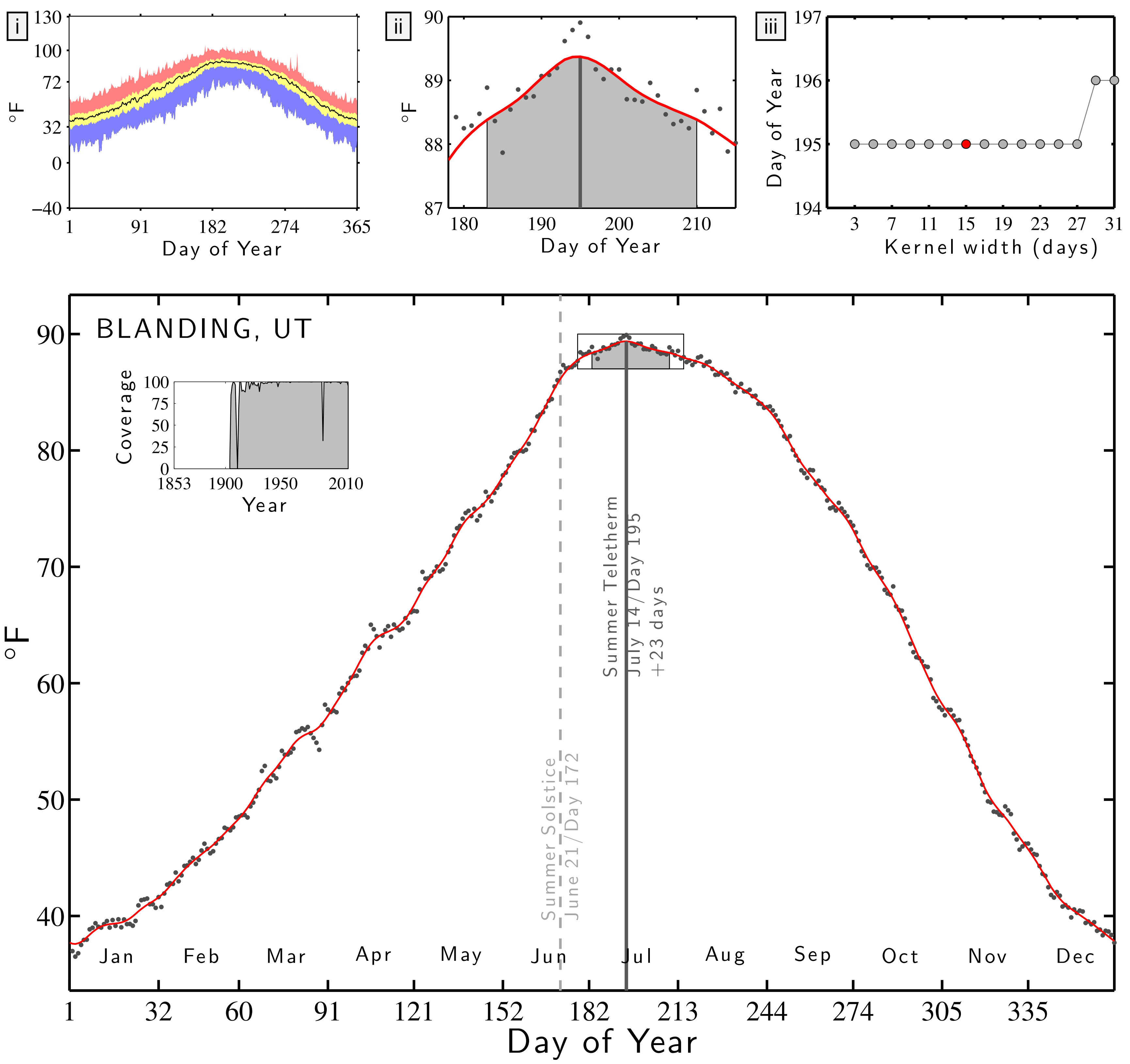

i
ii
iii
BLANDING, UT
Day of Year
°F
Kernel width (days)
Coverage
Year
Summer Solstice
June 21/Day 172
Summer Teletherm
July 14/Day 195
+23 days
Jan
Feb
Mar
Apr
May
Jun
Jul
Aug
Sep
Oct
Nov
Dec

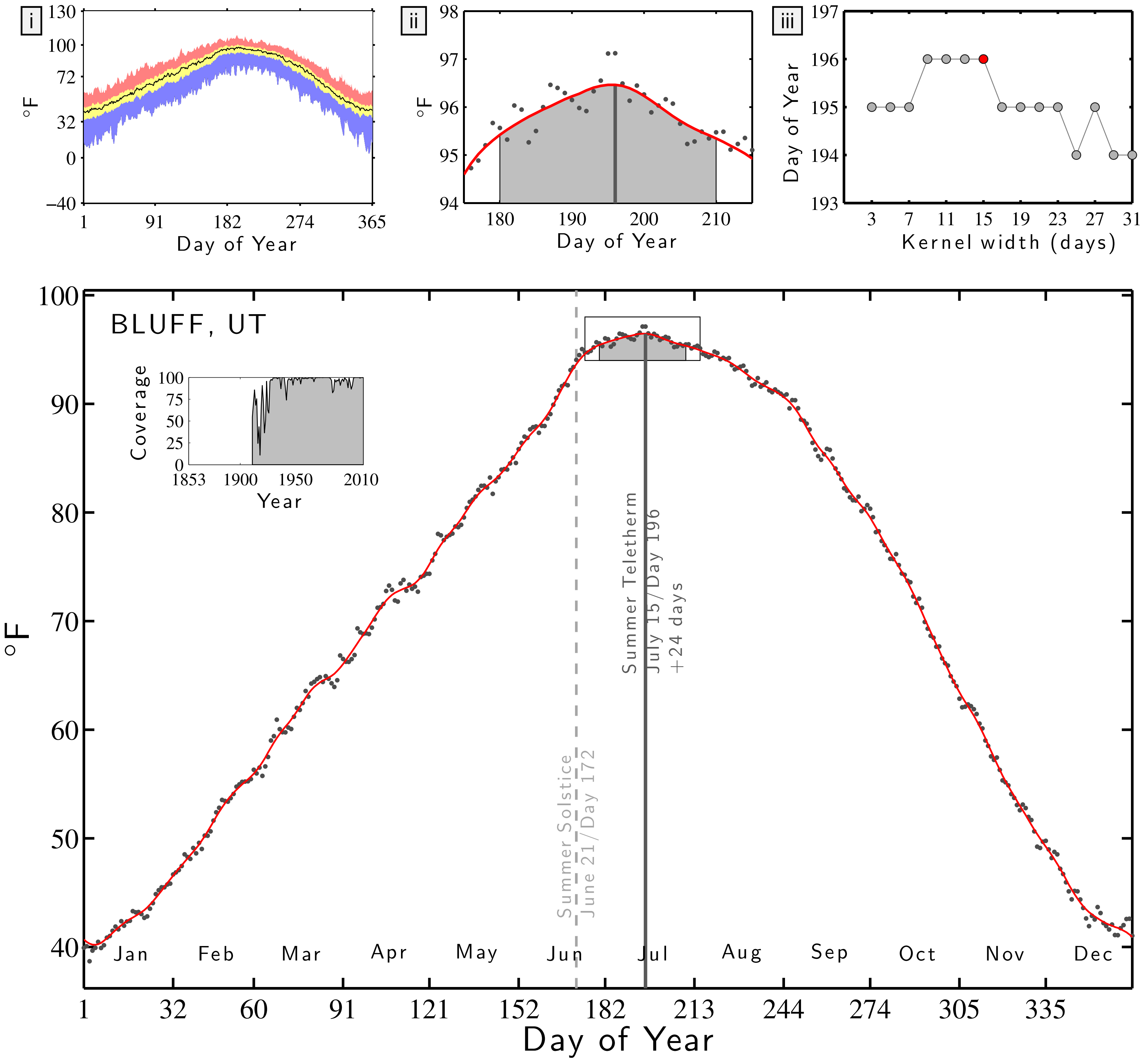

i
°F
Day of Year
ii
°F
Day of Year
iii
Day of Year
Kernel width (days)
BLUFF, UT
Coverage
Year
Summer Solstice
June 21/Day 172
Summer Teletherm
July 15/Day 196
+24 days
Jan
Feb
Mar
Apr
May
Jun
Jul
Aug
Sep
Oct
Nov
Dec
°F
Day of Year

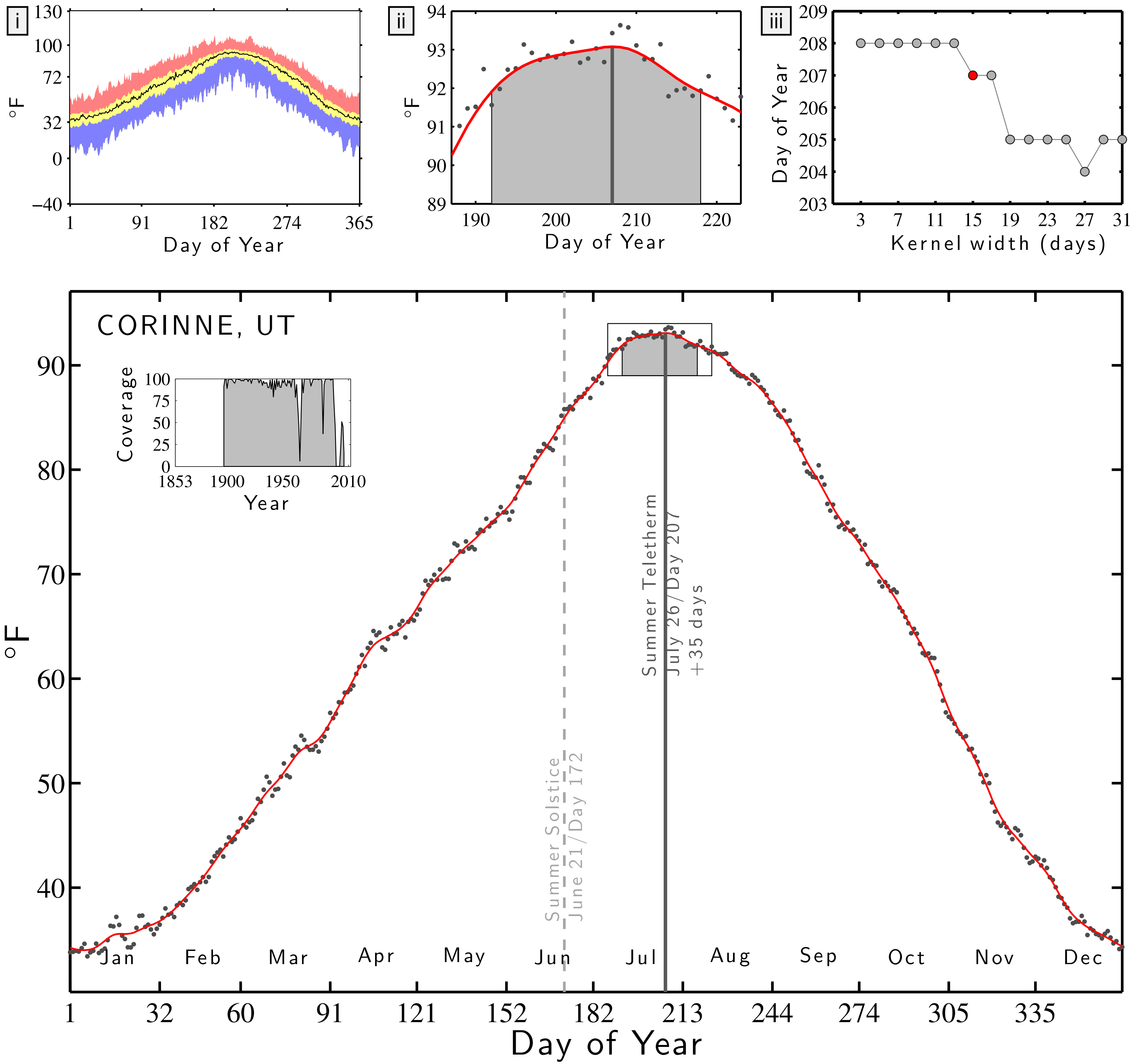

i
ii
iii
°F
Day of Year
Day of Year
Kernel width (days)
CORINNE, UT
Coverage
Year
Summer Solstice
June 21/Day 172
Summer Teletherm
July 26/Day 207
+35 days
Jan
Feb
Mar
Apr
May
Jun
Jul
Aug
Sep
Oct
Nov
Dec
Day of Year

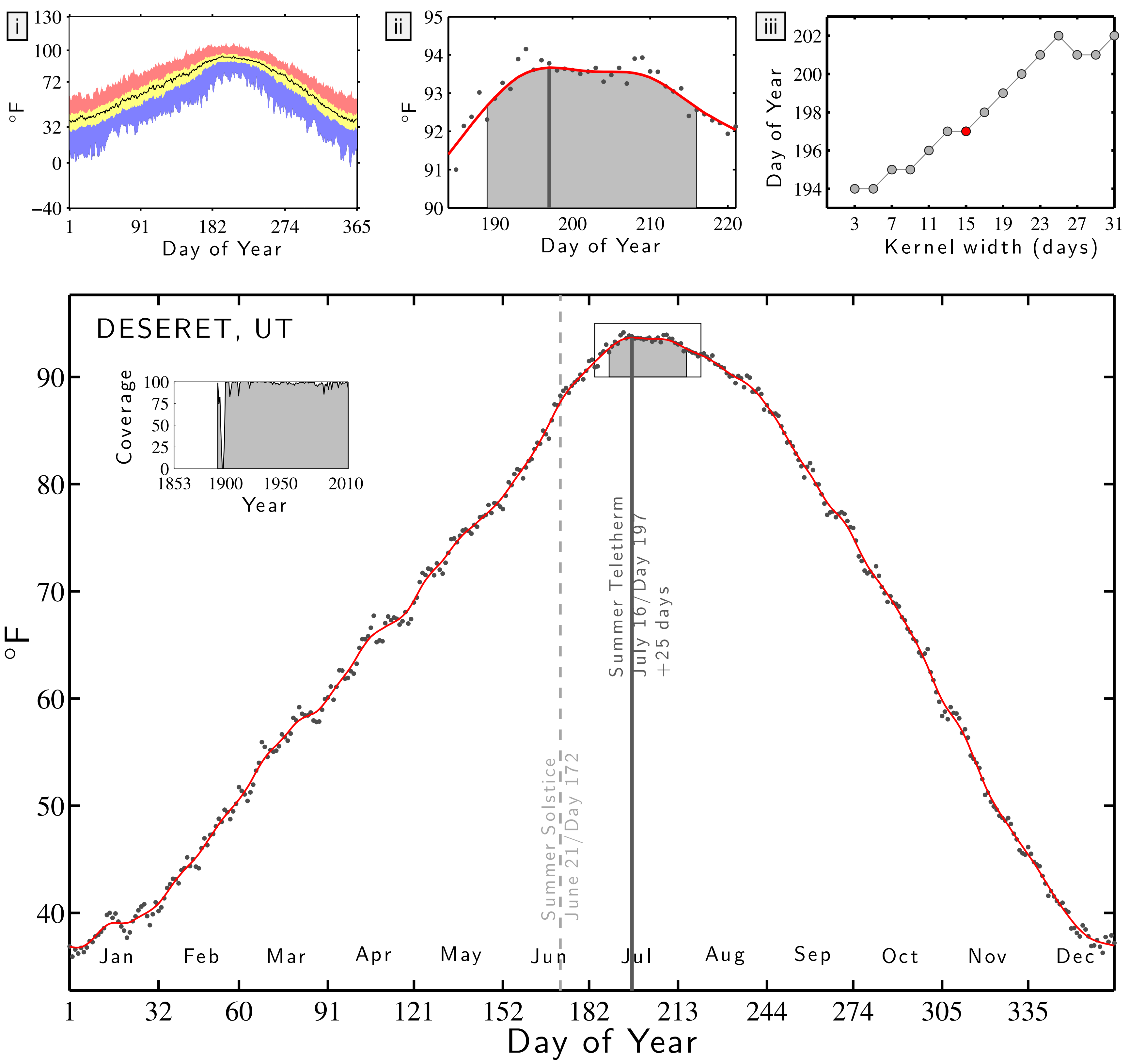

i
°F
130
100
72
32
0
−40
1
91
182
274
365
Day of Year
ii
95
94
93
92
91
90
190
200
210
220
iii
Day of Year
202
200
198
196
194
3
7
11
15
19
23
27
31
Kernel width (days)
DESERET, UT
Coverage
100
75
50
25
0
1853
1900
1950
2010
Year
Summer Solstice
June 21/Day 172
Summer Teletherm
July 16/Day 197
+25 days
Jan
Feb
Mar
Apr
May
Jun
Jul
Aug
Sep
Oct
Nov
Dec
40
50
60
70
80
90
32
60
121
152
213
244
305
335

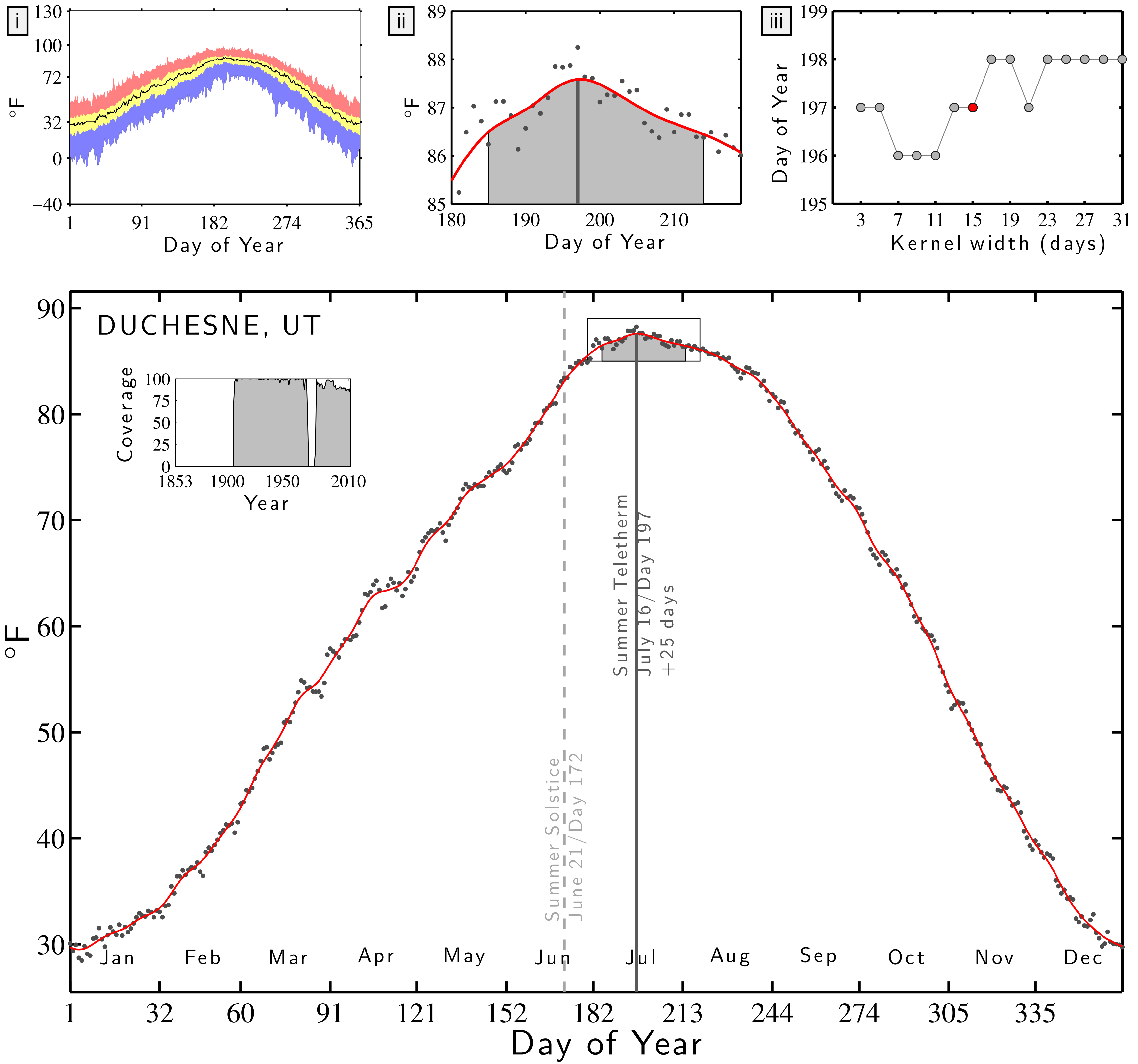

i
ii
iii
°F
Day of Year
Day of Year
Kernel width (days)
DUCHESNE, UT
Coverage
Year
Summer Solstice
June 21/Day 172
Summer Teletherm
July 16/Day 197
+25 days
Jan
Feb
Mar
Apr
May
Jun
Jul
Aug
Sep
Oct
Nov
Dec
Day of Year

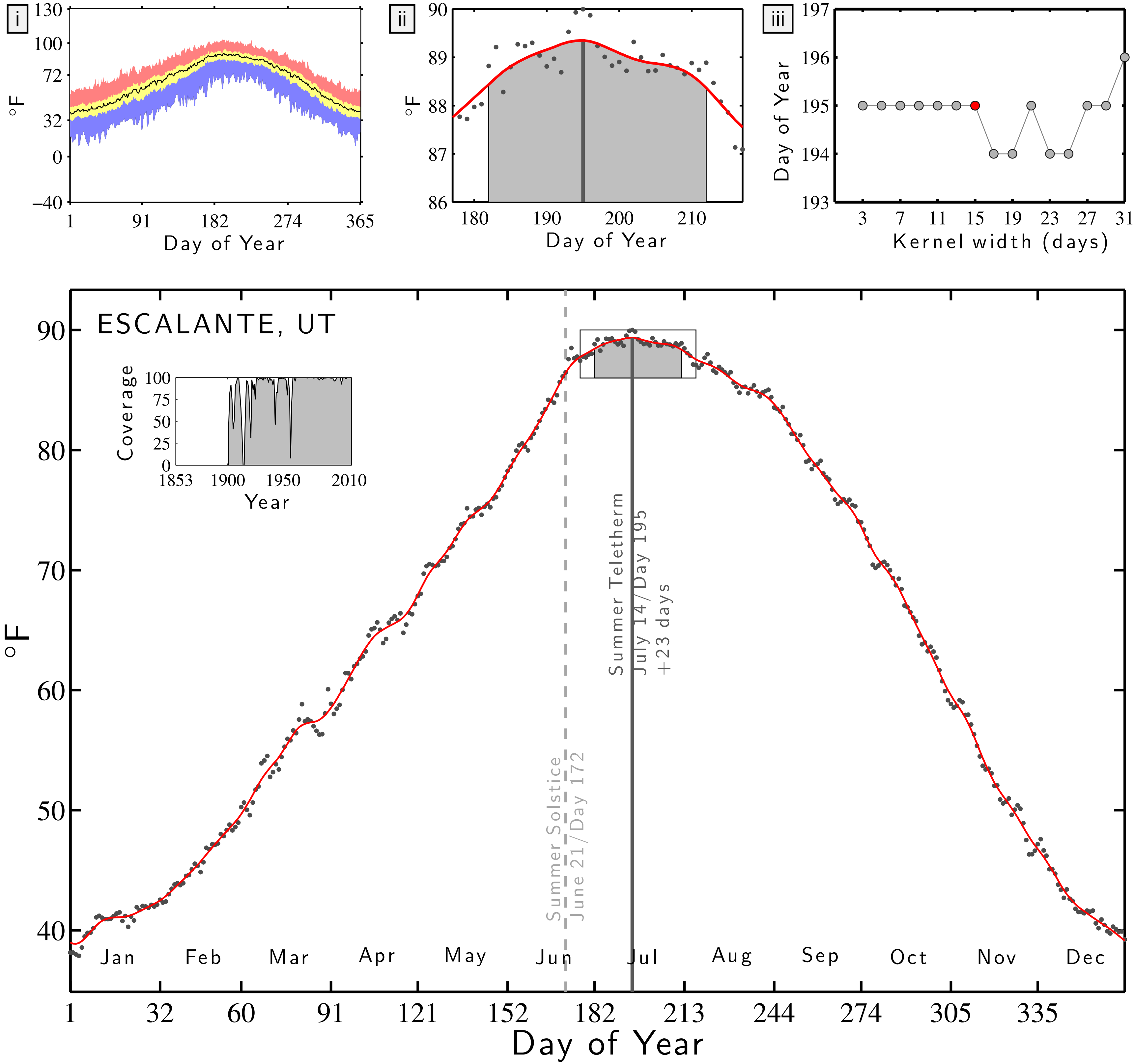

i
ii
iii
°F
Day of Year
Kernel width (days)
ESCALANTE, UT
Coverage
Year
Summer Solstice
June 21 / Day 172
Summer Teletherm
July 14 / Day 195
+23 days
Jan
Feb
Mar
Apr
May
Jun
Jul
Aug
Sep
Oct
Nov
Dec

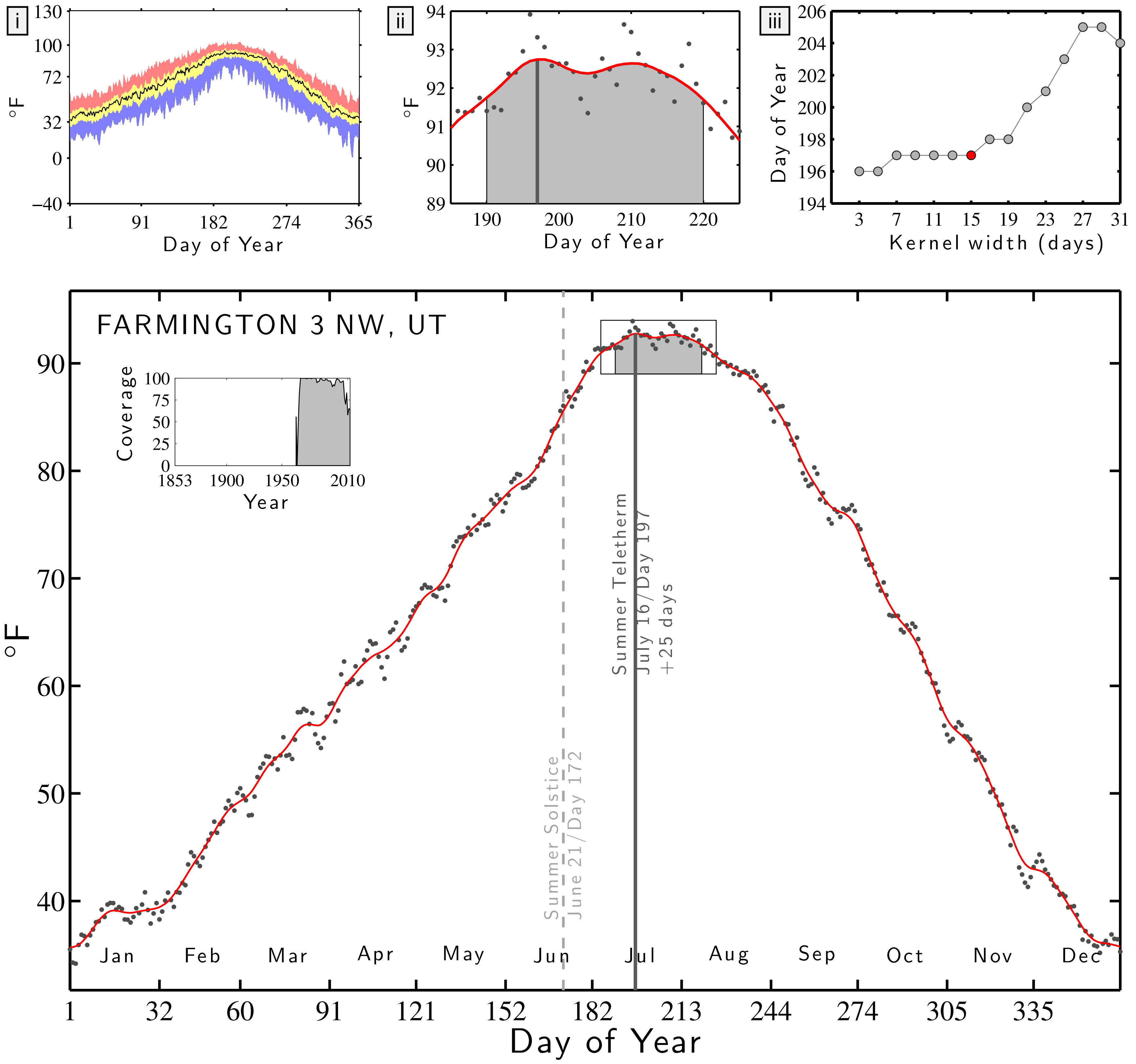

i
°F
Day of Year
ii
°F
Day of Year
iii
Day of Year
Kernel width (days)
FARMINGTON 3 NW, UT
Coverage
Year
Summer Solstice
June 21/Day 172
Summer Teletherm
July 16/Day 197
+25 days
Jan
Feb
Mar
Apr
May
Jun
Jul
Aug
Sep
Oct
Nov
Dec
°F
Day of Year

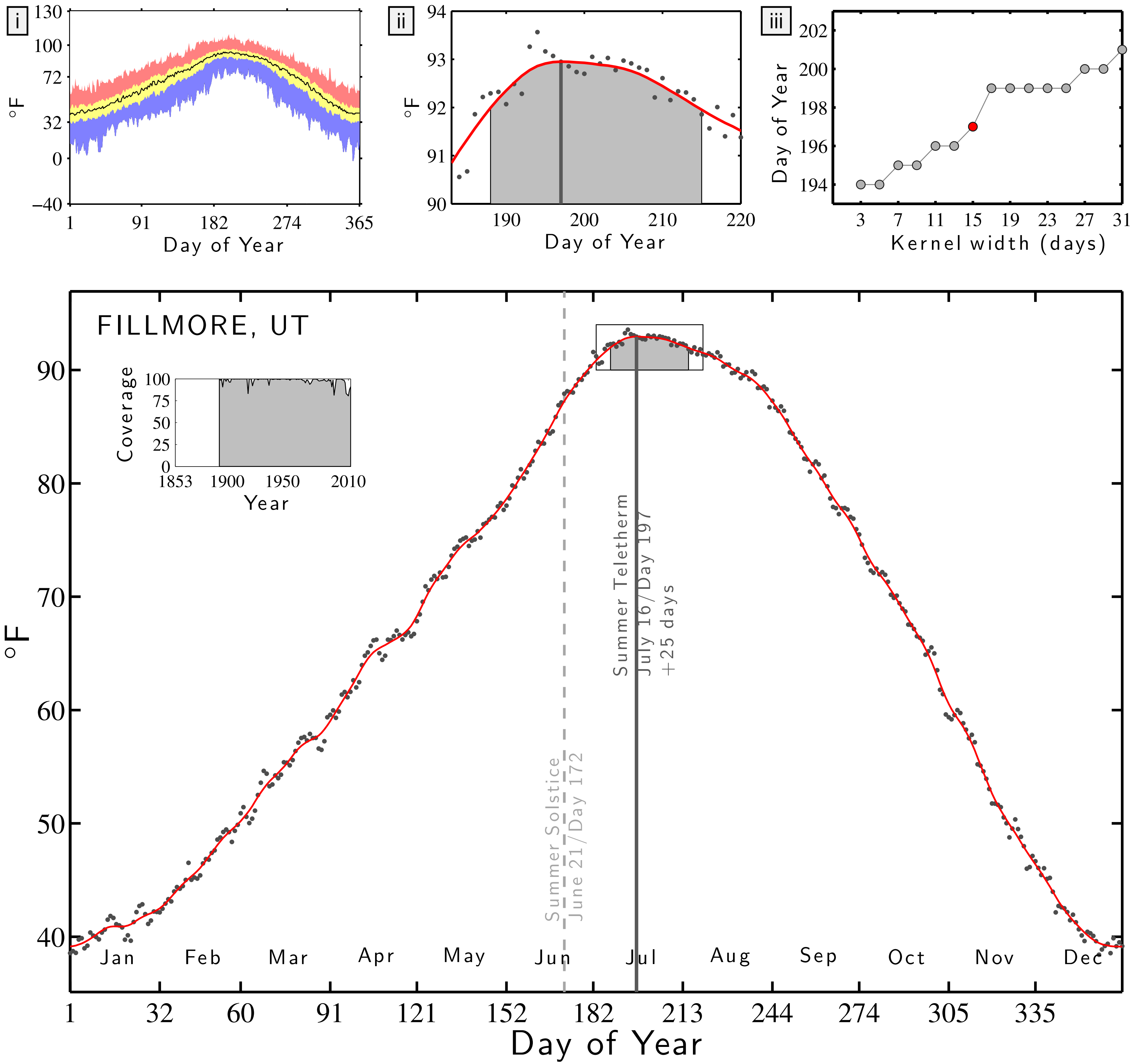

i
°F
130
100
72
32
0
−40
1
91
182
274
365
Day of Year
ii
°F
94
93
92
91
90
190
200
210
220
Day of Year
iii
Day of Year
202
200
198
196
194
3
7
11
15
19
23
27
31
Kernel width (days)
FILLMORE, UT
Coverage
100
75
50
25
0
1853
1900
1950
2010
Year
°F
90
80
70
60
50
40
Summer Solstice
June 21/Day 172
Summer Teletherm
July 16/Day 197
+25 days
Jan
Feb
Mar
Apr
May
Jun
Jul
Aug
Sep
Oct
Nov
Dec
1
32
60
91
121
152
182
213
244
274
305
335
Day of Year

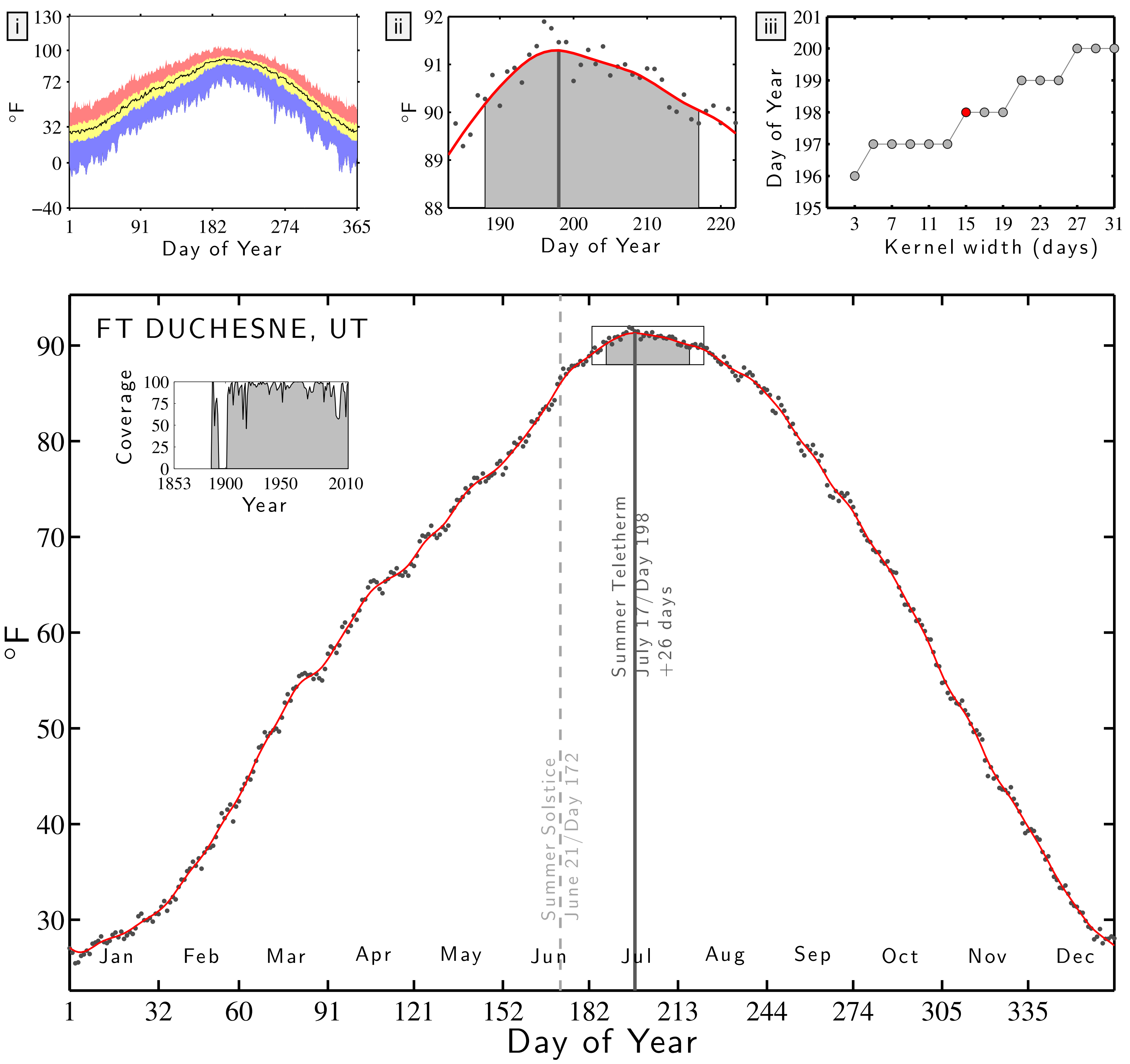

i
°F
Day of Year
ii
°F
Day of Year
iii
Day of Year
Kernel width (days)
FT DUCHESNE, UT
Coverage
Year
Summer Solstice
June 21/Day 172
Summer Teletherm
July 17/Day 198
+26 days
Jan
Feb
Mar
Apr
May
Jun
Jul
Aug
Sep
Oct
Nov
Dec
°F
Day of Year

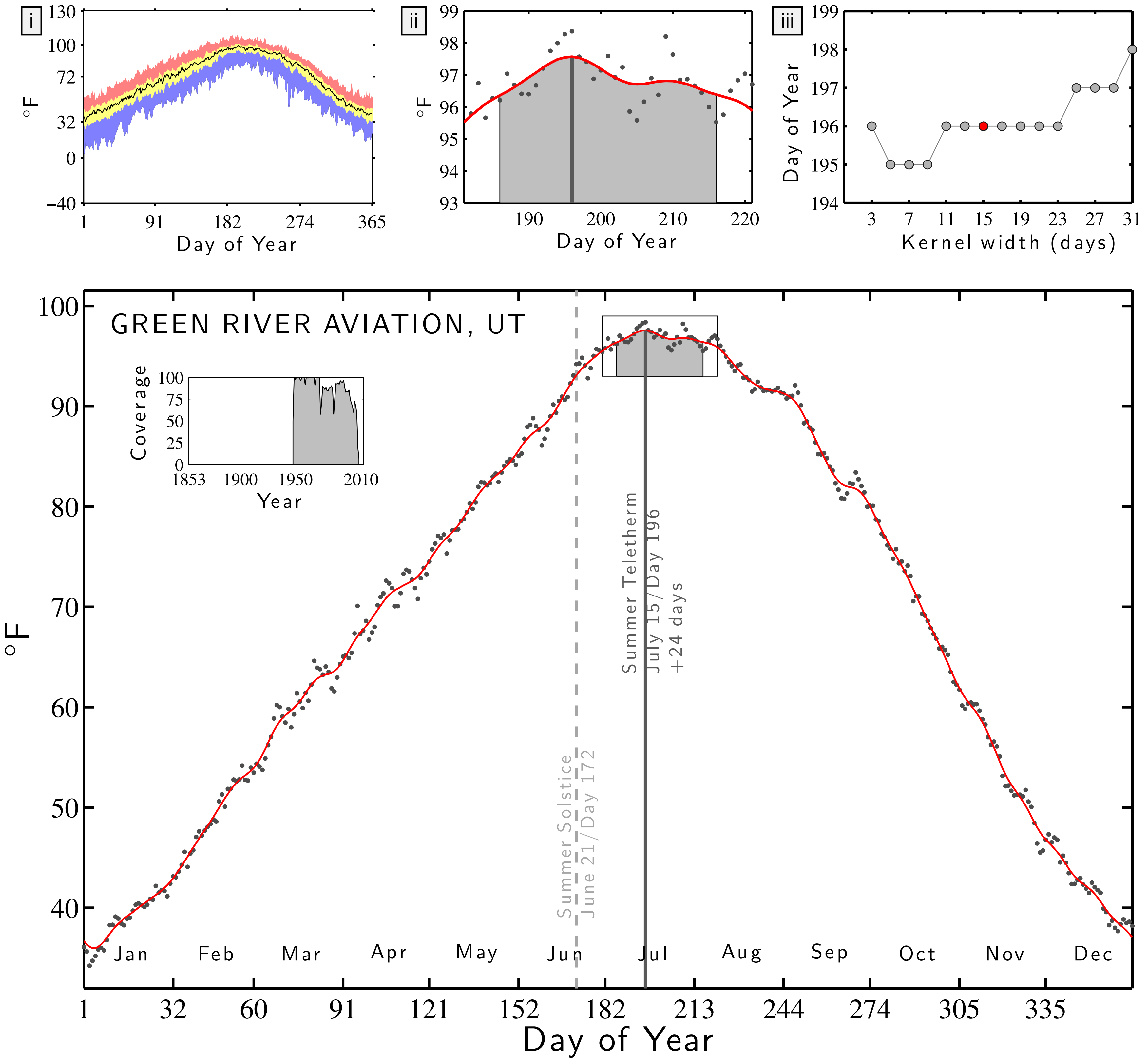

i
ii
iii
°F
Day of Year
Day of Year
Kernel width (days)
GREEN RIVER AVIATION, UT
Coverage
Year
Summer Solstice
June 21/Day 172
Summer Teletherm
July 15/Day 196
+24 days
Jan
Feb
Mar
Apr
May
Jun
Jul
Aug
Sep
Oct
Nov
Dec

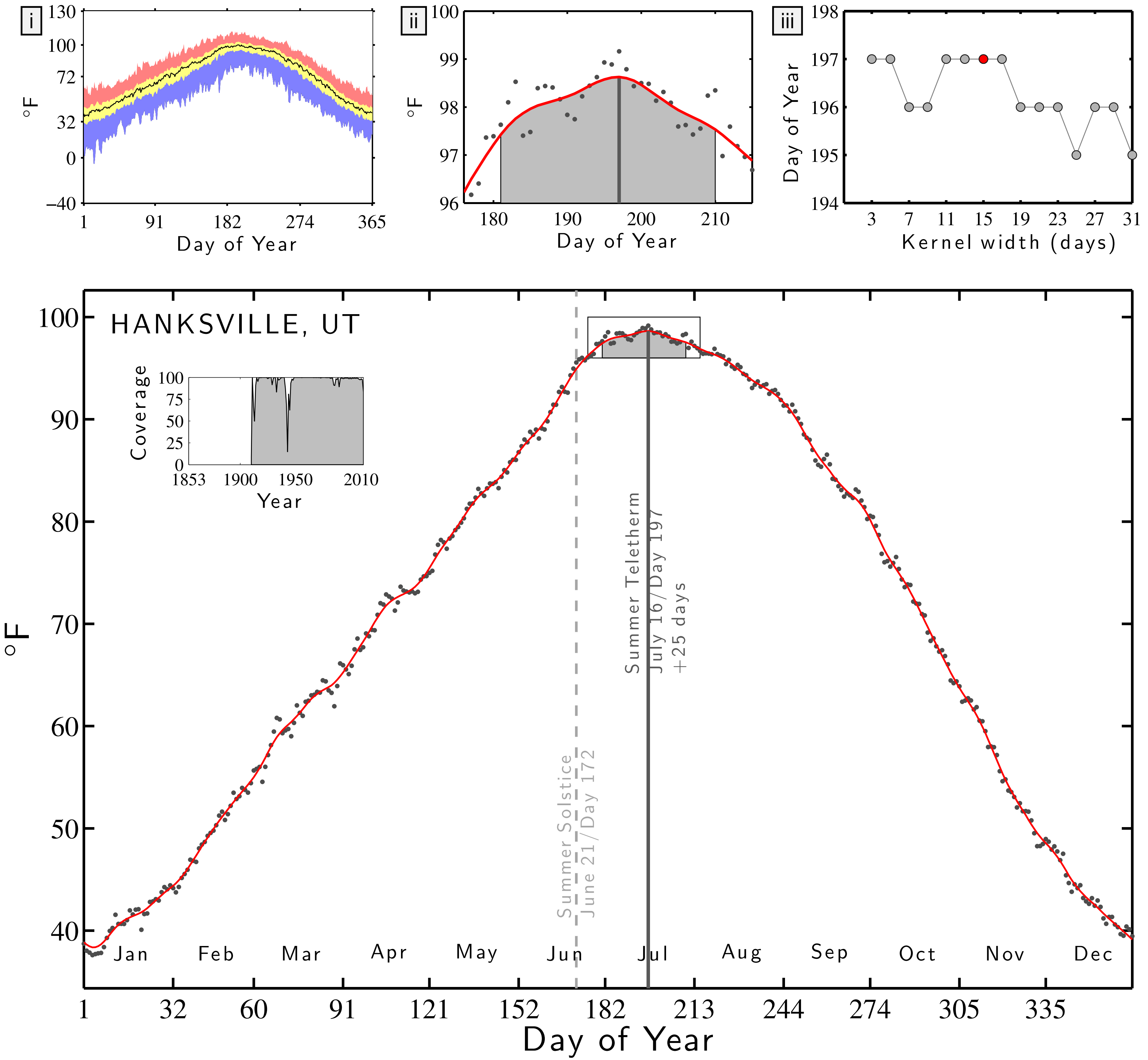

i
ii
iii
Day of Year
Day of Year
Kernel width (days)
HANKSVILLE, UT
Coverage
Year
Summer Solstice
June 21/Day 172
Summer Teletherm
July 16/Day 197
+25 days
Jan
Feb
Mar
Apr
May
Jun
Jul
Aug
Sep
Oct
Nov
Dec
°F
Day of Year

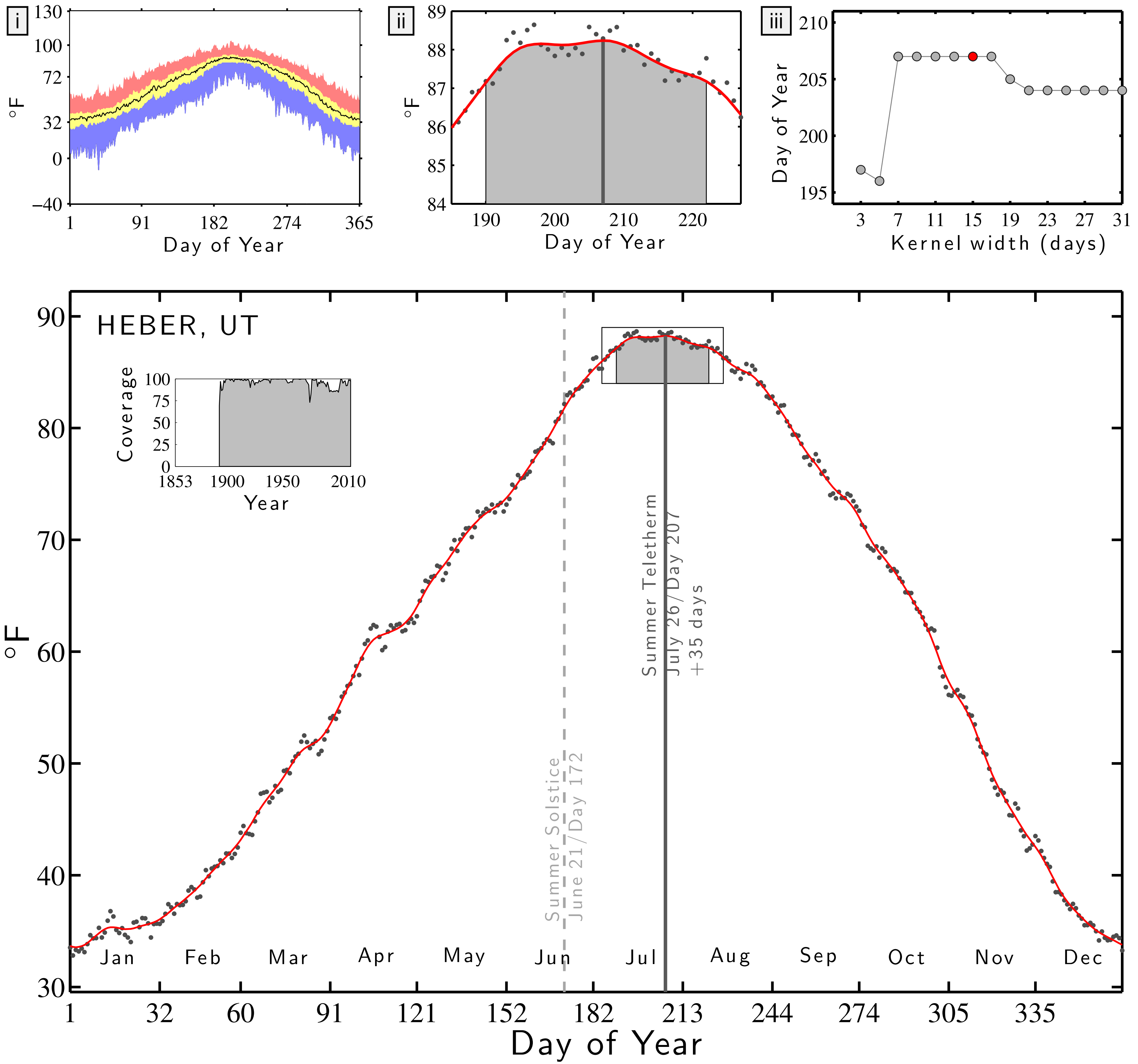

i
ii
iii
°F
Day of Year
Kernel width (days)
HEBER, UT
Coverage
Year
1853
1900
1950
2010
Summer Solstice
June 21 / Day 172
Summer Teletherm
July 26 / Day 207
+35 days
Jan
Feb
Mar
Apr
May
Jun
Jul
Aug
Sep
Oct
Nov
Dec

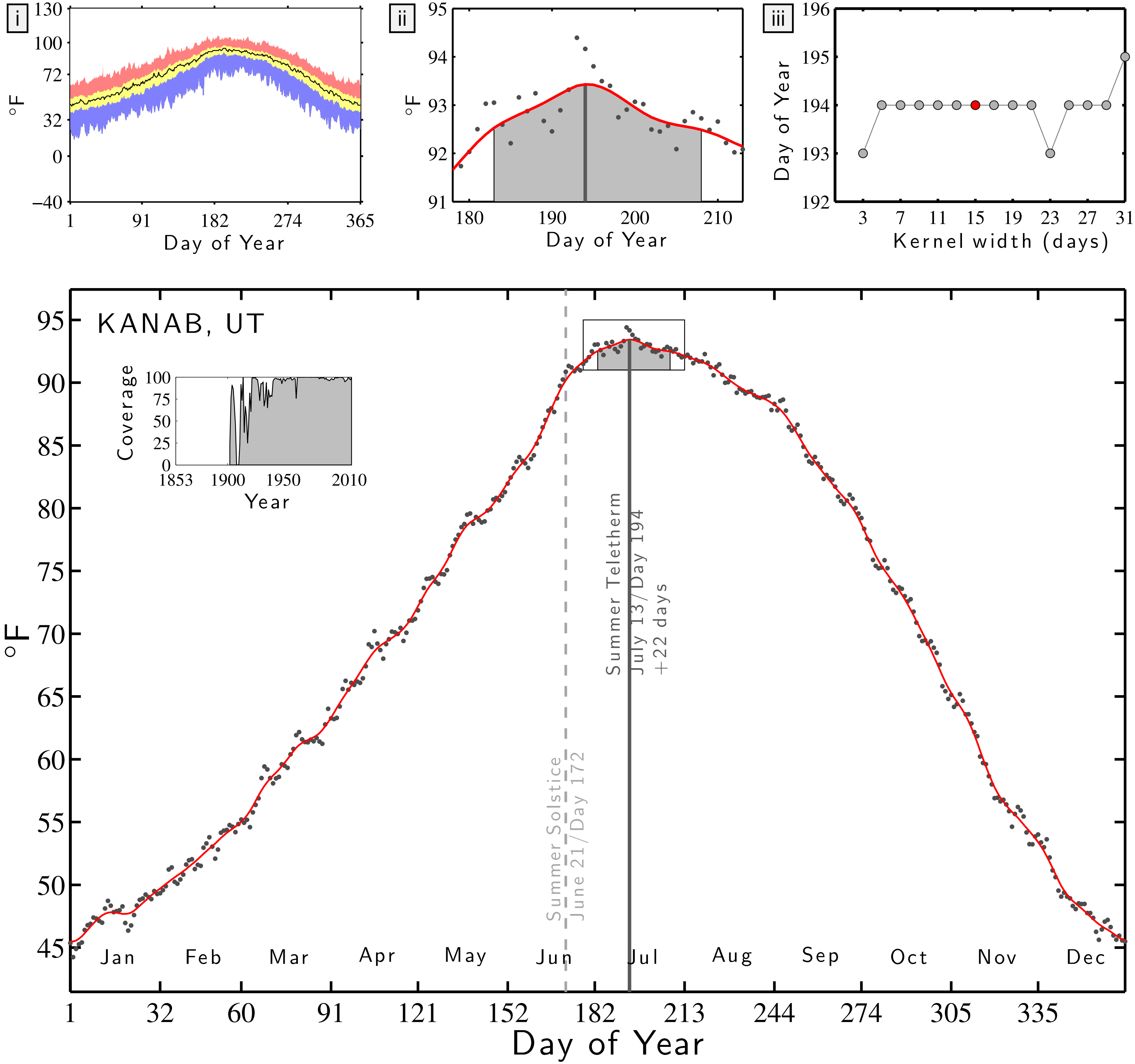

i
°F
Day of Year
ii
°F
Day of Year
iii
Day of Year
Kernel width (days)
KANAB, UT
Coverage
Year
Summer Solstice
June 21/Day 172
Summer Teletherm
July 13/Day 194
+22 days
Jan
Feb
Mar
Apr
May
Jun
Jul
Aug
Sep
Oct
Nov
Dec
°F
Day of Year

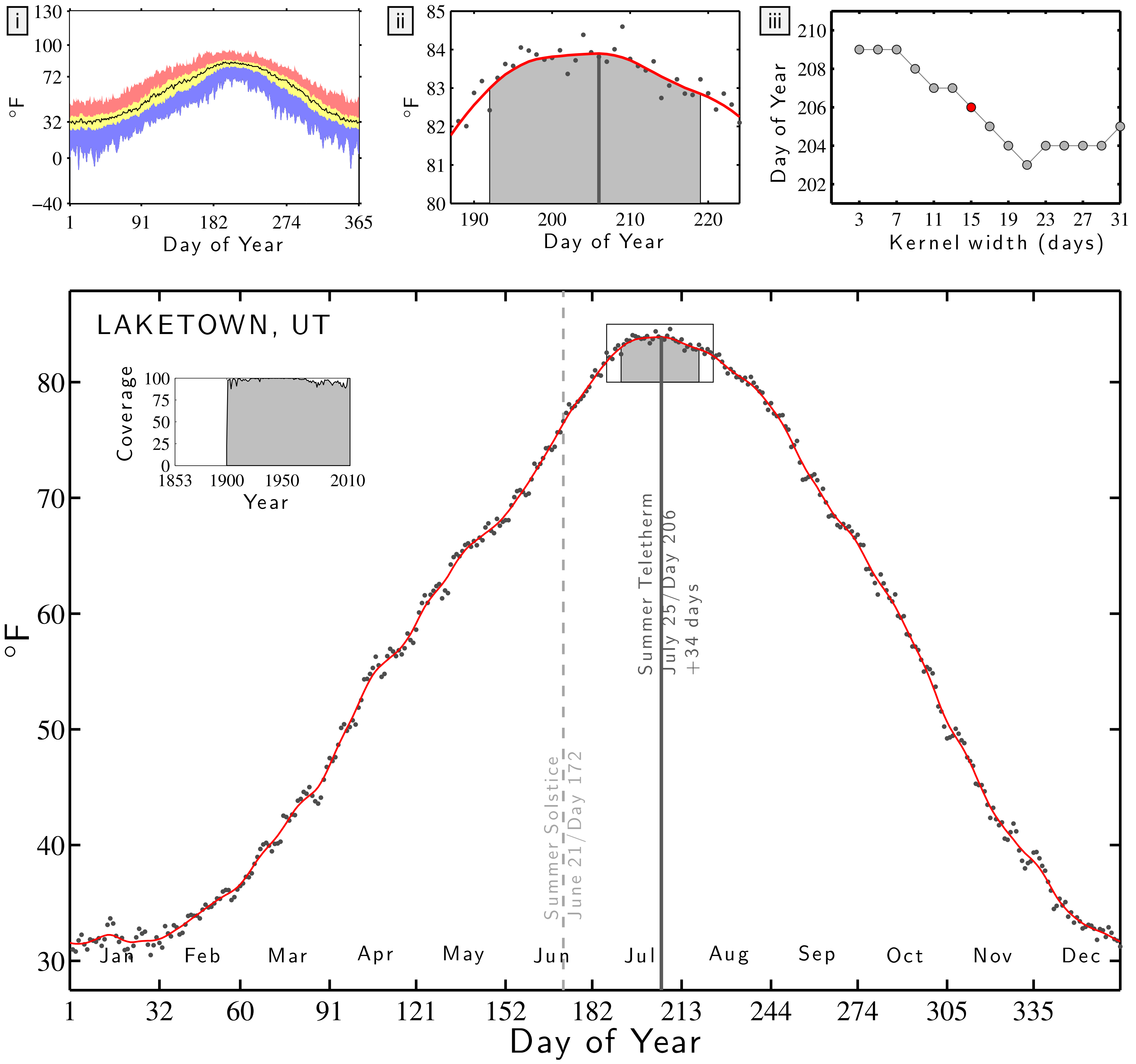

i
°F
Day of Year
ii
°F
Day of Year
iii
Day of Year
Kernel width (days)
LAKETOWN, UT
Coverage
Year
°F
Day of Year
Summer Solstice
June 21/Day 172
Summer Teletherm
July 25/Day 206
+34 days
Jan
Feb
Mar
Apr
May
Jun
Jul
Aug
Sep
Oct
Nov
Dec

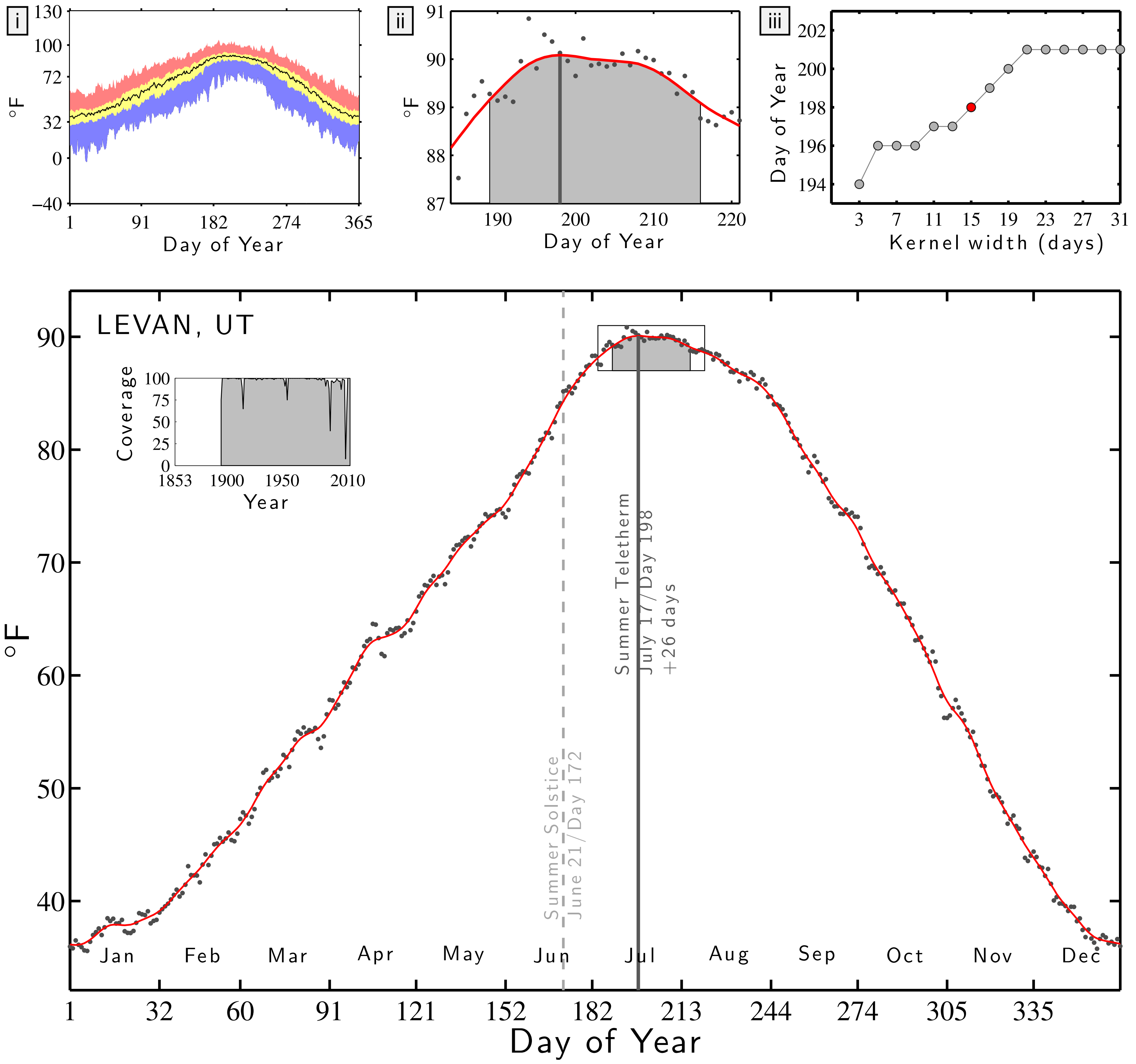

i
°F
Day of Year
ii
°F
Day of Year
iii
Day of Year
Kernel width (days)
LEVAN, UT
Coverage
Year
Summer Solstice
June 21/Day 172
Summer Teletherm
July 17/Day 198
+26 days
Jan
Feb
Mar
Apr
May
Jun
Jul
Aug
Sep
Oct
Nov
Dec
°F
Day of Year

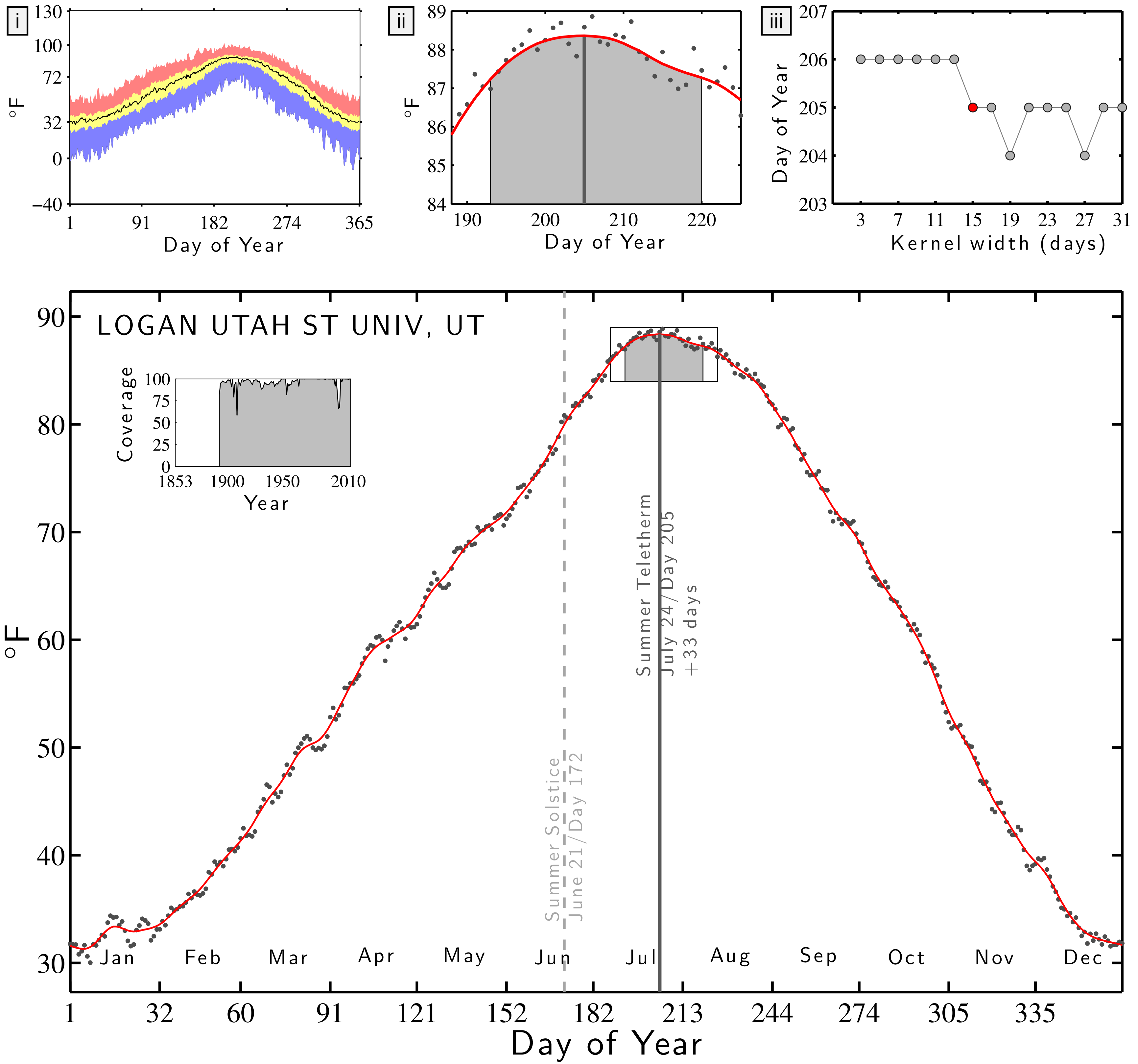

i
°F
Day of Year
ii
°F
Day of Year
iii
Day of Year
Kernel width (days)
LOGAN UTAH ST UNIV, UT
Coverage
Year
Summer Solstice
June 21/Day 172
Summer Teletherm
July 24/Day 205
+33 days
Jan
Feb
Mar
Apr
May
Jun
Jul
Aug
Sep
Oct
Nov
Dec
°F
Day of Year

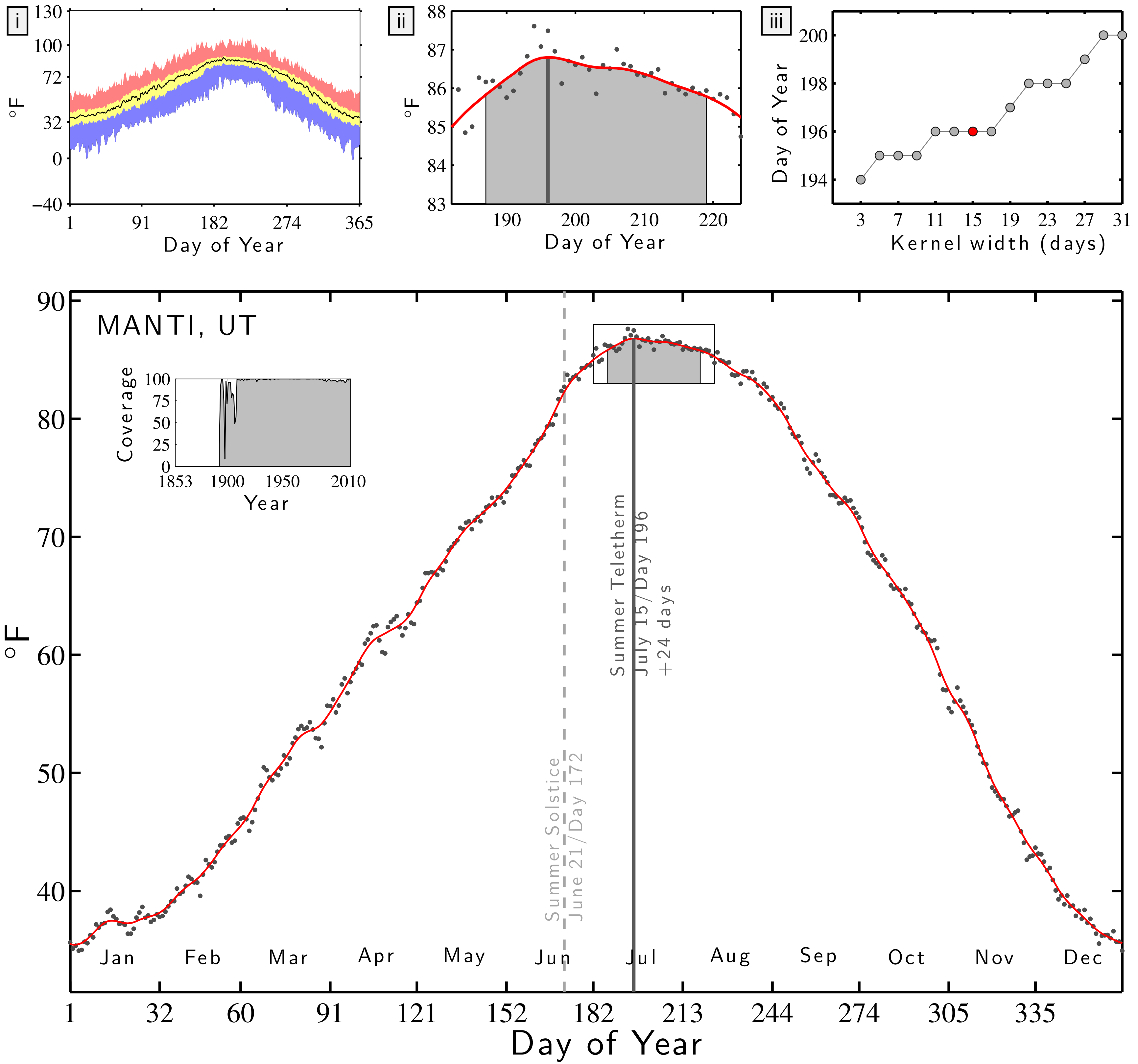

i
°F
Day of Year
ii
°F
Day of Year
iii
Day of Year
Kernel width (days)
MANTI, UT
Coverage
Year
Summer Solstice
June 21/Day 172
Summer Teletherm
July 15/Day 196
+24 days
Jan
Feb
Mar
Apr
May
Jun
Jul
Aug
Sep
Oct
Nov
Dec
°F
Day of Year

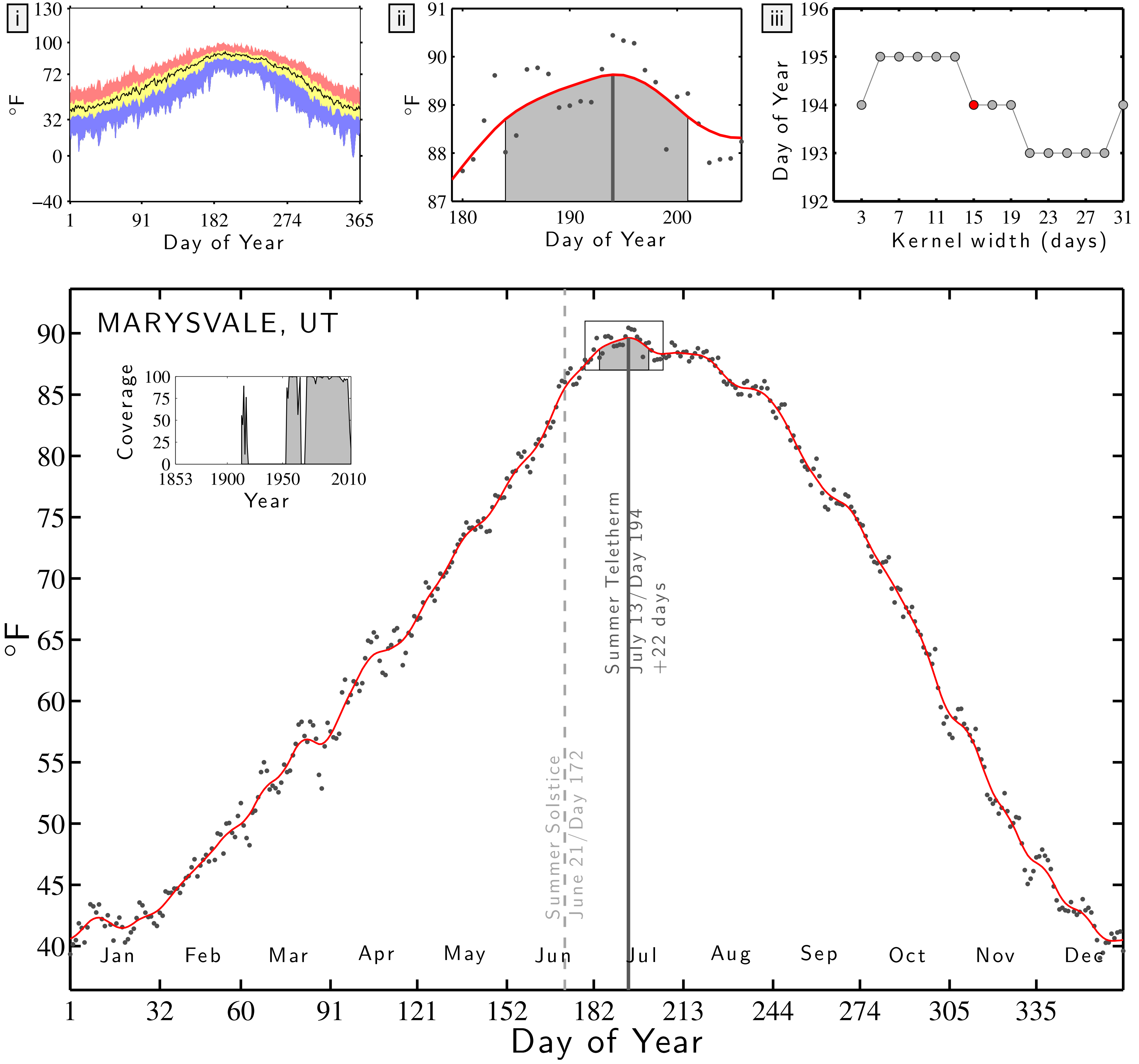

i
ii
iii
°F
Day of Year
Kernel width (days)
MARYSVALE, UT
Coverage
Year
Summer Solstice
June 21/Day 172
Summer Teletherm
July 13/Day 194
+22 days
Jan
Feb
Mar
Apr
May
Jun
Jul
Aug
Sep
Oct
Nov
Dec

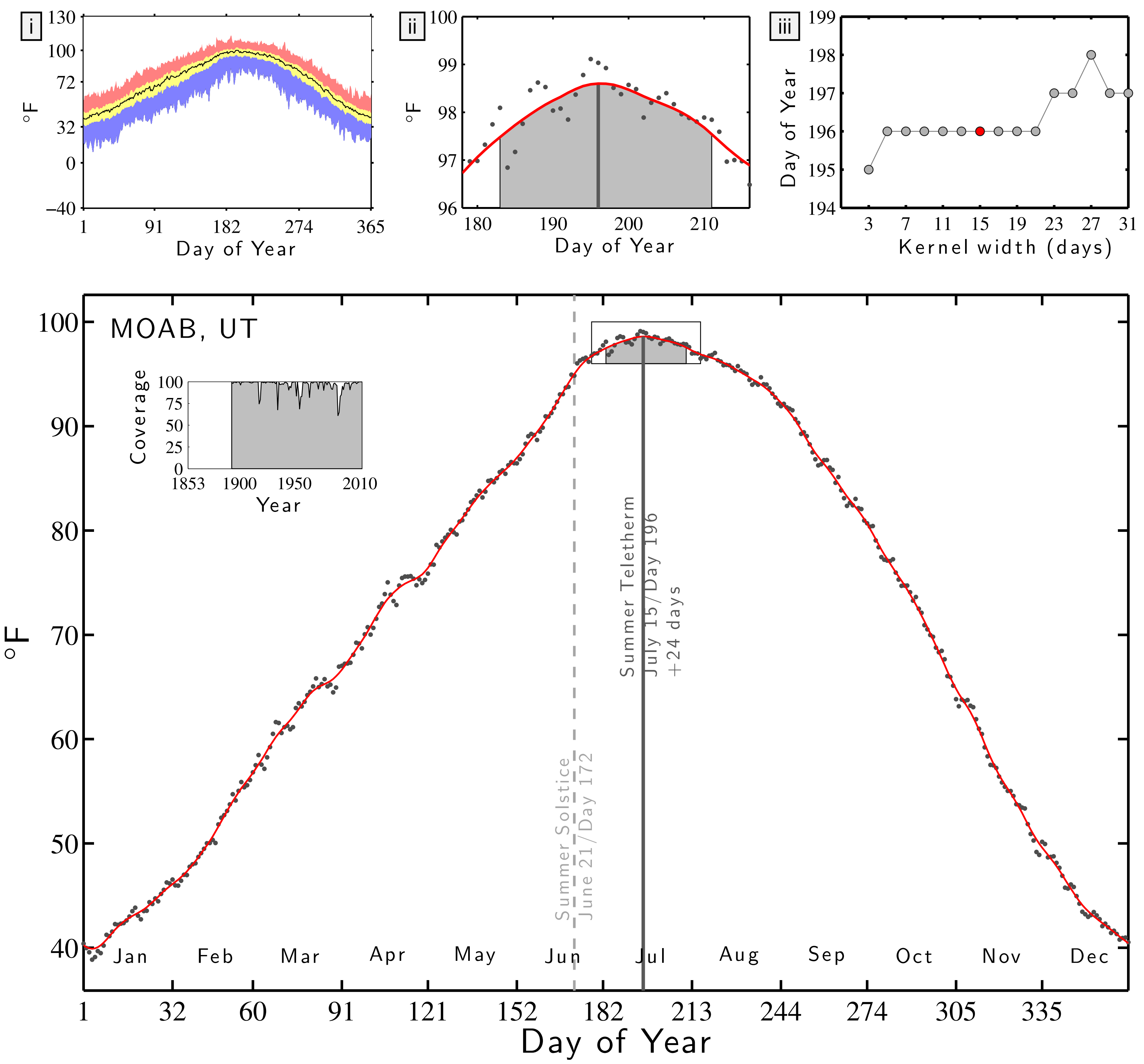

i
°F
Day of Year
ii
°F
Day of Year
iii
Day of Year
Kernel width (days)
MOAB, UT
Coverage
Year
Summer Solstice
June 21/Day 172
Summer Teletherm
July 15/Day 196
+24 days
Jan
Feb
Mar
Apr
May
Jun
Jul
Aug
Sep
Oct
Nov
Dec
°F
Day of Year

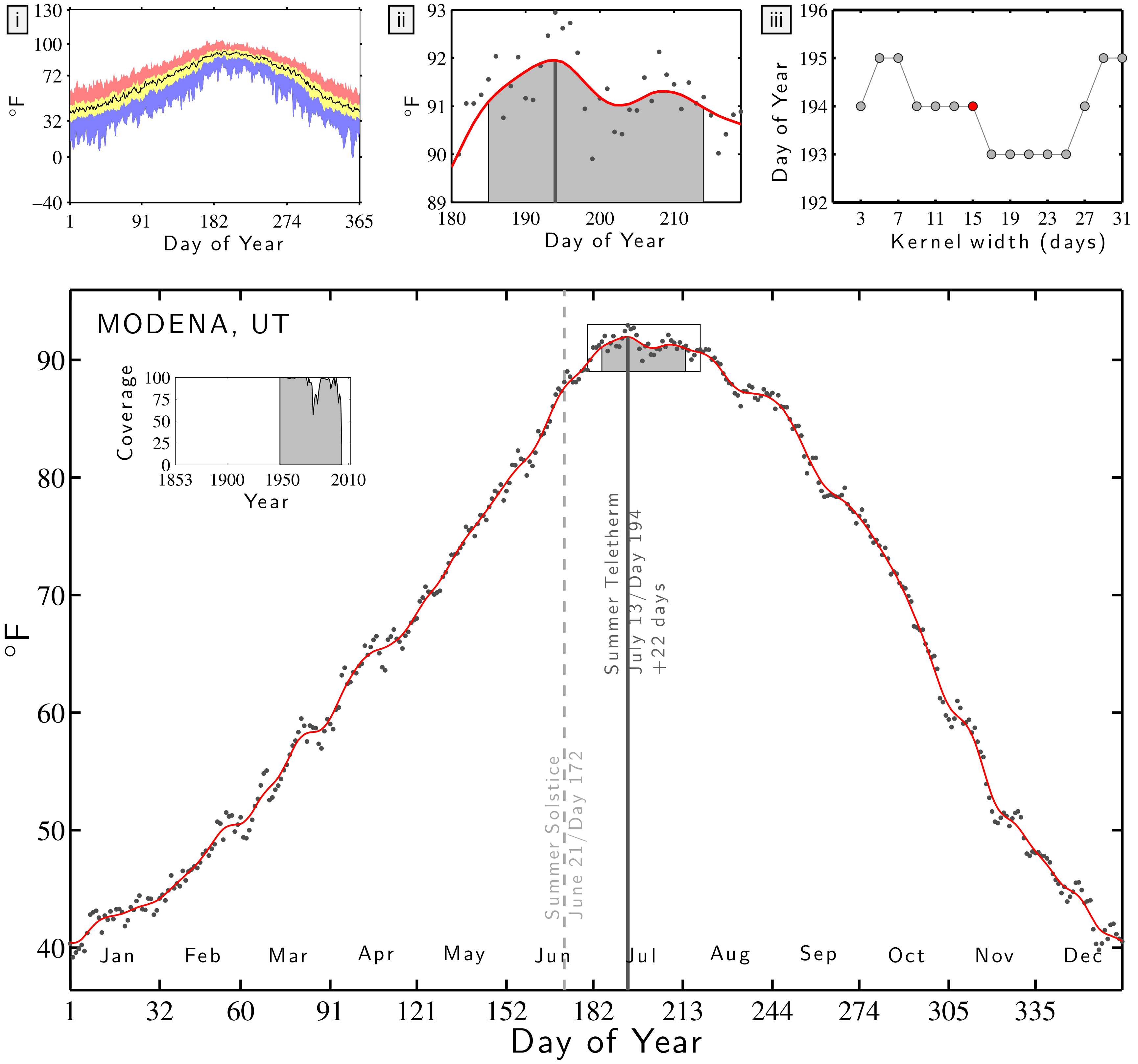

i
130
100
72
32
0
−40
°F
1
91
182
274
365
Day of Year
ii
93
92
91
90
89
°F
180
190
200
210
Day of Year
iii
196
195
194
193
192
Day of Year
3
7
11
15
19
23
27
31
Kernel width (days)
MODENA, UT
Coverage
100
75
50
25
0
1853
1900
1950
2010
Year
Summer Solstice
June 21/Day 172
Summer Teletherm
July 13/Day 194
+22 days
°F
90
80
70
60
50
40
Jan
Feb
Mar
Apr
May
Jun
Jul
Aug
Sep
Oct
Nov
Dec
1
32
60
91
121
152
182
213
244
274
305
335
Day of Year

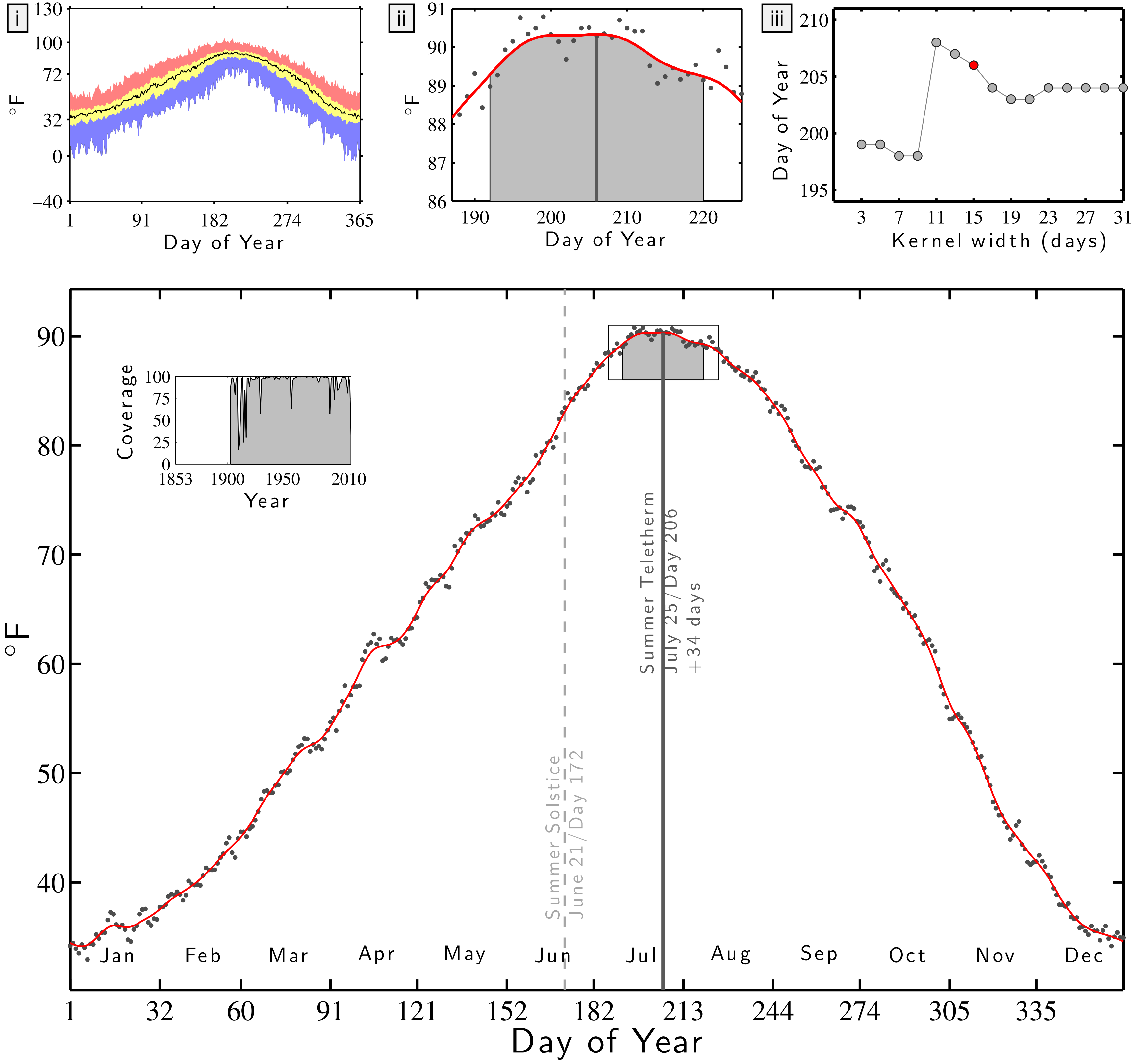

i
°F
130
100
72
32
0
−40
1
91
182
274
365
Day of Year
ii
91
90
89
88
87
86
190
200
210
220
Day of Year
iii
Day of Year
210
205
200
195
3
7
11
15
19
23
27
31
Kernel width (days)
Coverage
100
75
50
25
0
1853
1900
1950
2010
Year
Summer Solstice
June 21/Day 172
Summer Teletherm
July 25/Day 206
+34 days
Jan
Feb
Mar
Apr
May
Jun
Jul
Aug
Sep
Oct
Nov
Dec
90
80
70
60
50
40
1
32
60
91
121
152
182
213
244
274
305
335
Day of Year

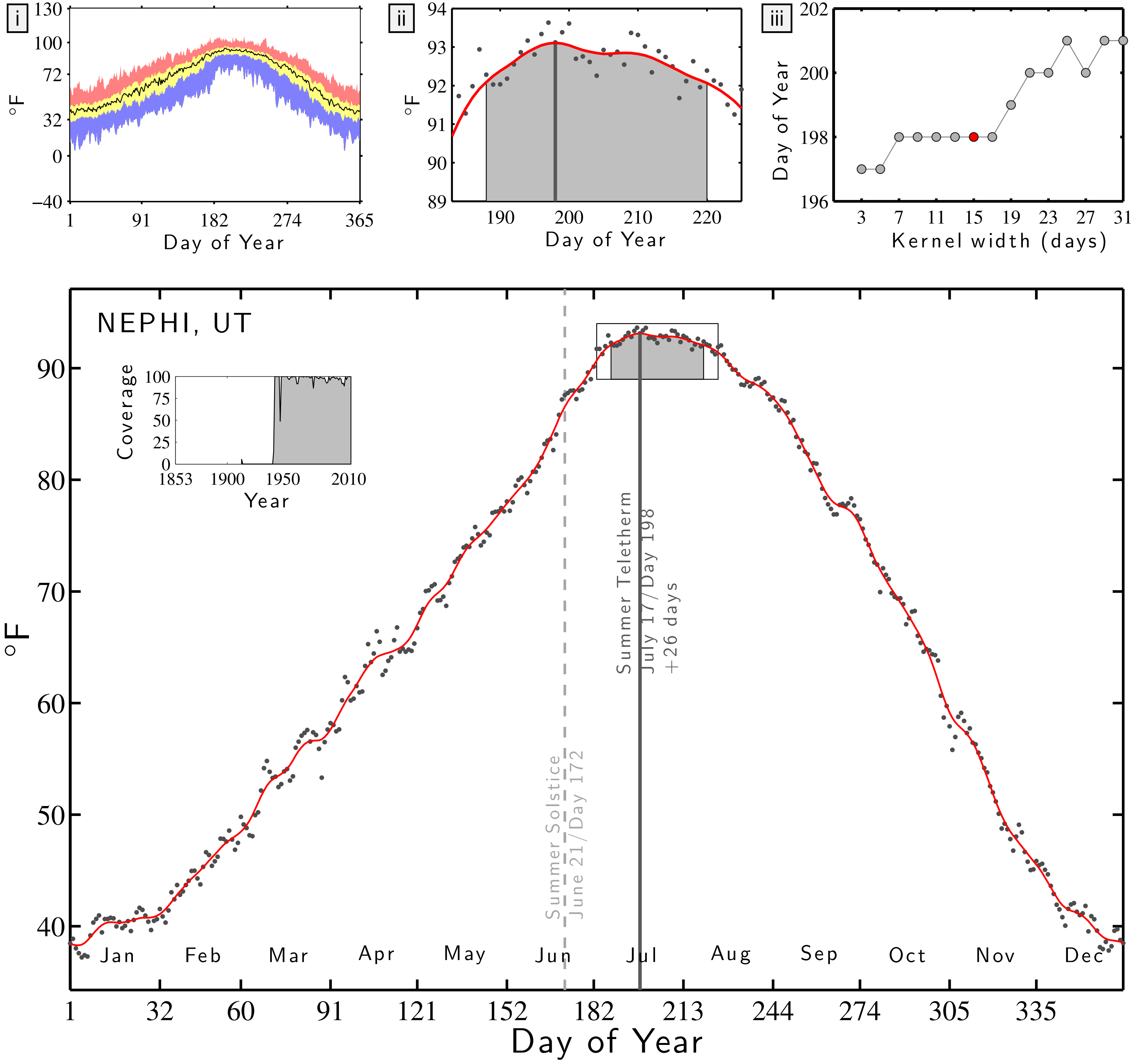

i
°F
Day of Year
ii
°F
Day of Year
iii
Day of Year
Kernel width (days)
NEPHI, UT
Coverage
Year
Summer Solstice
June 21/Day 172
Summer Teletherm
July 17/Day 198
+26 days
Jan
Feb
Mar
Apr
May
Jun
Jul
Aug
Sep
Oct
Nov
Dec
°F
Day of Year

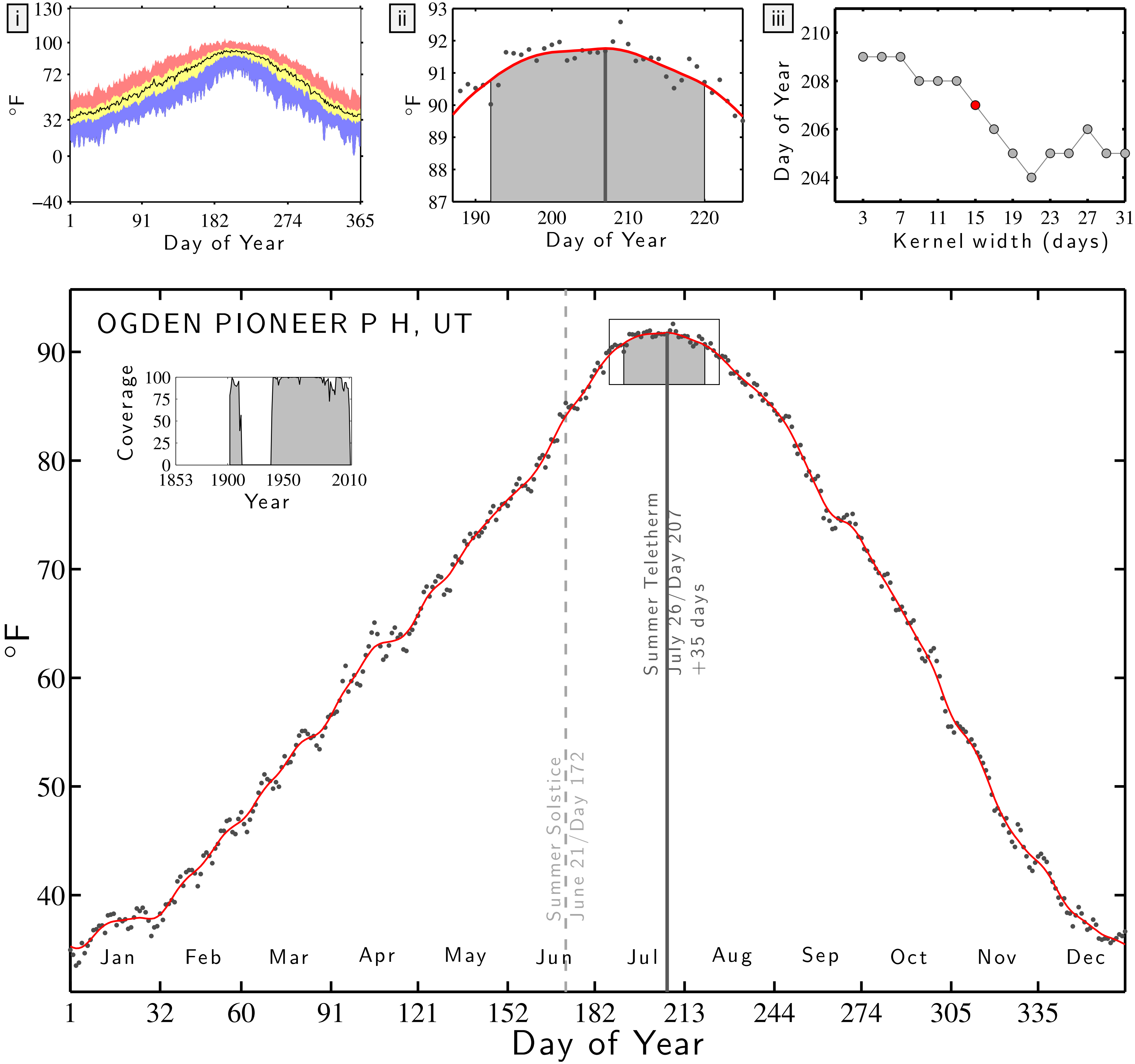

i
°F
Day of Year
ii
°F
Day of Year
iii
Day of Year
Kernel width (days)
OGDEN PIONEER P H, UT
Coverage
Year
Summer Solstice
June 21 / Day 172
Summer Teletherm
July 26 / Day 207
+35 days
Jan
Feb
Mar
Apr
May
Jun
Jul
Aug
Sep
Oct
Nov
Dec
°F
Day of Year

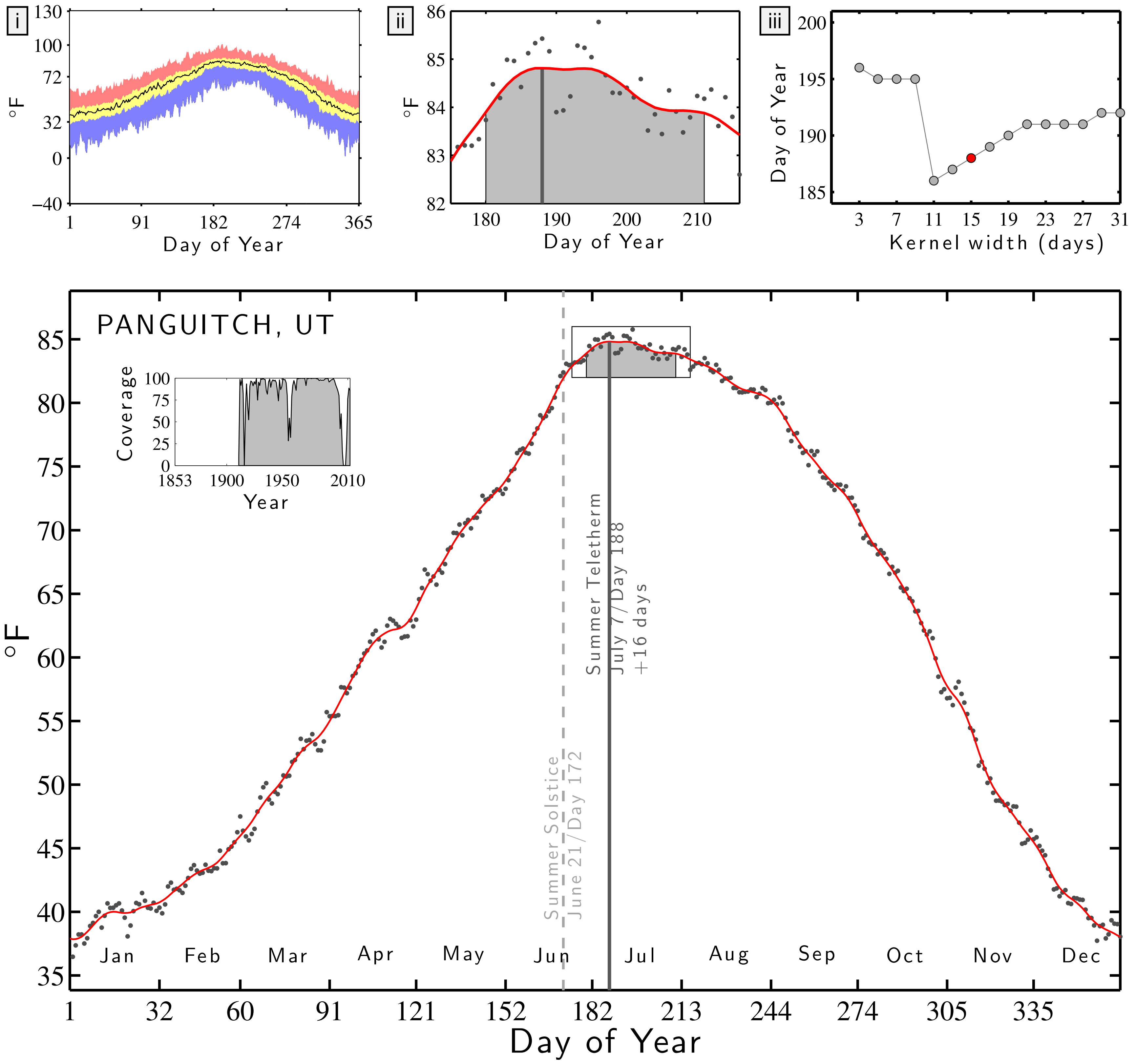

i
°F
Day of Year
ii
°F
Day of Year
iii
Day of Year
Kernel width (days)
PANGUITCH, UT
Coverage
Year
Summer Solstice
June 21/Day 172
Summer Teletherm
July 7/Day 188
+16 days
Jan
Feb
Mar
Apr
May
Jun
Jul
Aug
Sep
Oct
Nov
Dec
°F
Day of Year

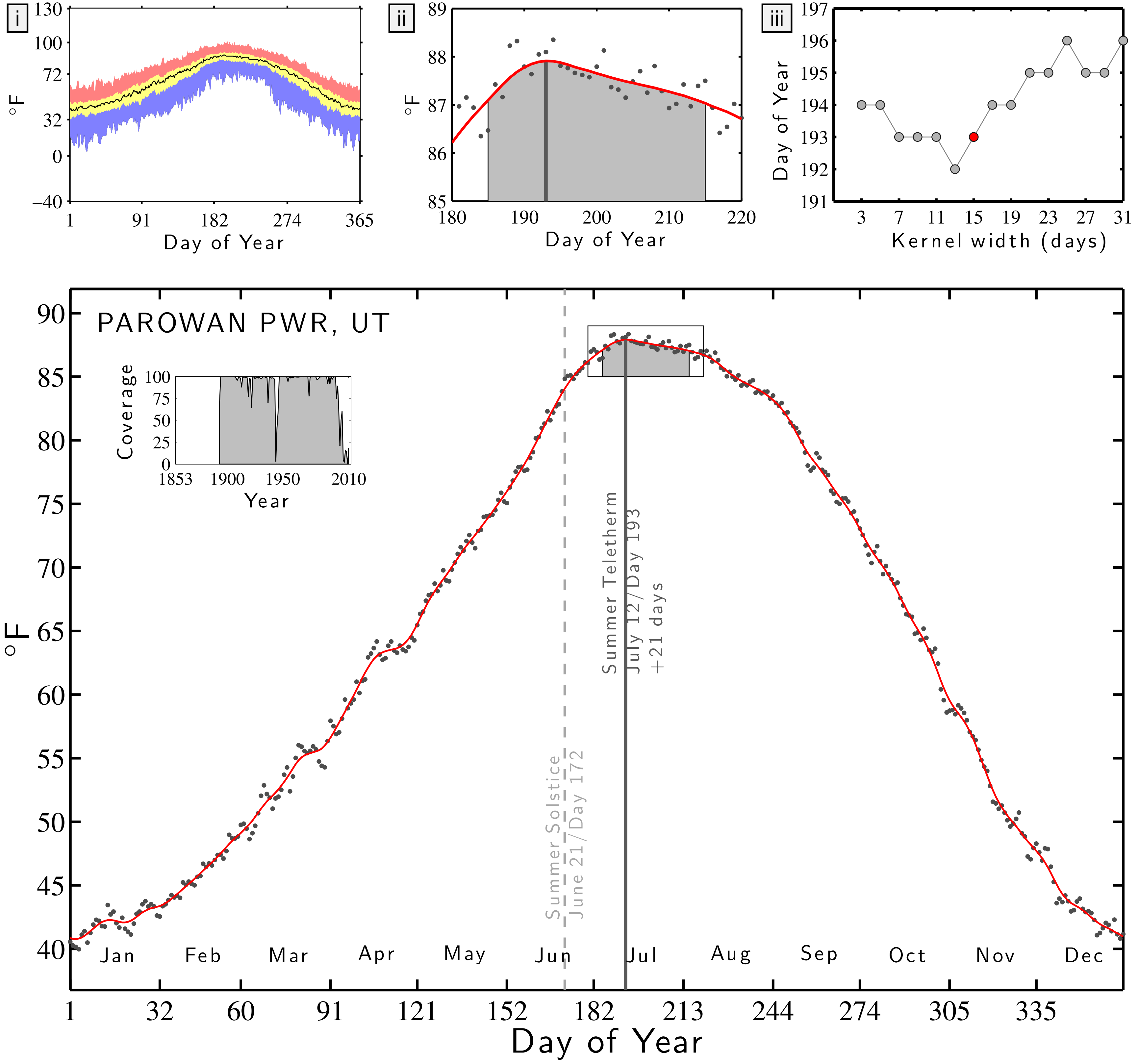

i
ii
iii
°F
Day of Year
Kernel width (days)
PAROWAN PWR, UT
Coverage
Year
Summer Solstice
June 21/Day 172
Summer Teletherm
July 12/Day 193
+21 days
Jan
Feb
Mar
Apr
May
Jun
Jul
Aug
Sep
Oct
Nov
Dec

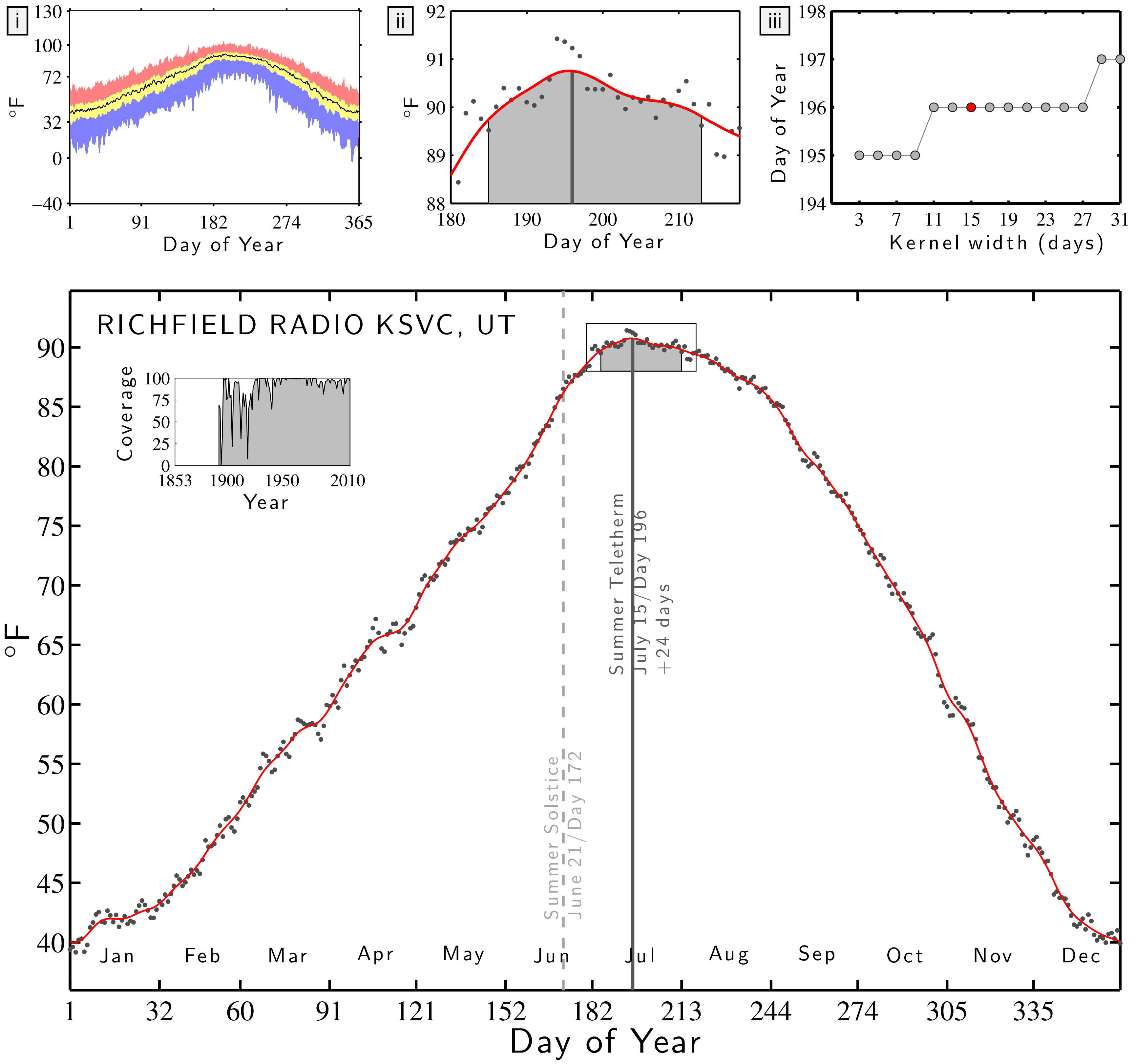

i
ii
iii
°F
Day of Year
Kernel width (days)
RICHFIELD RADIO KSVC, UT
Coverage
Year
Summer Solstice
June 21 / Day 172
Summer Teletherm
July 15 / Day 196
+24 days
Jan
Feb
Mar
Apr
May
Jun
Jul
Aug
Sep
Oct
Nov
Dec

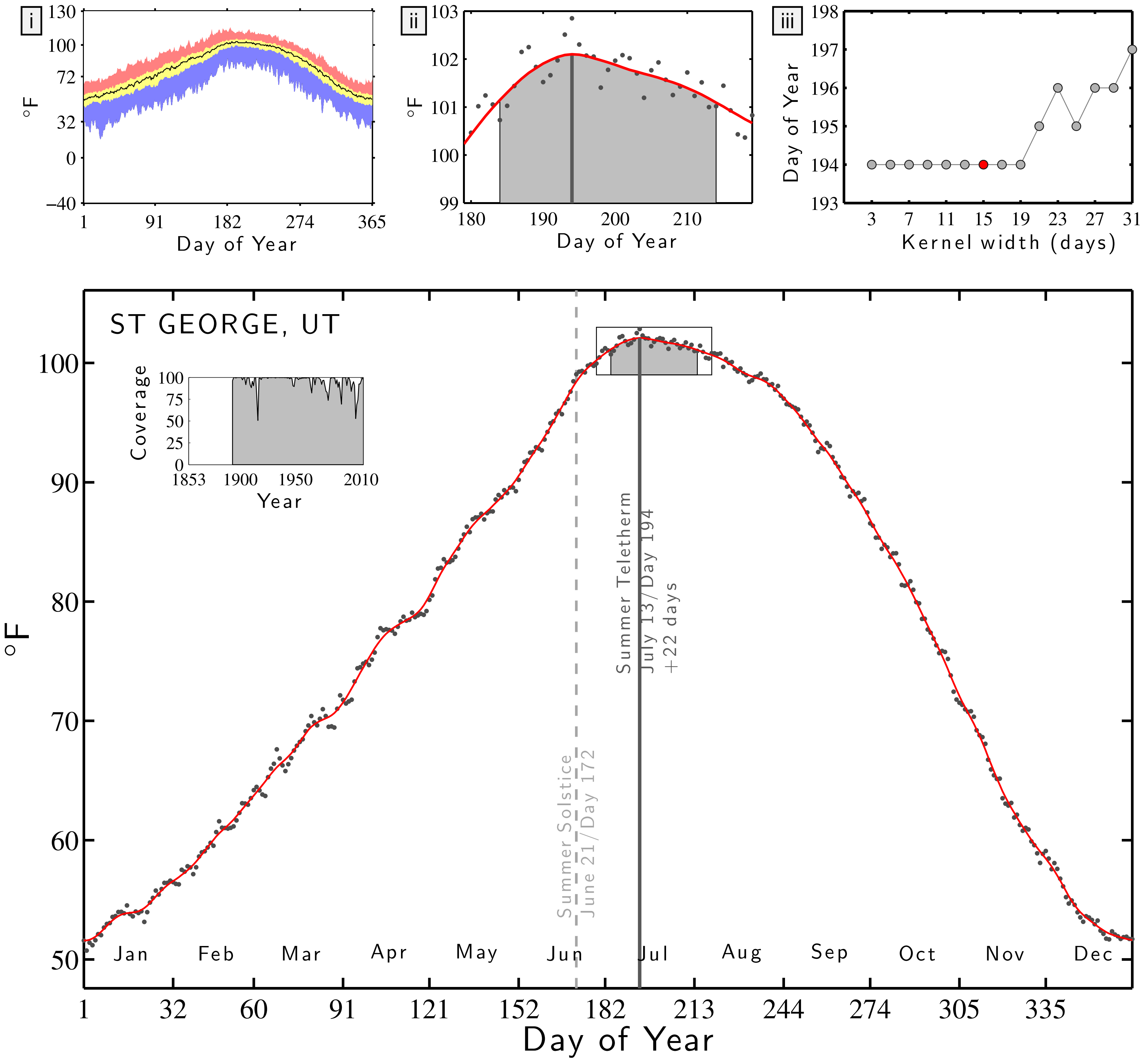
i
130
100
72
32
0
−40
°F
1
91
182
274
365
Day of Year
ii
103
102
101
100
99
°F
180
190
200
210
Day of Year
iii
198
197
196
195
194
193
Day of Year
3
7
11
15
19
23
27
31
Kernel width (days)
ST GEORGE, UT
Coverage
100
75
50
25
0
1853
1900
1950
2010
Year
Summer Solstice
June 21/Day 172
Summer Teletherm
July 13/Day 194
+22 days
100
90
80
70
60
50
°F
Jan
Feb
Mar
Apr
May
Jun
Jul
Aug
Sep
Oct
Nov
Dec
1
32
60
91
121
152
182
213
244
274
305
335
Day of Year

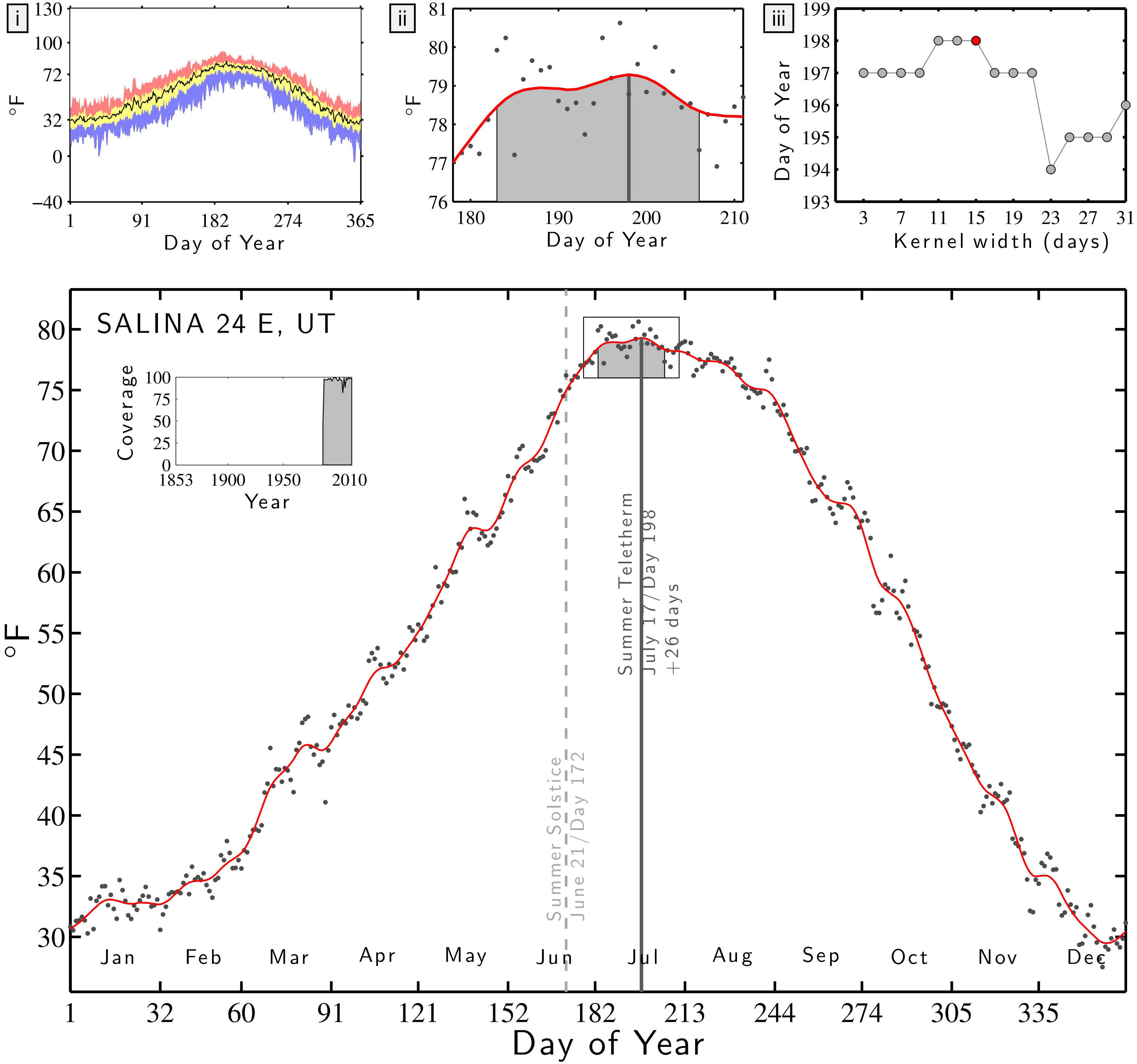

i
°F
Day of Year
ii
°F
Day of Year
iii
Day of Year
Kernel width (days)
SALINA 24 E, UT
Coverage
Year
Summer Solstice
June 21/Day 172
Summer Teletherm
July 17/Day 198
+26 days
Jan
Feb
Mar
Apr
May
Jun
Jul
Aug
Sep
Oct
Nov
Dec
°F
Day of Year

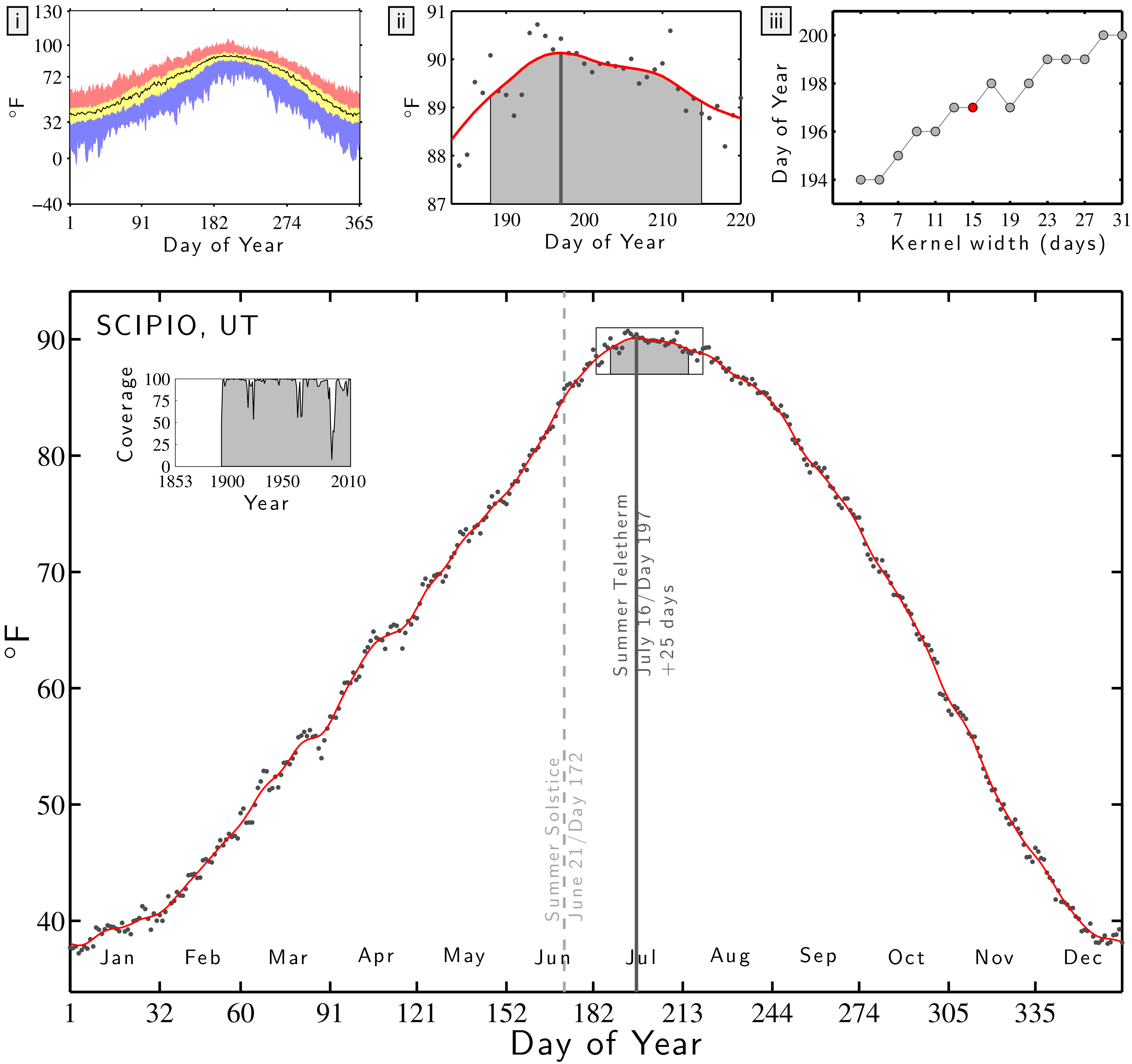

i
ii
iii
°F
Day of Year
Kernel width (days)
SCIPIO, UT
Coverage
Year
Summer Solstice
June 21/Day 172
Summer Teletherm
July 16/Day 197
+25 days
Jan
Feb
Mar
Apr
May
Jun
Jul
Aug
Sep
Oct
Nov
Dec

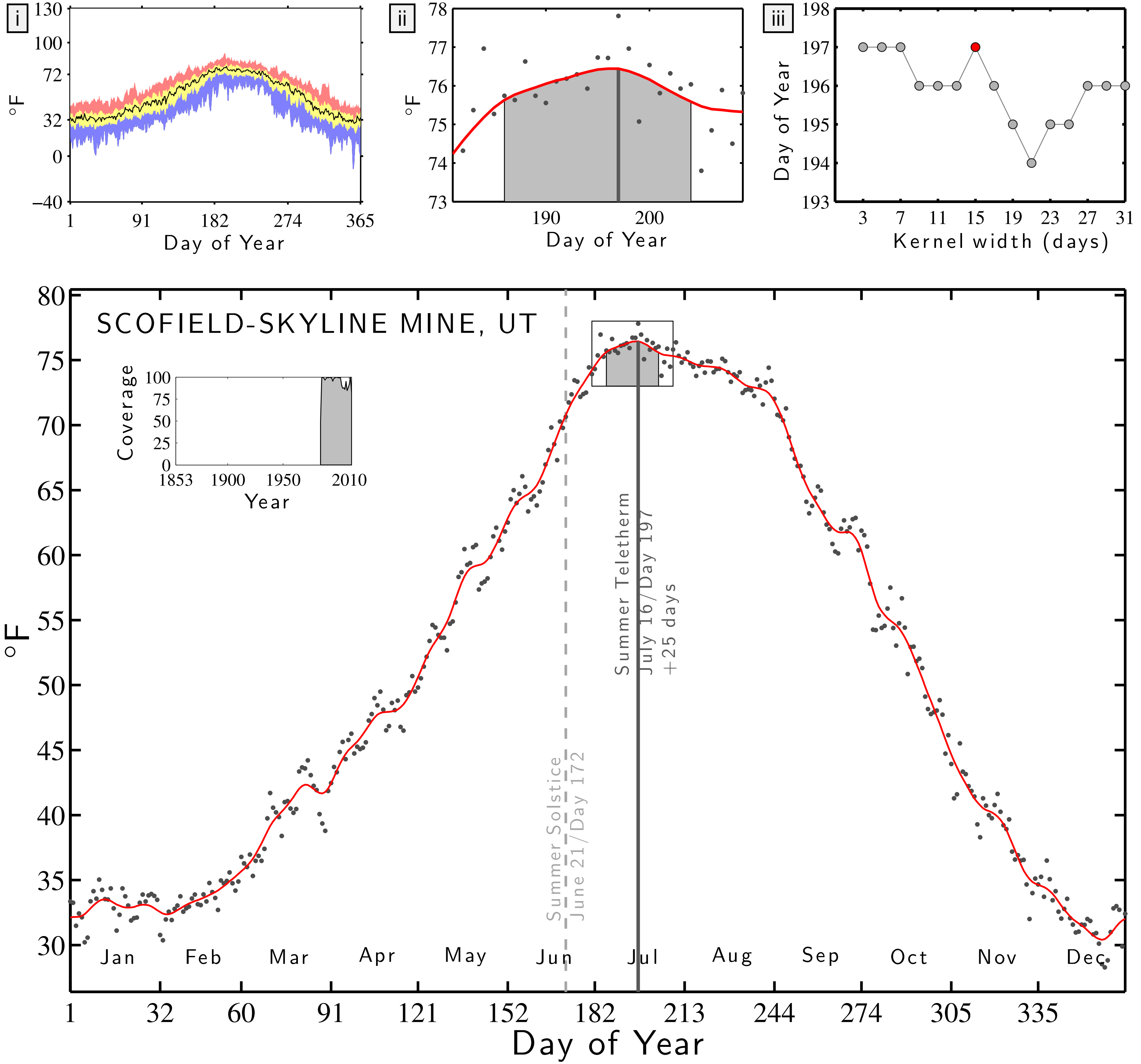

i
°F
130
100
72
32
0
−40
1
91
182
274
365
Day of Year
ii
°F
78
77
76
75
74
73
190
200
Day of Year
iii
Day of Year
198
197
196
195
194
193
3
7
11
15
19
23
27
31
Kernel width (days)
SCOFIELD-SKYLINE MINE, UT
Coverage
100
75
50
25
0
1853
1900
1950
2010
Year
Summer Solstice
June 21/Day 172
Summer Teletherm
July 16/Day 197
+25 days
Jan
Feb
Mar
Apr
May
Jun
Jul
Aug
Sep
Oct
Nov
Dec
°F
80
75
70
65
60
55
50
45
40
35
30
1
32
60
91
121
152
182
213
244
274
305
335
Day of Year

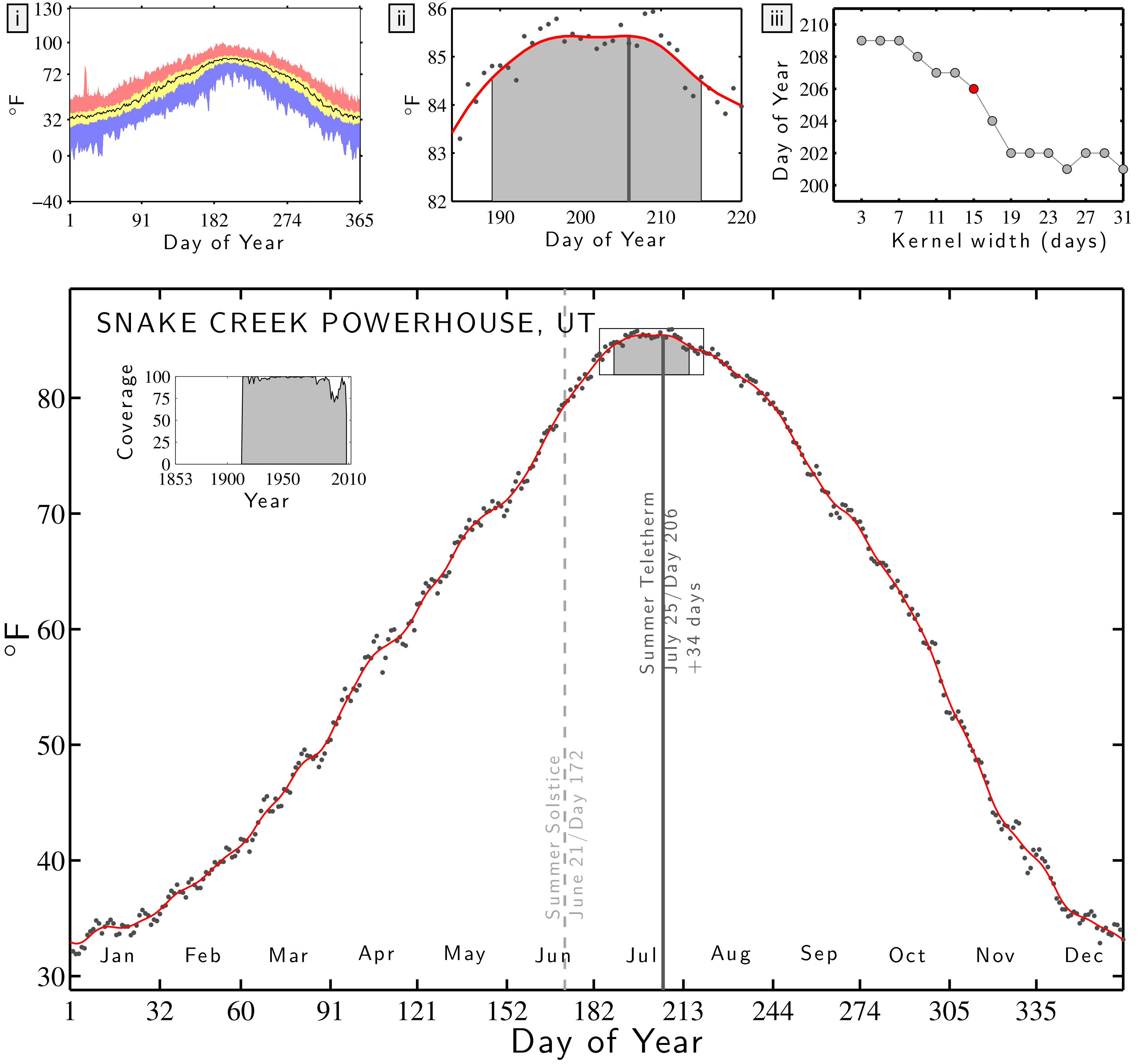

i
ii
iii
°F
Day of Year
Kernel width (days)
SNAKE CREEK POWERHOUSE, UT
Coverage
Year
Summer Solstice
June 21/Day 172
Summer Teletherm
July 25/Day 206
+34 days
Jan
Feb
Mar
Apr
May
Jun
Jul
Aug
Sep
Oct
Nov
Dec

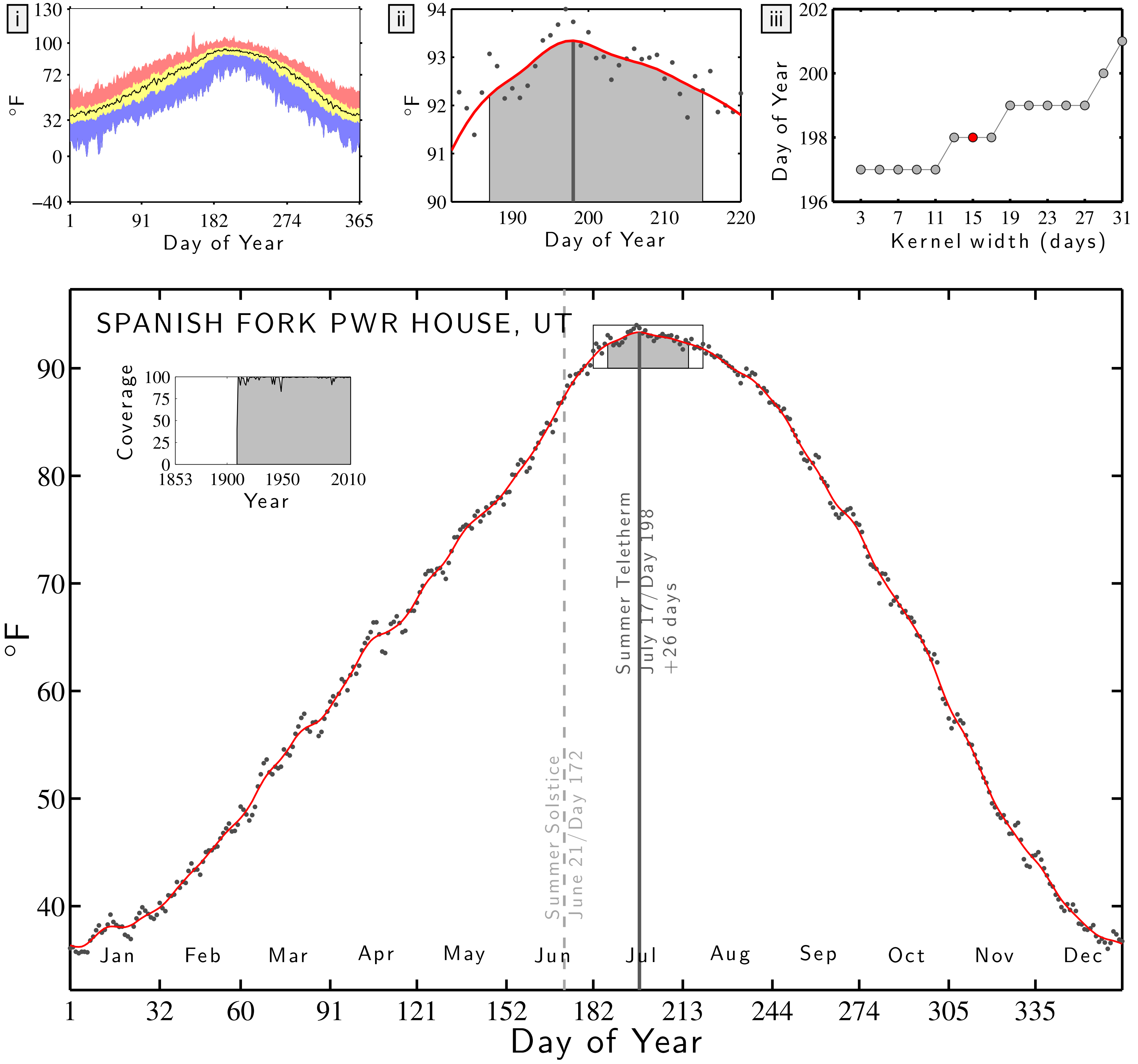

i
°F
Day of Year
ii
°F
Day of Year
iii
Day of Year
Kernel width (days)
SPANISH FORK PWR HOUSE, UT
Coverage
Year
Summer Solstice
June 21 / Day 172
Summer Teletherm
July 17 / Day 198
+26 days
°F
Jan
Feb
Mar
Apr
May
Jun
Jul
Aug
Sep
Oct
Nov
Dec
Day of Year

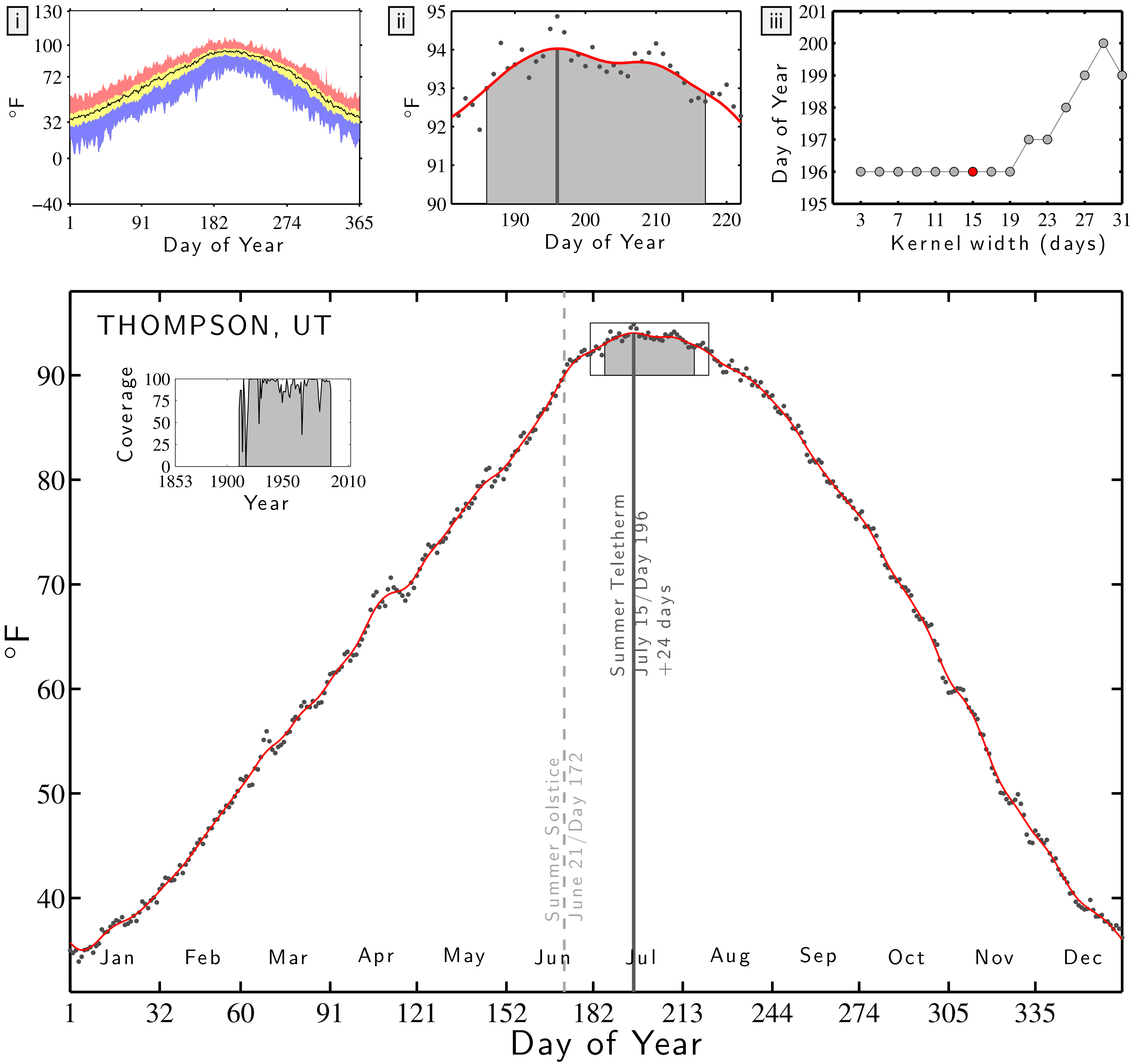

i
°F
Day of Year
ii
°F
Day of Year
iii
Day of Year
Kernel width (days)
THOMPSON, UT
Coverage
Year
Summer Solstice
June 21/Day 172
Summer Teletherm
July 15/Day 196
+24 days
Jan
Feb
Mar
Apr
May
Jun
Jul
Aug
Sep
Oct
Nov
Dec
°F
Day of Year

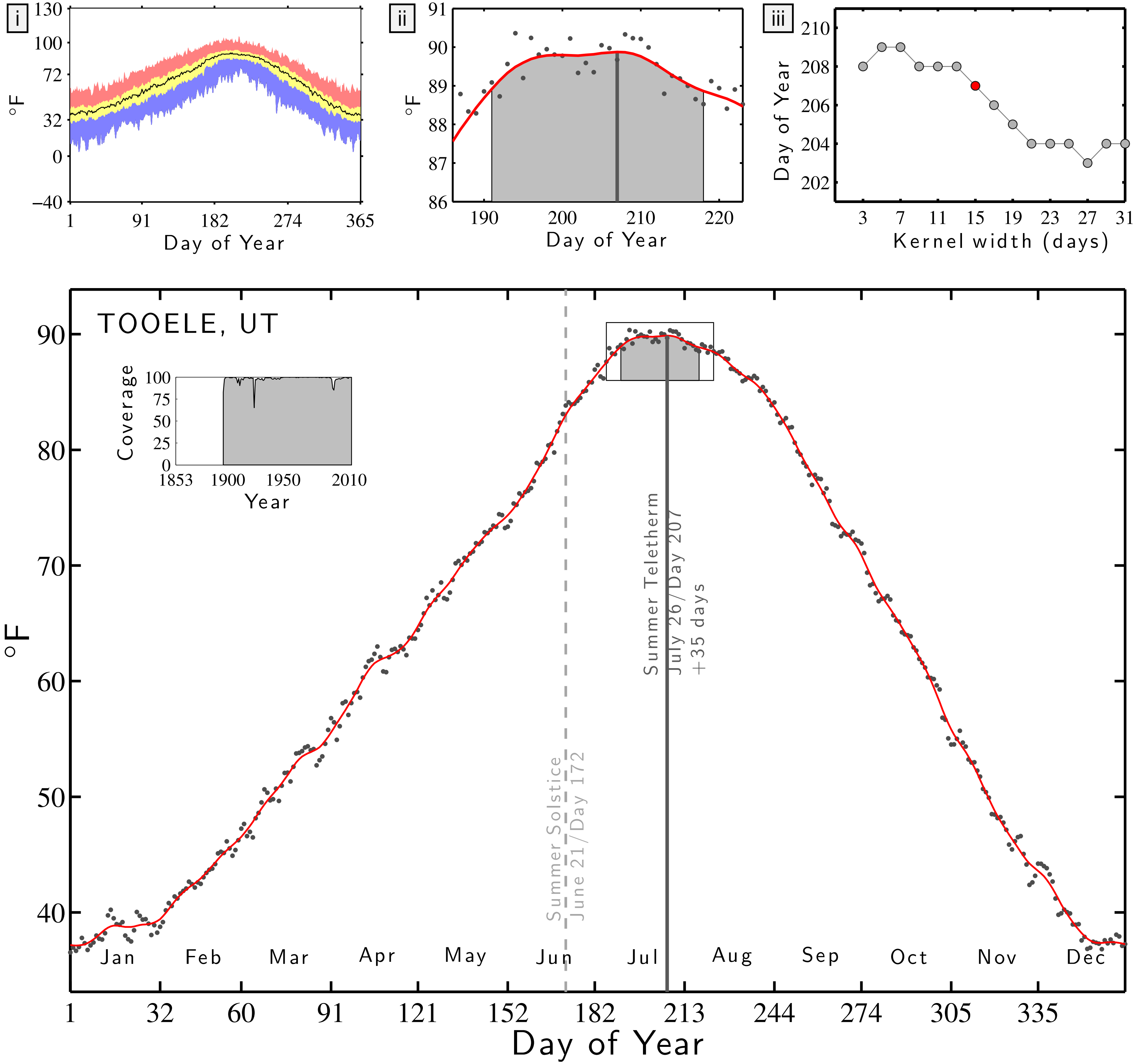

i
°F
Day of Year
ii
°F
Day of Year
iii
Day of Year
Kernel width (days)
TOOELE, UT
Coverage
Year
Summer Solstice
June 21 / Day 172
Summer Teletherm
July 26 / Day 207
+35 days
Jan
Feb
Mar
Apr
May
Jun
Jul
Aug
Sep
Oct
Nov
Dec
°F
Day of Year

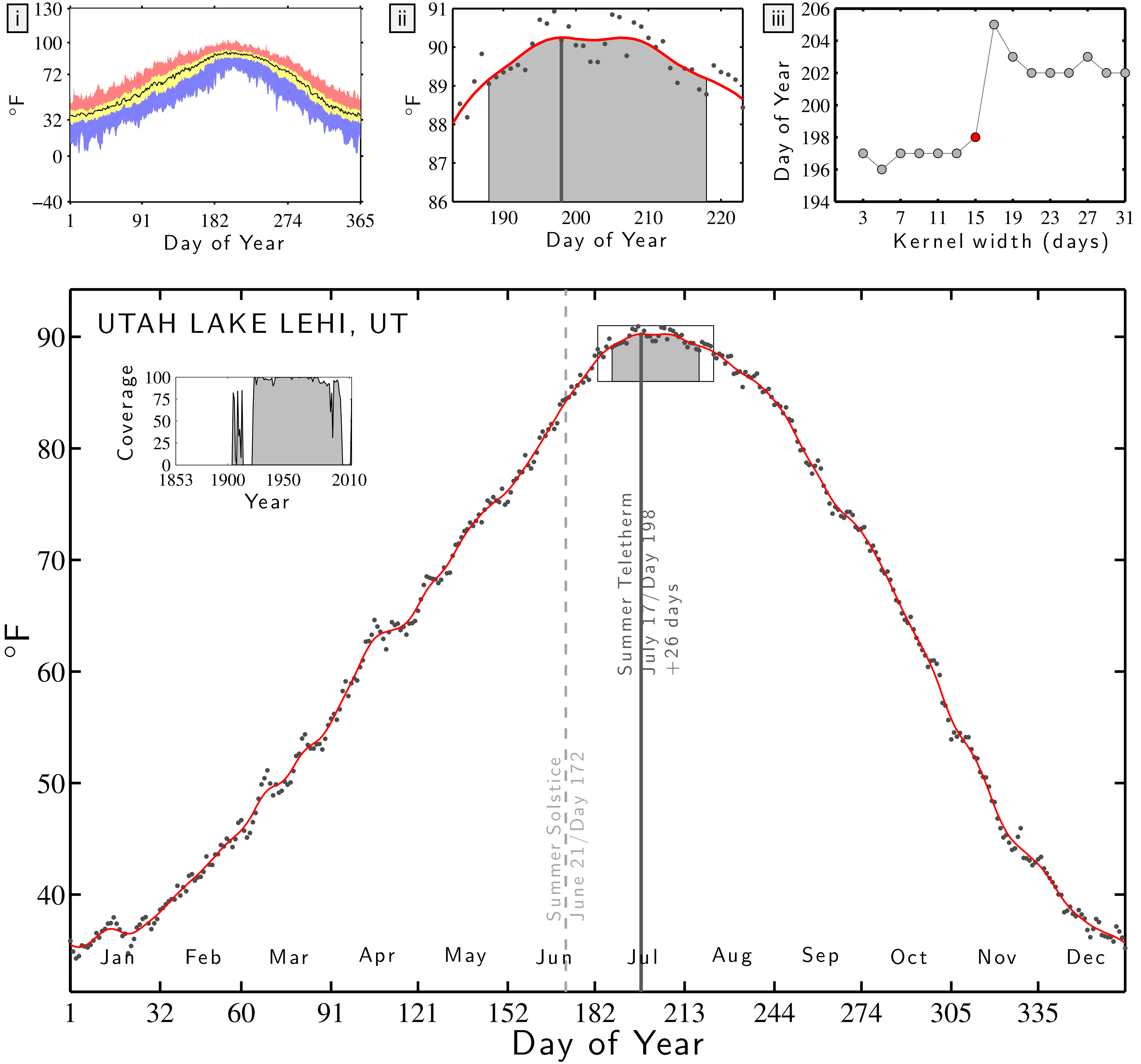

i
ii
iii
°F
Day of Year
Kernel width (days)
UTAH LAKE LEHI, UT
Coverage
Year
Summer Solstice
June 21 / Day 172
Summer Teletherm
July 17 / Day 198
+26 days
Jan
Feb
Mar
Apr
May
Jun
Jul
Aug
Sep
Oct
Nov
Dec

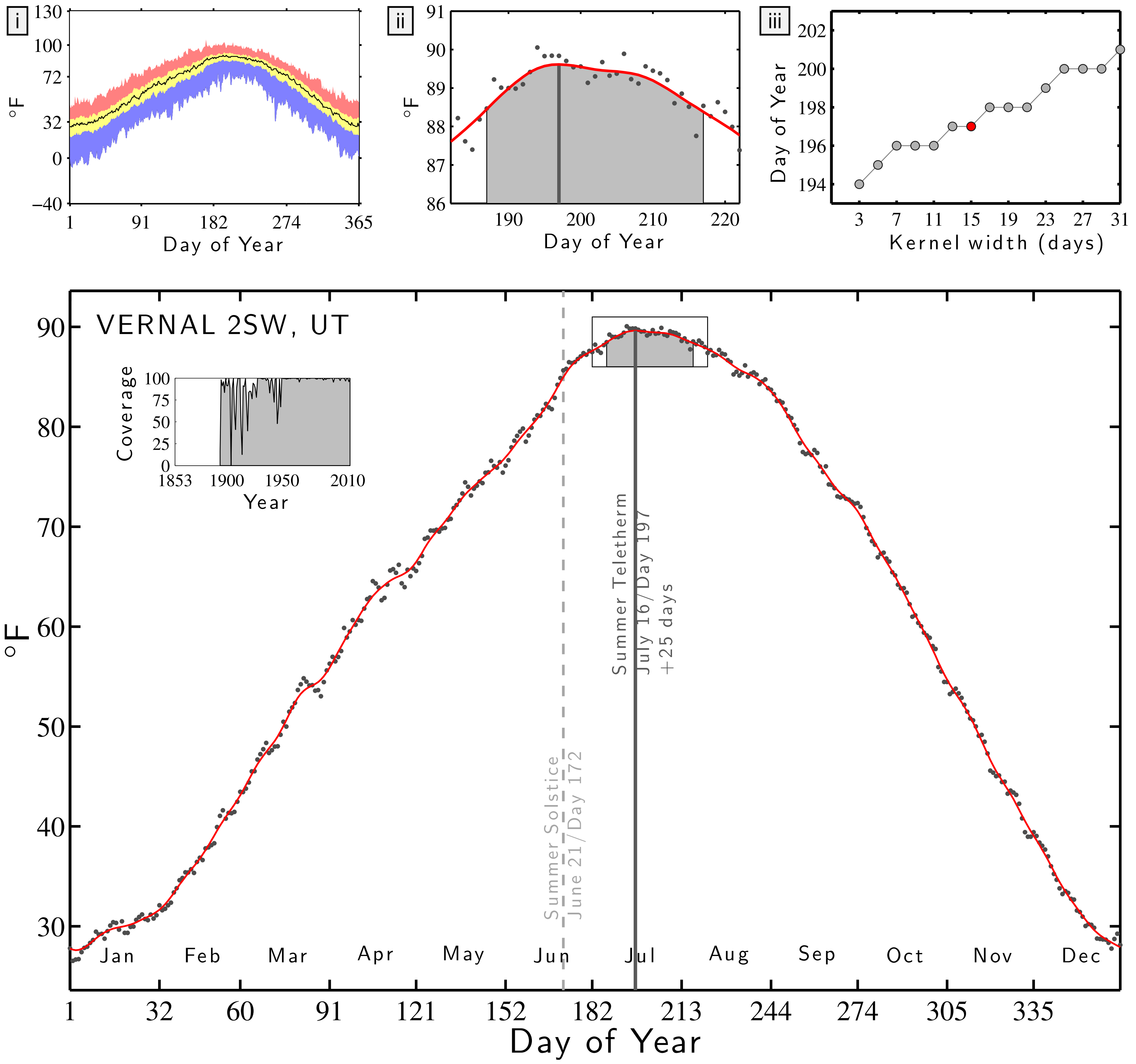

i
°F
Day of Year
ii
°F
Day of Year
iii
Day of Year
Kernel width (days)
VERNAL 2SW, UT
Coverage
Year
Summer Solstice
June 21/Day 172
Summer Teletherm
July 16/Day 197
+25 days
Jan Feb Mar Apr May Jun Jul Aug Sep Oct Nov Dec
°F
Day of Year

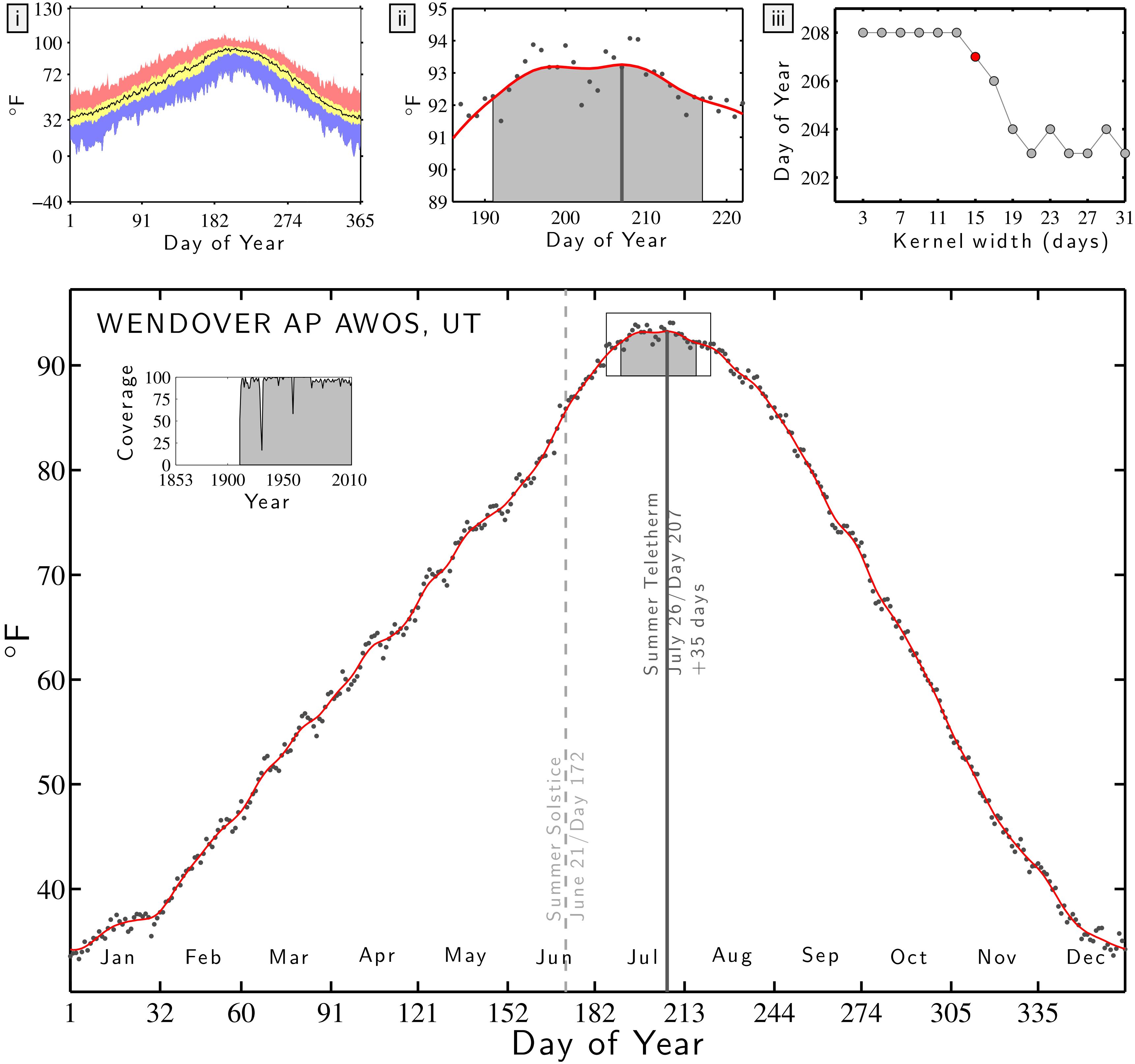

i
°F
Day of Year
ii
°F
Day of Year
iii
Day of Year
Kernel width (days)
WENDOVER AP AWOS, UT
Coverage
Year
Summer Solstice
June 21 / Day 172
Summer Teletherm
July 26 / Day 207
+35 days
Jan
Feb
Mar
Apr
May
Jun
Jul
Aug
Sep
Oct
Nov
Dec
°F
Day of Year

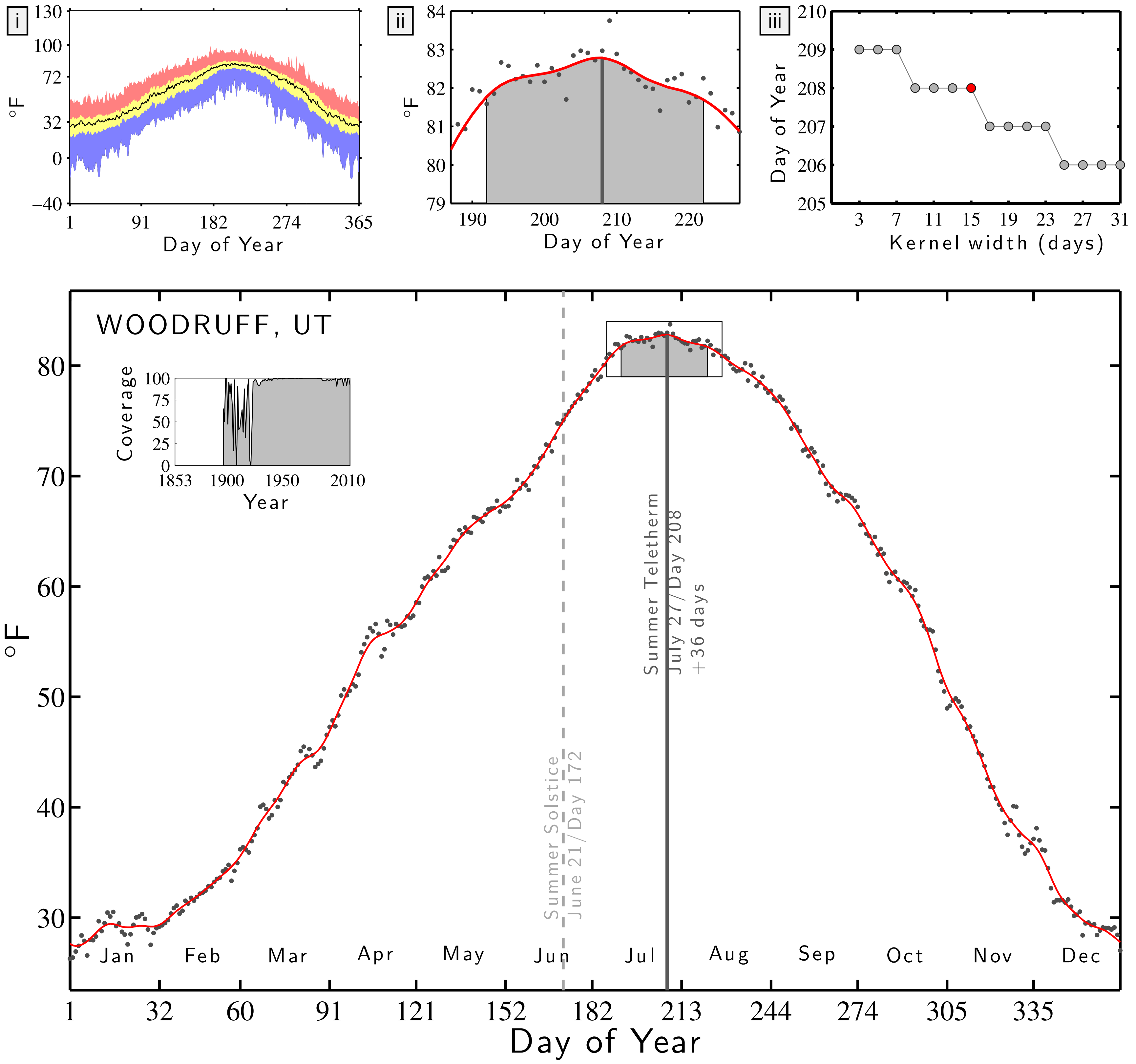

i
°F
Day of Year
ii
°F
Day of Year
iii
Day of Year
Kernel width (days)
WOODRUFF, UT
Coverage
Year
Summer Solstice
June 21/Day 172
Summer Teletherm
July 27/Day 208
+36 days
Jan
Feb
Mar
Apr
May
Jun
Jul
Aug
Sep
Oct
Nov
Dec
°F
Day of Year

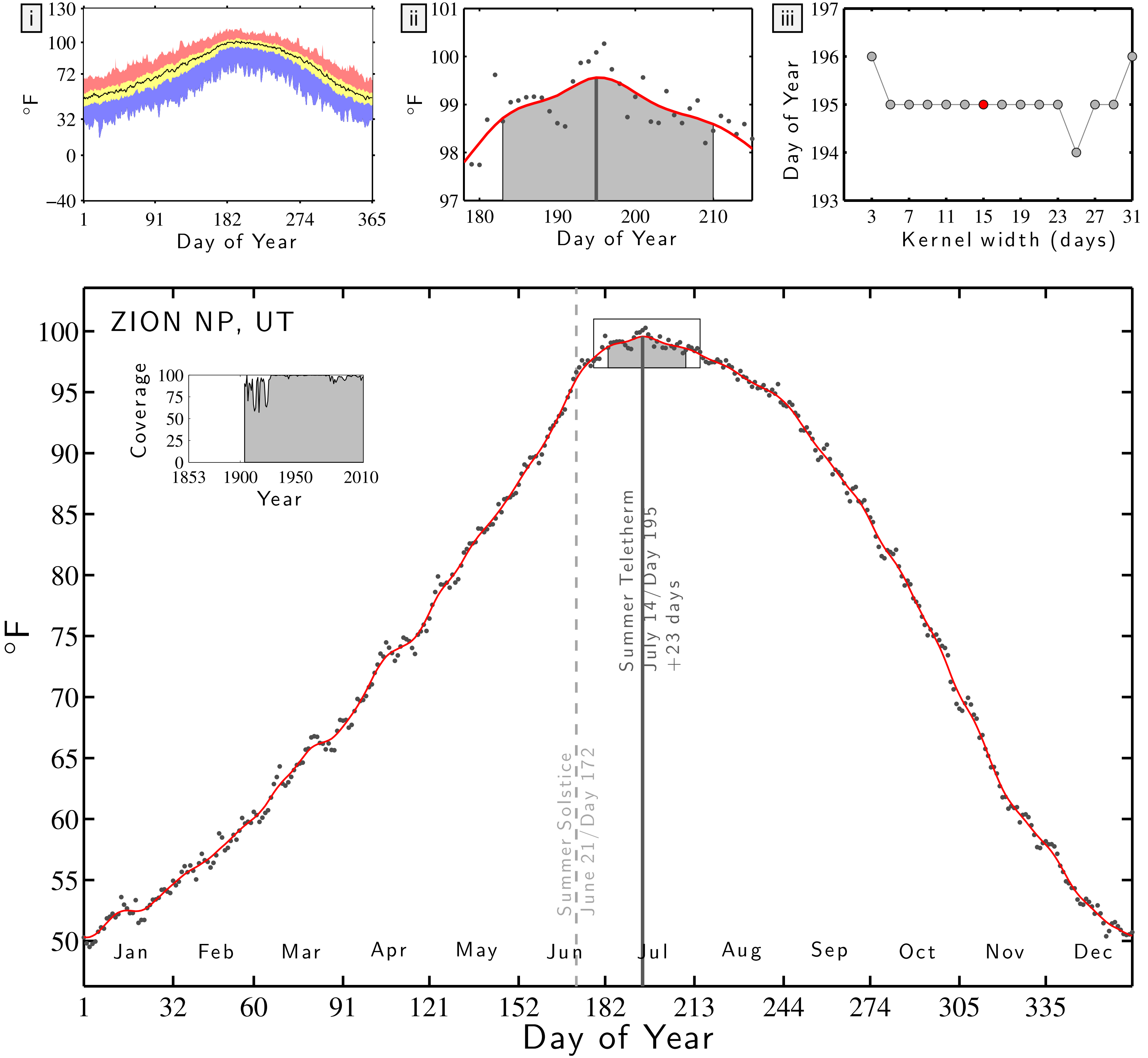

i
°F
Day of Year
ii
°F
Day of Year
iii
Day of Year
Kernel width (days)
ZION NP, UT
Coverage
Year
Summer Solstice
June 21/Day 172
Summer Teletherm
July 14/Day 195
+23 days
Jan
Feb
Mar
Apr
May
Jun
Jul
Aug
Sep
Oct
Nov
Dec
°F
Day of Year

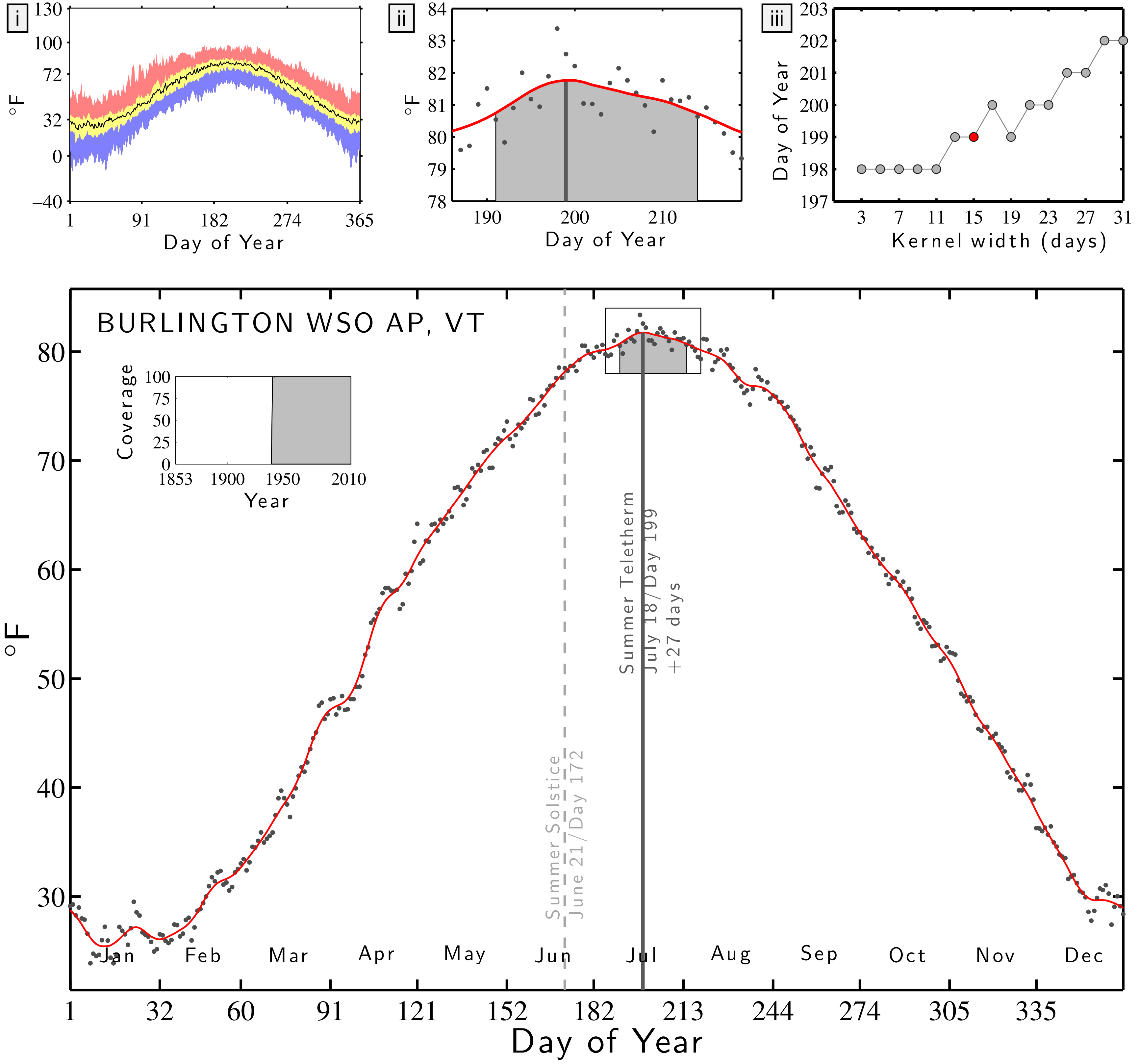

i
Day of Year
°F
ii
Day of Year
°F
iii
Kernel width (days)
Day of Year
BURLINGTON WSO AP, VT
Coverage
Year
Summer Solstice
June 21/Day 172
Summer Teletherm
July 18/Day 199
+27 days
Jan
Feb
Mar
Apr
May
Jun
Jul
Aug
Sep
Oct
Nov
Dec
Day of Year
°F

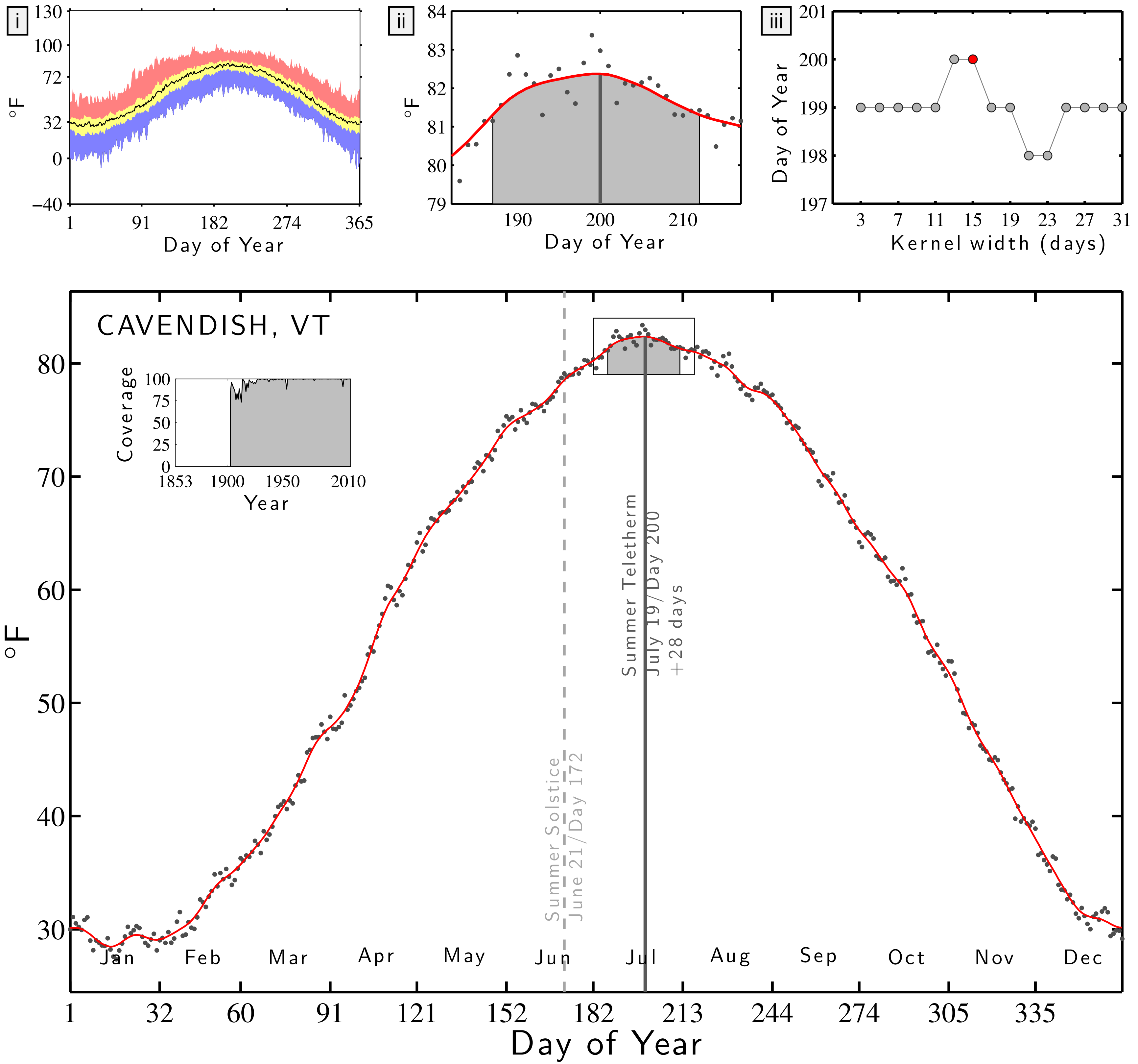

i
°F
Day of Year
ii
°F
Day of Year
iii
Day of Year
Kernel width (days)
CAVENDISH, VT
Coverage
Year
Summer Solstice
June 21/Day 172
Summer Teletherm
July 19/Day 200
+28 days
Jan
Feb
Mar
Apr
May
Jun
Jul
Aug
Sep
Oct
Nov
Dec
°F
Day of Year

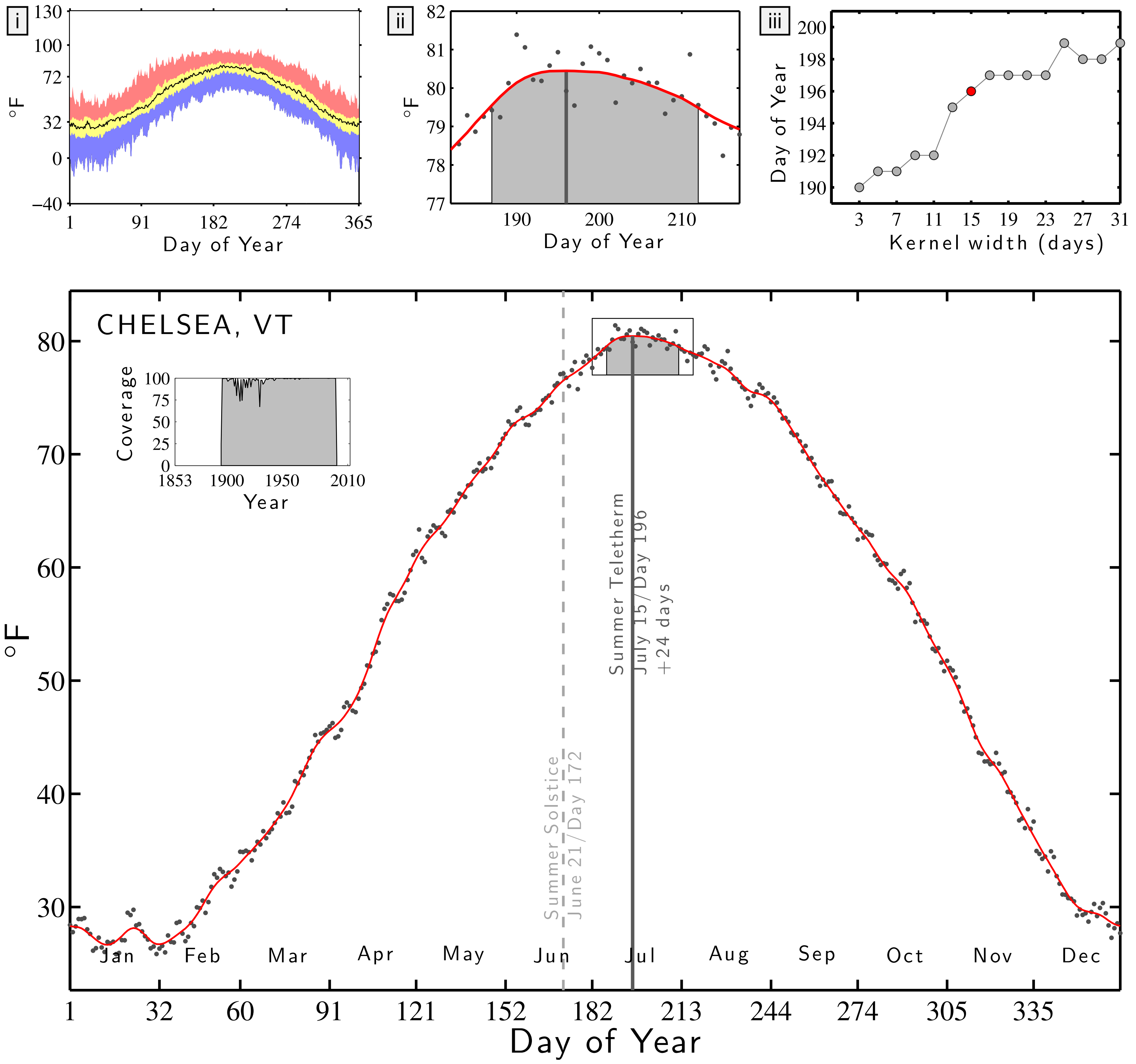

i
°F
Day of Year
ii
°F
Day of Year
iii
Day of Year
Kernel width (days)
CHELSEA, VT
Coverage
Year
Summer Solstice
June 21/Day 172
Summer Teletherm
July 15/Day 196
+24 days
Jan
Feb
Mar
Apr
May
Jun
Jul
Aug
Sep
Oct
Nov
Dec
°F
Day of Year

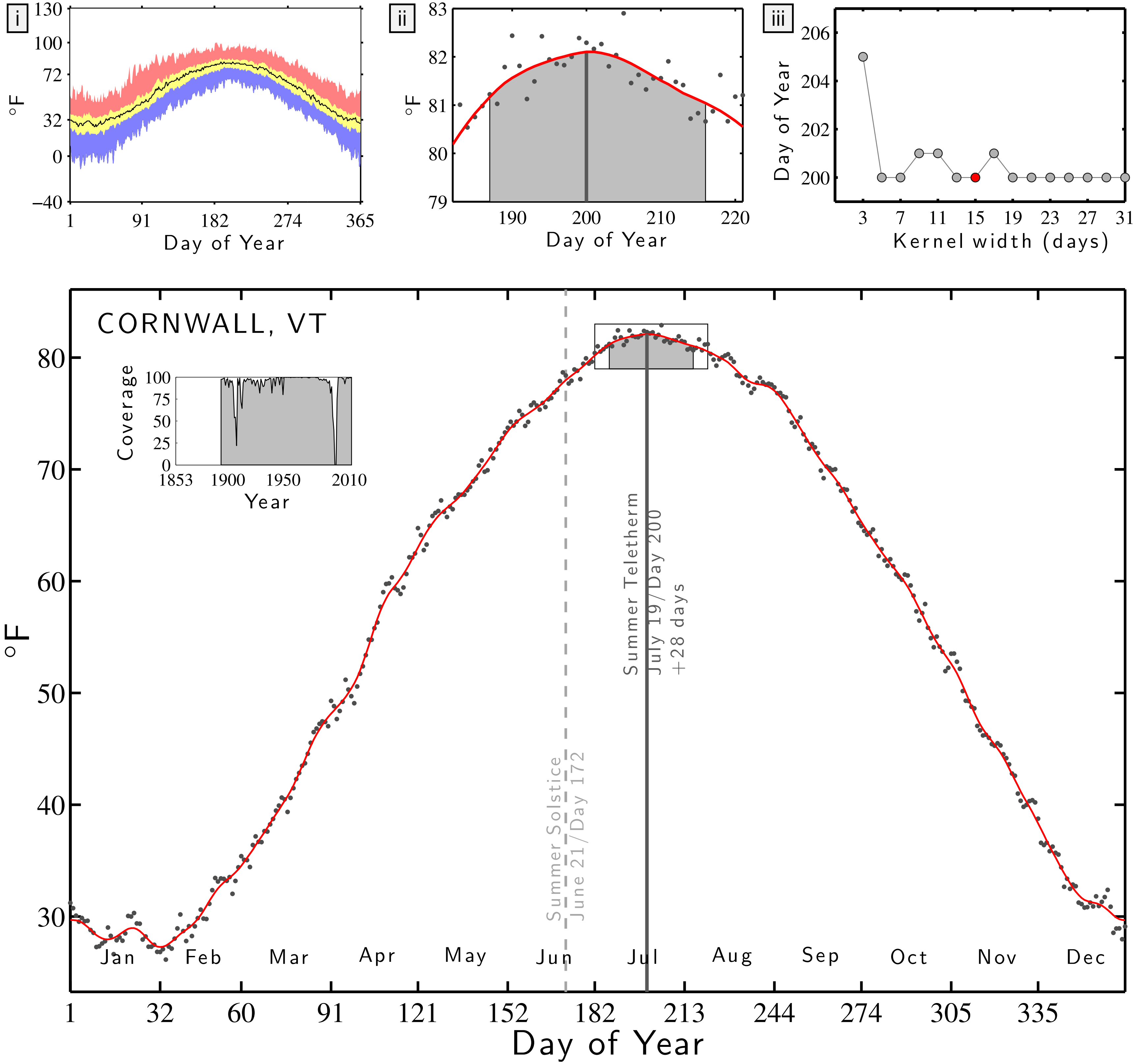

i
ii
iii
CORNWALL, VT
Coverage
Year
Summer Solstice
June 21 / Day 172
Summer Teletherm
July 19 / Day 200
+28 days
Day of Year
Kernel width (days)
°F
Jan
Feb
Mar
Apr
May
Jun
Jul
Aug
Sep
Oct
Nov
Dec

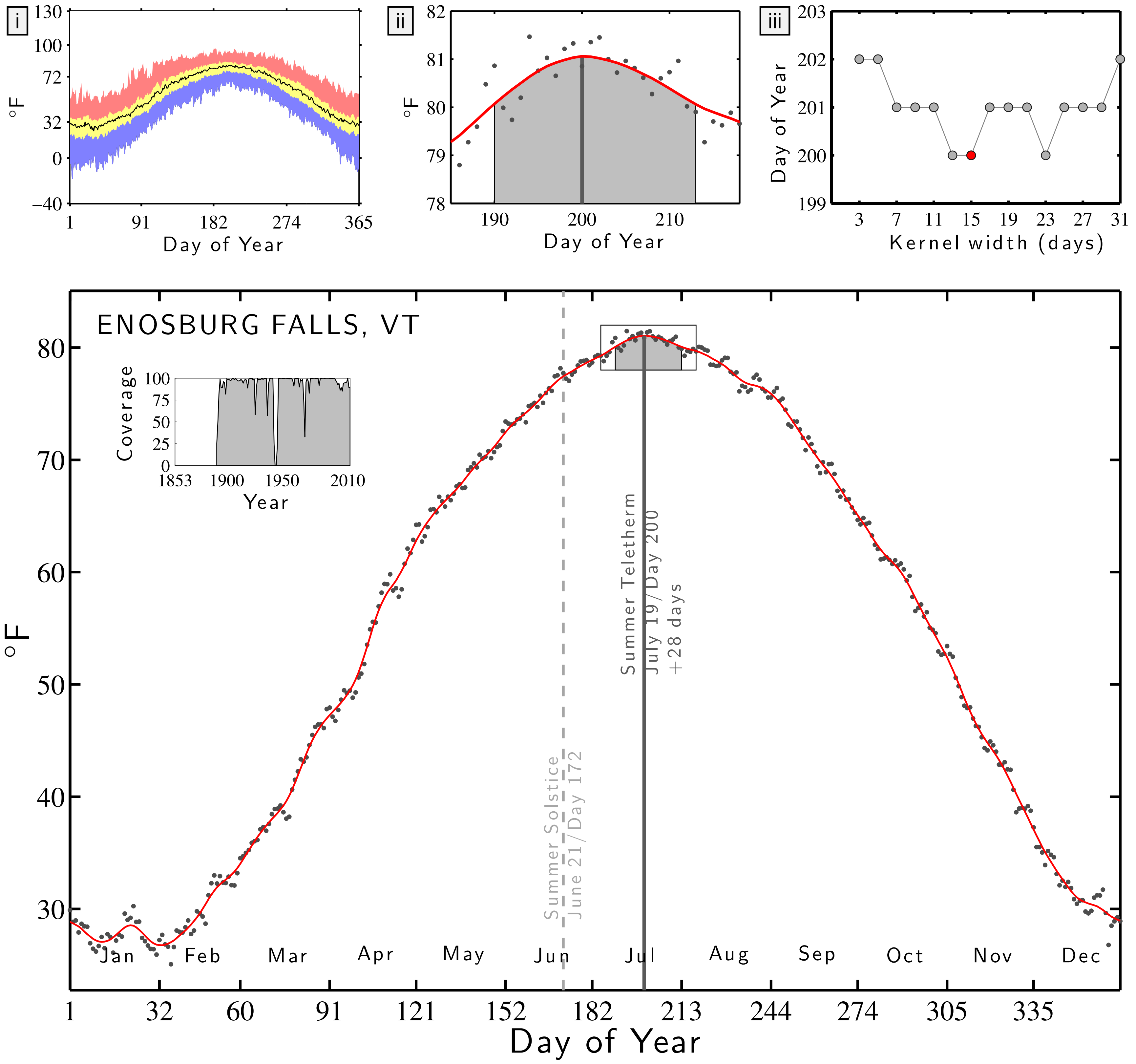
i
ii
iii
°F
Day of Year
Day of Year
Kernel width (days)
ENOSBURG FALLS, VT
Coverage
Year
Summer Solstice
June 21/Day 172
Summer Teletherm
July 19/Day 200
+28 days
Jan
Feb
Mar
Apr
May
Jun
Jul
Aug
Sep
Oct
Nov
Dec
Day of Year

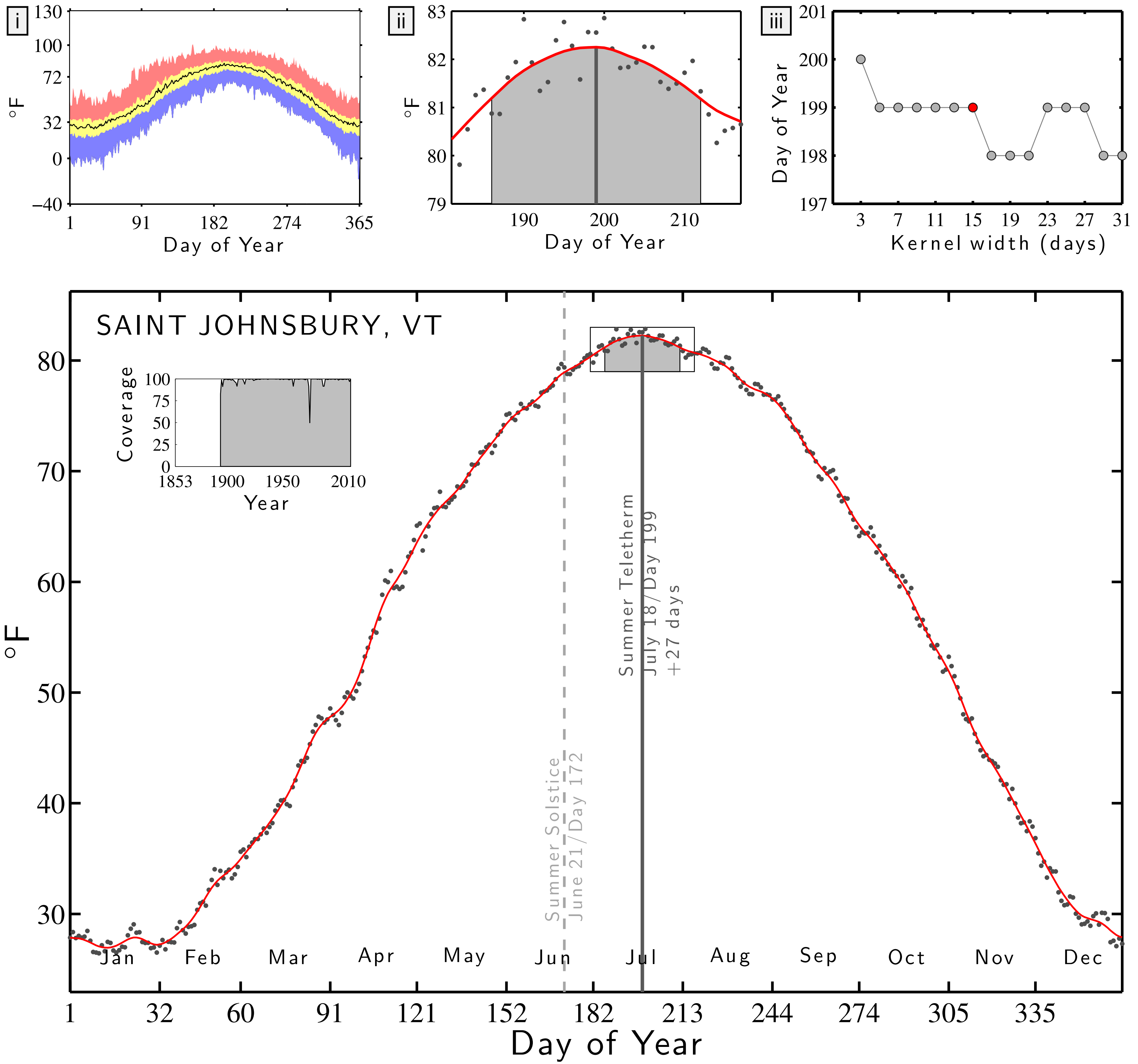

i
ii
iii
°F
Day of Year
Kernel width (days)
SAINT JOHNSBURY, VT
Coverage
Year
Summer Solstice
June 21/Day 172
Summer Teletherm
July 18/Day 199
+27 days
Jan
Feb
Mar
Apr
May
Jun
Jul
Aug
Sep
Oct
Nov
Dec

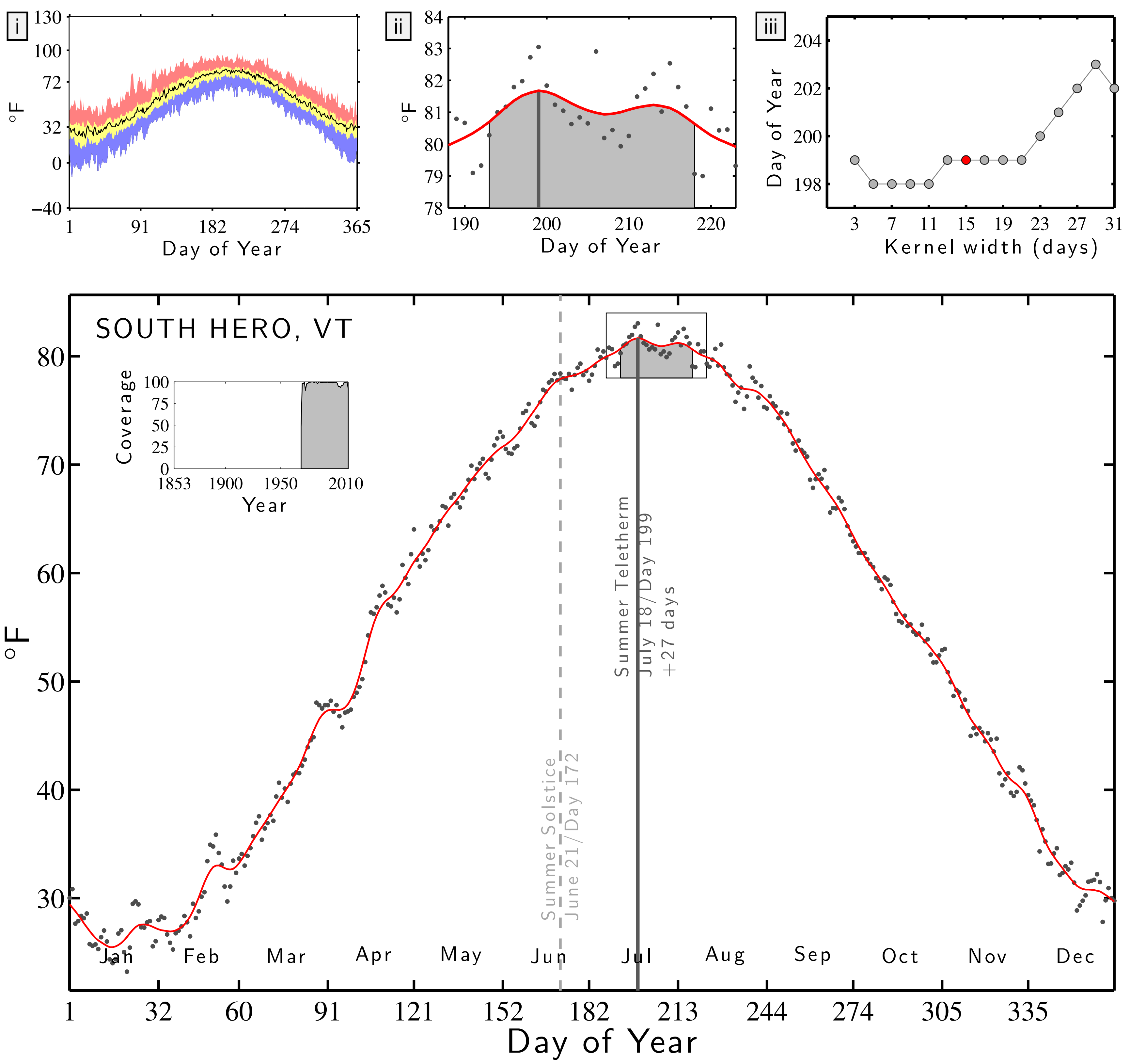

i
°F
Day of Year
ii
°F
Day of Year
iii
Day of Year
Kernel width (days)
SOUTH HERO, VT
Coverage
Year
1853
1900
1950
2010
Summer Solstice
June 21/Day 172
Summer Teletherm
July 18/Day 199
+27 days
Jan
Feb
Mar
Apr
May
Jun
Jul
Aug
Sep
Oct
Nov
Dec
°F
Day of Year

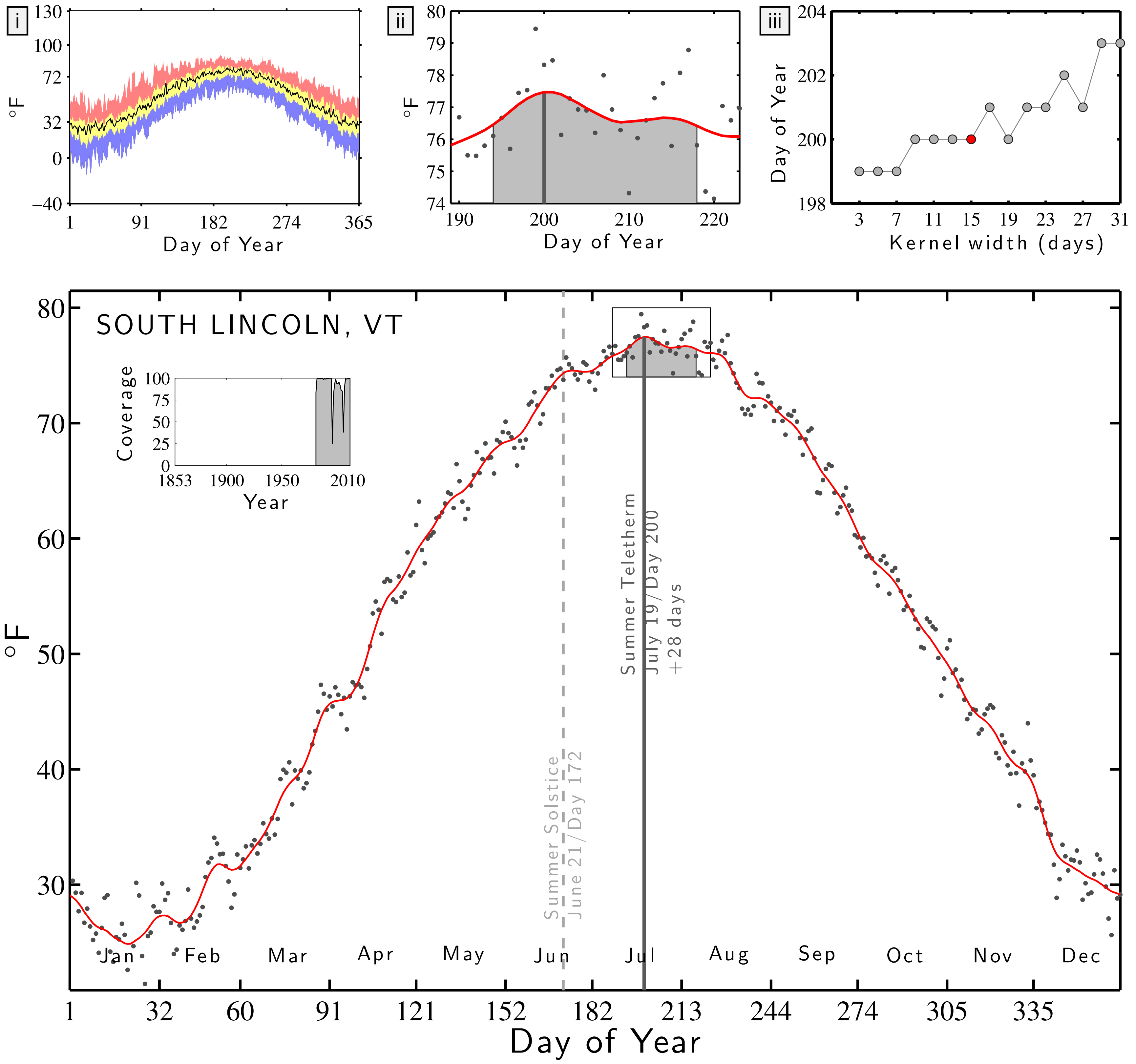

i
°F
Day of Year
ii
°F
Day of Year
iii
Day of Year
Kernel width (days)
SOUTH LINCOLN, VT
Coverage
Year
Summer Solstice
June 21/Day 172
Summer Teletherm
July 19/Day 200
+28 days
Jan
Feb
Mar
Apr
May
Jun
Jul
Aug
Sep
Oct
Nov
Dec
°F
Day of Year

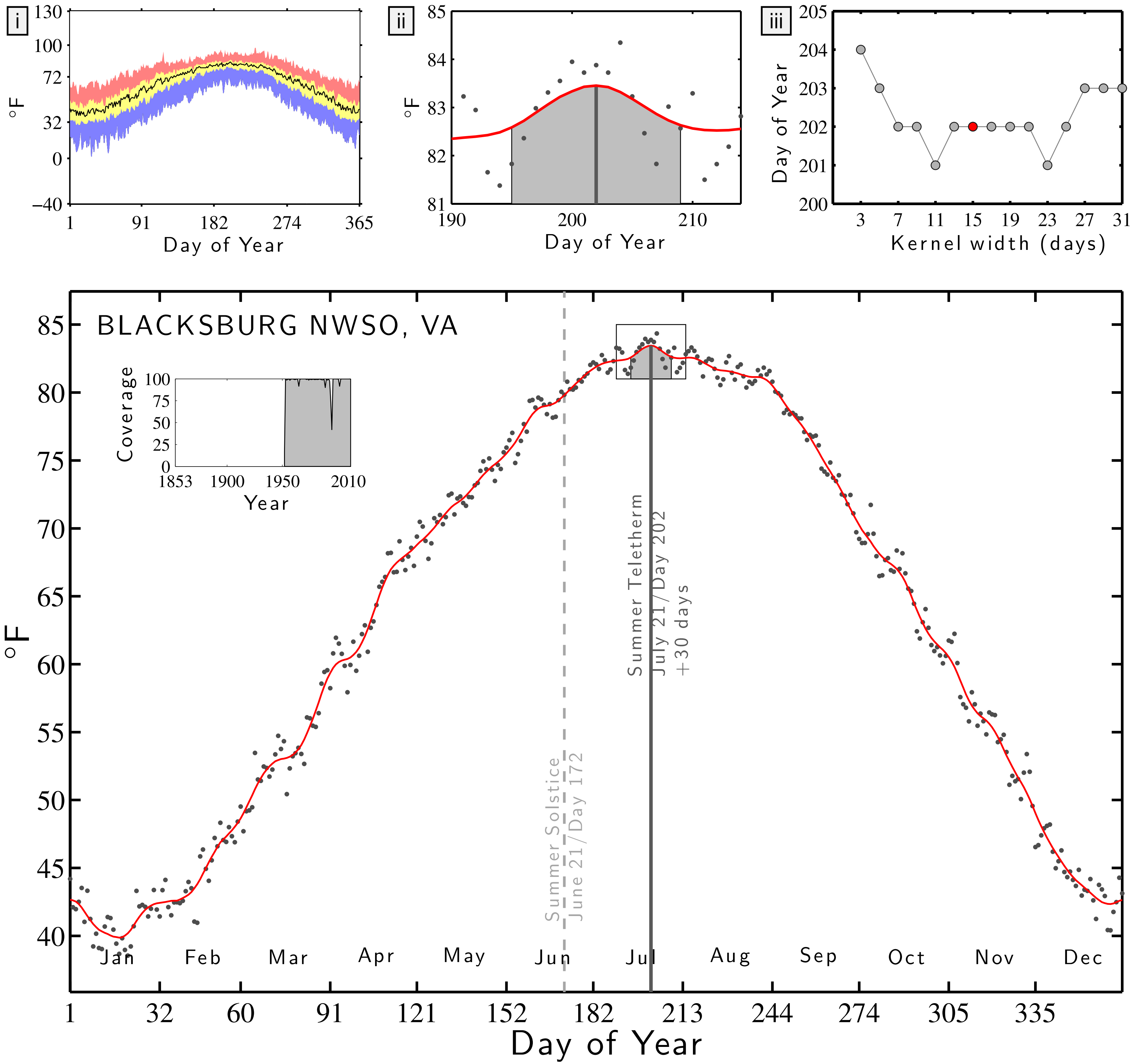

i
ii
iii
°F
Day of Year
Day of Year
Kernel width (days)
BLACKSBURG NWSO, VA
Coverage
Year
Summer Solstice
June 21 / Day 172
Summer Teletherm
July 21 / Day 202
+30 days
Jan
Feb
Mar
Apr
May
Jun
Jul
Aug
Sep
Oct
Nov
Dec

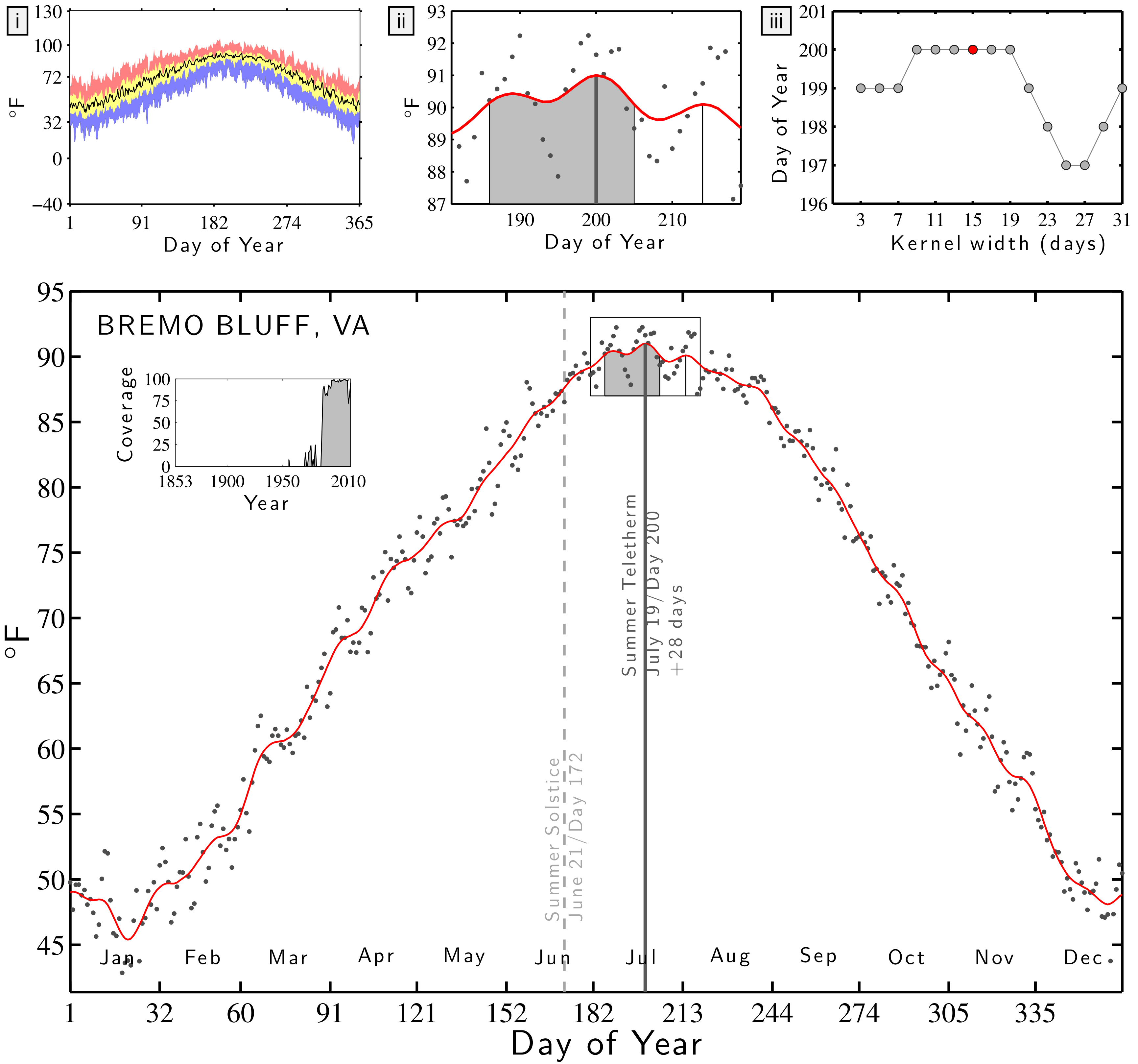

i
°F
Day of Year
ii
°F
Day of Year
iii
Day of Year
Kernel width (days)
BREMO BLUFF, VA
Coverage
Year
Summer Solstice
June 21/Day 172
Summer Teletherm
July 19/Day 200
+28 days
Jan Feb Mar Apr May Jun Jul Aug Sep Oct Nov Dec
°F
Day of Year

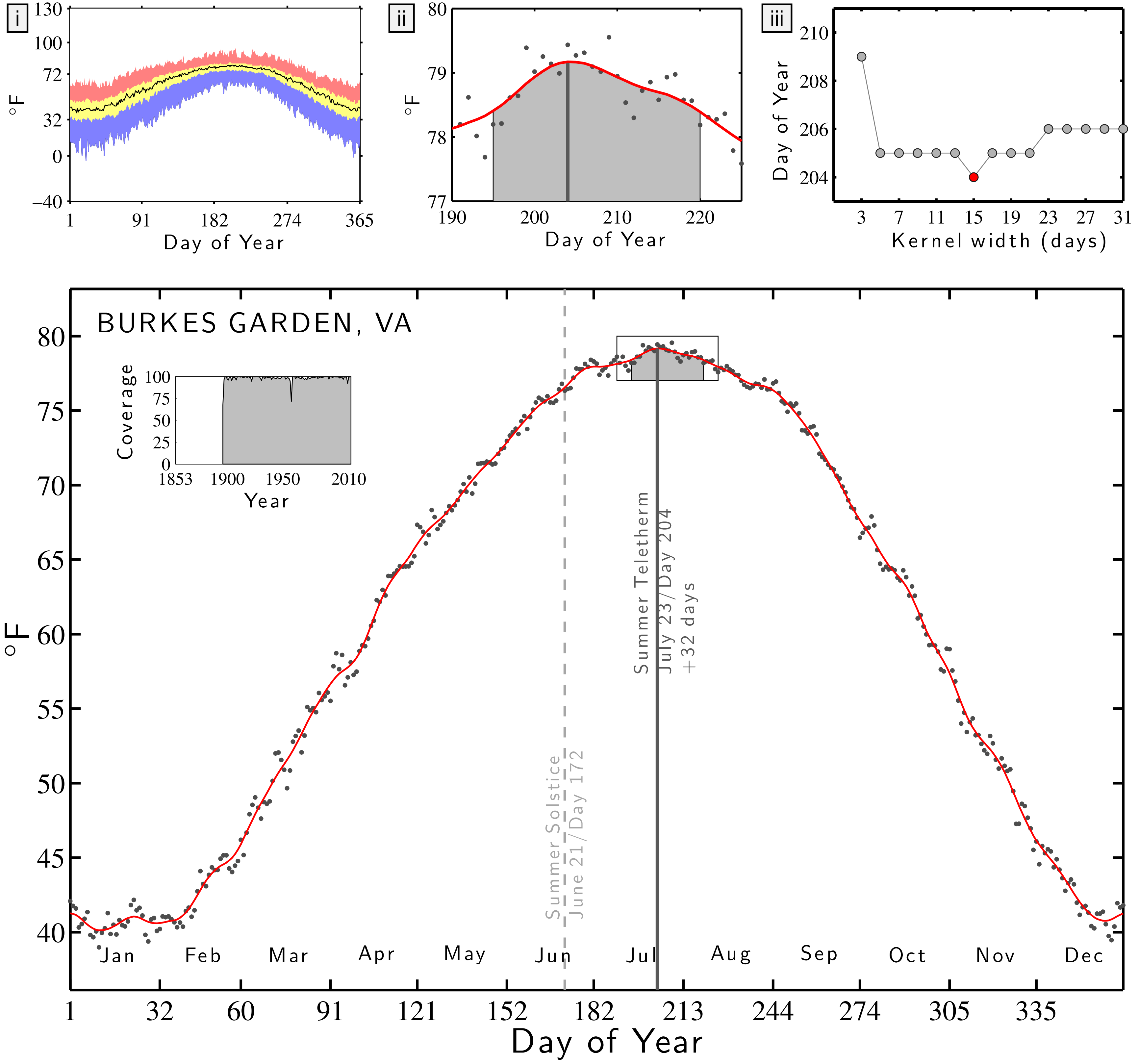

i
°F
Day of Year
ii
°F
Day of Year
iii
Day of Year
Kernel width (days)
BURKES GARDEN, VA
Coverage
Year
Summer Solstice
June 21 / Day 172
Summer Teletherm
July 23 / Day 204
+32 days
Jan
Feb
Mar
Apr
May
Jun
Jul
Aug
Sep
Oct
Nov
Dec
°F
Day of Year

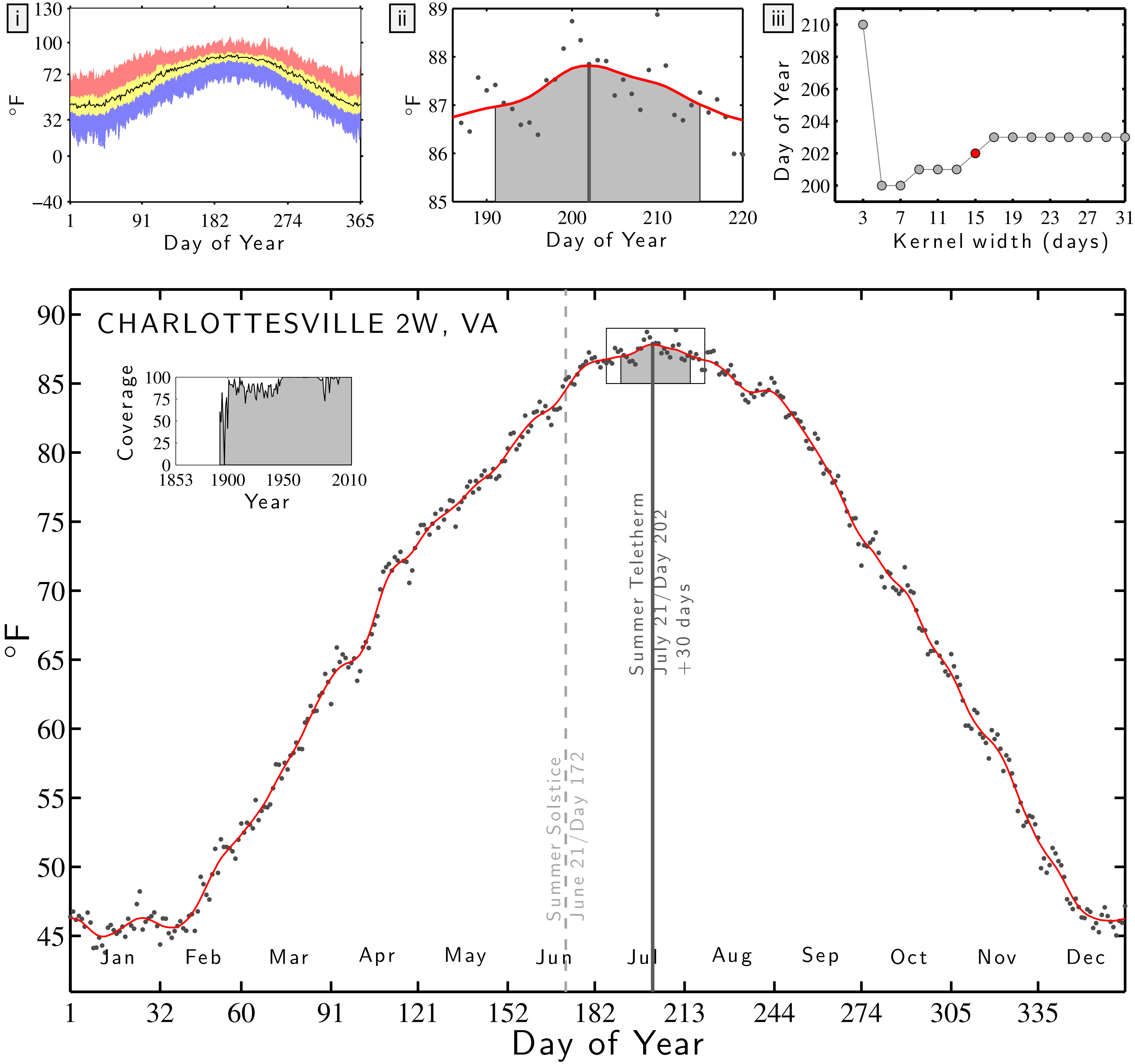

i
ii
iii
°F
Day of Year
Day of Year
Kernel width (days)
CHARLOTTESVILLE 2W, VA
Coverage
Year
Summer Solstice
June 21 / Day 172
Summer Teletherm
July 21 / Day 202
+30 days
Jan
Feb
Mar
Apr
May
Jun
Jul
Aug
Sep
Oct
Nov
Dec
°F
Day of Year

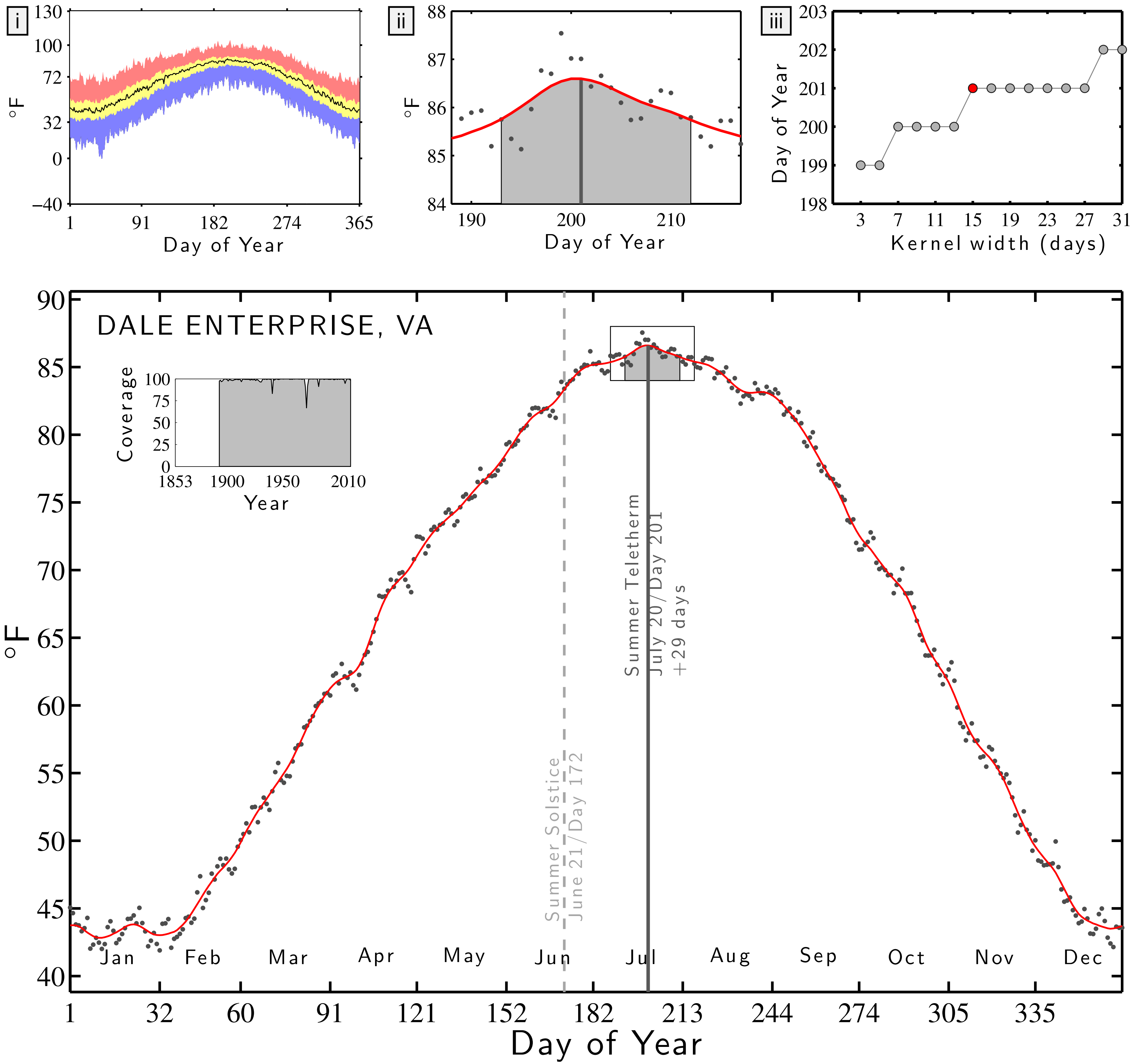

i
°F
Day of Year
ii
°F
Day of Year
iii
Day of Year
Kernel width (days)
DALE ENTERPRISE, VA
Coverage
Year
Summer Solstice
June 21/Day 172
Summer Teletherm
July 20/Day 201
+29 days
Jan
Feb
Mar
Apr
May
Jun
Jul
Aug
Sep
Oct
Nov
Dec
°F
Day of Year

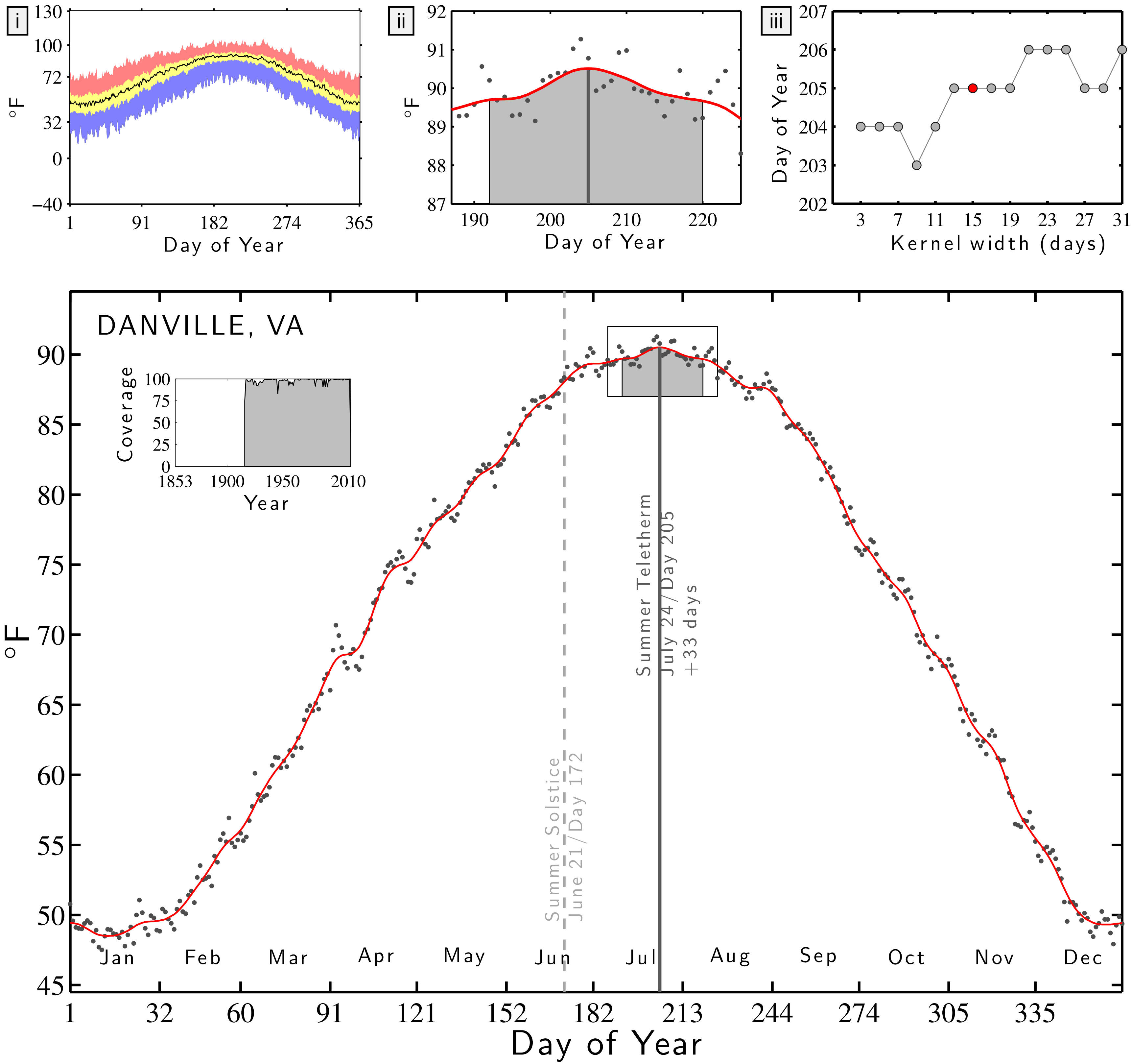

i
ii
iii
Day of Year
°F
Kernel width (days)
DANVILLE, VA
Coverage
Year
Summer Solstice
June 21 / Day 172
Summer Teletherm
July 24 / Day 205
+33 days
Jan
Feb
Mar
Apr
May
Jun
Jul
Aug
Sep
Oct
Nov
Dec

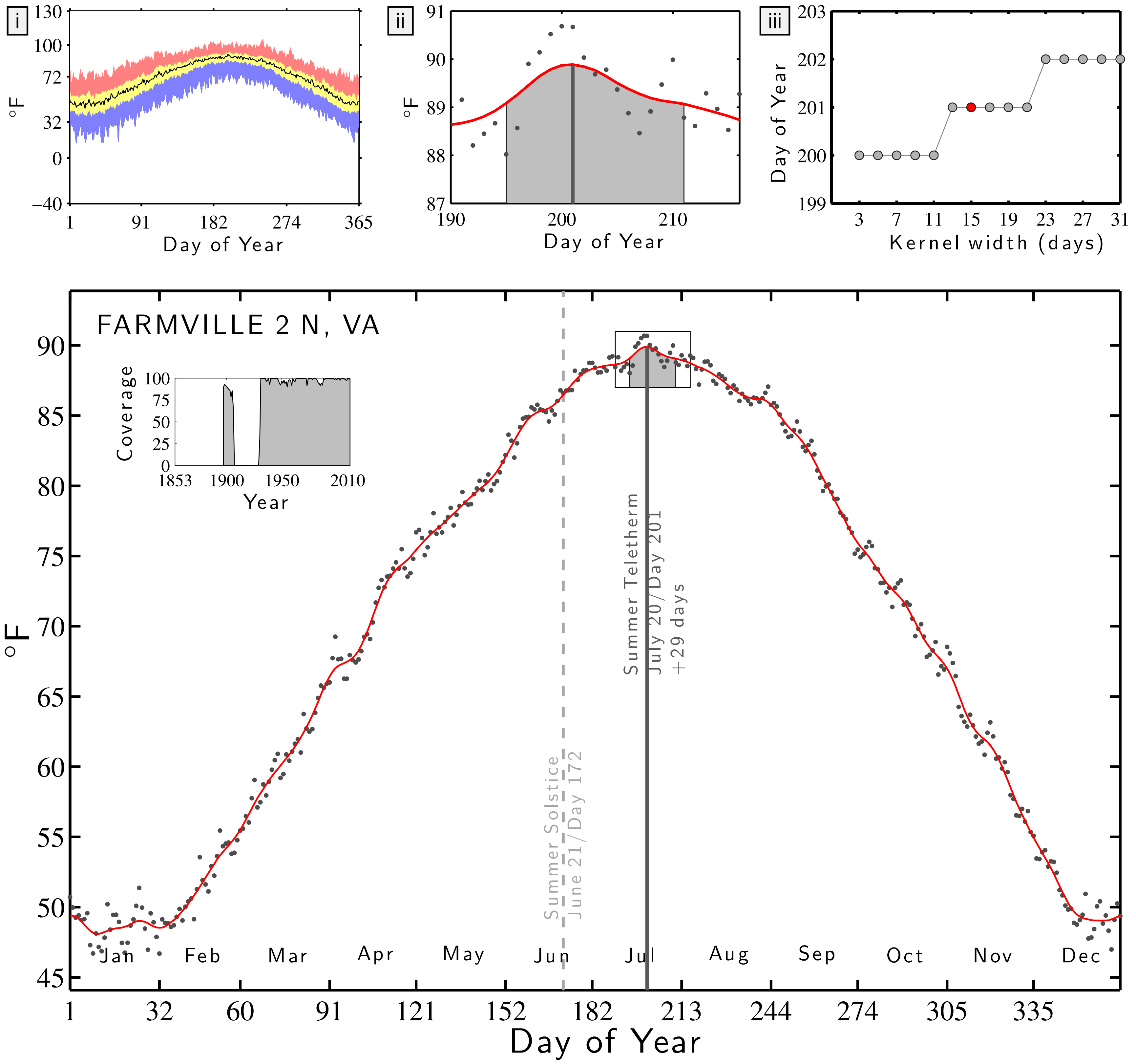

i
°F
Day of Year
ii
°F
Day of Year
iii
Day of Year
Kernel width (days)
FARMVILLE 2 N, VA
Coverage
Year
Summer Solstice
June 21/Day 172
Summer Teletherm
July 20/Day 201
+29 days
Jan
Feb
Mar
Apr
May
Jun
Jul
Aug
Sep
Oct
Nov
Dec
°F
Day of Year

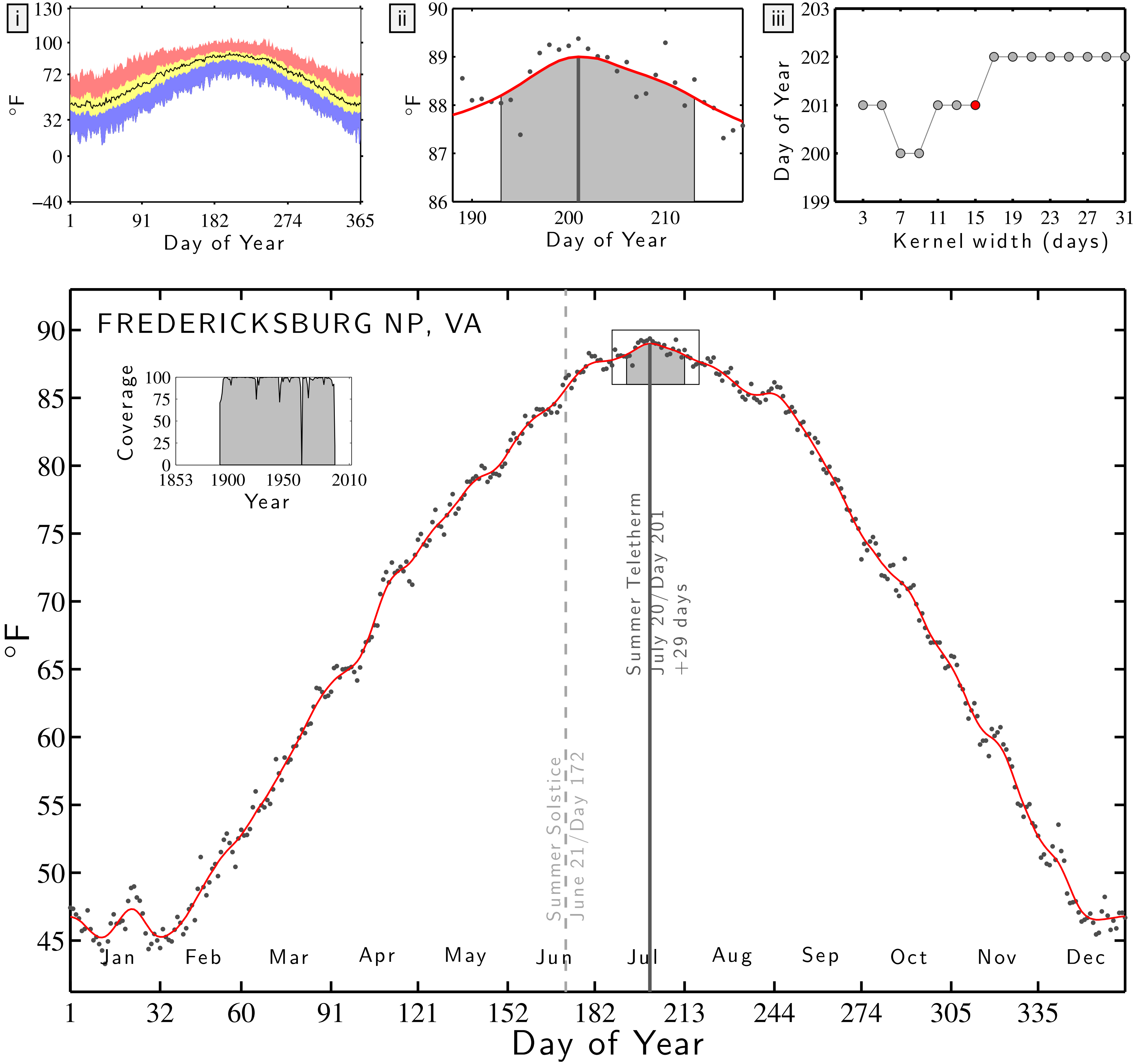

i
°F
Day of Year
ii
°F
Day of Year
iii
Day of Year
Kernel width (days)
FREDERICKSBURG NP, VA
Coverage
Year
Summer Solstice
June 21/Day 172
Summer Teletherm
July 20/Day 201
+29 days
Jan
Feb
Mar
Apr
May
Jun
Jul
Aug
Sep
Oct
Nov
Dec
°F
Day of Year

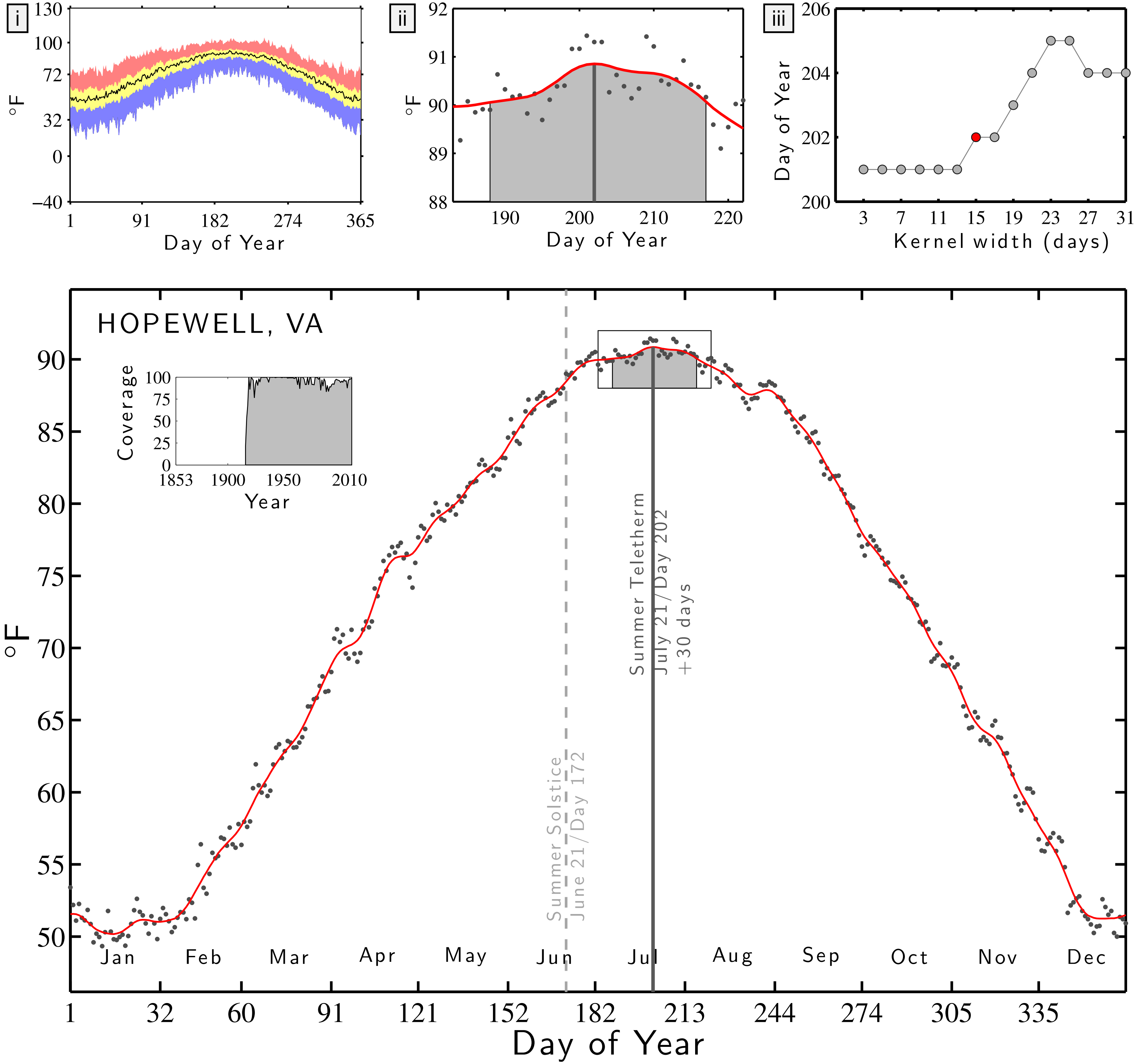
i
ii
iii
Day of Year
°F
Kernel width (days)
HOPEWELL, VA
Coverage
Year
Summer Solstice
June 21/Day 172
Summer Teletherm
July 21/Day 202
+30 days
Jan
Feb
Mar
Apr
May
Jun
Jul
Aug
Sep
Oct
Nov
Dec
Day of Year

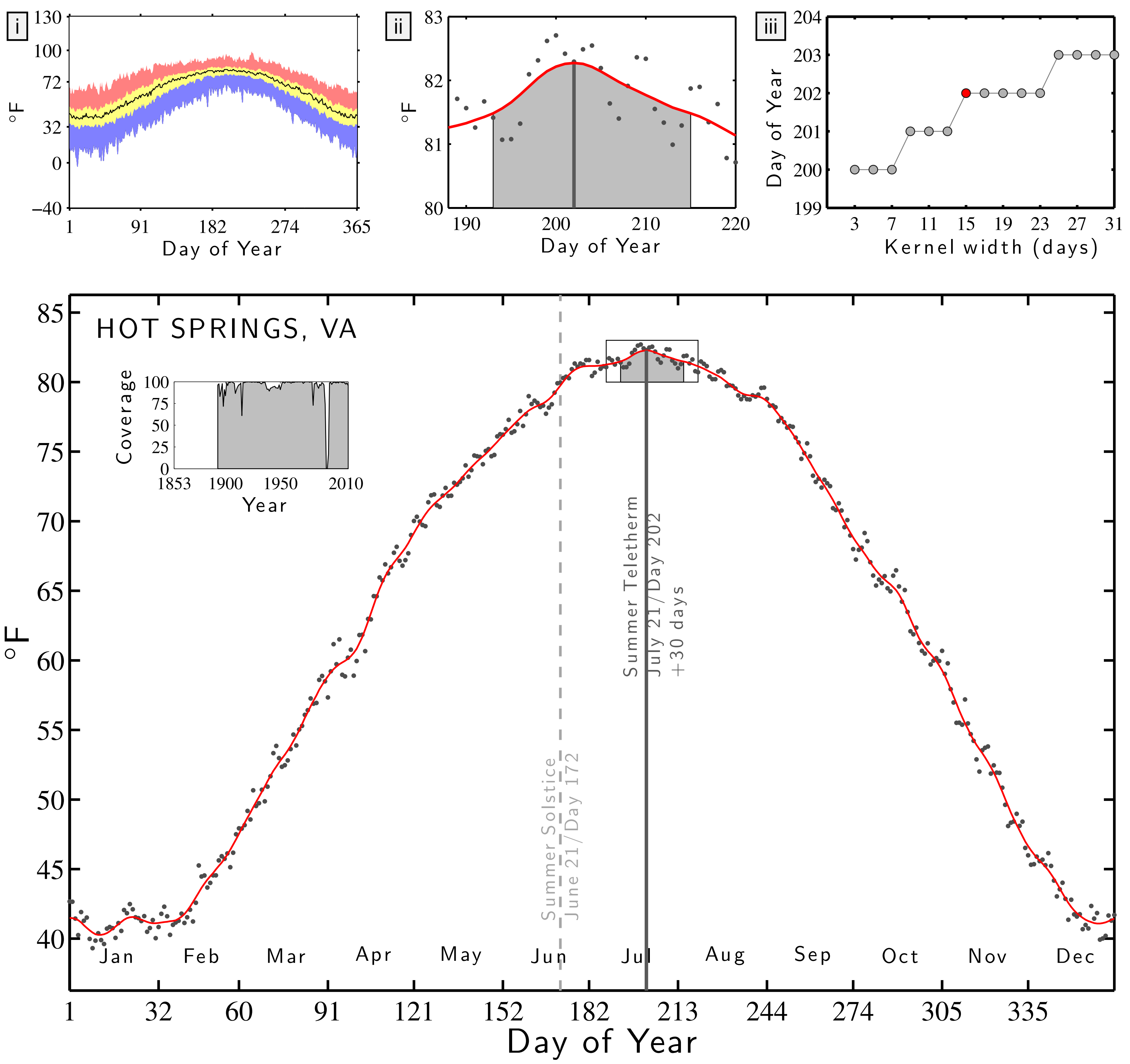

i
ii
iii
Day of Year
Kernel width (days)
HOT SPRINGS, VA
Coverage
Year
Summer Solstice
June 21 / Day 172
Summer Teletherm
July 21 / Day 202
+30 days
Jan
Feb
Mar
Apr
May
Jun
Jul
Aug
Sep
Oct
Nov
Dec
°F
Day of Year

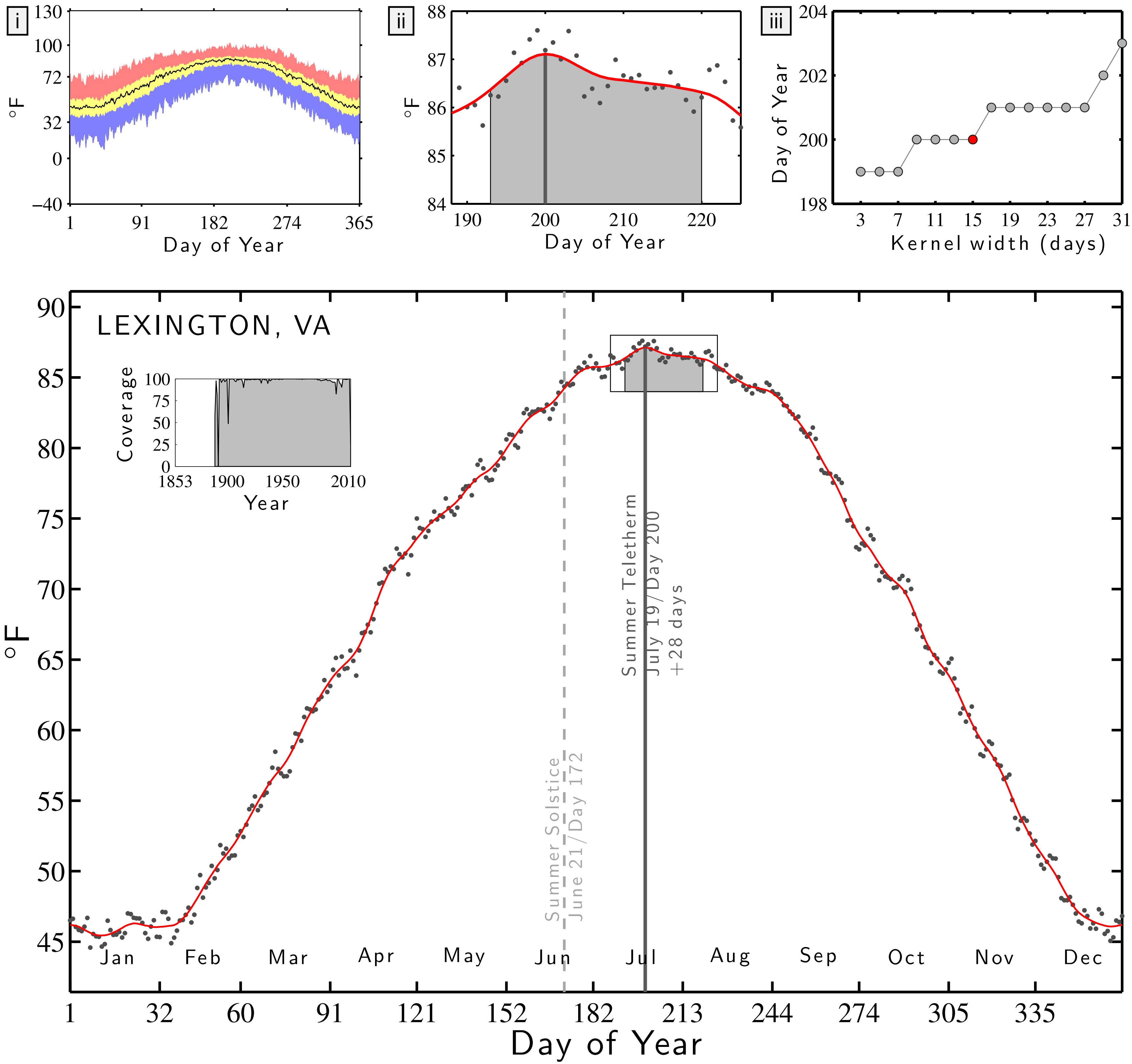

i
°F
Day of Year
ii
°F
Day of Year
iii
Day of Year
Kernel width (days)
LEXINGTON, VA
Coverage
Year
Summer Solstice
June 21/Day 172
Summer Teletherm
July 19/Day 200
+28 days
Jan
Feb
Mar
Apr
May
Jun
Jul
Aug
Sep
Oct
Nov
Dec
°F
Day of Year

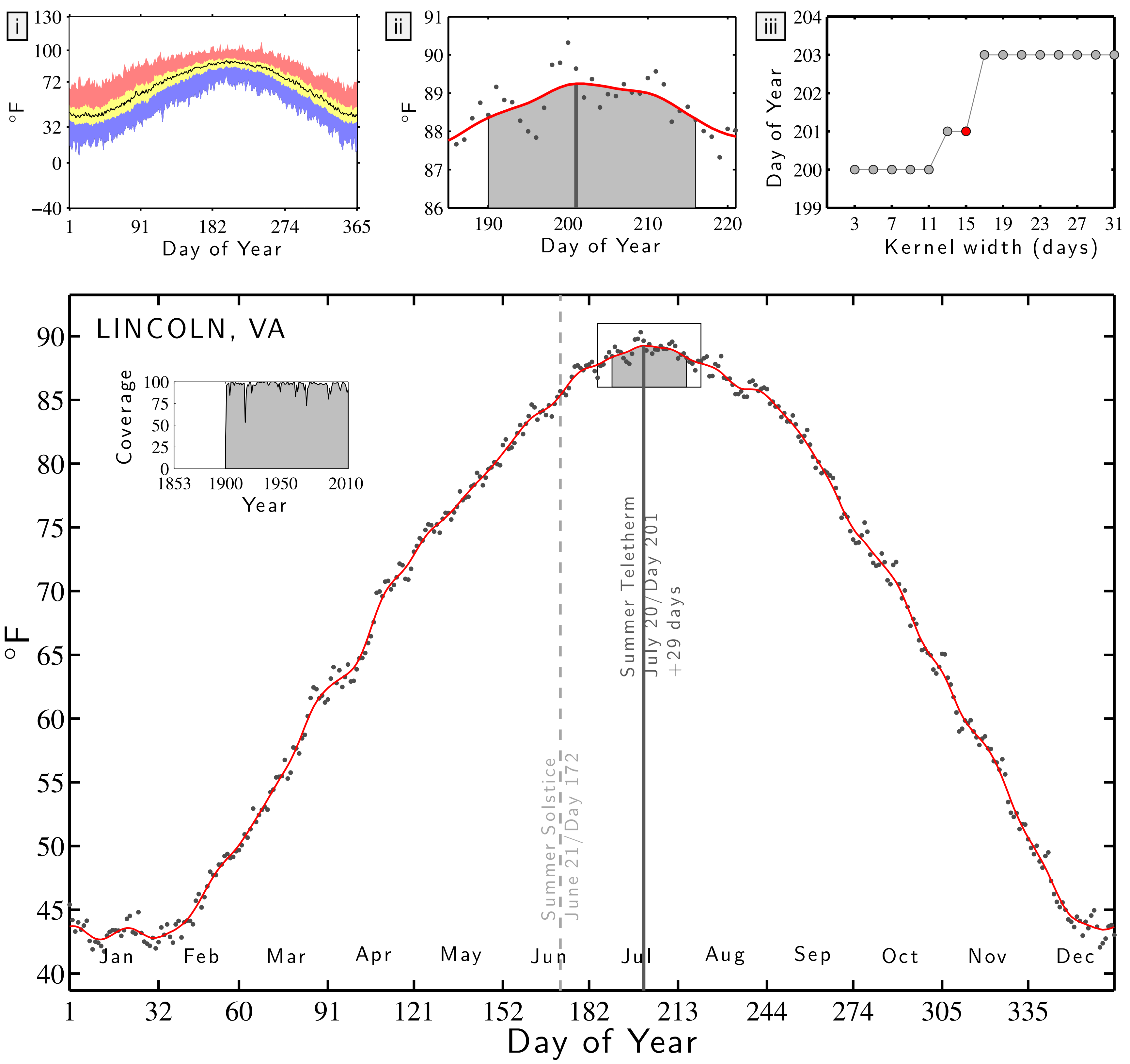

i
°F
Day of Year
ii
°F
Day of Year
iii
Day of Year
Kernel width (days)
LINCOLN, VA
Coverage
Year
Summer Solstice
June 21/Day 172
Summer Teletherm
July 20/Day 201
+29 days
Jan
Feb
Mar
Apr
May
Jun
Jul
Aug
Sep
Oct
Nov
Dec
°F
Day of Year

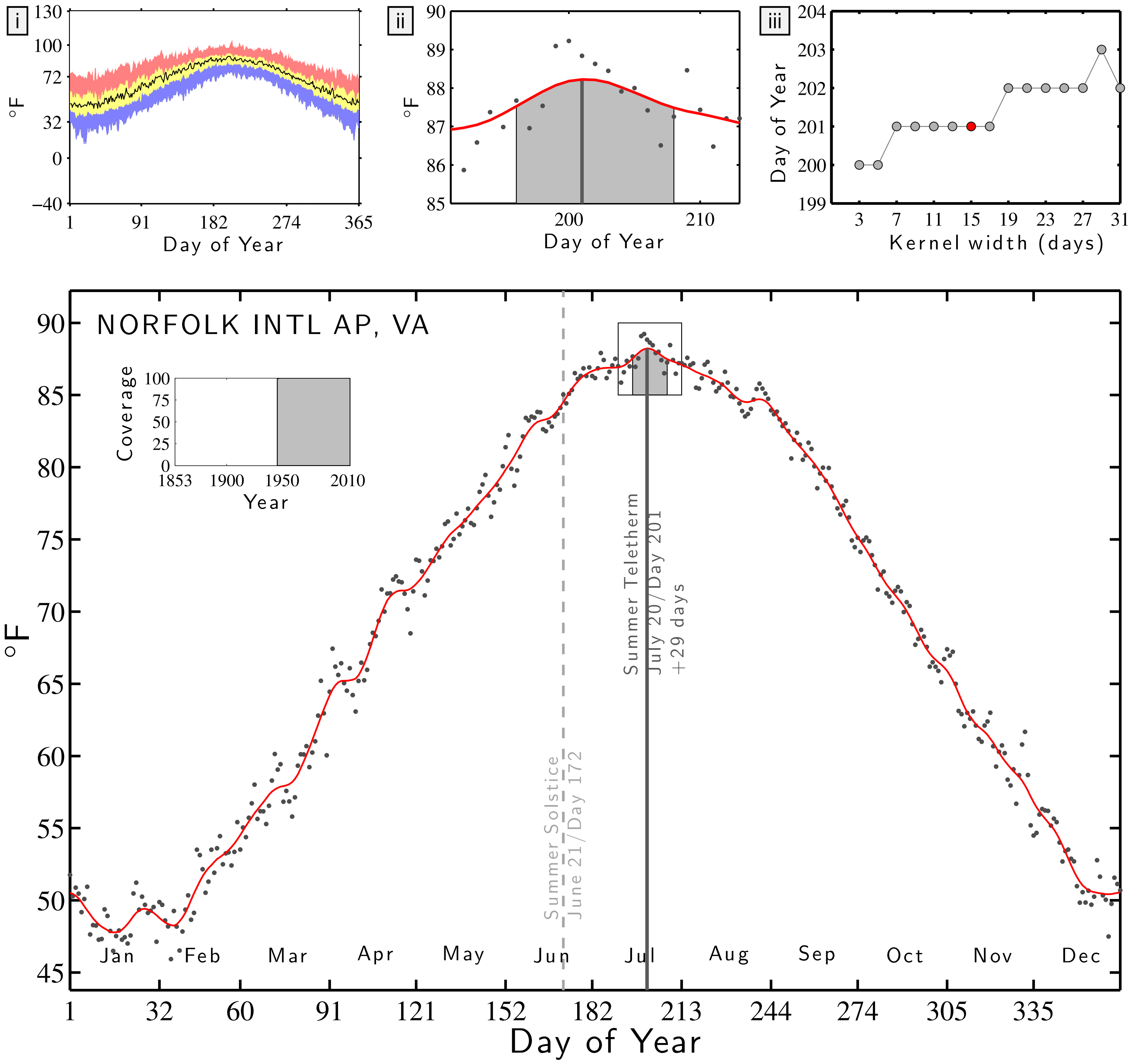

i
ii
iii
°F
Day of Year
Day of Year
Kernel width (days)
NORFOLK INTL AP, VA
Coverage
Year
Summer Solstice
June 21/Day 172
Summer Teletherm
July 20/Day 201
+29 days
Jan
Feb
Mar
Apr
May
Jun
Jul
Aug
Sep
Oct
Nov
Dec

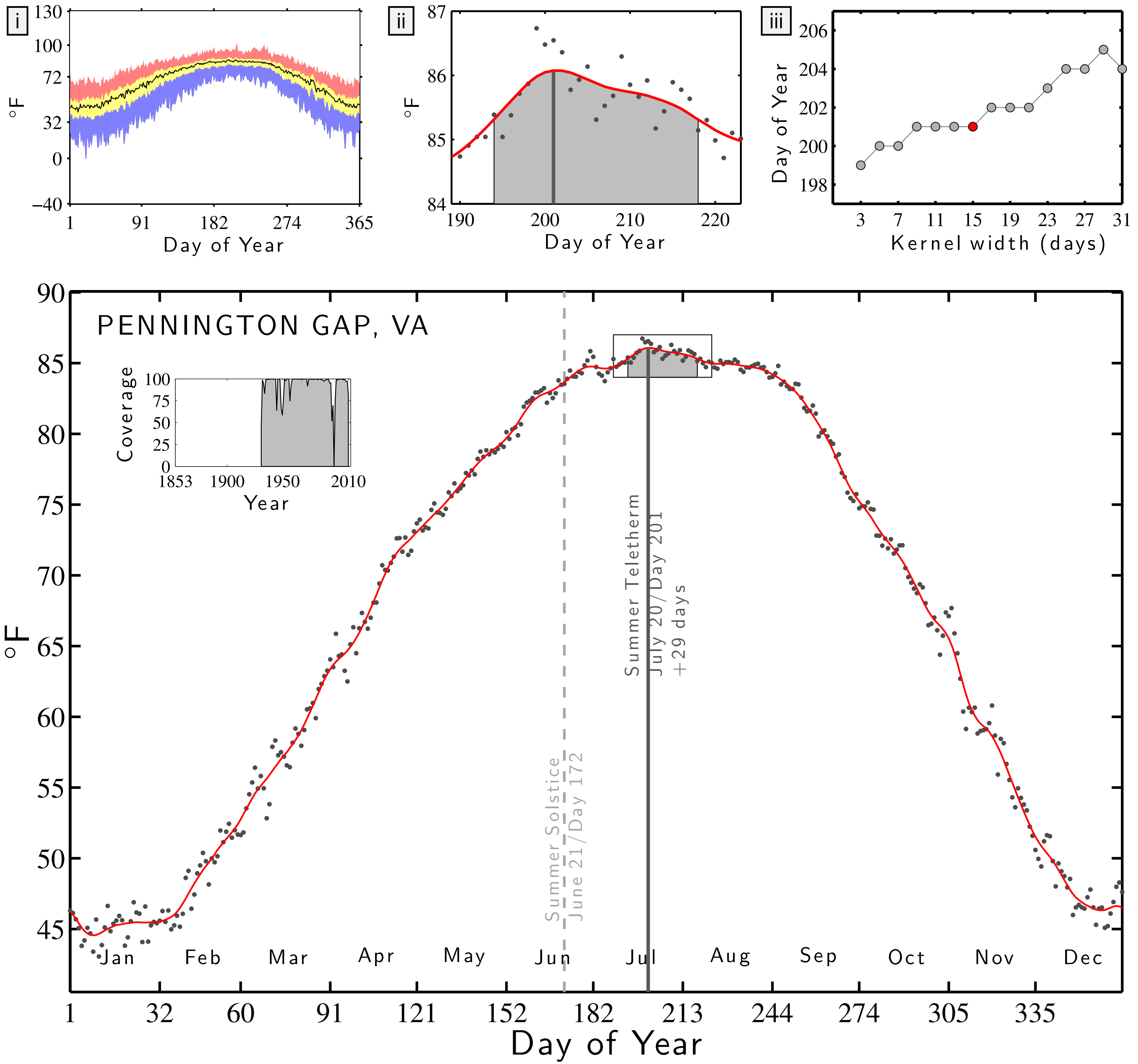

i
°F
Day of Year
ii
°F
Day of Year
iii
Day of Year
Kernel width (days)
PENNINGTON GAP, VA
Coverage
Year
Summer Solstice
June 21/Day 172
Summer Teletherm
July 20/Day 201
+29 days
Jan
Feb
Mar
Apr
May
Jun
Jul
Aug
Sep
Oct
Nov
Dec
°F
Day of Year

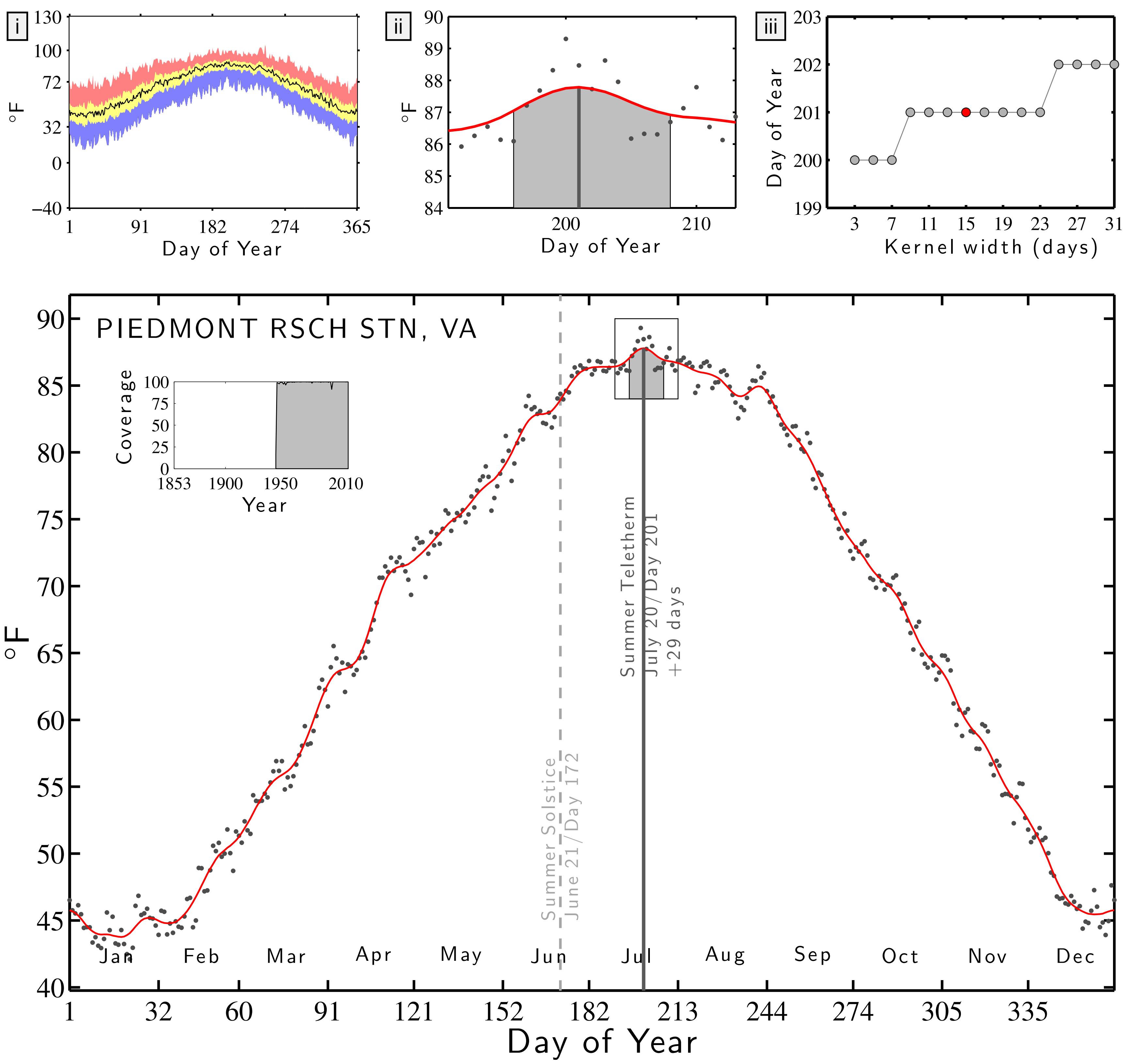

i
ii
iii
°F
Day of Year
Kernel width (days)
PIEDMONT RSCH STN, VA
Coverage
Year
Summer Solstice
June 21/Day 172
Summer Teletherm
July 20/Day 201
+29 days
Jan
Feb
Mar
Apr
May
Jun
Jul
Aug
Sep
Oct
Nov
Dec

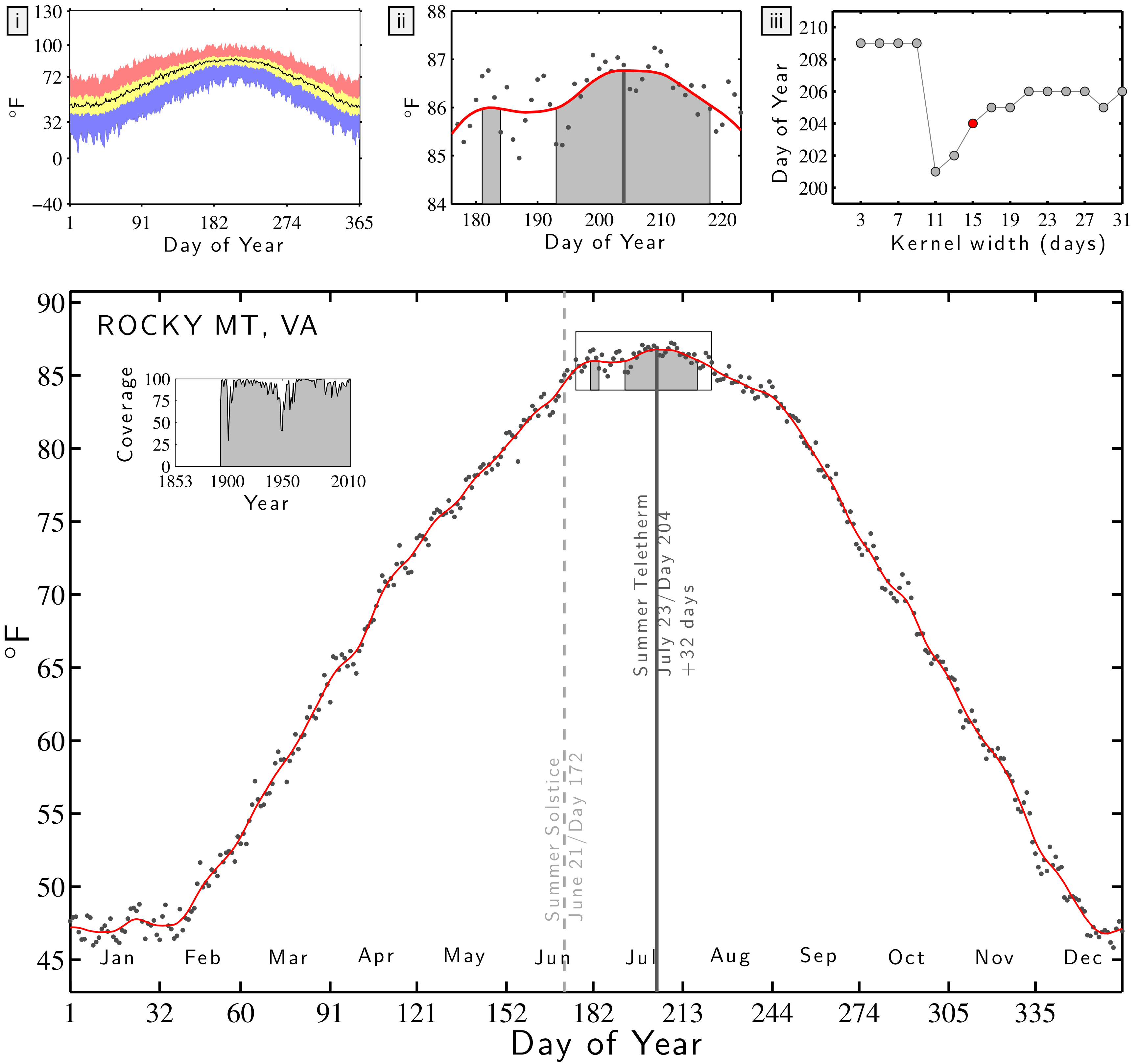

i
°F
Day of Year
ii
°F
Day of Year
iii
Day of Year
Kernel width (days)
ROCKY MT, VA
Coverage
Year
Summer Solstice
June 21 / Day 172
Summer Teletherm
July 23 / Day 204
+32 days
Jan
Feb
Mar
Apr
May
Jun
Jul
Aug
Sep
Oct
Nov
Dec
°F
Day of Year

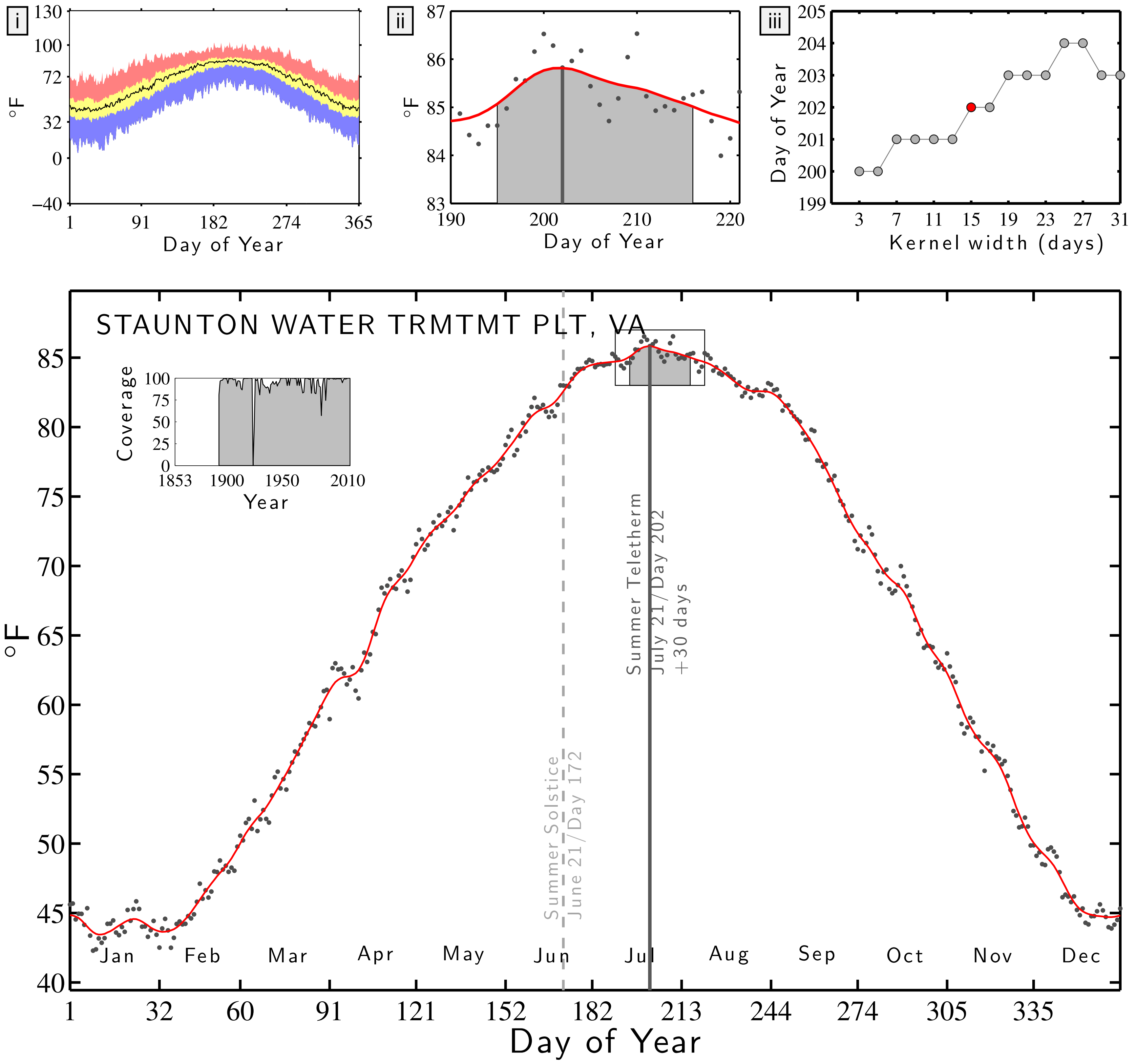

i
°F
Day of Year
ii
°F
Day of Year
iii
Day of Year
Kernel width (days)
STAUNTON WATER TRMTMT PLT, VA
Coverage
Year
Summer Solstice
June 21 / Day 172
Summer Teletherm
July 21 / Day 202
+30 days
Jan
Feb
Mar
Apr
May
Jun
Jul
Aug
Sep
Oct
Nov
Dec
°F
Day of Year

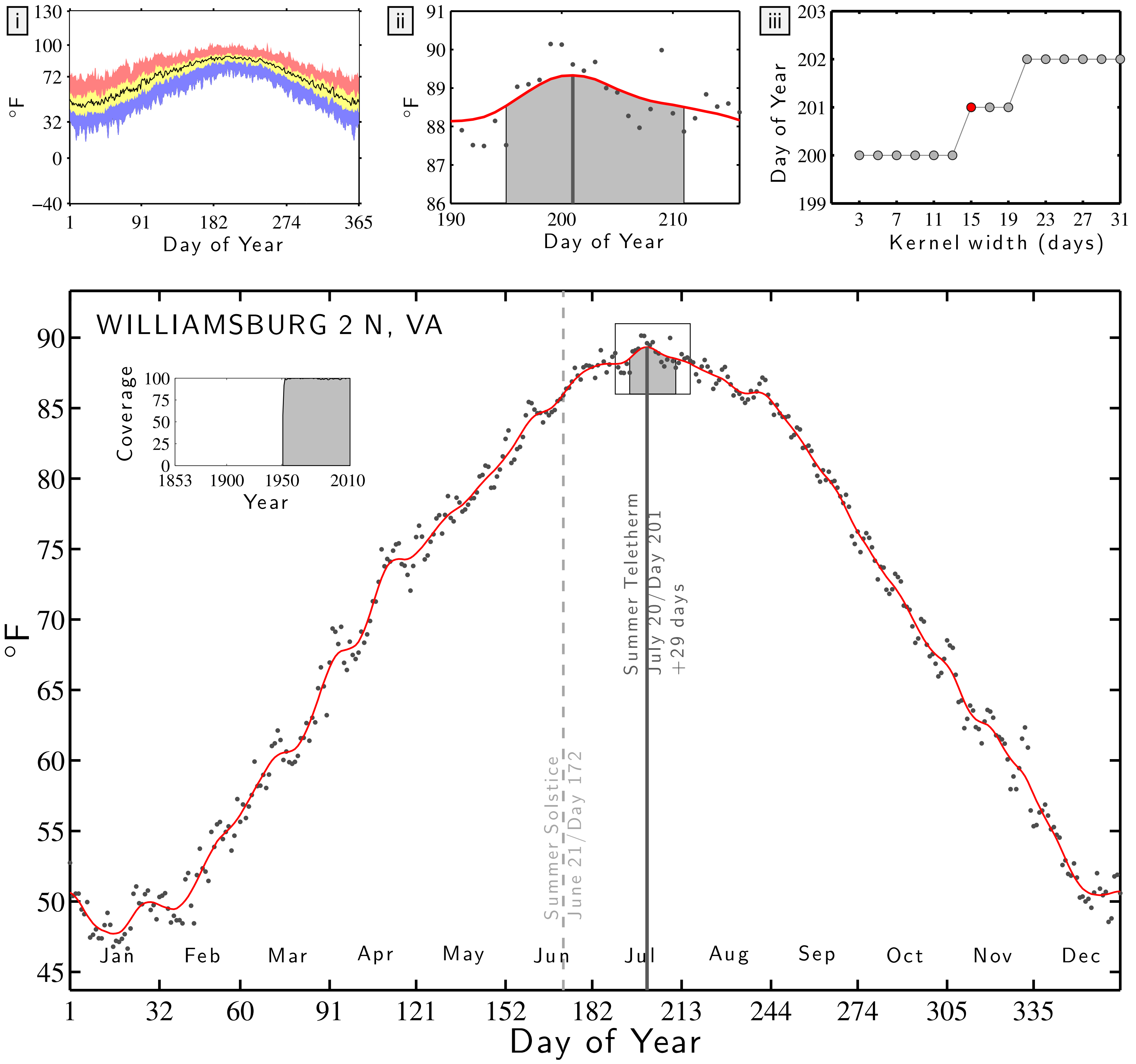

i
°F
Day of Year
ii
°F
Day of Year
iii
Day of Year
Kernel width (days)
WILLIAMSBURG 2 N, VA
Coverage
Year
Summer Solstice
June 21 / Day 172
Summer Teletherm
July 20 / Day 201
+29 days
Jan
Feb
Mar
Apr
May
Jun
Jul
Aug
Sep
Oct
Nov
Dec
°F
Day of Year

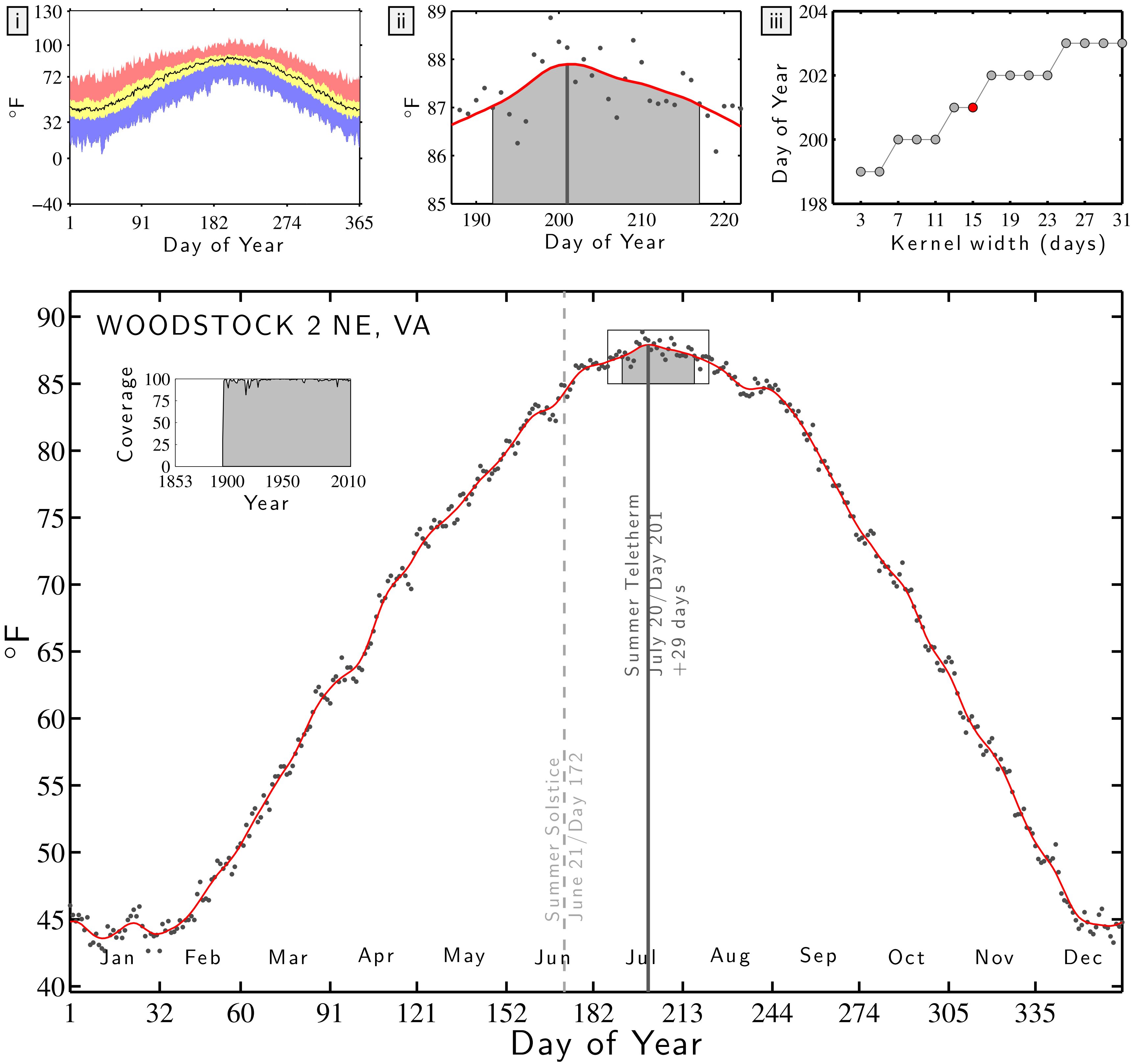

i
°F
Day of Year
ii
°F
Day of Year
iii
Day of Year
Kernel width (days)
WOODSTOCK 2 NE, VA
Coverage
Year
Summer Solstice
June 21/Day 172
Summer Teletherm
July 20/Day 201
+29 days
Jan Feb Mar Apr May Jun Jul Aug Sep Oct Nov Dec
°F
Day of Year

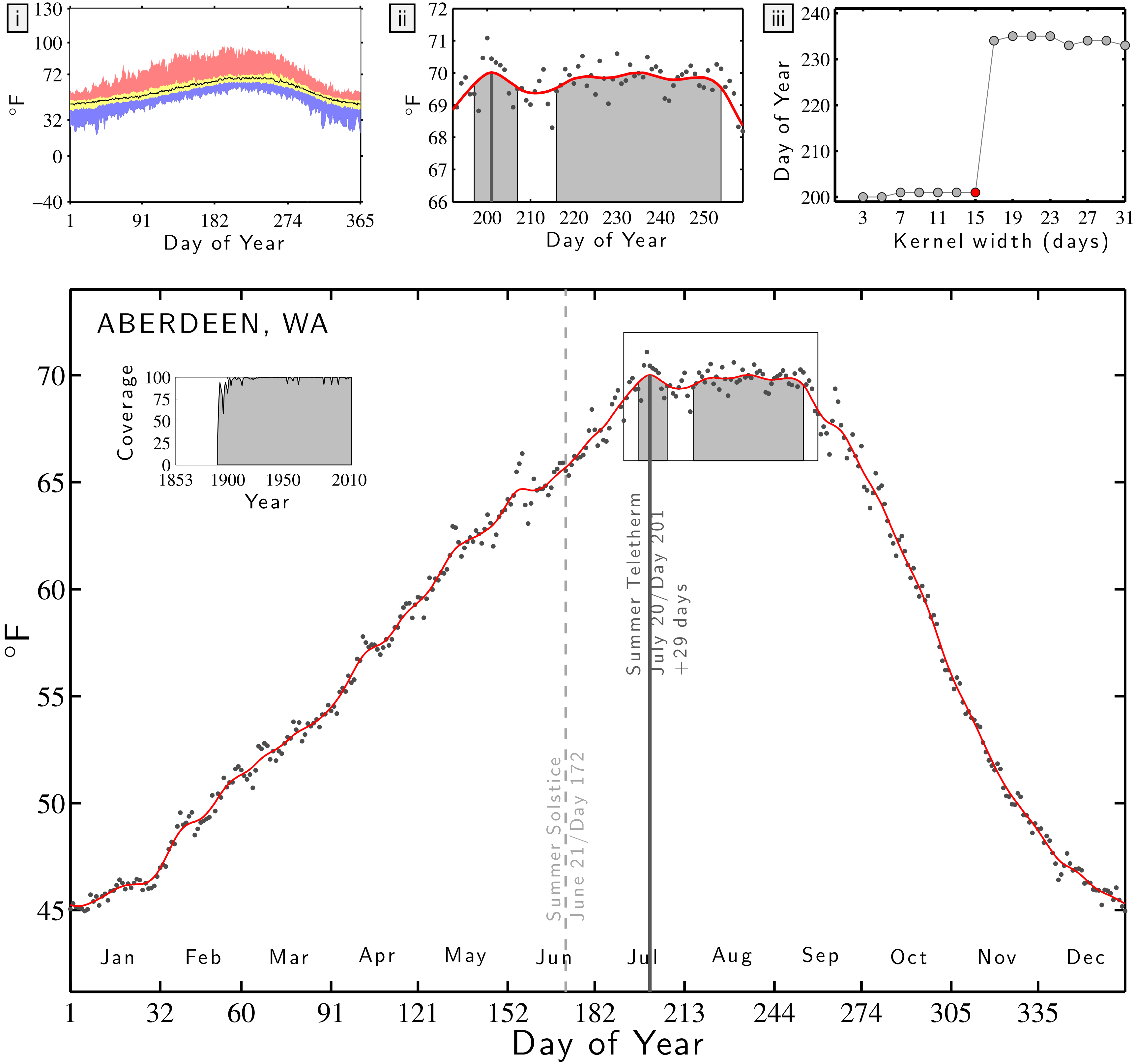

i
°F
Day of Year
ii
°F
Day of Year
iii
Day of Year
Kernel width (days)
ABERDEEN, WA
Coverage
Year
Summer Solstice
June 21/Day 172
Summer Teletherm
July 20/Day 201
+29 days
Jan
Feb
Mar
Apr
May
Jun
Jul
Aug
Sep
Oct
Nov
Dec
°F
Day of Year

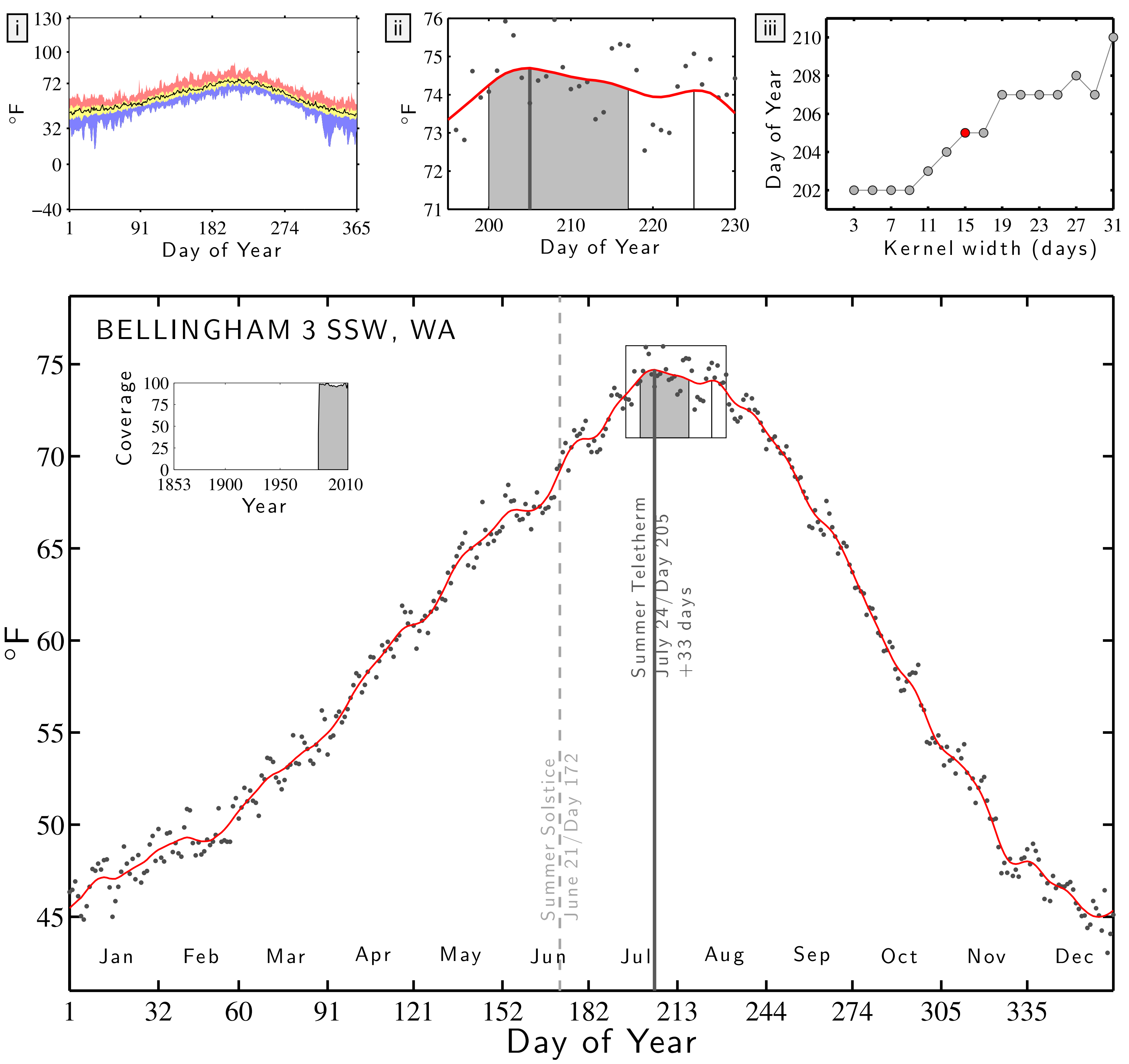

i
°F
Day of Year
ii
°F
Day of Year
iii
Day of Year
Kernel width (days)
BELLINGHAM 3 SSW, WA
Coverage
Year
Summer Solstice
June 21 / Day 172
Summer Teletherm
July 24 / Day 205
+33 days
Jan
Feb
Mar
Apr
May
Jun
Jul
Aug
Sep
Oct
Nov
Dec
°F
Day of Year

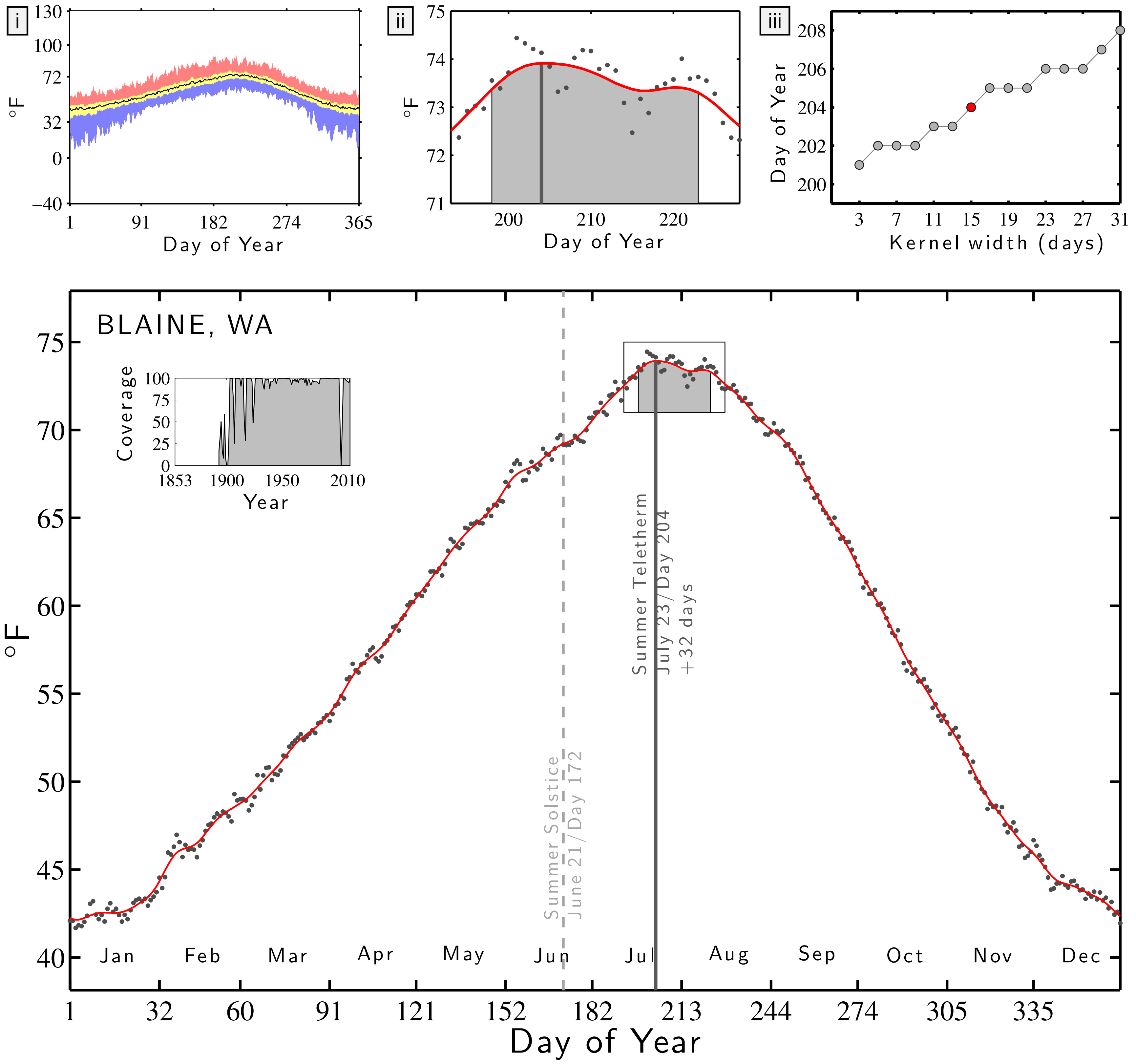

i
ii
iii
°F
Day of Year
Day of Year
Kernel width (days)
BLAINE, WA
Coverage
Year
Summer Solstice
June 21 / Day 172
Summer Teletherm
July 23 / Day 204
+32 days
Jan
Feb
Mar
Apr
May
Jun
Jul
Aug
Sep
Oct
Nov
Dec
Day of Year

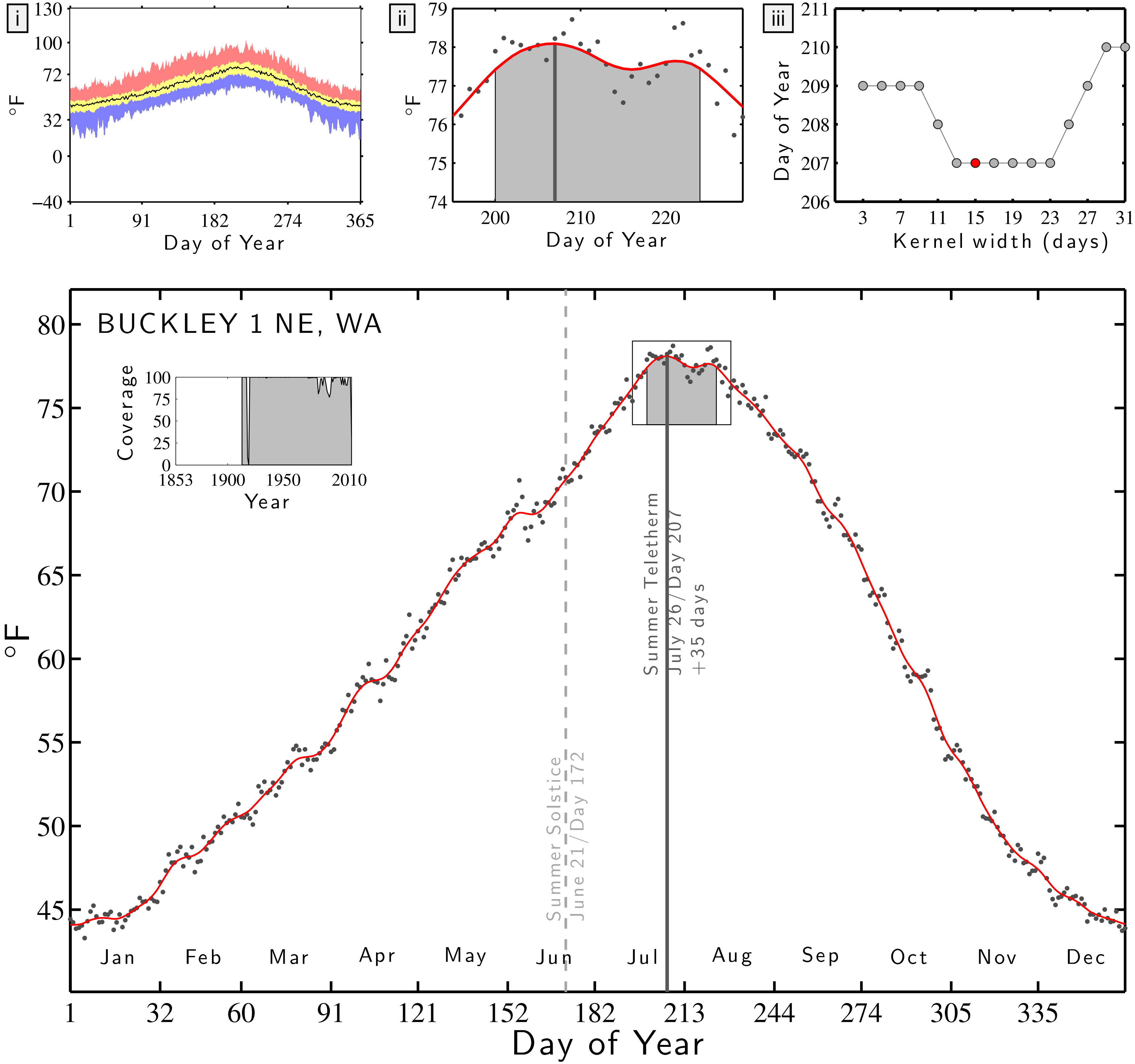

i
ii
iii
°F
Day of Year
Day of Year
Kernel width (days)
BUCKLEY 1 NE, WA
Coverage
Year
Summer Solstice
June 21 / Day 172
Summer Teletherm
July 26 / Day 207
+35 days
Jan
Feb
Mar
Apr
May
Jun
Jul
Aug
Sep
Oct
Nov
Dec
°F
Day of Year

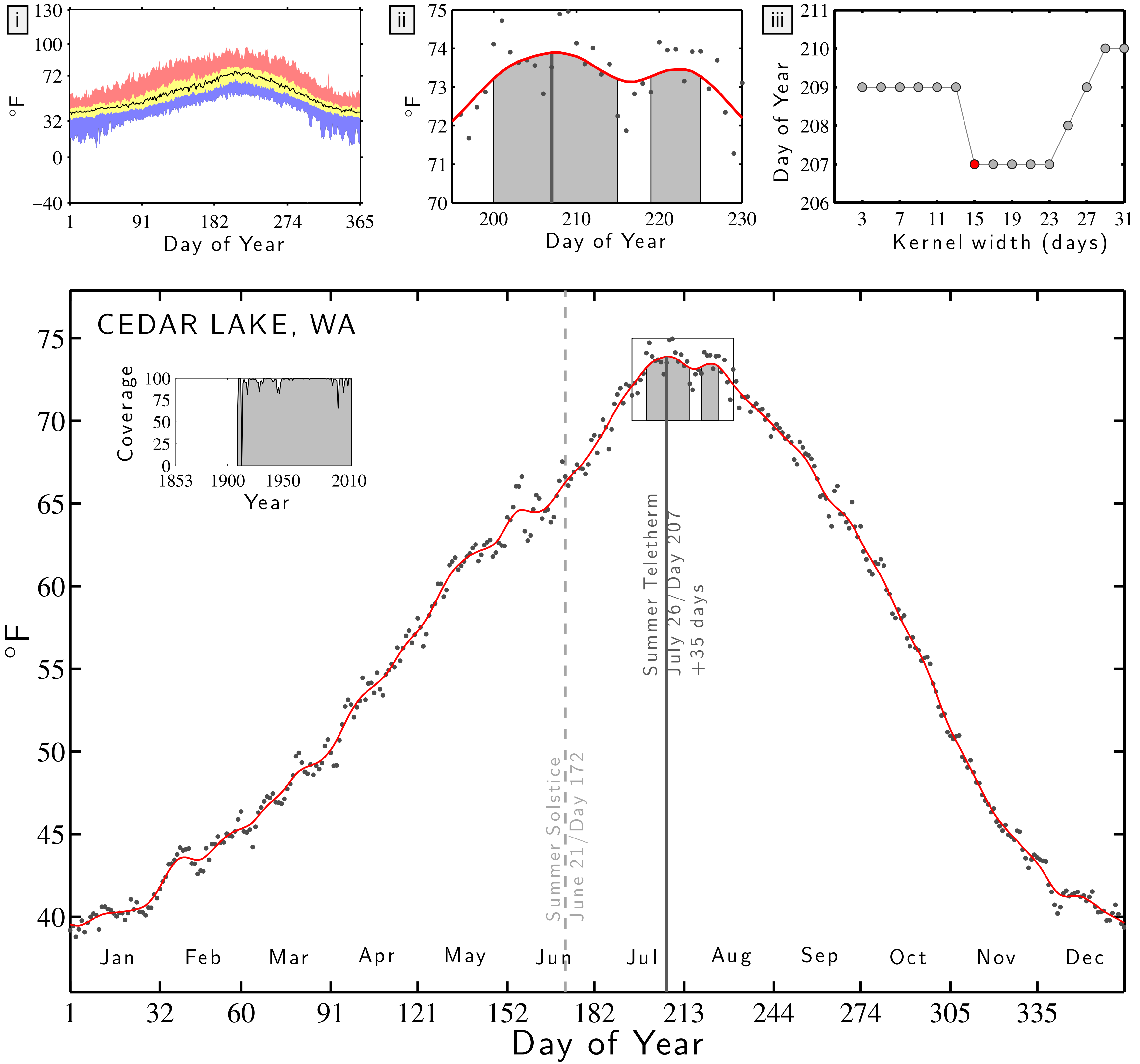

i
°F
Day of Year
ii
°F
Day of Year
iii
Day of Year
Kernel width (days)
CEDAR LAKE, WA
Coverage
Year
1853
1900
1950
2010
Summer Solstice
June 21 / Day 172
Summer Teletherm
July 26 / Day 207
+35 days
Jan
Feb
Mar
Apr
May
Jun
Jul
Aug
Sep
Oct
Nov
Dec
°F
Day of Year

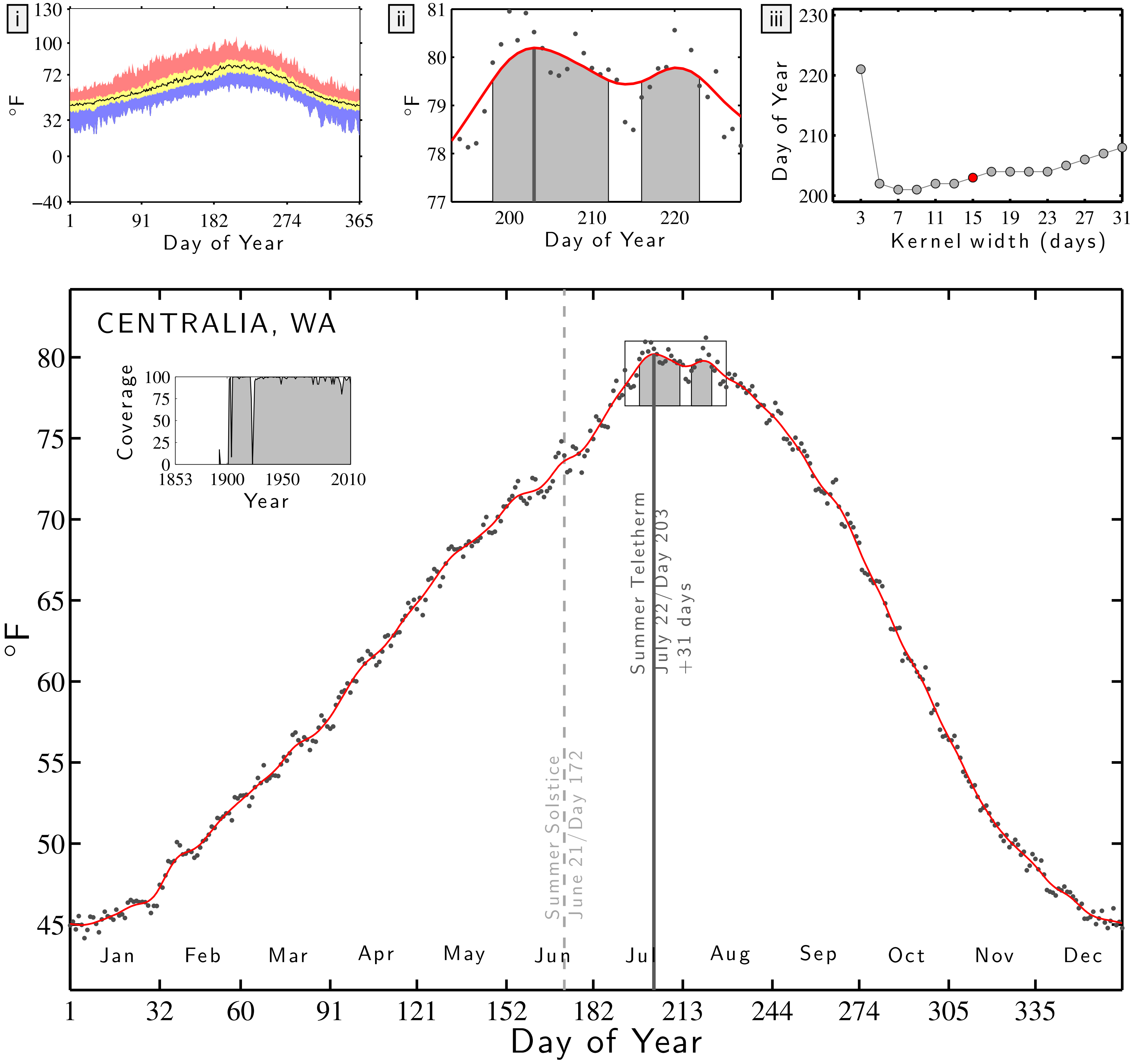

i
°F
Day of Year
ii
°F
Day of Year
iii
Day of Year
Kernel width (days)
CENTRALIA, WA
Coverage
Year
1853
1900
1950
2010
Summer Solstice
June 21/Day 172
Summer Teletherm
July 22/Day 203
+31 days
Jan
Feb
Mar
Apr
May
Jun
Jul
Aug
Sep
Oct
Nov
Dec
°F
Day of Year

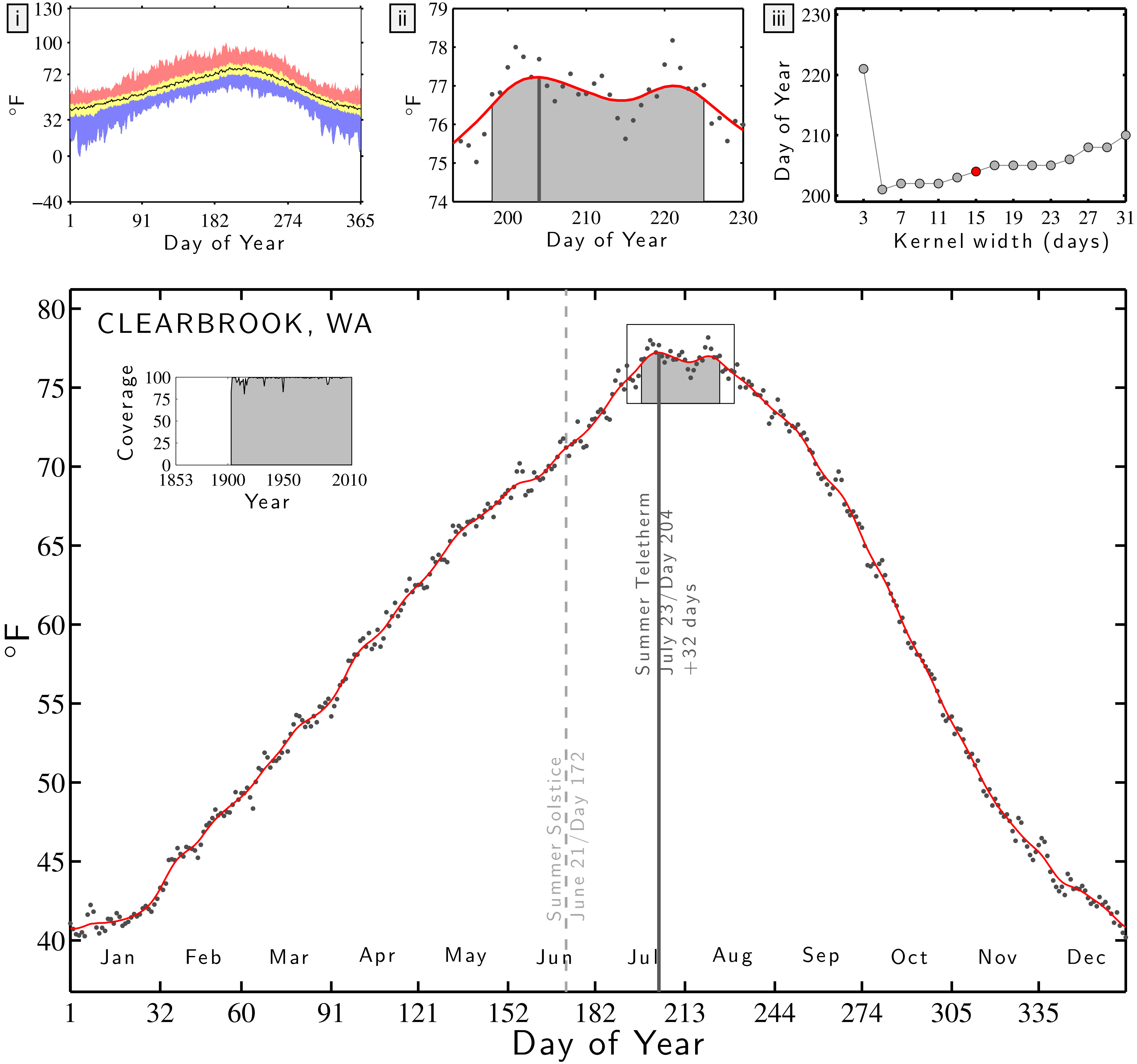
i
°F
Day of Year
ii
°F
Day of Year
iii
Day of Year
Kernel width (days)
CLEARBROOK, WA
Coverage
Year
Summer Solstice
June 21 / Day 172
Summer Teletherm
July 23 / Day 204
+32 days
Jan
Feb
Mar
Apr
May
Jun
Jul
Aug
Sep
Oct
Nov
Dec
°F
Day of Year

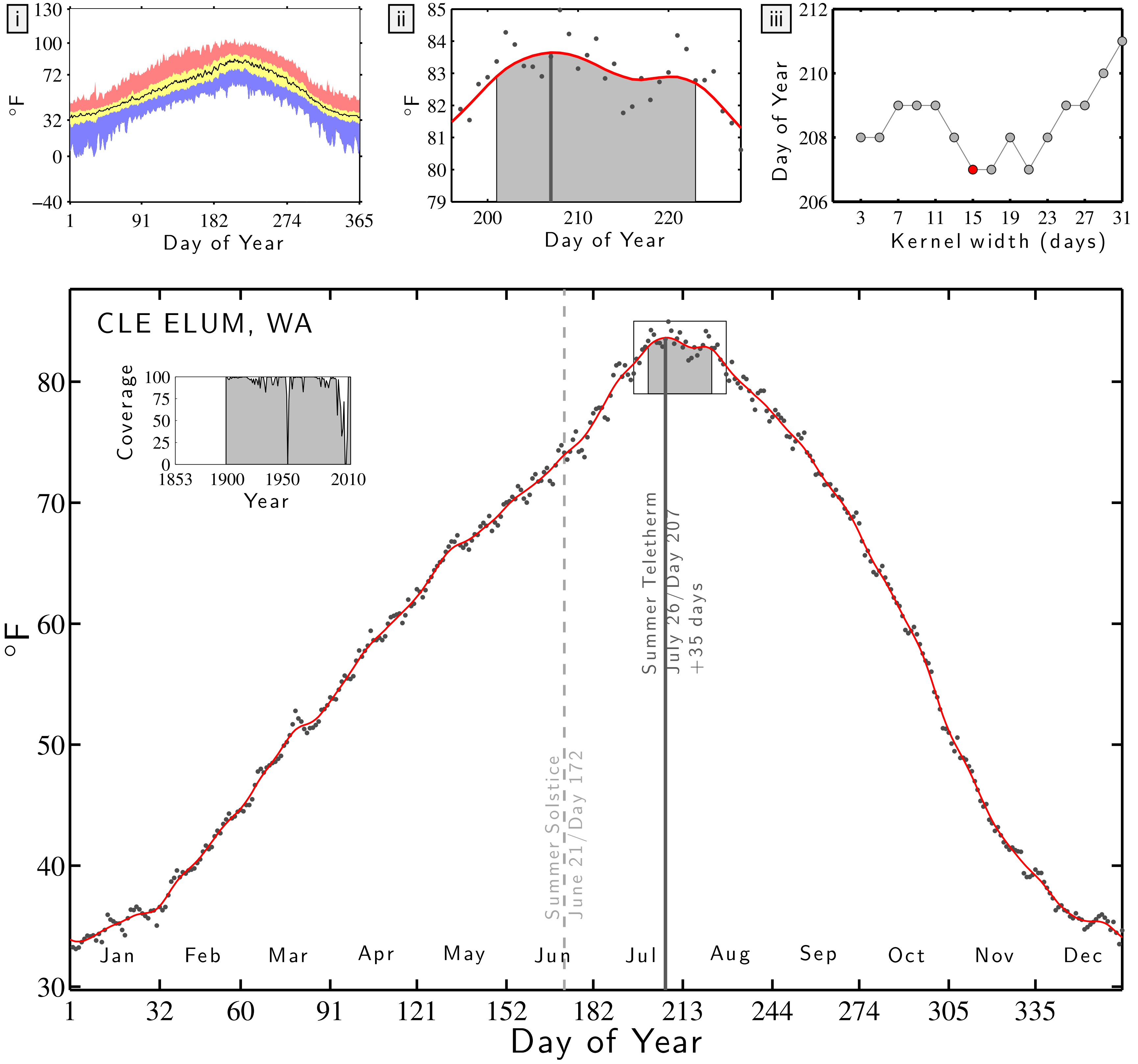

i
ii
iii
°F
Day of Year
Day of Year
Kernel width (days)
CLE ELUM, WA
Coverage
Year
Summer Solstice
June 21 / Day 172
Summer Teletherm
July 26 / Day 207
+35 days
Jan
Feb
Mar
Apr
May
Jun
Jul
Aug
Sep
Oct
Nov
Dec

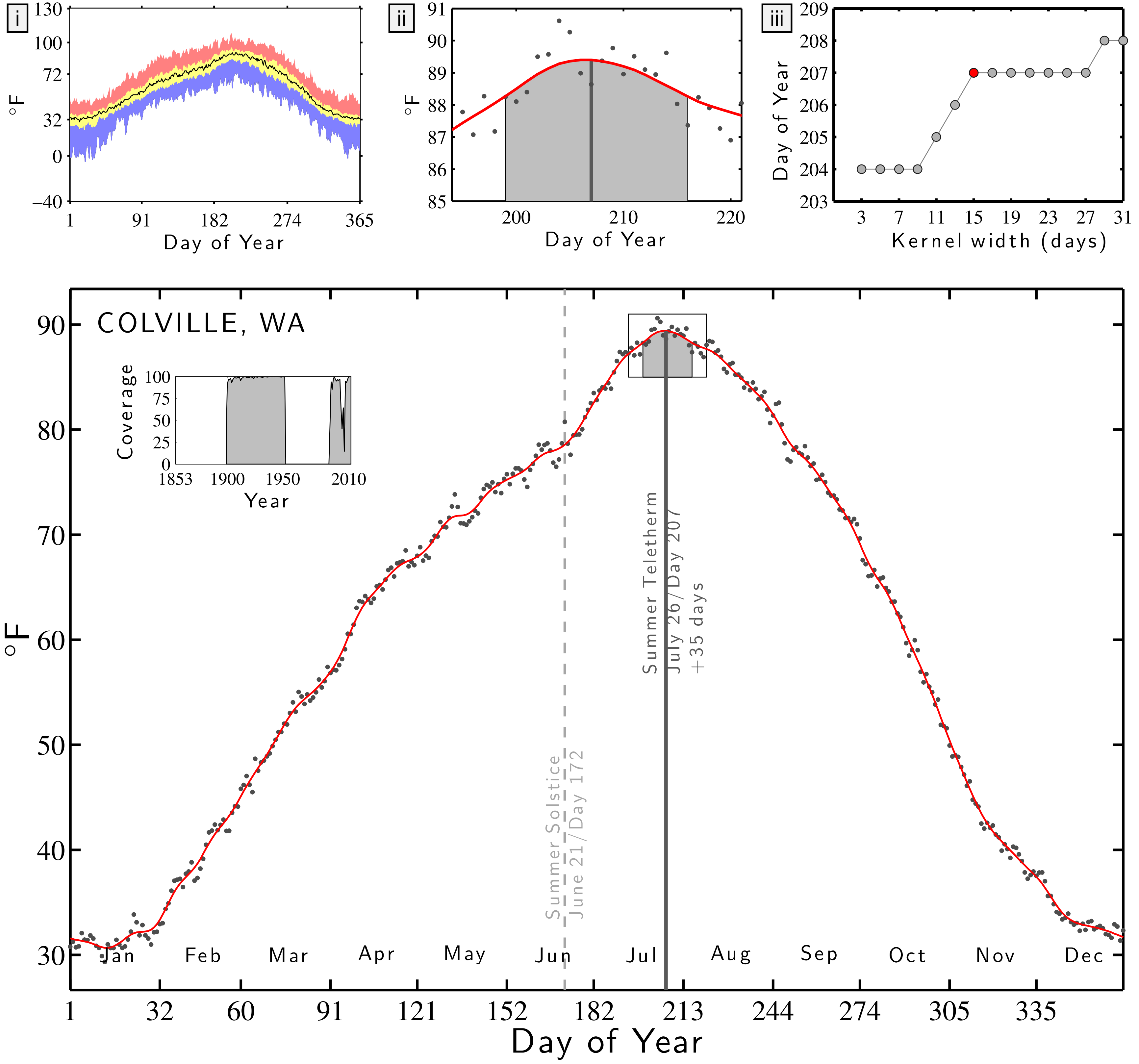

i
°F
Day of Year
ii
°F
Day of Year
iii
Day of Year
Kernel width (days)
COLVILLE, WA
Coverage
Year
Summer Solstice
June 21 / Day 172
Summer Teletherm
July 26 / Day 207
+35 days
Jan
Feb
Mar
Apr
May
Jun
Jul
Aug
Sep
Oct
Nov
Dec
°F
Day of Year

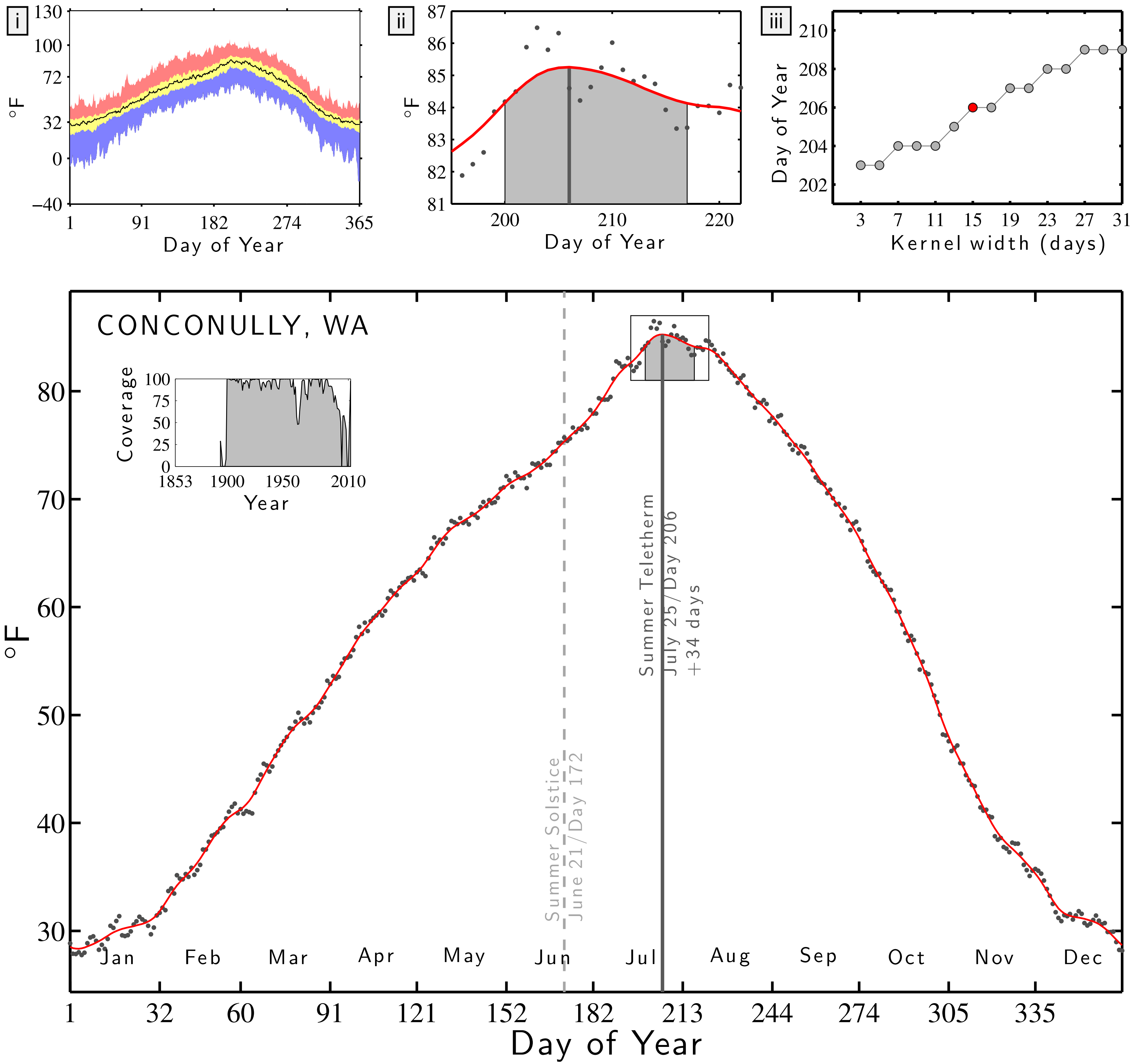

i
130
100
72
32
0
−40
°F
1
91
182
274
365
Day of Year
ii
87
86
85
84
83
82
81
°F
200
210
220
Day of Year
iii
210
208
206
204
202
Day of Year
3
7
11
15
19
23
27
31
Kernel width (days)
CONCONULLY, WA
Coverage
100
75
50
25
0
1853
1900
1950
2010
Year
80
70
60
50
40
30
°F
Summer Solstice
June 21/Day 172
Summer Teletherm
July 25/Day 206
+34 days
Jan
Feb
Mar
Apr
May
Jun
Jul
Aug
Sep
Oct
Nov
Dec
1
32
60
91
121
152
182
213
244
274
305
335
Day of Year

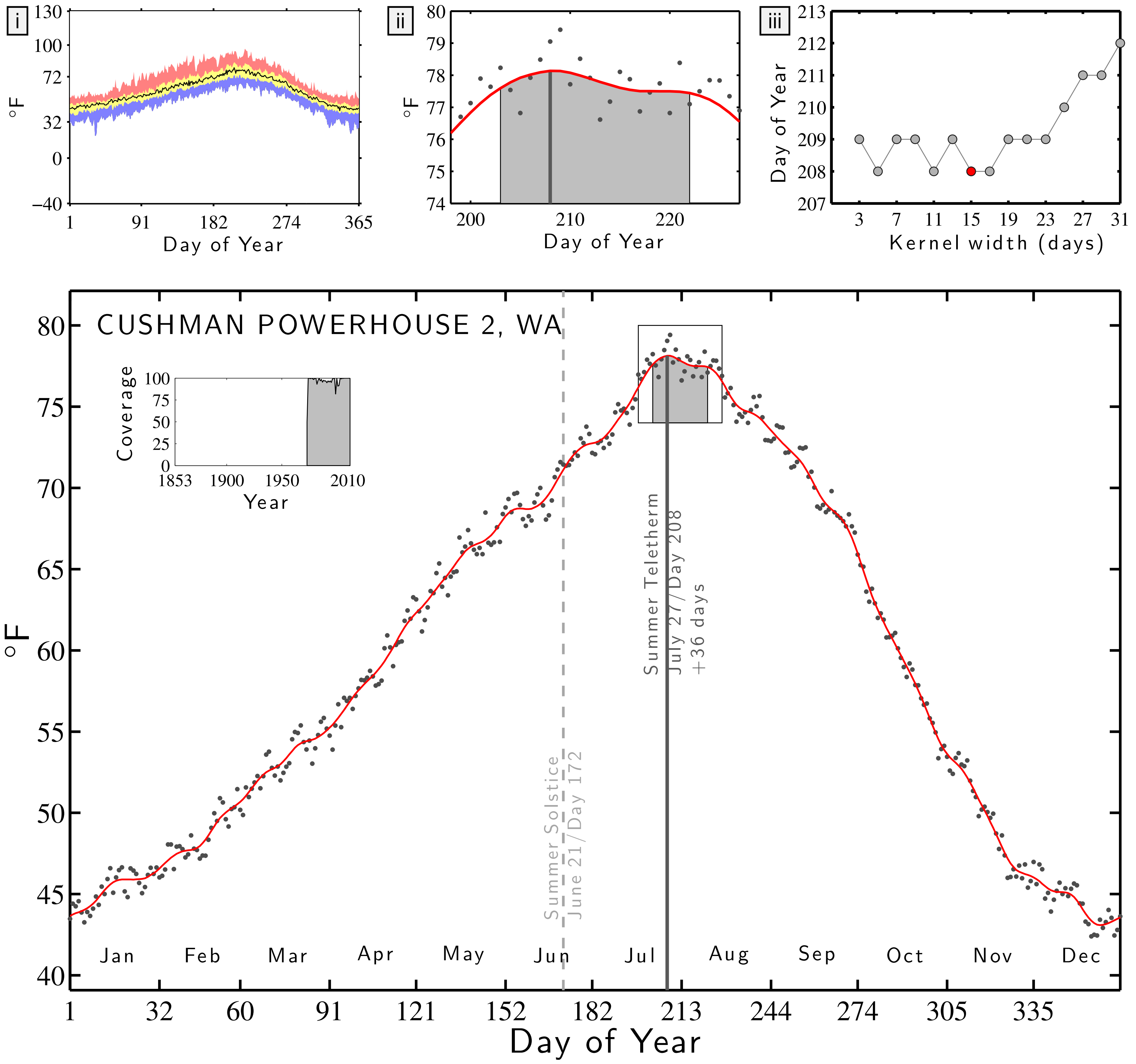
i
ii
iii
Day of Year
Kernel width (days)
CUSHMAN POWERHOUSE 2, WA
Coverage
Year
Summer Solstice
June 21 / Day 172
Summer Teletherm
July 27 / Day 208
+36 days
Jan
Feb
Mar
Apr
May
Jun
Jul
Aug
Sep
Oct
Nov
Dec
°F
Day of Year

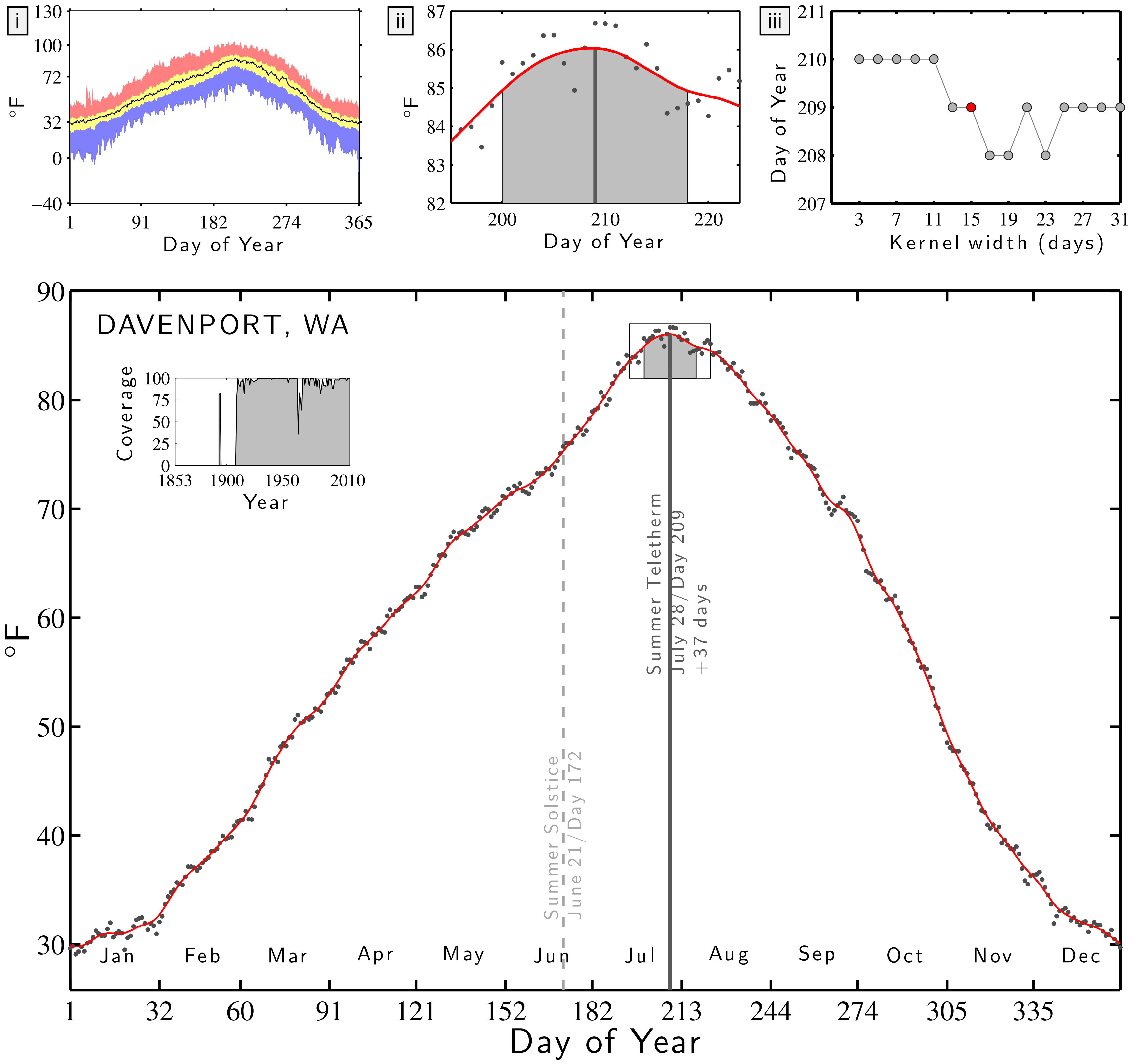

i
°F
Day of Year
ii
°F
Day of Year
iii
Day of Year
Kernel width (days)
DAVENPORT, WA
Coverage
Year
Summer Solstice
June 21/Day 172
Summer Teletherm
July 28/Day 209
+37 days
Jan
Feb
Mar
Apr
May
Jun
Jul
Aug
Sep
Oct
Nov
Dec
°F
Day of Year

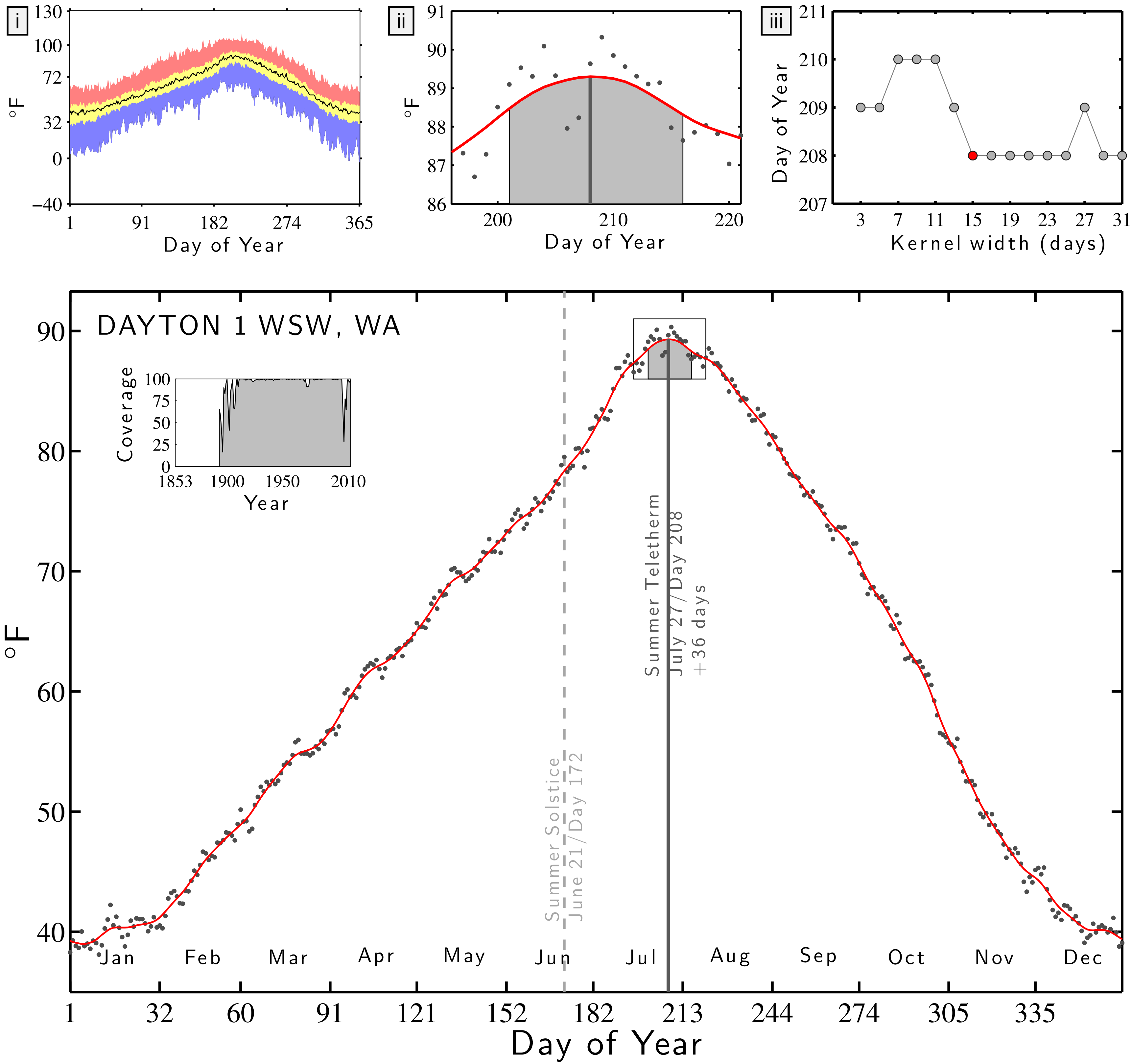

i
°F
Day of Year
ii
°F
Day of Year
iii
Day of Year
Kernel width (days)
DAYTON 1 WSW, WA
Coverage
Year
Summer Solstice
June 21/Day 172
Summer Teletherm
July 27/Day 208
+36 days
Jan
Feb
Mar
Apr
May
Jun
Jul
Aug
Sep
Oct
Nov
Dec
°F
Day of Year

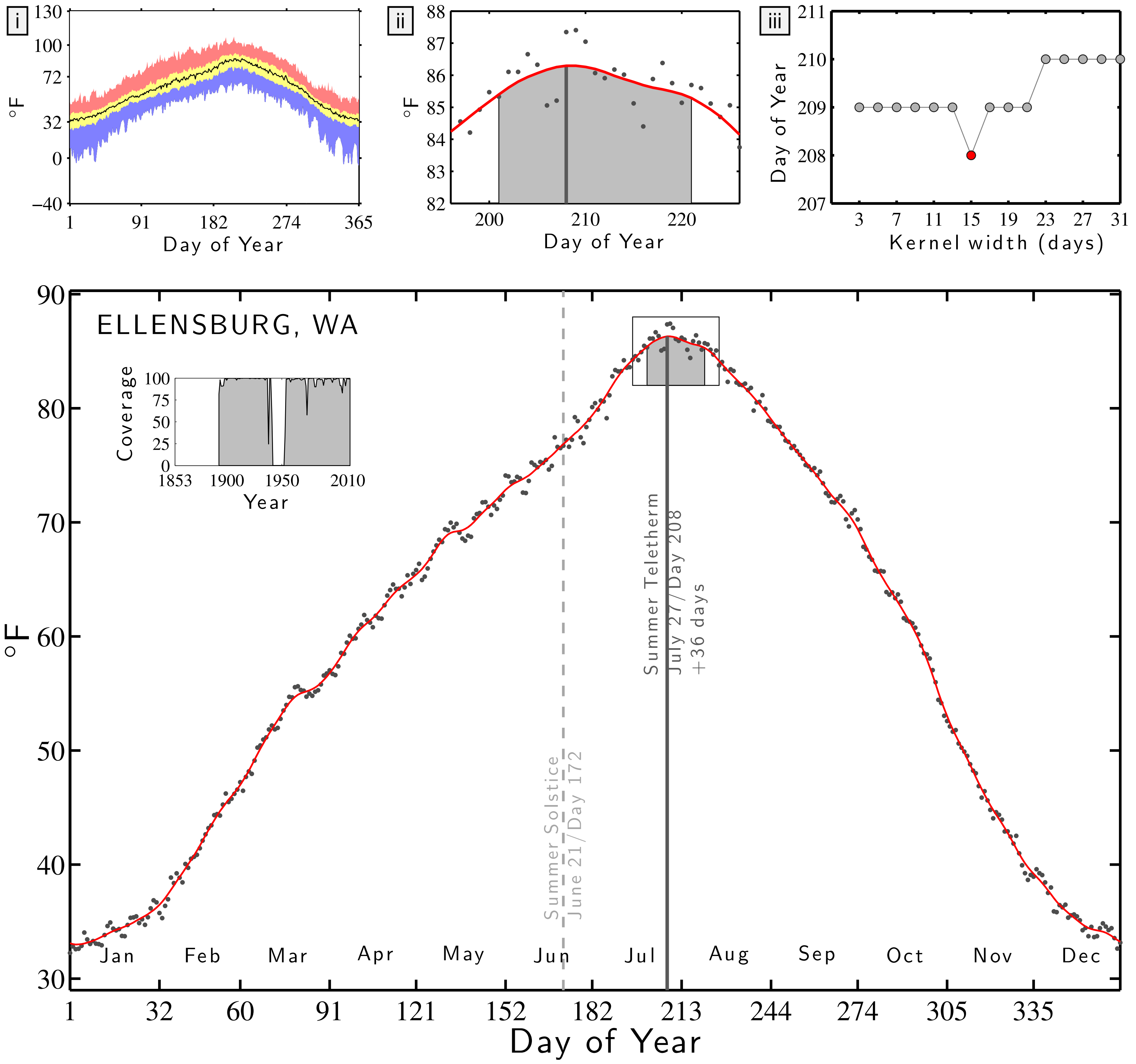

i
ii
iii
°F
Day of Year
Kernel width (days)
ELLENSBURG, WA
Coverage
Year
Summer Solstice
June 21 / Day 172
Summer Teletherm
July 27 / Day 208
+36 days
Jan
Feb
Mar
Apr
May
Jun
Jul
Aug
Sep
Oct
Nov
Dec

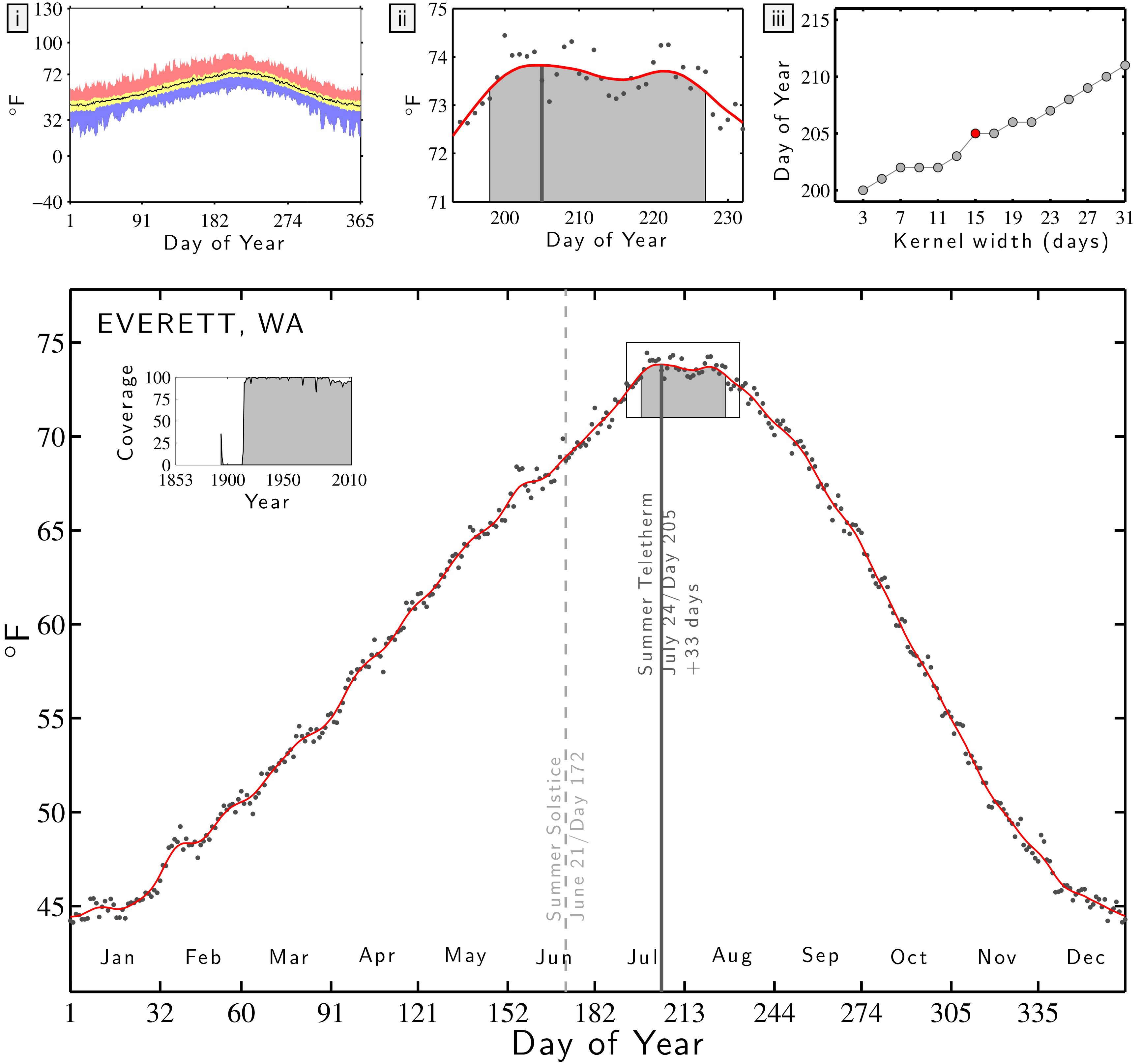

i
°F
Day of Year
ii
°F
Day of Year
iii
Day of Year
Kernel width (days)
EVERETT, WA
Coverage
Year
Summer Solstice
June 21/Day 172
Summer Teletherm
July 24/Day 205
+33 days
Jan
Feb
Mar
Apr
May
Jun
Jul
Aug
Sep
Oct
Nov
Dec
°F
Day of Year

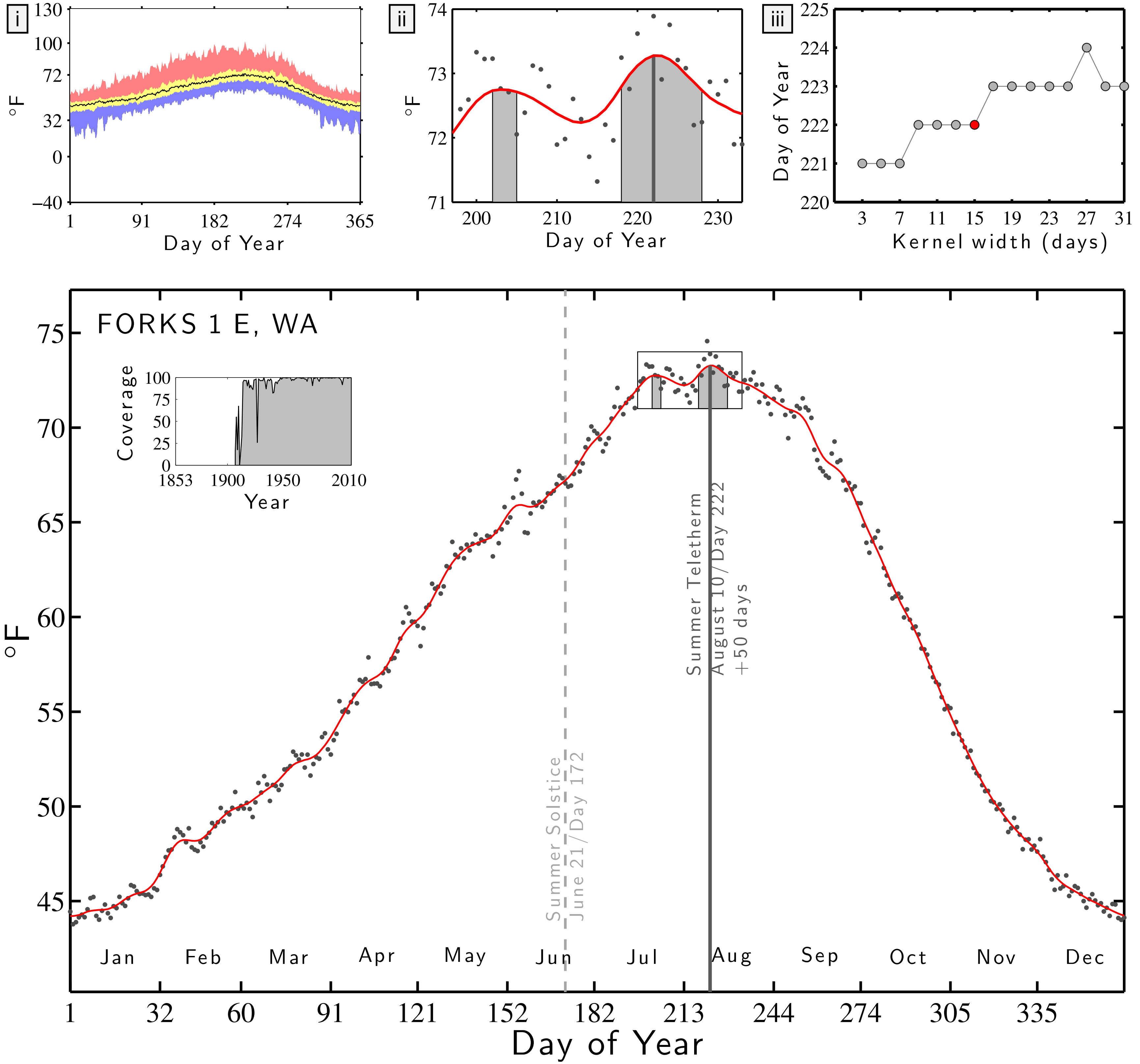

i
°F
Day of Year
ii
°F
Day of Year
iii
Day of Year
Kernel width (days)
FORKS 1 E, WA
Coverage
Year
Summer Solstice
June 21 / Day 172
Summer Teletherm
August 10 / Day 222
+50 days
Jan
Feb
Mar
Apr
May
Jun
Jul
Aug
Sep
Oct
Nov
Dec
°F
Day of Year

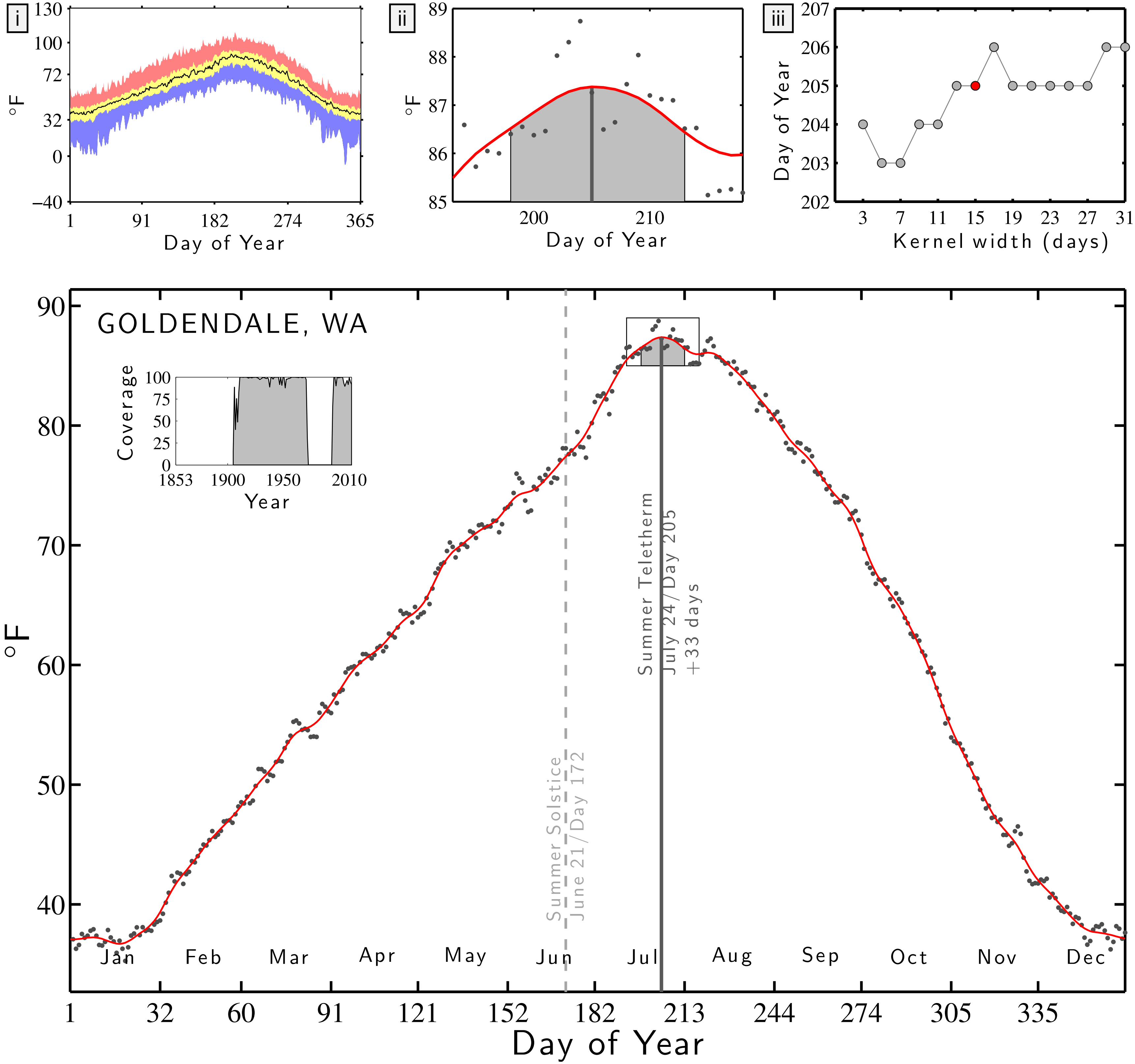

i
ii
iii
°F
Day of Year
Day of Year
Kernel width (days)
GOLDENDALE, WA
Coverage
Year
Summer Solstice
June 21 / Day 172
Summer Teletherm
July 24 / Day 205
+33 days
Jan
Feb
Mar
Apr
May
Jun
Jul
Aug
Sep
Oct
Nov
Dec
°F
Day of Year

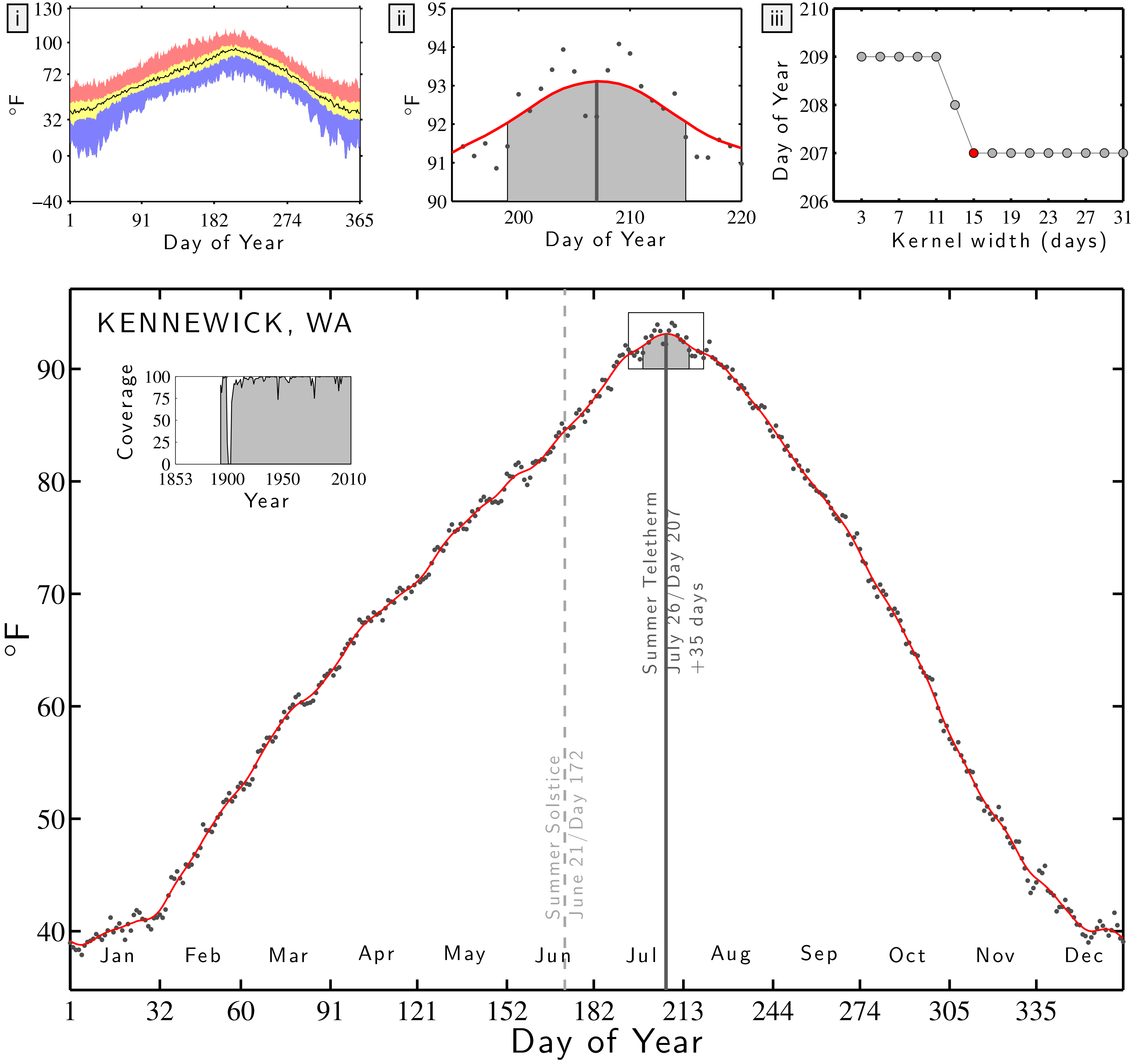

i
°F
Day of Year
ii
°F
Day of Year
iii
Day of Year
Kernel width (days)
KENNEWICK, WA
Coverage
Year
Summer Solstice
June 21 / Day 172
Summer Teletherm
July 26 / Day 207
+35 days
Jan
Feb
Mar
Apr
May
Jun
Jul
Aug
Sep
Oct
Nov
Dec
°F
Day of Year

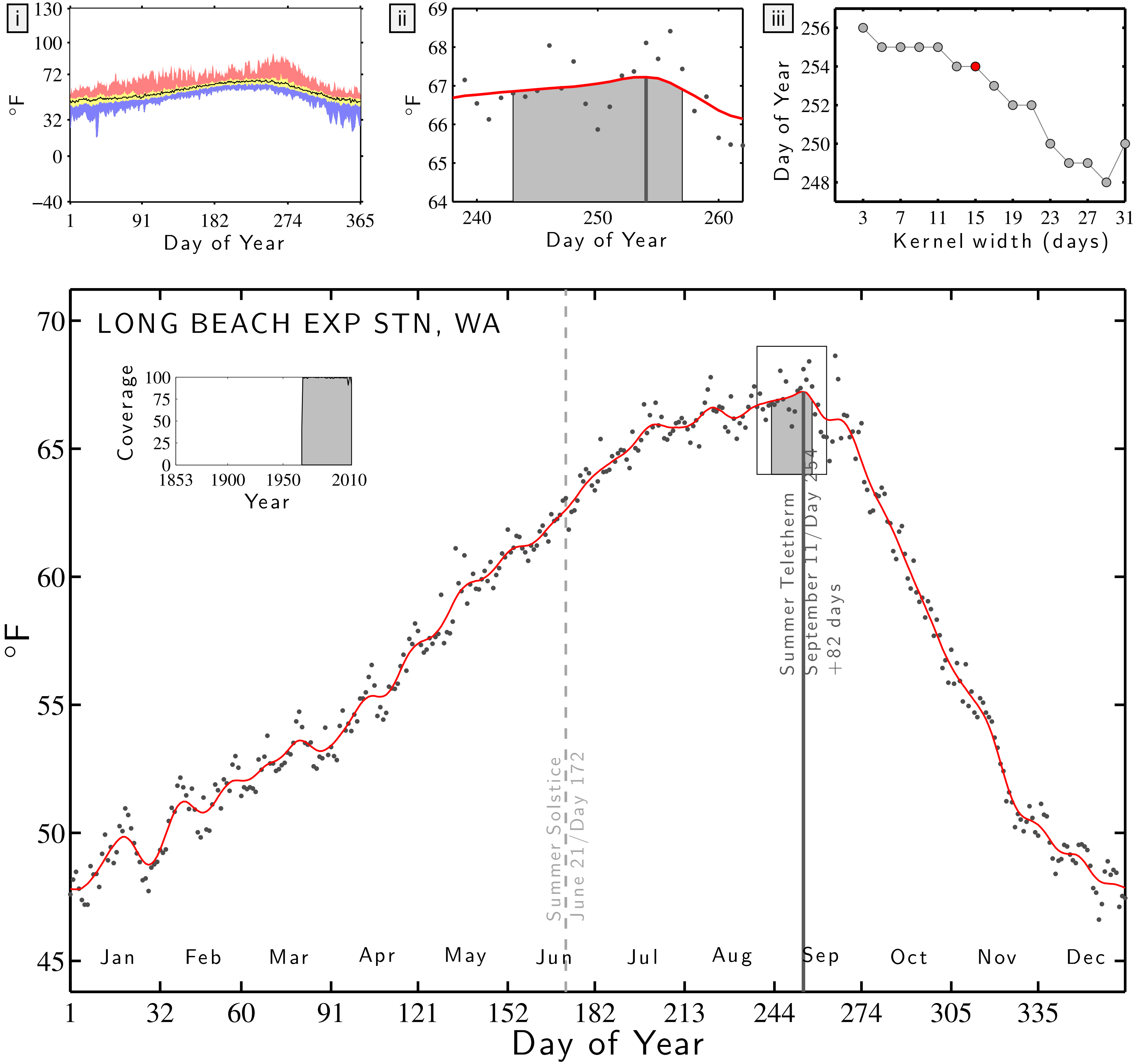

i
°F
Day of Year
ii
°F
Day of Year
iii
Day of Year
Kernel width (days)
LONG BEACH EXP STN, WA
Coverage
Year
Summer Solstice
June 21 / Day 172
Summer Teletherm
September 11 / Day 254
+82 days
Jan
Feb
Mar
Apr
May
Jun
Jul
Aug
Sep
Oct
Nov
Dec
°F
Day of Year

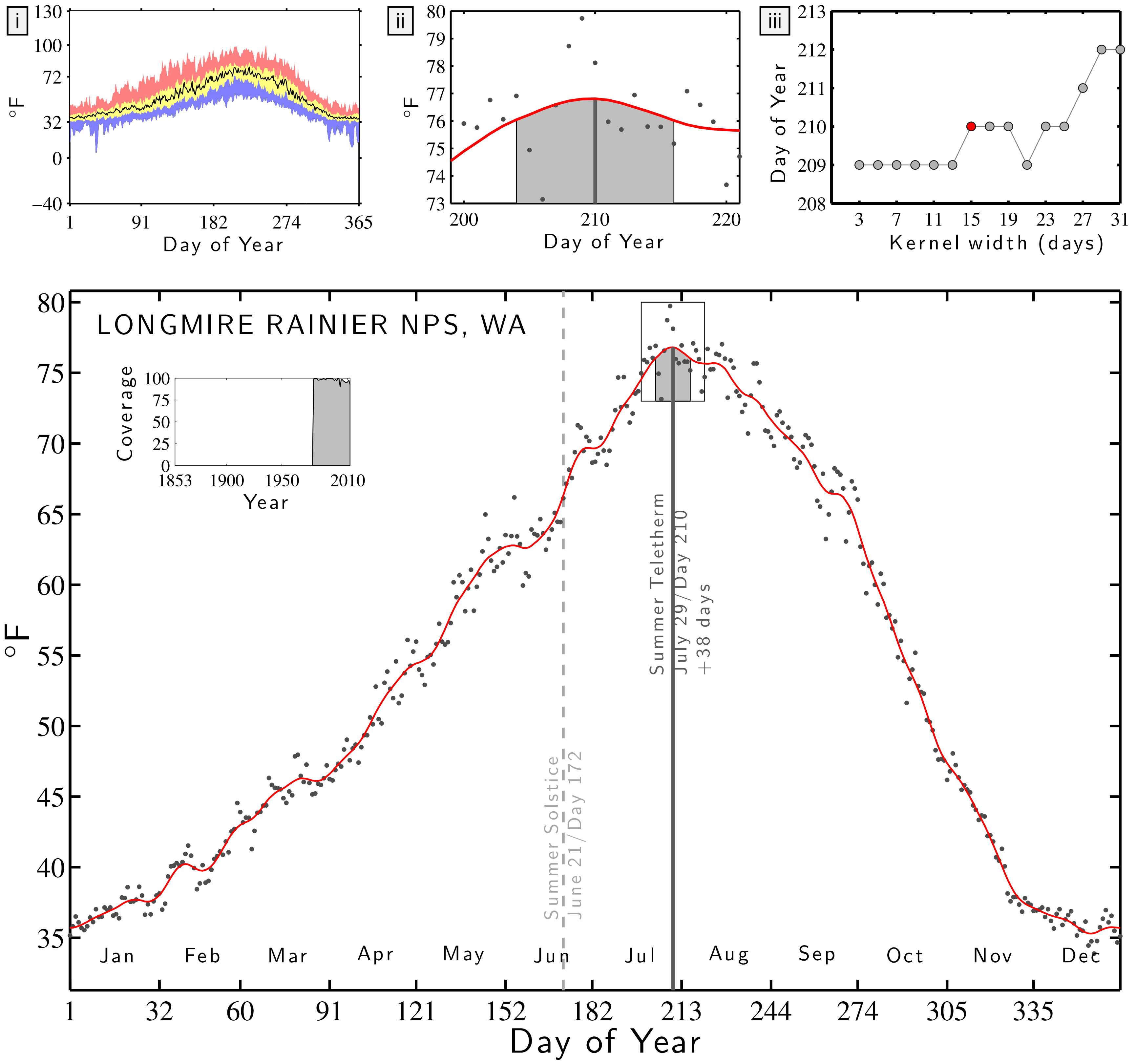

i
°F
Day of Year
ii
°F
Day of Year
iii
Day of Year
Kernel width (days)
LONGMIRE RAINIER NPS, WA
Coverage
Year
Summer Solstice
June 21 / Day 172
Summer Teletherm
July 29 / Day 210
+38 days
Jan
Feb
Mar
Apr
May
Jun
Jul
Aug
Sep
Oct
Nov
Dec
°F
Day of Year

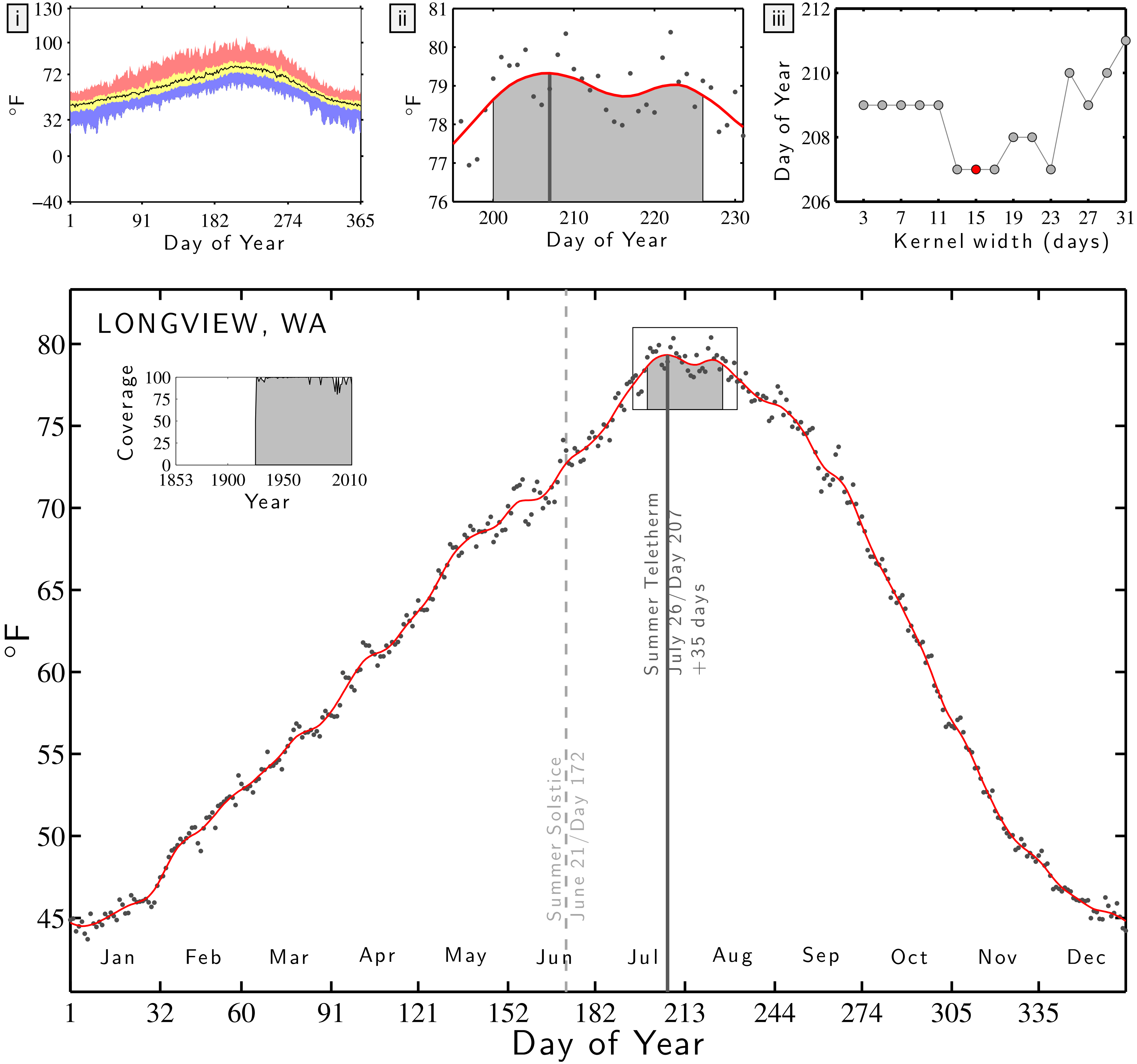

i
°F
Day of Year
ii
°F
Day of Year
iii
Day of Year
Kernel width (days)
LONGVIEW, WA
Coverage
Year
Summer Solstice
June 21 / Day 172
Summer Teletherm
July 26 / Day 207
+35 days
Jan
Feb
Mar
Apr
May
Jun
Jul
Aug
Sep
Oct
Nov
Dec
°F
Day of Year

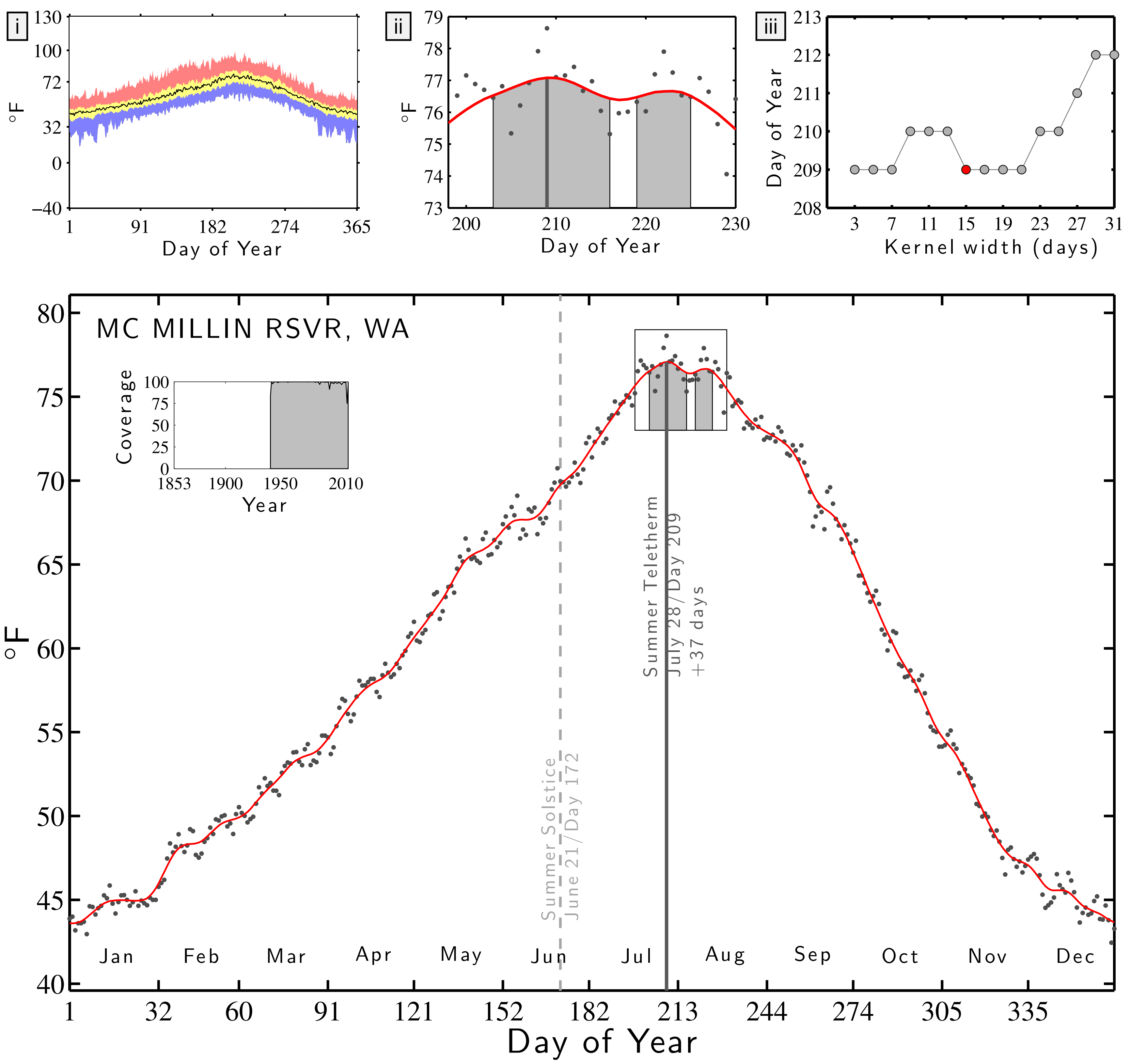

i
°F
Day of Year
ii
°F
Day of Year
iii
Day of Year
Kernel width (days)
MC MILLIN RSVR, WA
Coverage
Year
Summer Solstice
June 21/Day 172
Summer Teletherm
July 28/Day 209
+37 days
Jan
Feb
Mar
Apr
May
Jun
Jul
Aug
Sep
Oct
Nov
Dec
°F
Day of Year

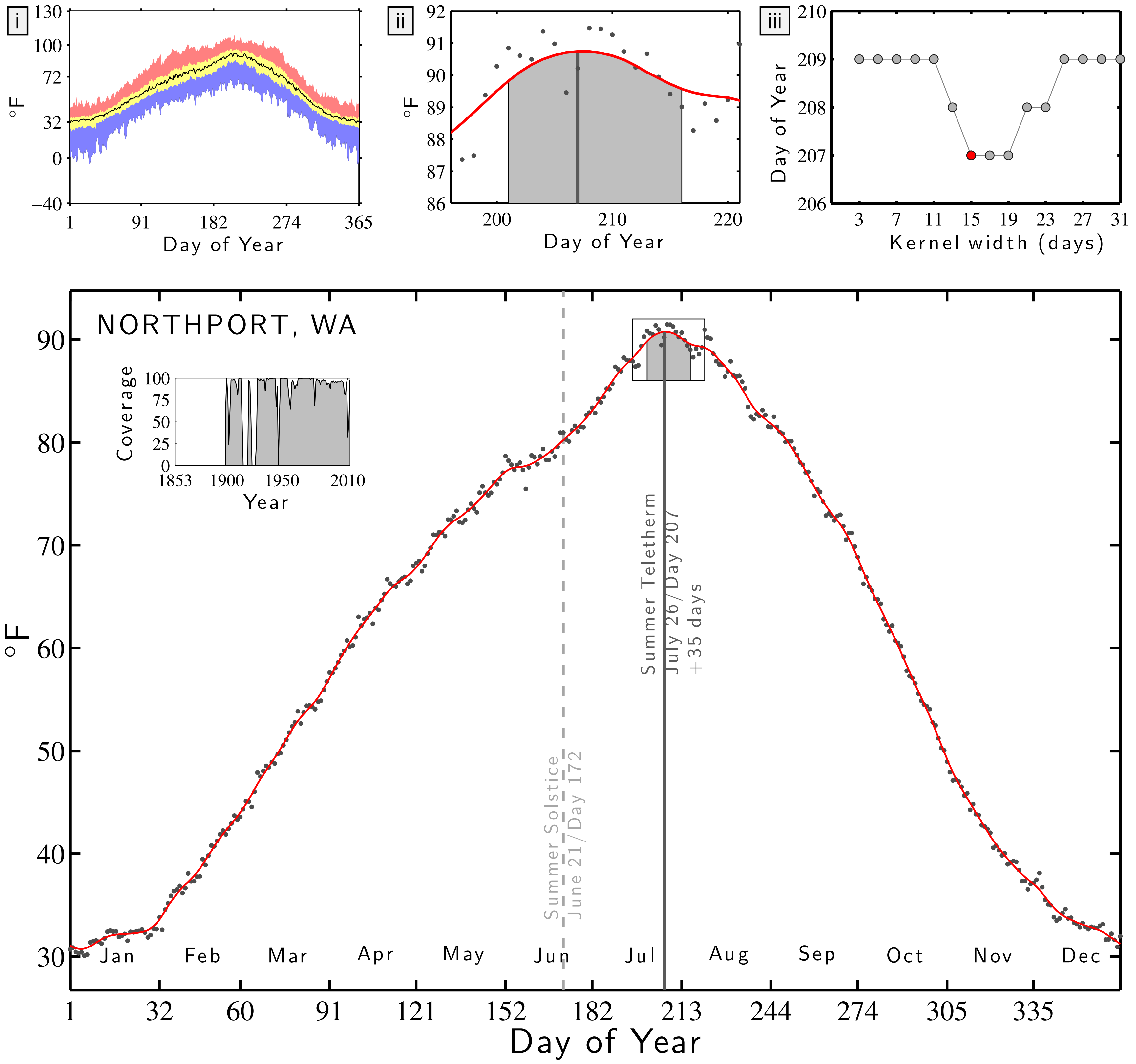

i
ii
iii
°F
Day of Year
Kernel width (days)
NORTHPORT, WA
Coverage
Year
Summer Solstice
June 21 / Day 172
Summer Teletherm
July 26 / Day 207
+35 days
Jan
Feb
Mar
Apr
May
Jun
Jul
Aug
Sep
Oct
Nov
Dec

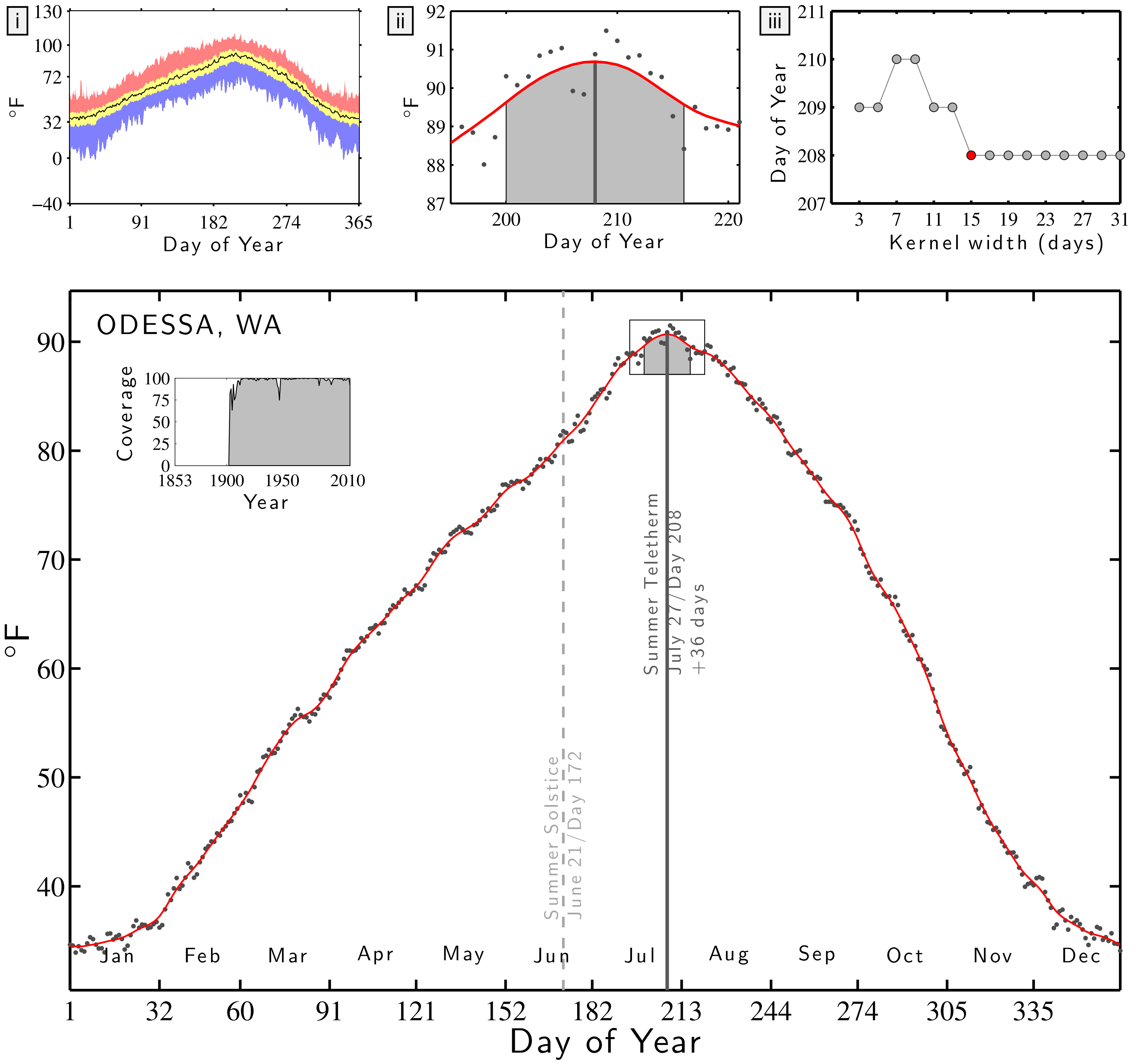

i
130
100
72
32
0
−40
°F
1
91
182
274
365
Day of Year
ii
92
91
90
89
88
87
°F
200
210
220
Day of Year
iii
211
210
209
208
207
Day of Year
3
7
11
15
19
23
27
31
Kernel width (days)
ODESSA, WA
Coverage
100
75
50
25
0
1853
1900
1950
2010
Year
Summer Solstice
June 21/Day 172
Summer Teletherm
July 27/Day 208
+36 days
90
80
70
60
50
40
°F
Jan
Feb
Mar
Apr
May
Jun
Jul
Aug
Sep
Oct
Nov
Dec
1
32
60
91
121
152
182
213
244
274
305
335
Day of Year

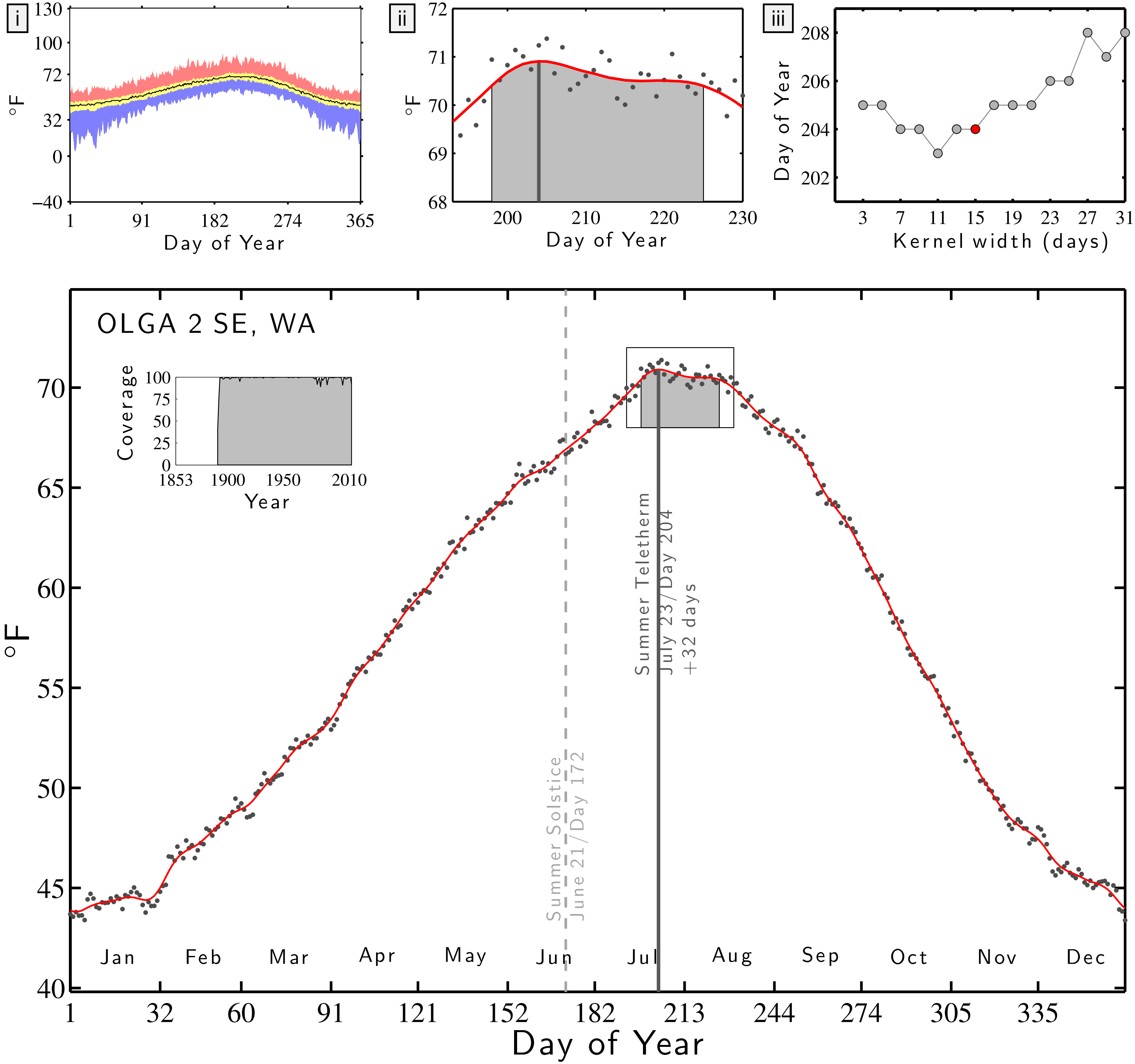

i
°F
Day of Year
ii
°F
Day of Year
iii
Day of Year
Kernel width (days)
OLGA 2 SE, WA
Coverage
Year
Summer Solstice
June 21 / Day 172
Summer Teletherm
July 23 / Day 204
+32 days
Jan
Feb
Mar
Apr
May
Jun
Jul
Aug
Sep
Oct
Nov
Dec
°F
Day of Year

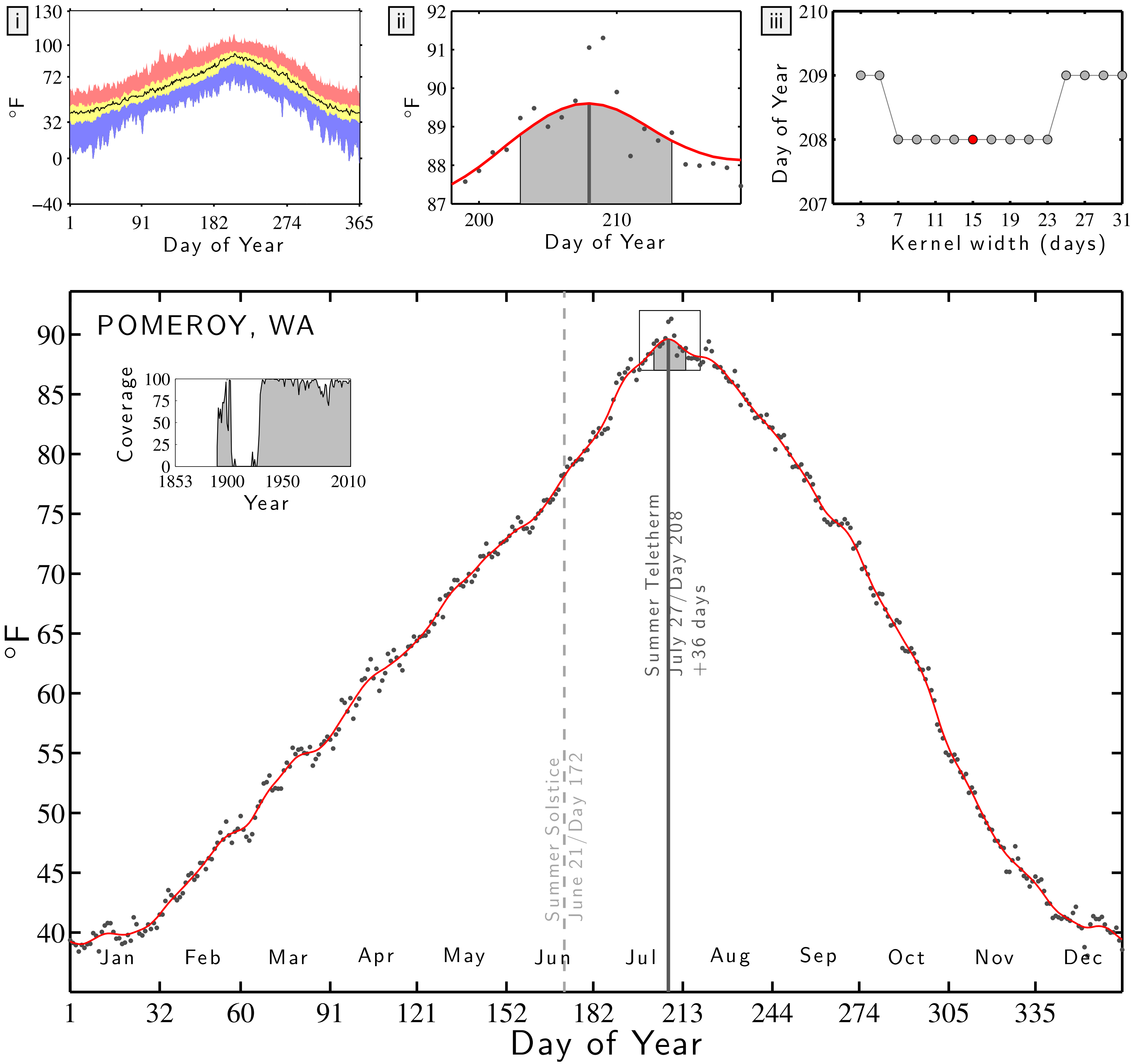

i
°F
Day of Year
ii
Day of Year
iii
Day of Year
Kernel width (days)
POMEROY, WA
Coverage
Year
Summer Solstice
June 21 / Day 172
Summer Teletherm
July 27 / Day 208
+36 days
Jan
Feb
Mar
Apr
May
Jun
Jul
Aug
Sep
Oct
Nov
Dec
°F
Day of Year

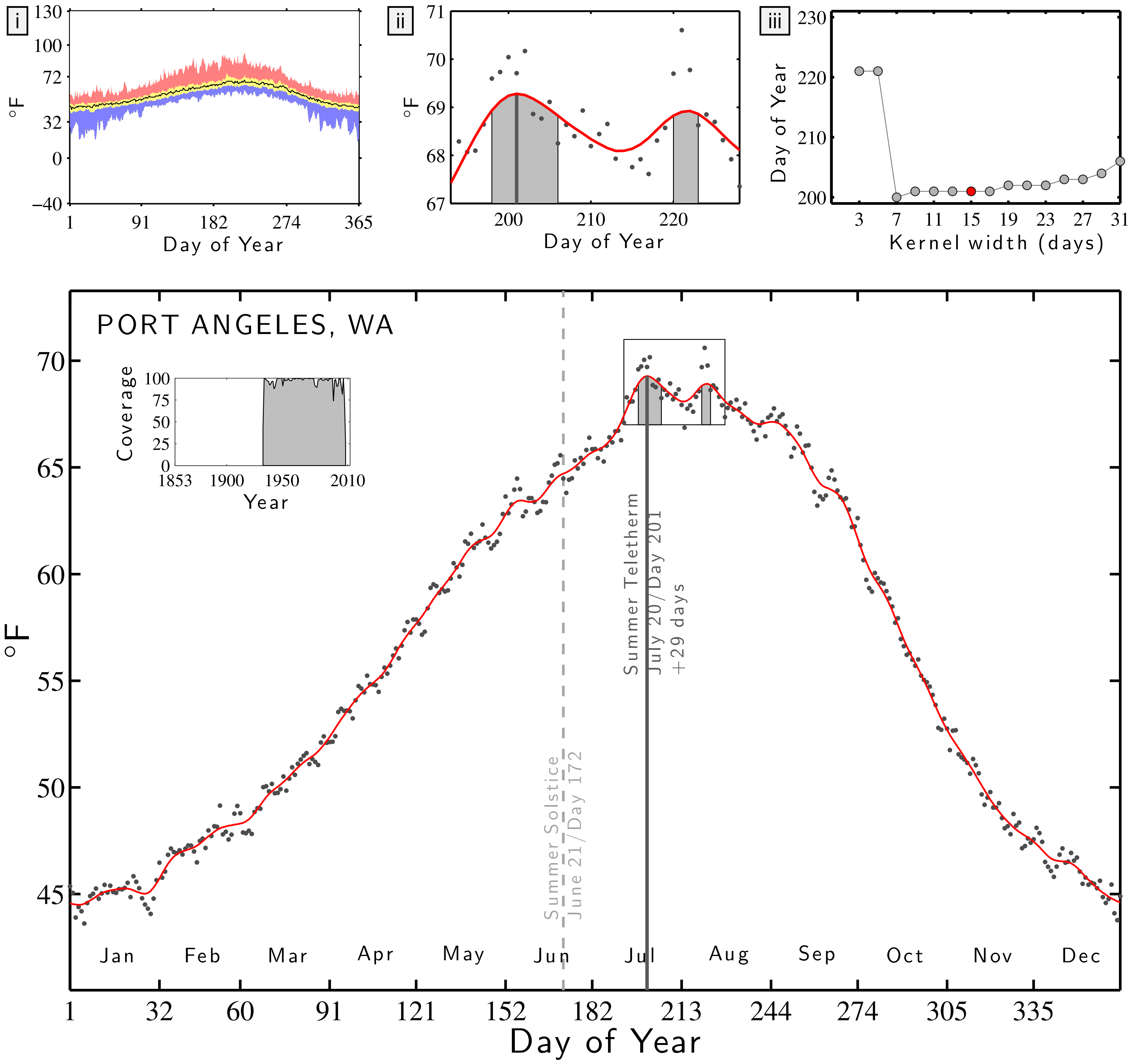

i
°F
Day of Year
ii
°F
Day of Year
iii
Day of Year
Kernel width (days)
PORT ANGELES, WA
Coverage
Year
Summer Solstice
June 21 / Day 172
Summer Teletherm
July 20 / Day 201
+29 days
Jan
Feb
Mar
Apr
May
Jun
Jul
Aug
Sep
Oct
Nov
Dec
°F
Day of Year

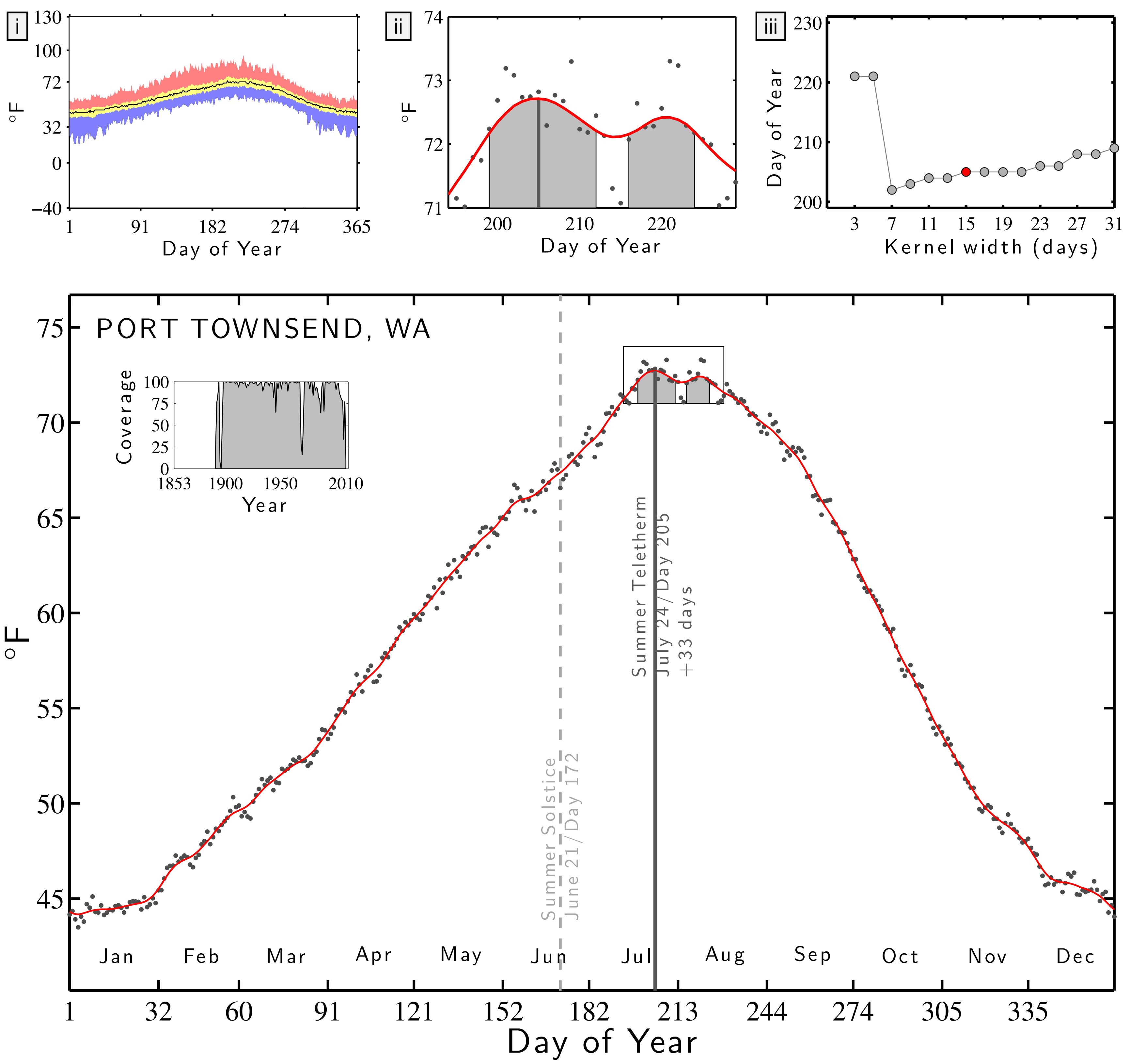

i
ii
iii
°F
Day of Year
Kernel width (days)
PORT TOWNSEND, WA
Coverage
Year
Summer Solstice
June 21 / Day 172
Summer Teletherm
July 24 / Day 205
+33 days
Jan
Feb
Mar
Apr
May
Jun
Jul
Aug
Sep
Oct
Nov
Dec

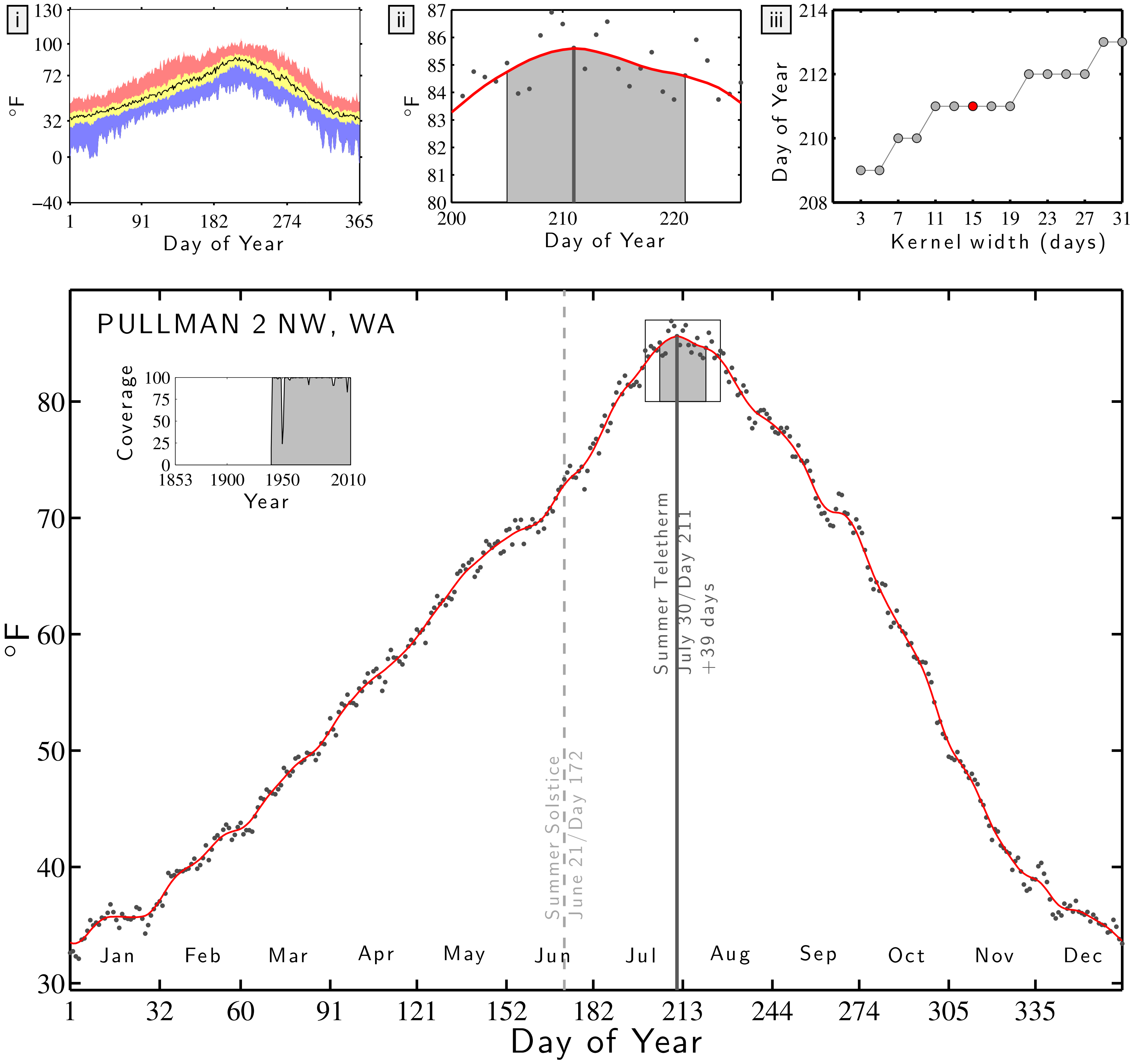

i
°F
Day of Year
ii
°F
Day of Year
iii
Day of Year
Kernel width (days)
PULLMAN 2 NW, WA
Coverage
Year
Summer Solstice
June 21/Day 172
Summer Teletherm
July 30/Day 211
+39 days
°F
Jan
Feb
Mar
Apr
May
Jun
Jul
Aug
Sep
Oct
Nov
Dec
Day of Year

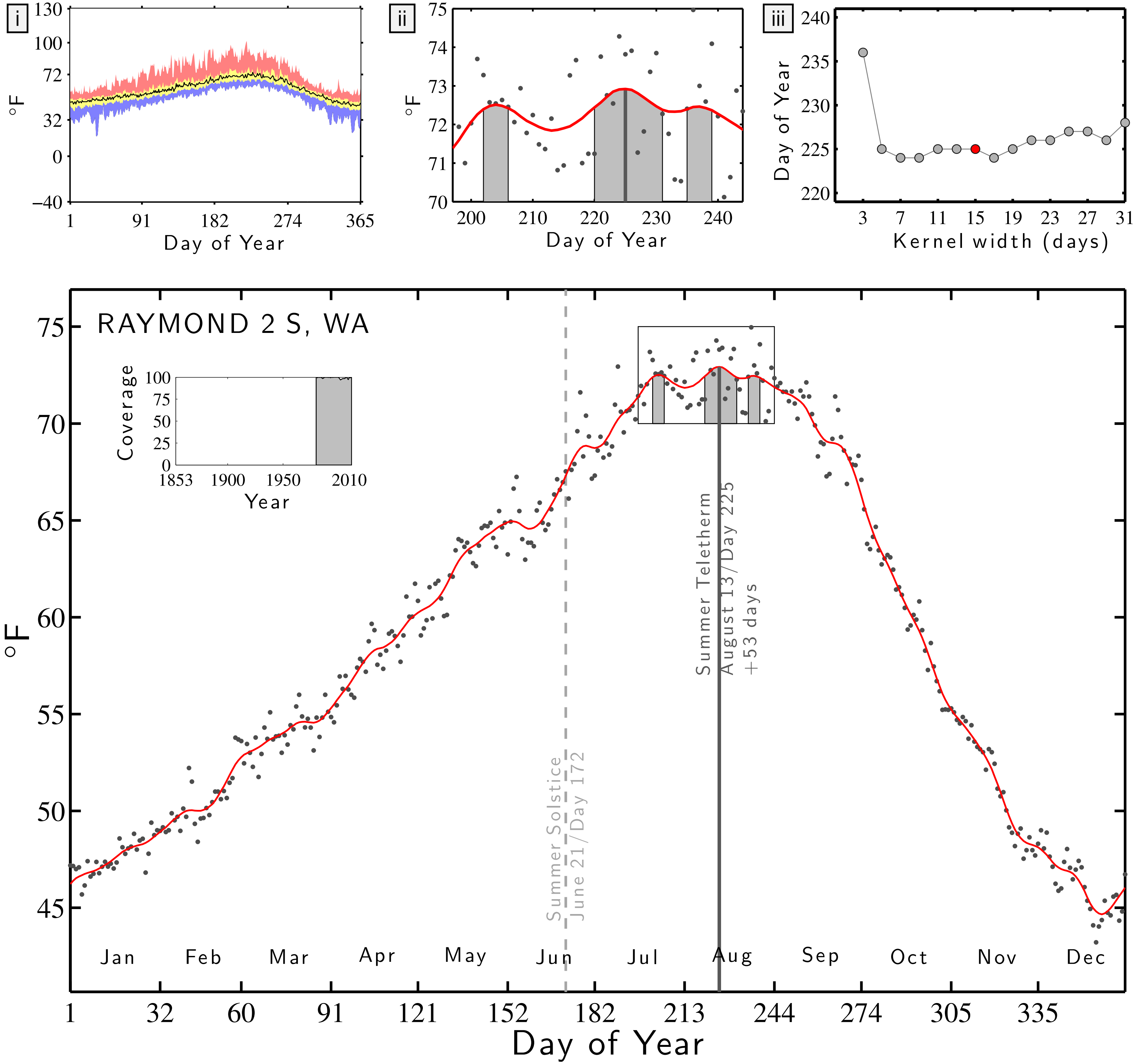

i
°F
130
100
72
32
0
−40
1
91
182
274
365
Day of Year
ii
°F
75
74
73
72
71
70
200
210
220
230
240
Day of Year
iii
Day of Year
240
235
230
225
220
3
7
11
15
19
23
27
31
Kernel width (days)
RAYMOND 2 S, WA
Coverage
100
75
50
25
0
1853
1900
1950
2010
Year
Summer Solstice
June 21/Day 172
Summer Teletherm
August 13/Day 225
+53 days
°F
75
70
65
60
55
50
45
Jan
Feb
Mar
Apr
May
Jun
Jul
Aug
Sep
Oct
Nov
Dec
1
32
60
91
121
152
182
213
244
274
305
335
Day of Year

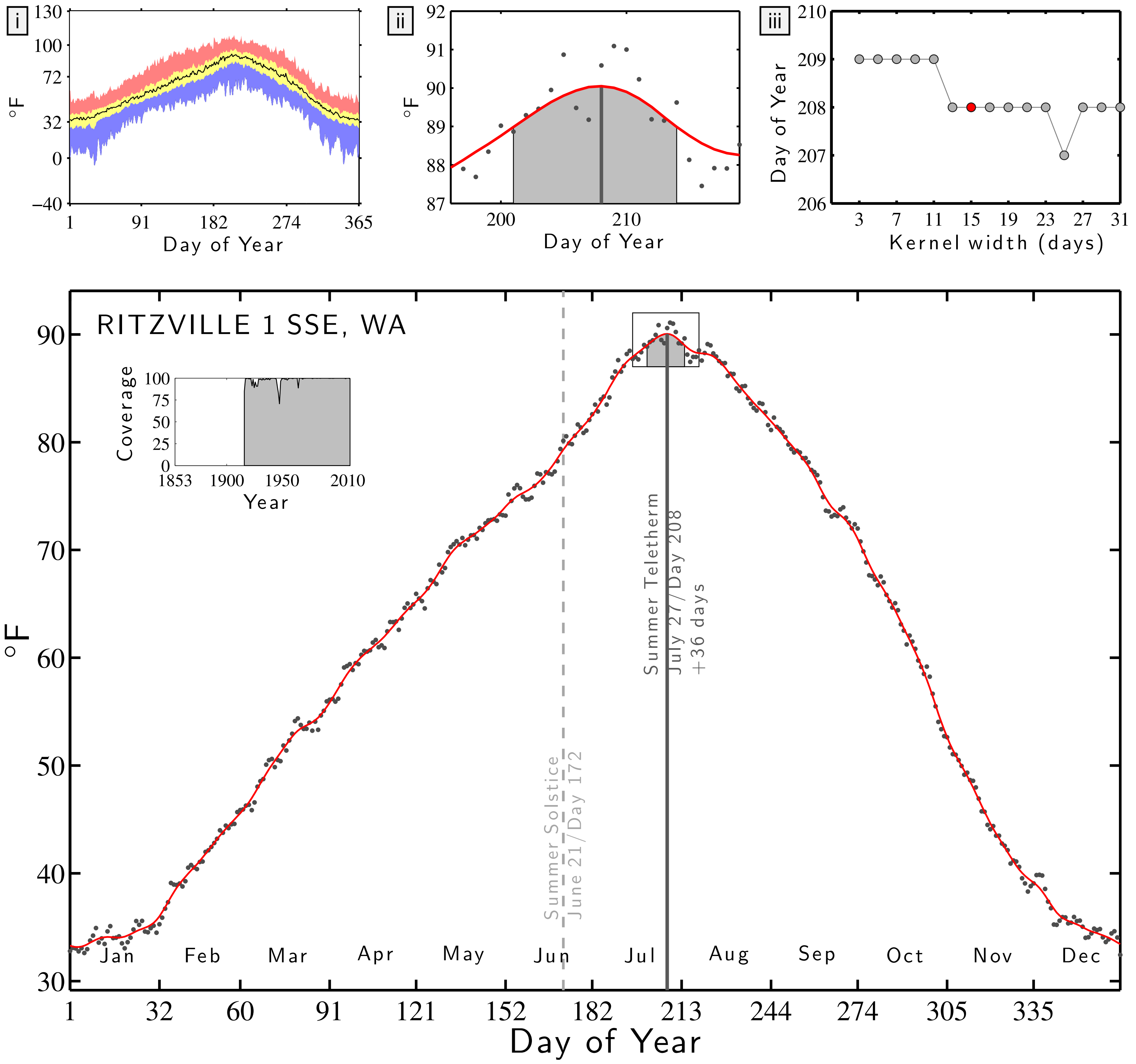

i
°F
Day of Year
ii
°F
Day of Year
iii
Day of Year
Kernel width (days)
RITZVILLE 1 SSE, WA
Coverage
Year
Summer Solstice
June 21 / Day 172
Summer Teletherm
July 27 / Day 208
+36 days
Jan
Feb
Mar
Apr
May
Jun
Jul
Aug
Sep
Oct
Nov
Dec
°F
Day of Year

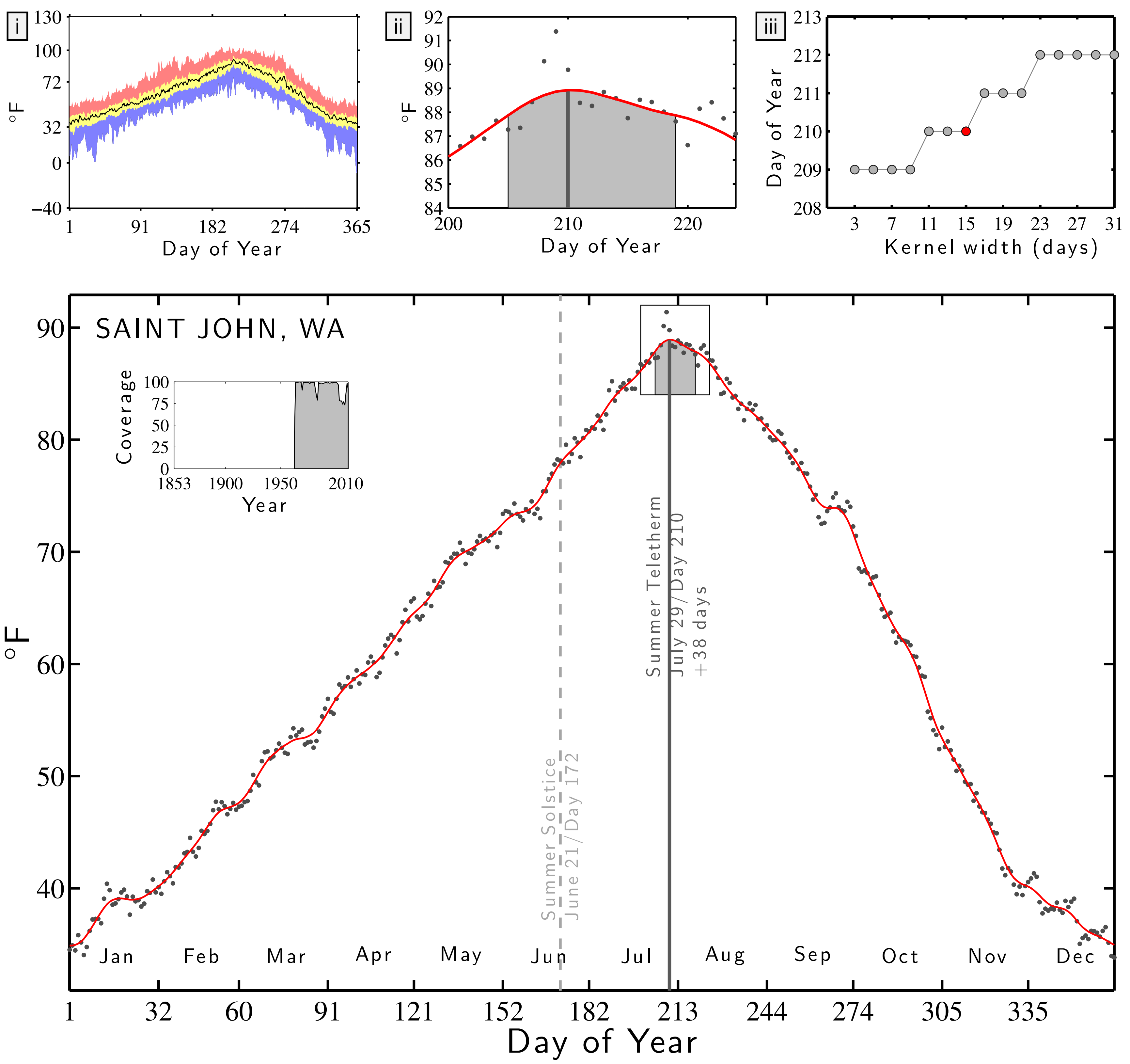

i
°F
Day of Year
ii
°F
Day of Year
iii
Day of Year
Kernel width (days)
SAINT JOHN, WA
Coverage
Year
Summer Solstice
June 21 / Day 172
Summer Teletherm
July 29 / Day 210
+38 days
Jan
Feb
Mar
Apr
May
Jun
Jul
Aug
Sep
Oct
Nov
Dec
°F
Day of Year

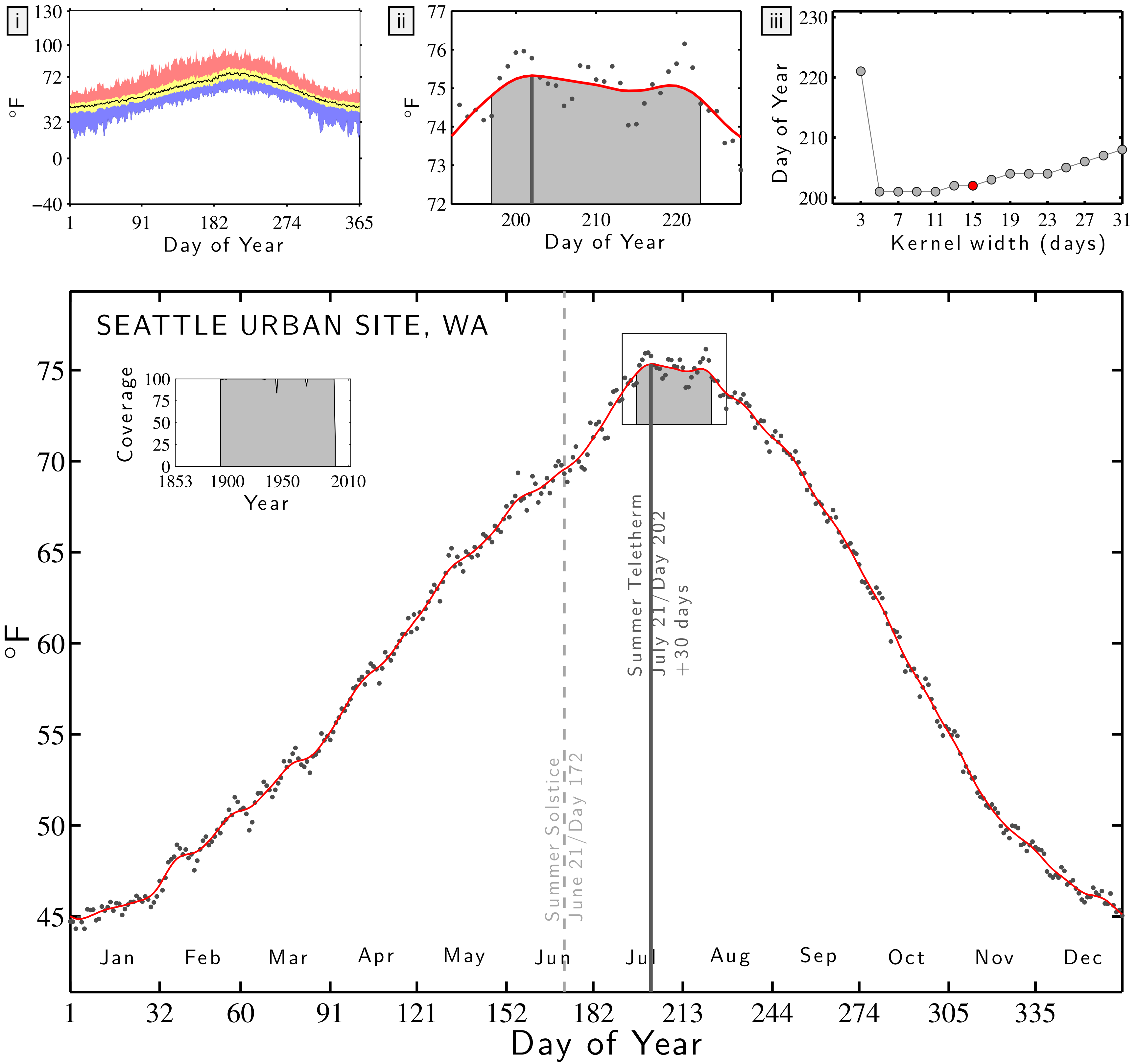

i
°F
Day of Year
ii
°F
Day of Year
iii
Day of Year
Kernel width (days)
SEATTLE URBAN SITE, WA
Coverage
Year
Summer Solstice
June 21 / Day 172
Summer Teletherm
July 21 / Day 202
+30 days
Jan
Feb
Mar
Apr
May
Jun
Jul
Aug
Sep
Oct
Nov
Dec
°F
Day of Year

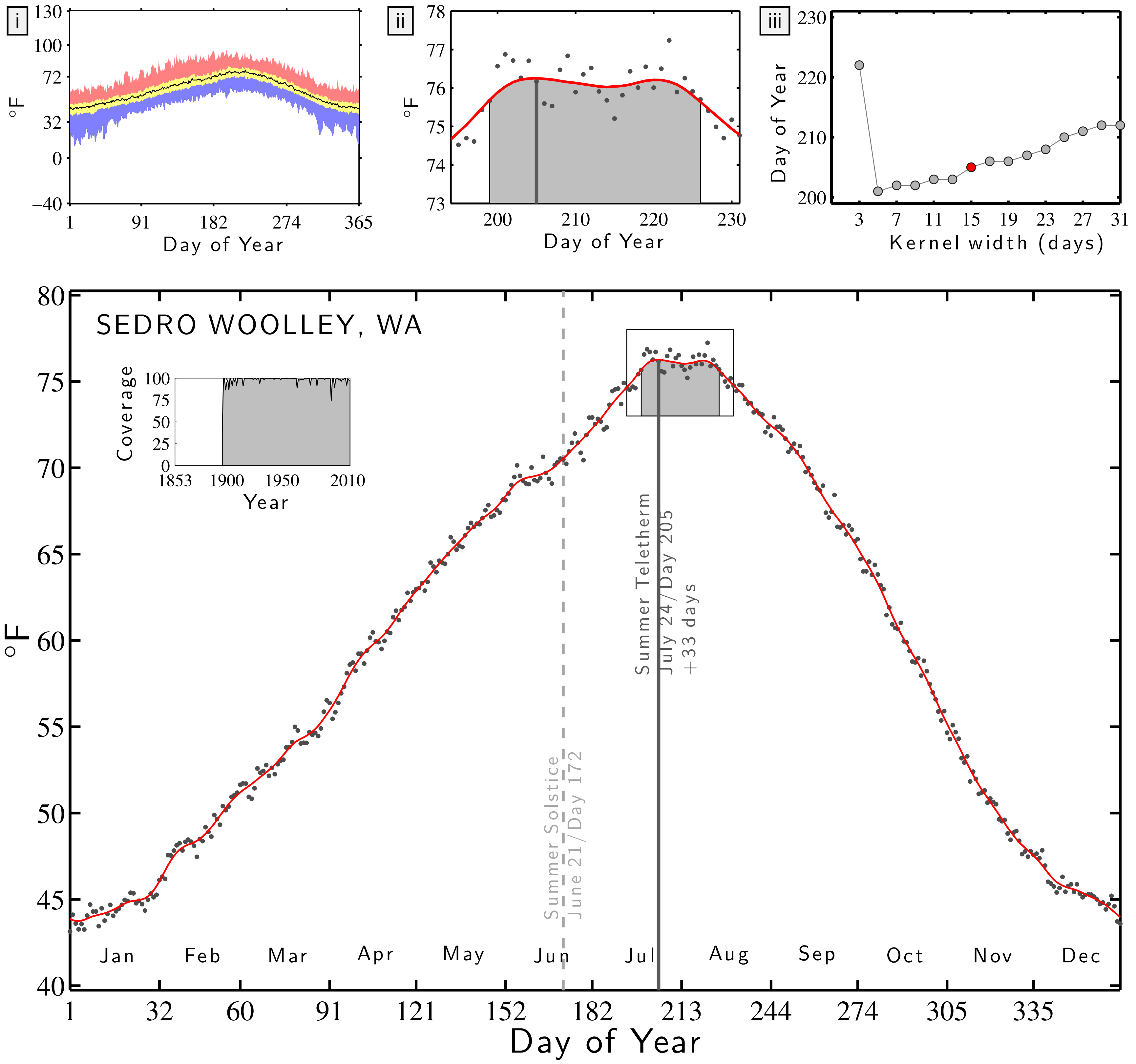

i
ii
iii
°F
Day of Year
Day of Year
Kernel width (days)
SEDRO WOOLLEY, WA
Coverage
Year
1853
1900
1950
2010
Summer Solstice
June 21/Day 172
Summer Teletherm
July 24/Day 205
+33 days
Jan
Feb
Mar
Apr
May
Jun
Jul
Aug
Sep
Oct
Nov
Dec

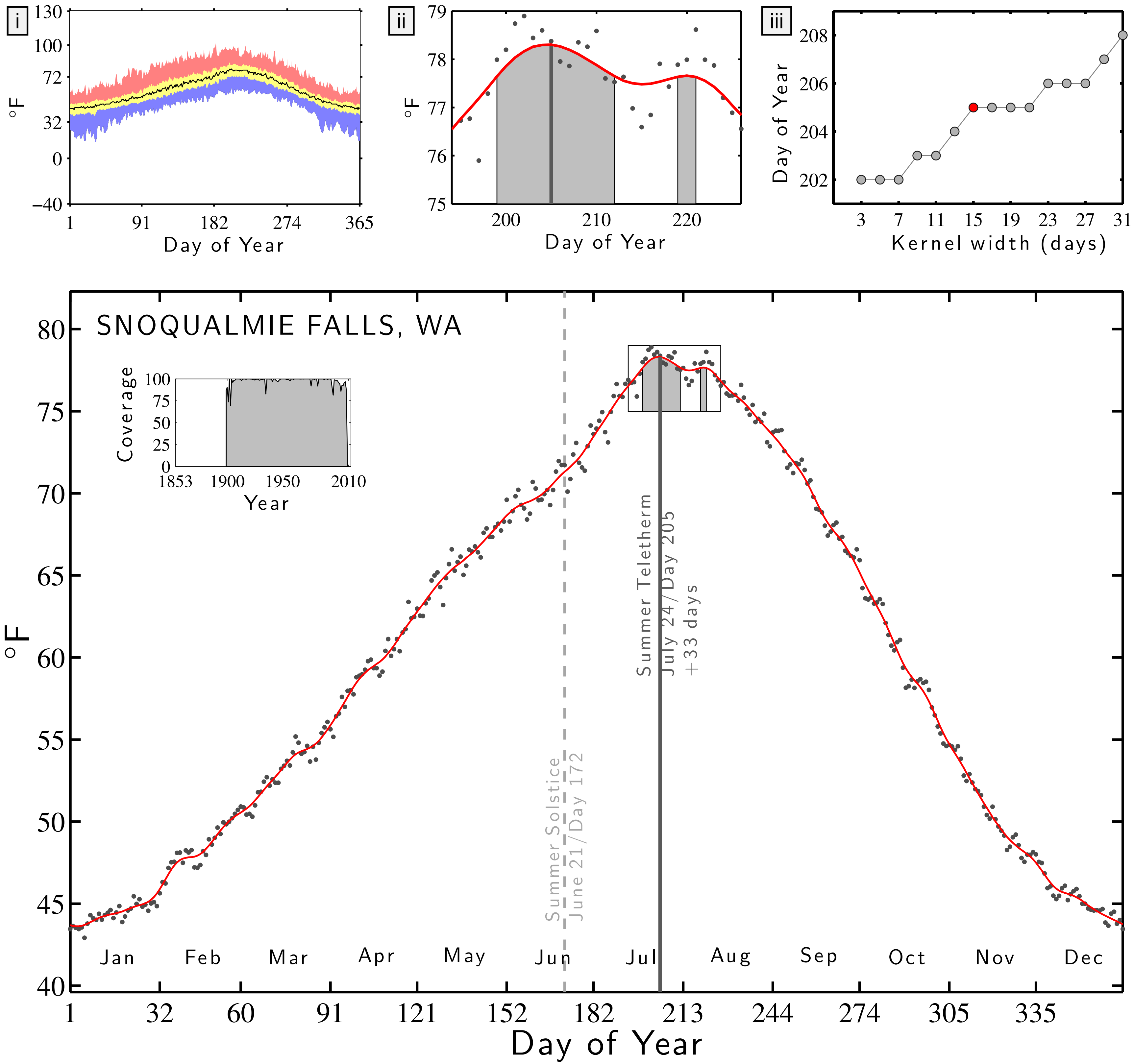

i
°F
130
100
72
32
0
−40
1
91
182
274
365
Day of Year
ii
79
78
77
76
75
200
210
220
iii
Day of Year
208
206
204
202
3
7
11
15
19
23
27
31
Kernel width (days)
SNOQUALMIE FALLS, WA
Coverage
25
50
75
1853
1900
1950
2010
Year
80
70
65
60
55
50
45
40
Summer Solstice
June 21 / Day 172
Summer Teletherm
July 24 / Day 205
+33 days
Jan
Feb
Mar
Apr
May
Jun
Jul
Aug
Sep
Oct
Nov
Dec
32
60
121
152
213
244
305
335

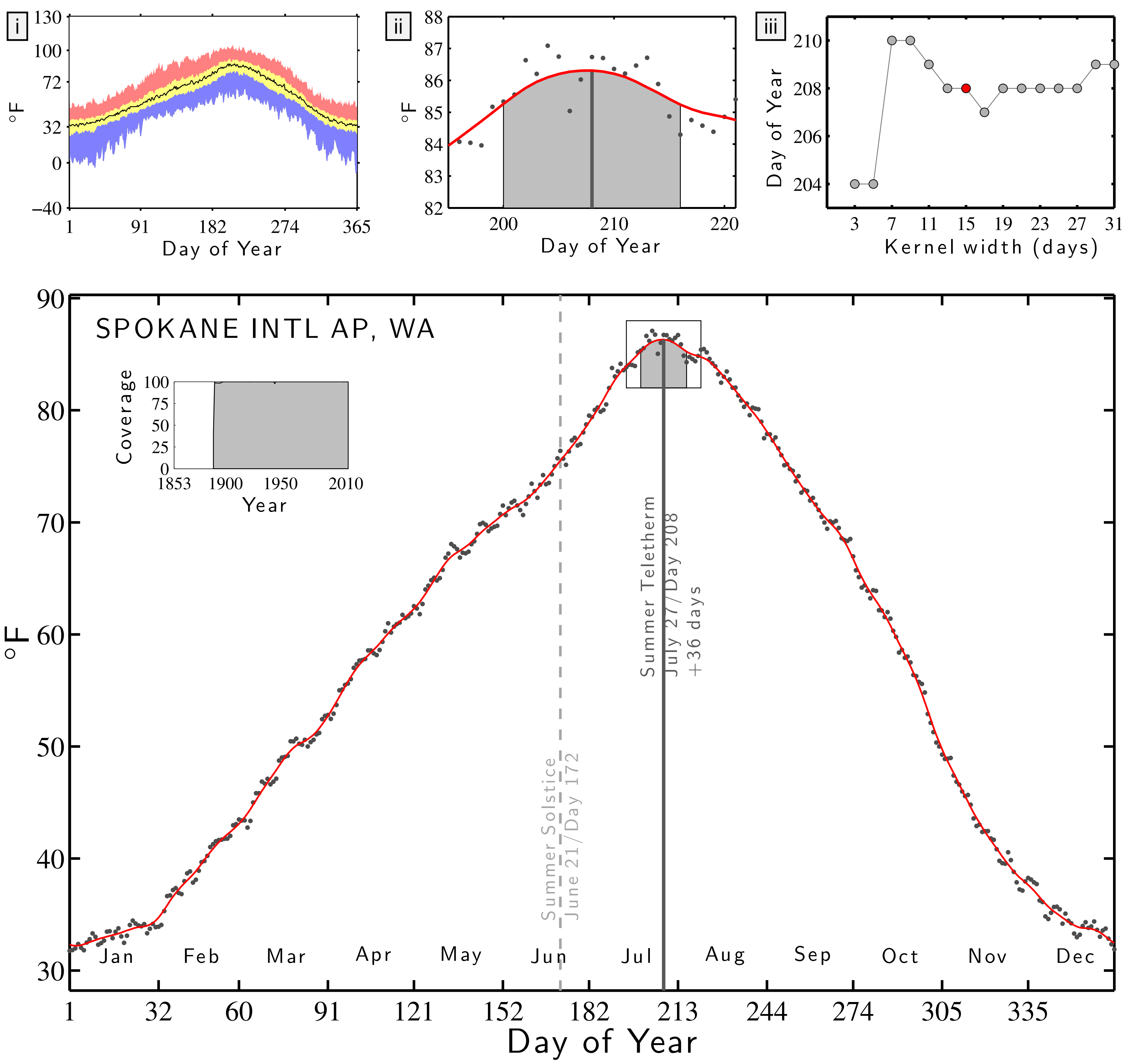
i
°F
Day of Year
ii
°F
Day of Year
iii
Day of Year
Kernel width (days)
SPOKANE INTL AP, WA
Coverage
Year
Summer Solstice
June 21/Day 172
Summer Teletherm
July 27/Day 208
+36 days
Jan
Feb
Mar
Apr
May
Jun
Jul
Aug
Sep
Oct
Nov
Dec
°F
Day of Year

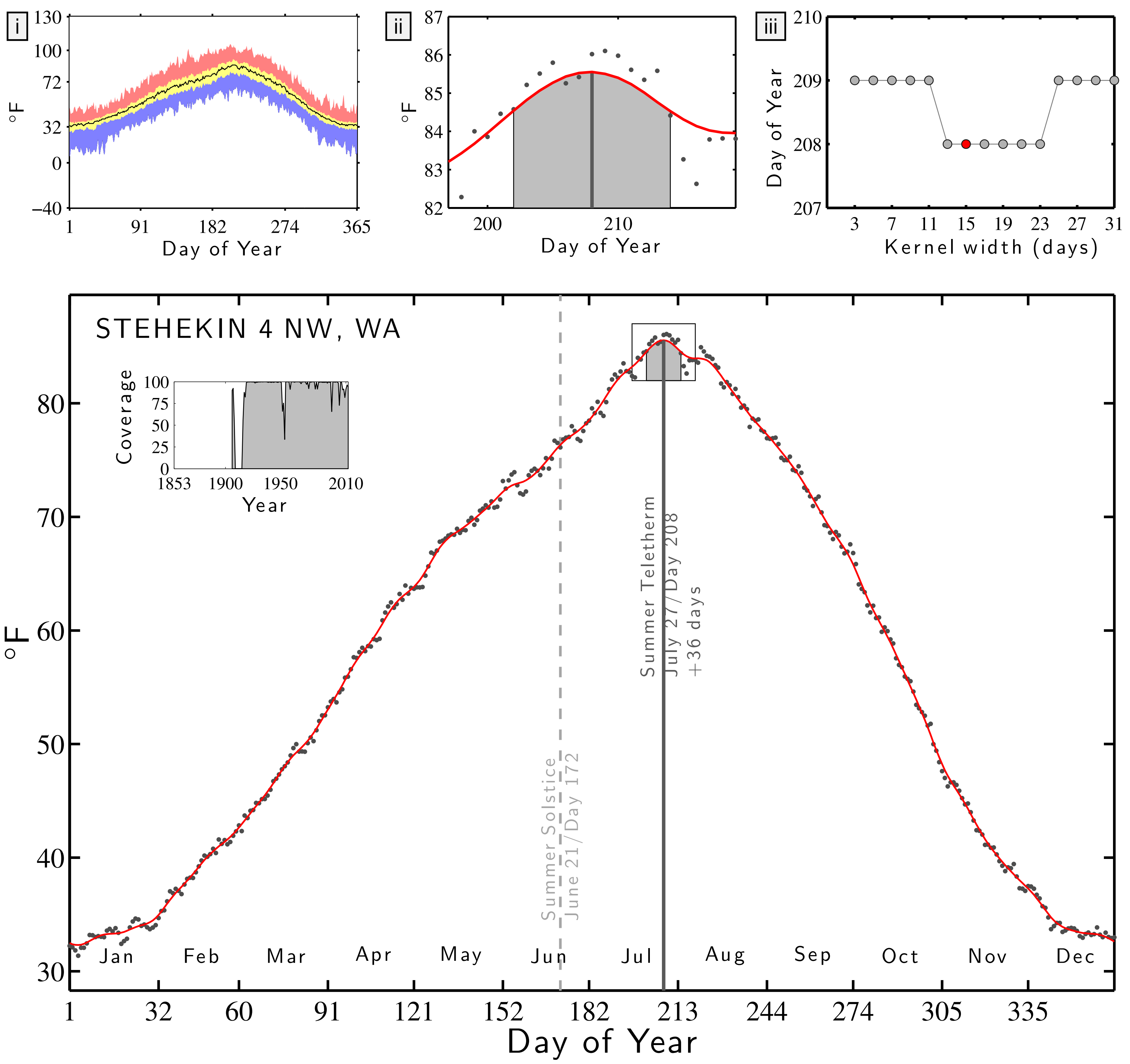

i
°F
Day of Year
ii
°F
Day of Year
iii
Day of Year
Kernel width (days)
STEHEKIN 4 NW, WA
Coverage
Year
Summer Solstice
June 21 / Day 172
Summer Teletherm
July 27 / Day 208
+36 days
Jan
Feb
Mar
Apr
May
Jun
Jul
Aug
Sep
Oct
Nov
Dec
°F
Day of Year

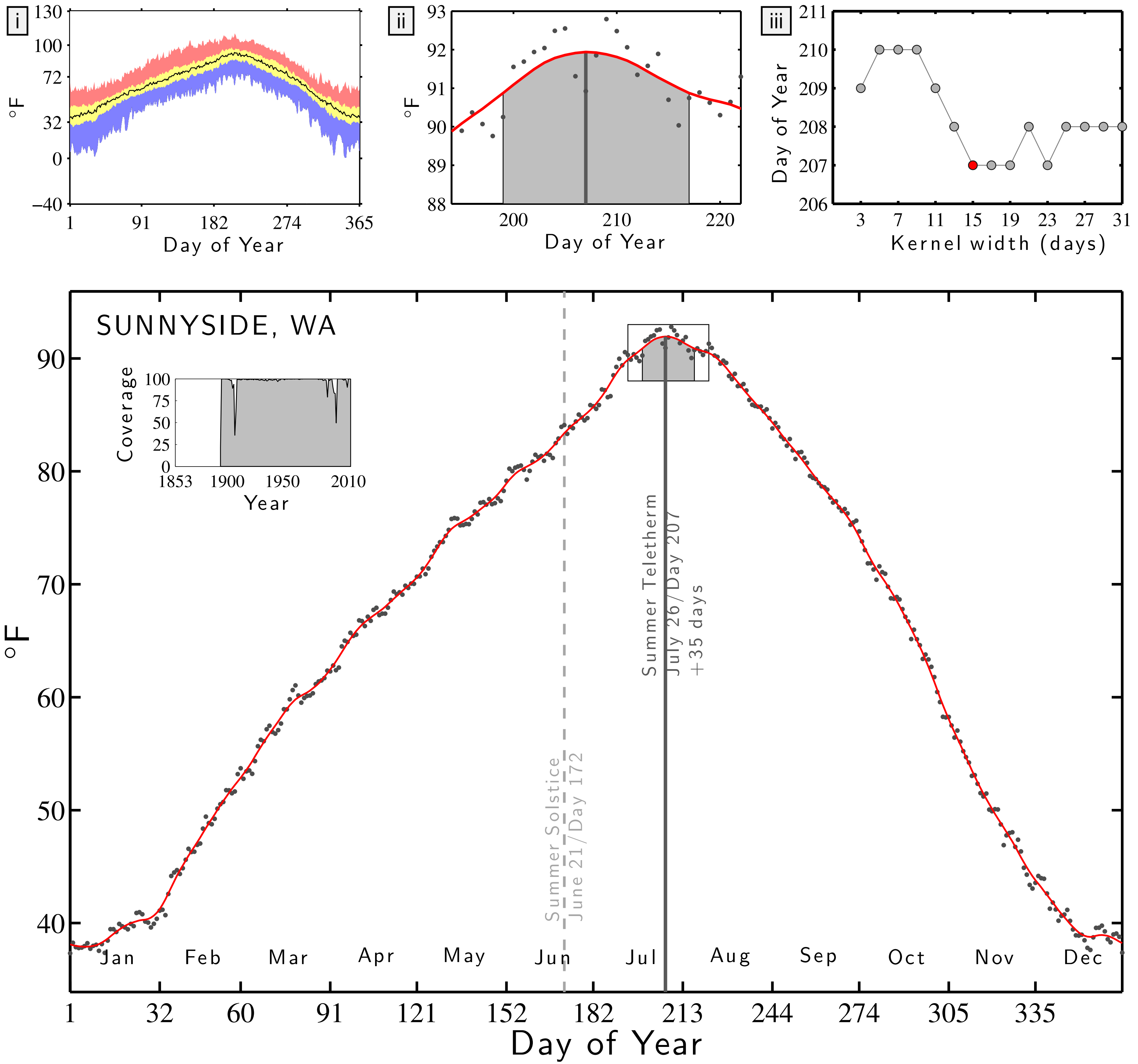

i
°F
Day of Year
ii
°F
Day of Year
iii
Day of Year
Kernel width (days)
SUNNYSIDE, WA
Coverage
Year
Summer Solstice
June 21 / Day 172
Summer Teletherm
July 26 / Day 207
+35 days
Jan
Feb
Mar
Apr
May
Jun
Jul
Aug
Sep
Oct
Nov
Dec
°F
Day of Year

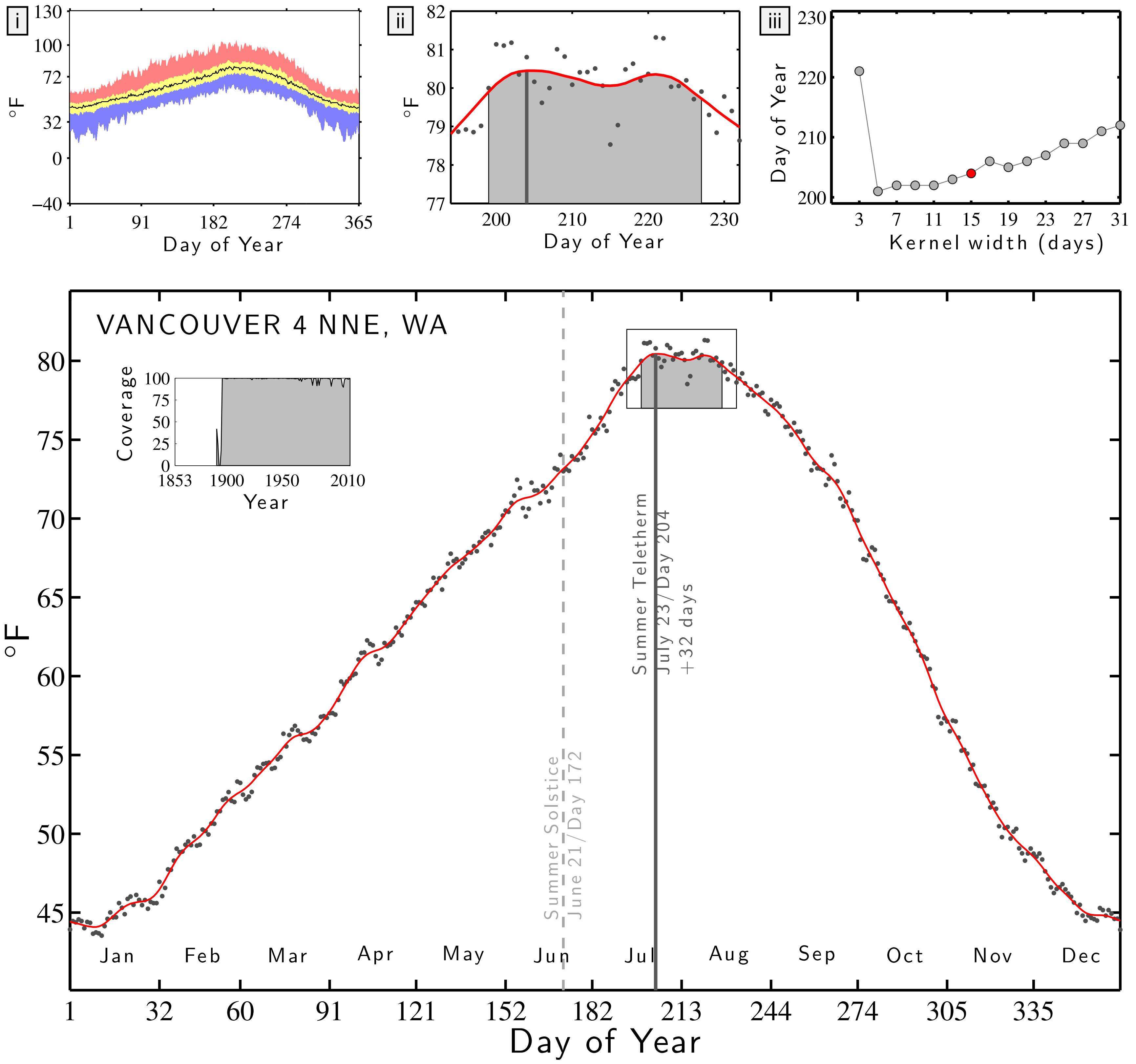

i
ii
iii
°F
Day of Year
Kernel width (days)
Day of Year
VANCOUVER 4 NNE, WA
Coverage
Year
Summer Solstice
June 21 / Day 172
Summer Teletherm
July 23 / Day 204
+32 days
Jan
Feb
Mar
Apr
May
Jun
Jul
Aug
Sep
Oct
Nov
Dec

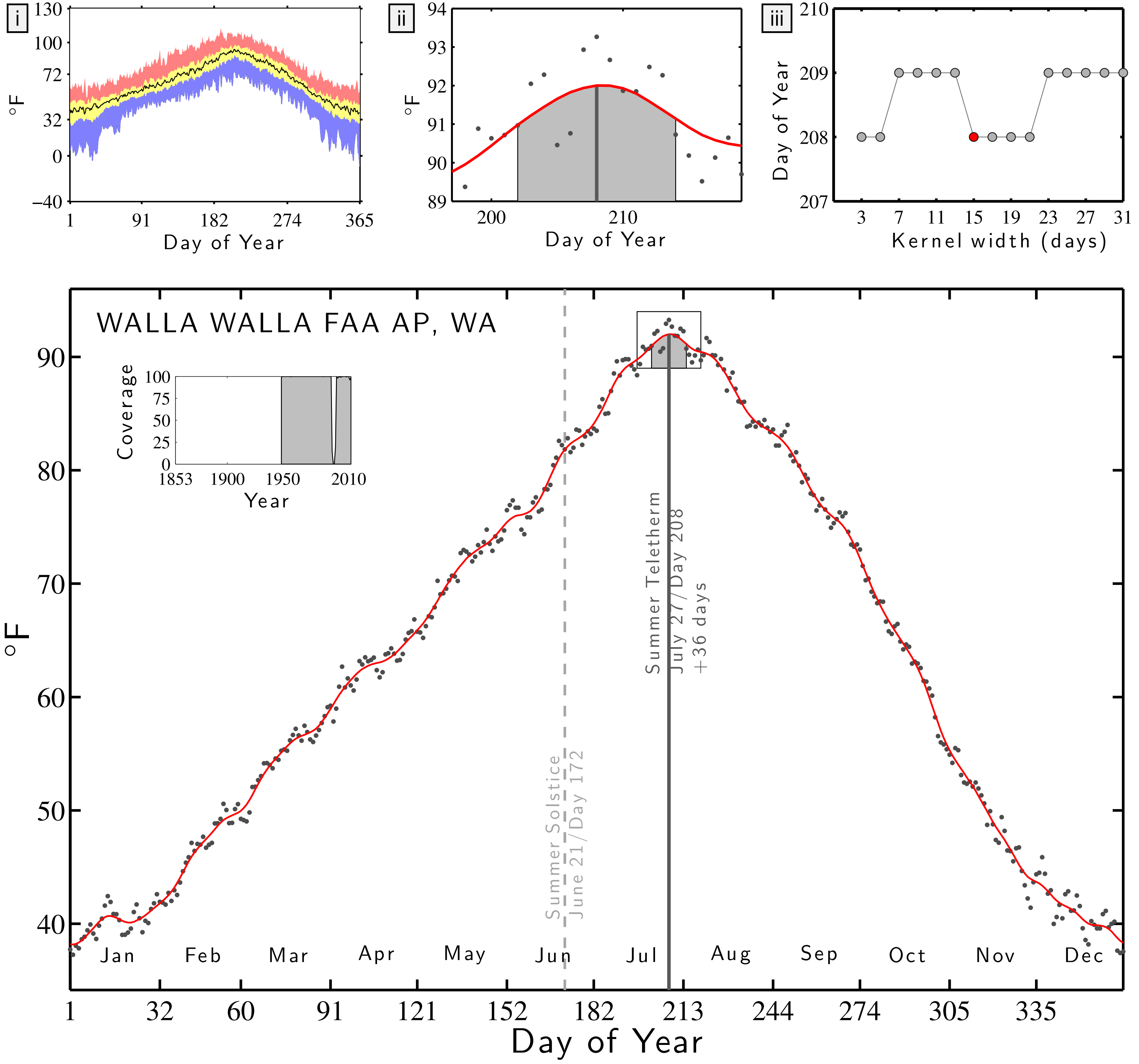

i
ii
iii
°F
Day of Year
Day of Year
Kernel width (days)
WALLA WALLA FAA AP, WA
Coverage
Year
Summer Solstice
June 21/Day 172
Summer Teletherm
July 27/Day 208
+36 days
Jan
Feb
Mar
Apr
May
Jun
Jul
Aug
Sep
Oct
Nov
Dec
°F
Day of Year

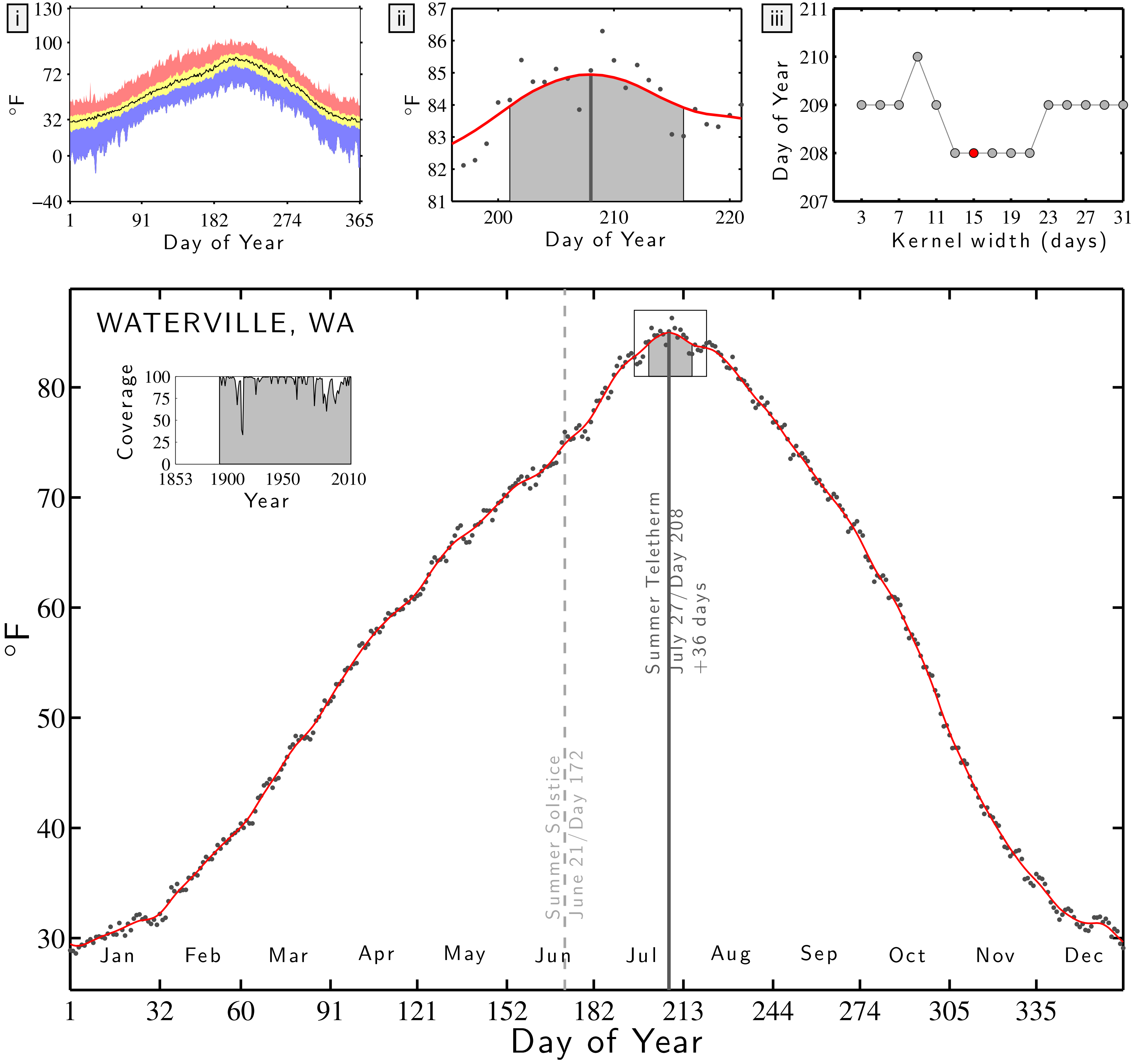

i
°F
Day of Year
ii
°F
Day of Year
iii
Day of Year
Kernel width (days)
WATERVILLE, WA
Coverage
Year
Summer Solstice
June 21 / Day 172
Summer Teletherm
July 27 / Day 208
+36 days
Jan
Feb
Mar
Apr
May
Jun
Jul
Aug
Sep
Oct
Nov
Dec
°F
Day of Year

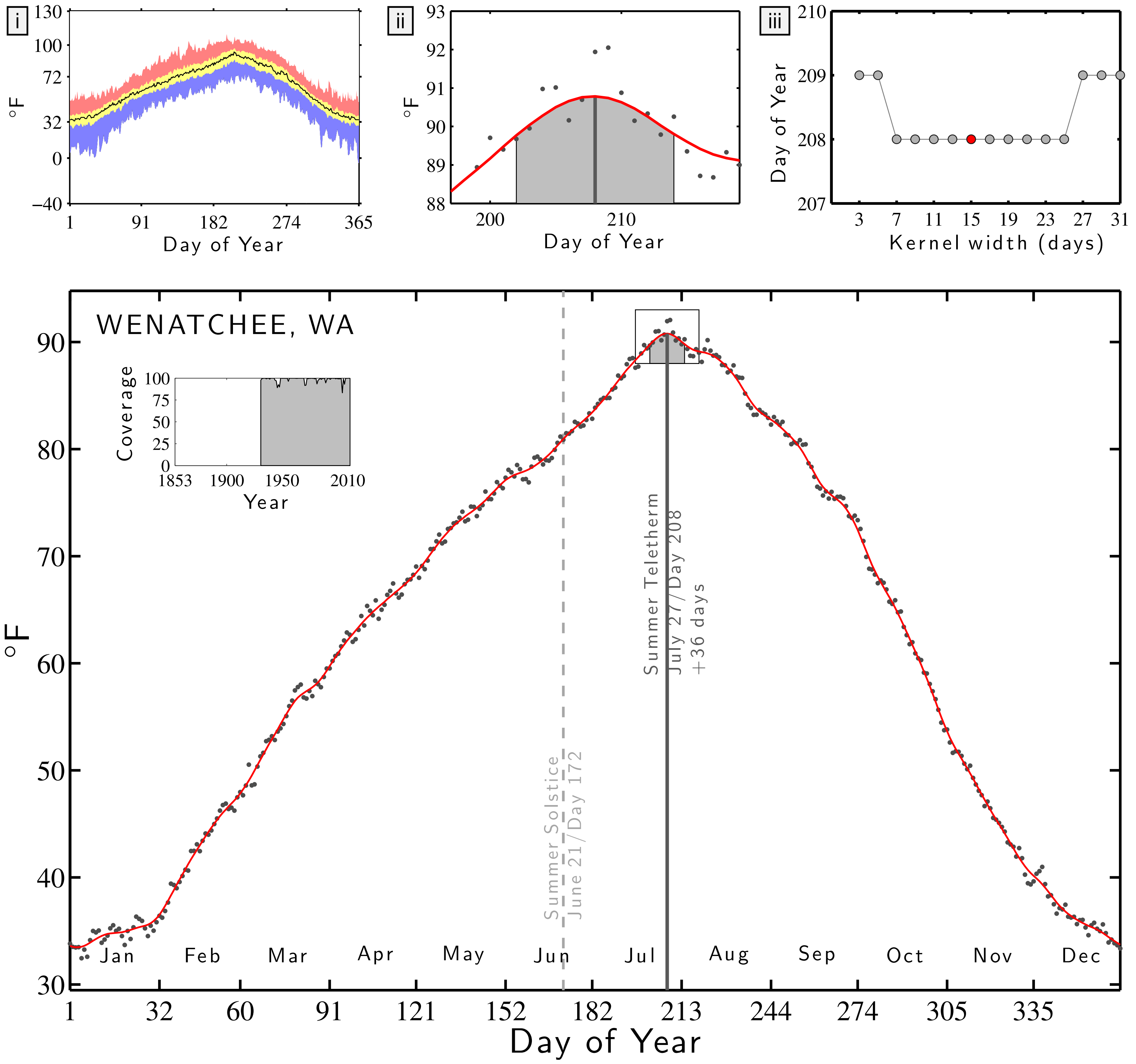

i
ii
iii
°F
Day of Year
Day of Year
Kernel width (days)
WENATCHEE, WA
Coverage
Year
1853
1900
1950
2010
Summer Solstice
June 21/Day 172
Summer Teletherm
July 27/Day 208
+36 days
Jan
Feb
Mar
Apr
May
Jun
Jul
Aug
Sep
Oct
Nov
Dec
Day of Year

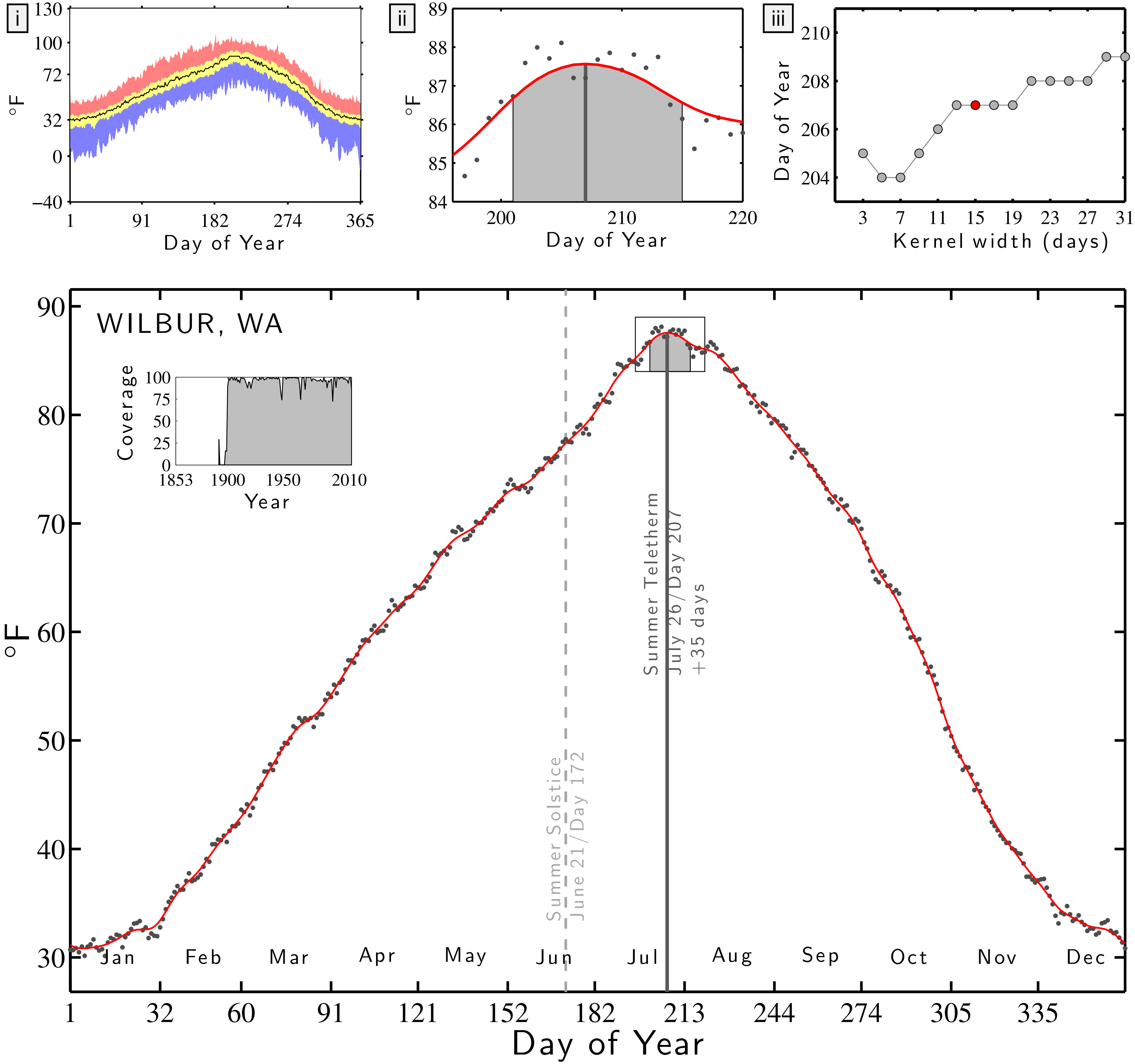

i
°F
Day of Year
ii
°F
Day of Year
iii
Day of Year
Kernel width (days)
WILBUR, WA
Coverage
Year
Summer Solstice
June 21 / Day 172
Summer Teletherm
July 26 / Day 207
+35 days
Jan
Feb
Mar
Apr
May
Jun
Jul
Aug
Sep
Oct
Nov
Dec
°F
Day of Year

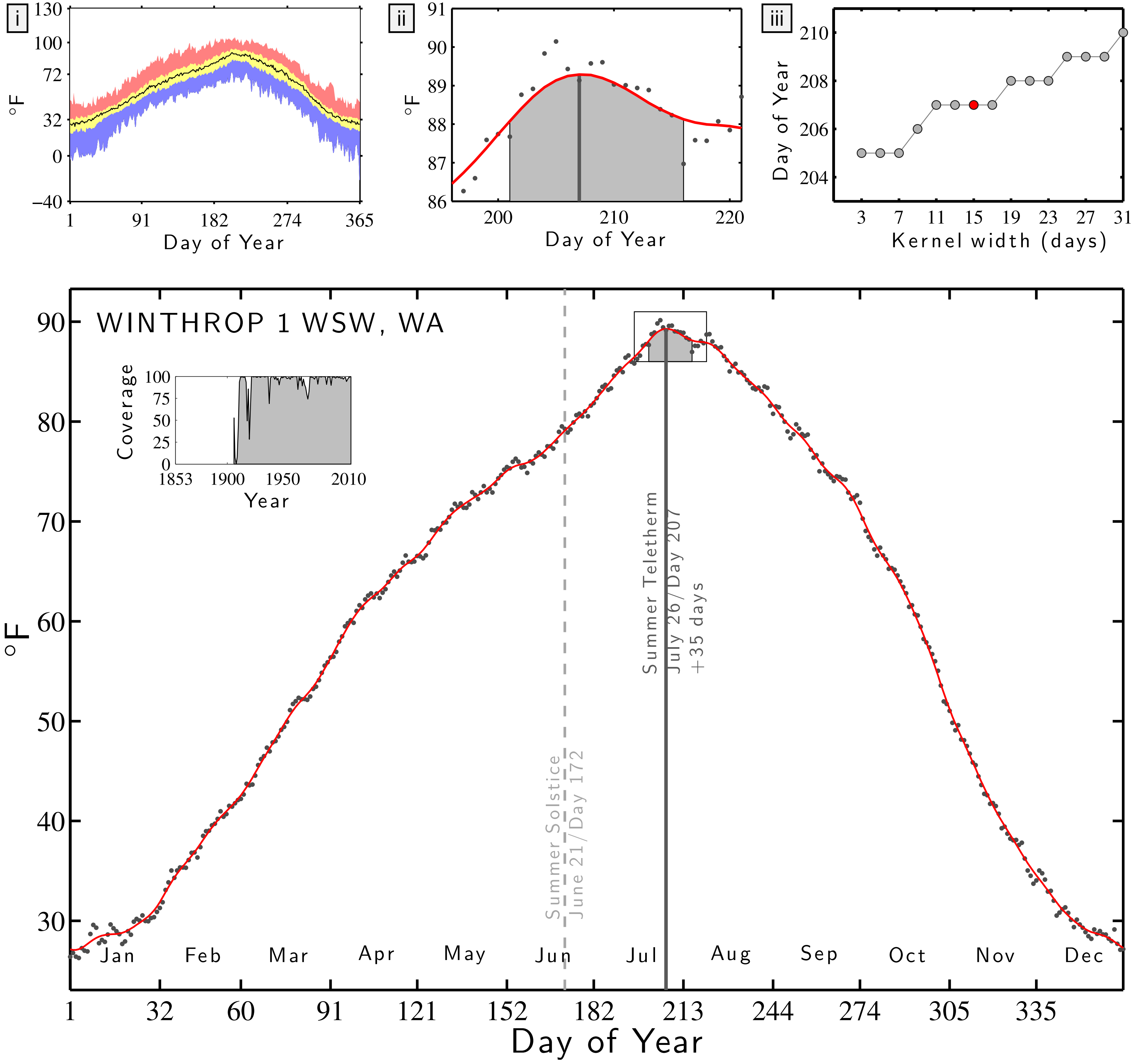

i
°F
Day of Year
ii
°F
Day of Year
iii
Day of Year
Kernel width (days)
WINTHROP 1 WSW, WA
Coverage
Year
Summer Solstice
June 21/Day 172
Summer Teletherm
July 26/Day 207
+35 days
Jan
Feb
Mar
Apr
May
Jun
Jul
Aug
Sep
Oct
Nov
Dec
°F
Day of Year

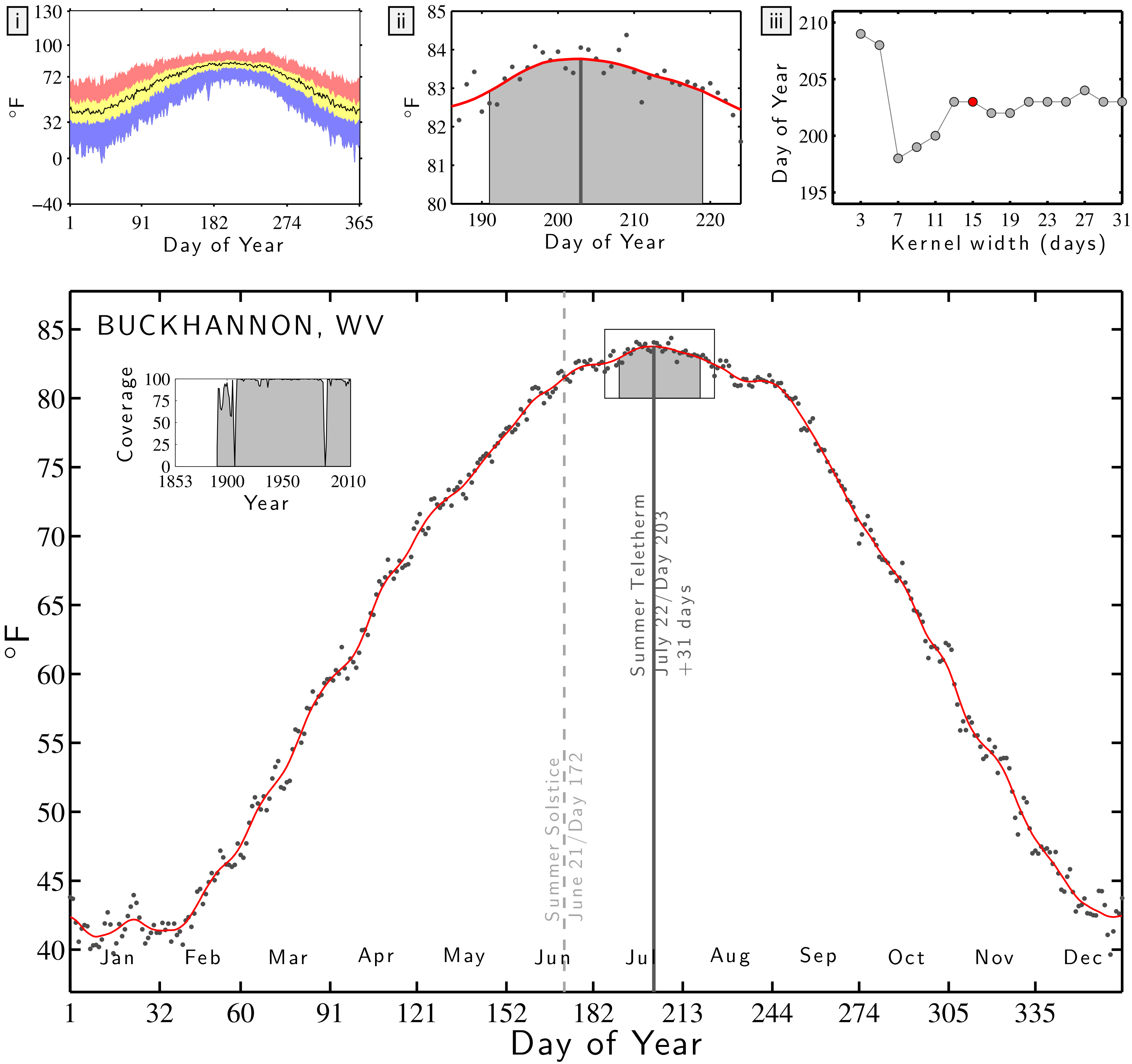

i
ii
iii
Day of Year
Kernel width (days)
°F
BUCKHANNON, WV
Coverage
Year
Summer Solstice
June 21/Day 172
Summer Teletherm
July 22/Day 203
+31 days
Jan
Feb
Mar
Apr
May
Jun
Jul
Aug
Sep
Oct
Nov
Dec
Day of Year

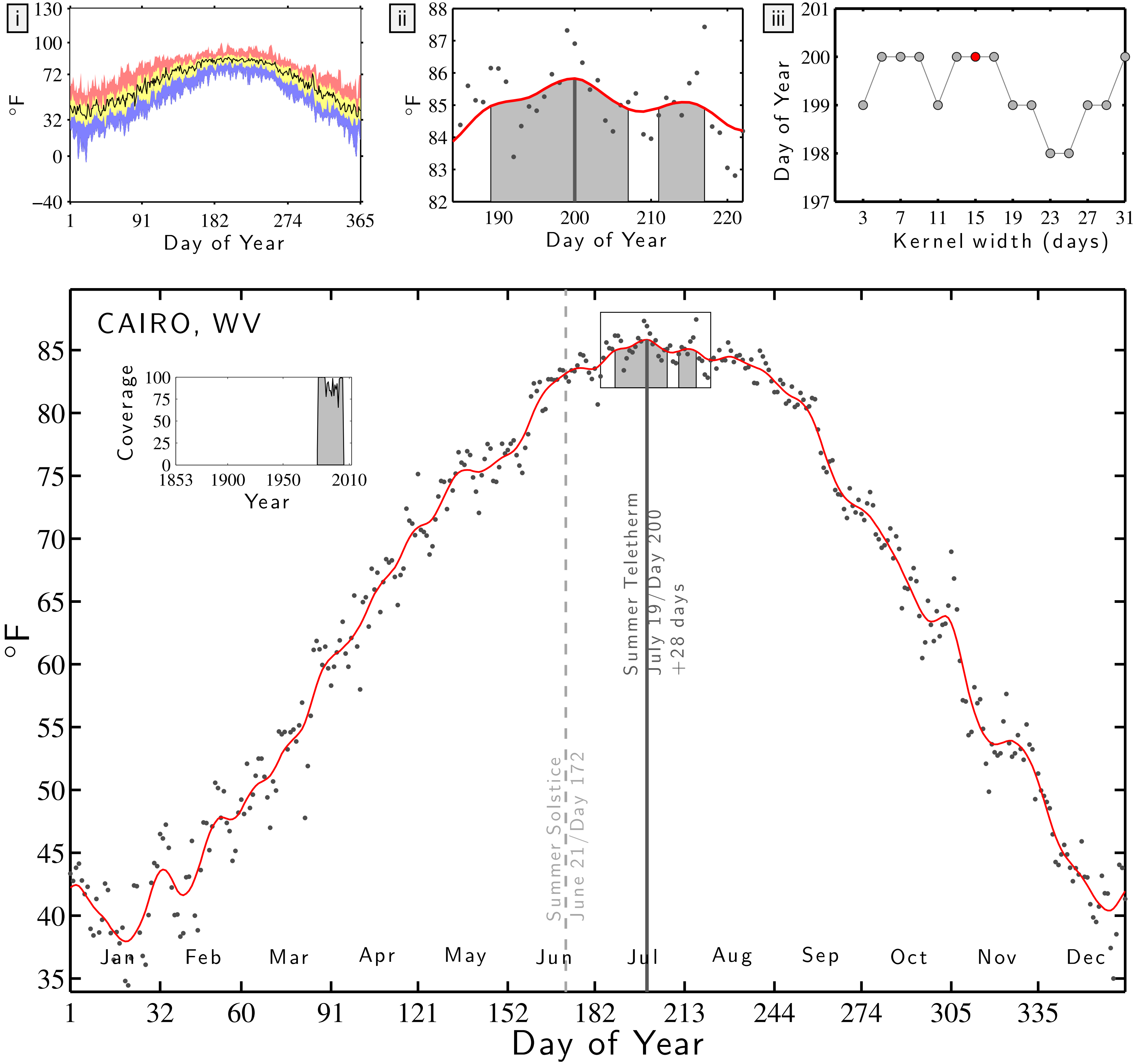

i
°F
130
100
72
32
0
−40
1
91
182
274
365
Day of Year
ii
°F
88
87
86
85
84
83
82
190
200
210
220
Day of Year
iii
Day of Year
201
200
199
198
197
3
7
11
15
19
23
27
31
Kernel width (days)
CAIRO, WV
°F
85
80
75
70
65
60
55
50
45
40
35
Coverage
100
75
50
25
0
1853
1900
1950
2010
Year
Summer Solstice
June 21/Day 172
Summer Teletherm
July 19/Day 200
+28 days
Jan
Feb
Mar
Apr
May
Jun
Jul
Aug
Sep
Oct
Nov
Dec
1
32
60
91
121
152
182
213
244
274
305
335
Day of Year

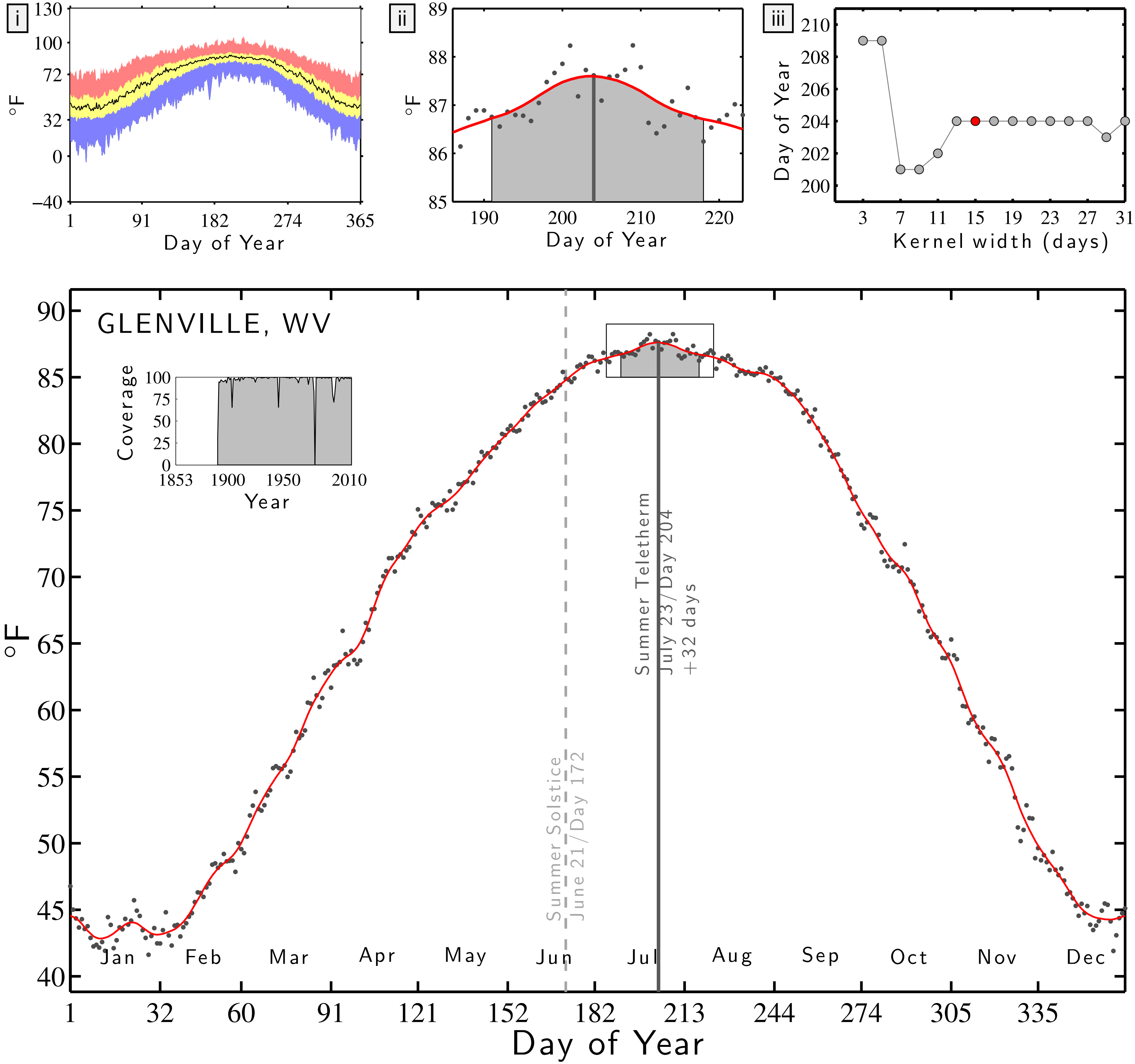

i
°F
130
100
72
32
0
−40
1
91
182
274
365
Day of Year
ii
89
88
87
86
85
190
200
210
220
Day of Year
iii
Day of Year
210
208
206
204
202
200
3
7
11
15
19
23
27
31
Kernel width (days)
GLENVILLE, WV
Coverage
100
75
50
25
0
1853
1900
1950
2010
Year
Summer Solstice
June 21/Day 172
Summer Teletherm
July 23/Day 204
+32 days
90
85
80
75
70
65
60
55
50
45
40
Jan
Feb
Mar
Apr
May
Jun
Jul
Aug
Sep
Oct
Nov
Dec
1
32
60
91
121
152
182
213
244
274
305
335
Day of Year

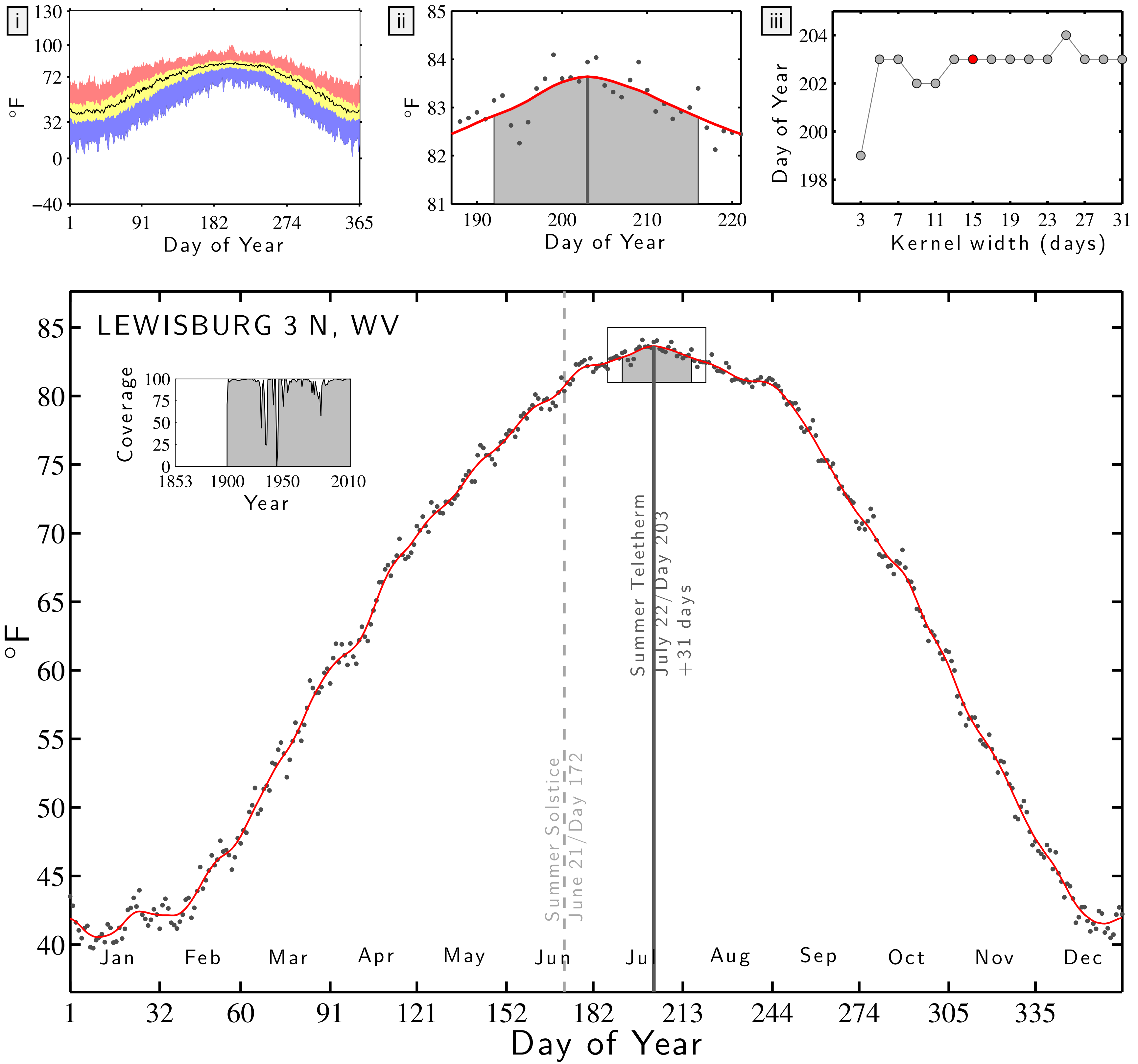

i
°F
Day of Year
ii
°F
Day of Year
iii
Day of Year
Kernel width (days)
LEWISBURG 3 N, WV
Coverage
Year
Summer Solstice
June 21/Day 172
Summer Teletherm
July 22/Day 203
+31 days
Jan
Feb
Mar
Apr
May
Jun
Jul
Aug
Sep
Oct
Nov
Dec
°F
Day of Year

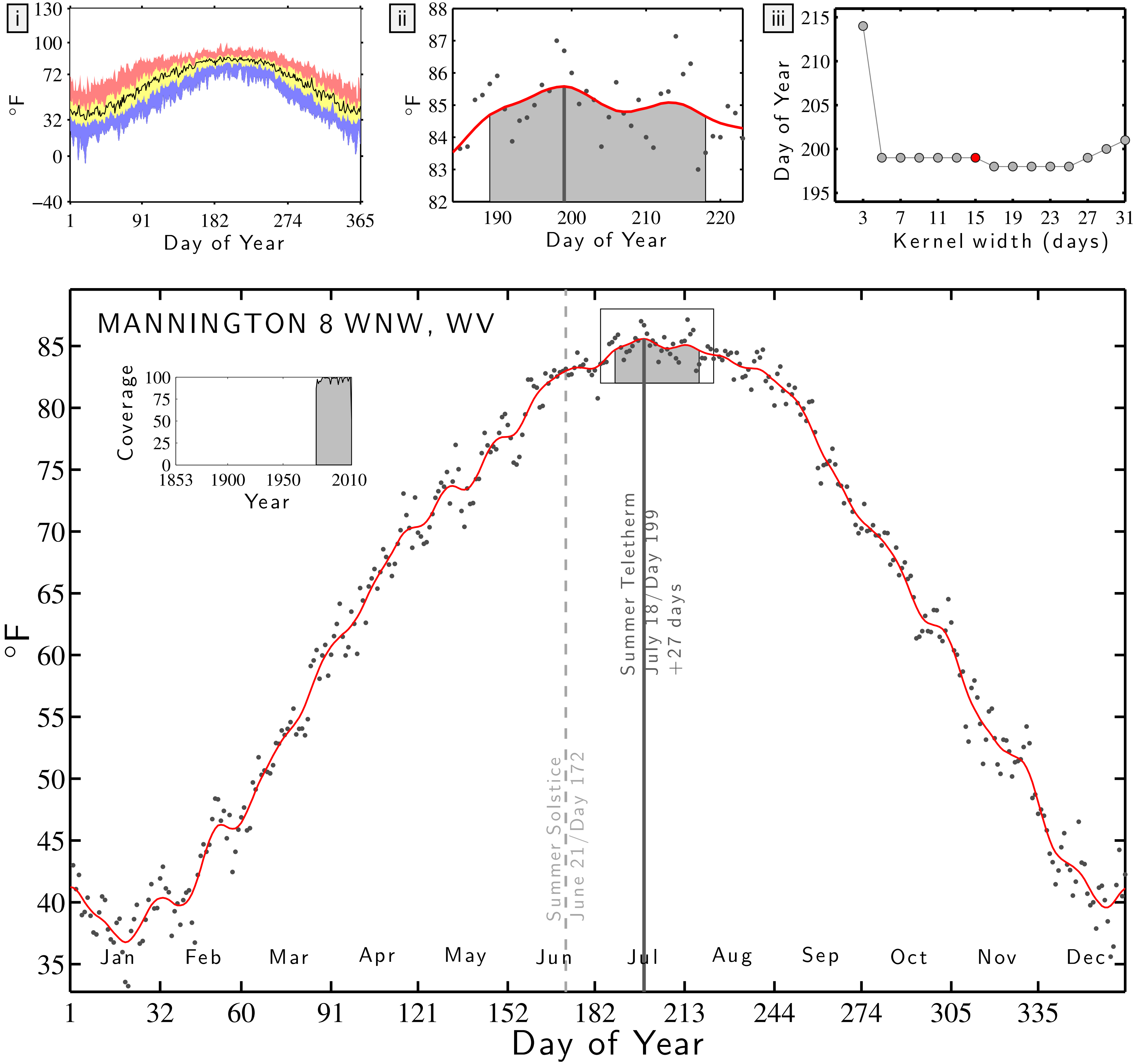

i
ii
iii
°F
Day of Year
Day of Year
Kernel width (days)
MANNINGTON 8 WNW, WV
Coverage
Year
Summer Solstice
June 21 / Day 172
Summer Teletherm
July 18 / Day 199
+27 days
Jan
Feb
Mar
Apr
May
Jun
Jul
Aug
Sep
Oct
Nov
Dec
°F
Day of Year

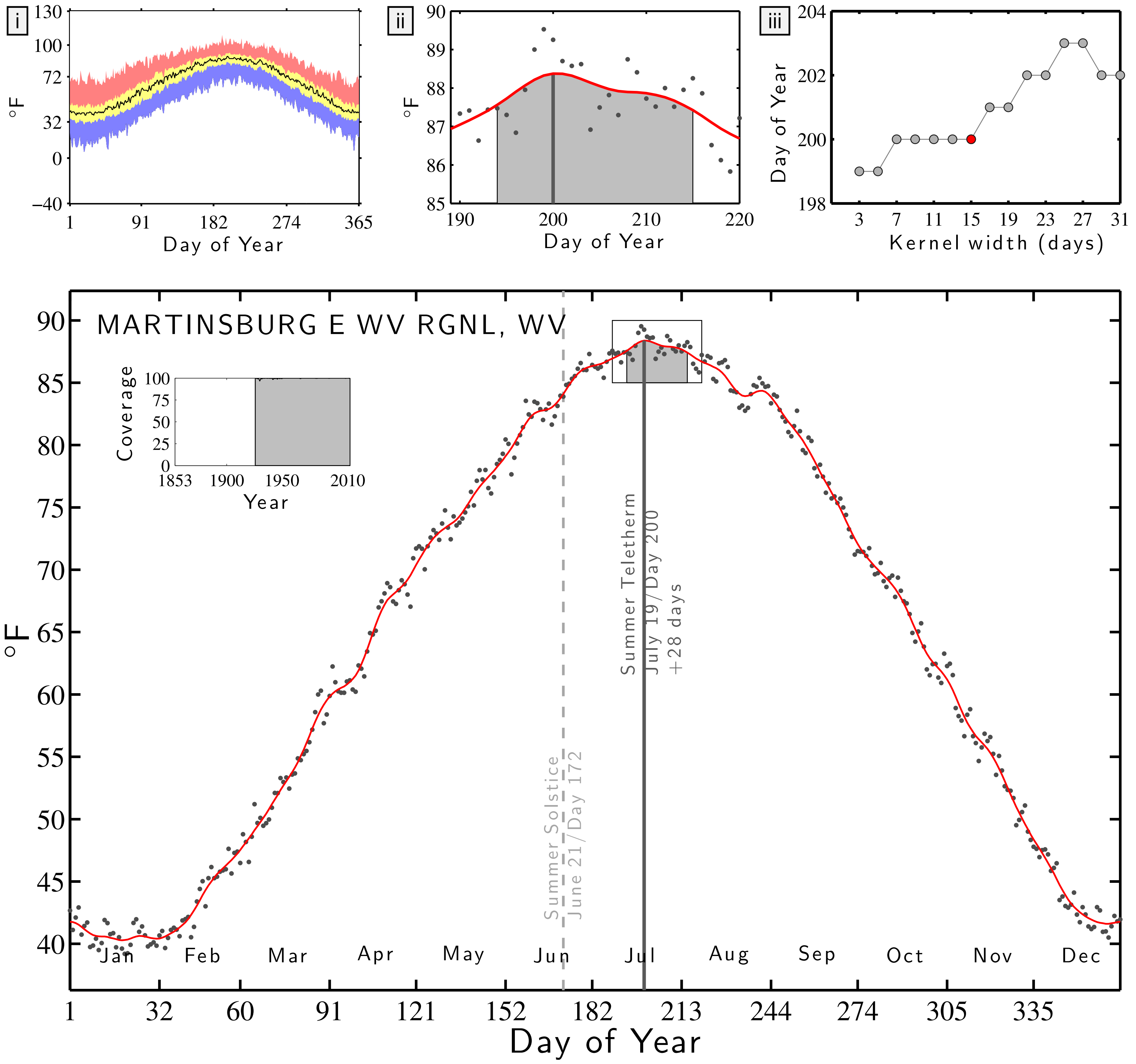

i
°F
Day of Year
ii
°F
Day of Year
iii
Day of Year
Kernel width (days)
MARTINSBURG E WV RGNL, WV
Coverage
Year
Summer Solstice
June 21/Day 172
Summer Teletherm
July 19/Day 200
+28 days
Jan
Feb
Mar
Apr
May
Jun
Jul
Aug
Sep
Oct
Nov
Dec
°F
Day of Year

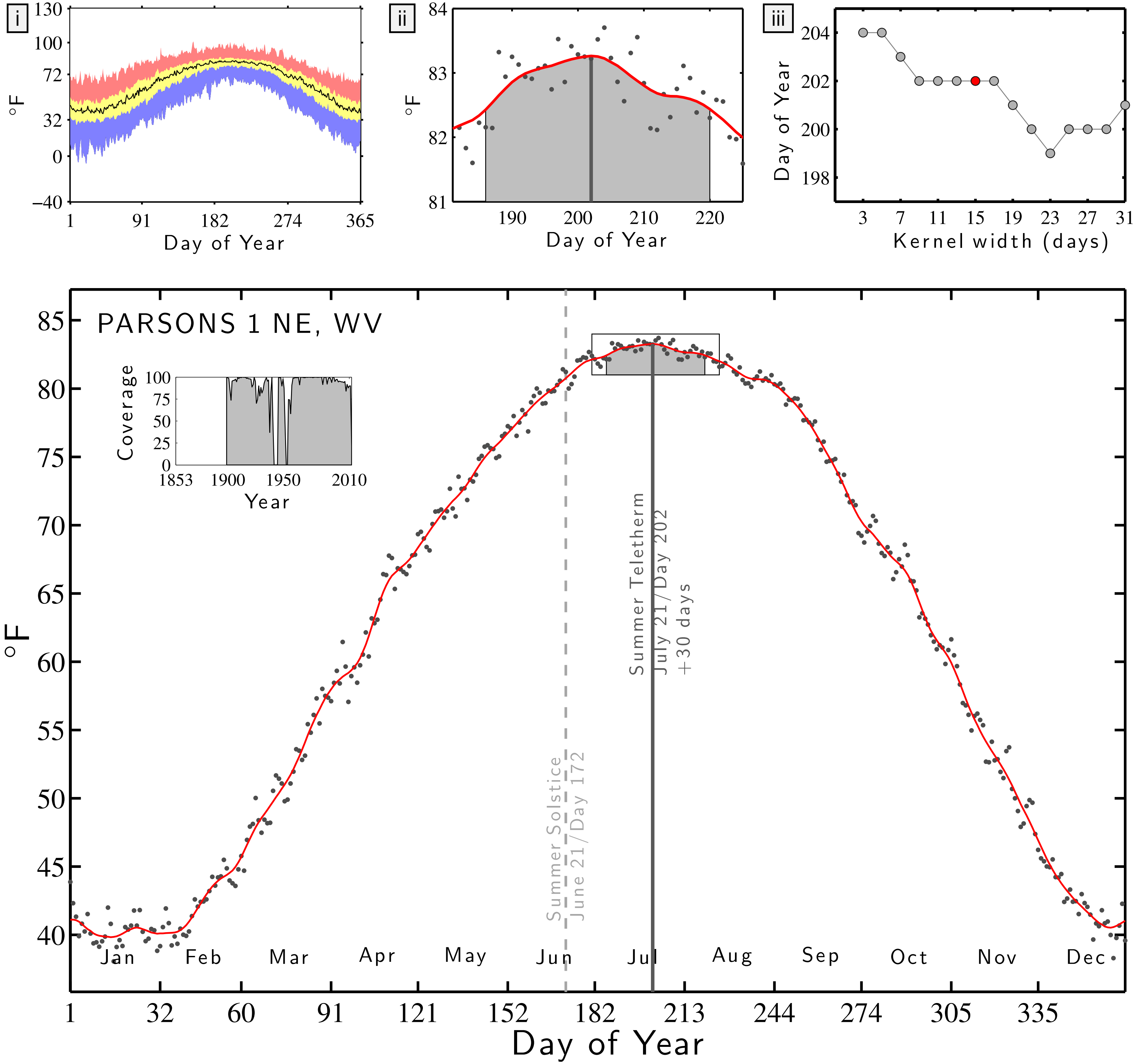

i
ii
iii
Day of Year
°F
Kernel width (days)
PARSONS 1 NE, WV
Coverage
Year
Summer Solstice
June 21 / Day 172
Summer Teletherm
July 21 / Day 202
+30 days
Jan
Feb
Mar
Apr
May
Jun
Jul
Aug
Sep
Oct
Nov
Dec

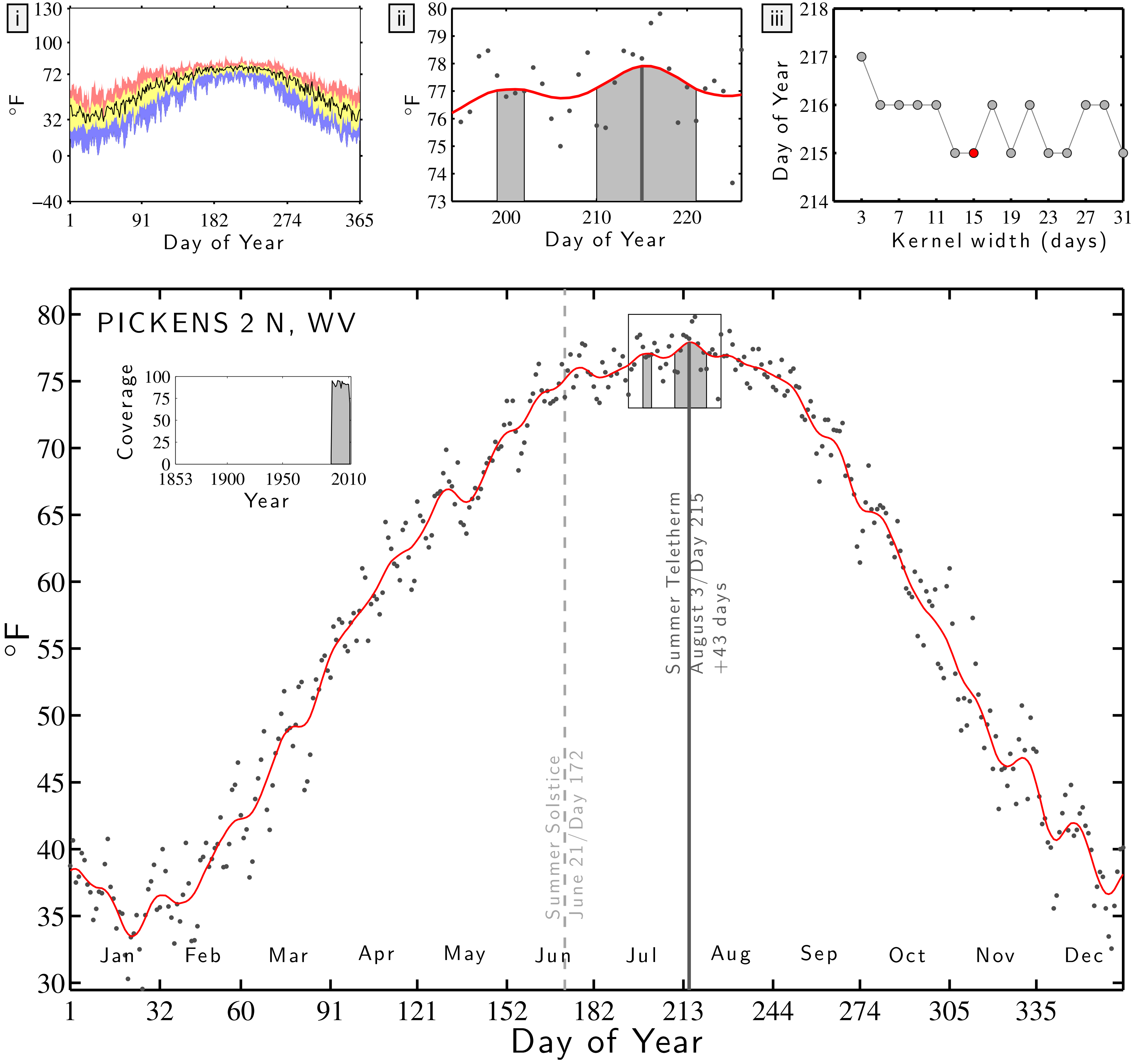

i
ii
iii
°F
Day of Year
Kernel width (days)
PICKENS 2 N, WV
Coverage
Year
Summer Solstice
June 21 / Day 172
Summer Teletherm
August 3 / Day 215
+43 days
Jan
Feb
Mar
Apr
May
Jun
Jul
Aug
Sep
Oct
Nov
Dec

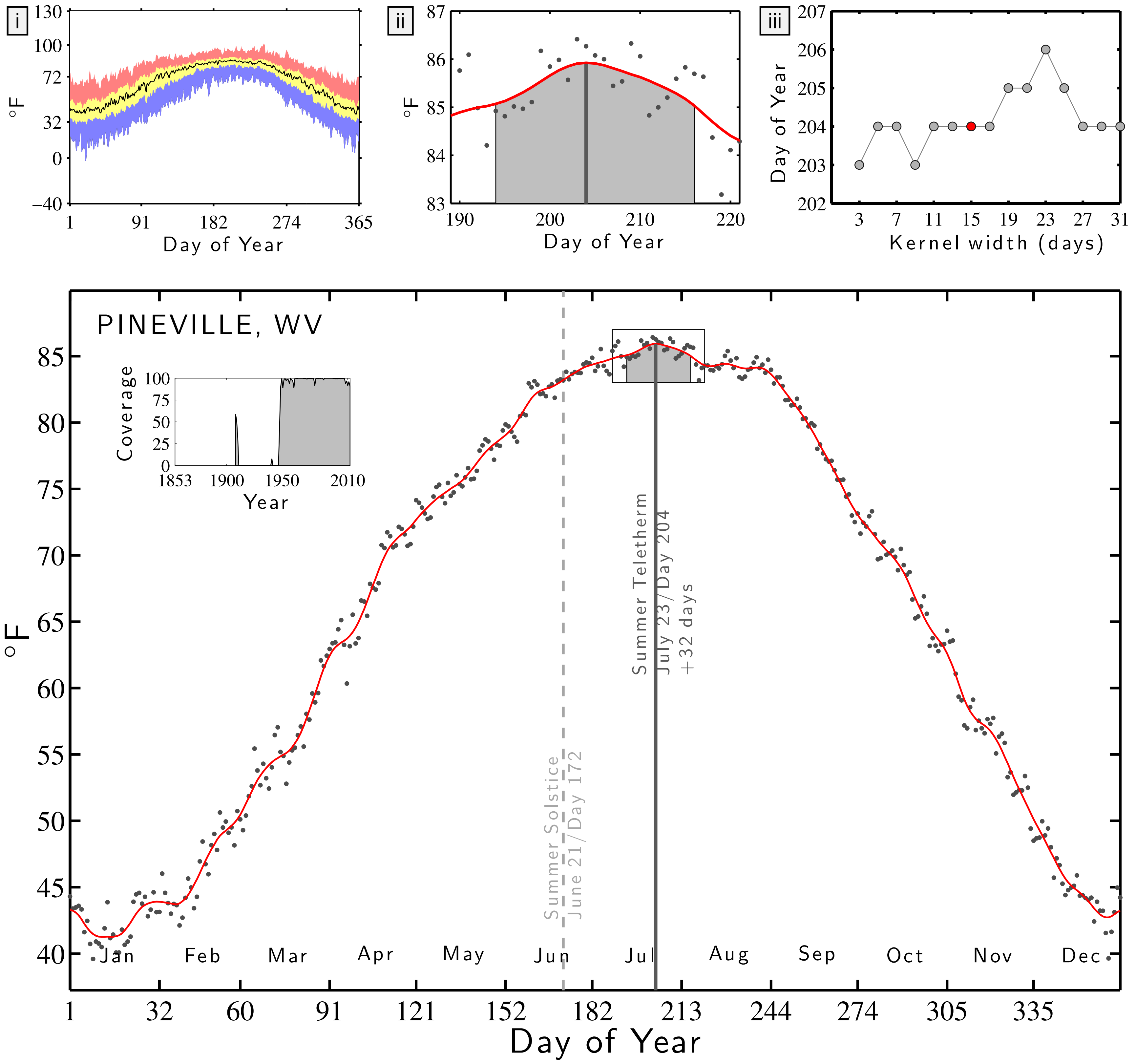

i
ii
iii
°F
Day of Year
Day of Year
Kernel width (days)
PINEVILLE, WV
Coverage
Year
Summer Solstice
June 21 / Day 172
Summer Teletherm
July 23 / Day 204
+32 days
Jan
Feb
Mar
Apr
May
Jun
Jul
Aug
Sep
Oct
Nov
Dec
°F
Day of Year

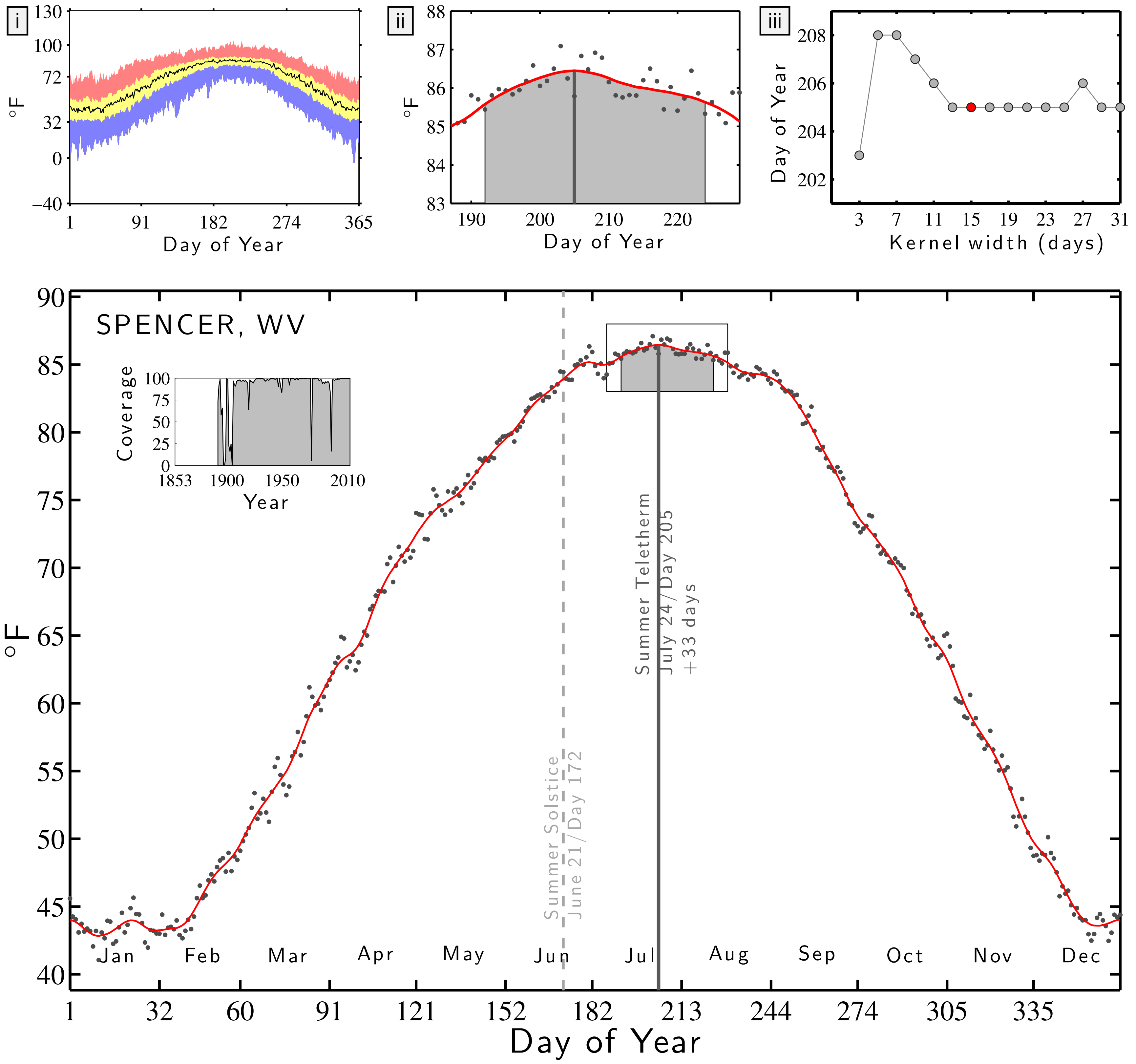

i
ii
iii
°F
Day of Year
Kernel width (days)
SPENCER, WV
Coverage
Year
Summer Solstice
June 21 / Day 172
Summer Teletherm
July 24 / Day 205
+33 days
Jan
Feb
Mar
Apr
May
Jun
Jul
Aug
Sep
Oct
Nov
Dec

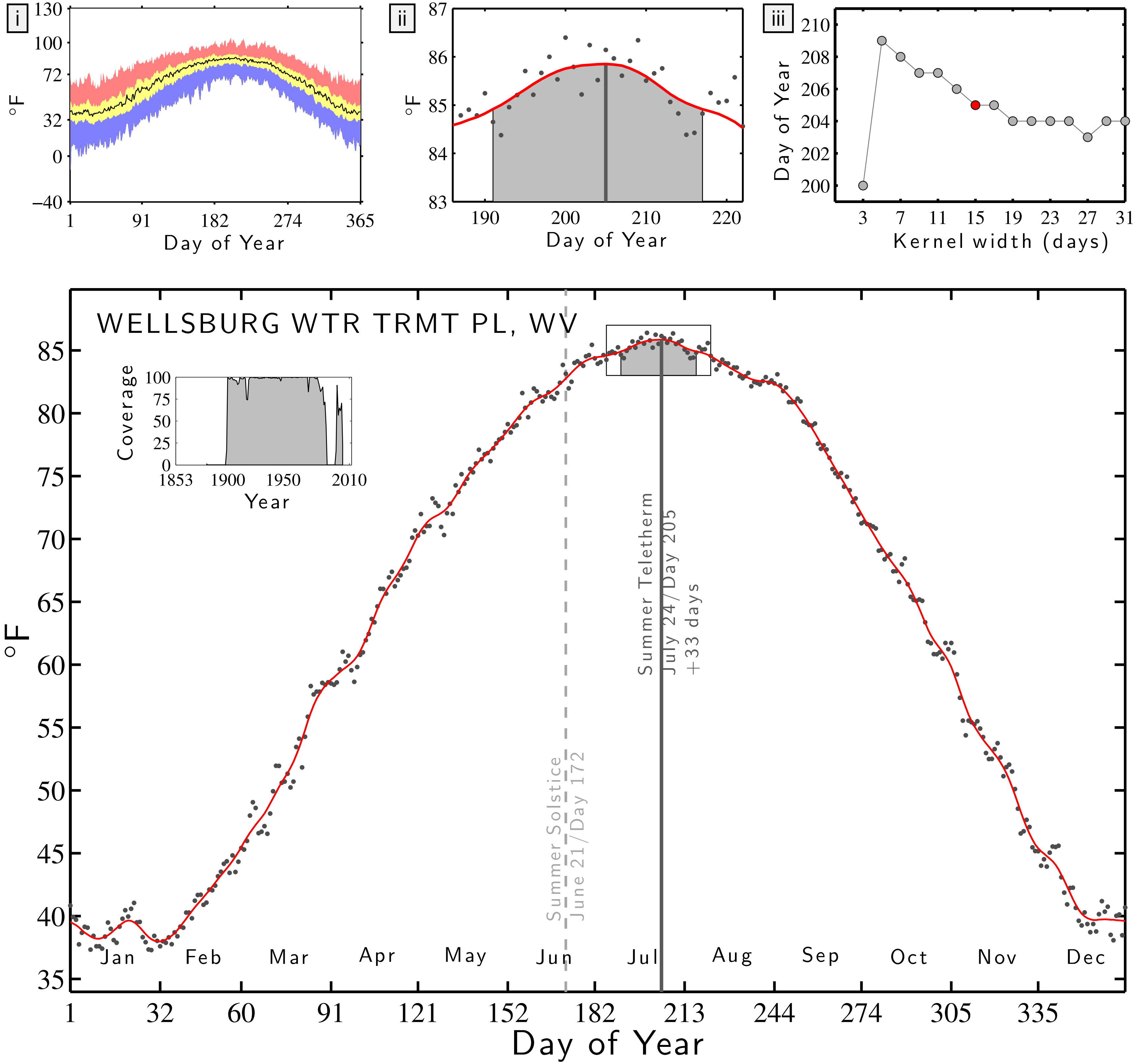

i
°F
Day of Year
ii
°F
Day of Year
iii
Day of Year
Kernel width (days)
WELLSBURG WTR TRMT PL, WV
Coverage
Year
Summer Solstice
June 21 / Day 172
Summer Teletherm
July 24 / Day 205
+33 days
Jan
Feb
Mar
Apr
May
Jun
Jul
Aug
Sep
Oct
Nov
Dec
°F
Day of Year

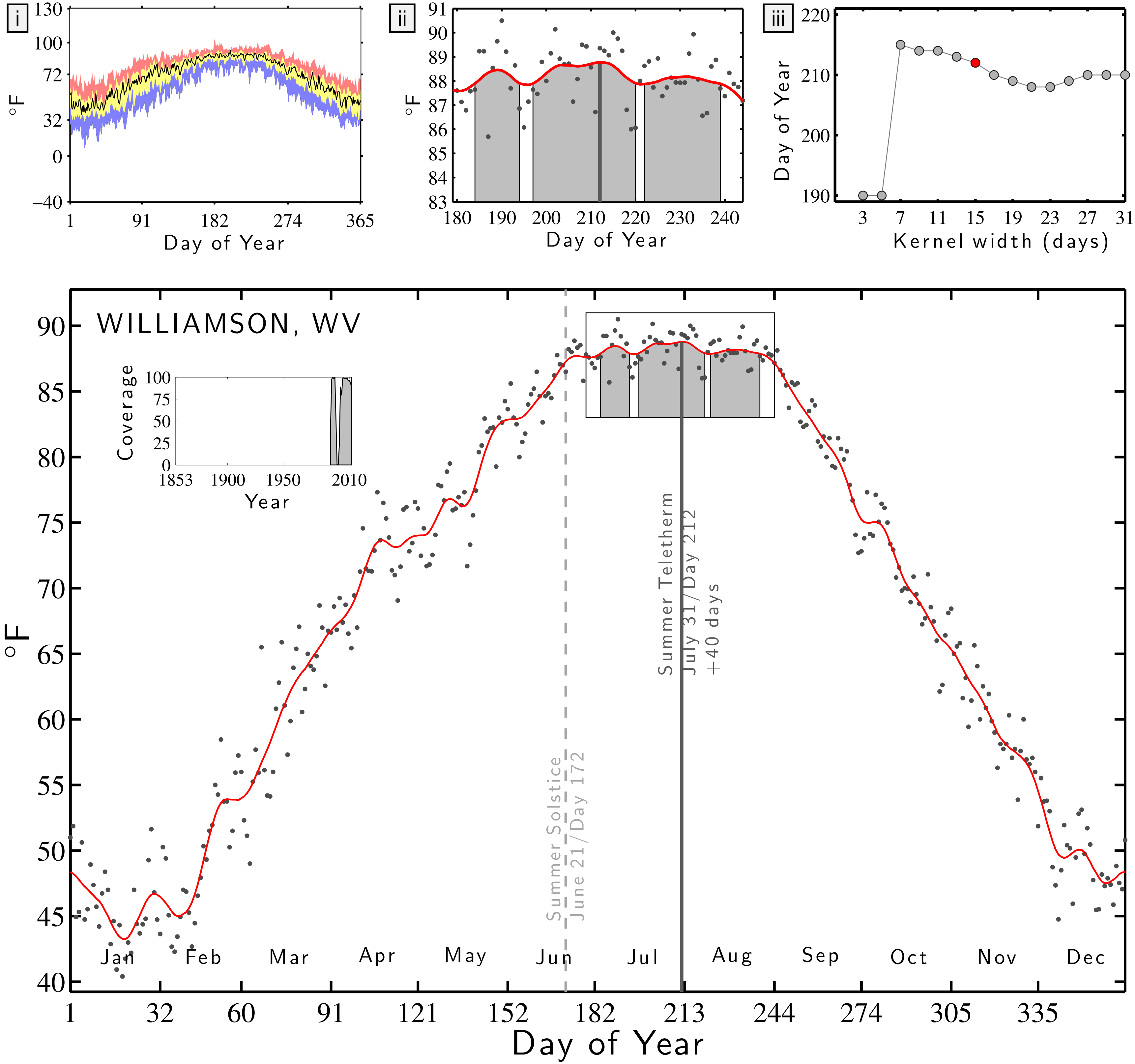

i
°F
Day of Year
ii
°F
Day of Year
iii
Day of Year
Kernel width (days)
WILLIAMSON, WV
Coverage
Year
Summer Solstice
June 21 / Day 172
Summer Teletherm
July 31 / Day 212
+40 days
Jan
Feb
Mar
Apr
May
Jun
Jul
Aug
Sep
Oct
Nov
Dec
°F
Day of Year

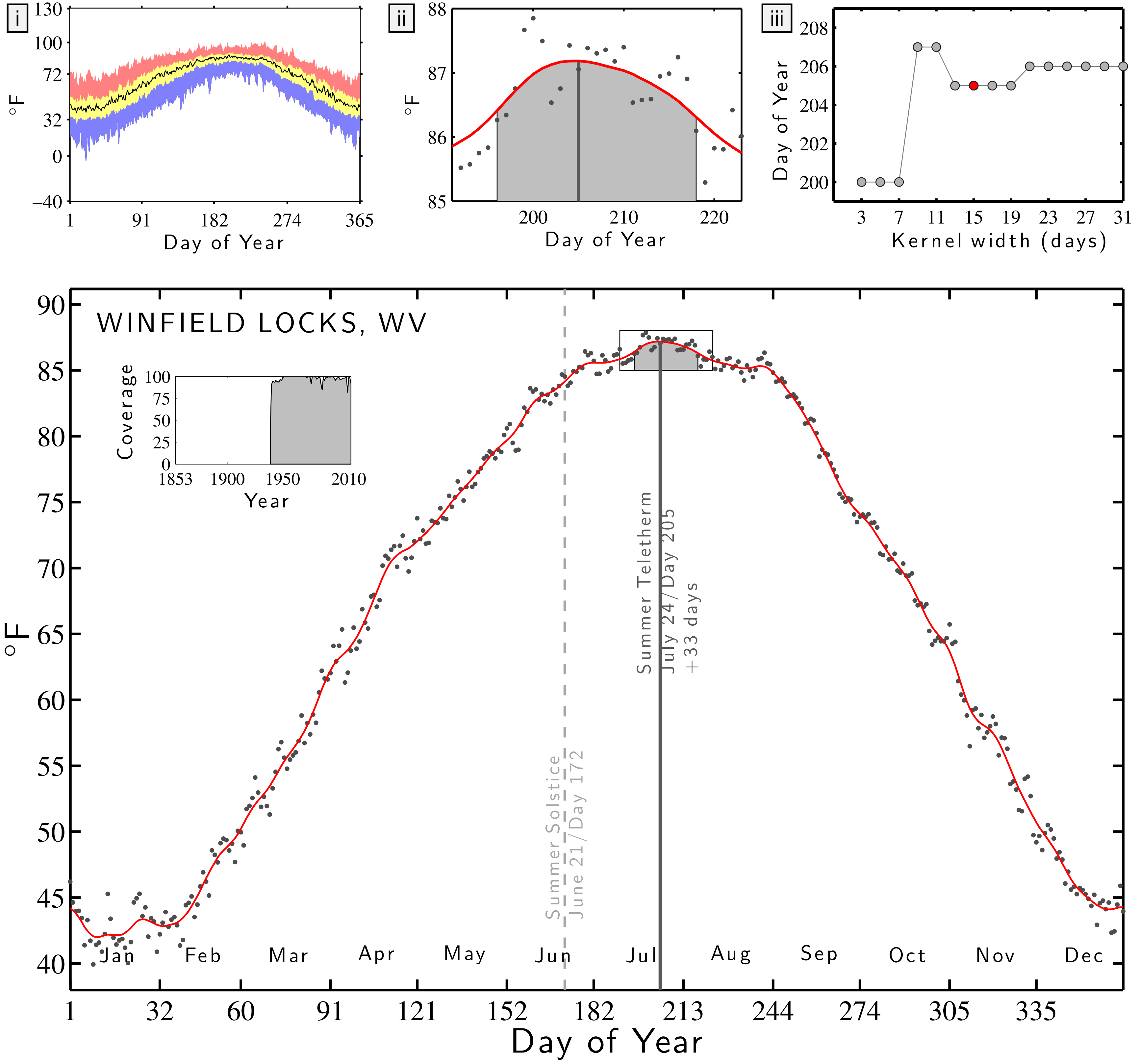

i
ii
iii
°F
Day of Year
Kernel width (days)
WINFIELD LOCKS, WV
Coverage
Year
Summer Solstice
June 21/Day 172
Summer Teletherm
July 24/Day 205
+33 days
Jan
Feb
Mar
Apr
May
Jun
Jul
Aug
Sep
Oct
Nov
Dec

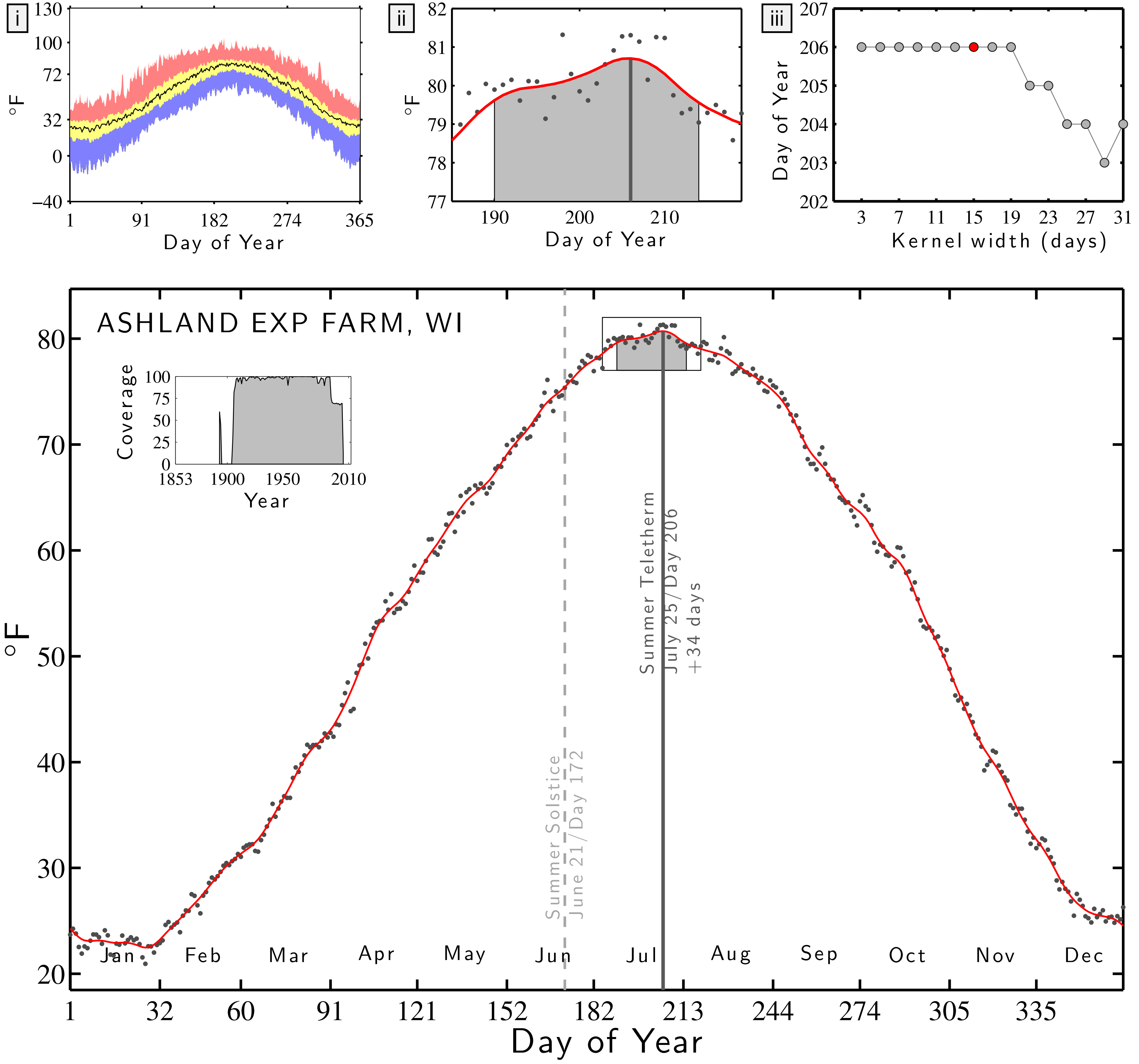
i
°F
Day of Year
ii
°F
Day of Year
iii
Day of Year
Kernel width (days)
ASHLAND EXP FARM, WI
Coverage
Year
Summer Solstice
June 21/Day 172
Summer Teletherm
July 25/Day 206
+34 days
Jan
Feb
Mar
Apr
May
Jun
Jul
Aug
Sep
Oct
Nov
Dec
°F
Day of Year

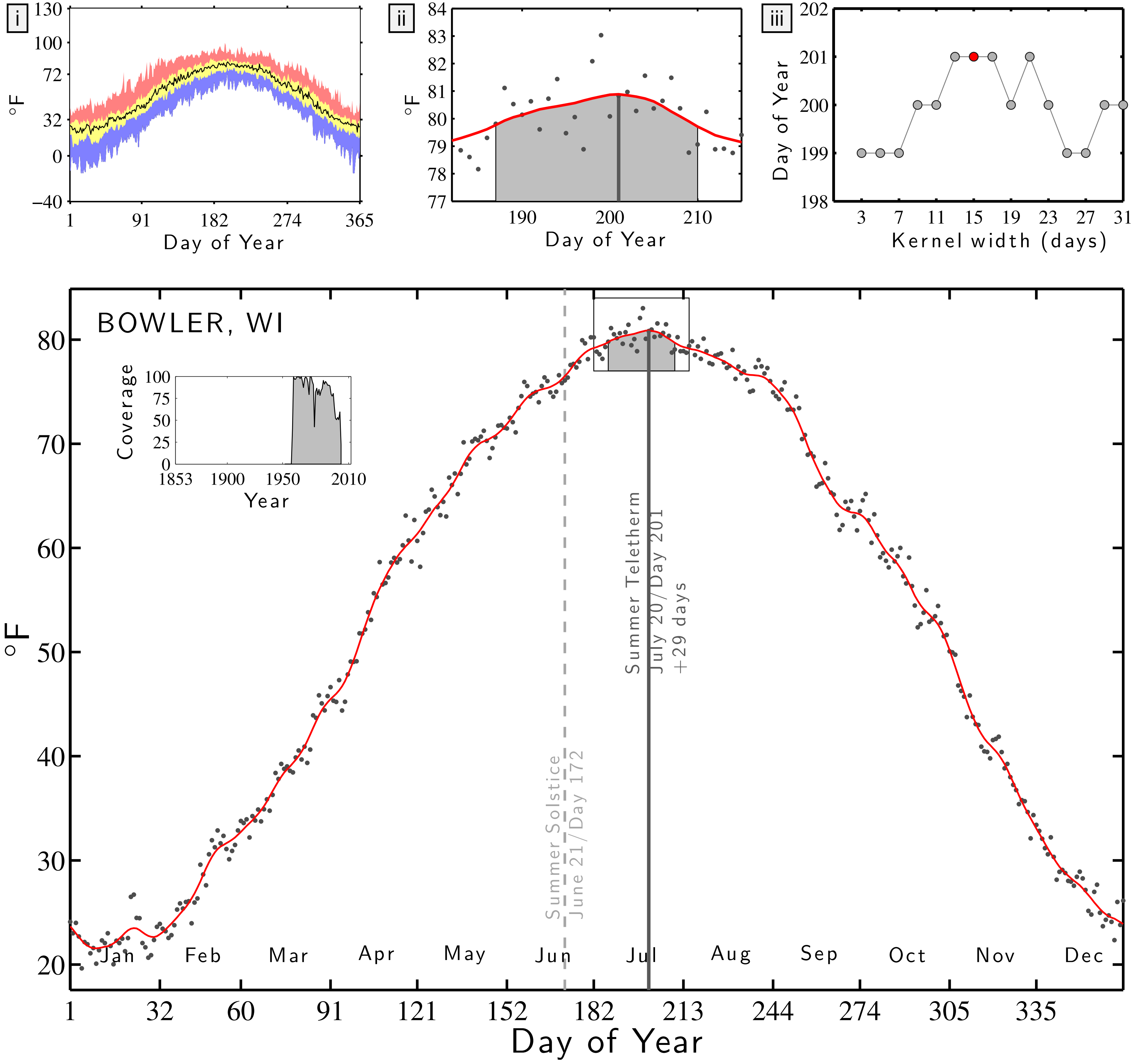

i
ii
iii
°F
Day of Year
Day of Year
Kernel width (days)
BOWLER, WI
Coverage
Year
Summer Solstice
June 21 / Day 172
Summer Teletherm
July 20 / Day 201
+29 days
Jan
Feb
Mar
Apr
May
Jun
Jul
Aug
Sep
Oct
Nov
Dec

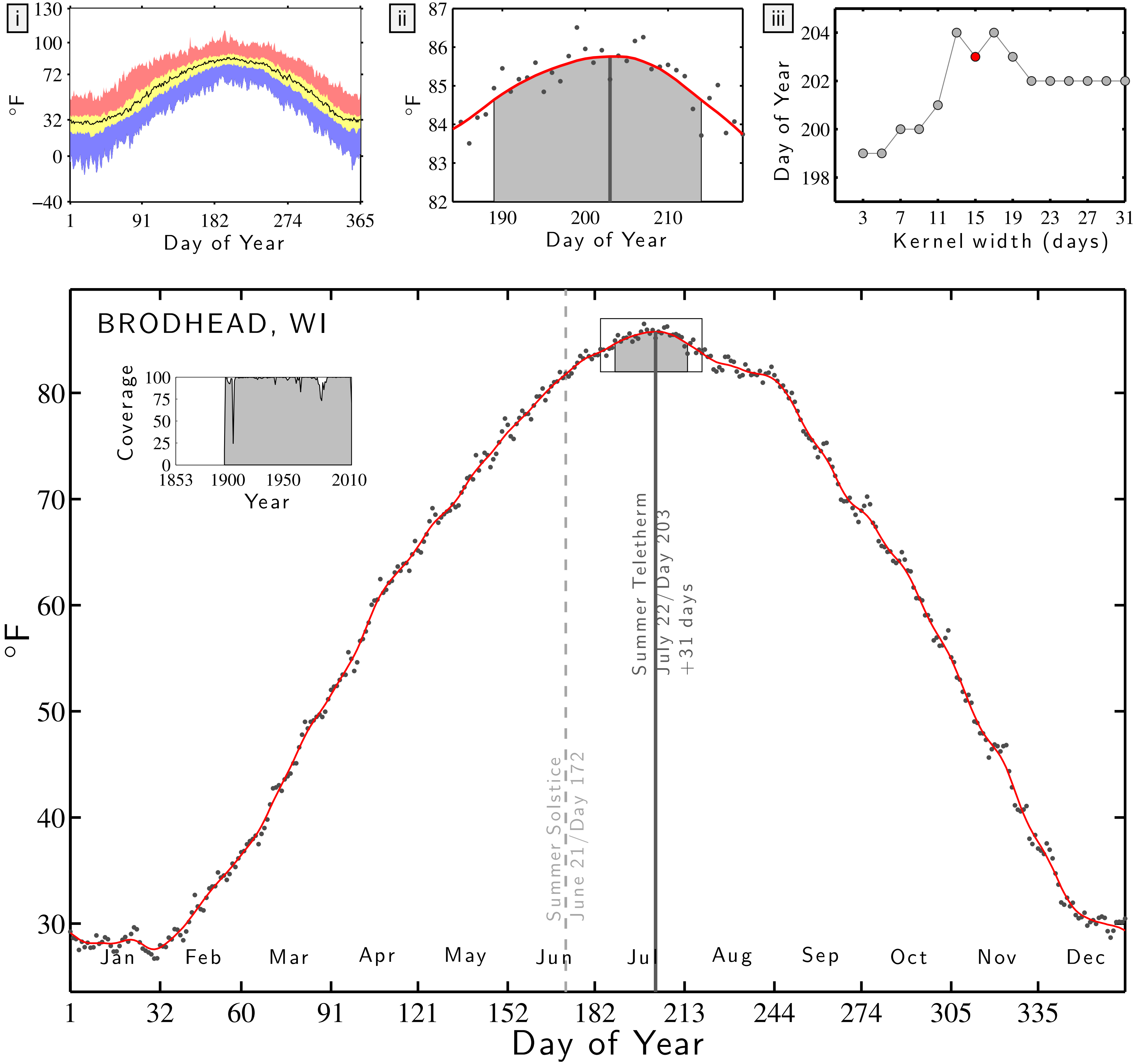

i
ii
iii
BRODHEAD, WI
Summer Solstice
June 21/Day 172
Summer Teletherm
July 22/Day 203
+31 days
Coverage
Year
Day of Year
°F
Kernel width (days)
Jan
Feb
Mar
Apr
May
Jun
Jul
Aug
Sep
Oct
Nov
Dec

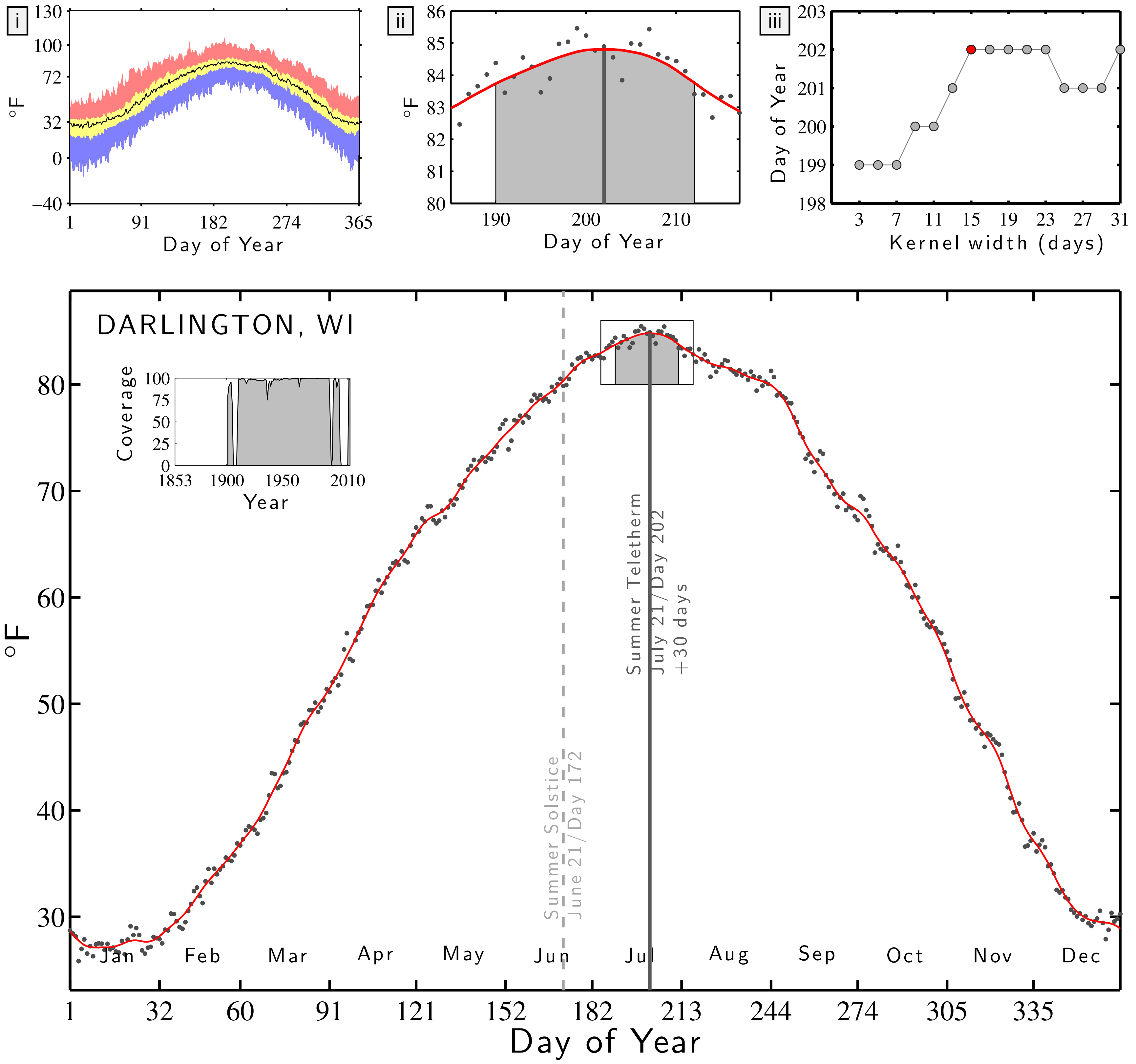

i
ii
iii
°F
Day of Year
Day of Year
Kernel width (days)
DARLINGTON, WI
Coverage
Year
Summer Solstice
June 21 / Day 172
Summer Teletherm
July 21 / Day 202
+30 days
Jan
Feb
Mar
Apr
May
Jun
Jul
Aug
Sep
Oct
Nov
Dec
°F
Day of Year

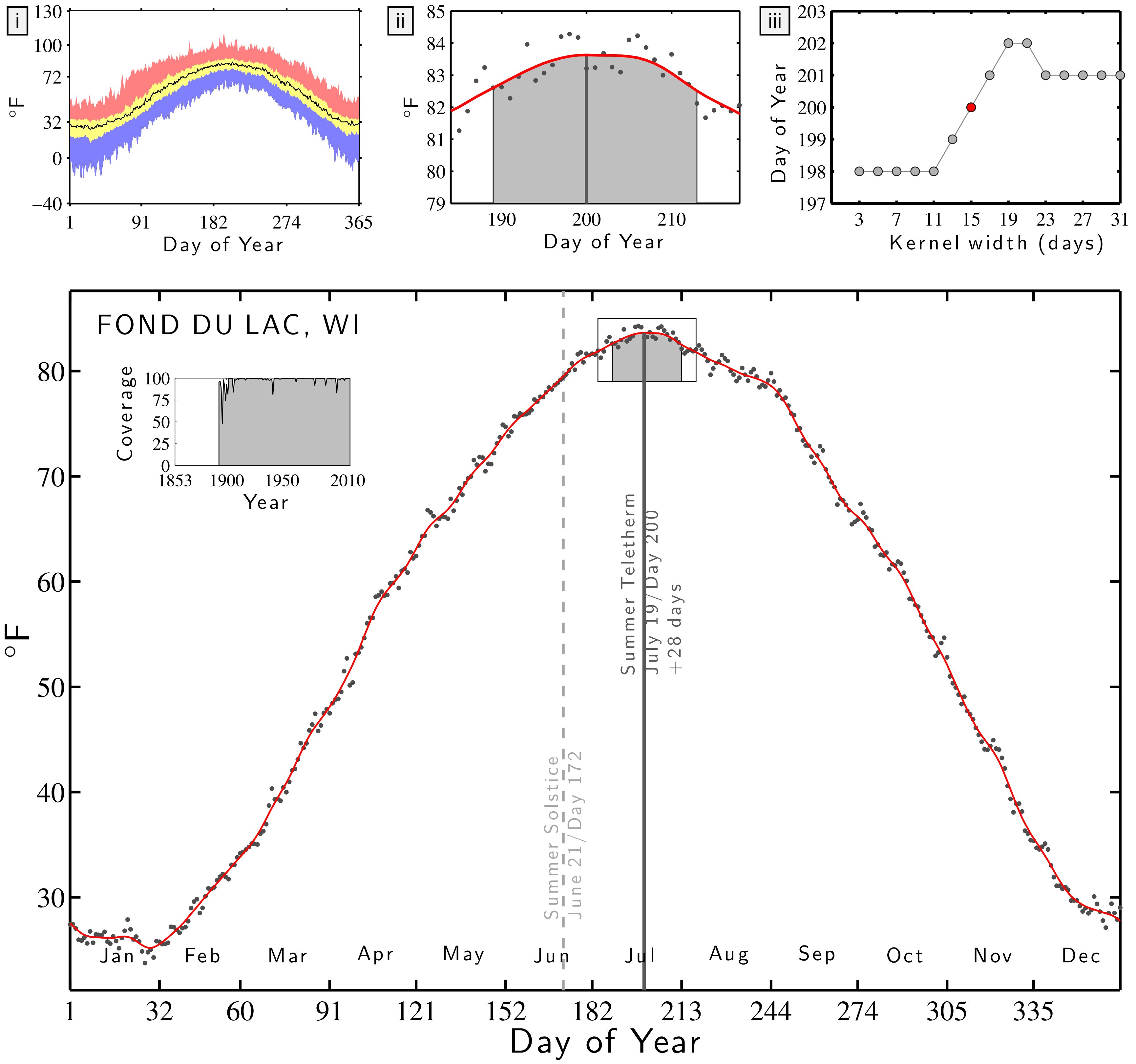

i
°F
Day of Year
ii
°F
Day of Year
iii
Day of Year
Kernel width (days)
FOND DU LAC, WI
Coverage
Year
Summer Solstice
June 21/Day 172
Summer Teletherm
July 19/Day 200
+28 days
Jan
Feb
Mar
Apr
May
Jun
Jul
Aug
Sep
Oct
Nov
Dec
°F
Day of Year

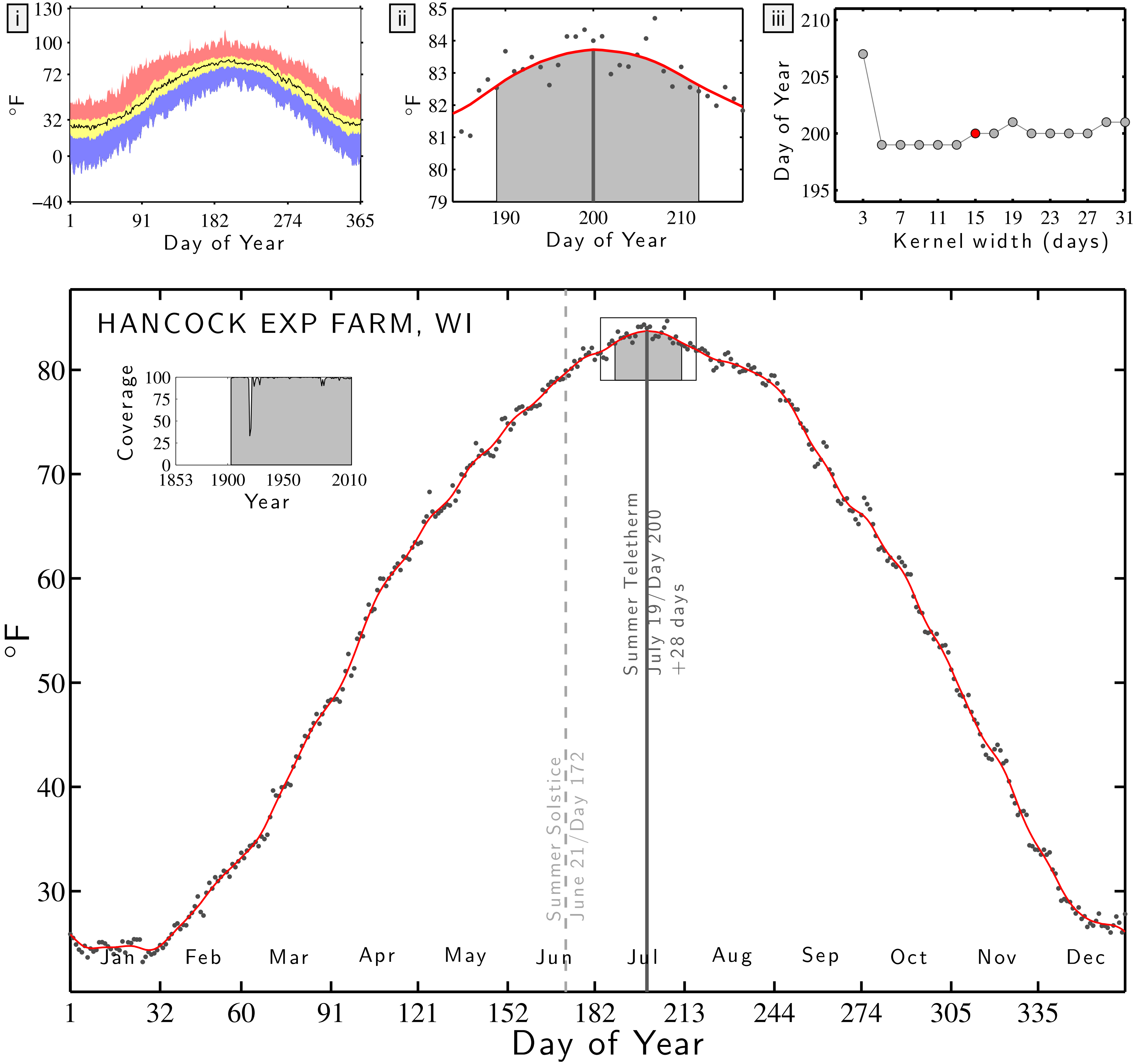

i
°F
Day of Year
ii
°F
Day of Year
iii
Day of Year
Kernel width (days)
HANCOCK EXP FARM, WI
Coverage
Year
Summer Solstice
June 21/Day 172
Summer Teletherm
July 19/Day 200
+28 days
Jan Feb Mar Apr May Jun Jul Aug Sep Oct Nov Dec
°F
Day of Year

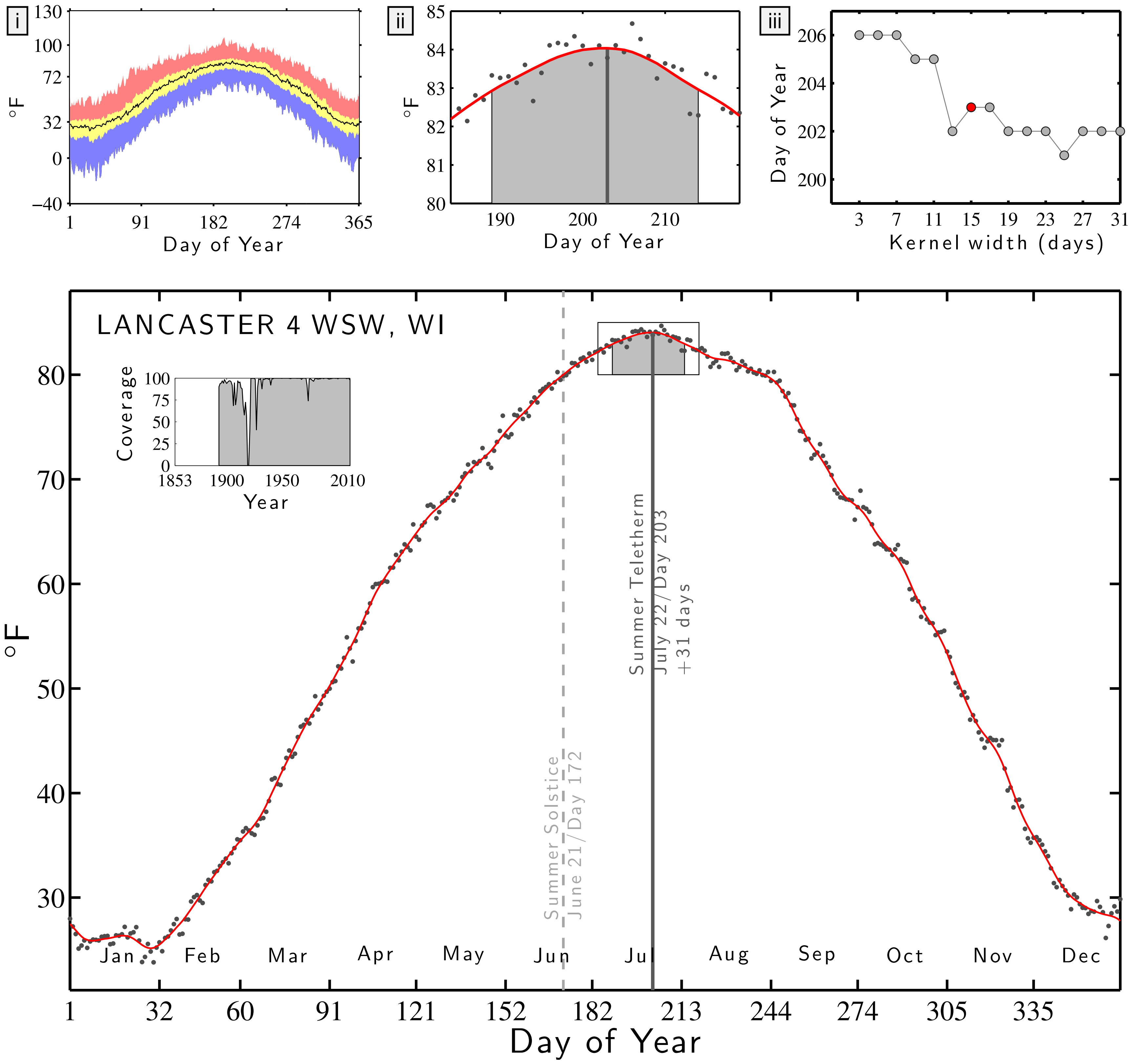

i
°F
Day of Year
ii
°F
Day of Year
iii
Day of Year
Kernel width (days)
LANCASTER 4 WSW, WI
Coverage
Year
1853
1900
1950
2010
Summer Solstice
June 21/Day 172
Summer Teletherm
July 22/Day 203
+31 days
Jan
Feb
Mar
Apr
May
Jun
Jul
Aug
Sep
Oct
Nov
Dec
°F
Day of Year

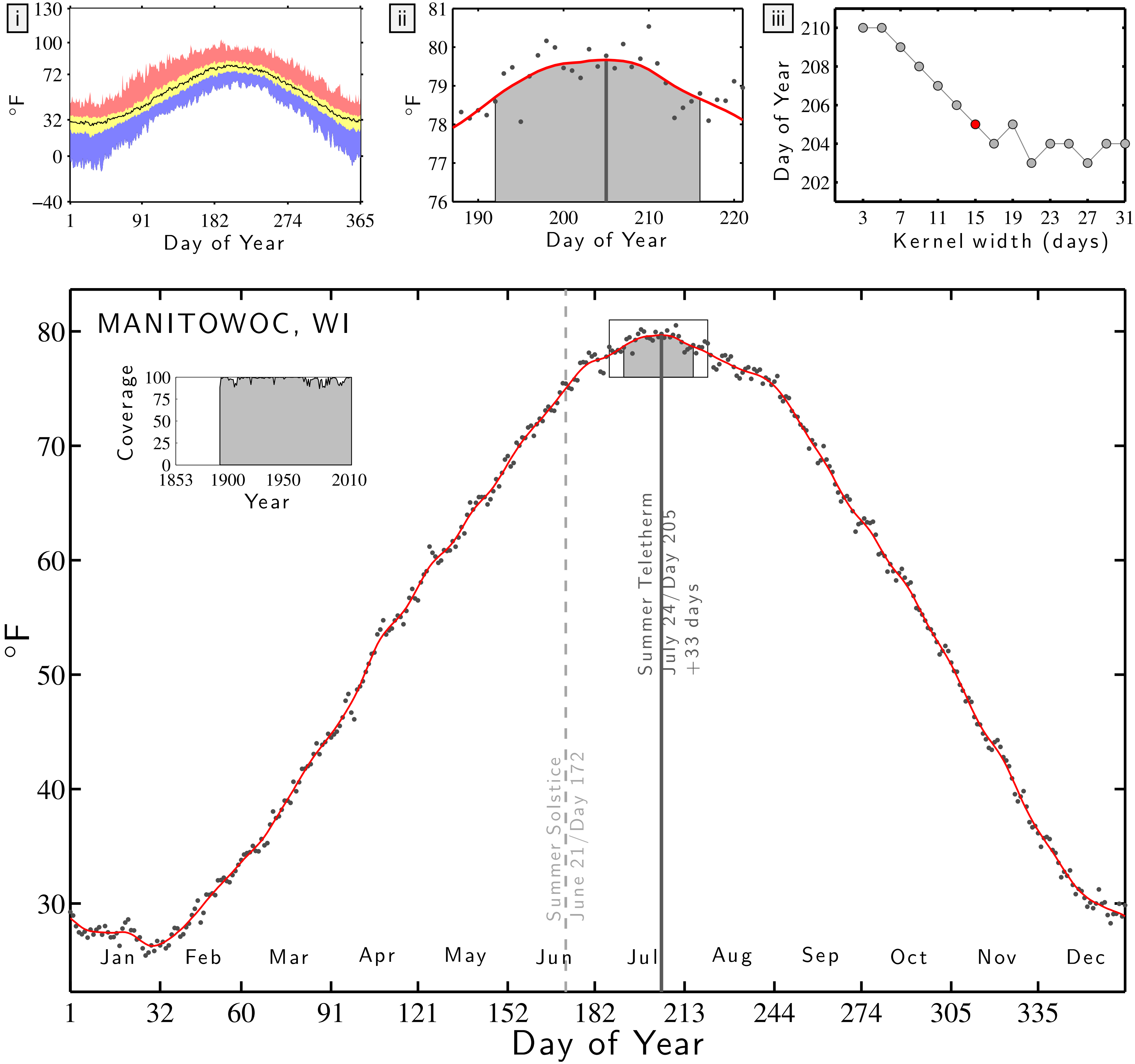

i
°F
Day of Year
ii
Day of Year
iii
Day of Year
Kernel width (days)
MANITOWOC, WI
Coverage
Year
Summer Solstice
June 21/Day 172
Summer Teletherm
July 24/Day 205
+33 days
Jan
Feb
Mar
Apr
May
Jun
Jul
Aug
Sep
Oct
Nov
Dec
Day of Year

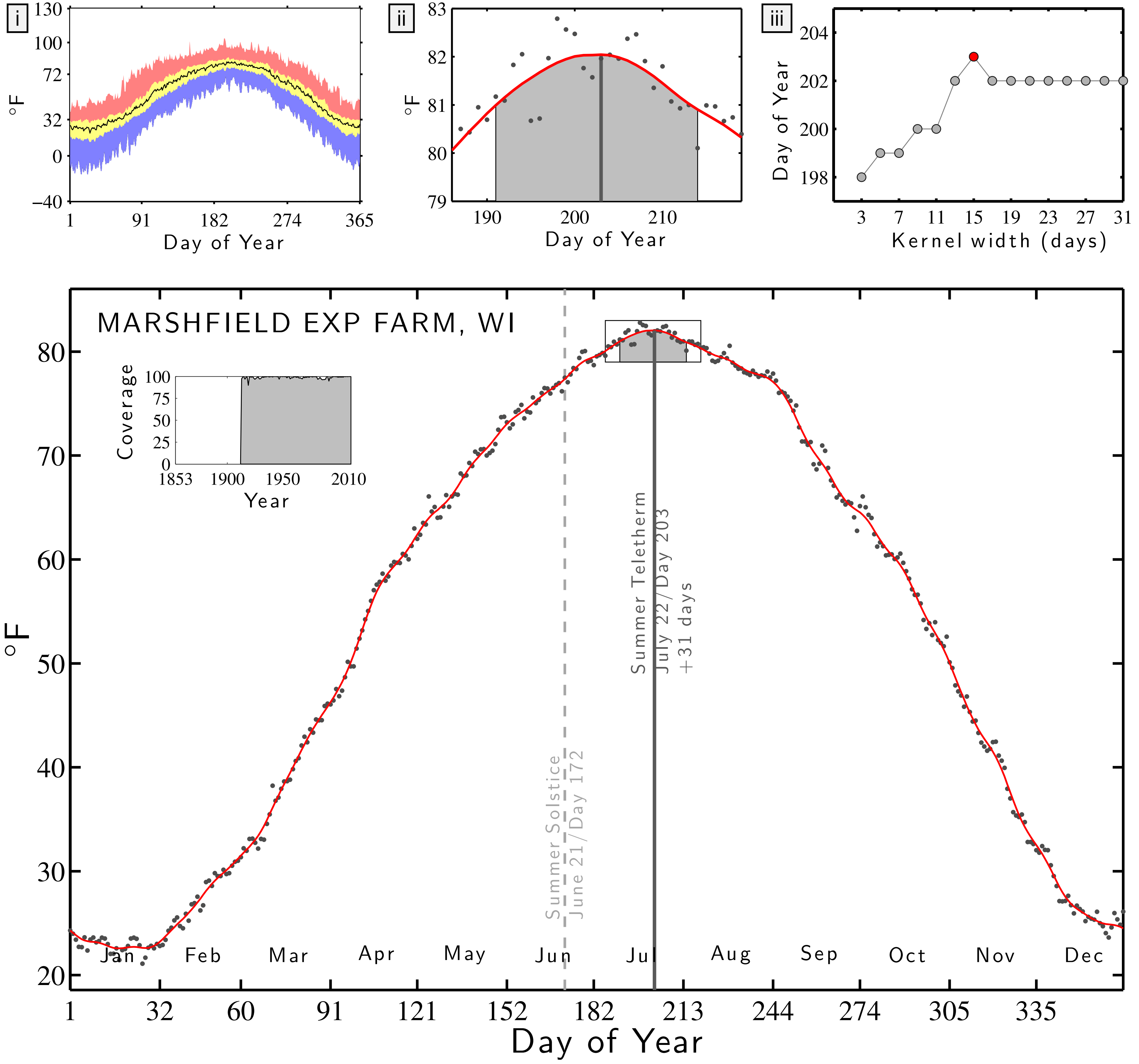

i
°F
Day of Year
ii
°F
Day of Year
iii
Day of Year
Kernel width (days)
MARSHFIELD EXP FARM, WI
Coverage
Year
Summer Solstice
June 21/Day 172
Summer Teletherm
July 22/Day 203
+31 days
Jan
Feb
Mar
Apr
May
Jun
Jul
Aug
Sep
Oct
Nov
Dec
°F
Day of Year

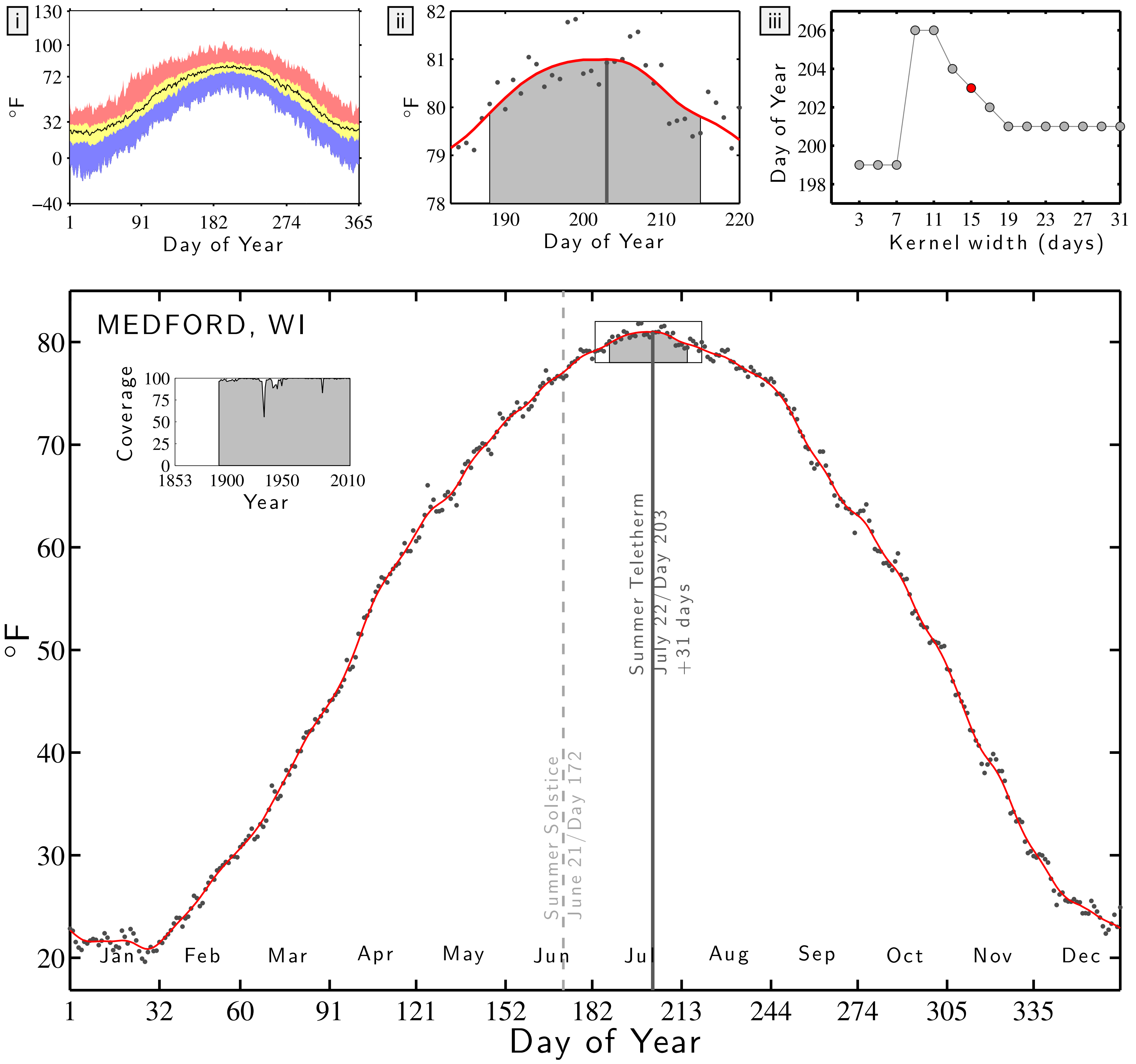

i
°F
Day of Year
ii
°F
Day of Year
iii
Day of Year
Kernel width (days)
MEDFORD, WI
Coverage
Year
Summer Solstice
June 21/Day 172
Summer Teletherm
July 22/Day 203
+31 days
Jan
Feb
Mar
Apr
May
Jun
Jul
Aug
Sep
Oct
Nov
Dec
°F
Day of Year

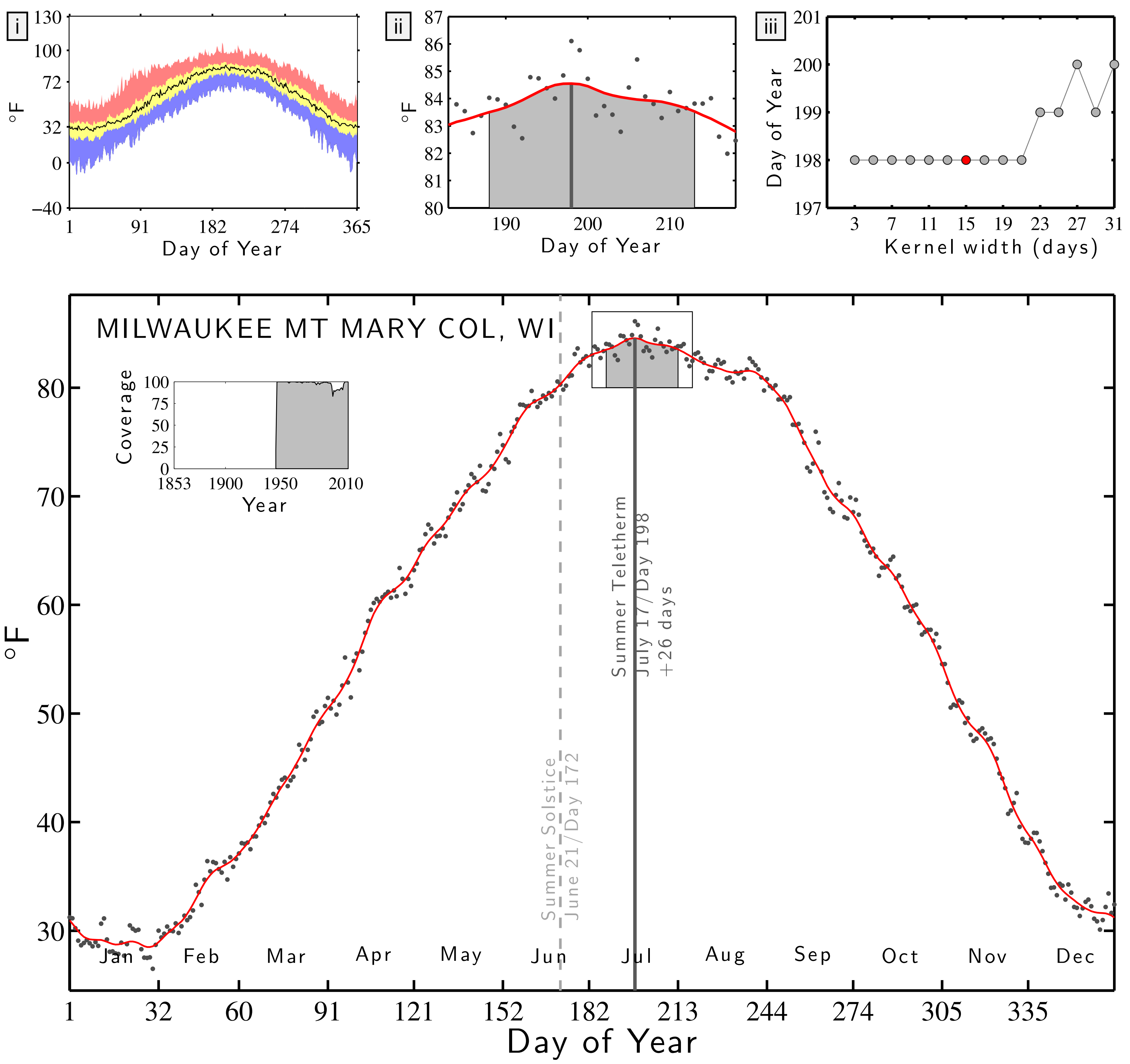

i
ii
iii
°F
Day of Year
Kernel width (days)
MILWAUKEE MT MARY COL, WI
Coverage
Year
Summer Solstice
June 21 / Day 172
Summer Teletherm
July 17 / Day 198
+26 days
Jan
Feb
Mar
Apr
May
Jun
Jul
Aug
Sep
Oct
Nov
Dec

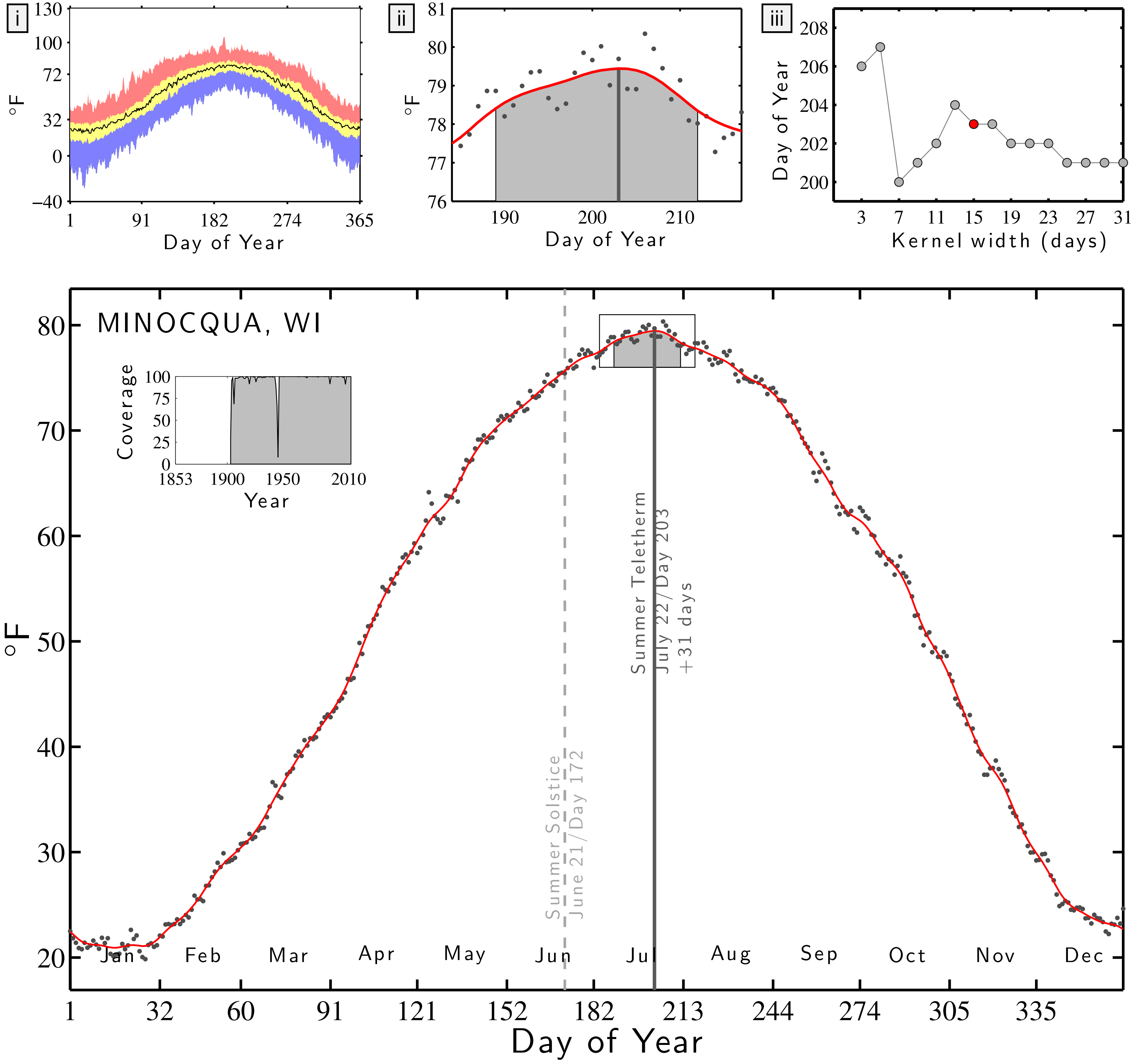

i
°F
Day of Year
ii
°F
Day of Year
iii
Day of Year
Kernel width (days)
MINOCQUA, WI
Coverage
Year
Summer Solstice
June 21/Day 172
Summer Teletherm
July 22/Day 203
+31 days
Jan
Feb
Mar
Apr
May
Jun
Jul
Aug
Sep
Oct
Nov
Dec
°F
Day of Year

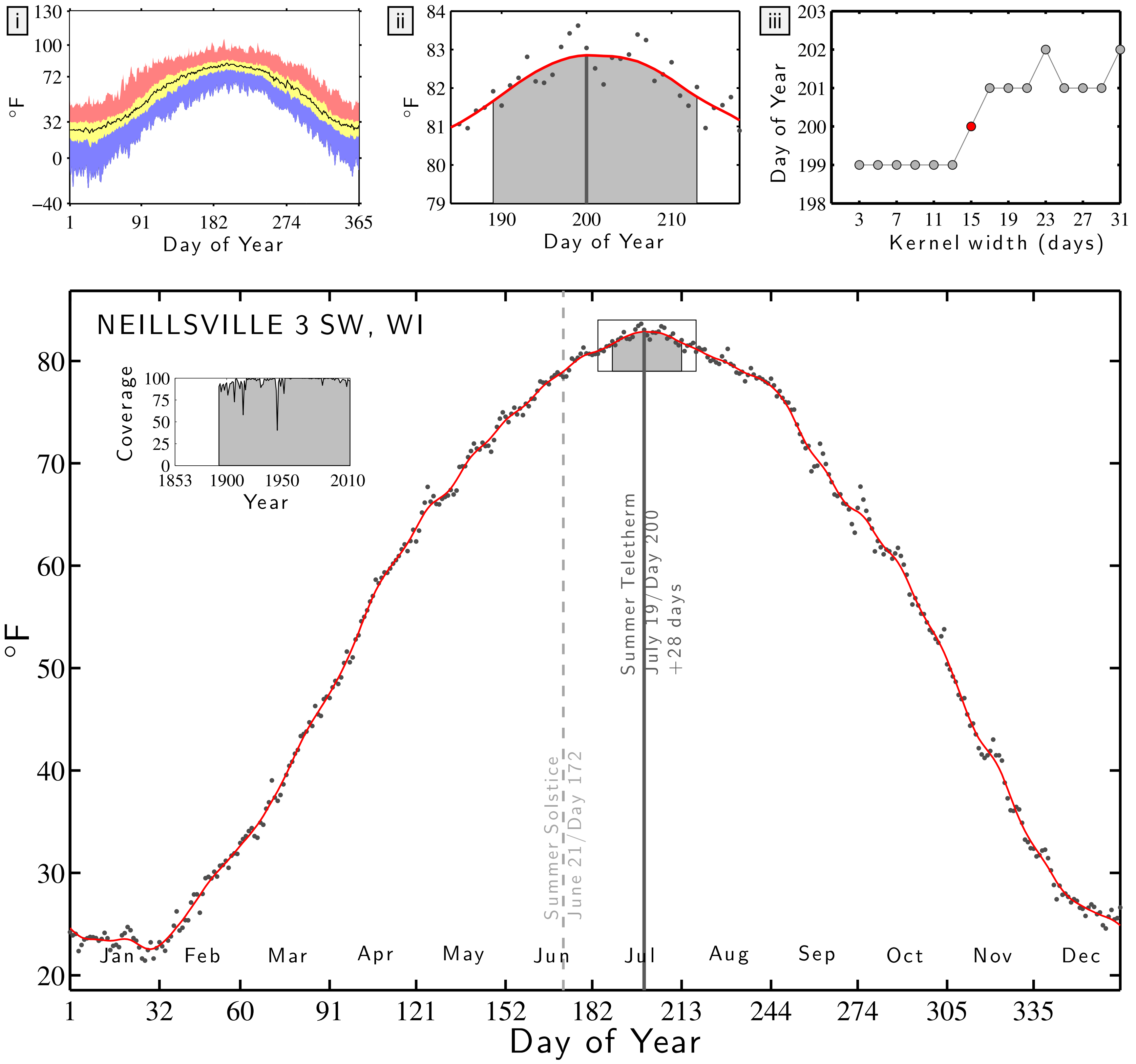

i
ii
iii
NEILLSVILLE 3 SW, WI
Coverage
Year
Summer Solstice
June 21/Day 172
Summer Teletherm
July 19/Day 200
+28 days
Day of Year
Kernel width (days)
°F
Jan
Feb
Mar
Apr
May
Jun
Jul
Aug
Sep
Oct
Nov
Dec

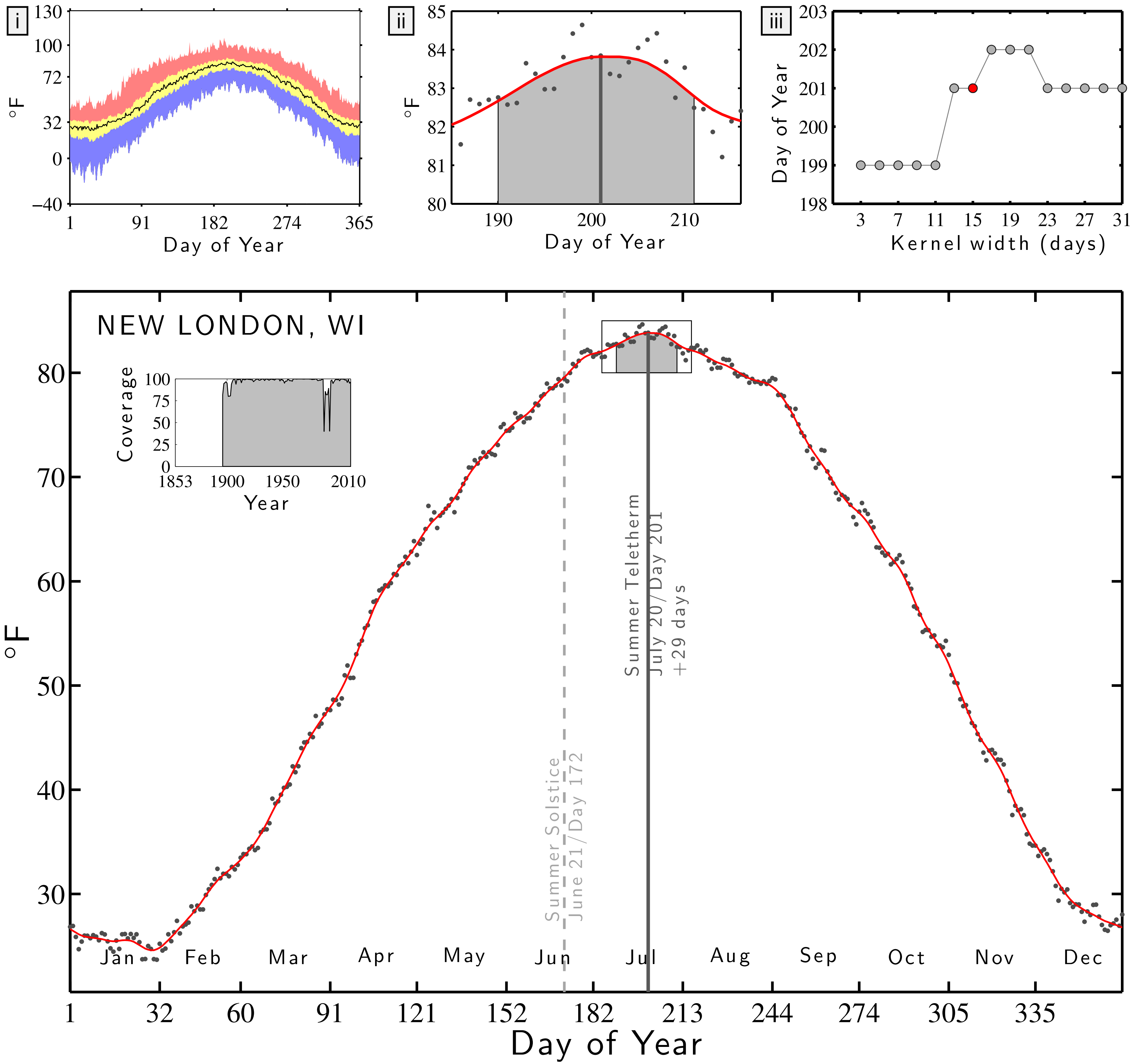

i
°F
Day of Year
ii
°F
Day of Year
iii
Day of Year
Kernel width (days)
NEW LONDON, WI
Coverage
Year
Summer Solstice
June 21/Day 172
Summer Teletherm
July 20/Day 201
+29 days
Jan
Feb
Mar
Apr
May
Jun
Jul
Aug
Sep
Oct
Nov
Dec
°F
Day of Year

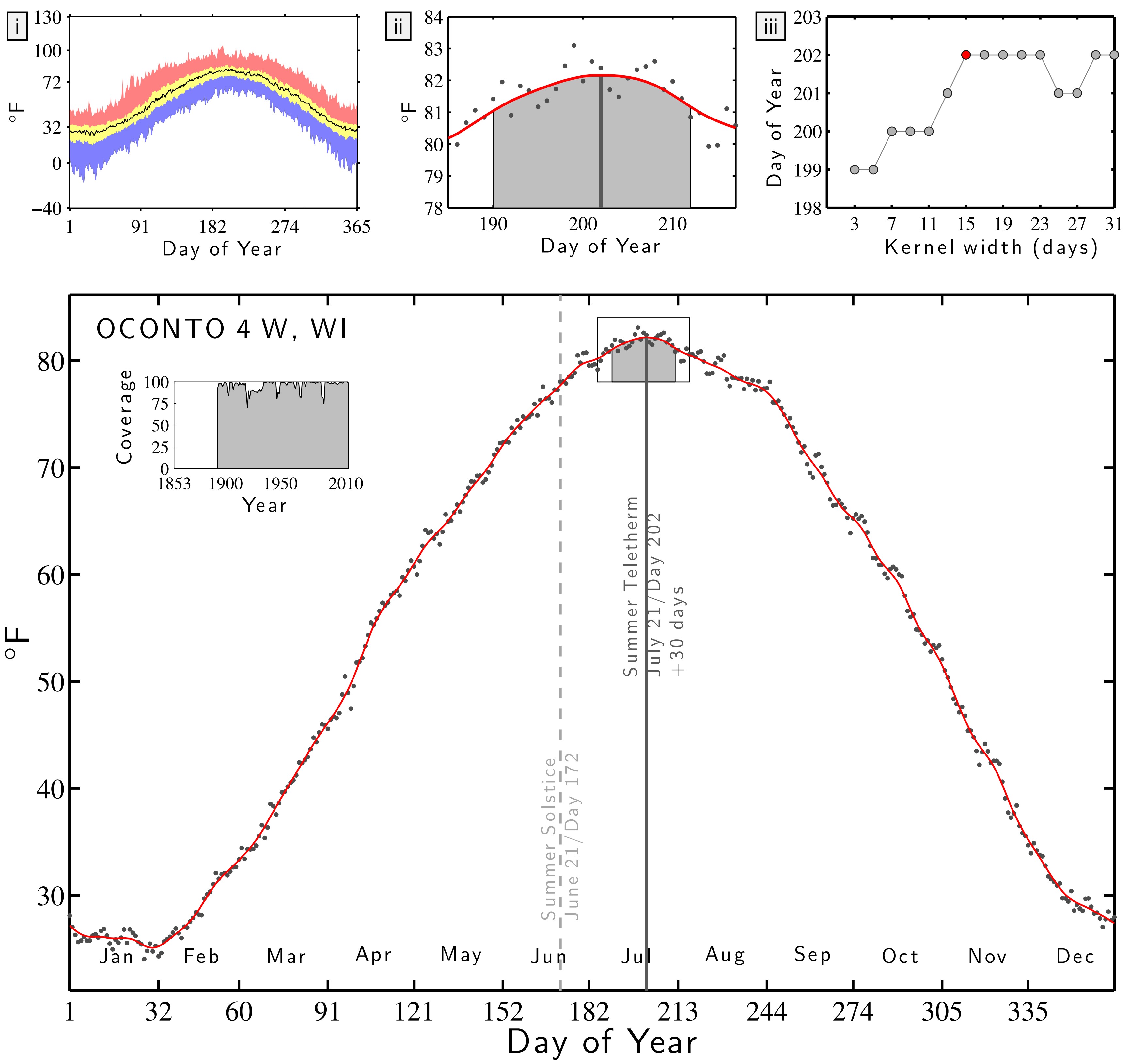

i
ii
iii
°F
Day of Year
Day of Year
Kernel width (days)
OCONTO 4 W, WI
Coverage
Year
Summer Solstice
June 21 / Day 172
Summer Teletherm
July 21 / Day 202
+30 days
Jan
Feb
Mar
Apr
May
Jun
Jul
Aug
Sep
Oct
Nov
Dec

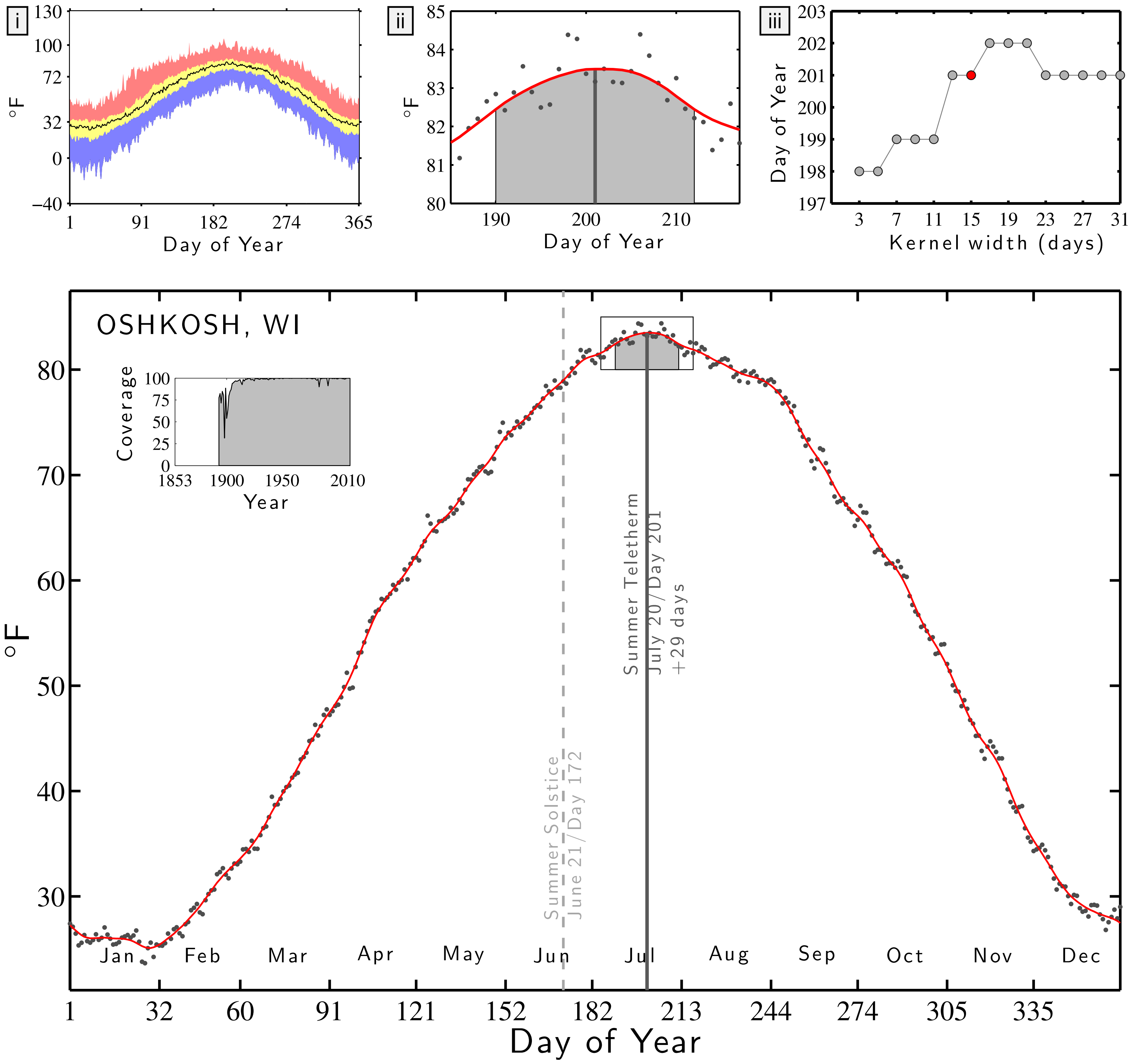

i
ii
iii
°F
Day of Year
Day of Year
Kernel width (days)
OSHKOSH, WI
Coverage
Year
Summer Solstice
June 21/Day 172
Summer Teletherm
July 20/Day 201
+29 days
Jan
Feb
Mar
Apr
May
Jun
Jul
Aug
Sep
Oct
Nov
Dec
Day of Year

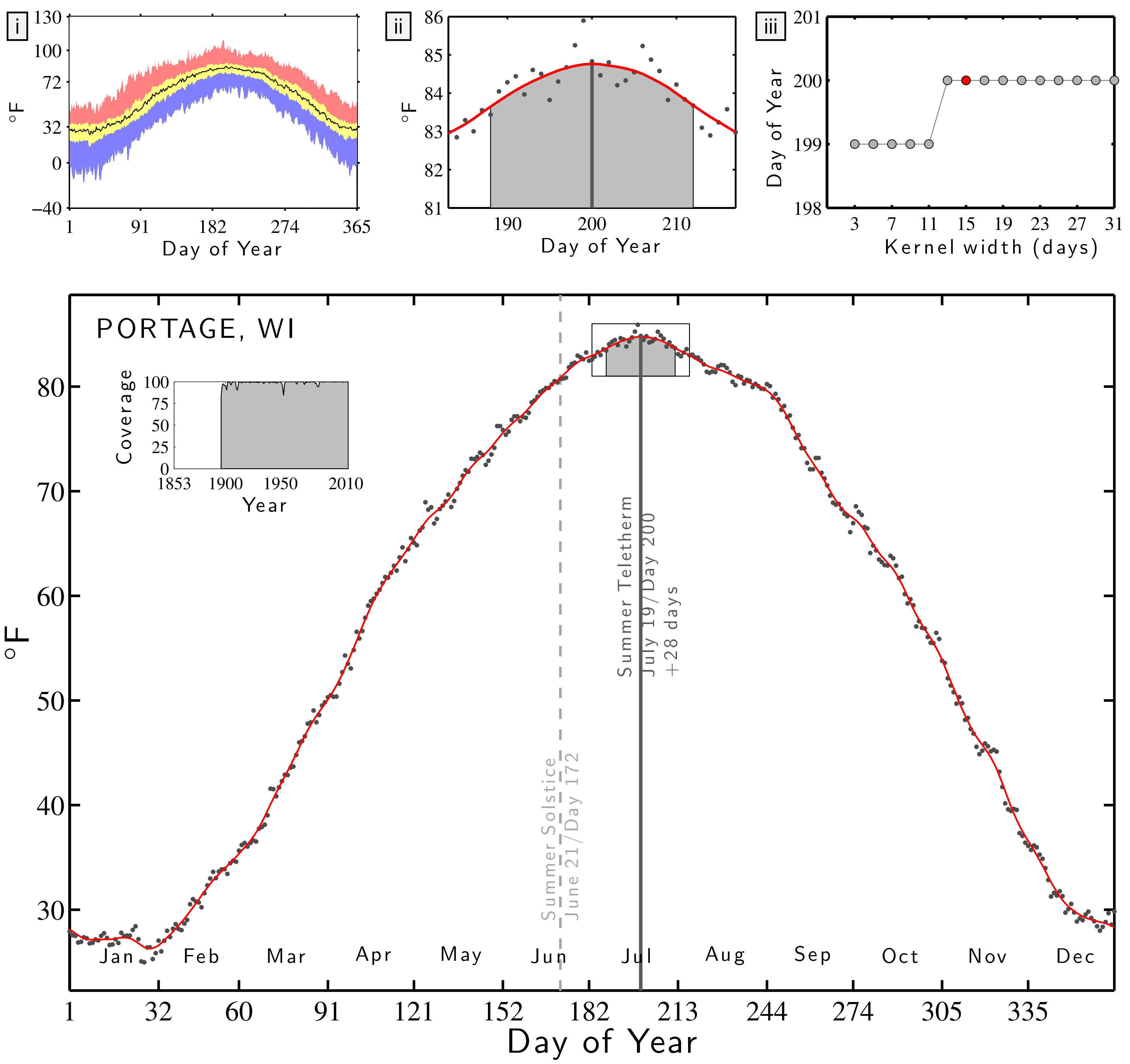

i
°F
130
100
72
32
0
−40
1
91
182
274
365
Day of Year
ii
°F
86
85
84
83
82
81
190
200
210
Day of Year
iii
Day of Year
201
200
199
198
3
7
11
15
19
23
27
31
Kernel width (days)
PORTAGE, WI
Coverage
100
75
50
25
0
1853
1900
1950
2010
Year
°F
80
70
60
50
40
30
Summer Solstice
June 21/Day 172
Summer Teletherm
July 19/Day 200
+28 days
Jan
Feb
Mar
Apr
May
Jun
Jul
Aug
Sep
Oct
Nov
Dec
1
32
60
91
121
152
182
213
244
274
305
335
Day of Year

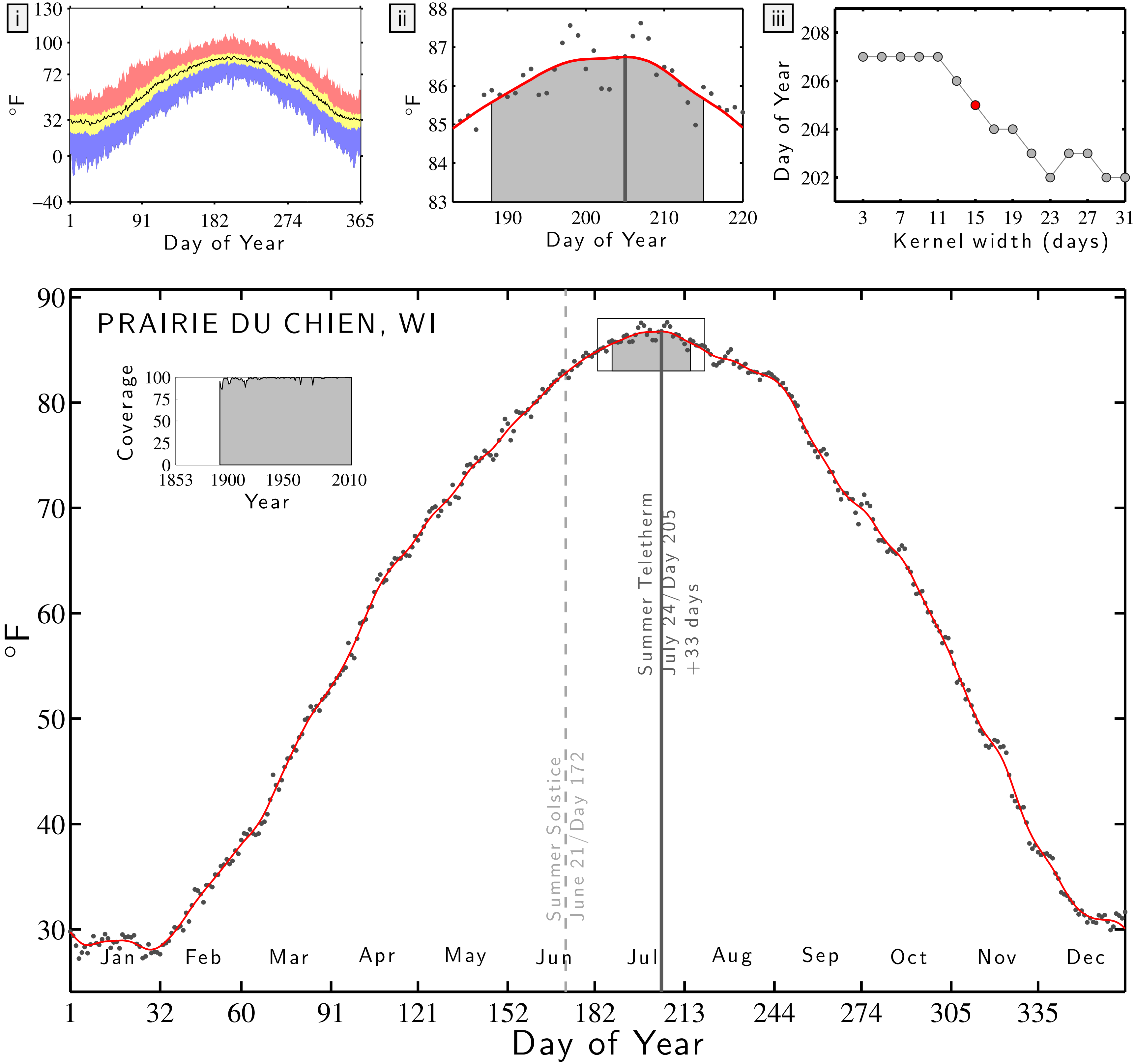

i
°F
Day of Year
ii
°F
Day of Year
iii
Day of Year
Kernel width (days)
PRAIRIE DU CHIEN, WI
Coverage
Year
Summer Solstice
June 21 / Day 172
Summer Teletherm
July 24 / Day 205
+33 days
Jan
Feb
Mar
Apr
May
Jun
Jul
Aug
Sep
Oct
Nov
Dec
°F
Day of Year

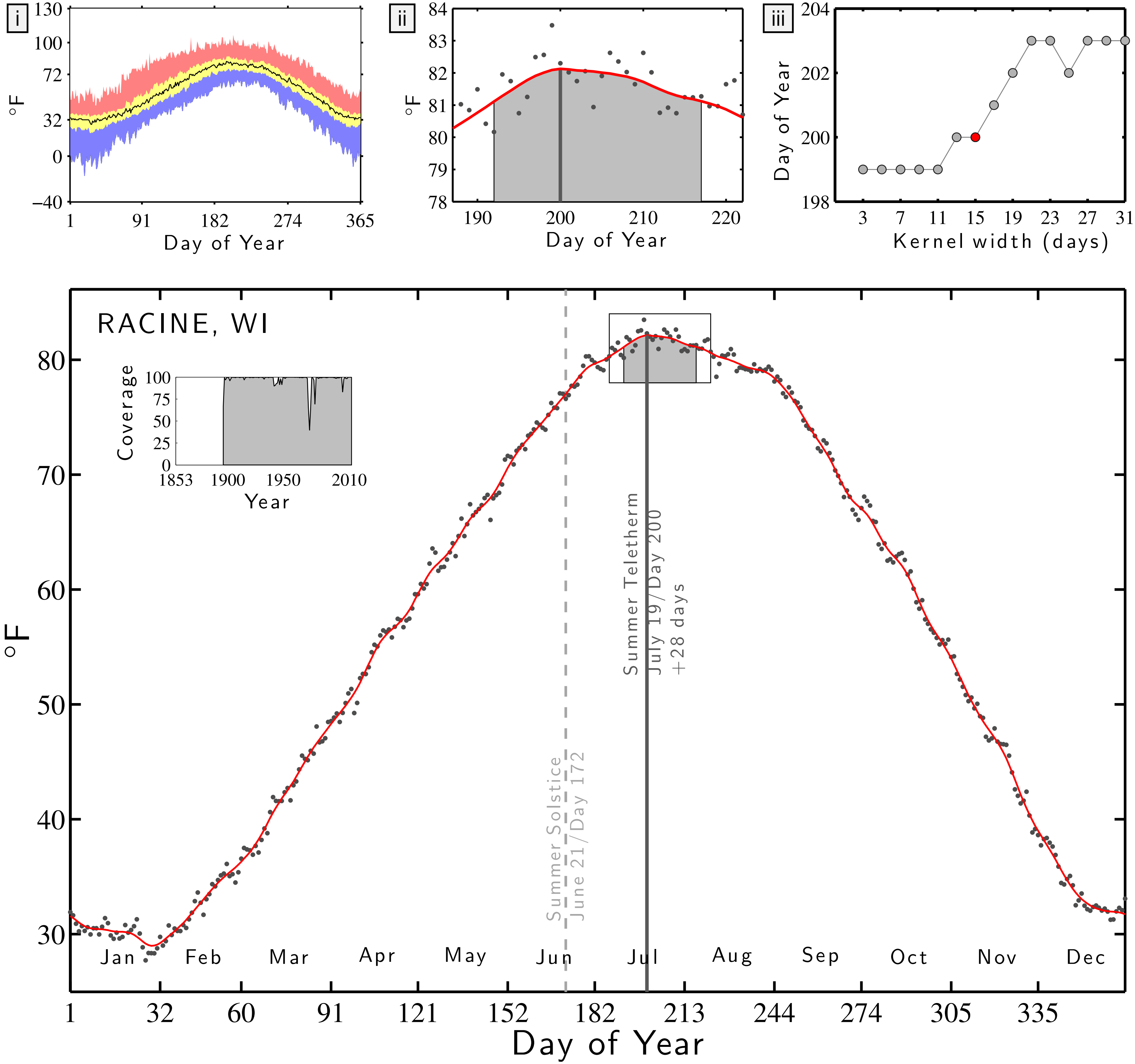

i
°F
Day of Year
ii
°F
Day of Year
iii
Day of Year
Kernel width (days)
RACINE, WI
Coverage
Year
Summer Solstice
June 21/Day 172
Summer Teletherm
July 19/Day 200
+28 days
Jan
Feb
Mar
Apr
May
Jun
Jul
Aug
Sep
Oct
Nov
Dec
°F
Day of Year

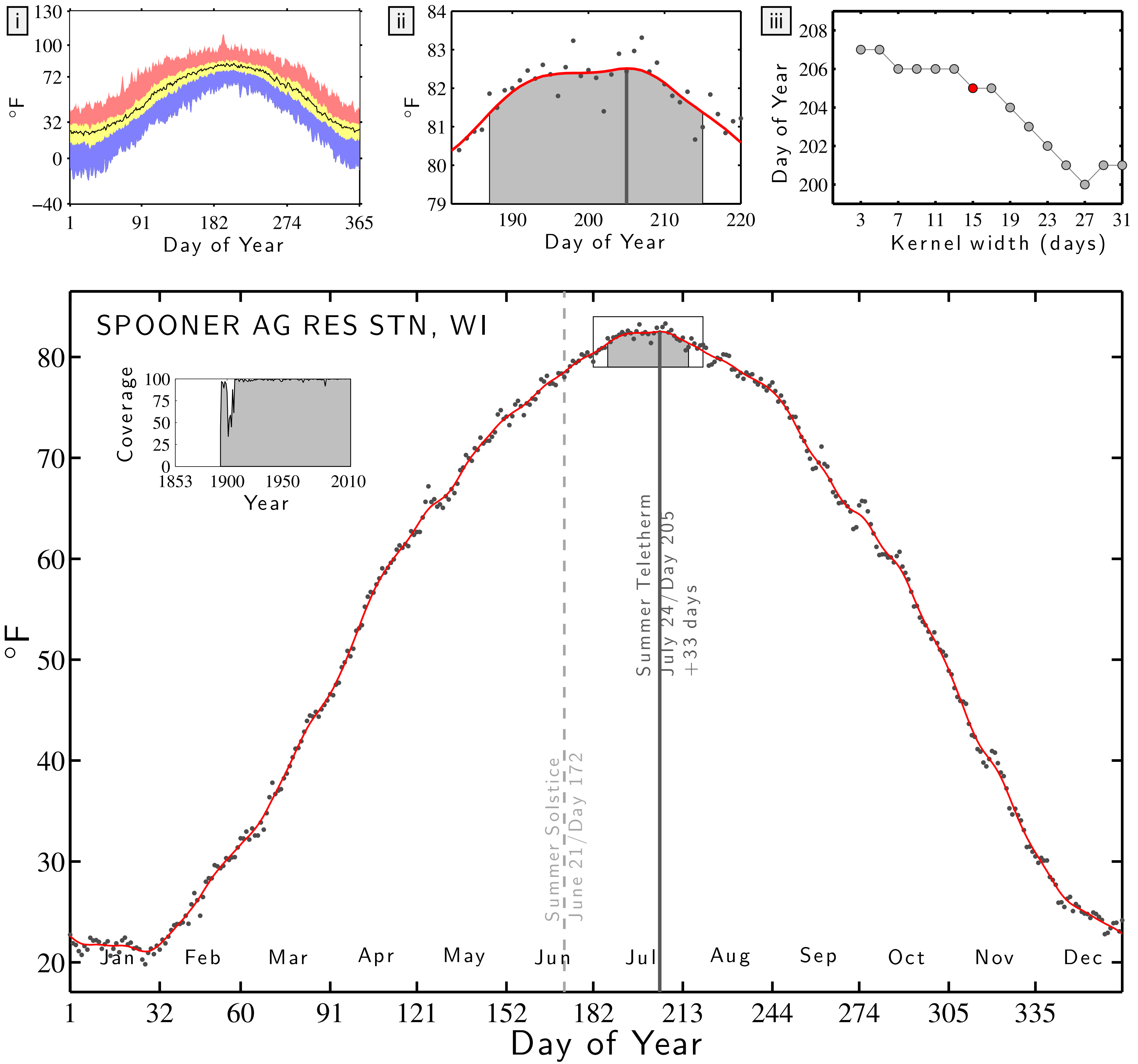

i
ii
iii
°F
Day of Year
Kernel width (days)
SPOONER AG RES STN, WI
Coverage
Year
Summer Solstice
June 21/Day 172
Summer Teletherm
July 24/Day 205
+33 days
Jan
Feb
Mar
Apr
May
Jun
Jul
Aug
Sep
Oct
Nov
Dec

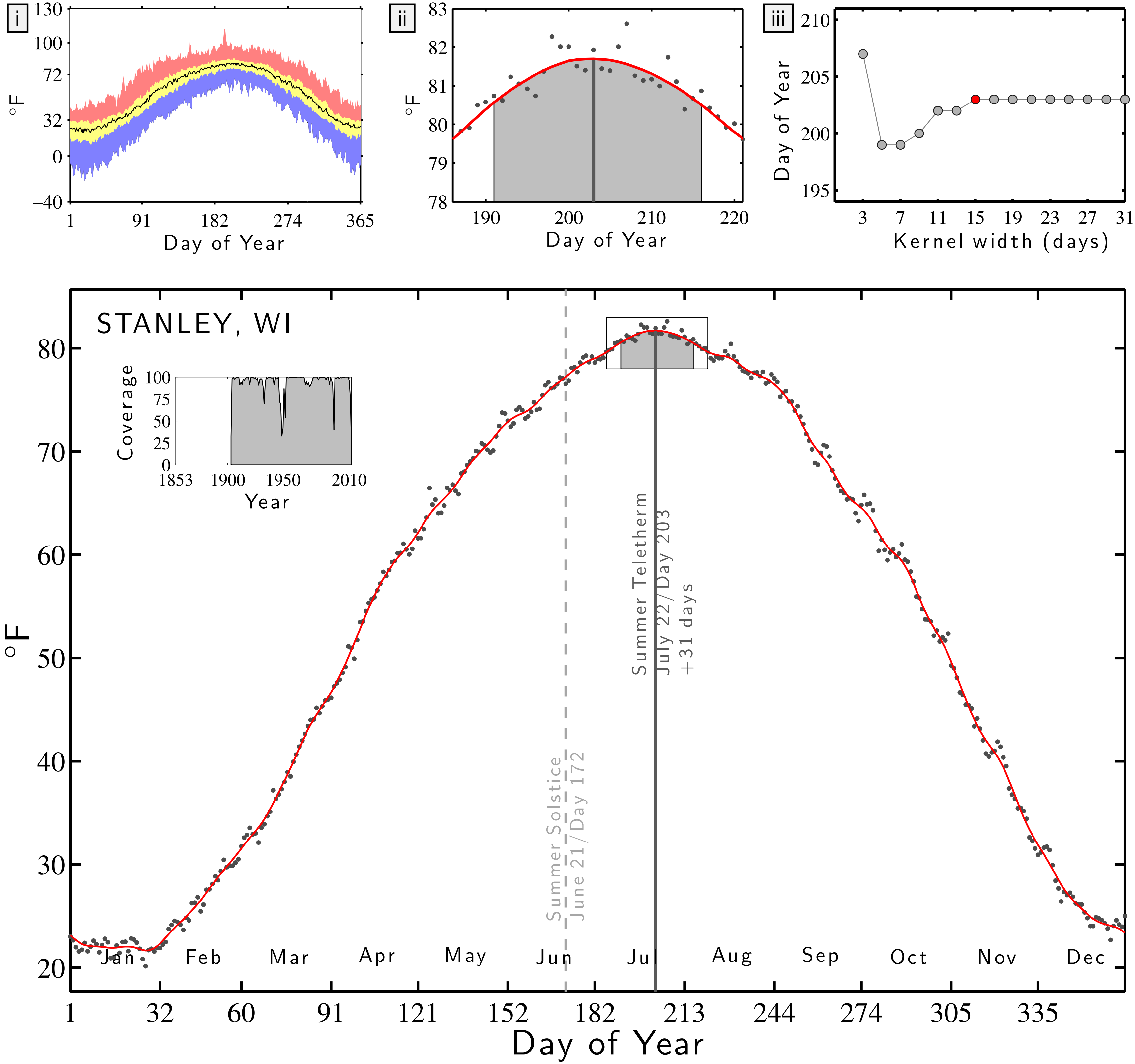

i
°F
Day of Year
ii
°F
Day of Year
iii
Day of Year
Kernel width (days)
STANLEY, WI
Coverage
Year
Summer Solstice
June 21/Day 172
Summer Teletherm
July 22/Day 203
+31 days
Jan
Feb
Mar
Apr
May
Jun
Jul
Aug
Sep
Oct
Nov
Dec
°F
Day of Year

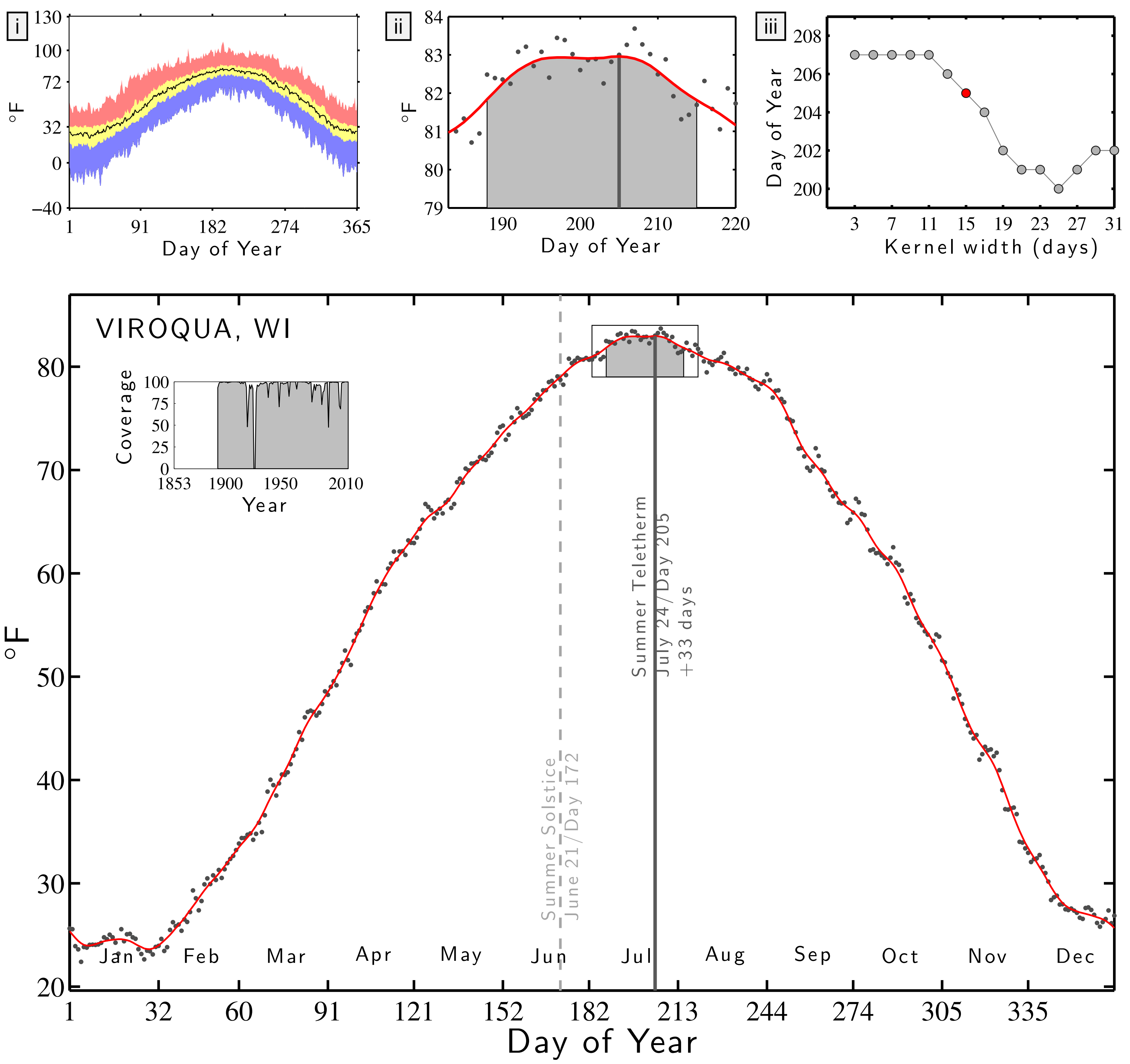
i
°F
Day of Year
ii
Day of Year
iii
Day of Year
Kernel width (days)
VIROQUA, WI
Coverage
Year
Summer Solstice
June 21 / Day 172
Summer Teletherm
July 24 / Day 205
+33 days
Jan
Feb
Mar
Apr
May
Jun
Jul
Aug
Sep
Oct
Nov
Dec

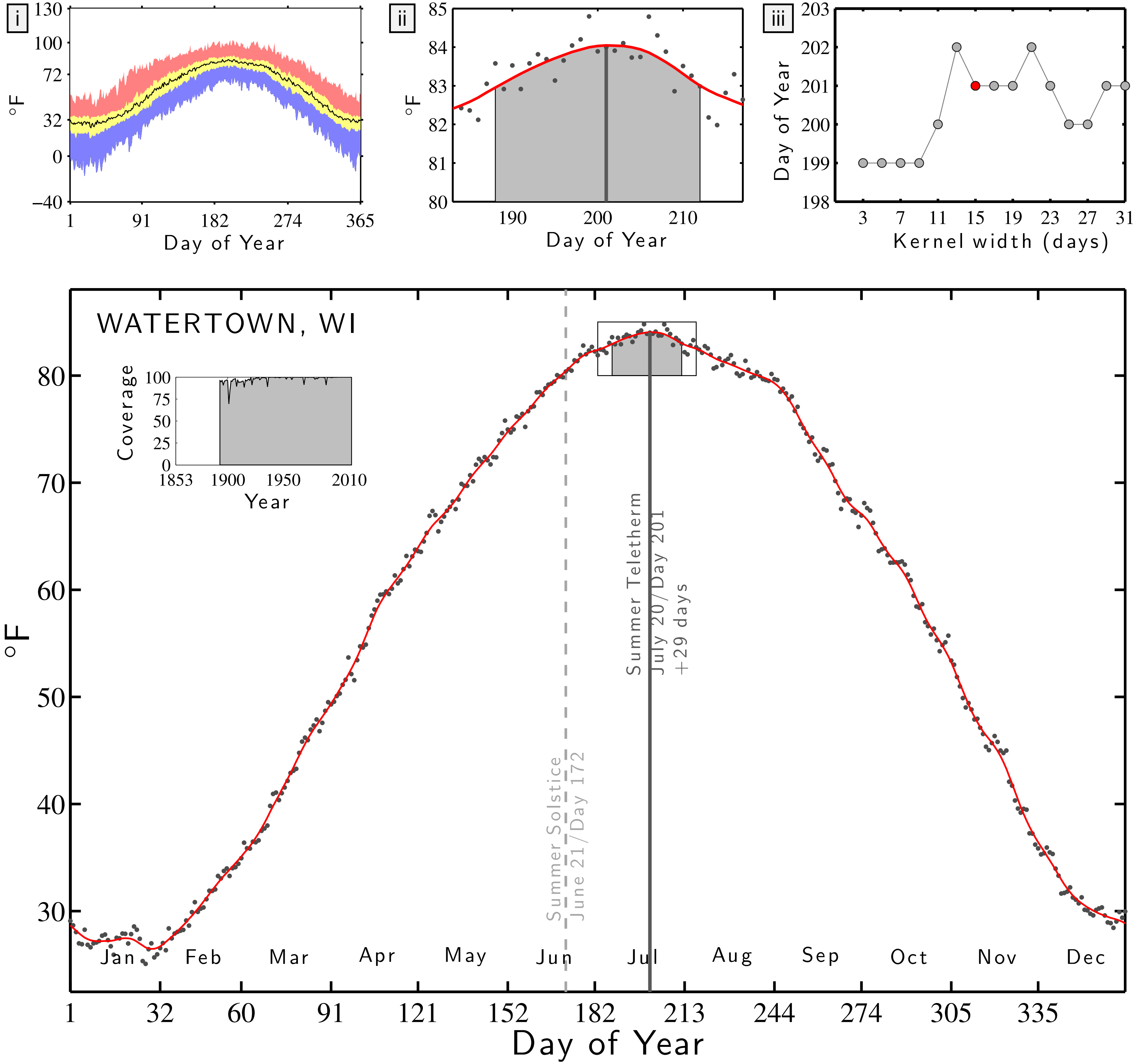

i
130
100
72
32
0
−40
°F
1
91
182
274
365
Day of Year
ii
85
84
83
82
81
80
°F
190
200
210
Day of Year
iii
203
202
201
200
199
198
Day of Year
3
7
11
15
19
23
27
31
Kernel width (days)
WATERTOWN, WI
Coverage
100
75
50
25
0
1853
1900
1950
2010
Year
Summer Solstice
June 21/Day 172
Summer Teletherm
July 20/Day 201
+29 days
80
70
60
50
40
30
°F
Jan
Feb
Mar
Apr
May
Jun
Jul
Aug
Sep
Oct
Nov
Dec
1
32
60
91
121
152
182
213
244
274
305
335
Day of Year

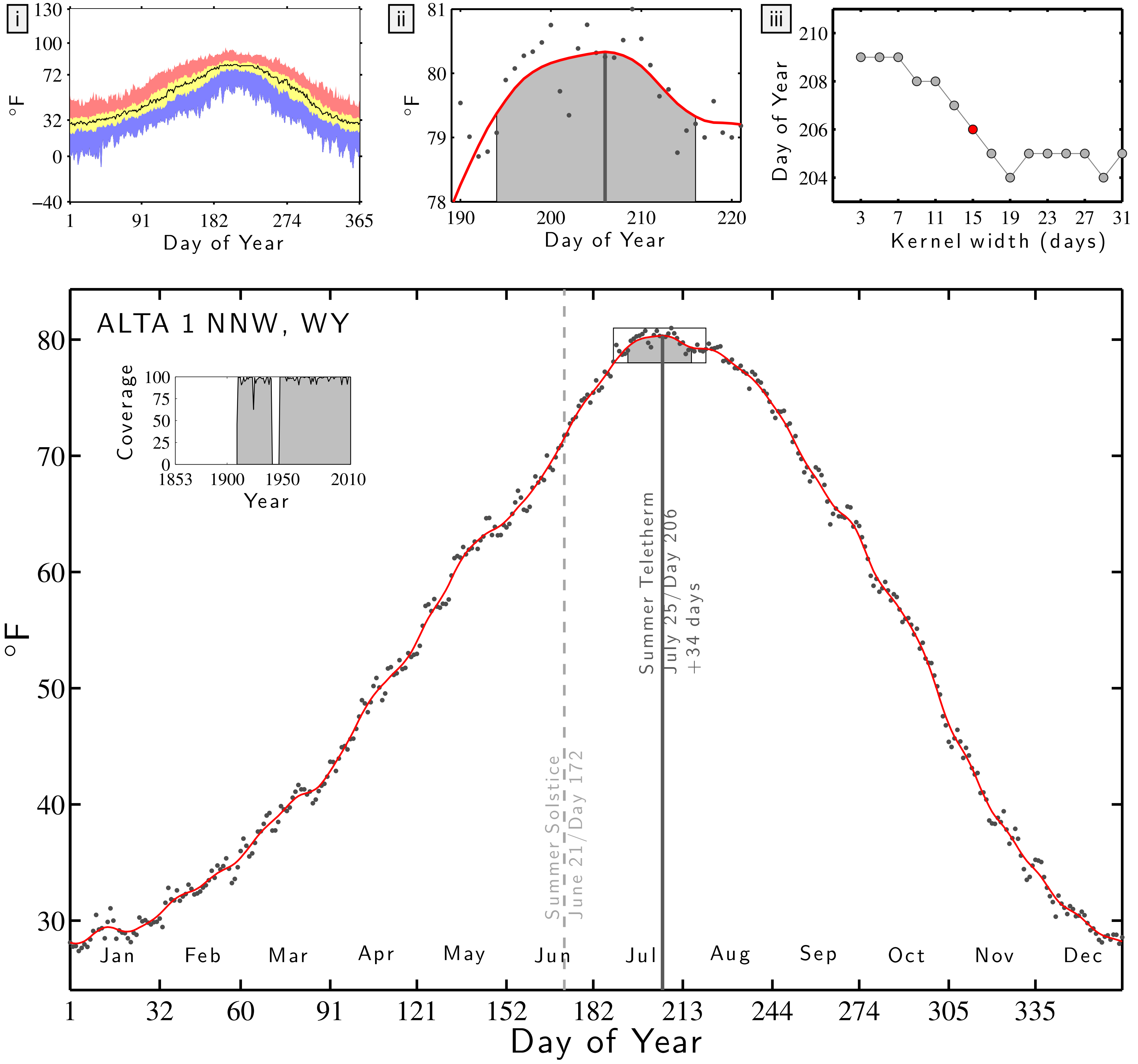

i
ii
iii
Day of Year
Kernel width (days)
ALTA 1 NNW, WY
Coverage
Year
Summer Solstice
June 21 / Day 172
Summer Teletherm
July 25 / Day 206
+34 days
Jan
Feb
Mar
Apr
May
Jun
Jul
Aug
Sep
Oct
Nov
Dec
°F
Day of Year

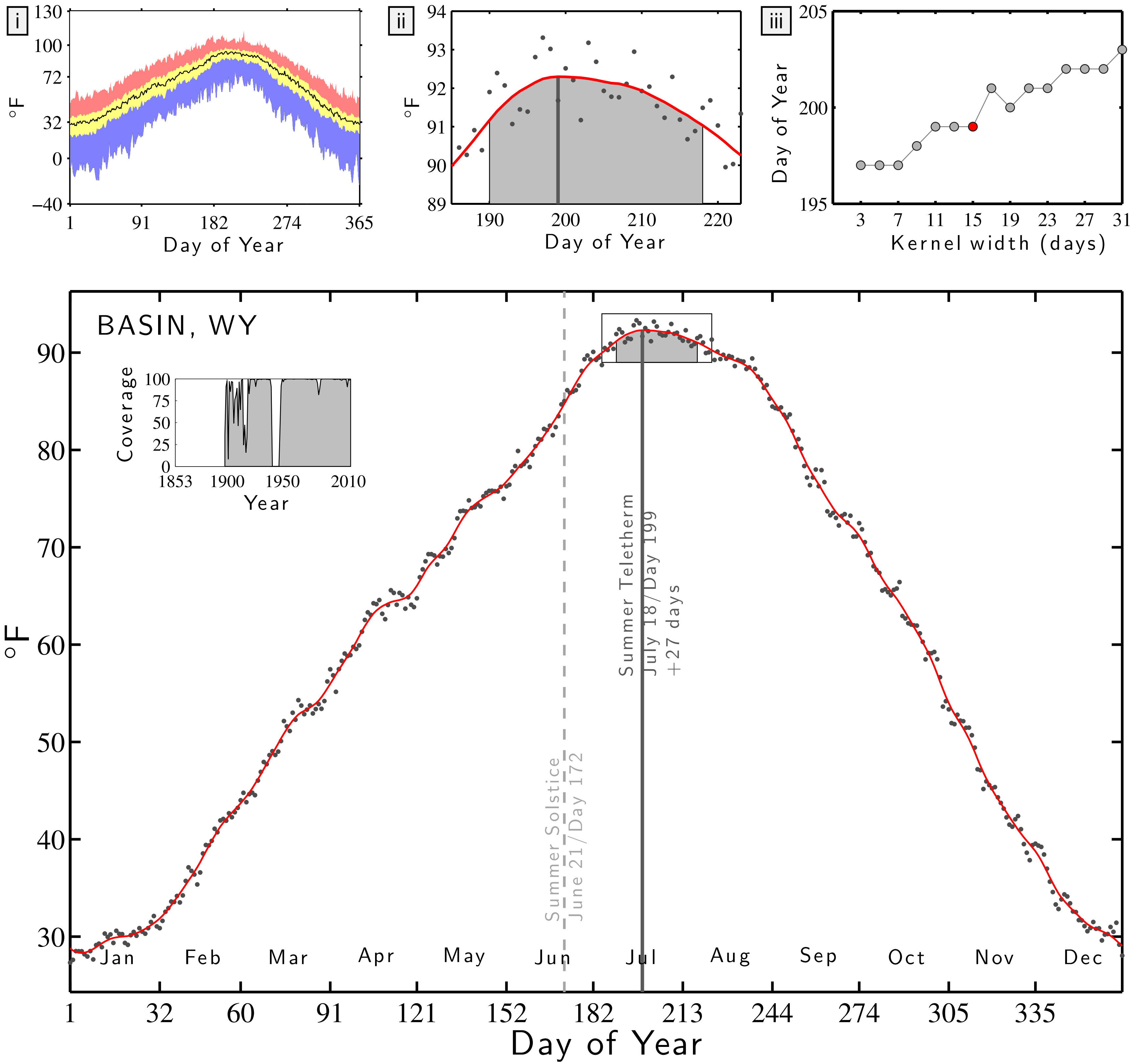
i
130
100
72
32
0
−40
°F
1
91
182
274
365
Day of Year
ii
94
93
92
91
90
89
°F
190
200
210
220
Day of Year
iii
205
200
195
Day of Year
3
7
11
15
19
23
27
31
Kernel width (days)
BASIN, WY
Coverage
100
75
50
25
0
1853
1900
1950
2010
Year
Summer Solstice
June 21/Day 172
Summer Teletherm
July 18/Day 199
+27 days
°F
90
80
70
60
50
40
30
Jan
Feb
Mar
Apr
May
Jun
Jul
Aug
Sep
Oct
Nov
Dec
1
32
60
91
121
152
182
213
244
274
305
335
Day of Year

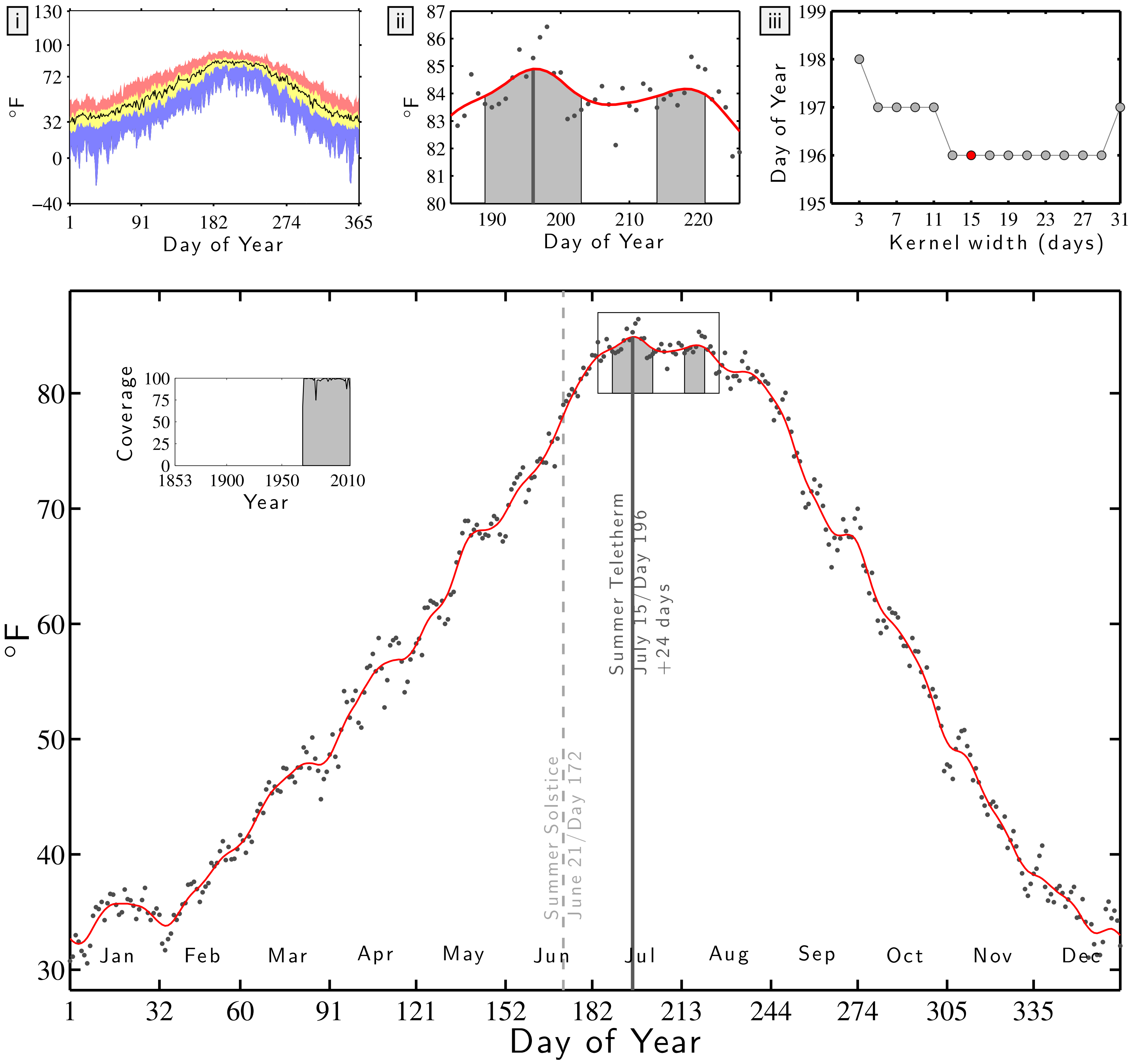

i
130
100
72
32
0
−40
°F
1
91
182
274
365
Day of Year
ii
87
86
85
84
83
82
81
80
°F
190
200
210
220
Day of Year
iii
199
198
197
196
195
Day of Year
3
7
11
15
19
23
27
31
Kernel width (days)
Coverage
100
75
50
25
0
1853
1900
1950
2010
Year
80
70
60
50
40
30
°F
Summer Solstice
June 21 / Day 172
Summer Teletherm
July 15 / Day 196
+24 days
Jan
Feb
Mar
Apr
May
Jun
Jul
Aug
Sep
Oct
Nov
Dec
1
32
60
91
121
152
182
213
244
274
305
335
Day of Year

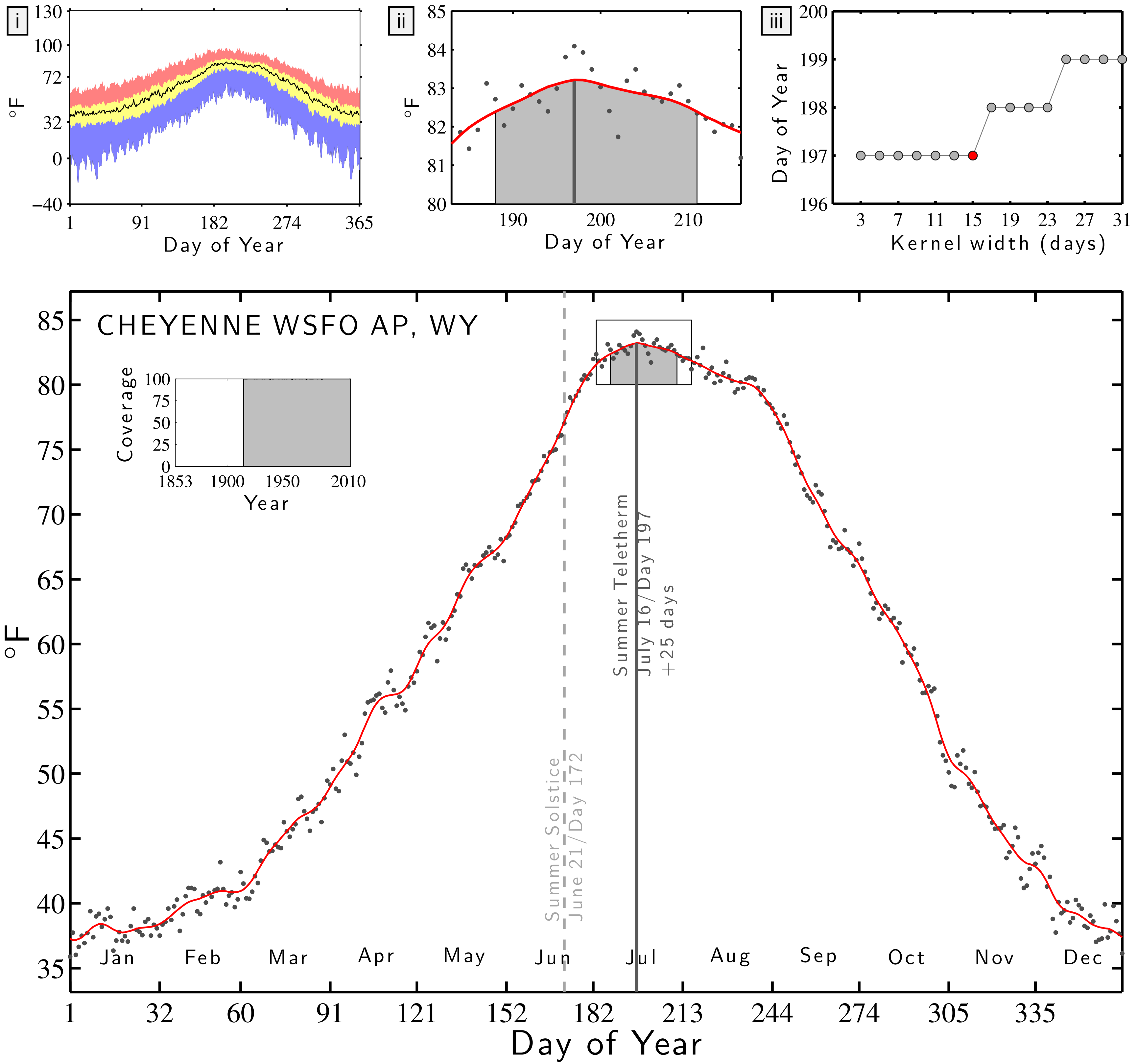

i
ii
iii
°F
Day of Year
Kernel width (days)
CHEYENNE WSFO AP, WY
Coverage
Year
Summer Solstice
June 21/Day 172
Summer Teletherm
July 16/Day 197
+25 days
Jan
Feb
Mar
Apr
May
Jun
Jul
Aug
Sep
Oct
Nov
Dec

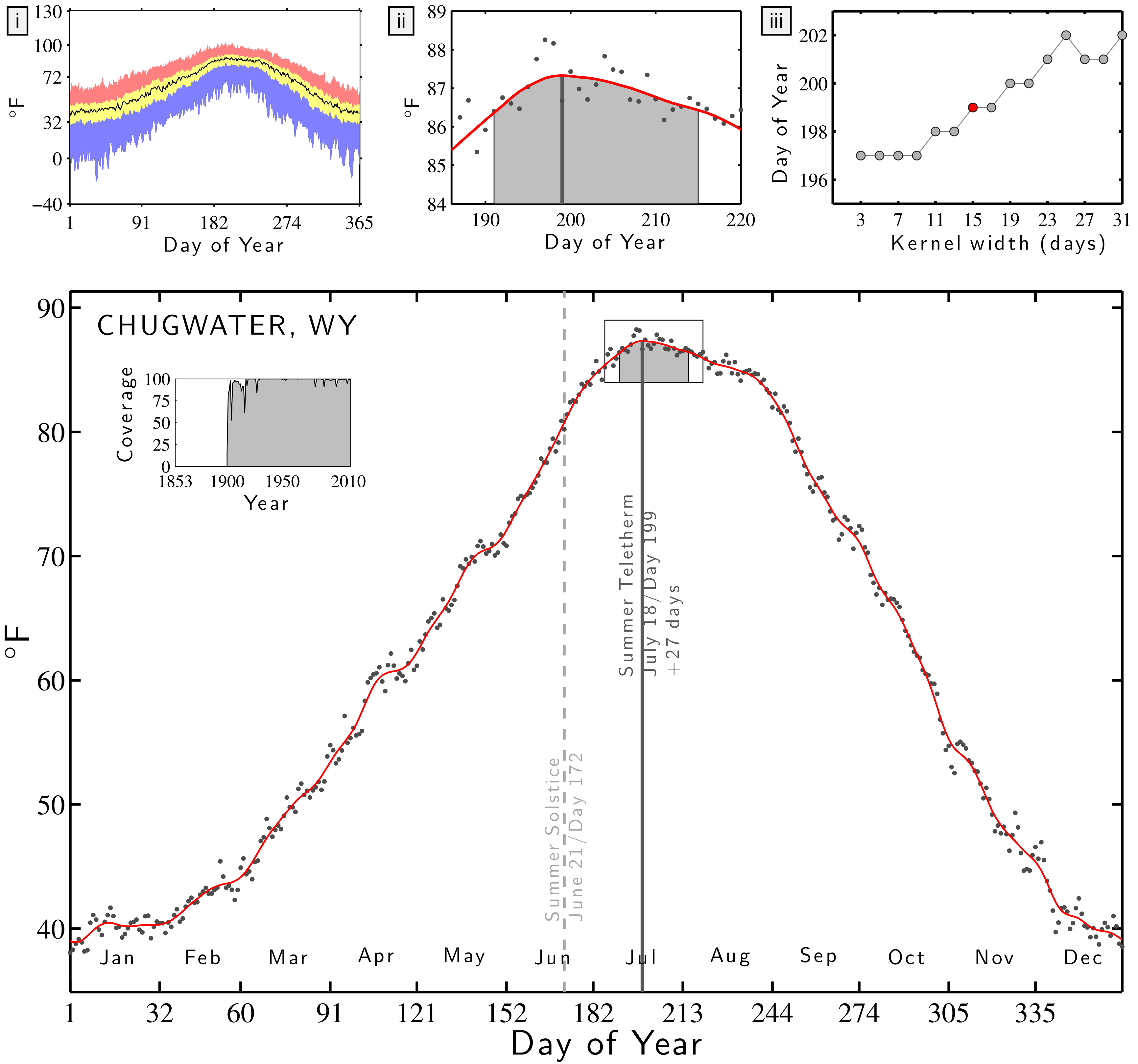

i
°F
Day of Year
ii
°F
Day of Year
iii
Day of Year
Kernel width (days)
CHUGWATER, WY
Coverage
Year
Summer Solstice
June 21 / Day 172
Summer Teletherm
July 18 / Day 199
+27 days
Jan
Feb
Mar
Apr
May
Jun
Jul
Aug
Sep
Oct
Nov
Dec
°F
Day of Year

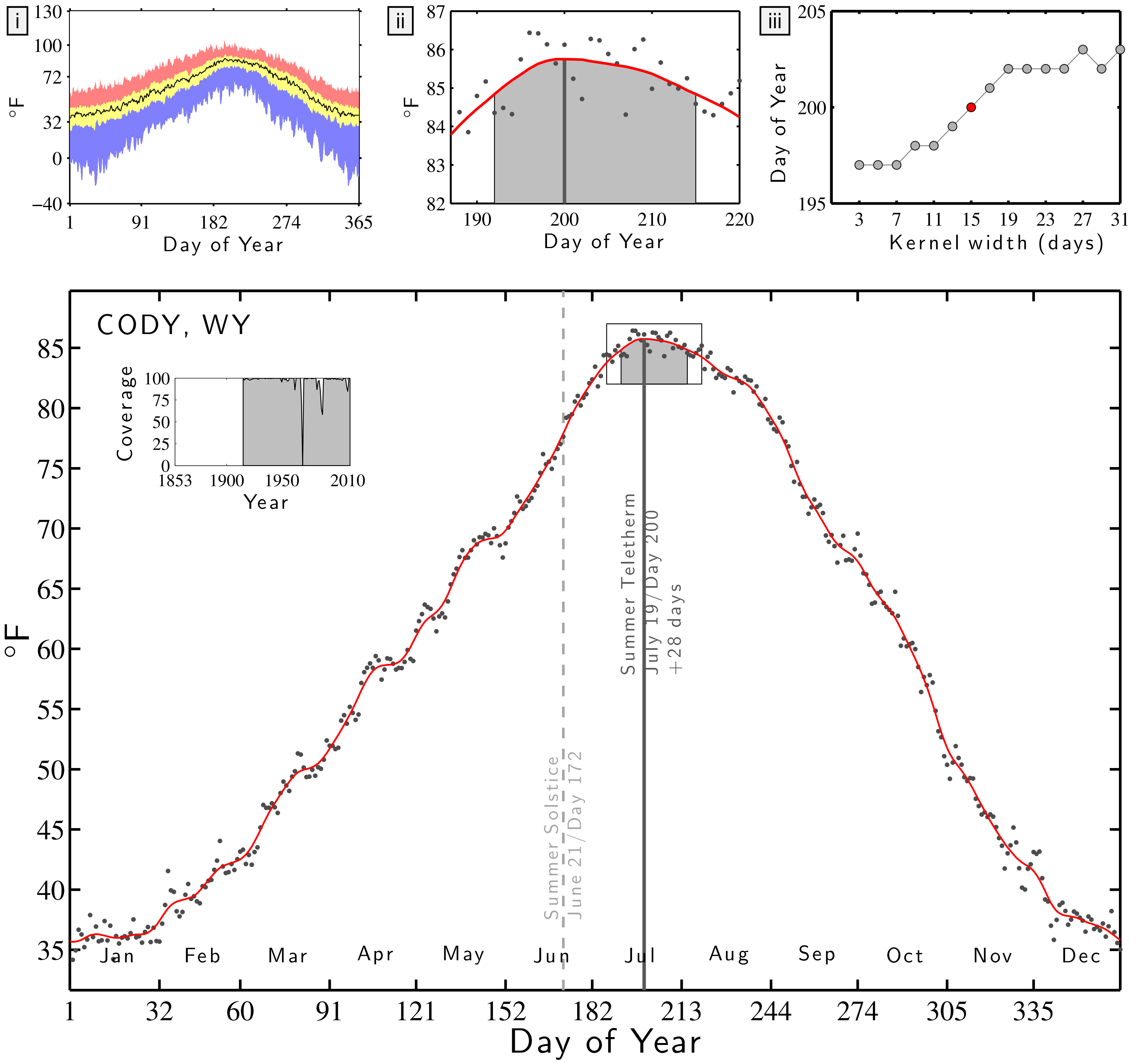

i
ii
iii
°F
Day of Year
Kernel width (days)
CODY, WY
Coverage
Year
Summer Solstice
June 21/Day 172
Summer Teletherm
July 19/Day 200
+28 days
Jan
Feb
Mar
Apr
May
Jun
Jul
Aug
Sep
Oct
Nov
Dec

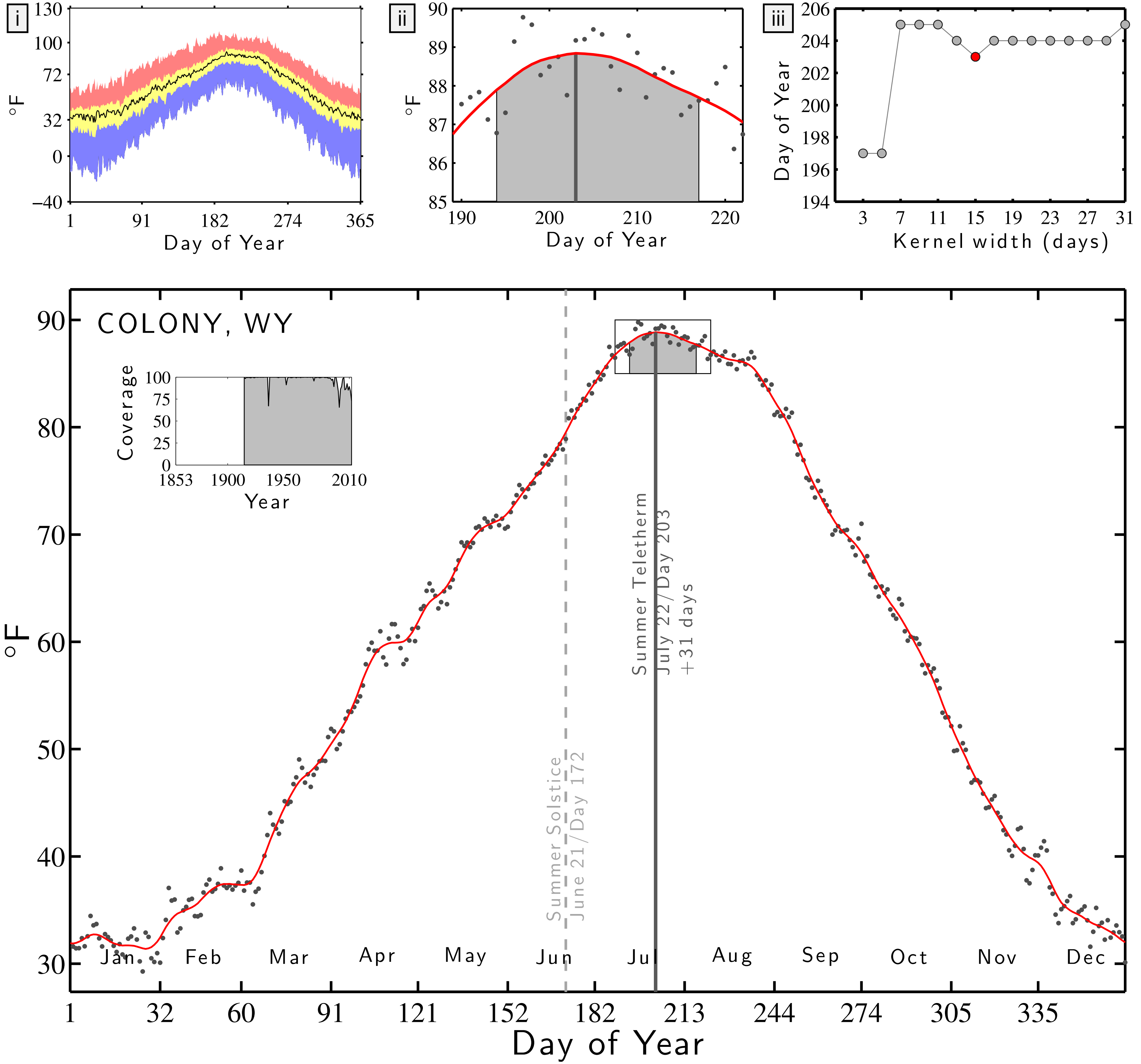

i
°F
Day of Year
ii
°F
Day of Year
iii
Day of Year
Kernel width (days)
COLONY, WY
Coverage
Year
Summer Solstice
June 21/Day 172
Summer Teletherm
July 22/Day 203
+31 days
Jan
Feb
Mar
Apr
May
Jun
Jul
Aug
Sep
Oct
Nov
Dec
°F
Day of Year

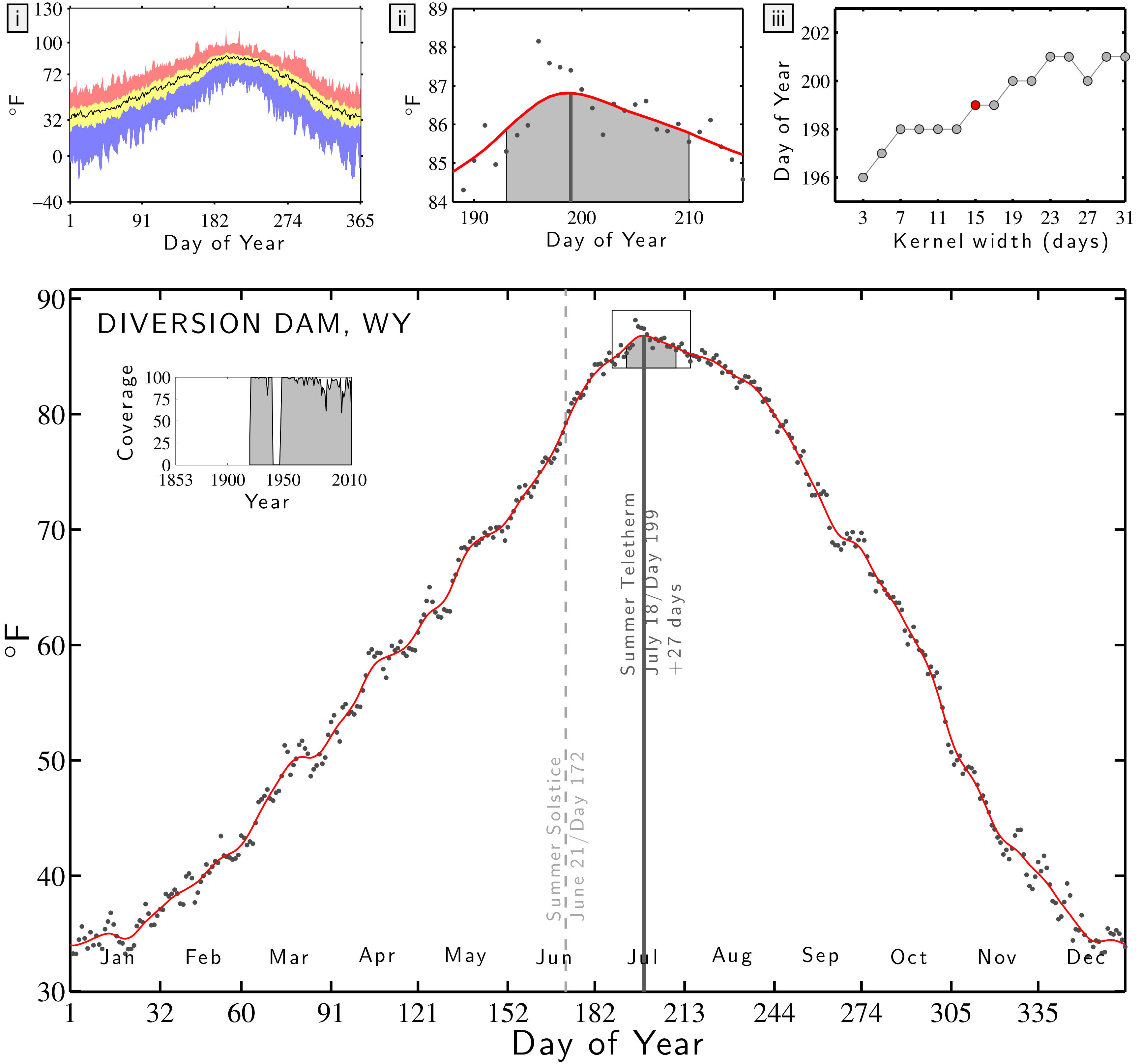

i
°F
Day of Year
ii
°F
Day of Year
iii
Day of Year
Kernel width (days)
DIVERSION DAM, WY
Coverage
Year
Summer Solstice
June 21/Day 172
Summer Teletherm
July 18/Day 199
+27 days
Jan
Feb
Mar
Apr
May
Jun
Jul
Aug
Sep
Oct
Nov
Dec
°F
Day of Year

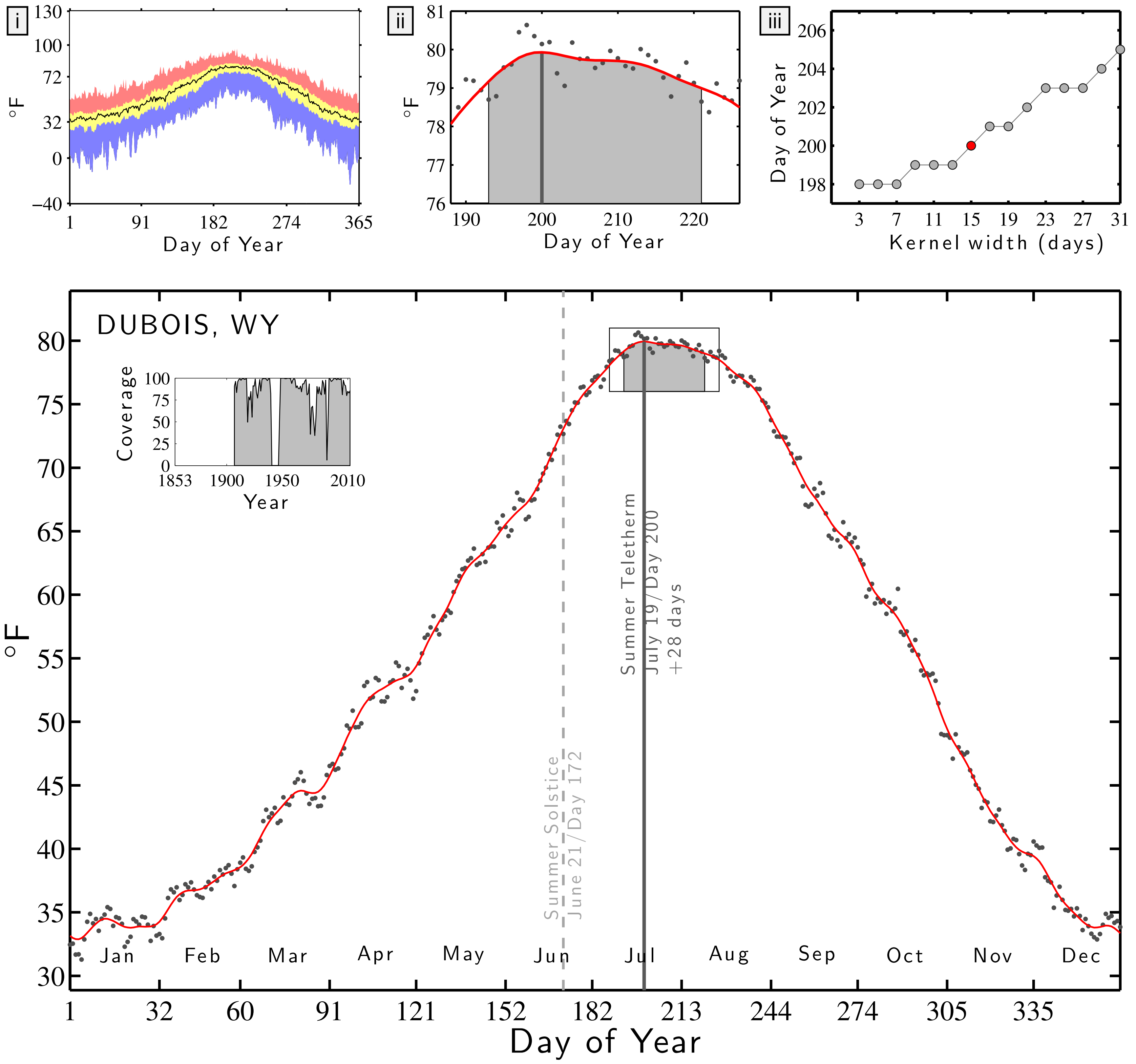

i
°F
Day of Year
ii
°F
Day of Year
iii
Day of Year
Kernel width (days)
DUBOIS, WY
Coverage
Year
Summer Solstice
June 21/Day 172
Summer Teletherm
July 19/Day 200
+28 days
Jan
Feb
Mar
Apr
May
Jun
Jul
Aug
Sep
Oct
Nov
Dec
°F
Day of Year

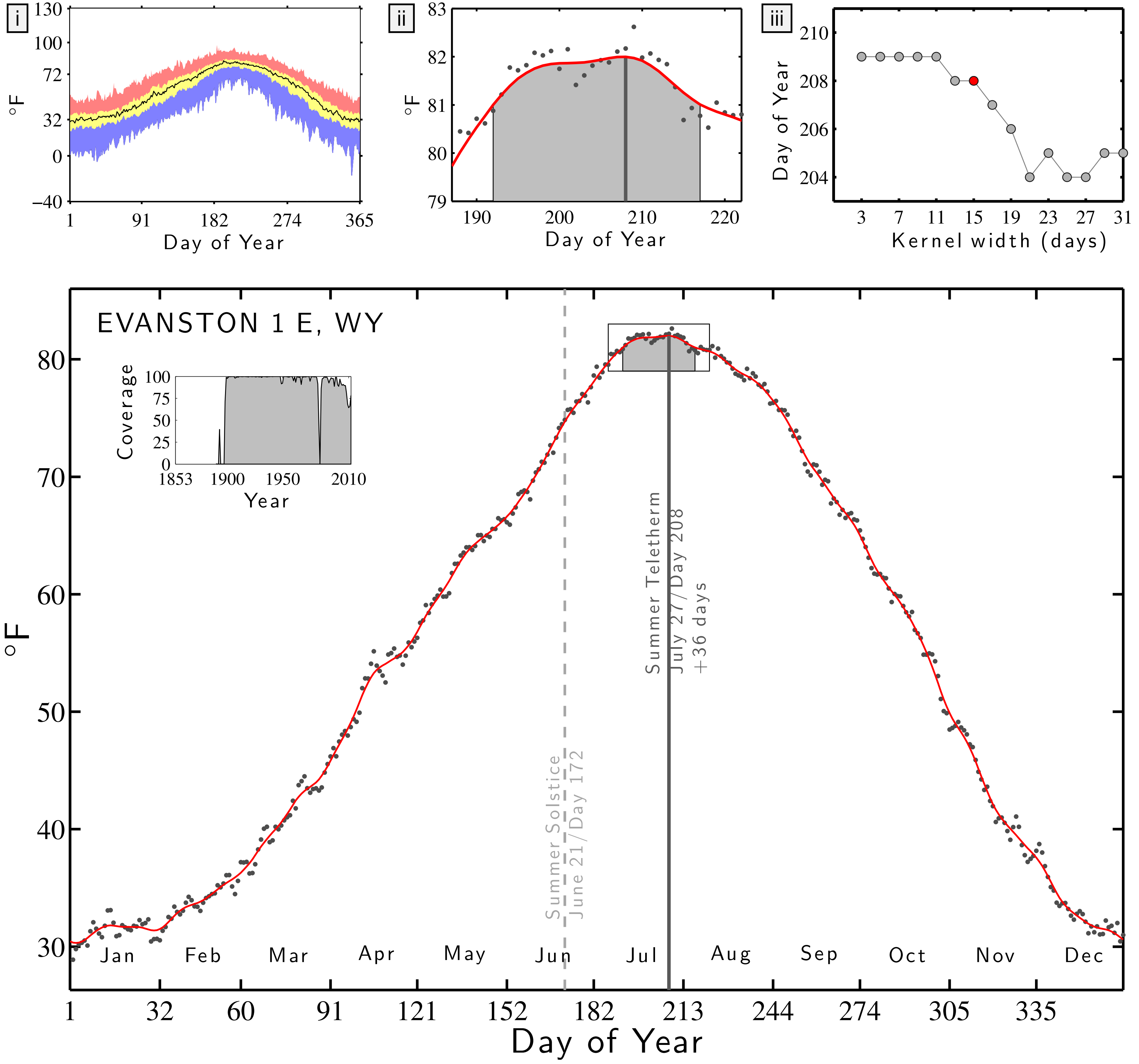

i
ii
iii
°F
Day of Year
Kernel width (days)
Day of Year
EVANSTON 1 E, WY
Coverage
Year
Summer Solstice
June 21 / Day 172
Summer Teletherm
July 27 / Day 208
+36 days
Jan
Feb
Mar
Apr
May
Jun
Jul
Aug
Sep
Oct
Nov
Dec

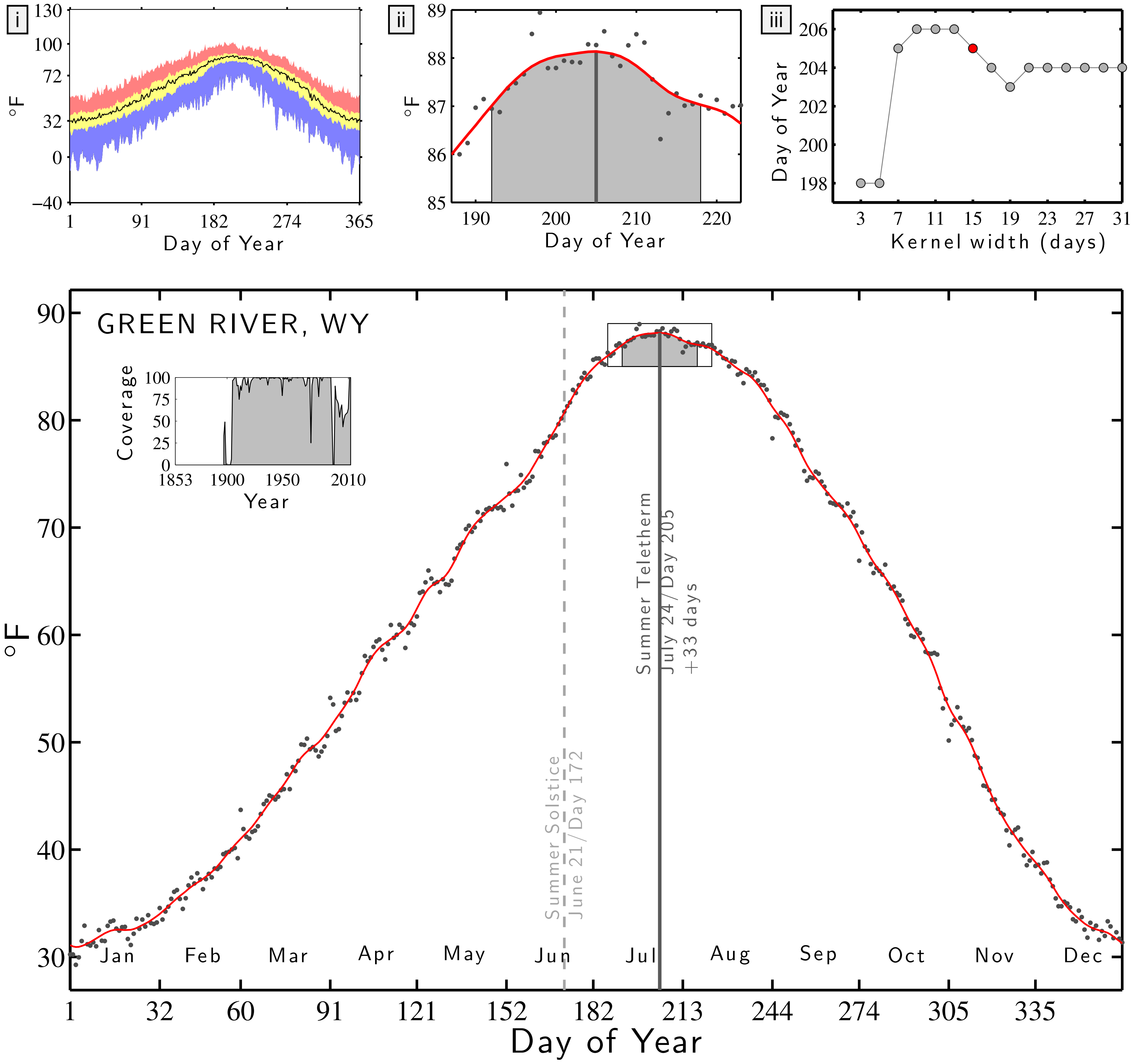

i
°F
Day of Year
ii
iii
Day of Year
Kernel width (days)
GREEN RIVER, WY
Coverage
Year
Summer Solstice
June 21 / Day 172
Summer Teletherm
July 24 / Day 205
+33 days
Jan
Feb
Mar
Apr
May
Jun
Jul
Aug
Sep
Oct
Nov
Dec

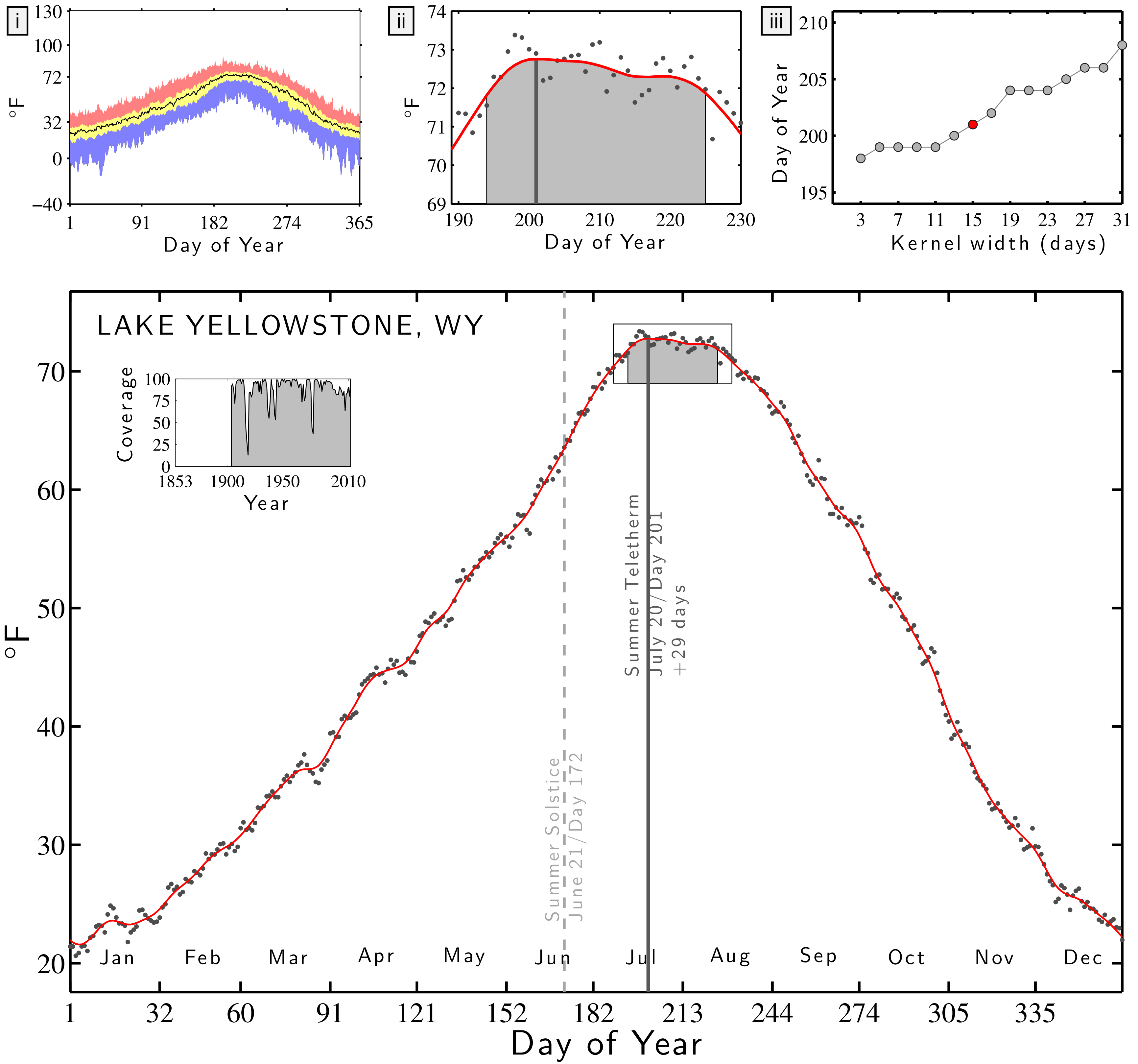

i
ii
iii
°F
Day of Year
Kernel width (days)
LAKE YELLOWSTONE, WY
Coverage
Year
Summer Solstice
June 21/Day 172
Summer Teletherm
July 20/Day 201
+29 days
Jan
Feb
Mar
Apr
May
Jun
Jul
Aug
Sep
Oct
Nov
Dec

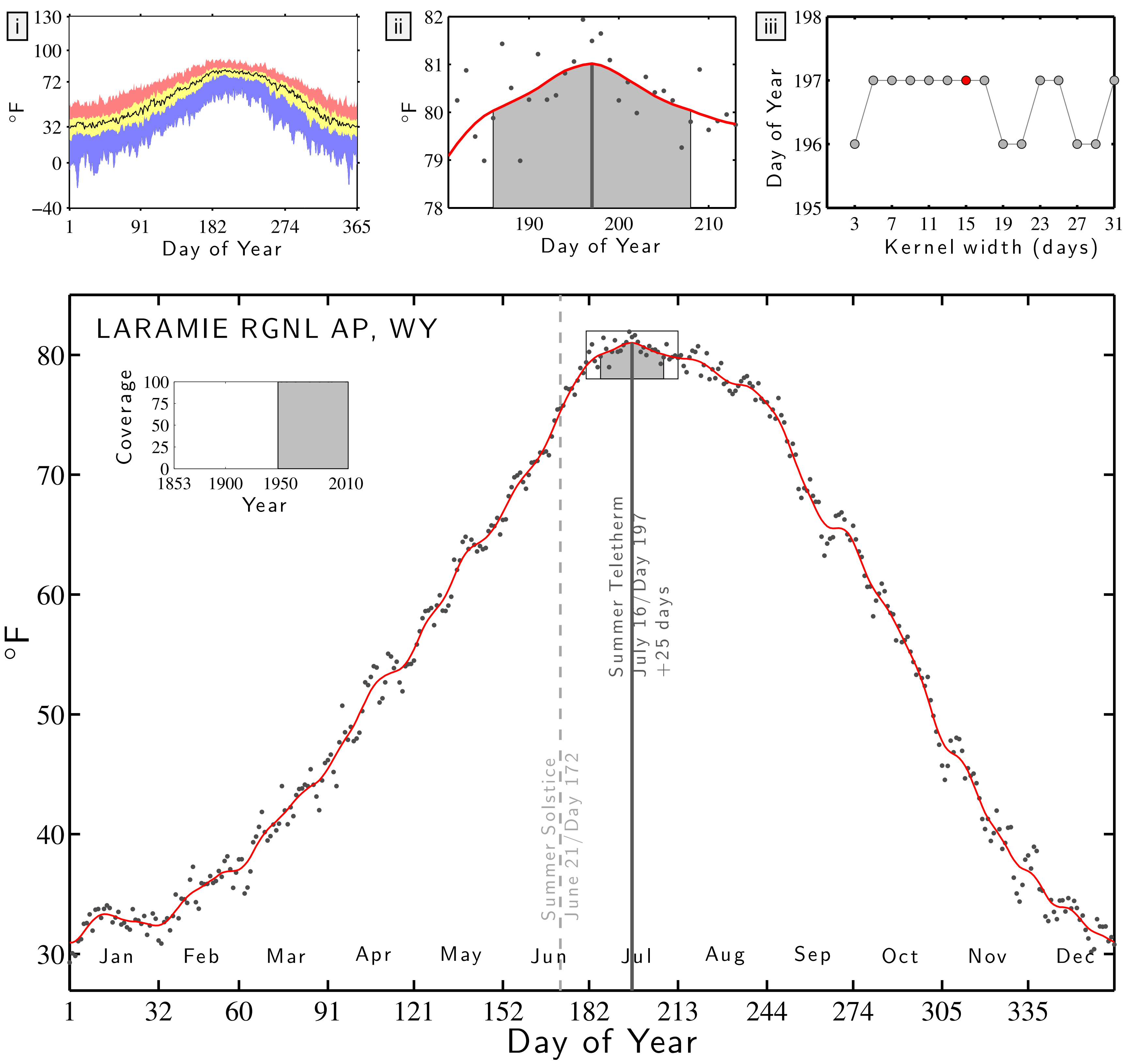

i
°F
Day of Year
ii
iii
Day of Year
Kernel width (days)
LARAMIE RGNL AP, WY
Coverage
Year
Summer Solstice
June 21/Day 172
Summer Teletherm
July 16/Day 197
+25 days
Jan
Feb
Mar
Apr
May
Jun
Jul
Aug
Sep
Oct
Nov
Dec

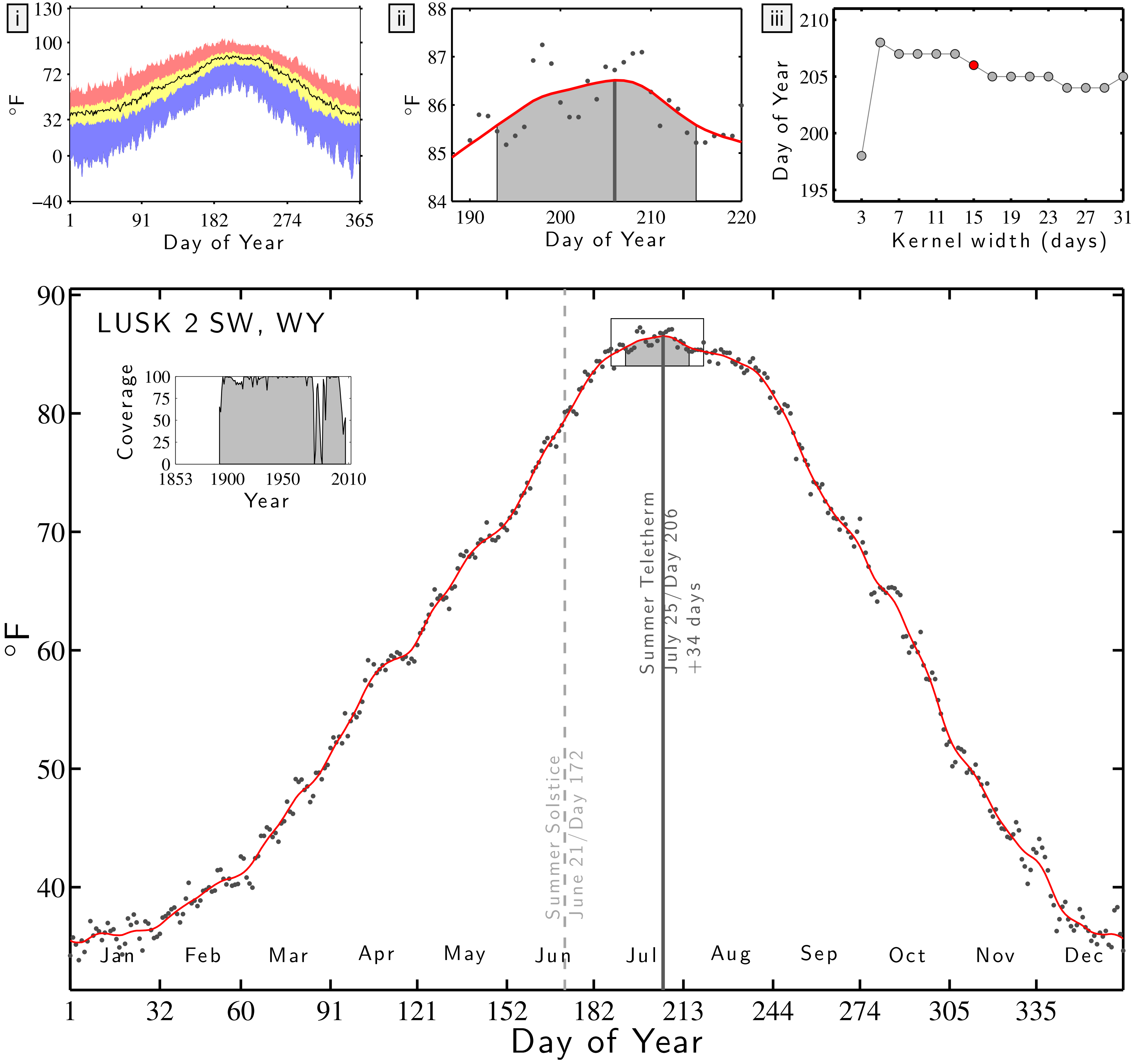

i
°F
Day of Year
ii
°F
Day of Year
iii
Day of Year
Kernel width (days)
LUSK 2 SW, WY
Coverage
Year
Summer Solstice
June 21/Day 172
Summer Teletherm
July 25/Day 206
+34 days
Jan
Feb
Mar
Apr
May
Jun
Jul
Aug
Sep
Oct
Nov
Dec
°F
Day of Year

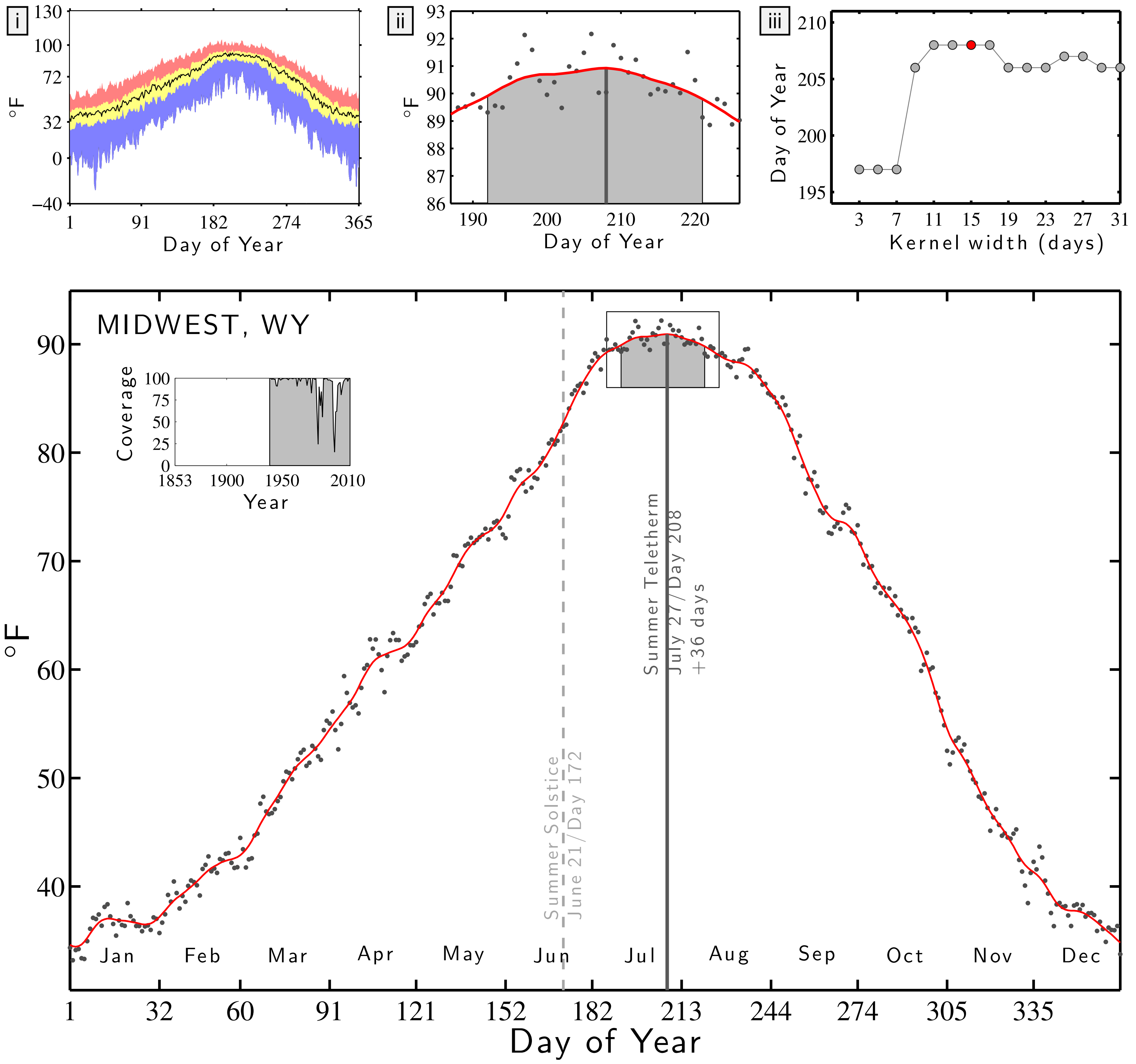

i
°F
Day of Year
ii
°F
Day of Year
iii
Day of Year
Kernel width (days)
MIDWEST, WY
Coverage
Year
Summer Solstice
June 21 / Day 172
Summer Teletherm
July 27 / Day 208
+36 days
Jan
Feb
Mar
Apr
May
Jun
Jul
Aug
Sep
Oct
Nov
Dec
°F
Day of Year

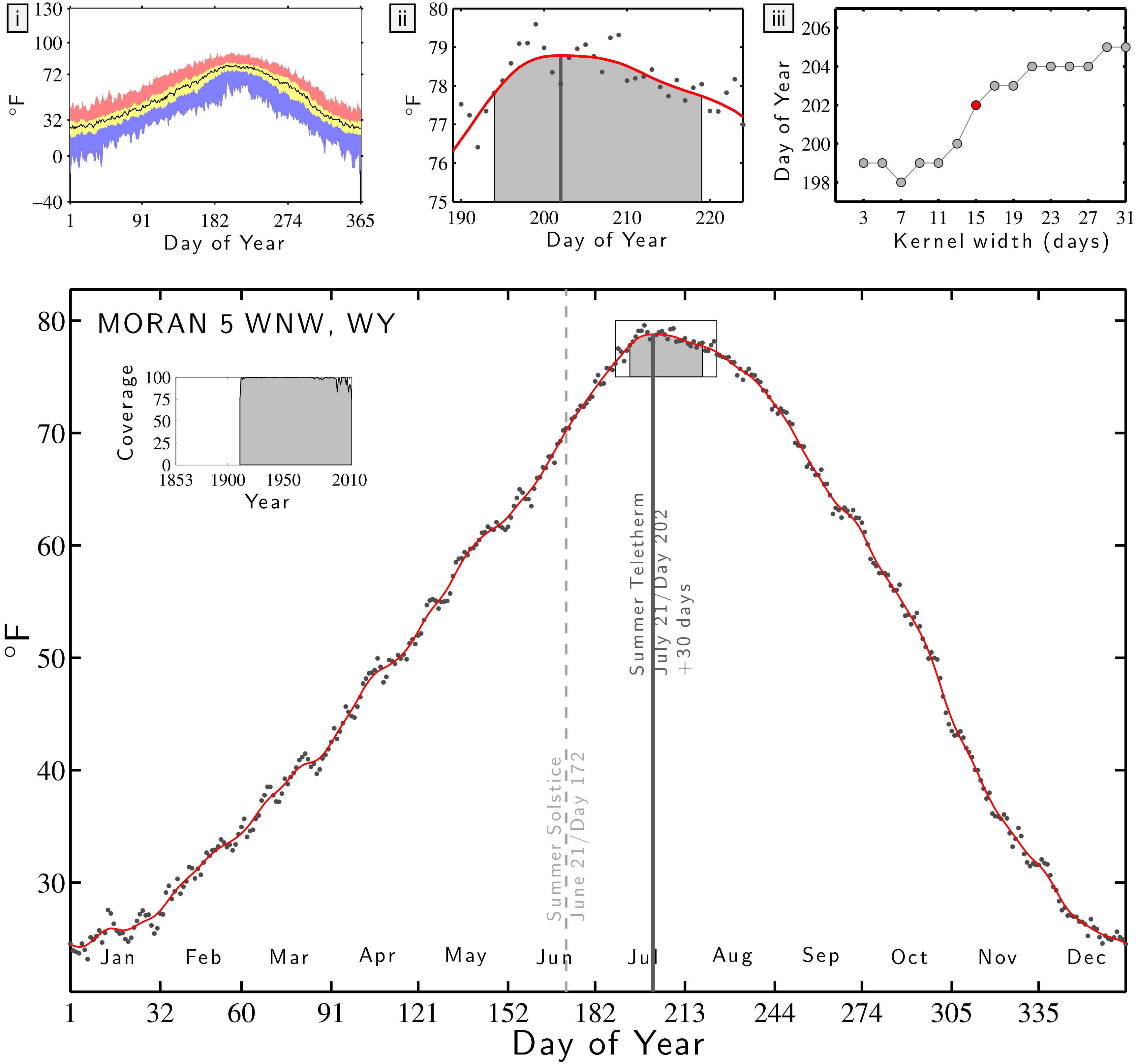

i
ii
iii
°F
Day of Year
Day of Year
Kernel width (days)
MORAN 5 WNW, WY
Coverage
Year
Summer Solstice
June 21 / Day 172
Summer Teletherm
July 21 / Day 202
+30 days
Jan
Feb
Mar
Apr
May
Jun
Jul
Aug
Sep
Oct
Nov
Dec

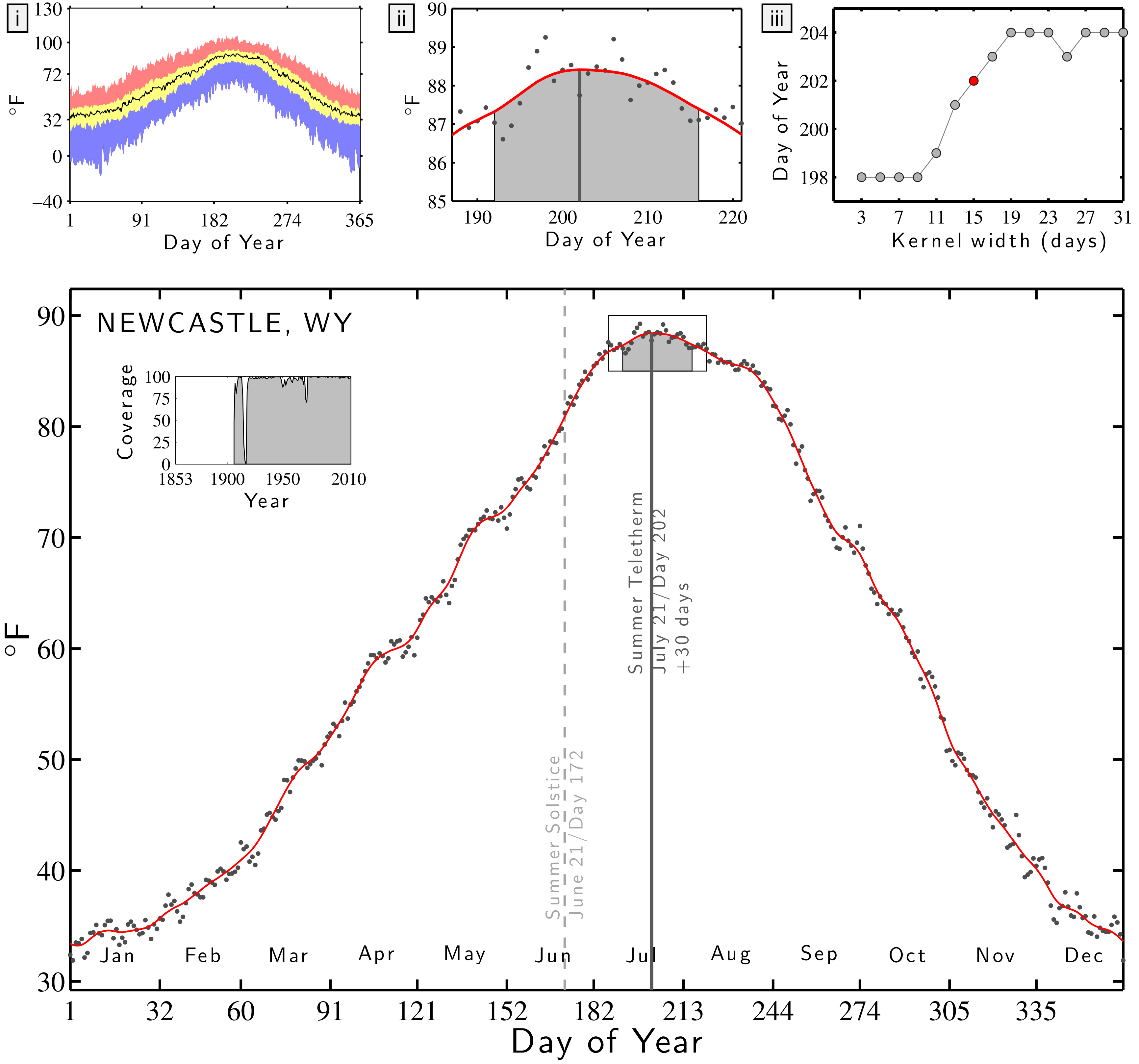

i
ii
iii
°F
Day of Year
Kernel width (days)
NEWCASTLE, WY
Coverage
Year
Summer Solstice
June 21 / Day 172
Summer Teletherm
July 21 / Day 202
+30 days
Jan
Feb
Mar
Apr
May
Jun
Jul
Aug
Sep
Oct
Nov
Dec

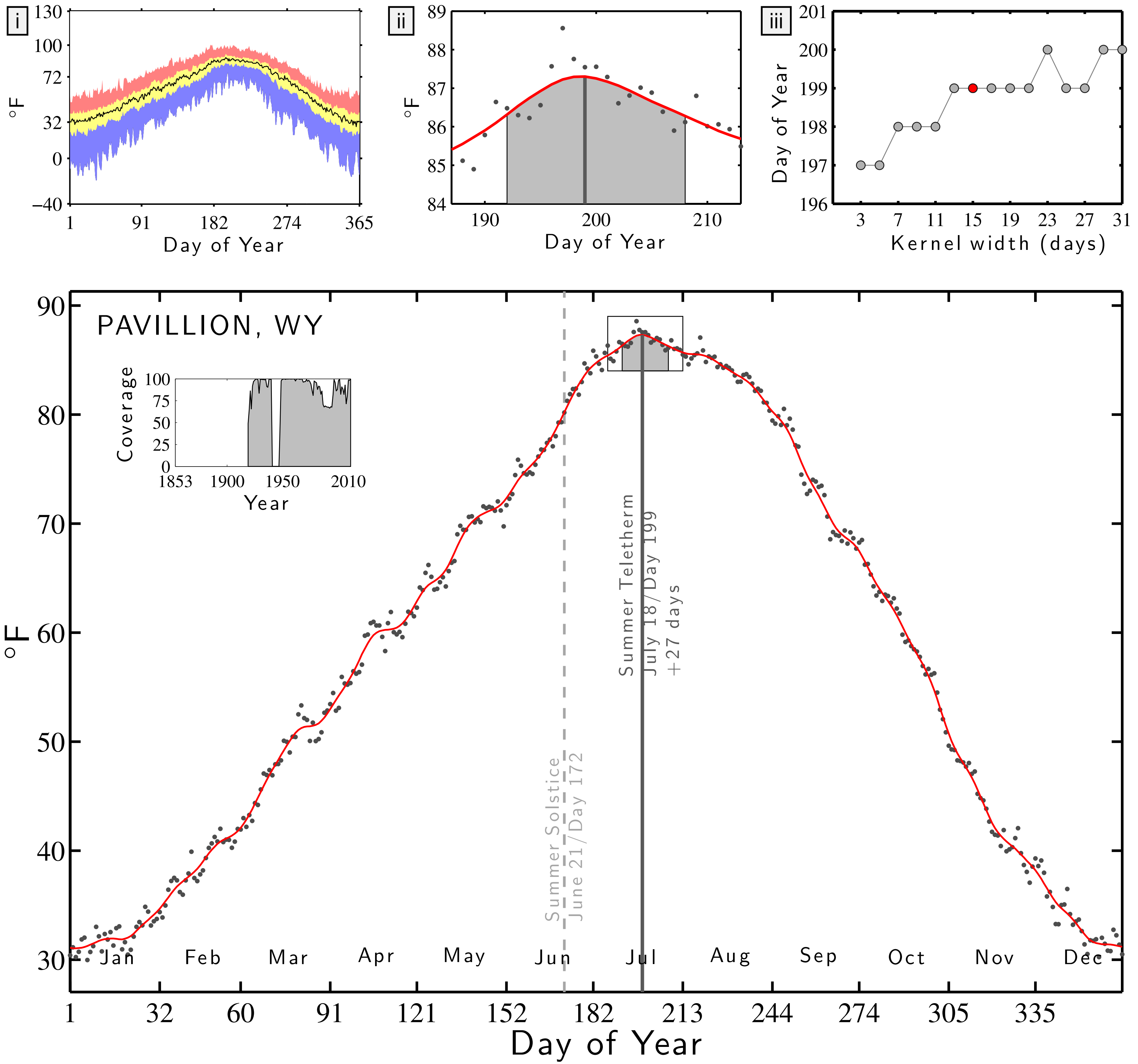

i
ii
iii
°F
Day of Year
Day of Year
Kernel width (days)
PAVILLION, WY
Coverage
Year
Summer Solstice
June 21/Day 172
Summer Teletherm
July 18/Day 199
+27 days
Jan
Feb
Mar
Apr
May
Jun
Jul
Aug
Sep
Oct
Nov
Dec

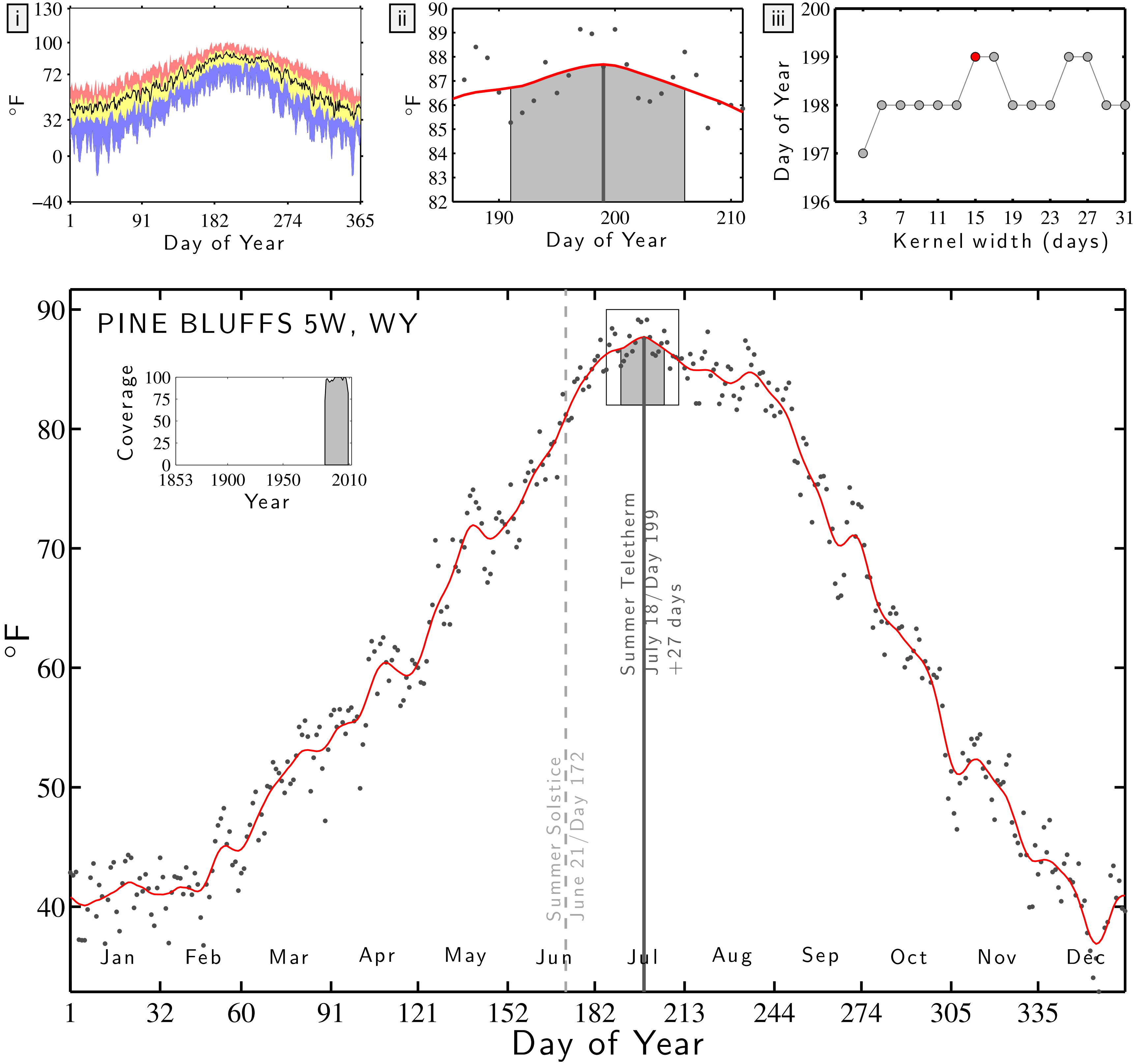

i
°F
130
100
72
32
0
−40
1
91
182
274
365
Day of Year
ii
°F
90
89
88
87
86
85
84
83
82
190
200
210
Day of Year
iii
Day of Year
200
199
198
197
196
3
7
11
15
19
23
27
31
Kernel width (days)
PINE BLUFFS 5W, WY
Coverage
100
75
50
25
0
1853
1900
1950
2010
Year
°F
90
80
70
60
50
40
Summer Solstice
June 21/Day 172
Summer Teletherm
July 18/Day 199
+27 days
Jan
Feb
Mar
Apr
May
Jun
Jul
Aug
Sep
Oct
Nov
Dec
1
32
60
91
121
152
182
213
244
274
305
335
Day of Year

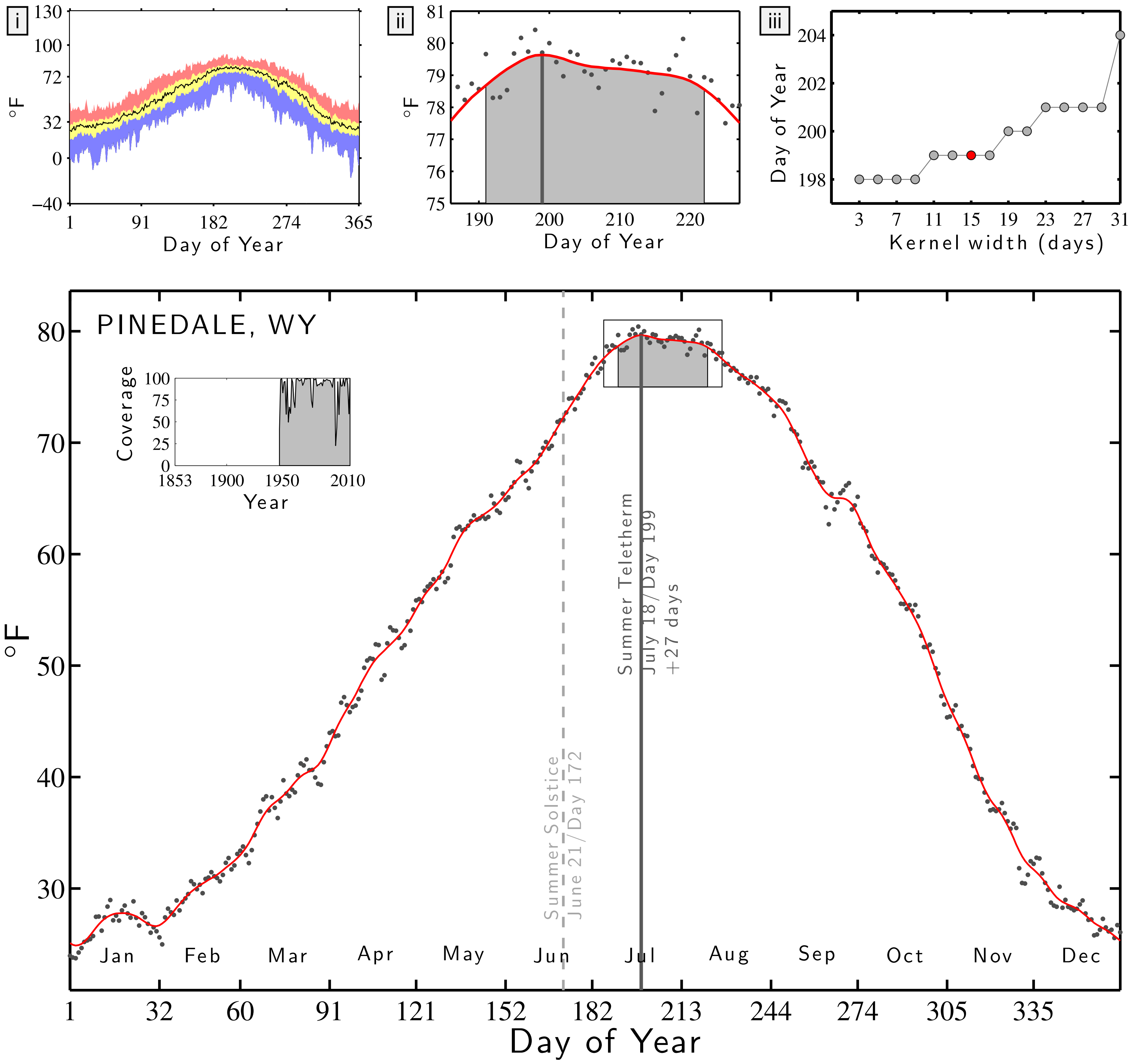

i
ii
iii
°F
Day of Year
Kernel width (days)
PINEDALE, WY
Coverage
Year
1853
1900
1950
2010
Summer Solstice
June 21/Day 172
Summer Teletherm
July 18/Day 199
+27 days
Jan
Feb
Mar
Apr
May
Jun
Jul
Aug
Sep
Oct
Nov
Dec

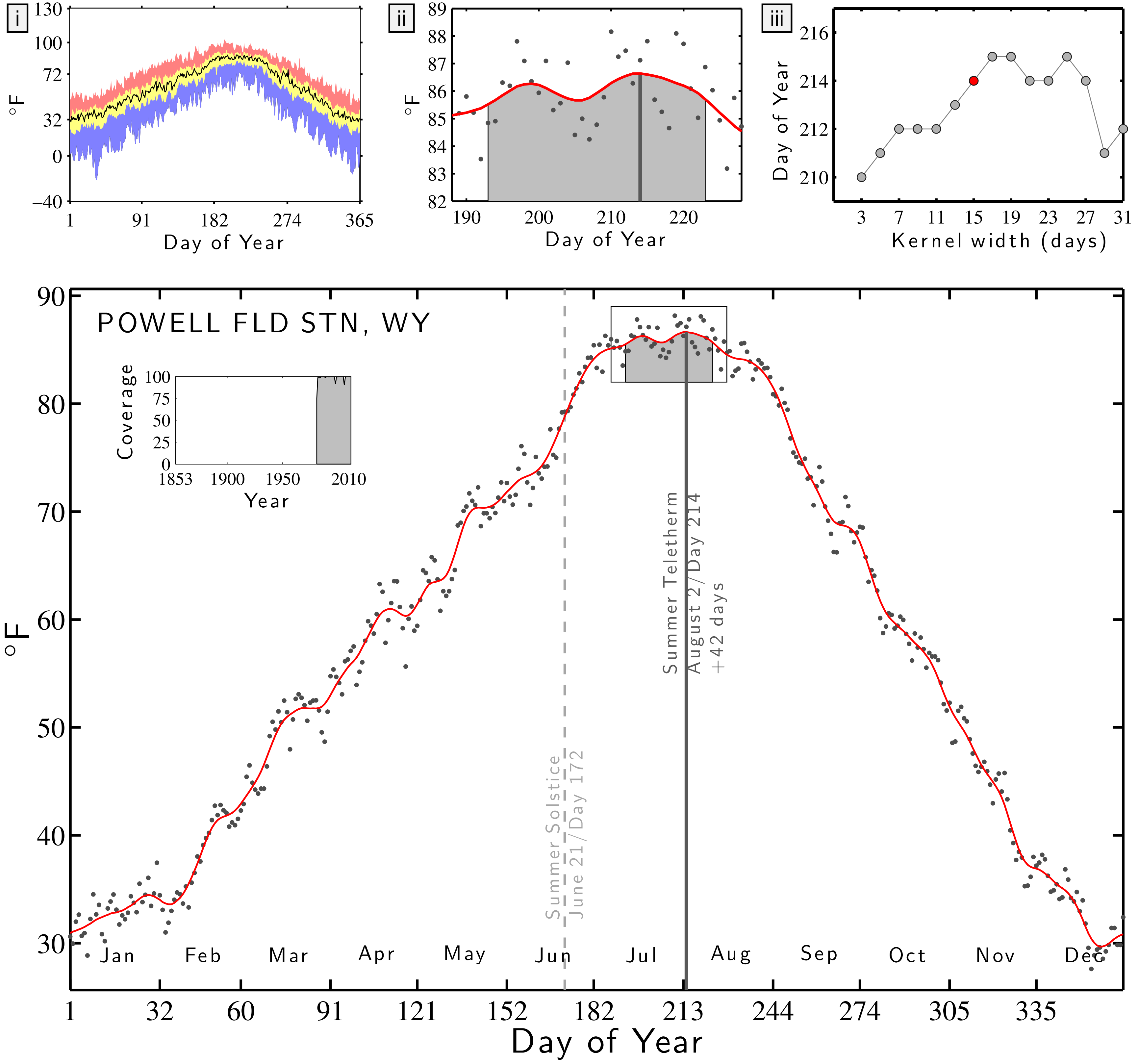

i
ii
iii
°F
Day of Year
Day of Year
Kernel width (days)
POWELL FLD STN, WY
Coverage
Year
Summer Solstice
June 21 / Day 172
Summer Teletherm
August 2 / Day 214
+42 days
Jan
Feb
Mar
Apr
May
Jun
Jul
Aug
Sep
Oct
Nov
Dec
°F
Day of Year

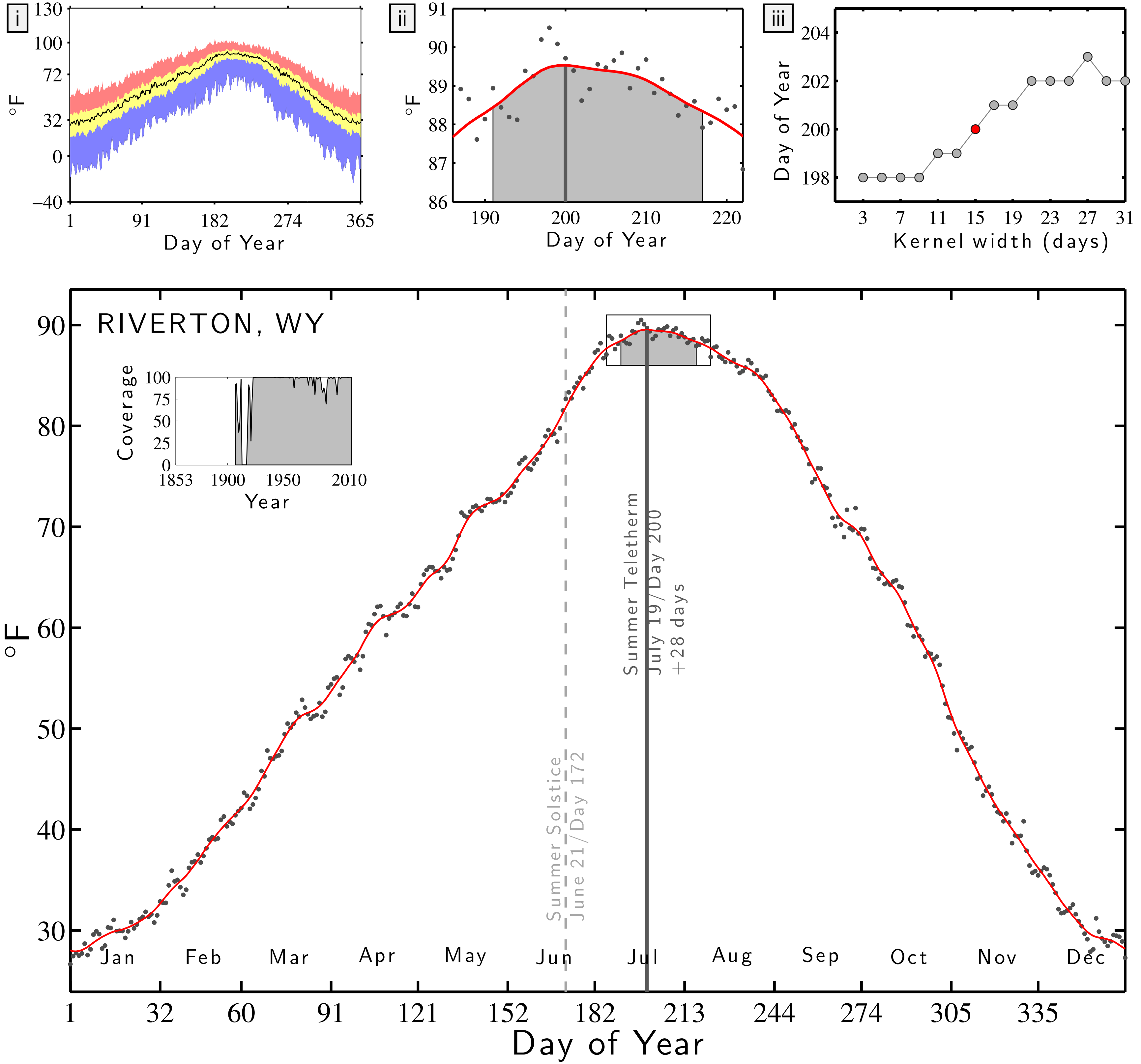

i
°F
130
100
72
32
0
−40
1
91
182
274
365
Day of Year
ii
°F
91
90
89
88
87
86
190
200
210
220
Day of Year
iii
Day of Year
204
202
200
198
3
7
11
15
19
23
27
31
Kernel width (days)
RIVERTON, WY
Coverage
100
75
50
25
0
1853
1900
1950
2010
Year
Summer Solstice
June 21/Day 172
Summer Teletherm
July 19/Day 200
+28 days
°F
90
80
70
60
50
40
30
Jan
Feb
Mar
Apr
May
Jun
Jul
Aug
Sep
Oct
Nov
Dec
1
32
60
91
121
152
182
213
244
274
305
335
Day of Year

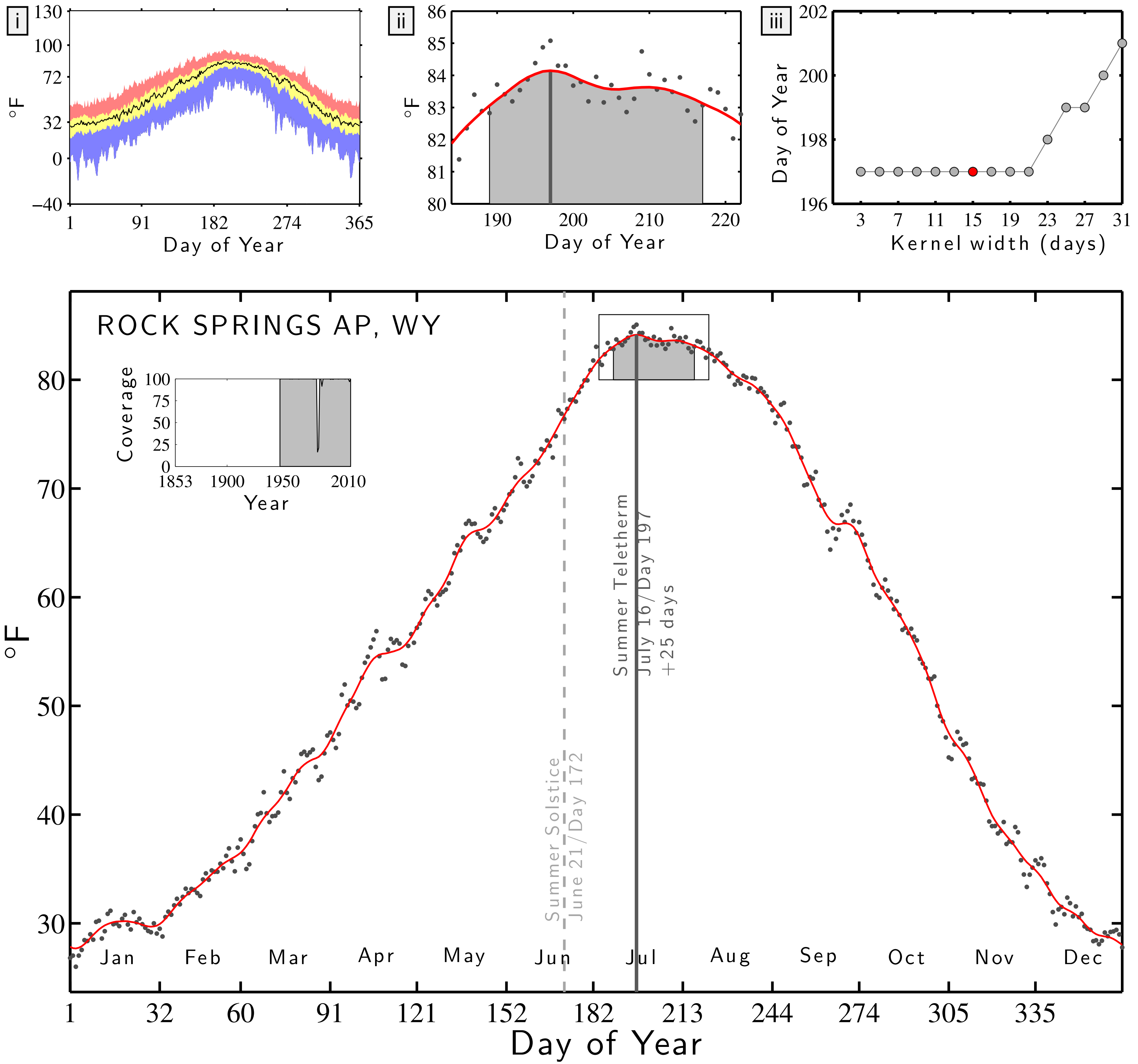

i
ii
iii
°F
Day of Year
Day of Year
Kernel width (days)
ROCK SPRINGS AP, WY
Coverage
Year
Summer Solstice
June 21/Day 172
Summer Teletherm
July 16/Day 197
+25 days
Jan
Feb
Mar
Apr
May
Jun
Jul
Aug
Sep
Oct
Nov
Dec
°F
Day of Year

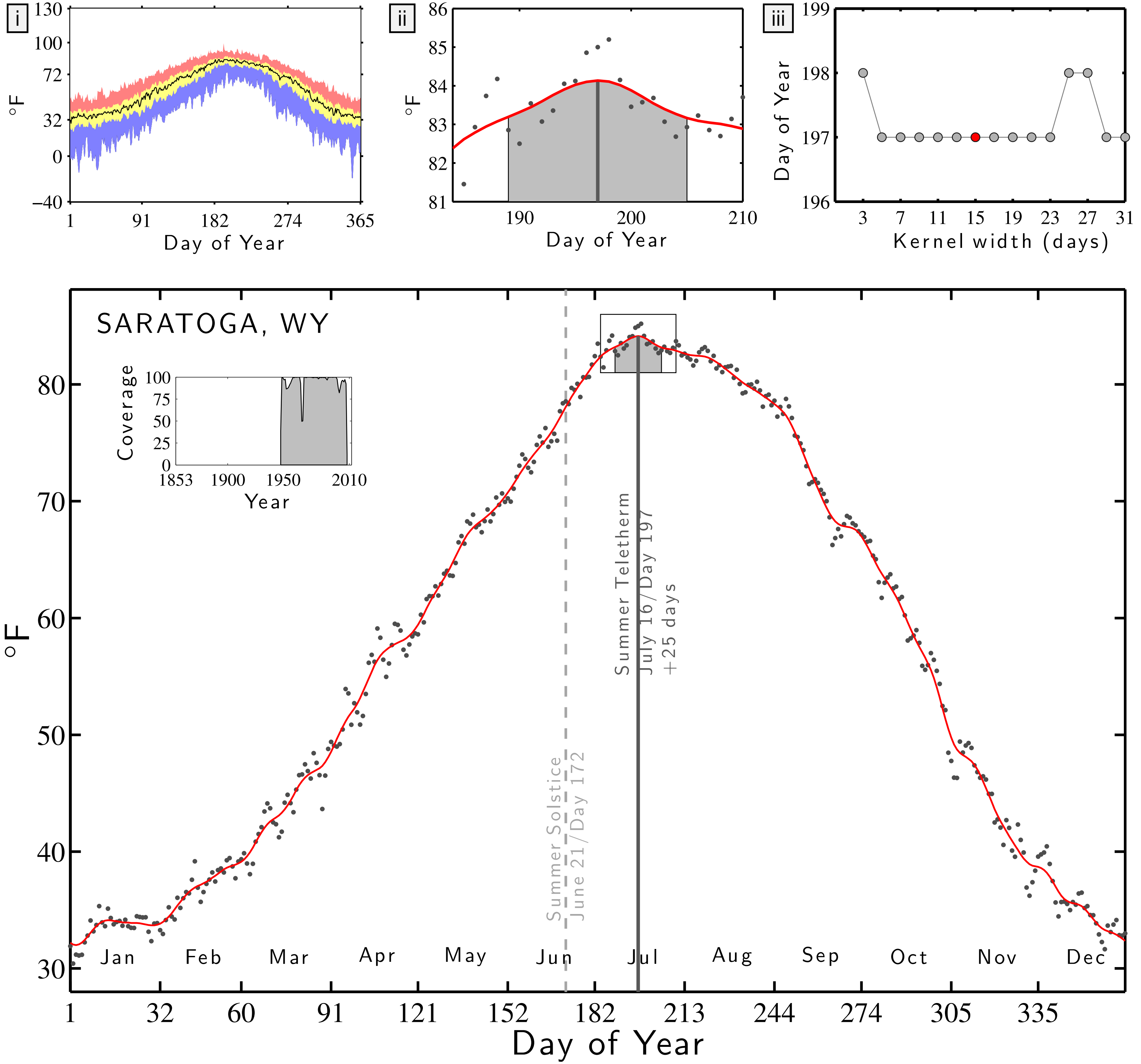

i
°F
Day of Year
ii
°F
Day of Year
iii
Day of Year
Kernel width (days)
SARATOGA, WY
Coverage
Year
Summer Solstice
June 21/Day 172
Summer Teletherm
July 16/Day 197
+25 days
Jan
Feb
Mar
Apr
May
Jun
Jul
Aug
Sep
Oct
Nov
Dec
°F
Day of Year

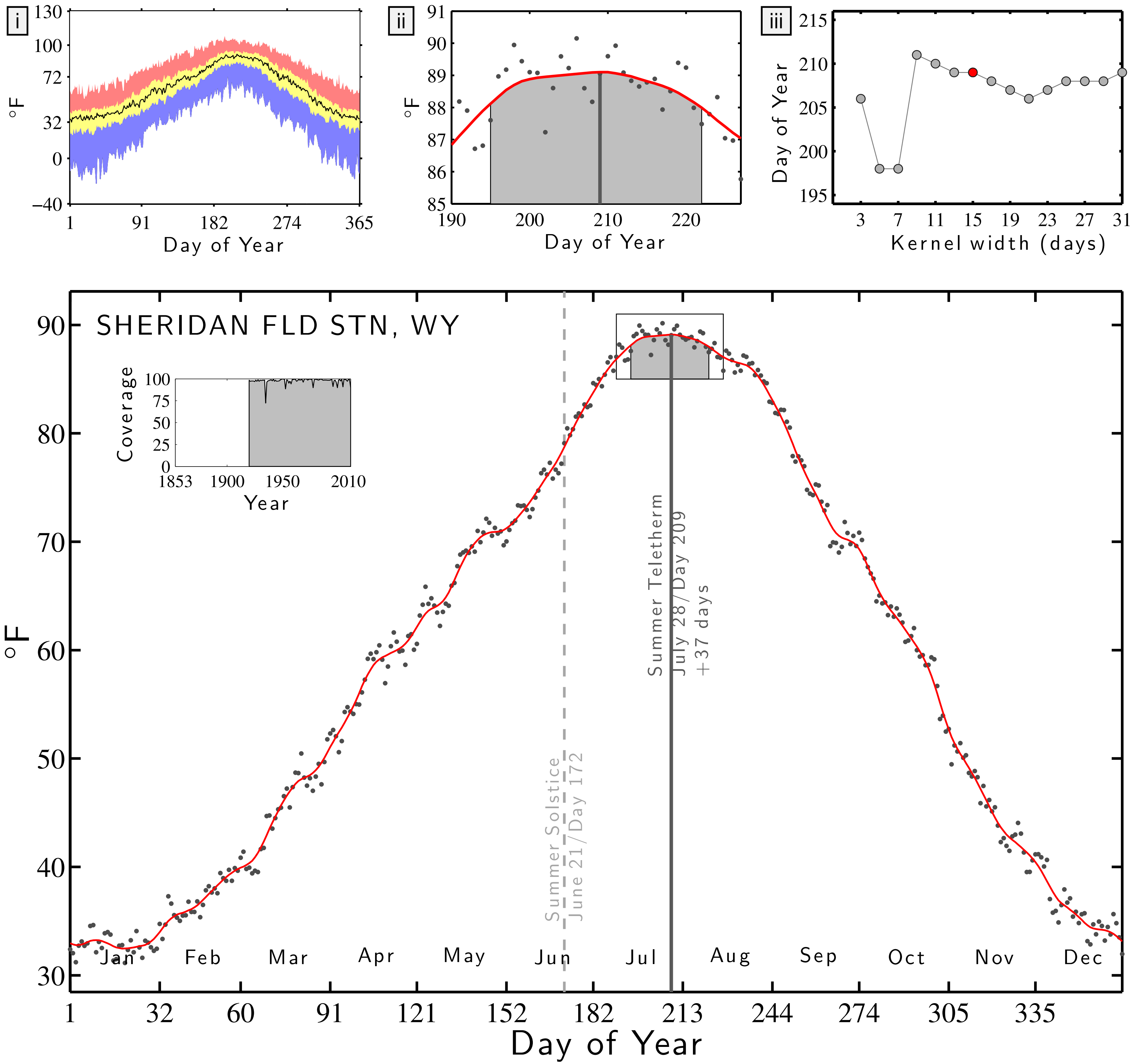

i
ii
iii
°F
Day of Year
Kernel width (days)
SHERIDAN FLD STN, WY
Coverage
Year
Summer Solstice
June 21/Day 172
Summer Teletherm
July 28/Day 209
+37 days
Jan
Feb
Mar
Apr
May
Jun
Jul
Aug
Sep
Oct
Nov
Dec

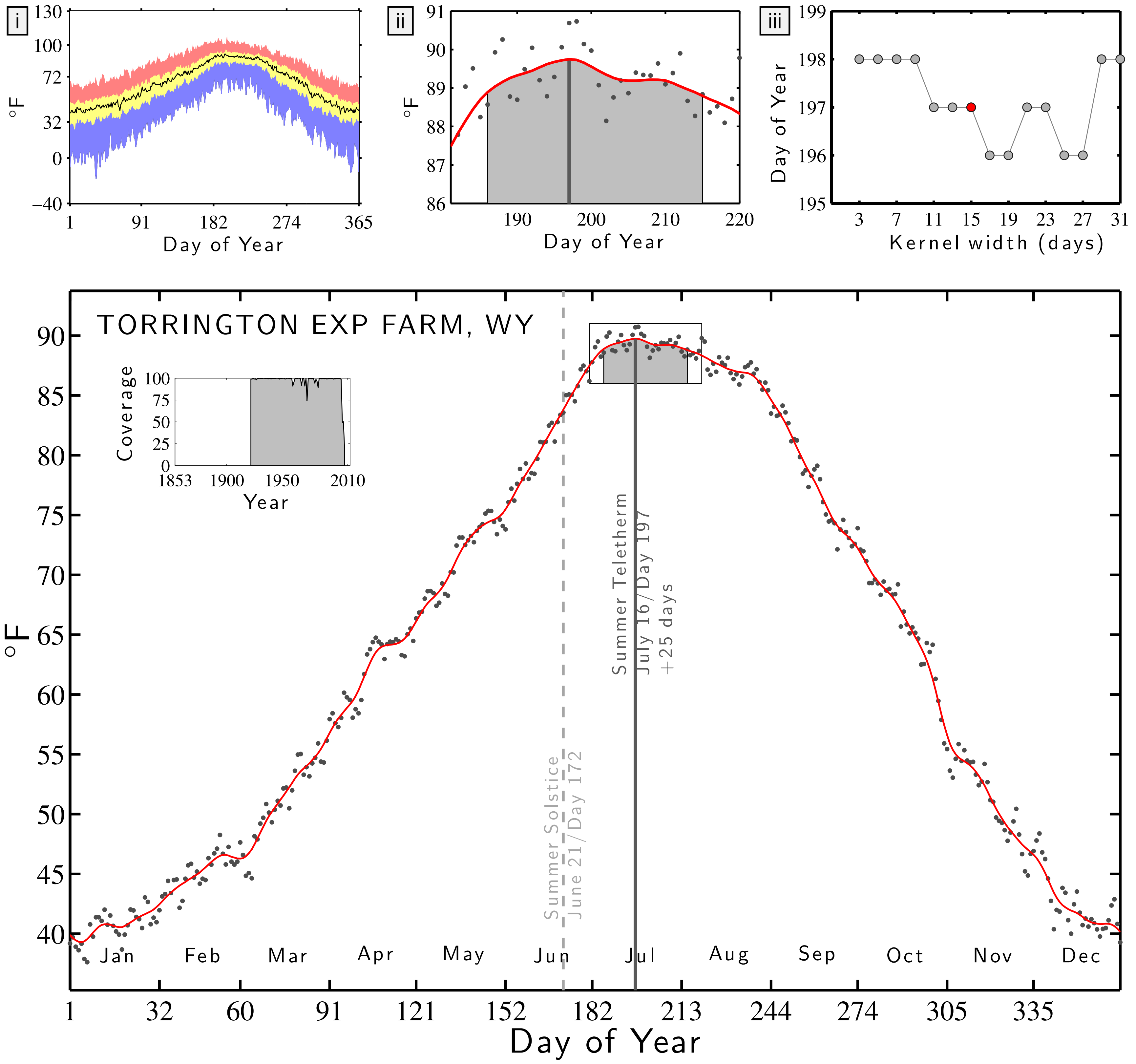

i
°F
Day of Year
ii
°F
Day of Year
iii
Day of Year
Kernel width (days)
TORRINGTON EXP FARM, WY
Coverage
Year
Summer Solstice
June 21/Day 172
Summer Teletherm
July 16/Day 197
+25 days
Jan
Feb
Mar
Apr
May
Jun
Jul
Aug
Sep
Oct
Nov
Dec
°F
Day of Year

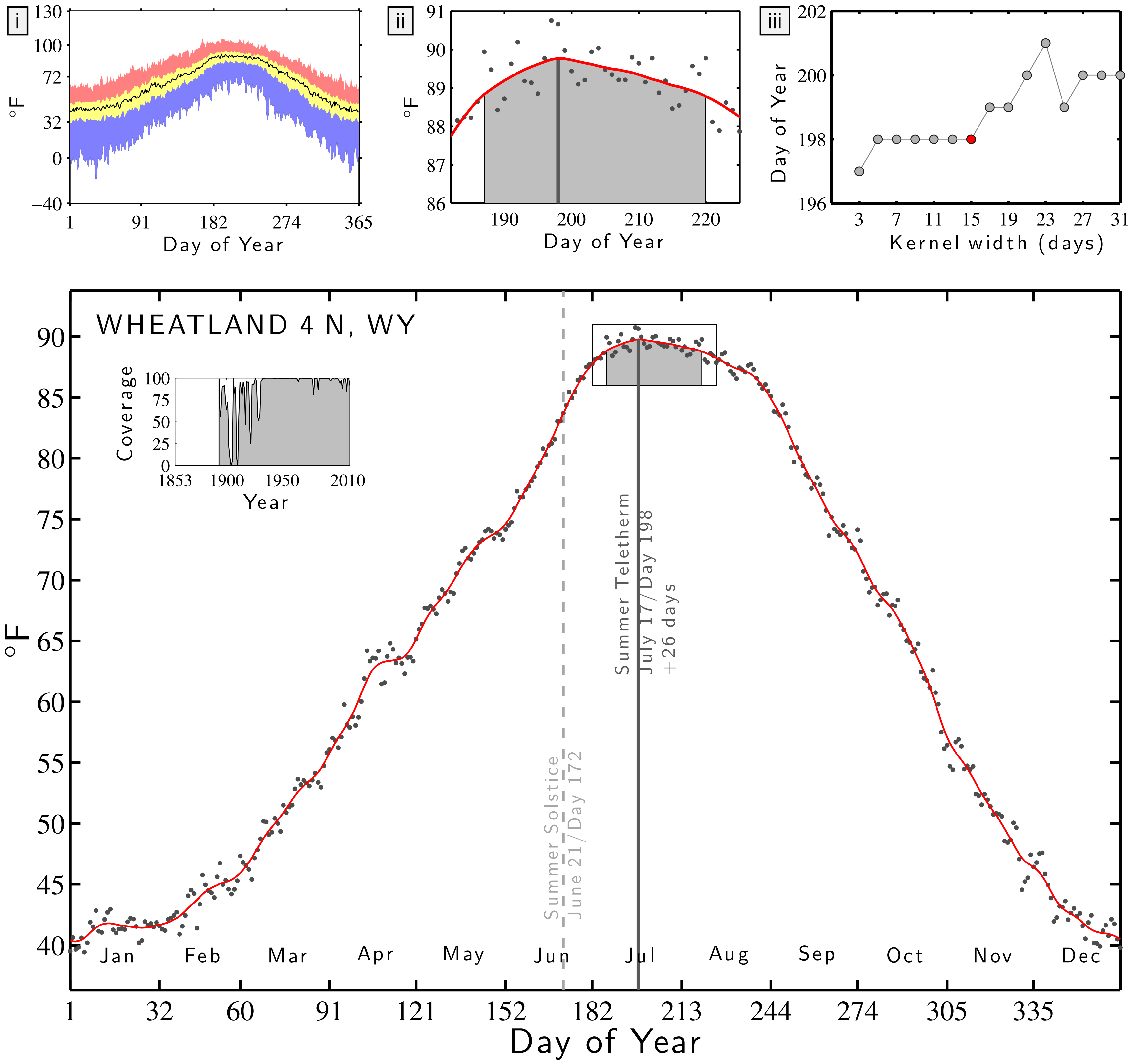

i
°F
Day of Year
ii
°F
Day of Year
iii
Day of Year
Kernel width (days)
WHEATLAND 4 N, WY
Coverage
Year
Summer Solstice
June 21/Day 172
Summer Teletherm
July 17/Day 198
+26 days
Jan
Feb
Mar
Apr
May
Jun
Jul
Aug
Sep
Oct
Nov
Dec
°F
Day of Year

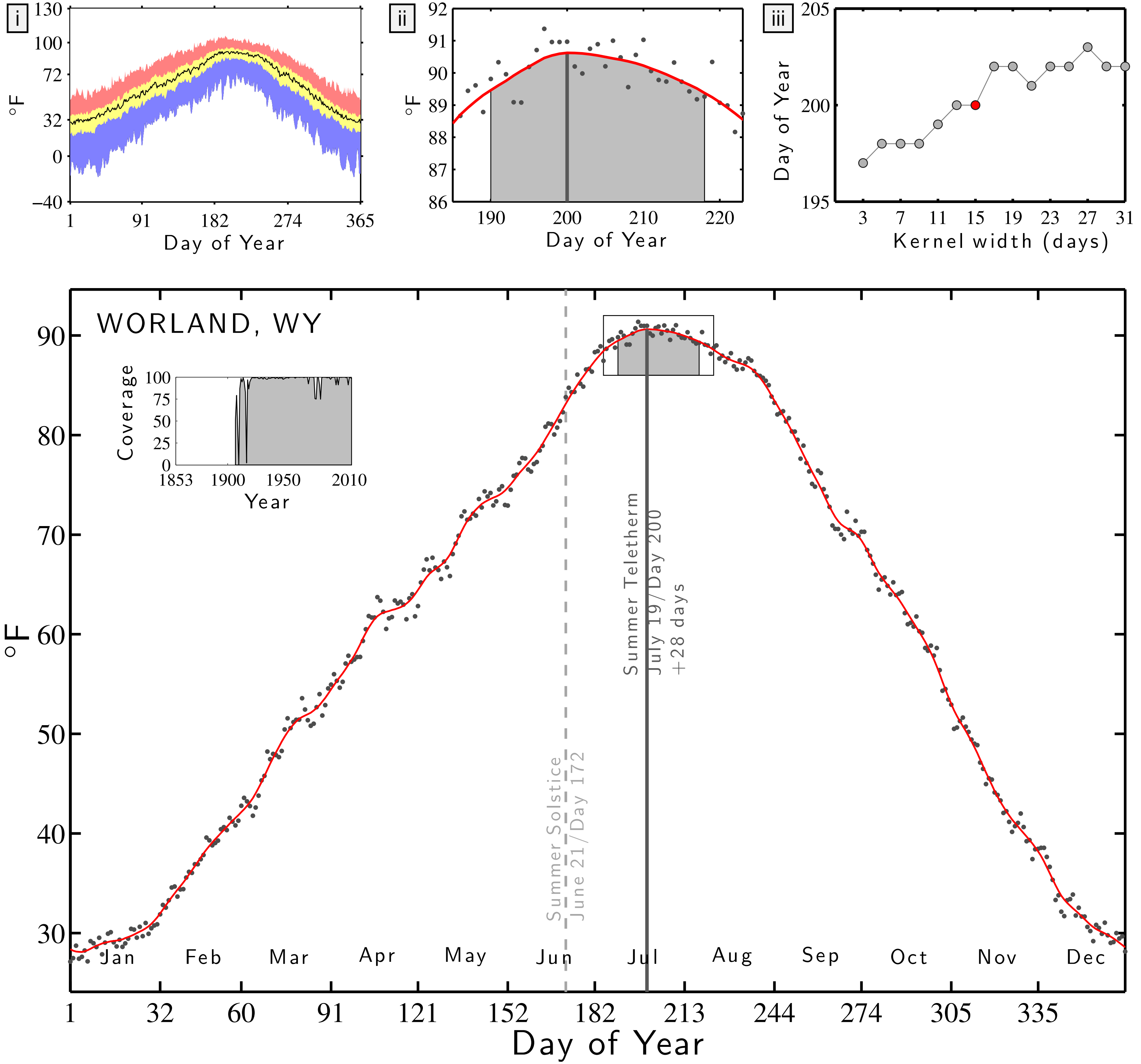

i
°F
130
100
72
32
0
−40
1
91
182
274
365
Day of Year
ii
°F
92
91
90
89
88
87
86
190
200
210
220
Day of Year
iii
Day of Year
205
200
195
3
7
11
15
19
23
27
31
Kernel width (days)
WORLAND, WY
Coverage
100
75
50
25
0
1853
1900
1950
2010
Year
Summer Solstice
June 21/Day 172
Summer Teletherm
July 19/Day 200
+28 days
Jan
Feb
Mar
Apr
May
Jun
Jul
Aug
Sep
Oct
Nov
Dec
°F
90
80
70
60
50
40
30
1
32
60
91
121
152
182
213
244
274
305
335
Day of Year

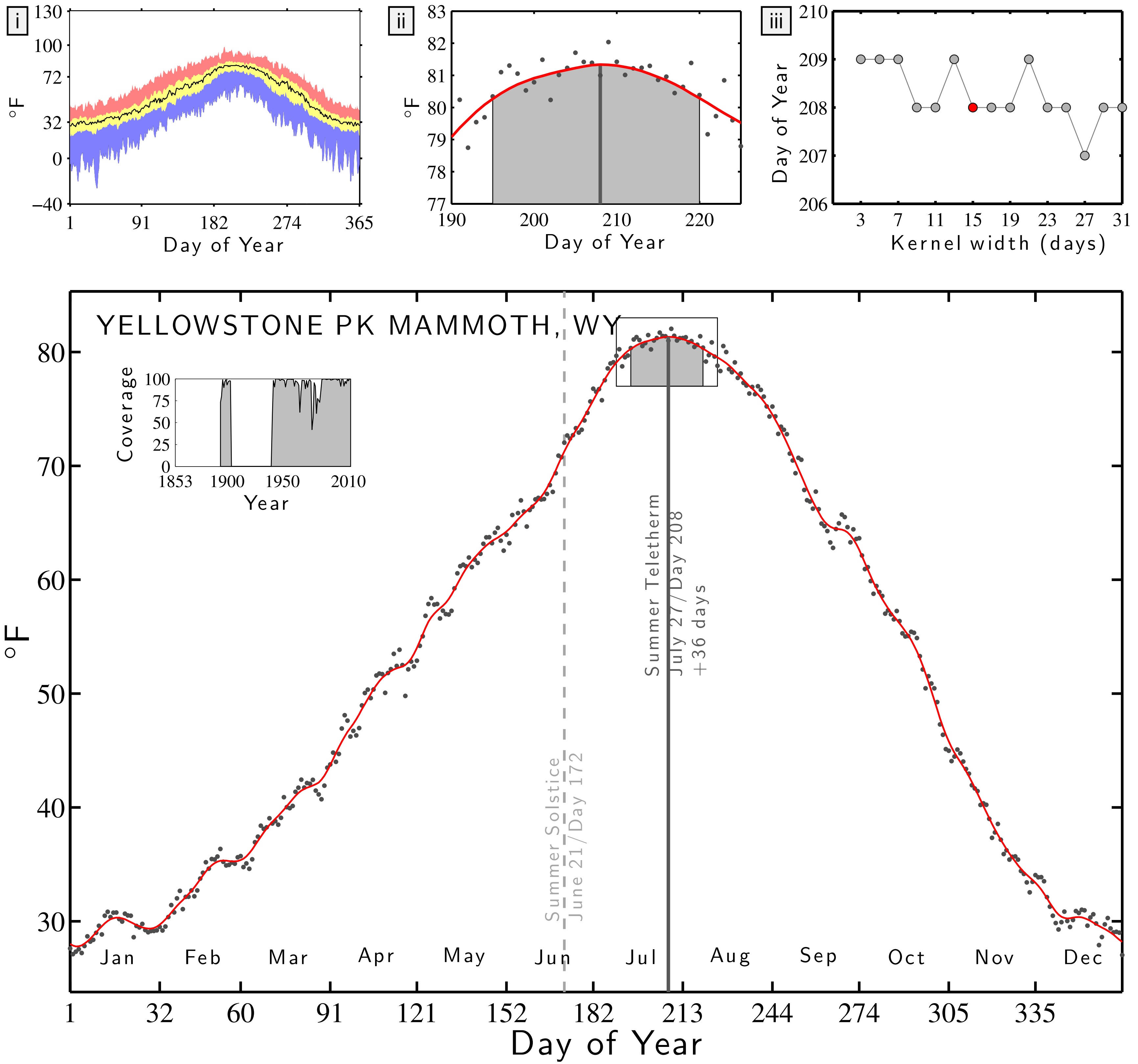

i
ii
iii
°F
Day of Year
Day of Year
Kernel width (days)
YELLOWSTONE PK MAMMOTH, WY
Coverage
Year
Summer Solstice
June 21/Day 172
Summer Teletherm
July 27/Day 208
+36 days
Jan
Feb
Mar
Apr
May
Jun
Jul
Aug
Sep
Oct
Nov
Dec
Day of Year
