# Supplementary material for: Tracking Climate Change through the Spatiotemporal Dynamics of the Teletherms, the Statistically Hottest and Coldest Days of the Year
Source: PLoS One. 2016 May 11;11(5):e0154184. doi: 10.1371/journal.pone.0154184 (PMC4864332; doi:10.1371/journal.pone.0154184)
Supplement: S2 File — (PDF) [file pone.0154184.s023.pdf]

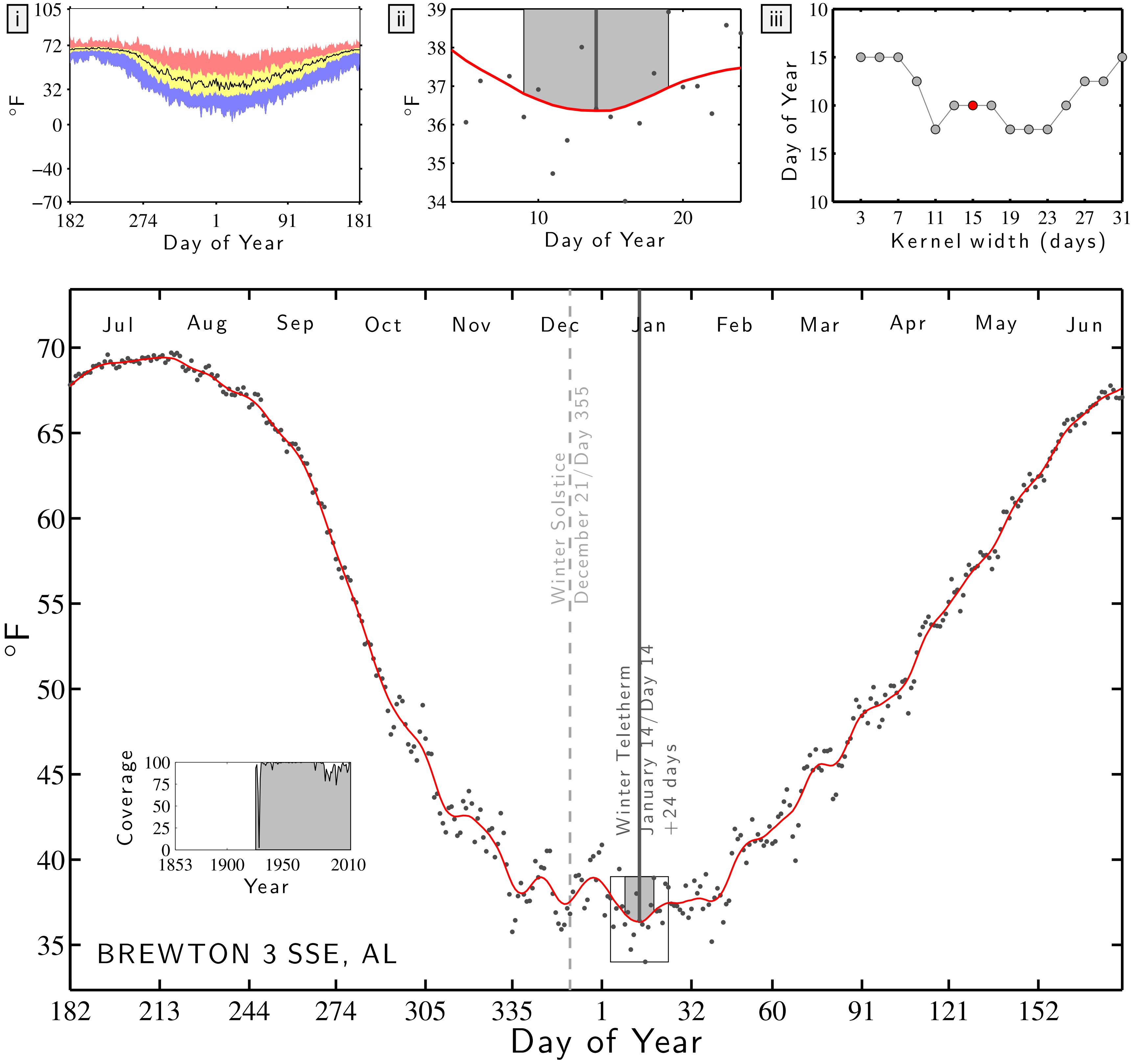

i
ii
iii
Day of Year
Kernel width (days)
Jul
Aug
Sep
Oct
Nov
Dec
Jan
Feb
Mar
Apr
May
Jun
Winter Solstice
December 21/Day 355
Winter Teletherm
January 14/Day 14
+24 days
Coverage
Year
BREWTON 3 SSE, AL
°F
Day of Year

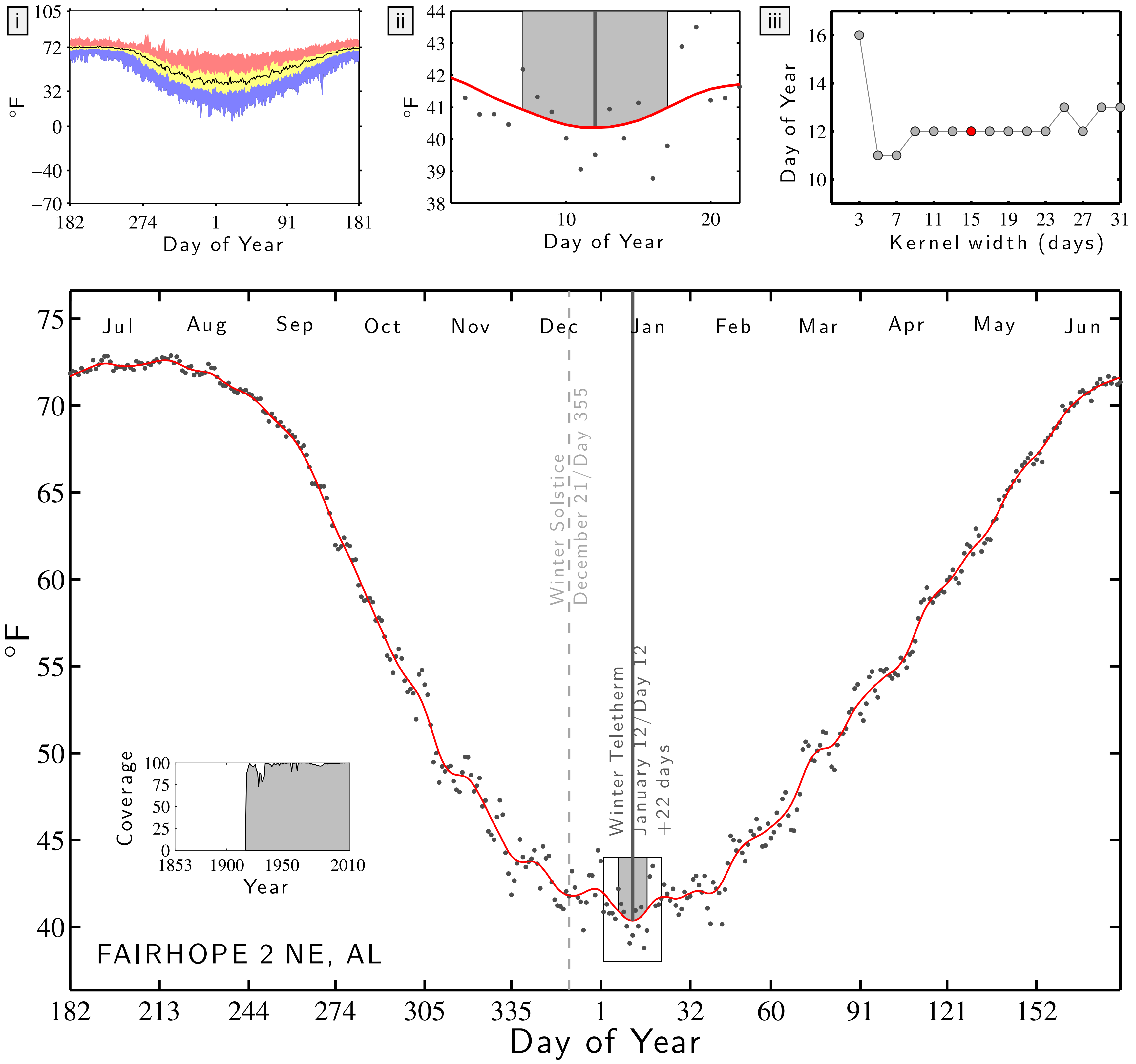

i
ii
iii
°F
Day of Year
Kernel width (days)
Jul
Aug
Sep
Oct
Nov
Dec
Jan
Feb
Mar
Apr
May
Jun
Winter Solstice
December 21/Day 355
Winter Teletherm
January 12/Day 12
+22 days
Coverage
Year
FAIRHOPE 2 NE, AL

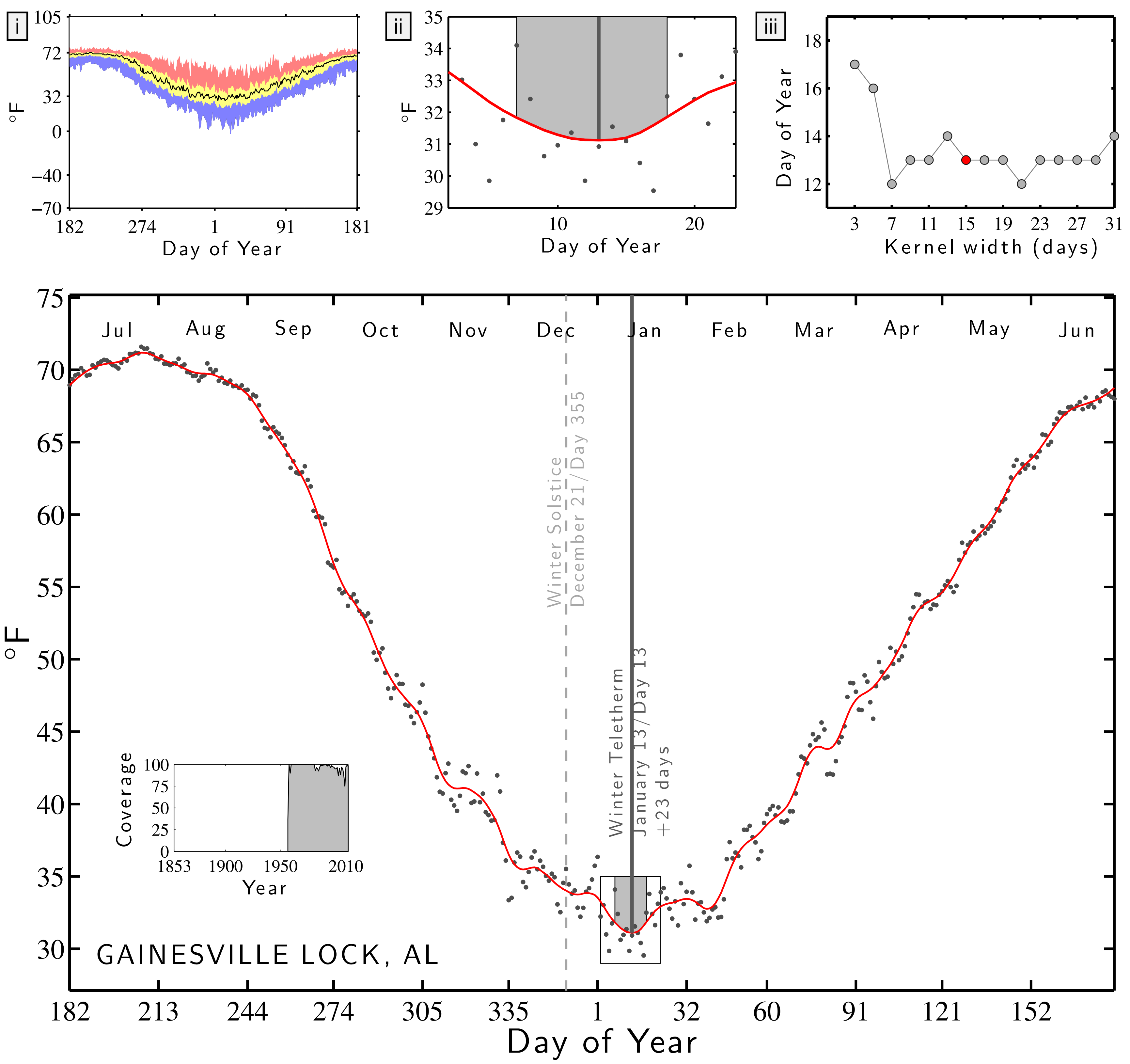

i
°F
Day of Year
ii
°F
Day of Year
iii
Day of Year
Kernel width (days)
Jul
Aug
Sep
Oct
Nov
Dec
Jan
Feb
Mar
Apr
May
Jun
Winter Solstice
December 21/Day 355
Winter Teletherm
January 13/Day 13
+23 days
Coverage
Year
GAINESVILLE LOCK, AL
°F
Day of Year

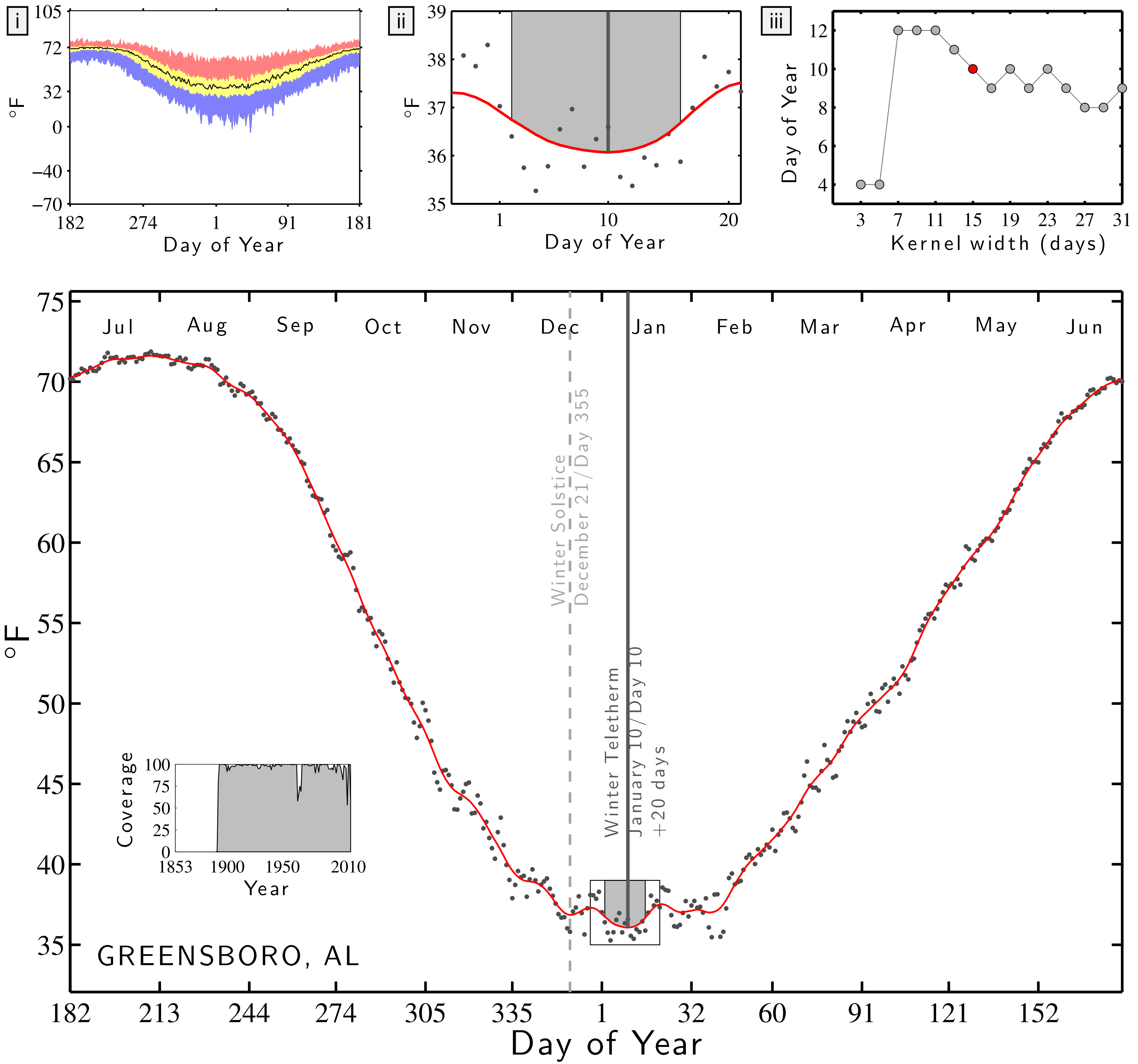
i
ii
iii
°F
Day of Year
Day of Year
Kernel width (days)
Jul
Aug
Sep
Oct
Nov
Dec
Jan
Feb
Mar
Apr
May
Jun
Winter Solstice
December 21/Day 355
Winter Teletherm
January 10/Day 10
+20 days
Coverage
Year
GREENSBORO, AL
Day of Year

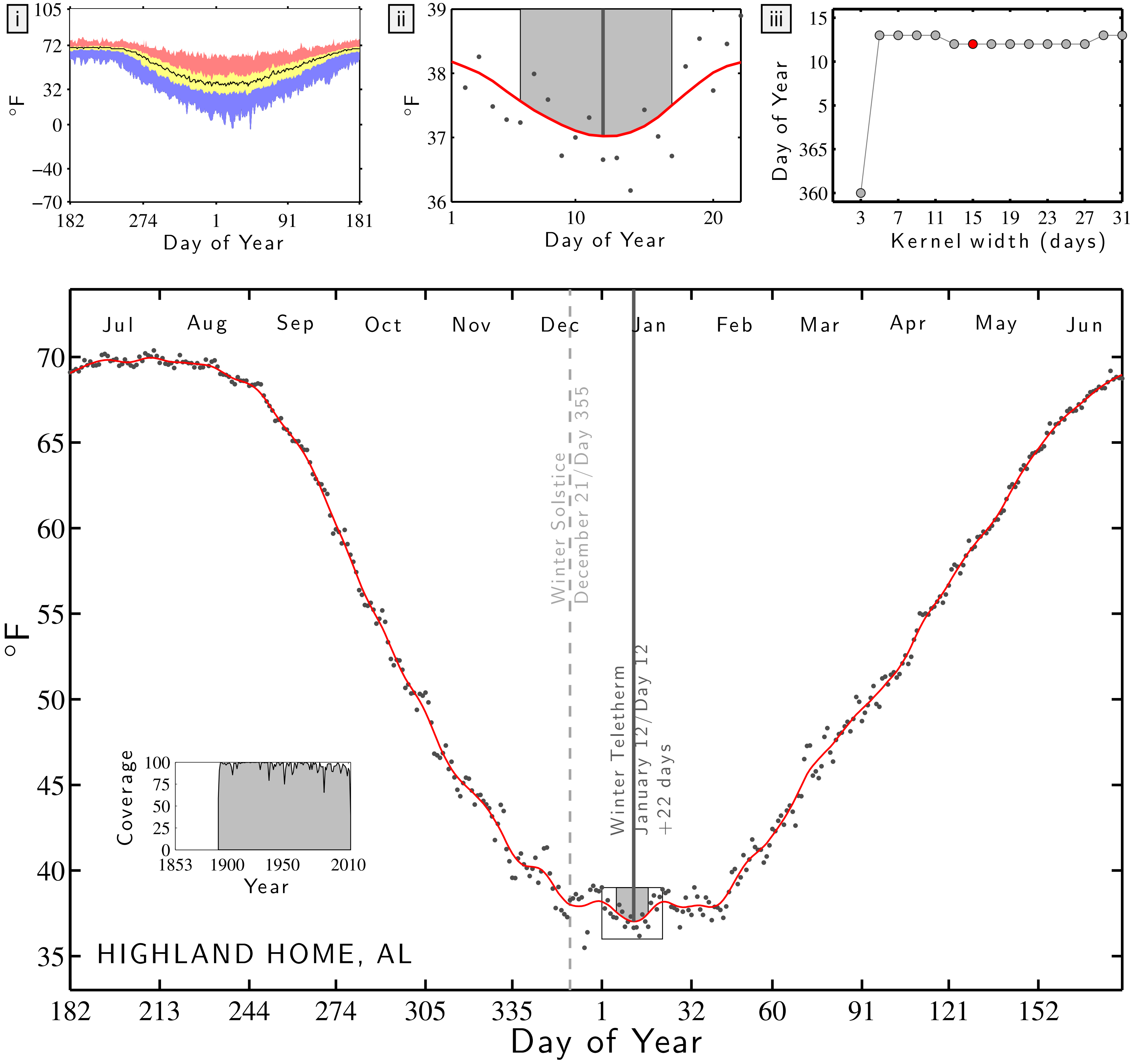

i
°F
Day of Year
ii
°F
Day of Year
iii
Day of Year
Kernel width (days)
Jul
Aug
Sep
Oct
Nov
Dec
Jan
Feb
Mar
Apr
May
Jun
Winter Solstice
December 21/Day 355
Winter Teletherm
January 12/Day 12
+22 days
Coverage
Year
HIGHLAND HOME, AL
°F
Day of Year

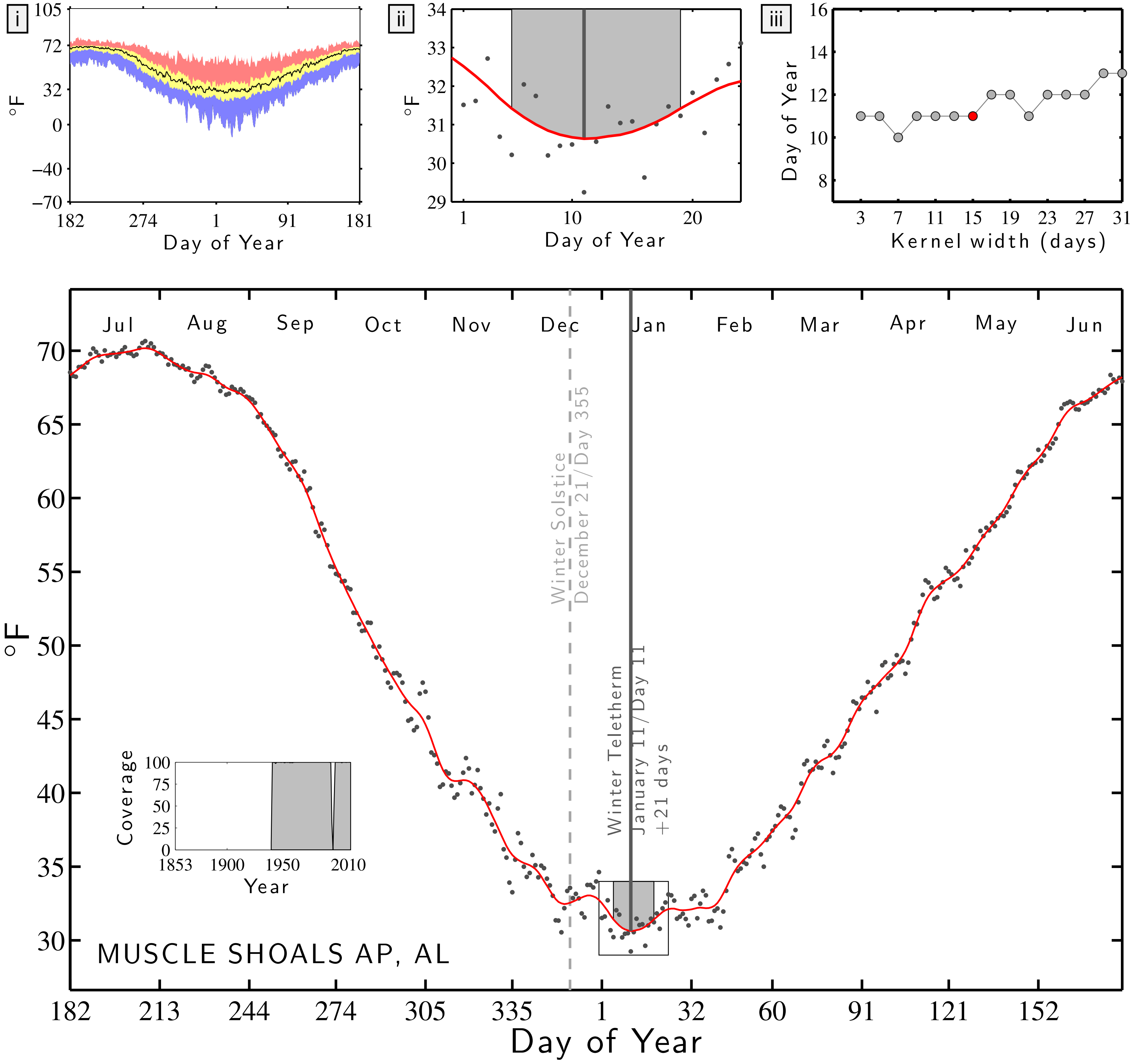

i
ii
iii
°F
Day of Year
Day of Year
Kernel width (days)
Jul
Aug
Sep
Oct
Nov
Dec
Jan
Feb
Mar
Apr
May
Jun
Winter Solstice
December 21/Day 355
Winter Teletherm
January 11/Day 11
+21 days
Coverage
Year
MUSCLE SHOALS AP, AL

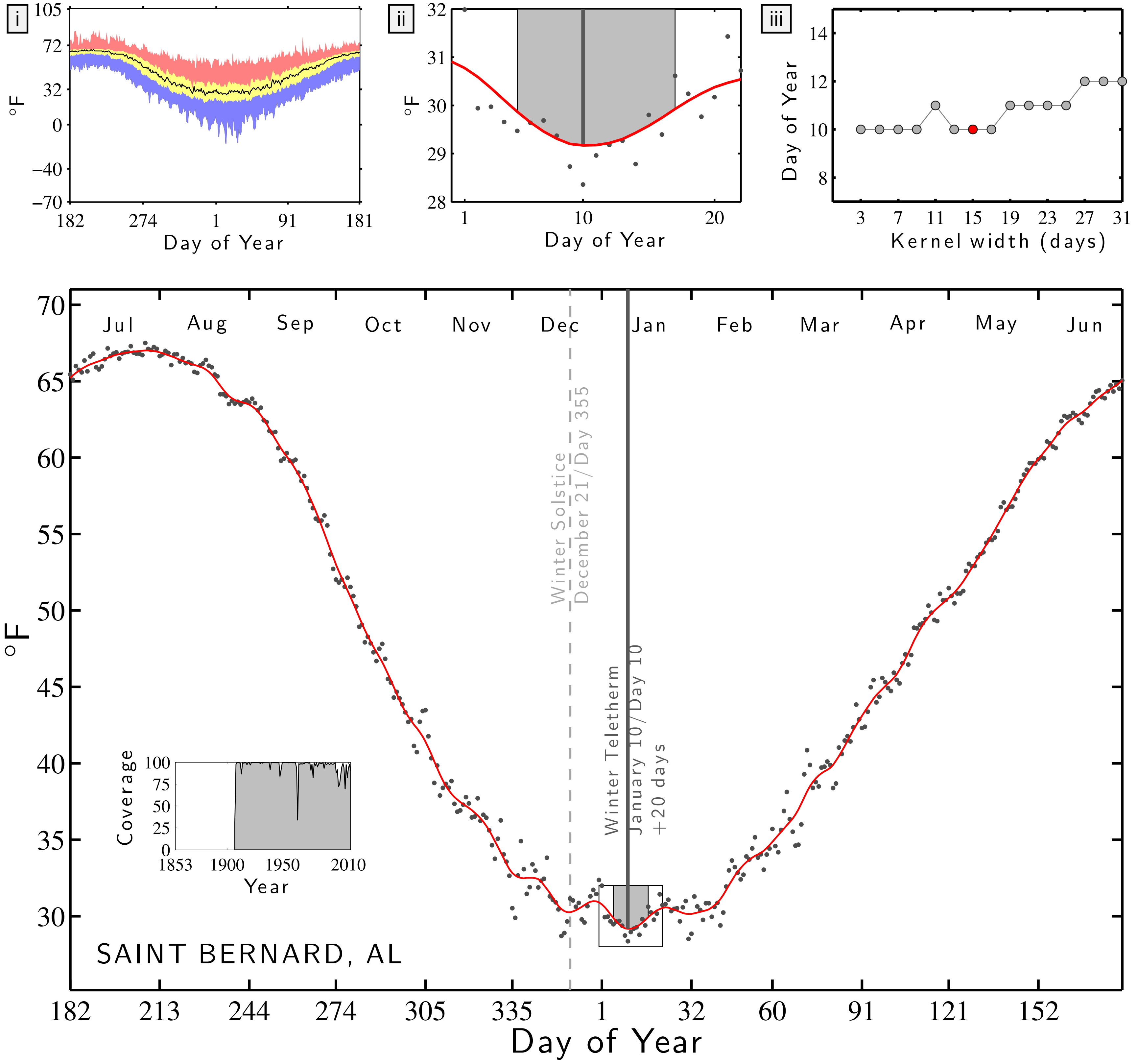
i
ii
iii
°F
Day of Year
Day of Year
Kernel width (days)
Jul
Aug
Sep
Oct
Nov
Dec
Jan
Feb
Mar
Apr
May
Jun
Winter Solstice
December 21/Day 355
Winter Teletherm
January 10/Day 10
+20 days
Coverage
Year
SAINT BERNARD, AL

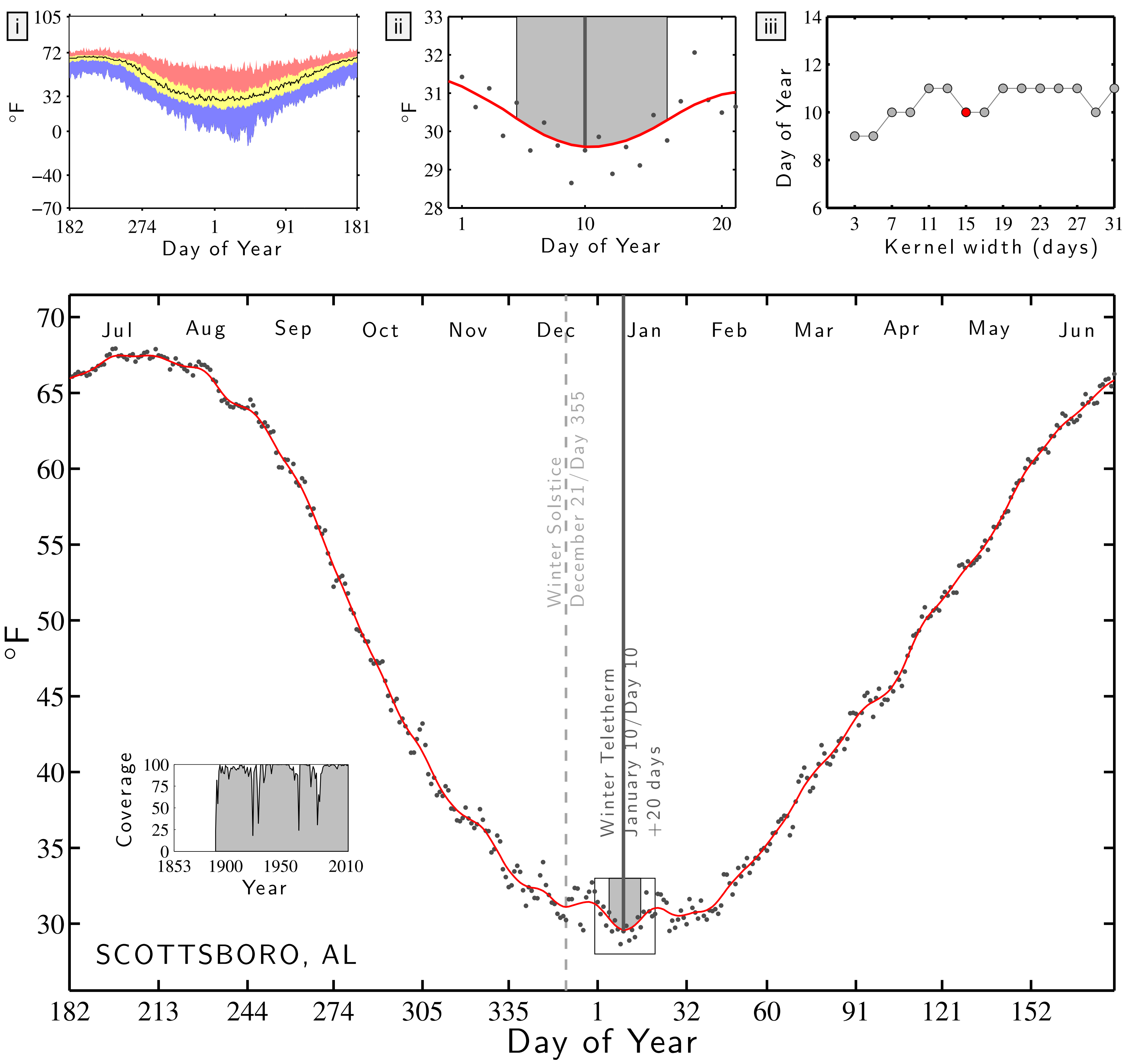
i
°F
Day of Year
ii
Day of Year
iii
Day of Year
Kernel width (days)
Jul
Aug
Sep
Oct
Nov
Dec
Jan
Feb
Mar
Apr
May
Jun
Winter Solstice
December 21/Day 355
Winter Teletherm
January 10/Day 10
+20 days
Coverage
Year
SCOTTSBORO, AL
Day of Year

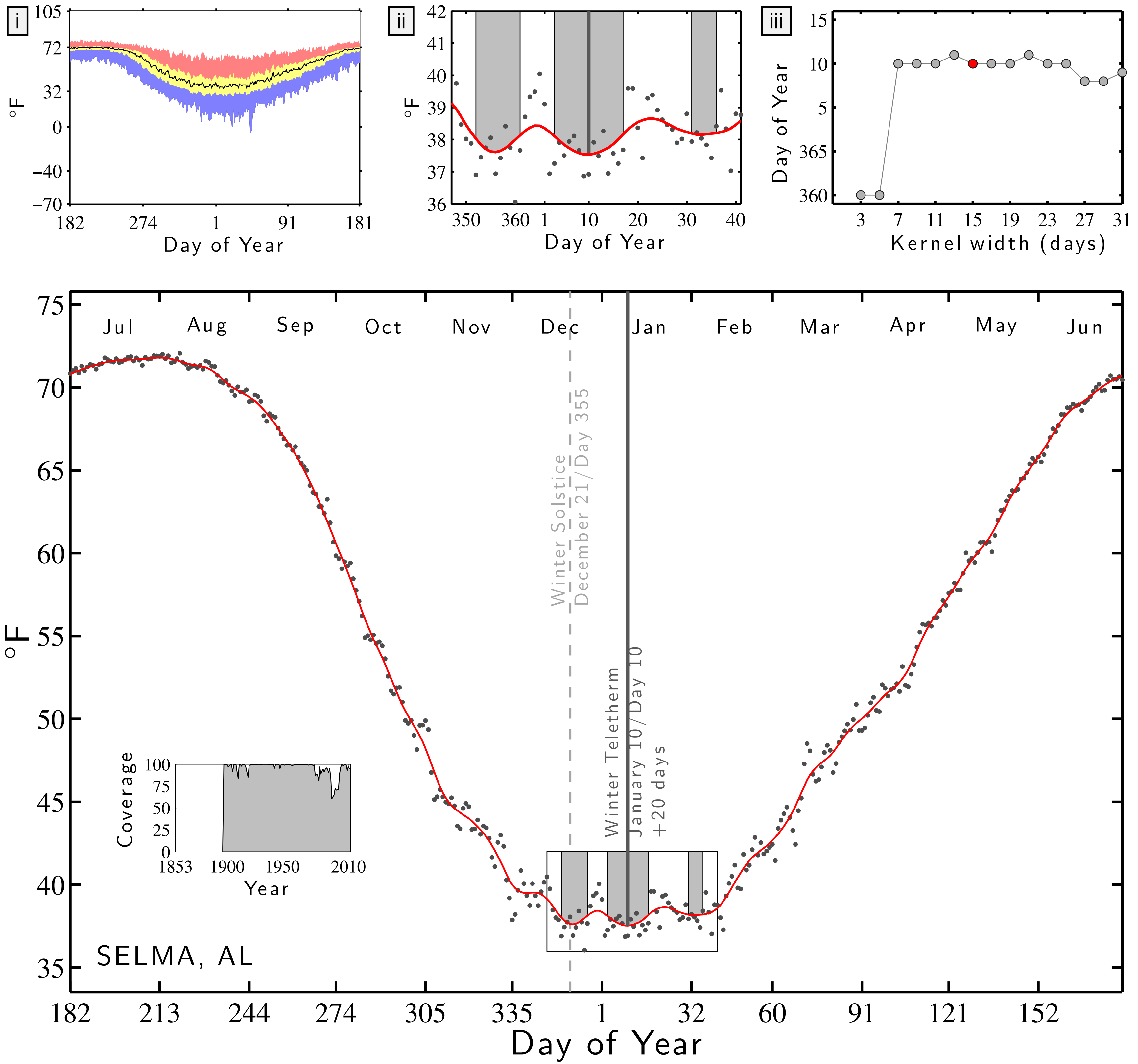

i
ii
iii
°F
Day of Year
Day of Year
Kernel width (days)
Jul
Aug
Sep
Oct
Nov
Dec
Jan
Feb
Mar
Apr
May
Jun
Winter Solstice
December 21/Day 355
Winter Teletherm
January 10/Day 10
+20 days
Coverage
Year
SELMA, AL

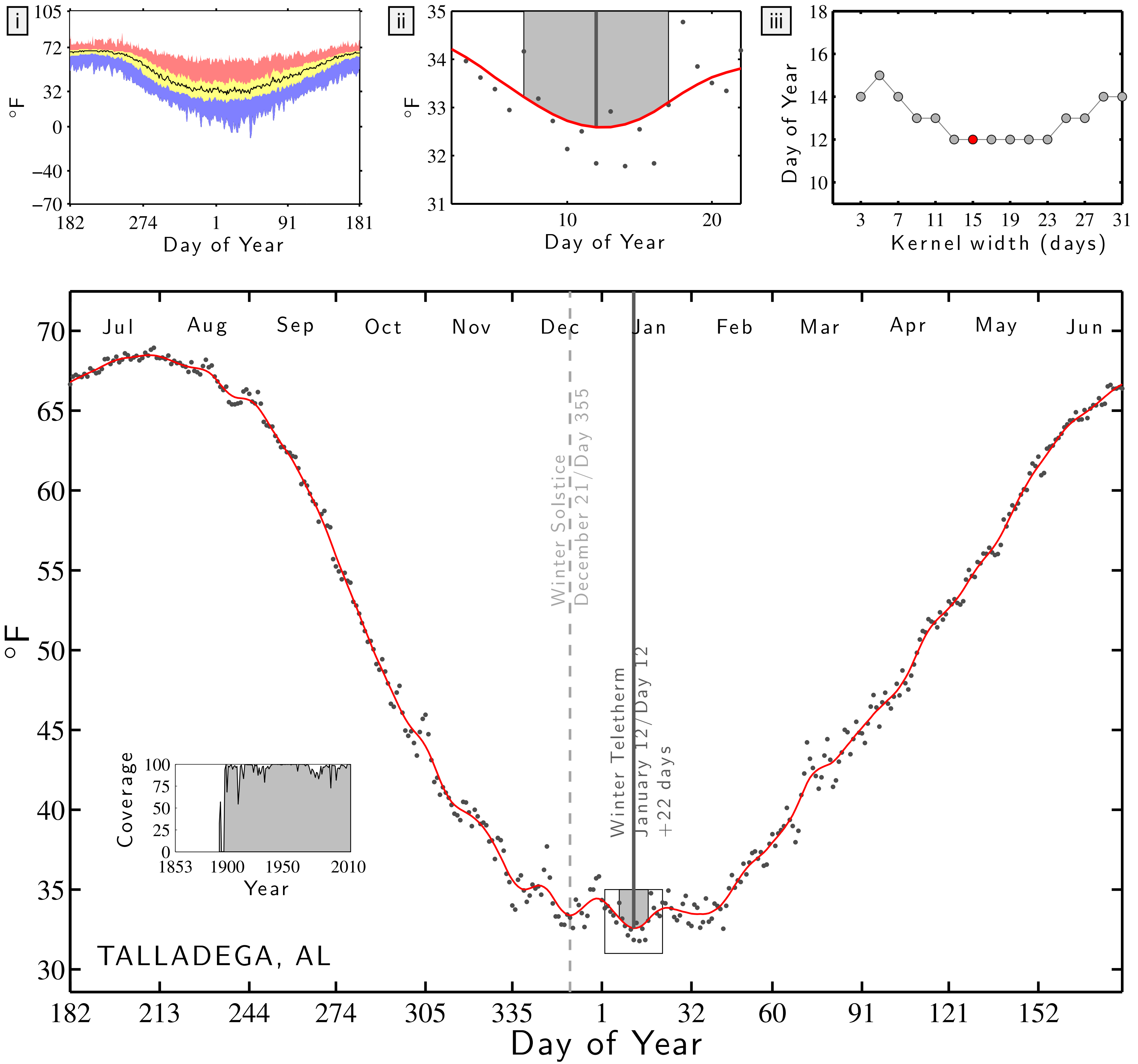

i
°F
Day of Year
ii
°F
Day of Year
iii
Day of Year
Kernel width (days)
Jul
Aug
Sep
Oct
Nov
Dec
Jan
Feb
Mar
Apr
May
Jun
Winter Solstice
December 21/Day 355
Winter Teletherm
January 12/Day 12
+22 days
Coverage
Year
TALLADEGA, AL
°F
Day of Year

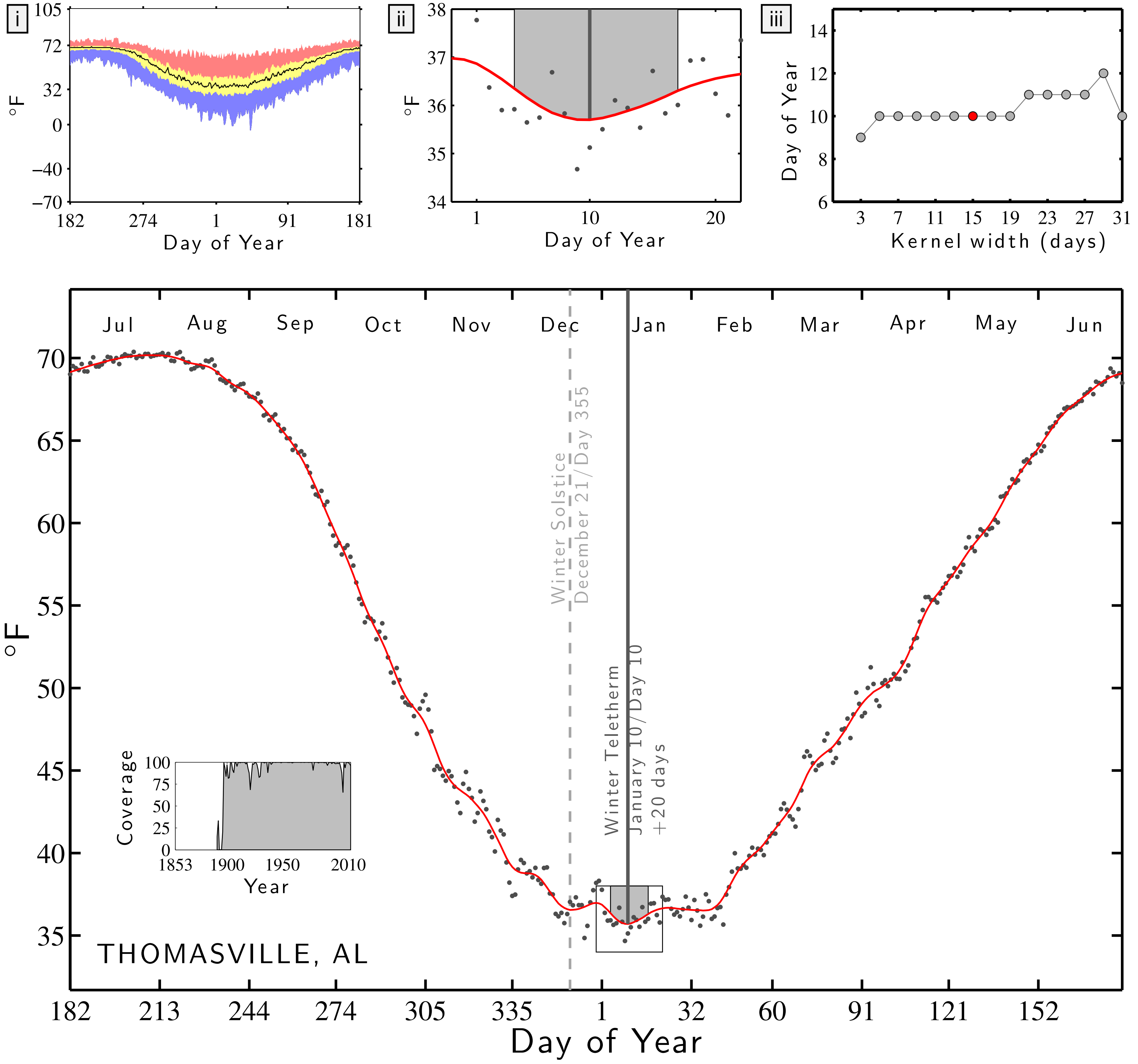

i
°F
Day of Year
ii
°F
Day of Year
iii
Day of Year
Kernel width (days)
Jul
Aug
Sep
Oct
Nov
Dec
Jan
Feb
Mar
Apr
May
Jun
Winter Solstice
December 21/Day 355
Winter Teletherm
January 10/Day 10
+20 days
Coverage
Year
THOMASVILLE, AL
°F
Day of Year

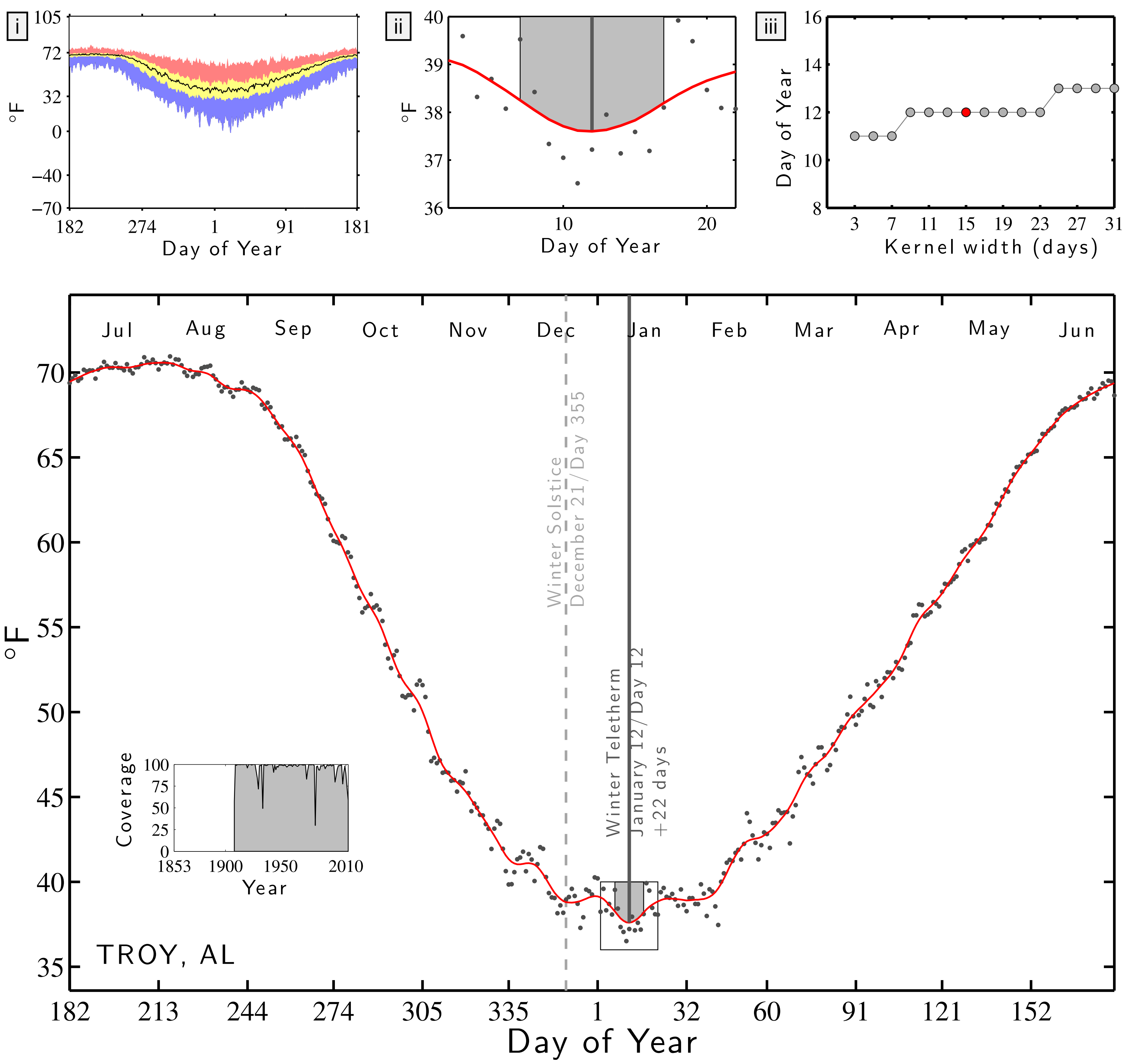

i
ii
iii
Day of Year
°F
Kernel width (days)
Jul
Aug
Sep
Oct
Nov
Dec
Jan
Feb
Mar
Apr
May
Jun
Winter Solstice
December 21/Day 355
Winter Teletherm
January 12/Day 12
+22 days
Coverage
Year
TROY, AL

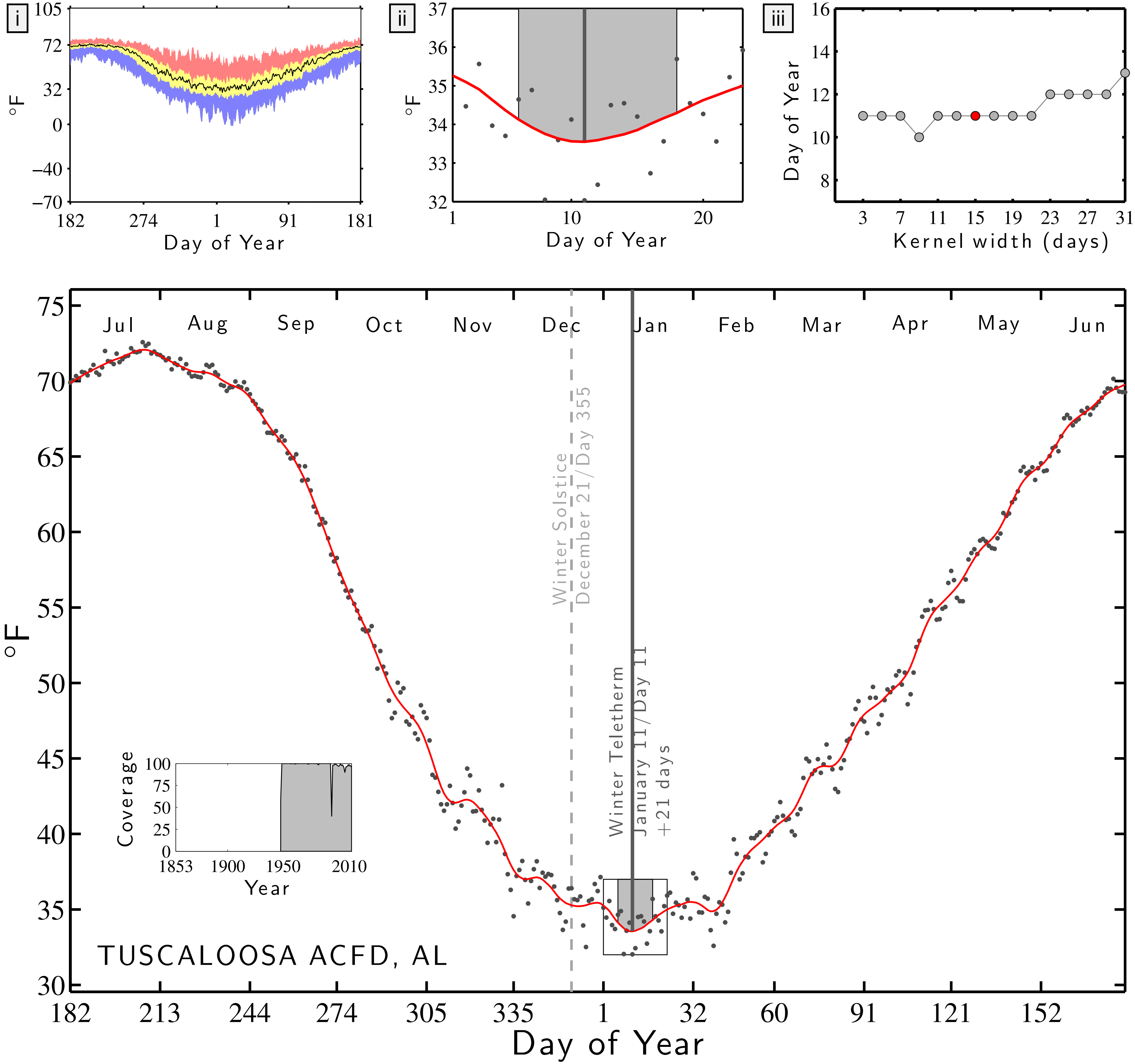

i
ii
iii
°F
Day of Year
Kernel width (days)
Jul
Aug
Sep
Oct
Nov
Dec
Jan
Feb
Mar
Apr
May
Jun
Winter Solstice
December 21/Day 355
Winter Teletherm
January 11/Day 11
+21 days
Coverage
Year
TUSCALOOSA ACFD, AL

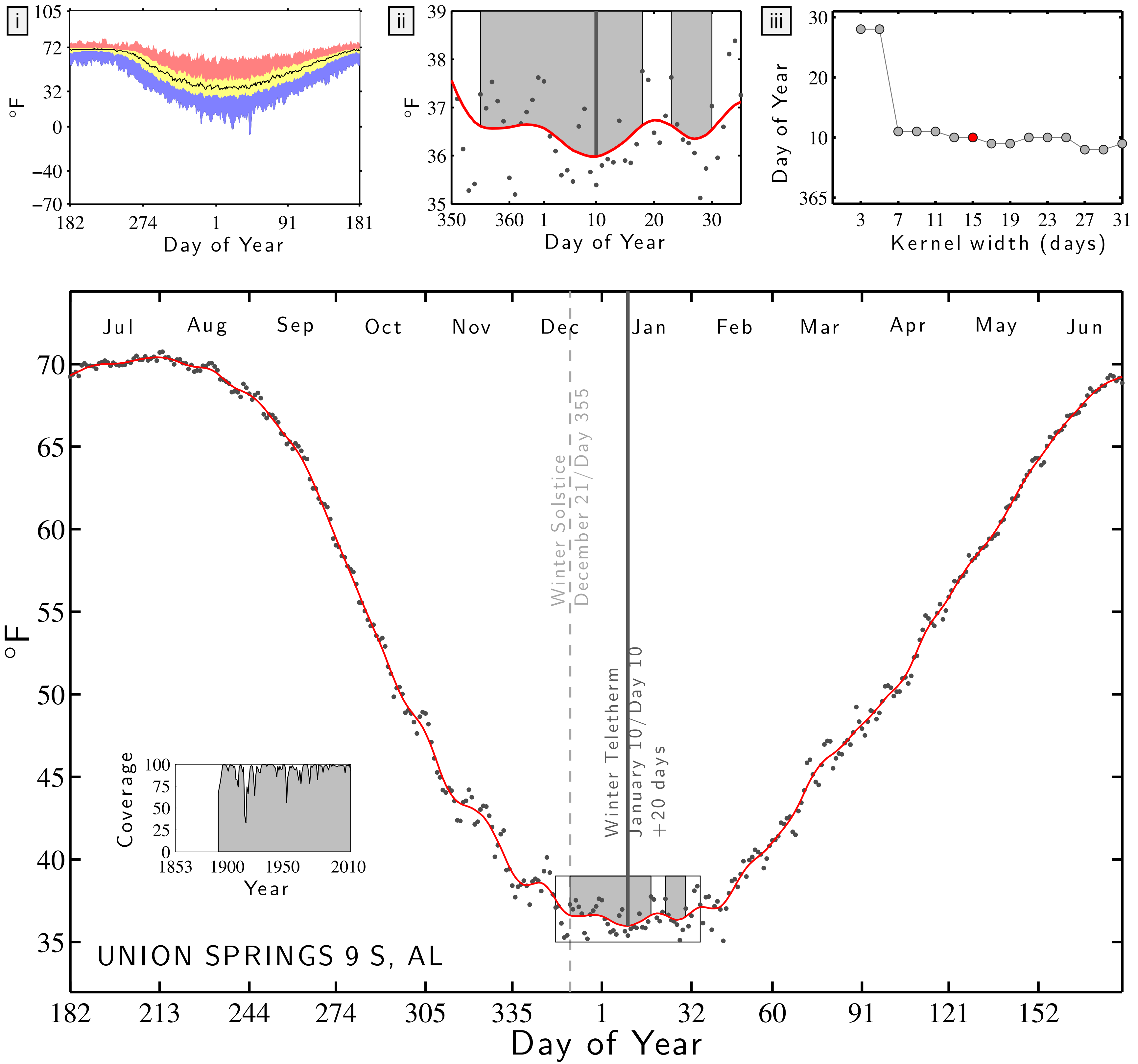

i
ii
iii
Day of Year
Kernel width (days)
Winter Solstice
December 21/Day 355
Winter Teletherm
January 10/Day 10
+20 days
Coverage
Year
UNION SPRINGS 9 S, AL
Jul
Aug
Sep
Oct
Nov
Dec
Jan
Feb
Mar
Apr
May
Jun
°F

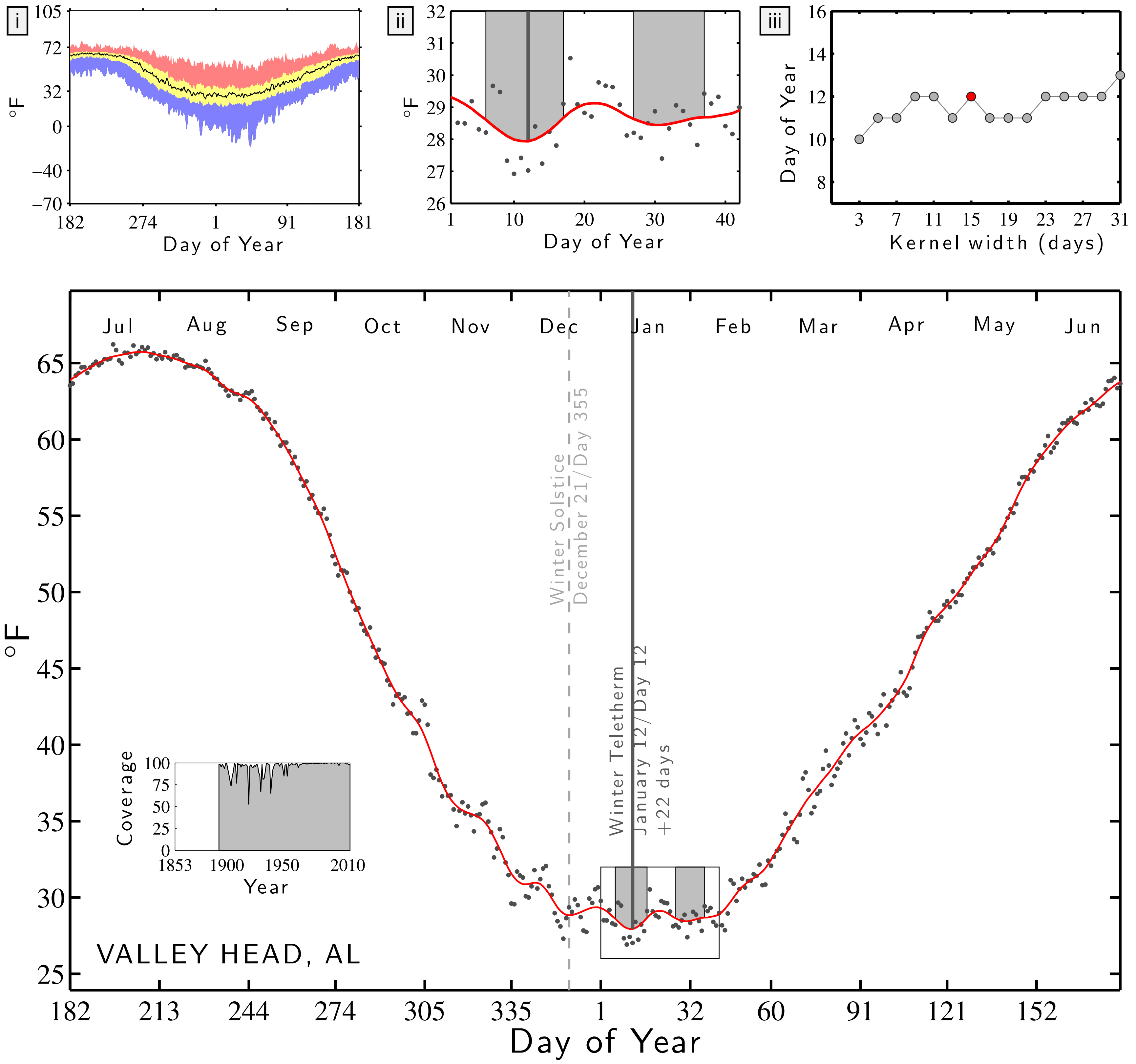

i
ii
iii
°F
Day of Year
Day of Year
Kernel width (days)
Jul
Aug
Sep
Oct
Nov
Dec
Jan
Feb
Mar
Apr
May
Jun
Winter Solstice
December 21/Day 355
Winter Teletherm
January 12/Day 12
+22 days
Coverage
Year
VALLEY HEAD, AL

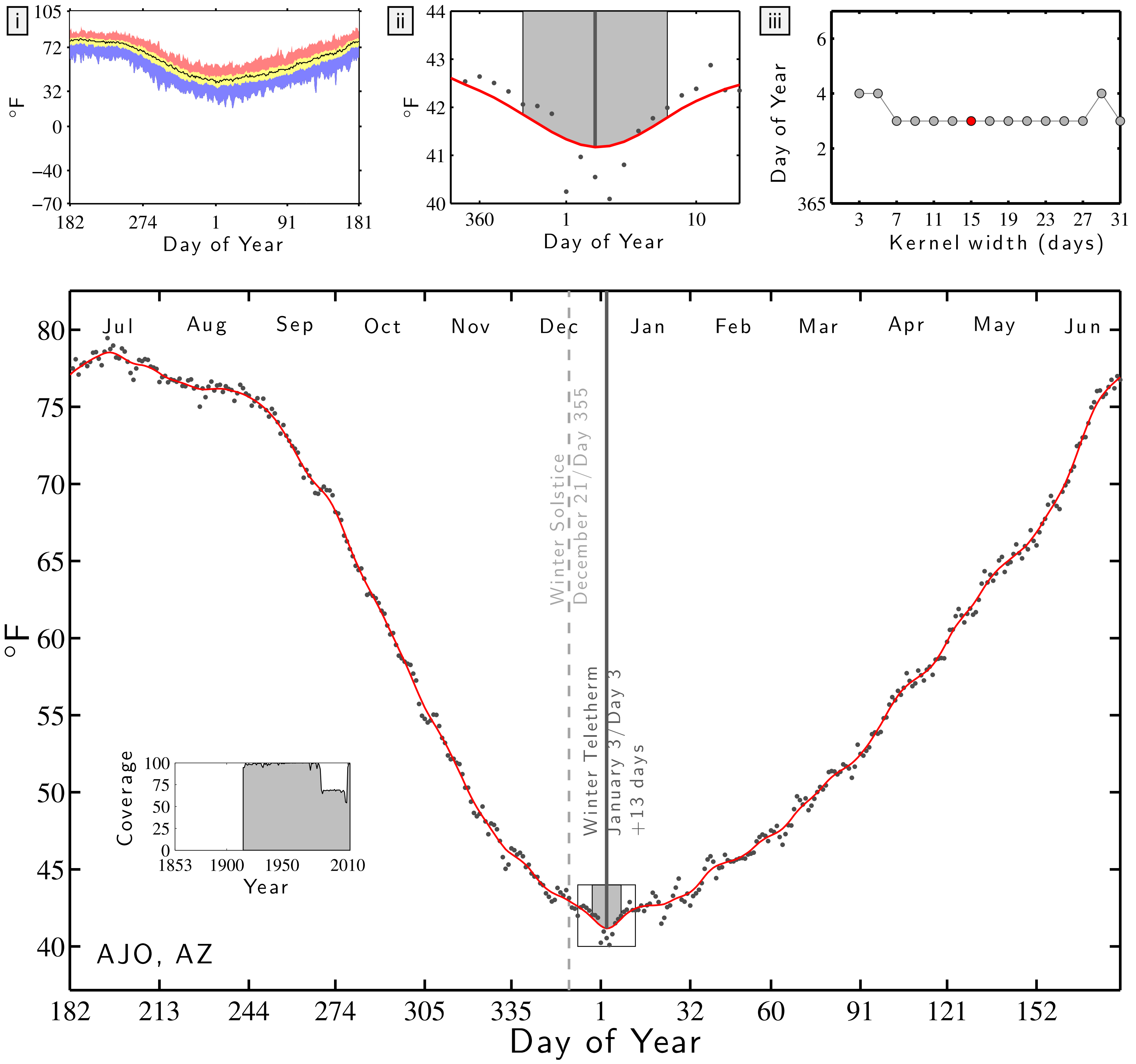

i
°F
Day of Year
ii
°F
Day of Year
iii
Day of Year
Kernel width (days)
Jul
Aug
Sep
Oct
Nov
Dec
Jan
Feb
Mar
Apr
May
Jun
Winter Solstice
December 21/Day 355
Winter Teletherm
January 3/Day 3
+13 days
Coverage
Year
AJO, AZ
°F
Day of Year

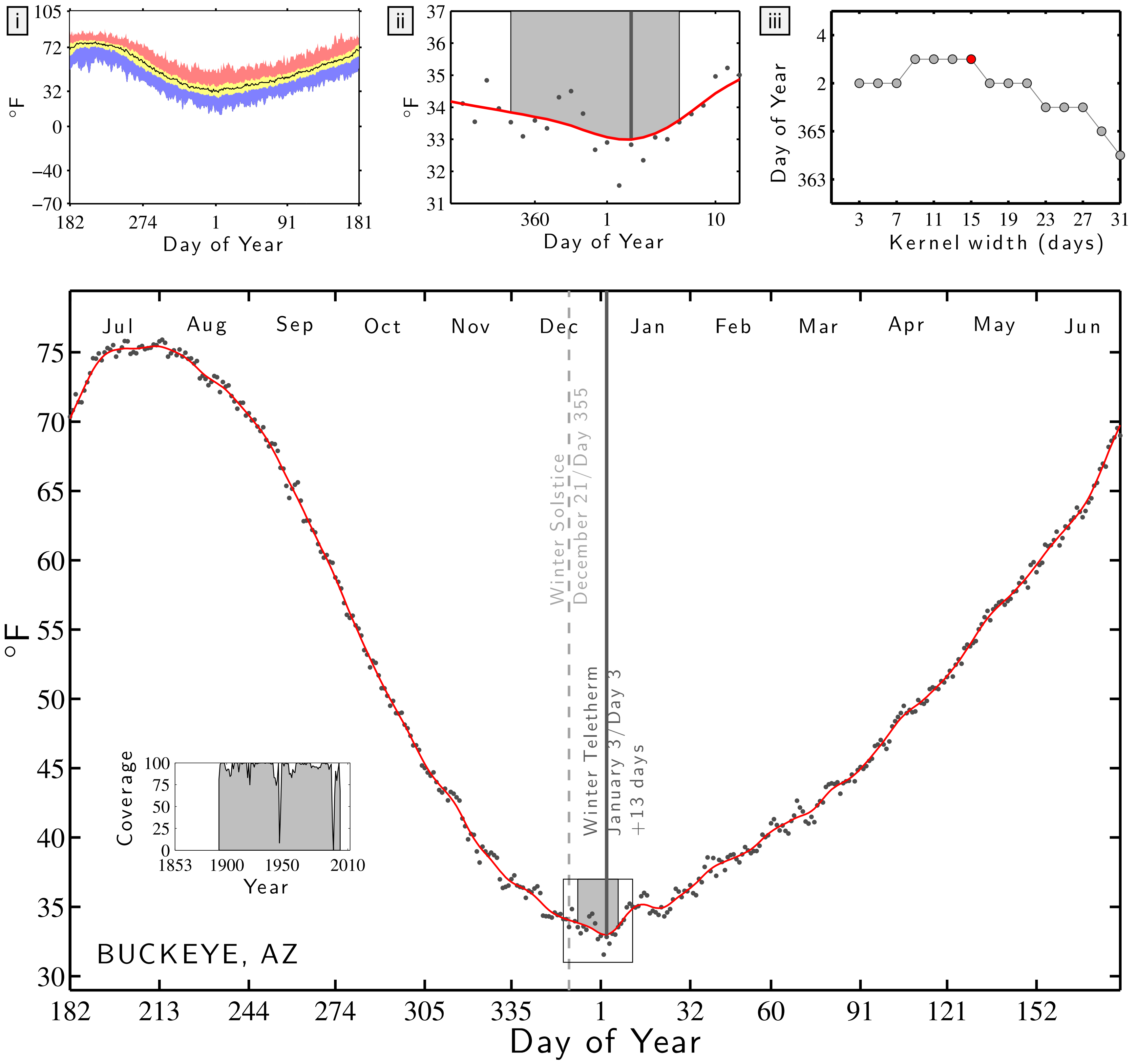
i
°F
Day of Year
ii
°F
Day of Year
iii
Day of Year
Kernel width (days)
Jul
Aug
Sep
Oct
Nov
Dec
Jan
Feb
Mar
Apr
May
Jun
Winter Solstice
December 21/Day 355
Winter Teletherm
January 3/Day 3
+13 days
Coverage
Year
BUCKEYE, AZ
°F
Day of Year

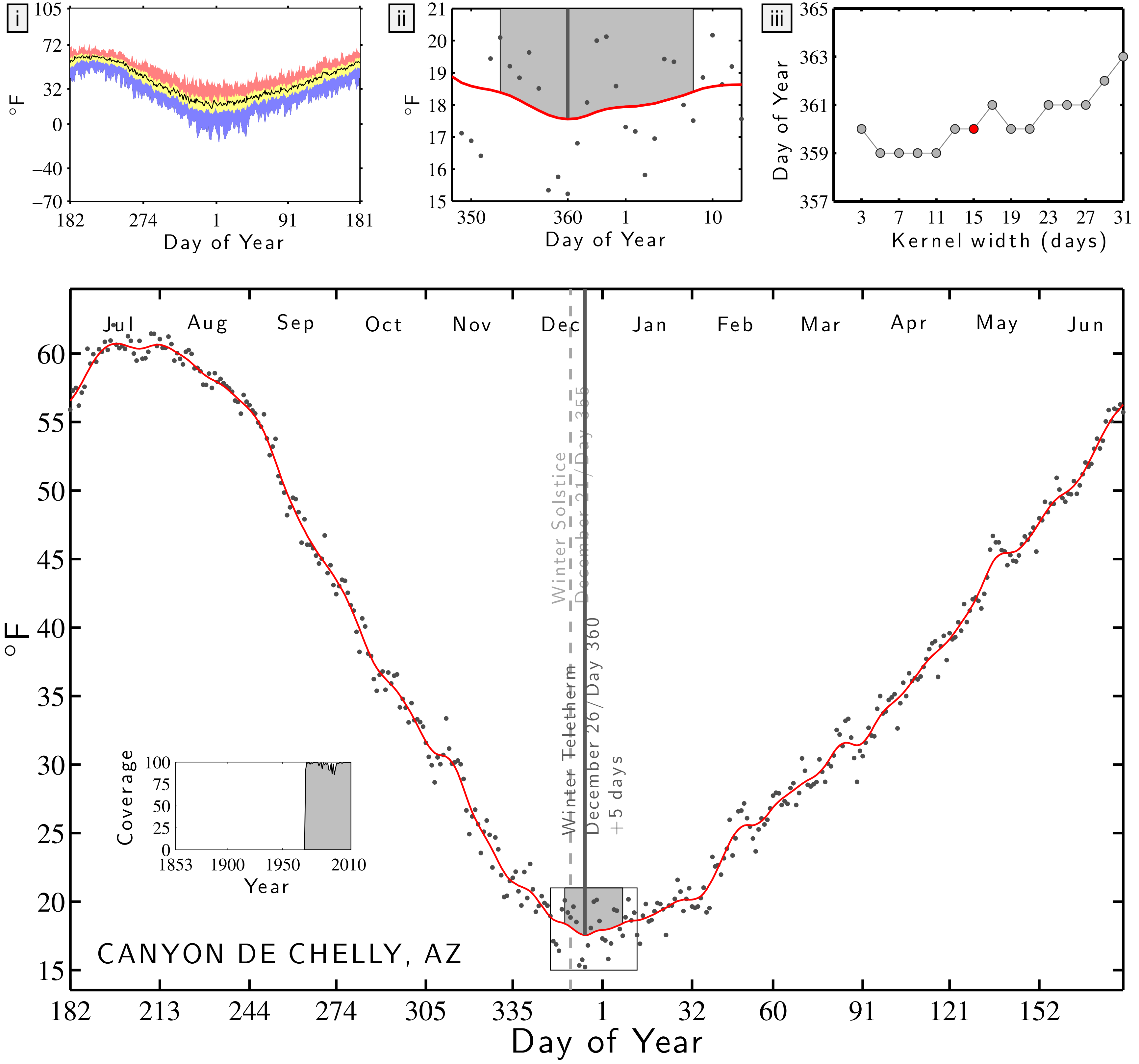

i
ii
iii
°F
Day of Year
Kernel width (days)
Jul
Aug
Sep
Oct
Nov
Dec
Jan
Feb
Mar
Apr
May
Jun
Winter Solstice
December 21/Day 355
Winter Teletherm
December 26/Day 360
+5 days
Coverage
Year
CANYON DE CHELLY, AZ

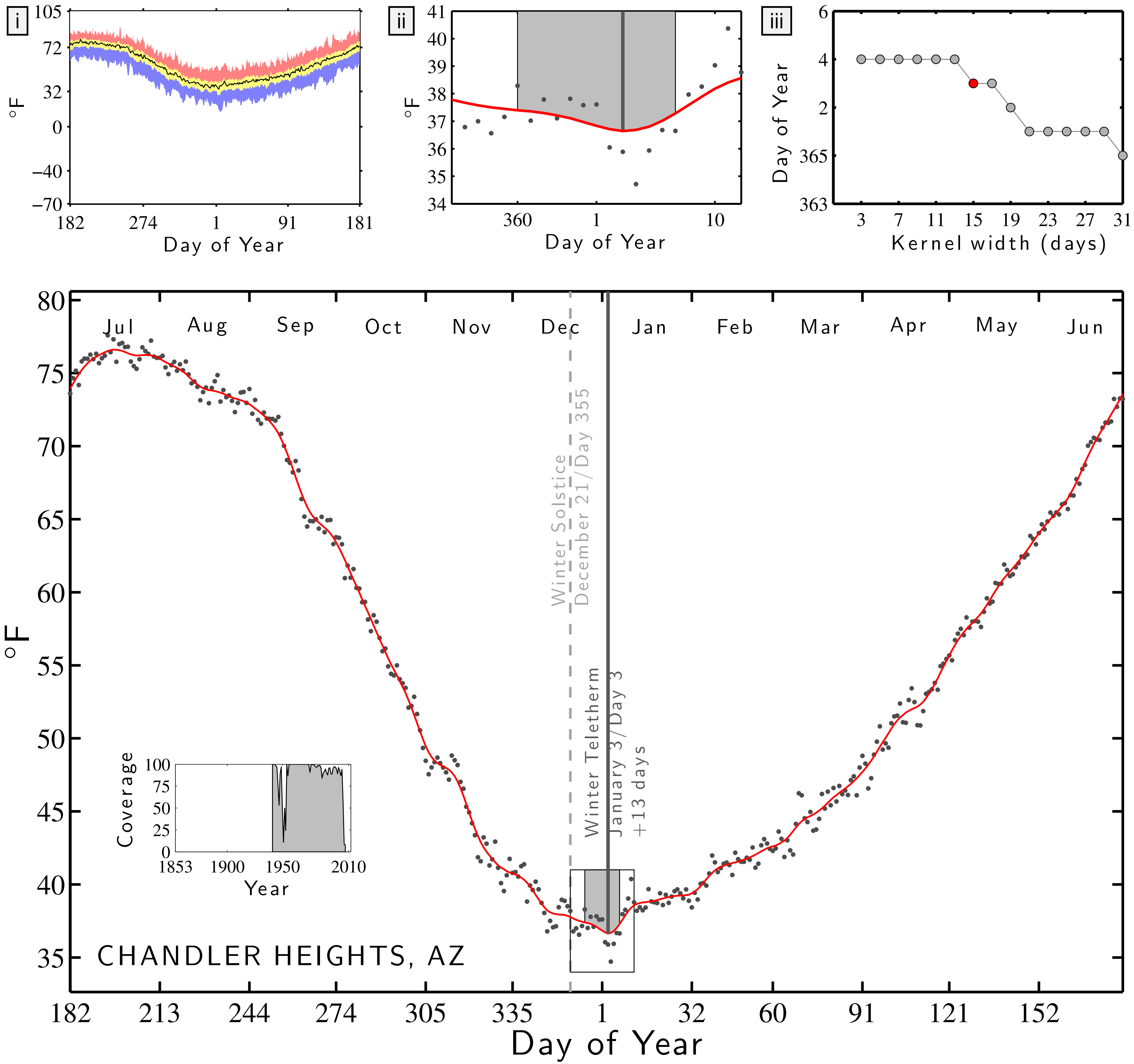

i
ii
iii
°F
Day of Year
Kernel width (days)
Jul
Aug
Sep
Oct
Nov
Dec
Jan
Feb
Mar
Apr
May
Jun
Winter Solstice
December 21/Day 355
Winter Teletherm
January 3/Day 3
+13 days
Coverage
Year
CHANDLER HEIGHTS, AZ

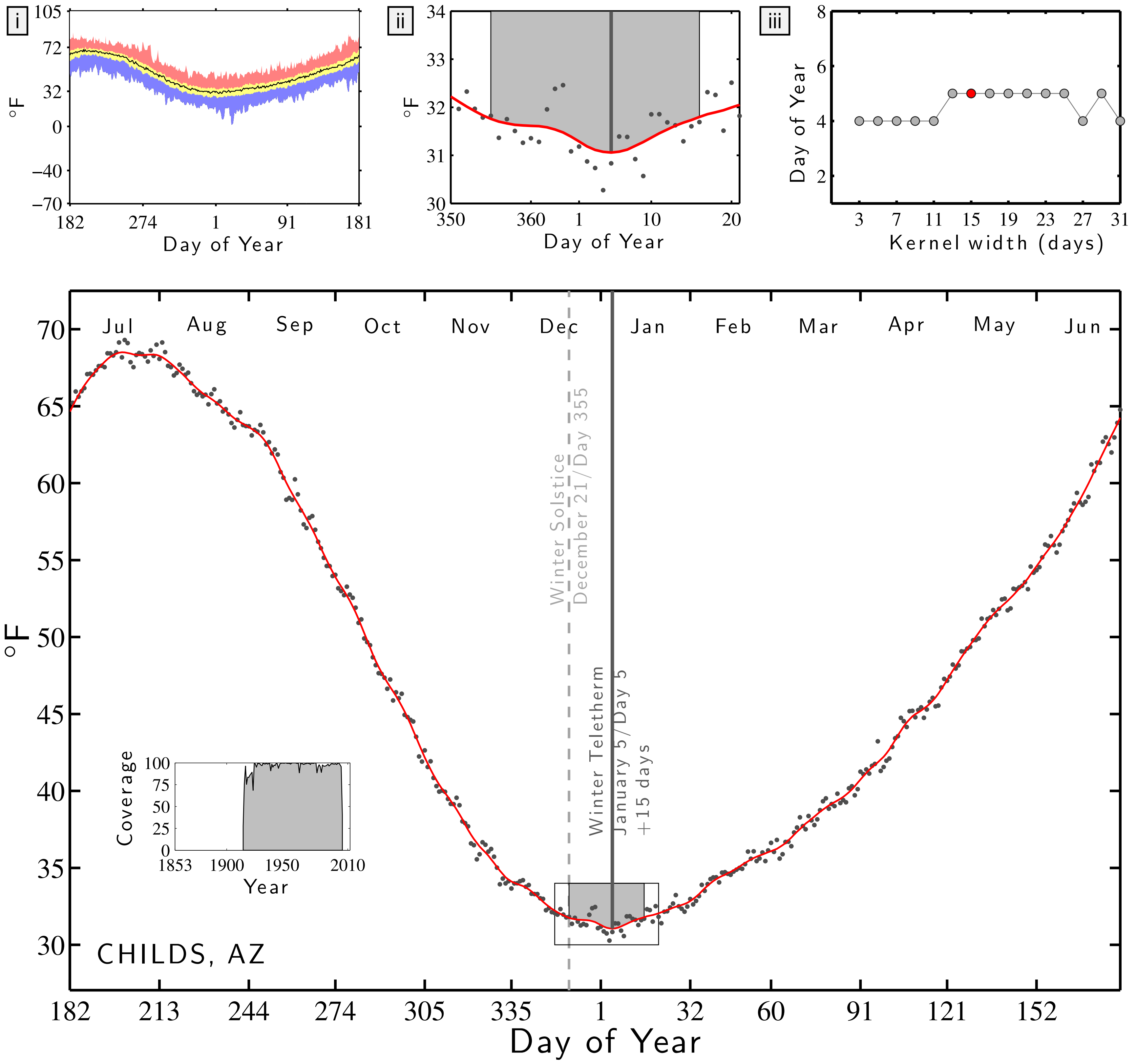

i
°F
Day of Year
ii
°F
Day of Year
iii
Day of Year
Kernel width (days)
Jul
Aug
Sep
Oct
Nov
Dec
Jan
Feb
Mar
Apr
May
Jun
Winter Solstice
December 21/Day 355
Winter Teletherm
January 5/Day 5
+15 days
Coverage
Year
CHILDS, AZ
°F
Day of Year

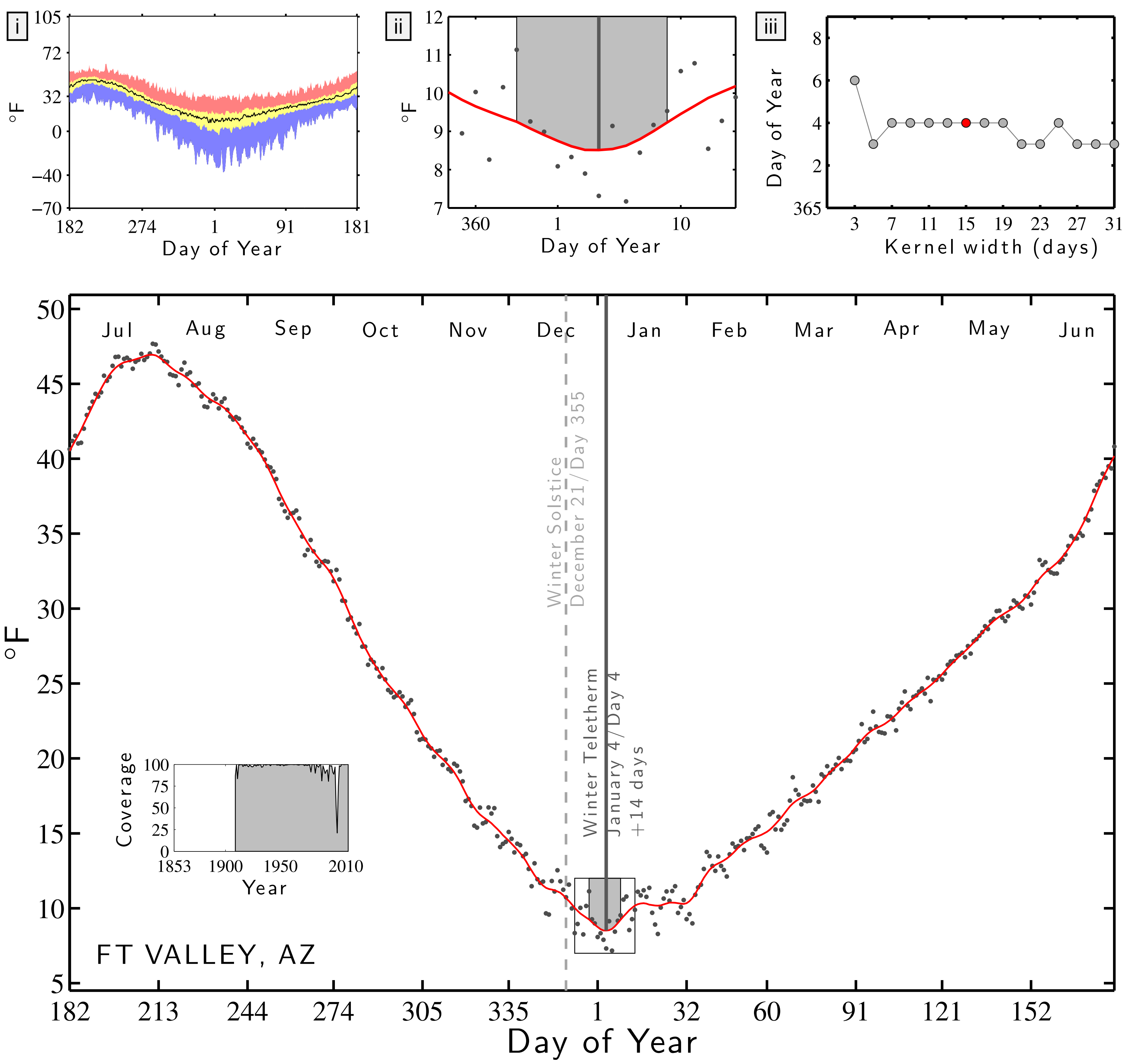

i
ii
iii
°F
Day of Year
Day of Year
Kernel width (days)
Winter Solstice
December 21/Day 355
Winter Teletherm
January 4/Day 4
+14 days
Jul
Aug
Sep
Oct
Nov
Dec
Jan
Feb
Mar
Apr
May
Jun
Coverage
Year
FT VALLEY, AZ

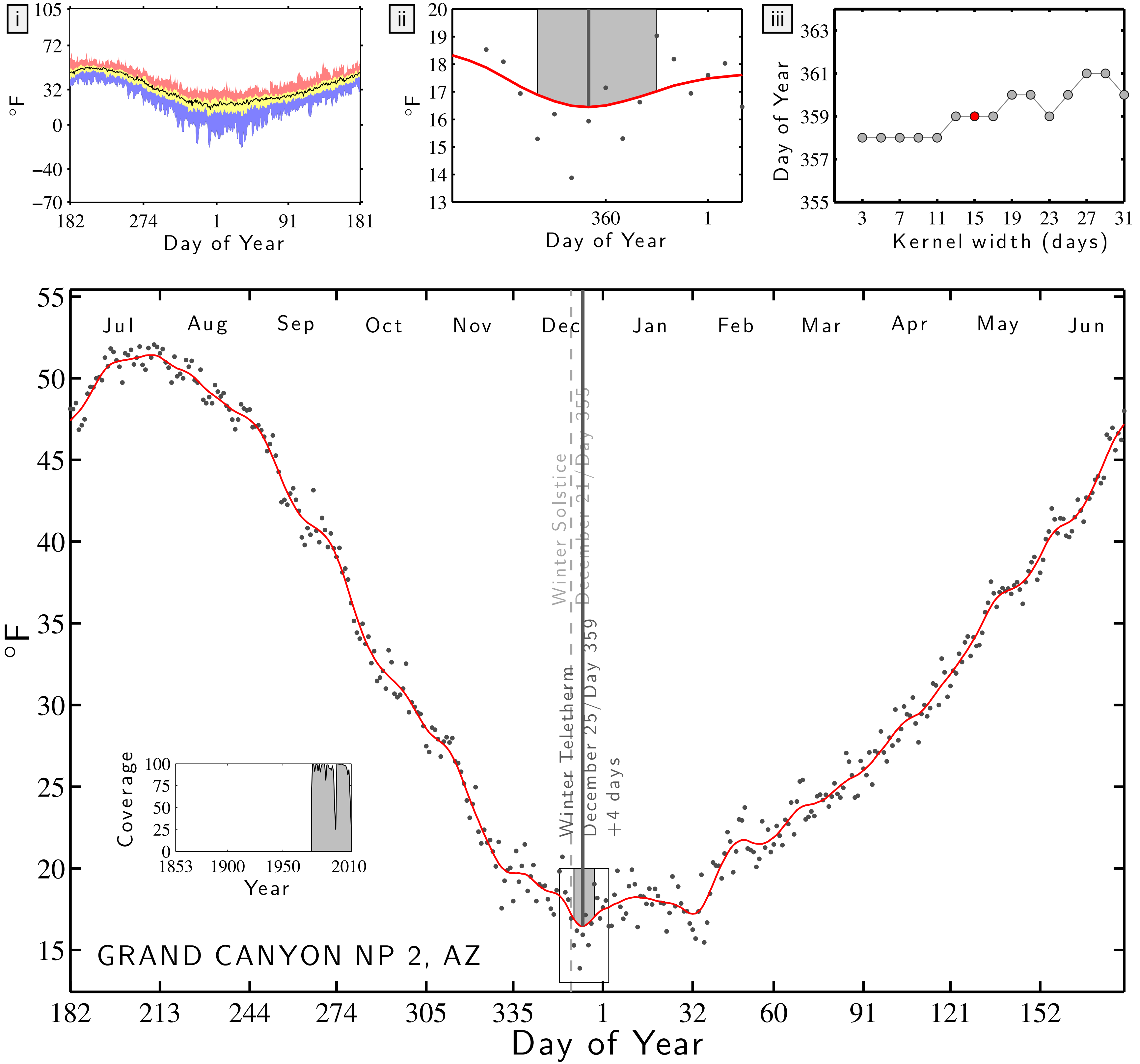

i
°F
Day of Year
ii
Day of Year
iii
Day of Year
Kernel width (days)
Jul
Aug
Sep
Oct
Nov
Dec
Jan
Feb
Mar
Apr
May
Jun
Winter Solstice
December 21/Day 355
Winter Teletherm
December 25/Day 359
+4 days
Coverage
Year
GRAND CANYON NP 2, AZ
°F
Day of Year

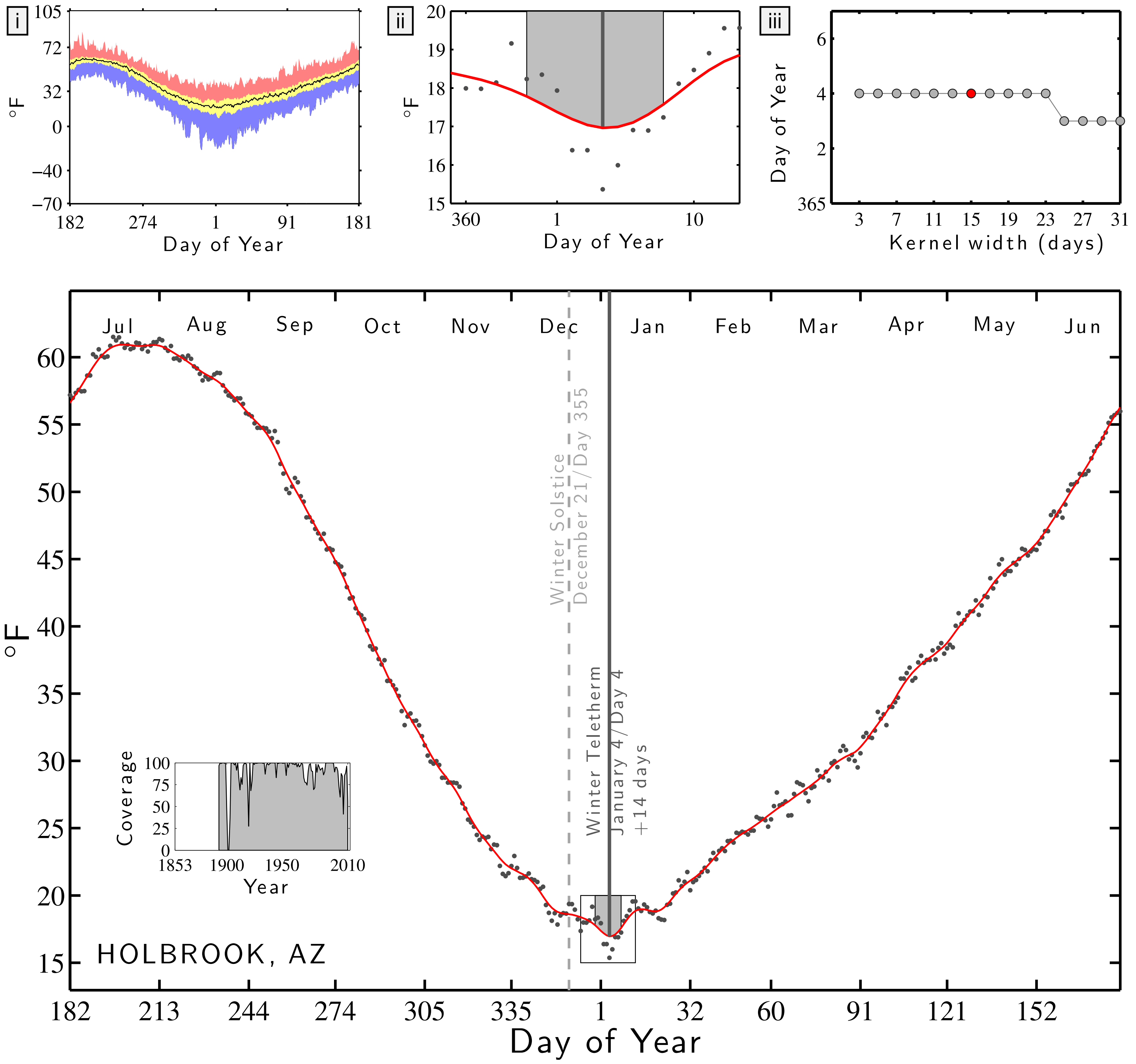

i
ii
iii
°F
Day of Year
Day of Year
Kernel width (days)
Winter Solstice
December 21/Day 355
Winter Teletherm
January 4/Day 4
+14 days
Coverage
Year
HOLBROOK, AZ
Jul
Aug
Sep
Oct
Nov
Dec
Jan
Feb
Mar
Apr
May
Jun

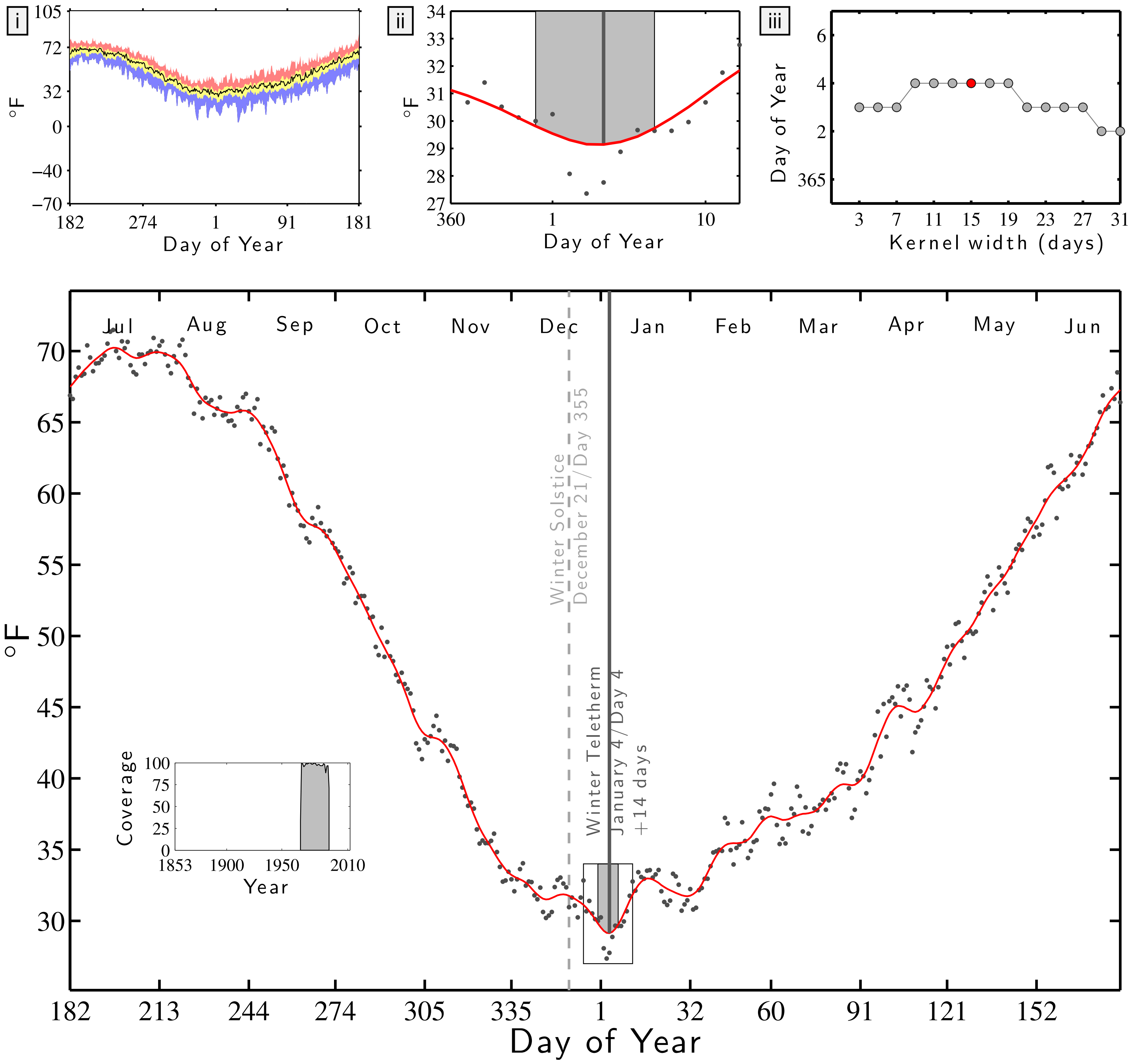

i
ii
iii
Day of Year
°F
Kernel width (days)
Jul
Aug
Sep
Oct
Nov
Dec
Jan
Feb
Mar
Apr
May
Jun
Winter Solstice
December 21/Day 355
Winter Teletherm
January 4/Day 4
+14 days
Coverage
Year

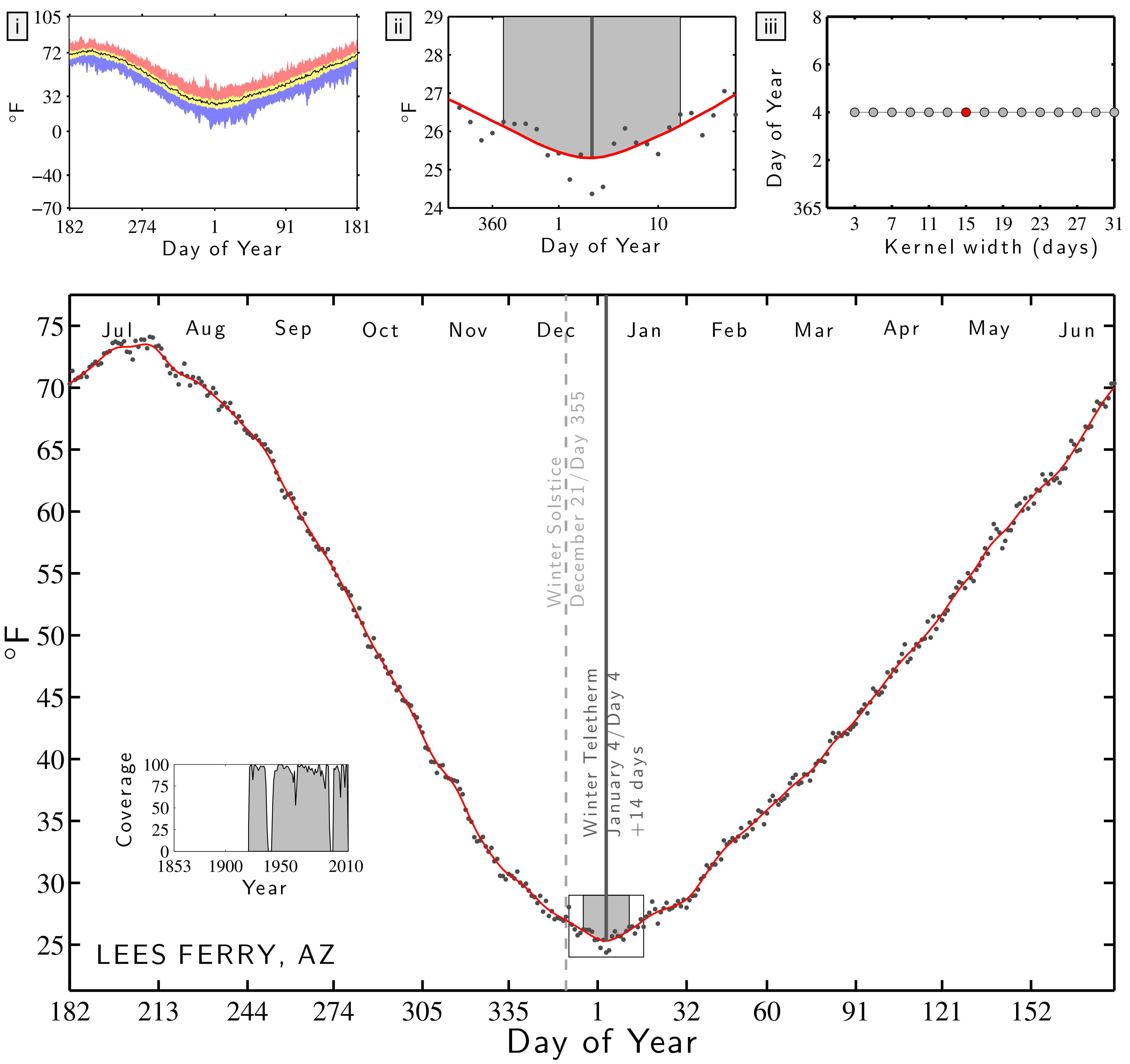

i
ii
iii
°F
Day of Year
Kernel width (days)
Jul
Aug
Sep
Oct
Nov
Dec
Jan
Feb
Mar
Apr
May
Jun
Winter Solstice
December 21/Day 355
Winter Teletherm
January 4/Day 4
+14 days
Coverage
Year
LEES FERRY, AZ

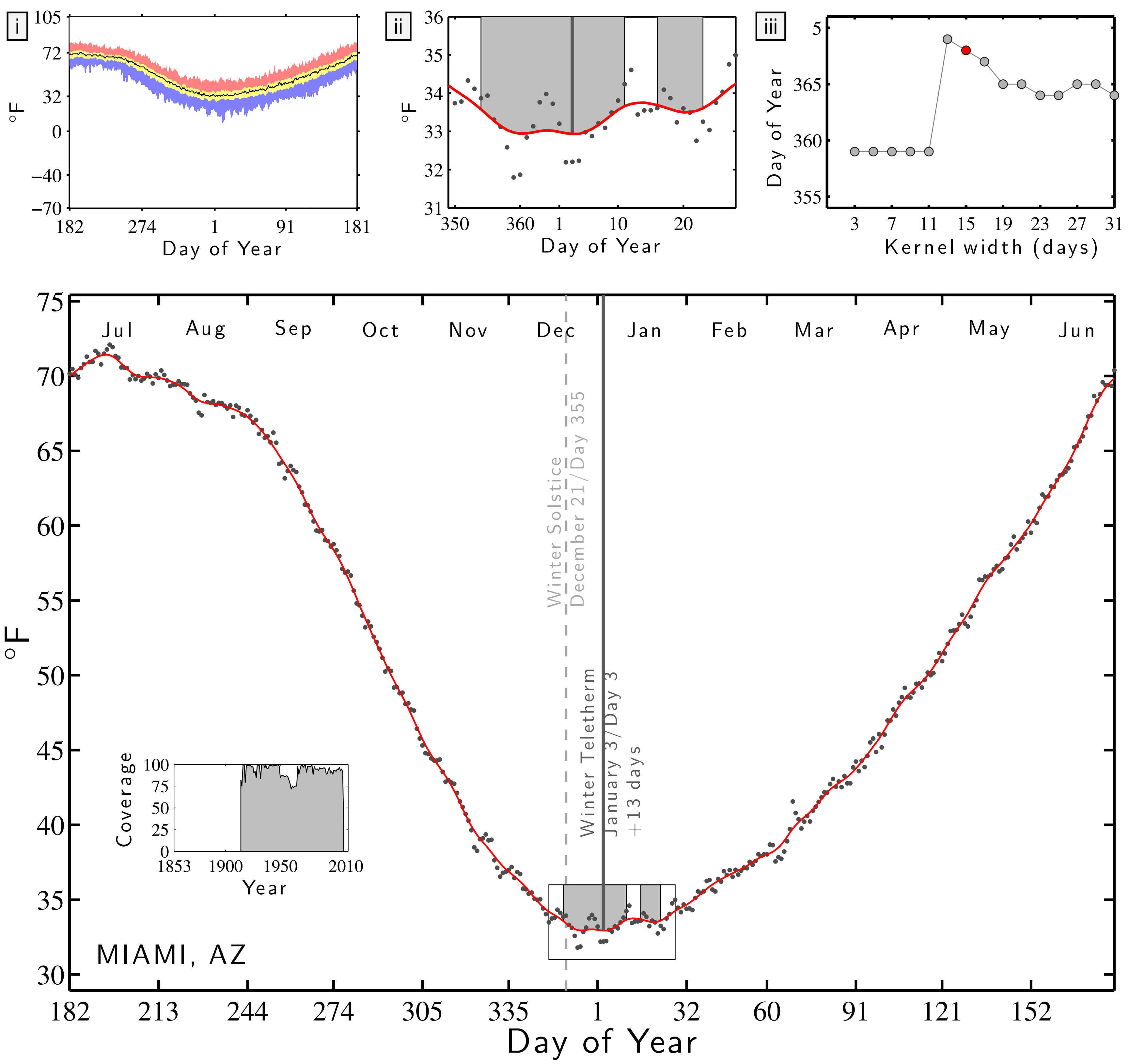

i
ii
iii
°F
Day of Year
Day of Year
Kernel width (days)
Jul
Aug
Sep
Oct
Nov
Dec
Jan
Feb
Mar
Apr
May
Jun
Winter Solstice
December 21/Day 355
Winter Teletherm
January 3/Day 3
+13 days
Coverage
Year
MIAMI, AZ
Day of Year

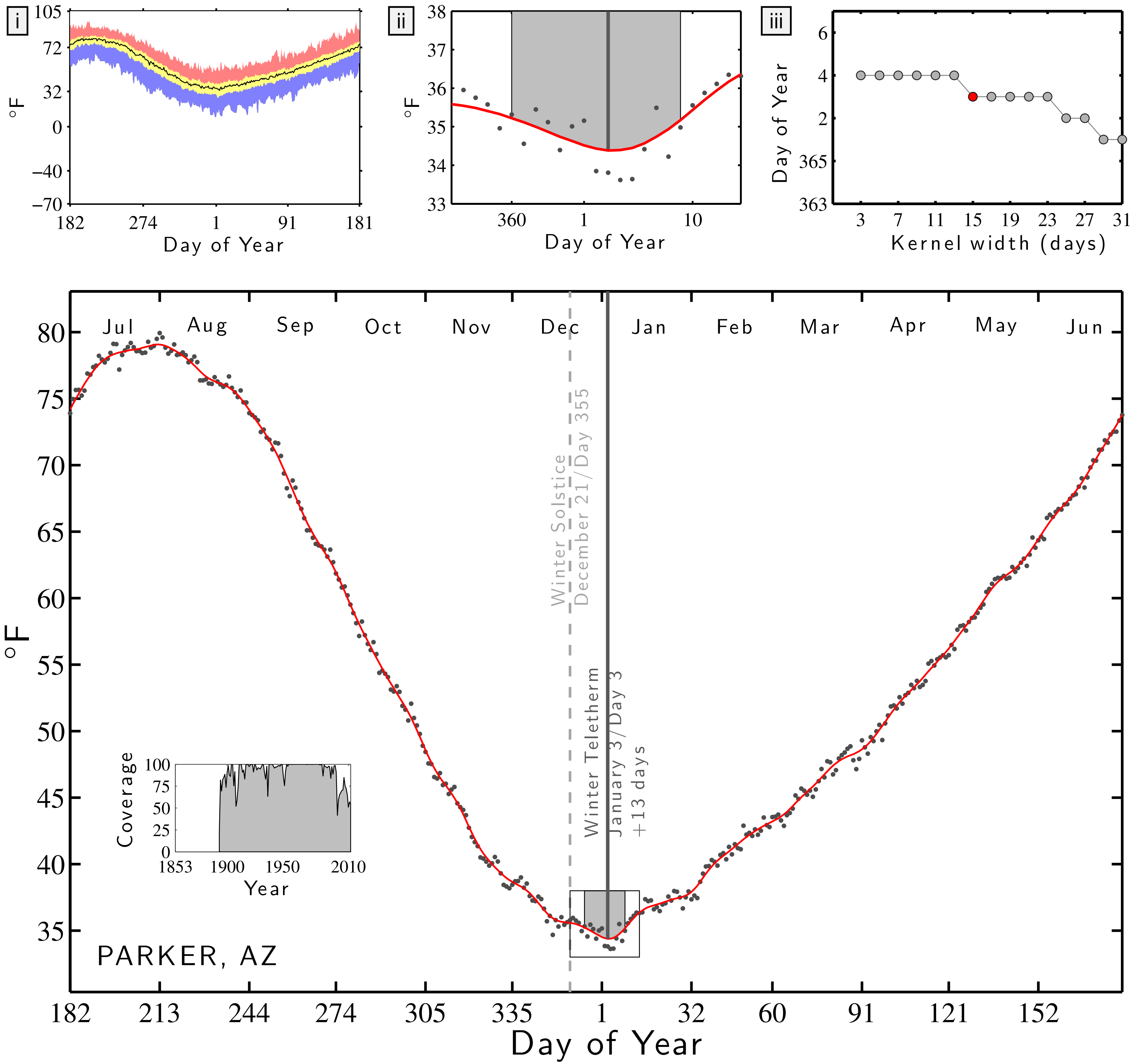

i
105
72
32
0
−40
−70
°F
182
274
1
91
181
Day of Year
ii
38
37
36
35
34
33
°F
360
1
10
Day of Year
iii
6
4
2
365
363
Day of Year
3
7
11
15
19
23
27
31
Kernel width (days)
Jul
Aug
Sep
Oct
Nov
Dec
Jan
Feb
Mar
Apr
May
Jun
80
75
70
65
60
55
50
45
40
35
°F
Winter Solstice
December 21/Day 355
Winter Teletherm
January 3/Day 3
+13 days
Coverage
100
75
50
25
0
1853
1900
1950
2010
Year
PARKER, AZ
182
213
244
274
305
335
1
32
60
91
121
152
Day of Year

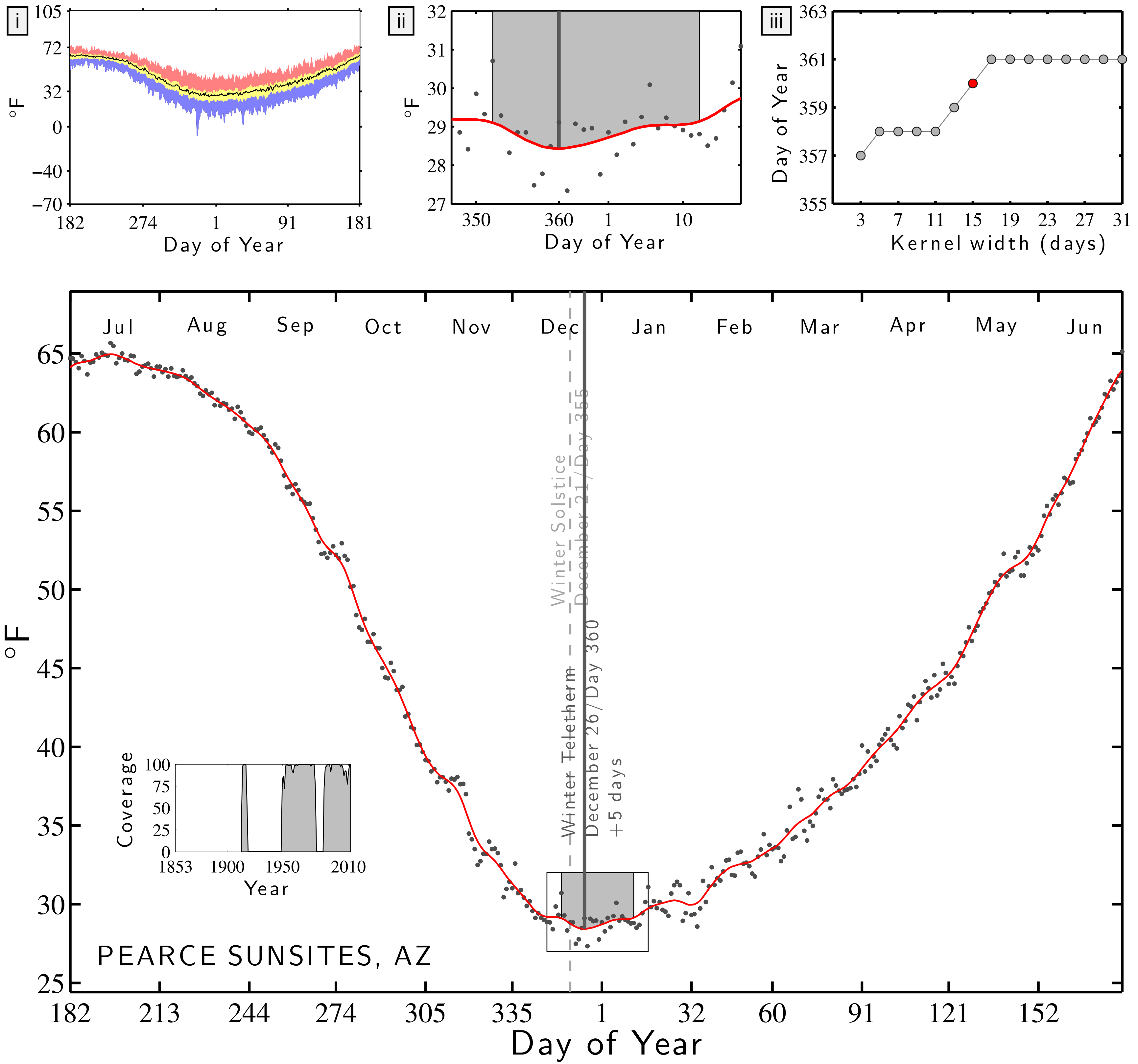

i
°F
Day of Year
ii
°F
Day of Year
iii
Day of Year
Kernel width (days)
Jul
Aug
Sep
Oct
Nov
Dec
Jan
Feb
Mar
Apr
May
Jun
Winter Solstice
December 21/Day 355
Winter Teletherm
December 26/Day 360
+5 days
Coverage
Year
PEARCE SUNSITES, AZ
°F
Day of Year

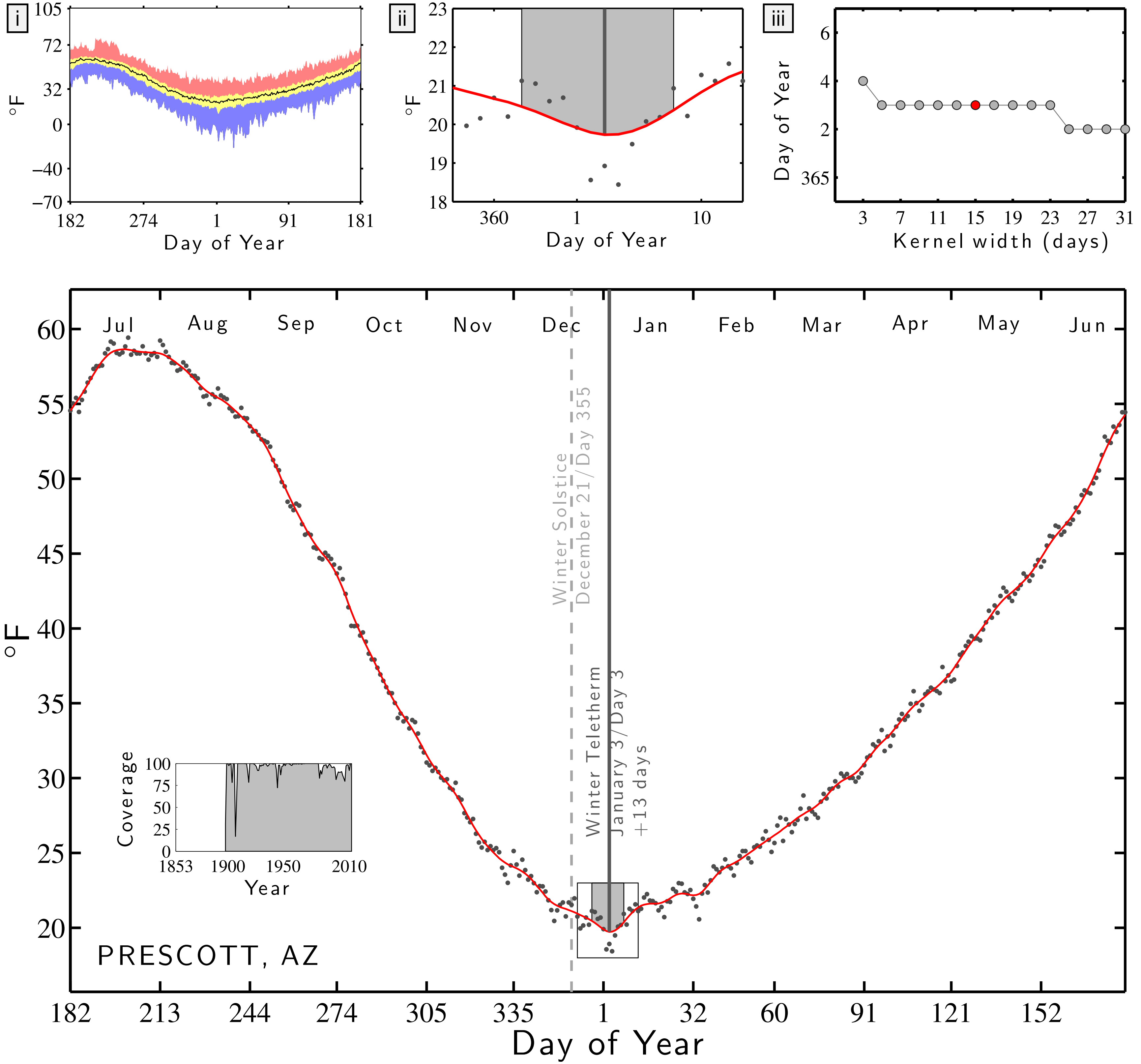

i
ii
iii
°F
Day of Year
Kernel width (days)
Jul
Aug
Sep
Oct
Nov
Dec
Jan
Feb
Mar
Apr
May
Jun
Winter Solstice
December 21/Day 355
Winter Teletherm
January 3/Day 3
+13 days
Coverage
Year
PRESCOTT, AZ

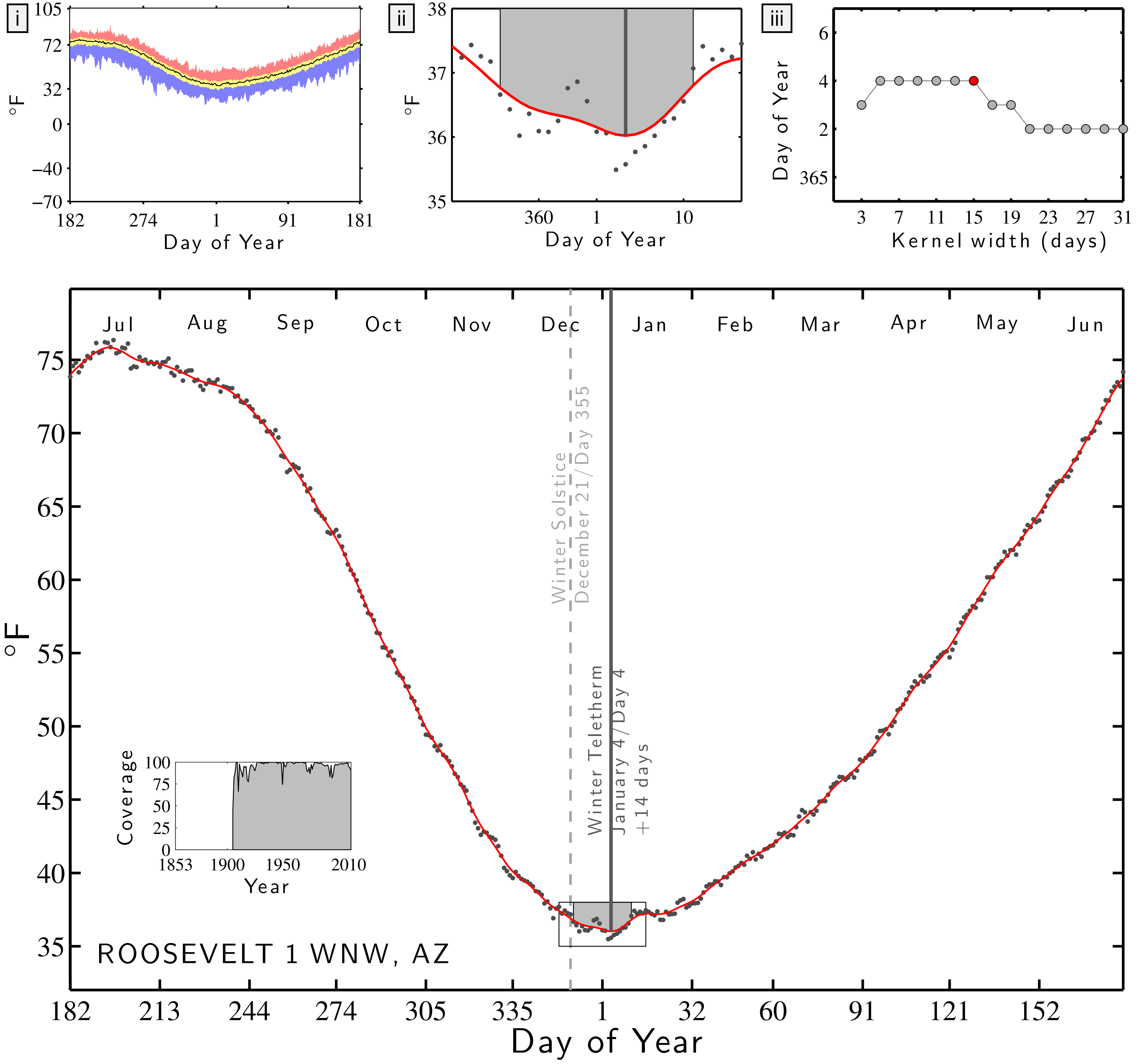

i
ii
iii
°F
Day of Year
Kernel width (days)
Day of Year
Jul
Aug
Sep
Oct
Nov
Dec
Jan
Feb
Mar
Apr
May
Jun
Winter Solstice
December 21/Day 355
Winter Teletherm
January 4/Day 4
+14 days
Coverage
Year
ROOSEVELT 1 WNW, AZ

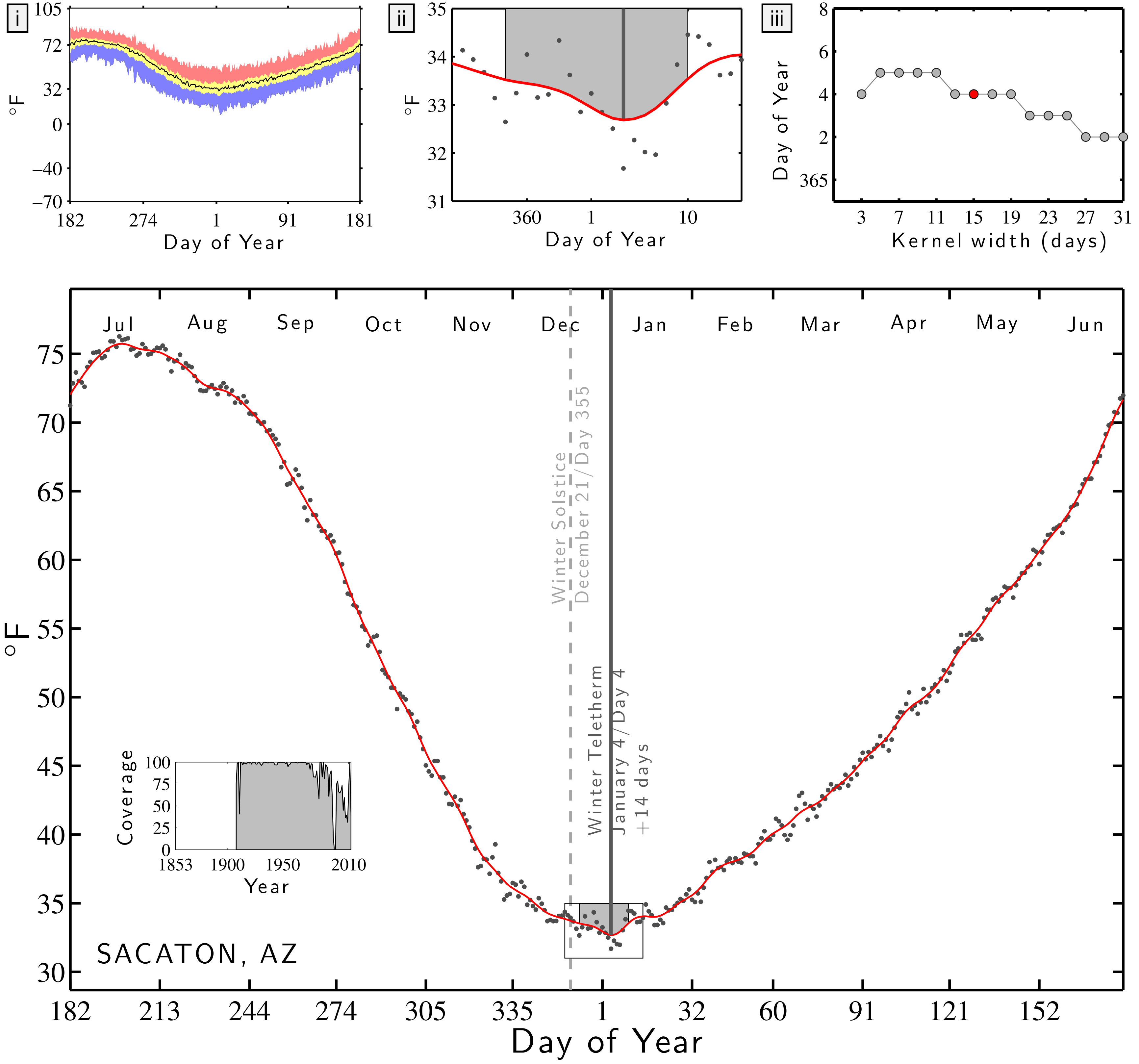

i
°F
Day of Year
ii
°F
Day of Year
iii
Day of Year
Kernel width (days)
Jul
Aug
Sep
Oct
Nov
Dec
Jan
Feb
Mar
Apr
May
Jun
Winter Solstice
December 21/Day 355
Winter Teletherm
January 4/Day 4
+14 days
Coverage
Year
SACATON, AZ
°F
Day of Year

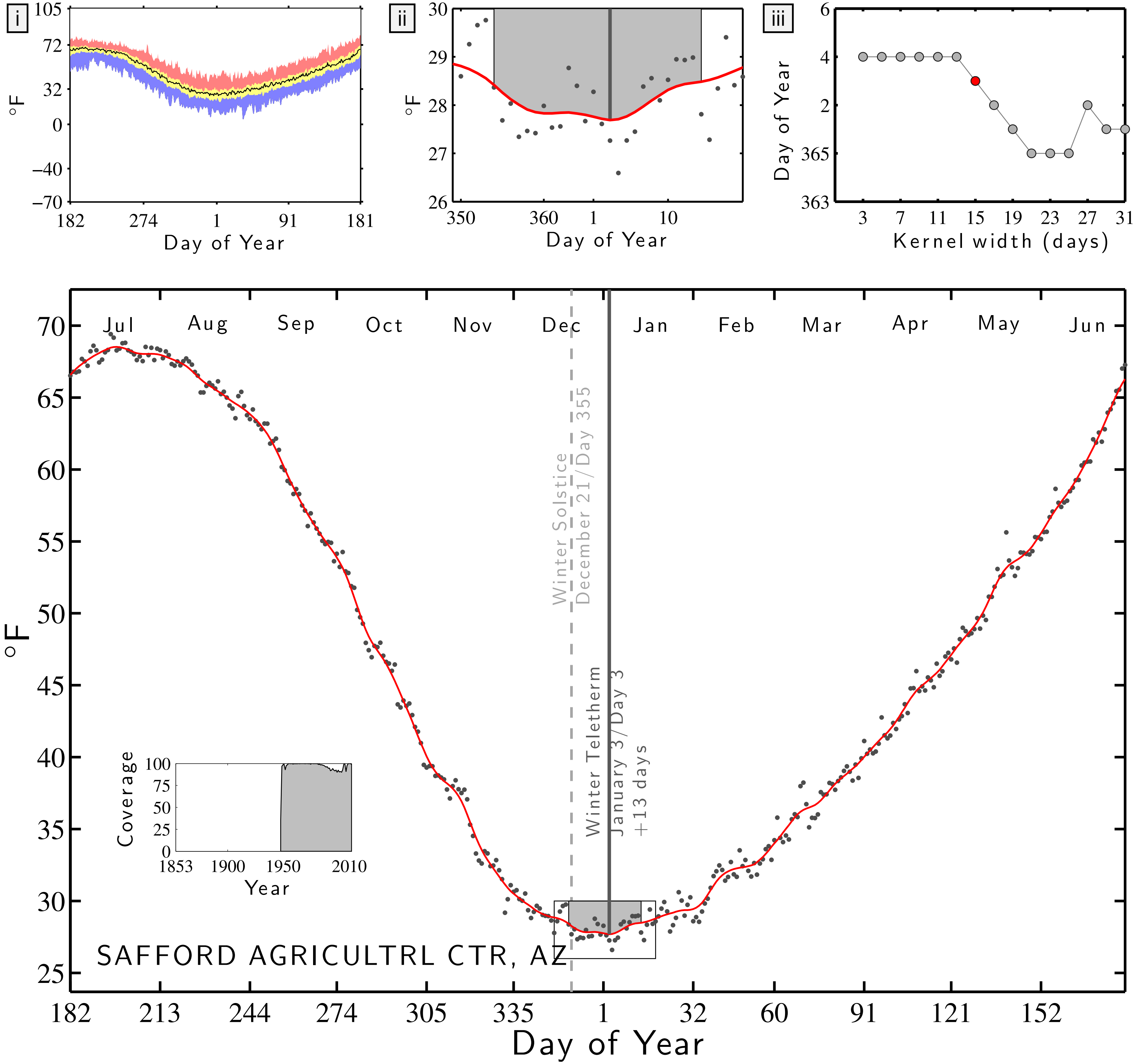

i
ii
iii
Day of Year
°F
Kernel width (days)
Jul
Aug
Sep
Oct
Nov
Dec
Jan
Feb
Mar
Apr
May
Jun
Winter Solstice
December 21/Day 355
Winter Teletherm
January 3/Day 3
+13 days
Coverage
Year
SAFFORD AGRICULTRL CTR, AZ

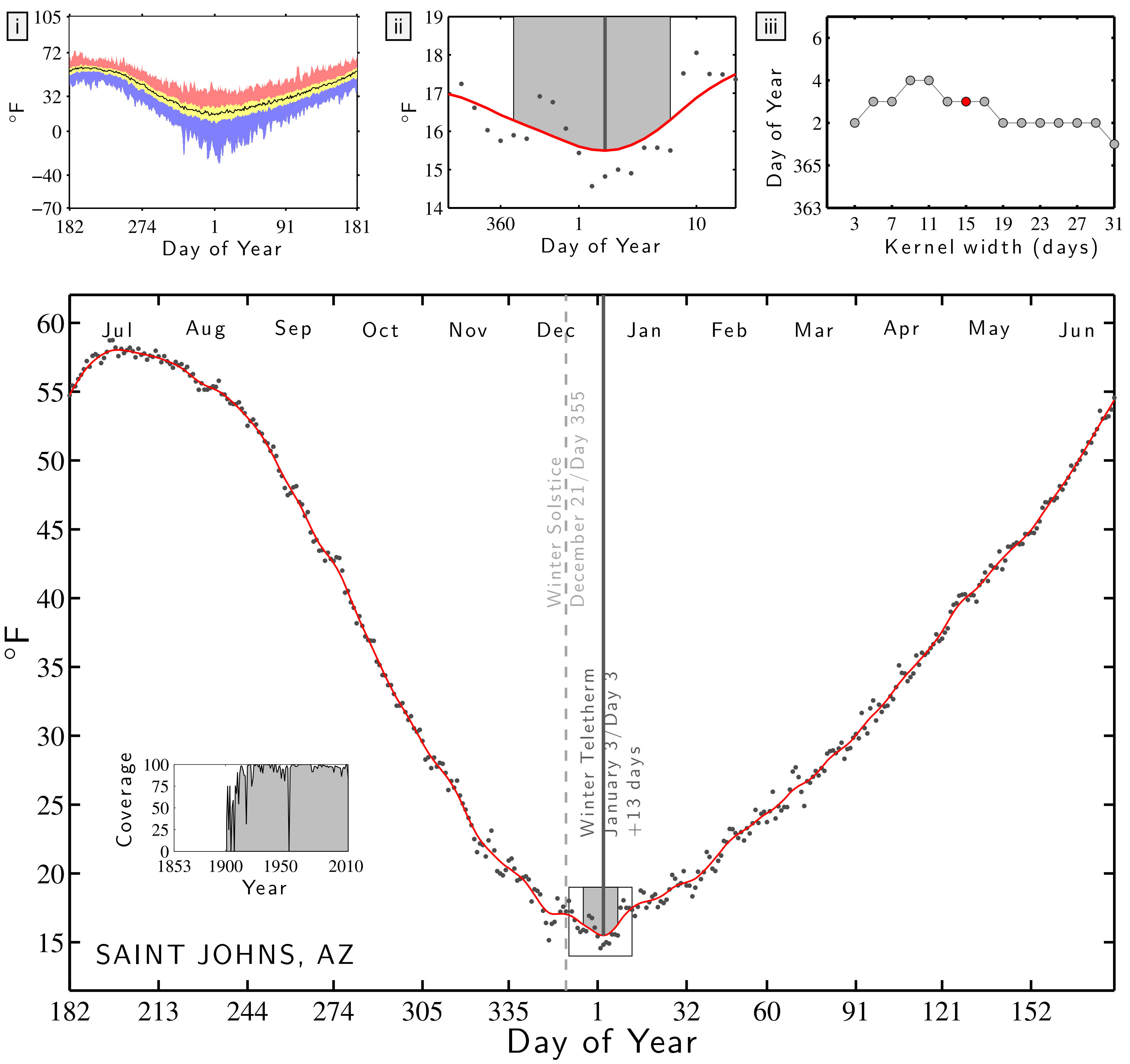

i
ii
iii
Day of Year
Kernel width (days)
°F
Jul
Aug
Sep
Oct
Nov
Dec
Jan
Feb
Mar
Apr
May
Jun
Winter Solstice
December 21/Day 355
Winter Teletherm
January 3/Day 3
+13 days
Coverage
Year
SAINT JOHNS, AZ

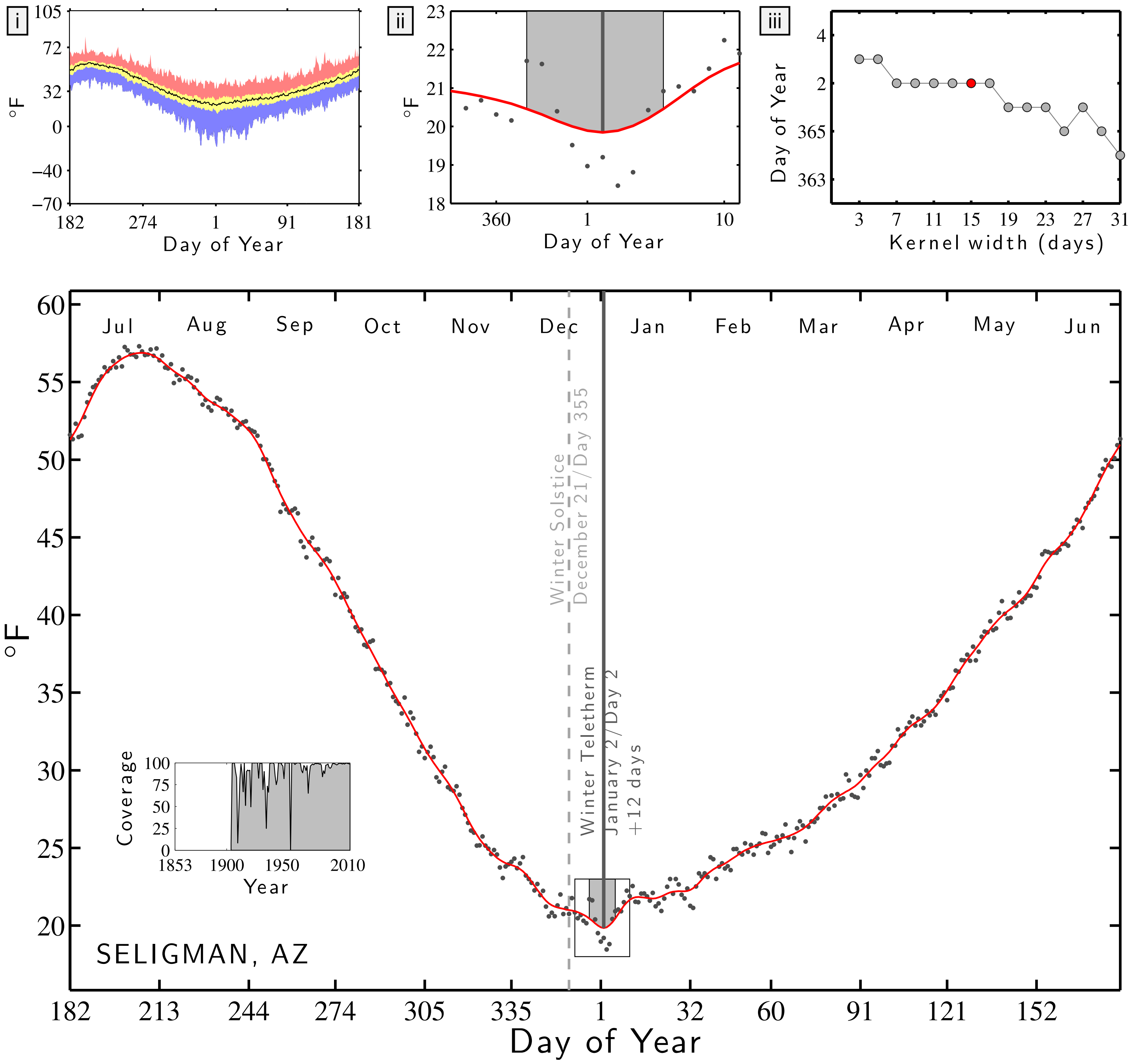

i
ii
iii
°F
Day of Year
Kernel width (days)
Jul
Aug
Sep
Oct
Nov
Dec
Jan
Feb
Mar
Apr
May
Jun
Winter Solstice
December 21/Day 355
Winter Teletherm
January 2/Day 2
+12 days
Coverage
Year
SELIGMAN, AZ

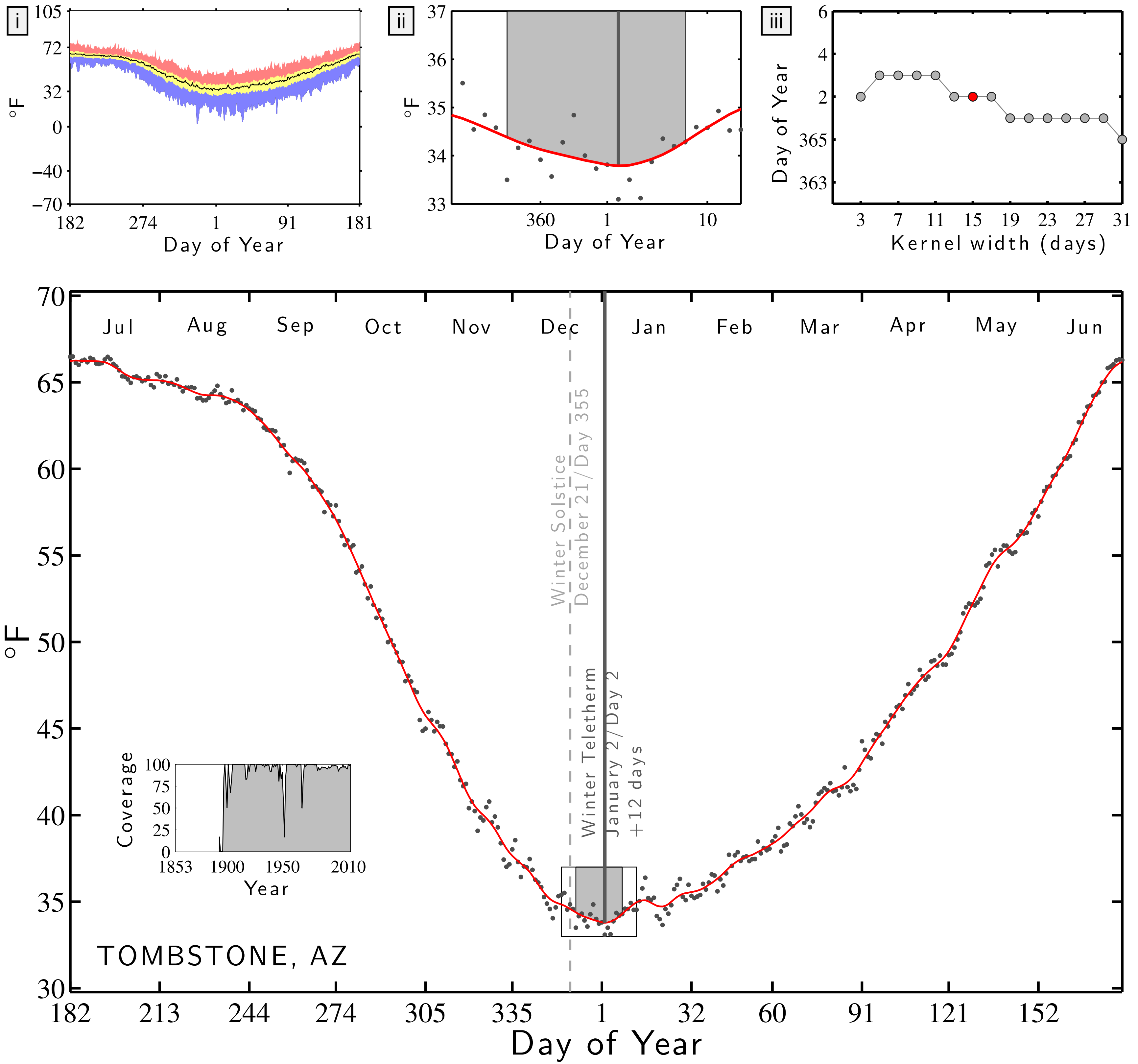

i
ii
iii
°F
Day of Year
Day of Year
Kernel width (days)
Jul
Aug
Sep
Oct
Nov
Dec
Jan
Feb
Mar
Apr
May
Jun
Winter Solstice
December 21/Day 355
Winter Teletherm
January 2/Day 2
+12 days
Coverage
Year
TOMBSTONE, AZ

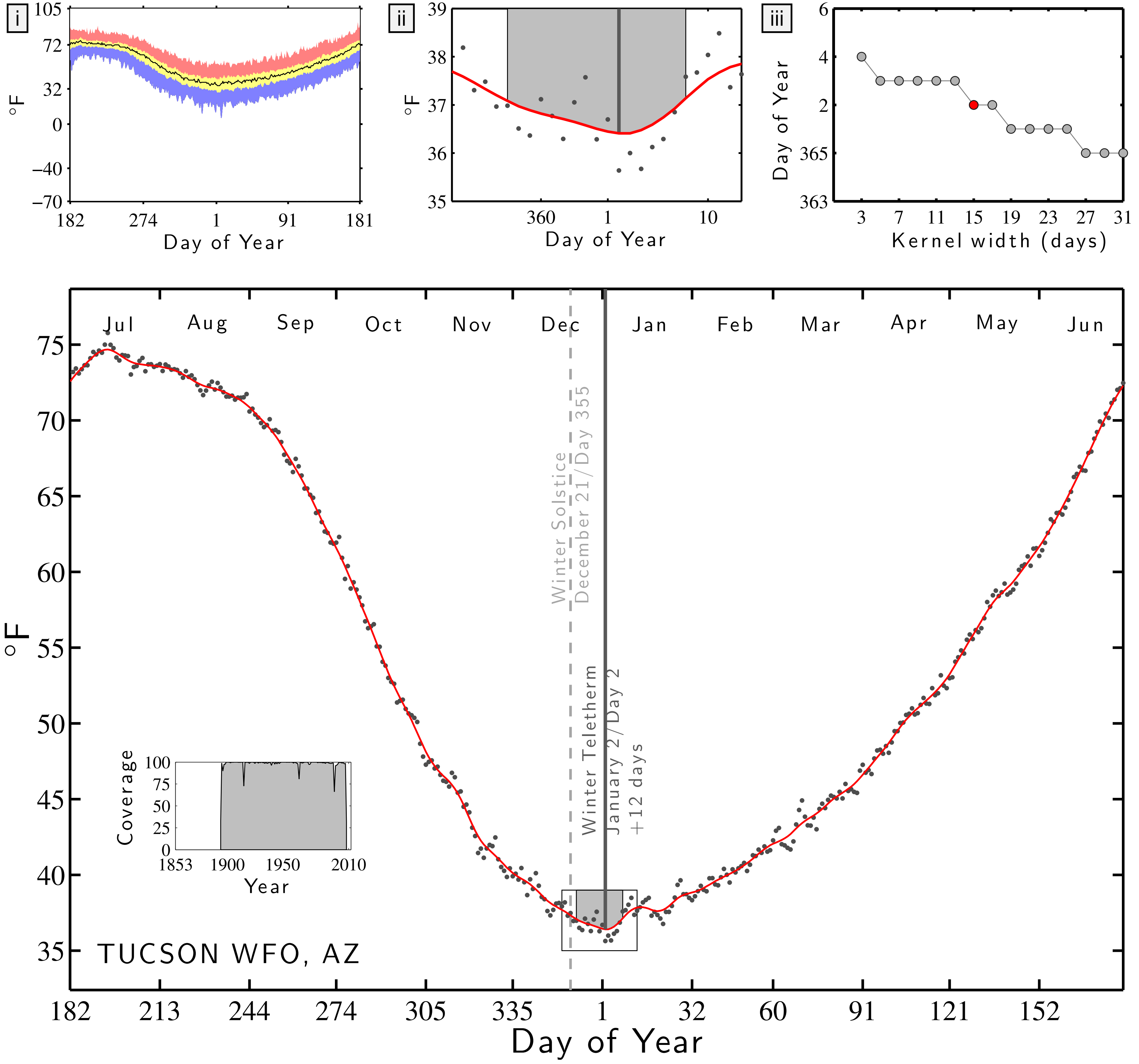

i
°F
Day of Year
ii
°F
Day of Year
iii
Day of Year
Kernel width (days)
Jul Aug Sep Oct Nov Dec Jan Feb Mar Apr May Jun
Winter Solstice
December 21/Day 355
Winter Teletherm
January 2/Day 2
+12 days
Coverage
Year
TUCSON WFO, AZ
°F
Day of Year

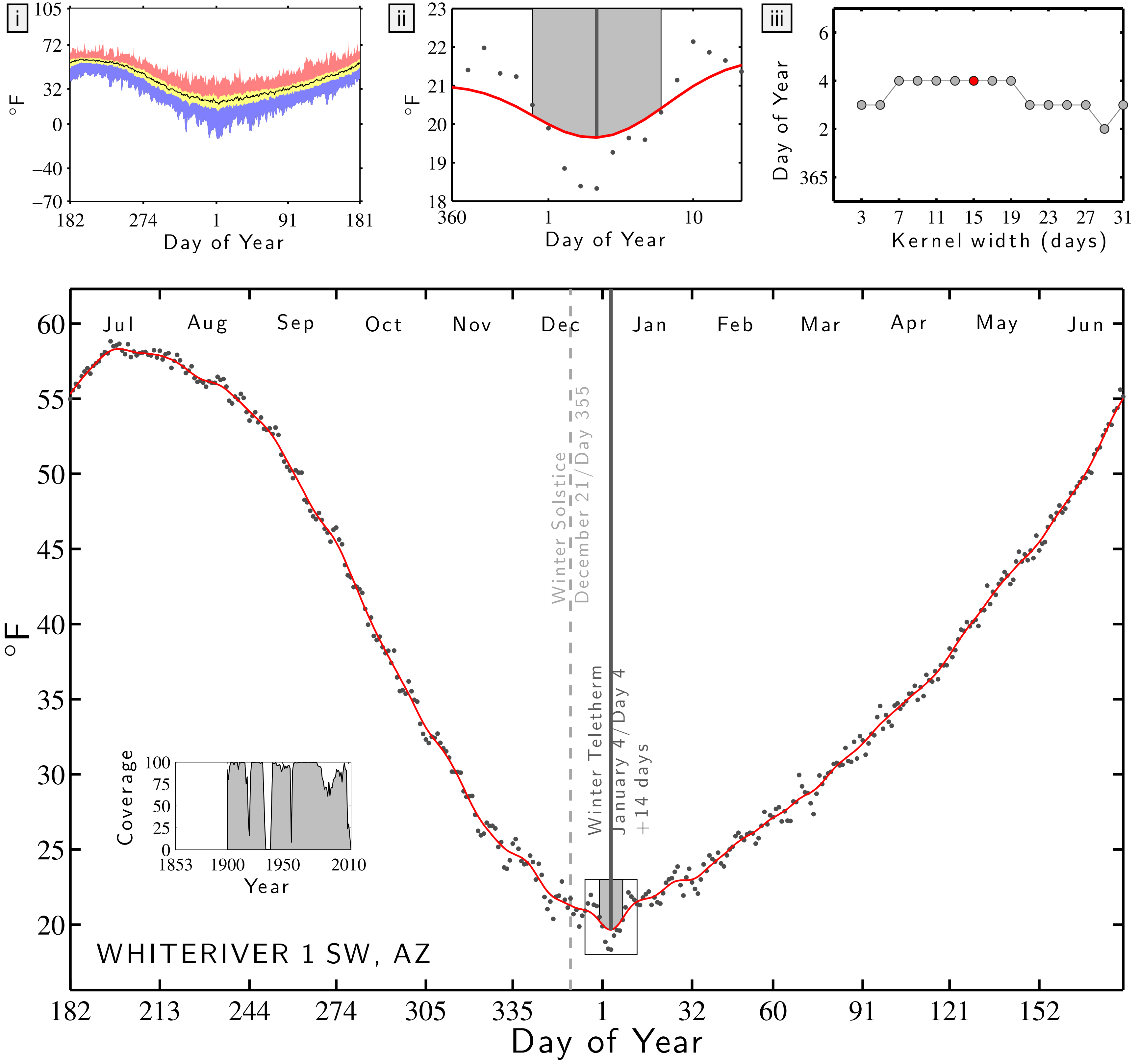

i
ii
iii
°F
Day of Year
Kernel width (days)
Jul
Aug
Sep
Oct
Nov
Dec
Jan
Feb
Mar
Apr
May
Jun
Winter Solstice
December 21/Day 355
Winter Teletherm
January 4/Day 4
+14 days
Coverage
Year
WHITERIVER 1 SW, AZ

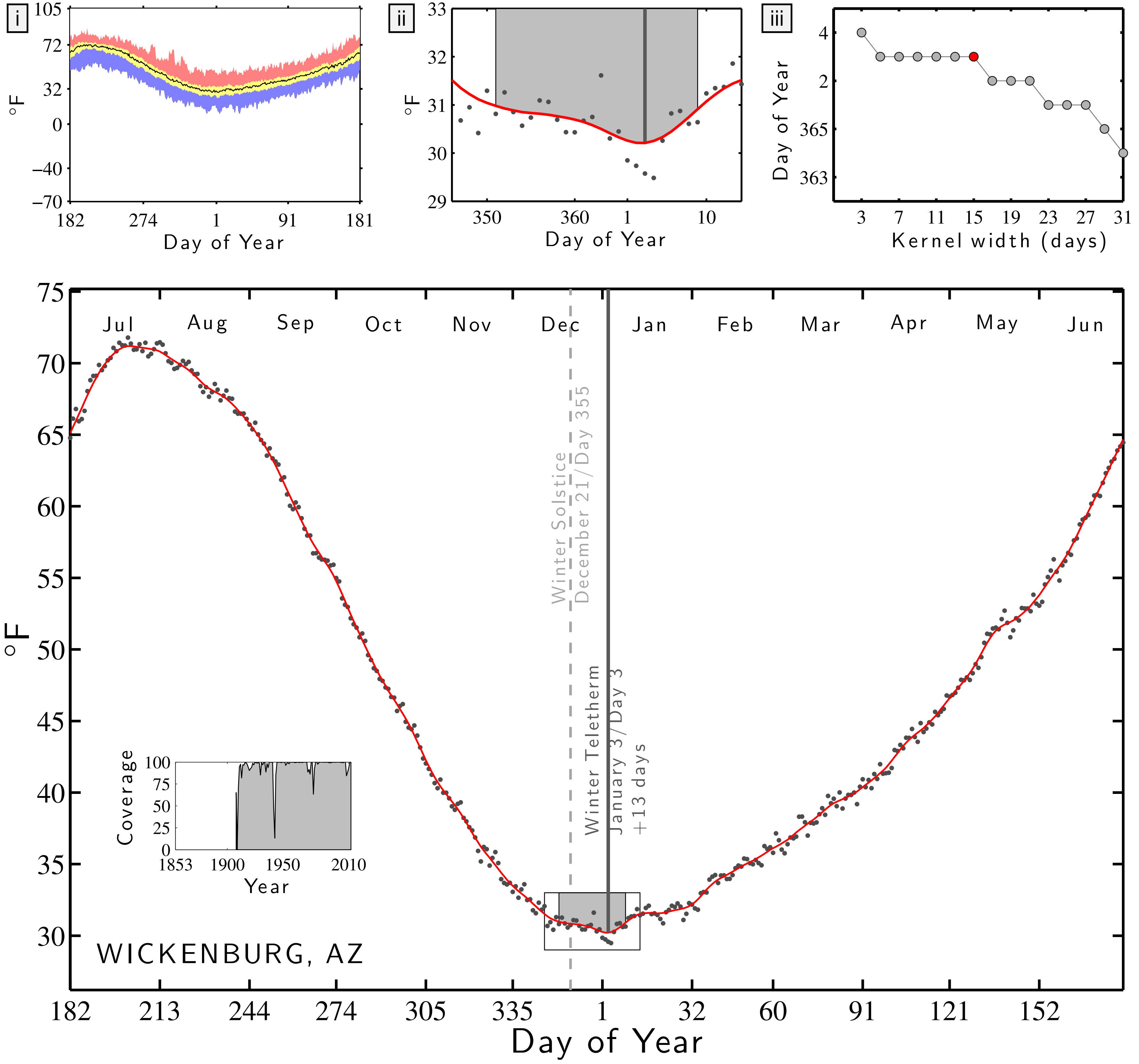

i
°F
Day of Year
ii
Day of Year
iii
Day of Year
Kernel width (days)
Jul
Aug
Sep
Oct
Nov
Dec
Jan
Feb
Mar
Apr
May
Jun
Winter Solstice
December 21/Day 355
Winter Teletherm
January 3/Day 3
+13 days
Coverage
Year
WICKENBURG, AZ
Day of Year

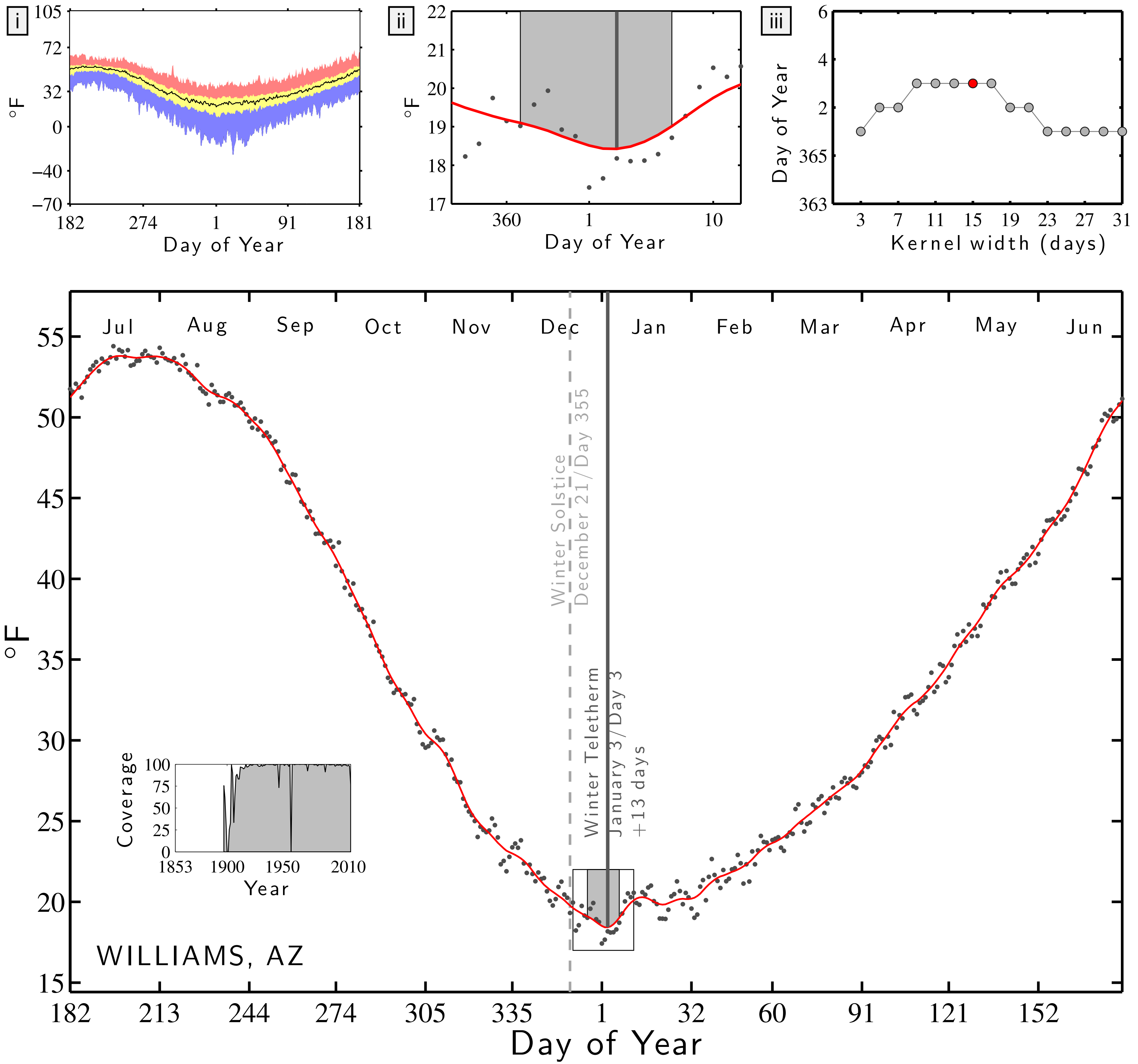

i
°F
Day of Year
ii
iii
Day of Year
Kernel width (days)
Jul
Aug
Sep
Oct
Nov
Dec
Jan
Feb
Mar
Apr
May
Jun
Winter Solstice
December 21/Day 355
Winter Teletherm
January 3/Day 3
+13 days
Coverage
Year
WILLIAMS, AZ
°F
Day of Year

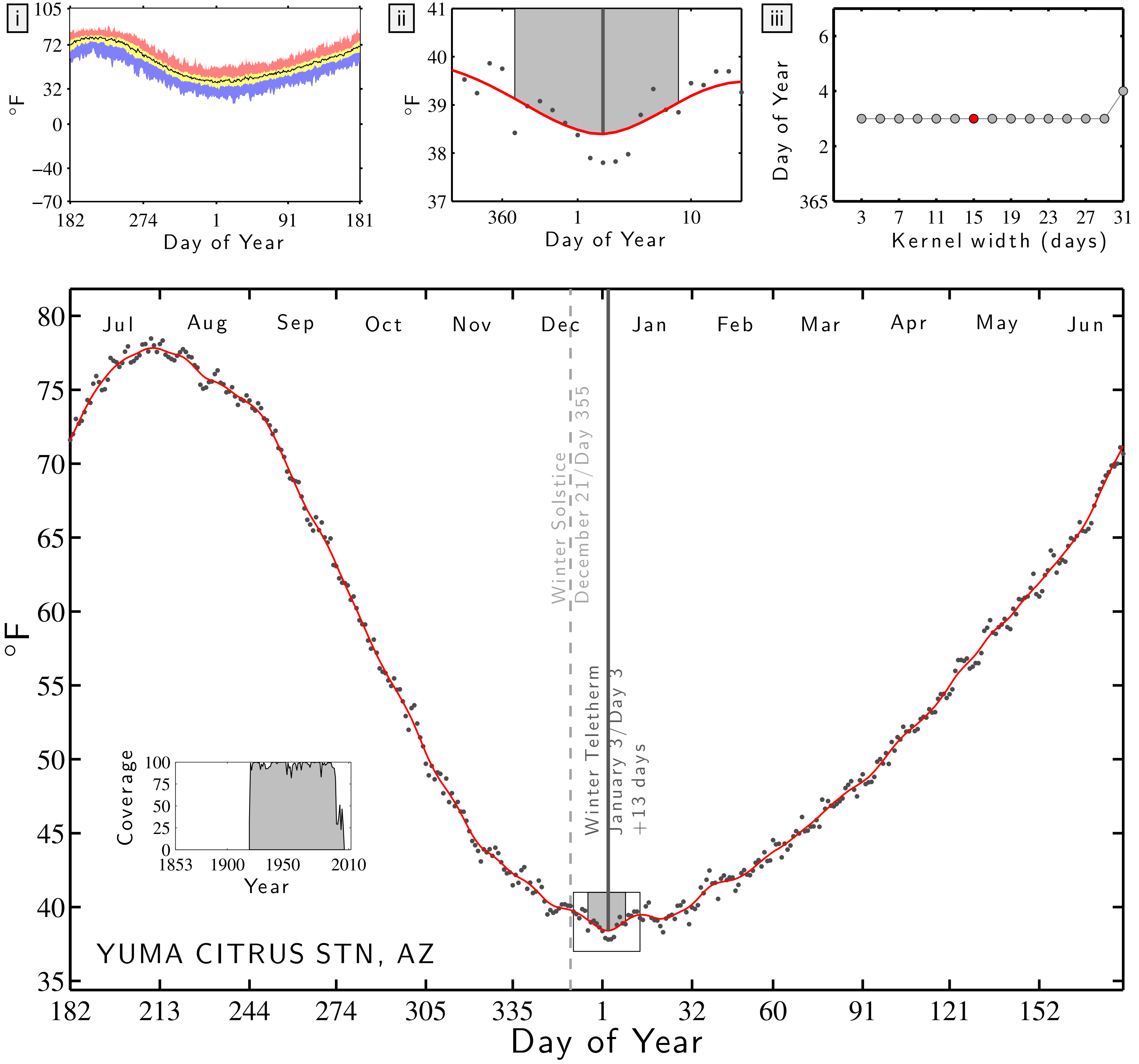

i
ii
iii
°F
Day of Year
Day of Year
Kernel width (days)
Jul
Aug
Sep
Oct
Nov
Dec
Jan
Feb
Mar
Apr
May
Jun
Winter Solstice
December 21/Day 355
Winter Teletherm
January 3/Day 3
+13 days
Coverage
Year
YUMA CITRUS STN, AZ

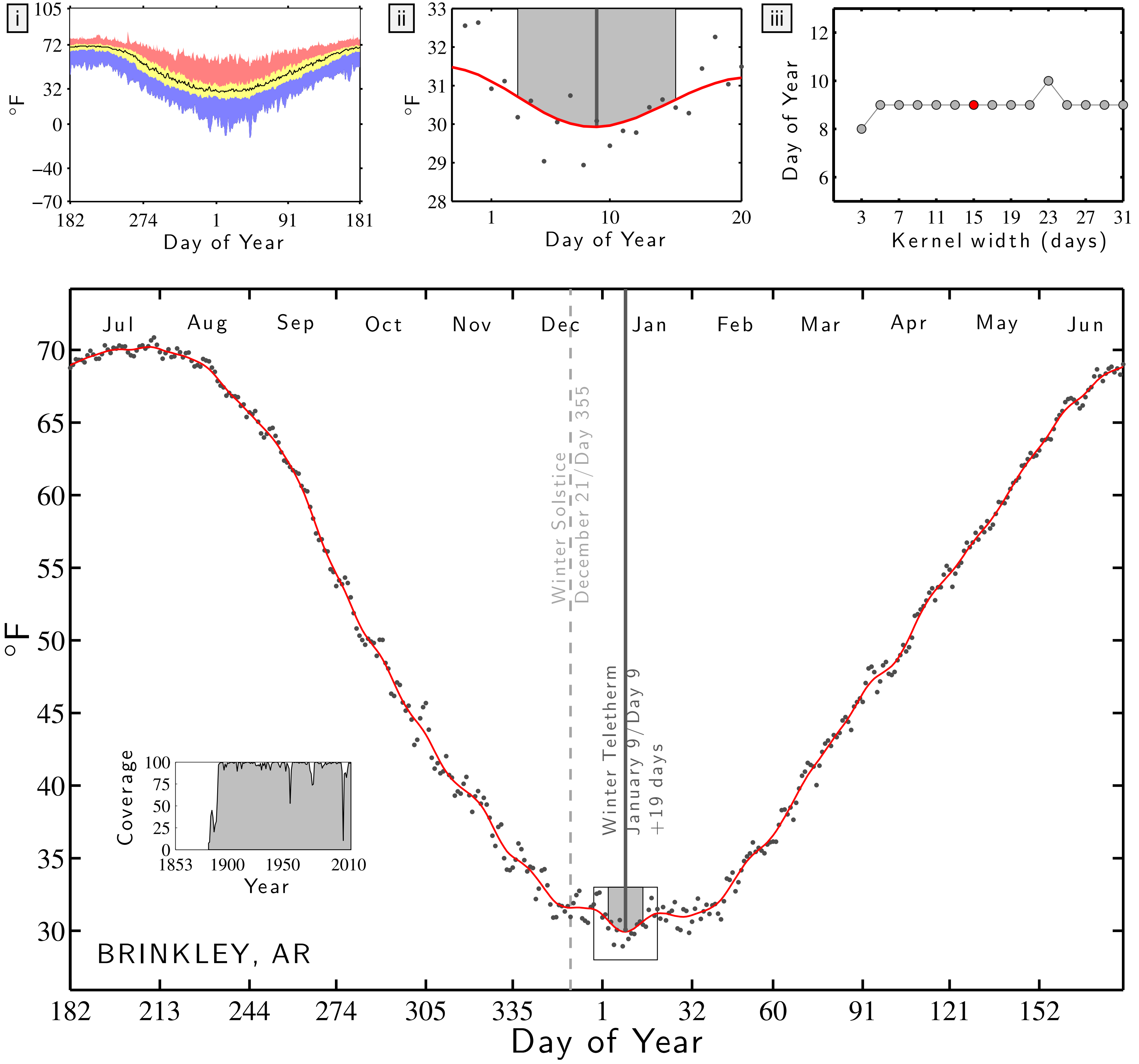

i
ii
iii
Day of Year
Kernel width (days)
°F
Jul
Aug
Sep
Oct
Nov
Dec
Jan
Feb
Mar
Apr
May
Jun
Winter Solstice
December 21/Day 355
Winter Teletherm
January 9/Day 9
+19 days
Coverage
Year
BRINKLEY, AR

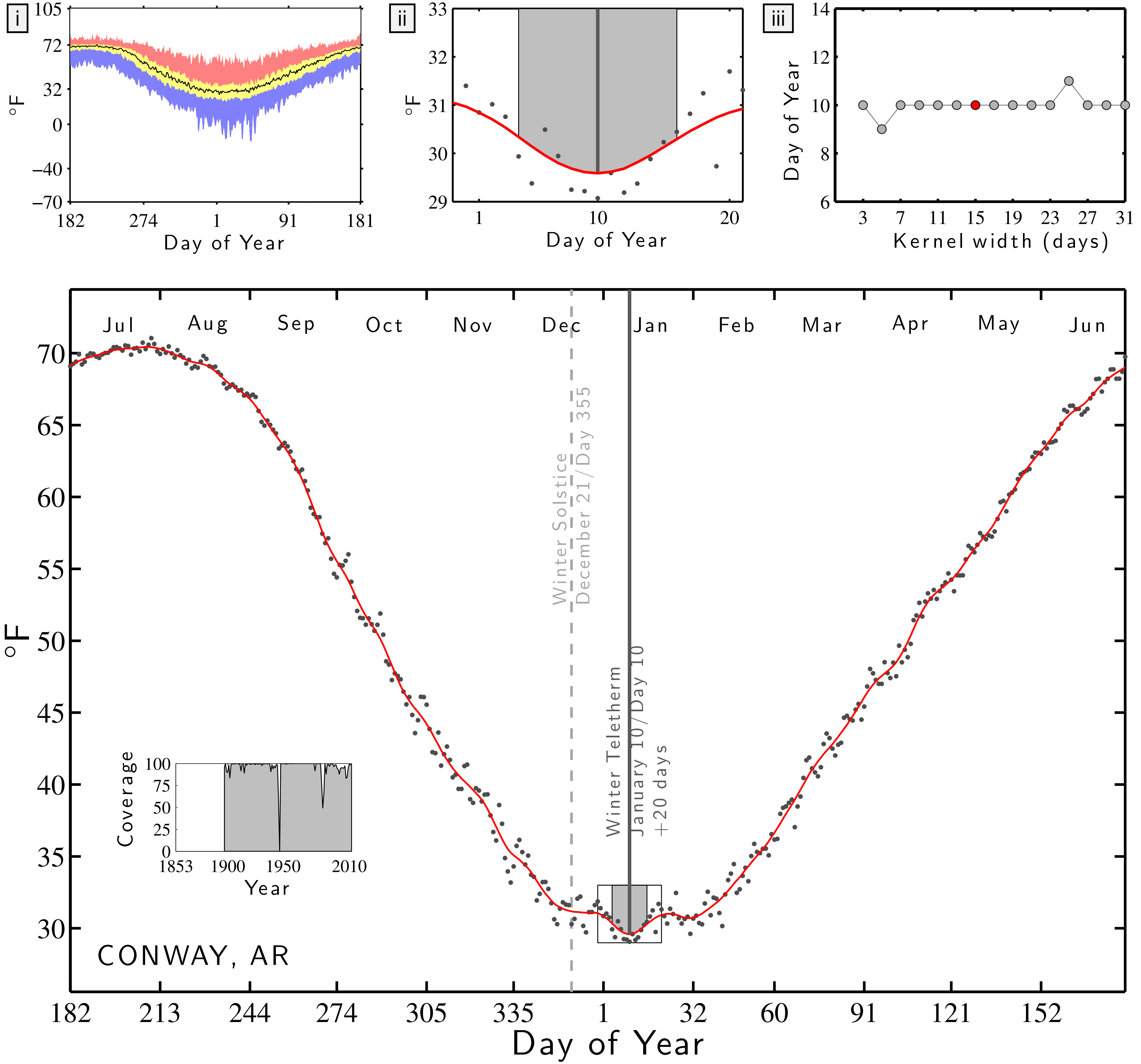

i
°F
Day of Year
ii
iii
Day of Year
Kernel width (days)
Jul
Aug
Sep
Oct
Nov
Dec
Jan
Feb
Mar
Apr
May
Jun
Winter Solstice
December 21/Day 355
Winter Teletherm
January 10/Day 10
+20 days
Coverage
Year
CONWAY, AR
Day of Year

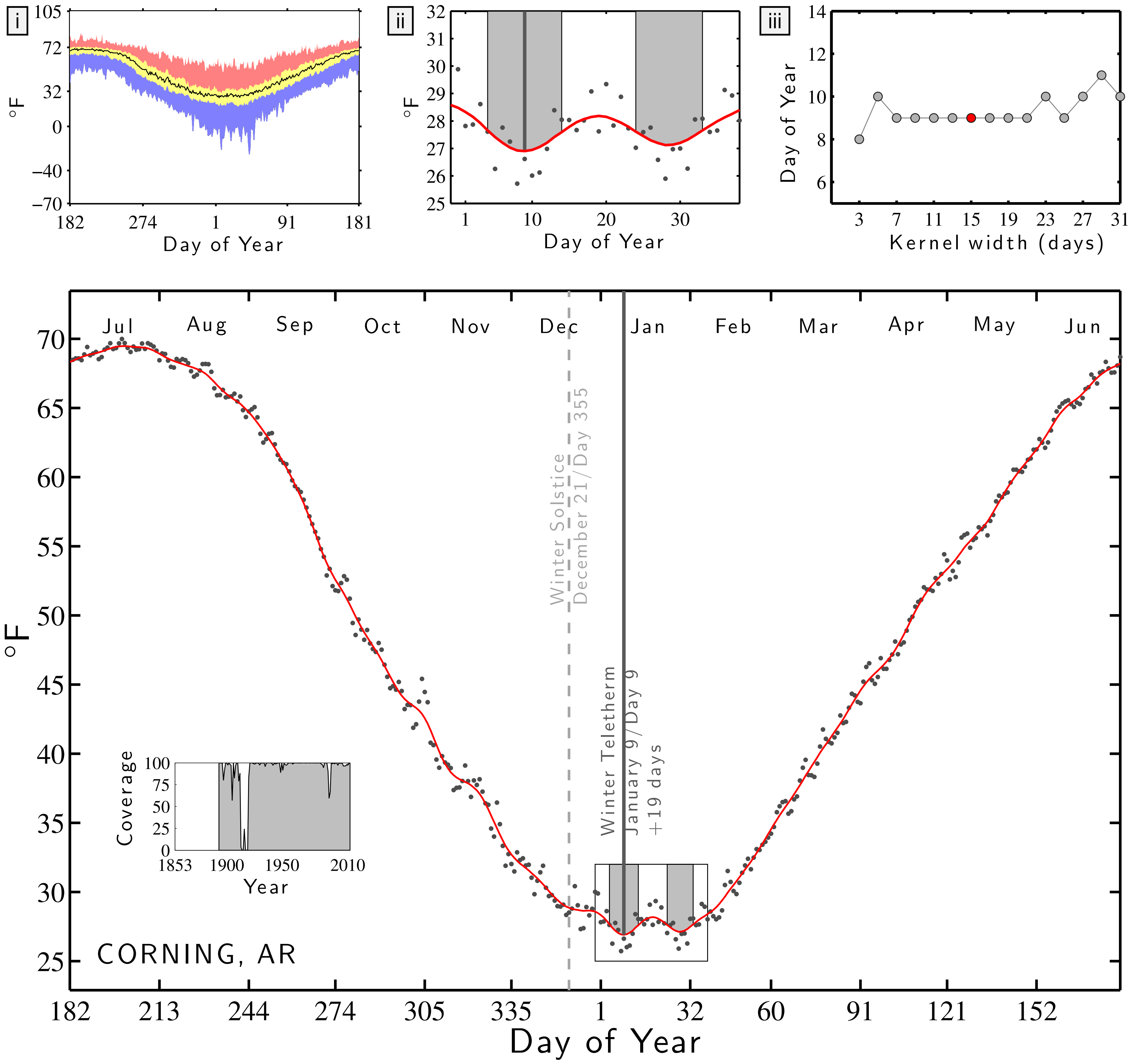

i
ii
iii
°F
Day of Year
Kernel width (days)
Jul
Aug
Sep
Oct
Nov
Dec
Jan
Feb
Mar
Apr
May
Jun
Winter Solstice
December 21/Day 355
Winter Teletherm
January 9/Day 9
+19 days
Coverage
Year
CORNING, AR

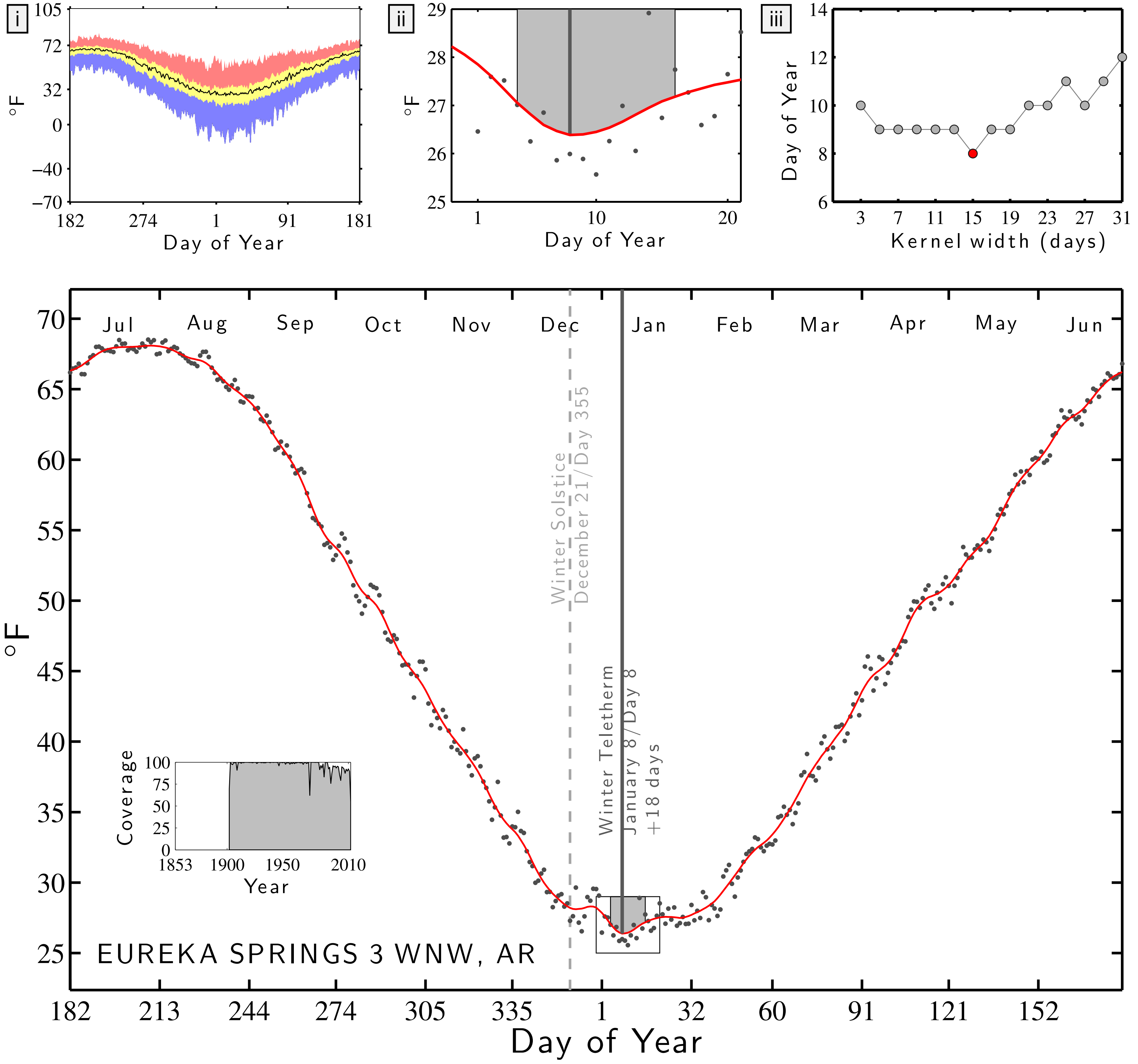

i
ii
iii
°F
Day of Year
Day of Year
Day of Year
Kernel width (days)
Jul
Aug
Sep
Oct
Nov
Dec
Jan
Feb
Mar
Apr
May
Jun
Winter Solstice
December 21/Day 355
Winter Teletherm
January 8/Day 8
+18 days
Coverage
Year
EUREKA SPRINGS 3 WNW, AR

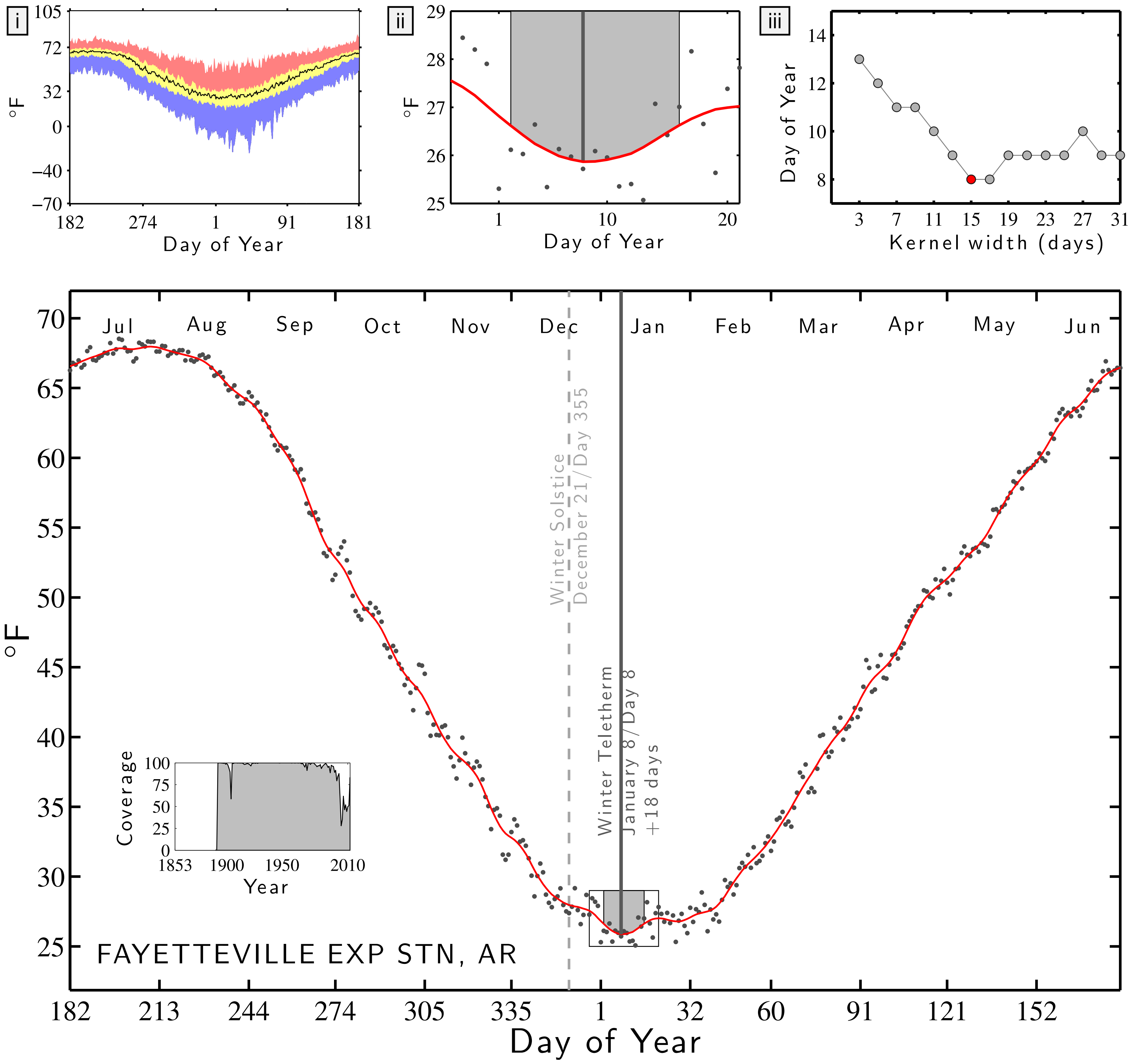
i
ii
iii
°F
Day of Year
Kernel width (days)
Day of Year
Jul
Aug
Sep
Oct
Nov
Dec
Jan
Feb
Mar
Apr
May
Jun
Winter Solstice
December 21/Day 355
Winter Teletherm
January 8/Day 8
+18 days
Coverage
Year
FAYETTEVILLE EXP STN, AR

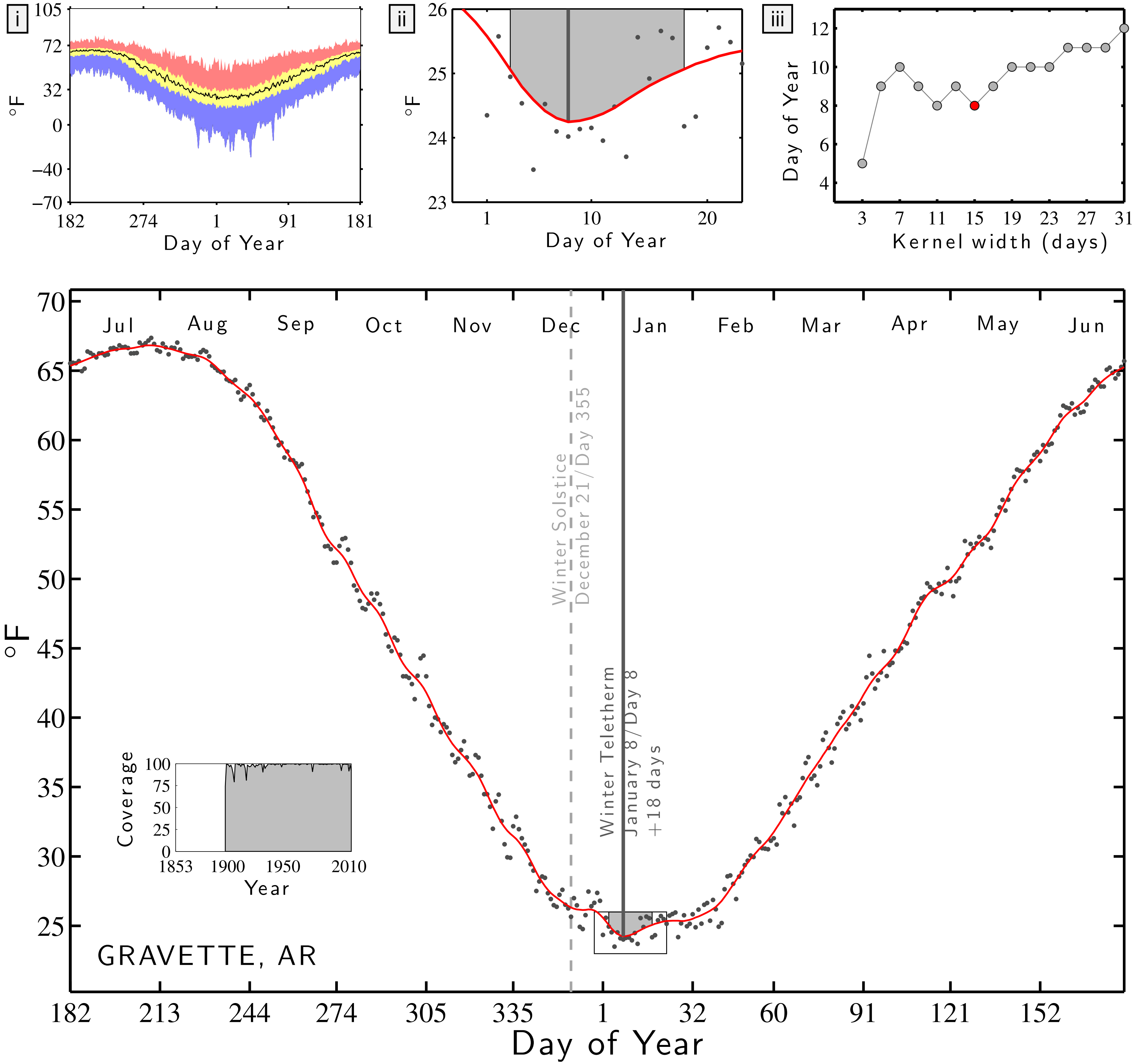

i
ii
iii
°F
Day of Year
Kernel width (days)
Jul
Aug
Sep
Oct
Nov
Dec
Jan
Feb
Mar
Apr
May
Jun
Winter Solstice
December 21/Day 355
Winter Teletherm
January 8/Day 8
+18 days
Coverage
Year
GRAVETTE, AR

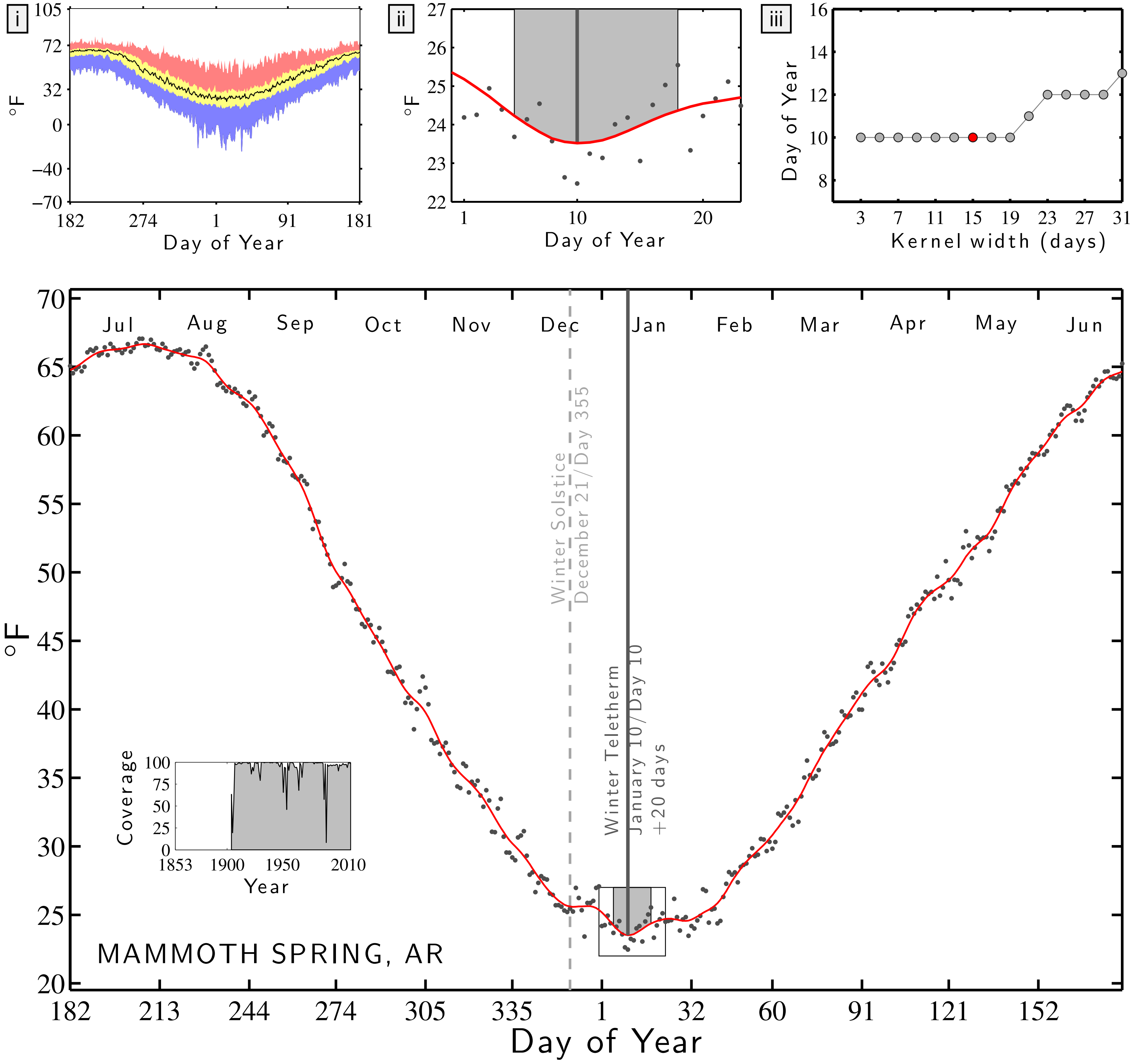
i
°F
Day of Year
ii
°F
Day of Year
iii
Day of Year
Kernel width (days)
Jul
Aug
Sep
Oct
Nov
Dec
Jan
Feb
Mar
Apr
May
Jun
Winter Solstice
December 21/Day 355
Winter Teletherm
January 10/Day 10
+20 days
Coverage
Year
MAMMOTH SPRING, AR
°F
Day of Year

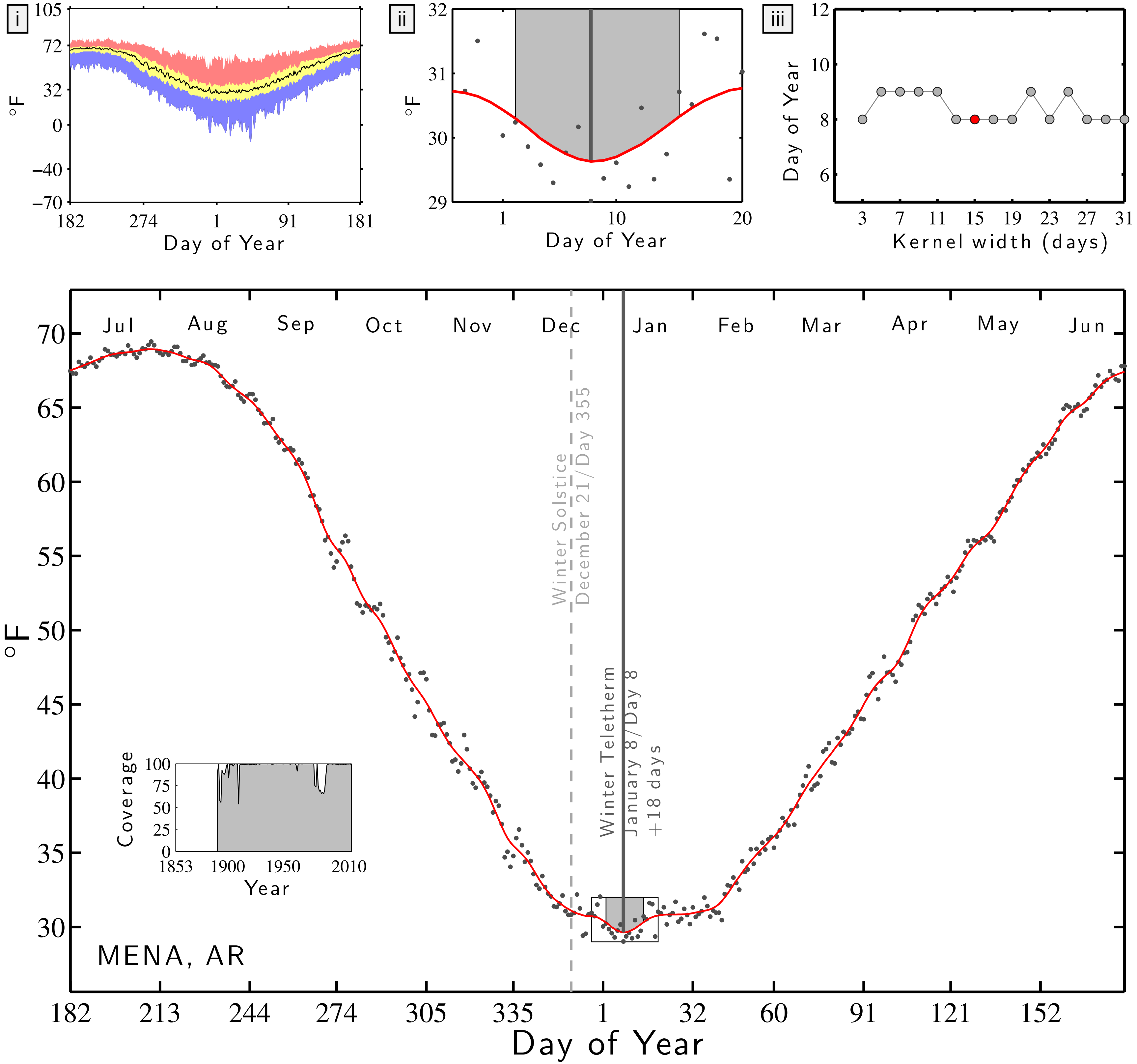

i
ii
iii
Day of Year
Kernel width (days)
°F
Jul
Aug
Sep
Oct
Nov
Dec
Jan
Feb
Mar
Apr
May
Jun
Winter Solstice
December 21/Day 355
Winter Teletherm
January 8/Day 8
+18 days
Coverage
Year
MENA, AR

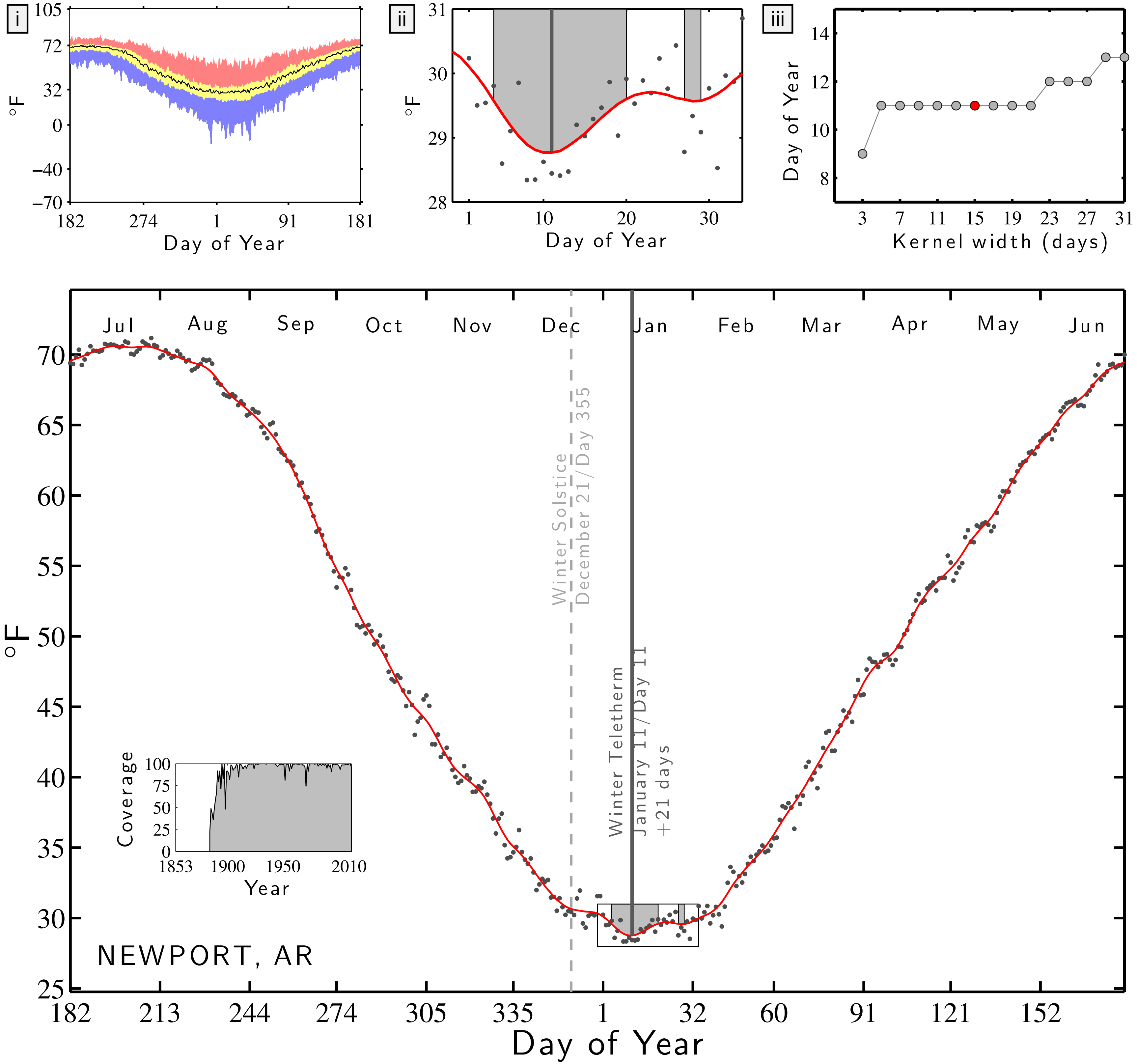
i
ii
iii
°F
Day of Year
Kernel width (days)
Jul
Aug
Sep
Oct
Nov
Dec
Jan
Feb
Mar
Apr
May
Jun
Winter Solstice
December 21/Day 355
Winter Teletherm
January 11/Day 11
+21 days
Coverage
Year
NEWPORT, AR

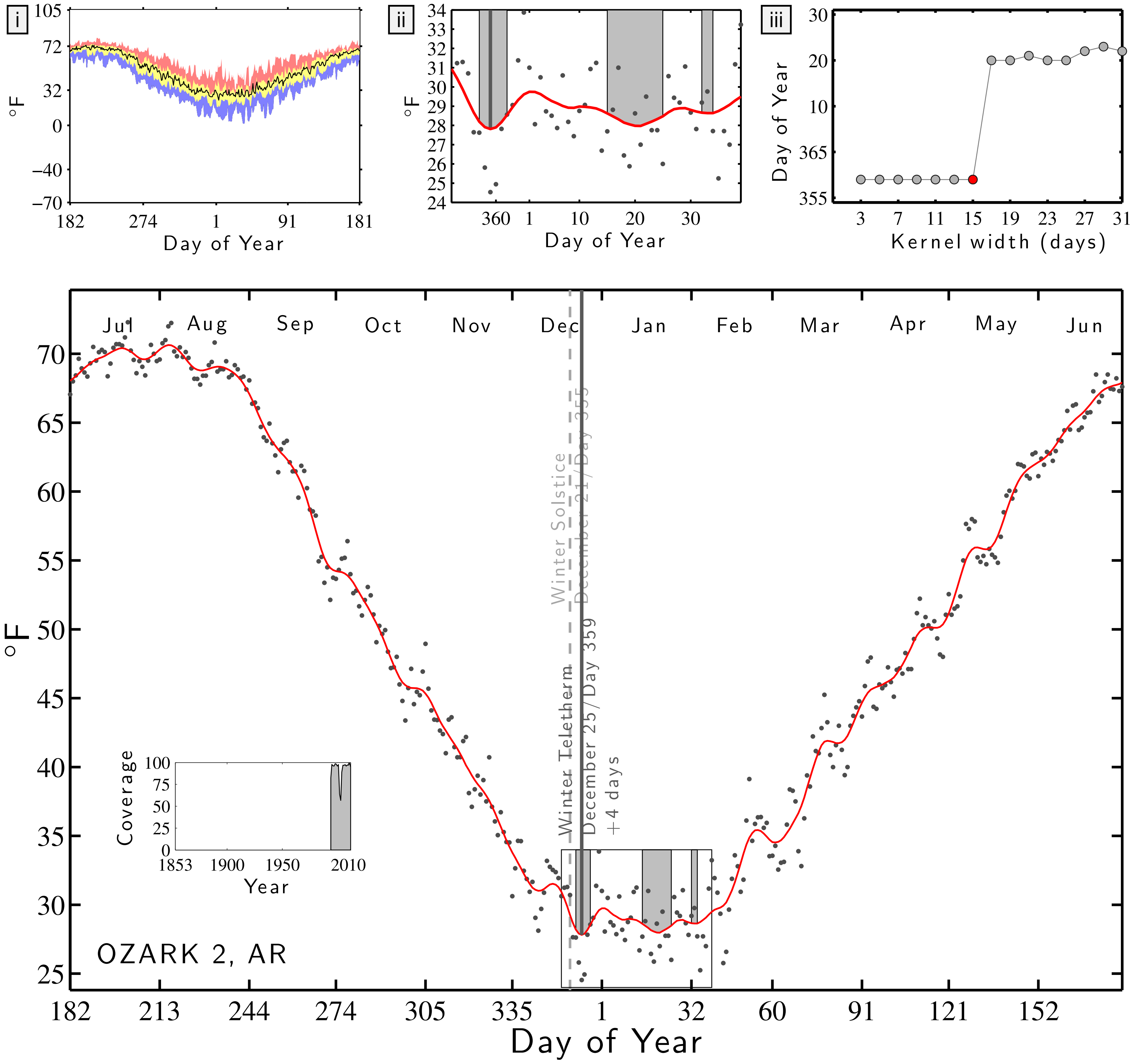

i
ii
iii
°F
Day of Year
Kernel width (days)
Jul
Aug
Sep
Oct
Nov
Dec
Jan
Feb
Mar
Apr
May
Jun
Winter Solstice
December 21/Day 355
Winter Teletherm
December 25/Day 359
+4 days
Coverage
Year
OZARK 2, AR

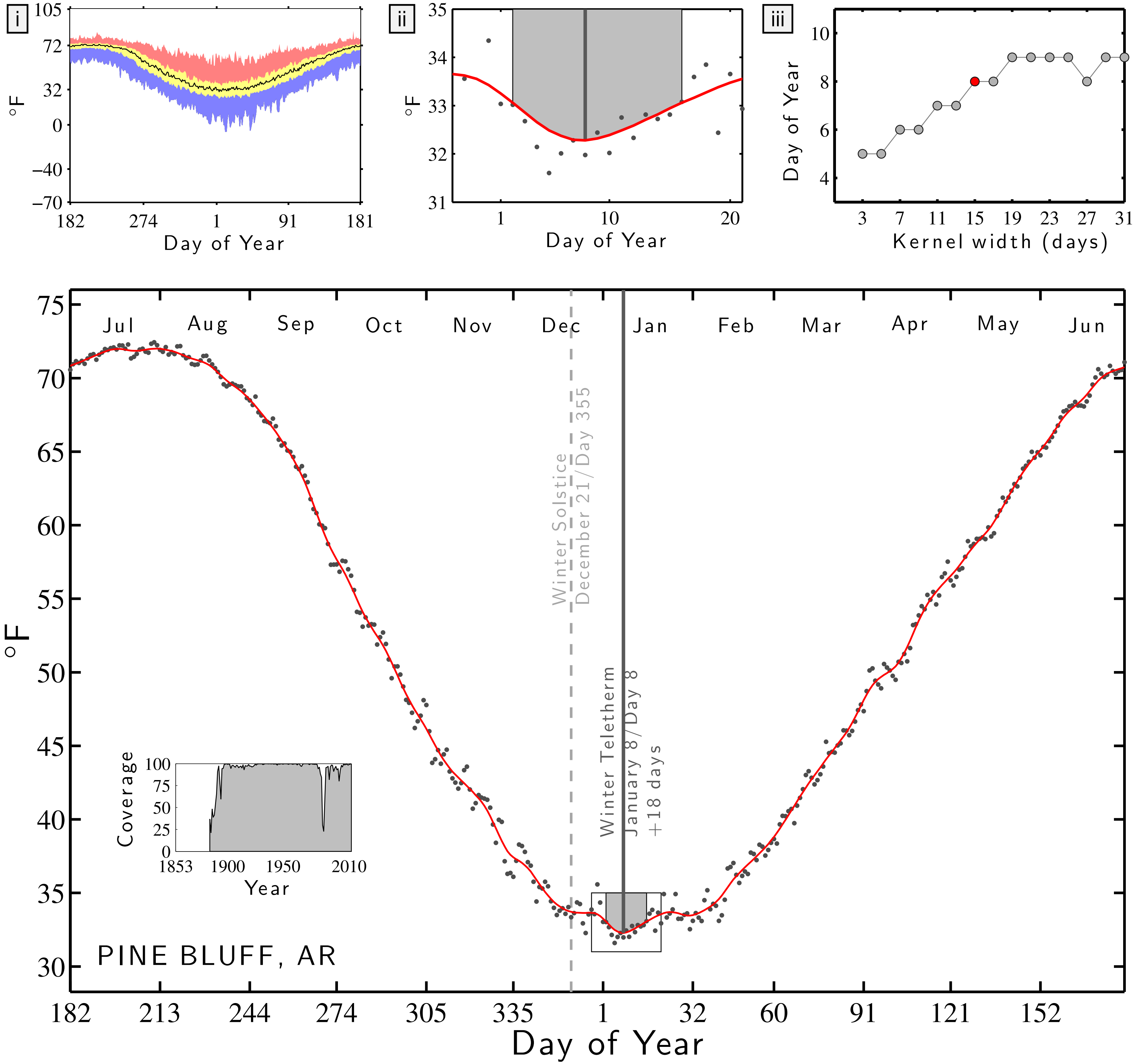

i
°F
Day of Year
ii
°F
Day of Year
iii
Day of Year
Kernel width (days)
Jul
Aug
Sep
Oct
Nov
Dec
Jan
Feb
Mar
Apr
May
Jun
Winter Solstice
December 21/Day 355
Winter Teletherm
January 8/Day 8
+18 days
Coverage
Year
PINE BLUFF, AR
°F
Day of Year

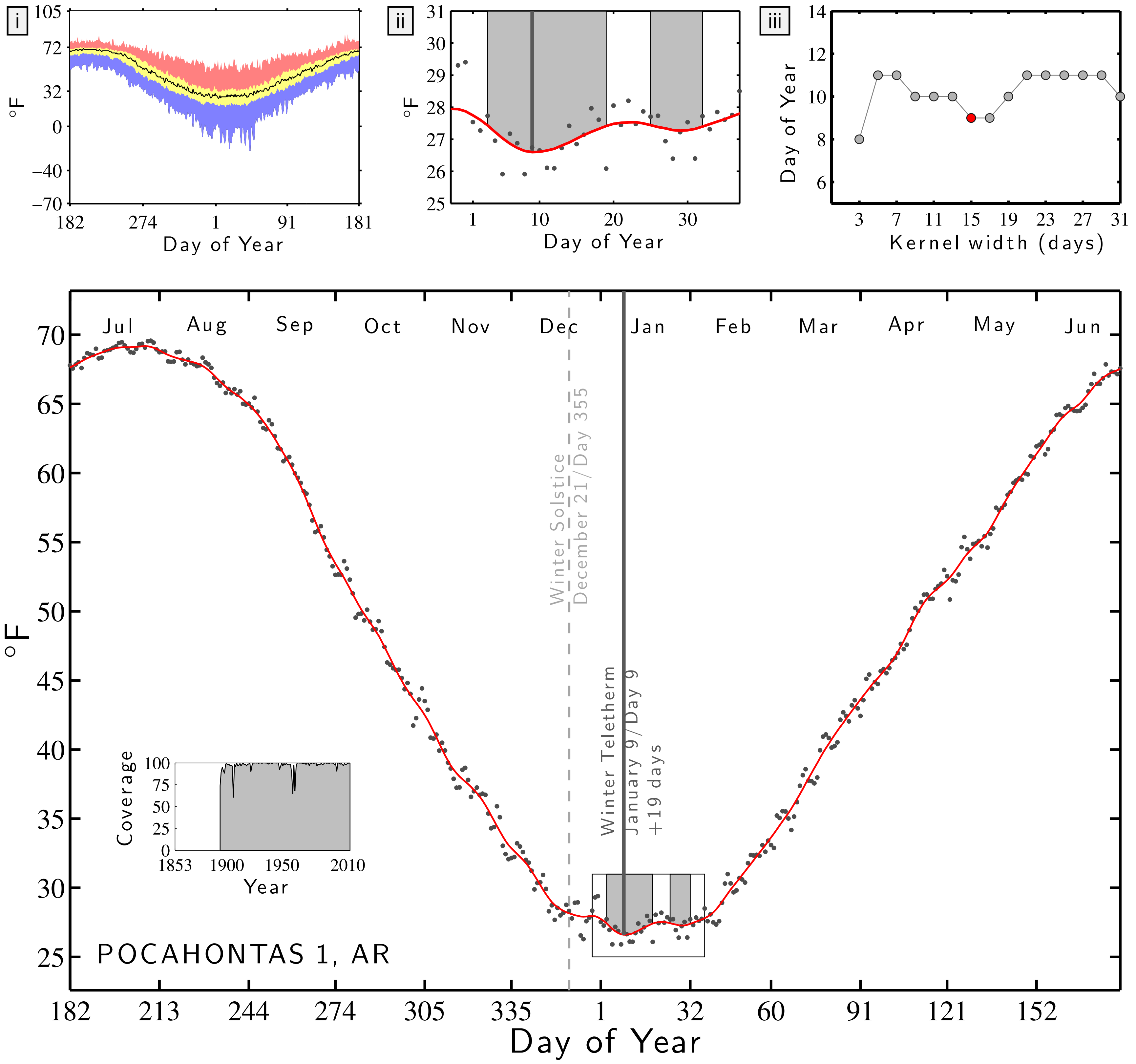

i
ii
iii
Day of Year
Kernel width (days)
°F
Jul
Aug
Sep
Oct
Nov
Dec
Jan
Feb
Mar
Apr
May
Jun
Winter Solstice
December 21/Day 355
Winter Teletherm
January 9/Day 9
+19 days
Coverage
Year
POCAHONTAS 1, AR

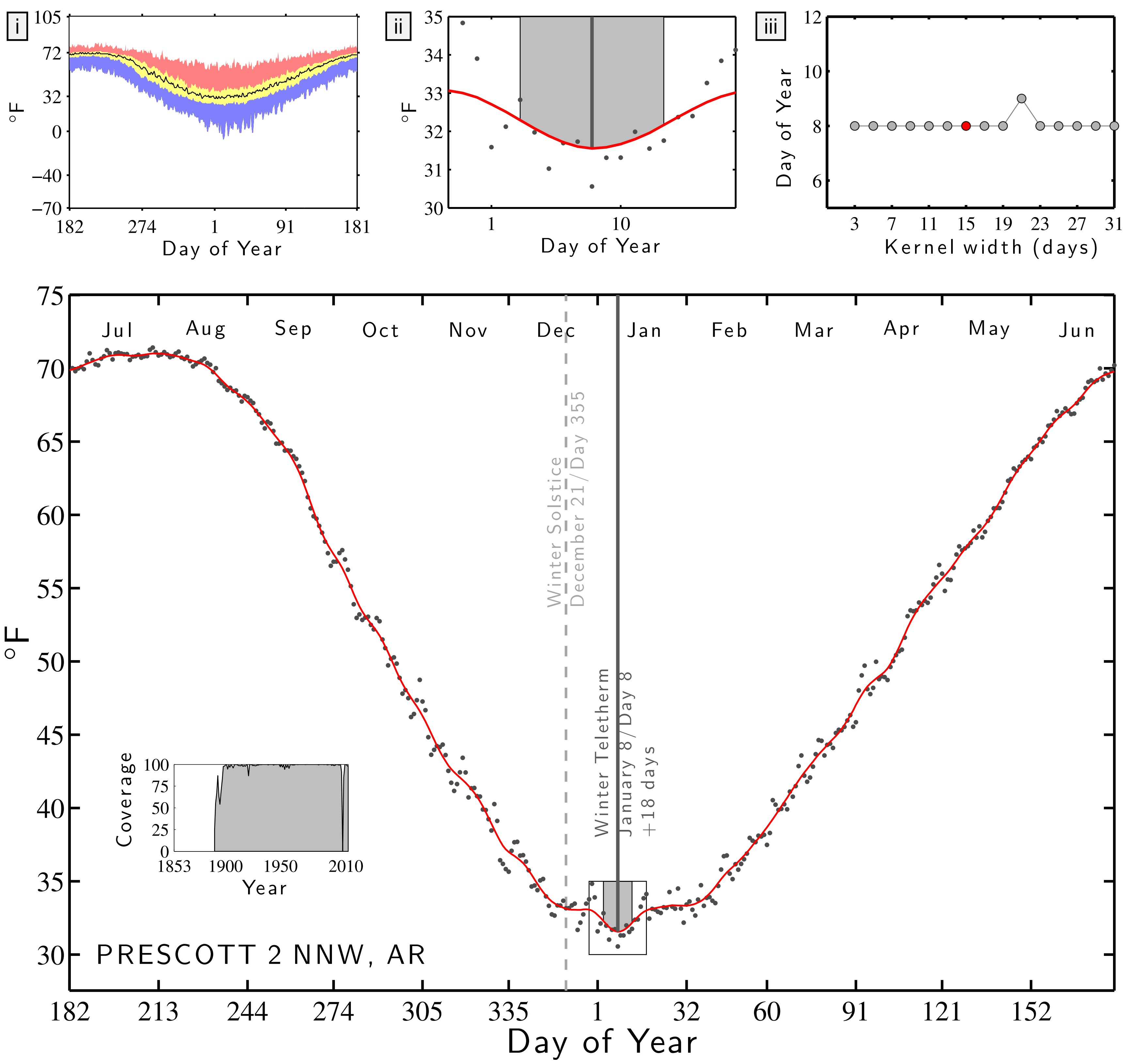

i
°F
Day of Year
ii
°F
Day of Year
iii
Day of Year
Kernel width (days)
Jul Aug Sep Oct Nov Dec Jan Feb Mar Apr May Jun
Winter Solstice
December 21/Day 355
Winter Teletherm
January 8/Day 8
+18 days
Coverage
Year
PRESCOTT 2 NNW, AR
°F
Day of Year

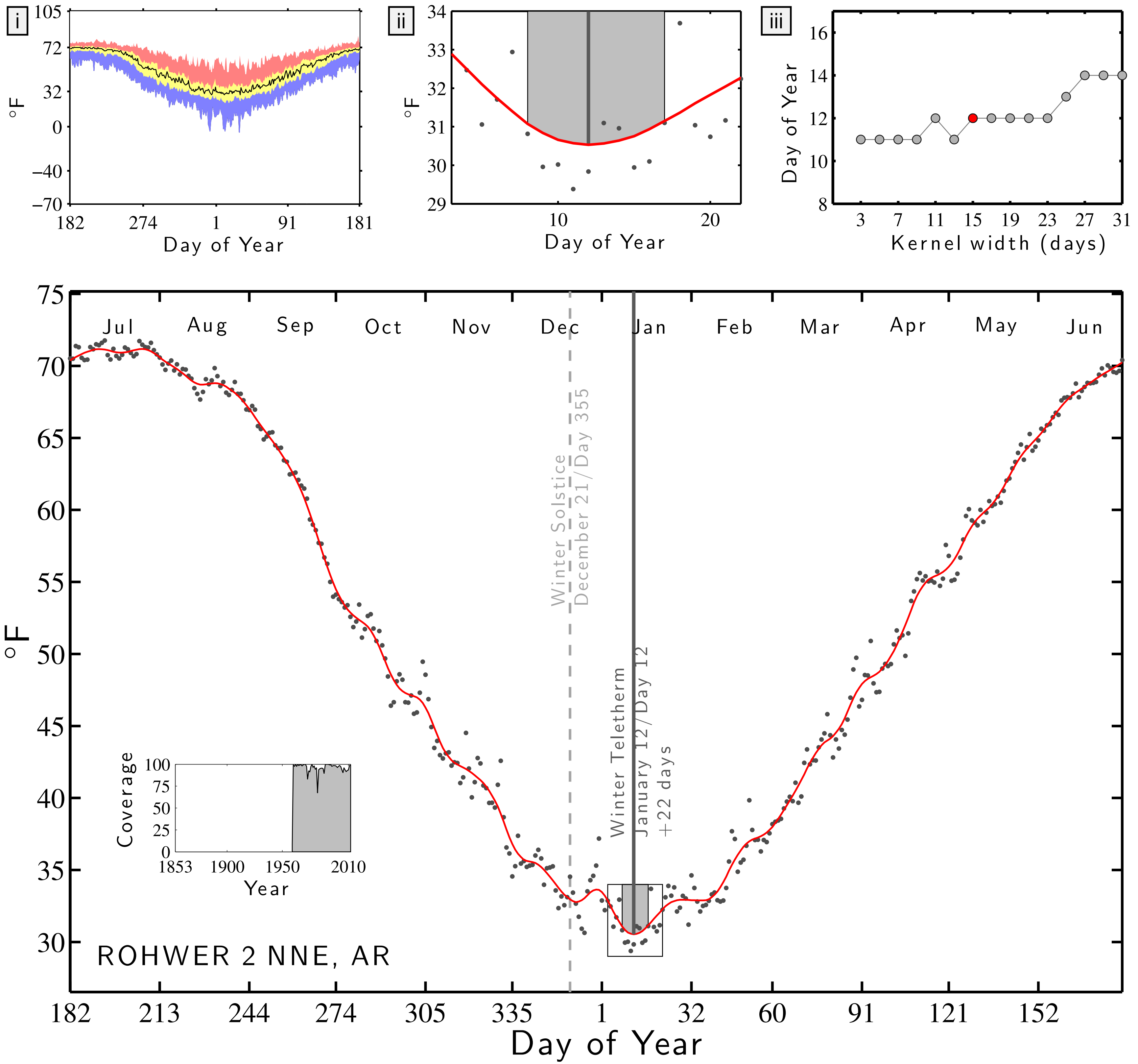

i
ii
iii
°F
Day of Year
Day of Year
Kernel width (days)
Jul
Aug
Sep
Oct
Nov
Dec
Jan
Feb
Mar
Apr
May
Jun
Winter Solstice
December 21/Day 355
Winter Teletherm
January 12/Day 12
+22 days
Coverage
Year
ROHWER 2 NNE, AR

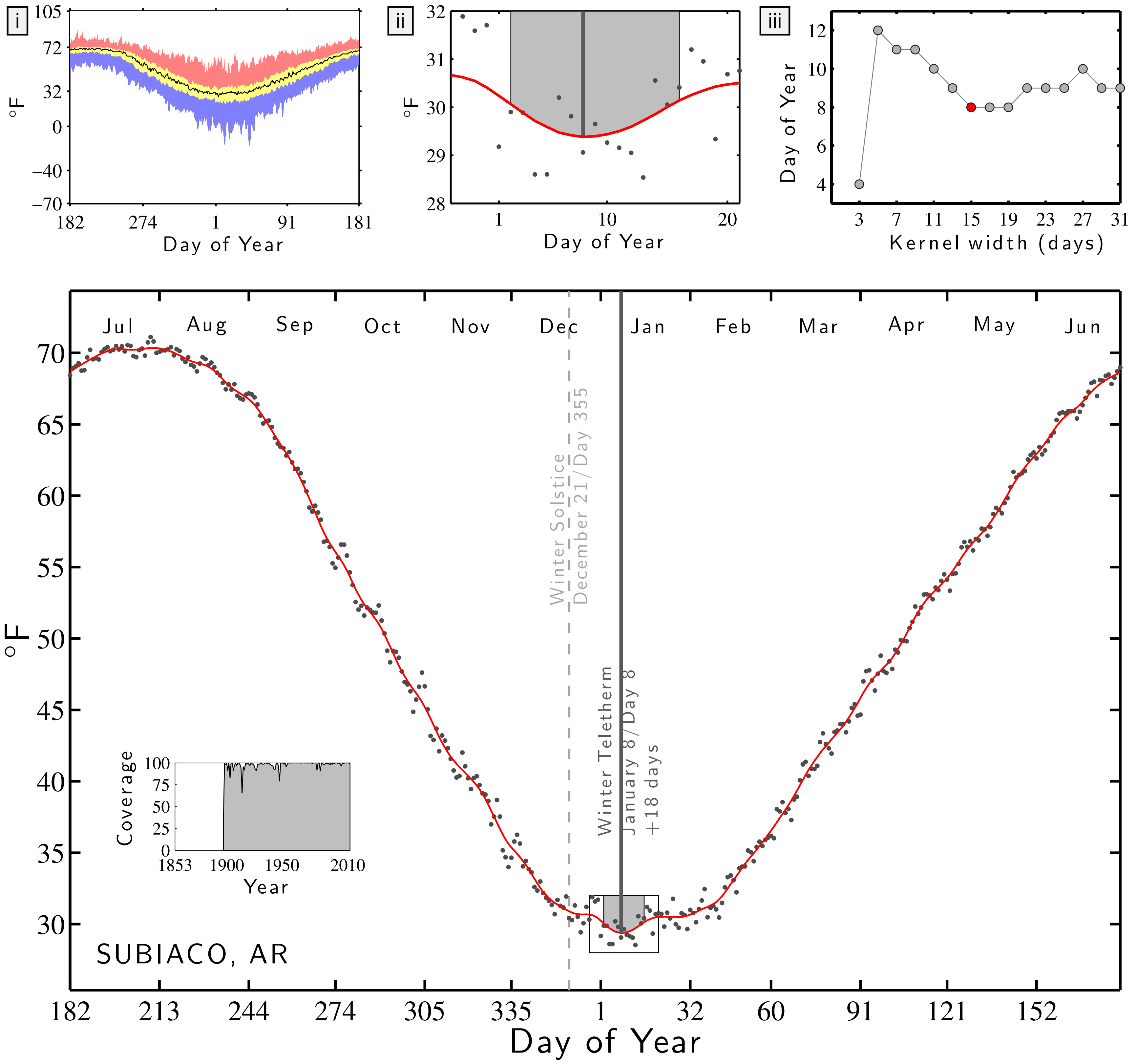

i
°F
Day of Year
ii
°F
Day of Year
iii
Day of Year
Kernel width (days)
Jul
Aug
Sep
Oct
Nov
Dec
Jan
Feb
Mar
Apr
May
Jun
Winter Solstice
December 21/Day 355
Winter Teletherm
January 8/Day 8
+18 days
Coverage
Year
SUBIACO, AR
°F
Day of Year

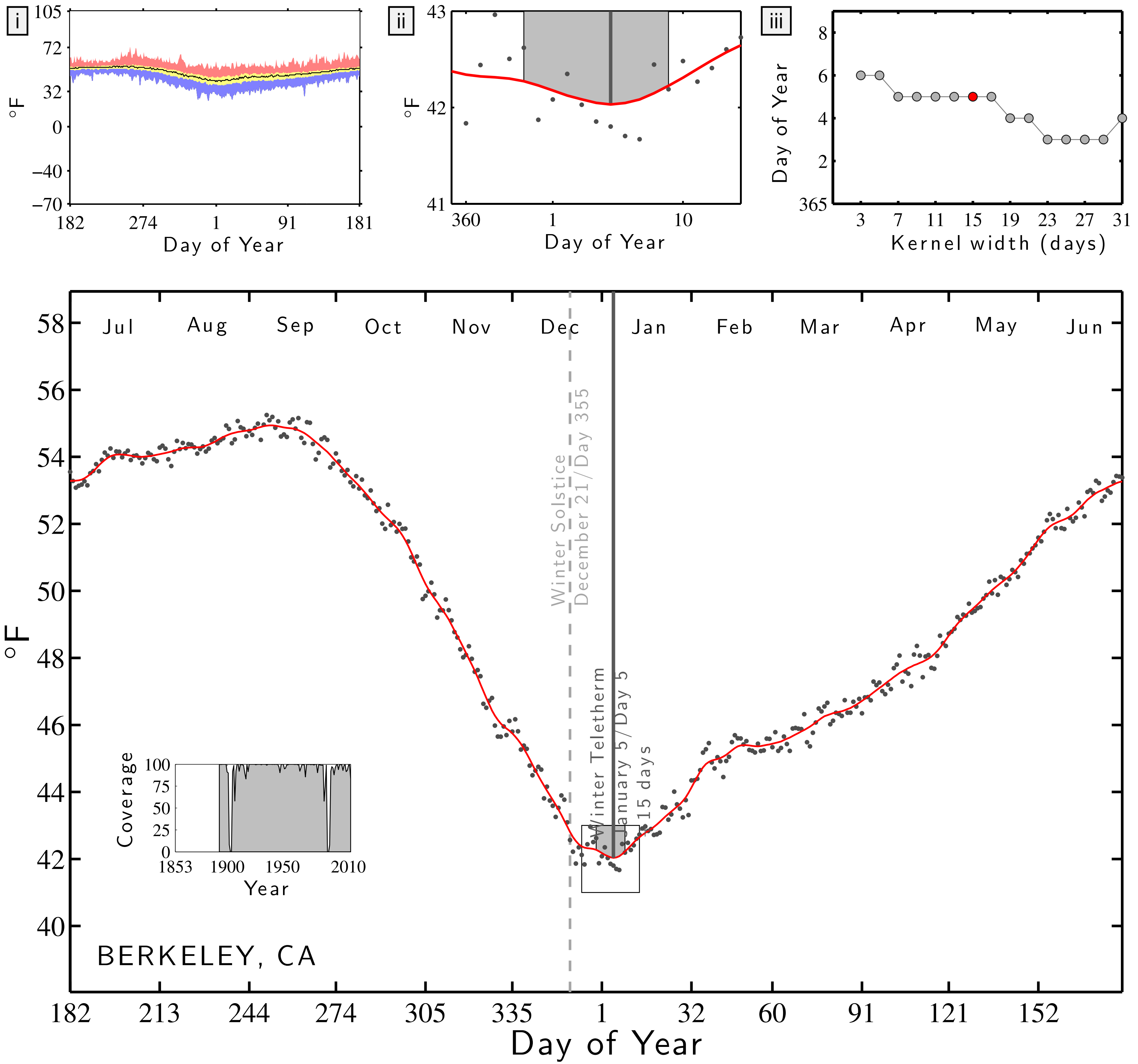
i
105
72
32
0
−40
−70
°F
182
274
1
91
181
Day of Year
ii
43
42
41
°F
360
1
10
Day of Year
iii
8
6
4
2
365
Day of Year
3
7
11
15
19
23
27
31
Kernel width (days)
Jul
Aug
Sep
Oct
Nov
Dec
Jan
Feb
Mar
Apr
May
Jun
58
56
54
52
50
48
46
44
42
40
°F
Winter Solstice
December 21/Day 355
Winter Teletherm
January 5/Day 5
15 days
Coverage
100
75
50
25
0
1853
1900
1950
2010
Year
BERKELEY, CA
182
213
244
274
305
335
1
32
60
91
121
152
Day of Year

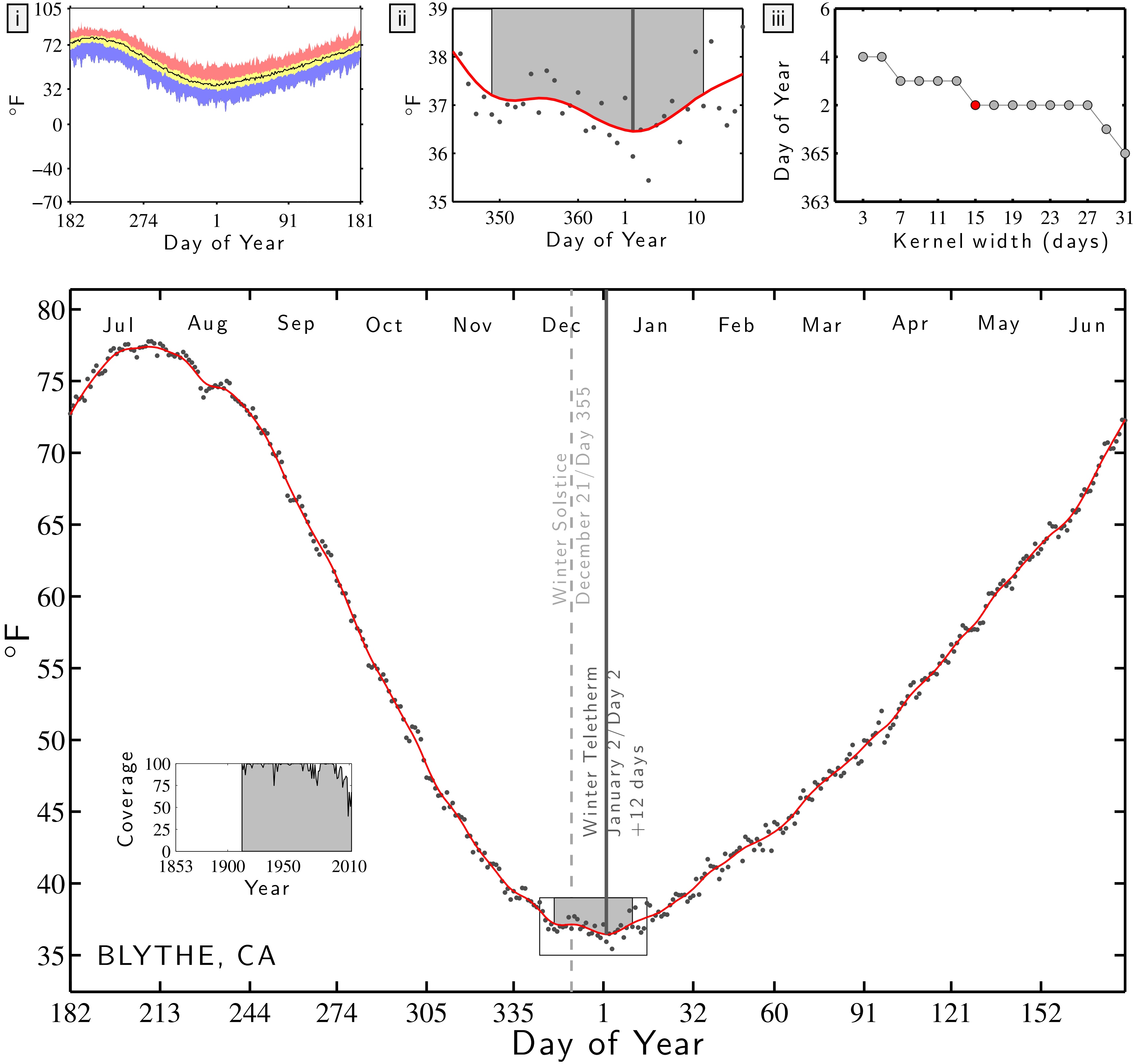

i
ii
iii
°F
Day of Year
Day of Year
Kernel width (days)
Jul
Aug
Sep
Oct
Nov
Dec
Jan
Feb
Mar
Apr
May
Jun
Winter Solstice
December 21/Day 355
Winter Teletherm
January 2/Day 2
+12 days
Coverage
Year
BLYTHE, CA

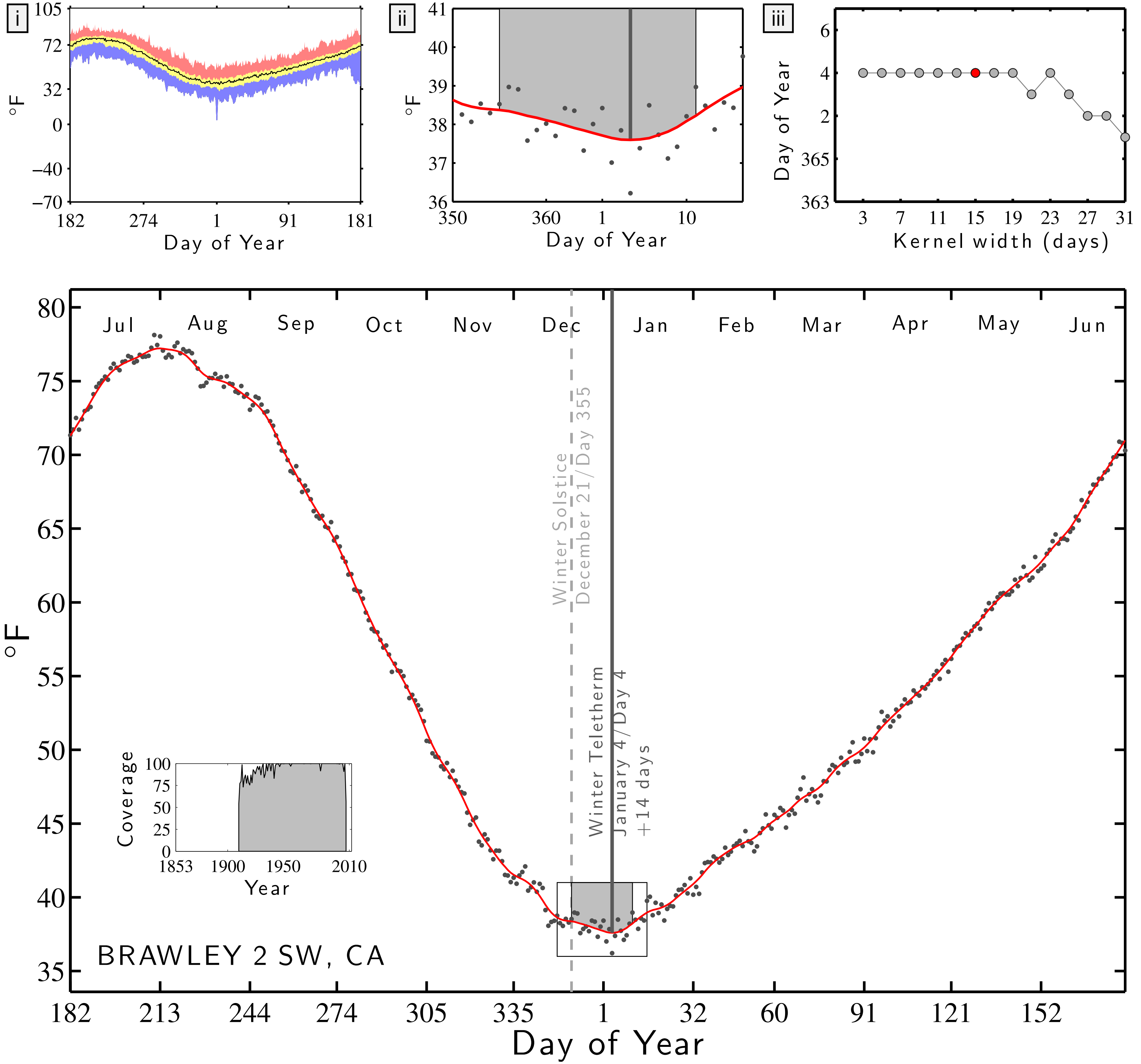

i
Day of Year
°F
ii
Day of Year
°F
iii
Kernel width (days)
Day of Year
Jul
Aug
Sep
Oct
Nov
Dec
Jan
Feb
Mar
Apr
May
Jun
Winter Solstice
December 21/Day 355
Winter Teletherm
January 4/Day 4
+14 days
Coverage
Year
BRAWLEY 2 SW, CA
Day of Year
°F

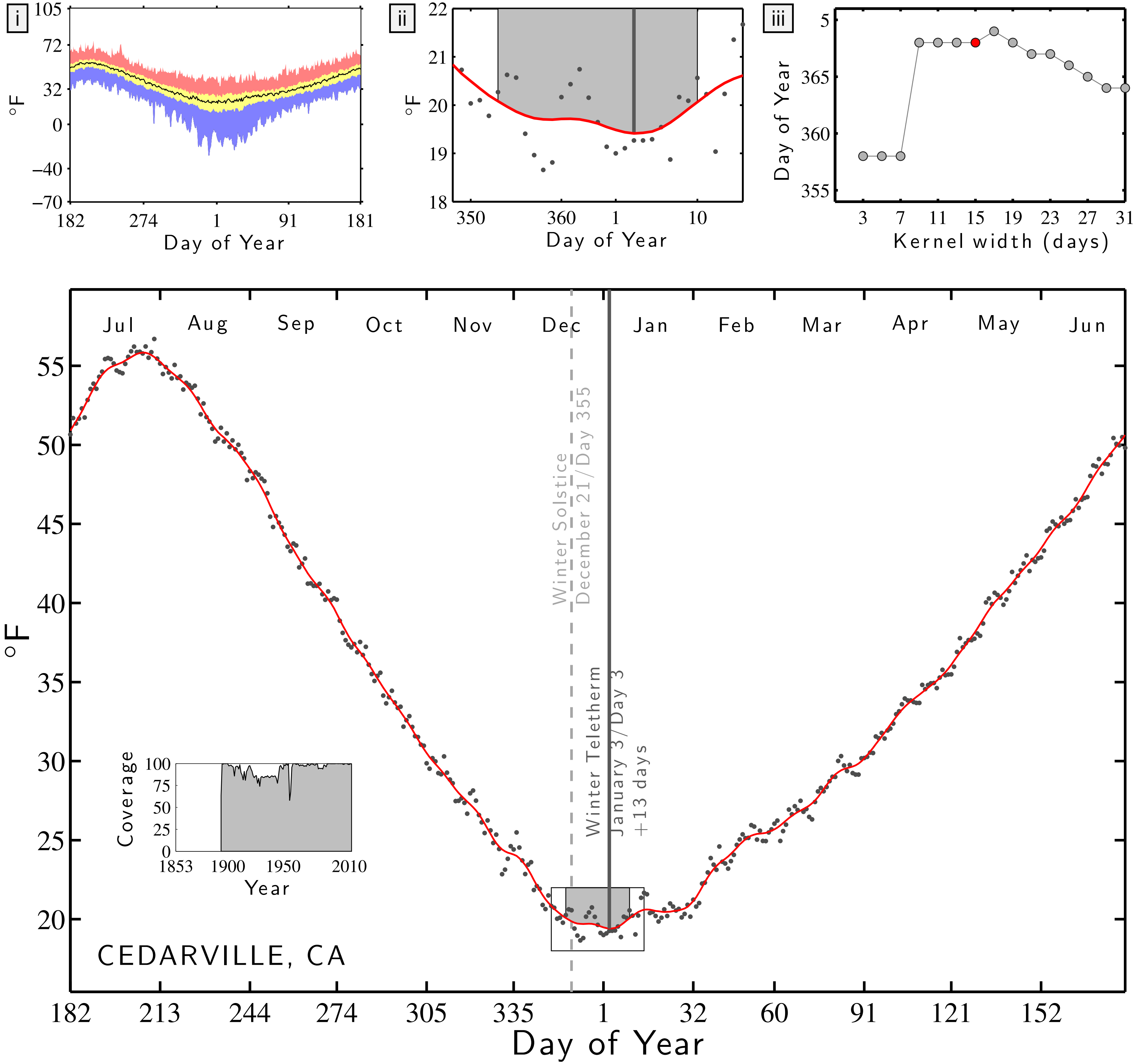

i
°F
Day of Year
ii
iii
Day of Year
Kernel width (days)
Jul
Aug
Sep
Oct
Nov
Dec
Jan
Feb
Mar
Apr
May
Jun
Winter Solstice
December 21/Day 355
Winter Teletherm
January 3/Day 3
+13 days
Coverage
Year
CEDARVILLE, CA

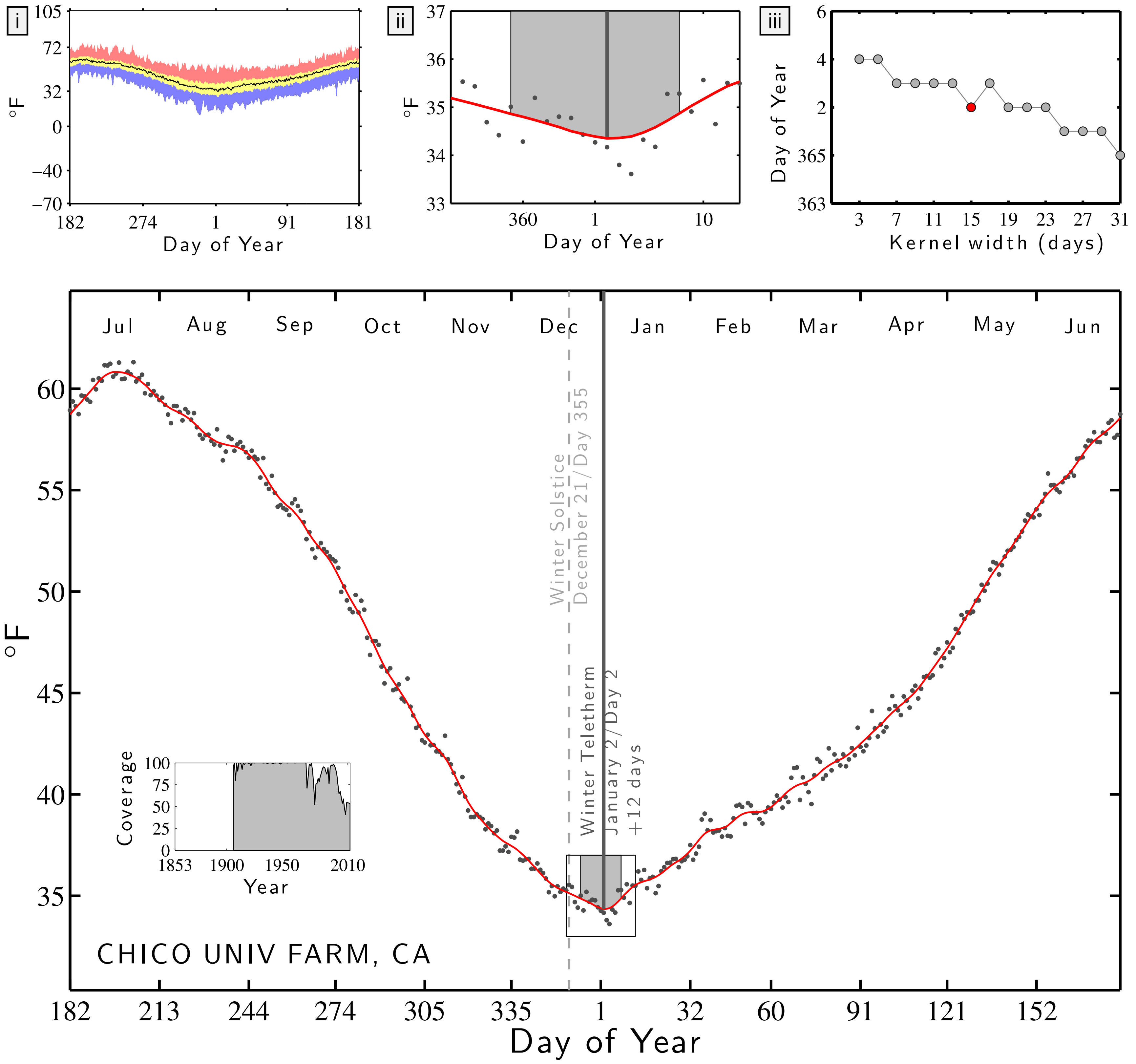

i
ii
iii
°F
Day of Year
Day of Year
Kernel width (days)
Jul
Aug
Sep
Oct
Nov
Dec
Jan
Feb
Mar
Apr
May
Jun
Winter Solstice
December 21/Day 355
Winter Teletherm
January 2/Day 2
+12 days
Coverage
Year
CHICO UNIV FARM, CA
Day of Year

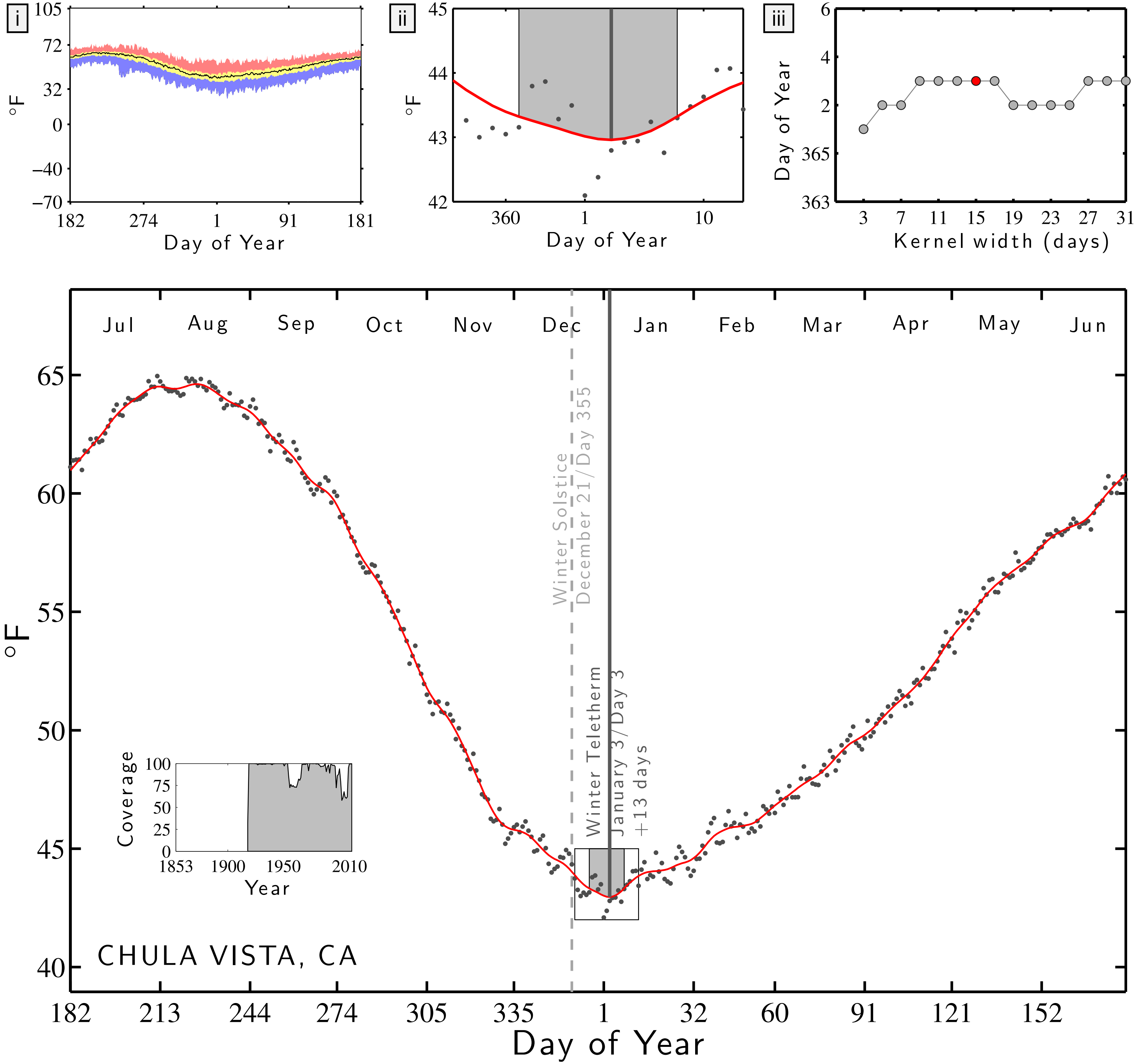

i
ii
iii
°F
Day of Year
Kernel width (days)
Jul
Aug
Sep
Oct
Nov
Dec
Jan
Feb
Mar
Apr
May
Jun
Winter Solstice
December 21/Day 355
Winter Teletherm
January 3/Day 3
+13 days
Coverage
Year
CHULA VISTA, CA

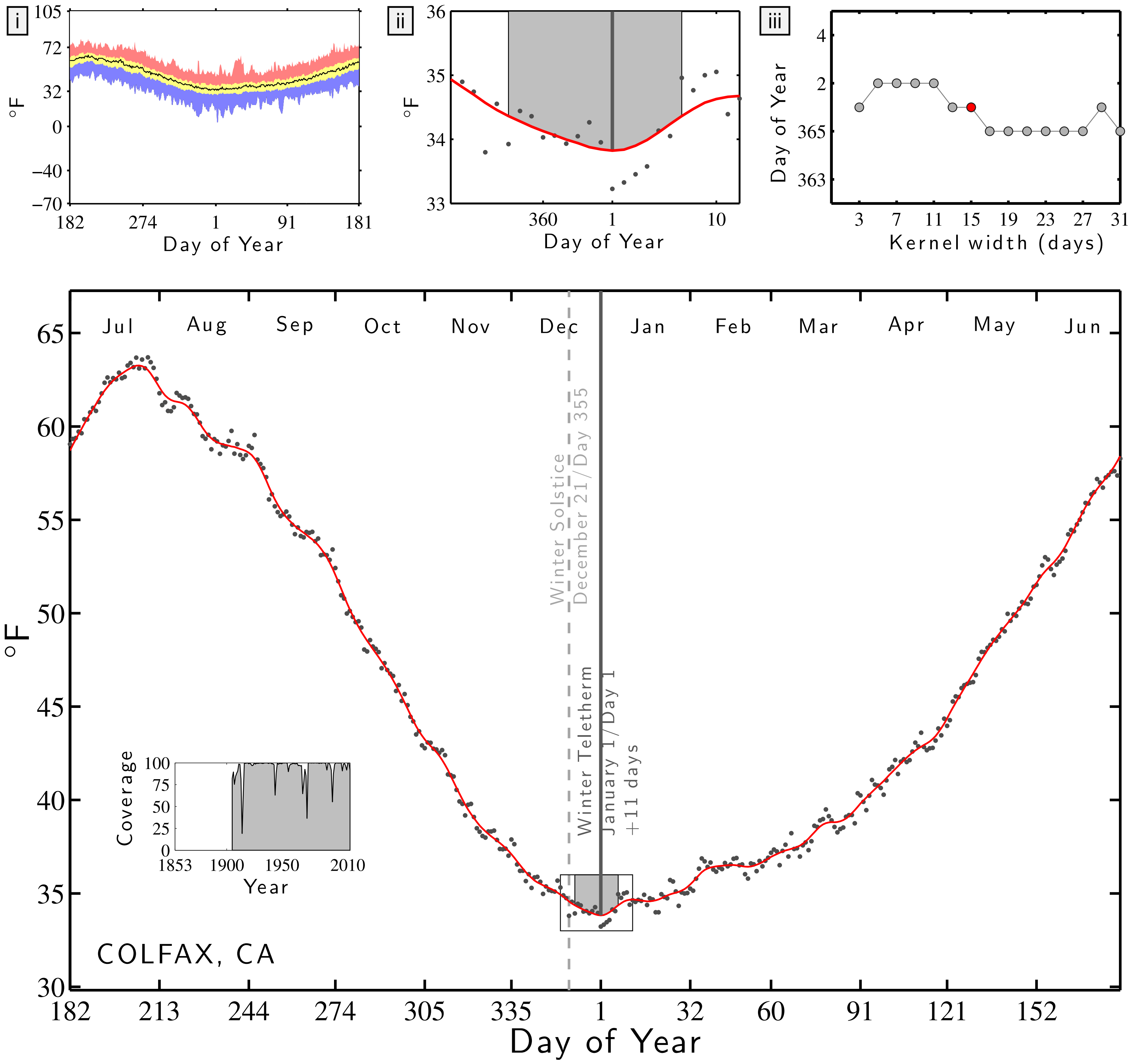

i
°F
Day of Year
ii
Day of Year
iii
Day of Year
Kernel width (days)
Jul
Aug
Sep
Oct
Nov
Dec
Jan
Feb
Mar
Apr
May
Jun
Winter Solstice
December 21/Day 355
Winter Teletherm
January 1/Day 1
+11 days
Coverage
Year
COLFAX, CA
Day of Year

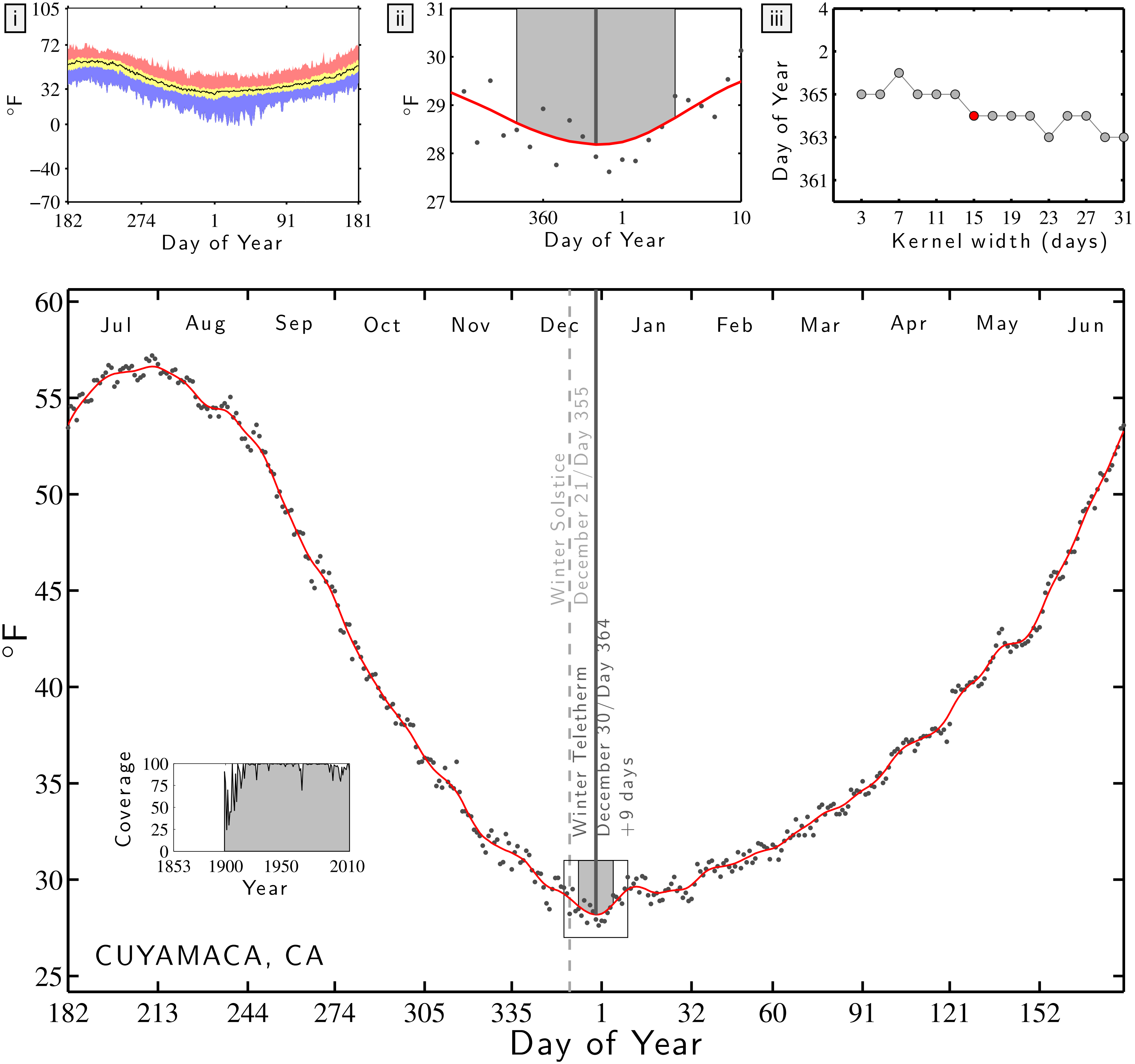
i
ii
iii
°F
Day of Year
Day of Year
Kernel width (days)
Jul
Aug
Sep
Oct
Nov
Dec
Jan
Feb
Mar
Apr
May
Jun
Winter Solstice
December 21/Day 355
Winter Teletherm
December 30/Day 364
+9 days
Coverage
Year
CUYAMACA, CA
Day of Year

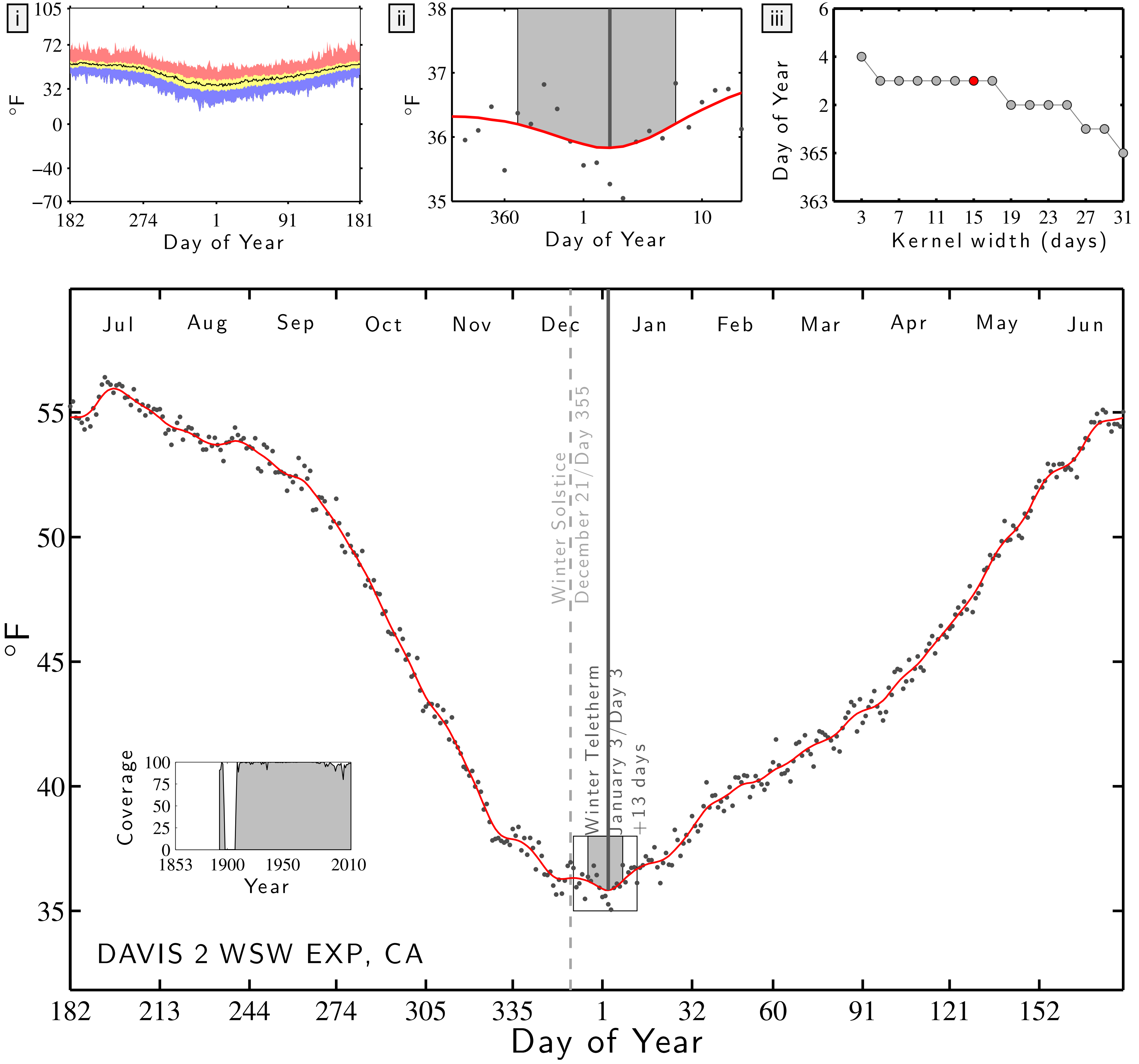

i
ii
iii
°F
Day of Year
Day of Year
Kernel width (days)
Jul
Aug
Sep
Oct
Nov
Dec
Jan
Feb
Mar
Apr
May
Jun
Winter Solstice
December 21/Day 355
Winter Teletherm
January 3/Day 3
+13 days
Coverage
Year
DAVIS 2 WSW EXP, CA
Day of Year

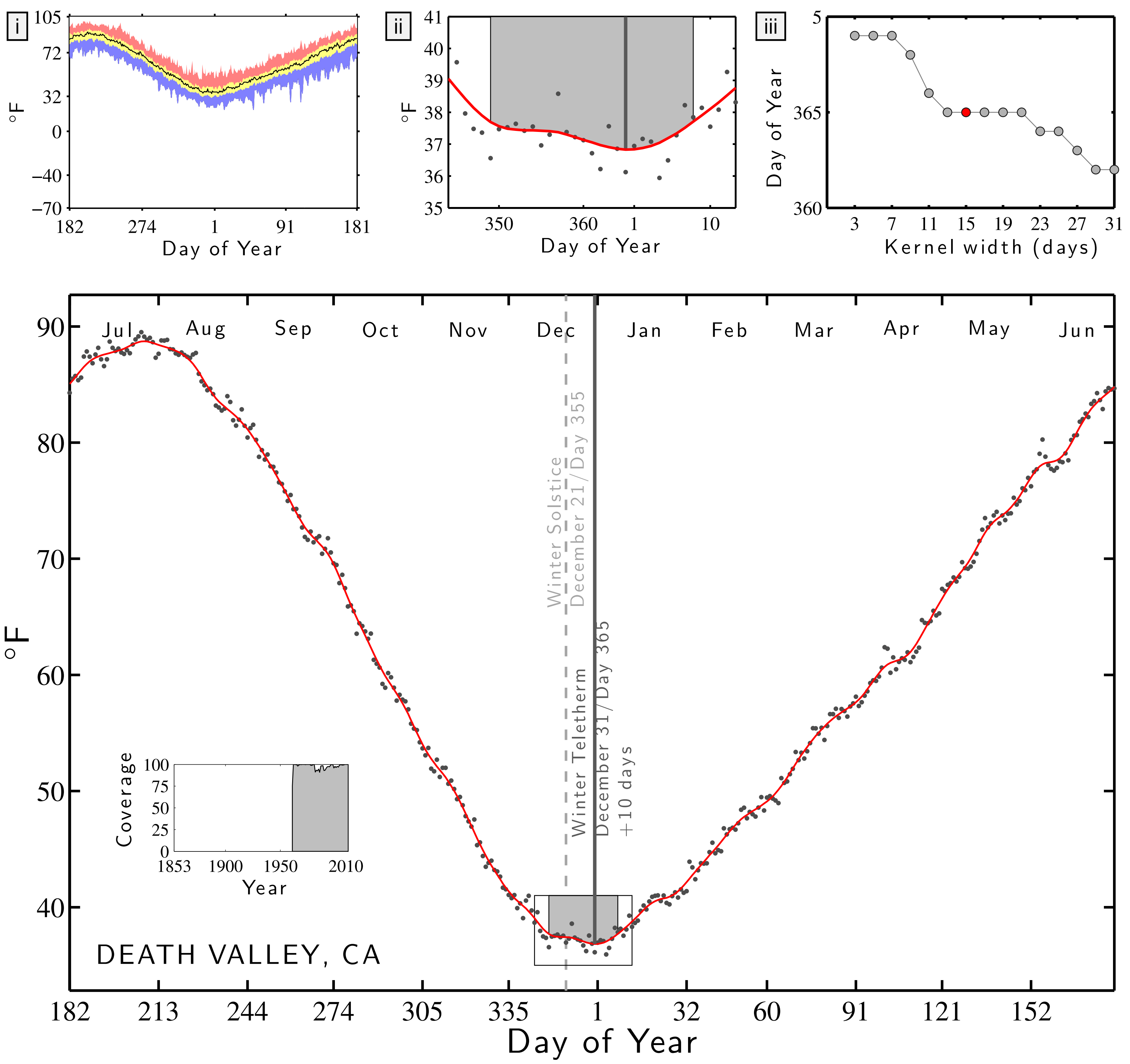

i
105
72
32
0
−40
−70
°F
182
274
1
91
181
Day of Year
ii
41
40
39
38
37
36
35
°F
350
360
1
10
Day of Year
iii
5
365
360
Day of Year
3
7
11
15
19
23
27
31
Kernel width (days)
90
80
70
60
50
40
°F
Jul
Aug
Sep
Oct
Nov
Dec
Jan
Feb
Mar
Apr
May
Jun
Winter Solstice
December 21/Day 355
Winter Teletherm
December 31/Day 365
+10 days
Coverage
100
75
50
25
0
1853
1900
1950
2010
Year
DEATH VALLEY, CA
182
213
244
274
305
335
1
32
60
91
121
152
Day of Year

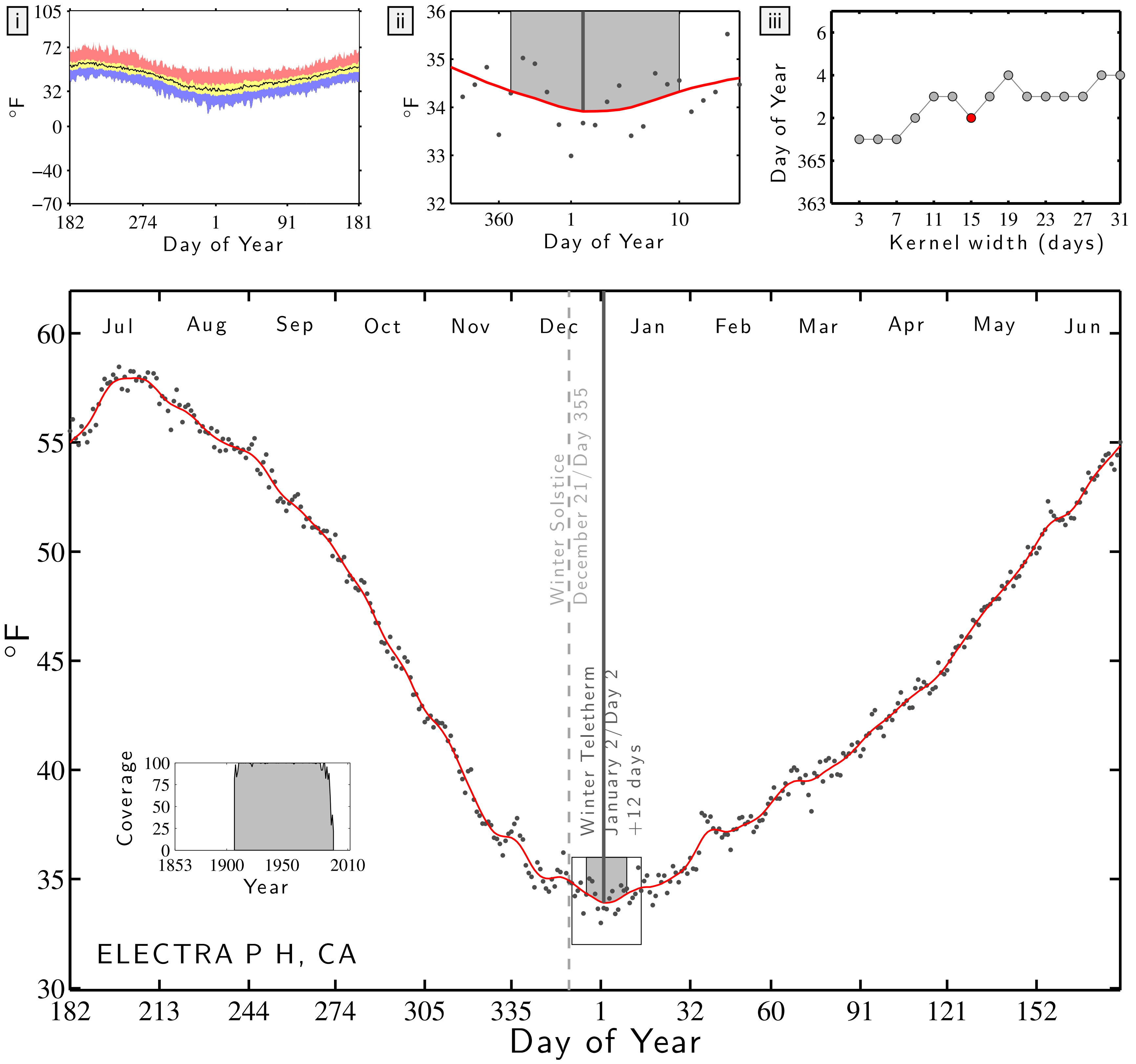

i
ii
iii
°F
Day of Year
Kernel width (days)
Jul
Aug
Sep
Oct
Nov
Dec
Jan
Feb
Mar
Apr
May
Jun
Winter Solstice
December 21/Day 355
Winter Teletherm
January 2/Day 2
+12 days
Coverage
Year
ELECTRA P H, CA

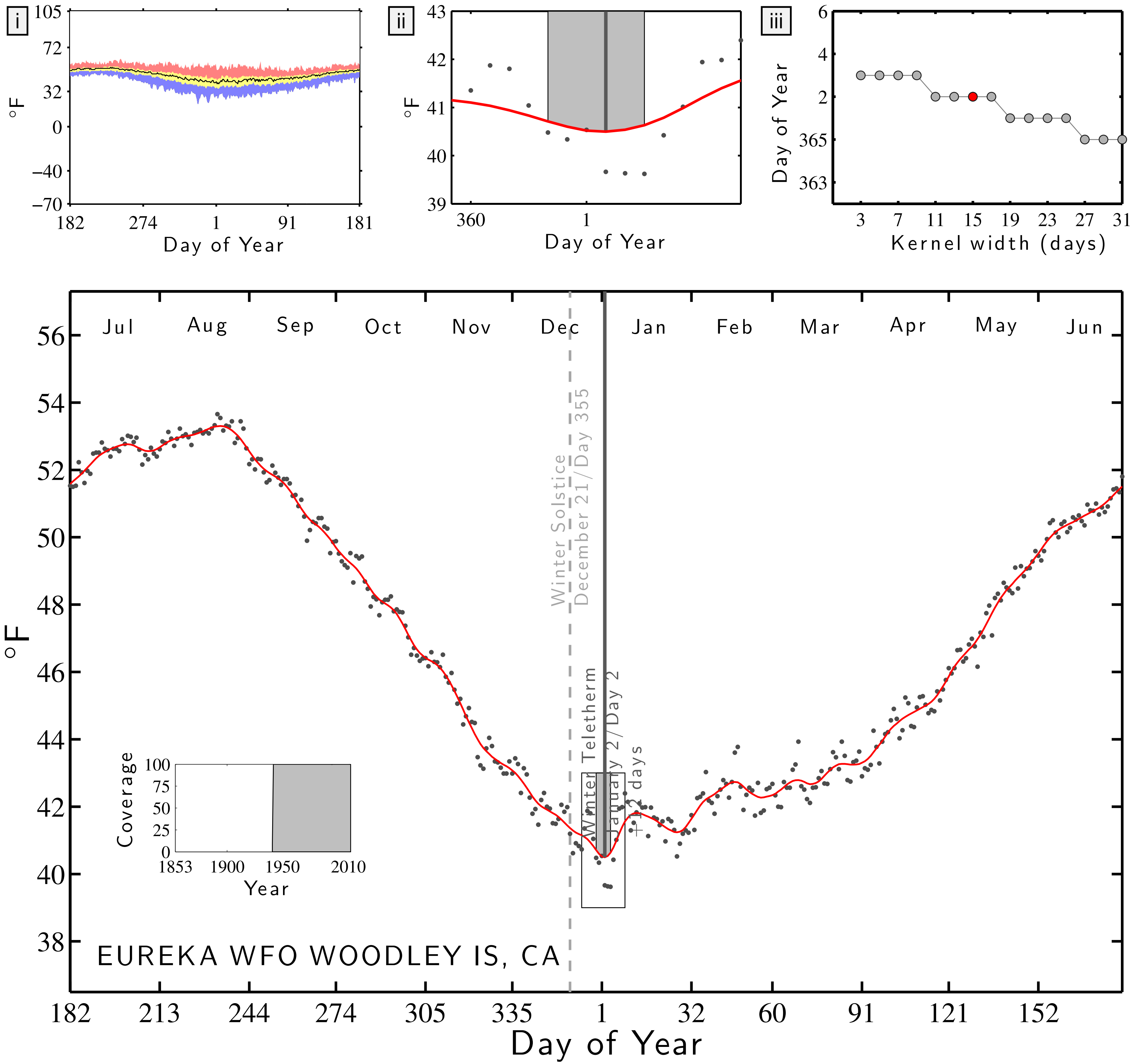

i
°F
Day of Year
ii
°F
Day of Year
iii
Day of Year
Kernel width (days)
Jul
Aug
Sep
Oct
Nov
Dec
Jan
Feb
Mar
Apr
May
Jun
Winter Solstice
December 21/Day 355
Winter Teletherm
January 2/Day 2
±12 days
Coverage
Year
EUREKA WFO WOODLEY IS, CA
°F
Day of Year

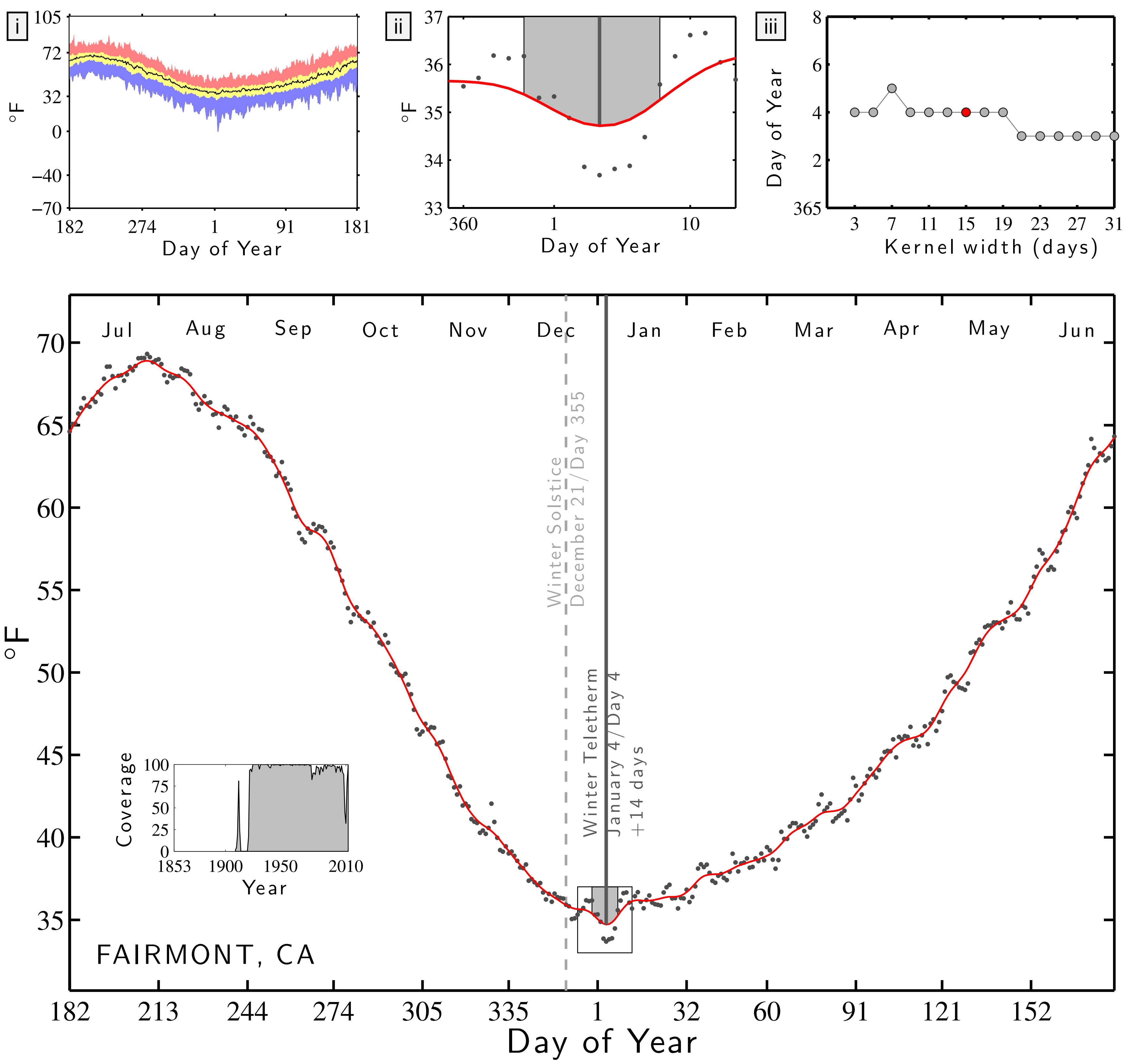

i
ii
iii
Day of Year
Kernel width (days)
°F
Jul
Aug
Sep
Oct
Nov
Dec
Jan
Feb
Mar
Apr
May
Jun
Winter Solstice
December 21/Day 355
Winter Teletherm
January 4/Day 4
+14 days
Coverage
Year
FAIRMONT, CA

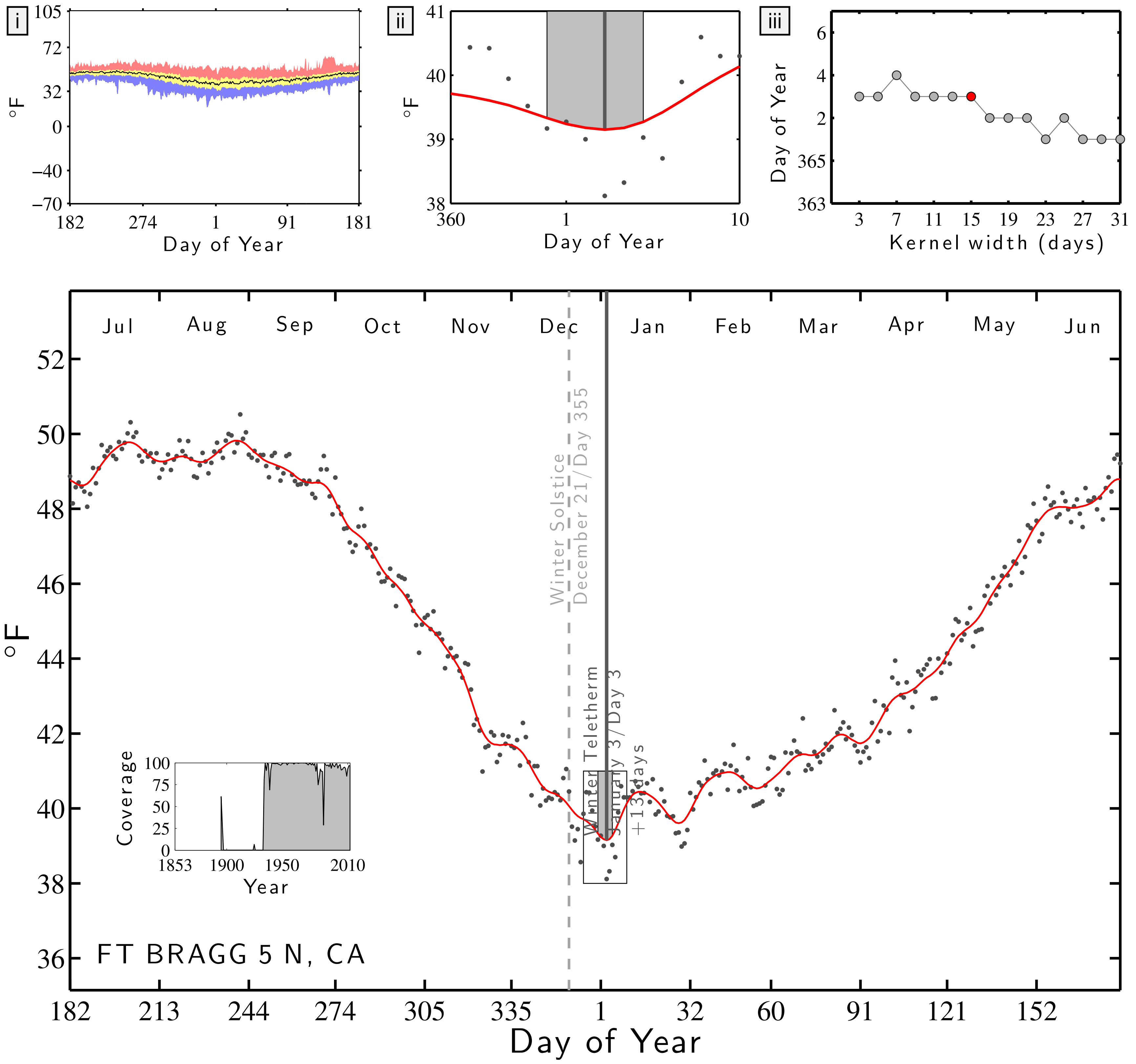

i
ii
iii
Day of Year
Kernel width (days)
Winter Solstice
December 21/Day 355
Winter Teletherm
January 3/Day 3
+13 days
Jul
Aug
Sep
Oct
Nov
Dec
Jan
Feb
Mar
Apr
May
Jun
Coverage
Year
FT BRAGG 5 N, CA
°F

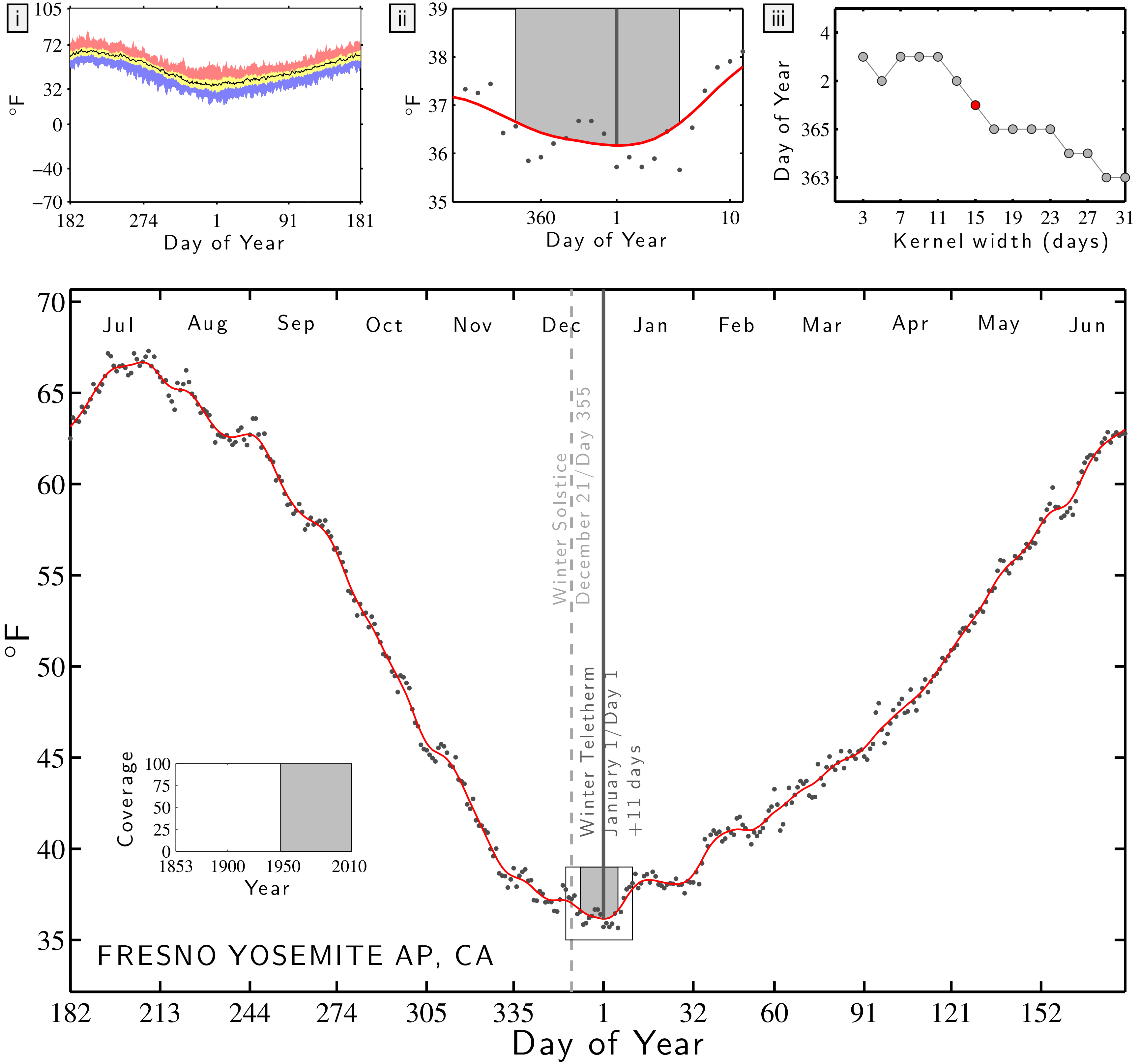

i
°F
105
72
32
0
−40
−70
182
274
1
91
181
Day of Year
ii
39
38
37
36
35
360
1
10
Day of Year
iii
Day of Year
4
2
365
363
3
7
11
15
19
23
27
31
Kernel width (days)
Jul
Aug
Sep
Oct
Nov
Dec
Jan
Feb
Mar
Apr
May
Jun
Winter Solstice
December 21/Day 355
Winter Teletherm
January 1/Day 1
+11 days
Coverage
100
75
50
25
0
1853
1900
1950
2010
Year
FRESNO YOSEMITE AP, CA
70
65
60
55
50
45
40
35
182
213
244
274
305
335
1
32
60
91
121
152
Day of Year

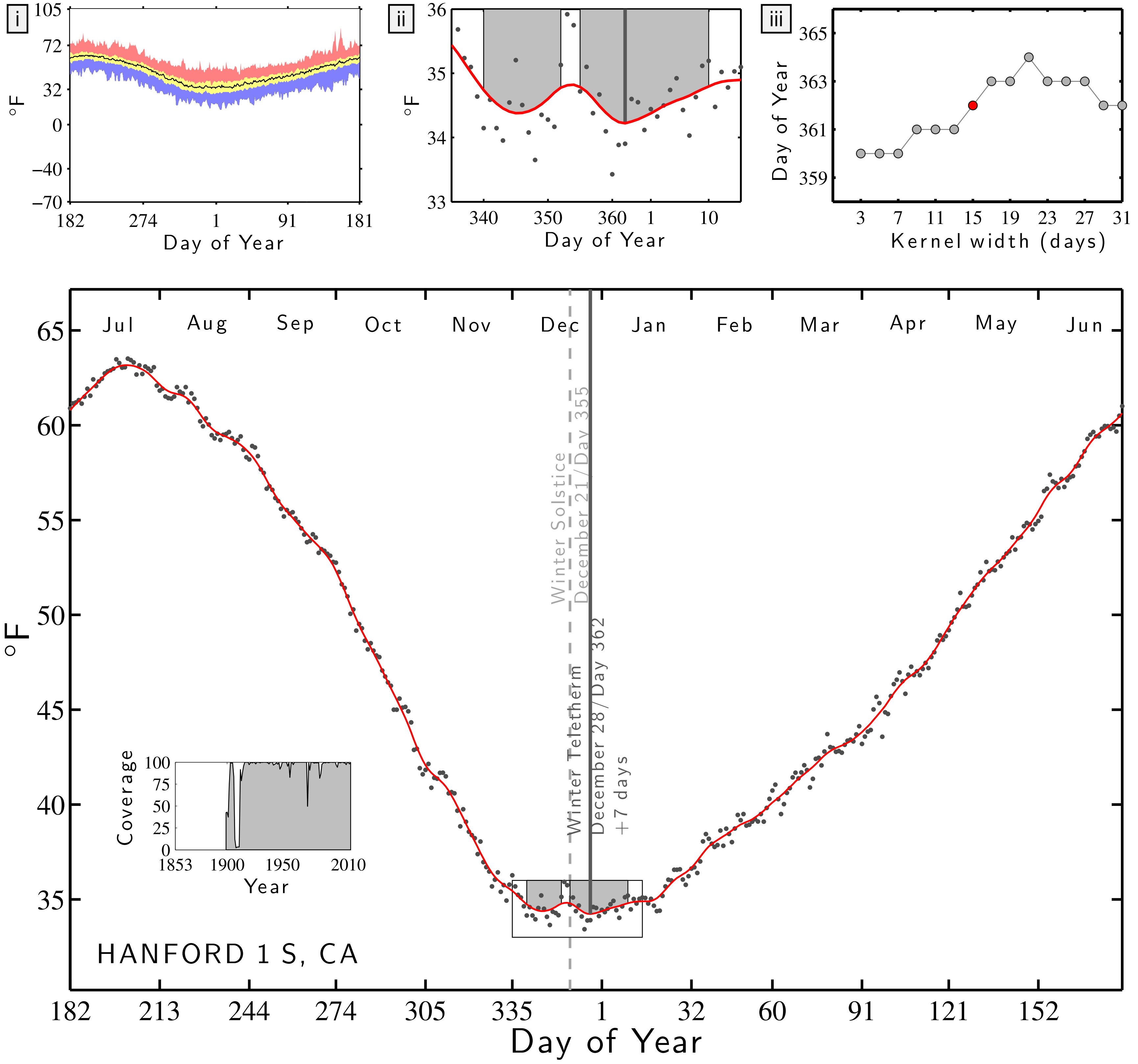

i
ii
iii
Day of Year
Kernel width (days)
°F
Jul
Aug
Sep
Oct
Nov
Dec
Jan
Feb
Mar
Apr
May
Jun
Winter Solstice
December 21/Day 355
Winter Teletherm
December 28/Day 362
+7 days
Coverage
Year
HANFORD 1 S, CA

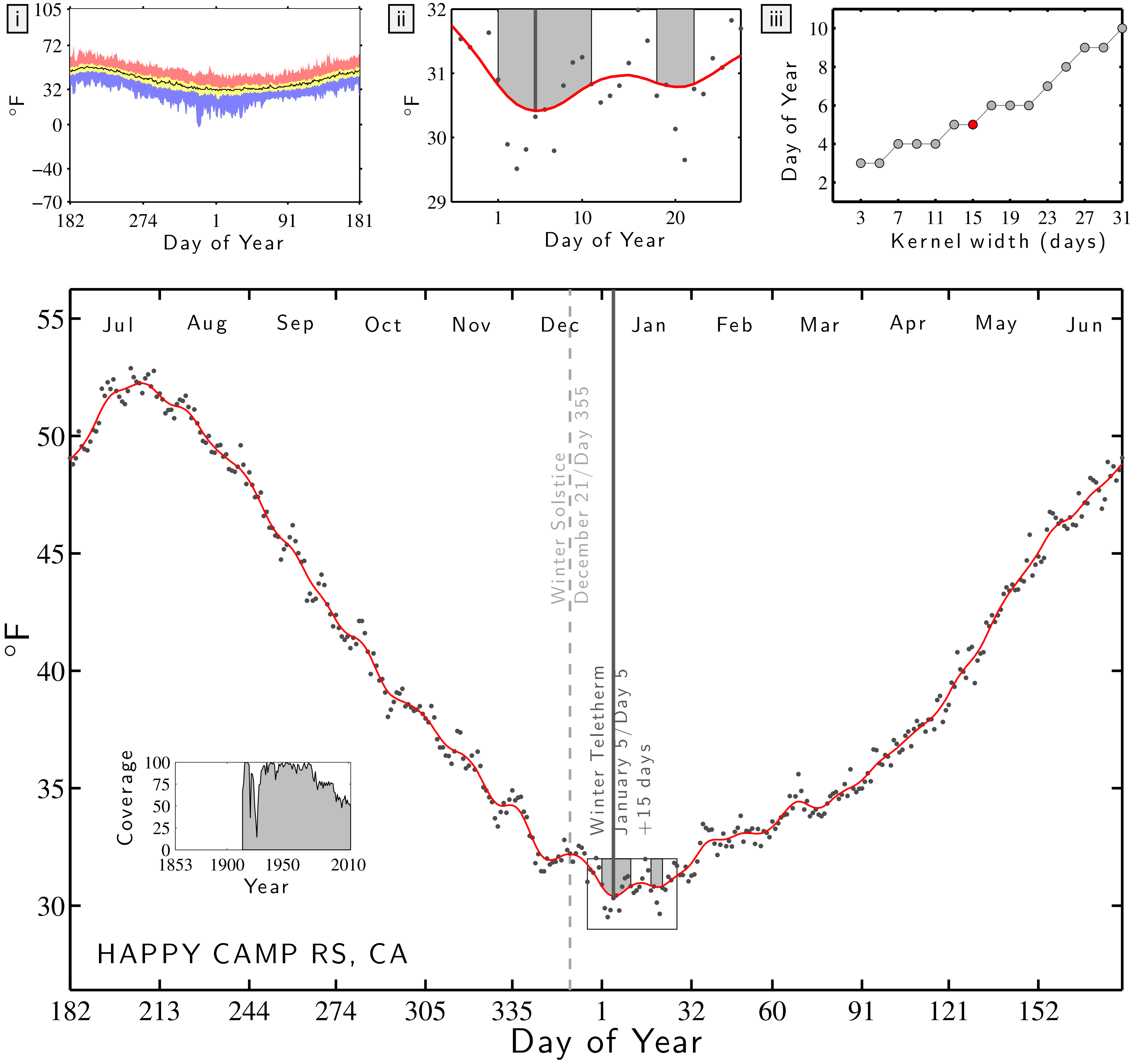
i
ii
iii
°F
Day of Year
Kernel width (days)
Jul
Aug
Sep
Oct
Nov
Dec
Jan
Feb
Mar
Apr
May
Jun
Winter Solstice
December 21/Day 355
Winter Teletherm
January 5/Day 5
+15 days
Coverage
Year
HAPPY CAMP RS, CA

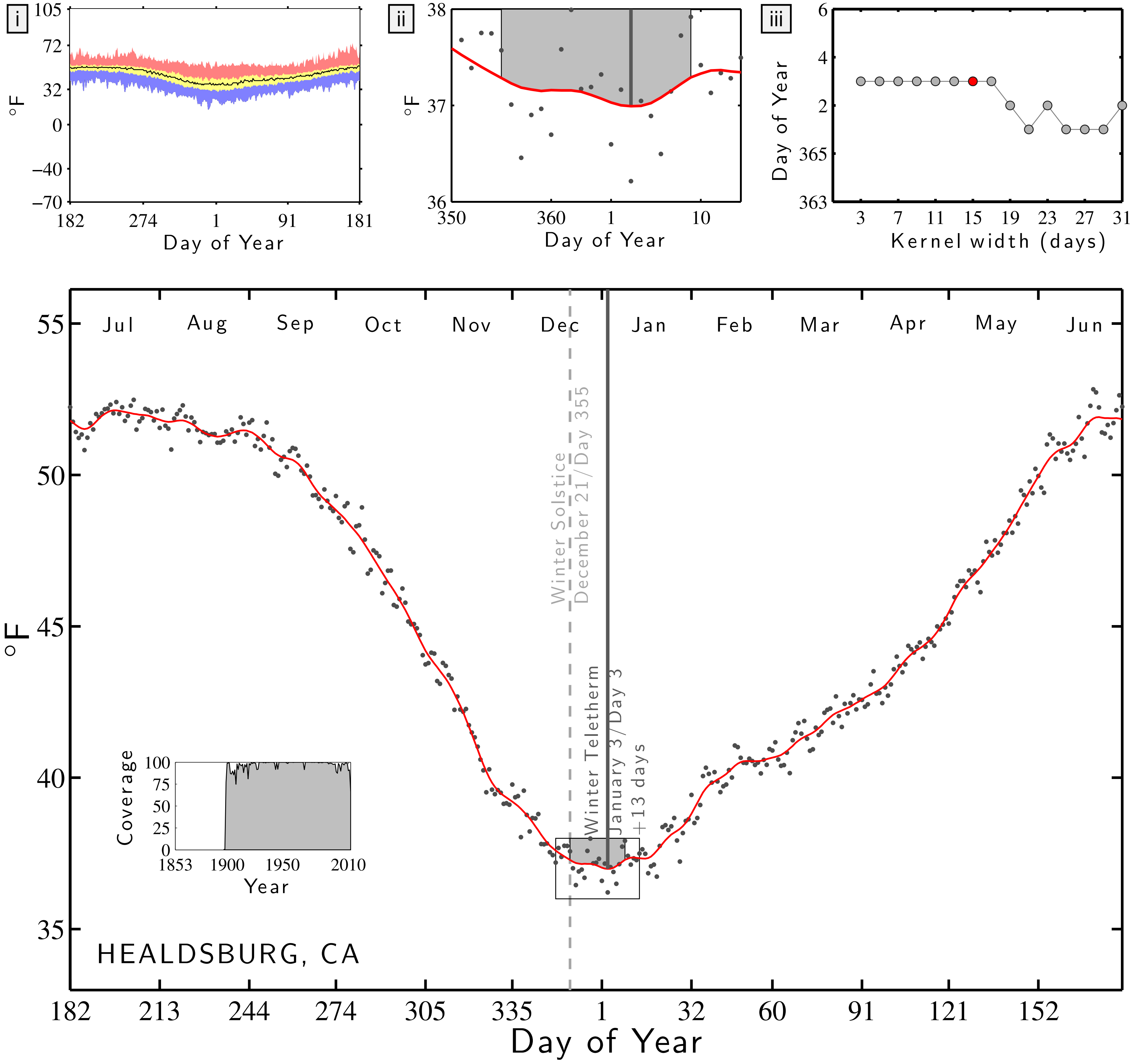

i
ii
iii
°F
Day of Year
Kernel width (days)
Day of Year
Winter Solstice
December 21/Day 355
Winter Teletherm
January 3/Day 3
+13 days
Coverage
Year
HEALDSBURG, CA
Jul
Aug
Sep
Oct
Nov
Dec
Jan
Feb
Mar
Apr
May
Jun

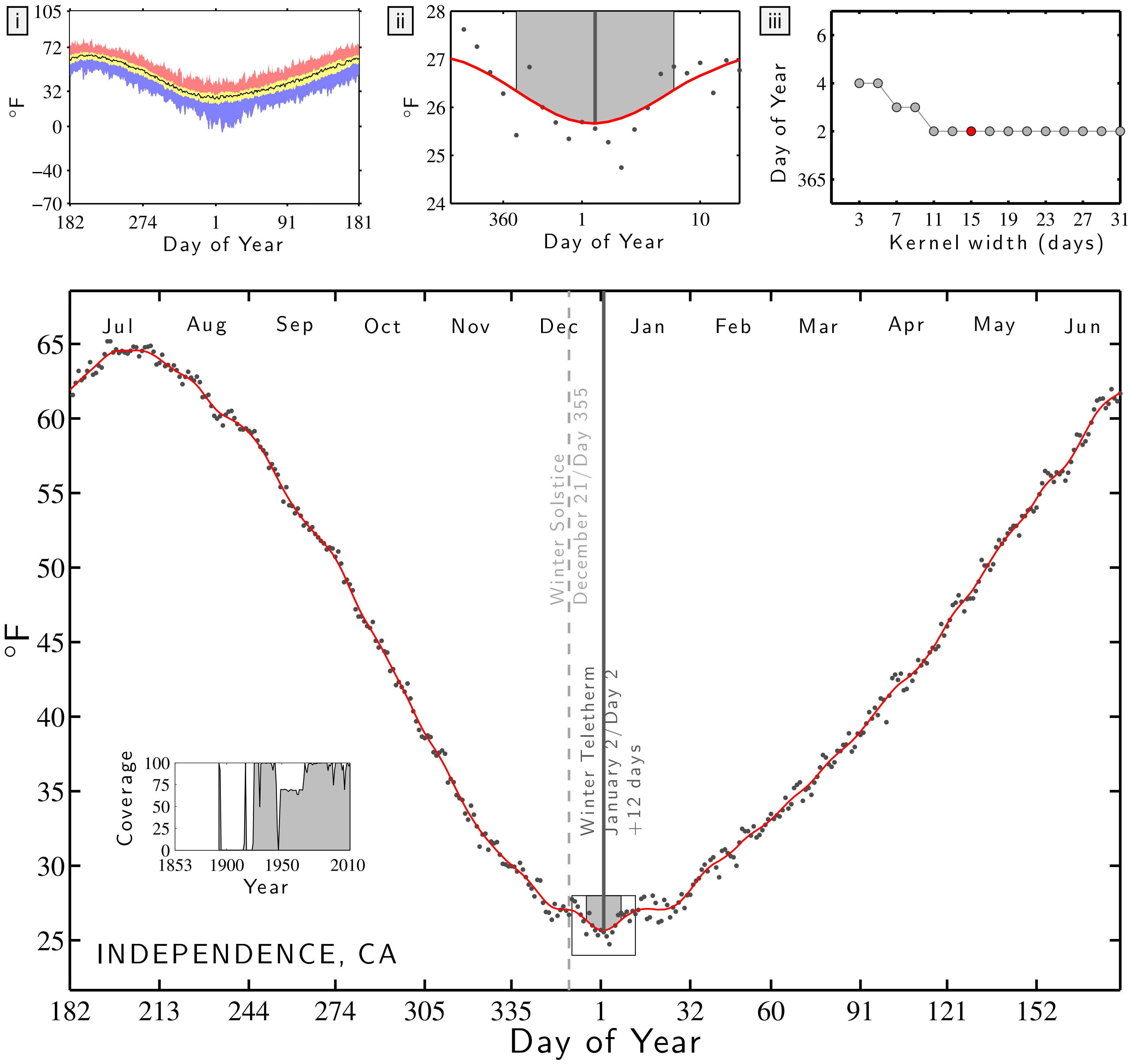
i
ii
iii
Day of Year
Kernel width (days)
Jul
Aug
Sep
Oct
Nov
Dec
Jan
Feb
Mar
Apr
May
Jun
Winter Solstice
December 21/Day 355
Winter Teletherm
January 2/Day 2
+12 days
Coverage
Year
INDEPENDENCE, CA
°F

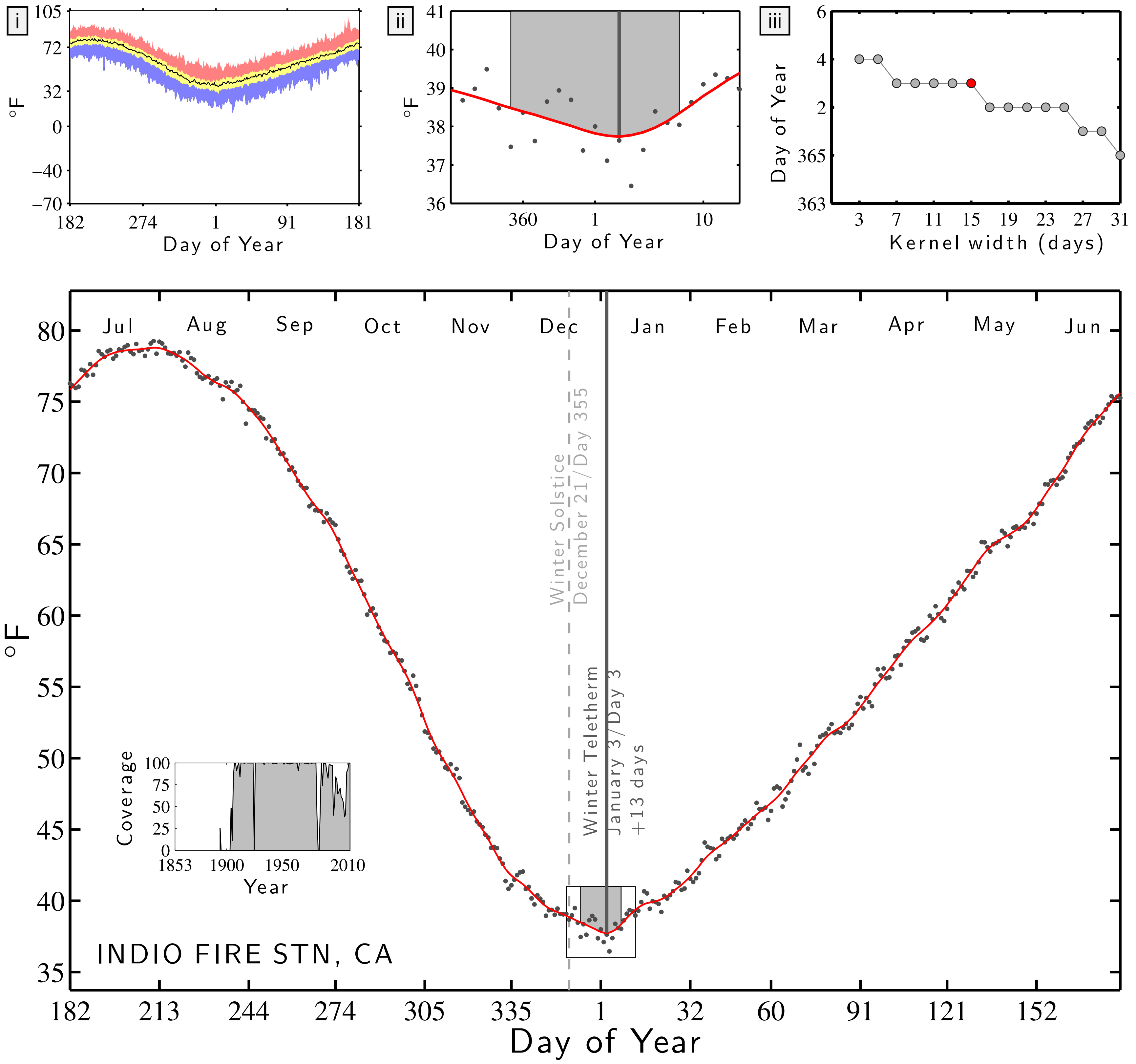

i
°F
Day of Year
ii
°F
Day of Year
iii
Day of Year
Kernel width (days)
Jul
Aug
Sep
Oct
Nov
Dec
Jan
Feb
Mar
Apr
May
Jun
Winter Solstice
December 21/Day 355
Winter Teletherm
January 3/Day 3
+13 days
Coverage
Year
INDIO FIRE STN, CA
°F
Day of Year

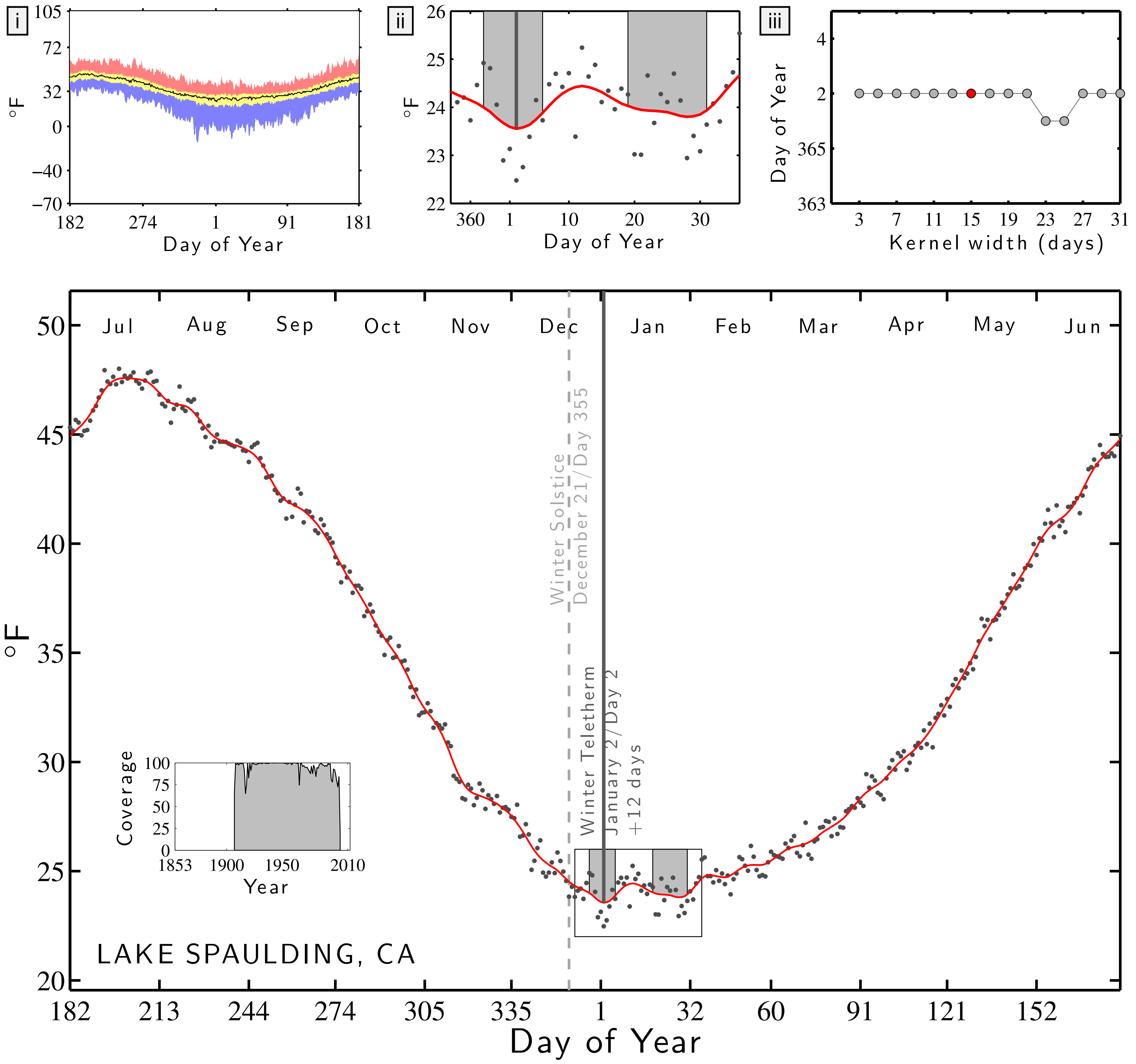

i
ii
iii
Day of Year
°F
Kernel width (days)
Winter Solstice
December 21/Day 355
Winter Teletherm
January 2/Day 2
+12 days
Coverage
Year
LAKE SPAULDING, CA
Jul
Aug
Sep
Oct
Nov
Dec
Jan
Feb
Mar
Apr
May
Jun

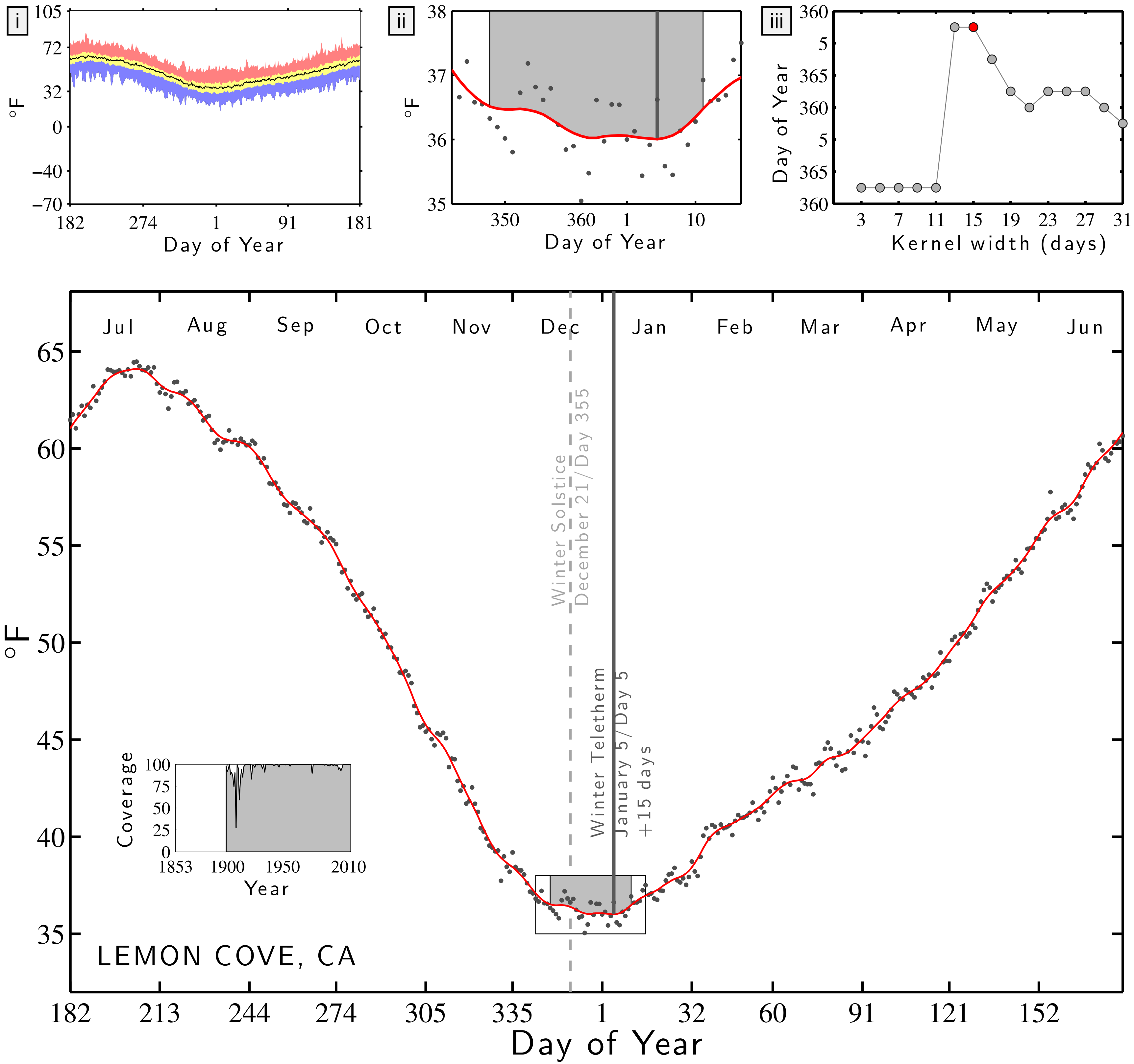

i
°F
Day of Year
ii
°F
Day of Year
iii
Day of Year
Kernel width (days)
Jul
Aug
Sep
Oct
Nov
Dec
Jan
Feb
Mar
Apr
May
Jun
Winter Solstice
December 21/Day 355
Winter Teletherm
January 5/Day 5
+15 days
Coverage
Year
LEMON COVE, CA
°F
Day of Year

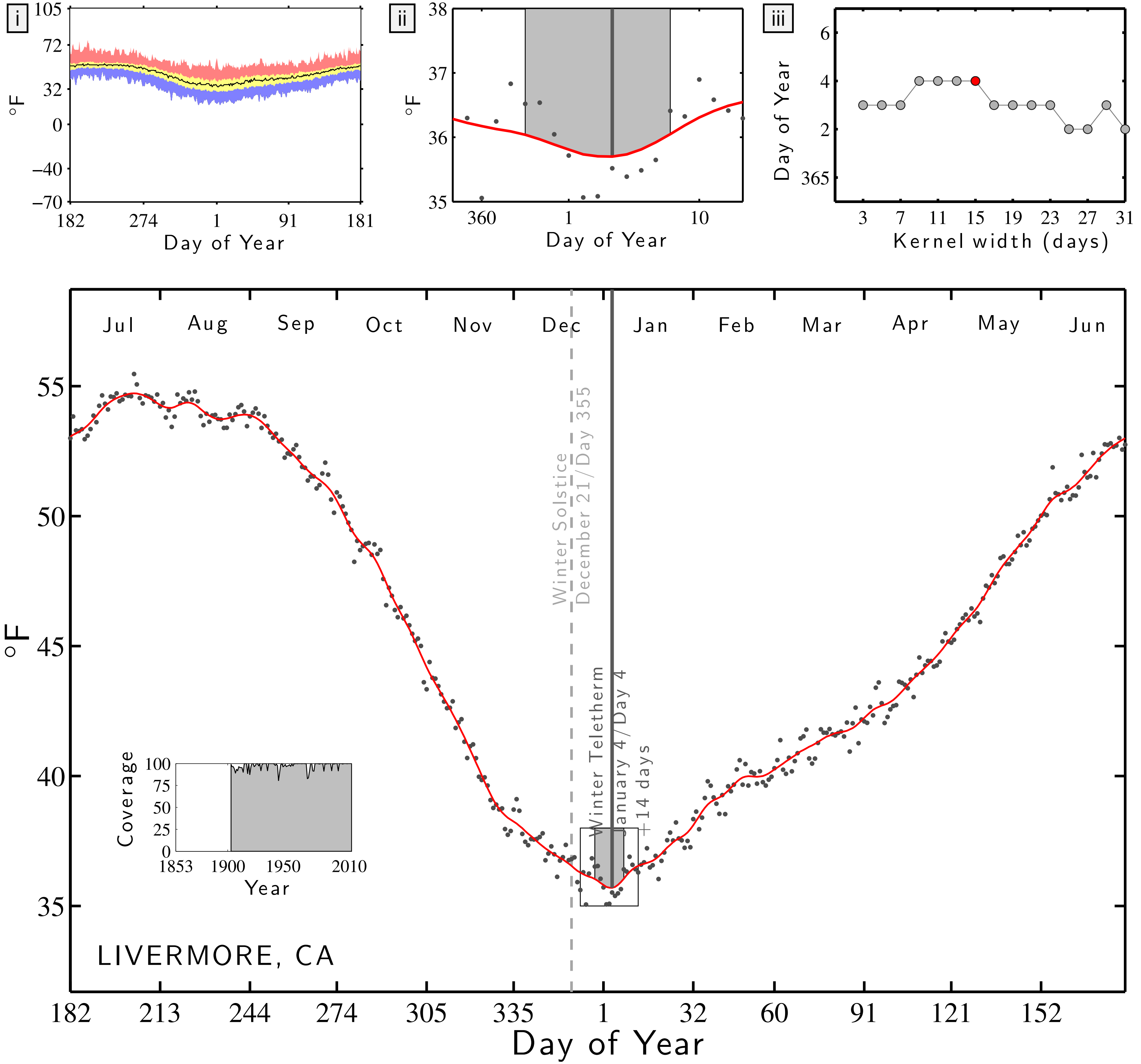
i
°F
Day of Year
ii
°F
Day of Year
iii
Day of Year
Kernel width (days)
Jul
Aug
Sep
Oct
Nov
Dec
Jan
Feb
Mar
Apr
May
Jun
Winter Solstice
December 21/Day 355
Winter Teletherm
January 4/Day 4
+14 days
Coverage
Year
LIVERMORE, CA
°F
Day of Year

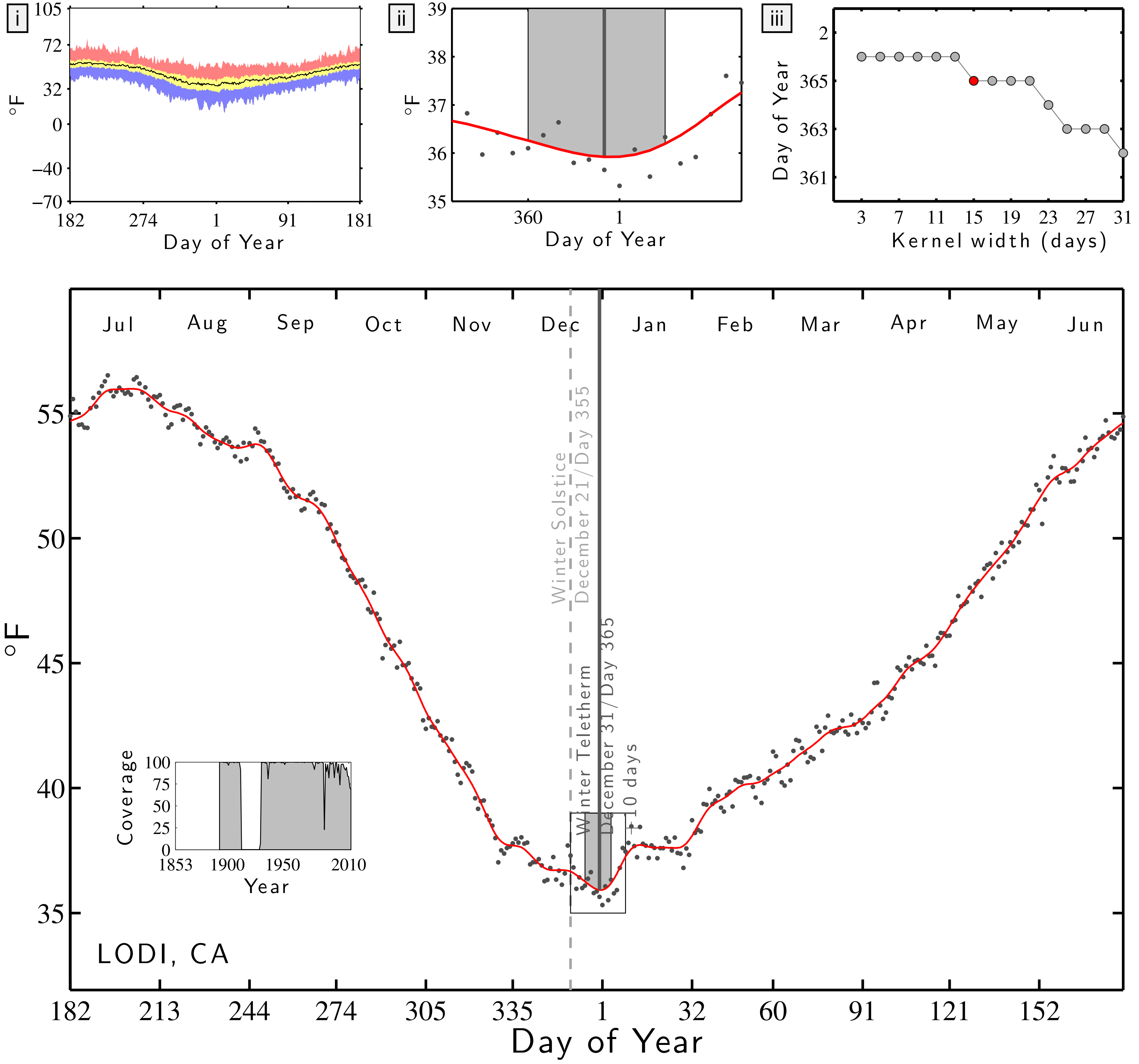

i
ii
iii
°F
Day of Year
Kernel width (days)
Jul
Aug
Sep
Oct
Nov
Dec
Jan
Feb
Mar
Apr
May
Jun
Winter Solstice
December 21/Day 355
Winter Teletherm
December 31/Day 365
±10 days
Coverage
Year
LODI, CA

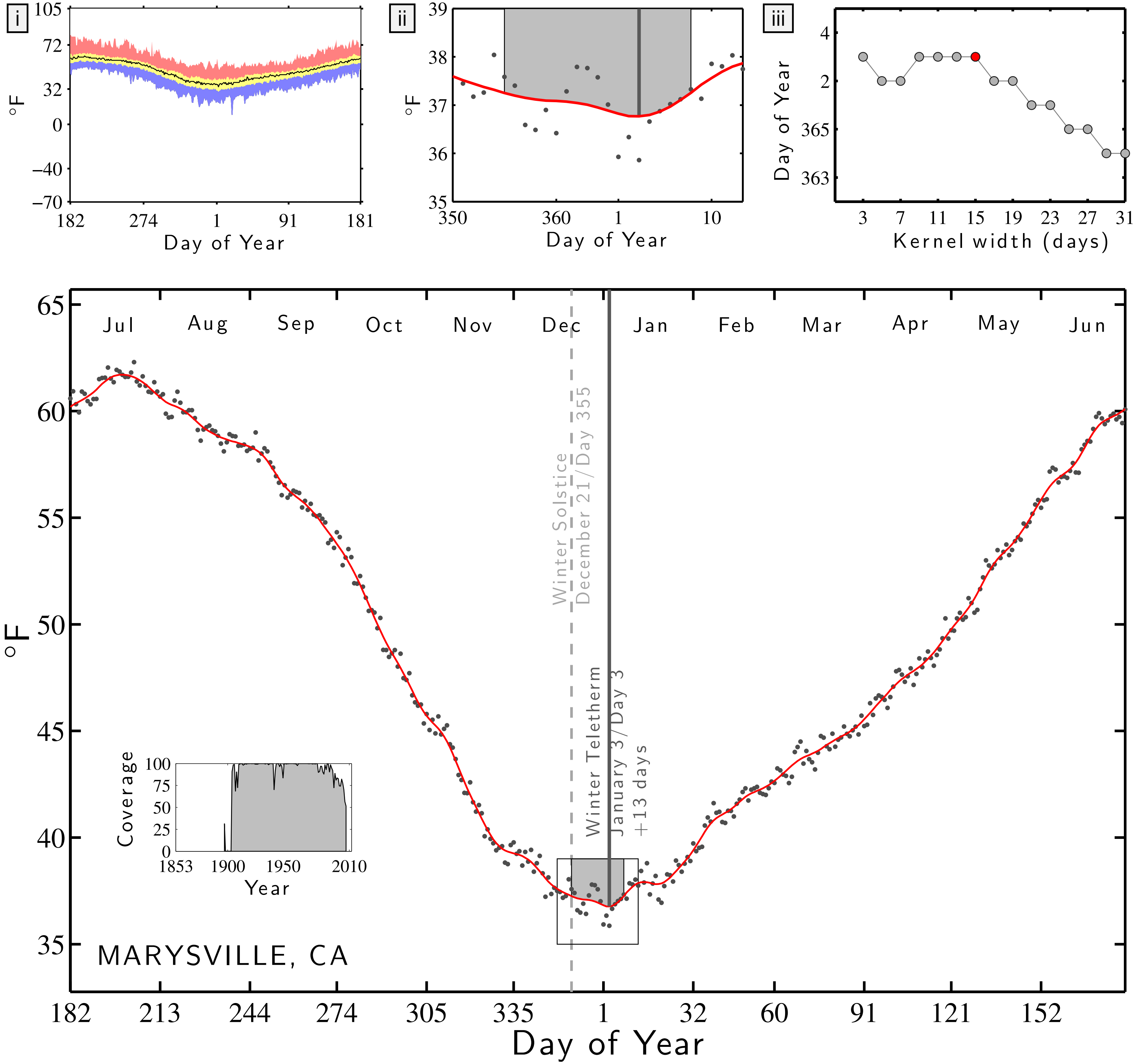

i
ii
iii
°F
Day of Year
Kernel width (days)
Jul
Aug
Sep
Oct
Nov
Dec
Jan
Feb
Mar
Apr
May
Jun
Winter Solstice
December 21/Day 355
Winter Teletherm
January 3/Day 3
+13 days
Coverage
Year
MARYSVILLE, CA

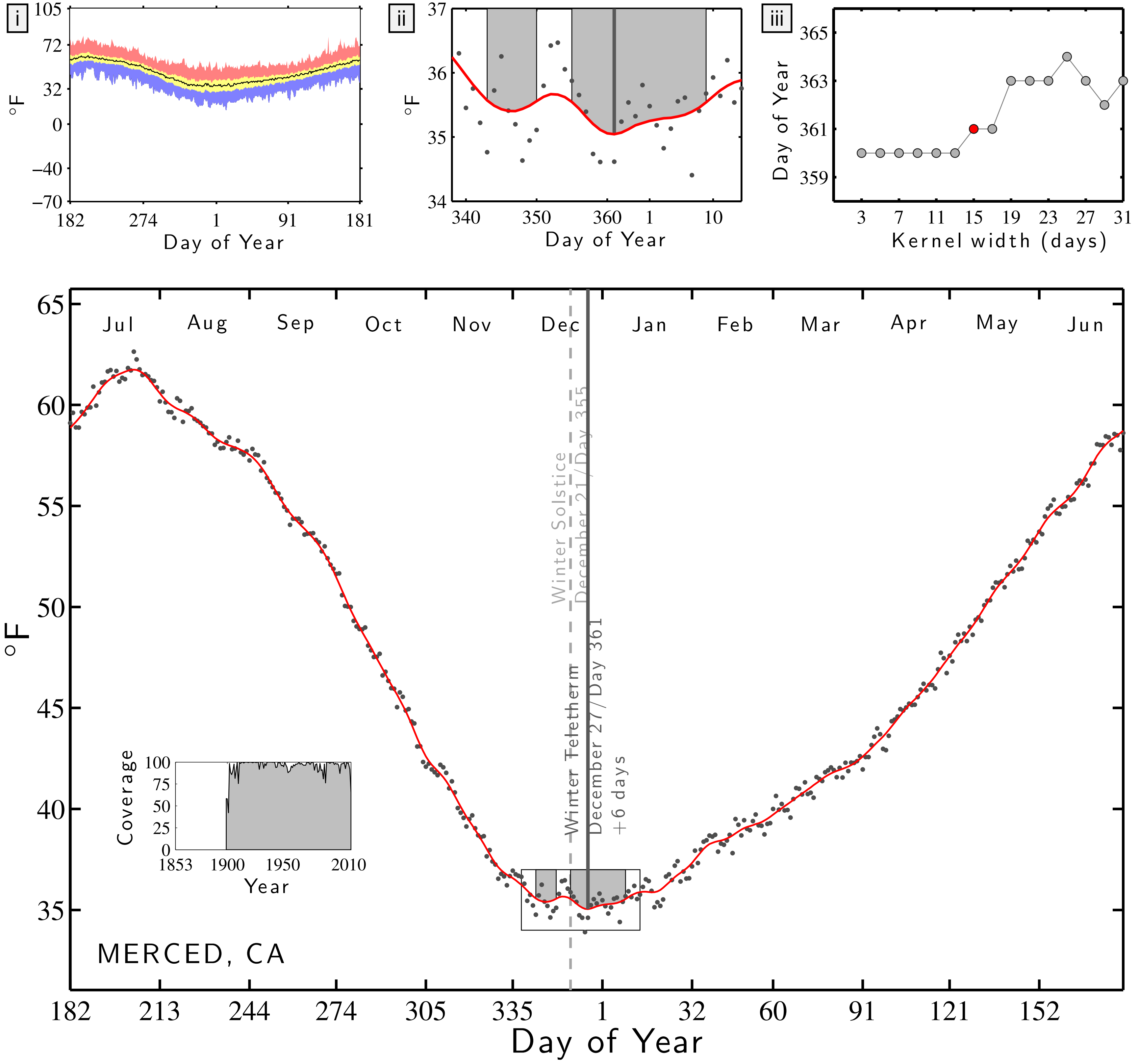

i
ii
iii
°F
Day of Year
Kernel width (days)
Jul
Aug
Sep
Oct
Nov
Dec
Jan
Feb
Mar
Apr
May
Jun
Winter Solstice
December 21/Day 355
Winter Teletherm
December 27/Day 361
+6 days
Coverage
Year
MERCED, CA

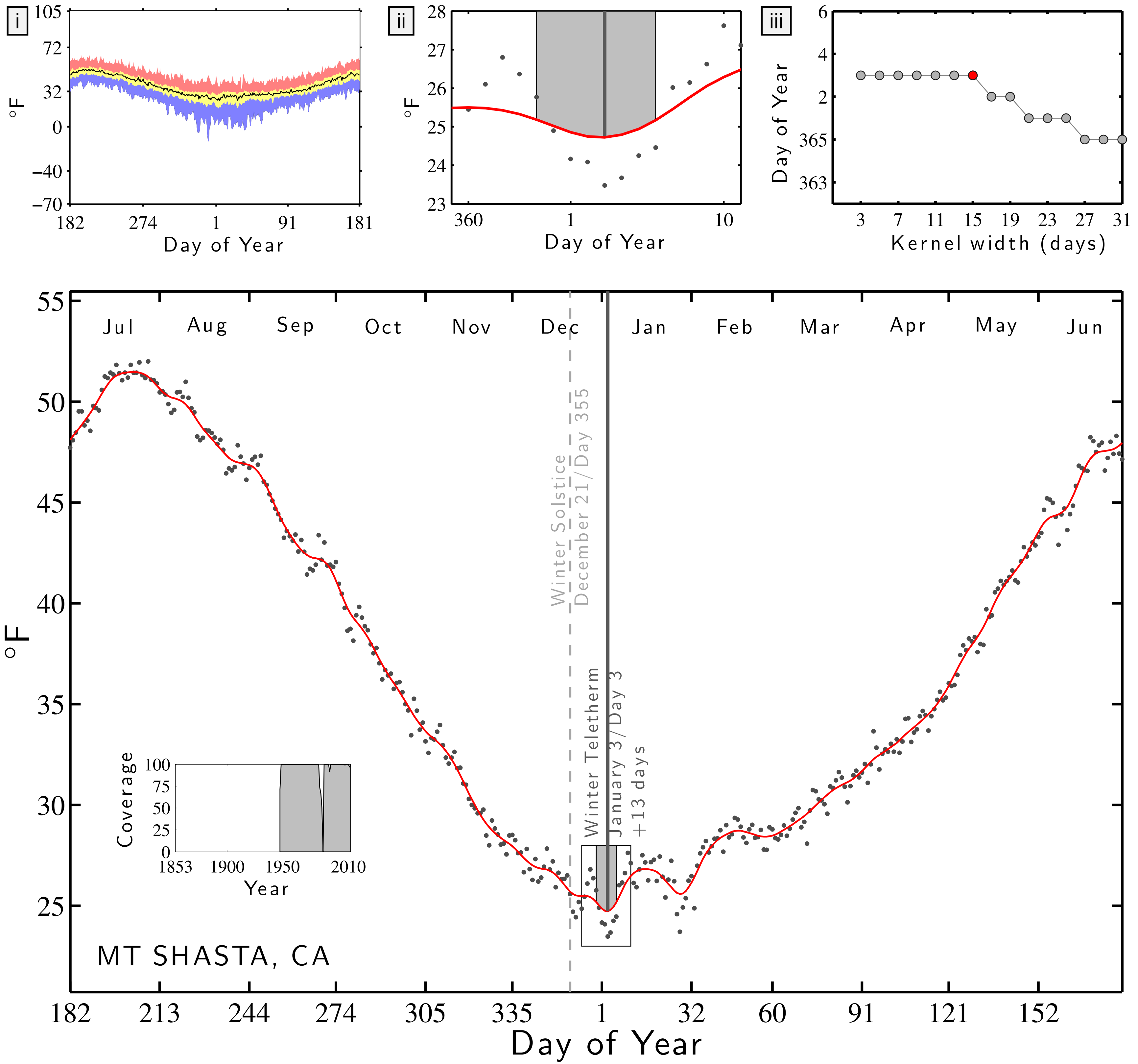

i
ii
iii
°F
Day of Year
Kernel width (days)
Jul
Aug
Sep
Oct
Nov
Dec
Jan
Feb
Mar
Apr
May
Jun
Winter Solstice
December 21/Day 355
Winter Teletherm
January 3/Day 3
+13 days
Coverage
Year
MT SHASTA, CA

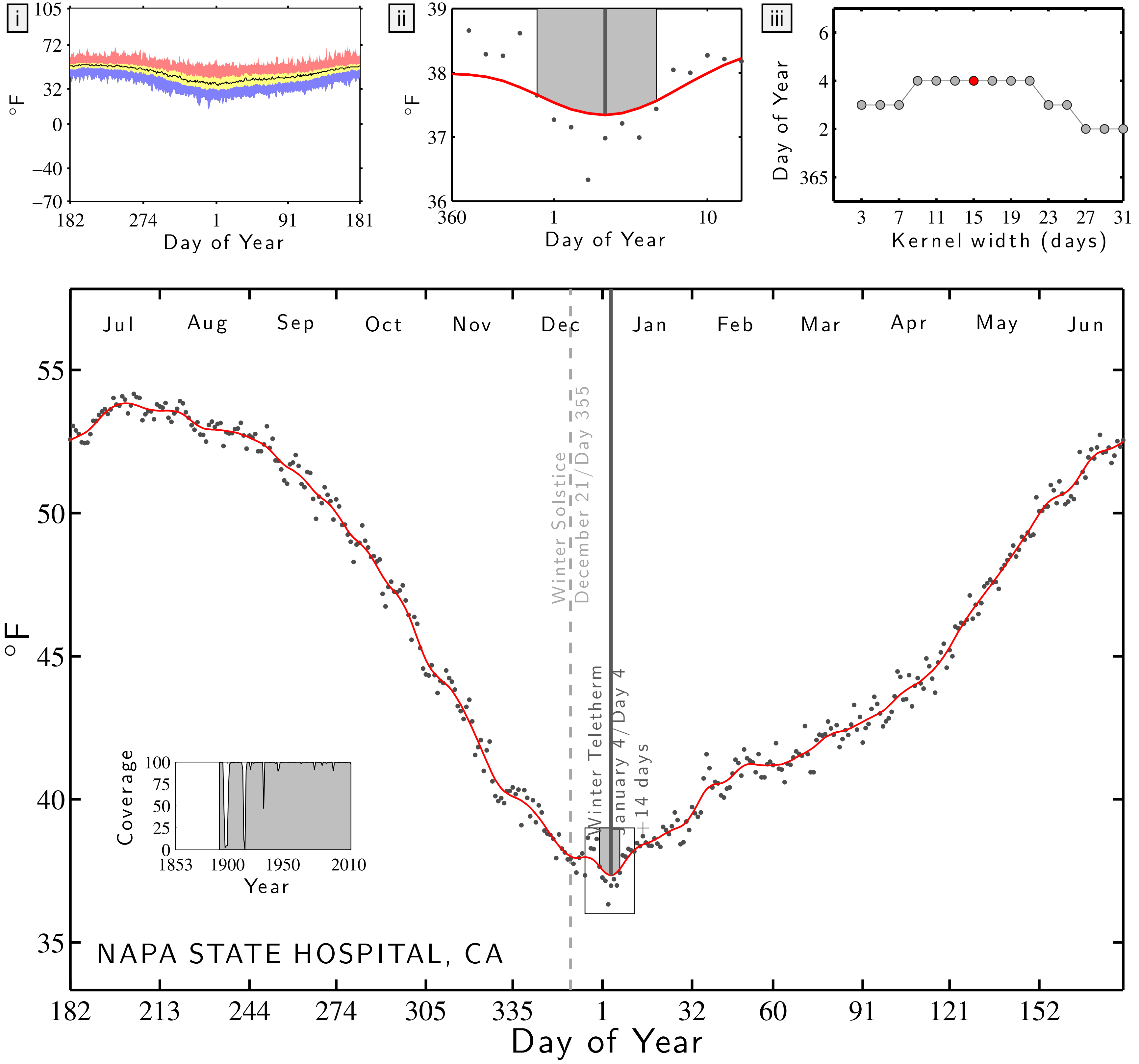

i
ii
iii
°F
Day of Year
Day of Year
Kernel width (days)
Jul
Aug
Sep
Oct
Nov
Dec
Jan
Feb
Mar
Apr
May
Jun
Winter Solstice
December 21/Day 355
Winter Teletherm
January 4/Day 4
±14 days
Coverage
Year
NAPA STATE HOSPITAL, CA
Day of Year

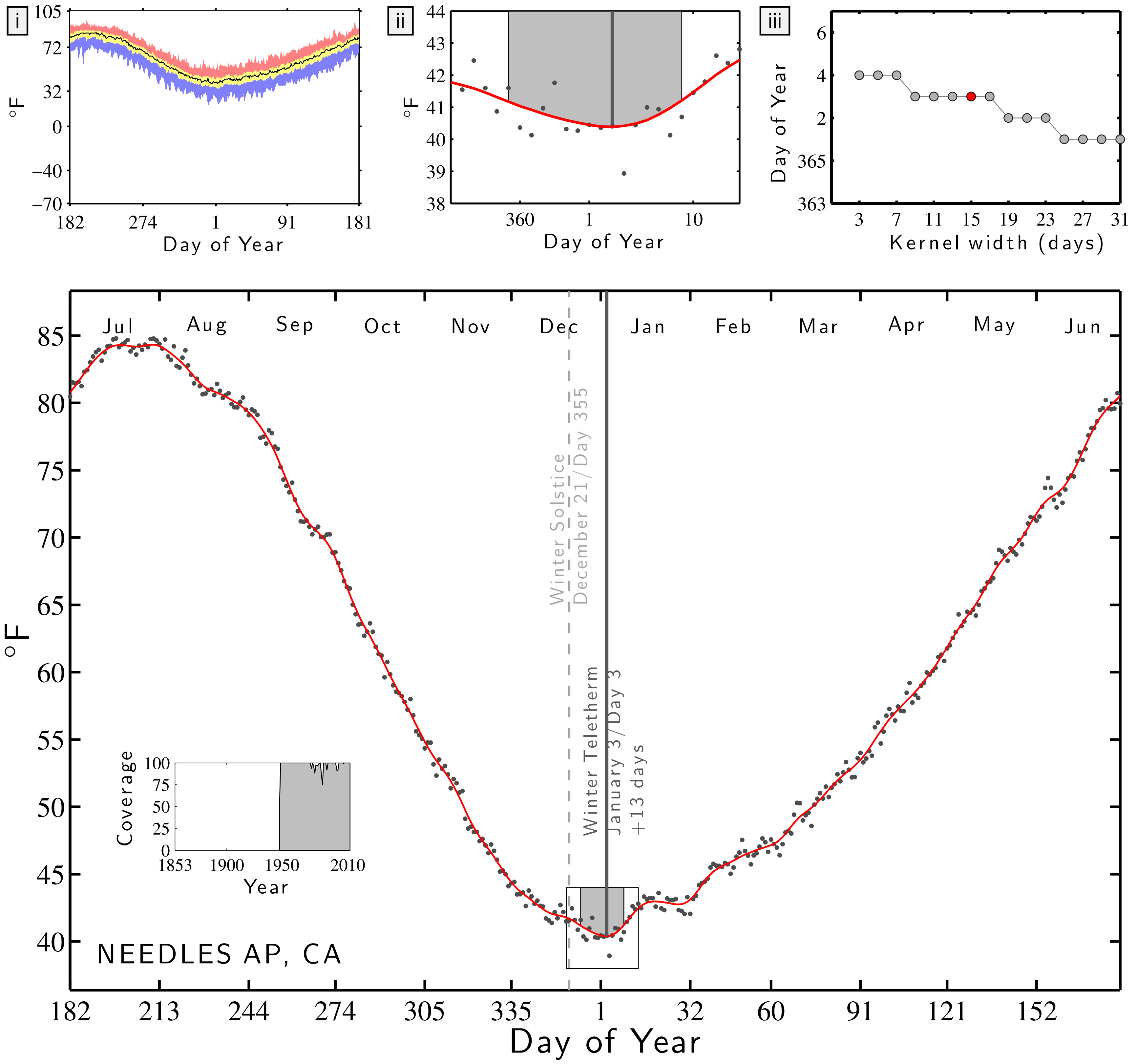

i
°F
Day of Year
ii
°F
Day of Year
iii
Day of Year
Kernel width (days)
Jul
Aug
Sep
Oct
Nov
Dec
Jan
Feb
Mar
Apr
May
Jun
Winter Solstice
December 21/Day 355
Winter Teletherm
January 3/Day 3
+13 days
Coverage
Year
NEEDLES AP, CA
°F
Day of Year

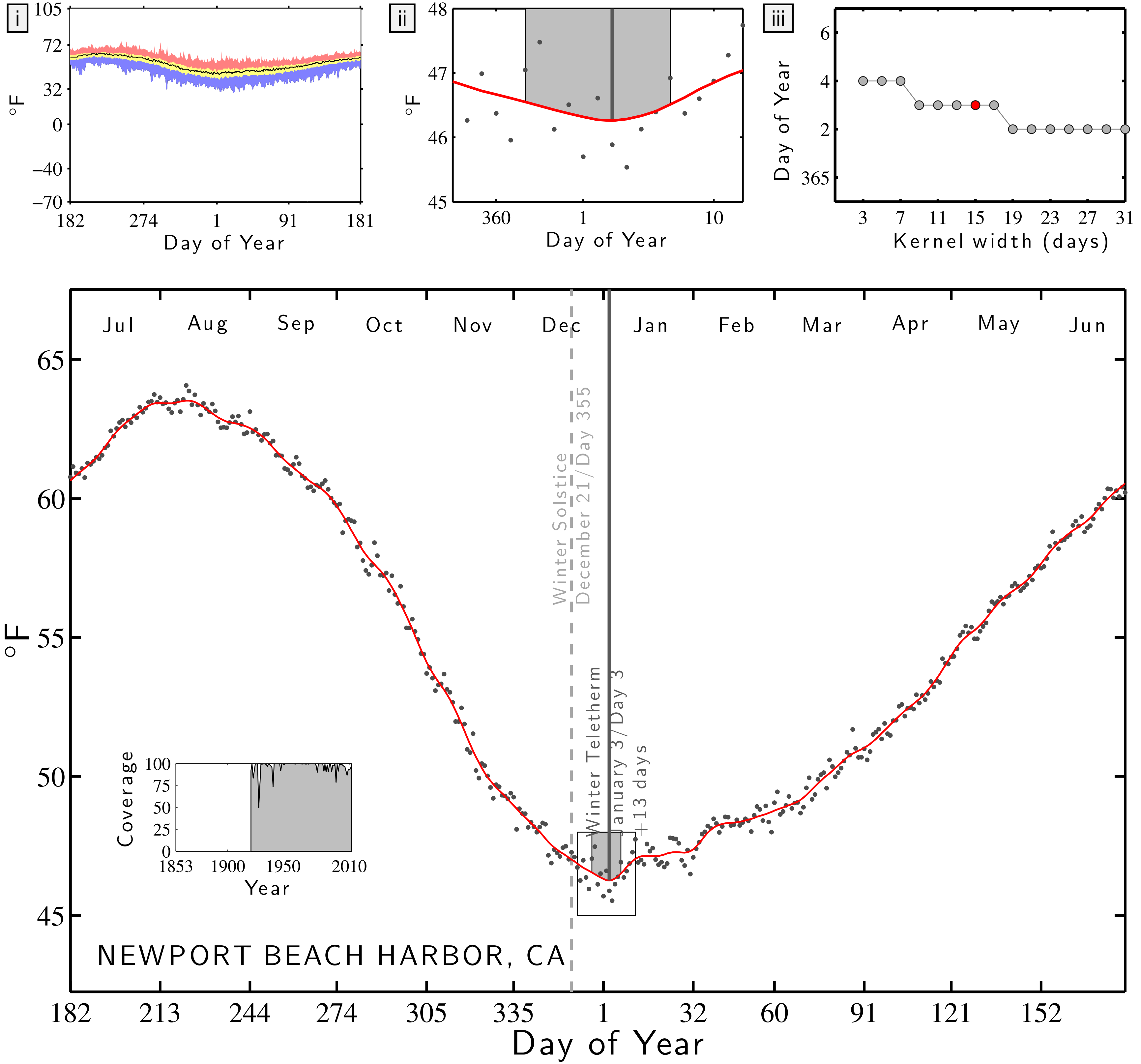

i
ii
iii
Day of Year
Kernel width (days)
°F
Jul
Aug
Sep
Oct
Nov
Dec
Jan
Feb
Mar
Apr
May
Jun
Winter Solstice
December 21/Day 355
Winter Teletherm
January 3/Day 3
±13 days
Coverage
Year
NEWPORT BEACH HARBOR, CA

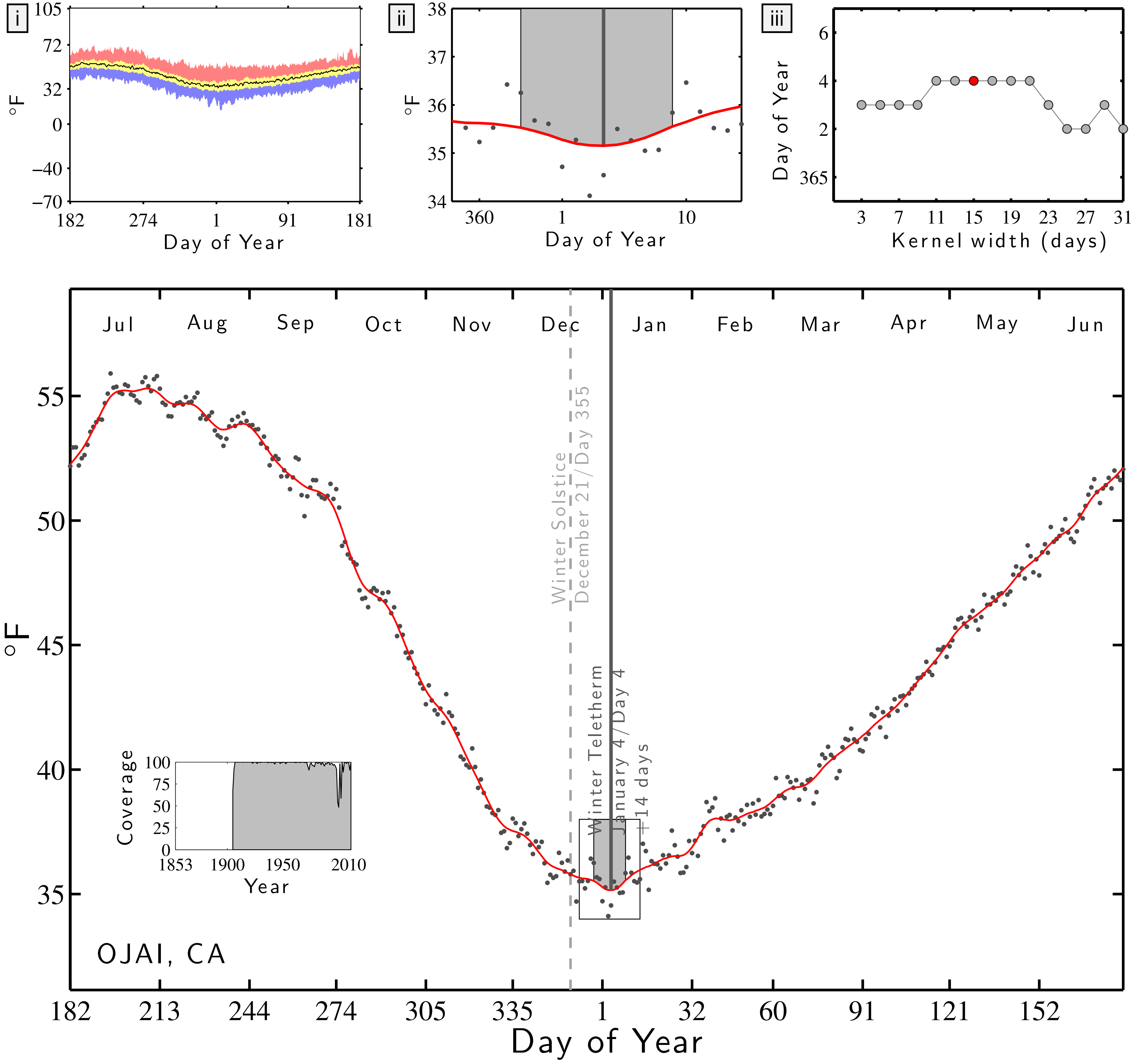

i
°F
Day of Year
ii
iii
Day of Year
Kernel width (days)
Jul
Aug
Sep
Oct
Nov
Dec
Jan
Feb
Mar
Apr
May
Jun
Winter Solstice
December 21/Day 355
Winter Teletherm
January 4/Day 4
±14 days
Coverage
Year
OJAI, CA

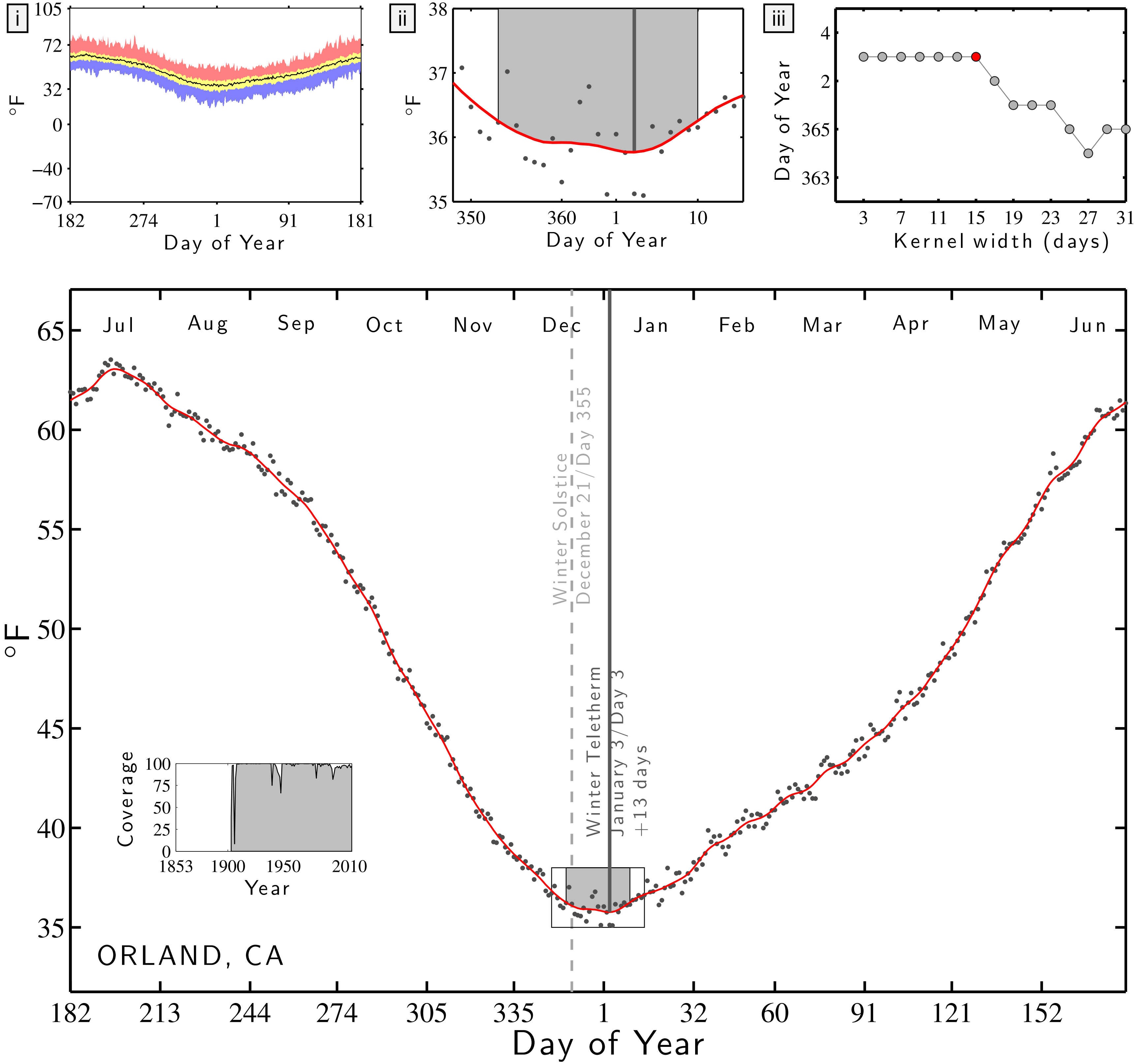
i
ii
iii
°F
Day of Year
Kernel width (days)
Jul
Aug
Sep
Oct
Nov
Dec
Jan
Feb
Mar
Apr
May
Jun
Winter Solstice
December 21/Day 355
Winter Teletherm
January 3/Day 3
+13 days
Coverage
Year
ORLAND, CA

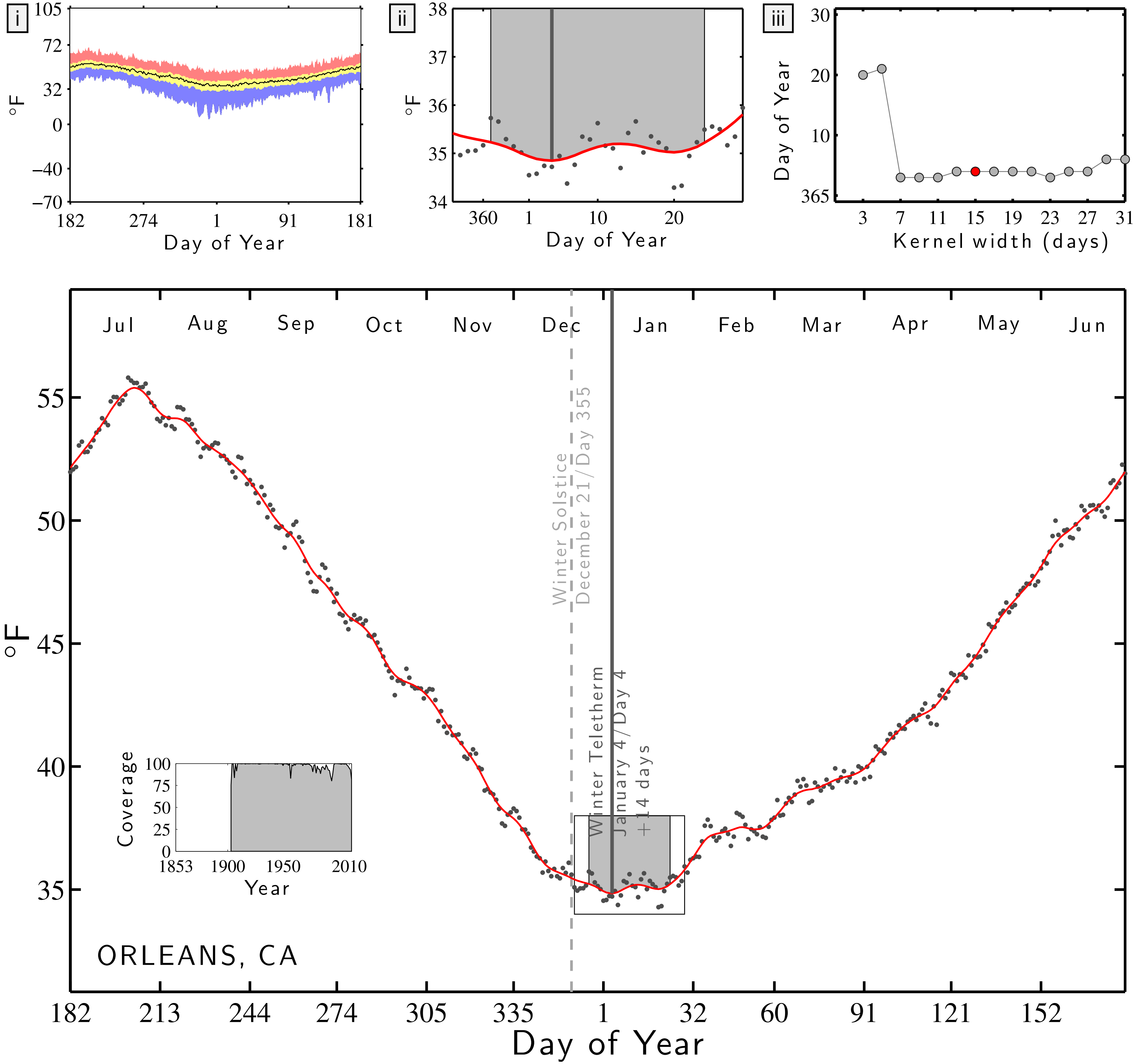

i
ii
iii
°F
Day of Year
Kernel width (days)
Jul
Aug
Sep
Oct
Nov
Dec
Jan
Feb
Mar
Apr
May
Jun
Winter Solstice
December 21/Day 355
Winter Teletherm
January 4/Day 4
± 14 days
Coverage
Year
ORLEANS, CA

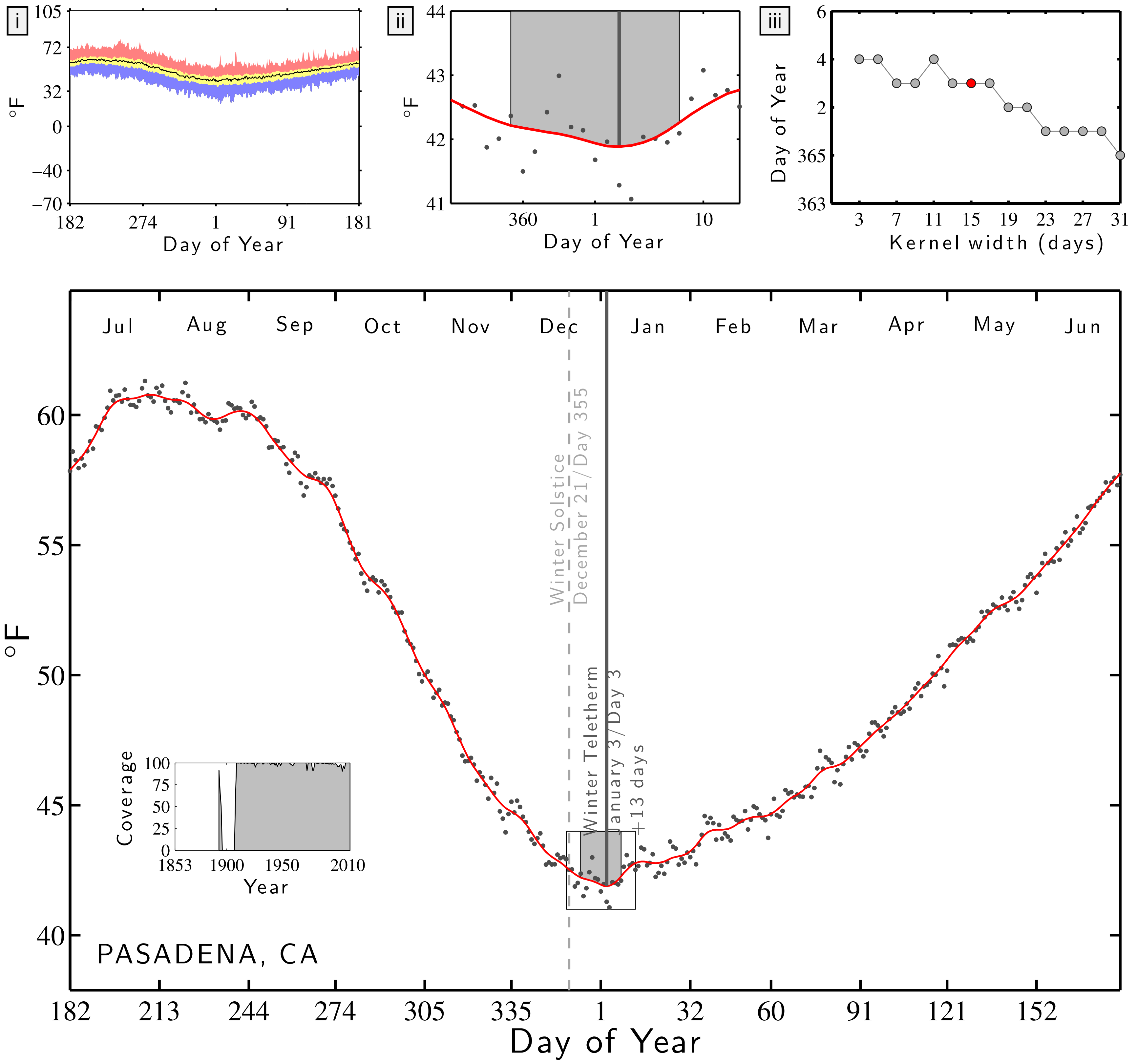

i
°F
Day of Year
ii
°F
Day of Year
iii
Day of Year
Kernel width (days)
Jul
Aug
Sep
Oct
Nov
Dec
Jan
Feb
Mar
Apr
May
Jun
Winter Solstice
December 21/Day 355
Winter Teletherm
January 3/Day 3
±13 days
Coverage
Year
PASADENA, CA
°F
Day of Year

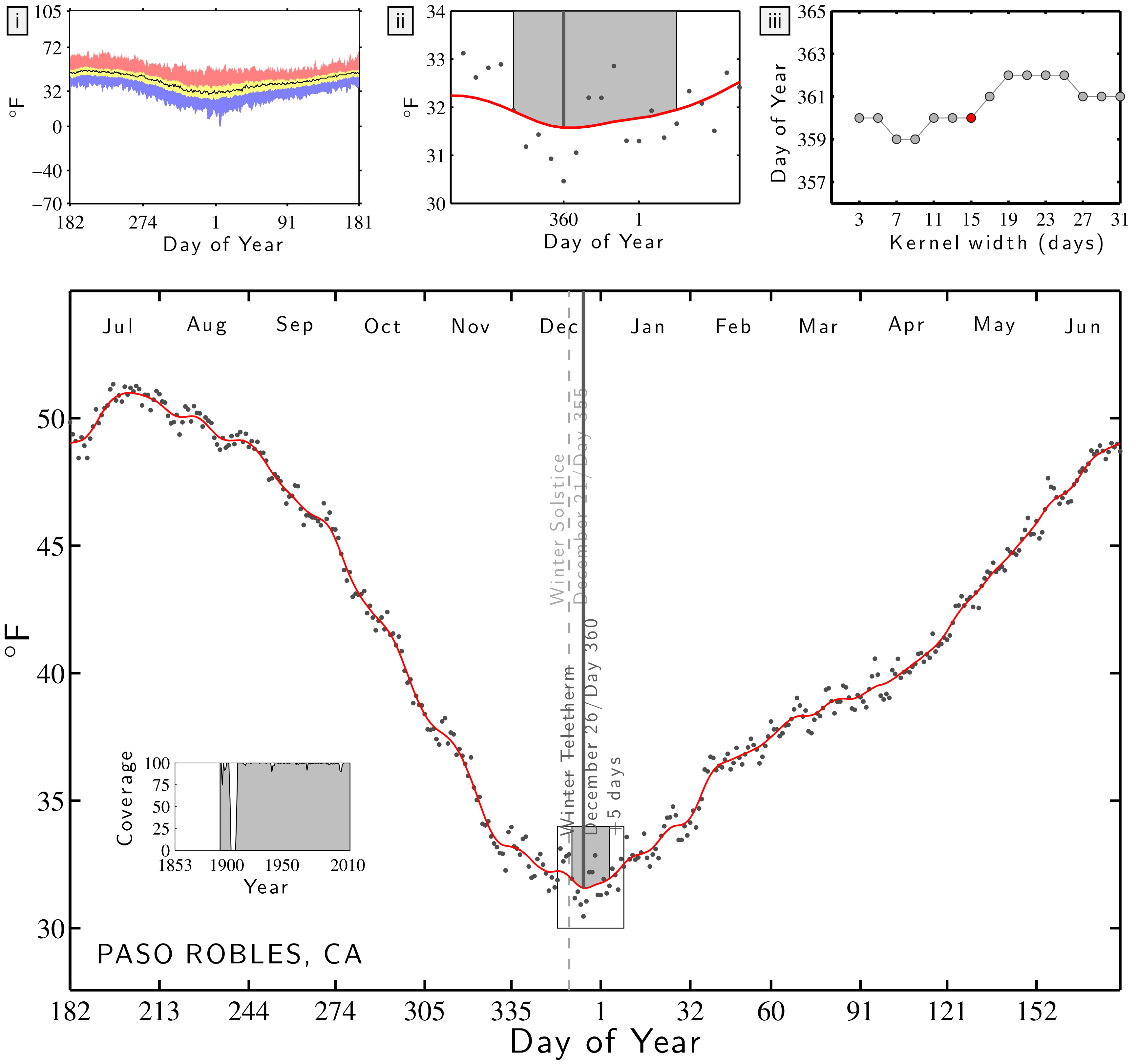

i
ii
iii
°F
Day of Year
Kernel width (days)
Jul
Aug
Sep
Oct
Nov
Dec
Jan
Feb
Mar
Apr
May
Jun
Winter Solstice
December 21/Day 355
Winter Teletherm
December 26/Day 360
±5 days
Coverage
Year
PASO ROBLES, CA

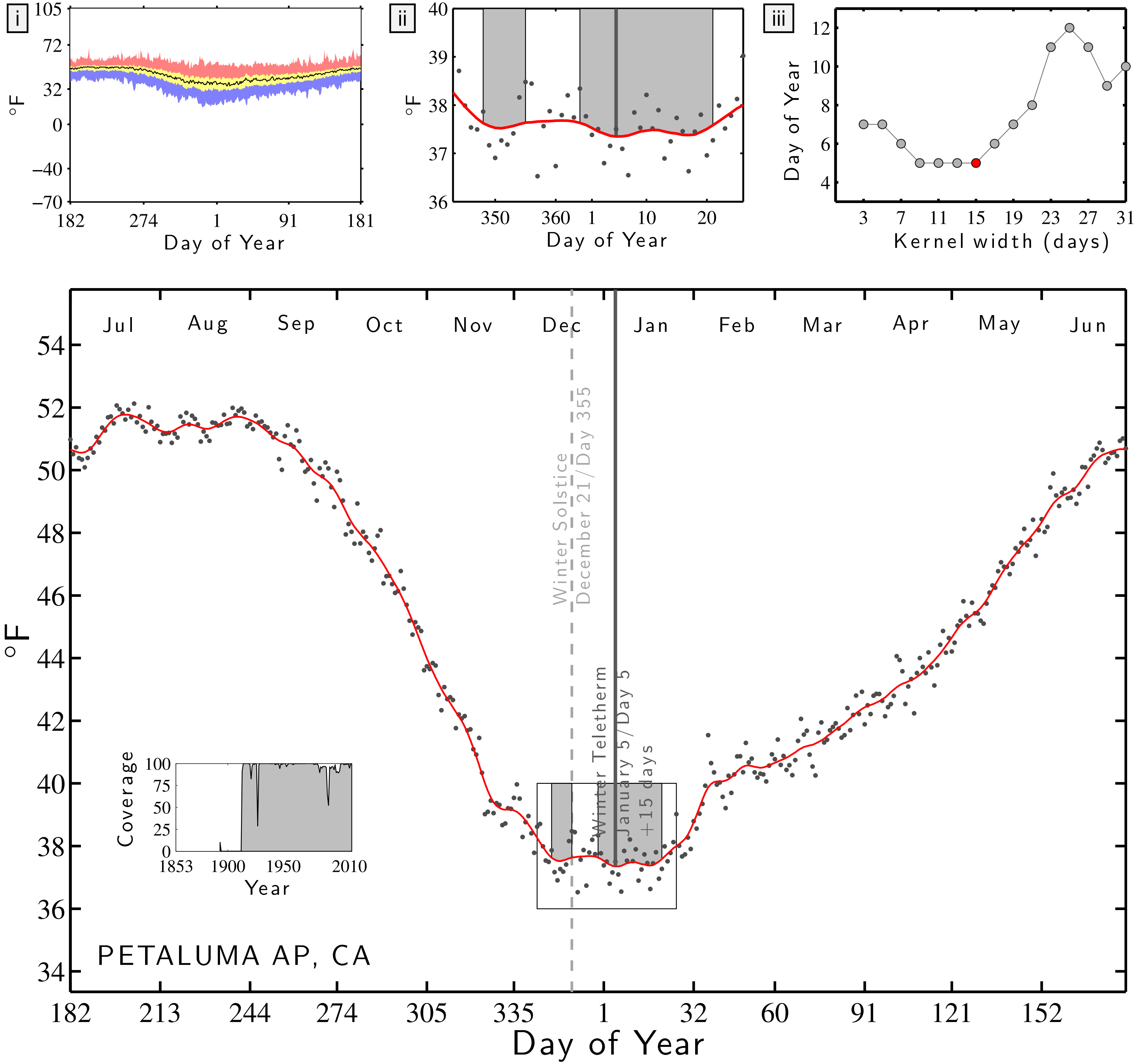

i
°F
105
72
32
0
−40
−70
182
274
1
91
181
Day of Year
ii
40
39
38
37
36
350
360
1
10
20
Day of Year
iii
Day of Year
12
10
8
6
4
3
7
11
15
19
23
27
31
Kernel width (days)
Jul
Aug
Sep
Oct
Nov
Dec
Jan
Feb
Mar
Apr
May
Jun
Winter Solstice
December 21/Day 355
Winter Teletherm
January 5/Day 5
+15 days
54
52
50
48
46
44
42
40
38
36
34
Coverage
100
75
50
25
0
1853
1900
1950
2010
Year
PETALUMA AP, CA
182
213
244
274
305
335
1
32
60
91
121
152
Day of Year

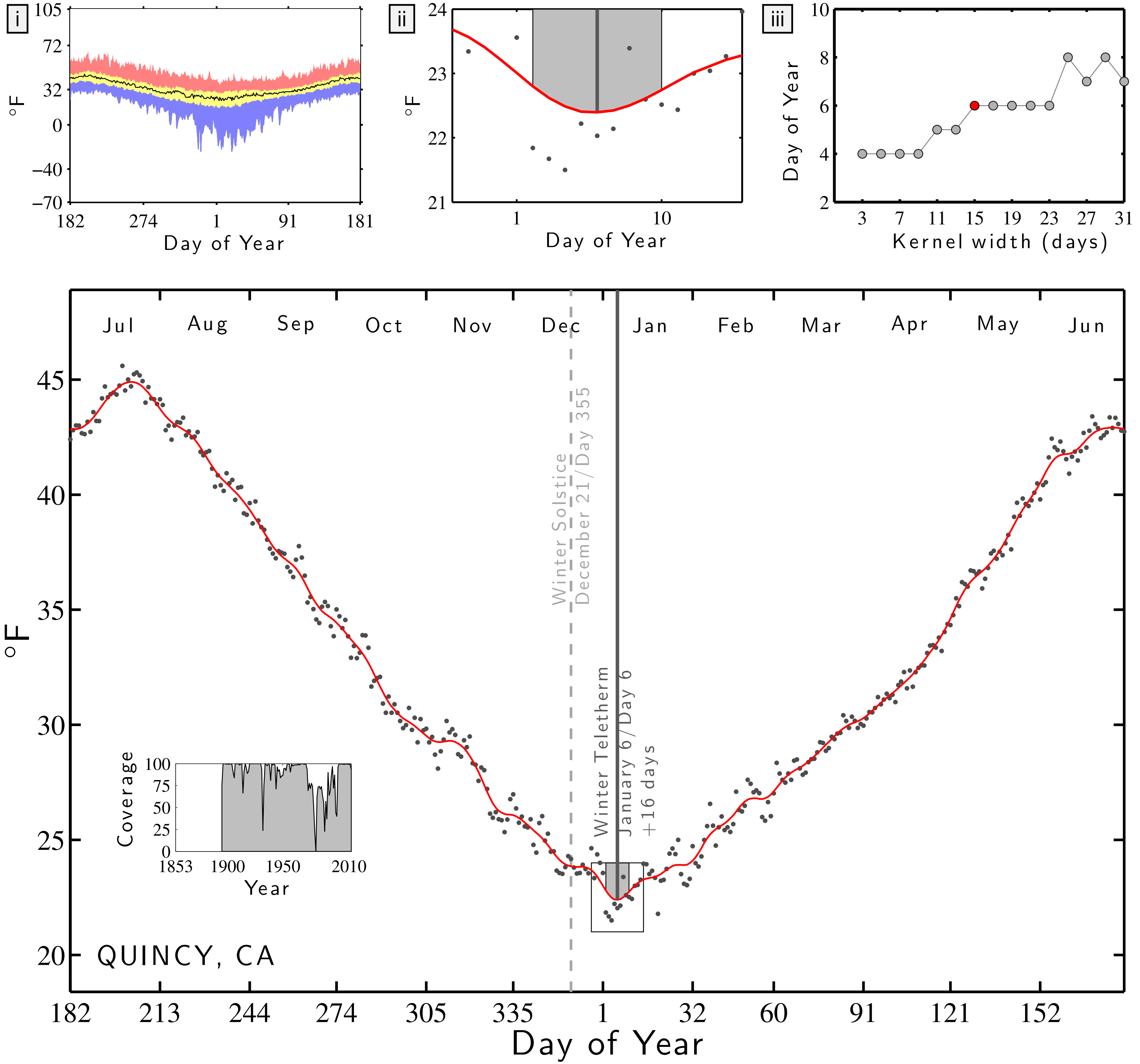

i
°F
Day of Year
ii
°F
Day of Year
iii
Day of Year
Kernel width (days)
Jul
Aug
Sep
Oct
Nov
Dec
Jan
Feb
Mar
Apr
May
Jun
Winter Solstice
December 21/Day 355
Winter Teletherm
January 6/Day 6
+16 days
Coverage
Year
QUINCY, CA
°F
Day of Year

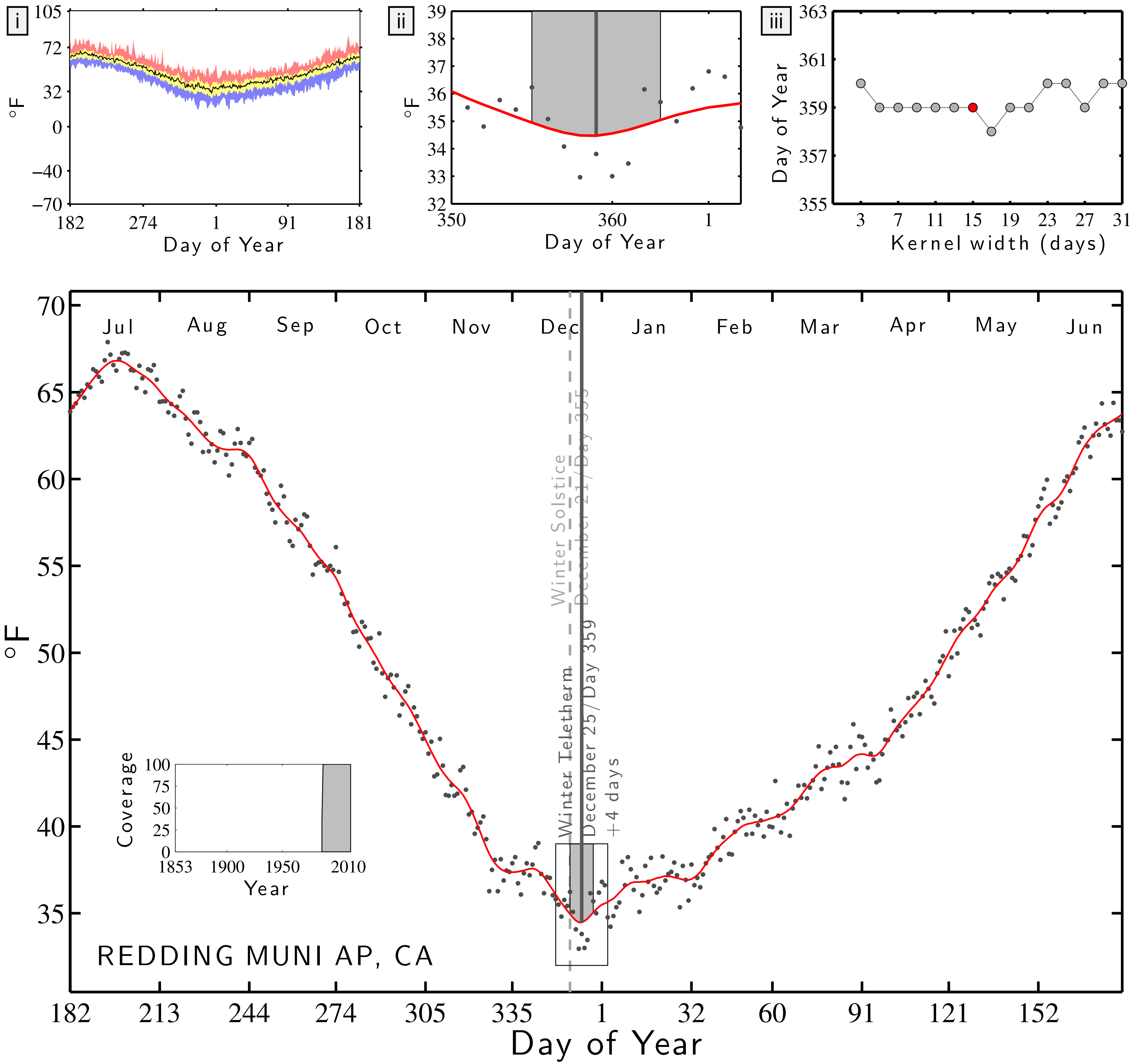

i
°F
105
72
32
0
−40
−70
182
274
1
91
181
Day of Year
ii
39
38
37
36
35
34
33
32
350
360
1
Day of Year
iii
Day of Year
363
361
359
357
355
3
7
11
15
19
23
27
31
Kernel width (days)
Jul
Aug
Sep
Oct
Nov
Dec
Jan
Feb
Mar
Apr
May
Jun
Winter Solstice
December 21/Day 355
Winter Teletherm
December 25/Day 359
+4 days
70
65
60
55
50
45
40
35
Coverage
100
75
50
25
0
1853
1900
1950
2010
Year
REDDING MUNI AP, CA
182
213
244
274
305
335
1
32
60
91
121
152
Day of Year

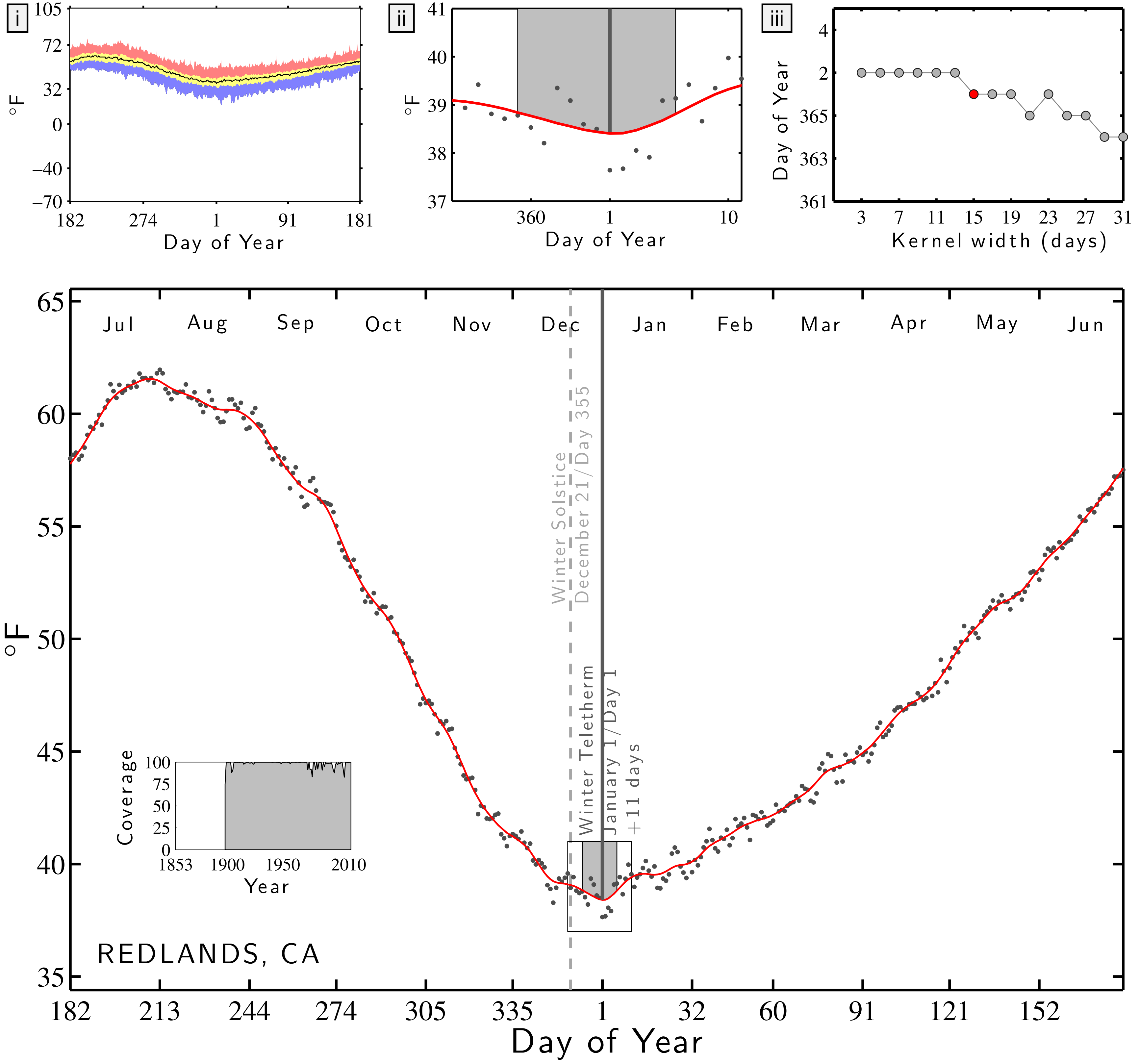

i
ii
iii
Day of Year
Kernel width (days)
°F
Jul
Aug
Sep
Oct
Nov
Dec
Jan
Feb
Mar
Apr
May
Jun
Winter Solstice
December 21/Day 355
Winter Teletherm
January 1/Day 1
+11 days
Coverage
Year
REDLANDS, CA

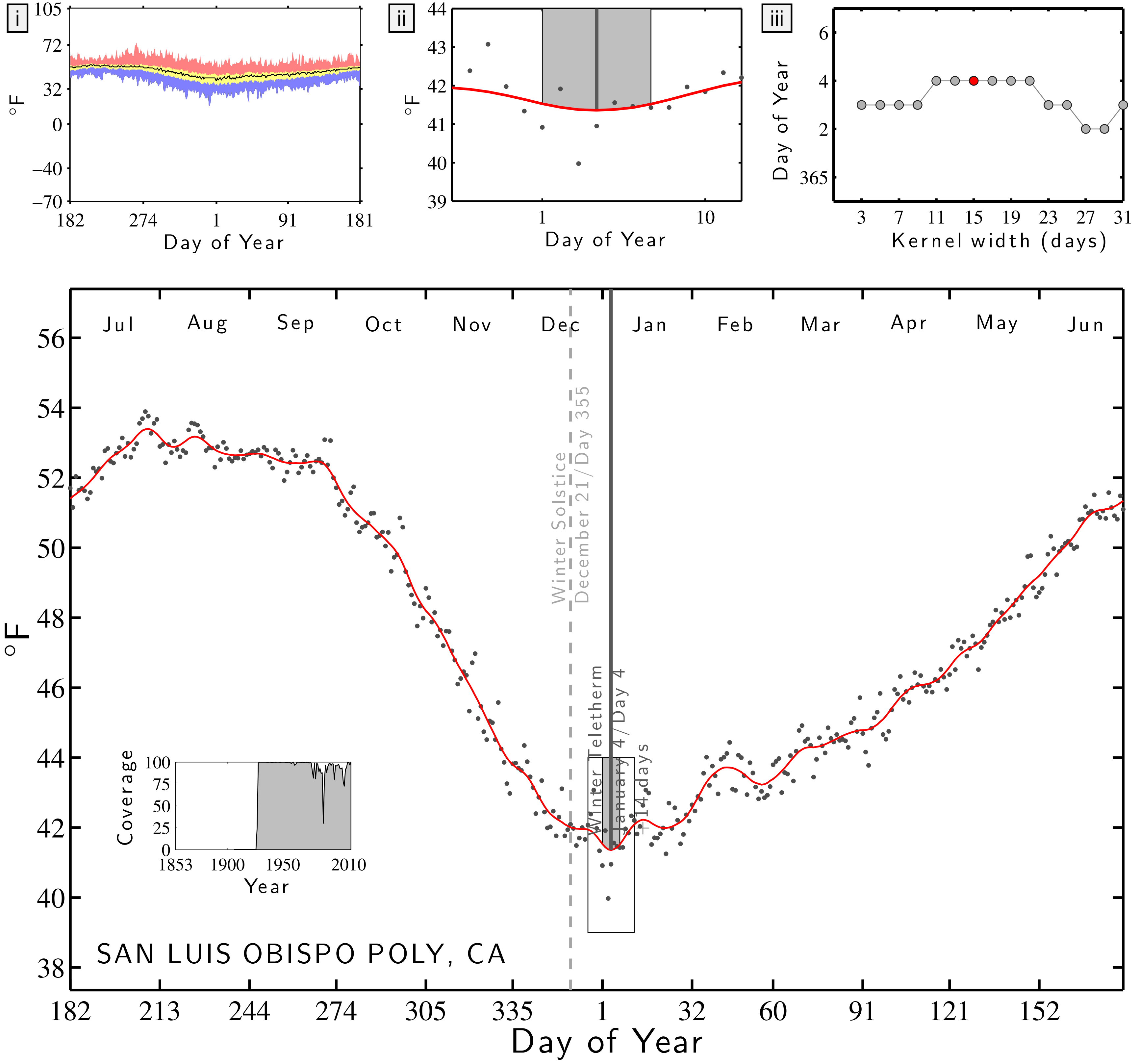

i
ii
iii
°F
Day of Year
Kernel width (days)
Jul
Aug
Sep
Oct
Nov
Dec
Jan
Feb
Mar
Apr
May
Jun
Winter Solstice
December 21/Day 355
Winter Teletherm
January 4/Day 4
±14 days
Coverage
Year
SAN LUIS OBISPO POLY, CA

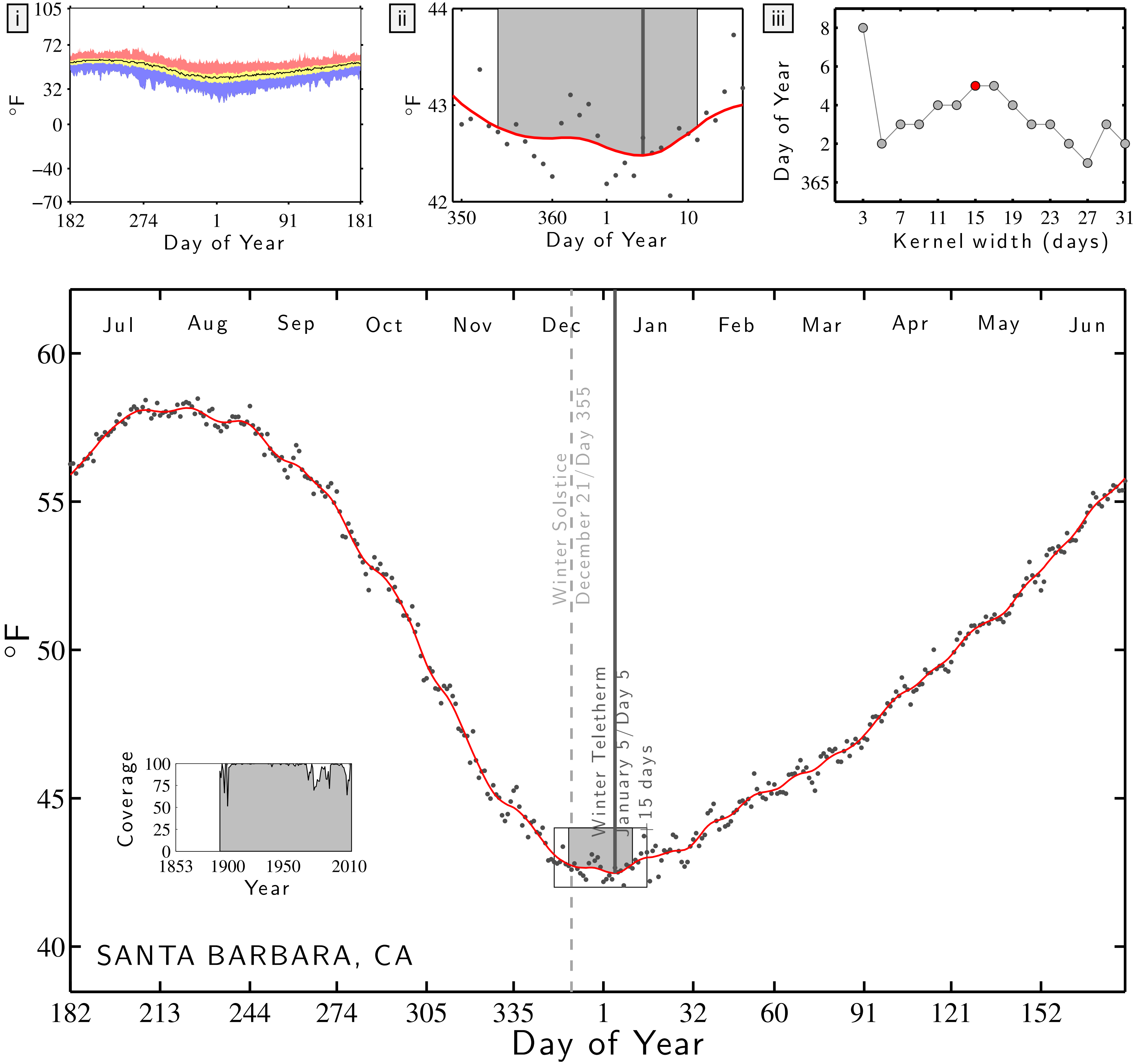

i
°F
Day of Year
182 274 1 91 181
ii
Day of Year
350 360 1 10
iii
Day of Year
Kernel width (days)
3 7 11 15 19 23 27 31
Jul Aug Sep Oct Nov Dec Jan Feb Mar Apr May Jun
Winter Solstice
December 21/Day 355
Winter Teletherm
January 5/Day 5
±15 days
Coverage
Year
1853 1900 1950 2010
SANTA BARBARA, CA
°F
Day of Year
182 213 244 274 305 335 1 32 60 91 121 152

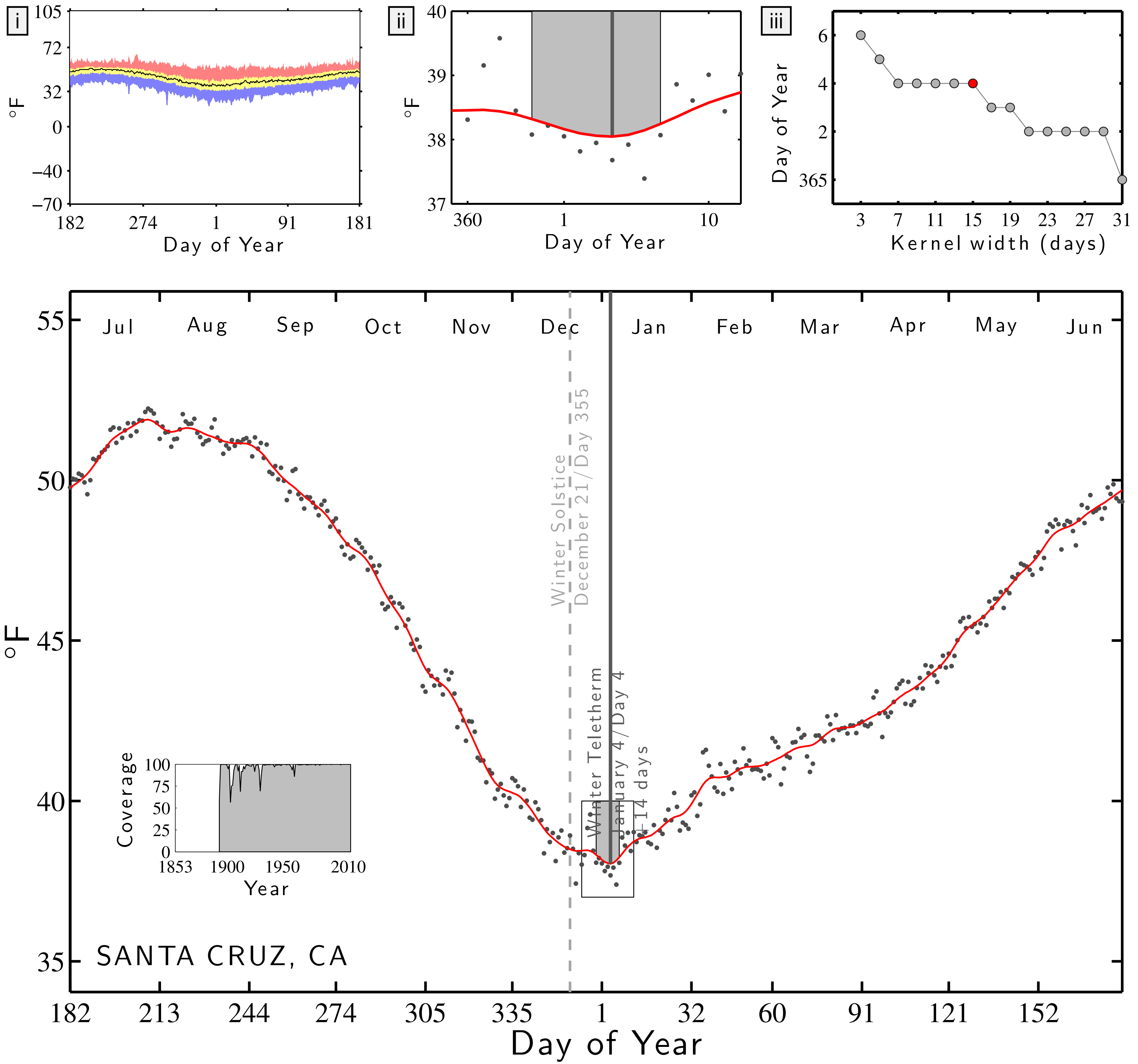

i
°F
Day of Year
ii
Day of Year
iii
Day of Year
Kernel width (days)
Jul
Aug
Sep
Oct
Nov
Dec
Jan
Feb
Mar
Apr
May
Jun
Winter Solstice
December 21/Day 355
Winter Teletherm
January 4/Day 4
±14 days
Coverage
Year
SANTA CRUZ, CA
Day of Year

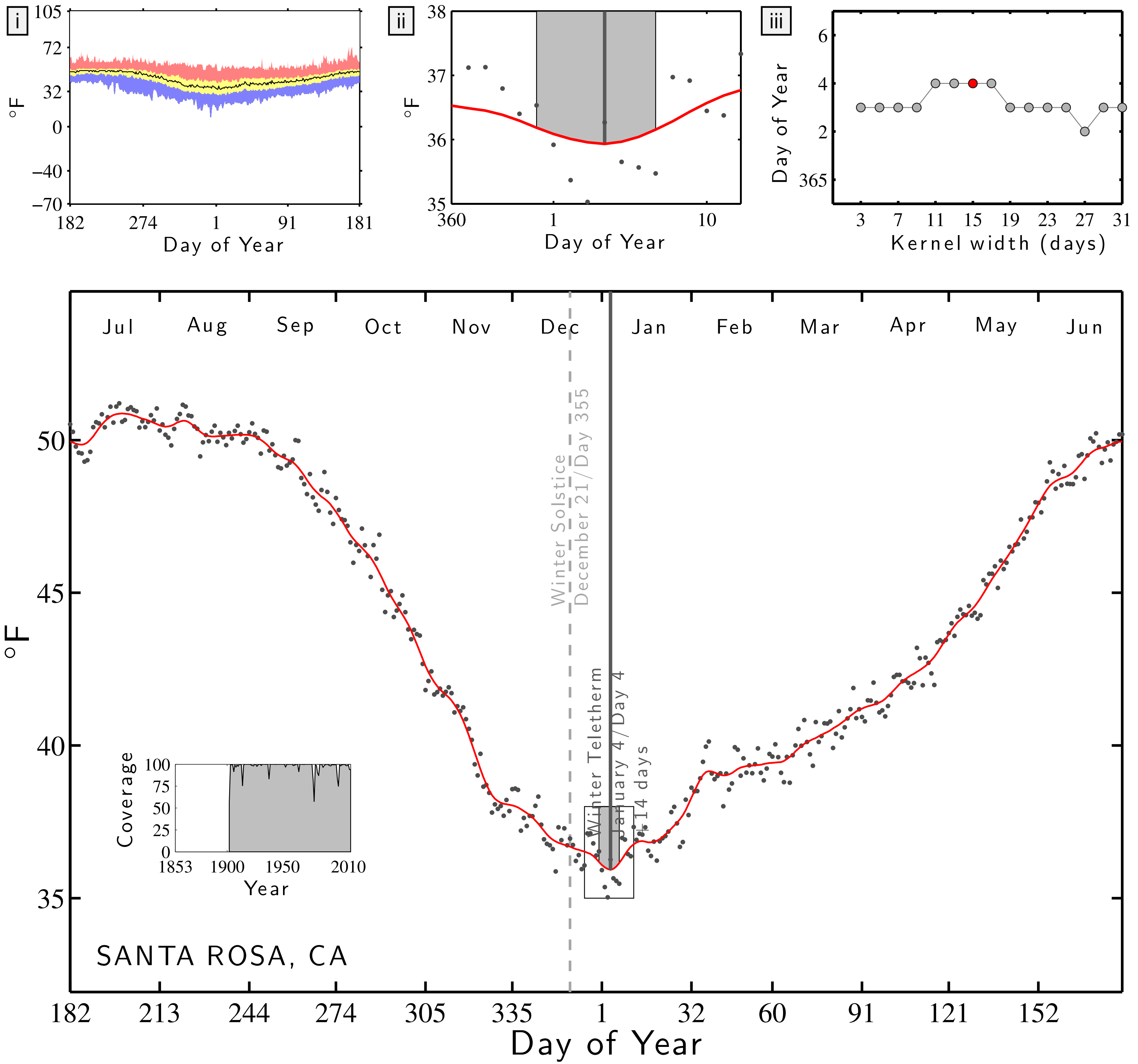

i
ii
iii
°F
Day of Year
Kernel width (days)
Jul
Aug
Sep
Oct
Nov
Dec
Jan
Feb
Mar
Apr
May
Jun
Winter Solstice
December 21/Day 355
Winter Teletherm
January 4/Day 4
±14 days
Coverage
Year
SANTA ROSA, CA

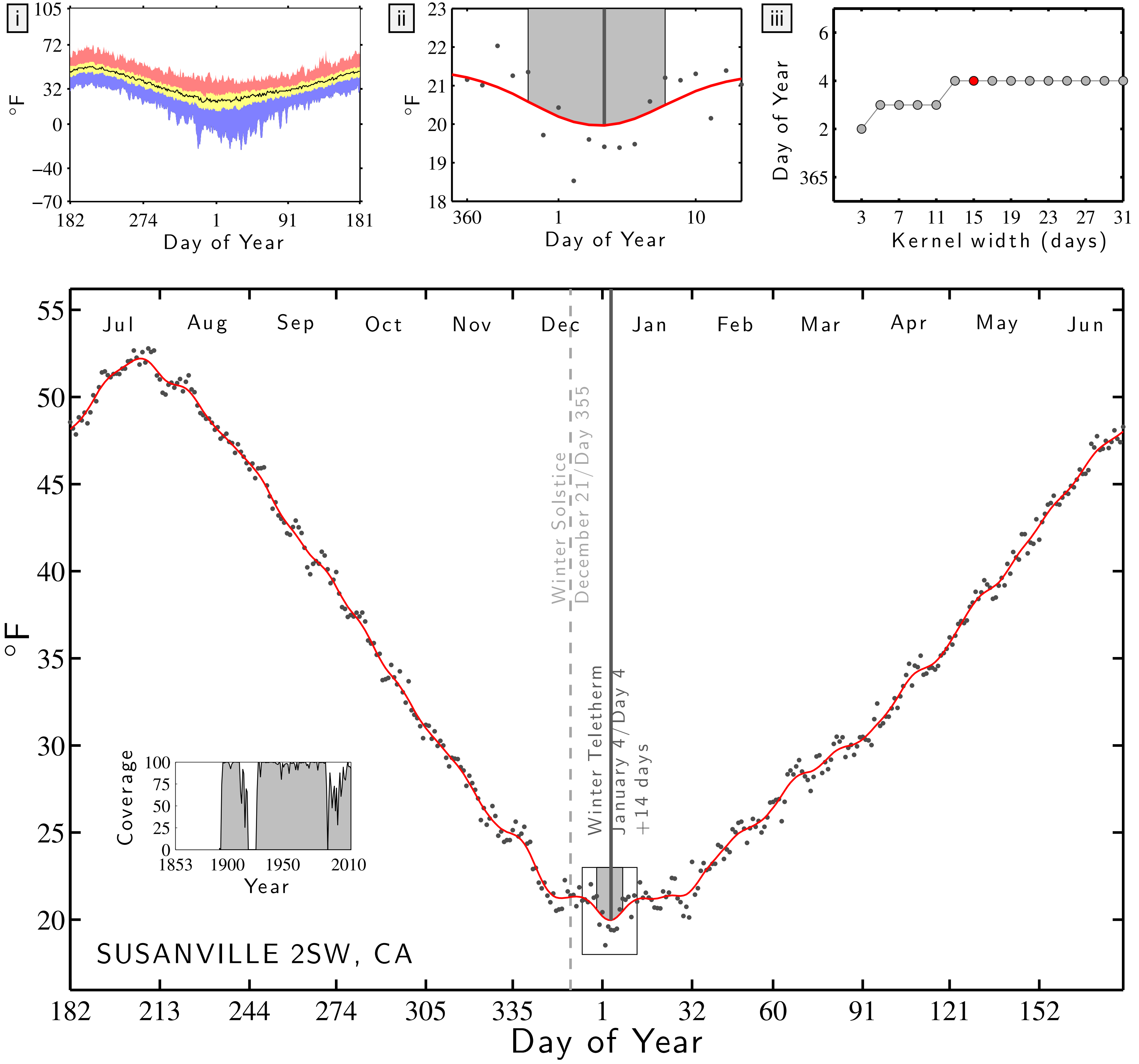

i
ii
iii
°F
Day of Year
Kernel width (days)
Jul
Aug
Sep
Oct
Nov
Dec
Jan
Feb
Mar
Apr
May
Jun
Winter Solstice
December 21/Day 355
Winter Teletherm
January 4/Day 4
+14 days
Coverage
Year
SUSANVILLE 2SW, CA

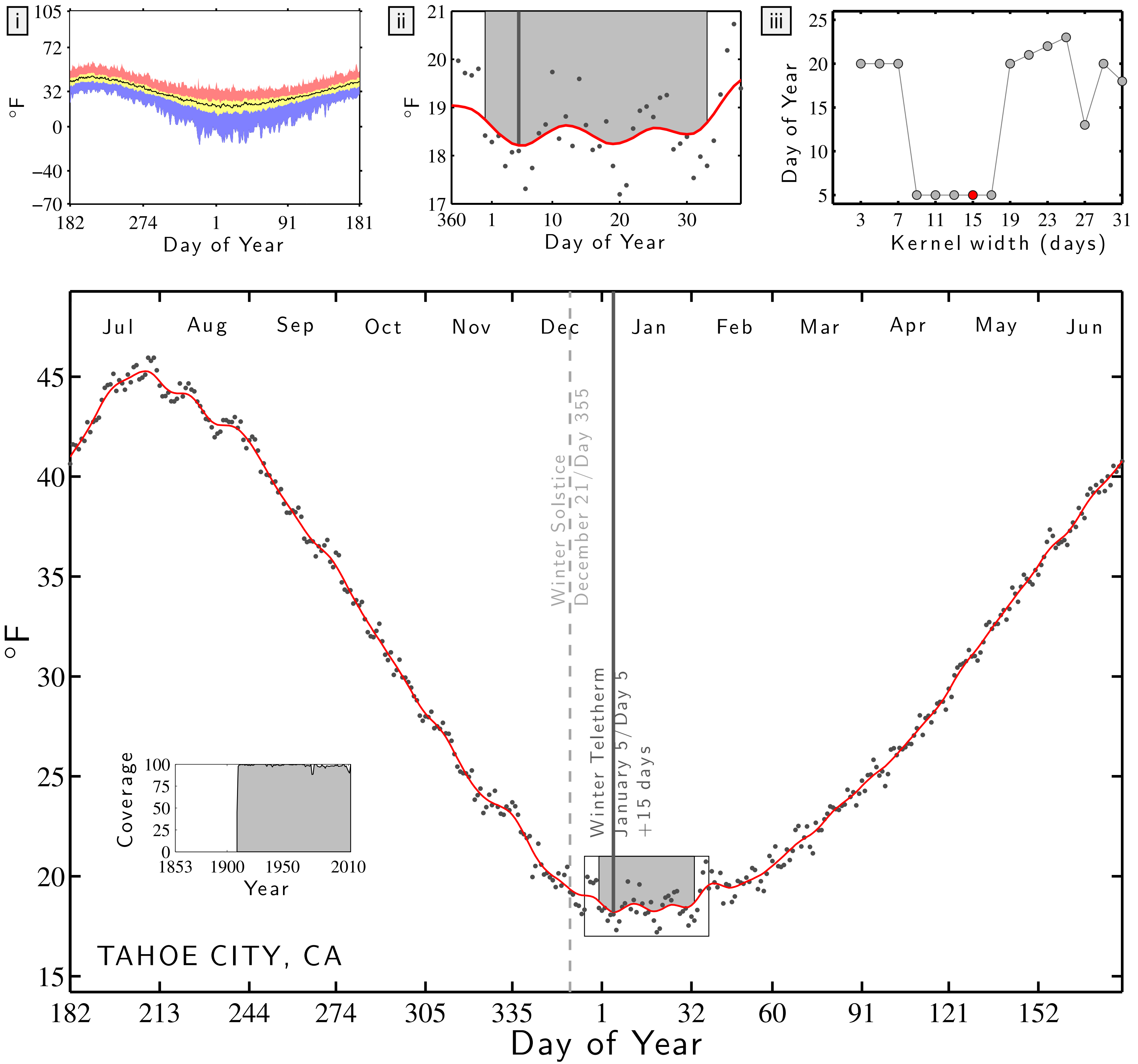

i
°F
Day of Year
ii
°F
Day of Year
iii
Day of Year
Kernel width (days)
Jul
Aug
Sep
Oct
Nov
Dec
Jan
Feb
Mar
Apr
May
Jun
Winter Solstice
December 21/Day 355
Winter Teletherm
January 5/Day 5
+15 days
Coverage
Year
TAHOE CITY, CA
°F
Day of Year

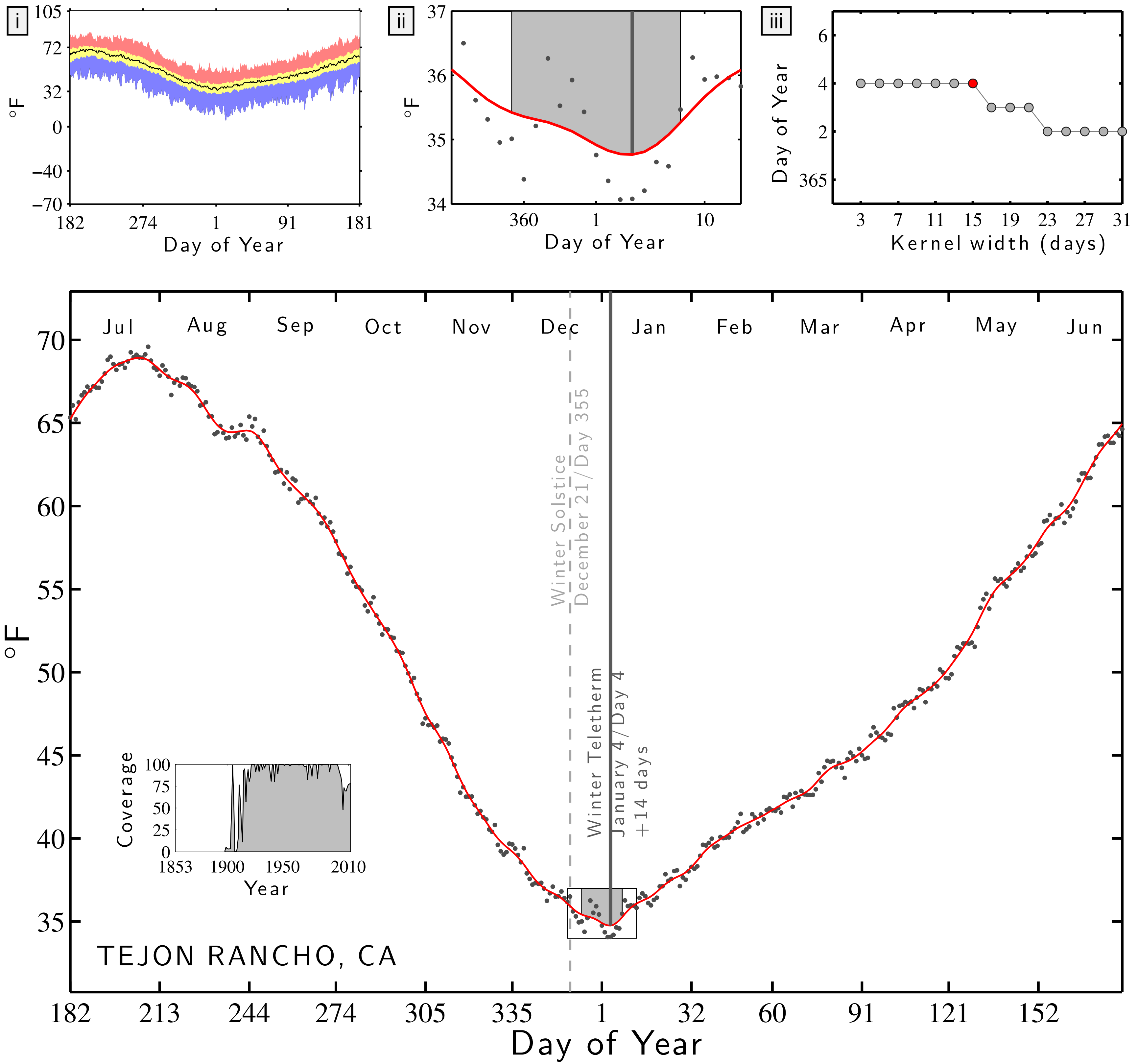

i
ii
iii
°F
Day of Year
Day of Year
Kernel width (days)
Jul
Aug
Sep
Oct
Nov
Dec
Jan
Feb
Mar
Apr
May
Jun
Winter Solstice
December 21/Day 355
Winter Teletherm
January 4/Day 4
+14 days
Coverage
Year
TEJON RANCHO, CA
Day of Year

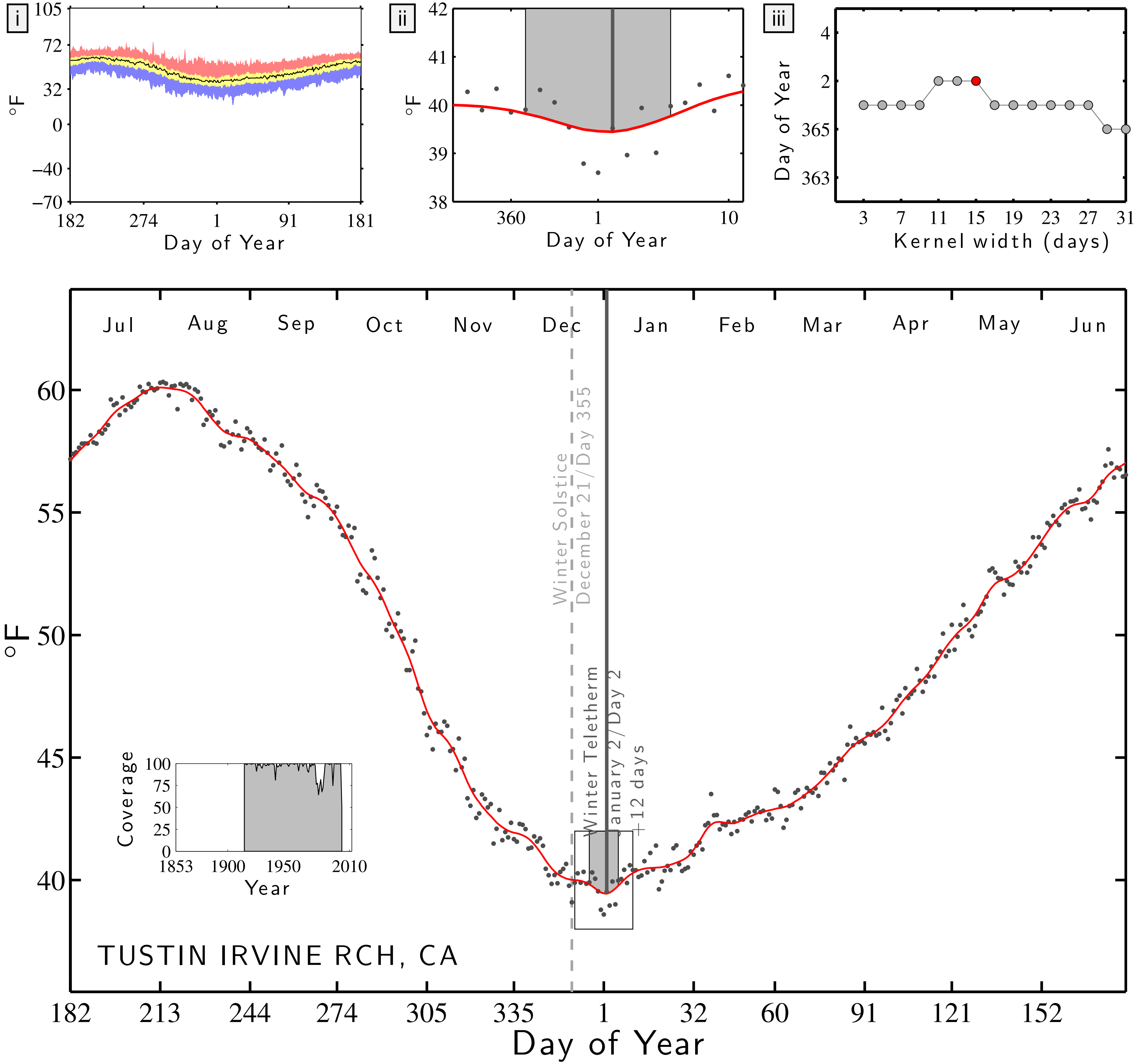

i
ii
iii
Day of Year
Kernel width (days)
°F
Day of Year
Jul
Aug
Sep
Oct
Nov
Dec
Jan
Feb
Mar
Apr
May
Jun
Winter Solstice
December 21/Day 355
Winter Teletherm
January 2/Day 2
+12 days
Coverage
Year
TUSTIN IRVINE RCH, CA

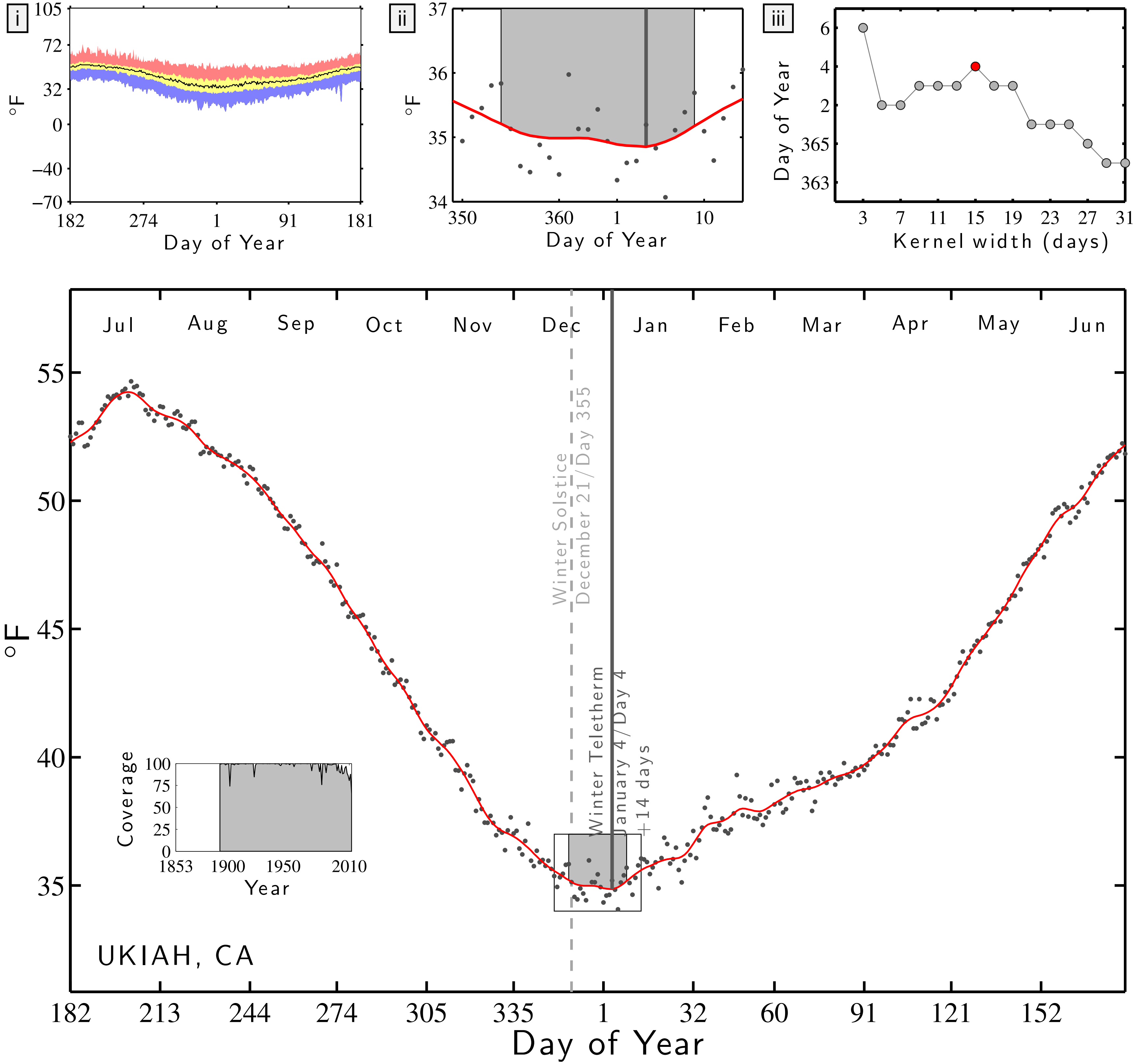

i
°F
Day of Year
ii
°F
Day of Year
iii
Day of Year
Kernel width (days)
Jul
Aug
Sep
Oct
Nov
Dec
Jan
Feb
Mar
Apr
May
Jun
Winter Solstice
December 21/Day 355
Winter Teletherm
January 4/Day 4
+14 days
Coverage
Year
UKIAH, CA
°F
Day of Year

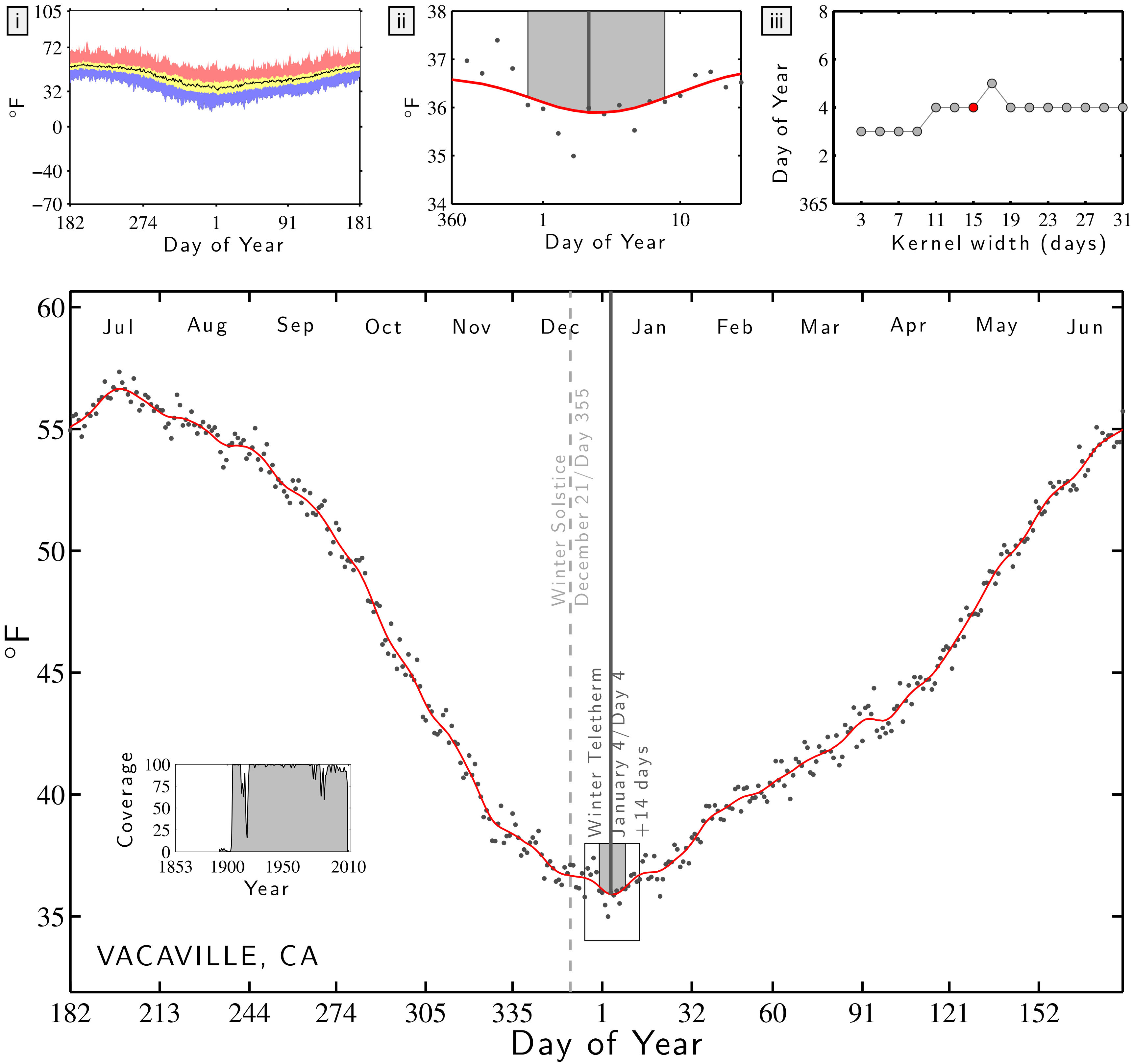

i
ii
iii
°F
Day of Year
Kernel width (days)
Jul
Aug
Sep
Oct
Nov
Dec
Jan
Feb
Mar
Apr
May
Jun
Winter Solstice
December 21/Day 355
Winter Teletherm
January 4/Day 4
+14 days
Coverage
Year
VACAVILLE, CA

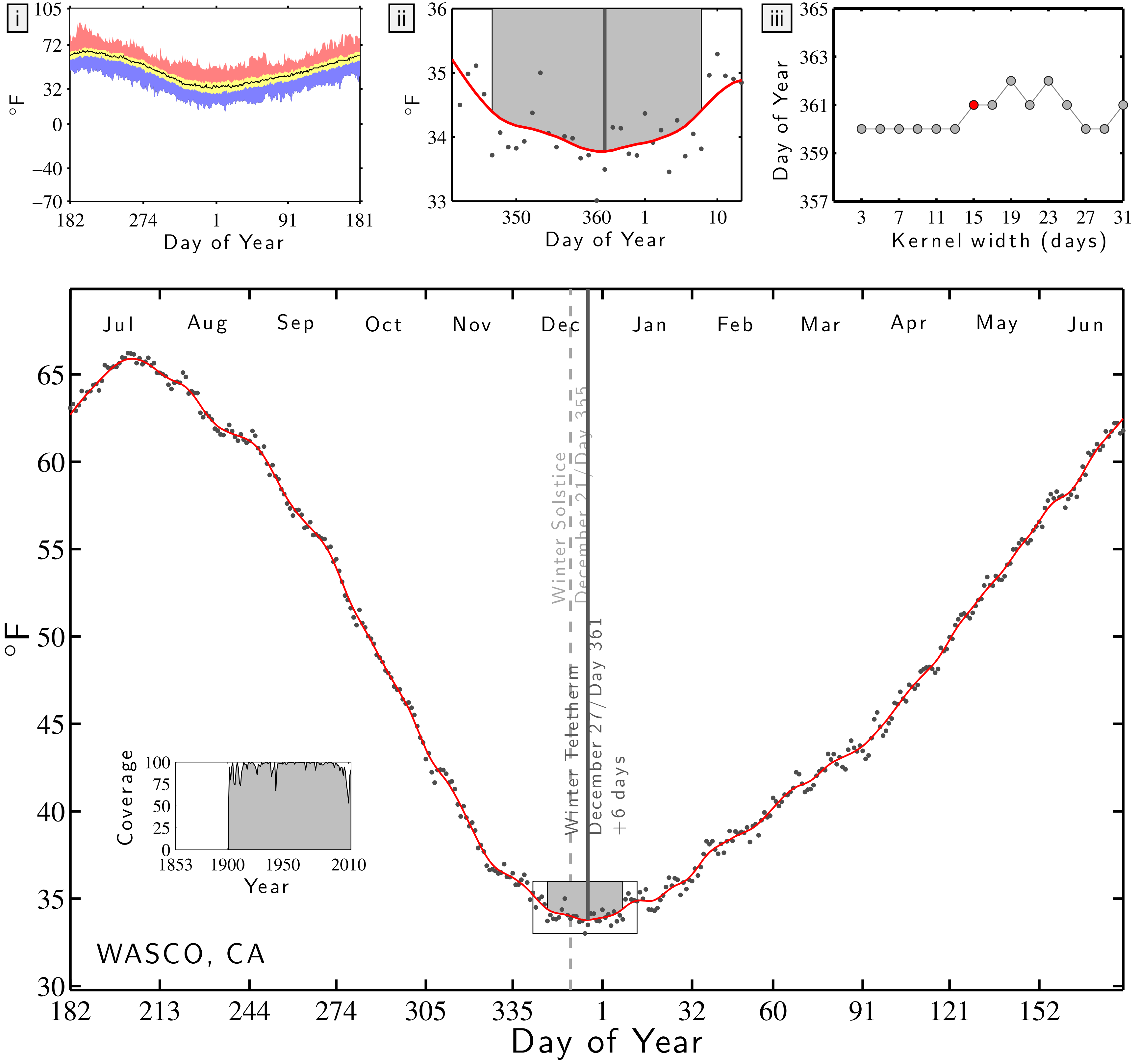

i
ii
iii
Day of Year
°F
Day of Year
Kernel width (days)
Jul
Aug
Sep
Oct
Nov
Dec
Jan
Feb
Mar
Apr
May
Jun
Winter Solstice
December 21/Day 355
Winter Teletherm
December 27/Day 361
+6 days
Coverage
Year
WASCO, CA

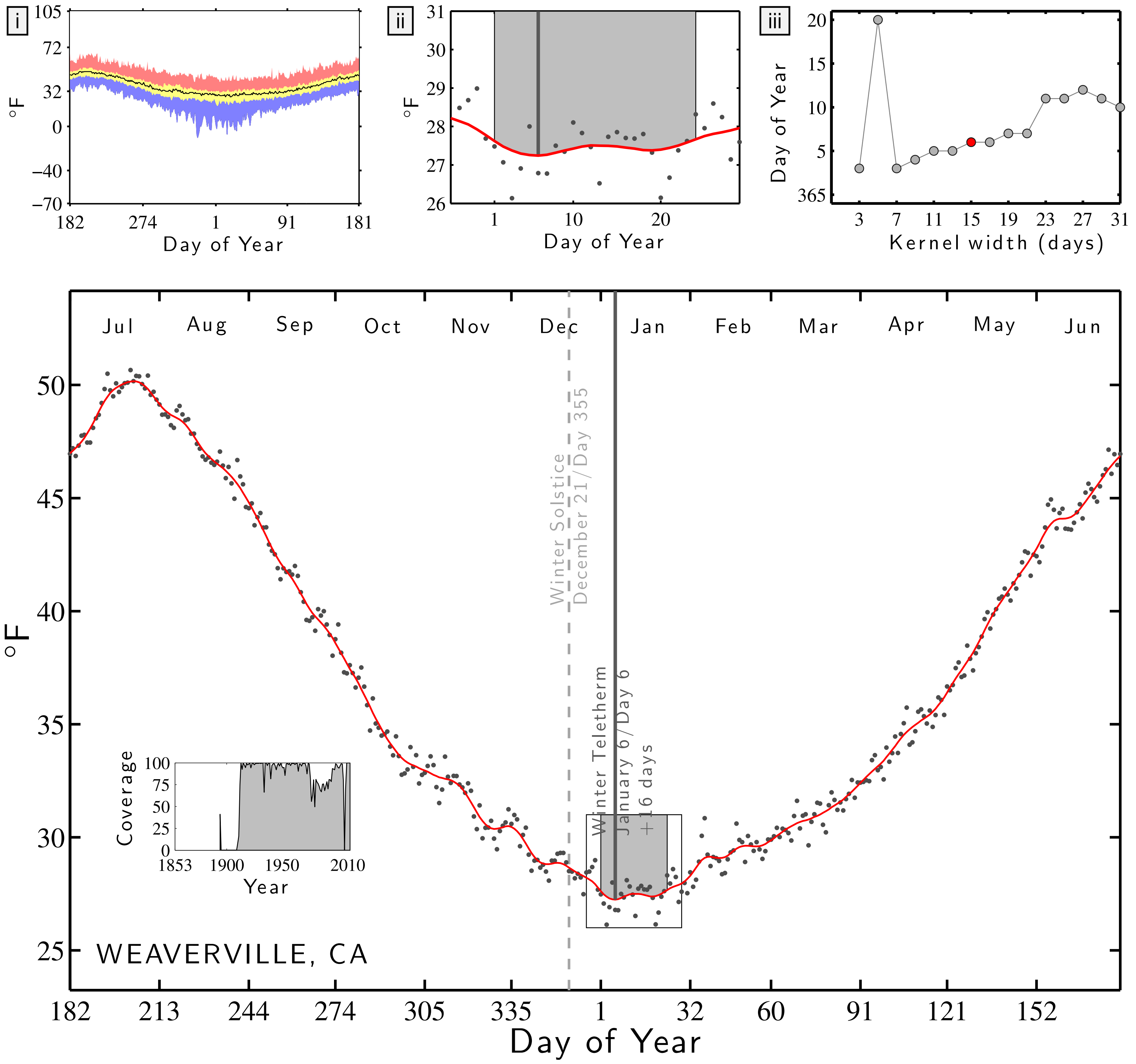

i
105
72
32
0
−40
−70
°F
182
274
1
91
181
Day of Year
ii
31
30
29
28
27
26
°F
1
10
20
Day of Year
iii
20
15
10
5
365
Day of Year
3
7
11
15
19
23
27
31
Kernel width (days)
Jul
Aug
Sep
Oct
Nov
Dec
Jan
Feb
Mar
Apr
May
Jun
50
45
40
35
30
25
°F
Winter Solstice
December 21/Day 355
Winter Teletherm
January 6/Day 6
±16 days
Coverage
100
75
50
25
0
1853
1900
1950
2010
Year
WEAVERVILLE, CA
182
213
244
274
305
335
1
32
60
91
121
152
Day of Year

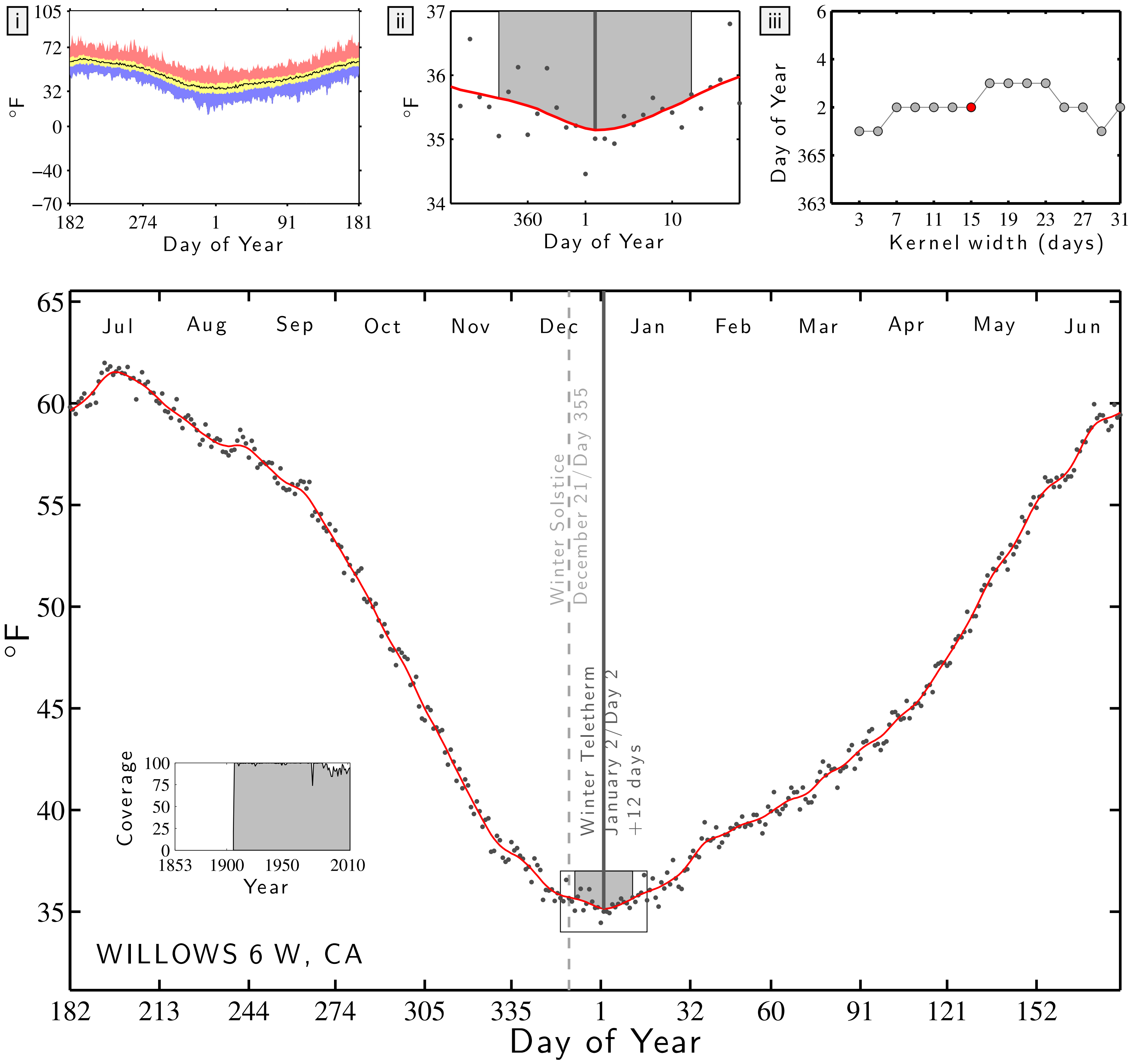

i
ii
iii
°F
Day of Year
Day of Year
Kernel width (days)
Jul
Aug
Sep
Oct
Nov
Dec
Jan
Feb
Mar
Apr
May
Jun
Winter Solstice
December 21/Day 355
Winter Teletherm
January 2/Day 2
+12 days
Coverage
Year
WILLOWS 6 W, CA

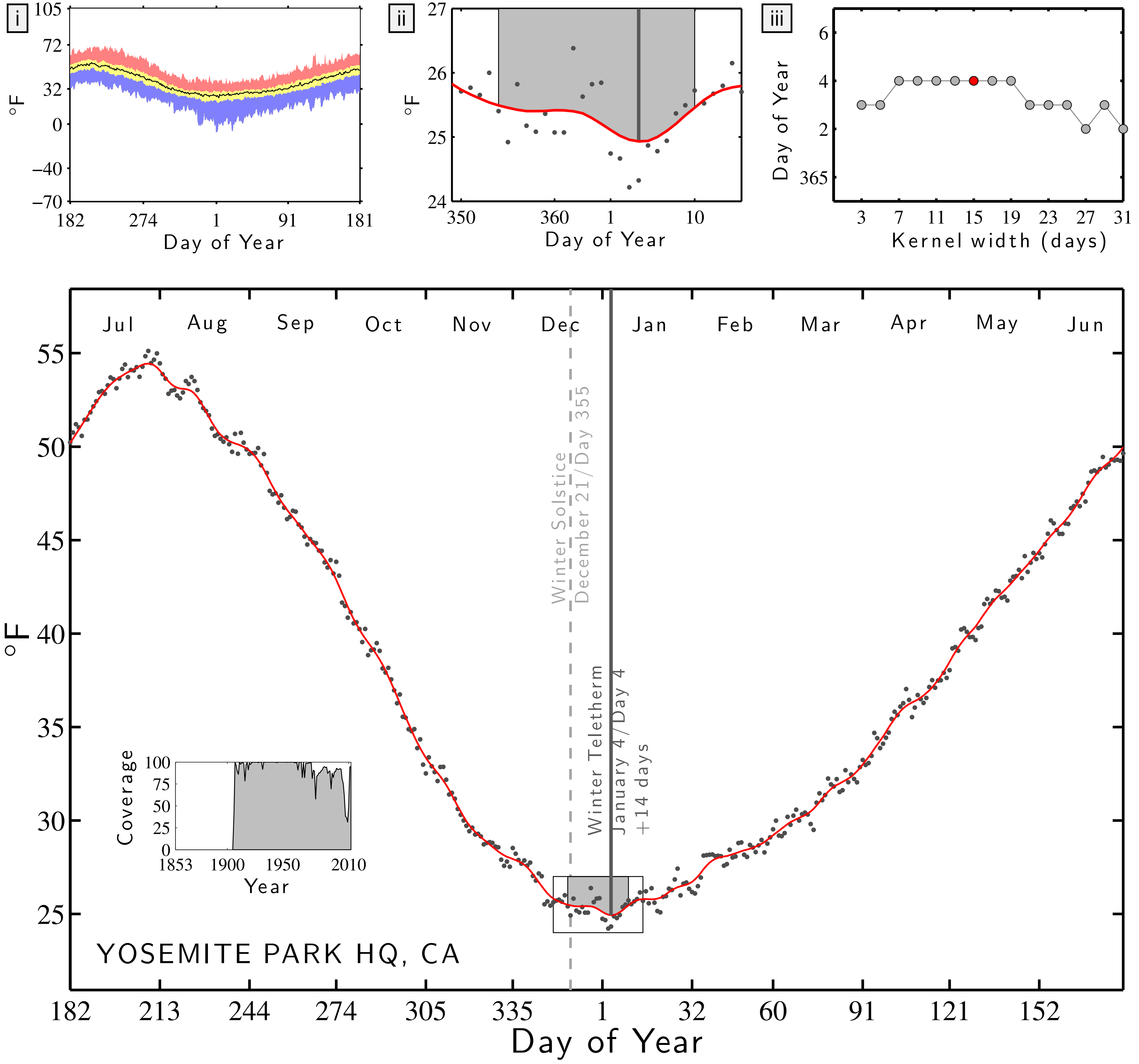

i
ii
iii
°F
Day of Year
Kernel width (days)
Jul
Aug
Sep
Oct
Nov
Dec
Jan
Feb
Mar
Apr
May
Jun
Winter Solstice
December 21/Day 355
Winter Teletherm
January 4/Day 4
+14 days
Coverage
Year
YOSEMITE PARK HQ, CA

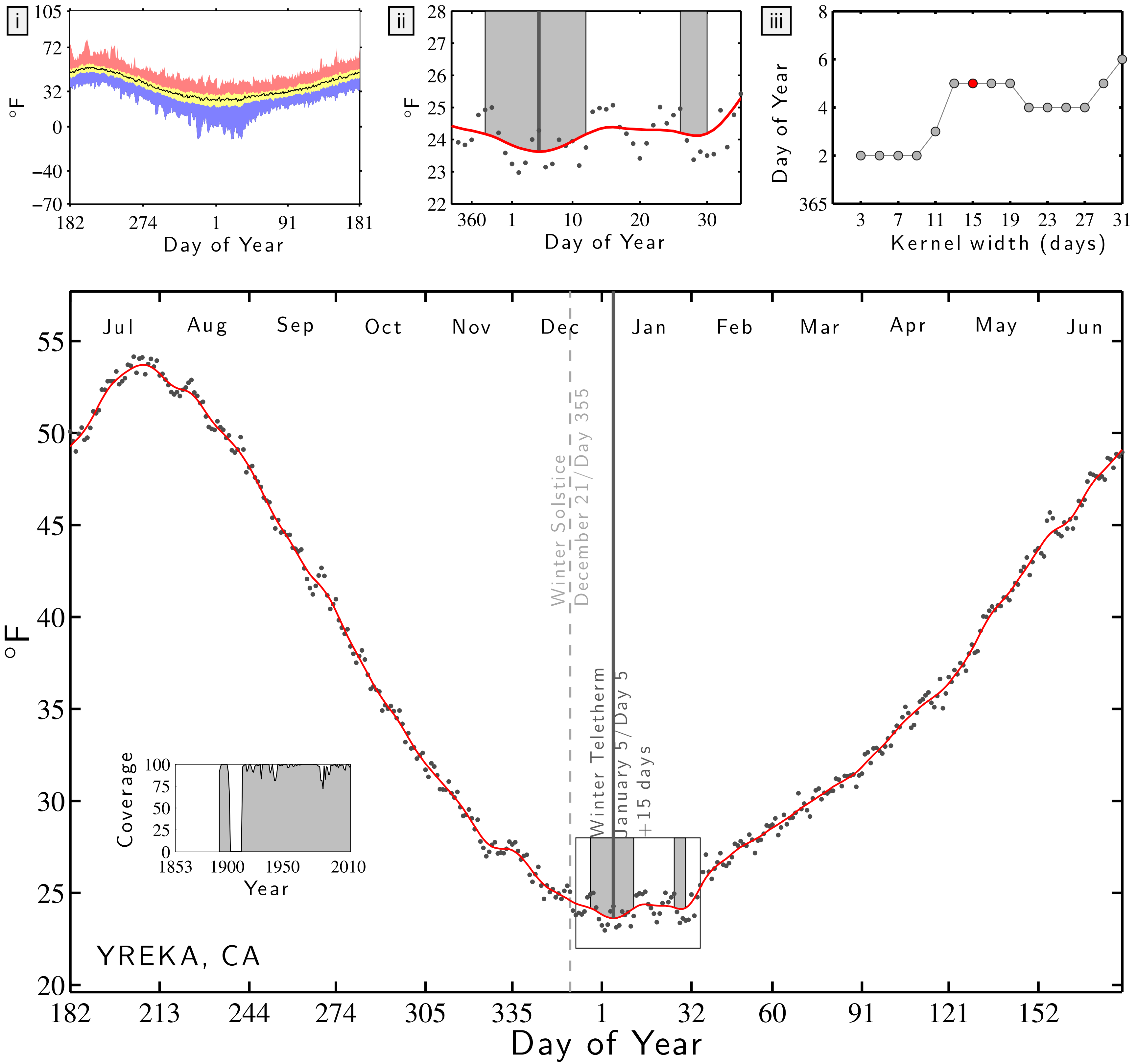

i
ii
iii
°F
Day of Year
Kernel width (days)
Jul
Aug
Sep
Oct
Nov
Dec
Jan
Feb
Mar
Apr
May
Jun
Winter Solstice
December 21/Day 355
Winter Teletherm
January 5/Day 5
+15 days
Coverage
Year
YREKA, CA

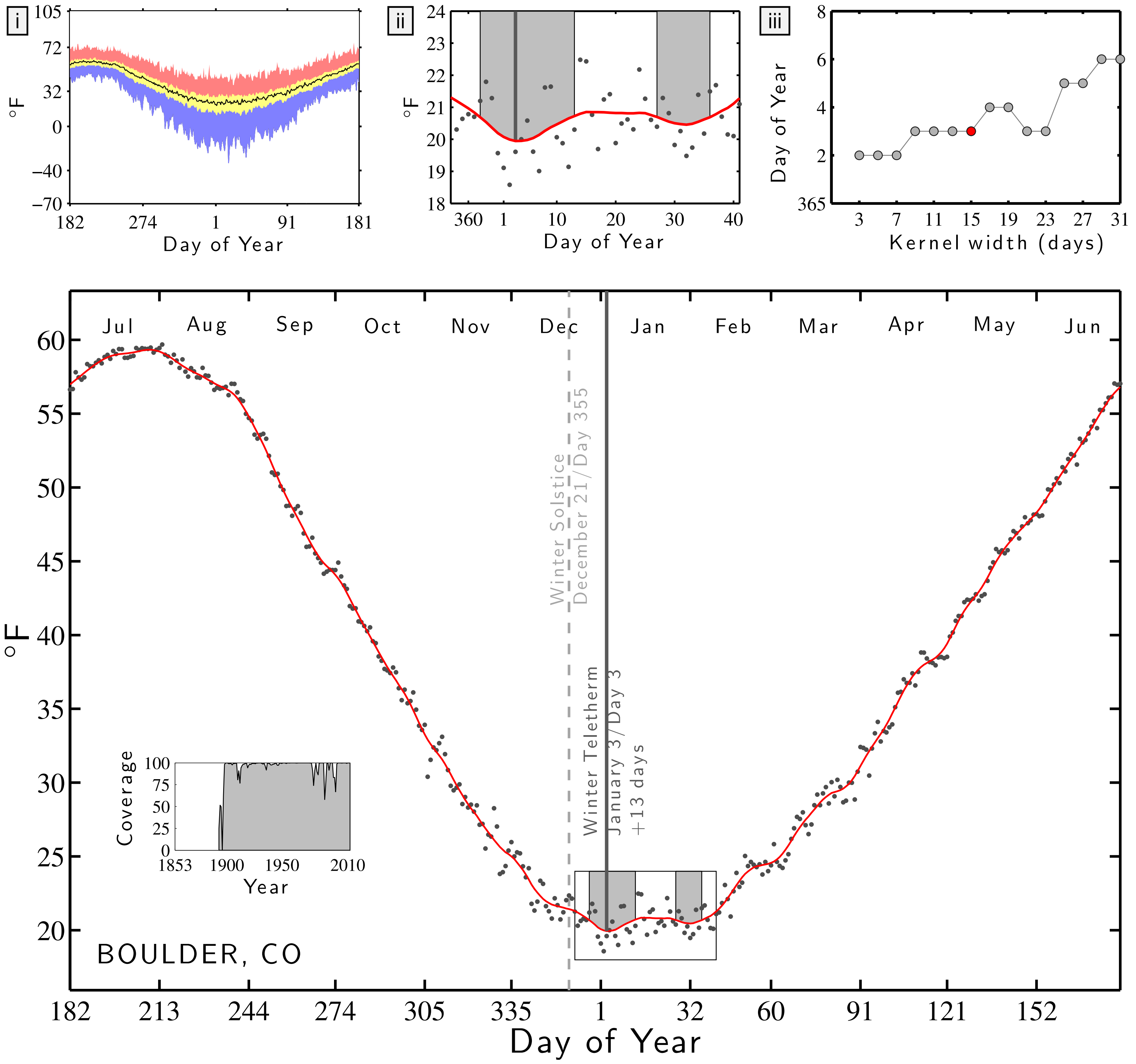

i
°F
105
72
32
0
−40
−70
182
274
1
91
181
Day of Year
ii
24
23
22
21
20
19
18
360
1
10
20
30
40
Day of Year
iii
Day of Year
8
6
4
2
365
3
7
11
15
19
23
27
31
Kernel width (days)
Jul
Aug
Sep
Oct
Nov
Dec
Jan
Feb
Mar
Apr
May
Jun
60
55
50
45
40
35
30
25
20
Winter Solstice
December 21/Day 355
Winter Teletherm
January 3/Day 3
+13 days
Coverage
100
75
50
25
0
1853
1900
1950
2010
Year
BOULDER, CO
182
213
244
274
305
335
1
32
60
91
121
152
Day of Year

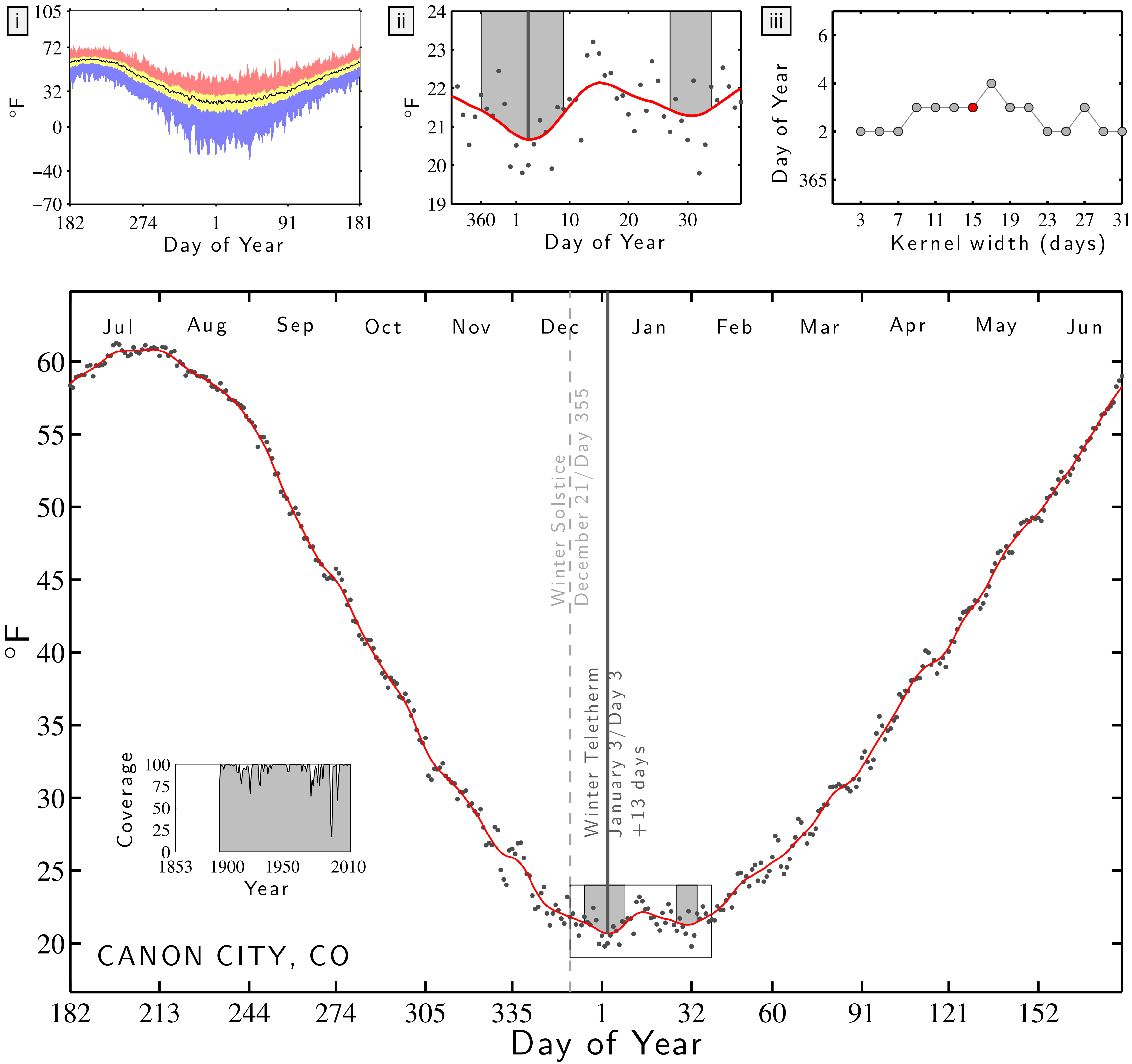

i
ii
iii
°F
Day of Year
Kernel width (days)
Jul
Aug
Sep
Oct
Nov
Dec
Jan
Feb
Mar
Apr
May
Jun
Winter Solstice
December 21/Day 355
Winter Teletherm
January 3/Day 3
+13 days
Coverage
Year
CANON CITY, CO

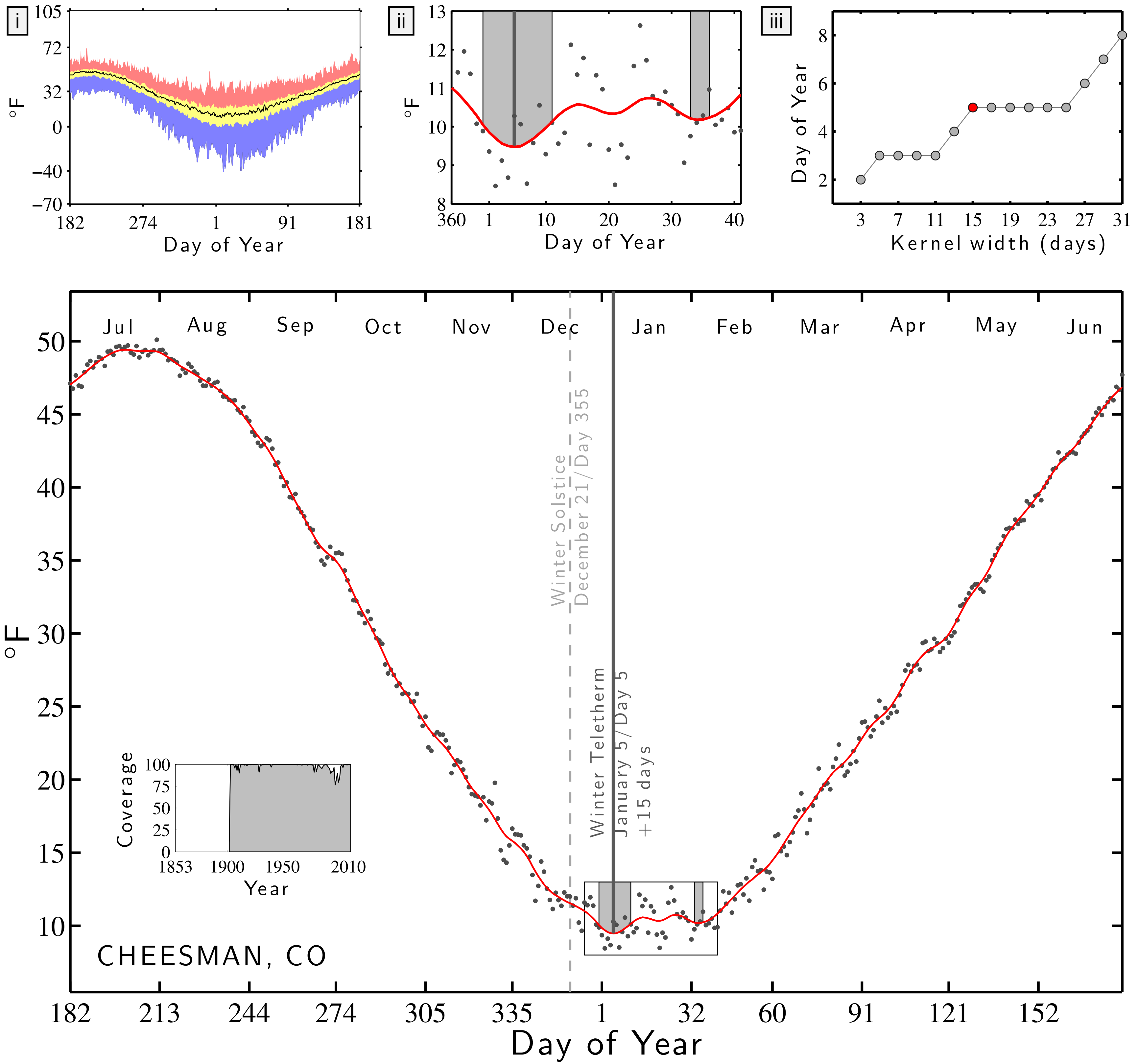

i
°F
Day of Year
ii
°F
Day of Year
iii
Day of Year
Kernel width (days)
Jul
Aug
Sep
Oct
Nov
Dec
Jan
Feb
Mar
Apr
May
Jun
Winter Solstice
December 21/Day 355
Winter Teletherm
January 5/Day 5
+15 days
Coverage
Year
CHEESMAN, CO
°F
Day of Year

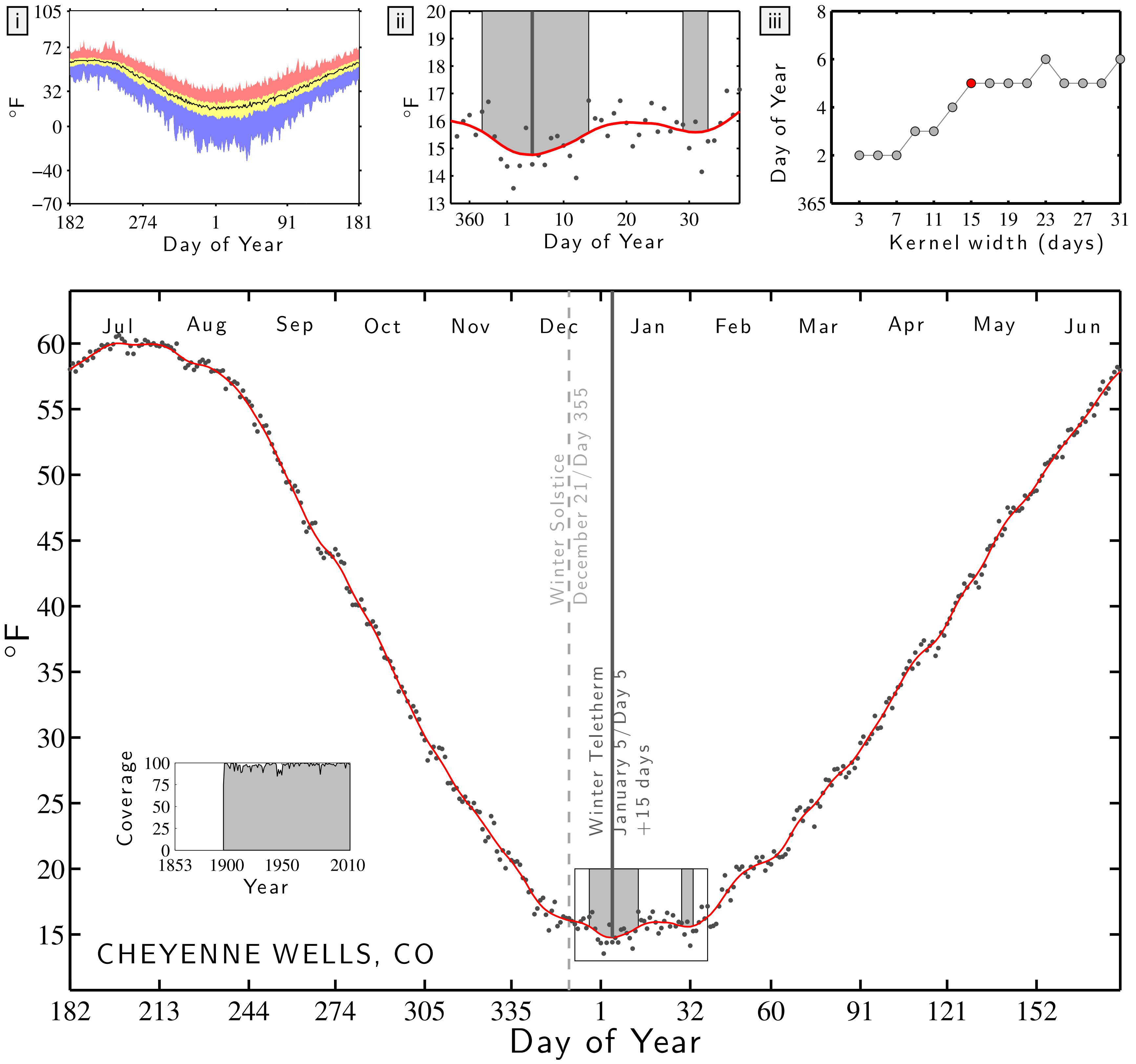

i
°F
Day of Year
ii
°F
Day of Year
iii
Day of Year
Kernel width (days)
Jul
Aug
Sep
Oct
Nov
Dec
Jan
Feb
Mar
Apr
May
Jun
Winter Solstice
December 21/Day 355
Winter Teletherm
January 5/Day 5
+15 days
Coverage
Year
CHEYENNE WELLS, CO
°F
Day of Year

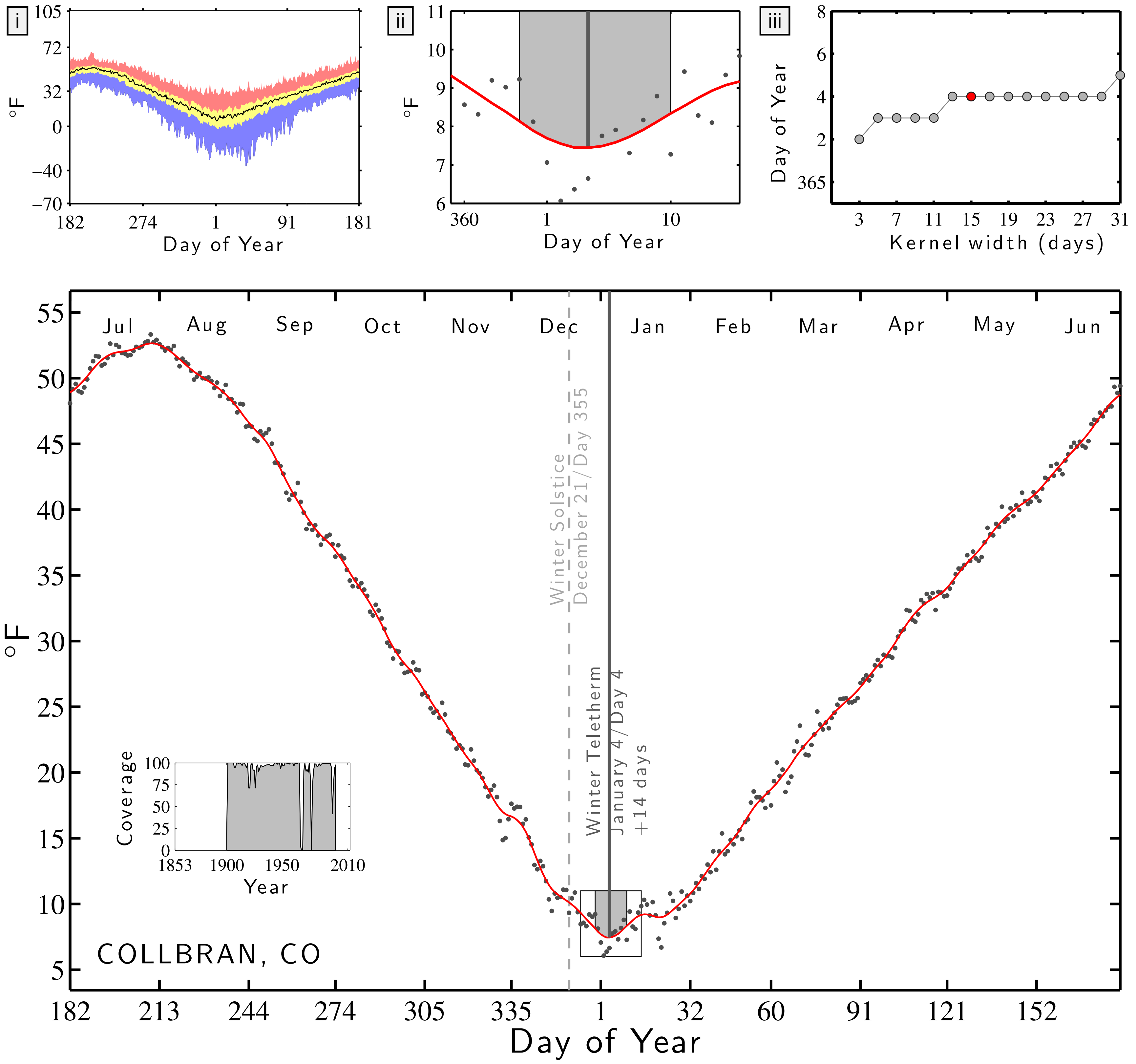

i
ii
iii
°F
Day of Year
Day of Year
Kernel width (days)
Jul
Aug
Sep
Oct
Nov
Dec
Jan
Feb
Mar
Apr
May
Jun
Winter Solstice
December 21/Day 355
Winter Teletherm
January 4/Day 4
+14 days
Coverage
Year
COLLBRAN, CO
Day of Year

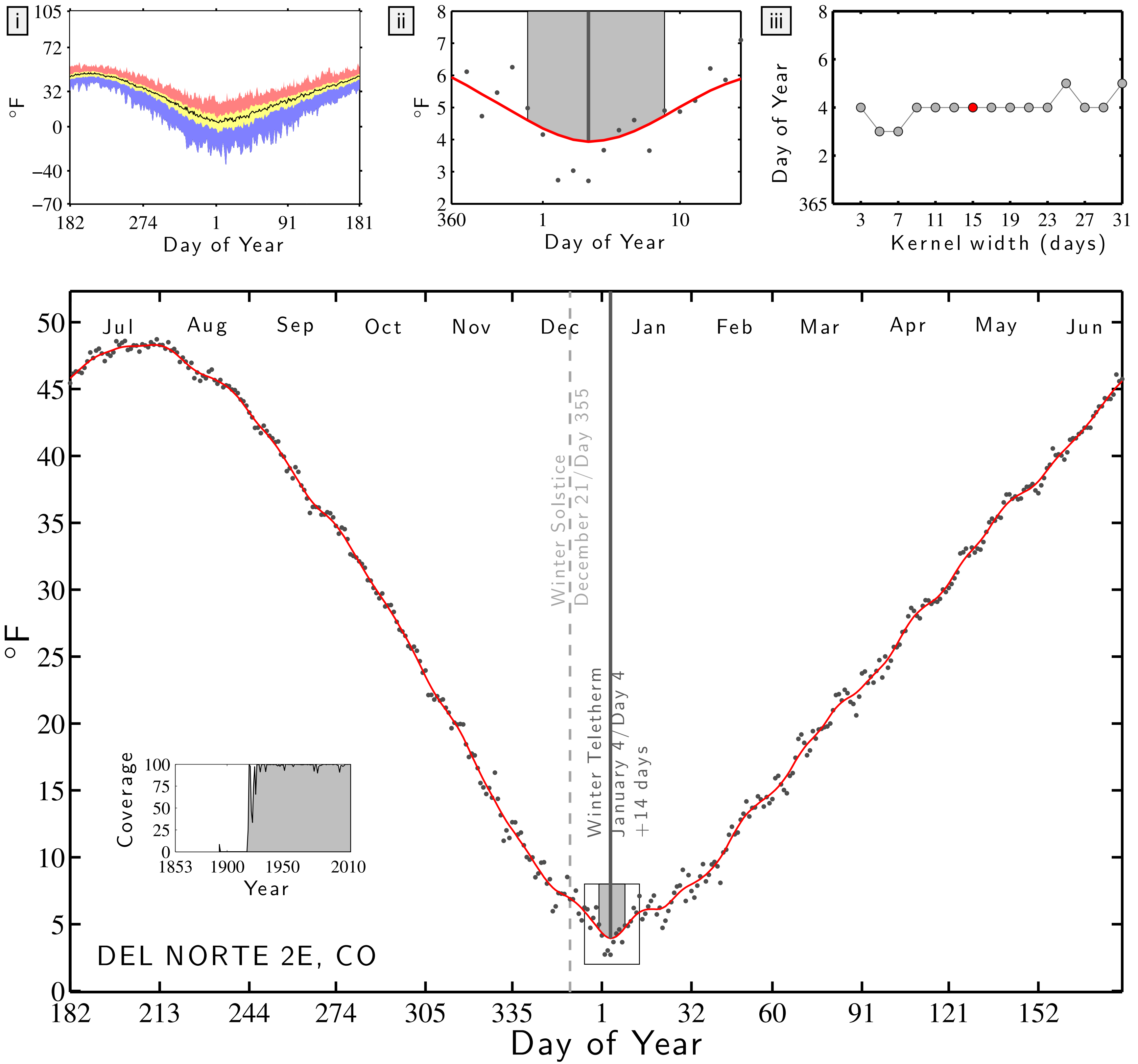

i
ii
iii
°F
Day of Year
Kernel width (days)
Jul
Aug
Sep
Oct
Nov
Dec
Jan
Feb
Mar
Apr
May
Jun
Winter Solstice
December 21/Day 355
Winter Teletherm
January 4/Day 4
+14 days
Coverage
Year
DEL NORTE 2E, CO

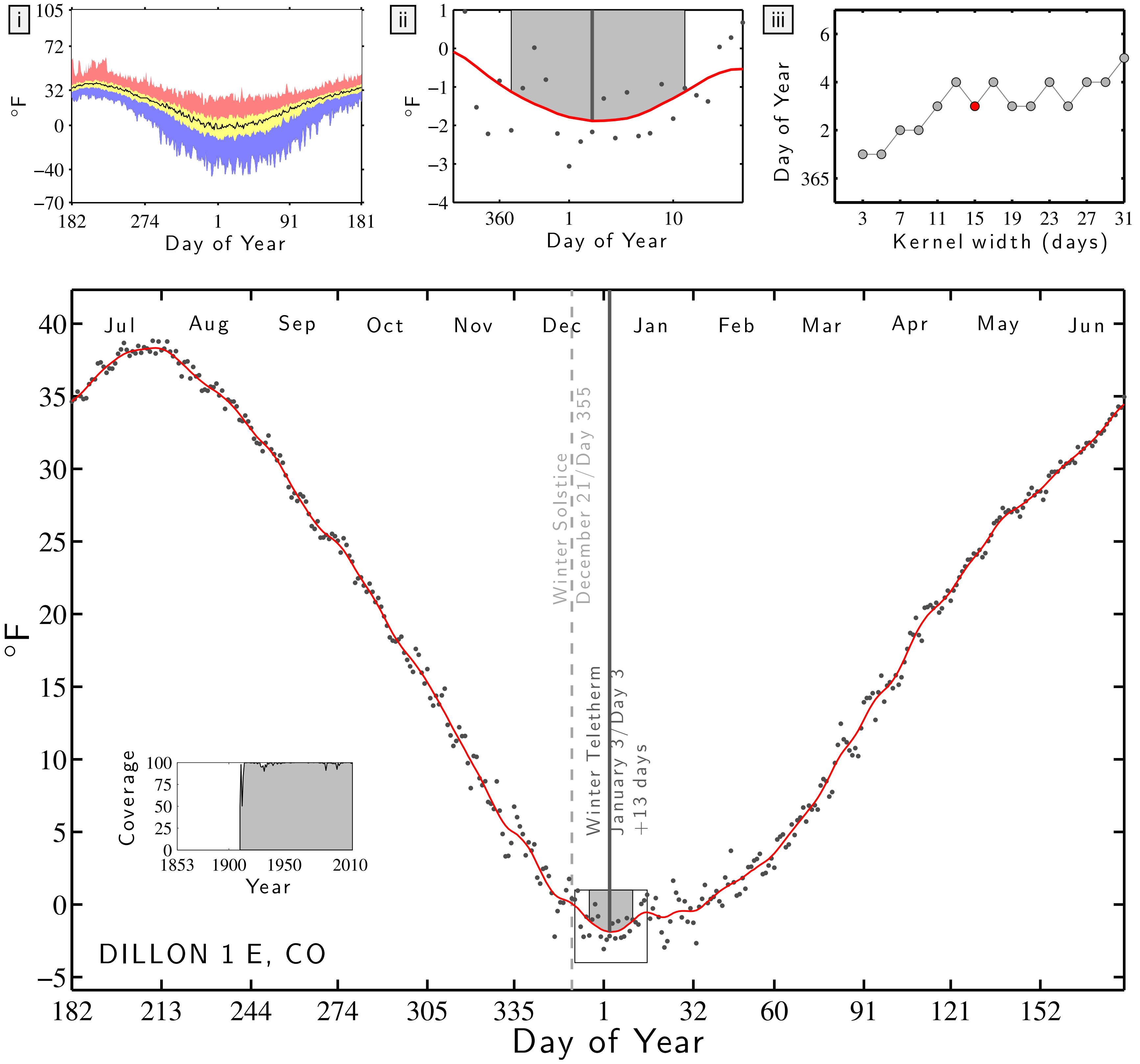

i
ii
iii
°F
Day of Year
Kernel width (days)
Jul
Aug
Sep
Oct
Nov
Dec
Jan
Feb
Mar
Apr
May
Jun
Winter Solstice
December 21/Day 355
Winter Teletherm
January 3/Day 3
+13 days
Coverage
Year
DILLON 1 E, CO

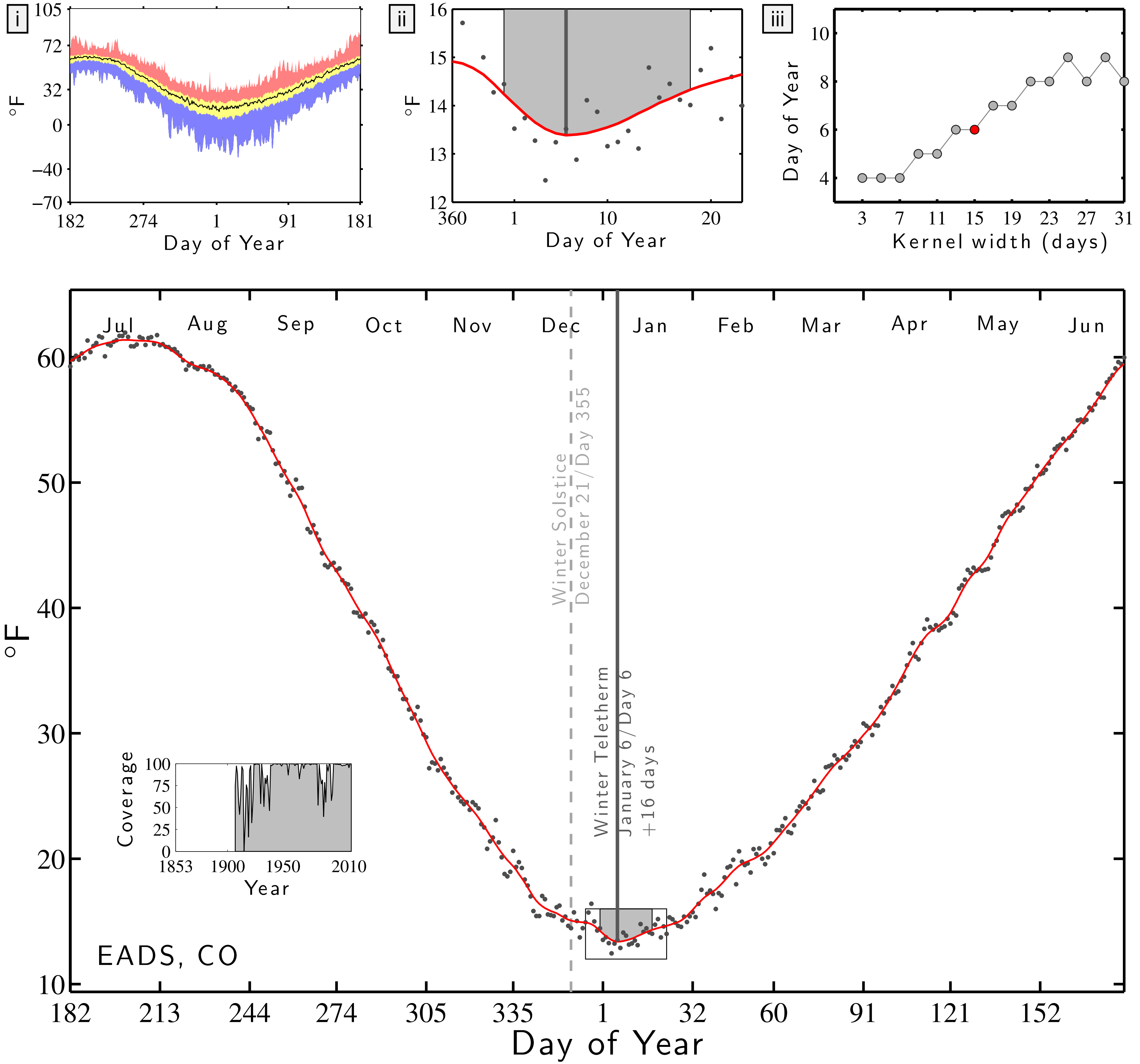

i
°F
Day of Year
ii
°F
Day of Year
iii
Day of Year
Kernel width (days)
Jul
Aug
Sep
Oct
Nov
Dec
Jan
Feb
Mar
Apr
May
Jun
Winter Solstice
December 21/Day 355
Winter Teletherm
January 6/Day 6
+16 days
Coverage
Year
EADS, CO
°F
Day of Year

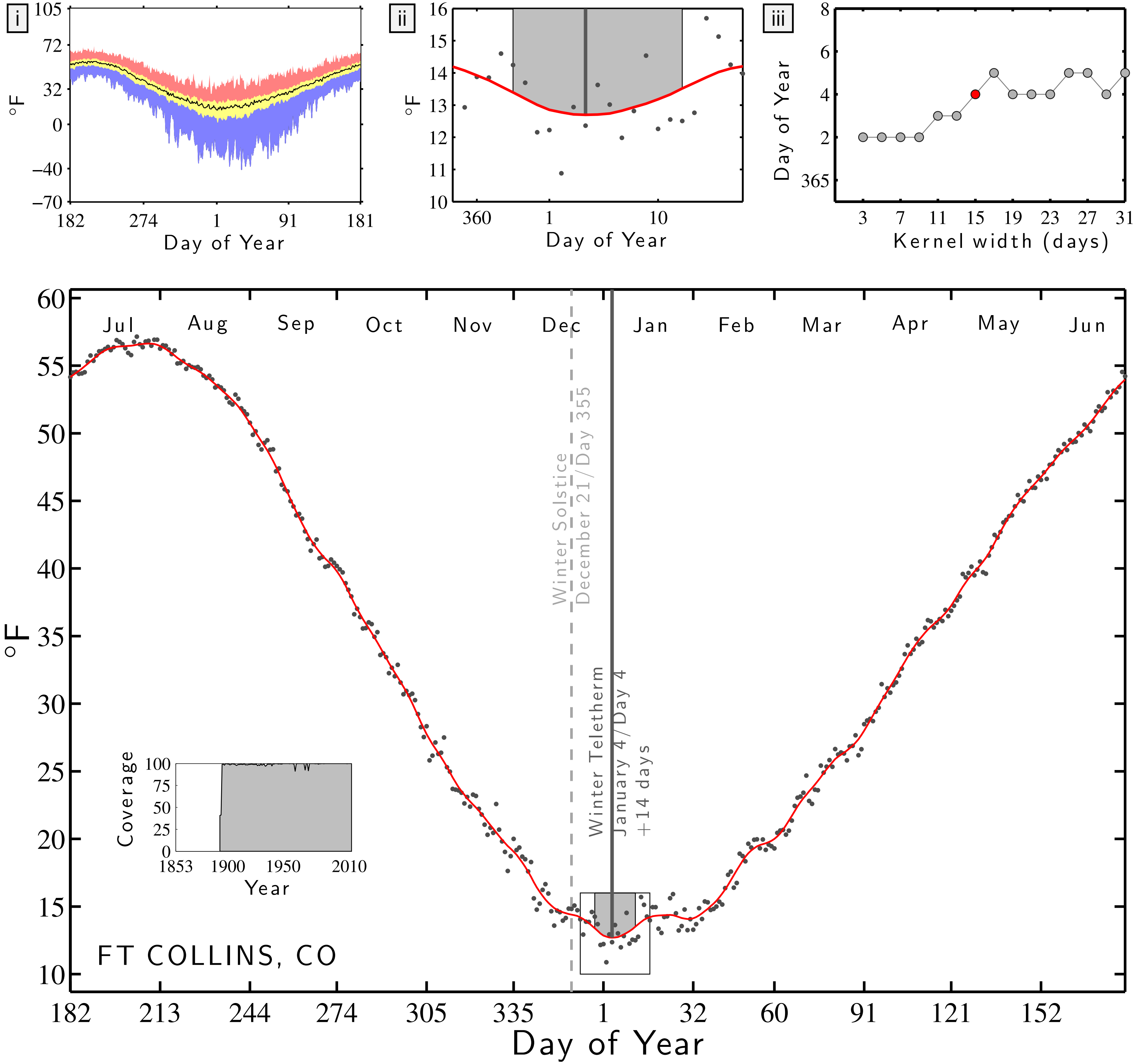

i
°F
Day of Year
ii
°F
Day of Year
iii
Day of Year
Kernel width (days)
Jul
Aug
Sep
Oct
Nov
Dec
Jan
Feb
Mar
Apr
May
Jun
Winter Solstice
December 21/Day 355
Winter Teletherm
January 4/Day 4
+14 days
Coverage
Year
FT COLLINS, CO
°F
Day of Year

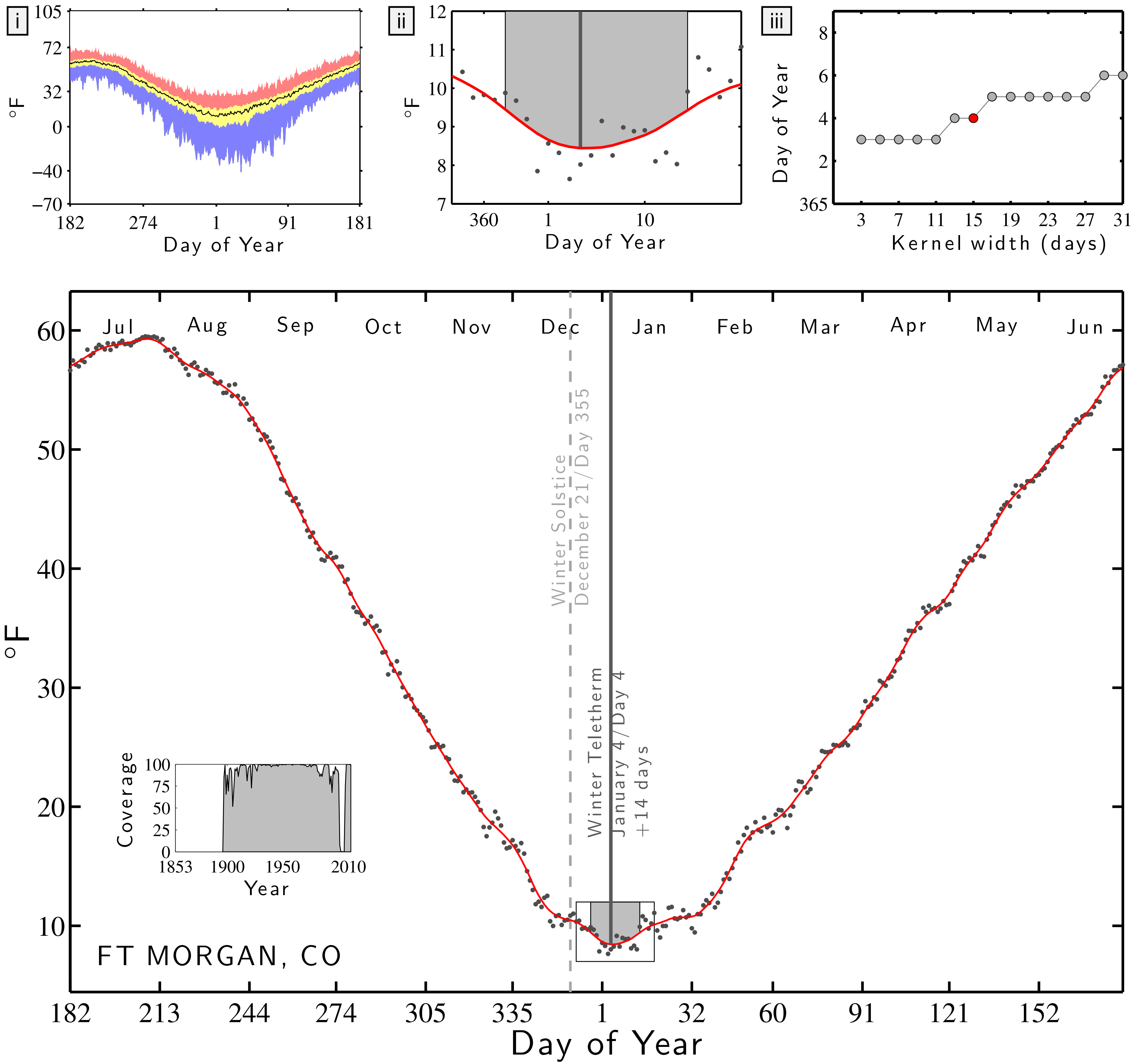

i
°F
Day of Year
ii
°F
Day of Year
iii
Day of Year
Kernel width (days)
Jul
Aug
Sep
Oct
Nov
Dec
Jan
Feb
Mar
Apr
May
Jun
Winter Solstice
December 21/Day 355
Winter Teletherm
January 4/Day 4
+14 days
Coverage
Year
FT MORGAN, CO
°F
Day of Year

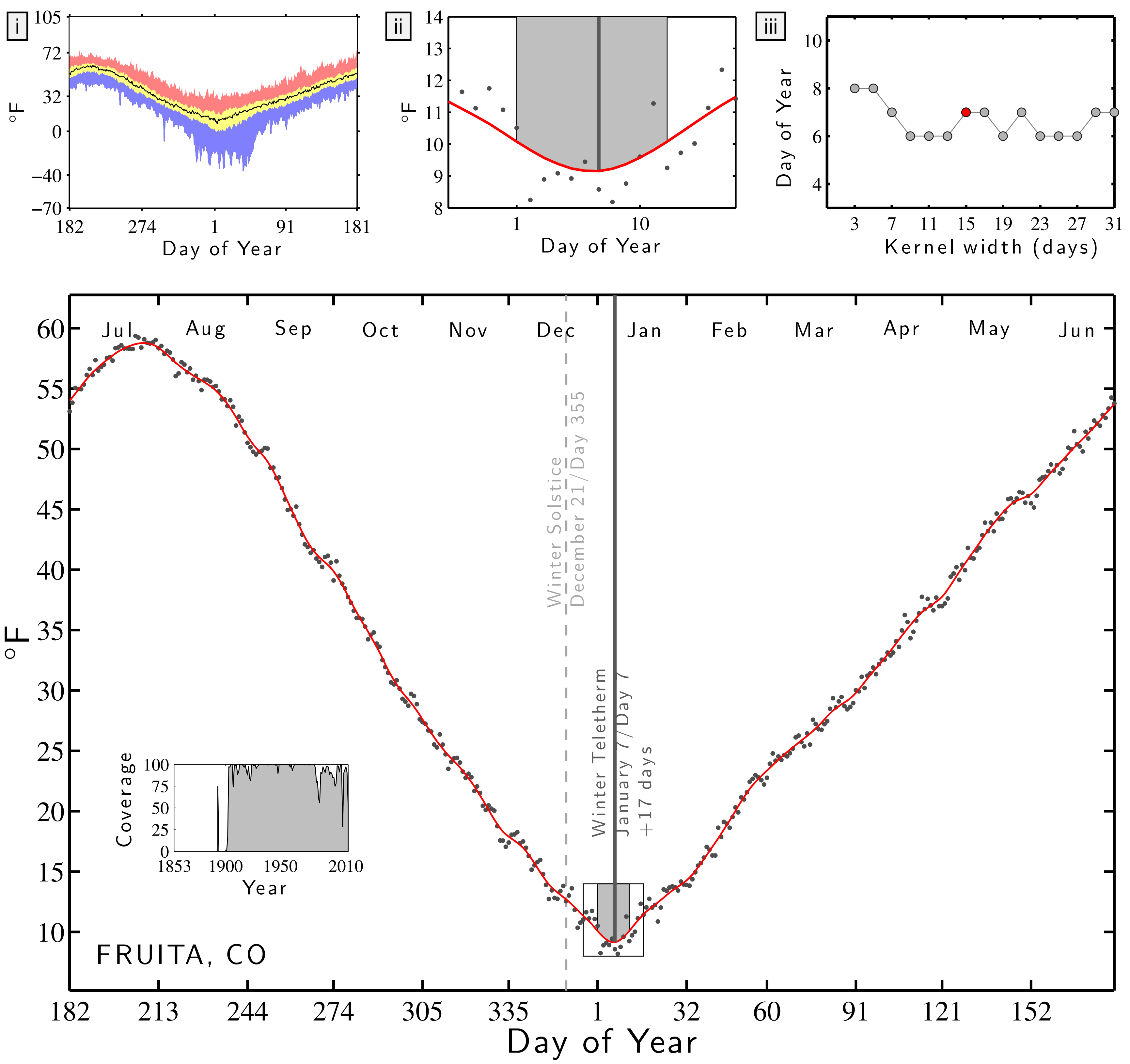

i
°F
Day of Year
ii
°F
Day of Year
iii
Day of Year
Kernel width (days)
Jul
Aug
Sep
Oct
Nov
Dec
Jan
Feb
Mar
Apr
May
Jun
Winter Solstice
December 21/Day 355
Winter Teletherm
January 7/Day 7
+17 days
Coverage
Year
FRUITA, CO
°F
Day of Year

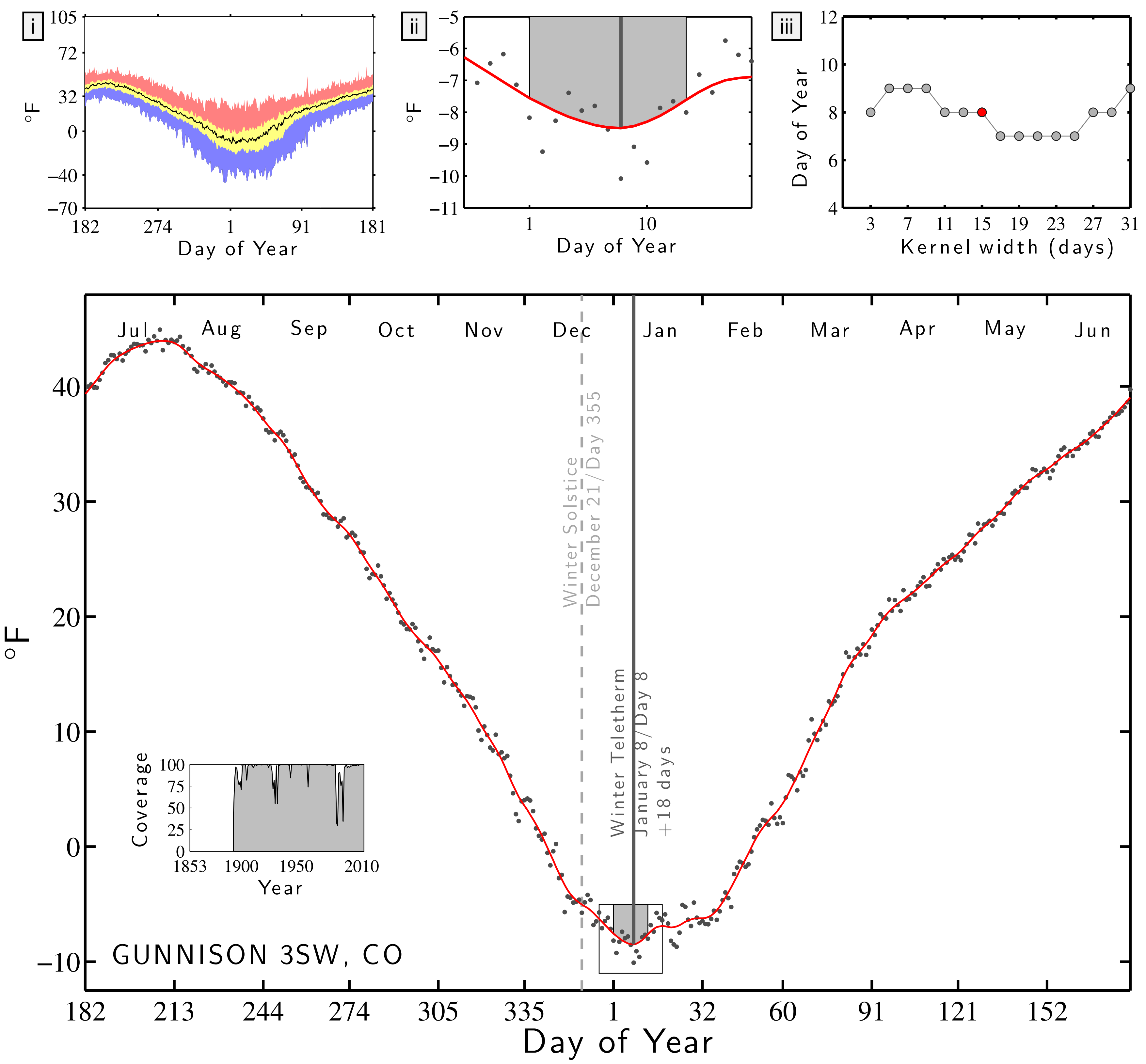

i
°F
Day of Year
ii
°F
Day of Year
iii
Day of Year
Kernel width (days)
Jul
Aug
Sep
Oct
Nov
Dec
Jan
Feb
Mar
Apr
May
Jun
Winter Solstice
December 21/Day 355
Winter Teletherm
January 8/Day 8
+18 days
Coverage
Year
GUNNISON 3SW, CO
°F
Day of Year

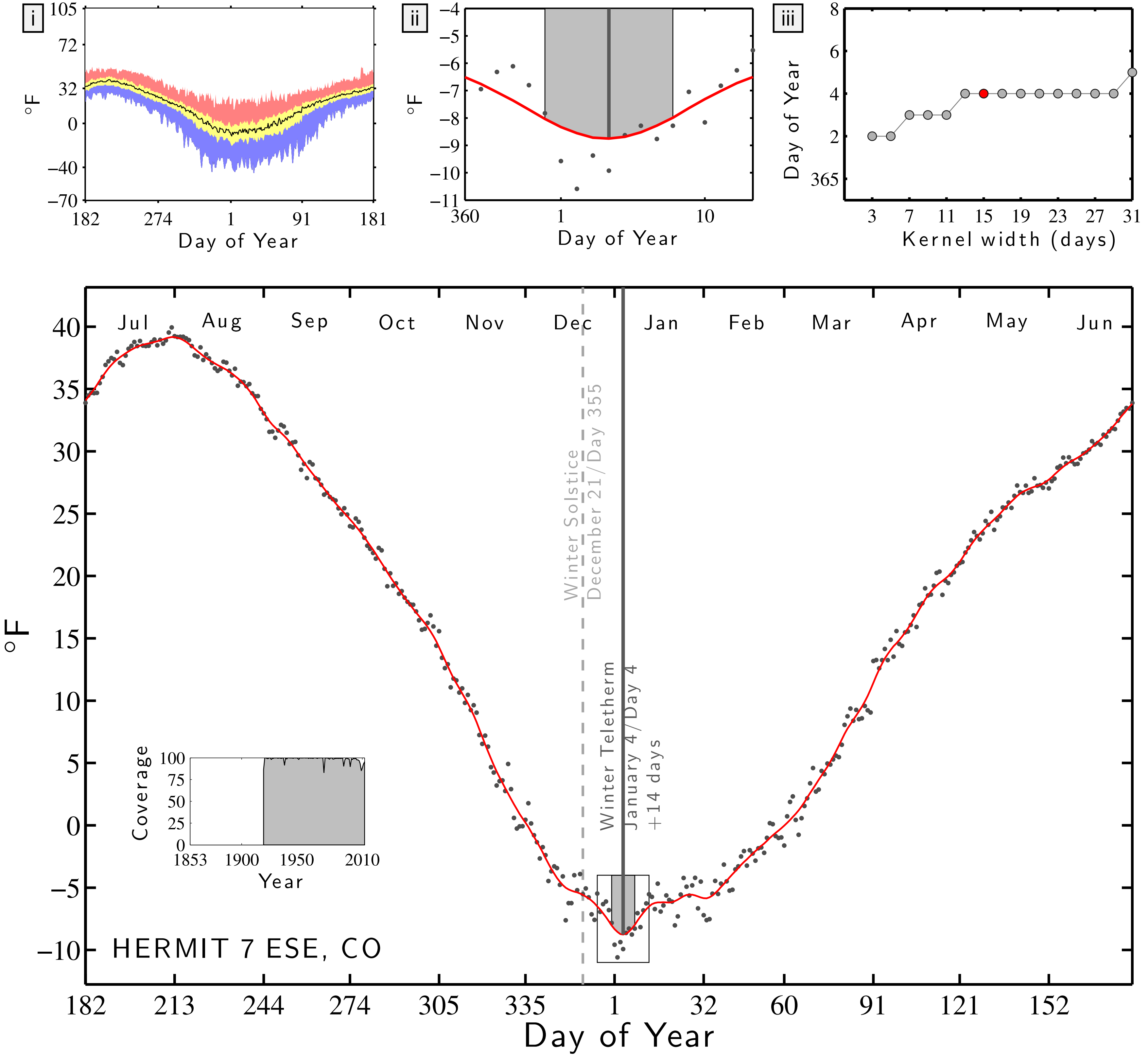

i
ii
iii
°F
Day of Year
Kernel width (days)
Jul
Aug
Sep
Oct
Nov
Dec
Jan
Feb
Mar
Apr
May
Jun
Winter Solstice
December 21/Day 355
Winter Teletherm
January 4/Day 4
+14 days
Coverage
Year
HERMIT 7 ESE, CO

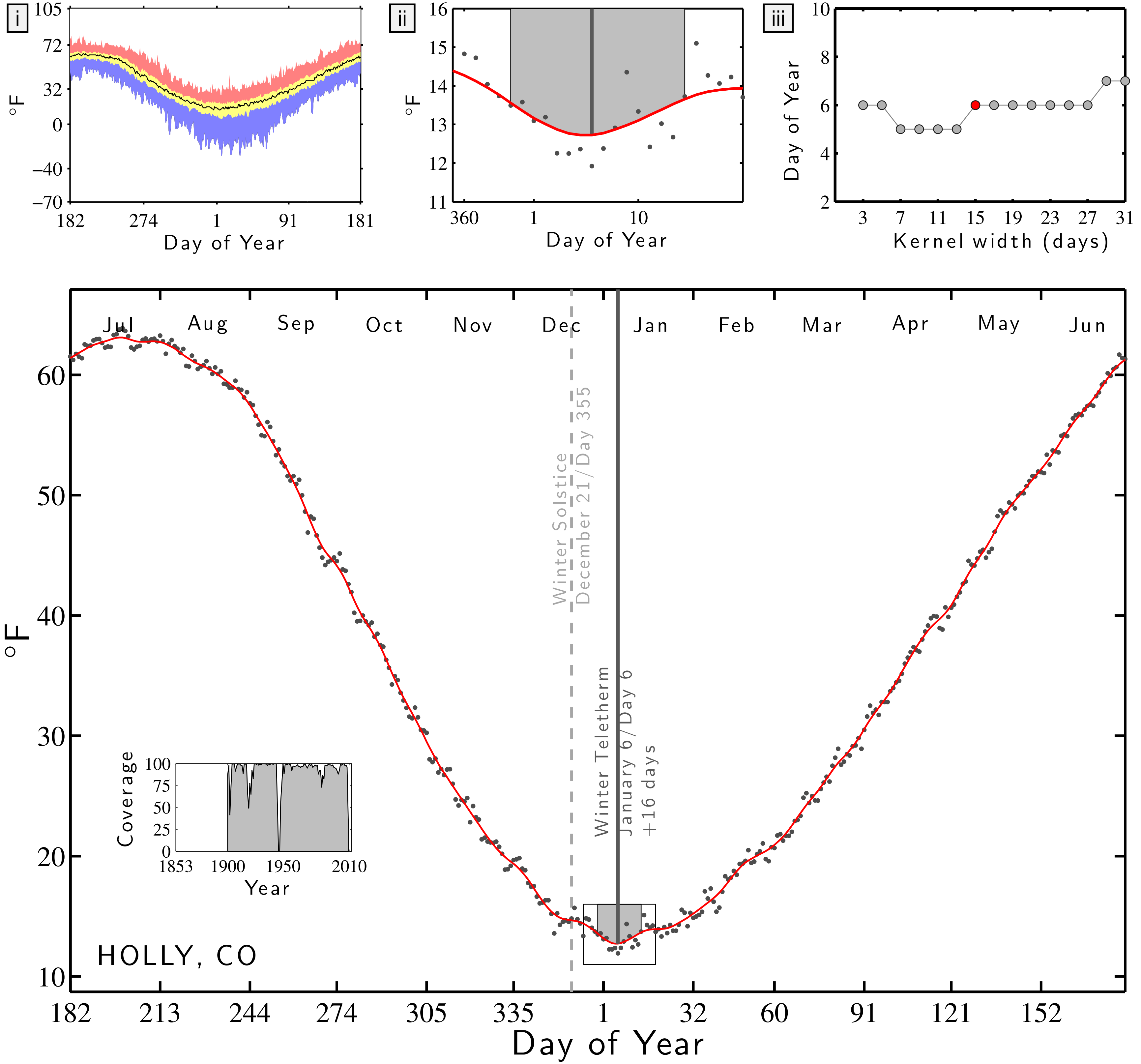

i
°F
Day of Year
ii
°F
Day of Year
iii
Day of Year
Kernel width (days)
Jul
Aug
Sep
Oct
Nov
Dec
Jan
Feb
Mar
Apr
May
Jun
Winter Solstice
December 21/Day 355
Winter Teletherm
January 6/Day 6
+16 days
Coverage
Year
HOLLY, CO
°F
Day of Year

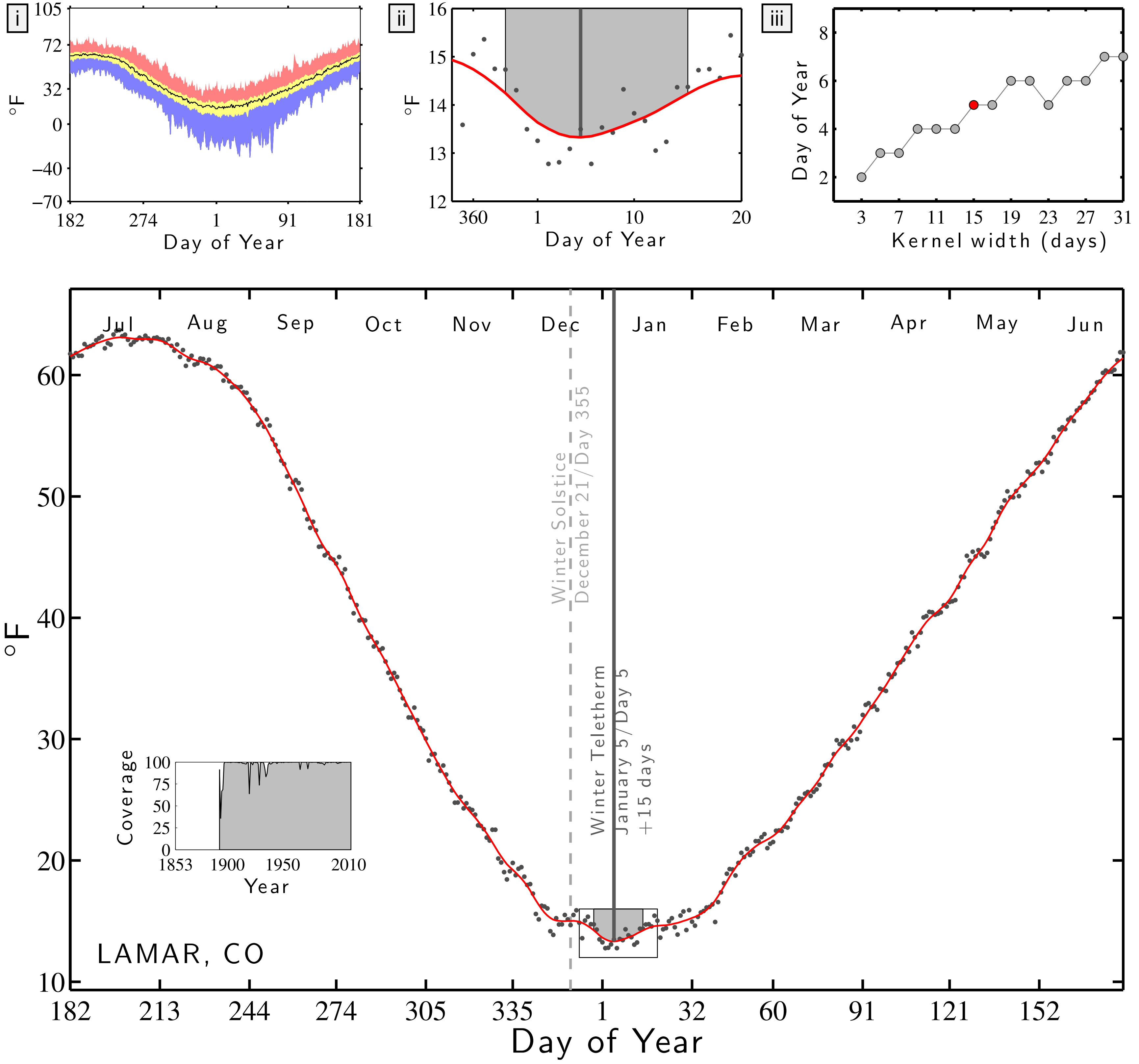

i
ii
iii
Day of Year
Kernel width (days)
°F
Day of Year
Jul
Aug
Sep
Oct
Nov
Dec
Jan
Feb
Mar
Apr
May
Jun
Winter Solstice
December 21/Day 355
Winter Teletherm
January 5/Day 5
+15 days
Coverage
Year
LAMAR, CO

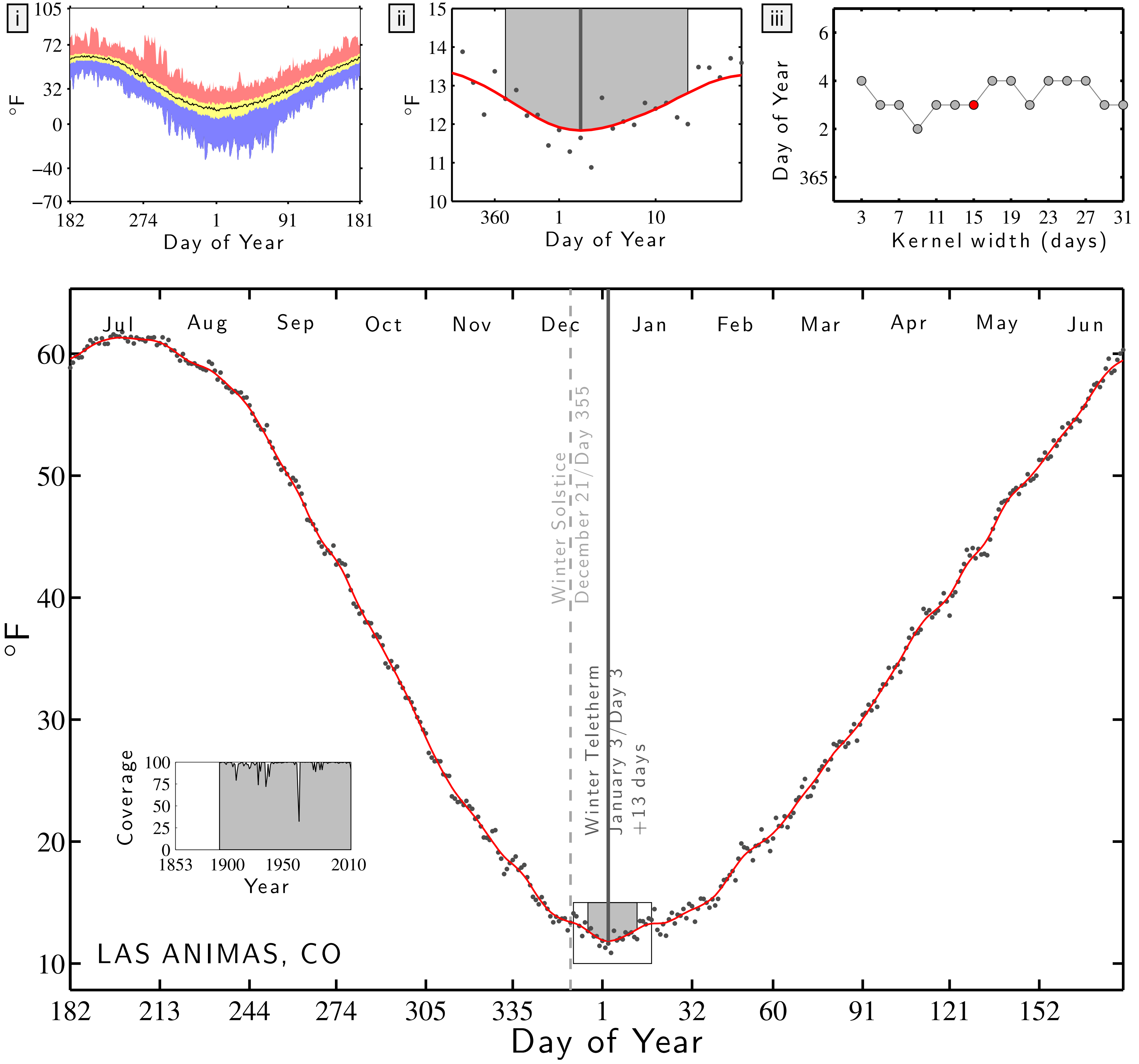

i
ii
iii
°F
Day of Year
Kernel width (days)
Jul
Aug
Sep
Oct
Nov
Dec
Jan
Feb
Mar
Apr
May
Jun
Winter Solstice
December 21/Day 355
Winter Teletherm
January 3/Day 3
+13 days
Coverage
Year
LAS ANIMAS, CO

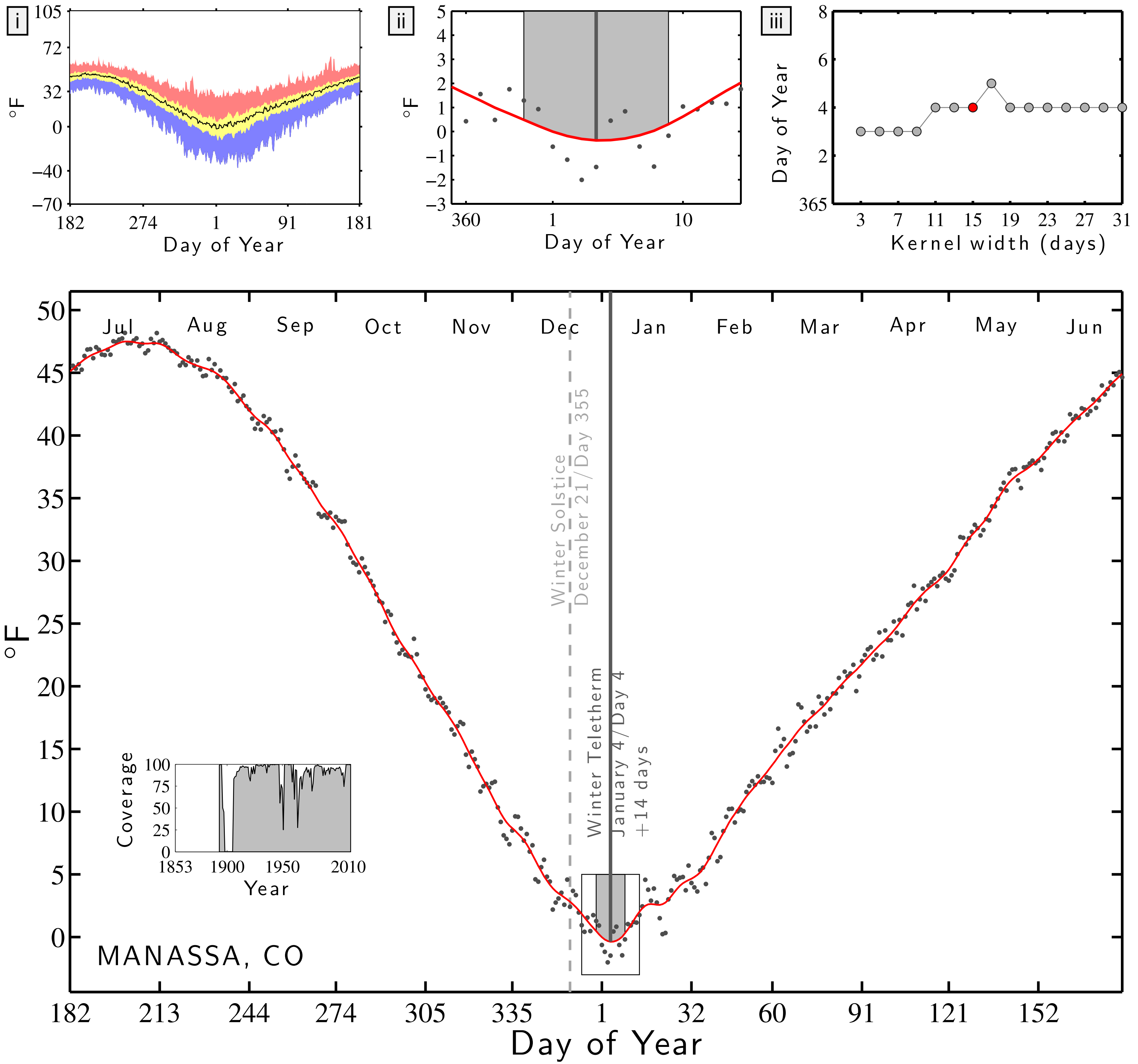

i
°F
Day of Year
ii
iii
Day of Year
Kernel width (days)
Jul
Aug
Sep
Oct
Nov
Dec
Jan
Feb
Mar
Apr
May
Jun
Winter Solstice
December 21/Day 355
Winter Teletherm
January 4/Day 4
+14 days
Coverage
Year
MANASSA, CO

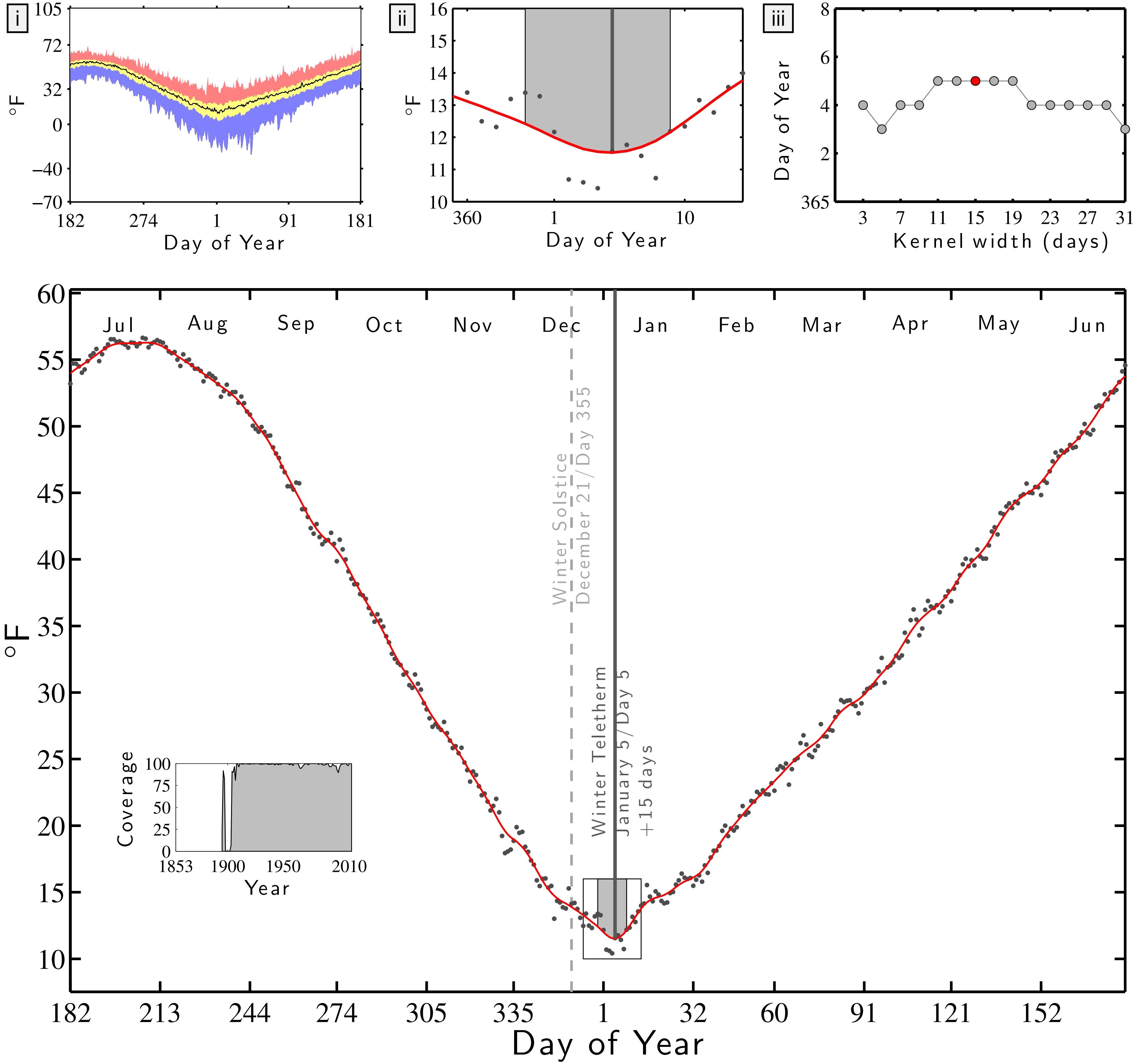

i
°F
Day of Year
ii
°F
Day of Year
iii
Day of Year
Kernel width (days)
Jul
Aug
Sep
Oct
Nov
Dec
Jan
Feb
Mar
Apr
May
Jun
Winter Solstice
December 21/Day 355
Winter Teletherm
January 5/Day 5
+15 days
Coverage
Year
°F
Day of Year

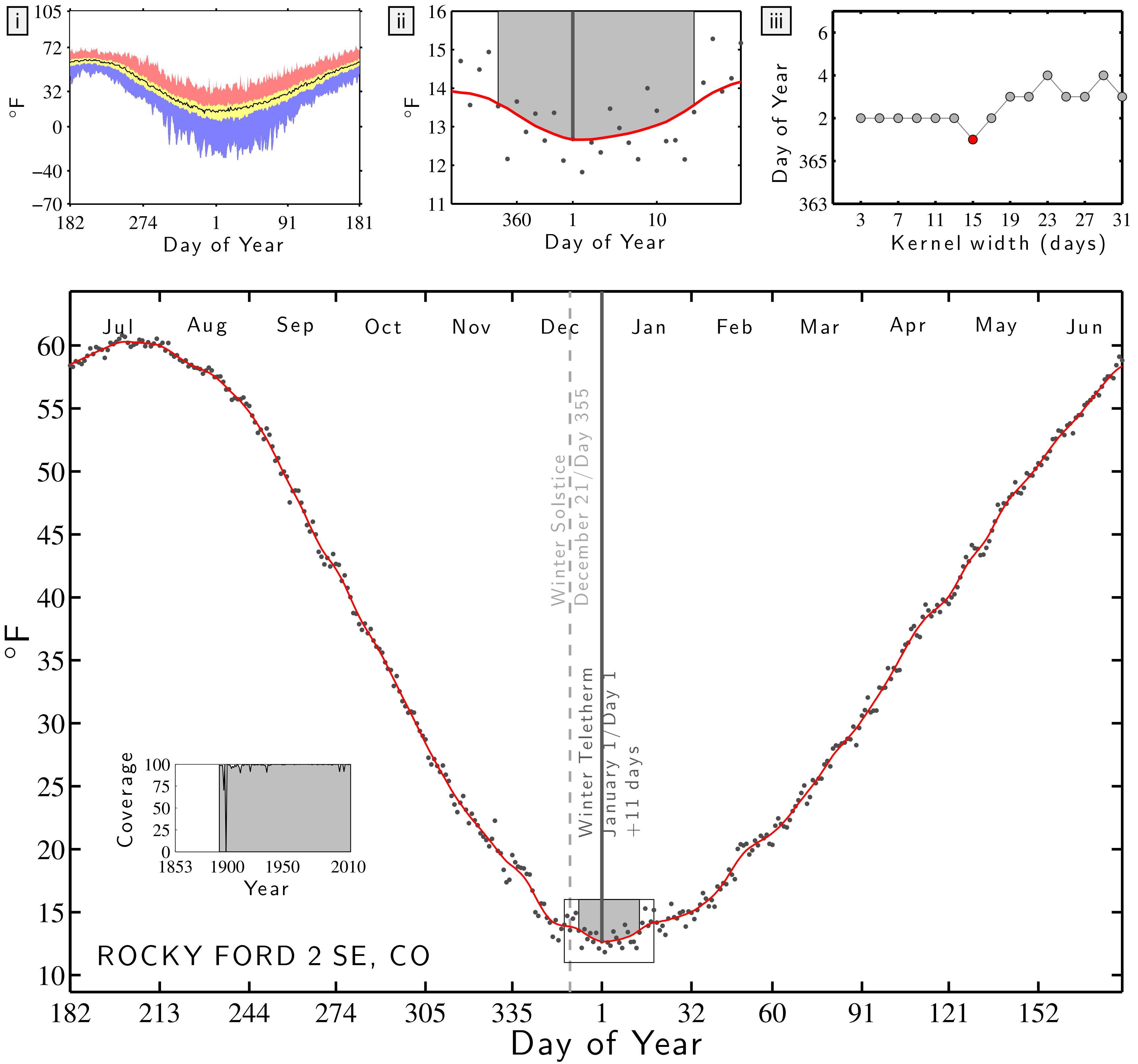

i
ii
iii
°F
Day of Year
Day of Year
Kernel width (days)
Jul
Aug
Sep
Oct
Nov
Dec
Jan
Feb
Mar
Apr
May
Jun
Winter Solstice
December 21/Day 355
Winter Teletherm
January 1/Day 1
+11 days
Coverage
Year
ROCKY FORD 2 SE, CO

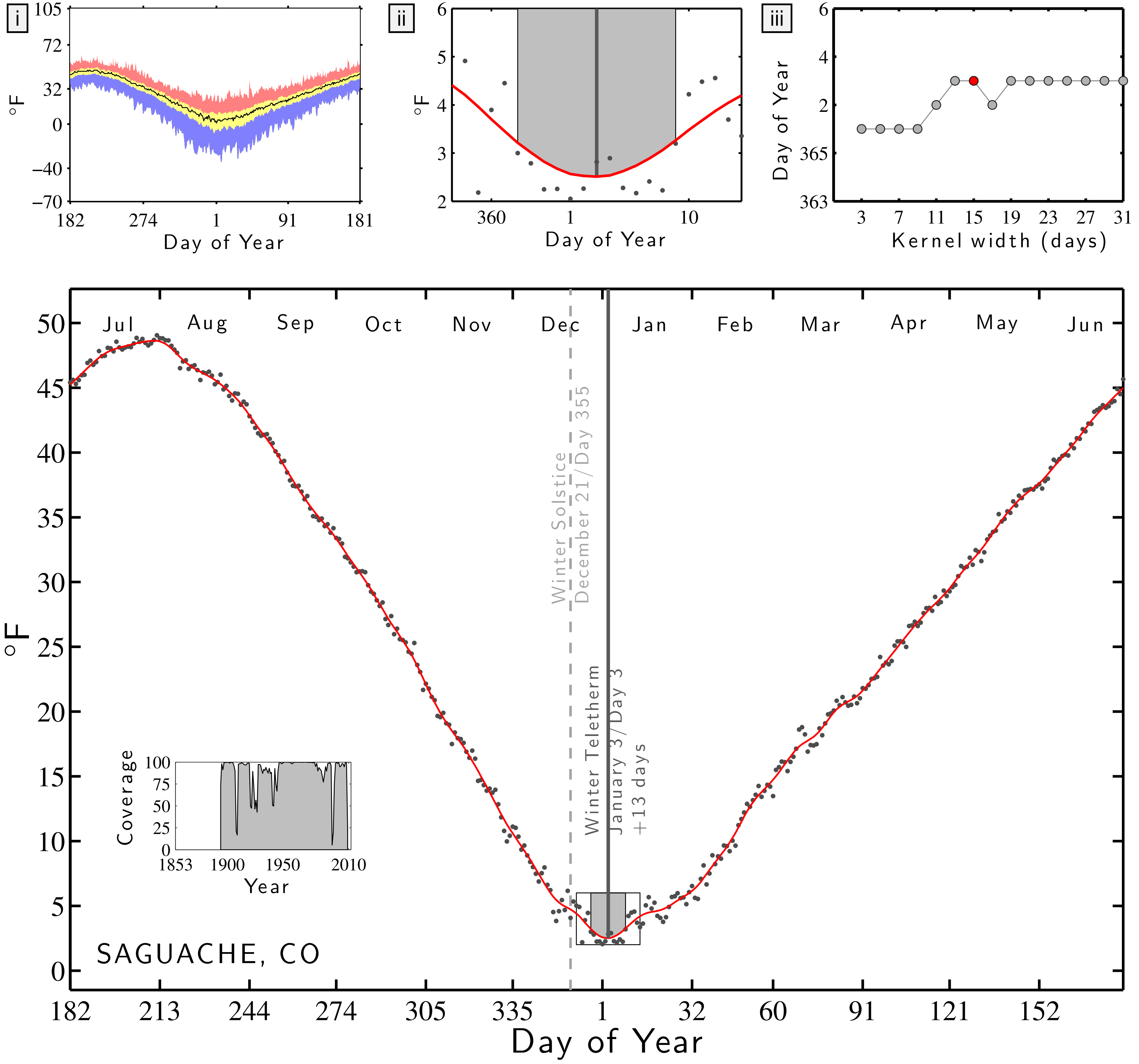

i
ii
iii
°F
Day of Year
Kernel width (days)
Jul
Aug
Sep
Oct
Nov
Dec
Jan
Feb
Mar
Apr
May
Jun
Winter Solstice
December 21/Day 355
Winter Teletherm
January 3/Day 3
+13 days
Coverage
Year
SAGUACHE, CO

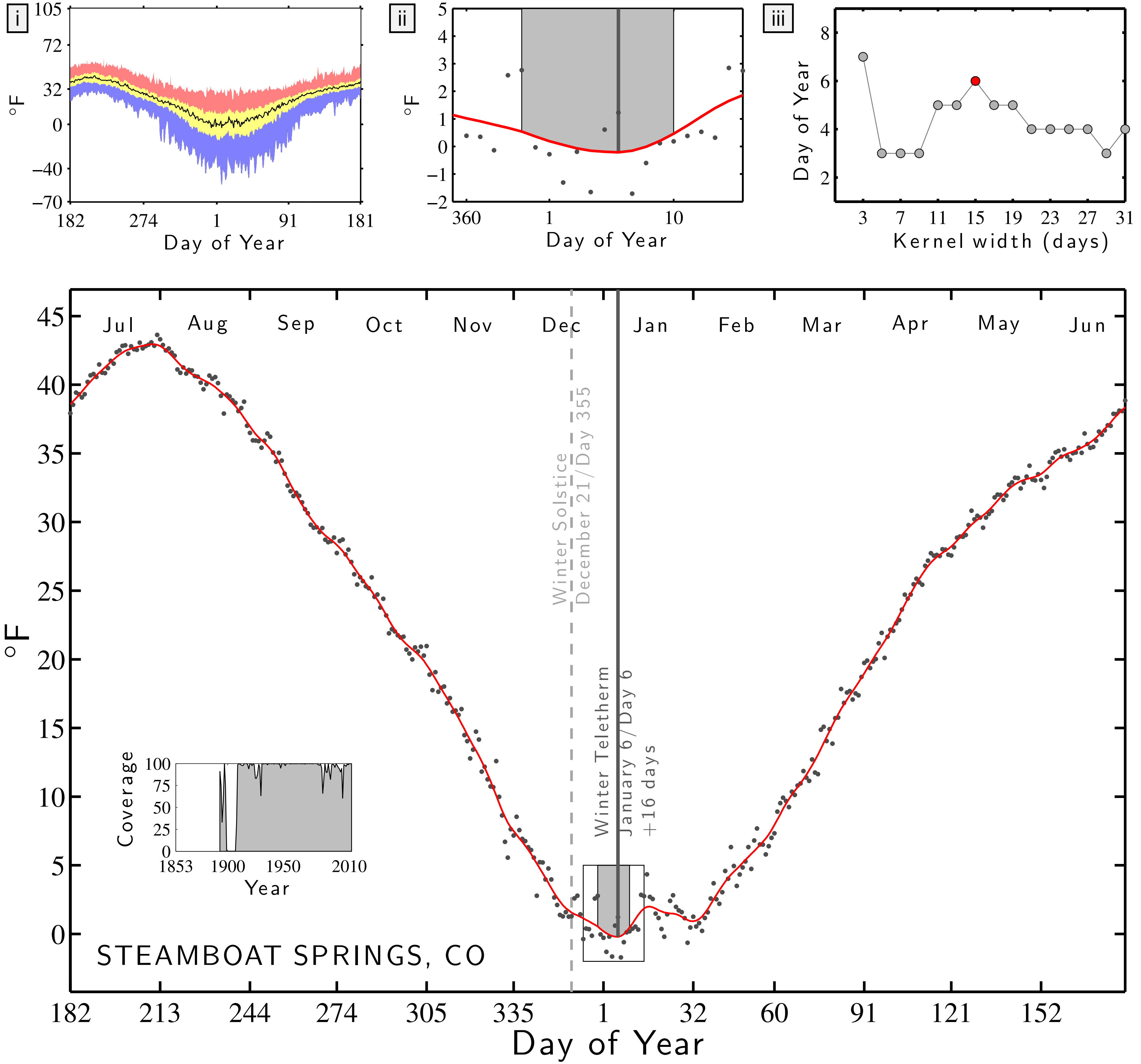

i
ii
iii
Day of Year
Kernel width (days)
Jul
Aug
Sep
Oct
Nov
Dec
Jan
Feb
Mar
Apr
May
Jun
Winter Solstice
December 21/Day 355
Winter Teletherm
January 6/Day 6
+16 days
Coverage
Year
STEAMBOAT SPRINGS, CO
°F

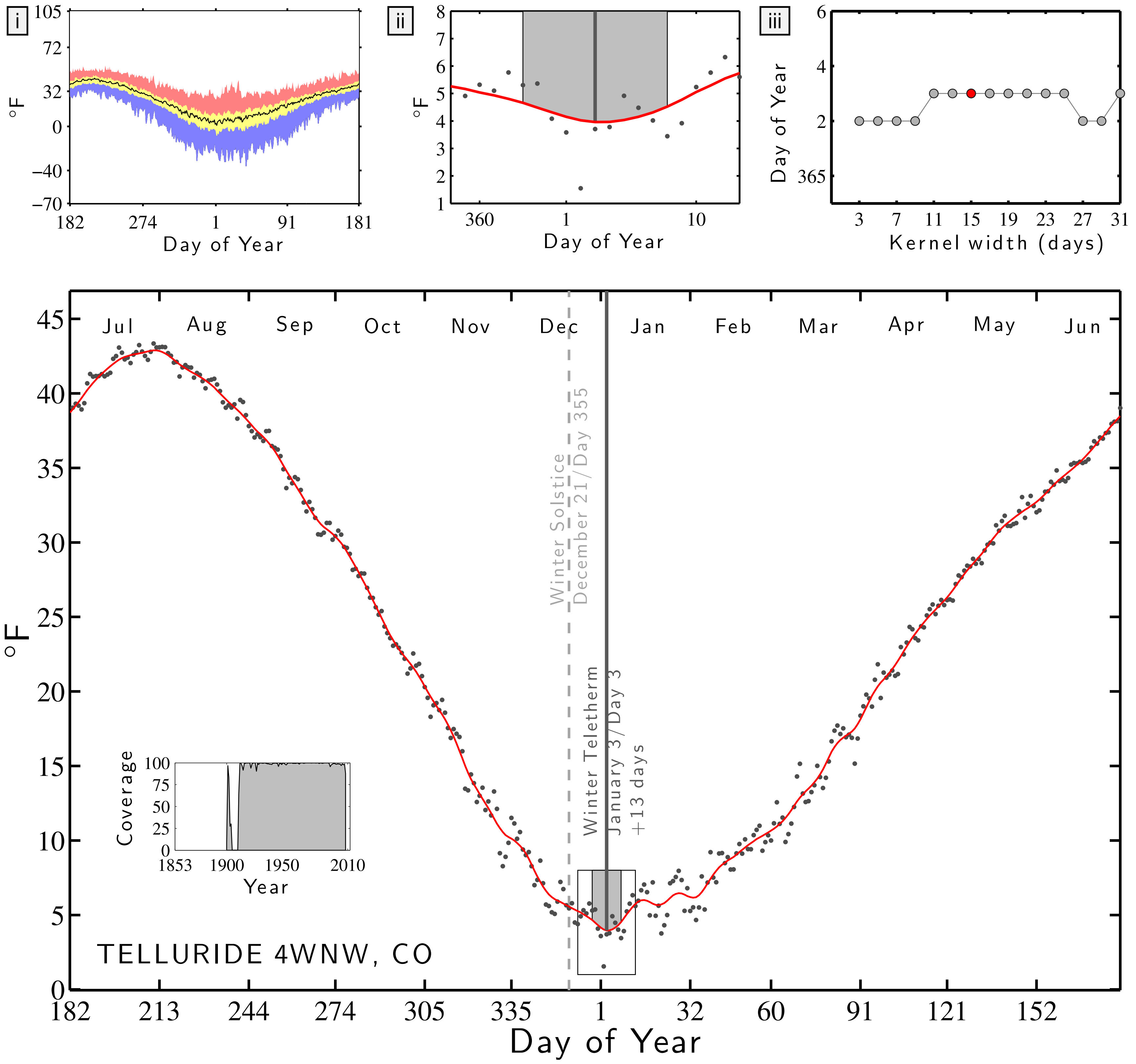

i
ii
iii
°F
Day of Year
Kernel width (days)
Jul
Aug
Sep
Oct
Nov
Dec
Jan
Feb
Mar
Apr
May
Jun
Winter Solstice
December 21/Day 355
Winter Teletherm
January 3/Day 3
+13 days
Coverage
Year
TELLURIDE 4WNW, CO

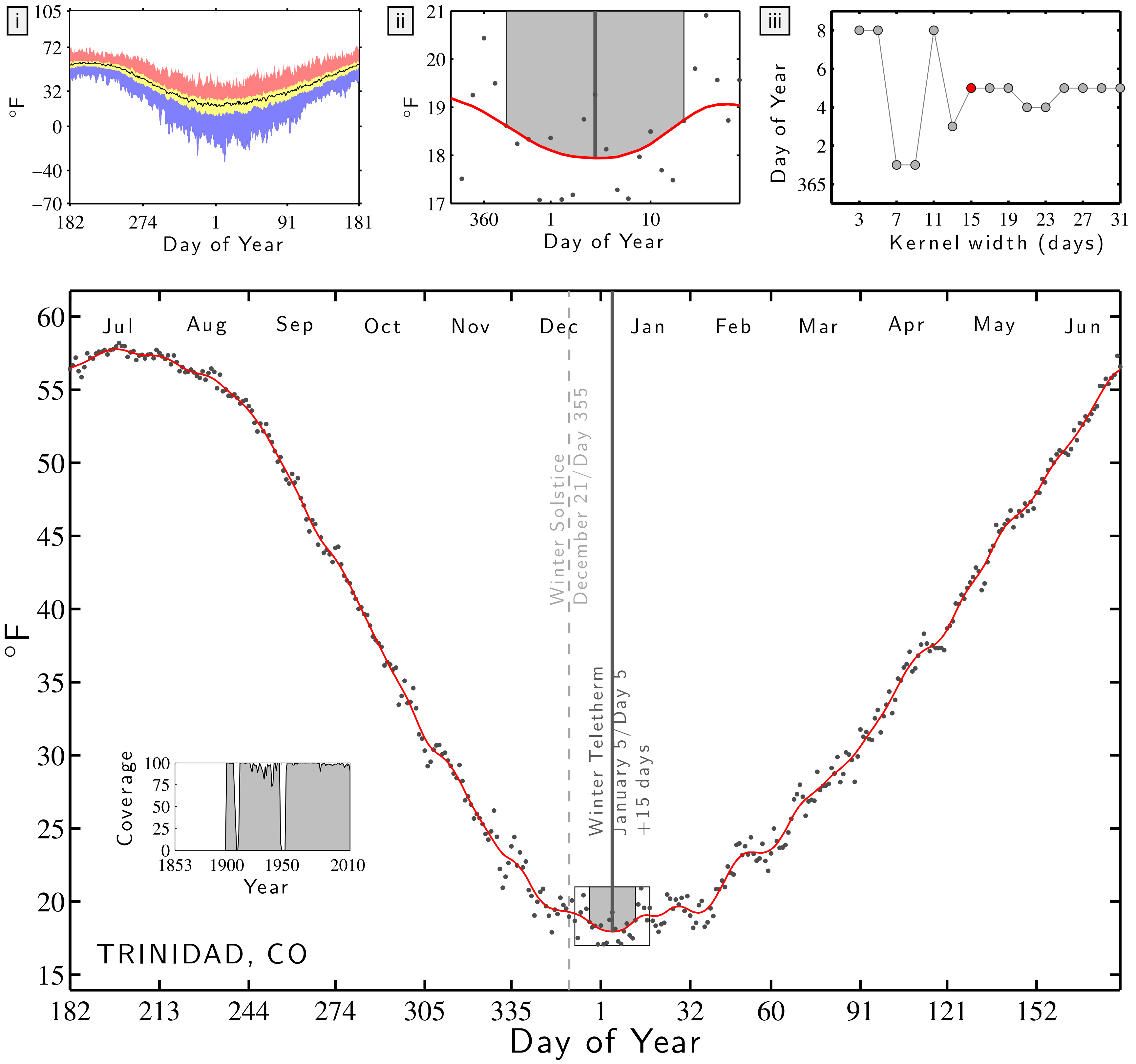

i
ii
iii
°F
Day of Year
Kernel width (days)
Jul
Aug
Sep
Oct
Nov
Dec
Jan
Feb
Mar
Apr
May
Jun
Winter Solstice
December 21/Day 355
Winter Teletherm
January 5/Day 5
+15 days
Coverage
Year
TRINIDAD, CO

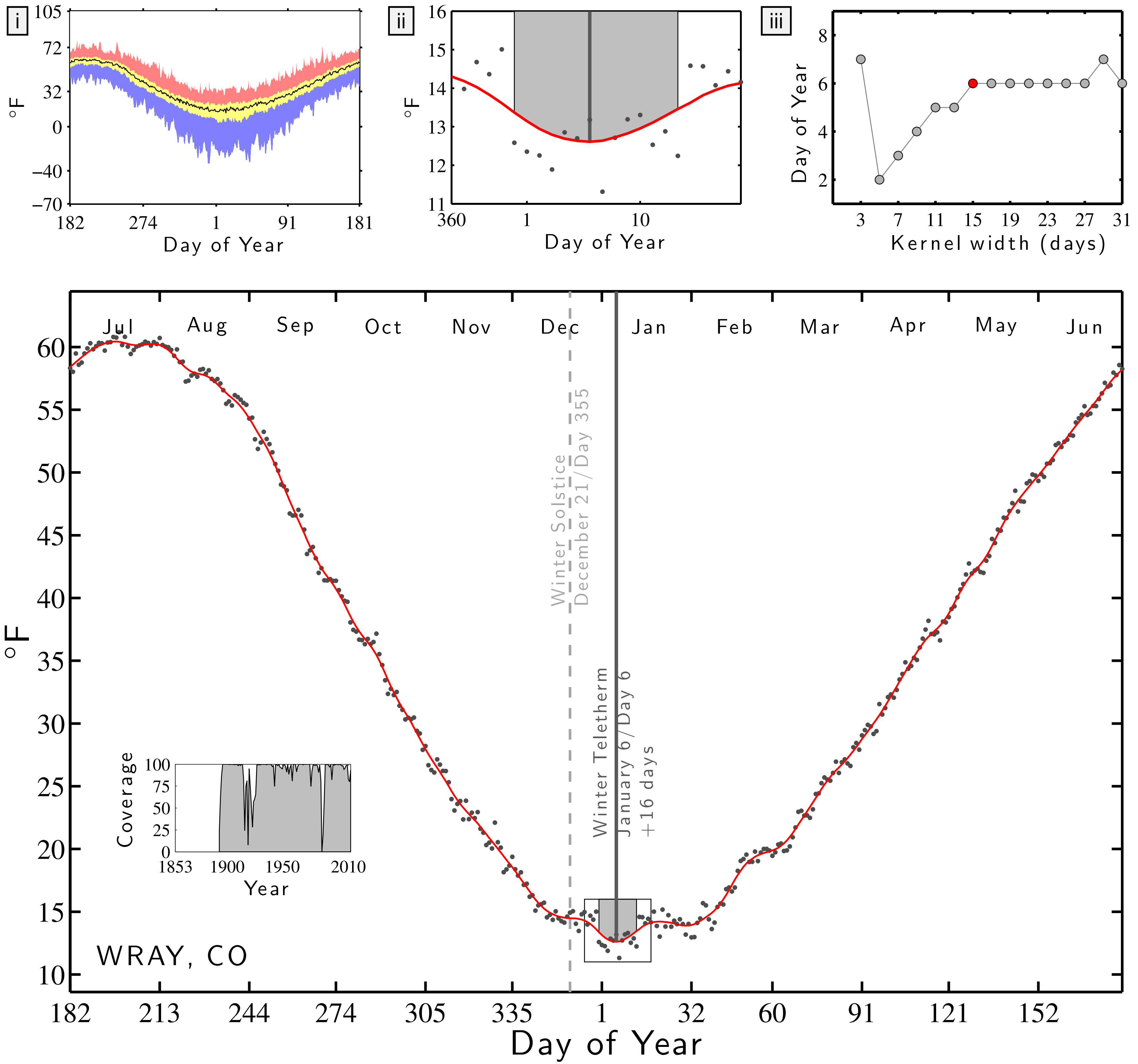

i
ii
iii
°F
Day of Year
Day of Year
Kernel width (days)
Jul
Aug
Sep
Oct
Nov
Dec
Jan
Feb
Mar
Apr
May
Jun
Winter Solstice
December 21/Day 355
Winter Teletherm
January 6/Day 6
+16 days
Coverage
Year
WRAY, CO

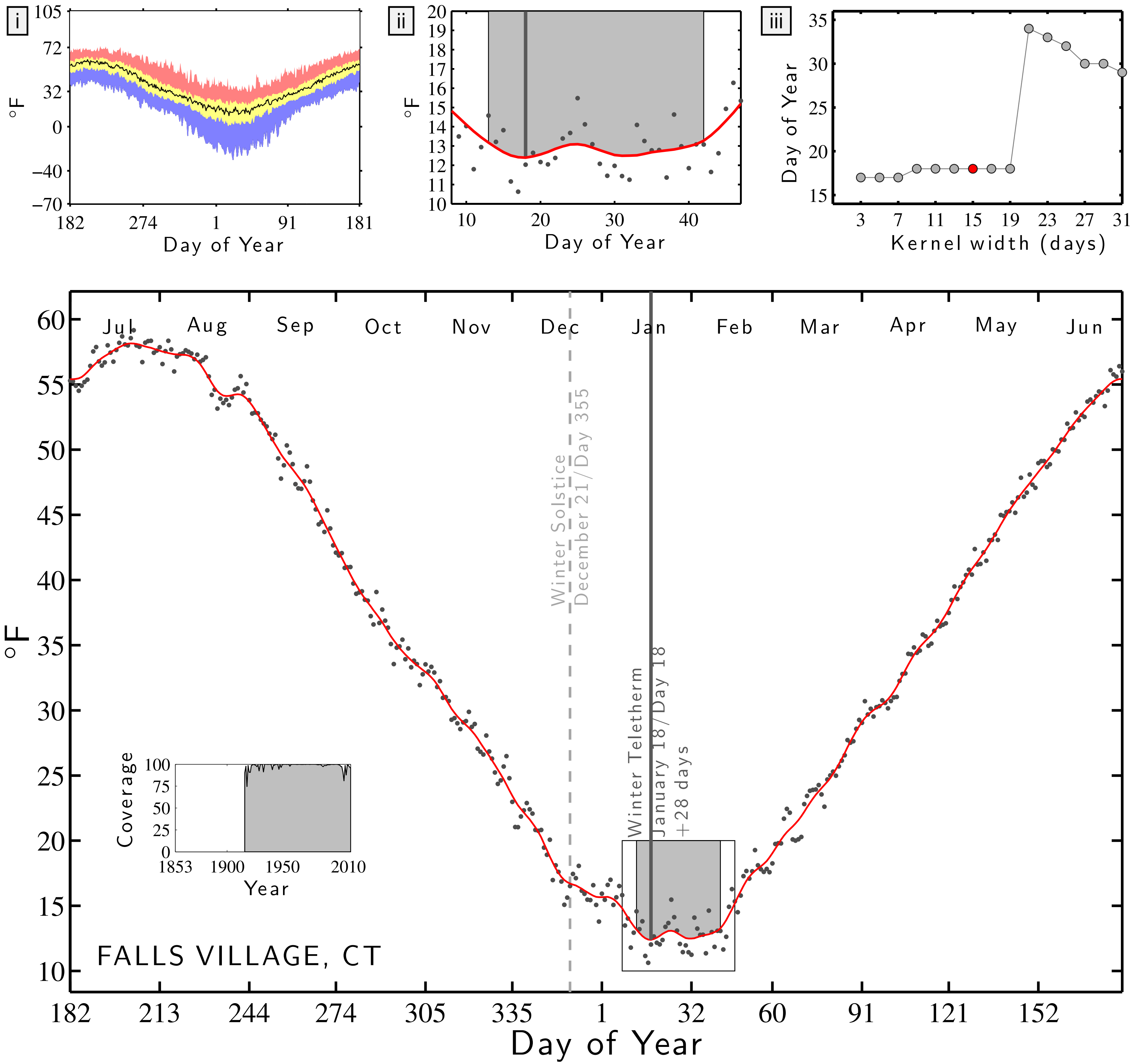

i
ii
iii
°F
Day of Year
Day of Year
Kernel width (days)
Jul
Aug
Sep
Oct
Nov
Dec
Jan
Feb
Mar
Apr
May
Jun
Winter Solstice
December 21/Day 355
Winter Teletherm
January 18/Day 18
+28 days
Coverage
Year
FALLS VILLAGE, CT

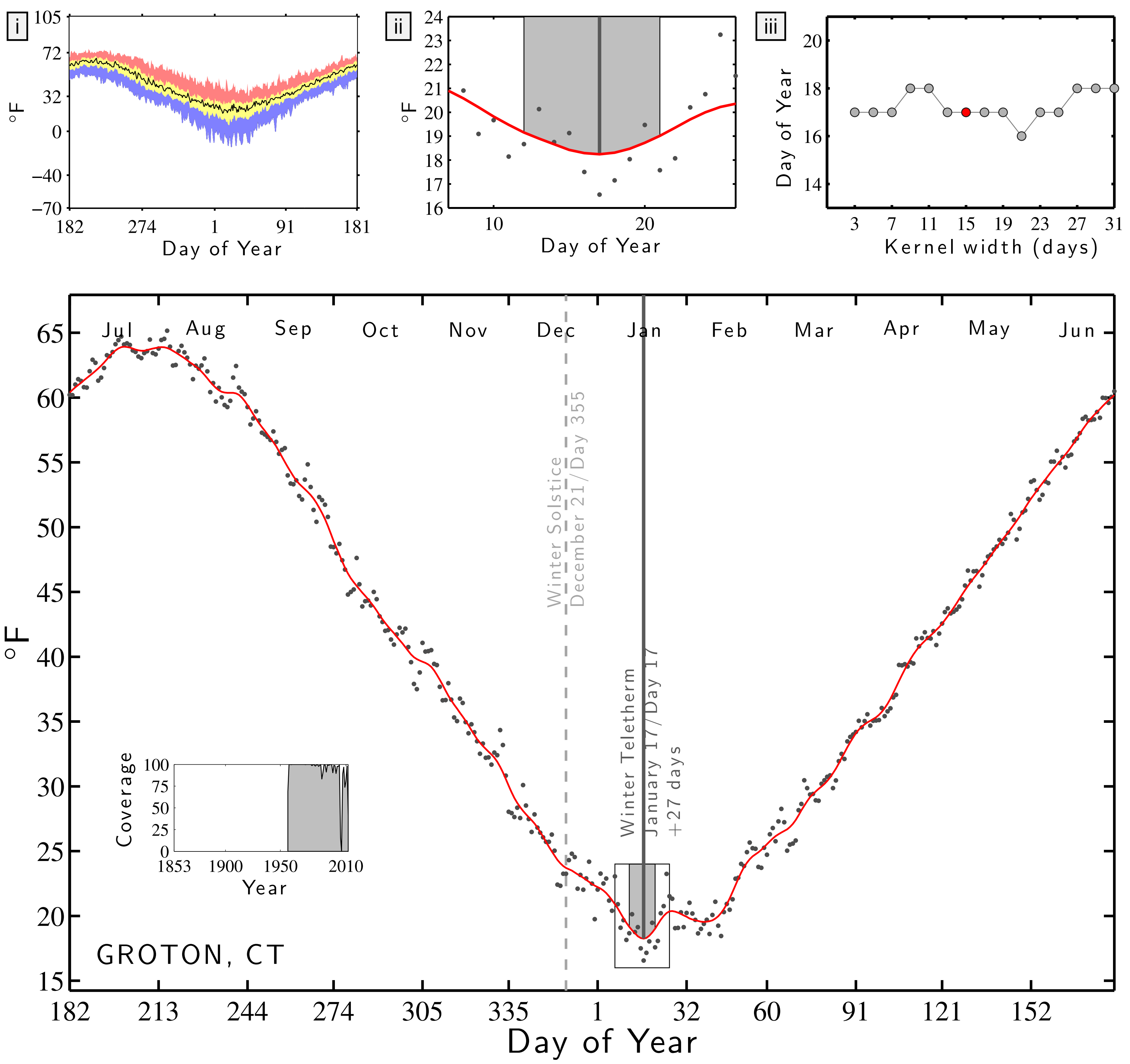

i
ii
iii
°F
Day of Year
Kernel width (days)
Jul
Aug
Sep
Oct
Nov
Dec
Jan
Feb
Mar
Apr
May
Jun
Winter Solstice
December 21/Day 355
Winter Teletherm
January 17/Day 17
+27 days
Coverage
Year
GROTON, CT

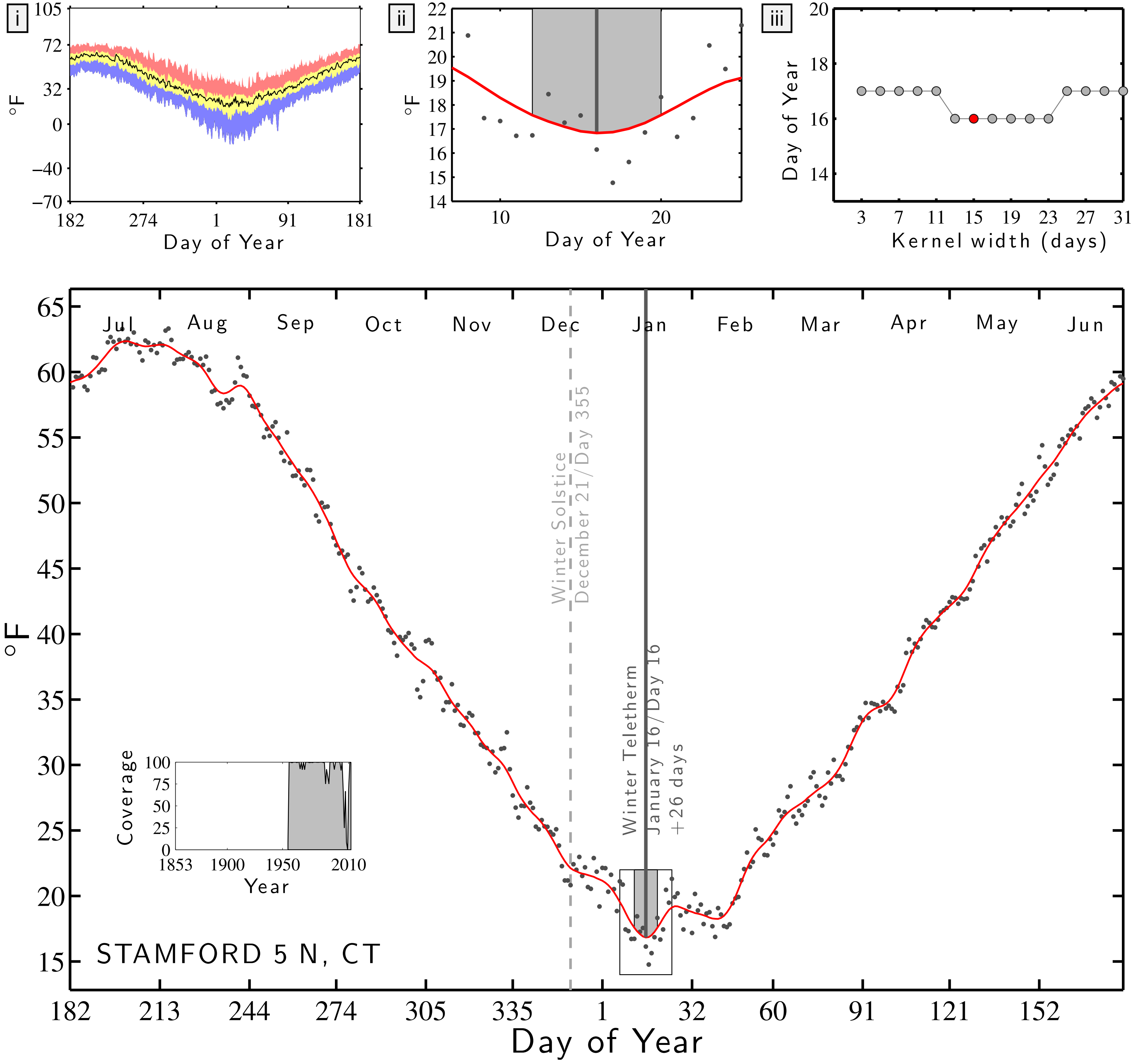

i
°F
Day of Year
ii
°F
Day of Year
iii
Day of Year
Kernel width (days)
Jul
Aug
Sep
Oct
Nov
Dec
Jan
Feb
Mar
Apr
May
Jun
Winter Solstice
December 21/Day 355
Winter Teletherm
January 16/Day 16
+26 days
Coverage
Year
STAMFORD 5 N, CT
°F
Day of Year

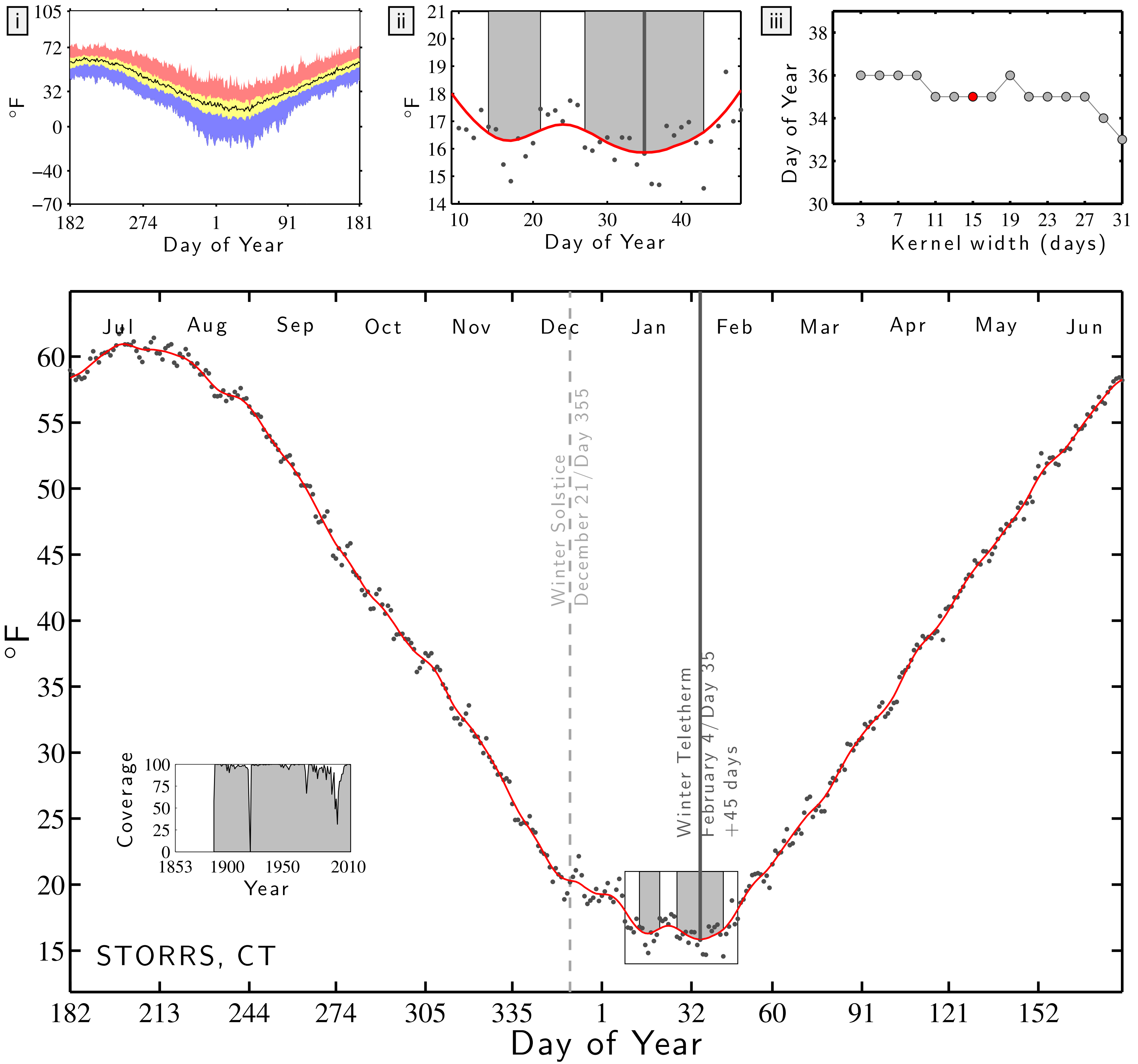

i
°F
Day of Year
ii
°F
Day of Year
iii
Day of Year
Kernel width (days)
Jul
Aug
Sep
Oct
Nov
Dec
Jan
Feb
Mar
Apr
May
Jun
Winter Solstice
December 21/Day 355
Winter Teletherm
February 4/Day 35
+45 days
Coverage
Year
STORRS, CT
°F
Day of Year

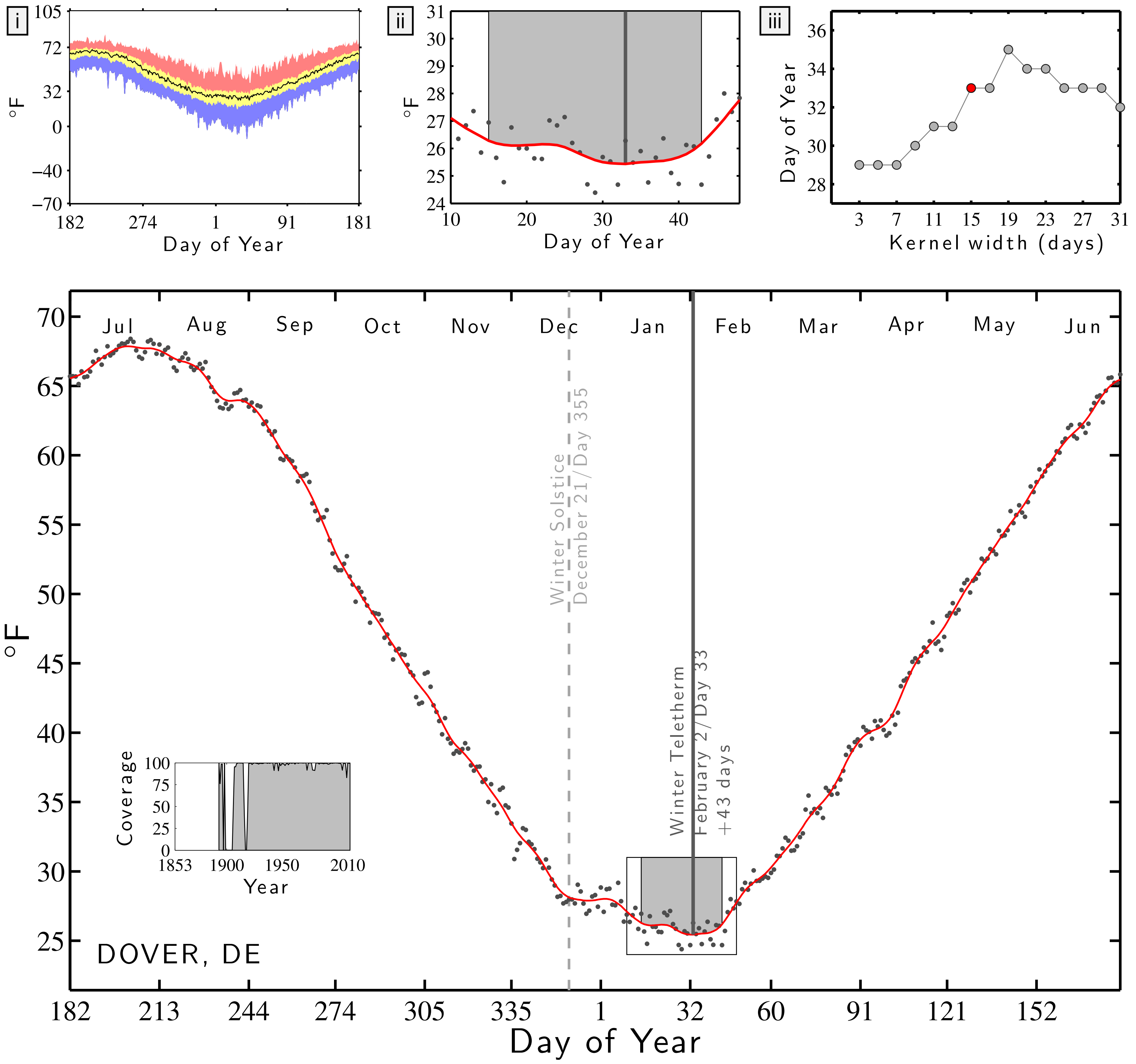
i
ii
iii
°F
Day of Year
Day of Year
Kernel width (days)
Jul
Aug
Sep
Oct
Nov
Dec
Jan
Feb
Mar
Apr
May
Jun
Winter Solstice
December 21/Day 355
Winter Teletherm
February 2/Day 33
+43 days
Coverage
Year
DOVER, DE

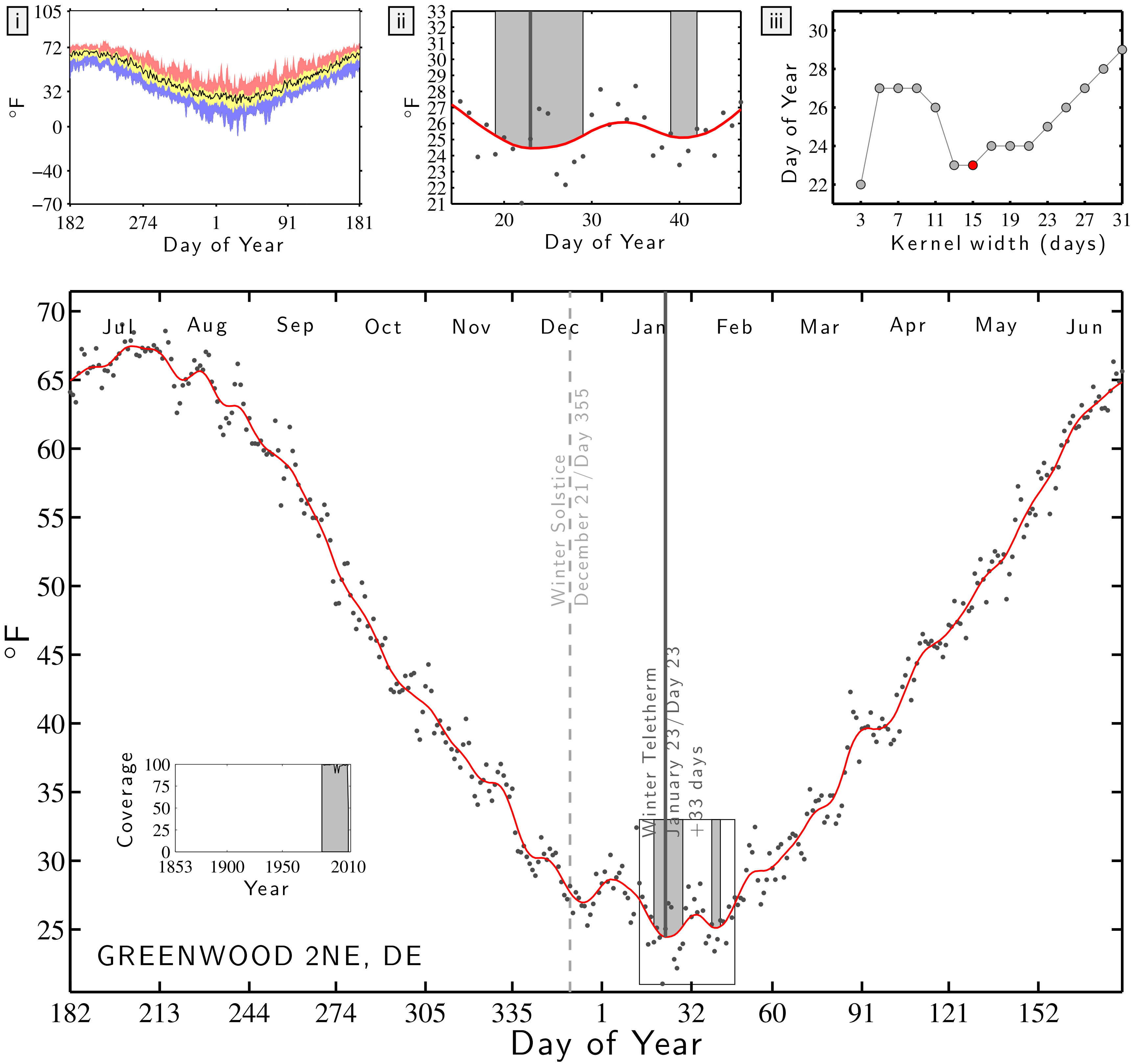

i
°F
Day of Year
ii
°F
Day of Year
iii
Day of Year
Kernel width (days)
Jul
Aug
Sep
Oct
Nov
Dec
Jan
Feb
Mar
Apr
May
Jun
Winter Solstice
December 21/Day 355
Winter Teletherm
January 23/Day 23
+33 days
Coverage
Year
GREENWOOD 2NE, DE
°F
Day of Year

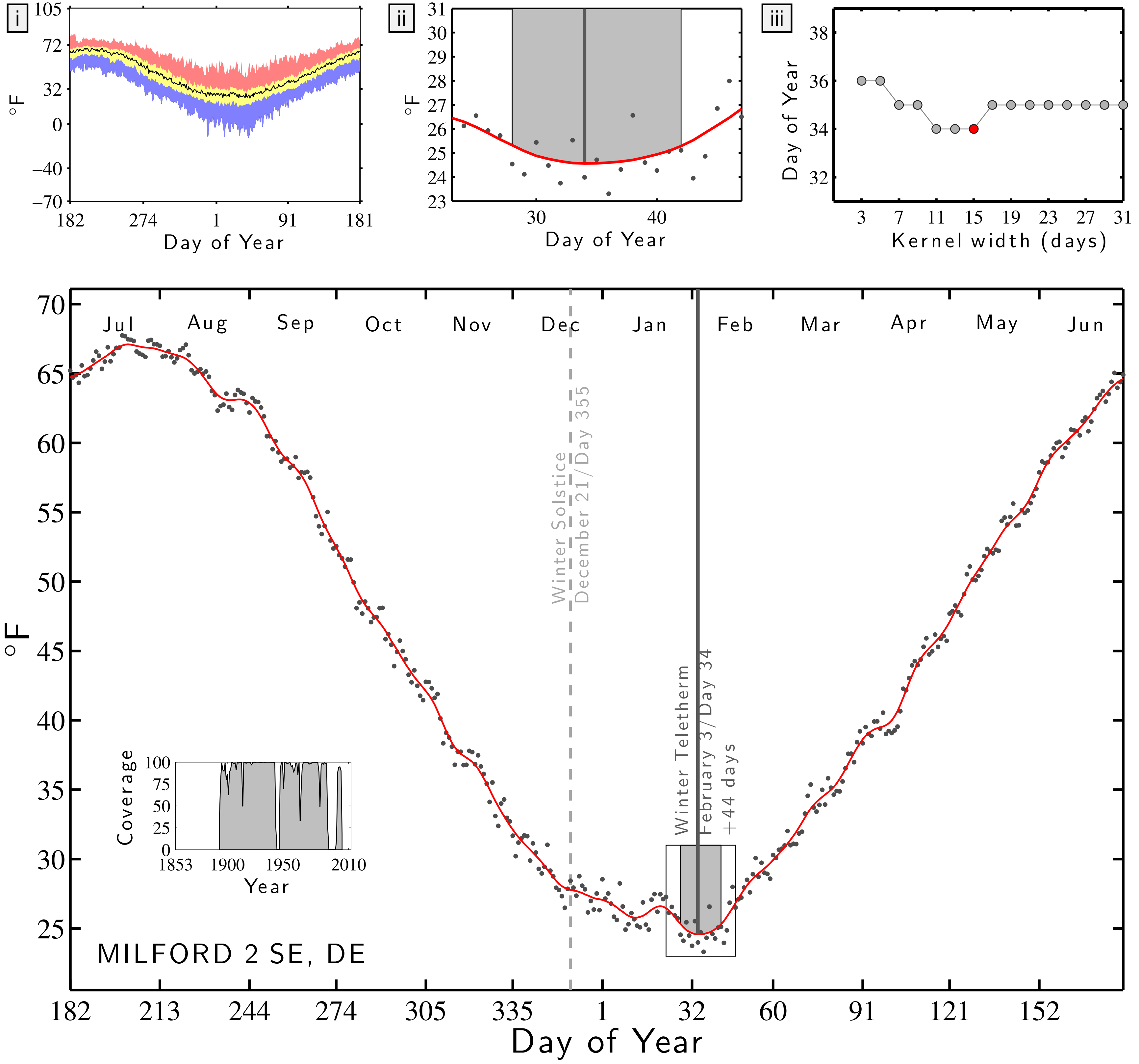

i
ii
iii
°F
Day of Year
Kernel width (days)
Jul
Aug
Sep
Oct
Nov
Dec
Jan
Feb
Mar
Apr
May
Jun
Winter Solstice
December 21/Day 355
Winter Teletherm
February 3/Day 34
+44 days
Coverage
Year
MILFORD 2 SE, DE

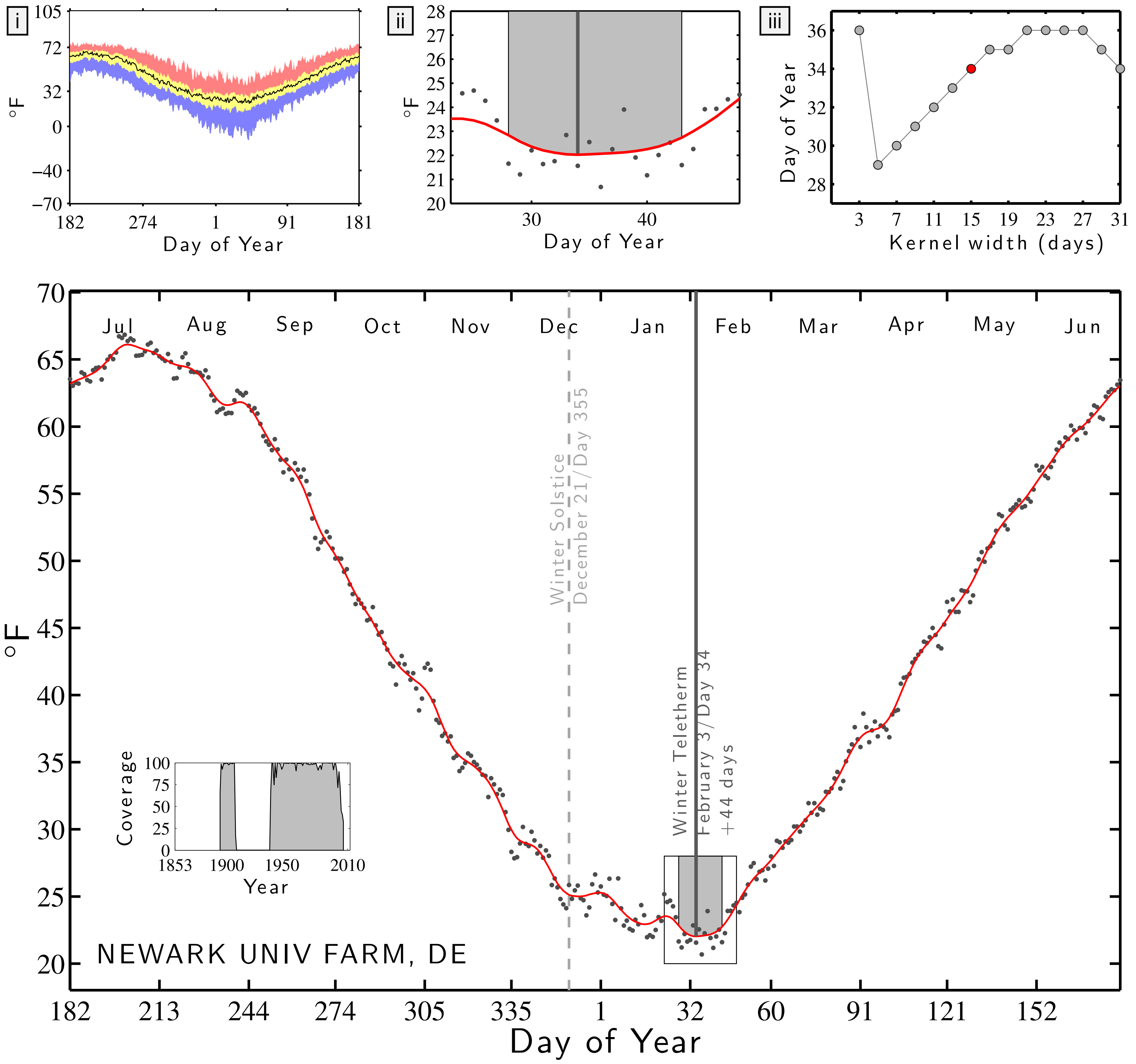

i
°F
Day of Year
ii
°F
Day of Year
iii
Day of Year
Kernel width (days)
Jul
Aug
Sep
Oct
Nov
Dec
Jan
Feb
Mar
Apr
May
Jun
Winter Solstice
December 21/Day 355
Winter Teletherm
February 3/Day 34
+44 days
Coverage
Year
NEWARK UNIV FARM, DE
°F
Day of Year

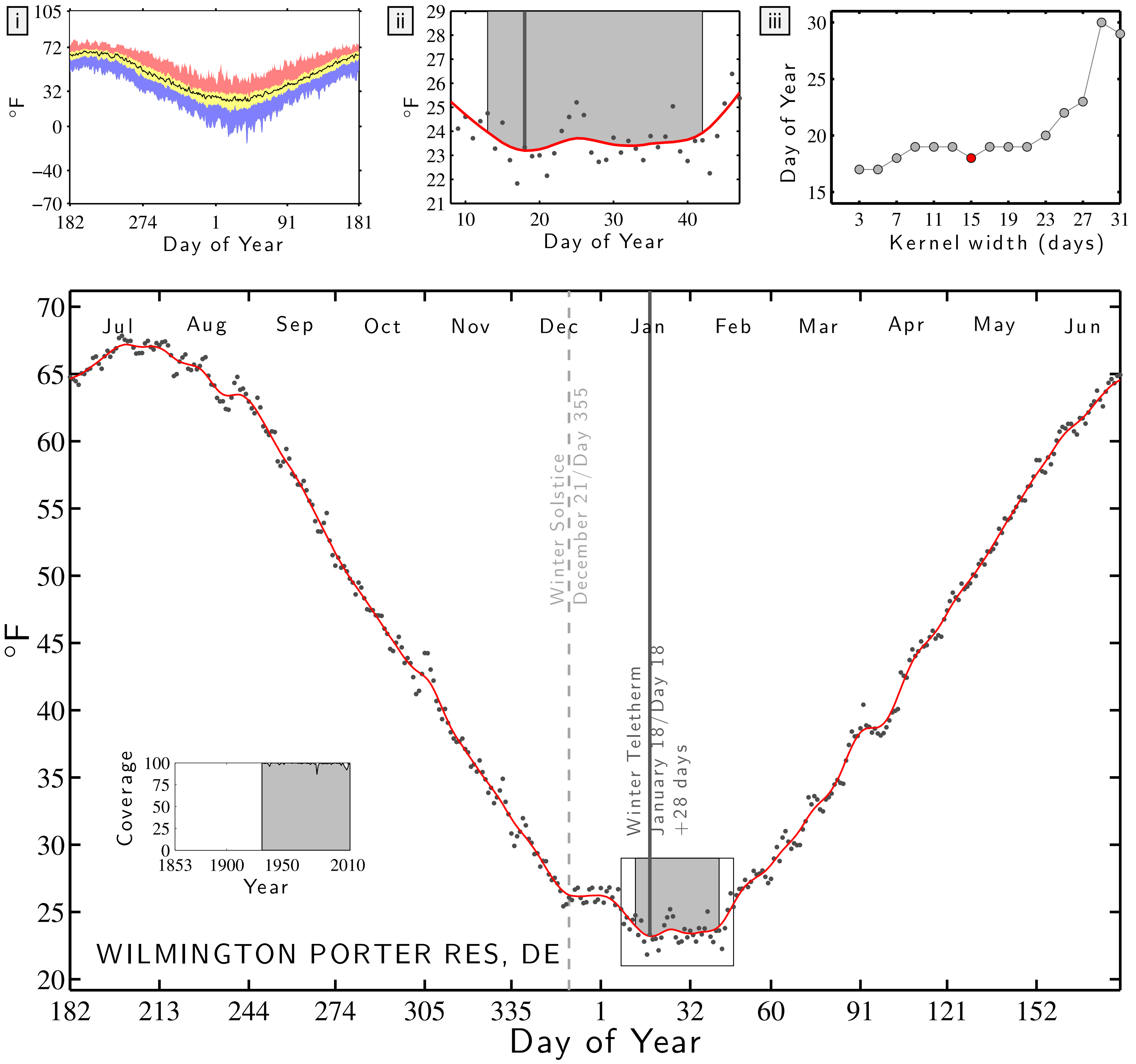

i
°F
Day of Year
ii
°F
Day of Year
iii
Day of Year
Kernel width (days)
Jul
Aug
Sep
Oct
Nov
Dec
Jan
Feb
Mar
Apr
May
Jun
Winter Solstice
December 21/Day 355
Winter Teletherm
January 18/Day 18
+28 days
Coverage
Year
WILMINGTON PORTER RES, DE
°F
Day of Year

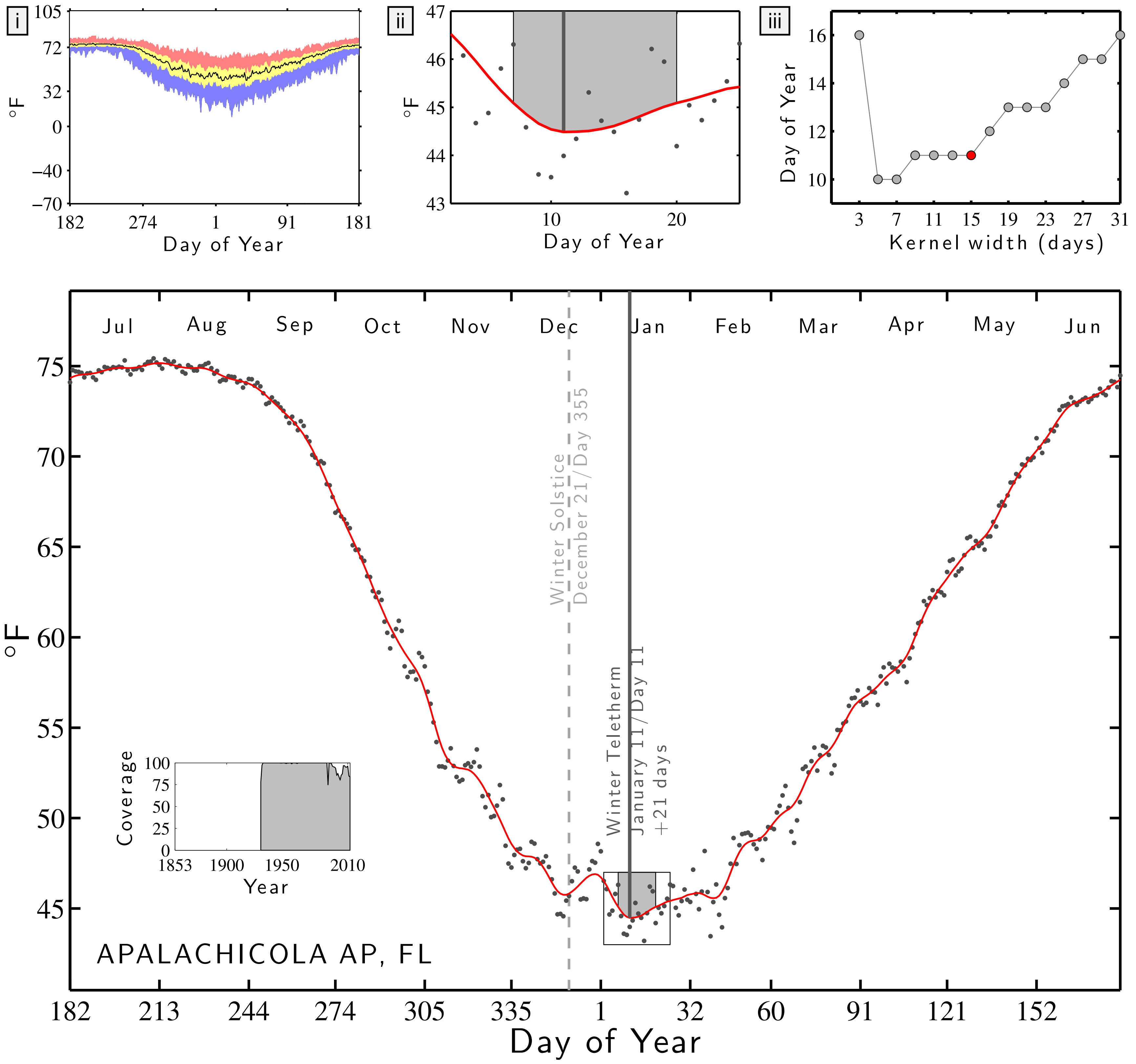

i
°F
105
72
32
0
−40
−70
182
274
1
91
181
Day of Year
ii
47
46
45
44
43
10
20
Day of Year
iii
Day of Year
16
14
12
10
3
7
11
15
19
23
27
31
Kernel width (days)
Jul
Aug
Sep
Oct
Nov
Dec
Jan
Feb
Mar
Apr
May
Jun
75
70
65
60
55
50
45
Winter Solstice
December 21/Day 355
Winter Teletherm
January 11/Day 11
+21 days
Coverage
100
75
50
25
0
1853
1900
1950
2010
Year
APALACHICOLA AP, FL
182
213
244
274
305
335
1
32
60
91
121
152
Day of Year

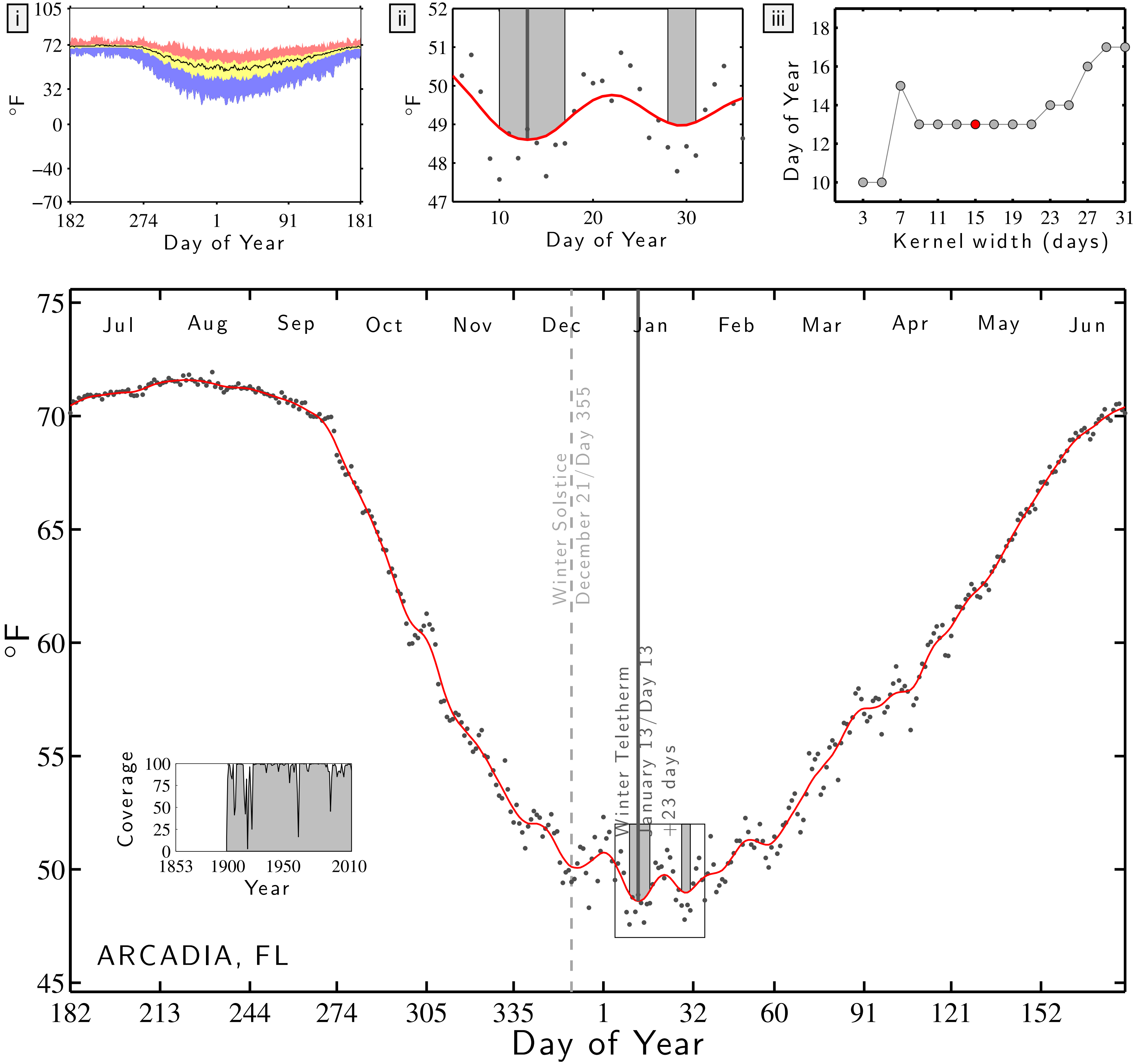

i
ii
iii
°F
Day of Year
Kernel width (days)
Jul
Aug
Sep
Oct
Nov
Dec
Jan
Feb
Mar
Apr
May
Jun
Winter Solstice
December 21/Day 355
Winter Teletherm
January 13/Day 13
+23 days
Coverage
Year
ARCADIA, FL

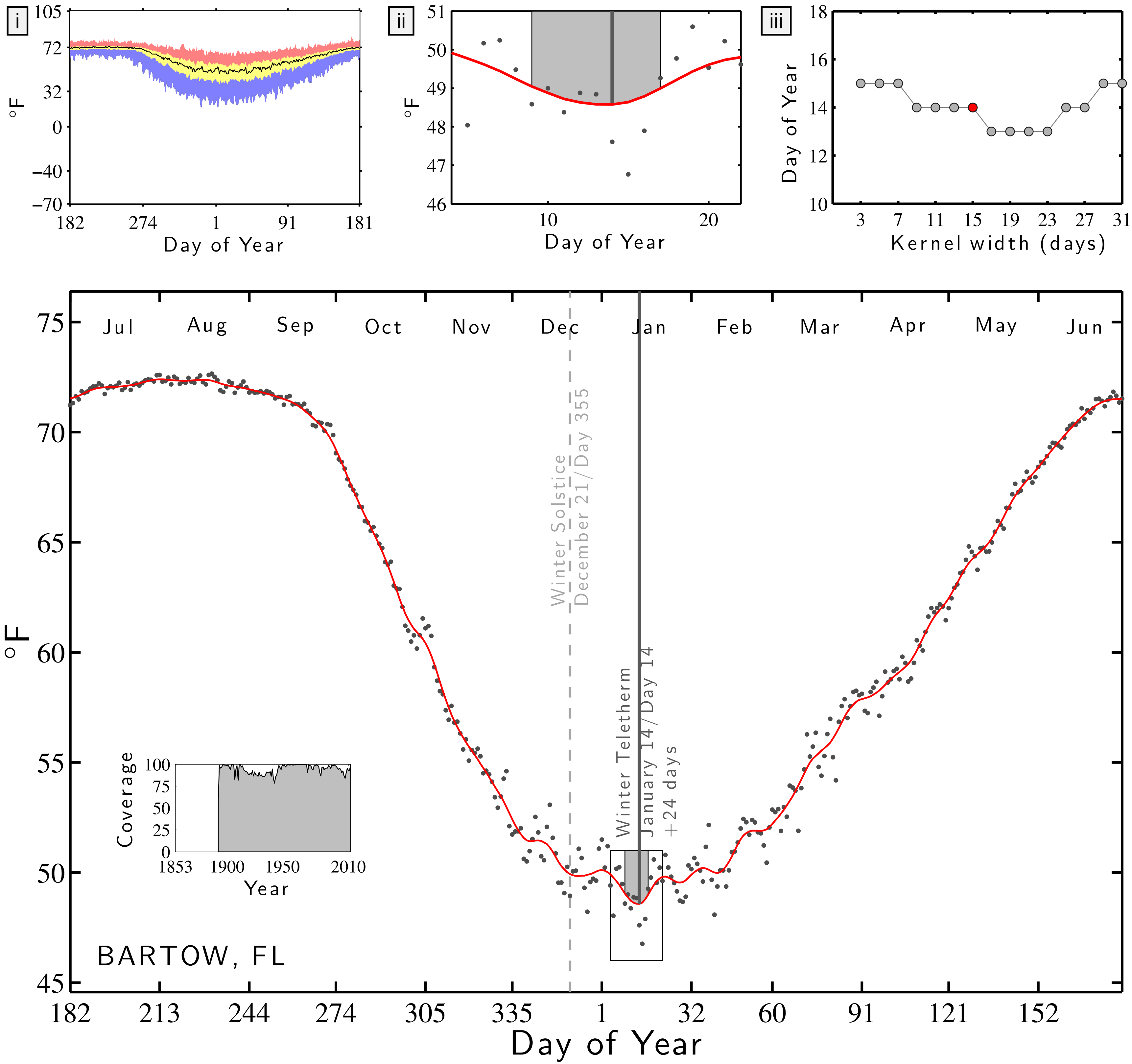

i
°F
Day of Year
ii
°F
Day of Year
iii
Day of Year
Kernel width (days)
Jul
Aug
Sep
Oct
Nov
Dec
Jan
Feb
Mar
Apr
May
Jun
Winter Solstice
December 21/Day 355
Winter Teletherm
January 14/Day 14
+24 days
Coverage
Year
BARTOW, FL
°F
Day of Year

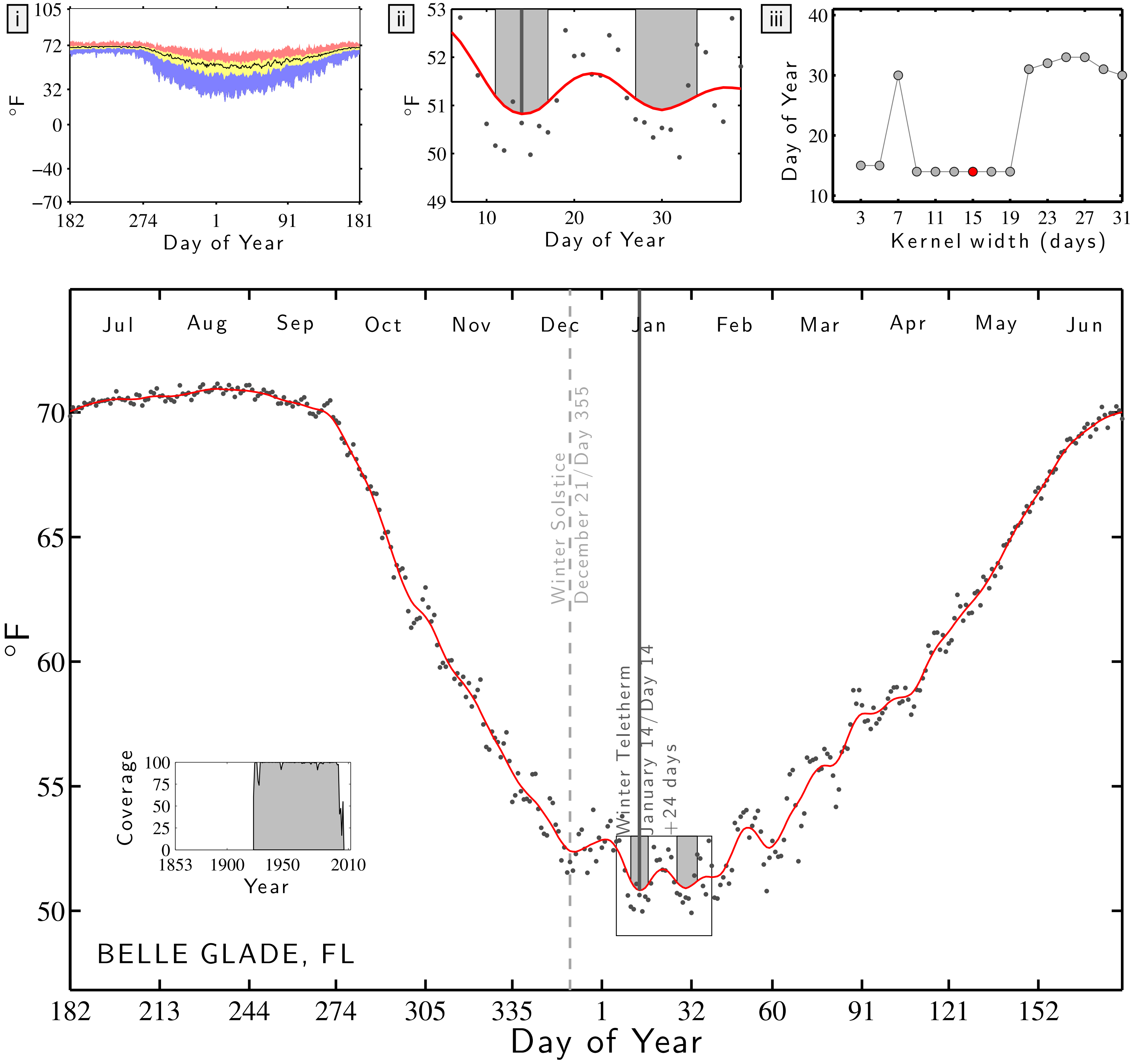

i
ii
iii
°F
Day of Year
Day of Year
Kernel width (days)
Jul
Aug
Sep
Oct
Nov
Dec
Jan
Feb
Mar
Apr
May
Jun
Winter Solstice
December 21/Day 355
Winter Teletherm
January 14/Day 14
+24 days
Coverage
Year
BELLE GLADE, FL
Day of Year

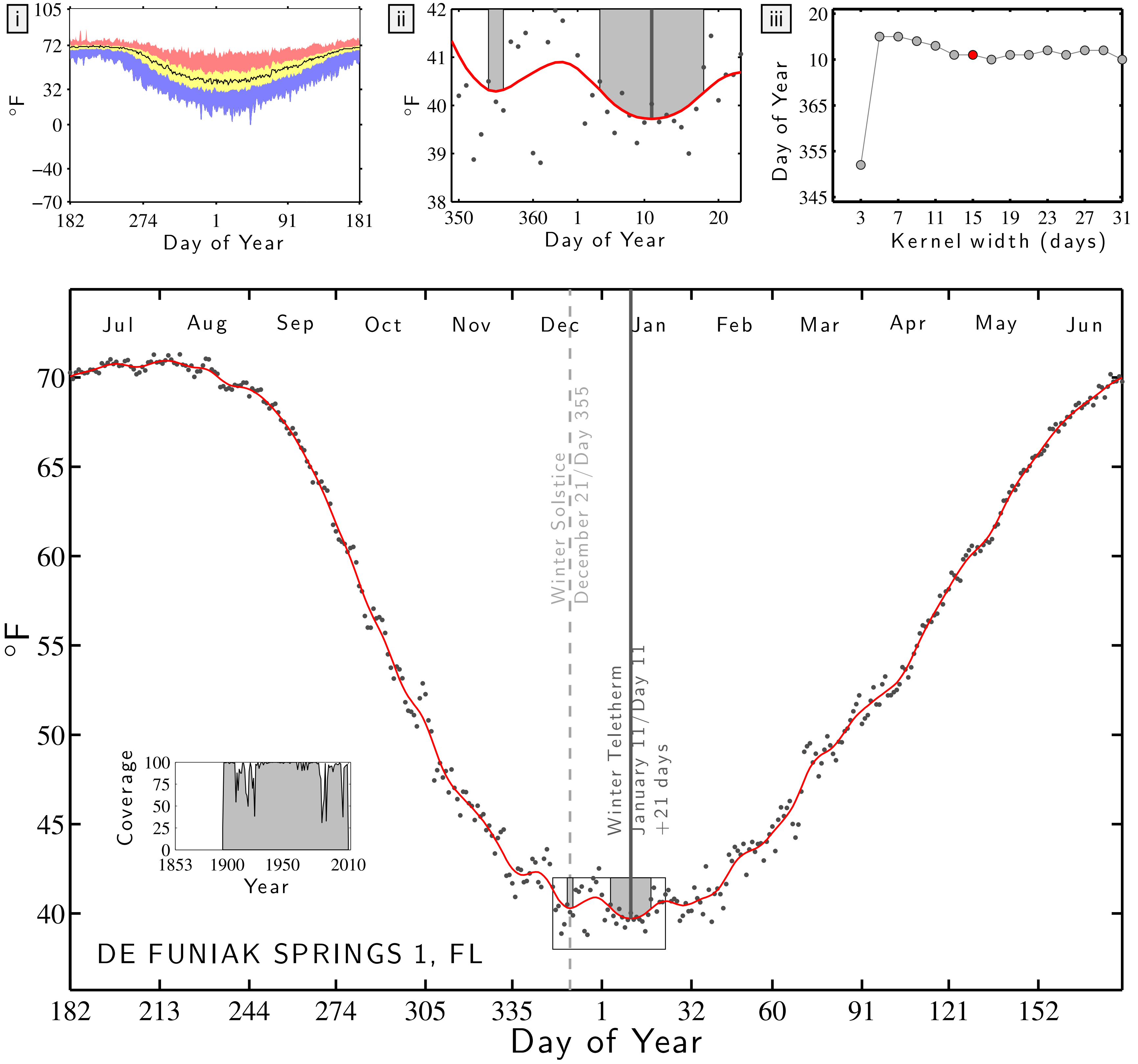

i
ii
iii
°F
Day of Year
Kernel width (days)
Jul
Aug
Sep
Oct
Nov
Dec
Jan
Feb
Mar
Apr
May
Jun
Winter Solstice
December 21/Day 355
Winter Teletherm
January 11/Day 11
+21 days
Coverage
Year
DE FUNIAK SPRINGS 1, FL

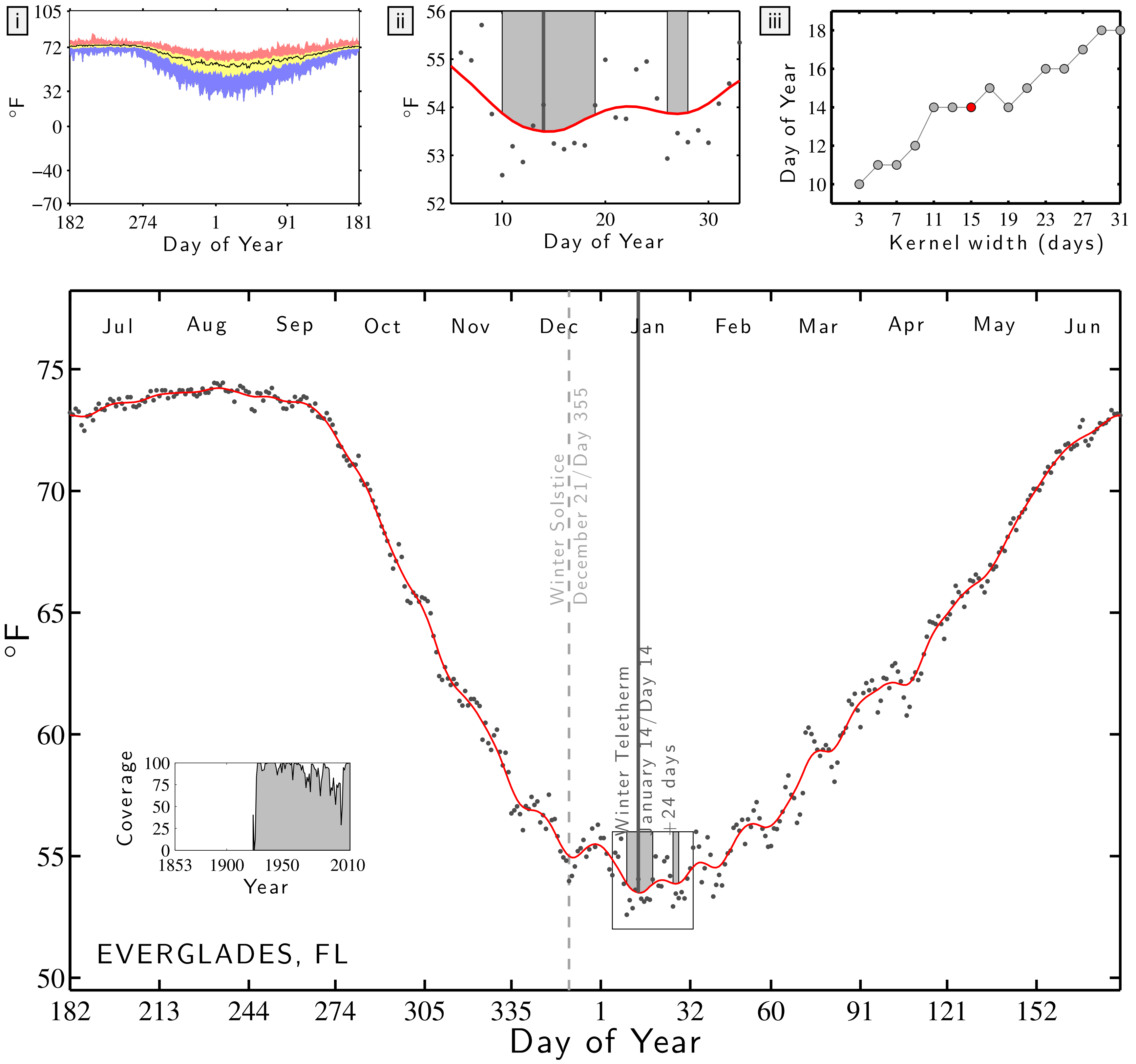

i
°F
Day of Year
ii
°F
Day of Year
iii
Day of Year
Kernel width (days)
Jul
Aug
Sep
Oct
Nov
Dec
Jan
Feb
Mar
Apr
May
Jun
Winter Solstice
December 21/Day 355
Winter Teletherm
January 14/Day 14
± 24 days
Coverage
Year
EVERGLADES, FL
°F
Day of Year

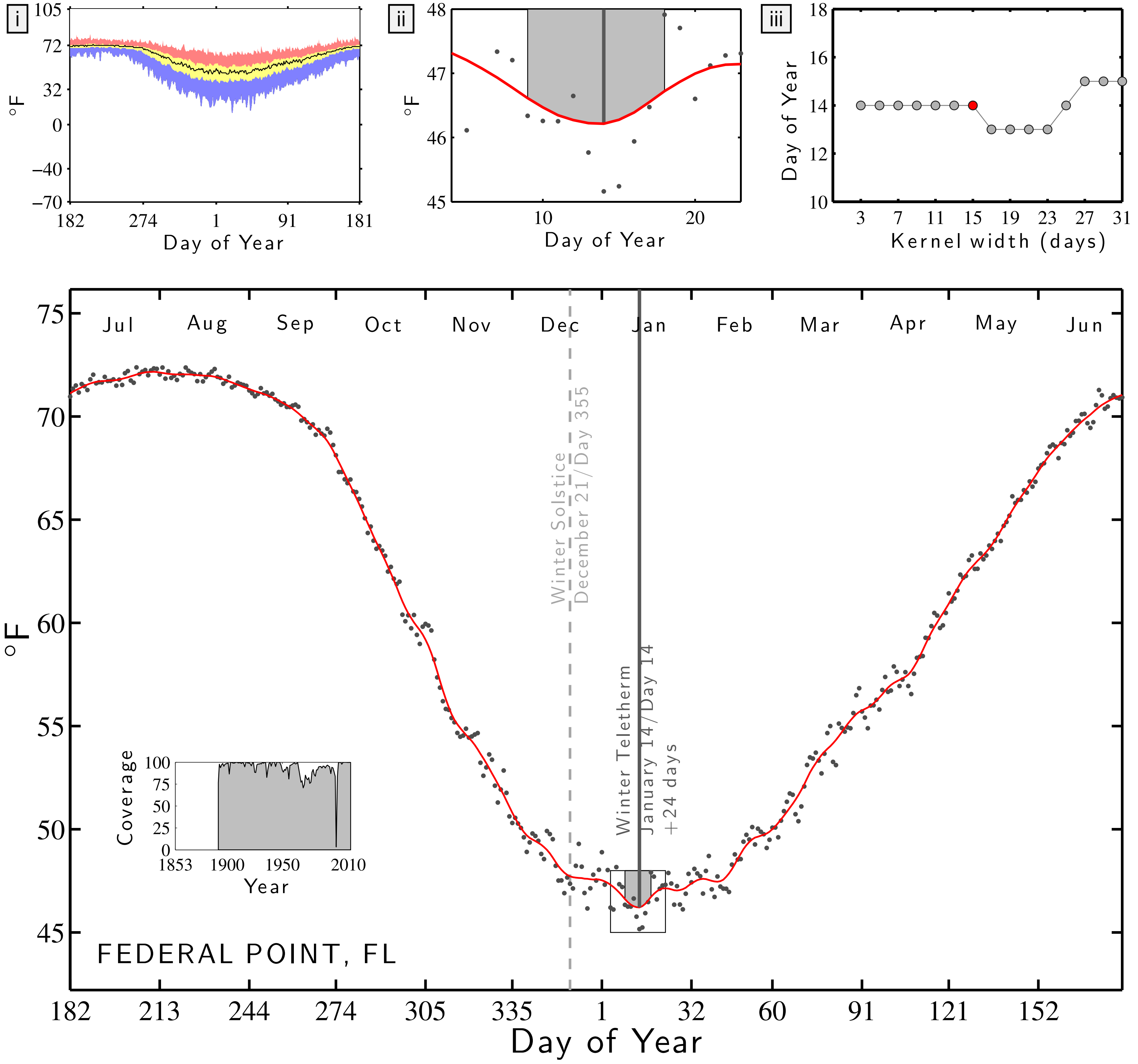

i
ii
iii
°F
Day of Year
Day of Year
Kernel width (days)
Jul
Aug
Sep
Oct
Nov
Dec
Jan
Feb
Mar
Apr
May
Jun
Winter Solstice
December 21/Day 355
Winter Teletherm
January 14/Day 14
+24 days
Coverage
Year
FEDERAL POINT, FL
Day of Year

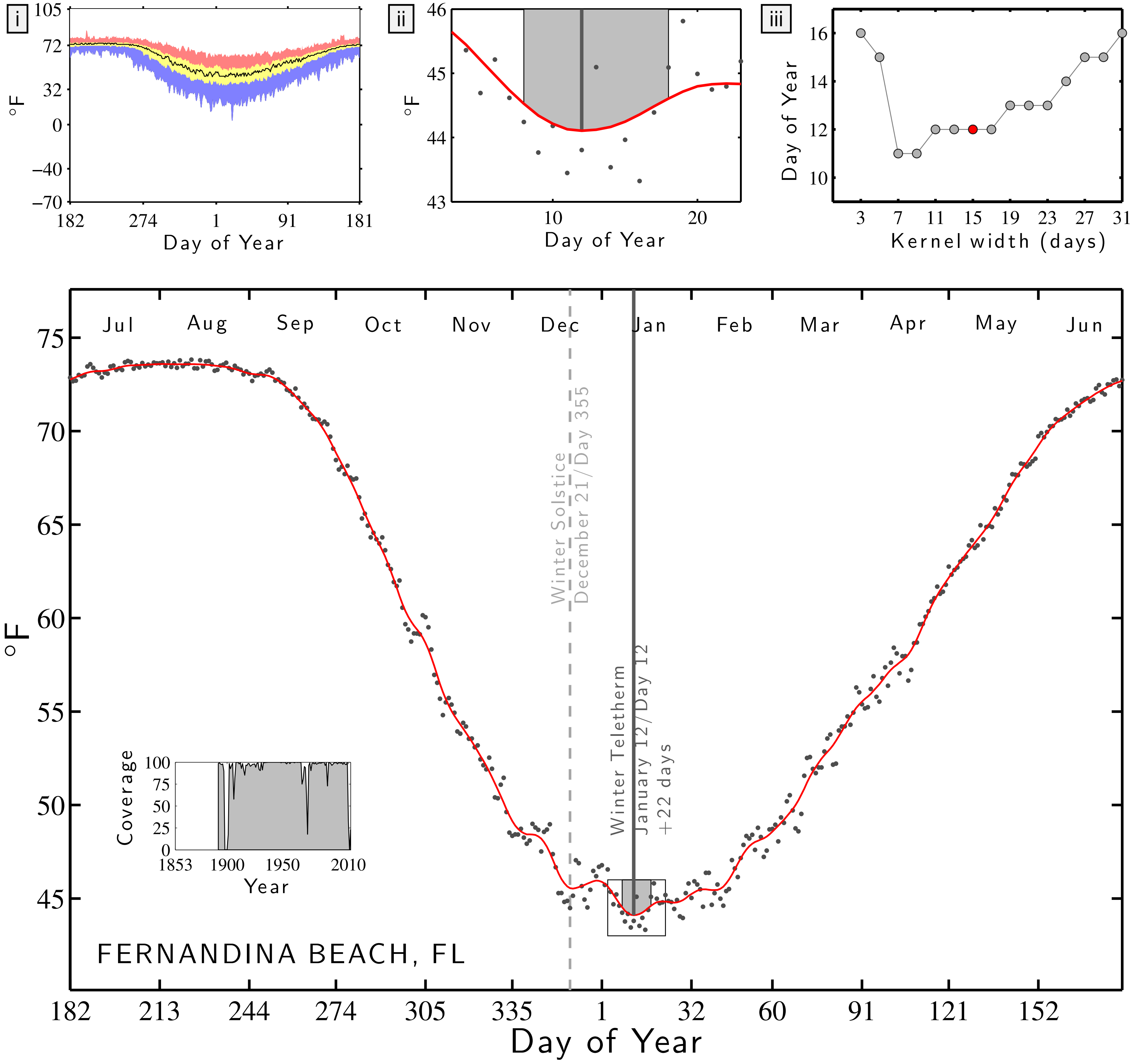

i
°F
Day of Year
ii
Day of Year
iii
Day of Year
Kernel width (days)
Jul
Aug
Sep
Oct
Nov
Dec
Jan
Feb
Mar
Apr
May
Jun
Winter Solstice
December 21/Day 355
Winter Teletherm
January 12/Day 12
+22 days
Coverage
Year
FERNANDINA BEACH, FL
Day of Year

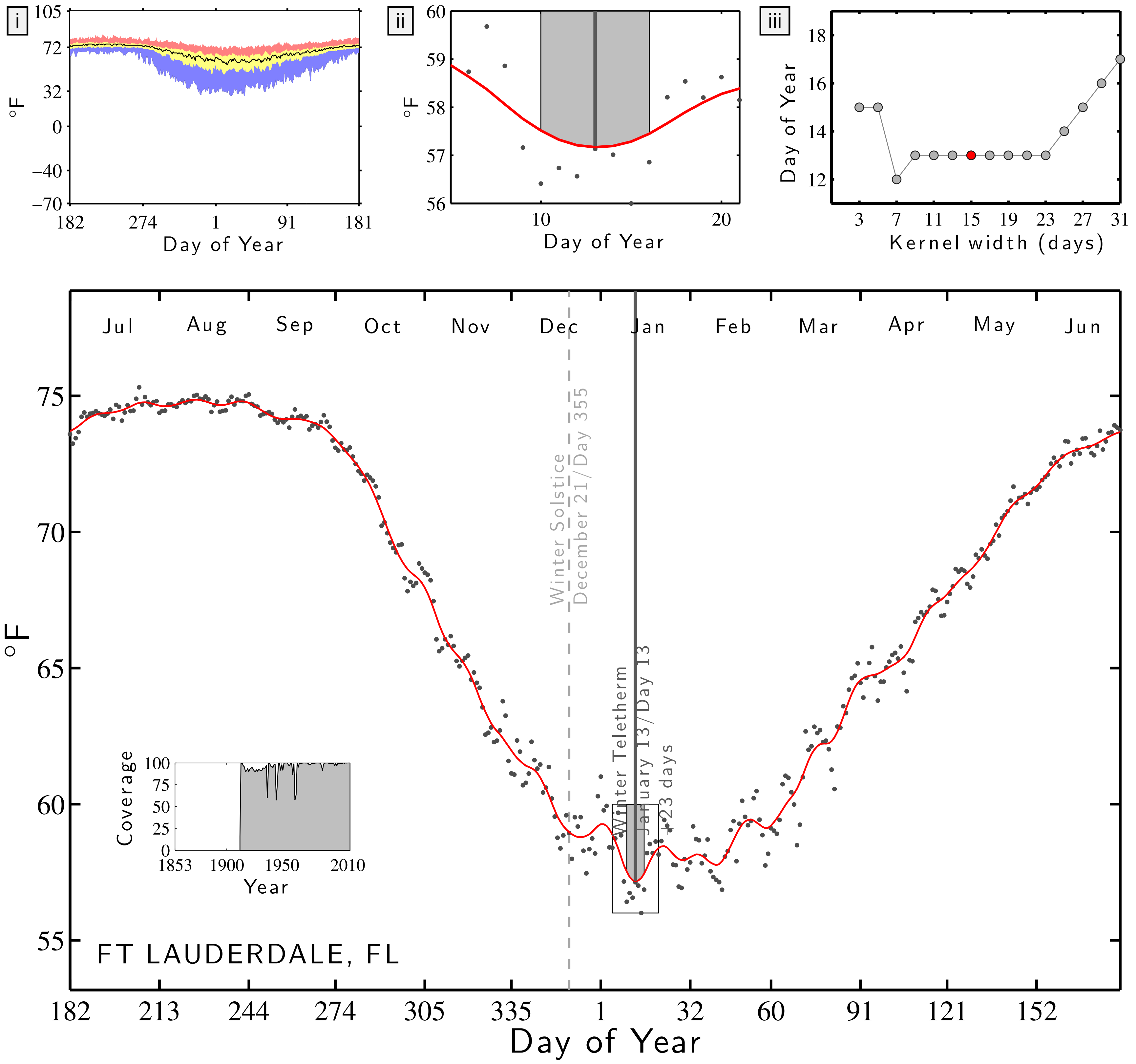

i
ii
iii
°F
Day of Year
Day of Year
Kernel width (days)
Jul
Aug
Sep
Oct
Nov
Dec
Jan
Feb
Mar
Apr
May
Jun
Winter Solstice
December 21/Day 355
Winter Teletherm
January 13/Day 13
±23 days
Coverage
Year
FT LAUDERDALE, FL
Day of Year

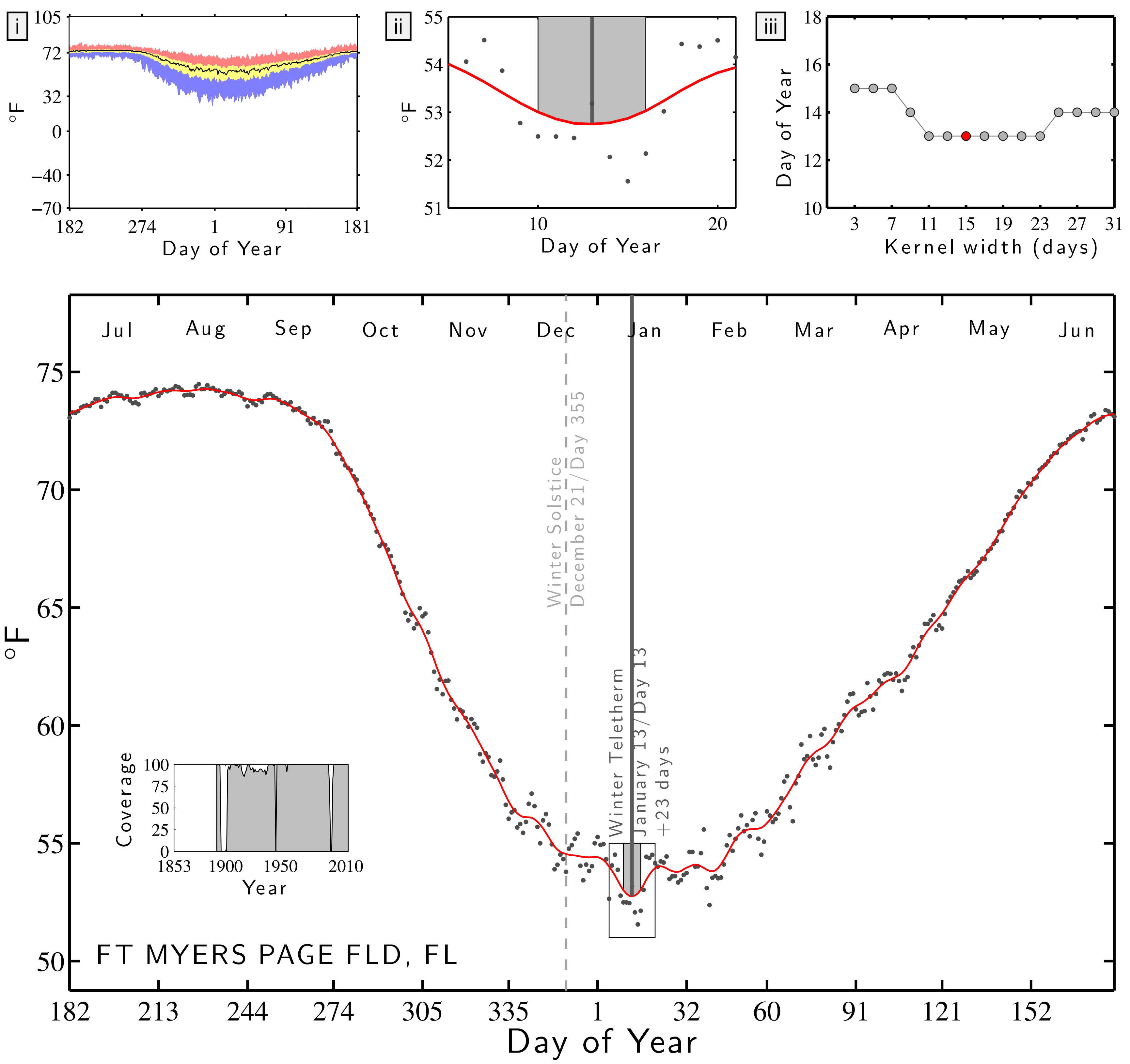

i
ii
iii
°F
Day of Year
Day of Year
Kernel width (days)
Jul
Aug
Sep
Oct
Nov
Dec
Jan
Feb
Mar
Apr
May
Jun
Winter Solstice
December 21/Day 355
Winter Teletherm
January 13/Day 13
+23 days
Coverage
Year
FT MYERS PAGE FLD, FL
Day of Year

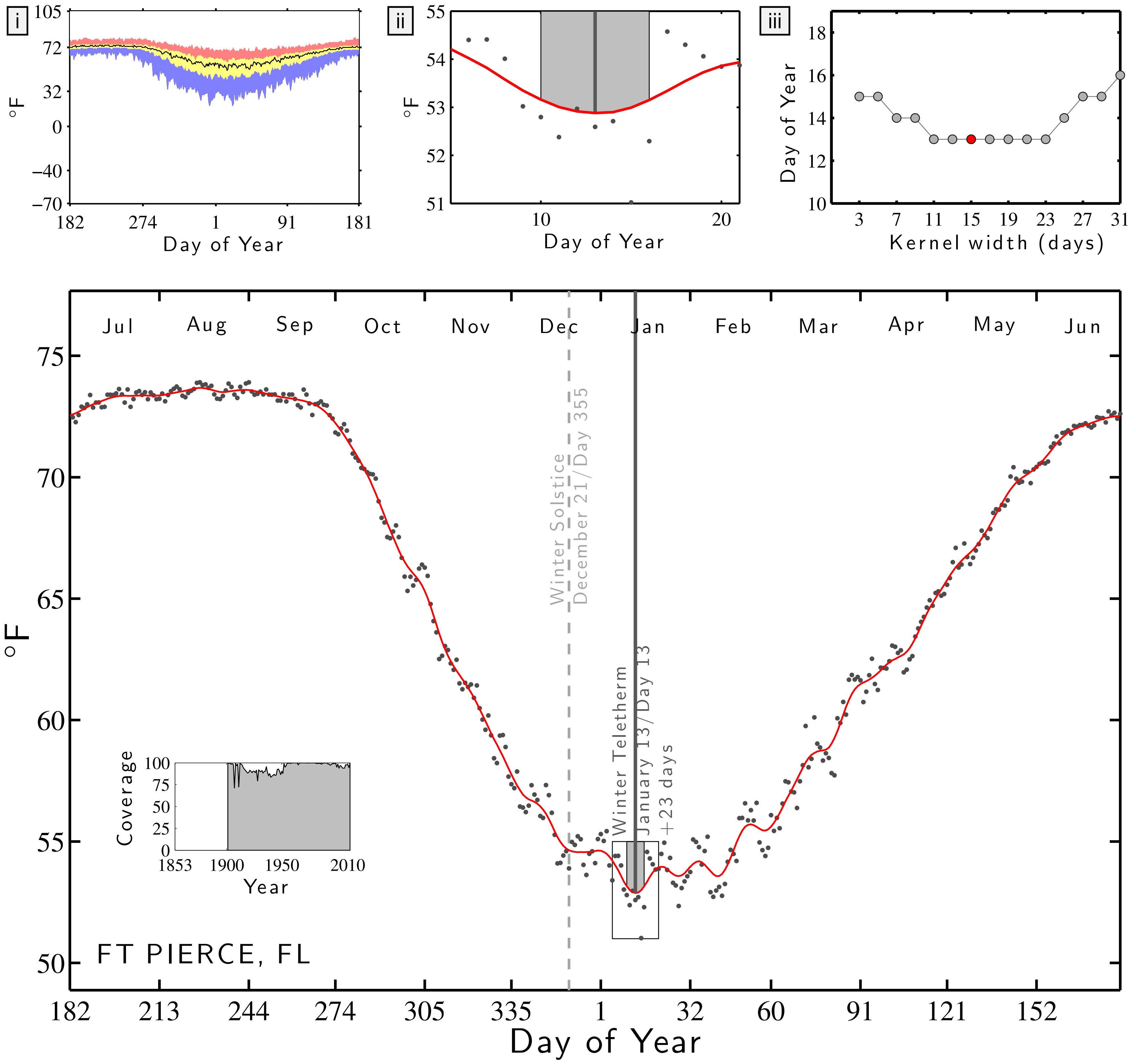

i
ii
iii
°F
Day of Year
Day of Year
Kernel width (days)
Jul
Aug
Sep
Oct
Nov
Dec
Jan
Feb
Mar
Apr
May
Jun
Winter Solstice
December 21/Day 355
Winter Teletherm
January 13/Day 13
+23 days
Coverage
Year
FT PIERCE, FL

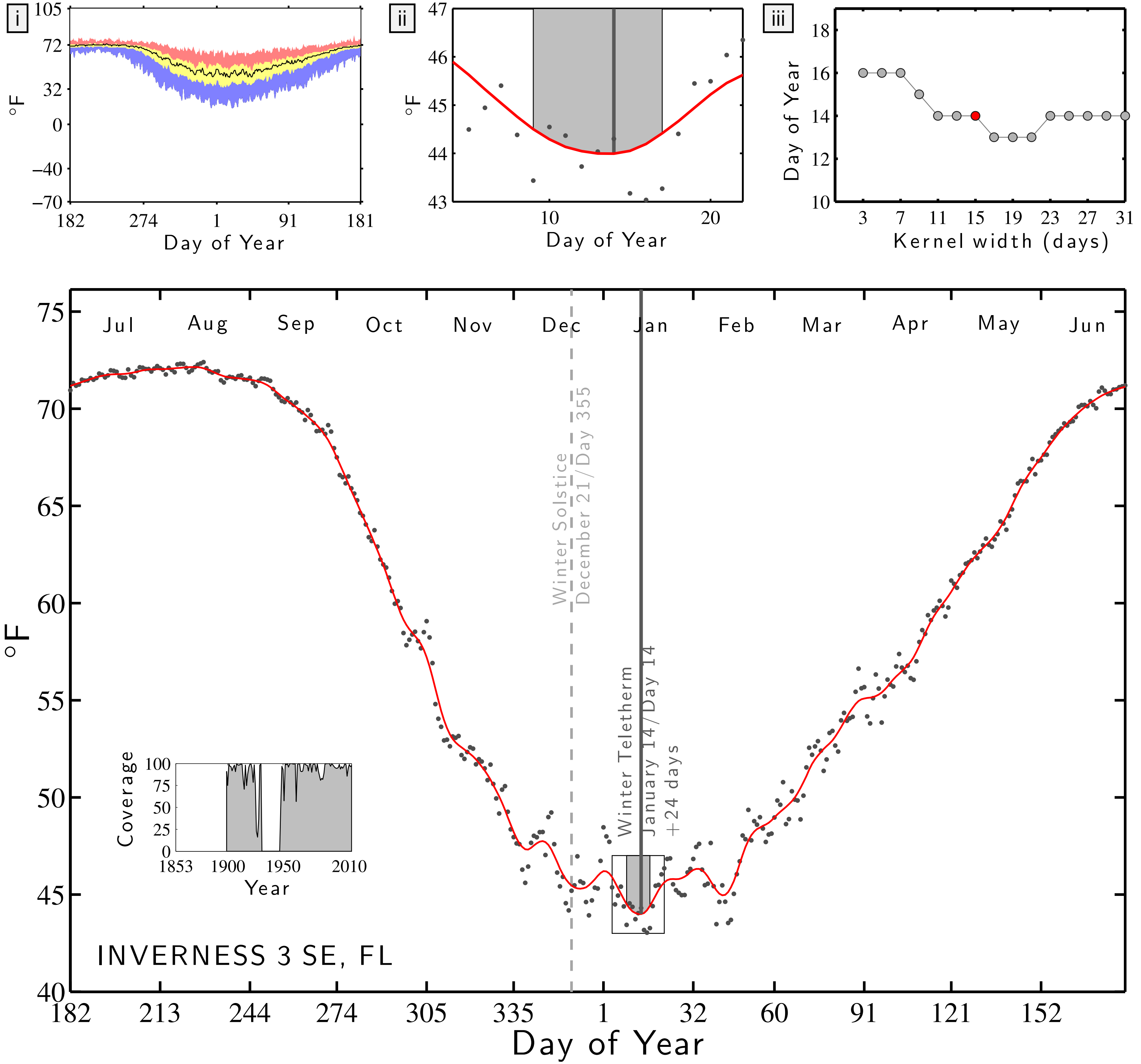

i
°F
Day of Year
ii
°F
Day of Year
iii
Day of Year
Kernel width (days)
Jul
Aug
Sep
Oct
Nov
Dec
Jan
Feb
Mar
Apr
May
Jun
Winter Solstice
December 21/Day 355
Winter Teletherm
January 14/Day 14
+24 days
Coverage
Year
INVERNESS 3 SE, FL
°F
Day of Year

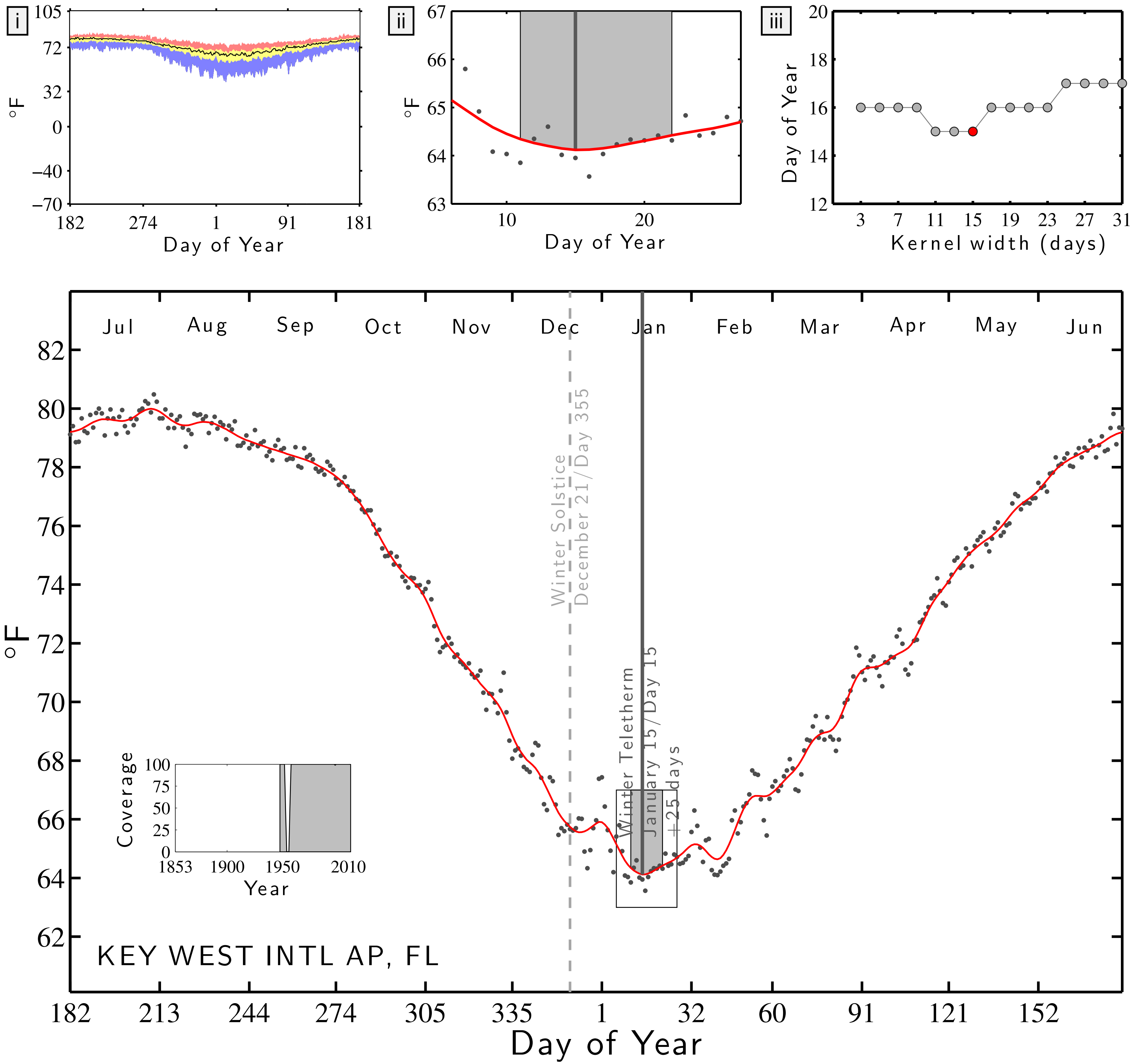

i
°F
Day of Year
ii
Day of Year
iii
Day of Year
Kernel width (days)
Jul
Aug
Sep
Oct
Nov
Dec
Jan
Feb
Mar
Apr
May
Jun
Winter Solstice
December 21/Day 355
Winter Teletherm
January 15/Day 15
±25 days
Coverage
Year
KEY WEST INTL AP, FL
Day of Year

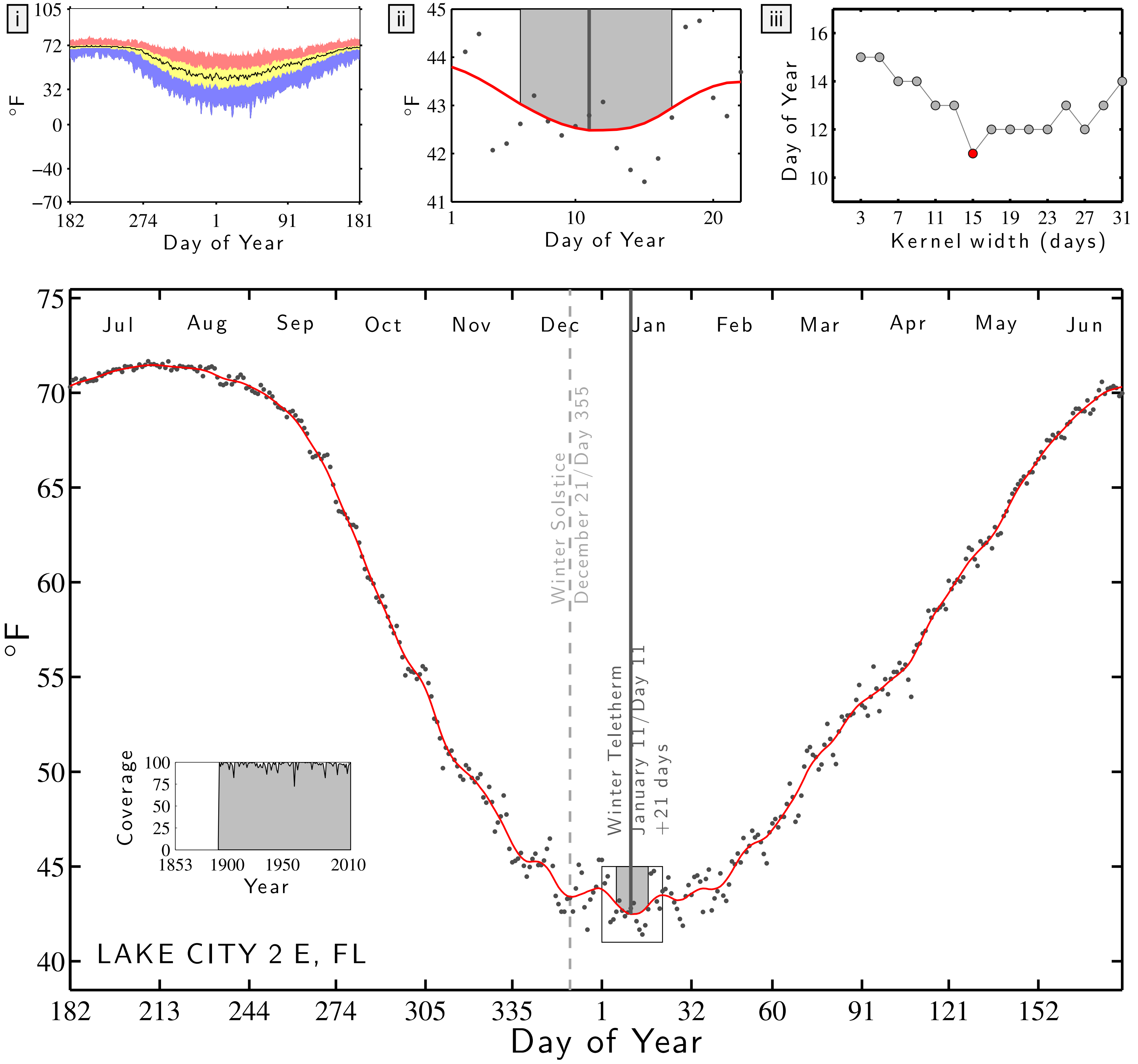

i
ii
iii
Day of Year
Kernel width (days)
°F
Jul
Aug
Sep
Oct
Nov
Dec
Jan
Feb
Mar
Apr
May
Jun
Winter Solstice
December 21/Day 355
Winter Teletherm
January 11/Day 11
+21 days
Coverage
Year
LAKE CITY 2 E, FL

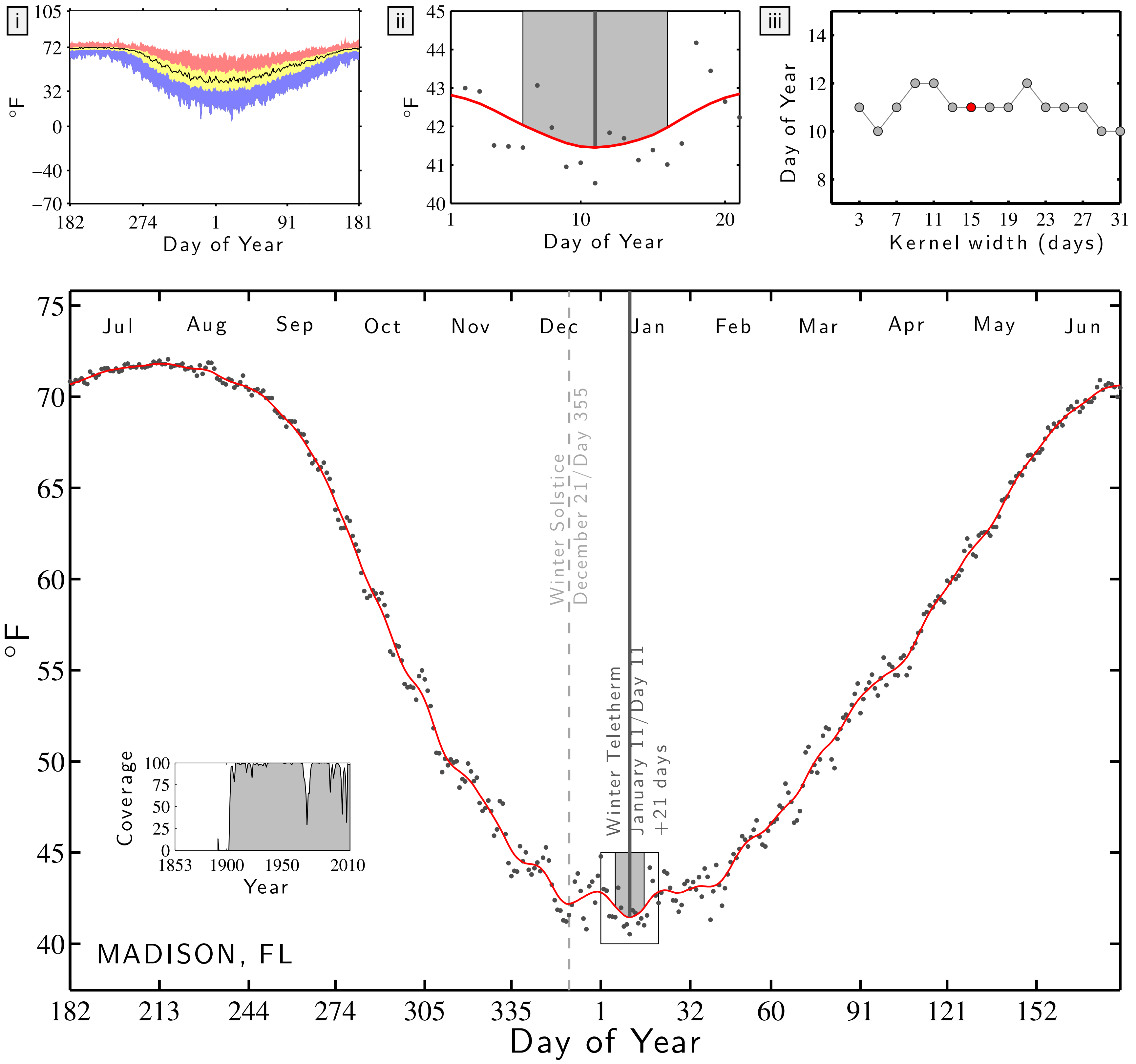

i
ii
iii
°F
Day of Year
Kernel width (days)
Jul
Aug
Sep
Oct
Nov
Dec
Jan
Feb
Mar
Apr
May
Jun
Winter Solstice
December 21/Day 355
Winter Teletherm
January 11/Day 11
+21 days
Coverage
Year
MADISON, FL

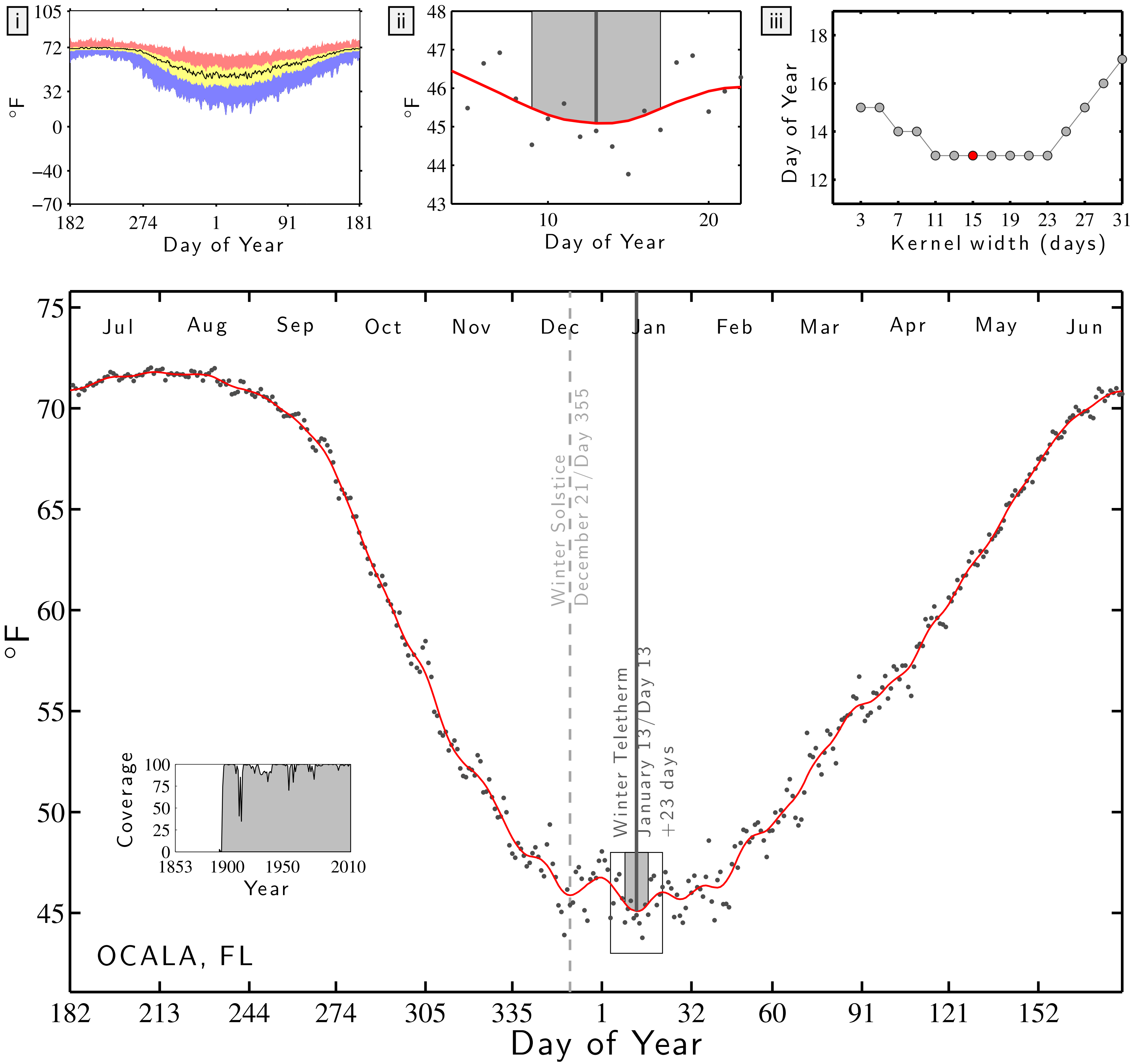

i
ii
iii
°F
Day of Year
Day of Year
Kernel width (days)
Jul
Aug
Sep
Oct
Nov
Dec
Jan
Feb
Mar
Apr
May
Jun
Winter Solstice
December 21/Day 355
Winter Teletherm
January 13/Day 13
+23 days
Coverage
Year
OCALA, FL

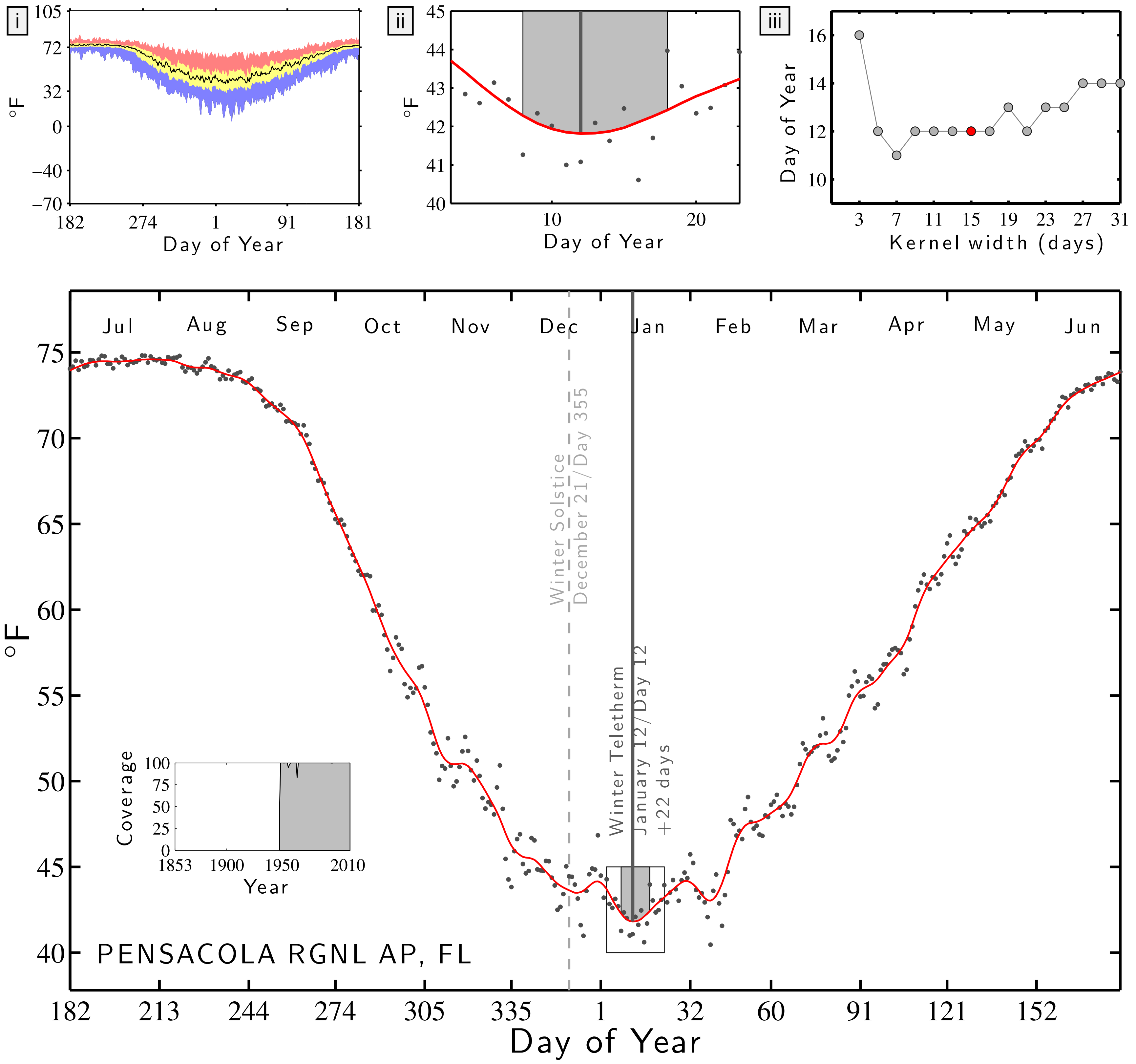

i
ii
iii
Day of Year
Kernel width (days)
°F
Day of Year
Jul
Aug
Sep
Oct
Nov
Dec
Jan
Feb
Mar
Apr
May
Jun
Winter Solstice
December 21/Day 355
Winter Teletherm
January 12/Day 12
+22 days
Coverage
Year
PENSACOLA RGNL AP, FL

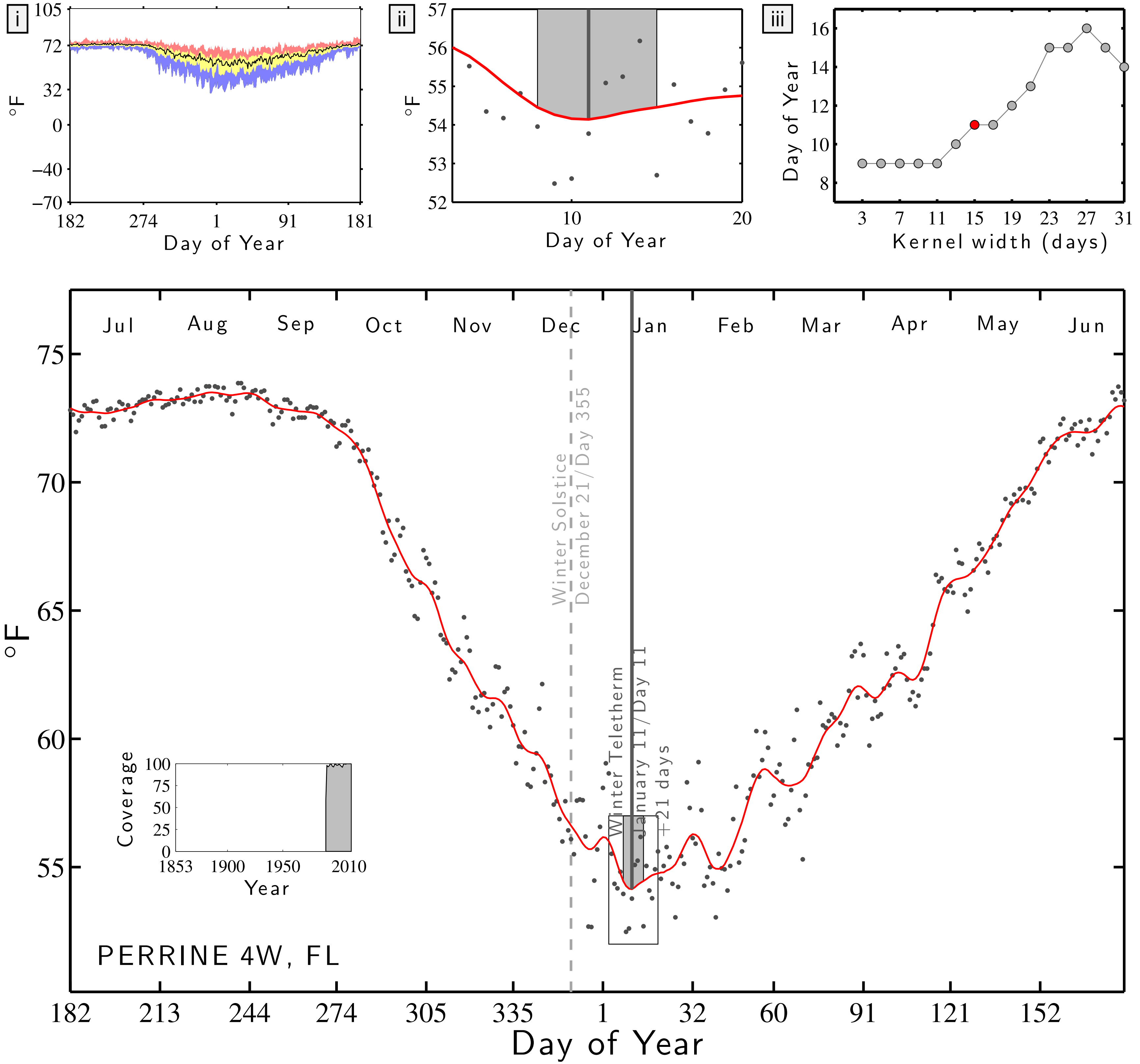

i
ii
iii
°F
Day of Year
Kernel width (days)
Winter Solstice
December 21/Day 355
Winter Teletherm
January 11/Day 11
±21 days
Coverage
Year
Jul
Aug
Sep
Oct
Nov
Dec
Jan
Feb
Mar
Apr
May
Jun
PERRINE 4W, FL

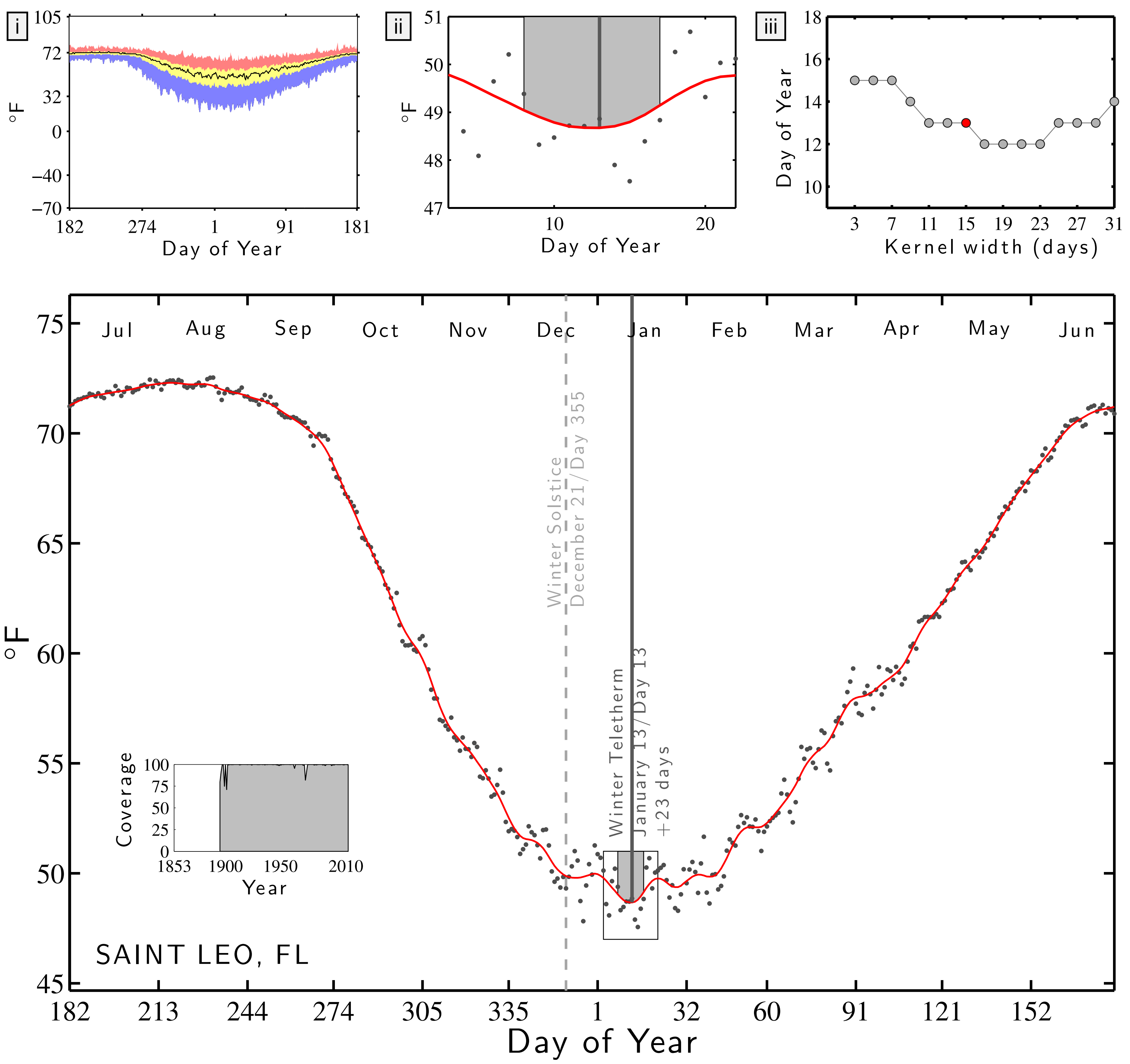

i
ii
iii
°F
Day of Year
Day of Year
Day of Year
Kernel width (days)
Jul
Aug
Sep
Oct
Nov
Dec
Jan
Feb
Mar
Apr
May
Jun
Winter Solstice
December 21/Day 355
Winter Teletherm
January 13/Day 13
+23 days
Coverage
Year
SAINT LEO, FL

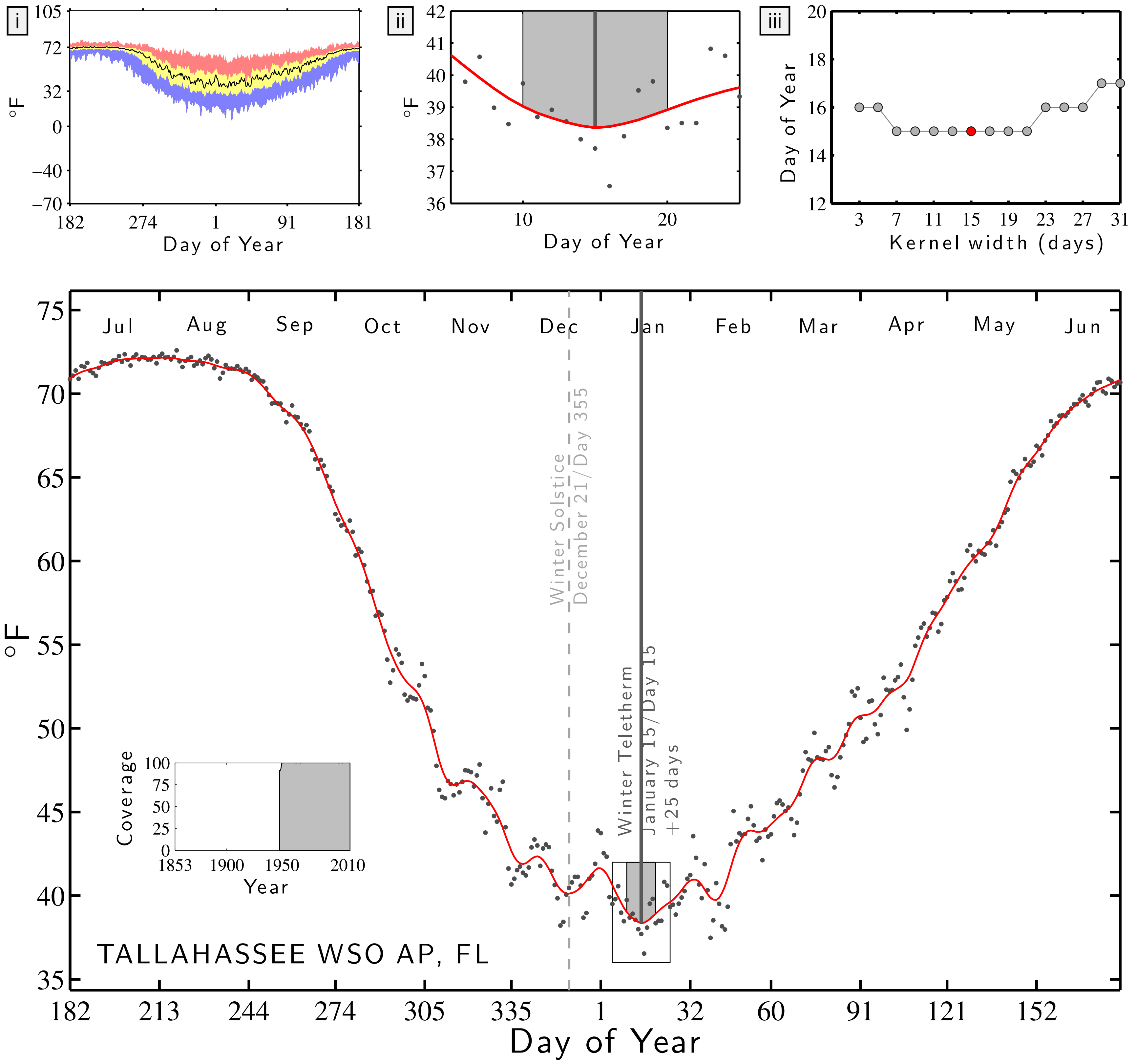

i
°F
Day of Year
ii
Day of Year
iii
Day of Year
Kernel width (days)
Jul
Aug
Sep
Oct
Nov
Dec
Jan
Feb
Mar
Apr
May
Jun
Winter Solstice
December 21/Day 355
Winter Teletherm
January 15/Day 15
+25 days
Coverage
Year
TALLAHASSEE WSO AP, FL
°F
Day of Year

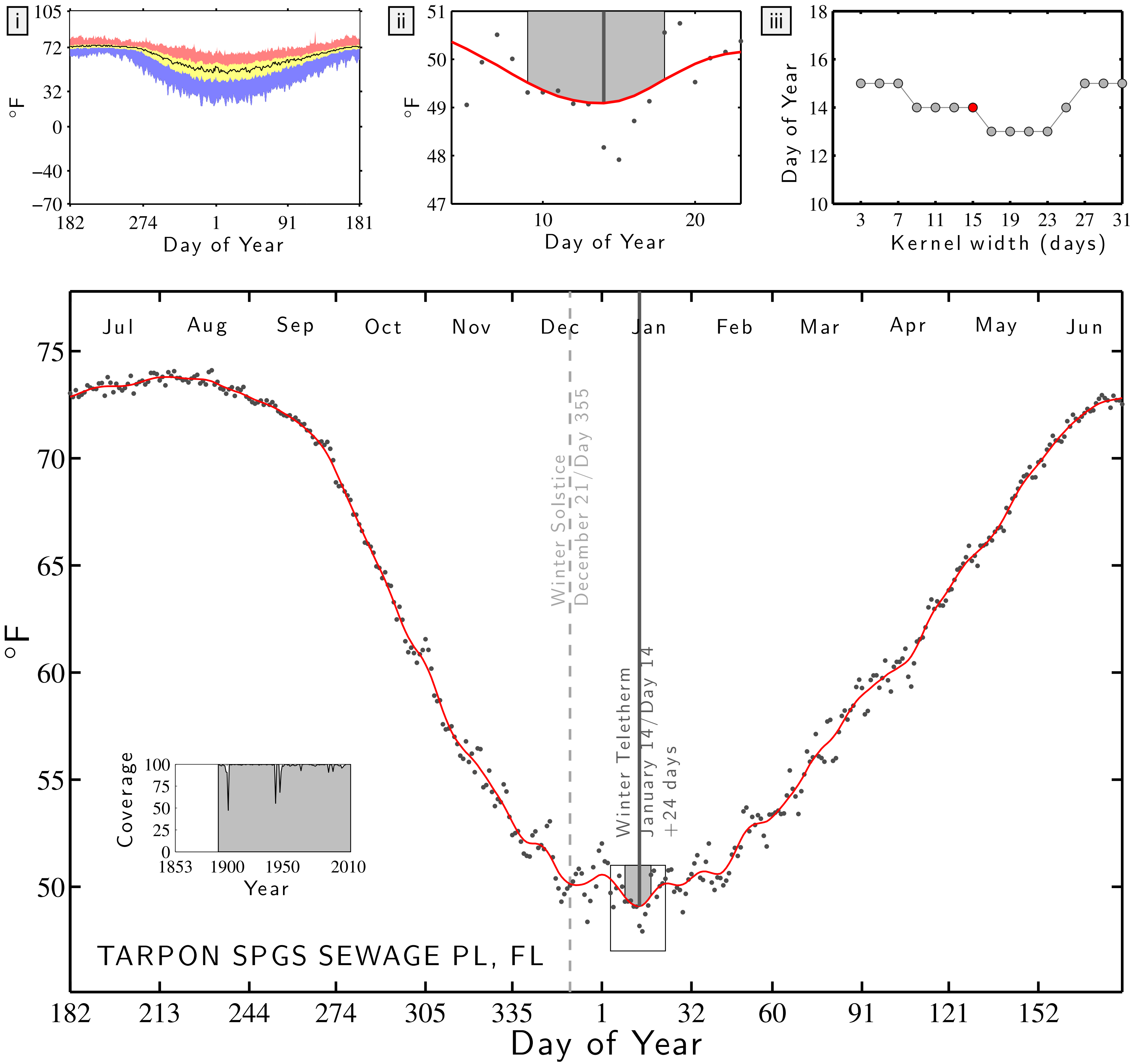

i
°F
Day of Year
ii
Day of Year
iii
Day of Year
Kernel width (days)
Jul
Aug
Sep
Oct
Nov
Dec
Jan
Feb
Mar
Apr
May
Jun
Winter Solstice
December 21/Day 355
Winter Teletherm
January 14/Day 14
+24 days
Coverage
Year
TARPON SPGS SEWAGE PL, FL
°F
Day of Year

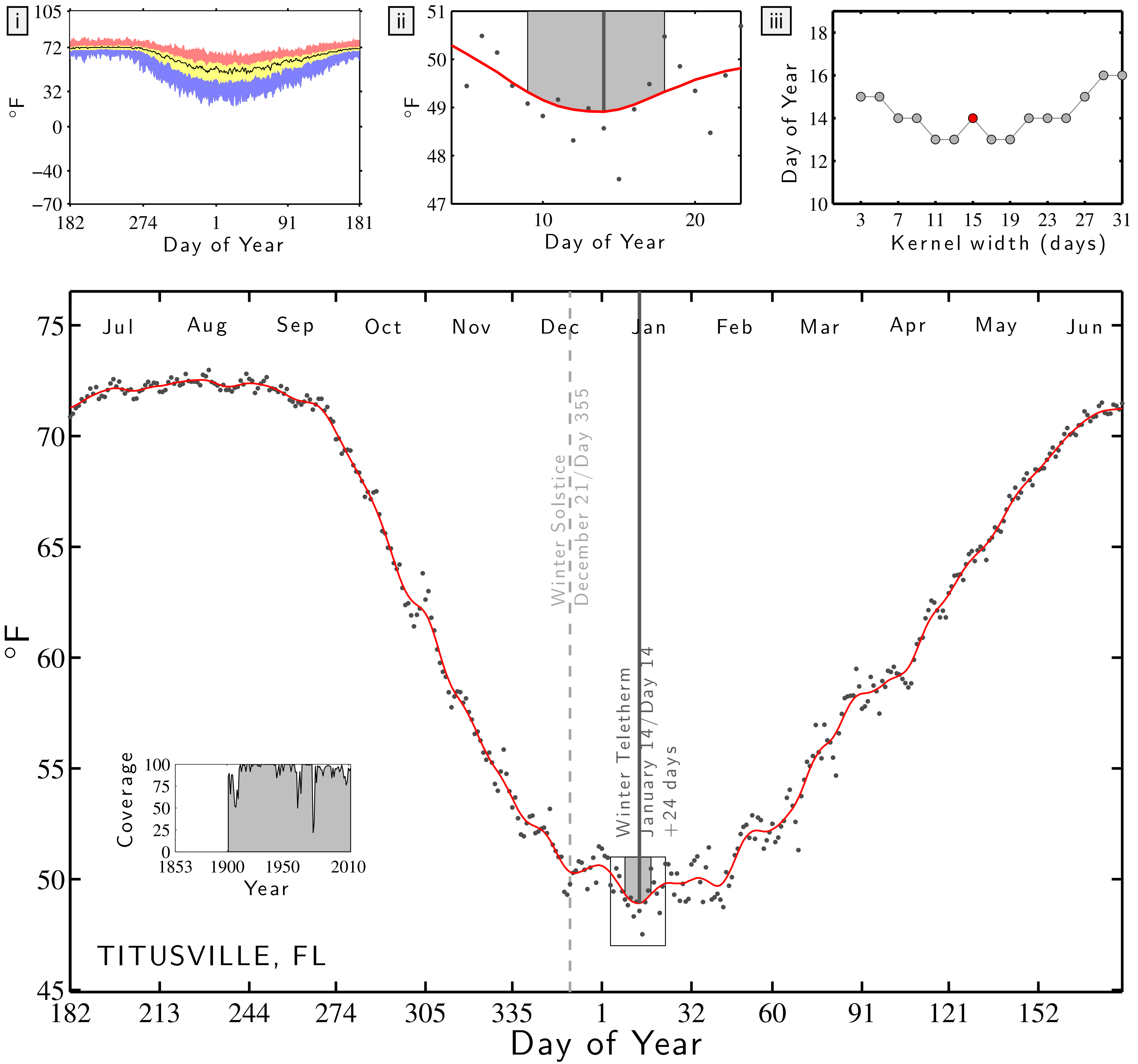

i
ii
iii
°F
Day of Year
Kernel width (days)
Jul
Aug
Sep
Oct
Nov
Dec
Jan
Feb
Mar
Apr
May
Jun
Winter Solstice
December 21/Day 355
Winter Teletherm
January 14/Day 14
+24 days
Coverage
Year
TITUSVILLE, FL

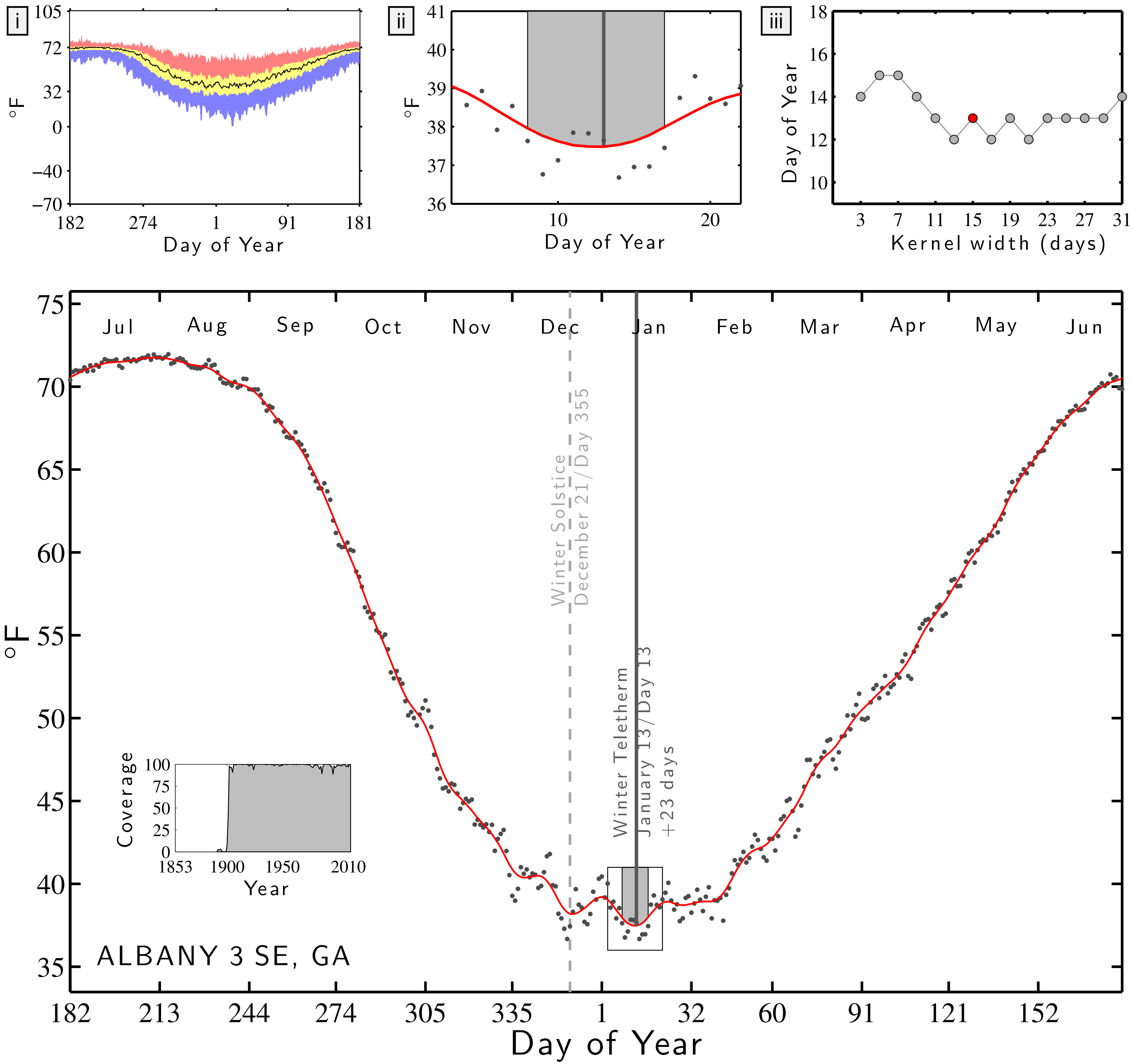

i
°F
105
72
32
0
−40
−70
182
274
1
91
181
Day of Year
ii
°F
41
40
39
38
37
36
10
20
Day of Year
iii
Day of Year
18
16
14
12
10
3
7
11
15
19
23
27
31
Kernel width (days)
Jul
Aug
Sep
Oct
Nov
Dec
Jan
Feb
Mar
Apr
May
Jun
Winter Solstice
December 21/Day 355
Winter Teletherm
January 13/Day 13
+23 days
Coverage
100
75
50
25
0
1853
1900
1950
2010
Year
ALBANY 3 SE, GA
°F
75
70
65
60
55
50
45
40
35
182
213
244
274
305
335
1
32
60
91
121
152
Day of Year

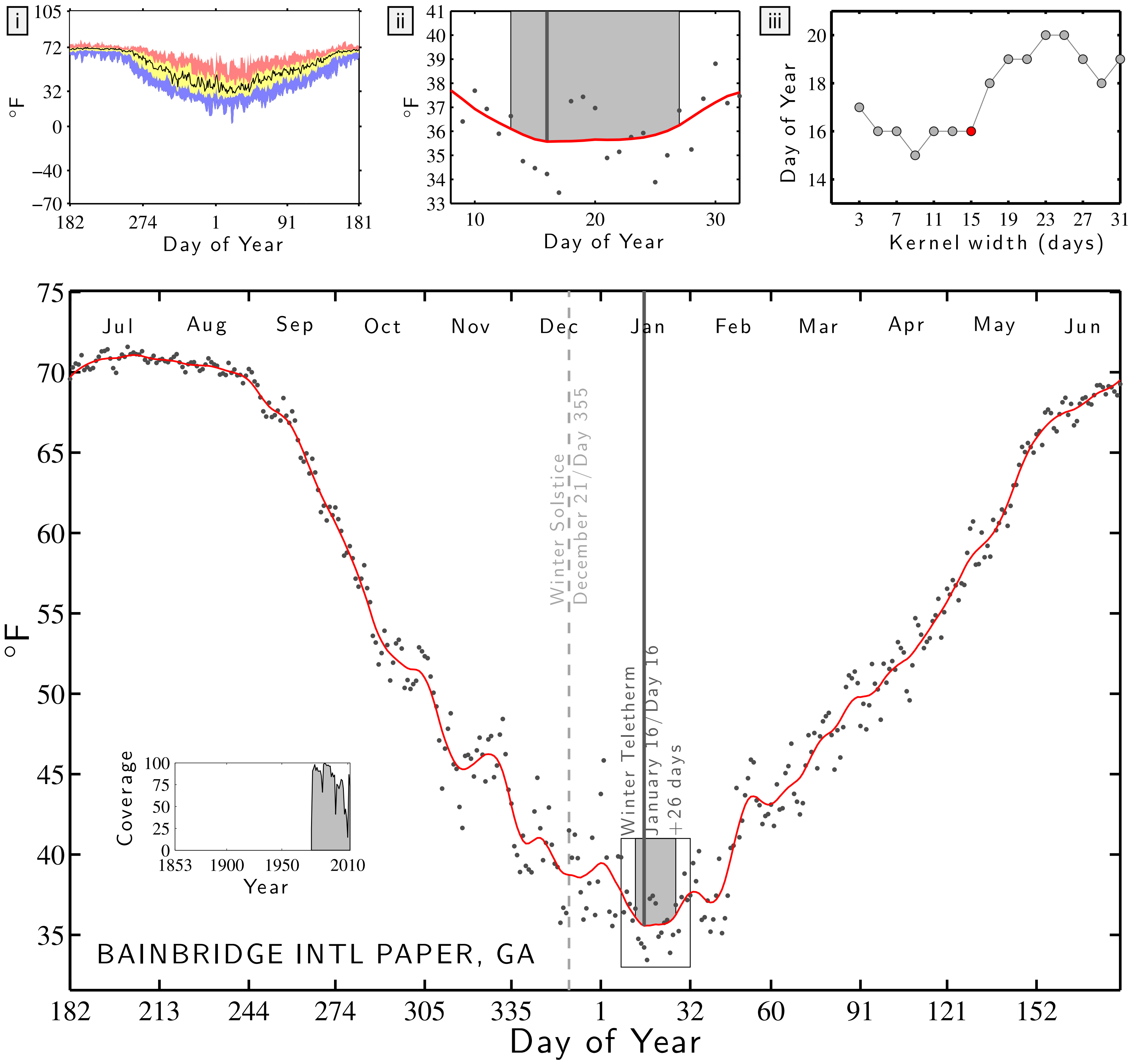

i
ii
iii
°F
Day of Year
Kernel width (days)
Jul
Aug
Sep
Oct
Nov
Dec
Jan
Feb
Mar
Apr
May
Jun
Winter Solstice
December 21/Day 355
Winter Teletherm
January 16/Day 16
+26 days
Coverage
Year
BAINBRIDGE INTL PAPER, GA

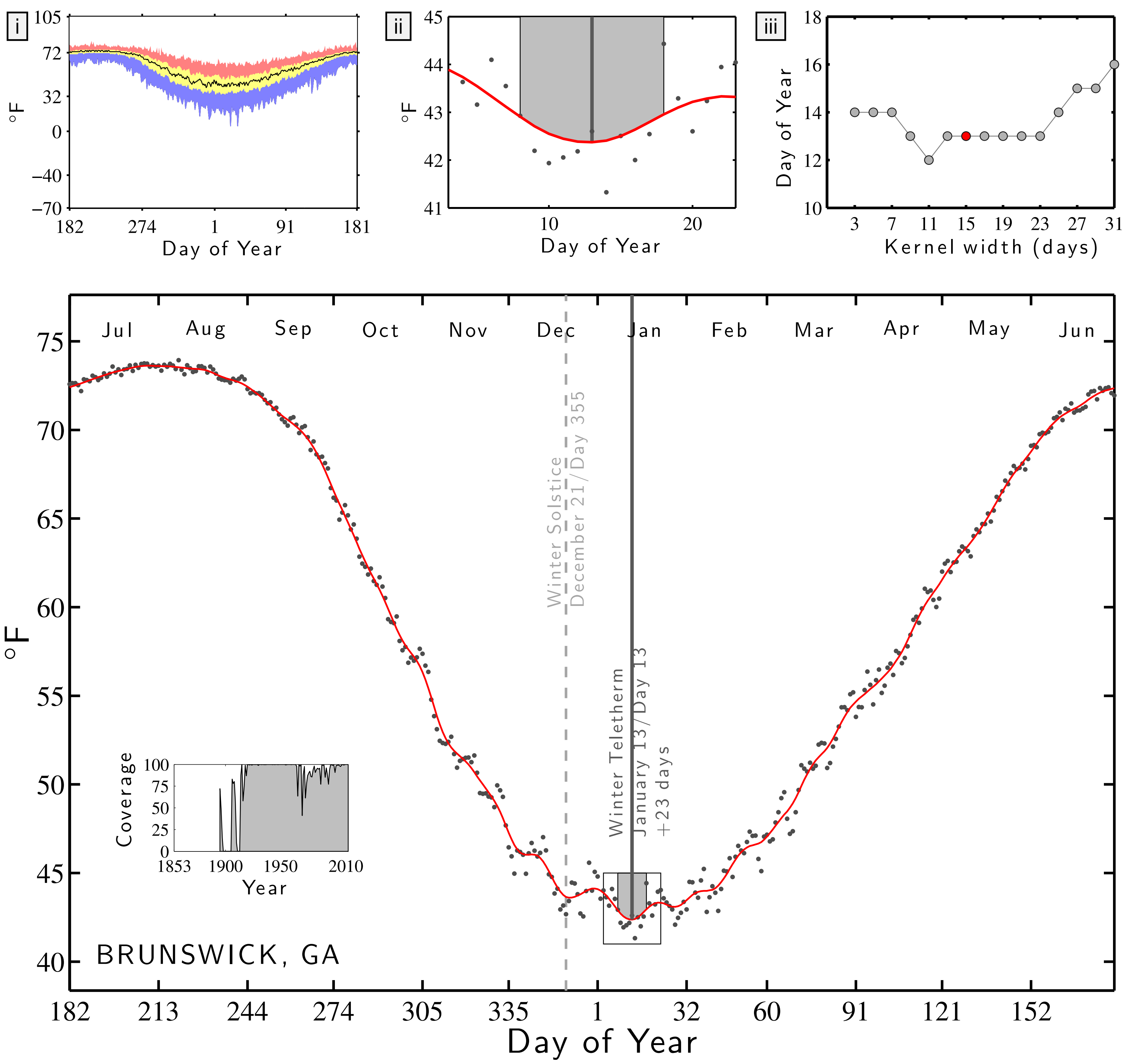

i
ii
iii
°F
Day of Year
Day of Year
Kernel width (days)
Jul
Aug
Sep
Oct
Nov
Dec
Jan
Feb
Mar
Apr
May
Jun
Winter Solstice
December 21/Day 355
Winter Teletherm
January 13/Day 13
+23 days
Coverage
Year
BRUNSWICK, GA

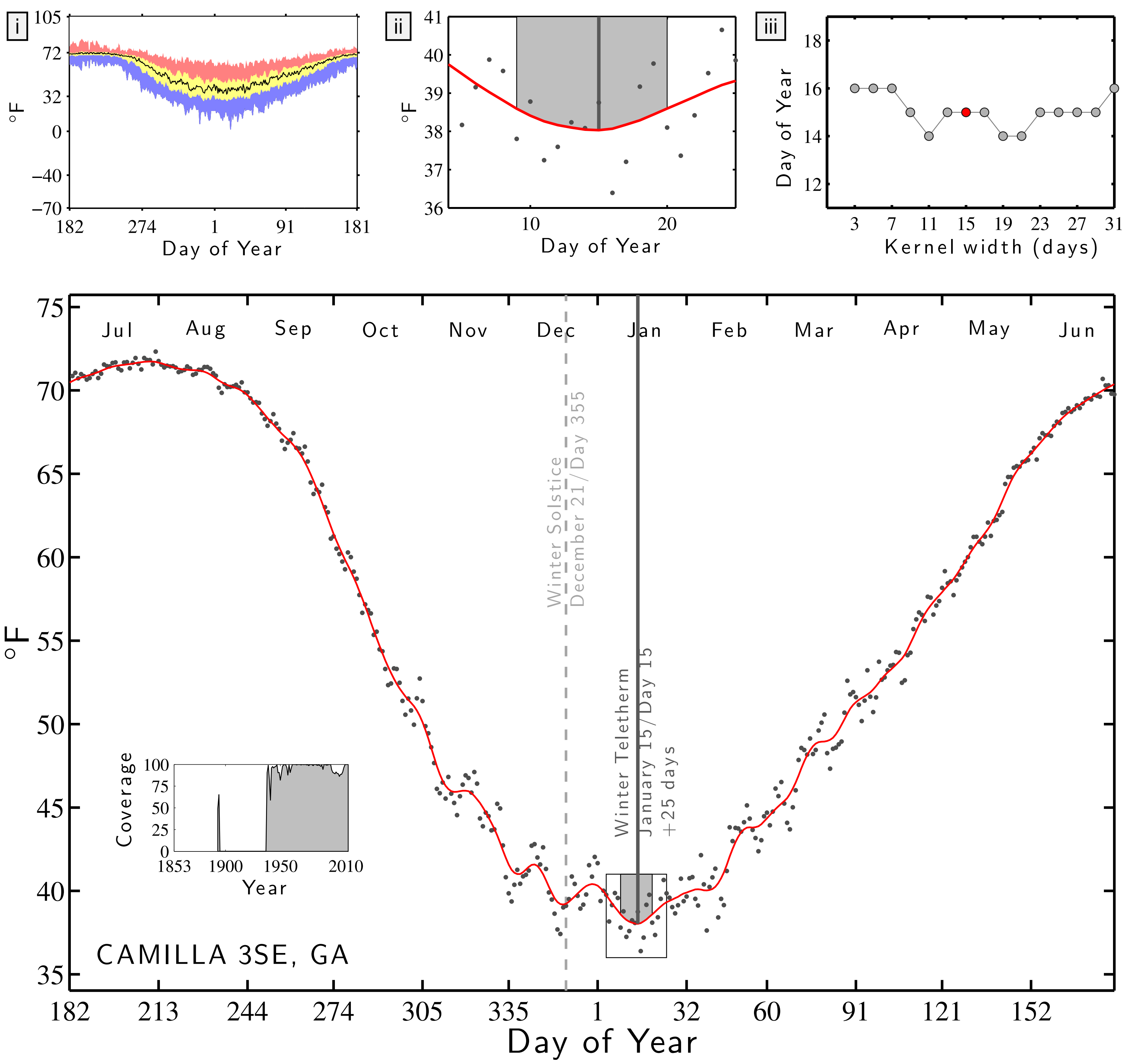

i
ii
iii
°F
Day of Year
Kernel width (days)
Jul
Aug
Sep
Oct
Nov
Dec
Jan
Feb
Mar
Apr
May
Jun
Winter Solstice
December 21/Day 355
Winter Teletherm
January 15/Day 15
+25 days
Coverage
Year
CAMILLA 3SE, GA

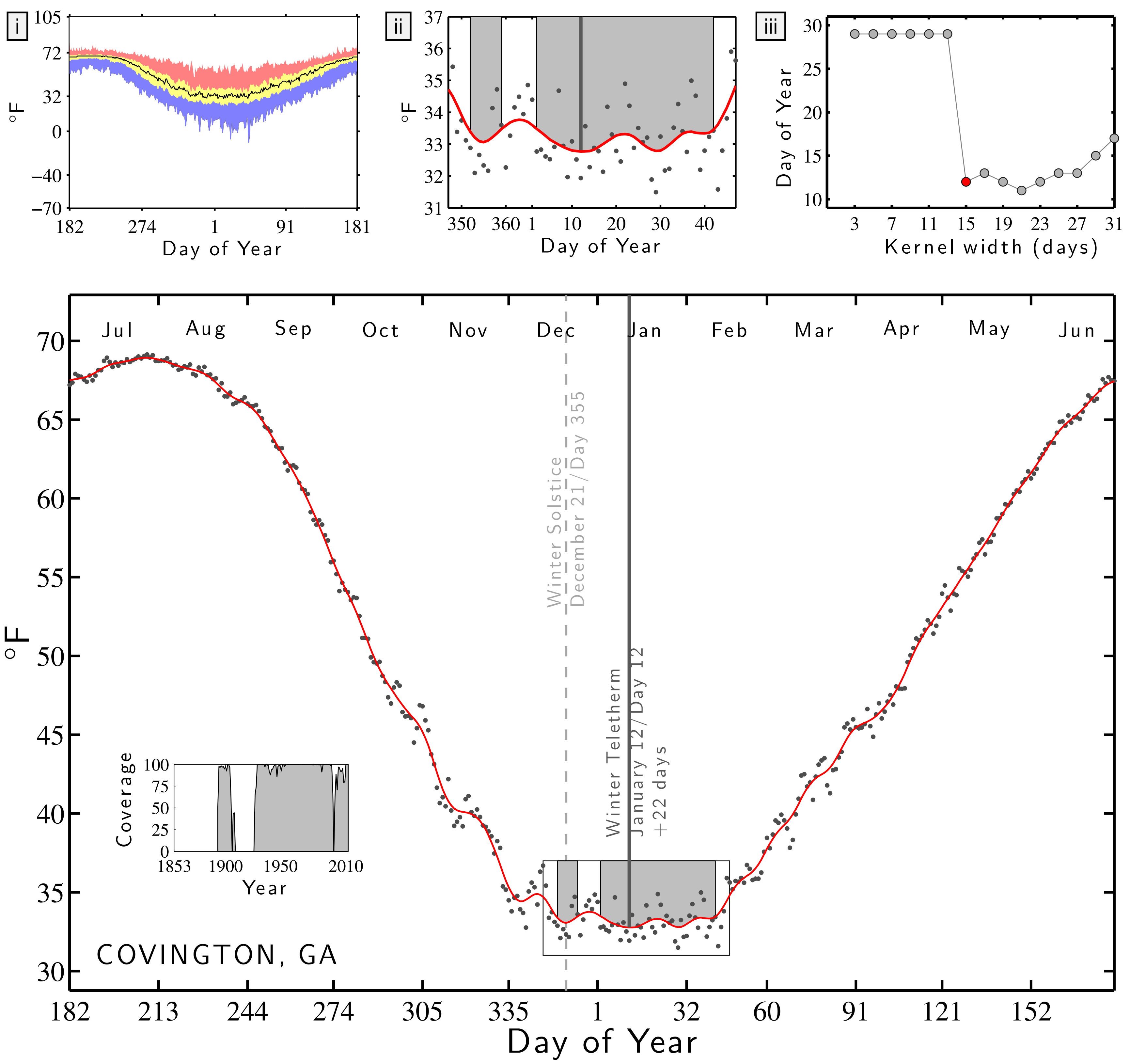

i
ii
iii
°F
Day of Year
Day of Year
Kernel width (days)
Jul
Aug
Sep
Oct
Nov
Dec
Jan
Feb
Mar
Apr
May
Jun
Winter Solstice
December 21/Day 355
Winter Teletherm
January 12/Day 12
+22 days
Coverage
Year
COVINGTON, GA
Day of Year

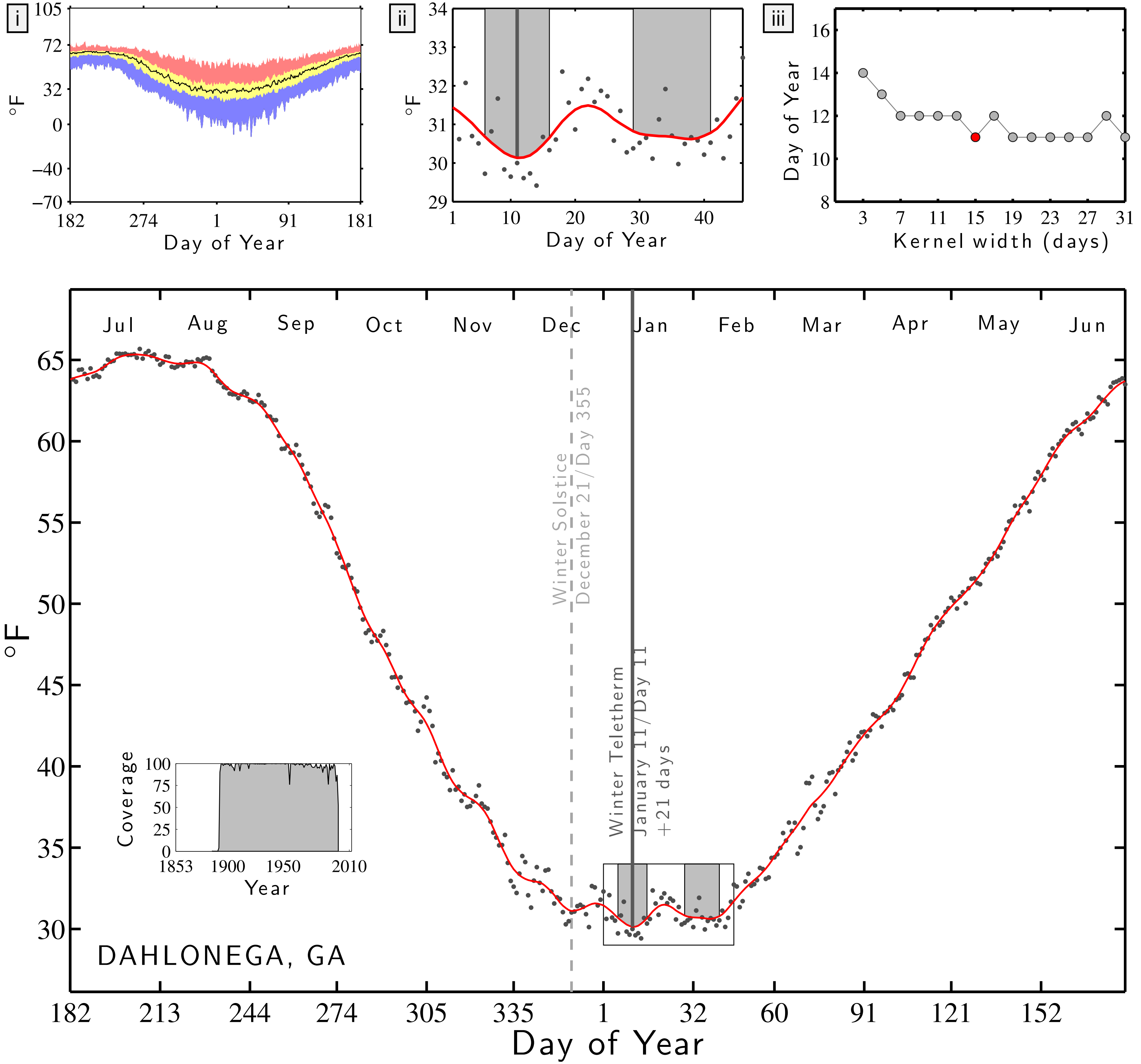

i
°F
105
72
32
0
−40
−70
182
274
1
91
181
Day of Year
ii
34
33
32
31
30
29
10
20
30
40
iii
Day of Year
16
14
12
10
8
3
7
11
15
19
23
27
31
Kernel width (days)
Jul
Aug
Sep
Oct
Nov
Dec
Jan
Feb
Mar
Apr
May
Jun
65
60
55
50
45
40
35
30
Winter Solstice
December 21/Day 355
Winter Teletherm
January 11/Day 11
+21 days
Coverage
100
75
50
25
1853
1900
1950
2010
Year
DAHLONEGA, GA
213
244
305
335
32
60
121
152

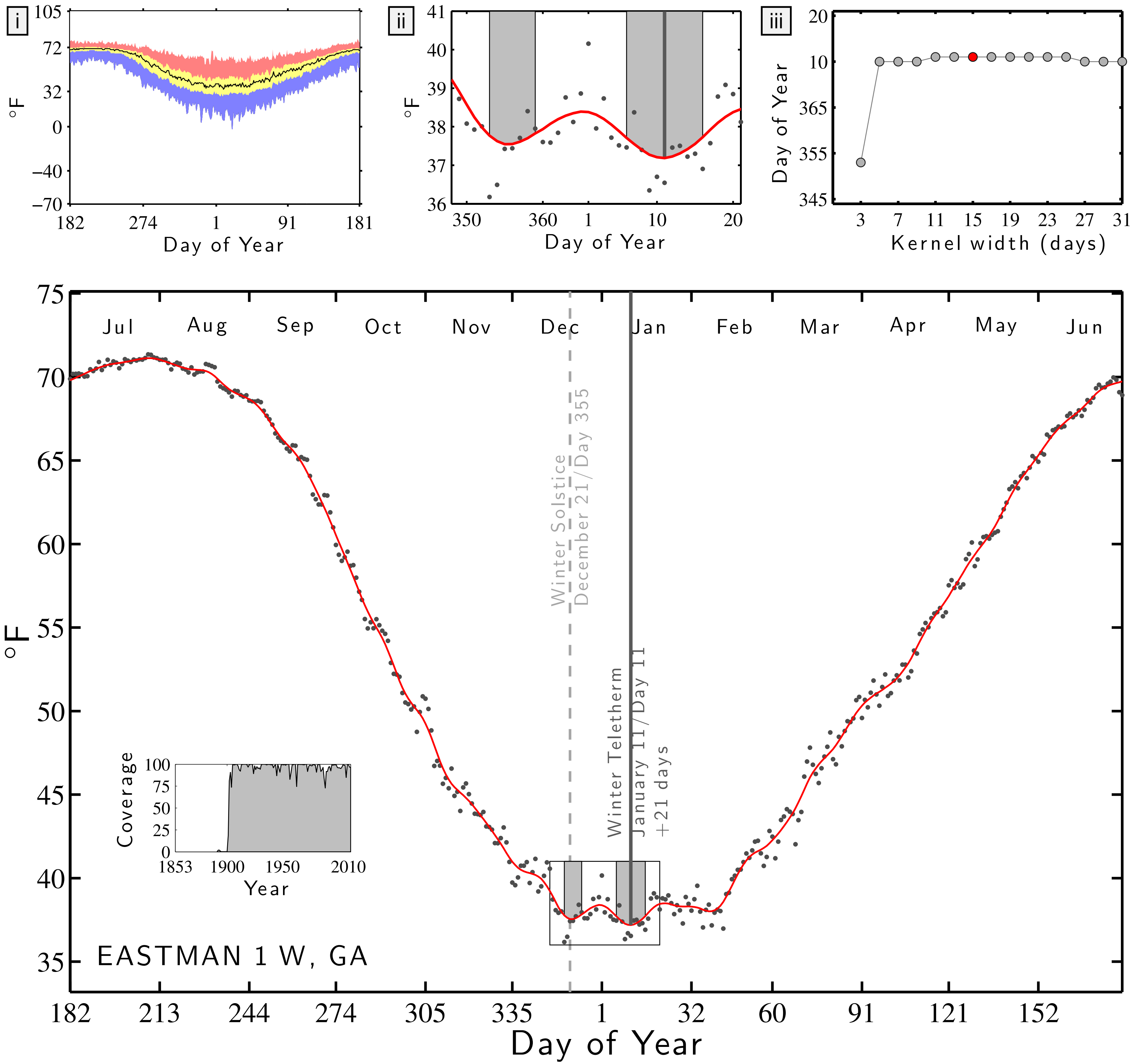
i
ii
iii
°F
Day of Year
Kernel width (days)
Jul
Aug
Sep
Oct
Nov
Dec
Jan
Feb
Mar
Apr
May
Jun
Winter Solstice
December 21/Day 355
Winter Teletherm
January 11/Day 11
+21 days
Coverage
Year
EASTMAN 1 W, GA

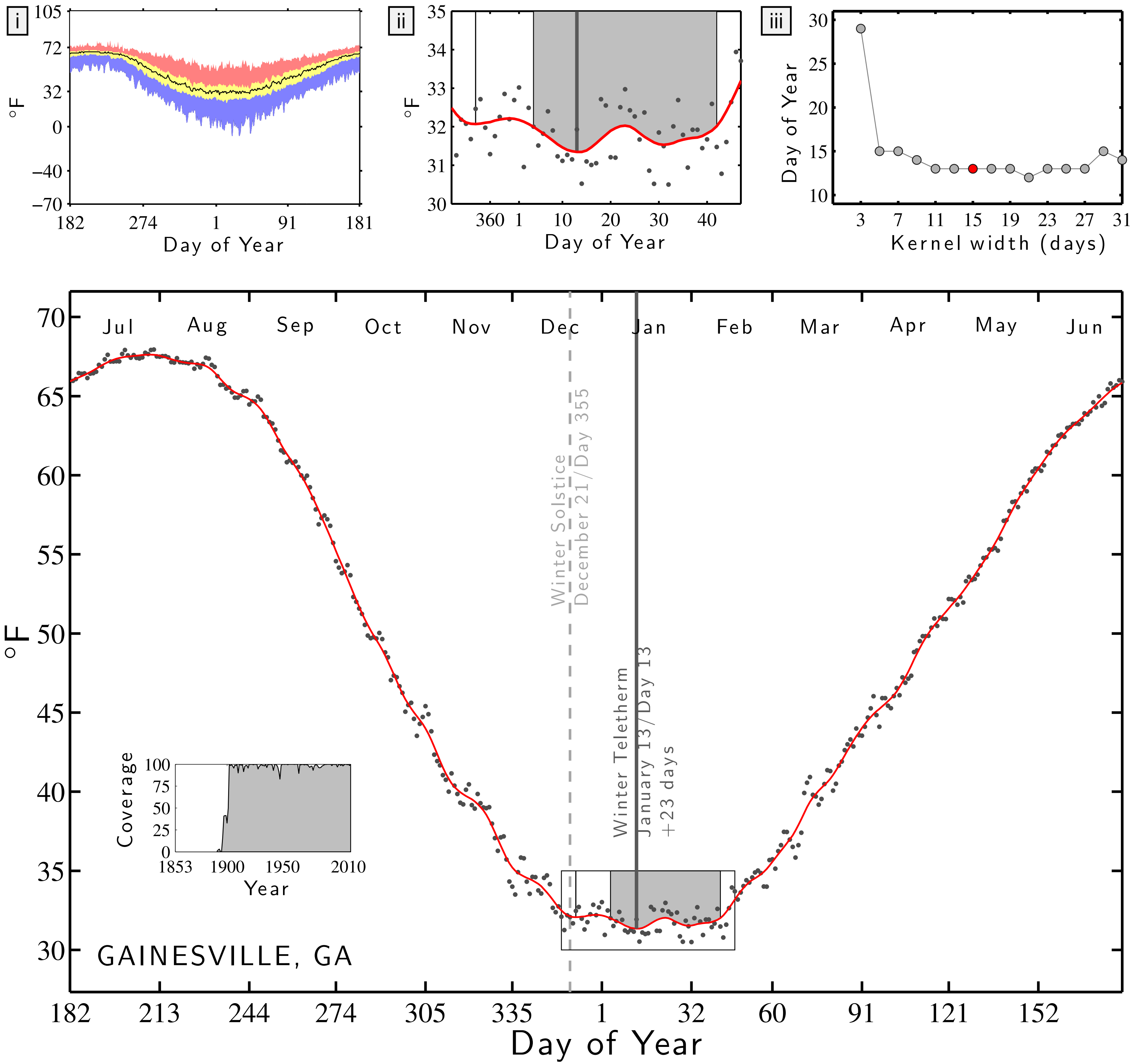

i
°F
Day of Year
ii
°F
Day of Year
iii
Day of Year
Kernel width (days)
Jul
Aug
Sep
Oct
Nov
Dec
Jan
Feb
Mar
Apr
May
Jun
Winter Solstice
December 21/Day 355
Winter Teletherm
January 13/Day 13
+23 days
Coverage
Year
GAINESVILLE, GA
°F
Day of Year

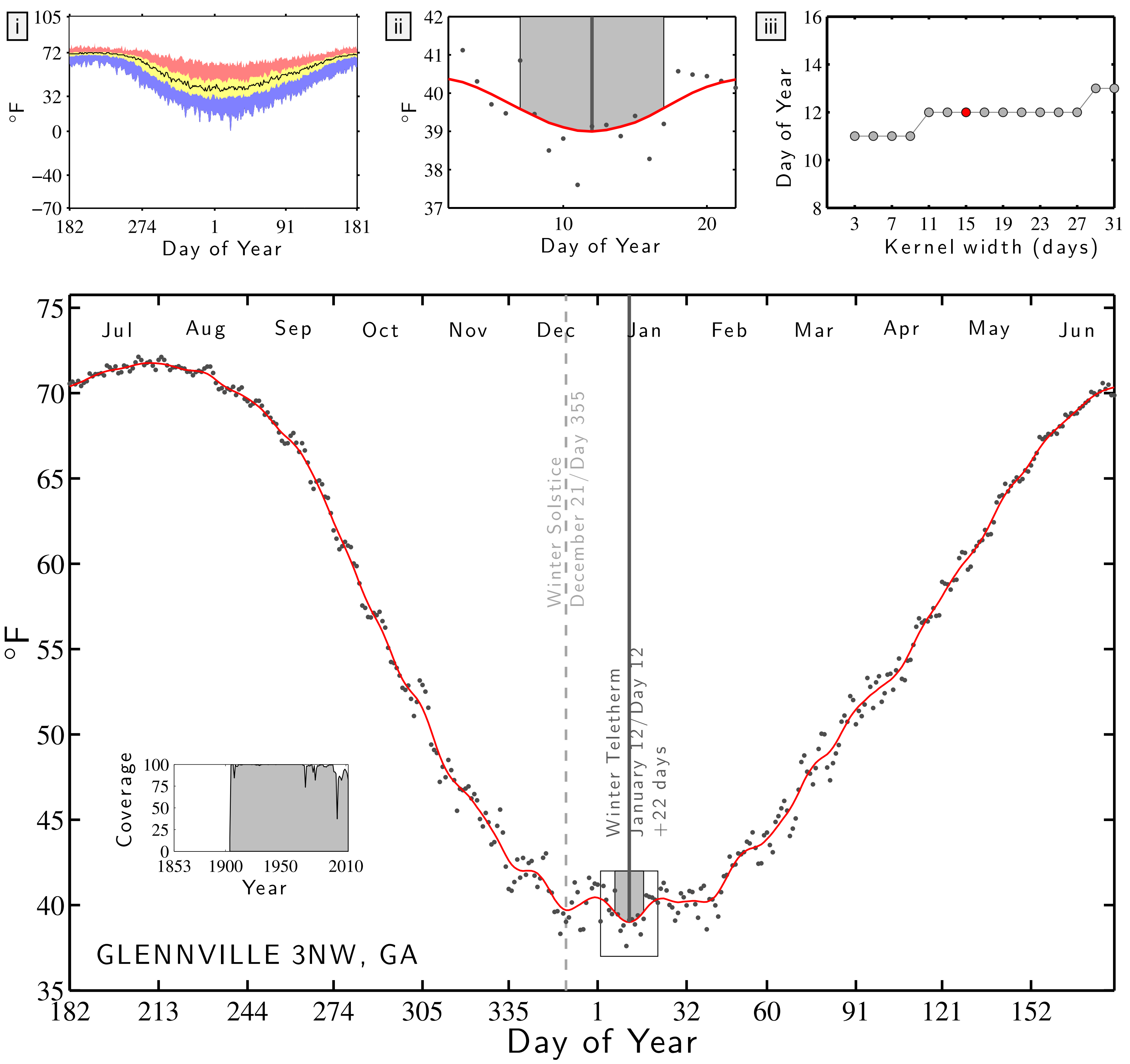

i
ii
iii
Day of Year
°F
Kernel width (days)
Jul
Aug
Sep
Oct
Nov
Dec
Jan
Feb
Mar
Apr
May
Jun
Winter Solstice
December 21/Day 355
Winter Teletherm
January 12/Day 12
+22 days
Coverage
Year
GLENNVILLE 3NW, GA

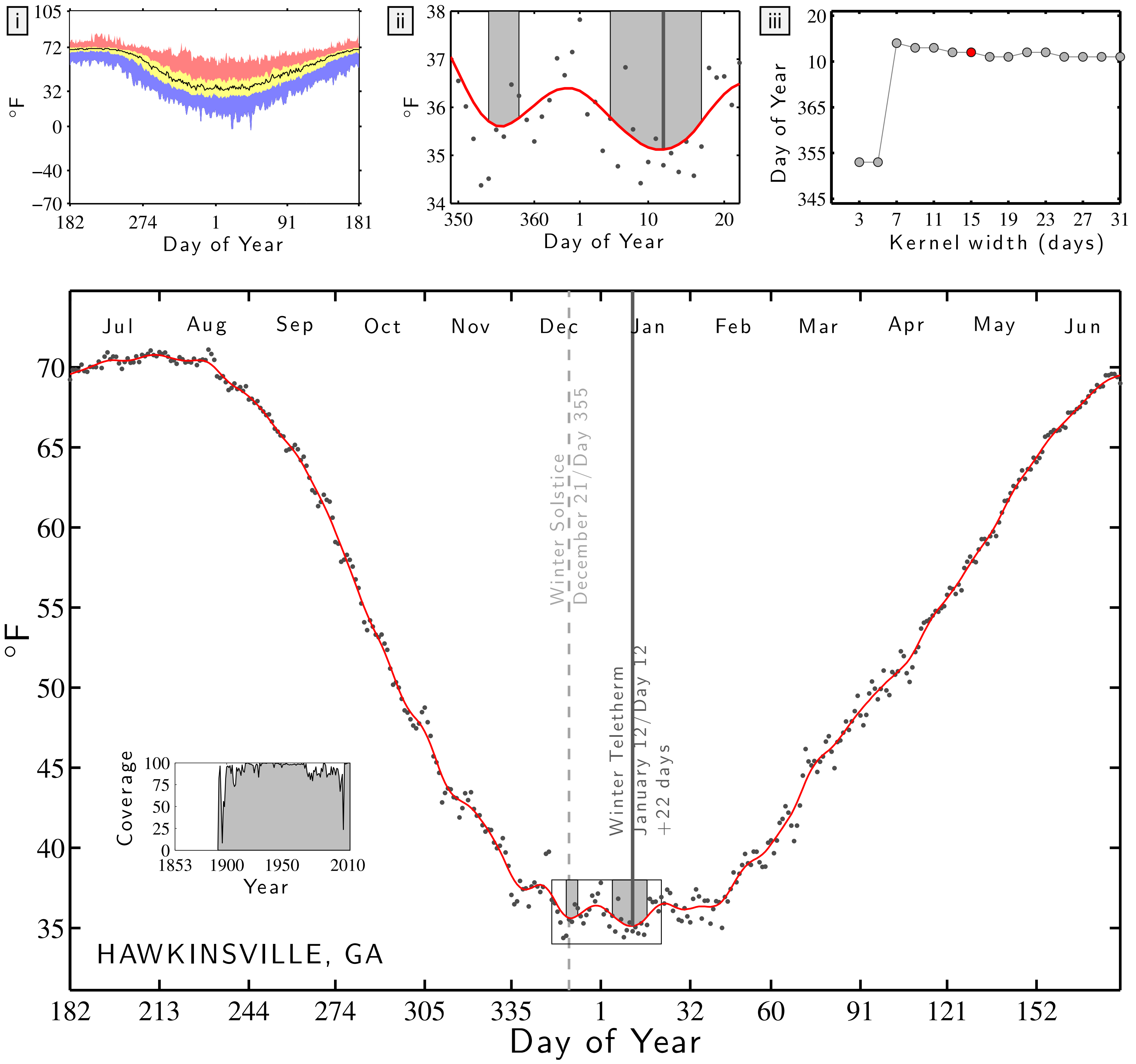
i
ii
iii
Day of Year
Kernel width (days)
°F
Day of Year
Jul
Aug
Sep
Oct
Nov
Dec
Jan
Feb
Mar
Apr
May
Jun
Winter Solstice
December 21/Day 355
Winter Teletherm
January 12/Day 12
+22 days
Coverage
Year
HAWKINSVILLE, GA

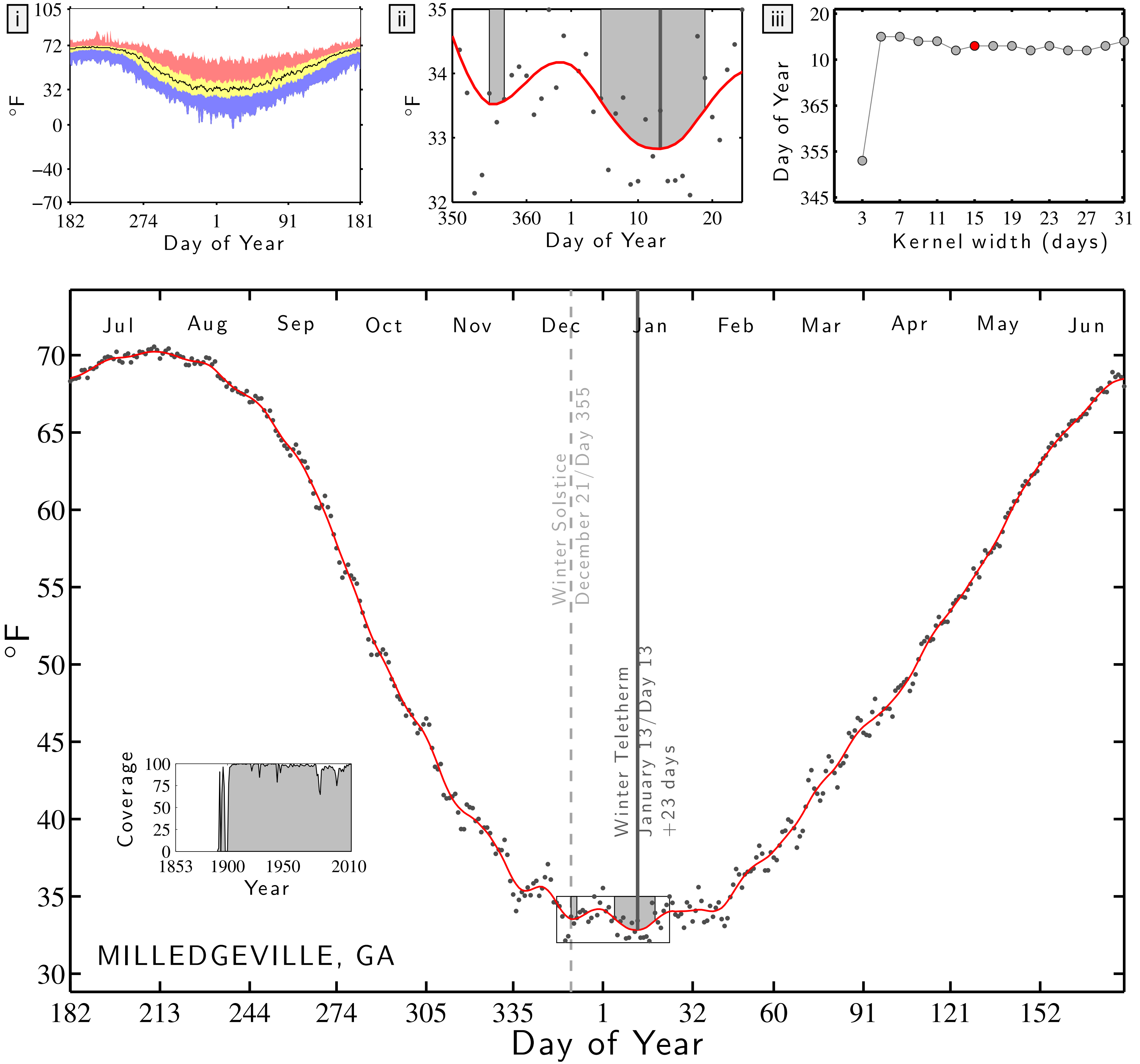

i
ii
iii
°F
Day of Year
Kernel width (days)
Jul
Aug
Sep
Oct
Nov
Dec
Jan
Feb
Mar
Apr
May
Jun
Winter Solstice
December 21/Day 355
Winter Teletherm
January 13/Day 13
+23 days
Coverage
Year
MILLEDGEVILLE, GA

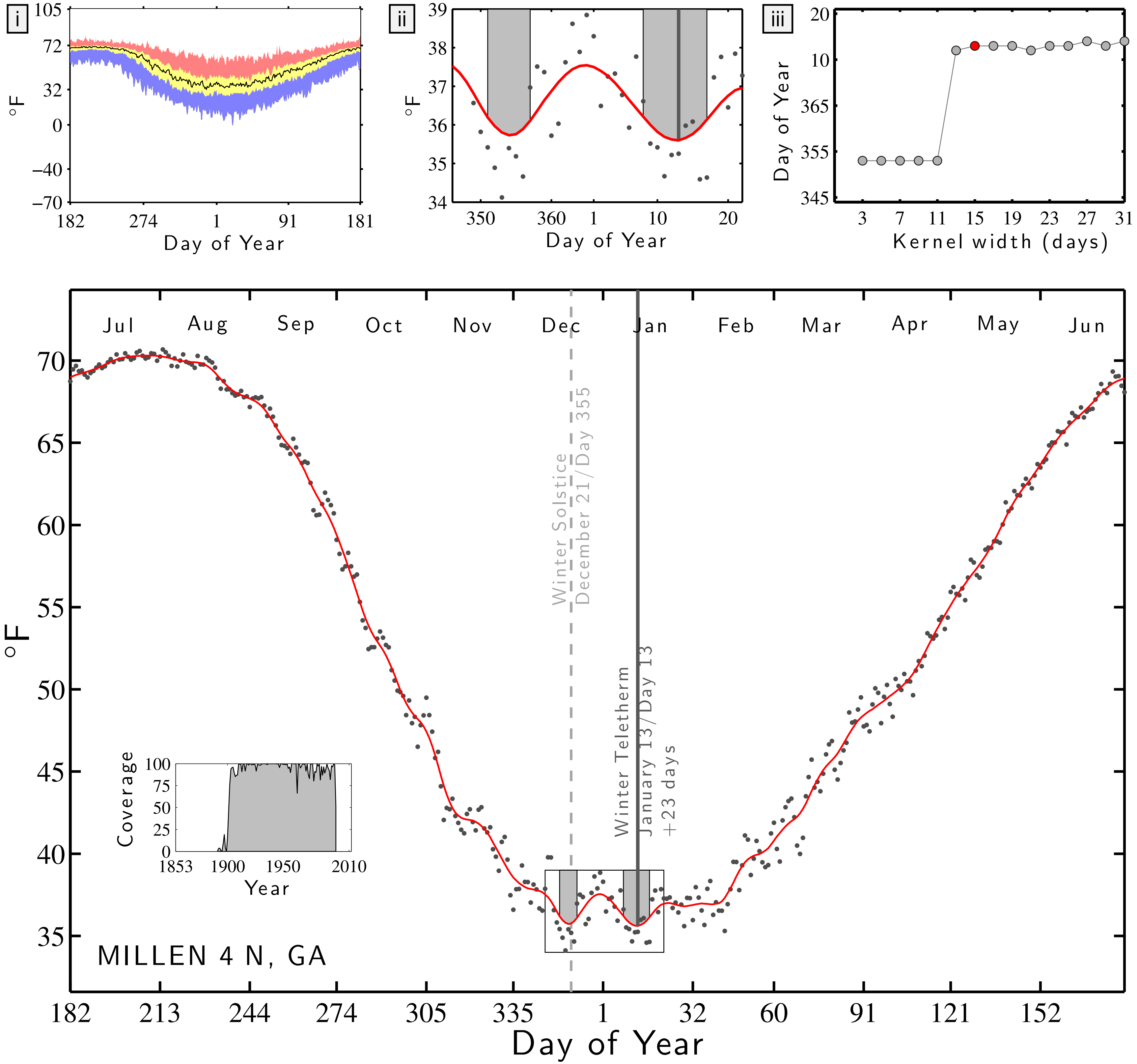

i
°F
Day of Year
ii
°F
Day of Year
iii
Day of Year
Kernel width (days)
Jul
Aug
Sep
Oct
Nov
Dec
Jan
Feb
Mar
Apr
May
Jun
Winter Solstice
December 21/Day 355
Winter Teletherm
January 13/Day 13
+23 days
Coverage
Year
MILLEN 4 N, GA
°F
Day of Year

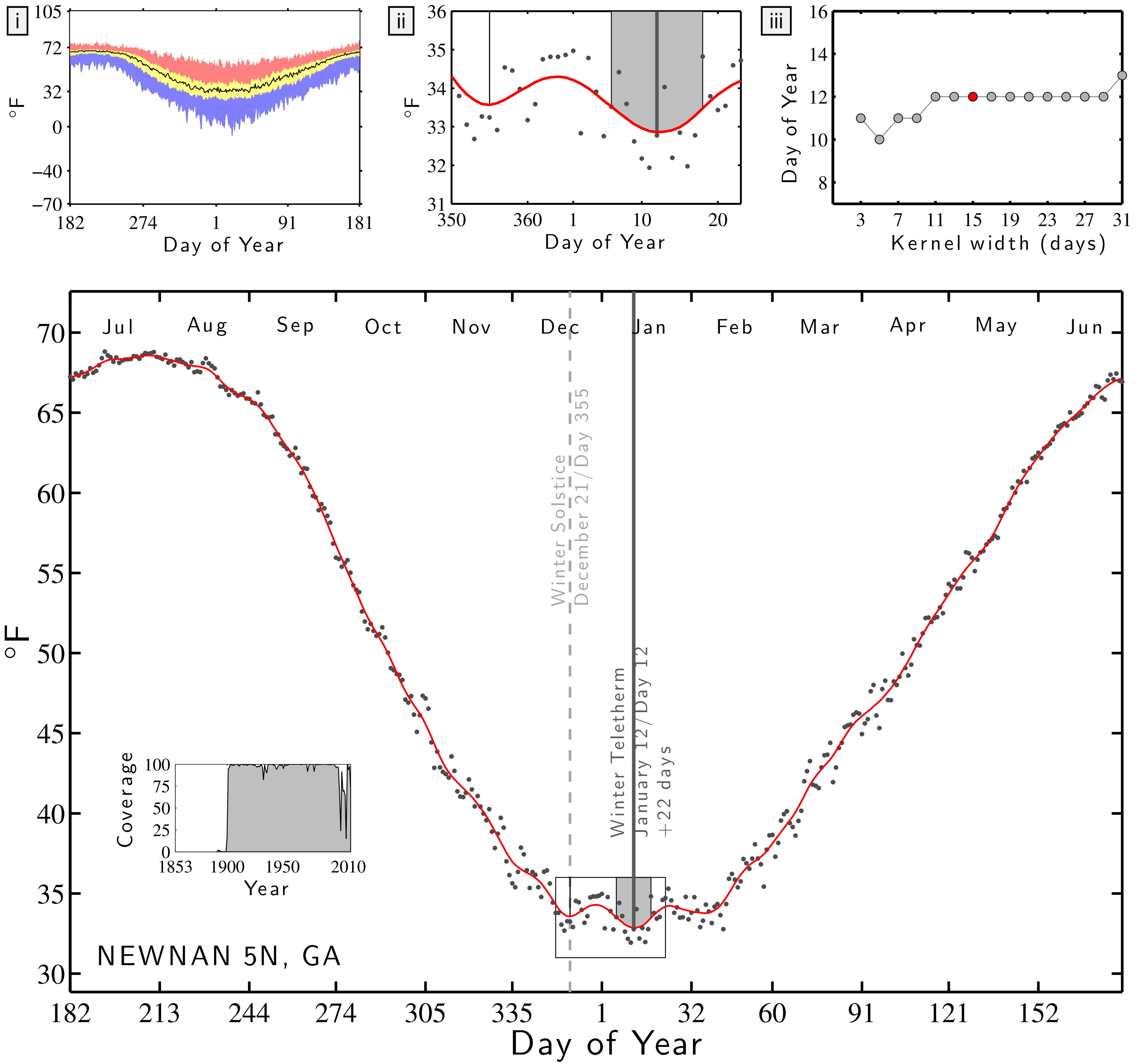
i
°F
Day of Year
ii
°F
Day of Year
iii
Day of Year
Kernel width (days)
Jul
Aug
Sep
Oct
Nov
Dec
Jan
Feb
Mar
Apr
May
Jun
Winter Solstice
December 21/Day 355
Winter Teletherm
January 12/Day 12
+22 days
Coverage
Year
NEWNAN 5N, GA
°F
Day of Year

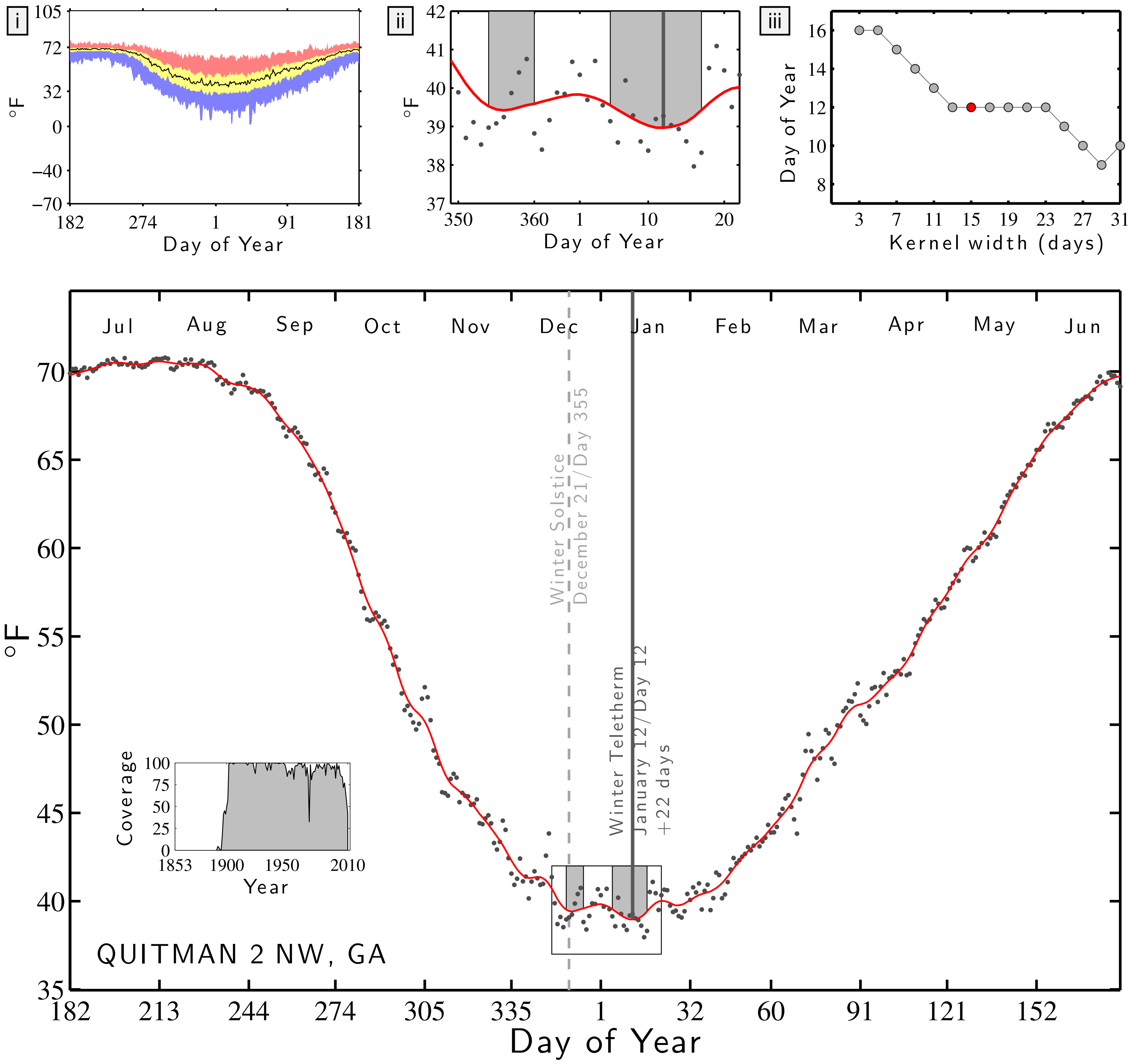

i
ii
iii
°F
Day of Year
Day of Year
Kernel width (days)
Jul
Aug
Sep
Oct
Nov
Dec
Jan
Feb
Mar
Apr
May
Jun
Winter Solstice
December 21/Day 355
Winter Teletherm
January 12/Day 12
+22 days
Coverage
Year
QUITMAN 2 NW, GA
Day of Year

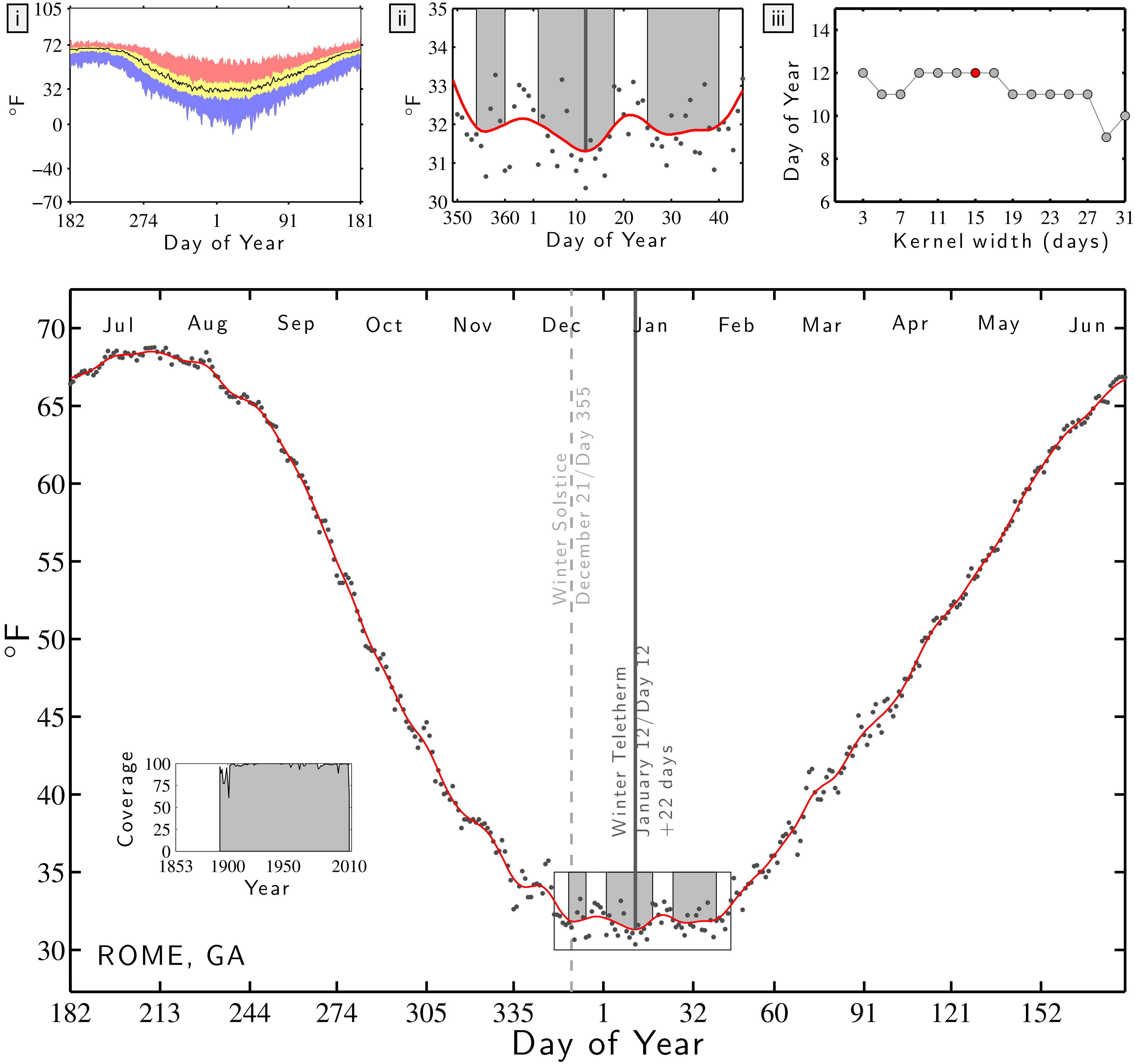

i
ii
iii
°F
Day of Year
Day of Year
Kernel width (days)
Jul
Aug
Sep
Oct
Nov
Dec
Jan
Feb
Mar
Apr
May
Jun
Winter Solstice
December 21/Day 355
Winter Teletherm
January 12/Day 12
+22 days
Coverage
Year
ROME, GA
Day of Year

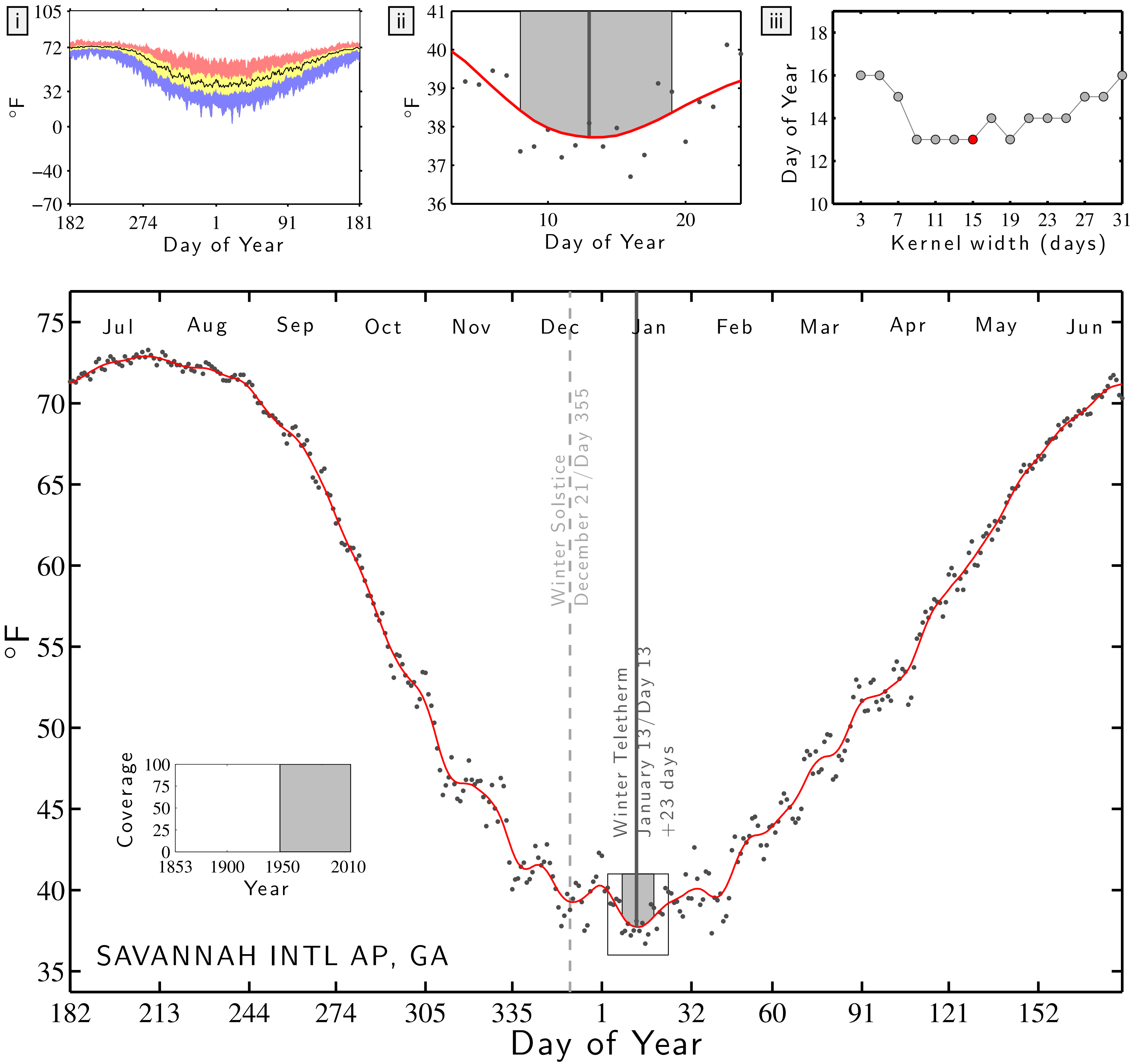

i
ii
iii
Day of Year
Kernel width (days)
°F
Winter Solstice
December 21/Day 355
Winter Teletherm
January 13/Day 13
+23 days
Coverage
Year
SAVANNAH INTL AP, GA
Jul
Aug
Sep
Oct
Nov
Dec
Jan
Feb
Mar
Apr
May
Jun

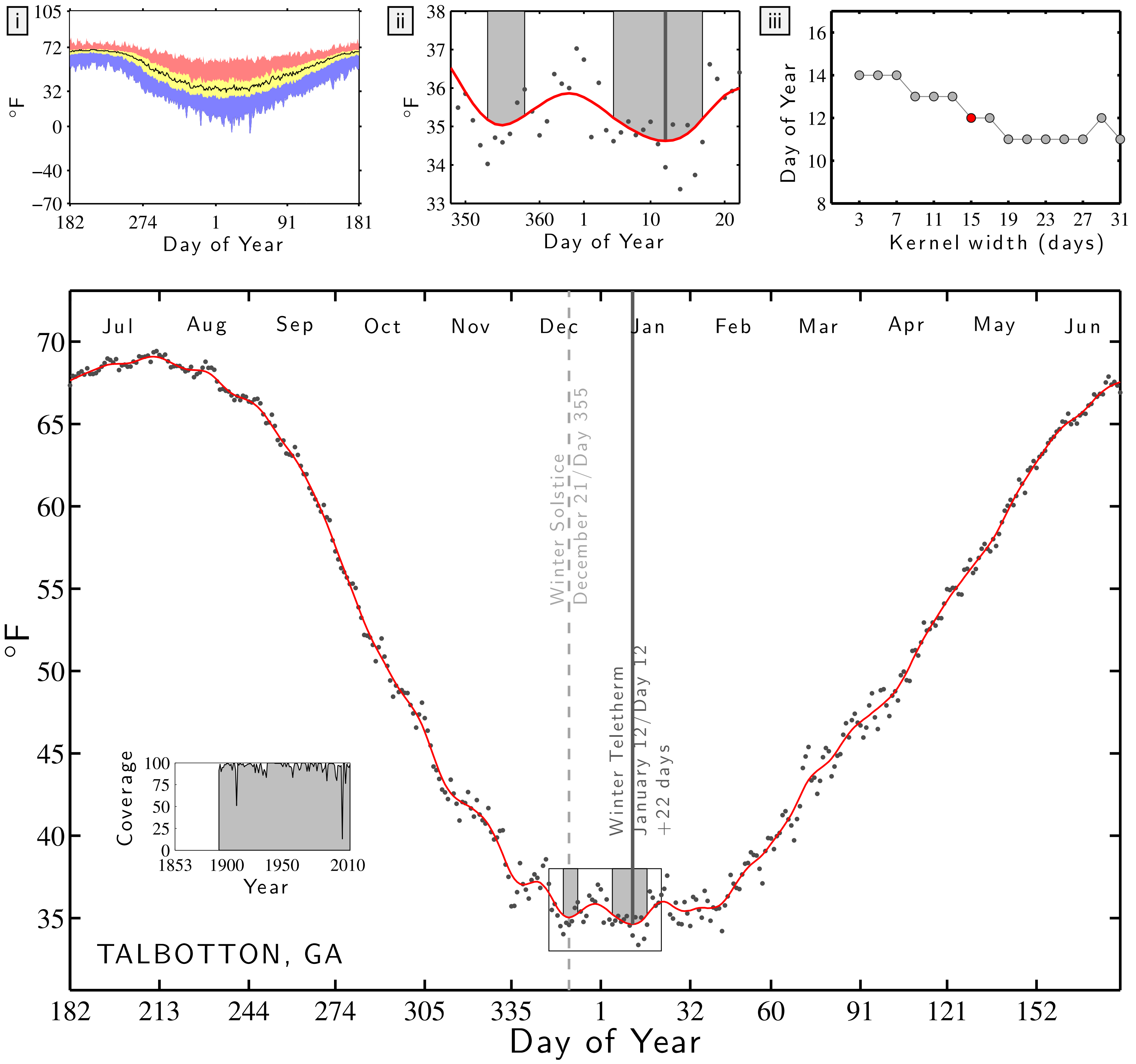

i
ii
iii
°F
Day of Year
Kernel width (days)
Jul Aug Sep Oct Nov Dec Jan Feb Mar Apr May Jun
Winter Solstice
December 21/Day 355
Winter Teletherm
January 12/Day 12
+22 days
Coverage
Year
TALBOTTON, GA

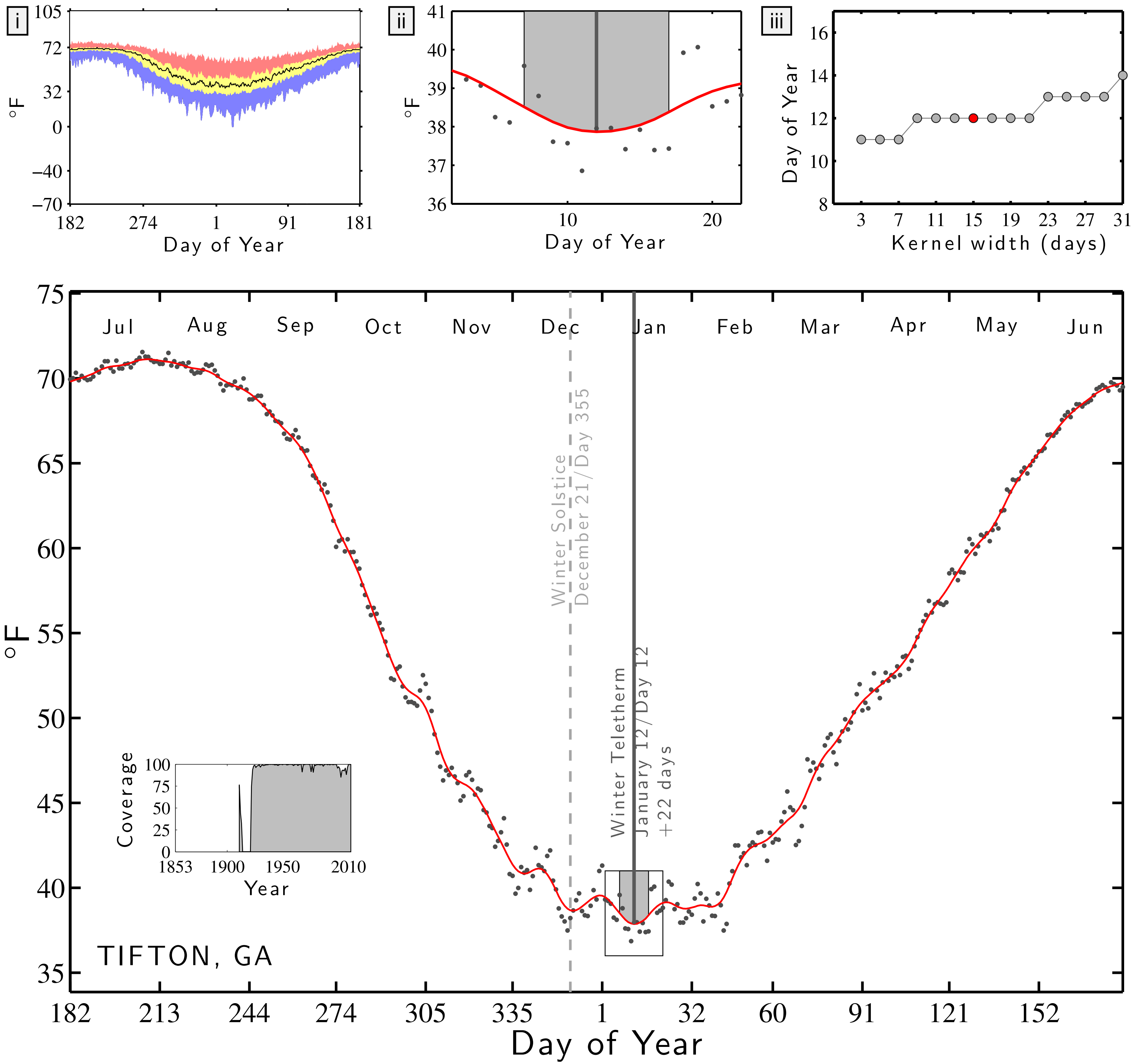

i
ii
iii
°F
Day of Year
Kernel width (days)
Jul
Aug
Sep
Oct
Nov
Dec
Jan
Feb
Mar
Apr
May
Jun
Winter Solstice
December 21/Day 355
Winter Teletherm
January 12/Day 12
+22 days
Coverage
Year
TIFTON, GA

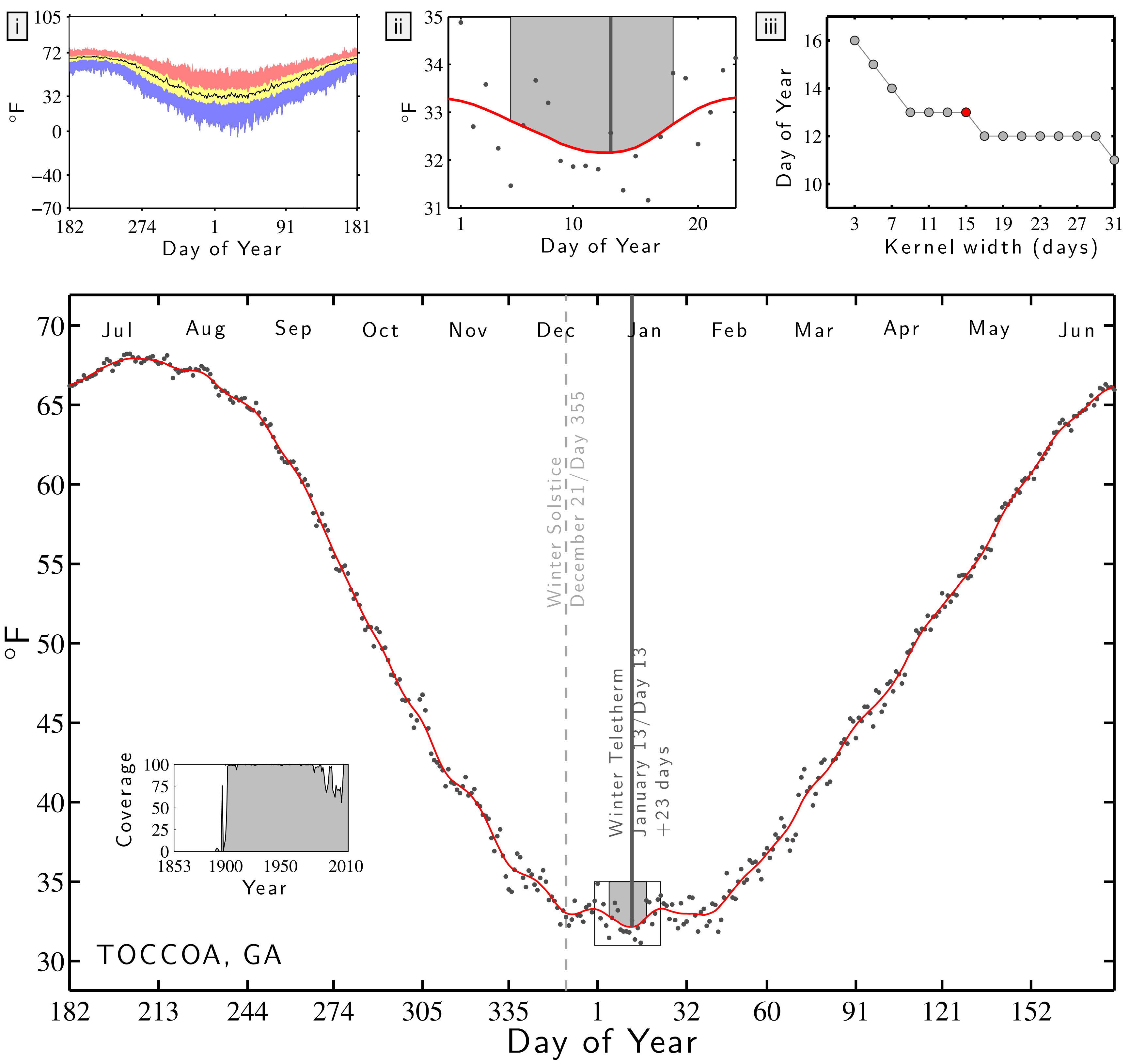

i
°F
Day of Year
ii
Day of Year
iii
Day of Year
Kernel width (days)
Jul
Aug
Sep
Oct
Nov
Dec
Jan
Feb
Mar
Apr
May
Jun
Winter Solstice
December 21/Day 355
Winter Teletherm
January 13/Day 13
+23 days
Coverage
Year
TOCCOA, GA
Day of Year

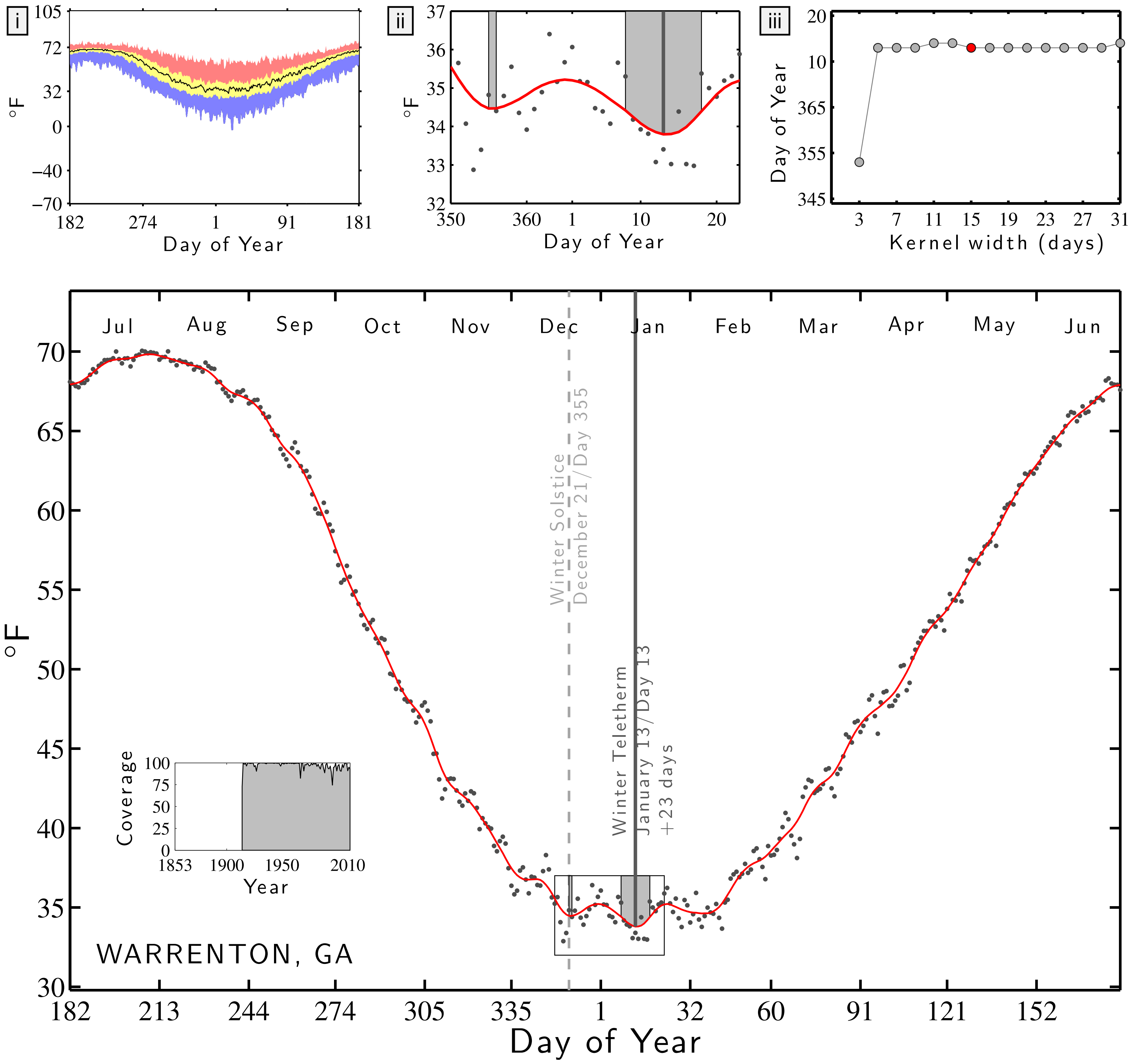

i
°F
Day of Year
ii
°F
Day of Year
iii
Day of Year
Kernel width (days)
Jul
Aug
Sep
Oct
Nov
Dec
Jan
Feb
Mar
Apr
May
Jun
Winter Solstice
December 21/Day 355
Winter Teletherm
January 13/Day 13
+23 days
Coverage
Year
WARRENTON, GA
°F
Day of Year

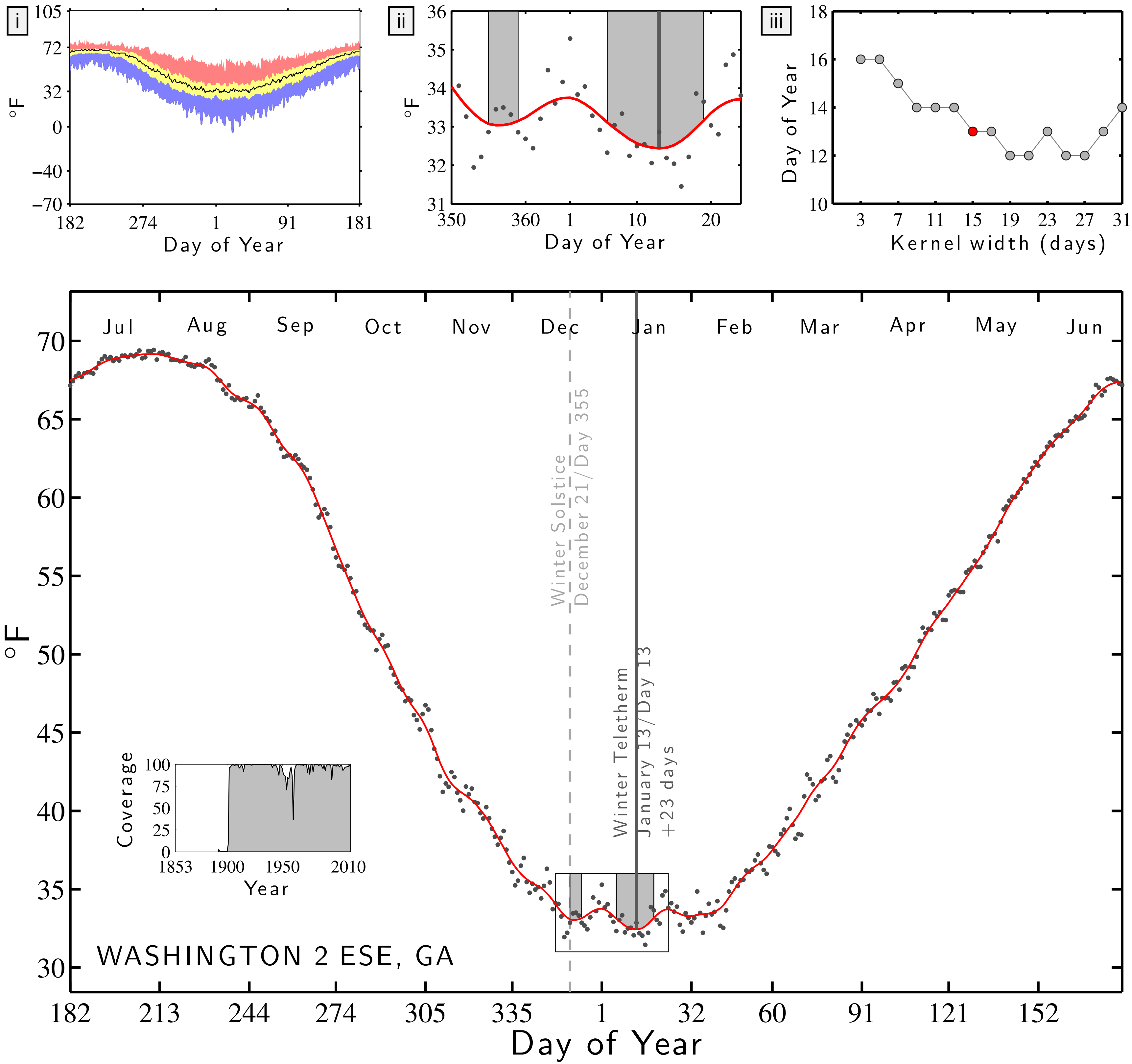

i
105
72
32
0
−40
−70
°F
182
274
1
91
181
Day of Year
ii
36
35
34
33
32
31
°F
350
360
1
10
20
Day of Year
iii
18
16
14
12
10
Day of Year
3
7
11
15
19
23
27
31
Kernel width (days)
Jul
Aug
Sep
Oct
Nov
Dec
Jan
Feb
Mar
Apr
May
Jun
70
65
60
55
50
45
40
35
30
°F
Winter Solstice
December 21/Day 355
Winter Teletherm
January 13/Day 13
+23 days
Coverage
100
75
50
25
0
1853
1900
1950
2010
Year
WASHINGTON 2 ESE, GA
182
213
244
274
305
335
1
32
60
91
121
152
Day of Year

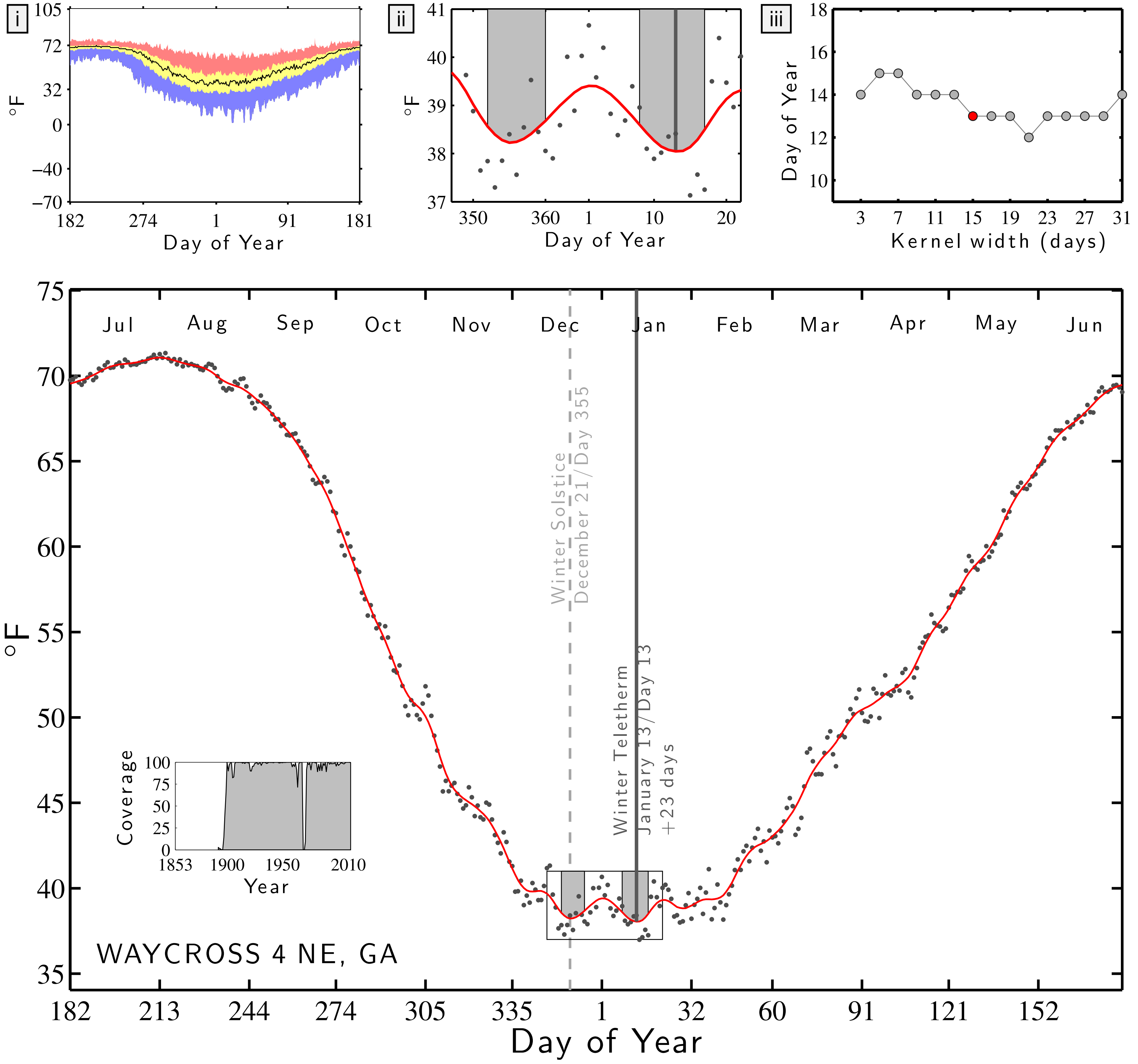

i
ii
iii
°F
Day of Year
Day of Year
Kernel width (days)
Jul
Aug
Sep
Oct
Nov
Dec
Jan
Feb
Mar
Apr
May
Jun
Winter Solstice
December 21/Day 355
Winter Teletherm
January 13/Day 13
+23 days
Coverage
Year
WAYCROSS 4 NE, GA
Day of Year

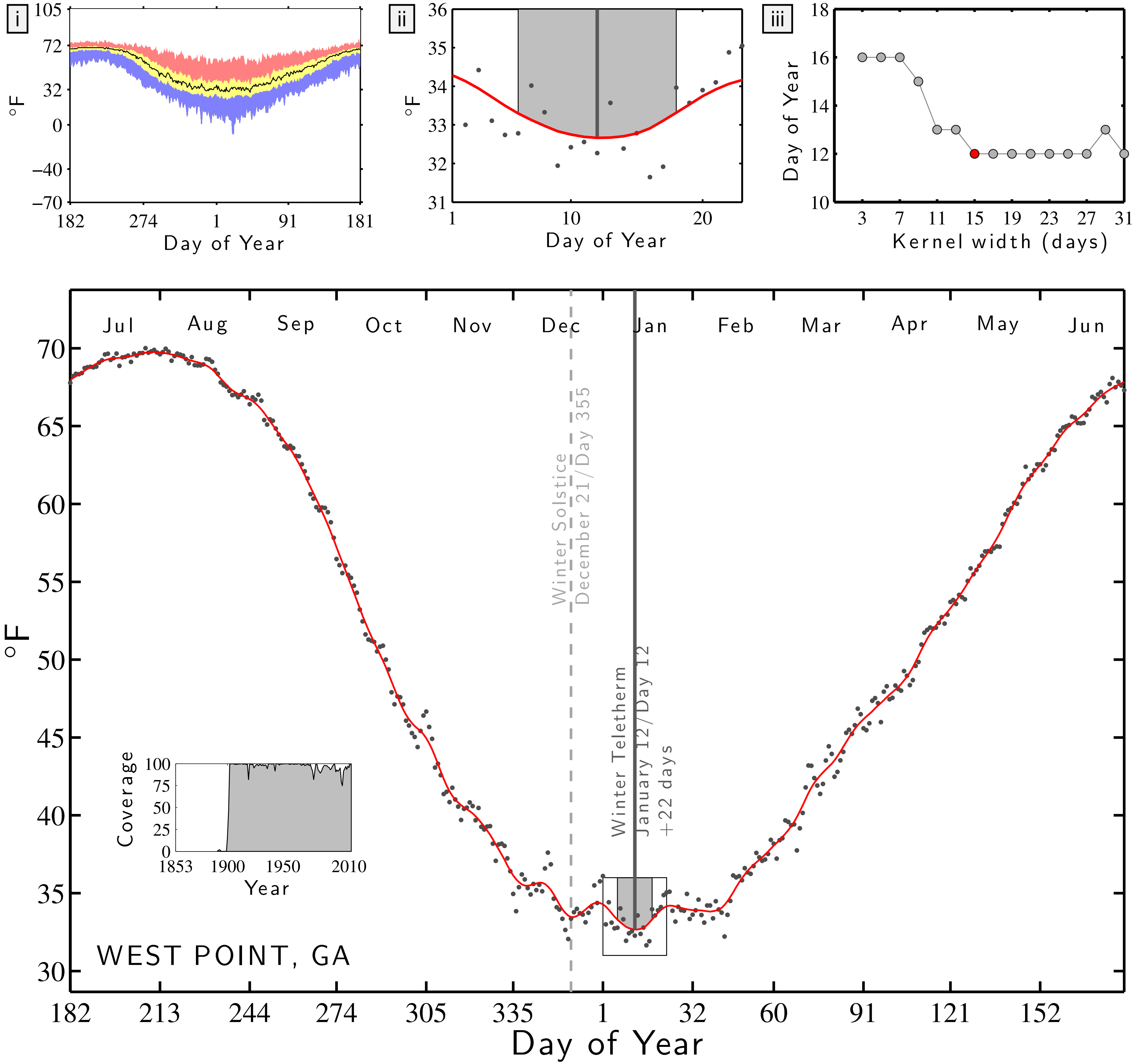

i
°F
Day of Year
ii
°F
Day of Year
iii
Day of Year
Kernel width (days)
Jul
Aug
Sep
Oct
Nov
Dec
Jan
Feb
Mar
Apr
May
Jun
Winter Solstice
December 21/Day 355
Winter Teletherm
January 12/Day 12
+22 days
Coverage
Year
WEST POINT, GA
°F
Day of Year

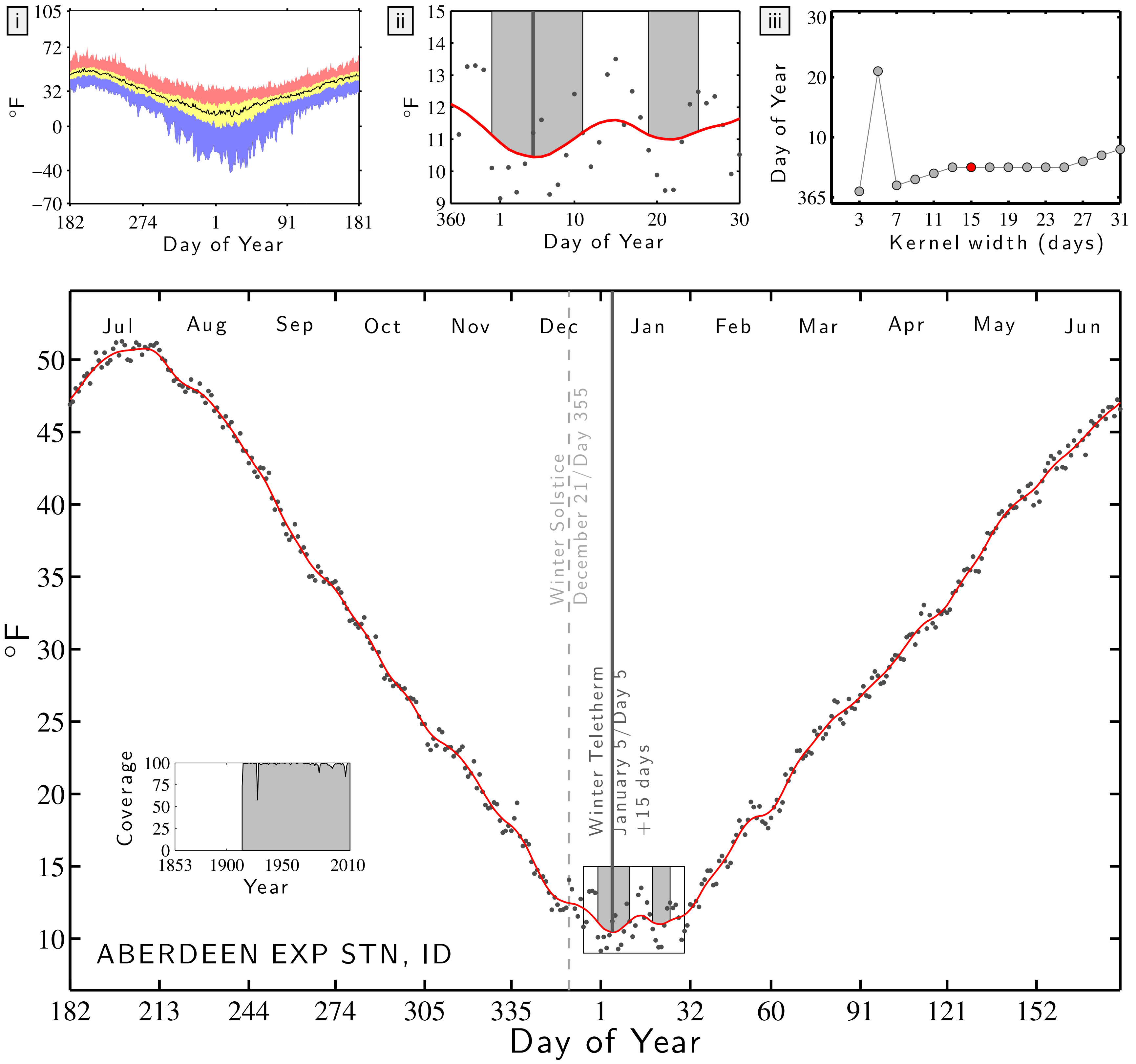

i
ii
iii
°F
Day of Year
Kernel width (days)
Jul
Aug
Sep
Oct
Nov
Dec
Jan
Feb
Mar
Apr
May
Jun
Winter Solstice
December 21/Day 355
Winter Teletherm
January 5/Day 5
+15 days
Coverage
Year
ABERDEEN EXP STN, ID

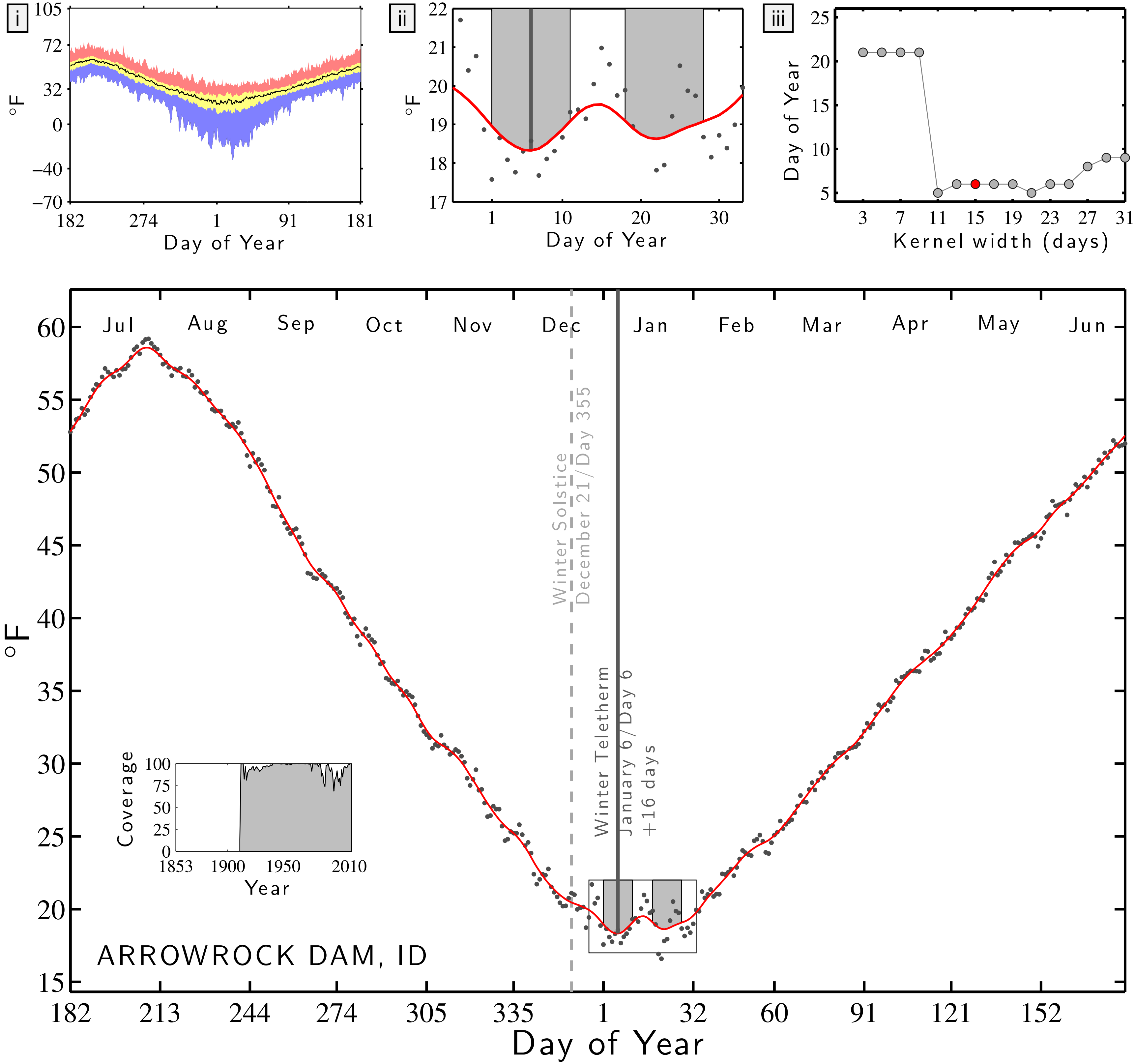

i
°F
Day of Year
ii
°F
Day of Year
iii
Day of Year
Kernel width (days)
Jul
Aug
Sep
Oct
Nov
Dec
Jan
Feb
Mar
Apr
May
Jun
Winter Solstice
December 21/Day 355
Winter Teletherm
January 6/Day 6
+16 days
Coverage
Year
ARROWROCK DAM, ID
°F
Day of Year

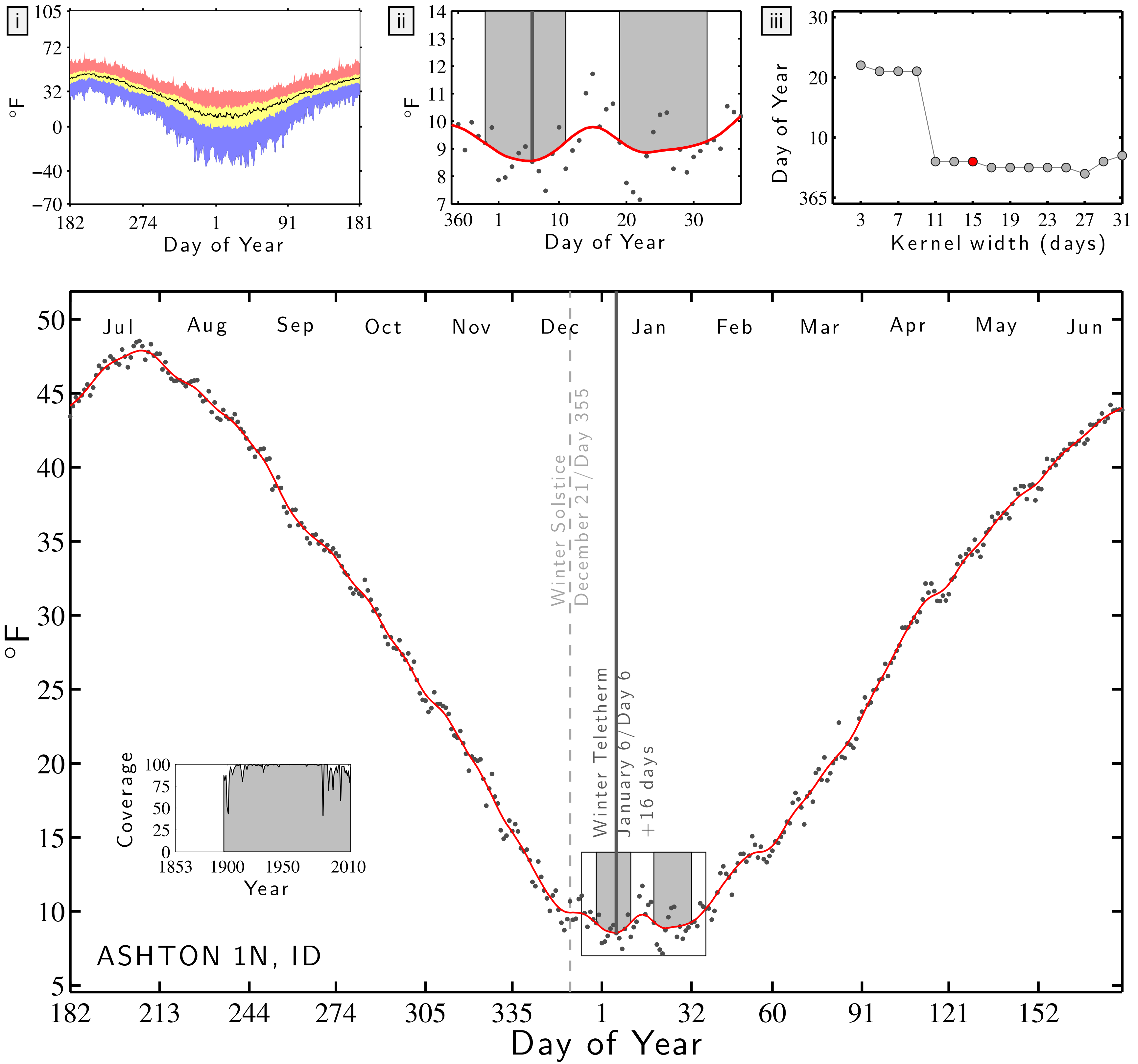

i
°F
Day of Year
ii
°F
Day of Year
iii
Day of Year
Kernel width (days)
Jul
Aug
Sep
Oct
Nov
Dec
Jan
Feb
Mar
Apr
May
Jun
Winter Solstice
December 21/Day 355
Winter Teletherm
January 6/Day 6
+16 days
°F
Coverage
Year
ASHTON 1N, ID
Day of Year

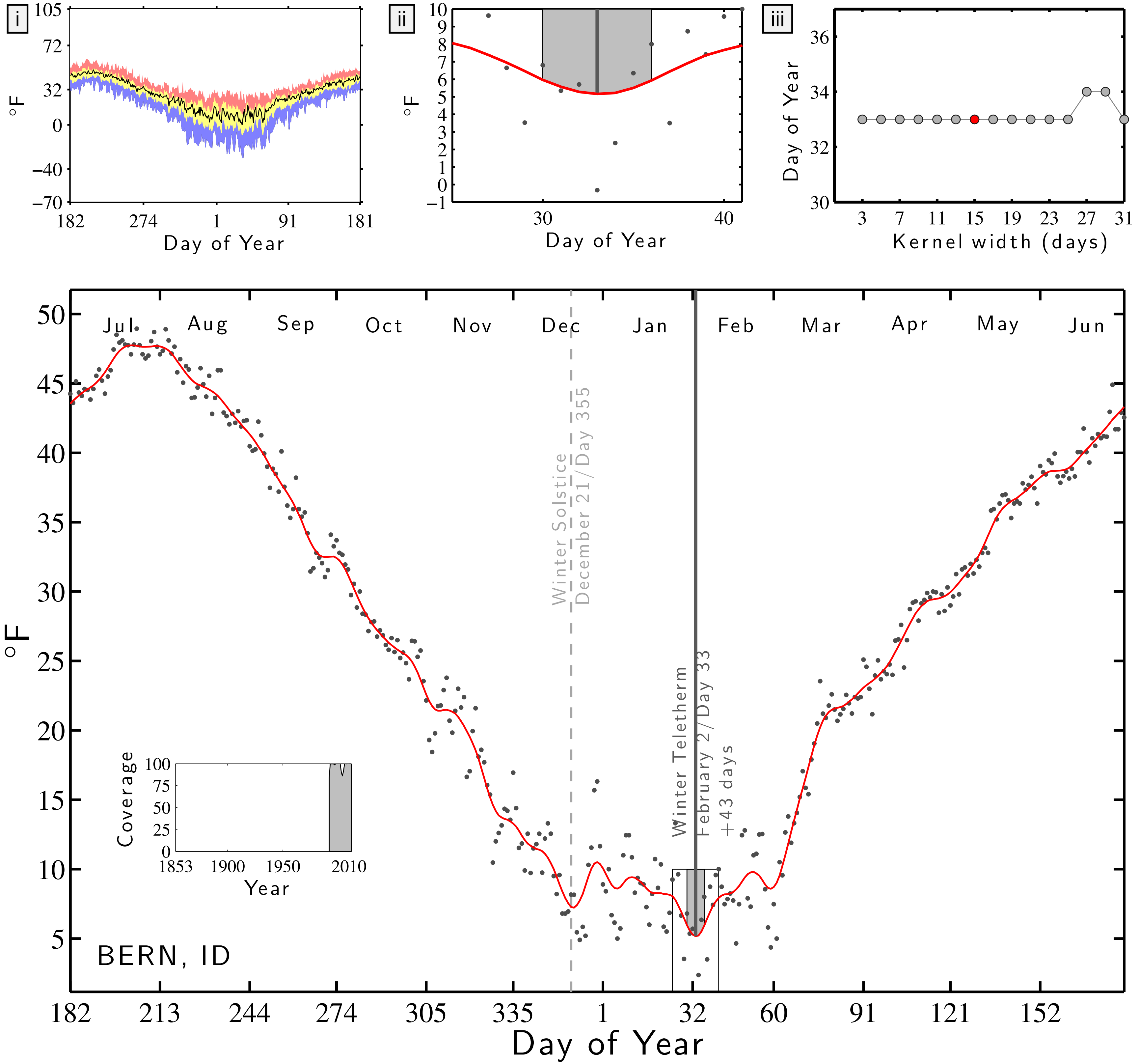

i
°F
Day of Year
ii
°F
Day of Year
iii
Day of Year
Kernel width (days)
Jul
Aug
Sep
Oct
Nov
Dec
Jan
Feb
Mar
Apr
May
Jun
Winter Solstice
December 21/Day 355
Winter Teletherm
February 2/Day 33
+43 days
Coverage
Year
BERN, ID
°F
Day of Year

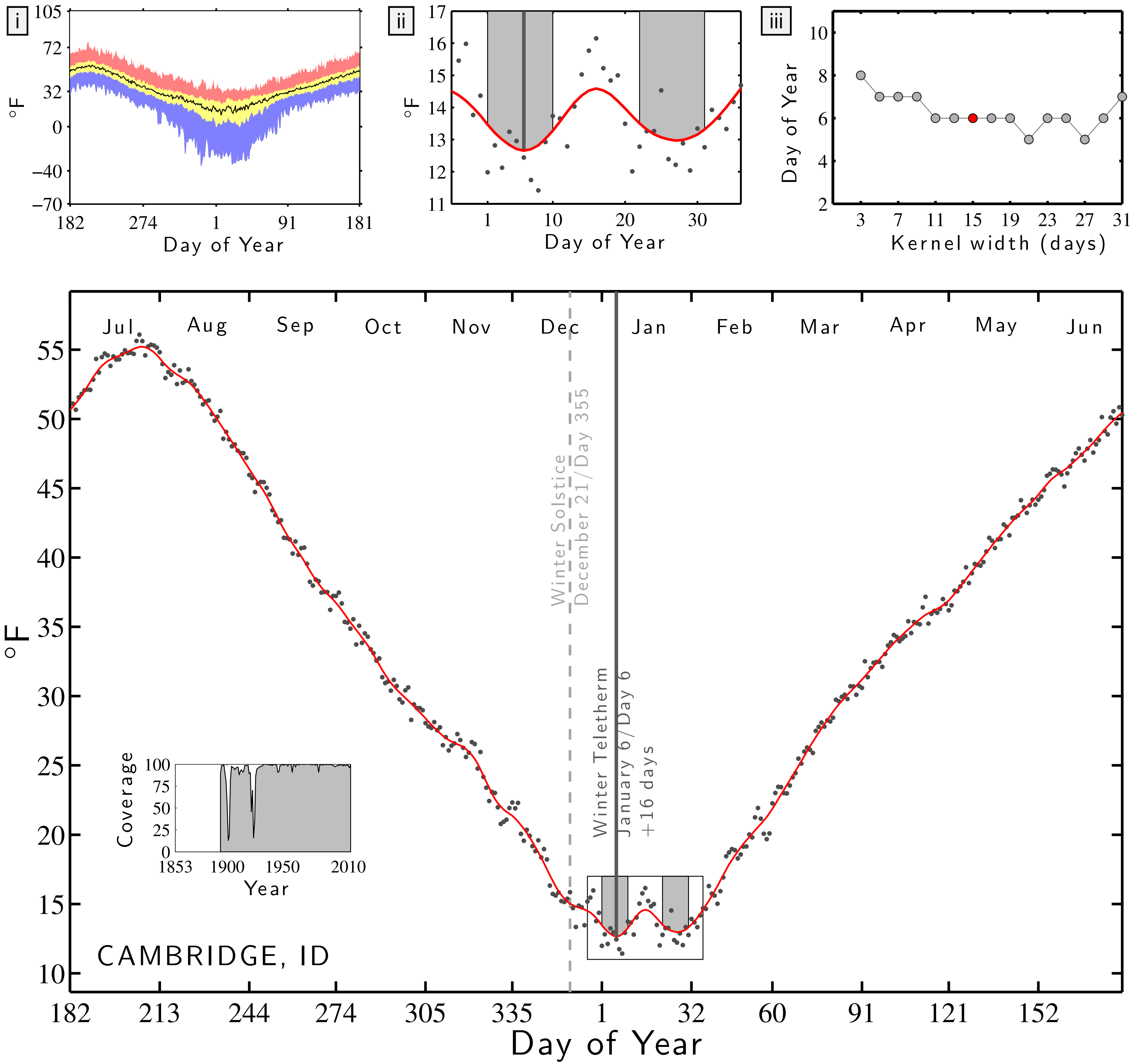

i
ii
iii
°F
Day of Year
Kernel width (days)
Jul
Aug
Sep
Oct
Nov
Dec
Jan
Feb
Mar
Apr
May
Jun
Winter Solstice
December 21/Day 355
Winter Teletherm
January 6/Day 6
+16 days
Coverage
Year
CAMBRIDGE, ID

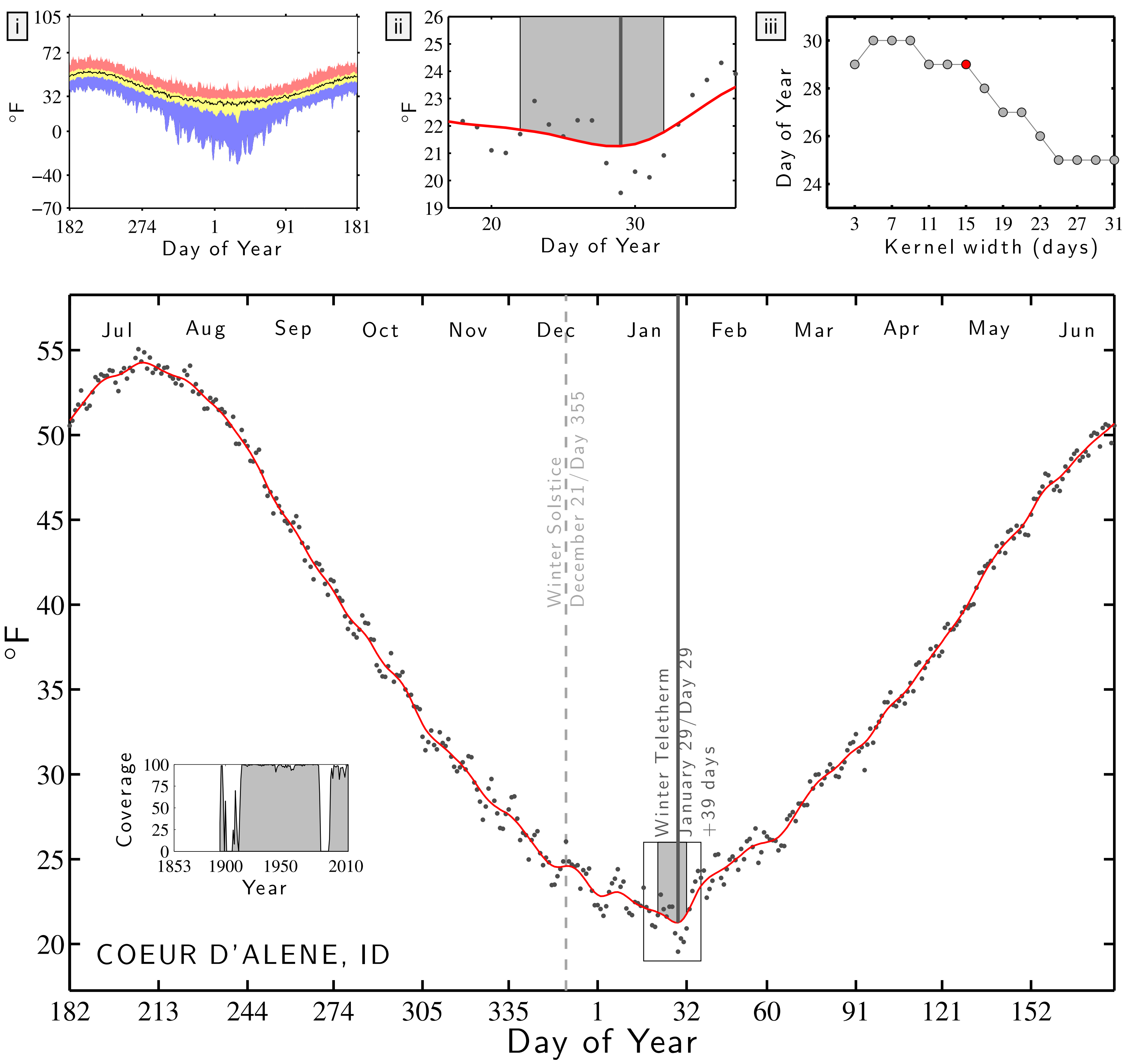

i
ii
iii
°F
Day of Year
Kernel width (days)
Jul
Aug
Sep
Oct
Nov
Dec
Jan
Feb
Mar
Apr
May
Jun
Winter Solstice
December 21/Day 355
Winter Teletherm
January 29/Day 29
+39 days
Coverage
Year
COEUR D'ALENE, ID

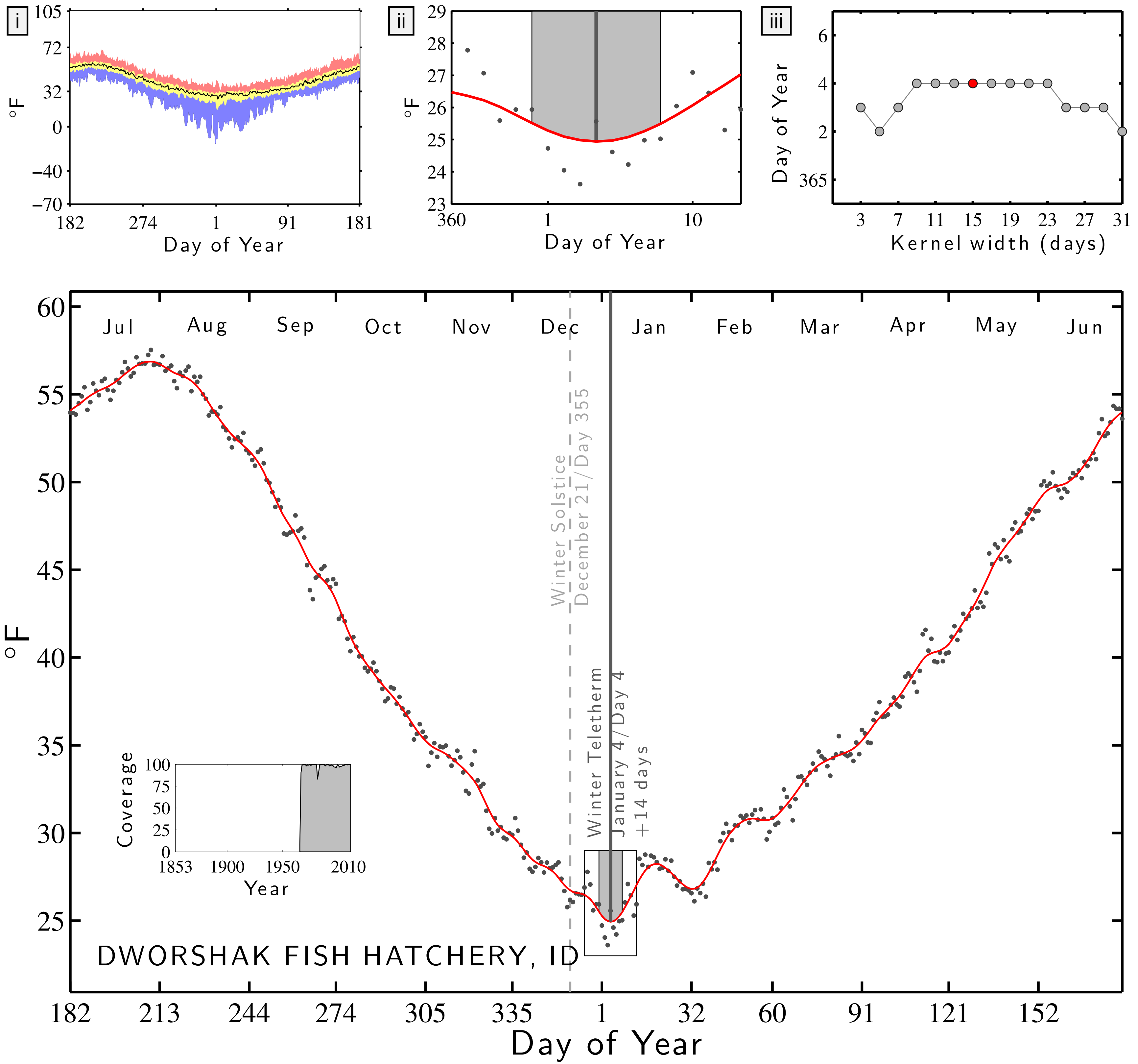

i
°F
Day of Year
ii
Day of Year
iii
Day of Year
Kernel width (days)
Jul
Aug
Sep
Oct
Nov
Dec
Jan
Feb
Mar
Apr
May
Jun
Winter Solstice
December 21/Day 355
Winter Teletherm
January 4/Day 4
+14 days
Coverage
Year
DWORSHAK FISH HATCHERY, ID
°F
Day of Year

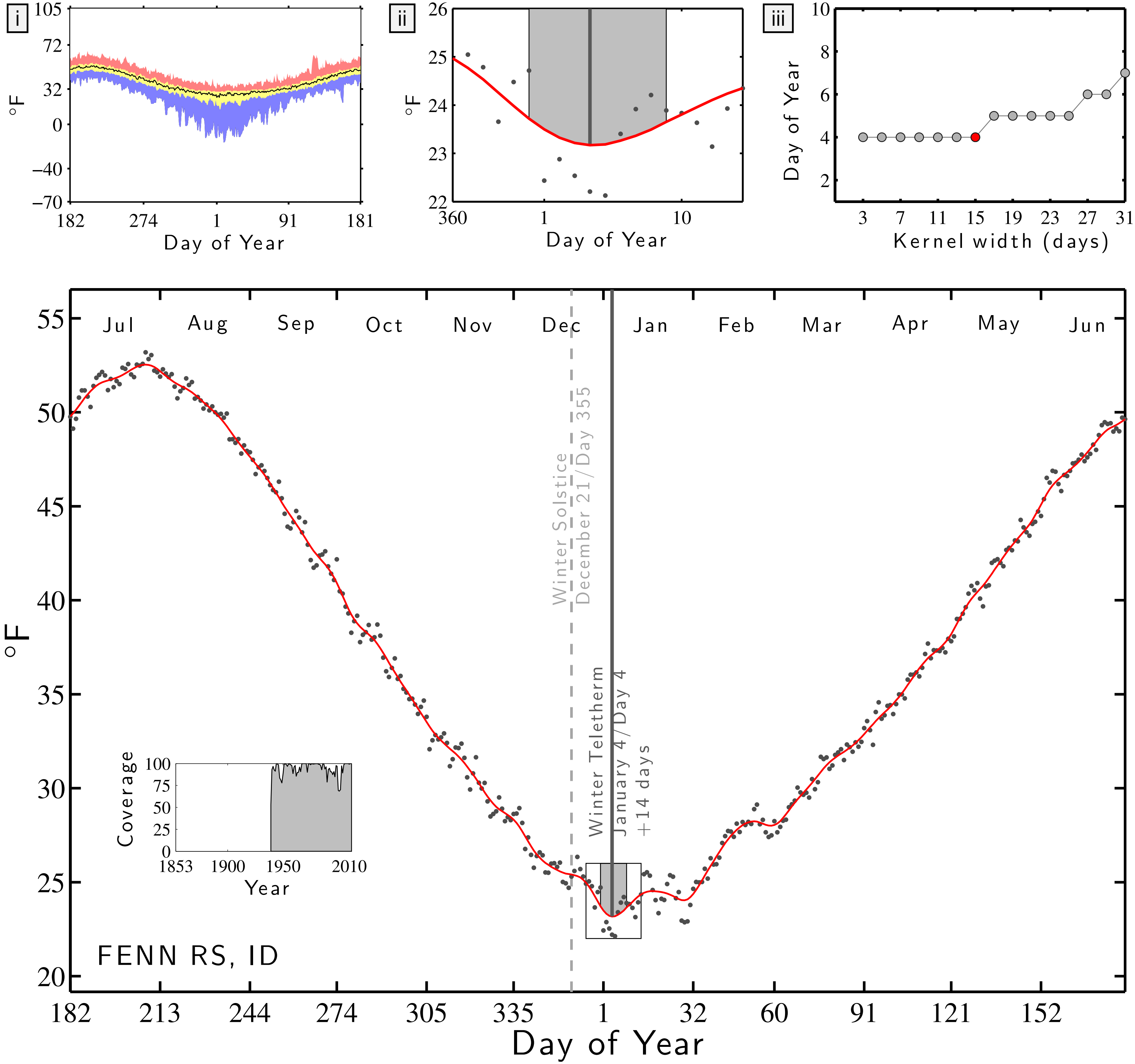

i
°F
105
72
32
0
−40
−70
182
274
1
91
181
Day of Year
ii
26
25
24
23
22
360
1
10
Day of Year
iii
Day of Year
10
8
6
4
2
3
7
11
15
19
23
27
31
Kernel width (days)
Jul
Aug
Sep
Oct
Nov
Dec
Jan
Feb
Mar
Apr
May
Jun
55
50
45
40
35
30
25
20
Winter Solstice
December 21/Day 355
Winter Teletherm
January 4/Day 4
+14 days
Coverage
100
75
50
25
0
1853
1900
1950
2010
Year
FENN RS, ID
182
213
244
274
305
335
1
32
60
91
121
152
Day of Year

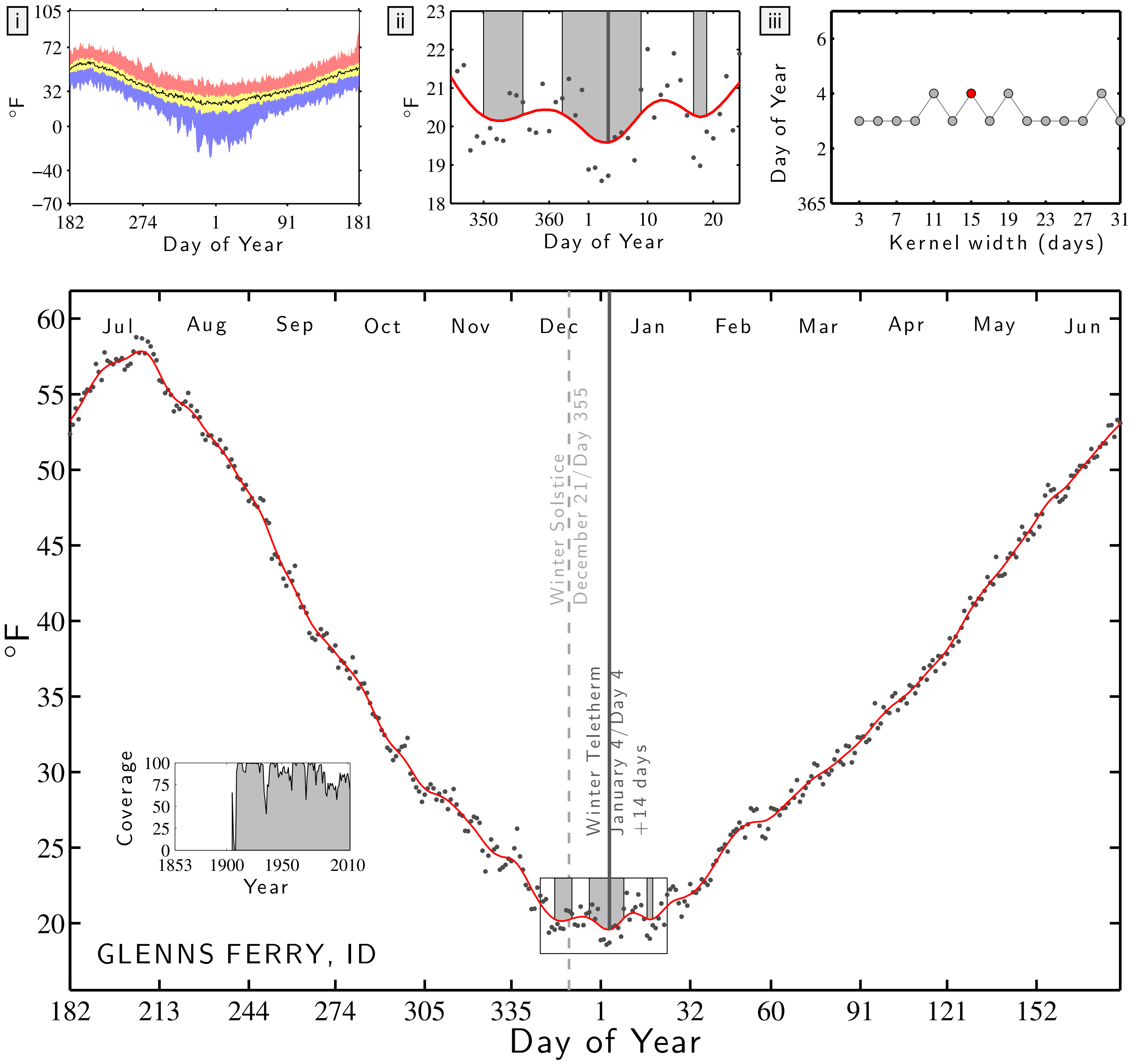

i
ii
iii
°F
Day of Year
Day of Year
Kernel width (days)
Jul
Aug
Sep
Oct
Nov
Dec
Jan
Feb
Mar
Apr
May
Jun
Winter Solstice
December 21/Day 355
Winter Teletherm
January 4/Day 4
+14 days
Coverage
Year
GLENNS FERRY, ID

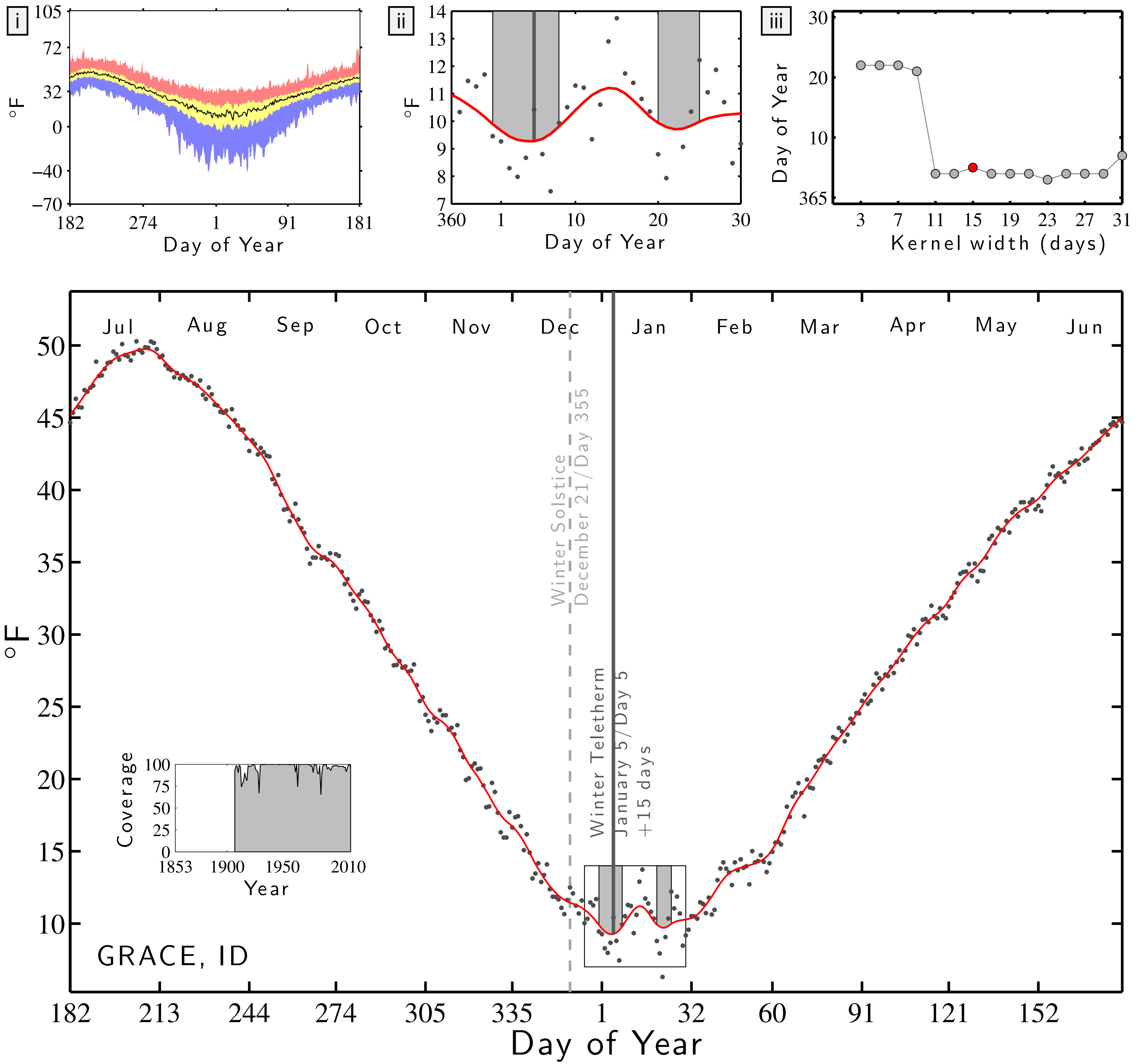
i
°F
Day of Year
ii
°F
Day of Year
iii
Day of Year
Kernel width (days)
Jul
Aug
Sep
Oct
Nov
Dec
Jan
Feb
Mar
Apr
May
Jun
Winter Solstice
December 21/Day 355
Winter Teletherm
January 5/Day 5
+15 days
Coverage
Year
GRACE, ID
°F
Day of Year

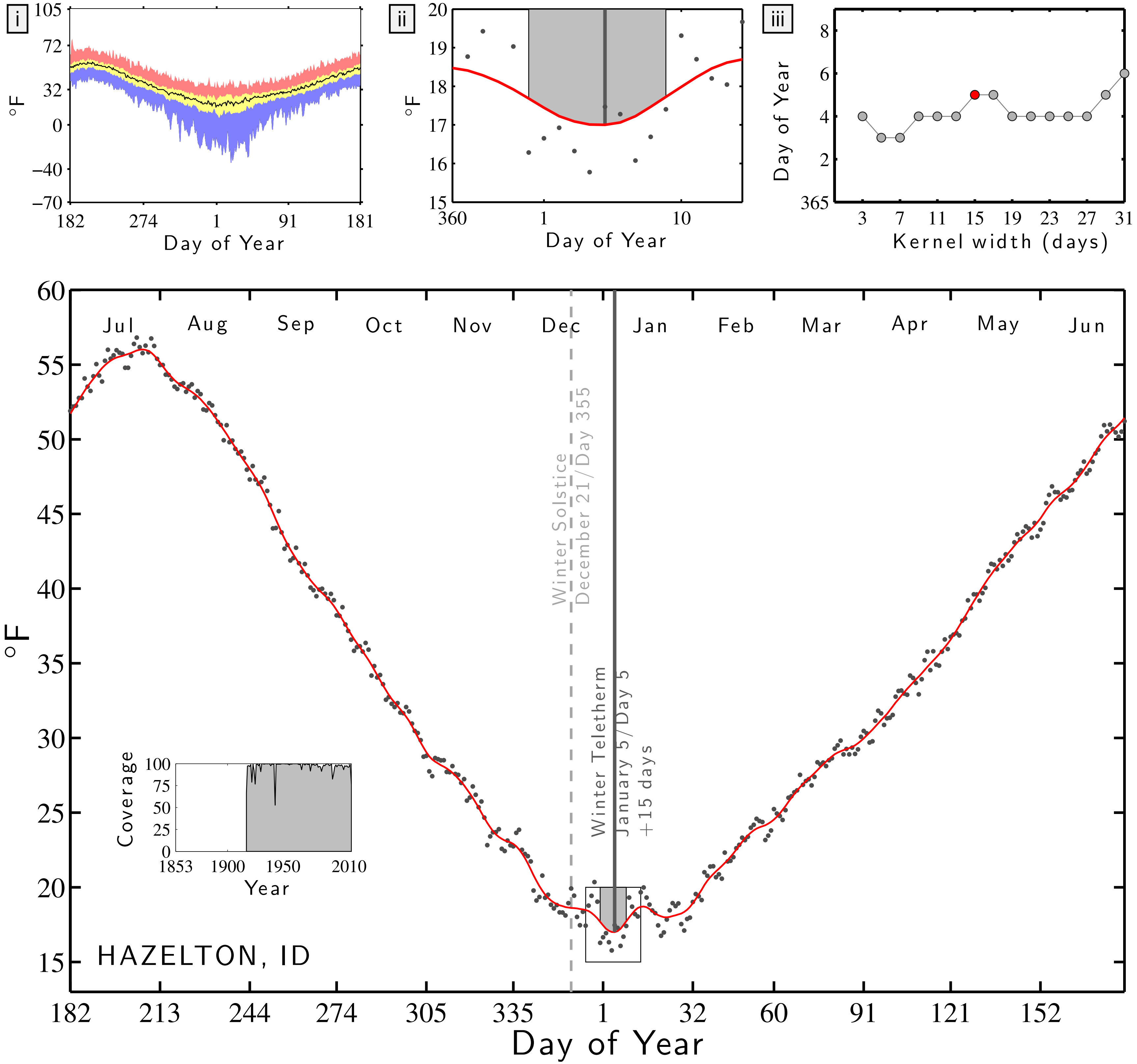

i
105
72
32
0
−40
−70
°F
182
274
1
91
181
Day of Year
ii
20
19
18
17
16
15
°F
360
1
10
Day of Year
iii
8
6
4
2
365
Day of Year
3
7
11
15
19
23
27
31
Kernel width (days)
60
55
50
45
40
35
30
25
20
15
°F
Jul
Aug
Sep
Oct
Nov
Dec
Jan
Feb
Mar
Apr
May
Jun
Winter Solstice
December 21/Day 355
Winter Teletherm
January 5/Day 5
+15 days
Coverage
100
75
50
25
0
1853
1900
1950
2010
Year
HAZELTON, ID
182
213
244
274
305
335
1
32
60
91
121
152
Day of Year

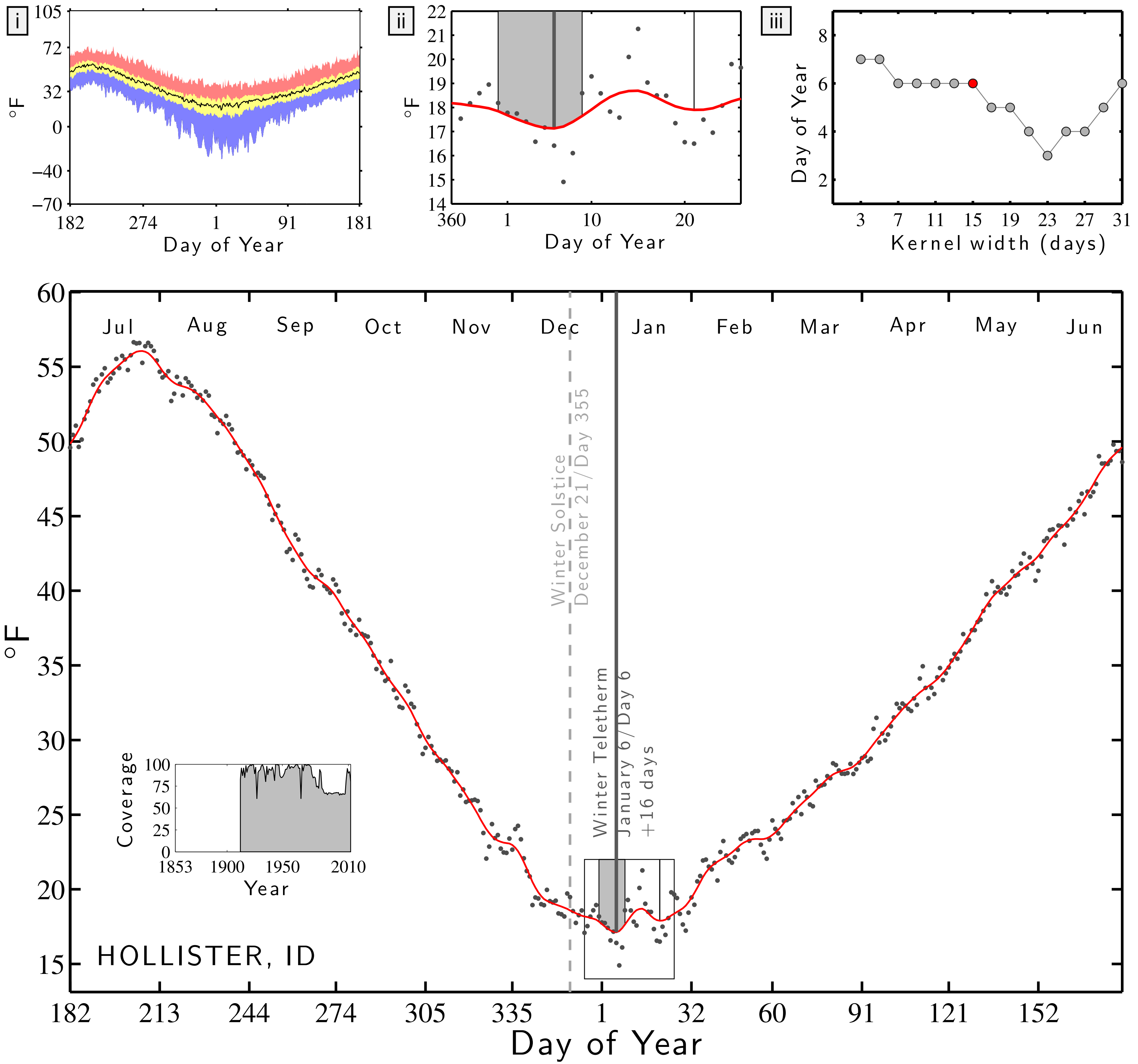

i
ii
iii
°F
Day of Year
Kernel width (days)
Jul
Aug
Sep
Oct
Nov
Dec
Jan
Feb
Mar
Apr
May
Jun
Winter Solstice
December 21/Day 355
Winter Teletherm
January 6/Day 6
+16 days
Coverage
Year
HOLLISTER, ID

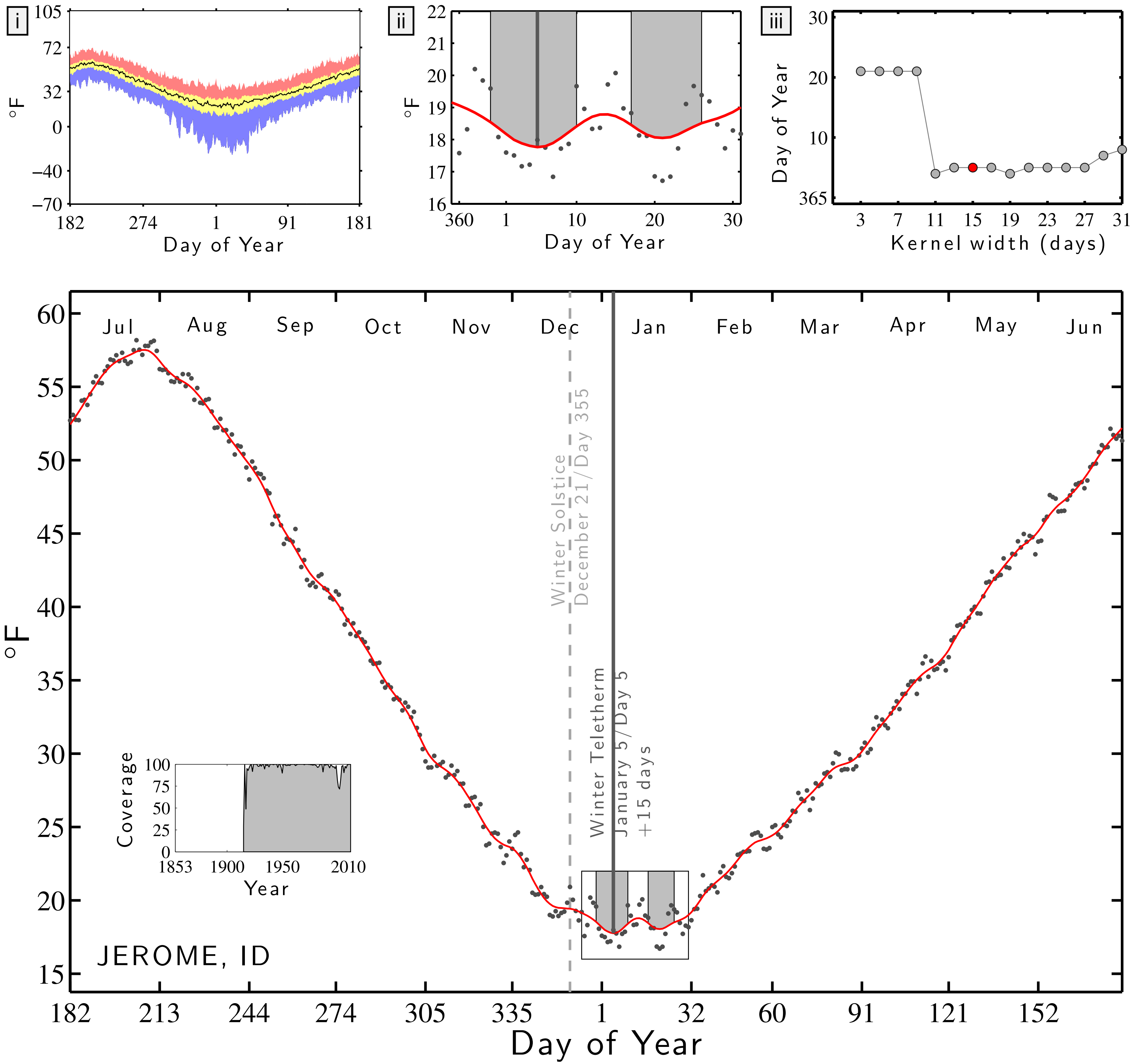

i
105
72
32
0
−40
−70
°F
182
274
1
91
181
Day of Year
ii
22
21
20
19
18
17
16
°F
360
1
10
20
30
Day of Year
iii
30
20
10
365
Day of Year
3
7
11
15
19
23
27
31
Kernel width (days)
60
55
50
45
40
35
30
25
20
15
°F
Jul
Aug
Sep
Oct
Nov
Dec
Jan
Feb
Mar
Apr
May
Jun
Winter Solstice
December 21/Day 355
Winter Teletherm
January 5/Day 5
+15 days
Coverage
100
75
50
25
0
1853
1900
1950
2010
Year
JEROME, ID
182
213
244
274
305
335
1
32
60
91
121
152
Day of Year

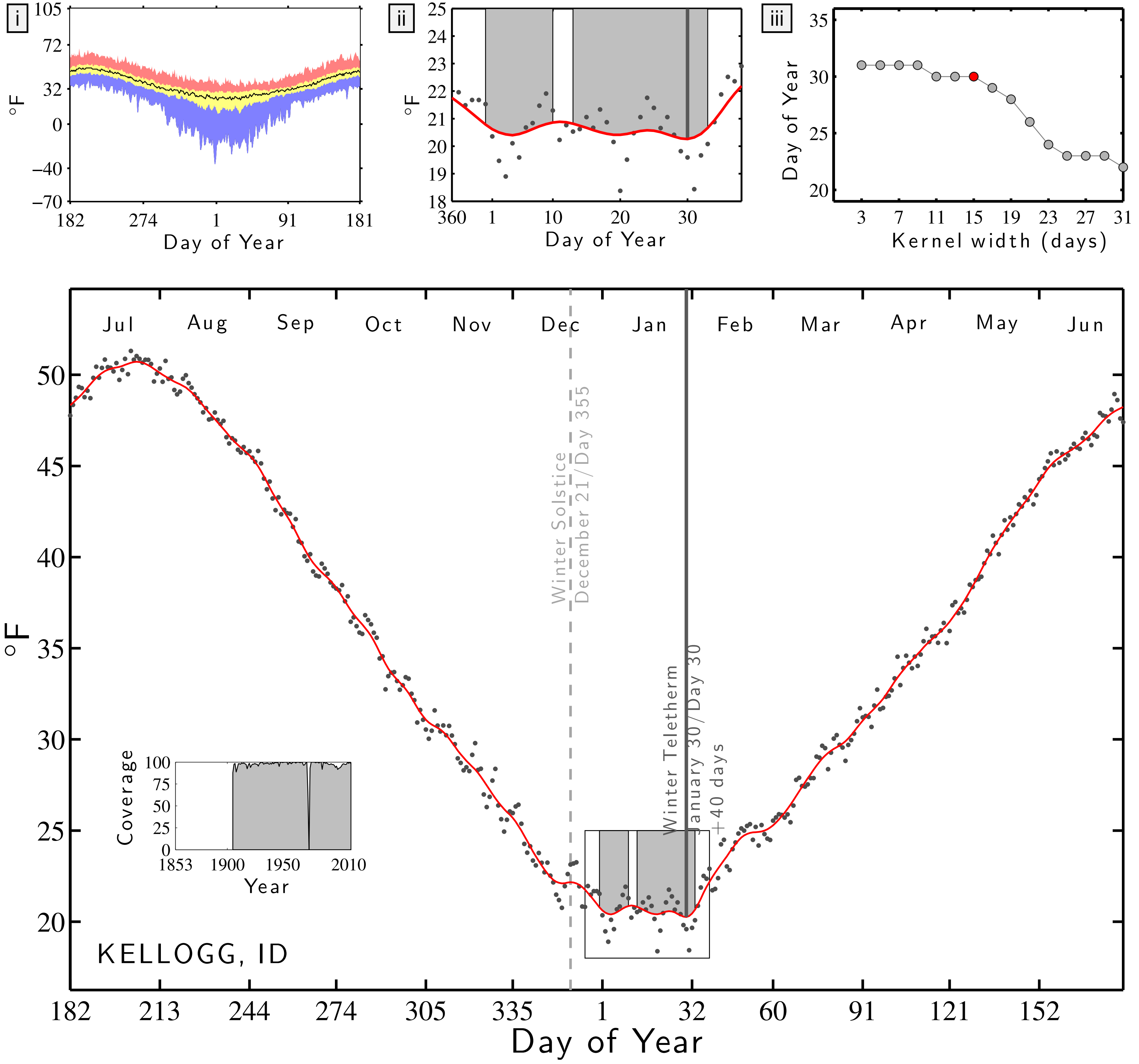

i
°F
Day of Year
ii
°F
Day of Year
iii
Day of Year
Kernel width (days)
Jul
Aug
Sep
Oct
Nov
Dec
Jan
Feb
Mar
Apr
May
Jun
Winter Solstice
December 21/Day 355
Winter Teletherm
January 30/Day 30
+40 days
°F
Coverage
Year
KELLOGG, ID
Day of Year

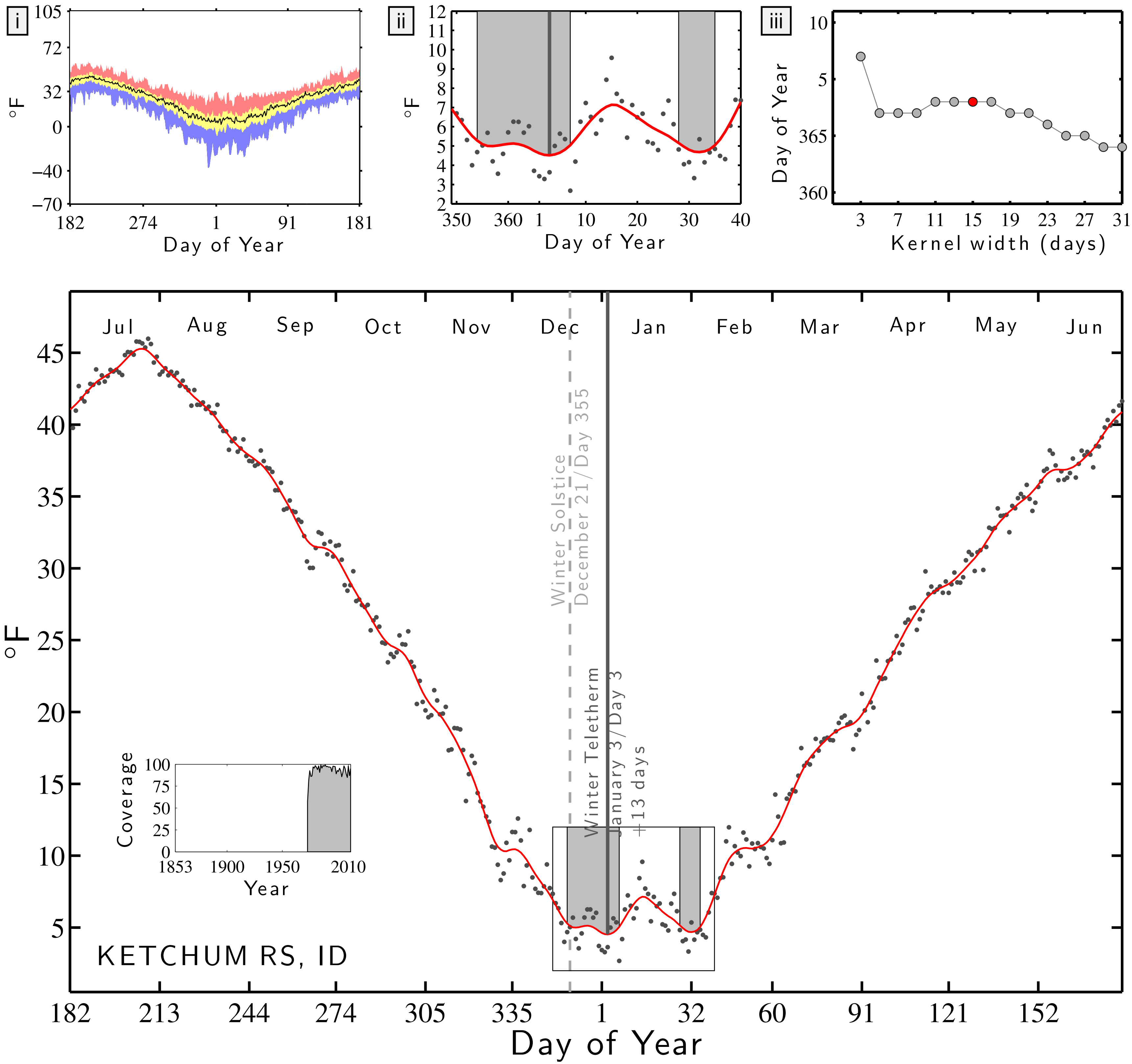

i
ii
iii
°F
Day of Year
Kernel width (days)
Day of Year
Jul
Aug
Sep
Oct
Nov
Dec
Jan
Feb
Mar
Apr
May
Jun
Winter Solstice
December 21/Day 355
Winter Teletherm
January 3/Day 3
+13 days
Coverage
Year
KETCHUM RS, ID

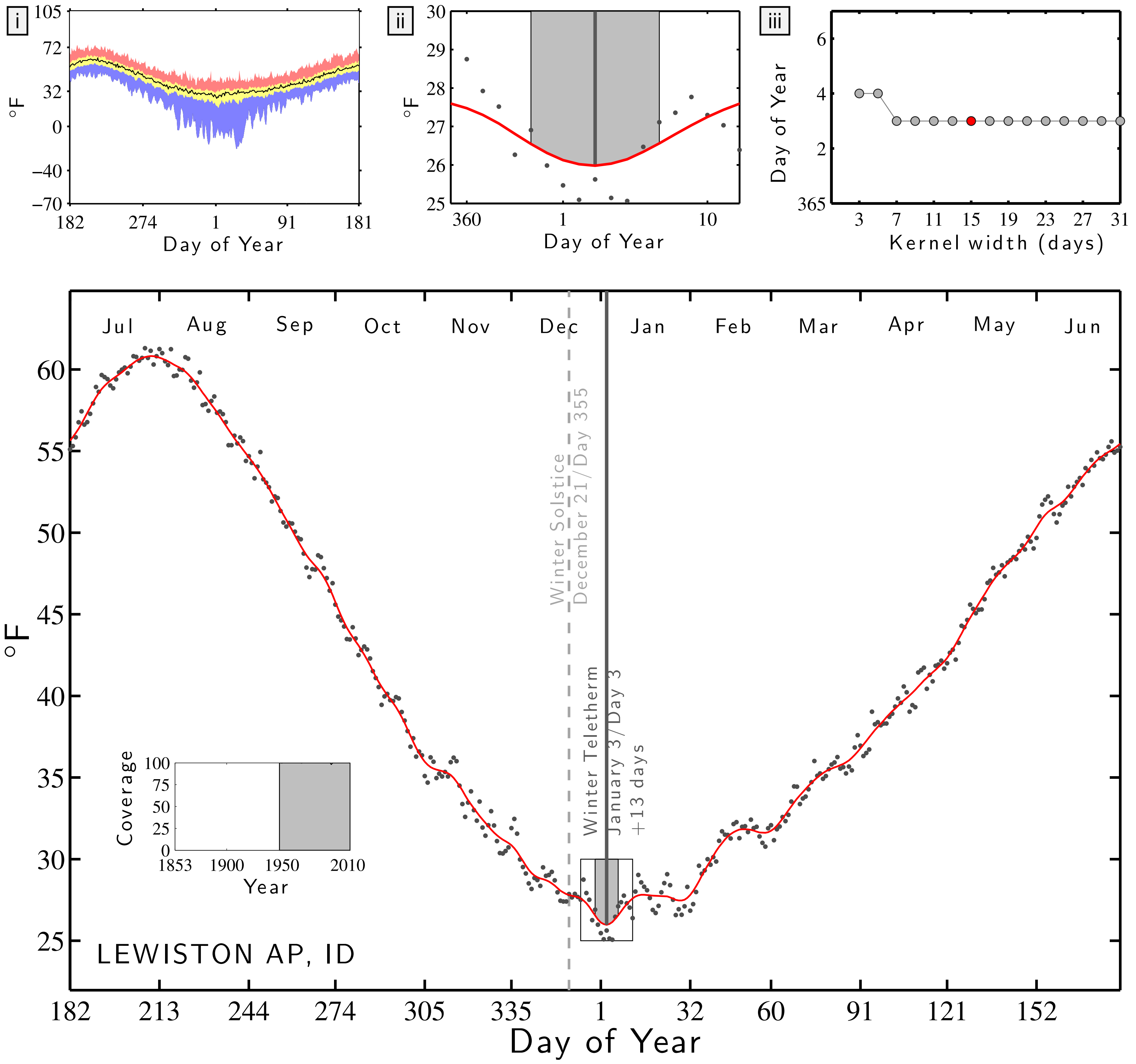

i
ii
iii
°F
Day of Year
Kernel width (days)
Day of Year
Jul
Aug
Sep
Oct
Nov
Dec
Jan
Feb
Mar
Apr
May
Jun
Winter Solstice
December 21/Day 355
Winter Teletherm
January 3/Day 3
+13 days
Coverage
Year
LEWISTON AP, ID

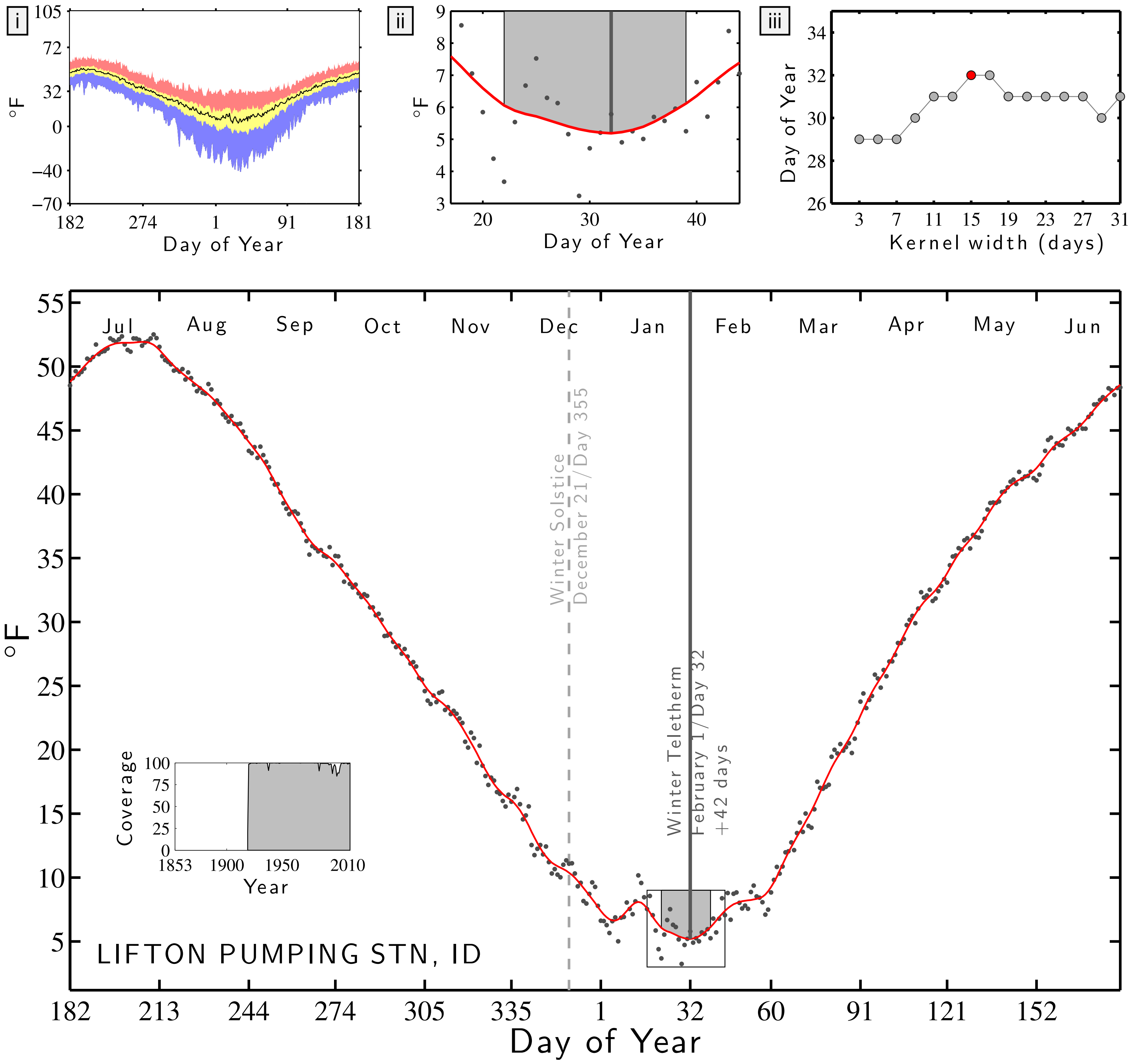

i
°F
Day of Year
ii
°F
Day of Year
iii
Day of Year
Kernel width (days)
Jul
Aug
Sep
Oct
Nov
Dec
Jan
Feb
Mar
Apr
May
Jun
Winter Solstice
December 21/Day 355
Winter Teletherm
February 1/Day 32
+42 days
Coverage
Year
LIFTON PUMPING STN, ID
°F
Day of Year

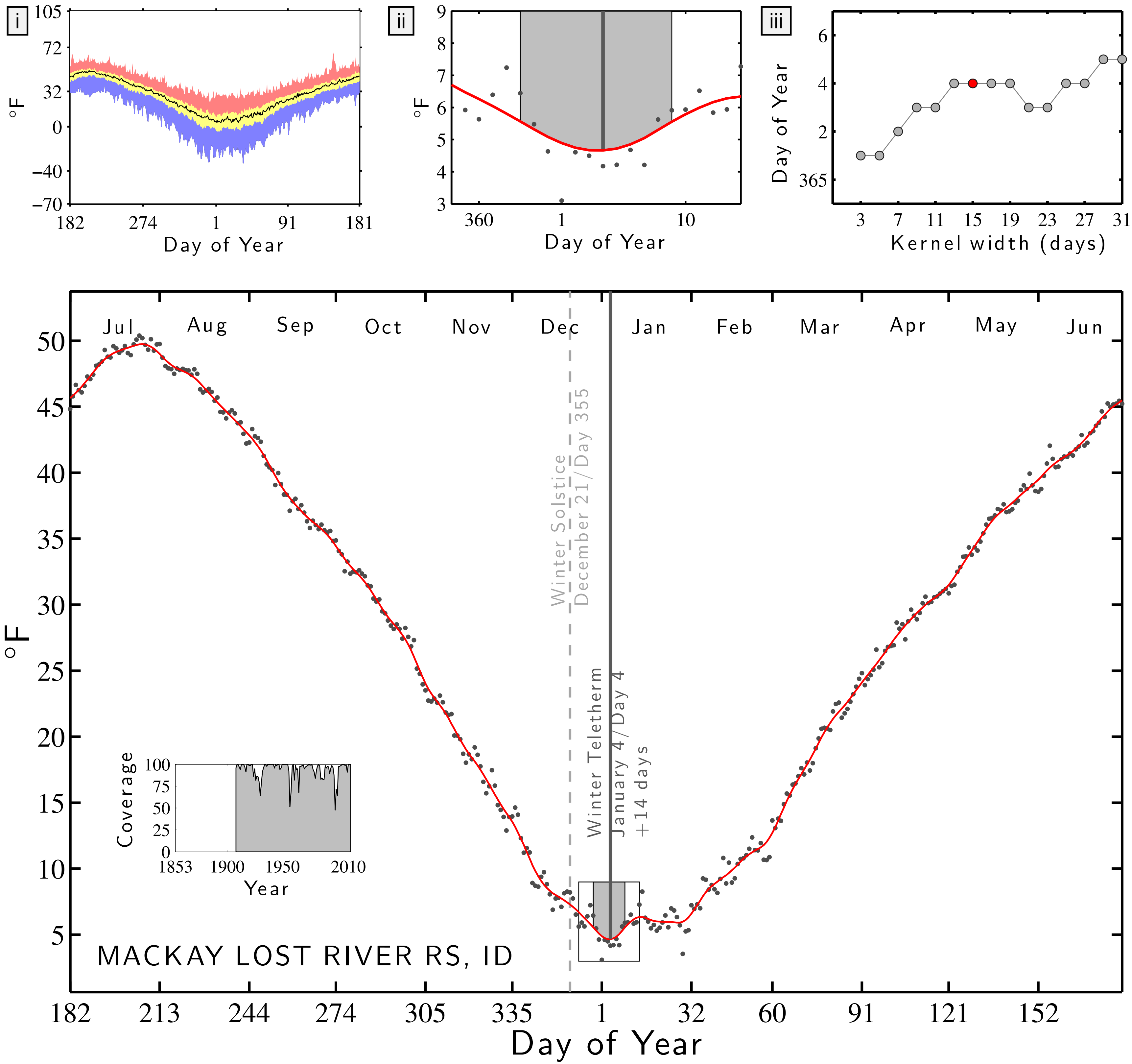

i
°F
105
72
32
0
−40
−70
182
274
1
91
181
Day of Year
ii
°F
360
1
10
Day of Year
iii
Day of Year
365
2
4
6
Kernel width (days)
3
7
11
15
19
23
27
31
Jul
Aug
Sep
Oct
Nov
Dec
Jan
Feb
Mar
Apr
May
Jun
Winter Solstice
December 21/Day 355
Winter Teletherm
January 4/Day 4
+14 days
Coverage
100
75
50
25
0
1853
1900
1950
2010
Year
MACKAY LOST RIVER RS, ID
°F
182
213
244
274
305
335
1
32
60
91
121
152
Day of Year

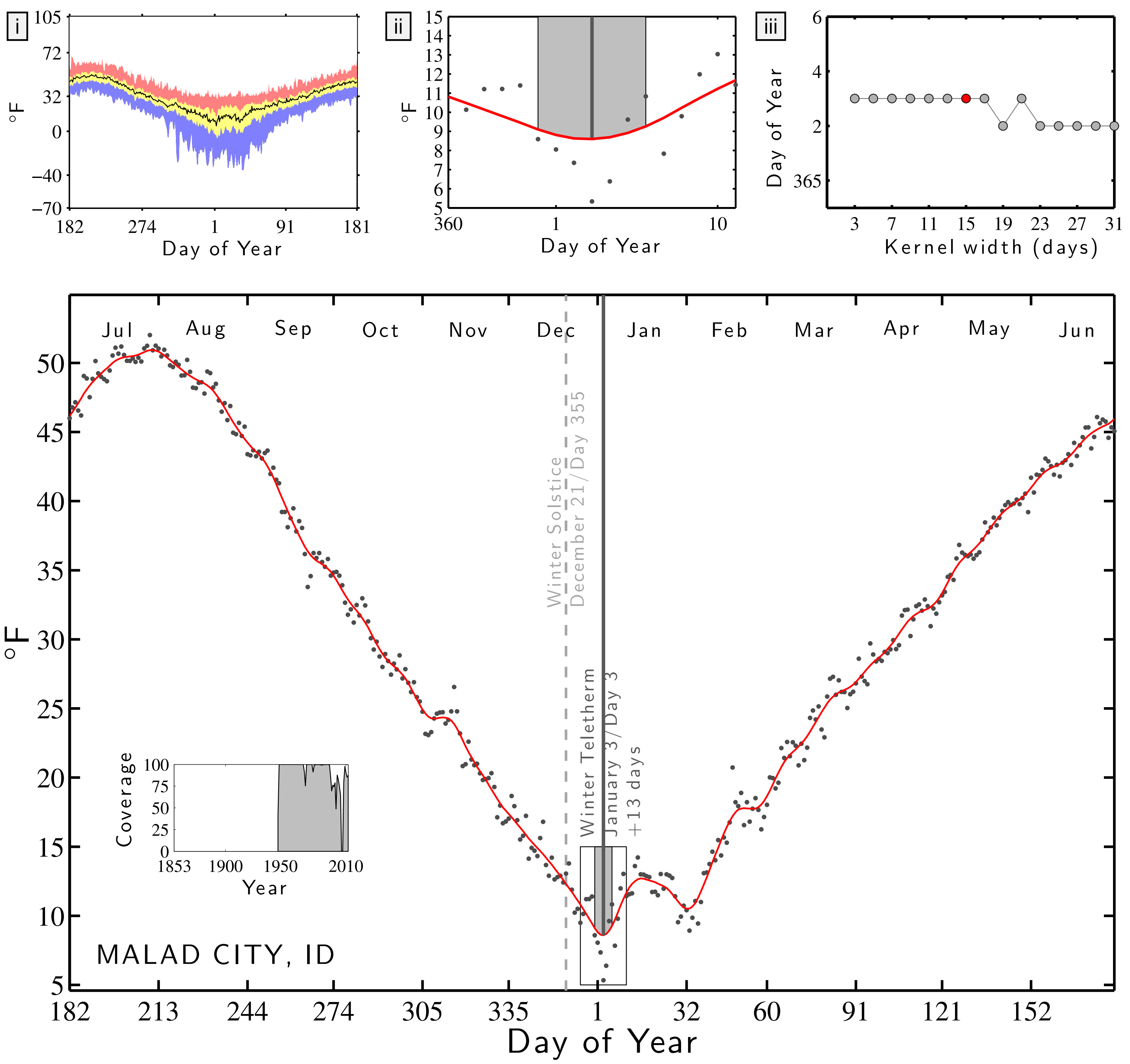

i
ii
iii
Day of Year
°F
Kernel width (days)
Jul
Aug
Sep
Oct
Nov
Dec
Jan
Feb
Mar
Apr
May
Jun
Winter Solstice
December 21/Day 355
Winter Teletherm
January 3/Day 3
+13 days
Coverage
Year
MALAD CITY, ID

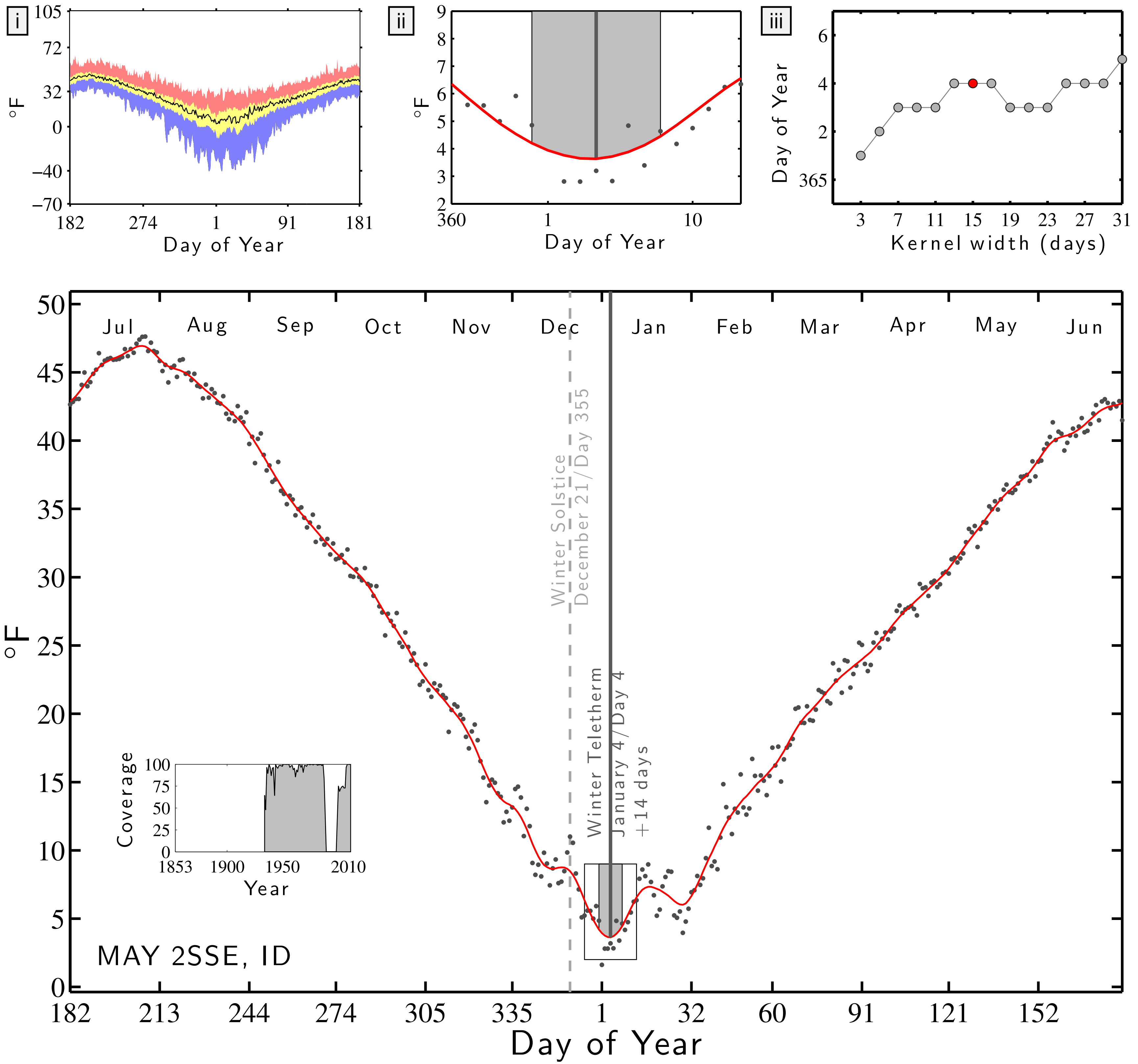

i
ii
iii
°F
Day of Year
Day of Year
Kernel width (days)
Jul
Aug
Sep
Oct
Nov
Dec
Jan
Feb
Mar
Apr
May
Jun
Winter Solstice
December 21/Day 355
Winter Teletherm
January 4/Day 4
+14 days
Coverage
Year
MAY 2SSE, ID

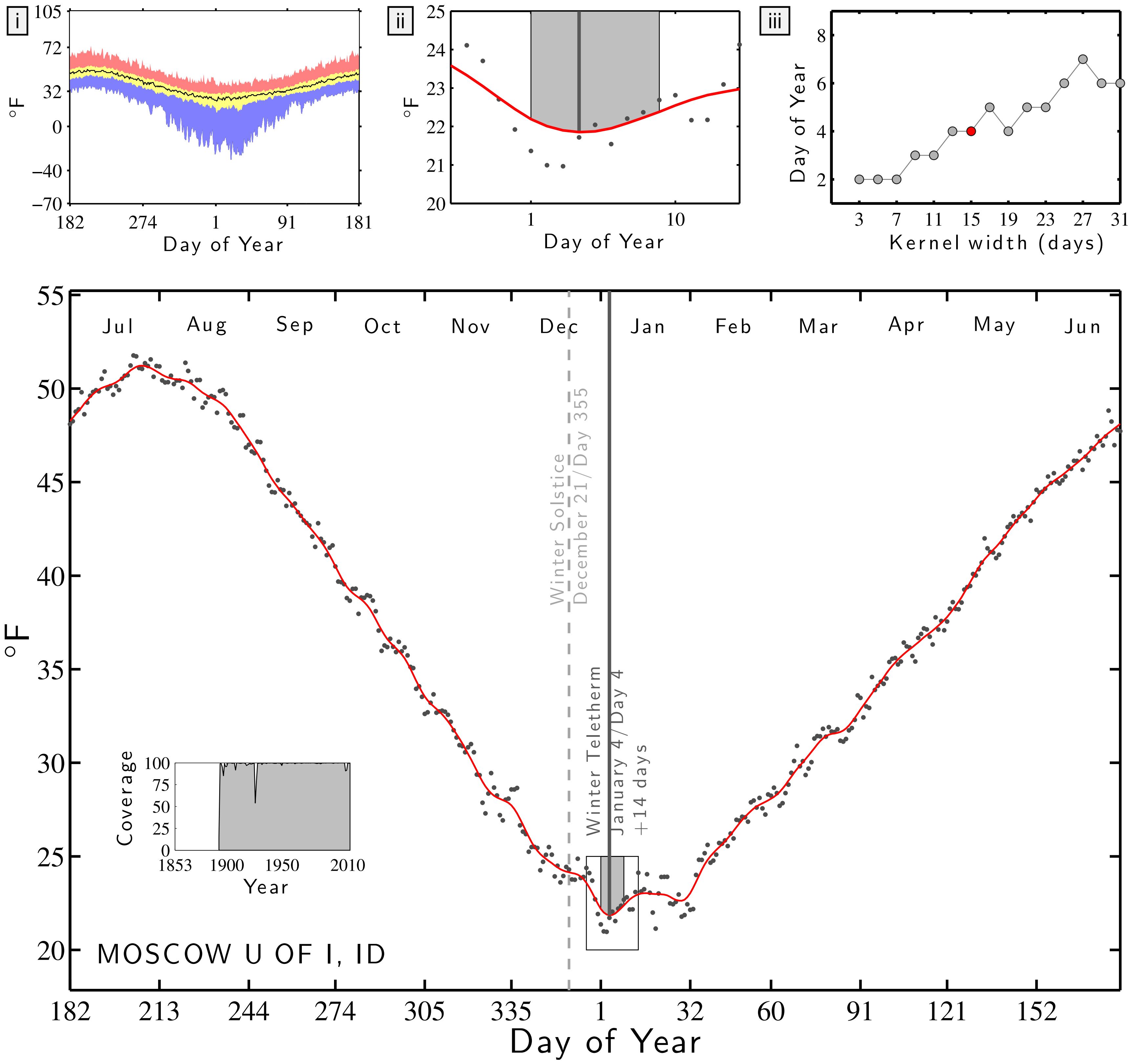

i
105
72
32
0
−40
−70
°F
182 274 1 91 181
Day of Year
ii
25
24
23
22
21
20
°F
1 10
Day of Year
iii
8
6
4
2
Day of Year
3 7 11 15 19 23 27 31
Kernel width (days)
55
50
45
40
35
30
25
20
°F
Jul Aug Sep Oct Nov Dec Jan Feb Mar Apr May Jun
Winter Solstice
December 21/Day 355
Winter Teletherm
January 4/Day 4
+14 days
Coverage
100
75
50
25
0
1853 1900 1950 2010
Year
MOSCOW U OF I, ID
182 213 244 274 305 335 1 32 60 91 121 152
Day of Year

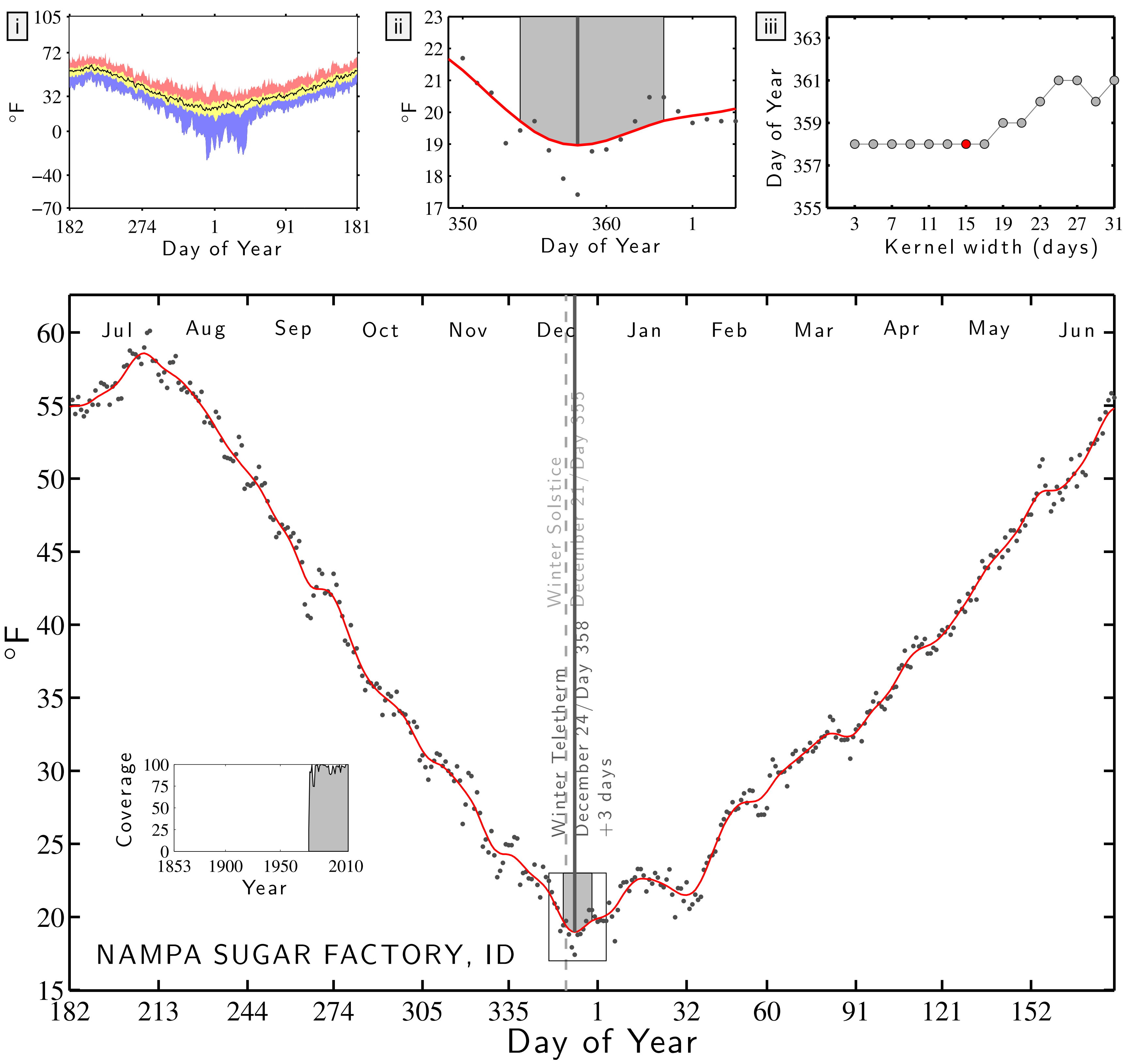

i
105
72
32
0
−40
−70
°F
182
274
1
91
181
Day of Year
ii
23
22
21
20
19
18
17
°F
350
360
1
Day of Year
iii
363
361
359
357
355
Day of Year
3
7
11
15
19
23
27
31
Kernel width (days)
Jul
Aug
Sep
Oct
Nov
Dec
Jan
Feb
Mar
Apr
May
Jun
60
55
50
45
40
35
30
25
20
15
°F
Winter Solstice
December 21/Day 355
Winter Teletherm
December 24/Day 358
+3 days
Coverage
100
75
50
25
0
1853
1900
1950
2010
Year
NAMPA SUGAR FACTORY, ID
182
213
244
274
305
335
1
32
60
91
121
152
Day of Year

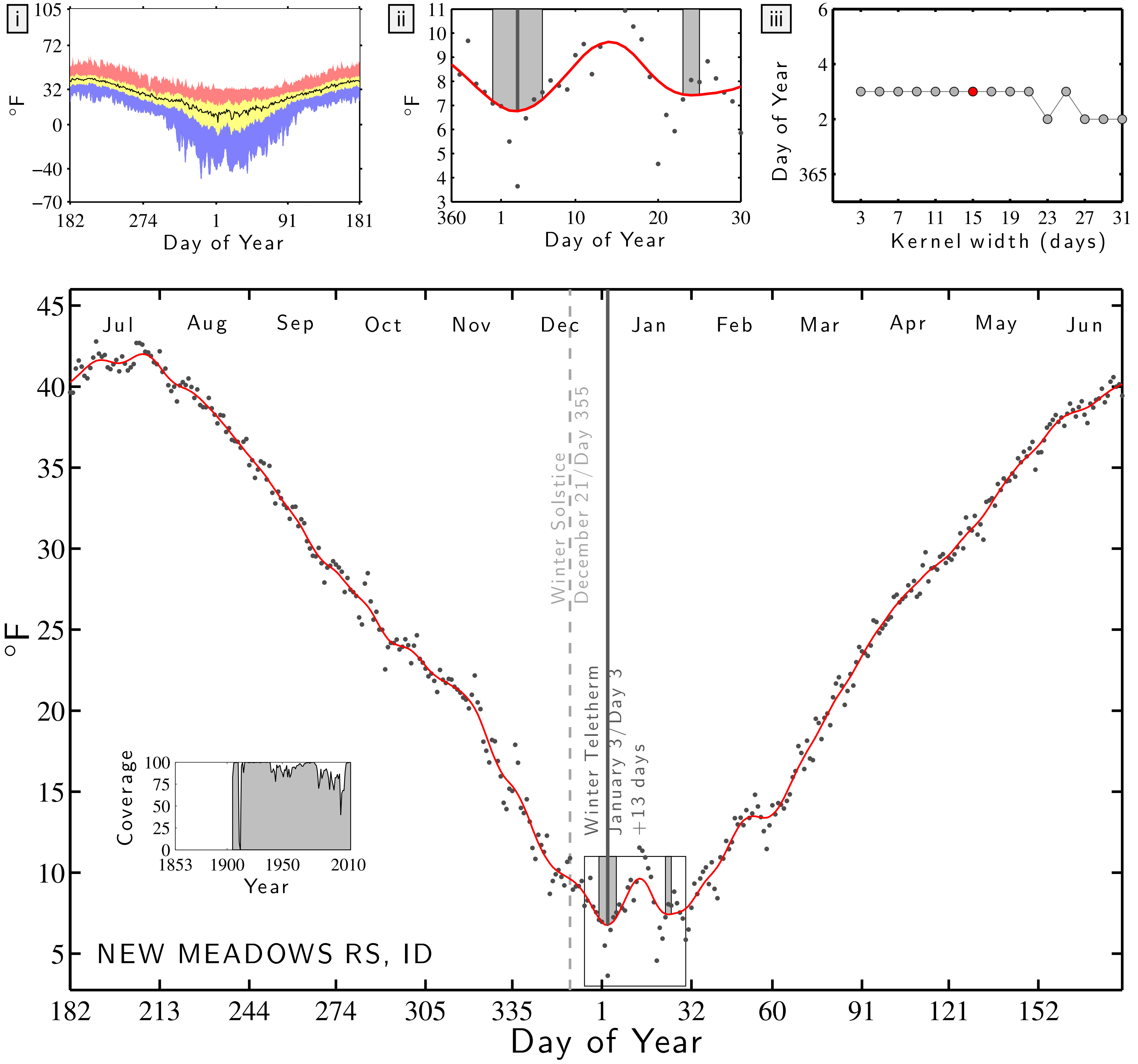

i
ii
iii
Day of Year
Kernel width (days)
°F
Day of Year
Jul
Aug
Sep
Oct
Nov
Dec
Jan
Feb
Mar
Apr
May
Jun
Winter Solstice
December 21/Day 355
Winter Teletherm
January 3/Day 3
+13 days
Coverage
Year
NEW MEADOWS RS, ID

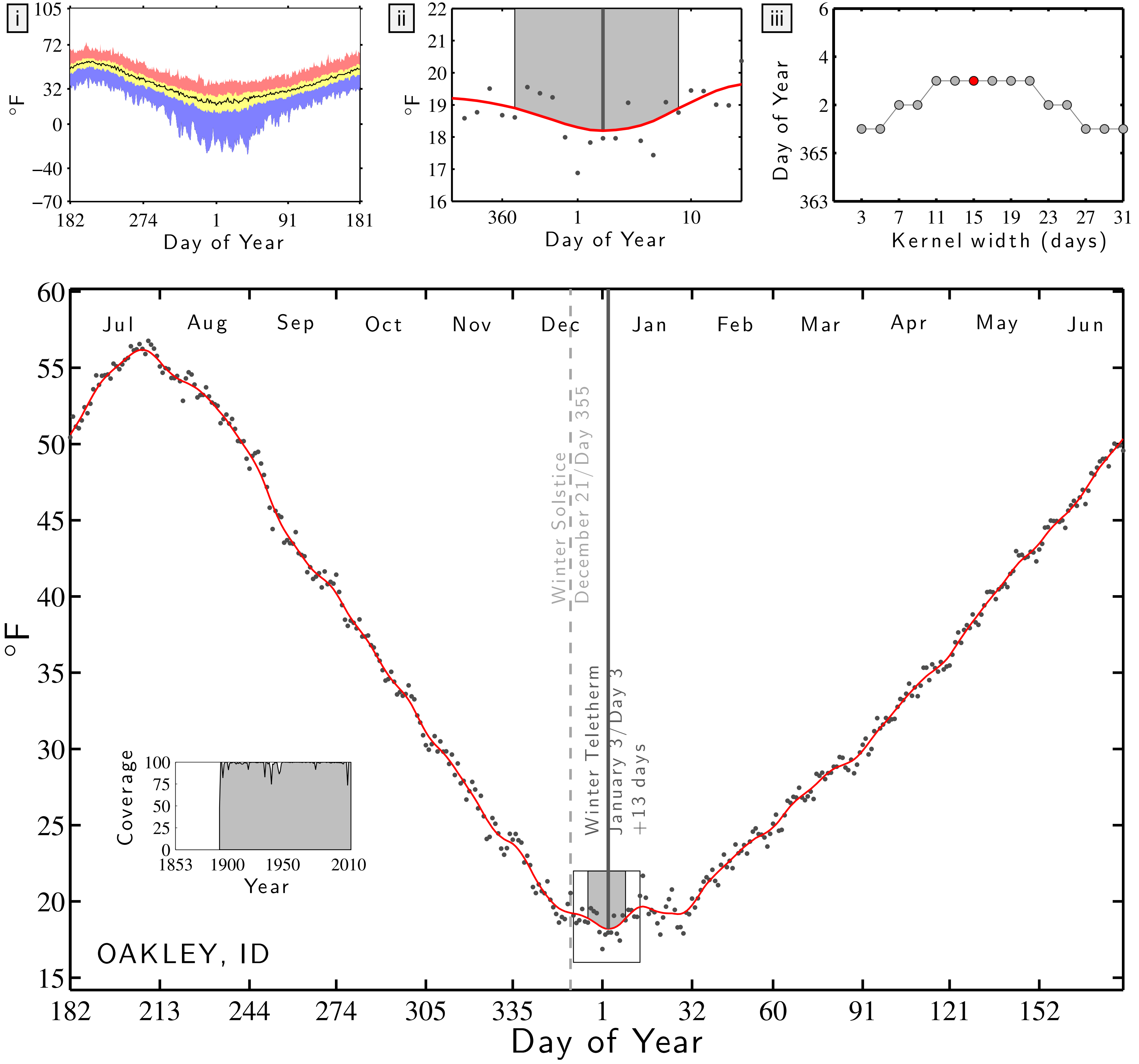

i
°F
Day of Year
182 274 1 91 181
105 72 32 0 −40 −70
ii
°F
Day of Year
360 1 10
22 21 20 19 18 17 16
iii
Day of Year
Kernel width (days)
3 7 11 15 19 23 27 31
6 4 2 365 363
Jul Aug Sep Oct Nov Dec Jan Feb Mar Apr May Jun
Winter Solstice
December 21/Day 355
Winter Teletherm
January 3/Day 3
+13 days
Coverage
100 75 50 25 0
1853 1900 1950 2010
Year
OAKLEY, ID
°F
60 55 50 45 40 35 30 25 20 15
182 213 244 274 305 335 1 32 60 91 121 152
Day of Year

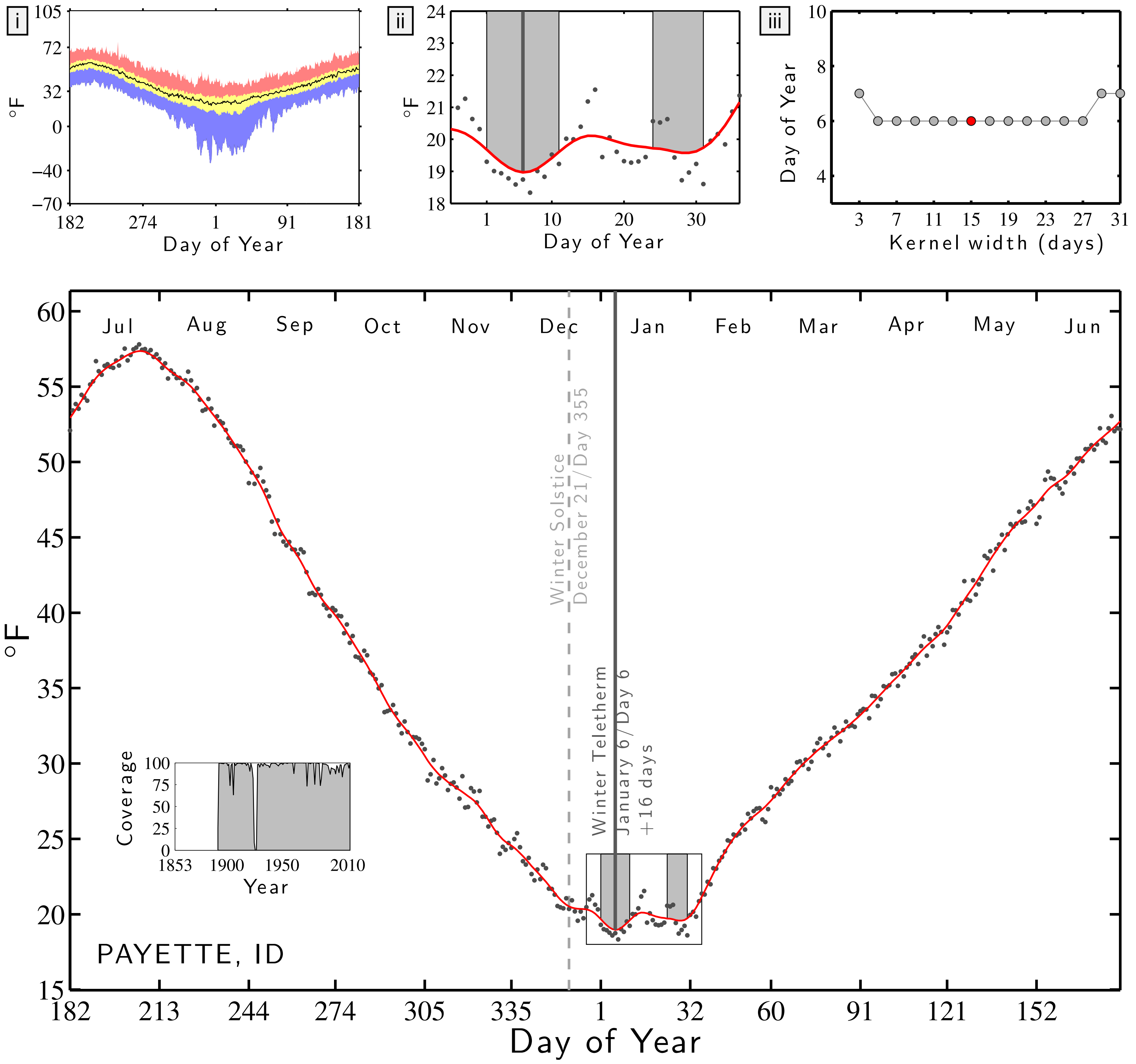

i
°F
Day of Year
ii
°F
Day of Year
iii
Day of Year
Kernel width (days)
Jul
Aug
Sep
Oct
Nov
Dec
Jan
Feb
Mar
Apr
May
Jun
Winter Solstice
December 21/Day 355
Winter Teletherm
January 6/Day 6
+16 days
Coverage
Year
PAYETTE, ID
°F
Day of Year

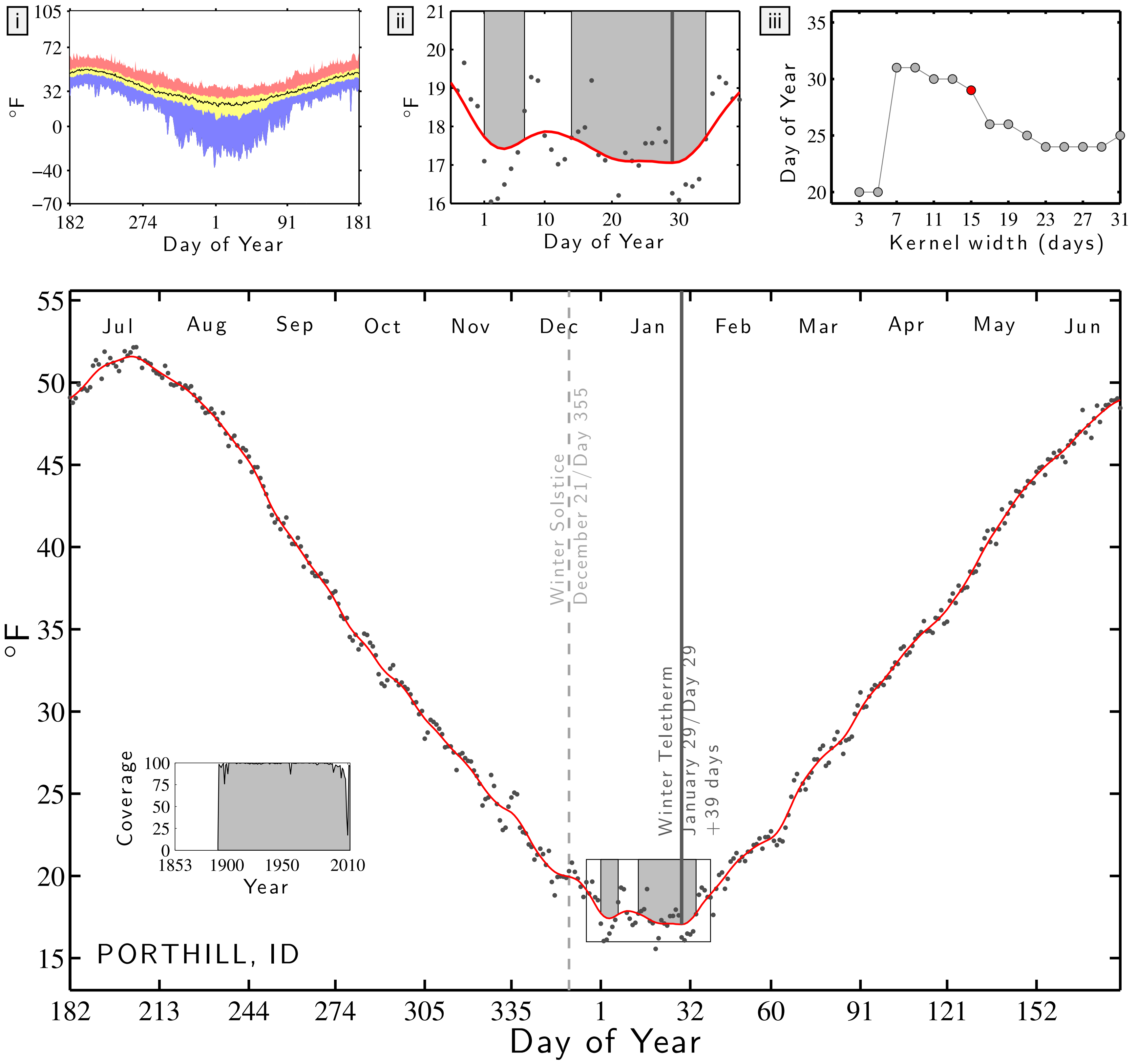

i
°F
Day of Year
ii
Day of Year
iii
Day of Year
Kernel width (days)
Jul
Aug
Sep
Oct
Nov
Dec
Jan
Feb
Mar
Apr
May
Jun
Winter Solstice
December 21/Day 355
Winter Teletherm
January 29/Day 29
+39 days
Coverage
Year
PORTHILL, ID
Day of Year

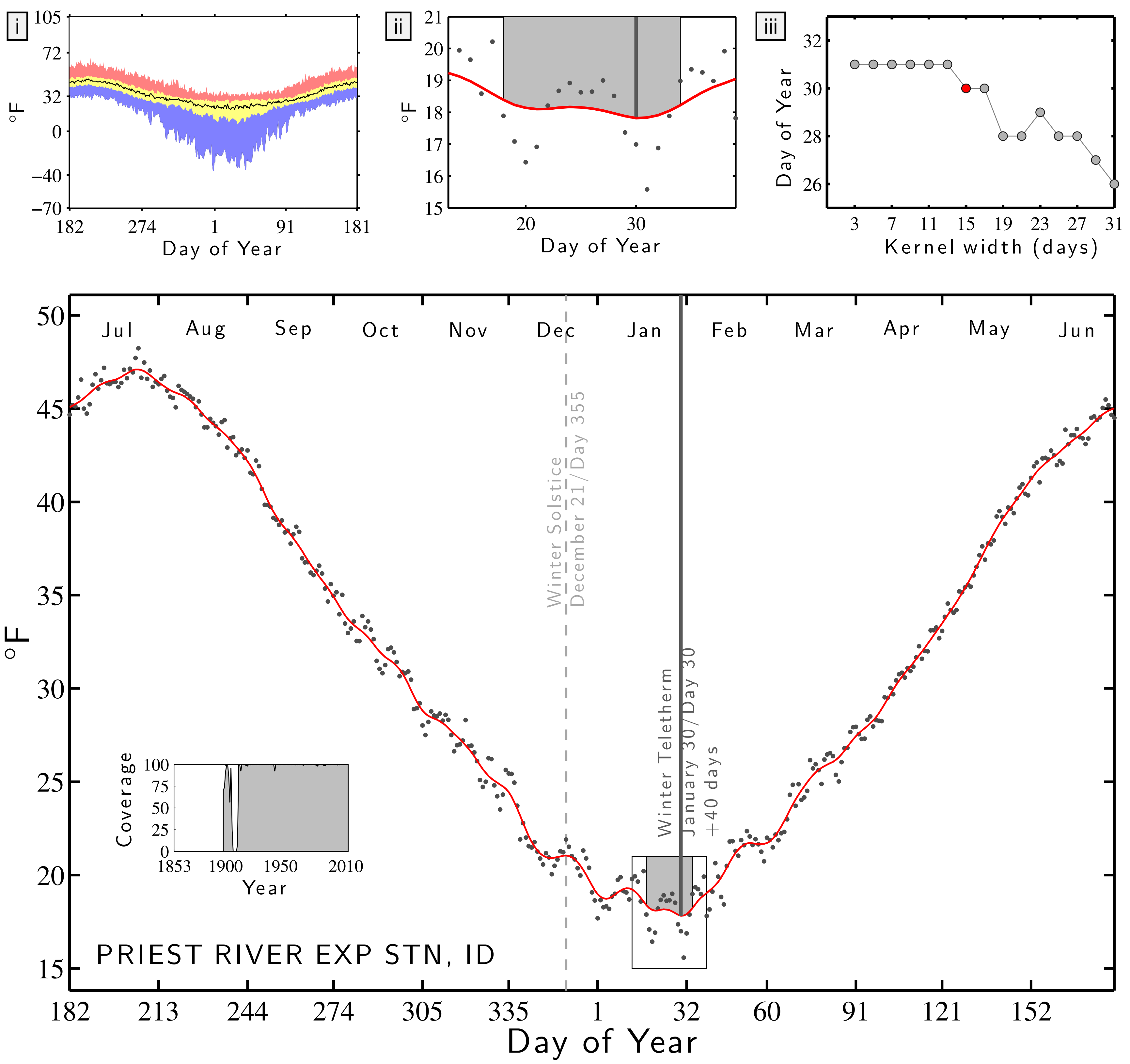

i
ii
iii
°F
Day of Year
Day of Year
Kernel width (days)
Jul
Aug
Sep
Oct
Nov
Dec
Jan
Feb
Mar
Apr
May
Jun
Winter Solstice
December 21/Day 355
Winter Teletherm
January 30/Day 30
+40 days
Coverage
Year
PRIEST RIVER EXP STN, ID
°F
Day of Year

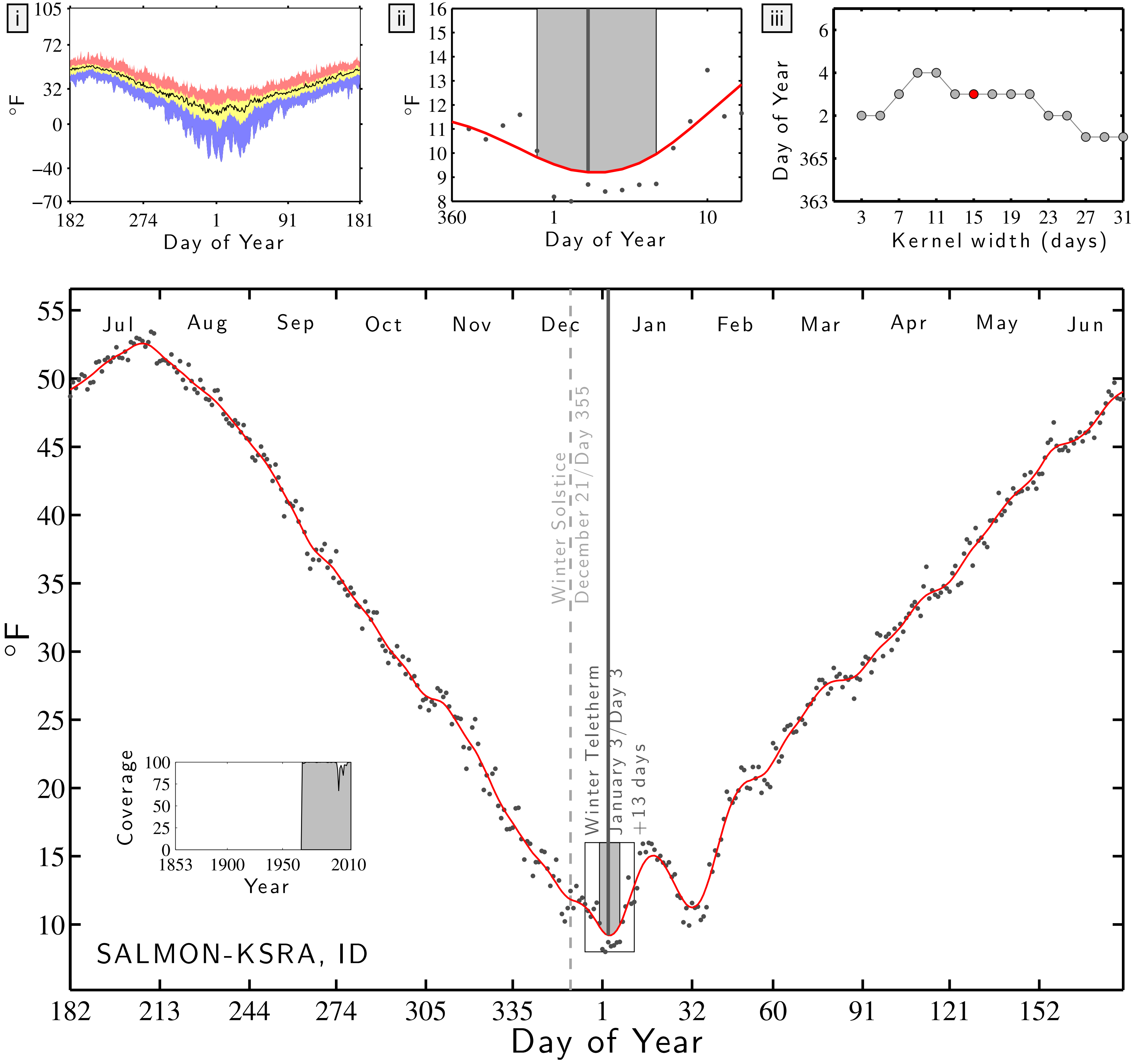

i
°F
Day of Year
ii
°F
Day of Year
iii
Day of Year
Kernel width (days)
Jul
Aug
Sep
Oct
Nov
Dec
Jan
Feb
Mar
Apr
May
Jun
Winter Solstice
December 21/Day 355
Winter Teletherm
January 3/Day 3
+13 days
Coverage
Year
SALMON-KSRA, ID
°F
Day of Year

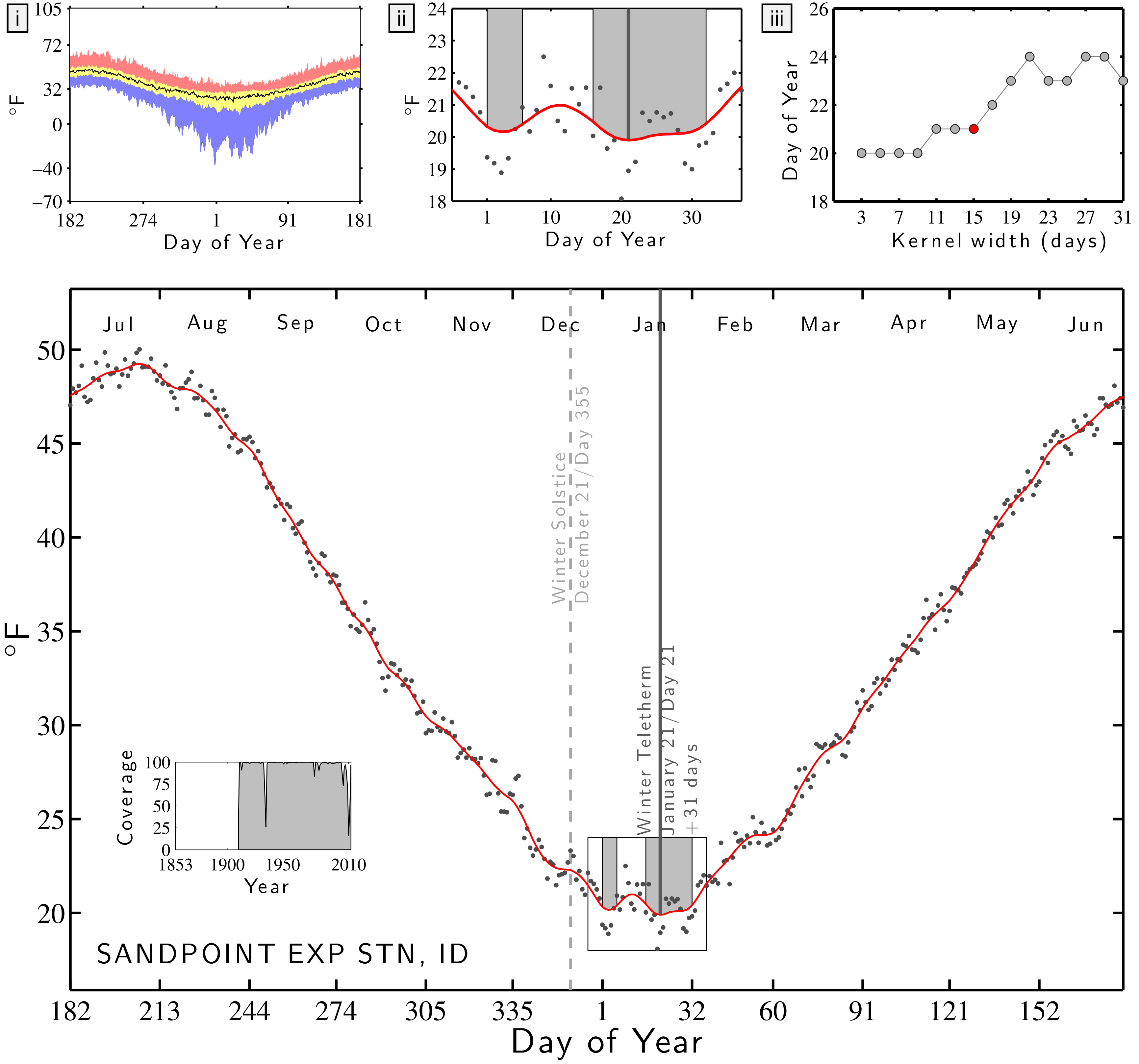
i
ii
iii
°F
Day of Year
Kernel width (days)
Day of Year
Jul
Aug
Sep
Oct
Nov
Dec
Jan
Feb
Mar
Apr
May
Jun
Winter Solstice
December 21/Day 355
Winter Teletherm
January 21/Day 21
+31 days
Coverage
Year
SANDPOINT EXP STN, ID

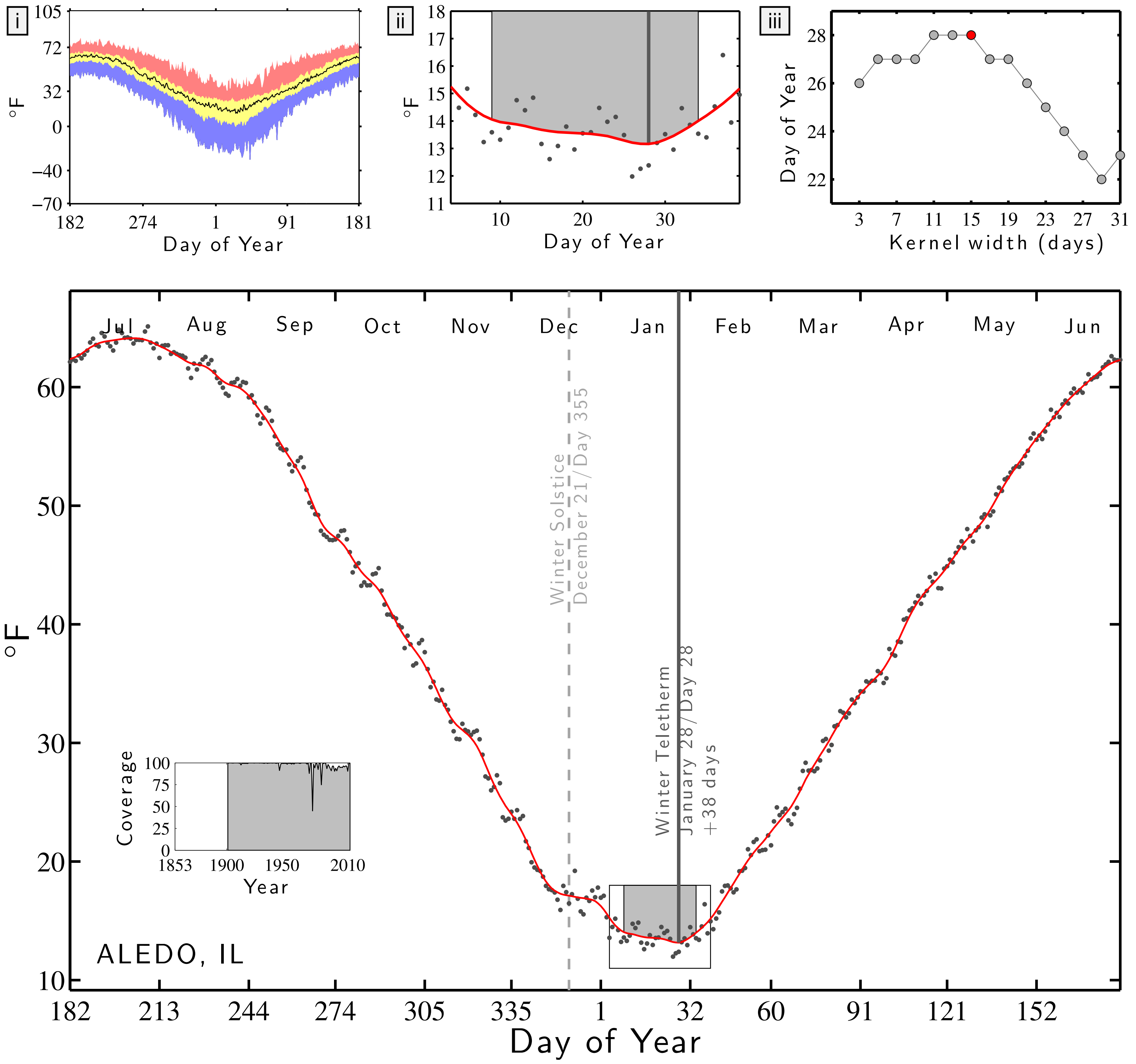
i
°F
Day of Year
ii
°F
Day of Year
iii
Day of Year
Kernel width (days)
Jul
Aug
Sep
Oct
Nov
Dec
Jan
Feb
Mar
Apr
May
Jun
Winter Solstice
December 21/Day 355
Winter Teletherm
January 28/Day 28
+38 days
Coverage
Year
ALEDO, IL
°F
Day of Year

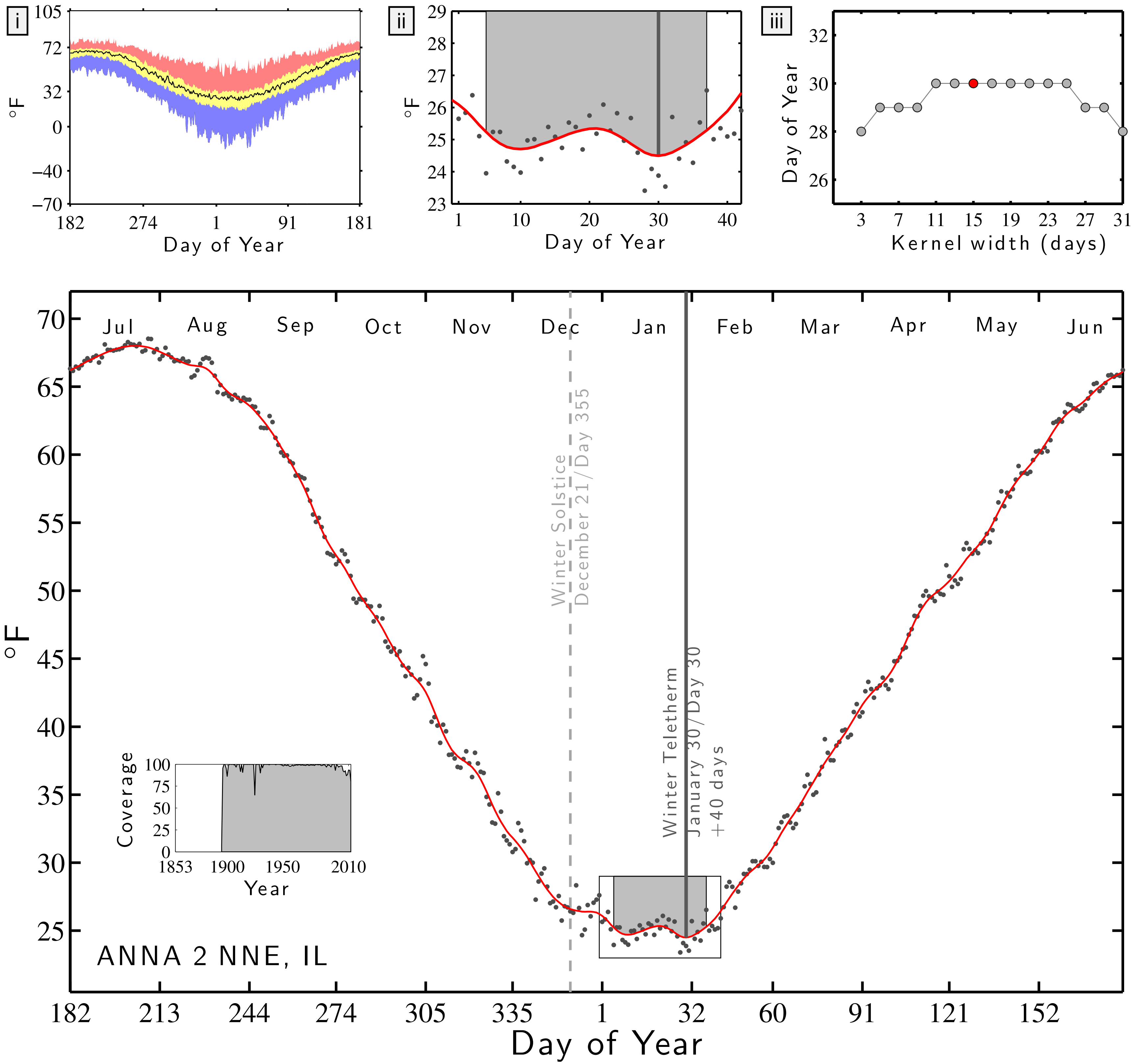

i
°F
Day of Year
ii
°F
Day of Year
iii
Day of Year
Kernel width (days)
Jul
Aug
Sep
Oct
Nov
Dec
Jan
Feb
Mar
Apr
May
Jun
Winter Solstice
December 21/Day 355
Winter Teletherm
January 30/Day 30
+40 days
Coverage
Year
ANNA 2 NNE, IL
°F
Day of Year

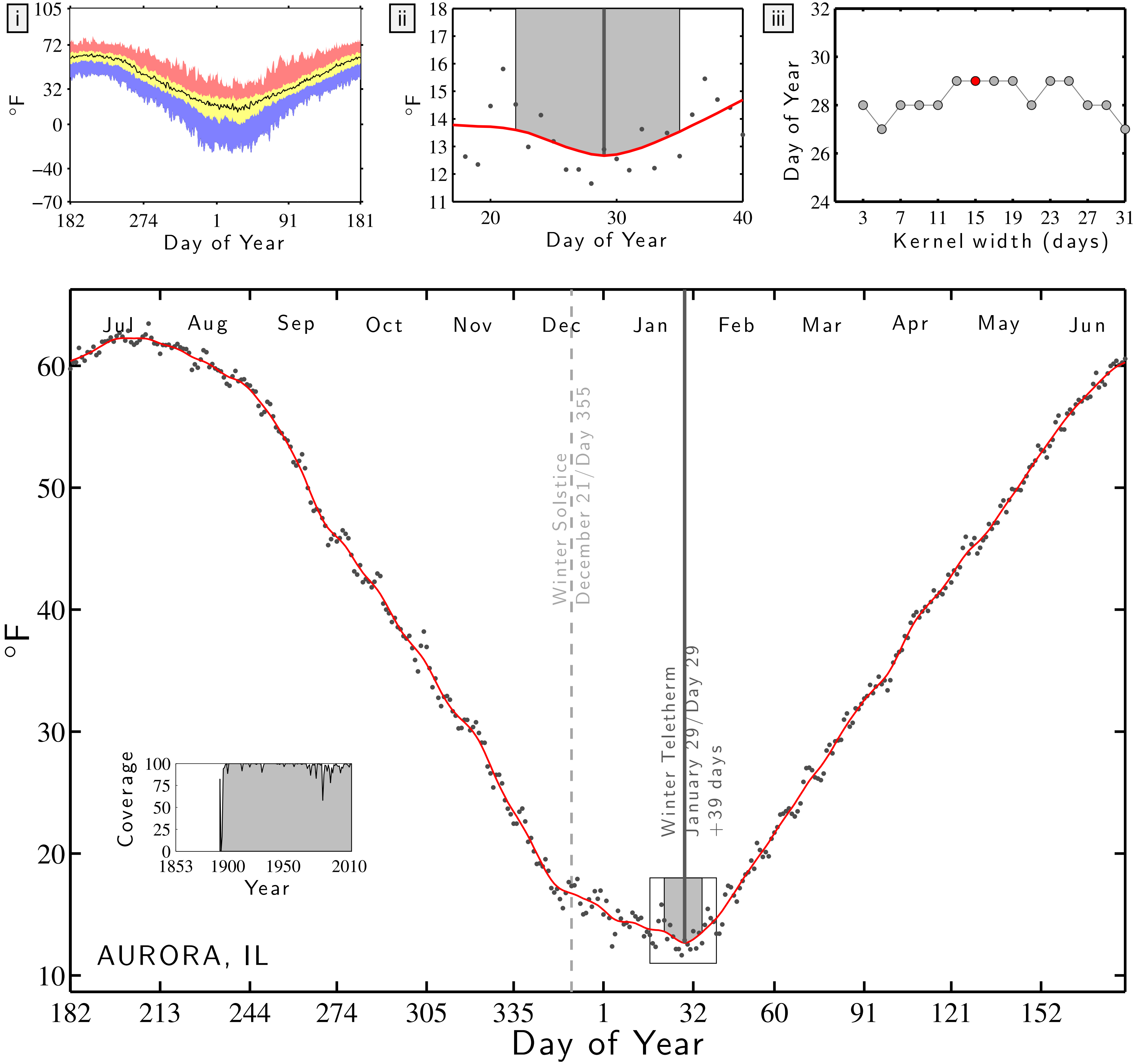

i
°F
Day of Year
ii
°F
Day of Year
iii
Day of Year
Kernel width (days)
Jul
Aug
Sep
Oct
Nov
Dec
Jan
Feb
Mar
Apr
May
Jun
Winter Solstice
December 21/Day 355
Winter Teletherm
January 29/Day 29
+39 days
Coverage
Year
AURORA, IL
°F
Day of Year

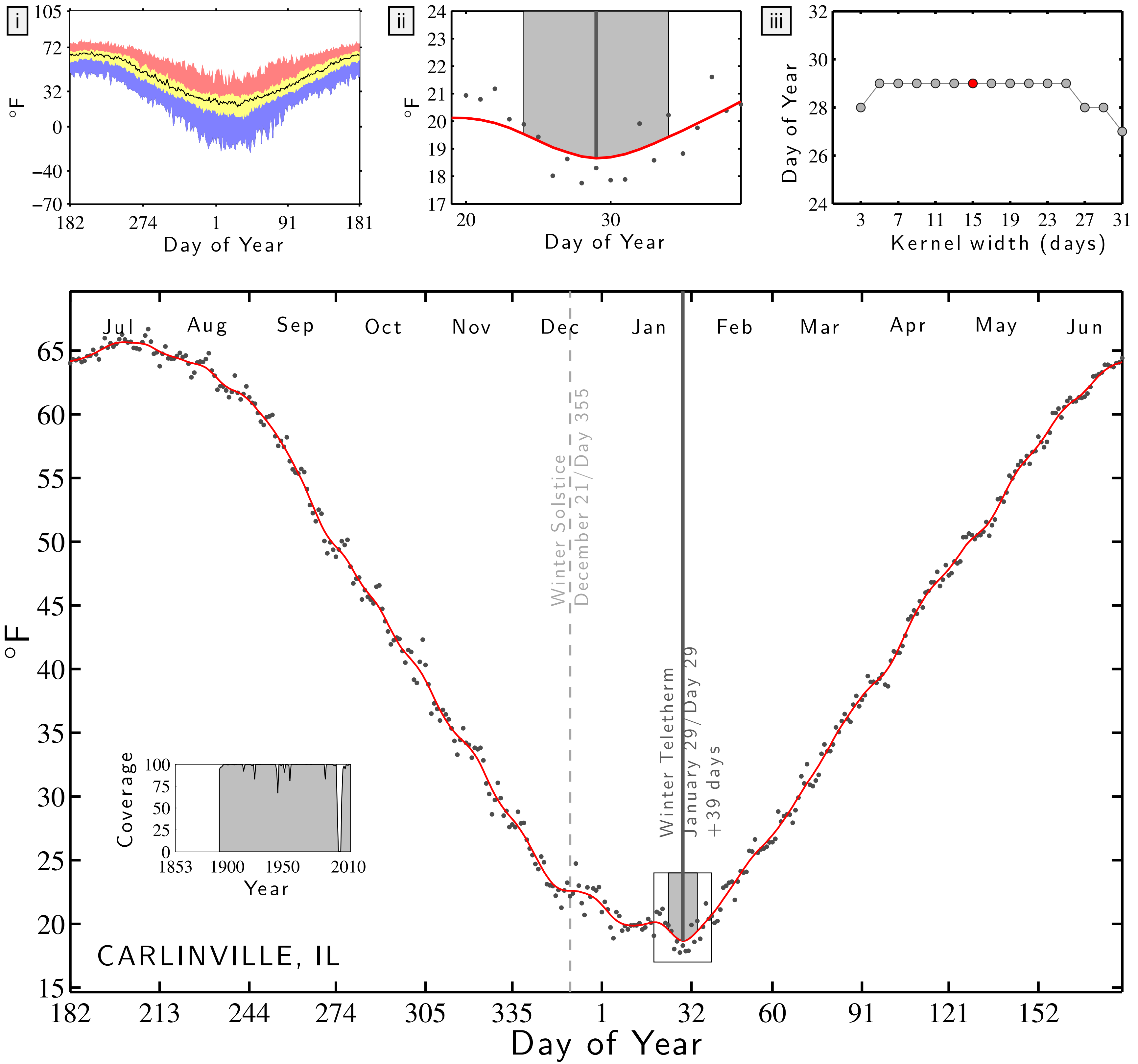

i
ii
iii
°F
Day of Year
Day of Year
Kernel width (days)
Winter Solstice
December 21/Day 355
Winter Teletherm
January 29/Day 29
+39 days
Jul
Aug
Sep
Oct
Nov
Dec
Jan
Feb
Mar
Apr
May
Jun
Coverage
Year
CARLINVILLE, IL

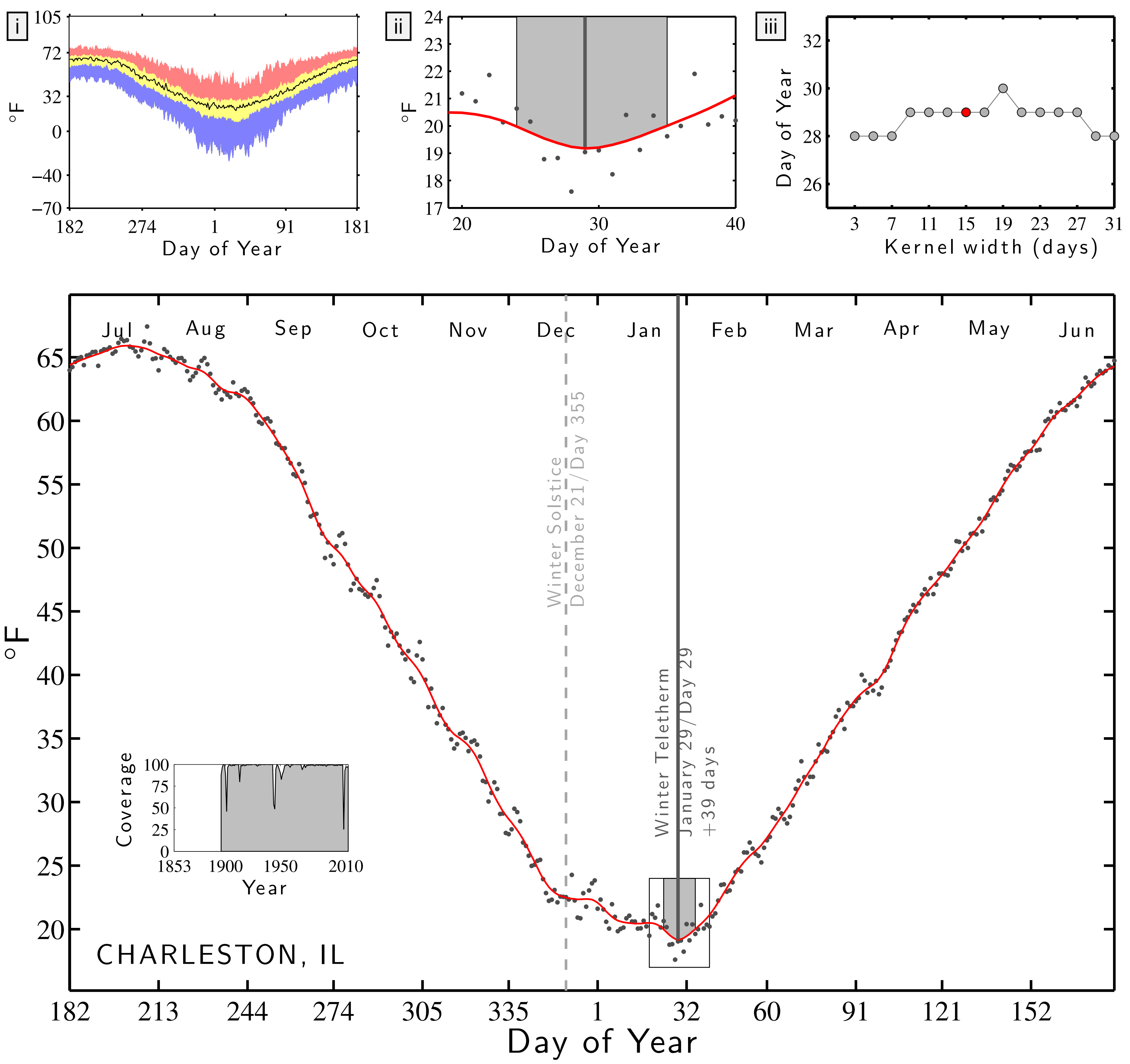

i
°F
Day of Year
ii
Day of Year
iii
Day of Year
Kernel width (days)
Jul
Aug
Sep
Oct
Nov
Dec
Jan
Feb
Mar
Apr
May
Jun
Winter Solstice
December 21/Day 355
Winter Teletherm
January 29/Day 29
+39 days
Coverage
Year
CHARLESTON, IL
Day of Year

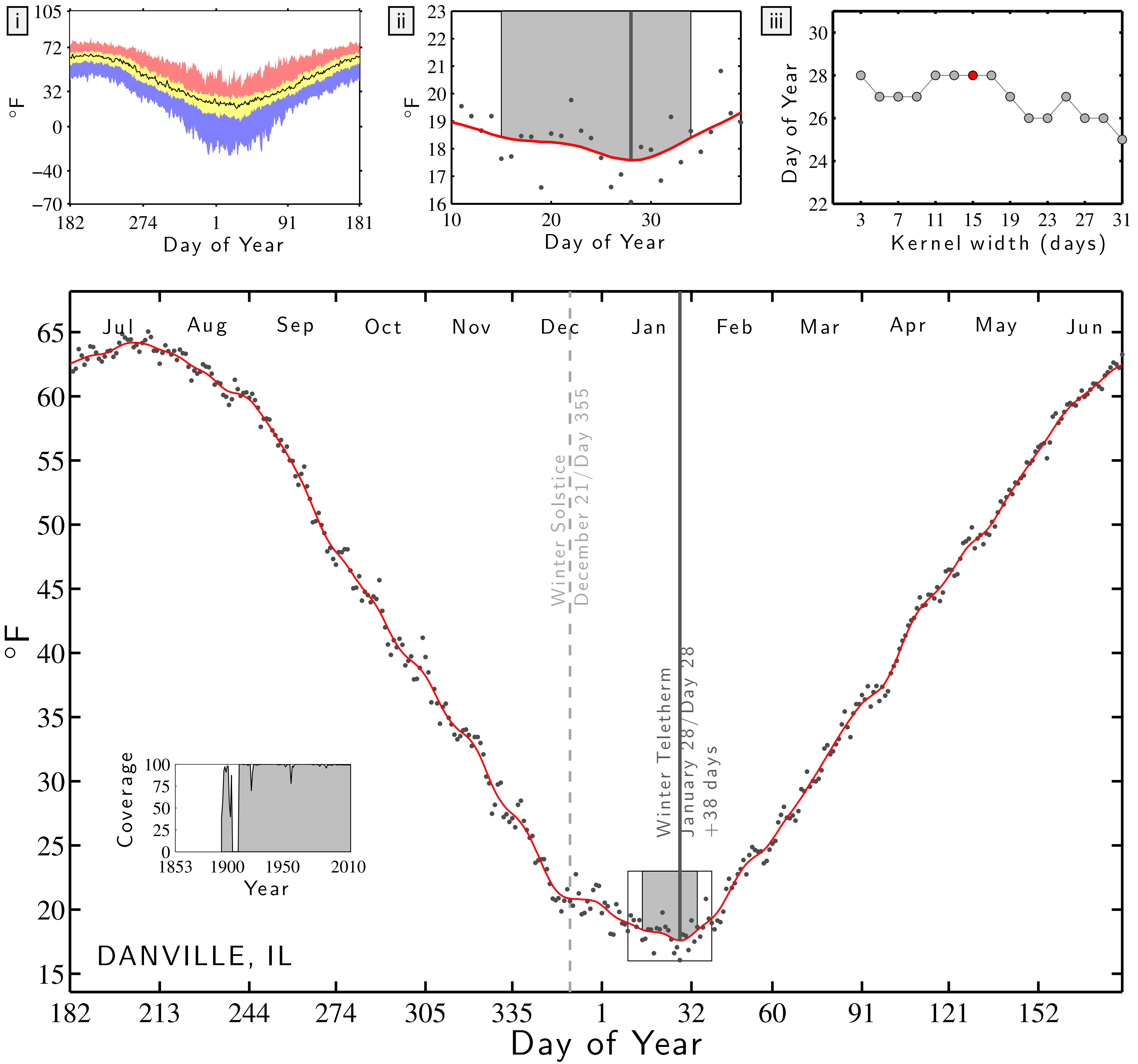

i
ii
iii
°F
Day of Year
Day of Year
Kernel width (days)
Jul
Aug
Sep
Oct
Nov
Dec
Jan
Feb
Mar
Apr
May
Jun
Winter Solstice
December 21/Day 355
Winter Teletherm
January 28/Day 28
+38 days
Coverage
Year
DANVILLE, IL

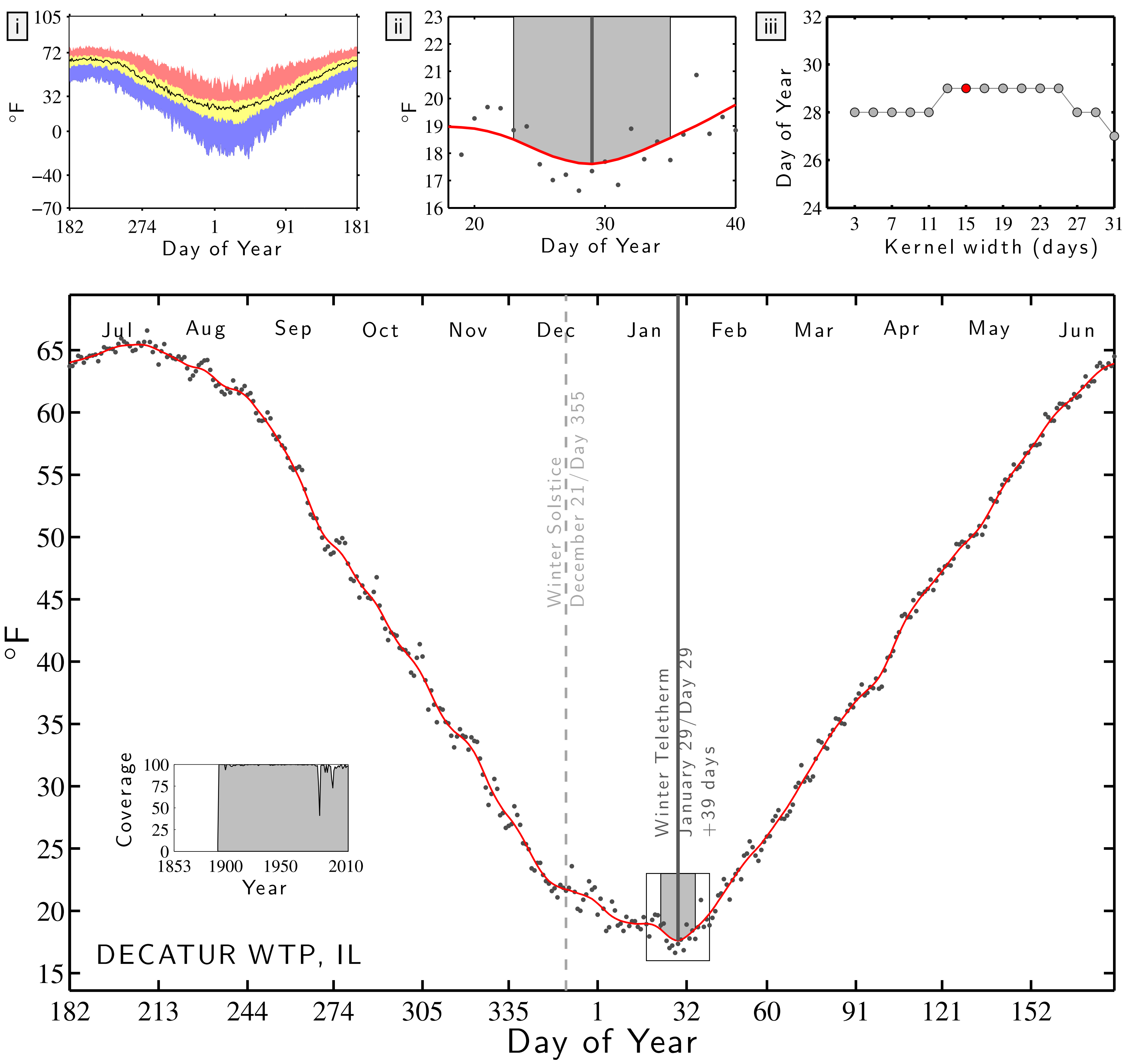
i
°F
Day of Year
ii
°F
Day of Year
iii
Day of Year
Kernel width (days)
Jul
Aug
Sep
Oct
Nov
Dec
Jan
Feb
Mar
Apr
May
Jun
Winter Solstice
December 21/Day 355
Winter Teletherm
January 29/Day 29
+39 days
Coverage
Year
DECATUR WTP, IL
°F
Day of Year

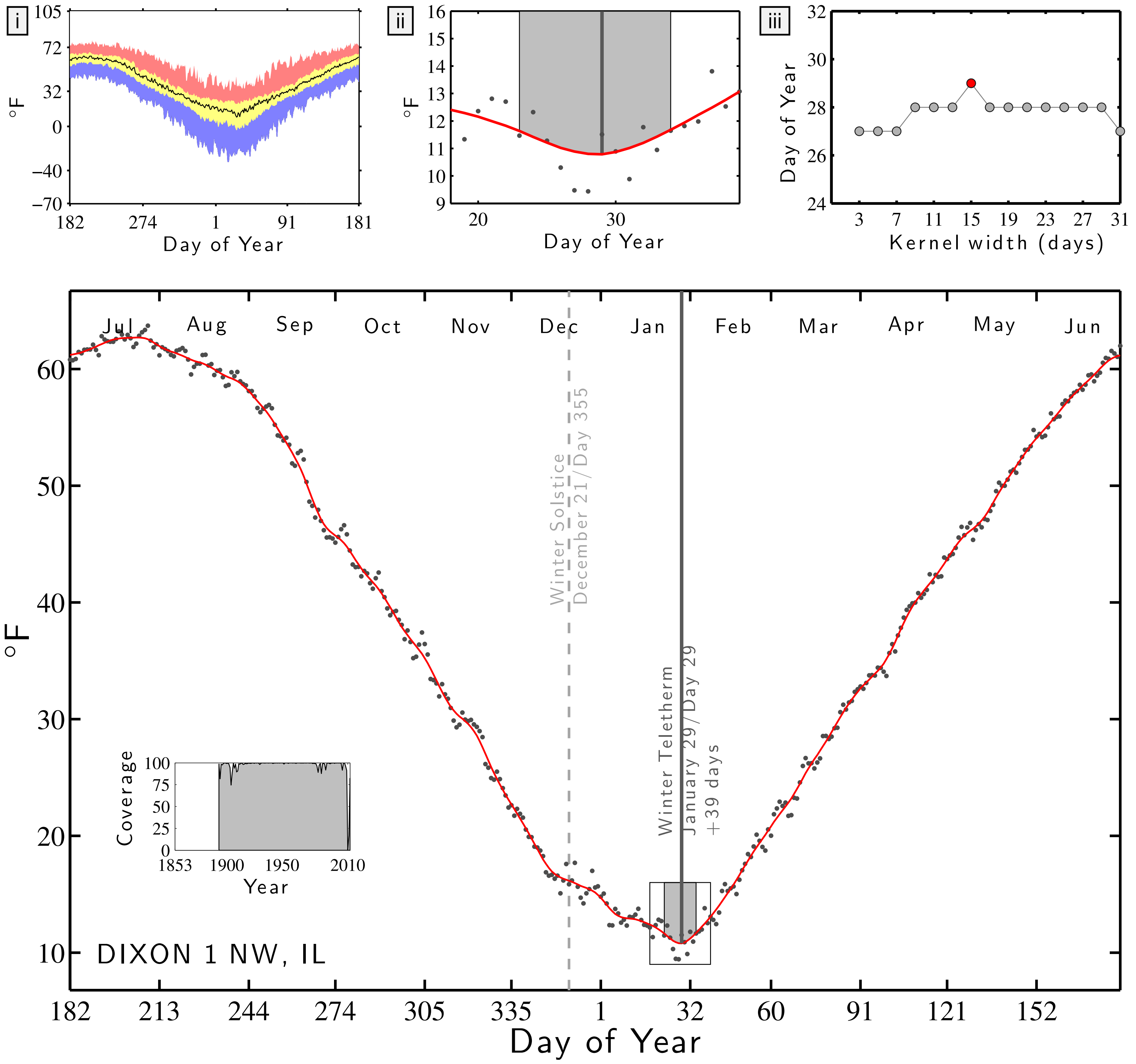

i
ii
iii
°F
Day of Year
Day of Year
Kernel width (days)
Jul
Aug
Sep
Oct
Nov
Dec
Jan
Feb
Mar
Apr
May
Jun
Winter Solstice
December 21/Day 355
Winter Teletherm
January 29/Day 29
+39 days
Coverage
Year
DIXON 1 NW, IL
Day of Year

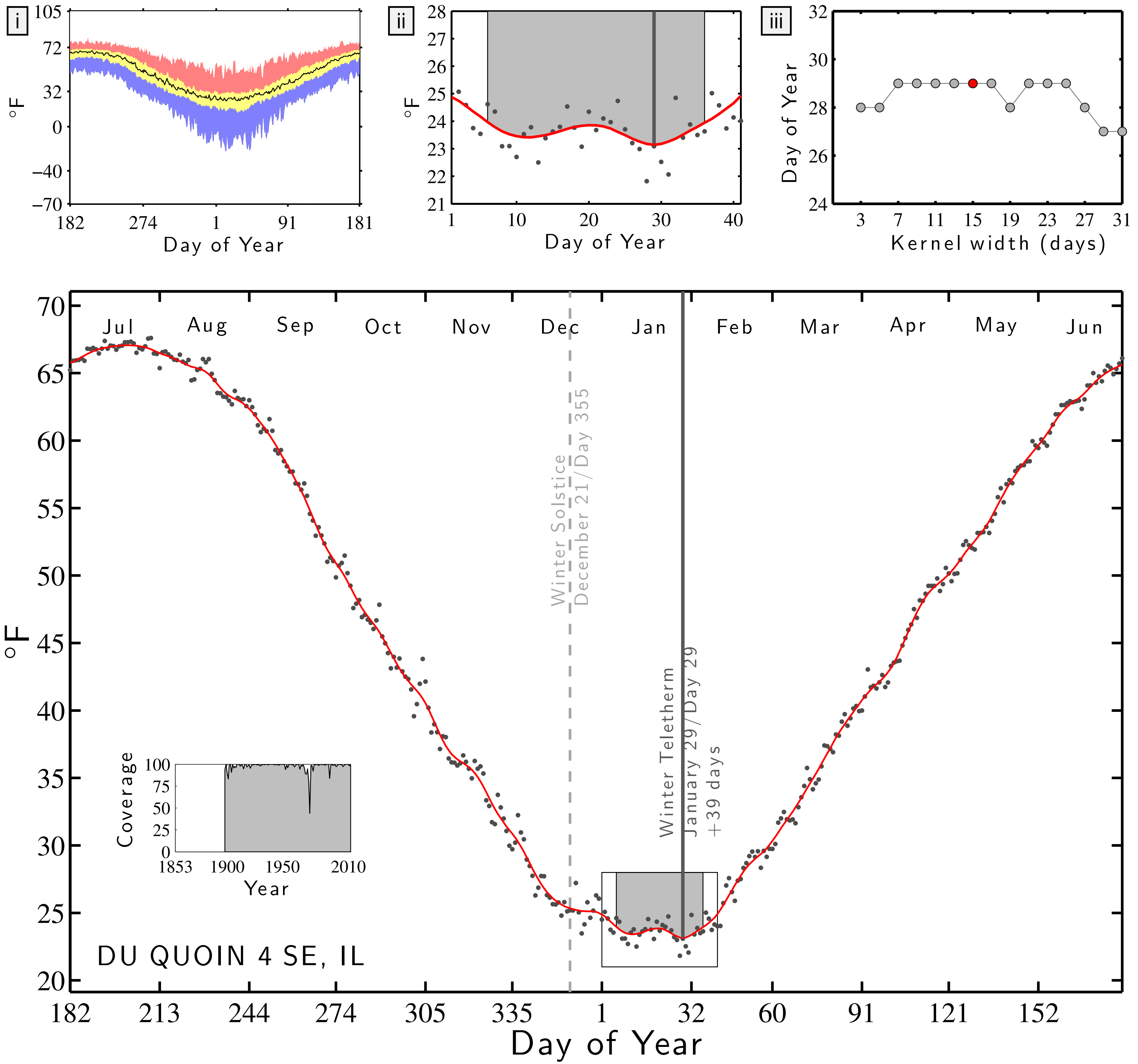

i
ii
iii
Day of Year
Kernel width (days)
°F
Jul
Aug
Sep
Oct
Nov
Dec
Jan
Feb
Mar
Apr
May
Jun
Winter Solstice
December 21/Day 355
Winter Teletherm
January 29/Day 29
+39 days
Coverage
Year
DU QUOIN 4 SE, IL

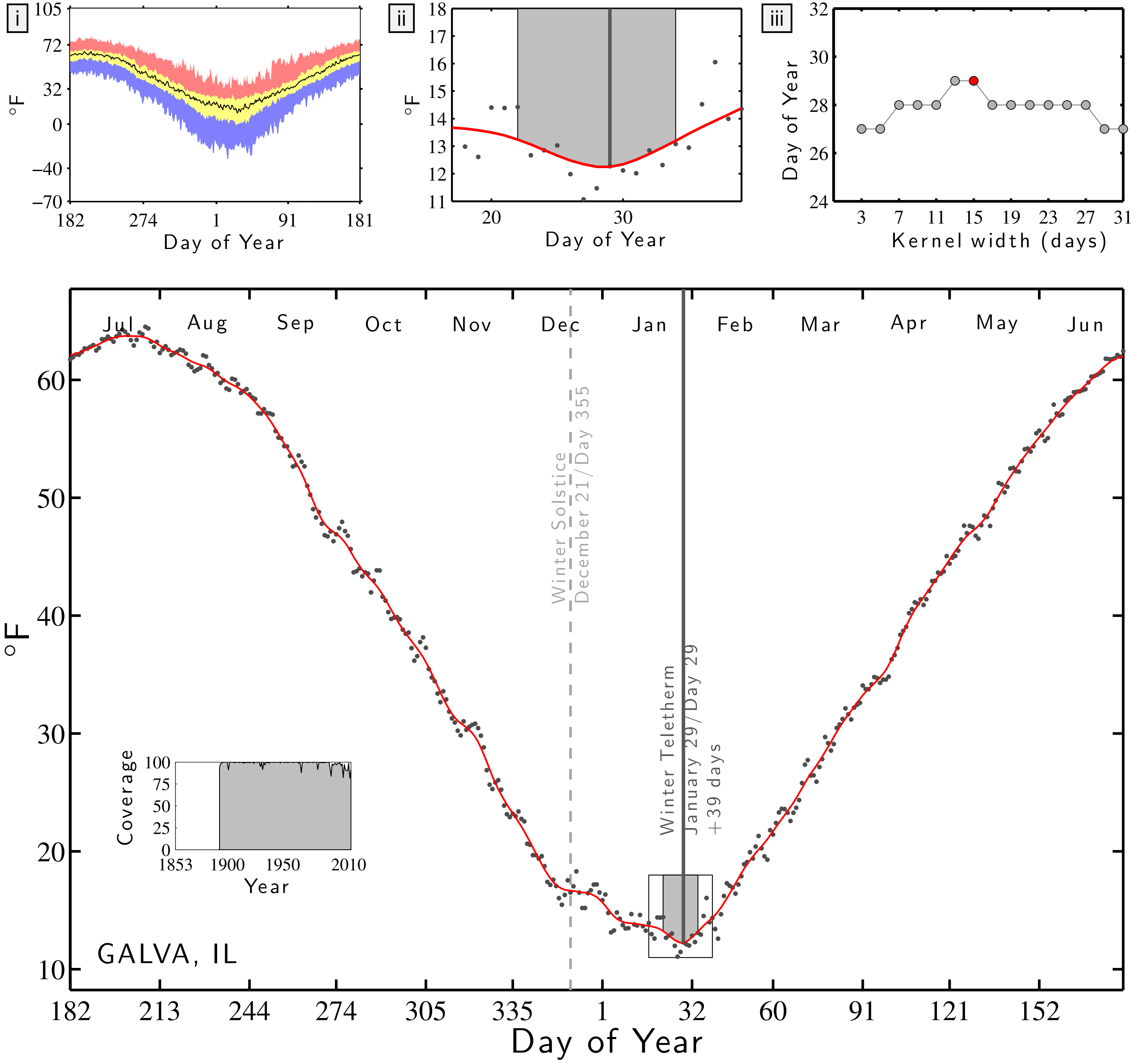

i
°F
Day of Year
ii
°F
Day of Year
iii
Day of Year
Kernel width (days)
Jul
Aug
Sep
Oct
Nov
Dec
Jan
Feb
Mar
Apr
May
Jun
Winter Solstice
December 21/Day 355
Winter Teletherm
January 29/Day 29
+39 days
Coverage
Year
GALVA, IL
°F
Day of Year

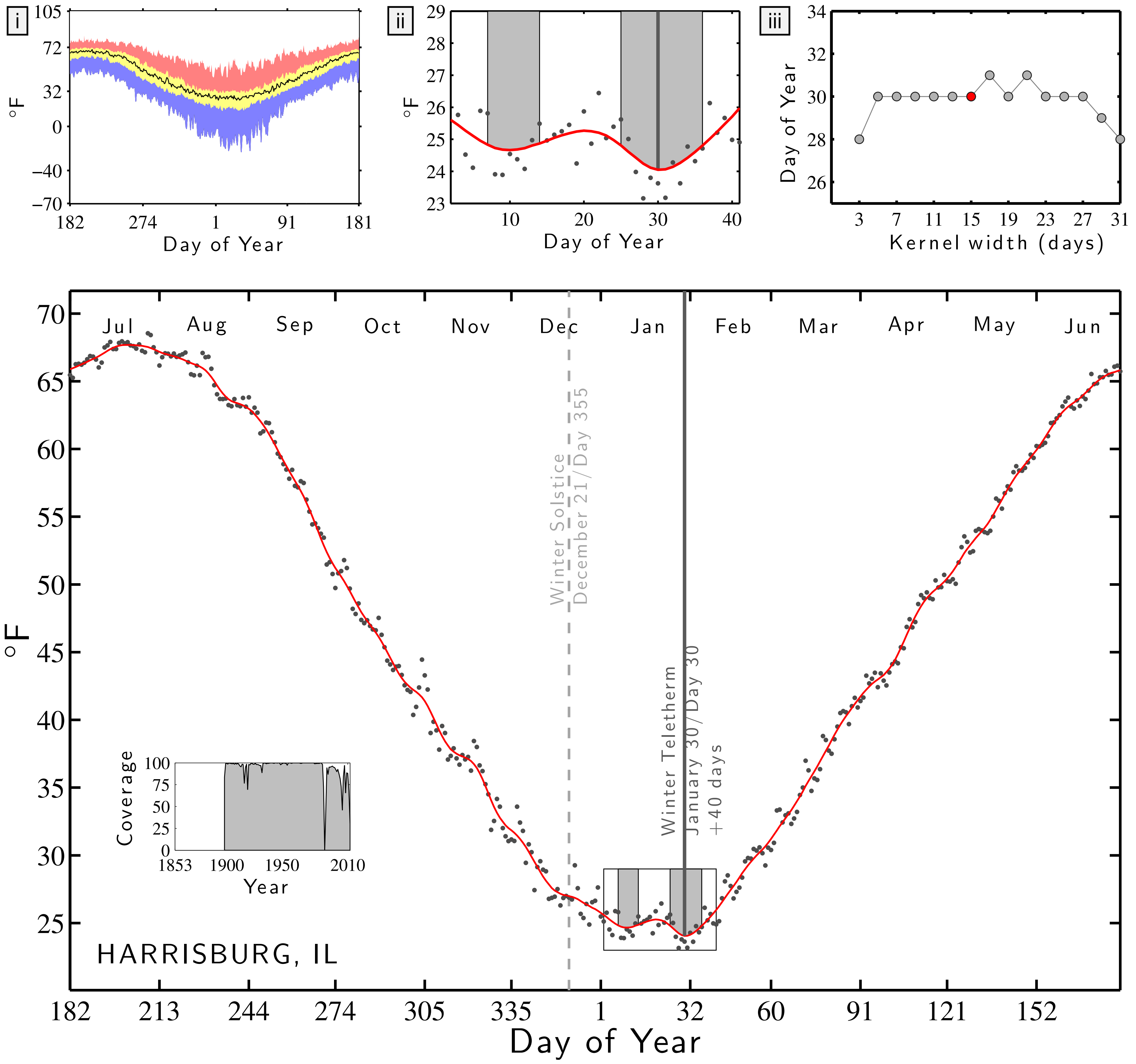

i
°F
Day of Year
ii
°F
Day of Year
iii
Day of Year
Kernel width (days)
Jul
Aug
Sep
Oct
Nov
Dec
Jan
Feb
Mar
Apr
May
Jun
Winter Solstice
December 21/Day 355
Winter Teletherm
January 30/Day 30
+40 days
Coverage
Year
HARRISBURG, IL
°F
Day of Year

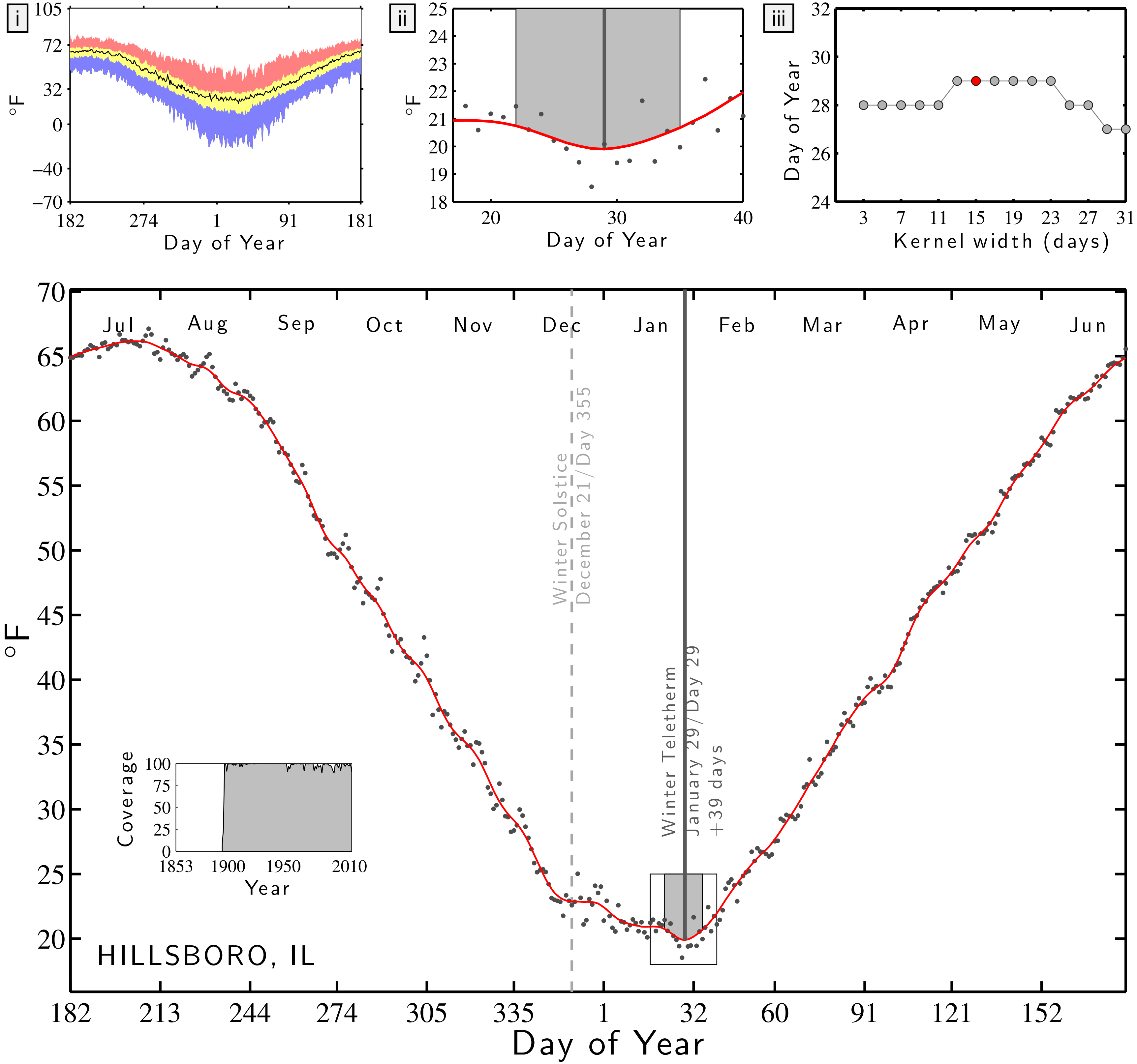

i
°F
Day of Year
ii
°F
Day of Year
iii
Day of Year
Kernel width (days)
Jul
Aug
Sep
Oct
Nov
Dec
Jan
Feb
Mar
Apr
May
Jun
Winter Solstice
December 21/Day 355
Winter Teletherm
January 29/Day 29
+39 days
Coverage
Year
HILLSBORO, IL
°F
Day of Year

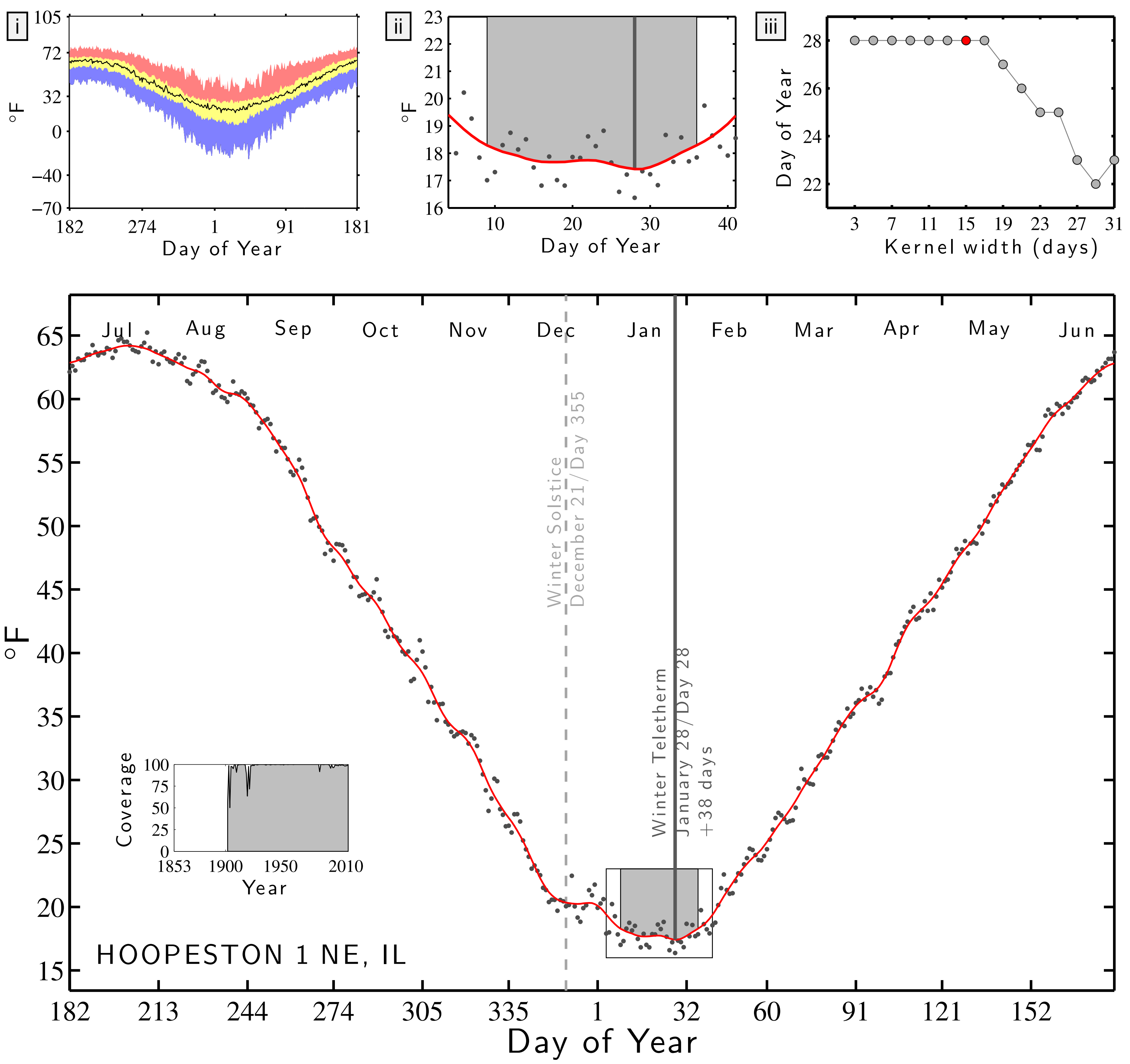

i
ii
iii
Day of Year
Kernel width (days)
°F
Day of Year
Jul
Aug
Sep
Oct
Nov
Dec
Jan
Feb
Mar
Apr
May
Jun
Winter Solstice
December 21/Day 355
Winter Teletherm
January 28/Day 28
+38 days
Coverage
Year
HOOPESTON 1 NE, IL

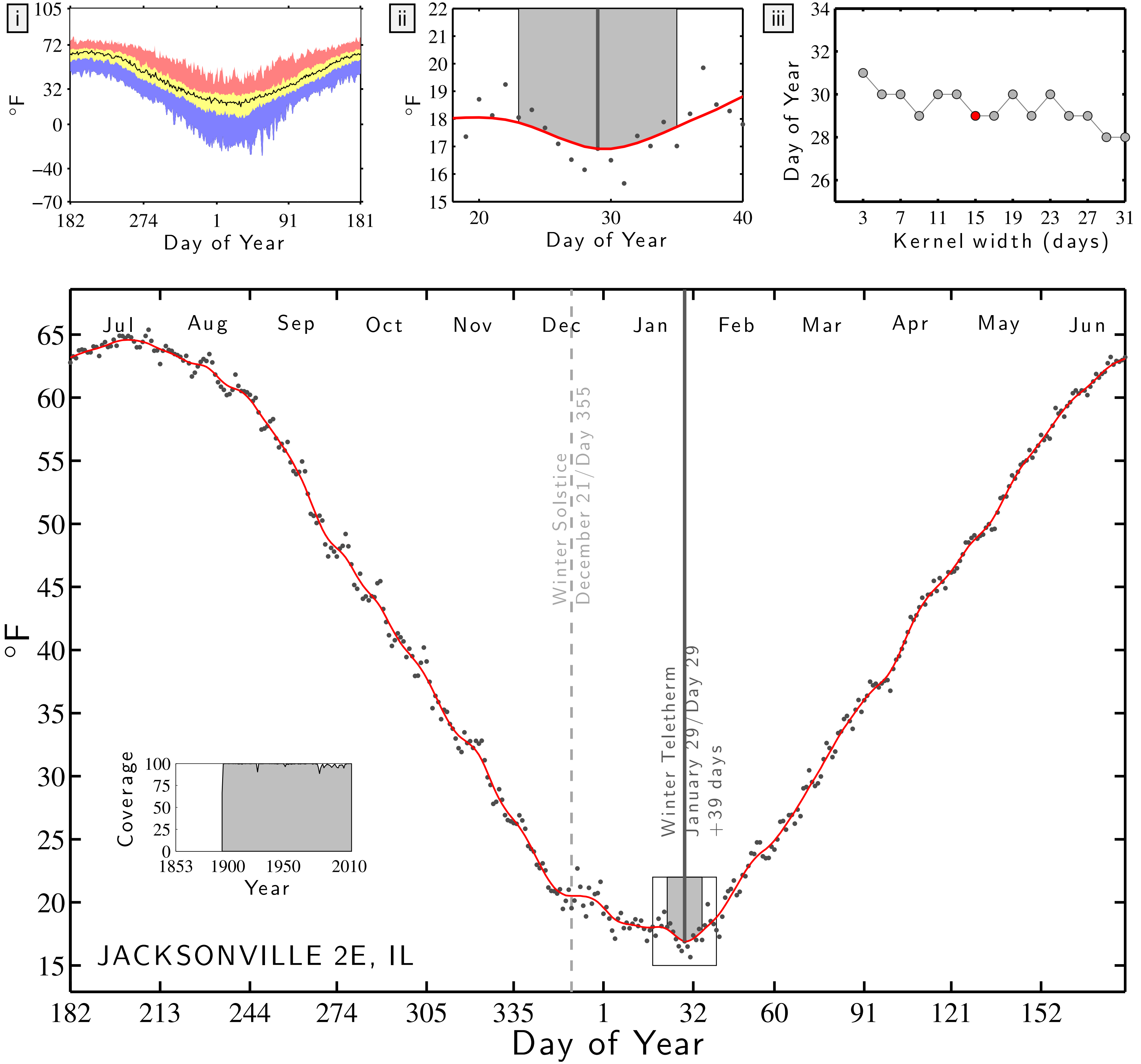

i
ii
iii
°F
Day of Year
Day of Year
Kernel width (days)
Jul
Aug
Sep
Oct
Nov
Dec
Jan
Feb
Mar
Apr
May
Jun
Winter Solstice
December 21/Day 355
Winter Teletherm
January 29/Day 29
+39 days
Coverage
Year
JACKSONVILLE 2E, IL

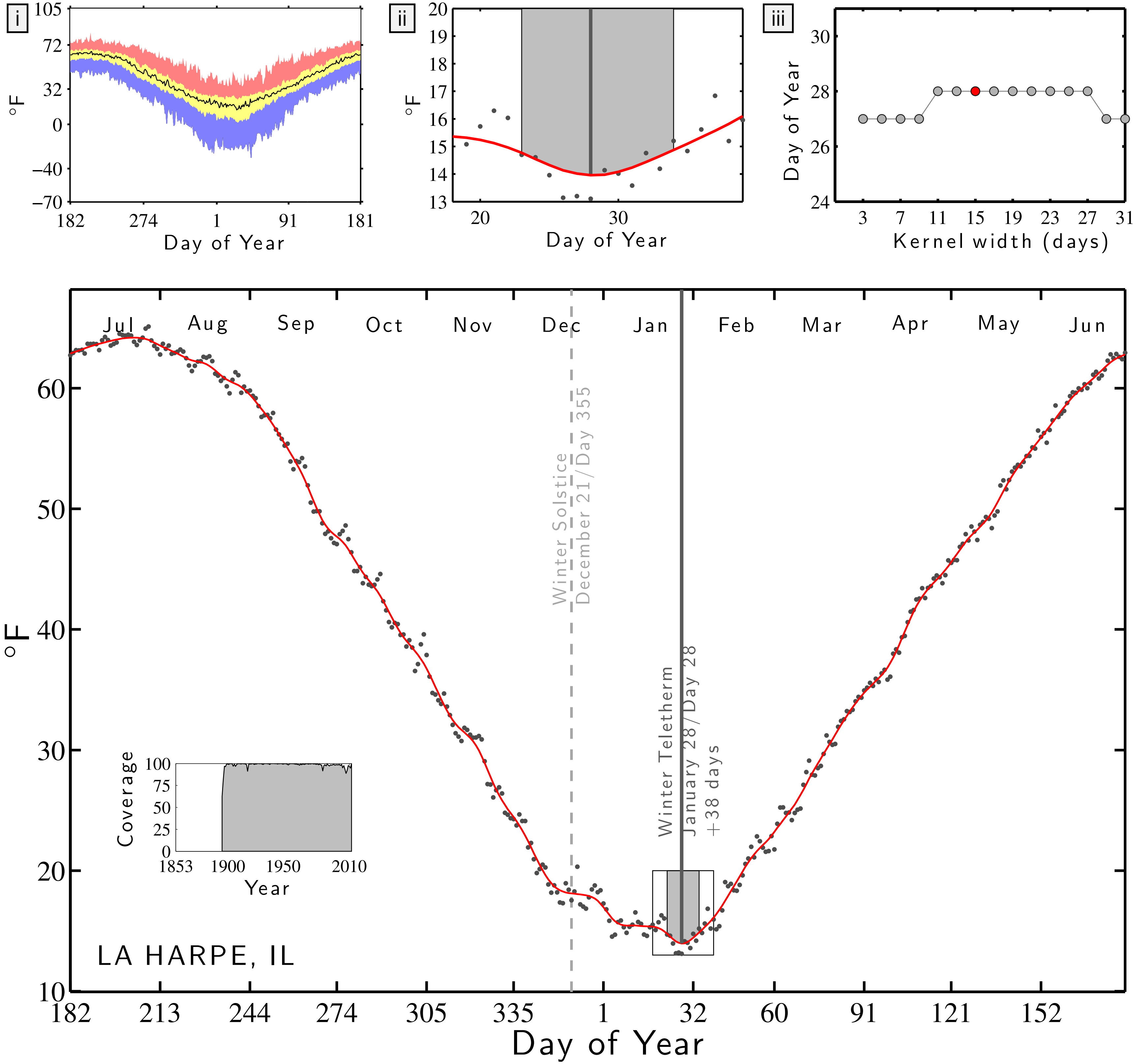

i
105
72
32
0
−40
−70
°F
182
274
1
91
181
Day of Year
ii
20
19
18
17
16
15
14
13
°F
20
30
Day of Year
iii
30
28
26
24
Day of Year
3
7
11
15
19
23
27
31
Kernel width (days)
Jul
Aug
Sep
Oct
Nov
Dec
Jan
Feb
Mar
Apr
May
Jun
60
50
40
30
20
10
°F
Winter Solstice
December 21/Day 355
Winter Teletherm
January 28/Day 28
+38 days
Coverage
100
75
50
25
0
1853
1900
1950
2010
Year
LA HARPE, IL
182
213
244
274
305
335
1
32
60
91
121
152
Day of Year

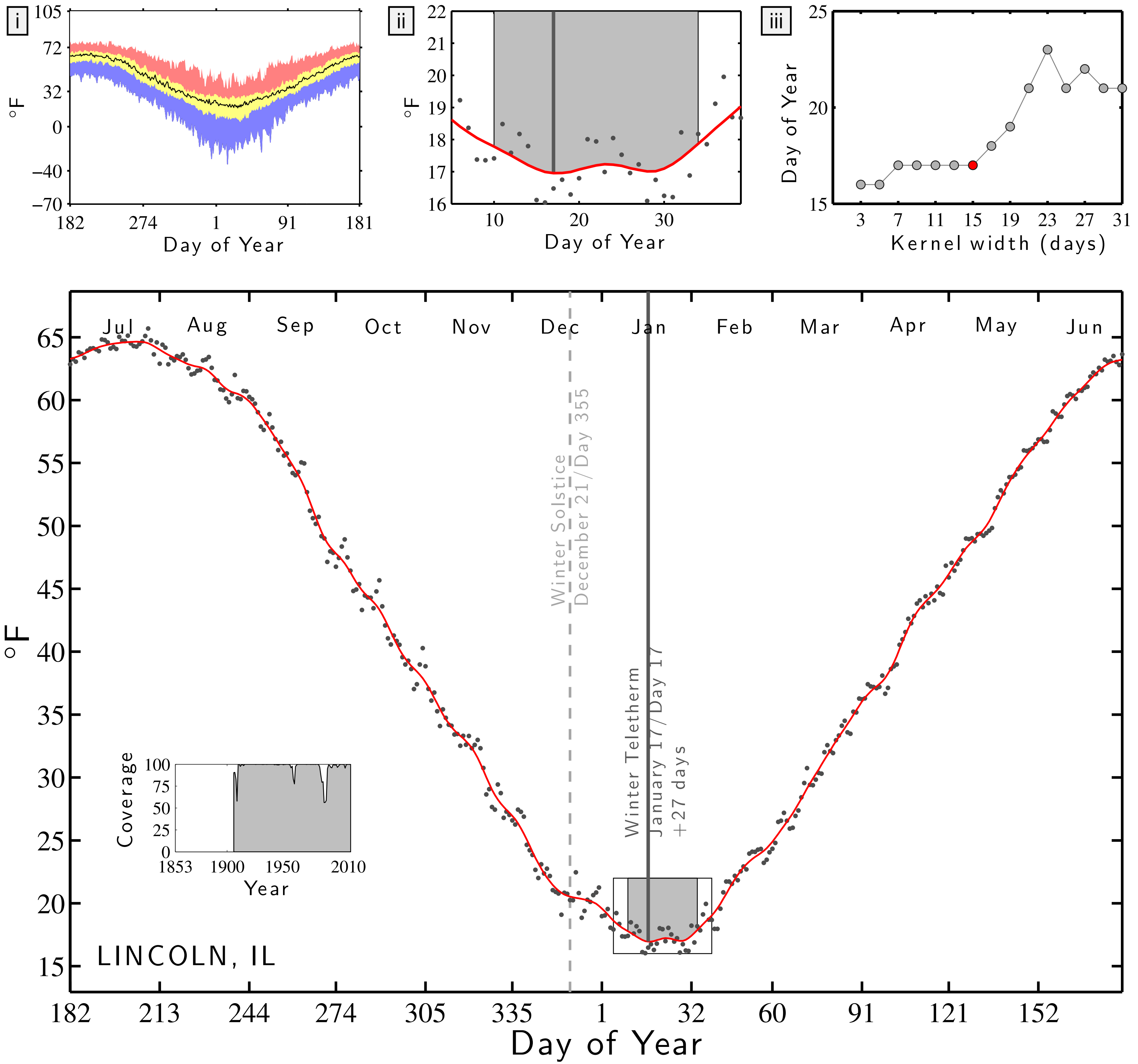

i
ii
iii
Day of Year
Kernel width (days)
Winter Solstice
December 21/Day 355
Winter Teletherm
January 17/Day 17
+27 days
Coverage
Year
LINCOLN, IL
Jul
Aug
Sep
Oct
Nov
Dec
Jan
Feb
Mar
Apr
May
Jun
°F

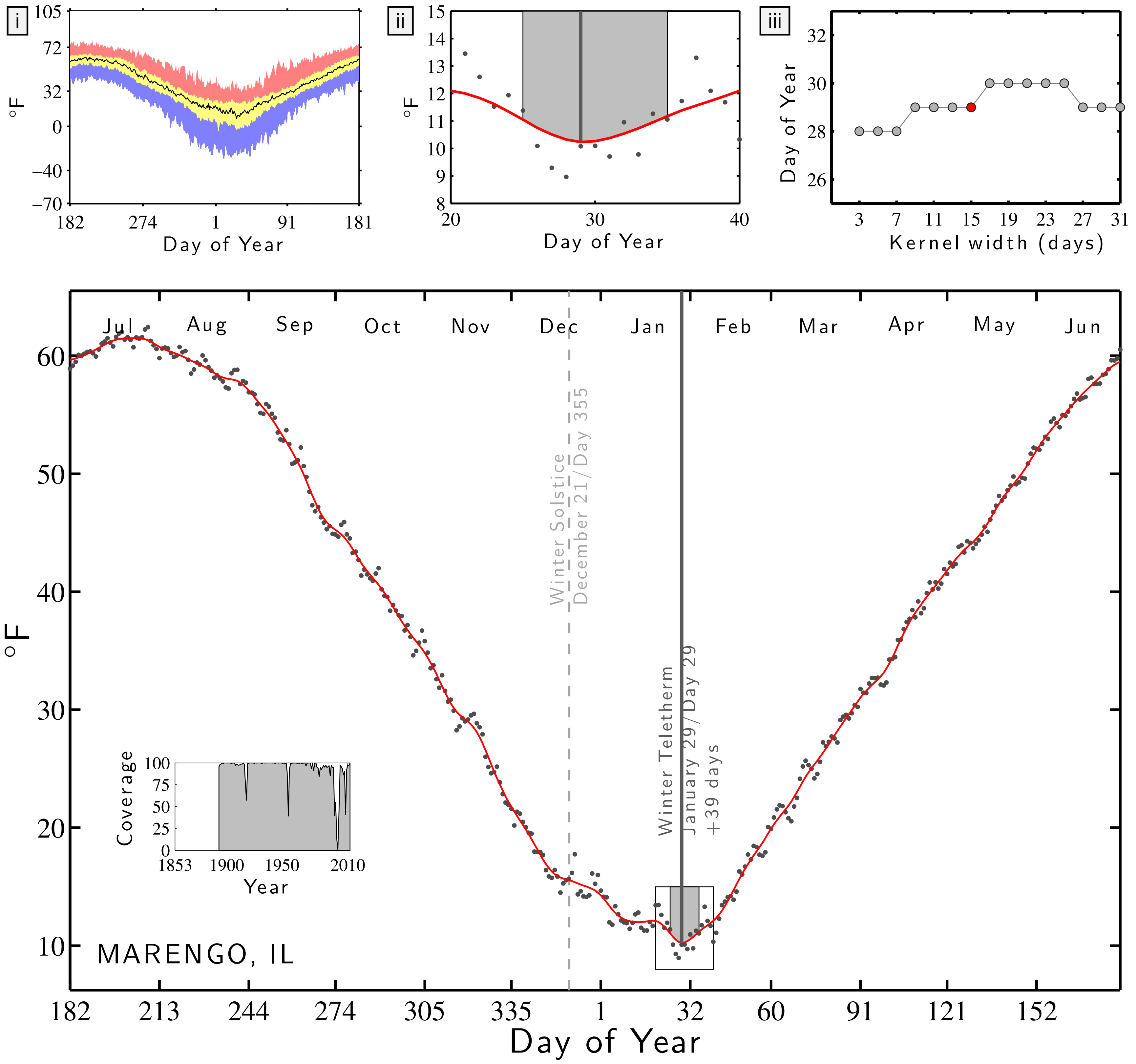

i
ii
iii
Day of Year
°F
Kernel width (days)
Jul
Aug
Sep
Oct
Nov
Dec
Jan
Feb
Mar
Apr
May
Jun
Winter Solstice
December 21/Day 355
Winter Teletherm
January 29/Day 29
+39 days
Coverage
Year
MARENGO, IL

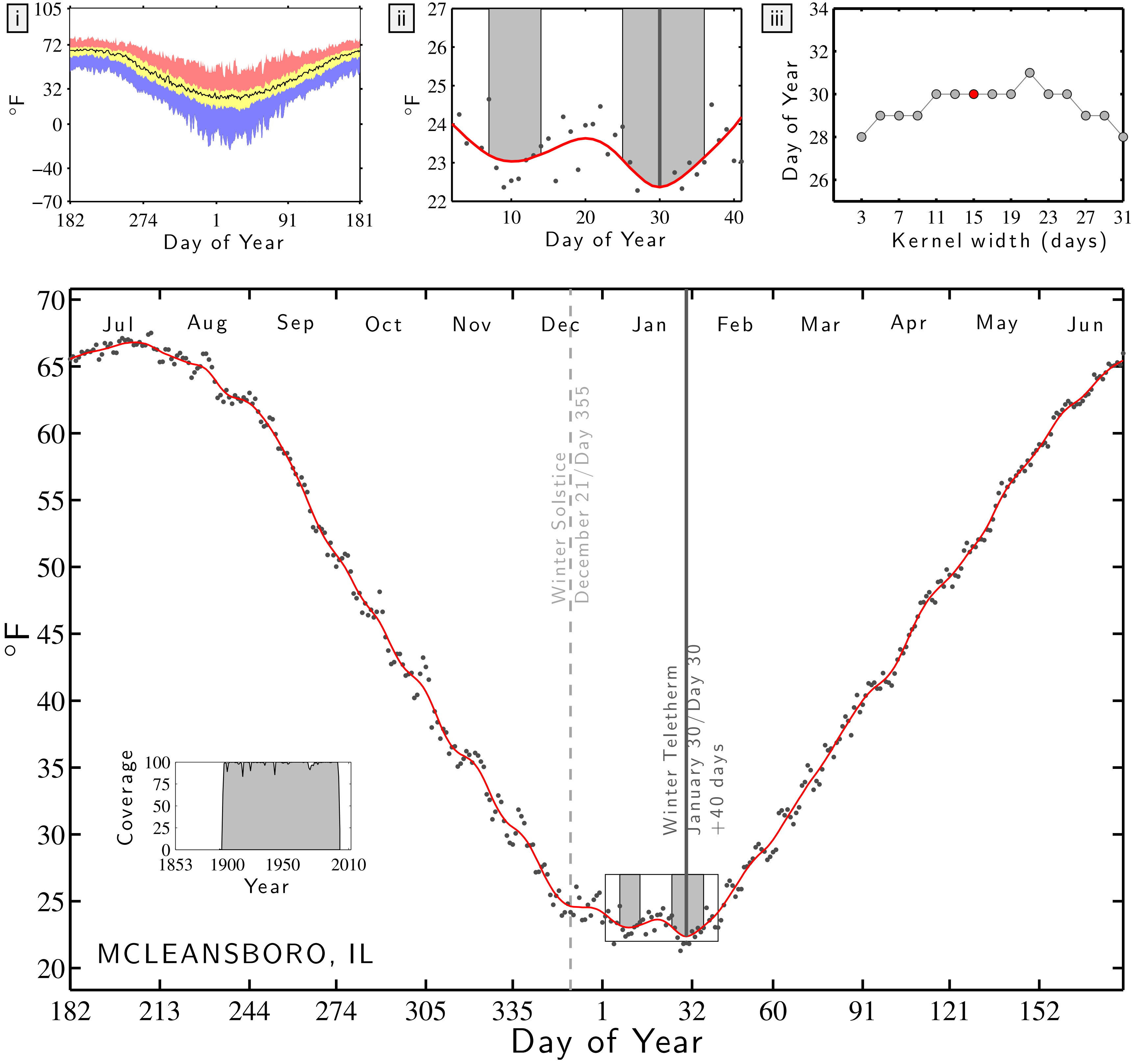

i
ii
iii
°F
Day of Year
Day of Year
Kernel width (days)
Jul
Aug
Sep
Oct
Nov
Dec
Jan
Feb
Mar
Apr
May
Jun
Winter Solstice
December 21/Day 355
Winter Teletherm
January 30/Day 30
+40 days
Coverage
Year
MCLEANSBORO, IL

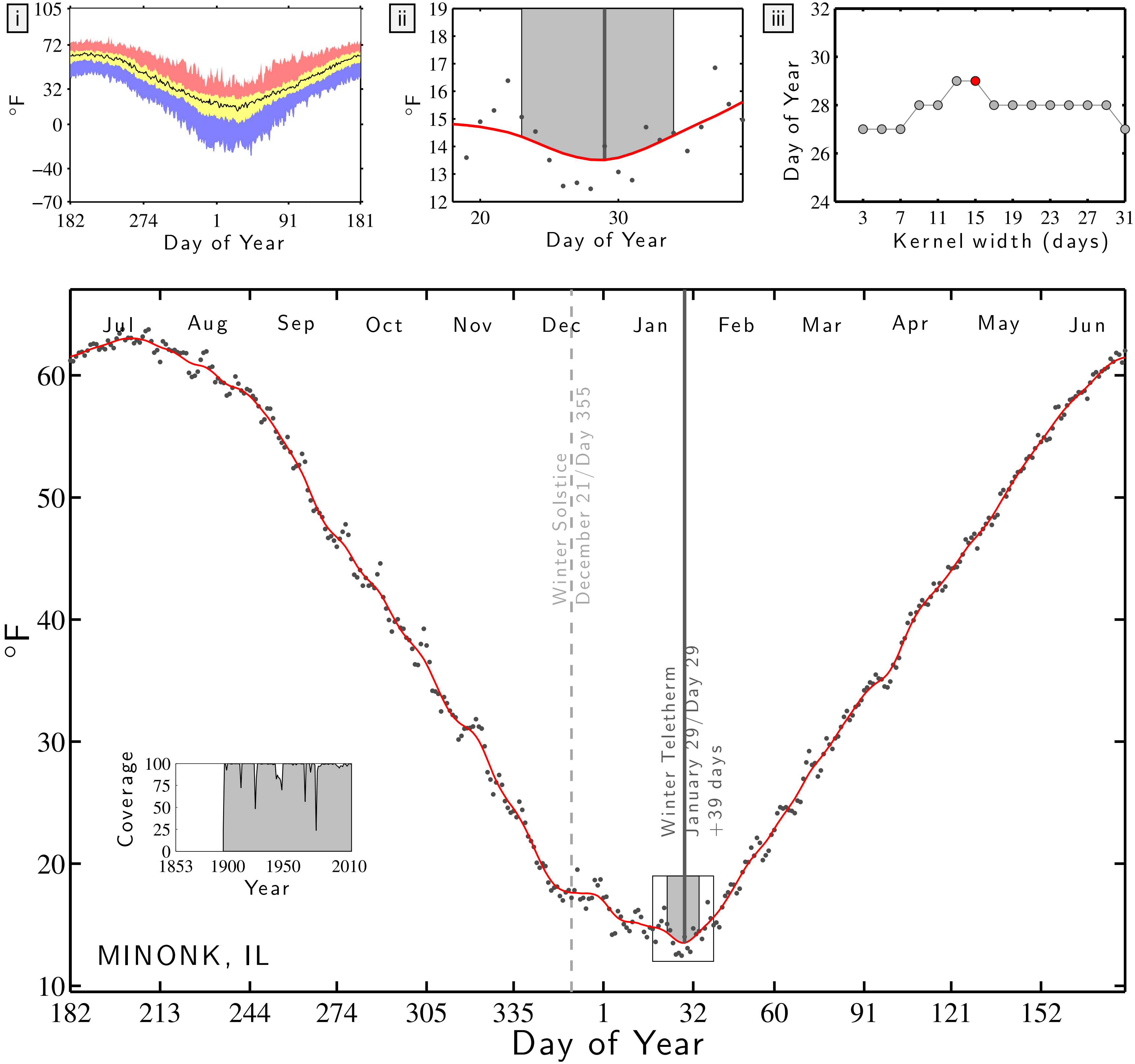

i
°F
Day of Year
ii
iii
Day of Year
Kernel width (days)
Jul
Aug
Sep
Oct
Nov
Dec
Jan
Feb
Mar
Apr
May
Jun
Winter Solstice
December 21/Day 355
Winter Teletherm
January 29/Day 29
+39 days
Coverage
Year
MINONK, IL

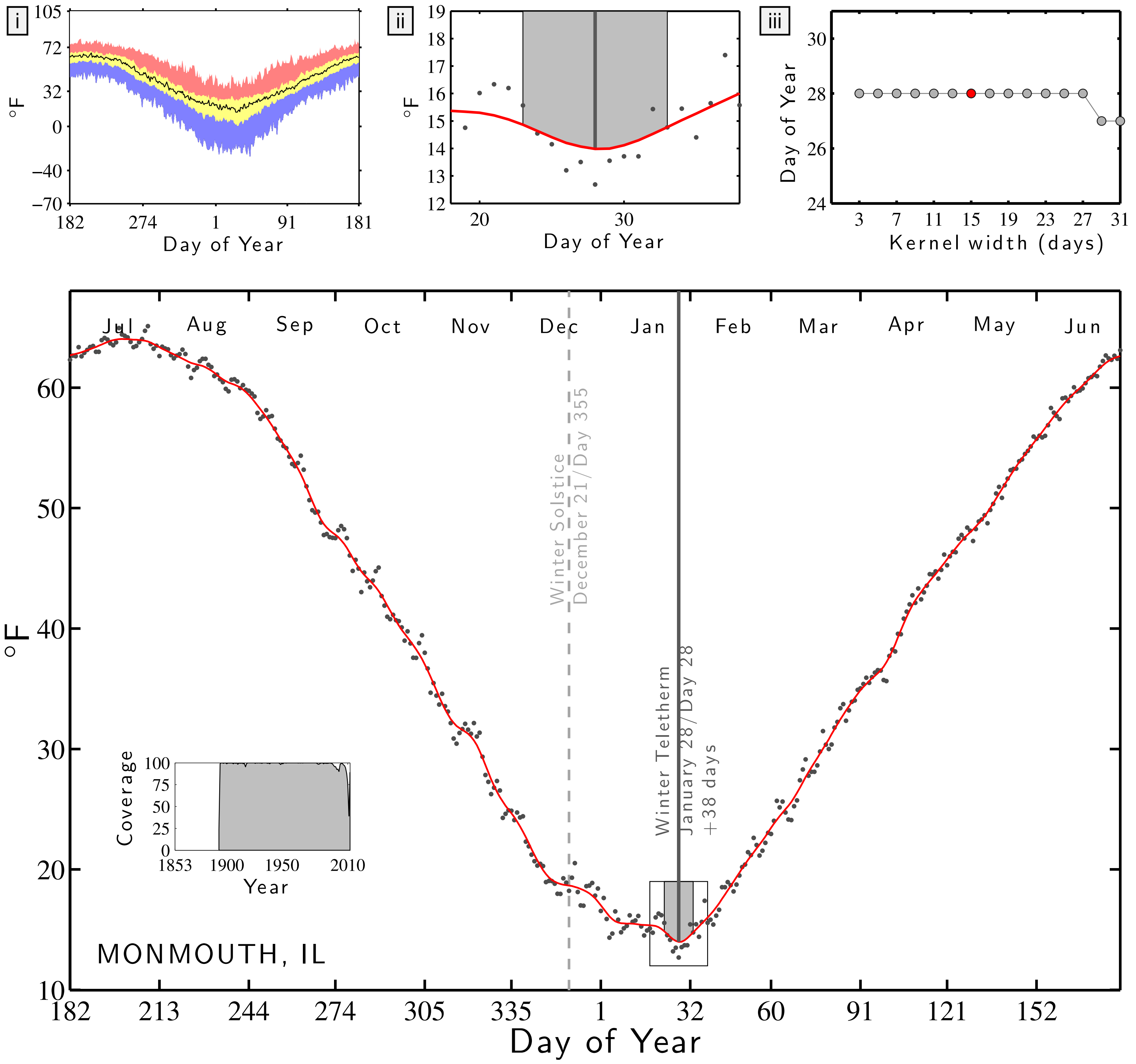

i
Day of Year
°F
ii
Day of Year
°F
iii
Kernel width (days)
Day of Year
Jul
Aug
Sep
Oct
Nov
Dec
Jan
Feb
Mar
Apr
May
Jun
Winter Solstice
December 21/Day 355
Winter Teletherm
January 28/Day 28
+38 days
Coverage
Year
MONMOUTH, IL
Day of Year
°F

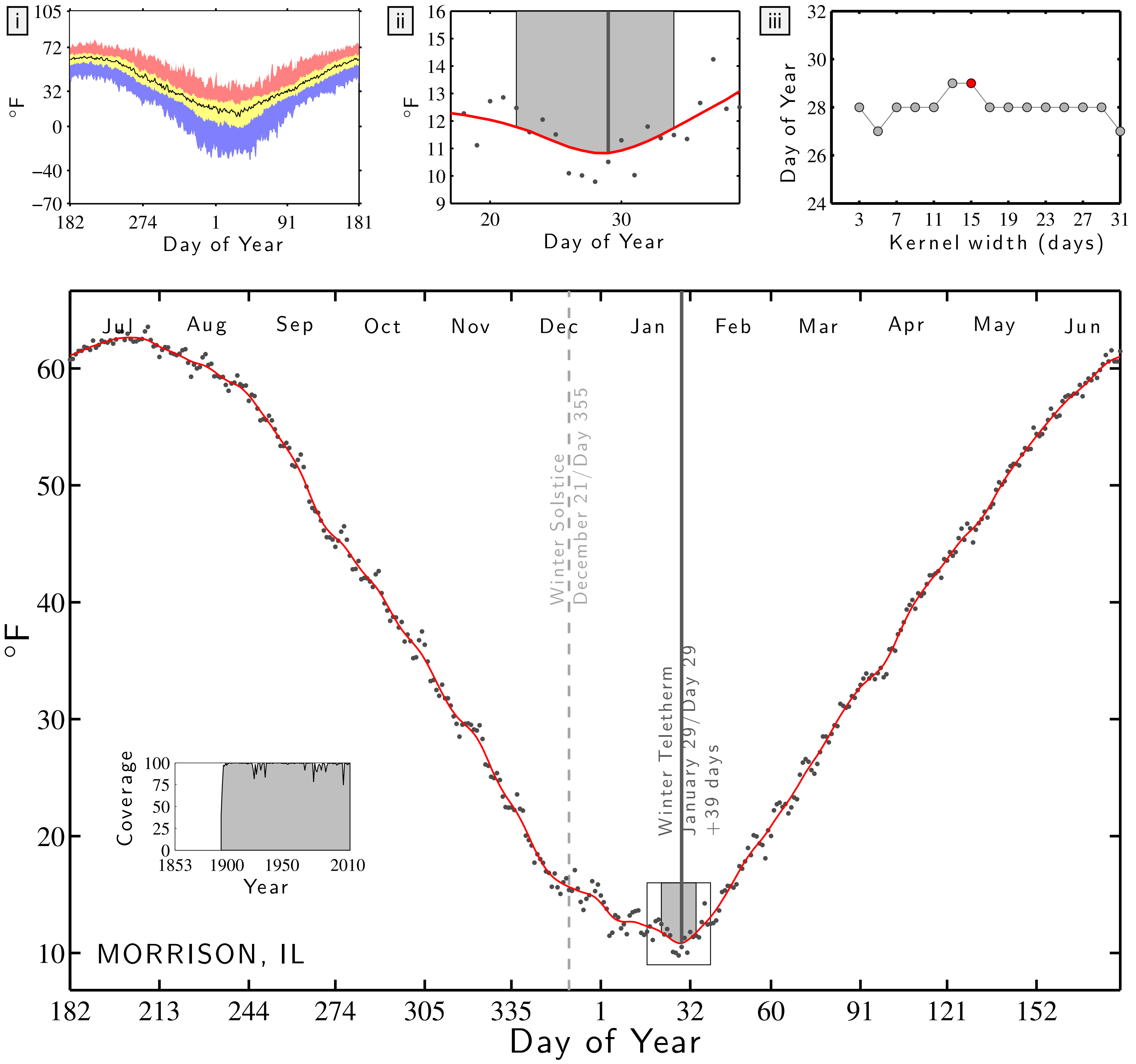

i
ii
iii
°F
Day of Year
Kernel width (days)
Jul
Aug
Sep
Oct
Nov
Dec
Jan
Feb
Mar
Apr
May
Jun
Winter Solstice
December 21/Day 355
Winter Teletherm
January 29/Day 29
+39 days
Coverage
Year
MORRISON, IL

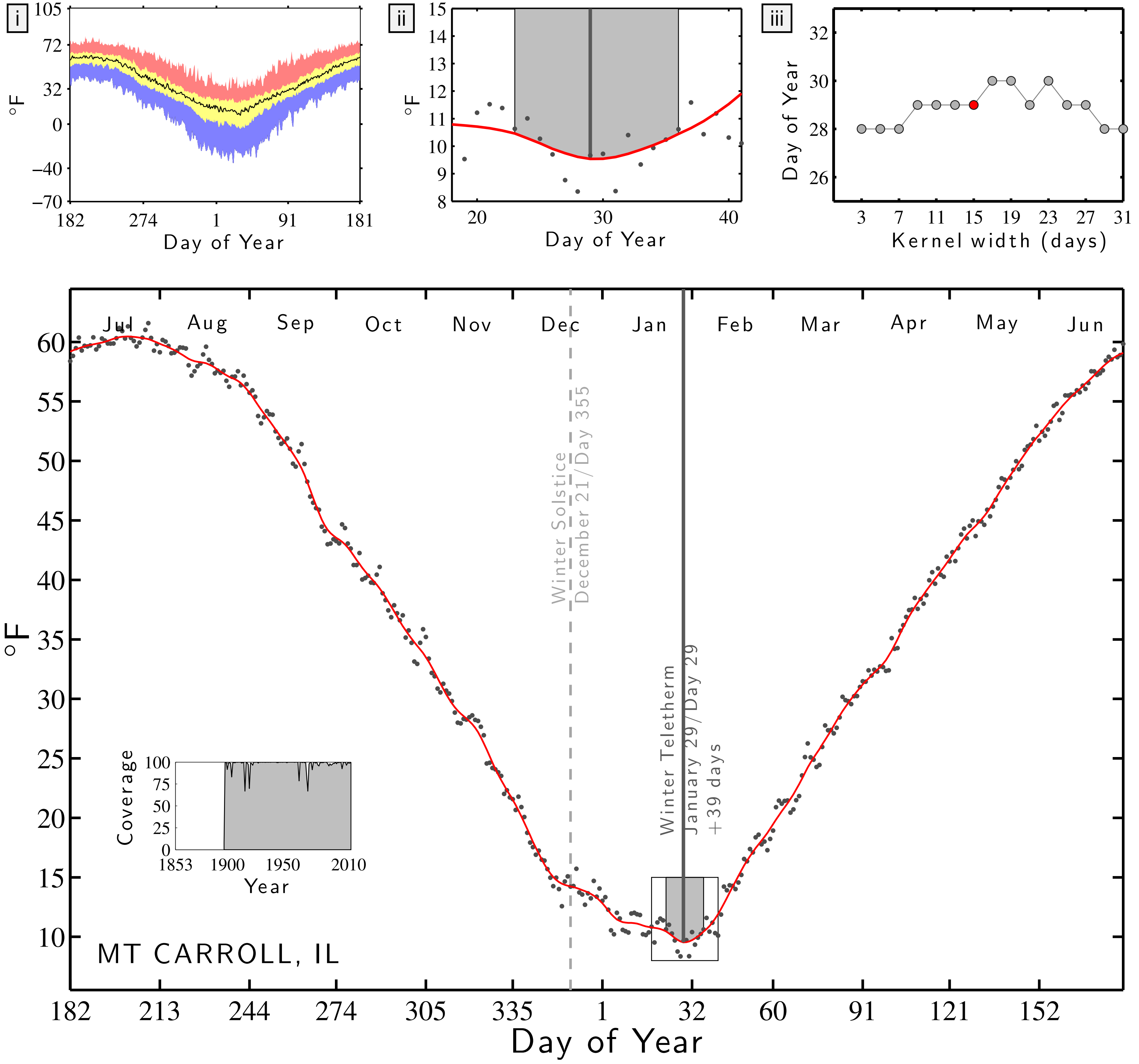

i
ii
iii
°F
Day of Year
Kernel width (days)
Jul
Aug
Sep
Oct
Nov
Dec
Jan
Feb
Mar
Apr
May
Jun
Winter Solstice
December 21/Day 355
Winter Teletherm
January 29/Day 29
+39 days
Coverage
Year
MT CARROLL, IL

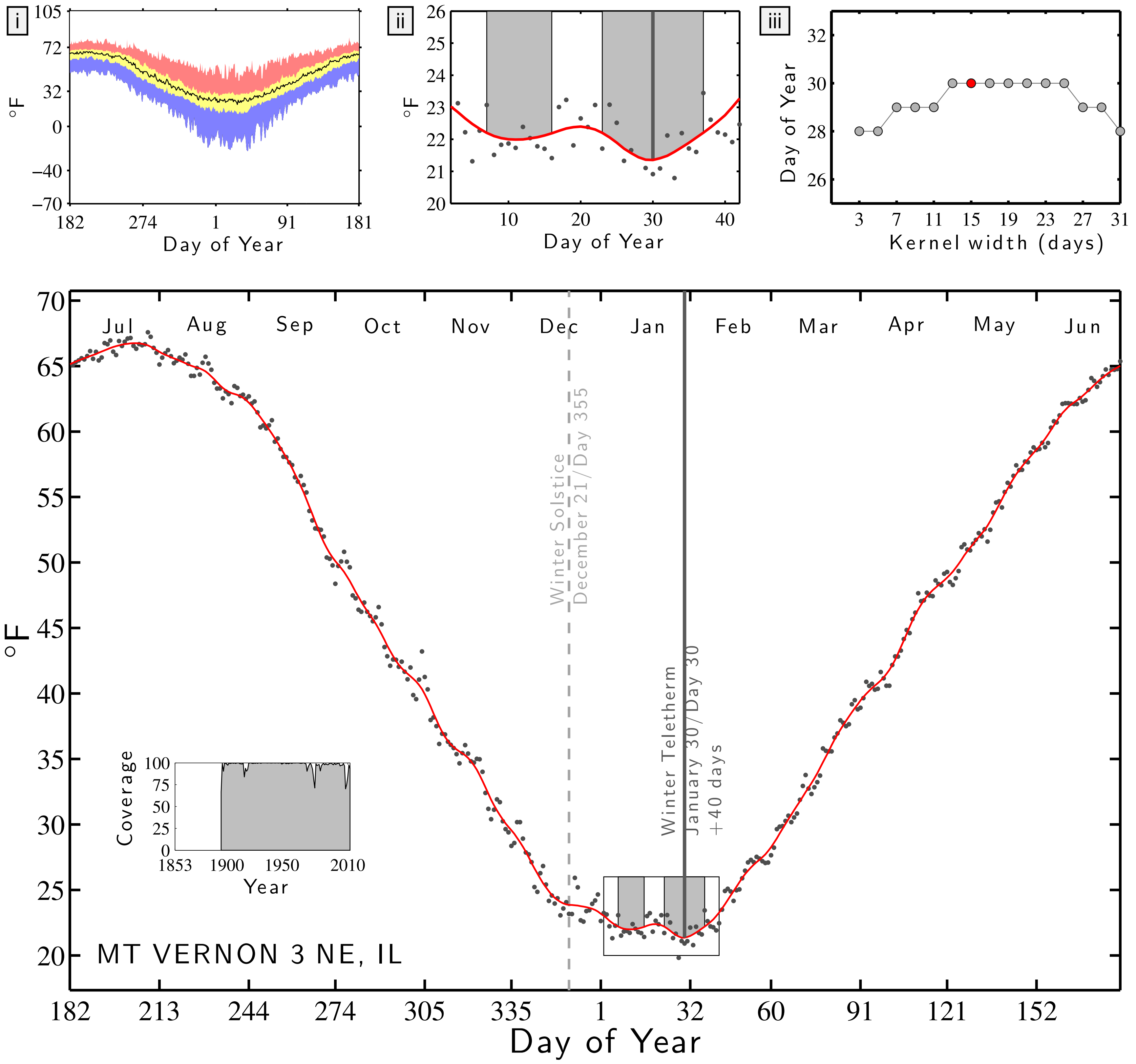

i
°F
Day of Year
ii
°F
Day of Year
iii
Day of Year
Kernel width (days)
Jul
Aug
Sep
Oct
Nov
Dec
Jan
Feb
Mar
Apr
May
Jun
Winter Solstice
December 21/Day 355
Winter Teletherm
January 30/Day 30
+40 days
Coverage
Year
MT VERNON 3 NE, IL
°F
Day of Year

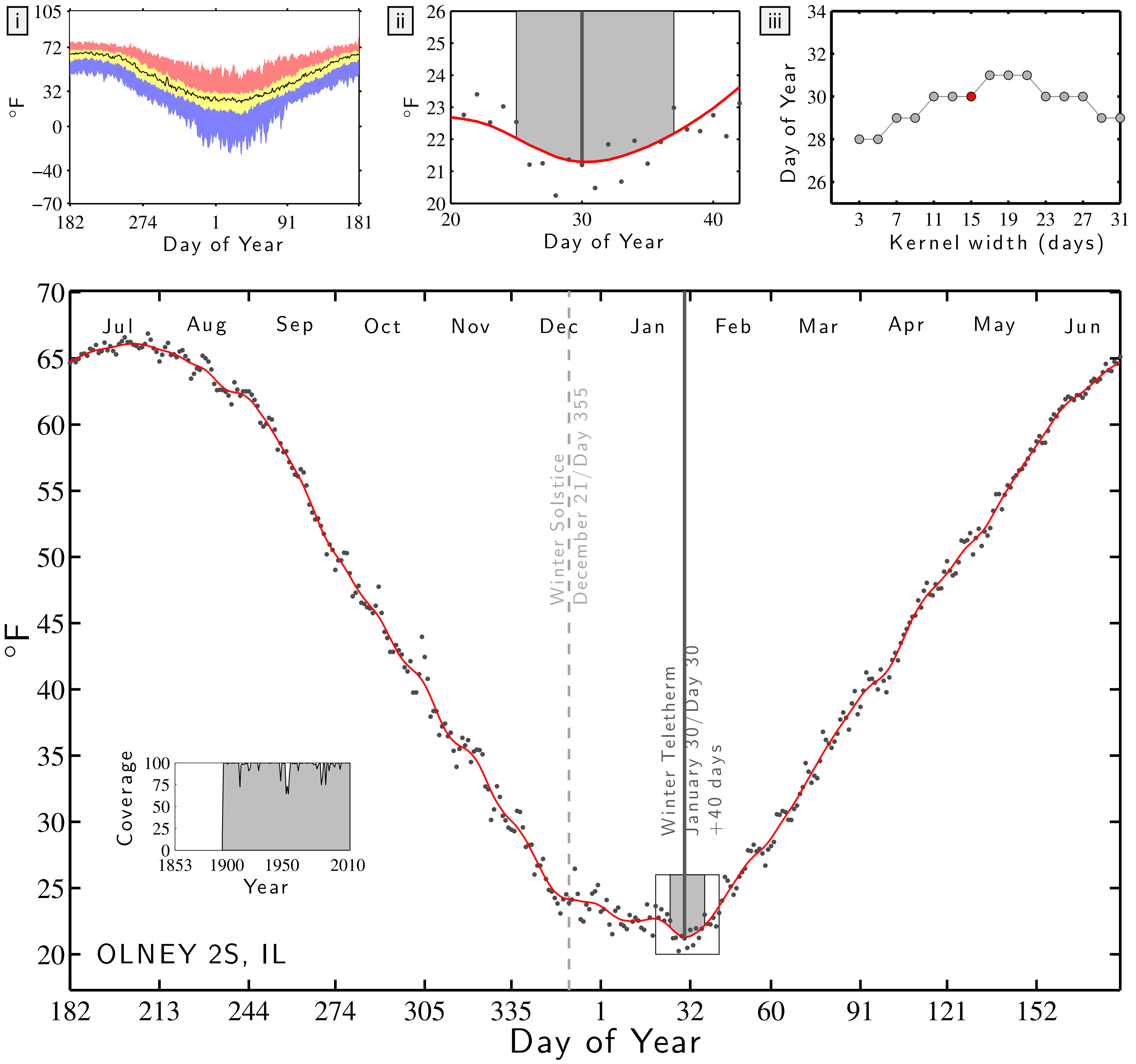

i
°F
Day of Year
ii
°F
Day of Year
iii
Day of Year
Kernel width (days)
Jul
Aug
Sep
Oct
Nov
Dec
Jan
Feb
Mar
Apr
May
Jun
Winter Solstice
December 21/Day 355
Winter Teletherm
January 30/Day 30
+40 days
Coverage
Year
OLNEY 2S, IL
°F
Day of Year

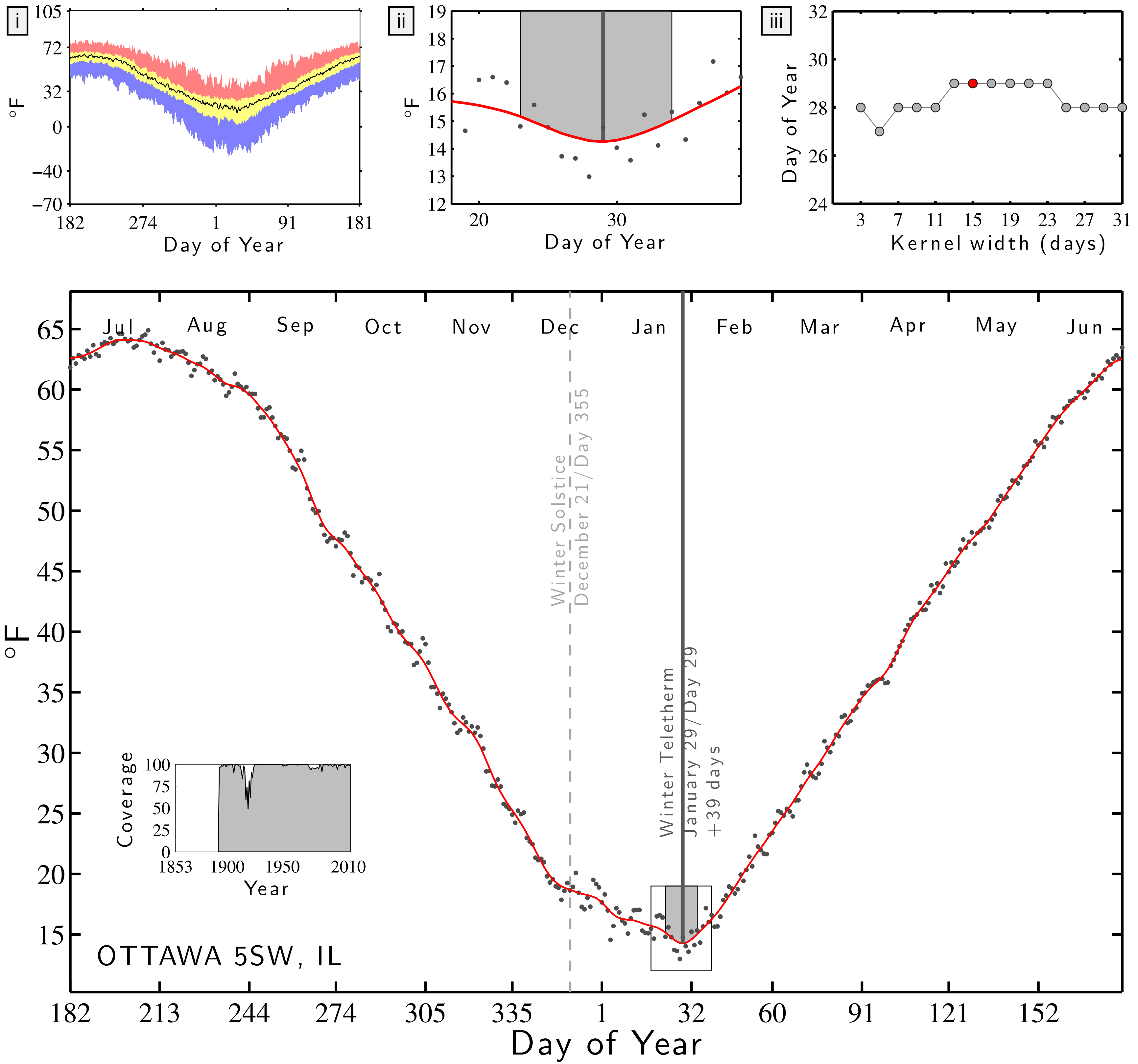

i
ii
iii
°F
Day of Year
Kernel width (days)
Jul
Aug
Sep
Oct
Nov
Dec
Jan
Feb
Mar
Apr
May
Jun
Winter Solstice
December 21/Day 355
Winter Teletherm
January 29/Day 29
+39 days
Coverage
Year
OTTAWA 5SW, IL

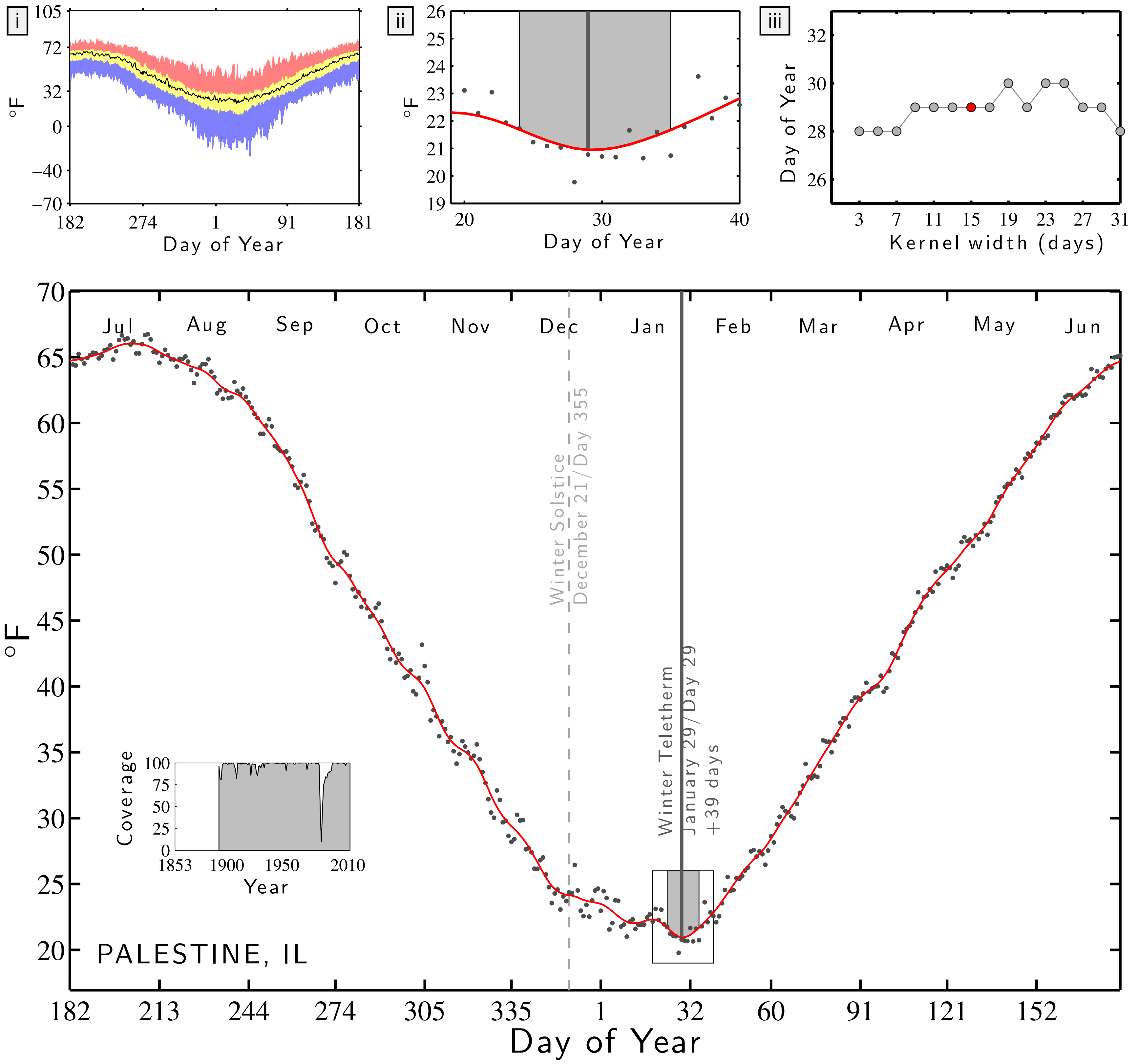

i
°F
Day of Year
ii
°F
Day of Year
iii
Day of Year
Kernel width (days)
Jul
Aug
Sep
Oct
Nov
Dec
Jan
Feb
Mar
Apr
May
Jun
Winter Solstice
December 21/Day 355
Winter Teletherm
January 29/Day 29
+39 days
Coverage
Year
PALESTINE, IL
°F
Day of Year

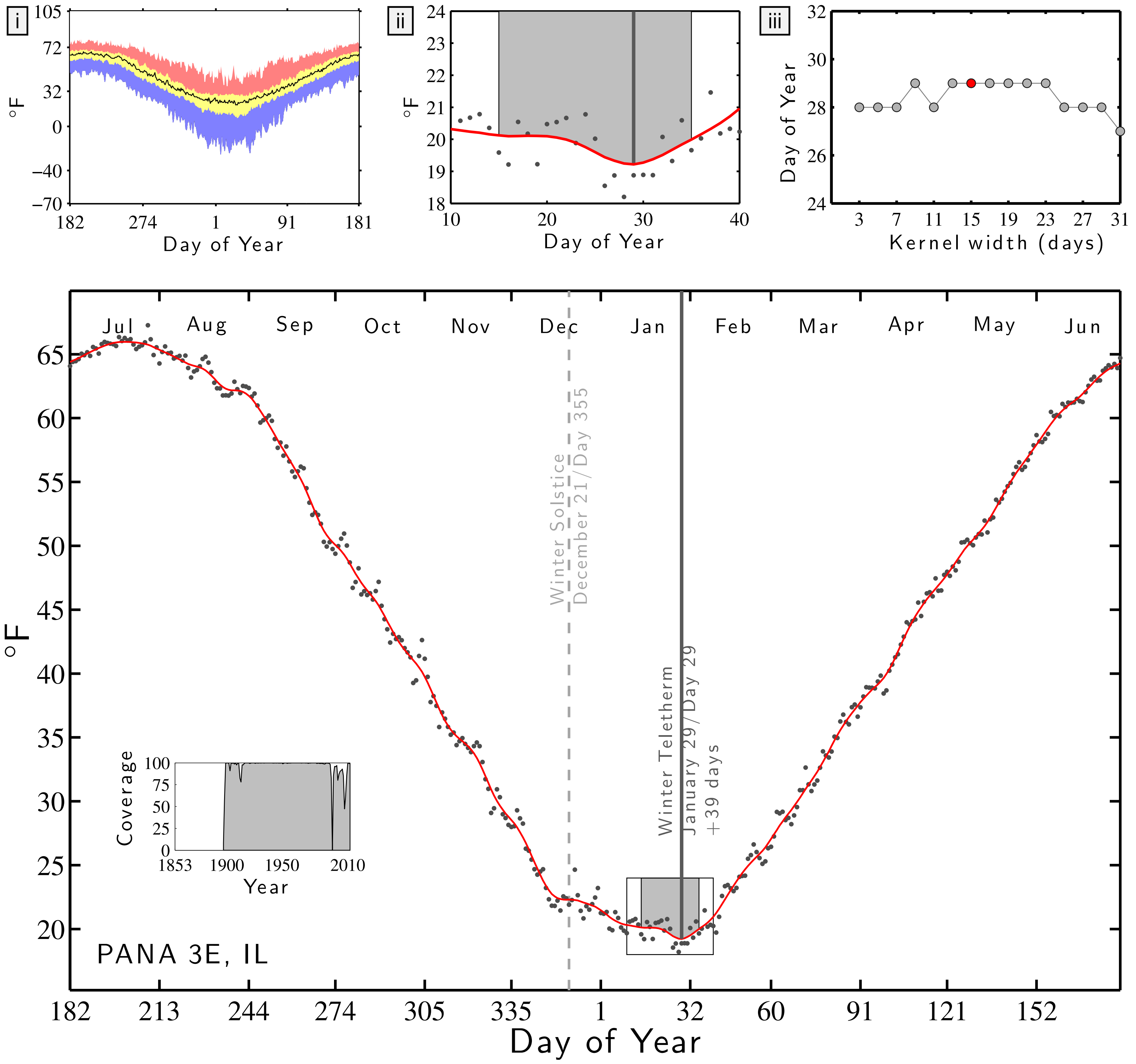

i
°F
Day of Year
ii
°F
Day of Year
iii
Day of Year
Kernel width (days)
Jul
Aug
Sep
Oct
Nov
Dec
Jan
Feb
Mar
Apr
May
Jun
Winter Solstice
December 21/Day 355
Winter Teletherm
January 29/Day 29
+39 days
Coverage
Year
PANA 3E, IL
°F
Day of Year

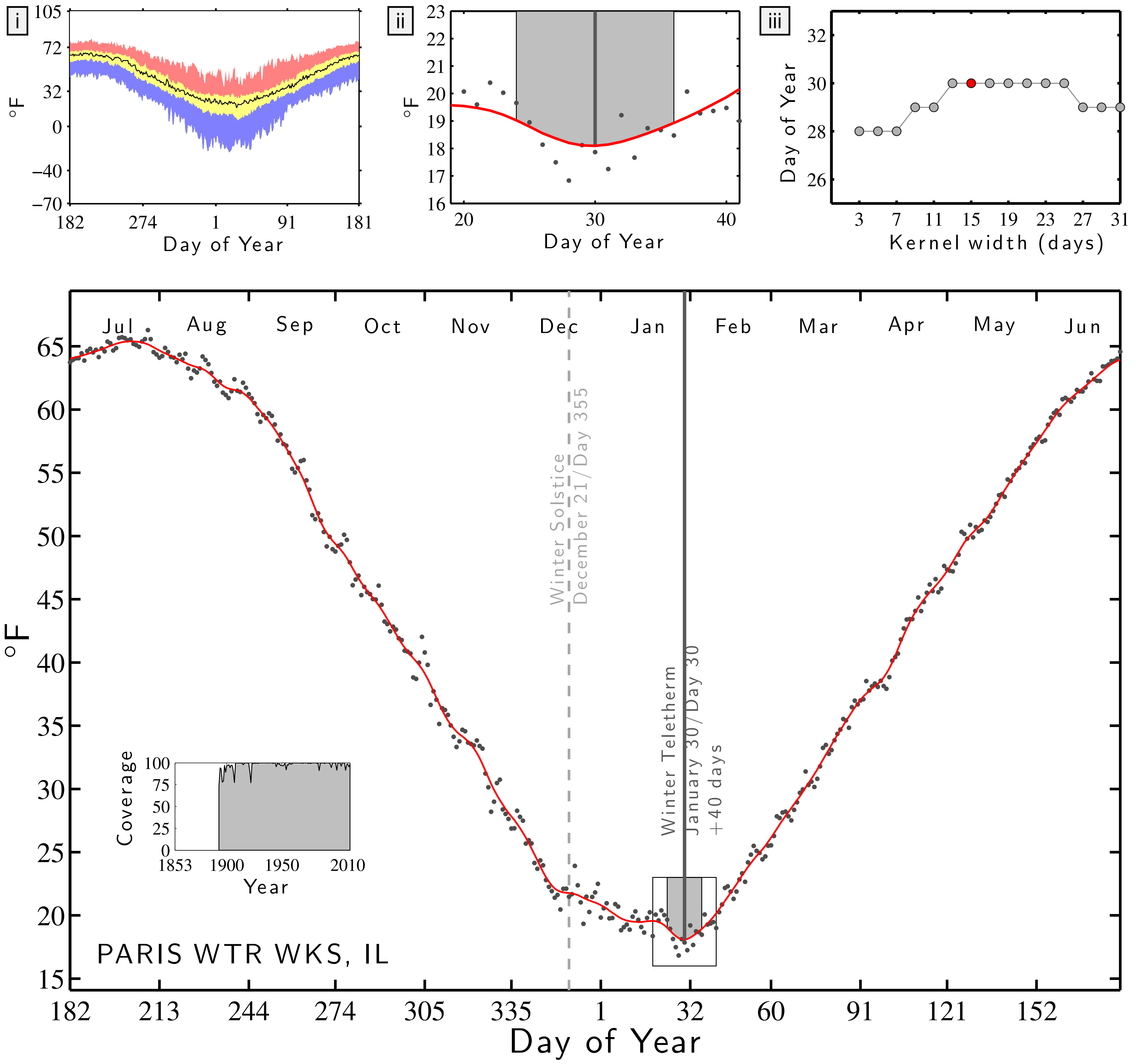

i
°F
Day of Year
ii
°F
Day of Year
iii
Day of Year
Kernel width (days)
Jul
Aug
Sep
Oct
Nov
Dec
Jan
Feb
Mar
Apr
May
Jun
Winter Solstice
December 21/Day 355
Winter Teletherm
January 30/Day 30
+40 days
Coverage
Year
PARIS WTR WKS, IL
°F
Day of Year

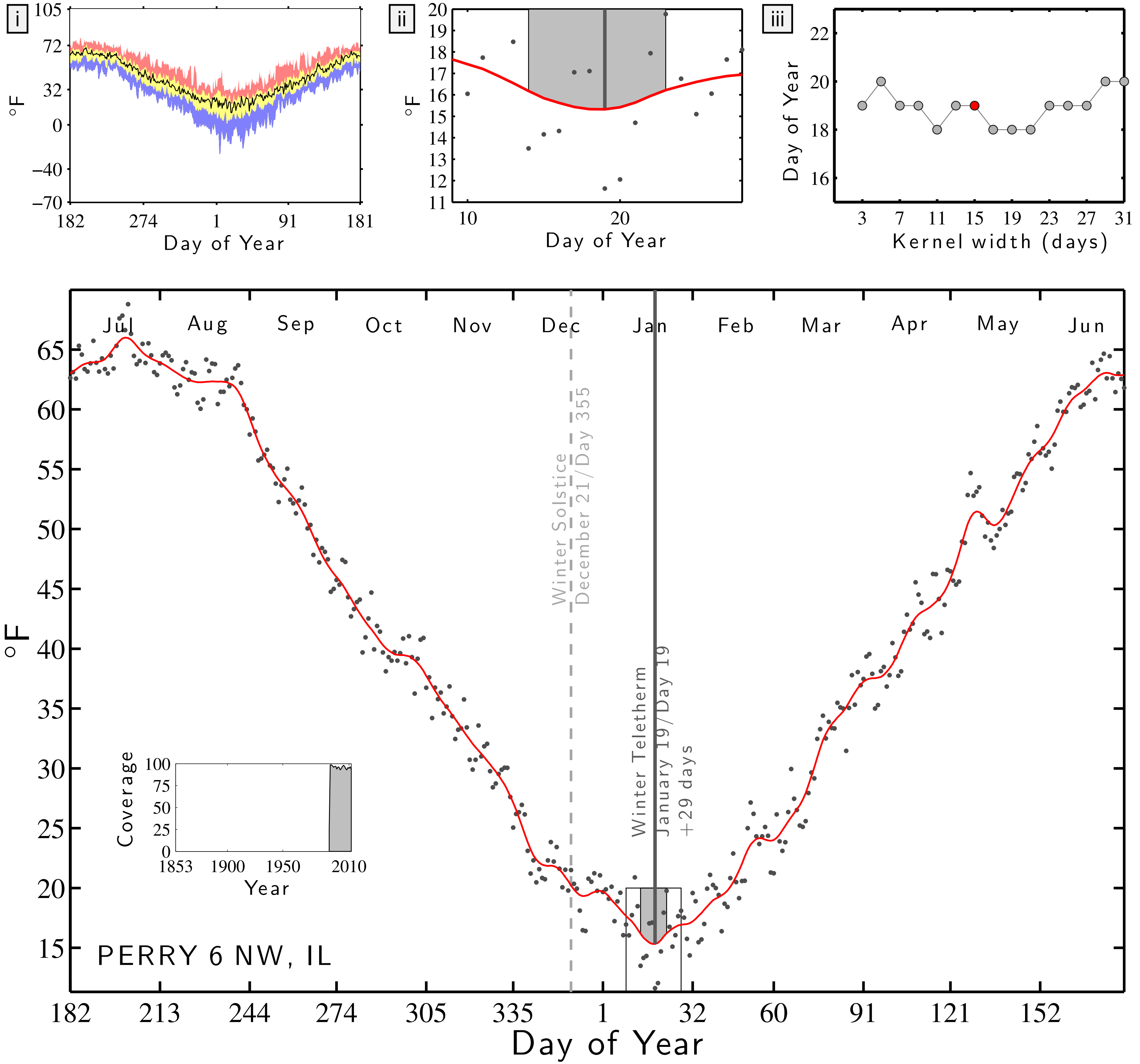

i
°F
Day of Year
ii
°F
Day of Year
iii
Day of Year
Kernel width (days)
Jul
Aug
Sep
Oct
Nov
Dec
Jan
Feb
Mar
Apr
May
Jun
Winter Solstice
December 21/Day 355
Winter Teletherm
January 19/Day 19
+29 days
Coverage
Year
PERRY 6 NW, IL
°F
Day of Year

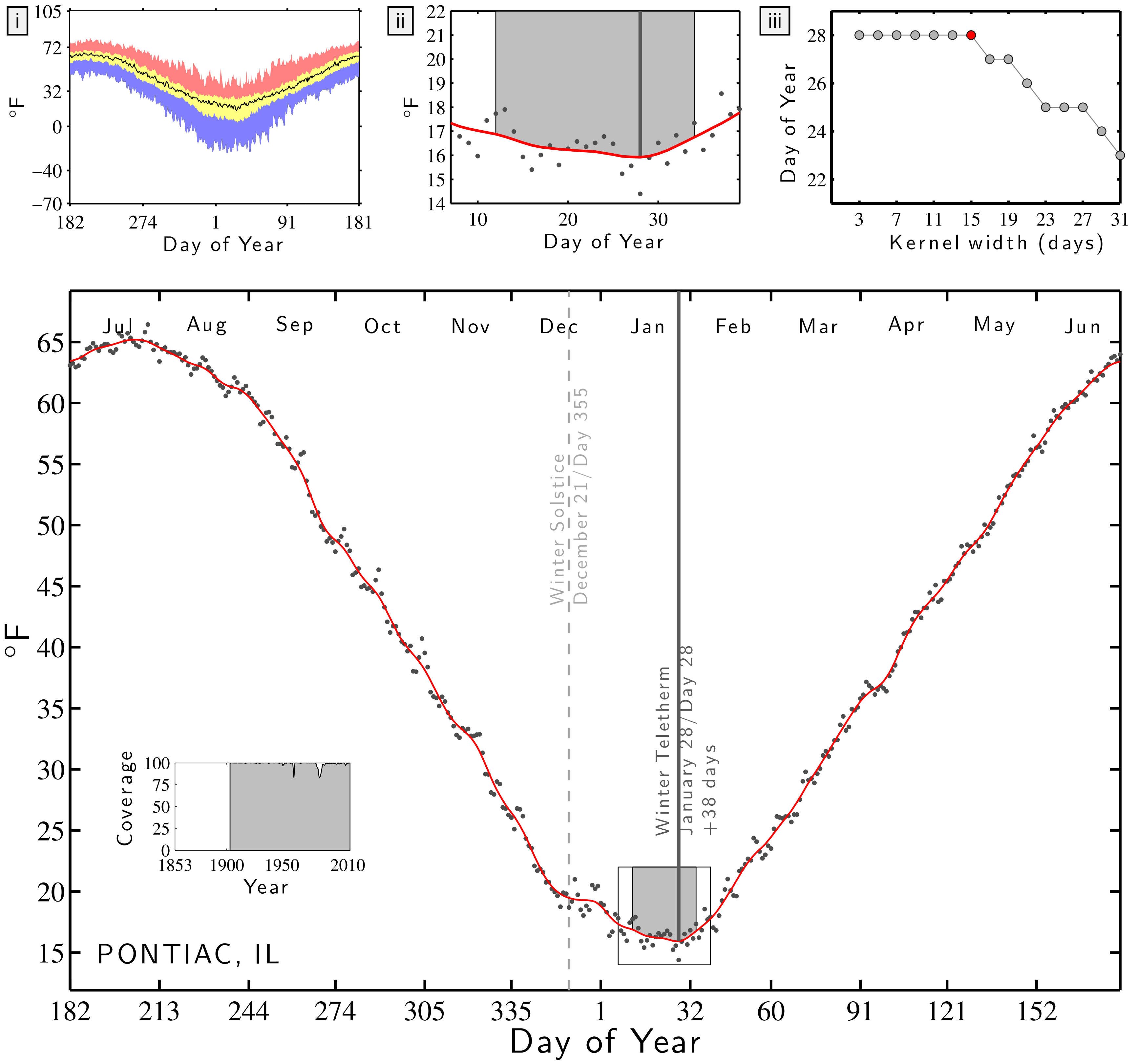

i
°F
Day of Year
ii
°F
Day of Year
iii
Day of Year
Kernel width (days)
Jul
Aug
Sep
Oct
Nov
Dec
Jan
Feb
Mar
Apr
May
Jun
Winter Solstice
December 21/Day 355
Winter Teletherm
January 28/Day 28
+38 days
Coverage
Year
PONTIAC, IL
°F
Day of Year

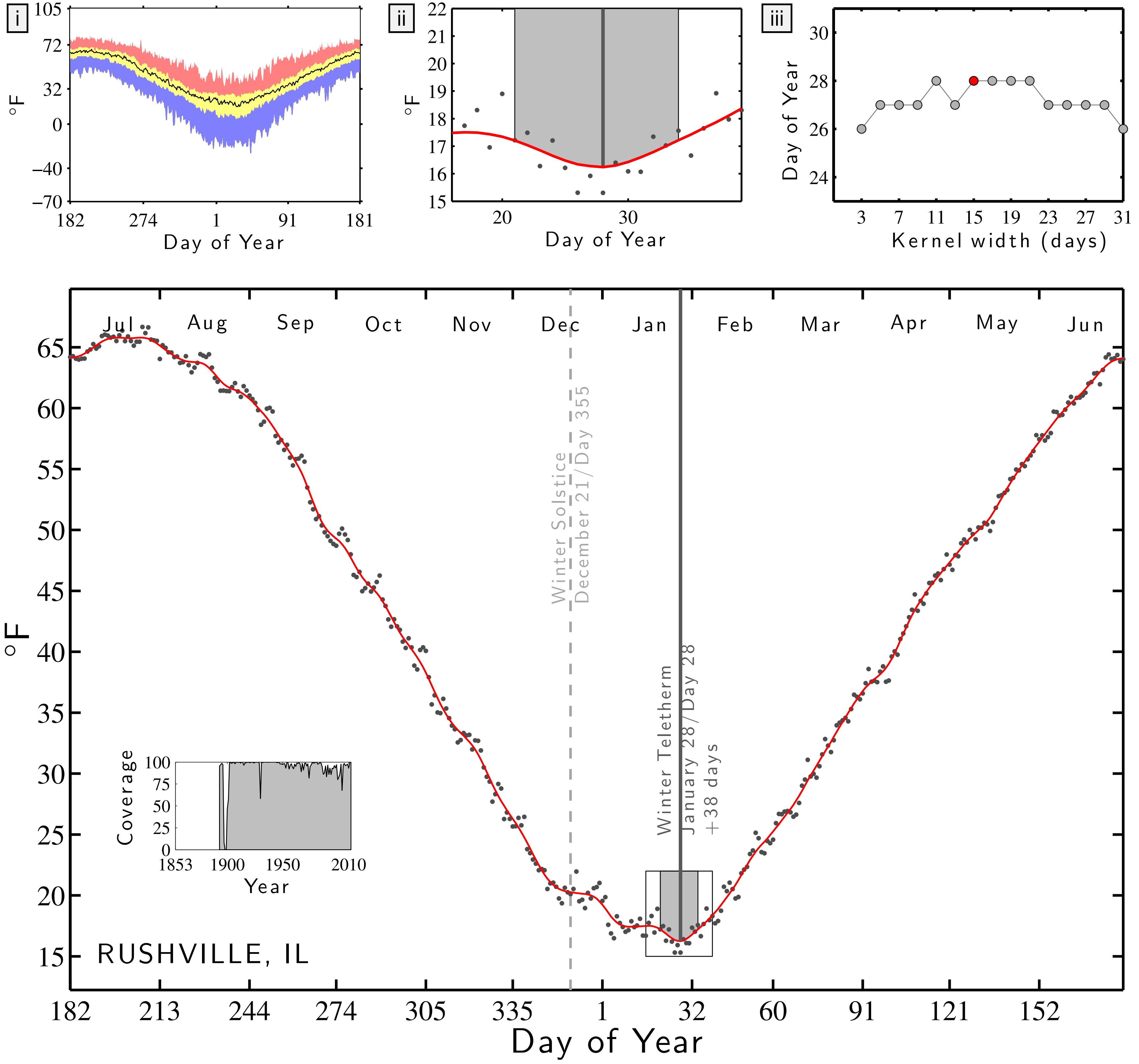

i
°F
Day of Year
ii
°F
Day of Year
iii
Day of Year
Kernel width (days)
Jul
Aug
Sep
Oct
Nov
Dec
Jan
Feb
Mar
Apr
May
Jun
Winter Solstice
December 21/Day 355
Winter Teletherm
January 28/Day 28
+38 days
Coverage
Year
RUSHVILLE, IL
°F
Day of Year

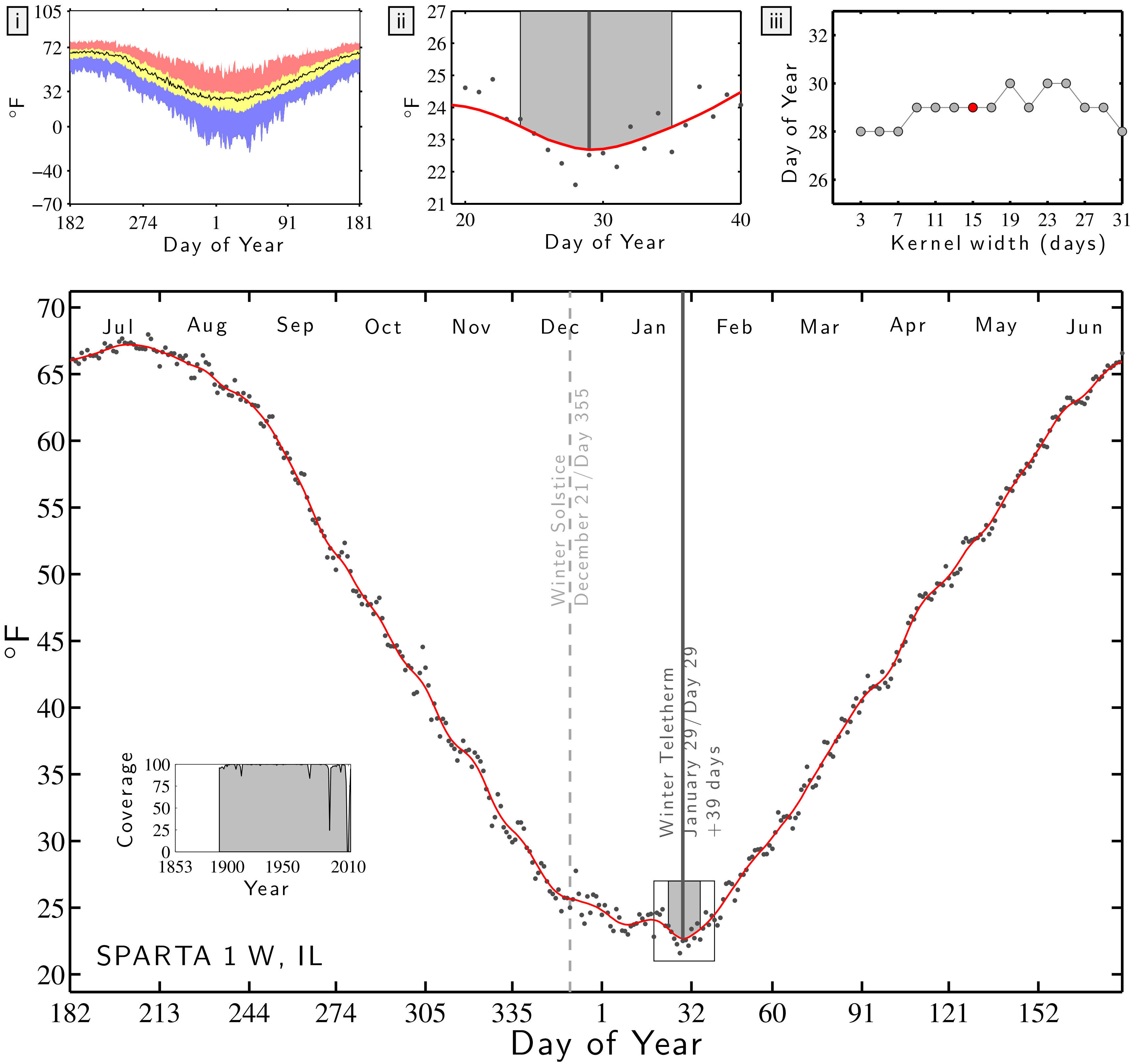

i
105
72
32
0
−40
−70
°F
182
274
1
91
181
Day of Year
ii
27
26
25
24
23
22
21
°F
20
30
40
Day of Year
iii
32
30
28
26
Day of Year
3
7
11
15
19
23
27
31
Kernel width (days)
Jul
Aug
Sep
Oct
Nov
Dec
Jan
Feb
Mar
Apr
May
Jun
70
65
60
55
50
45
40
35
30
25
20
°F
Winter Solstice
December 21/Day 355
Winter Teletherm
January 29/Day 29
+39 days
Coverage
100
75
50
25
0
1853
1900
1950
2010
Year
SPARTA 1 W, IL
182
213
244
274
305
335
1
32
60
91
121
152
Day of Year

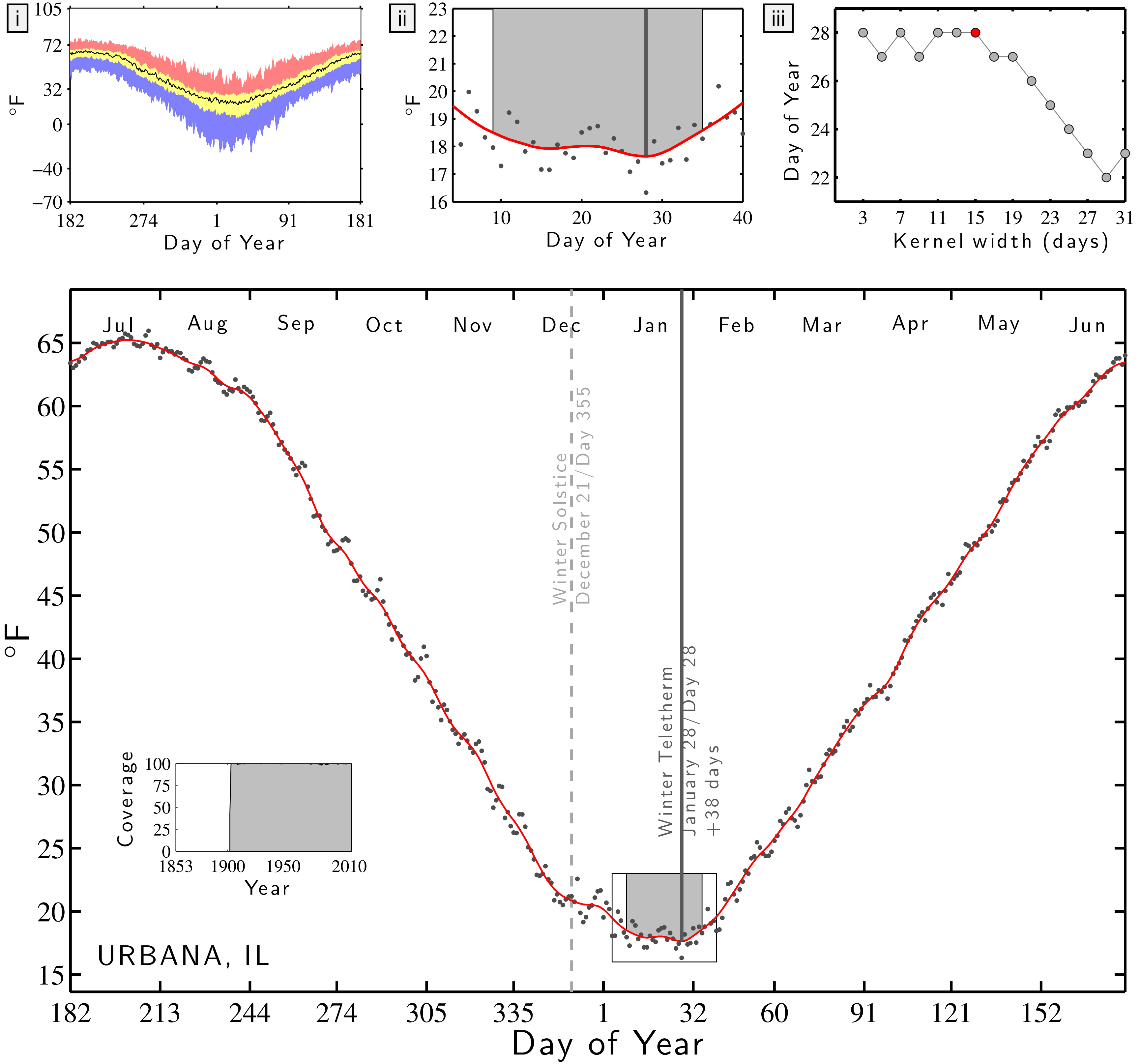

i
°F
Day of Year
ii
°F
Day of Year
iii
Day of Year
Kernel width (days)
Jul
Aug
Sep
Oct
Nov
Dec
Jan
Feb
Mar
Apr
May
Jun
Winter Solstice
December 21/Day 355
Winter Teletherm
January 28/Day 28
+38 days
Coverage
Year
URBANA, IL
°F
Day of Year

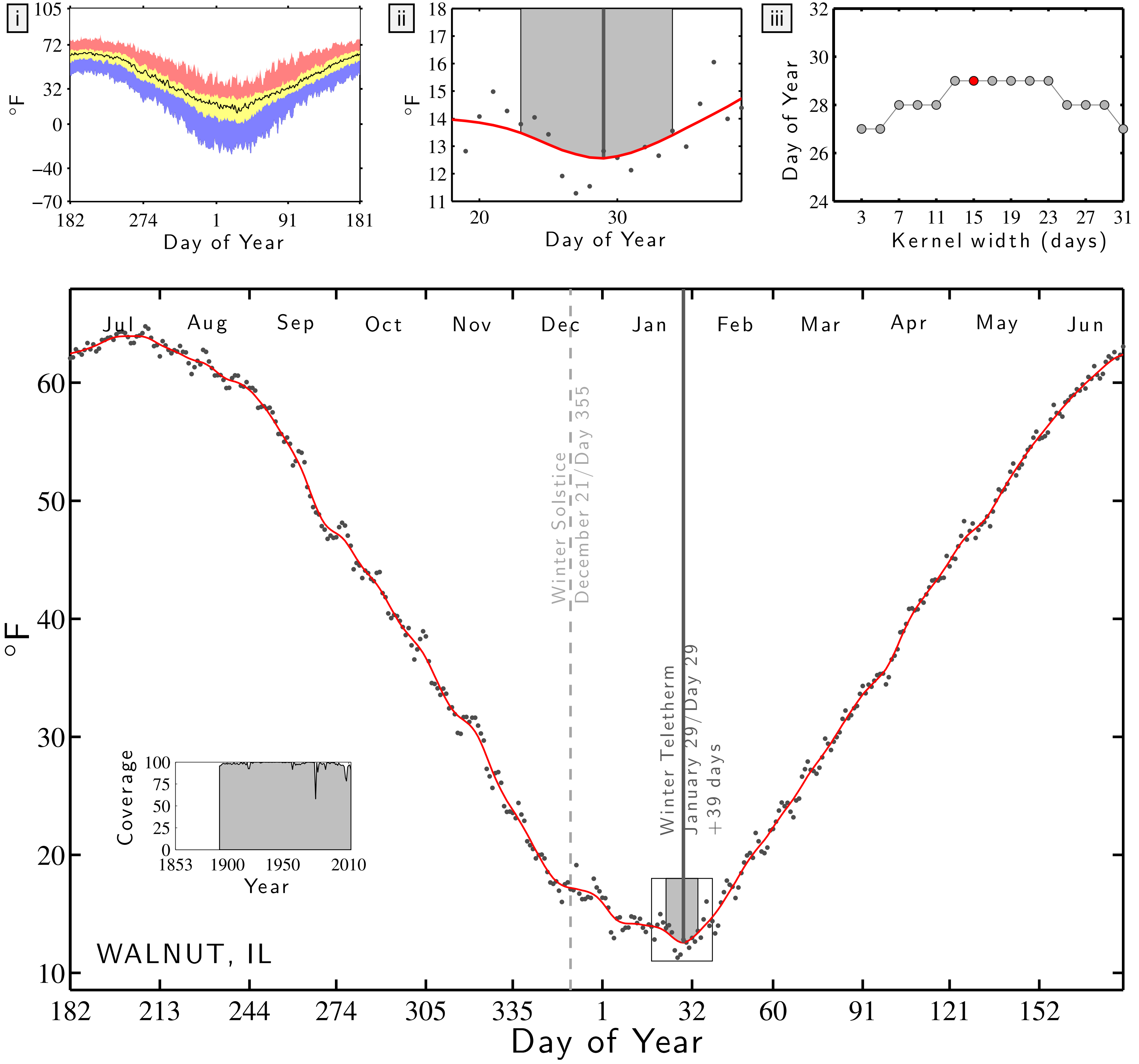

i
°F
Day of Year
ii
°F
Day of Year
iii
Day of Year
Kernel width (days)
Jul
Aug
Sep
Oct
Nov
Dec
Jan
Feb
Mar
Apr
May
Jun
Winter Solstice
December 21/Day 355
Winter Teletherm
January 29/Day 29
+39 days
Coverage
Year
WALNUT, IL
°F
Day of Year

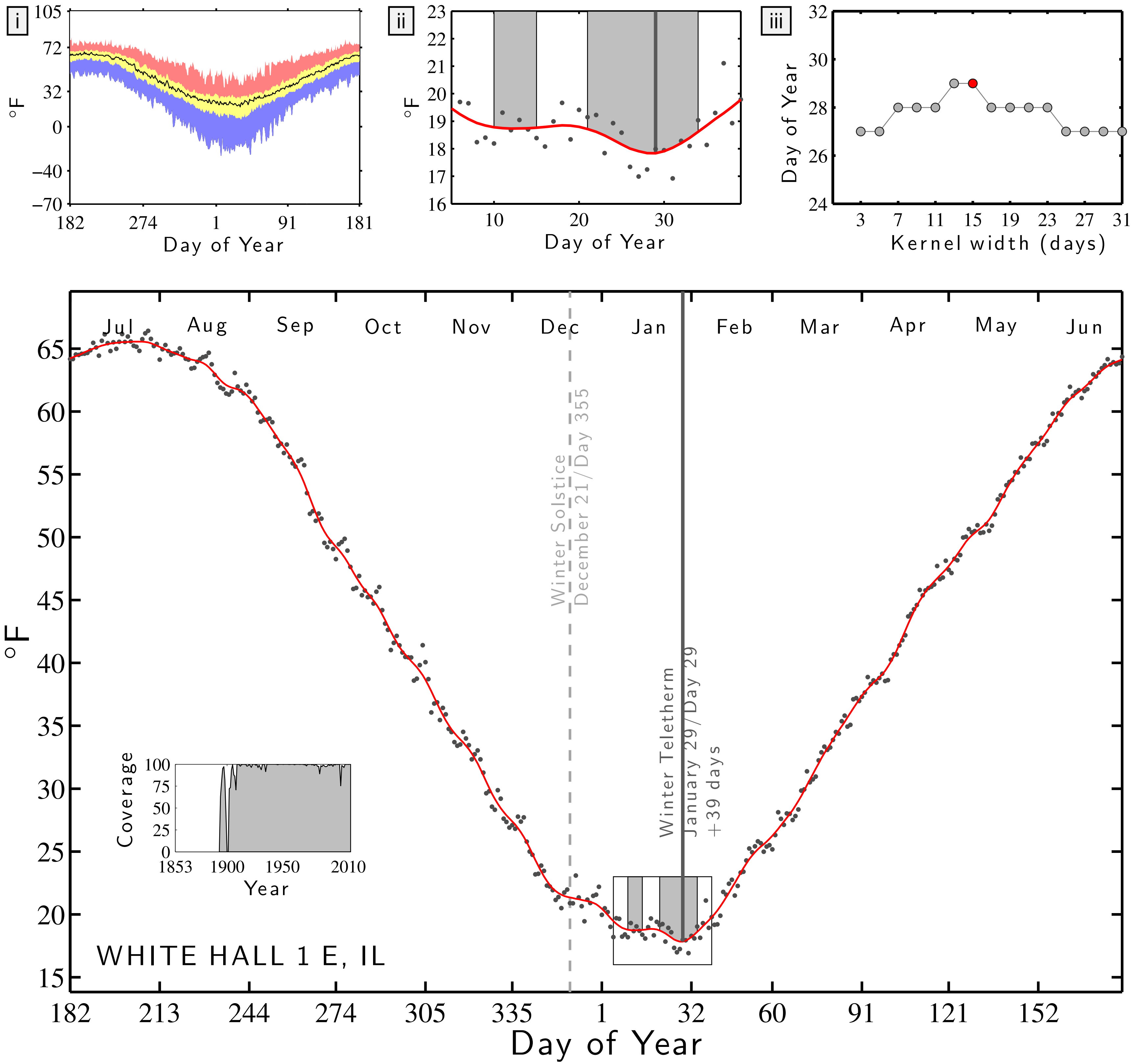

i
105
72
32
0
−40
−70
°F
182
274
1
91
181
Day of Year
ii
23
22
21
20
19
18
17
16
°F
10
20
30
Day of Year
iii
32
30
28
26
24
Day of Year
3
7
11
15
19
23
27
31
Kernel width (days)
Jul
Aug
Sep
Oct
Nov
Dec
Jan
Feb
Mar
Apr
May
Jun
65
60
55
50
45
40
35
30
25
20
15
°F
Winter Solstice
December 21/Day 355
Winter Teletherm
January 29/Day 29
+39 days
Coverage
100
75
50
25
0
1853
1900
1950
2010
Year
WHITE HALL 1 E, IL
182
213
244
274
305
335
1
32
60
91
121
152
Day of Year

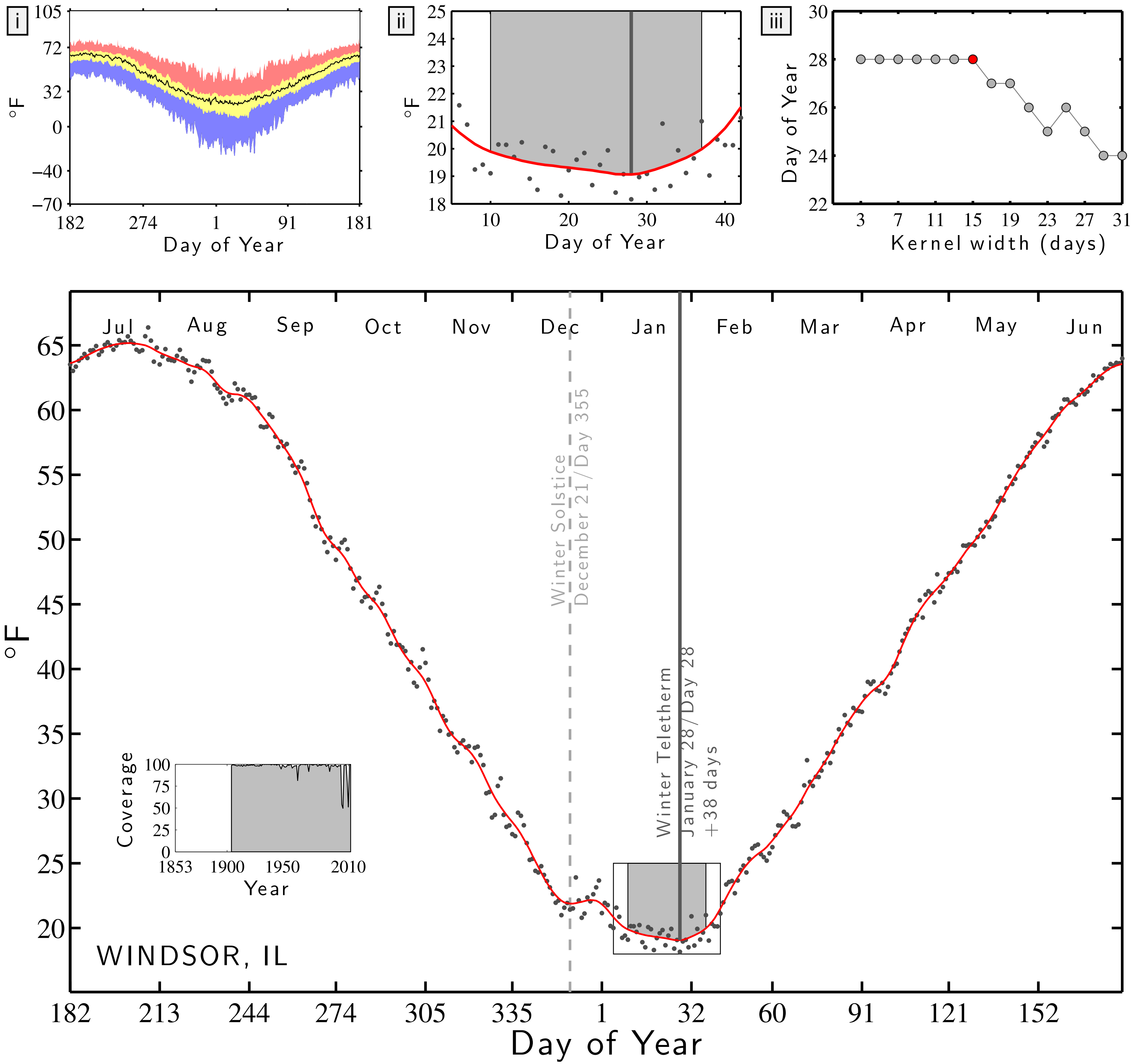

i
ii
iii
°F
Day of Year
Day of Year
Kernel width (days)
Jul
Aug
Sep
Oct
Nov
Dec
Jan
Feb
Mar
Apr
May
Jun
Winter Solstice
December 21/Day 355
Winter Teletherm
January 28/Day 28
+38 days
Coverage
Year
WINDSOR, IL
Day of Year

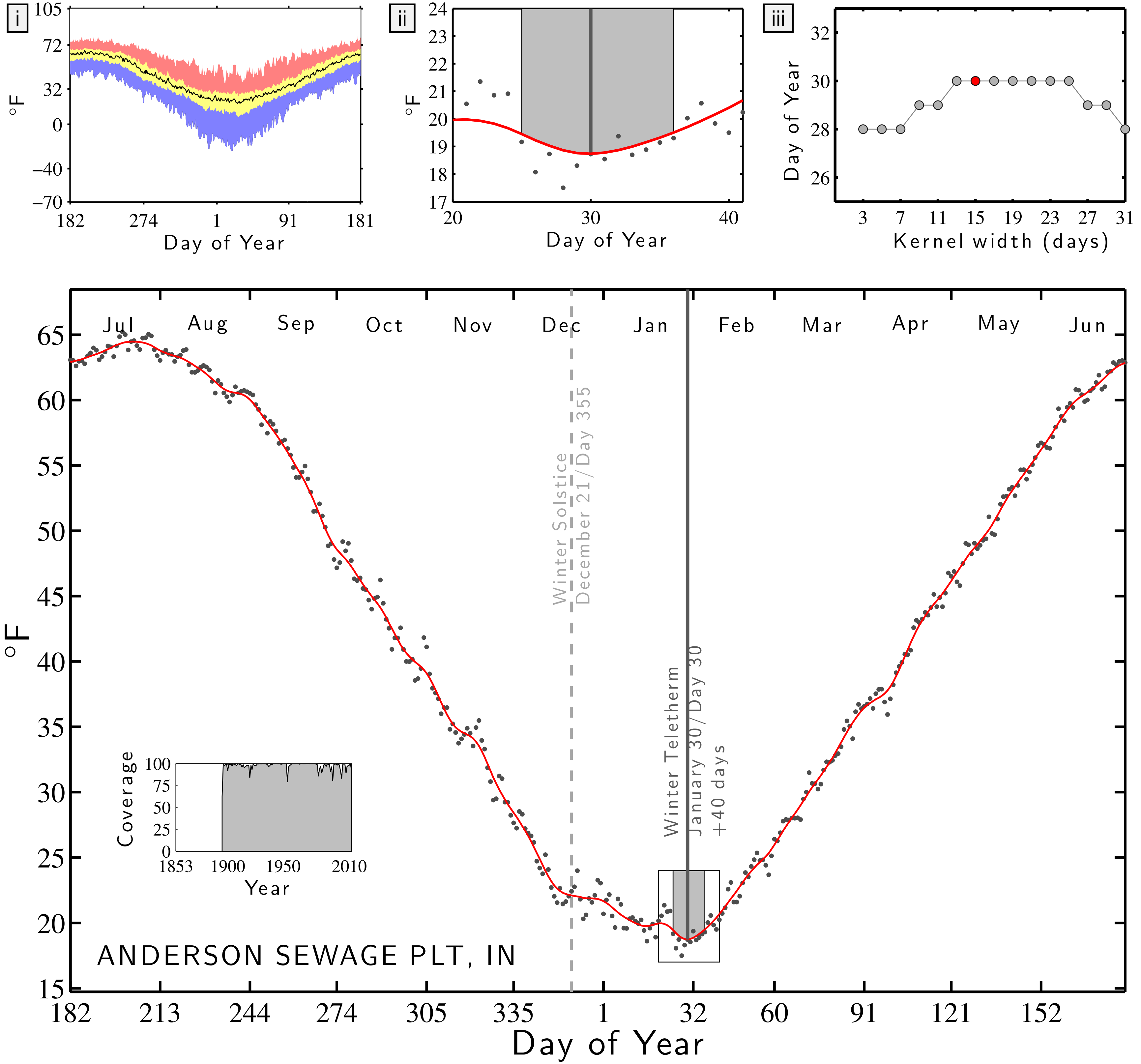

i
ii
iii
°F
Day of Year
Day of Year
Kernel width (days)
Jul
Aug
Sep
Oct
Nov
Dec
Jan
Feb
Mar
Apr
May
Jun
Winter Solstice
December 21/Day 355
Winter Teletherm
January 30/Day 30
+40 days
Coverage
Year
ANDERSON SEWAGE PLT, IN

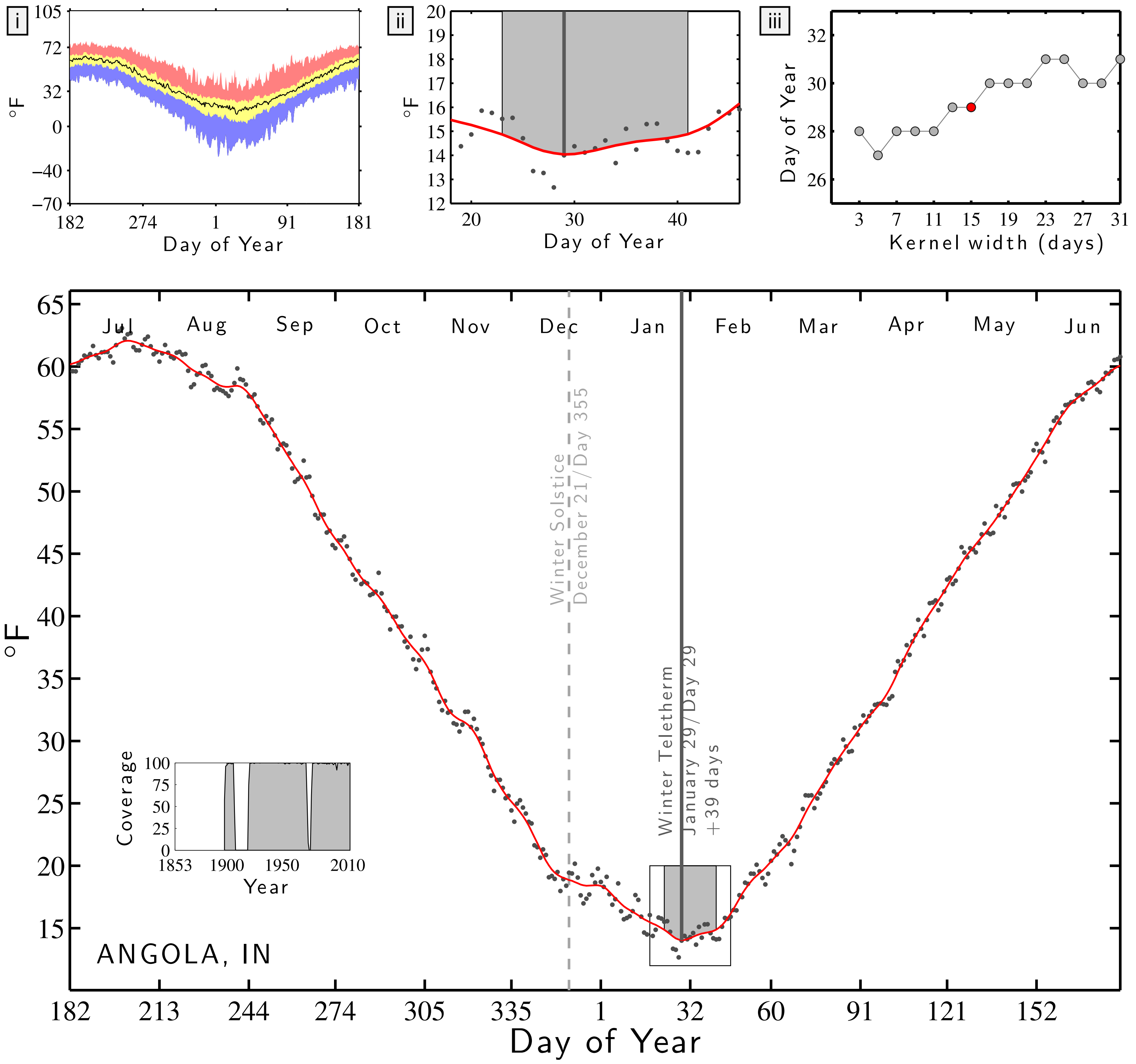

i
°F
Day of Year
ii
°F
Day of Year
iii
Day of Year
Kernel width (days)
Jul
Aug
Sep
Oct
Nov
Dec
Jan
Feb
Mar
Apr
May
Jun
Winter Solstice
December 21/Day 355
Winter Teletherm
January 29/Day 29
+39 days
Coverage
Year
ANGOLA, IN
°F
Day of Year

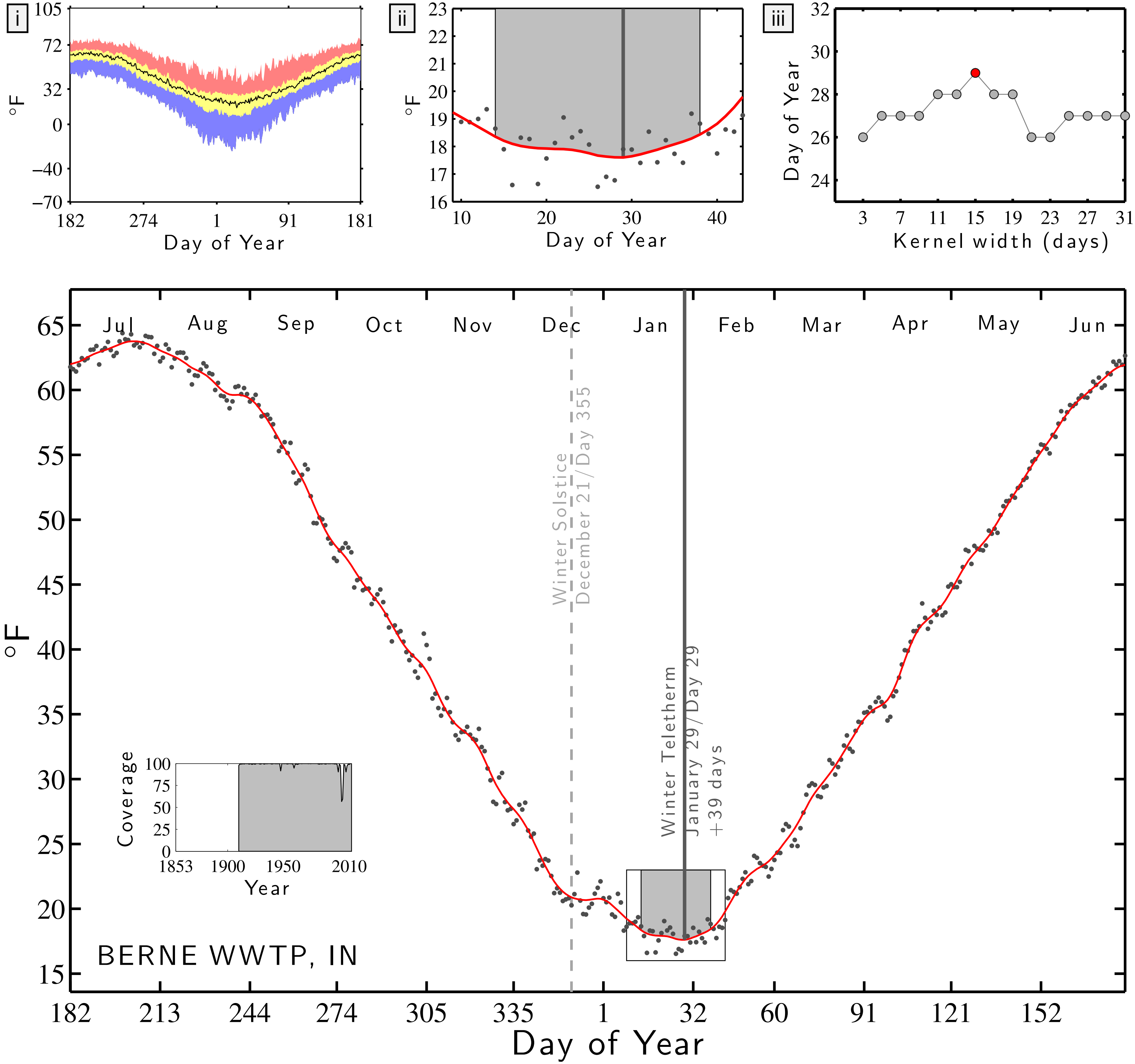

i
ii
iii
Day of Year
Kernel width (days)
°F
Jul
Aug
Sep
Oct
Nov
Dec
Jan
Feb
Mar
Apr
May
Jun
Winter Solstice
December 21/Day 355
Winter Teletherm
January 29/Day 29
+39 days
Coverage
Year
BERNE WWTP, IN

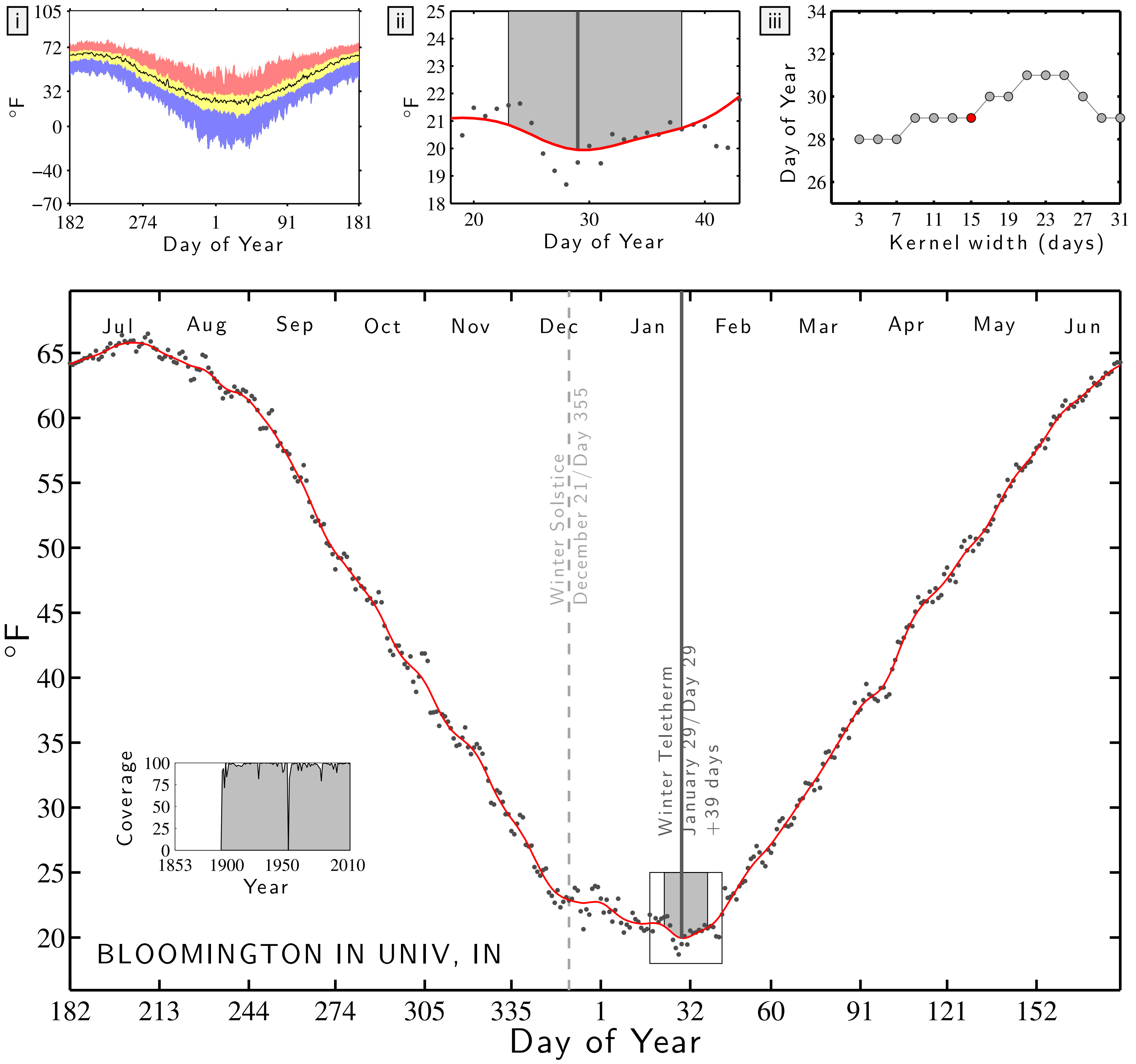

i
ii
iii
°F
Day of Year
Day of Year
Kernel width (days)
Jul
Aug
Sep
Oct
Nov
Dec
Jan
Feb
Mar
Apr
May
Jun
Winter Solstice
December 21/Day 355
Winter Teletherm
January 29/Day 29
+39 days
Coverage
Year
BLOOMINGTON IN UNIV, IN
Day of Year

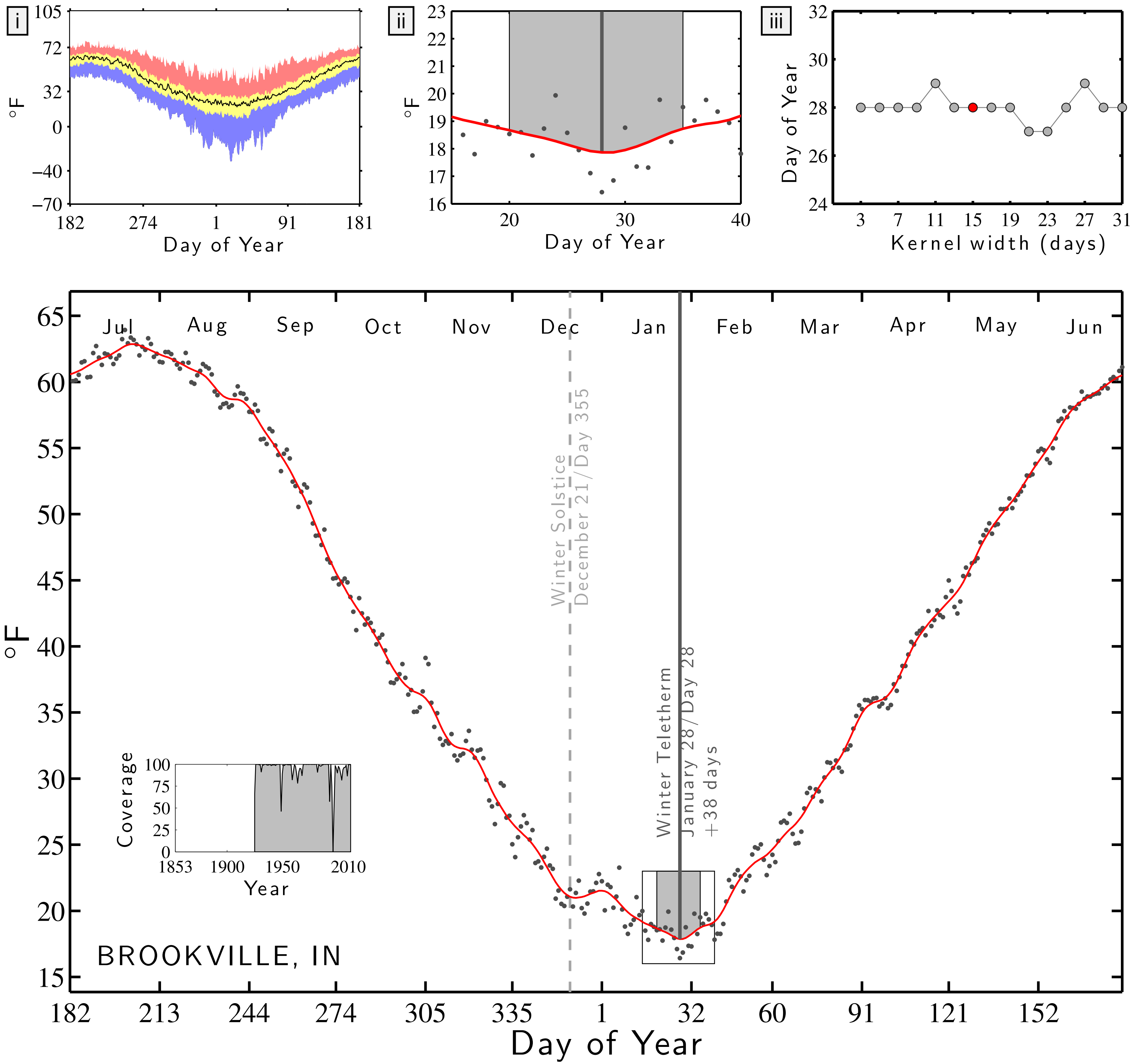

i
°F
Day of Year
ii
°F
Day of Year
iii
Day of Year
Kernel width (days)
Jul
Aug
Sep
Oct
Nov
Dec
Jan
Feb
Mar
Apr
May
Jun
Winter Solstice
December 21/Day 355
Winter Teletherm
January 28/Day 28
+38 days
Coverage
Year
BROOKVILLE, IN
°F
Day of Year

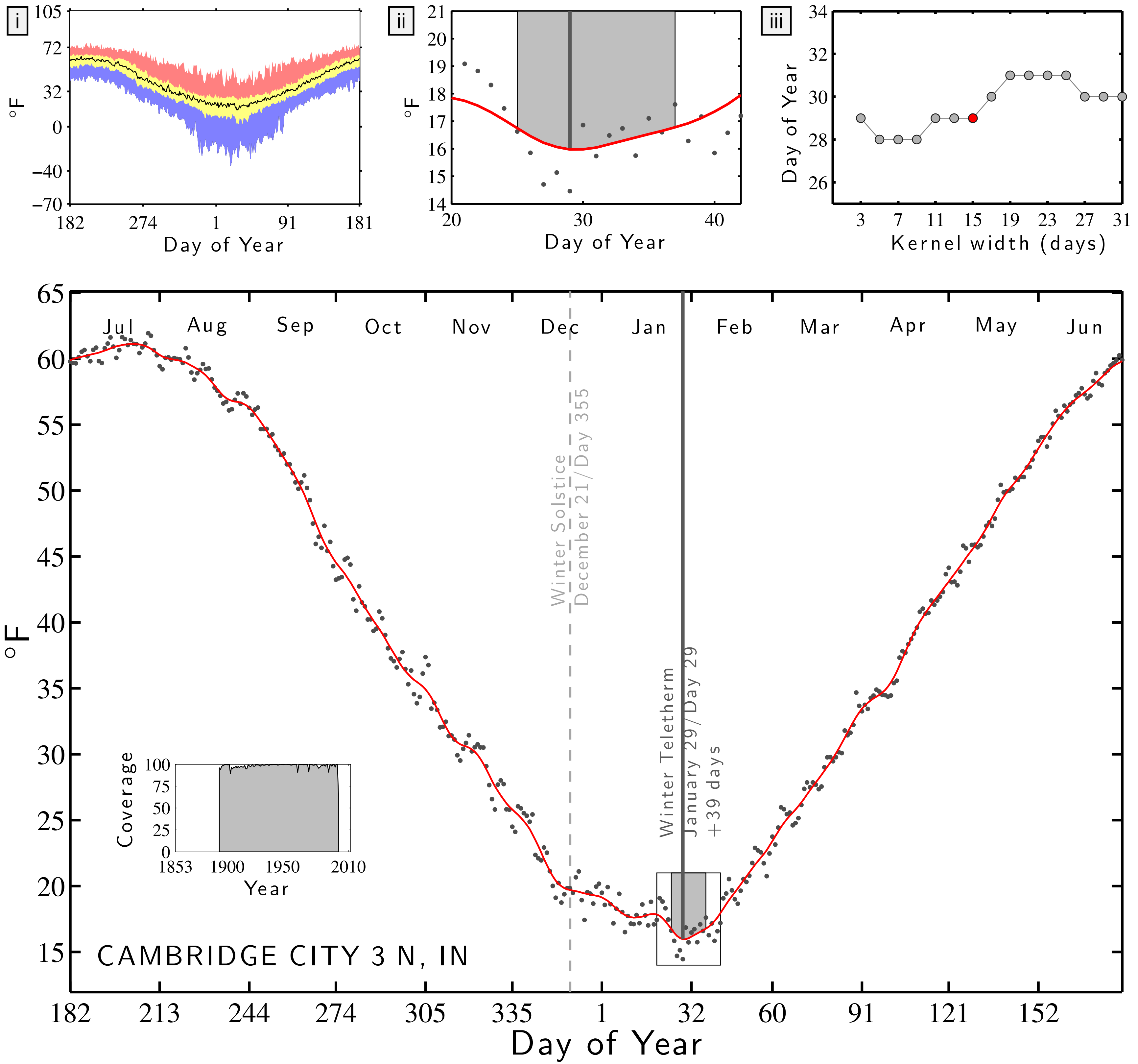

i
ii
iii
Day of Year
°F
Kernel width (days)
Jul
Aug
Sep
Oct
Nov
Dec
Jan
Feb
Mar
Apr
May
Jun
Winter Solstice
December 21/Day 355
Winter Teletherm
January 29/Day 29
+39 days
Coverage
Year
CAMBRIDGE CITY 3 N, IN

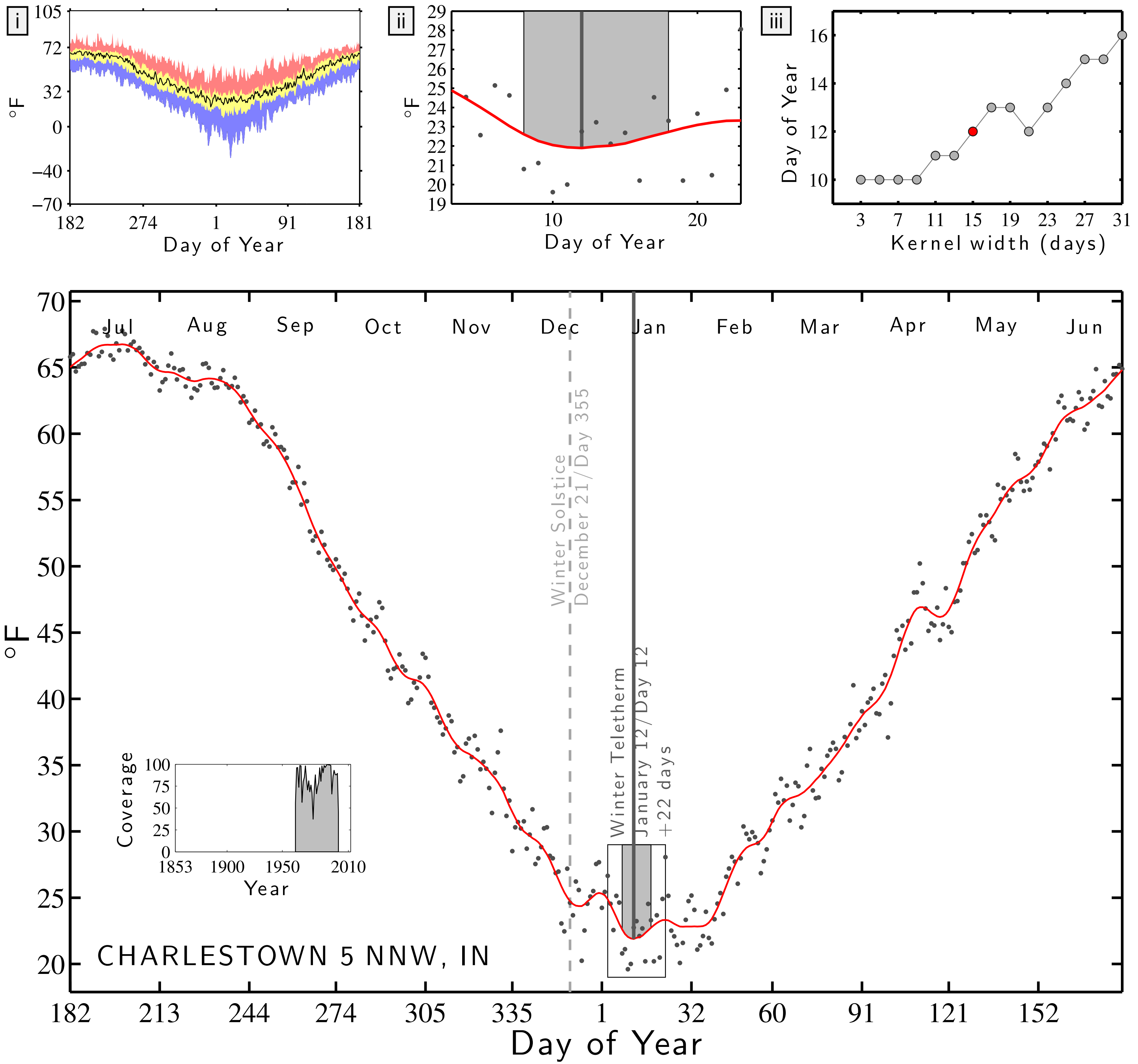

i
°F
Day of Year
ii
°F
Day of Year
iii
Day of Year
Kernel width (days)
°F
Day of Year
Jul
Aug
Sep
Oct
Nov
Dec
Jan
Feb
Mar
Apr
May
Jun
Winter Solstice
December 21/Day 355
Winter Teletherm
January 12/Day 12
+22 days
Coverage
Year
CHARLESTOWN 5 NNW, IN

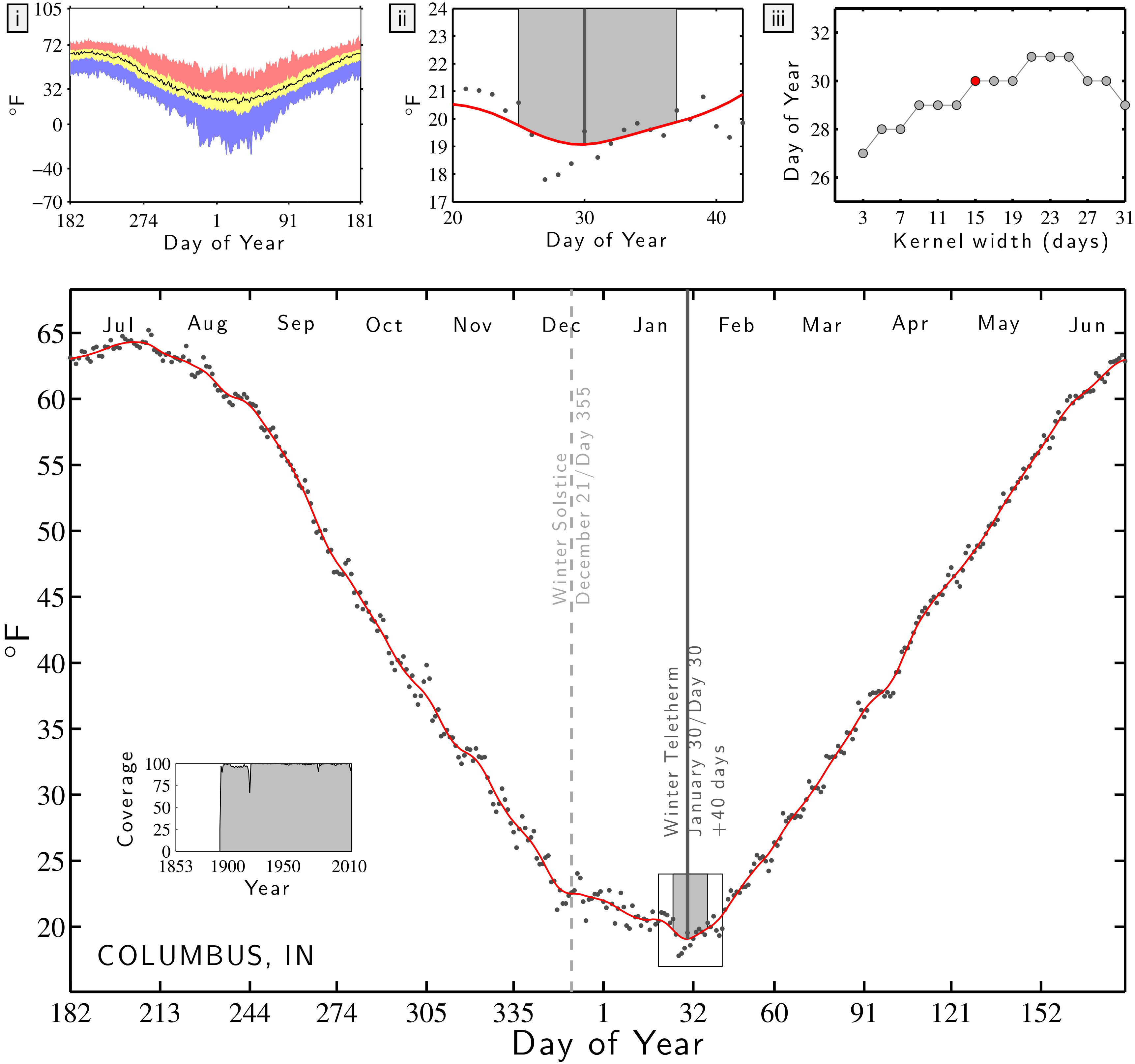

i
ii
iii
°F
Day of Year
Kernel width (days)
Jul
Aug
Sep
Oct
Nov
Dec
Jan
Feb
Mar
Apr
May
Jun
Winter Solstice
December 21/Day 355
Winter Teletherm
January 30/Day 30
+40 days
Coverage
Year
COLUMBUS, IN

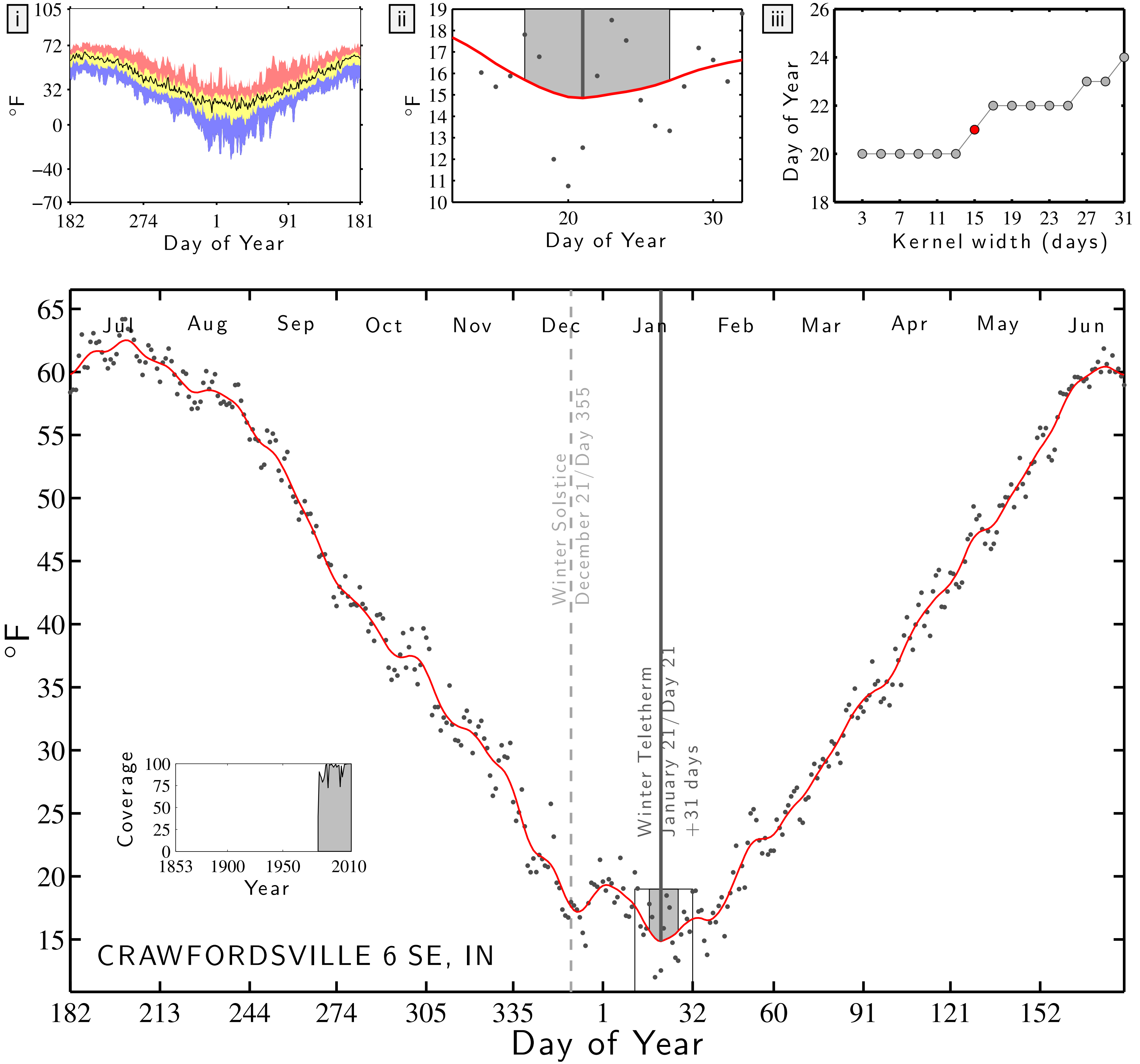

i
°F
Day of Year
ii
°F
Day of Year
iii
Day of Year
Kernel width (days)
Jul
Aug
Sep
Oct
Nov
Dec
Jan
Feb
Mar
Apr
May
Jun
Winter Solstice
December 21/Day 355
Winter Teletherm
January 21/Day 21
+31 days
Coverage
Year
CRAWFORDSVILLE 6 SE, IN
°F
Day of Year

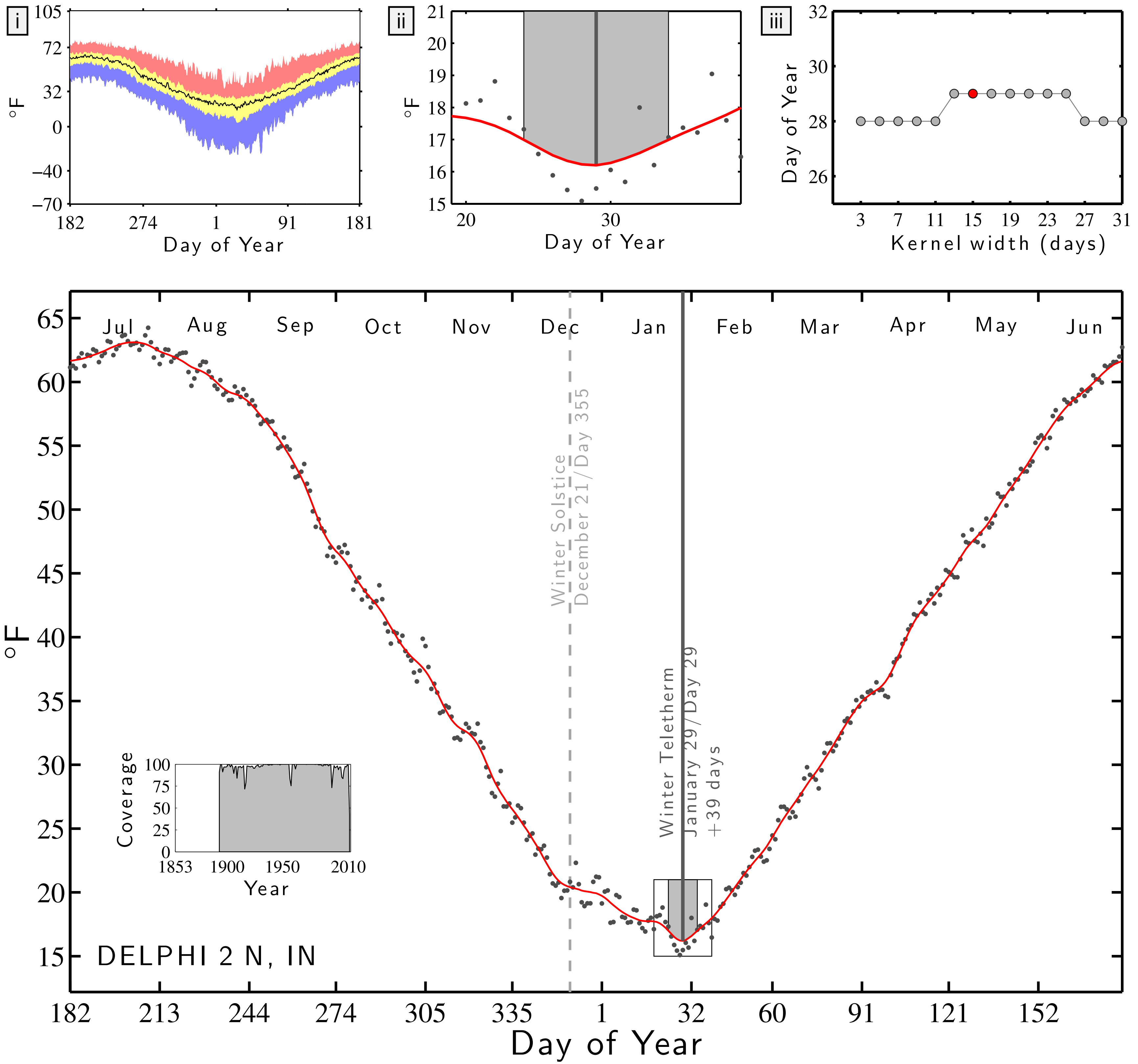

i
°F
Day of Year
ii
°F
Day of Year
iii
Day of Year
Kernel width (days)
Jul
Aug
Sep
Oct
Nov
Dec
Jan
Feb
Mar
Apr
May
Jun
Winter Solstice
December 21/Day 355
Winter Teletherm
January 29/Day 29
+39 days
Coverage
Year
DELPHI 2 N, IN
°F
Day of Year

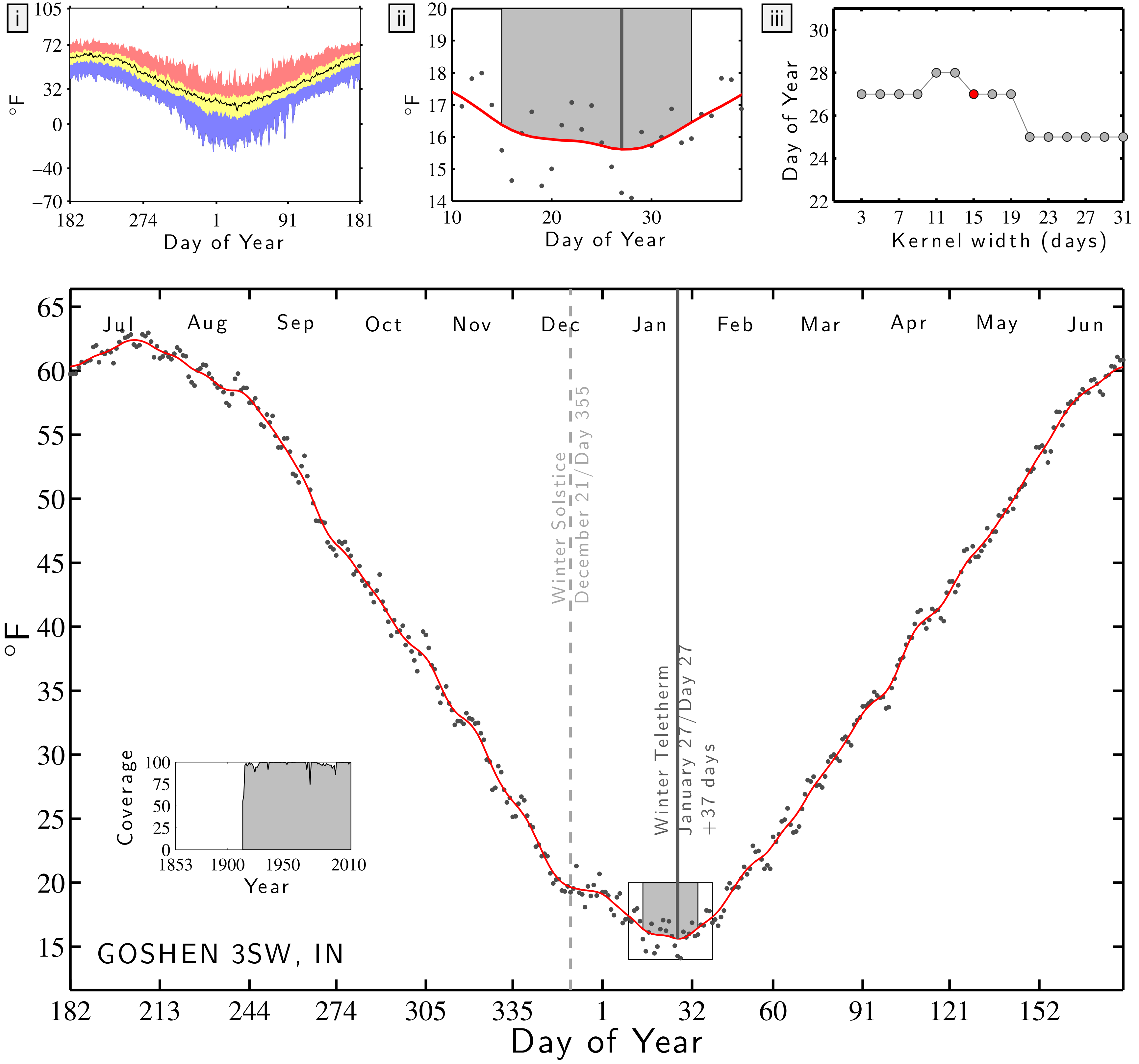

i
ii
iii
°F
Day of Year
Kernel width (days)
Jul
Aug
Sep
Oct
Nov
Dec
Jan
Feb
Mar
Apr
May
Jun
Winter Solstice
December 21/Day 355
Winter Teletherm
January 27/Day 27
+37 days
Coverage
Year
GOSHEN 3SW, IN

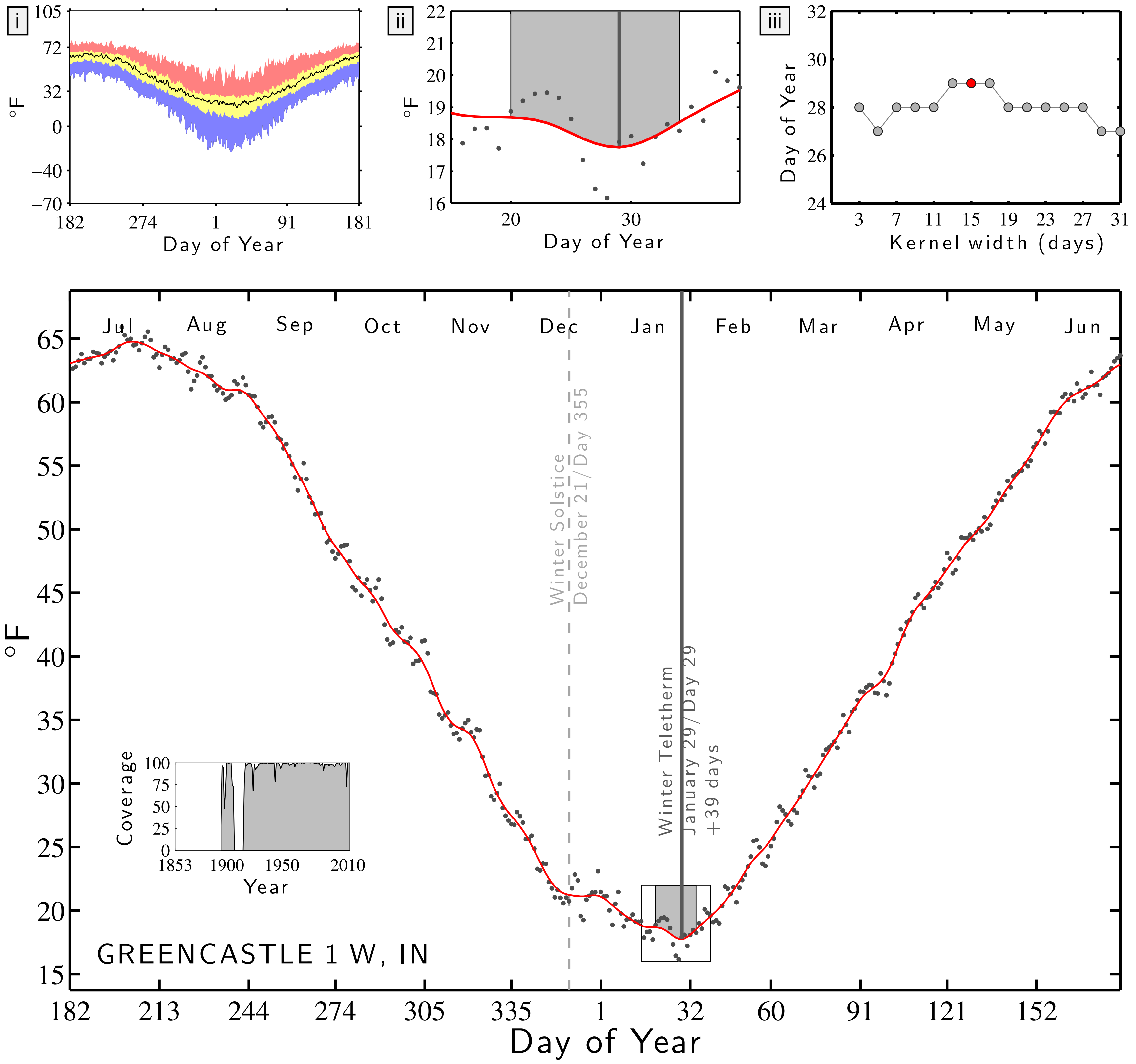

i
ii
iii
°F
Day of Year
Kernel width (days)
Jul
Aug
Sep
Oct
Nov
Dec
Jan
Feb
Mar
Apr
May
Jun
Winter Solstice
December 21/Day 355
Winter Teletherm
January 29/Day 29
+39 days
Coverage
Year
GREENCASTLE 1 W, IN

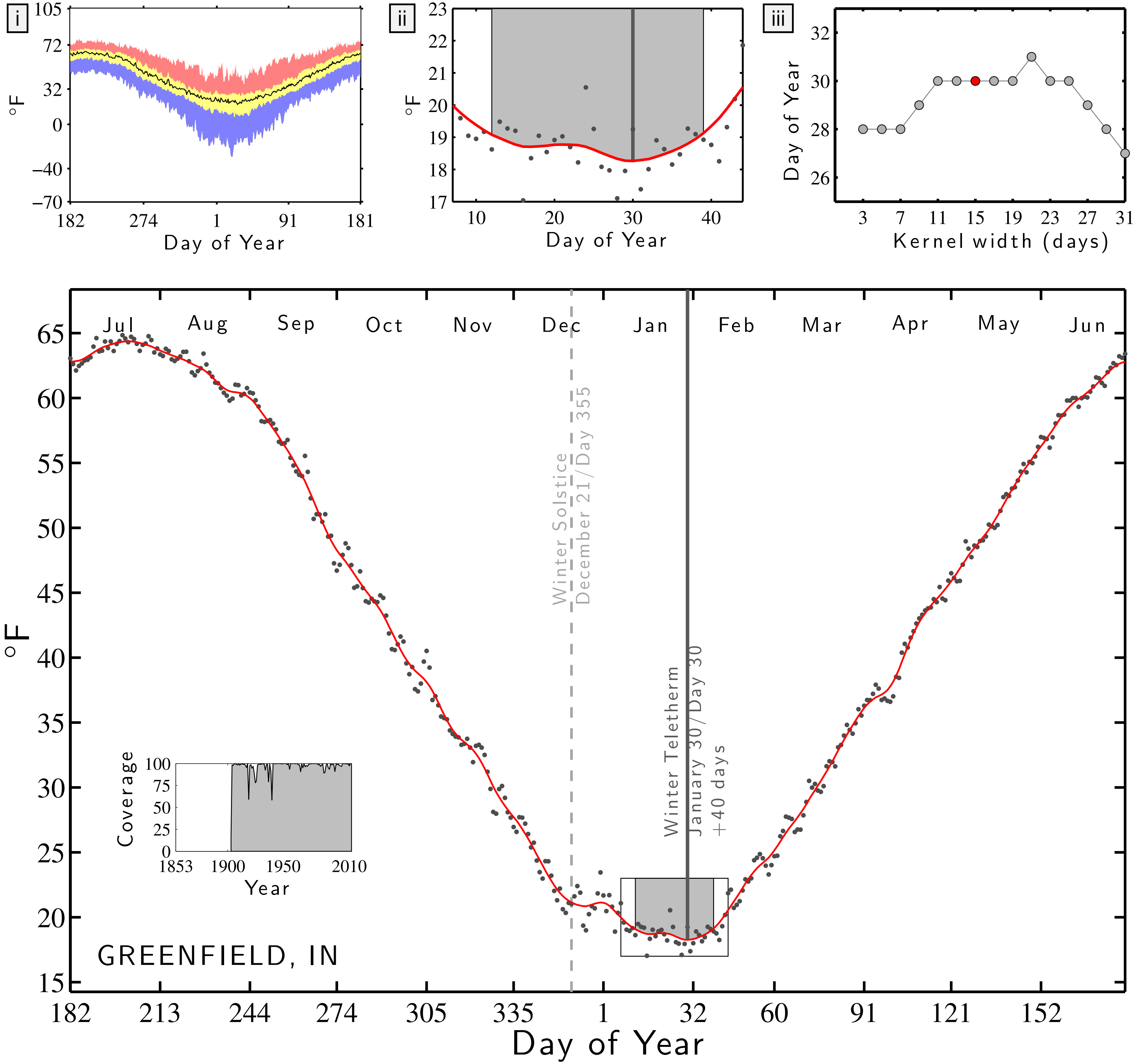

i
°F
Day of Year
ii
°F
Day of Year
iii
Day of Year
Kernel width (days)
Jul
Aug
Sep
Oct
Nov
Dec
Jan
Feb
Mar
Apr
May
Jun
Winter Solstice
December 21/Day 355
Winter Teletherm
January 30/Day 30
+40 days
Coverage
Year
GREENFIELD, IN
°F
Day of Year

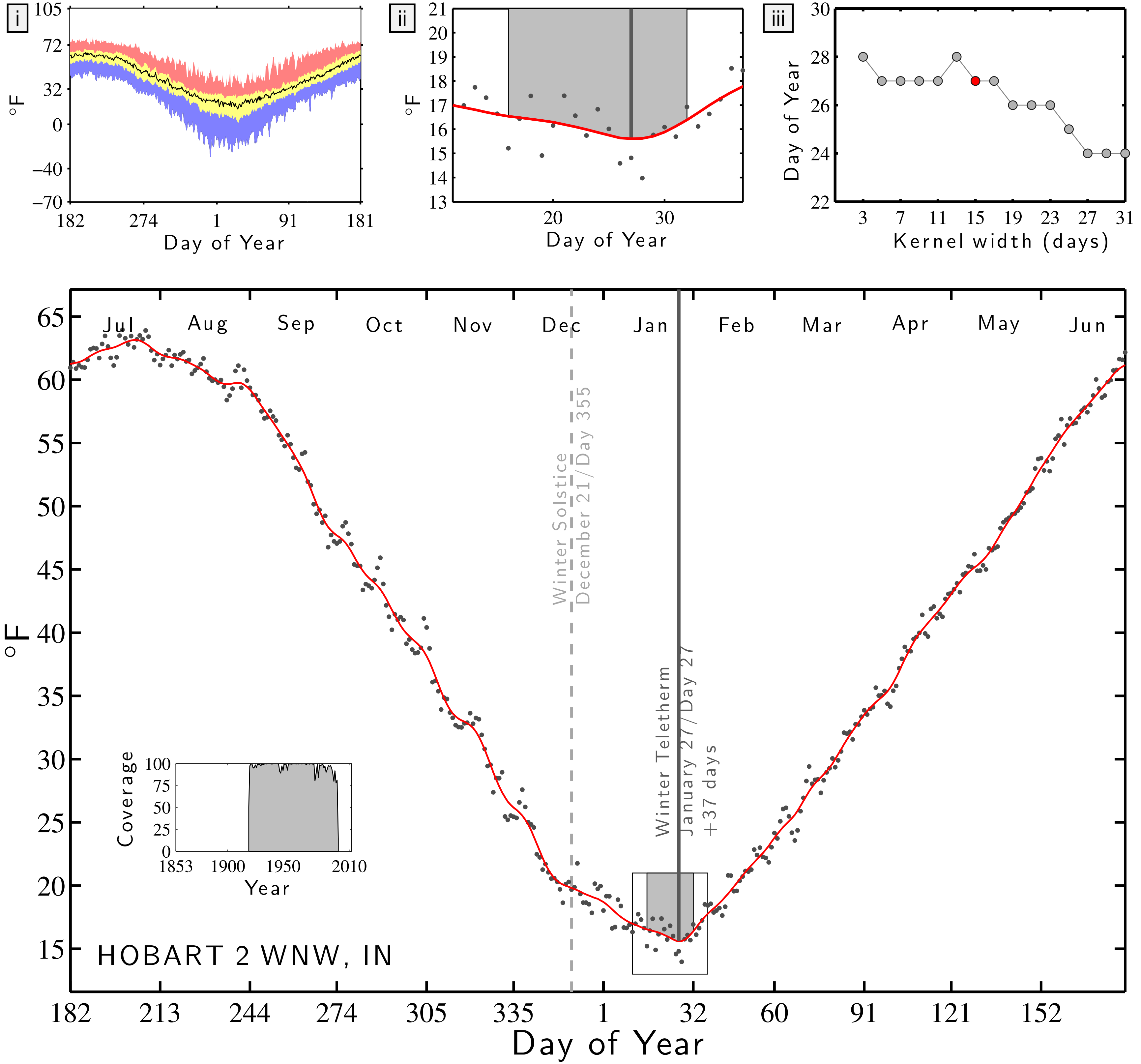

i
°F
Day of Year
ii
°F
Day of Year
iii
Day of Year
Kernel width (days)
Jul
Aug
Sep
Oct
Nov
Dec
Jan
Feb
Mar
Apr
May
Jun
Winter Solstice
December 21/Day 355
Winter Teletherm
January 27/Day 27
+37 days
Coverage
Year
HOBART 2 WNW, IN
°F
Day of Year

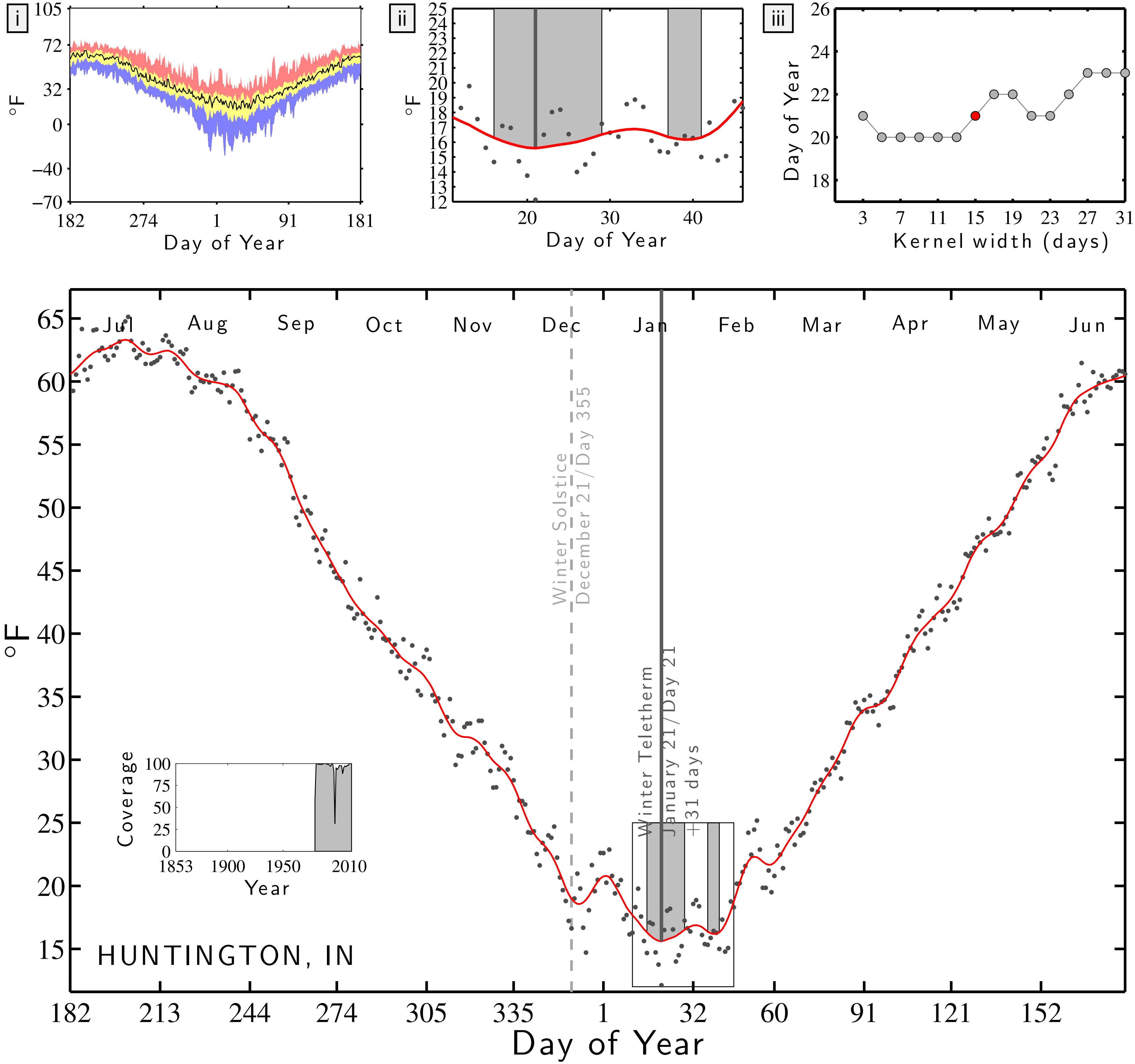

i
°F
Day of Year
ii
°F
Day of Year
iii
Day of Year
Kernel width (days)
Jul
Aug
Sep
Oct
Nov
Dec
Jan
Feb
Mar
Apr
May
Jun
Winter Solstice
December 21/Day 355
Winter Teletherm
January 21/Day 21
+31 days
Coverage
Year
HUNTINGTON, IN
°F
Day of Year

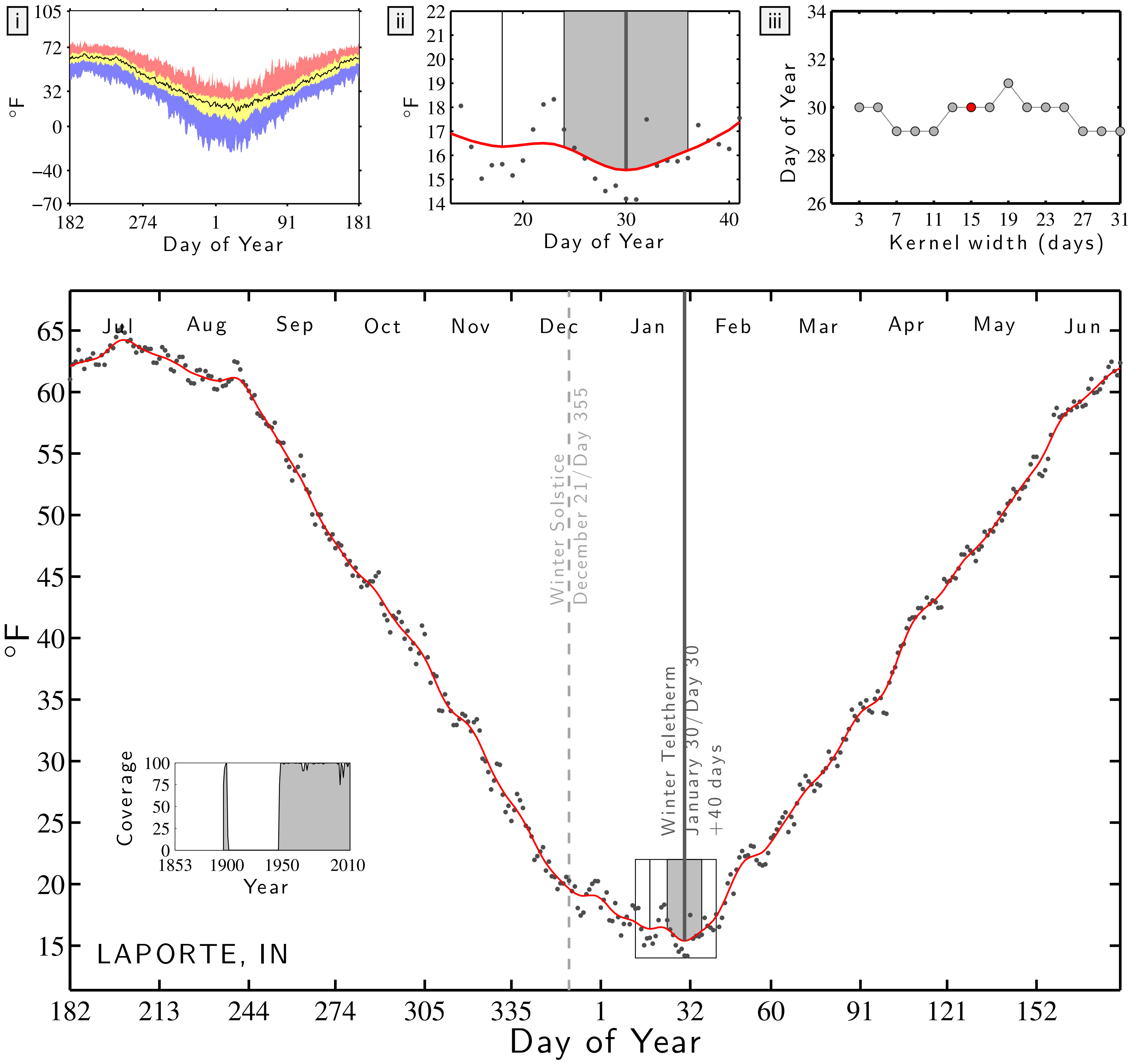

i
°F
Day of Year
ii
°F
Day of Year
iii
Day of Year
Kernel width (days)
Jul
Aug
Sep
Oct
Nov
Dec
Jan
Feb
Mar
Apr
May
Jun
Winter Solstice
December 21/Day 355
Winter Teletherm
January 30/Day 30
+40 days
Coverage
Year
LAPORTE, IN
°F
Day of Year

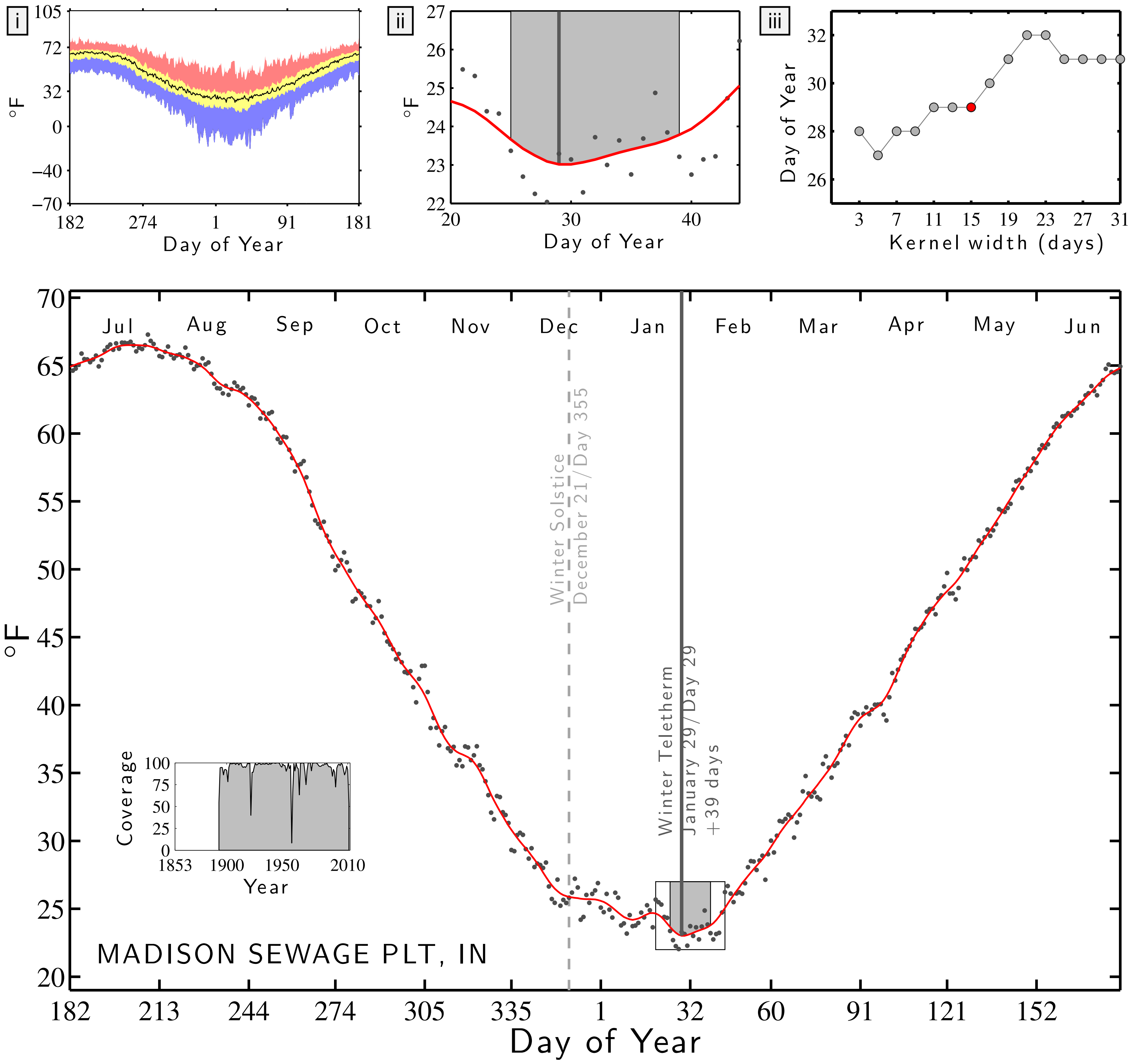

i
ii
iii
°F
Day of Year
Kernel width (days)
Jul
Aug
Sep
Oct
Nov
Dec
Jan
Feb
Mar
Apr
May
Jun
Winter Solstice
December 21/Day 355
Winter Teletherm
January 29/Day 29
+39 days
Coverage
Year
MADISON SEWAGE PLT, IN

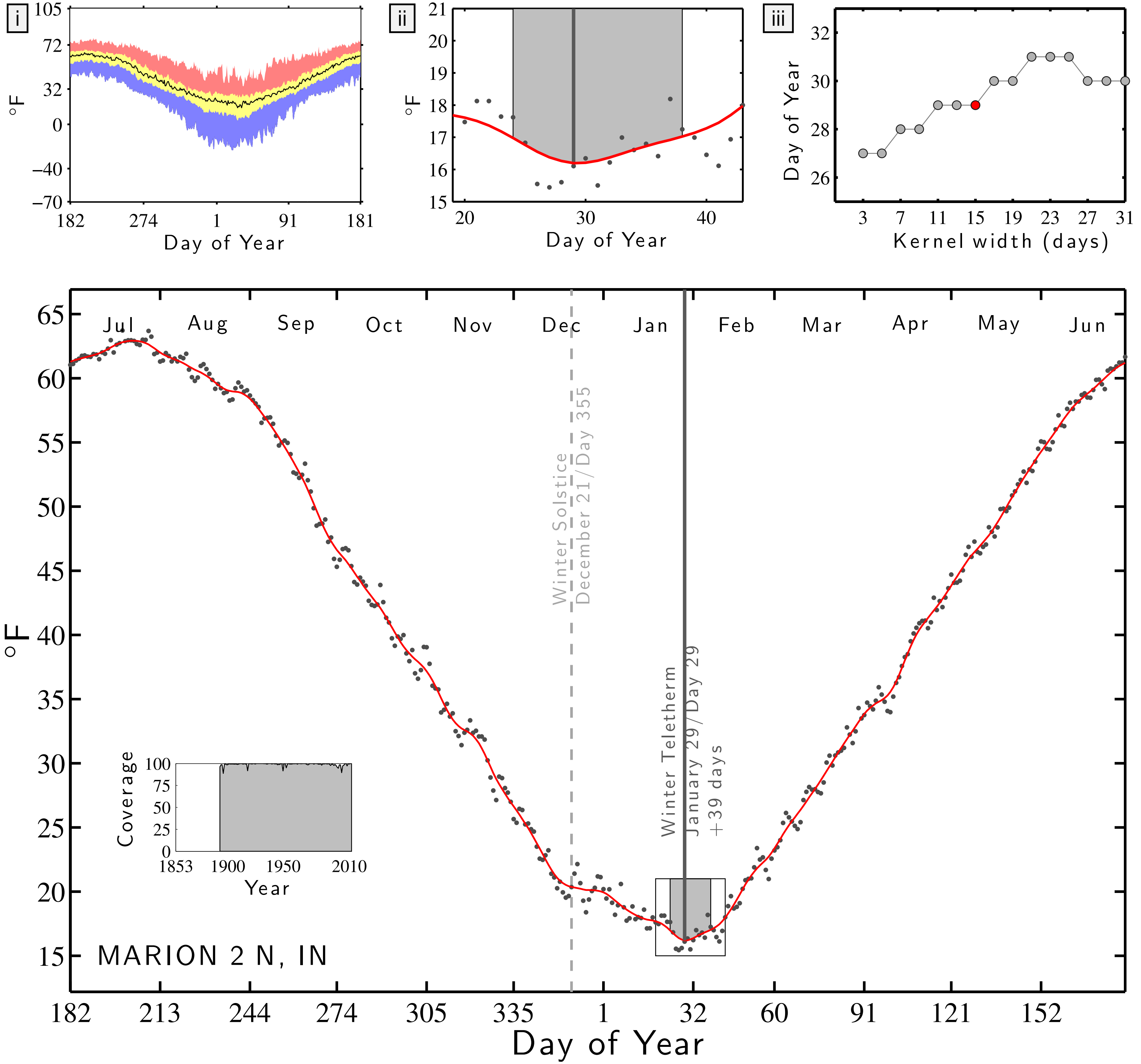

i
°F
Day of Year
ii
°F
Day of Year
iii
Day of Year
Kernel width (days)
Jul
Aug
Sep
Oct
Nov
Dec
Jan
Feb
Mar
Apr
May
Jun
Winter Solstice
December 21/Day 355
Winter Teletherm
January 29/Day 29
+39 days
Coverage
Year
MARION 2 N, IN
°F
Day of Year

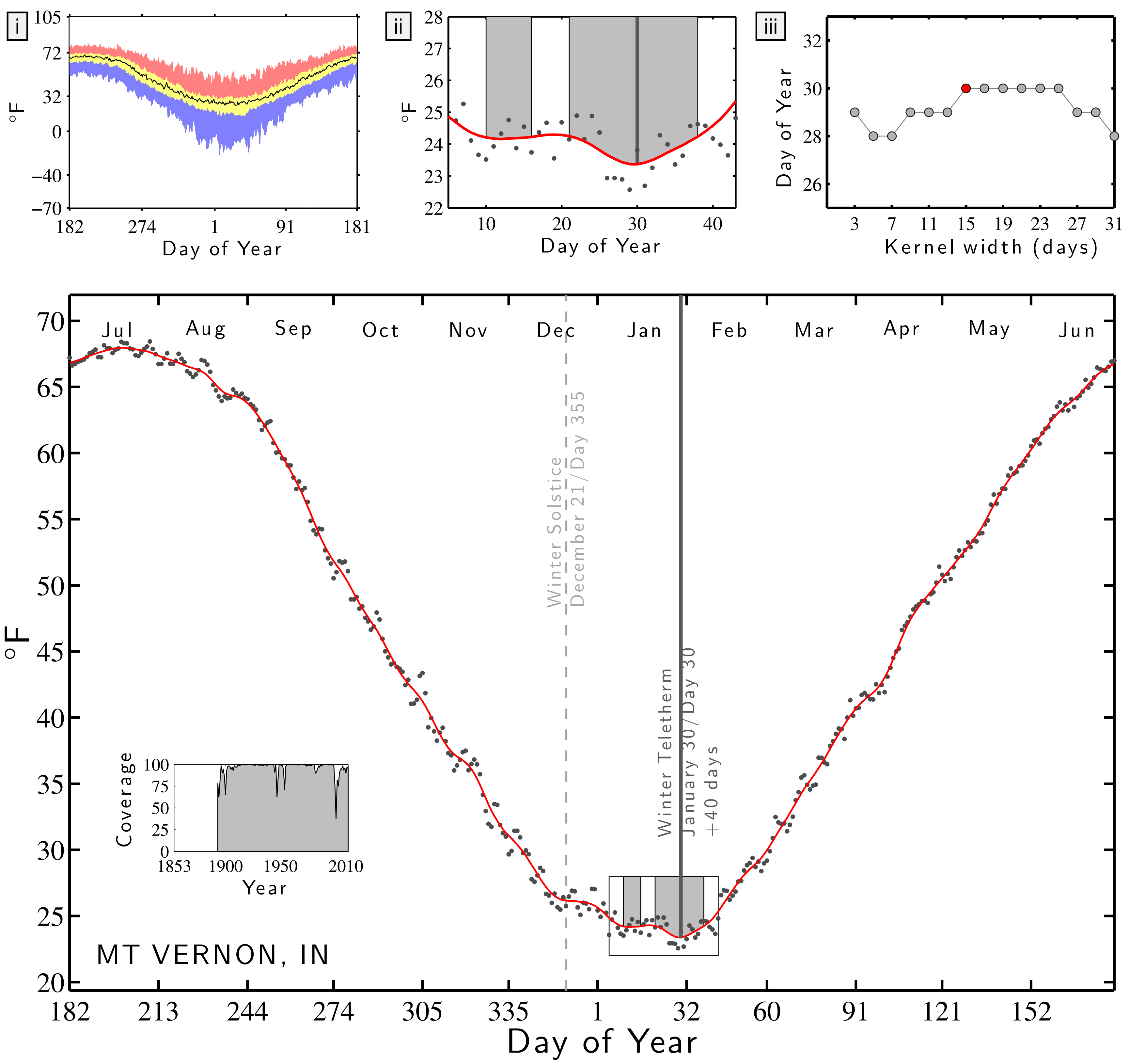

i
°F
Day of Year
ii
°F
Day of Year
iii
Day of Year
Kernel width (days)
°F
Jul
Aug
Sep
Oct
Nov
Dec
Jan
Feb
Mar
Apr
May
Jun
Winter Solstice
December 21/Day 355
Winter Teletherm
January 30/Day 30
+40 days
Coverage
Year
MT VERNON, IN
Day of Year

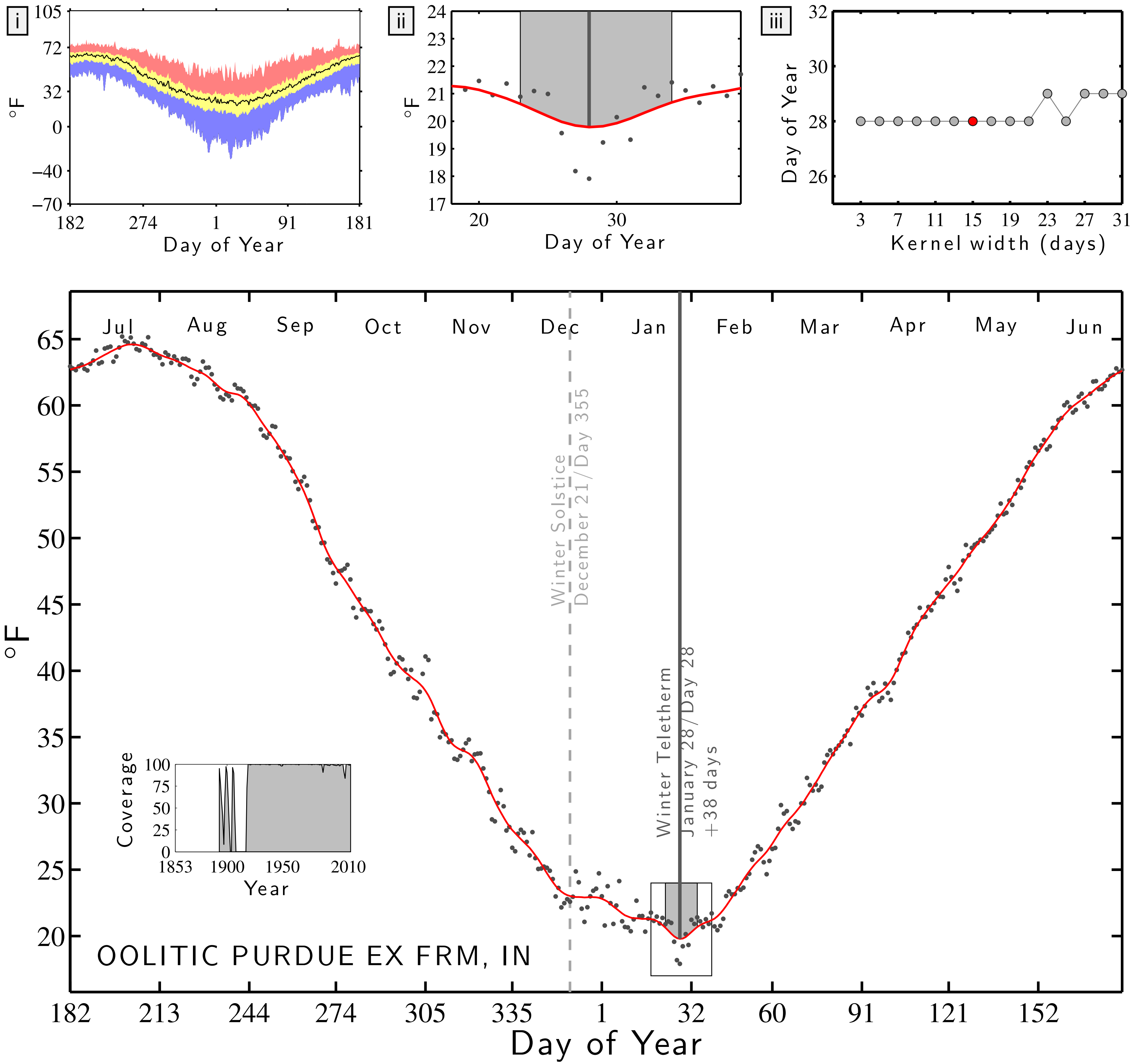

i
ii
iii
°F
Day of Year
Day of Year
Kernel width (days)
Jul
Aug
Sep
Oct
Nov
Dec
Jan
Feb
Mar
Apr
May
Jun
Winter Solstice
December 21/Day 355
Winter Teletherm
January 28/Day 28
+38 days
Coverage
Year
OOLITIC PURDUE EX FRM, IN

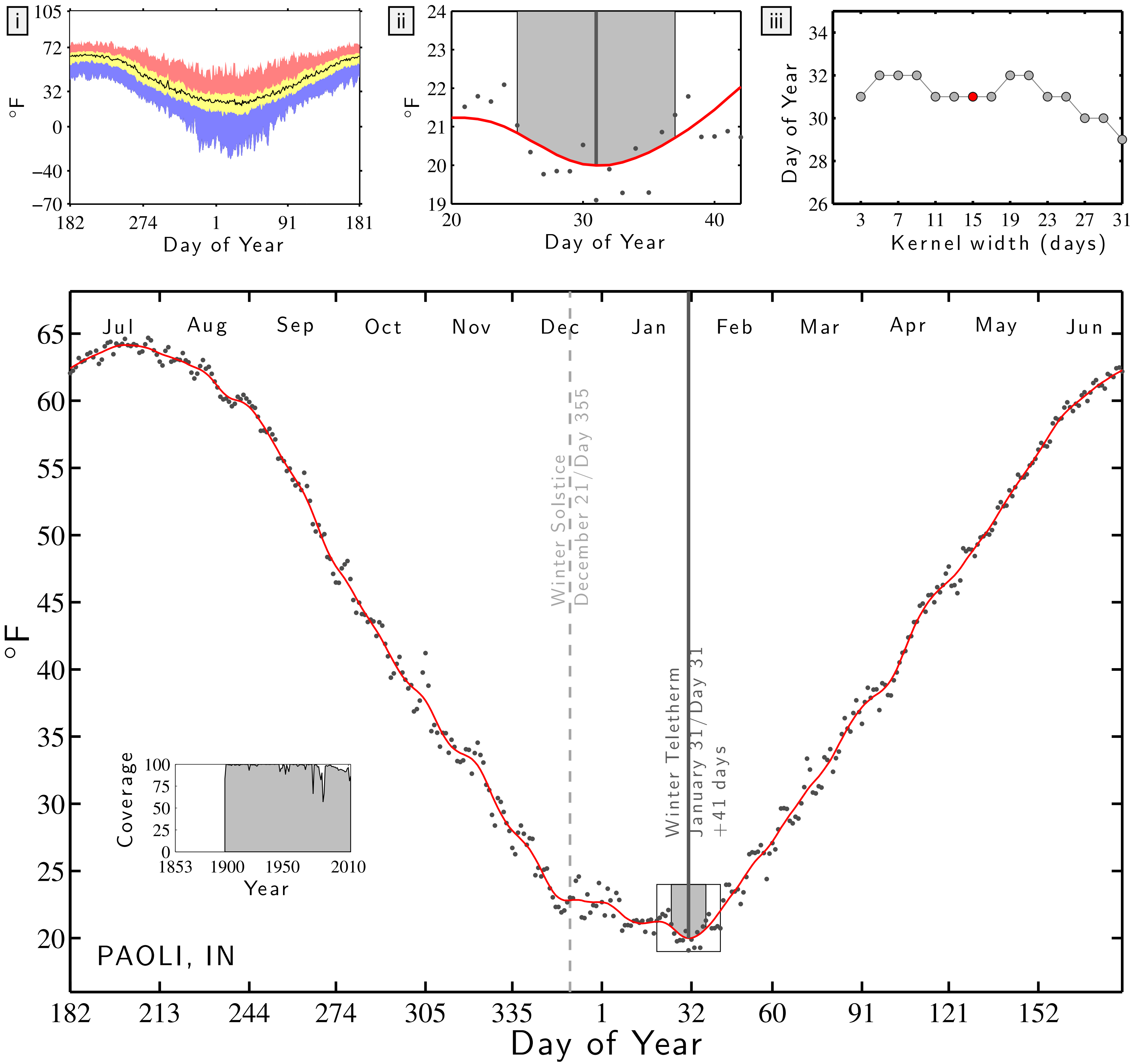

i
°F
Day of Year
ii
°F
Day of Year
iii
Day of Year
Kernel width (days)
Jul
Aug
Sep
Oct
Nov
Dec
Jan
Feb
Mar
Apr
May
Jun
Winter Solstice
December 21/Day 355
Winter Teletherm
January 31/Day 31
+41 days
Coverage
Year
PAOLI, IN
°F
Day of Year

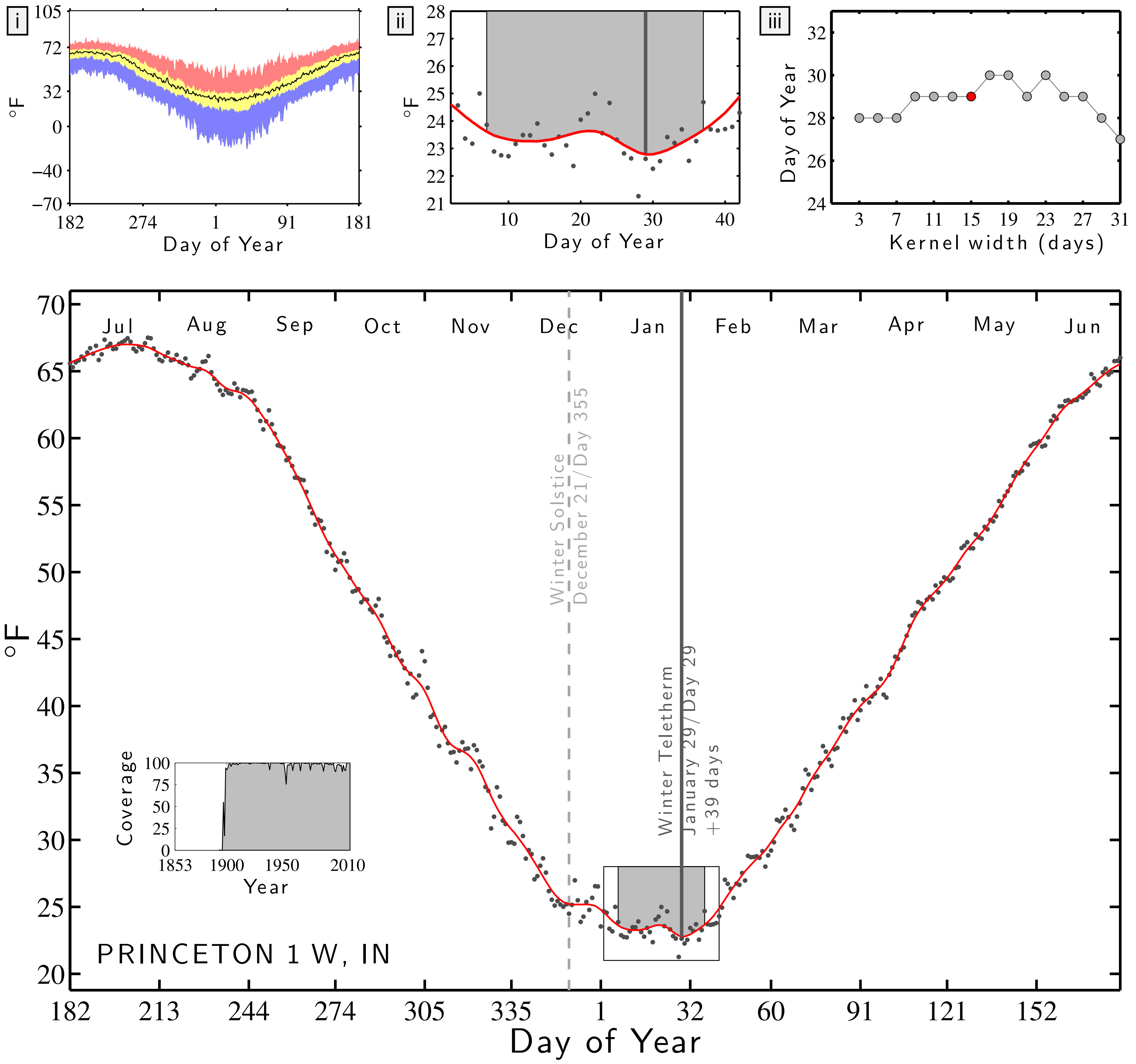

i
ii
iii
°F
Day of Year
Day of Year
Kernel width (days)
Jul
Aug
Sep
Oct
Nov
Dec
Jan
Feb
Mar
Apr
May
Jun
Winter Solstice
December 21/Day 355
Winter Teletherm
January 29/Day 29
+39 days
Coverage
Year
PRINCETON 1 W, IN

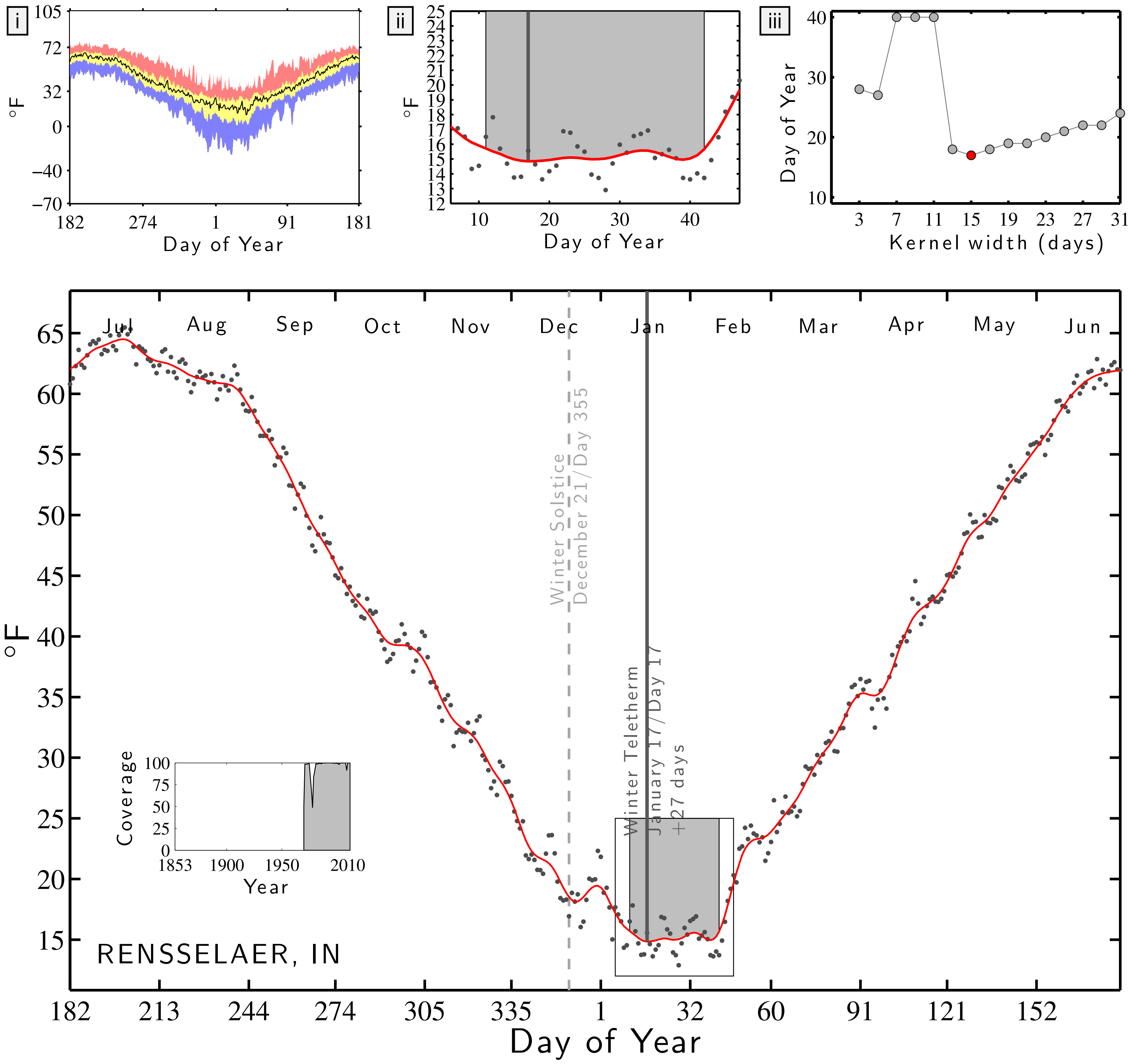

i
ii
iii
°F
Day of Year
Kernel width (days)
Jul
Aug
Sep
Oct
Nov
Dec
Jan
Feb
Mar
Apr
May
Jun
Winter Solstice
December 21/Day 355
Winter Teletherm
January 17/Day 17
+27 days
Coverage
Year
RENSSELAER, IN

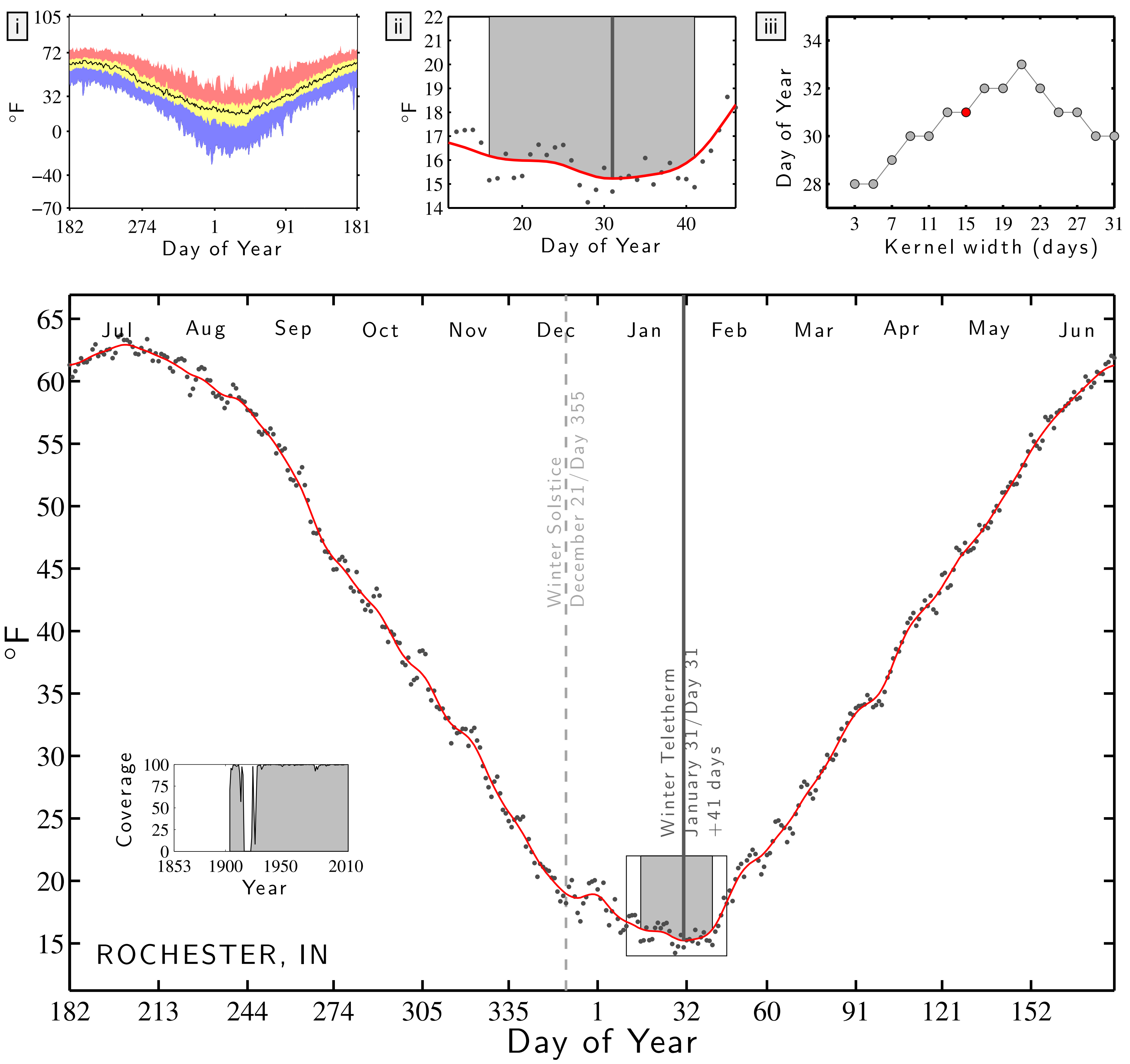

i
ii
iii
Day of Year
°F
Kernel width (days)
Jul
Aug
Sep
Oct
Nov
Dec
Jan
Feb
Mar
Apr
May
Jun
Winter Solstice
December 21/Day 355
Winter Teletherm
January 31/Day 31
+41 days
Coverage
Year
ROCHESTER, IN

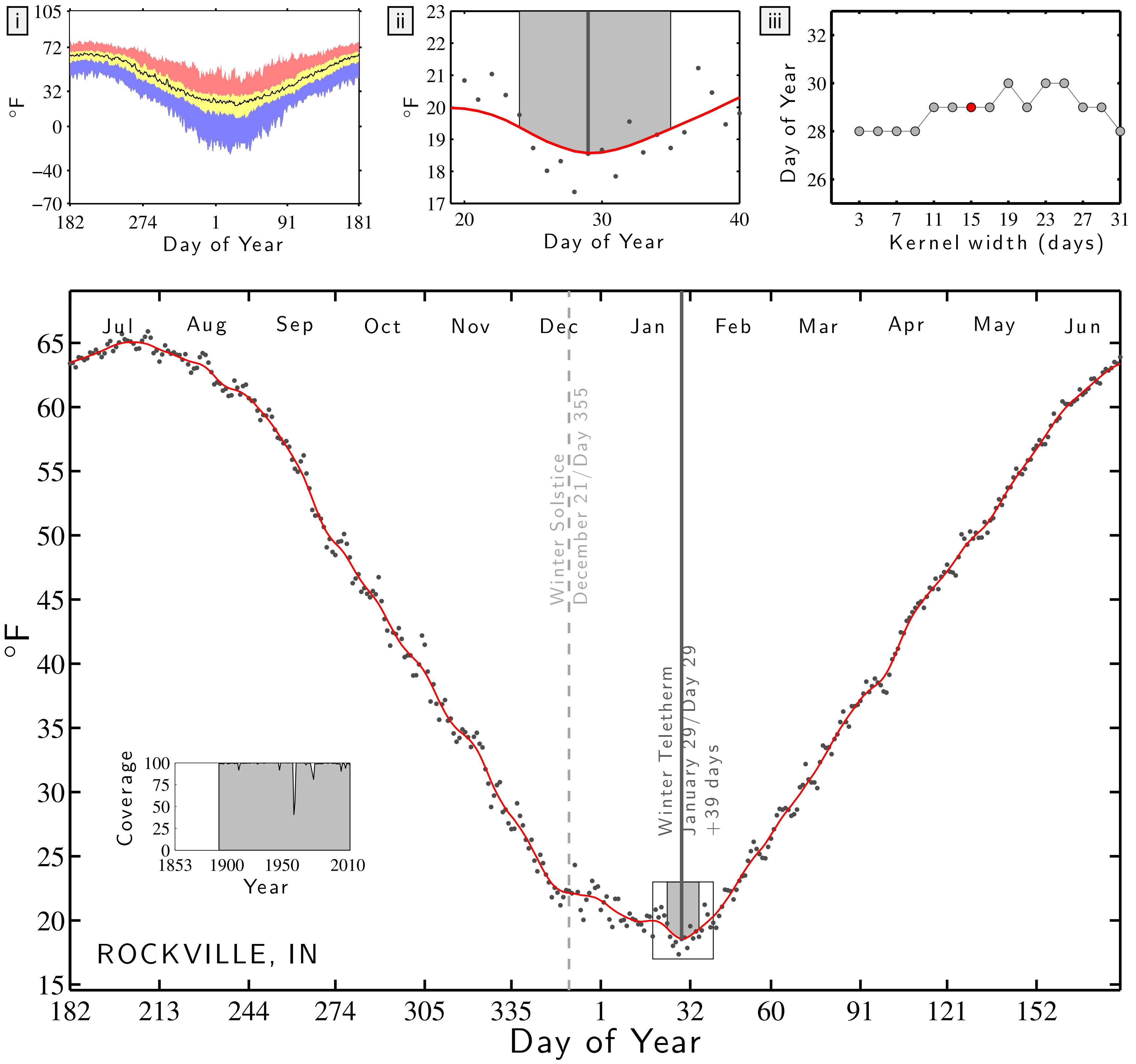

i
ii
iii
°F
Day of Year
Kernel width (days)
Winter Solstice
December 21/Day 355
Winter Teletherm
January 29/Day 29
+39 days
Coverage
Year
ROCKVILLE, IN
Jul
Aug
Sep
Oct
Nov
Dec
Jan
Feb
Mar
Apr
May
Jun

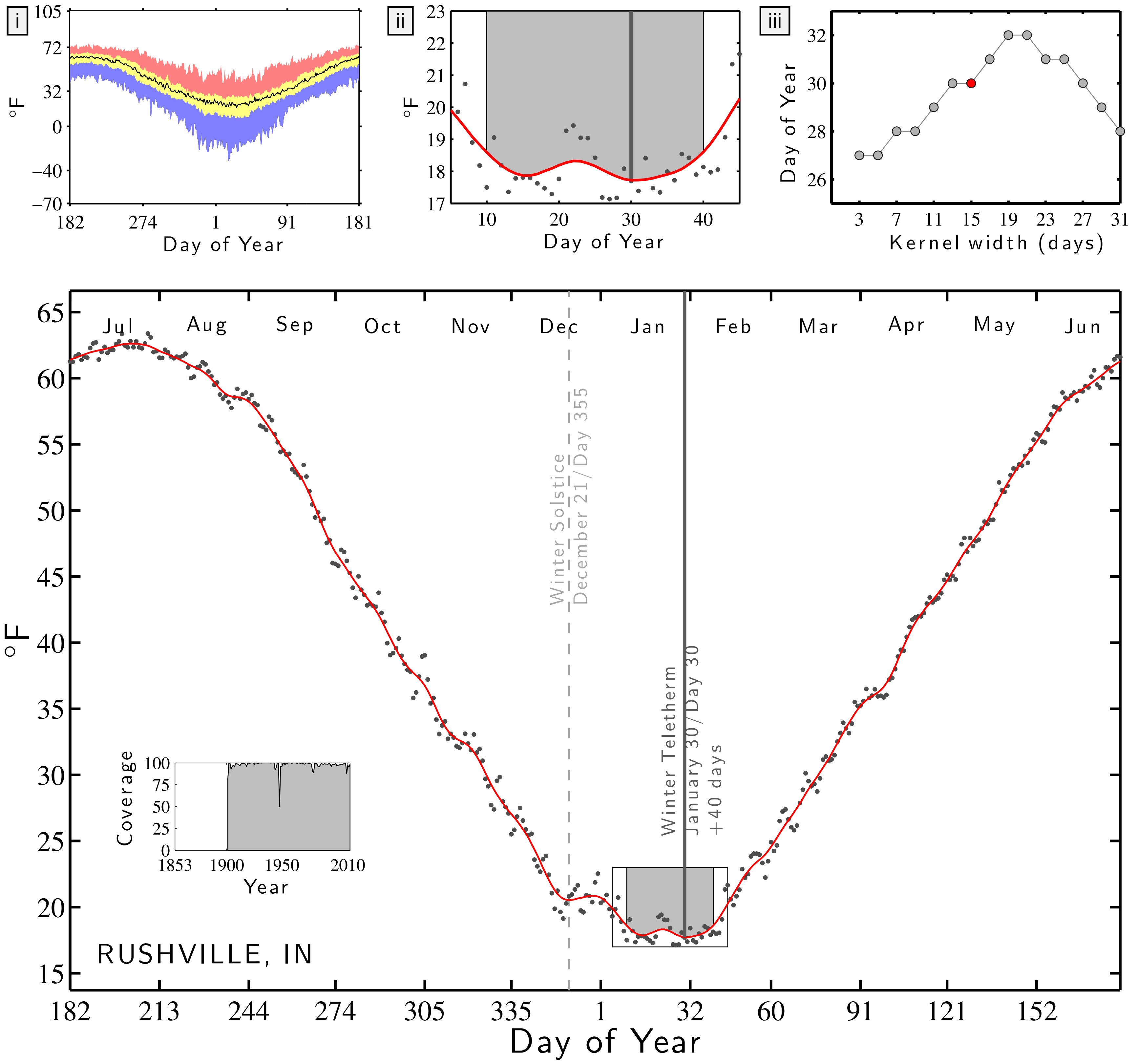

i
°F
Day of Year
ii
°F
Day of Year
iii
Day of Year
Kernel width (days)
Jul
Aug
Sep
Oct
Nov
Dec
Jan
Feb
Mar
Apr
May
Jun
Winter Solstice
December 21/Day 355
Winter Teletherm
January 30/Day 30
+40 days
Coverage
Year
RUSHVILLE, IN
°F
Day of Year

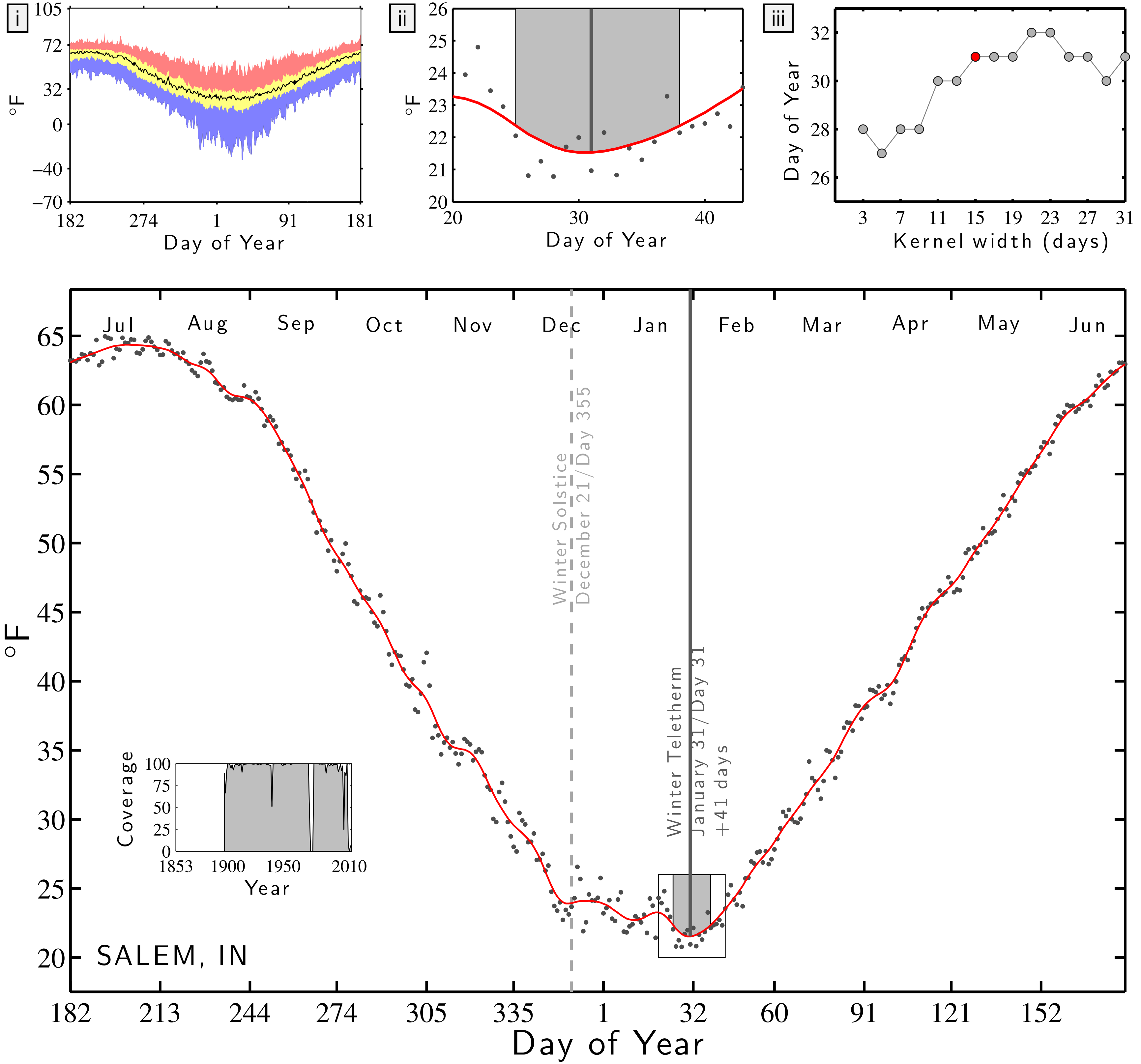

i
°F
Day of Year
ii
°F
Day of Year
iii
Day of Year
Kernel width (days)
Jul
Aug
Sep
Oct
Nov
Dec
Jan
Feb
Mar
Apr
May
Jun
Winter Solstice
December 21/Day 355
Winter Teletherm
January 31/Day 31
+41 days
Coverage
Year
SALEM, IN
°F
Day of Year

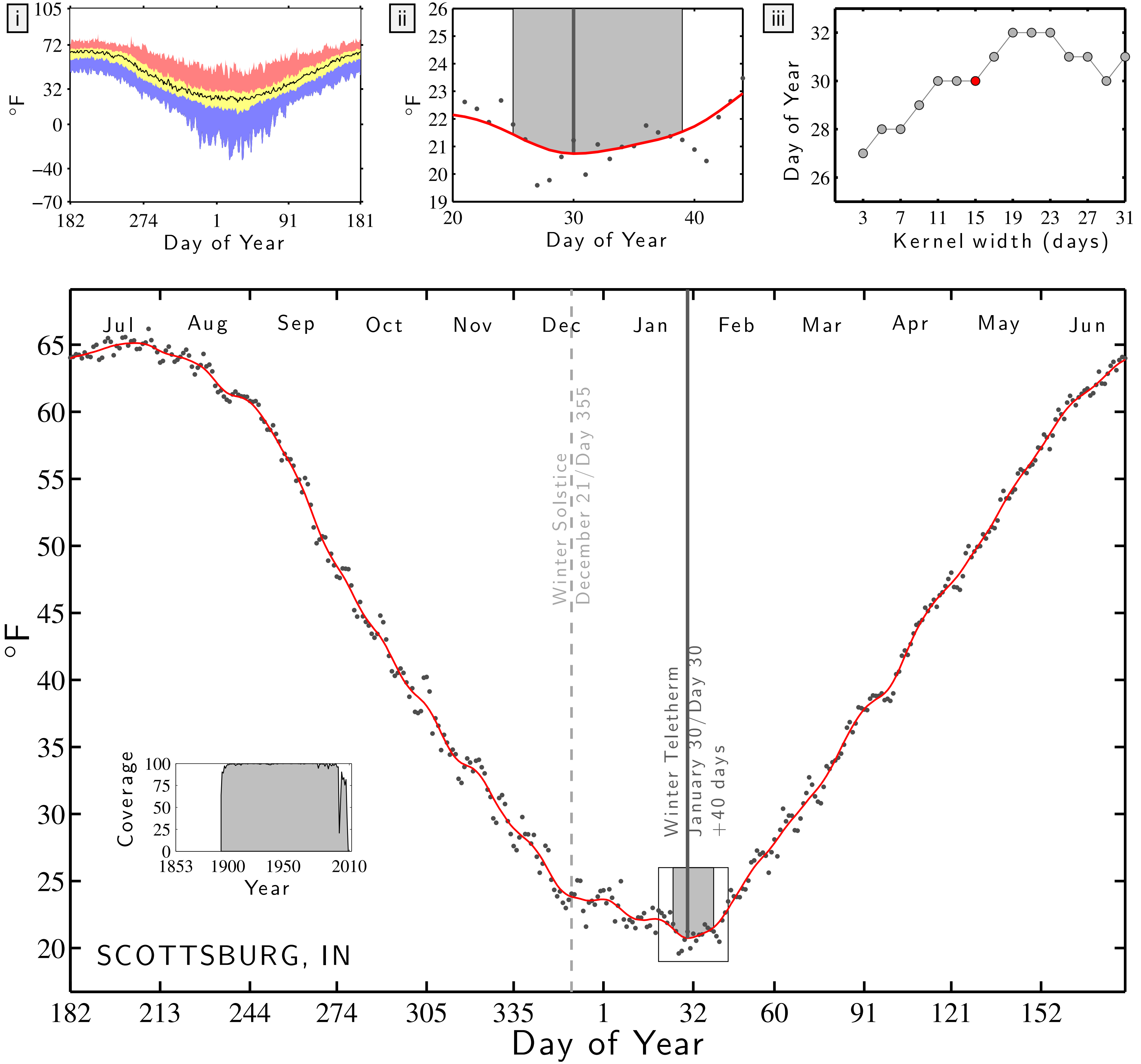

i
°F
Day of Year
ii
°F
Day of Year
iii
Day of Year
Kernel width (days)
Jul
Aug
Sep
Oct
Nov
Dec
Jan
Feb
Mar
Apr
May
Jun
Winter Solstice
December 21/Day 355
Winter Teletherm
January 30/Day 30
+40 days
Coverage
Year
SCOTTSBURG, IN
°F
Day of Year

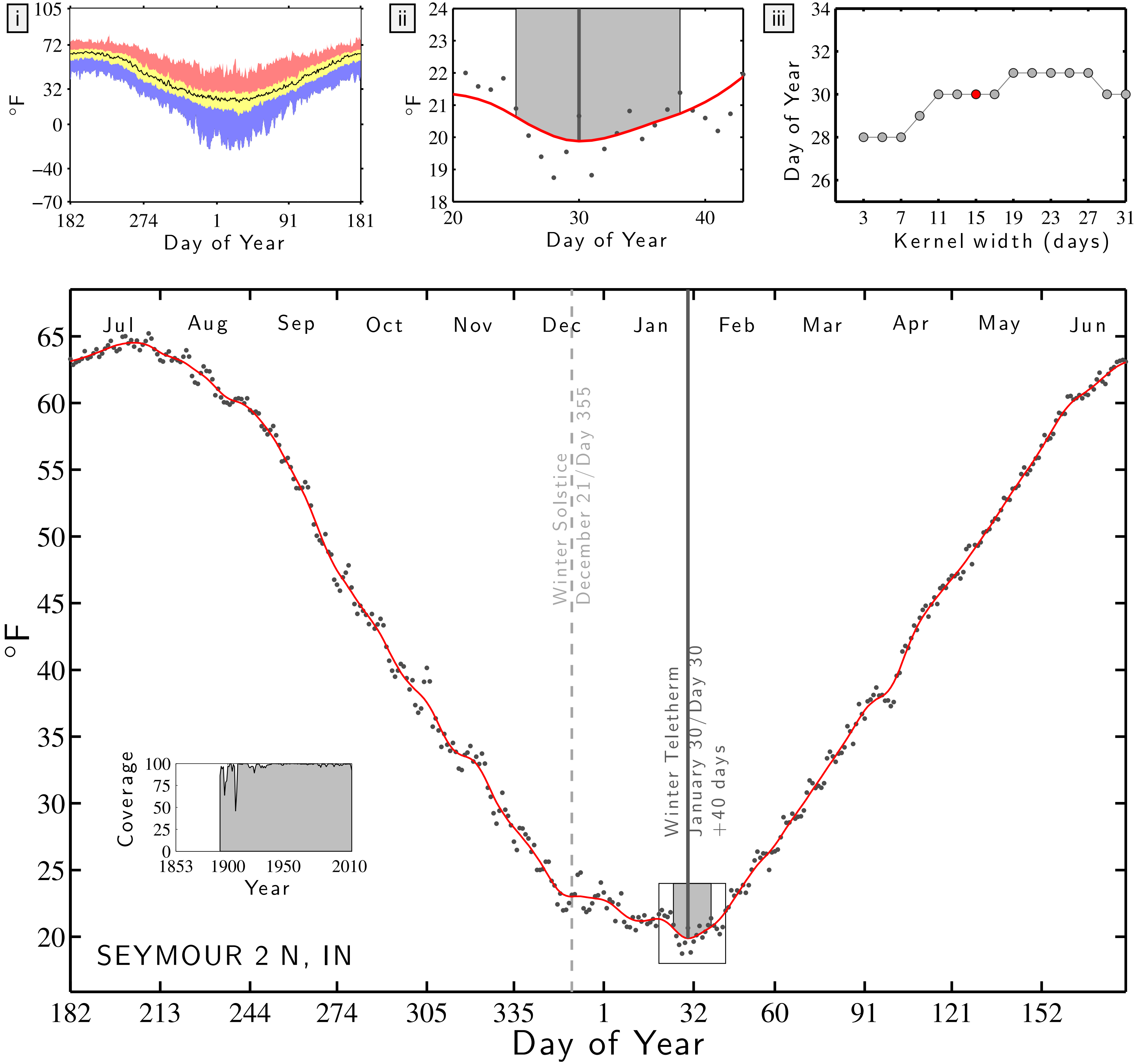

i
°F
Day of Year
ii
°F
Day of Year
iii
Day of Year
Kernel width (days)
Jul
Aug
Sep
Oct
Nov
Dec
Jan
Feb
Mar
Apr
May
Jun
Winter Solstice
December 21/Day 355
Winter Teletherm
January 30/Day 30
+40 days
Coverage
Year
SEYMOUR 2 N, IN
°F
Day of Year

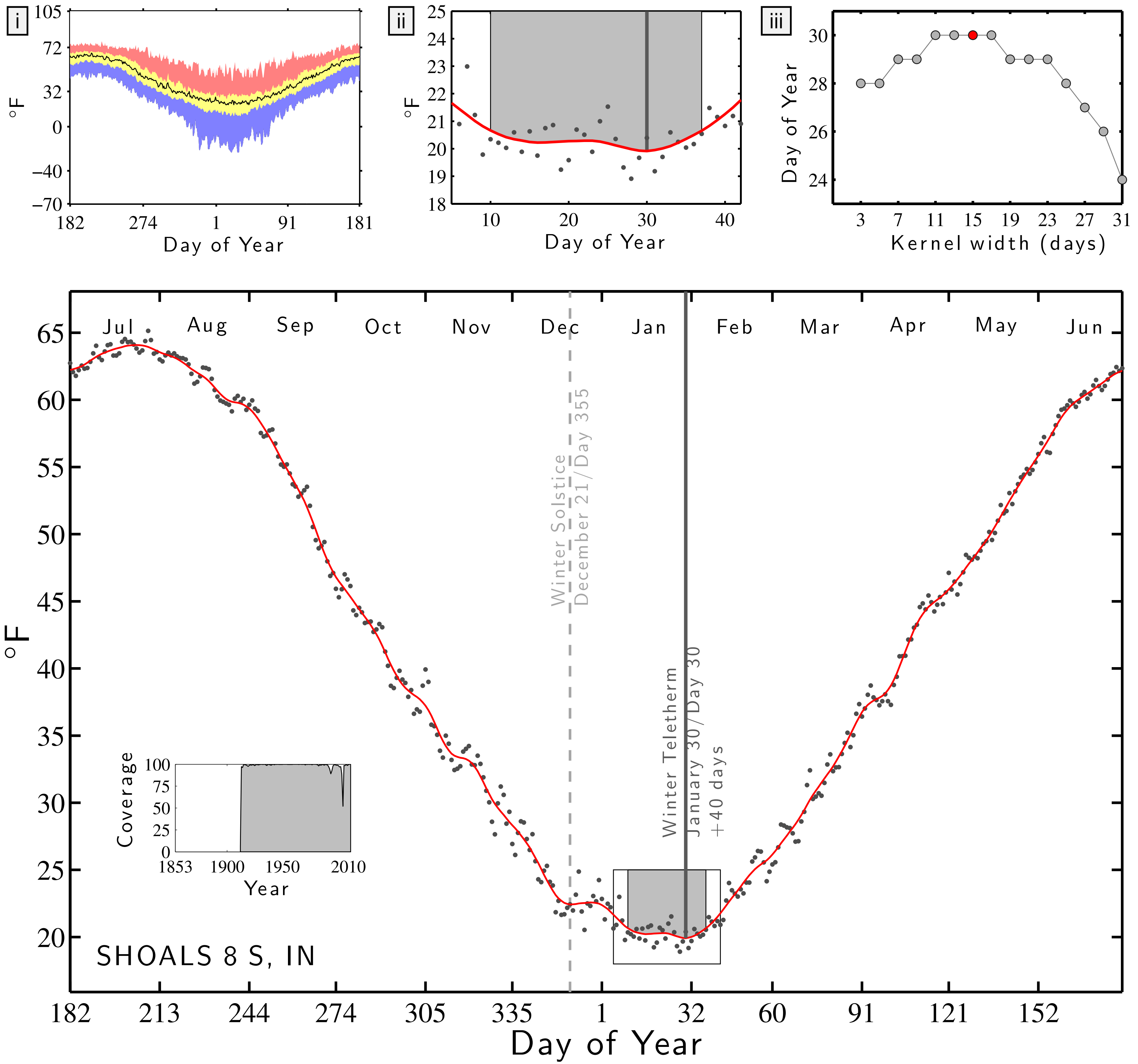

i
°F
Day of Year
ii
°F
Day of Year
iii
Day of Year
Kernel width (days)
Jul
Aug
Sep
Oct
Nov
Dec
Jan
Feb
Mar
Apr
May
Jun
Winter Solstice
December 21/Day 355
Winter Teletherm
January 30/Day 30
+40 days
Coverage
Year
SHOALS 8 S, IN
°F
Day of Year

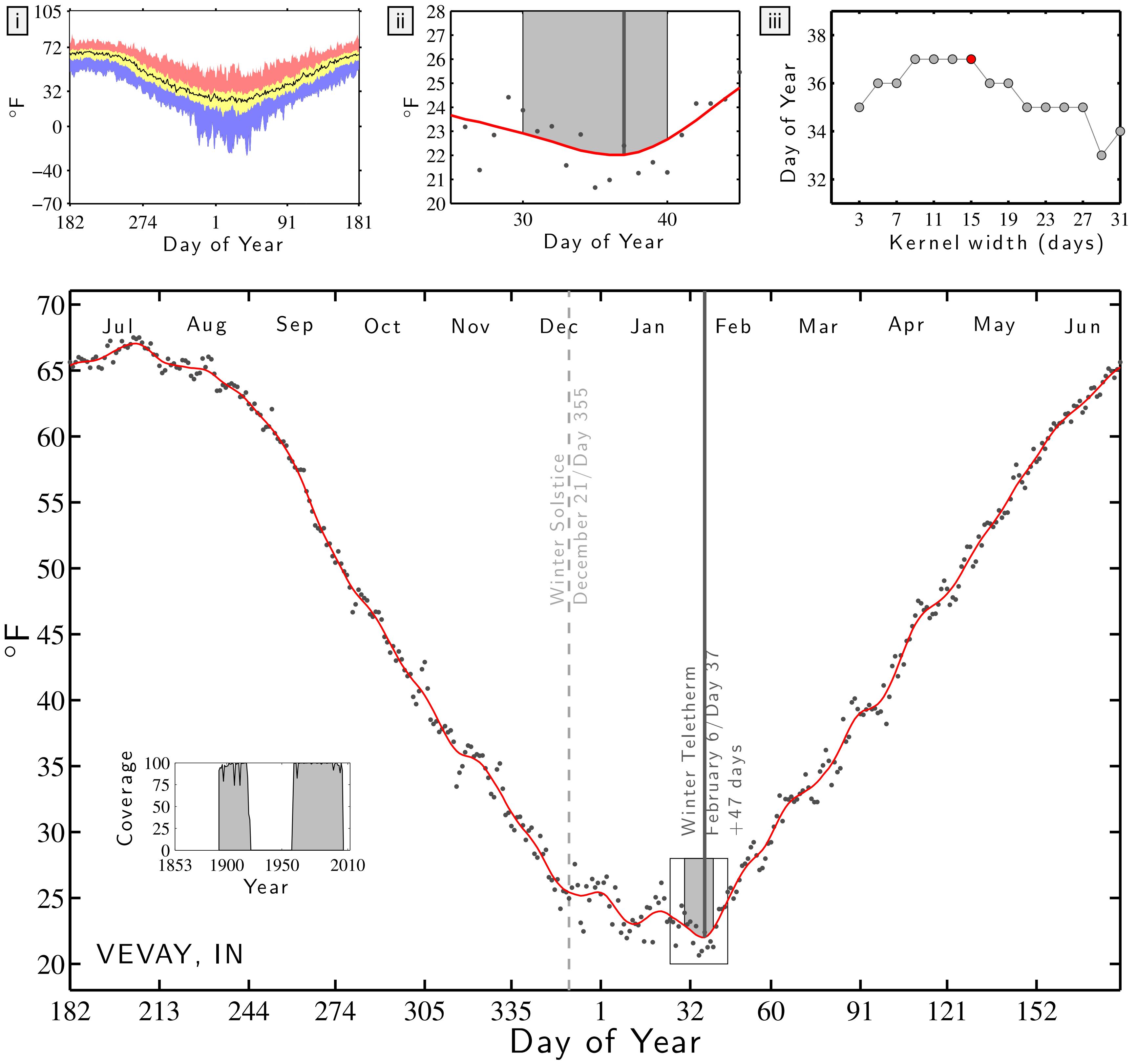

i
ii
iii
°F
Day of Year
Kernel width (days)
Jul
Aug
Sep
Oct
Nov
Dec
Jan
Feb
Mar
Apr
May
Jun
Winter Solstice
December 21/Day 355
Winter Teletherm
February 6/Day 37
+47 days
Coverage
Year
VEVAY, IN

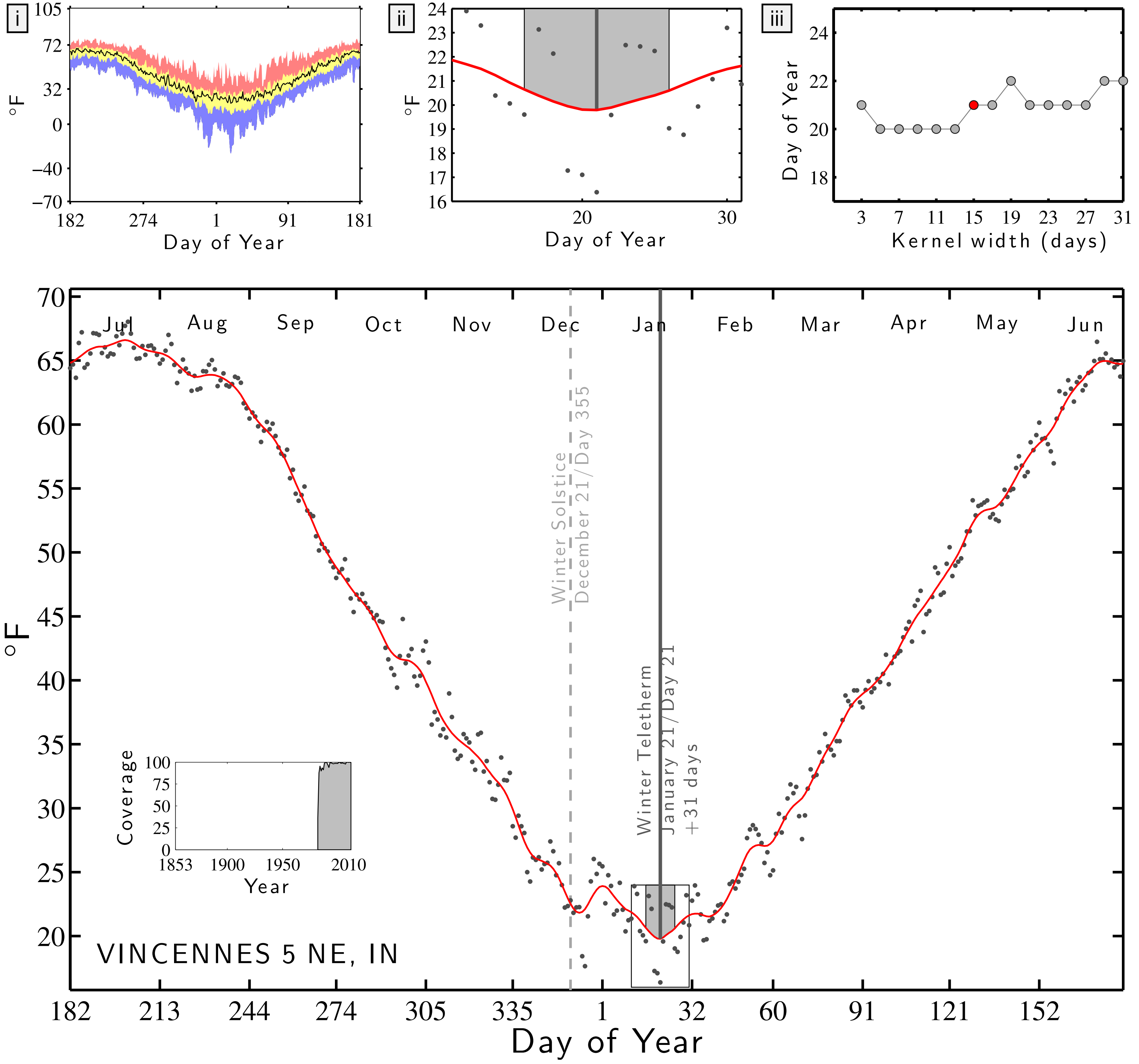

i
105
72
32
0
–40
–70
°F
182
274
1
91
181
Day of Year
ii
24
23
22
21
20
19
18
17
16
°F
20
30
Day of Year
iii
24
22
20
18
Day of Year
3
7
11
15
19
23
27
31
Kernel width (days)
70
65
60
55
50
45
40
35
30
25
20
°F
Jul
Aug
Sep
Oct
Nov
Dec
Jan
Feb
Mar
Apr
May
Jun
Winter Solstice
December 21/Day 355
Winter Teletherm
January 21/Day 21
+31 days
Coverage
100
75
50
25
0
1853
1900
1950
2010
Year
VINCENNES 5 NE, IN
182
213
244
274
305
335
1
32
60
91
121
152
Day of Year

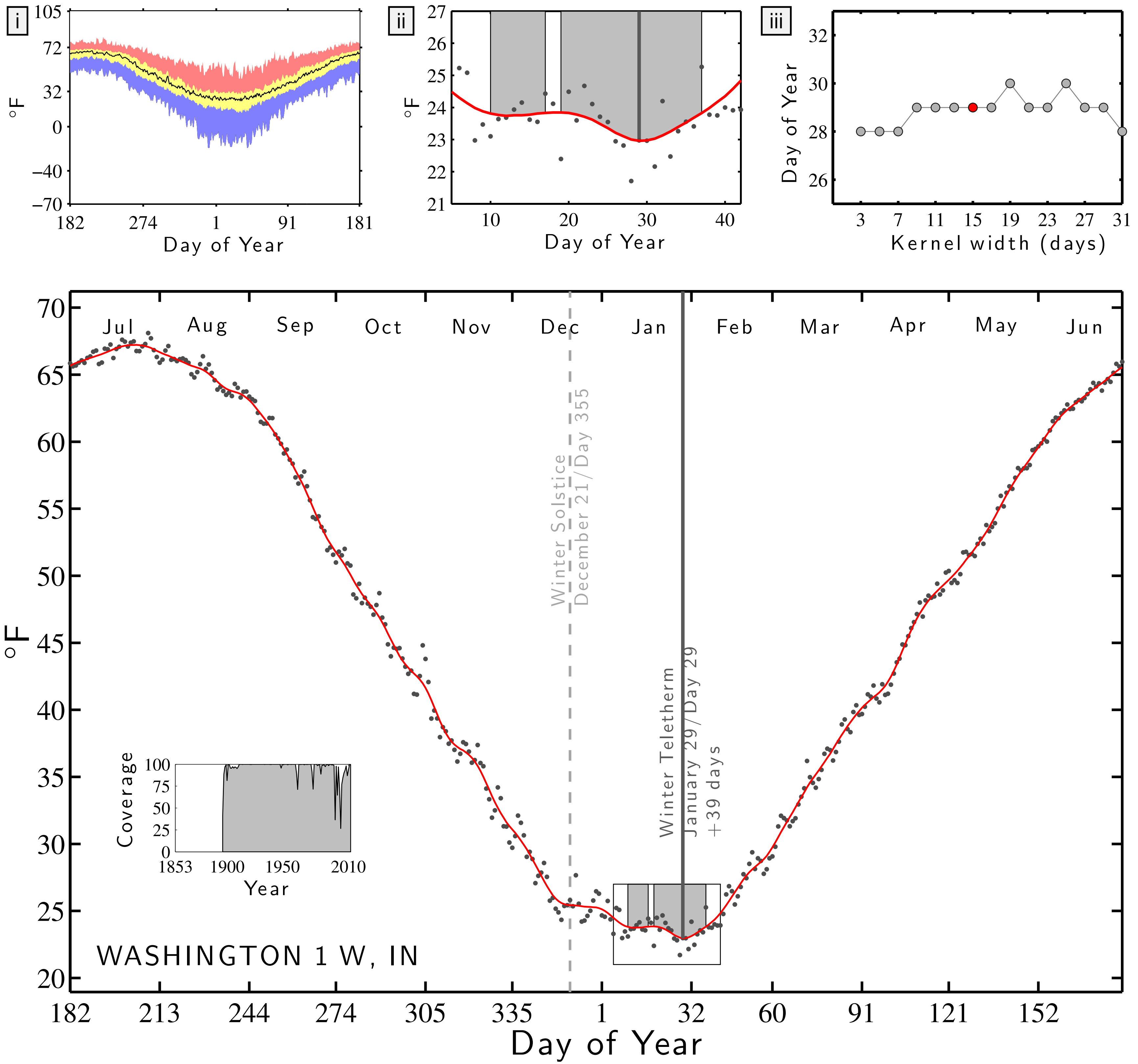

i
ii
iii
°F
Day of Year
Day of Year
Kernel width (days)
Jul
Aug
Sep
Oct
Nov
Dec
Jan
Feb
Mar
Apr
May
Jun
Winter Solstice
December 21/Day 355
Winter Teletherm
January 29/Day 29
+39 days
Coverage
Year
WASHINGTON 1 W, IN
Day of Year

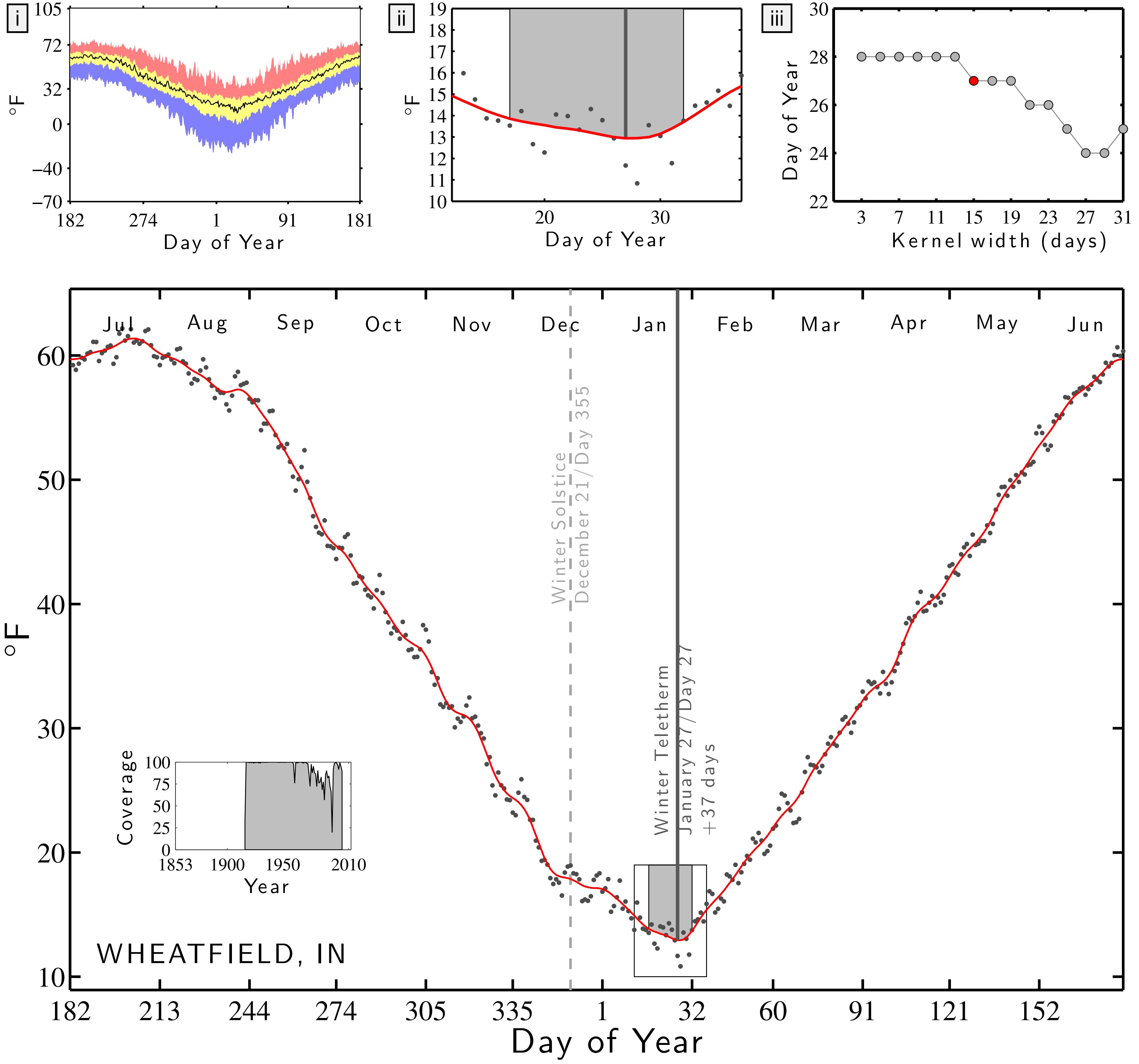

i
105
72
32
0
−40
−70
°F
182
274
1
91
181
Day of Year
ii
°F
20
30
Day of Year
iii
Day of Year
Kernel width (days)
Jul
Aug
Sep
Oct
Nov
Dec
Jan
Feb
Mar
Apr
May
Jun
Winter Solstice
December 21/Day 355
Winter Teletherm
January 27/Day 27
+37 days
Coverage
Year
1853
1900
1950
2010
WHEATFIELD, IN
°F
182
213
244
274
305
335
1
32
60
91
121
152
Day of Year

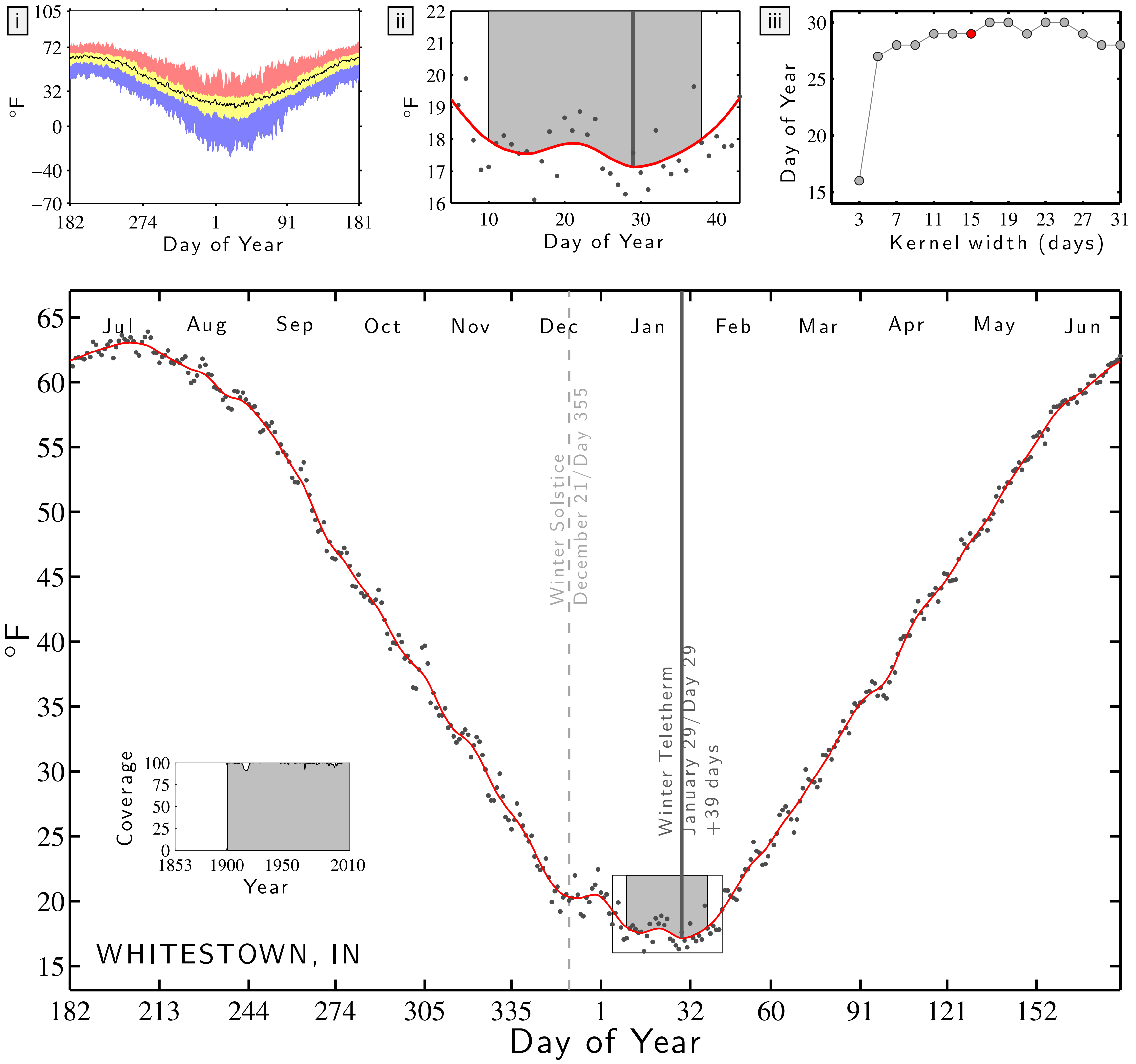

i
°F
Day of Year
ii
Day of Year
iii
Day of Year
Kernel width (days)
Jul
Aug
Sep
Oct
Nov
Dec
Jan
Feb
Mar
Apr
May
Jun
Winter Solstice
December 21/Day 355
Winter Teletherm
January 29/Day 29
+39 days
Coverage
Year
WHITESTOWN, IN
°F
Day of Year

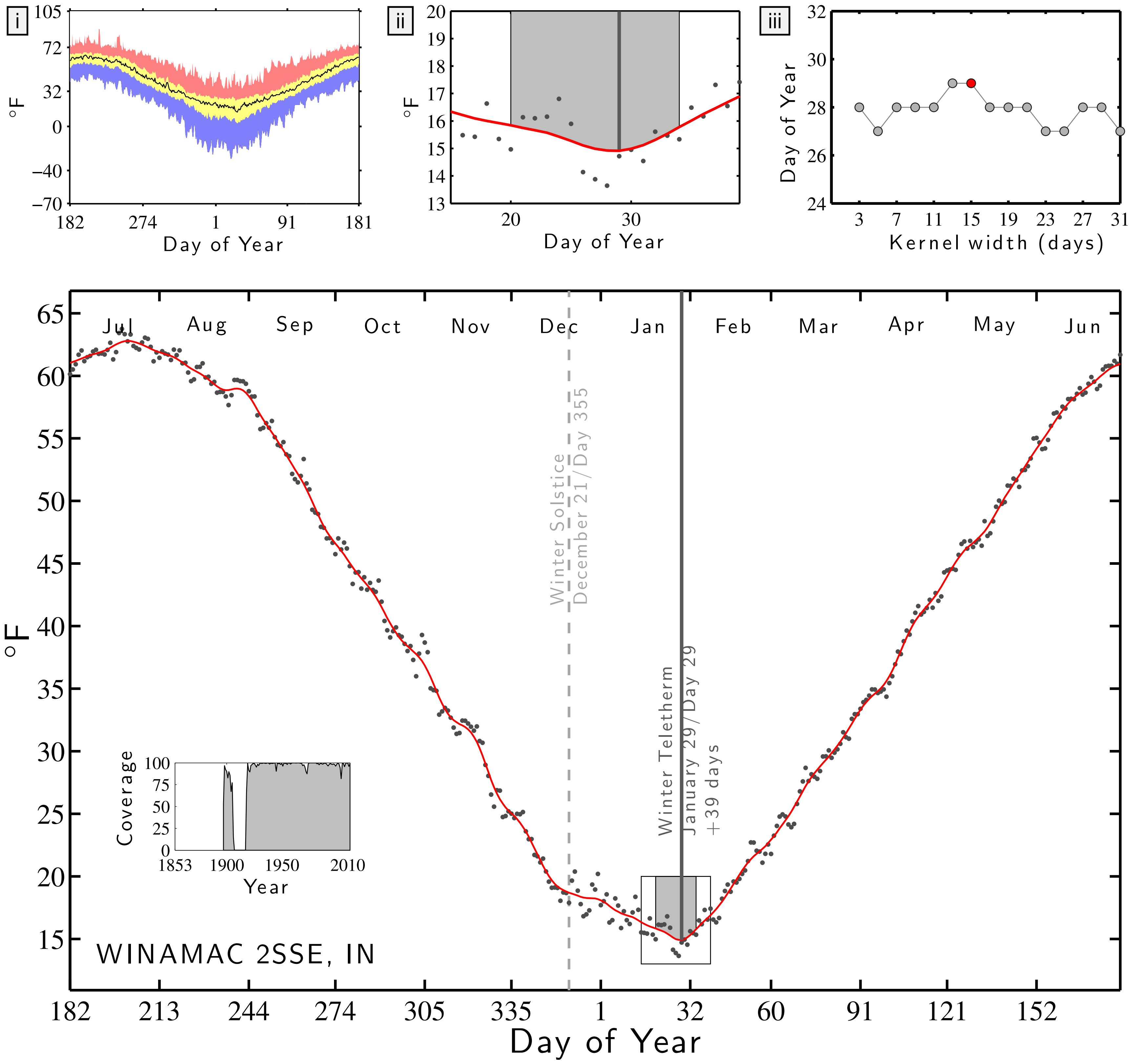

i
°F
Day of Year
ii
°F
Day of Year
iii
Day of Year
Kernel width (days)
Jul
Aug
Sep
Oct
Nov
Dec
Jan
Feb
Mar
Apr
May
Jun
Winter Solstice
December 21/Day 355
Winter Teletherm
January 29/Day 29
+39 days
Coverage
Year
WINAMAC 2SSE, IN
°F
Day of Year

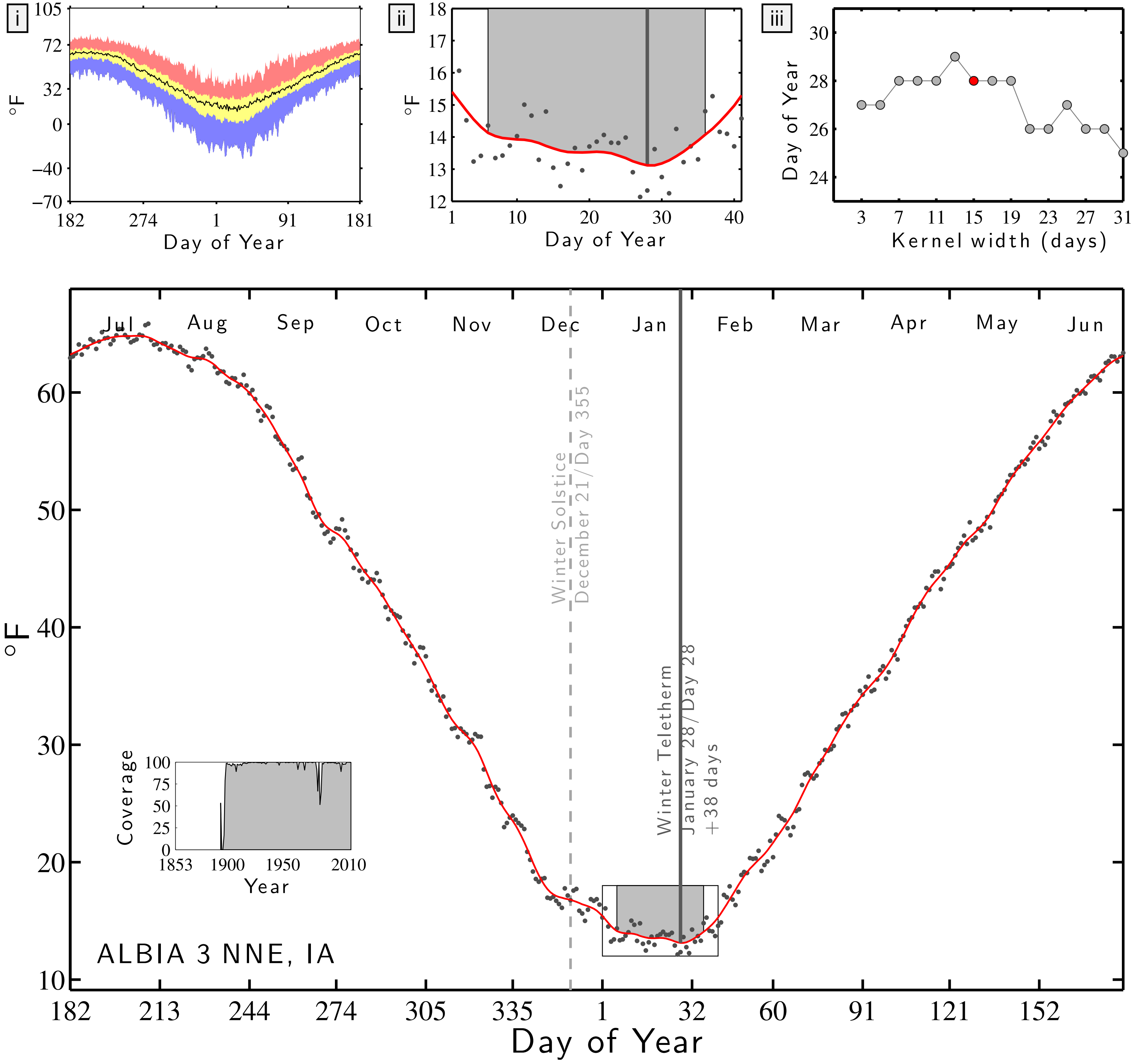

i
ii
iii
°F
Day of Year
Day of Year
Kernel width (days)
Jul
Aug
Sep
Oct
Nov
Dec
Jan
Feb
Mar
Apr
May
Jun
Winter Solstice
December 21/Day 355
Winter Teletherm
January 28/Day 28
+38 days
Coverage
Year
ALBIA 3 NNE, IA
Day of Year

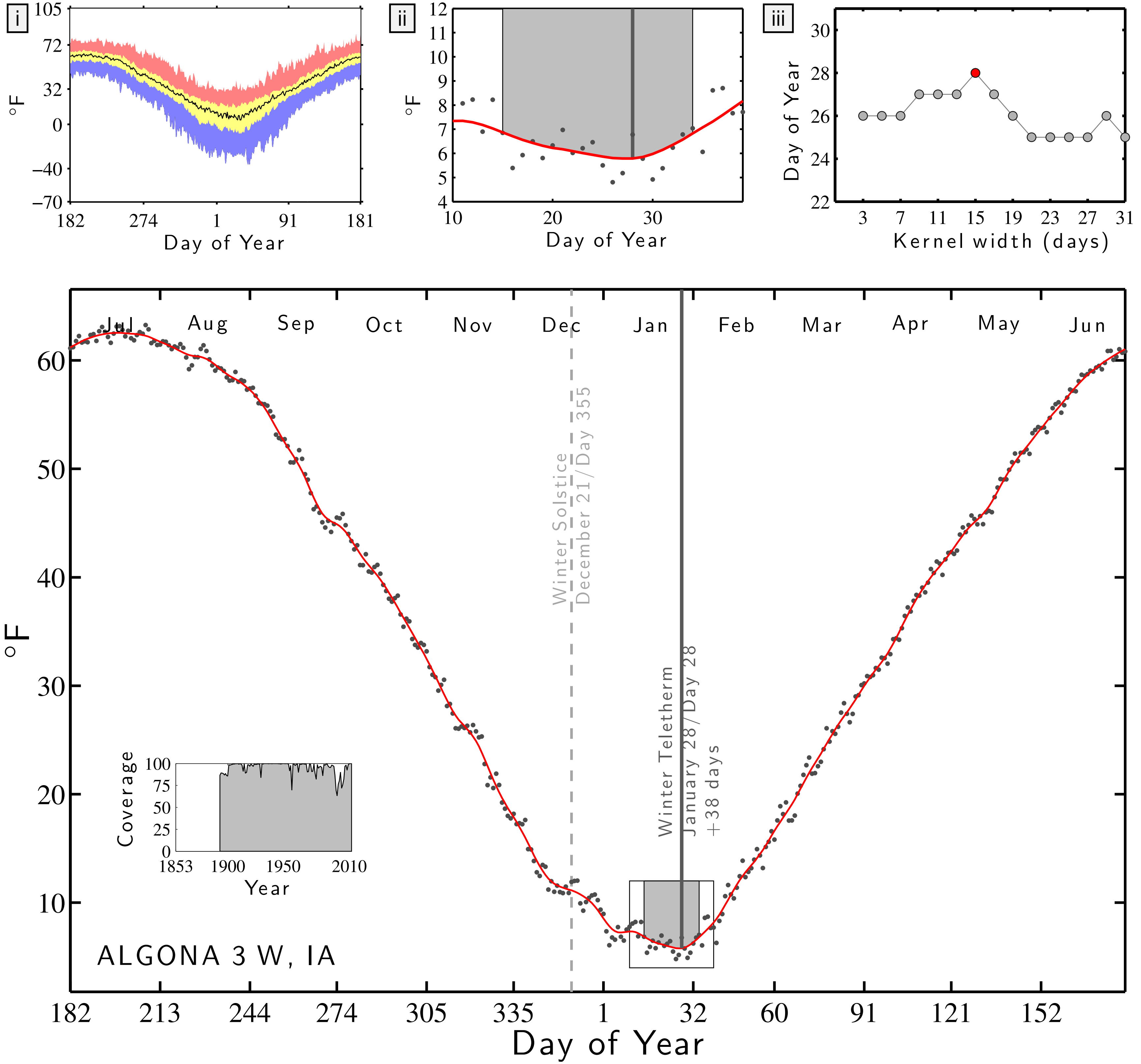

i
ii
iii
Day of Year
Kernel width (days)
Jul
Aug
Sep
Oct
Nov
Dec
Jan
Feb
Mar
Apr
May
Jun
Winter Solstice
December 21/Day 355
Winter Teletherm
January 28/Day 28
+38 days
Coverage
Year
ALGONA 3 W, IA
°F

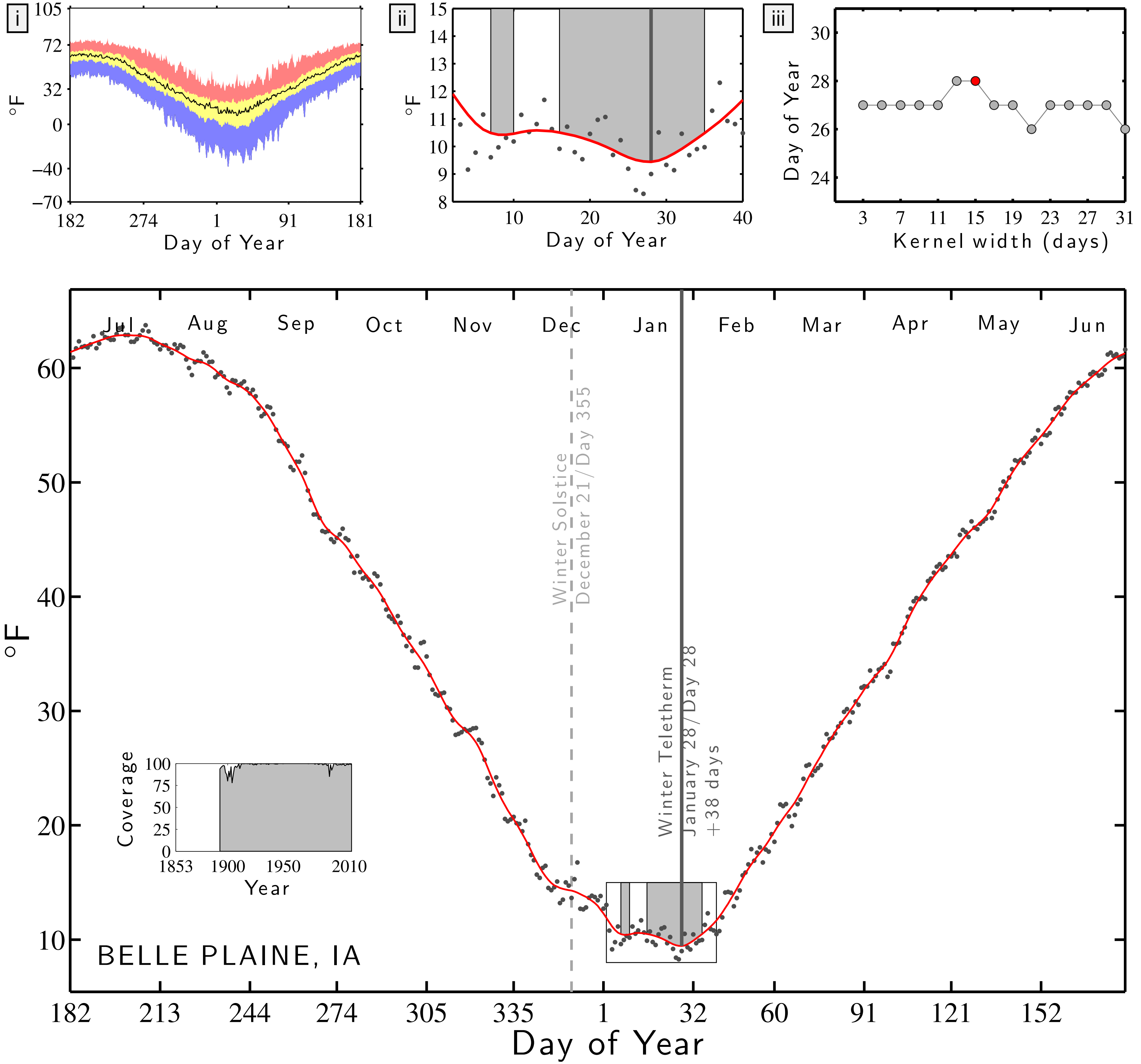

i
°F
Day of Year
ii
Day of Year
iii
Day of Year
Kernel width (days)
Jul
Aug
Sep
Oct
Nov
Dec
Jan
Feb
Mar
Apr
May
Jun
Winter Solstice
December 21/Day 355
Winter Teletherm
January 28/Day 28
+38 days
Coverage
Year
BELLE PLAINE, IA
°F
Day of Year

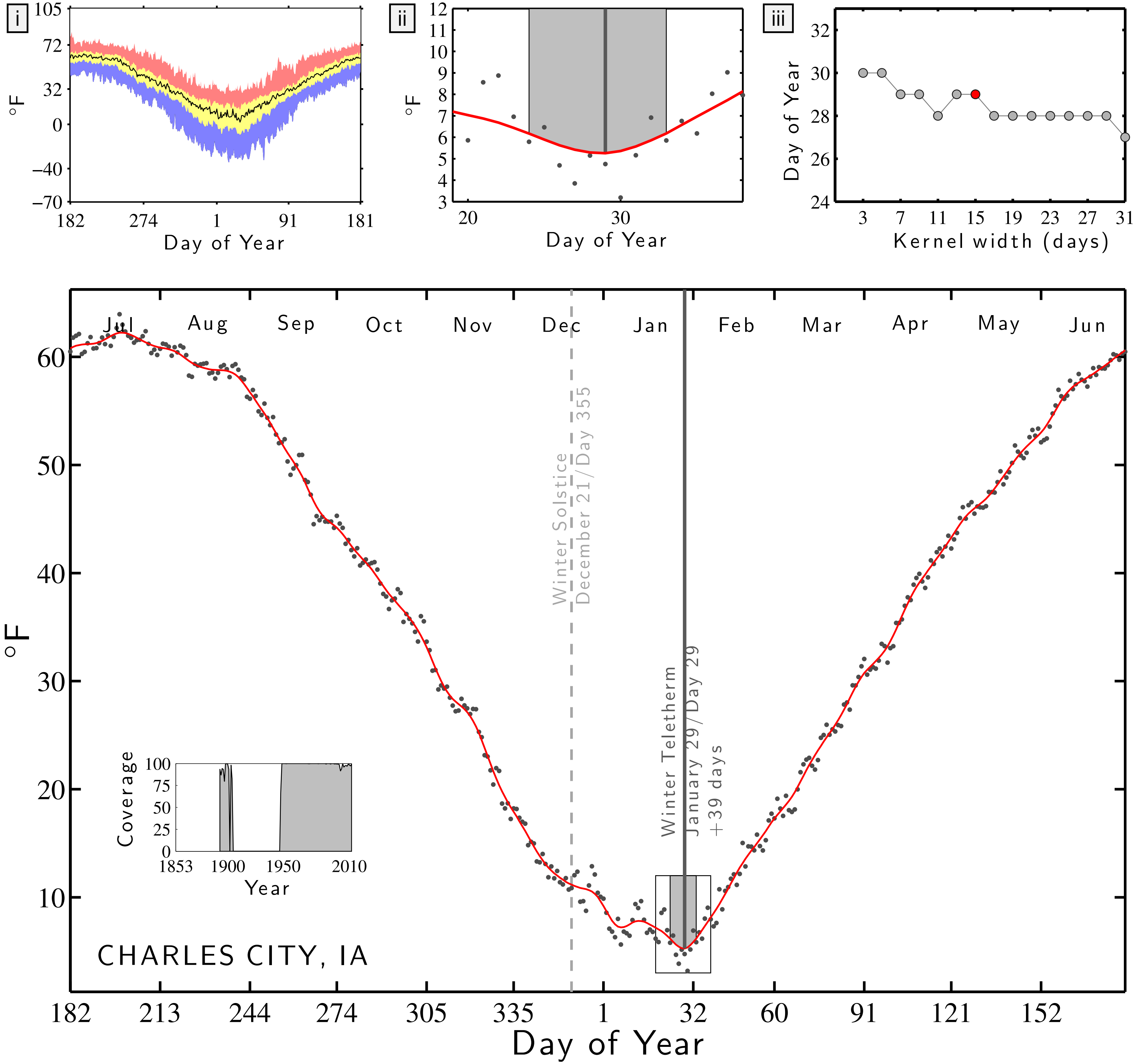

i
°F
Day of Year
ii
°F
Day of Year
iii
Day of Year
Kernel width (days)
Jul
Aug
Sep
Oct
Nov
Dec
Jan
Feb
Mar
Apr
May
Jun
Winter Solstice
December 21/Day 355
Winter Teletherm
January 29/Day 29
+39 days
Coverage
Year
CHARLES CITY, IA
°F
Day of Year

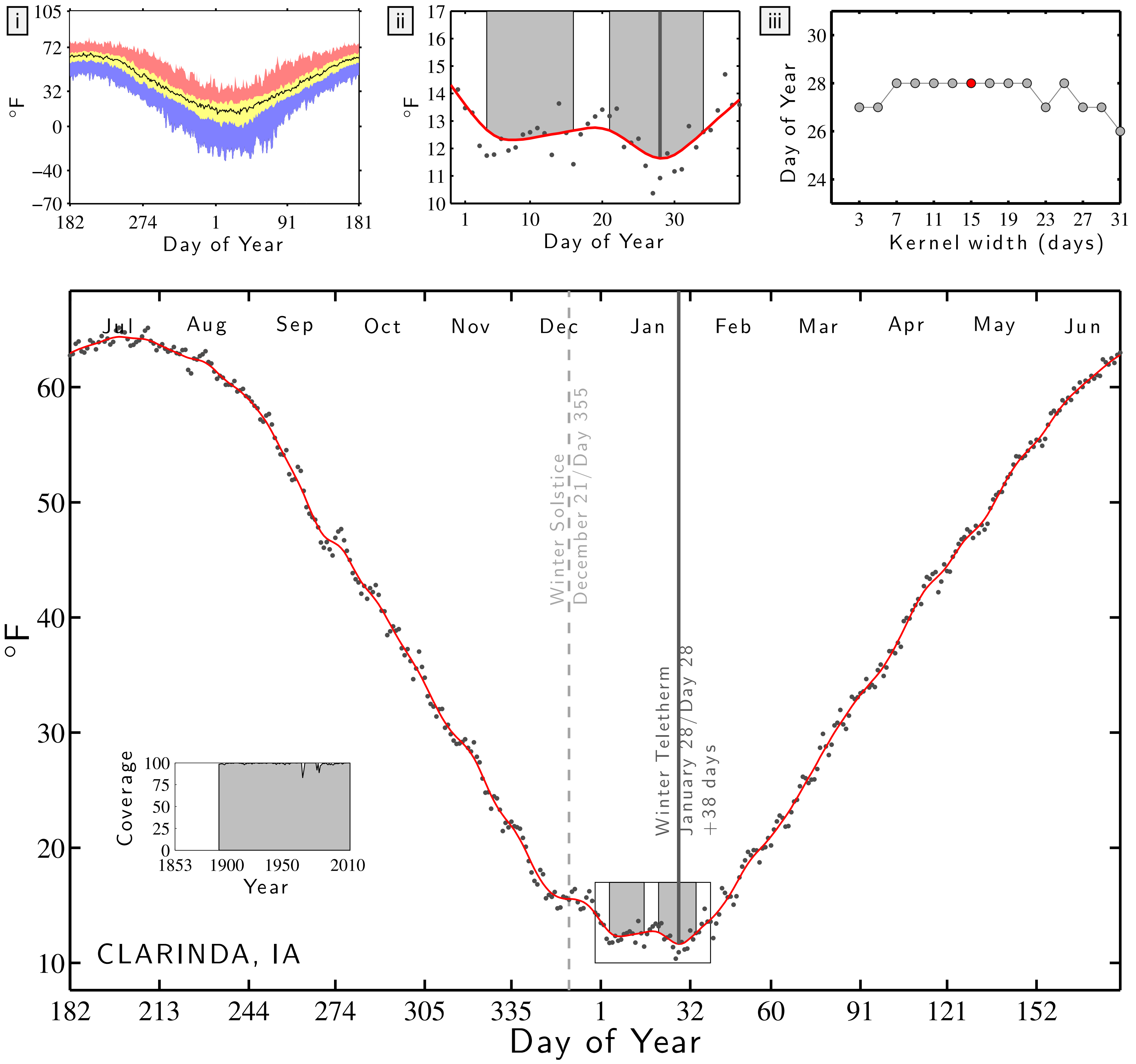

i
°F
Day of Year
ii
iii
Day of Year
Kernel width (days)
Jul
Aug
Sep
Oct
Nov
Dec
Jan
Feb
Mar
Apr
May
Jun
Winter Solstice
December 21/Day 355
Winter Teletherm
January 28/Day 28
+38 days
Coverage
Year
CLARINDA, IA

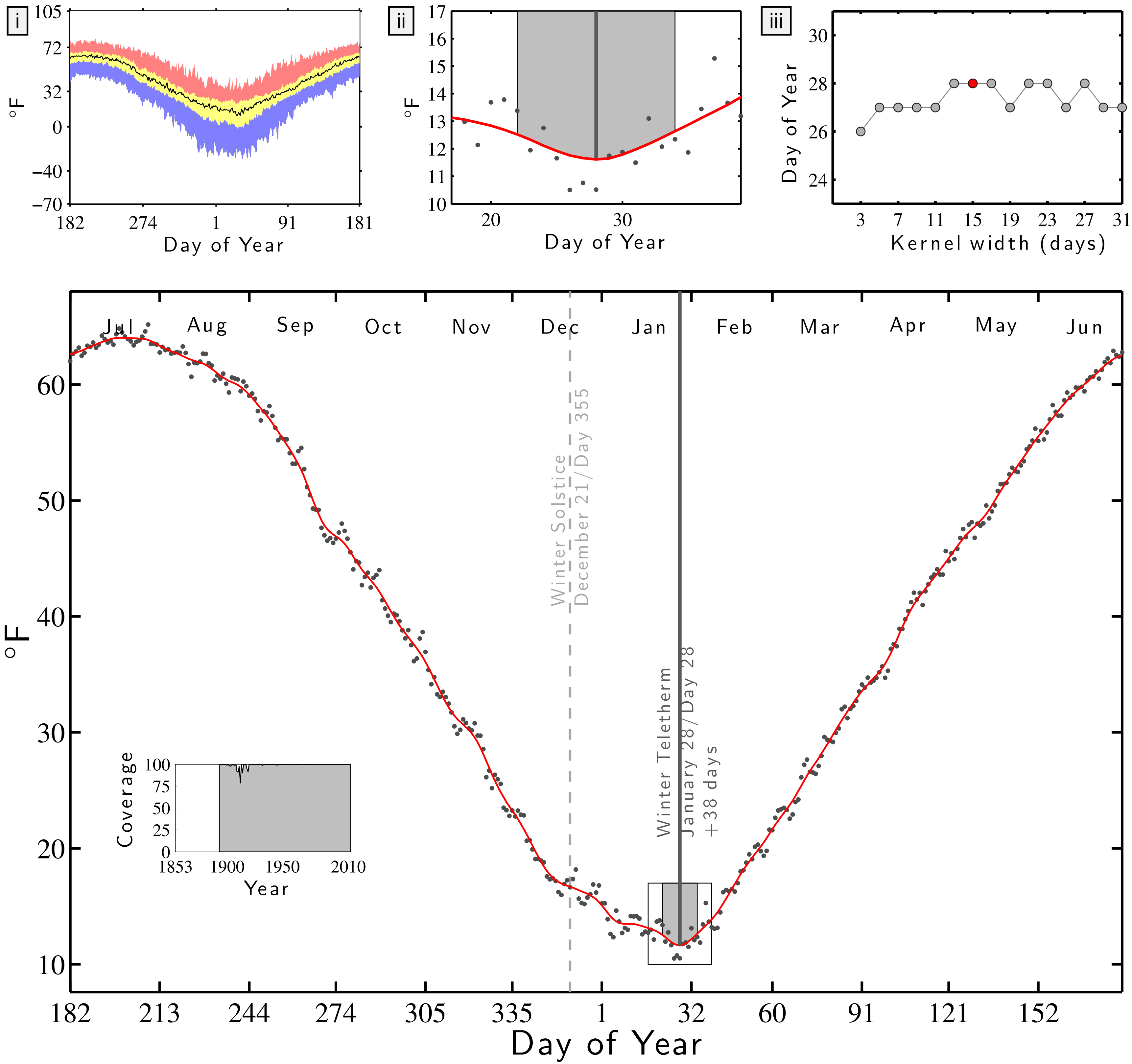
i
°F
Day of Year
ii
°F
Day of Year
iii
Day of Year
Kernel width (days)
Jul
Aug
Sep
Oct
Nov
Dec
Jan
Feb
Mar
Apr
May
Jun
Winter Solstice
December 21/Day 355
Winter Teletherm
January 28/Day 28
+38 days
Coverage
Year
°F
Day of Year

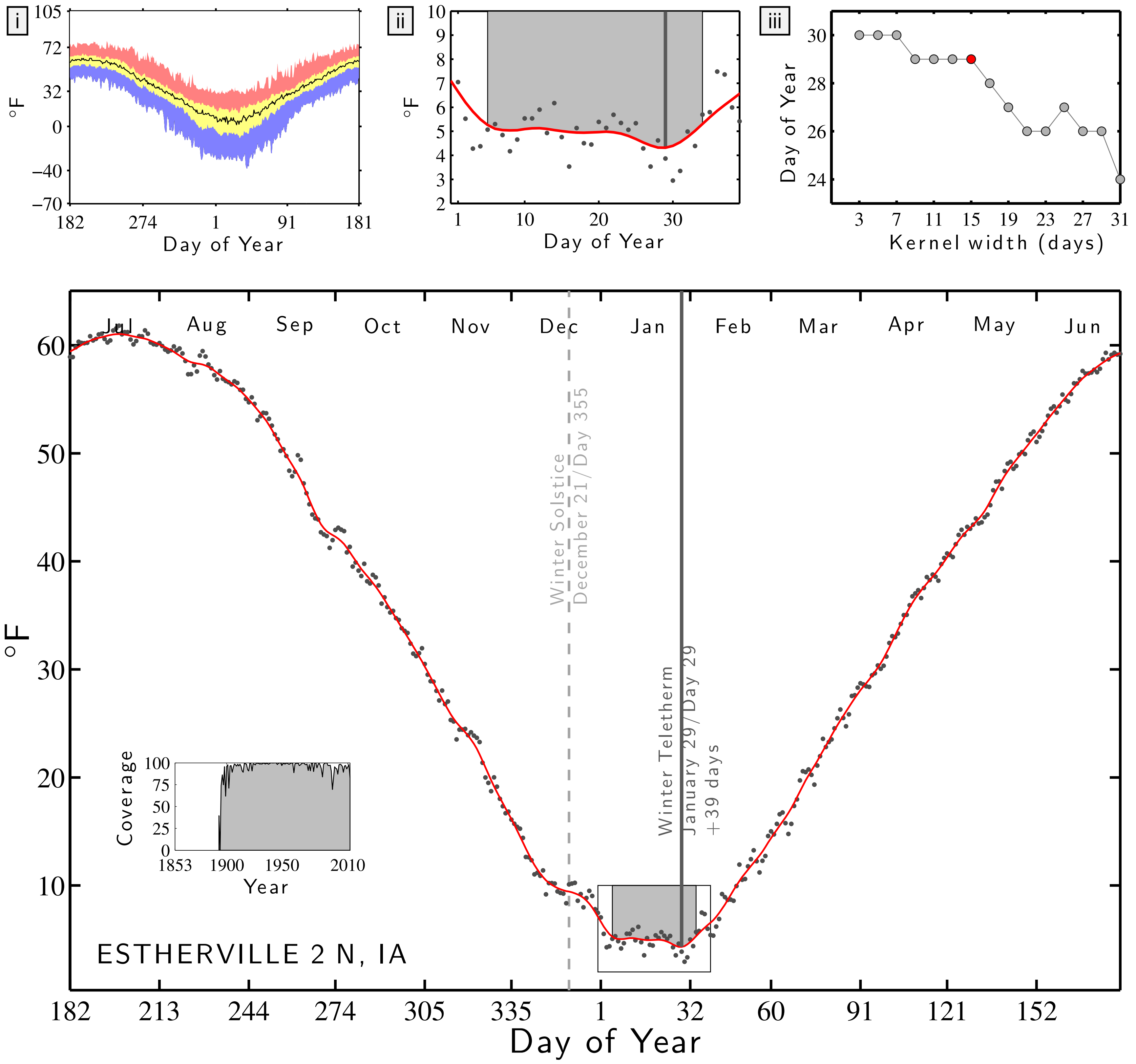

i
ii
iii
Day of Year
°F
Kernel width (days)
Jul
Aug
Sep
Oct
Nov
Dec
Jan
Feb
Mar
Apr
May
Jun
Winter Solstice
December 21/Day 355
Winter Teletherm
January 29/Day 29
+39 days
Coverage
Year
ESTHERVILLE 2 N, IA

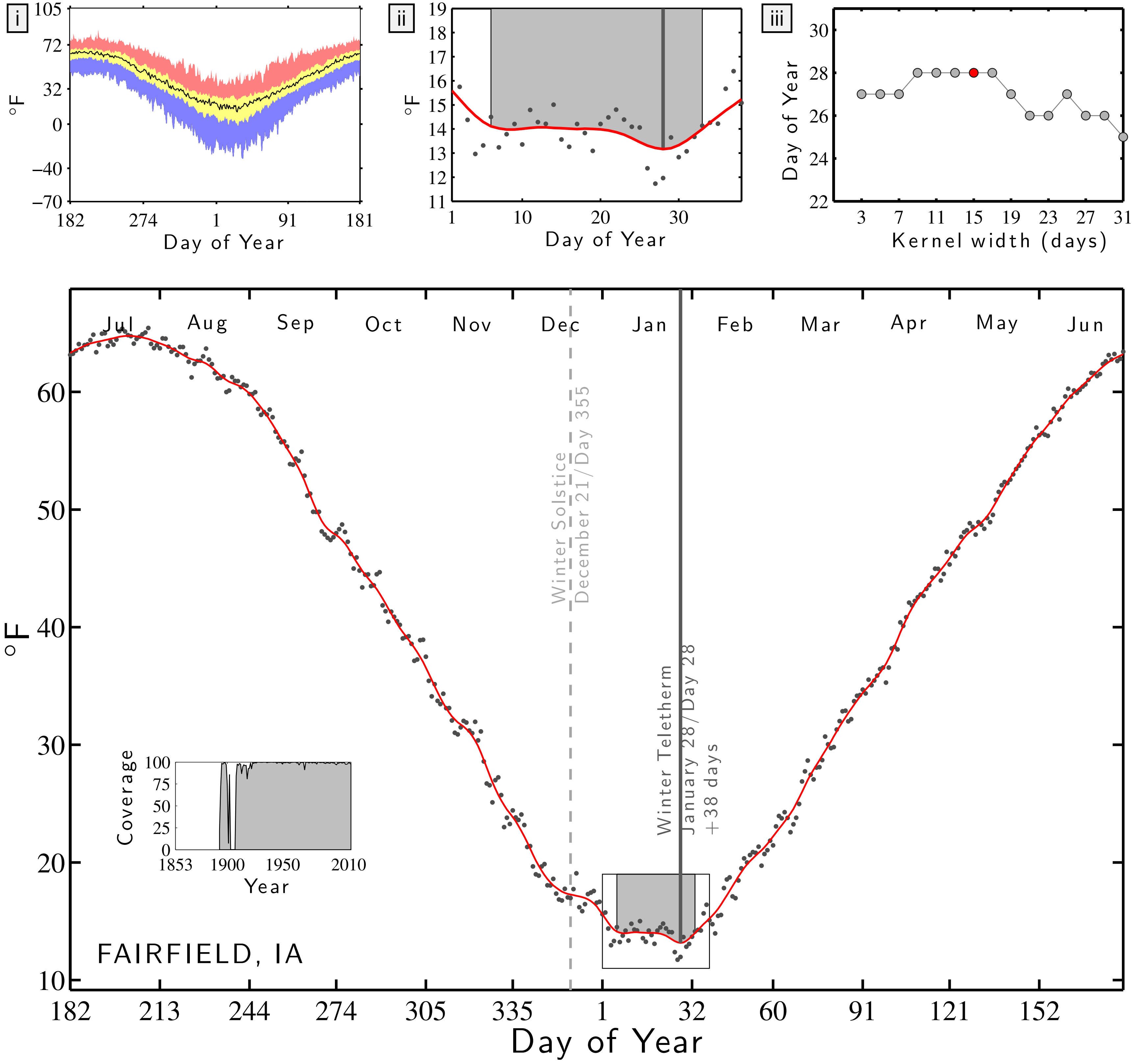

i
ii
iii
°F
Day of Year
Kernel width (days)
Jul
Aug
Sep
Oct
Nov
Dec
Jan
Feb
Mar
Apr
May
Jun
Winter Solstice
December 21/Day 355
Winter Teletherm
January 28/Day 28
+38 days
Coverage
Year
FAIRFIELD, IA

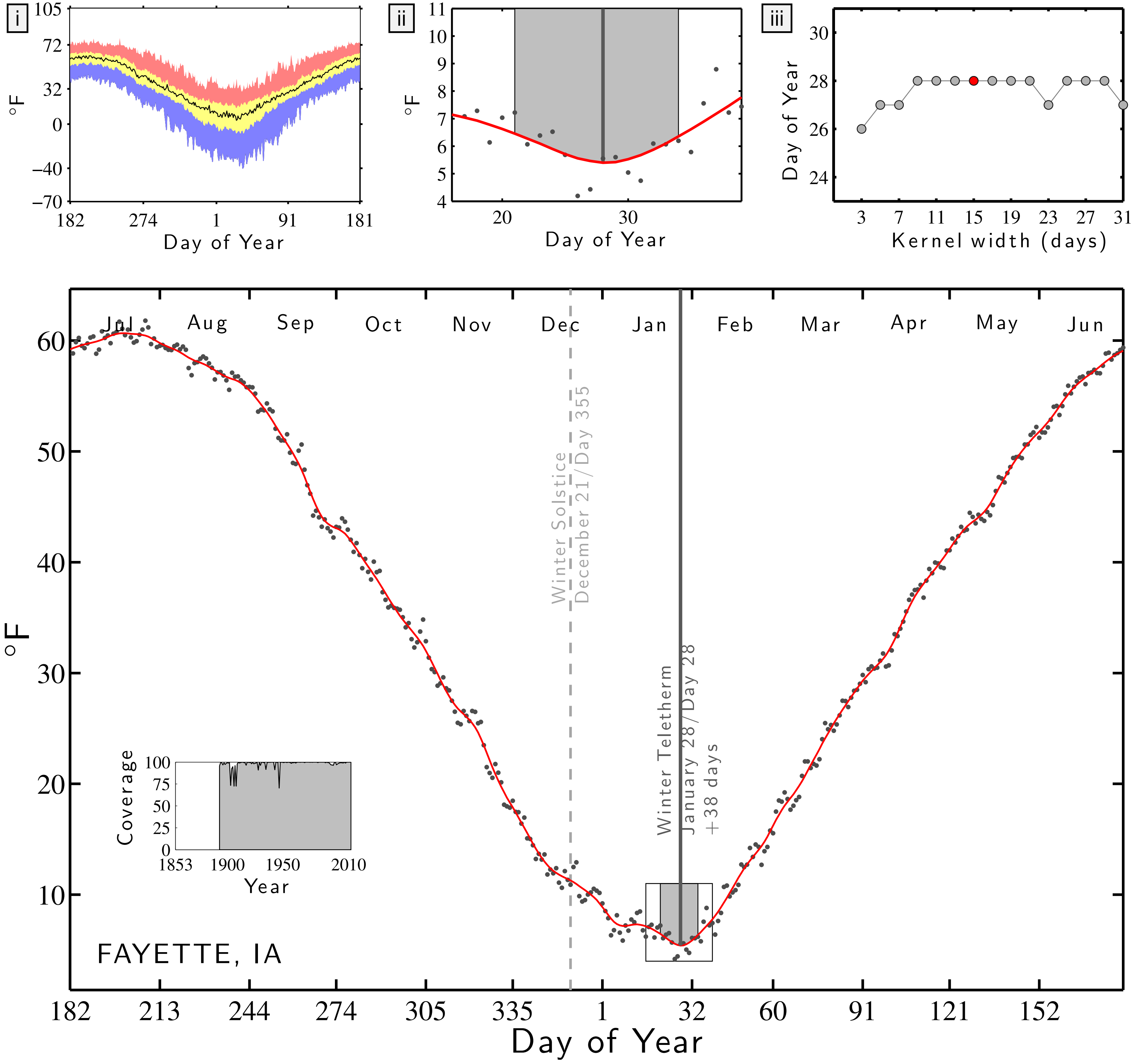

i
°F
Day of Year
ii
Day of Year
iii
Day of Year
Kernel width (days)
Jul
Aug
Sep
Oct
Nov
Dec
Jan
Feb
Mar
Apr
May
Jun
Winter Solstice
December 21/Day 355
Winter Teletherm
January 28/Day 28
+38 days
Coverage
Year
FAYETTE, IA
Day of Year

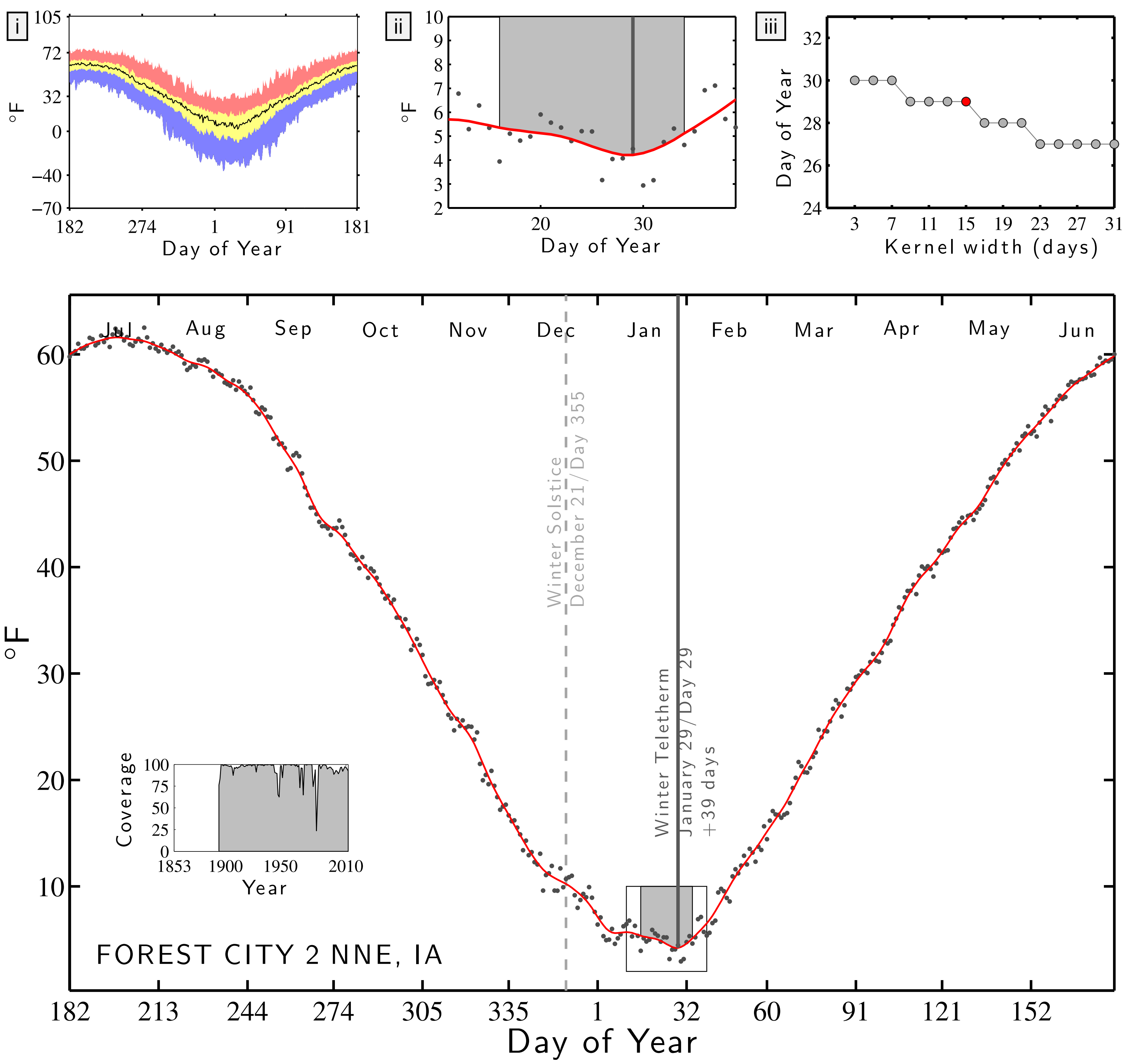

i
°F
Day of Year
ii
Day of Year
iii
Day of Year
Kernel width (days)
Jul
Aug
Sep
Oct
Nov
Dec
Jan
Feb
Mar
Apr
May
Jun
Winter Solstice
December 21/Day 355
Winter Teletherm
January 29/Day 29
+39 days
Coverage
Year
FOREST CITY 2 NNE, IA
Day of Year

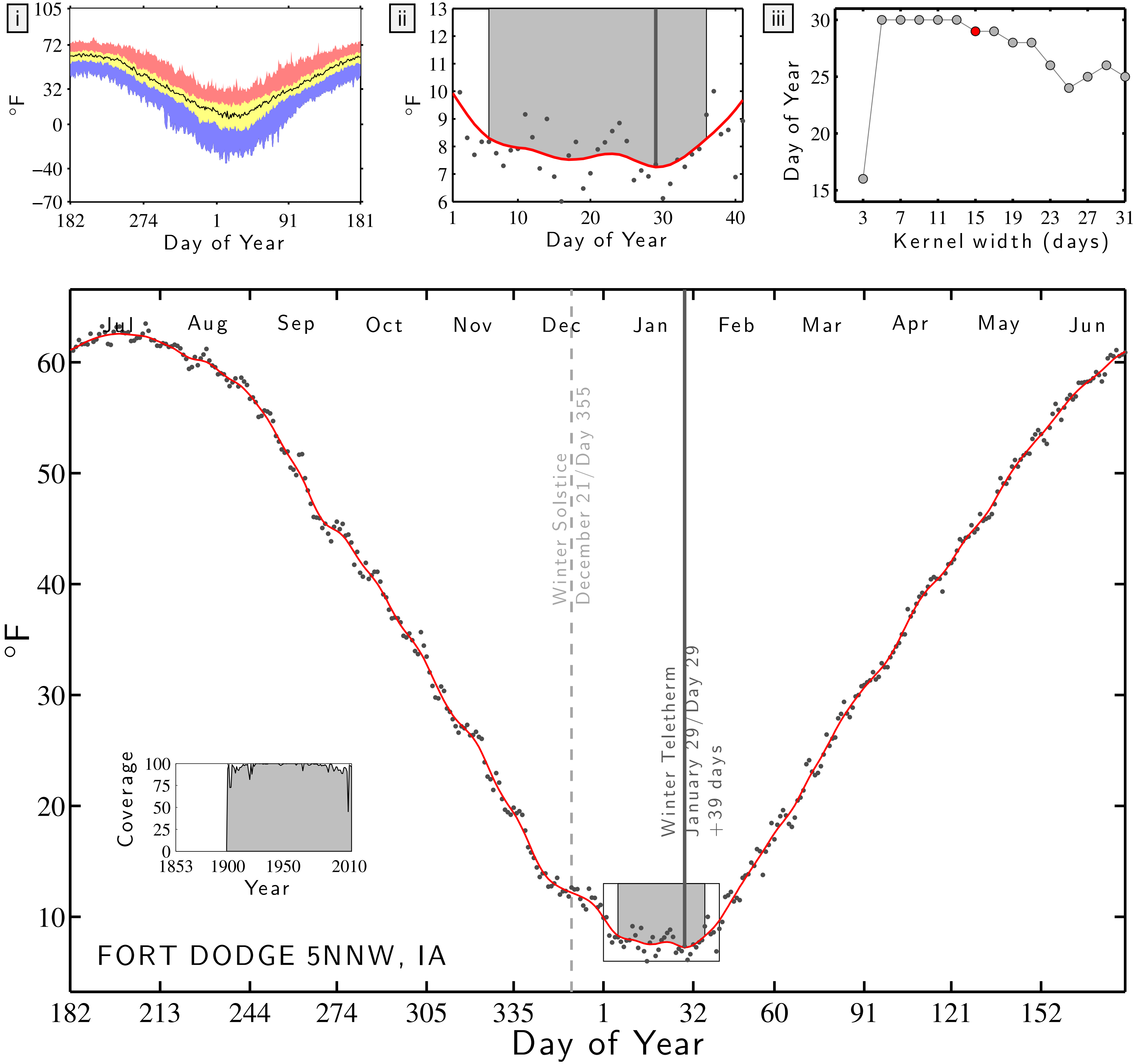

i
ii
iii
°F
Day of Year
Kernel width (days)
Jul
Aug
Sep
Oct
Nov
Dec
Jan
Feb
Mar
Apr
May
Jun
Winter Solstice
December 21/Day 355
Winter Teletherm
January 29/Day 29
+39 days
Coverage
Year
FORT DODGE 5NNW, IA

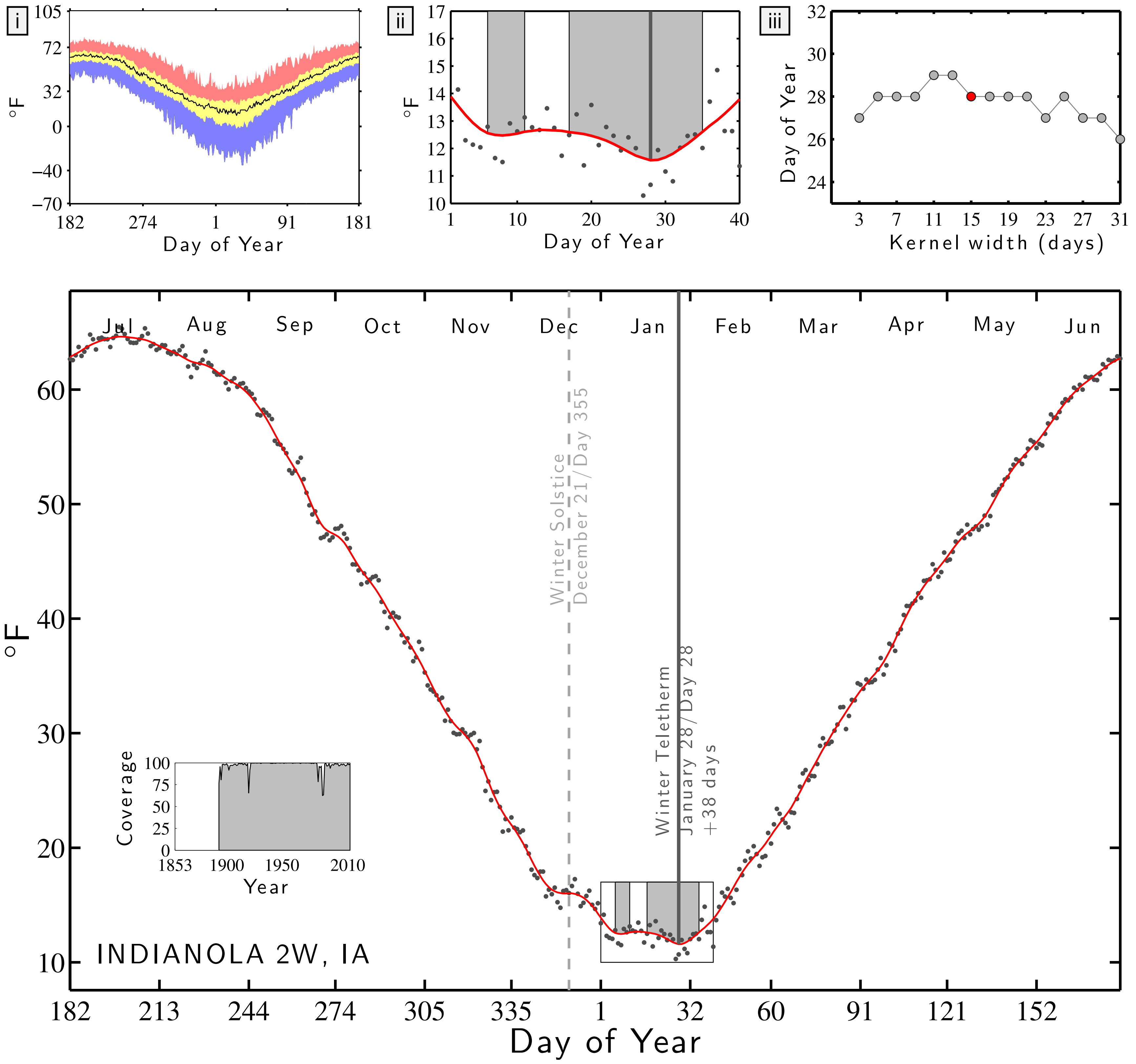

i
°F
Day of Year
ii
°F
Day of Year
iii
Day of Year
Kernel width (days)
Jul
Aug
Sep
Oct
Nov
Dec
Jan
Feb
Mar
Apr
May
Jun
Winter Solstice
December 21/Day 355
Winter Teletherm
January 28/Day 28
+38 days
Coverage
Year
INDIANOLA 2W, IA
°F
Day of Year

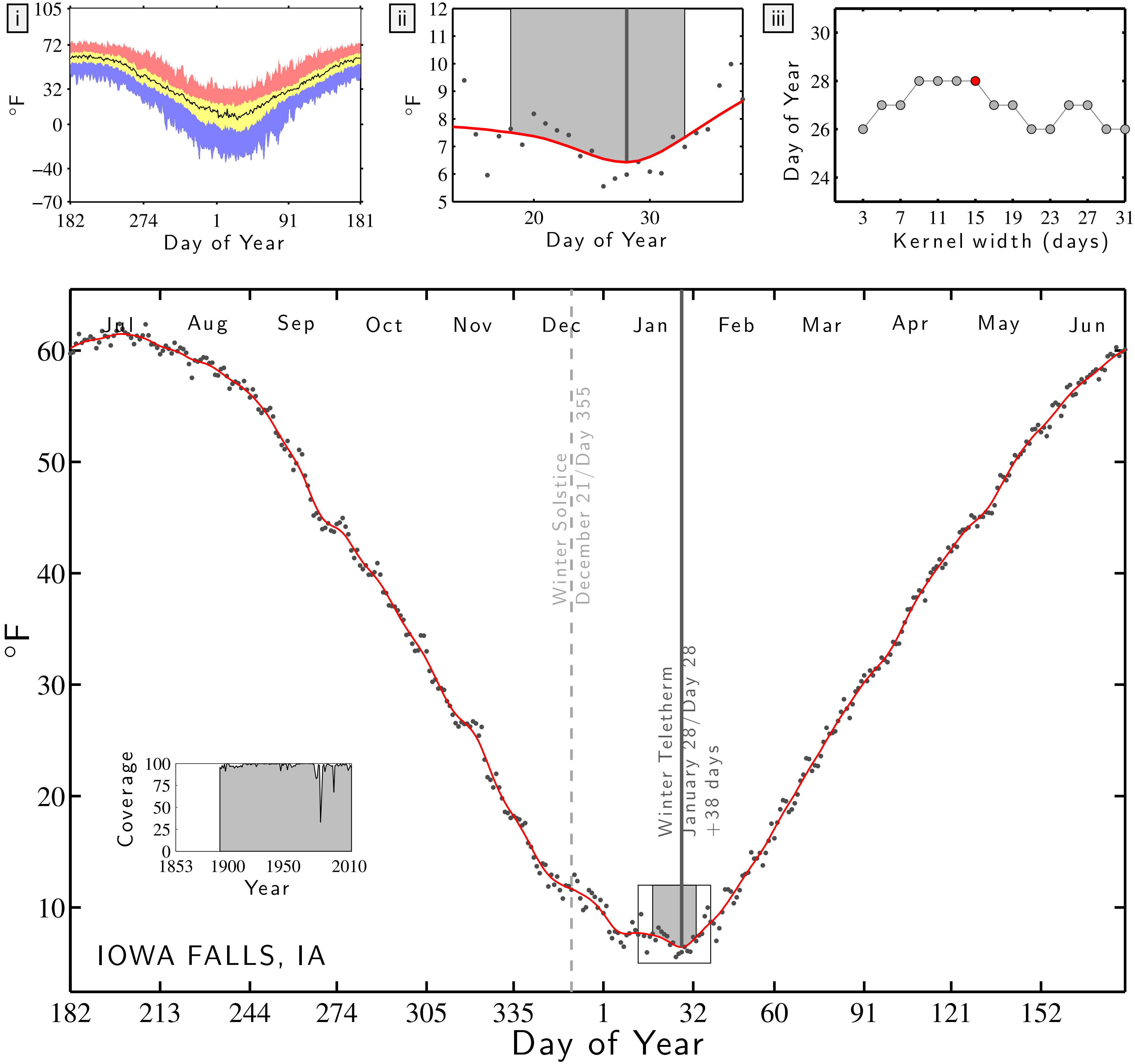

i
ii
iii
°F
Day of Year
Kernel width (days)
Jul
Aug
Sep
Oct
Nov
Dec
Jan
Feb
Mar
Apr
May
Jun
Winter Solstice
December 21/Day 355
Winter Teletherm
January 28/Day 28
+38 days
Coverage
Year
IOWA FALLS, IA

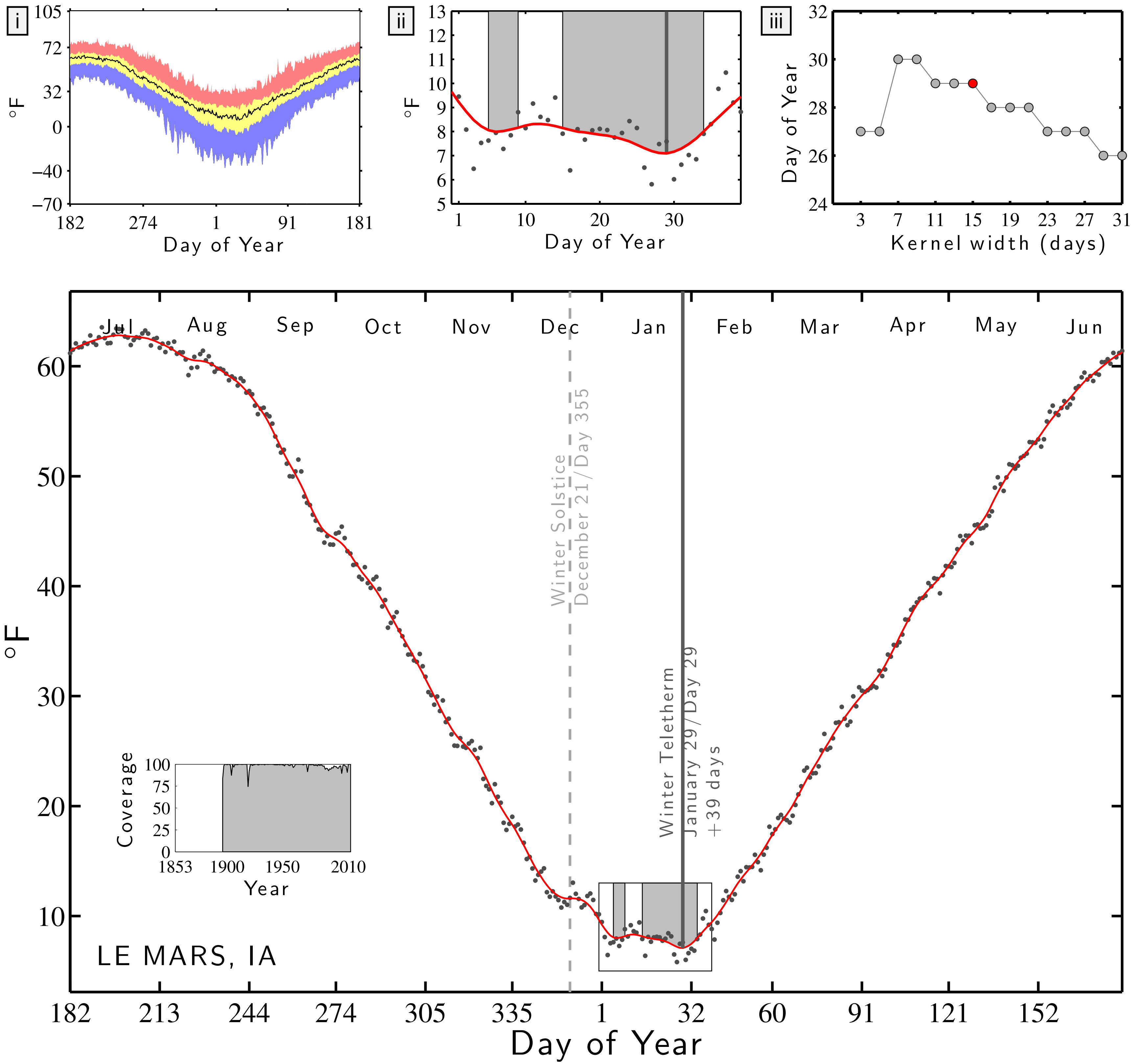

i
°F
Day of Year
ii
°F
Day of Year
iii
Day of Year
Kernel width (days)
Jul
Aug
Sep
Oct
Nov
Dec
Jan
Feb
Mar
Apr
May
Jun
Winter Solstice
December 21/Day 355
Winter Teletherm
January 29/Day 29
+39 days
Coverage
Year
LE MARS, IA
°F
Day of Year

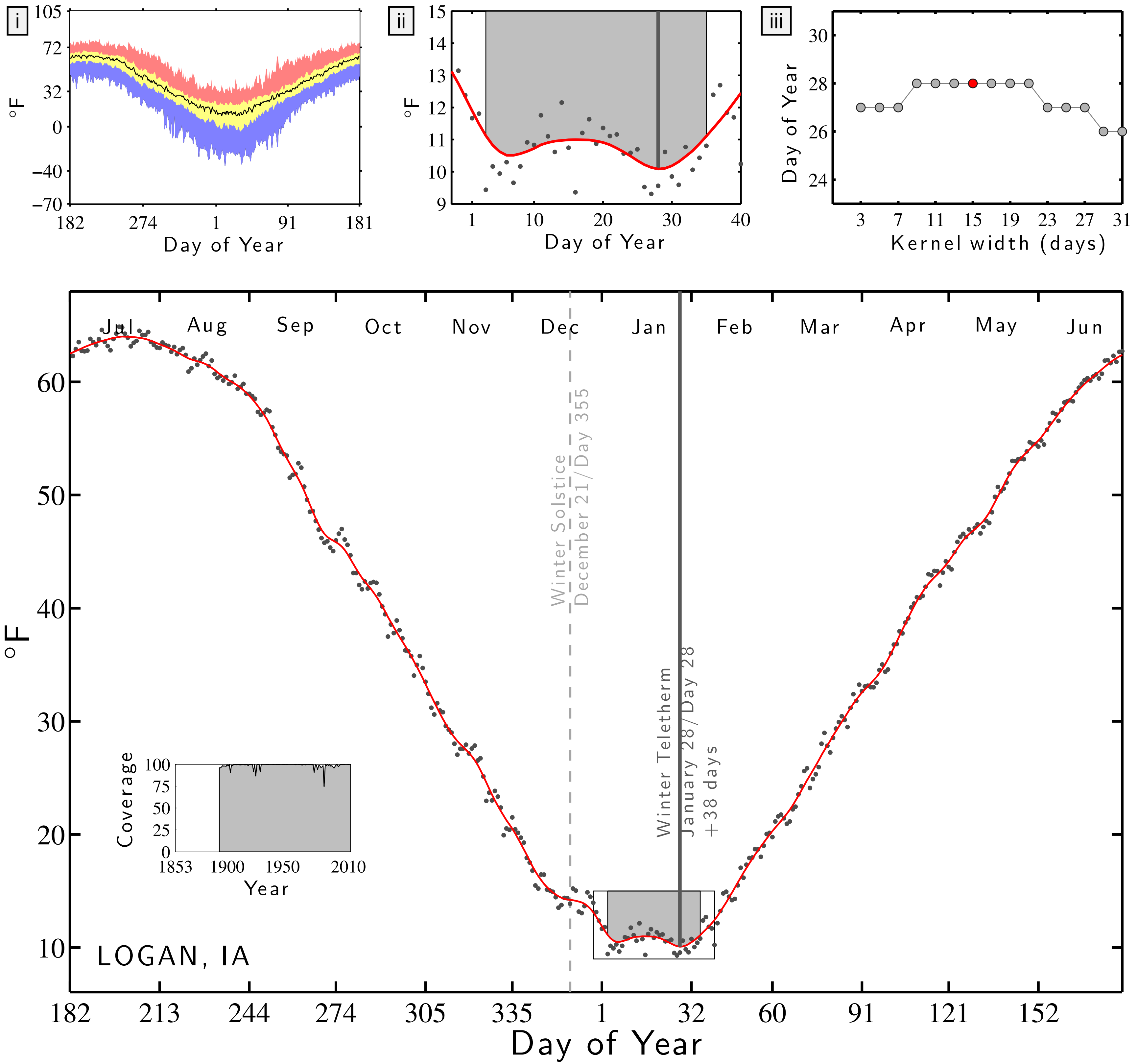

i
°F
Day of Year
ii
iii
Day of Year
Kernel width (days)
Jul
Aug
Sep
Oct
Nov
Dec
Jan
Feb
Mar
Apr
May
Jun
Winter Solstice
December 21/Day 355
Winter Teletherm
January 28/Day 28
+38 days
Coverage
Year
LOGAN, IA
Day of Year

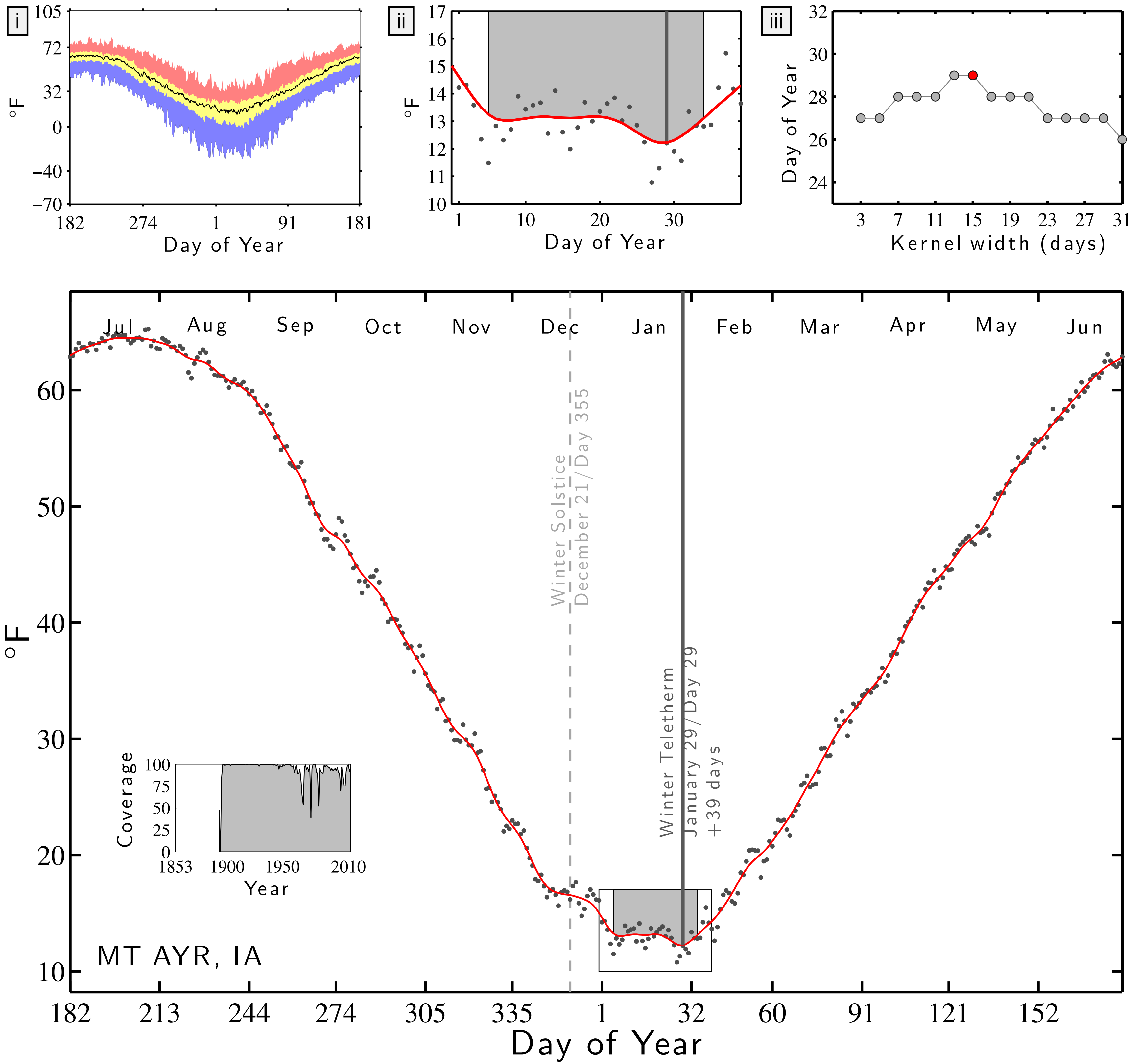

i
°F
Day of Year
ii
°F
Day of Year
iii
Day of Year
Kernel width (days)
Jul
Aug
Sep
Oct
Nov
Dec
Jan
Feb
Mar
Apr
May
Jun
Winter Solstice
December 21/Day 355
Winter Teletherm
January 29/Day 29
+39 days
Coverage
Year
MT AYR, IA
°F
Day of Year

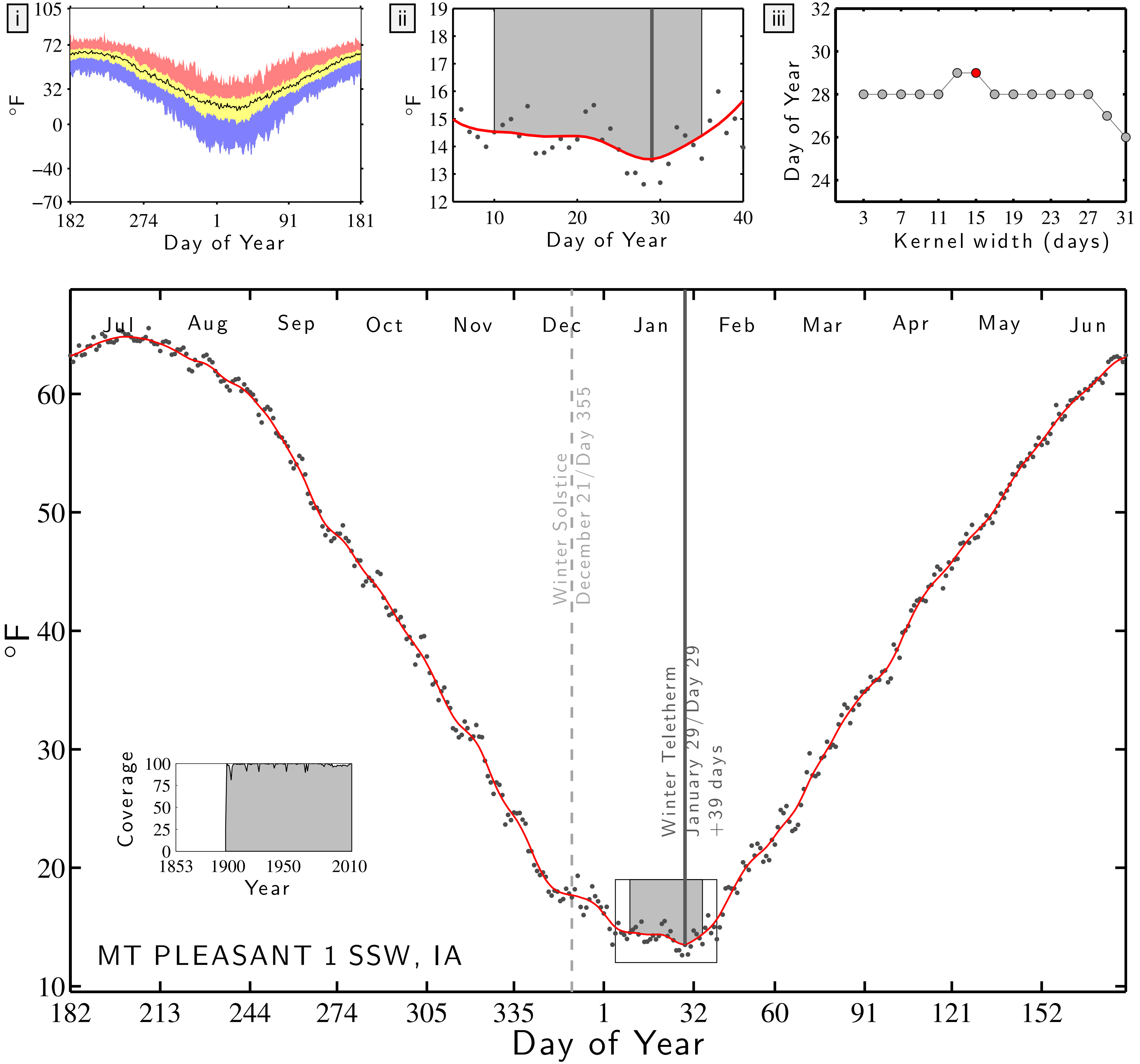

i
°F
Day of Year
ii
°F
Day of Year
iii
Day of Year
Kernel width (days)
Jul
Aug
Sep
Oct
Nov
Dec
Jan
Feb
Mar
Apr
May
Jun
Winter Solstice
December 21/Day 355
Winter Teletherm
January 29/Day 29
+39 days
Coverage
Year
MT PLEASANT 1 SSW, IA
°F
Day of Year

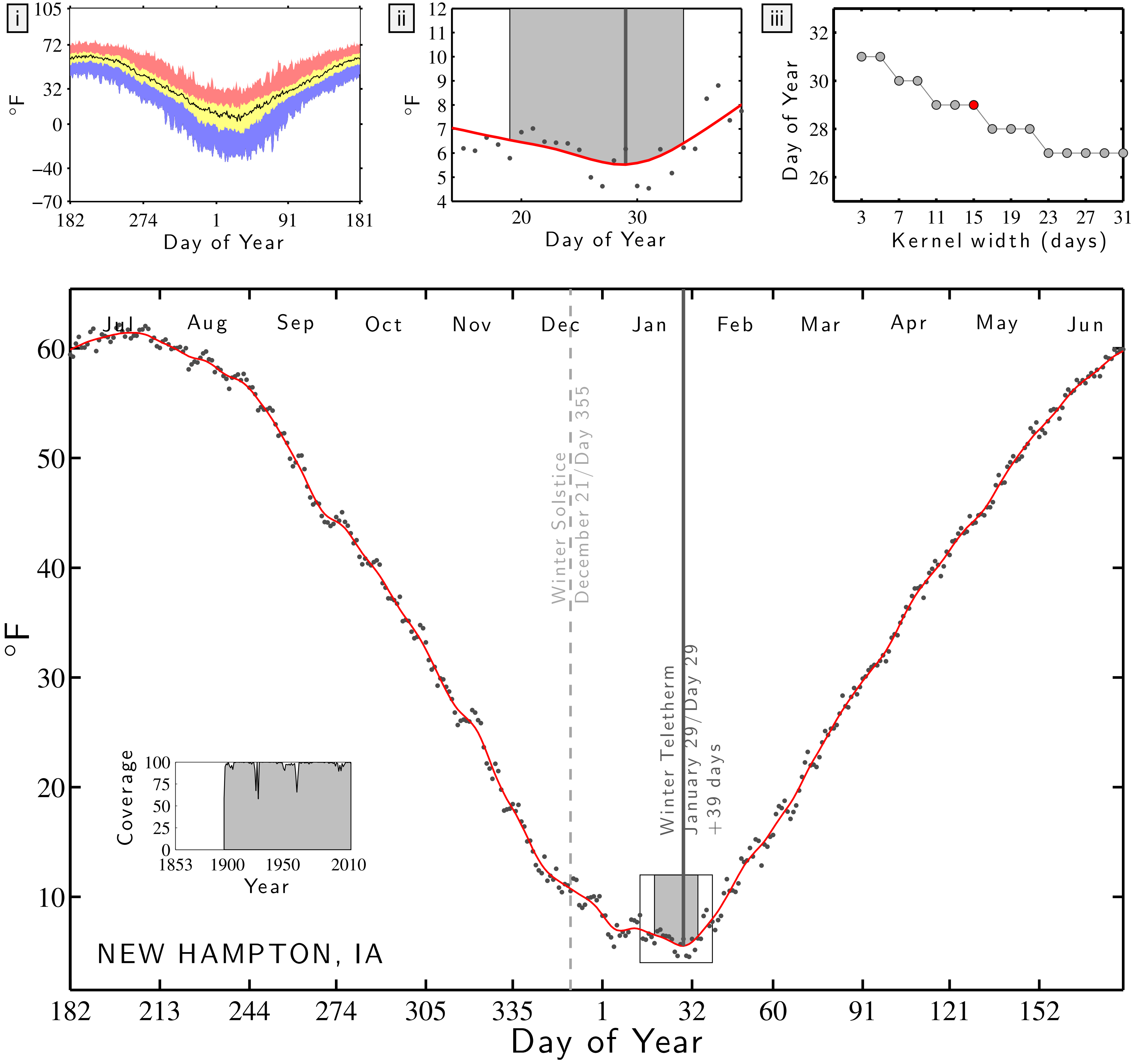

i
°F
Day of Year
ii
iii
Day of Year
Kernel width (days)
Jul
Aug
Sep
Oct
Nov
Dec
Jan
Feb
Mar
Apr
May
Jun
Winter Solstice
December 21/Day 355
Winter Teletherm
January 29/Day 29
+39 days
Coverage
Year
NEW HAMPTON, IA

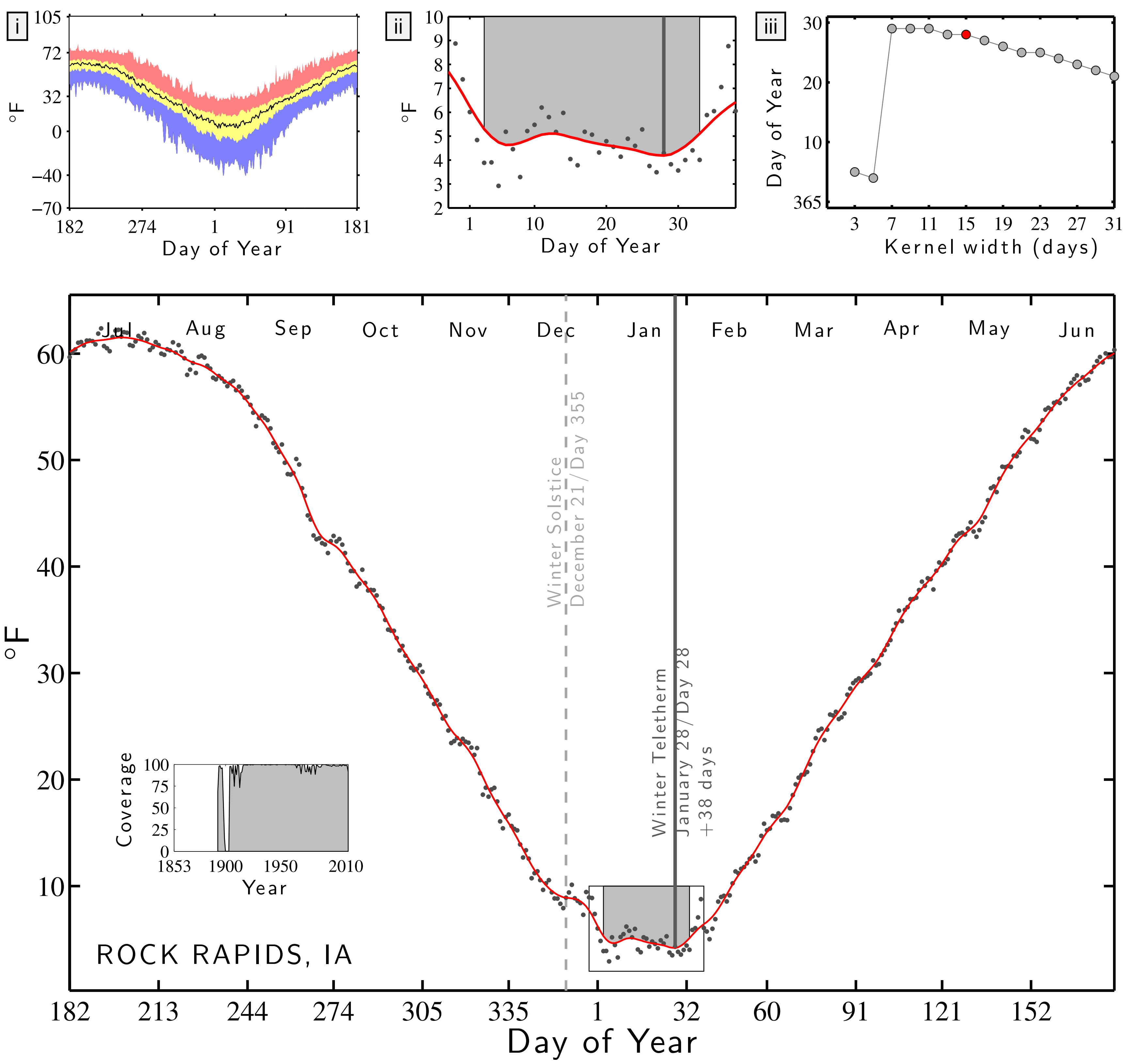

i
°F
Day of Year
ii
°F
Day of Year
iii
Day of Year
Kernel width (days)
Jul
Aug
Sep
Oct
Nov
Dec
Jan
Feb
Mar
Apr
May
Jun
Winter Solstice
December 21/Day 355
Winter Teletherm
January 28/Day 28
+38 days
Coverage
Year
ROCK RAPIDS, IA
°F
Day of Year

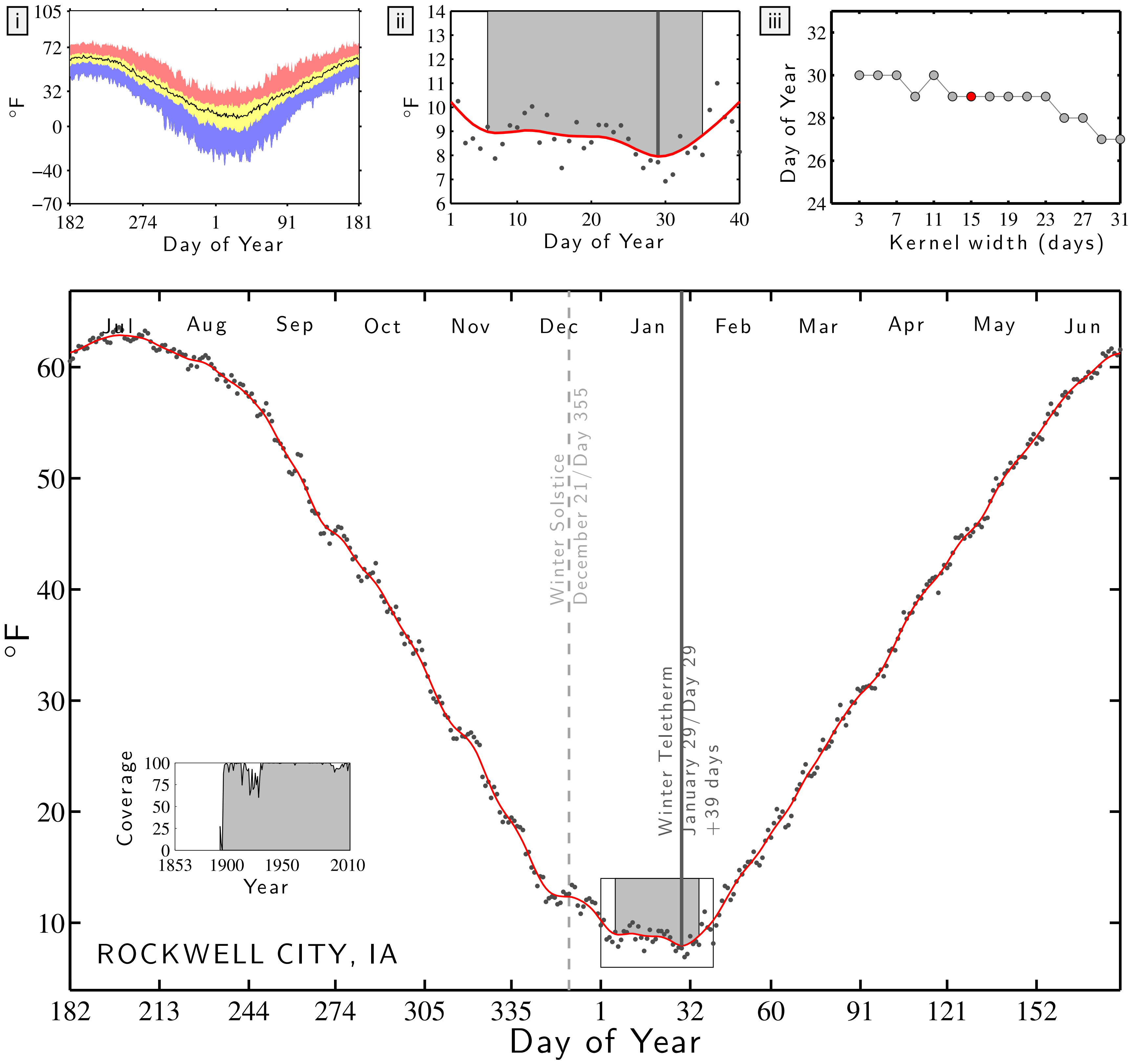

i
ii
iii
°F
Day of Year
Day of Year
Kernel width (days)
Jul
Aug
Sep
Oct
Nov
Dec
Jan
Feb
Mar
Apr
May
Jun
Winter Solstice
December 21/Day 355
Winter Teletherm
January 29/Day 29
+39 days
Coverage
Year
ROCKWELL CITY, IA
°F
Day of Year

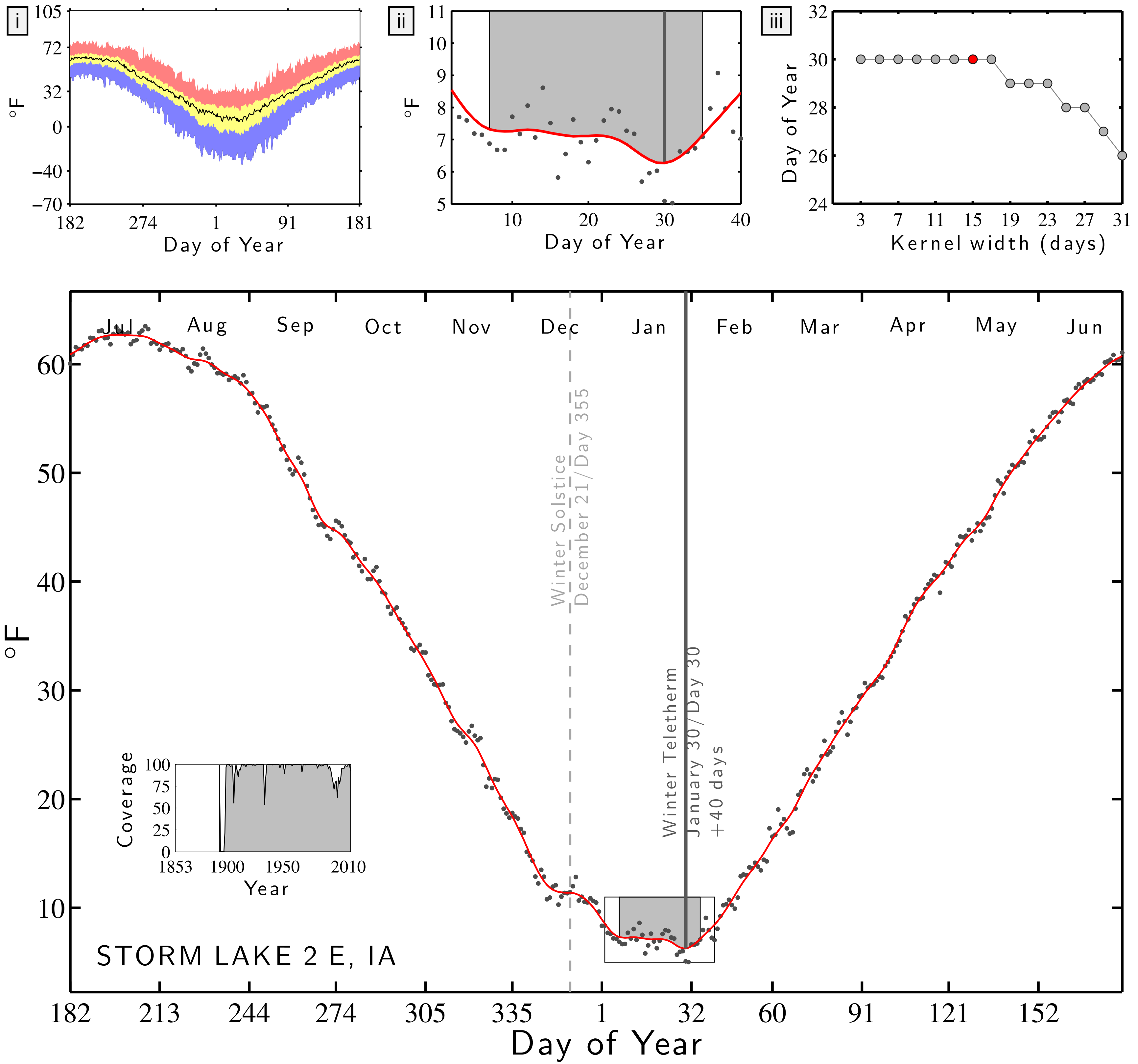

i
°F
Day of Year
ii
°F
Day of Year
iii
Day of Year
Kernel width (days)
Jul
Aug
Sep
Oct
Nov
Dec
Jan
Feb
Mar
Apr
May
Jun
Winter Solstice
December 21/Day 355
Winter Teletherm
January 30/Day 30
+40 days
Coverage
Year
STORM LAKE 2 E, IA
°F
Day of Year

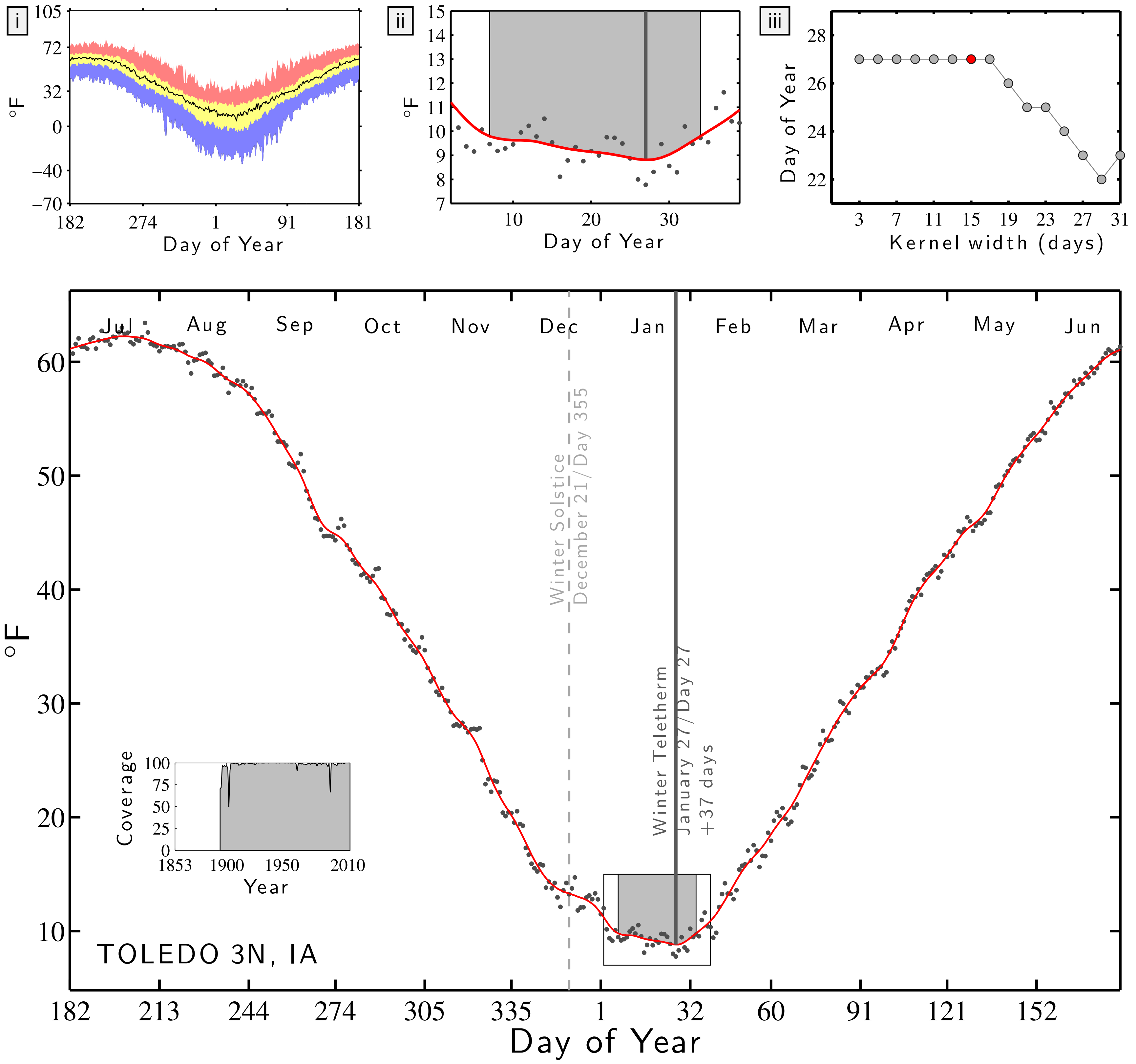

i
°F
Day of Year
ii
°F
Day of Year
iii
Day of Year
Kernel width (days)
Jul
Aug
Sep
Oct
Nov
Dec
Jan
Feb
Mar
Apr
May
Jun
Winter Solstice
December 21/Day 355
Winter Teletherm
January 27/Day 27
+37 days
Coverage
Year
TOLEDO 3N, IA
°F
Day of Year

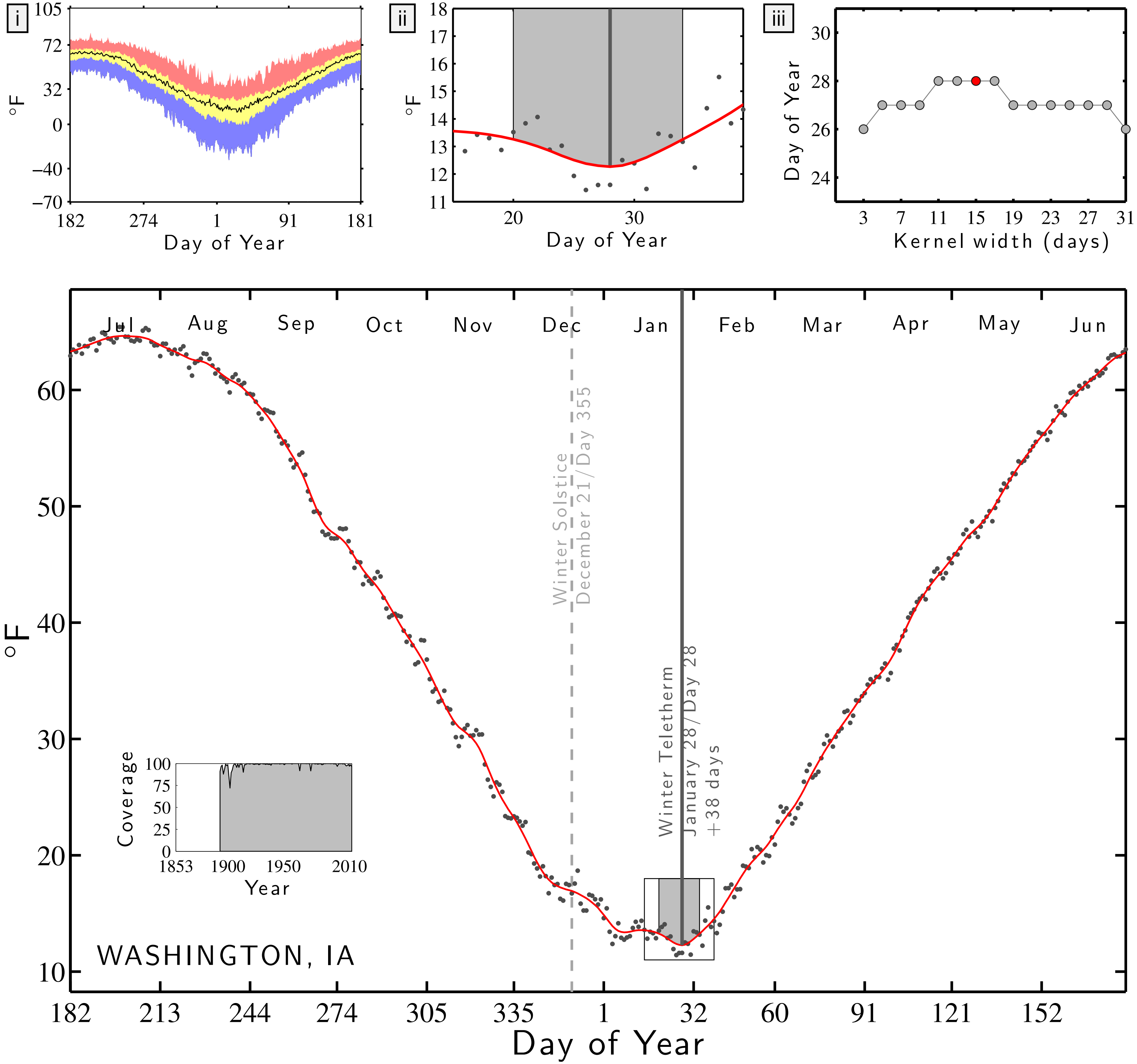

i
°F
Day of Year
ii
°F
Day of Year
iii
Day of Year
Kernel width (days)
Jul
Aug
Sep
Oct
Nov
Dec
Jan
Feb
Mar
Apr
May
Jun
Winter Solstice
December 21/Day 355
Winter Teletherm
January 28/Day 28
+38 days
Coverage
Year
WASHINGTON, IA
°F
Day of Year

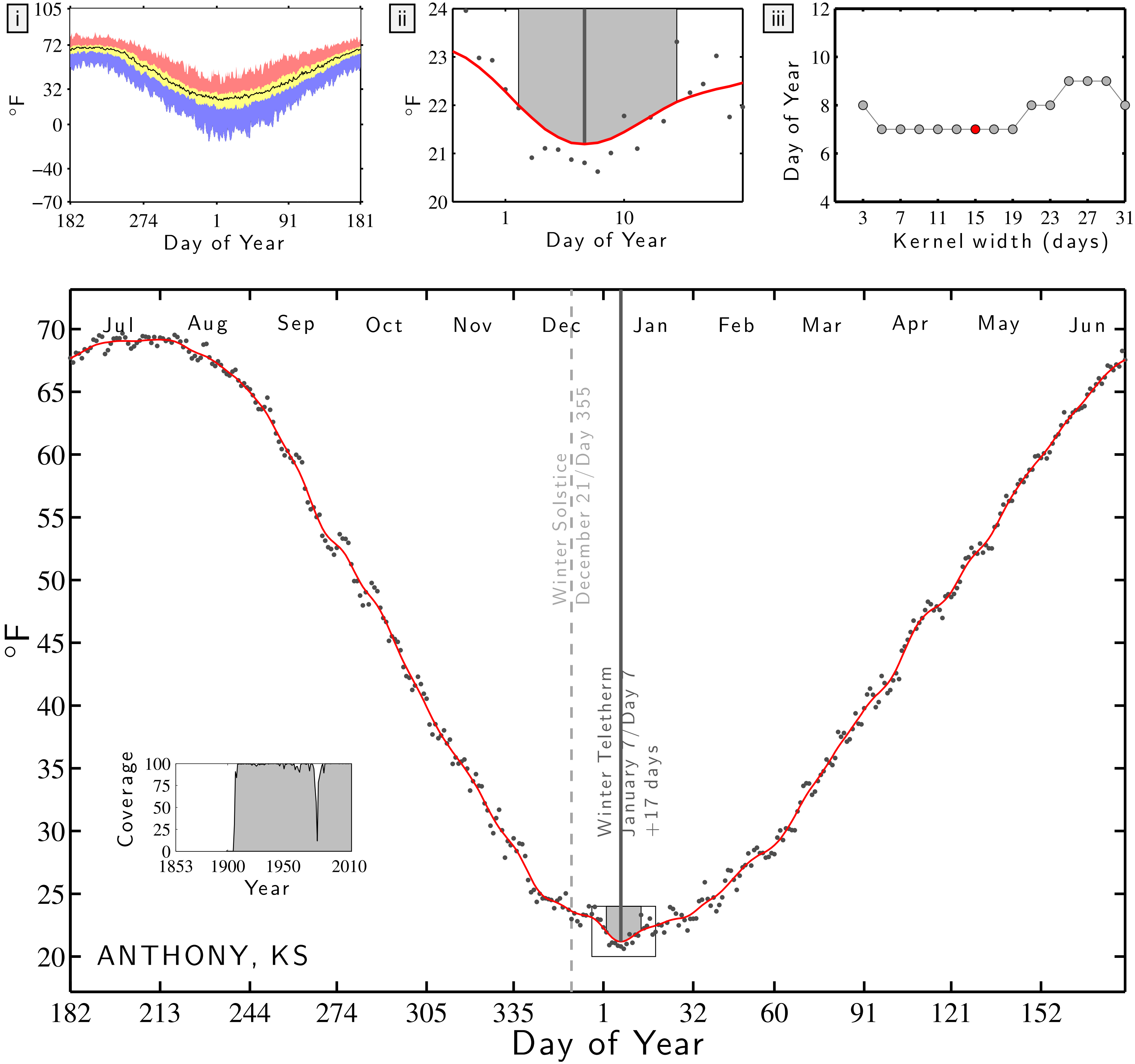

i
105
72
32
0
−40
−70
°F
182
274
1
91
181
Day of Year
ii
24
23
22
21
20
°F
1
10
Day of Year
iii
12
10
8
6
4
Day of Year
3
7
11
15
19
23
27
31
Kernel width (days)
70
65
60
55
50
45
40
35
30
25
20
°F
Jul
Aug
Sep
Oct
Nov
Dec
Jan
Feb
Mar
Apr
May
Jun
Winter Solstice
December 21/Day 355
Winter Teletherm
January 7/Day 7
+17 days
Coverage
100
75
50
25
0
1853
1900
1950
2010
Year
ANTHONY, KS
182
213
244
274
305
335
1
32
60
91
121
152
Day of Year

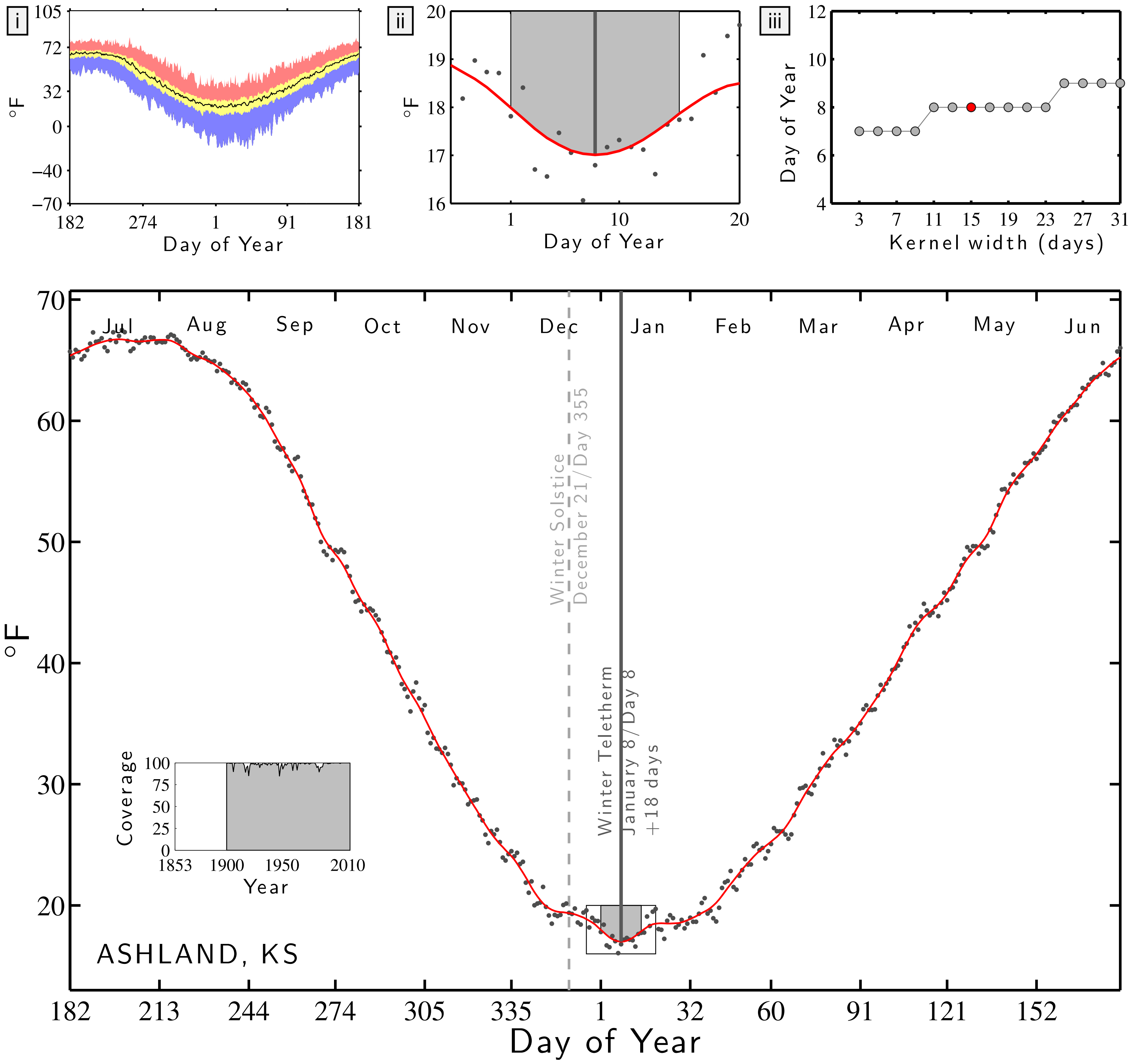

i
°F
Day of Year
ii
°F
Day of Year
iii
Day of Year
Kernel width (days)
Jul
Aug
Sep
Oct
Nov
Dec
Jan
Feb
Mar
Apr
May
Jun
Winter Solstice
December 21/Day 355
Winter Teletherm
January 8/Day 8
+18 days
Coverage
Year
ASHLAND, KS
°F
Day of Year

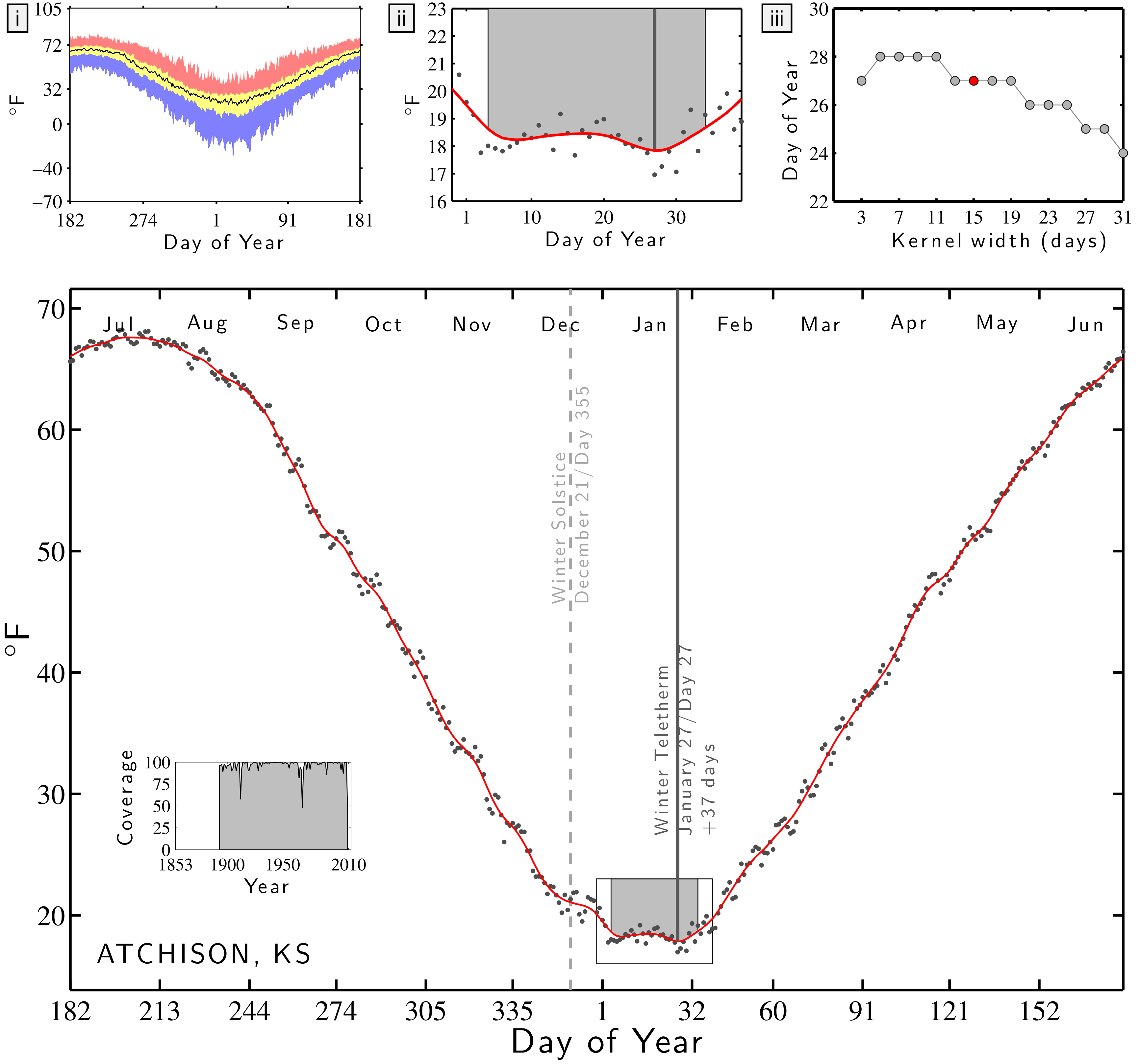

i
°F
Day of Year
ii
°F
Day of Year
iii
Day of Year
Kernel width (days)
Jul
Aug
Sep
Oct
Nov
Dec
Jan
Feb
Mar
Apr
May
Jun
Winter Solstice
December 21/Day 355
Winter Teletherm
January 27/Day 27
+37 days
Coverage
Year
ATCHISON, KS
°F
Day of Year

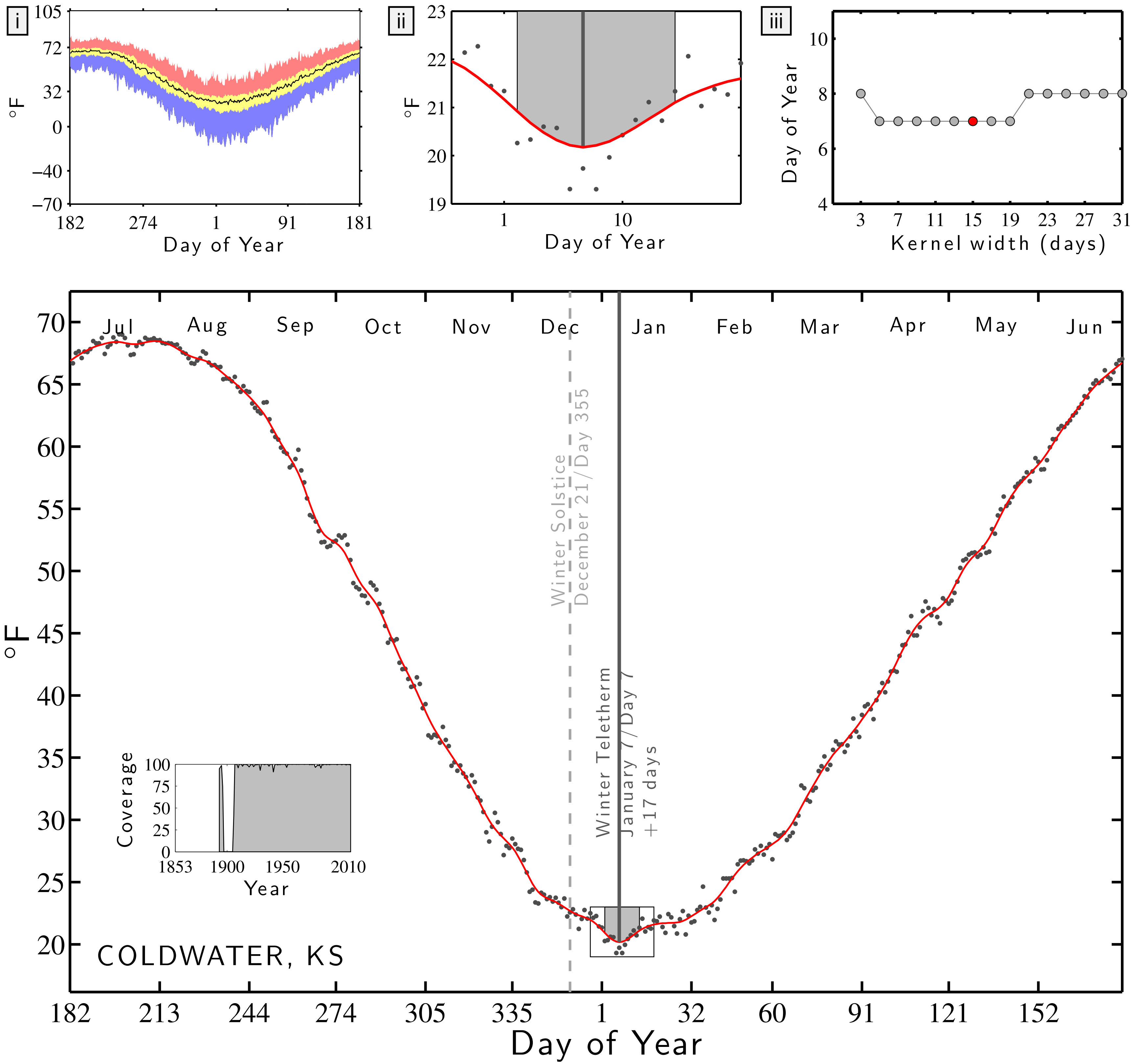

i
°F
Day of Year
ii
°F
Day of Year
iii
Day of Year
Kernel width (days)
Jul
Aug
Sep
Oct
Nov
Dec
Jan
Feb
Mar
Apr
May
Jun
Winter Solstice
December 21/Day 355
Winter Teletherm
January 7/Day 7
+17 days
Coverage
Year
COLDWATER, KS
°F
Day of Year

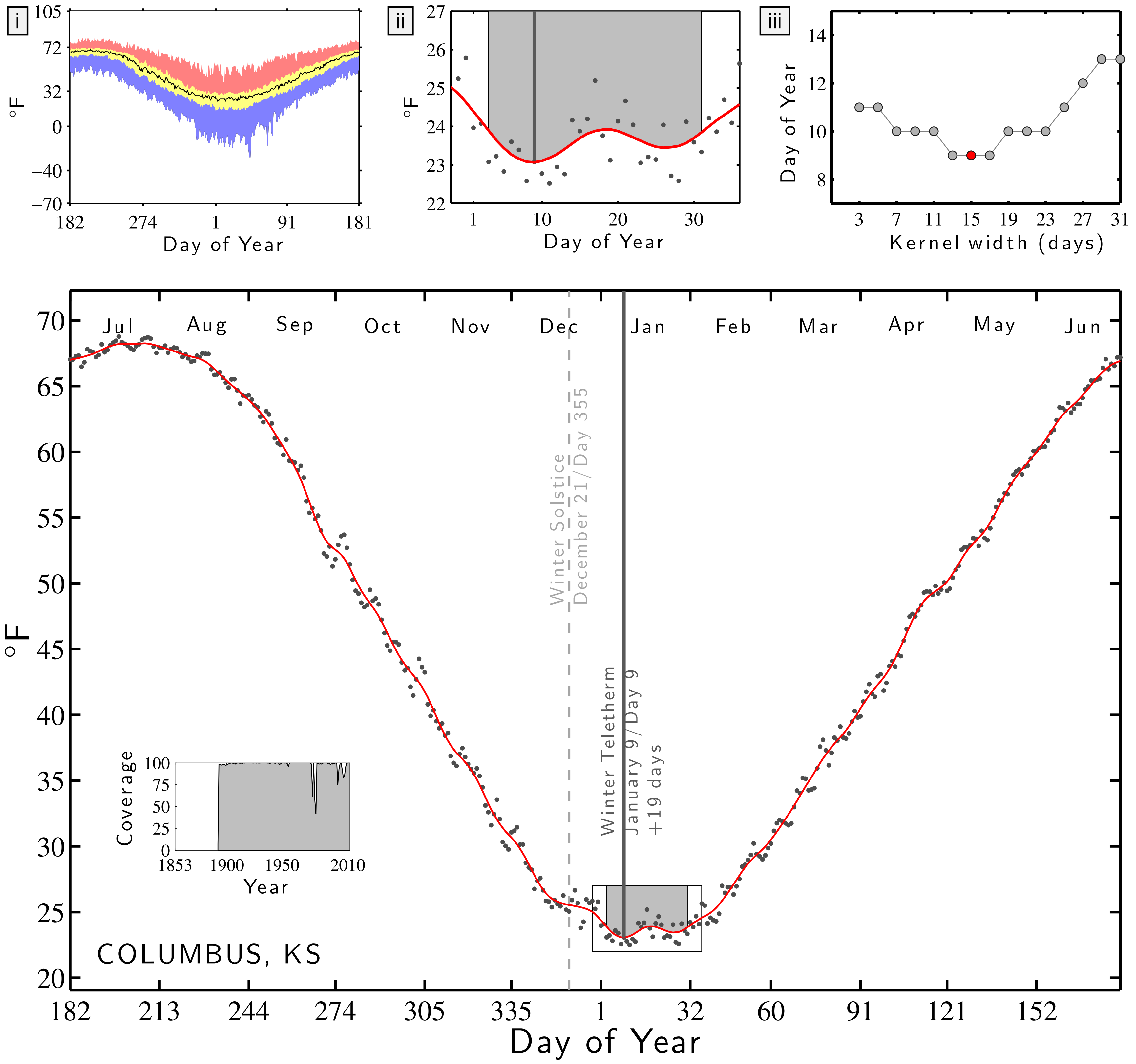

i
°F
Day of Year
ii
°F
Day of Year
iii
Day of Year
Kernel width (days)
Jul
Aug
Sep
Oct
Nov
Dec
Jan
Feb
Mar
Apr
May
Jun
Winter Solstice
December 21/Day 355
Winter Teletherm
January 9/Day 9
+19 days
Coverage
Year
COLUMBUS, KS
°F
Day of Year

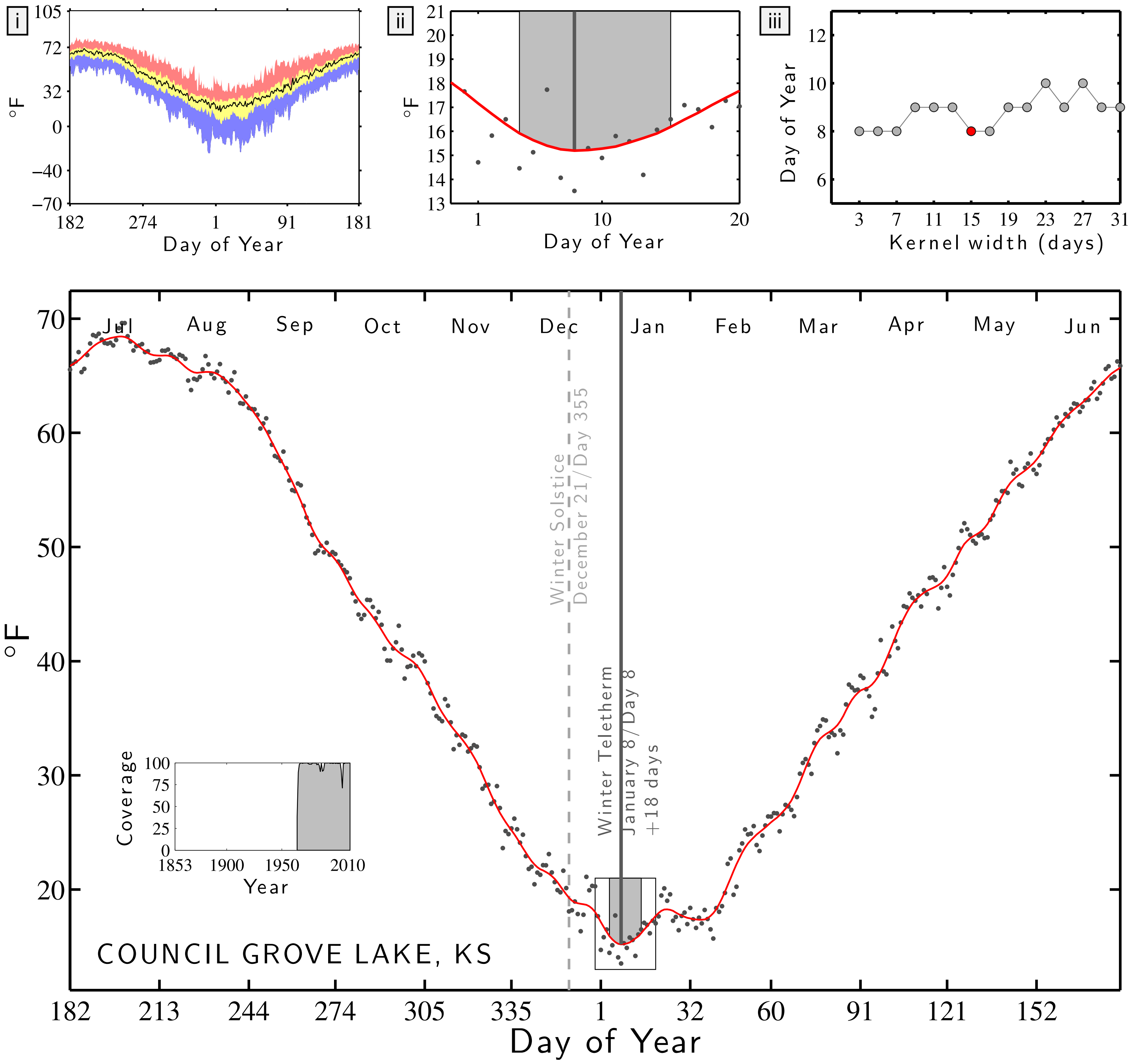

i
ii
iii
Day of Year
Kernel width (days)
°F
Jul
Aug
Sep
Oct
Nov
Dec
Jan
Feb
Mar
Apr
May
Jun
Winter Solstice
December 21/Day 355
Winter Teletherm
January 8/Day 8
+18 days
Coverage
Year
COUNCIL GROVE LAKE, KS

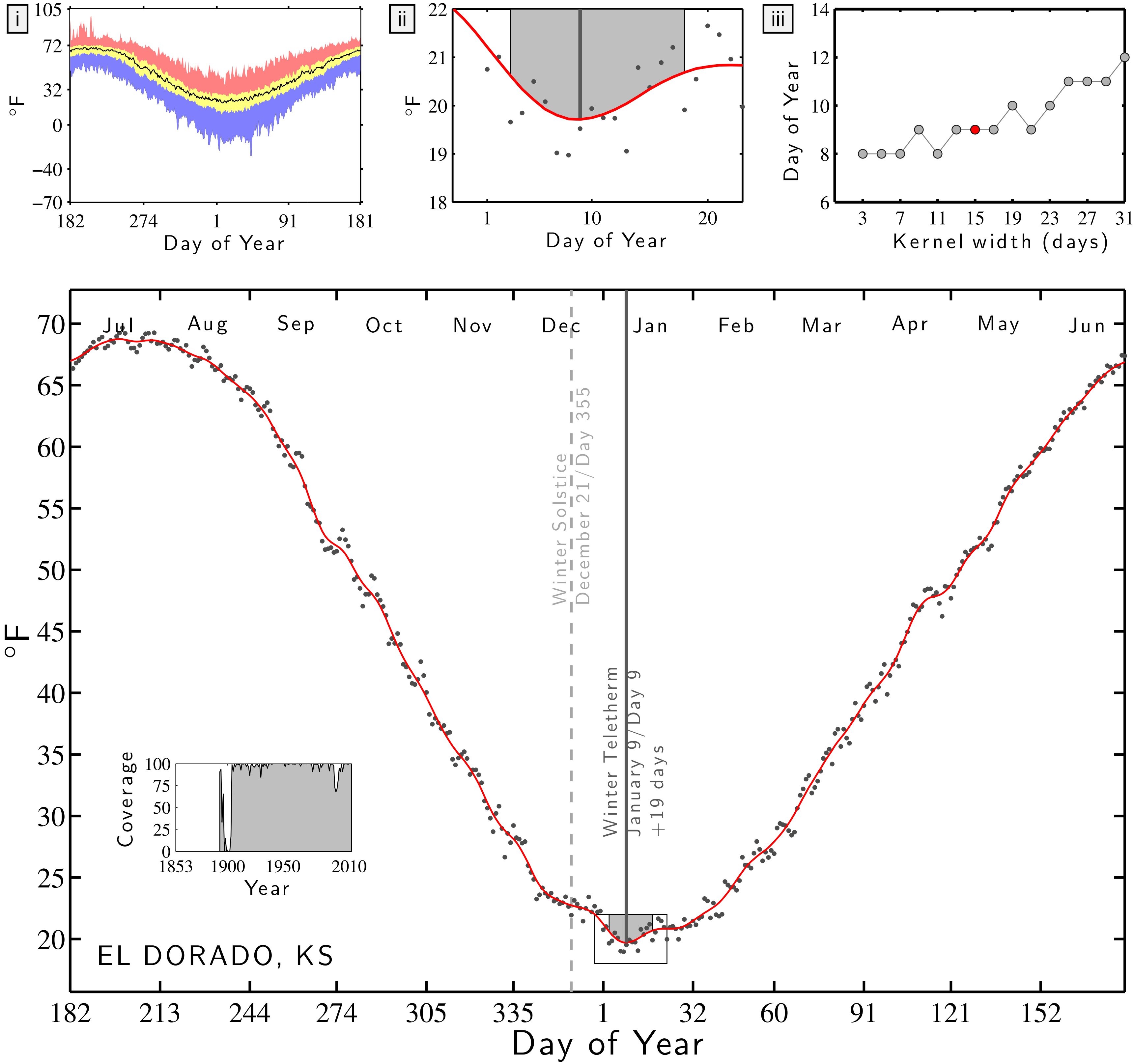

i
°F
Day of Year
ii
°F
Day of Year
iii
Day of Year
Kernel width (days)
Jul
Aug
Sep
Oct
Nov
Dec
Jan
Feb
Mar
Apr
May
Jun
Winter Solstice
December 21/Day 355
Winter Teletherm
January 9/Day 9
+19 days
Coverage
Year
EL DORADO, KS
°F
Day of Year

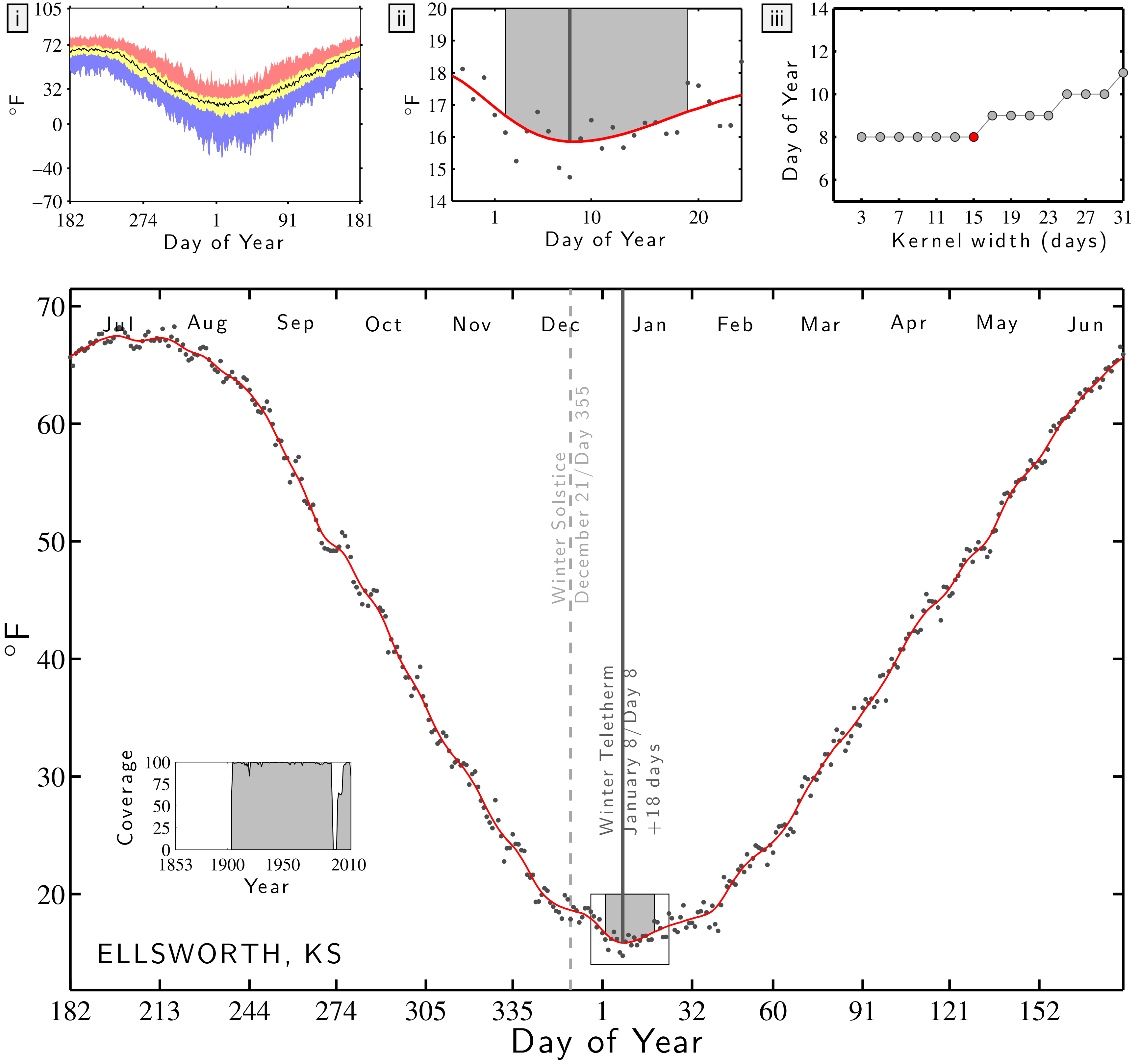

i
105
72
32
0
−40
−70
°F
182
274
1
91
181
Day of Year
ii
20
19
18
17
16
15
14
°F
1
10
20
Day of Year
iii
14
12
10
8
6
Day of Year
3
7
11
15
19
23
27
31
Kernel width (days)
70
60
50
40
30
20
°F
Jul
Aug
Sep
Oct
Nov
Dec
Jan
Feb
Mar
Apr
May
Jun
Winter Solstice
December 21/Day 355
Winter Teletherm
January 8/Day 8
+18 days
Coverage
100
75
50
25
0
1853
1900
1950
2010
Year
ELLSWORTH, KS
182
213
244
274
305
335
1
32
60
91
121
152
Day of Year

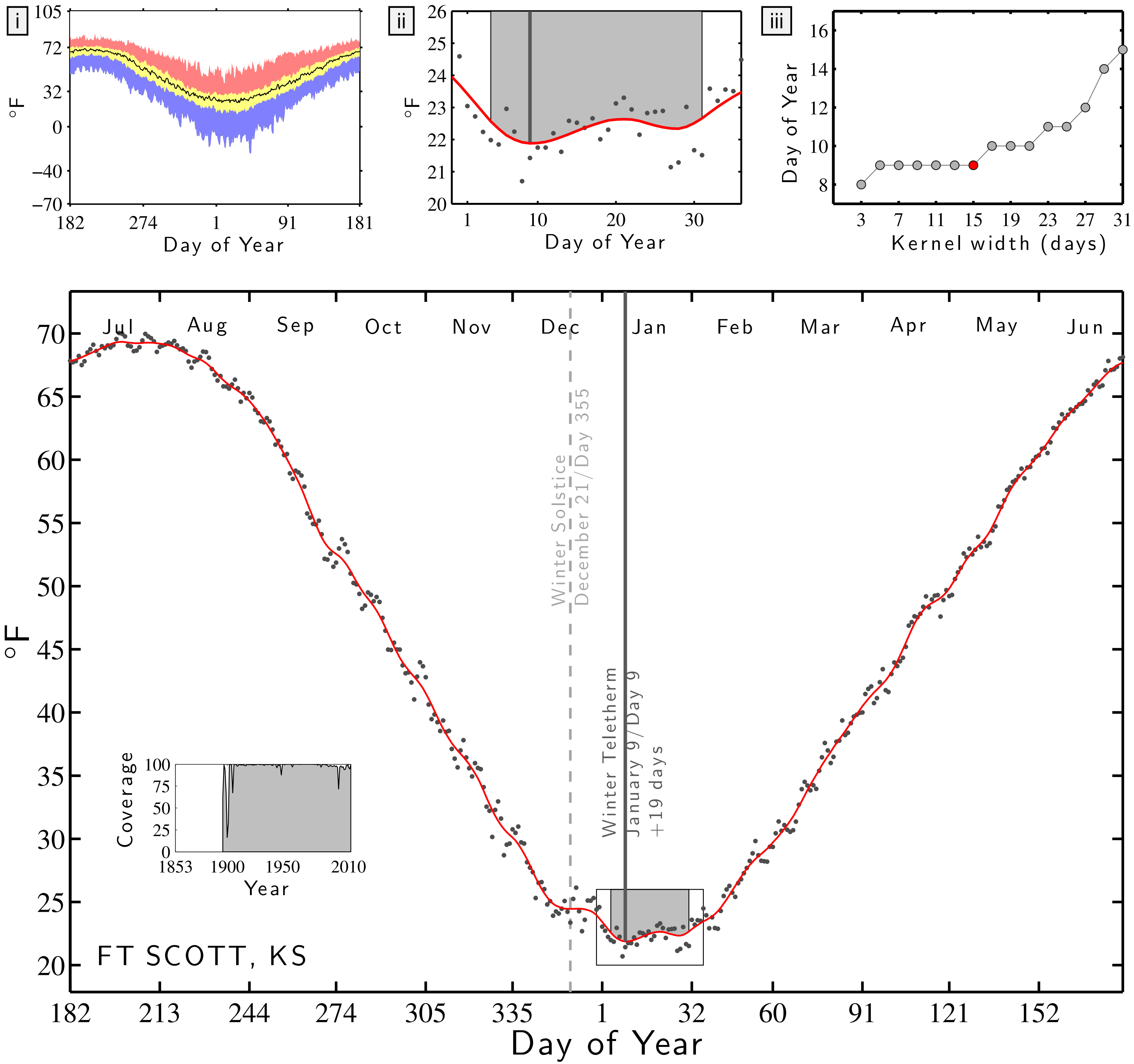
i
°F
Day of Year
ii
°F
Day of Year
iii
Day of Year
Kernel width (days)
Jul
Aug
Sep
Oct
Nov
Dec
Jan
Feb
Mar
Apr
May
Jun
Winter Solstice
December 21/Day 355
Winter Teletherm
January 9/Day 9
+19 days
Coverage
Year
FT SCOTT, KS
°F
Day of Year

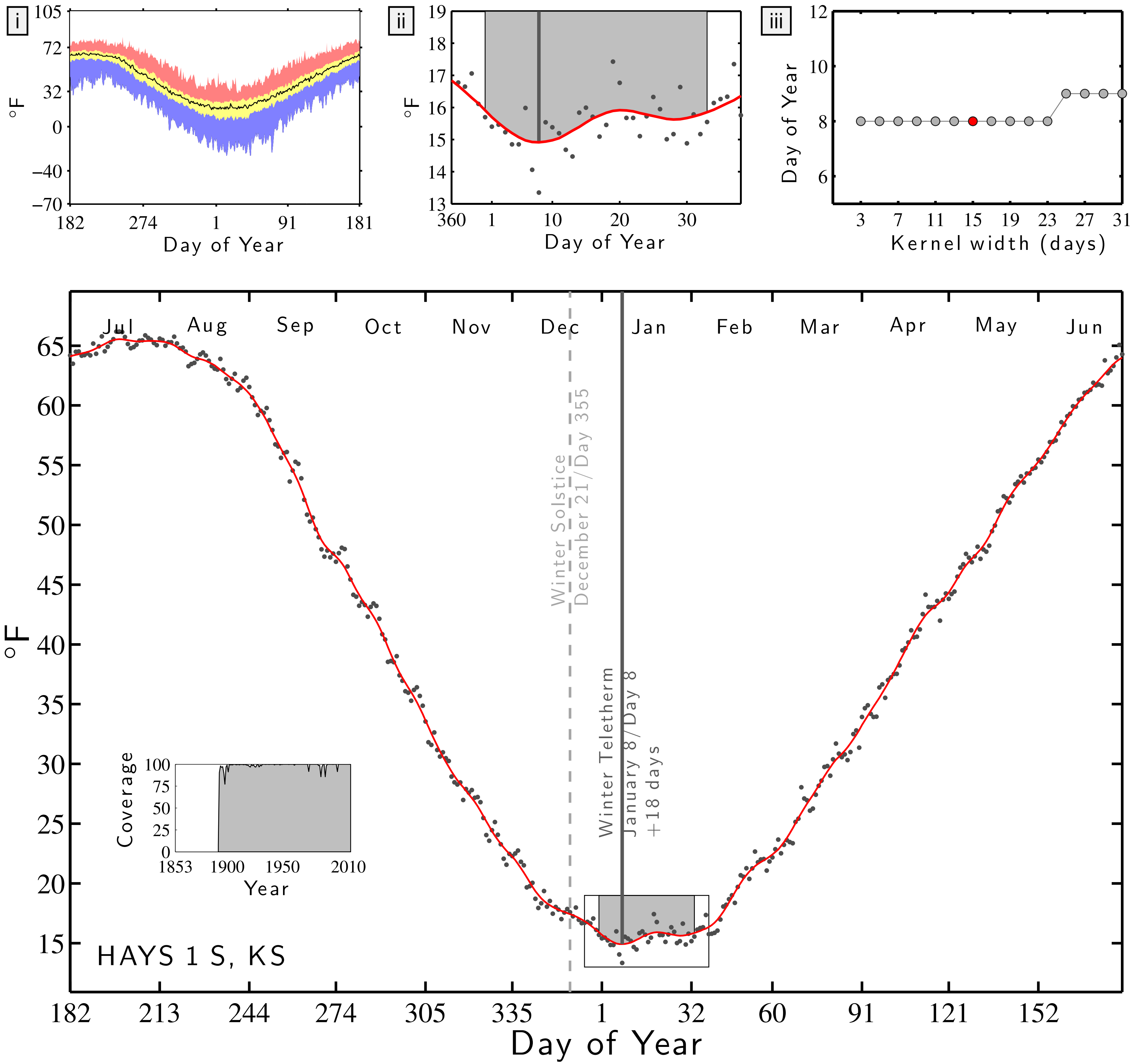

i
ii
iii
°F
Day of Year
Kernel width (days)
Jul
Aug
Sep
Oct
Nov
Dec
Jan
Feb
Mar
Apr
May
Jun
Winter Solstice
December 21/Day 355
Winter Teletherm
January 8/Day 8
+18 days
Coverage
Year
HAYS 1 S, KS

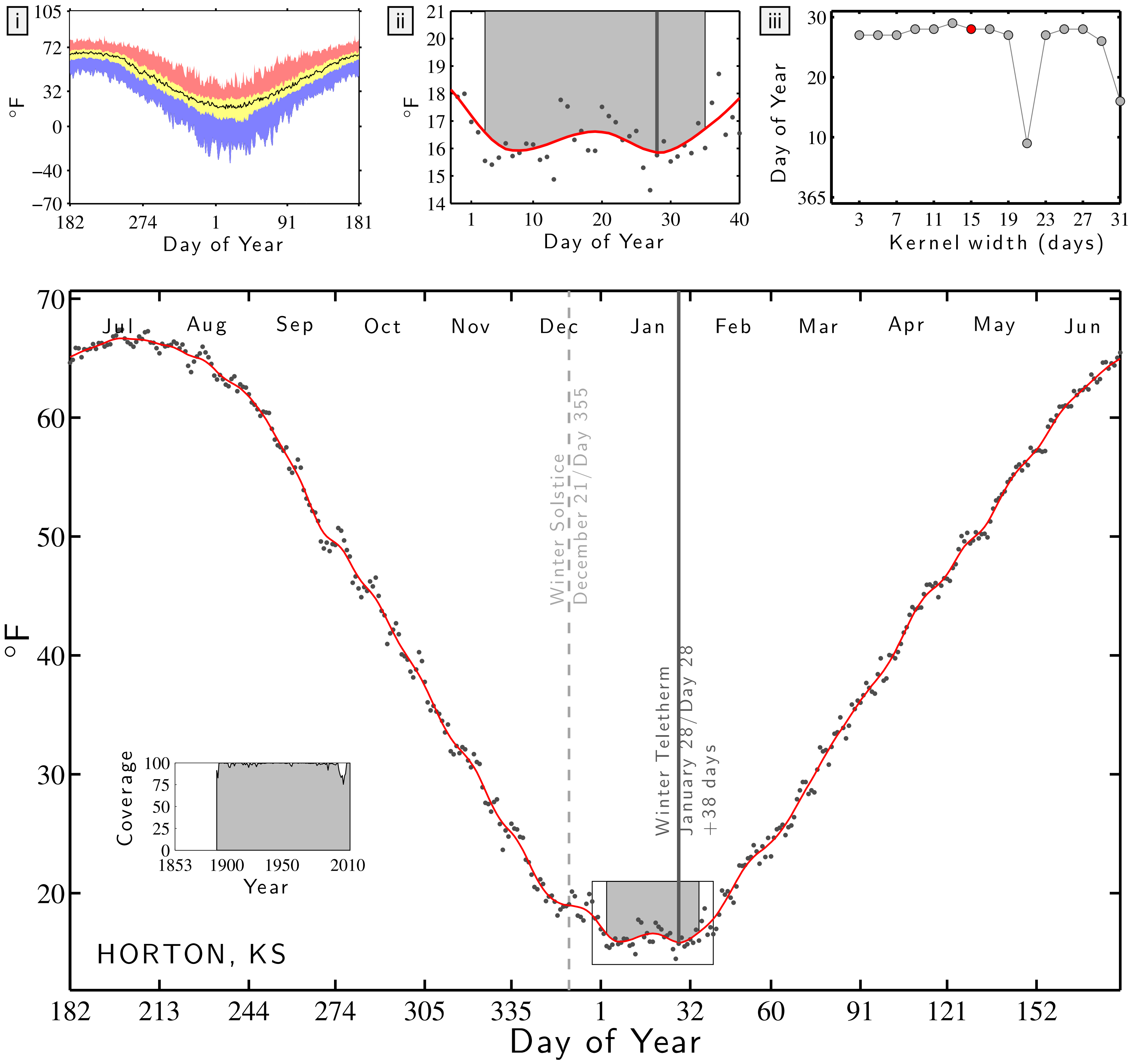
i
°F
105
72
32
0
−40
−70
182
274
1
91
181
Day of Year
ii
°F
21
20
19
18
17
16
15
14
1
10
20
30
40
Day of Year
iii
Day of Year
30
20
10
365
3
7
11
15
19
23
27
31
Kernel width (days)
°F
70
60
50
40
30
20
Jul
Aug
Sep
Oct
Nov
Dec
Jan
Feb
Mar
Apr
May
Jun
Winter Solstice
December 21/Day 355
Winter Teletherm
January 28/Day 28
+38 days
Coverage
100
75
50
25
0
1853
1900
1950
2010
Year
HORTON, KS
182
213
244
274
305
335
1
32
60
91
121
152
Day of Year

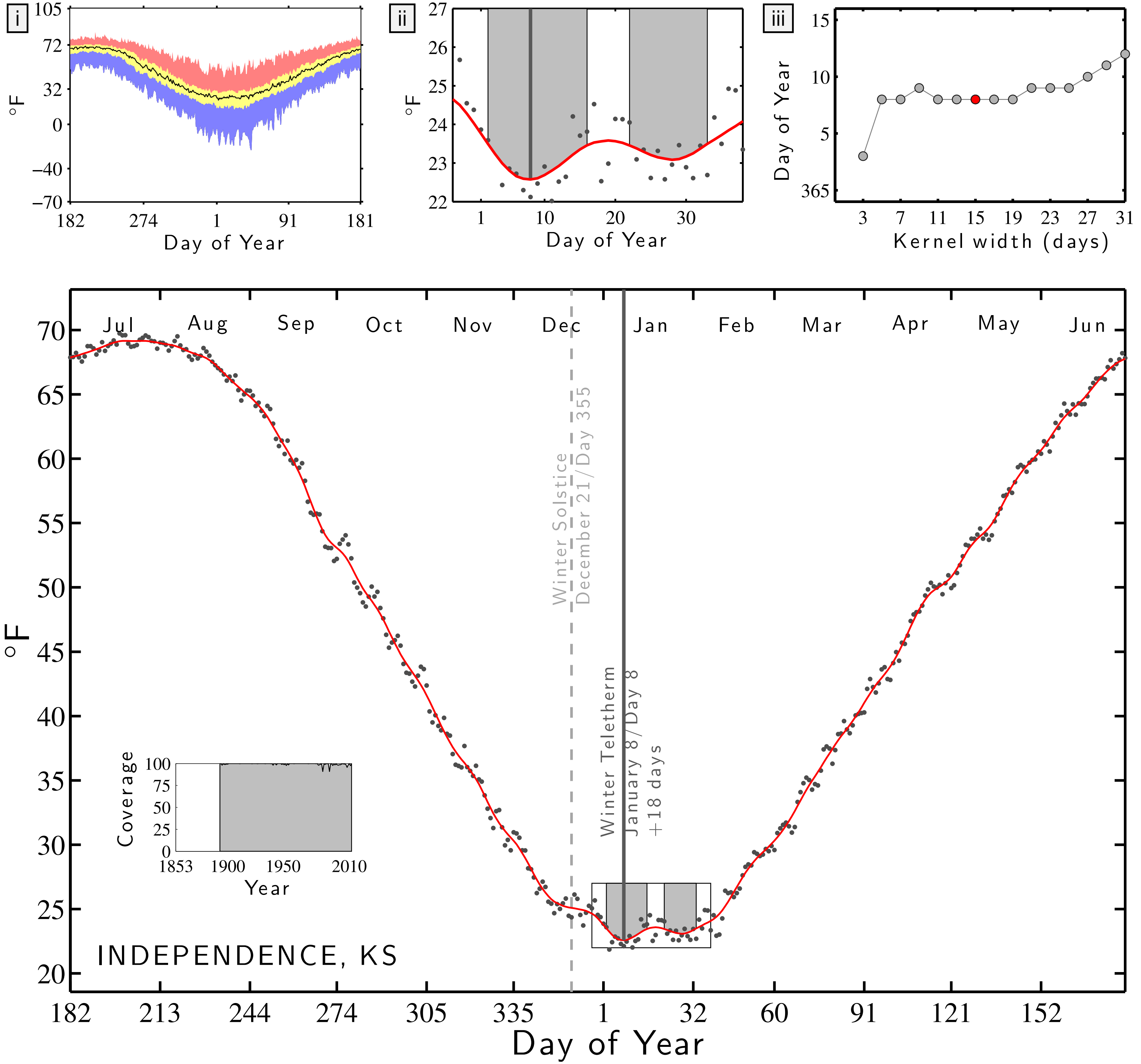

i
ii
iii
°F
Day of Year
Day of Year
Kernel width (days)
Jul
Aug
Sep
Oct
Nov
Dec
Jan
Feb
Mar
Apr
May
Jun
Winter Solstice
December 21/Day 355
Winter Teletherm
January 8/Day 8
+18 days
Coverage
Year
INDEPENDENCE, KS

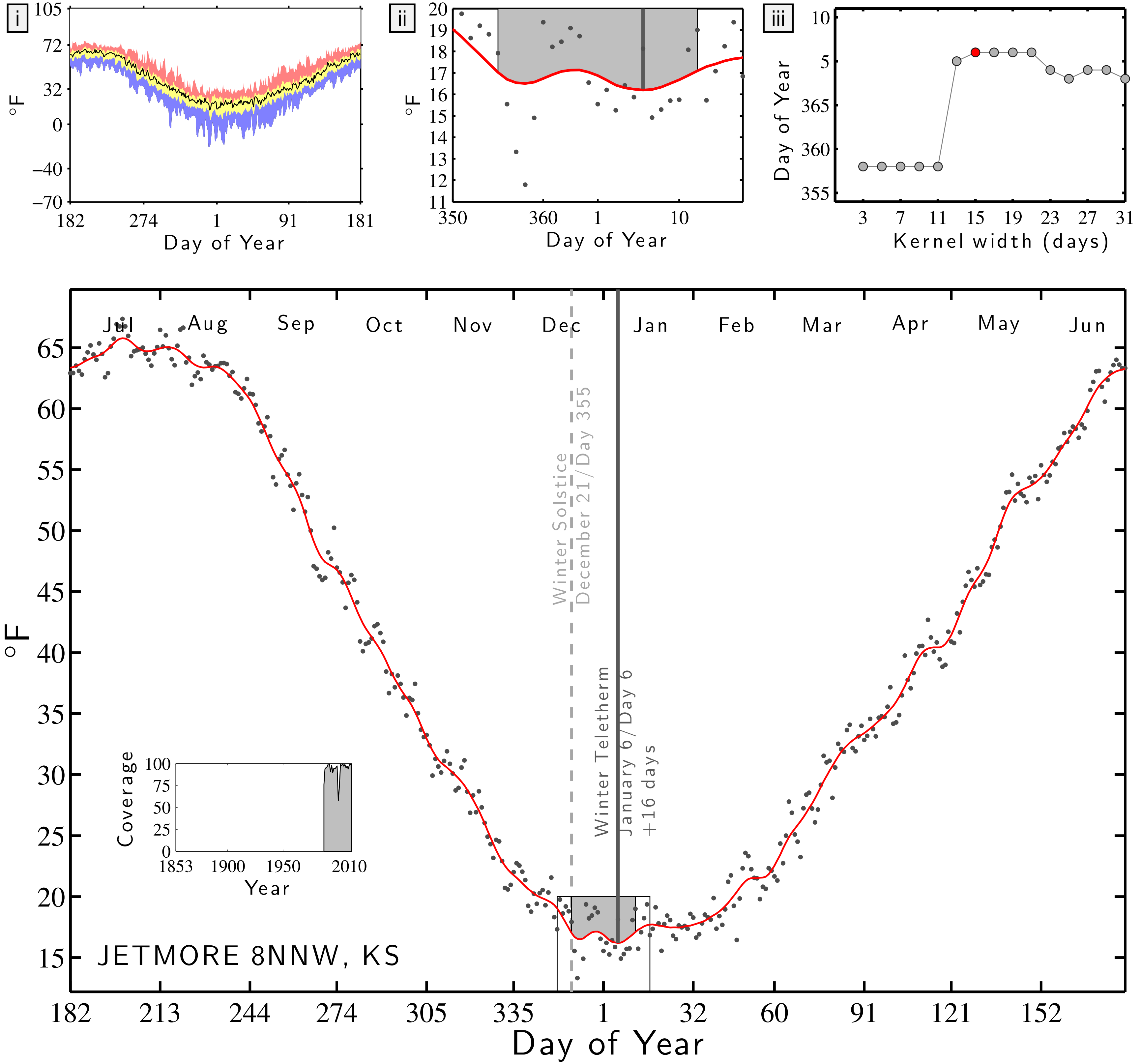
i
ii
iii
°F
Day of Year
Kernel width (days)
Jul
Aug
Sep
Oct
Nov
Dec
Jan
Feb
Mar
Apr
May
Jun
Winter Solstice
December 21/Day 355
Winter Teletherm
January 6/Day 6
+16 days
Coverage
Year
JETMORE 8NNW, KS

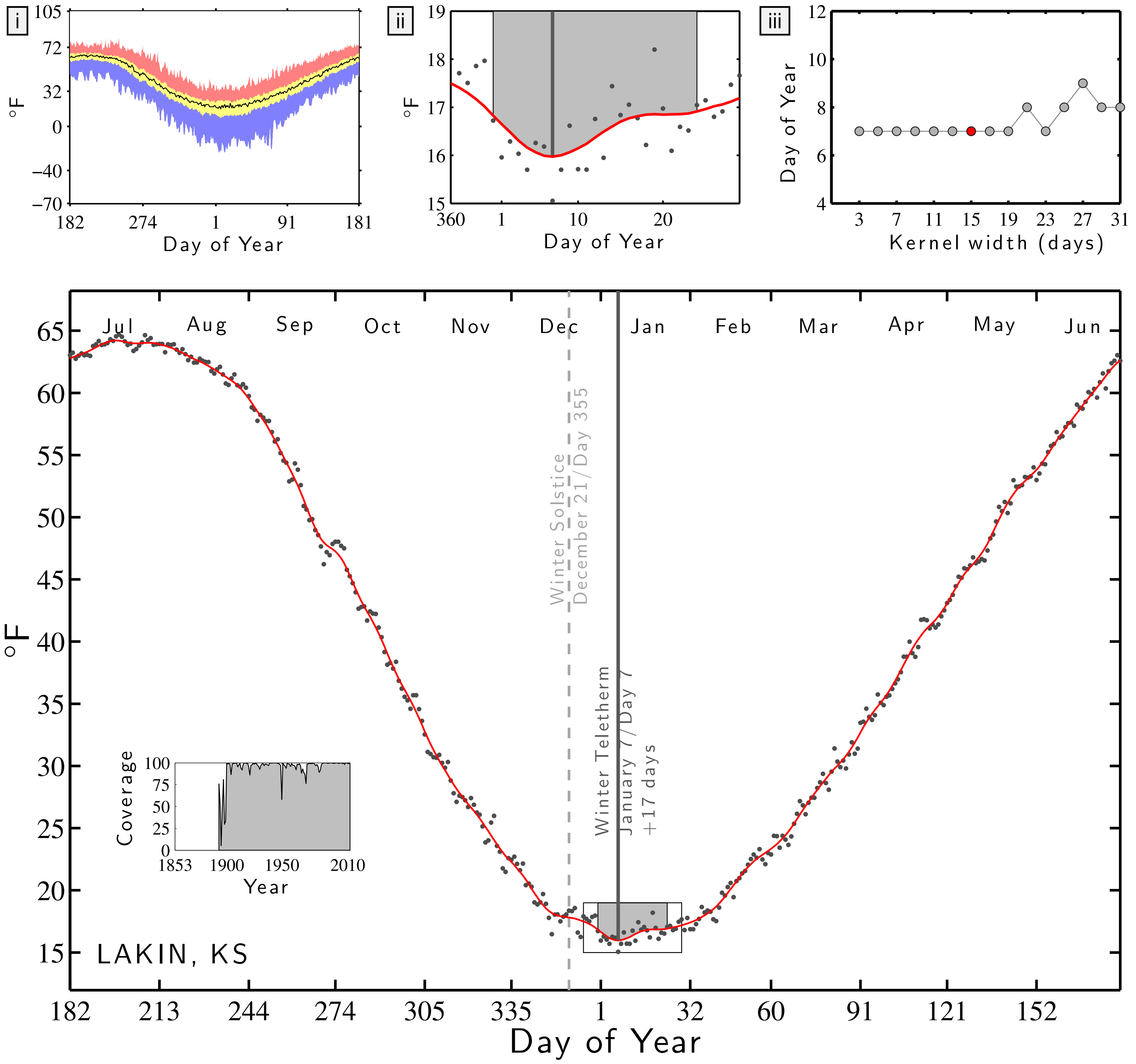

i
ii
iii
°F
Day of Year
Kernel width (days)
Jul
Aug
Sep
Oct
Nov
Dec
Jan
Feb
Mar
Apr
May
Jun
Winter Solstice
December 21/Day 355
Winter Teletherm
January 7/Day 7
+17 days
Coverage
Year
LAKIN, KS

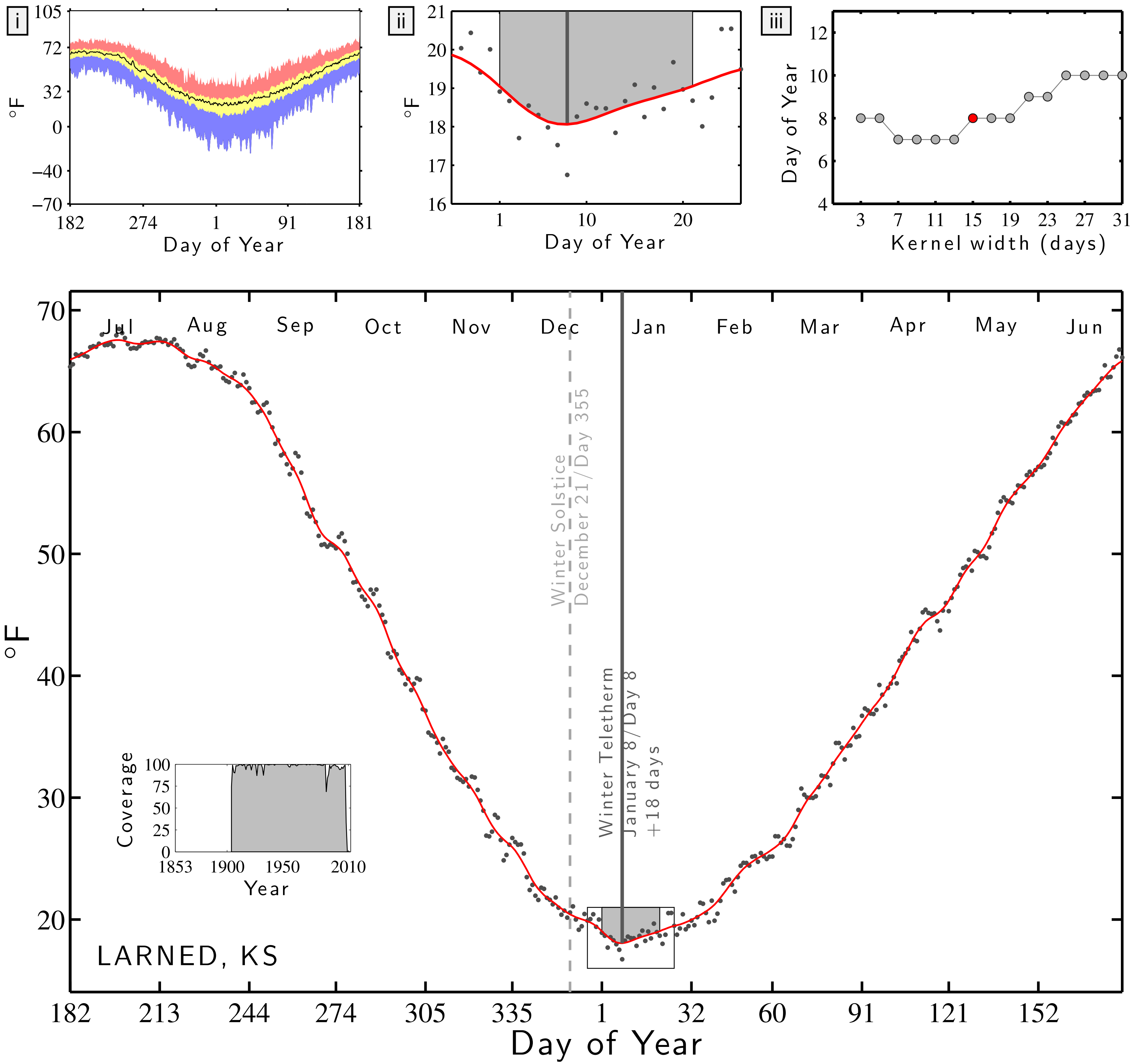

i
°F
Day of Year
ii
Day of Year
iii
Day of Year
Kernel width (days)
Jul
Aug
Sep
Oct
Nov
Dec
Jan
Feb
Mar
Apr
May
Jun
Winter Solstice
December 21/Day 355
Winter Teletherm
January 8/Day 8
+18 days
Coverage
Year
LARNED, KS
Day of Year

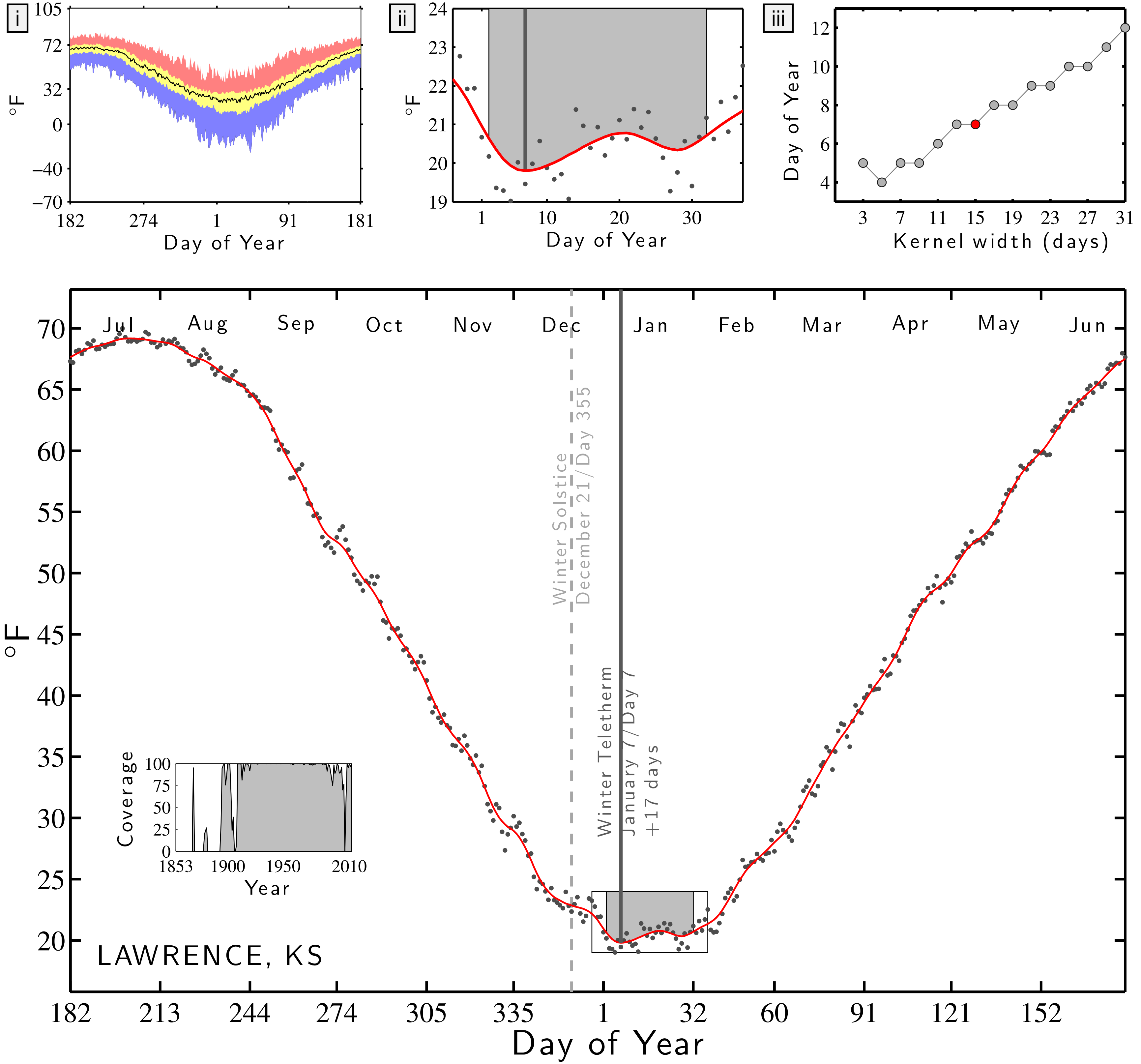

i
ii
iii
°F
Day of Year
Kernel width (days)
Jul
Aug
Sep
Oct
Nov
Dec
Jan
Feb
Mar
Apr
May
Jun
Winter Solstice
December 21/Day 355
Winter Teletherm
January 7/Day 7
+17 days
Coverage
Year
LAWRENCE, KS

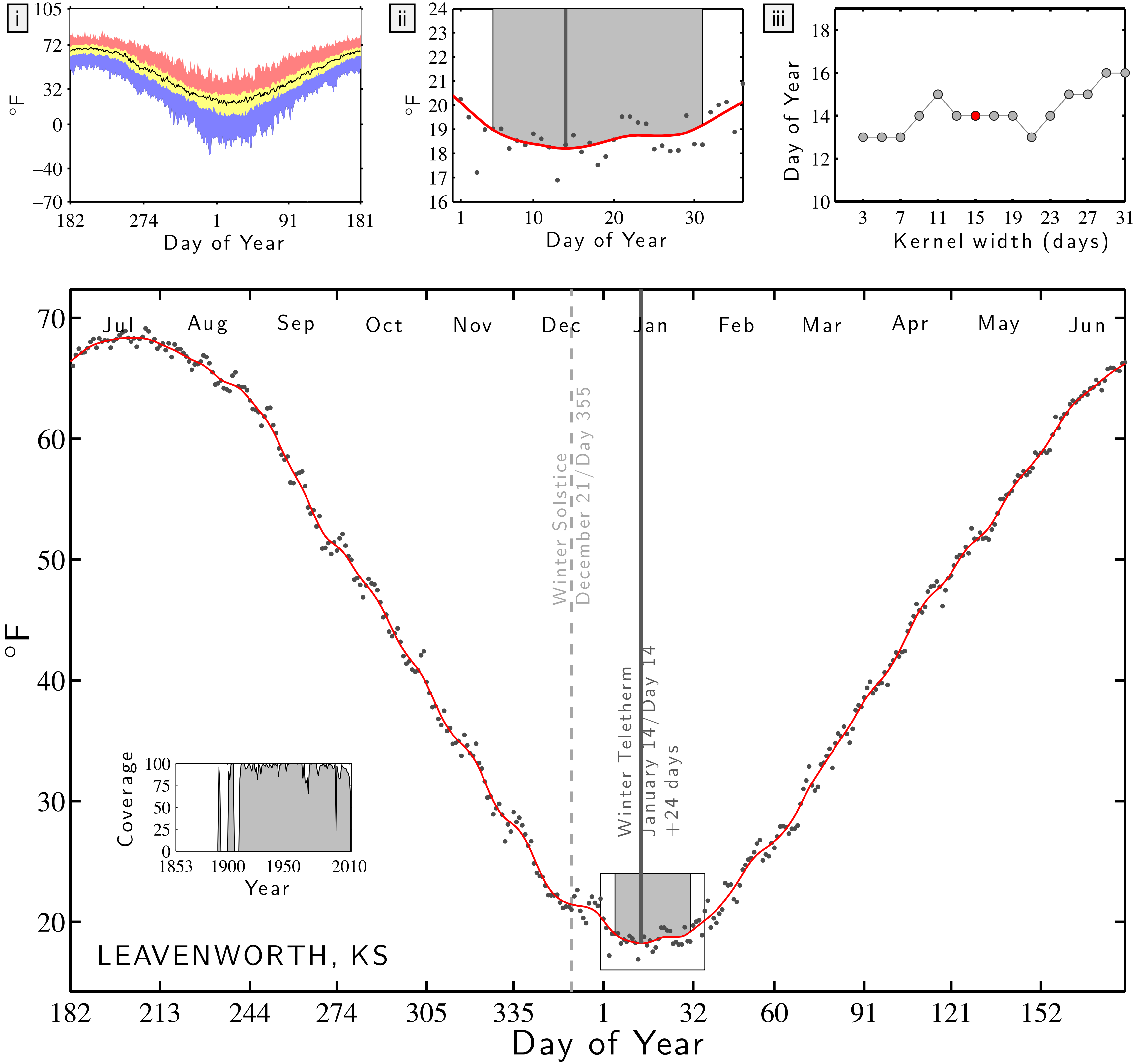

i
ii
iii
Day of Year
Kernel width (days)
Jul
Aug
Sep
Oct
Nov
Dec
Jan
Feb
Mar
Apr
May
Jun
Winter Solstice
December 21/Day 355
Winter Teletherm
January 14/Day 14
+24 days
Coverage
Year
LEAVENWORTH, KS
°F

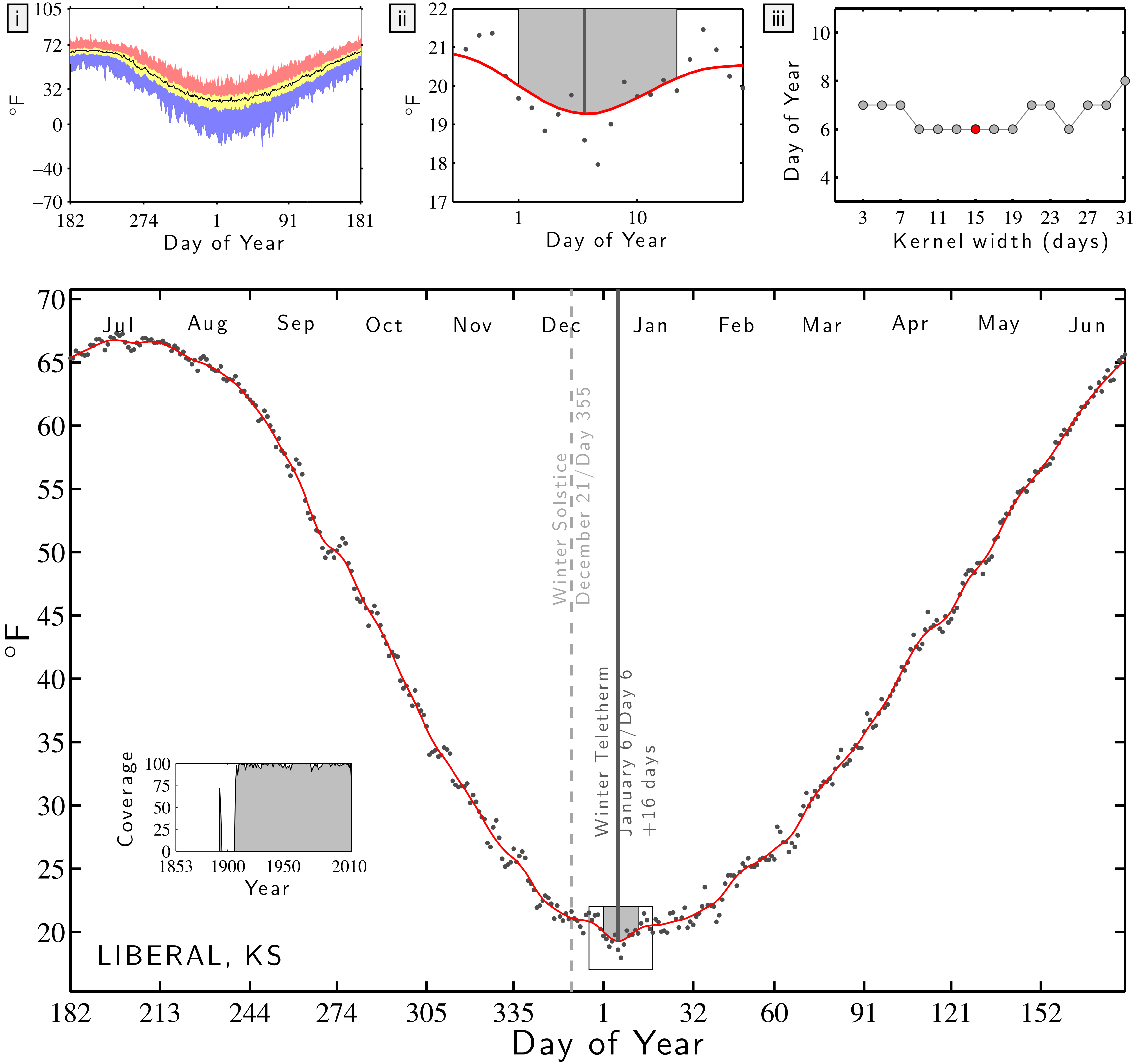
i
ii
iii
Day of Year
°F
Kernel width (days)
Winter Solstice
December 21/Day 355
Winter Teletherm
January 6/Day 6
+16 days
Jul
Aug
Sep
Oct
Nov
Dec
Jan
Feb
Mar
Apr
May
Jun
Coverage
Year
LIBERAL, KS

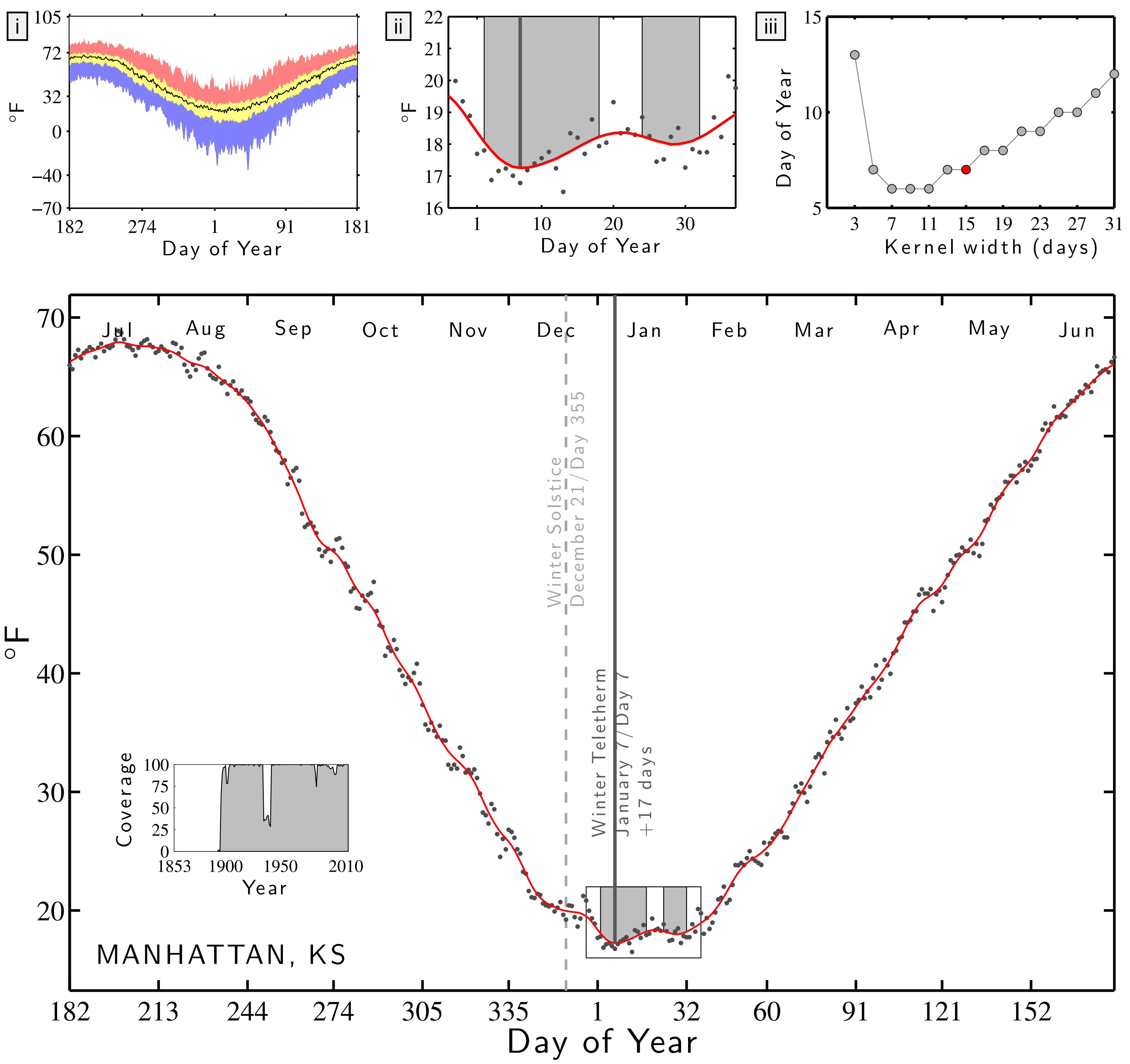

i
ii
iii
°F
Day of Year
Kernel width (days)
Jul
Aug
Sep
Oct
Nov
Dec
Jan
Feb
Mar
Apr
May
Jun
Winter Solstice
December 21/Day 355
Winter Teletherm
January 7/Day 7
+17 days
Coverage
Year
MANHATTAN, KS

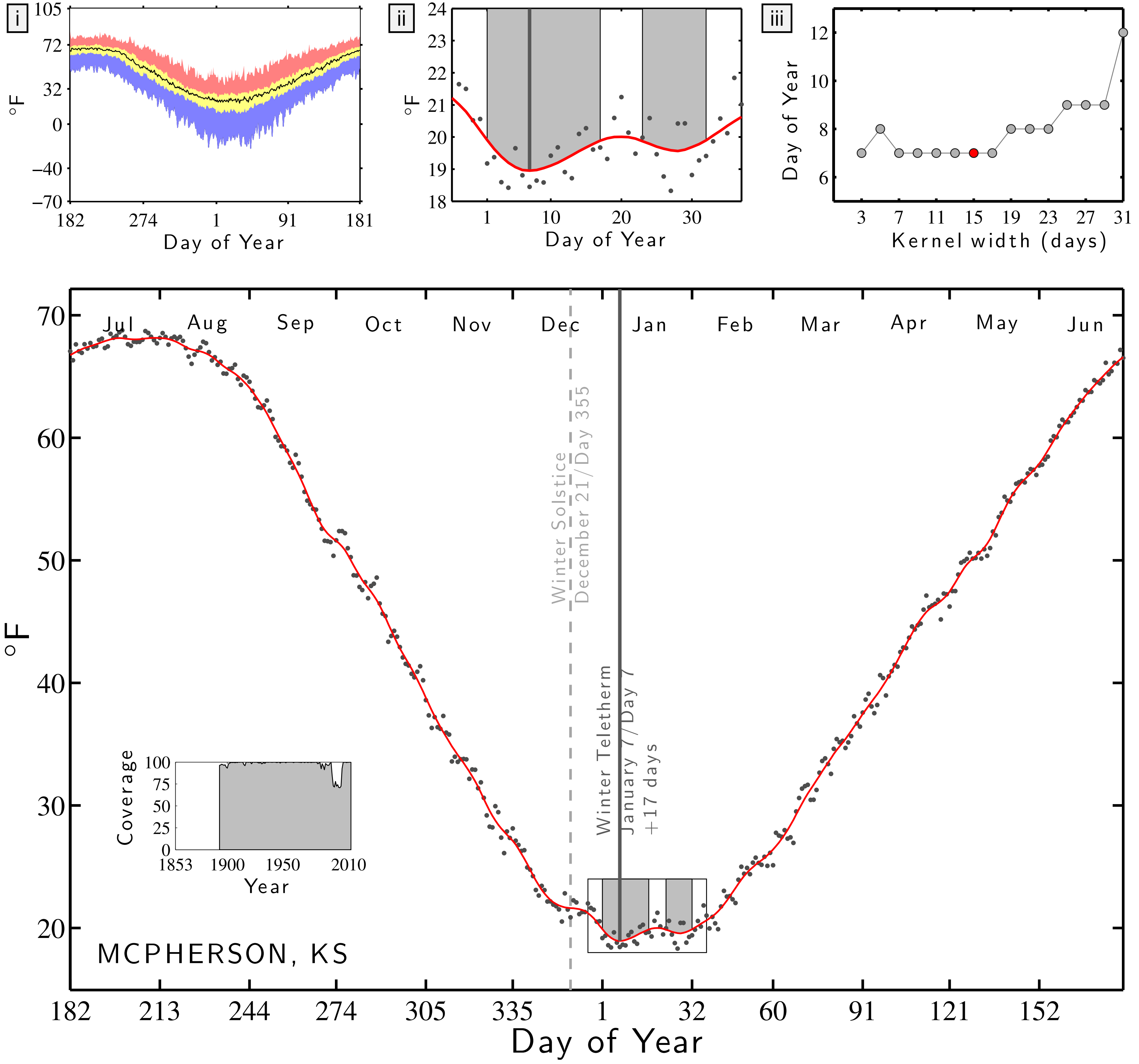

i
ii
iii
Day of Year
Kernel width (days)
°F
Jul
Aug
Sep
Oct
Nov
Dec
Jan
Feb
Mar
Apr
May
Jun
Winter Solstice
December 21/Day 355
Winter Teletherm
January 7/Day 7
+17 days
Coverage
Year
MCPHERSON, KS
Day of Year

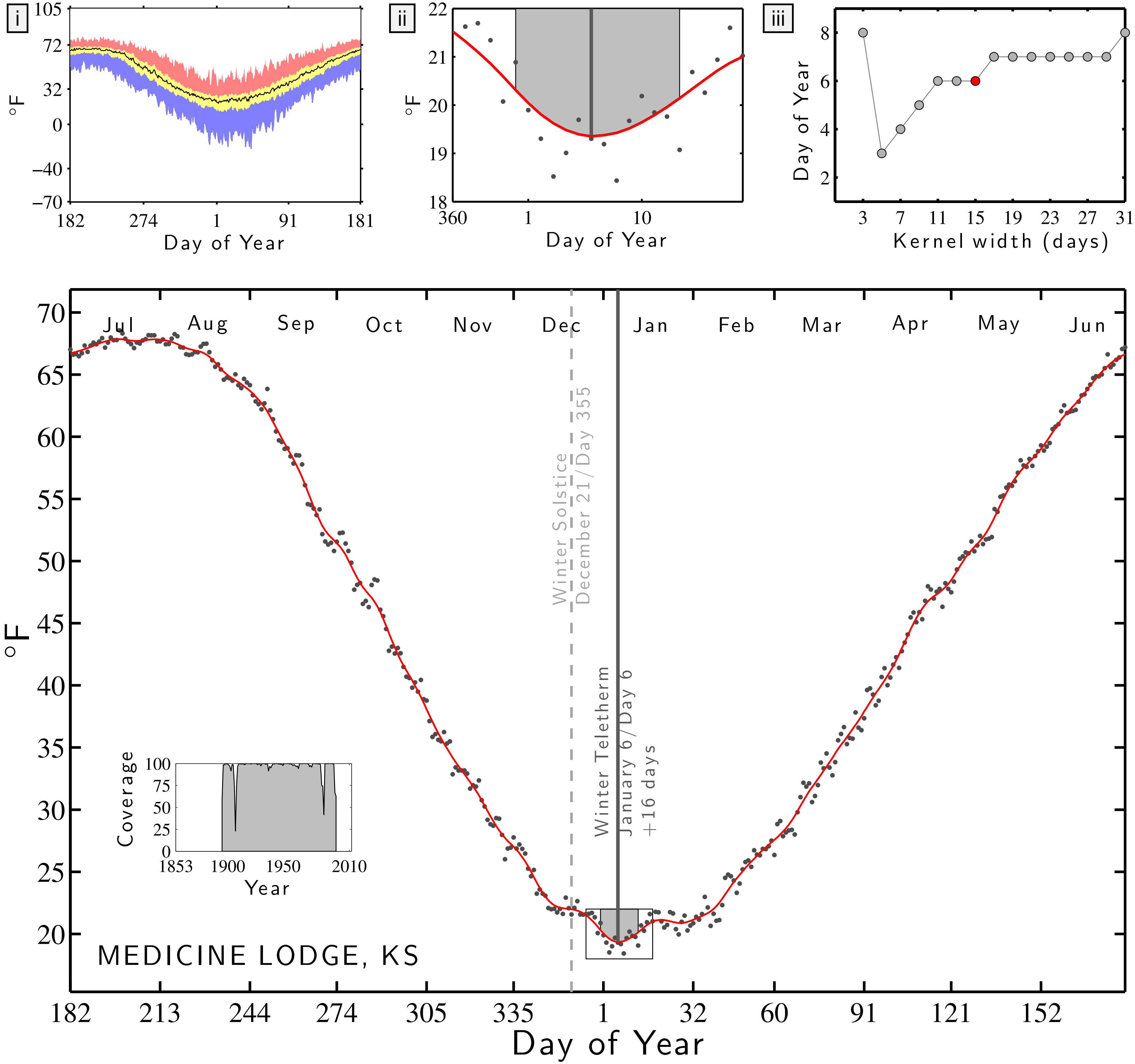

i
105
72
32
0
−40
−70
°F
182
274
1
91
181
Day of Year
ii
22
21
20
19
18
°F
360
1
10
Day of Year
iii
8
6
4
2
Day of Year
3
7
11
15
19
23
27
31
Kernel width (days)
70
65
60
55
50
45
40
35
30
25
20
°F
Jul
Aug
Sep
Oct
Nov
Dec
Jan
Feb
Mar
Apr
May
Jun
Winter Solstice
December 21/Day 355
Winter Teletherm
January 6/Day 6
+16 days
Coverage
100
75
50
25
0
1853
1900
1950
2010
Year
MEDICINE LODGE, KS
182
213
244
274
305
335
1
32
60
91
121
152
Day of Year

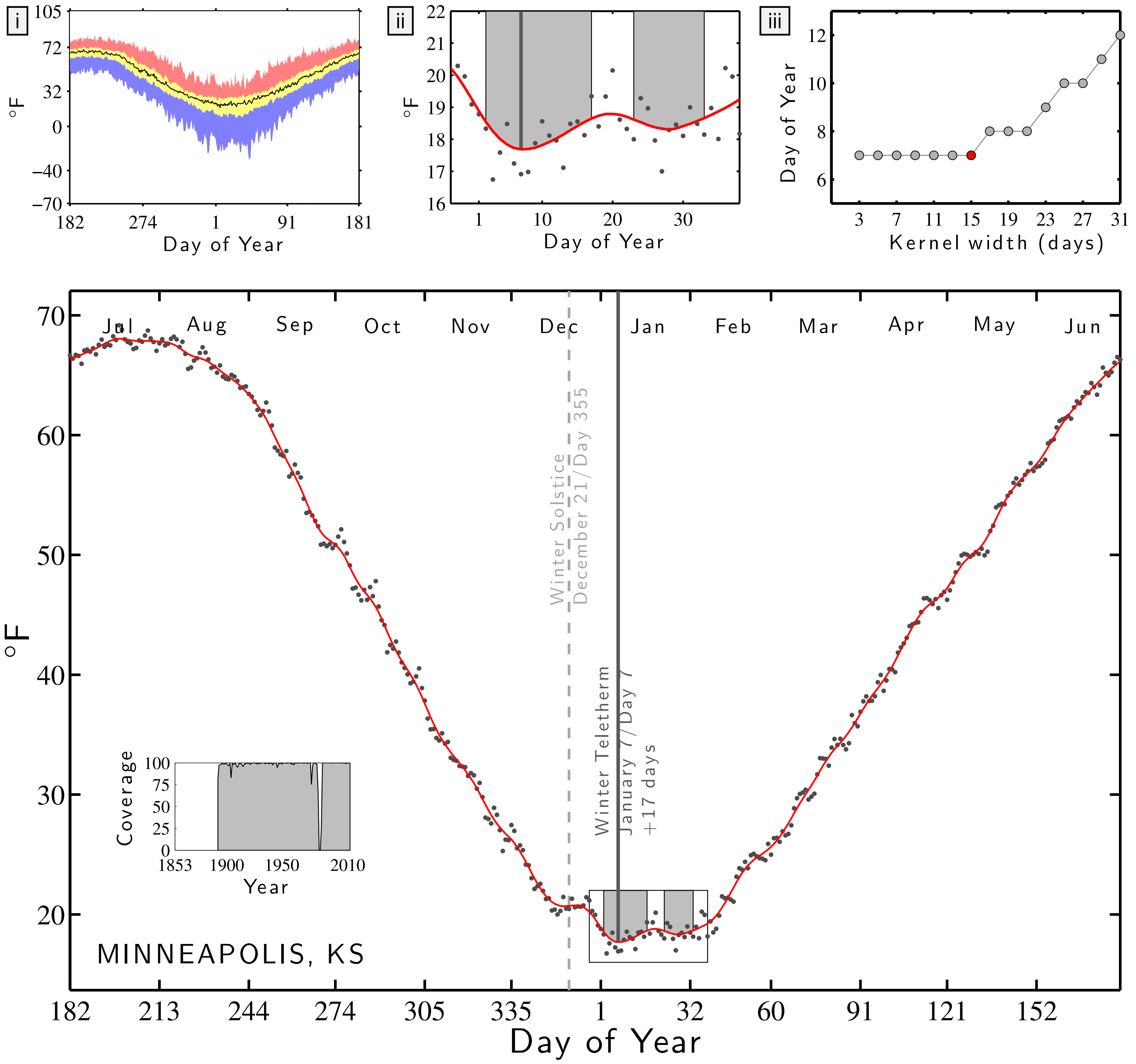

i
ii
iii
°F
Day of Year
Kernel width (days)
Jul
Aug
Sep
Oct
Nov
Dec
Jan
Feb
Mar
Apr
May
Jun
Winter Solstice
December 21/Day 355
Winter Teletherm
January 7/Day 7
+17 days
Coverage
Year
MINNEAPOLIS, KS

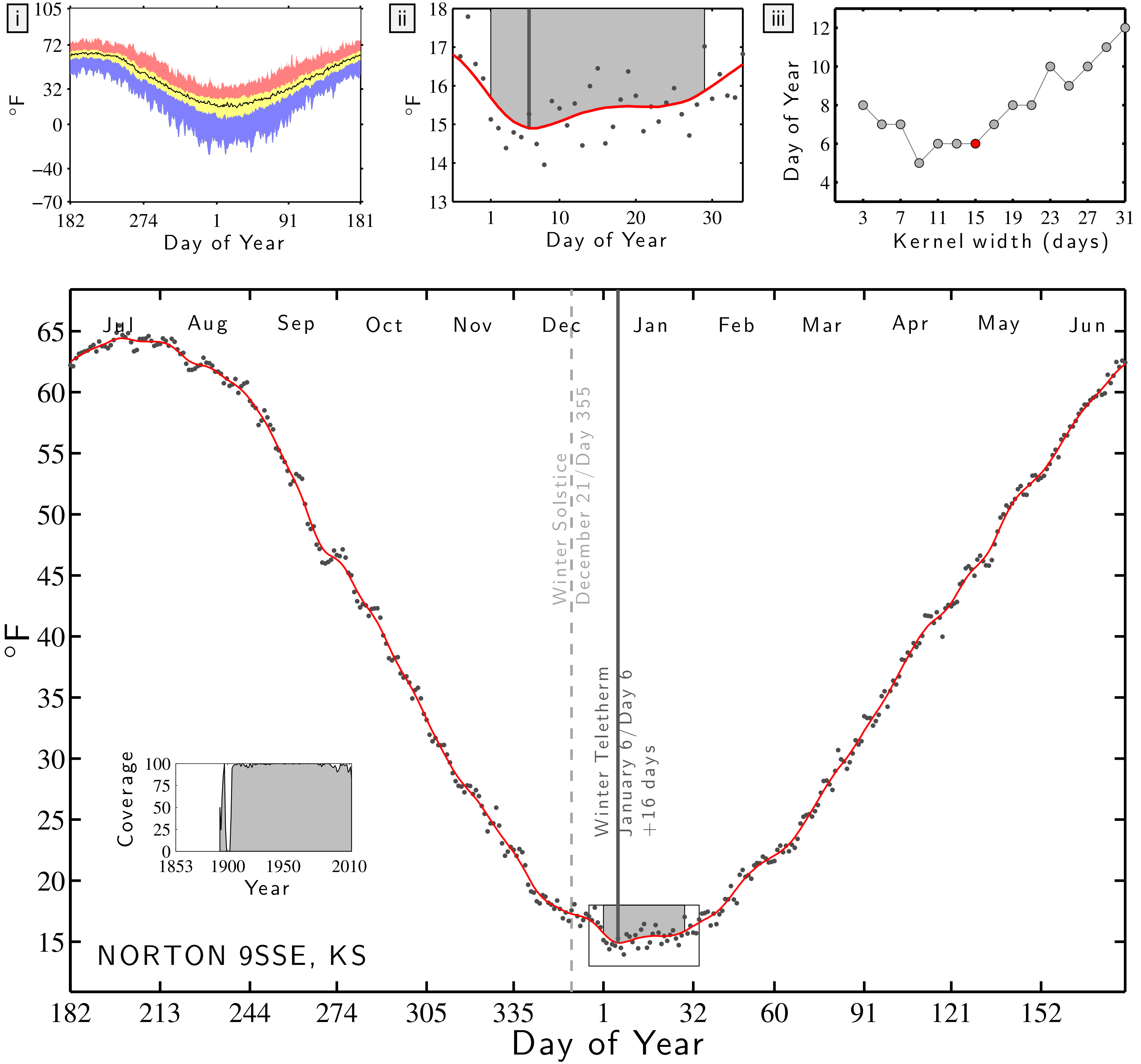

i
°F
Day of Year
ii
°F
Day of Year
iii
Day of Year
Kernel width (days)
Jul
Aug
Sep
Oct
Nov
Dec
Jan
Feb
Mar
Apr
May
Jun
Winter Solstice
December 21/Day 355
Winter Teletherm
January 6/Day 6
+16 days
Coverage
Year
NORTON 9SSE, KS
°F
Day of Year

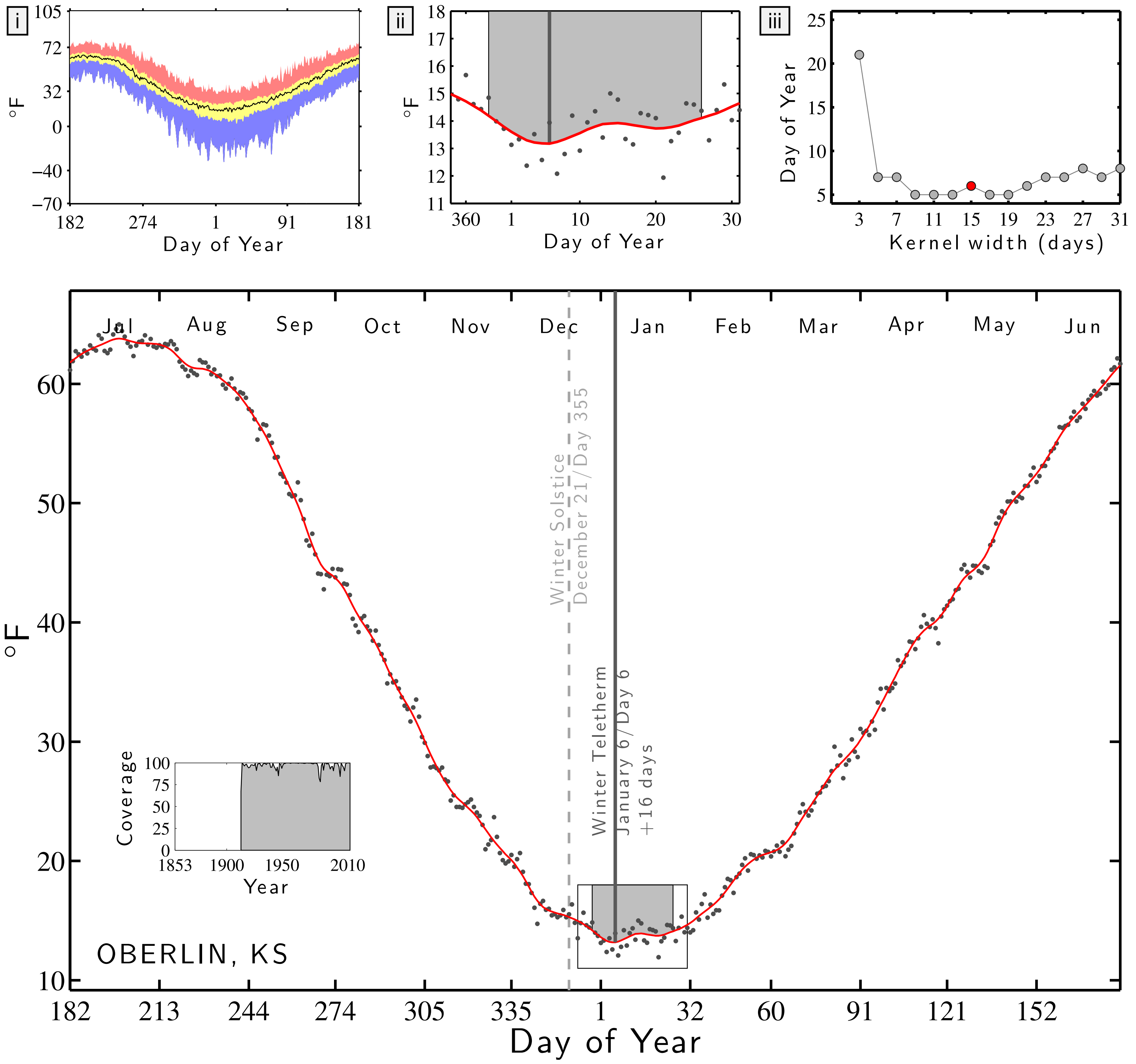

i
°F
Day of Year
ii
°F
Day of Year
iii
Day of Year
Kernel width (days)
Jul
Aug
Sep
Oct
Nov
Dec
Jan
Feb
Mar
Apr
May
Jun
Winter Solstice
December 21/Day 355
Winter Teletherm
January 6/Day 6
+16 days
Coverage
Year
OBERLIN, KS
°F
Day of Year

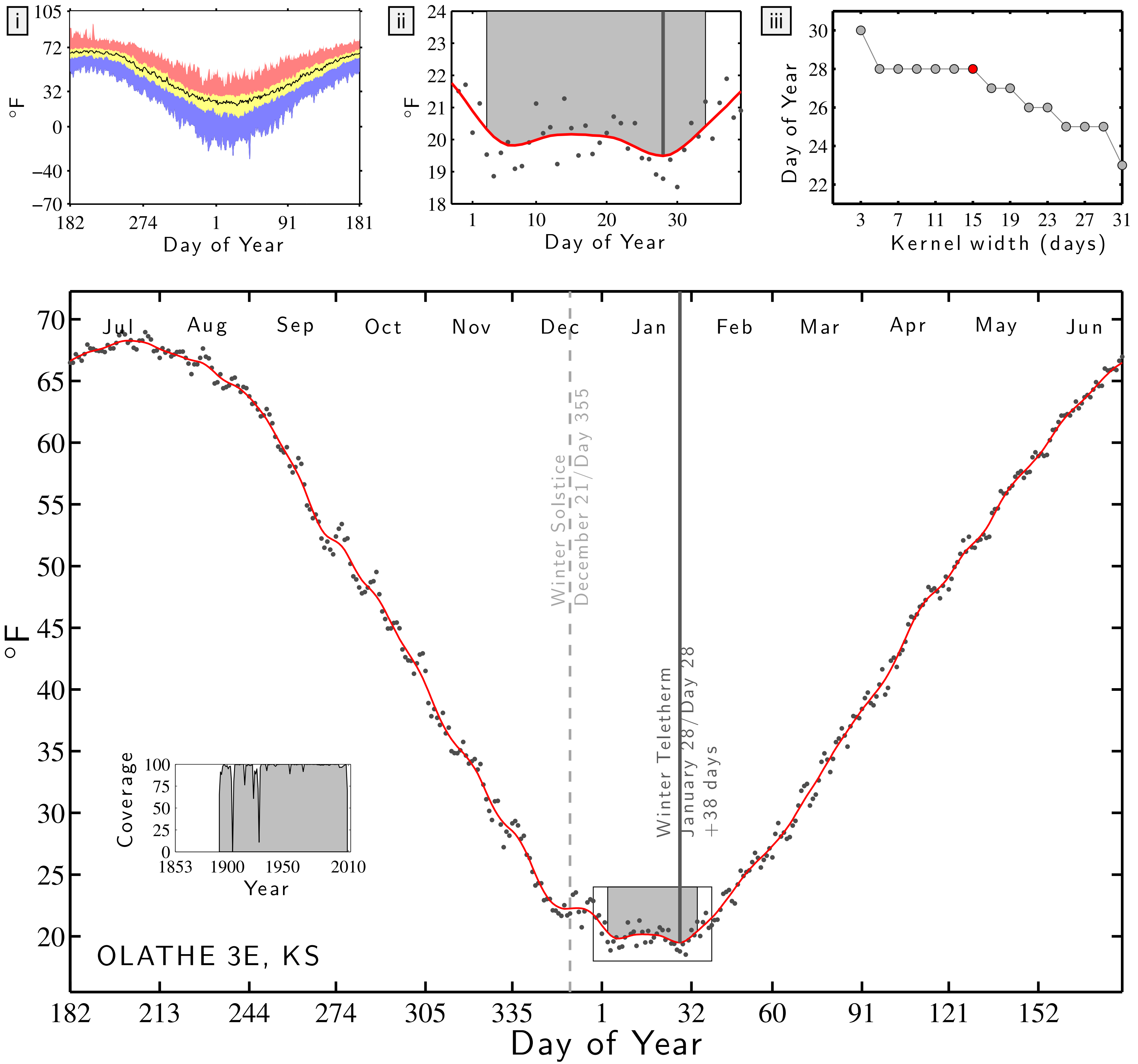

i
°F
Day of Year
ii
°F
Day of Year
iii
Day of Year
Kernel width (days)
Jul
Aug
Sep
Oct
Nov
Dec
Jan
Feb
Mar
Apr
May
Jun
Winter Solstice
December 21/Day 355
Winter Teletherm
January 28/Day 28
+38 days
Coverage
Year
OLATHE 3E, KS
°F
Day of Year

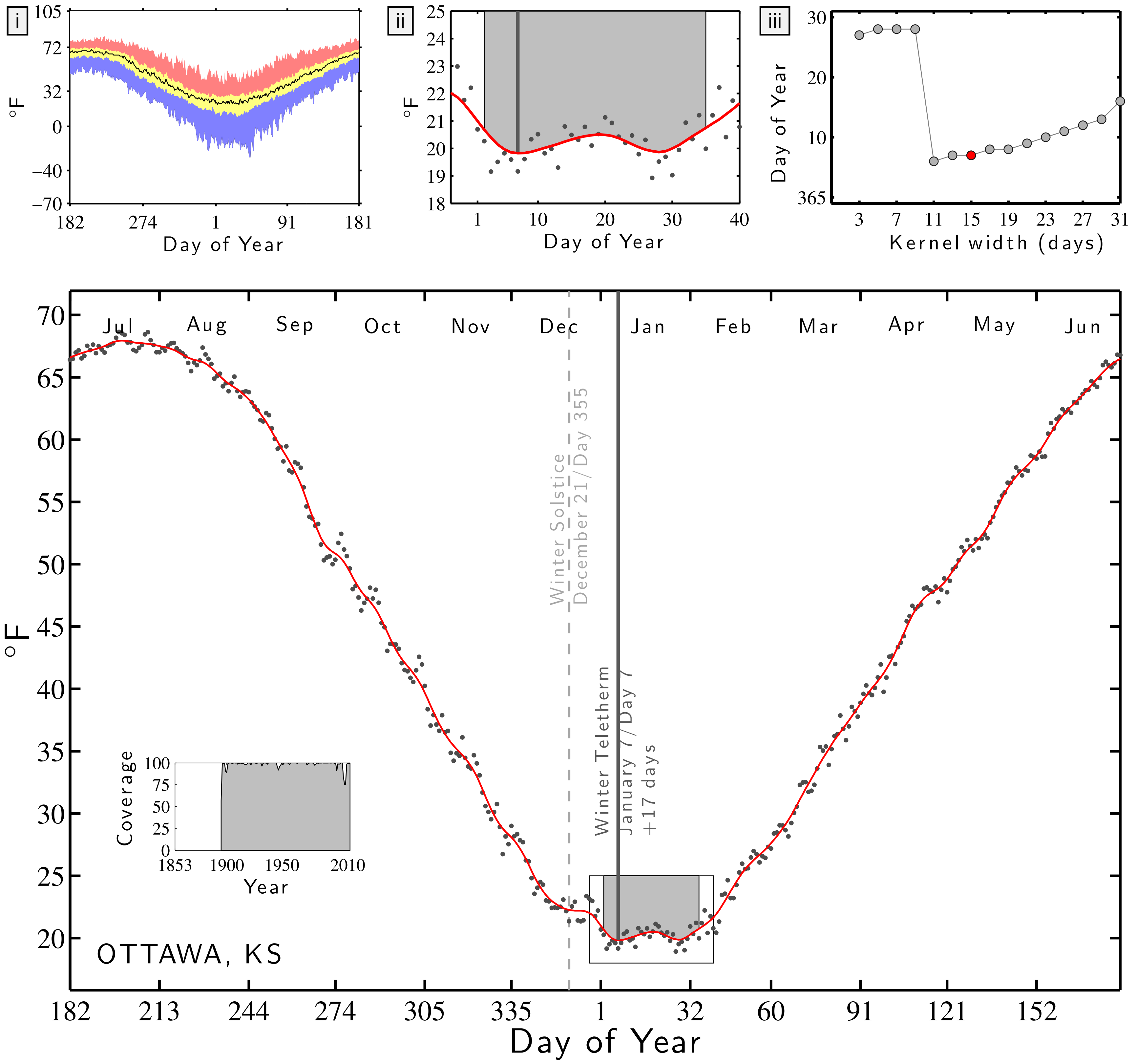
i
°F
Day of Year
ii
°F
Day of Year
iii
Day of Year
Kernel width (days)
Jul
Aug
Sep
Oct
Nov
Dec
Jan
Feb
Mar
Apr
May
Jun
Winter Solstice
December 21/Day 355
Winter Teletherm
January 7/Day 7
+17 days
Coverage
Year
OTTAWA, KS
°F
Day of Year

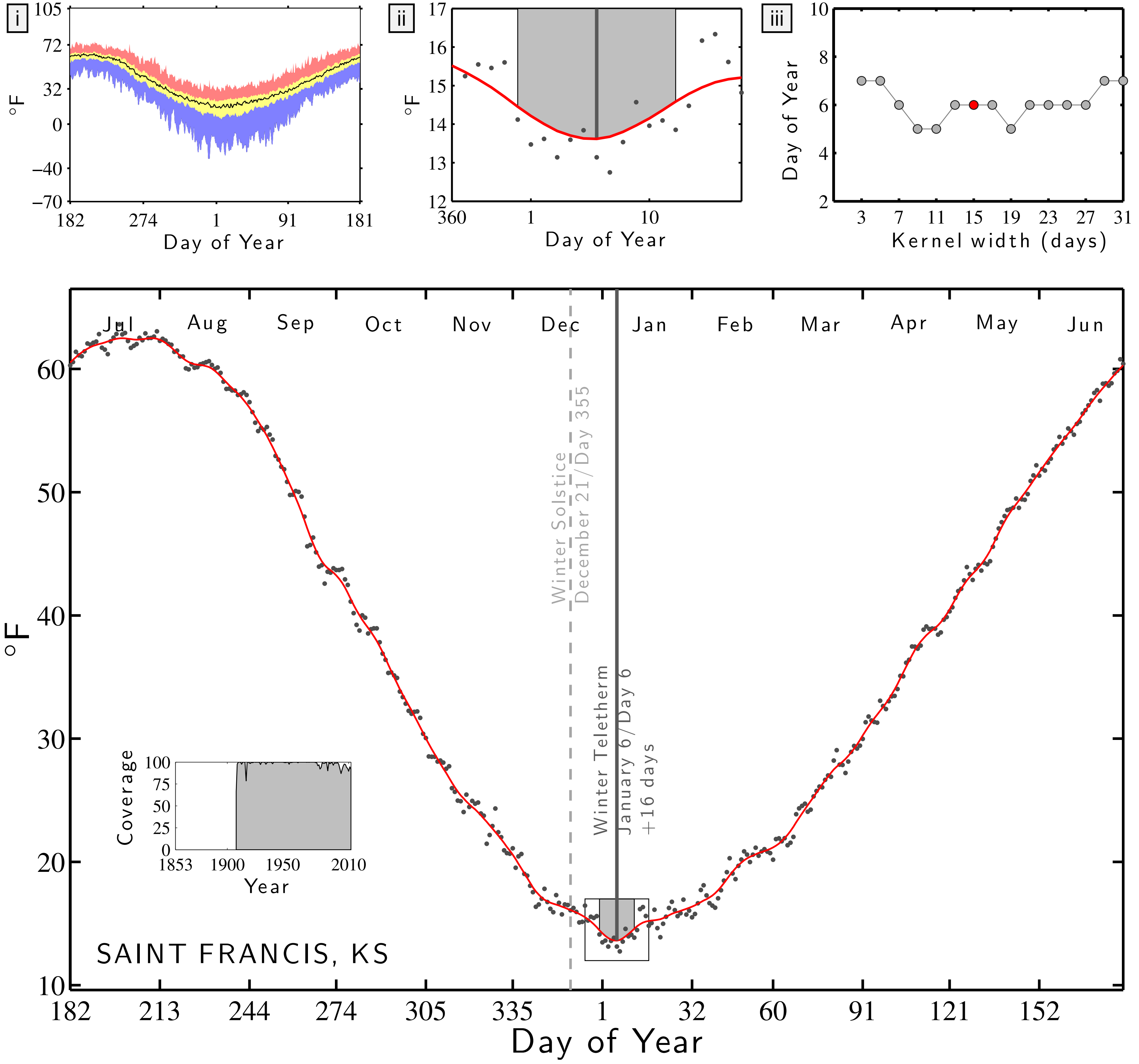

i
ii
iii
°F
Day of Year
Kernel width (days)
Jul
Aug
Sep
Oct
Nov
Dec
Jan
Feb
Mar
Apr
May
Jun
Winter Solstice
December 21/Day 355
Winter Teletherm
January 6/Day 6
+16 days
Coverage
Year
SAINT FRANCIS, KS

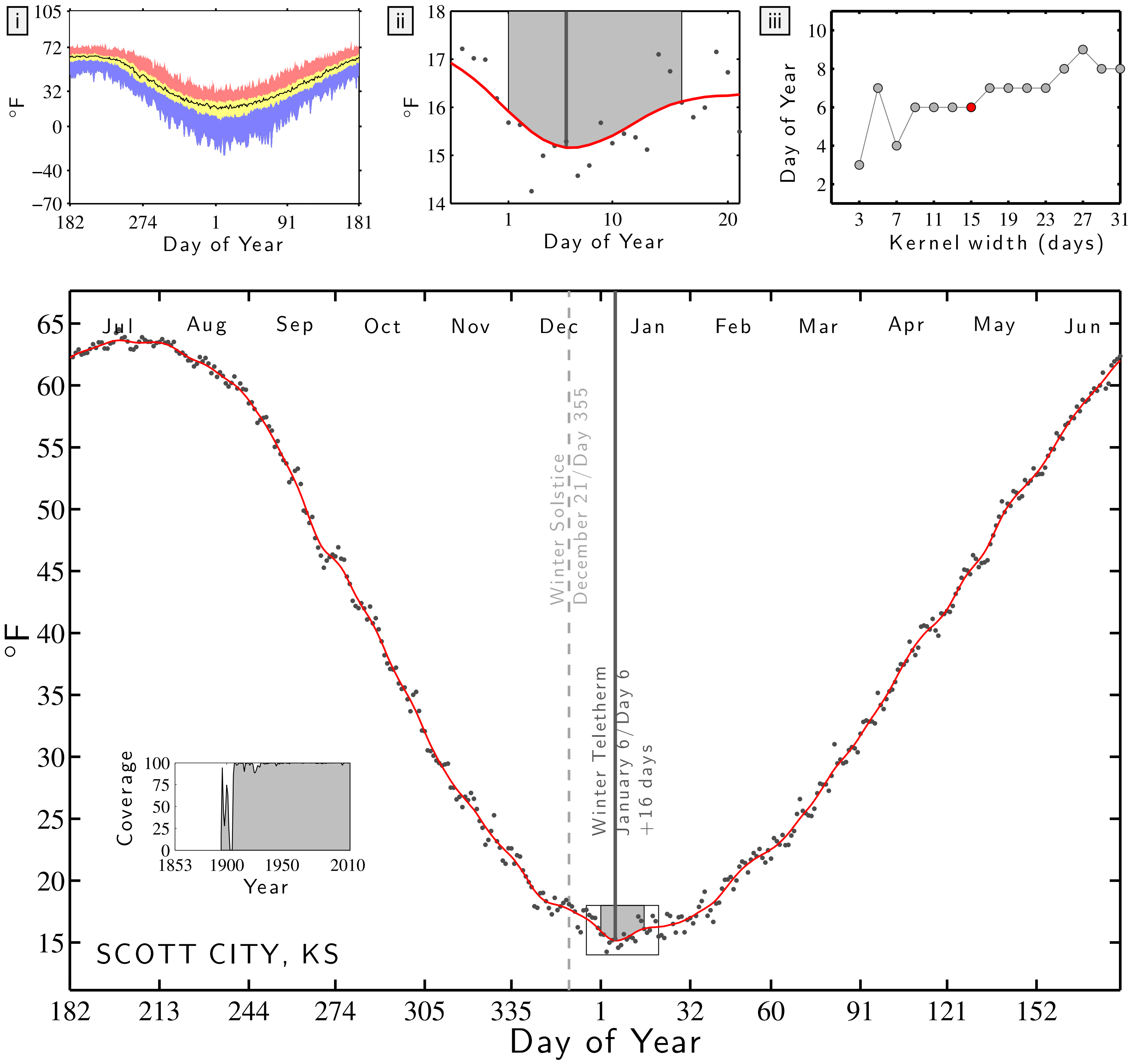

i
ii
iii
°F
Day of Year
Kernel width (days)
Jul
Aug
Sep
Oct
Nov
Dec
Jan
Feb
Mar
Apr
May
Jun
Winter Solstice
December 21/Day 355
Winter Teletherm
January 6/Day 6
+16 days
Coverage
Year
SCOTT CITY, KS

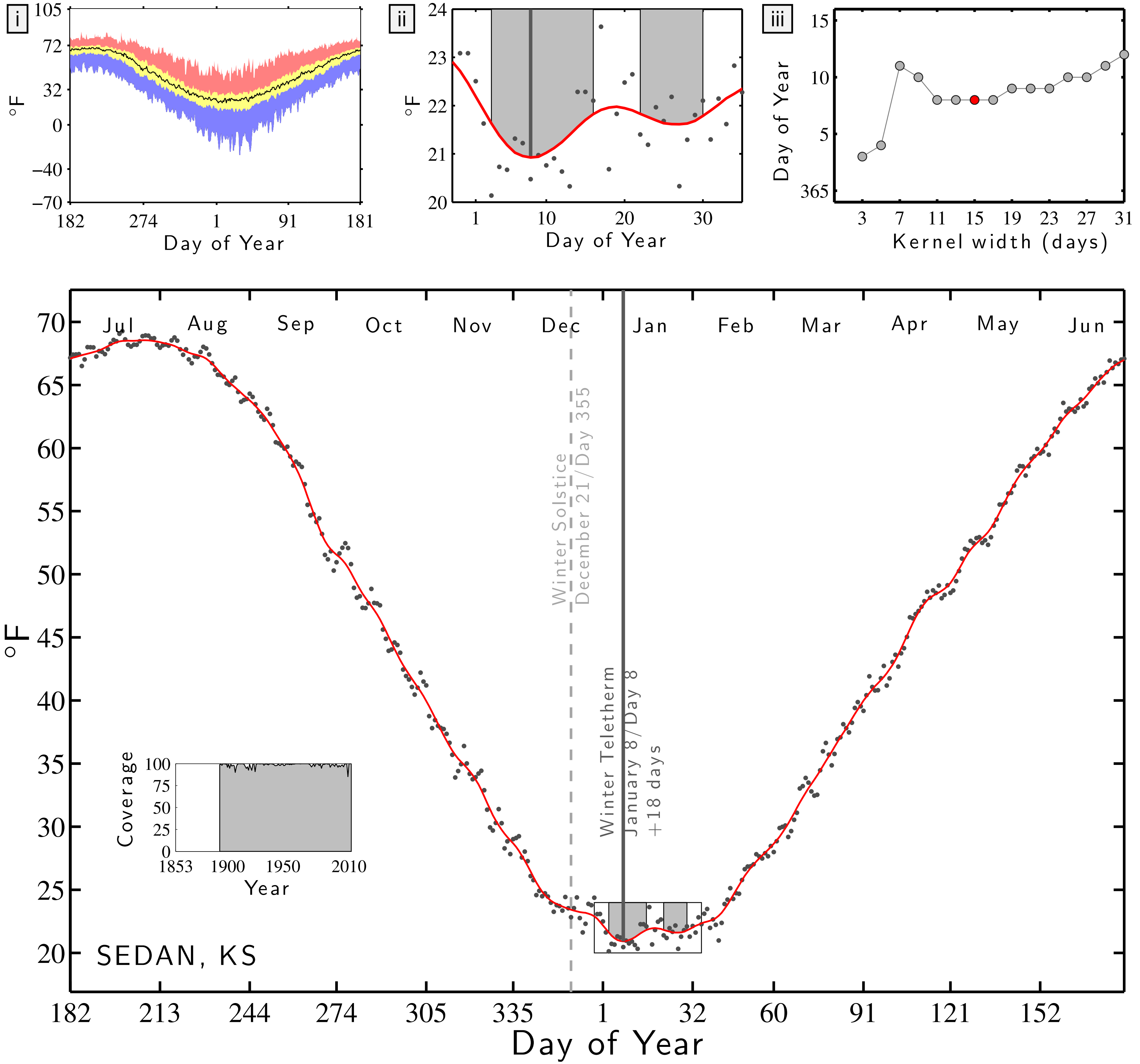

i
°F
Day of Year
ii
iii
Day of Year
Kernel width (days)
Jul
Aug
Sep
Oct
Nov
Dec
Jan
Feb
Mar
Apr
May
Jun
Winter Solstice
December 21/Day 355
Winter Teletherm
January 8/Day 8
+18 days
Coverage
Year
SEDAN, KS

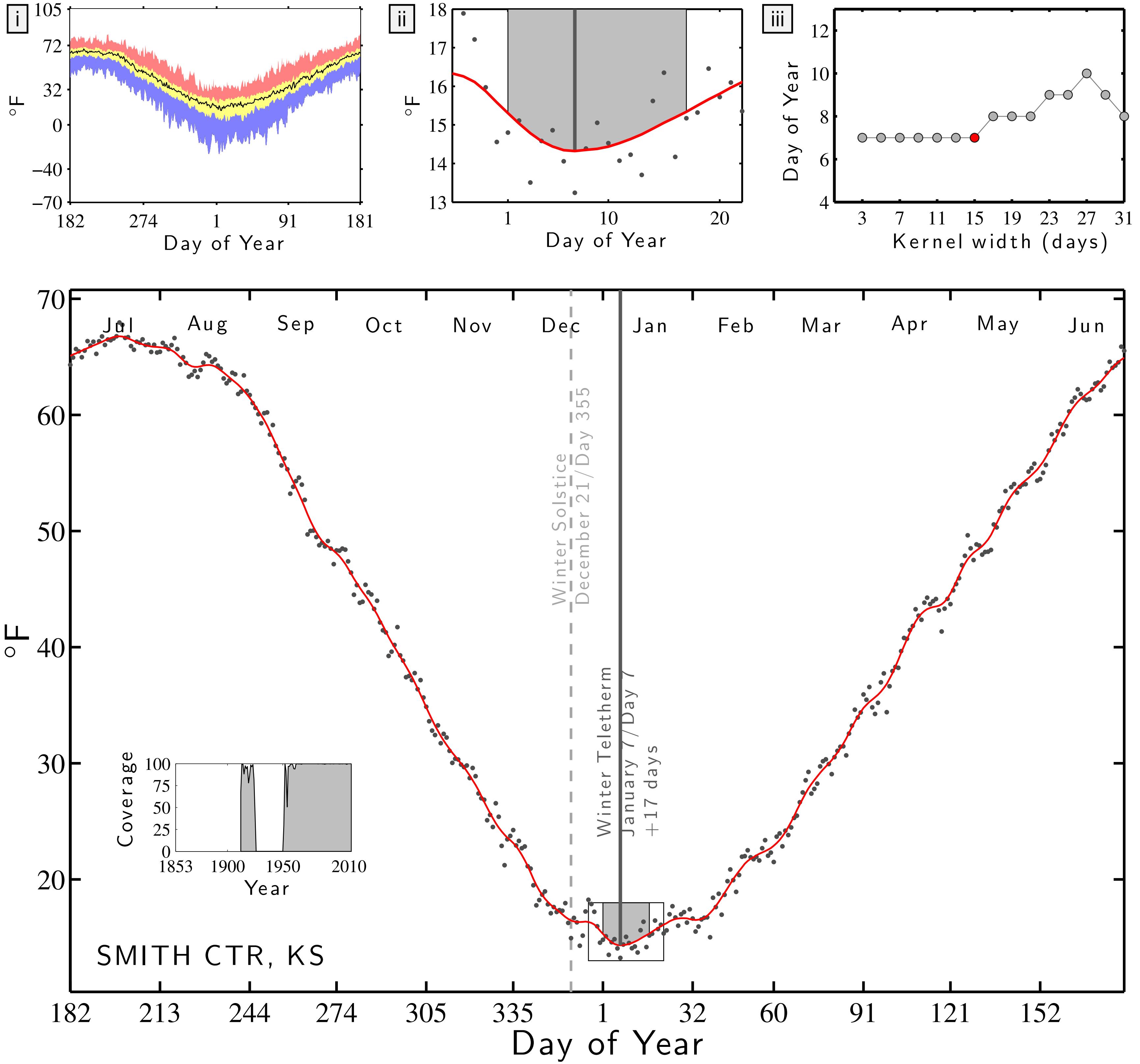

i
ii
iii
°F
Day of Year
Kernel width (days)
Jul
Aug
Sep
Oct
Nov
Dec
Jan
Feb
Mar
Apr
May
Jun
Winter Solstice
December 21/Day 355
Winter Teletherm
January 7/Day 7
+17 days
Coverage
Year
SMITH CTR, KS

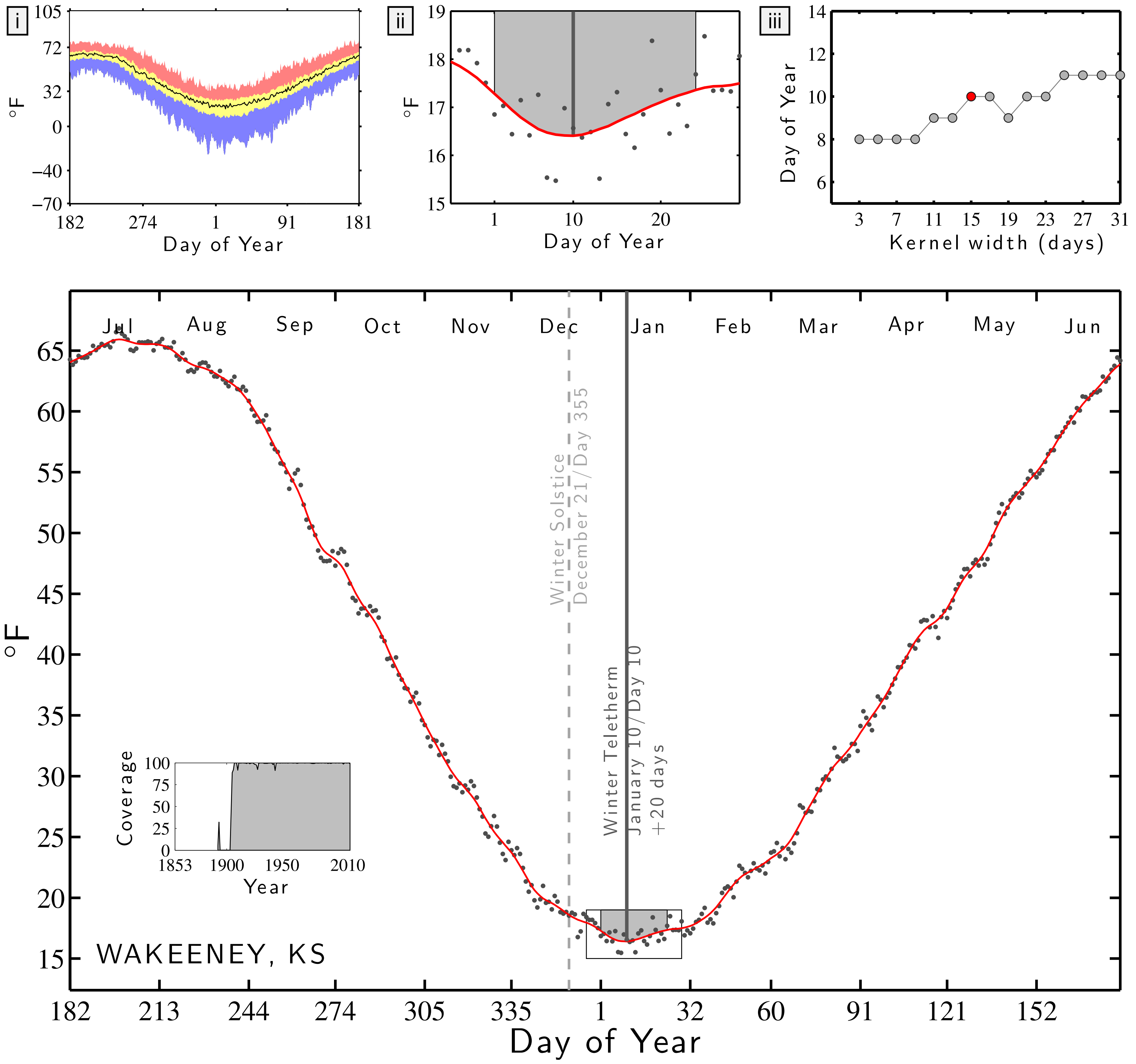

i
°F
Day of Year
ii
°F
Day of Year
iii
Day of Year
Kernel width (days)
Jul
Aug
Sep
Oct
Nov
Dec
Jan
Feb
Mar
Apr
May
Jun
Winter Solstice
December 21/Day 355
Winter Teletherm
January 10/Day 10
+20 days
Coverage
Year
WAKEENEY, KS
°F
Day of Year

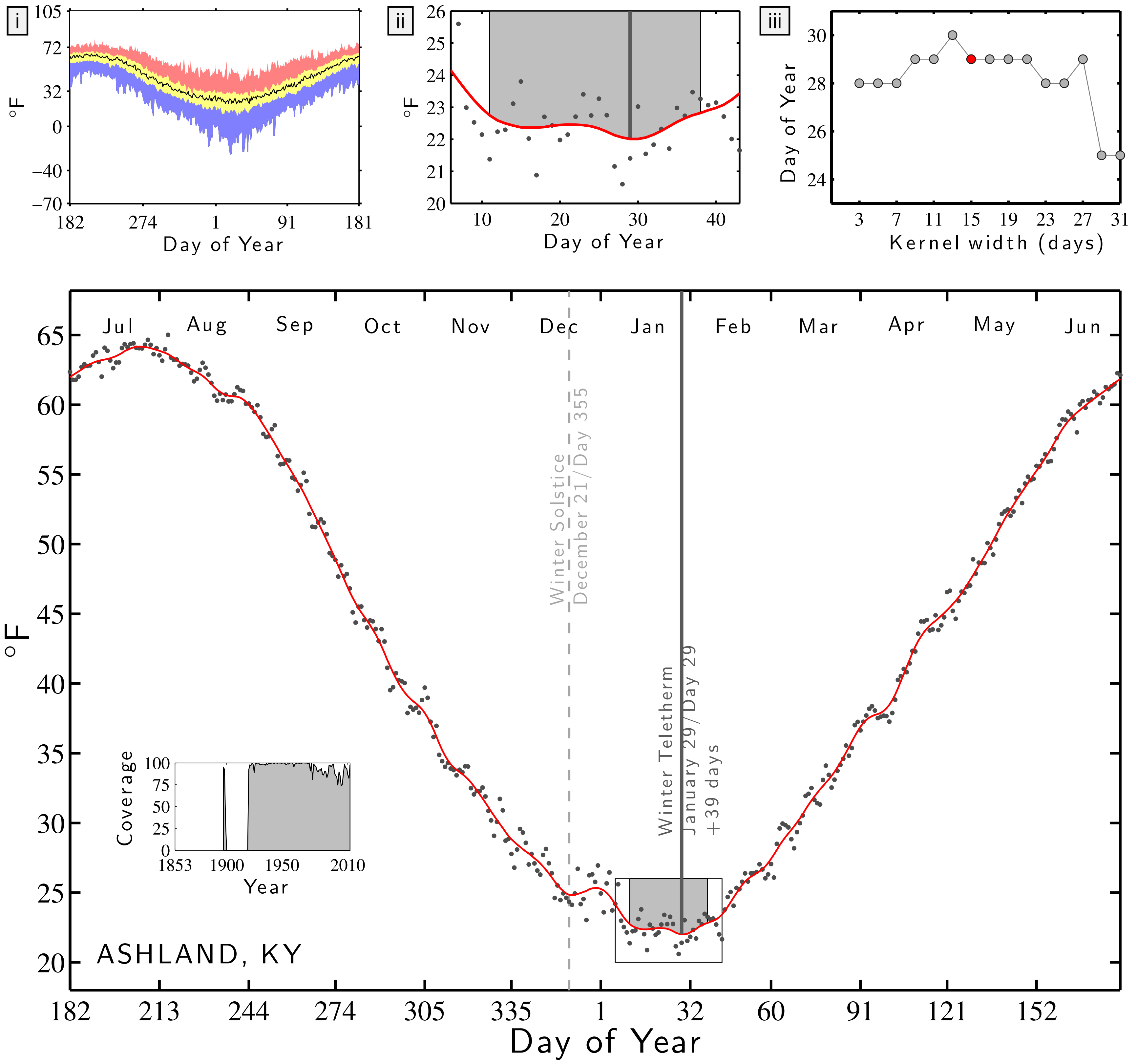

i
°F
Day of Year
ii
°F
Day of Year
iii
Day of Year
Kernel width (days)
Jul
Aug
Sep
Oct
Nov
Dec
Jan
Feb
Mar
Apr
May
Jun
Winter Solstice
December 21/Day 355
Winter Teletherm
January 29/Day 29
+39 days
Coverage
Year
ASHLAND, KY
°F
Day of Year

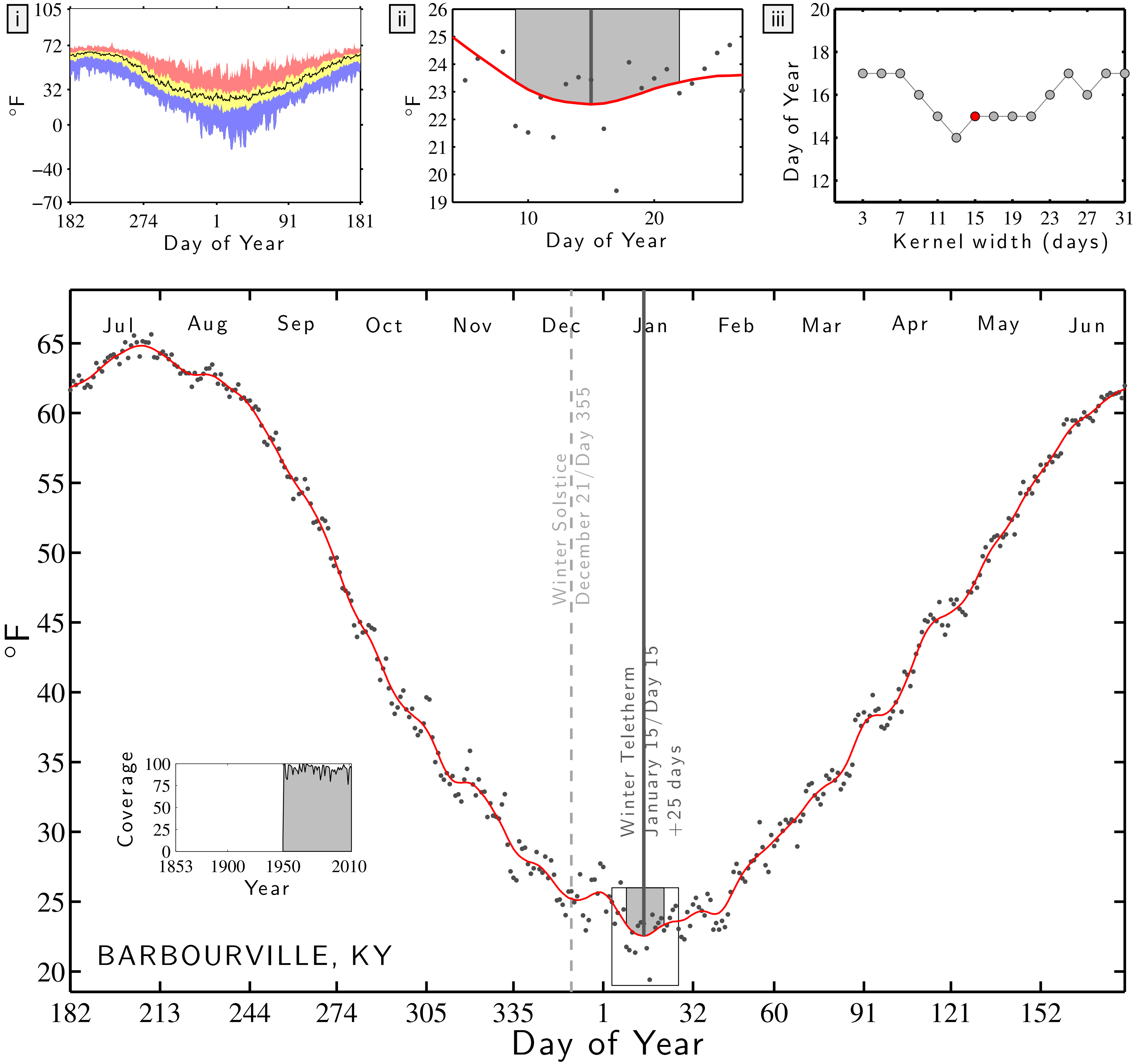
i
°F
105
72
32
0
−40
−70
182
274
1
91
181
Day of Year
ii
26
25
24
23
22
21
20
19
10
20
Day of Year
iii
Day of Year
20
18
16
14
12
3
7
11
15
19
23
27
31
Kernel width (days)
Jul
Aug
Sep
Oct
Nov
Dec
Jan
Feb
Mar
Apr
May
Jun
Winter Solstice
December 21/Day 355
Winter Teletherm
January 15/Day 15
+25 days
Coverage
100
75
50
25
0
1853
1900
1950
2010
Year
BARBOURVILLE, KY
65
60
55
50
45
40
35
30
25
20
182
213
244
274
305
335
1
32
60
91
121
152
Day of Year

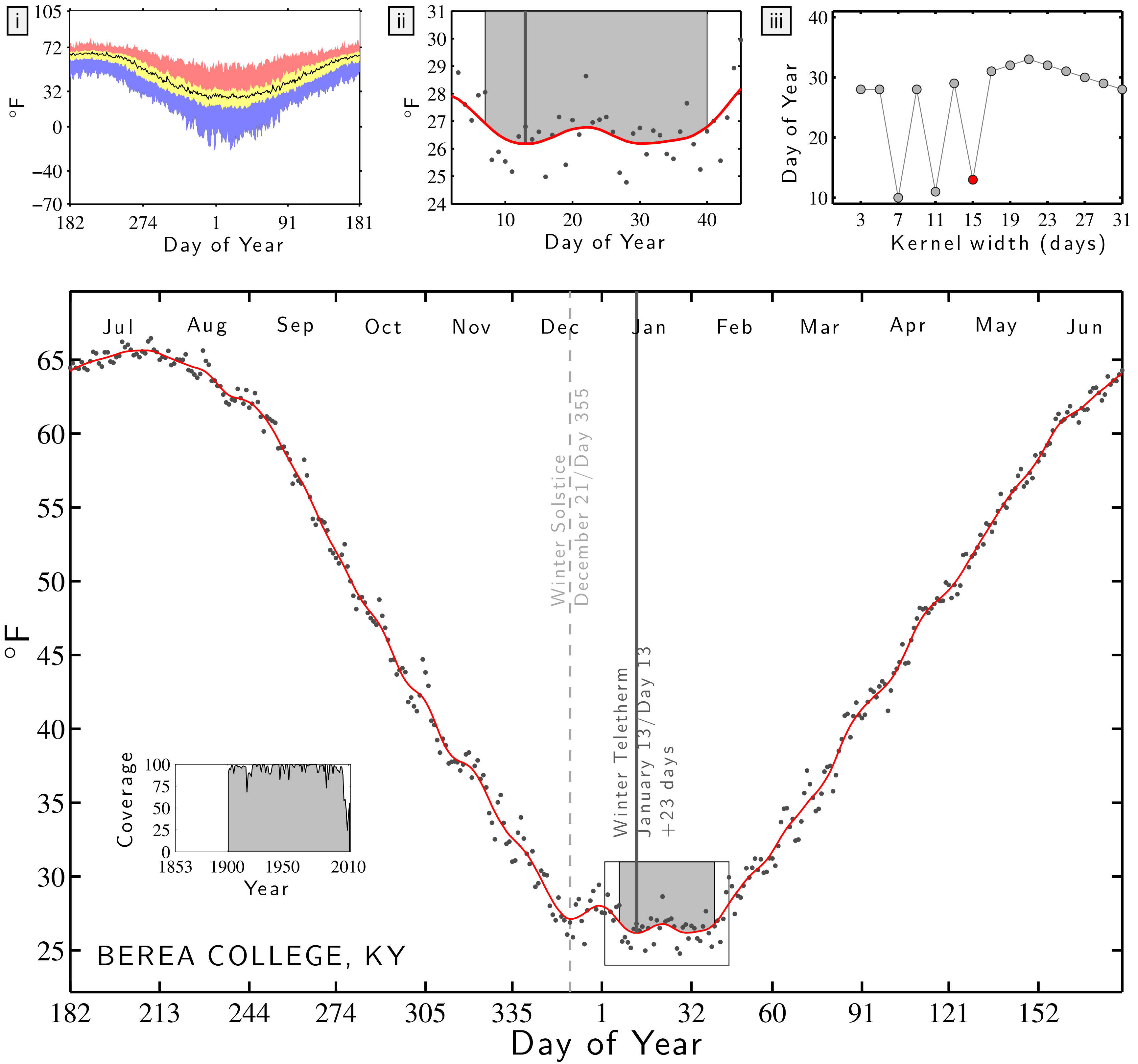

i
ii
iii
°F
Day of Year
Day of Year
Kernel width (days)
Jul
Aug
Sep
Oct
Nov
Dec
Jan
Feb
Mar
Apr
May
Jun
Winter Solstice
December 21/Day 355
Winter Teletherm
January 13/Day 13
+23 days
Coverage
Year
BEREA COLLEGE, KY

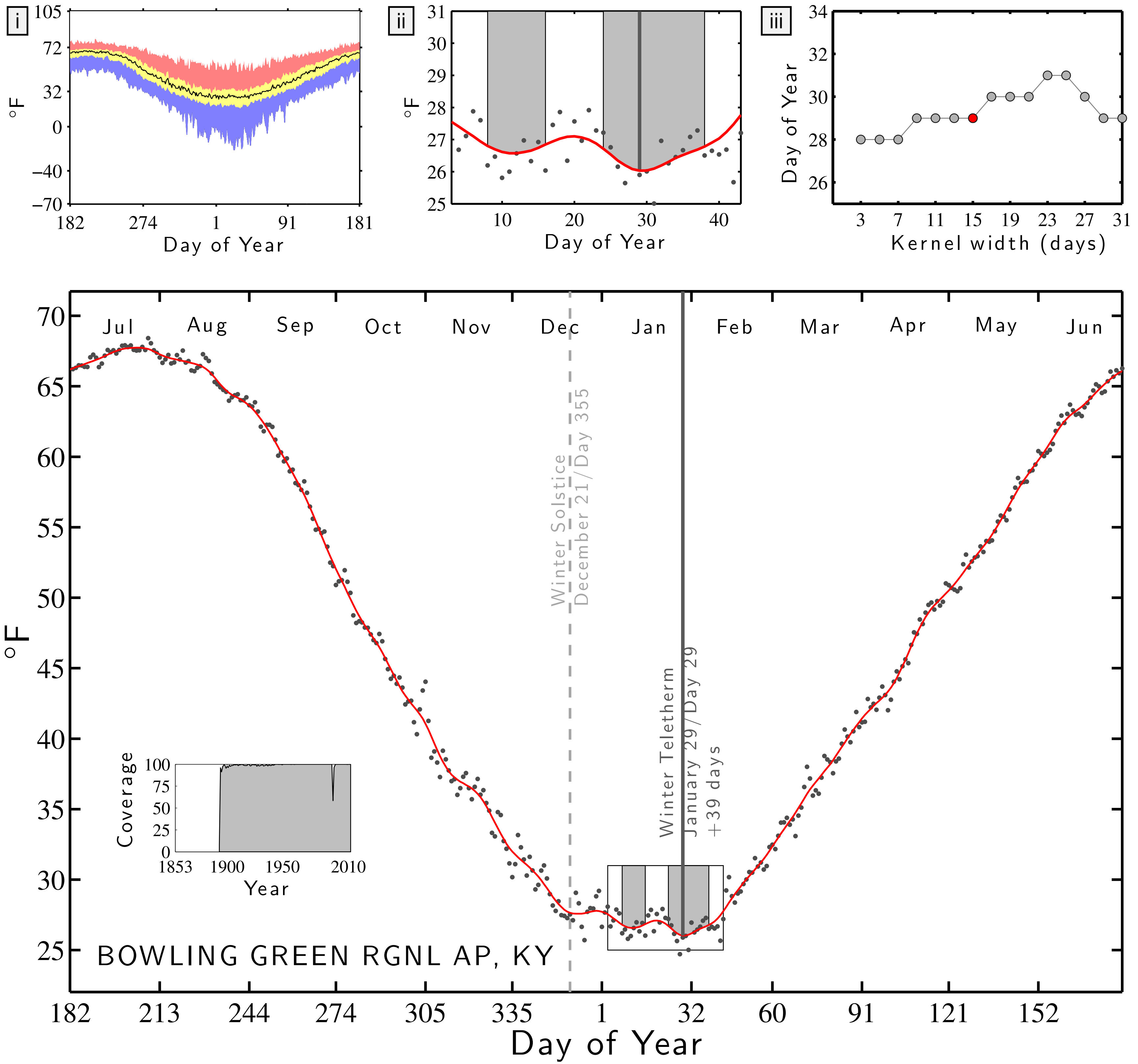

i
ii
iii
°F
Day of Year
Kernel width (days)
Jul
Aug
Sep
Oct
Nov
Dec
Jan
Feb
Mar
Apr
May
Jun
Winter Solstice
December 21/Day 355
Winter Teletherm
January 29/Day 29
+39 days
Coverage
Year
BOWLING GREEN RGNL AP, KY

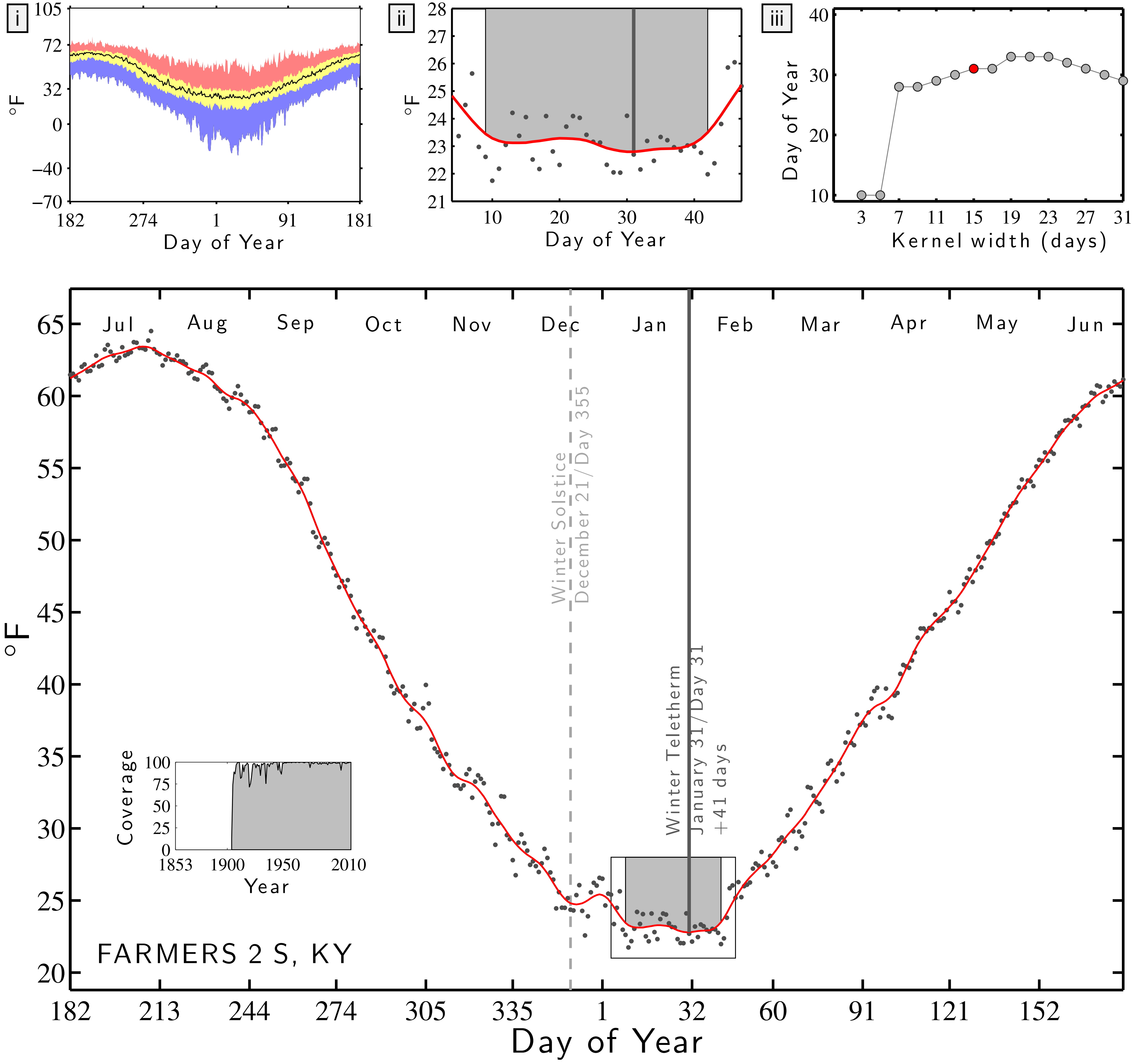

i
ii
iii
°F
Day of Year
Kernel width (days)
Jul
Aug
Sep
Oct
Nov
Dec
Jan
Feb
Mar
Apr
May
Jun
Winter Solstice
December 21/Day 355
Winter Teletherm
January 31/Day 31
+41 days
Coverage
Year
FARMERS 2 S, KY

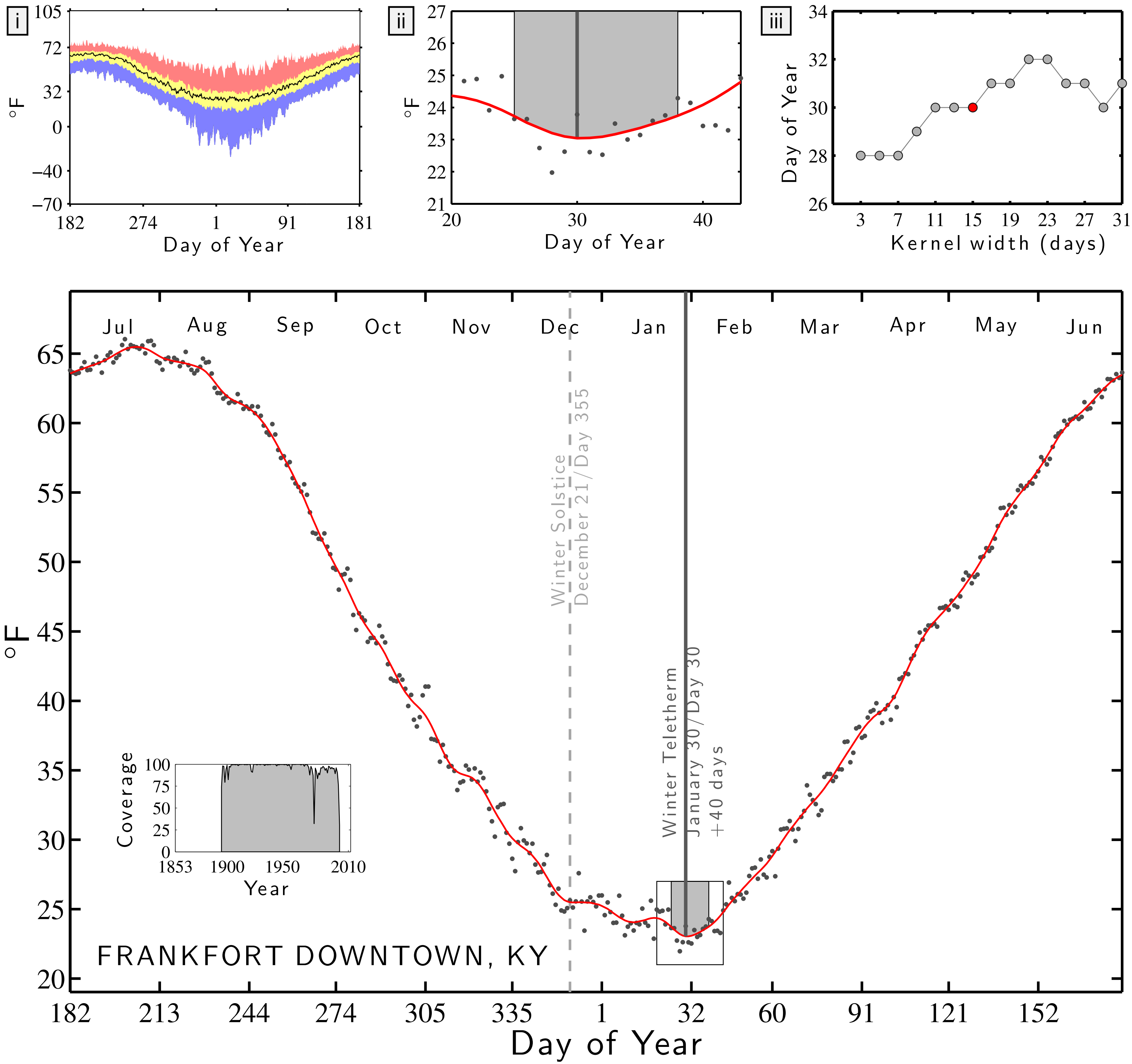

i
ii
iii
°F
Day of Year
Day of Year
Kernel width (days)
Jul
Aug
Sep
Oct
Nov
Dec
Jan
Feb
Mar
Apr
May
Jun
Winter Solstice
December 21/Day 355
Winter Teletherm
January 30/Day 30
+40 days
Coverage
Year
FRANKFORT DOWNTOWN, KY

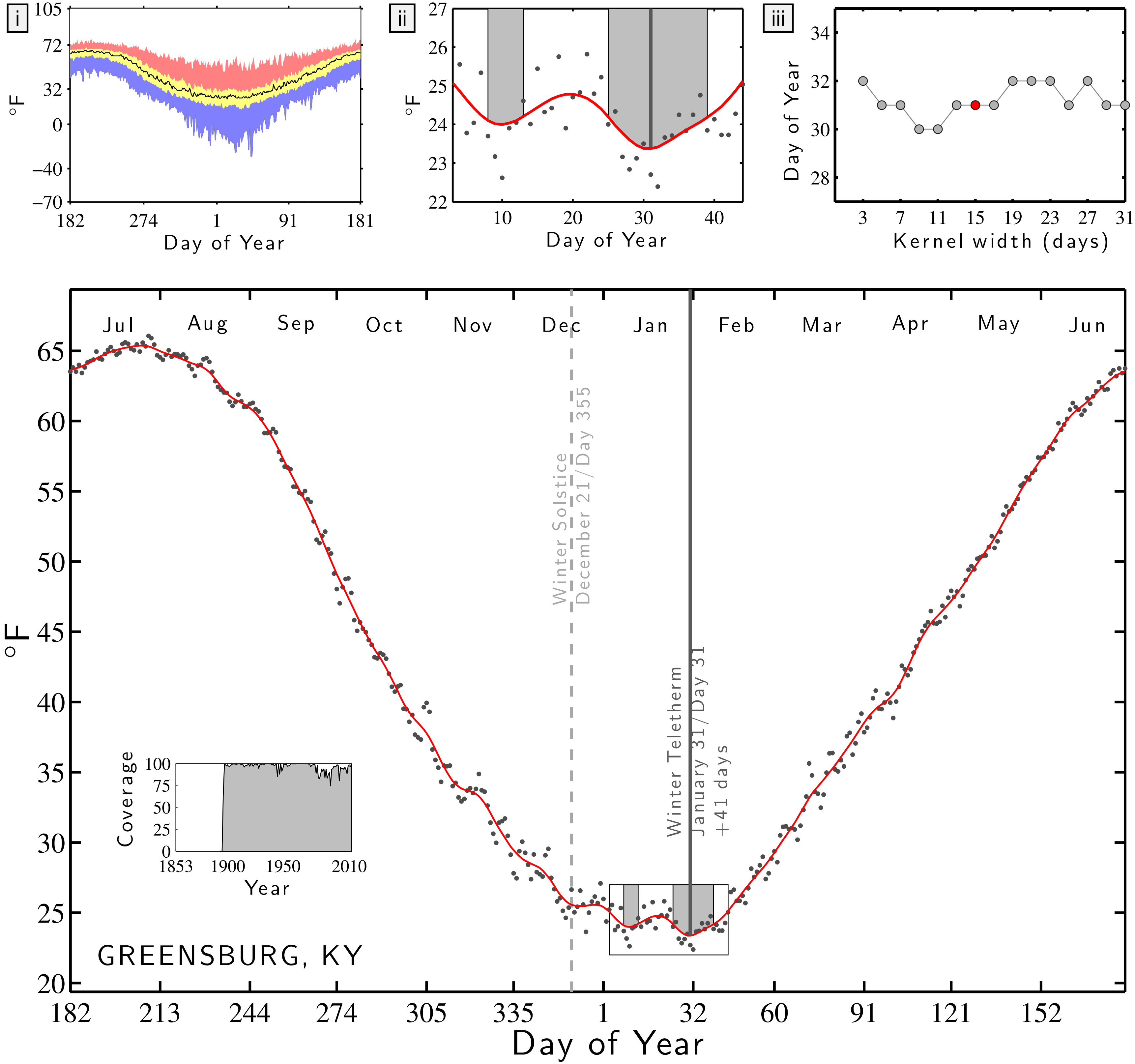

i
°F
Day of Year
ii
°F
Day of Year
iii
Day of Year
Kernel width (days)
Jul
Aug
Sep
Oct
Nov
Dec
Jan
Feb
Mar
Apr
May
Jun
Winter Solstice
December 21/Day 355
Winter Teletherm
January 31/Day 31
+41 days
Coverage
Year
GREENSBURG, KY
°F
Day of Year

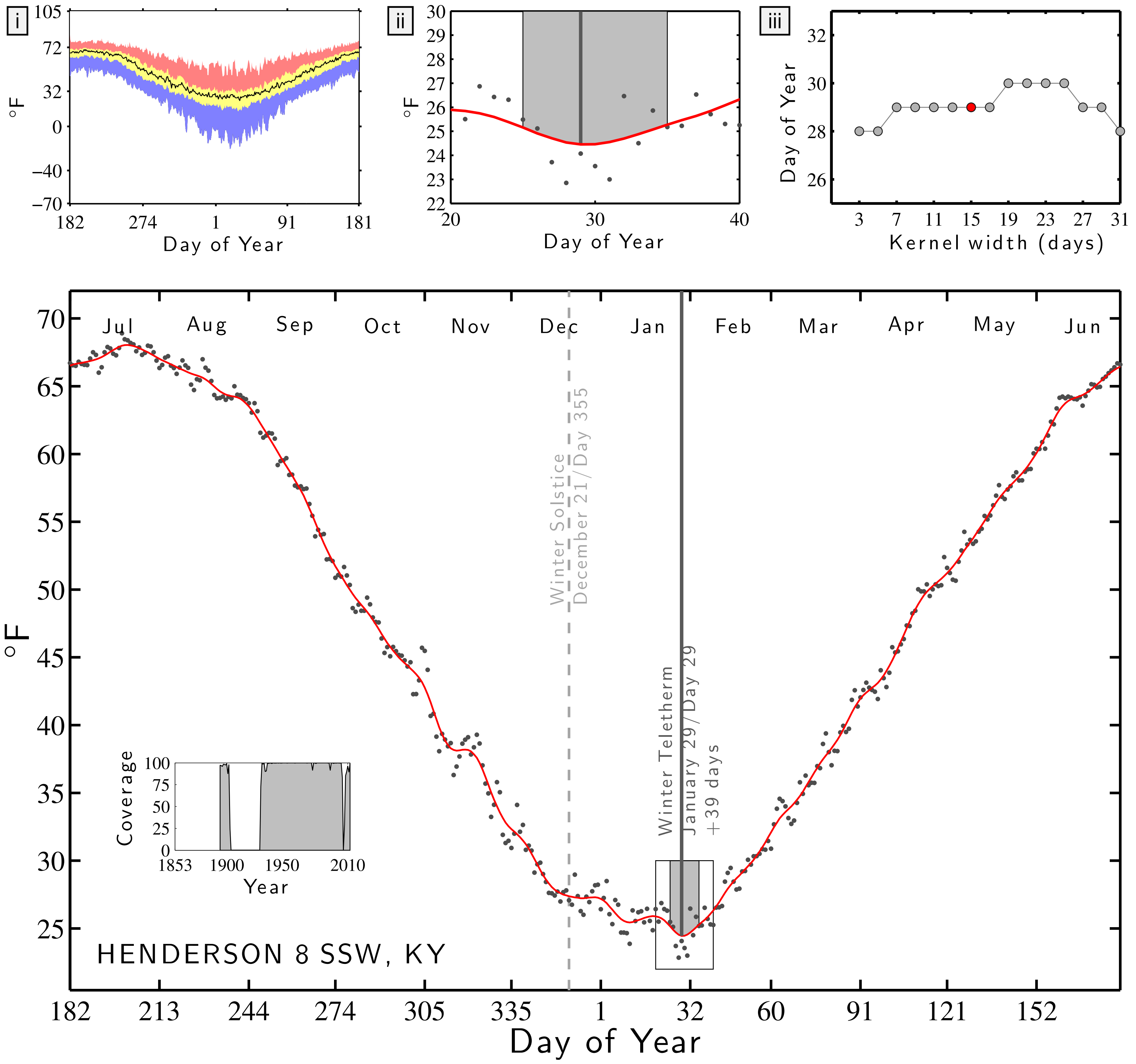

i
°F
Day of Year
ii
°F
Day of Year
iii
Day of Year
Kernel width (days)
Jul Aug Sep Oct Nov Dec Jan Feb Mar Apr May Jun
Winter Solstice
December 21/Day 355
Winter Teletherm
January 29/Day 29
+39 days
Coverage
Year
HENDERSON 8 SSW, KY
°F
Day of Year

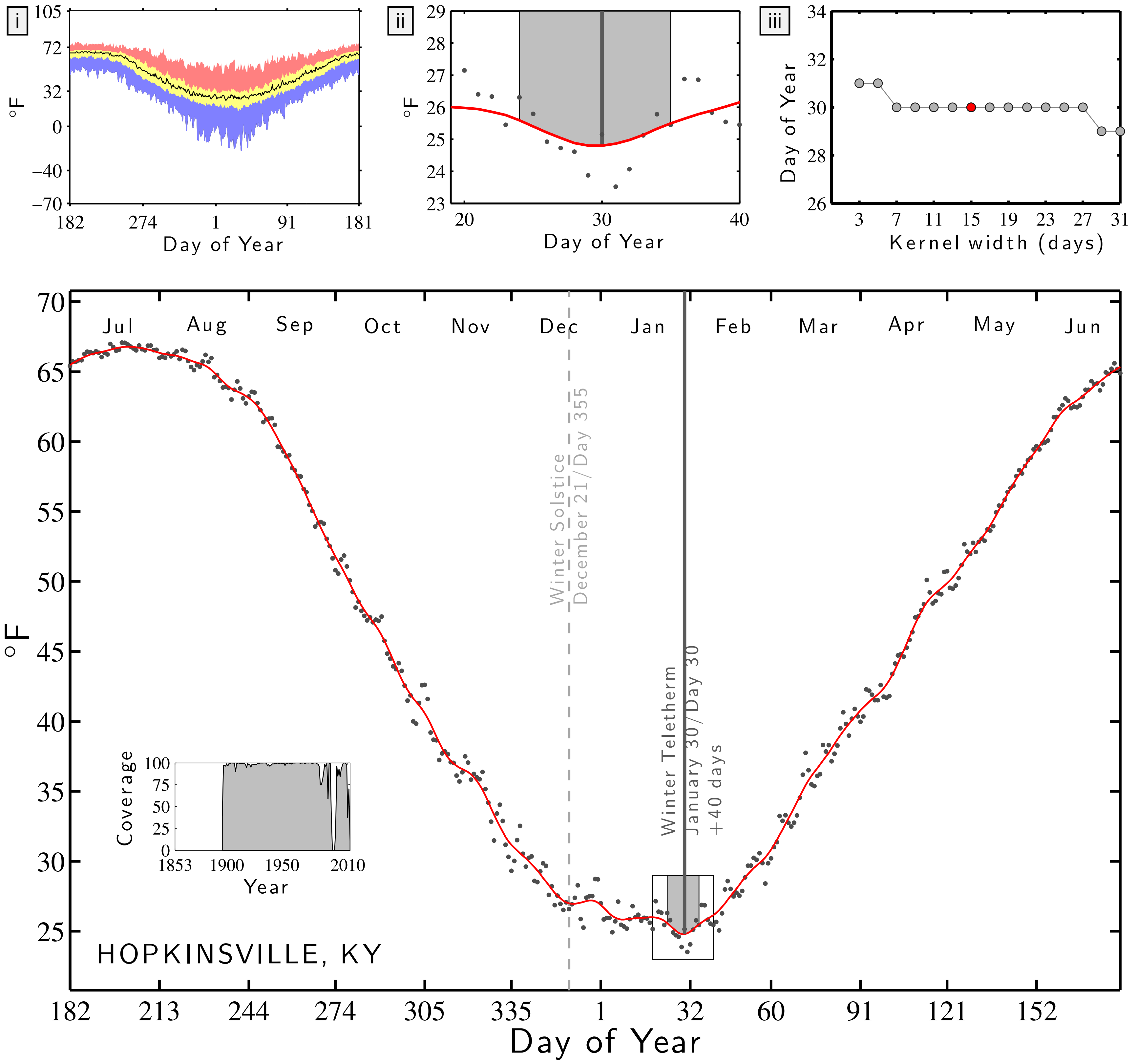

i
°F
Day of Year
ii
°F
Day of Year
iii
Day of Year
Kernel width (days)
Jul
Aug
Sep
Oct
Nov
Dec
Jan
Feb
Mar
Apr
May
Jun
Winter Solstice
December 21/Day 355
Winter Teletherm
January 30/Day 30
+40 days
Coverage
Year
HOPKINSVILLE, KY
°F
Day of Year

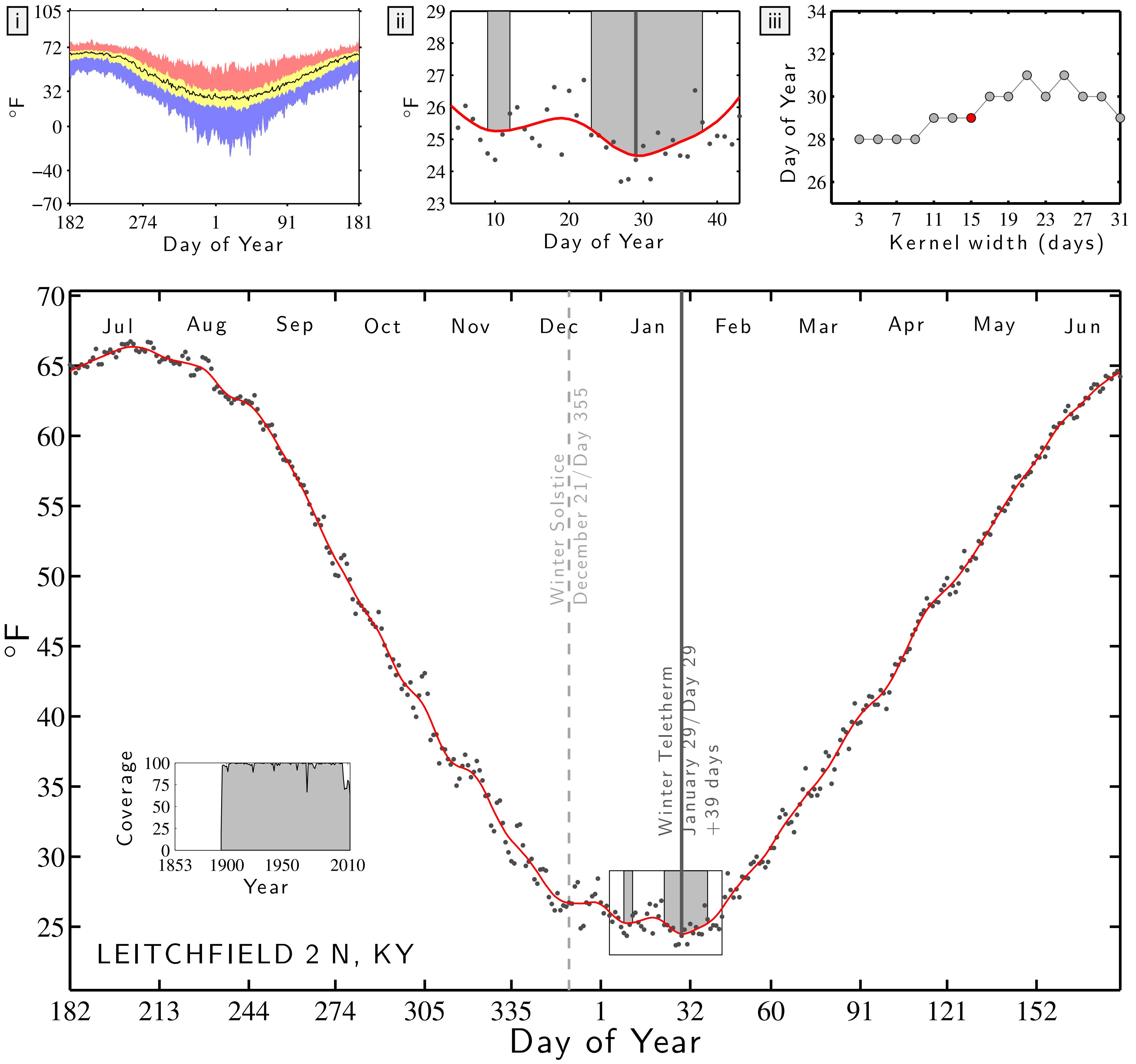

i
ii
iii
°F
Day of Year
Day of Year
Kernel width (days)
Jul
Aug
Sep
Oct
Nov
Dec
Jan
Feb
Mar
Apr
May
Jun
Winter Solstice
December 21/Day 355
Winter Teletherm
January 29/Day 29
+39 days
Coverage
Year
LEITCHFIELD 2 N, KY
Day of Year

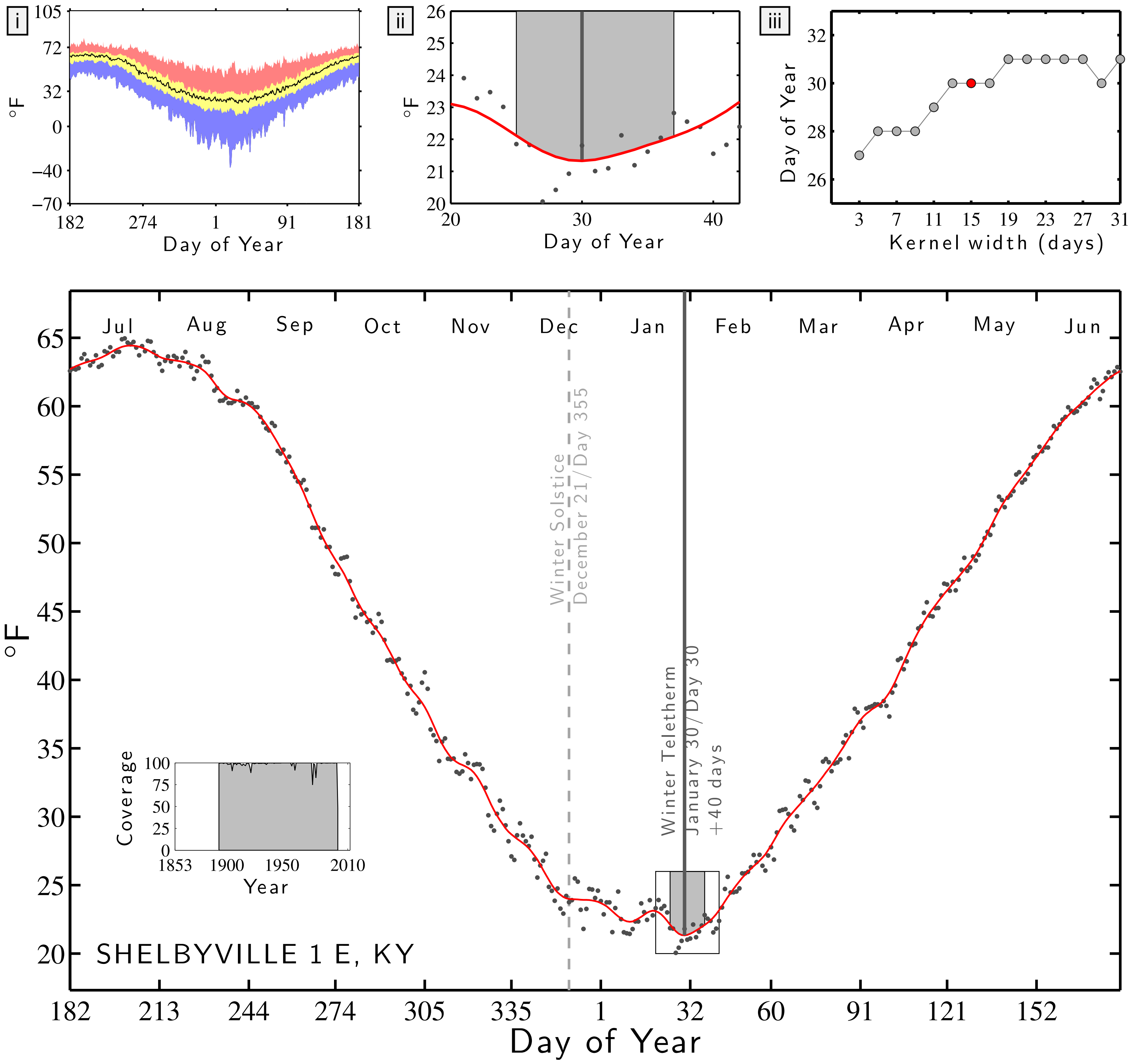

i
105
72
32
0
−40
−70
°F
182
274
1
91
181
Day of Year
ii
26
25
24
23
22
21
20
°F
20
30
40
Day of Year
iii
32
30
28
26
Day of Year
3
7
11
15
19
23
27
31
Kernel width (days)
Jul
Aug
Sep
Oct
Nov
Dec
Jan
Feb
Mar
Apr
May
Jun
65
60
55
50
45
40
35
30
25
20
°F
Winter Solstice
December 21/Day 355
Winter Teletherm
January 30/Day 30
+40 days
Coverage
100
75
50
25
0
1853
1900
1950
2010
Year
SHELBYVILLE 1 E, KY
182
213
244
274
305
335
1
32
60
91
121
152
Day of Year

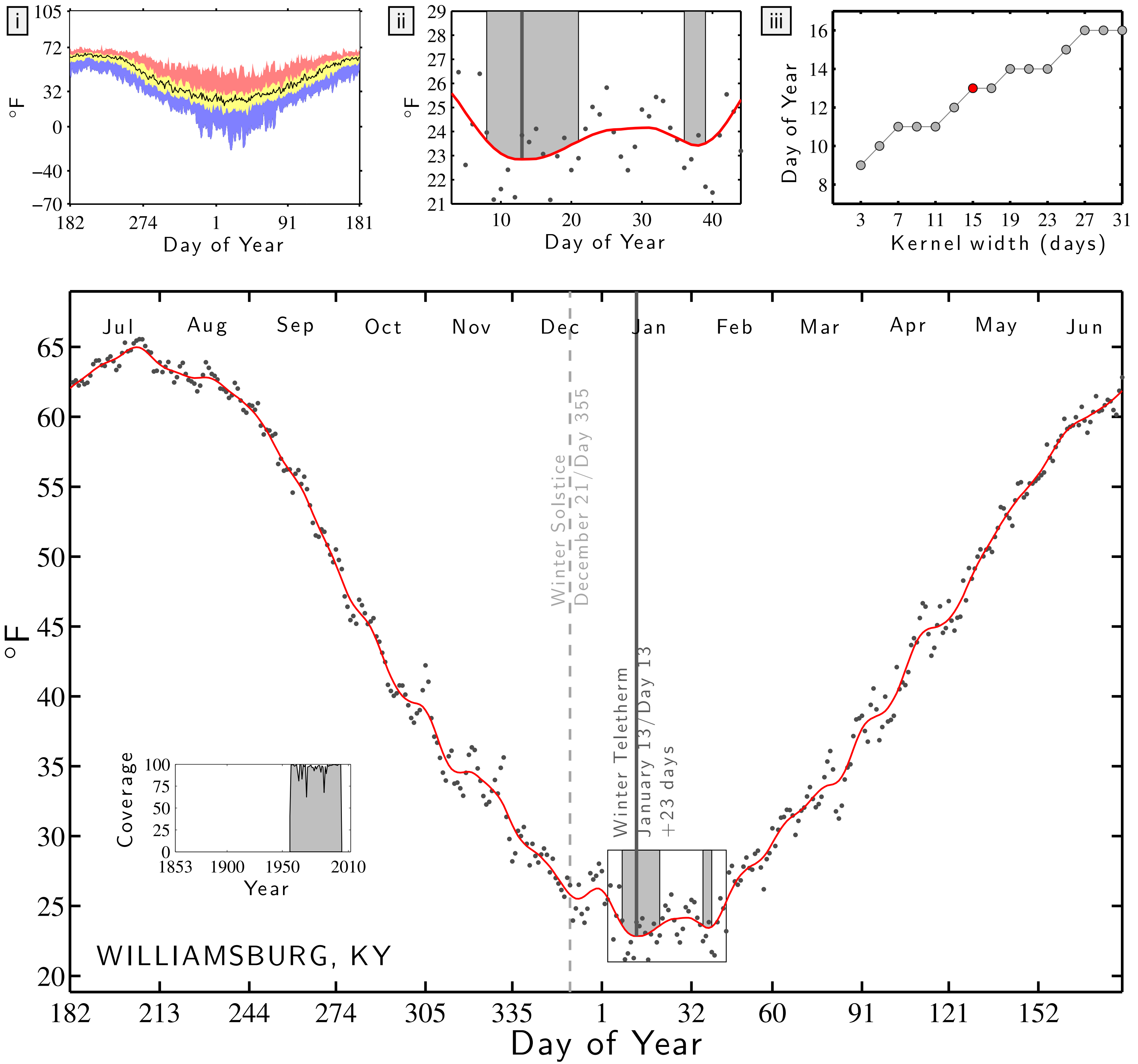

i
°F
Day of Year
ii
°F
Day of Year
iii
Day of Year
Kernel width (days)
Jul
Aug
Sep
Oct
Nov
Dec
Jan
Feb
Mar
Apr
May
Jun
Winter Solstice
December 21/Day 355
Winter Teletherm
January 13/Day 13
+23 days
Coverage
Year
WILLIAMSBURG, KY
°F
Day of Year

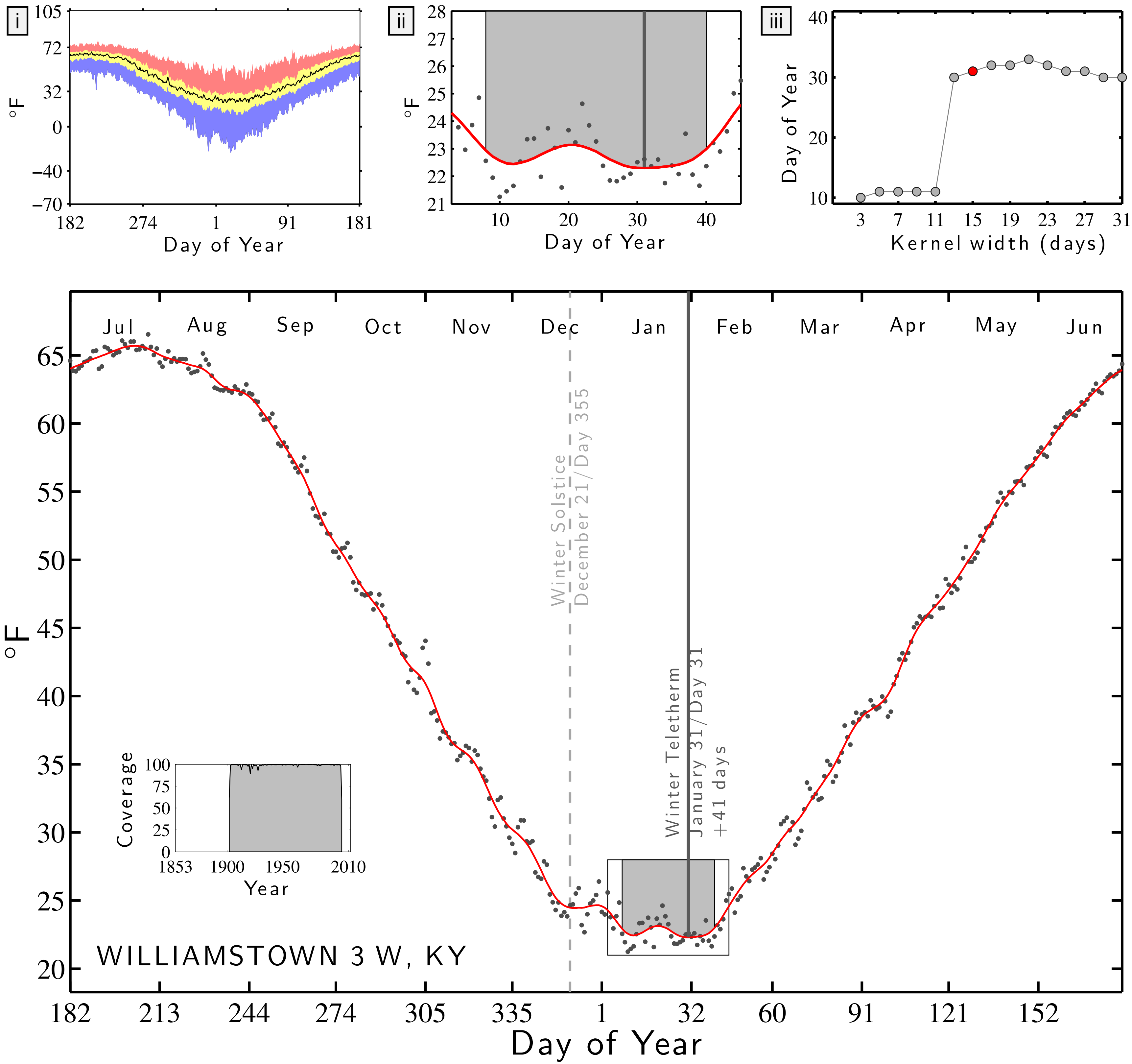

i
ii
iii
°F
Day of Year
Kernel width (days)
Jul
Aug
Sep
Oct
Nov
Dec
Jan
Feb
Mar
Apr
May
Jun
Winter Solstice
December 21/Day 355
Winter Teletherm
January 31/Day 31
+41 days
Coverage
Year
WILLIAMSTOWN 3 W, KY

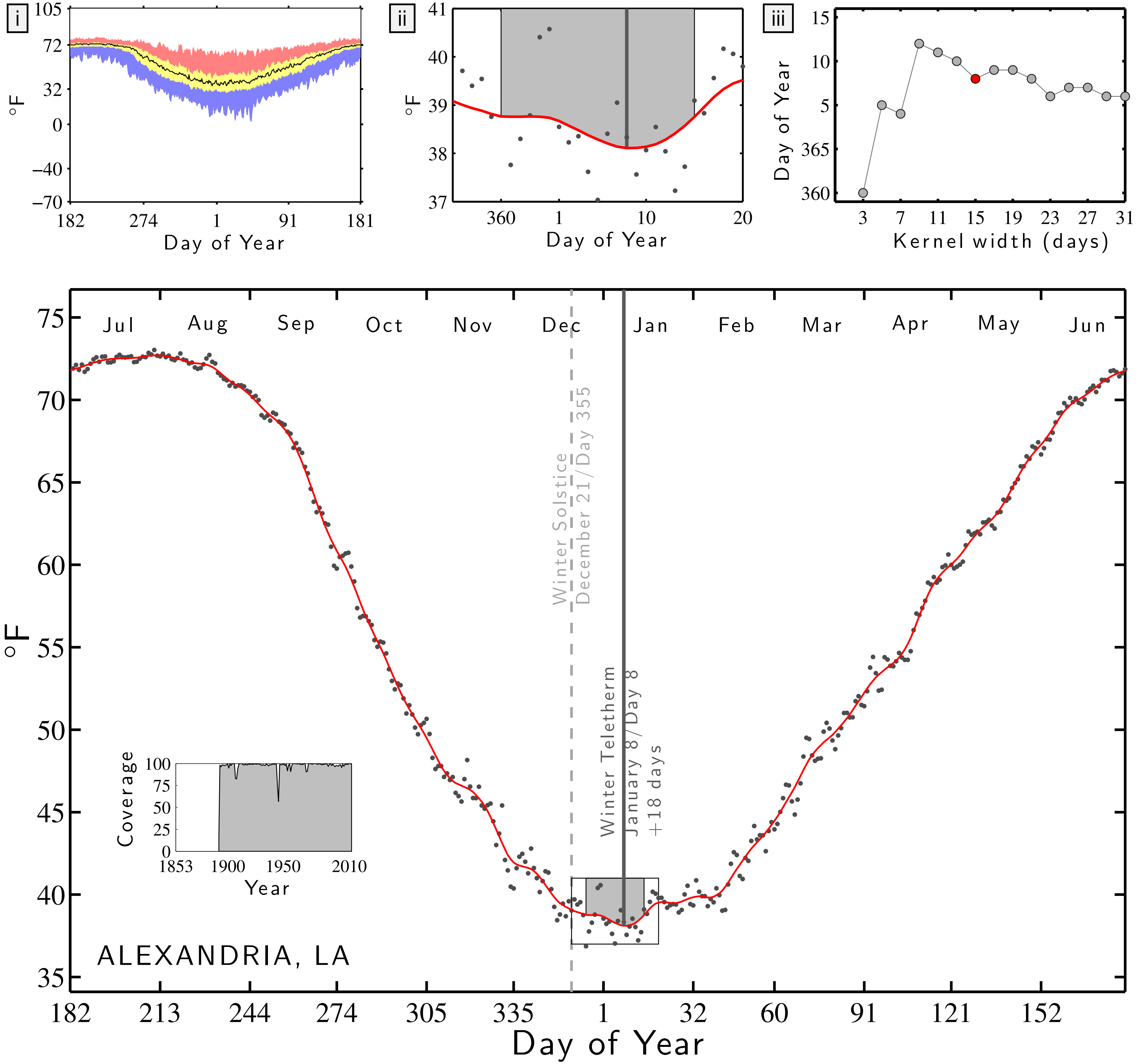

i
°F
Day of Year
ii
iii
Day of Year
Kernel width (days)
Jul
Aug
Sep
Oct
Nov
Dec
Jan
Feb
Mar
Apr
May
Jun
Winter Solstice
December 21/Day 355
Winter Teletherm
January 8/Day 8
+18 days
Coverage
Year
ALEXANDRIA, LA

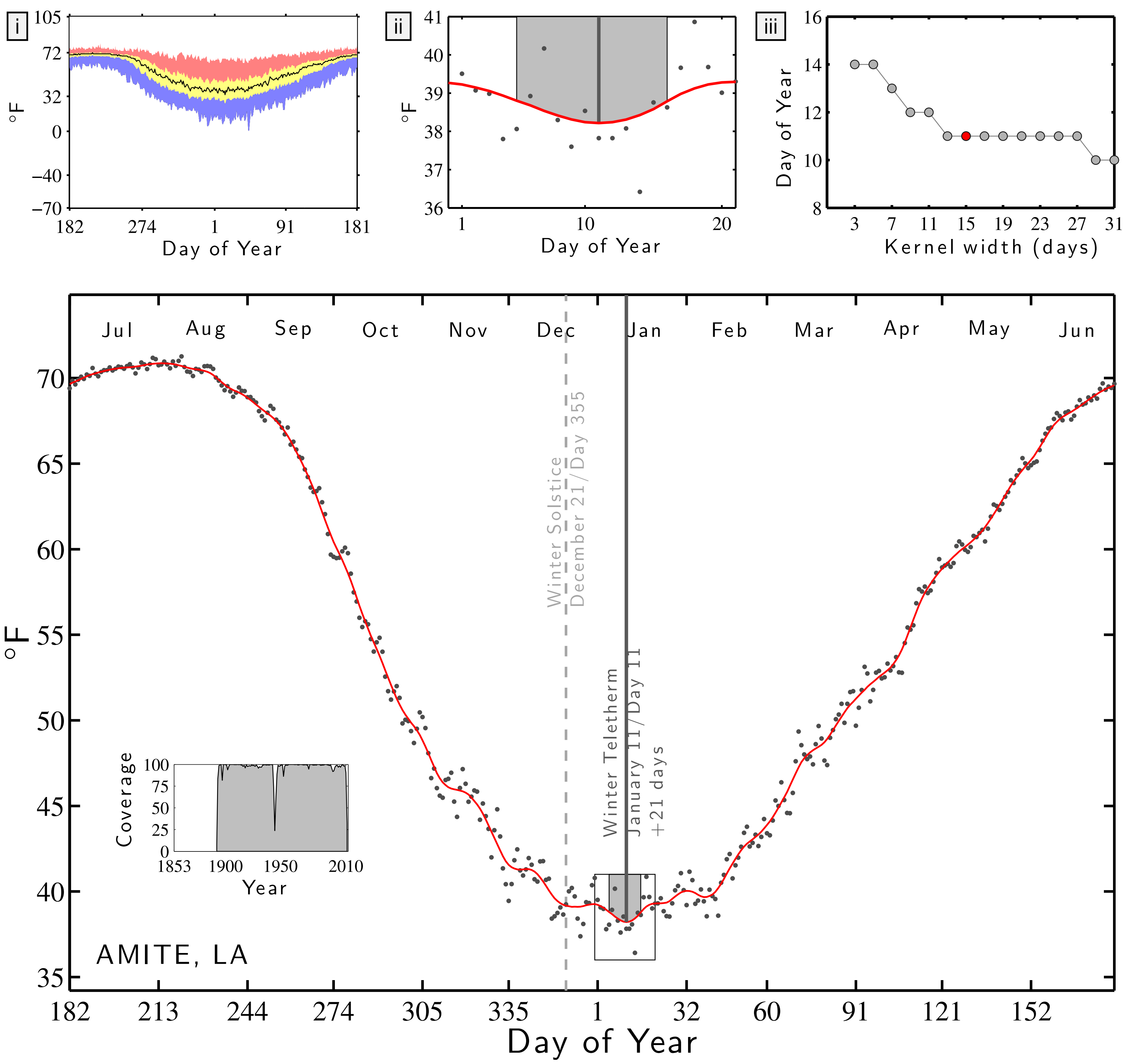

i
°F
Day of Year
ii
Day of Year
iii
Day of Year
Kernel width (days)
Jul
Aug
Sep
Oct
Nov
Dec
Jan
Feb
Mar
Apr
May
Jun
Winter Solstice
December 21/Day 355
Winter Teletherm
January 11/Day 11
+21 days
Coverage
Year
AMITE, LA
°F
Day of Year

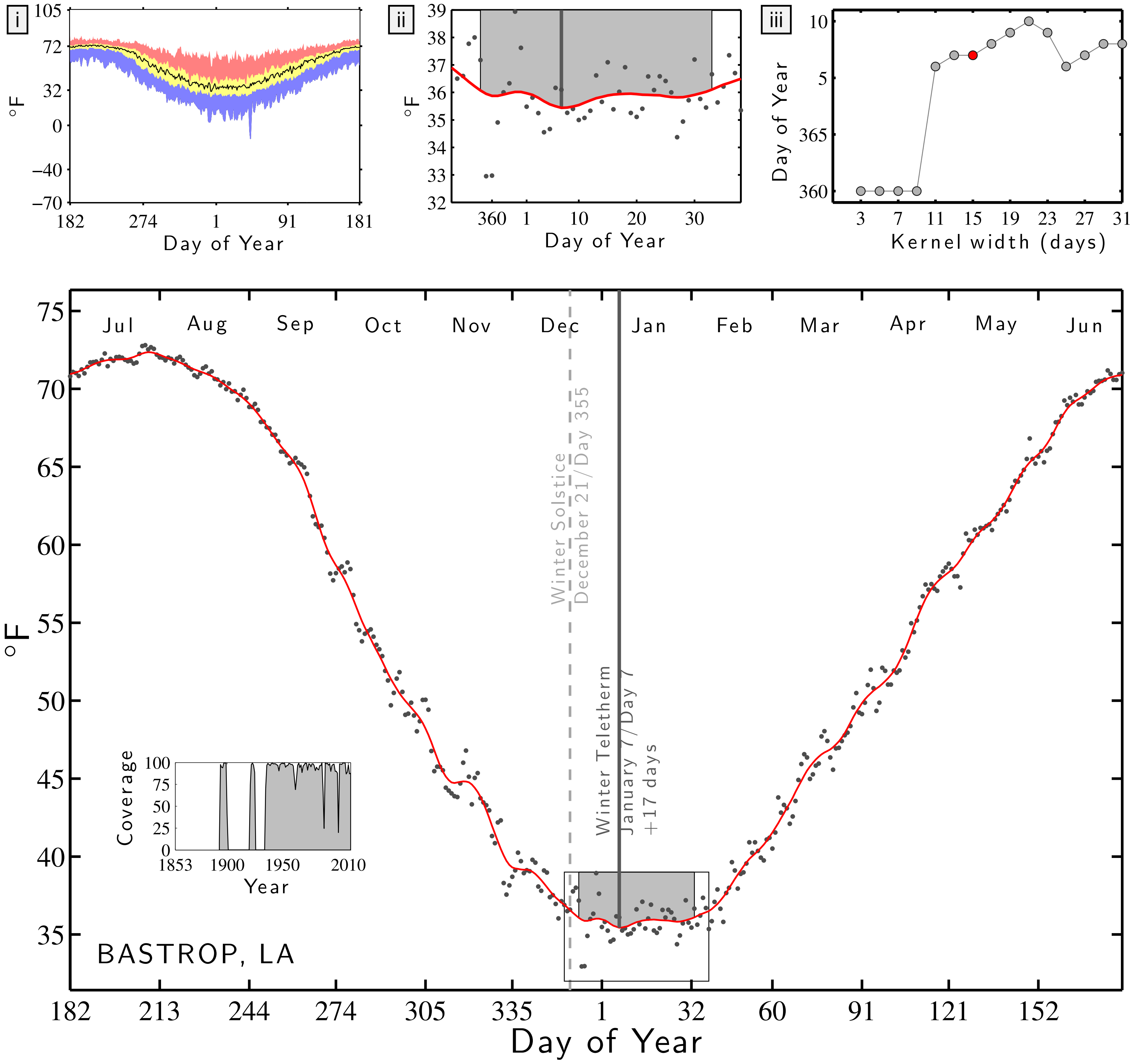

i
°F
Day of Year
ii
°F
Day of Year
iii
Day of Year
Kernel width (days)
Jul
Aug
Sep
Oct
Nov
Dec
Jan
Feb
Mar
Apr
May
Jun
Winter Solstice
December 21/Day 355
Winter Teletherm
January 7/Day 7
+17 days
Coverage
Year
BASTROP, LA
°F
Day of Year

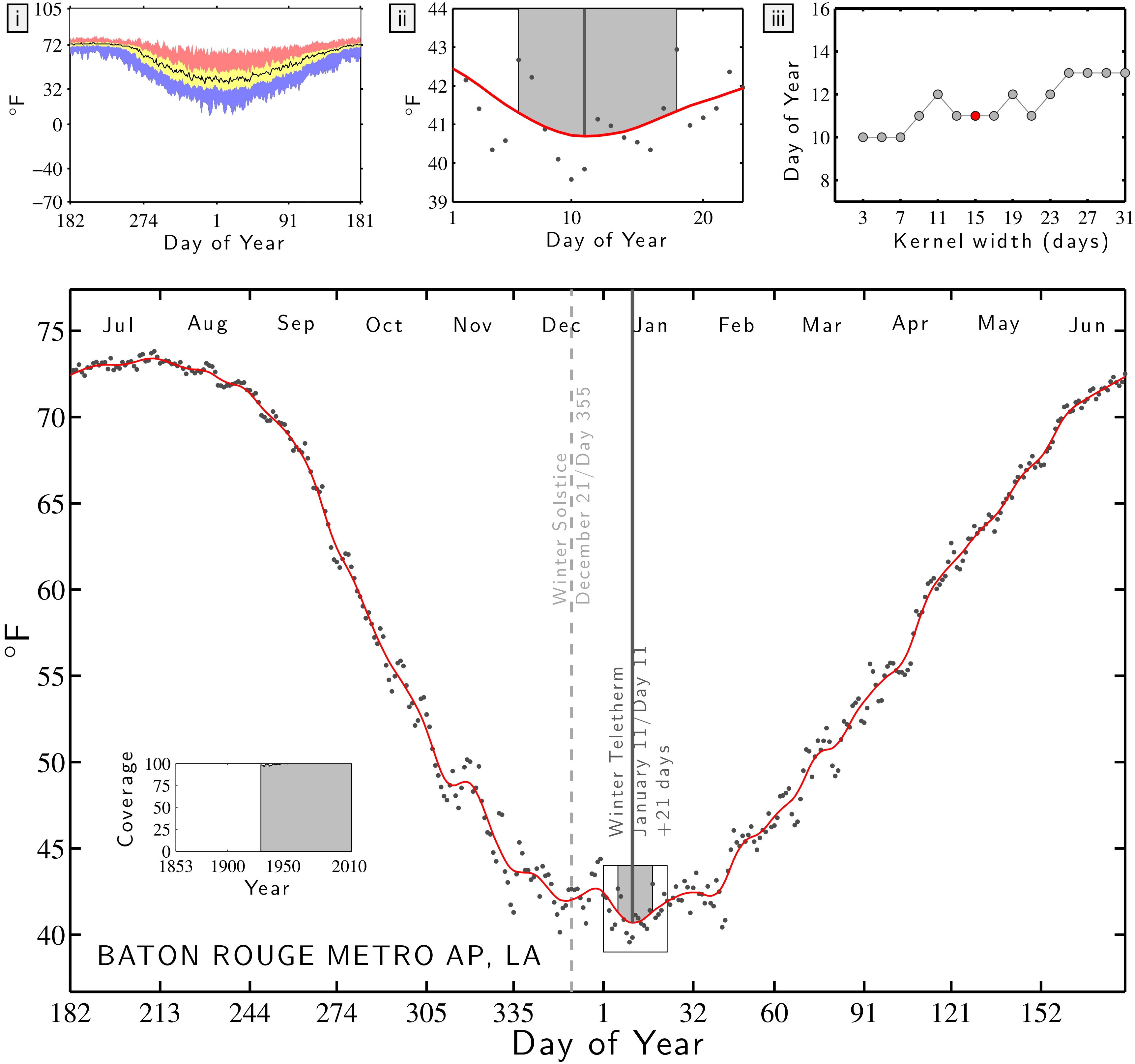

i
ii
iii
°F
Day of Year
Day of Year
Kernel width (days)
Jul
Aug
Sep
Oct
Nov
Dec
Jan
Feb
Mar
Apr
May
Jun
Winter Solstice
December 21/Day 355
Winter Teletherm
January 11/Day 11
+21 days
Coverage
Year
BATON ROUGE METRO AP, LA
Day of Year

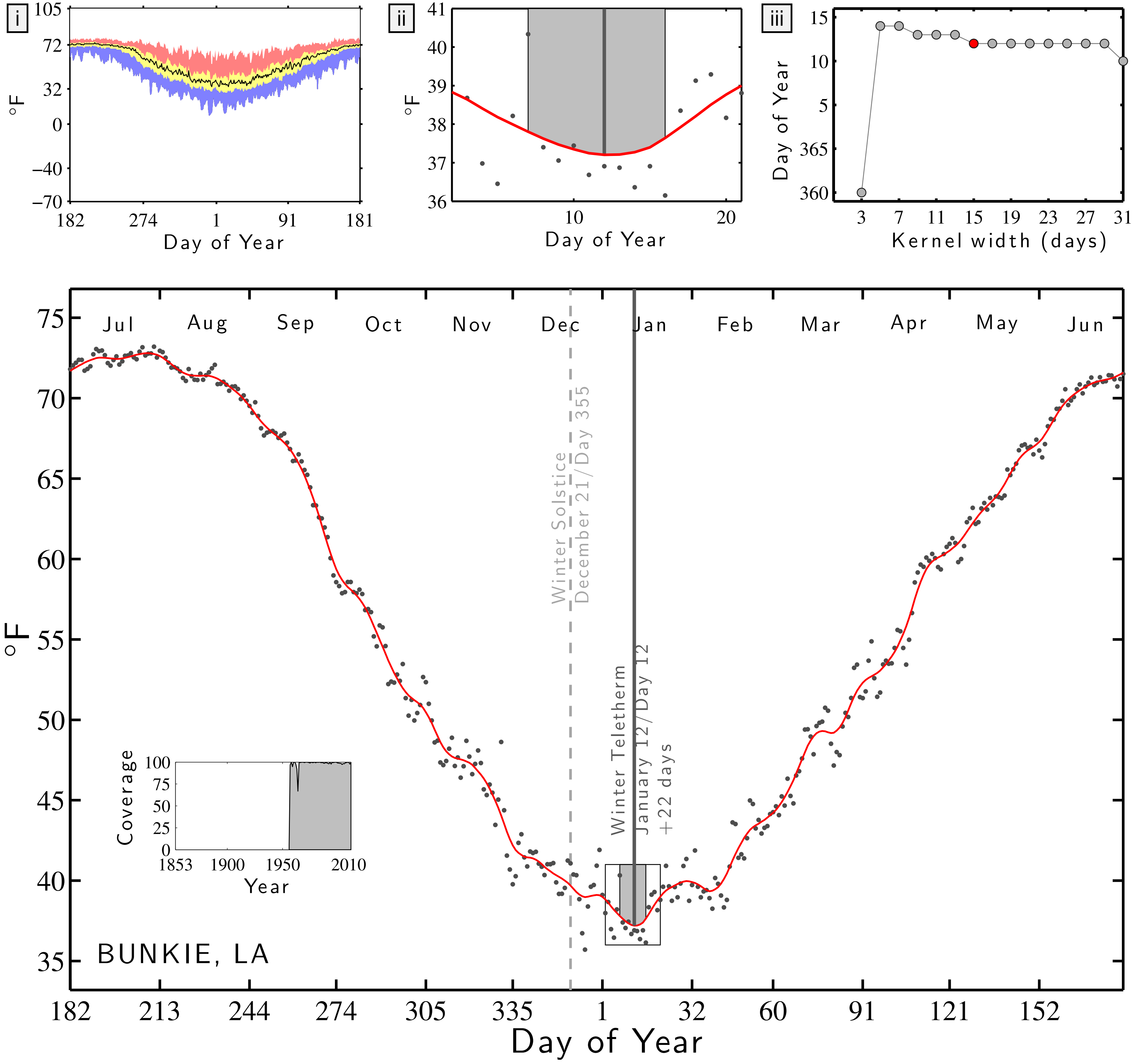
i
°F
Day of Year
ii
Day of Year
iii
Day of Year
Kernel width (days)
Jul
Aug
Sep
Oct
Nov
Dec
Jan
Feb
Mar
Apr
May
Jun
Winter Solstice
December 21/Day 355
Winter Teletherm
January 12/Day 12
+22 days
Coverage
Year
BUNKIE, LA
Day of Year

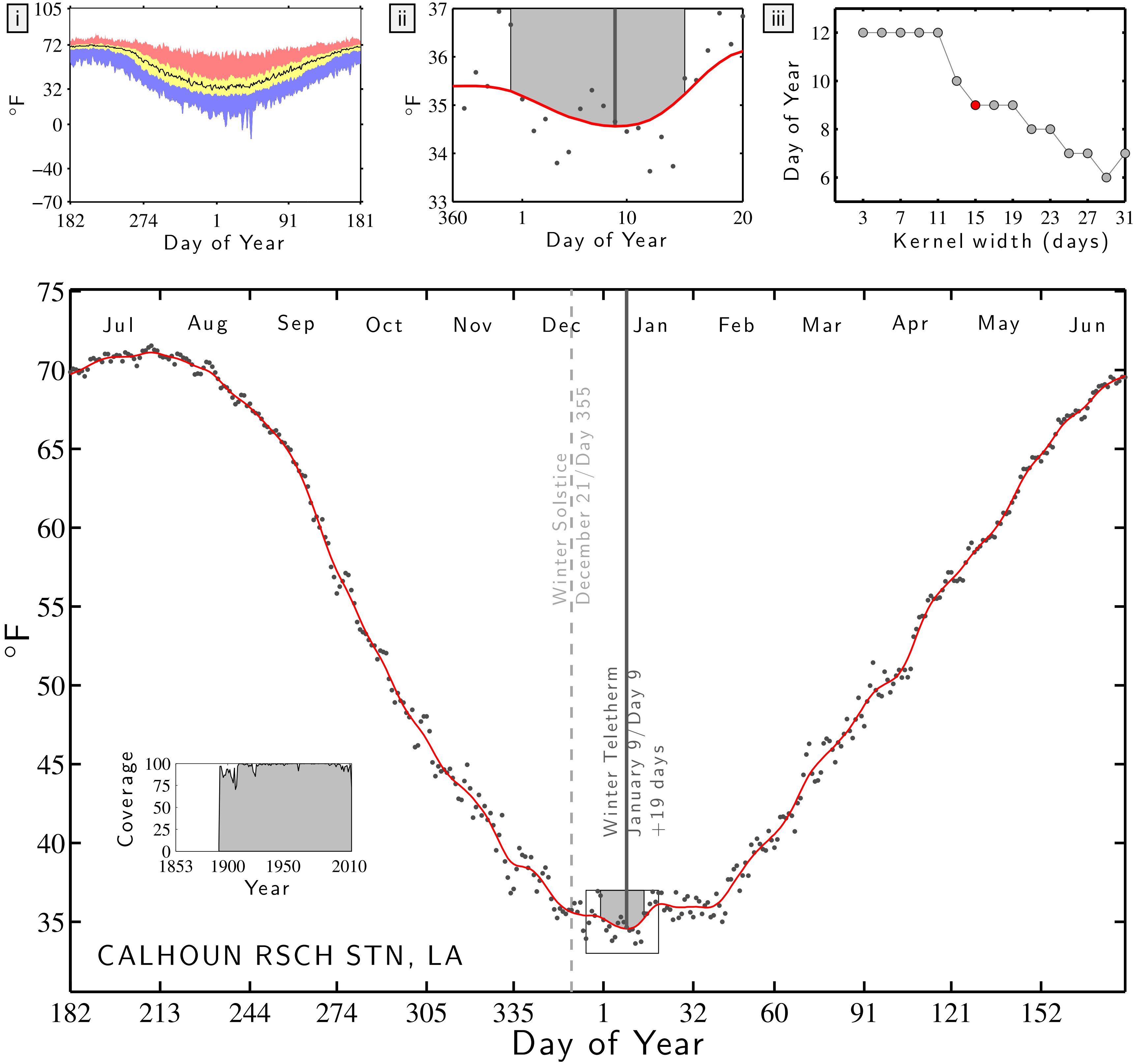

i
105
72
32
0
−40
−70
°F
182
274
1
91
181
Day of Year
ii
37
36
35
34
33
°F
360
1
10
20
Day of Year
iii
12
10
8
6
Day of Year
3
7
11
15
19
23
27
31
Kernel width (days)
75
70
65
60
55
50
45
40
35
°F
Jul
Aug
Sep
Oct
Nov
Dec
Jan
Feb
Mar
Apr
May
Jun
Winter Solstice
December 21/Day 355
Winter Teletherm
January 9/Day 9
+19 days
Coverage
100
75
50
25
0
1853
1900
1950
2010
Year
CALHOUN RSCH STN, LA
182
213
244
274
305
335
1
32
60
91
121
152
Day of Year

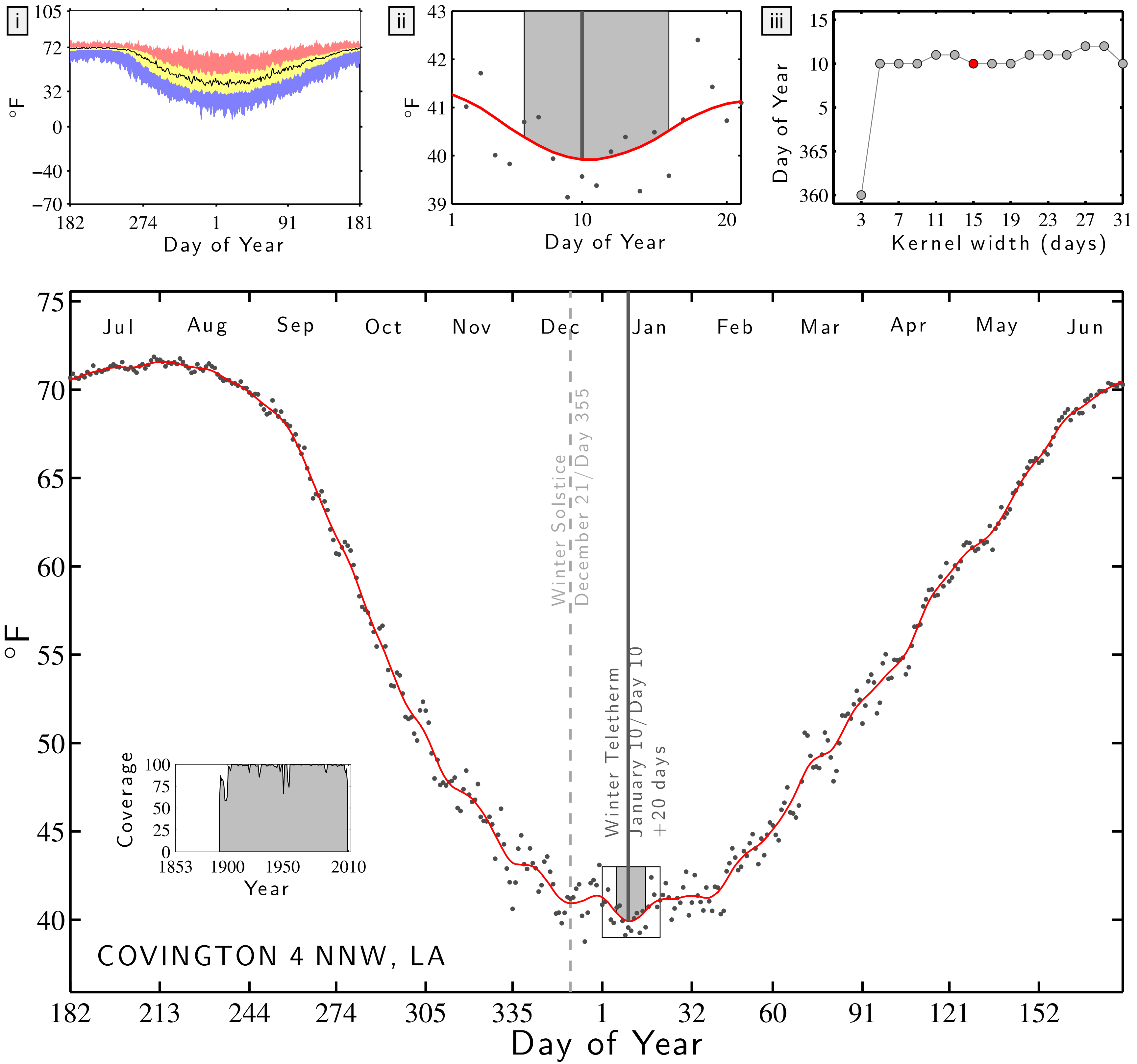

i
°F
105
72
32
0
−40
−70
182
274
1
91
181
Day of Year
ii
43
42
41
40
39
1
10
20
Day of Year
iii
15
10
5
365
360
Day of Year
3
7
11
15
19
23
27
31
Kernel width (days)
Jul
Aug
Sep
Oct
Nov
Dec
Jan
Feb
Mar
Apr
May
Jun
75
70
65
60
55
50
45
40
Winter Solstice
December 21/Day 355
Winter Teletherm
January 10/Day 10
+20 days
Coverage
100
75
50
25
0
1853
1900
1950
2010
Year
COVINGTON 4 NNW, LA
182
213
244
274
305
335
1
32
60
91
121
152
Day of Year

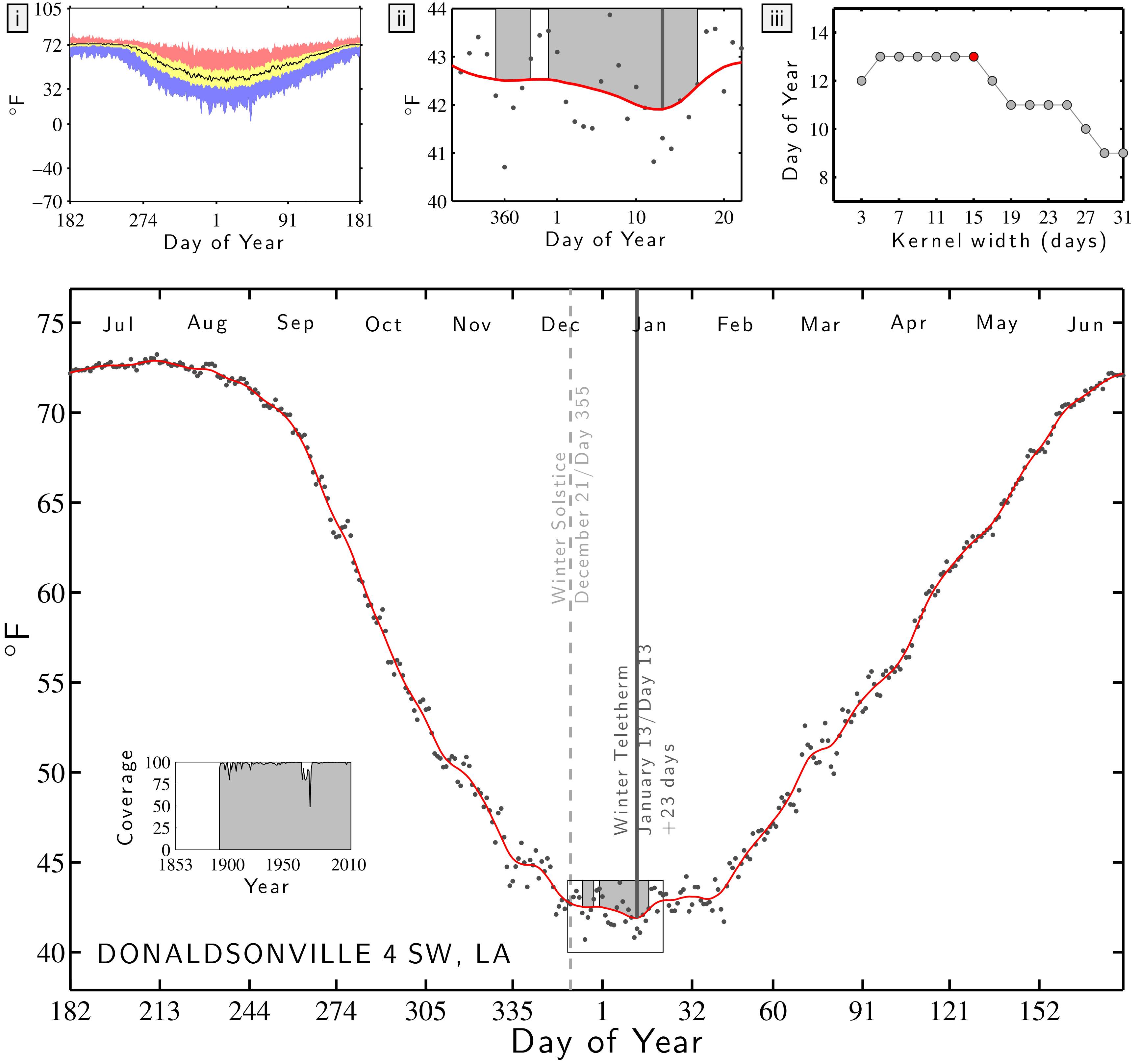

i
°F
Day of Year
ii
iii
Day of Year
Kernel width (days)
Jul
Aug
Sep
Oct
Nov
Dec
Jan
Feb
Mar
Apr
May
Jun
Winter Solstice
December 21/Day 355
Winter Teletherm
January 13/Day 13
+23 days
Coverage
Year
DONALDSONVILLE 4 SW, LA

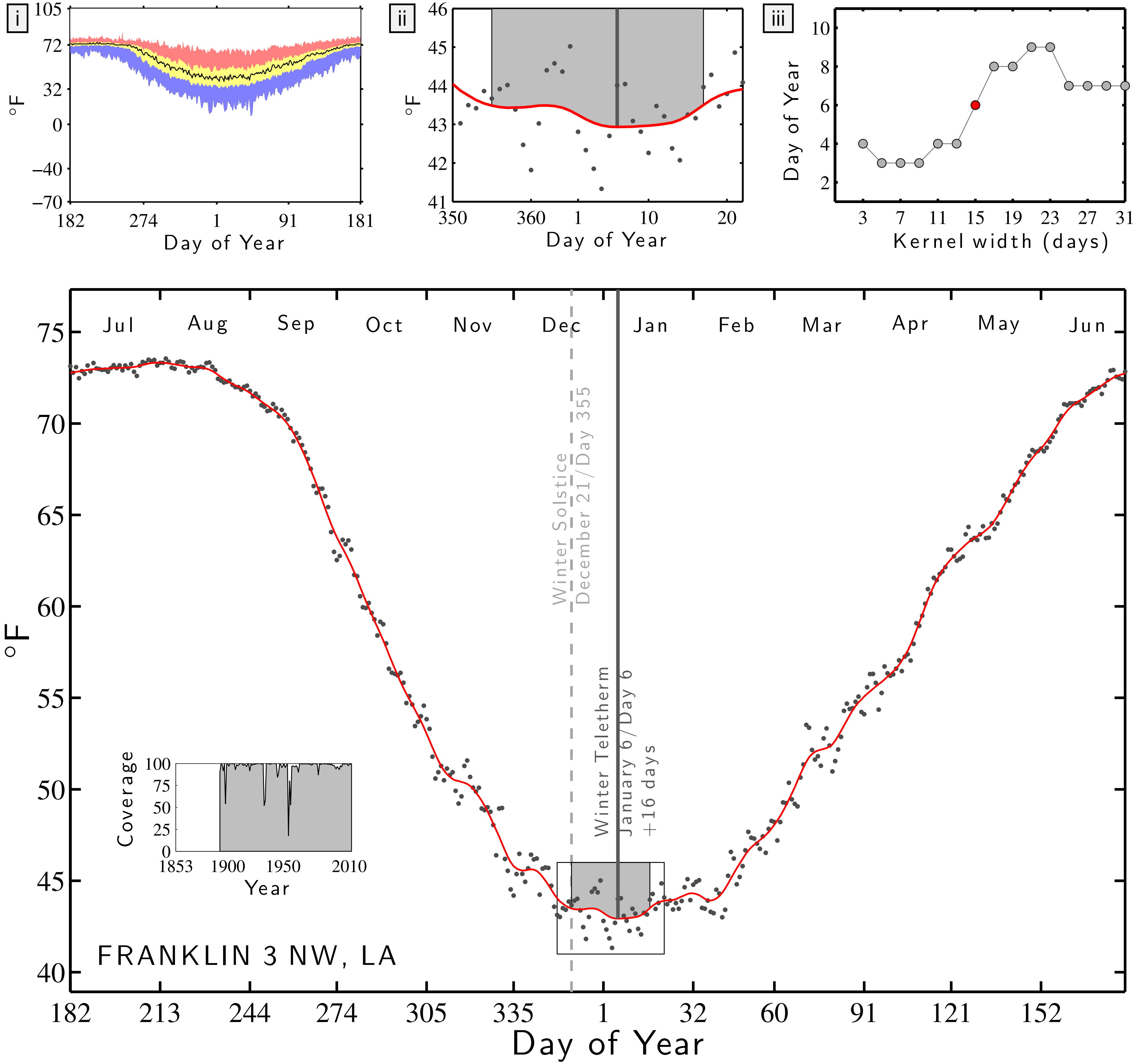

i
°F
Day of Year
ii
°F
Day of Year
iii
Day of Year
Kernel width (days)
Jul
Aug
Sep
Oct
Nov
Dec
Jan
Feb
Mar
Apr
May
Jun
Winter Solstice
December 21/Day 355
Winter Teletherm
January 6/Day 6
+16 days
Coverage
Year
FRANKLIN 3 NW, LA
°F
Day of Year

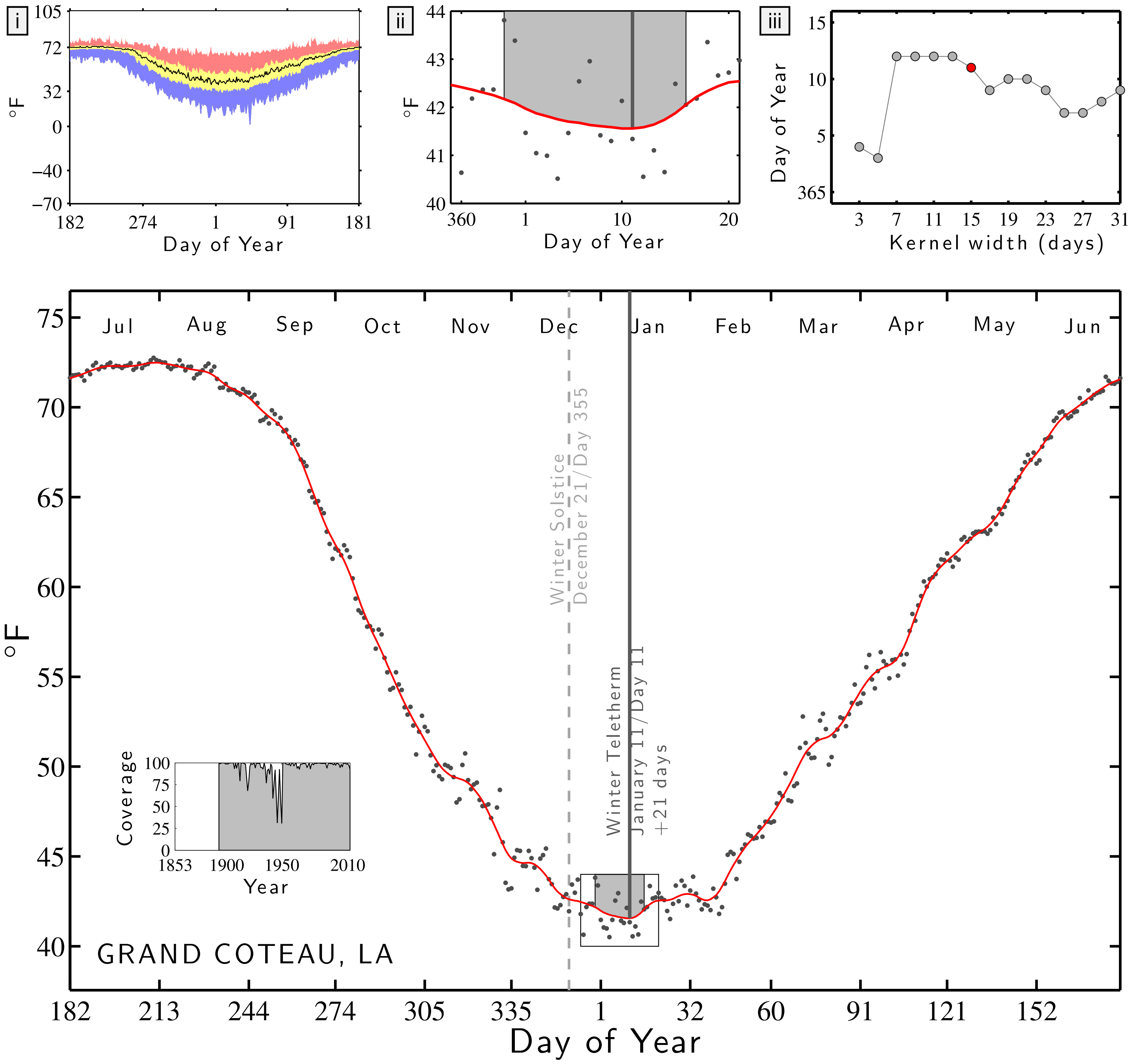

i
°F
105
72
32
0
−40
−70
182
274
1
91
181
Day of Year
ii
44
43
42
41
40
360
1
10
20
Day of Year
iii
Day of Year
15
10
5
365
3
7
11
15
19
23
27
31
Kernel width (days)
Jul
Aug
Sep
Oct
Nov
Dec
Jan
Feb
Mar
Apr
May
Jun
Winter Solstice
December 21/Day 355
Winter Teletherm
January 11/Day 11
+21 days
Coverage
100
75
50
25
0
1853
1900
1950
2010
Year
GRAND COTEAU, LA
°F
75
70
65
60
55
50
45
40
182
213
244
274
305
335
1
32
60
91
121
152
Day of Year

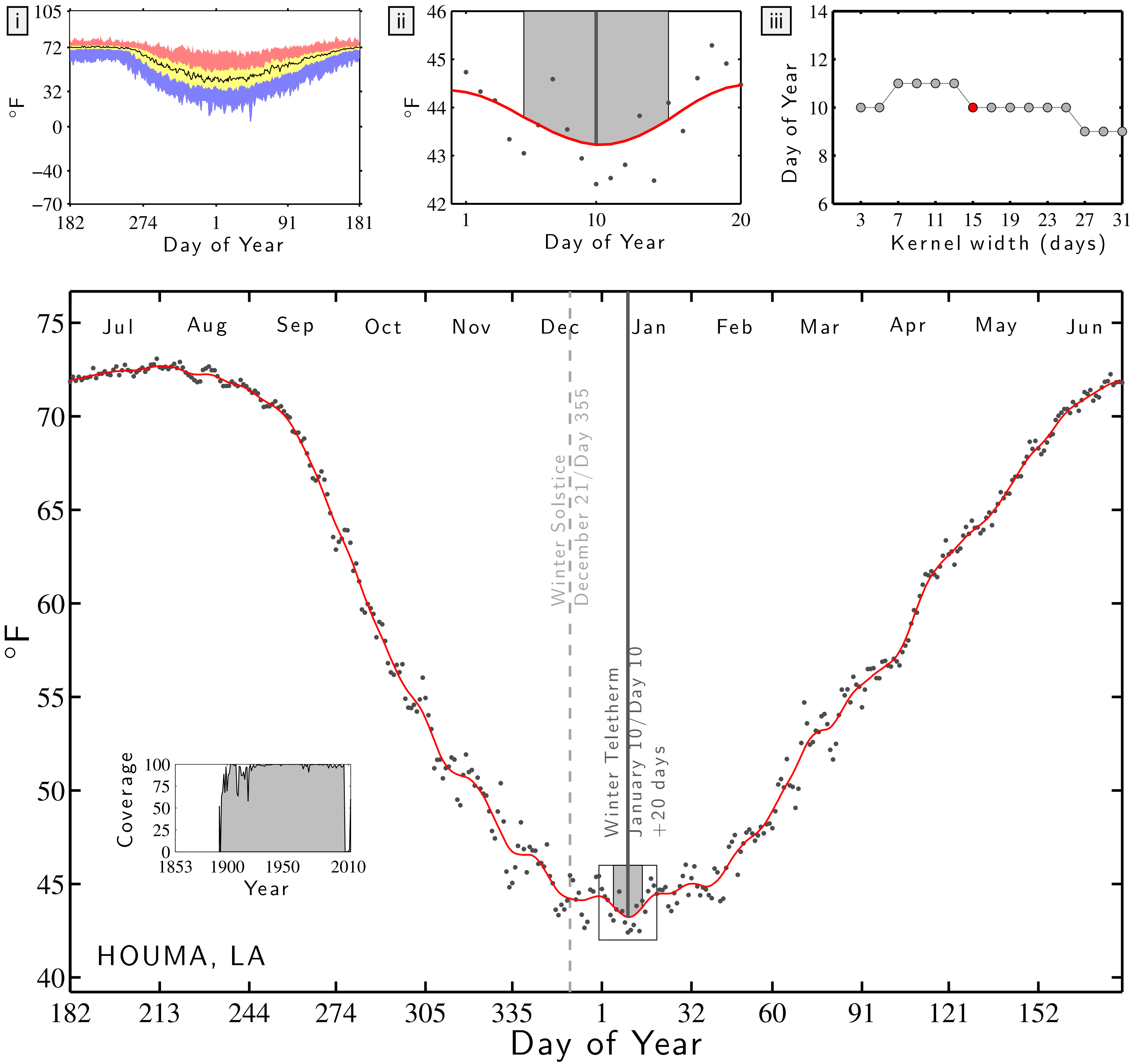

i
ii
iii
°F
Day of Year
Day of Year
Kernel width (days)
Jul
Aug
Sep
Oct
Nov
Dec
Jan
Feb
Mar
Apr
May
Jun
Winter Solstice
December 21/Day 355
Winter Teletherm
January 10/Day 10
+20 days
Coverage
Year
HOUMA, LA
Day of Year

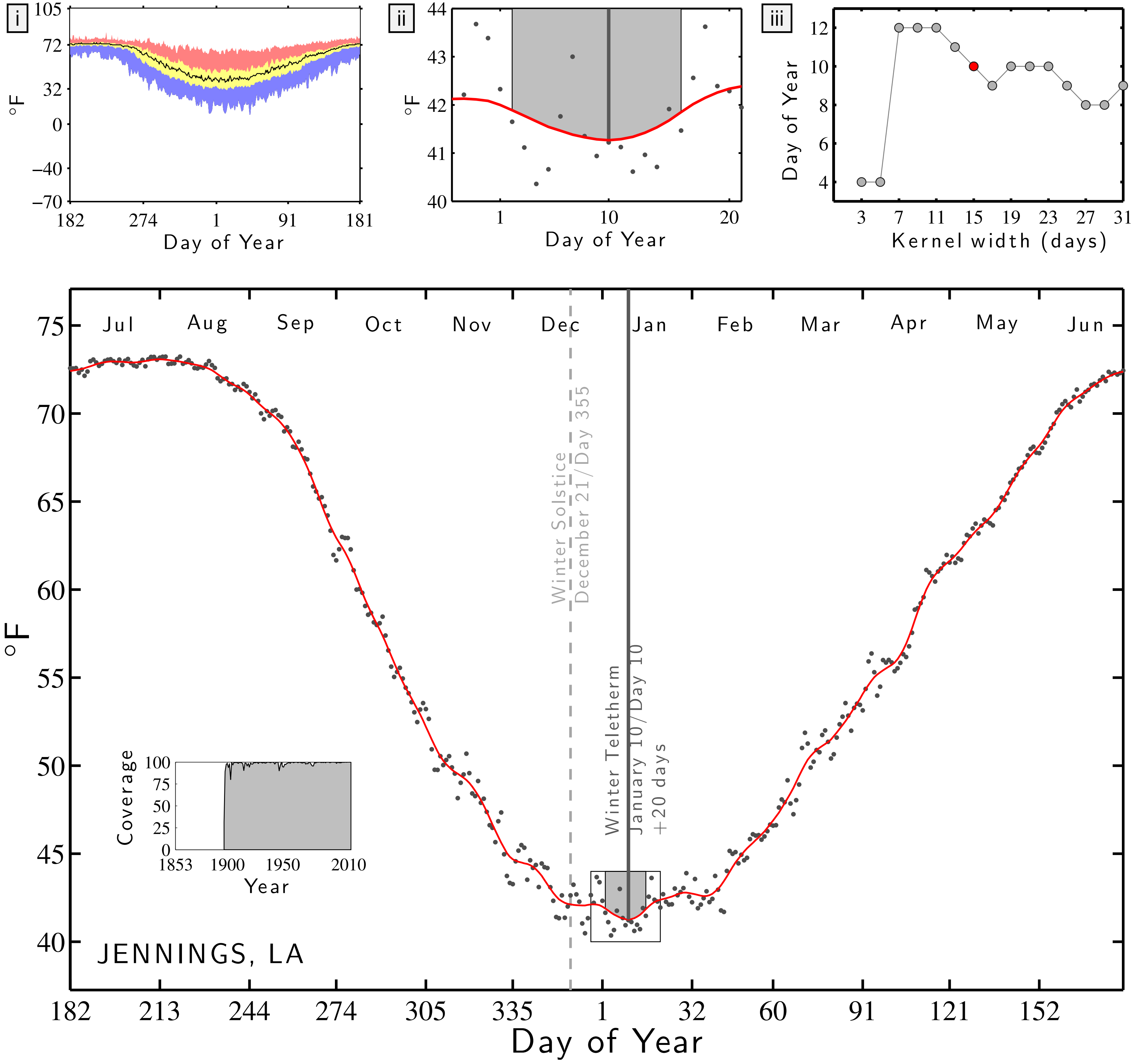

i
°F
Day of Year
ii
°F
Day of Year
iii
Day of Year
Kernel width (days)
Jul
Aug
Sep
Oct
Nov
Dec
Jan
Feb
Mar
Apr
May
Jun
Winter Solstice
December 21/Day 355
Winter Teletherm
January 10/Day 10
+20 days
Coverage
Year
JENNINGS, LA
°F
Day of Year

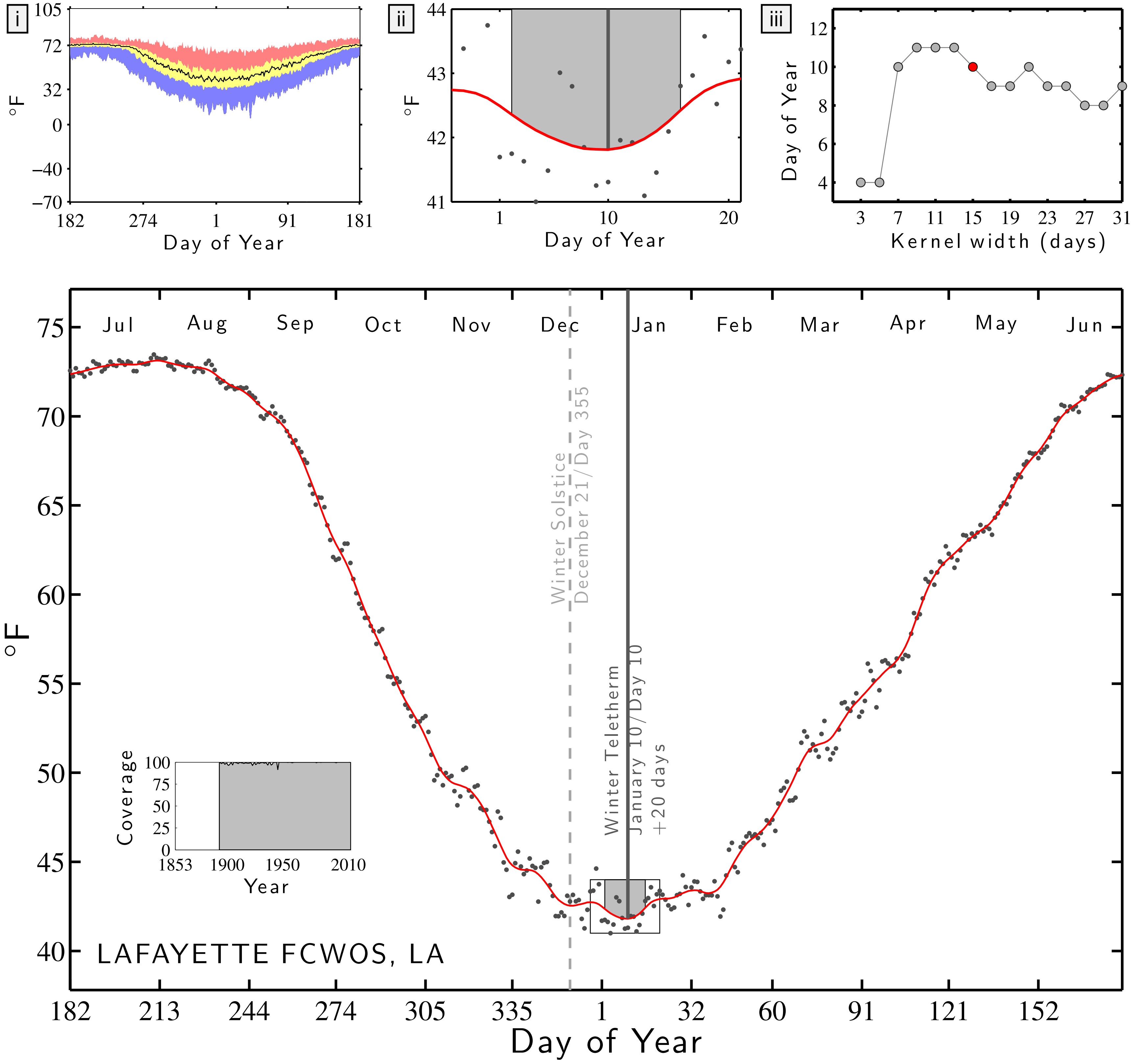

i
ii
iii
°F
Day of Year
Day of Year
Kernel width (days)
Jul
Aug
Sep
Oct
Nov
Dec
Jan
Feb
Mar
Apr
May
Jun
Winter Solstice
December 21/Day 355
Winter Teletherm
January 10/Day 10
+20 days
Coverage
Year
LAFAYETTE FCWOS, LA

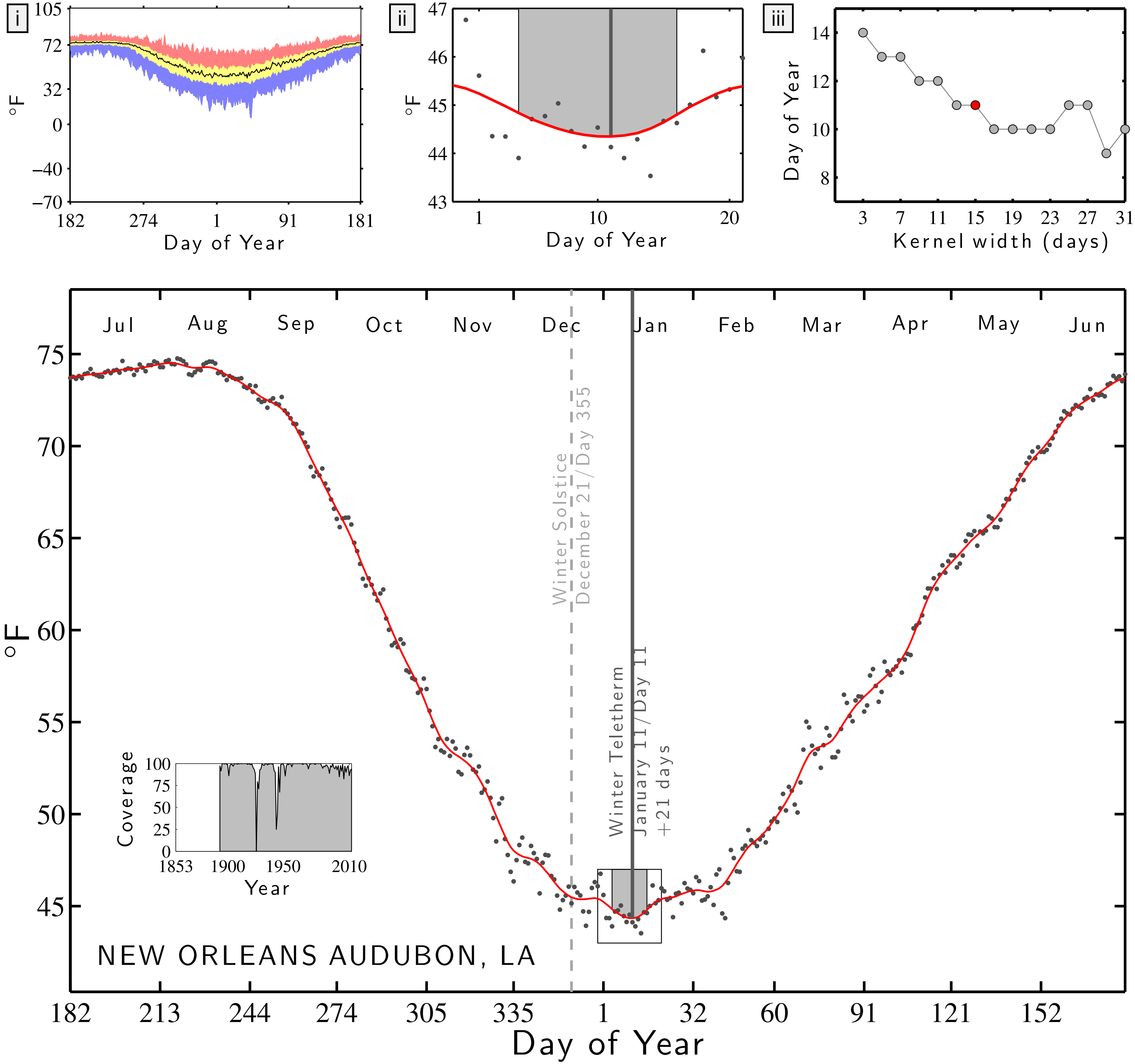

i
105
72
32
0
−40
−70
°F
182
274
1
91
181
Day of Year
ii
47
46
45
44
43
°F
1
10
20
Day of Year
iii
14
12
10
8
Day of Year
3
7
11
15
19
23
27
31
Kernel width (days)
Jul
Aug
Sep
Oct
Nov
Dec
Jan
Feb
Mar
Apr
May
Jun
75
70
65
60
55
50
45
°F
Winter Solstice
December 21/Day 355
Winter Teletherm
January 11/Day 11
+21 days
Coverage
100
75
50
25
0
1853
1900
1950
2010
Year
NEW ORLEANS AUDUBON, LA
182
213
244
274
305
335
1
32
60
91
121
152
Day of Year

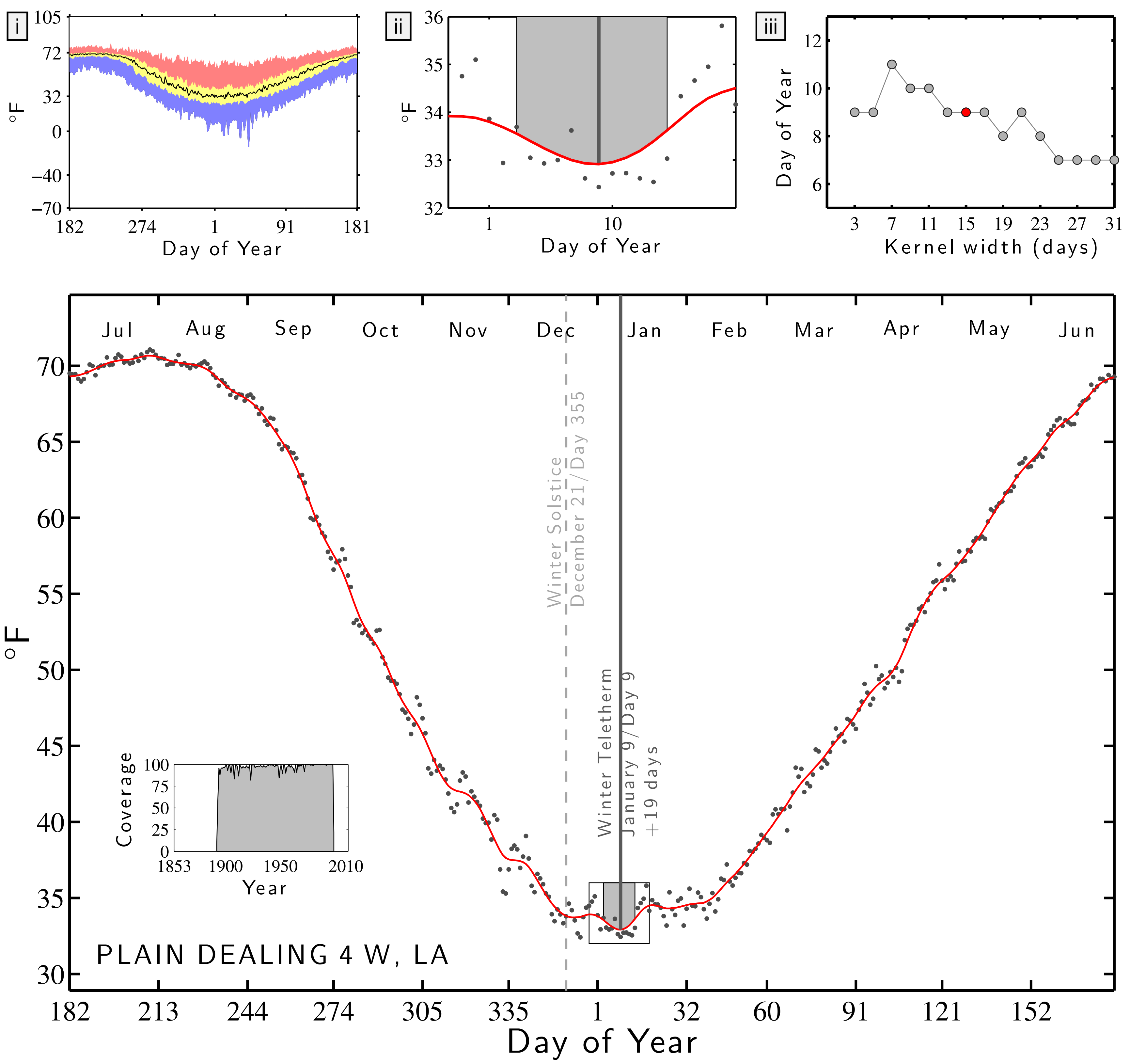

i
ii
iii
°F
Day of Year
Kernel width (days)
Jul
Aug
Sep
Oct
Nov
Dec
Jan
Feb
Mar
Apr
May
Jun
Winter Solstice
December 21/Day 355
Winter Teletherm
January 9/Day 9
+19 days
Coverage
Year
PLAIN DEALING 4 W, LA

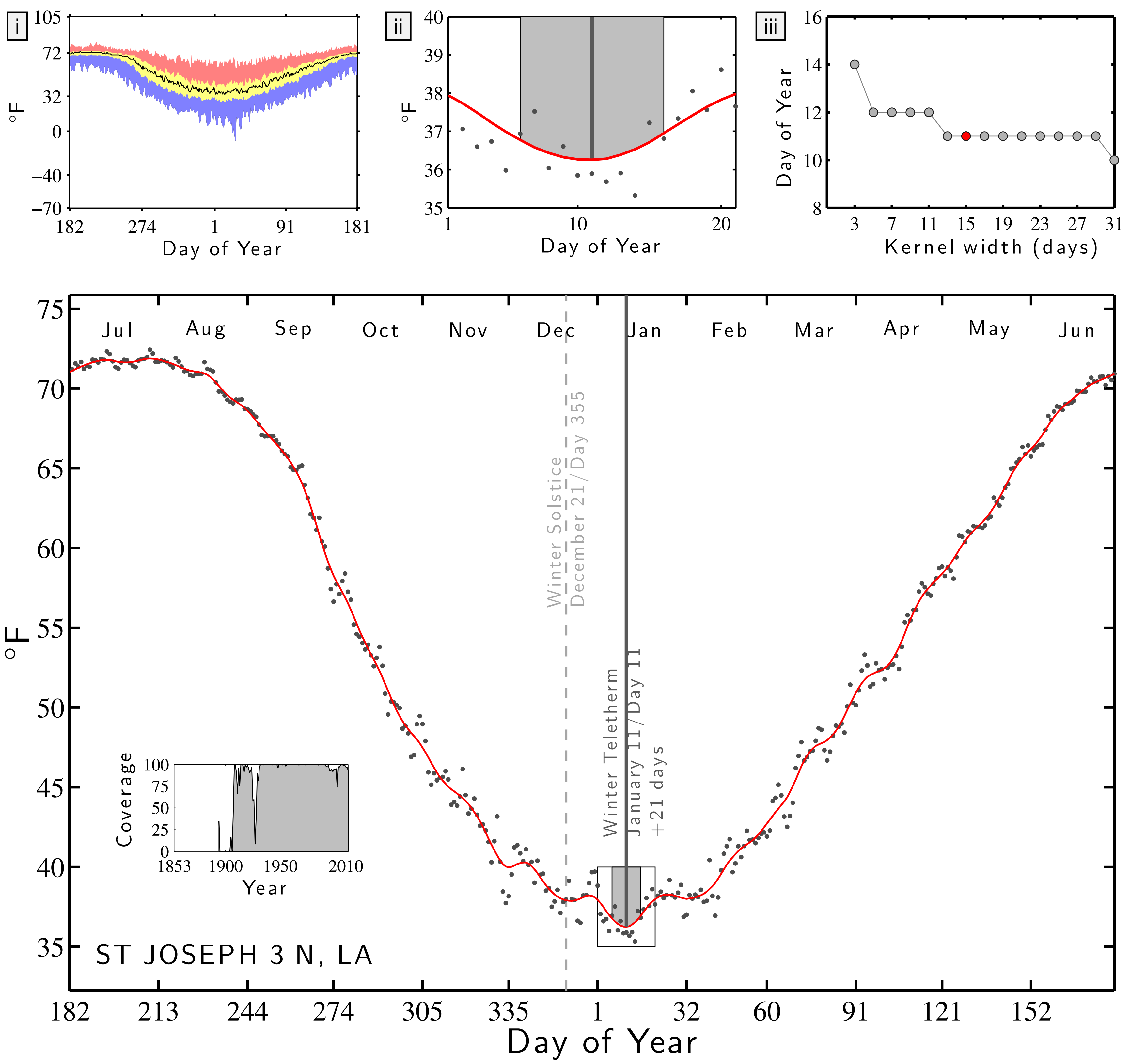

i
Day of Year
°F
ii
Day of Year
iii
Day of Year
Kernel width (days)
Jul
Aug
Sep
Oct
Nov
Dec
Jan
Feb
Mar
Apr
May
Jun
Winter Solstice
December 21/Day 355
Winter Teletherm
January 11/Day 11
+21 days
Coverage
Year
ST JOSEPH 3 N, LA

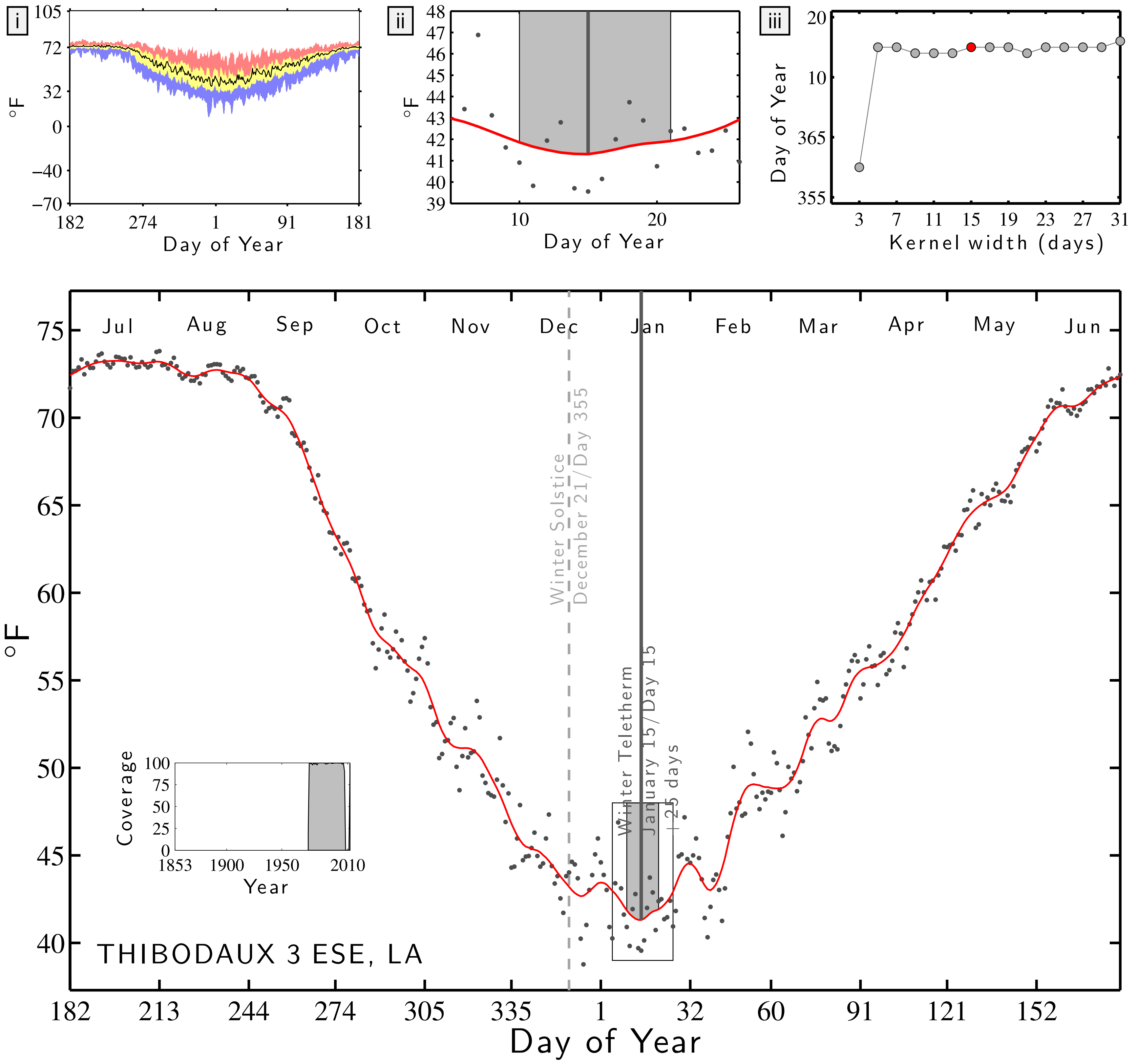

i
°F
Day of Year
ii
°F
Day of Year
iii
Day of Year
Kernel width (days)
Jul
Aug
Sep
Oct
Nov
Dec
Jan
Feb
Mar
Apr
May
Jun
Winter Solstice
December 21/Day 355
Winter Teletherm
January 15/Day 15
±25 days
Coverage
Year
THIBODAUX 3 ESE, LA
°F
Day of Year

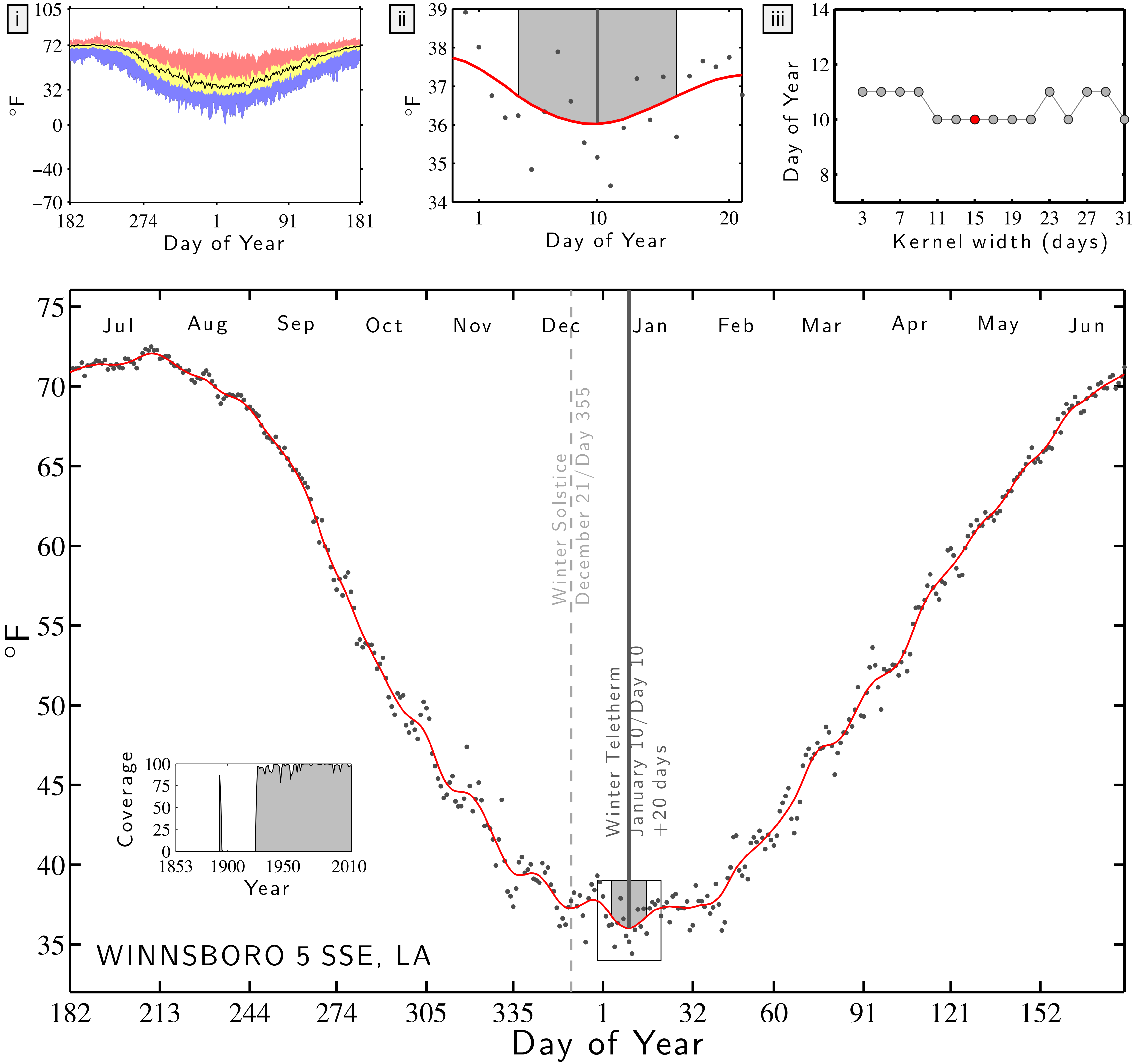

i
ii
iii
°F
Day of Year
Kernel width (days)
Jul
Aug
Sep
Oct
Nov
Dec
Jan
Feb
Mar
Apr
May
Jun
Winter Solstice
December 21/Day 355
Winter Teletherm
January 10/Day 10
+20 days
Coverage
Year
WINNSBORO 5 SSE, LA

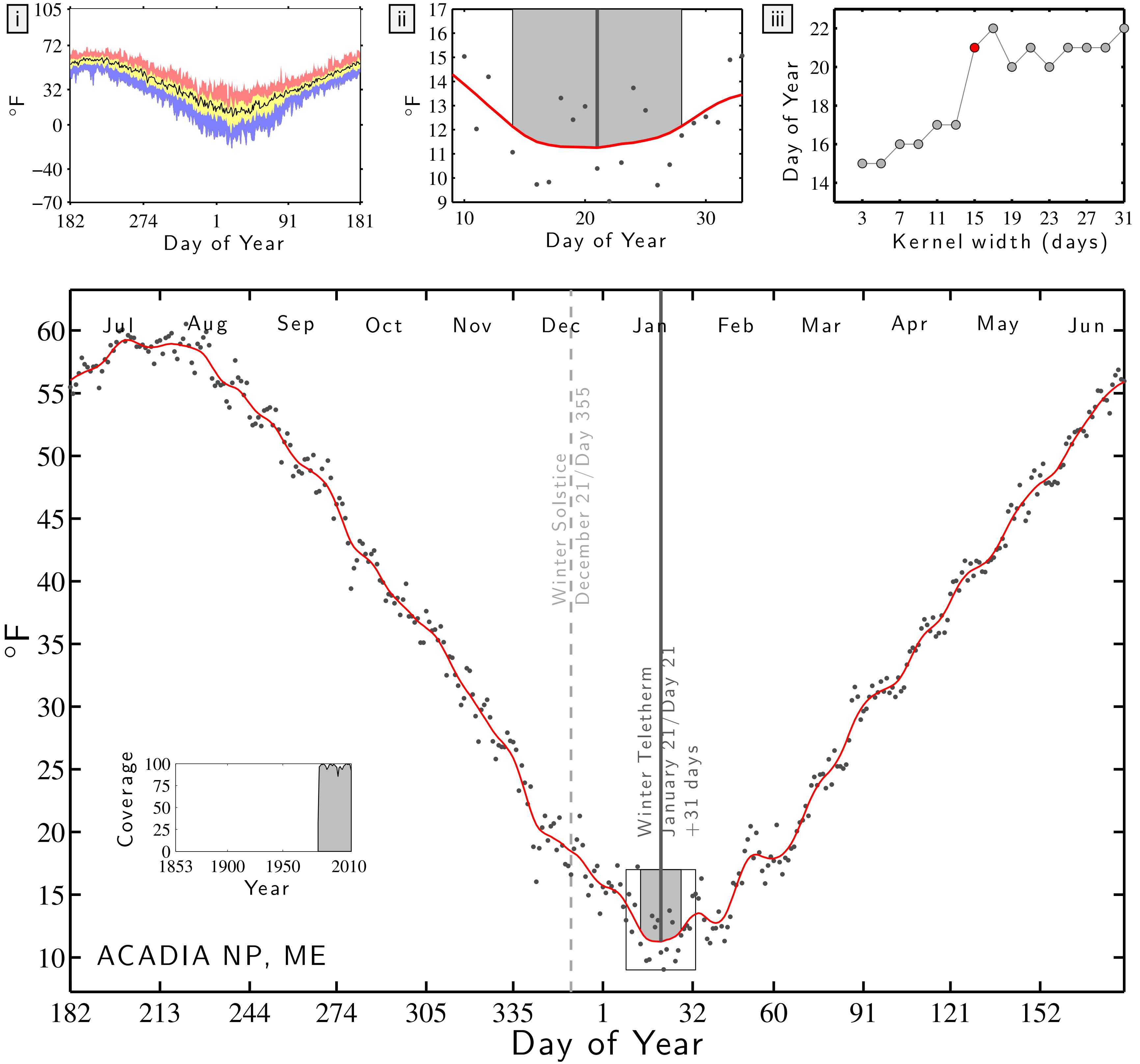

i
°F
Day of Year
ii
°F
Day of Year
iii
Day of Year
Kernel width (days)
Jul
Aug
Sep
Oct
Nov
Dec
Jan
Feb
Mar
Apr
May
Jun
Winter Solstice
December 21/Day 355
Winter Teletherm
January 21/Day 21
+31 days
Coverage
Year
ACADIA NP, ME
°F
Day of Year

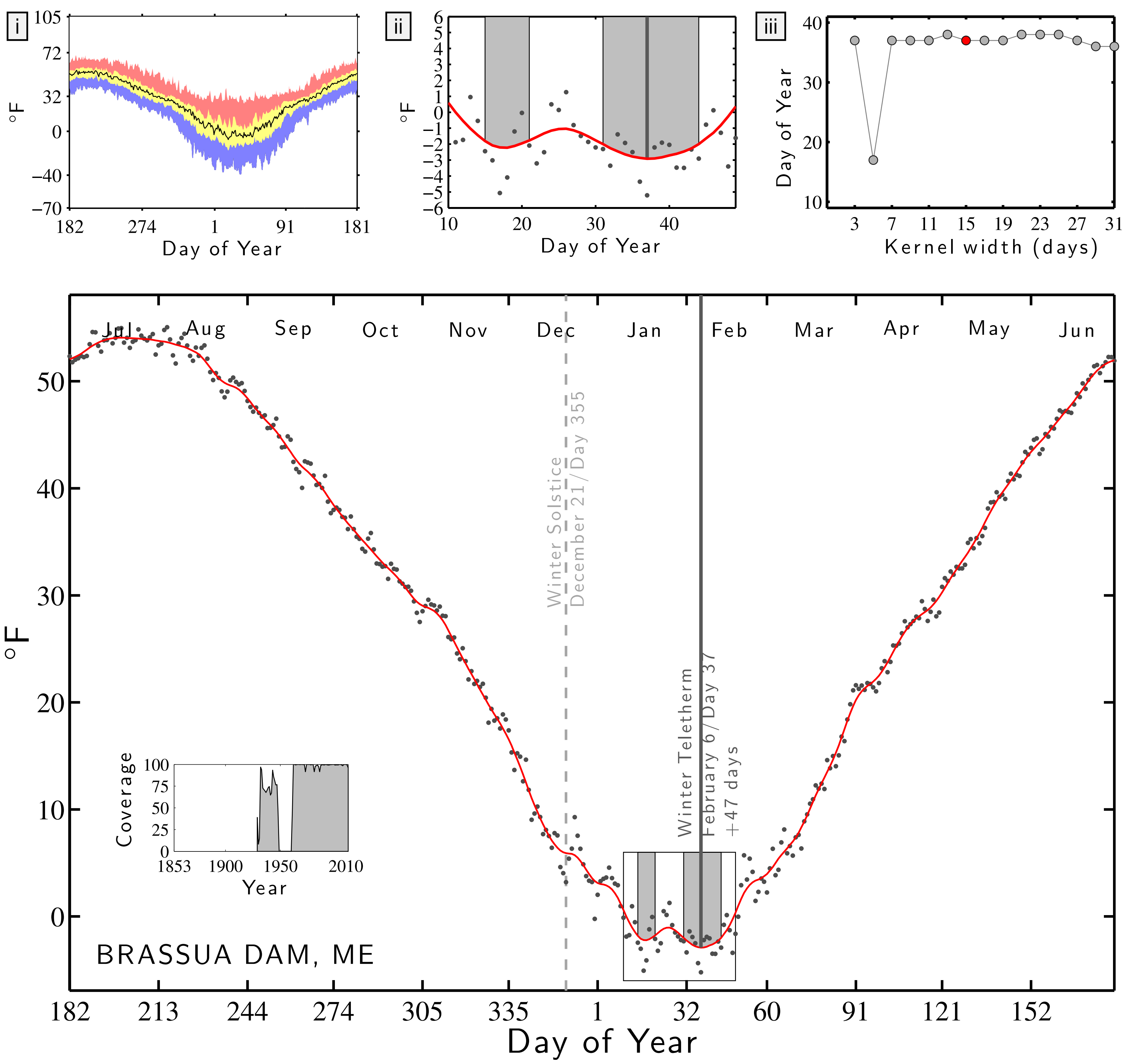

i
ii
iii
Day of Year
°F
Kernel width (days)
Jul
Aug
Sep
Oct
Nov
Dec
Jan
Feb
Mar
Apr
May
Jun
Winter Solstice
December 21/Day 355
Winter Teletherm
February 6/Day 37
+47 days
Coverage
Year
BRASSUA DAM, ME

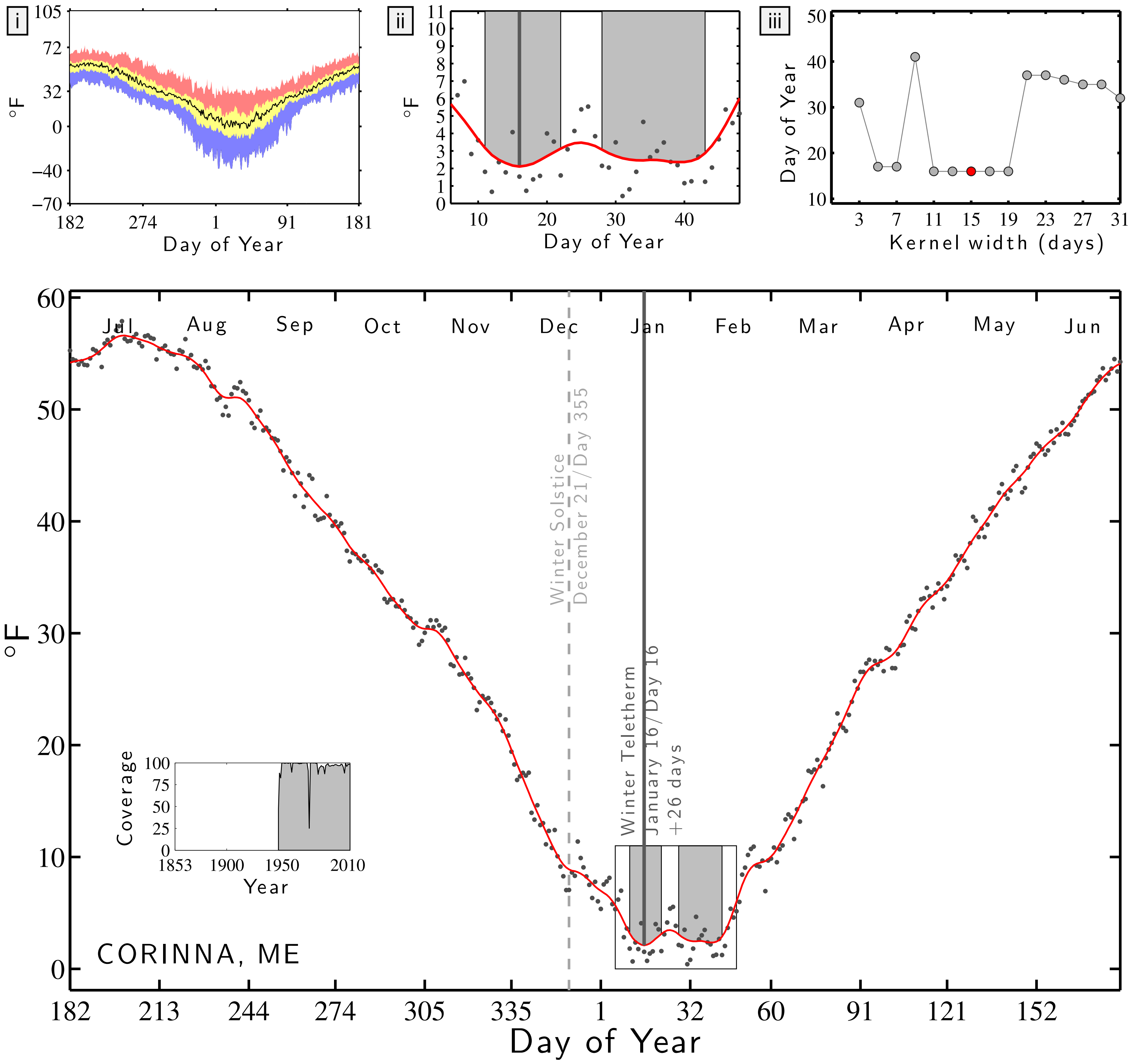

i
°F
Day of Year
ii
°F
Day of Year
iii
Day of Year
Kernel width (days)
Jul
Aug
Sep
Oct
Nov
Dec
Jan
Feb
Mar
Apr
May
Jun
Winter Solstice
December 21/Day 355
Winter Teletherm
January 16/Day 16
+26 days
Coverage
Year
CORINNA, ME
°F
Day of Year

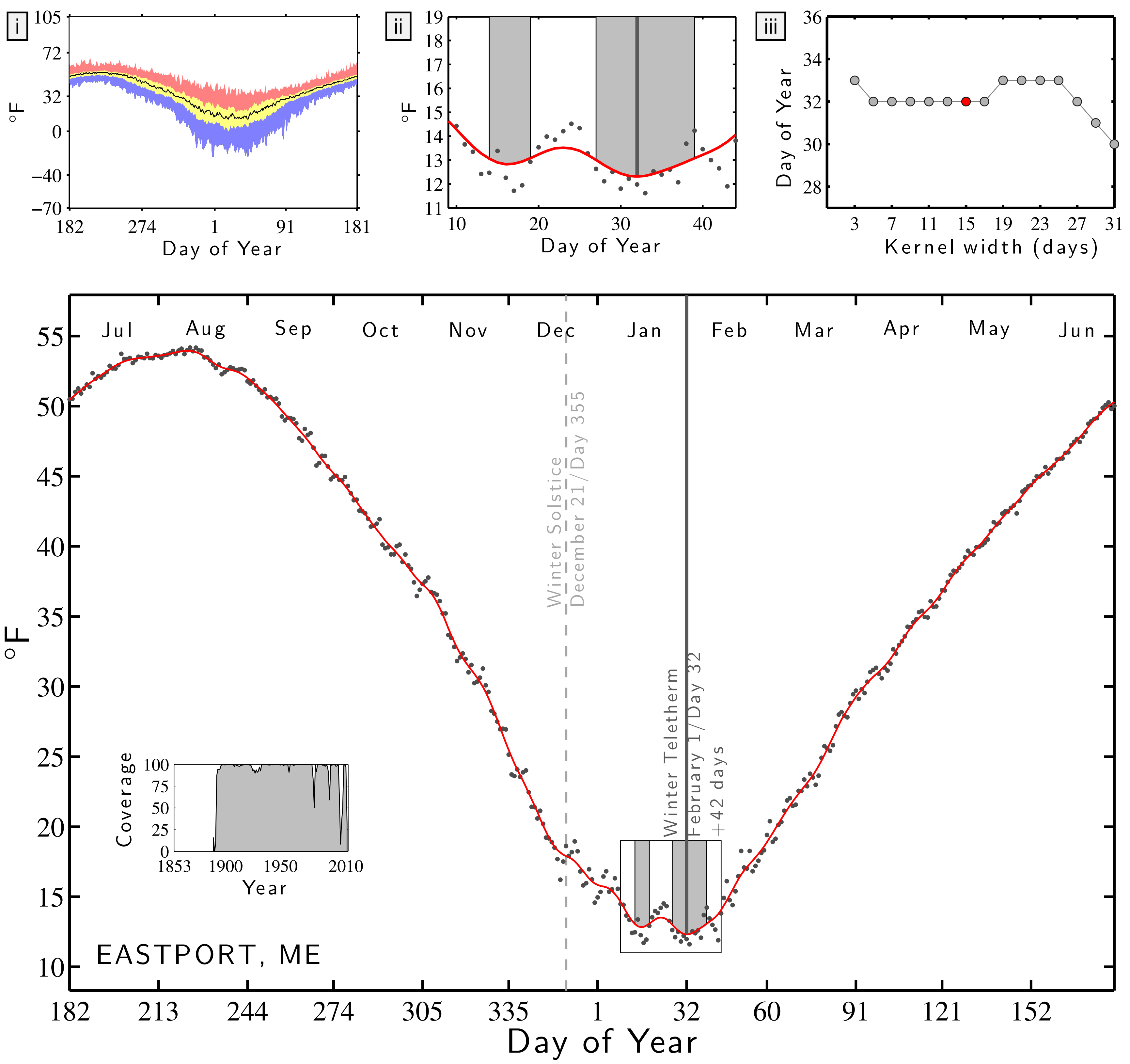
i
ii
iii
°F
Day of Year
Day of Year
Kernel width (days)
Jul
Aug
Sep
Oct
Nov
Dec
Jan
Feb
Mar
Apr
May
Jun
Winter Solstice
December 21/Day 355
Winter Teletherm
February 1/Day 32
+42 days
Coverage
Year
EASTPORT, ME

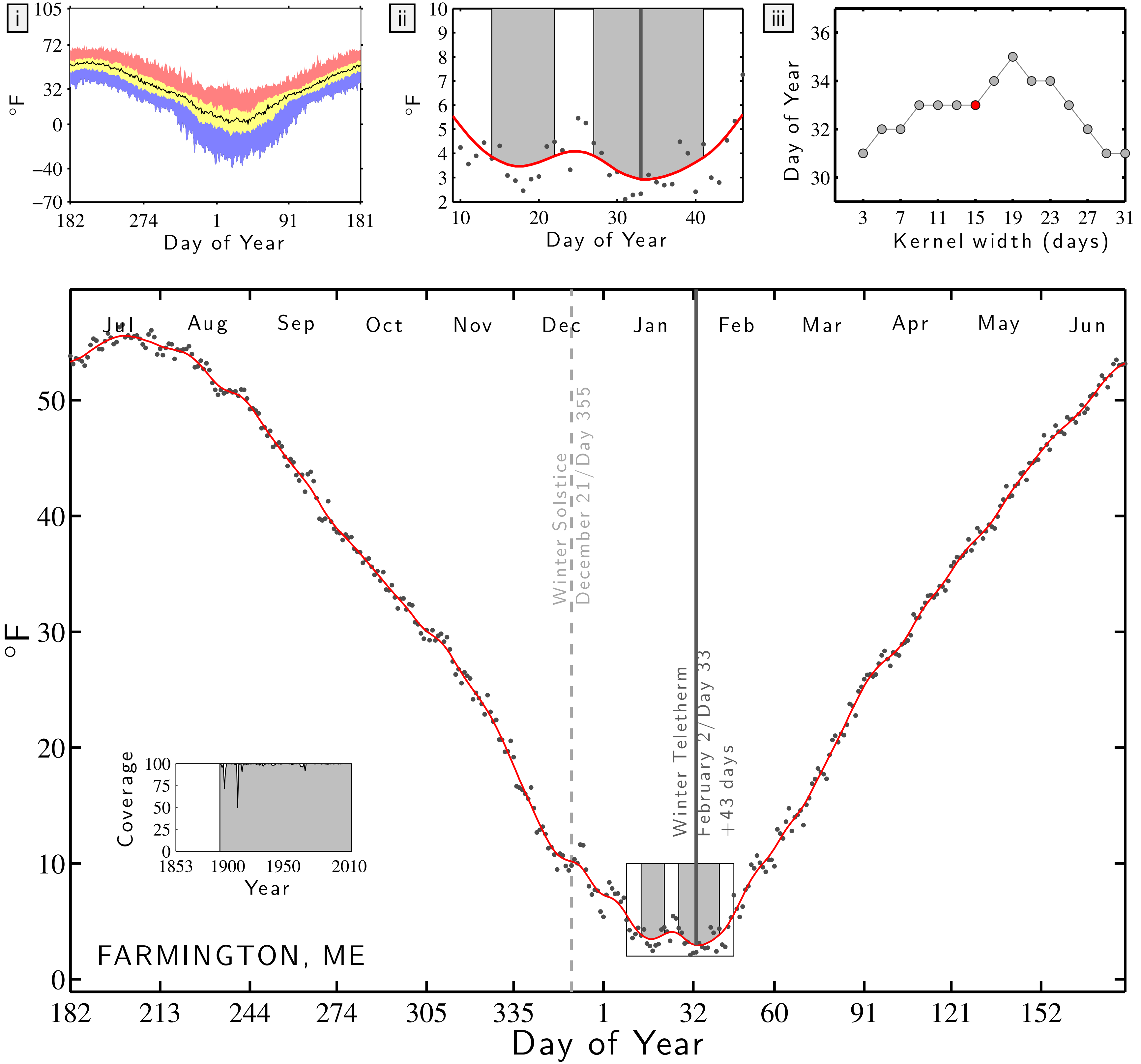

i
ii
iii
°F
Day of Year
Kernel width (days)
Jul
Aug
Sep
Oct
Nov
Dec
Jan
Feb
Mar
Apr
May
Jun
Winter Solstice
December 21/Day 355
Winter Teletherm
February 2/Day 33
+43 days
Coverage
Year
FARMINGTON, ME

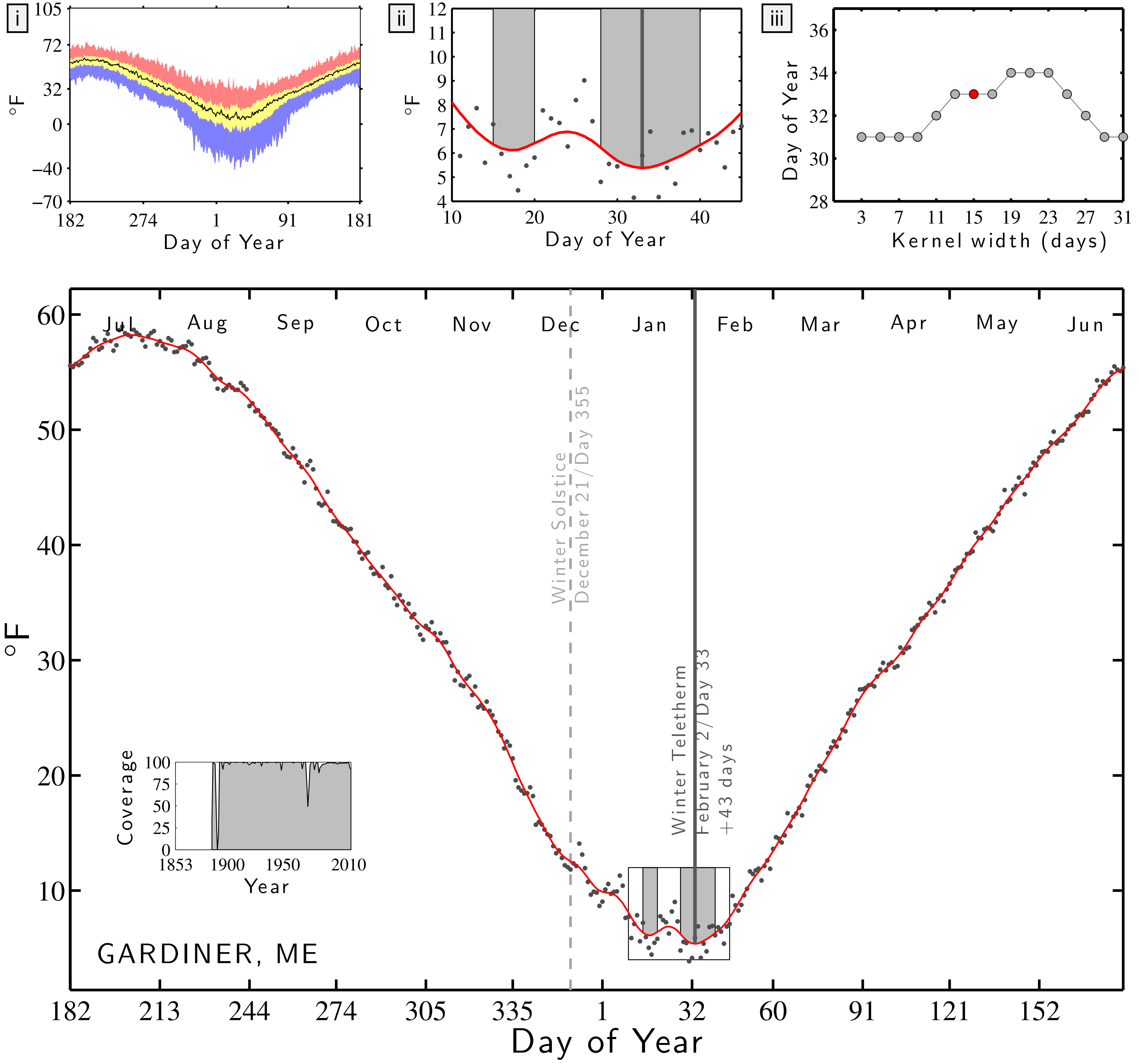

i
°F
Day of Year
ii
°F
Day of Year
iii
Day of Year
Kernel width (days)
Jul
Aug
Sep
Oct
Nov
Dec
Jan
Feb
Mar
Apr
May
Jun
Winter Solstice
December 21/Day 355
Winter Teletherm
February 2/Day 33
+43 days
Coverage
Year
GARDINER, ME
°F
Day of Year

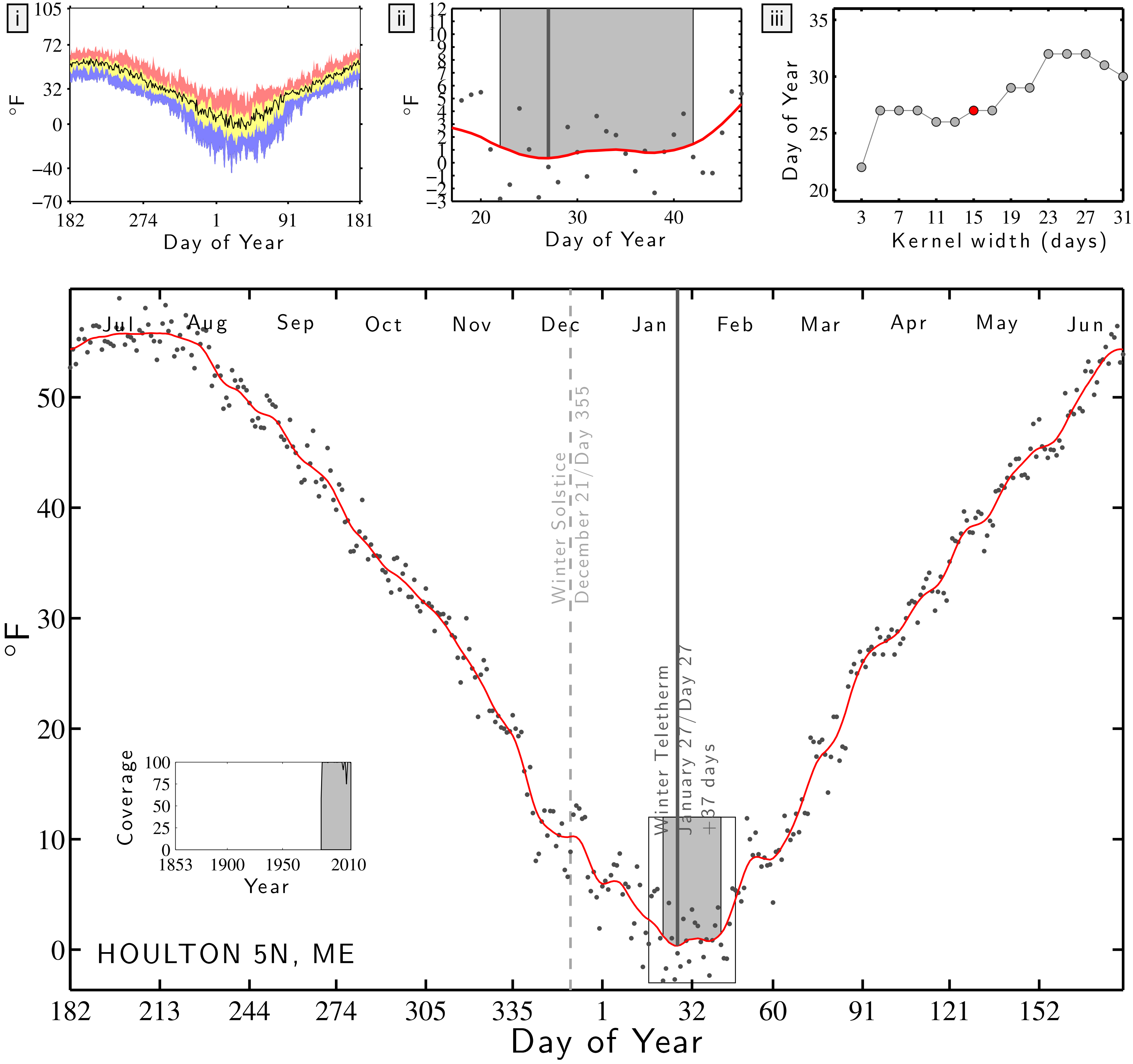

i
ii
iii
°F
Day of Year
Kernel width (days)
Jul
Aug
Sep
Oct
Nov
Dec
Jan
Feb
Mar
Apr
May
Jun
Winter Solstice
December 21/Day 355
Winter Teletherm
January 27/Day 27
+37 days
Coverage
Year
HOULTON 5N, ME

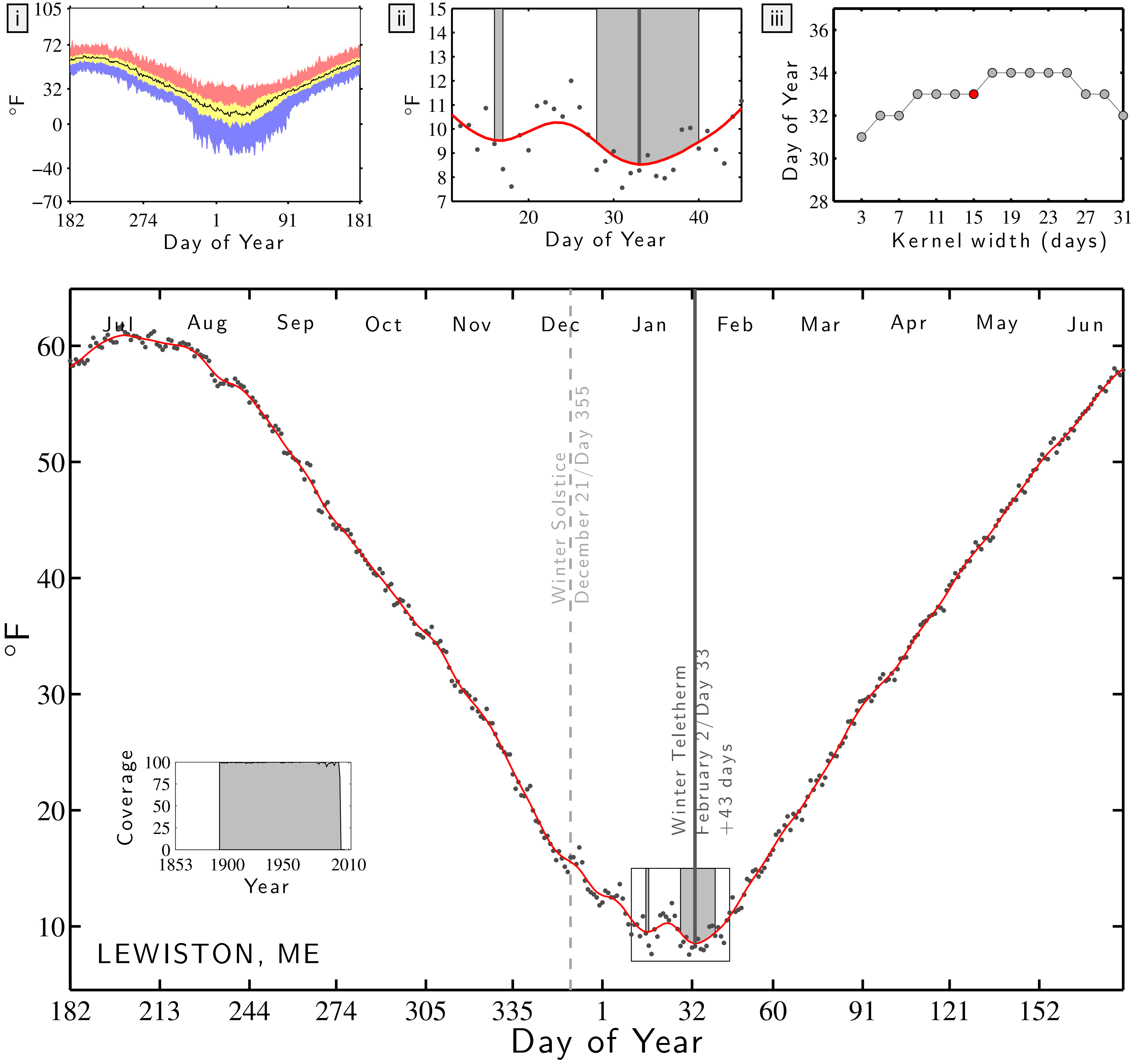

i
°F
Day of Year
ii
°F
Day of Year
iii
Day of Year
Kernel width (days)
Jul
Aug
Sep
Oct
Nov
Dec
Jan
Feb
Mar
Apr
May
Jun
Winter Solstice
December 21/Day 355
Winter Teletherm
February 2/Day 33
+43 days
Coverage
Year
LEWISTON, ME
°F
Day of Year

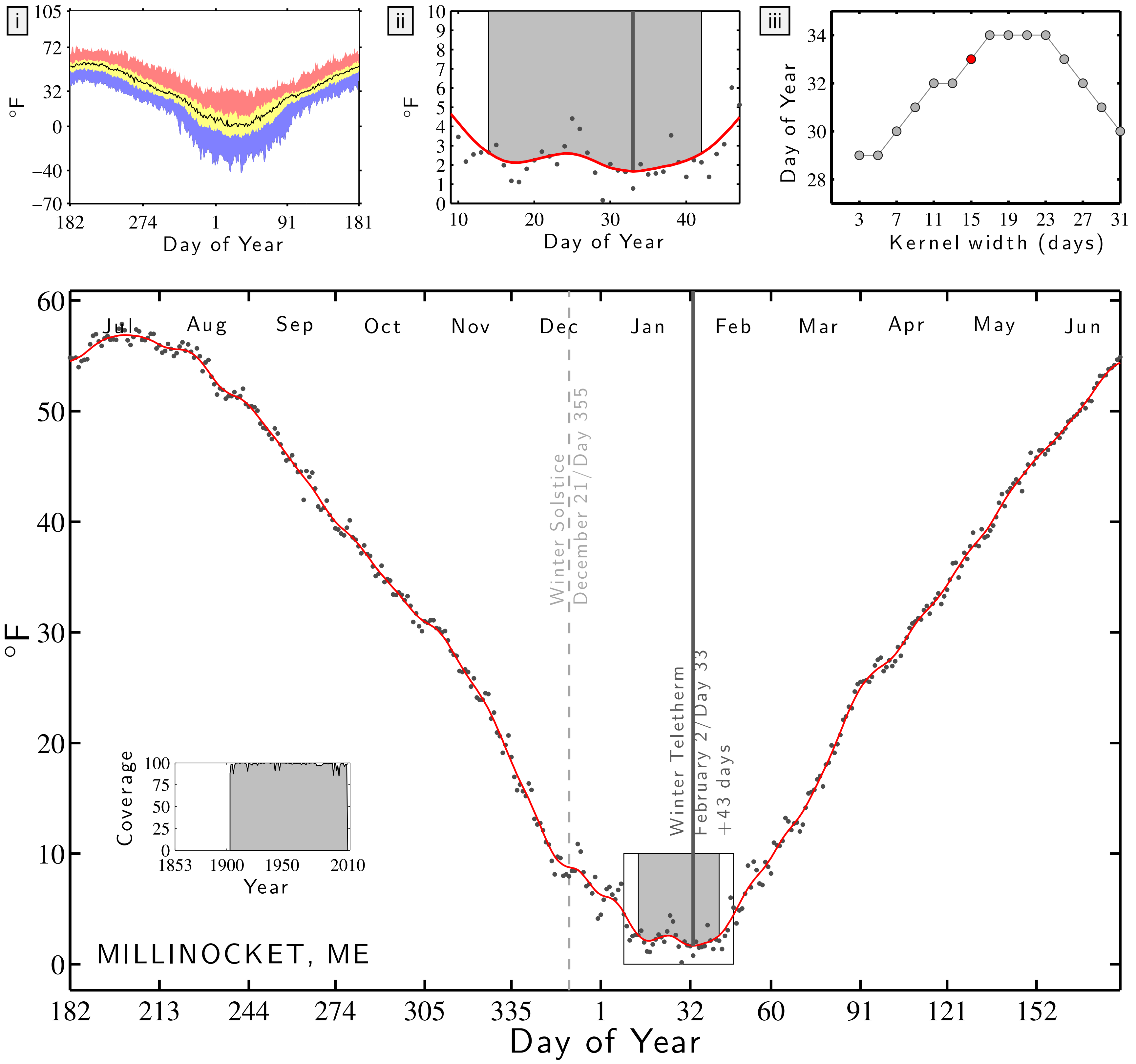

i
°F
Day of Year
ii
°F
Day of Year
iii
Day of Year
Kernel width (days)
Jul
Aug
Sep
Oct
Nov
Dec
Jan
Feb
Mar
Apr
May
Jun
Winter Solstice
December 21/Day 355
Winter Teletherm
February 2/Day 33
+43 days
Coverage
Year
MILLINOCKET, ME
°F
Day of Year

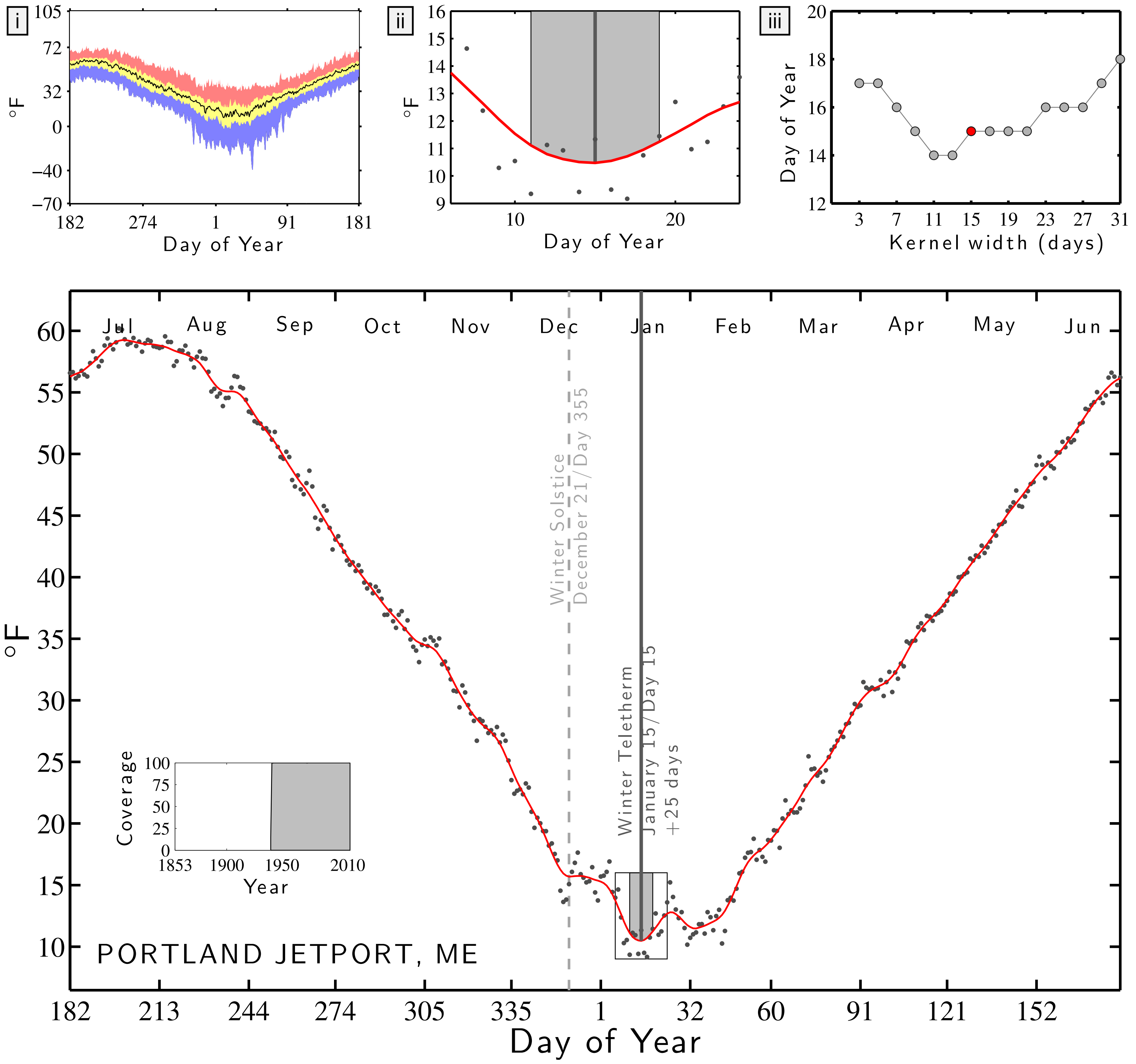

i
°F
Day of Year
ii
°F
Day of Year
iii
Day of Year
Kernel width (days)
Jul
Aug
Sep
Oct
Nov
Dec
Jan
Feb
Mar
Apr
May
Jun
Winter Solstice
December 21/Day 355
Winter Teletherm
January 15/Day 15
+25 days
Coverage
Year
PORTLAND JETPORT, ME
°F
Day of Year

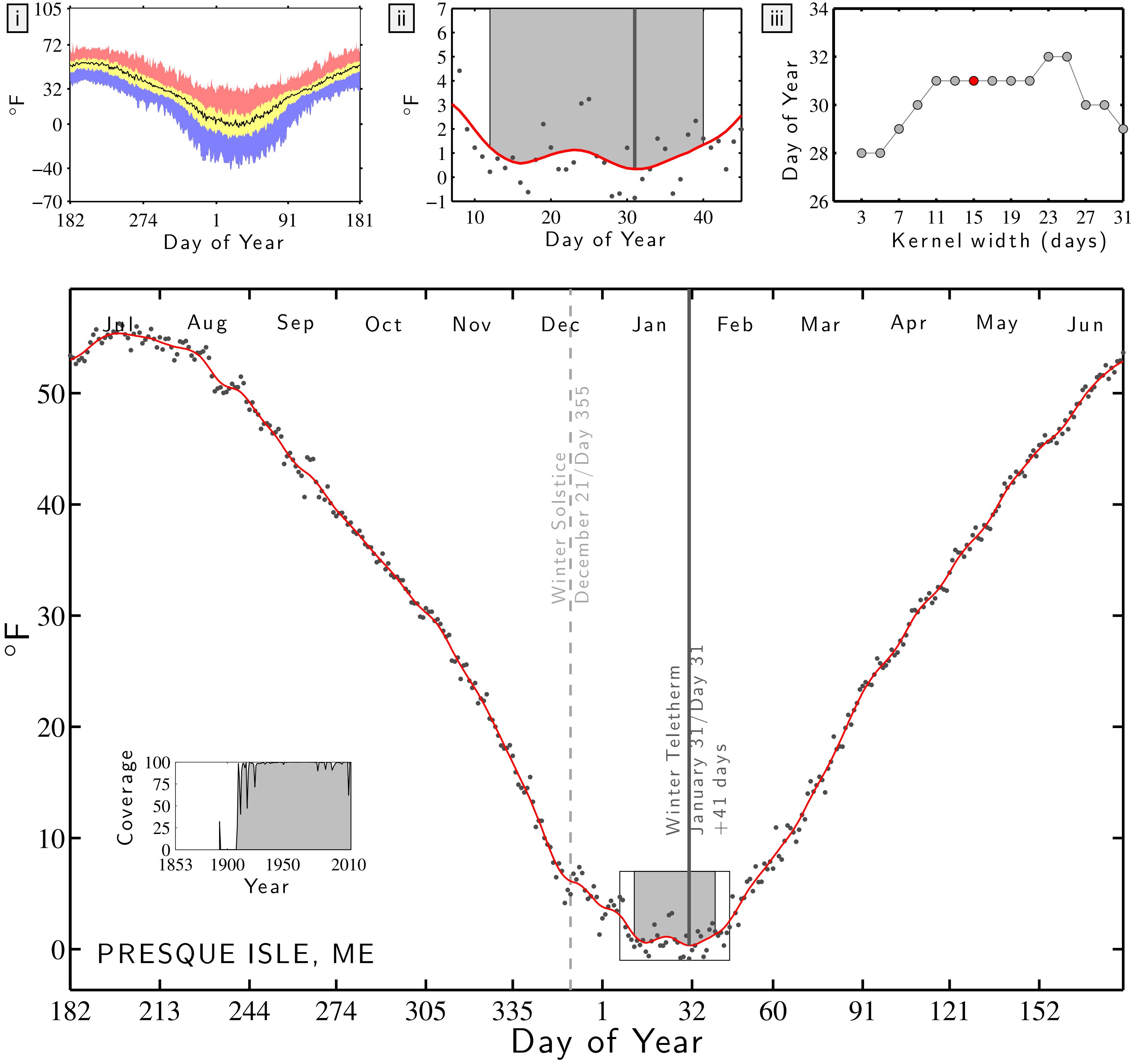

i
°F
Day of Year
ii
°F
Day of Year
iii
Day of Year
Kernel width (days)
Jul
Aug
Sep
Oct
Nov
Dec
Jan
Feb
Mar
Apr
May
Jun
Winter Solstice
December 21/Day 355
Winter Teletherm
January 31/Day 31
+41 days
Coverage
Year
PRESQUE ISLE, ME
°F
Day of Year

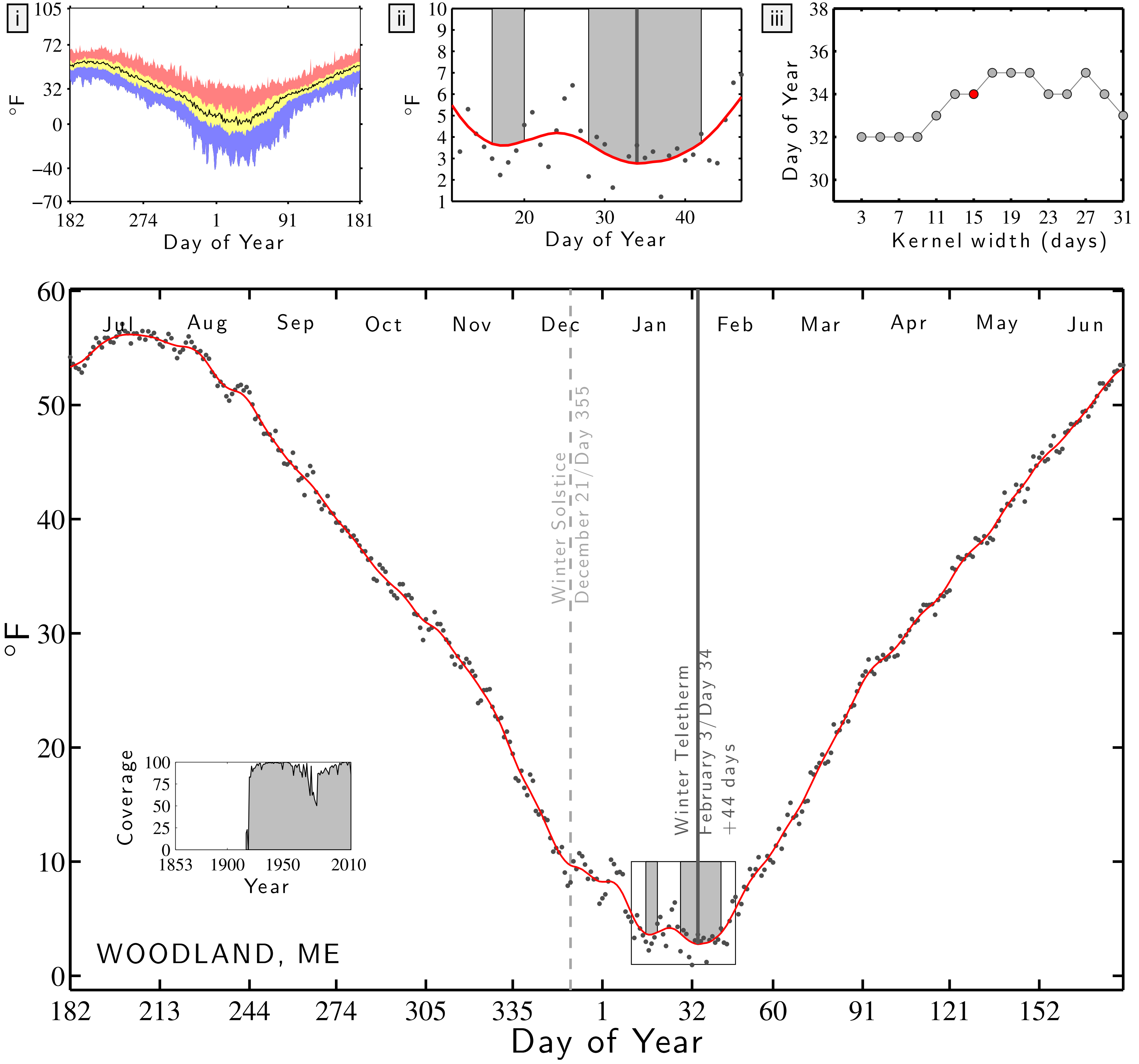
i
°F
Day of Year
ii
°F
Day of Year
iii
Day of Year
Kernel width (days)
Jul
Aug
Sep
Oct
Nov
Dec
Jan
Feb
Mar
Apr
May
Jun
Winter Solstice
December 21/Day 355
Winter Teletherm
February 3/Day 34
+44 days
Coverage
Year
WOODLAND, ME
°F
Day of Year

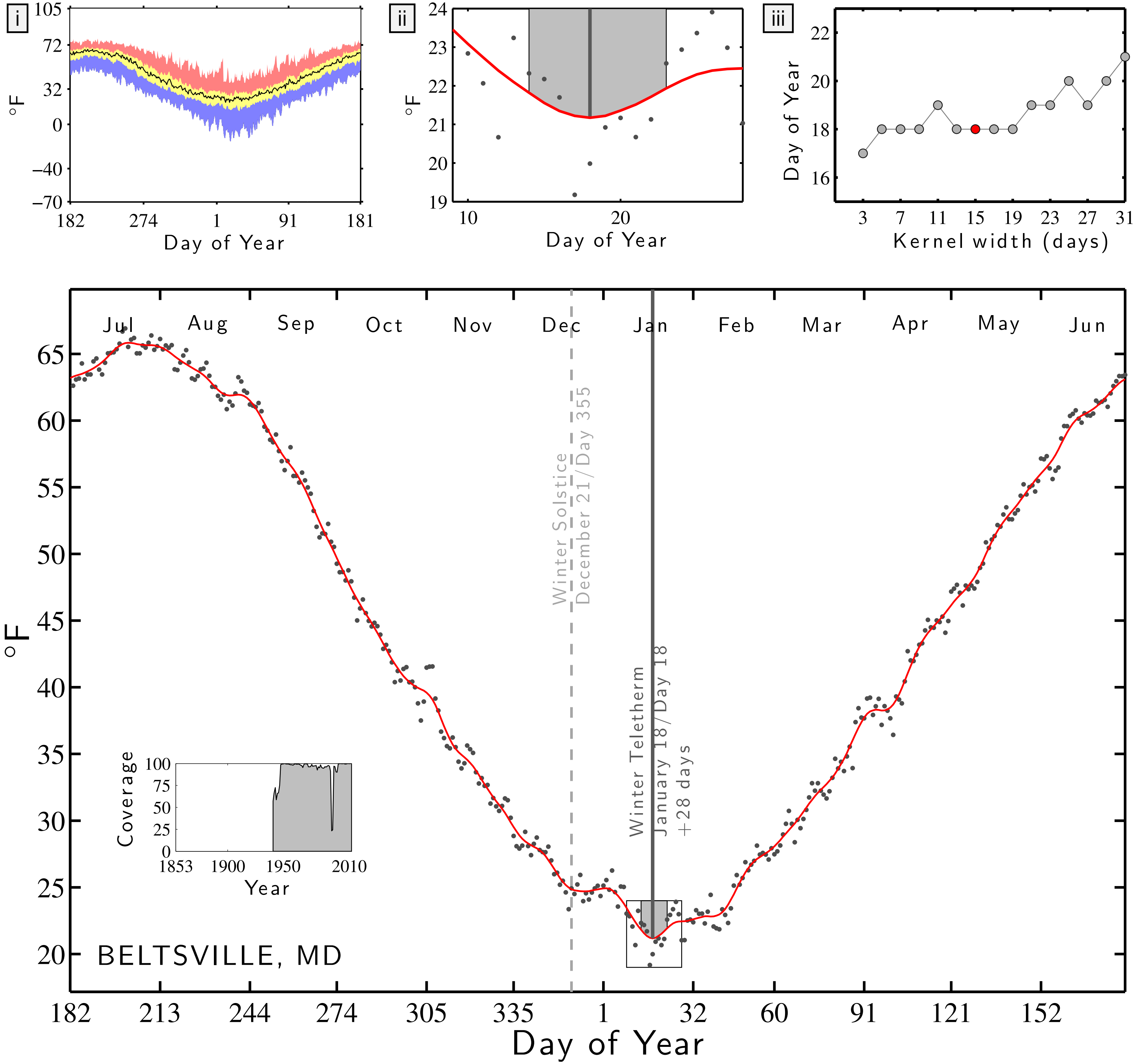

i
ii
iii
°F
Day of Year
Kernel width (days)
Jul
Aug
Sep
Oct
Nov
Dec
Jan
Feb
Mar
Apr
May
Jun
Winter Solstice
December 21/Day 355
Winter Teletherm
January 18/Day 18
+28 days
Coverage
Year
BELTSVILLE, MD

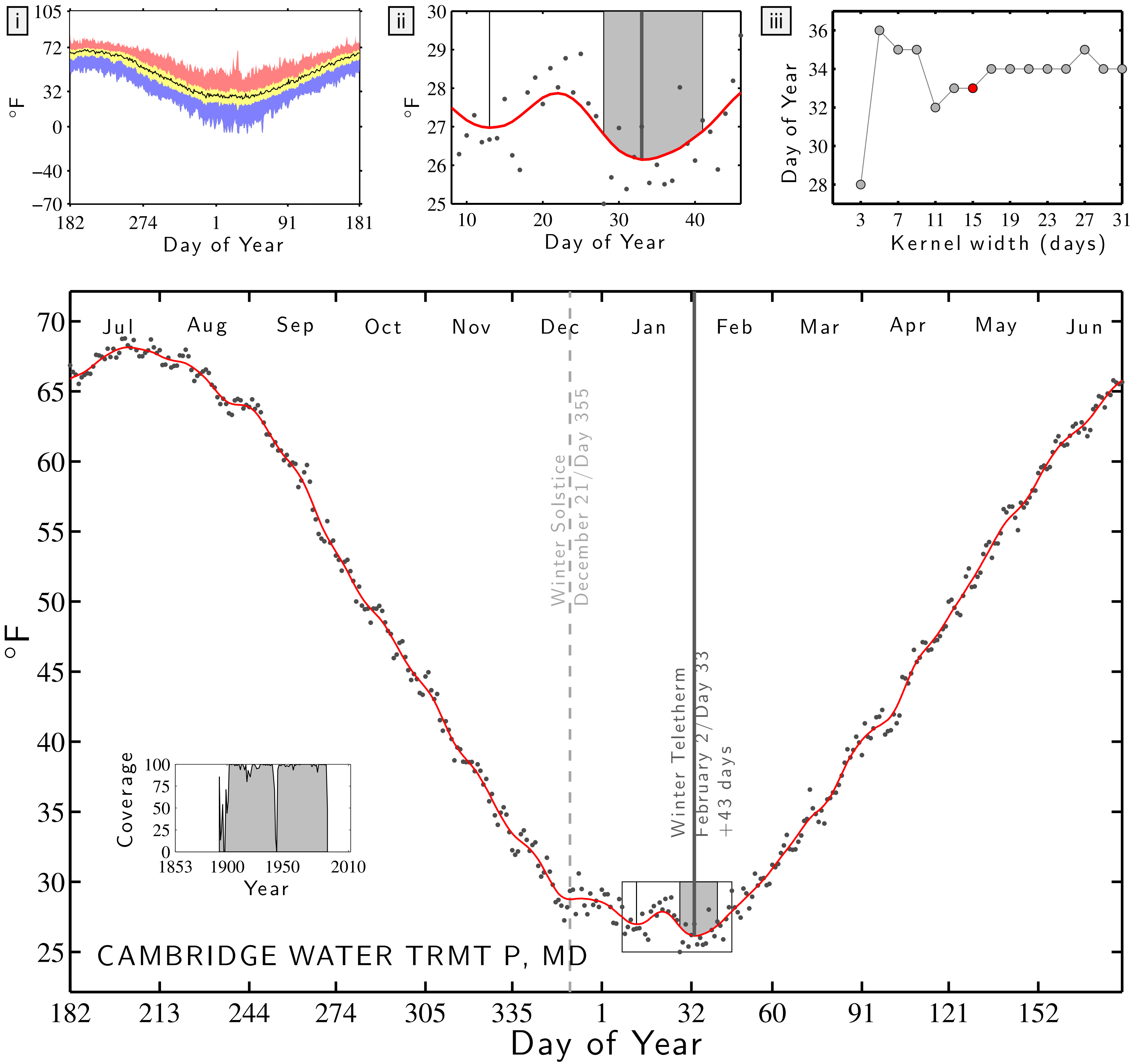

i
ii
iii
°F
Day of Year
Day of Year
Kernel width (days)
Jul
Aug
Sep
Oct
Nov
Dec
Jan
Feb
Mar
Apr
May
Jun
Winter Solstice
December 21/Day 355
Winter Teletherm
February 2/Day 33
+43 days
Coverage
Year
CAMBRIDGE WATER TRMT P, MD

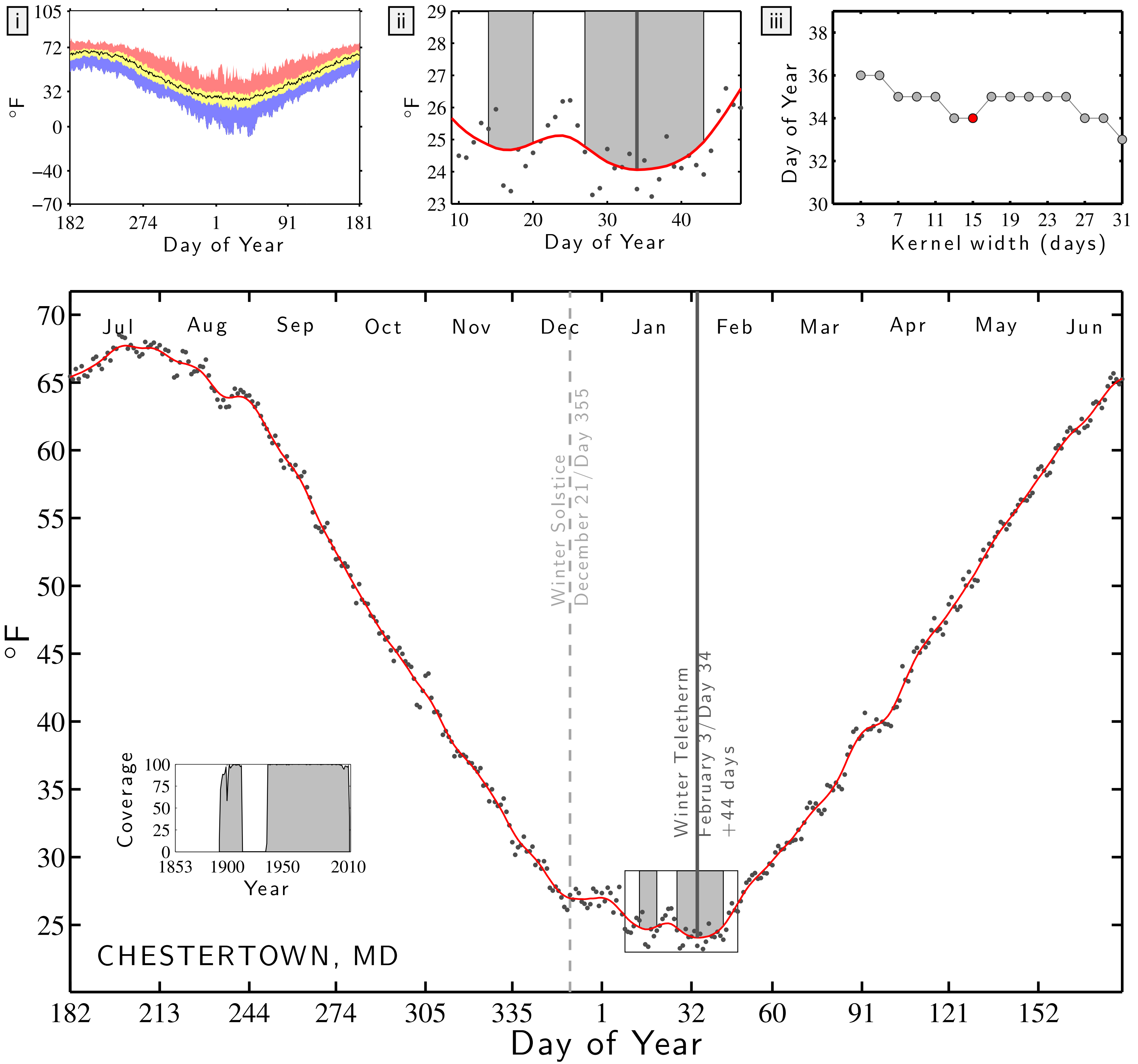

i
°F
Day of Year
ii
Day of Year
iii
Day of Year
Kernel width (days)
Jul
Aug
Sep
Oct
Nov
Dec
Jan
Feb
Mar
Apr
May
Jun
Winter Solstice
December 21/Day 355
Winter Teletherm
February 3/Day 34
+44 days
Coverage
Year
CHESTERTOWN, MD
°F
Day of Year

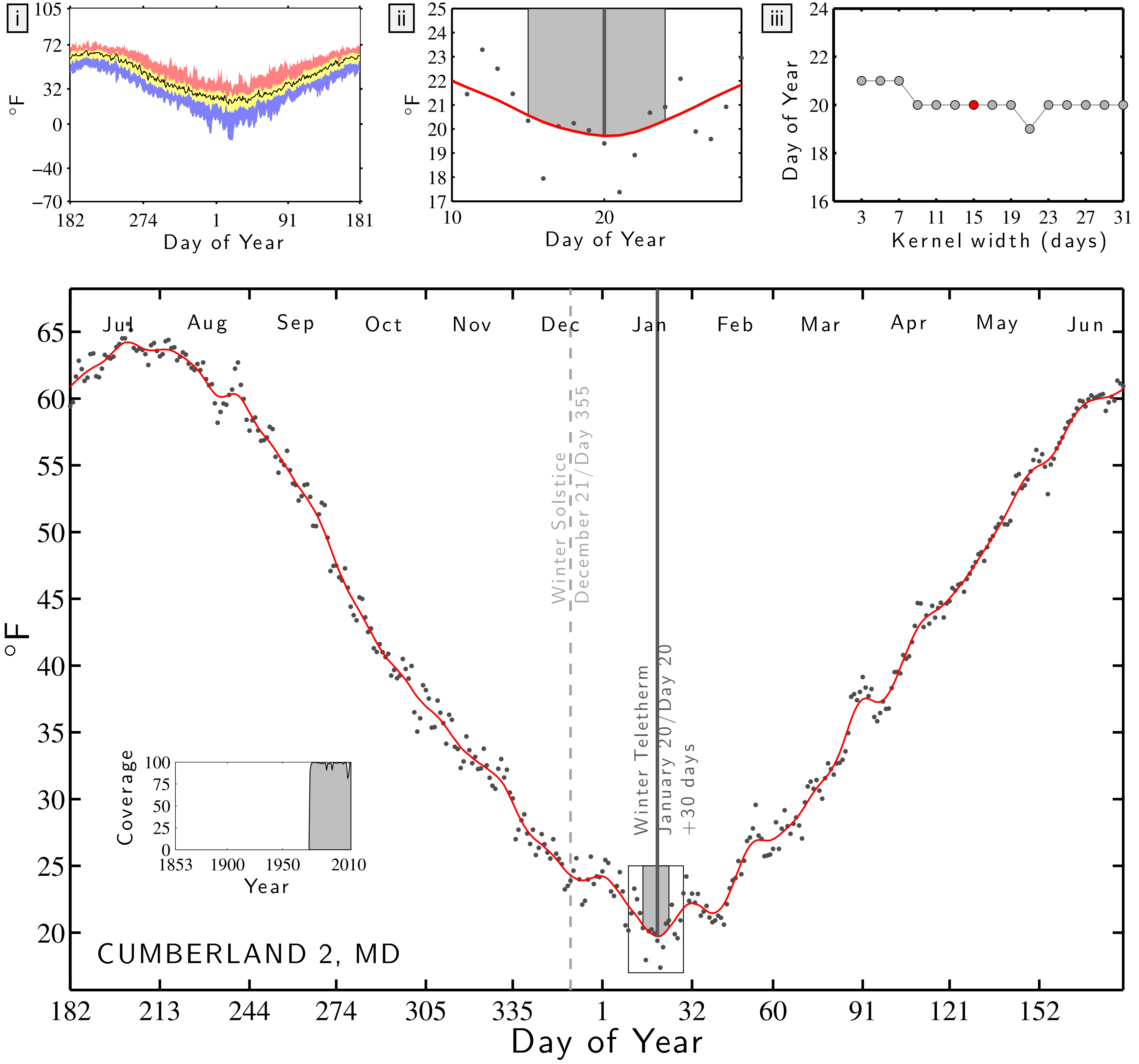

i
ii
iii
°F
Day of Year
Kernel width (days)
Jul
Aug
Sep
Oct
Nov
Dec
Jan
Feb
Mar
Apr
May
Jun
Winter Solstice
December 21/Day 355
Winter Teletherm
January 20/Day 20
+30 days
Coverage
Year
CUMBERLAND 2, MD

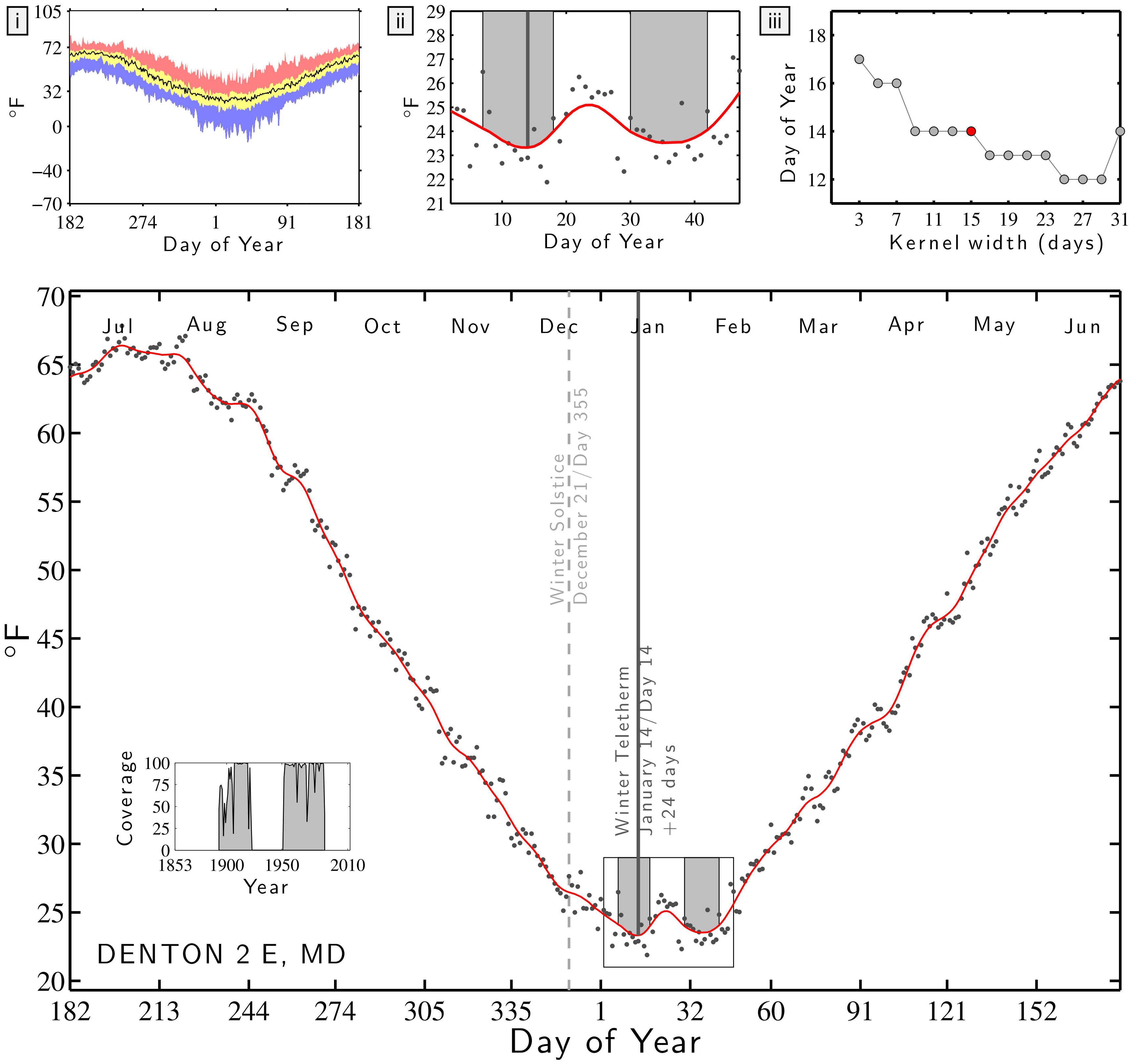

i
ii
iii
°F
Day of Year
Kernel width (days)
Jul
Aug
Sep
Oct
Nov
Dec
Jan
Feb
Mar
Apr
May
Jun
Winter Solstice
December 21/Day 355
Winter Teletherm
January 14/Day 14
+24 days
Coverage
Year
DENTON 2 E, MD

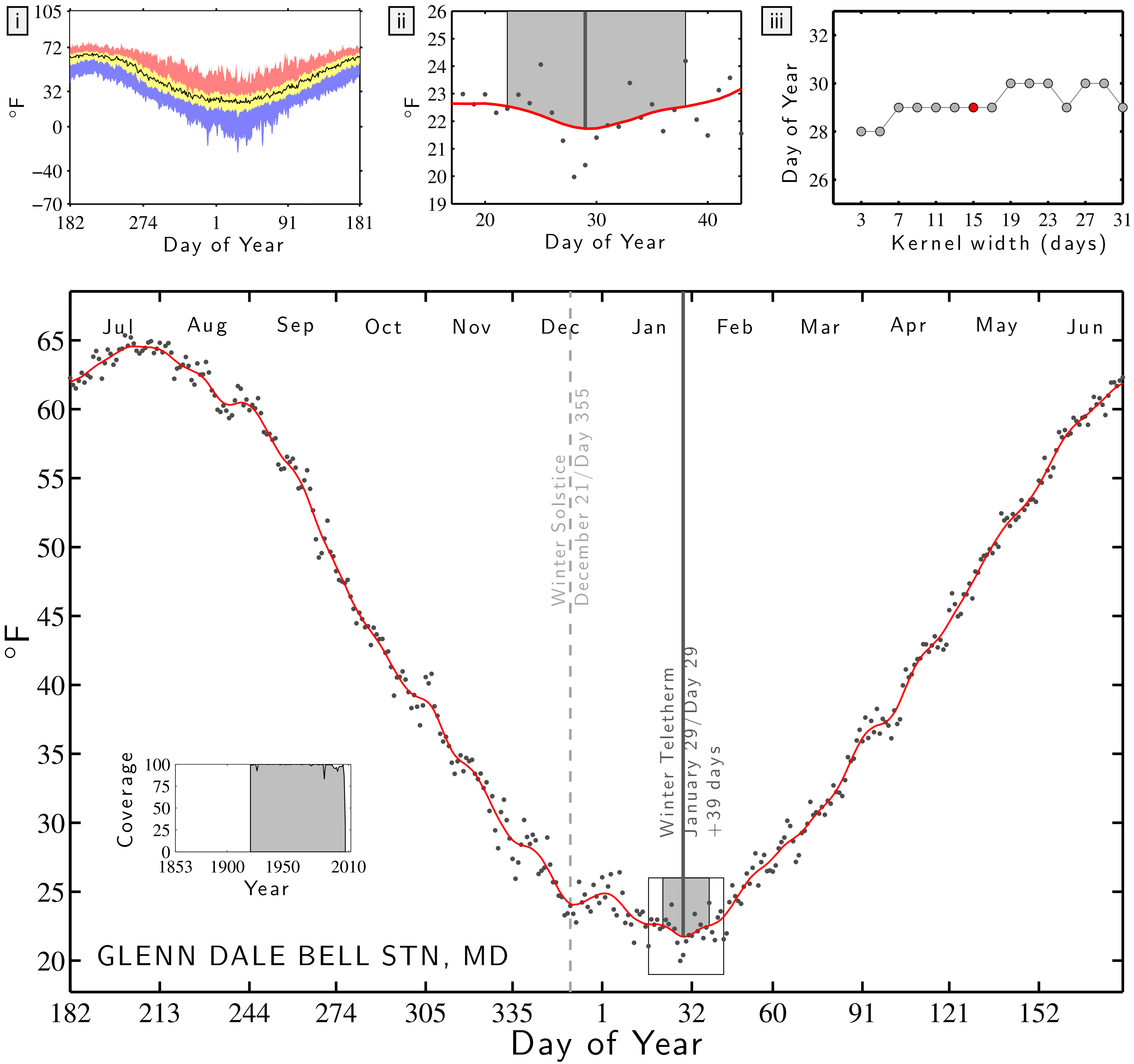

i
ii
iii
Day of Year
Kernel width (days)
°F
Jul
Aug
Sep
Oct
Nov
Dec
Jan
Feb
Mar
Apr
May
Jun
Winter Solstice
December 21/Day 355
Winter Teletherm
January 29/Day 29
+39 days
Coverage
Year
GLENN DALE BELL STN, MD

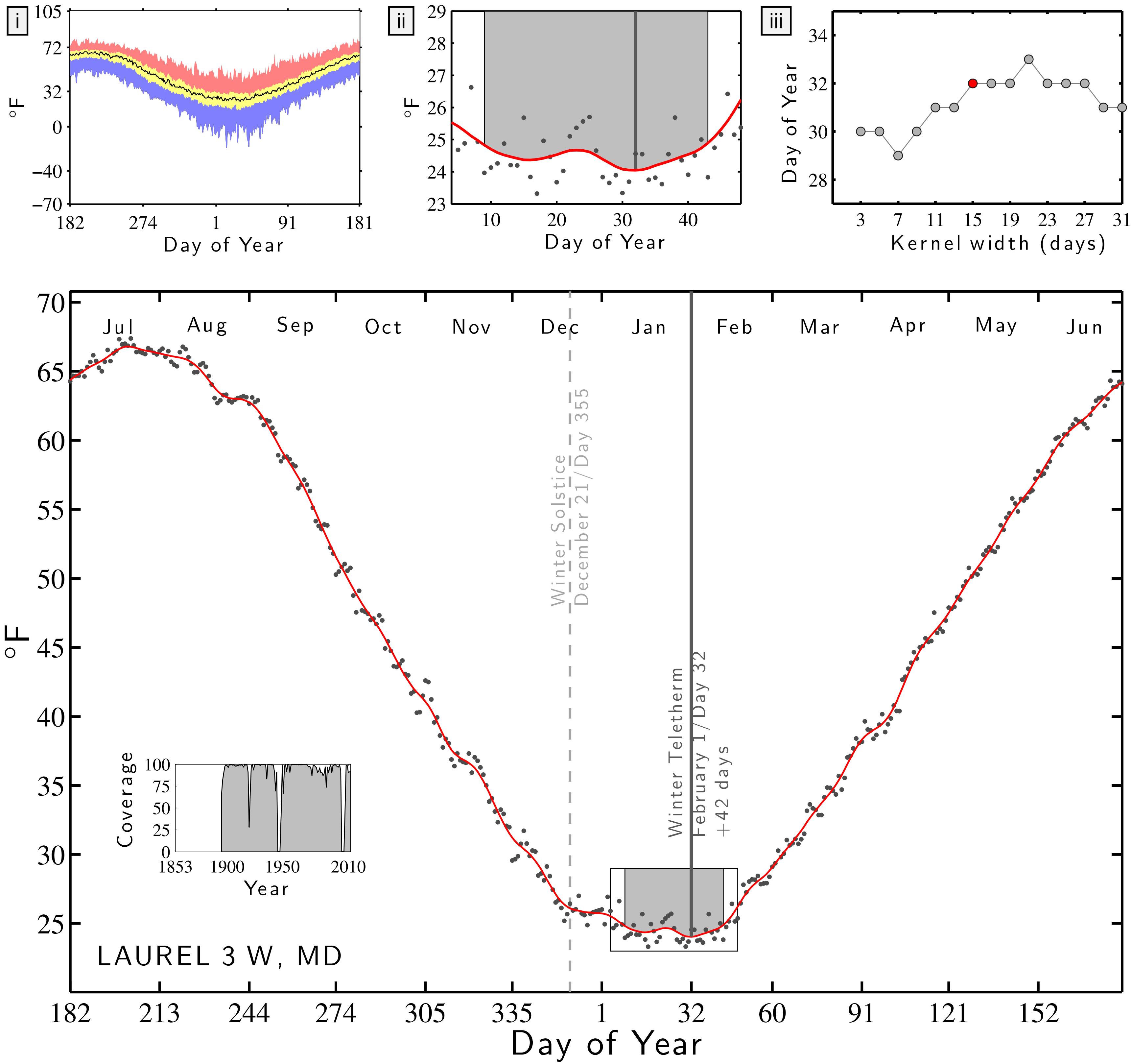

i
°F
105
72
32
0
−40
−70
182
274
1
91
181
Day of Year
ii
29
28
27
26
25
24
23
10
20
30
40
iii
Day of Year
34
32
30
28
3
7
11
15
19
23
27
31
Kernel width (days)
Jul
Aug
Sep
Oct
Nov
Dec
Jan
Feb
Mar
Apr
May
Jun
70
65
60
55
50
45
40
35
30
25
Winter Solstice
December 21/Day 355
Winter Teletherm
February 1/Day 32
+42 days
Coverage
100
75
50
25
0
1853
1900
1950
2010
Year
LAUREL 3 W, MD
182
213
244
274
305
335
1
32
60
91
121
152
Day of Year

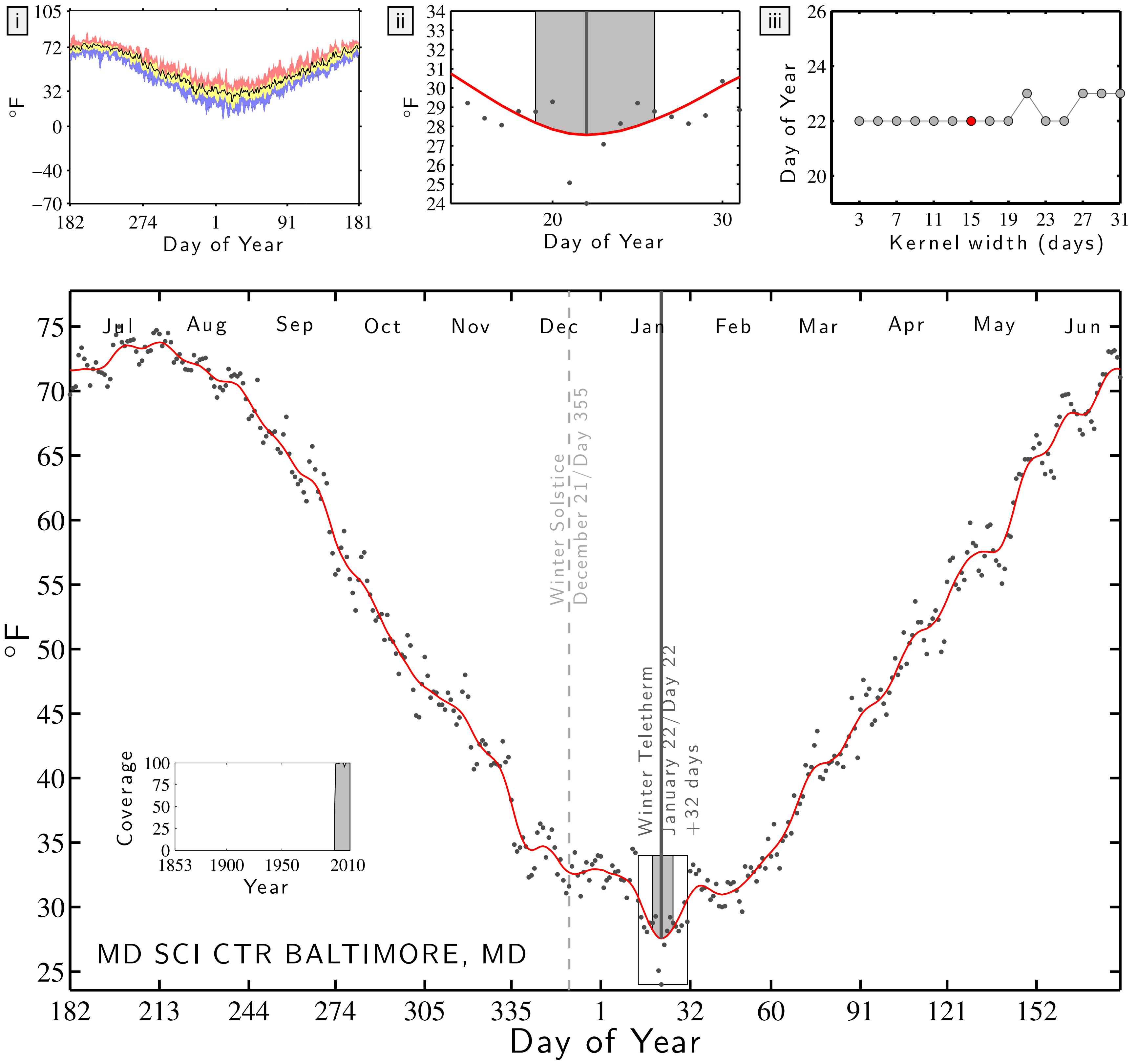

i
ii
iii
Day of Year
Kernel width (days)
Winter Solstice
December 21/Day 355
Winter Teletherm
January 22/Day 22
+32 days
Coverage
Year
MD SCI CTR BALTIMORE, MD
Jul
Aug
Sep
Oct
Nov
Dec
Jan
Feb
Mar
Apr
May
Jun
°F
Day of Year

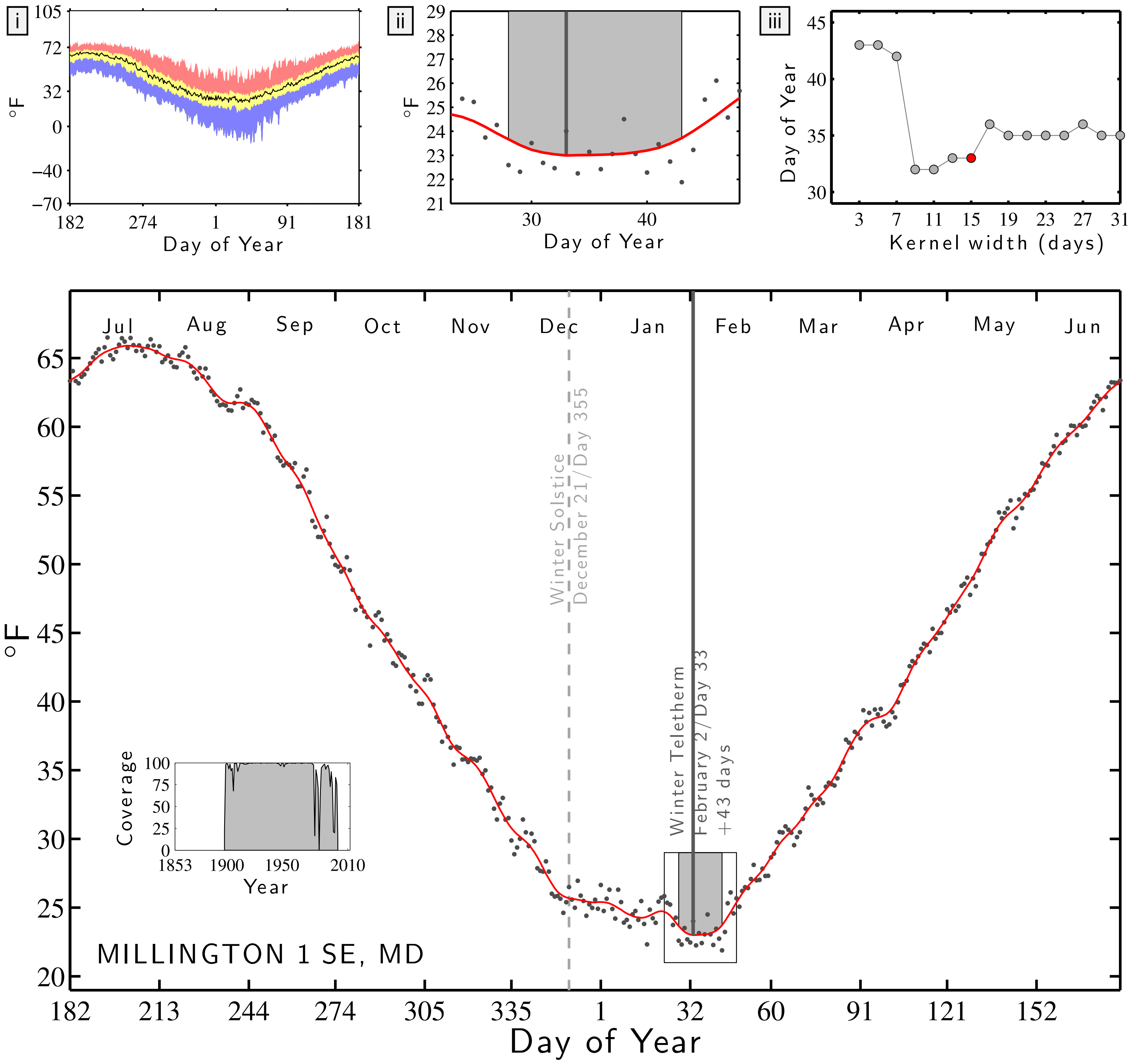

i
°F
Day of Year
ii
°F
Day of Year
iii
Day of Year
Kernel width (days)
Jul
Aug
Sep
Oct
Nov
Dec
Jan
Feb
Mar
Apr
May
Jun
Winter Solstice
December 21/Day 355
Winter Teletherm
February 2/Day 33
+43 days
Coverage
Year
MILLINGTON 1 SE, MD
°F
Day of Year

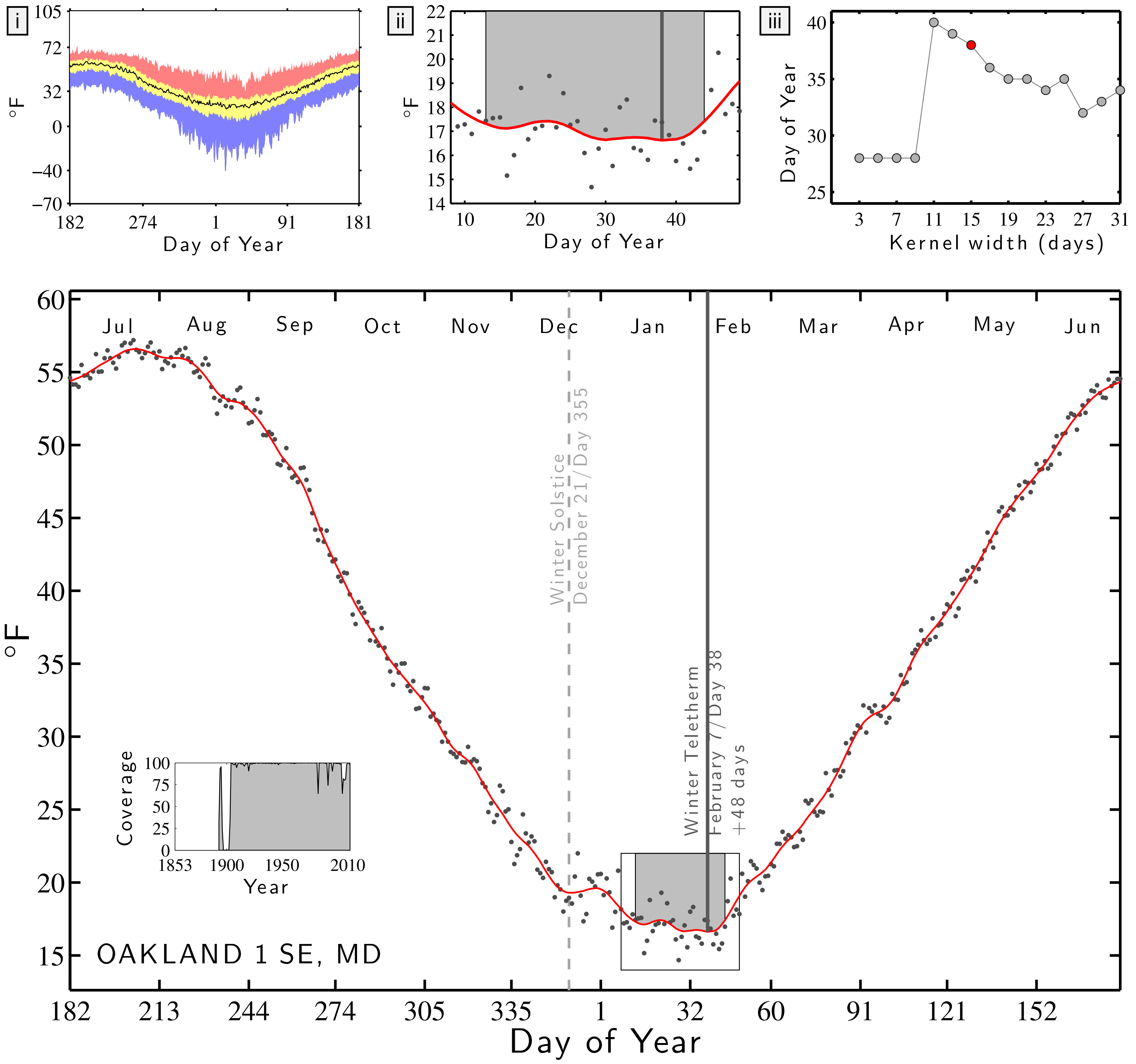

i
°F
Day of Year
ii
°F
Day of Year
iii
Day of Year
Kernel width (days)
Jul
Aug
Sep
Oct
Nov
Dec
Jan
Feb
Mar
Apr
May
Jun
Winter Solstice
December 21/Day 355
Winter Teletherm
February 7/Day 38
+48 days
Coverage
Year
OAKLAND 1 SE, MD
°F
Day of Year

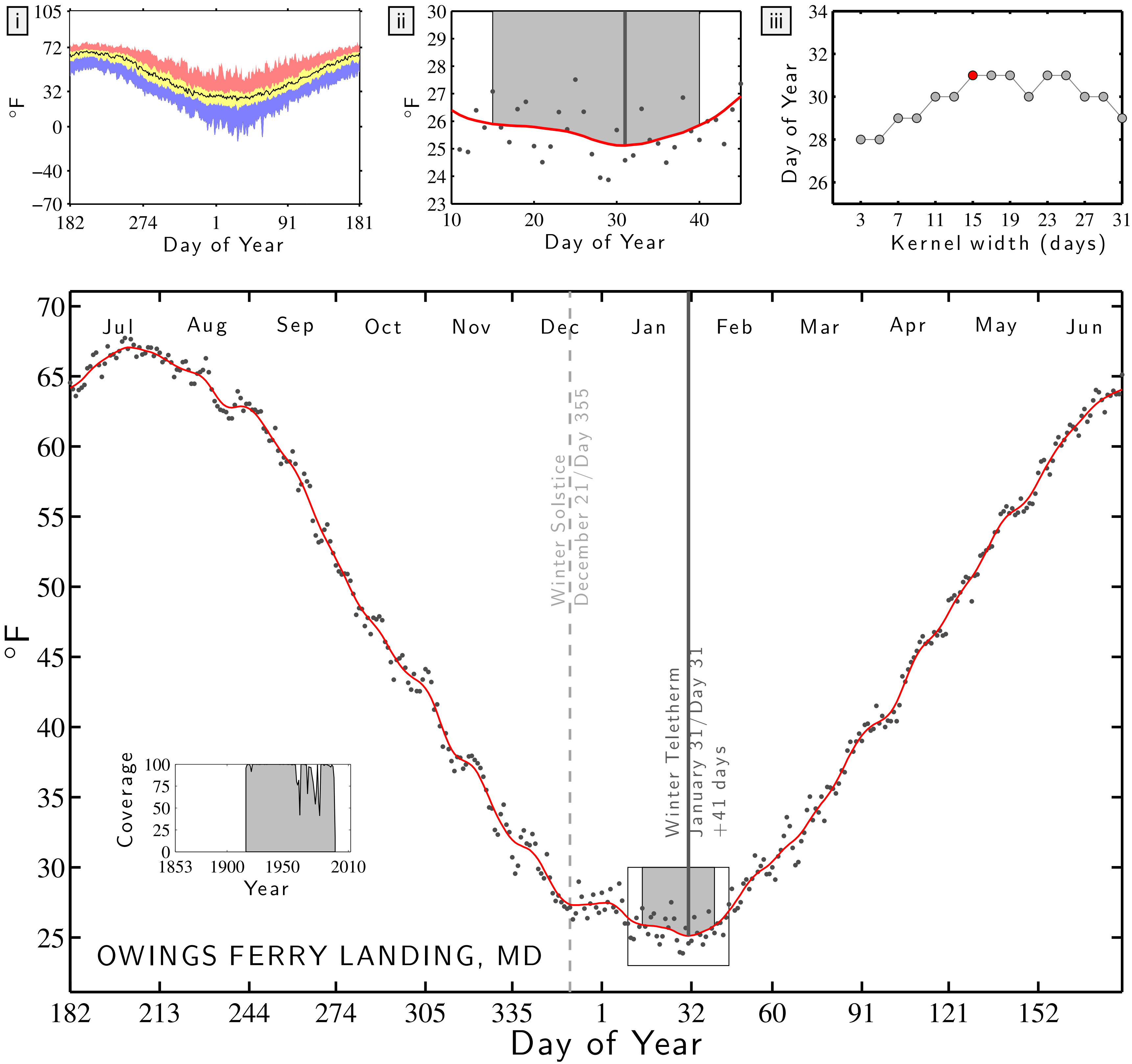
i
°F
Day of Year
ii
°F
Day of Year
iii
Day of Year
Kernel width (days)
Jul
Aug
Sep
Oct
Nov
Dec
Jan
Feb
Mar
Apr
May
Jun
Winter Solstice
December 21/Day 355
Winter Teletherm
January 31/Day 31
+41 days
Coverage
Year
OWINGS FERRY LANDING, MD
°F
Day of Year

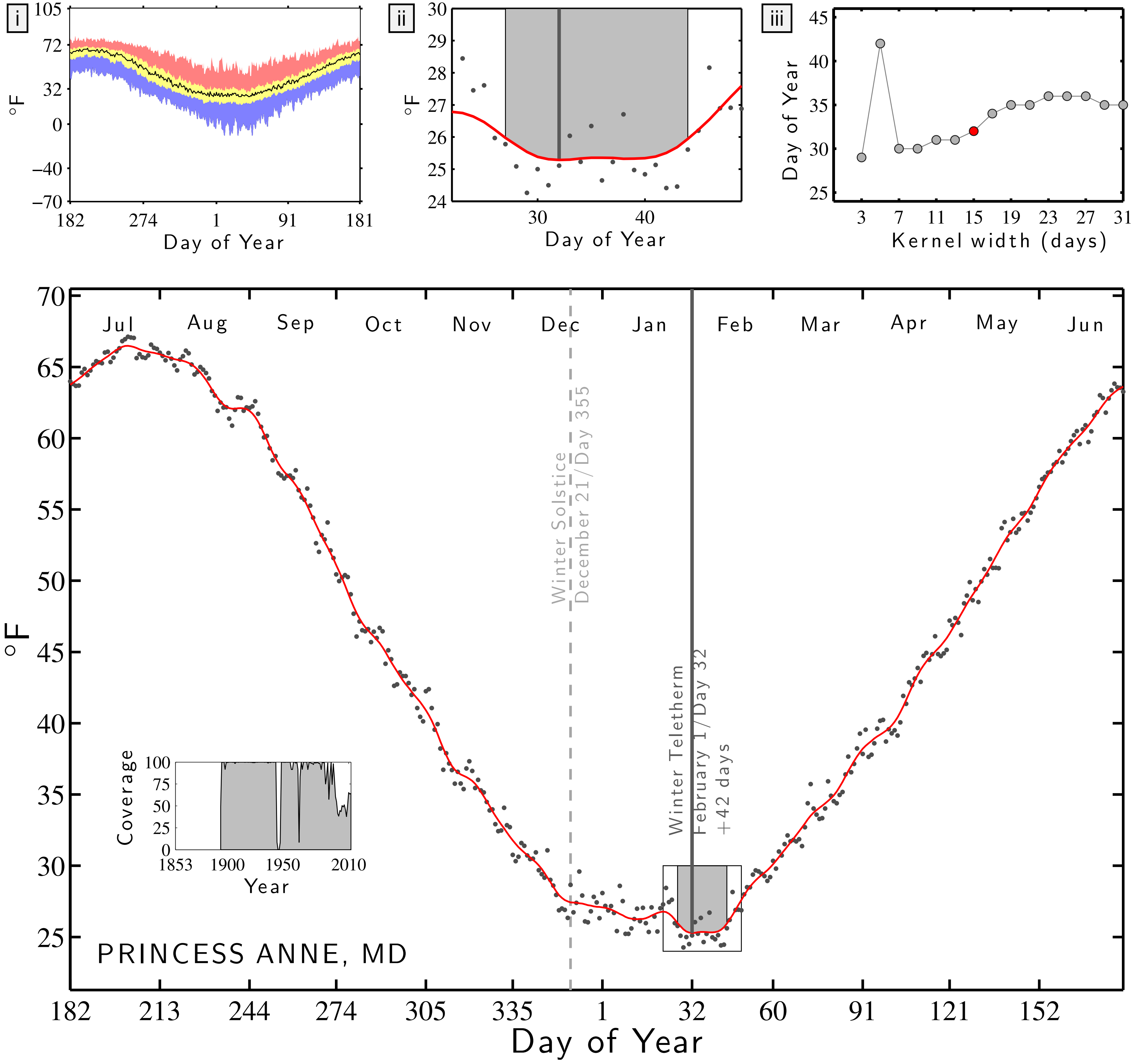

i
°F
105
72
32
0
−40
−70
182
274
1
91
181
Day of Year
ii
30
29
28
27
26
25
24
30
40
Day of Year
iii
Day of Year
45
40
35
30
25
3
7
11
15
19
23
27
31
Kernel width (days)
Jul
Aug
Sep
Oct
Nov
Dec
Jan
Feb
Mar
Apr
May
Jun
70
65
60
55
50
45
40
35
30
25
Winter Solstice
December 21/Day 355
Winter Teletherm
February 1/Day 32
+42 days
Coverage
100
75
50
25
0
1853
1900
1950
2010
Year
PRINCESS ANNE, MD
182
213
244
274
305
335
1
32
60
91
121
152
Day of Year

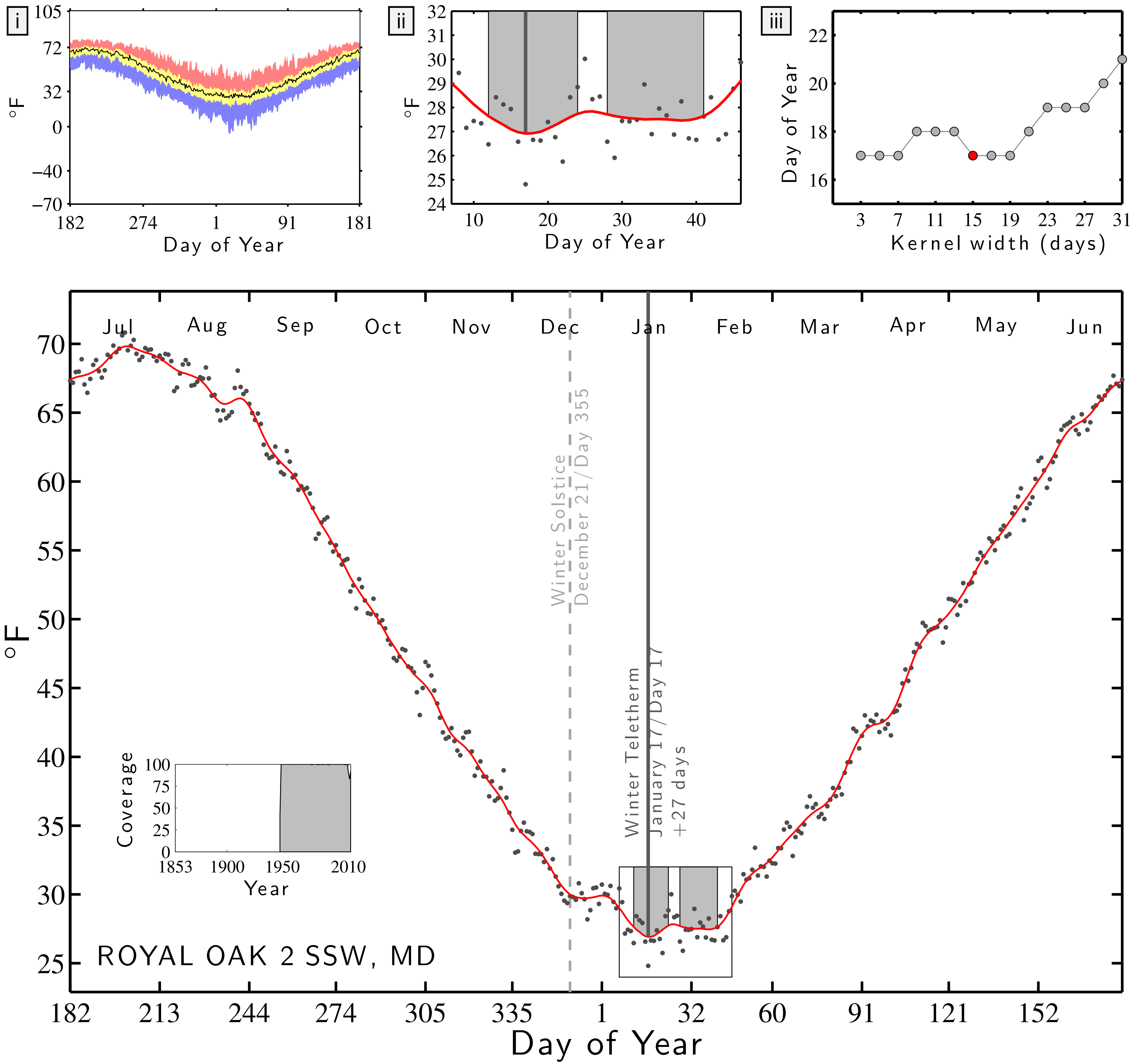

i
ii
iii
°F
Day of Year
Kernel width (days)
Jul
Aug
Sep
Oct
Nov
Dec
Jan
Feb
Mar
Apr
May
Jun
Winter Solstice
December 21/Day 355
Winter Teletherm
January 17/Day 17
+27 days
Coverage
Year
ROYAL OAK 2 SSW, MD

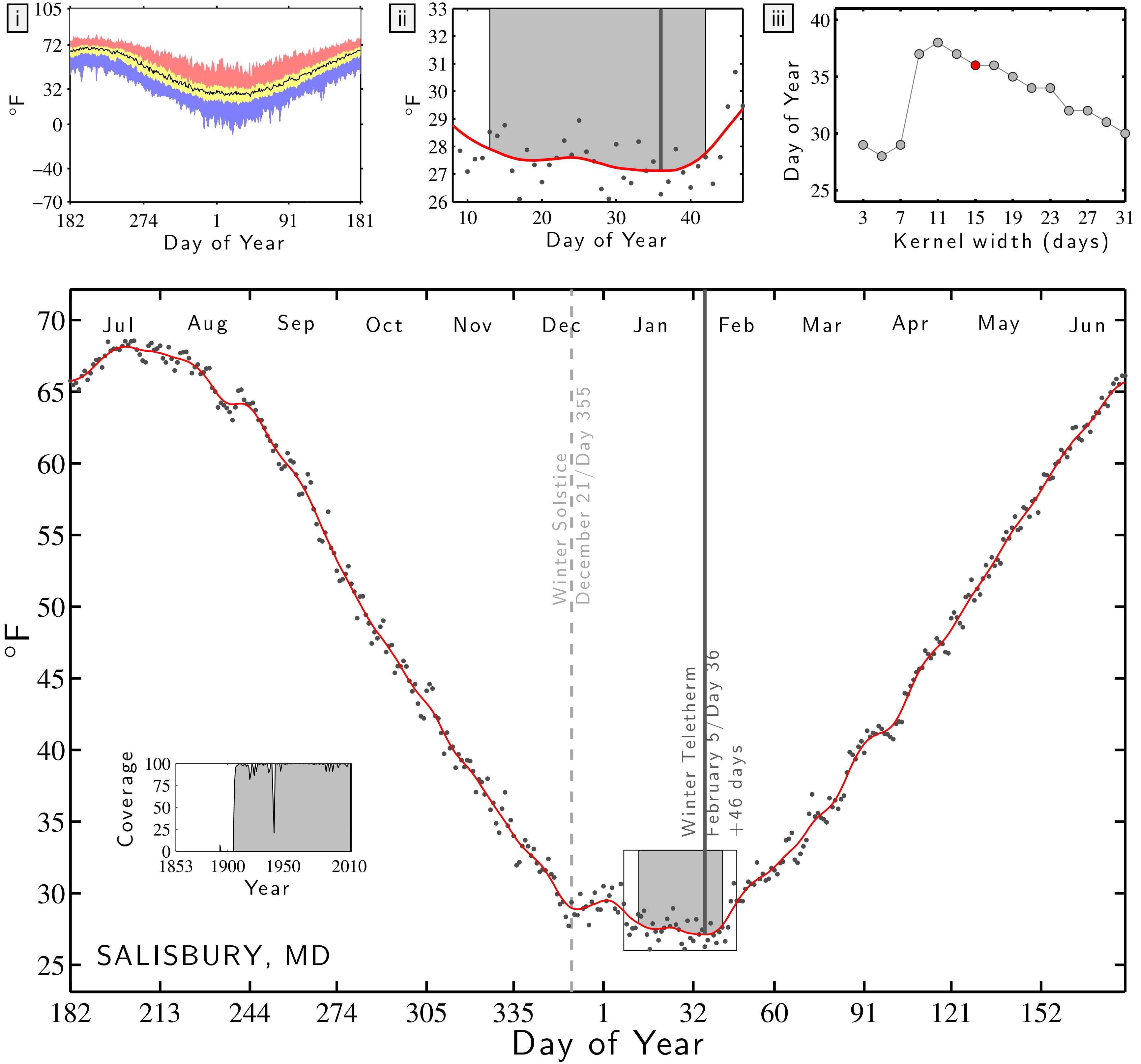

i
ii
iii
Day of Year
°F
Kernel width (days)
Jul
Aug
Sep
Oct
Nov
Dec
Jan
Feb
Mar
Apr
May
Jun
Winter Solstice
December 21/Day 355
Winter Teletherm
February 5/Day 36
+46 days
Coverage
Year
SALISBURY, MD

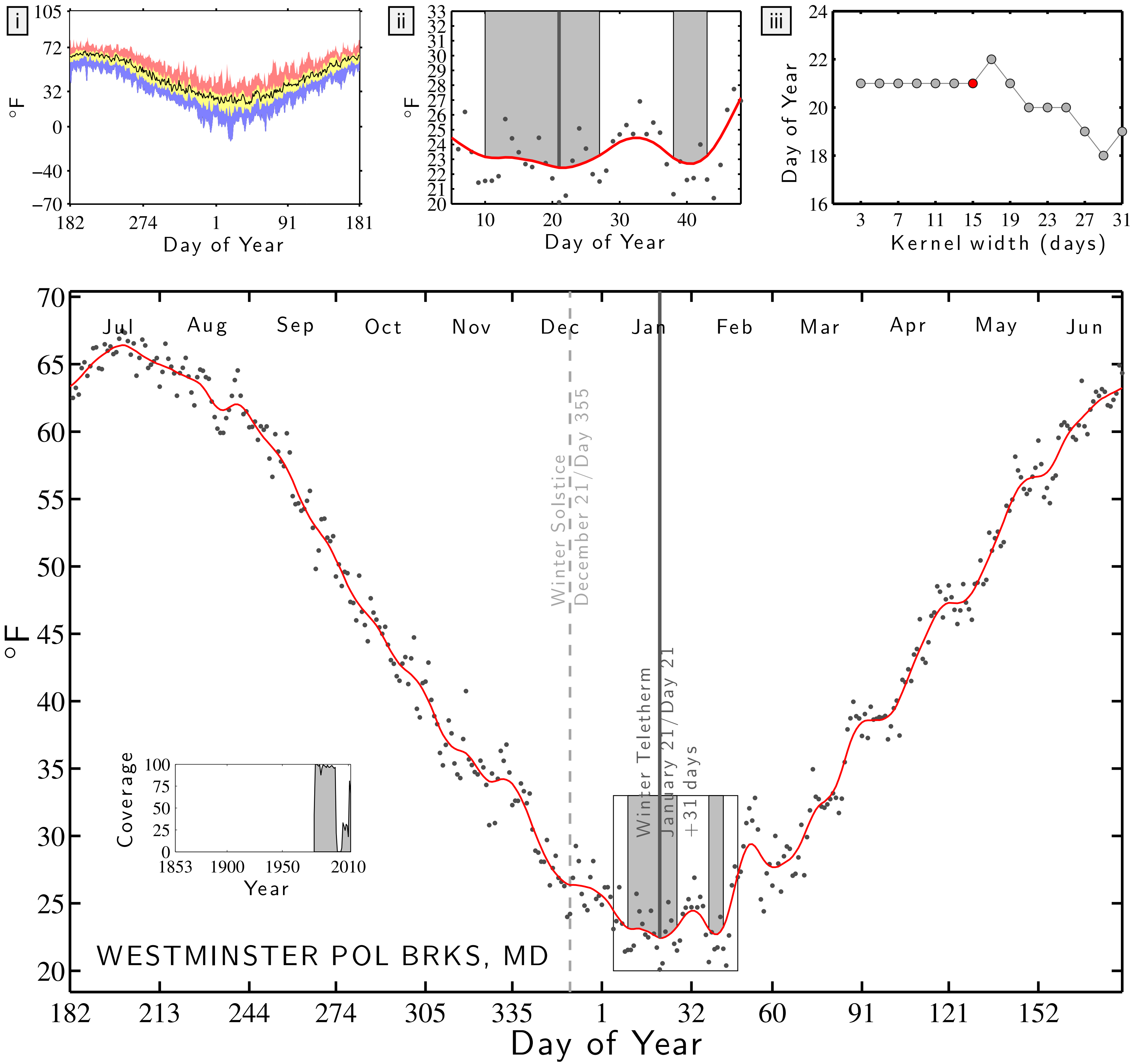

i
105
72
32
0
−40
−70
°F
182
274
1
91
181
Day of Year
ii
33
32
31
30
29
28
27
26
25
24
23
22
21
20
°F
10
20
30
40
Day of Year
iii
24
22
20
18
16
Day of Year
3
7
11
15
19
23
27
31
Kernel width (days)
70
65
60
55
50
45
40
35
30
25
20
°F
Jul
Aug
Sep
Oct
Nov
Dec
Jan
Feb
Mar
Apr
May
Jun
Winter Solstice
December 21/Day 355
Winter Teletherm
January 21/Day 21
+31 days
Coverage
100
75
50
25
0
1853
1900
1950
2010
Year
WESTMINSTER POL BRKS, MD
182
213
244
274
305
335
1
32
60
91
121
152
Day of Year

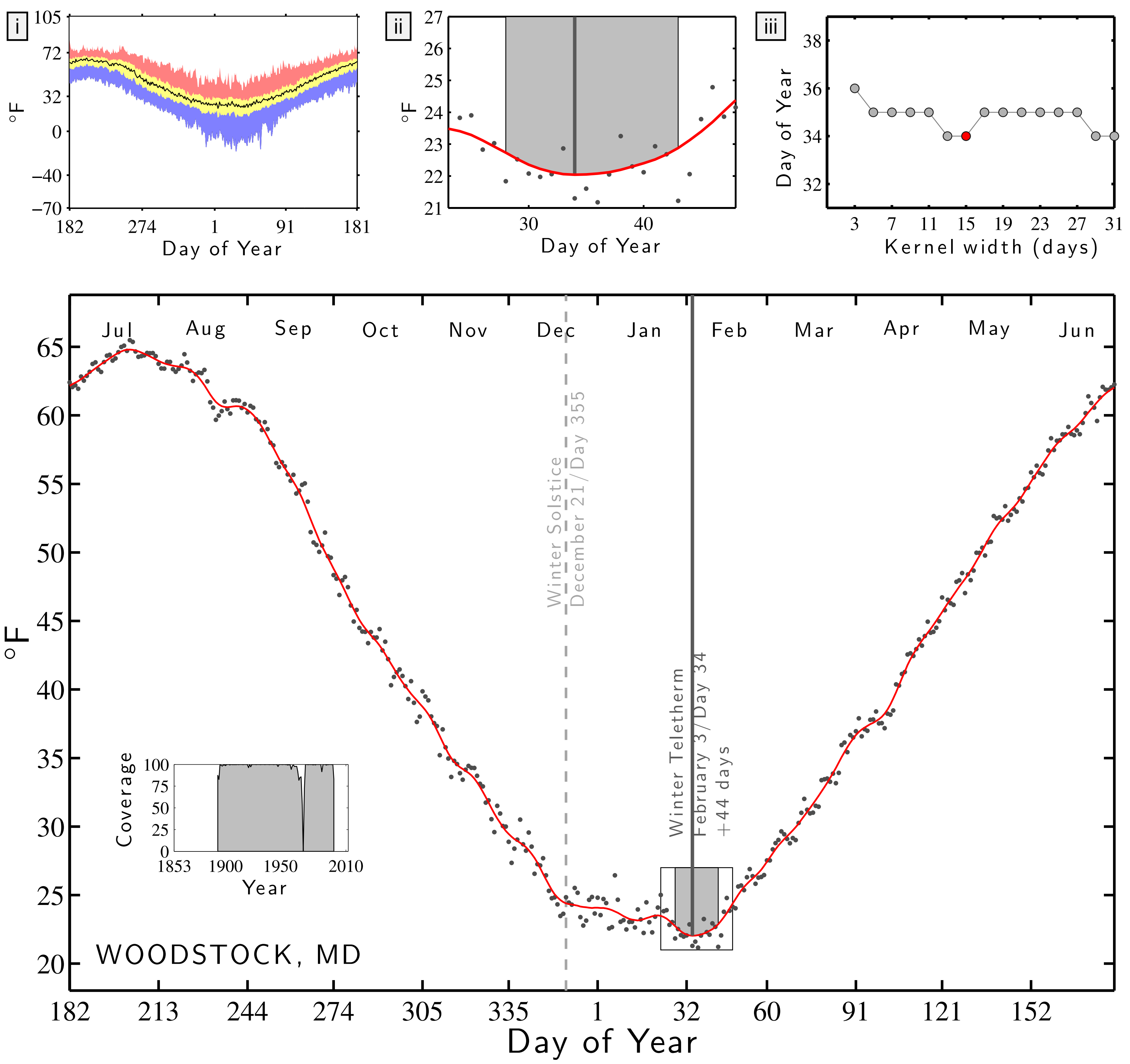

i
°F
Day of Year
ii
°F
Day of Year
iii
Day of Year
Kernel width (days)
Jul
Aug
Sep
Oct
Nov
Dec
Jan
Feb
Mar
Apr
May
Jun
Winter Solstice
December 21/Day 355
Winter Teletherm
February 3/Day 34
+44 days
Coverage
Year
WOODSTOCK, MD
°F
Day of Year

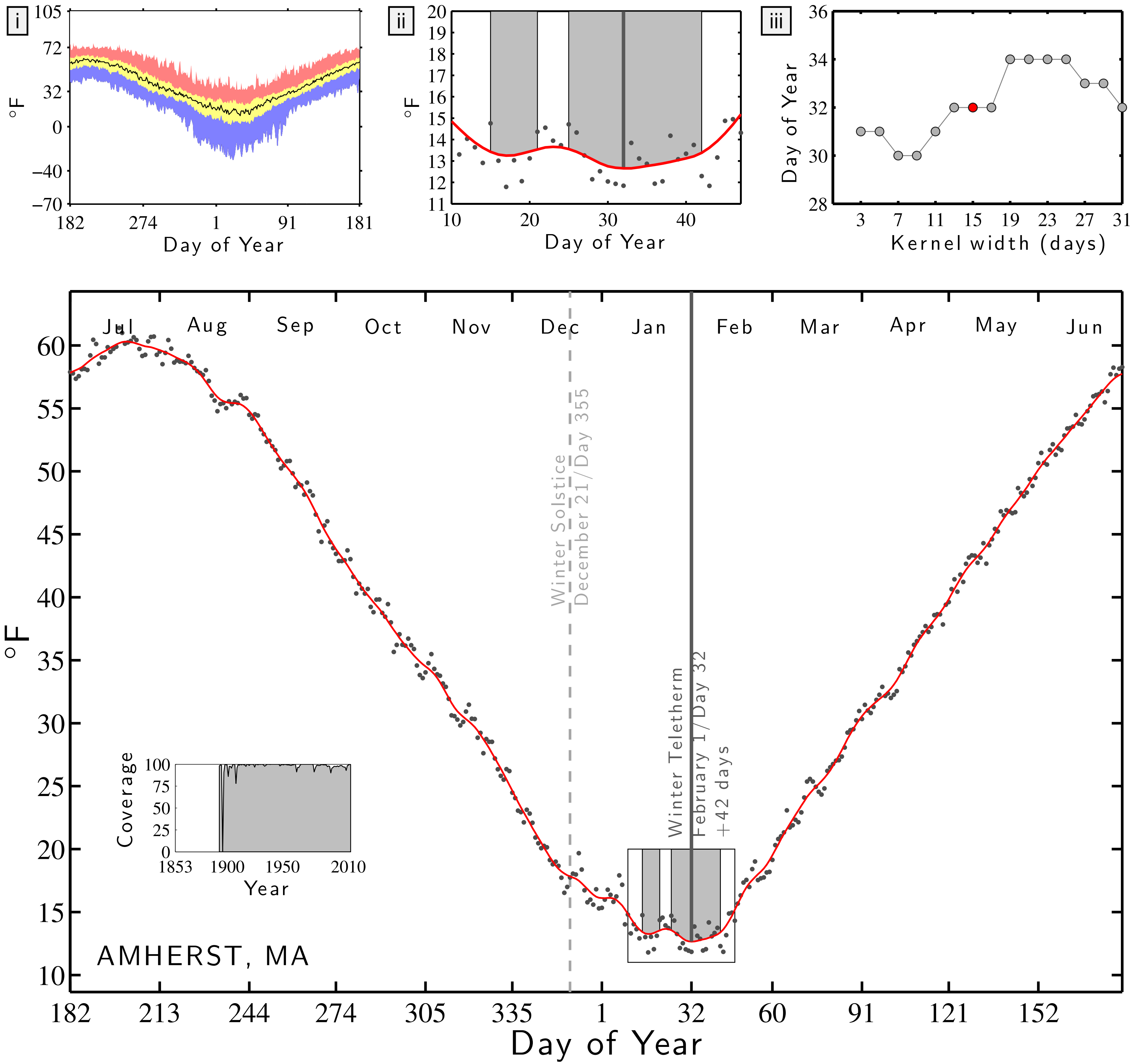

i
ii
iii
°F
Day of Year
Day of Year
Kernel width (days)
Winter Solstice
December 21/Day 355
Winter Teletherm
February 1/Day 32
+42 days
Coverage
Year
AMHERST, MA
Jul
Aug
Sep
Oct
Nov
Dec
Jan
Feb
Mar
Apr
May
Jun

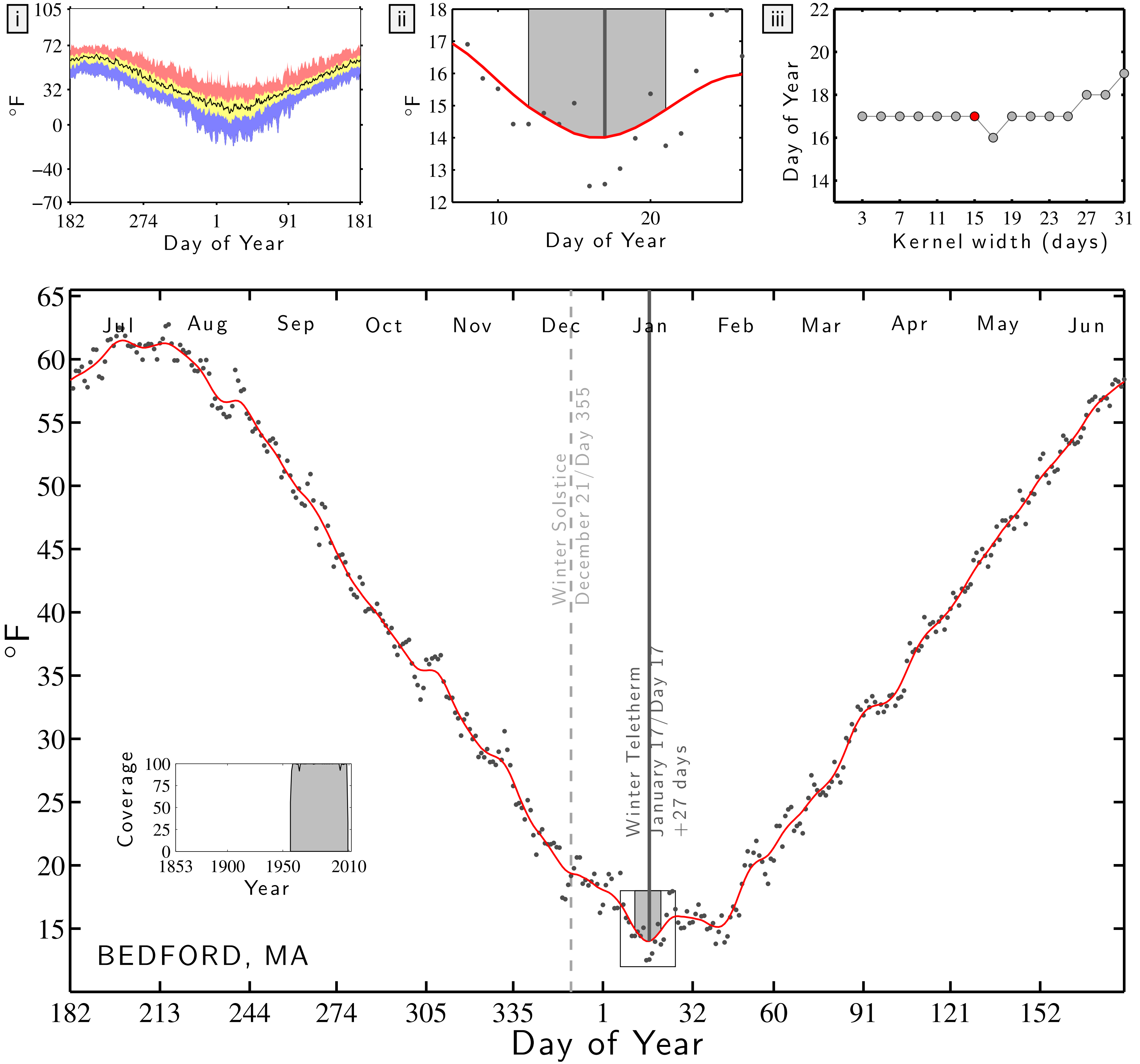

i
°F
Day of Year
ii
°F
Day of Year
iii
Day of Year
Kernel width (days)
Jul
Aug
Sep
Oct
Nov
Dec
Jan
Feb
Mar
Apr
May
Jun
Winter Solstice
December 21/Day 355
Winter Teletherm
January 17/Day 17
+27 days
Coverage
Year
BEDFORD, MA
°F
Day of Year

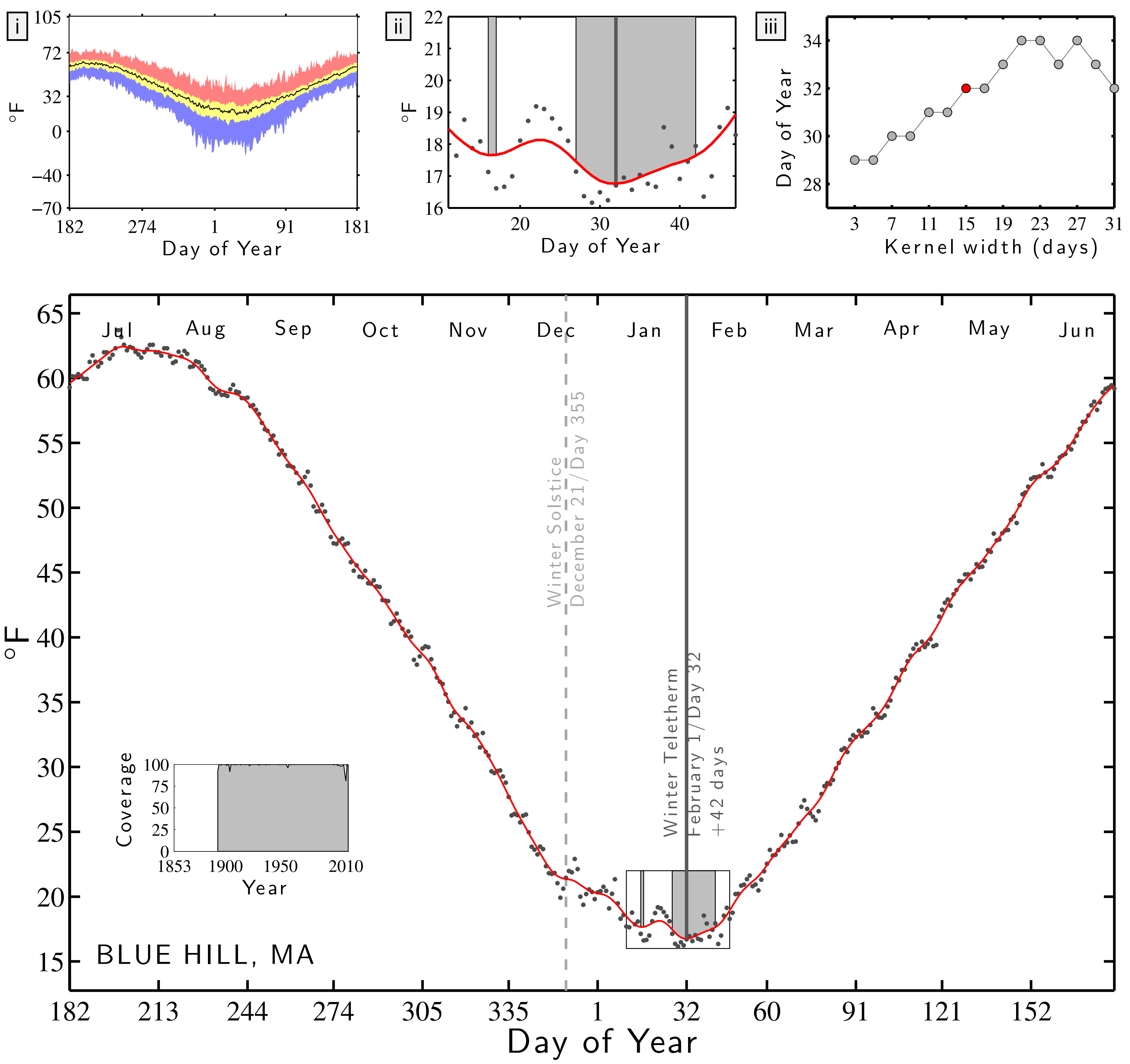

i
°F
Day of Year
ii
°F
Day of Year
iii
Day of Year
Kernel width (days)
Jul
Aug
Sep
Oct
Nov
Dec
Jan
Feb
Mar
Apr
May
Jun
Winter Solstice
December 21/Day 355
Winter Teletherm
February 1/Day 32
+42 days
Coverage
Year
BLUE HILL, MA
°F
Day of Year

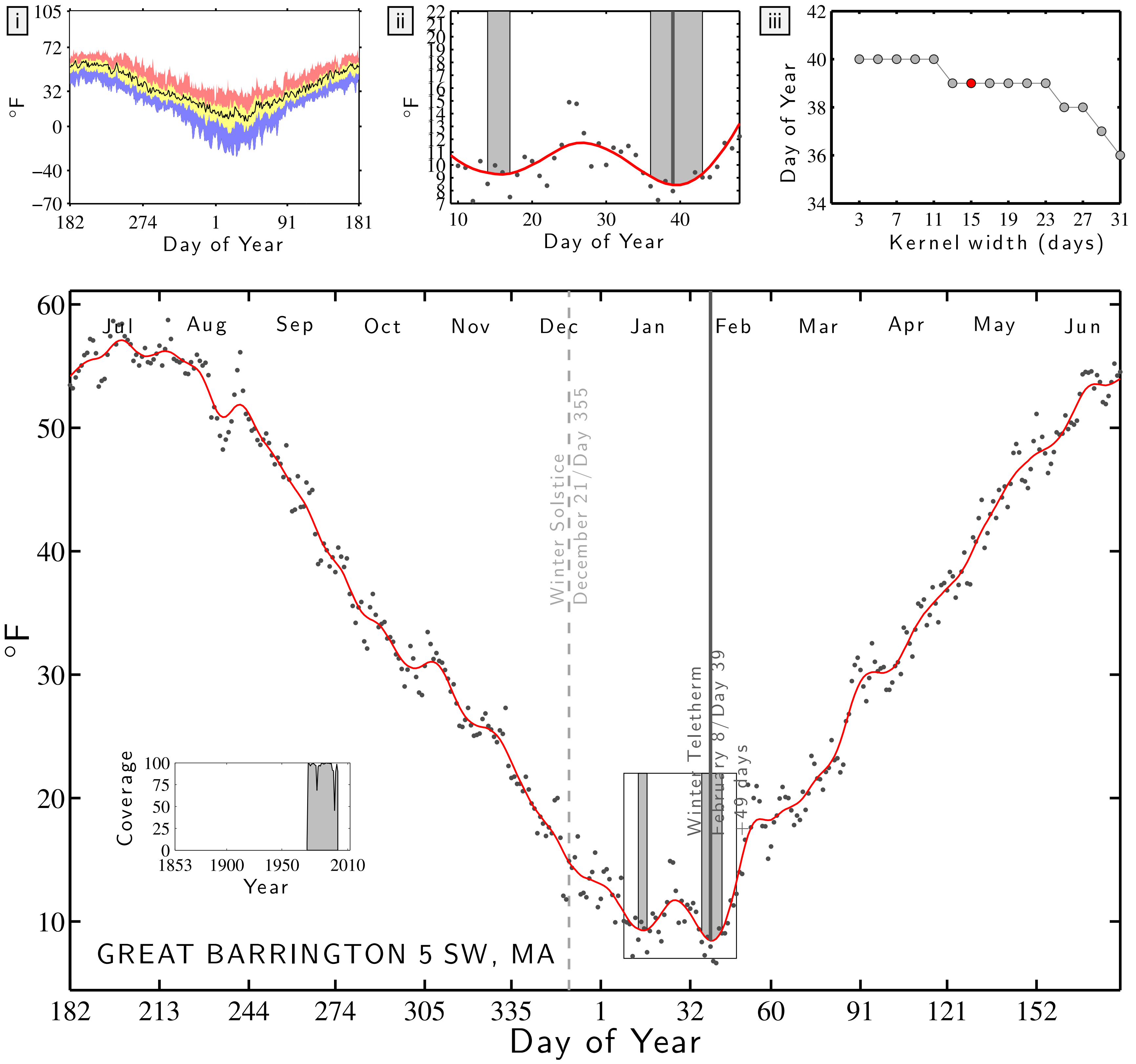

i
°F
Day of Year
ii
°F
Day of Year
iii
Day of Year
Kernel width (days)
Jul
Aug
Sep
Oct
Nov
Dec
Jan
Feb
Mar
Apr
May
Jun
Winter Solstice
December 21/Day 355
Winter Teletherm
February 8/Day 39
+49 days
Coverage
Year
GREAT BARRINGTON 5 SW, MA
°F
Day of Year

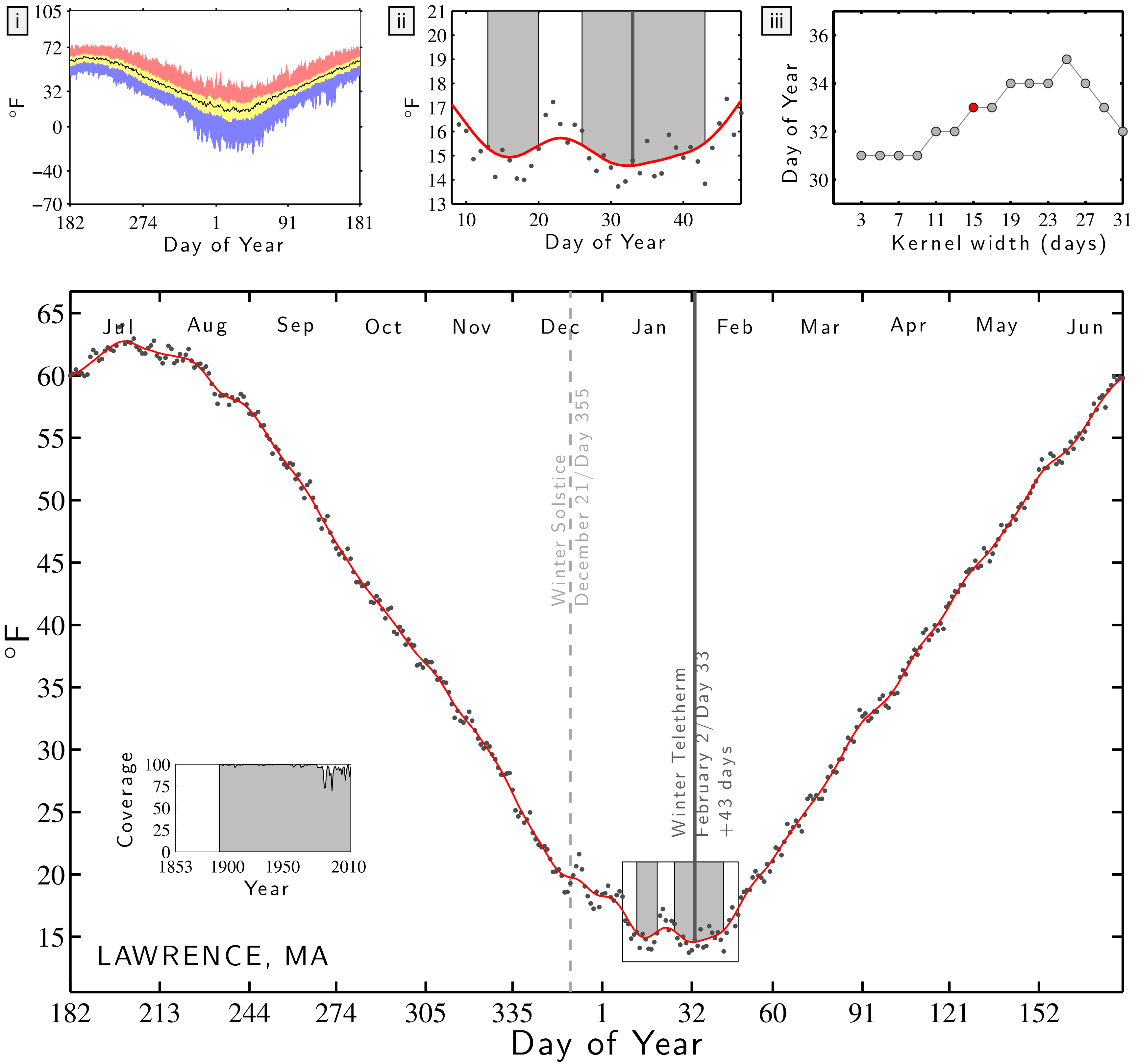

i
ii
iii
°F
Day of Year
Kernel width (days)
Jul
Aug
Sep
Oct
Nov
Dec
Jan
Feb
Mar
Apr
May
Jun
Winter Solstice
December 21/Day 355
Winter Teletherm
February 2/Day 33
+43 days
Coverage
Year
LAWRENCE, MA

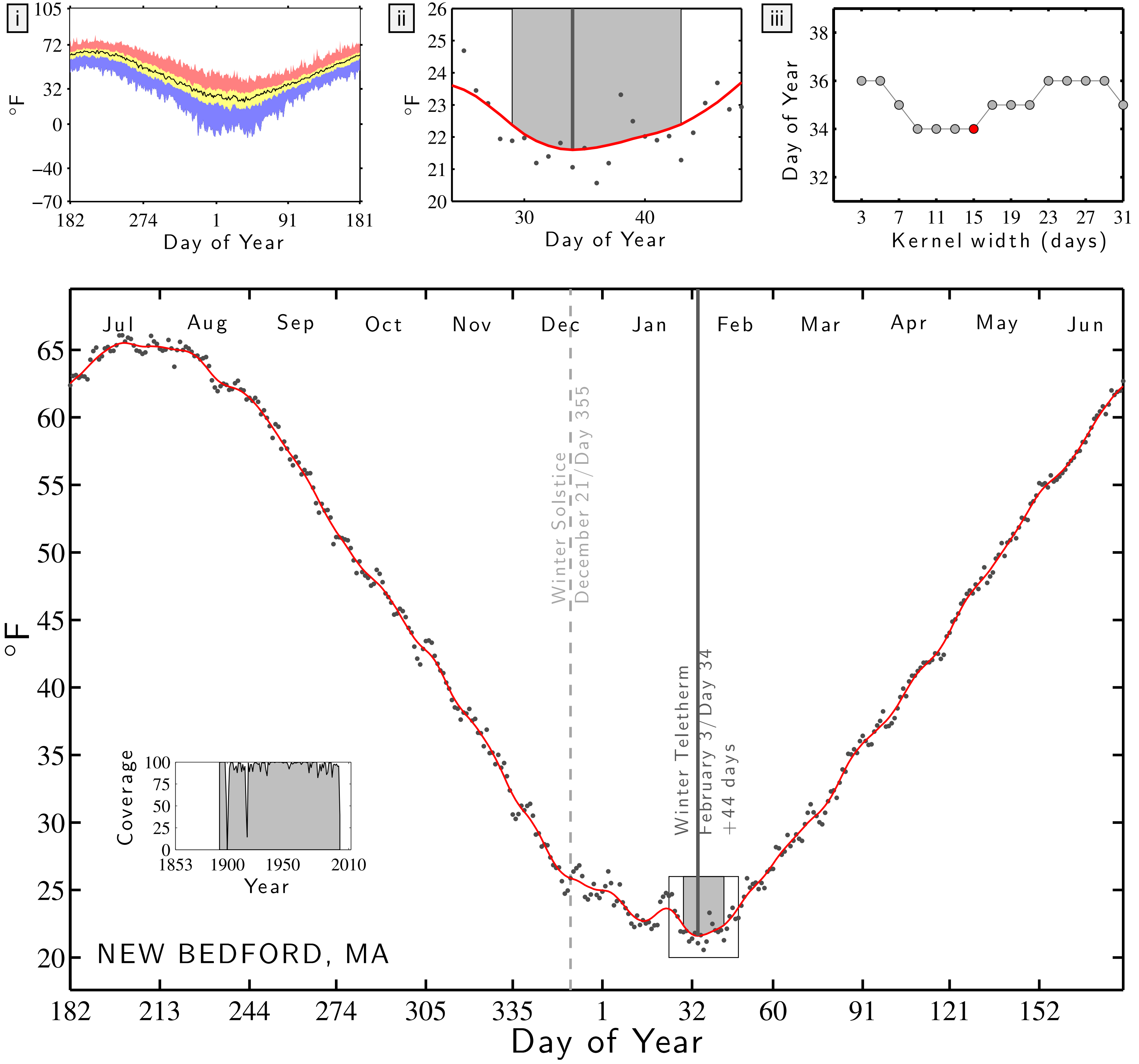

i
°F
Day of Year
ii
°F
Day of Year
iii
Day of Year
Kernel width (days)
Jul
Aug
Sep
Oct
Nov
Dec
Jan
Feb
Mar
Apr
May
Jun
Winter Solstice
December 21/Day 355
Winter Teletherm
February 3/Day 34
+44 days
Coverage
Year
NEW BEDFORD, MA
°F
Day of Year

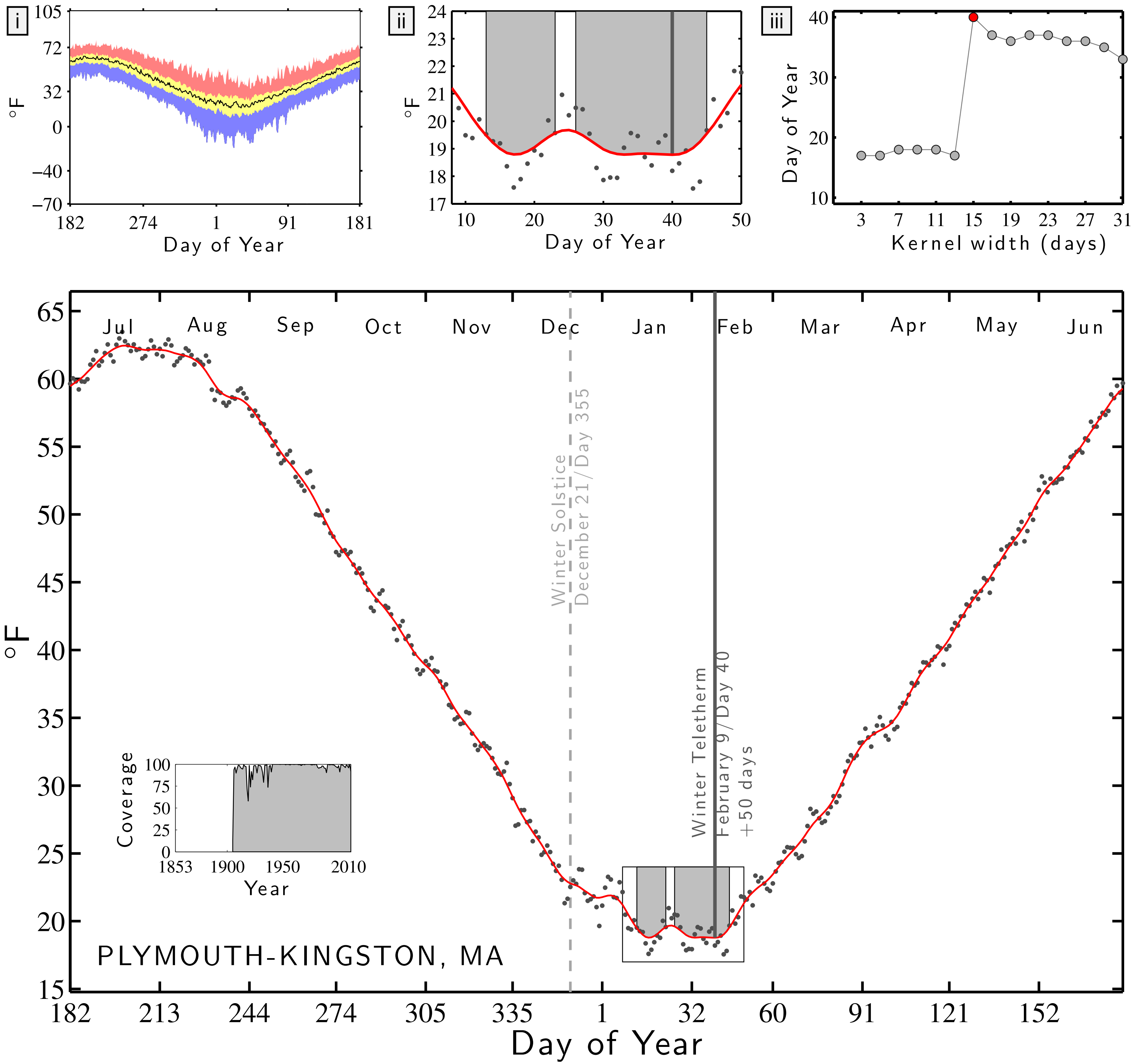

i
°F
105
72
32
0
−40
−70
182
274
1
91
181
Day of Year
ii
°F
24
23
22
21
20
19
18
17
10
20
30
40
50
Day of Year
iii
Day of Year
40
30
20
10
3
7
11
15
19
23
27
31
Kernel width (days)
°F
Jul
Aug
Sep
Oct
Nov
Dec
Jan
Feb
Mar
Apr
May
Jun
Winter Solstice
December 21/Day 355
Winter Teletherm
February 9/Day 40
+50 days
Coverage
100
75
50
25
0
1853
1900
1950
2010
Year
PLYMOUTH-KINGSTON, MA
65
60
55
50
45
40
35
30
25
20
15
182
213
244
274
305
335
1
32
60
91
121
152
Day of Year

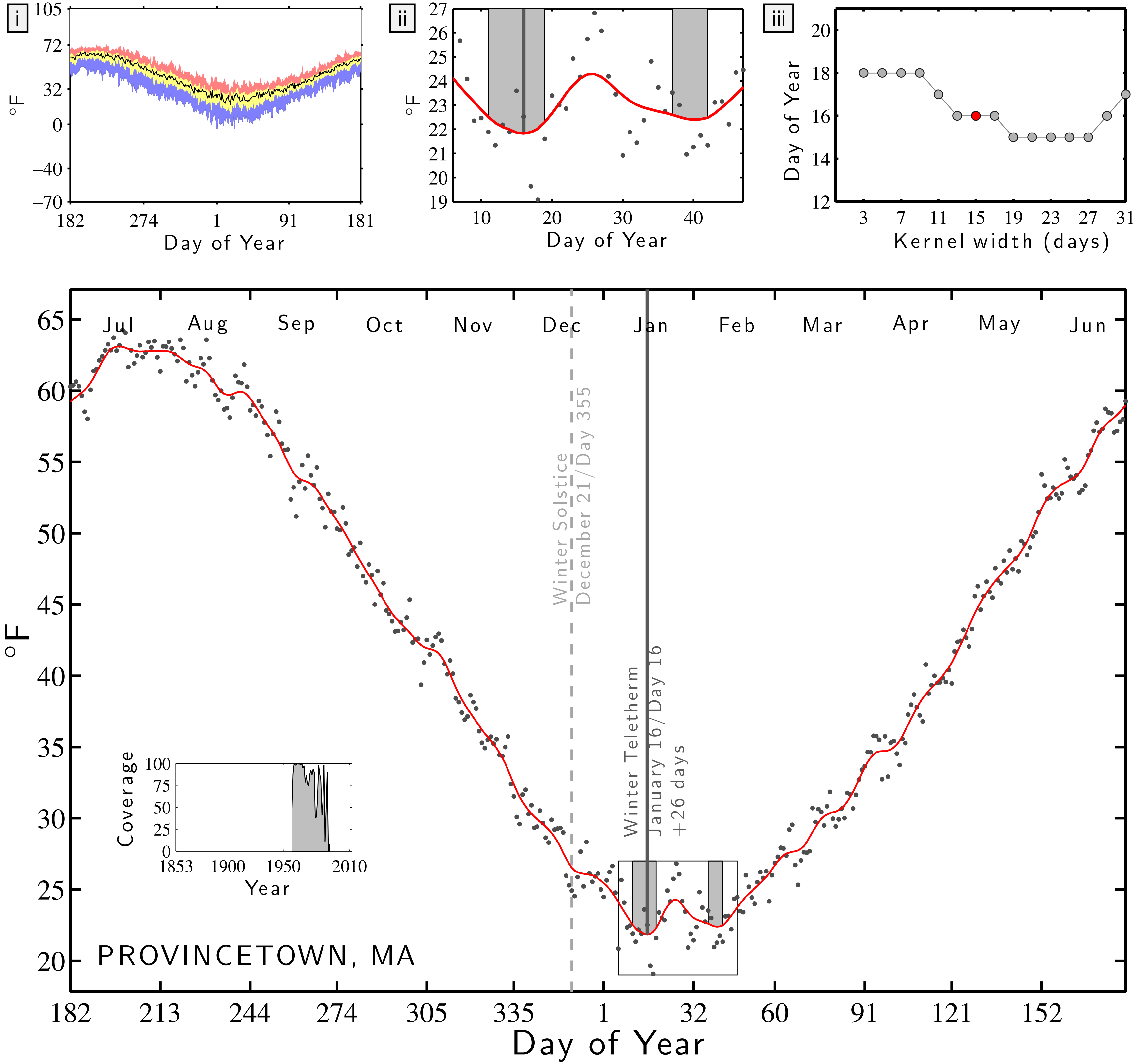

i
°F
Day of Year
ii
°F
Day of Year
iii
Day of Year
Kernel width (days)
Jul
Aug
Sep
Oct
Nov
Dec
Jan
Feb
Mar
Apr
May
Jun
Winter Solstice
December 21/Day 355
Winter Teletherm
January 16/Day 16
+26 days
Coverage
Year
PROVINCETOWN, MA
°F
Day of Year

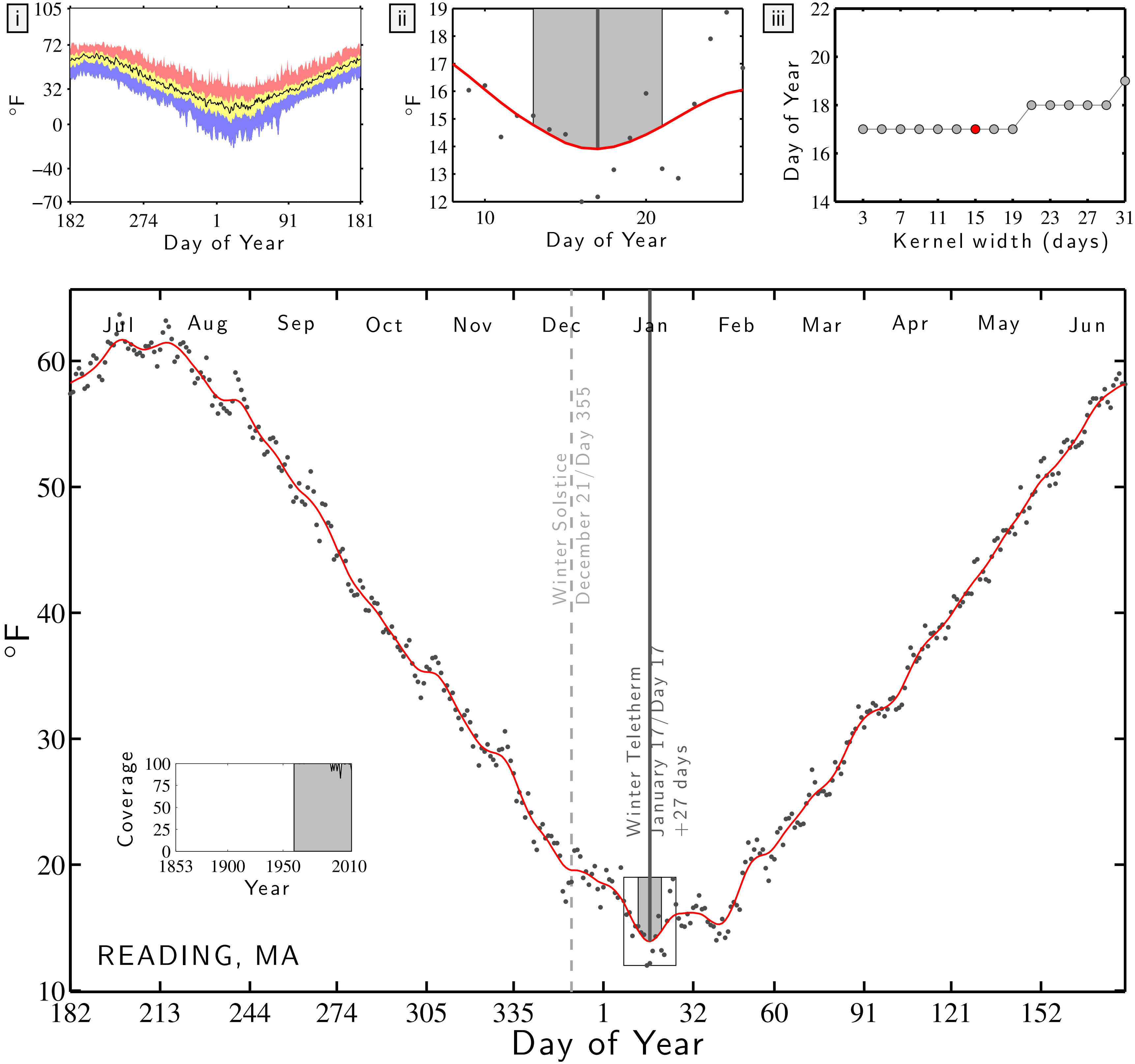

i
ii
iii
°F
Day of Year
Kernel width (days)
Jul
Aug
Sep
Oct
Nov
Dec
Jan
Feb
Mar
Apr
May
Jun
Winter Solstice
December 21/Day 355
Winter Teletherm
January 17/Day 17
+27 days
Coverage
Year
READING, MA

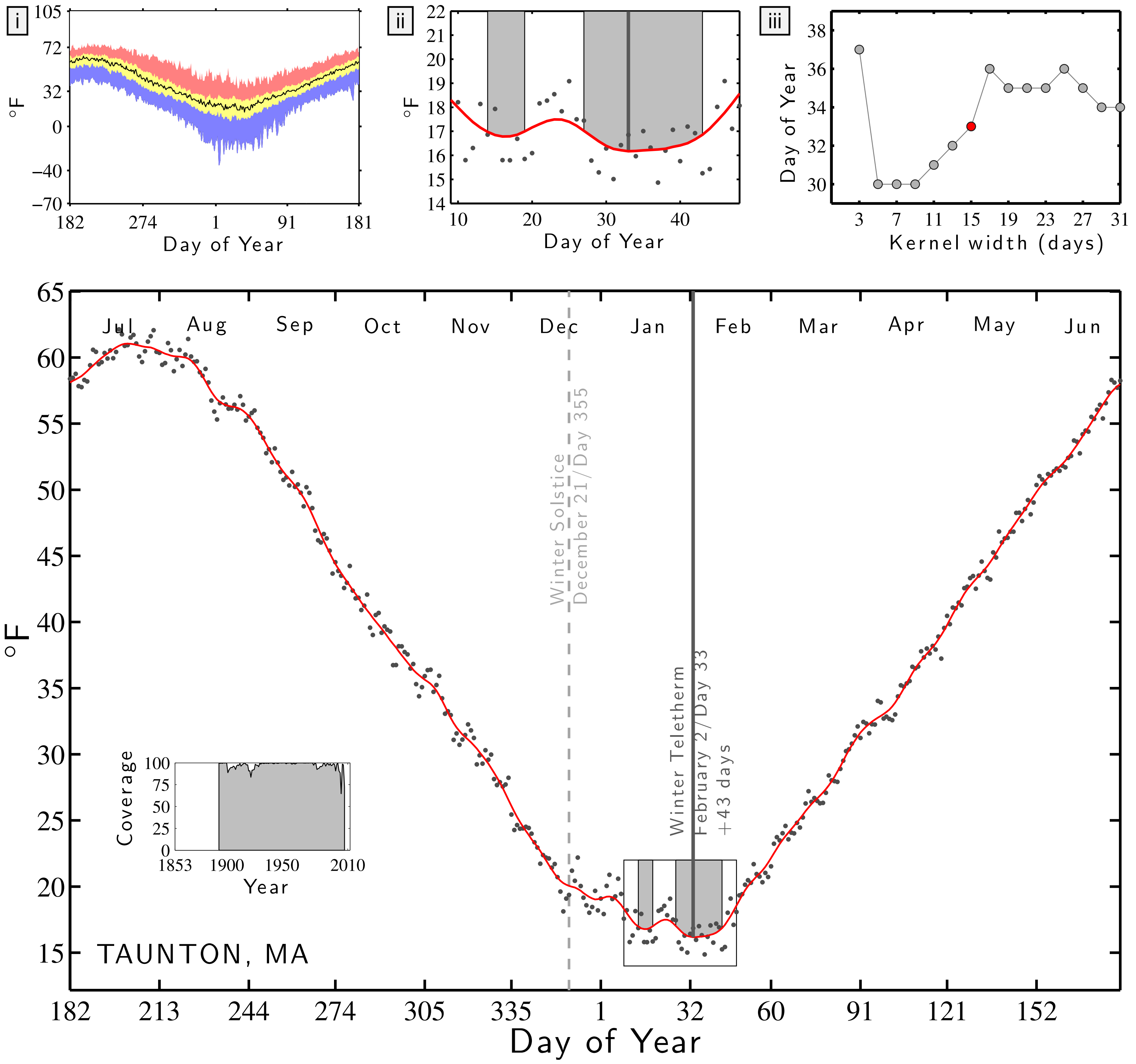

i
°F
Day of Year
ii
Day of Year
iii
Day of Year
Kernel width (days)
Jul
Aug
Sep
Oct
Nov
Dec
Jan
Feb
Mar
Apr
May
Jun
Winter Solstice
December 21/Day 355
Winter Teletherm
February 2/Day 33
+43 days
Coverage
Year
TAUNTON, MA
Day of Year

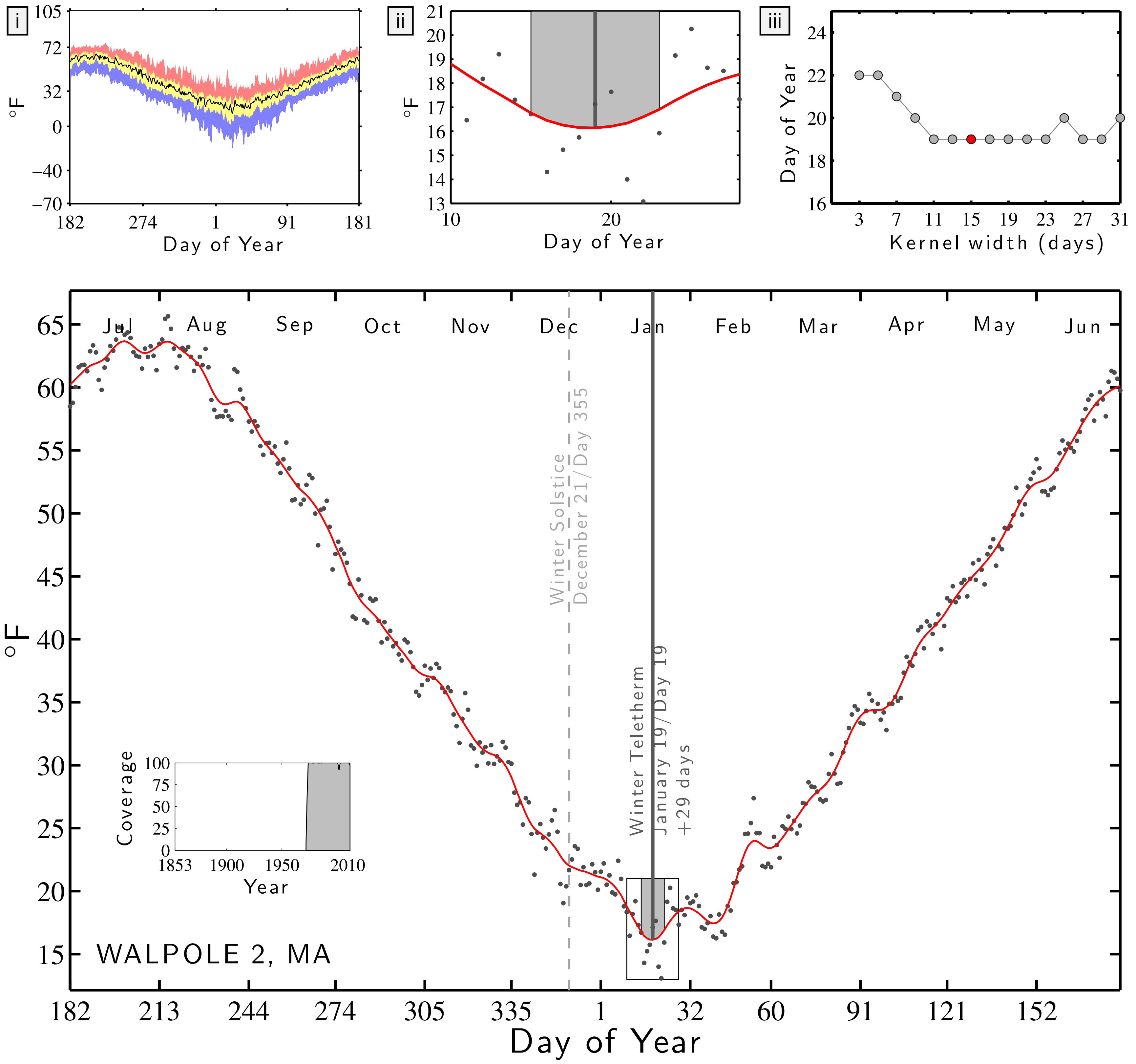

i
ii
iii
°F
Day of Year
Day of Year
Kernel width (days)
Jul
Aug
Sep
Oct
Nov
Dec
Jan
Feb
Mar
Apr
May
Jun
Winter Solstice
December 21/Day 355
Winter Teletherm
January 19/Day 19
+29 days
Coverage
Year
WALPOLE 2, MA

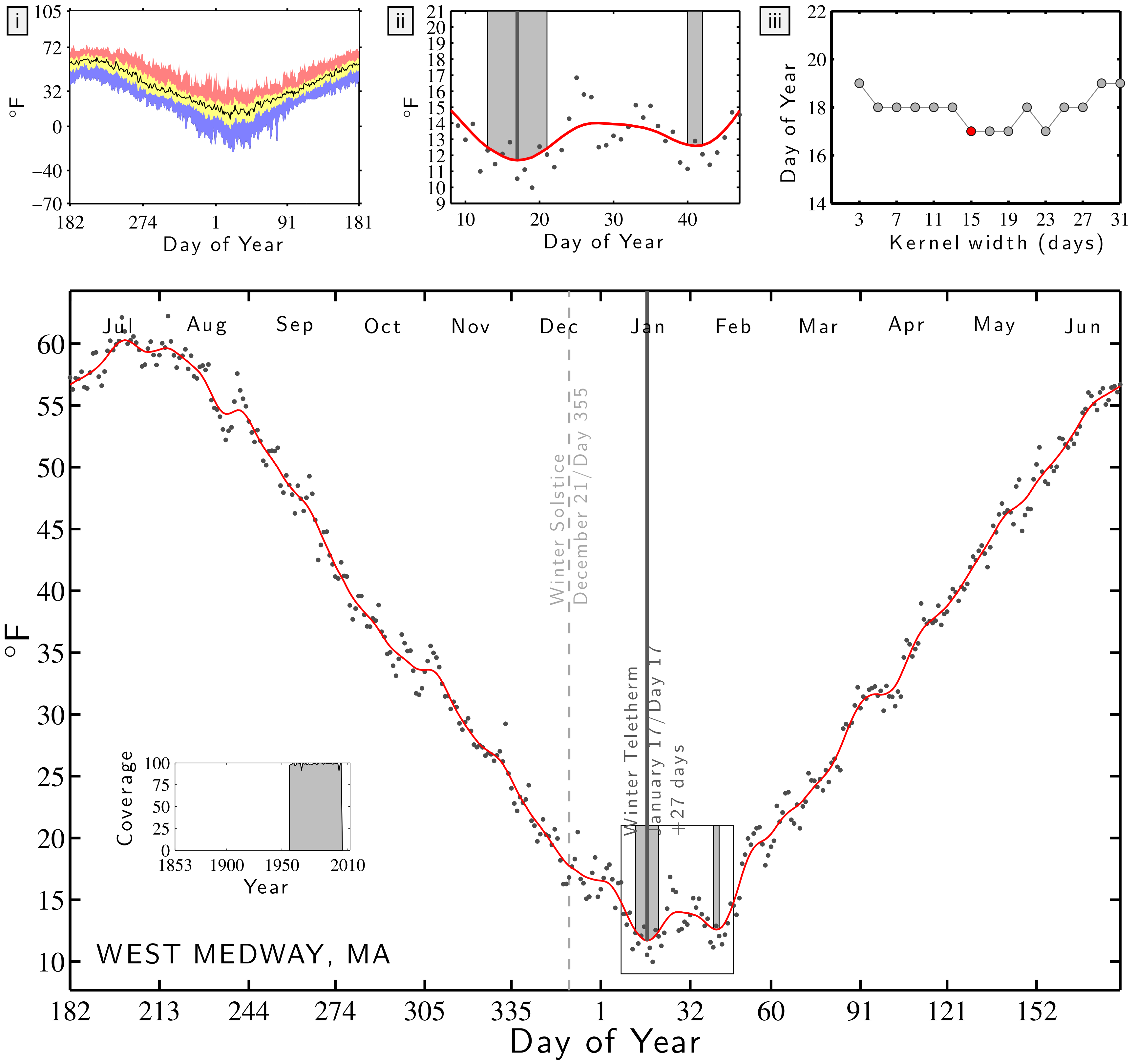

i
°F
Day of Year
ii
°F
Day of Year
iii
Day of Year
Kernel width (days)
Jul
Aug
Sep
Oct
Nov
Dec
Jan
Feb
Mar
Apr
May
Jun
Winter Solstice
December 21/Day 355
Winter Teletherm
January 17/Day 17
±27 days
Coverage
Year
WEST MEDWAY, MA
°F
Day of Year

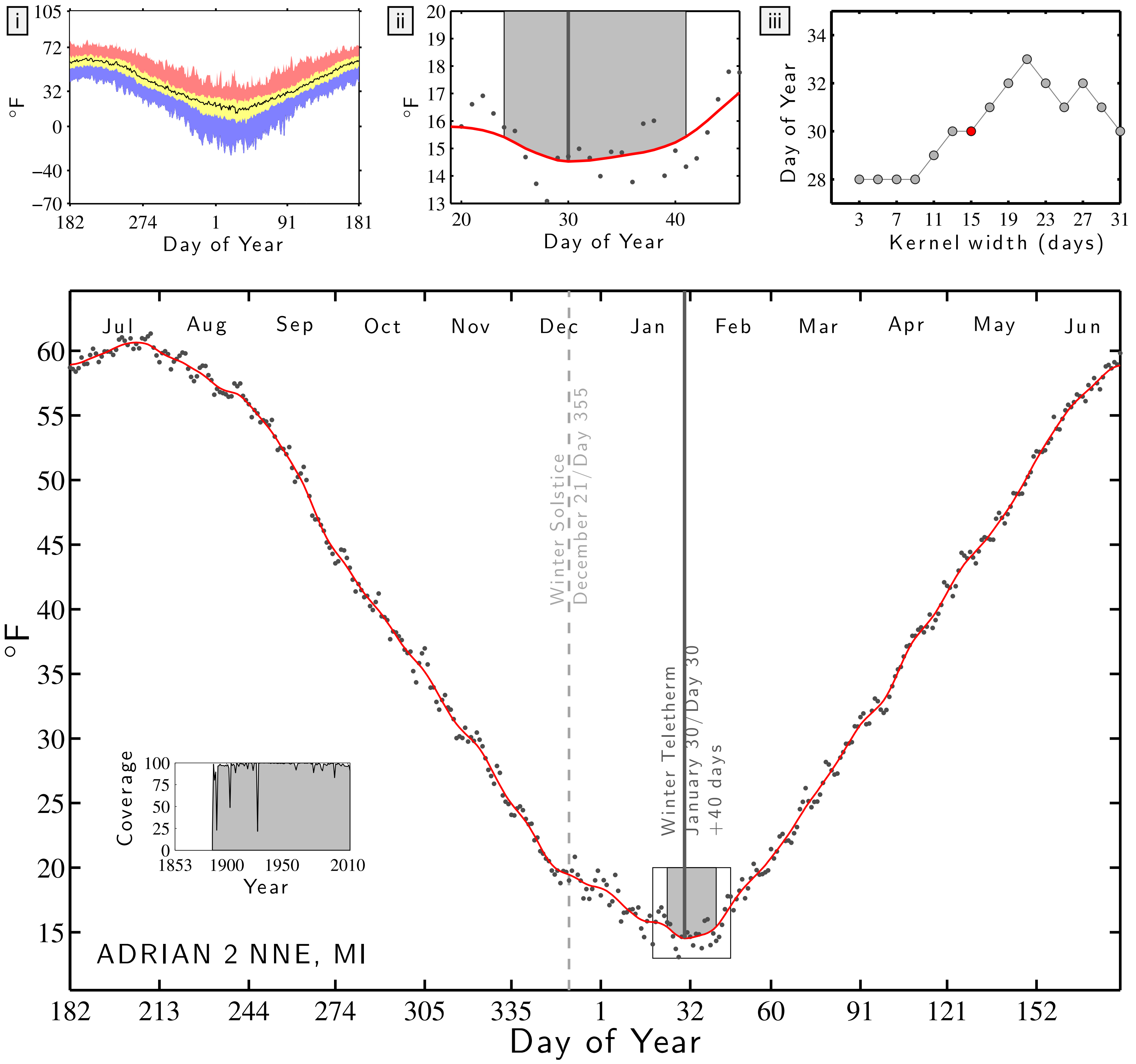

i
°F
Day of Year
ii
°F
Day of Year
iii
Day of Year
Kernel width (days)
Jul
Aug
Sep
Oct
Nov
Dec
Jan
Feb
Mar
Apr
May
Jun
Winter Solstice
December 21/Day 355
Winter Teletherm
January 30/Day 30
+40 days
Coverage
Year
ADRIAN 2 NNE, MI
°F
Day of Year

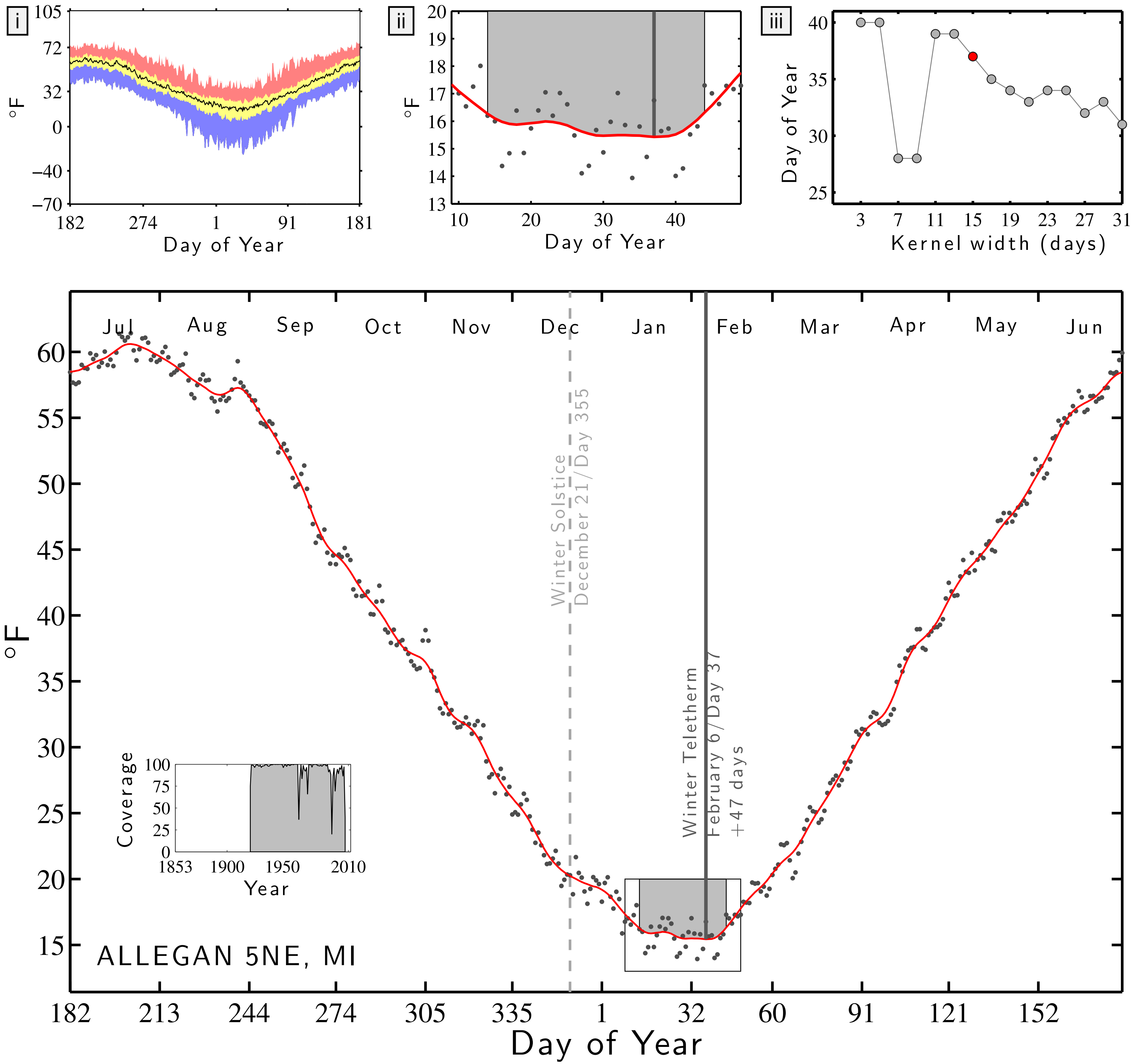

i
Day of Year
°F
ii
Day of Year
°F
iii
Kernel width (days)
Day of Year
Jul
Aug
Sep
Oct
Nov
Dec
Jan
Feb
Mar
Apr
May
Jun
Winter Solstice
December 21/Day 355
Winter Teletherm
February 6/Day 37
+47 days
Coverage
Year
ALLEGAN 5NE, MI
°F
Day of Year

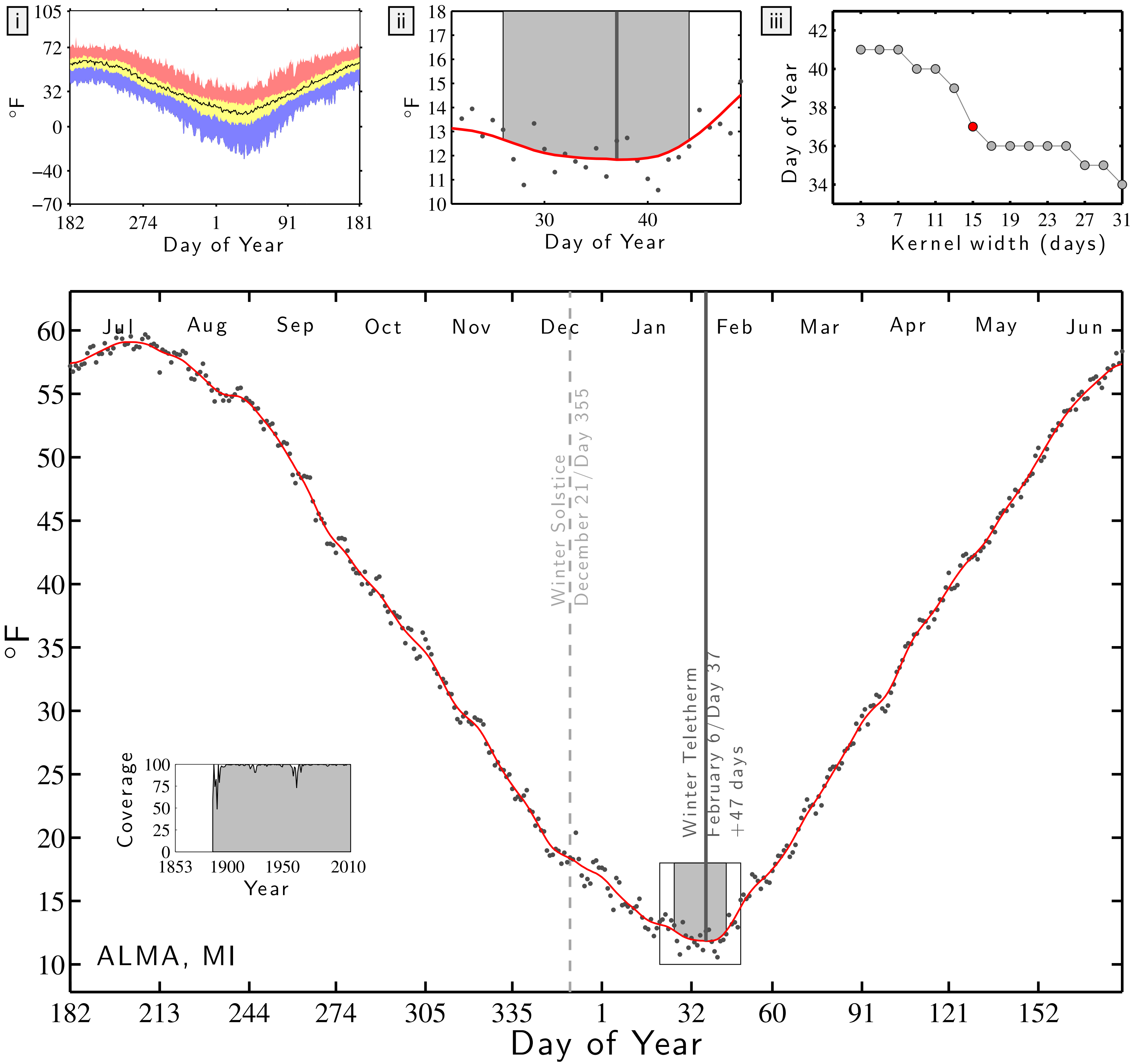
i
ii
iii
°F
Day of Year
Kernel width (days)
Jul
Aug
Sep
Oct
Nov
Dec
Jan
Feb
Mar
Apr
May
Jun
Winter Solstice
December 21/Day 355
Winter Teletherm
February 6/Day 37
+47 days
Coverage
Year
ALMA, MI

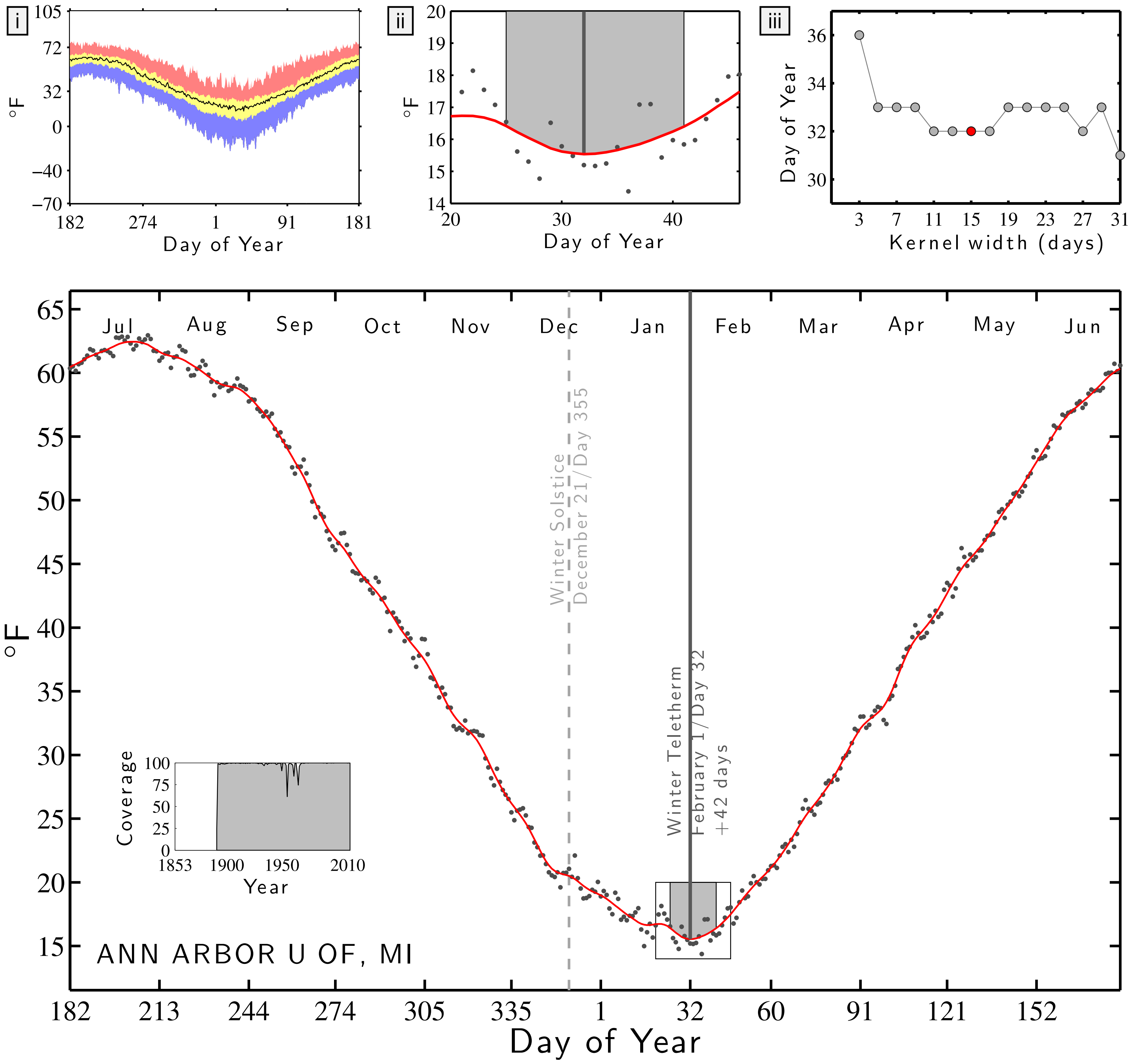

i
°F
Day of Year
ii
°F
Day of Year
iii
Day of Year
Kernel width (days)
Jul
Aug
Sep
Oct
Nov
Dec
Jan
Feb
Mar
Apr
May
Jun
Winter Solstice
December 21/Day 355
Winter Teletherm
February 1/Day 32
+42 days
Coverage
Year
ANN ARBOR U OF, MI
°F
Day of Year

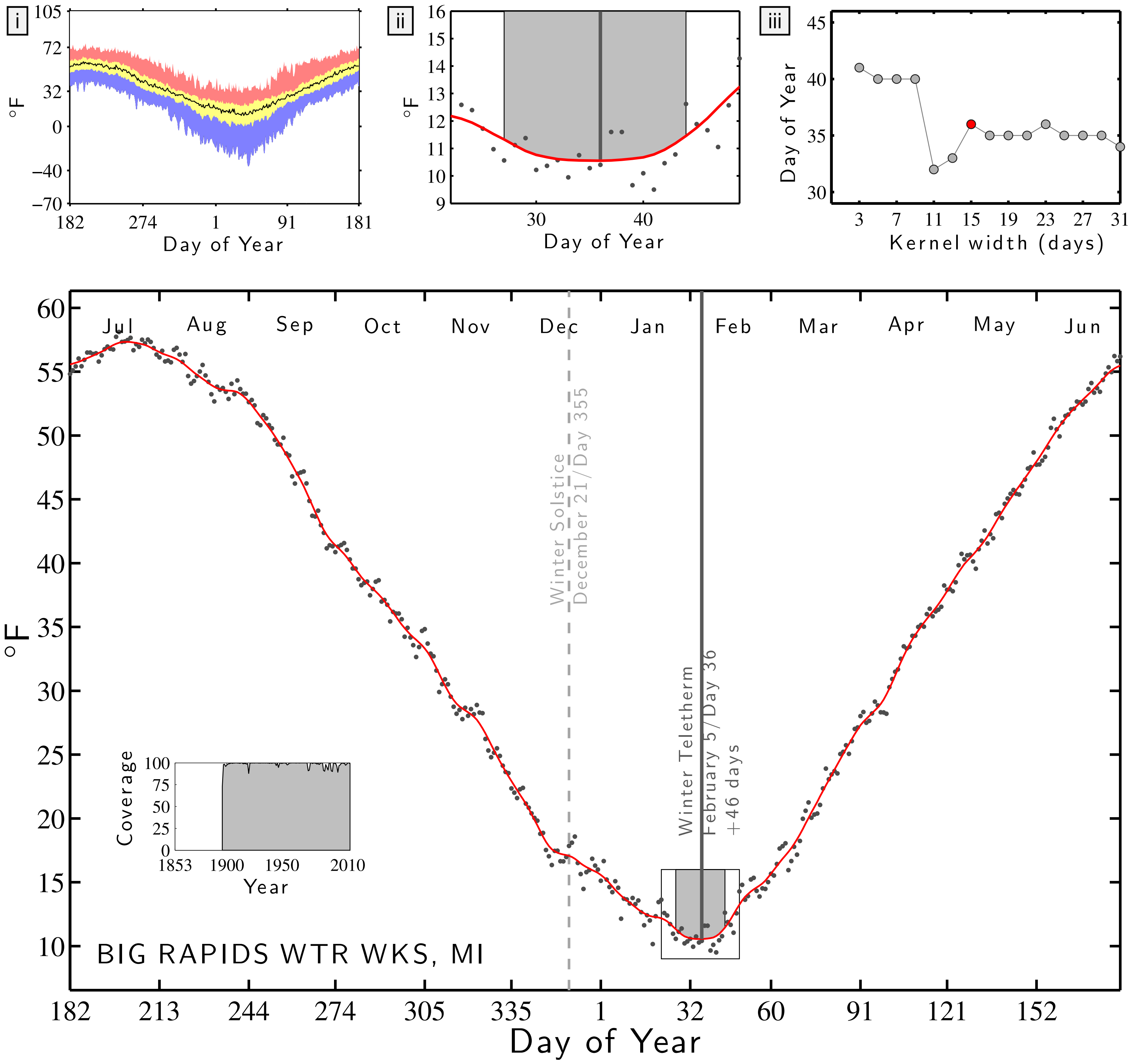

i
ii
iii
°F
Day of Year
Kernel width (days)
Jul
Aug
Sep
Oct
Nov
Dec
Jan
Feb
Mar
Apr
May
Jun
Winter Solstice
December 21/Day 355
Winter Teletherm
February 5/Day 36
+46 days
Coverage
Year
BIG RAPIDS WTR WKS, MI

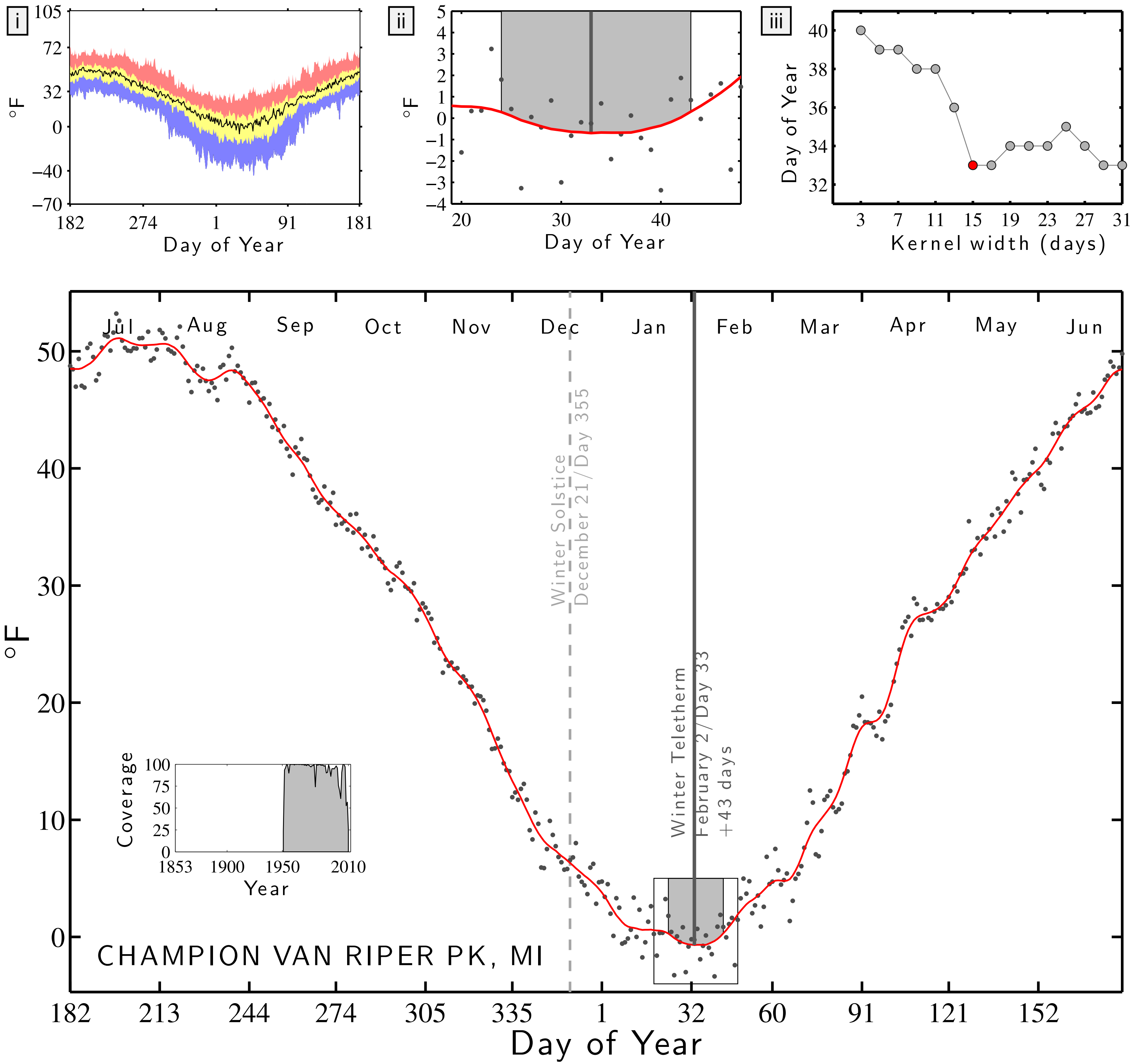

i
°F
Day of Year
ii
°F
Day of Year
iii
Day of Year
Kernel width (days)
Jul Aug Sep Oct Nov Dec Jan Feb Mar Apr May Jun
Winter Solstice
December 21/Day 355
Winter Teletherm
February 2/Day 33
+43 days
Coverage
Year
CHAMPION VAN RIPER PK, MI
°F
Day of Year

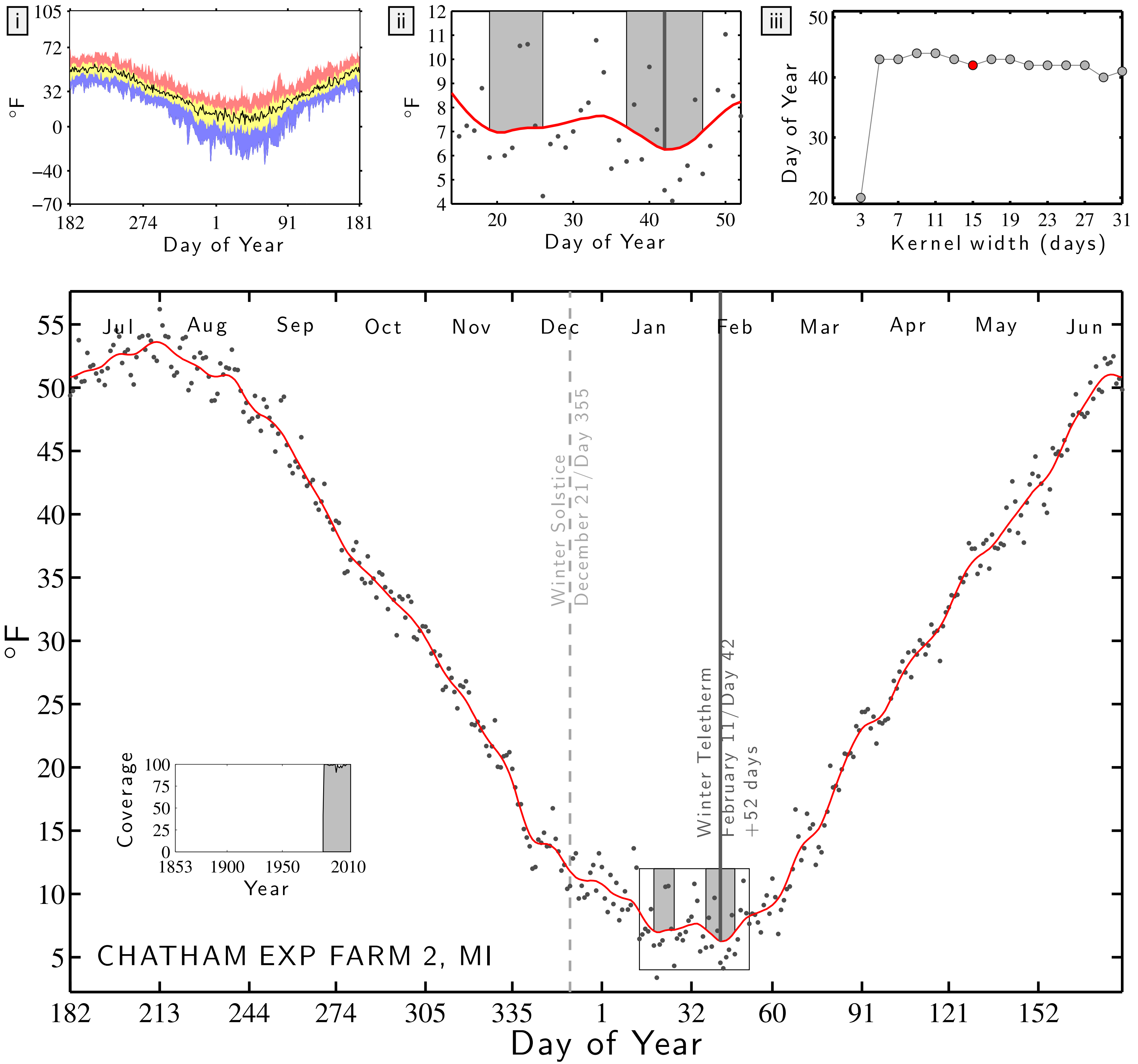

i
ii
iii
°F
Day of Year
Day of Year
Kernel width (days)
Jul
Aug
Sep
Oct
Nov
Dec
Jan
Feb
Mar
Apr
May
Jun
Winter Solstice
December 21/Day 355
Winter Teletherm
February 11/Day 42
+52 days
Coverage
Year
CHATHAM EXP FARM 2, MI
Day of Year

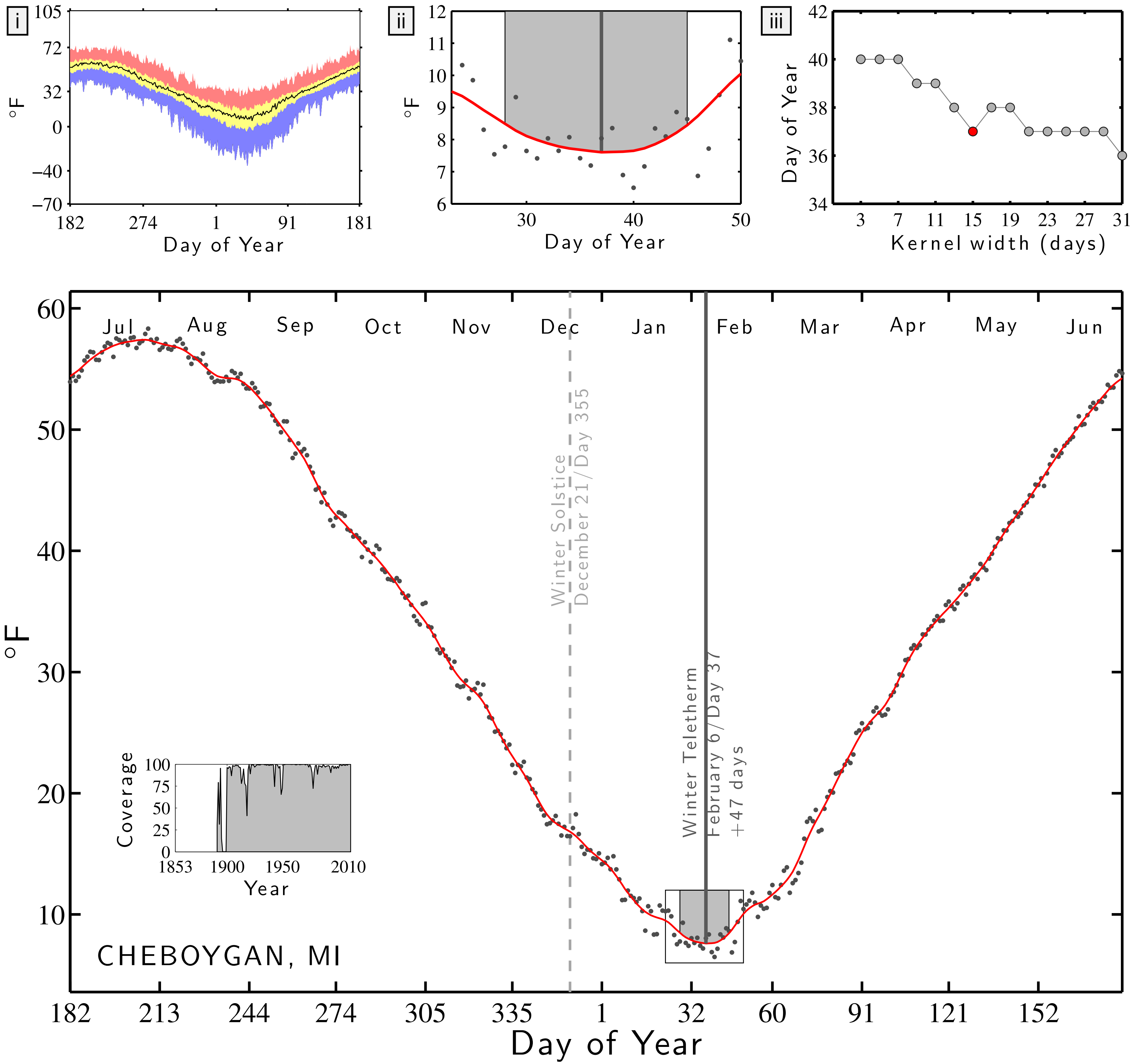

i
°F
Day of Year
ii
°F
Day of Year
iii
Day of Year
Kernel width (days)
Jul Aug Sep Oct Nov Dec Jan Feb Mar Apr May Jun
Winter Solstice
December 21/Day 355
Winter Teletherm
February 6/Day 37
+47 days
Coverage
Year
CHEBOYGAN, MI
°F
Day of Year

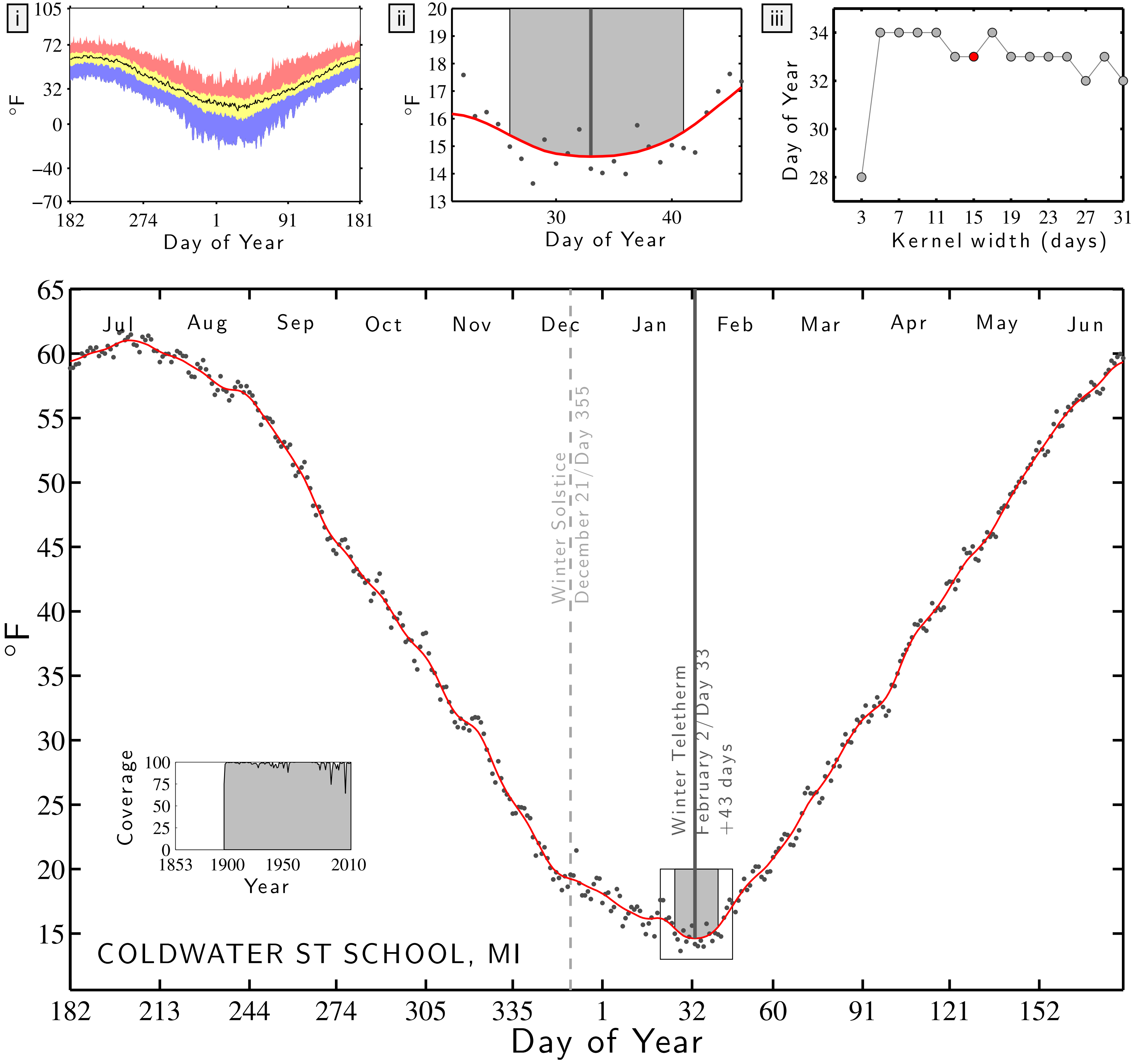

i
ii
iii
°F
Day of Year
Day of Year
Kernel width (days)
Jul
Aug
Sep
Oct
Nov
Dec
Jan
Feb
Mar
Apr
May
Jun
Winter Solstice
December 21/Day 355
Winter Teletherm
February 2/Day 33
+43 days
Coverage
Year
COLDWATER ST SCHOOL, MI

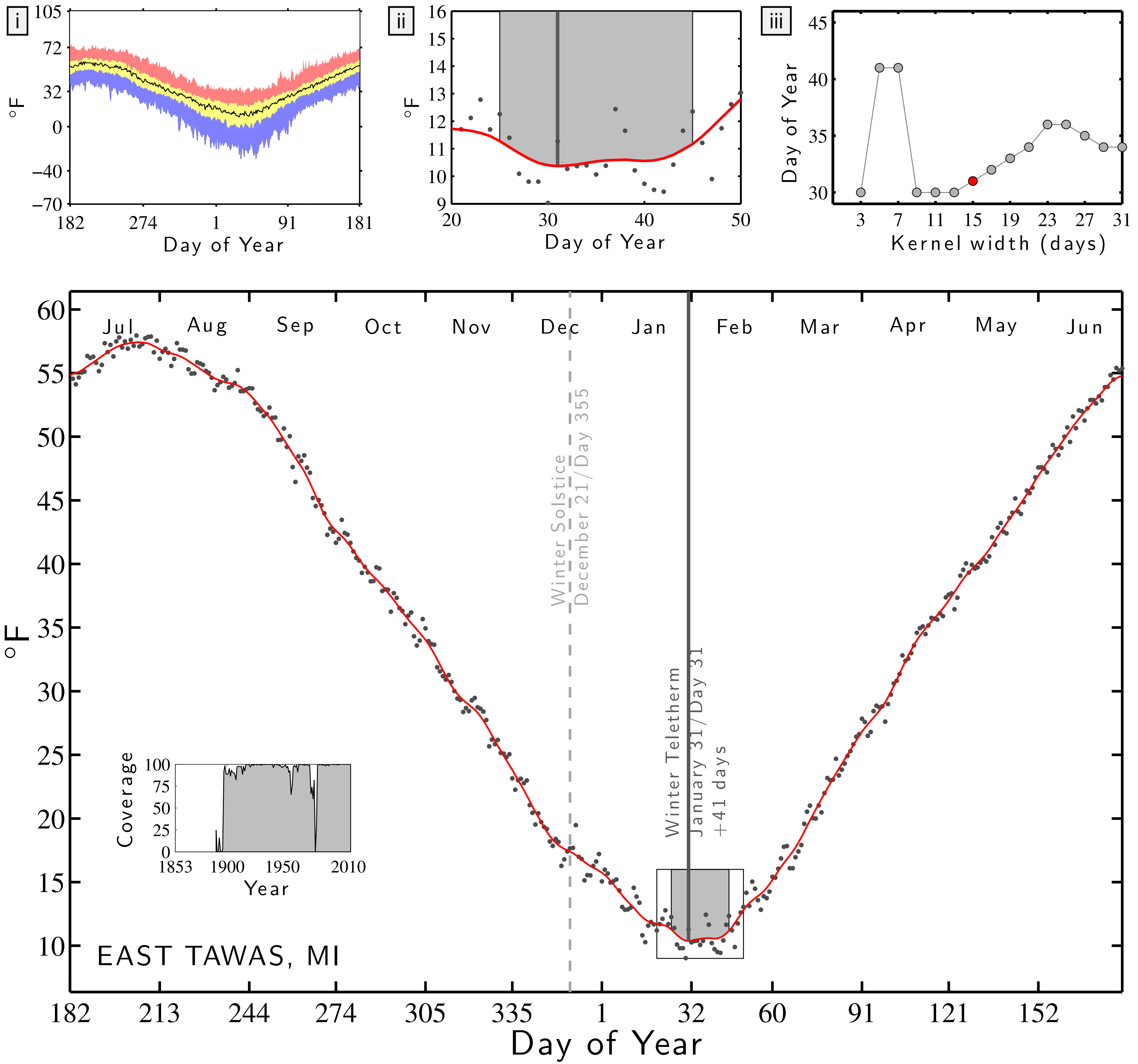

i
ii
iii
°F
Day of Year
Kernel width (days)
Jul
Aug
Sep
Oct
Nov
Dec
Jan
Feb
Mar
Apr
May
Jun
Winter Solstice
December 21/Day 355
Winter Teletherm
January 31/Day 31
+41 days
Coverage
Year
EAST TAWAS, MI

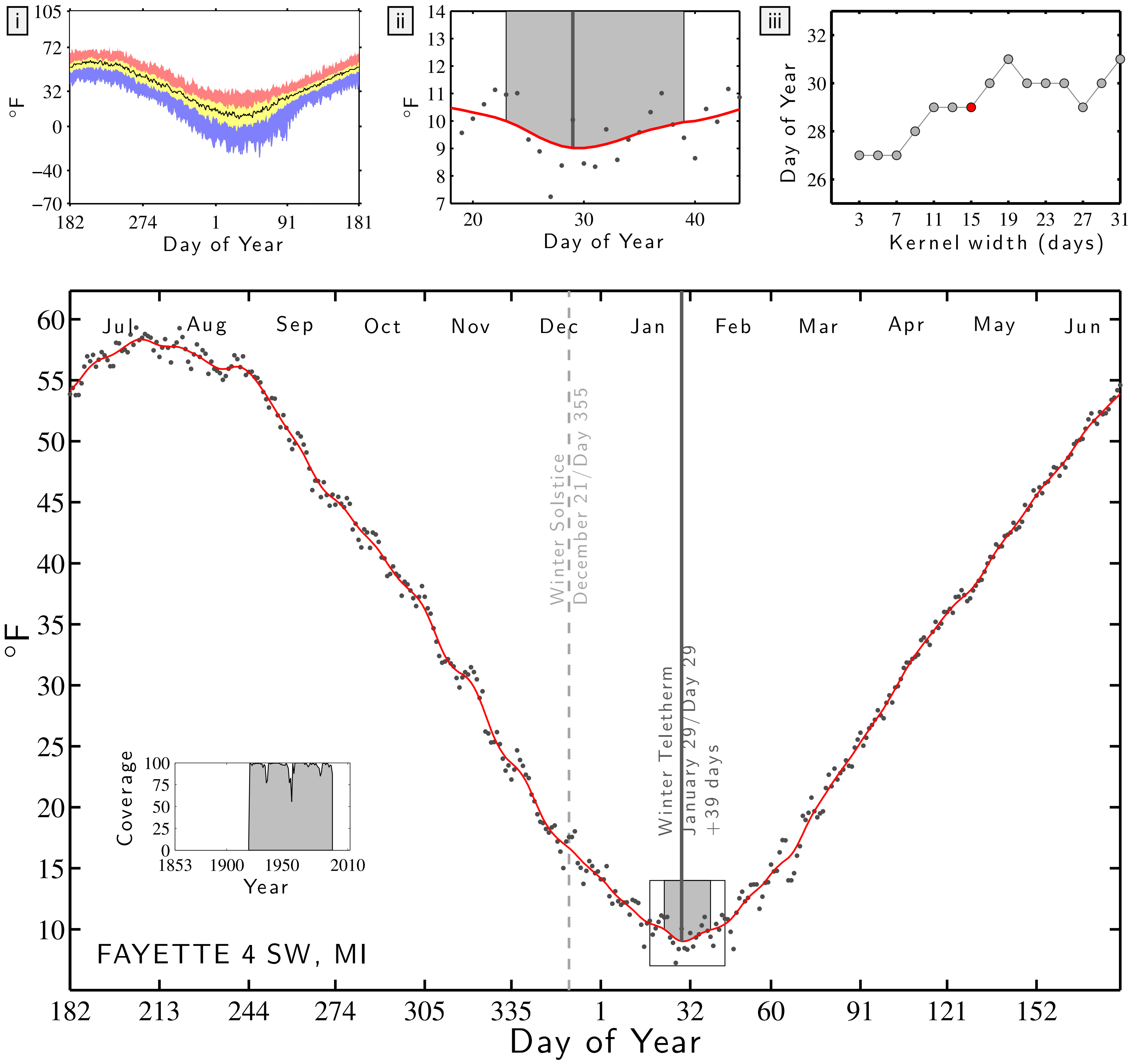

i
Day of Year
°F
ii
Day of Year
°F
iii
Kernel width (days)
Day of Year
Jul
Aug
Sep
Oct
Nov
Dec
Jan
Feb
Mar
Apr
May
Jun
Winter Solstice
December 21/Day 355
Winter Teletherm
January 29/Day 29
+39 days
Coverage
Year
FAYETTE 4 SW, MI
Day of Year
°F

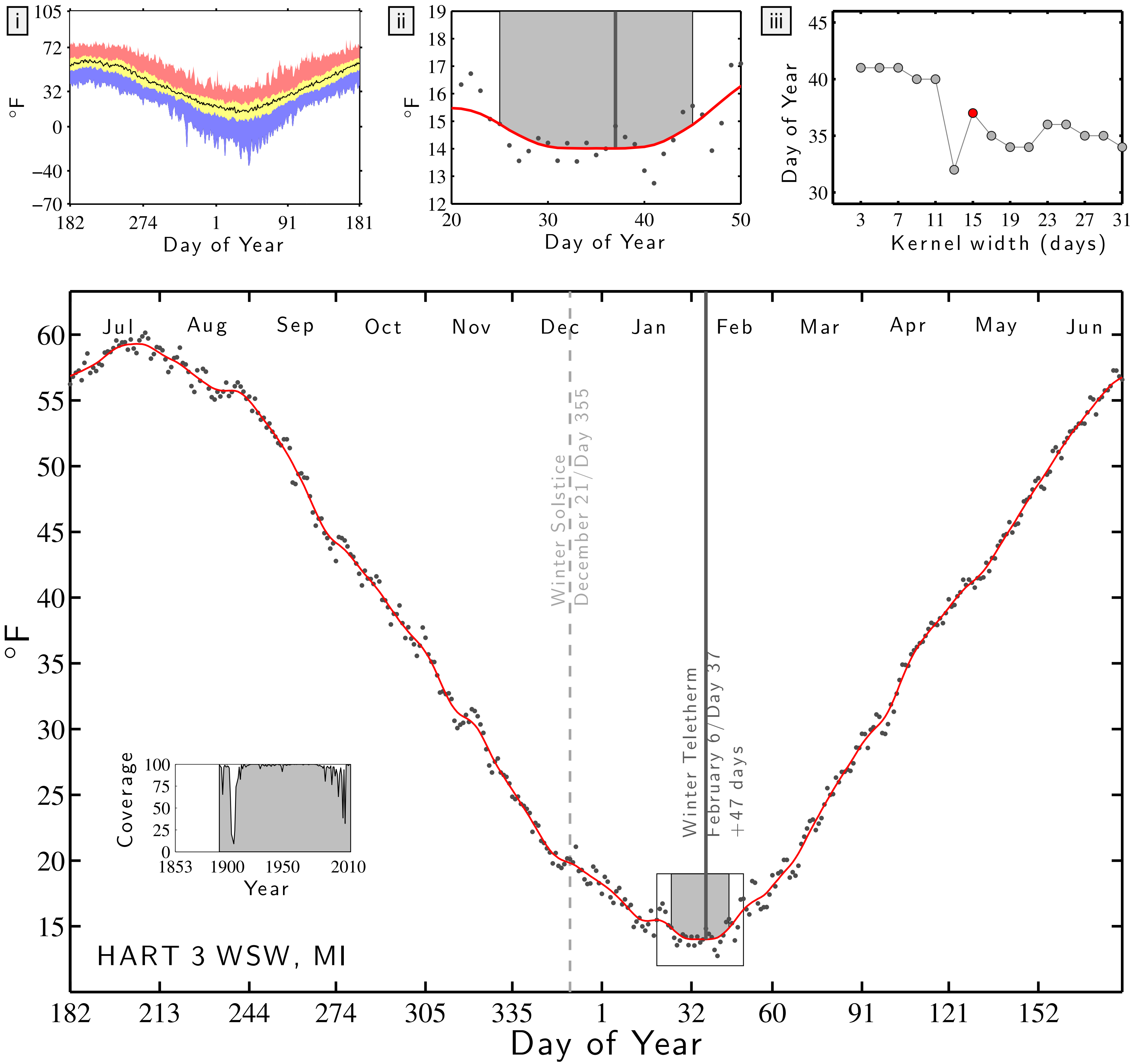
i
ii
iii
°F
Day of Year
Day of Year
Kernel width (days)
Jul
Aug
Sep
Oct
Nov
Dec
Jan
Feb
Mar
Apr
May
Jun
Winter Solstice
December 21/Day 355
Winter Teletherm
February 6/Day 37
+47 days
Coverage
Year
HART 3 WSW, MI

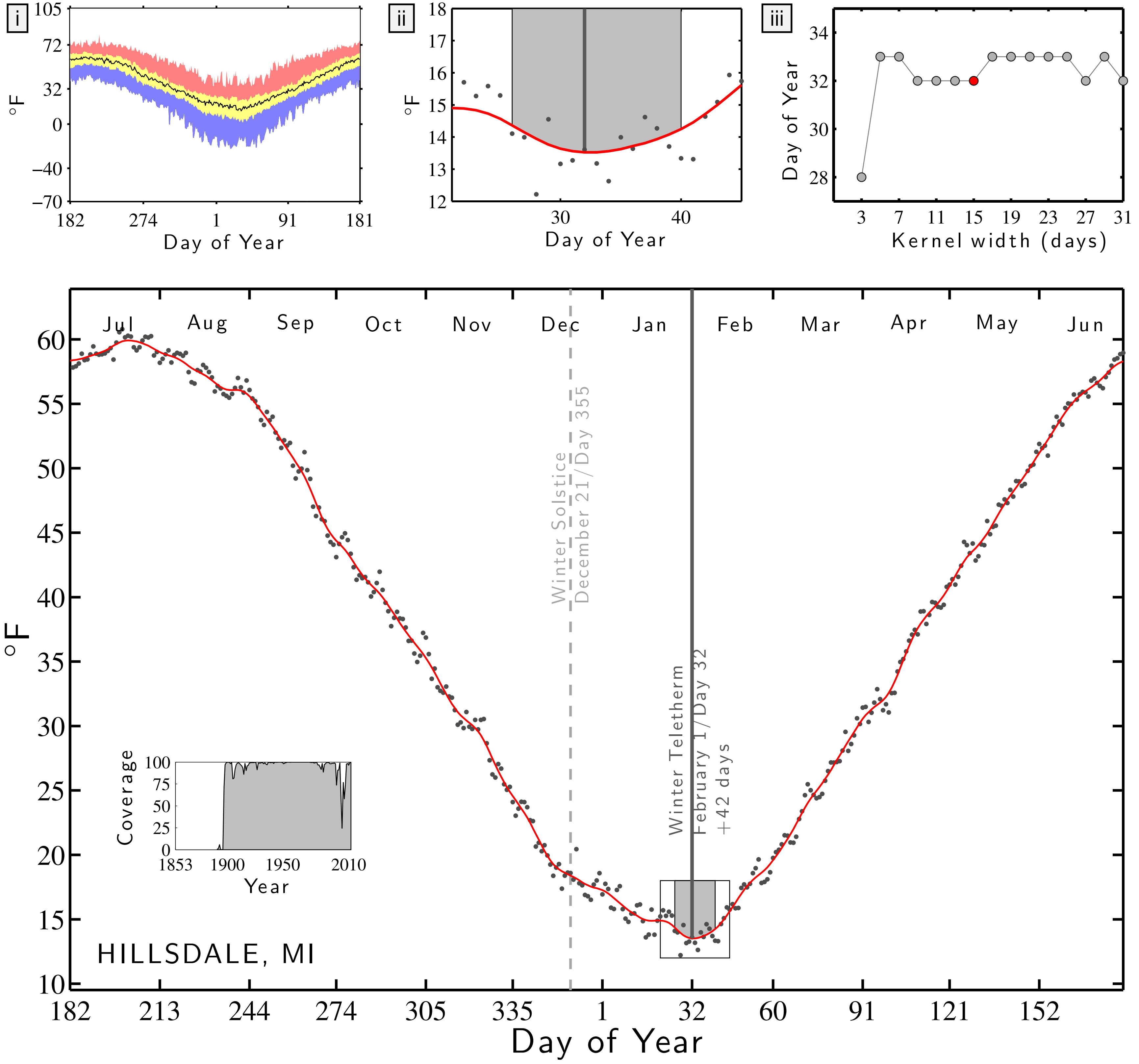
i
ii
iii
Day of Year
°F
Kernel width (days)
Jul
Aug
Sep
Oct
Nov
Dec
Jan
Feb
Mar
Apr
May
Jun
Winter Solstice
December 21/Day 355
Winter Teletherm
February 1/Day 32
+42 days
Coverage
Year
HILLSDALE, MI

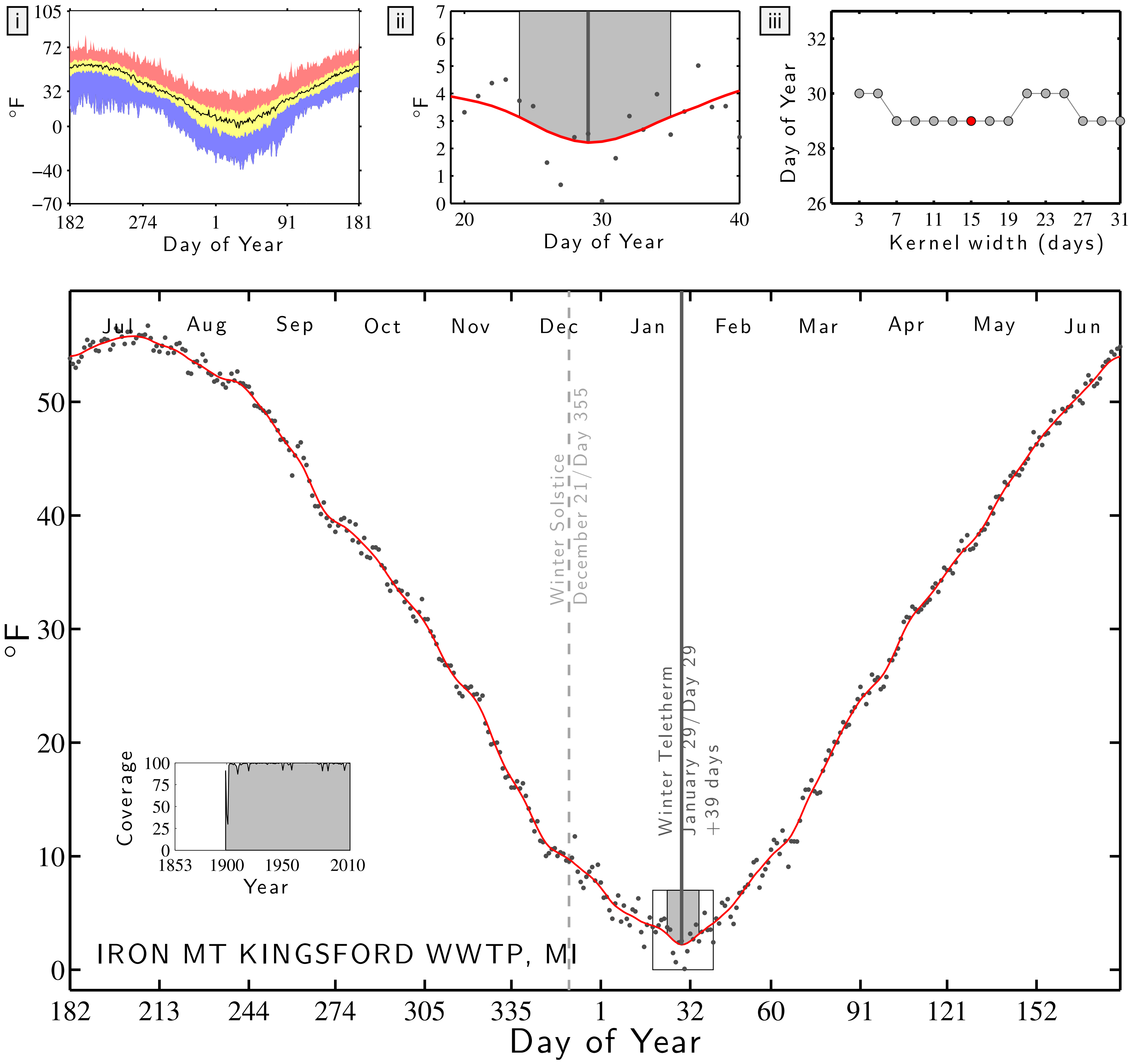

i
ii
iii
°F
Day of Year
Day of Year
Kernel width (days)
Jul
Aug
Sep
Oct
Nov
Dec
Jan
Feb
Mar
Apr
May
Jun
Winter Solstice
December 21/Day 355
Winter Teletherm
January 29/Day 29
+39 days
Coverage
Year
IRON MT KINGSFORD WWTP, MI

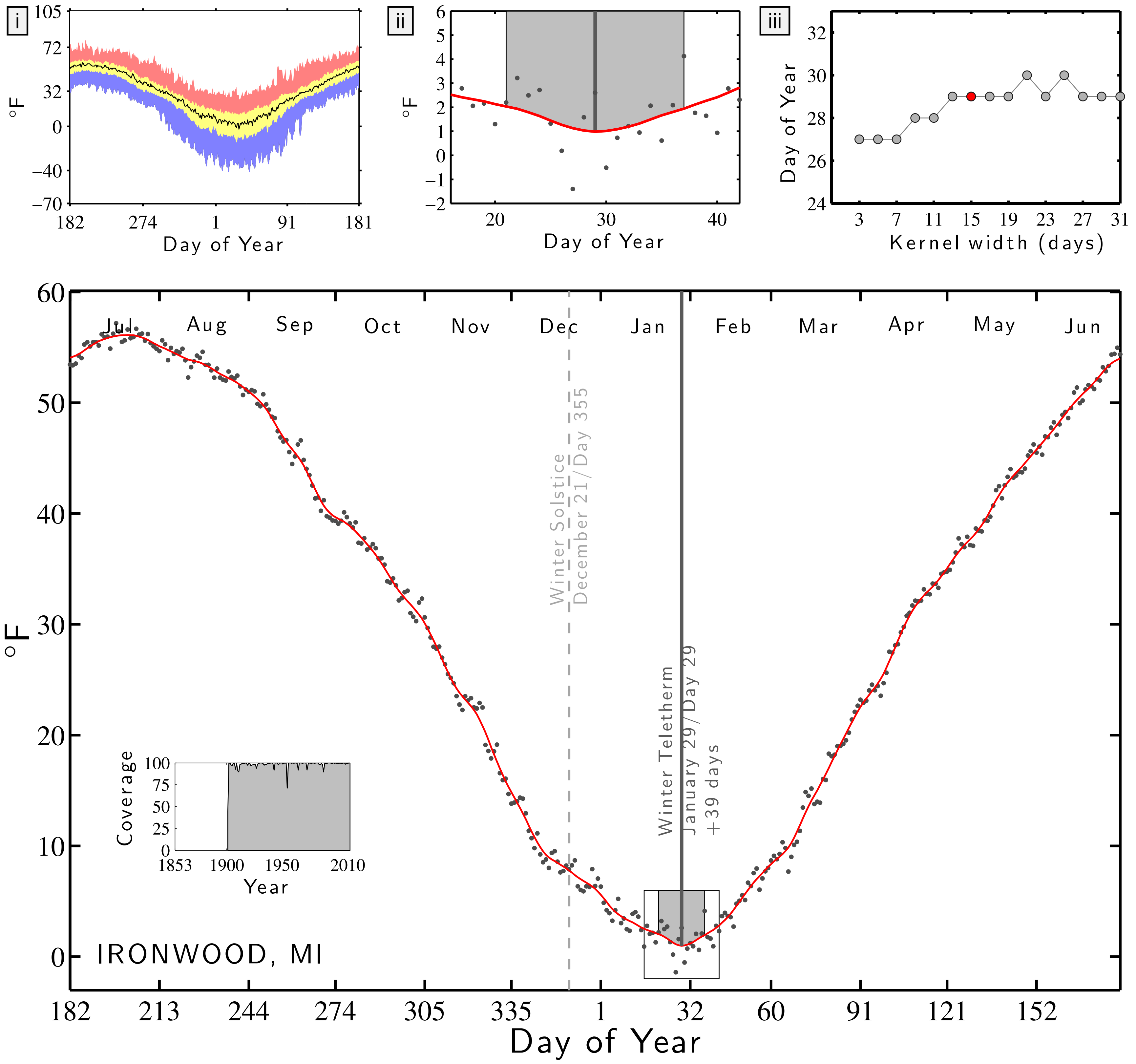

i
ii
iii
°F
Day of Year
Day of Year
Kernel width (days)
Jul
Aug
Sep
Oct
Nov
Dec
Jan
Feb
Mar
Apr
May
Jun
Winter Solstice
December 21/Day 355
Winter Teletherm
January 29/Day 29
+39 days
Coverage
Year
IRONWOOD, MI
Day of Year

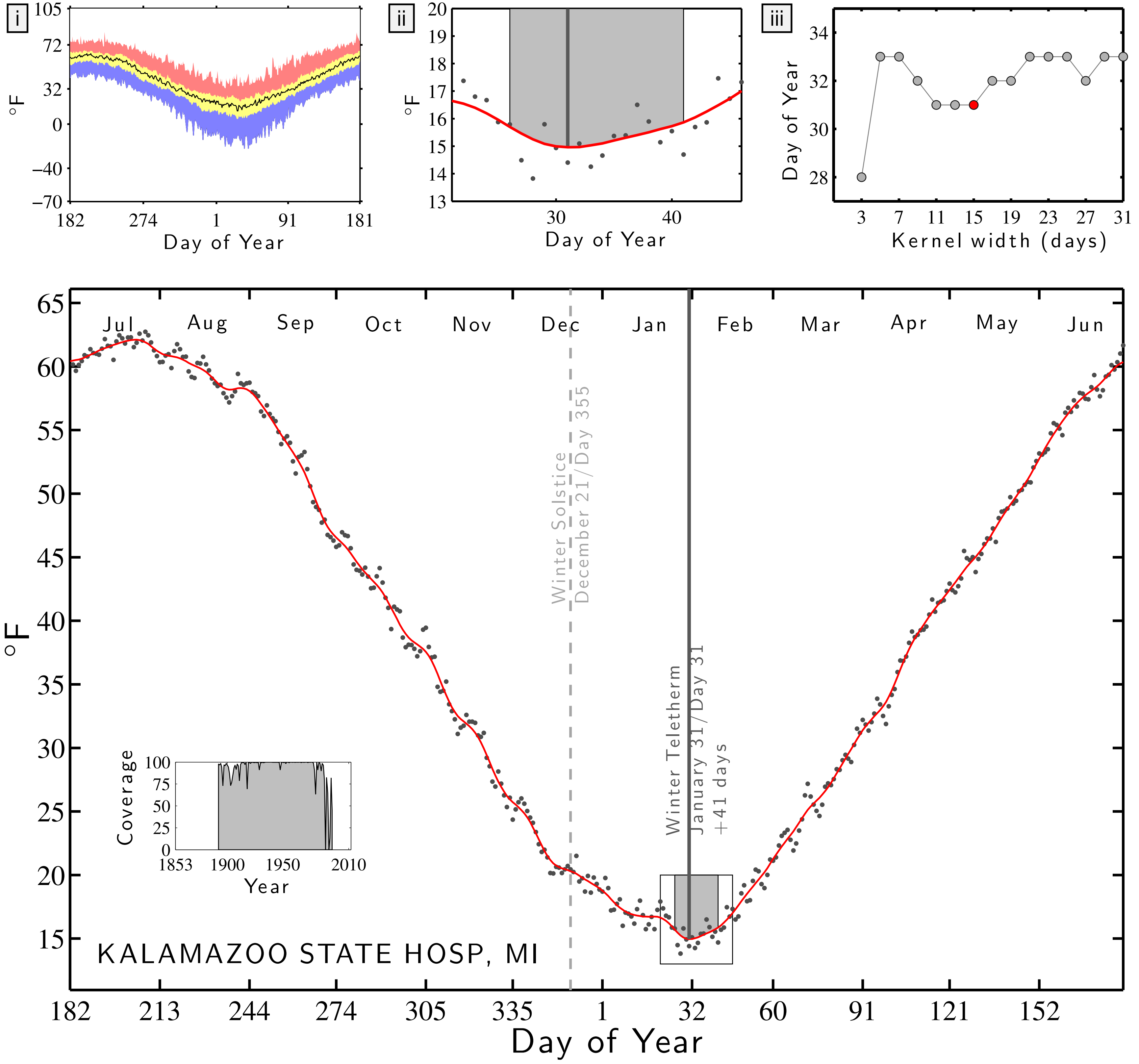

i
ii
iii
°F
Day of Year
Day of Year
Kernel width (days)
Winter Solstice
December 21/Day 355
Winter Teletherm
January 31/Day 31
+41 days
Jul
Aug
Sep
Oct
Nov
Dec
Jan
Feb
Mar
Apr
May
Jun
Coverage
Year
KALAMAZOO STATE HOSP, MI

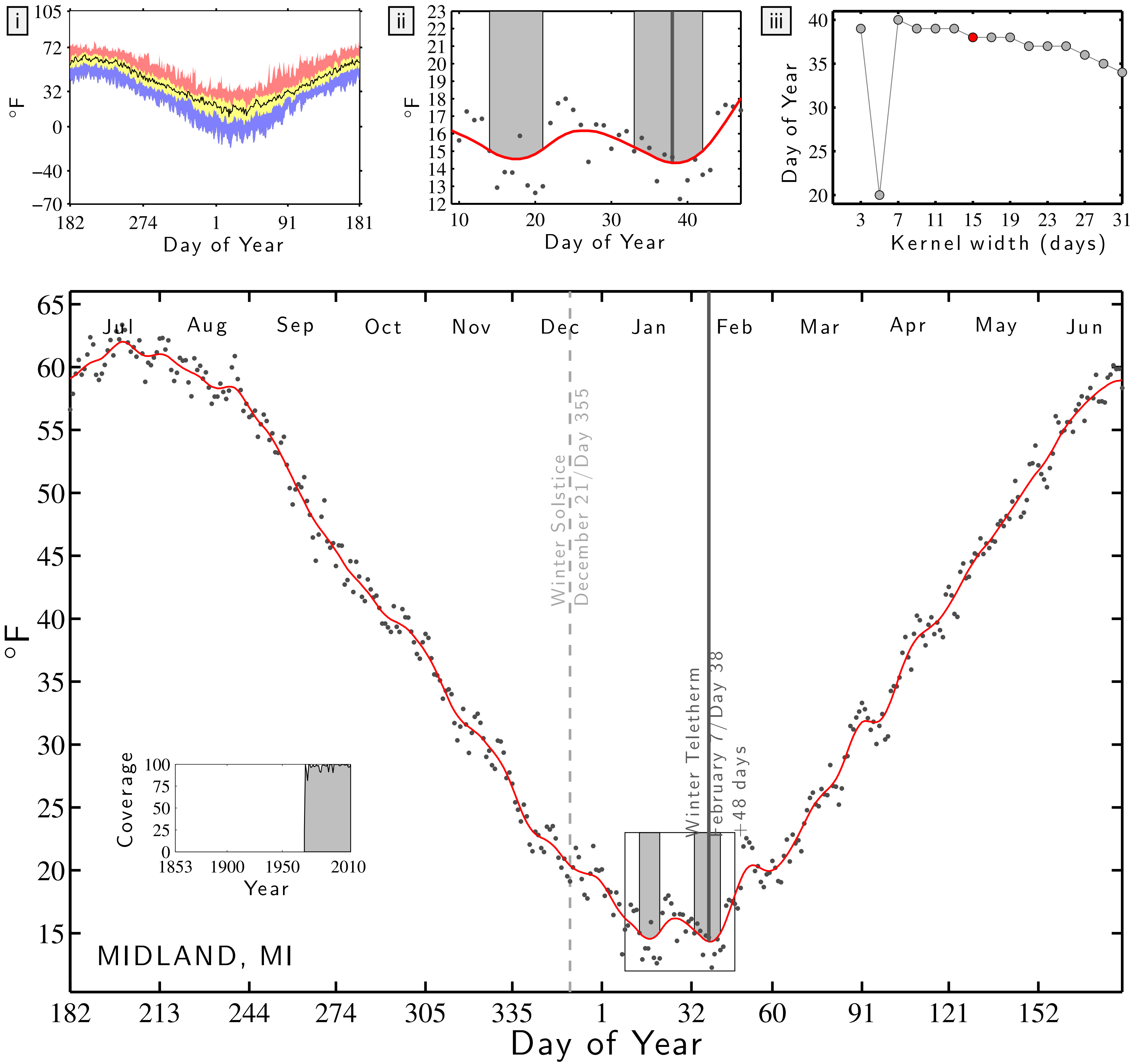

i
ii
iii
°F
Day of Year
Day of Year
Kernel width (days)
Jul
Aug
Sep
Oct
Nov
Dec
Jan
Feb
Mar
Apr
May
Jun
Winter Solstice
December 21/Day 355
Winter Teletherm
February 7/Day 38
+48 days
Coverage
Year
MIDLAND, MI
Day of Year

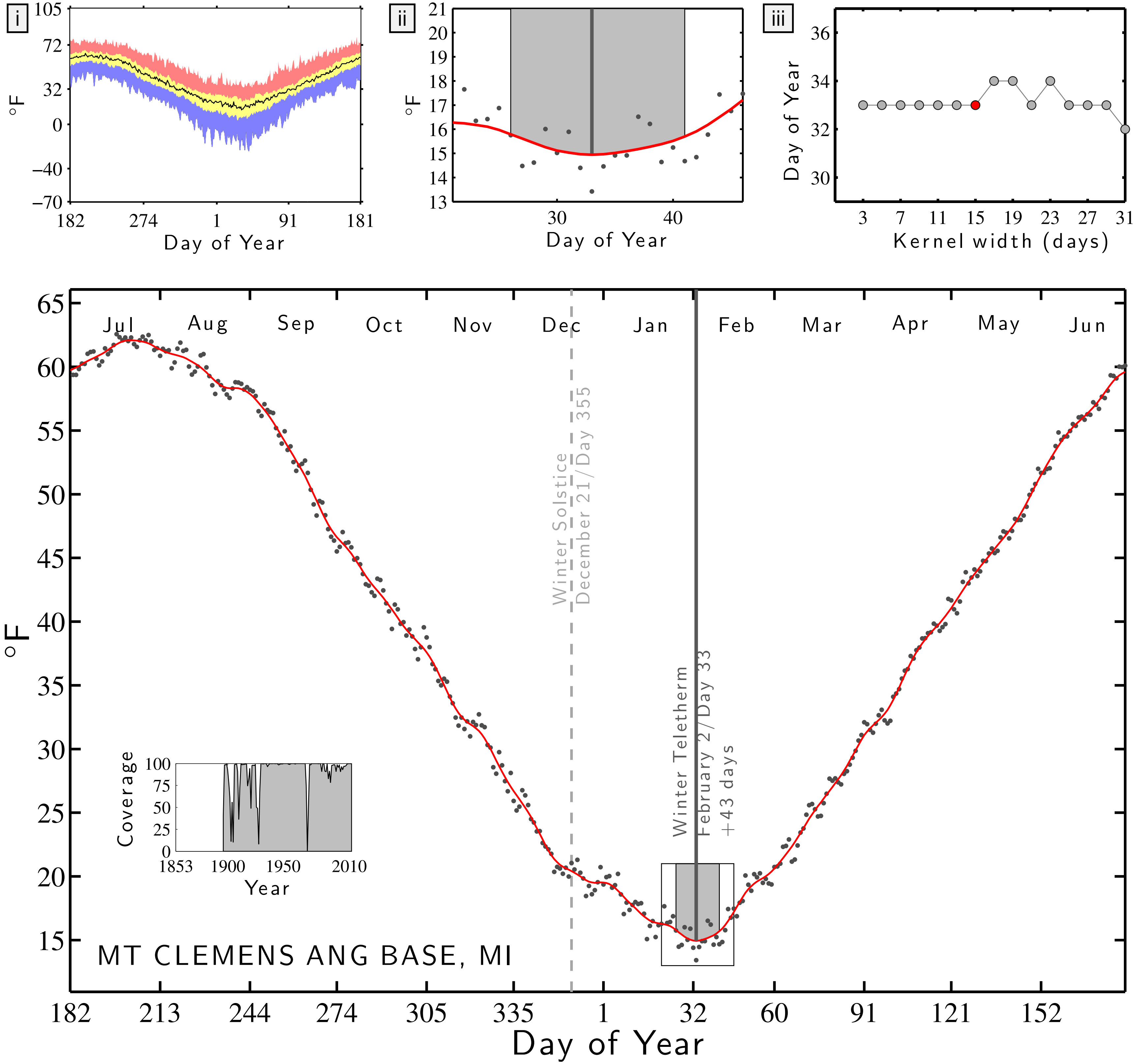

i
°F
Day of Year
ii
°F
Day of Year
iii
Day of Year
Kernel width (days)
Jul
Aug
Sep
Oct
Nov
Dec
Jan
Feb
Mar
Apr
May
Jun
Winter Solstice
December 21/Day 355
Winter Teletherm
February 2/Day 33
+43 days
Coverage
Year
MT CLEMENS ANG BASE, MI
°F
Day of Year

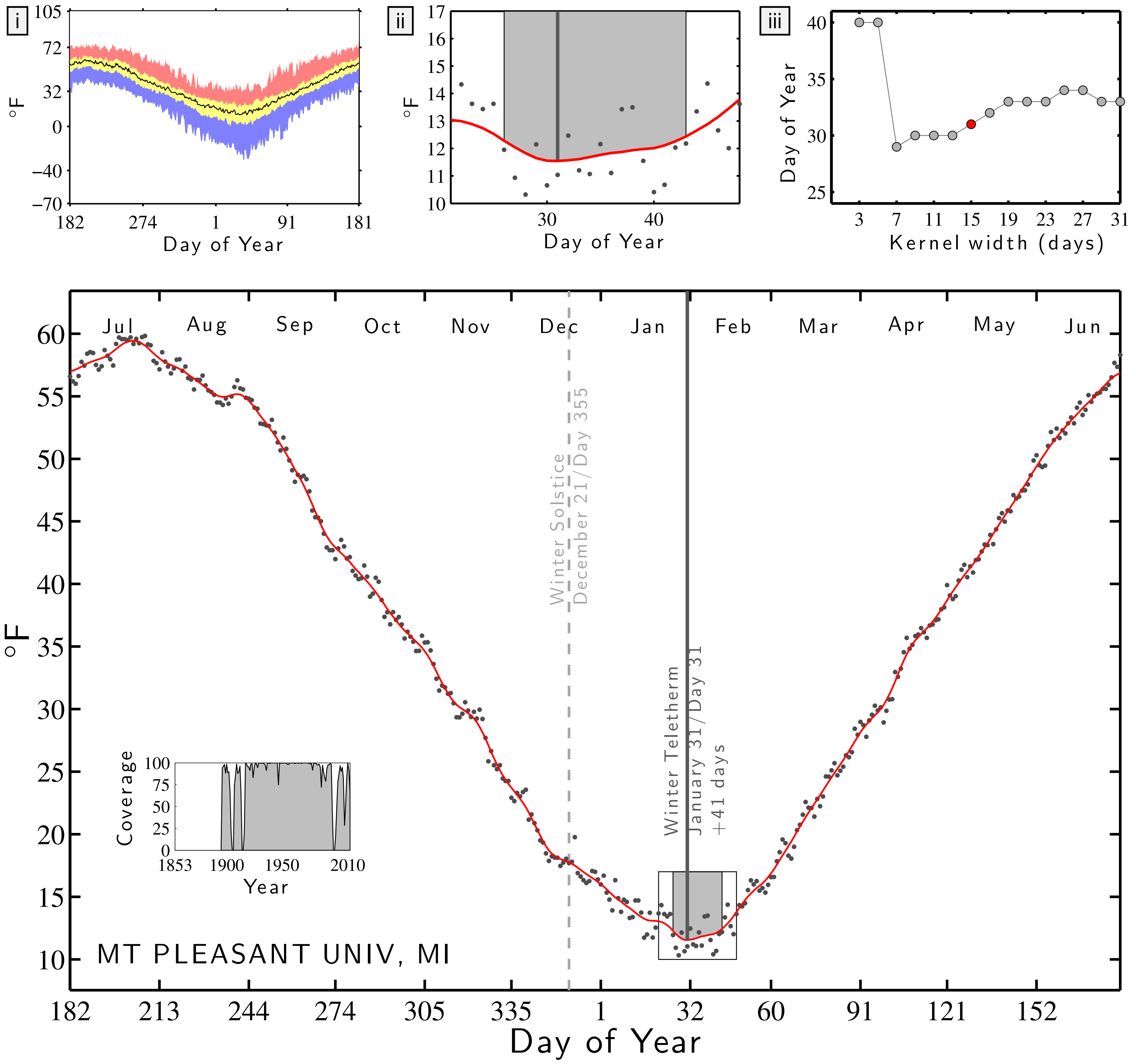

i
ii
iii
°F
Day of Year
Kernel width (days)
Jul
Aug
Sep
Oct
Nov
Dec
Jan
Feb
Mar
Apr
May
Jun
Winter Solstice
December 21/Day 355
Winter Teletherm
January 31/Day 31
+41 days
Coverage
Year
MT PLEASANT UNIV, MI

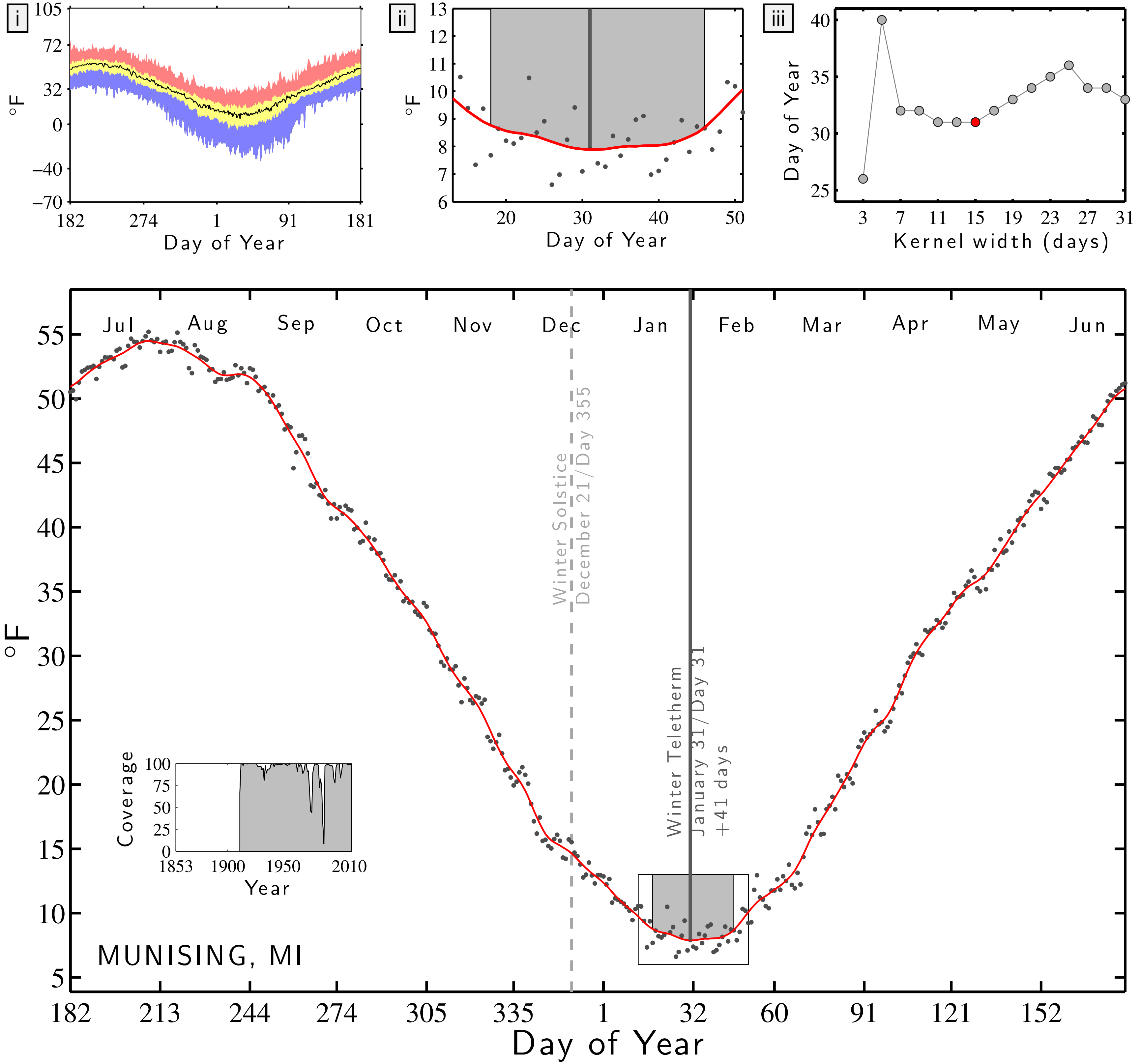

i
ii
iii
°F
Day of Year
Kernel width (days)
Jul
Aug
Sep
Oct
Nov
Dec
Jan
Feb
Mar
Apr
May
Jun
Winter Solstice
December 21/Day 355
Winter Teletherm
January 31/Day 31
+41 days
Coverage
Year
MUNISING, MI

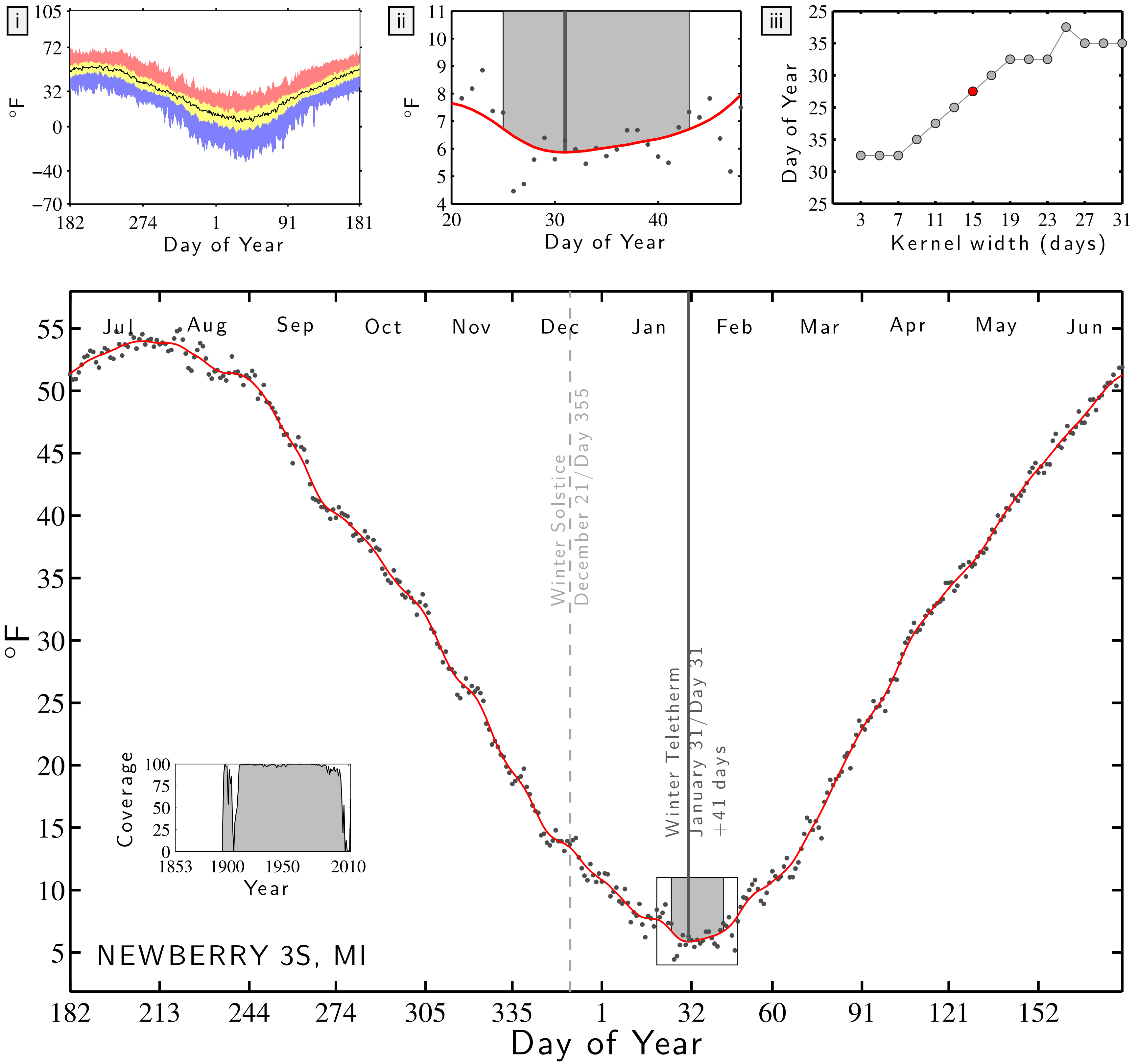

i
ii
iii
°F
Day of Year
Kernel width (days)
Jul
Aug
Sep
Oct
Nov
Dec
Jan
Feb
Mar
Apr
May
Jun
Winter Solstice
December 21/Day 355
Winter Teletherm
January 31/Day 31
+41 days
Coverage
Year
NEWBERRY 3S, MI

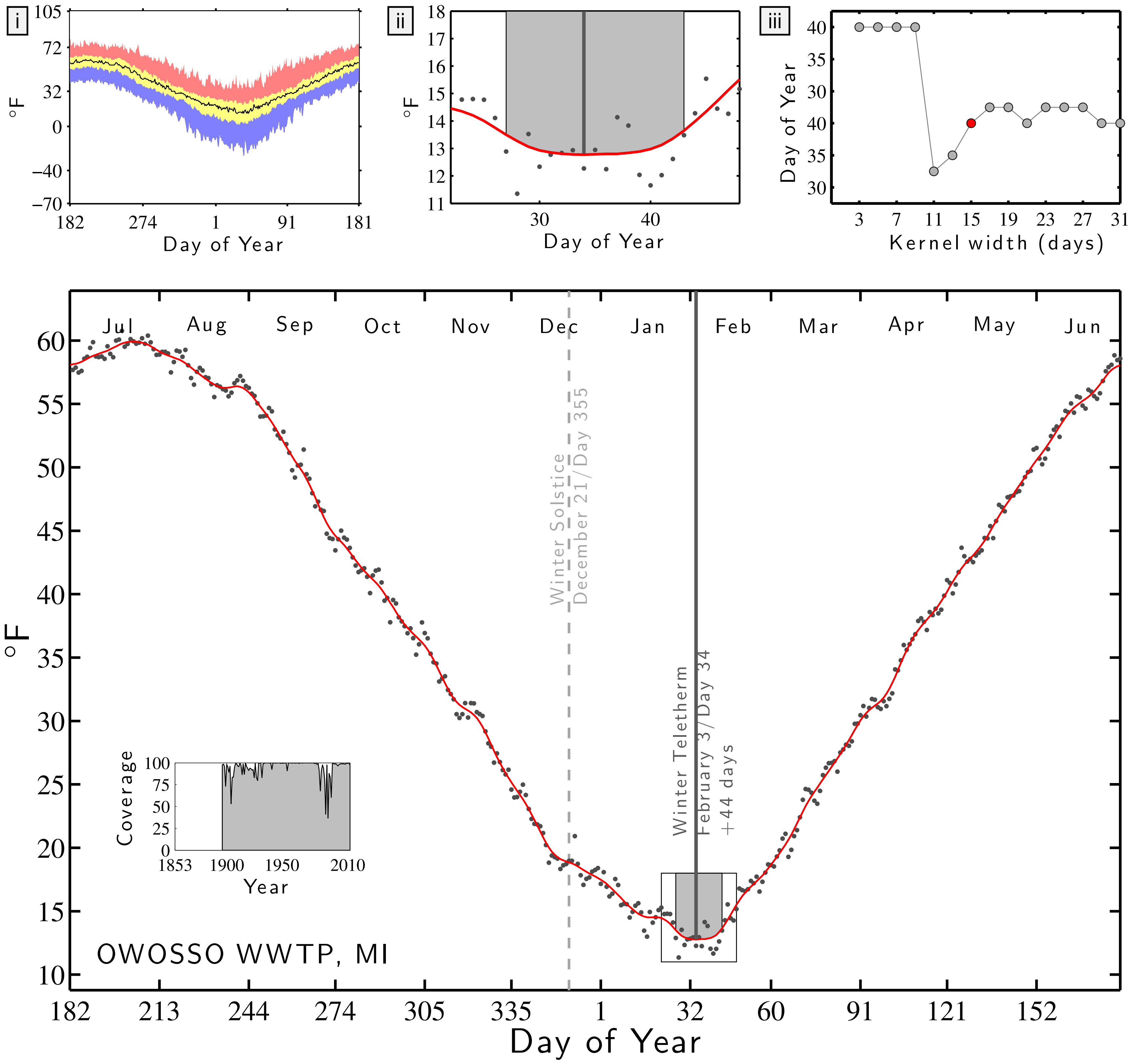

i
°F
Day of Year
ii
°F
Day of Year
iii
Day of Year
Kernel width (days)
Jul
Aug
Sep
Oct
Nov
Dec
Jan
Feb
Mar
Apr
May
Jun
Winter Solstice
December 21/Day 355
Winter Teletherm
February 3/Day 34
+44 days
Coverage
Year
OWOSSO WWTP, MI
°F
Day of Year

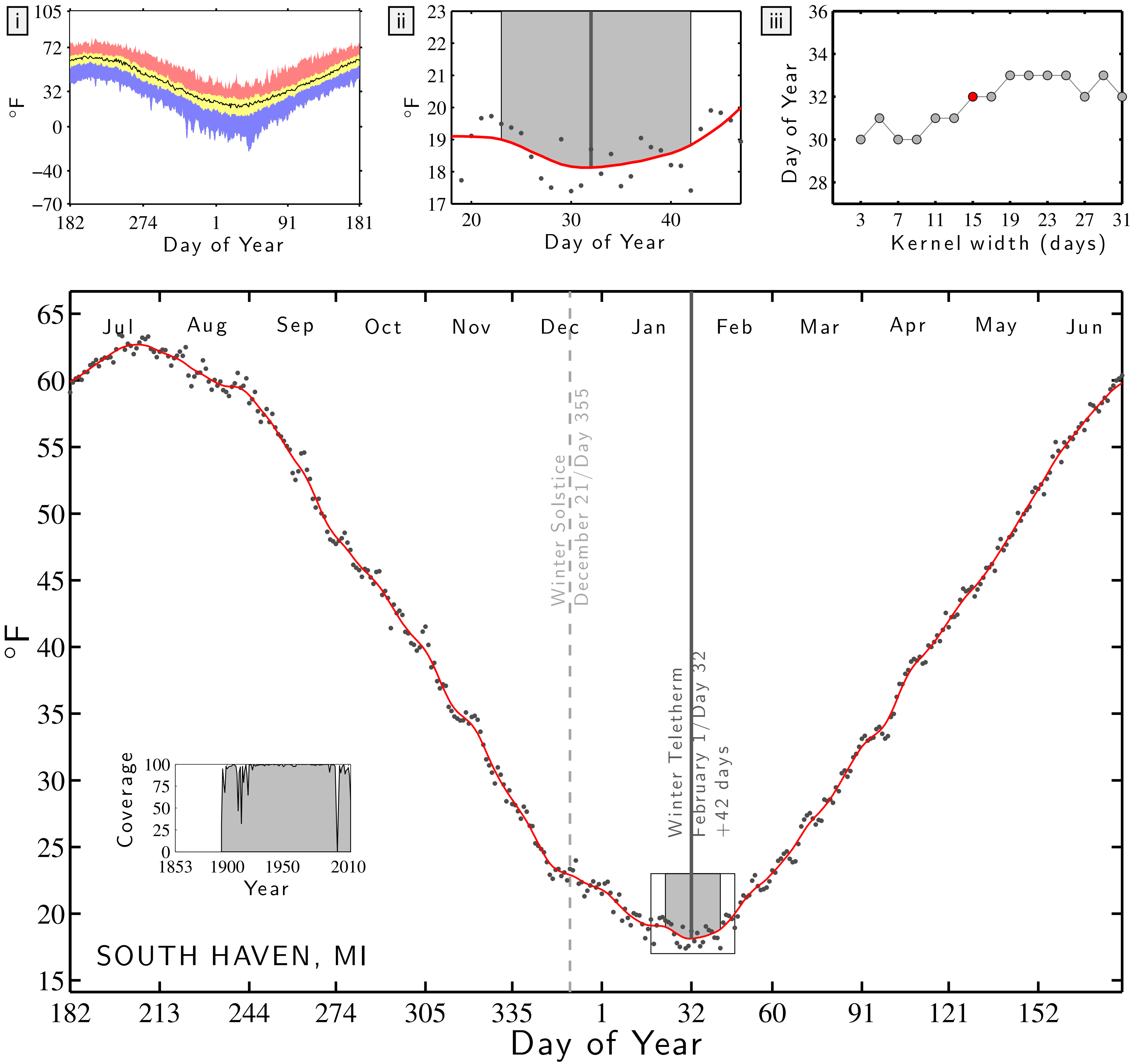

i
°F
Day of Year
ii
°F
Day of Year
iii
Day of Year
Kernel width (days)
Jul
Aug
Sep
Oct
Nov
Dec
Jan
Feb
Mar
Apr
May
Jun
Winter Solstice
December 21/Day 355
Winter Teletherm
February 1/Day 32
+42 days
Coverage
Year
SOUTH HAVEN, MI
°F
Day of Year

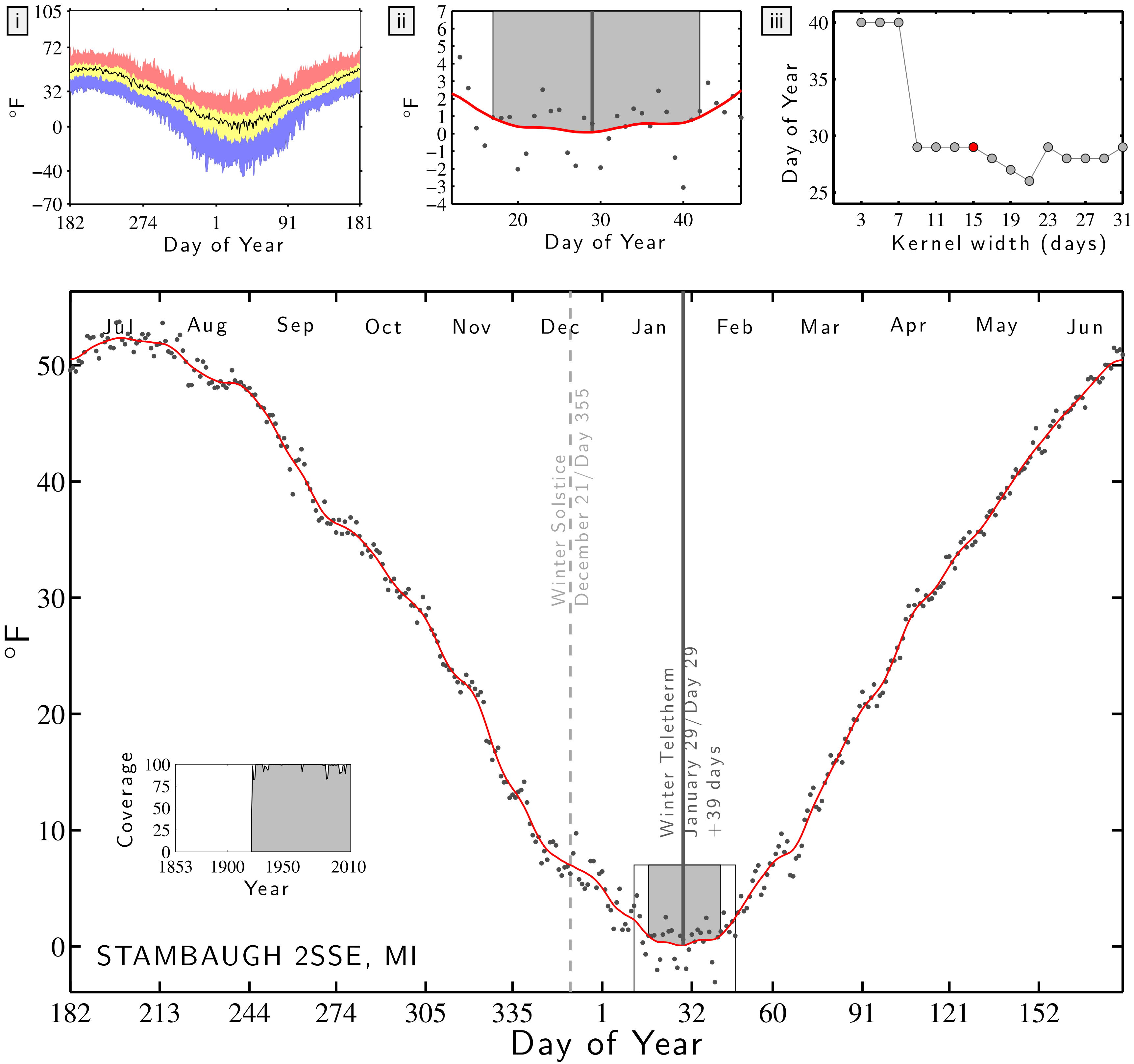

i
°F
Day of Year
ii
°F
Day of Year
iii
Day of Year
Kernel width (days)
Jul
Aug
Sep
Oct
Nov
Dec
Jan
Feb
Mar
Apr
May
Jun
Winter Solstice
December 21/Day 355
Winter Teletherm
January 29/Day 29
+39 days
Coverage
Year
STAMBAUGH 2SSE, MI
°F
Day of Year

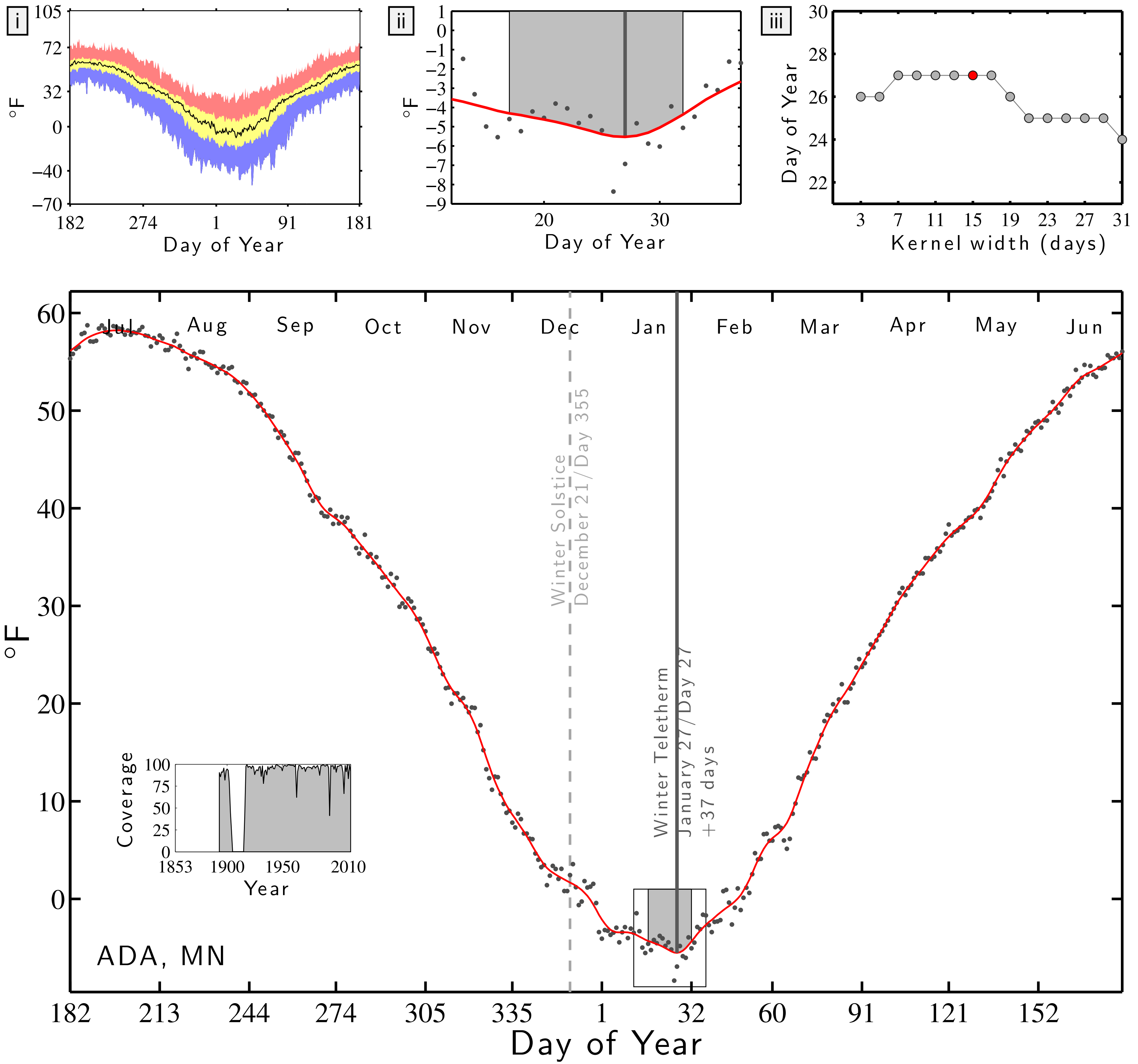

i
ii
iii
°F
Day of Year
Day of Year
Kernel width (days)
Jul
Aug
Sep
Oct
Nov
Dec
Jan
Feb
Mar
Apr
May
Jun
Winter Solstice
December 21/Day 355
Winter Teletherm
January 27/Day 27
+37 days
Coverage
Year
ADA, MN

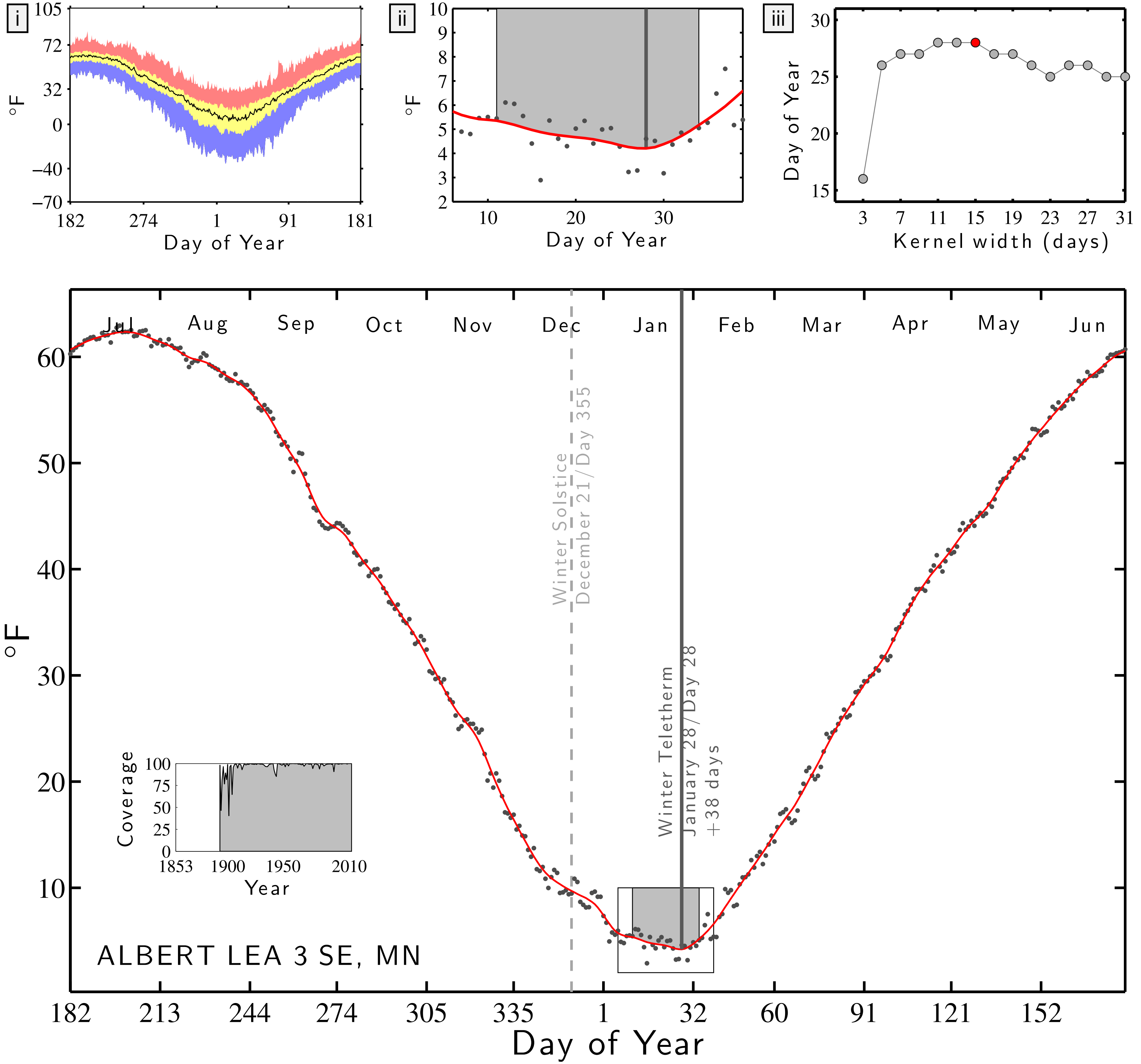

i
ii
iii
Day of Year
Kernel width (days)
°F
Day of Year
Jul
Aug
Sep
Oct
Nov
Dec
Jan
Feb
Mar
Apr
May
Jun
Winter Solstice
December 21/Day 355
Winter Teletherm
January 28/Day 28
+38 days
Coverage
Year
ALBERT LEA 3 SE, MN
Day of Year

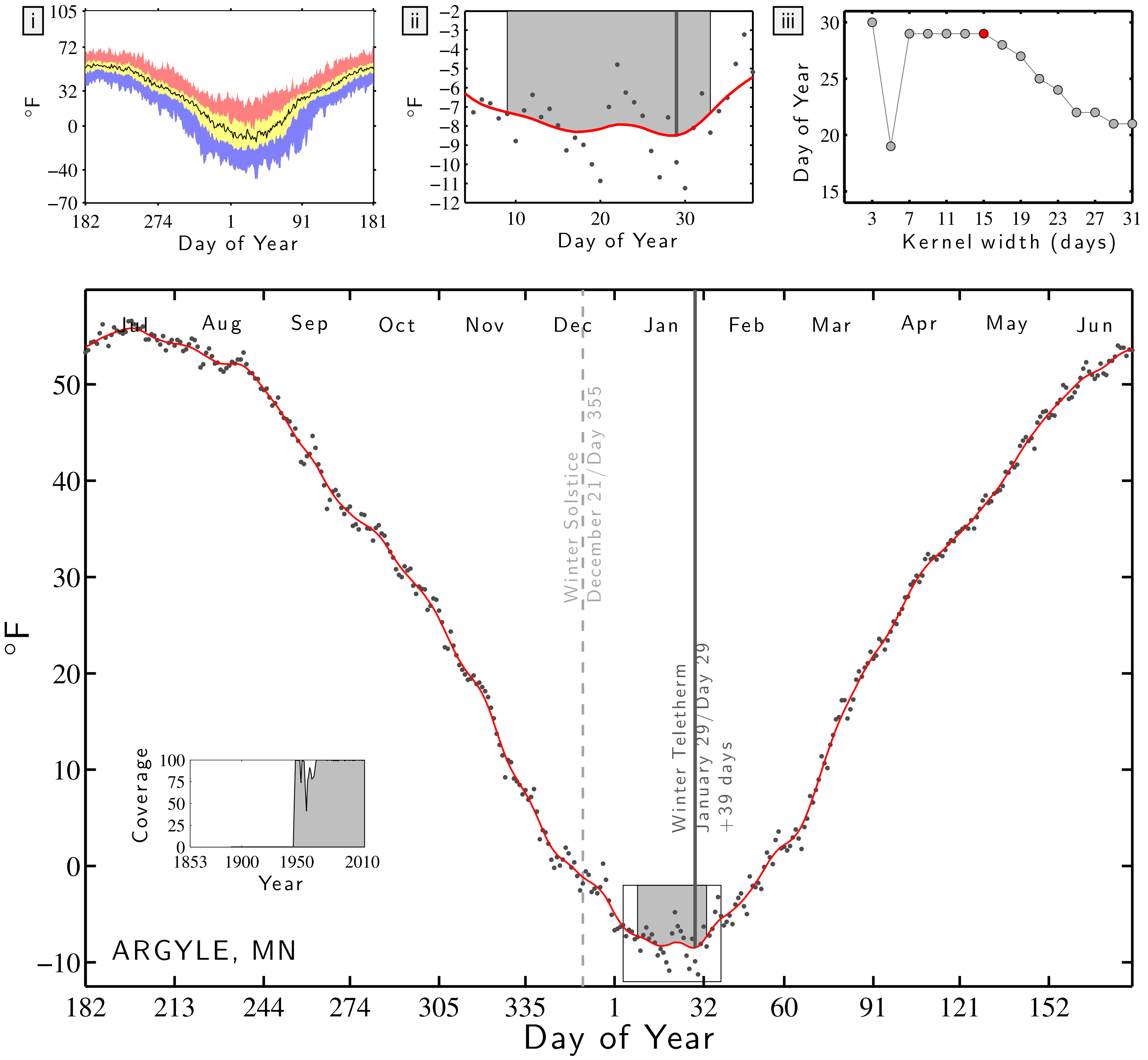

i
°F
Day of Year
ii
°F
Day of Year
iii
Day of Year
Kernel width (days)
Jul
Aug
Sep
Oct
Nov
Dec
Jan
Feb
Mar
Apr
May
Jun
Winter Solstice
December 21/Day 355
Winter Teletherm
January 29/Day 29
+39 days
Coverage
Year
ARGYLE, MN
°F
Day of Year

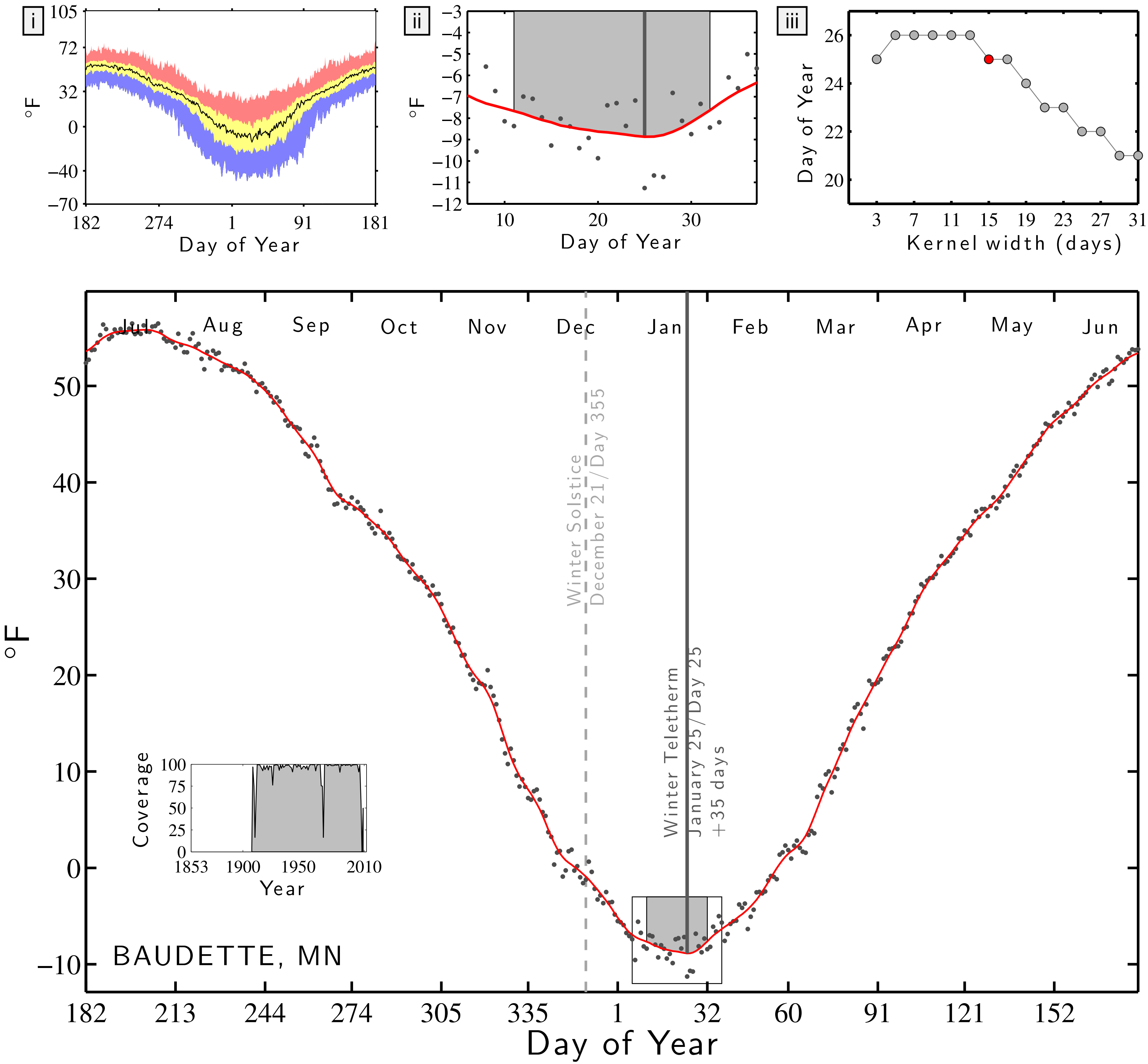

i
°F
Day of Year
ii
°F
Day of Year
iii
Day of Year
Kernel width (days)
Jul
Aug
Sep
Oct
Nov
Dec
Jan
Feb
Mar
Apr
May
Jun
Winter Solstice
December 21/Day 355
Winter Teletherm
January 25/Day 25
+35 days
Coverage
Year
BAUDETTE, MN
°F
Day of Year

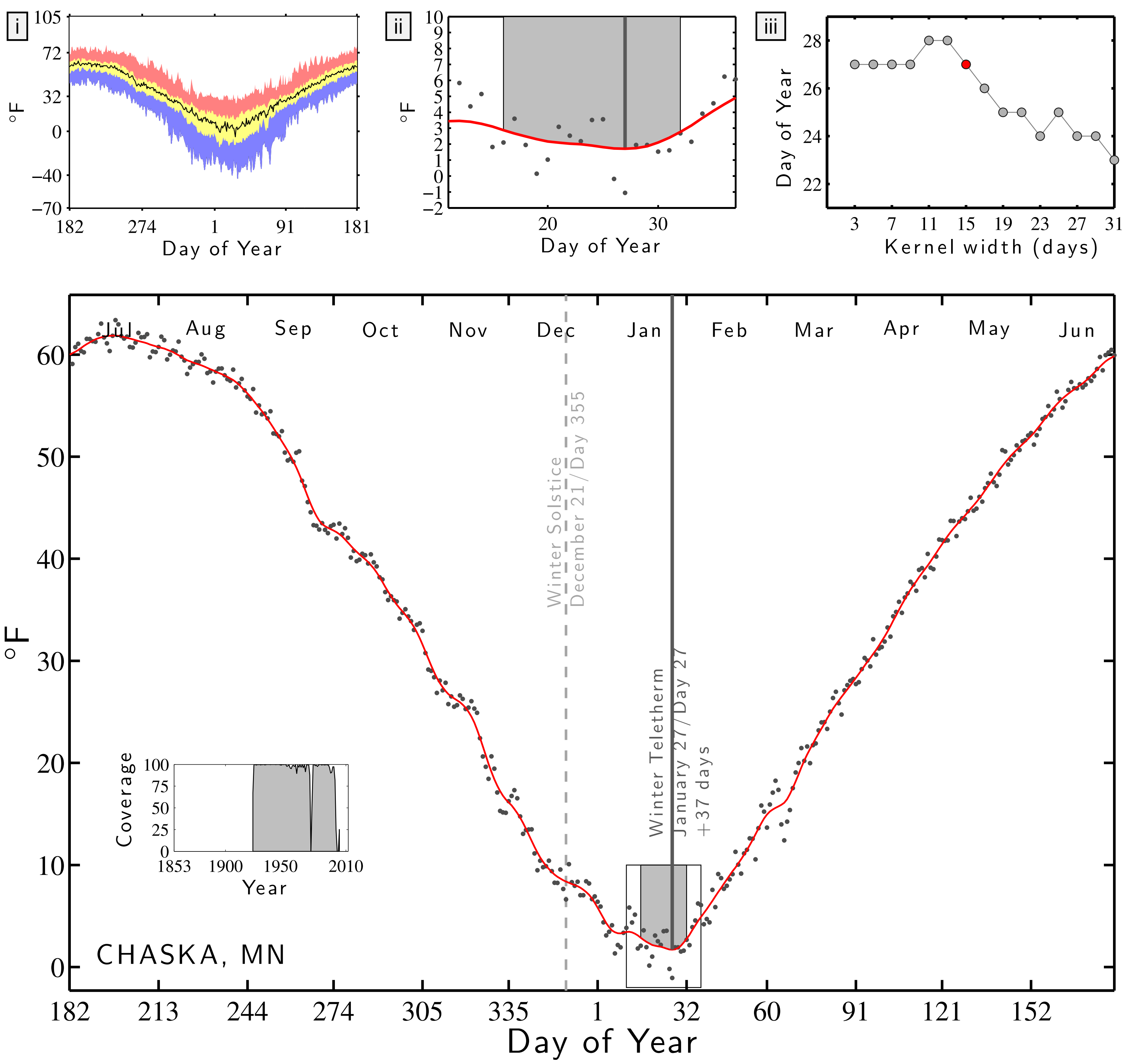

i
ii
iii
°F
Day of Year
Day of Year
Kernel width (days)
Jul
Aug
Sep
Oct
Nov
Dec
Jan
Feb
Mar
Apr
May
Jun
Winter Solstice
December 21/Day 355
Winter Teletherm
January 27/Day 27
+37 days
Coverage
Year
CHASKA, MN

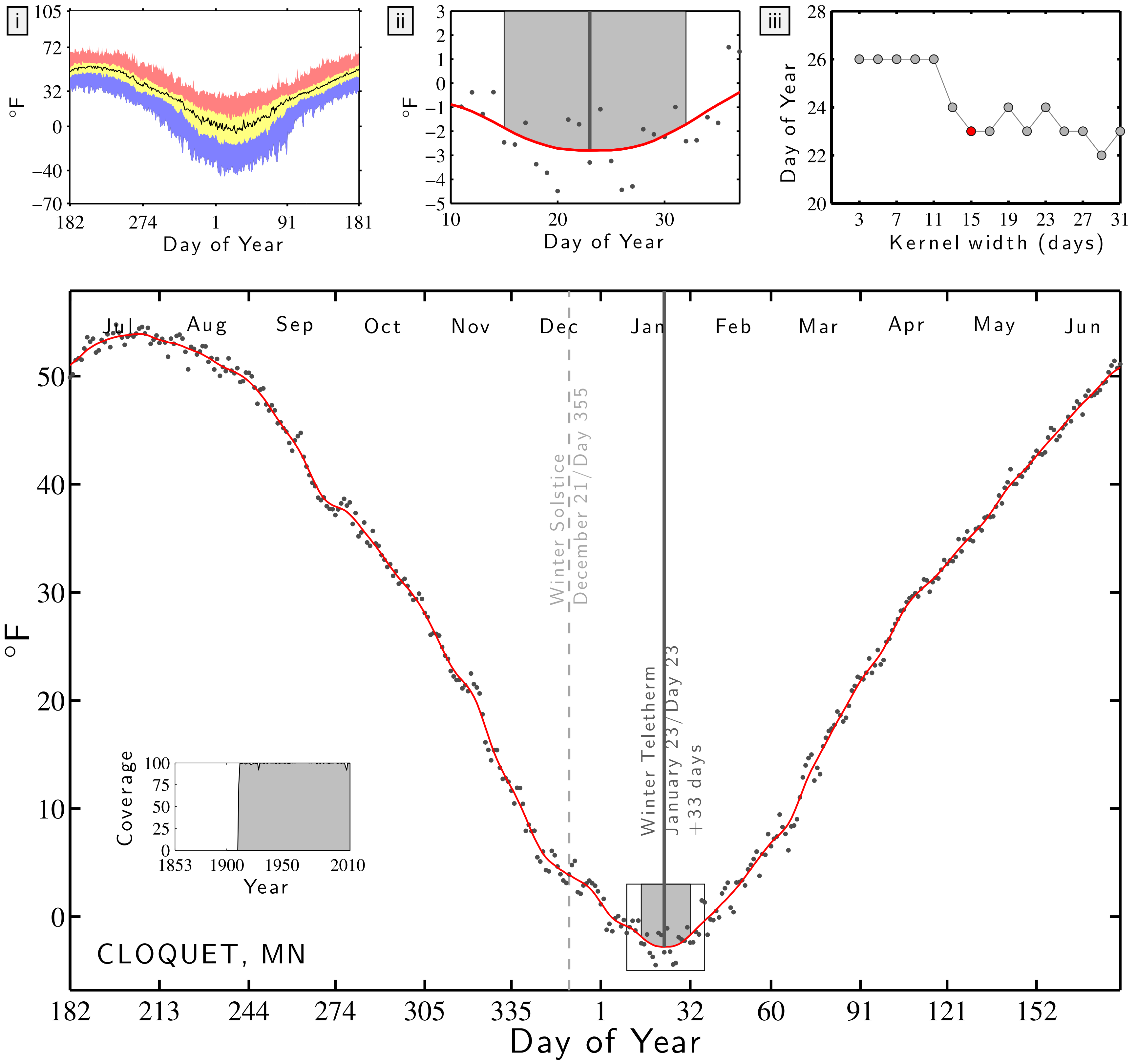

i
°F
Day of Year
ii
°F
Day of Year
iii
Day of Year
Kernel width (days)
Jul
Aug
Sep
Oct
Nov
Dec
Jan
Feb
Mar
Apr
May
Jun
Winter Solstice
December 21/Day 355
Winter Teletherm
January 23/Day 23
+33 days
Coverage
Year
CLOQUET, MN
°F
Day of Year

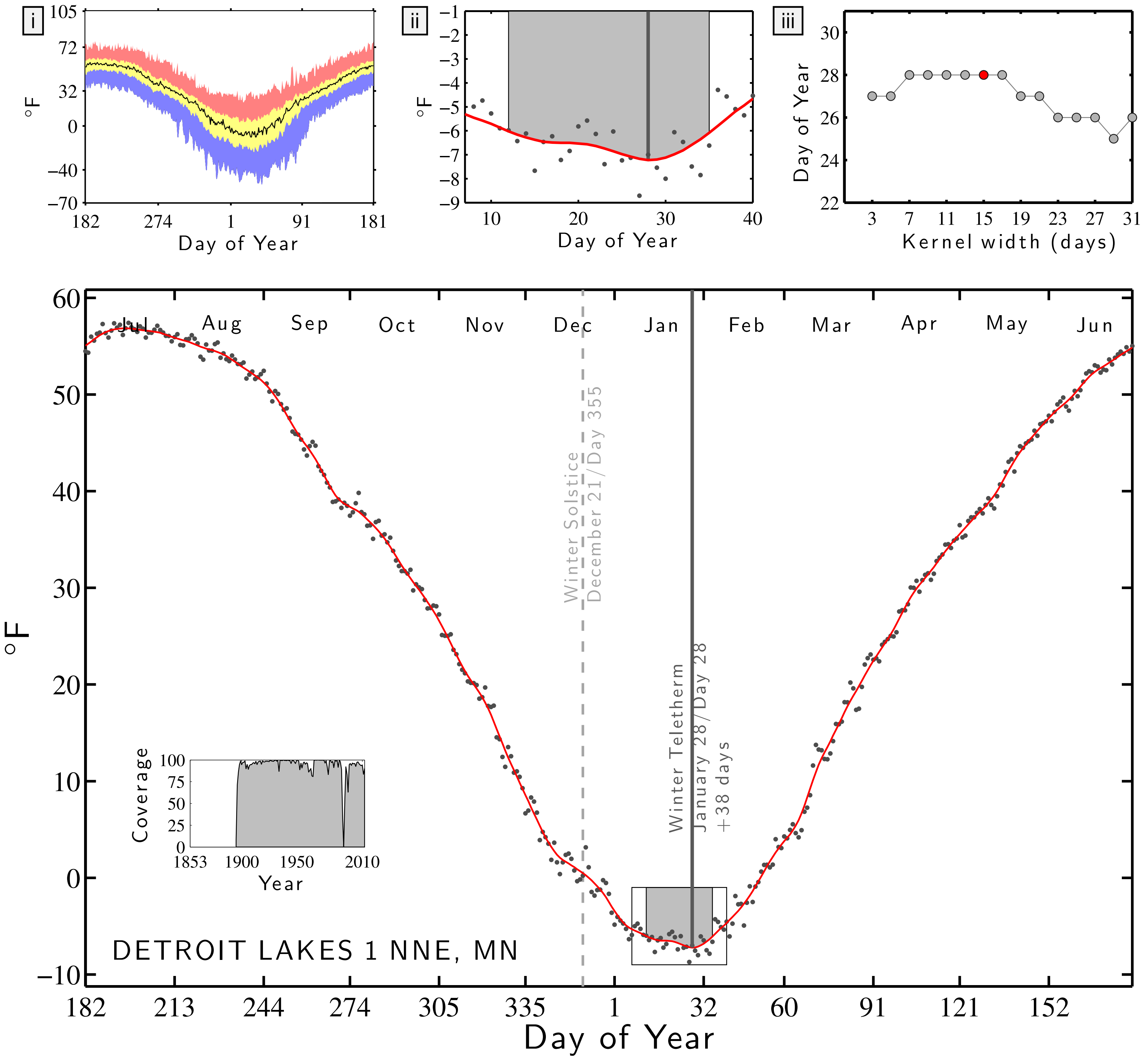
i
ii
iii
°F
Day of Year
Kernel width (days)
Jul
Aug
Sep
Oct
Nov
Dec
Jan
Feb
Mar
Apr
May
Jun
Winter Solstice
December 21/Day 355
Winter Teletherm
January 28/Day 28
+38 days
Coverage
Year
DETROIT LAKES 1 NNE, MN

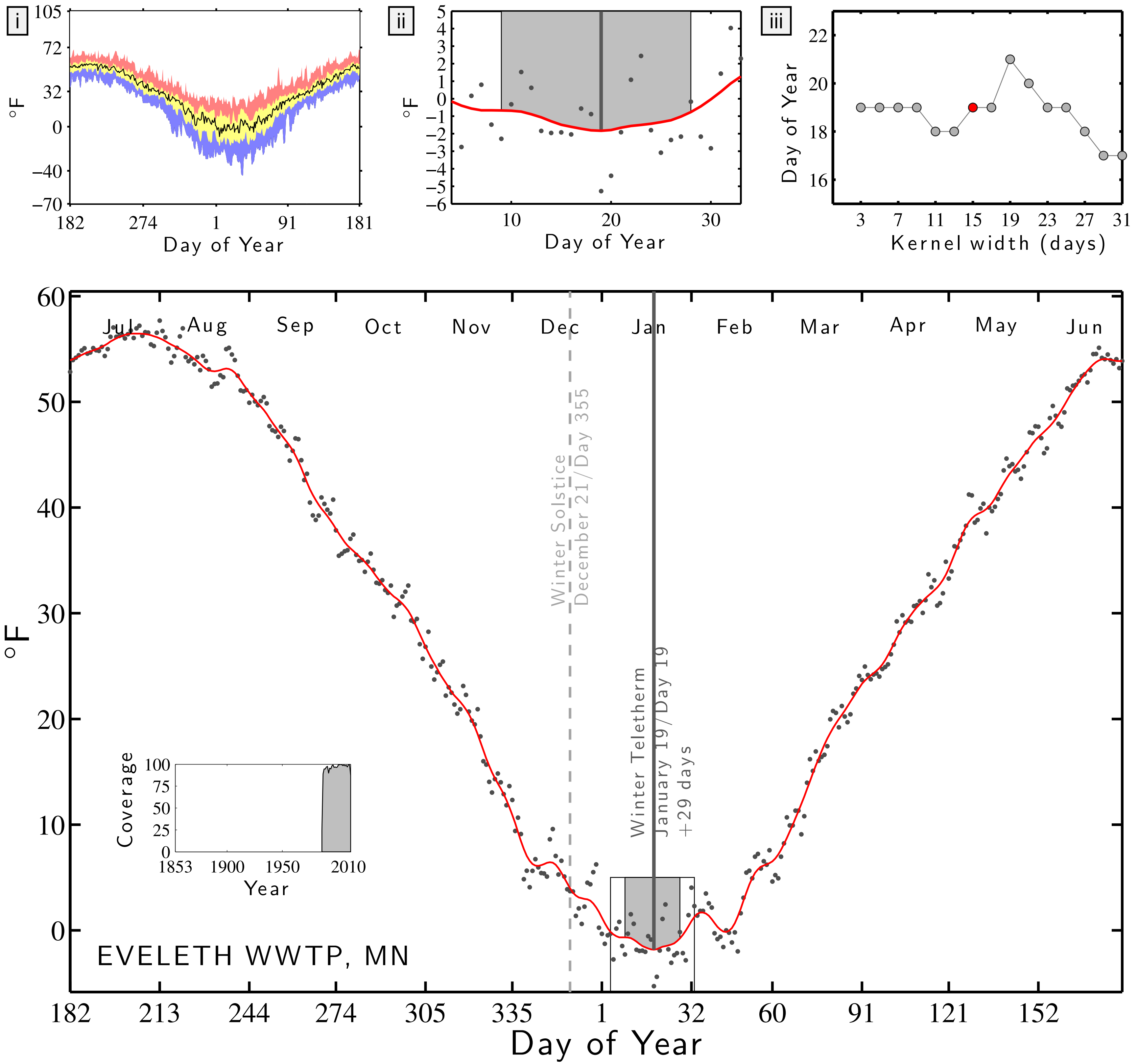

i
ii
iii
°F
Day of Year
Kernel width (days)
Jul
Aug
Sep
Oct
Nov
Dec
Jan
Feb
Mar
Apr
May
Jun
Winter Solstice
December 21/Day 355
Winter Teletherm
January 19/Day 19
+29 days
Coverage
Year
EVELETH WWTP, MN

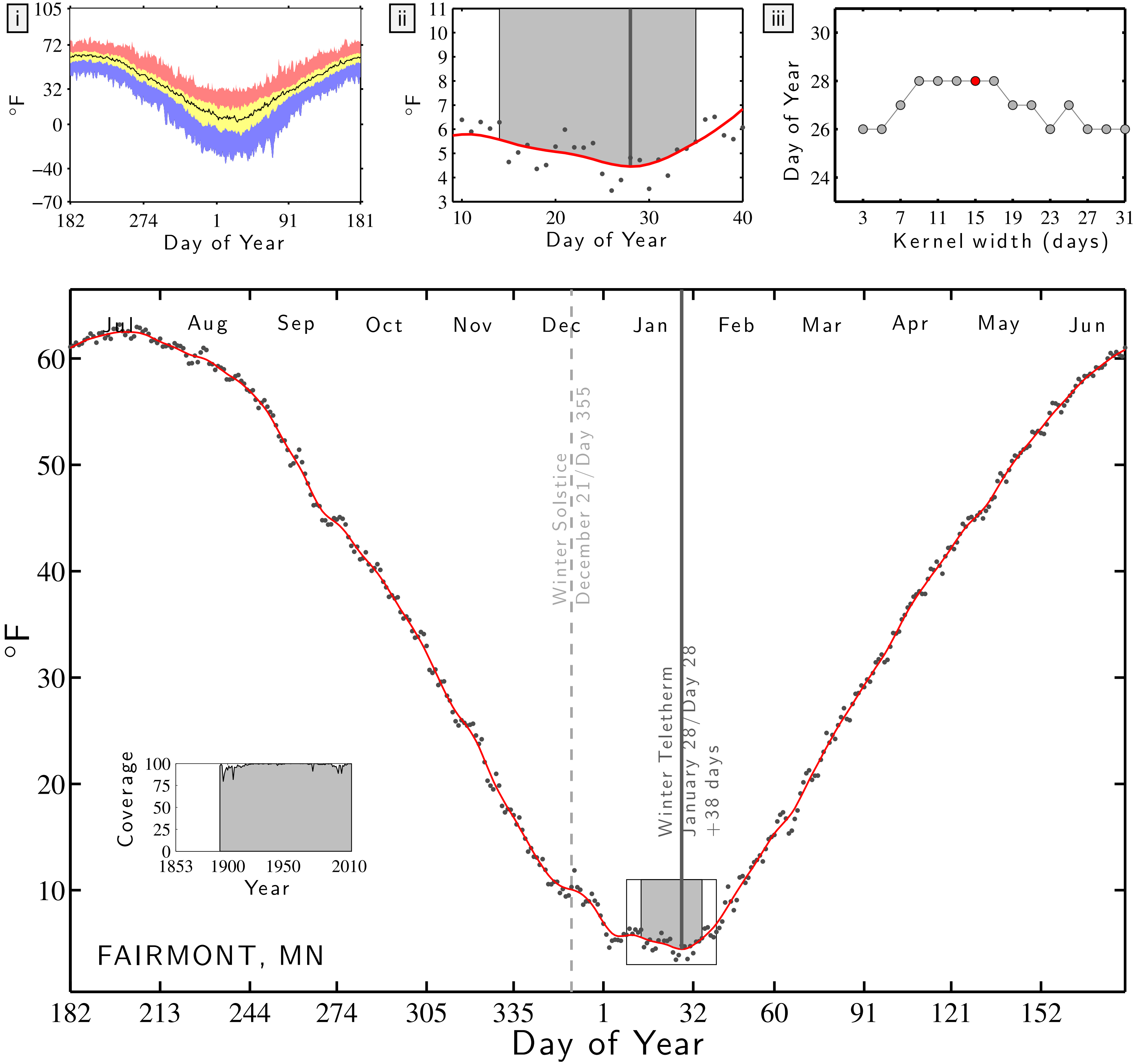
i
°F
Day of Year
ii
Day of Year
iii
Day of Year
Kernel width (days)
Jul
Aug
Sep
Oct
Nov
Dec
Jan
Feb
Mar
Apr
May
Jun
Winter Solstice
December 21/Day 355
Winter Teletherm
January 28/Day 28
+38 days
Coverage
Year
FAIRMONT, MN
°F
Day of Year

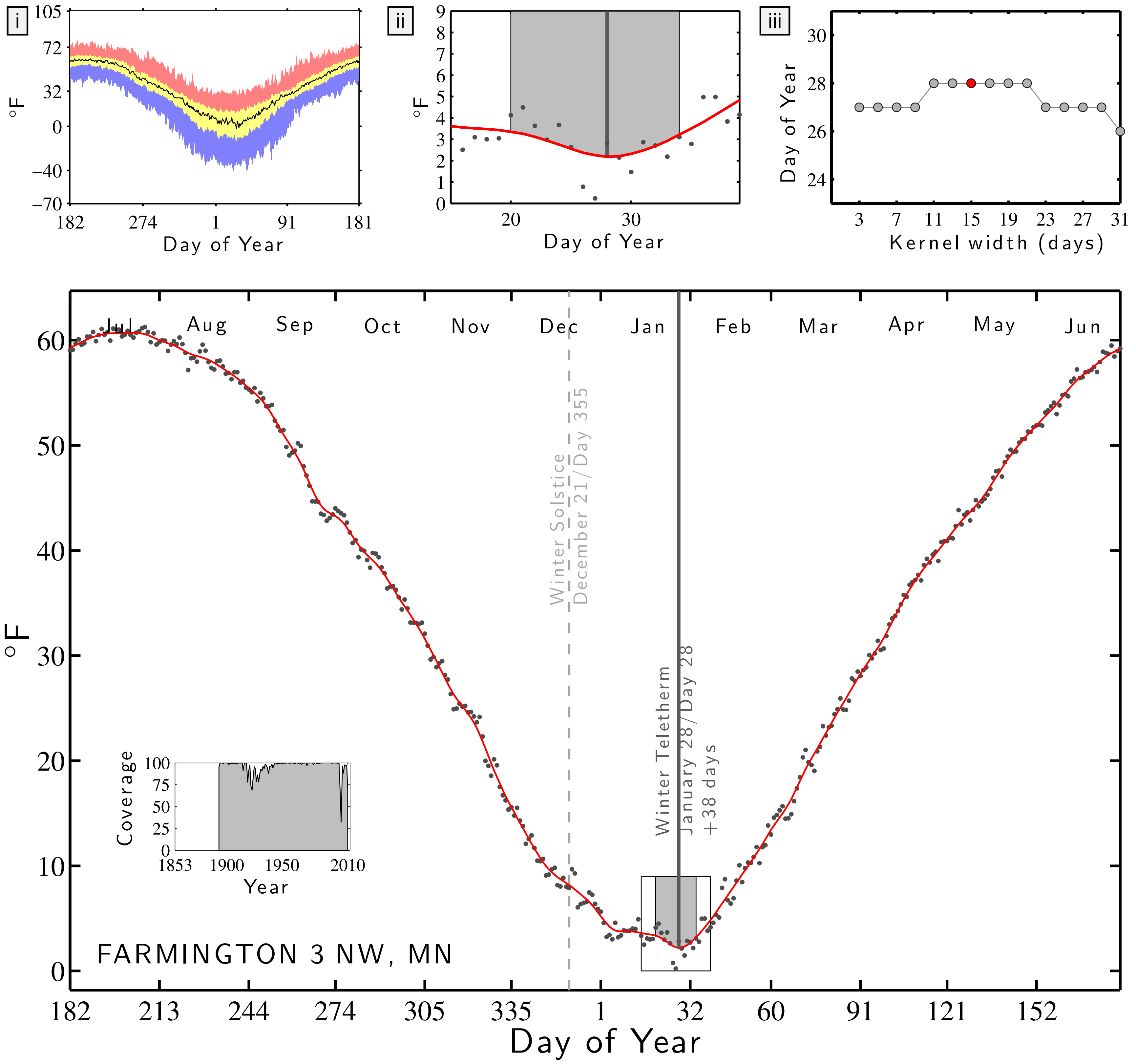

i
ii
iii
°F
Day of Year
Kernel width (days)
Jul
Aug
Sep
Oct
Nov
Dec
Jan
Feb
Mar
Apr
May
Jun
Winter Solstice
December 21/Day 355
Winter Teletherm
January 28/Day 28
+38 days
Coverage
Year
FARMINGTON 3 NW, MN

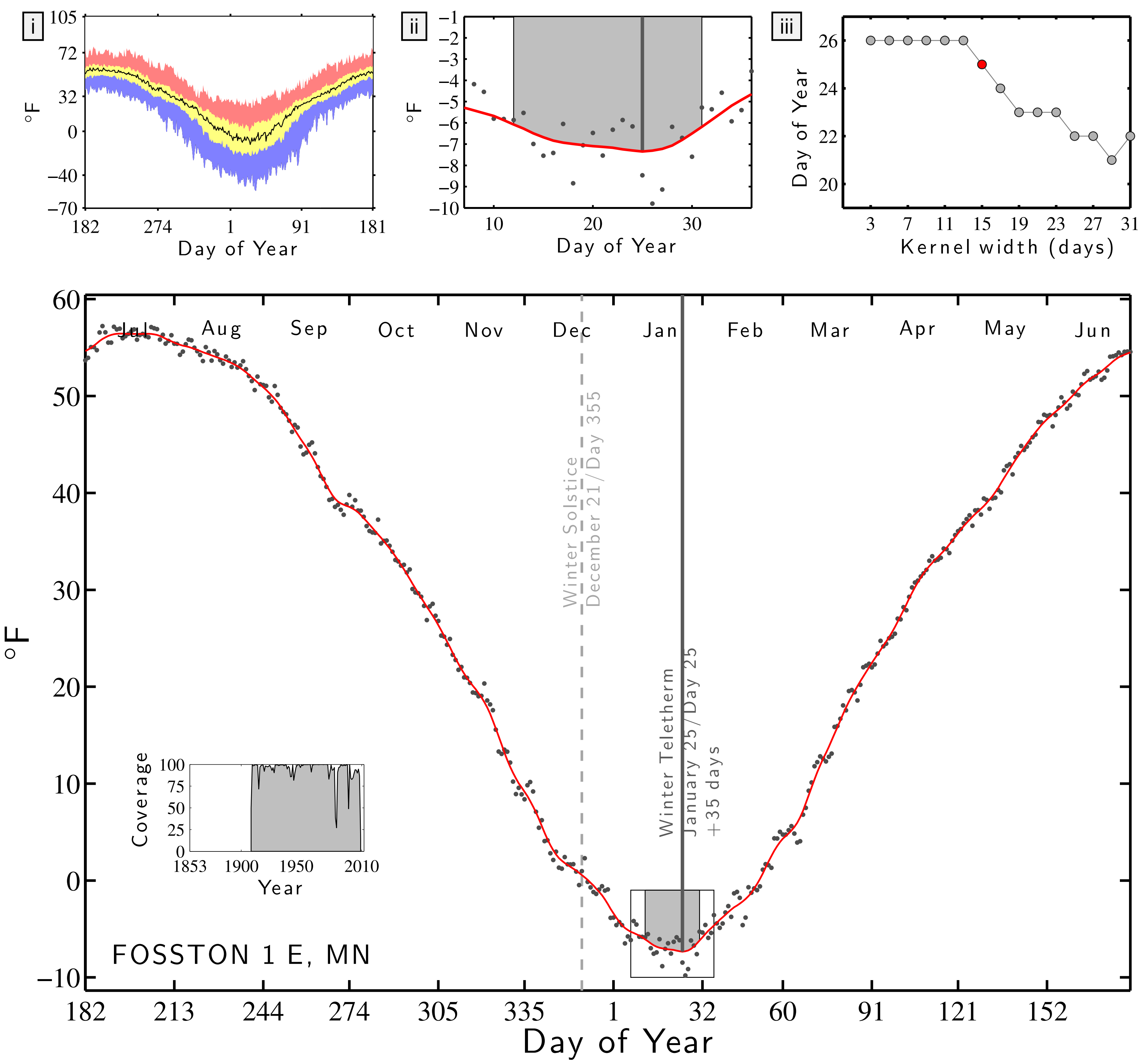

i
°F
Day of Year
ii
°F
Day of Year
iii
Day of Year
Kernel width (days)
Jul
Aug
Sep
Oct
Nov
Dec
Jan
Feb
Mar
Apr
May
Jun
Winter Solstice
December 21/Day 355
Winter Teletherm
January 25/Day 25
+35 days
Coverage
Year
FOSSTON 1 E, MN
°F
Day of Year

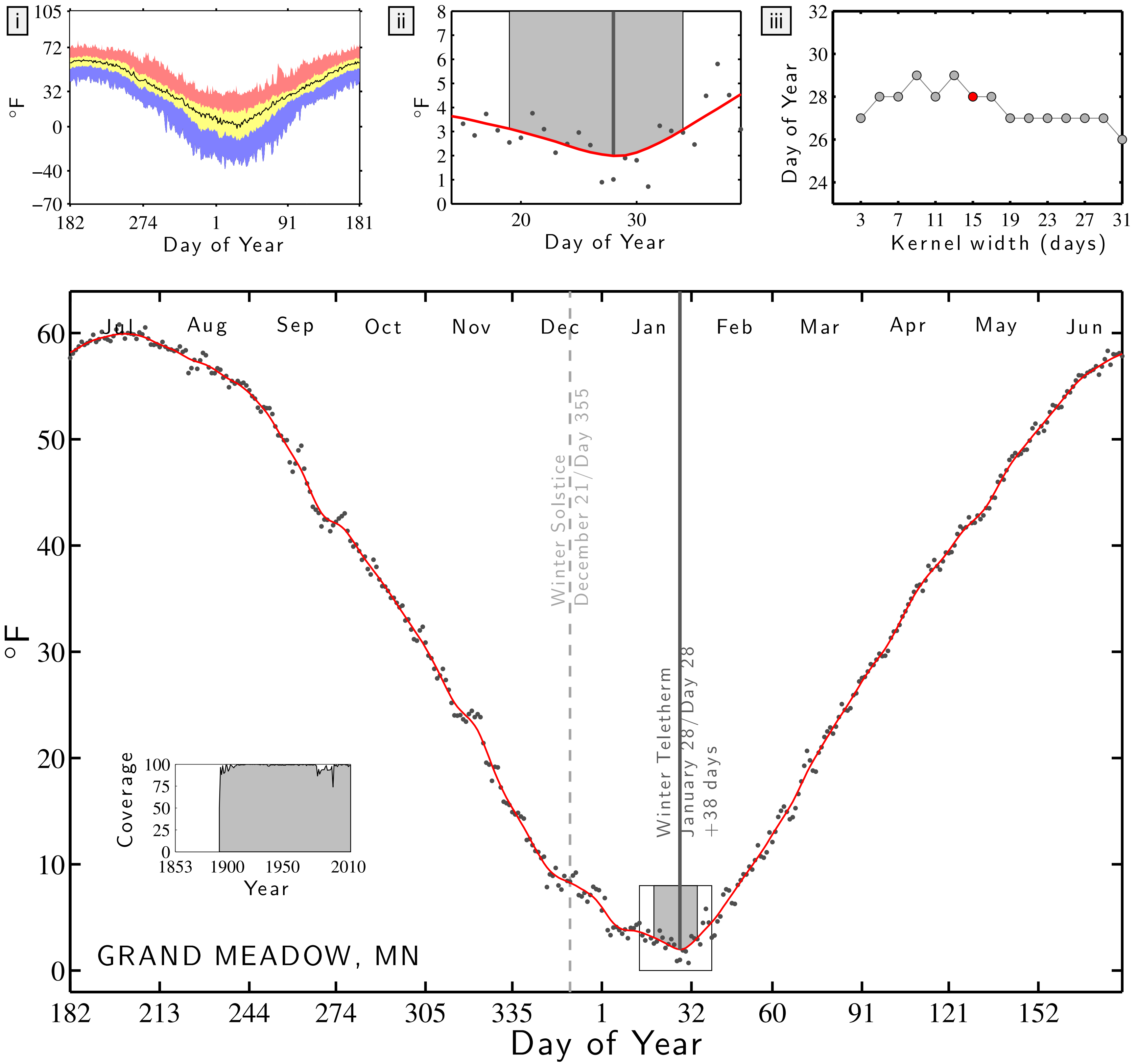

i
°F
Day of Year
ii
°F
Day of Year
iii
Day of Year
Kernel width (days)
Jul
Aug
Sep
Oct
Nov
Dec
Jan
Feb
Mar
Apr
May
Jun
Winter Solstice
December 21/Day 355
Winter Teletherm
January 28/Day 28
+38 days
Coverage
Year
GRAND MEADOW, MN
°F
Day of Year

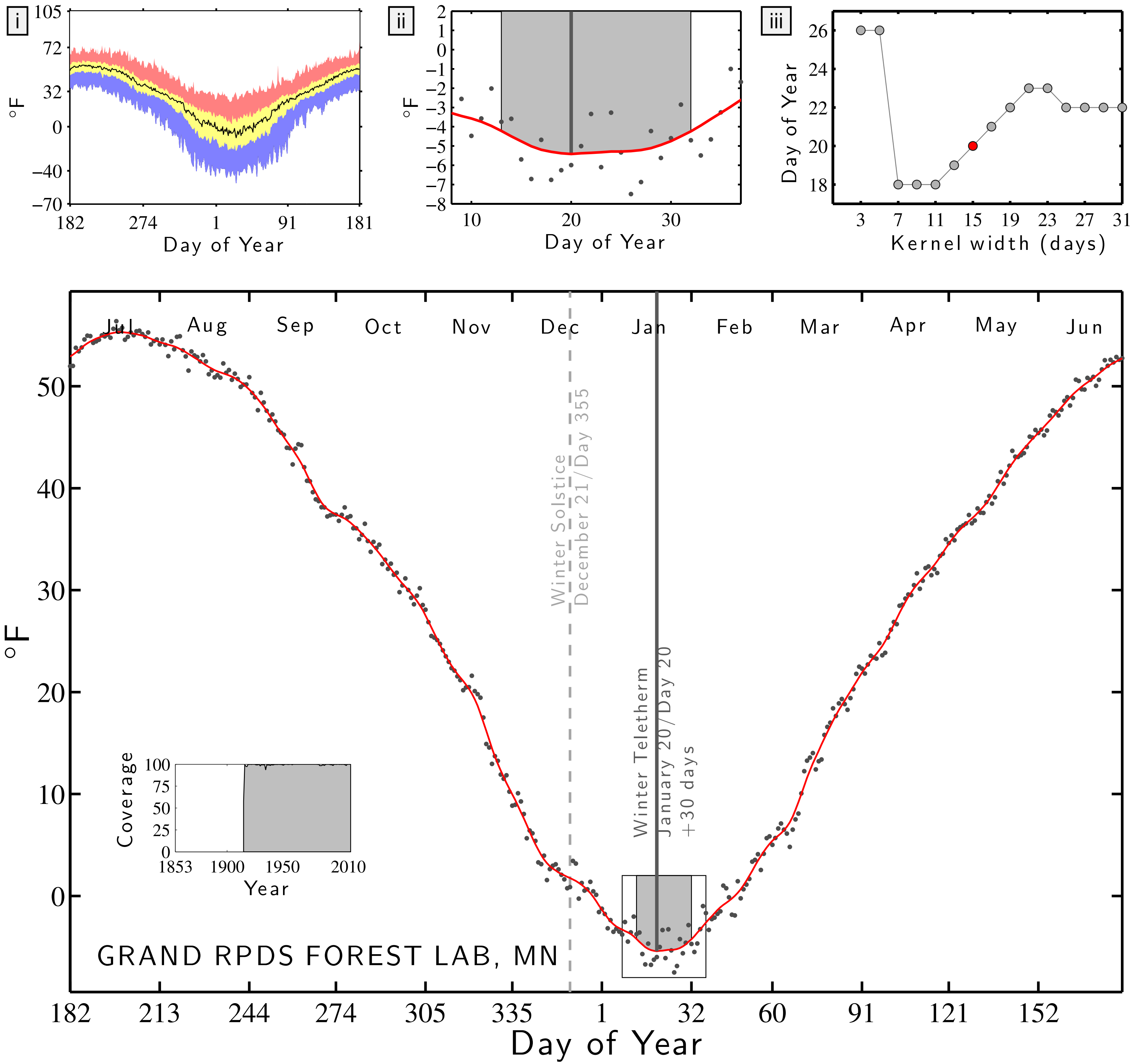

i
°F
Day of Year
ii
°F
Day of Year
iii
Day of Year
Kernel width (days)
Jul
Aug
Sep
Oct
Nov
Dec
Jan
Feb
Mar
Apr
May
Jun
Winter Solstice
December 21/Day 355
Winter Teletherm
January 20/Day 20
+30 days
Coverage
Year
GRAND RPDS FOREST LAB, MN
°F
Day of Year

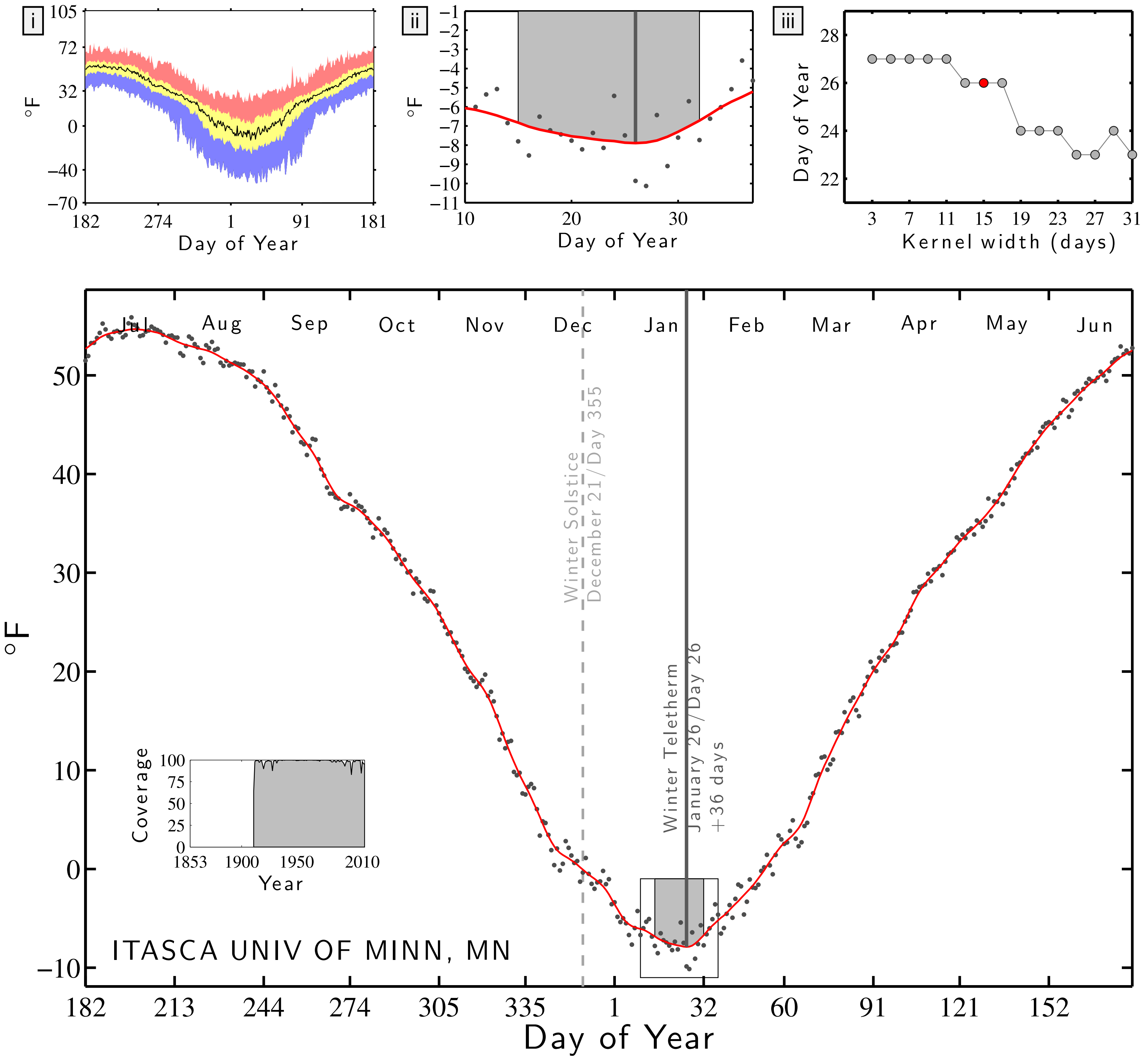

i
ii
iii
°F
Day of Year
Kernel width (days)
Jul
Aug
Sep
Oct
Nov
Dec
Jan
Feb
Mar
Apr
May
Jun
Winter Solstice
December 21/Day 355
Winter Teletherm
January 26/Day 26
+36 days
Coverage
Year
ITASCA UNIV OF MINN, MN

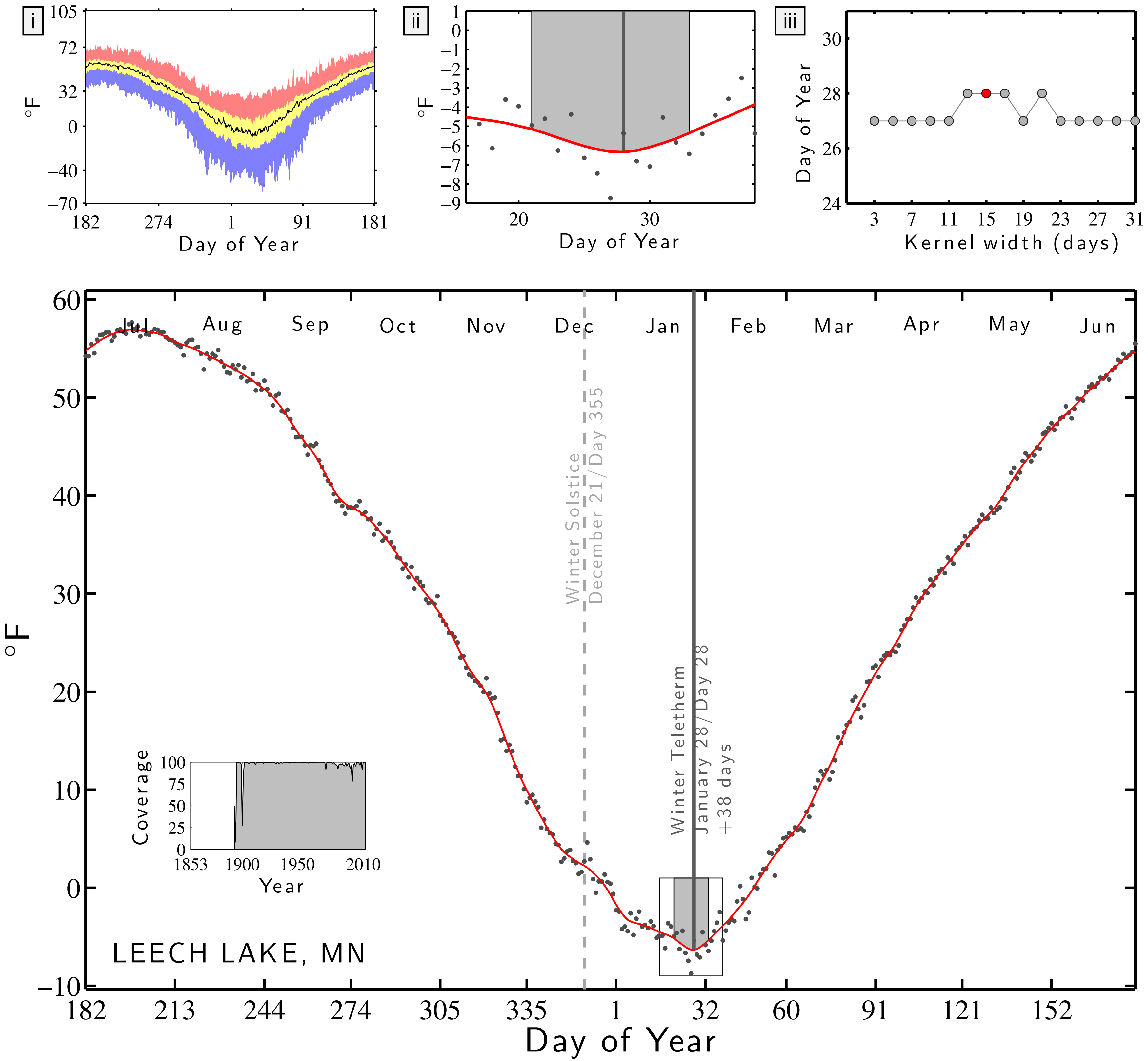

i
°F
Day of Year
ii
°F
Day of Year
iii
Day of Year
Kernel width (days)
Jul
Aug
Sep
Oct
Nov
Dec
Jan
Feb
Mar
Apr
May
Jun
Winter Solstice
December 21/Day 355
Winter Teletherm
January 28/Day 28
+38 days
Coverage
Year
LEECH LAKE, MN
°F
Day of Year

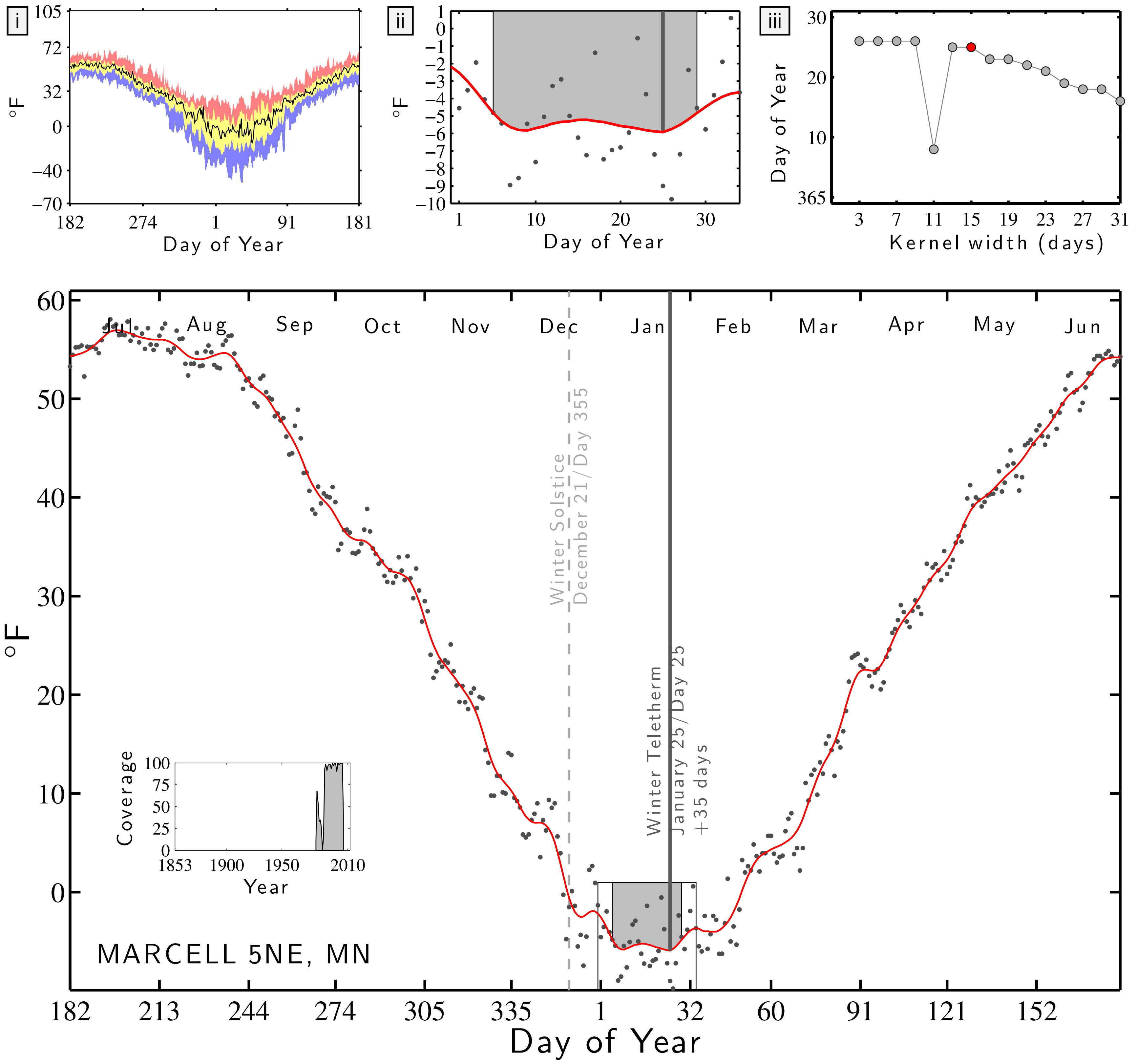
i
ii
iii
°F
Day of Year
Kernel width (days)
MARCELL 5NE, MN
Jul
Aug
Sep
Oct
Nov
Dec
Jan
Feb
Mar
Apr
May
Jun
Winter Solstice
December 21/Day 355
Winter Teletherm
January 25/Day 25
+35 days
Coverage
Year
1853
1900
1950
2010

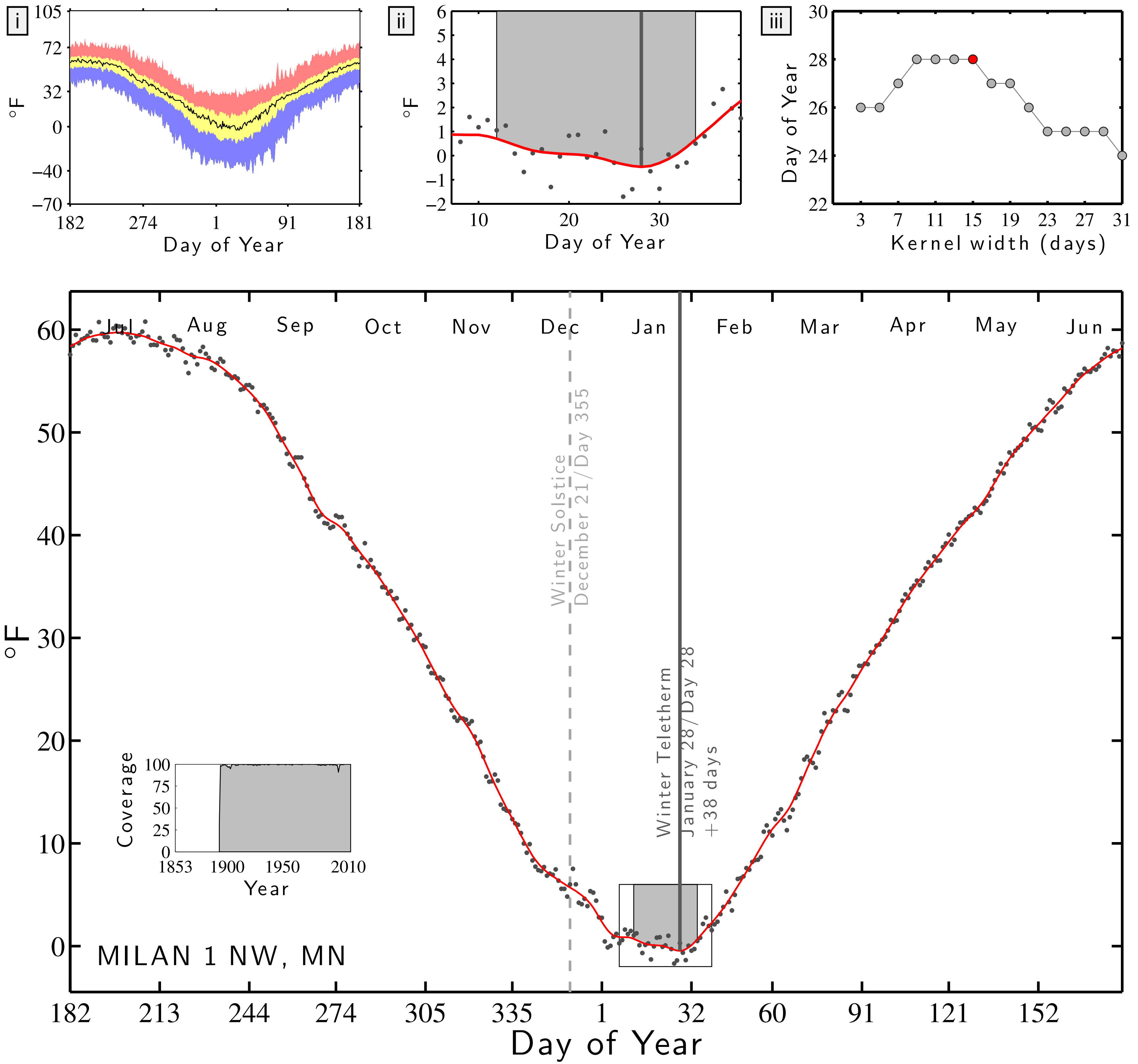

i
ii
iii
°F
Day of Year
Kernel width (days)
Day of Year
Jul Aug Sep Oct Nov Dec Jan Feb Mar Apr May Jun
Winter Solstice
December 21/Day 355
Winter Teletherm
January 28/Day 28
+38 days
Coverage
Year
MILAN 1 NW, MN

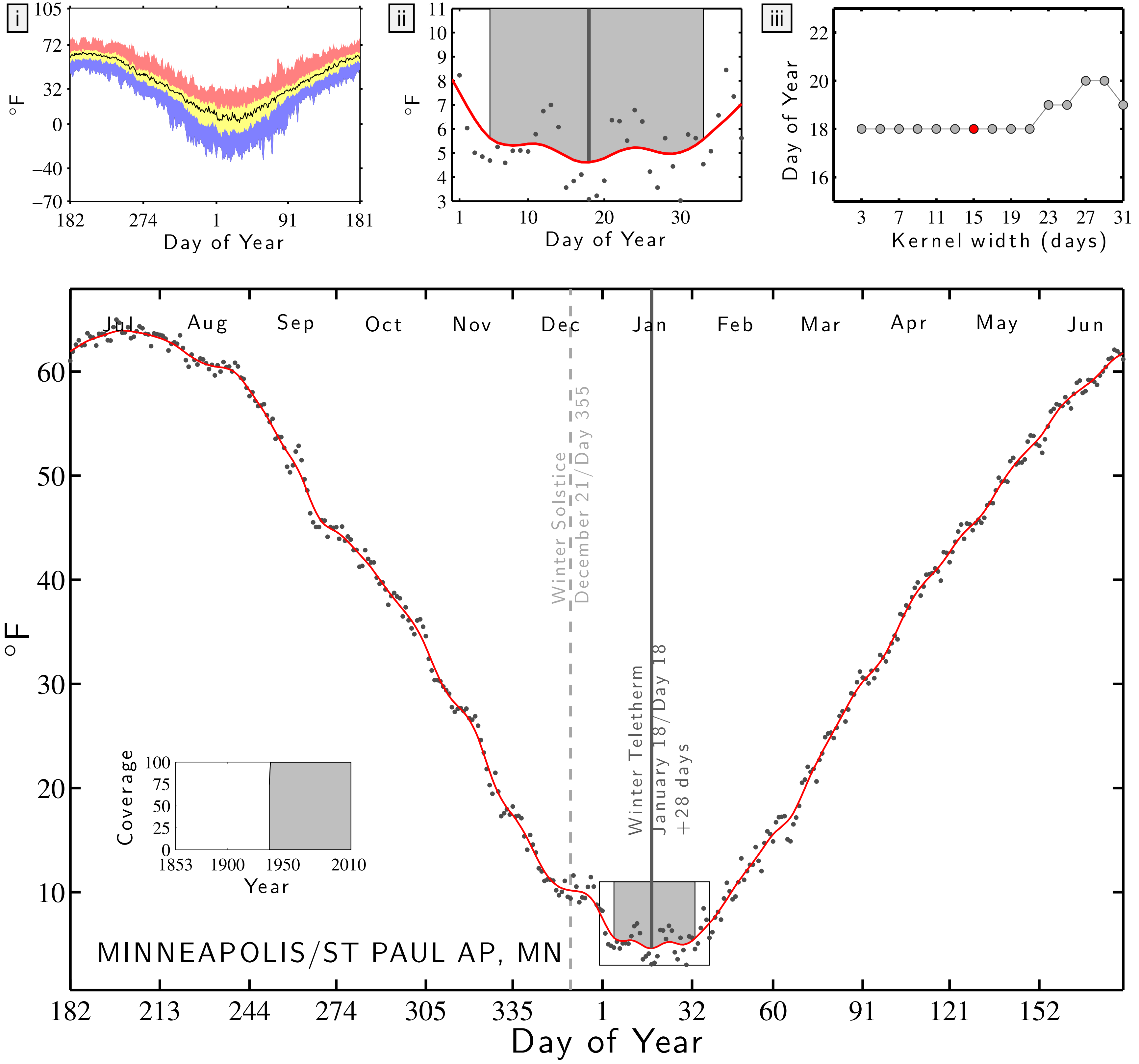

i
ii
iii
°F
Day of Year
Day of Year
Kernel width (days)
Jul
Aug
Sep
Oct
Nov
Dec
Jan
Feb
Mar
Apr
May
Jun
Winter Solstice
December 21/Day 355
Winter Teletherm
January 18/Day 18
+28 days
Coverage
Year
MINNEAPOLIS/ST PAUL AP, MN
Day of Year

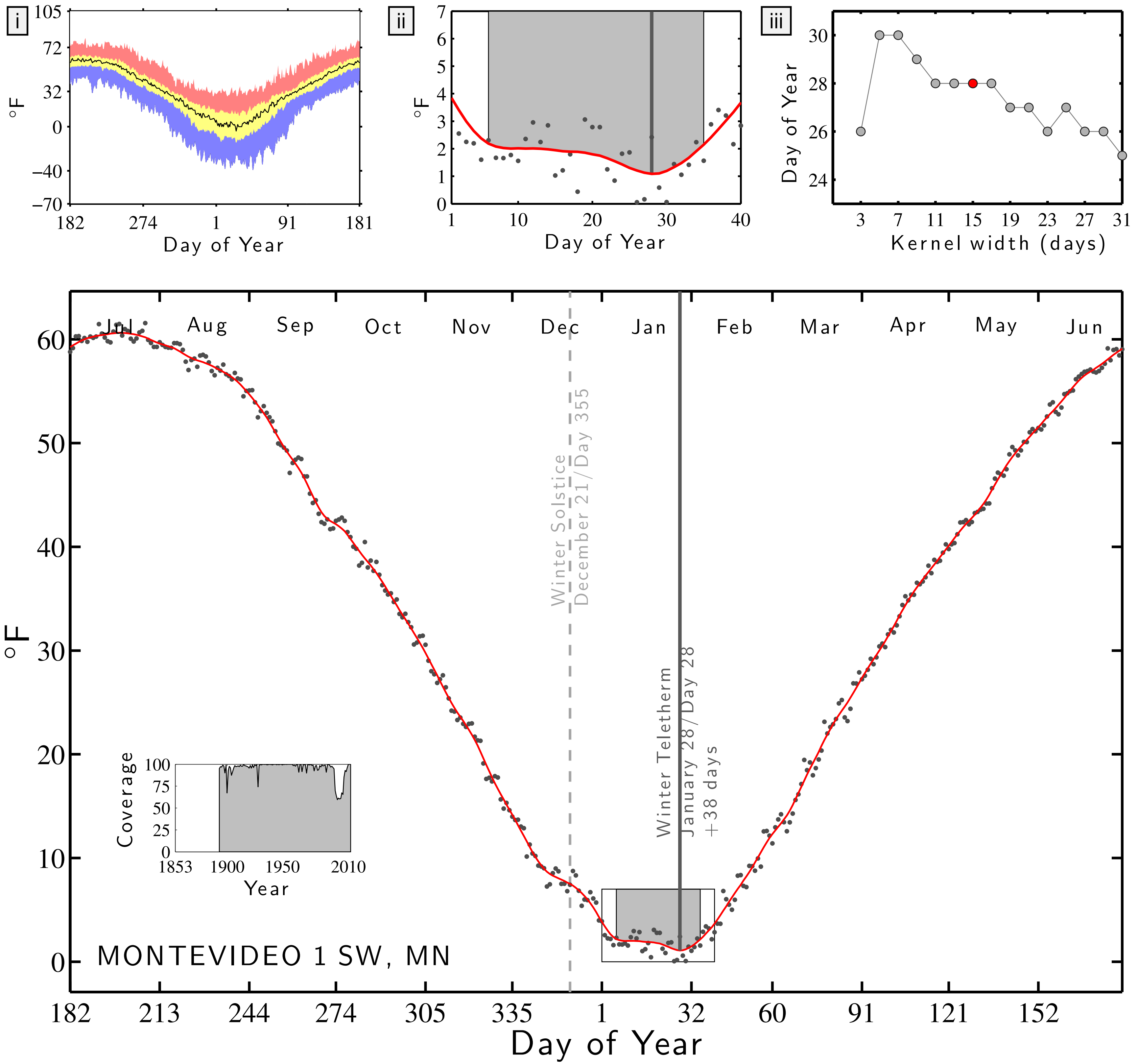

i
ii
iii
°F
Day of Year
Kernel width (days)
Jul
Aug
Sep
Oct
Nov
Dec
Jan
Feb
Mar
Apr
May
Jun
Winter Solstice
December 21/Day 355
Winter Teletherm
January 28/Day 28
+38 days
Coverage
Year
MONTEVIDEO 1 SW, MN

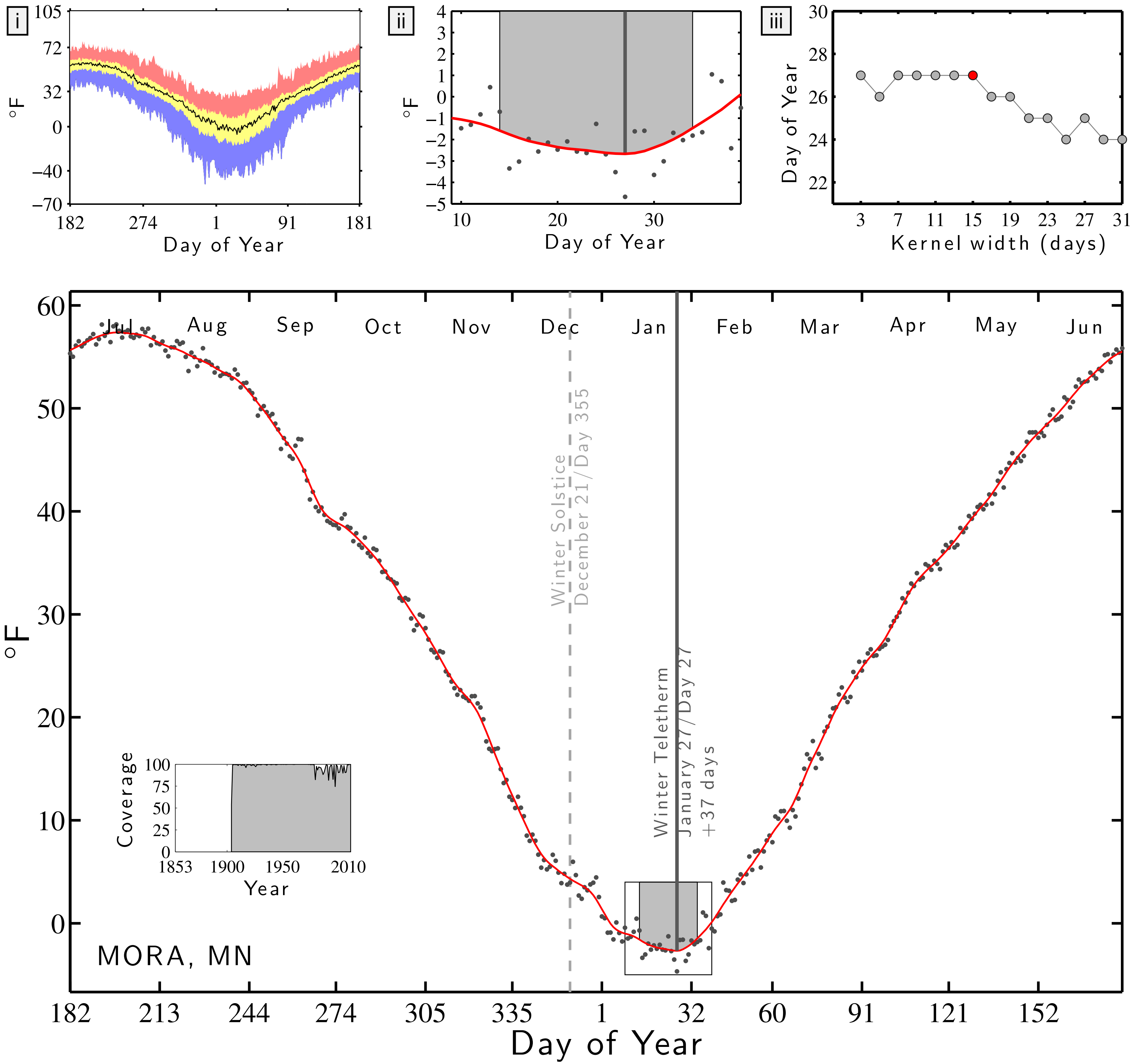

i
°F
Day of Year
ii
°F
Day of Year
iii
Day of Year
Kernel width (days)
Jul
Aug
Sep
Oct
Nov
Dec
Jan
Feb
Mar
Apr
May
Jun
Winter Solstice
December 21/Day 355
Winter Teletherm
January 27/Day 27
+37 days
Coverage
Year
MORA, MN
°F
Day of Year

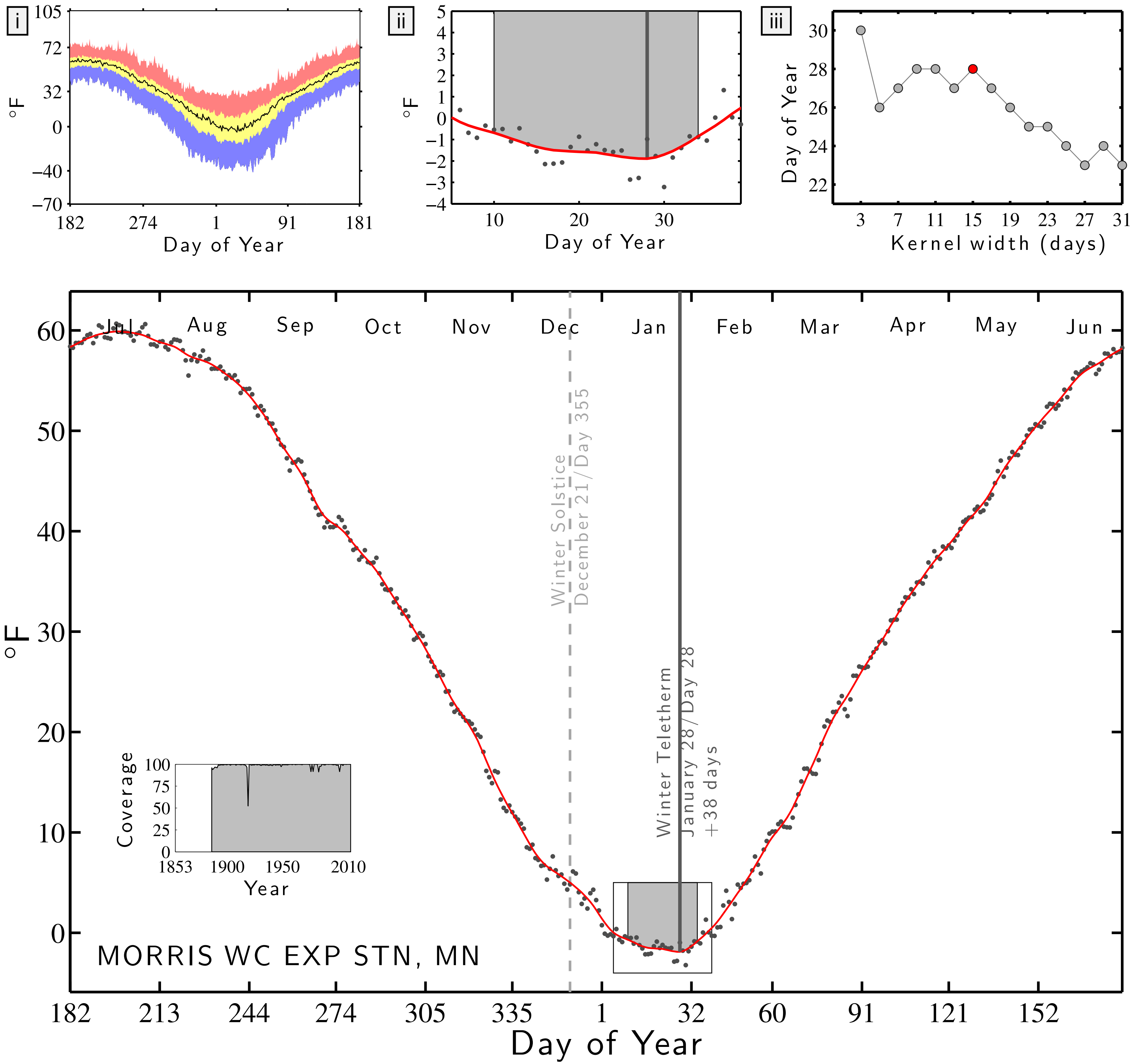

i
ii
iii
°F
Day of Year
Kernel width (days)
Jul
Aug
Sep
Oct
Nov
Dec
Jan
Feb
Mar
Apr
May
Jun
Winter Solstice
December 21/Day 355
Winter Teletherm
January 28/Day 28
+38 days
Coverage
Year
MORRIS WC EXP STN, MN

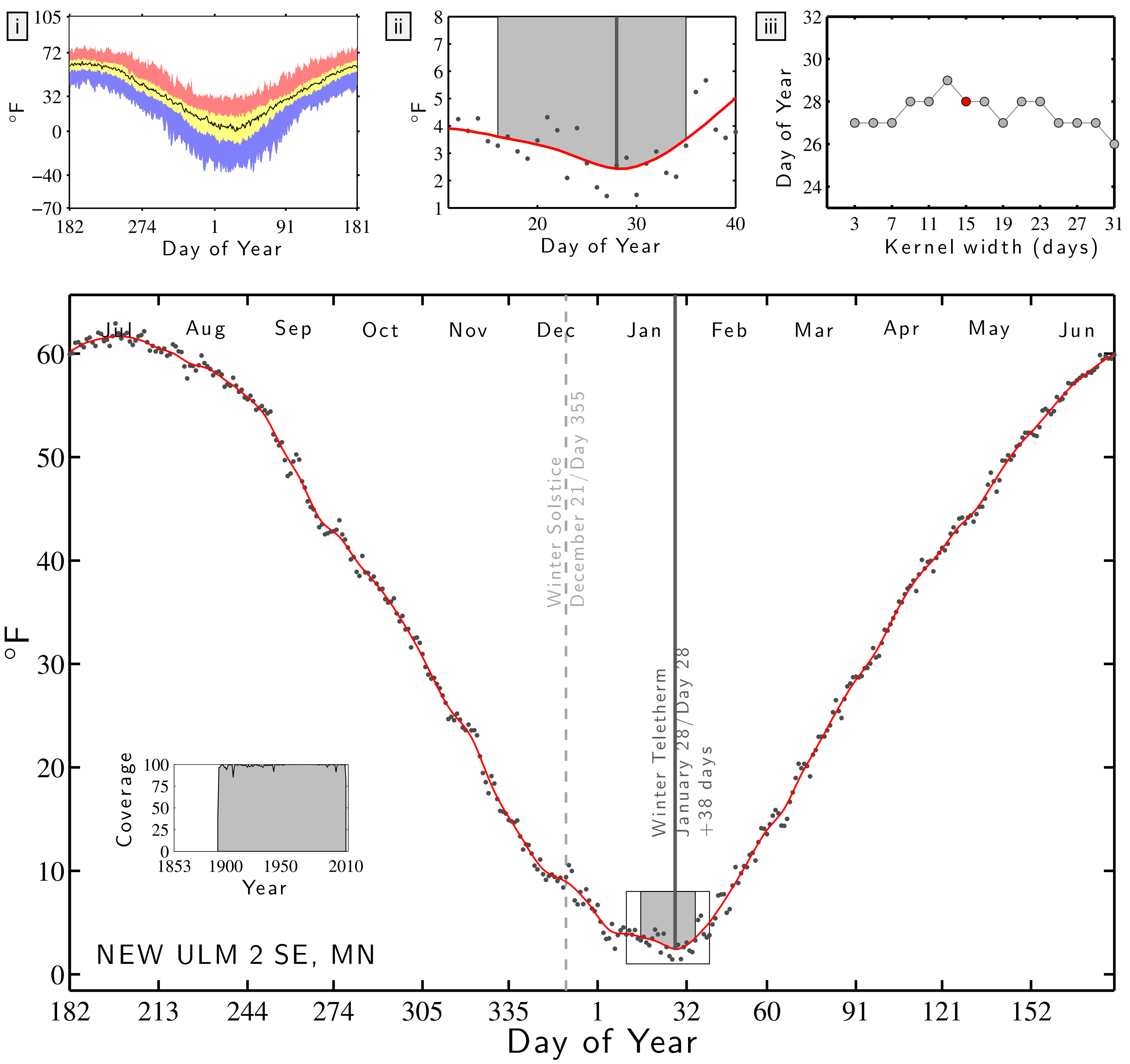

i
°F
Day of Year
ii
°F
Day of Year
iii
Day of Year
Kernel width (days)
Jul
Aug
Sep
Oct
Nov
Dec
Jan
Feb
Mar
Apr
May
Jun
Winter Solstice
December 21/Day 355
Winter Teletherm
January 28/Day 28
+38 days
Coverage
Year
NEW ULM 2 SE, MN
°F
Day of Year

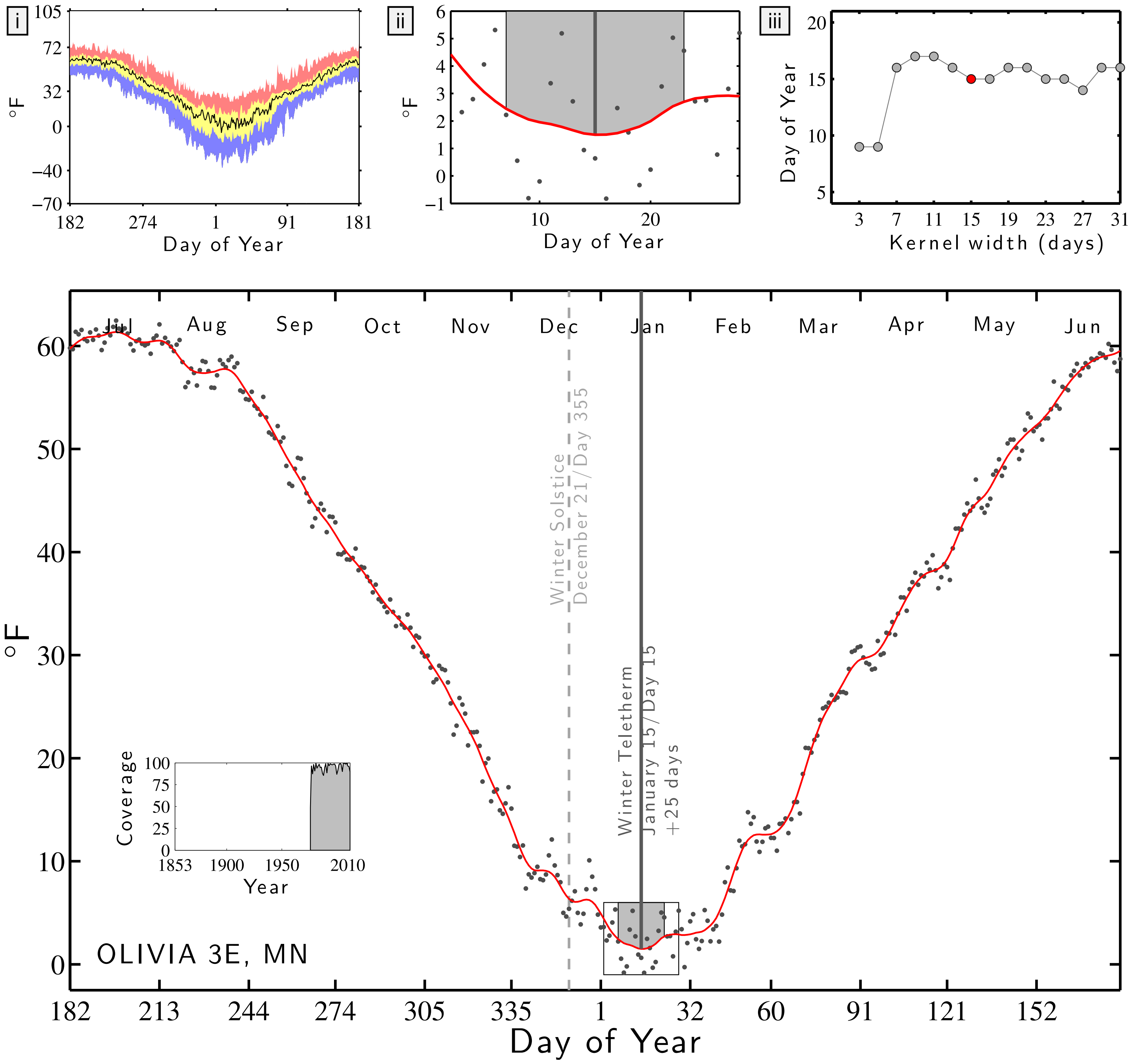

i
°F
Day of Year
ii
°F
Day of Year
iii
Day of Year
Kernel width (days)
Jul
Aug
Sep
Oct
Nov
Dec
Jan
Feb
Mar
Apr
May
Jun
Winter Solstice
December 21/Day 355
Winter Teletherm
January 15/Day 15
+25 days
Coverage
Year
OLIVIA 3E, MN
°F
Day of Year

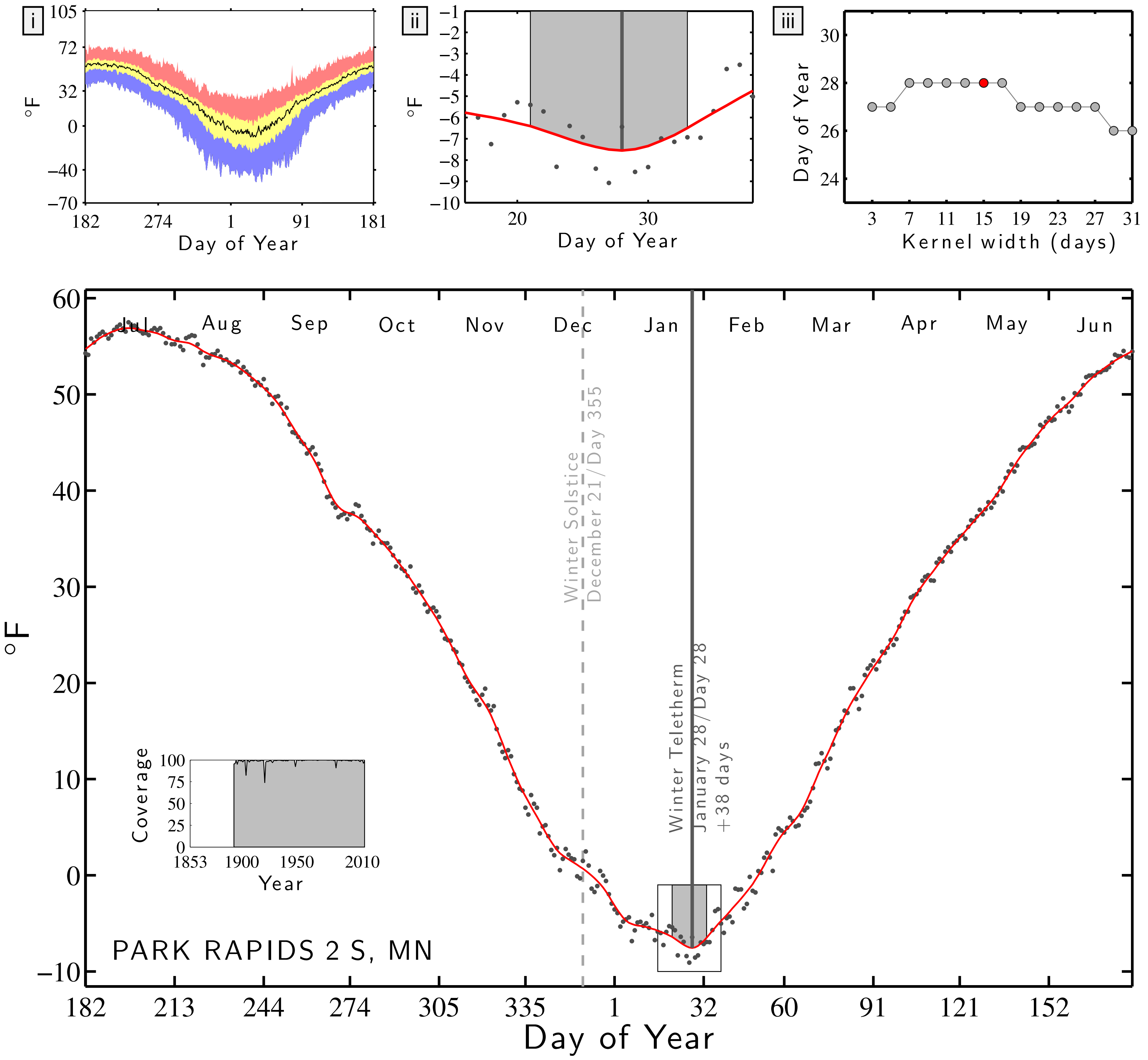

i
ii
iii
°F
Day of Year
Kernel width (days)
Jul
Aug
Sep
Oct
Nov
Dec
Jan
Feb
Mar
Apr
May
Jun
Winter Solstice
December 21/Day 355
Winter Teletherm
January 28/Day 28
+38 days
Coverage
Year
PARK RAPIDS 2 S, MN

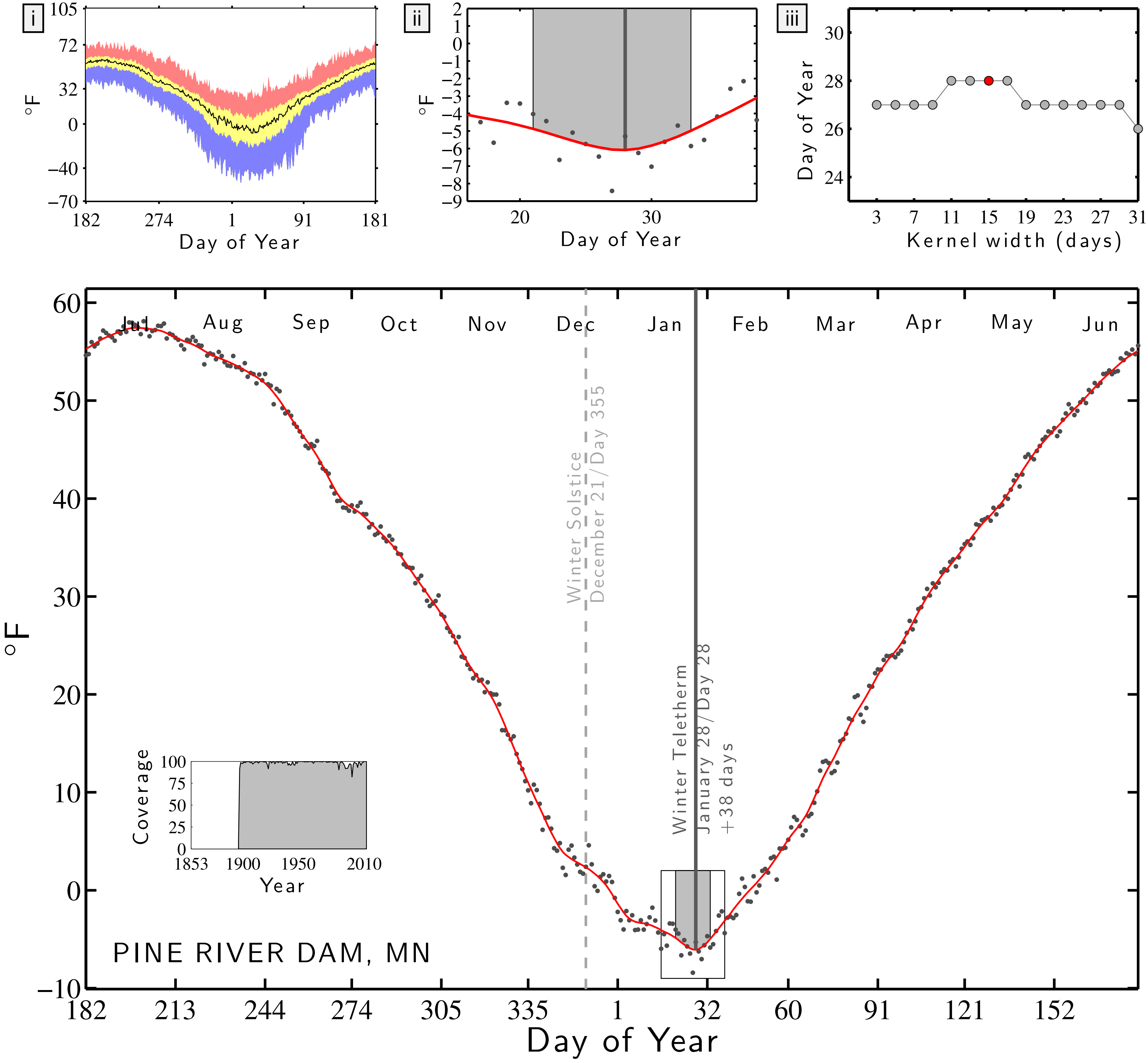

i
ii
iii
Day of Year
Kernel width (days)
Winter Solstice
December 21/Day 355
Winter Teletherm
January 28/Day 28
+38 days
Coverage
Year
PINE RIVER DAM, MN
Jul
Aug
Sep
Oct
Nov
Dec
Jan
Feb
Mar
Apr
May
Jun
°F

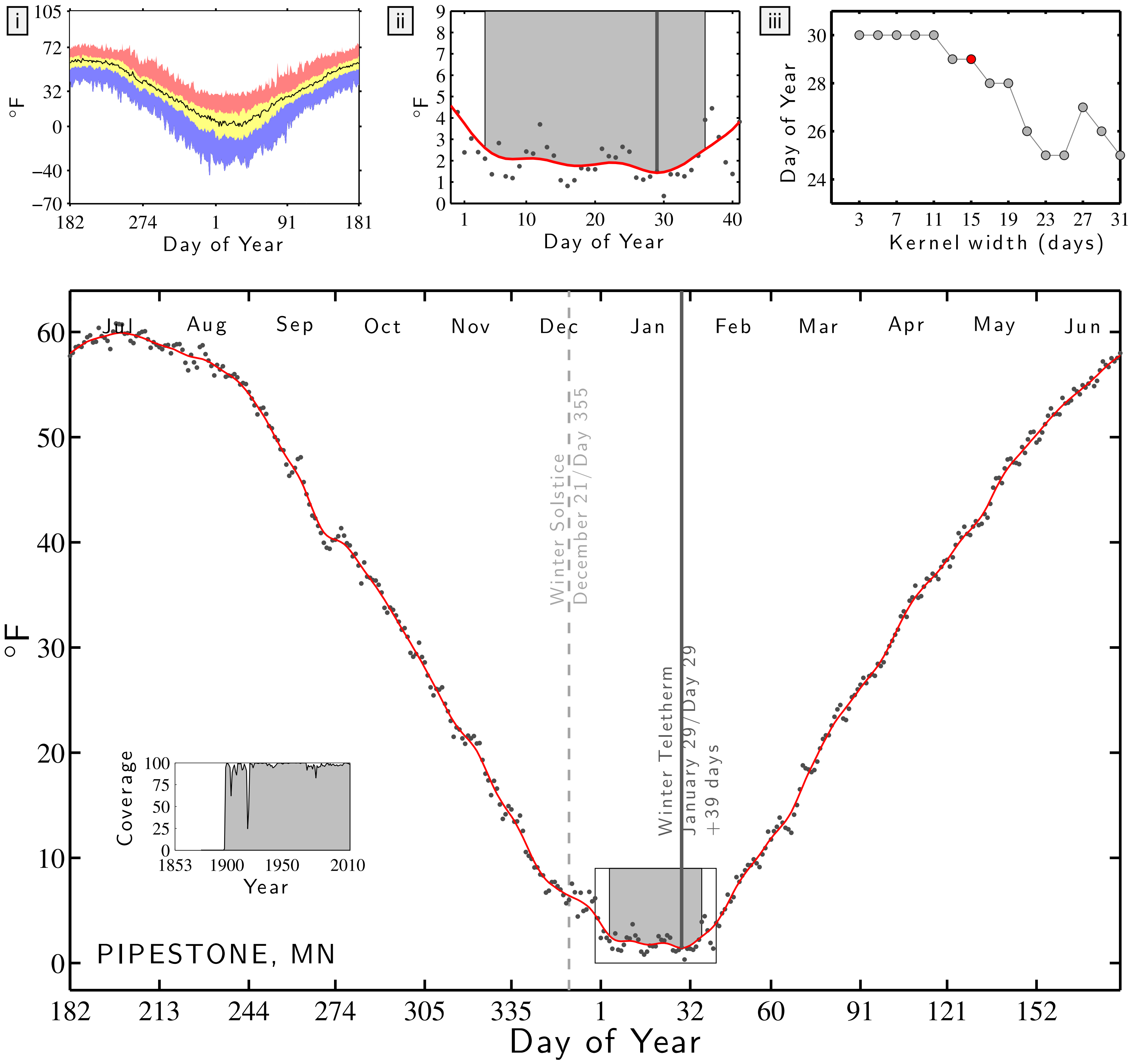

i
°F
Day of Year
ii
°F
Day of Year
iii
Day of Year
Kernel width (days)
Jul
Aug
Sep
Oct
Nov
Dec
Jan
Feb
Mar
Apr
May
Jun
Winter Solstice
December 21/Day 355
Winter Teletherm
January 29/Day 29
+39 days
Coverage
Year
PIPESTONE, MN
°F
Day of Year

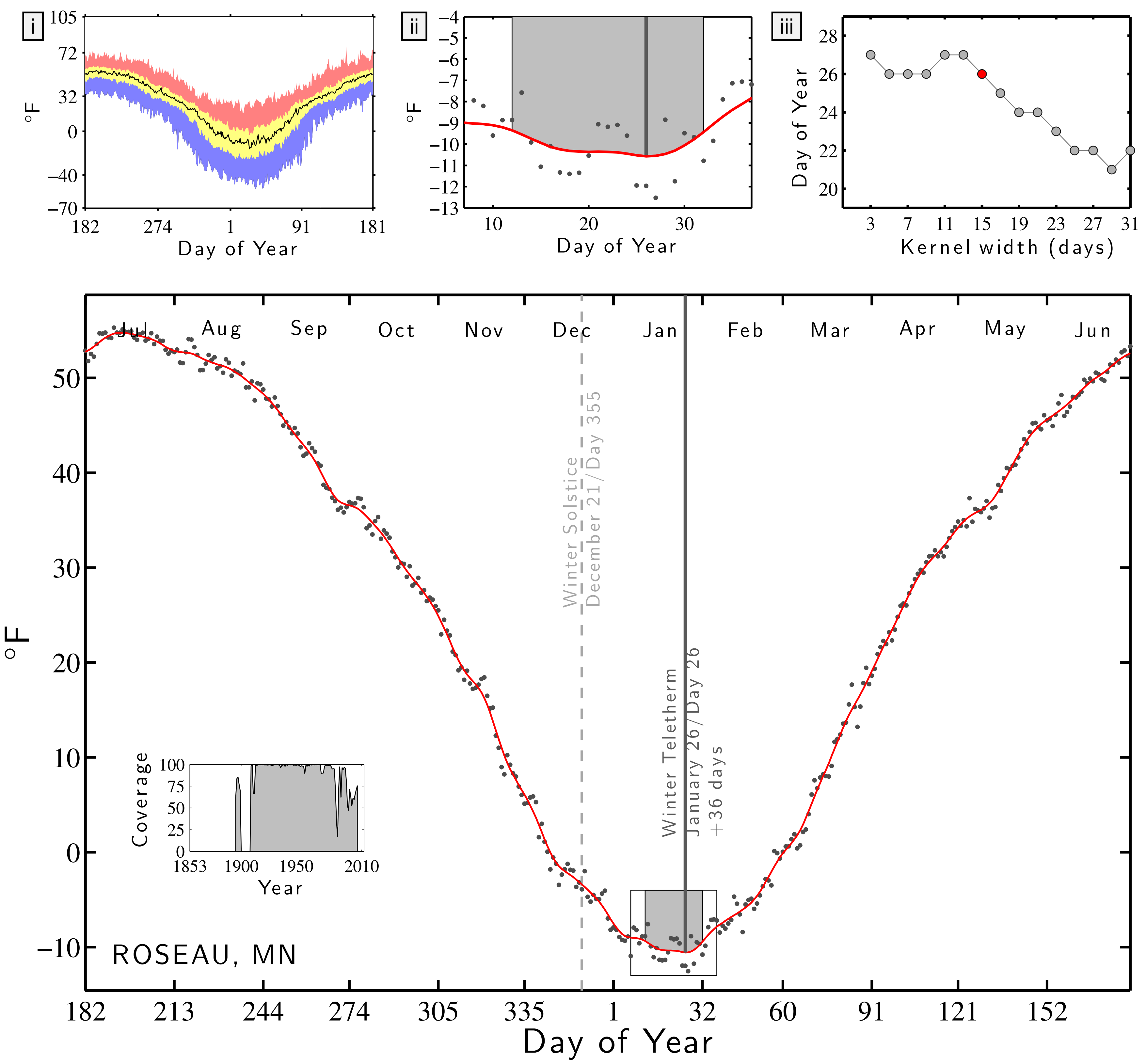

i
°F
Day of Year
ii
°F
Day of Year
iii
Day of Year
Kernel width (days)
Jul
Aug
Sep
Oct
Nov
Dec
Jan
Feb
Mar
Apr
May
Jun
Winter Solstice
December 21/Day 355
Winter Teletherm
January 26/Day 26
+36 days
Coverage
Year
ROSEAU, MN
°F
Day of Year

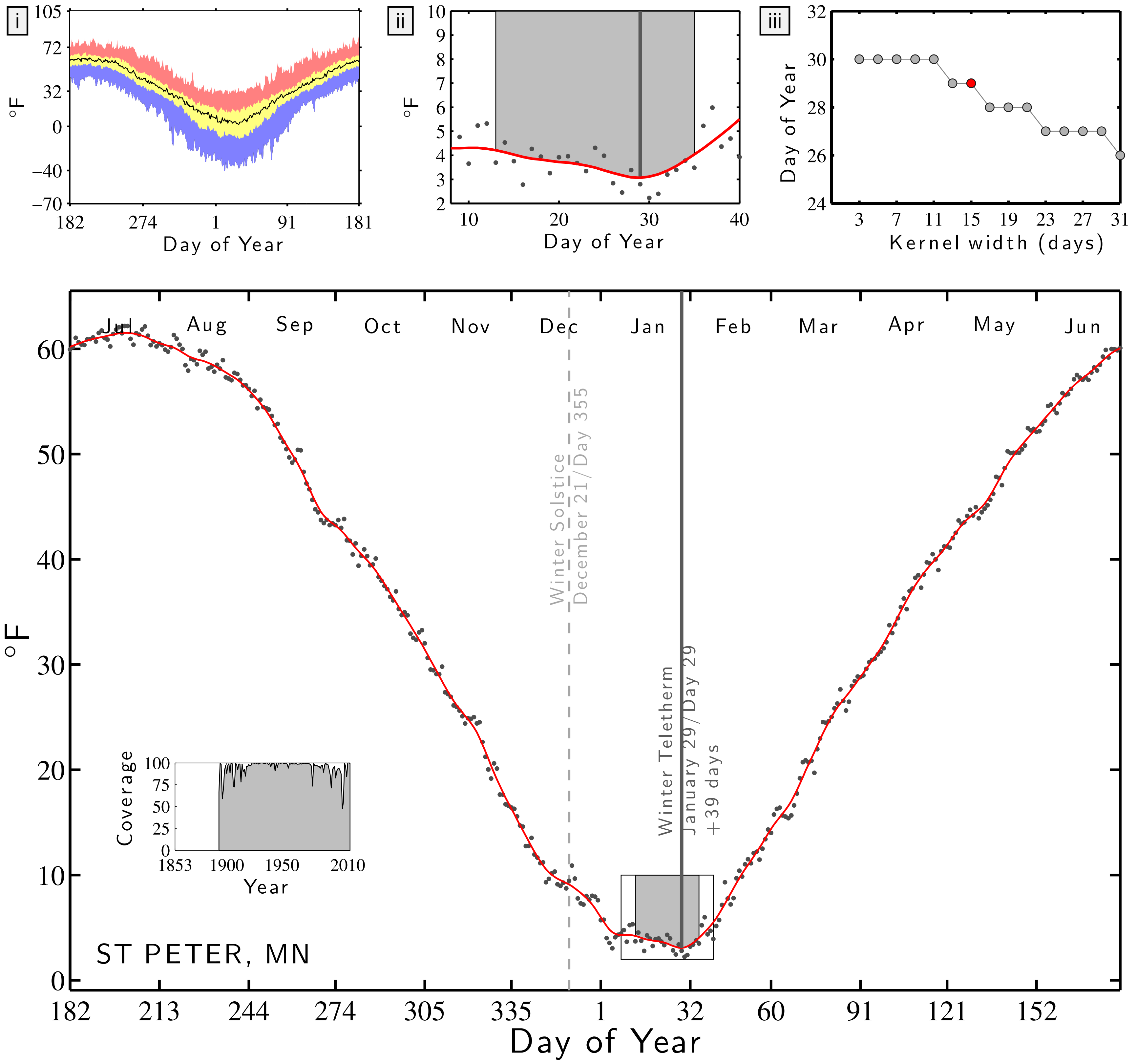
i
ii
iii
°F
Day of Year
Kernel width (days)
Day of Year
Jul
Aug
Sep
Oct
Nov
Dec
Jan
Feb
Mar
Apr
May
Jun
Winter Solstice
December 21/Day 355
Winter Teletherm
January 29/Day 29
+39 days
Coverage
Year
ST PETER, MN

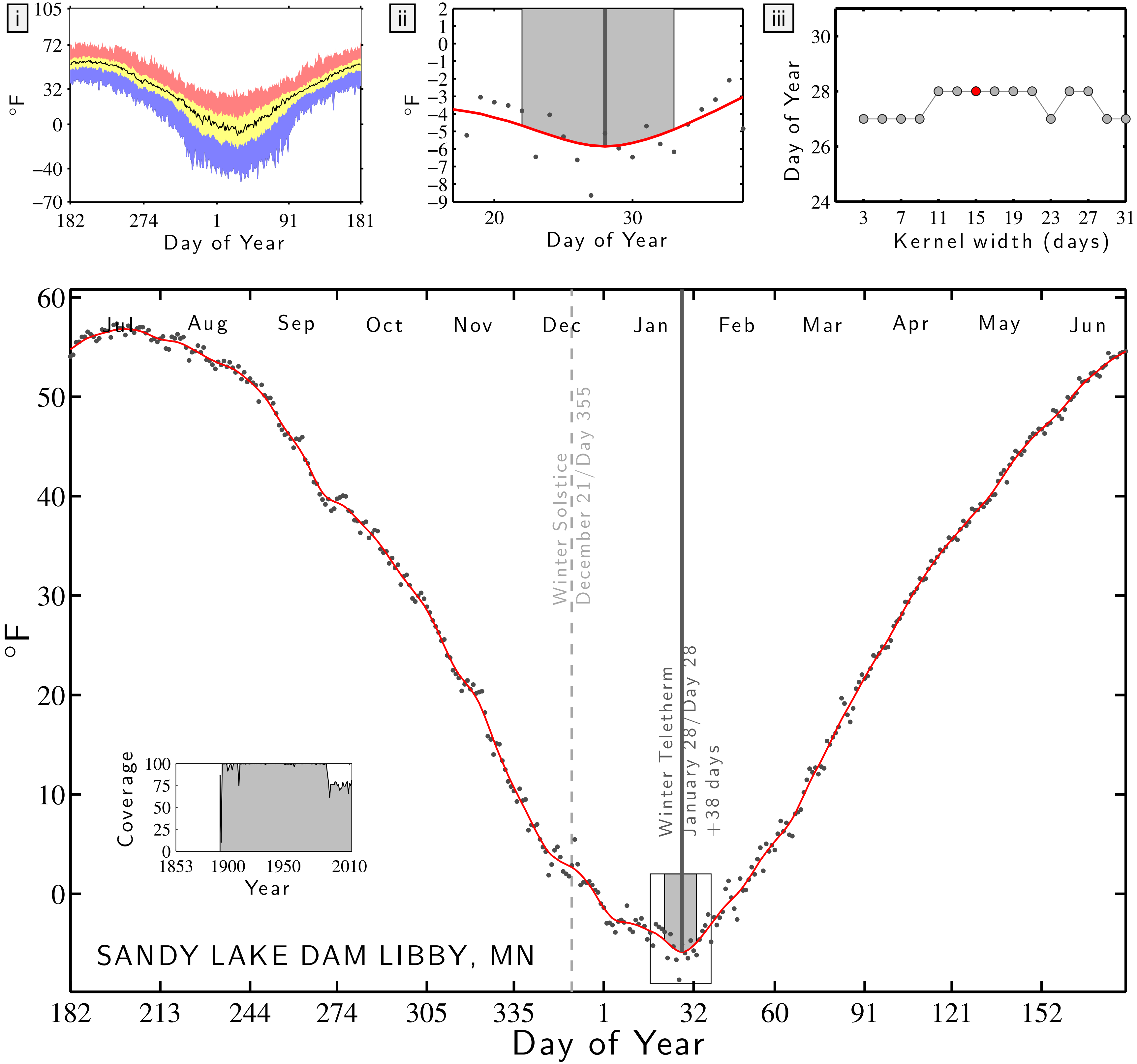

i
ii
iii
°F
Day of Year
Kernel width (days)
Jul
Aug
Sep
Oct
Nov
Dec
Jan
Feb
Mar
Apr
May
Jun
Winter Solstice
December 21/Day 355
Winter Teletherm
January 28/Day 28
+38 days
Coverage
Year
SANDY LAKE DAM LIBBY, MN

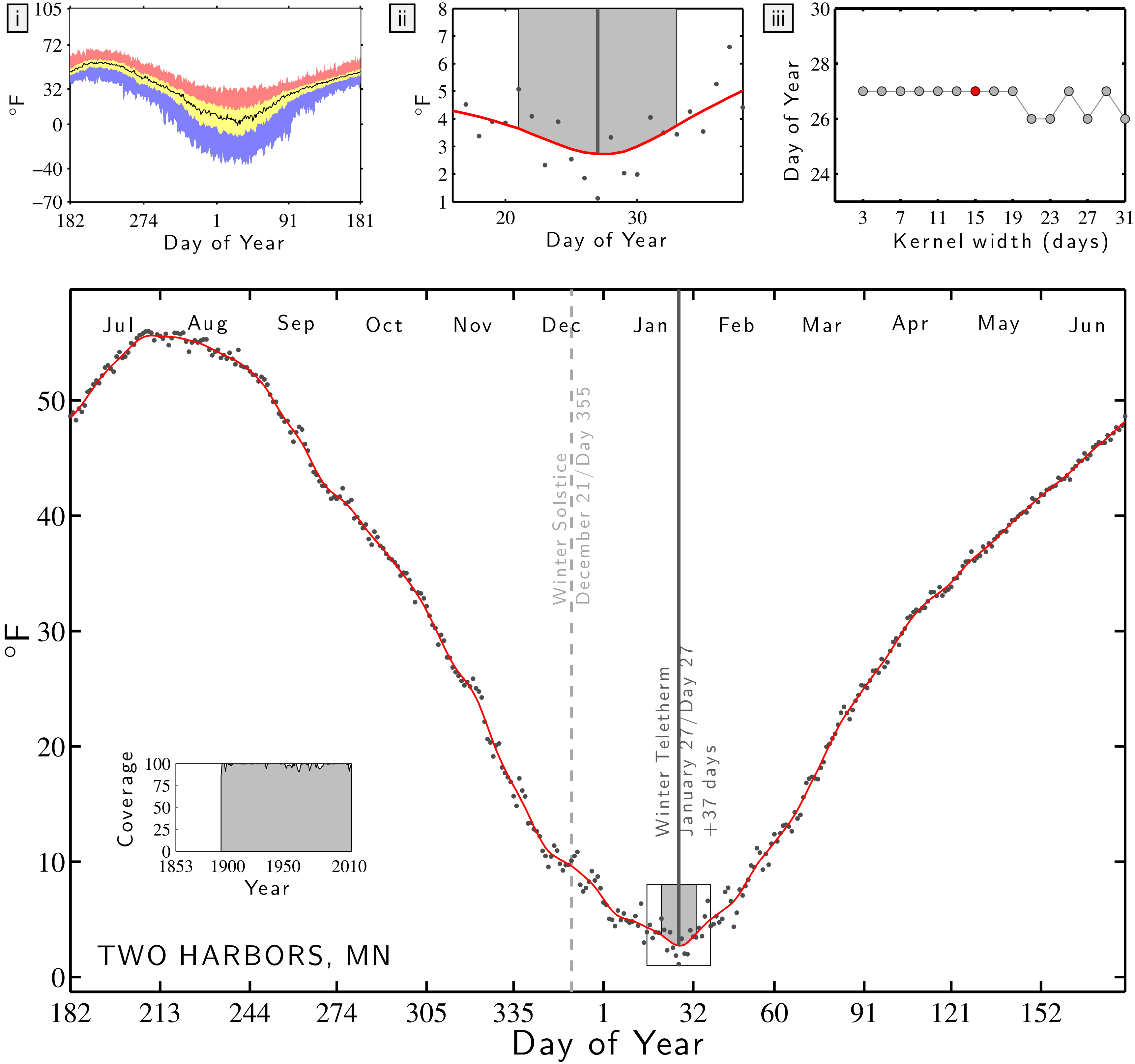

i
°F
Day of Year
ii
°F
Day of Year
iii
Day of Year
Kernel width (days)
Jul
Aug
Sep
Oct
Nov
Dec
Jan
Feb
Mar
Apr
May
Jun
Winter Solstice
December 21/Day 355
Winter Teletherm
January 27/Day 27
+37 days
Coverage
Year
TWO HARBORS, MN
°F
Day of Year

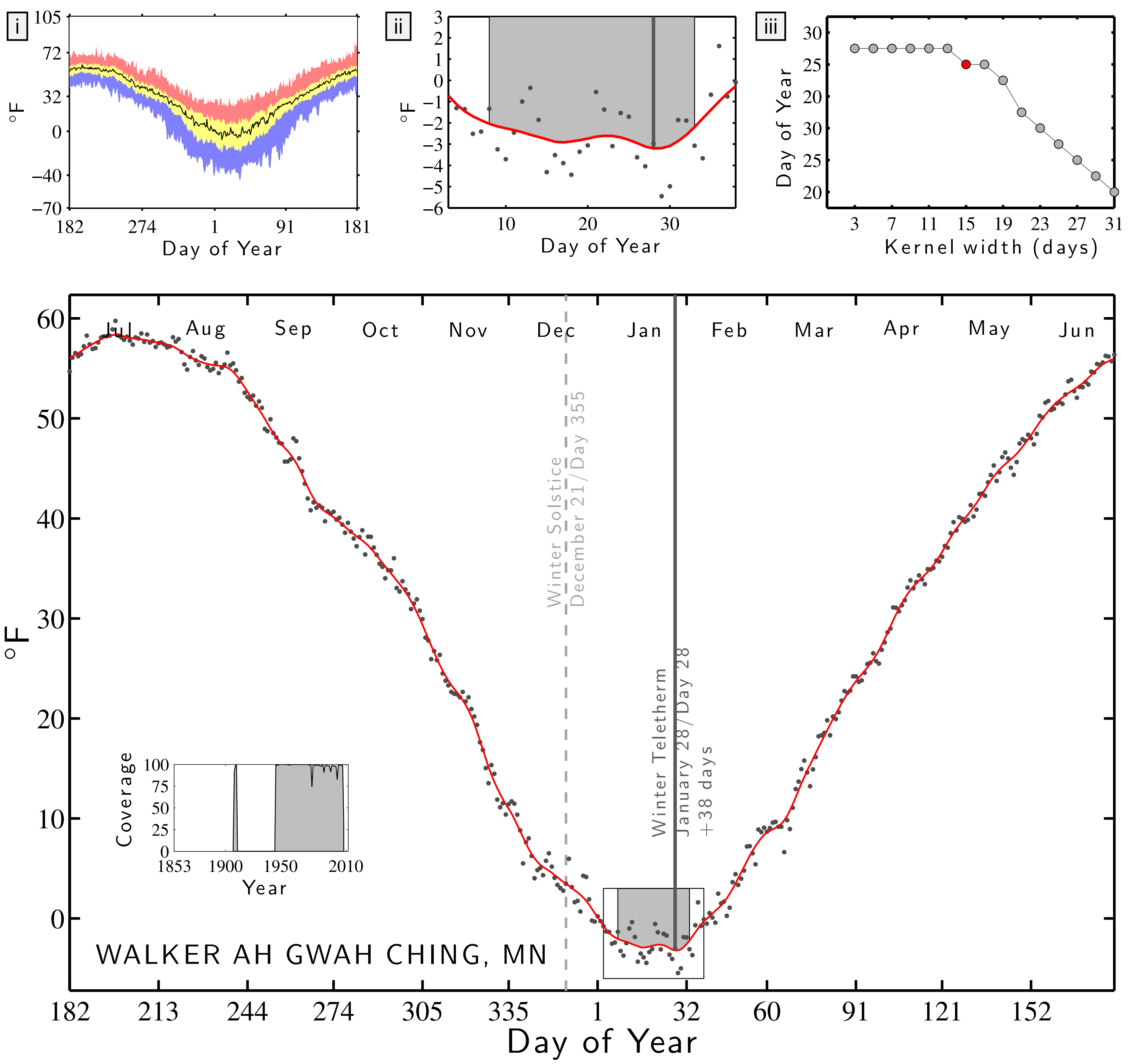
i
ii
iii
Day of Year
Kernel width (days)
Jul
Aug
Sep
Oct
Nov
Dec
Jan
Feb
Mar
Apr
May
Jun
Winter Solstice
December 21/Day 355
Winter Teletherm
January 28/Day 28
+38 days
Coverage
Year
WALKER AH GWAH CHING, MN
°F

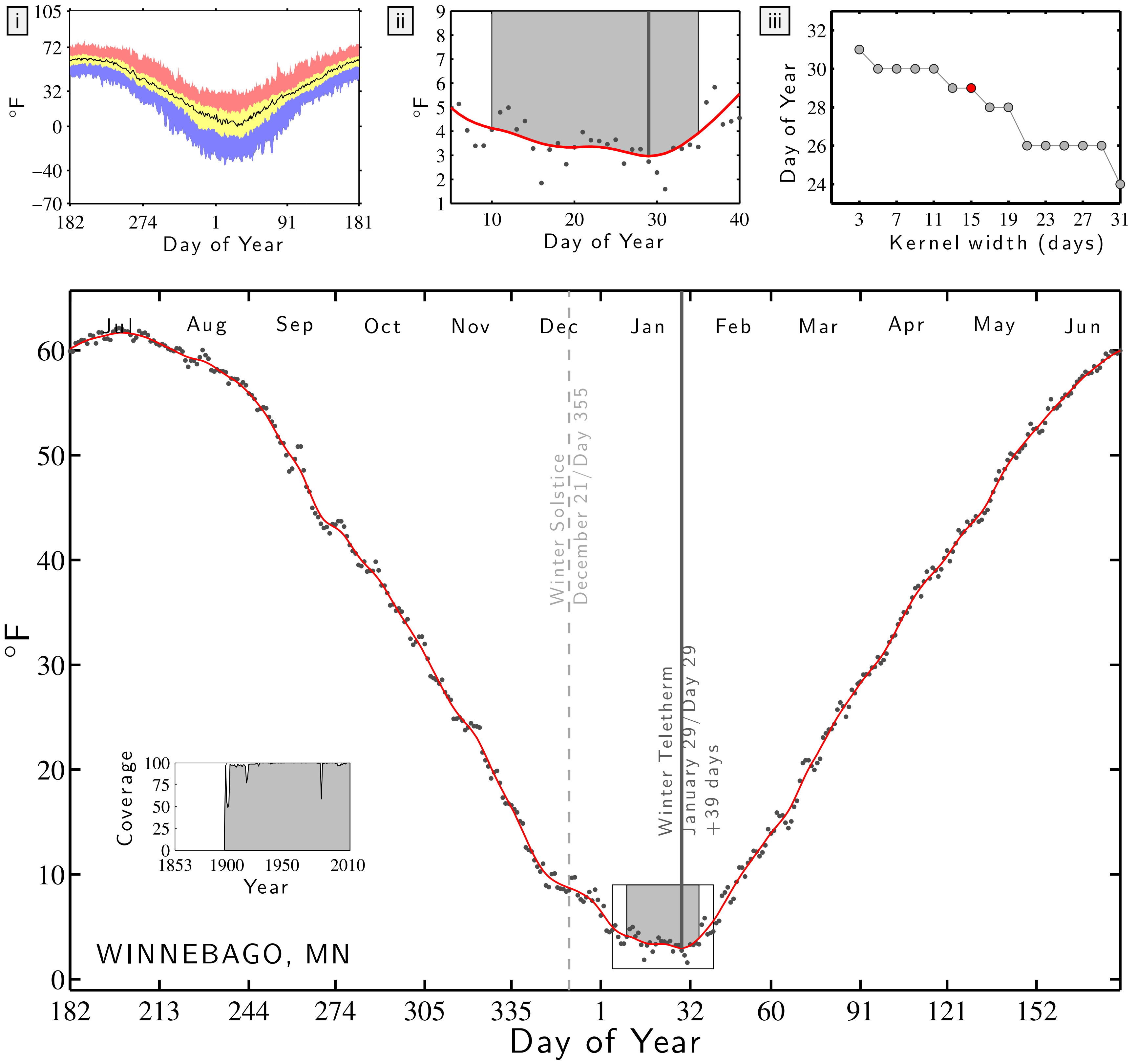

i
ii
iii
Day of Year
°F
Kernel width (days)
Jul
Aug
Sep
Oct
Nov
Dec
Jan
Feb
Mar
Apr
May
Jun
Winter Solstice
December 21/Day 355
Winter Teletherm
January 29/Day 29
+39 days
Coverage
Year
WINNEBAGO, MN

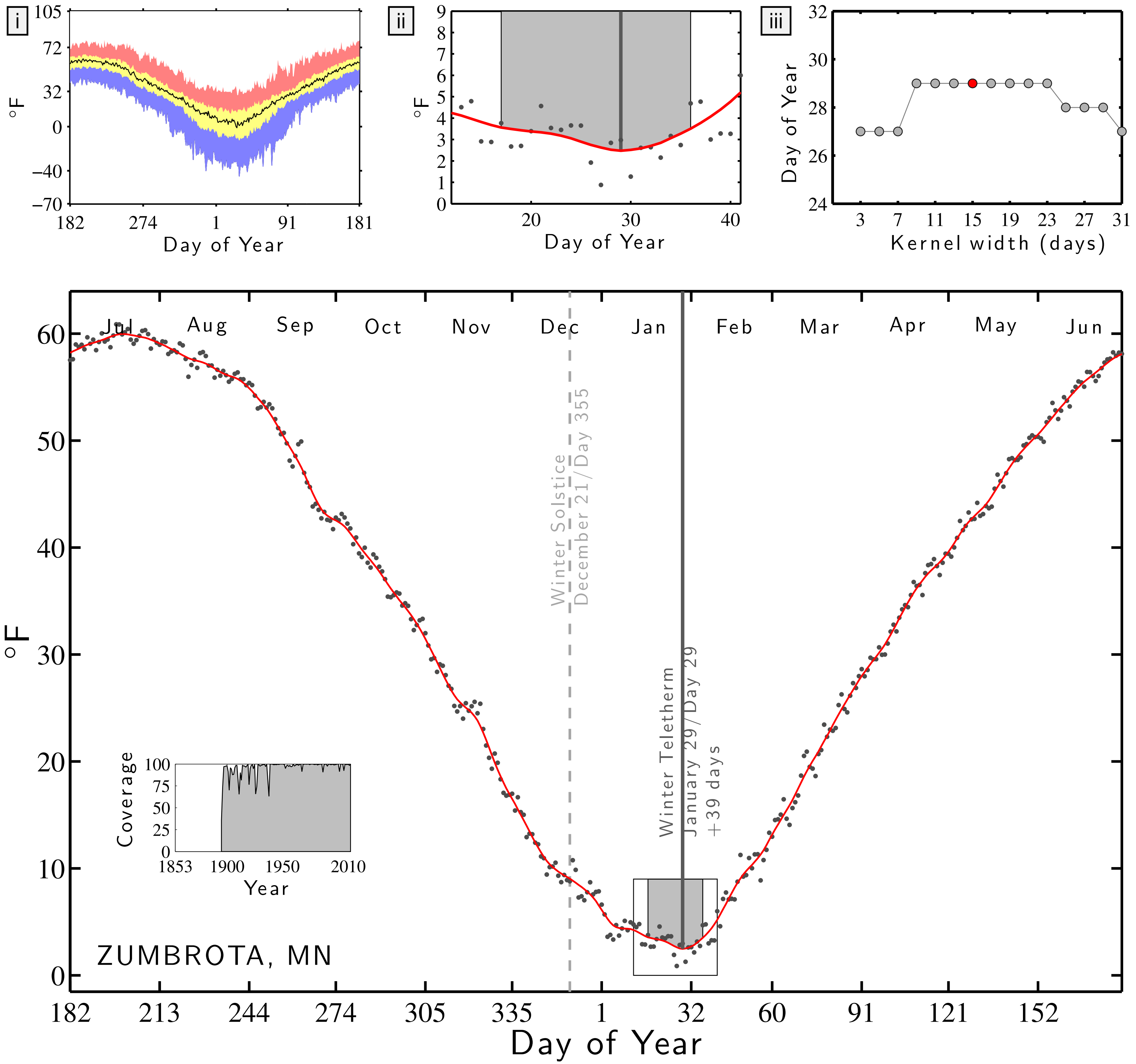

i
ii
iii
°F
Day of Year
Day of Year
Kernel width (days)
Jul
Aug
Sep
Oct
Nov
Dec
Jan
Feb
Mar
Apr
May
Jun
Winter Solstice
December 21/Day 355
Winter Teletherm
January 29/Day 29
+39 days
Coverage
Year
ZUMBROTA, MN
°F
Day of Year

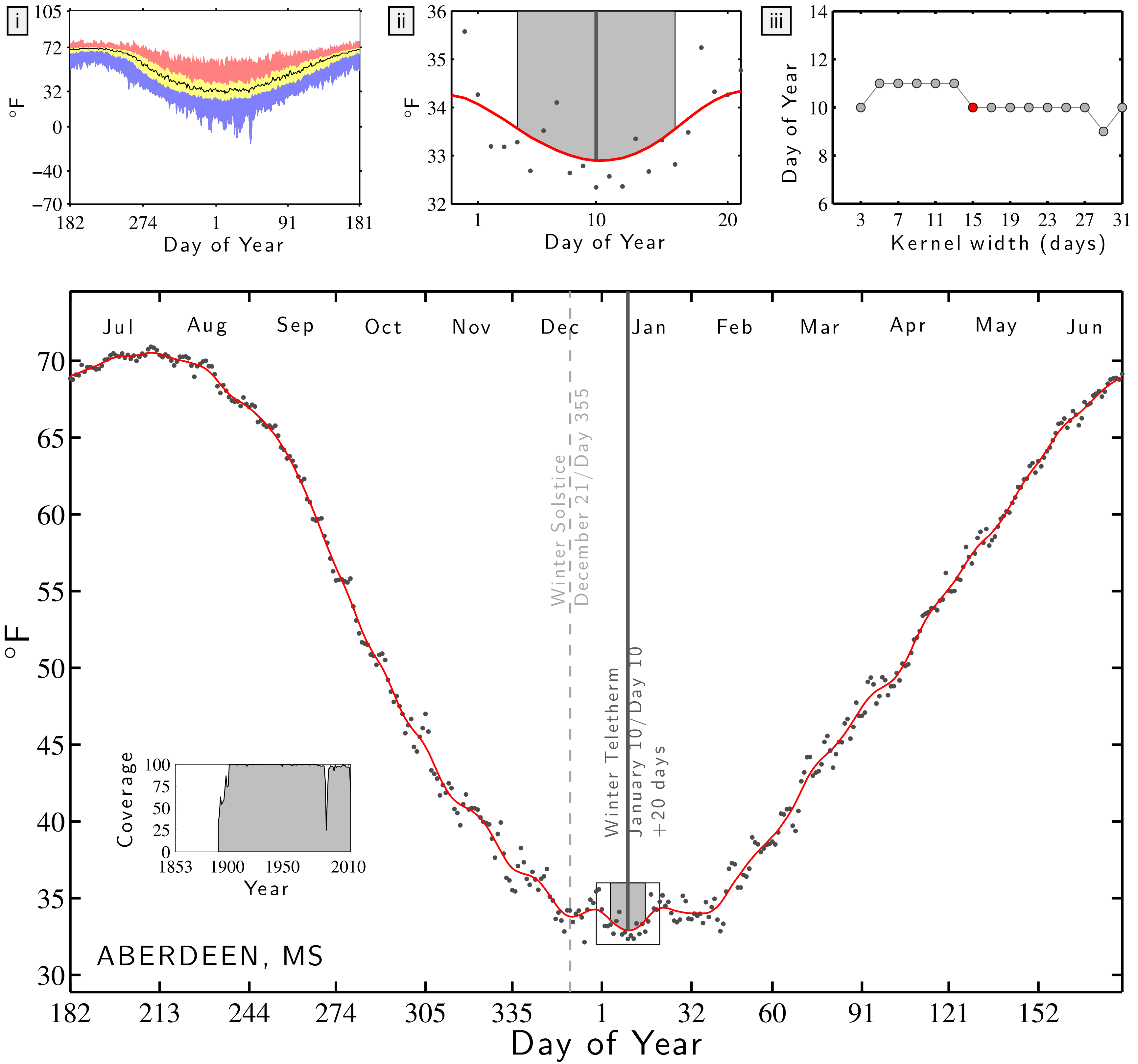

i
ii
iii
°F
Day of Year
Kernel width (days)
Jul
Aug
Sep
Oct
Nov
Dec
Jan
Feb
Mar
Apr
May
Jun
Winter Solstice
December 21/Day 355
Winter Teletherm
January 10/Day 10
+20 days
Coverage
Year
ABERDEEN, MS

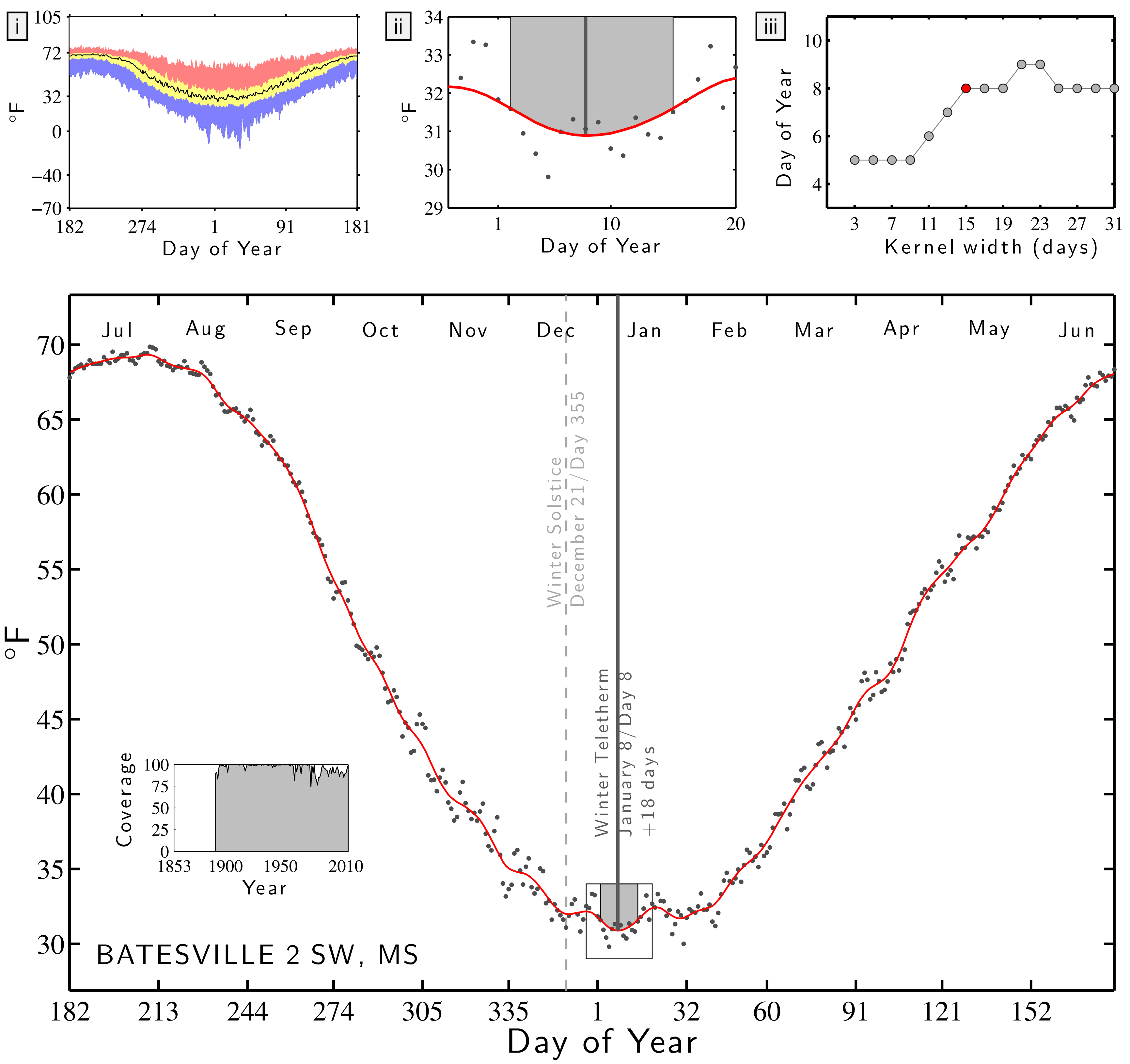

i
ii
iii
Day of Year
Kernel width (days)
Jul
Aug
Sep
Oct
Nov
Dec
Jan
Feb
Mar
Apr
May
Jun
Winter Solstice
December 21/Day 355
Winter Teletherm
January 8/Day 8
+18 days
Coverage
Year
BATESVILLE 2 SW, MS
°F
Day of Year

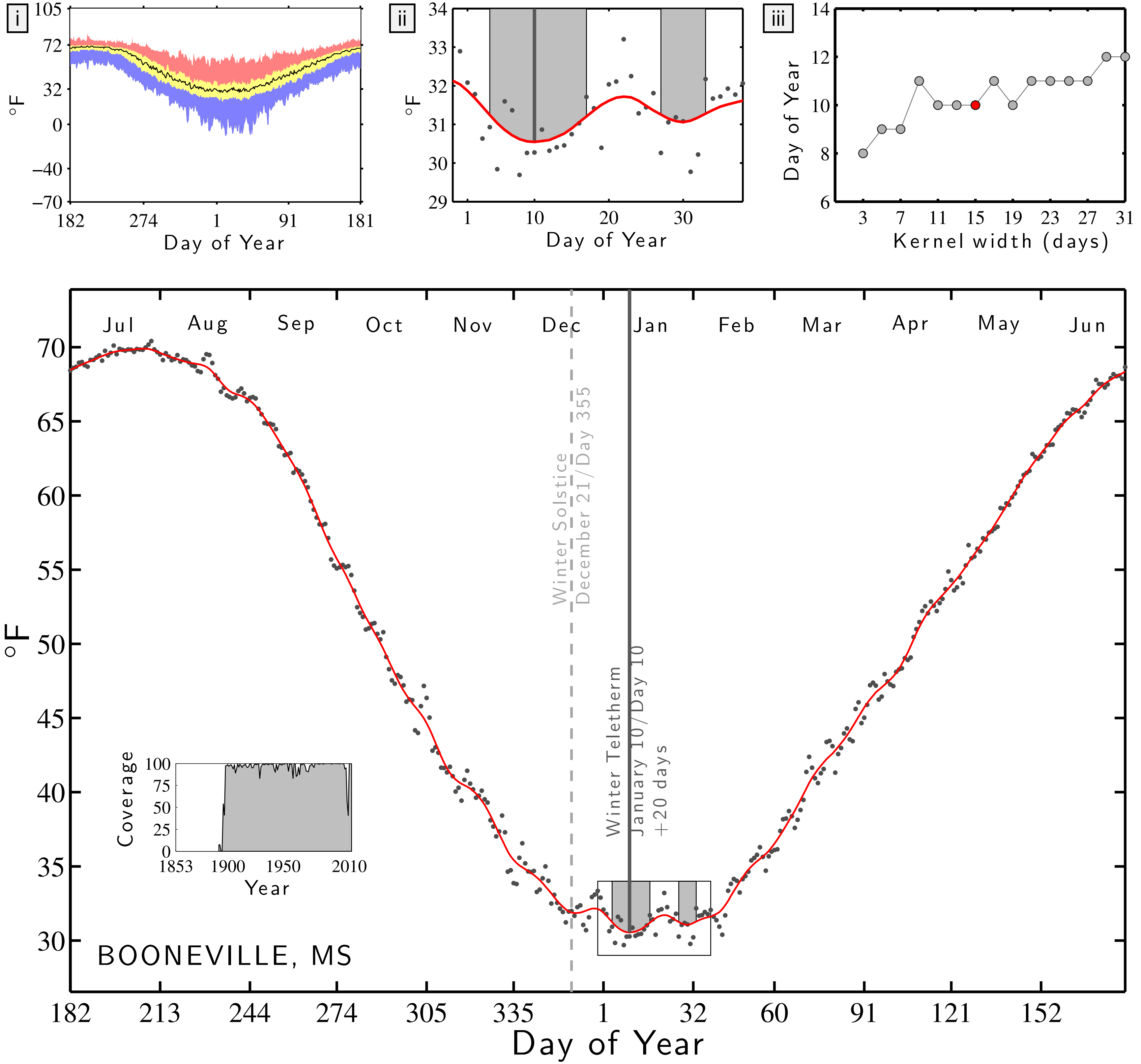

i
°F
Day of Year
ii
Day of Year
iii
Day of Year
Kernel width (days)
Jul
Aug
Sep
Oct
Nov
Dec
Jan
Feb
Mar
Apr
May
Jun
Winter Solstice
December 21/Day 355
Winter Teletherm
January 10/Day 10
+20 days
Coverage
Year
BOONEVILLE, MS
Day of Year

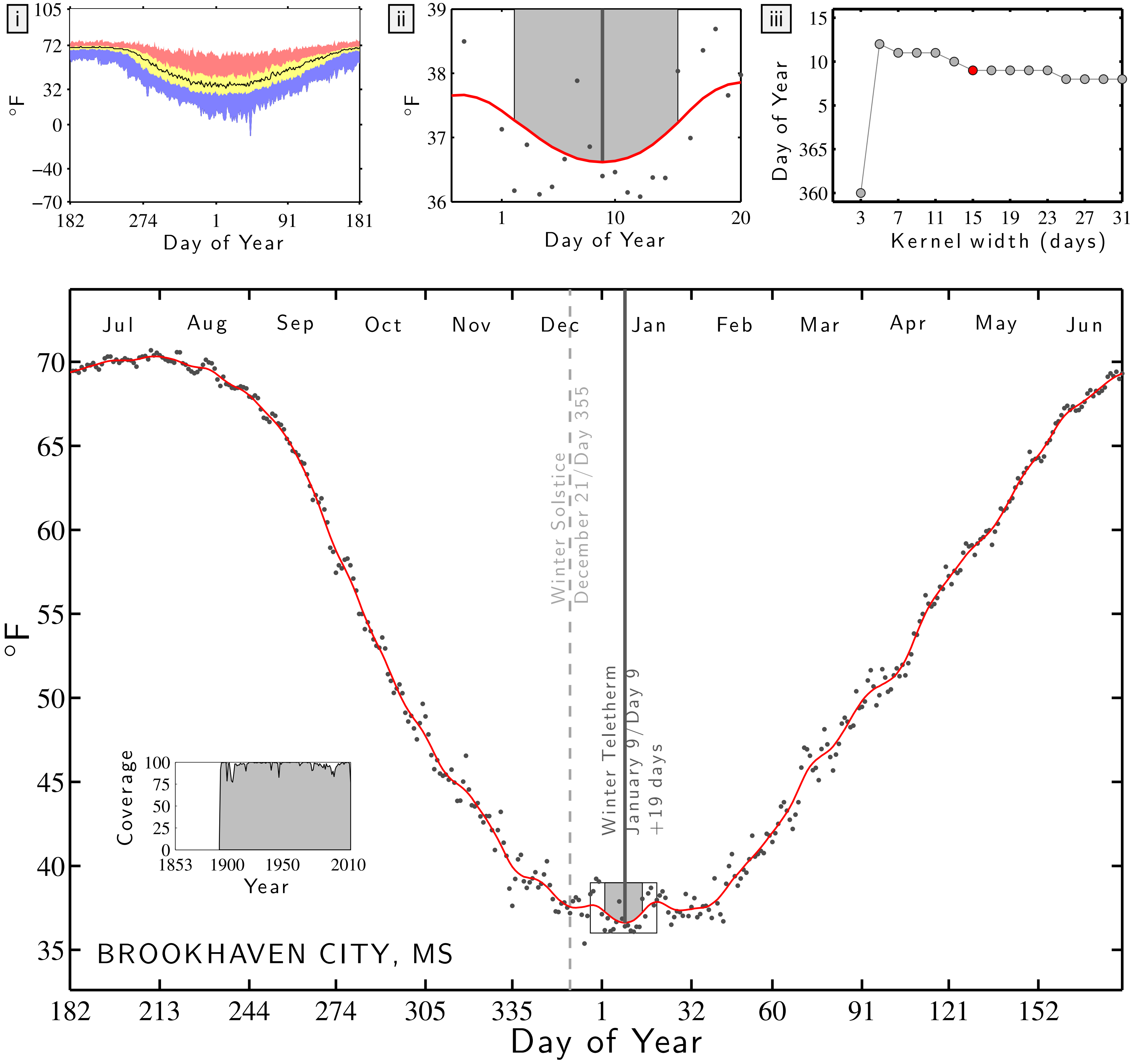

i
ii
iii
°F
Day of Year
Kernel width (days)
Jul
Aug
Sep
Oct
Nov
Dec
Jan
Feb
Mar
Apr
May
Jun
Winter Solstice
December 21/Day 355
Winter Teletherm
January 9/Day 9
+19 days
Coverage
Year
BROOKHAVEN CITY, MS

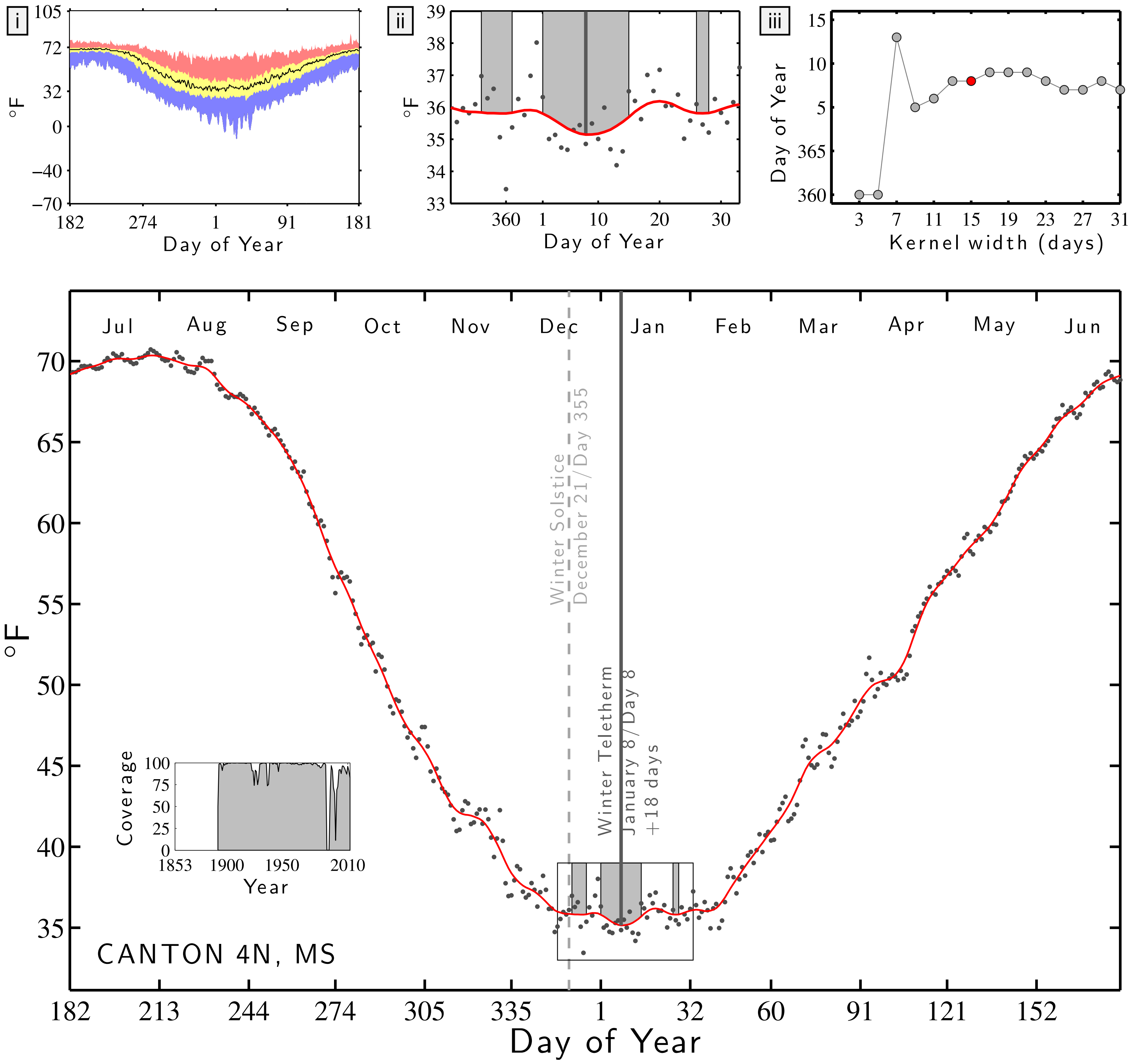

i
°F
Day of Year
ii
°F
Day of Year
iii
Day of Year
Kernel width (days)
Jul
Aug
Sep
Oct
Nov
Dec
Jan
Feb
Mar
Apr
May
Jun
Winter Solstice
December 21/Day 355
Winter Teletherm
January 8/Day 8
+18 days
Coverage
Year
CANTON 4N, MS
°F
Day of Year

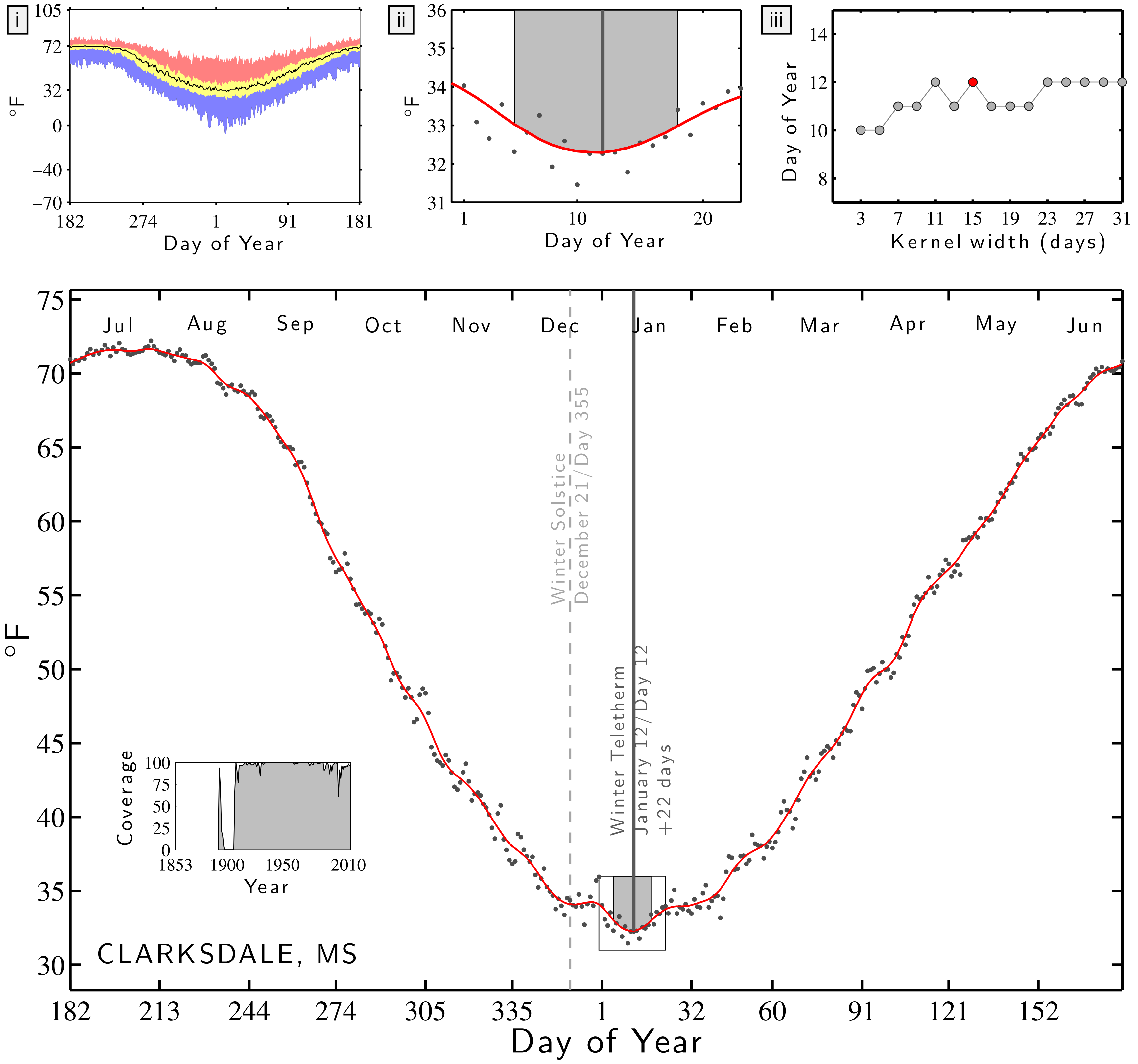

i
ii
iii
°F
Day of Year
Kernel width (days)
Jul
Aug
Sep
Oct
Nov
Dec
Jan
Feb
Mar
Apr
May
Jun
Winter Solstice
December 21/Day 355
Winter Teletherm
January 12/Day 12
+22 days
Coverage
Year
CLARKSDALE, MS

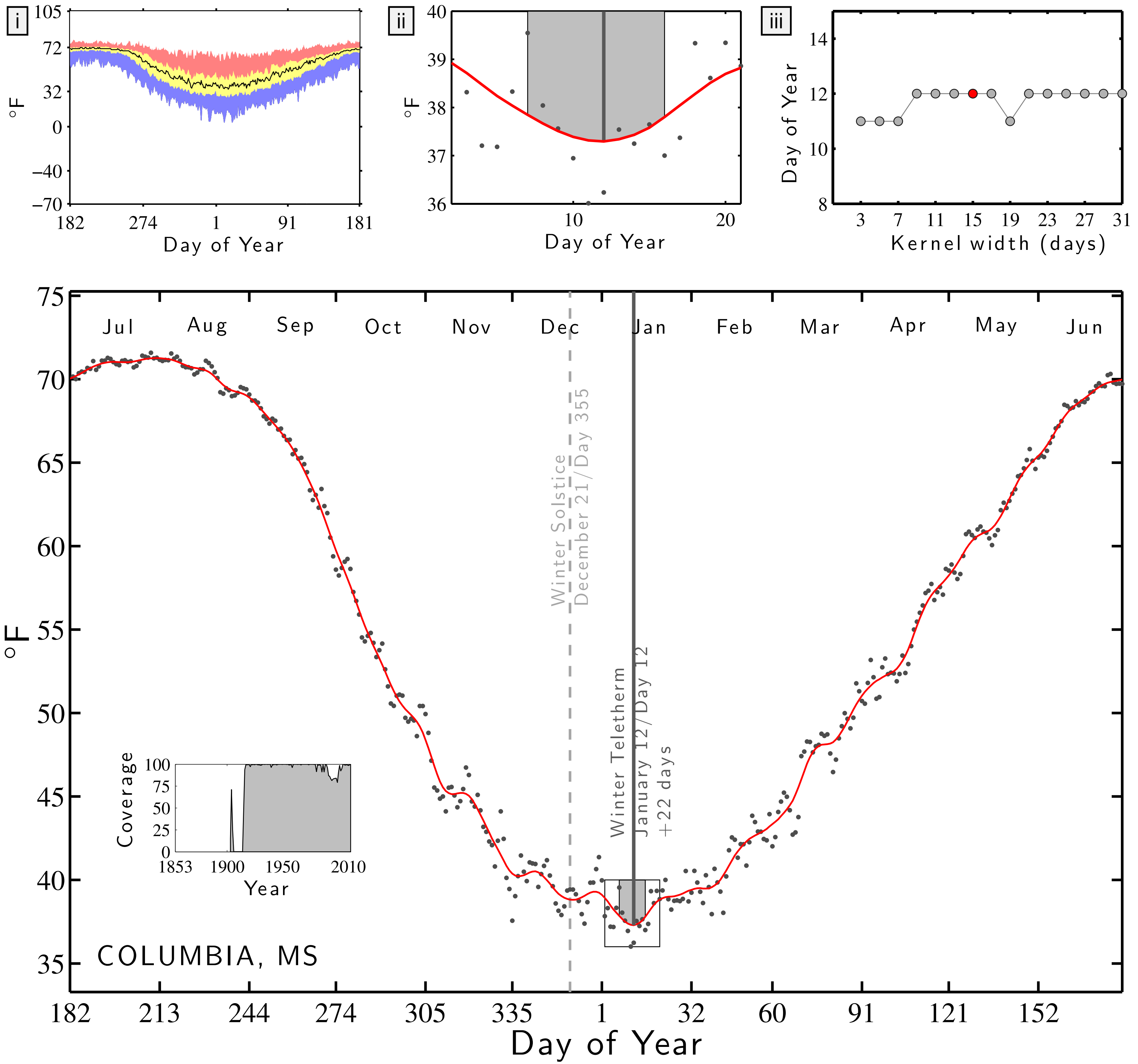

i
ii
iii
°F
Day of Year
Kernel width (days)
Jul
Aug
Sep
Oct
Nov
Dec
Jan
Feb
Mar
Apr
May
Jun
Winter Solstice
December 21/Day 355
Winter Teletherm
January 12/Day 12
+22 days
Coverage
Year
COLUMBIA, MS

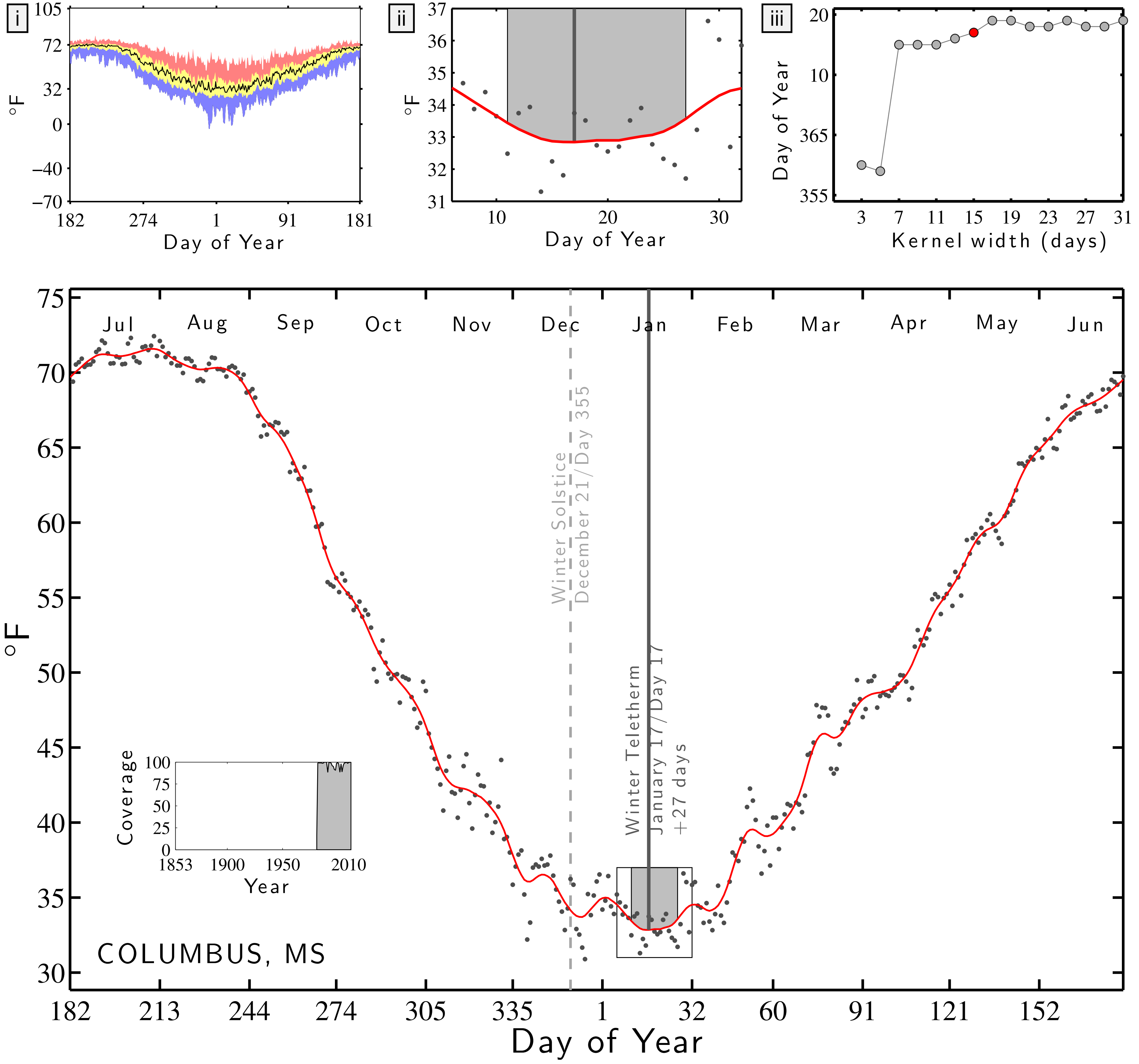

i
105
72
32
0
−40
−70
°F
182
274
1
91
181
Day of Year
ii
37
36
35
34
33
32
31
°F
10
20
30
Day of Year
iii
20
10
365
355
Day of Year
3
7
11
15
19
23
27
31
Kernel width (days)
Jul
Aug
Sep
Oct
Nov
Dec
Jan
Feb
Mar
Apr
May
Jun
75
70
65
60
55
50
45
40
35
30
°F
Winter Solstice
December 21/Day 355
Winter Teletherm
January 17/Day 17
+27 days
Coverage
100
75
50
25
0
1853
1900
1950
2010
Year
COLUMBUS, MS
182
213
244
274
305
335
1
32
60
91
121
152
Day of Year

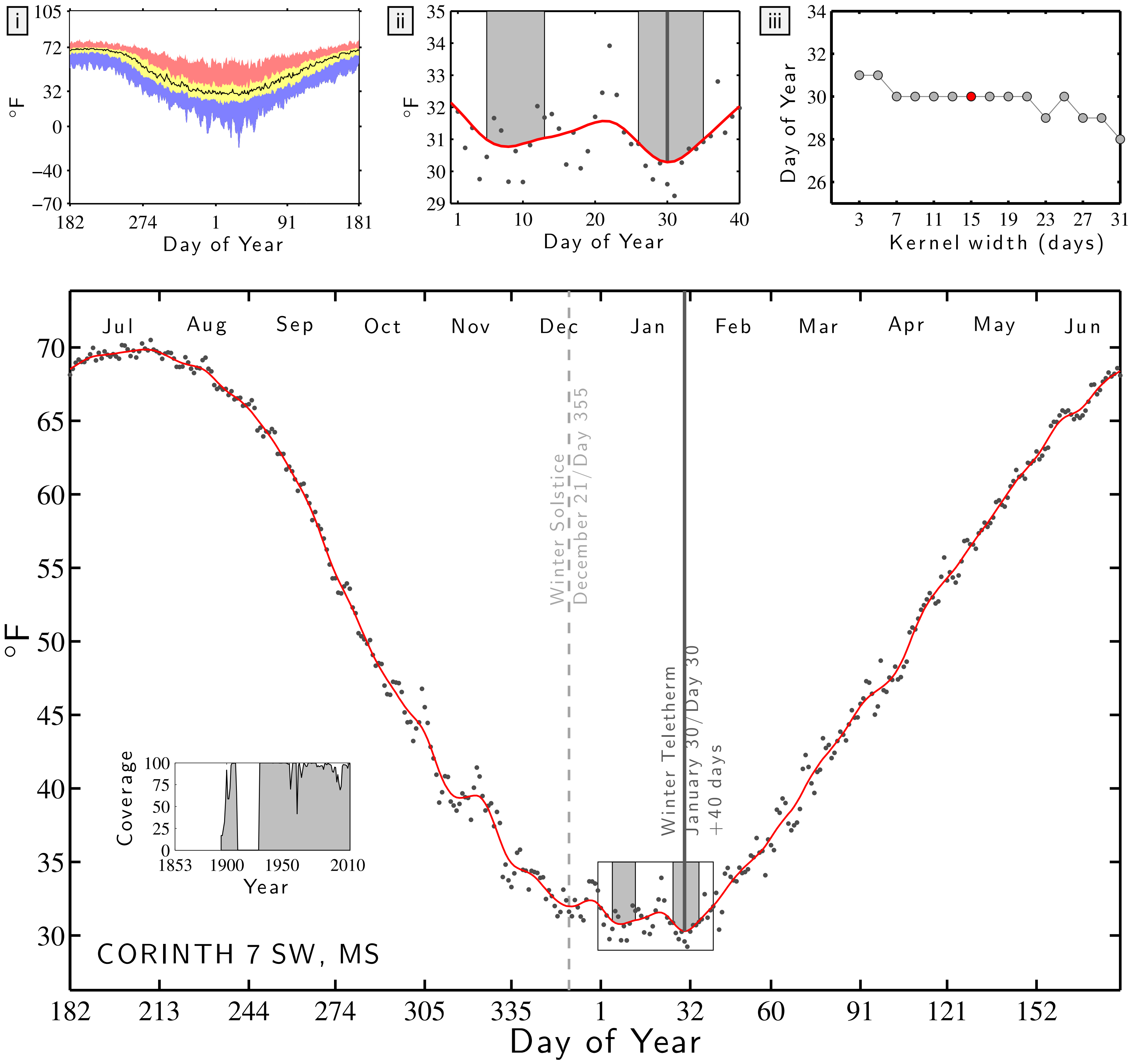

i
°F
Day of Year
ii
°F
Day of Year
iii
Day of Year
Kernel width (days)
Jul
Aug
Sep
Oct
Nov
Dec
Jan
Feb
Mar
Apr
May
Jun
Winter Solstice
December 21/Day 355
Winter Teletherm
January 30/Day 30
+40 days
Coverage
Year
CORINTH 7 SW, MS
°F
Day of Year

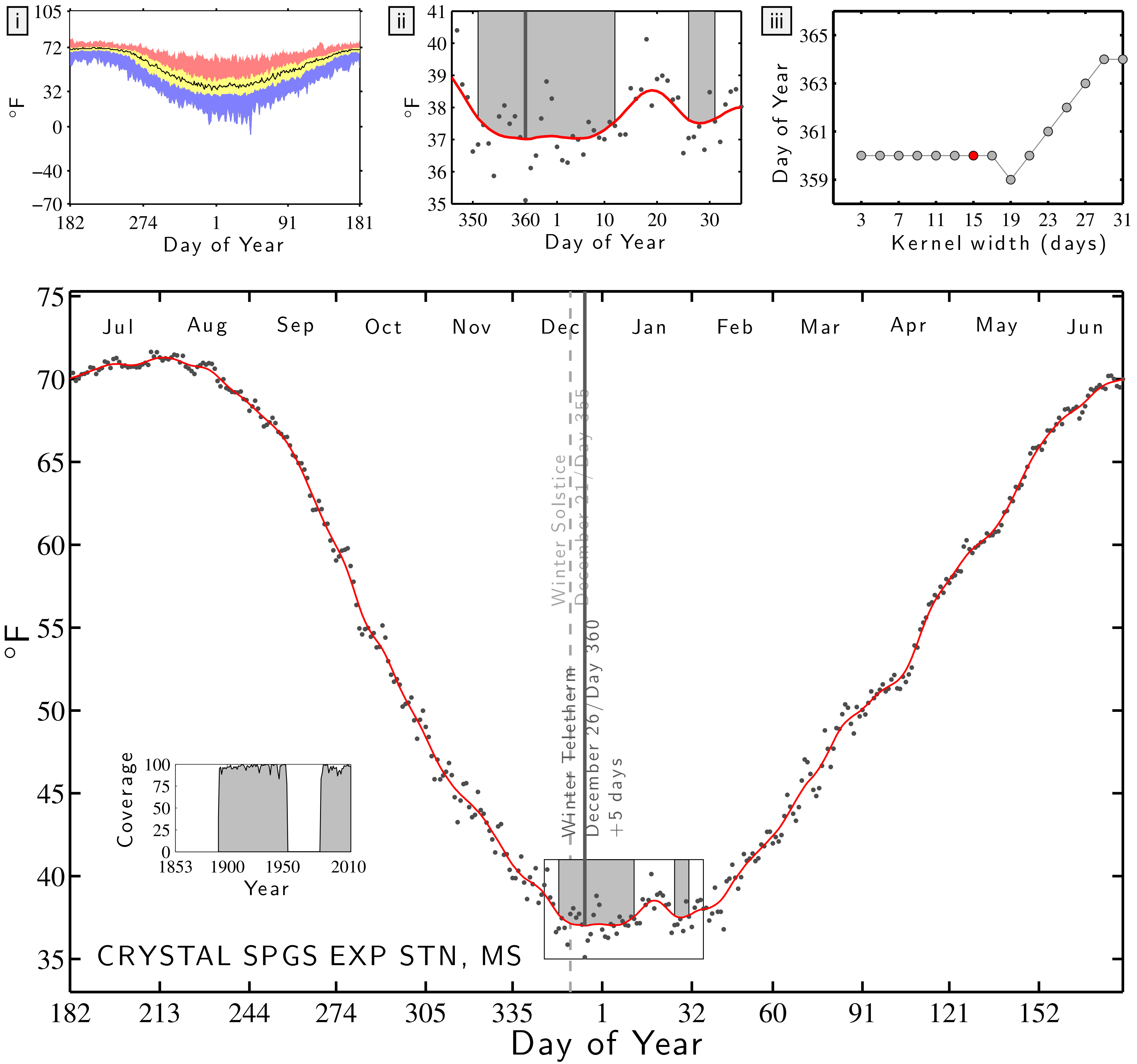

i
°F
Day of Year
ii
°F
Day of Year
iii
Day of Year
Kernel width (days)
Jul
Aug
Sep
Oct
Nov
Dec
Jan
Feb
Mar
Apr
May
Jun
Winter Solstice
December 21/Day 355
Winter Teletherm
December 26/Day 360
+5 days
Coverage
Year
CRYSTAL SPGS EXP STN, MS
°F
Day of Year

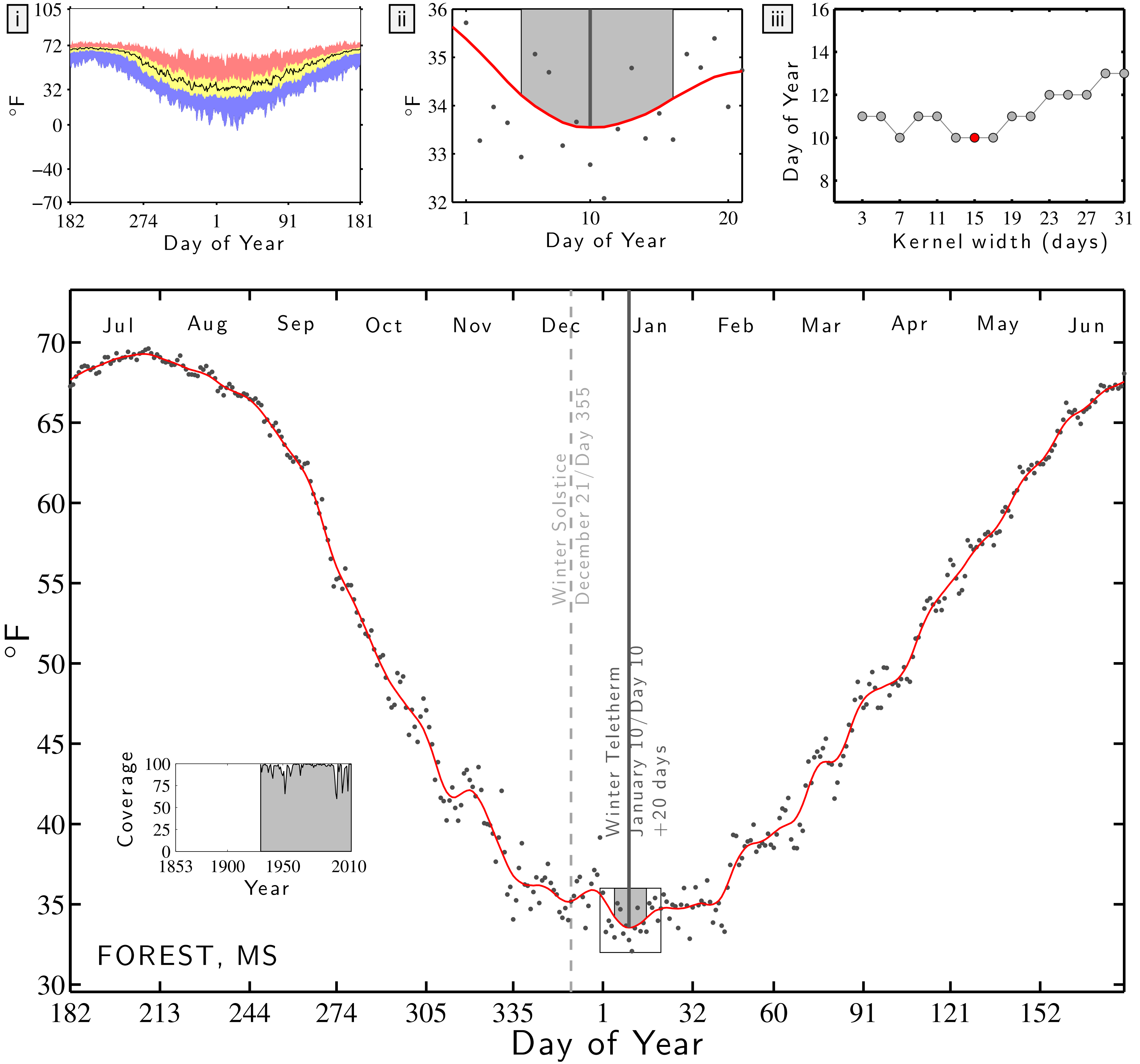

i
°F
105
72
32
0
−40
−70
182
274
1
91
181
Day of Year
ii
°F
36
35
34
33
32
1
10
20
Day of Year
iii
Day of Year
16
14
12
10
8
3
7
11
15
19
23
27
31
Kernel width (days)
Jul
Aug
Sep
Oct
Nov
Dec
Jan
Feb
Mar
Apr
May
Jun
Winter Solstice
December 21/Day 355
Winter Teletherm
January 10/Day 10
+20 days
°F
70
65
60
55
50
45
40
35
30
Coverage
100
75
50
25
0
1853
1900
1950
2010
Year
FOREST, MS
182
213
244
274
305
335
1
32
60
91
121
152
Day of Year

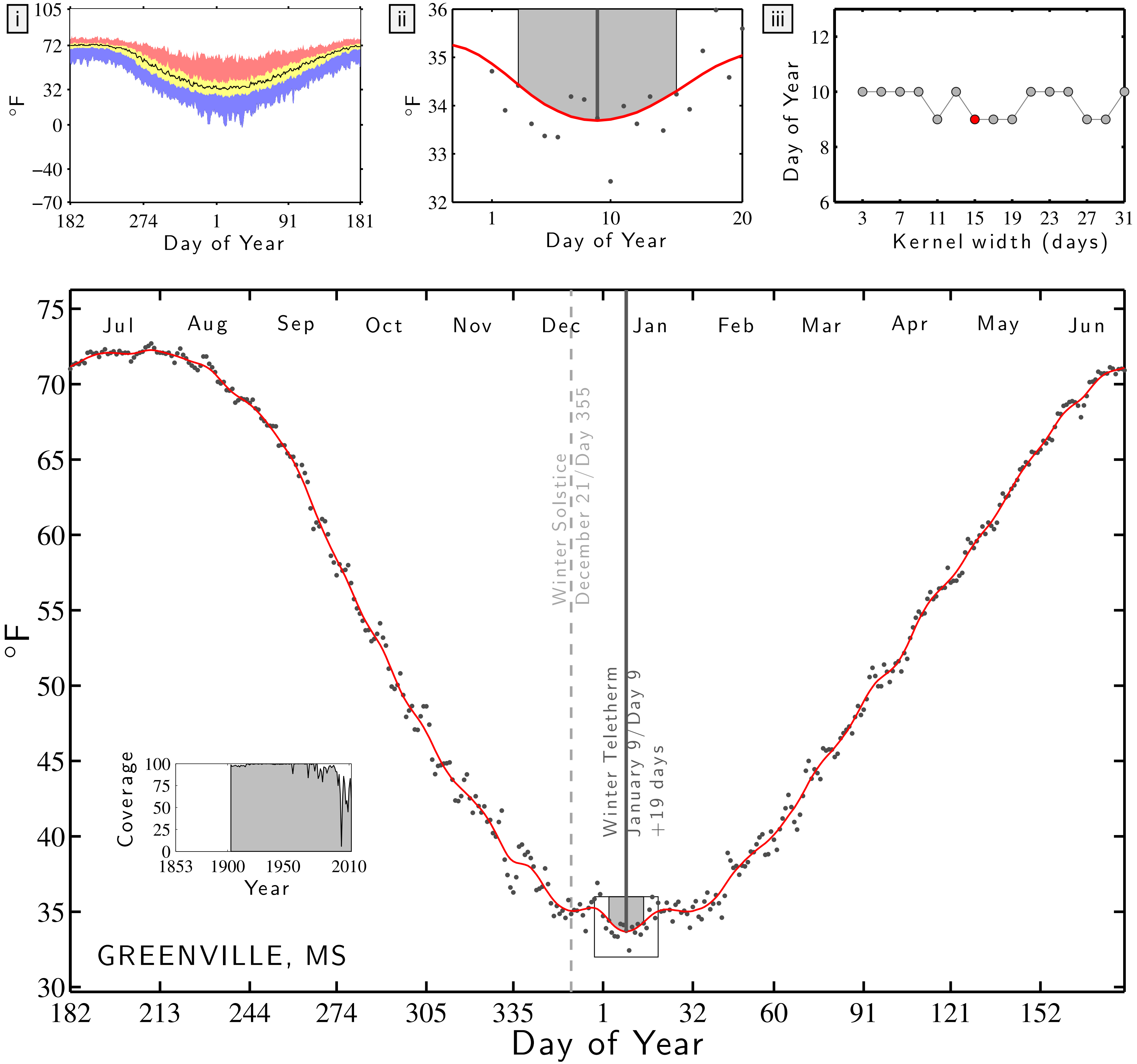

i
ii
iii
Day of Year
°F
Kernel width (days)
Jul
Aug
Sep
Oct
Nov
Dec
Jan
Feb
Mar
Apr
May
Jun
Winter Solstice
December 21/Day 355
Winter Teletherm
January 9/Day 9
+19 days
Coverage
Year
GREENVILLE, MS

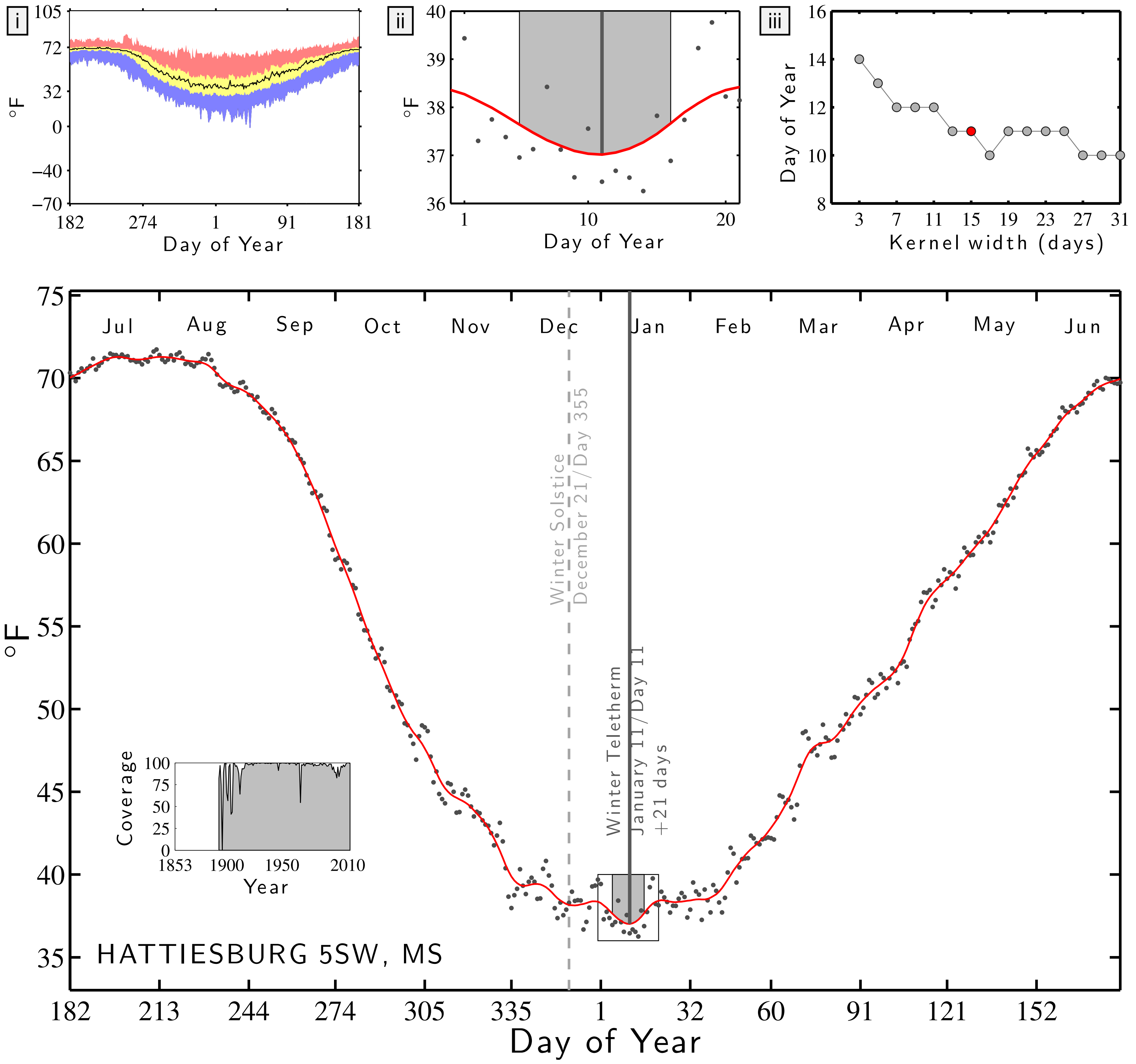

i
°F
Day of Year
ii
°F
Day of Year
iii
Day of Year
Kernel width (days)
Jul
Aug
Sep
Oct
Nov
Dec
Jan
Feb
Mar
Apr
May
Jun
Winter Solstice
December 21/Day 355
Winter Teletherm
January 11/Day 11
+21 days
Coverage
Year
HATTIESBURG 5SW, MS
°F
Day of Year

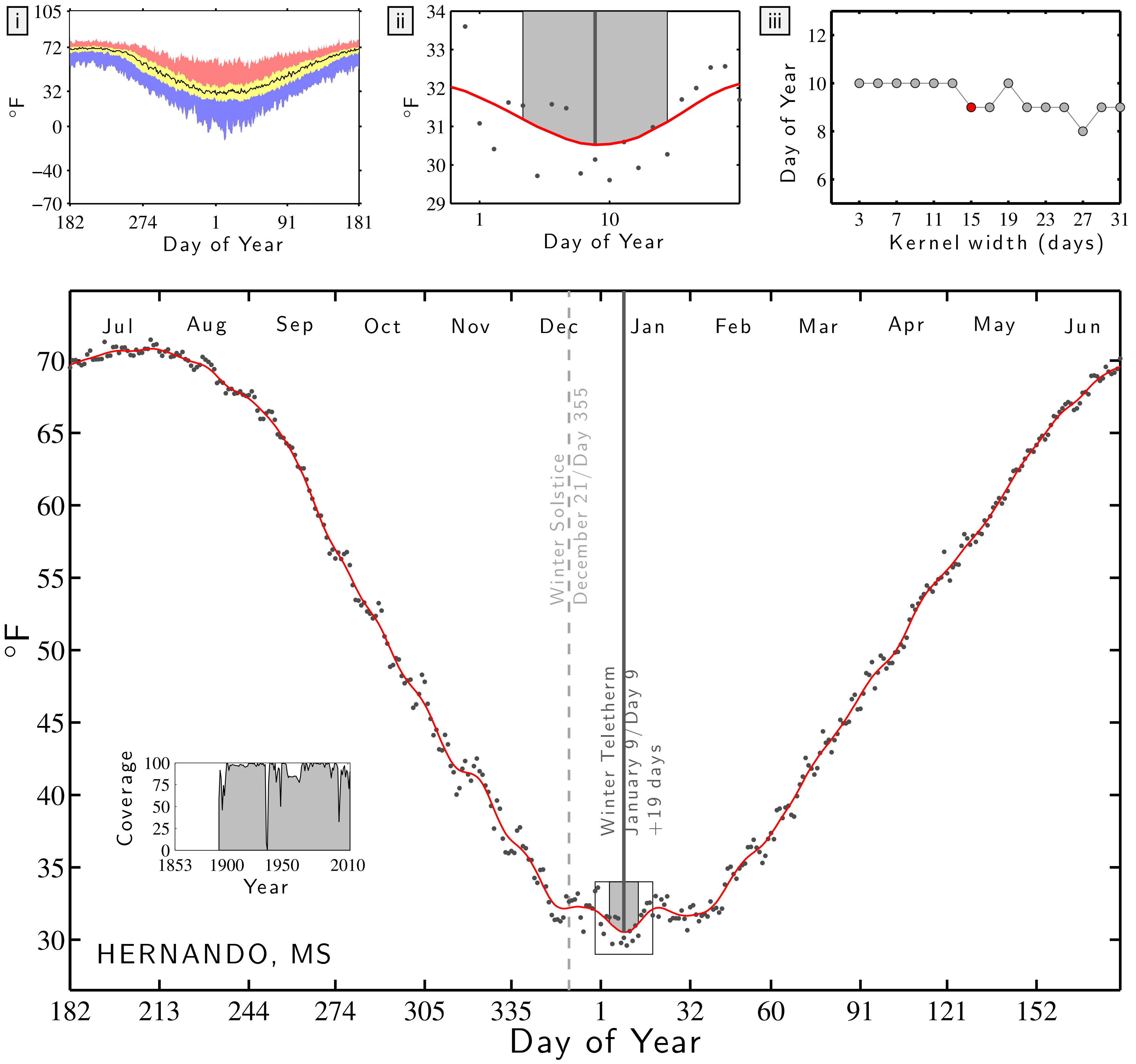

i
ii
iii
°F
Day of Year
Kernel width (days)
Jul
Aug
Sep
Oct
Nov
Dec
Jan
Feb
Mar
Apr
May
Jun
Winter Solstice
December 21/Day 355
Winter Teletherm
January 9/Day 9
+19 days
Coverage
Year
HERNANDO, MS

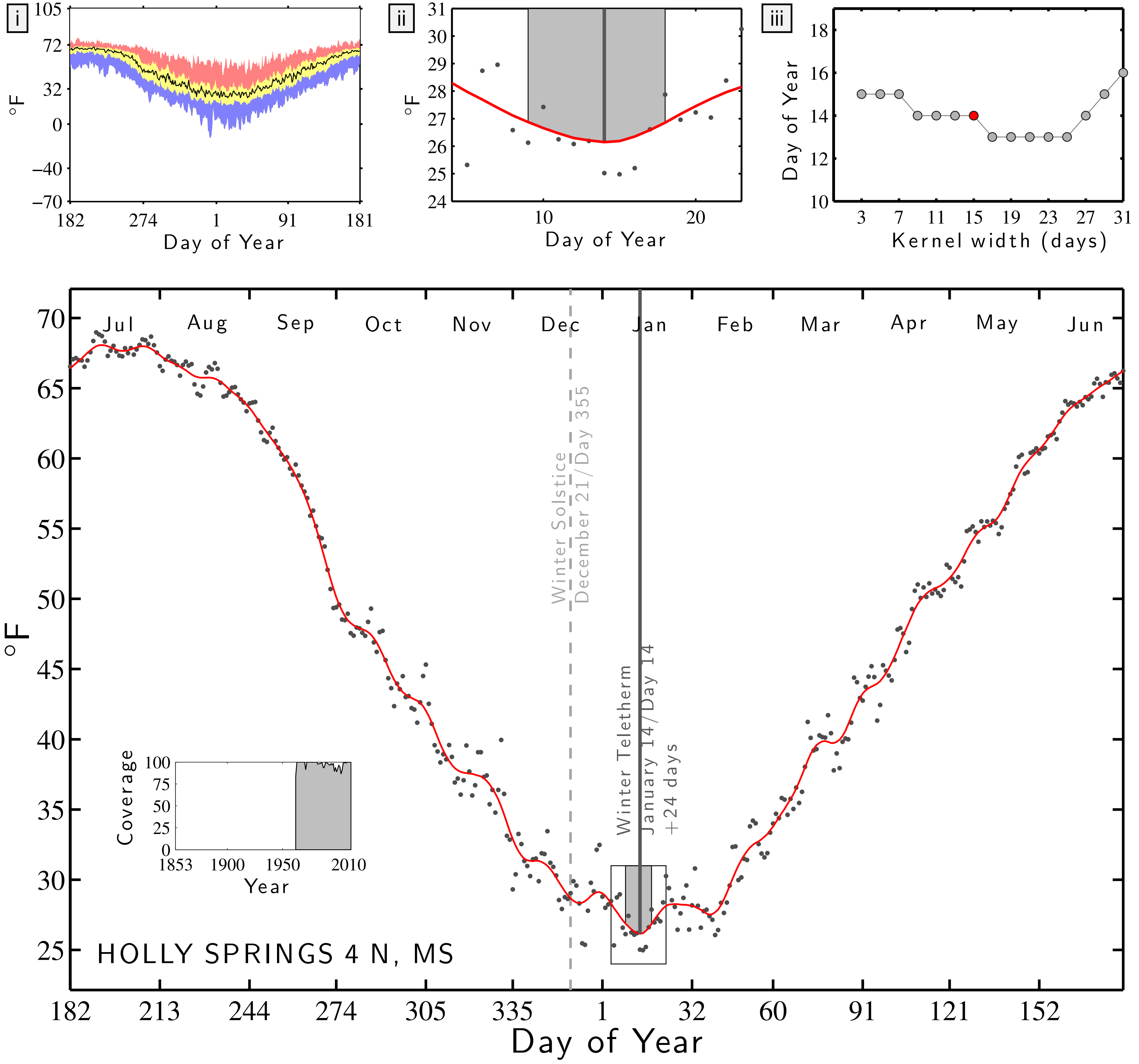

i
°F
Day of Year
ii
°F
Day of Year
iii
Day of Year
Kernel width (days)
Jul
Aug
Sep
Oct
Nov
Dec
Jan
Feb
Mar
Apr
May
Jun
Winter Solstice
December 21/Day 355
Winter Teletherm
January 14/Day 14
+24 days
Coverage
Year
HOLLY SPRINGS 4 N, MS
°F
Day of Year

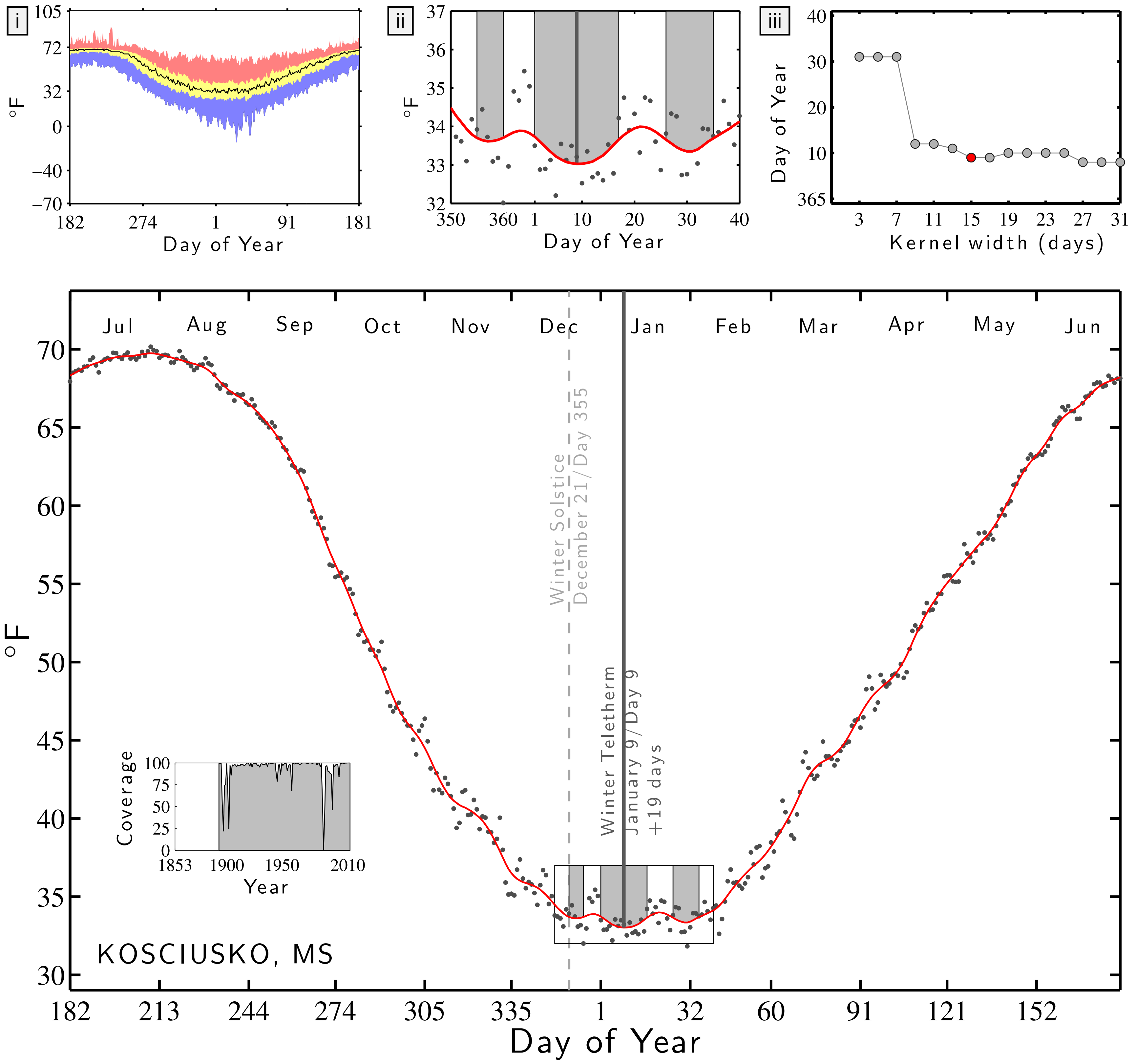

i
ii
iii
°F
Day of Year
Day of Year
Kernel width (days)
Jul
Aug
Sep
Oct
Nov
Dec
Jan
Feb
Mar
Apr
May
Jun
Winter Solstice
December 21/Day 355
Winter Teletherm
January 9/Day 9
+19 days
Coverage
Year
KOSCIUSKO, MS
°F
Day of Year

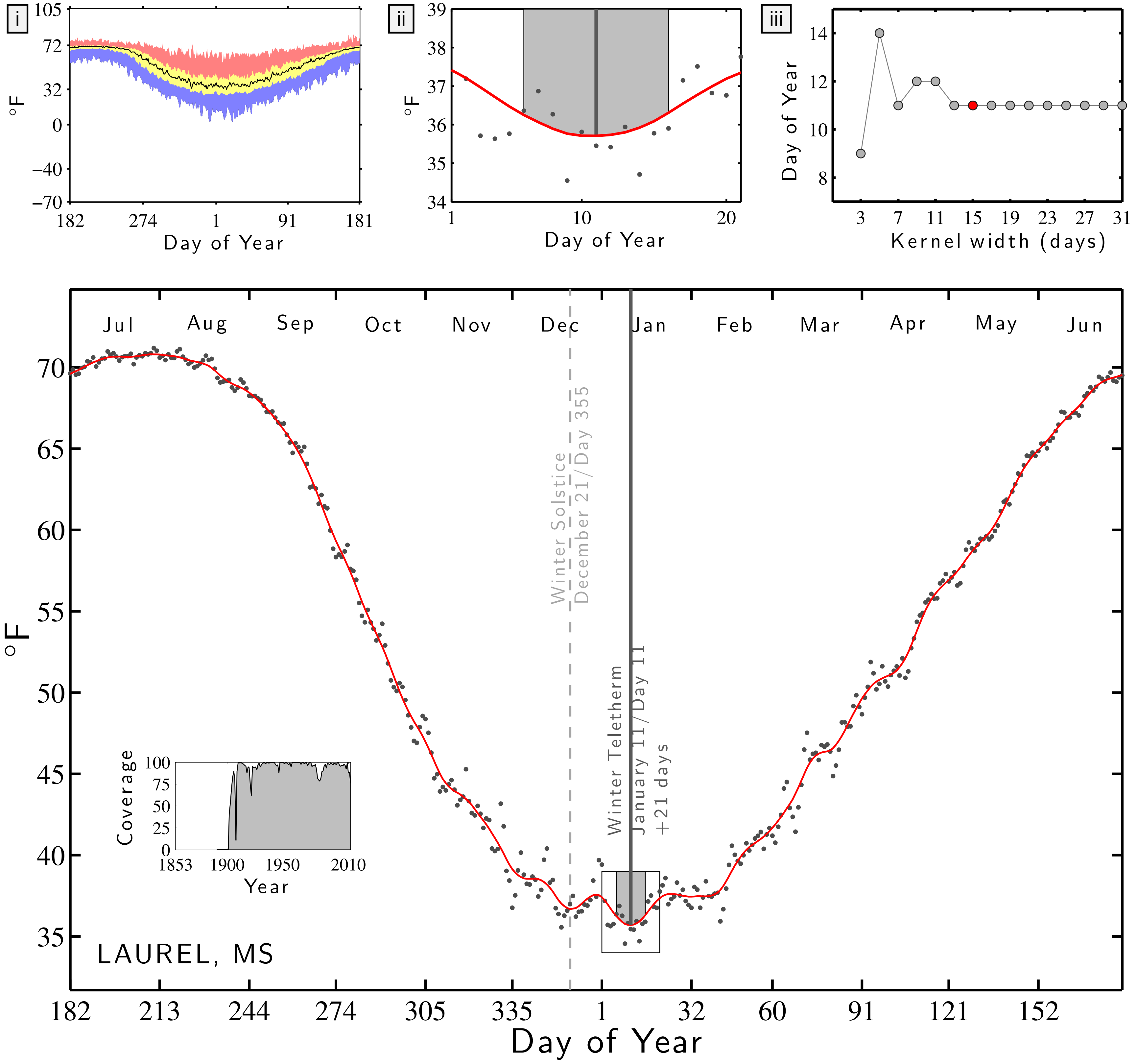

i
°F
Day of Year
ii
°F
Day of Year
iii
Day of Year
Kernel width (days)
Jul
Aug
Sep
Oct
Nov
Dec
Jan
Feb
Mar
Apr
May
Jun
Winter Solstice
December 21/Day 355
Winter Teletherm
January 11/Day 11
+21 days
Coverage
Year
LAUREL, MS
°F
Day of Year

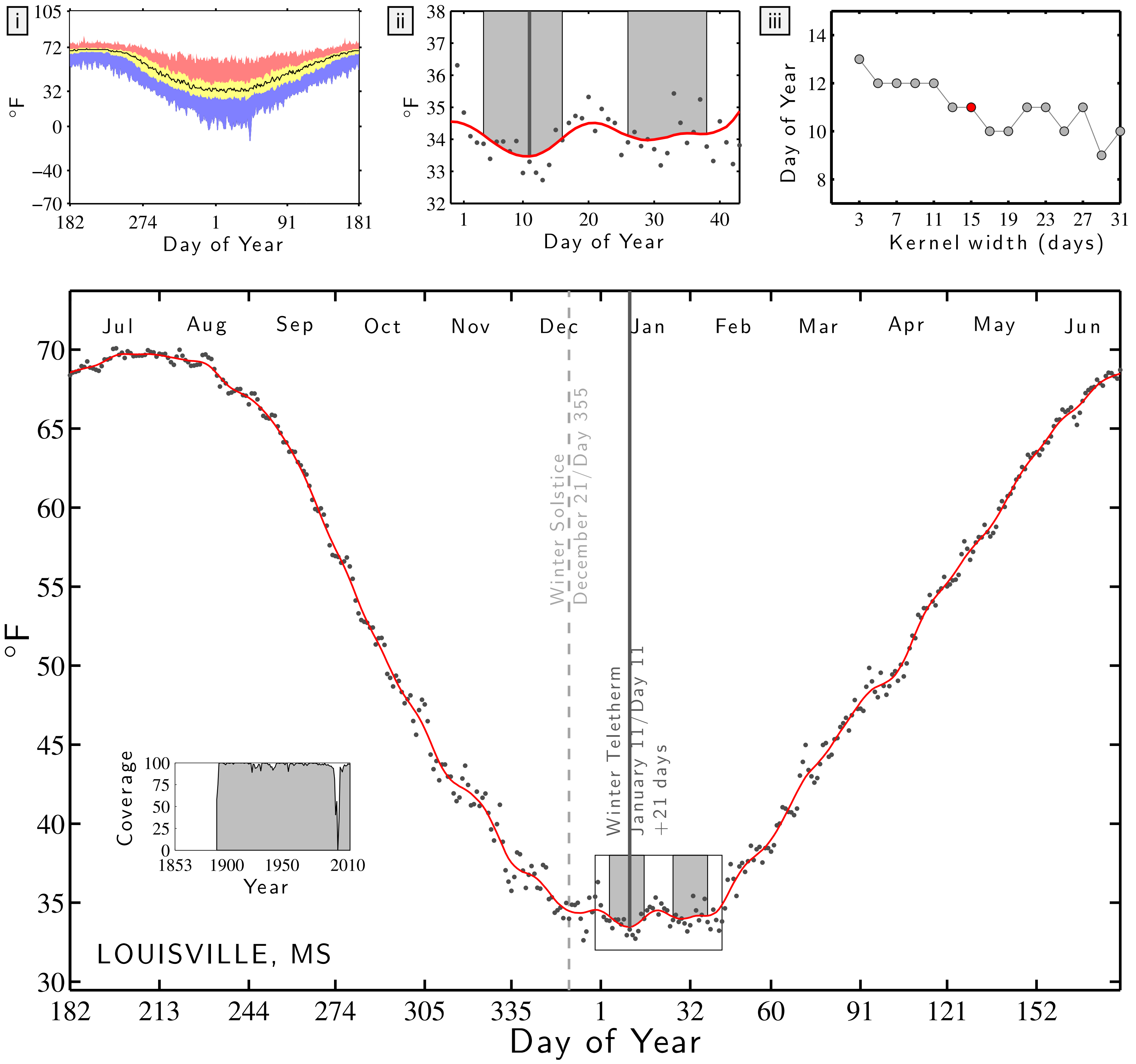

i
ii
iii
°F
Day of Year
Kernel width (days)
Winter Solstice
December 21/Day 355
Winter Teletherm
January 11/Day 11
+21 days
Jul
Aug
Sep
Oct
Nov
Dec
Jan
Feb
Mar
Apr
May
Jun
Coverage
Year
LOUISVILLE, MS

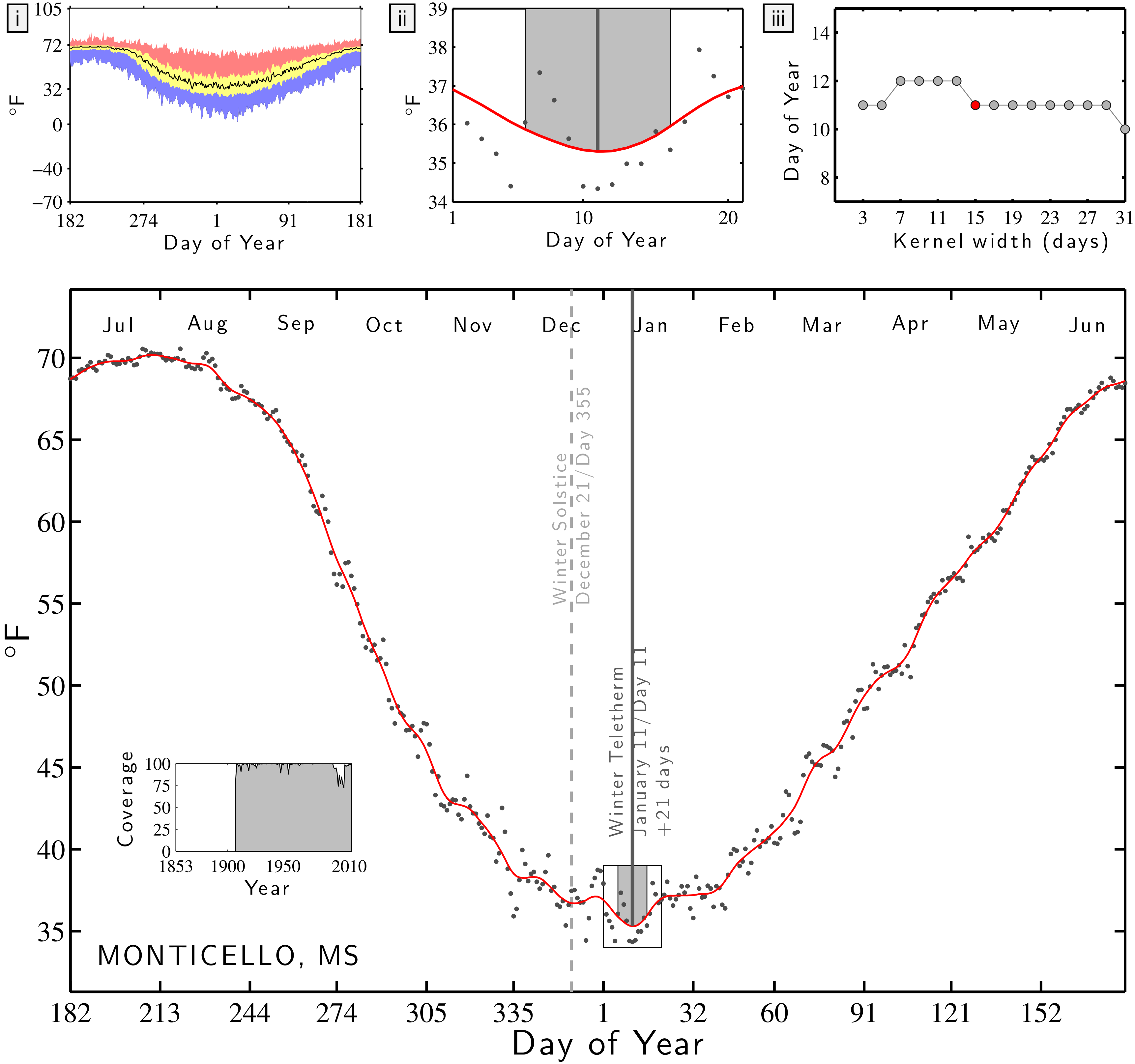

i
°F
Day of Year
ii
°F
Day of Year
iii
Day of Year
Kernel width (days)
Jul
Aug
Sep
Oct
Nov
Dec
Jan
Feb
Mar
Apr
May
Jun
Winter Solstice
December 21/Day 355
Winter Teletherm
January 11/Day 11
+21 days
Coverage
Year
MONTICELLO, MS
°F
Day of Year

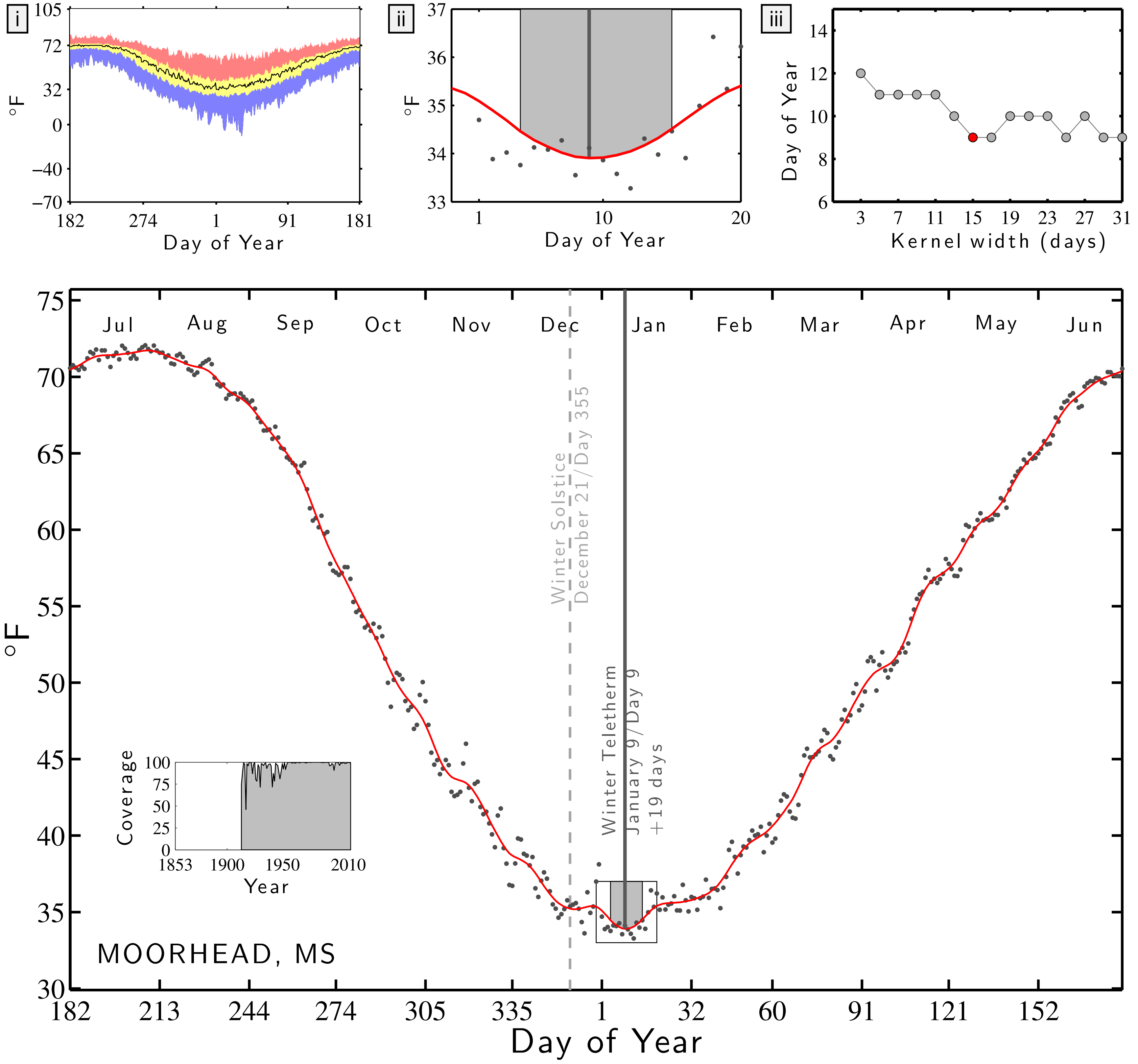

i
°F
Day of Year
ii
Day of Year
iii
Day of Year
Kernel width (days)
Jul
Aug
Sep
Oct
Nov
Dec
Jan
Feb
Mar
Apr
May
Jun
Winter Solstice
December 21/Day 355
Winter Teletherm
January 9/Day 9
+19 days
Coverage
Year
MOORHEAD, MS
Day of Year

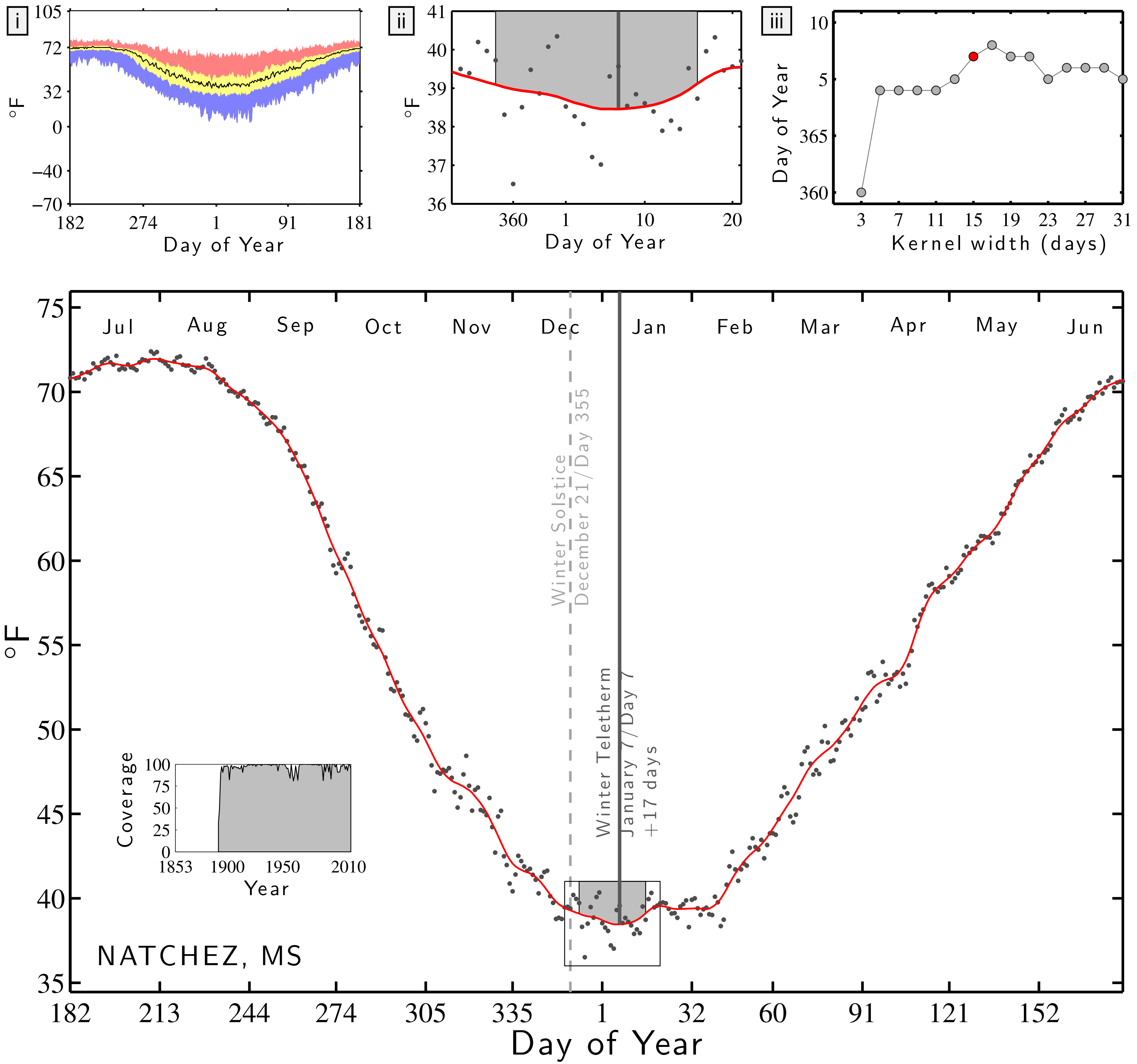

i
ii
iii
°F
Day of Year
Day of Year
Kernel width (days)
Winter Solstice
December 21/Day 355
Winter Teletherm
January 7/Day 7
+17 days
Coverage
Year
NATCHEZ, MS
Jul
Aug
Sep
Oct
Nov
Dec
Jan
Feb
Mar
Apr
May
Jun

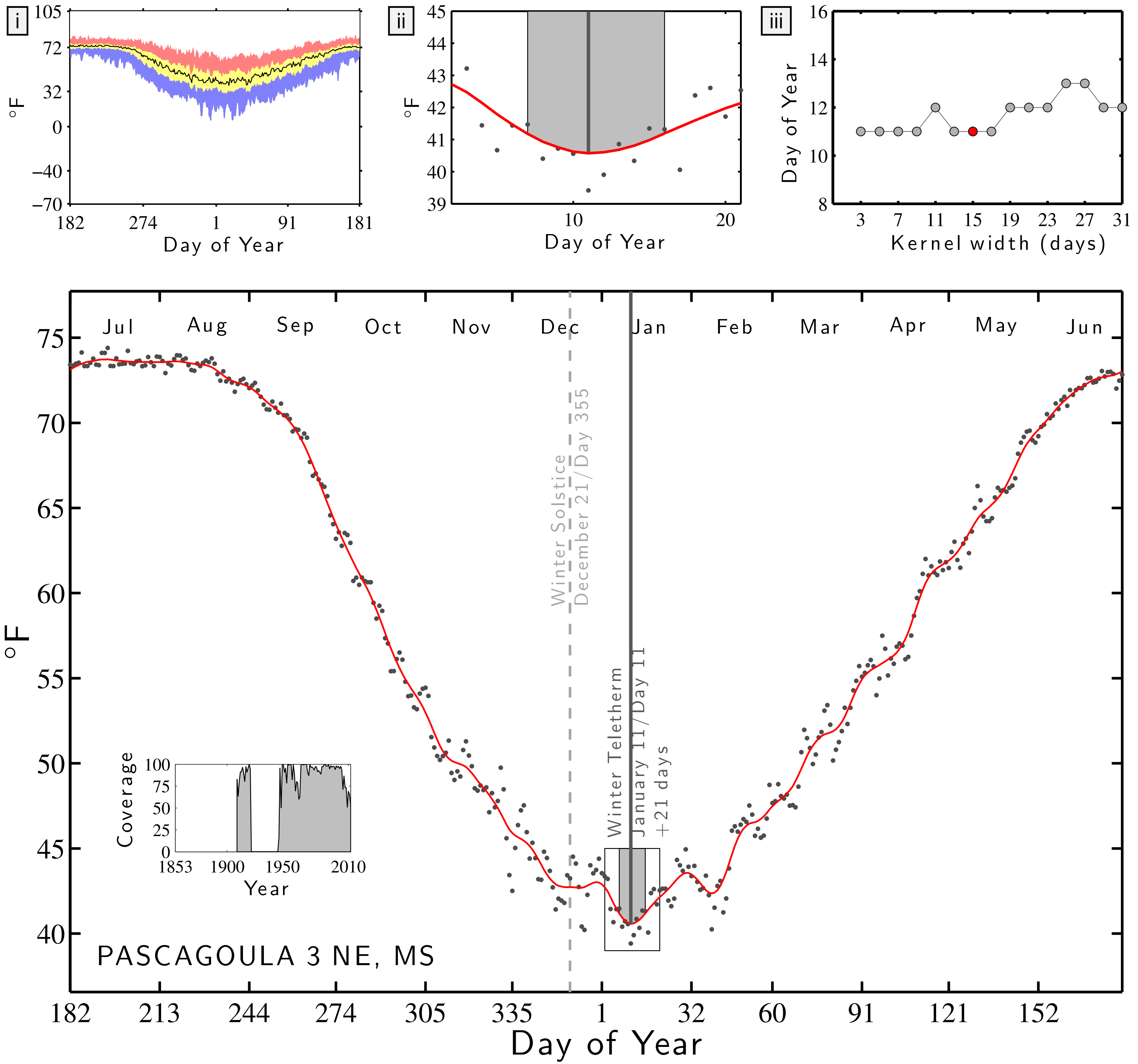

i
ii
iii
°F
Day of Year
Day of Year
Kernel width (days)
Jul
Aug
Sep
Oct
Nov
Dec
Jan
Feb
Mar
Apr
May
Jun
Winter Solstice
December 21/Day 355
Winter Teletherm
January 11/Day 11
+21 days
Coverage
Year
PASCAGOULA 3 NE, MS

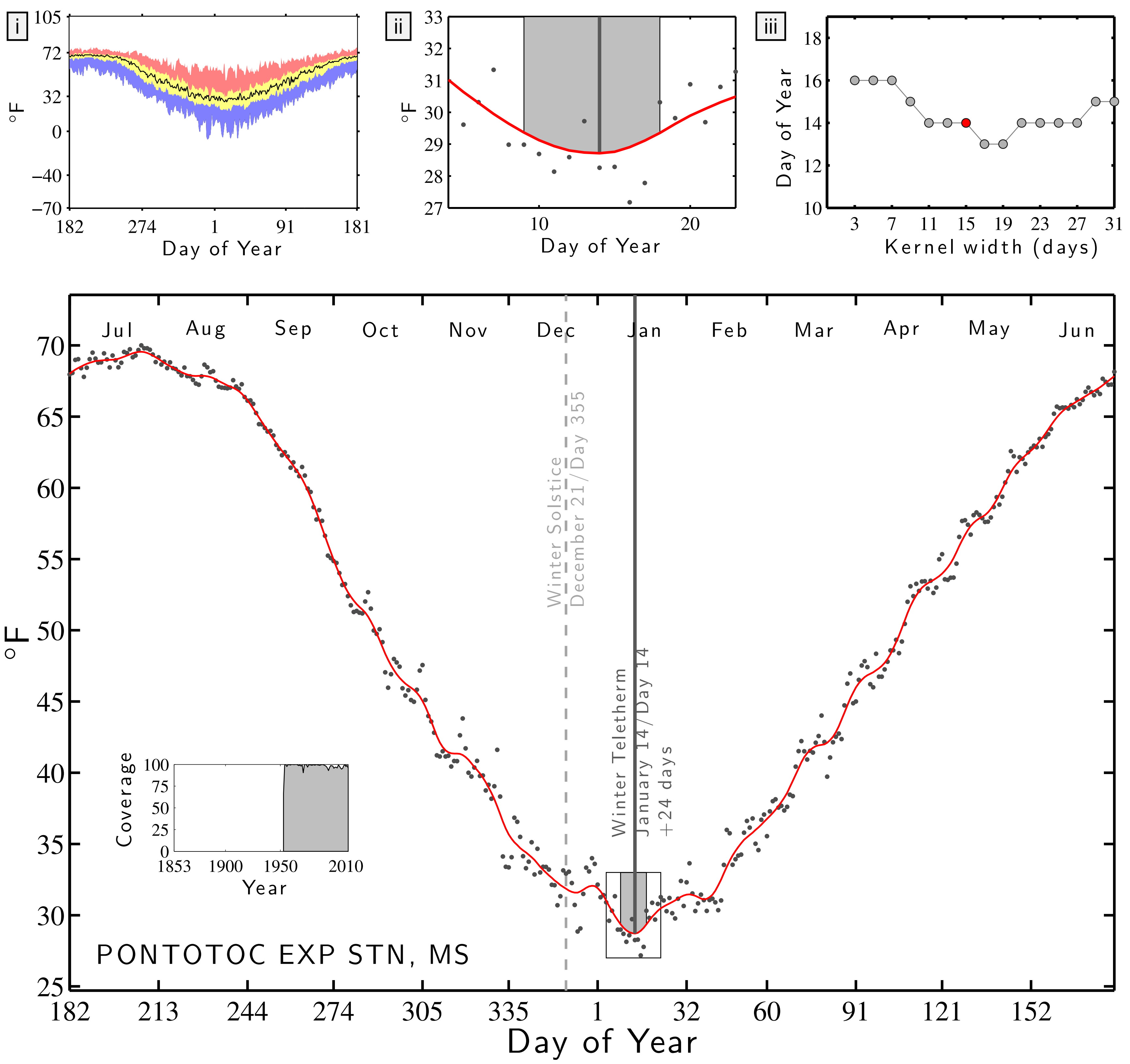

i
ii
iii
°F
Day of Year
Kernel width (days)
Jul
Aug
Sep
Oct
Nov
Dec
Jan
Feb
Mar
Apr
May
Jun
Winter Solstice
December 21/Day 355
Winter Teletherm
January 14/Day 14
+24 days
Coverage
Year
PONTOTOC EXP STN, MS

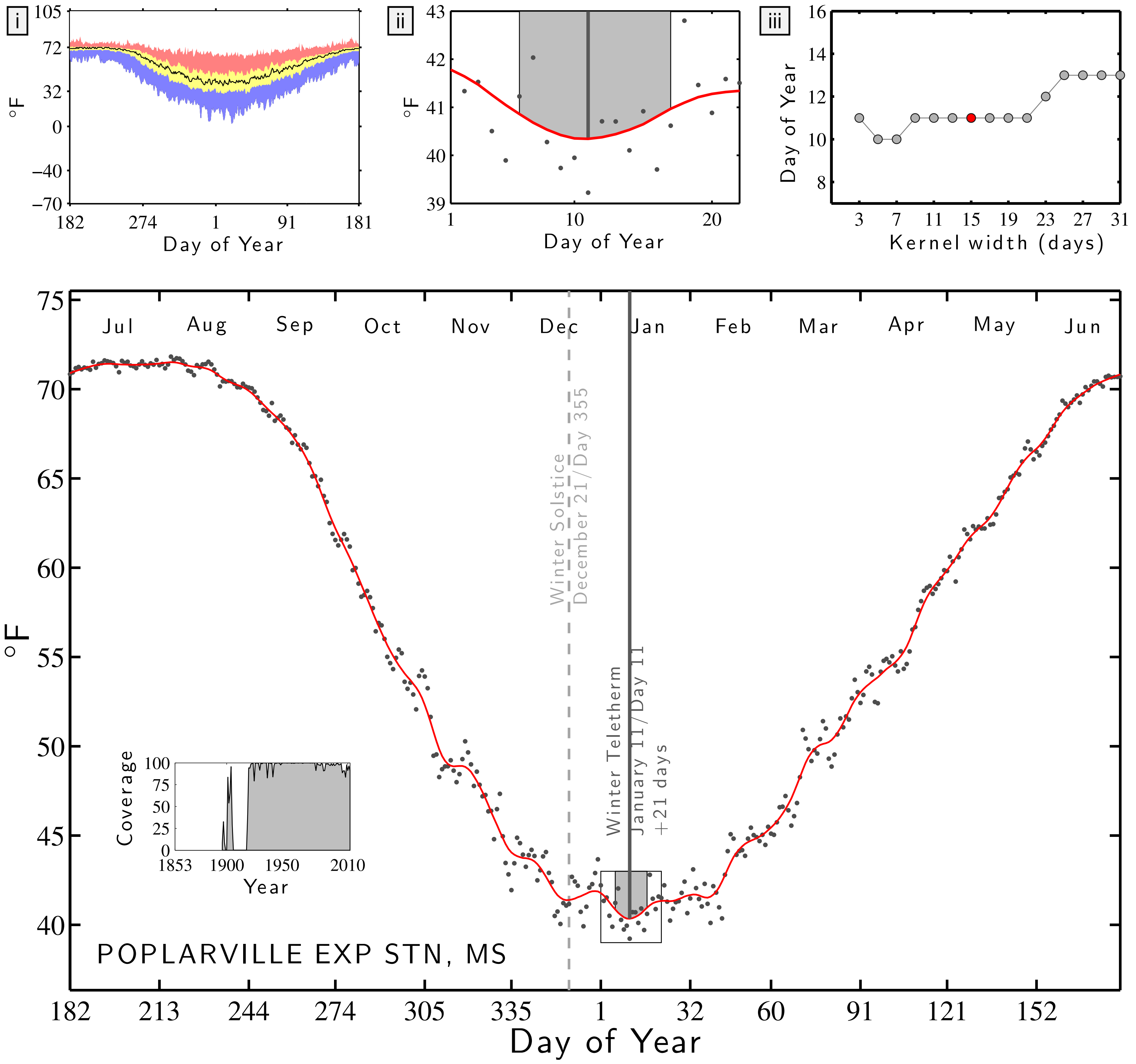

i
ii
iii
°F
Day of Year
Kernel width (days)
Jul
Aug
Sep
Oct
Nov
Dec
Jan
Feb
Mar
Apr
May
Jun
Winter Solstice
December 21/Day 355
Winter Teletherm
January 11/Day 11
+21 days
Coverage
Year
POPLARVILLE EXP STN, MS

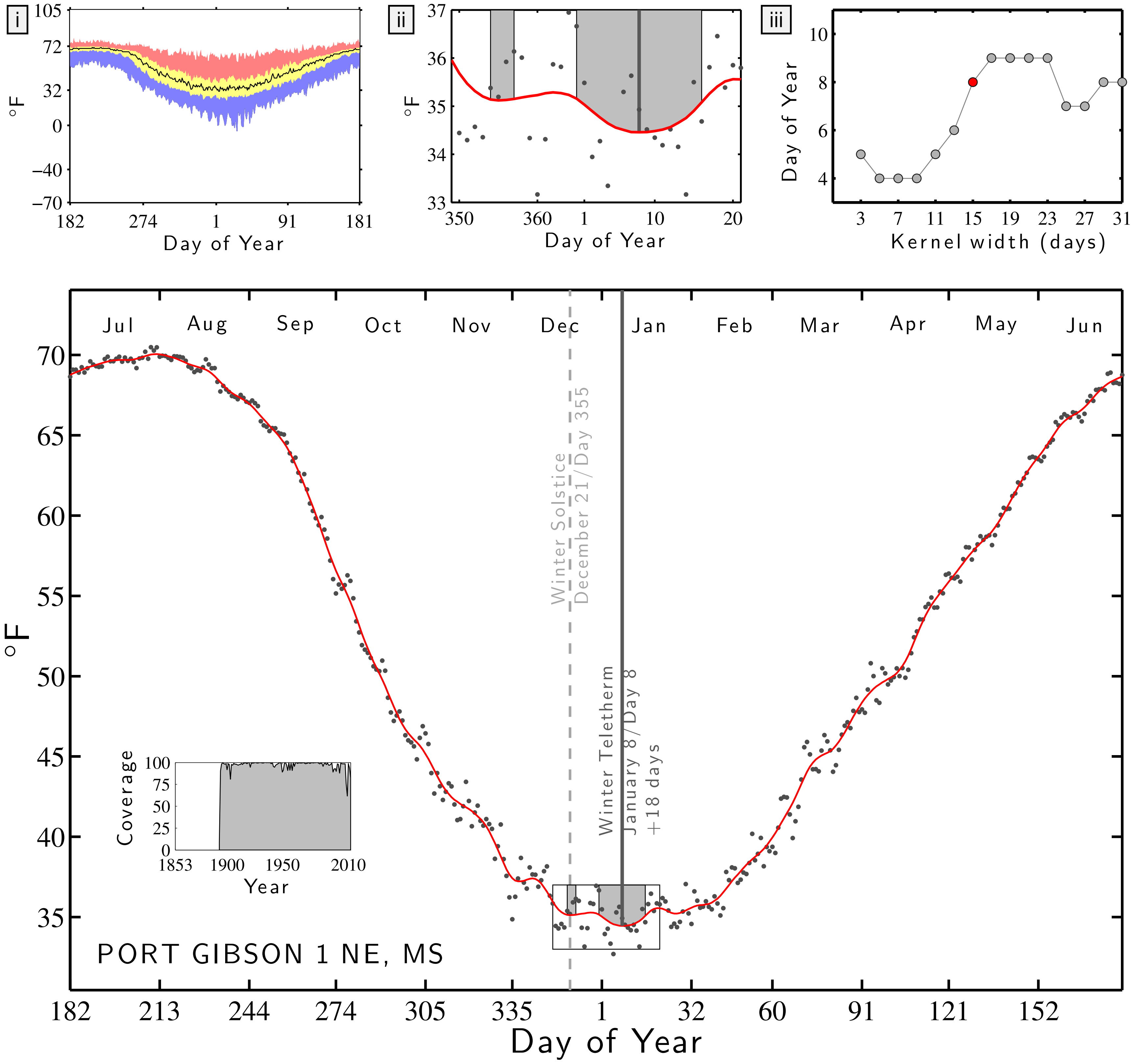

i
°F
Day of Year
ii
°F
Day of Year
iii
Day of Year
Kernel width (days)
Jul
Aug
Sep
Oct
Nov
Dec
Jan
Feb
Mar
Apr
May
Jun
Winter Solstice
December 21/Day 355
Winter Teletherm
January 8/Day 8
+18 days
Coverage
Year
PORT GIBSON 1 NE, MS
°F
Day of Year

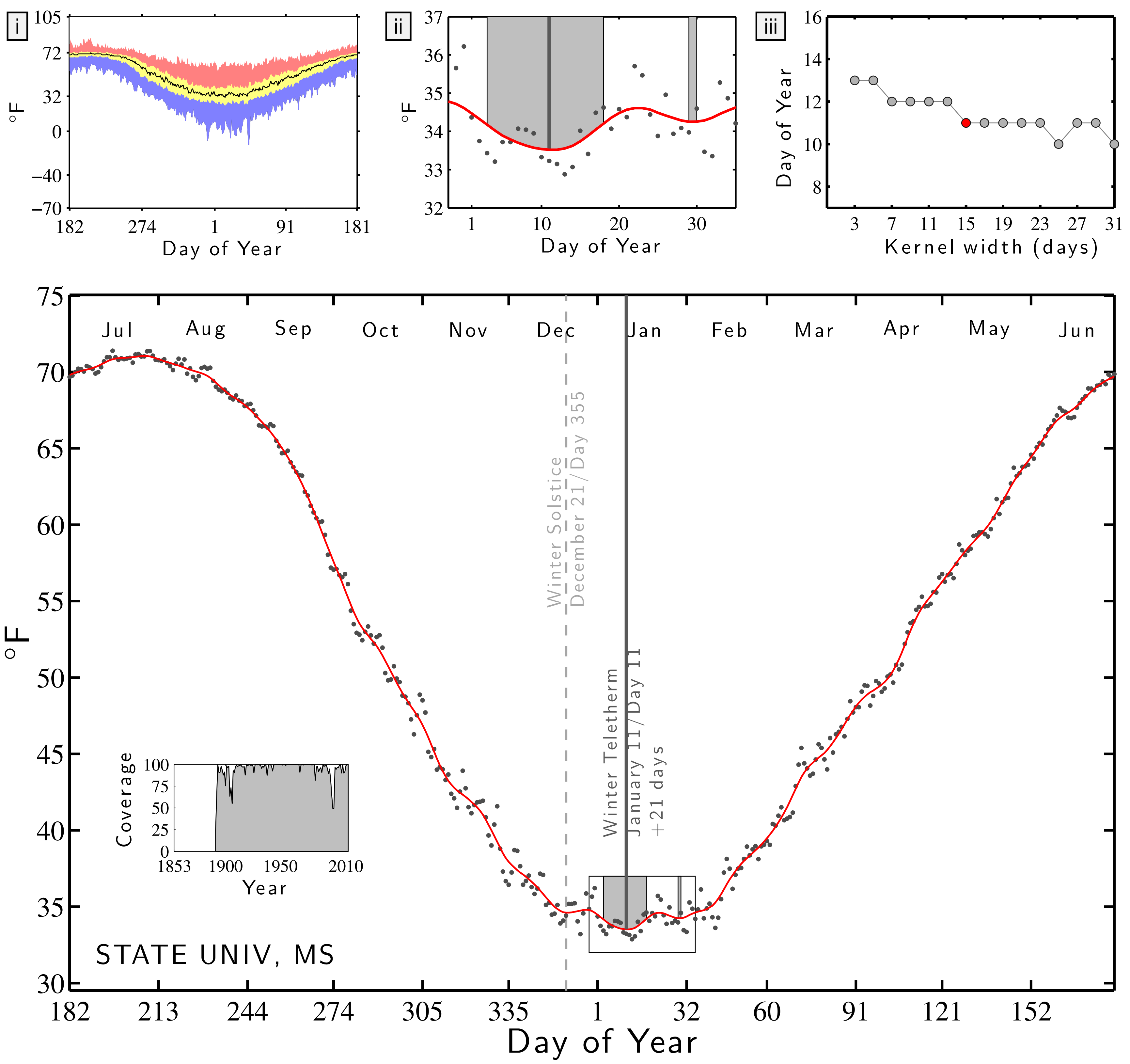

i
ii
iii
°F
Day of Year
Day of Year
Kernel width (days)
Jul
Aug
Sep
Oct
Nov
Dec
Jan
Feb
Mar
Apr
May
Jun
Winter Solstice
December 21/Day 355
Winter Teletherm
January 11/Day 11
+21 days
Coverage
Year
STATE UNIV, MS
°F
Day of Year

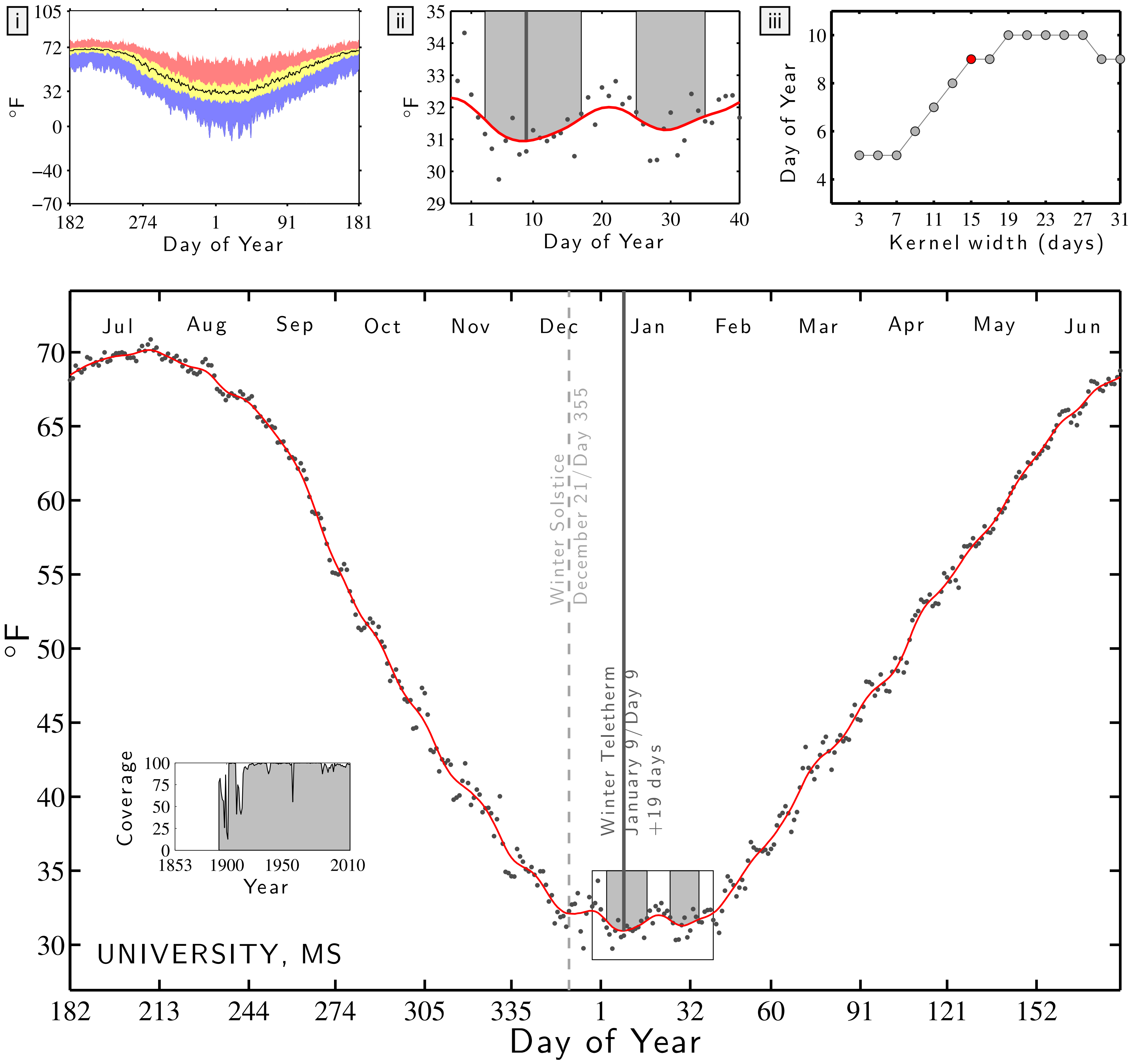

i
°F
Day of Year
ii
°F
Day of Year
iii
Day of Year
Kernel width (days)
Jul
Aug
Sep
Oct
Nov
Dec
Jan
Feb
Mar
Apr
May
Jun
Winter Solstice
December 21/Day 355
Winter Teletherm
January 9/Day 9
+19 days
Coverage
Year
UNIVERSITY, MS
°F
Day of Year

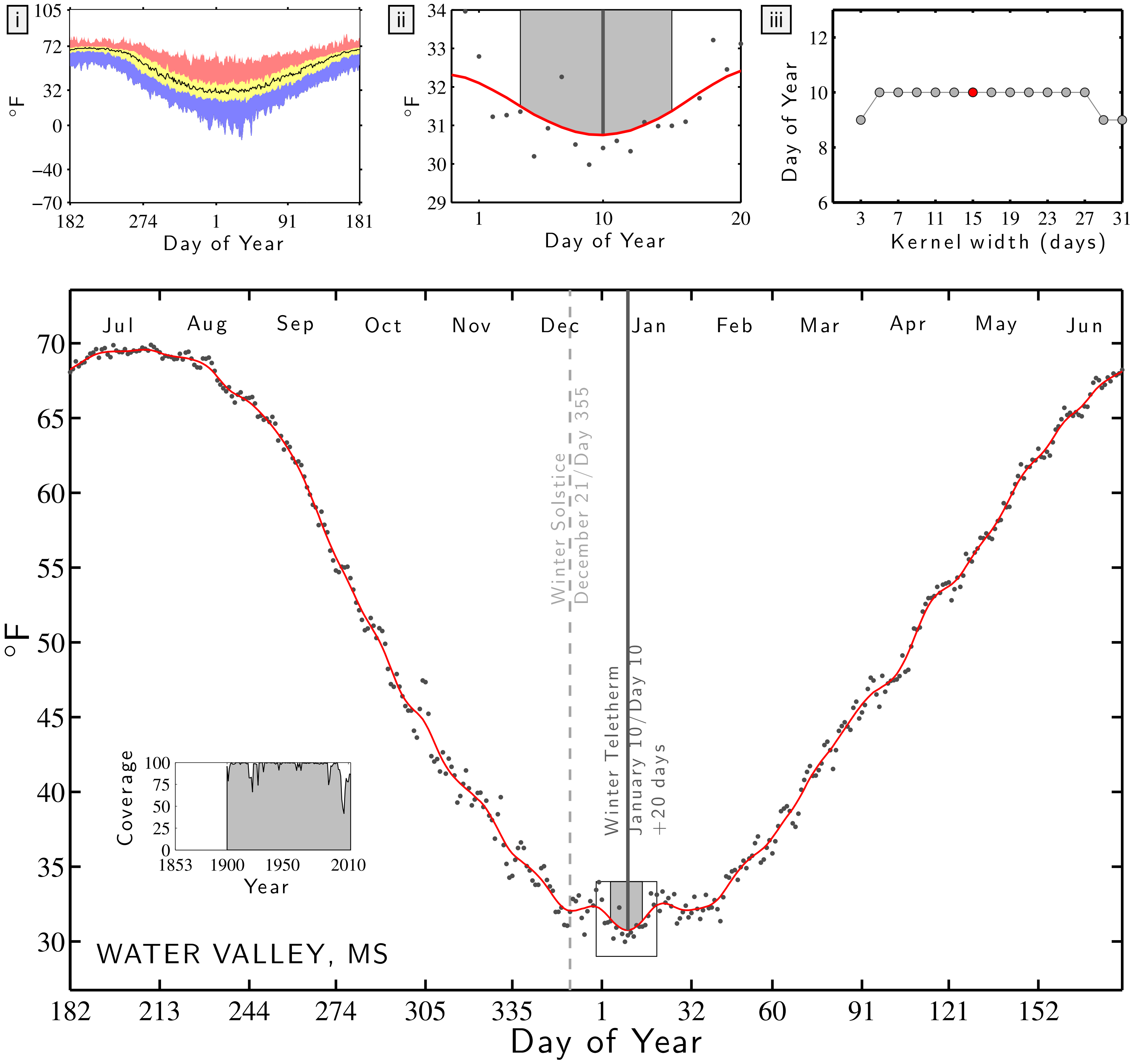

i
°F
Day of Year
ii
°F
Day of Year
iii
Day of Year
Kernel width (days)
Jul Aug Sep Oct Nov Dec Jan Feb Mar Apr May Jun
Winter Solstice
December 21/Day 355
Winter Teletherm
January 10/Day 10
+20 days
Coverage
Year
WATER VALLEY, MS
°F
Day of Year

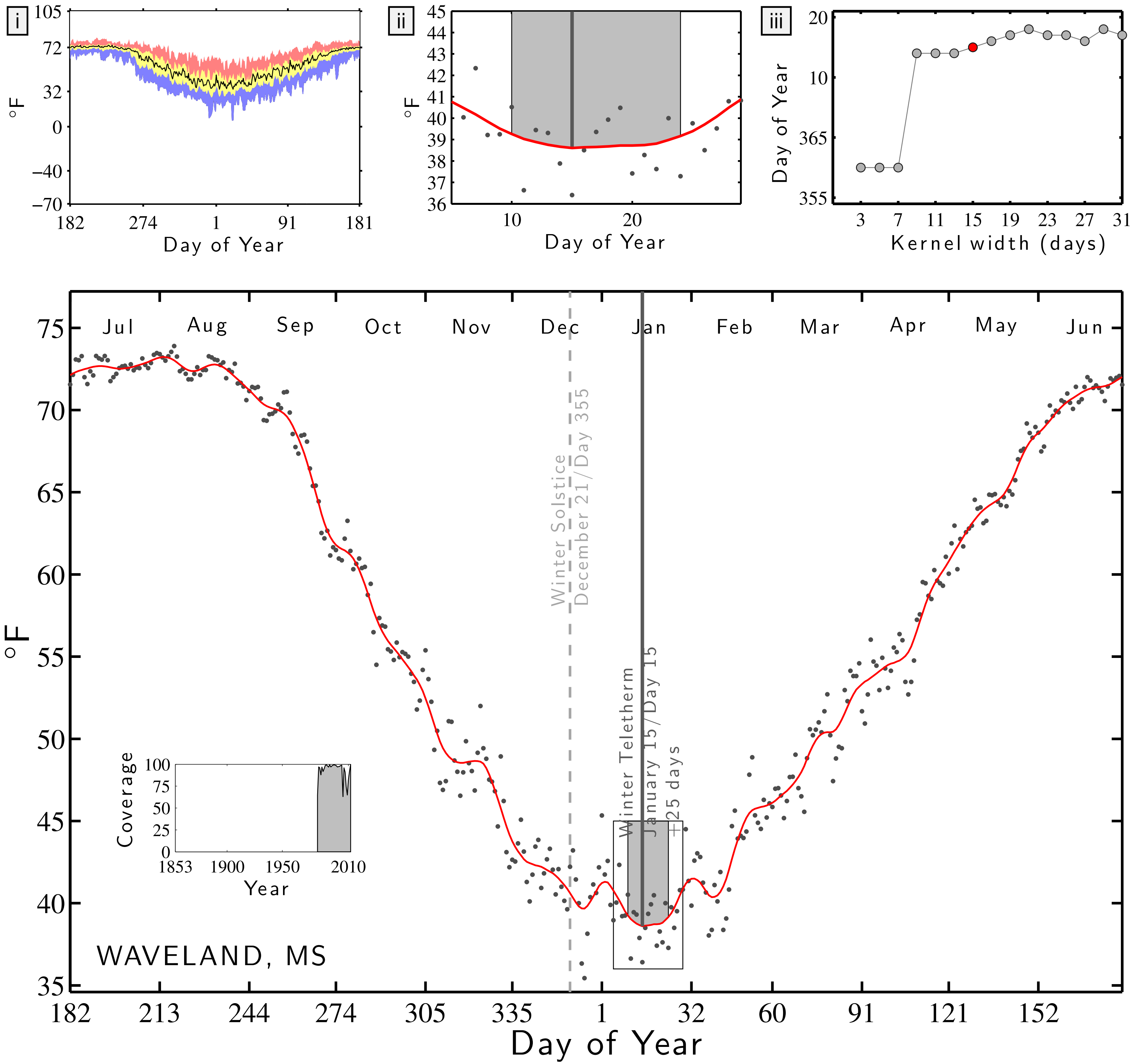

i
°F
Day of Year
ii
Day of Year
iii
Day of Year
Kernel width (days)
Jul
Aug
Sep
Oct
Nov
Dec
Jan
Feb
Mar
Apr
May
Jun
Winter Solstice
December 21/Day 355
Winter Teletherm
January 15/Day 15
±25 days
Coverage
Year
WAVELAND, MS
°F
Day of Year

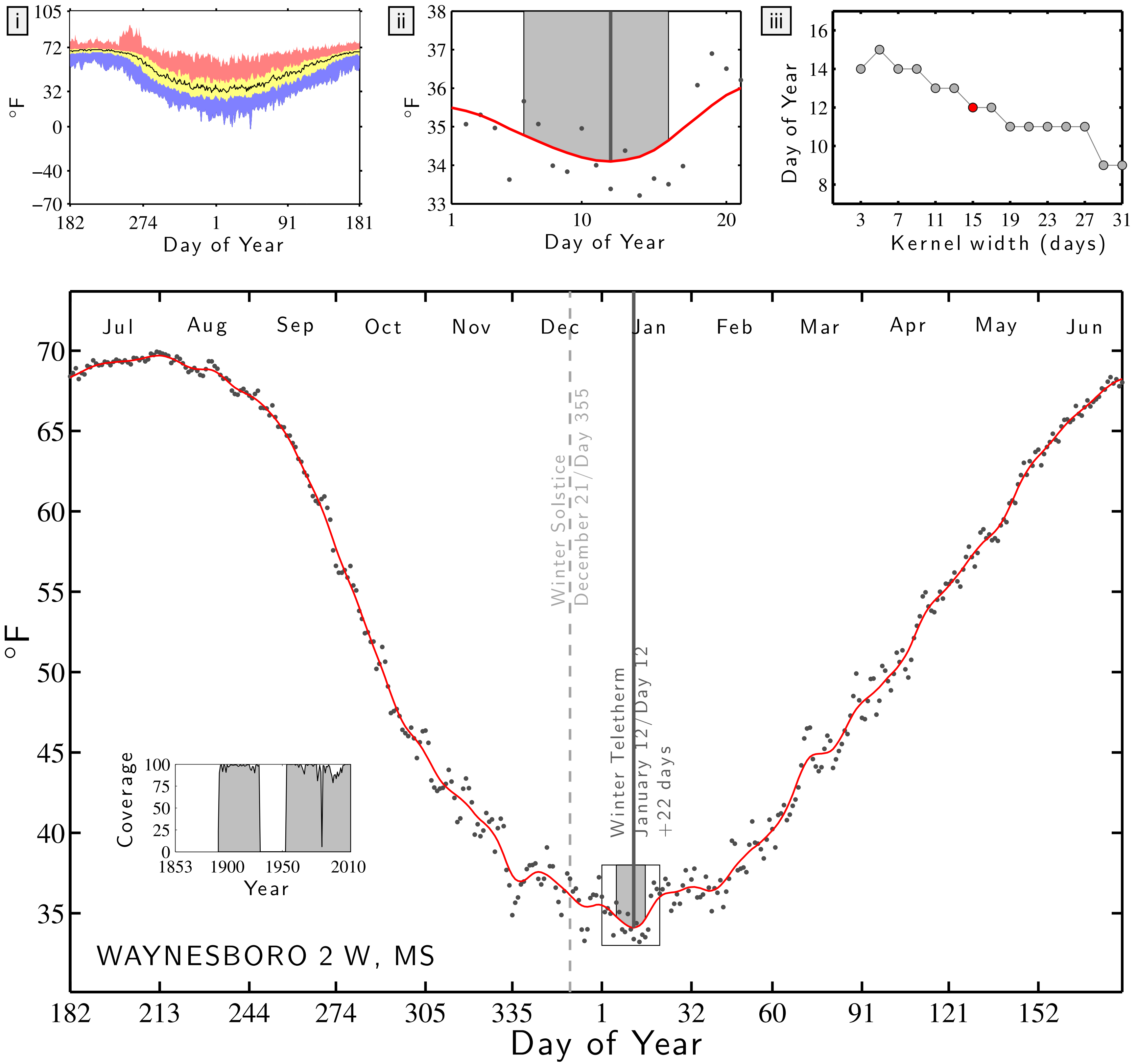

i
ii
iii
°F
Day of Year
Kernel width (days)
Jul
Aug
Sep
Oct
Nov
Dec
Jan
Feb
Mar
Apr
May
Jun
Winter Solstice
December 21/Day 355
Winter Teletherm
January 12/Day 12
+22 days
Coverage
Year
WAYNESBORO 2 W, MS

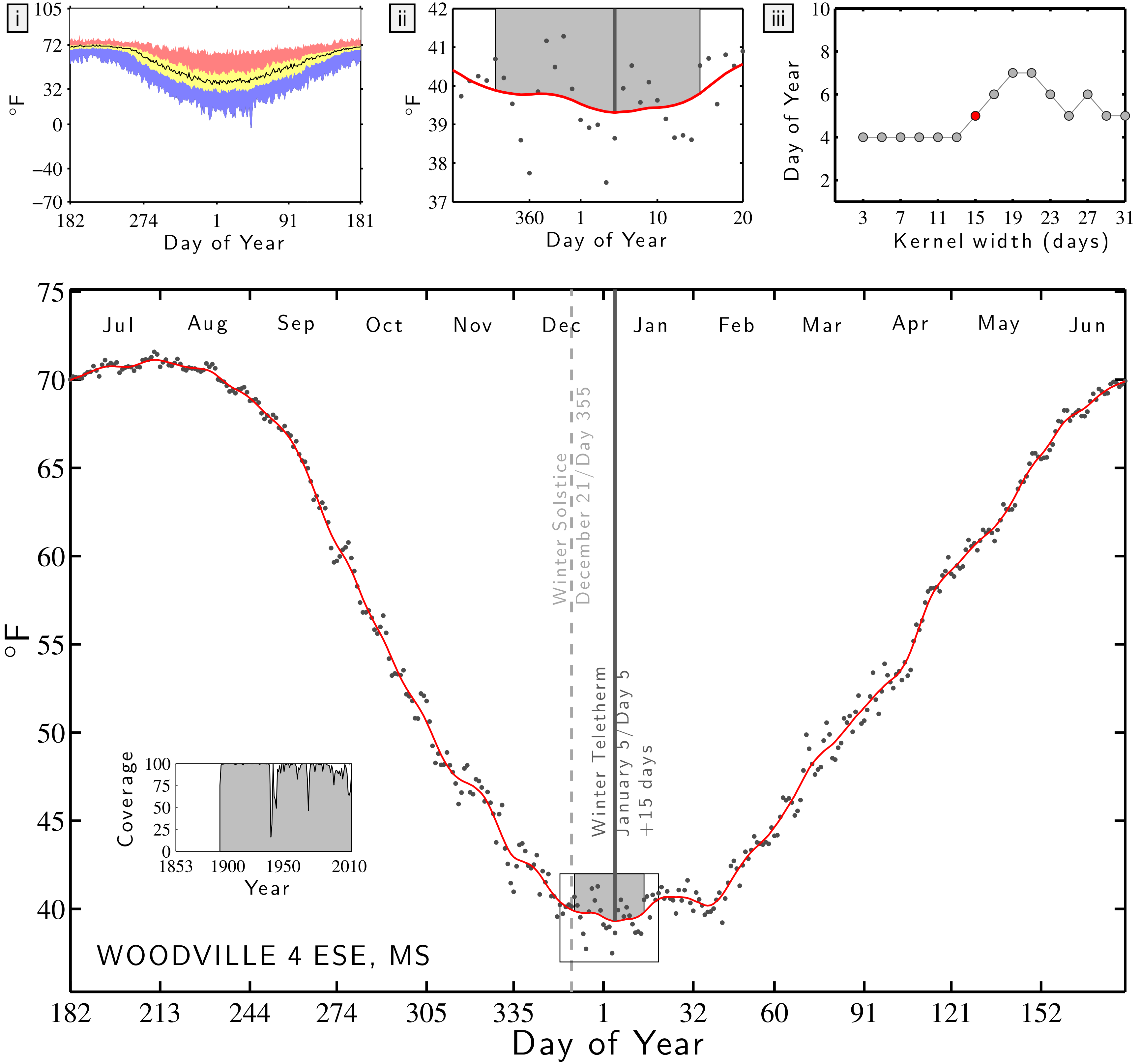

i
°F
Day of Year
ii
°F
Day of Year
iii
Day of Year
Kernel width (days)
Jul
Aug
Sep
Oct
Nov
Dec
Jan
Feb
Mar
Apr
May
Jun
Winter Solstice
December 21/Day 355
Winter Teletherm
January 5/Day 5
+15 days
Coverage
Year
WOODVILLE 4 ESE, MS
°F
Day of Year

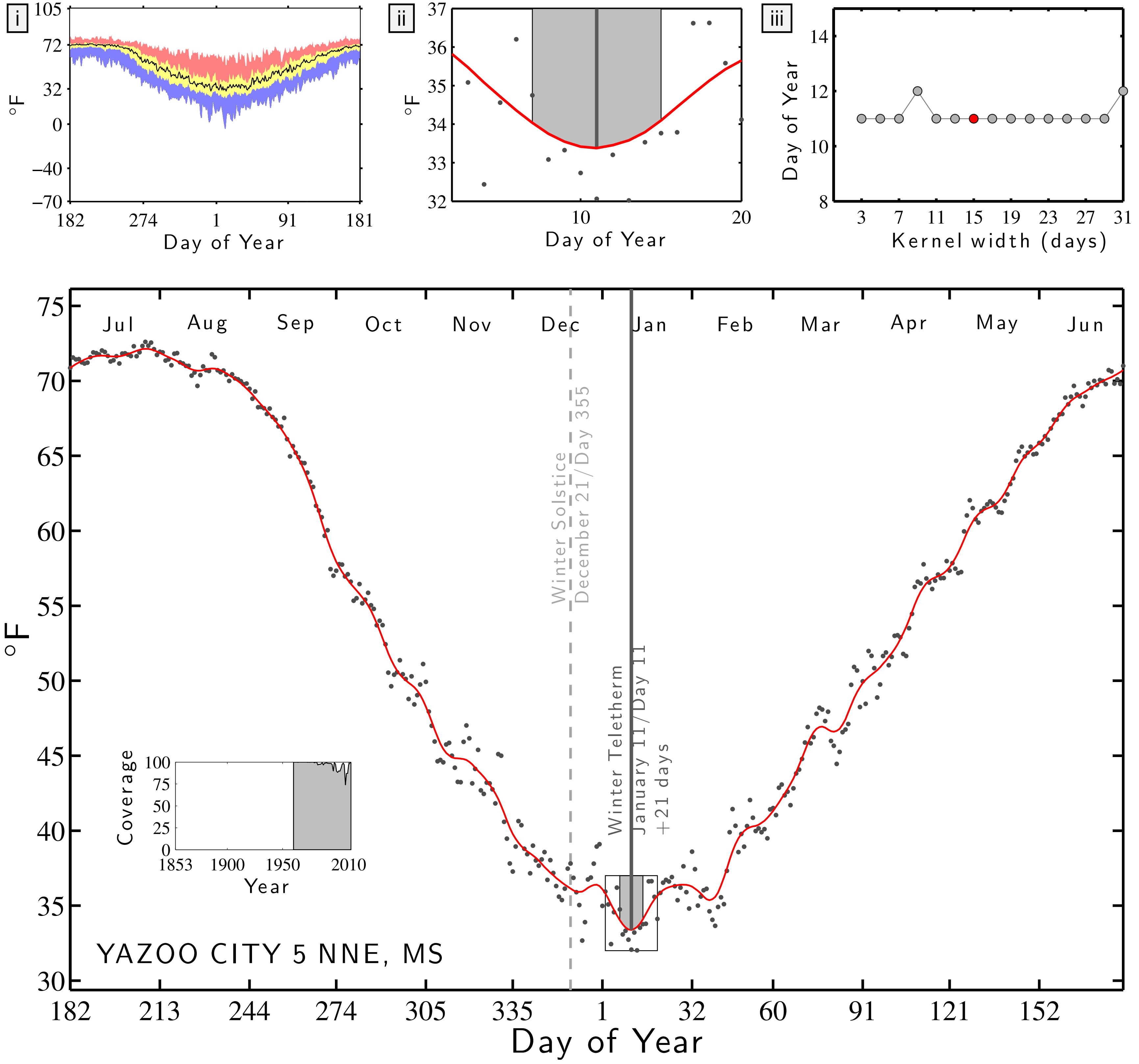

i
°F
Day of Year
ii
°F
Day of Year
iii
Day of Year
Kernel width (days)
Jul
Aug
Sep
Oct
Nov
Dec
Jan
Feb
Mar
Apr
May
Jun
Winter Solstice
December 21/Day 355
Winter Teletherm
January 11/Day 11
+21 days
Coverage
Year
YAZOO CITY 5 NNE, MS
°F
Day of Year

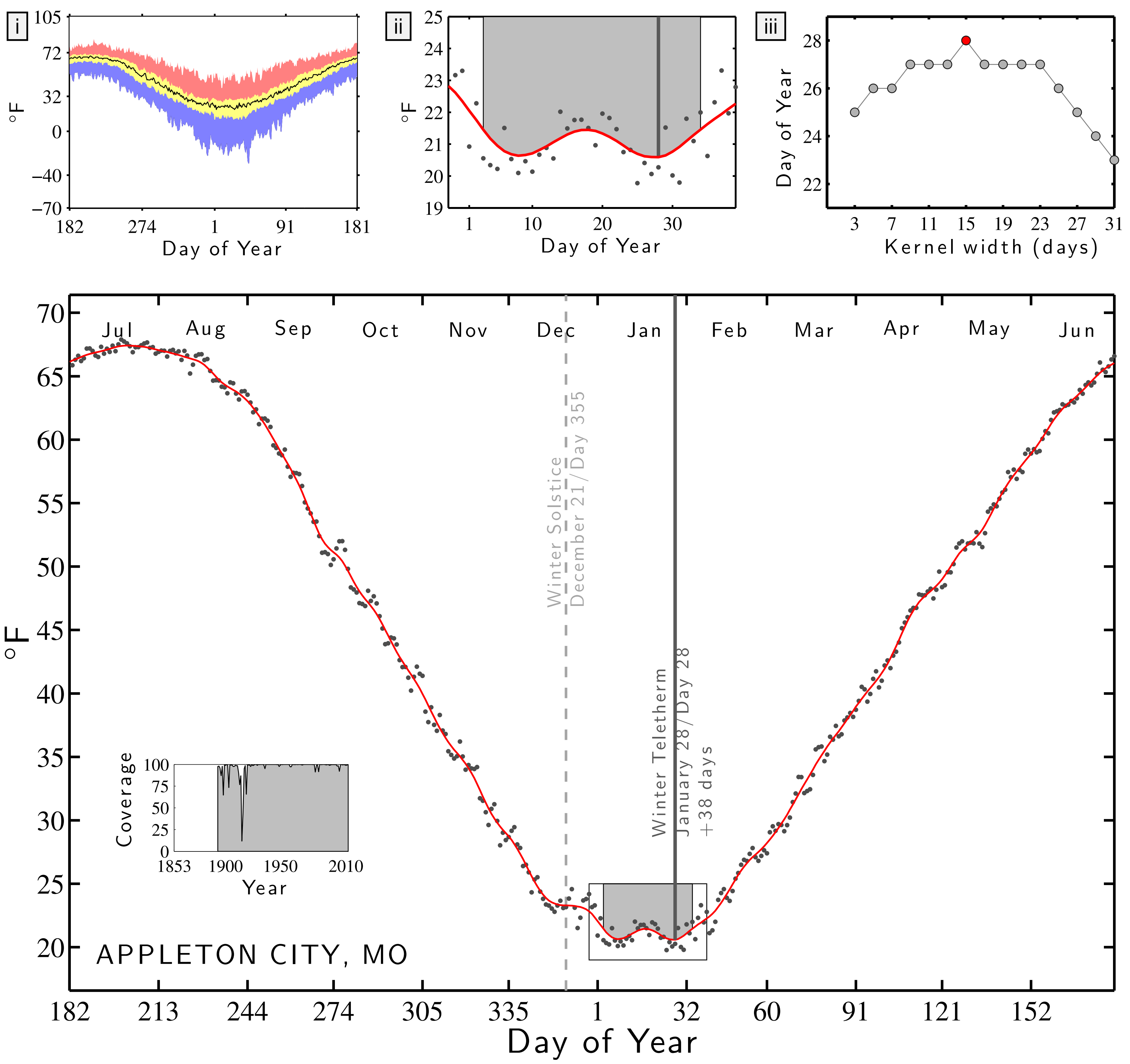

i
ii
iii
°F
Day of Year
Kernel width (days)
Jul
Aug
Sep
Oct
Nov
Dec
Jan
Feb
Mar
Apr
May
Jun
Winter Solstice
December 21/Day 355
Winter Teletherm
January 28/Day 28
+38 days
Coverage
Year
APPLETON CITY, MO

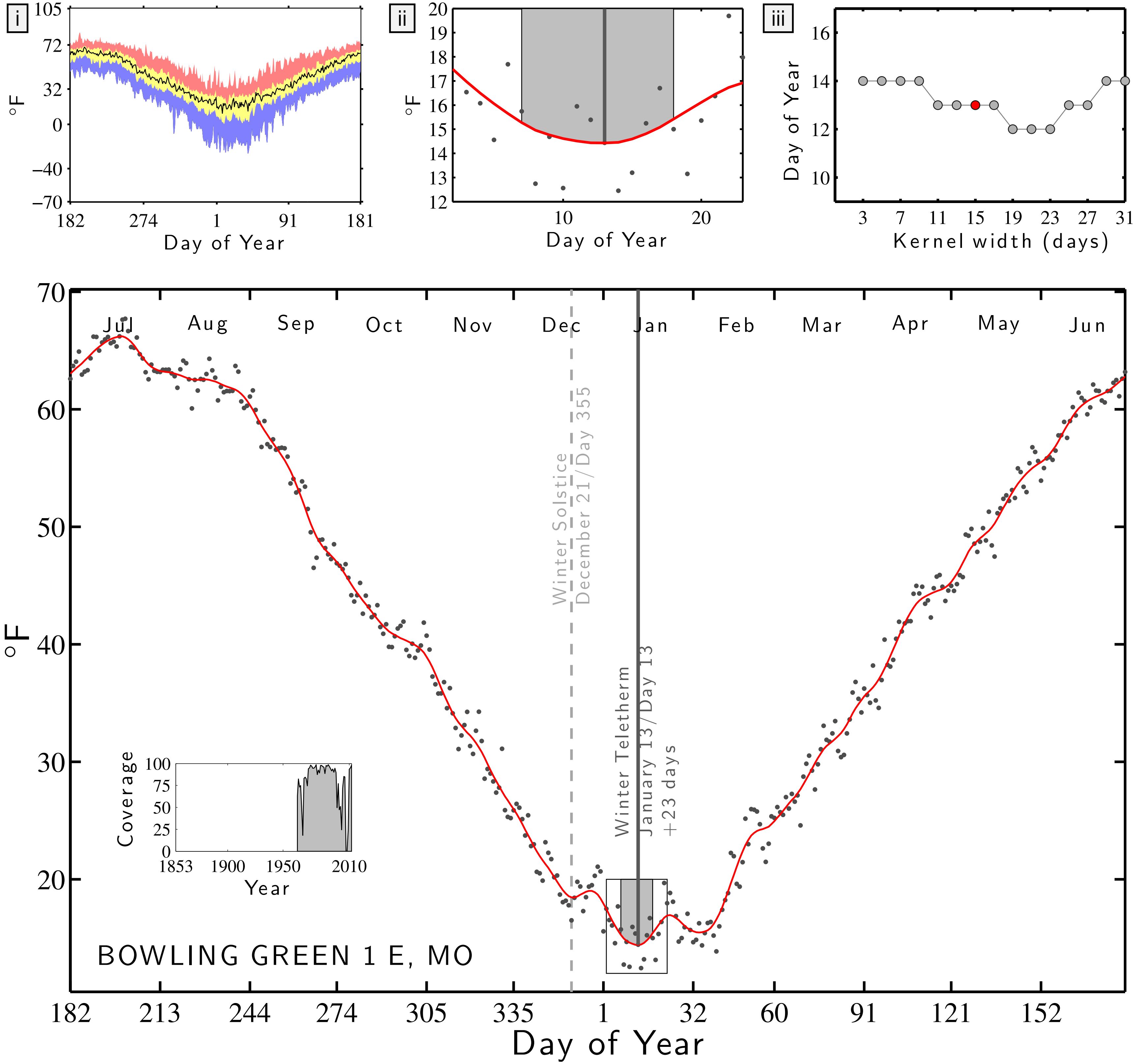

i
°F
Day of Year
ii
°F
Day of Year
iii
Day of Year
Kernel width (days)
Jul
Aug
Sep
Oct
Nov
Dec
Jan
Feb
Mar
Apr
May
Jun
Winter Solstice
December 21/Day 355
Winter Teletherm
January 13/Day 13
+23 days
Coverage
Year
BOWLING GREEN 1 E, MO
°F
Day of Year

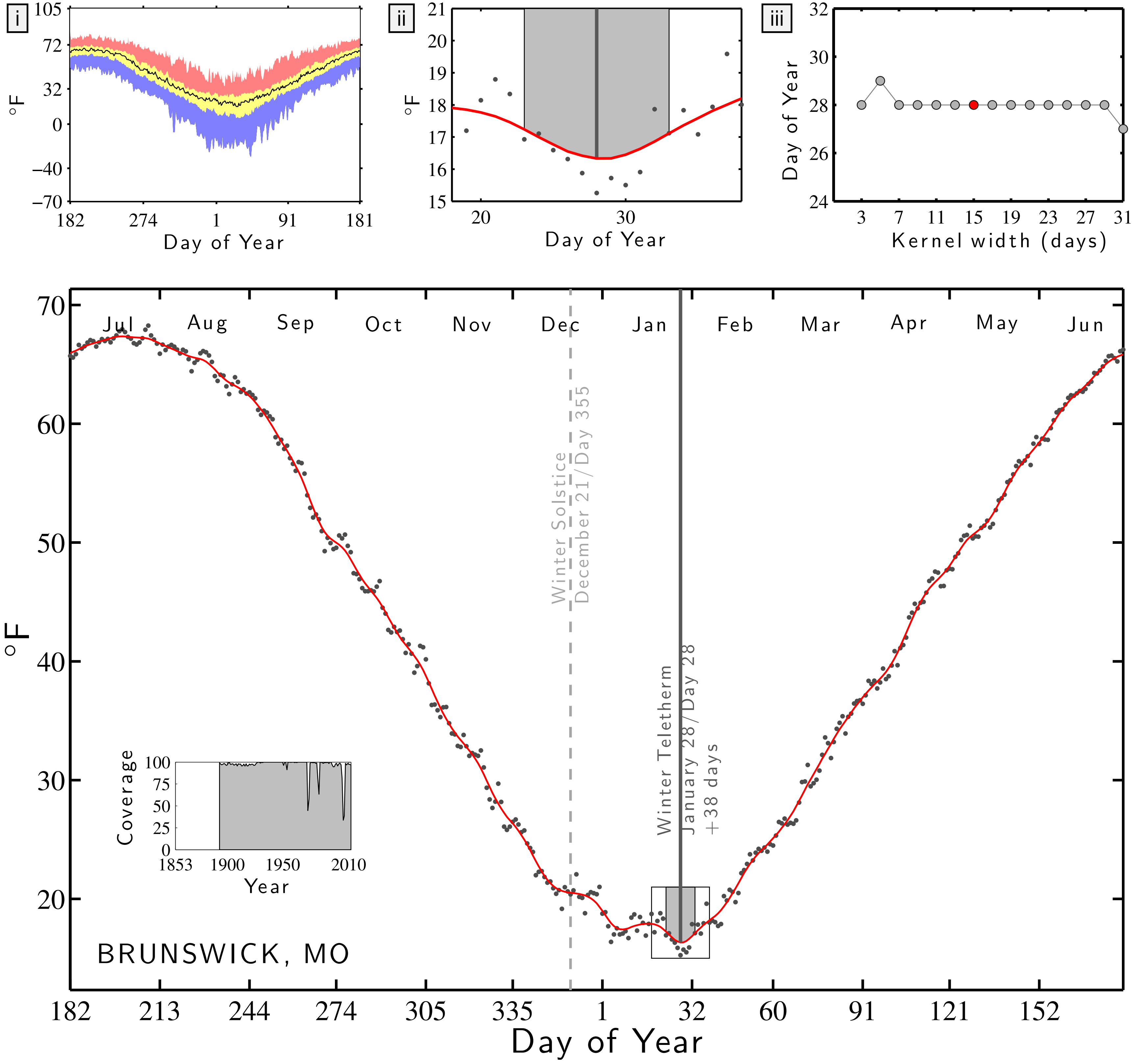

i
°F
Day of Year
ii
°F
Day of Year
iii
Day of Year
Kernel width (days)
Jul
Aug
Sep
Oct
Nov
Dec
Jan
Feb
Mar
Apr
May
Jun
Winter Solstice
December 21/Day 355
Winter Teletherm
January 28/Day 28
+38 days
Coverage
Year
BRUNSWICK, MO
°F
Day of Year

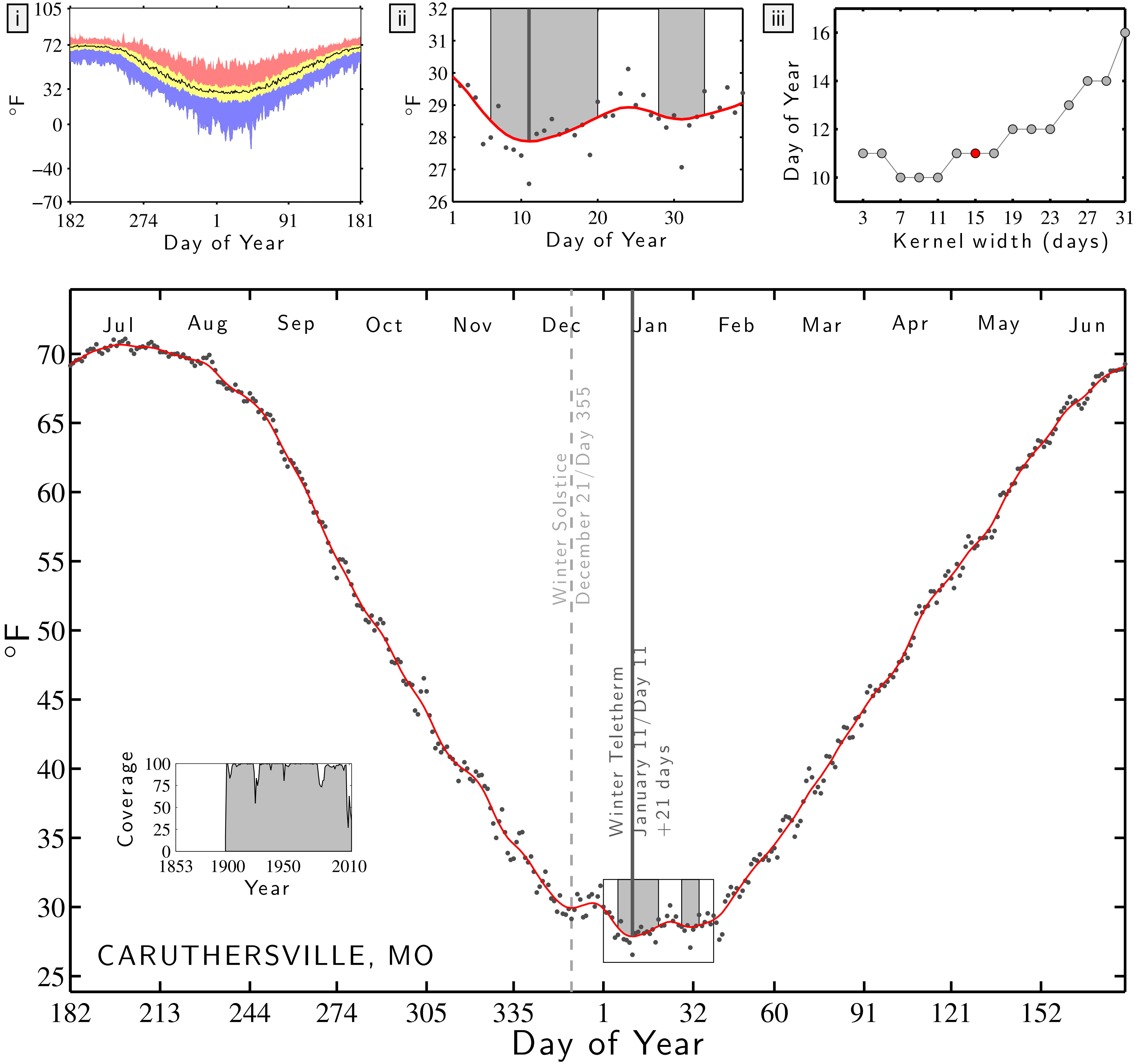

i
ii
iii
°F
Day of Year
Kernel width (days)
Jul
Aug
Sep
Oct
Nov
Dec
Jan
Feb
Mar
Apr
May
Jun
Winter Solstice
December 21/Day 355
Winter Teletherm
January 11/Day 11
+21 days
Coverage
Year
CARUTHERSVILLE, MO

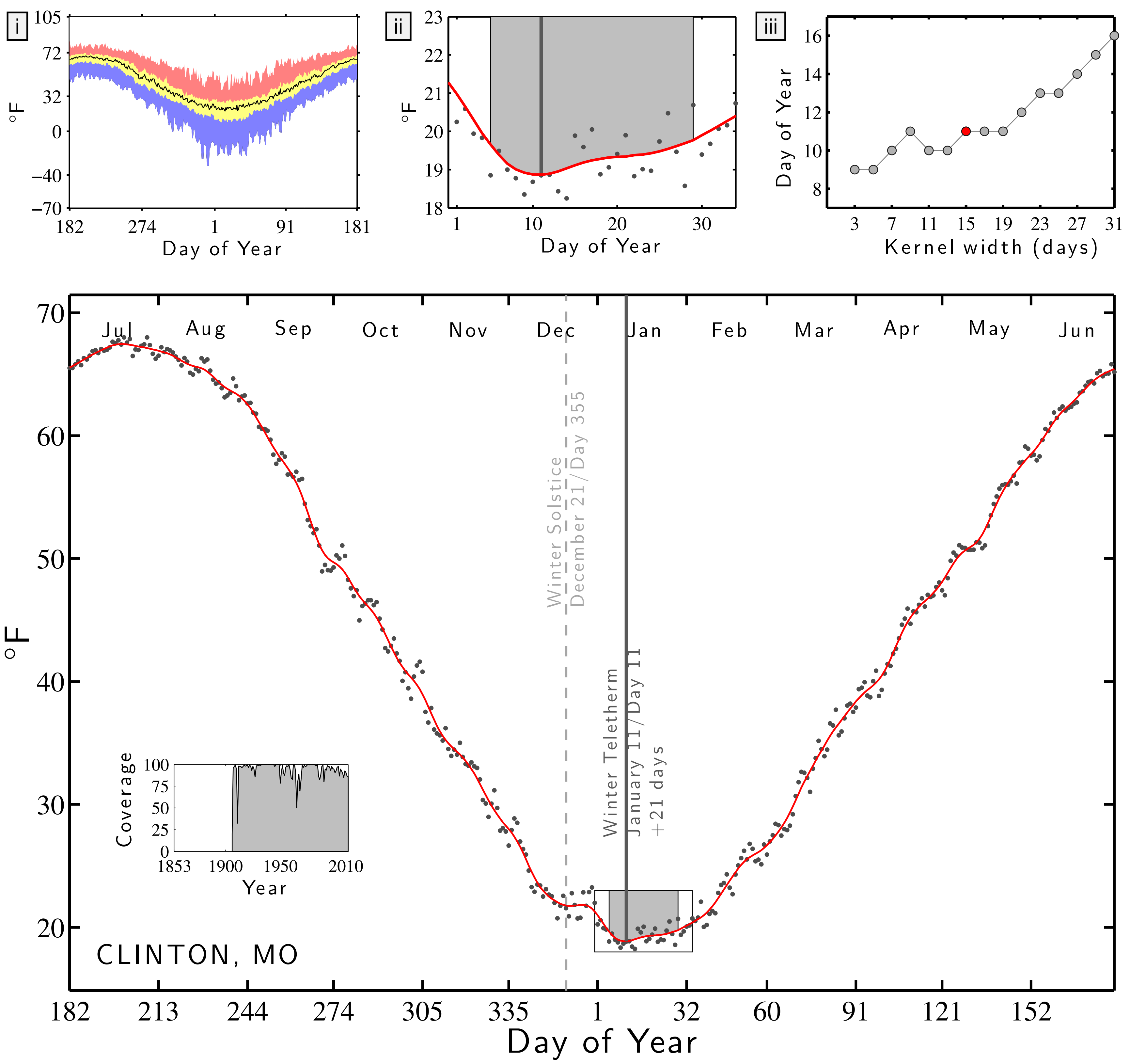

i
°F
105
72
32
0
−40
−70
182
274
1
91
181
Day of Year
ii
23
22
21
20
19
18
1
10
20
30
iii
16
14
12
10
8
3
7
11
15
19
23
27
31
Kernel width (days)
Jul
Aug
Sep
Oct
Nov
Dec
Jan
Feb
Mar
Apr
May
Jun
70
60
50
40
30
20
Winter Solstice
December 21/Day 355
Winter Teletherm
January 11/Day 11
+21 days
Coverage
100
75
50
25
0
1853
1900
1950
2010
Year
CLINTON, MO
182
213
244
274
305
335
1
32
60
91
121
152

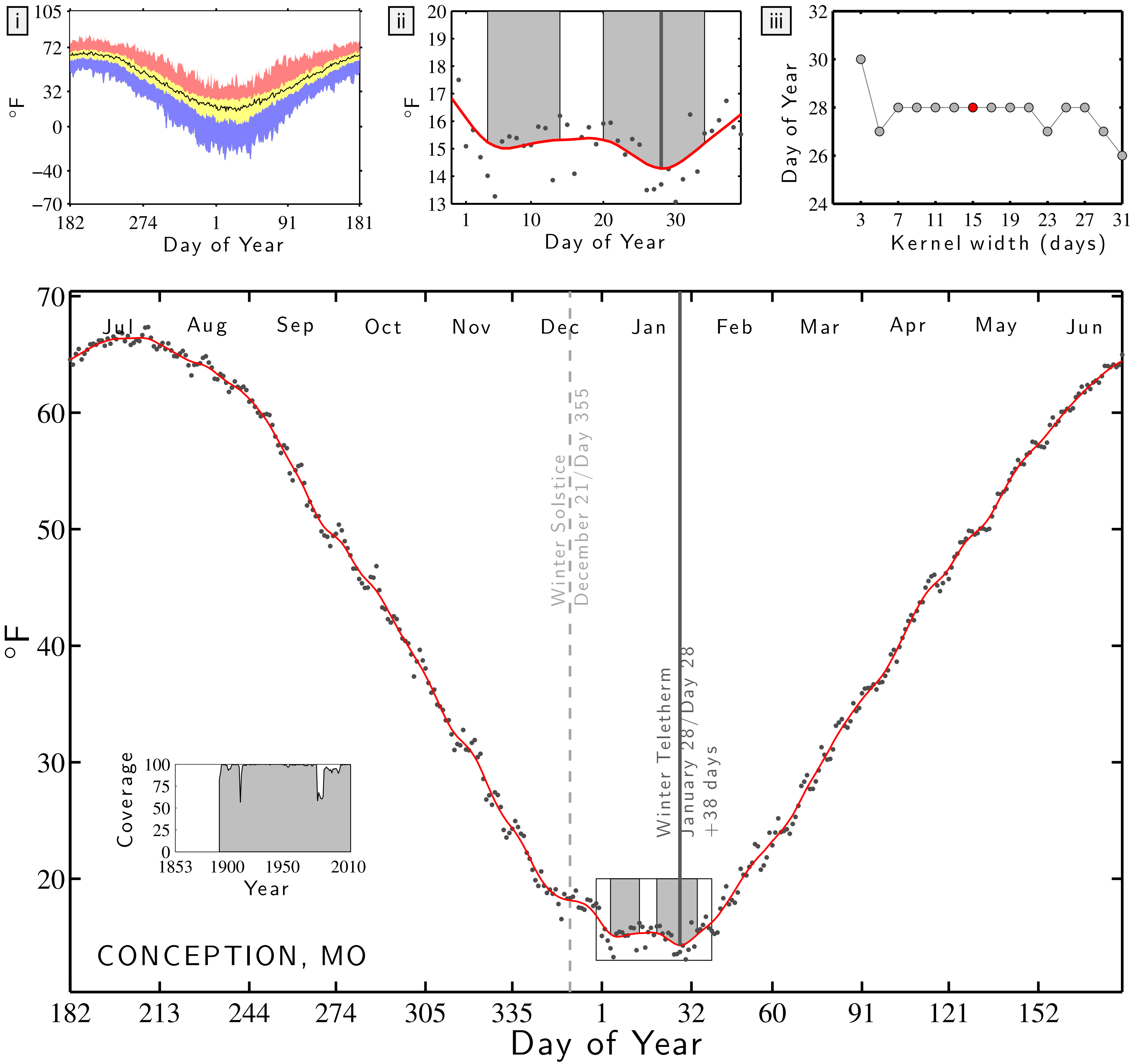

i
ii
iii
°F
Day of Year
Kernel width (days)
Day of Year
Jul
Aug
Sep
Oct
Nov
Dec
Jan
Feb
Mar
Apr
May
Jun
Winter Solstice
December 21/Day 355
Winter Teletherm
January 28/Day 28
+38 days
Coverage
Year
CONCEPTION, MO

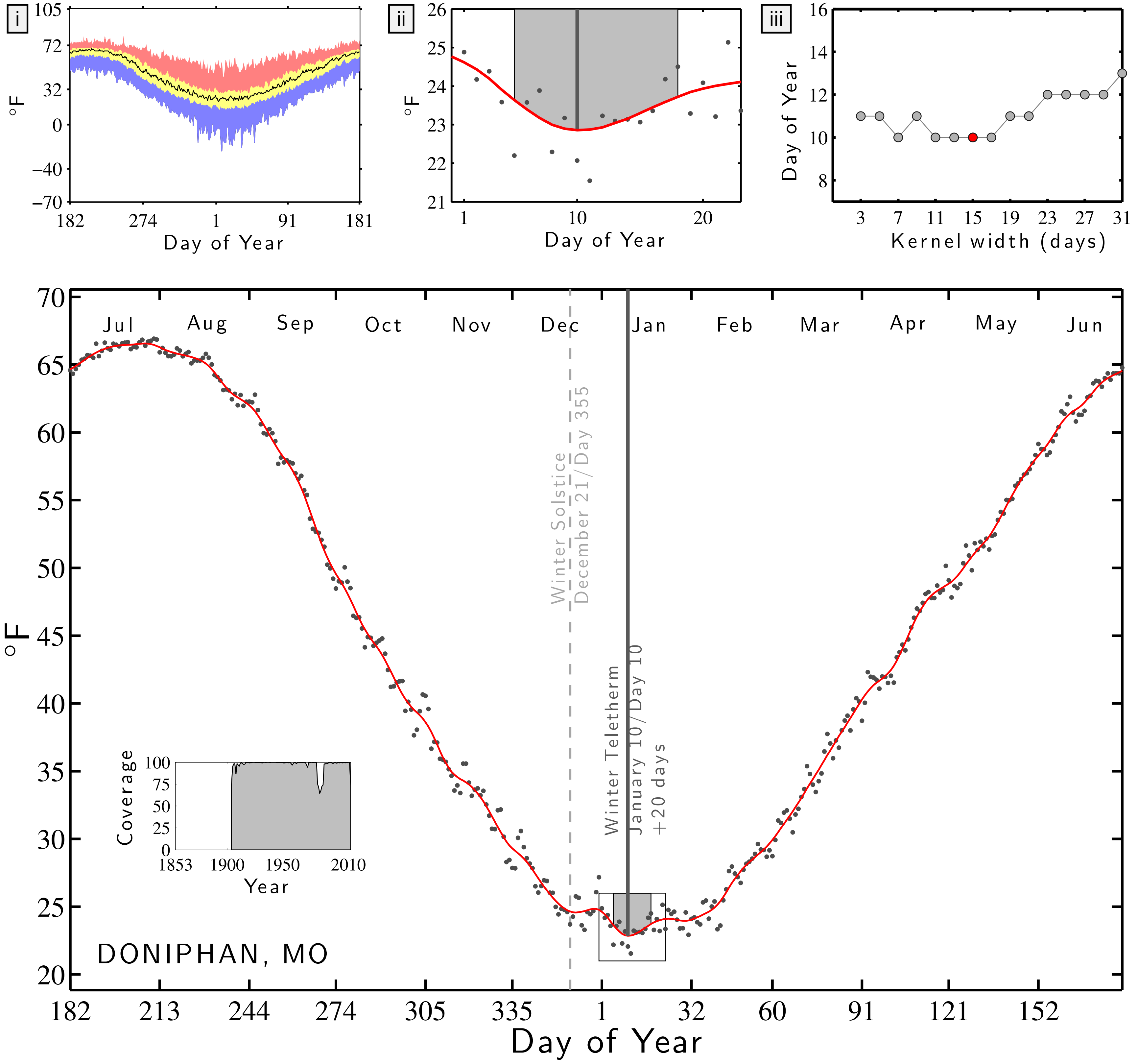

i
ii
iii
°F
Day of Year
Day of Year
Kernel width (days)
Winter Solstice
December 21/Day 355
Winter Teletherm
January 10/Day 10
+20 days
Coverage
Year
DONIPHAN, MO
Jul
Aug
Sep
Oct
Nov
Dec
Jan
Feb
Mar
Apr
May
Jun

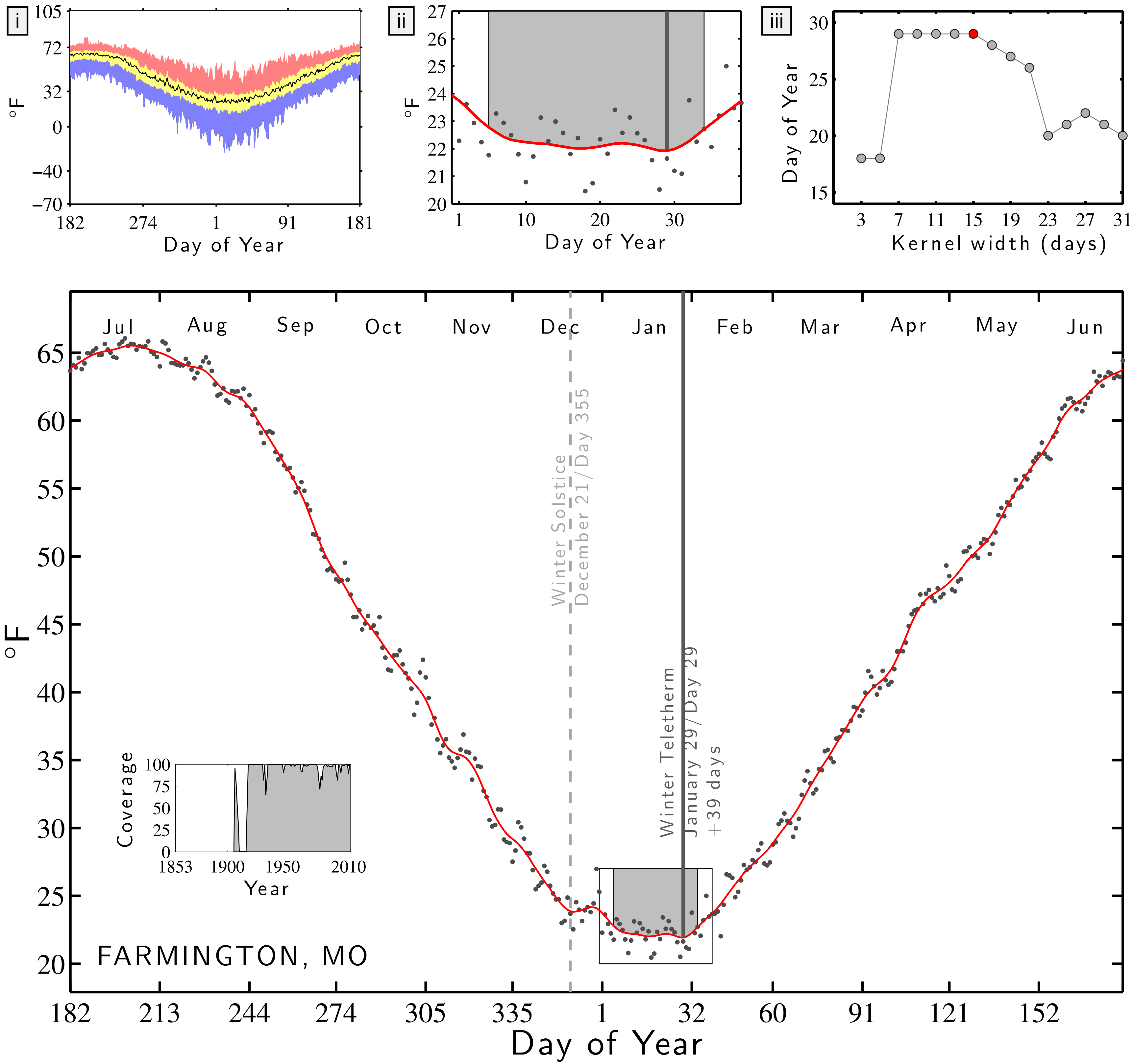

i
°F
Day of Year
ii
°F
Day of Year
iii
Day of Year
Kernel width (days)
Jul
Aug
Sep
Oct
Nov
Dec
Jan
Feb
Mar
Apr
May
Jun
Winter Solstice
December 21/Day 355
Winter Teletherm
January 29/Day 29
+39 days
Coverage
Year
FARMINGTON, MO
°F
Day of Year

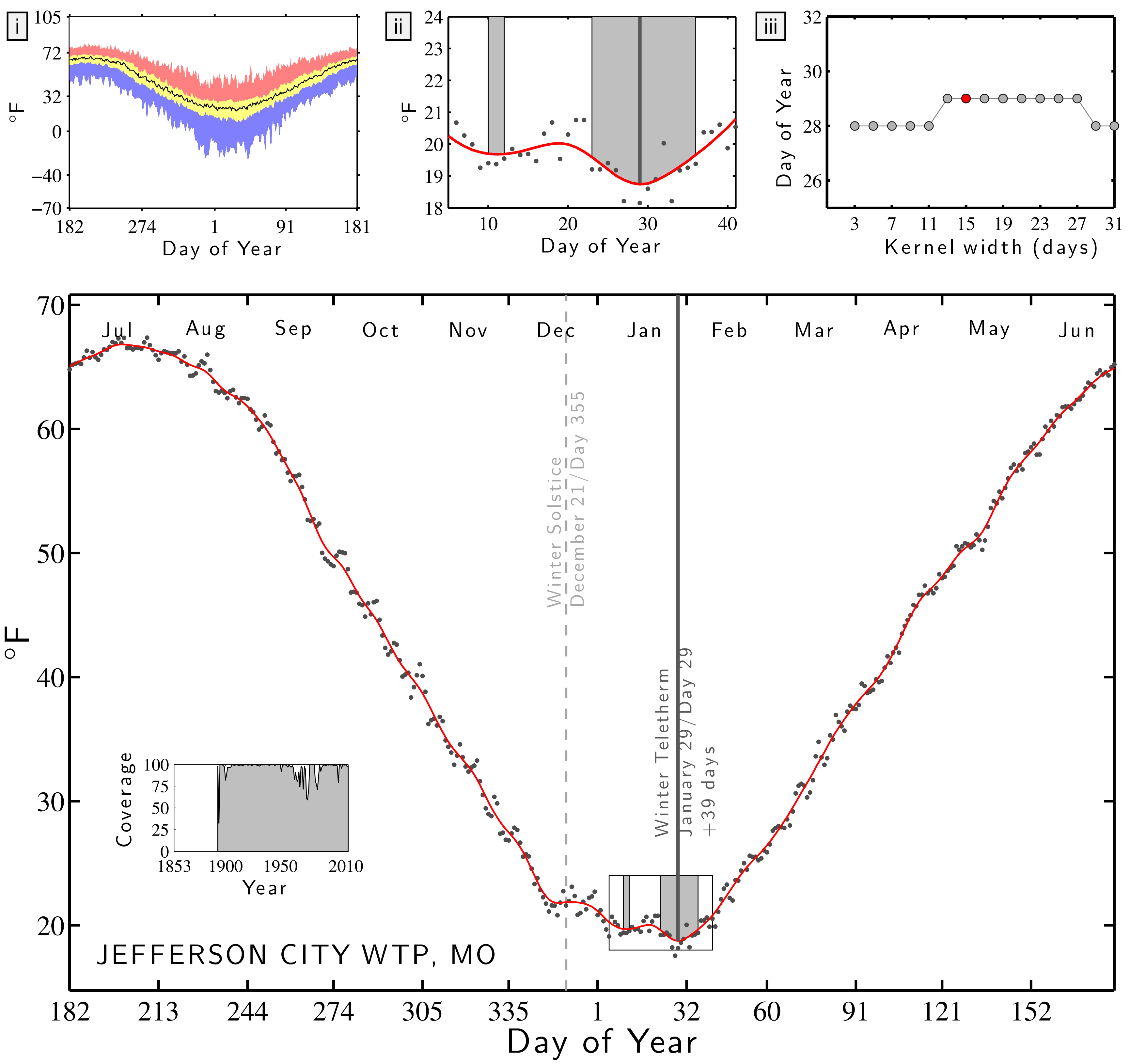

i
°F
105
72
32
0
−40
−70
182
274
1
91
181
Day of Year
ii
24
23
22
21
20
19
18
10
20
30
40
iii
32
30
28
26
Day of Year
3
7
11
15
19
23
27
31
Kernel width (days)
Jul
Aug
Sep
Oct
Nov
Dec
Jan
Feb
Mar
Apr
May
Jun
70
60
50
40
30
20
Winter Solstice
December 21/Day 355
Winter Teletherm
January 29/Day 29
+39 days
Coverage
100
75
50
25
0
1853
1900
1950
2010
Year
JEFFERSON CITY WTP, MO
182
213
244
274
305
335
1
32
60
91
121
152
Day of Year

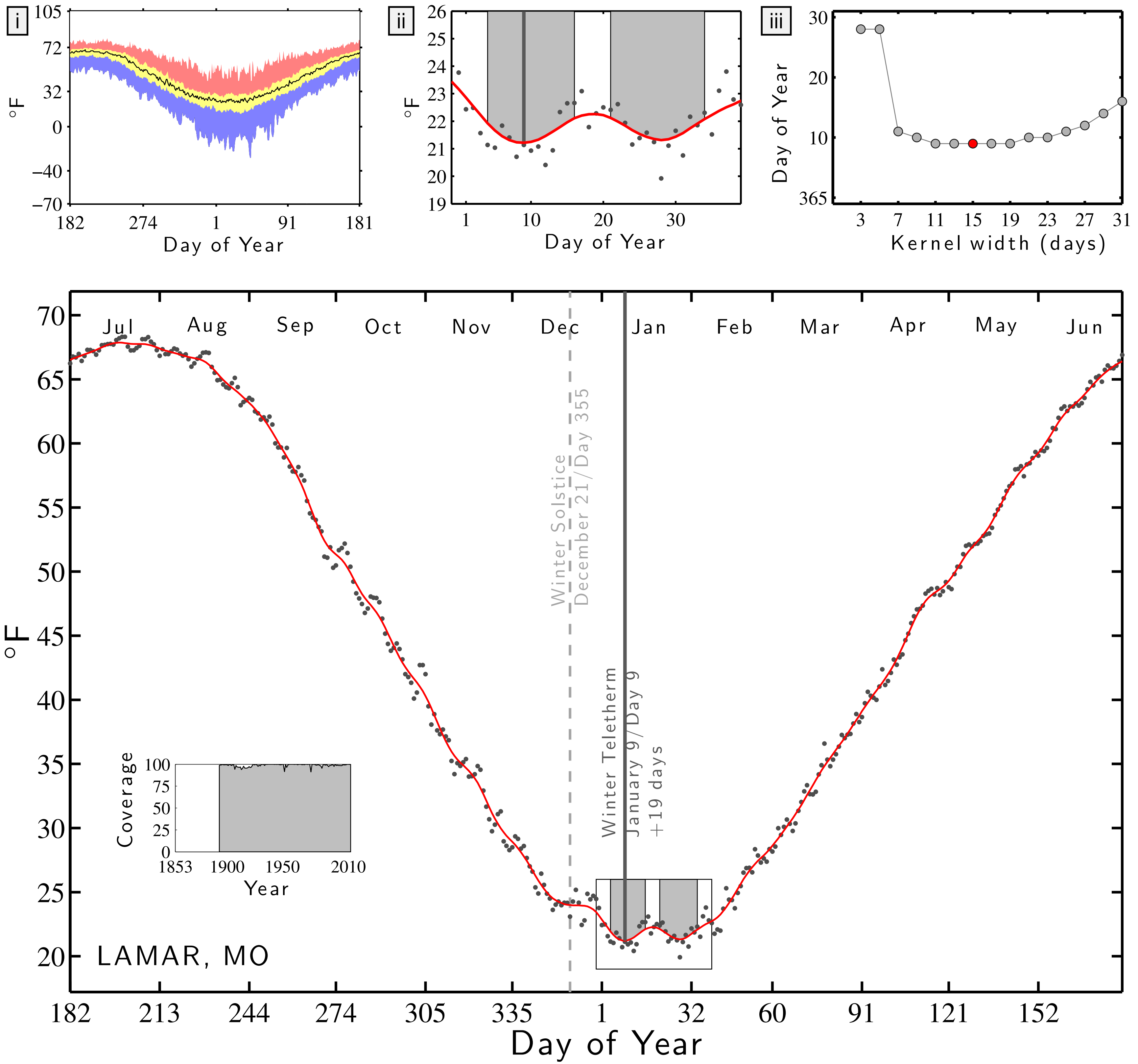

i
°F
Day of Year
ii
°F
Day of Year
iii
Day of Year
Kernel width (days)
Jul
Aug
Sep
Oct
Nov
Dec
Jan
Feb
Mar
Apr
May
Jun
Winter Solstice
December 21/Day 355
Winter Teletherm
January 9/Day 9
+19 days
Coverage
Year
LAMAR, MO
°F
Day of Year

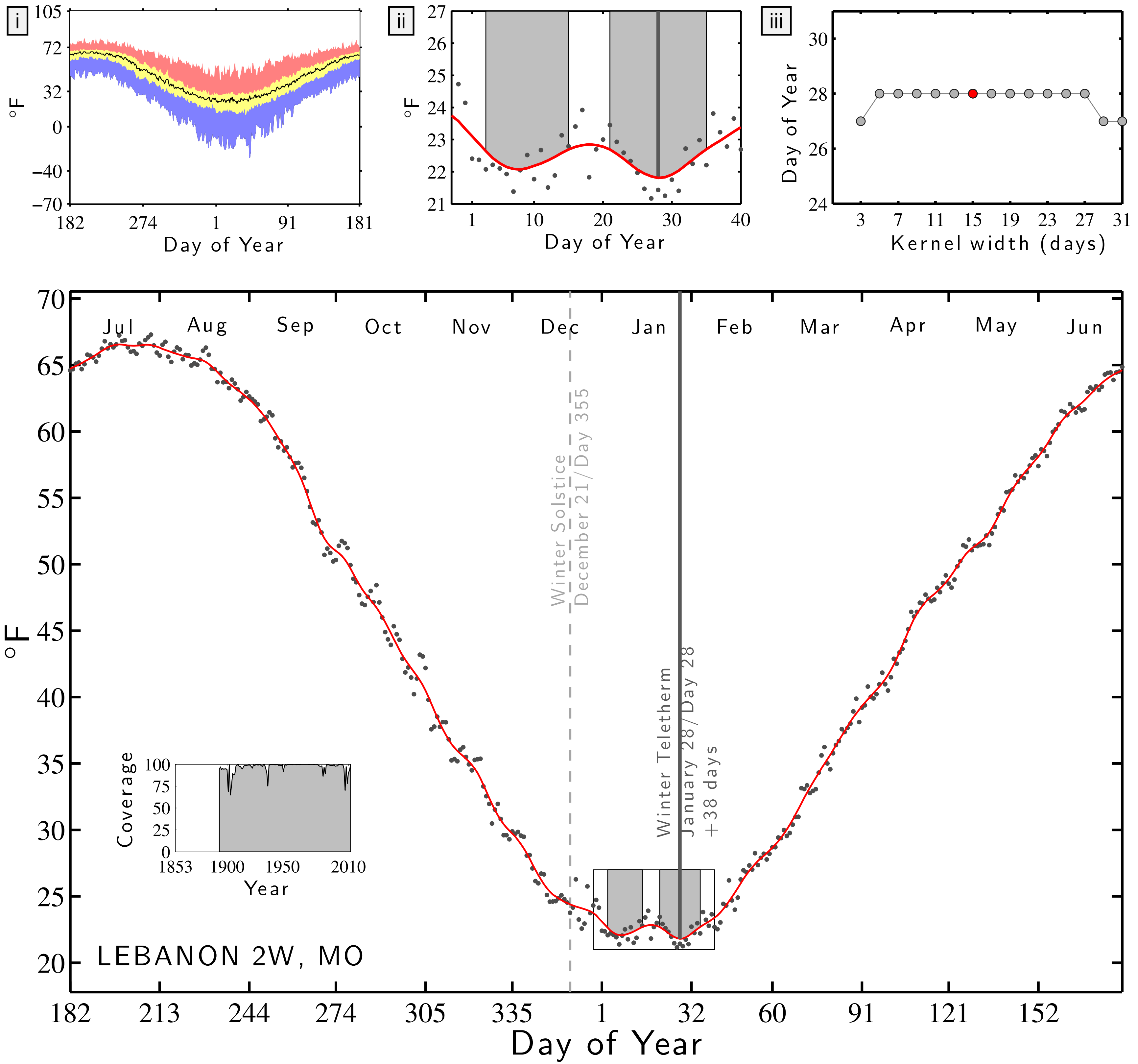

i
105
72
32
0
−40
−70
°F
182
274
1
91
181
Day of Year
ii
27
26
25
24
23
22
21
°F
1
10
20
30
40
Day of Year
iii
30
28
26
24
Day of Year
3
7
11
15
19
23
27
31
Kernel width (days)
Jul
Aug
Sep
Oct
Nov
Dec
Jan
Feb
Mar
Apr
May
Jun
Winter Solstice
December 21/Day 355
Winter Teletherm
January 28/Day 28
+38 days
Coverage
100
75
50
25
0
1853
1900
1950
2010
Year
LEBANON 2W, MO
70
65
60
55
50
45
40
35
30
25
20
°F
182
213
244
274
305
335
1
32
60
91
121
152
Day of Year

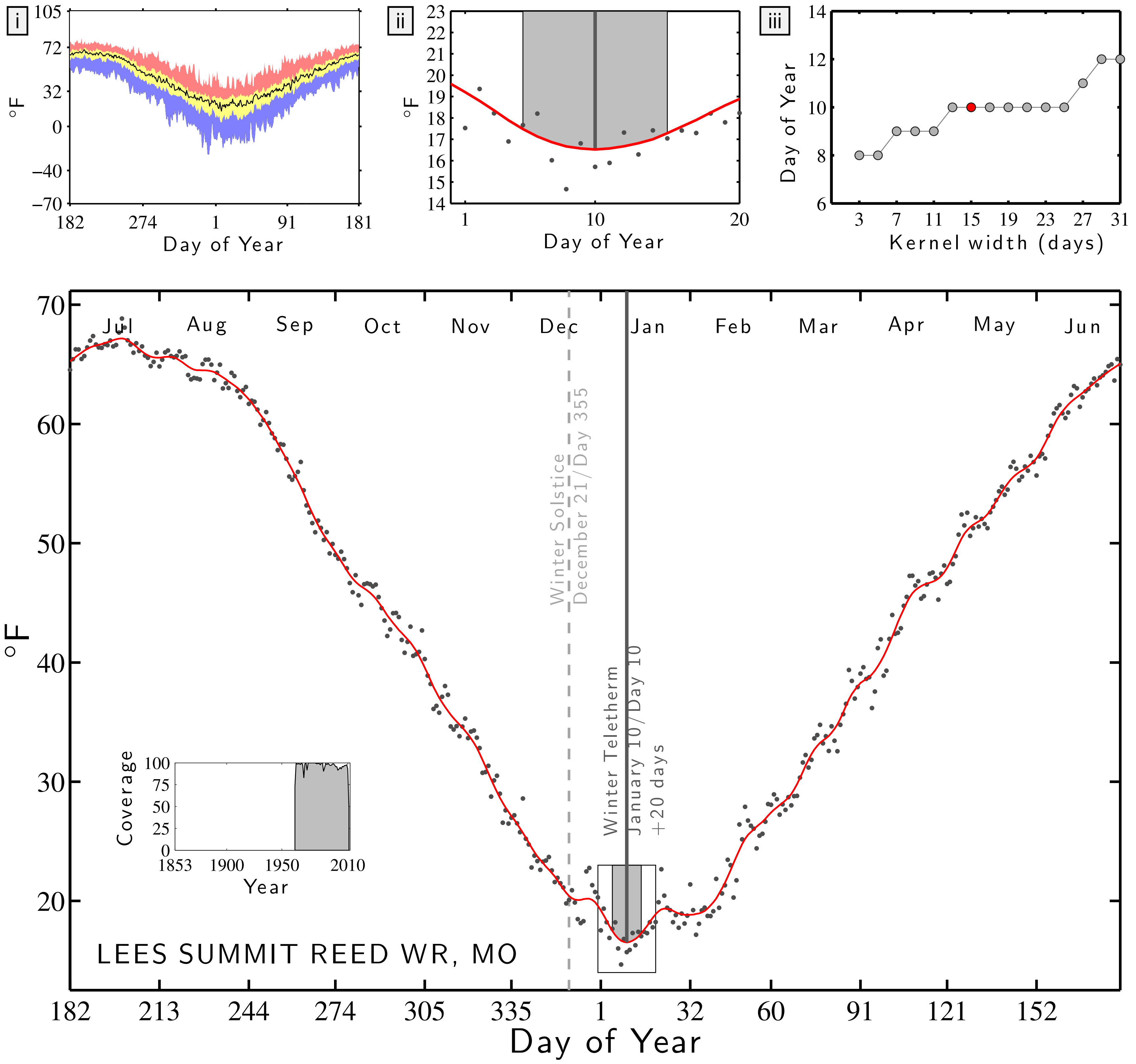

i
ii
iii
Day of Year
Kernel width (days)
Jul
Aug
Sep
Oct
Nov
Dec
Jan
Feb
Mar
Apr
May
Jun
Winter Solstice
December 21/Day 355
Winter Teletherm
January 10/Day 10
+20 days
Coverage
Year
LEES SUMMIT REED WR, MO
°F

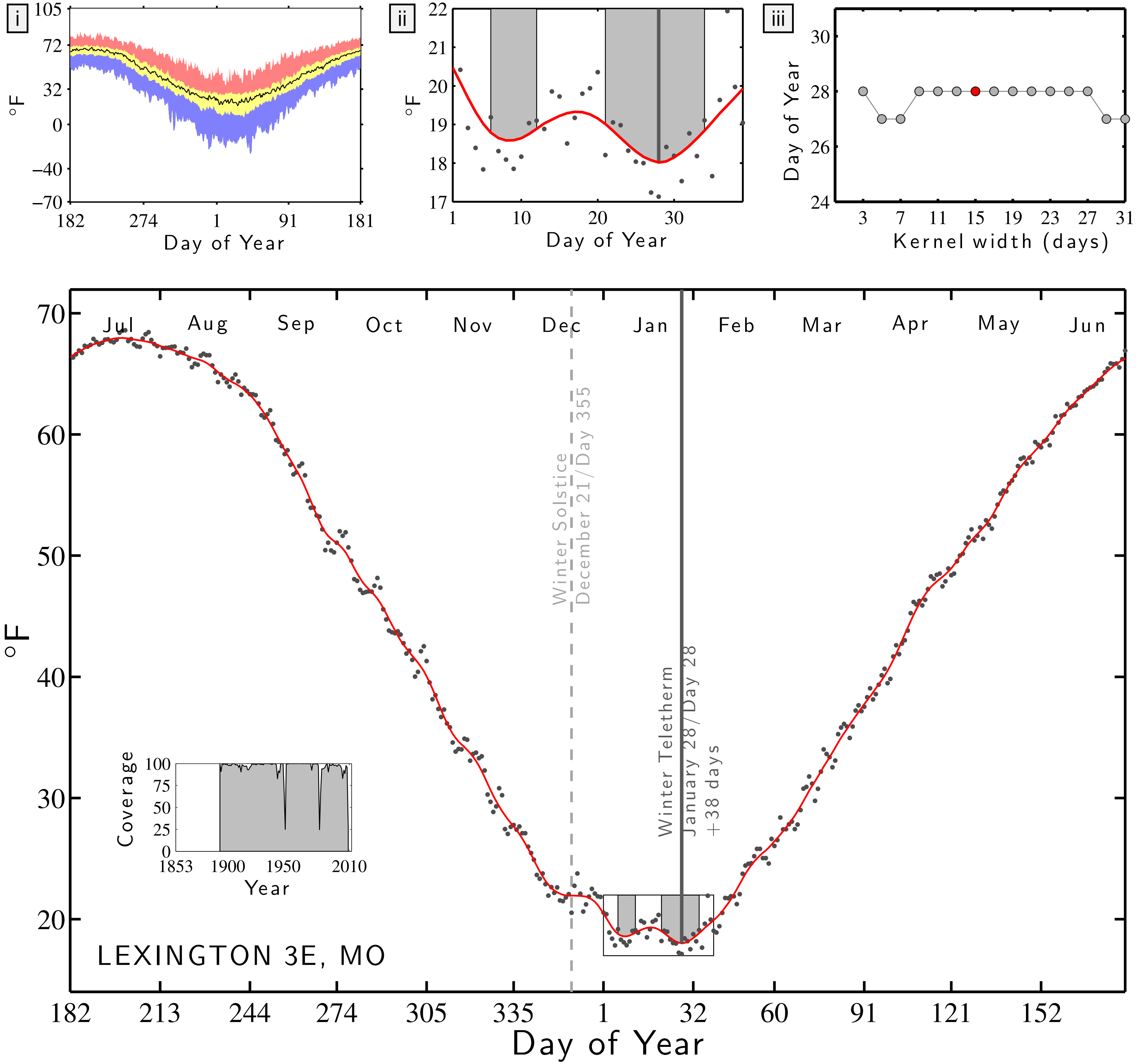

i
°F
Day of Year
ii
Day of Year
iii
Day of Year
Kernel width (days)
Jul
Aug
Sep
Oct
Nov
Dec
Jan
Feb
Mar
Apr
May
Jun
Winter Solstice
December 21/Day 355
Winter Teletherm
January 28/Day 28
+38 days
Coverage
Year
LEXINGTON 3E, MO
Day of Year

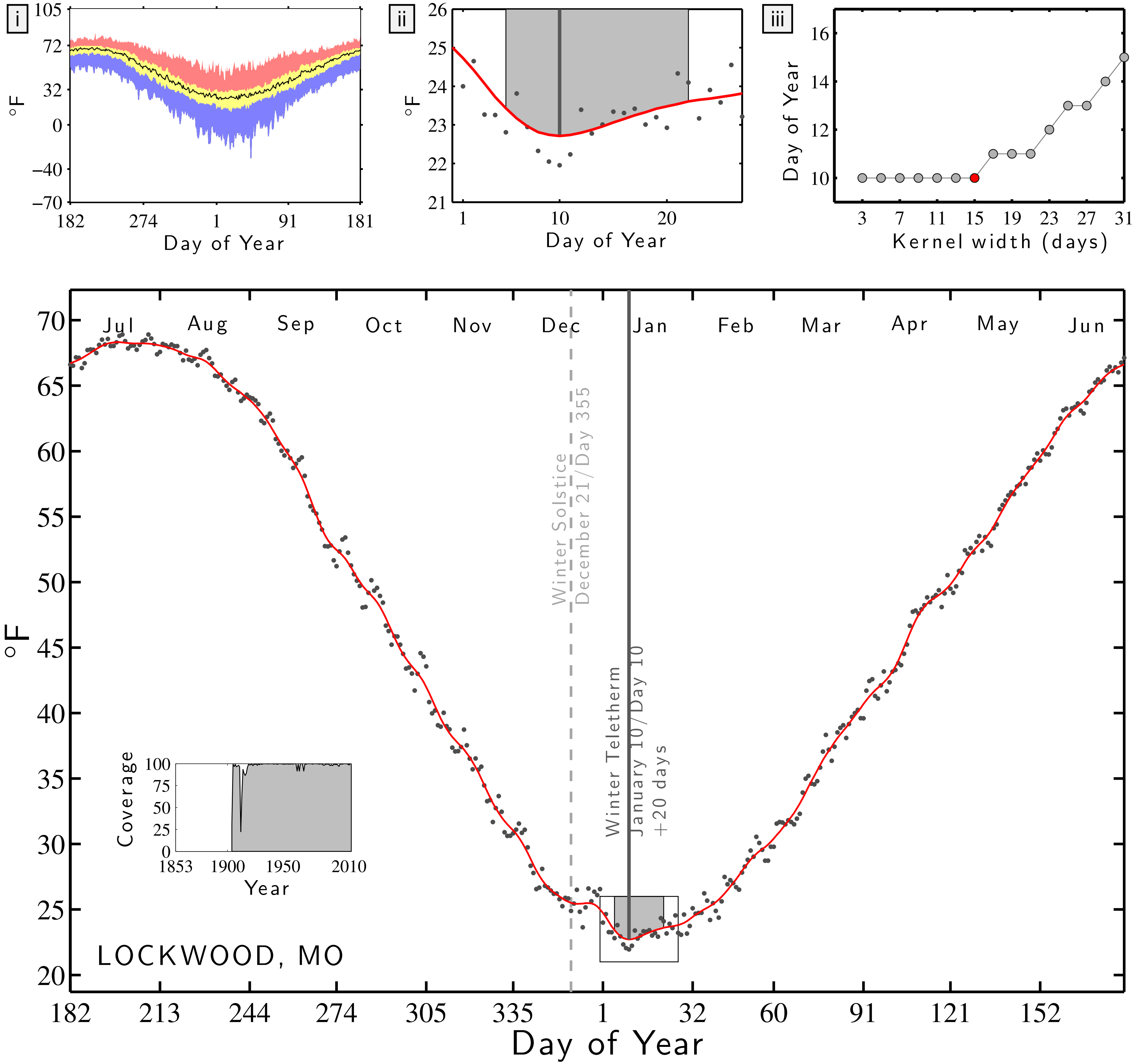
i
105
72
32
0
−40
−70
°F
182
274
1
91
181
Day of Year
ii
26
25
24
23
22
21
°F
1
10
20
Day of Year
iii
16
14
12
10
Day of Year
3
7
11
15
19
23
27
31
Kernel width (days)
70
65
60
55
50
45
40
35
30
25
20
°F
Jul
Aug
Sep
Oct
Nov
Dec
Jan
Feb
Mar
Apr
May
Jun
Winter Solstice
December 21/Day 355
Winter Teletherm
January 10/Day 10
+20 days
Coverage
100
75
50
25
0
1853
1900
1950
2010
Year
LOCKWOOD, MO
182
213
244
274
305
335
1
32
60
91
121
152
Day of Year

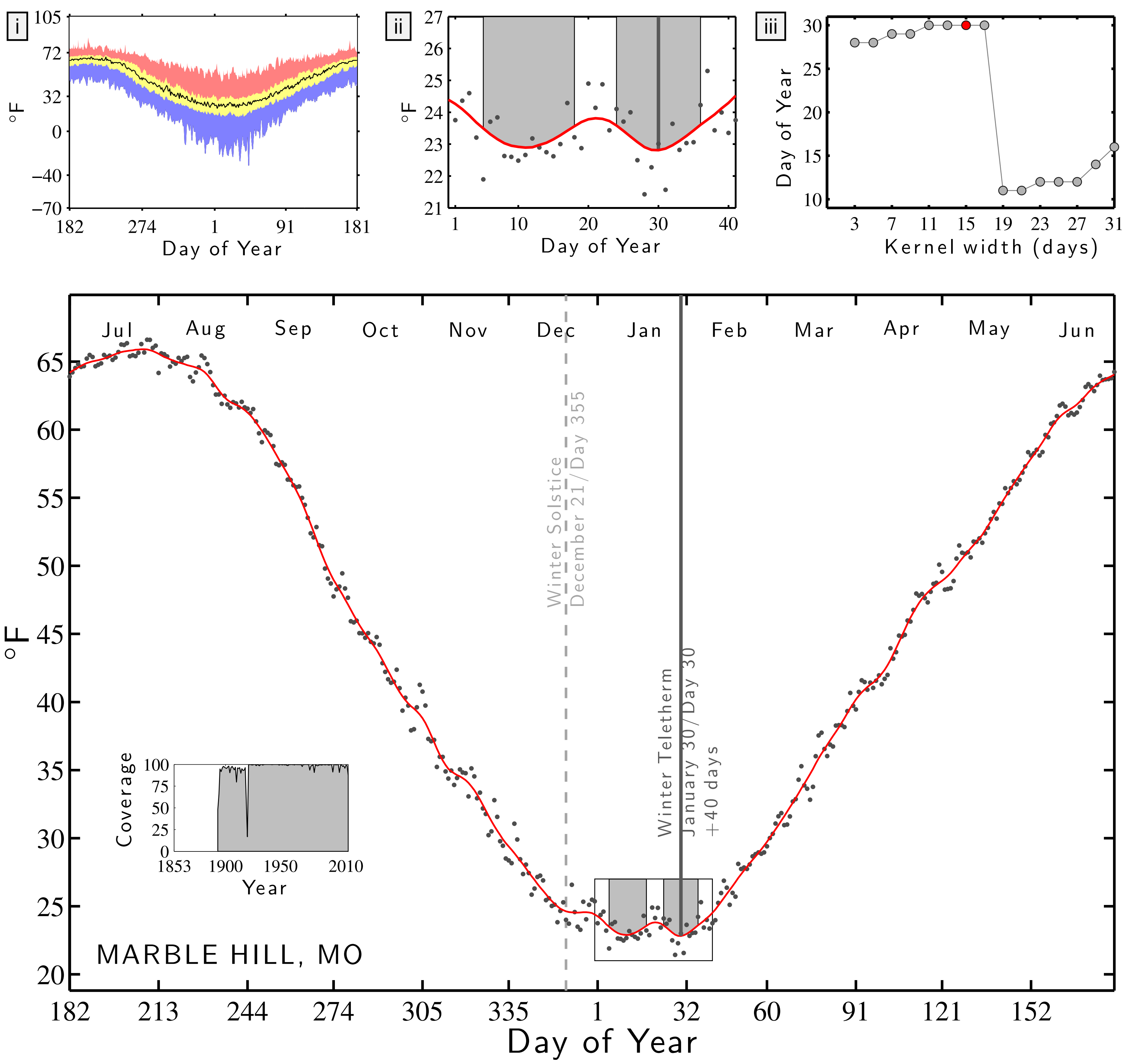
i
°F
Day of Year
ii
°F
Day of Year
iii
Day of Year
Kernel width (days)
Jul
Aug
Sep
Oct
Nov
Dec
Jan
Feb
Mar
Apr
May
Jun
Winter Solstice
December 21/Day 355
Winter Teletherm
January 30/Day 30
+40 days
Coverage
Year
MARBLE HILL, MO
°F
Day of Year

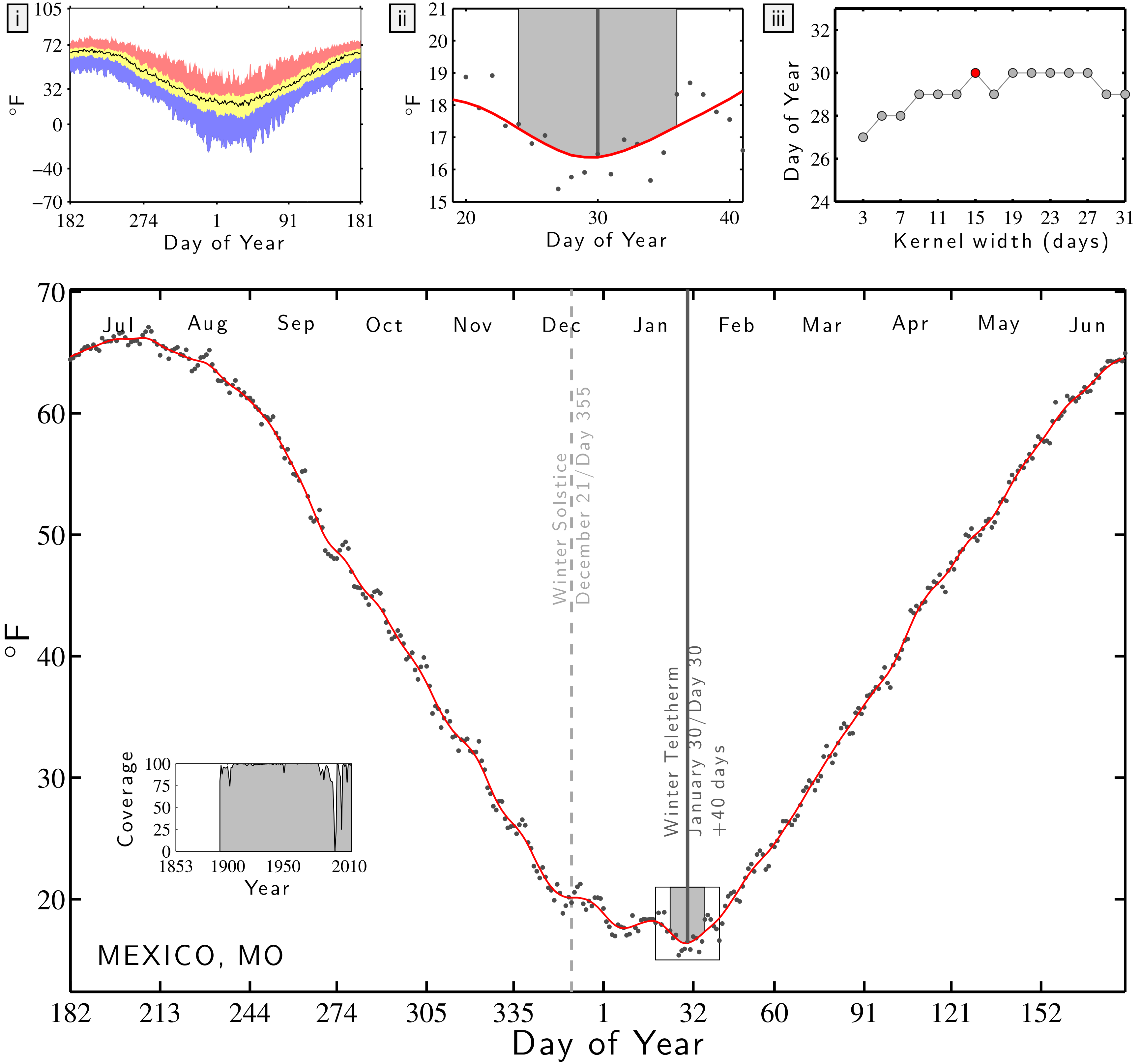

i
ii
iii
°F
Day of Year
Kernel width (days)
Jul
Aug
Sep
Oct
Nov
Dec
Jan
Feb
Mar
Apr
May
Jun
Winter Solstice
December 21/Day 355
Winter Teletherm
January 30/Day 30
+40 days
Coverage
Year
MEXICO, MO

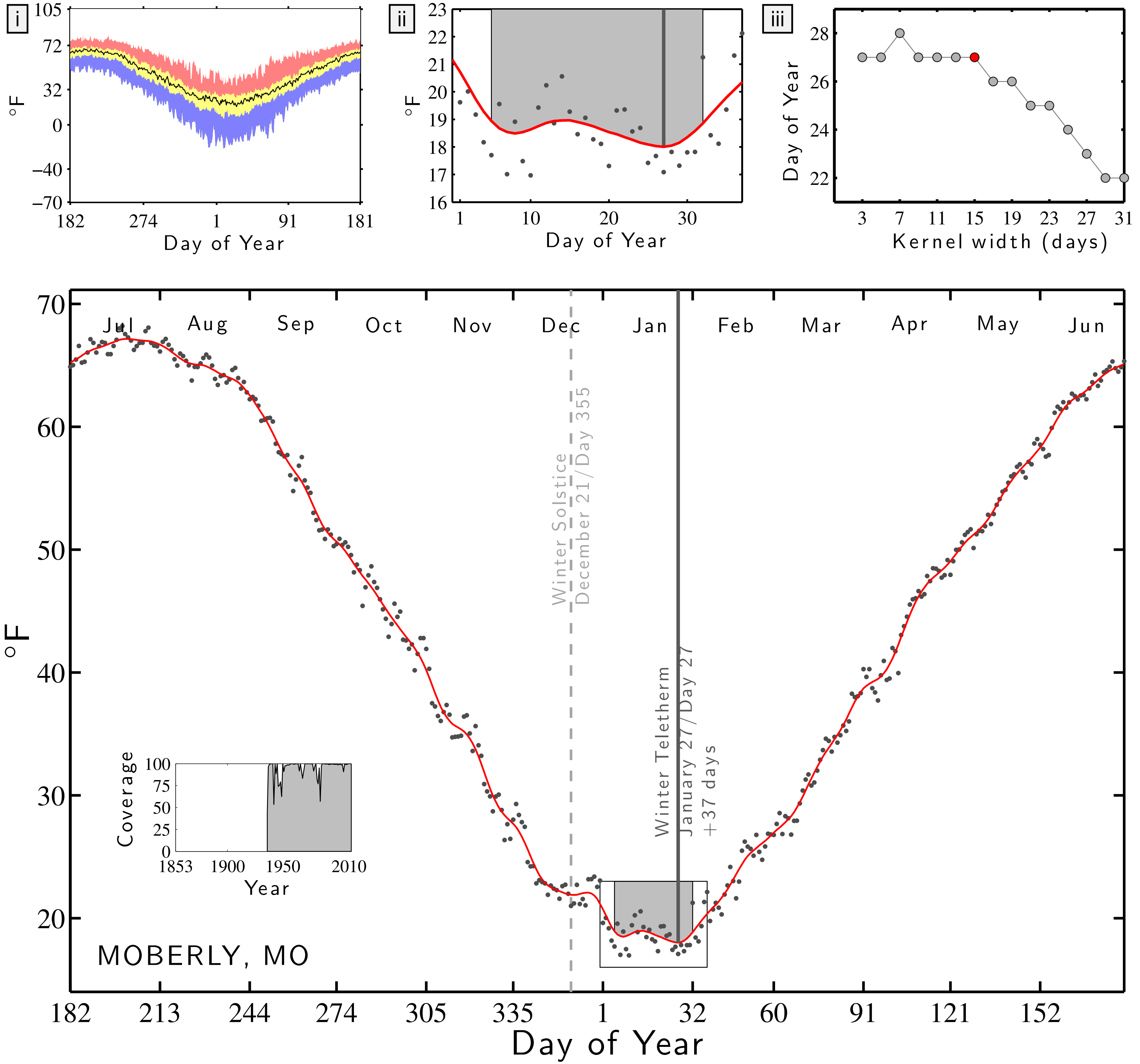

i
°F
Day of Year
ii
°F
Day of Year
iii
Day of Year
Kernel width (days)
Jul
Aug
Sep
Oct
Nov
Dec
Jan
Feb
Mar
Apr
May
Jun
Winter Solstice
December 21/Day 355
Winter Teletherm
January 27/Day 27
+37 days
Coverage
Year
MOBERLY, MO
°F
Day of Year

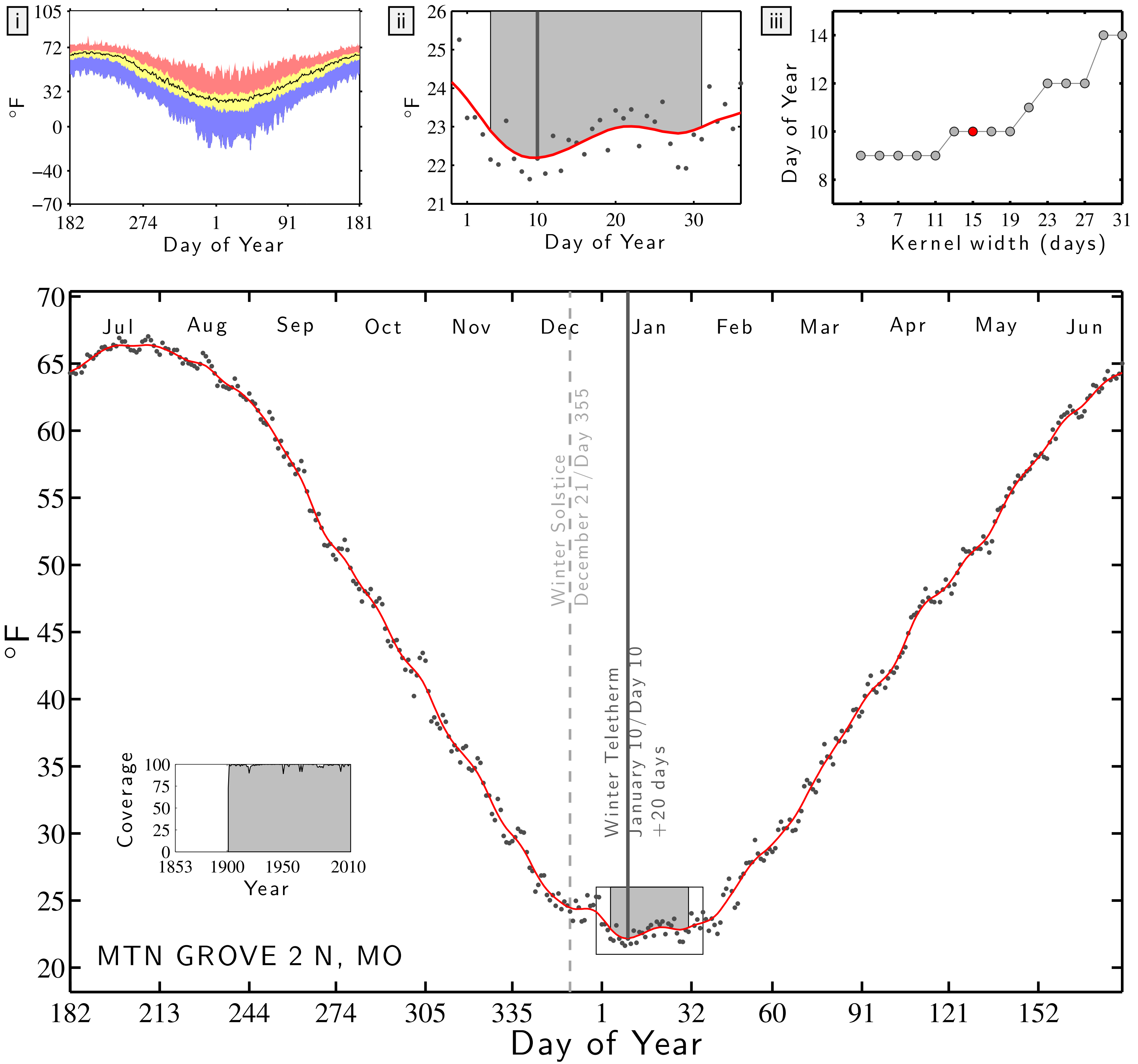

i
ii
iii
Day of Year
Kernel width (days)
°F
Winter Solstice
December 21/Day 355
Winter Teletherm
January 10/Day 10
+20 days
Coverage
Year
MTN GROVE 2 N, MO
Jul
Aug
Sep
Oct
Nov
Dec
Jan
Feb
Mar
Apr
May
Jun

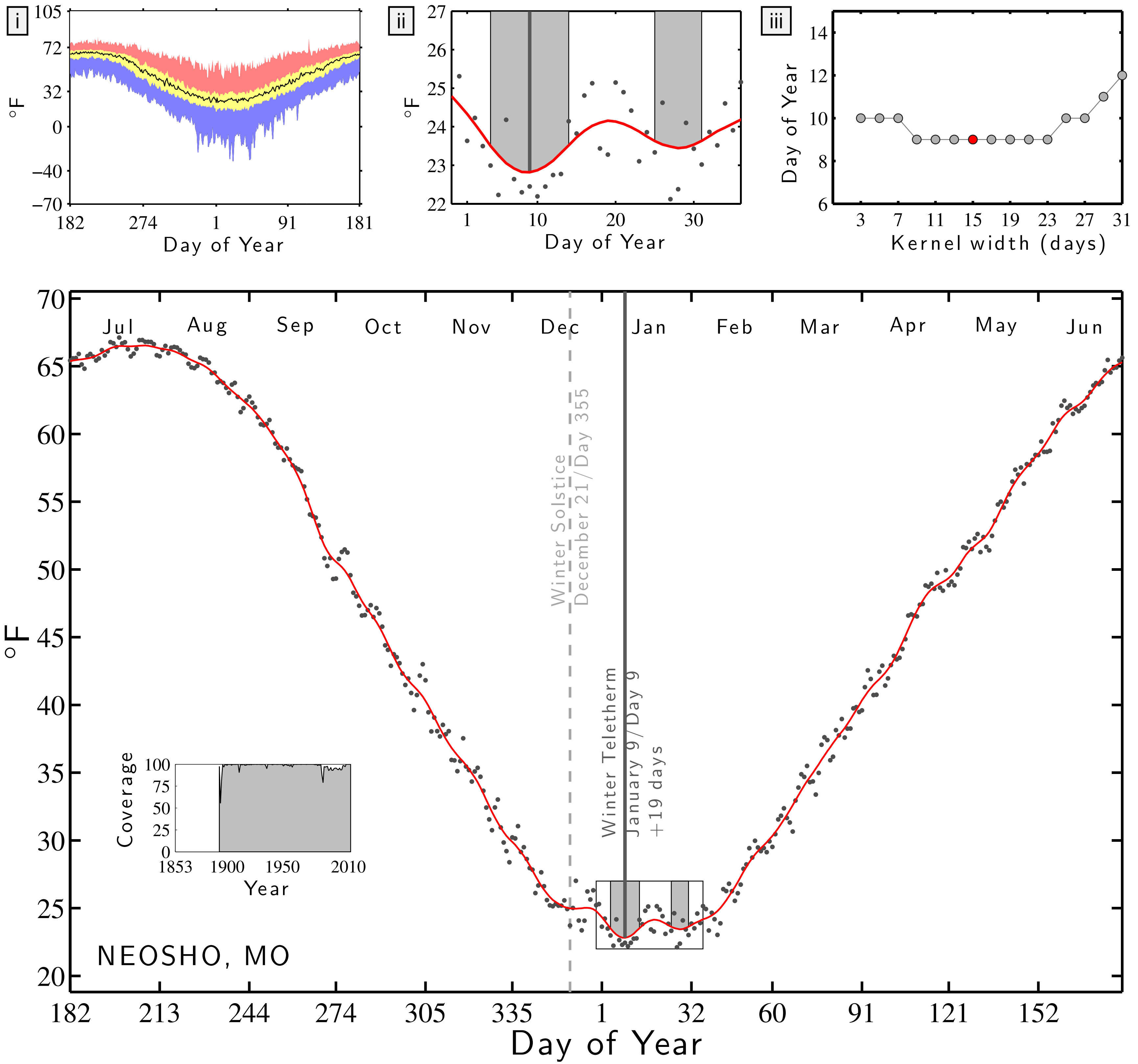

i
°F
Day of Year
ii
°F
Day of Year
iii
Day of Year
Kernel width (days)
Jul
Aug
Sep
Oct
Nov
Dec
Jan
Feb
Mar
Apr
May
Jun
Winter Solstice
December 21/Day 355
Winter Teletherm
January 9/Day 9
+19 days
Coverage
Year
NEOSHO, MO
°F
Day of Year

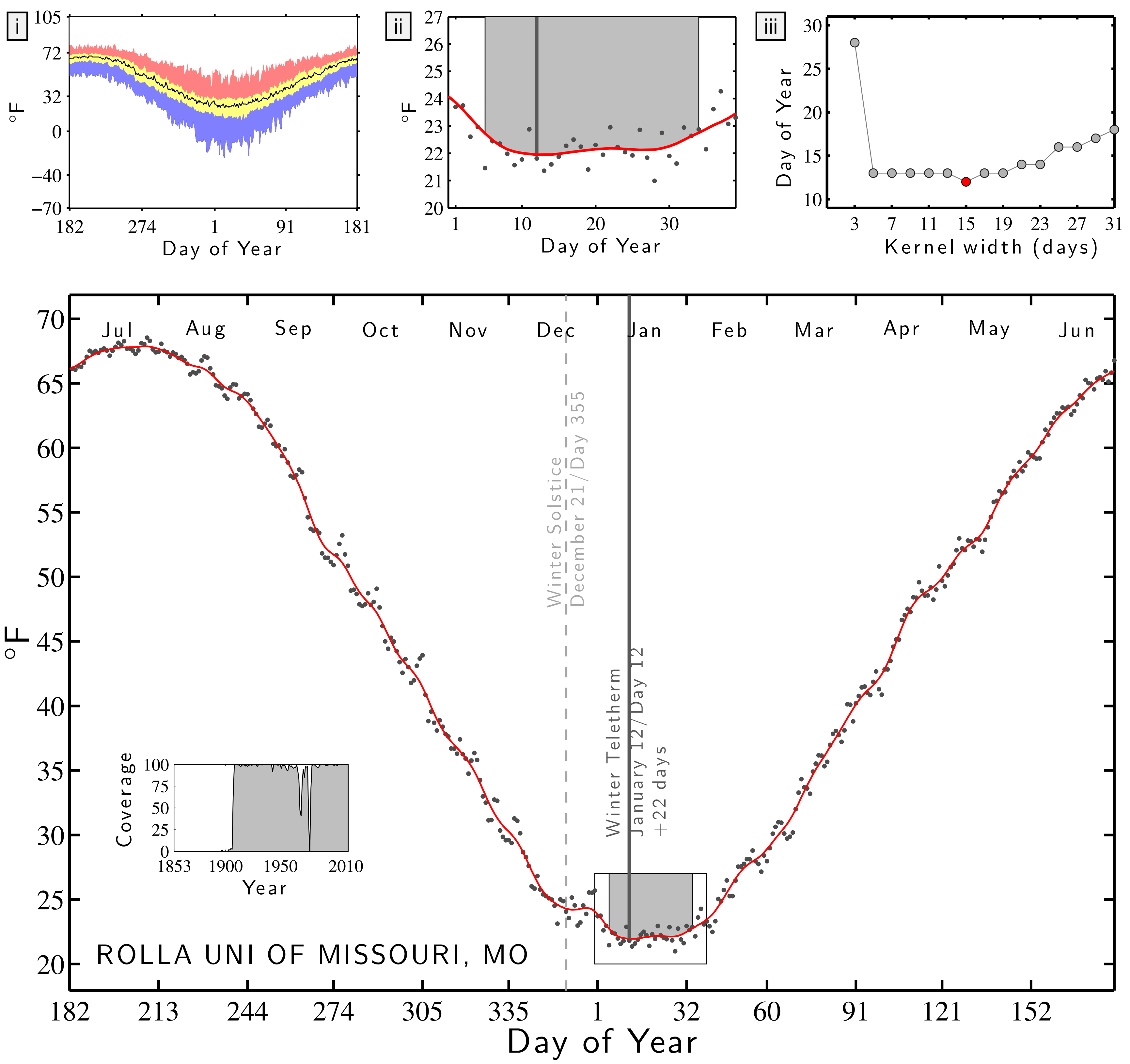

i
105
72
32
0
−40
−70
°F
182
274
1
91
181
Day of Year
ii
27
26
25
24
23
22
21
20
°F
1
10
20
30
Day of Year
iii
30
25
20
15
10
Day of Year
3
7
11
15
19
23
27
31
Kernel width (days)
70
65
60
55
50
45
40
35
30
25
20
°F
Jul
Aug
Sep
Oct
Nov
Dec
Jan
Feb
Mar
Apr
May
Jun
Winter Solstice
December 21/Day 355
Winter Teletherm
January 12/Day 12
+22 days
Coverage
100
75
50
25
0
1853
1900
1950
2010
Year
ROLLA UNI OF MISSOURI, MO
182
213
244
274
305
335
1
32
60
91
121
152
Day of Year

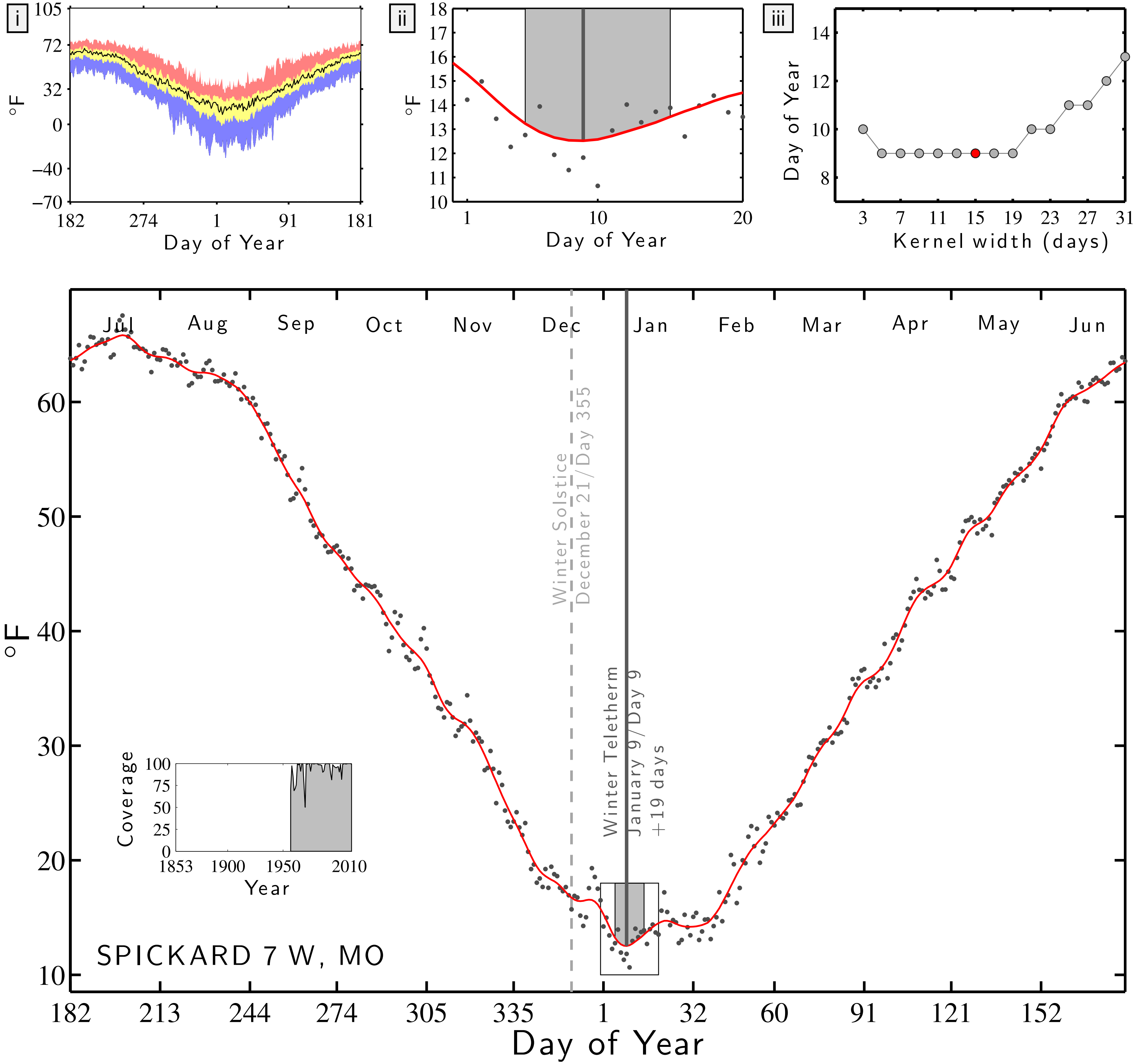
i
°F
Day of Year
ii
°F
Day of Year
iii
Day of Year
Kernel width (days)
Jul
Aug
Sep
Oct
Nov
Dec
Jan
Feb
Mar
Apr
May
Jun
Winter Solstice
December 21/Day 355
Winter Teletherm
January 9/Day 9
+19 days
Coverage
Year
SPICKARD 7 W, MO
°F
Day of Year

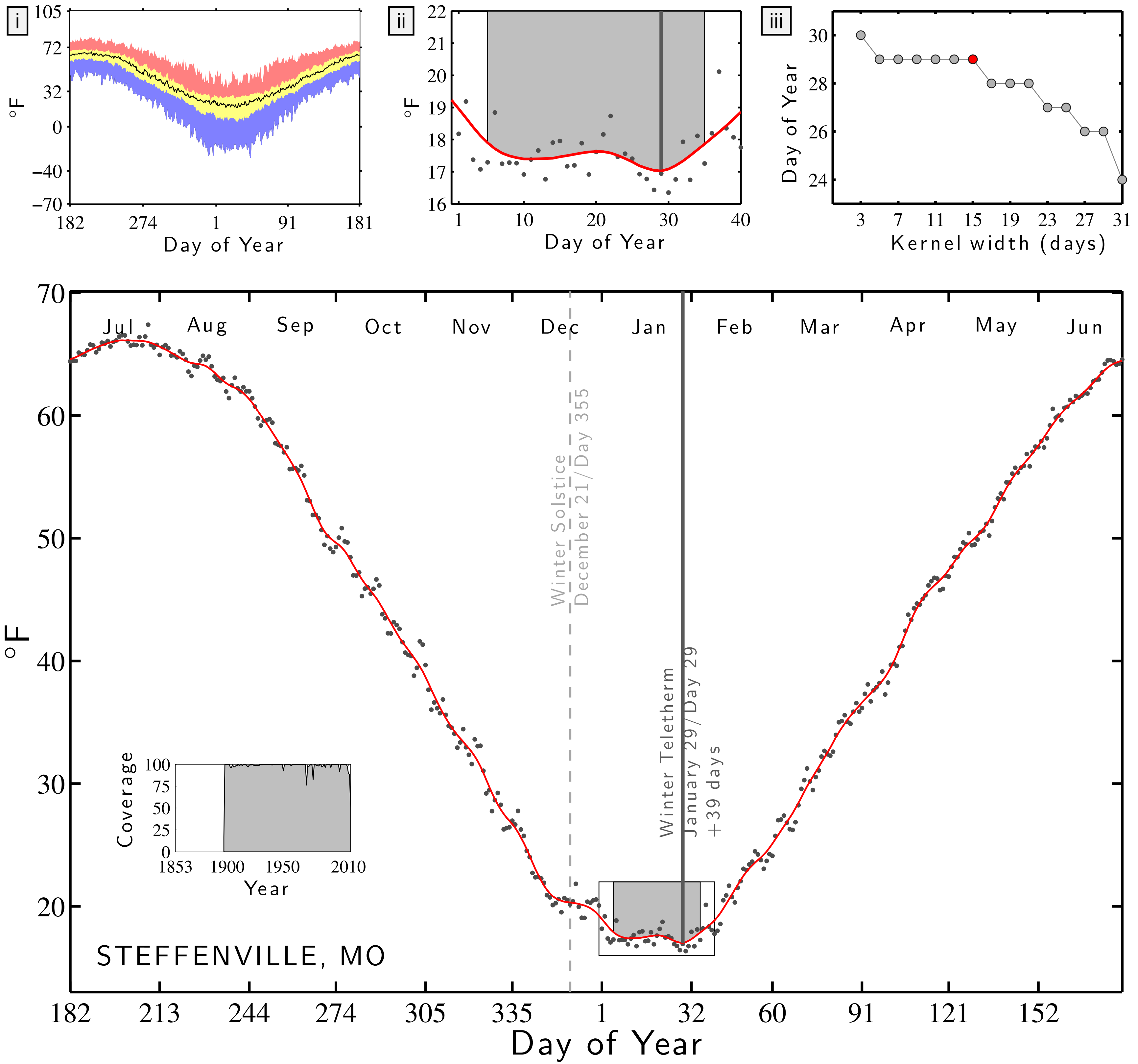

i
ii
iii
°F
Day of Year
Day of Year
Kernel width (days)
Jul
Aug
Sep
Oct
Nov
Dec
Jan
Feb
Mar
Apr
May
Jun
Winter Solstice
December 21/Day 355
Winter Teletherm
January 29/Day 29
+39 days
Coverage
Year
STEFFENVILLE, MO
Day of Year

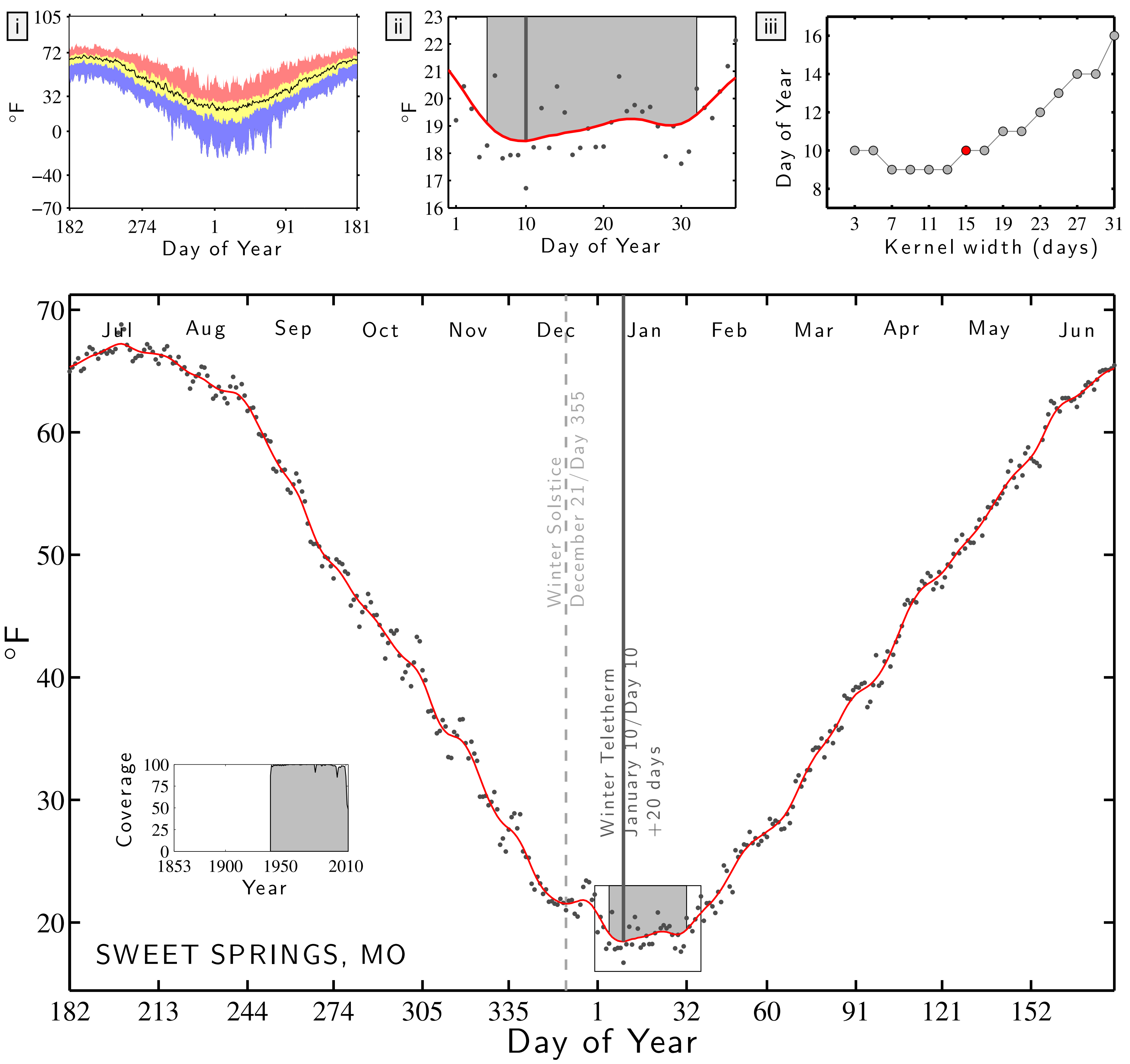

i
ii
iii
°F
Day of Year
Kernel width (days)
Jul
Aug
Sep
Oct
Nov
Dec
Jan
Feb
Mar
Apr
May
Jun
Winter Solstice
December 21/Day 355
Winter Teletherm
January 10/Day 10
+20 days
Coverage
Year
SWEET SPRINGS, MO

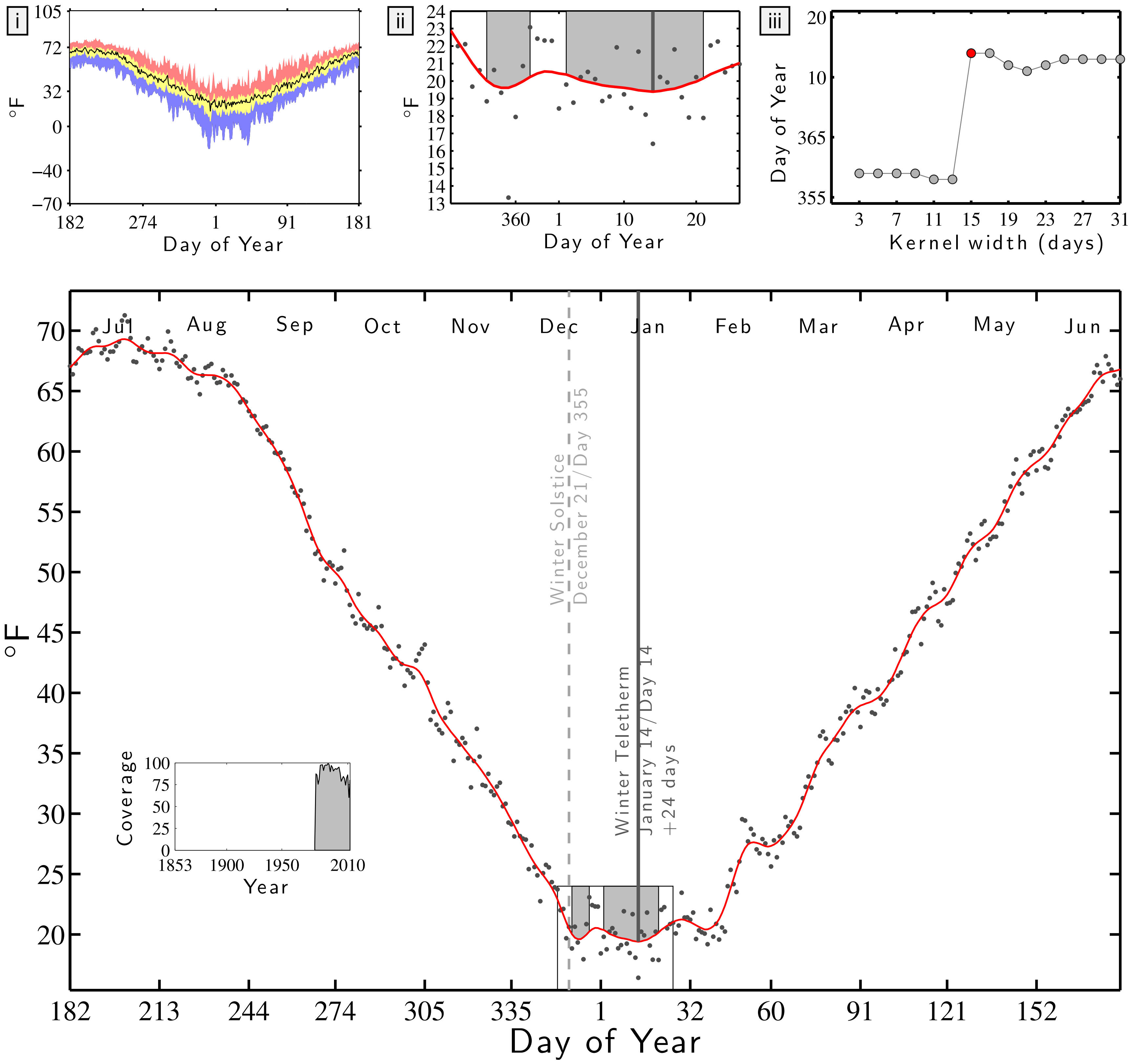

i
ii
iii
°F
Day of Year
Kernel width (days)
Jul
Aug
Sep
Oct
Nov
Dec
Jan
Feb
Mar
Apr
May
Jun
Winter Solstice
December 21/Day 355
Winter Teletherm
January 14/Day 14
+24 days
Coverage
Year

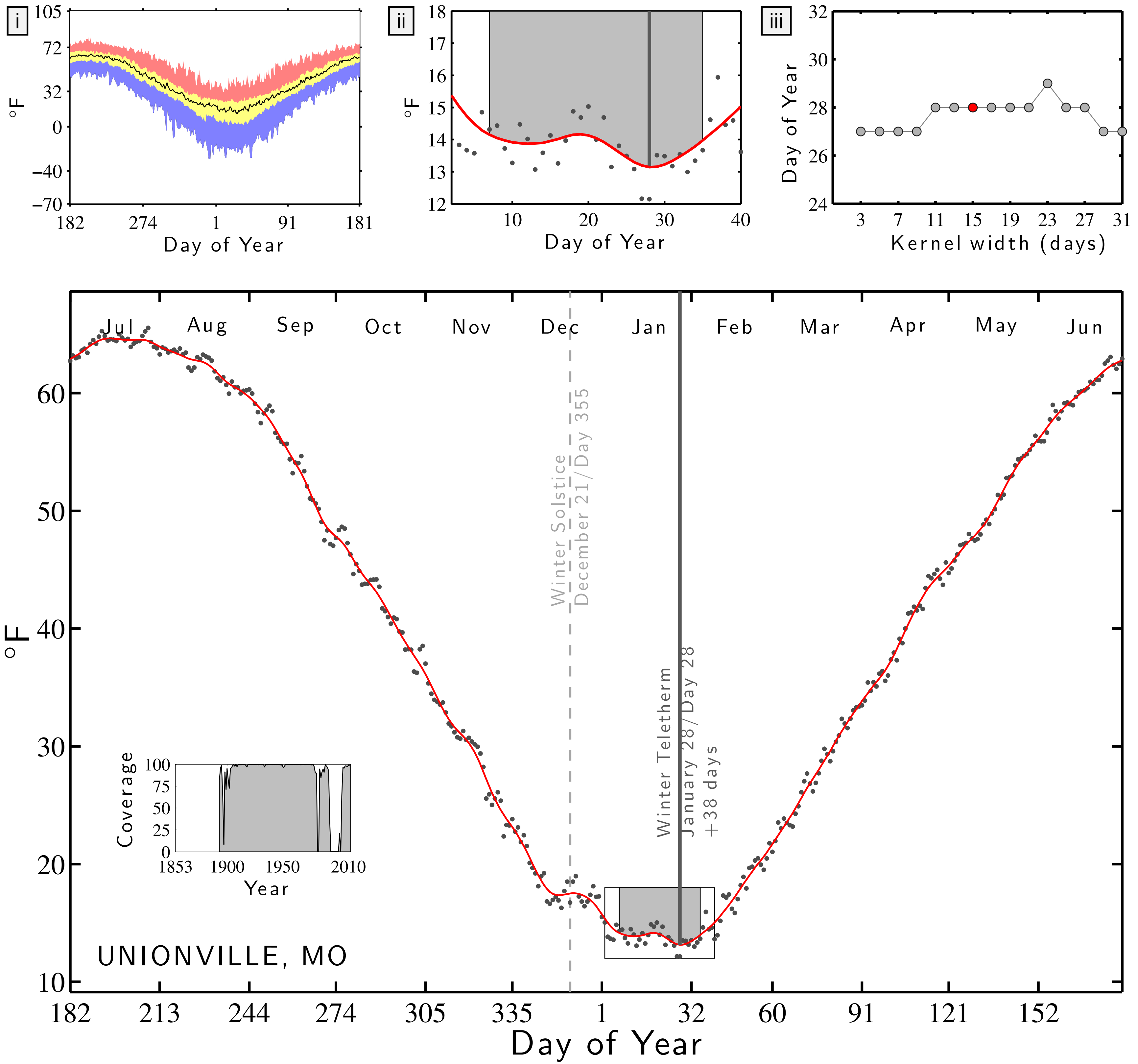

i
°F
Day of Year
ii
Day of Year
iii
Day of Year
Kernel width (days)
Jul
Aug
Sep
Oct
Nov
Dec
Jan
Feb
Mar
Apr
May
Jun
Winter Solstice
December 21/Day 355
Winter Teletherm
January 28/Day 28
+38 days
Coverage
Year
UNIONVILLE, MO
Day of Year

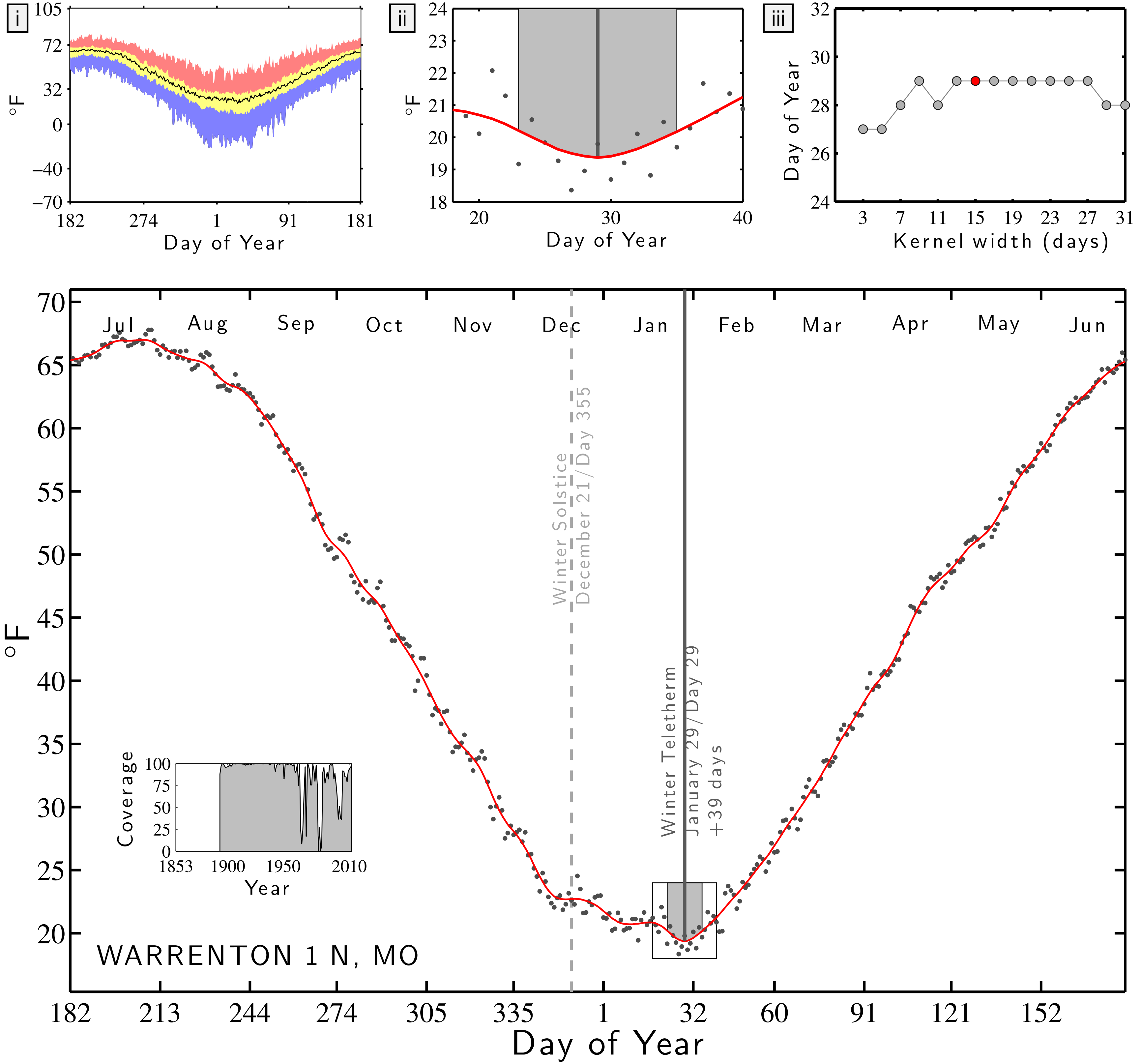

i
°F
Day of Year
ii
°F
Day of Year
iii
Day of Year
Kernel width (days)
Jul
Aug
Sep
Oct
Nov
Dec
Jan
Feb
Mar
Apr
May
Jun
Winter Solstice
December 21/Day 355
Winter Teletherm
January 29/Day 29
+39 days
Coverage
Year
WARRENTON 1 N, MO
°F
Day of Year

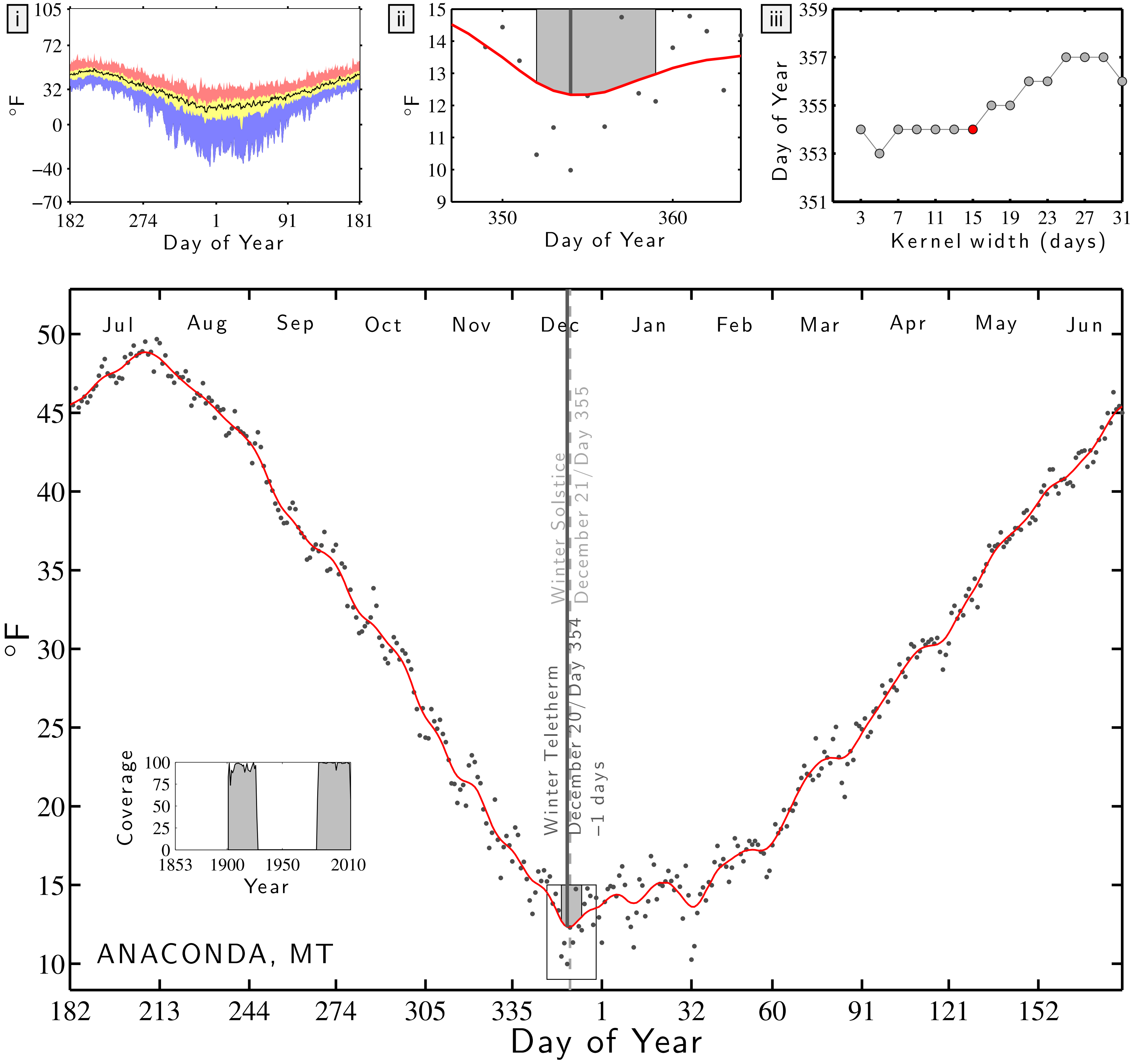

i
°F
Day of Year
ii
Day of Year
iii
Day of Year
Kernel width (days)
Jul
Aug
Sep
Oct
Nov
Dec
Jan
Feb
Mar
Apr
May
Jun
Winter Solstice
December 21/Day 355
Winter Teletherm
December 20/Day 354
−1 days
Coverage
Year
ANACONDA, MT
°F
Day of Year

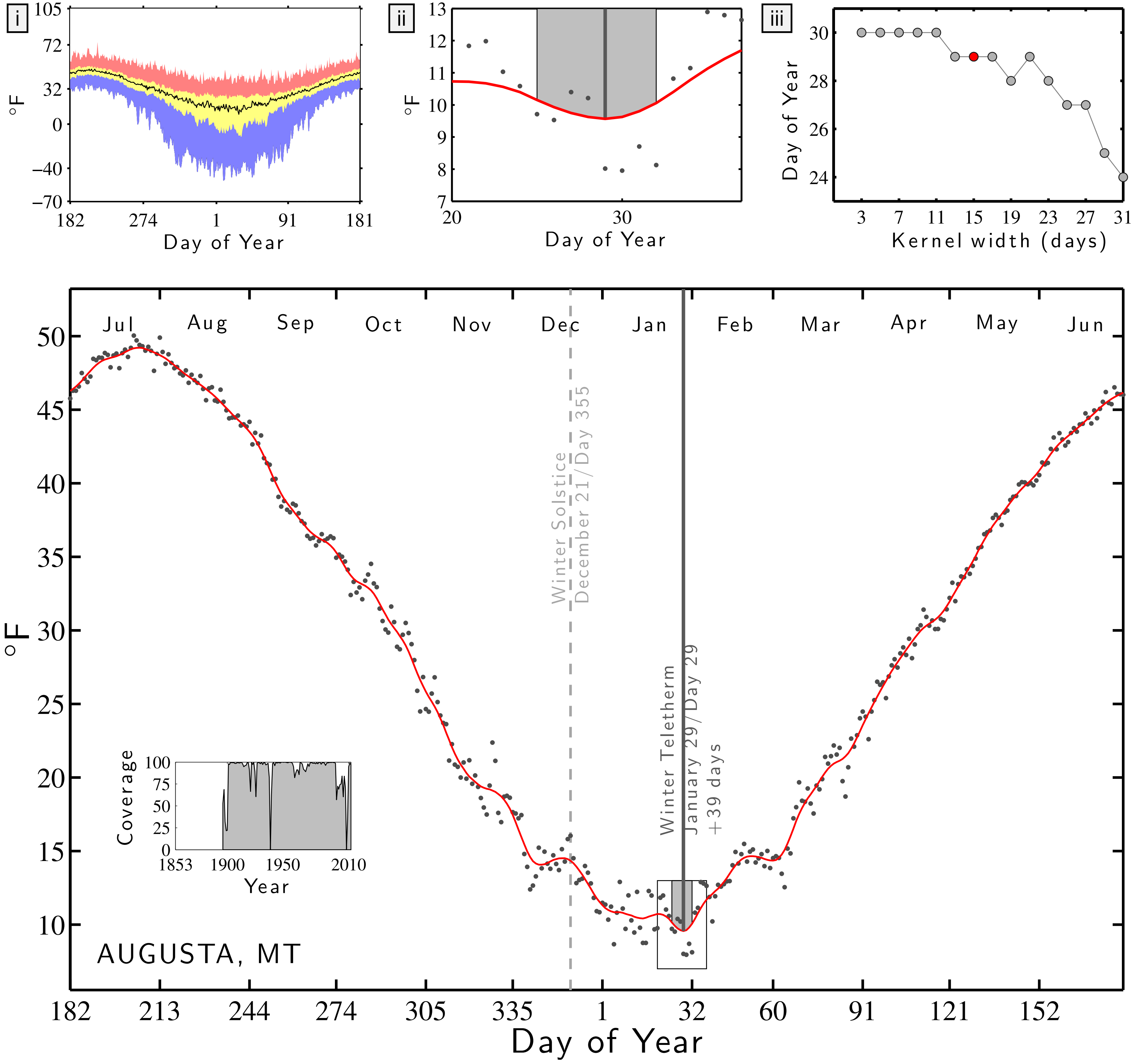

i
°F
Day of Year
ii
°F
Day of Year
iii
Day of Year
Kernel width (days)
Jul
Aug
Sep
Oct
Nov
Dec
Jan
Feb
Mar
Apr
May
Jun
Winter Solstice
December 21/Day 355
Winter Teletherm
January 29/Day 29
+39 days
Coverage
Year
AUGUSTA, MT
°F
Day of Year

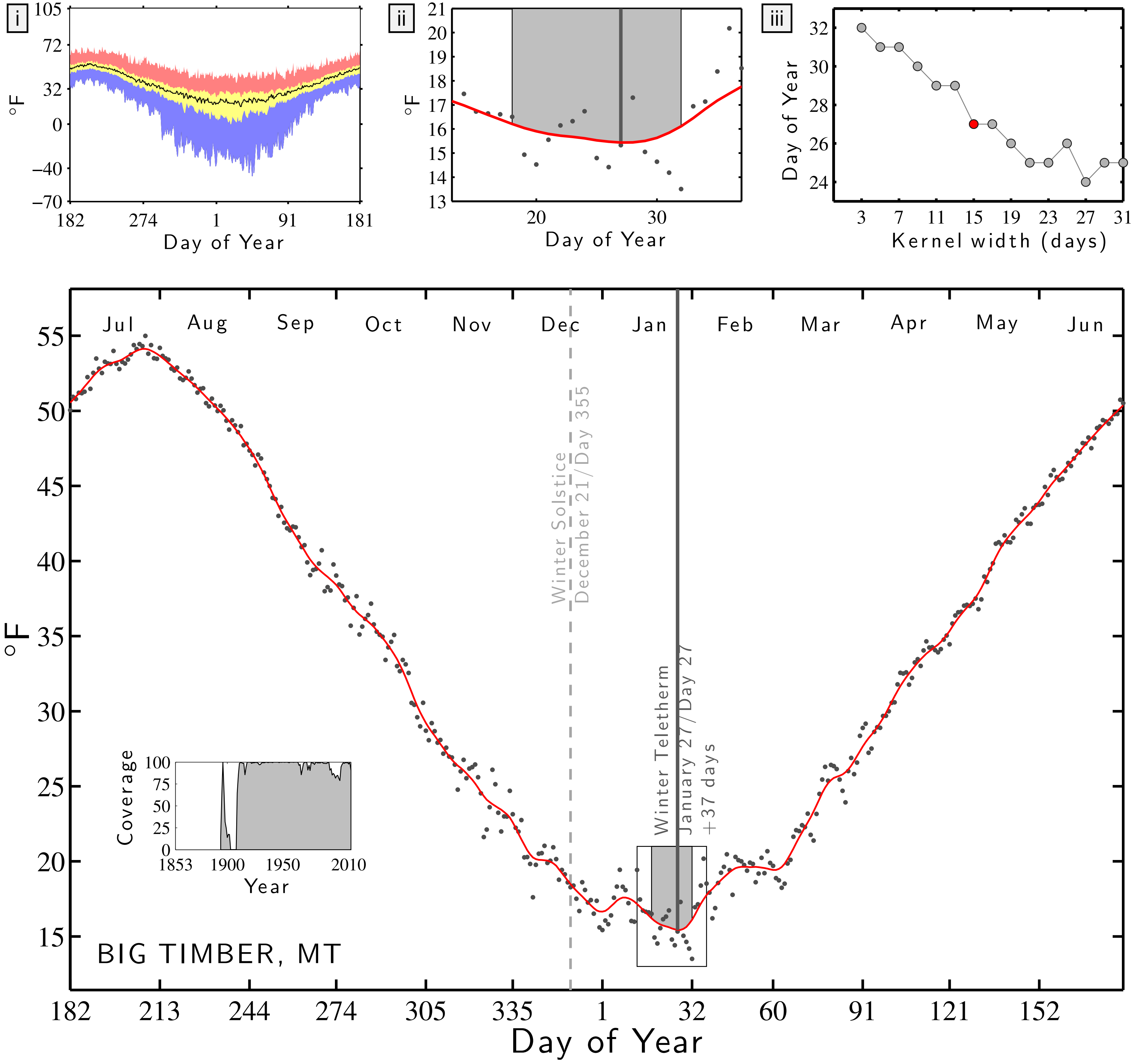

i
ii
iii
°F
Day of Year
Kernel width (days)
Jul
Aug
Sep
Oct
Nov
Dec
Jan
Feb
Mar
Apr
May
Jun
Winter Solstice
December 21/Day 355
Winter Teletherm
January 27/Day 27
+37 days
Coverage
Year
BIG TIMBER, MT

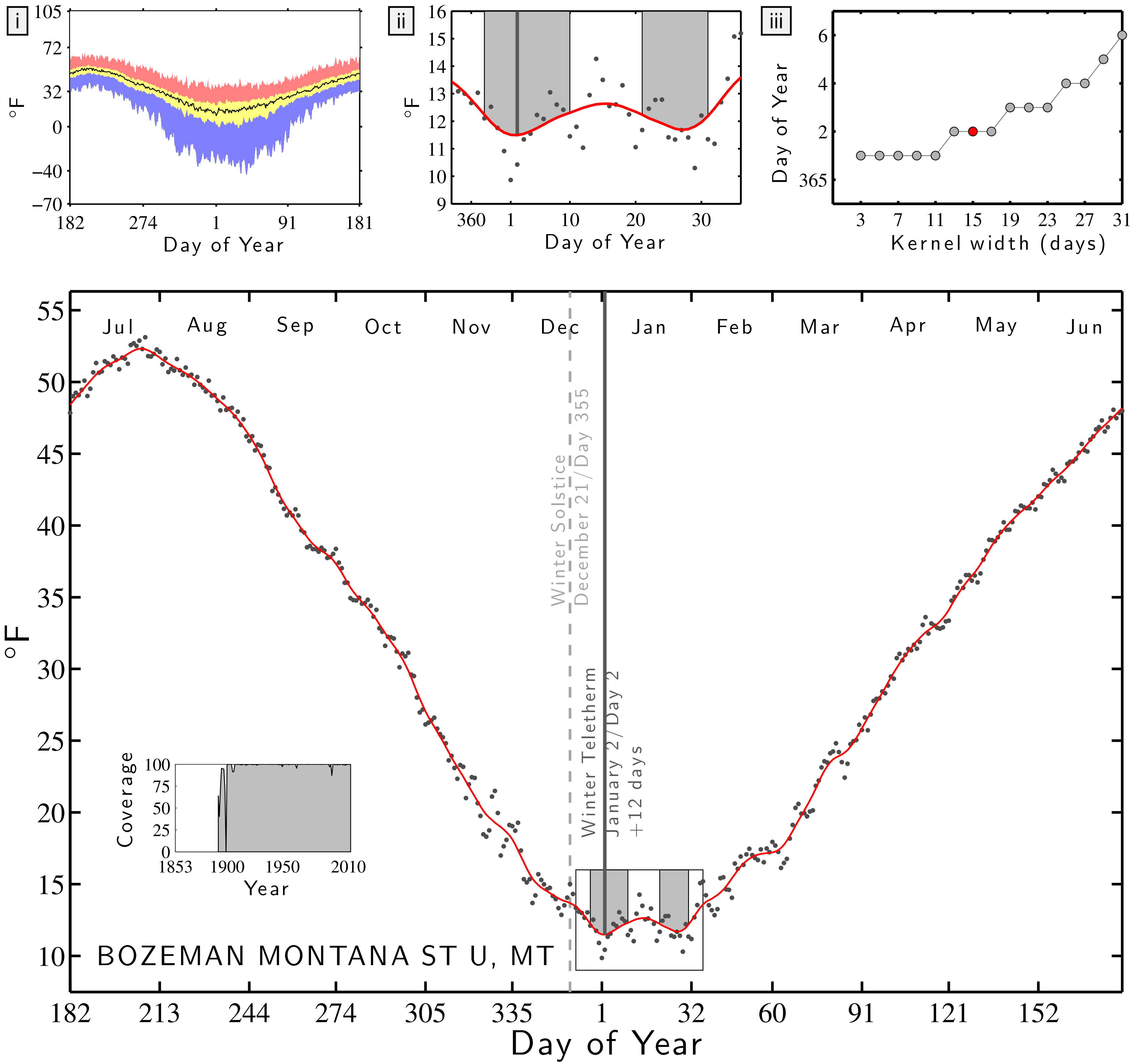

i
105
72
32
0
−40
−70
°F
182
274
1
91
181
Day of Year
ii
16
15
14
13
12
11
10
9
°F
360
1
10
20
30
Day of Year
iii
6
4
2
365
Day of Year
3
7
11
15
19
23
27
31
Kernel width (days)
55
50
45
40
35
30
25
20
15
10
°F
Jul
Aug
Sep
Oct
Nov
Dec
Jan
Feb
Mar
Apr
May
Jun
Winter Solstice
December 21/Day 355
Winter Teletherm
January 2/Day 2
+12 days
Coverage
100
75
50
25
0
1853
1900
1950
2010
Year
BOZEMAN MONTANA ST U, MT
182
213
244
274
305
335
1
32
60
91
121
152
Day of Year

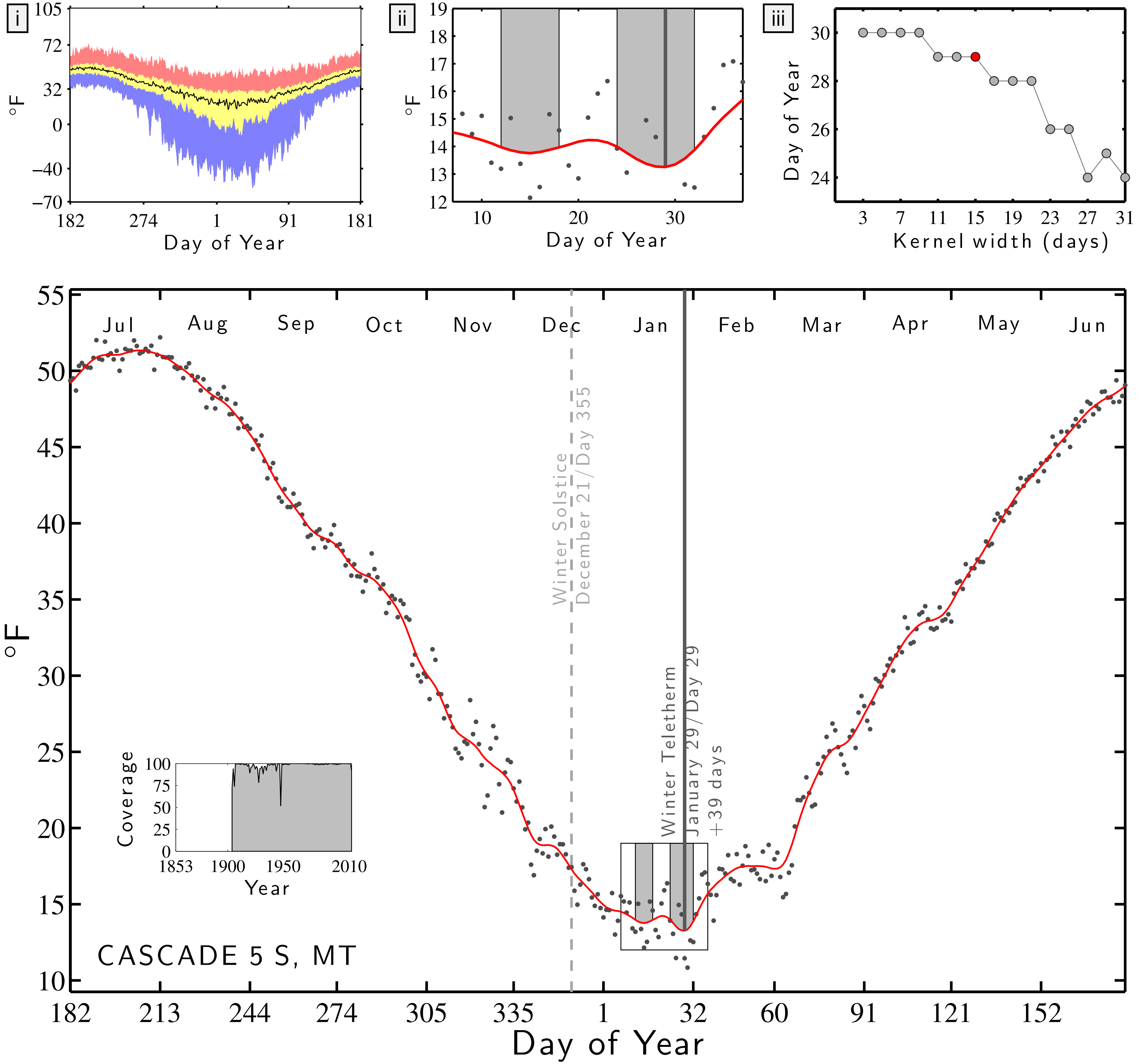

i
ii
iii
°F
Day of Year
Kernel width (days)
Jul
Aug
Sep
Oct
Nov
Dec
Jan
Feb
Mar
Apr
May
Jun
Winter Solstice
December 21/Day 355
Winter Teletherm
January 29/Day 29
+39 days
Coverage
Year
CASCADE 5 S, MT

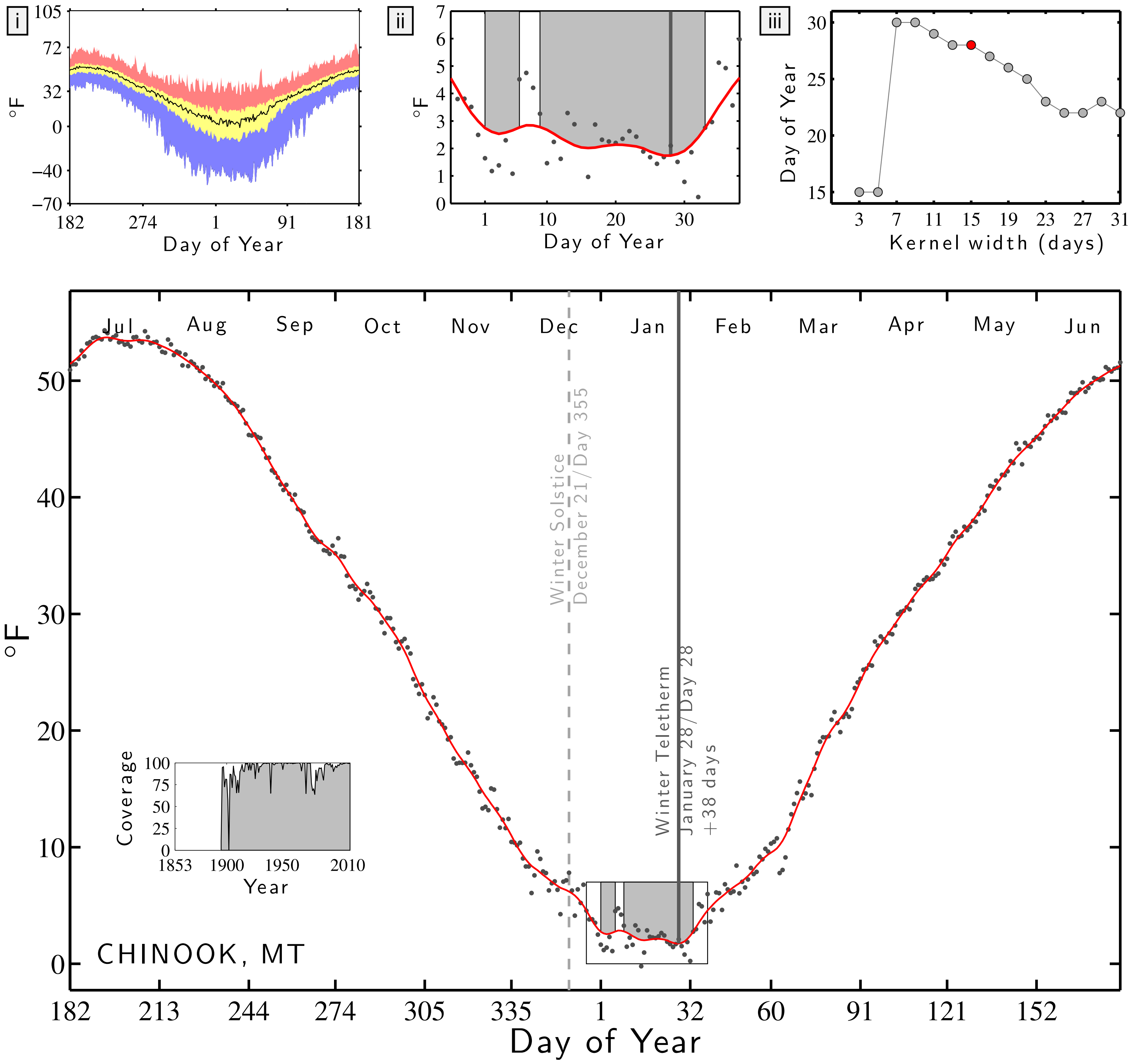

i
°F
Day of Year
ii
°F
Day of Year
iii
Day of Year
Kernel width (days)
Jul
Aug
Sep
Oct
Nov
Dec
Jan
Feb
Mar
Apr
May
Jun
Winter Solstice
December 21/Day 355
Winter Teletherm
January 28/Day 28
+38 days
Coverage
Year
CHINOOK, MT
°F
Day of Year

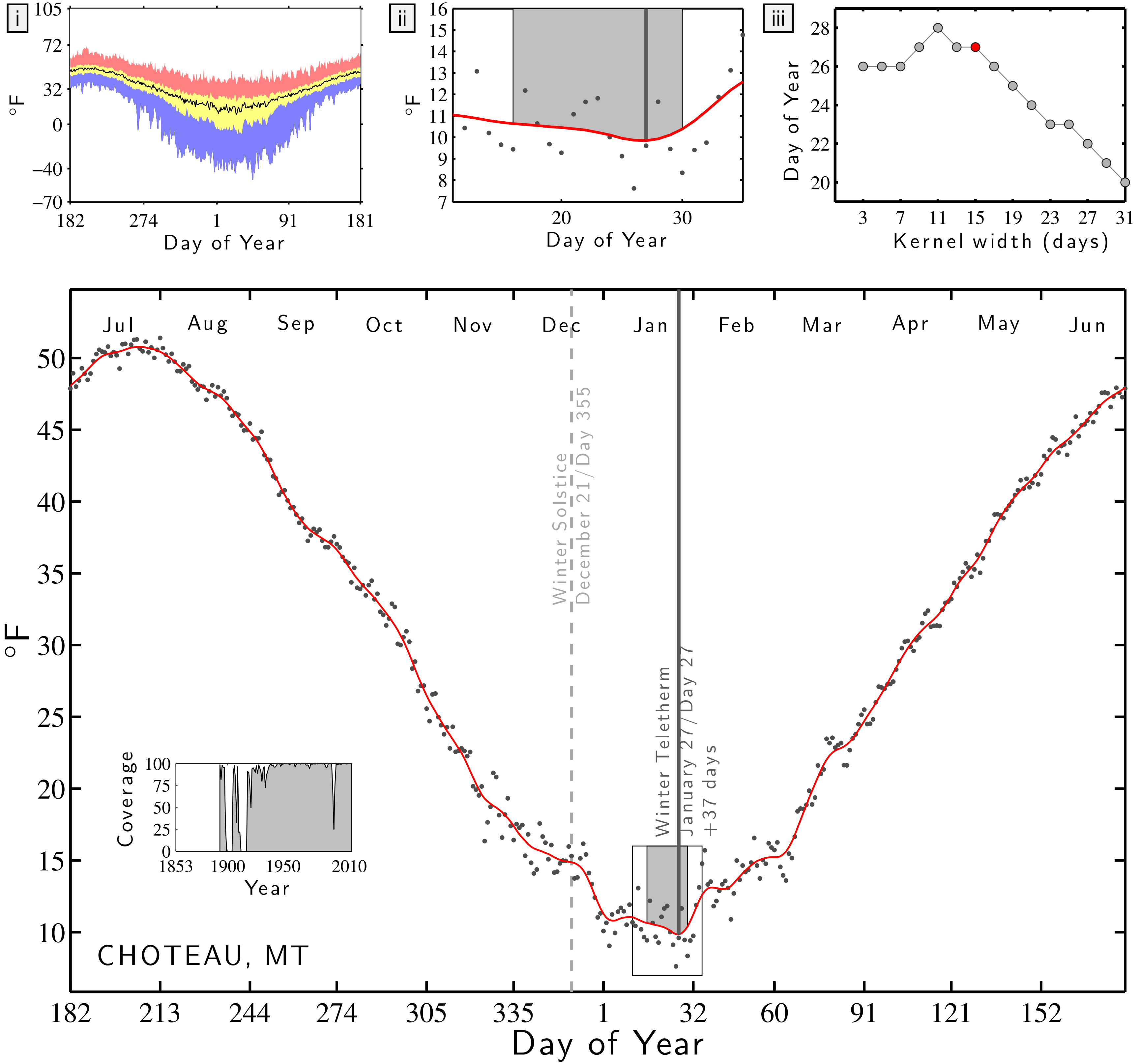

i
°F
Day of Year
ii
°F
Day of Year
iii
Day of Year
Kernel width (days)
Jul
Aug
Sep
Oct
Nov
Dec
Jan
Feb
Mar
Apr
May
Jun
Winter Solstice
December 21/Day 355
Winter Teletherm
January 27/Day 27
+37 days
Coverage
Year
CHOTEAU, MT
°F
Day of Year

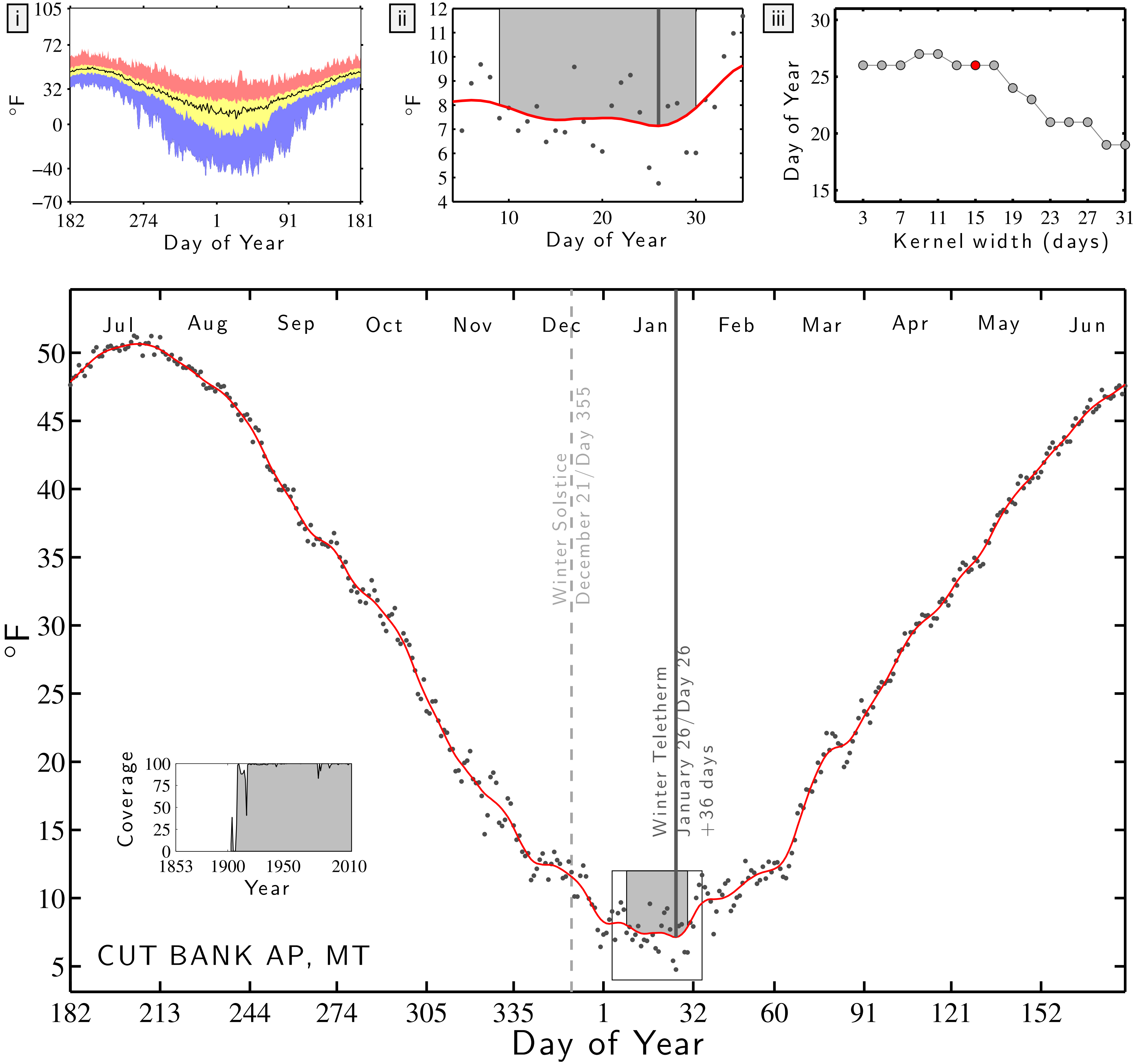

i
ii
iii
Day of Year
Kernel width (days)
Jul
Aug
Sep
Oct
Nov
Dec
Jan
Feb
Mar
Apr
May
Jun
Winter Solstice
December 21/Day 355
Winter Teletherm
January 26/Day 26
+36 days
Coverage
Year
CUT BANK AP, MT
°F
Day of Year

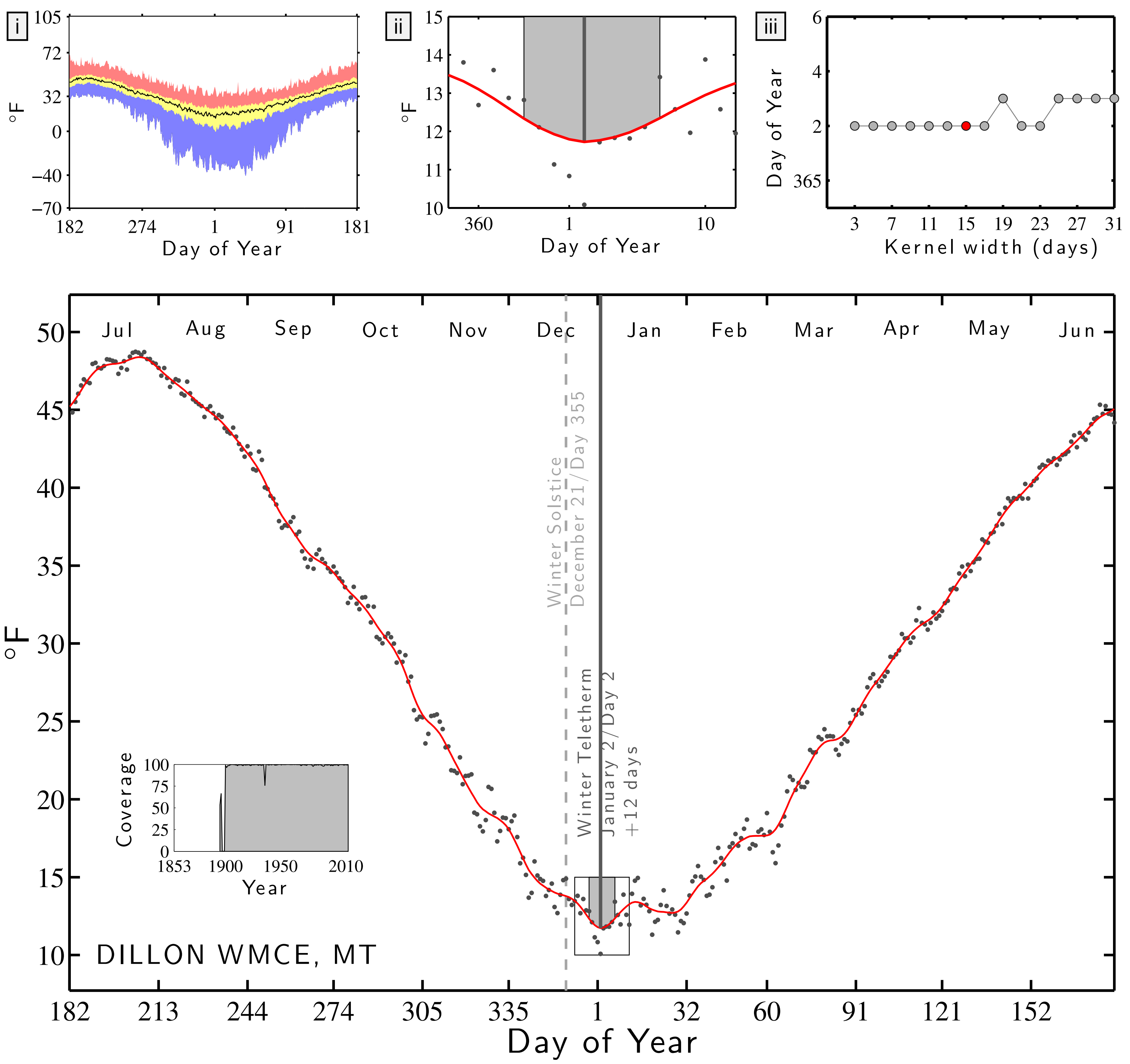

i
ii
iii
°F
Day of Year
Kernel width (days)
Jul
Aug
Sep
Oct
Nov
Dec
Jan
Feb
Mar
Apr
May
Jun
Winter Solstice
December 21/Day 355
Winter Teletherm
January 2/Day 2
+12 days
Coverage
Year
DILLON WMCE, MT

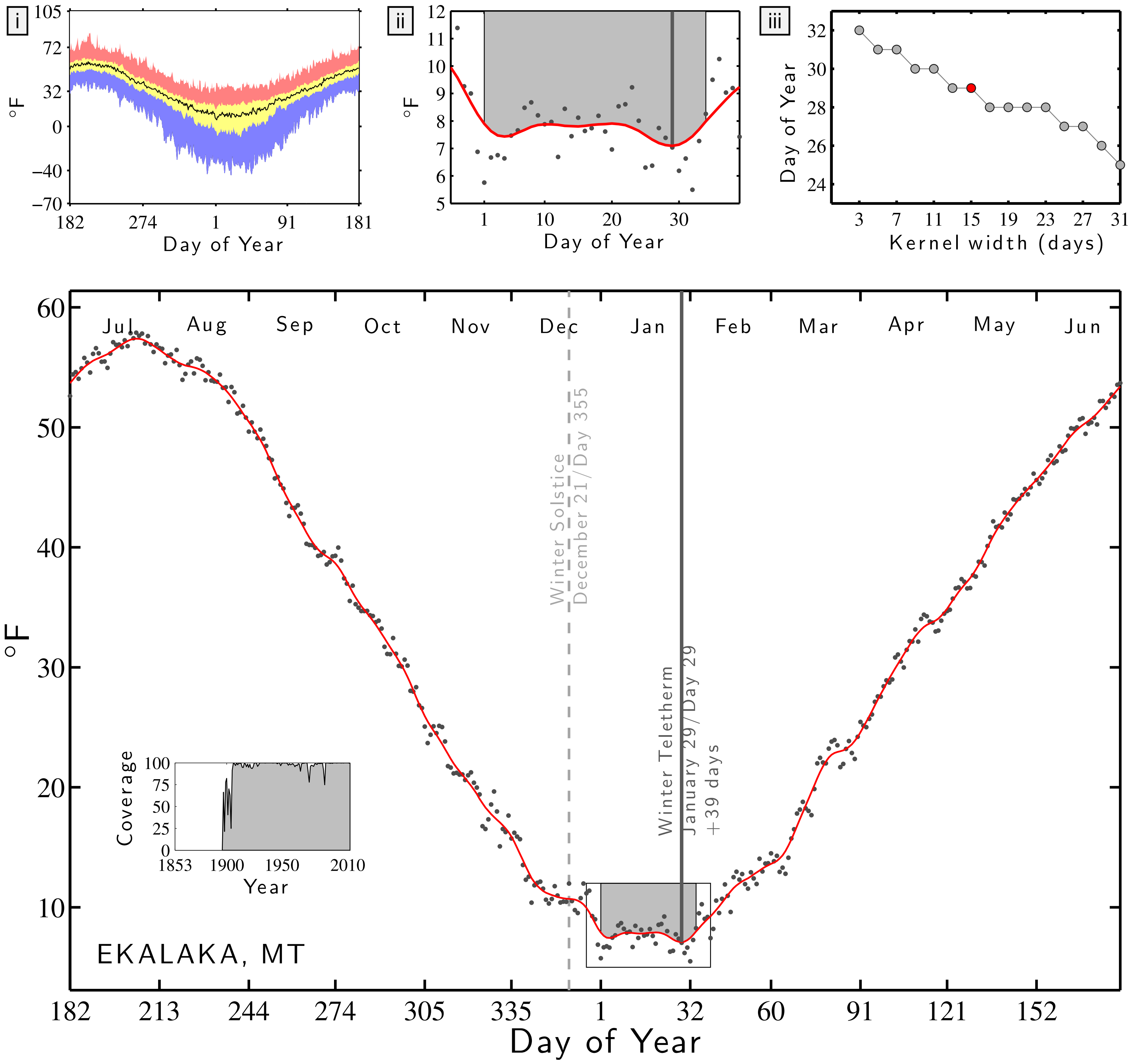

i
°F
Day of Year
ii
°F
Day of Year
iii
Day of Year
Kernel width (days)
Jul
Aug
Sep
Oct
Nov
Dec
Jan
Feb
Mar
Apr
May
Jun
Winter Solstice
December 21/Day 355
Winter Teletherm
January 29/Day 29
+39 days
Coverage
Year
EKALAKA, MT
°F
Day of Year

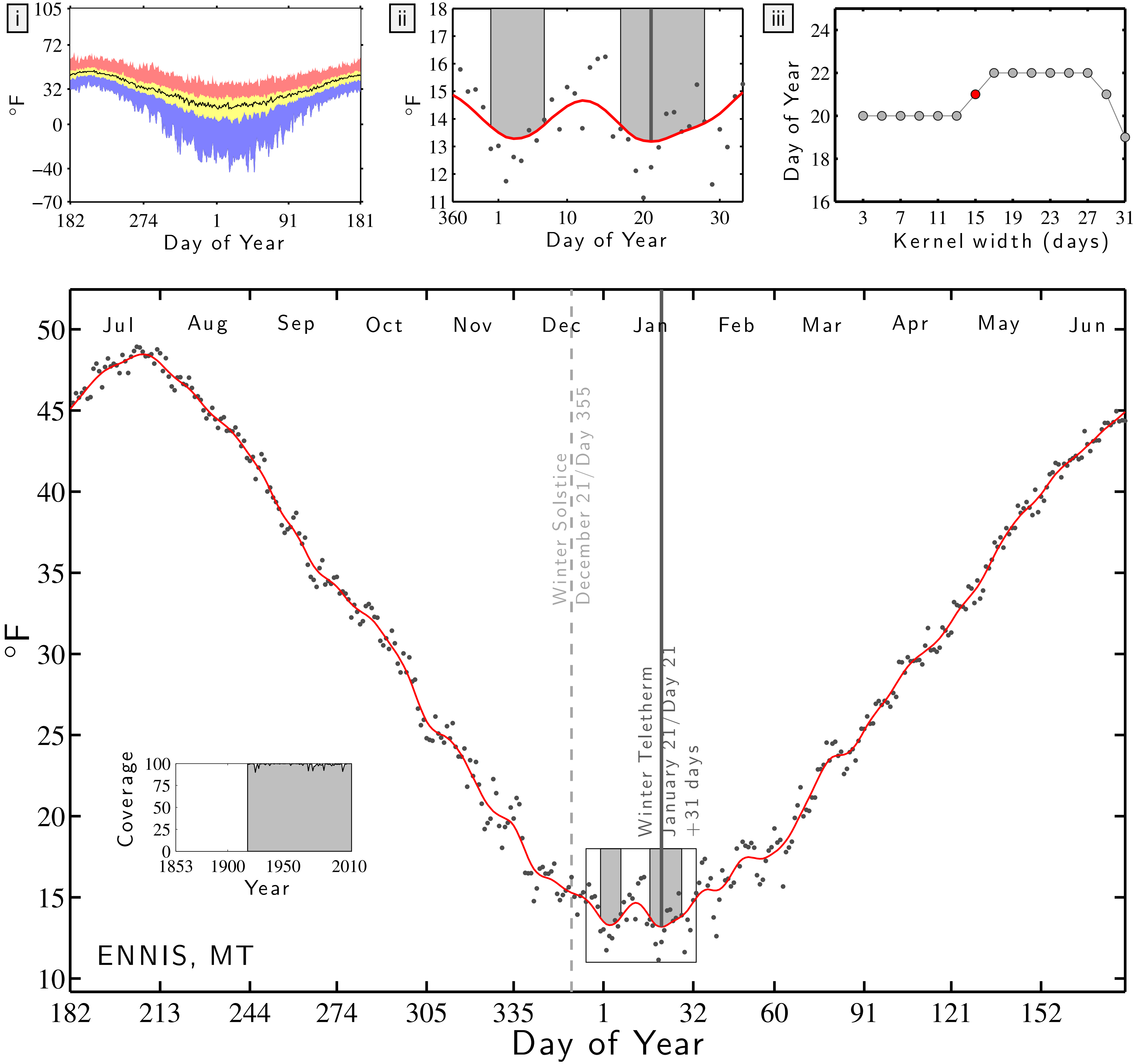

i
ii
iii
Day of Year
Kernel width (days)
°F
Day of Year
Jul
Aug
Sep
Oct
Nov
Dec
Jan
Feb
Mar
Apr
May
Jun
Winter Solstice
December 21/Day 355
Winter Teletherm
January 21/Day 21
+31 days
Coverage
Year
ENNIS, MT

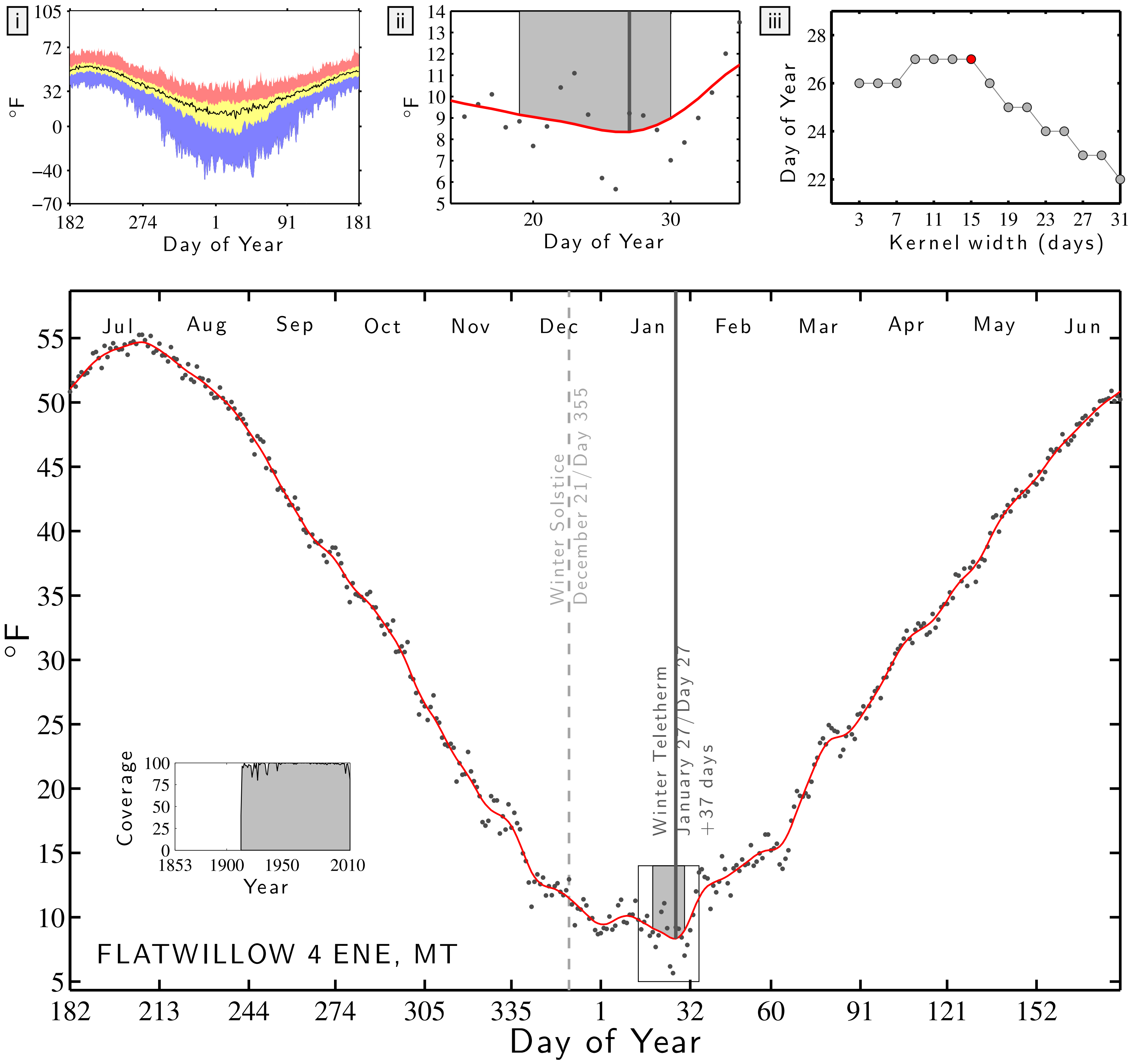

i
ii
iii
°F
Day of Year
Kernel width (days)
Jul
Aug
Sep
Oct
Nov
Dec
Jan
Feb
Mar
Apr
May
Jun
Winter Solstice
December 21/Day 355
Winter Teletherm
January 27/Day 27
+37 days
Coverage
Year
FLATWILLOW 4 ENE, MT

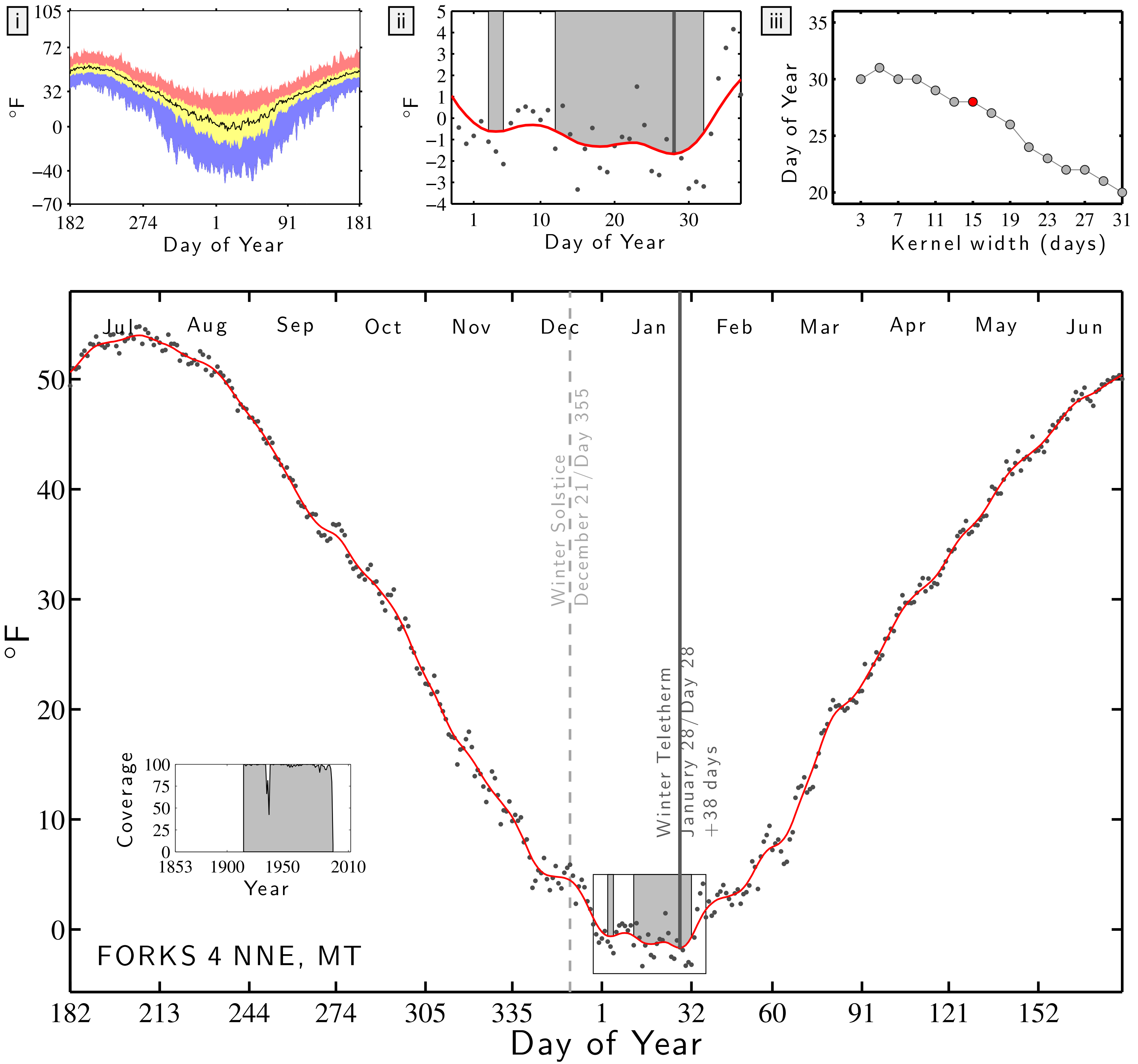

i
°F
Day of Year
ii
°F
Day of Year
iii
Day of Year
Kernel width (days)
Jul
Aug
Sep
Oct
Nov
Dec
Jan
Feb
Mar
Apr
May
Jun
Winter Solstice
December 21/Day 355
Winter Teletherm
January 28/Day 28
+38 days
Coverage
Year
FORKS 4 NNE, MT
°F
Day of Year

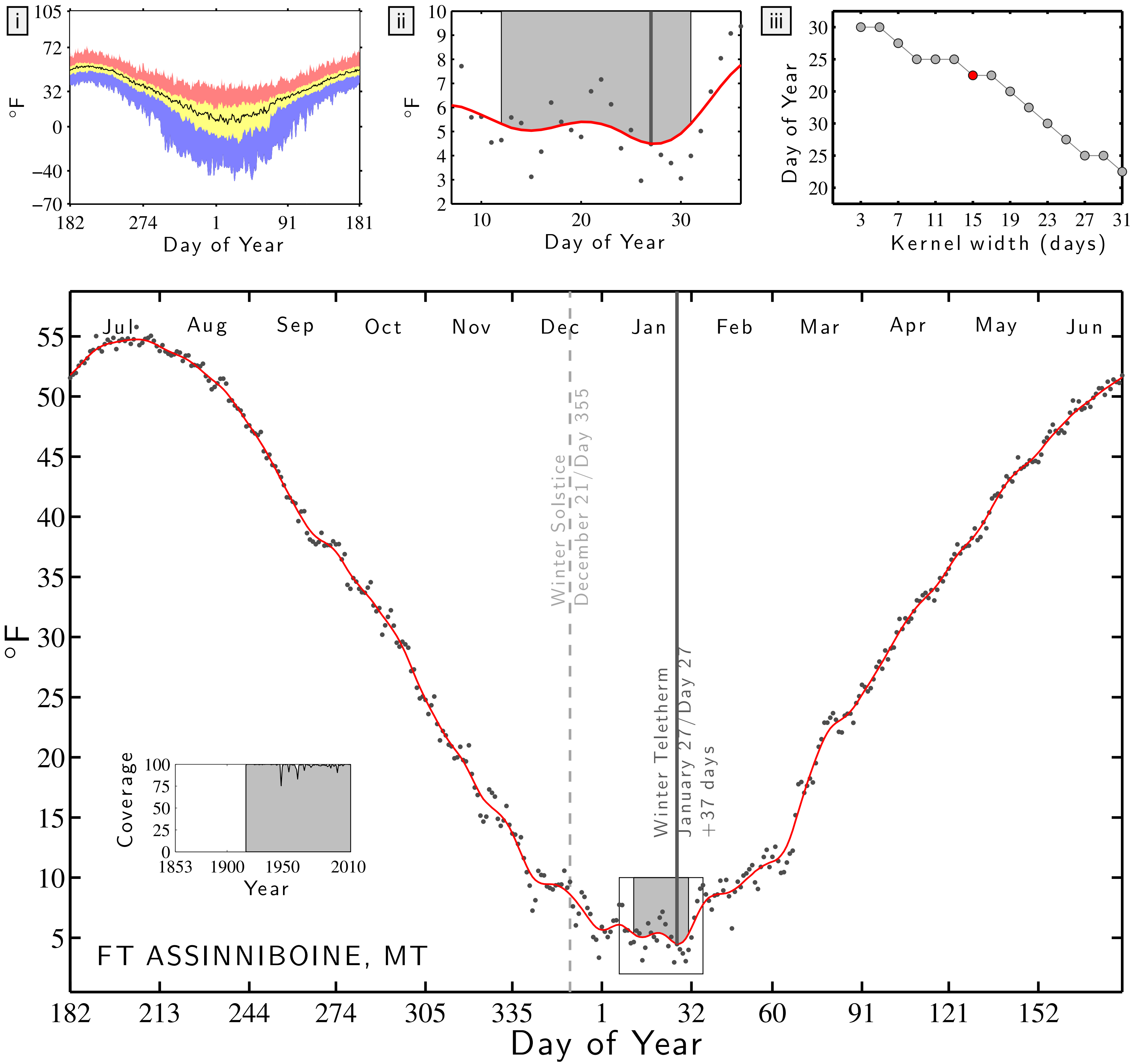

i
°F
Day of Year
ii
°F
Day of Year
iii
Day of Year
Kernel width (days)
Jul
Aug
Sep
Oct
Nov
Dec
Jan
Feb
Mar
Apr
May
Jun
Winter Solstice
December 21/Day 355
Winter Teletherm
January 27/Day 27
+37 days
Coverage
Year
FT ASSINNIBOINE, MT
°F
Day of Year

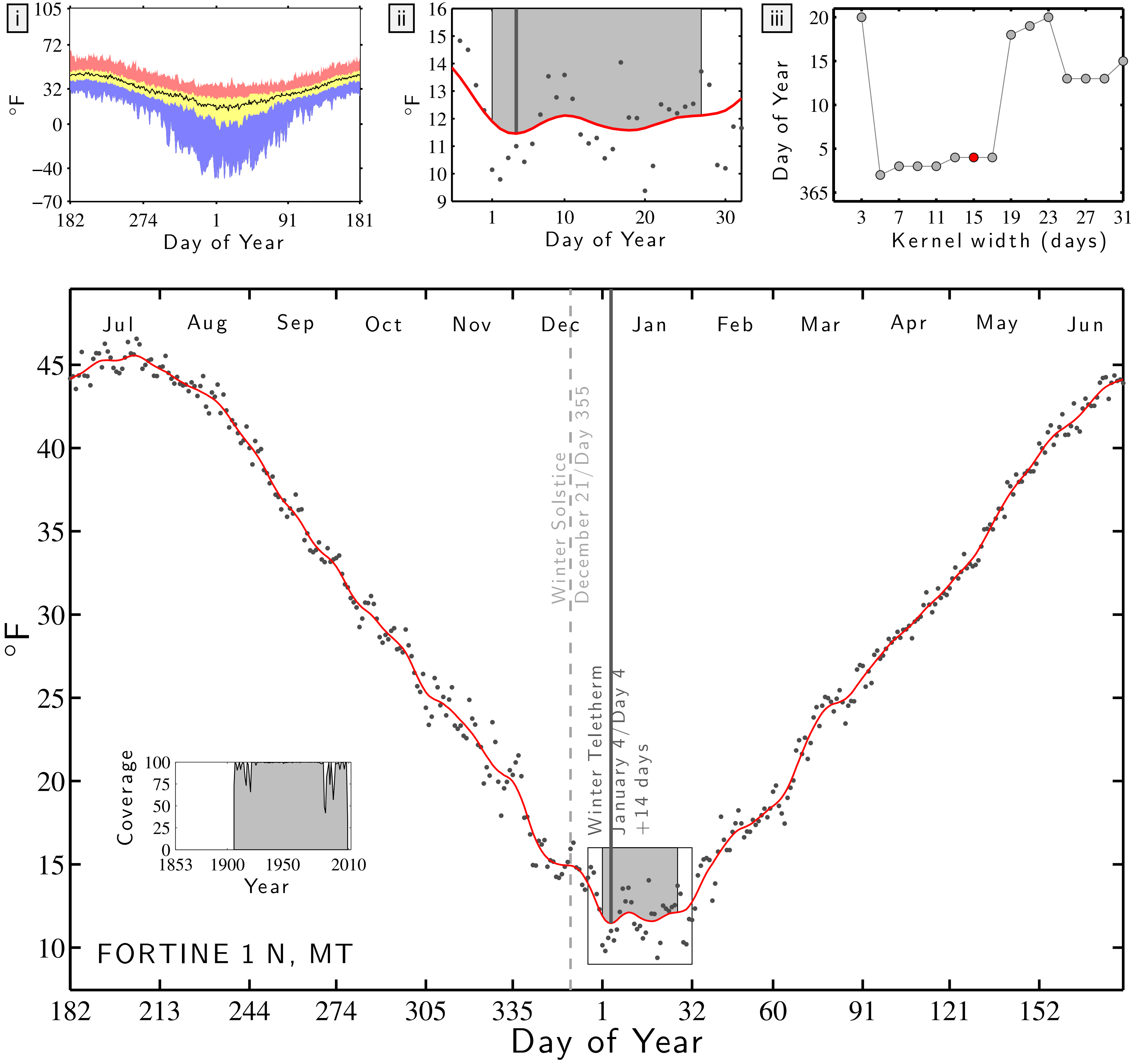

i
ii
iii
°F
Day of Year
Kernel width (days)
Jul
Aug
Sep
Oct
Nov
Dec
Jan
Feb
Mar
Apr
May
Jun
Winter Solstice
December 21/Day 355
Winter Teletherm
January 4/Day 4
+14 days
Coverage
Year
FORTINE 1 N, MT

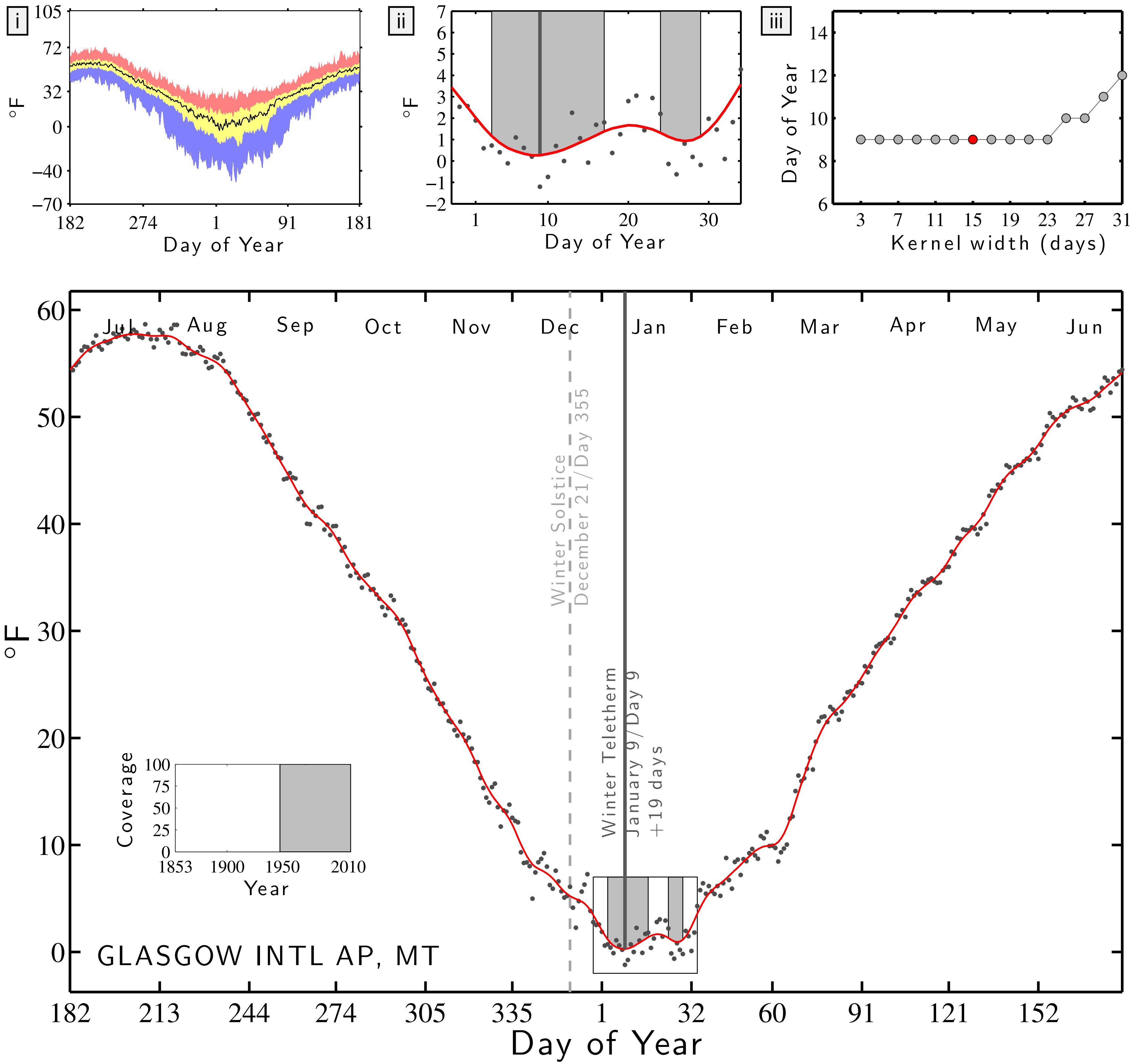

i
°F
Day of Year
ii
°F
Day of Year
iii
Day of Year
Kernel width (days)
Jul
Aug
Sep
Oct
Nov
Dec
Jan
Feb
Mar
Apr
May
Jun
Winter Solstice
December 21/Day 355
Winter Teletherm
January 9/Day 9
+19 days
Coverage
Year
GLASGOW INTL AP, MT
°F
Day of Year

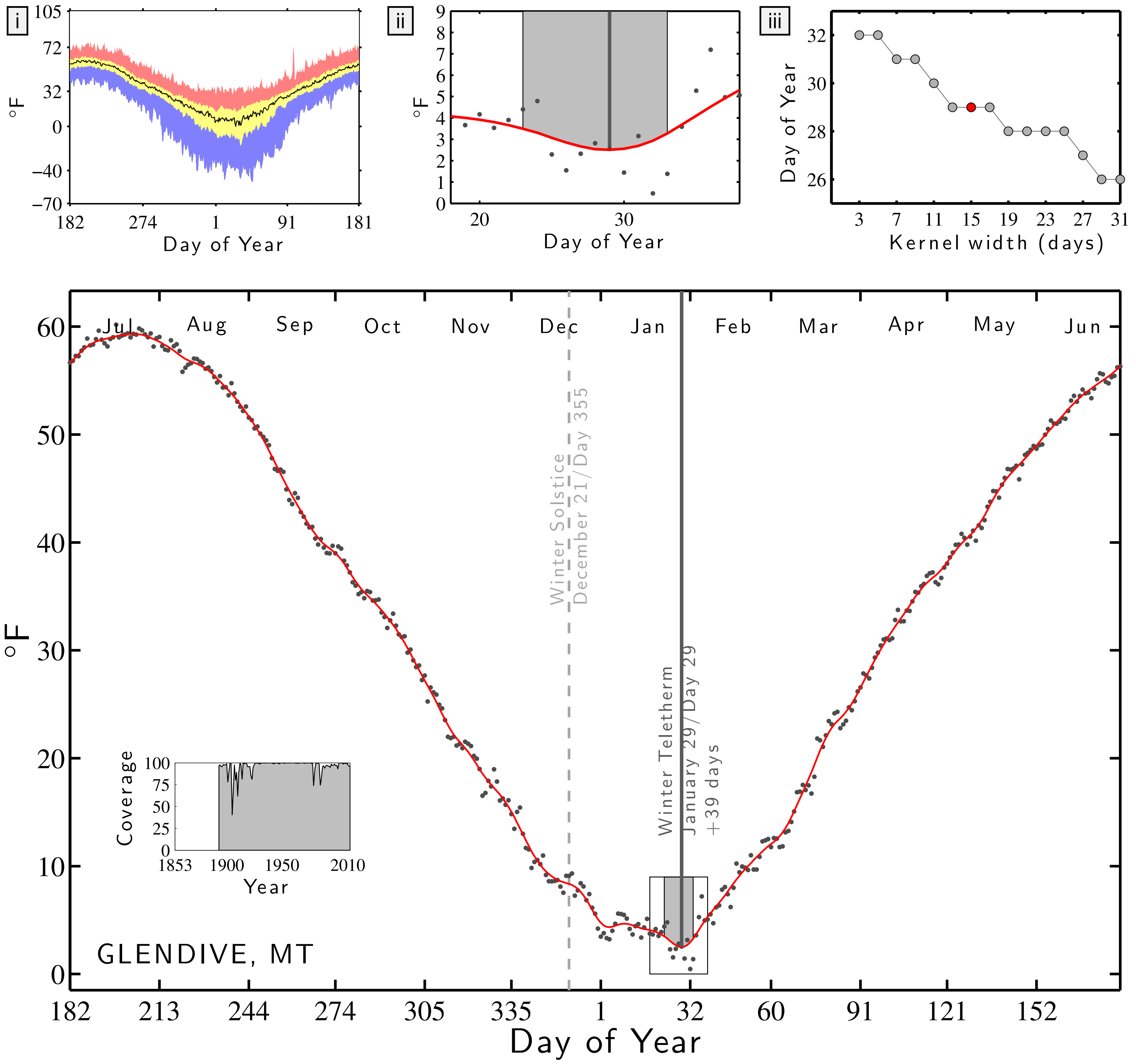

i
°F
Day of Year
ii
°F
Day of Year
iii
Day of Year
Kernel width (days)
Jul
Aug
Sep
Oct
Nov
Dec
Jan
Feb
Mar
Apr
May
Jun
Winter Solstice
December 21/Day 355
Winter Teletherm
January 29/Day 29
+39 days
Coverage
Year
GLENDIVE, MT
°F
Day of Year

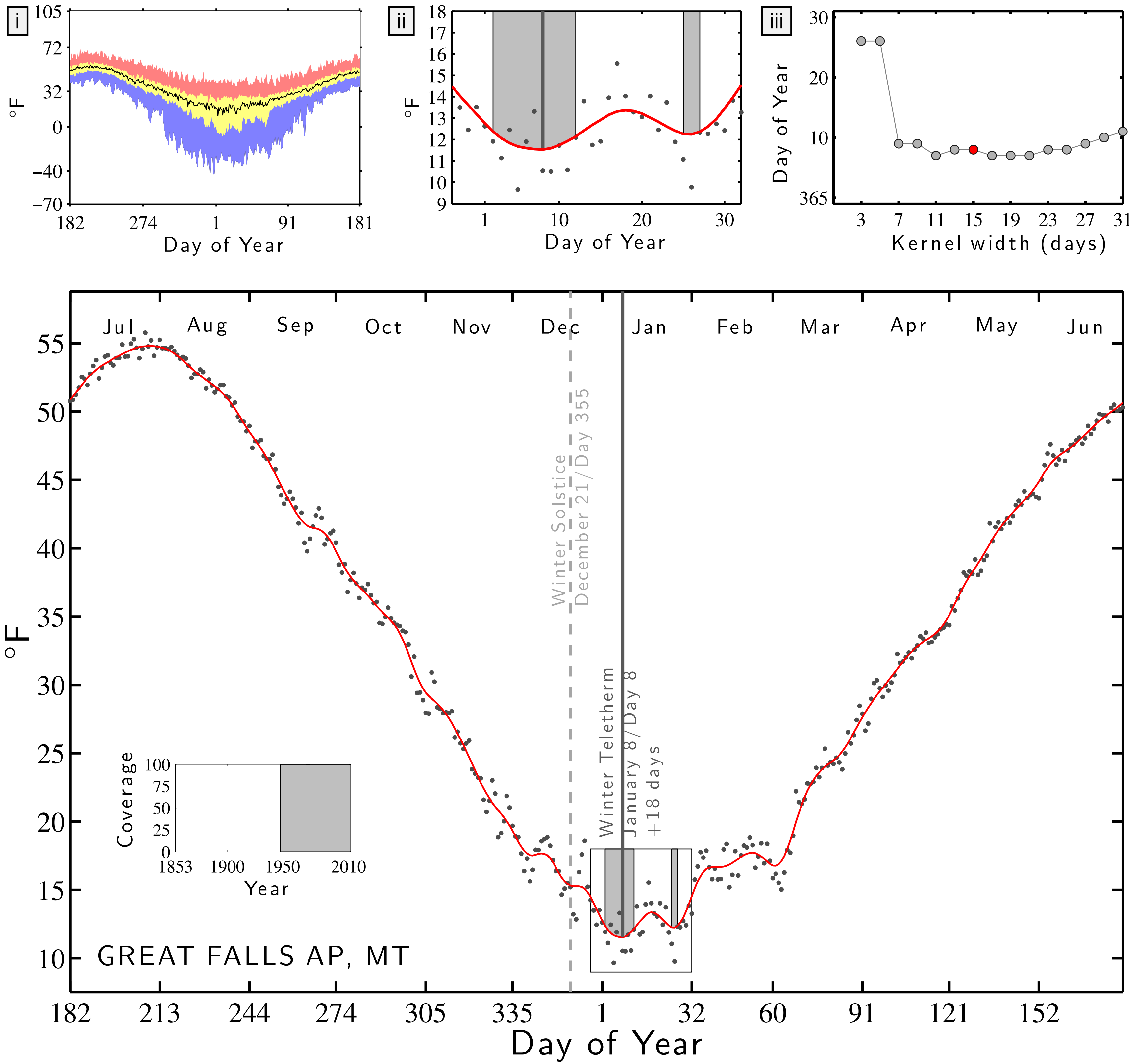
i
°F
Day of Year
ii
°F
Day of Year
iii
Day of Year
Kernel width (days)
Jul
Aug
Sep
Oct
Nov
Dec
Jan
Feb
Mar
Apr
May
Jun
Winter Solstice
December 21/Day 355
Winter Teletherm
January 8/Day 8
+18 days
Coverage
Year
GREAT FALLS AP, MT
°F
Day of Year

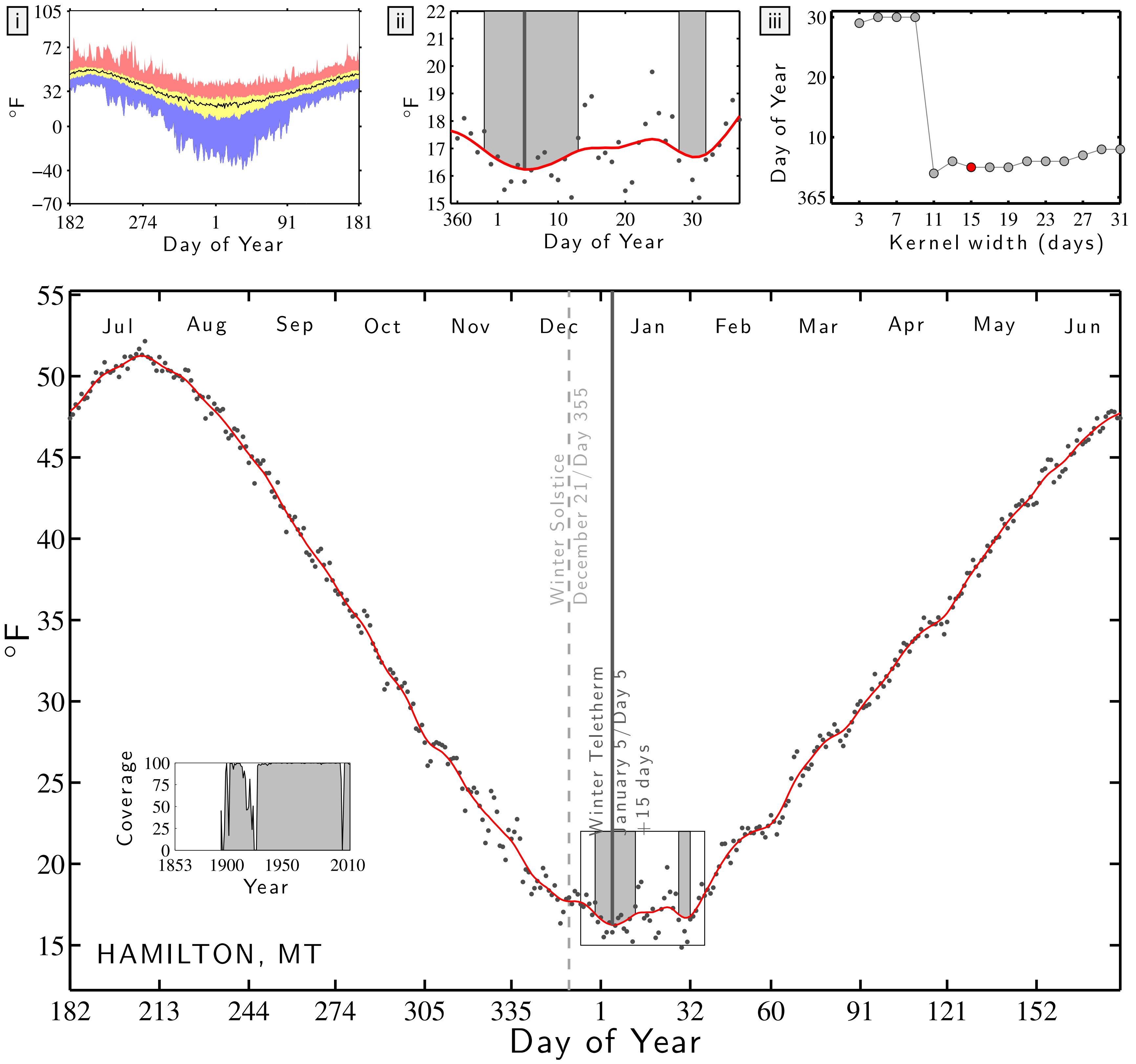

i
°F
Day of Year
ii
°F
Day of Year
iii
Day of Year
Kernel width (days)
Jul
Aug
Sep
Oct
Nov
Dec
Jan
Feb
Mar
Apr
May
Jun
Winter Solstice
December 21/Day 355
Winter Teletherm
January 5/Day 5
±15 days
Coverage
Year
HAMILTON, MT
°F
Day of Year

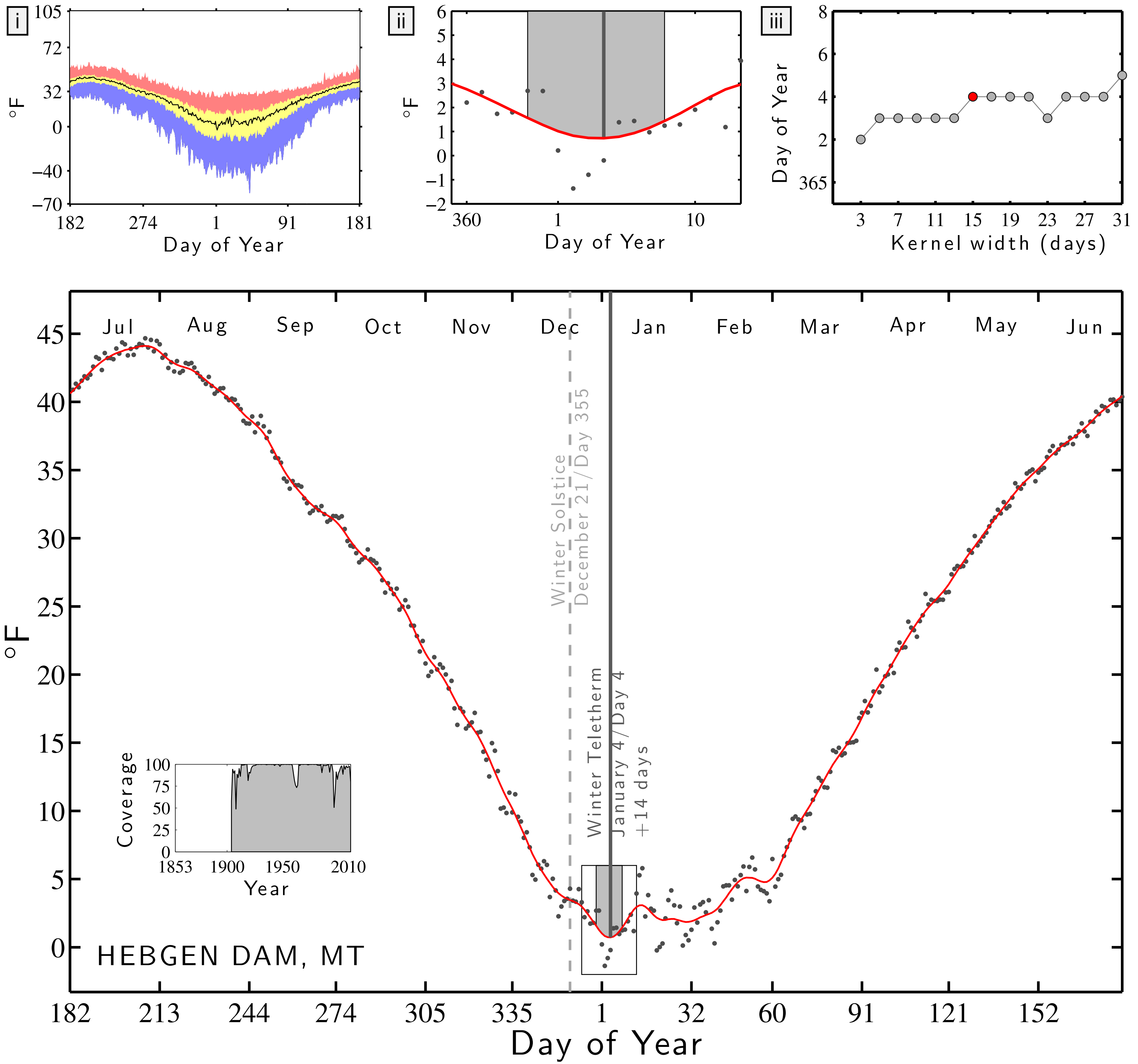

i
ii
iii
°F
Day of Year
Kernel width (days)
Jul
Aug
Sep
Oct
Nov
Dec
Jan
Feb
Mar
Apr
May
Jun
Winter Solstice
December 21/Day 355
Winter Teletherm
January 4/Day 4
+14 days
Coverage
Year
HEBGEN DAM, MT

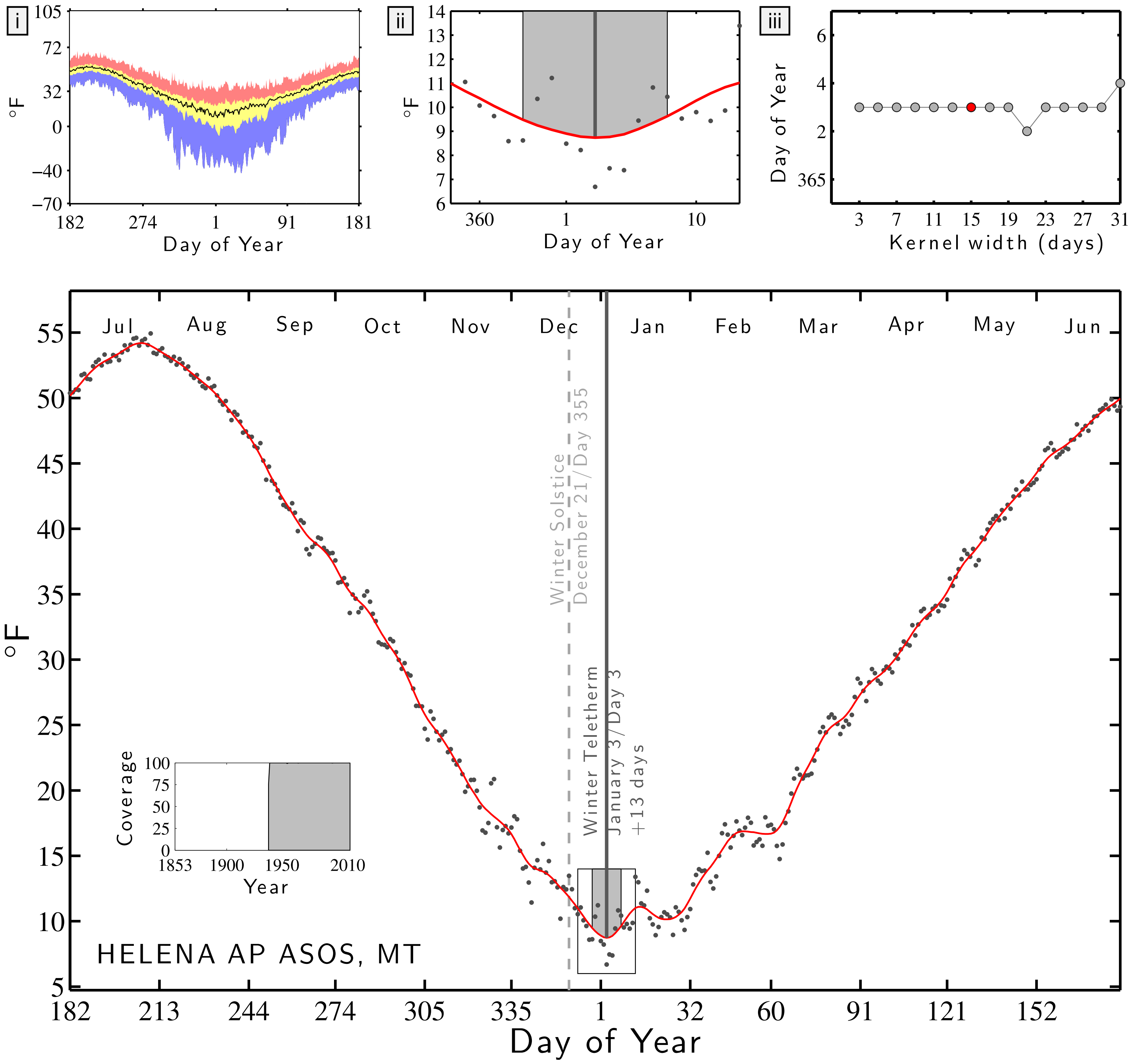

i
°F
Day of Year
ii
°F
Day of Year
iii
Day of Year
Kernel width (days)
Jul
Aug
Sep
Oct
Nov
Dec
Jan
Feb
Mar
Apr
May
Jun
Winter Solstice
December 21/Day 355
Winter Teletherm
January 3/Day 3
+13 days
Coverage
Year
HELENA AP ASOS, MT
°F
Day of Year

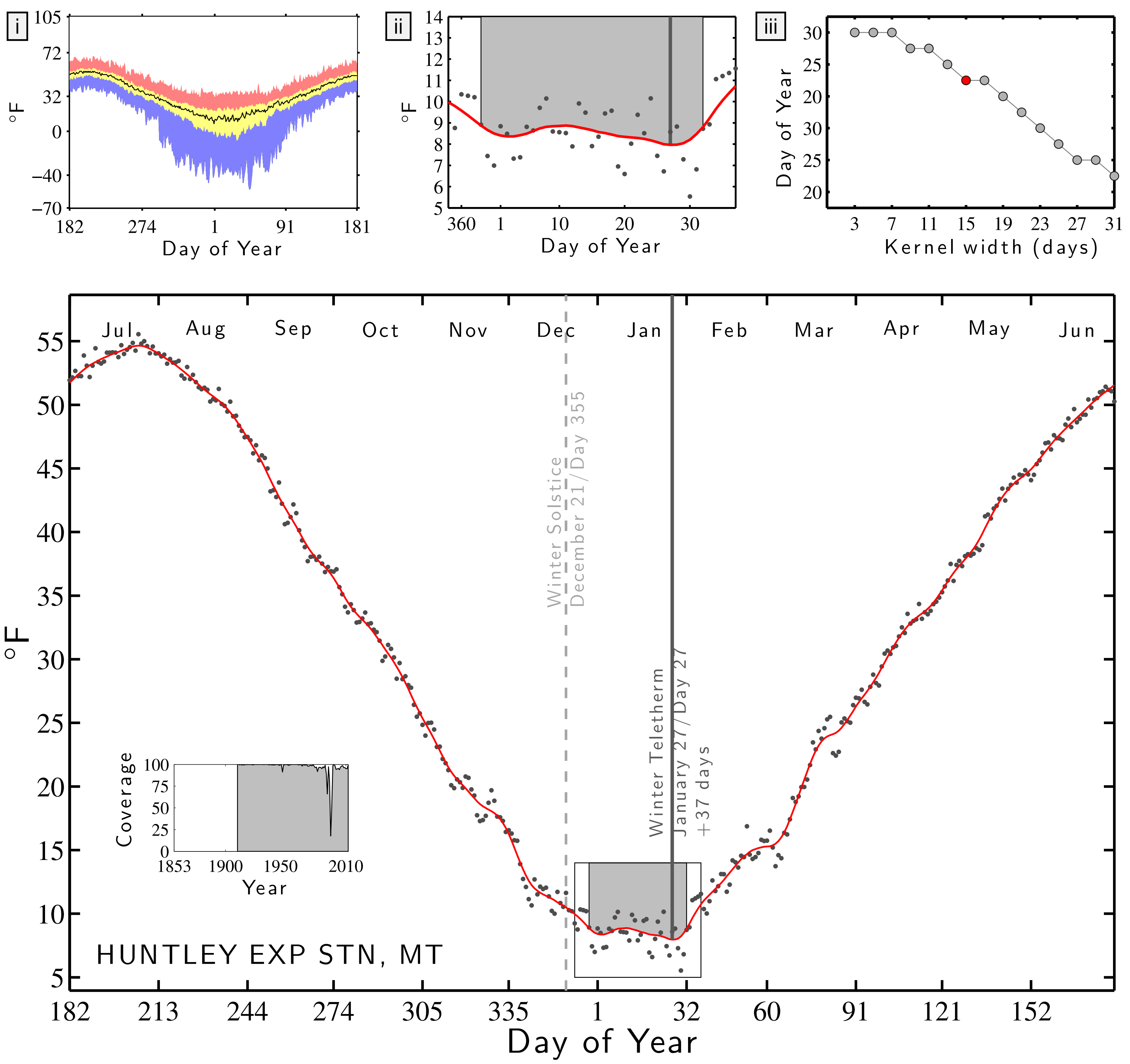

i
°F
Day of Year
ii
°F
Day of Year
iii
Day of Year
Kernel width (days)
Jul
Aug
Sep
Oct
Nov
Dec
Jan
Feb
Mar
Apr
May
Jun
Winter Solstice
December 21/Day 355
Winter Teletherm
January 27/Day 27
+37 days
Coverage
Year
HUNTLEY EXP STN, MT
°F
Day of Year

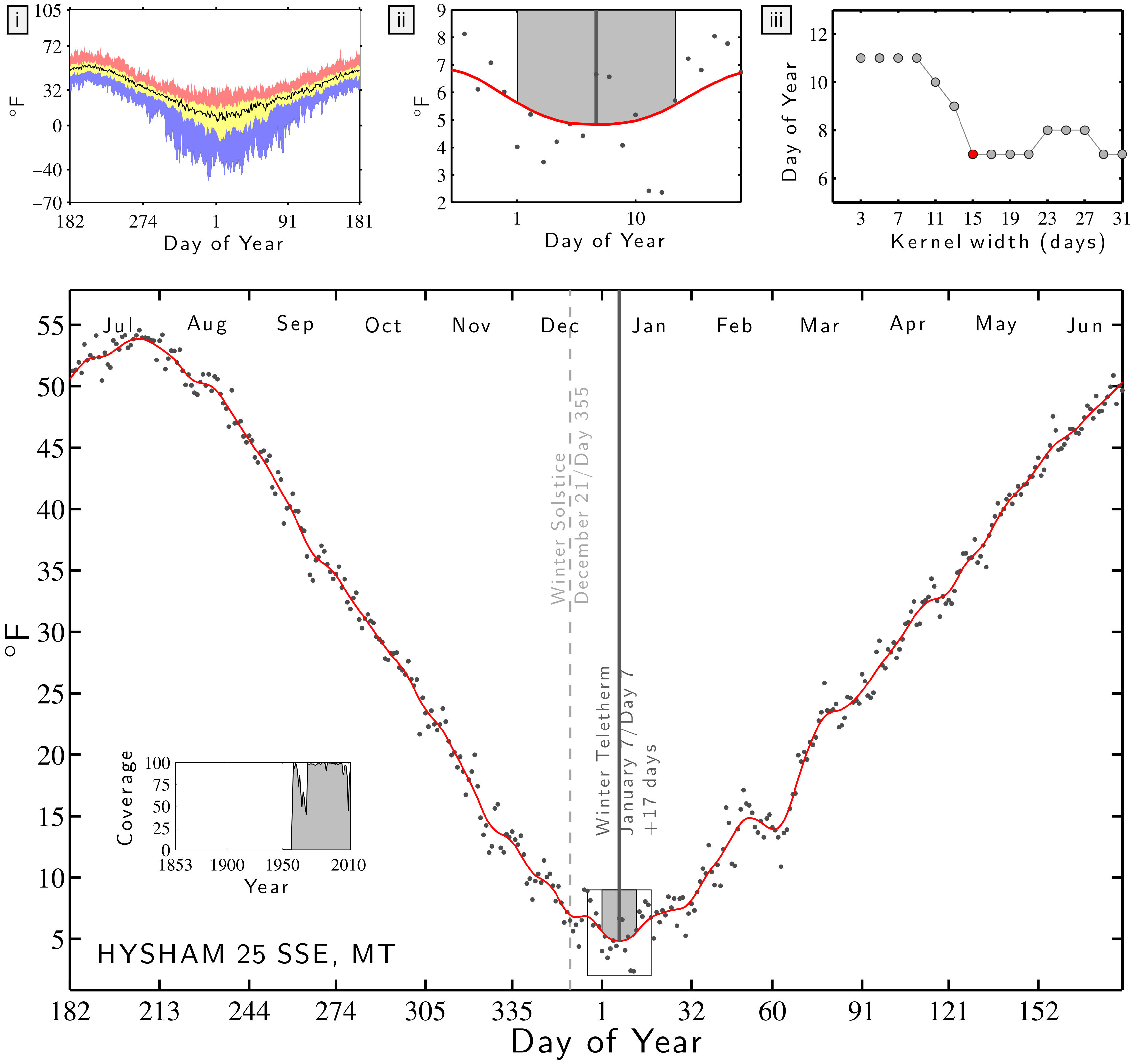

i
ii
iii
°F
Day of Year
Kernel width (days)
HYSHAM 25 SSE, MT
Winter Solstice
December 21/Day 355
Winter Teletherm
January 7/Day 7
+17 days
Jul
Aug
Sep
Oct
Nov
Dec
Jan
Feb
Mar
Apr
May
Jun
Coverage
Year

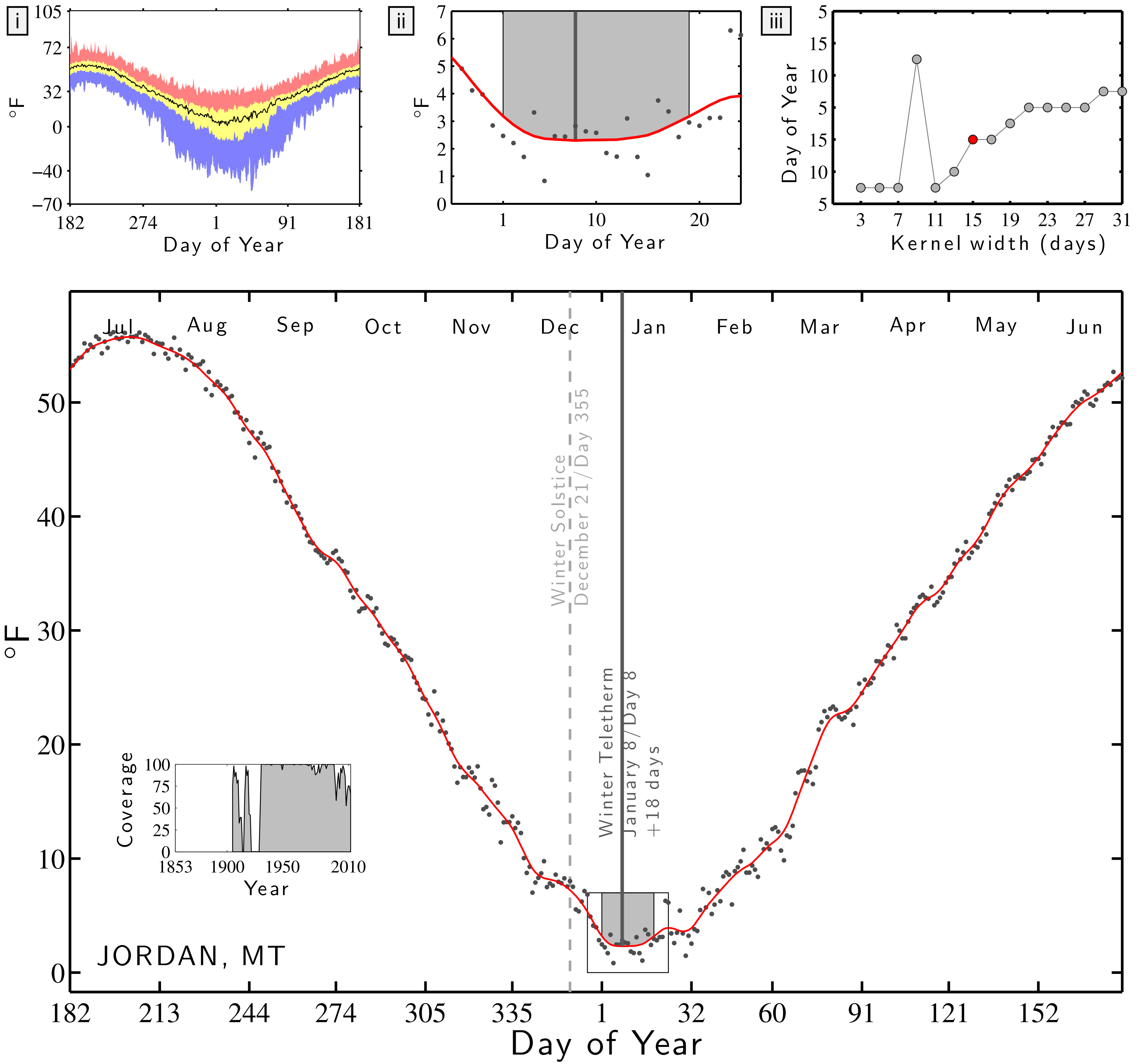

i
ii
iii
°F
Day of Year
Kernel width (days)
Jul
Aug
Sep
Oct
Nov
Dec
Jan
Feb
Mar
Apr
May
Jun
Winter Solstice
December 21/Day 355
Winter Teletherm
January 8/Day 8
+18 days
Coverage
Year
JORDAN, MT

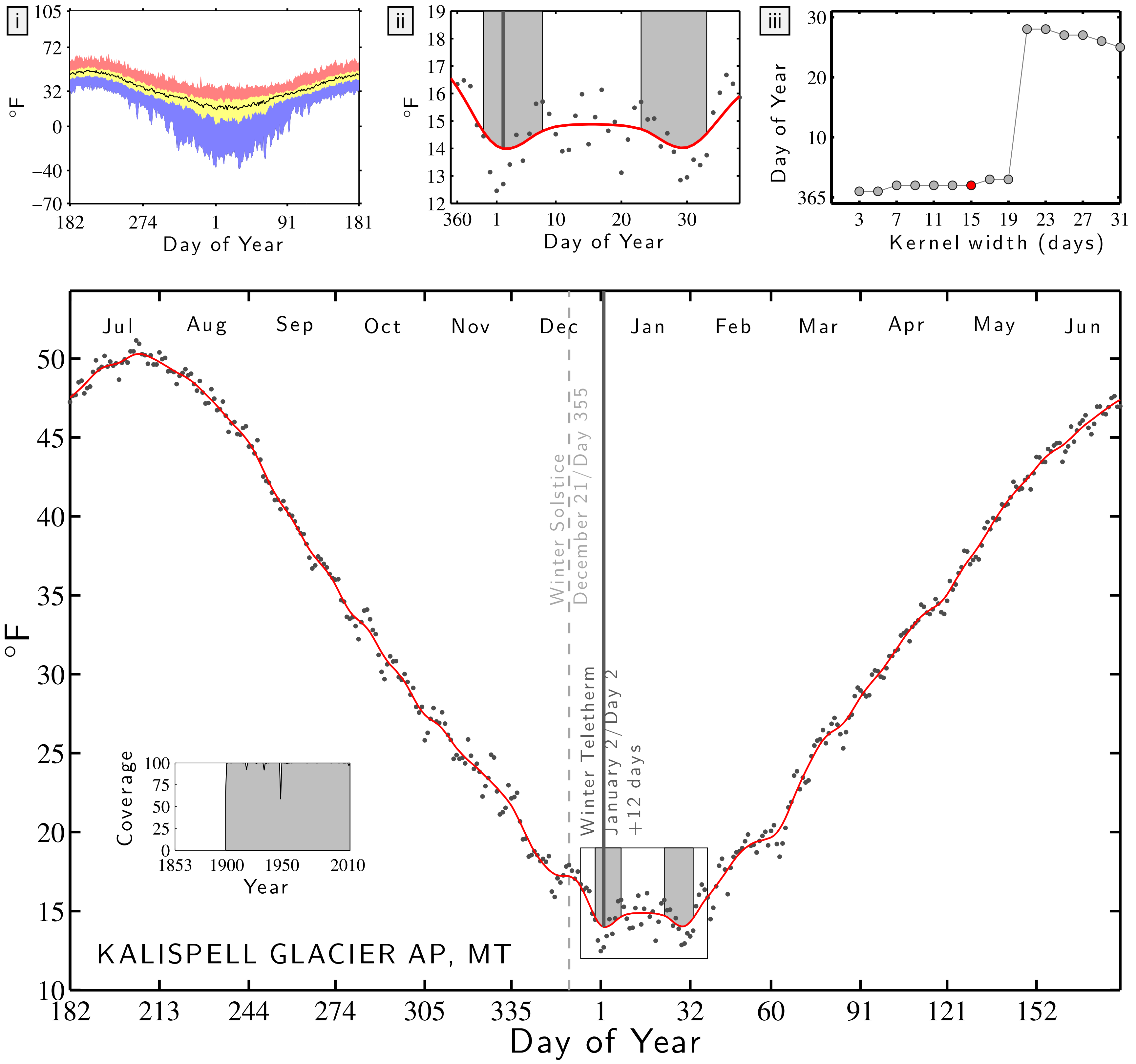

i
°F
Day of Year
ii
°F
Day of Year
iii
Day of Year
Kernel width (days)
Jul
Aug
Sep
Oct
Nov
Dec
Jan
Feb
Mar
Apr
May
Jun
Winter Solstice
December 21/Day 355
Winter Teletherm
January 2/Day 2
+12 days
Coverage
Year
KALISPELL GLACIER AP, MT
°F
Day of Year

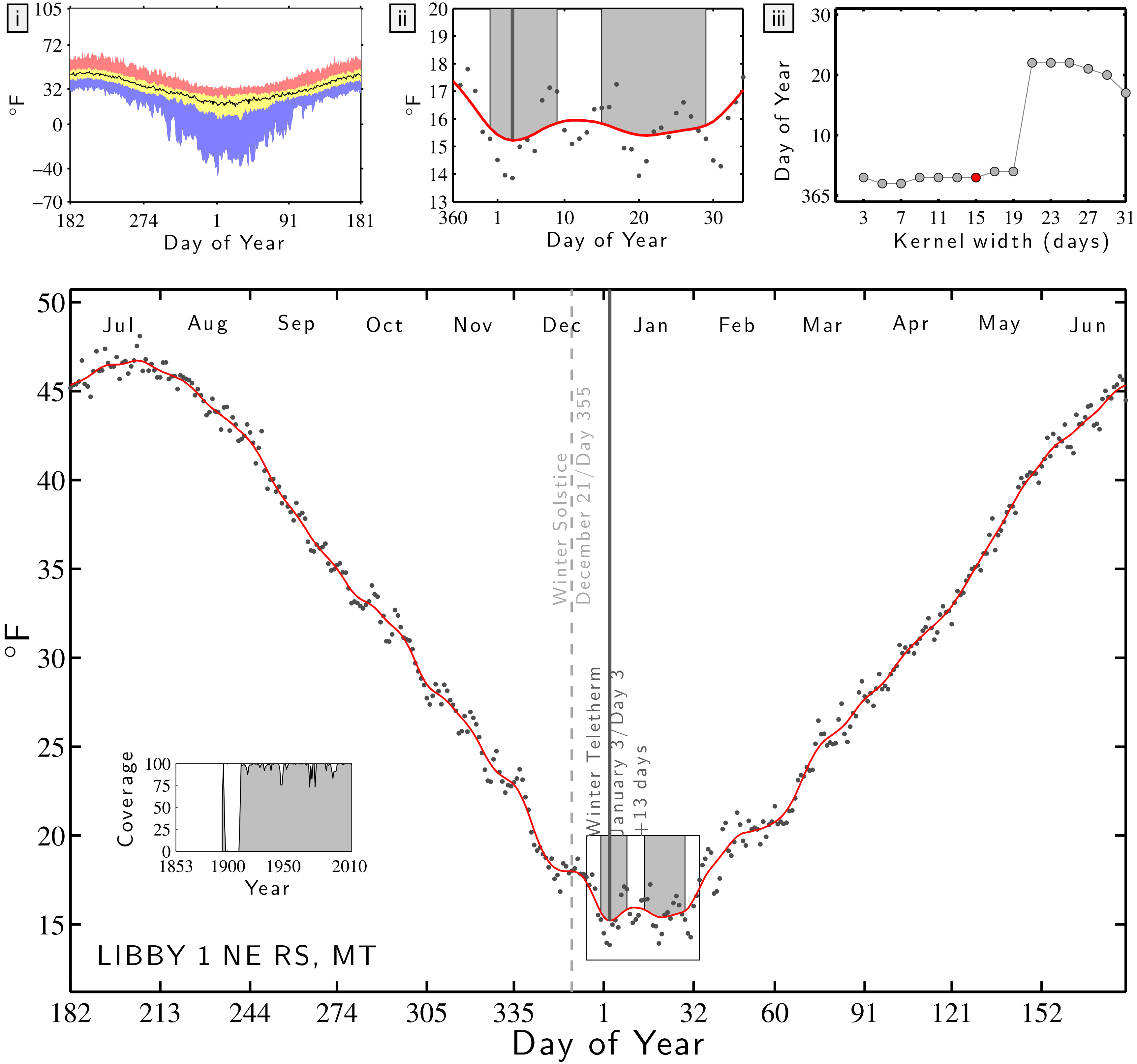

i
ii
iii
°F
Day of Year
Kernel width (days)
Jul
Aug
Sep
Oct
Nov
Dec
Jan
Feb
Mar
Apr
May
Jun
Winter Solstice
December 21/Day 355
Winter Teletherm
January 3/Day 3
+13 days
Coverage
Year
LIBBY 1 NE RS, MT

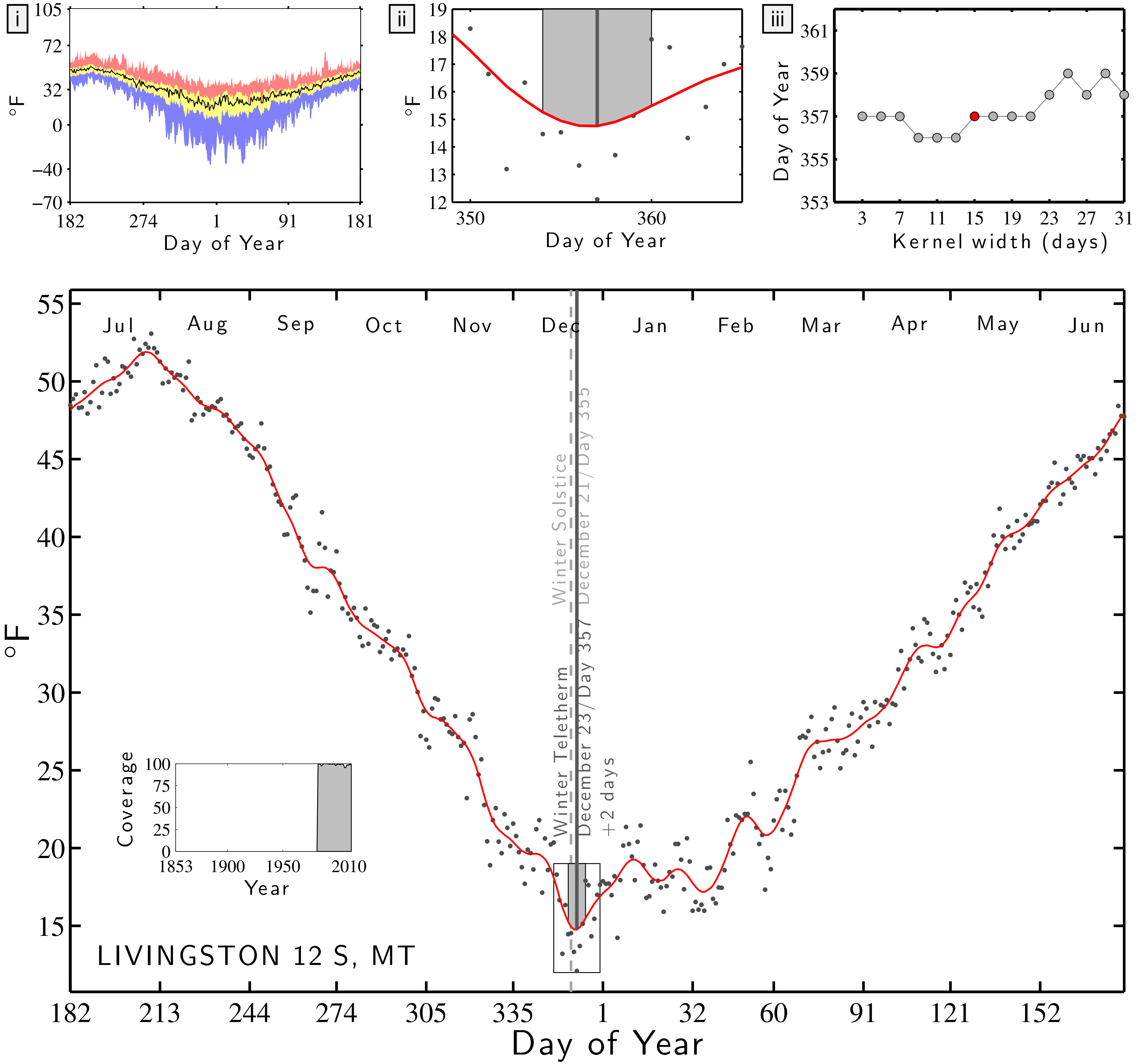

i
°F
Day of Year
ii
°F
Day of Year
iii
Day of Year
Kernel width (days)
Jul
Aug
Sep
Oct
Nov
Dec
Jan
Feb
Mar
Apr
May
Jun
Winter Solstice December 21 / Day 355
Winter Teletherm December 23 / Day 357
+2 days
Coverage
Year
LIVINGSTON 12 S, MT
°F
Day of Year

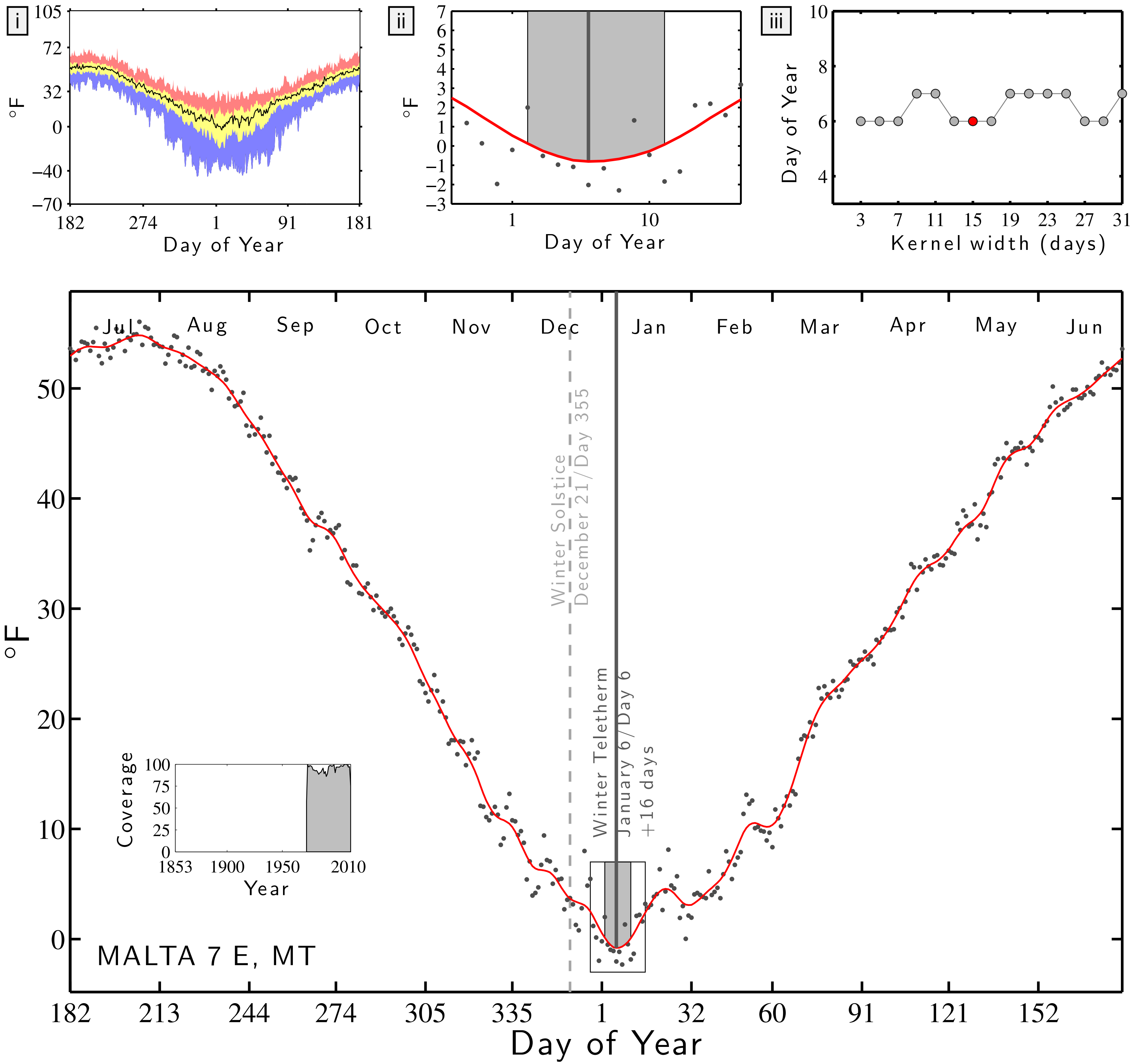

i
°F
Day of Year
ii
°F
Day of Year
iii
Day of Year
Kernel width (days)
Jul
Aug
Sep
Oct
Nov
Dec
Jan
Feb
Mar
Apr
May
Jun
Winter Solstice
December 21/Day 355
Winter Teletherm
January 6/Day 6
+16 days
Coverage
Year
MALTA 7 E, MT
°F
Day of Year

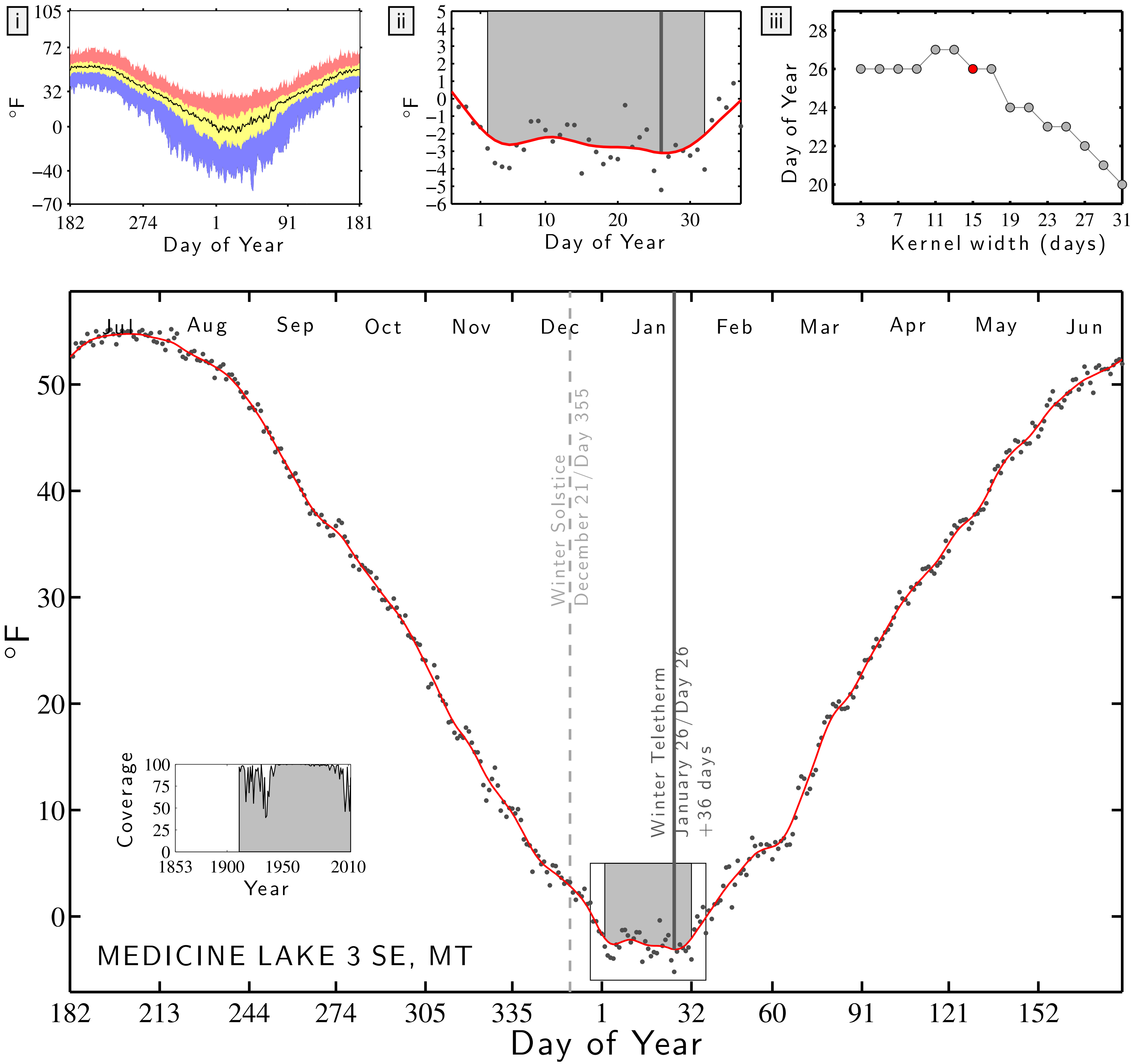

i
°F
Day of Year
ii
°F
Day of Year
iii
Day of Year
Kernel width (days)
Jul
Aug
Sep
Oct
Nov
Dec
Jan
Feb
Mar
Apr
May
Jun
Winter Solstice
December 21/Day 355
Winter Teletherm
January 26/Day 26
+36 days
Coverage
Year
MEDICINE LAKE 3 SE, MT
°F
Day of Year

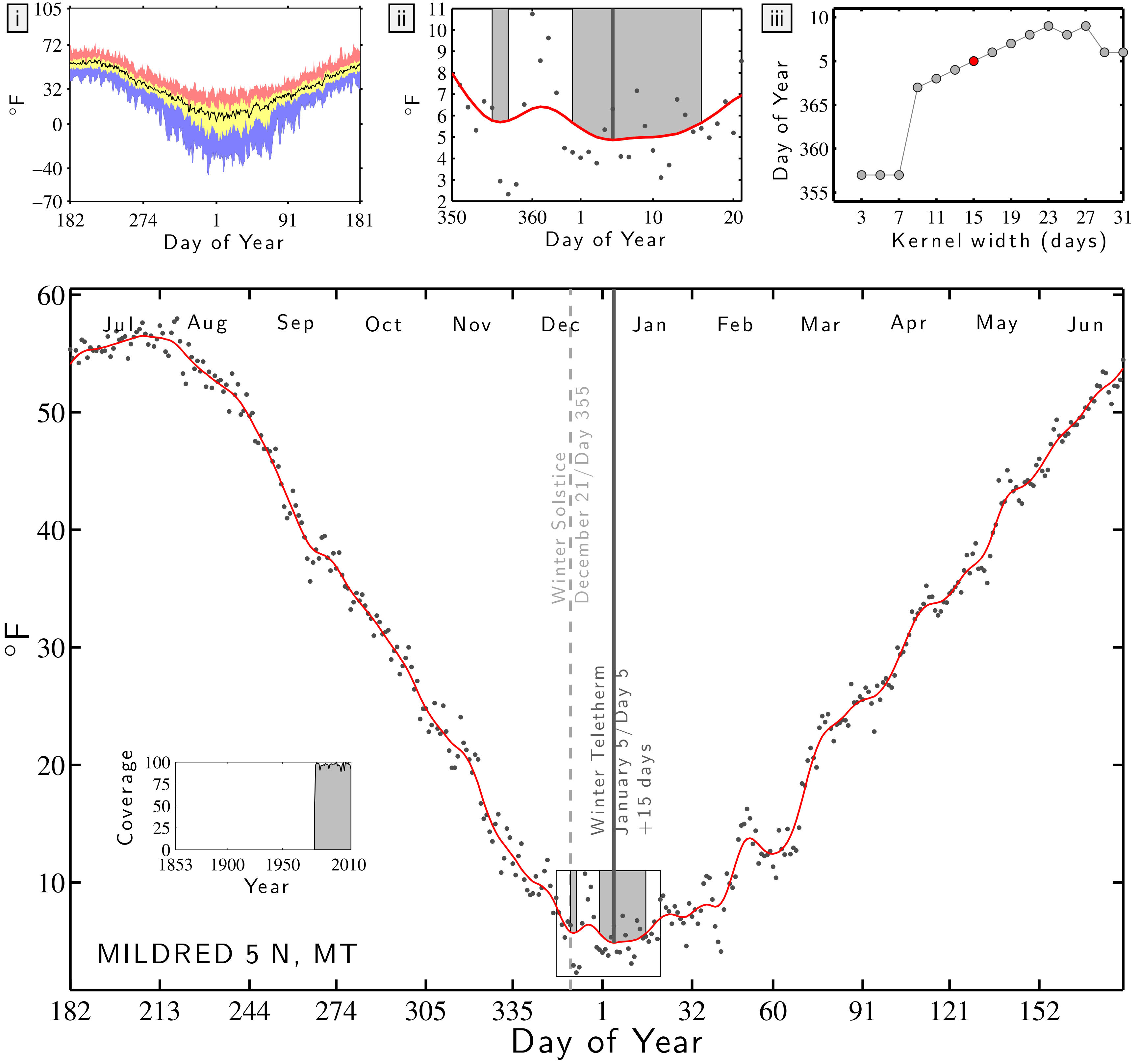

i
ii
iii
°F
Day of Year
Kernel width (days)
Day of Year
Jul
Aug
Sep
Oct
Nov
Dec
Jan
Feb
Mar
Apr
May
Jun
Winter Solstice
December 21/Day 355
Winter Teletherm
January 5/Day 5
+15 days
Coverage
Year
MILDRED 5 N, MT

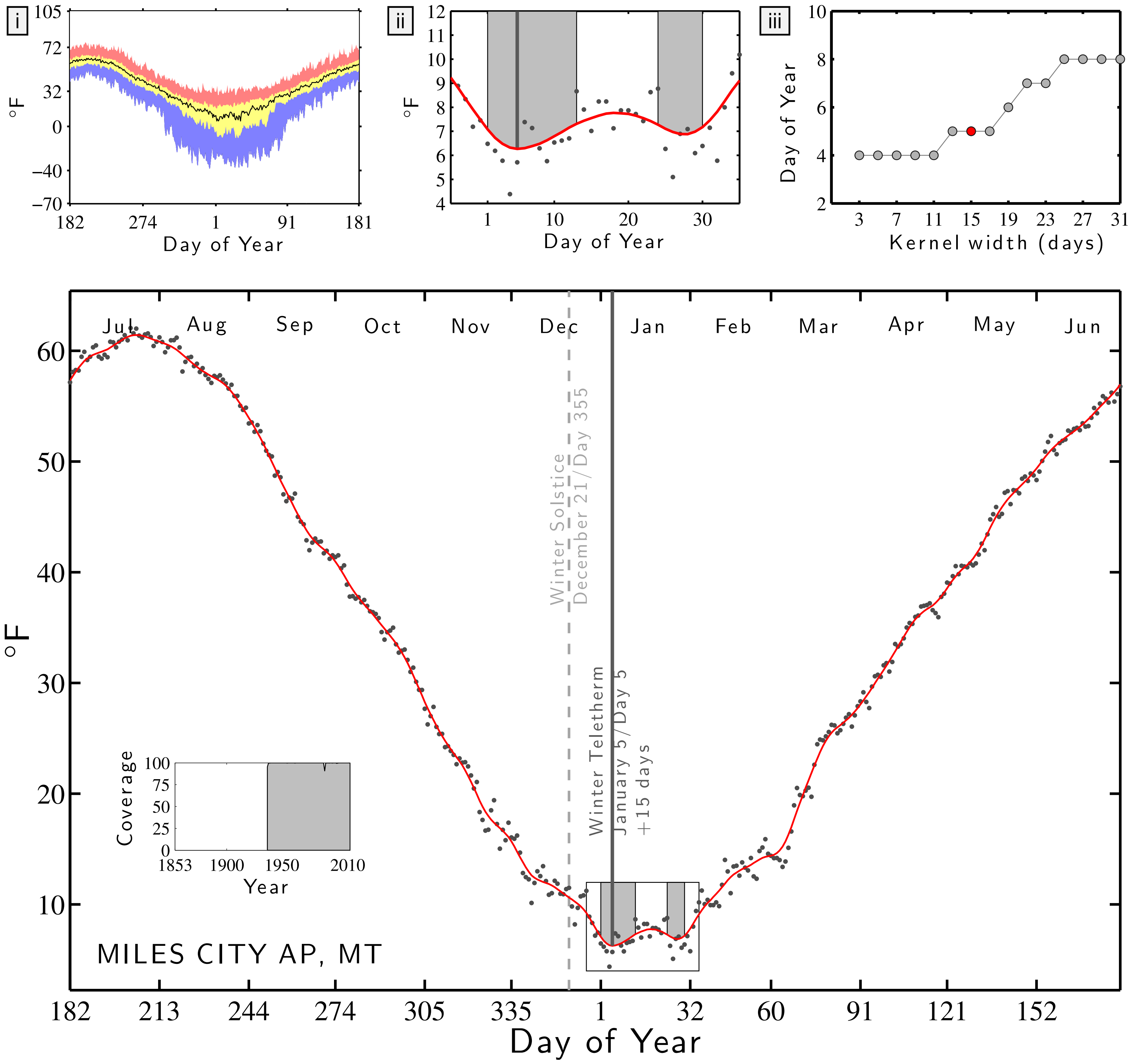

i
°F
Day of Year
ii
°F
Day of Year
iii
Day of Year
Kernel width (days)
Jul
Aug
Sep
Oct
Nov
Dec
Jan
Feb
Mar
Apr
May
Jun
Winter Solstice
December 21/Day 355
Winter Teletherm
January 5/Day 5
+15 days
Coverage
Year
MILES CITY AP, MT
°F
Day of Year

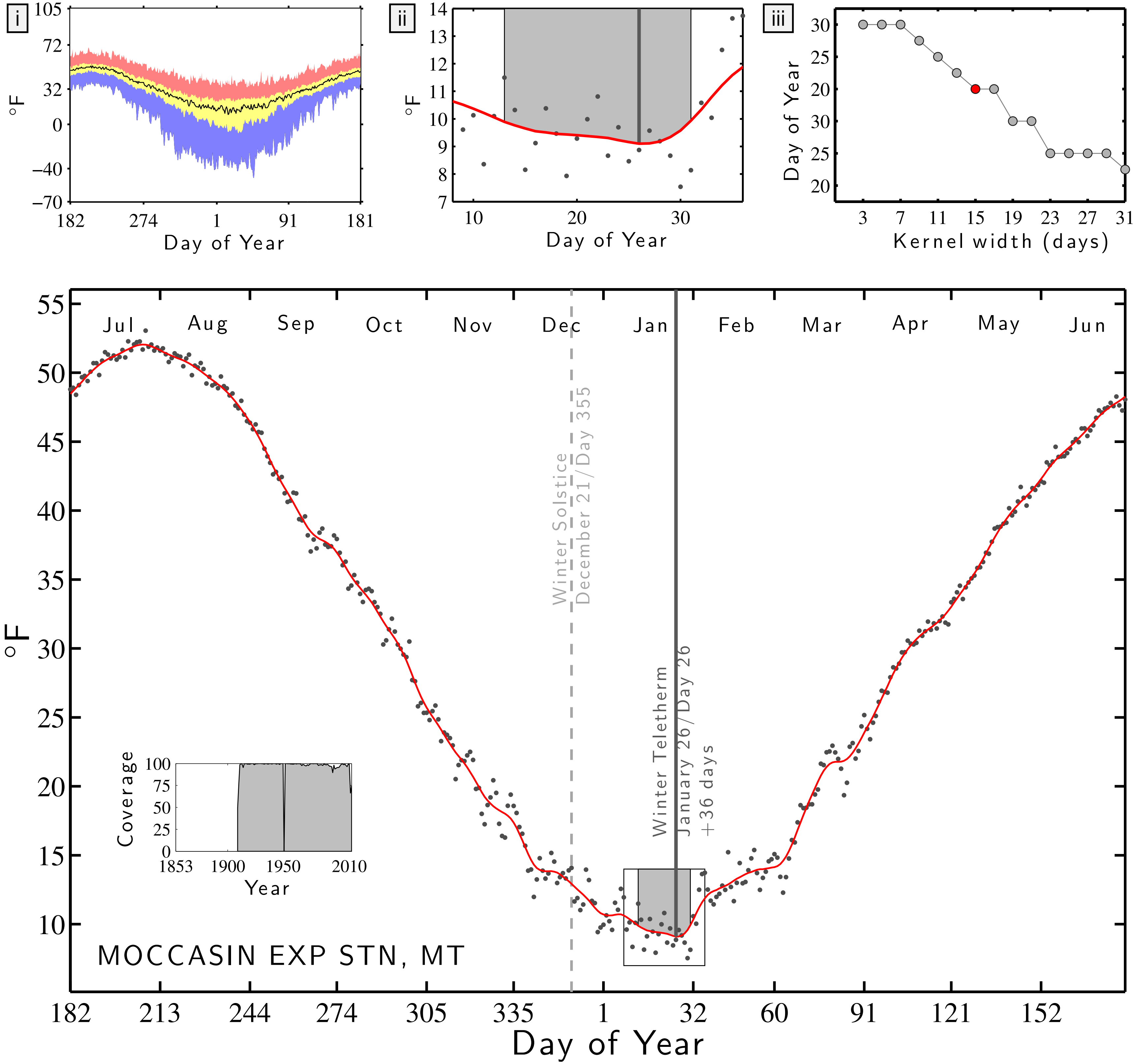

i
°F
Day of Year
ii
°F
Day of Year
iii
Day of Year
Kernel width (days)
Jul
Aug
Sep
Oct
Nov
Dec
Jan
Feb
Mar
Apr
May
Jun
Winter Solstice
December 21/Day 355
Winter Teletherm
January 26/Day 26
+36 days
Coverage
Year
MOCCASIN EXP STN, MT
°F
Day of Year

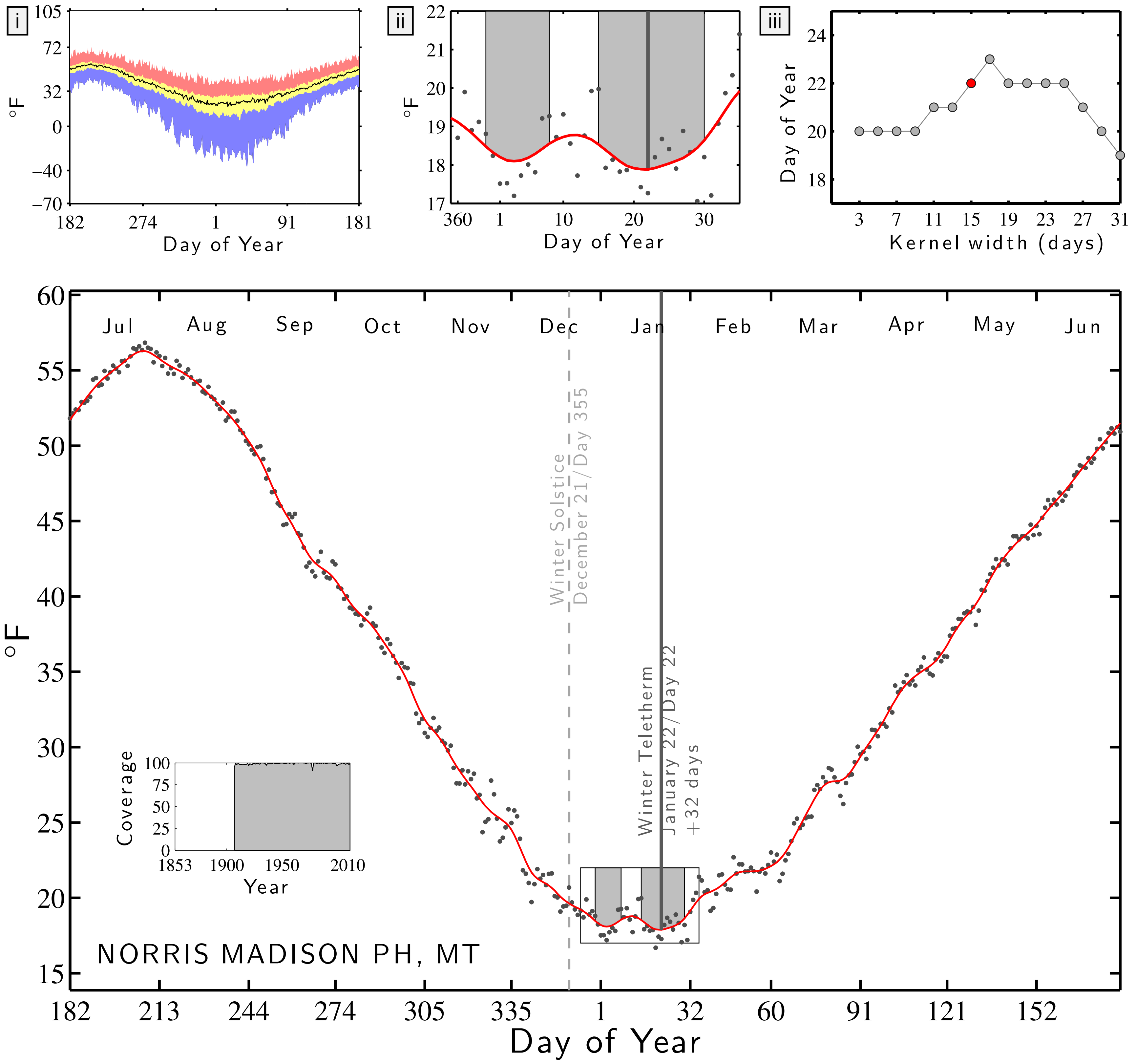

i
ii
iii
°F
Day of Year
Kernel width (days)
Jul
Aug
Sep
Oct
Nov
Dec
Jan
Feb
Mar
Apr
May
Jun
Winter Solstice
December 21/Day 355
Winter Teletherm
January 22/Day 22
+32 days
Coverage
Year
NORRIS MADISON PH, MT

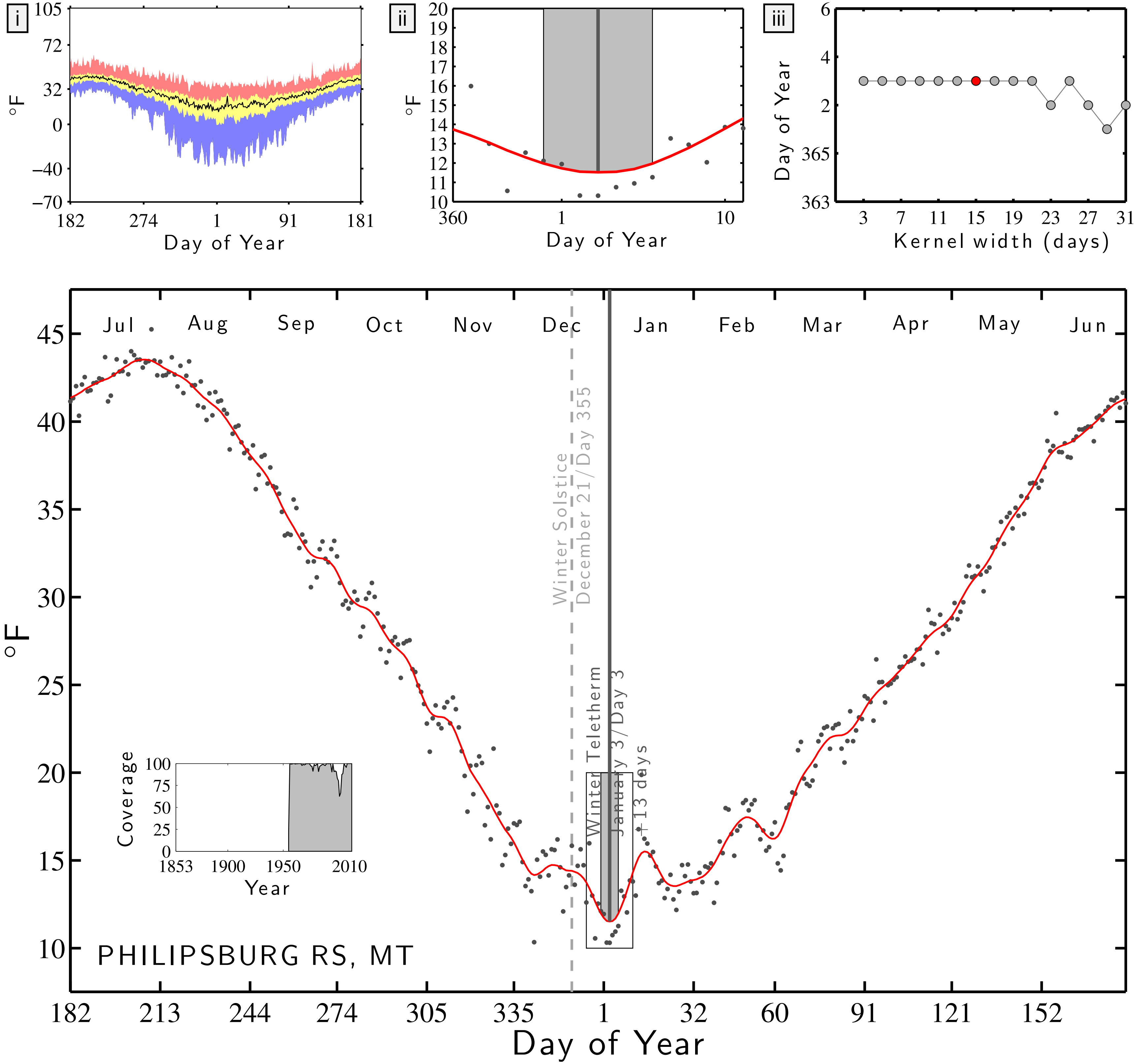

i
105
72
32
0
−40
−70
°F
182
274
1
91
181
Day of Year
ii
20
10
°F
360
1
10
Day of Year
iii
6
4
2
365
363
Day of Year
3
7
11
15
19
23
27
31
Kernel width (days)
Jul
Aug
Sep
Oct
Nov
Dec
Jan
Feb
Mar
Apr
May
Jun
Winter Solstice
December 21/Day 355
Winter Teletherm
January 3/Day 3
+13 days
Coverage
100
75
50
25
0
1853
1900
1950
2010
Year
PHILIPSBURG RS, MT
45
40
35
30
25
20
15
10
°F
182
213
244
274
305
335
1
32
60
91
121
152
Day of Year

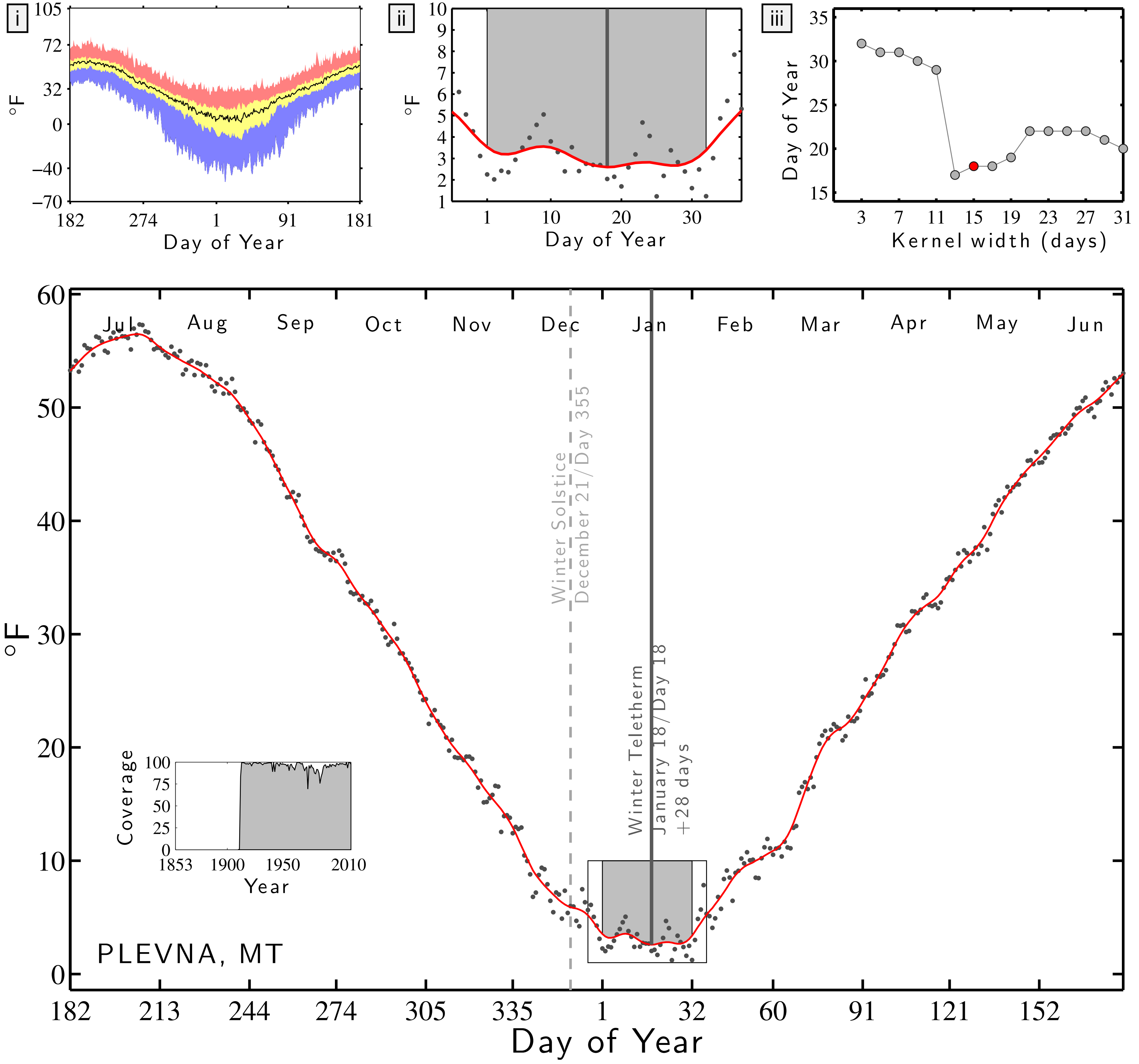

i
°F
Day of Year
ii
Day of Year
iii
Day of Year
Kernel width (days)
Jul
Aug
Sep
Oct
Nov
Dec
Jan
Feb
Mar
Apr
May
Jun
Winter Solstice
December 21/Day 355
Winter Teletherm
January 18/Day 18
+28 days
Coverage
Year
PLEVNA, MT
Day of Year

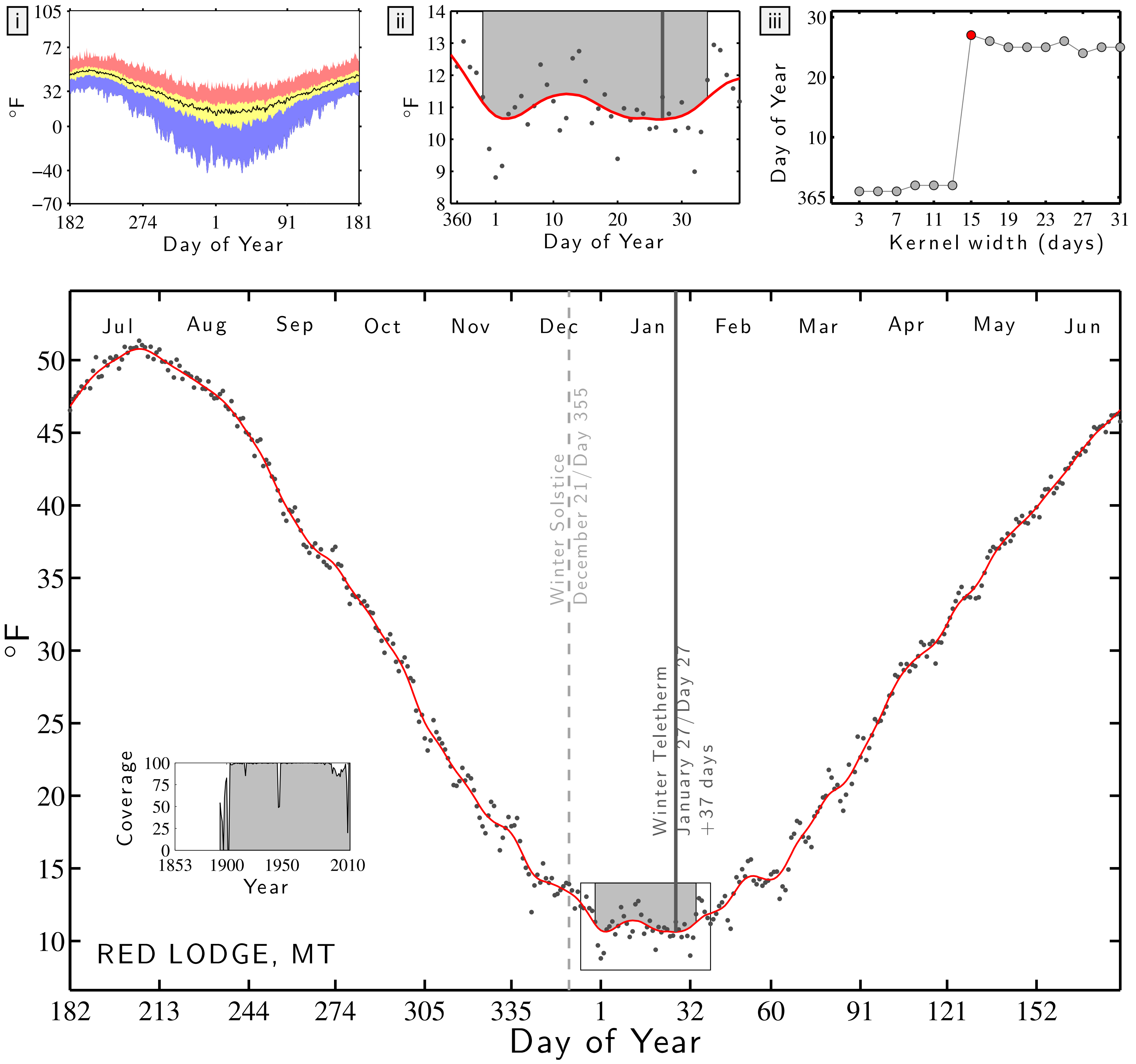

i
°F
Day of Year
ii
°F
Day of Year
iii
Day of Year
Kernel width (days)
Jul
Aug
Sep
Oct
Nov
Dec
Jan
Feb
Mar
Apr
May
Jun
Winter Solstice
December 21/Day 355
Winter Teletherm
January 27/Day 27
+37 days
Coverage
Year
RED LODGE, MT
°F
Day of Year

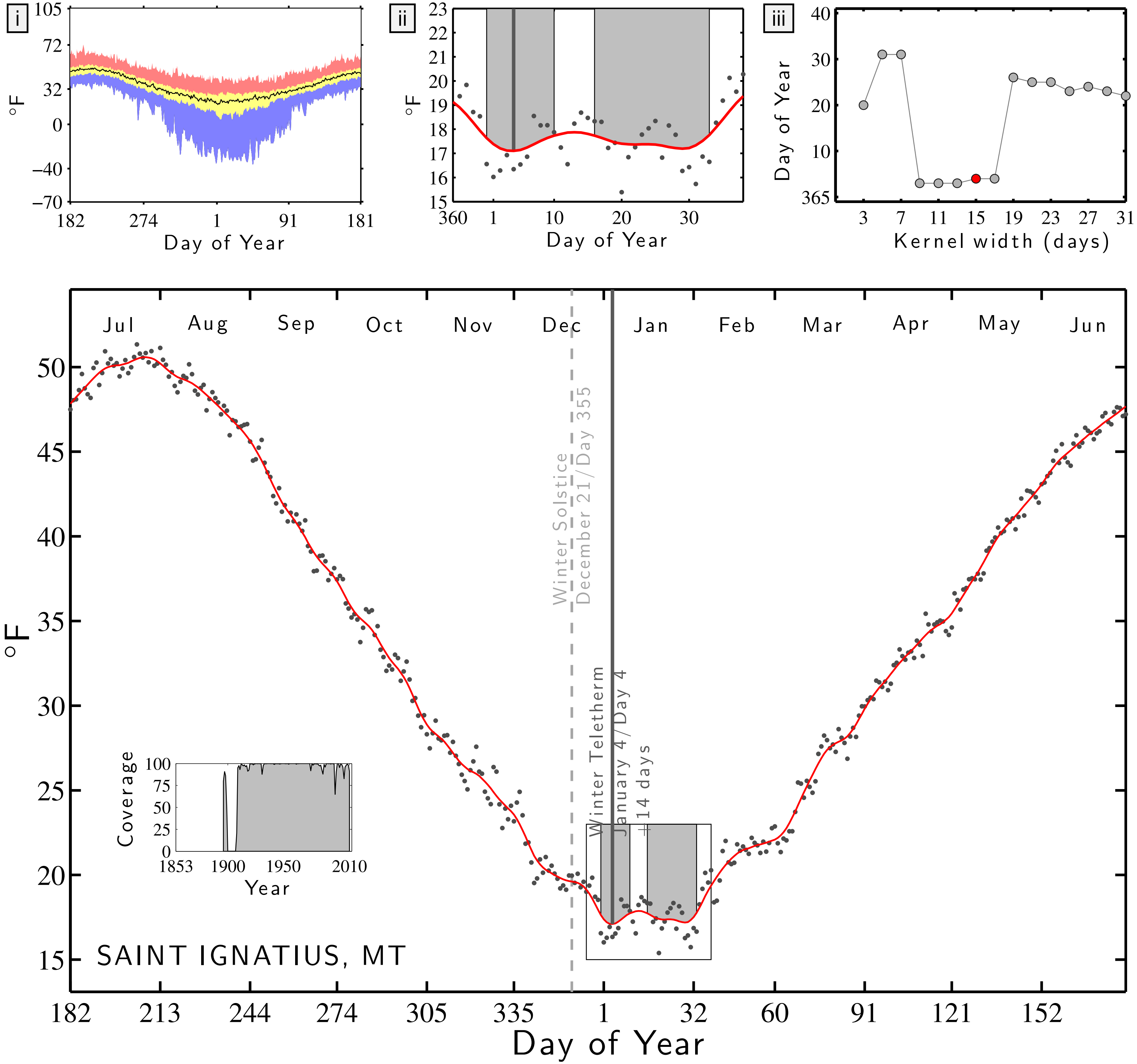

i
°F
Day of Year
ii
°F
Day of Year
iii
Day of Year
Kernel width (days)
Jul
Aug
Sep
Oct
Nov
Dec
Jan
Feb
Mar
Apr
May
Jun
Winter Solstice
December 21/Day 355
Winter Teletherm
January 4/Day 4
±14 days
Coverage
Year
SAINT IGNATIUS, MT
°F
Day of Year

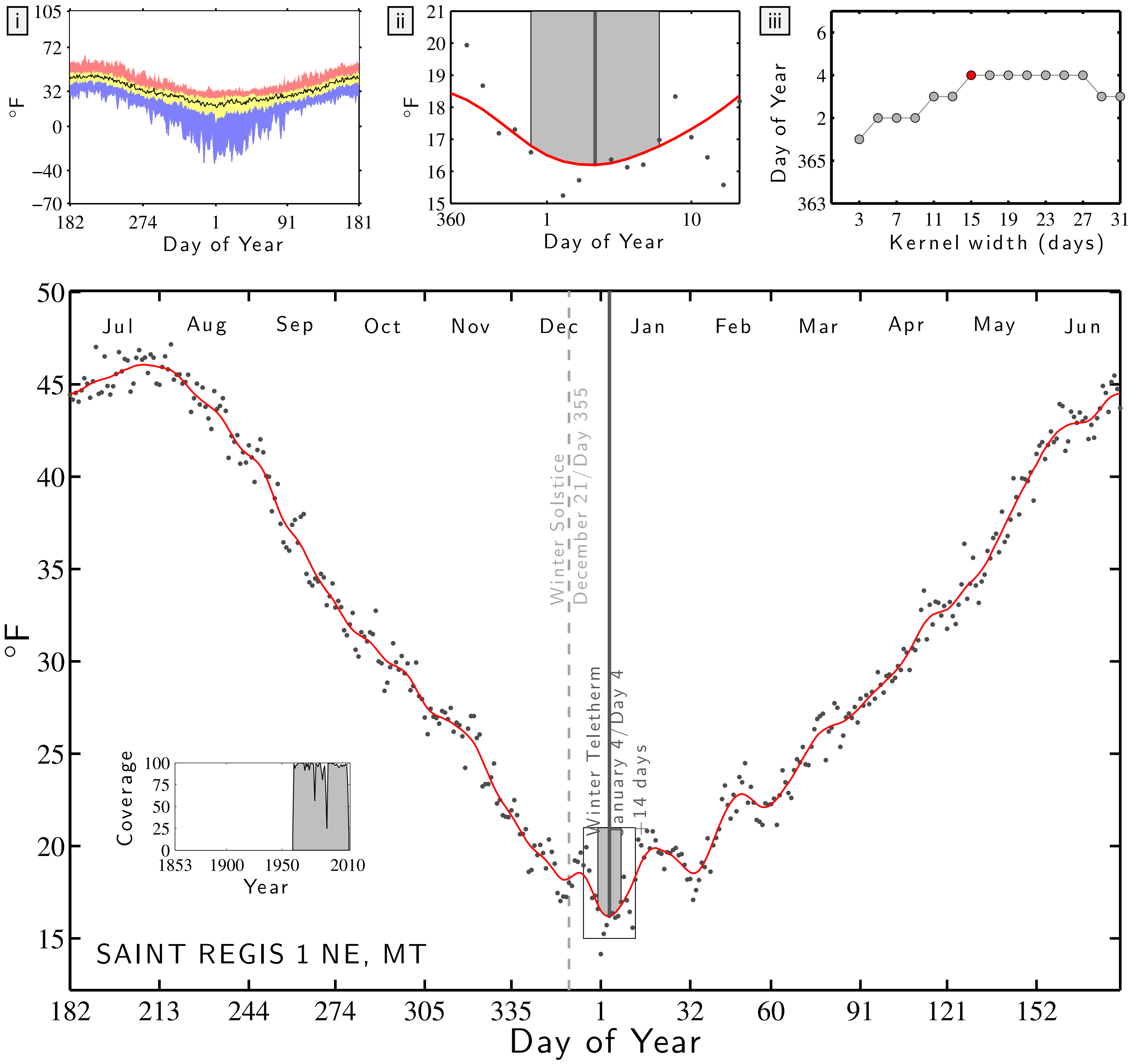

i
°F
Day of Year
ii
°F
Day of Year
iii
Day of Year
Kernel width (days)
Jul
Aug
Sep
Oct
Nov
Dec
Jan
Feb
Mar
Apr
May
Jun
Winter Solstice
December 21/Day 355
Winter Teletherm
January 4/Day 4
±14 days
Coverage
Year
SAINT REGIS 1 NE, MT
°F
Day of Year

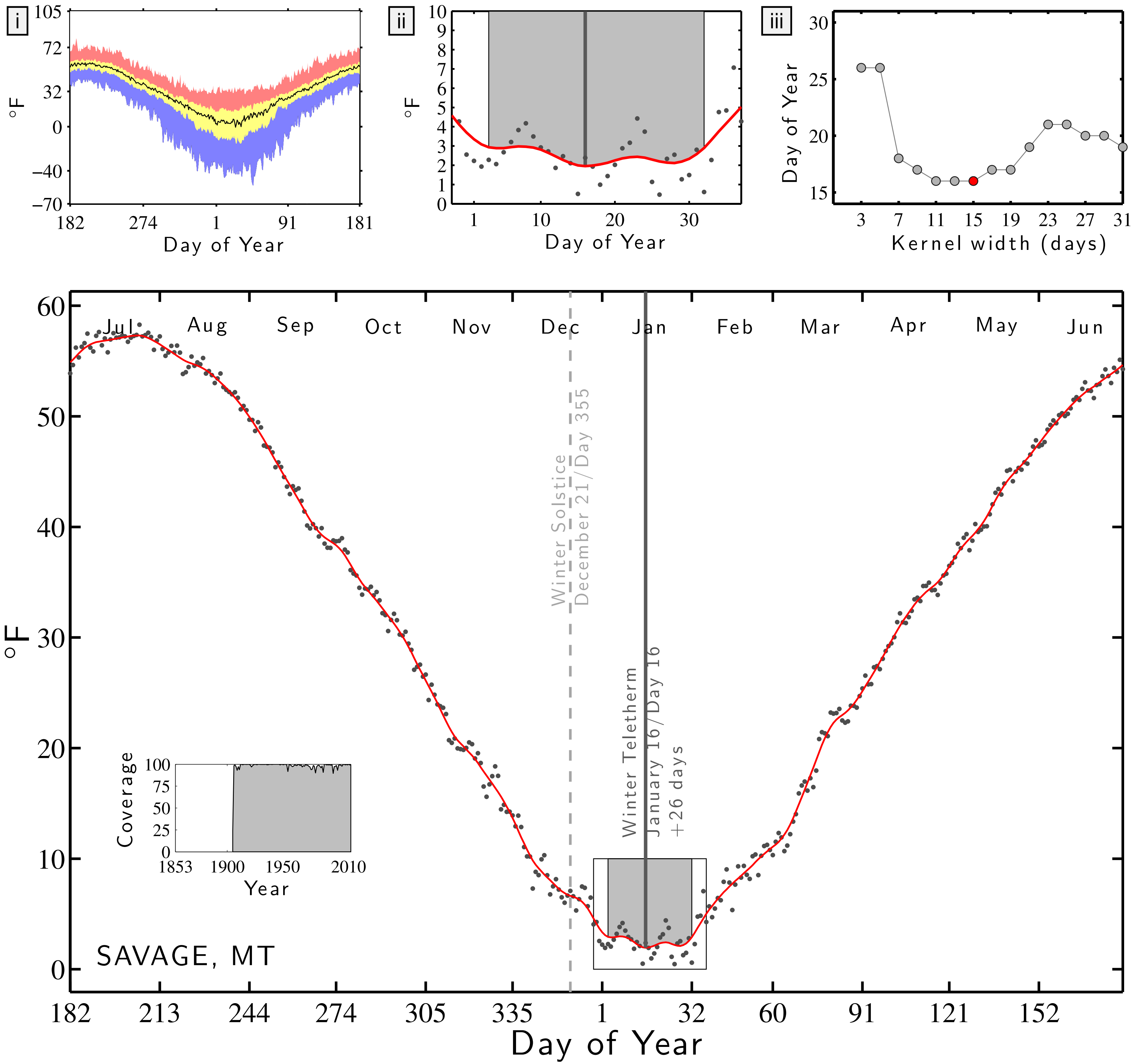

i
°F
Day of Year
ii
°F
Day of Year
iii
Day of Year
Kernel width (days)
Jul
Aug
Sep
Oct
Nov
Dec
Jan
Feb
Mar
Apr
May
Jun
Winter Solstice
December 21/Day 355
Winter Teletherm
January 16/Day 16
+26 days
Coverage
Year
SAVAGE, MT
°F
Day of Year

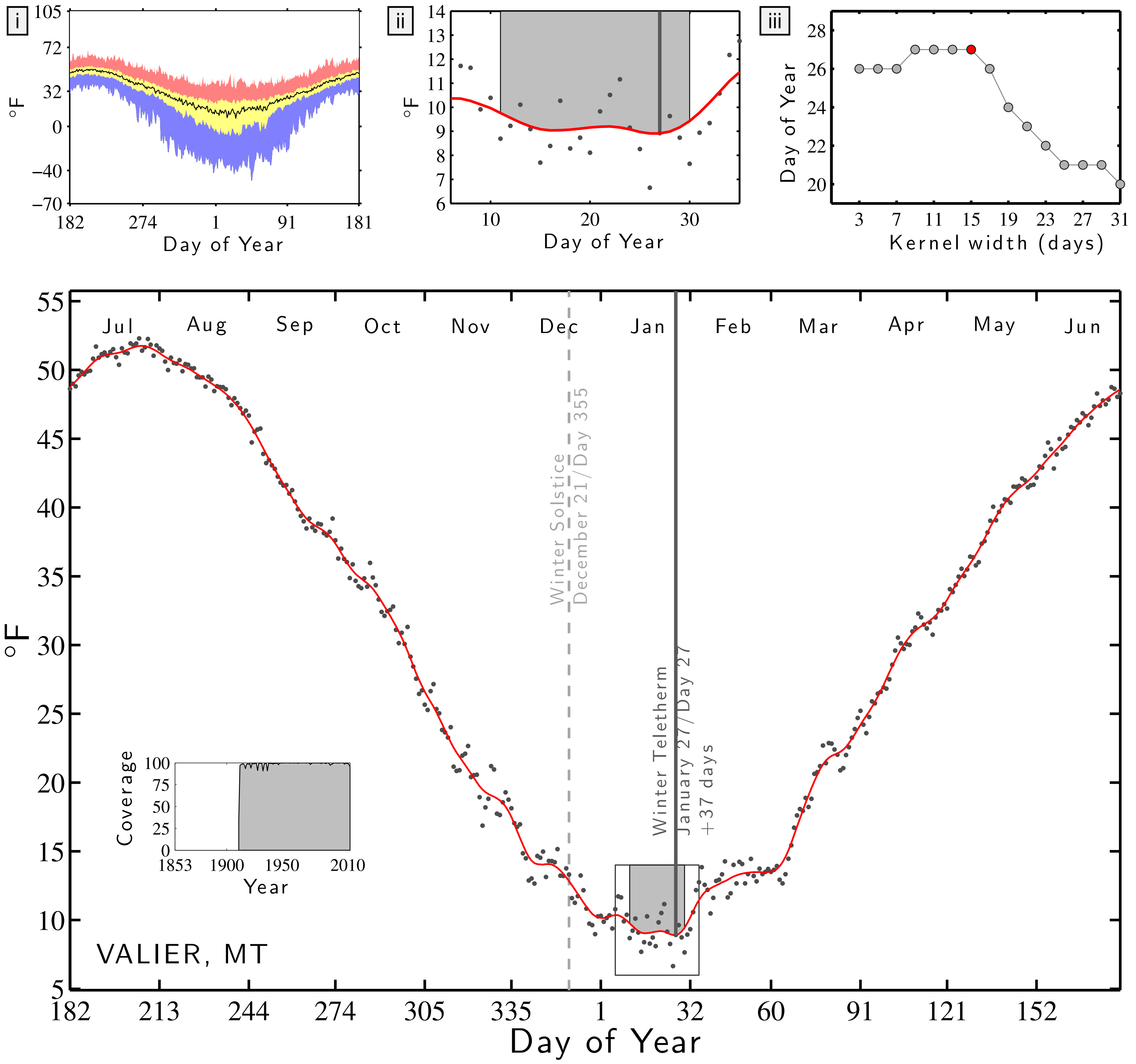

i
°F
Day of Year
ii
°F
Day of Year
iii
Day of Year
Kernel width (days)
Jul
Aug
Sep
Oct
Nov
Dec
Jan
Feb
Mar
Apr
May
Jun
Winter Solstice
December 21/Day 355
Winter Teletherm
January 27/Day 27
+37 days
Coverage
Year
VALIER, MT
°F
Day of Year

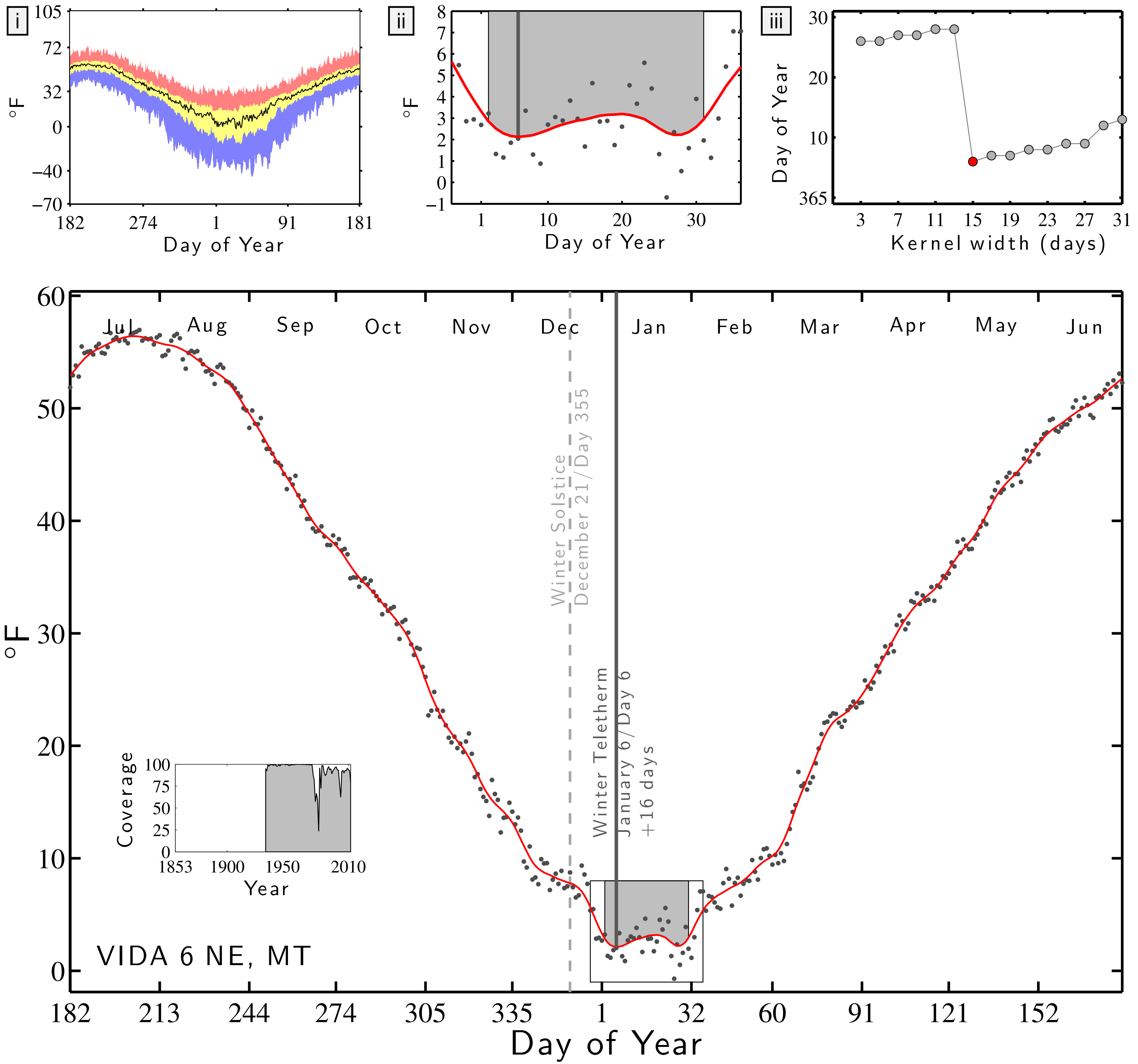

i
°F
Day of Year
ii
°F
Day of Year
iii
Day of Year
Kernel width (days)
Jul
Aug
Sep
Oct
Nov
Dec
Jan
Feb
Mar
Apr
May
Jun
Winter Solstice
December 21/Day 355
Winter Teletherm
January 6/Day 6
+16 days
Coverage
Year
VIDA 6 NE, MT
°F
Day of Year

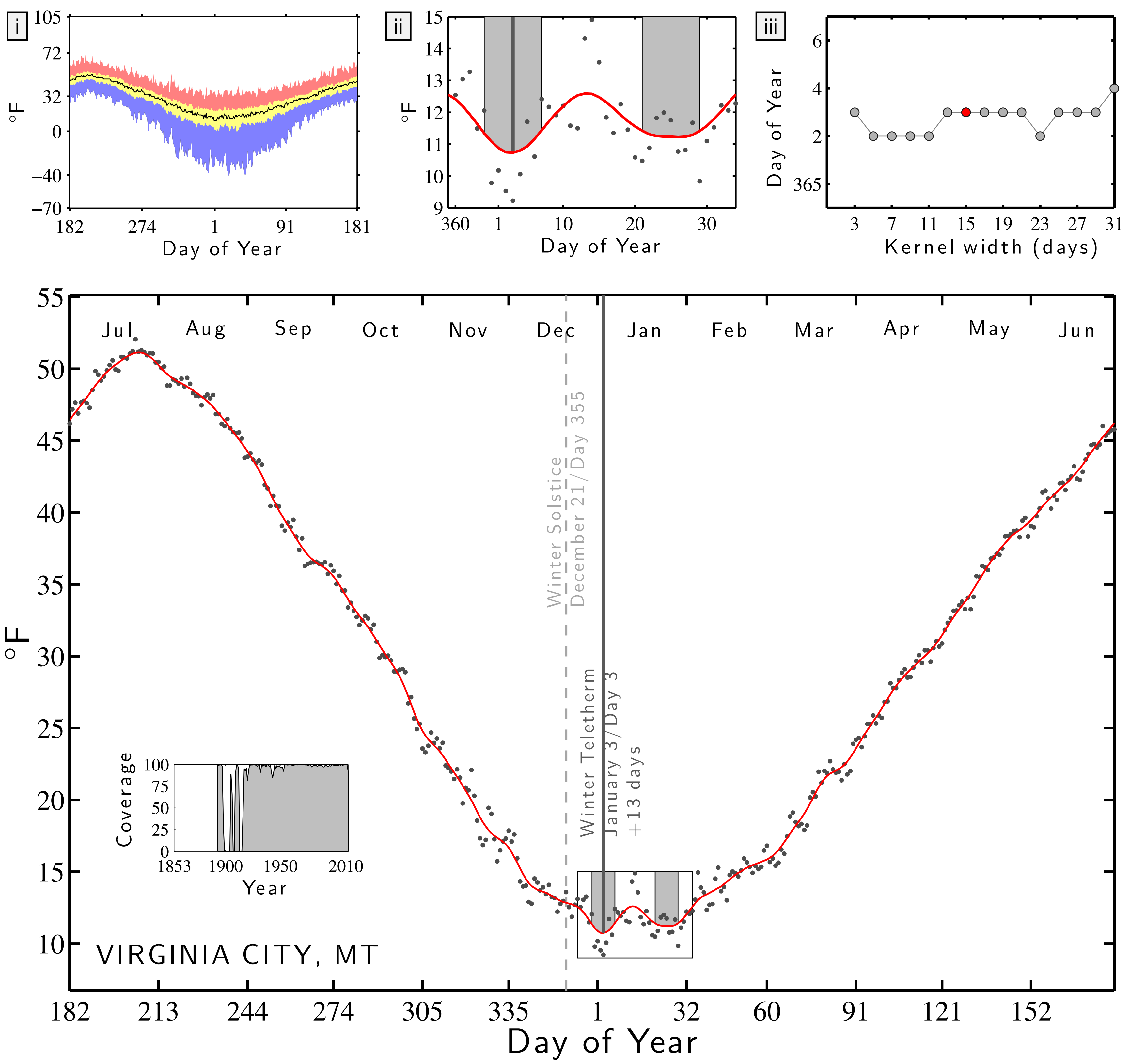

i
°F
105
72
32
0
−40
−70
182
274
1
91
181
Day of Year
ii
15
14
13
12
11
10
9
360
1
10
20
30
iii
6
4
2
365
Day of Year
3
7
11
15
19
23
27
31
Kernel width (days)
55
50
45
40
35
30
25
20
15
10
Jul
Aug
Sep
Oct
Nov
Dec
Jan
Feb
Mar
Apr
May
Jun
Winter Solstice
December 21/Day 355
Winter Teletherm
January 3/Day 3
+13 days
Coverage
100
75
50
25
0
1853
1900
1950
2010
Year
VIRGINIA CITY, MT
182
213
244
274
305
335
1
32
60
91
121
152
Day of Year

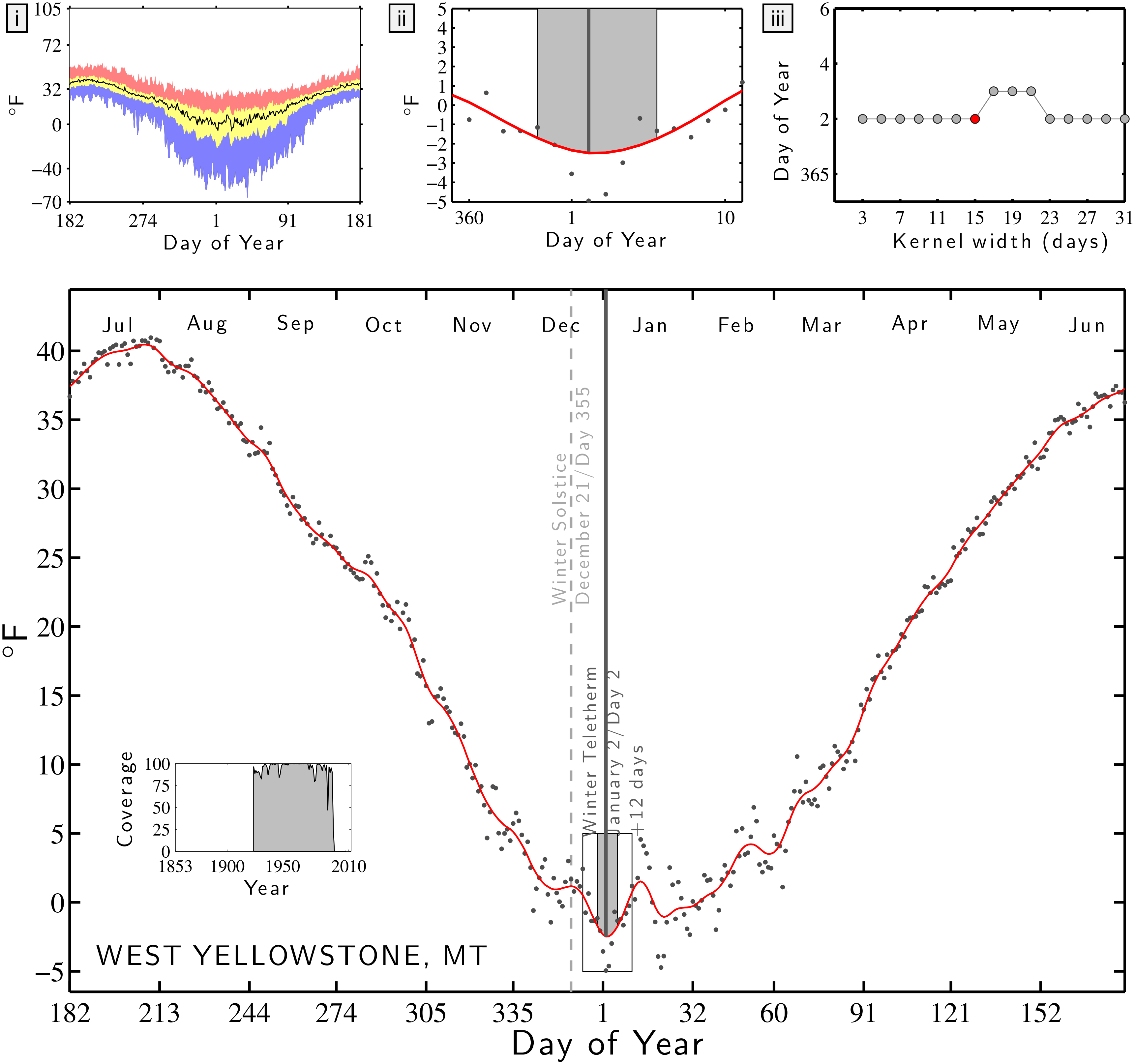

i
°F
Day of Year
ii
°F
Day of Year
iii
Day of Year
Kernel width (days)
Jul
Aug
Sep
Oct
Nov
Dec
Jan
Feb
Mar
Apr
May
Jun
Winter Solstice
December 21/Day 355
Winter Teletherm
January 2/Day 2
+12 days
Coverage
Year
WEST YELLOWSTONE, MT
°F
Day of Year

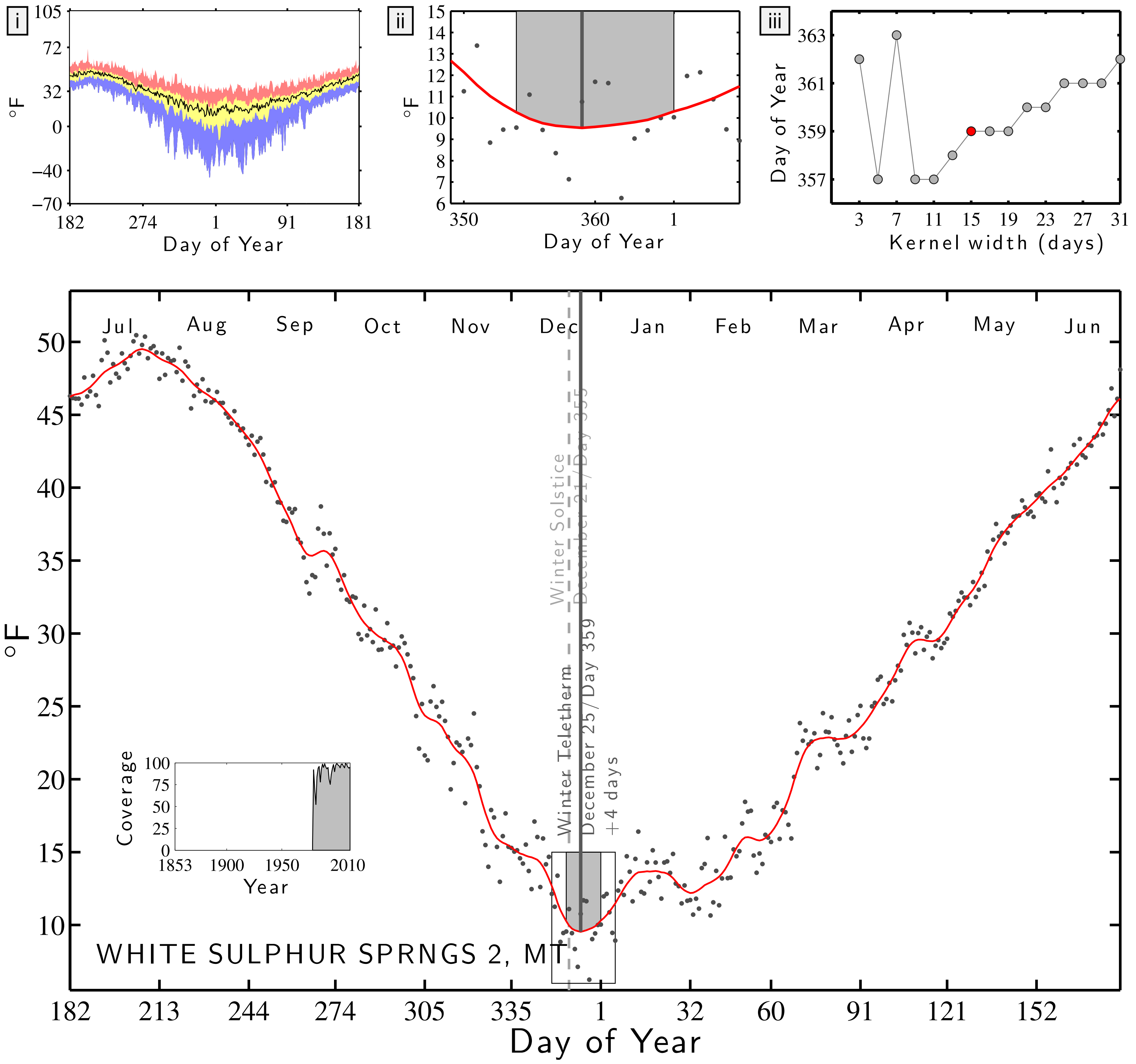
i
ii
iii
Day of Year
°F
Kernel width (days)
Jul
Aug
Sep
Oct
Nov
Dec
Jan
Feb
Mar
Apr
May
Jun
Winter Solstice
December 21/Day 355
Winter Teletherm
December 25/Day 359
+4 days
Coverage
Year
WHITE SULPHUR SPRNGS 2, MT

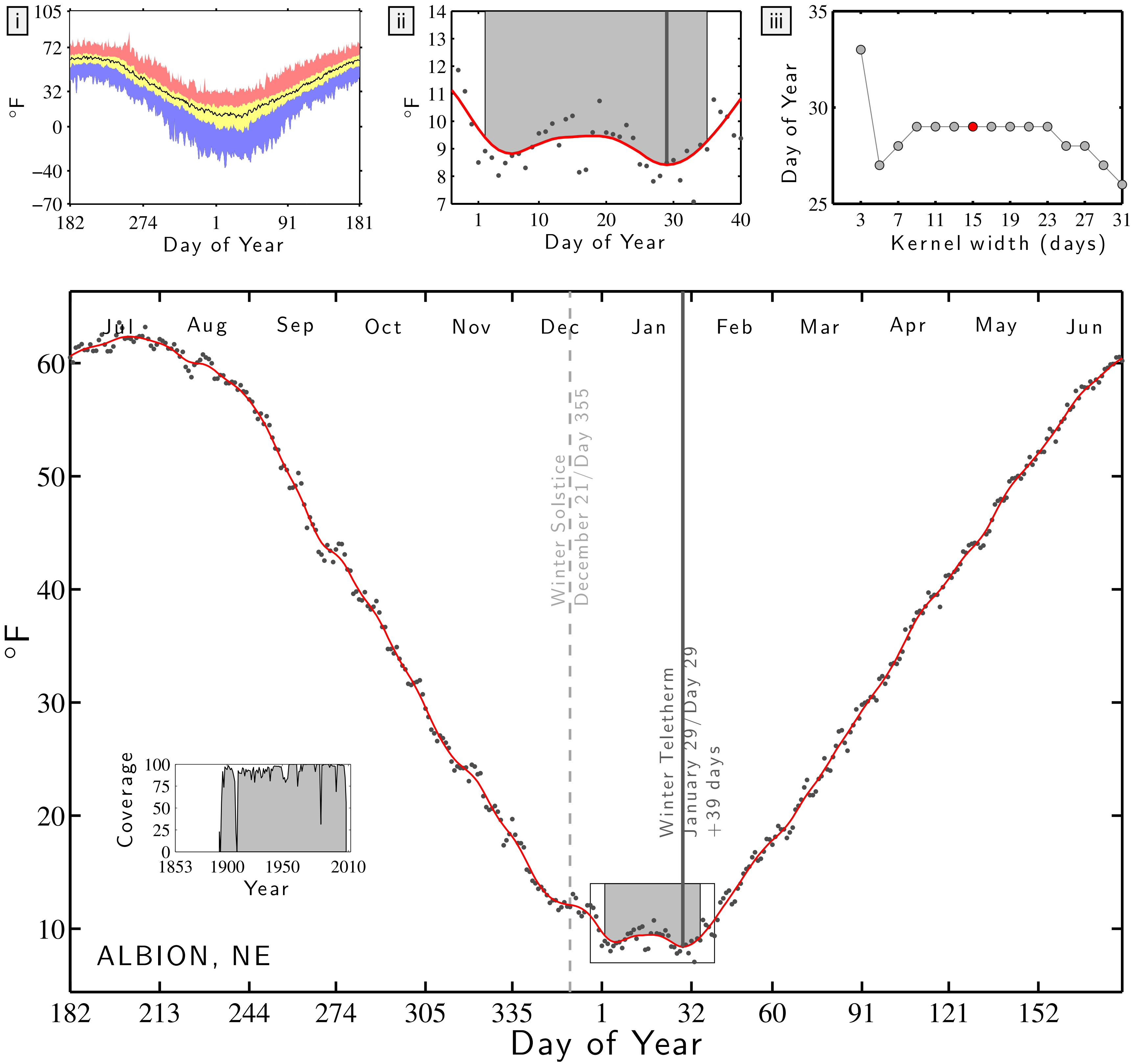

i
ii
iii
°F
Day of Year
Kernel width (days)
Jul
Aug
Sep
Oct
Nov
Dec
Jan
Feb
Mar
Apr
May
Jun
Winter Solstice
December 21/Day 355
Winter Teletherm
January 29/Day 29
+39 days
Coverage
Year
ALBION, NE

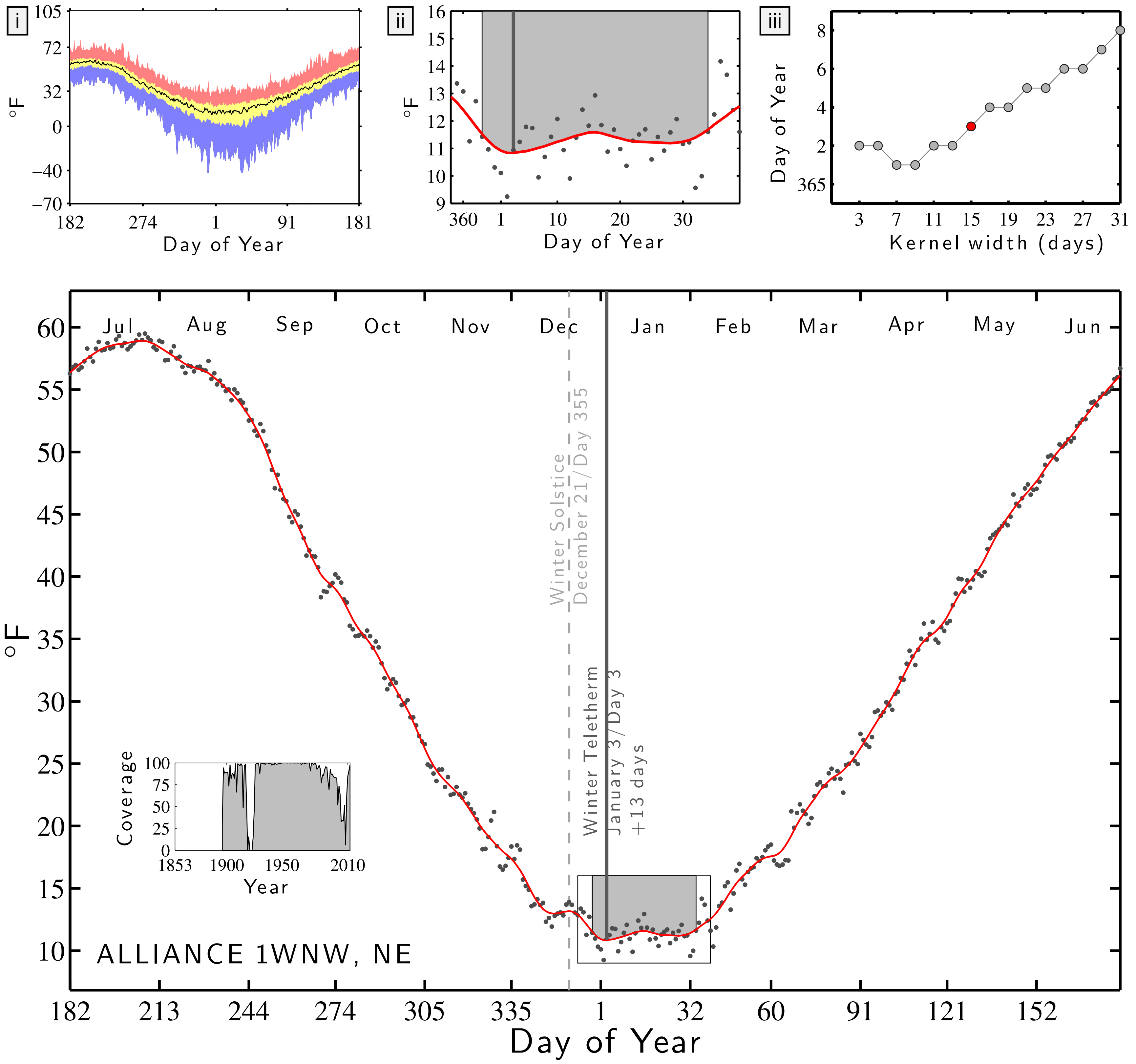

i
ii
iii
°F
Day of Year
Day of Year
Kernel width (days)
Jul
Aug
Sep
Oct
Nov
Dec
Jan
Feb
Mar
Apr
May
Jun
Winter Solstice
December 21/Day 355
Winter Teletherm
January 3/Day 3
+13 days
Coverage
Year
ALLIANCE 1WNW, NE

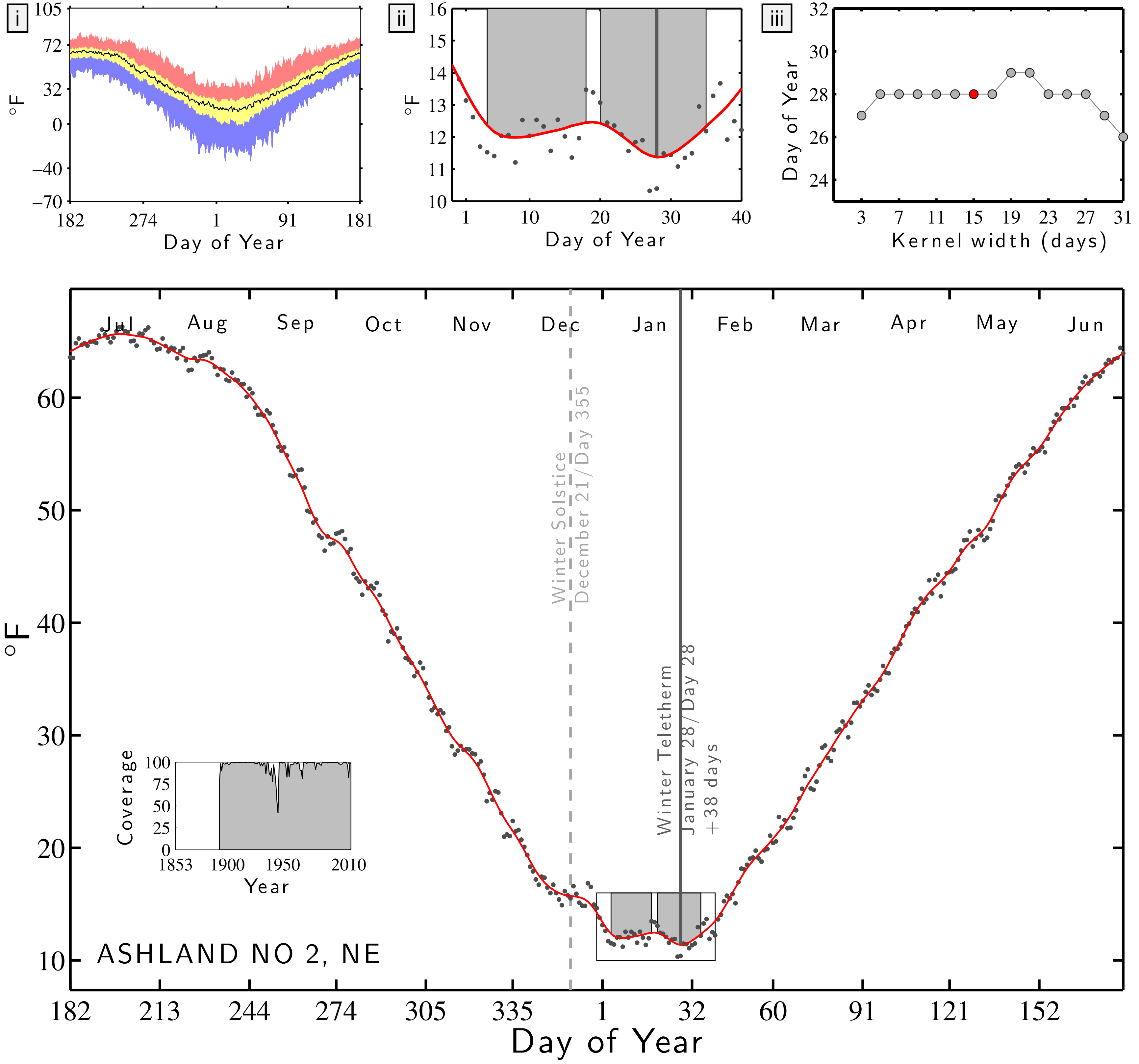

i
ii
iii
°F
Day of Year
Kernel width (days)
Jul
Aug
Sep
Oct
Nov
Dec
Jan
Feb
Mar
Apr
May
Jun
Winter Solstice
December 21/Day 355
Winter Teletherm
January 28/Day 28
+38 days
Coverage
Year
ASHLAND NO 2, NE

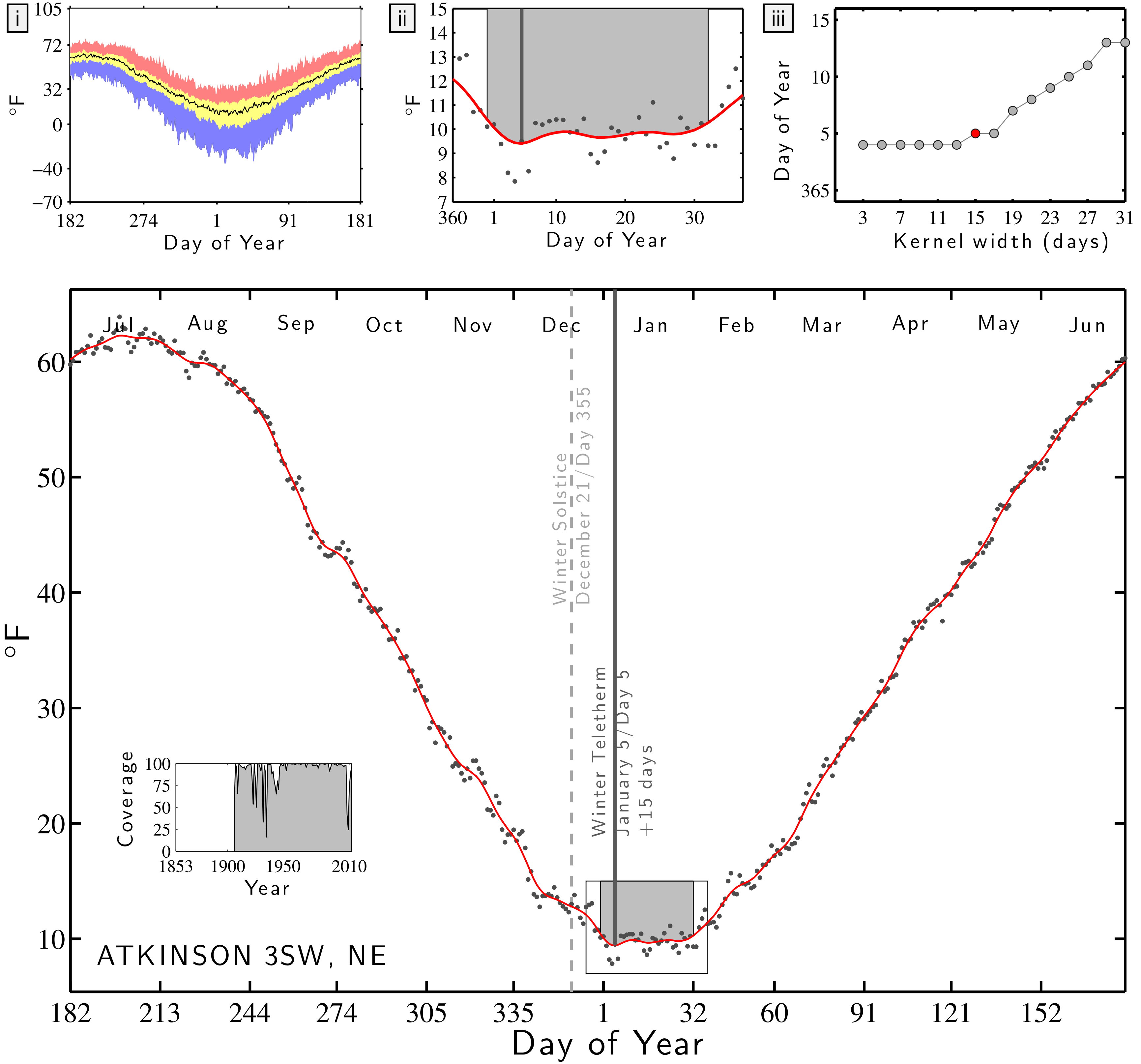

i
°F
Day of Year
ii
°F
Day of Year
iii
Day of Year
Kernel width (days)
Jul
Aug
Sep
Oct
Nov
Dec
Jan
Feb
Mar
Apr
May
Jun
Winter Solstice
December 21/Day 355
Winter Teletherm
January 5/Day 5
+15 days
Coverage
Year
ATKINSON 3SW, NE
°F
Day of Year

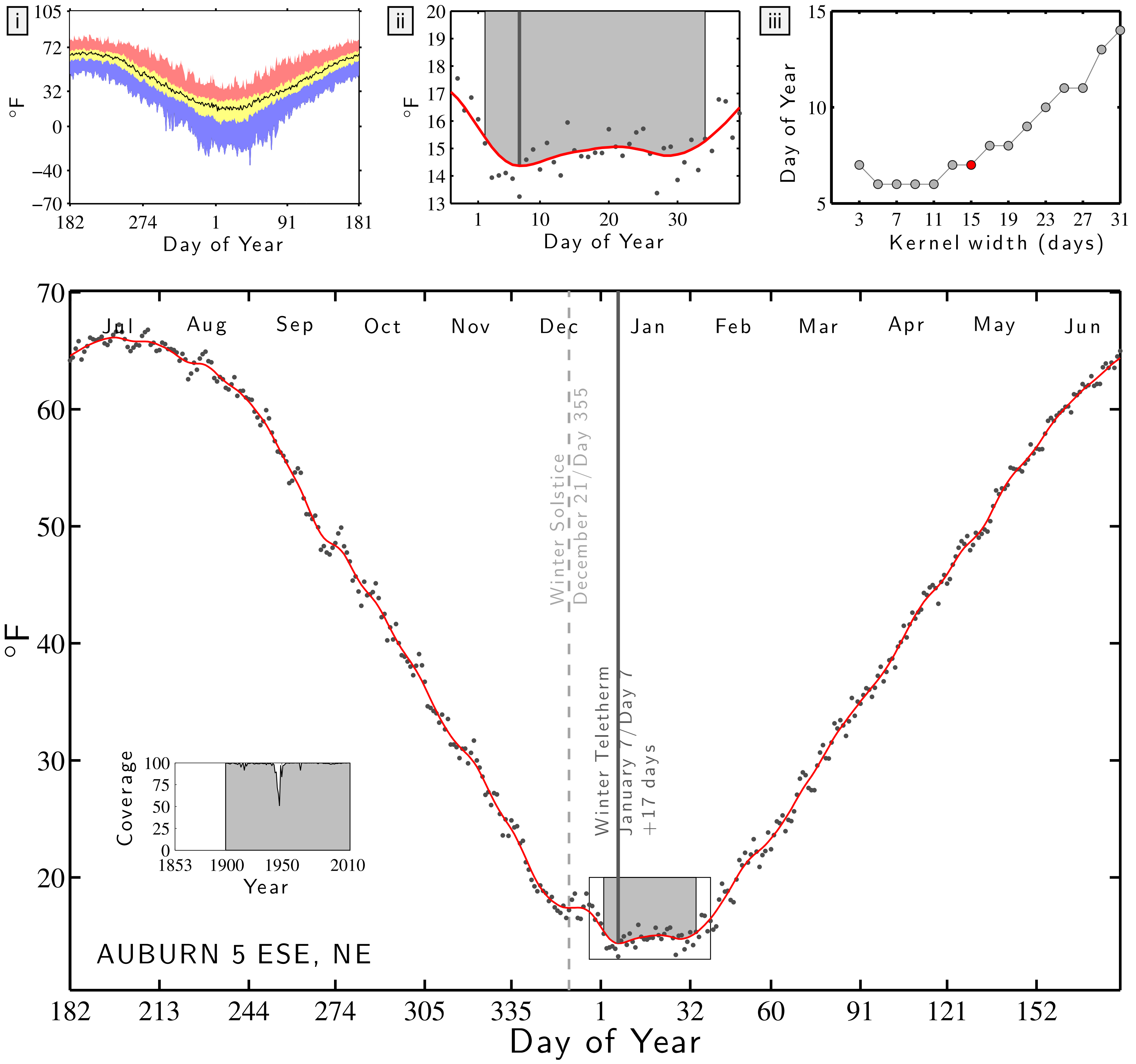

i
°F
Day of Year
ii
°F
Day of Year
iii
Day of Year
Kernel width (days)
Jul
Aug
Sep
Oct
Nov
Dec
Jan
Feb
Mar
Apr
May
Jun
Winter Solstice
December 21/Day 355
Winter Teletherm
January 7/Day 7
+17 days
Coverage
Year
AUBURN 5 ESE, NE
°F
Day of Year

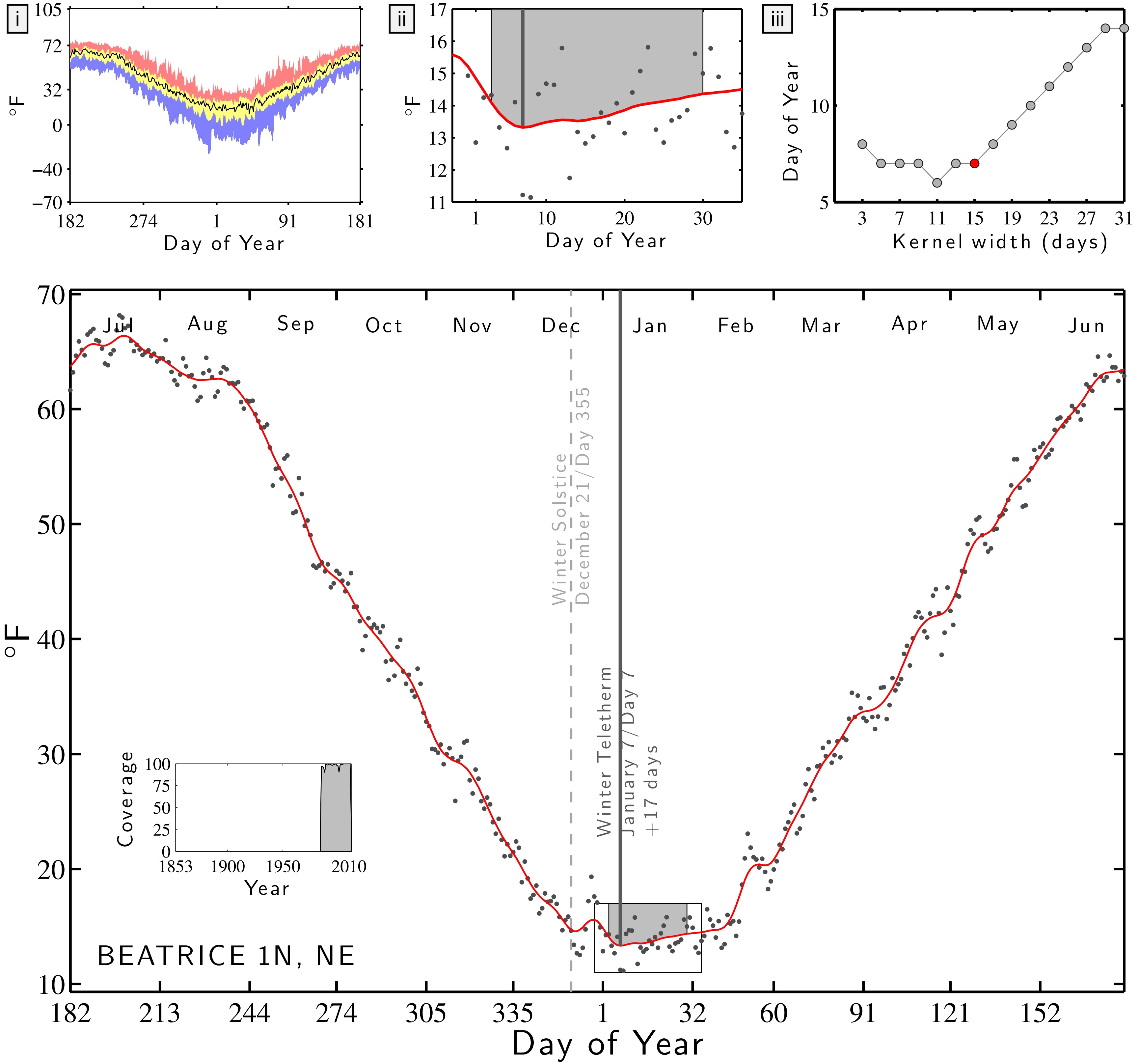

i
°F
Day of Year
ii
°F
Day of Year
iii
Day of Year
Kernel width (days)
Jul
Aug
Sep
Oct
Nov
Dec
Jan
Feb
Mar
Apr
May
Jun
Winter Solstice
December 21/Day 355
Winter Teletherm
January 7/Day 7
+17 days
Coverage
Year
BEATRICE 1N, NE
°F
Day of Year

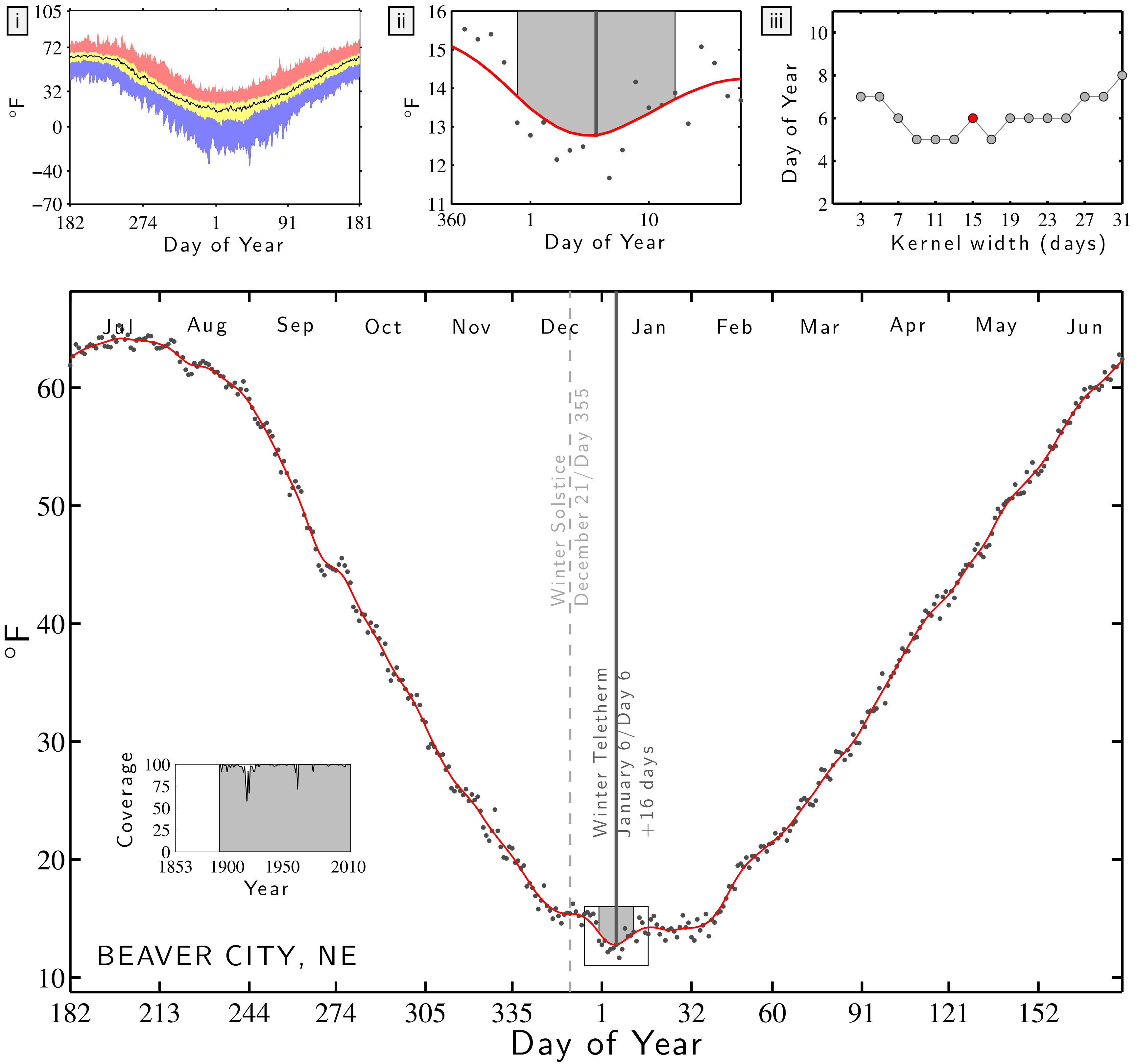

i
°F
Day of Year
ii
°F
Day of Year
iii
Day of Year
Kernel width (days)
Jul
Aug
Sep
Oct
Nov
Dec
Jan
Feb
Mar
Apr
May
Jun
Winter Solstice
December 21/Day 355
Winter Teletherm
January 6/Day 6
+16 days
Coverage
Year
BEAVER CITY, NE
°F
Day of Year

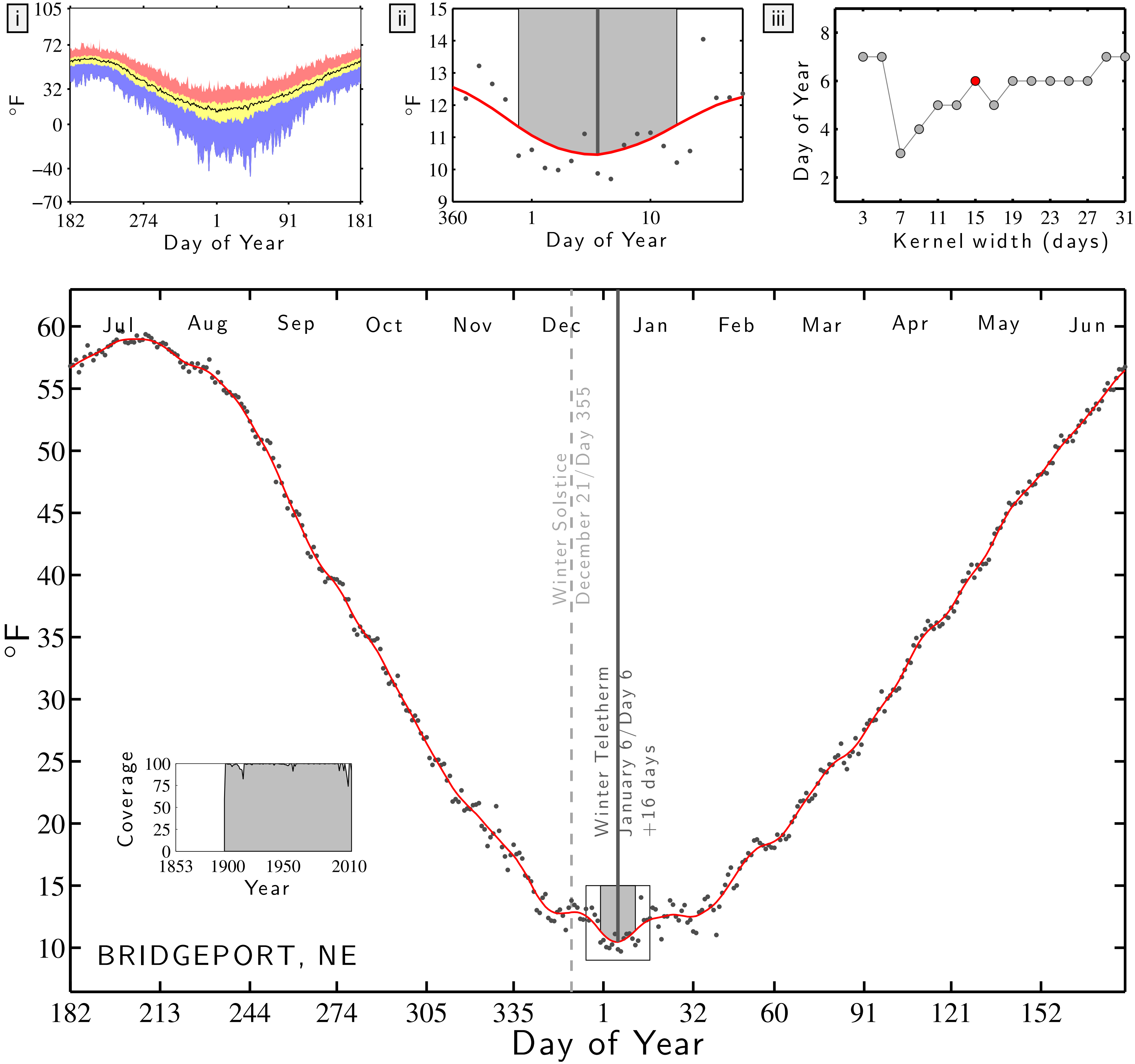

i
ii
iii
°F
Day of Year
Kernel width (days)
Winter Solstice
December 21/Day 355
Winter Teletherm
January 6/Day 6
+16 days
Coverage
Year
BRIDGEPORT, NE
Jul
Aug
Sep
Oct
Nov
Dec
Jan
Feb
Mar
Apr
May
Jun

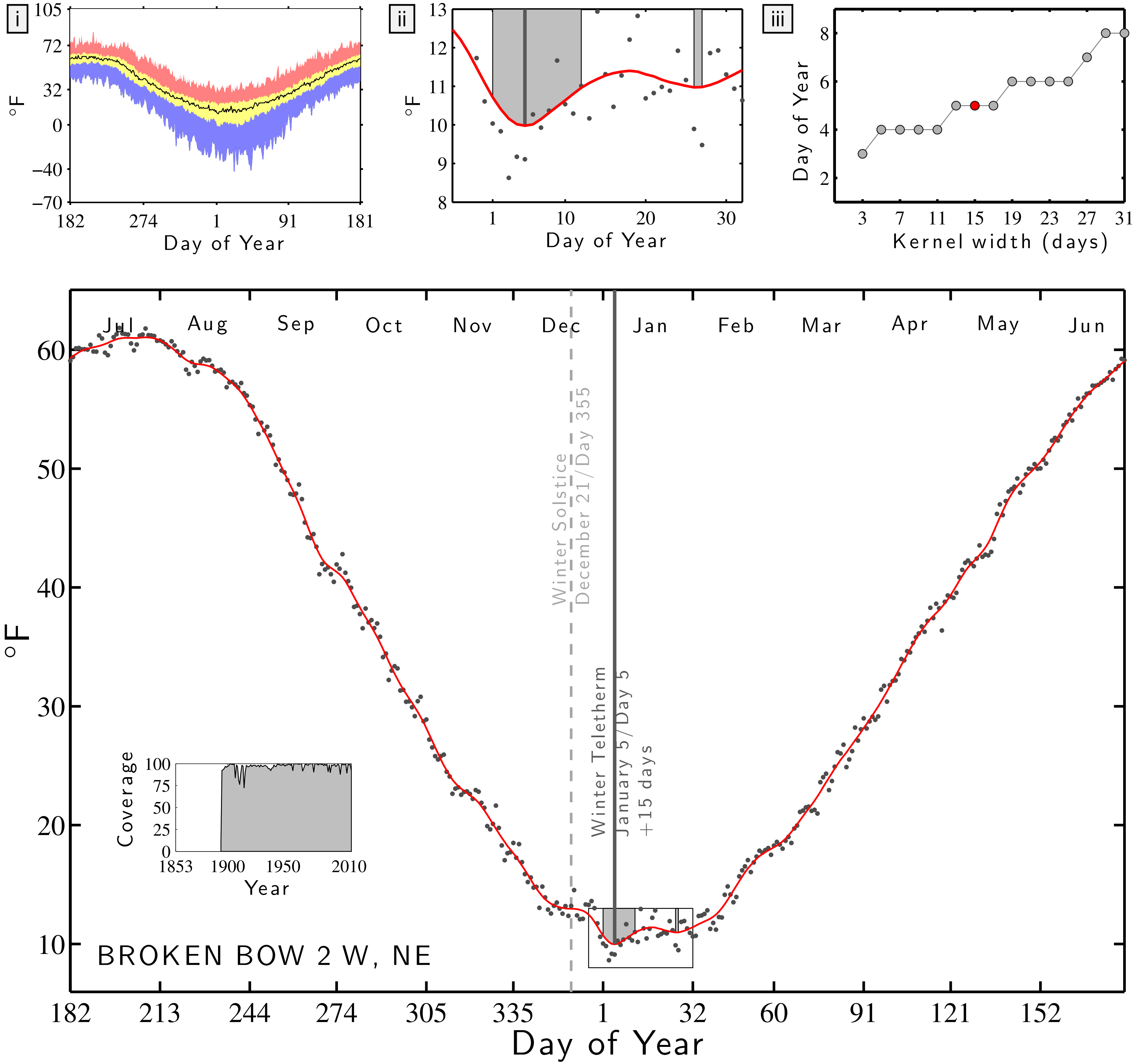

i
°F
Day of Year
ii
°F
Day of Year
iii
Day of Year
Kernel width (days)
Jul
Aug
Sep
Oct
Nov
Dec
Jan
Feb
Mar
Apr
May
Jun
Winter Solstice
December 21/Day 355
Winter Teletherm
January 5/Day 5
+15 days
Coverage
Year
BROKEN BOW 2 W, NE
°F
Day of Year

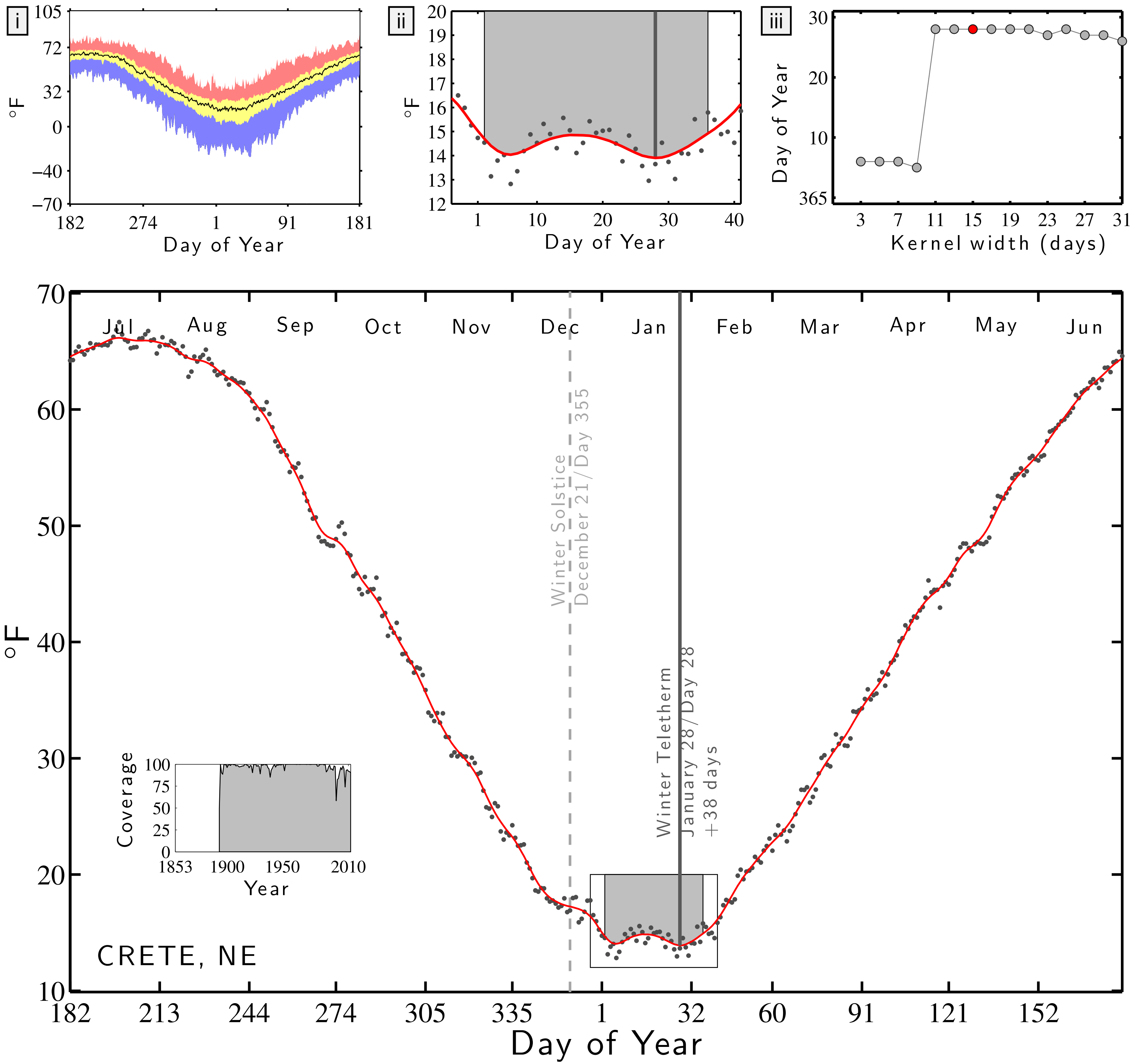
i
°F
Day of Year
ii
°F
Day of Year
iii
Day of Year
Kernel width (days)
Jul
Aug
Sep
Oct
Nov
Dec
Jan
Feb
Mar
Apr
May
Jun
Winter Solstice
December 21/Day 355
Winter Teletherm
January 28/Day 28
+38 days
Coverage
Year
CRETE, NE
°F
Day of Year

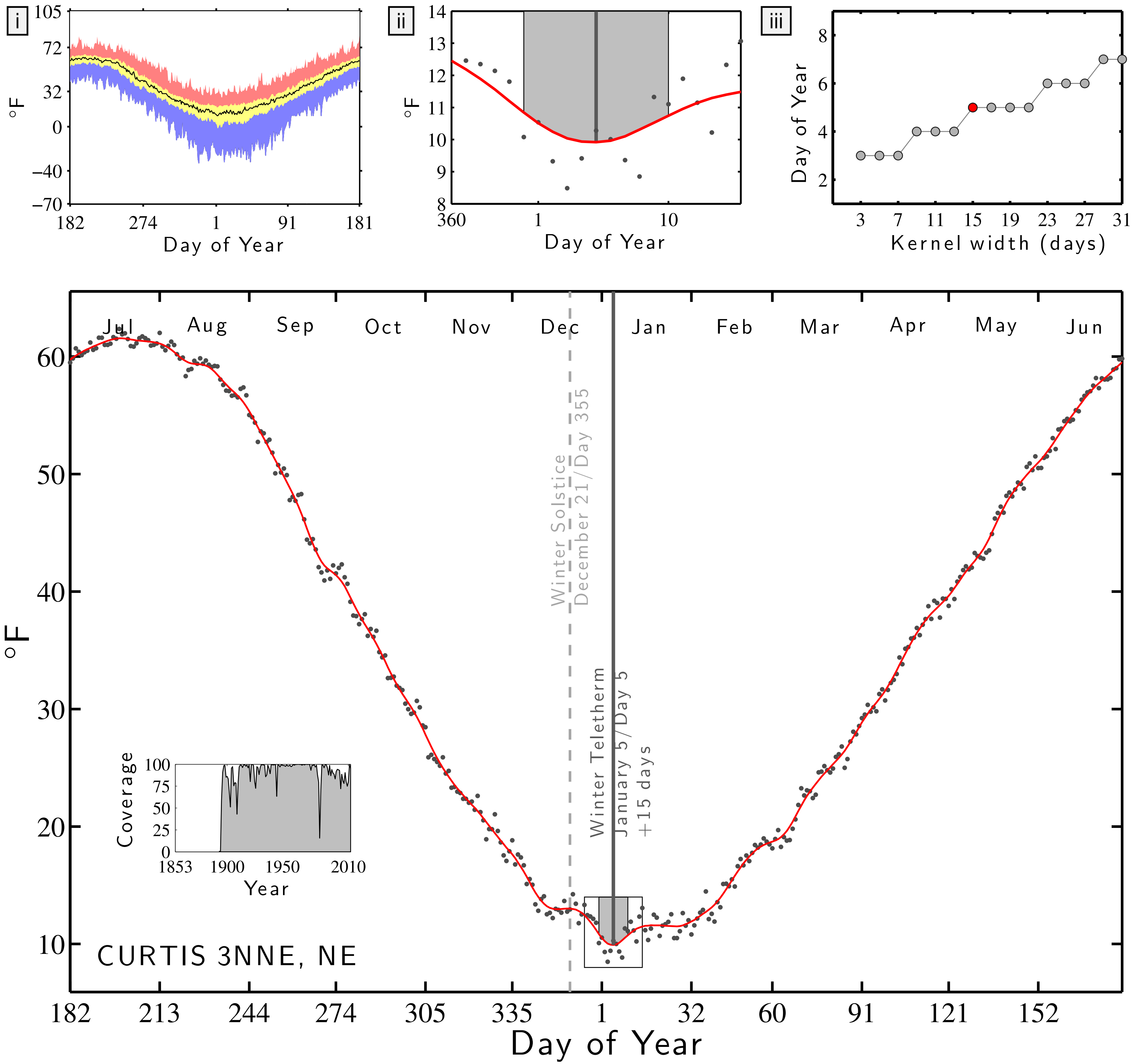

i
ii
iii
°F
Day of Year
Day of Year
Kernel width (days)
Jul
Aug
Sep
Oct
Nov
Dec
Jan
Feb
Mar
Apr
May
Jun
Winter Solstice
December 21/Day 355
Winter Teletherm
January 5/Day 5
+15 days
Coverage
Year
CURTIS 3NNE, NE
Day of Year

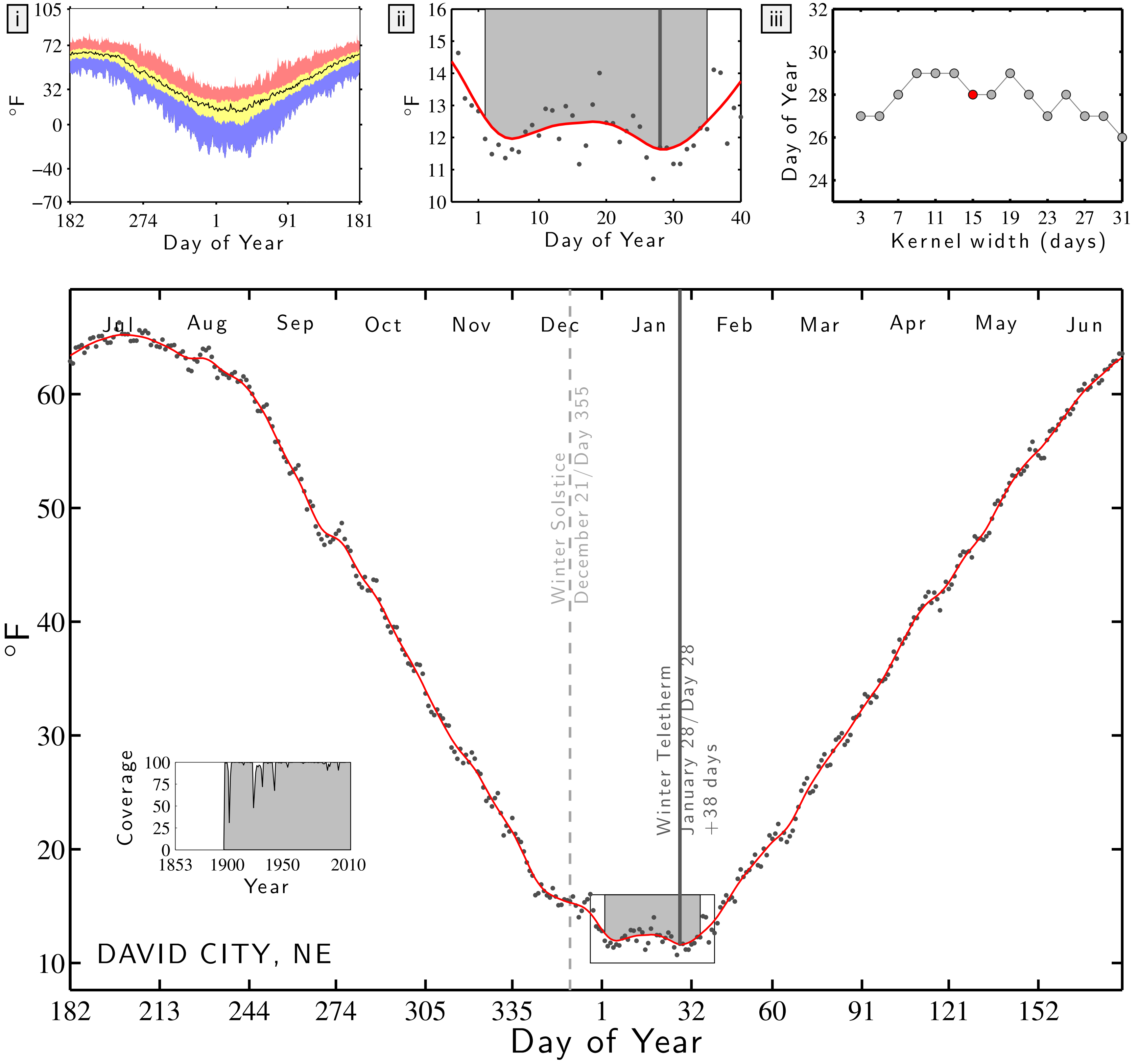

i
°F
Day of Year
ii
°F
Day of Year
iii
Day of Year
Kernel width (days)
Jul
Aug
Sep
Oct
Nov
Dec
Jan
Feb
Mar
Apr
May
Jun
Winter Solstice
December 21/Day 355
Winter Teletherm
January 28/Day 28
+38 days
Coverage
Year
DAVID CITY, NE
°F
Day of Year

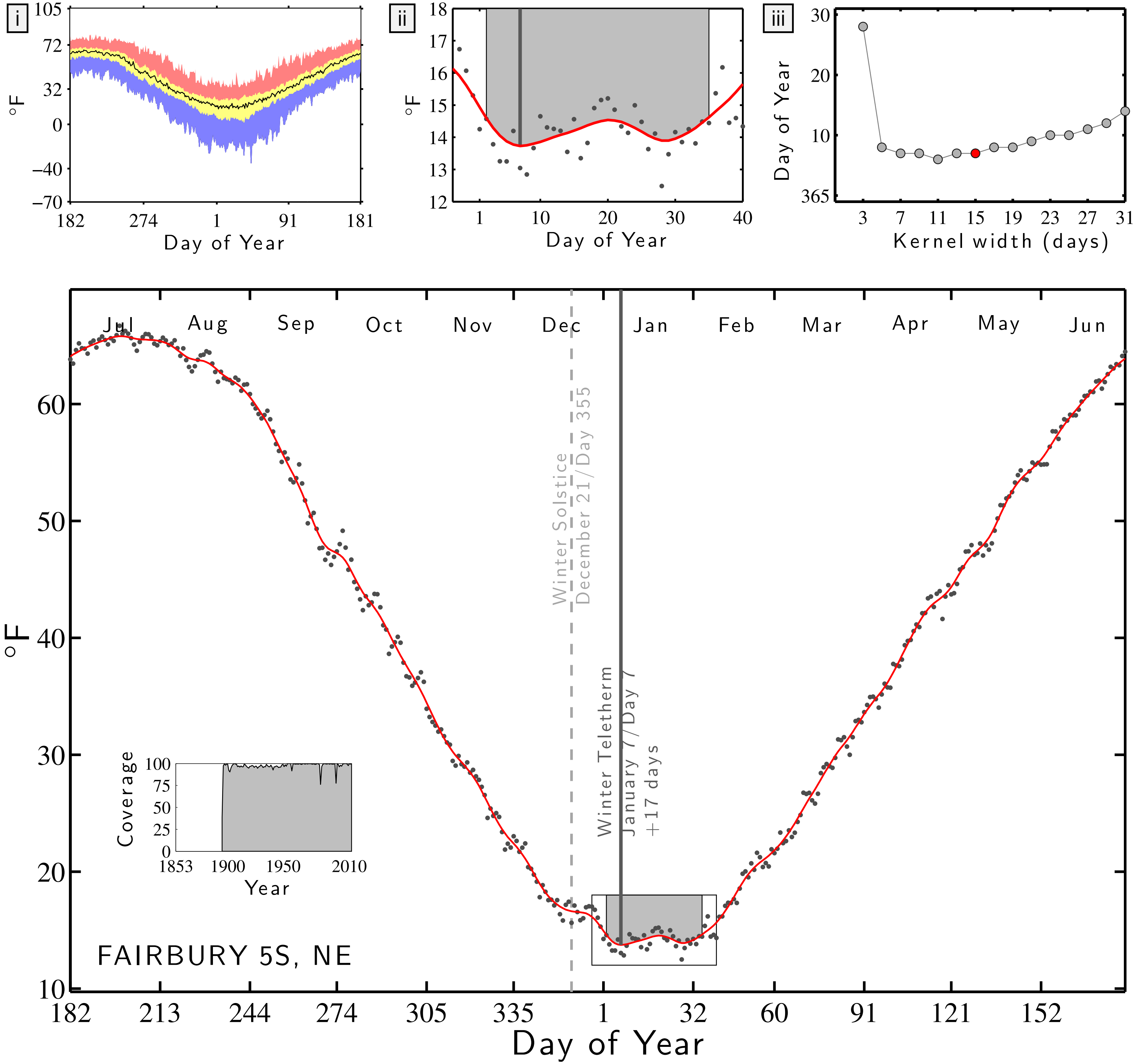

i
ii
iii
°F
Day of Year
Kernel width (days)
Jul
Aug
Sep
Oct
Nov
Dec
Jan
Feb
Mar
Apr
May
Jun
Winter Solstice
December 21/Day 355
Winter Teletherm
January 7/Day 7
+17 days
Coverage
Year
FAIRBURY 5S, NE

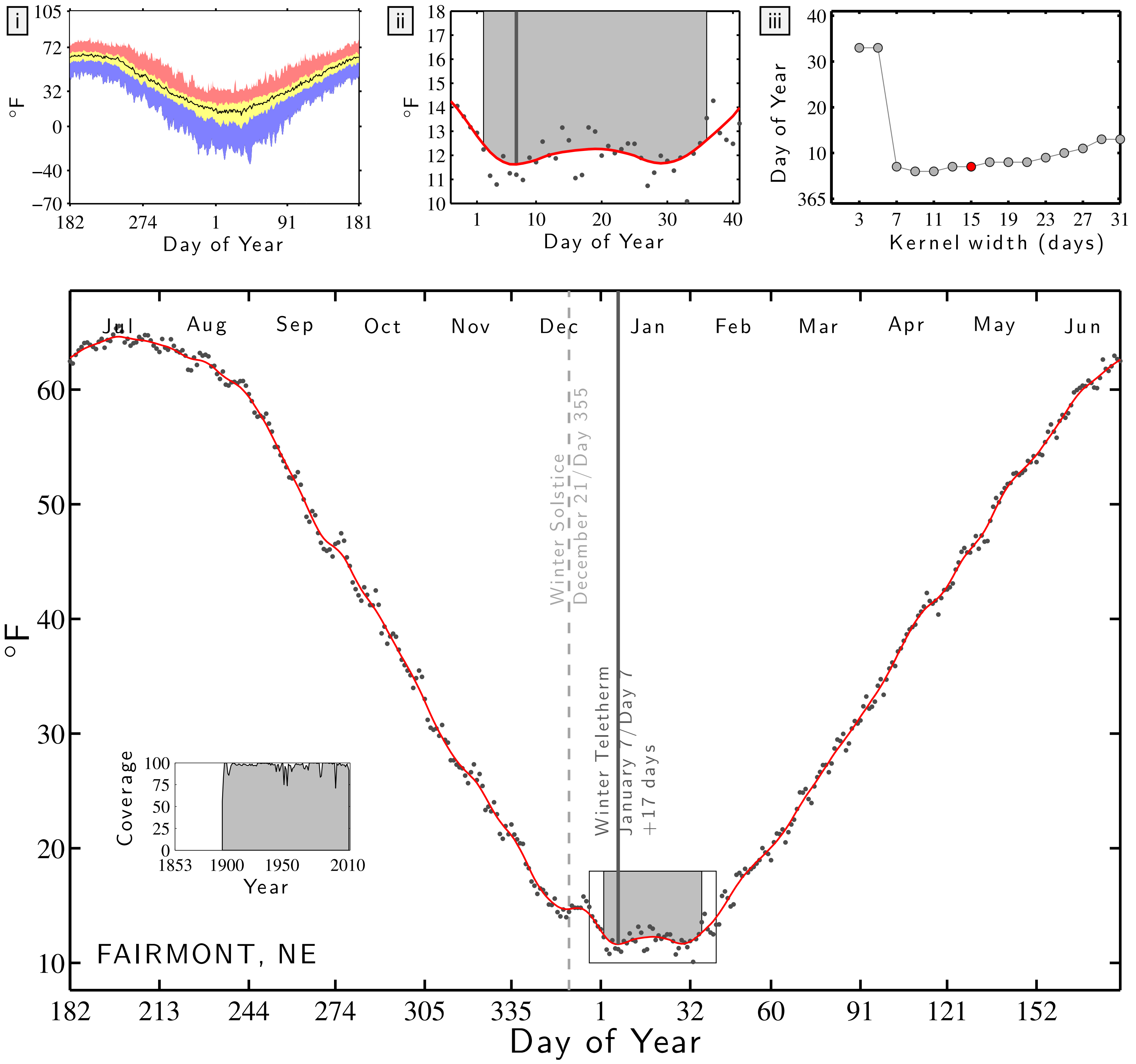

i
°F
Day of Year
ii
Day of Year
iii
Day of Year
Kernel width (days)
Jul
Aug
Sep
Oct
Nov
Dec
Jan
Feb
Mar
Apr
May
Jun
Winter Solstice
December 21/Day 355
Winter Teletherm
January 7/Day 7
+17 days
Coverage
Year
FAIRMONT, NE
Day of Year

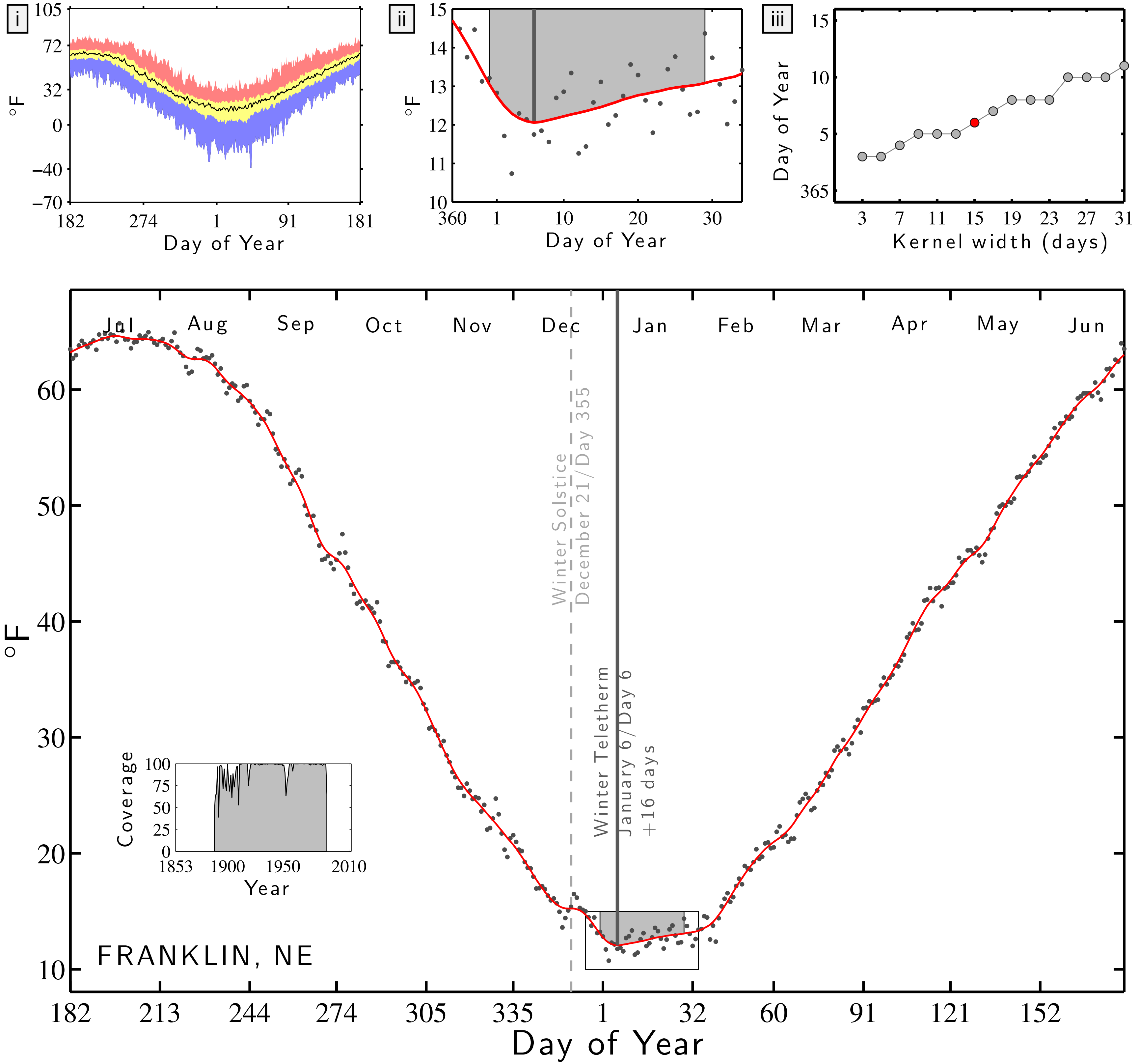

i
ii
iii
°F
Day of Year
Kernel width (days)
Jul
Aug
Sep
Oct
Nov
Dec
Jan
Feb
Mar
Apr
May
Jun
Winter Solstice
December 21/Day 355
Winter Teletherm
January 6/Day 6
+16 days
Coverage
Year
FRANKLIN, NE

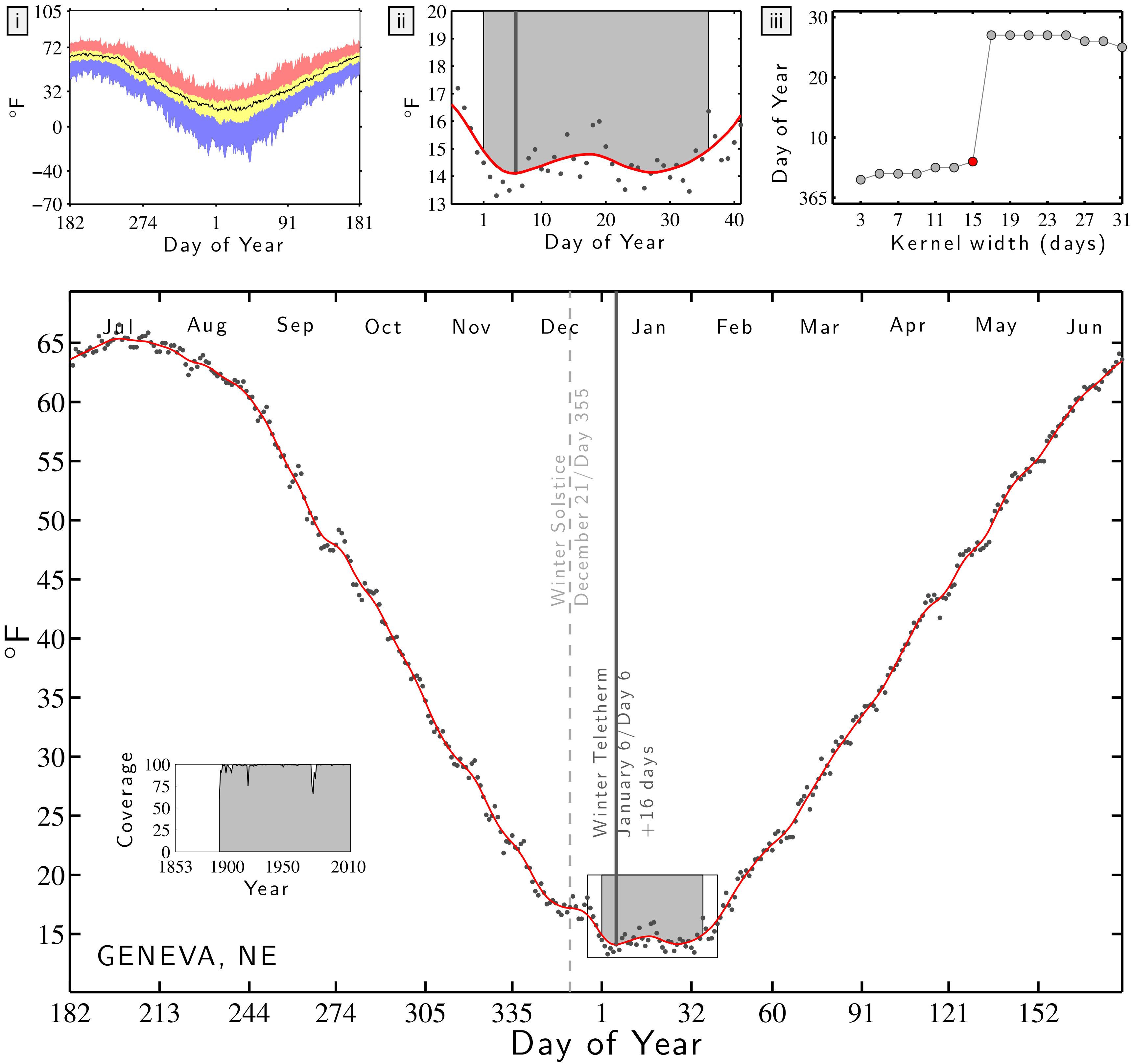

i
°F
Day of Year
ii
°F
Day of Year
iii
Day of Year
Kernel width (days)
Jul
Aug
Sep
Oct
Nov
Dec
Jan
Feb
Mar
Apr
May
Jun
Winter Solstice
December 21/Day 355
Winter Teletherm
January 6/Day 6
+16 days
Coverage
Year
GENEVA, NE
°F
Day of Year

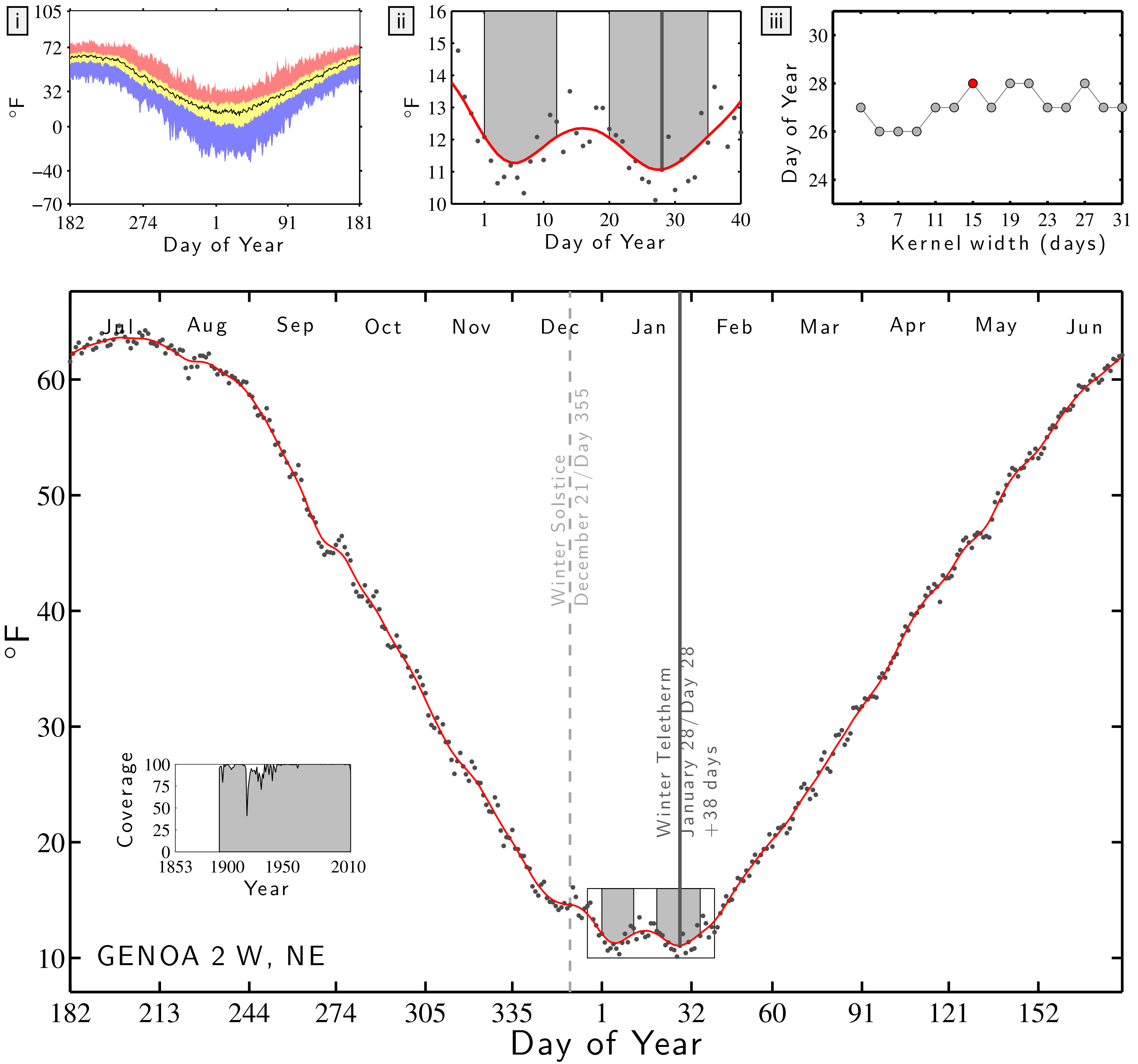

i
°F
Day of Year
ii
Day of Year
iii
Day of Year
Kernel width (days)
Jul
Aug
Sep
Oct
Nov
Dec
Jan
Feb
Mar
Apr
May
Jun
Winter Solstice
December 21/Day 355
Winter Teletherm
January 28/Day 28
+38 days
Coverage
Year
GENOA 2 W, NE
Day of Year

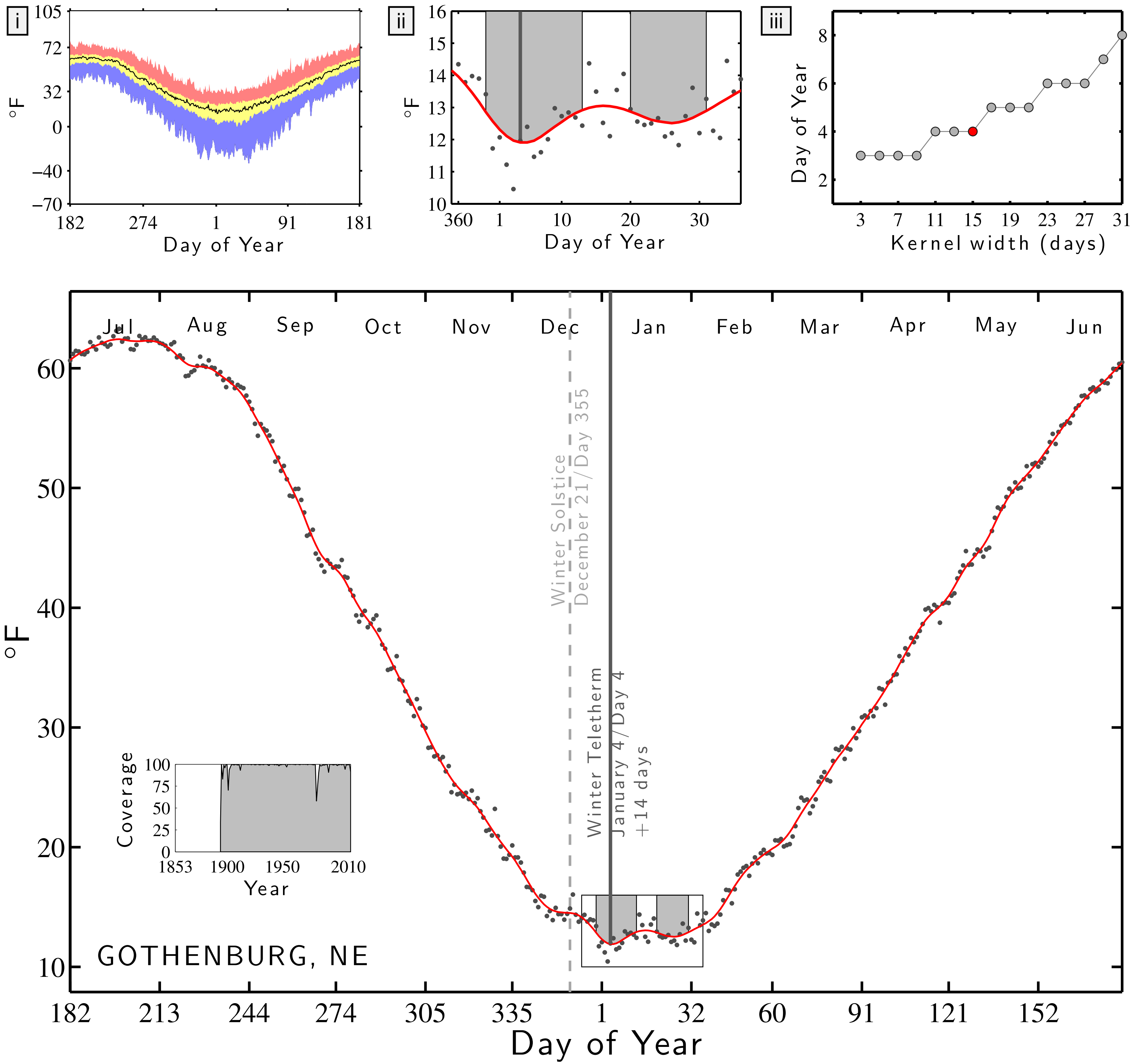

i
°F
105
72
32
0
−40
−70
182
274
1
91
181
Day of Year
ii
°F
16
15
14
13
12
11
10
360
1
10
20
30
Day of Year
iii
Day of Year
8
6
4
2
3
7
11
15
19
23
27
31
Kernel width (days)
Jul
Aug
Sep
Oct
Nov
Dec
Jan
Feb
Mar
Apr
May
Jun
Winter Solstice
December 21/Day 355
Winter Teletherm
January 4/Day 4
+14 days
Coverage
100
75
50
25
0
1853
1900
1950
2010
Year
GOTHENBURG, NE
°F
60
50
40
30
20
10
182
213
244
274
305
335
1
32
60
91
121
152
Day of Year

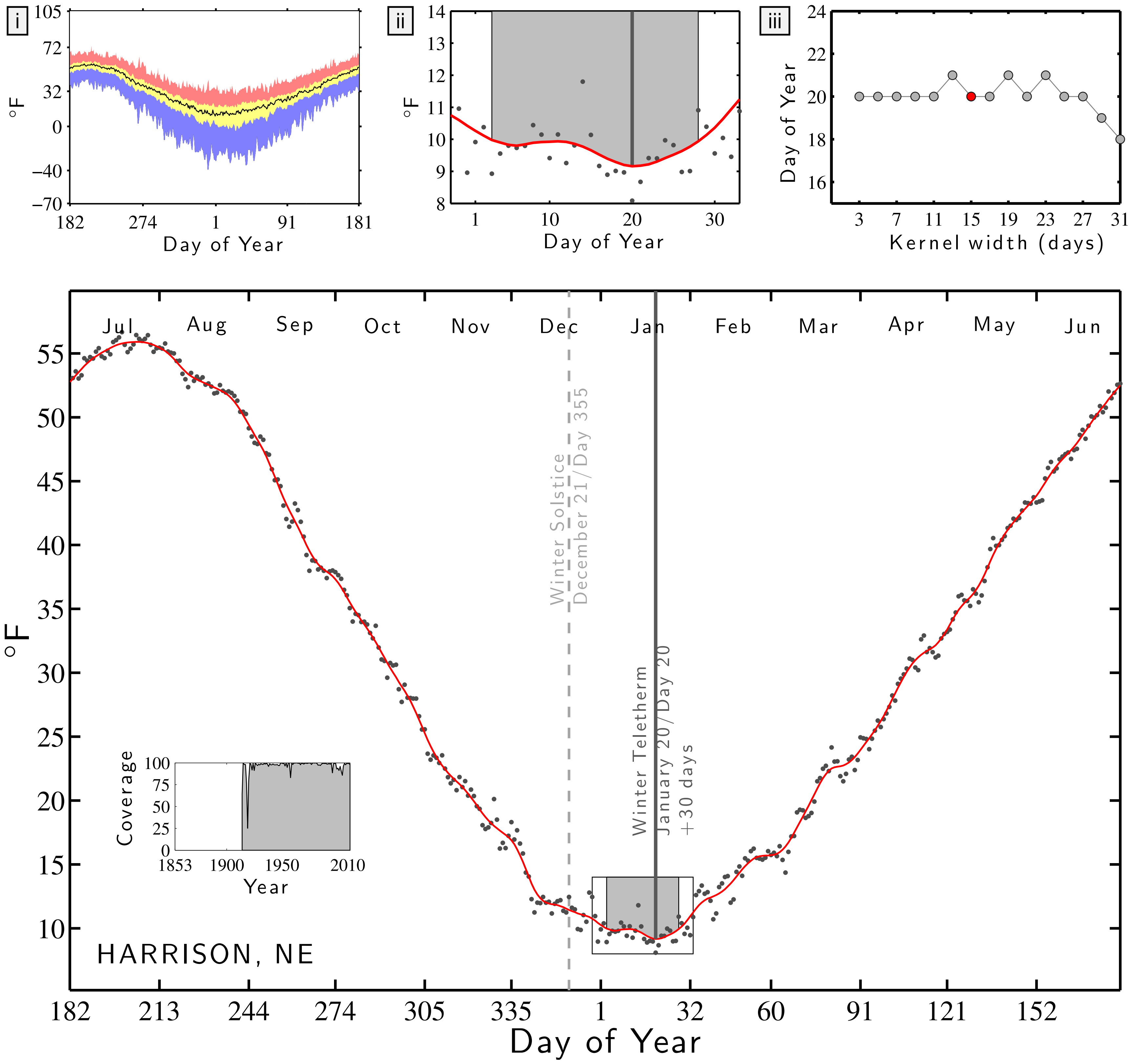

i
ii
iii
°F
Day of Year
Kernel width (days)
Jul
Aug
Sep
Oct
Nov
Dec
Jan
Feb
Mar
Apr
May
Jun
Winter Solstice
December 21/Day 355
Winter Teletherm
January 20/Day 20
+30 days
Coverage
Year
HARRISON, NE

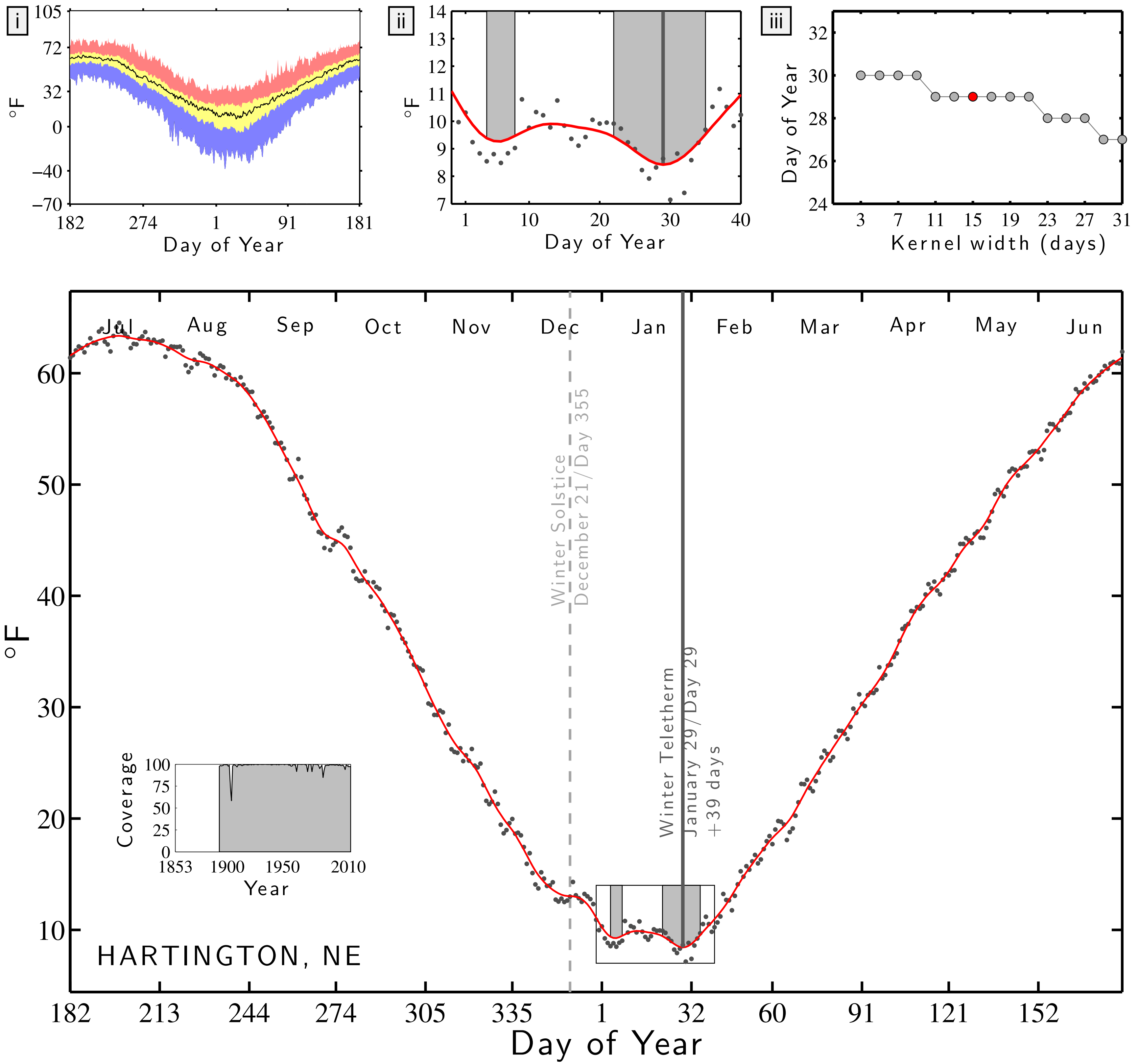
i
ii
iii
Day of Year
Kernel width (days)
°F
Jul
Aug
Sep
Oct
Nov
Dec
Jan
Feb
Mar
Apr
May
Jun
Winter Solstice
December 21/Day 355
Winter Teletherm
January 29/Day 29
+39 days
Coverage
Year
HARTINGTON, NE

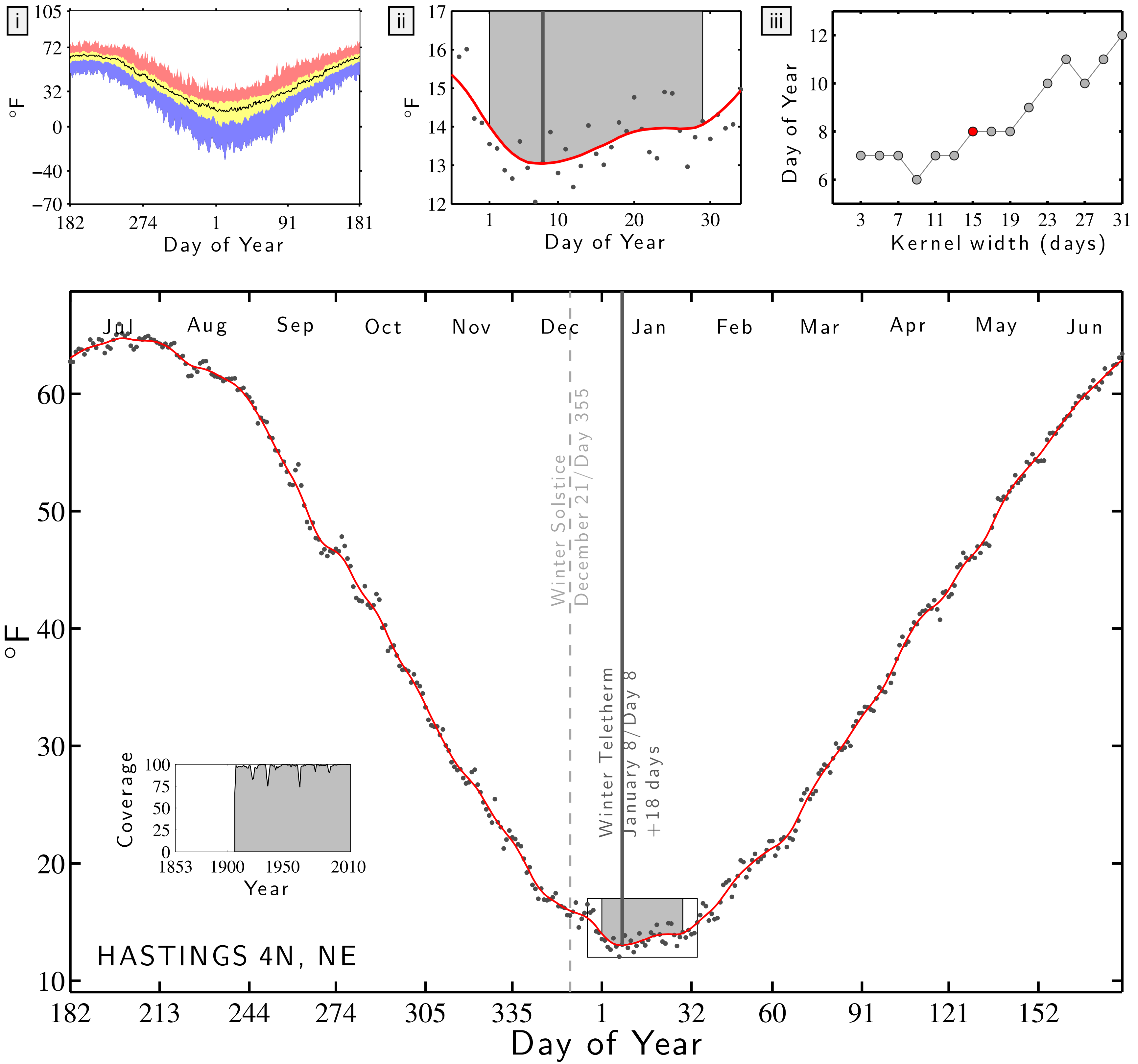

i
ii
iii
°F
Day of Year
Kernel width (days)
Jul
Aug
Sep
Oct
Nov
Dec
Jan
Feb
Mar
Apr
May
Jun
Winter Solstice
December 21/Day 355
Winter Teletherm
January 8/Day 8
+18 days
Coverage
Year
HASTINGS 4N, NE

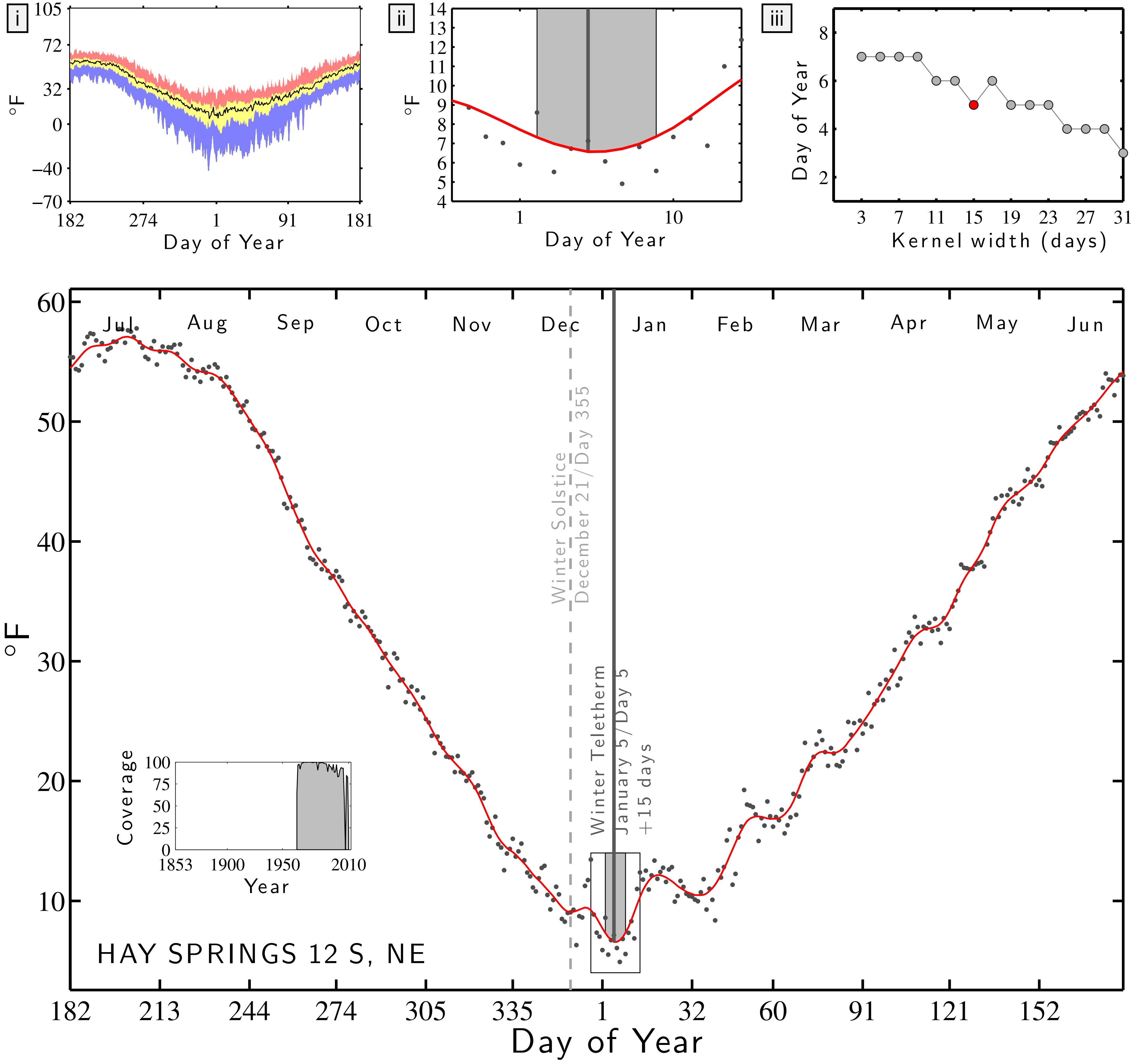

i
ii
iii
Day of Year
Kernel width (days)
Jul
Aug
Sep
Oct
Nov
Dec
Jan
Feb
Mar
Apr
May
Jun
Winter Solstice
December 21/Day 355
Winter Teletherm
January 5/Day 5
+15 days
Coverage
Year
HAY SPRINGS 12 S, NE
°F

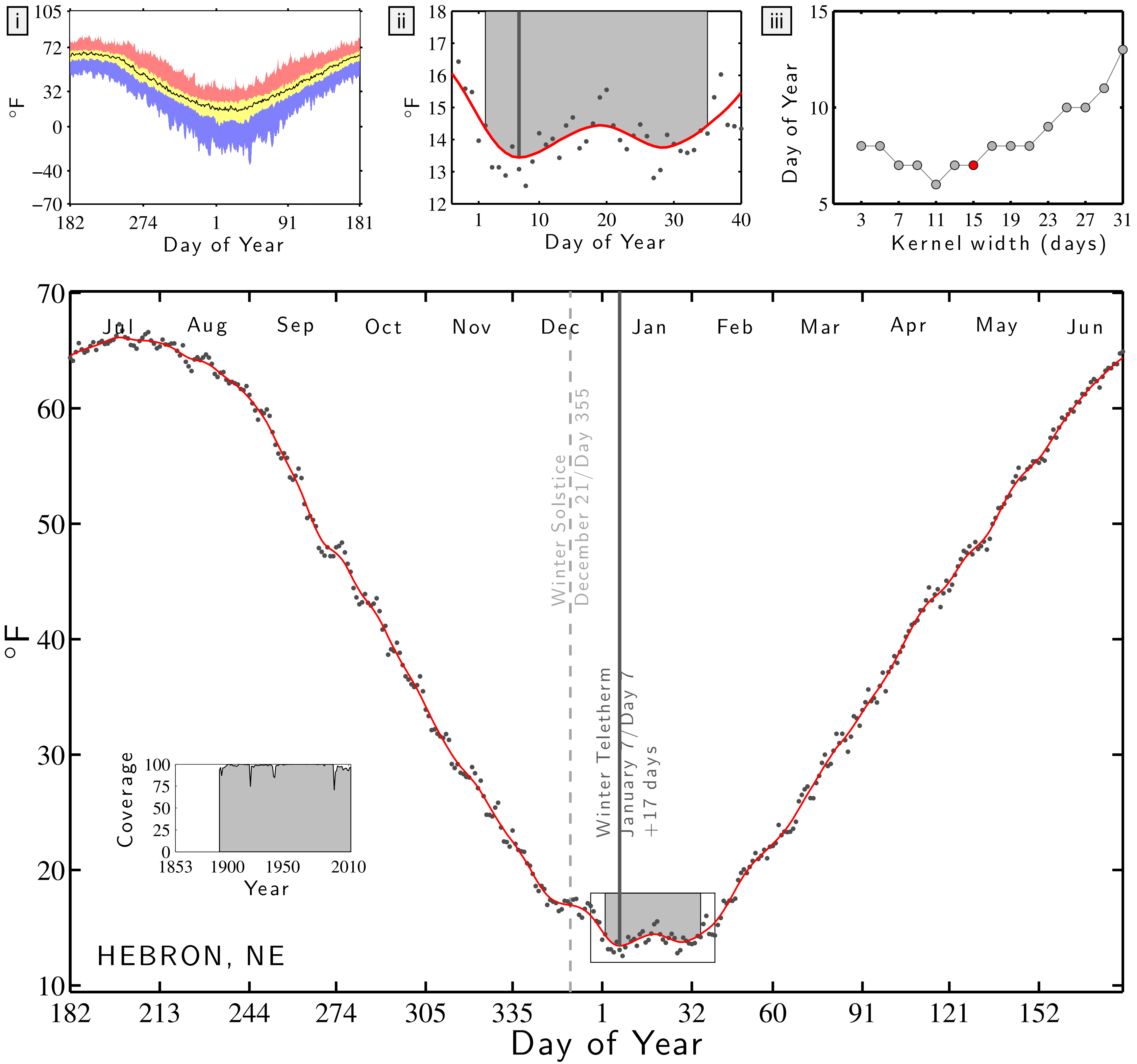
i
°F
105
72
32
0
−40
−70
182
274
1
91
181
Day of Year
ii
°F
18
17
16
15
14
13
12
1
10
20
30
40
Day of Year
iii
Day of Year
15
10
5
3
7
11
15
19
23
27
31
Kernel width (days)
°F
70
60
50
40
30
20
10
Jul
Aug
Sep
Oct
Nov
Dec
Jan
Feb
Mar
Apr
May
Jun
Winter Solstice
December 21/Day 355
Winter Teletherm
January 7/Day 7
+17 days
Coverage
100
75
50
25
0
1853
1900
1950
2010
Year
HEBRON, NE
182
213
244
274
305
335
1
32
60
91
121
152
Day of Year

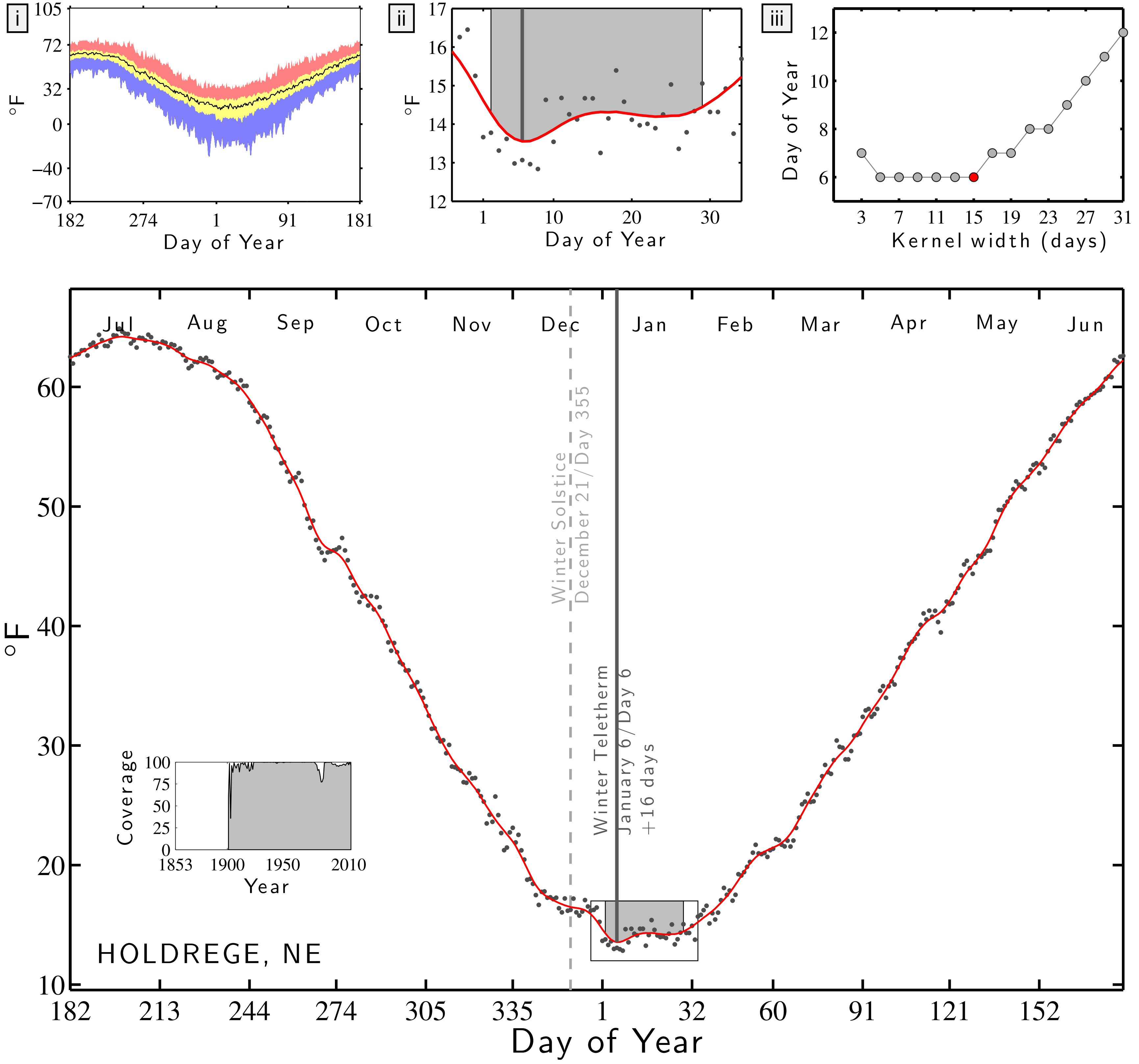

i
ii
iii
°F
Day of Year
Kernel width (days)
Jul
Aug
Sep
Oct
Nov
Dec
Jan
Feb
Mar
Apr
May
Jun
Winter Solstice
December 21/Day 355
Winter Teletherm
January 6/Day 6
+16 days
Coverage
Year
HOLDREGE, NE

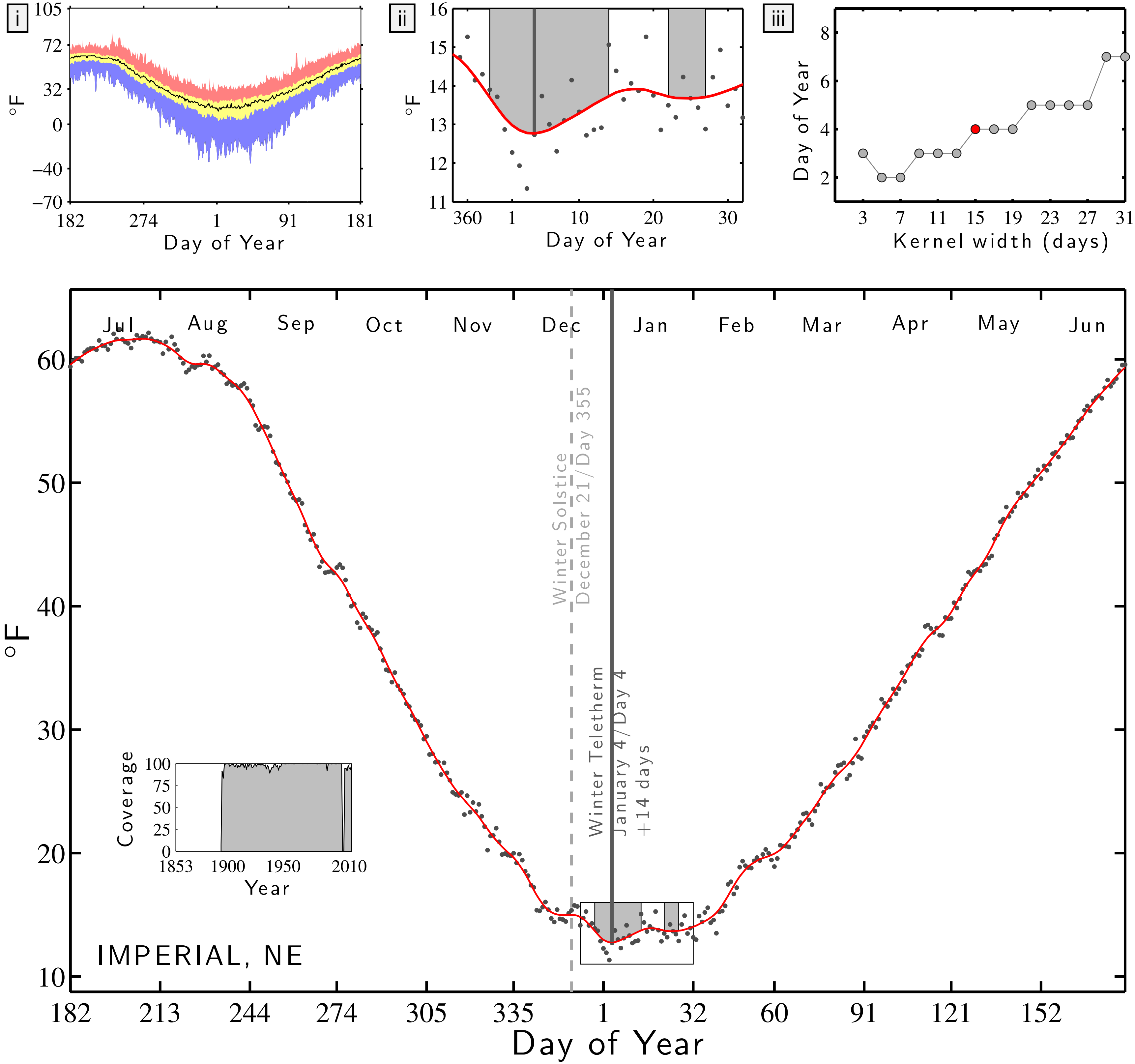
i
ii
iii
Day of Year
Kernel width (days)
Jul
Aug
Sep
Oct
Nov
Dec
Jan
Feb
Mar
Apr
May
Jun
Winter Solstice
December 21/Day 355
Winter Teletherm
January 4/Day 4
+14 days
Coverage
Year
IMPERIAL, NE
°F
Day of Year

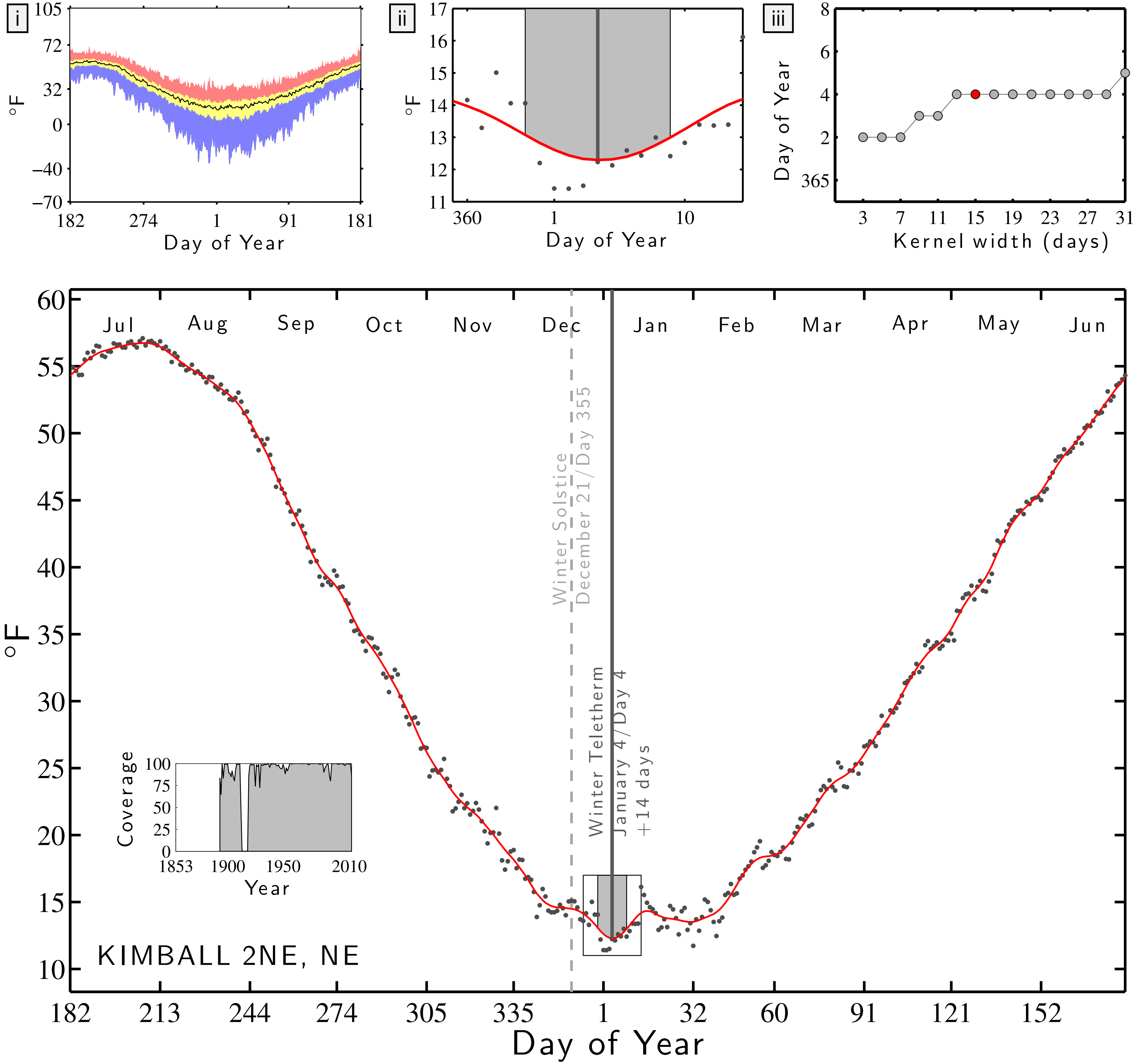

i
°F
Day of Year
ii
°F
Day of Year
iii
Day of Year
Kernel width (days)
Jul
Aug
Sep
Oct
Nov
Dec
Jan
Feb
Mar
Apr
May
Jun
Winter Solstice
December 21/Day 355
Winter Teletherm
January 4/Day 4
+14 days
Coverage
Year
KIMBALL 2NE, NE
°F
Day of Year

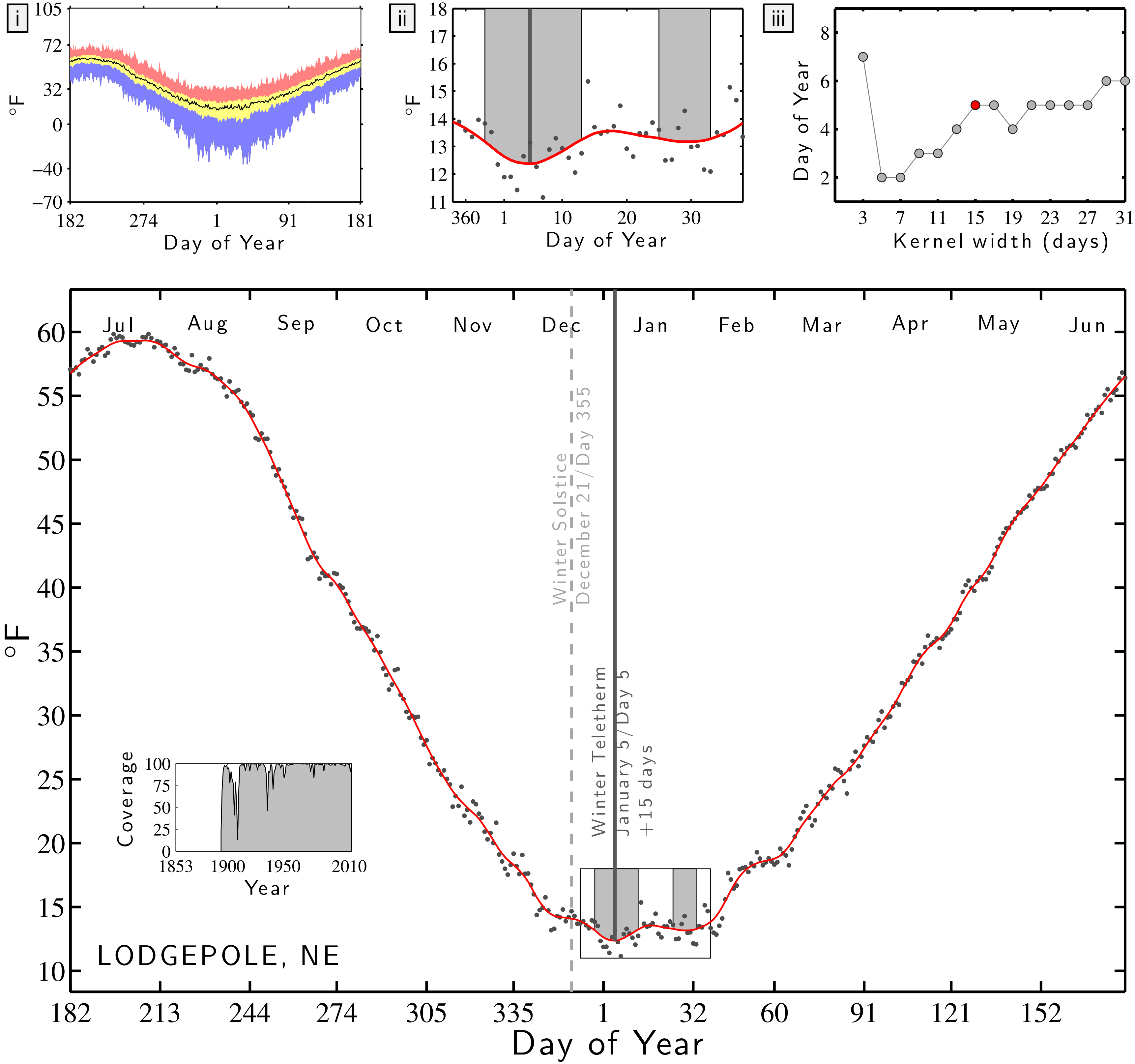

i
°F
Day of Year
ii
°F
Day of Year
iii
Day of Year
Kernel width (days)
Jul
Aug
Sep
Oct
Nov
Dec
Jan
Feb
Mar
Apr
May
Jun
Winter Solstice
December 21/Day 355
Winter Teletherm
January 5/Day 5
+15 days
Coverage
Year
LODGEPOLE, NE
°F
Day of Year

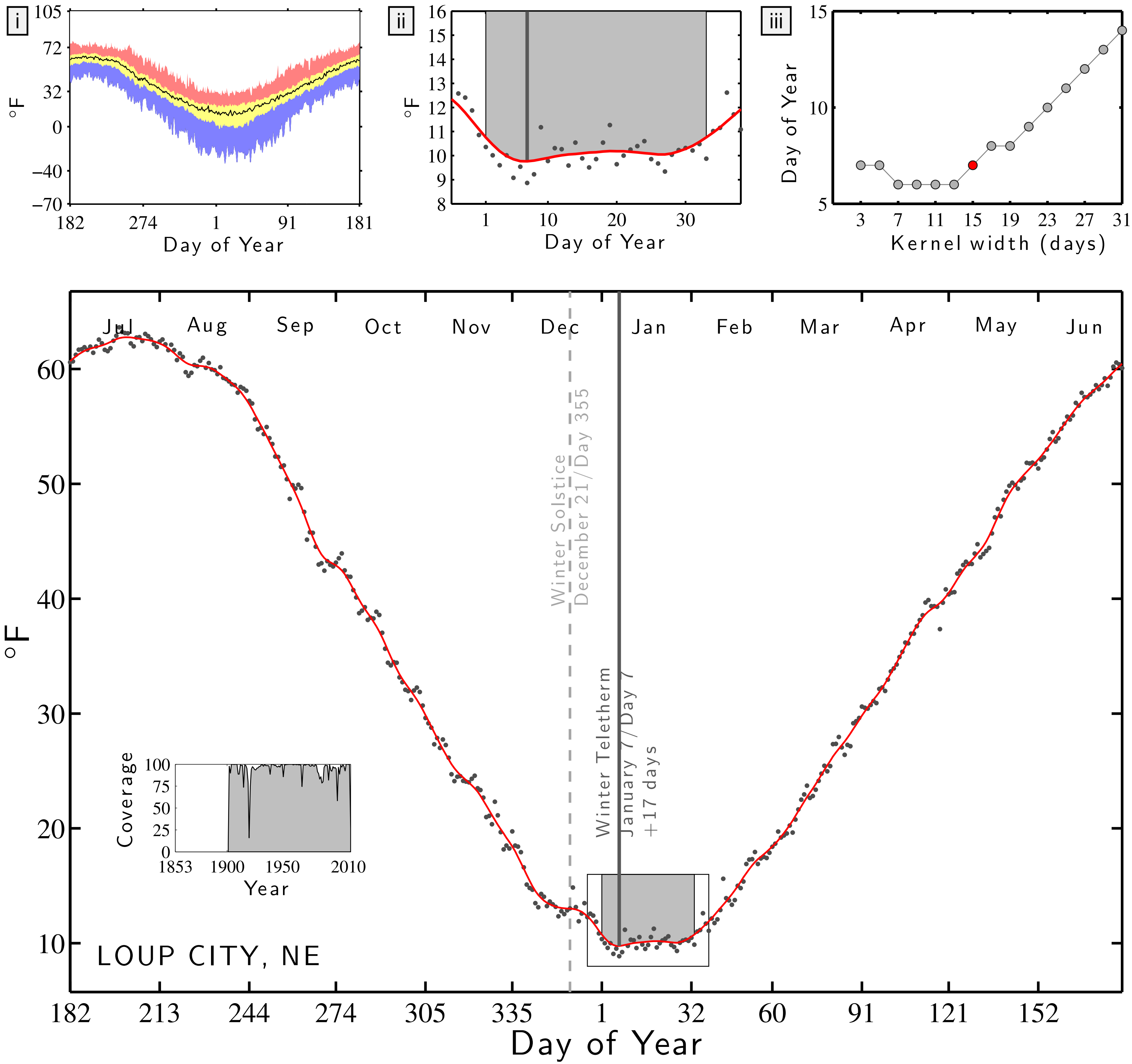

i
°F
Day of Year
ii
°F
Day of Year
iii
Day of Year
Kernel width (days)
Jul
Aug
Sep
Oct
Nov
Dec
Jan
Feb
Mar
Apr
May
Jun
Winter Solstice
December 21/Day 355
Winter Teletherm
January 7/Day 7
+17 days
Coverage
Year
LOUP CITY, NE
°F
Day of Year

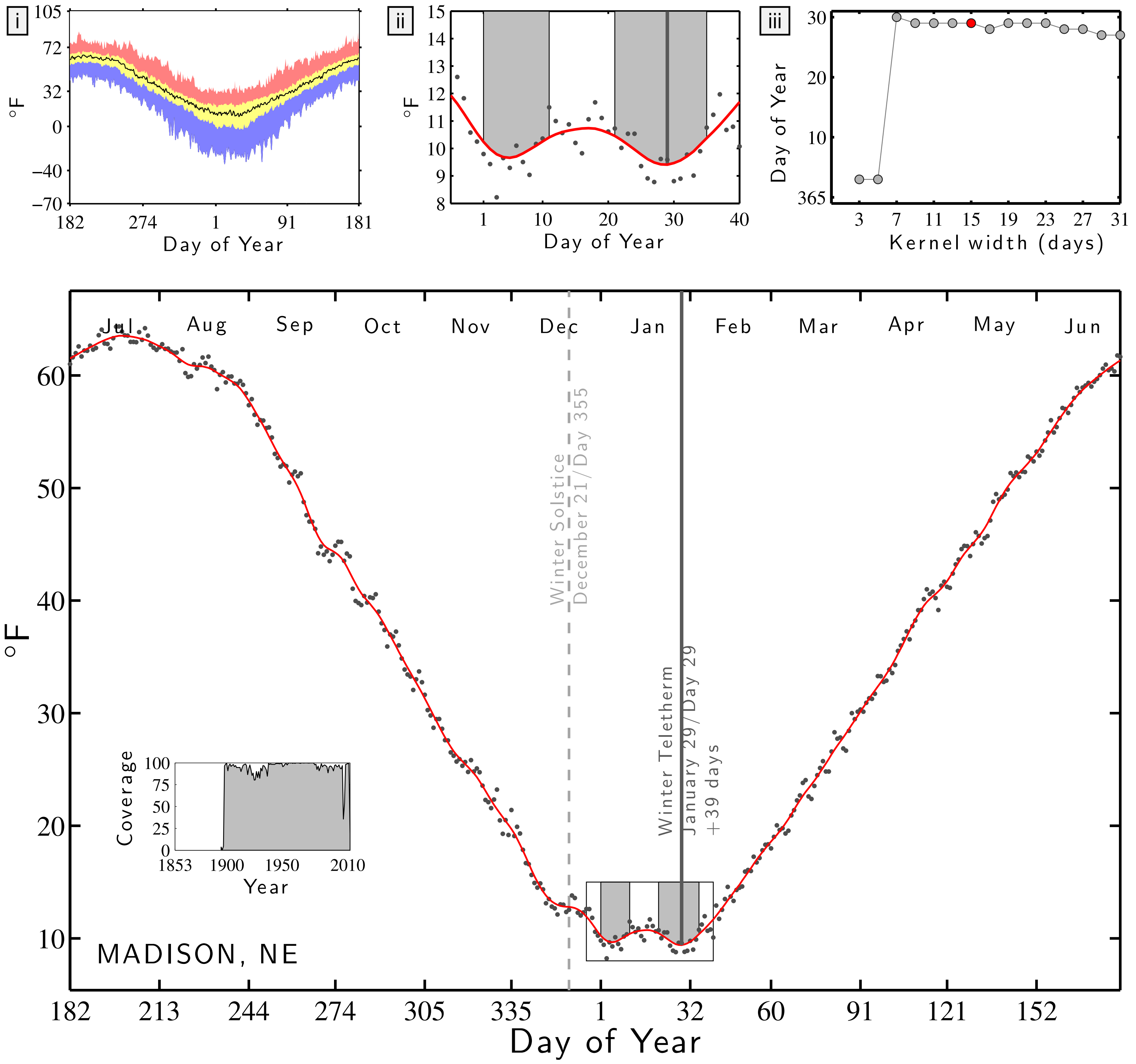

i
105
72
32
0
−40
−70
°F
182
274
1
91
181
Day of Year
ii
15
14
13
12
11
10
9
8
°F
1
10
20
30
40
Day of Year
iii
30
20
10
365
Day of Year
3
7
11
15
19
23
27
31
Kernel width (days)
Jul
Aug
Sep
Oct
Nov
Dec
Jan
Feb
Mar
Apr
May
Jun
60
50
40
30
20
10
°F
Winter Solstice
December 21/Day 355
Winter Teletherm
January 29/Day 29
+39 days
Coverage
100
75
50
25
0
1853
1900
1950
2010
Year
MADISON, NE
182
213
244
274
305
335
1
32
60
91
121
152
Day of Year

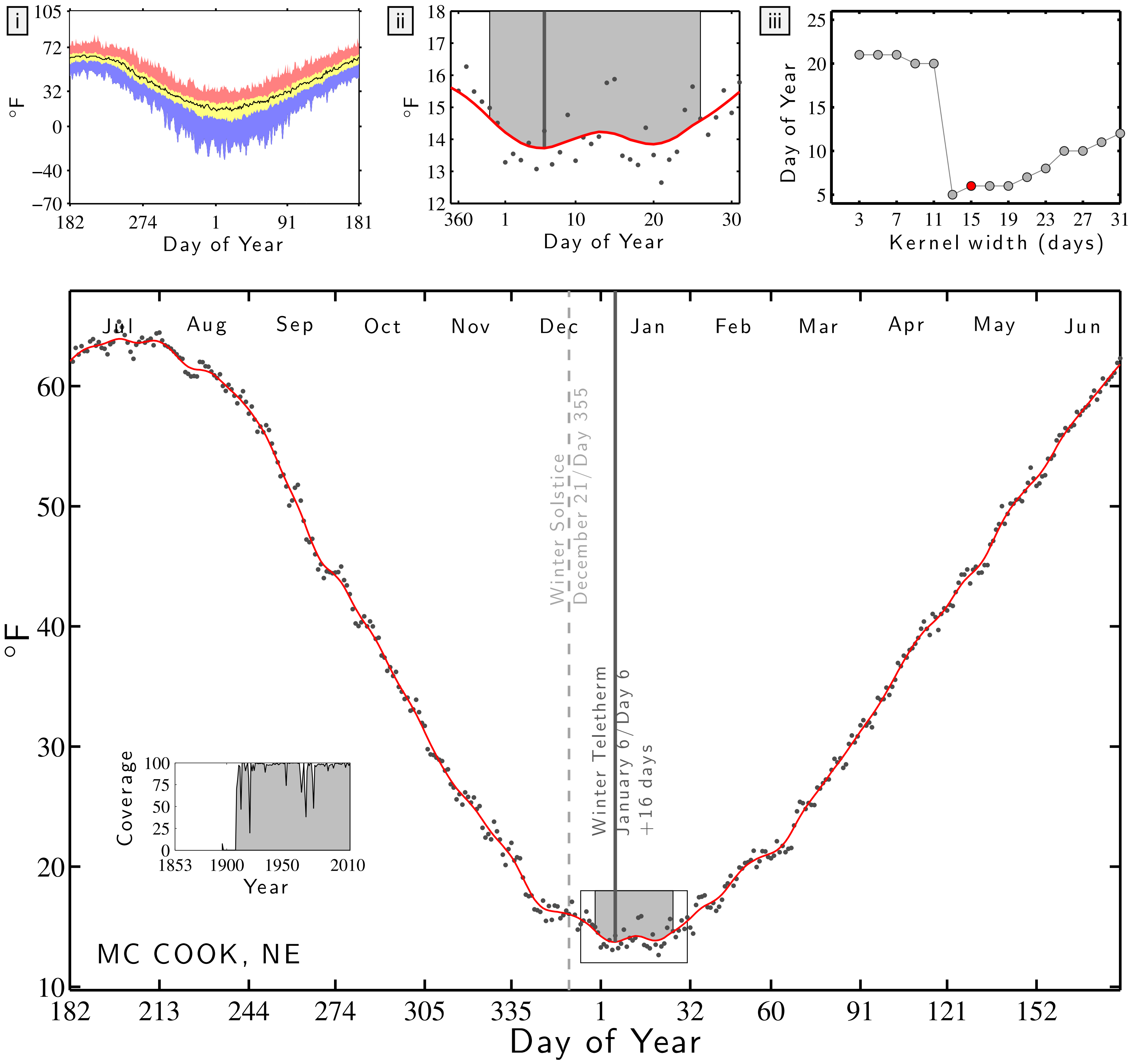

i
°F
Day of Year
ii
°F
Day of Year
iii
Day of Year
Kernel width (days)
Jul Aug Sep Oct Nov Dec Jan Feb Mar Apr May Jun
Winter Solstice
December 21/Day 355
Winter Teletherm
January 6/Day 6
+16 days
Coverage
Year
MC COOK, NE
°F
Day of Year

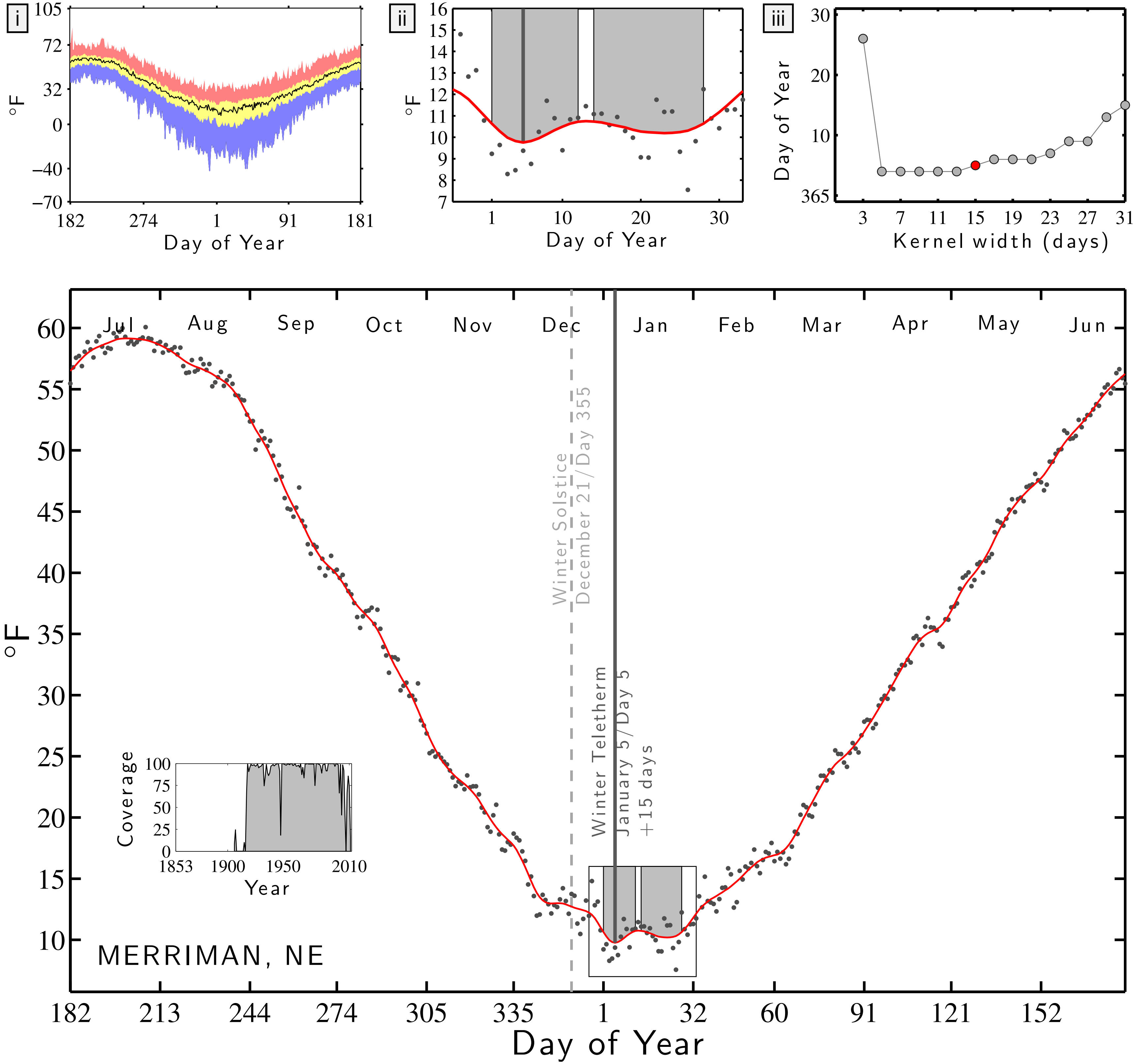

i
°F
105
72
32
0
−40
−70
182
274
1
91
181
Day of Year
ii
16
15
14
13
12
11
10
9
8
7
°F
1
10
20
30
Day of Year
iii
30
20
10
365
Day of Year
3
7
11
15
19
23
27
31
Kernel width (days)
Jul
Aug
Sep
Oct
Nov
Dec
Jan
Feb
Mar
Apr
May
Jun
60
55
50
45
40
35
30
25
20
15
10
°F
Winter Solstice
December 21/Day 355
Winter Teletherm
January 5/Day 5
+15 days
Coverage
100
75
50
25
0
1853
1900
1950
2010
Year
MERRIMAN, NE
182
213
244
274
305
335
1
32
60
91
121
152
Day of Year

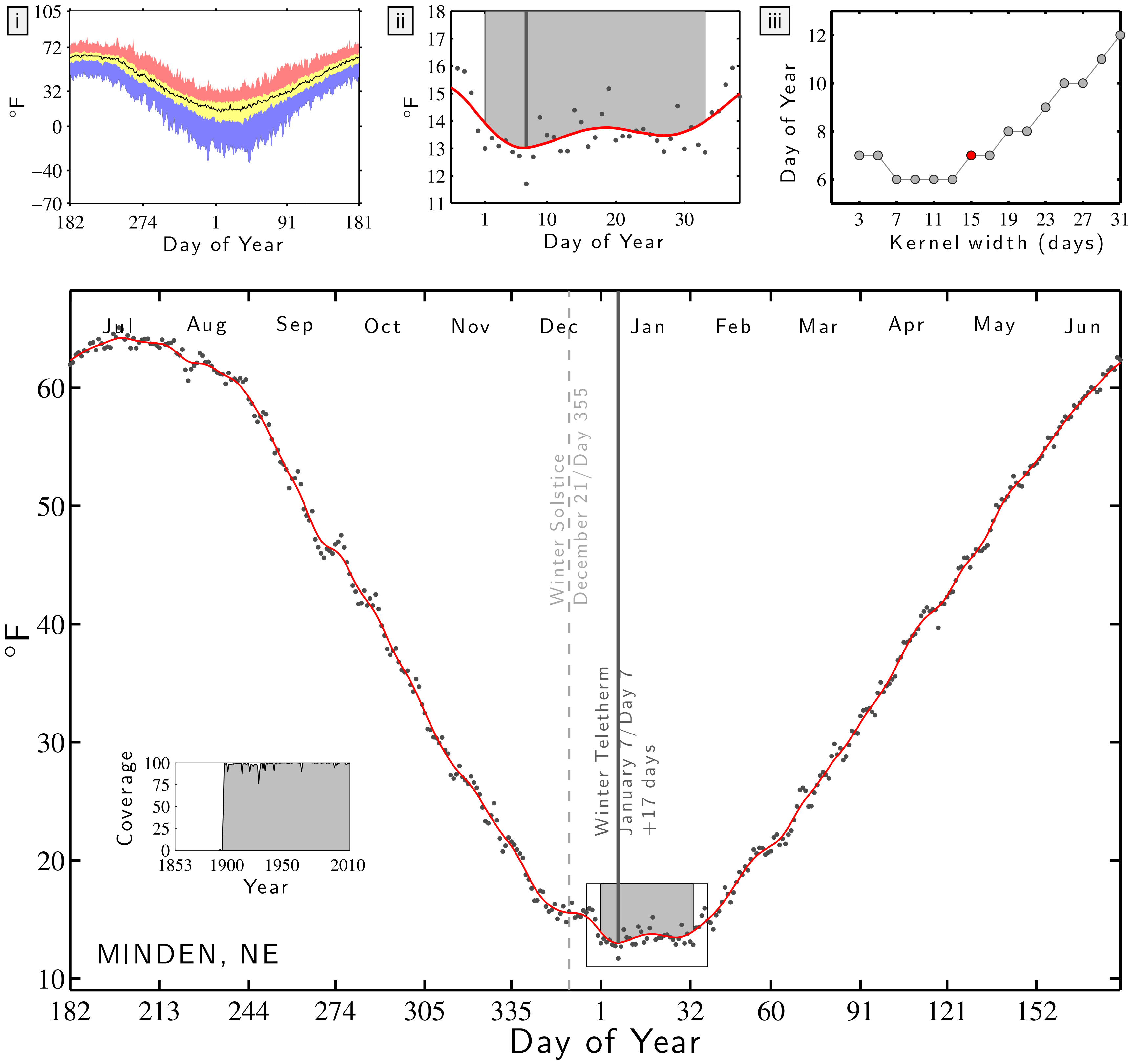

i
°F
Day of Year
ii
°F
Day of Year
iii
Day of Year
Kernel width (days)
Jul
Aug
Sep
Oct
Nov
Dec
Jan
Feb
Mar
Apr
May
Jun
Winter Solstice
December 21/Day 355
Winter Teletherm
January 7/Day 7
+17 days
Coverage
Year
MINDEN, NE
°F
Day of Year

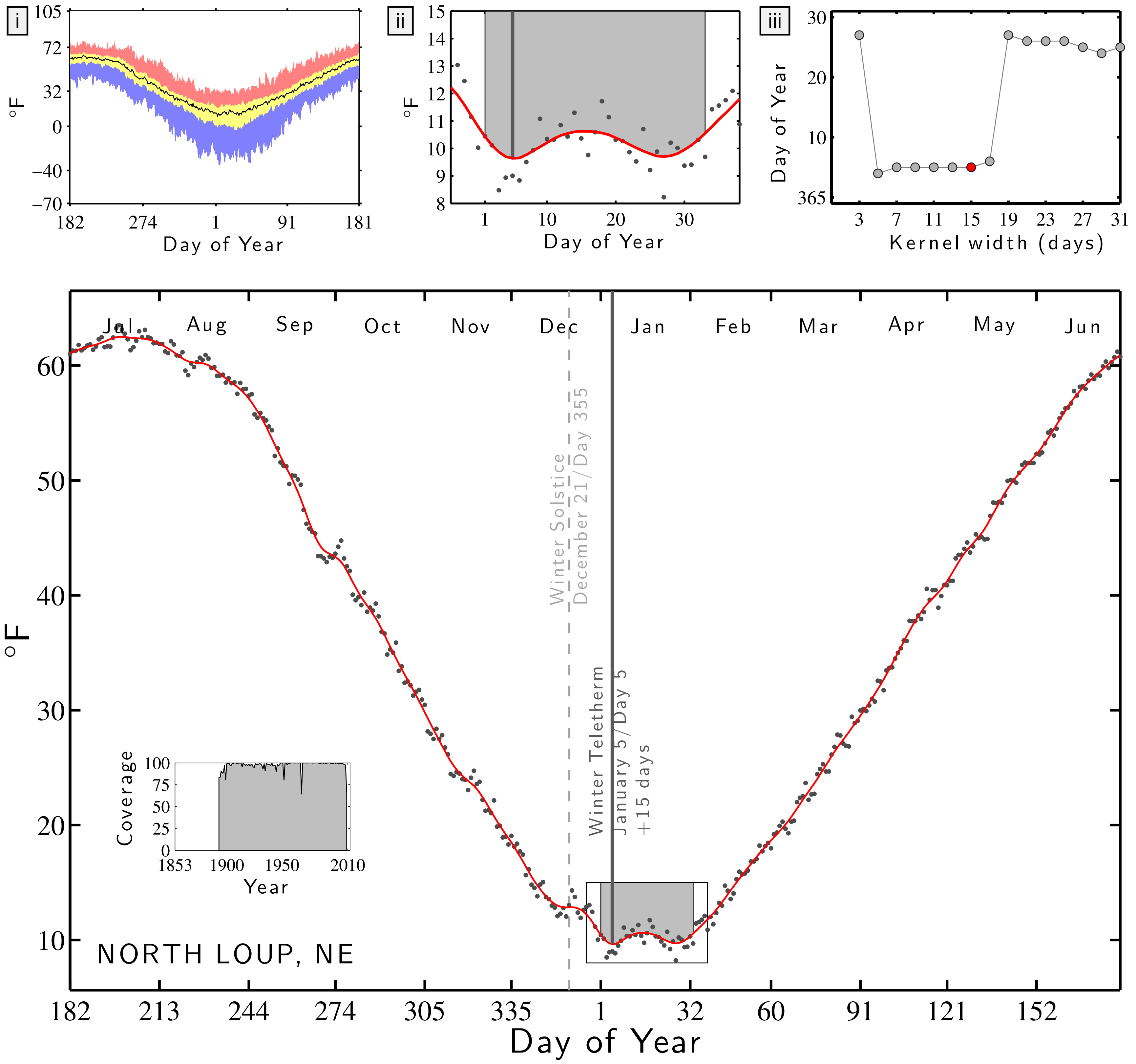

i
°F
105
72
32
0
−40
−70
182
274
1
91
181
Day of Year
ii
°F
Day of Year
iii
Day of Year
365
Kernel width (days)
Jul
Aug
Sep
Oct
Nov
Dec
Jan
Feb
Mar
Apr
May
Jun
Winter Solstice
December 21/Day 355
Winter Teletherm
January 5/Day 5
+15 days
Coverage
Year
NORTH LOUP, NE
182
213
244
274
305
335
1
32
60
91
121
152
Day of Year

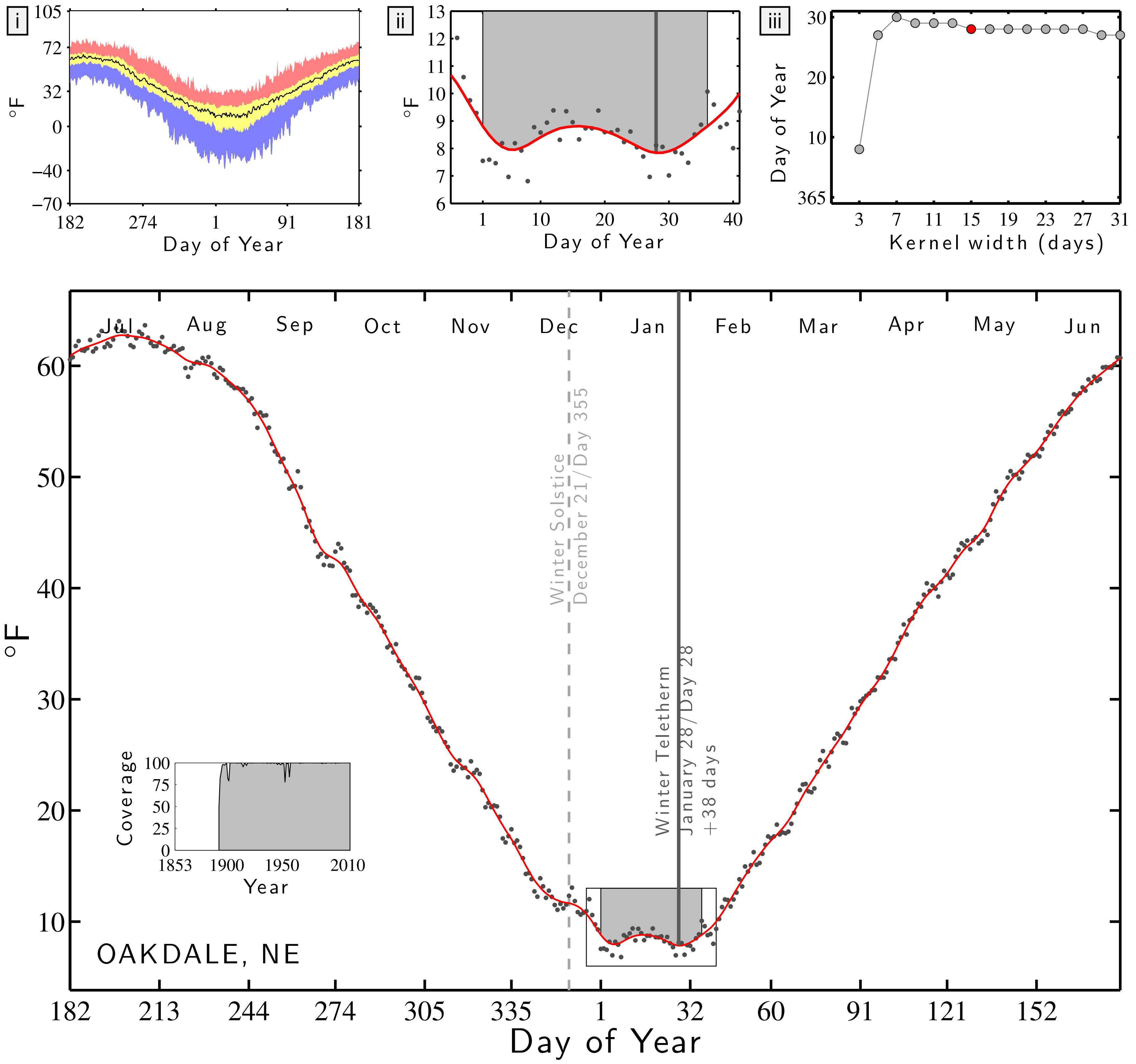

i
°F
Day of Year
ii
°F
Day of Year
iii
Day of Year
Kernel width (days)
Jul
Aug
Sep
Oct
Nov
Dec
Jan
Feb
Mar
Apr
May
Jun
Winter Solstice
December 21/Day 355
Winter Teletherm
January 28/Day 28
+38 days
Coverage
Year
OAKDALE, NE
°F
Day of Year

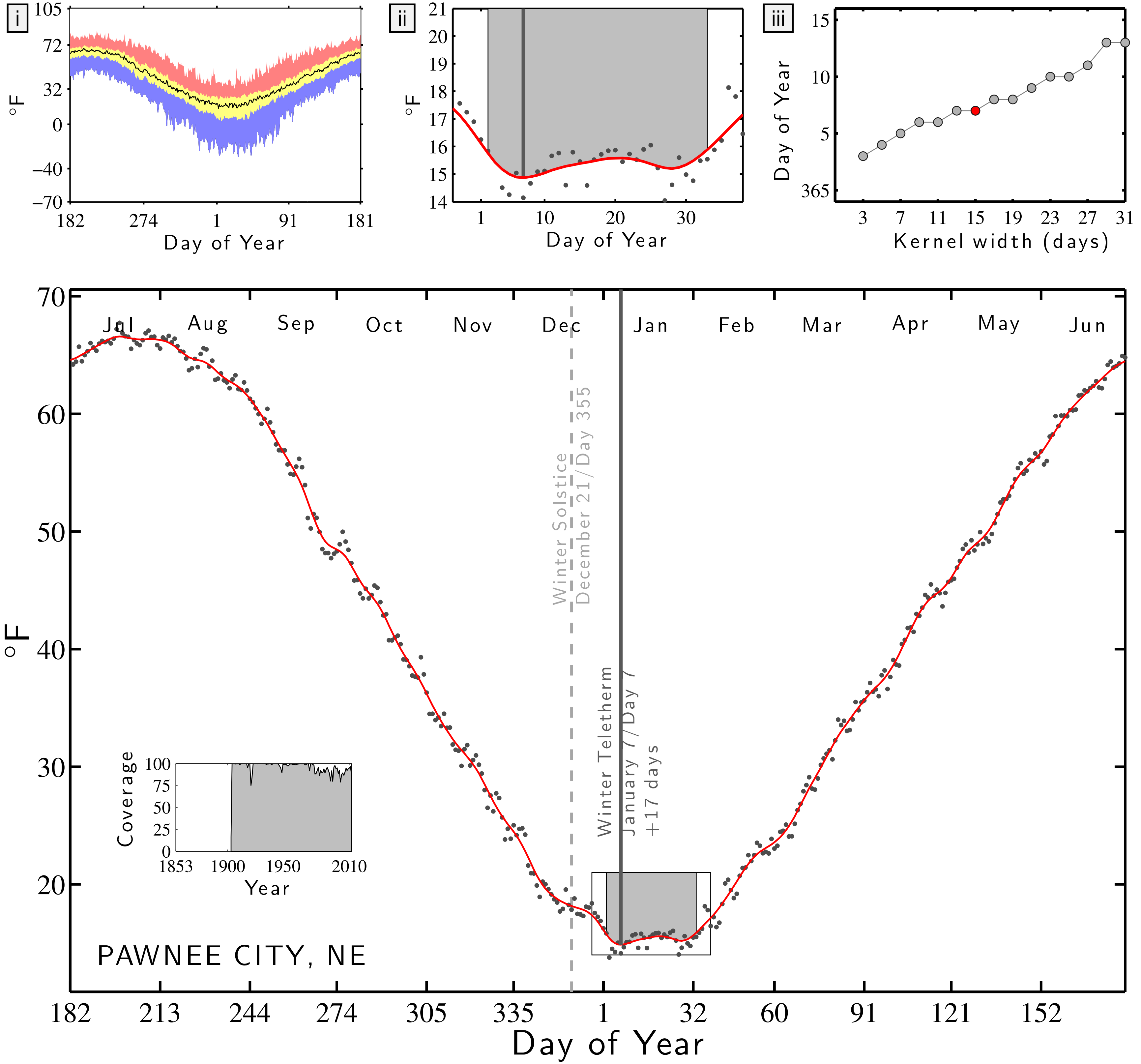

i
105
72
32
0
−40
−70
°F
182
274
1
91
181
Day of Year
ii
21
20
19
18
17
16
15
14
°F
1
10
20
30
Day of Year
iii
15
10
5
365
Day of Year
3
7
11
15
19
23
27
31
Kernel width (days)
70
60
50
40
30
20
°F
Jul
Aug
Sep
Oct
Nov
Dec
Jan
Feb
Mar
Apr
May
Jun
Winter Solstice
December 21/Day 355
Winter Teletherm
January 7/Day 7
+17 days
Coverage
100
75
50
25
0
1853
1900
1950
2010
Year
PAWNEE CITY, NE
182
213
244
274
305
335
1
32
60
91
121
152
Day of Year

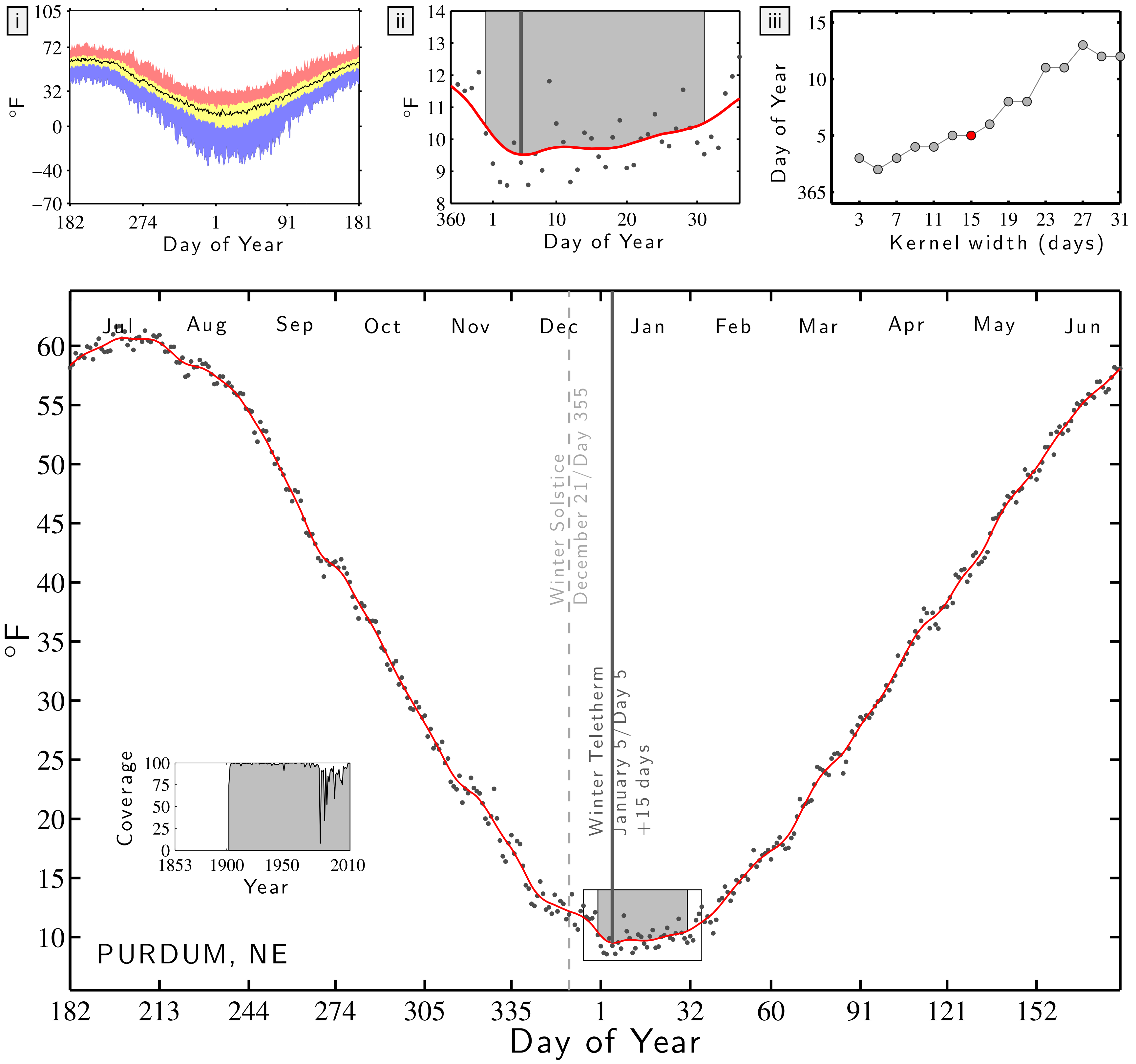

i
°F
Day of Year
ii
°F
Day of Year
iii
Day of Year
Kernel width (days)
Jul
Aug
Sep
Oct
Nov
Dec
Jan
Feb
Mar
Apr
May
Jun
Winter Solstice
December 21/Day 355
Winter Teletherm
January 5/Day 5
+15 days
Coverage
Year
PURDUM, NE
°F
Day of Year

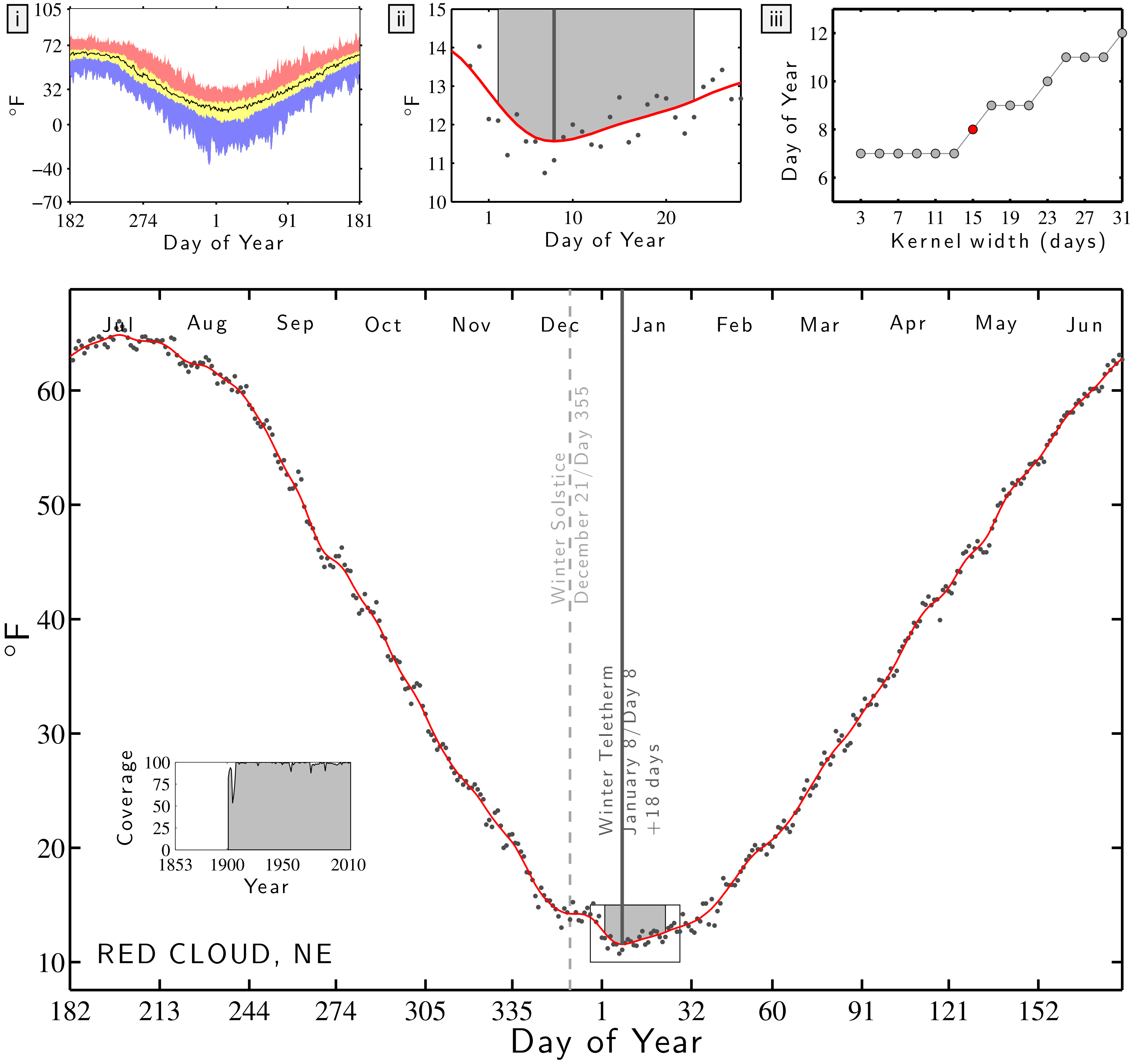

i
105
72
32
0
−40
−70
°F
182
274
1
91
181
Day of Year
ii
15
14
13
12
11
10
°F
1
10
20
Day of Year
iii
12
10
8
6
Day of Year
3
7
11
15
19
23
27
31
Kernel width (days)
Jul
Aug
Sep
Oct
Nov
Dec
Jan
Feb
Mar
Apr
May
Jun
60
50
40
30
20
10
°F
Winter Solstice
December 21/Day 355
Winter Teletherm
January 8/Day 8
+18 days
Coverage
100
75
50
25
0
1853
1900
1950
2010
Year
RED CLOUD, NE
182
213
244
274
305
335
1
32
60
91
121
152
Day of Year

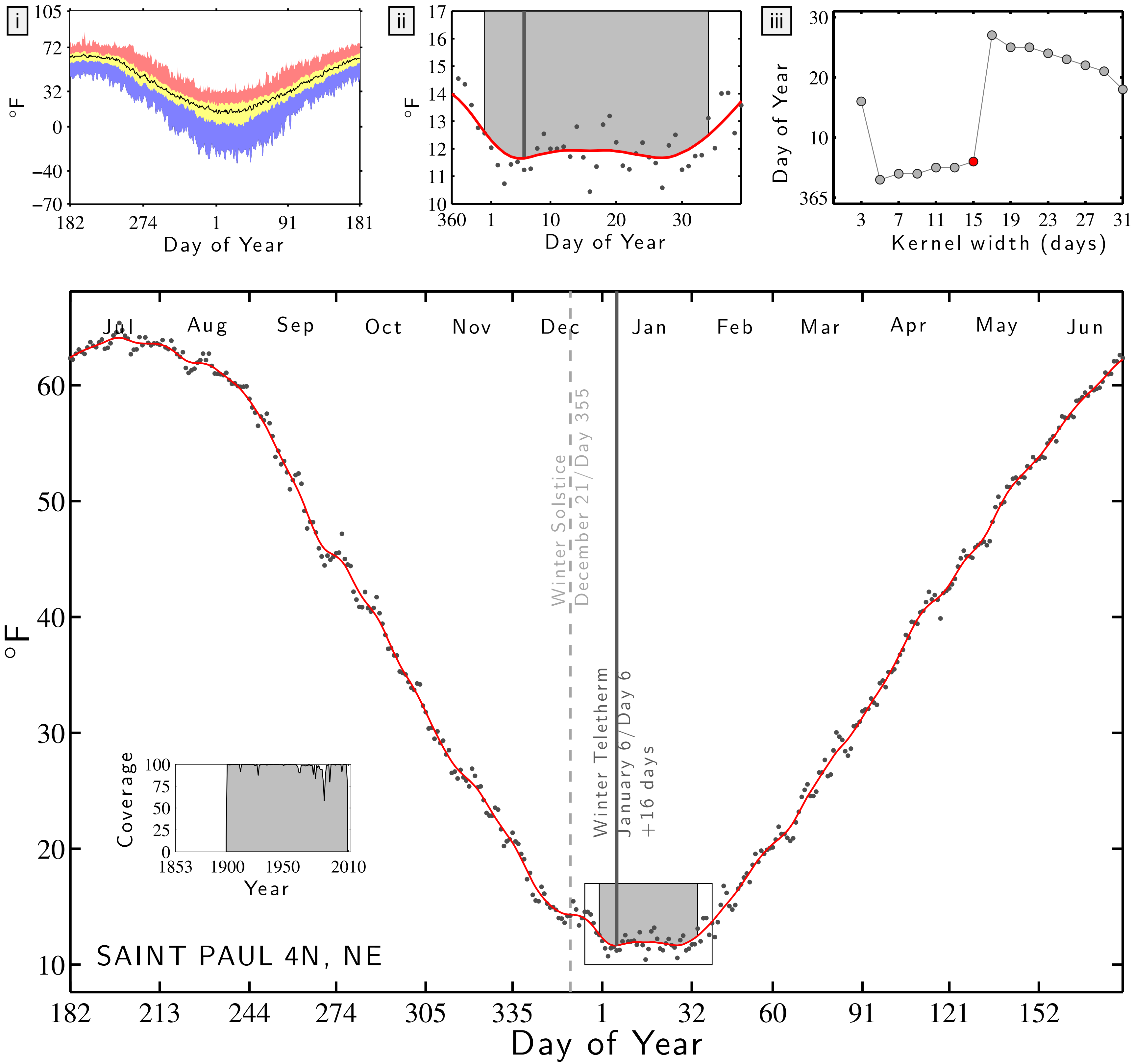

i
°F
Day of Year
ii
°F
Day of Year
iii
Day of Year
Kernel width (days)
Jul
Aug
Sep
Oct
Nov
Dec
Jan
Feb
Mar
Apr
May
Jun
Winter Solstice
December 21/Day 355
Winter Teletherm
January 6/Day 6
+16 days
Coverage
Year
SAINT PAUL 4N, NE
°F
Day of Year

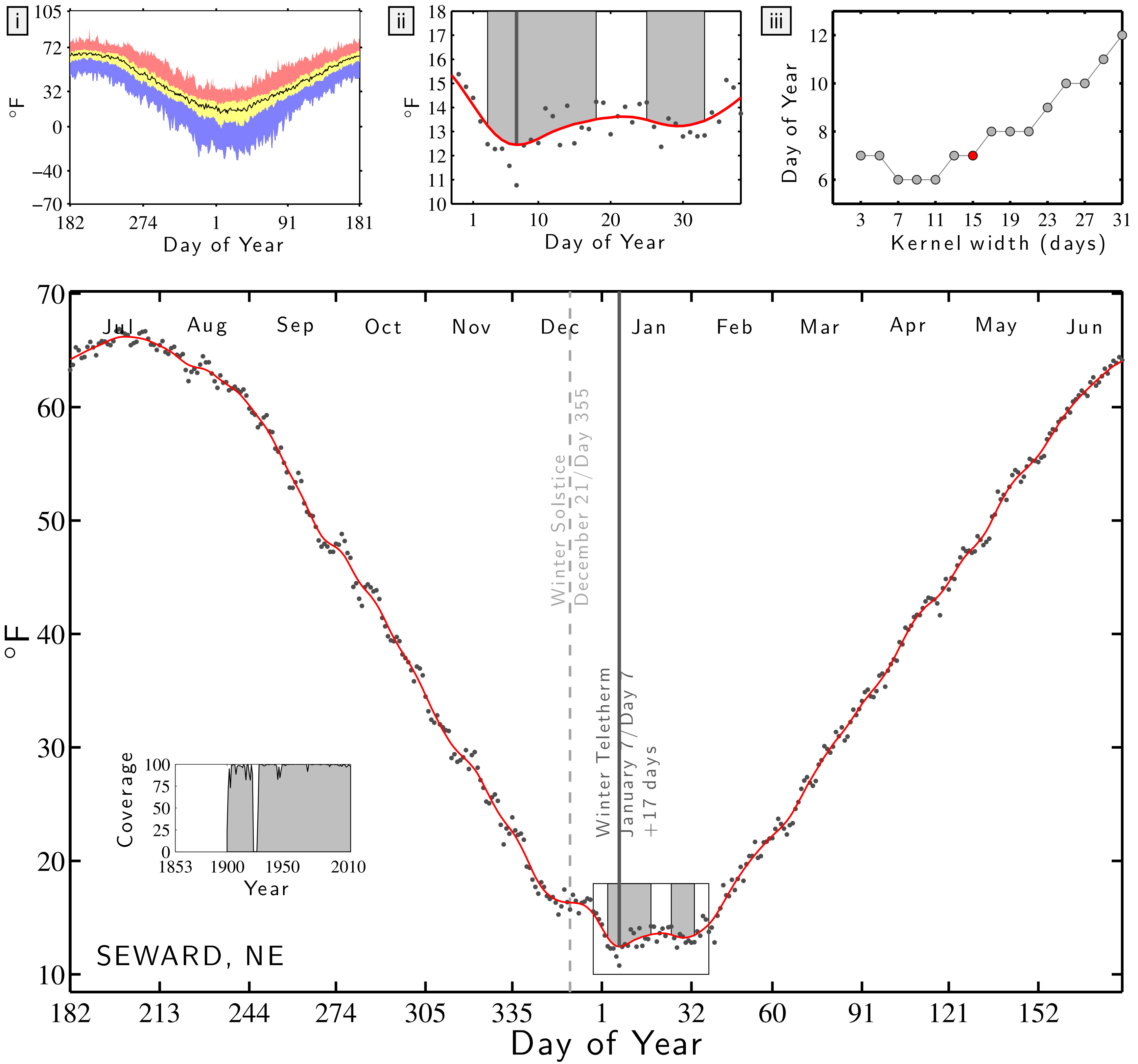

i
°F
Day of Year
ii
°F
Day of Year
iii
Day of Year
Kernel width (days)
Jul
Aug
Sep
Oct
Nov
Dec
Jan
Feb
Mar
Apr
May
Jun
Winter Solstice
December 21/Day 355
Winter Teletherm
January 7/Day 7
+17 days
Coverage
Year
SEWARD, NE
°F
Day of Year

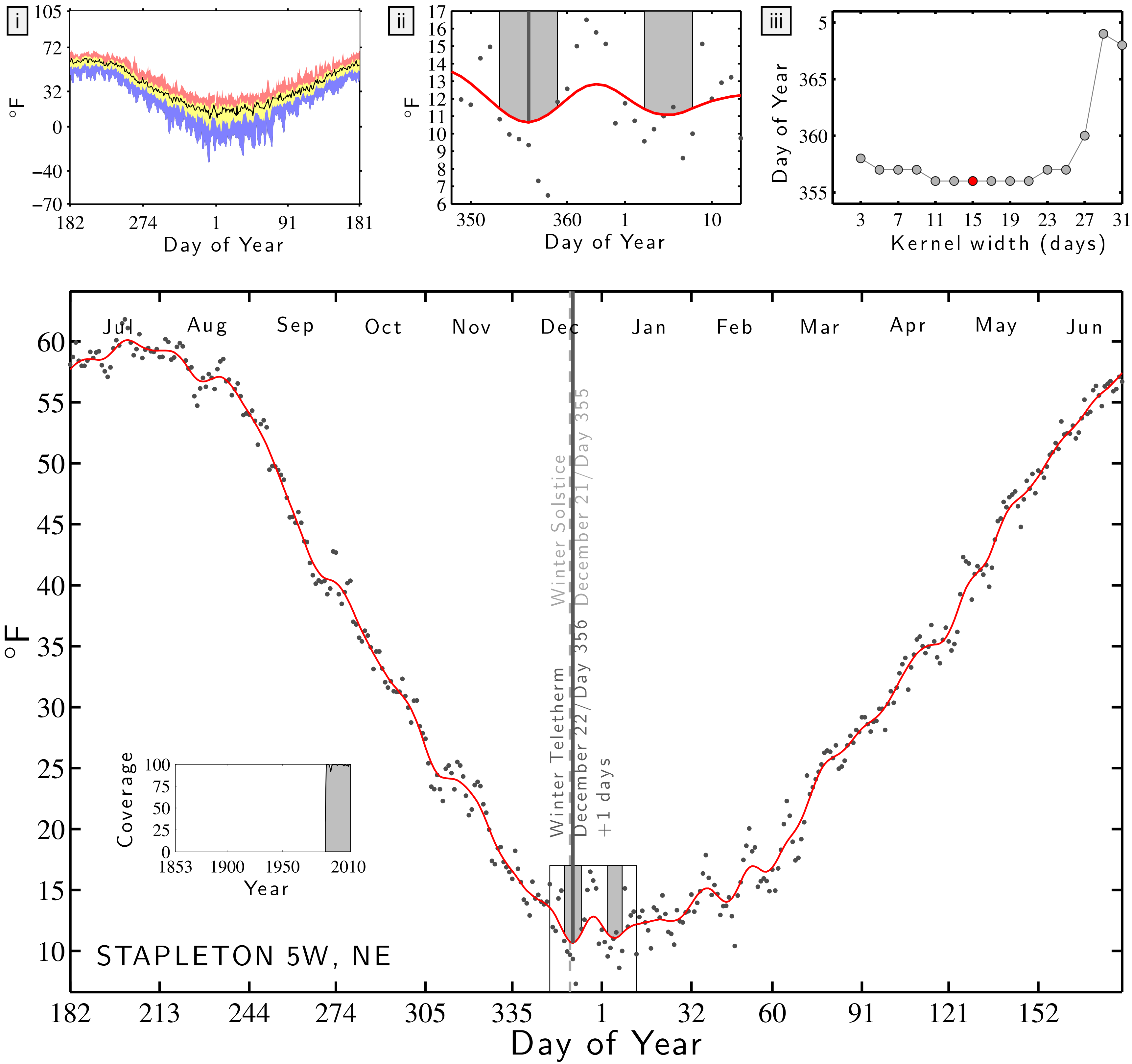

i
°F
Day of Year
ii
°F
Day of Year
iii
Day of Year
Kernel width (days)
Jul
Aug
Sep
Oct
Nov
Dec
Jan
Feb
Mar
Apr
May
Jun
Winter Solstice
December 21/Day 355
Winter Teletherm
December 22/Day 356
+1 days
Coverage
Year
STAPLETON 5W, NE
°F
Day of Year

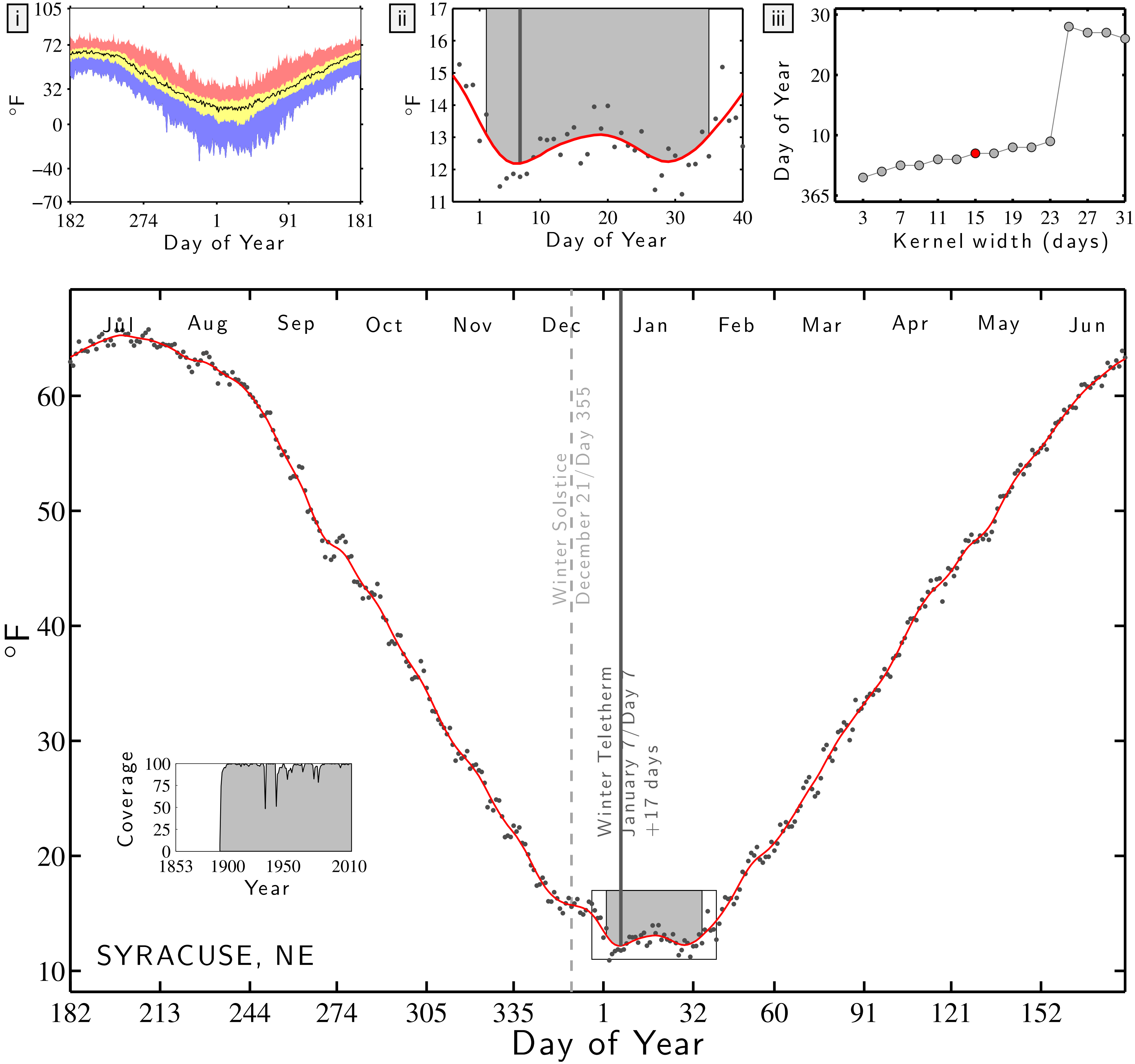

i
°F
Day of Year
ii
°F
Day of Year
iii
Day of Year
Kernel width (days)
Jul
Aug
Sep
Oct
Nov
Dec
Jan
Feb
Mar
Apr
May
Jun
Winter Solstice
December 21/Day 355
Winter Teletherm
January 7/Day 7
+17 days
Coverage
Year
SYRACUSE, NE
°F
Day of Year

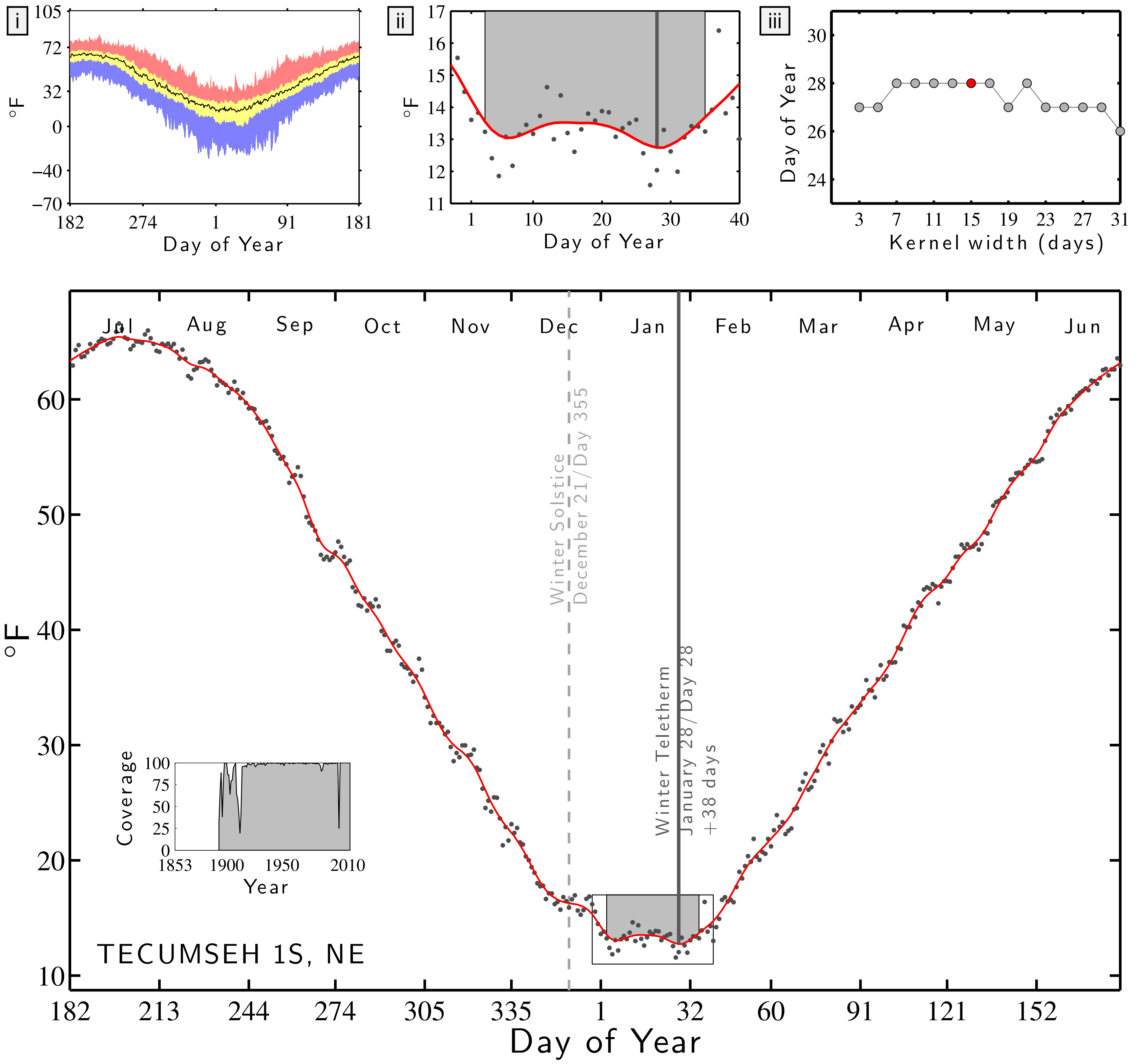

i
ii
iii
°F
Day of Year
Day of Year
Kernel width (days)
Jul
Aug
Sep
Oct
Nov
Dec
Jan
Feb
Mar
Apr
May
Jun
Winter Solstice
December 21/Day 355
Winter Teletherm
January 28/Day 28
+38 days
Coverage
Year
TECUMSEH 1S, NE

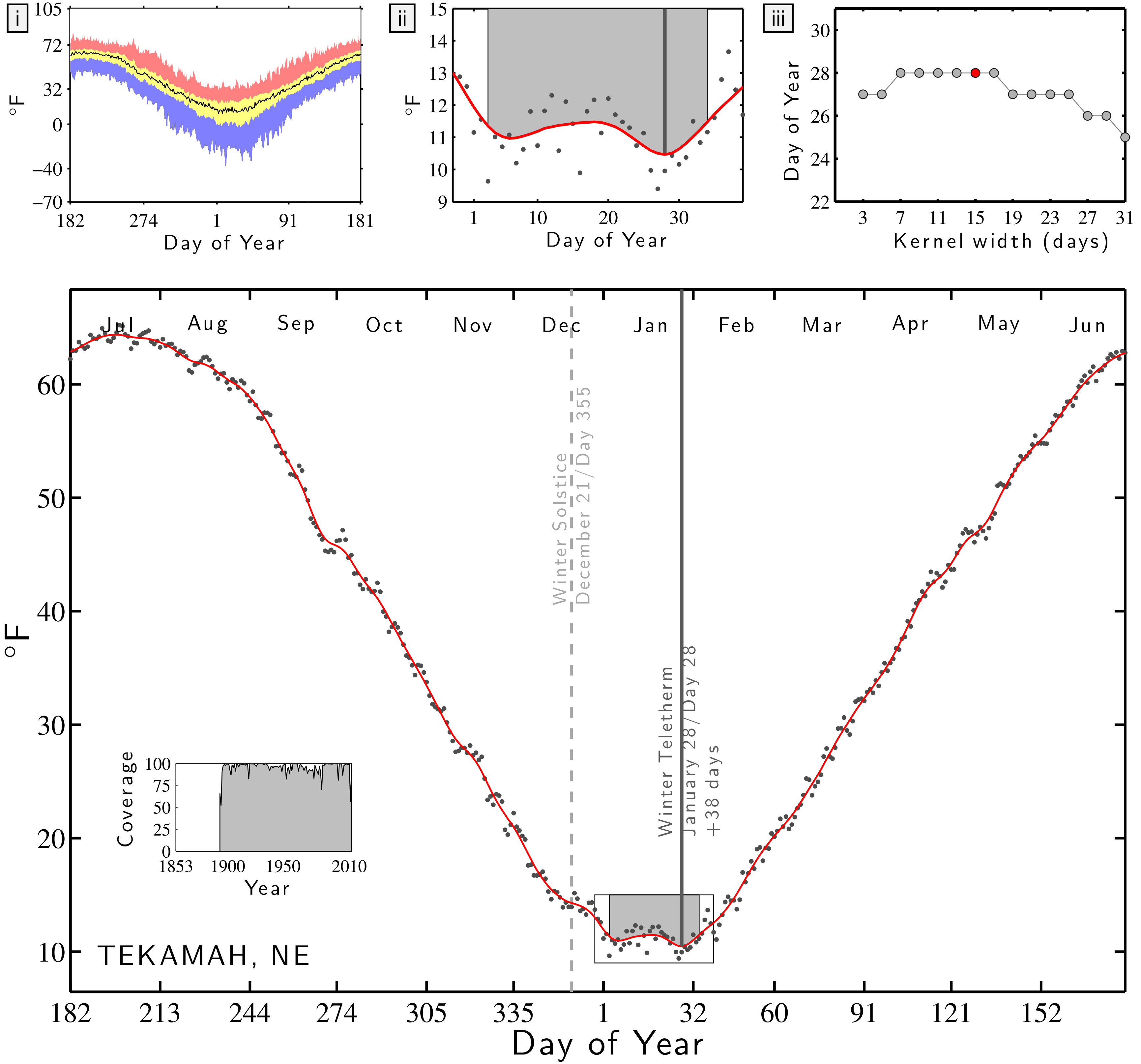

i
105
72
32
0
−40
−70
°F
182
274
1
91
181
Day of Year
ii
15
14
13
12
11
10
9
°F
1
10
20
30
Day of Year
iii
30
28
26
24
22
Day of Year
3
7
11
15
19
23
27
31
Kernel width (days)
Jul
Aug
Sep
Oct
Nov
Dec
Jan
Feb
Mar
Apr
May
Jun
60
50
40
30
20
10
°F
Winter Solstice
December 21/Day 355
Winter Teletherm
January 28/Day 28
+38 days
Coverage
100
75
50
25
0
1853
1900
1950
2010
Year
TEKAMAH, NE
182
213
244
274
305
335
1
32
60
91
121
152
Day of Year

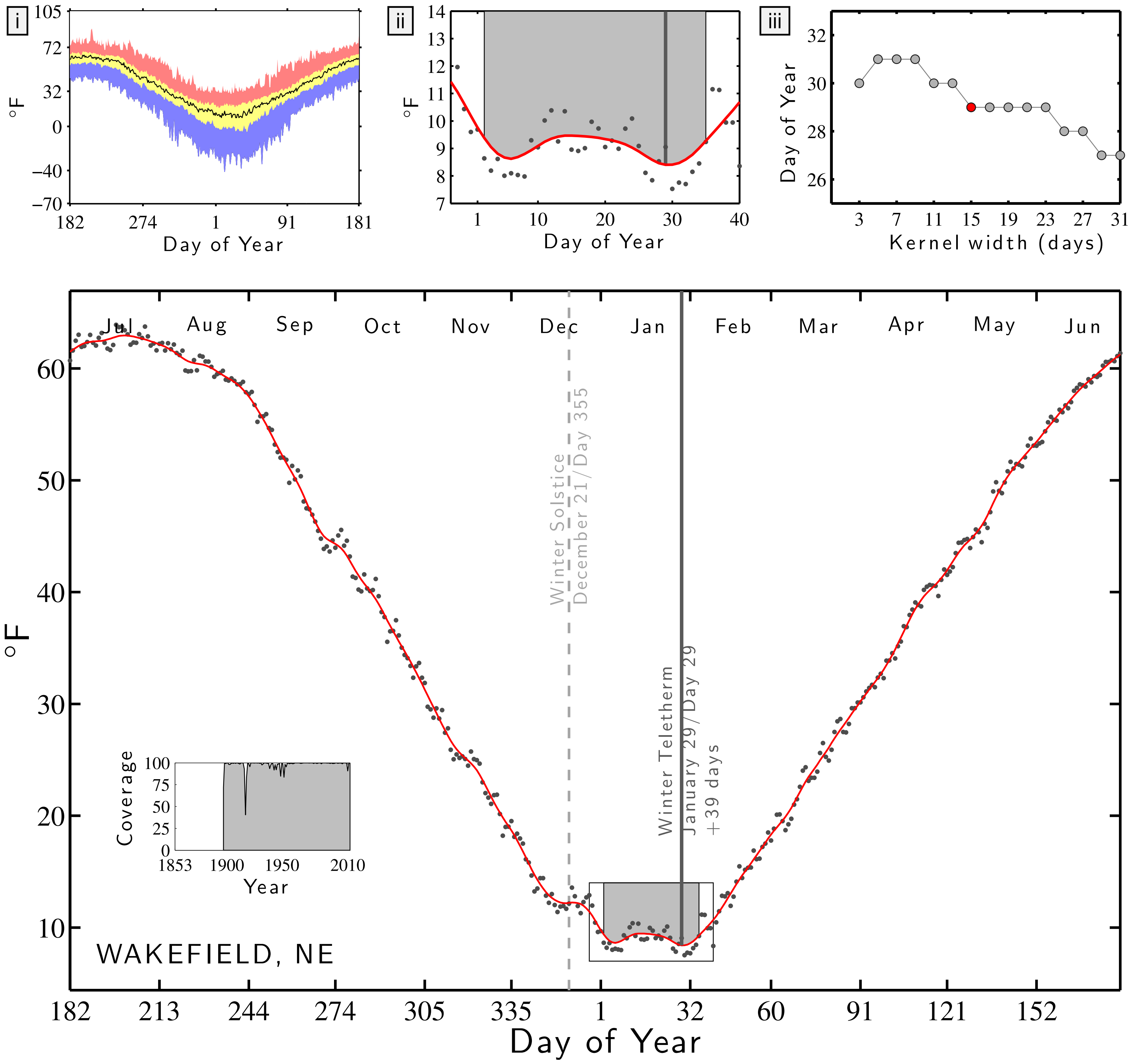

i
ii
iii
°F
Day of Year
Day of Year
Kernel width (days)
Jul
Aug
Sep
Oct
Nov
Dec
Jan
Feb
Mar
Apr
May
Jun
Winter Solstice
December 21/Day 355
Winter Teletherm
January 29/Day 29
+39 days
Coverage
Year
WAKEFIELD, NE
°F
Day of Year

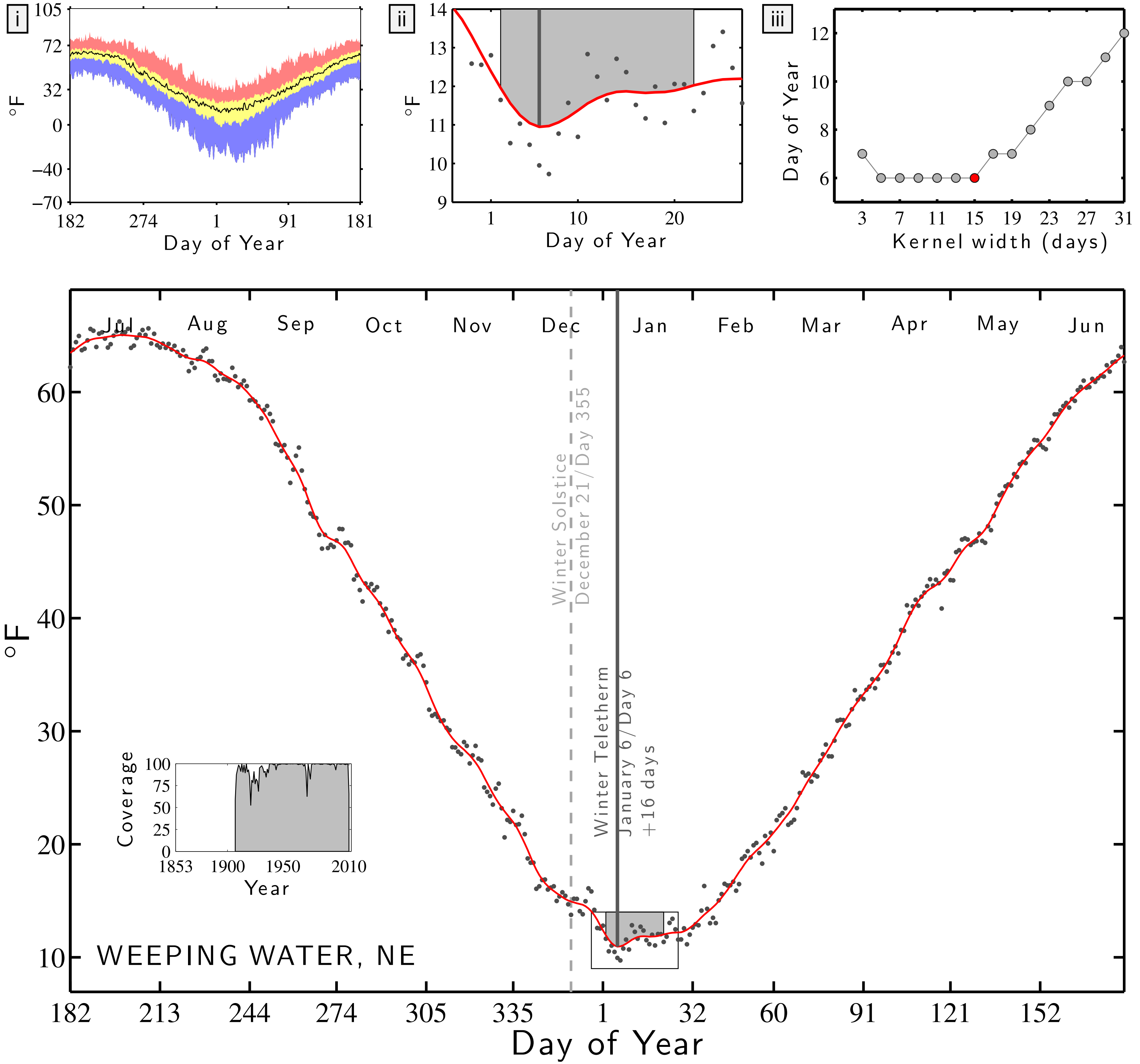

i
ii
iii
°F
Day of Year
Kernel width (days)
Jul
Aug
Sep
Oct
Nov
Dec
Jan
Feb
Mar
Apr
May
Jun
Winter Solstice
December 21/Day 355
Winter Teletherm
January 6/Day 6
+16 days
Coverage
Year
WEEPING WATER, NE

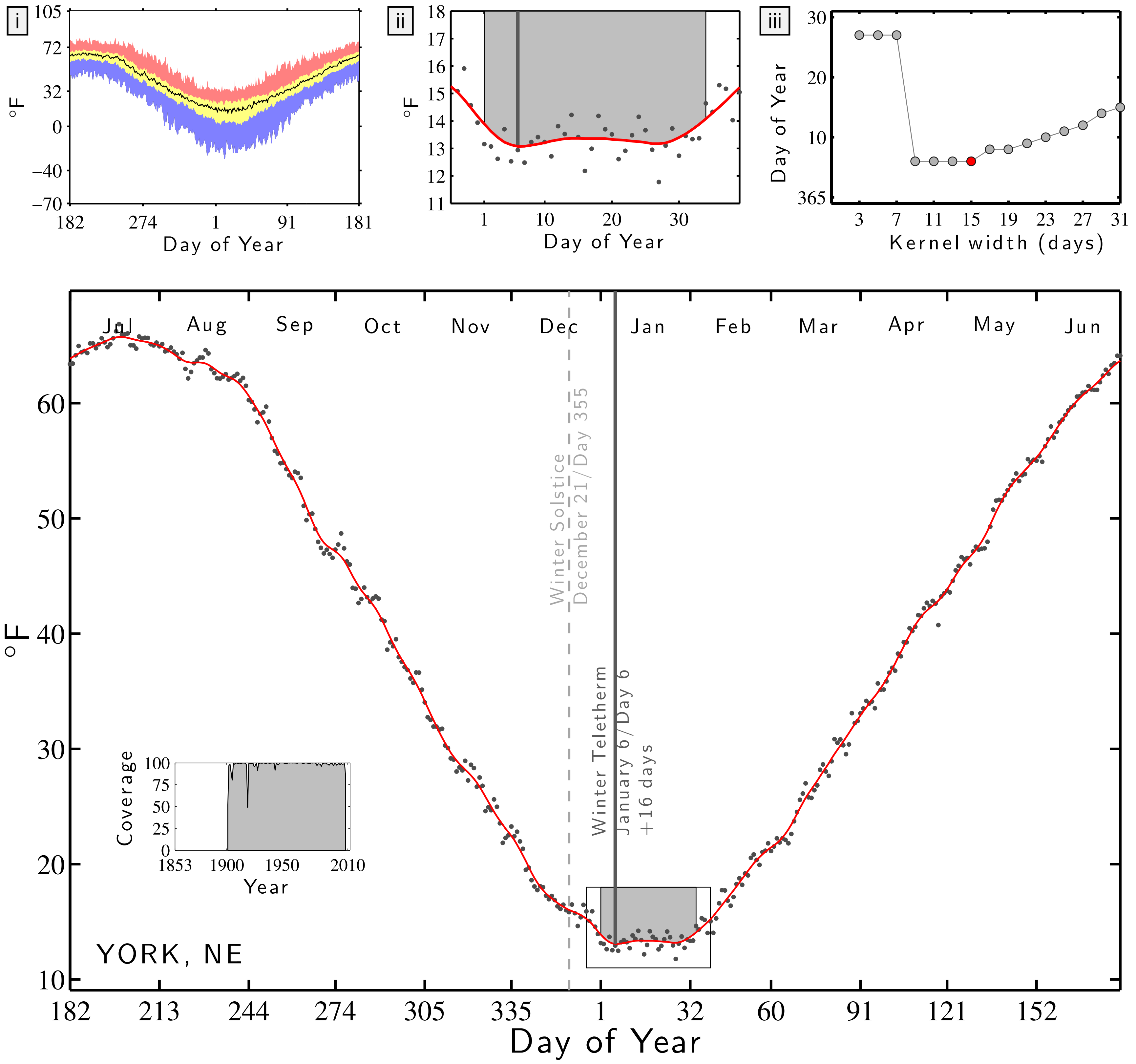

i
105
72
32
0
−40
−70
°F
182
274
1
91
181
Day of Year
ii
18
17
16
15
14
13
12
11
°F
1
10
20
30
Day of Year
iii
30
20
10
365
Day of Year
3
7
11
15
19
23
27
31
Kernel width (days)
Jul
Aug
Sep
Oct
Nov
Dec
Jan
Feb
Mar
Apr
May
Jun
Winter Solstice
December 21/Day 355
Winter Teletherm
January 6/Day 6
+16 days
60
50
40
30
20
10
°F
Coverage
100
75
50
25
0
1853
1900
1950
2010
Year
YORK, NE
182
213
244
274
305
335
1
32
60
91
121
152
Day of Year

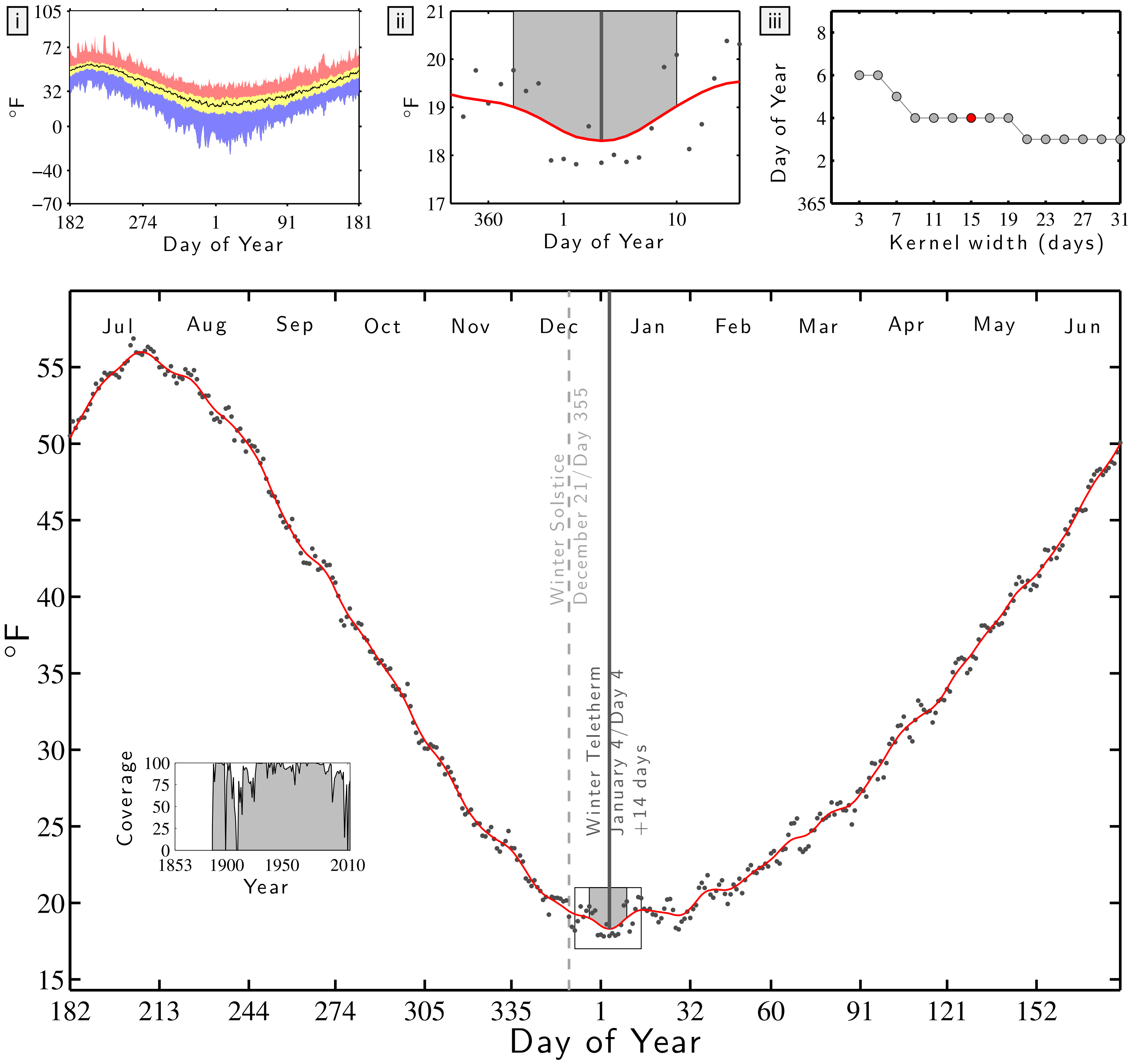

i
ii
iii
°F
Day of Year
Day of Year
Kernel width (days)
Jul
Aug
Sep
Oct
Nov
Dec
Jan
Feb
Mar
Apr
May
Jun
Winter Solstice
December 21/Day 355
Winter Teletherm
January 4/Day 4
+14 days
Coverage
Year
°F
Day of Year

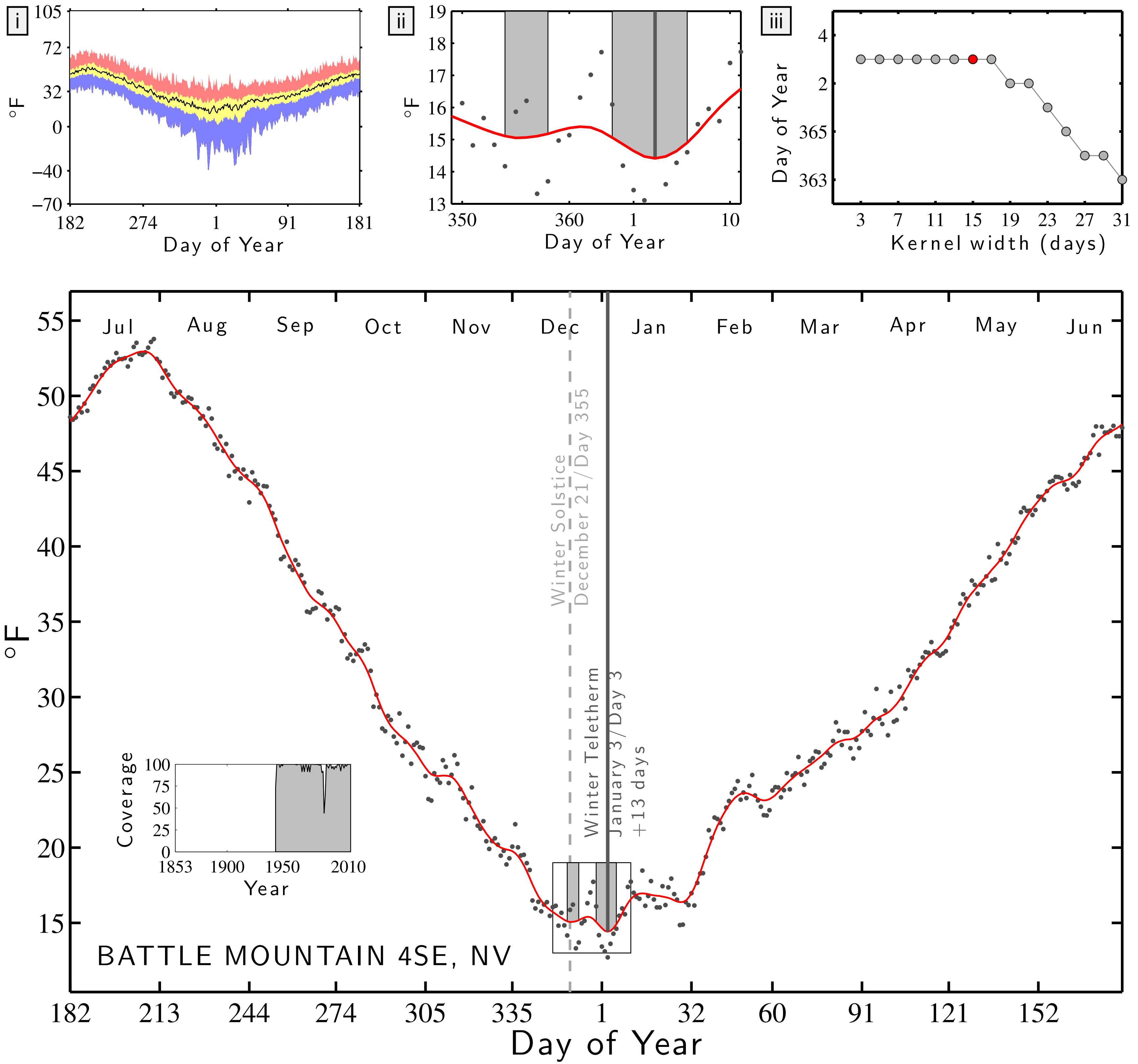

i
°F
Day of Year
ii
°F
Day of Year
iii
Day of Year
Kernel width (days)
Jul
Aug
Sep
Oct
Nov
Dec
Jan
Feb
Mar
Apr
May
Jun
Winter Solstice
December 21/Day 355
Winter Teletherm
January 3/Day 3
+13 days
Coverage
Year
BATTLE MOUNTAIN 4SE, NV
°F
Day of Year

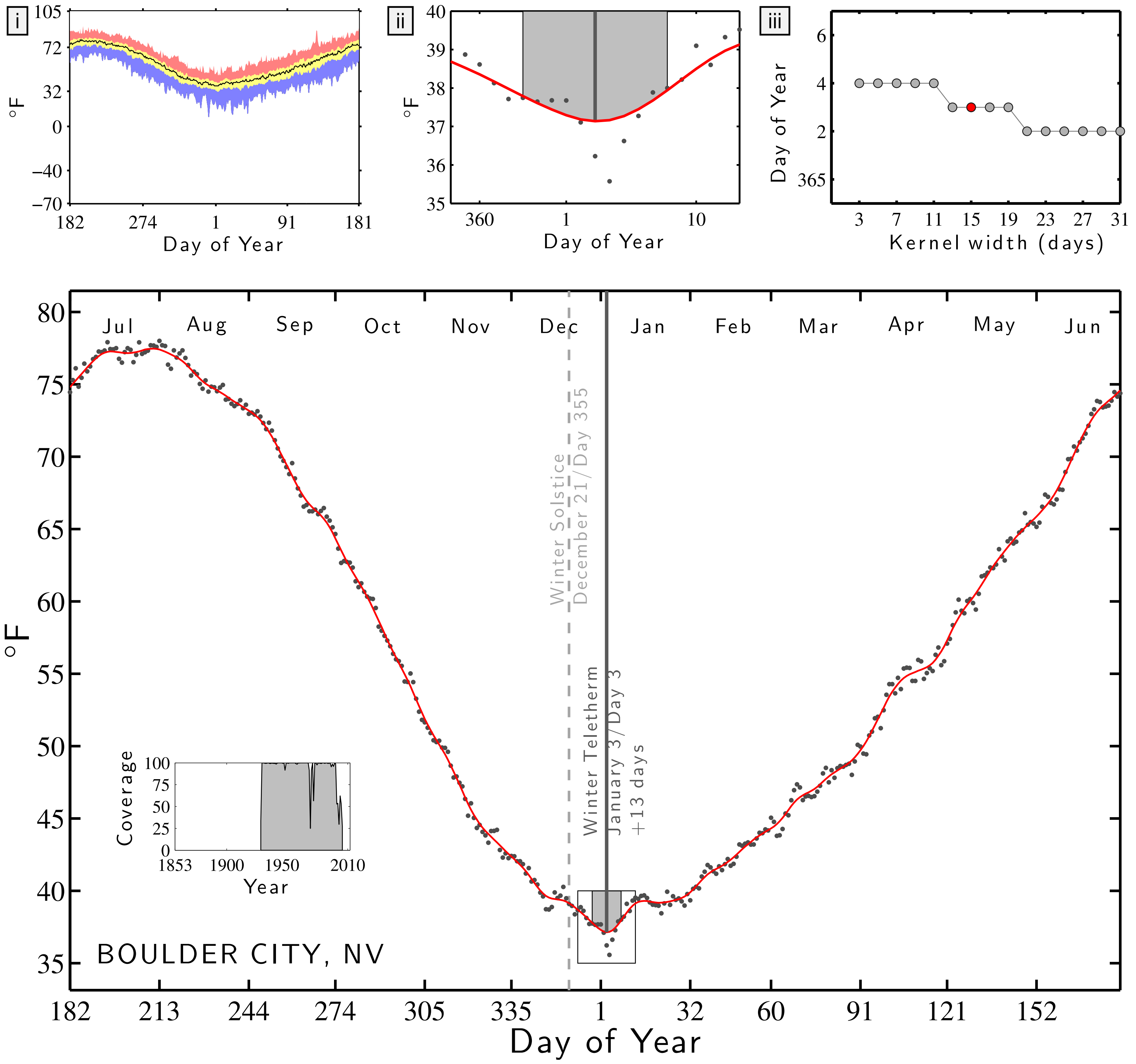

i
°F
Day of Year
ii
°F
Day of Year
iii
Day of Year
Kernel width (days)
Jul
Aug
Sep
Oct
Nov
Dec
Jan
Feb
Mar
Apr
May
Jun
Winter Solstice
December 21/Day 355
Winter Teletherm
January 3/Day 3
+13 days
Coverage
Year
BOULDER CITY, NV
°F
Day of Year

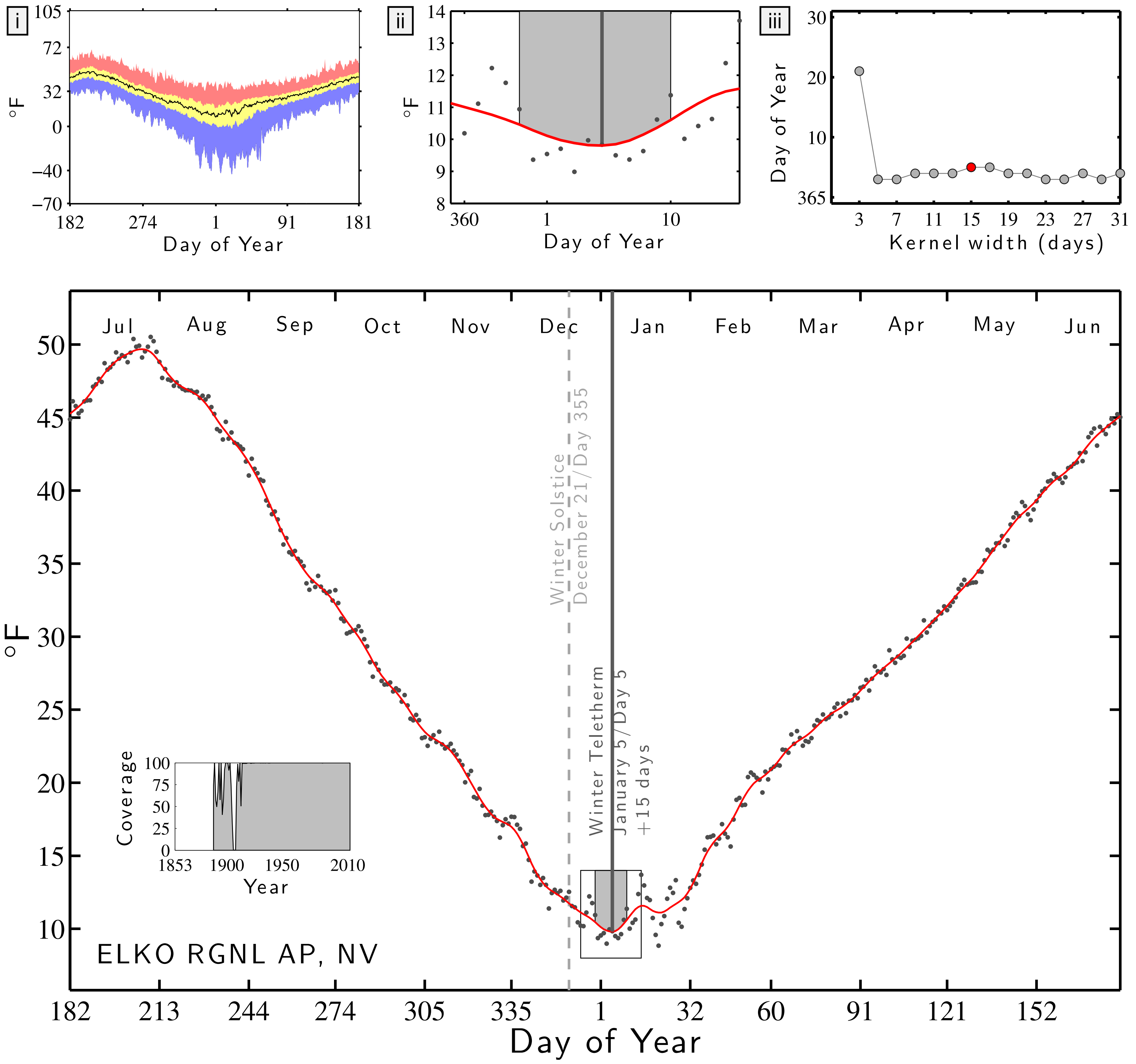

i
ii
iii
°F
Day of Year
Kernel width (days)
Jul
Aug
Sep
Oct
Nov
Dec
Jan
Feb
Mar
Apr
May
Jun
Winter Solstice
December 21/Day 355
Winter Teletherm
January 5/Day 5
+15 days
Coverage
Year
ELKO RGNL AP, NV

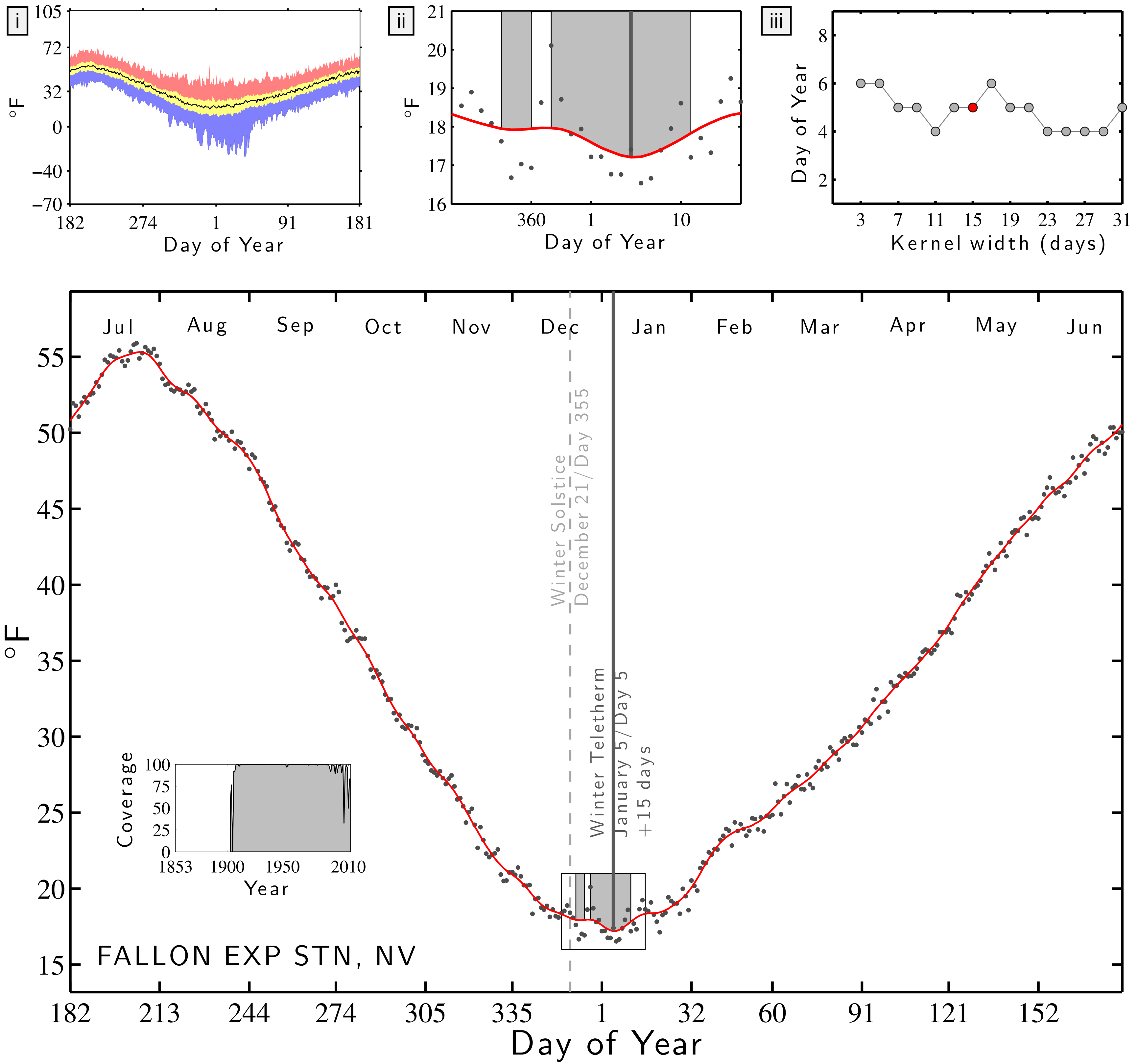

i
°F
Day of Year
ii
°F
Day of Year
iii
Day of Year
Kernel width (days)
Jul
Aug
Sep
Oct
Nov
Dec
Jan
Feb
Mar
Apr
May
Jun
Winter Solstice
December 21/Day 355
Winter Teletherm
January 5/Day 5
+15 days
Coverage
Year
FALLON EXP STN, NV
°F
Day of Year

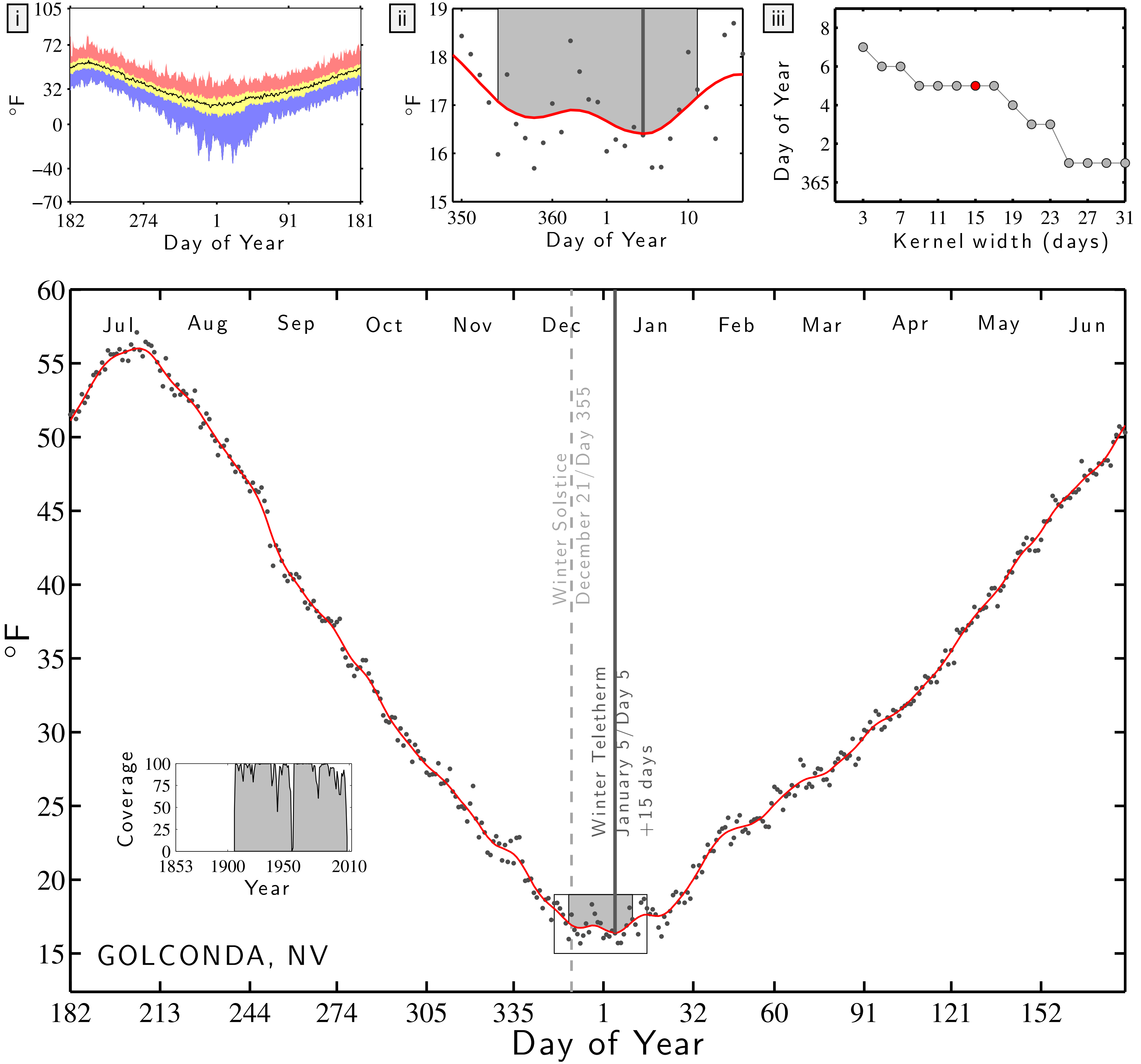

i
°F
Day of Year
ii
Day of Year
iii
Day of Year
Kernel width (days)
Jul
Aug
Sep
Oct
Nov
Dec
Jan
Feb
Mar
Apr
May
Jun
Winter Solstice
December 21/Day 355
Winter Teletherm
January 5/Day 5
+15 days
Coverage
Year
GOLCONDA, NV
Day of Year

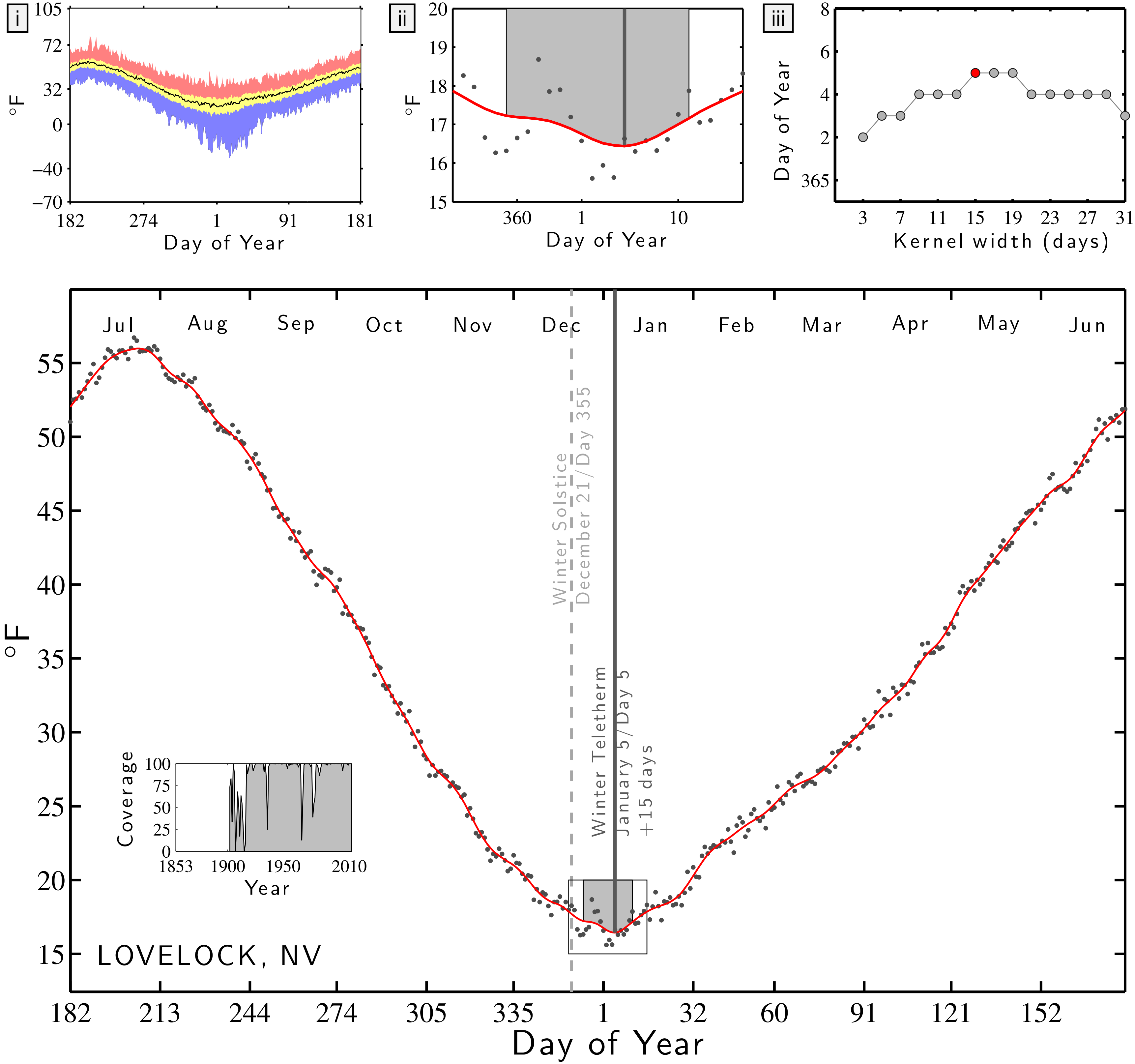

i
°F
105
72
32
0
−40
−70
182
274
1
91
181
Day of Year
ii
20
19
18
17
16
15
360
1
10
iii
Day of Year
8
6
4
2
365
3
7
11
15
19
23
27
31
Kernel width (days)
Jul
Aug
Sep
Oct
Nov
Dec
Jan
Feb
Mar
Apr
May
Jun
55
50
45
40
35
30
25
20
15
Winter Solstice
December 21/Day 355
Winter Teletherm
January 5/Day 5
+15 days
Coverage
100
75
50
25
0
1853
1900
1950
2010
Year
LOVELOCK, NV
182
213
244
274
305
335
1
32
60
91
121
152
Day of Year

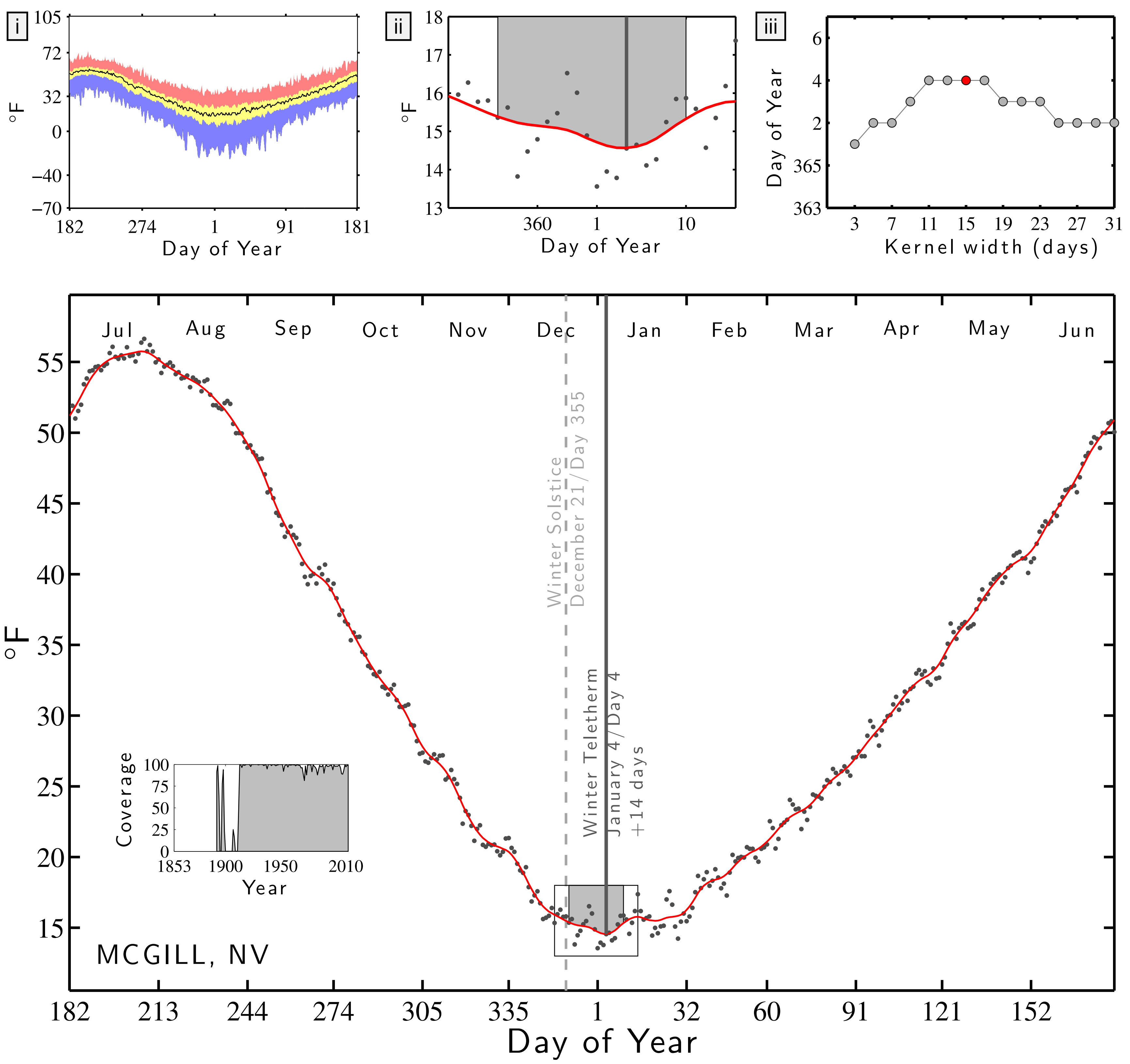

i
ii
iii
Day of Year
°F
Kernel width (days)
Jul
Aug
Sep
Oct
Nov
Dec
Jan
Feb
Mar
Apr
May
Jun
Winter Solstice
December 21/Day 355
Winter Teletherm
January 4/Day 4
+14 days
Coverage
Year
MCGILL, NV

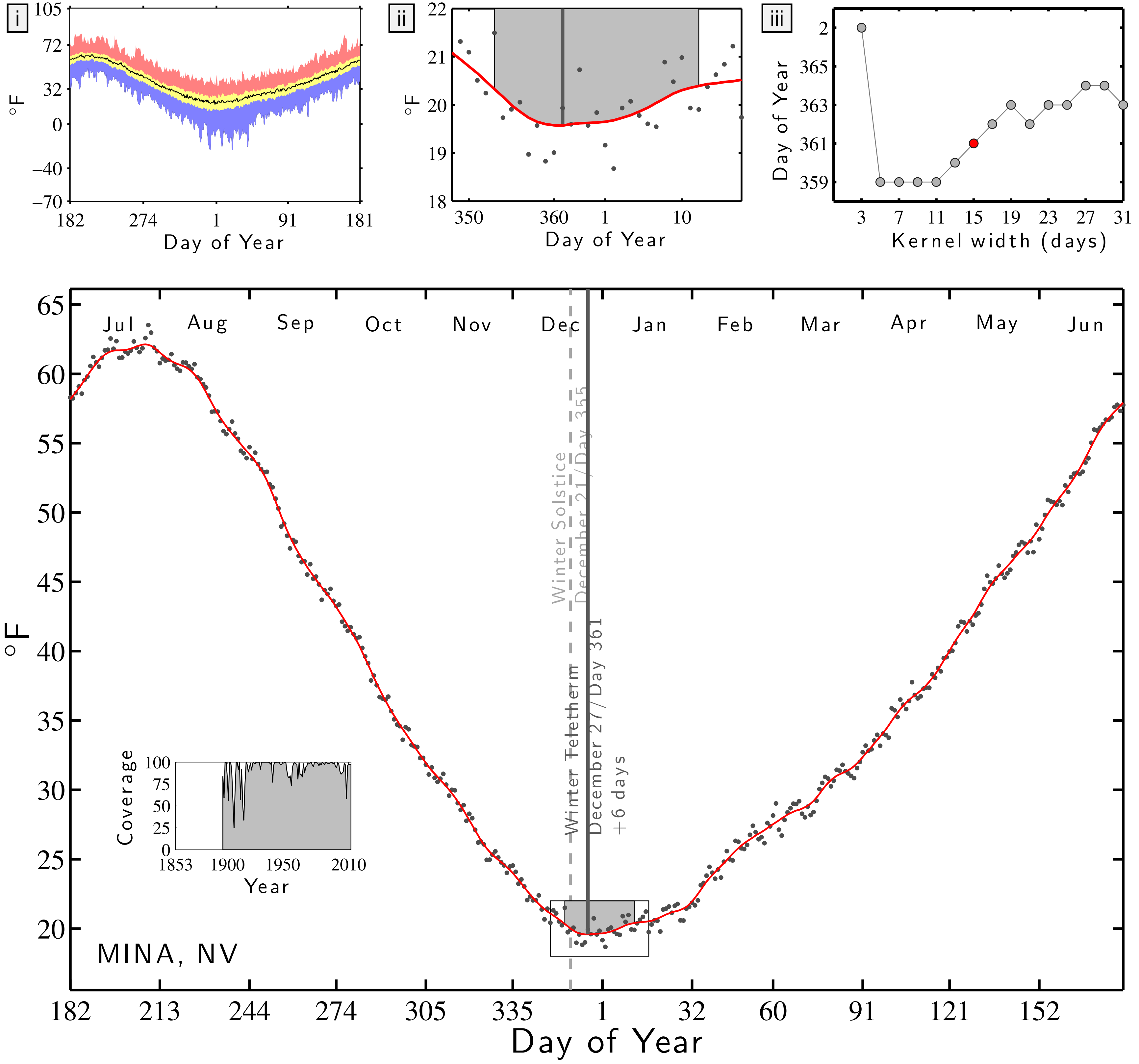

i
ii
iii
°F
Day of Year
Kernel width (days)
Jul
Aug
Sep
Oct
Nov
Dec
Jan
Feb
Mar
Apr
May
Jun
Winter Solstice
December 21/Day 355
Winter Teletherm
December 27/Day 361
+6 days
Coverage
Year
MINA, NV

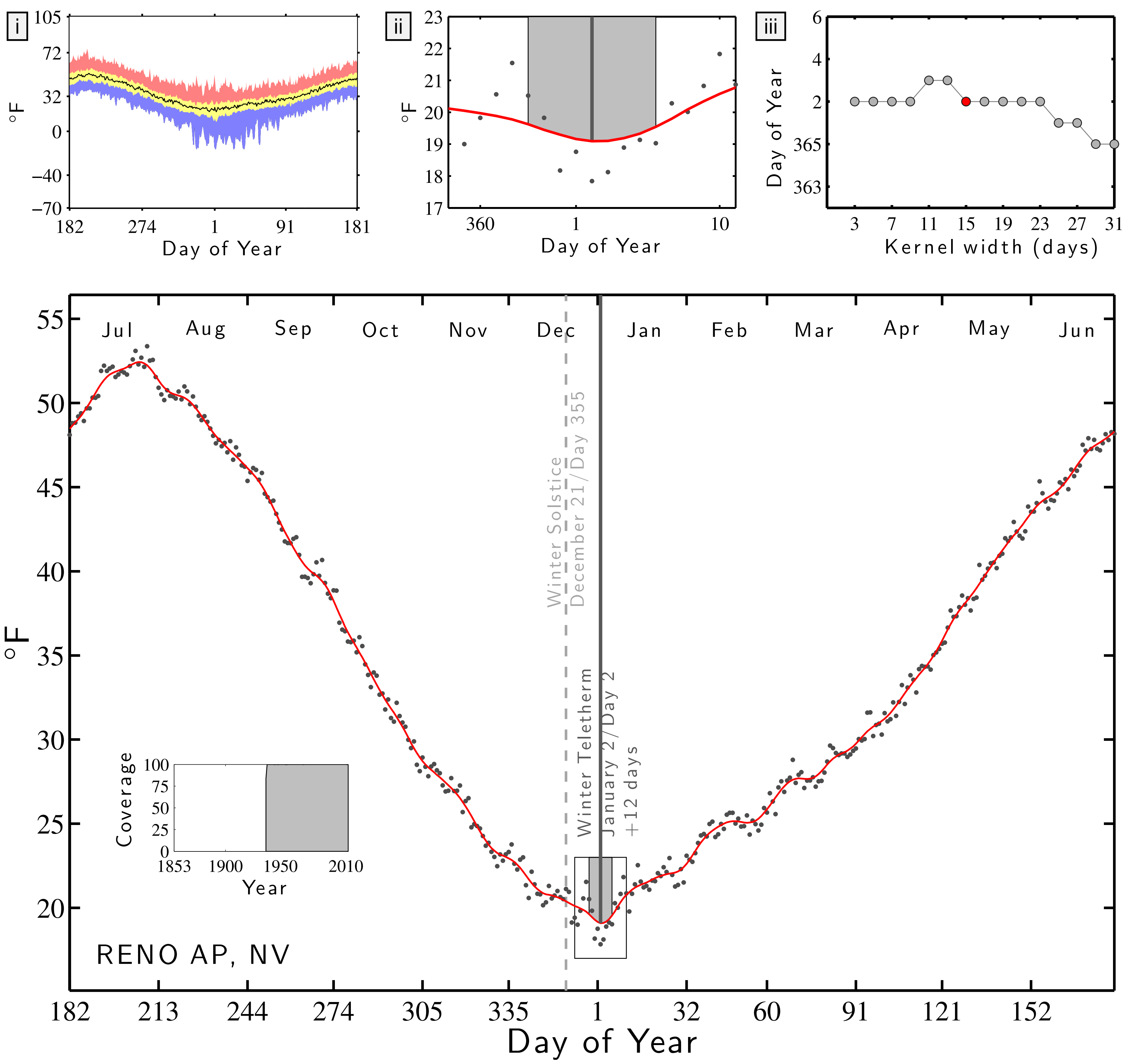

i
ii
iii
°F
Day of Year
Kernel width (days)
Jul
Aug
Sep
Oct
Nov
Dec
Jan
Feb
Mar
Apr
May
Jun
Winter Solstice
December 21/Day 355
Winter Teletherm
January 2/Day 2
+12 days
Coverage
Year
RENO AP, NV

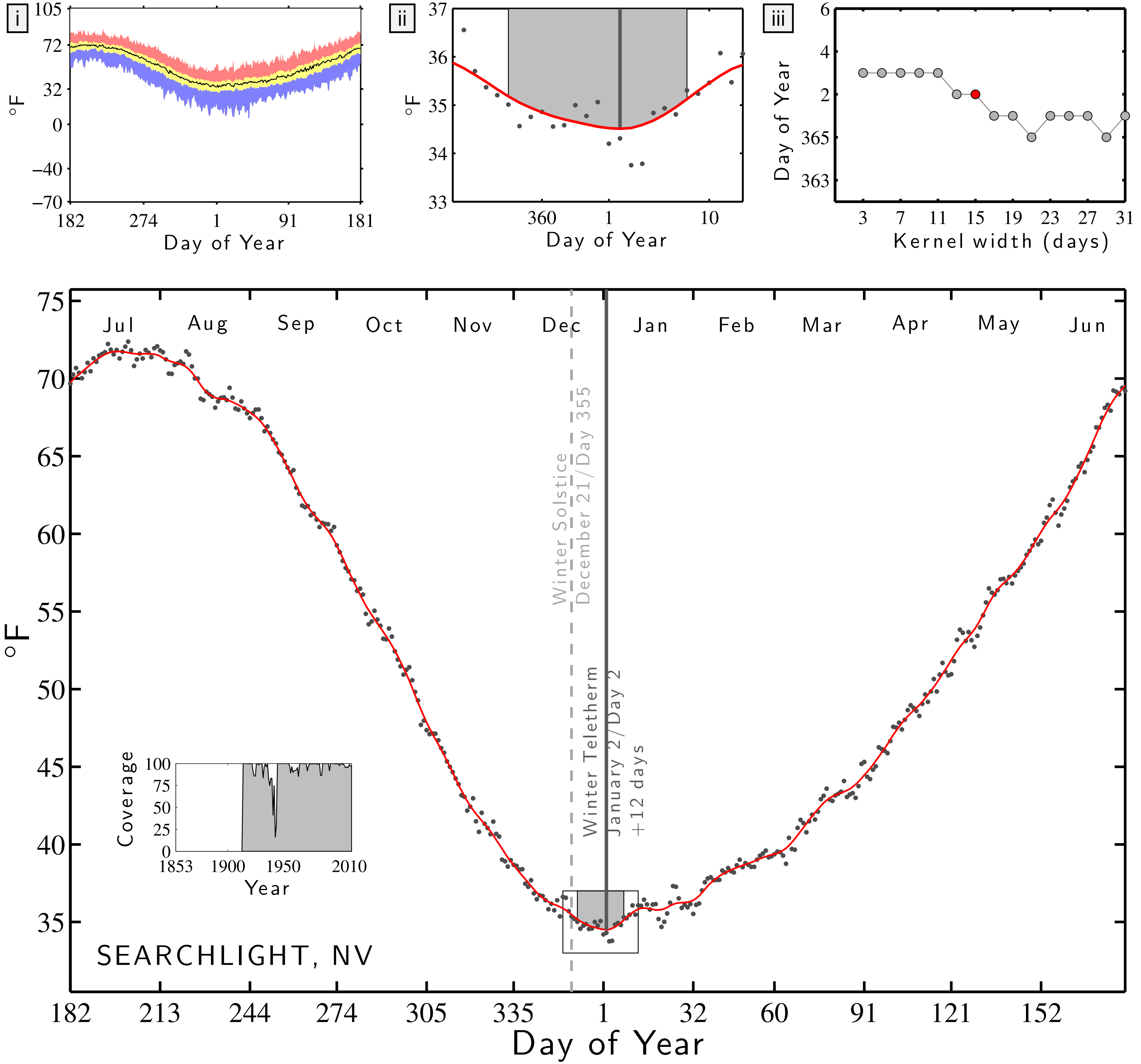

i
ii
iii
°F
Day of Year
Day of Year
Kernel width (days)
Jul
Aug
Sep
Oct
Nov
Dec
Jan
Feb
Mar
Apr
May
Jun
Winter Solstice
December 21/Day 355
Winter Teletherm
January 2/Day 2
+12 days
Coverage
Year
SEARCHLIGHT, NV

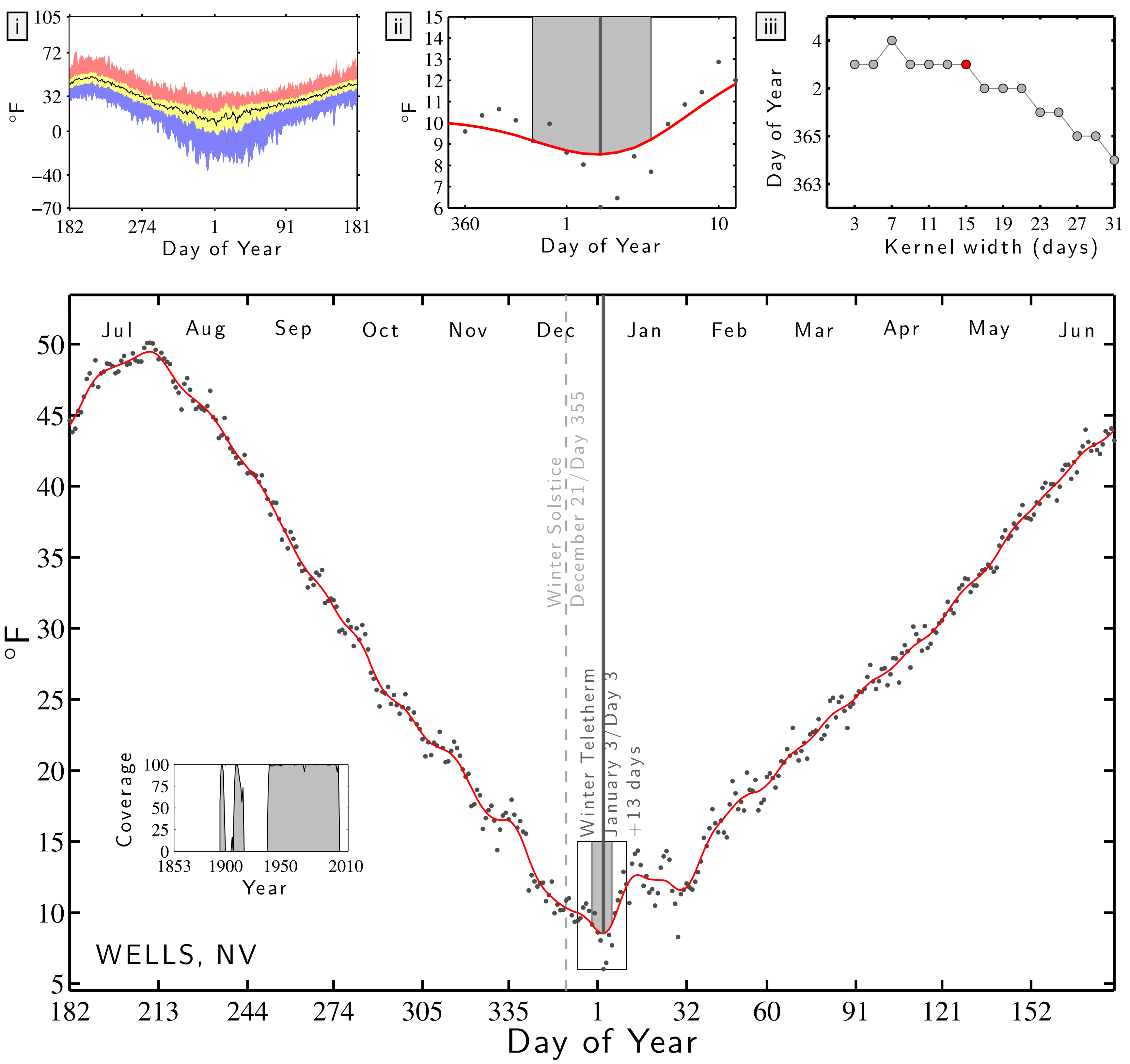

i
ii
iii
°F
Day of Year
Kernel width (days)
Jul
Aug
Sep
Oct
Nov
Dec
Jan
Feb
Mar
Apr
May
Jun
Winter Solstice
December 21/Day 355
Winter Teletherm
January 3/Day 3
+13 days
Coverage
Year
WELLS, NV

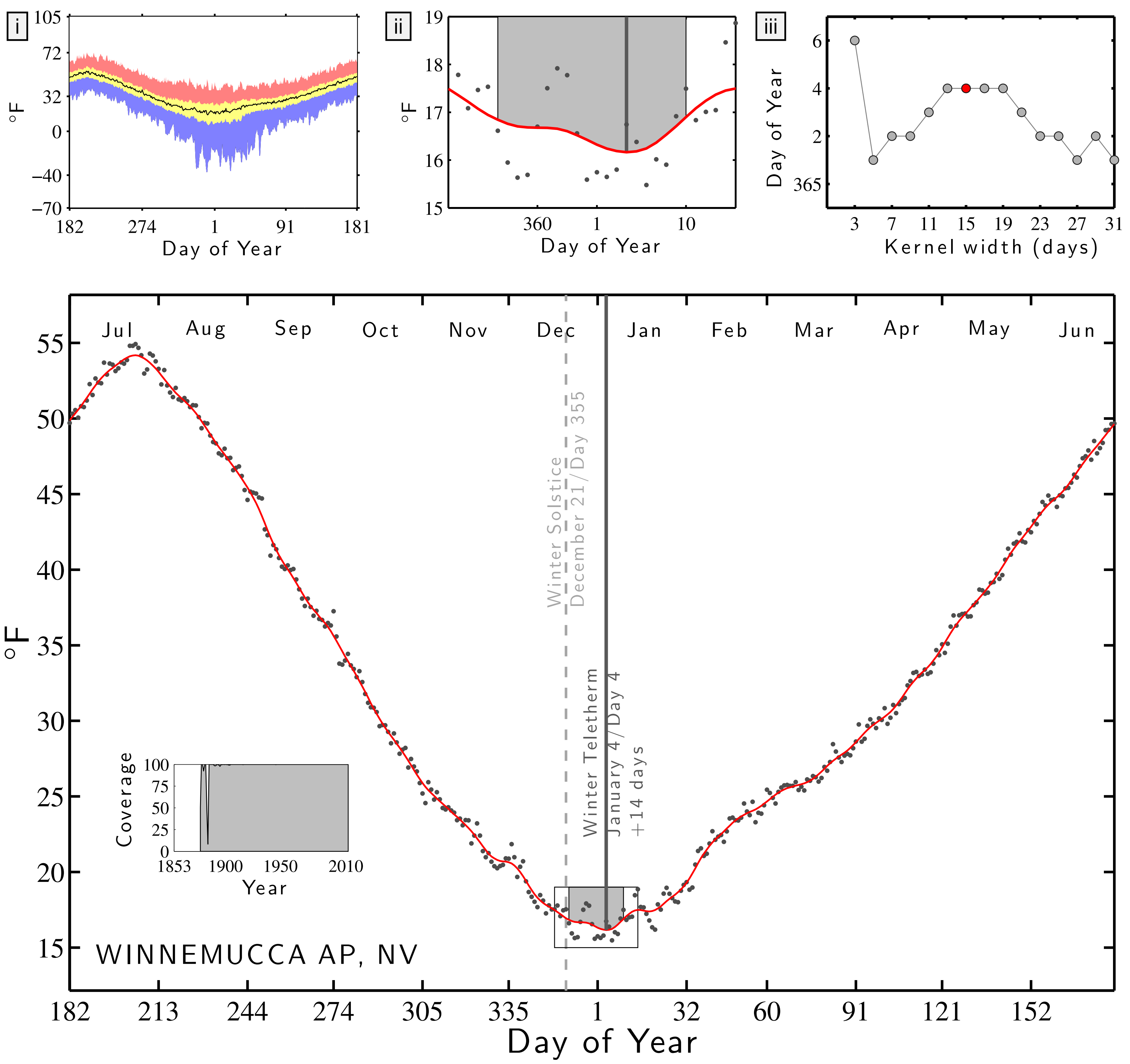

i
°F
Day of Year
ii
°F
Day of Year
iii
Day of Year
Kernel width (days)
Jul
Aug
Sep
Oct
Nov
Dec
Jan
Feb
Mar
Apr
May
Jun
Winter Solstice
December 21/Day 355
Winter Teletherm
January 4/Day 4
+14 days
Coverage
Year
WINNEMUCCA AP, NV
°F
Day of Year

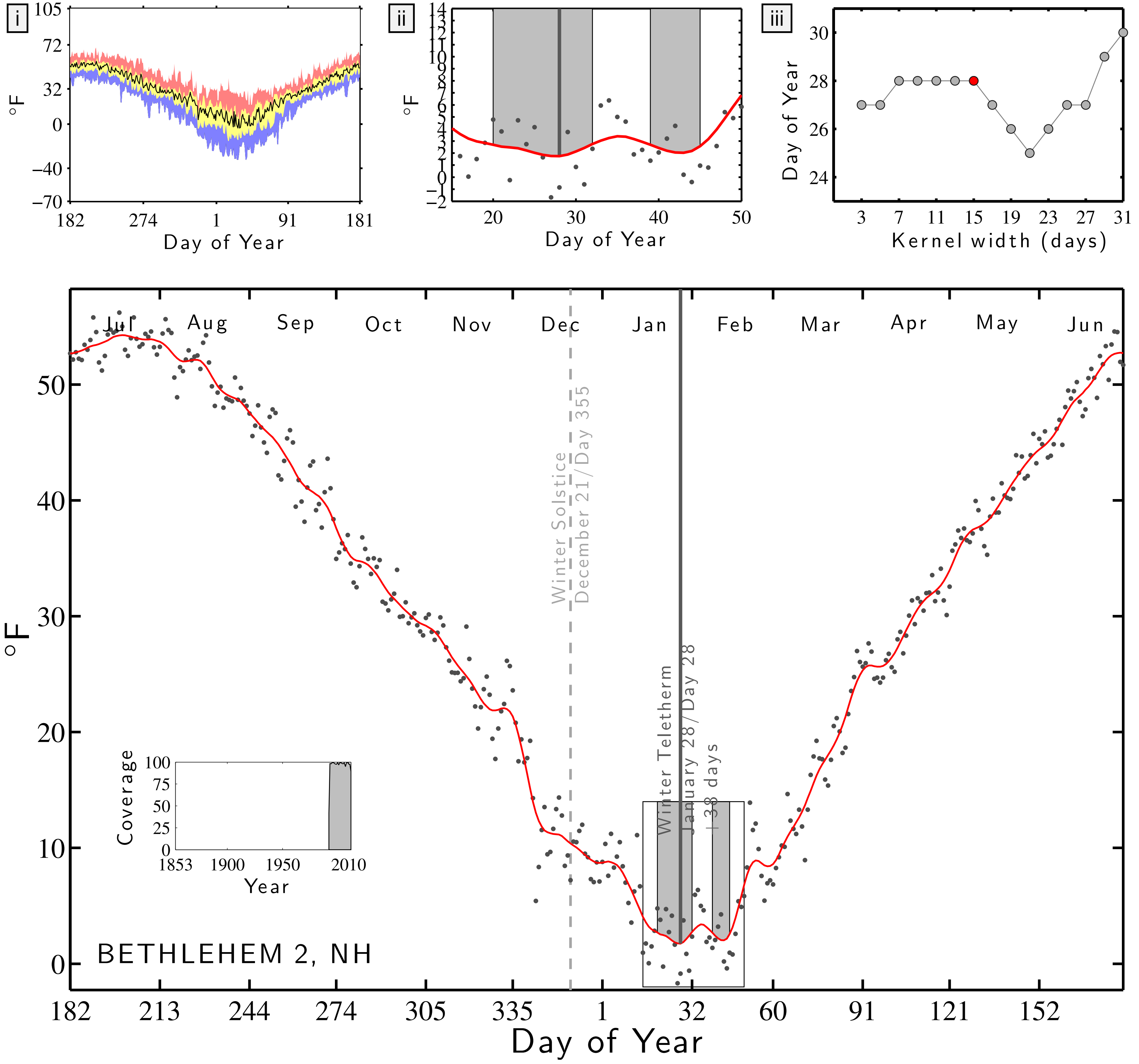

i
ii
iii
°F
Day of Year
Kernel width (days)
Jul
Aug
Sep
Oct
Nov
Dec
Jan
Feb
Mar
Apr
May
Jun
Winter Solstice
December 21/Day 355
Winter Teletherm
January 28/Day 28
±38 days
Coverage
Year
BETHLEHEM 2, NH

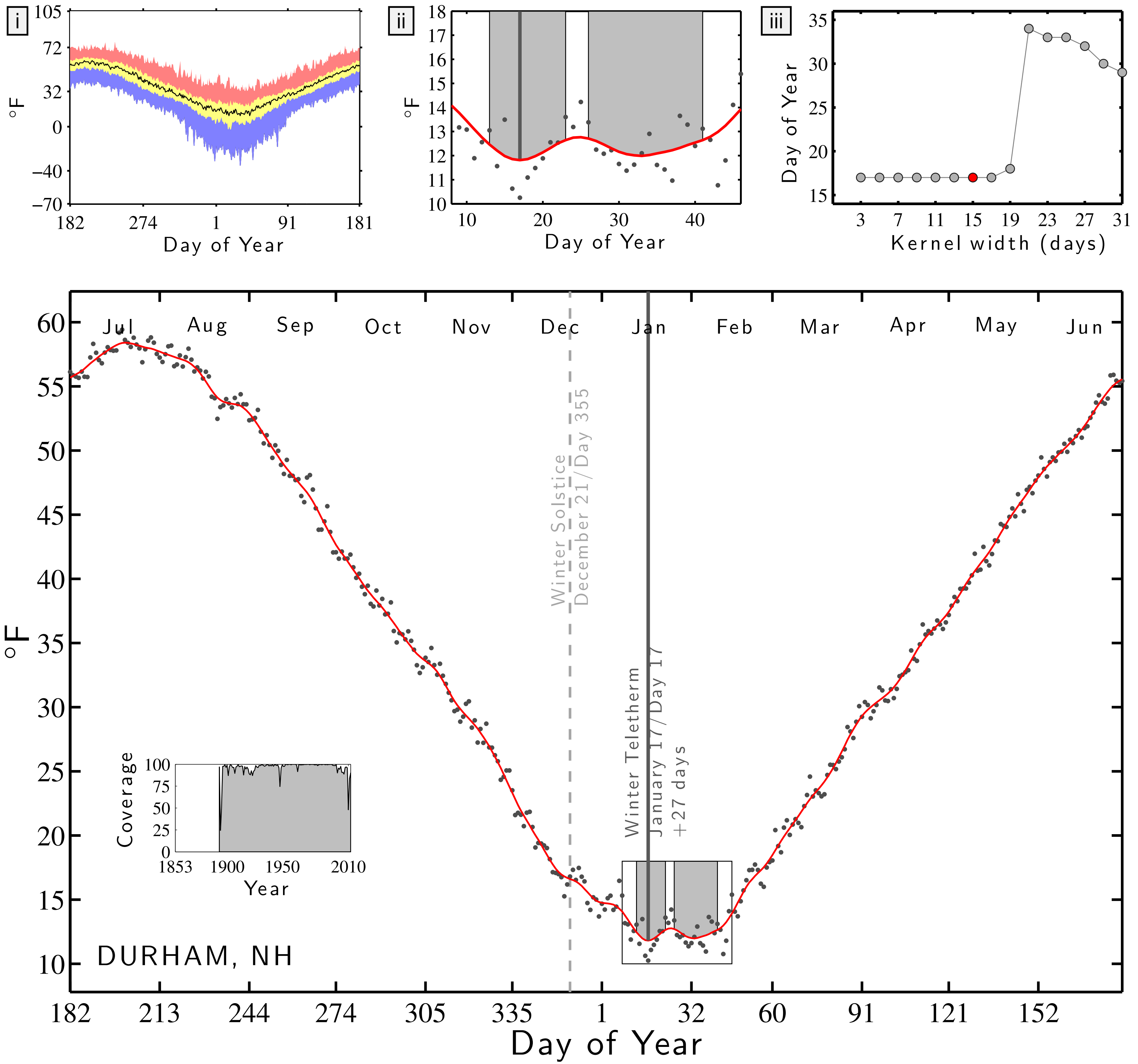

i
ii
iii
°F
Day of Year
Day of Year
Kernel width (days)
Jul
Aug
Sep
Oct
Nov
Dec
Jan
Feb
Mar
Apr
May
Jun
Winter Solstice
December 21/Day 355
Winter Teletherm
January 17/Day 17
+27 days
Coverage
Year
DURHAM, NH

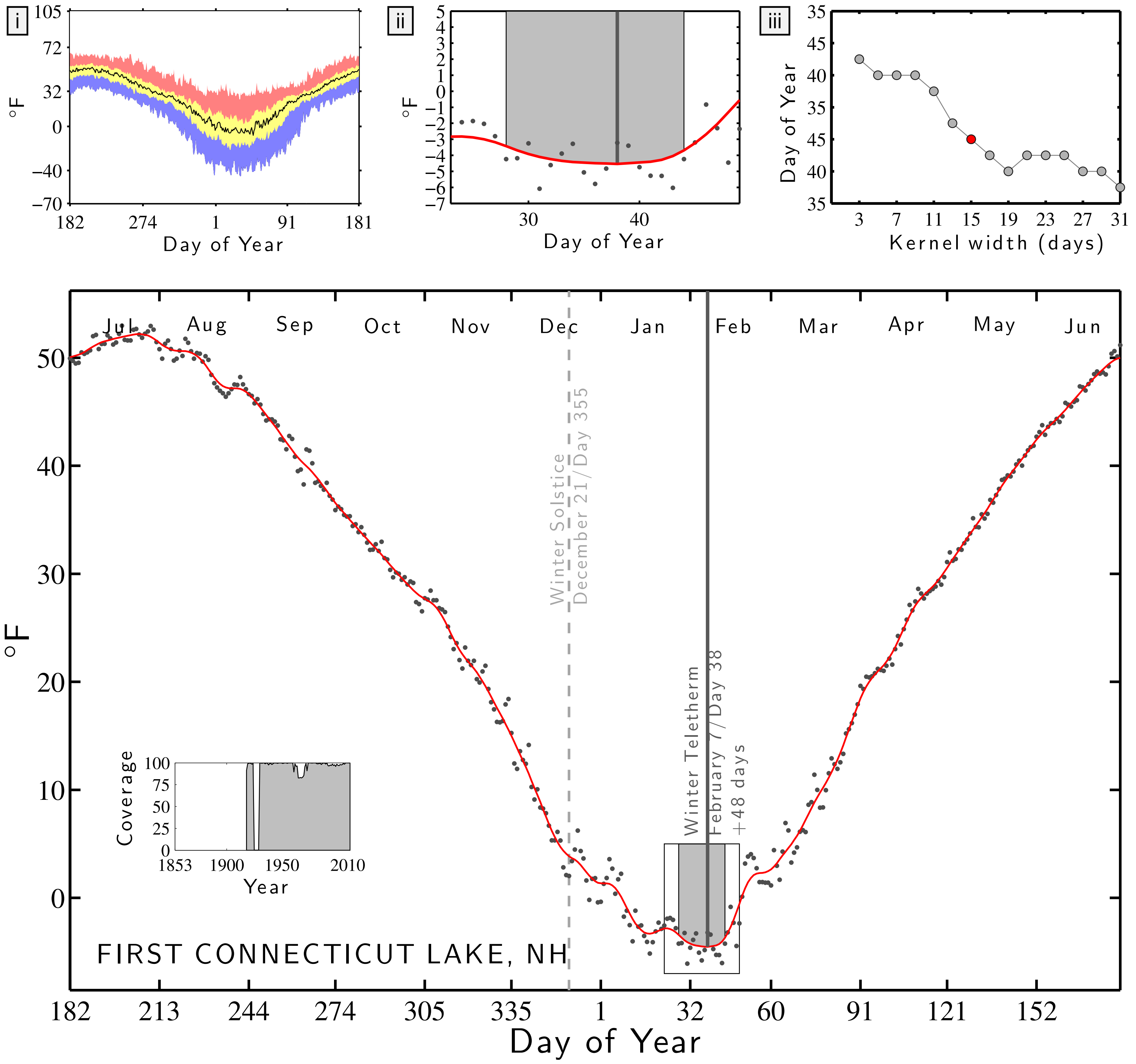

i
°F
Day of Year
ii
°F
Day of Year
iii
Day of Year
Kernel width (days)
Jul
Aug
Sep
Oct
Nov
Dec
Jan
Feb
Mar
Apr
May
Jun
Winter Solstice
December 21/Day 355
Winter Teletherm
February 7/Day 38
+48 days
Coverage
Year
FIRST CONNECTICUT LAKE, NH
°F
Day of Year

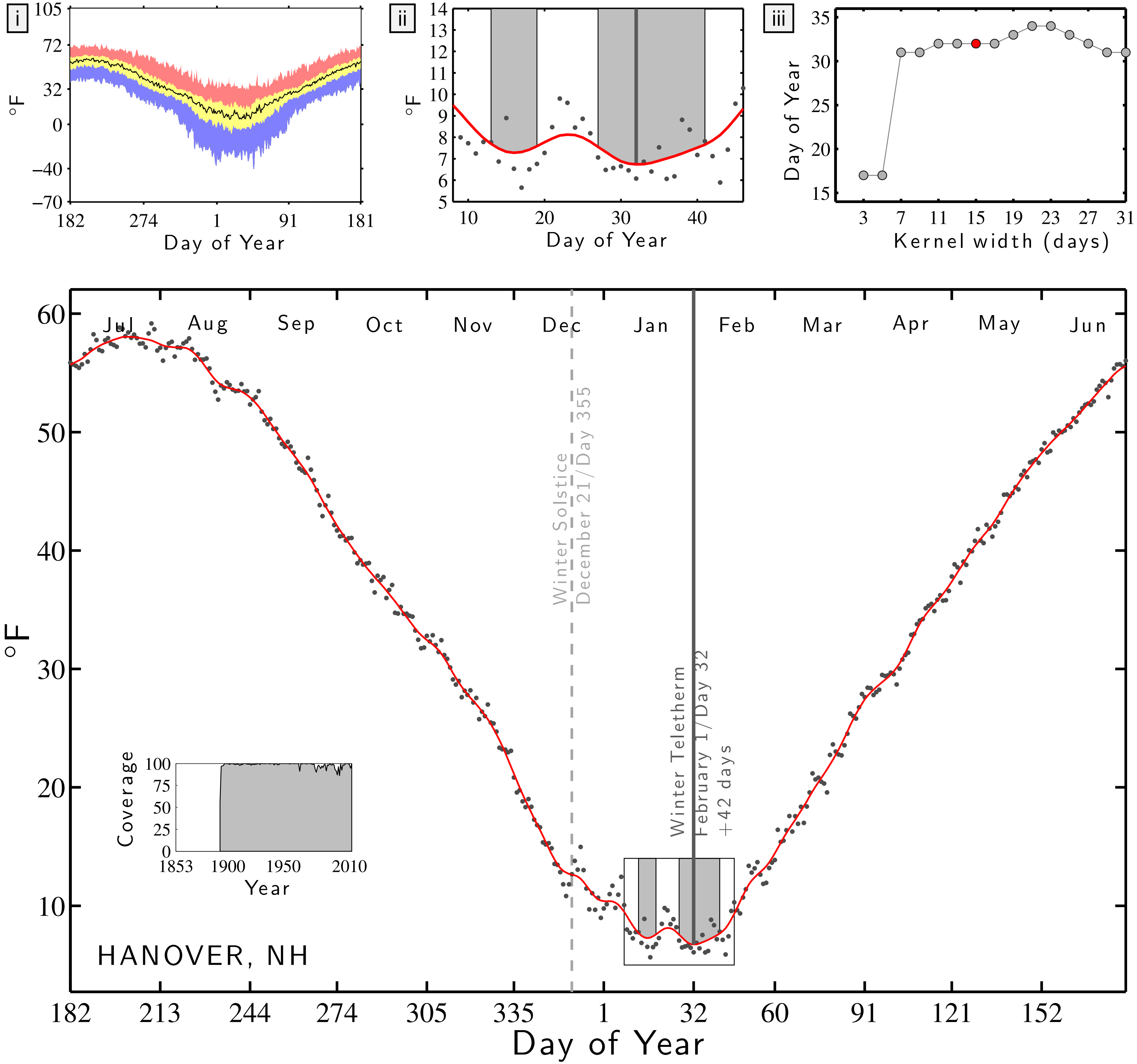

i
ii
iii
°F
Day of Year
Day of Year
Kernel width (days)
Jul
Aug
Sep
Oct
Nov
Dec
Jan
Feb
Mar
Apr
May
Jun
Winter Solstice
December 21/Day 355
Winter Teletherm
February 1/Day 32
+42 days
Coverage
Year
HANOVER, NH

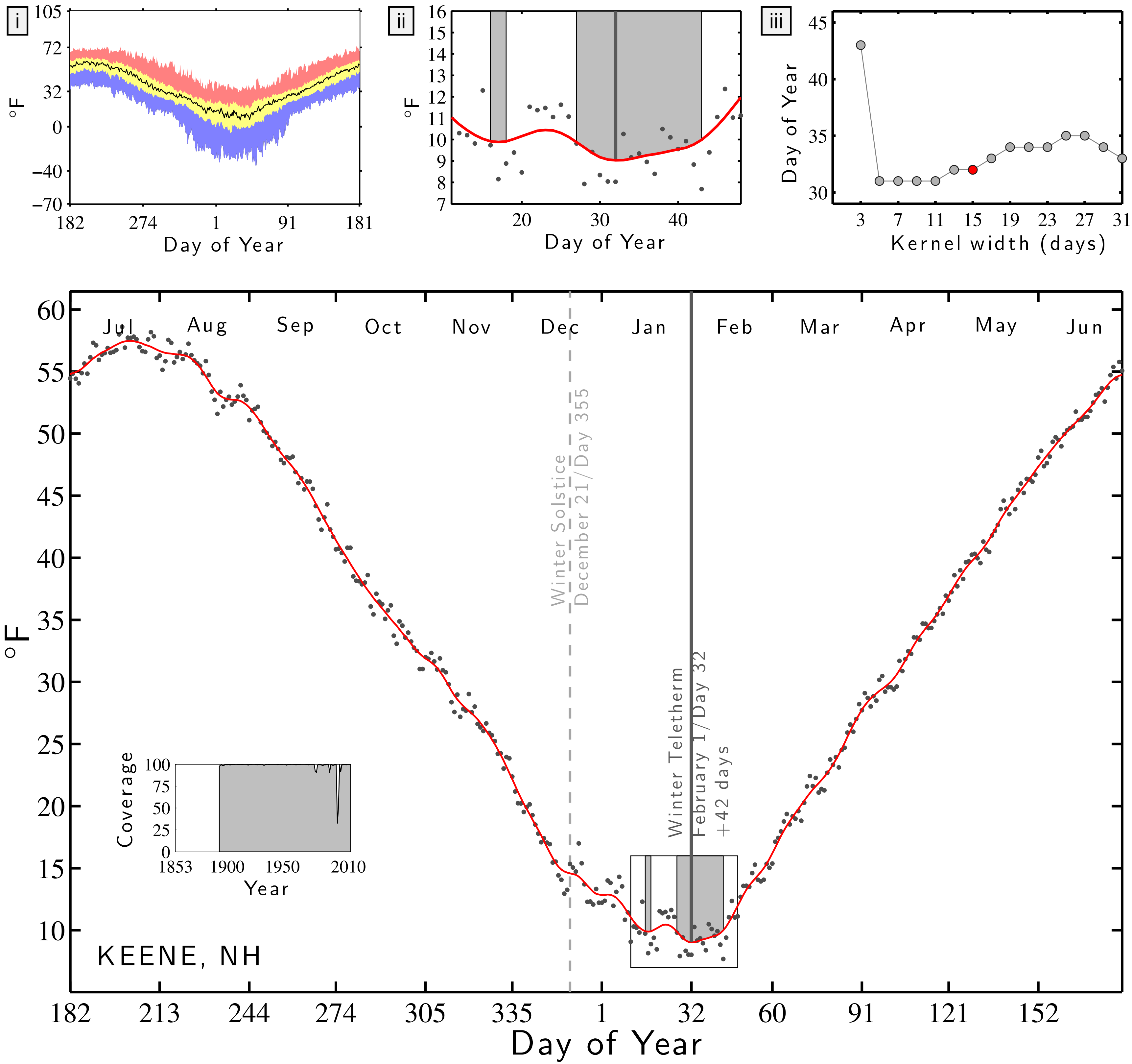

i
°F
Day of Year
ii
°F
Day of Year
iii
Day of Year
Kernel width (days)
Jul
Aug
Sep
Oct
Nov
Dec
Jan
Feb
Mar
Apr
May
Jun
Winter Solstice
December 21/Day 355
Winter Teletherm
February 1/Day 32
+42 days
Coverage
Year
KEENE, NH
°F
Day of Year

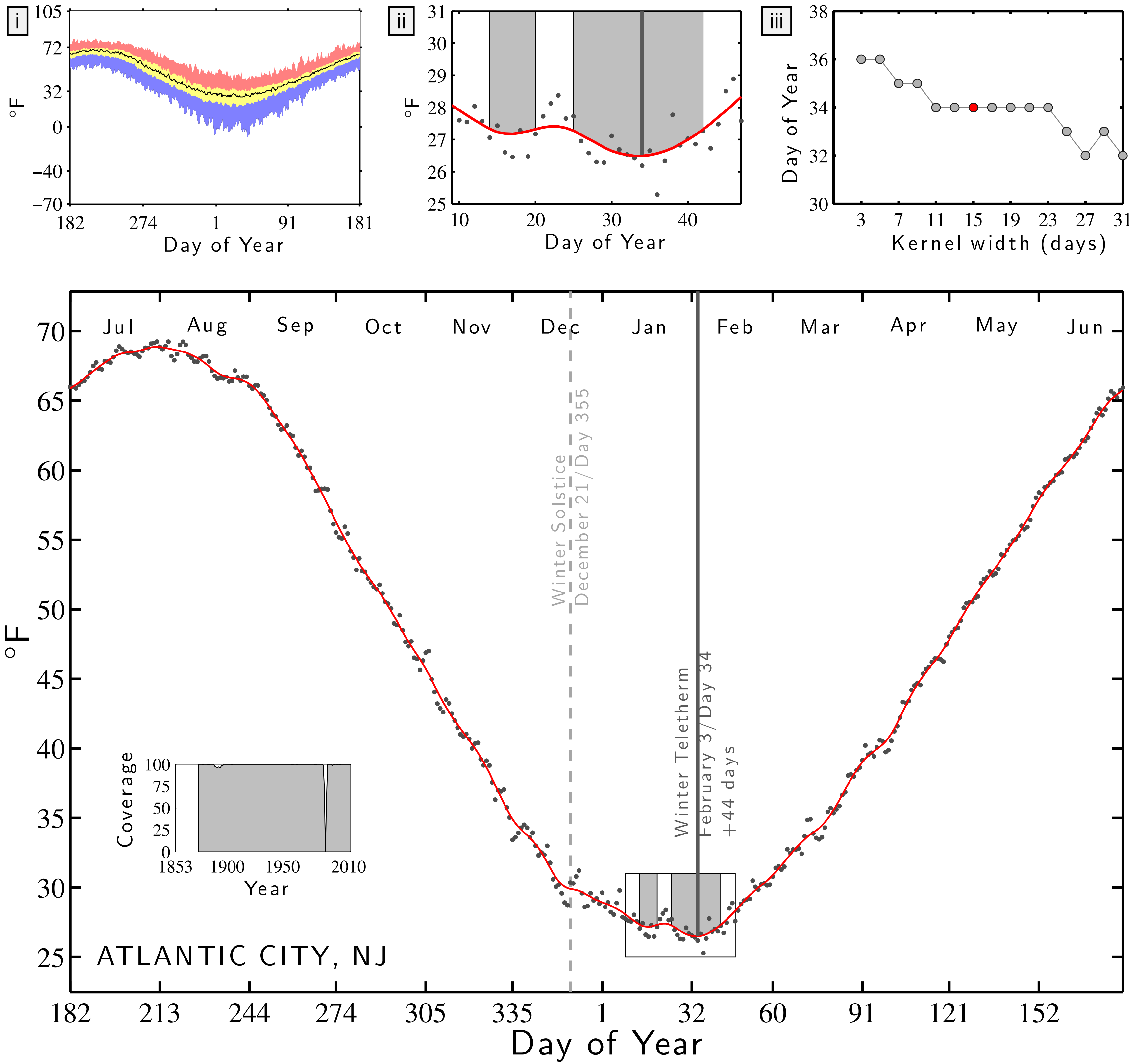

i
°F
Day of Year
ii
°F
Day of Year
iii
Day of Year
Kernel width (days)
Jul
Aug
Sep
Oct
Nov
Dec
Jan
Feb
Mar
Apr
May
Jun
Winter Solstice
December 21/Day 355
Winter Teletherm
February 3/Day 34
+44 days
Coverage
Year
ATLANTIC CITY, NJ
°F
Day of Year

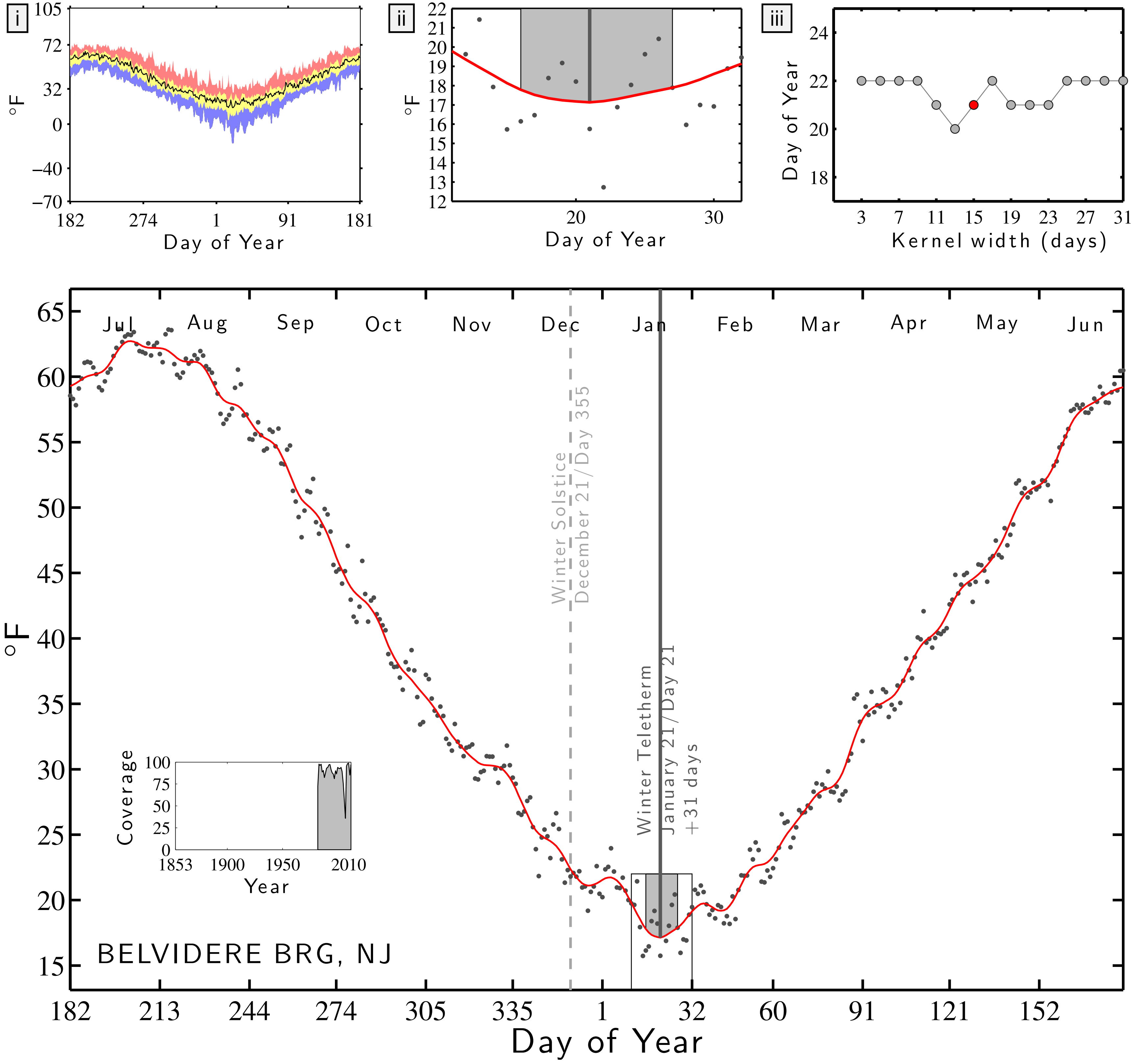

i
°F
Day of Year
ii
iii
Day of Year
Kernel width (days)
Jul
Aug
Sep
Oct
Nov
Dec
Jan
Feb
Mar
Apr
May
Jun
Winter Solstice
December 21/Day 355
Winter Teletherm
January 21/Day 21
+31 days
Coverage
Year
BELVIDERE BRG, NJ

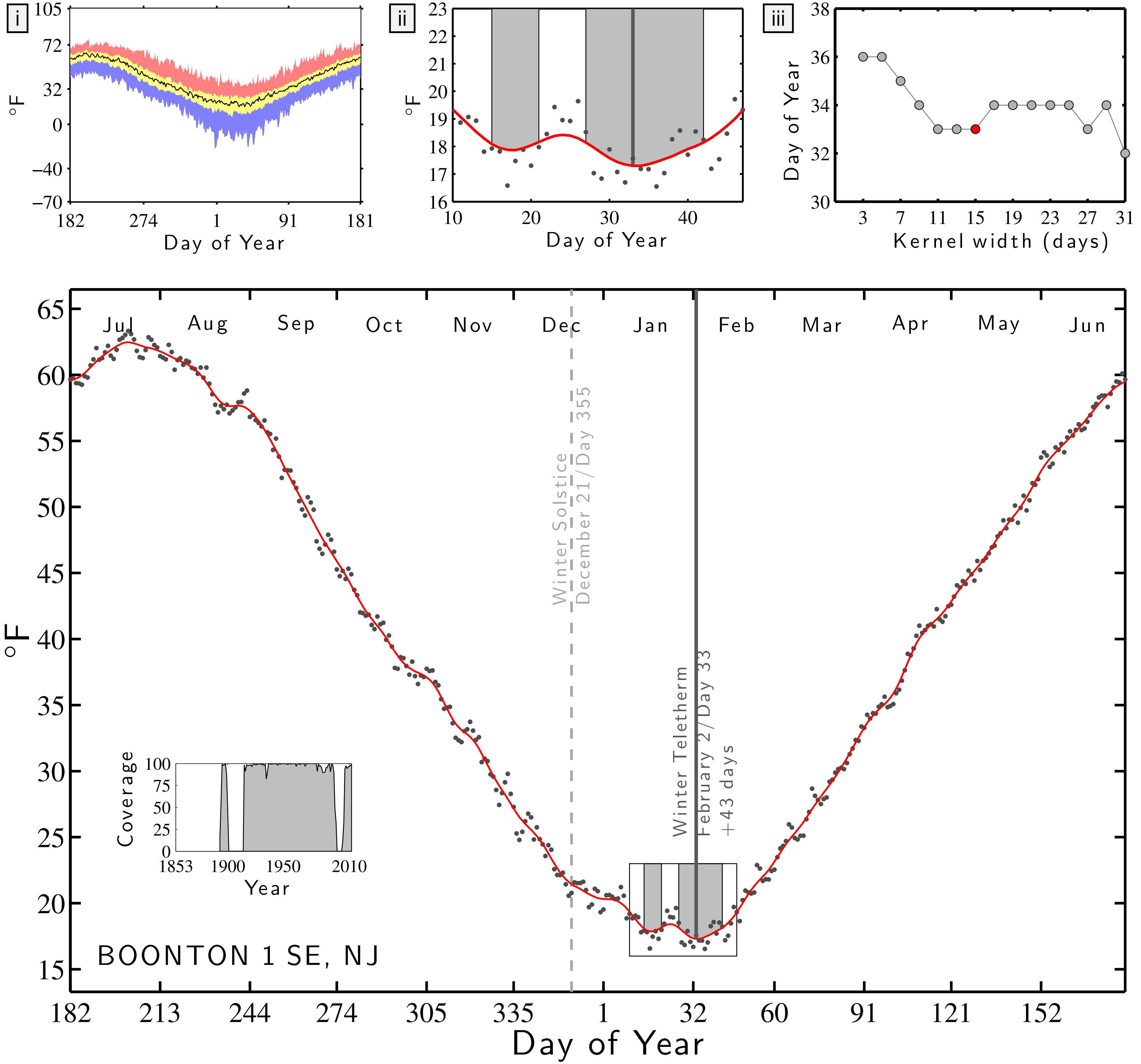

i
ii
iii
°F
Day of Year
Kernel width (days)
Winter Solstice
December 21/Day 355
Winter Teletherm
February 2/Day 33
+43 days
Coverage
Year
BOONTON 1 SE, NJ
Jul
Aug
Sep
Oct
Nov
Dec
Jan
Feb
Mar
Apr
May
Jun

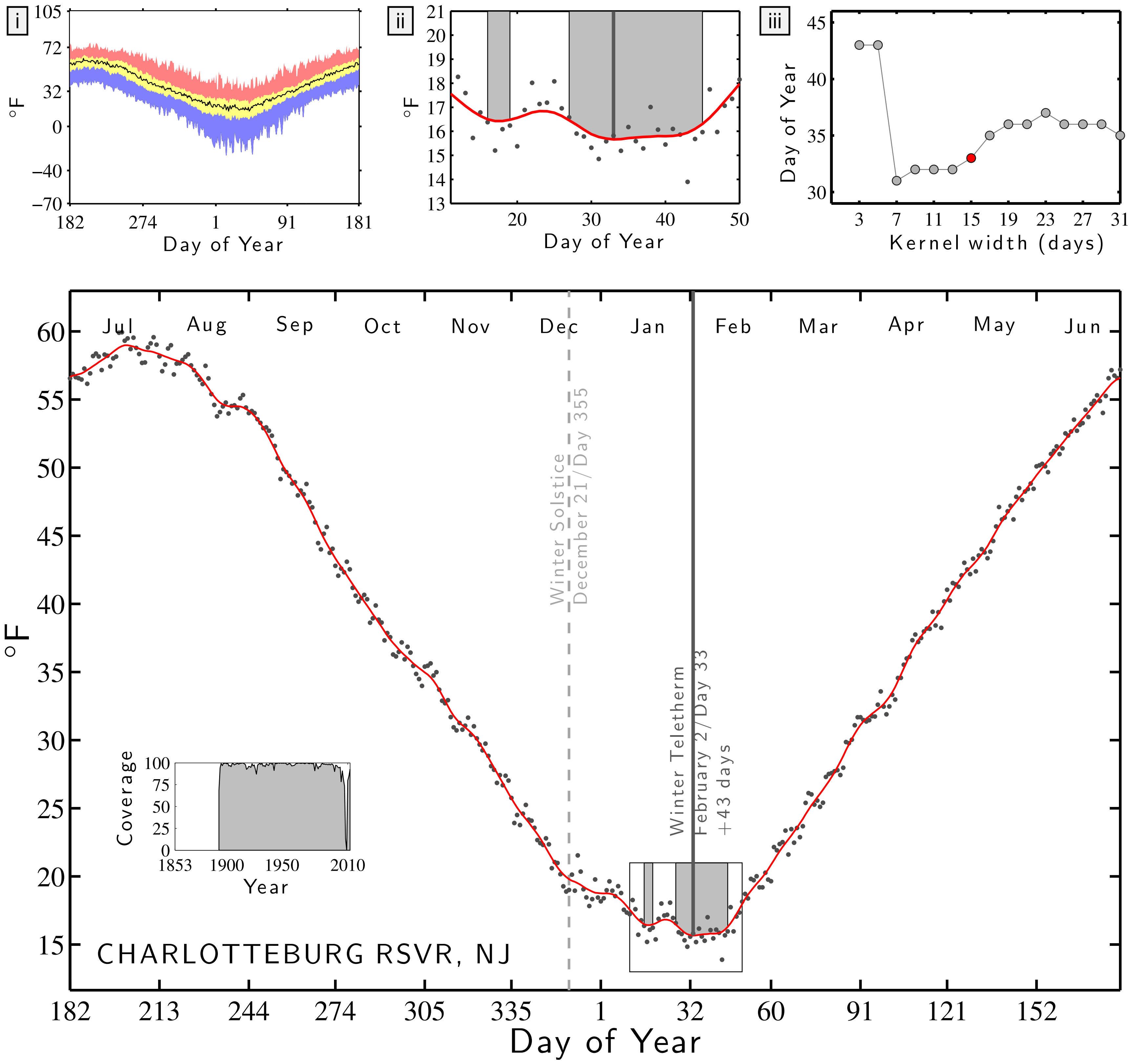

i
ii
iii
°F
Day of Year
Day of Year
Kernel width (days)
Winter Solstice
December 21/Day 355
Winter Teletherm
February 2/Day 33
+43 days
Coverage
Year
CHARLOTTEBURG RSVR, NJ
Jul
Aug
Sep
Oct
Nov
Dec
Jan
Feb
Mar
Apr
May
Jun

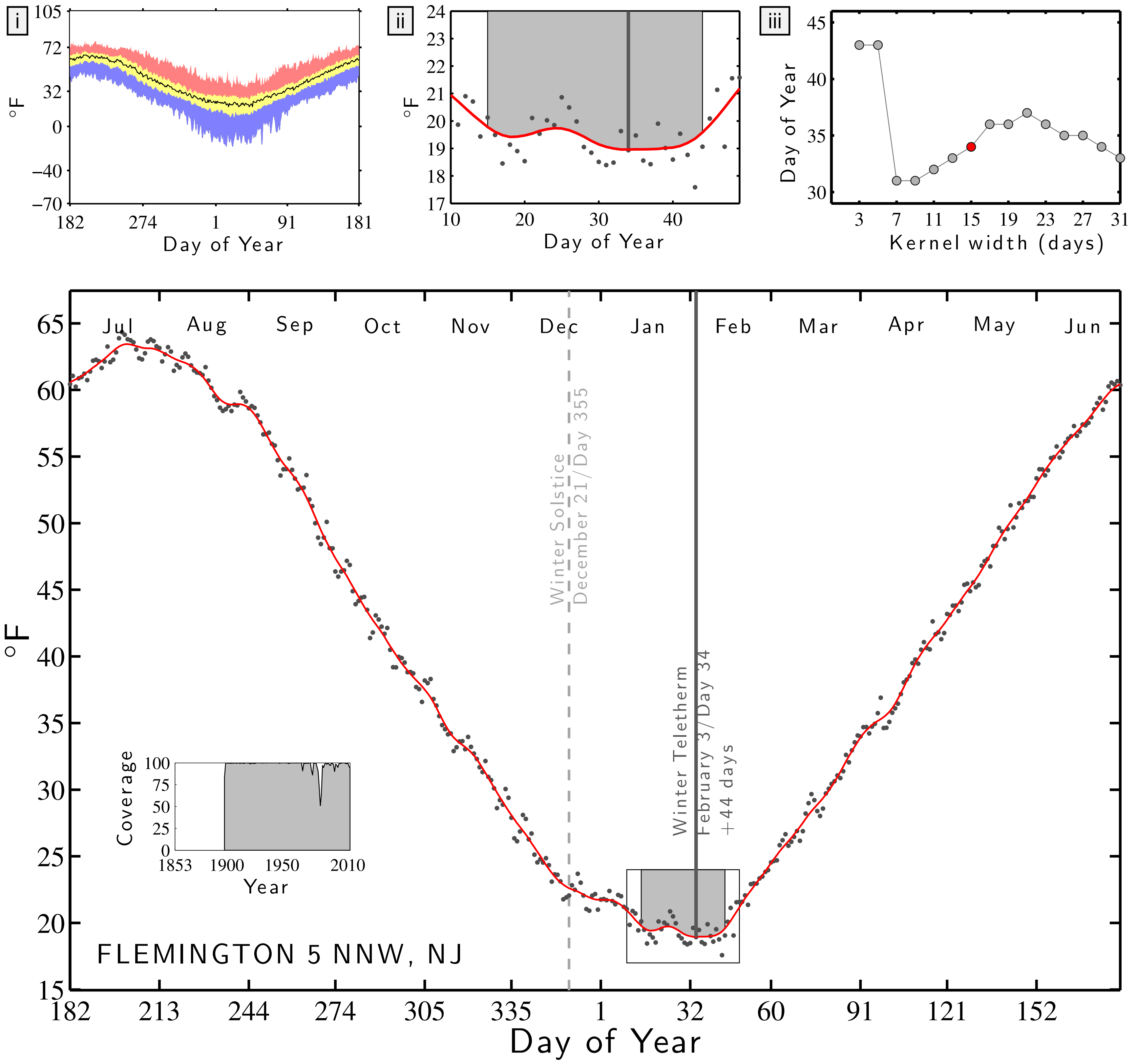

i
ii
iii
Day of Year
°F
Kernel width (days)
Jul
Aug
Sep
Oct
Nov
Dec
Jan
Feb
Mar
Apr
May
Jun
Winter Solstice
December 21/Day 355
Winter Teletherm
February 3/Day 34
+44 days
Coverage
Year
FLEMINGTON 5 NNW, NJ

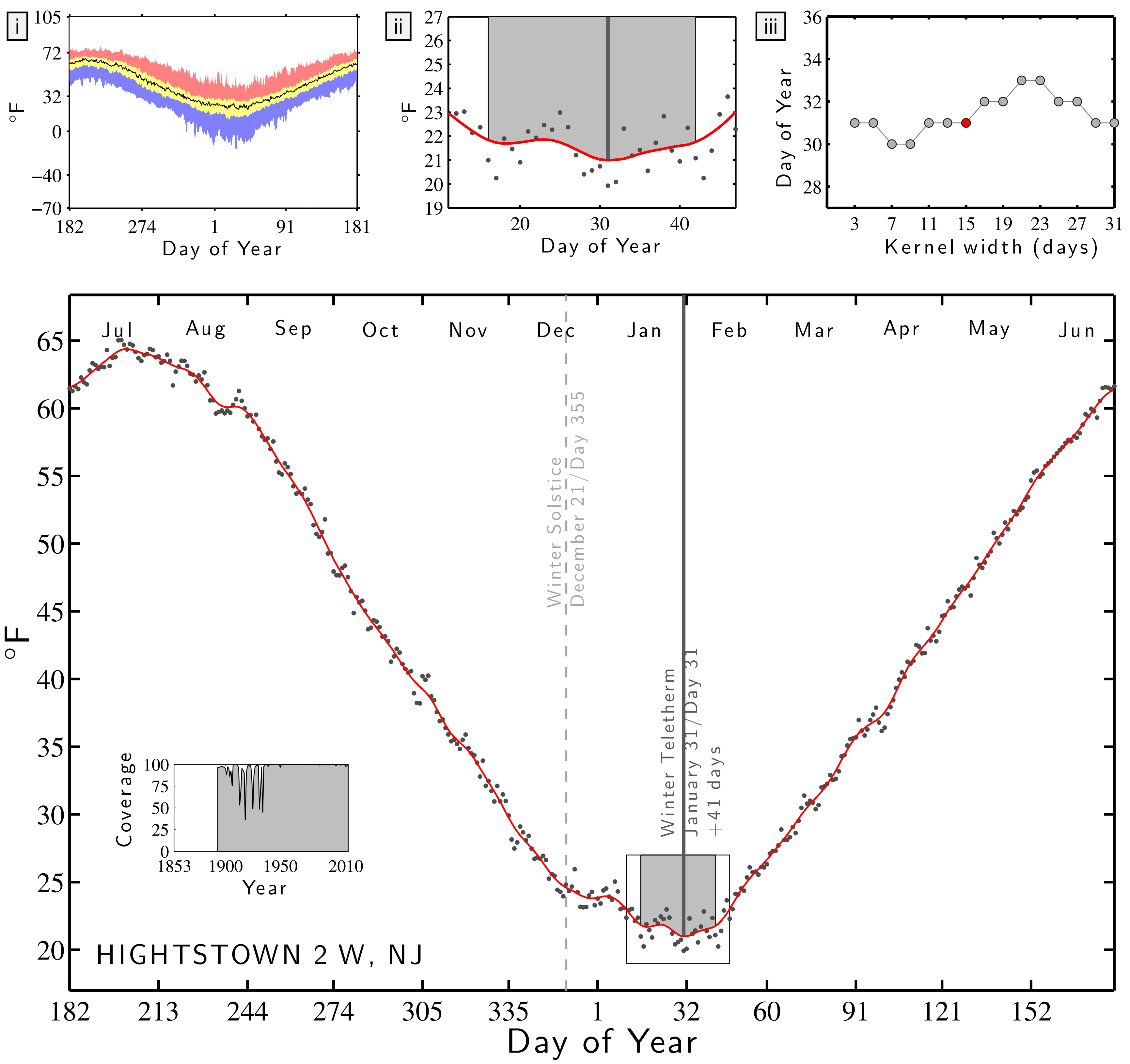

i
ii
iii
°F
Day of Year
Day of Year
Kernel width (days)
Winter Solstice
December 21/Day 355
Winter Teletherm
January 31/Day 31
+41 days
Jul
Aug
Sep
Oct
Nov
Dec
Jan
Feb
Mar
Apr
May
Jun
Coverage
Year
HIGHTSTOWN 2 W, NJ
Day of Year

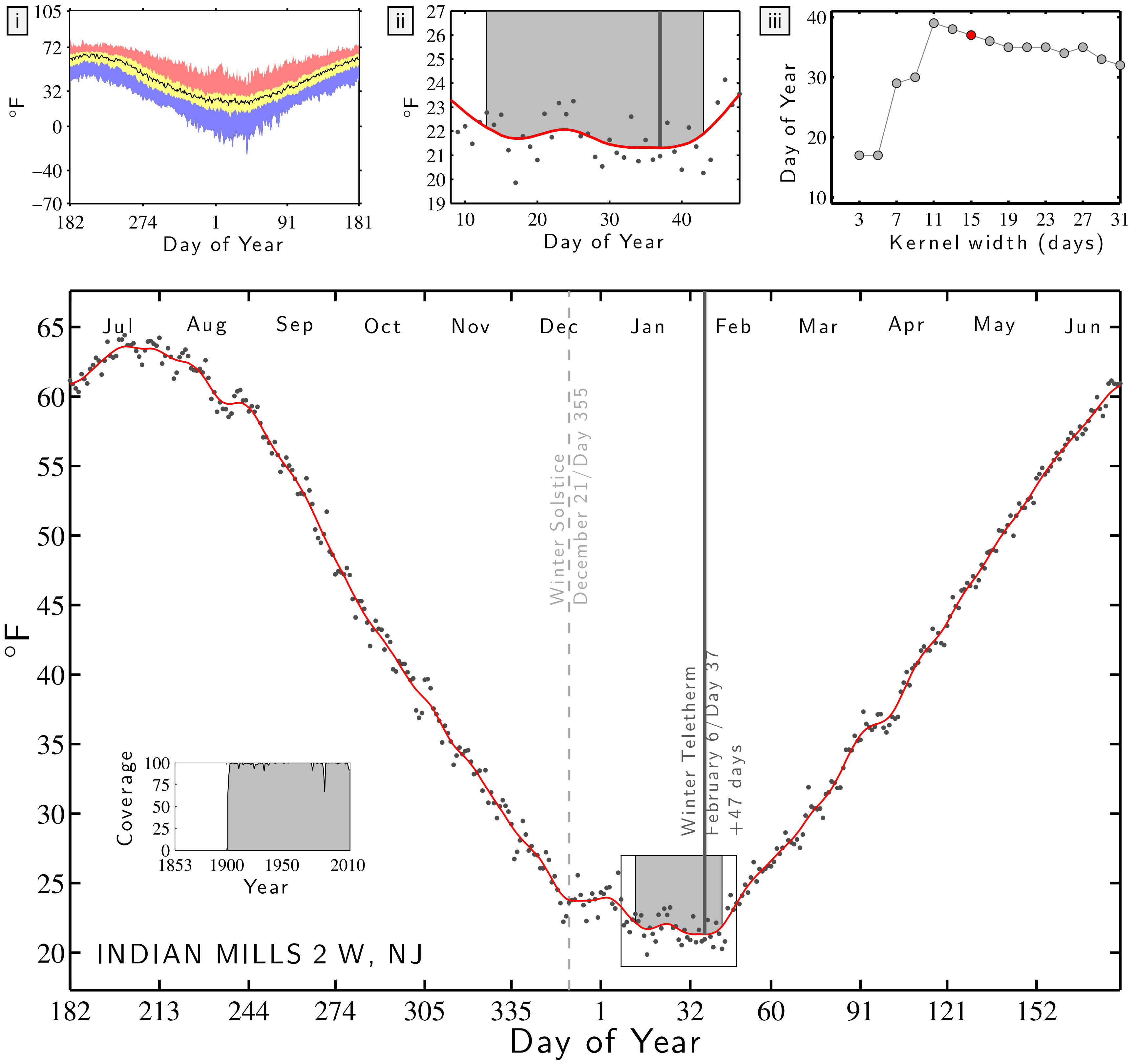

i
ii
iii
°F
Day of Year
Kernel width (days)
Jul
Aug
Sep
Oct
Nov
Dec
Jan
Feb
Mar
Apr
May
Jun
Winter Solstice
December 21/Day 355
Winter Teletherm
February 6/Day 37
+47 days
Coverage
Year
INDIAN MILLS 2 W, NJ

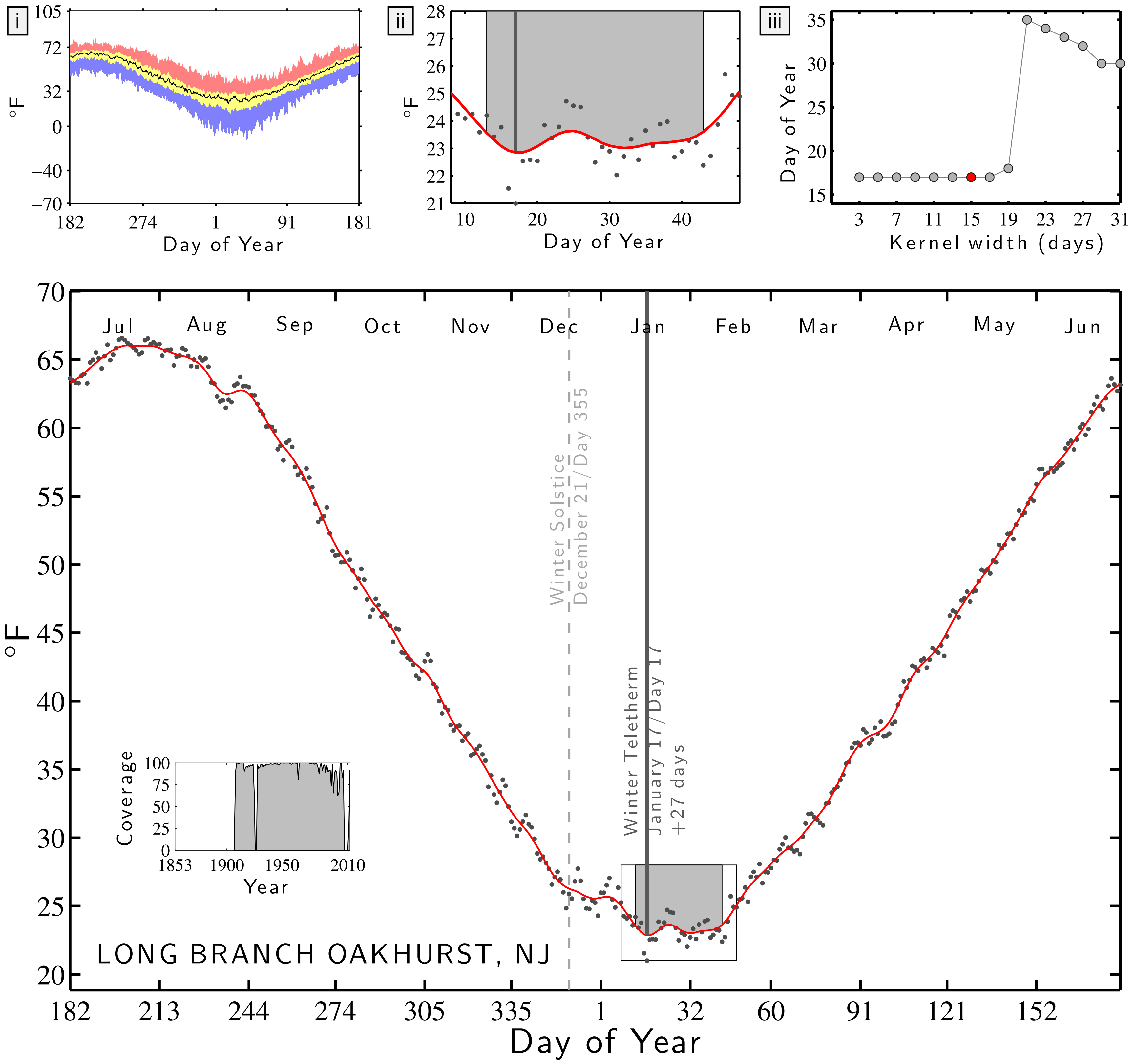

i
°F
Day of Year
ii
°F
Day of Year
iii
Day of Year
Kernel width (days)
Jul
Aug
Sep
Oct
Nov
Dec
Jan
Feb
Mar
Apr
May
Jun
Winter Solstice
December 21/Day 355
Winter Teletherm
January 17/Day 17
+27 days
Coverage
Year
LONG BRANCH OAKHURST, NJ
°F
Day of Year

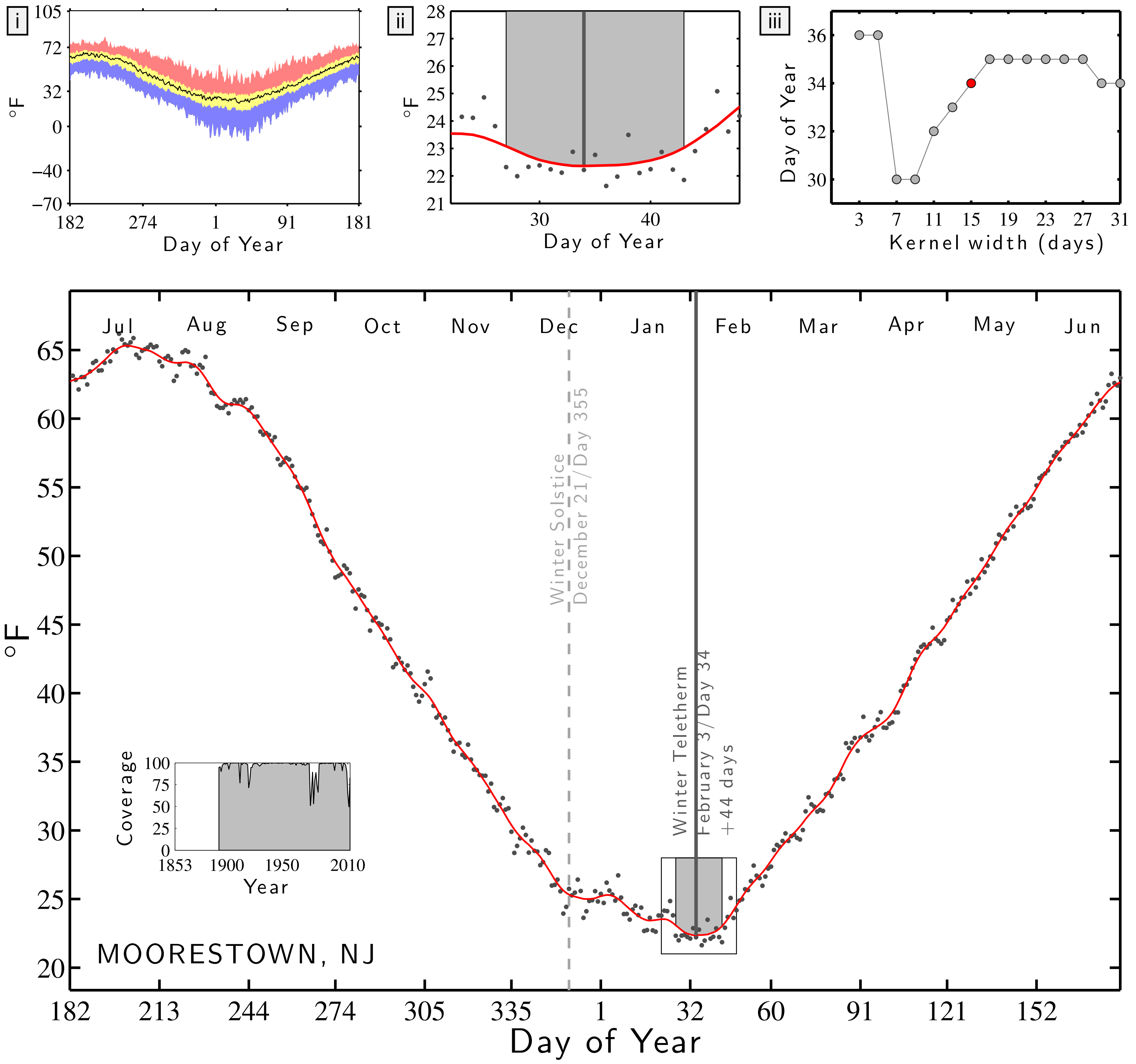

i
°F
Day of Year
ii
°F
Day of Year
iii
Day of Year
Kernel width (days)
Jul
Aug
Sep
Oct
Nov
Dec
Jan
Feb
Mar
Apr
May
Jun
Winter Solstice
December 21/Day 355
Winter Teletherm
February 3/Day 34
+44 days
Coverage
Year
MOORESTOWN, NJ
°F
Day of Year

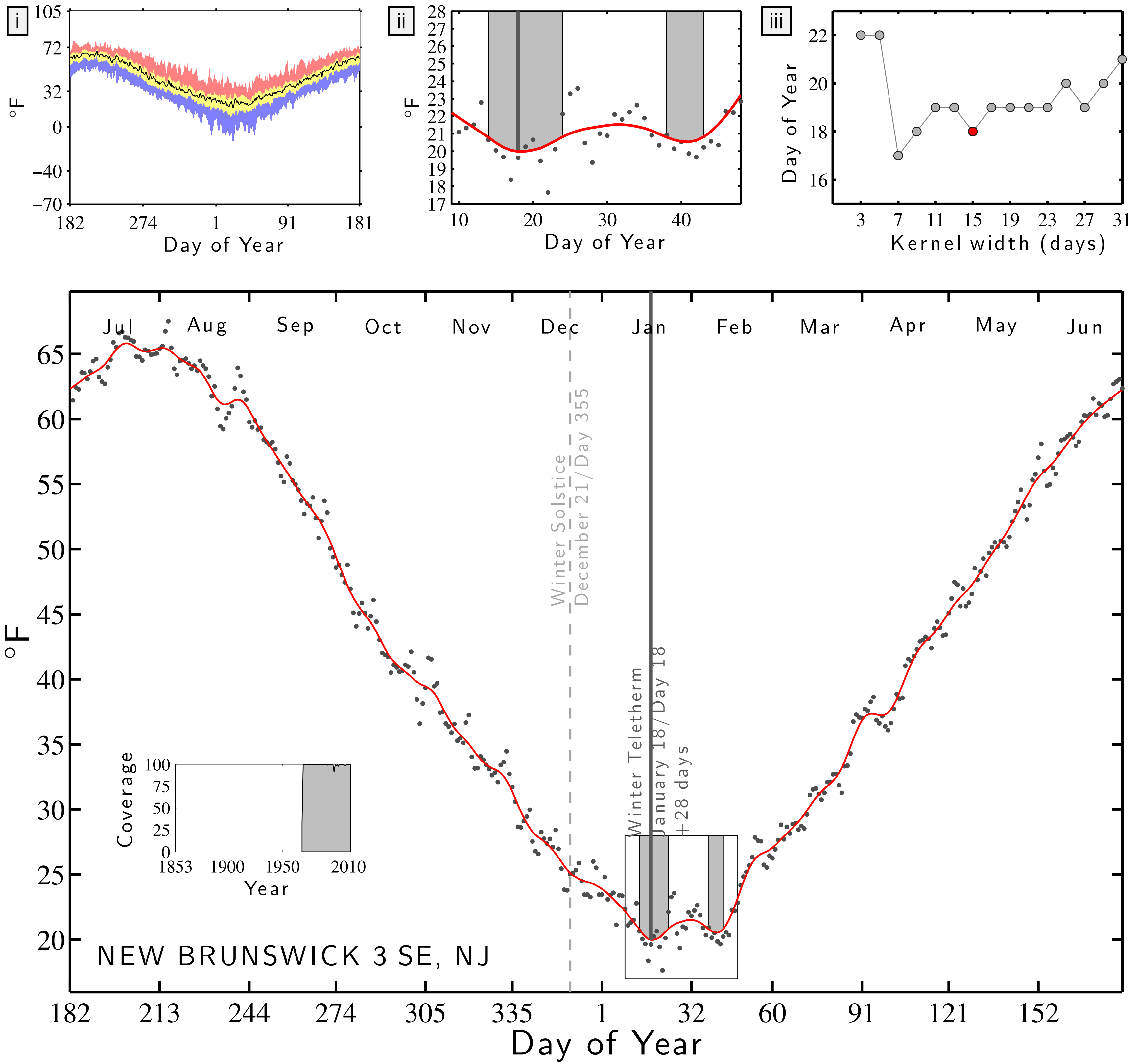

i
105
72
32
0
−40
−70
°F
182
274
1
91
181
Day of Year
ii
28
27
26
25
24
23
22
21
20
19
18
17
°F
10
20
30
40
Day of Year
iii
22
20
18
16
Day of Year
3
7
11
15
19
23
27
31
Kernel width (days)
Jul
Aug
Sep
Oct
Nov
Dec
Jan
Feb
Mar
Apr
May
Jun
Winter Solstice
December 21/Day 355
Winter Teletherm
January 18/Day 18
+28 days
65
60
55
50
45
40
35
30
25
20
°F
Coverage
100
75
50
25
0
1853
1900
1950
2010
Year
NEW BRUNSWICK 3 SE, NJ
182
213
244
274
305
335
1
32
60
91
121
152
Day of Year

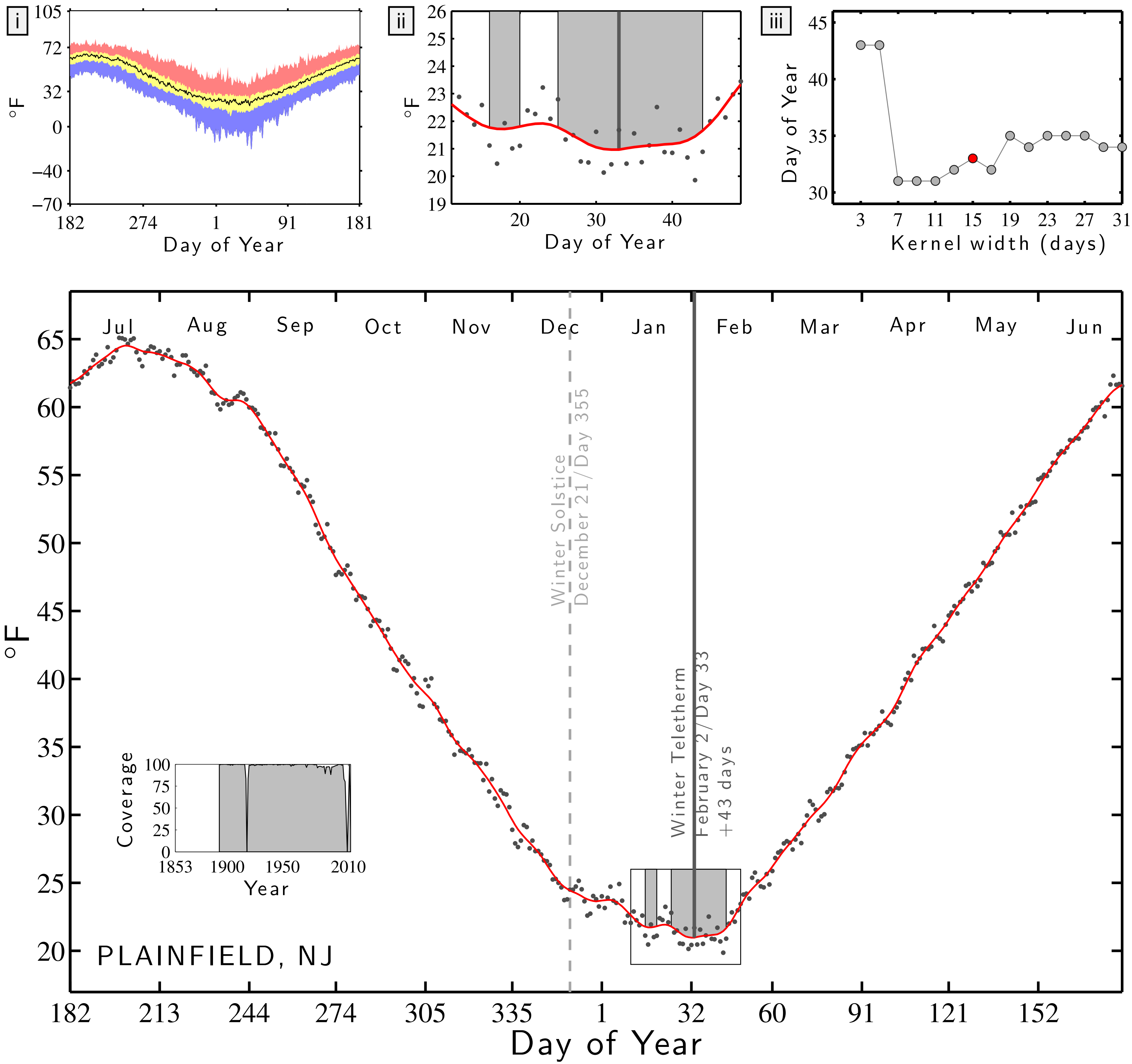

i
°F
Day of Year
ii
°F
Day of Year
iii
Day of Year
Kernel width (days)
Jul
Aug
Sep
Oct
Nov
Dec
Jan
Feb
Mar
Apr
May
Jun
Winter Solstice
December 21/Day 355
Winter Teletherm
February 2/Day 33
+43 days
Coverage
Year
PLAINFIELD, NJ
°F
Day of Year

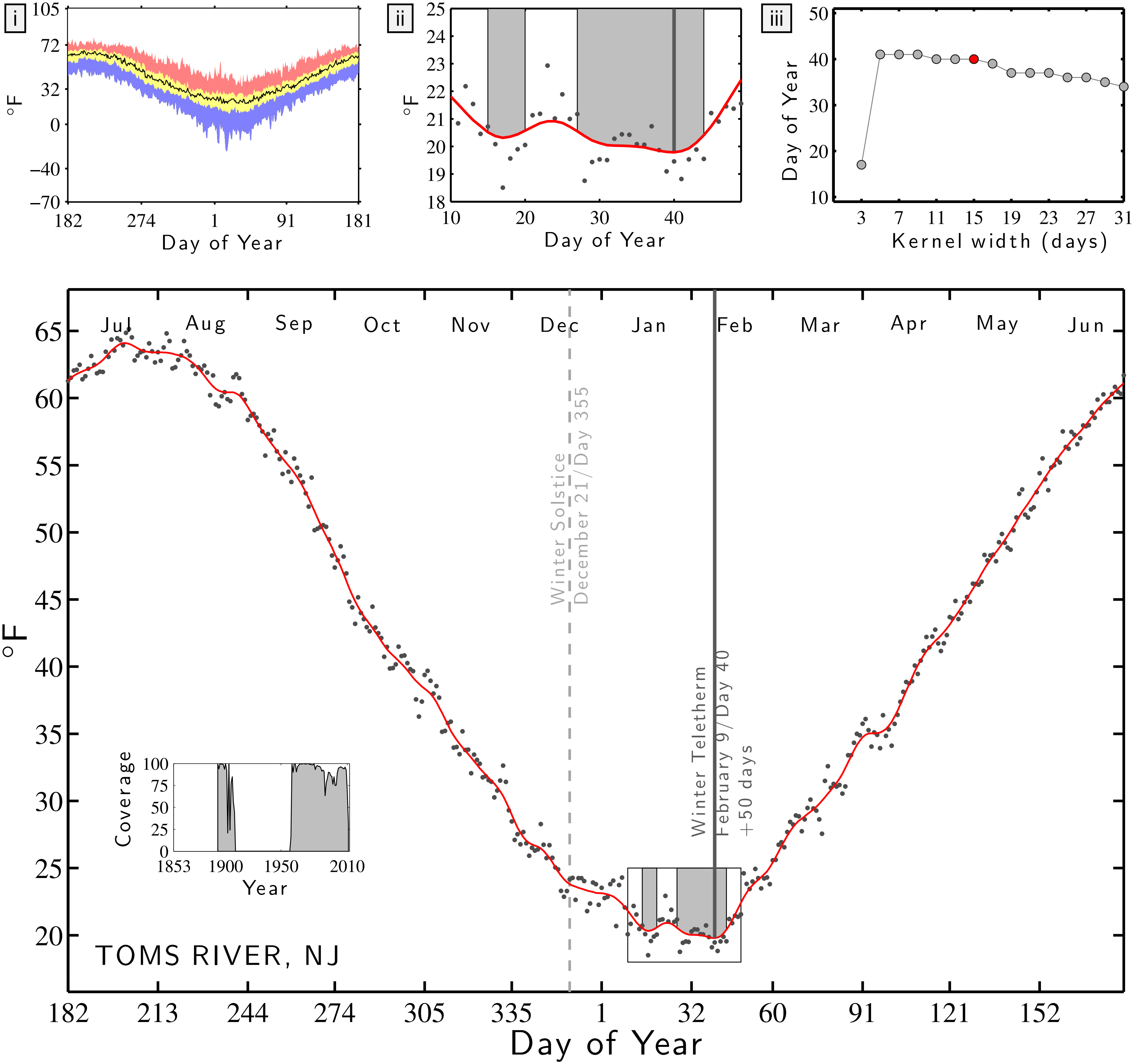

i
105
72
32
0
−40
−70
°F
182
274
1
91
181
Day of Year
ii
25
24
23
22
21
20
19
18
°F
10
20
30
40
Day of Year
iii
50
40
30
20
10
Day of Year
3
7
11
15
19
23
27
31
Kernel width (days)
Jul
Aug
Sep
Oct
Nov
Dec
Jan
Feb
Mar
Apr
May
Jun
65
60
55
50
45
40
35
30
25
20
°F
Winter Solstice
December 21/Day 355
Winter Teletherm
February 9/Day 40
+50 days
Coverage
100
75
50
25
0
1853
1900
1950
2010
Year
TOMS RIVER, NJ
182
213
244
274
305
335
1
32
60
91
121
152
Day of Year

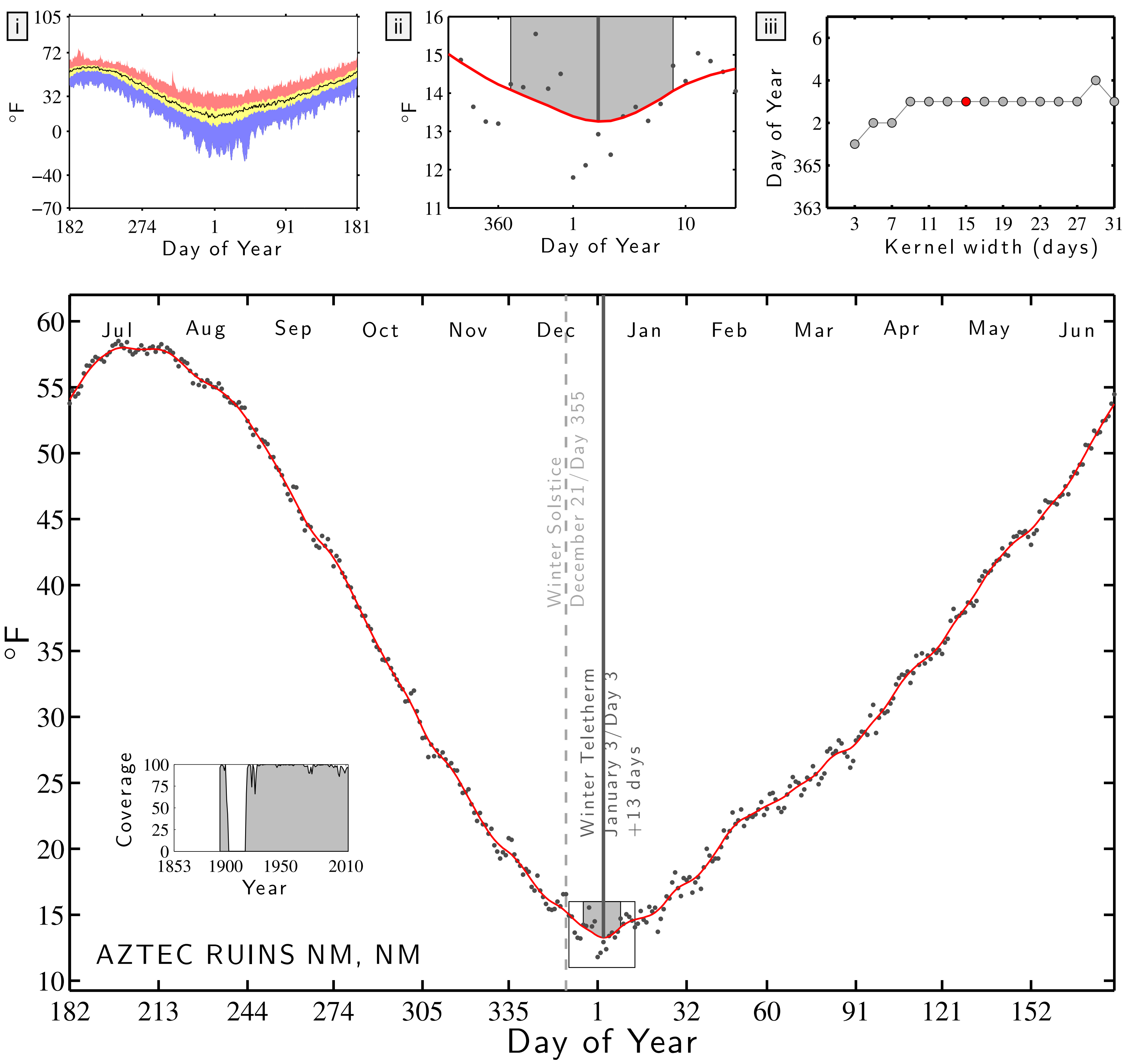

i
ii
iii
°F
Day of Year
Kernel width (days)
Jul
Aug
Sep
Oct
Nov
Dec
Jan
Feb
Mar
Apr
May
Jun
Winter Solstice
December 21/Day 355
Winter Teletherm
January 3/Day 3
+13 days
Coverage
Year
AZTEC RUINS NM, NM

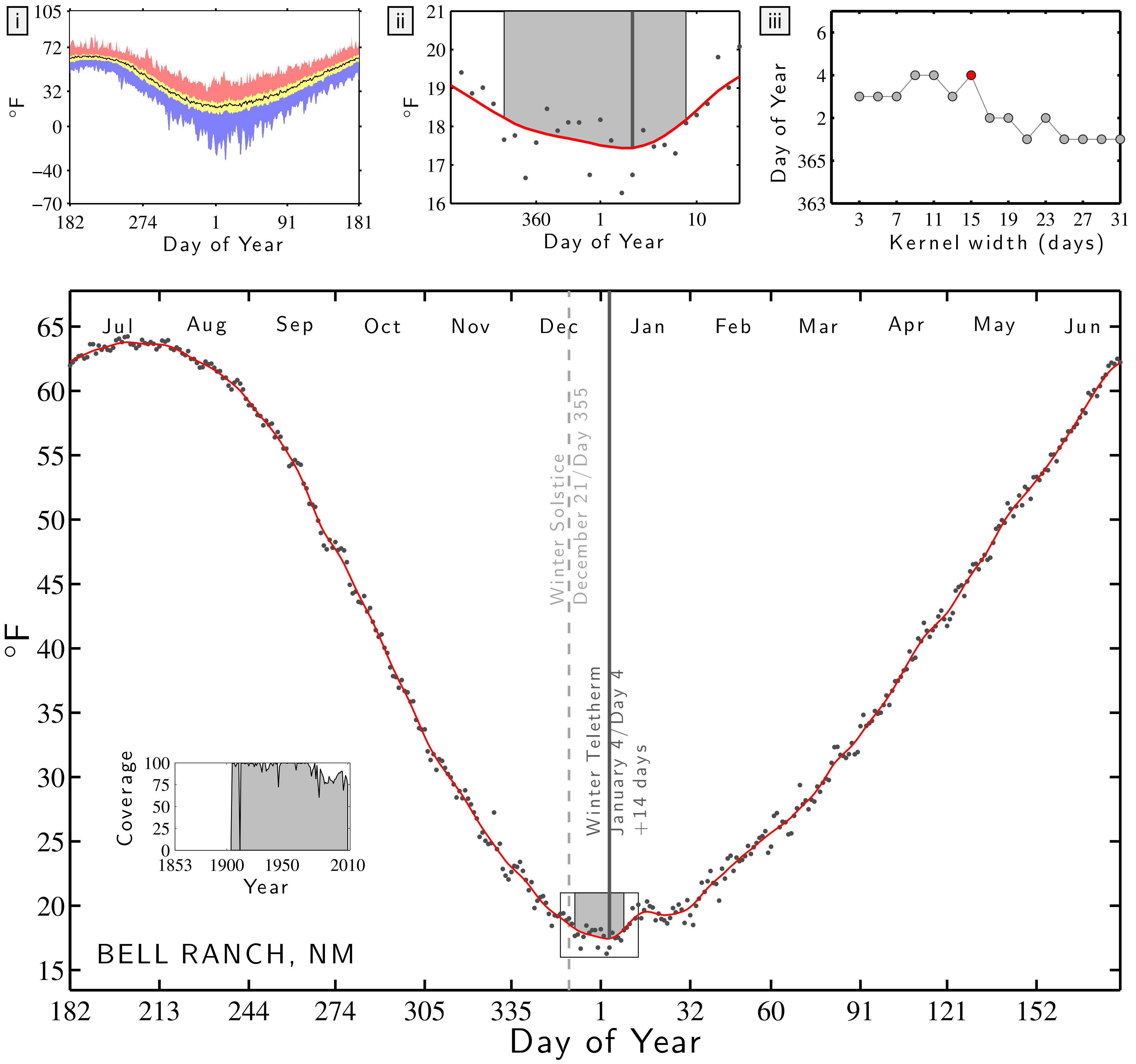

i
ii
iii
°F
Day of Year
Kernel width (days)
Jul
Aug
Sep
Oct
Nov
Dec
Jan
Feb
Mar
Apr
May
Jun
Winter Solstice
December 21/Day 355
Winter Teletherm
January 4/Day 4
+14 days
Coverage
Year
BELL RANCH, NM

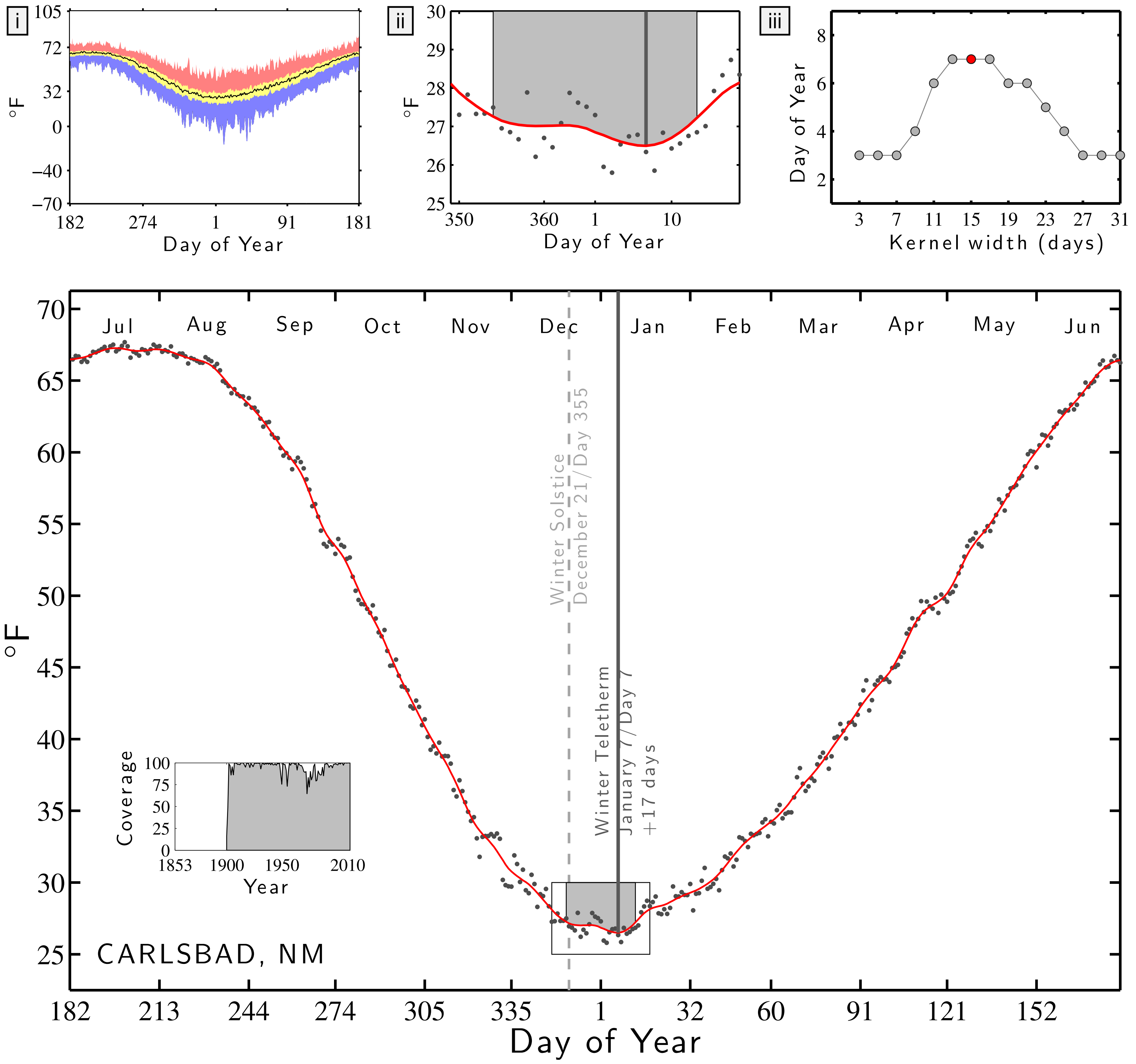

i
ii
iii
Day of Year
Kernel width (days)
°F
Jul
Aug
Sep
Oct
Nov
Dec
Jan
Feb
Mar
Apr
May
Jun
Winter Solstice
December 21/Day 355
Winter Teletherm
January 7/Day 7
+17 days
Coverage
Year
CARLSBAD, NM

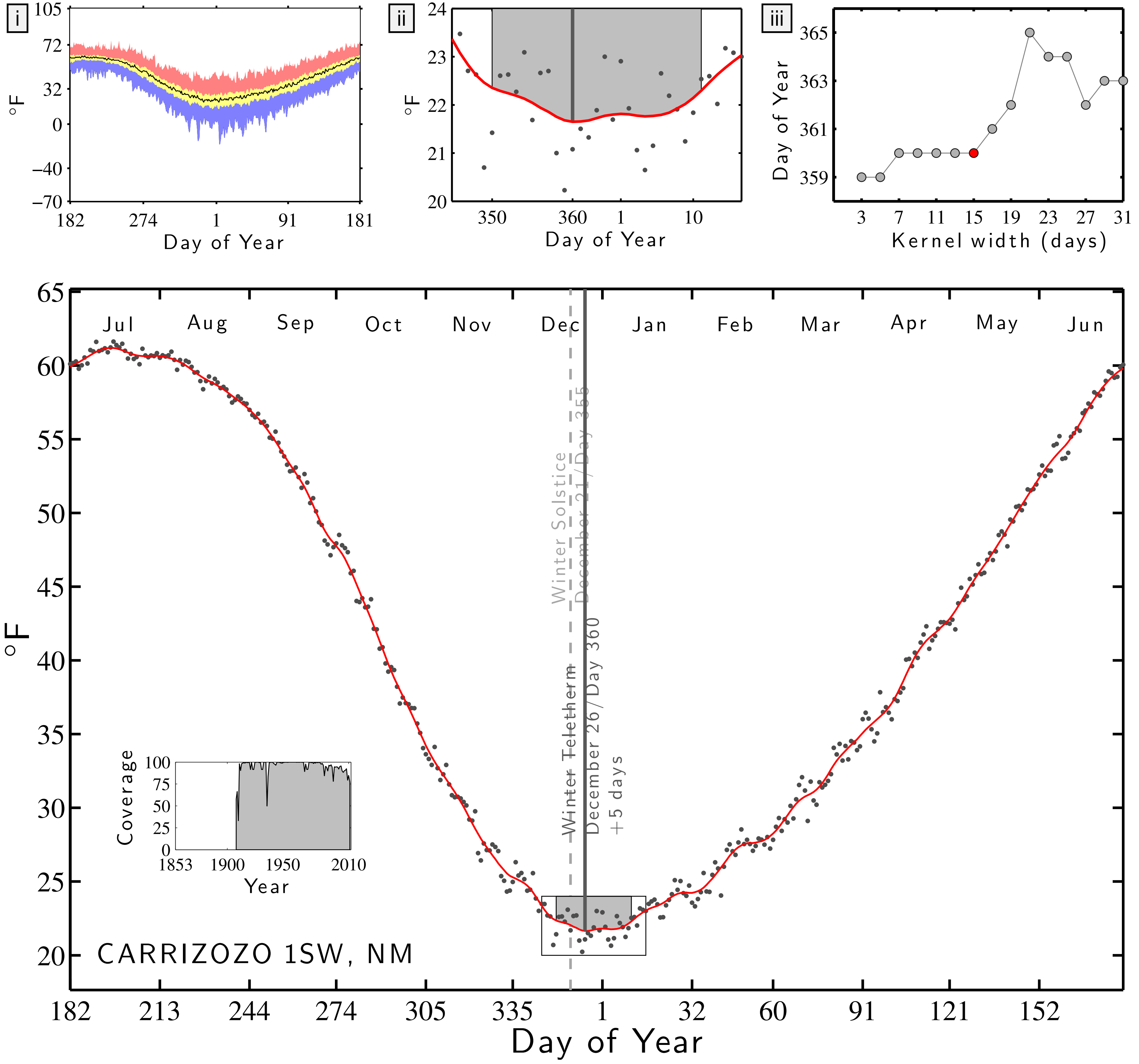

i
105
72
32
0
−40
−70
°F
182
274
1
91
181
Day of Year
ii
24
23
22
21
20
°F
350
360
1
10
Day of Year
iii
365
363
361
359
Day of Year
3
7
11
15
19
23
27
31
Kernel width (days)
65
60
55
50
45
40
35
30
25
20
°F
Jul
Aug
Sep
Oct
Nov
Dec
Jan
Feb
Mar
Apr
May
Jun
Winter Solstice
December 21/Day 355
Winter Teletherm
December 26/Day 360
+5 days
Coverage
100
75
50
25
0
1853
1900
1950
2010
Year
CARRIZOZO 1SW, NM
182
213
244
274
305
335
1
32
60
91
121
152
Day of Year

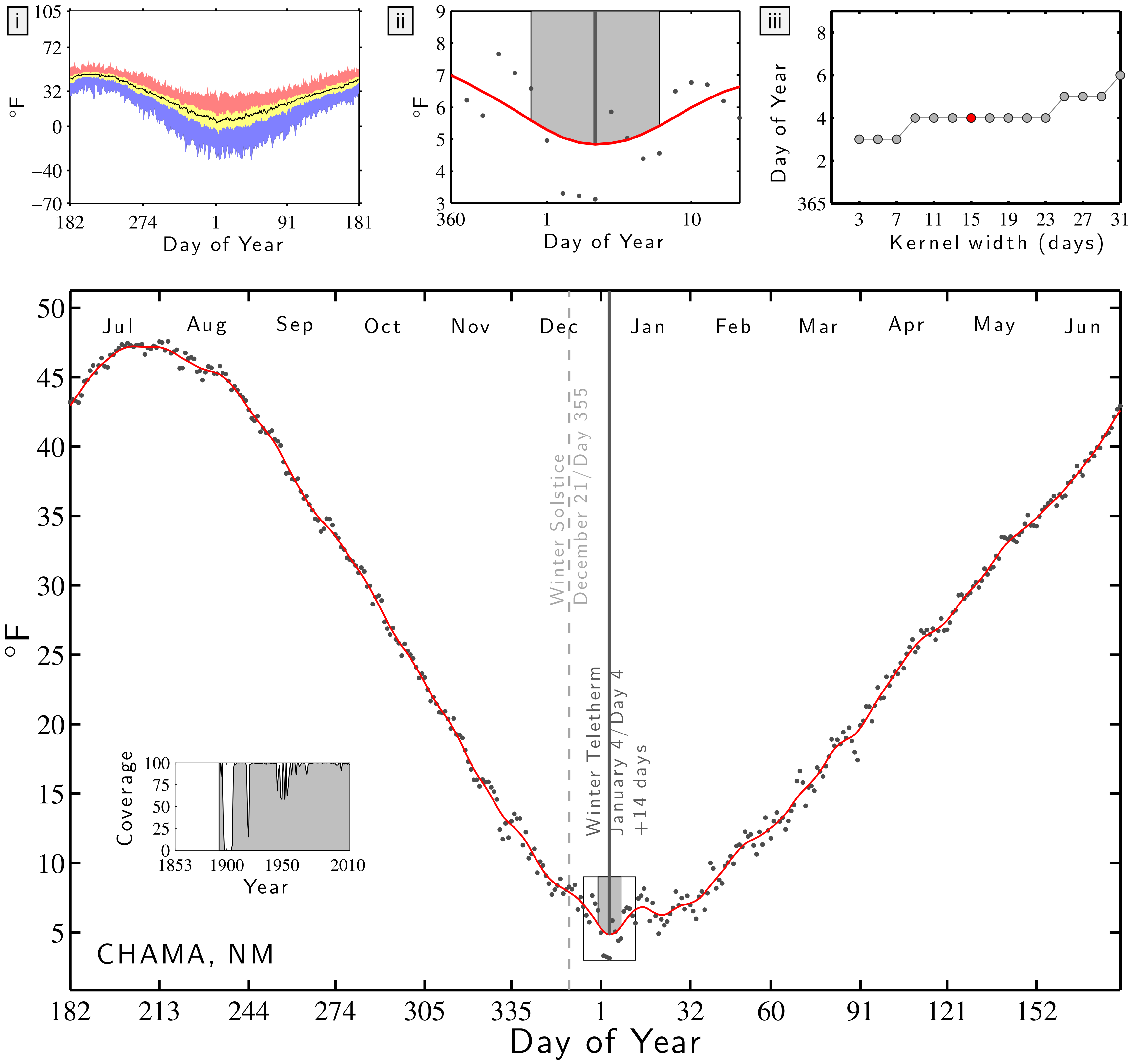

i
°F
Day of Year
ii
°F
Day of Year
iii
Day of Year
Kernel width (days)
Jul
Aug
Sep
Oct
Nov
Dec
Jan
Feb
Mar
Apr
May
Jun
Winter Solstice
December 21/Day 355
Winter Teletherm
January 4/Day 4
+14 days
Coverage
Year
CHAMA, NM
°F
Day of Year

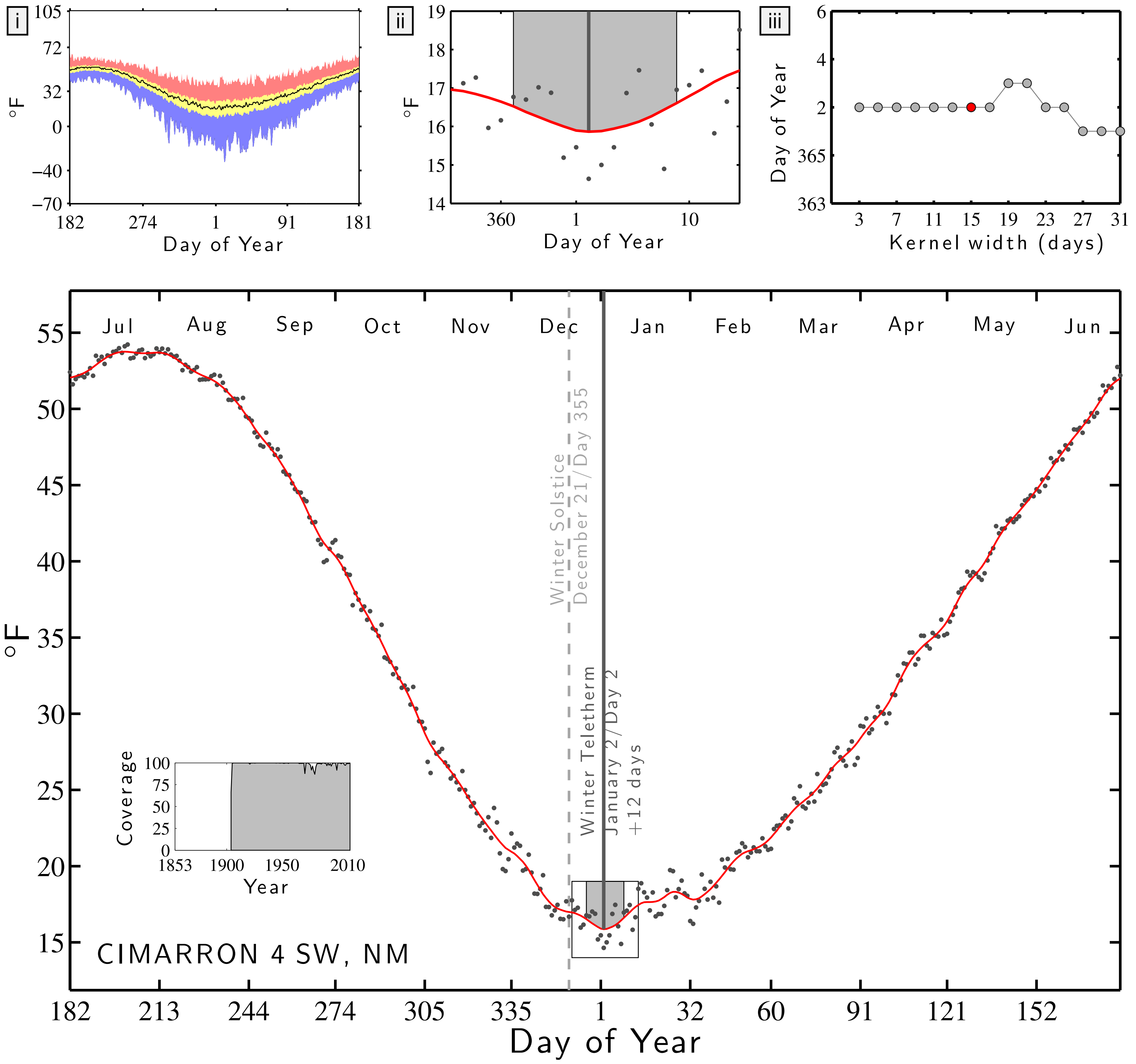

i
°F
Day of Year
ii
°F
Day of Year
iii
Day of Year
Kernel width (days)
Jul
Aug
Sep
Oct
Nov
Dec
Jan
Feb
Mar
Apr
May
Jun
Winter Solstice
December 21/Day 355
Winter Teletherm
January 2/Day 2
+12 days
Coverage
Year
CIMARRON 4 SW, NM
°F
Day of Year

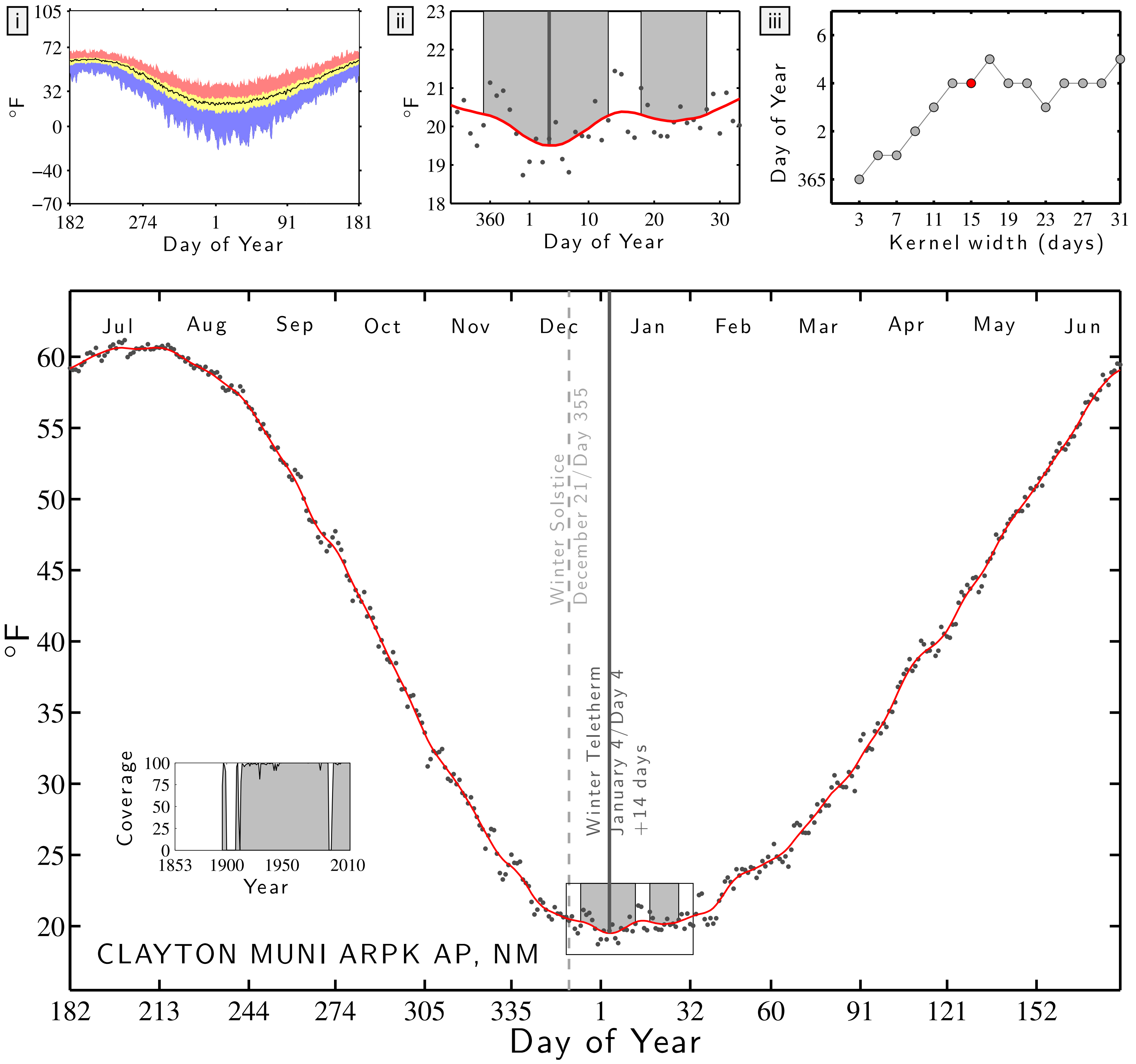

i
ii
iii
°F
Day of Year
Day of Year
Kernel width (days)
Jul
Aug
Sep
Oct
Nov
Dec
Jan
Feb
Mar
Apr
May
Jun
Winter Solstice
December 21/Day 355
Winter Teletherm
January 4/Day 4
+14 days
Coverage
Year
CLAYTON MUNI ARPK AP, NM

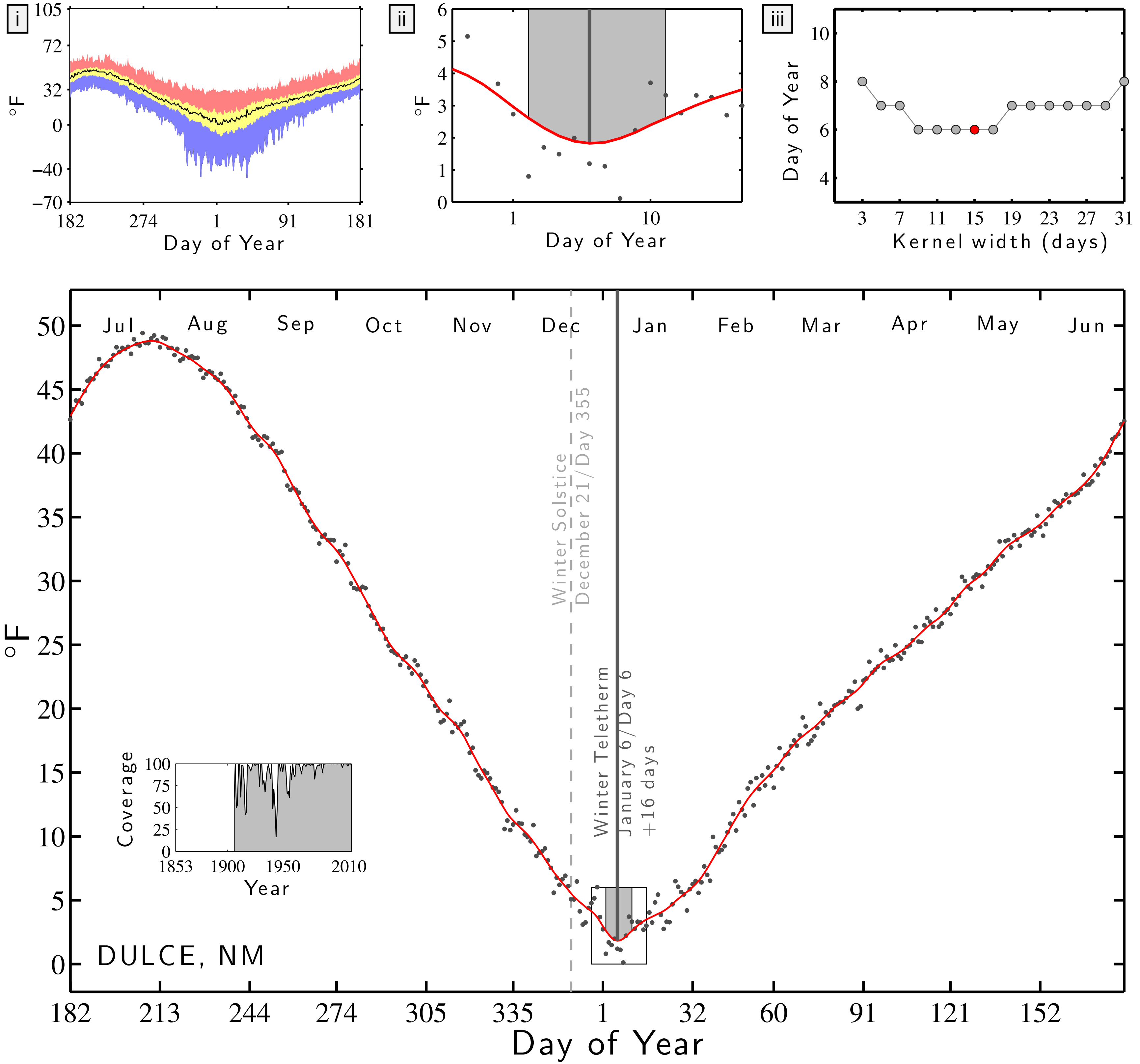

i
ii
iii
°F
Day of Year
Day of Year
Kernel width (days)
Jul
Aug
Sep
Oct
Nov
Dec
Jan
Feb
Mar
Apr
May
Jun
Winter Solstice
December 21/Day 355
Winter Teletherm
January 6/Day 6
+16 days
Coverage
Year
DULCE, NM

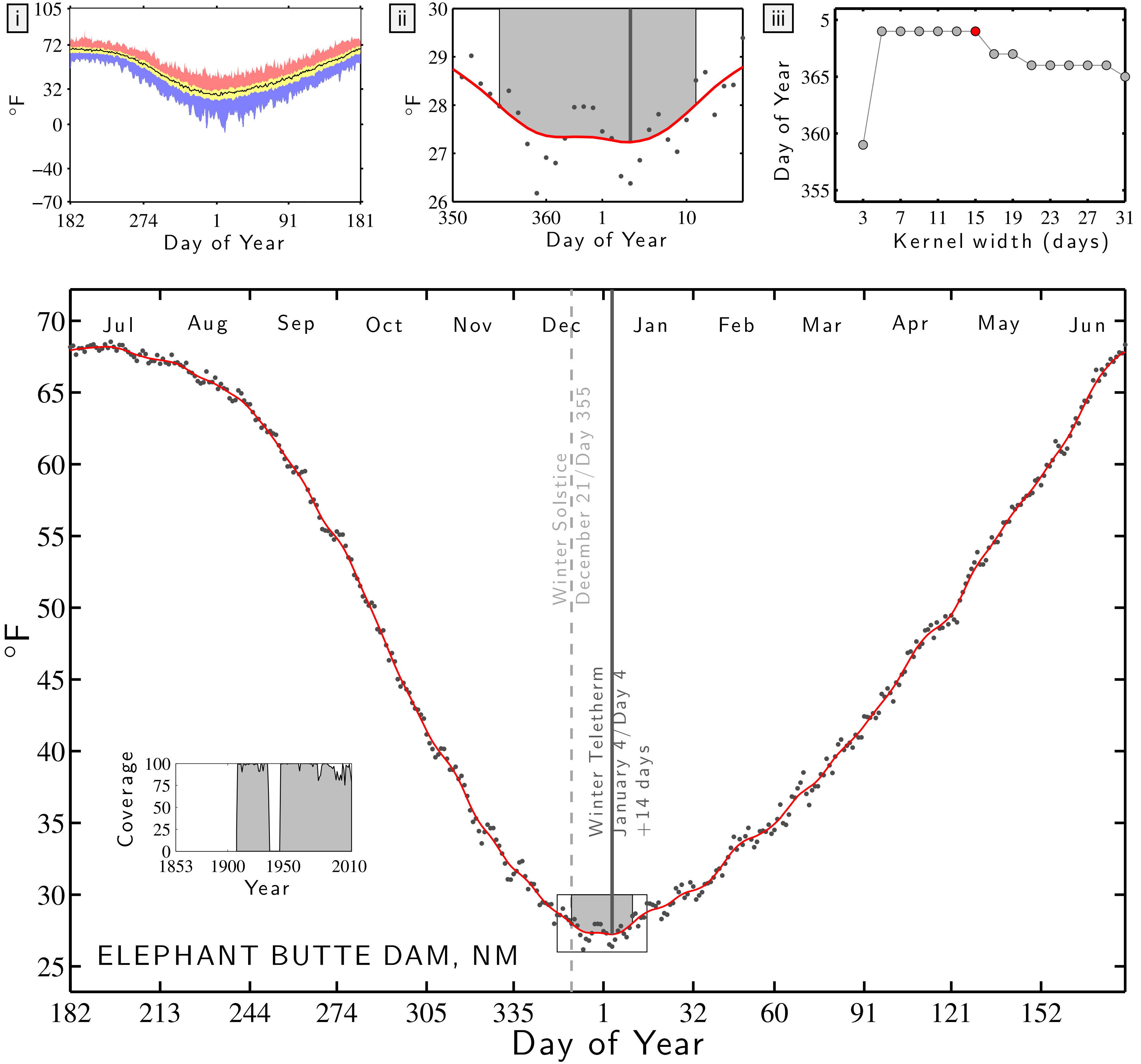

i
°F
Day of Year
ii
°F
Day of Year
iii
Day of Year
Kernel width (days)
Jul
Aug
Sep
Oct
Nov
Dec
Jan
Feb
Mar
Apr
May
Jun
Winter Solstice
December 21/Day 355
Winter Teletherm
January 4/Day 4
+14 days
Coverage
Year
ELEPHANT BUTTE DAM, NM
°F
Day of Year

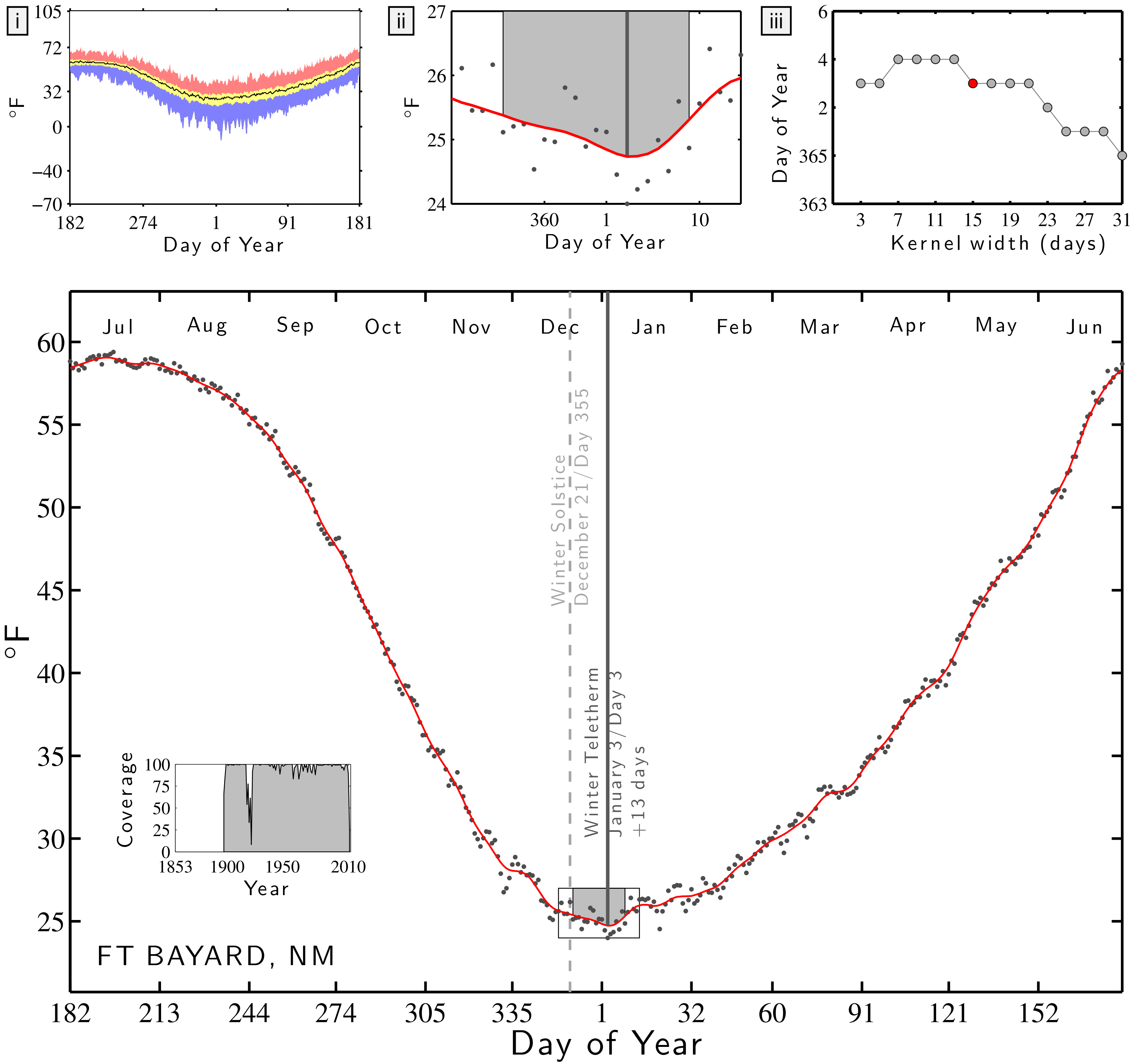

i
°F
Day of Year
ii
°F
Day of Year
iii
Day of Year
Kernel width (days)
Jul
Aug
Sep
Oct
Nov
Dec
Jan
Feb
Mar
Apr
May
Jun
Winter Solstice
December 21/Day 355
Winter Teletherm
January 3/Day 3
+13 days
Coverage
Year
FT BAYARD, NM
°F
Day of Year

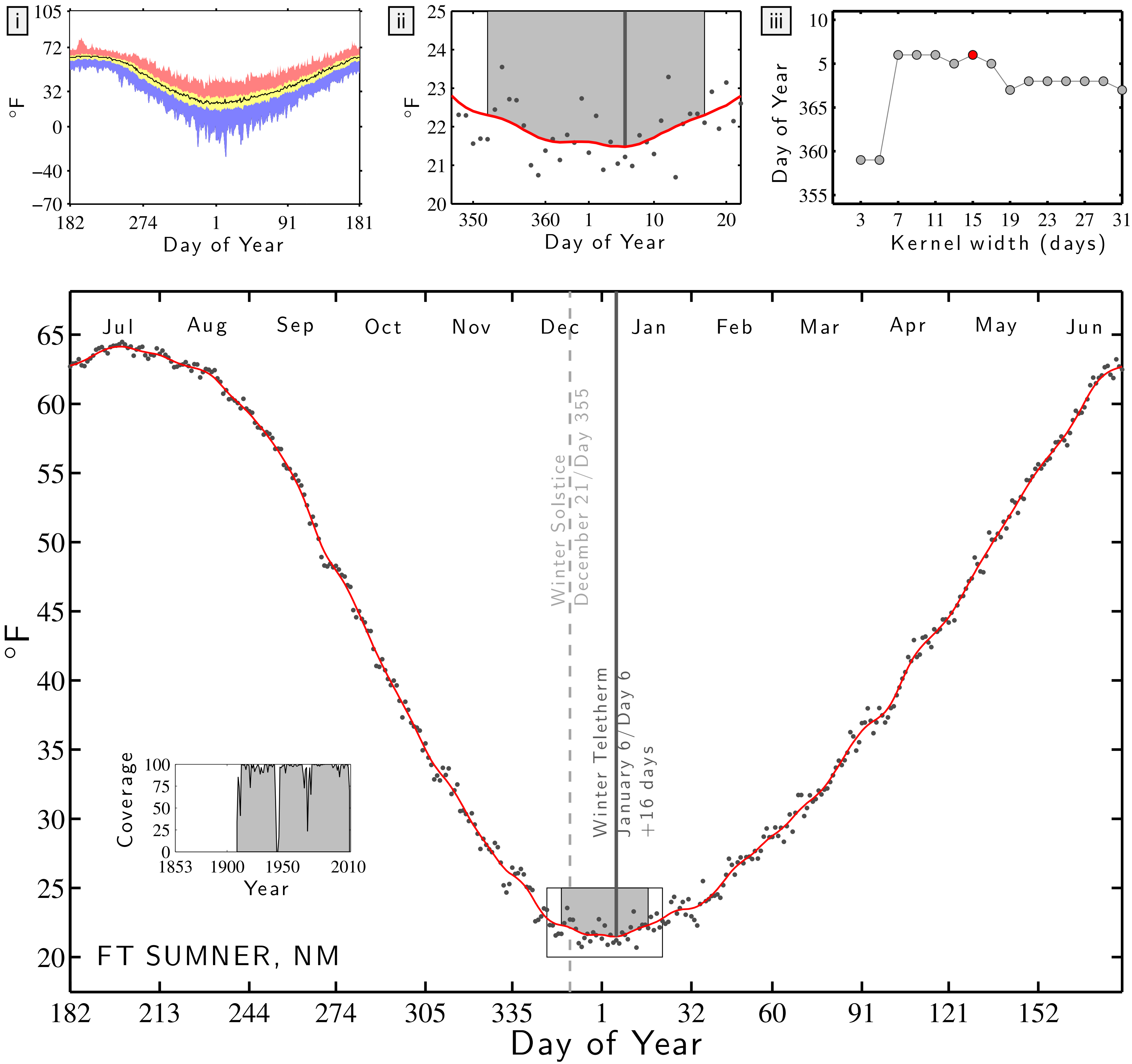
i
105
72
32
0
−40
−70
°F
182
274
1
91
181
Day of Year
ii
25
24
23
22
21
20
°F
350
360
1
10
20
Day of Year
iii
10
5
365
360
355
Day of Year
3
7
11
15
19
23
27
31
Kernel width (days)
Jul
Aug
Sep
Oct
Nov
Dec
Jan
Feb
Mar
Apr
May
Jun
65
60
55
50
45
40
35
30
25
20
°F
Winter Solstice
December 21/Day 355
Winter Teletherm
January 6/Day 6
+16 days
Coverage
100
75
50
25
0
1853
1900
1950
2010
Year
FT SUMNER, NM
182
213
244
274
305
335
1
32
60
91
121
152
Day of Year

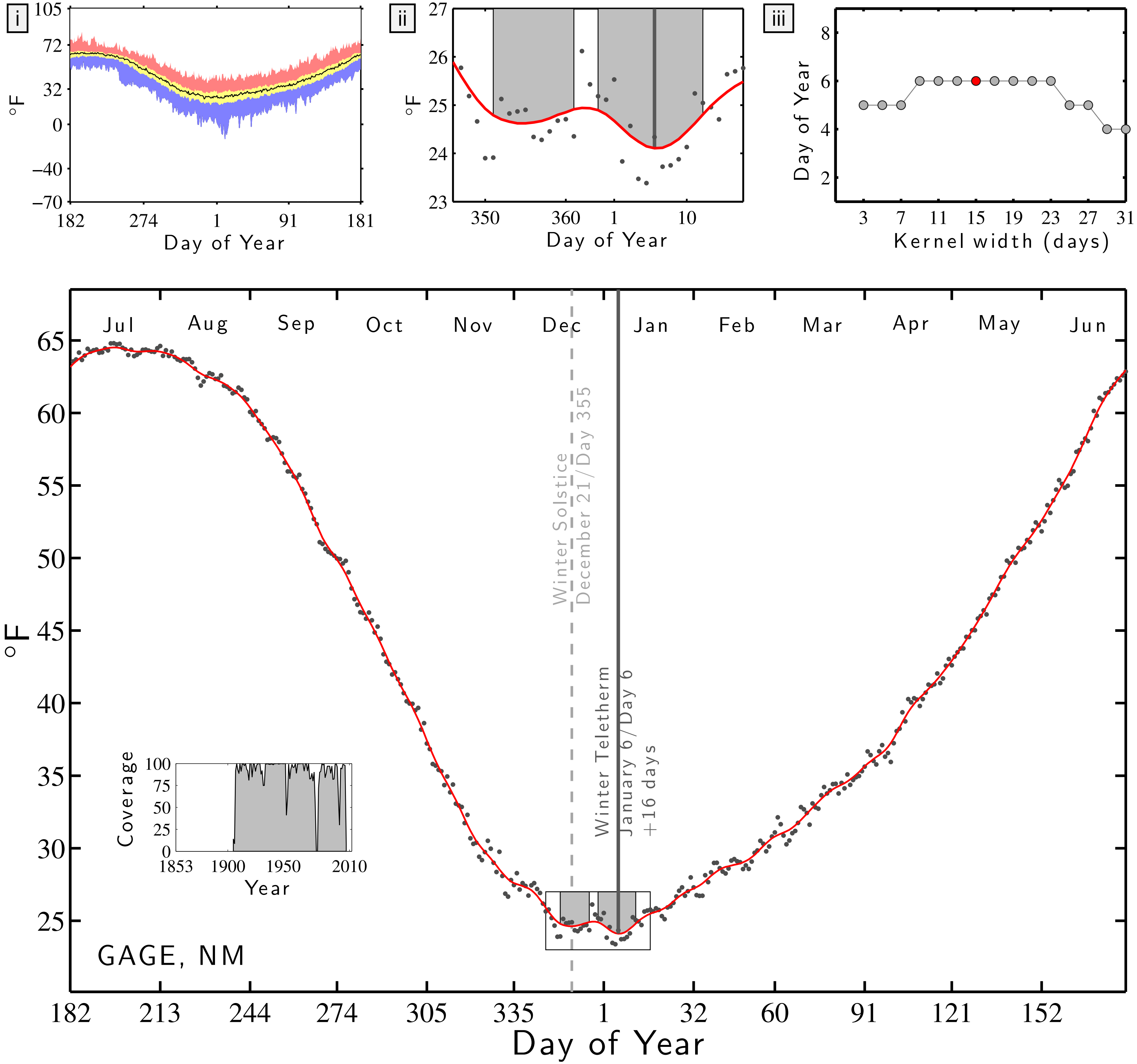

i
105
72
32
0
−40
−70
°F
182
274
1
91
181
Day of Year
ii
27
26
25
24
23
°F
350
360
1
10
Day of Year
iii
8
6
4
2
Day of Year
3
7
11
15
19
23
27
31
Kernel width (days)
Jul
Aug
Sep
Oct
Nov
Dec
Jan
Feb
Mar
Apr
May
Jun
65
60
55
50
45
40
35
30
25
°F
Winter Solstice
December 21/Day 355
Winter Teletherm
January 6/Day 6
+16 days
Coverage
100
75
50
25
0
1853
1900
1950
2010
Year
GAGE, NM
182
213
244
274
305
335
1
32
60
91
121
152
Day of Year

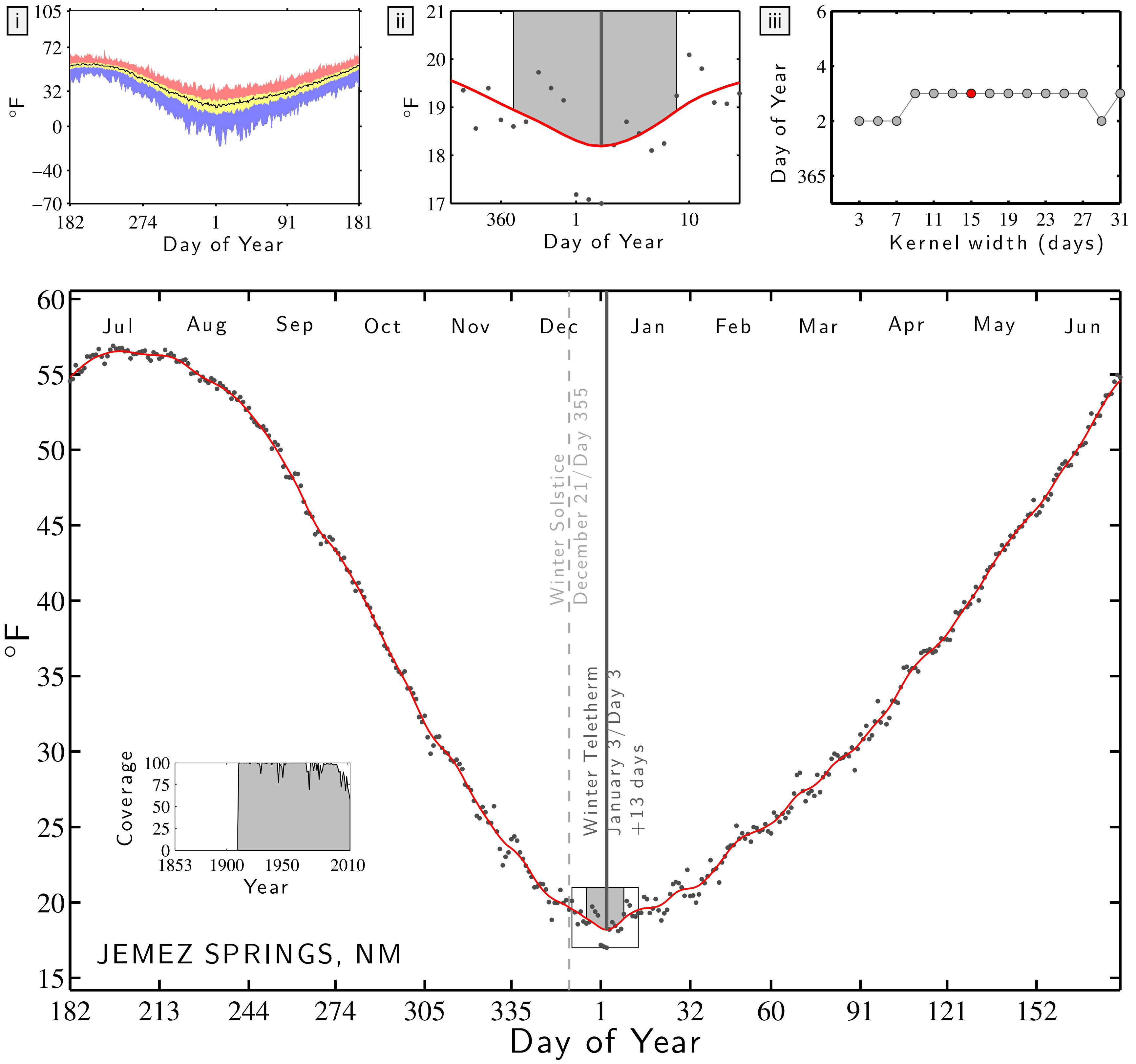

i
ii
iii
°F
Day of Year
Kernel width (days)
Jul
Aug
Sep
Oct
Nov
Dec
Jan
Feb
Mar
Apr
May
Jun
Winter Solstice
December 21/Day 355
Winter Teletherm
January 3/Day 3
+13 days
Coverage
Year
JEMEZ SPRINGS, NM

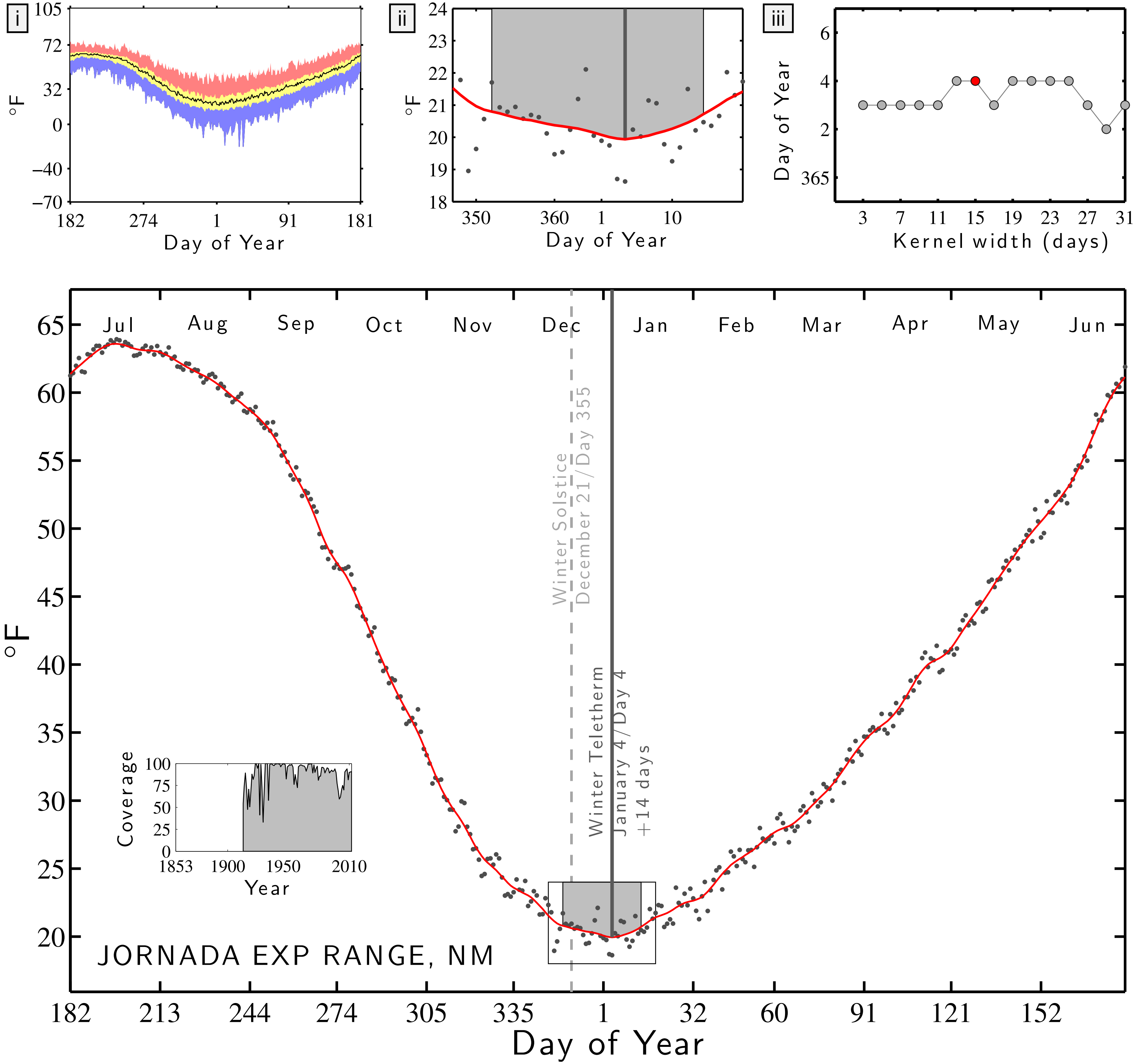

i
105
72
32
0
−40
−70
°F
182
274
1
91
181
Day of Year
ii
24
23
22
21
20
19
18
°F
350
360
1
10
Day of Year
iii
6
4
2
365
Day of Year
3
7
11
15
19
23
27
31
Kernel width (days)
Jul
Aug
Sep
Oct
Nov
Dec
Jan
Feb
Mar
Apr
May
Jun
65
60
55
50
45
40
35
30
25
20
°F
Winter Solstice
December 21/Day 355
Winter Teletherm
January 4/Day 4
+14 days
Coverage
100
75
50
25
0
1853
1900
1950
2010
Year
JORNADA EXP RANGE, NM
182
213
244
274
305
335
1
32
60
91
121
152
Day of Year

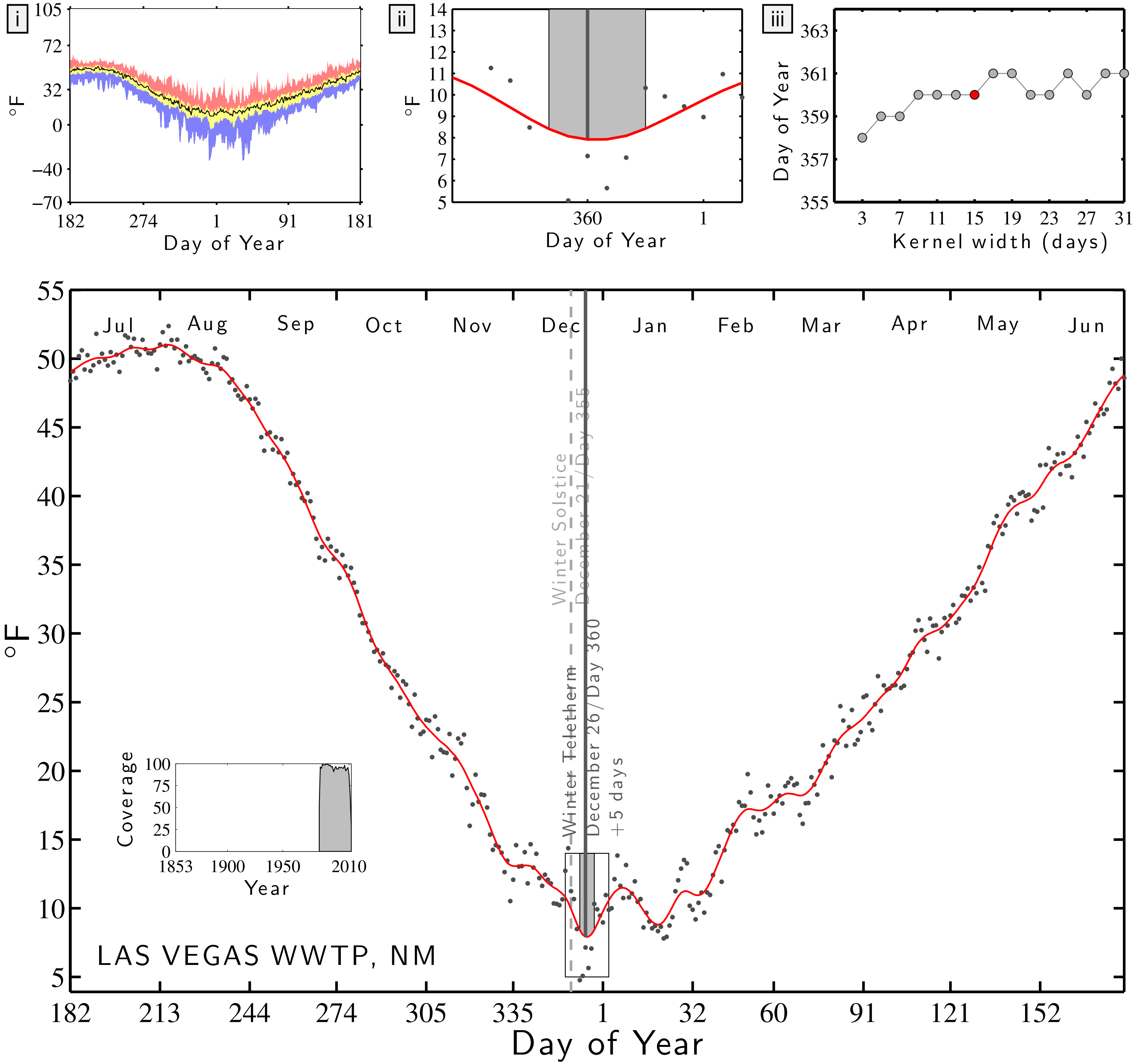

i
°F
Day of Year
ii
°F
Day of Year
iii
Day of Year
Kernel width (days)
Jul
Aug
Sep
Oct
Nov
Dec
Jan
Feb
Mar
Apr
May
Jun
Winter Solstice
December 21/Day 355
Winter Teletherm
December 26/Day 360
+5 days
Coverage
Year
LAS VEGAS WWTP, NM
°F
Day of Year

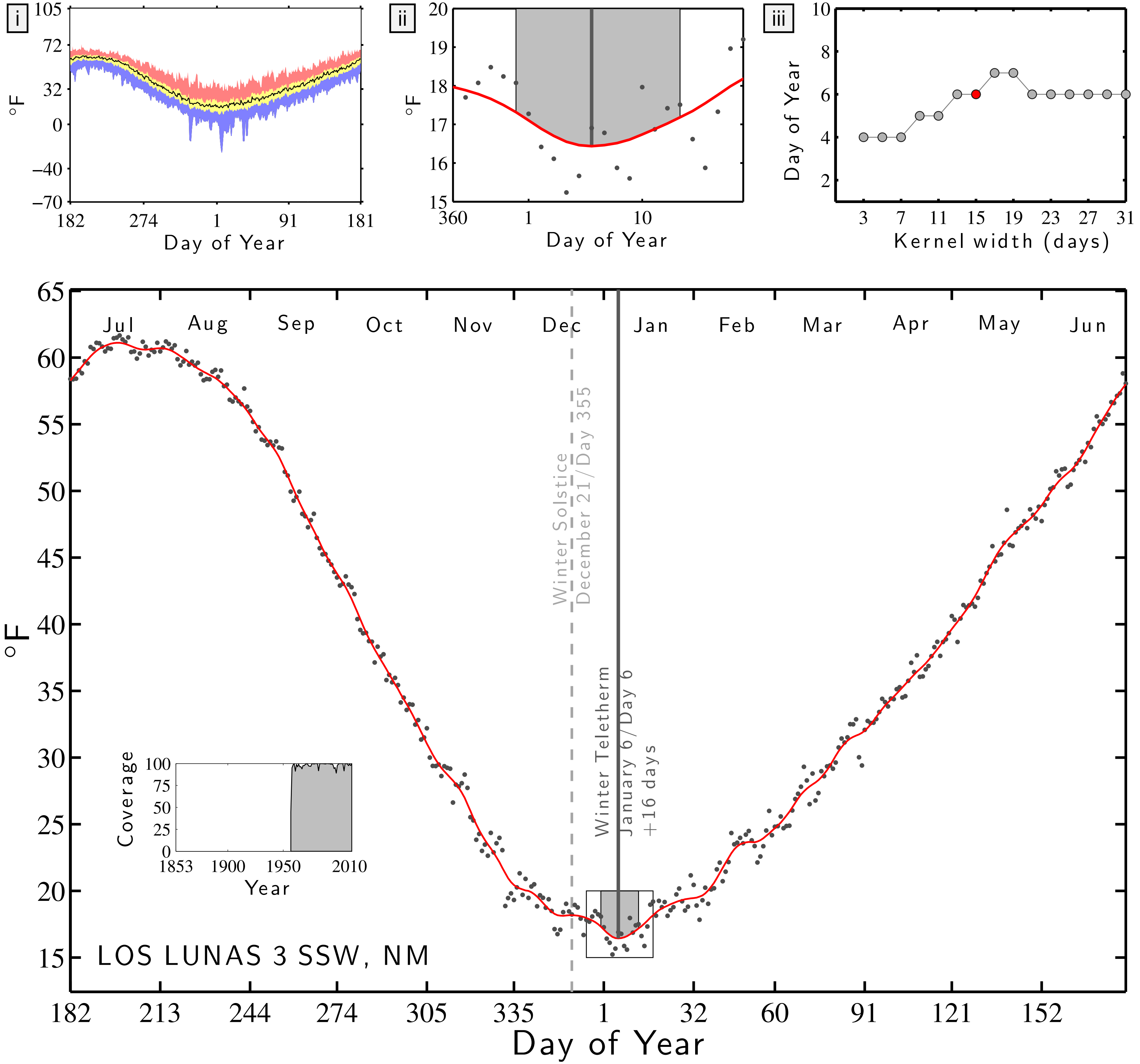

i
ii
iii
Day of Year
Kernel width (days)
Jul
Aug
Sep
Oct
Nov
Dec
Jan
Feb
Mar
Apr
May
Jun
Winter Solstice
December 21/Day 355
Winter Teletherm
January 6/Day 6
+16 days
Coverage
Year
LOS LUNAS 3 SSW, NM
°F
Day of Year

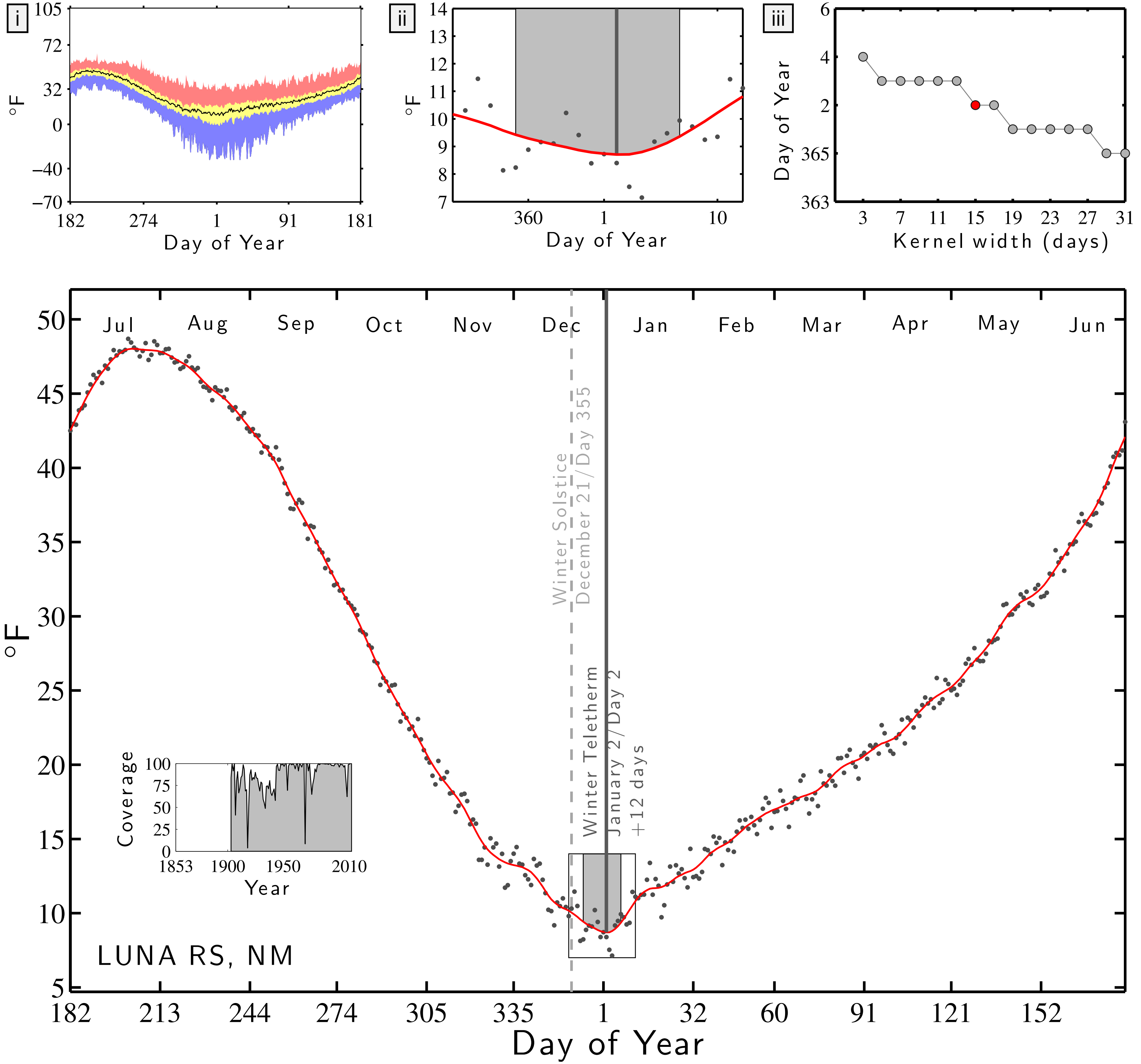

i
ii
iii
°F
Day of Year
Kernel width (days)
Winter Solstice
December 21/Day 355
Winter Teletherm
January 2/Day 2
+12 days
Jul
Aug
Sep
Oct
Nov
Dec
Jan
Feb
Mar
Apr
May
Jun
Coverage
Year
LUNA RS, NM

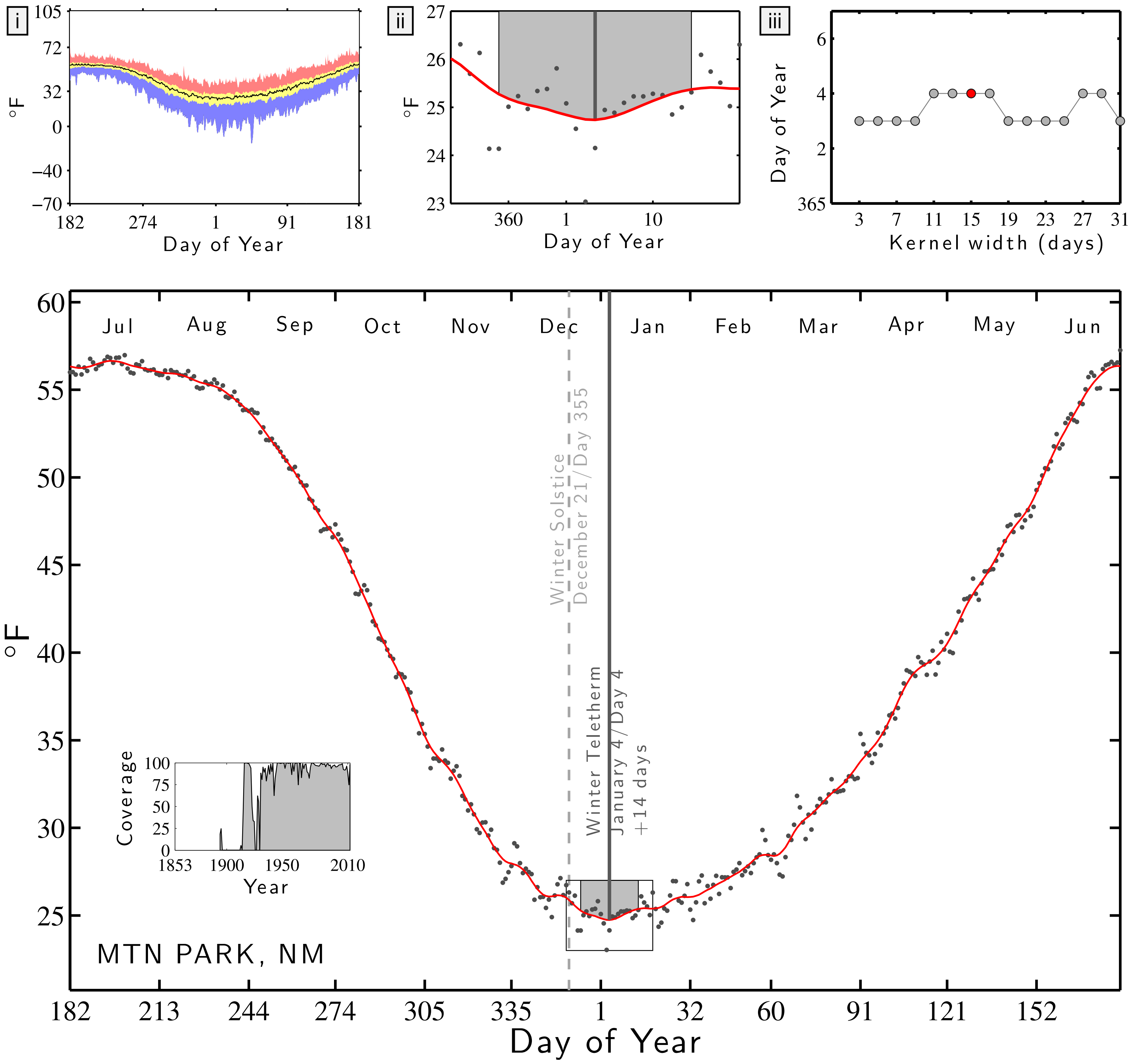

i
ii
iii
°F
Day of Year
Day of Year
Kernel width (days)
Jul
Aug
Sep
Oct
Nov
Dec
Jan
Feb
Mar
Apr
May
Jun
Winter Solstice
December 21/Day 355
Winter Teletherm
January 4/Day 4
+14 days
Coverage
Year
MTN PARK, NM

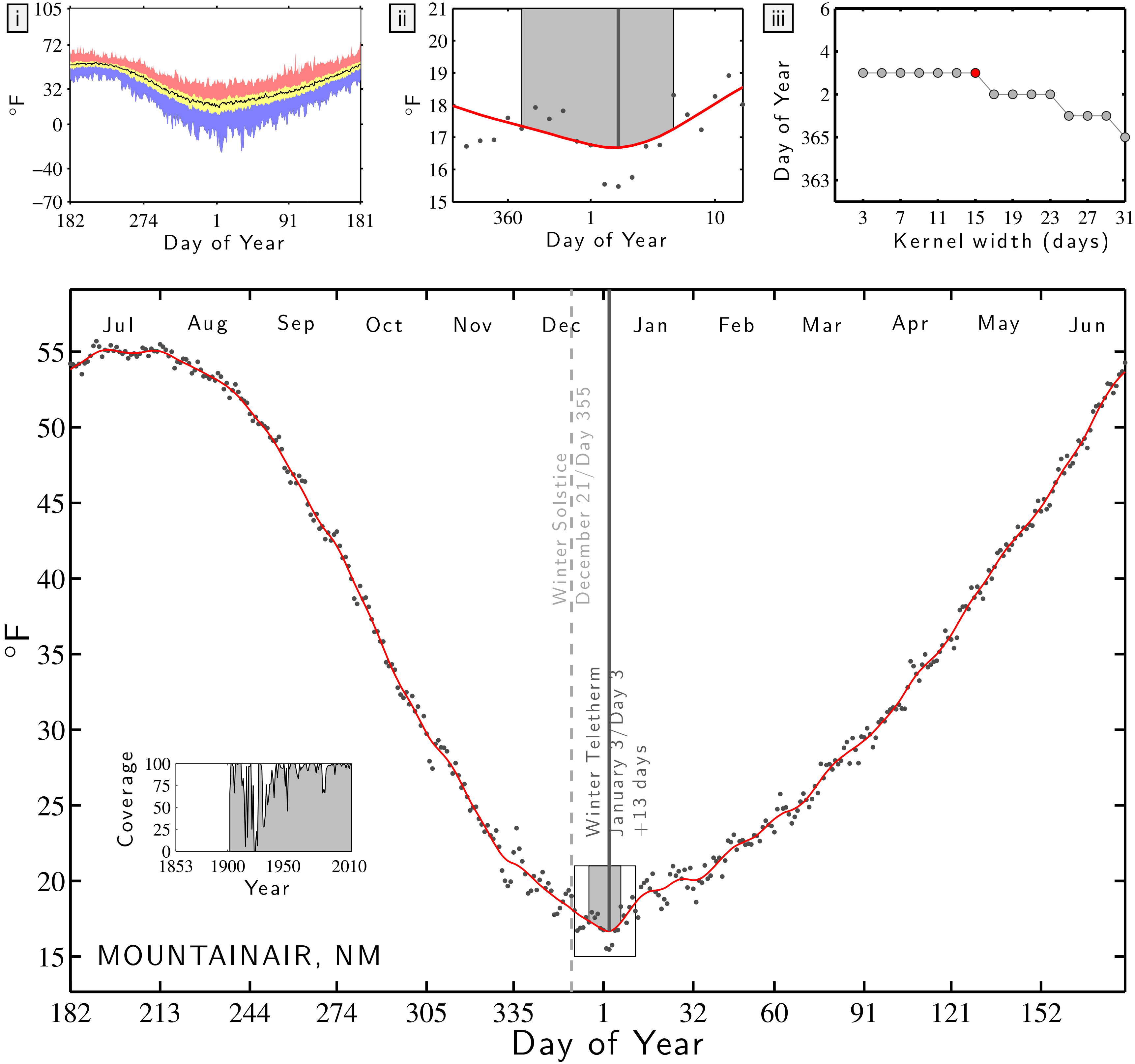

i
ii
iii
°F
Day of Year
Kernel width (days)
Jul
Aug
Sep
Oct
Nov
Dec
Jan
Feb
Mar
Apr
May
Jun
Winter Solstice
December 21/Day 355
Winter Teletherm
January 3/Day 3
+13 days
Coverage
Year
MOUNTAINAIR, NM

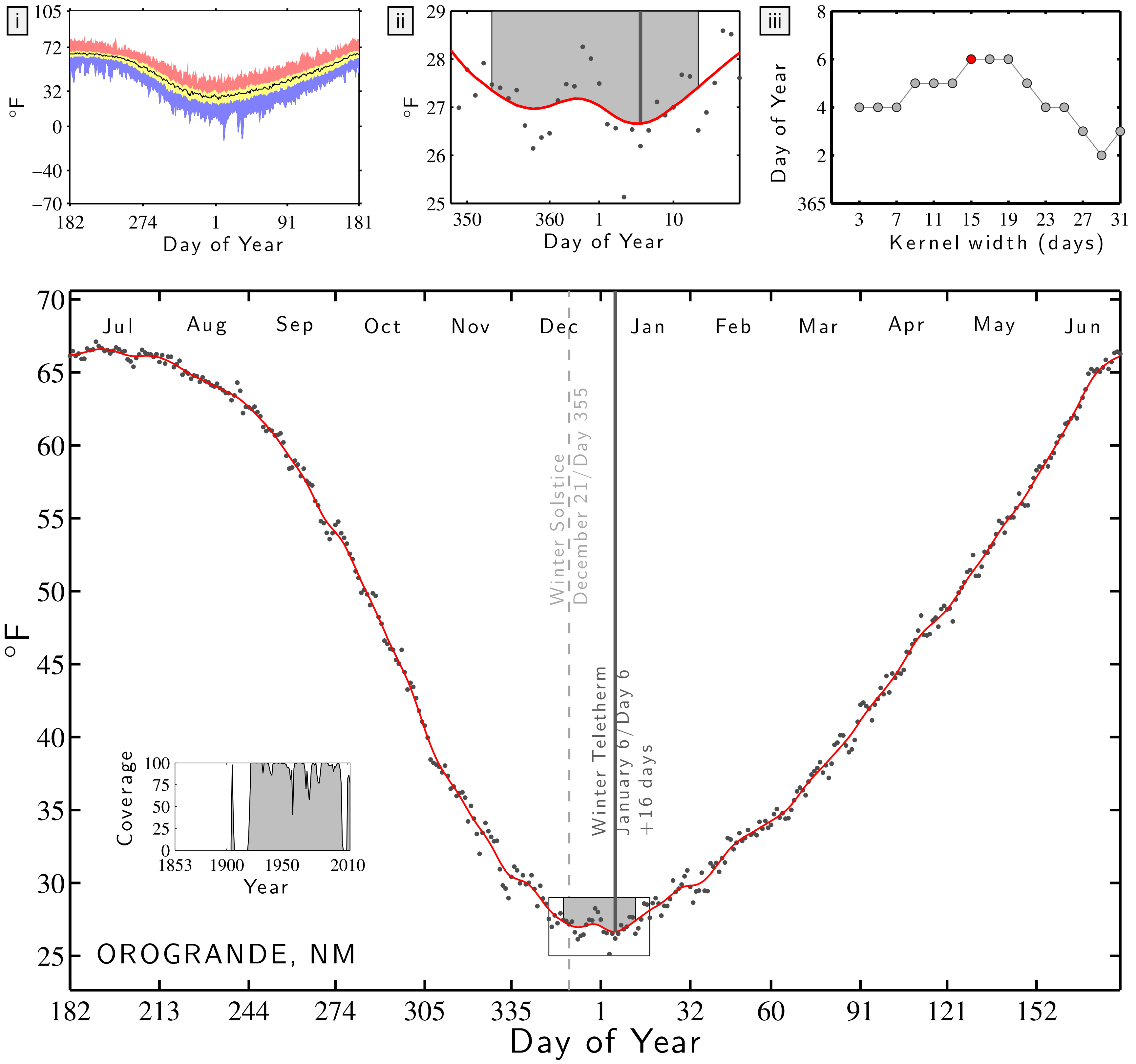

i
ii
iii
°F
Day of Year
Day of Year
Kernel width (days)
Jul
Aug
Sep
Oct
Nov
Dec
Jan
Feb
Mar
Apr
May
Jun
Winter Solstice
December 21/Day 355
Winter Teletherm
January 6/Day 6
+16 days
Coverage
Year
OROGRANDE, NM

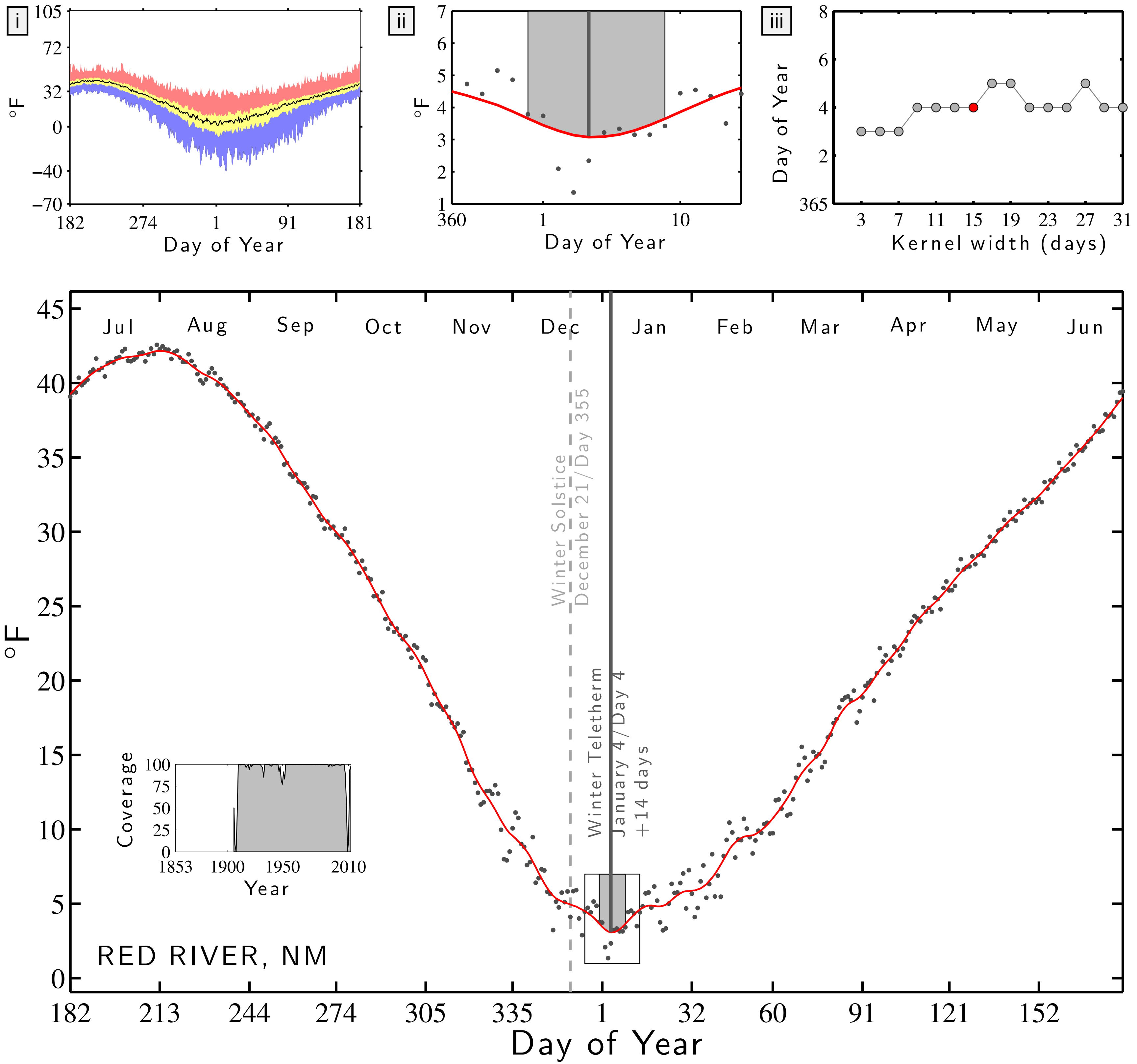

i
°F
Day of Year
ii
°F
Day of Year
iii
Day of Year
Kernel width (days)
Jul
Aug
Sep
Oct
Nov
Dec
Jan
Feb
Mar
Apr
May
Jun
Winter Solstice
December 21/Day 355
Winter Teletherm
January 4/Day 4
+14 days
Coverage
Year
RED RIVER, NM
°F
Day of Year

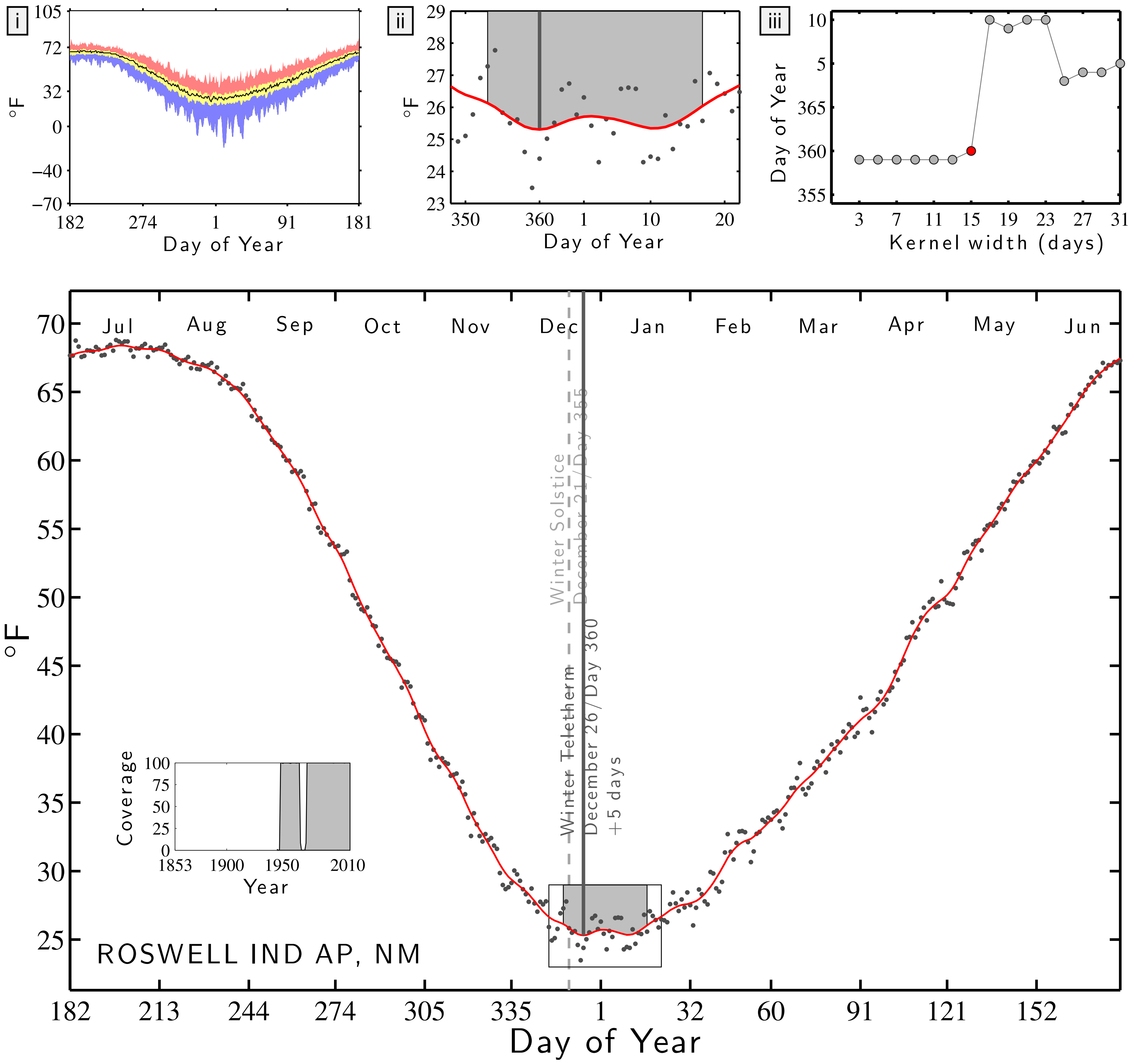

i
°F
Day of Year
ii
°F
Day of Year
iii
Day of Year
Kernel width (days)
Jul
Aug
Sep
Oct
Nov
Dec
Jan
Feb
Mar
Apr
May
Jun
Winter Solstice
December 21/Day 355
Winter Teletherm
December 26/Day 360
+5 days
Coverage
Year
ROSWELL IND AP, NM
°F
Day of Year

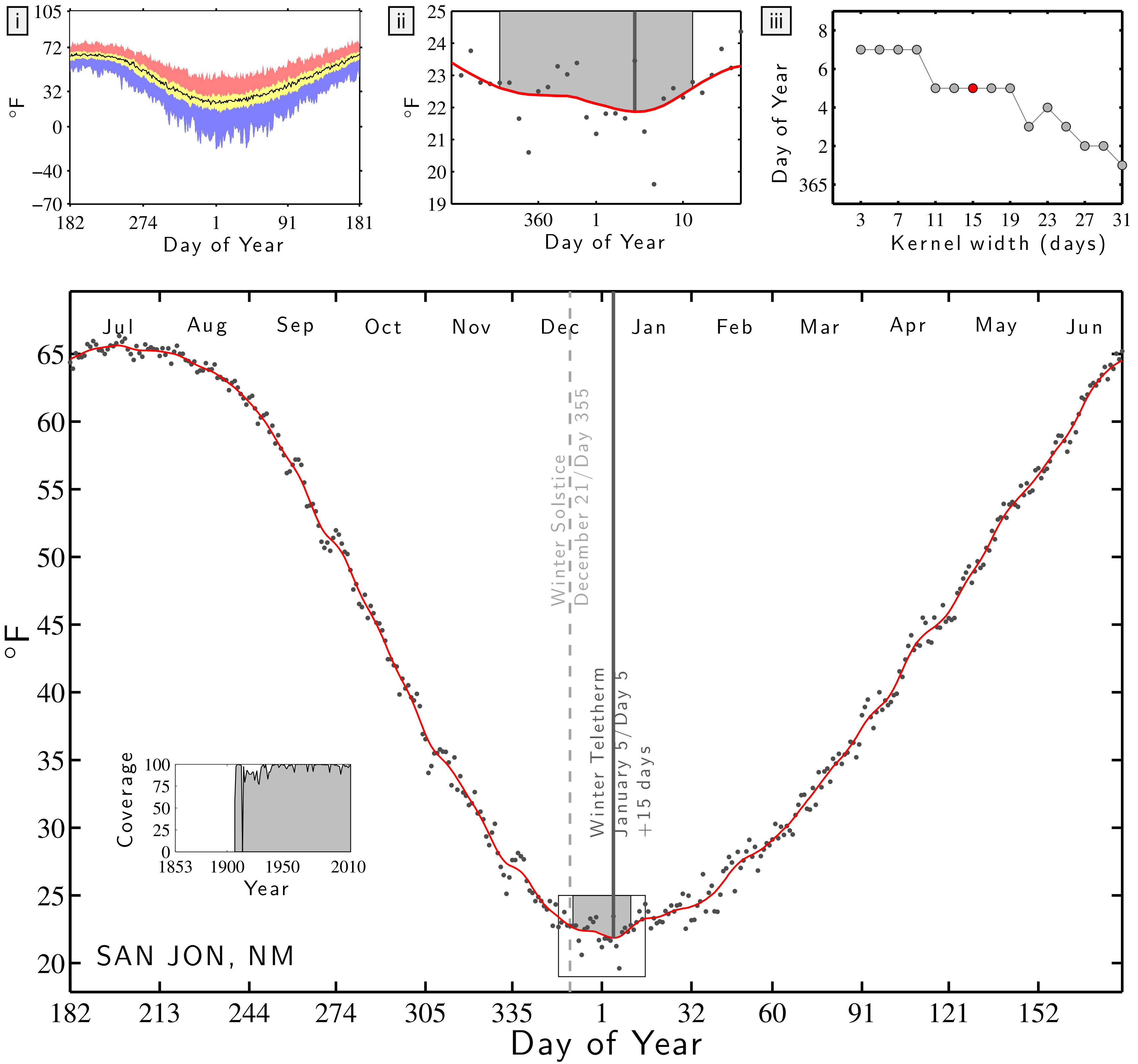

i
Day of Year
°F
ii
Day of Year
iii
Day of Year
Kernel width (days)
Jul
Aug
Sep
Oct
Nov
Dec
Jan
Feb
Mar
Apr
May
Jun
Winter Solstice
December 21/Day 355
Winter Teletherm
January 5/Day 5
+15 days
Coverage
Year
SAN JON, NM
Day of Year

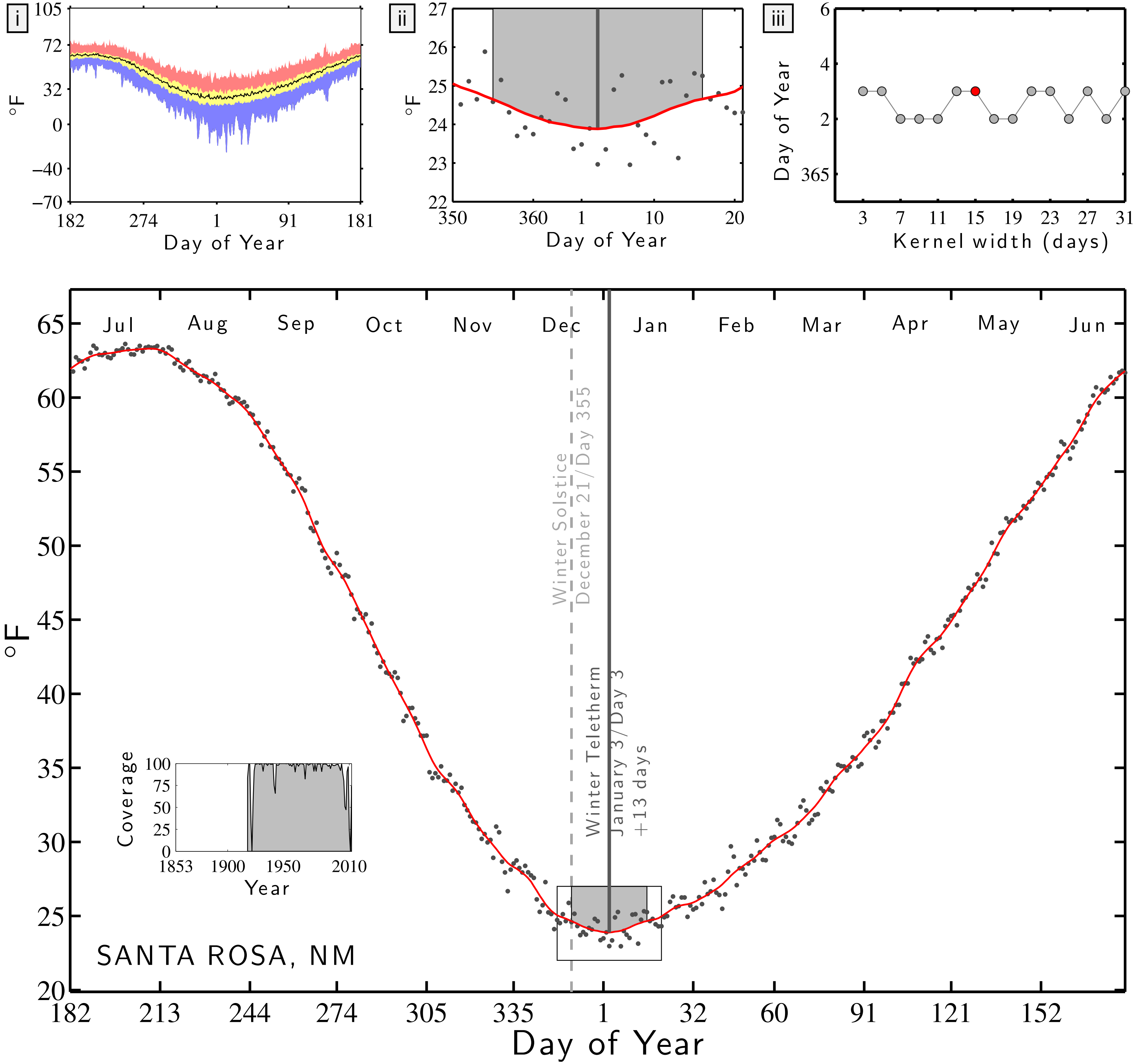

i
ii
iii
°F
Day of Year
Kernel width (days)
Jul
Aug
Sep
Oct
Nov
Dec
Jan
Feb
Mar
Apr
May
Jun
Winter Solstice
December 21/Day 355
Winter Teletherm
January 3/Day 3
+13 days
Coverage
Year
SANTA ROSA, NM

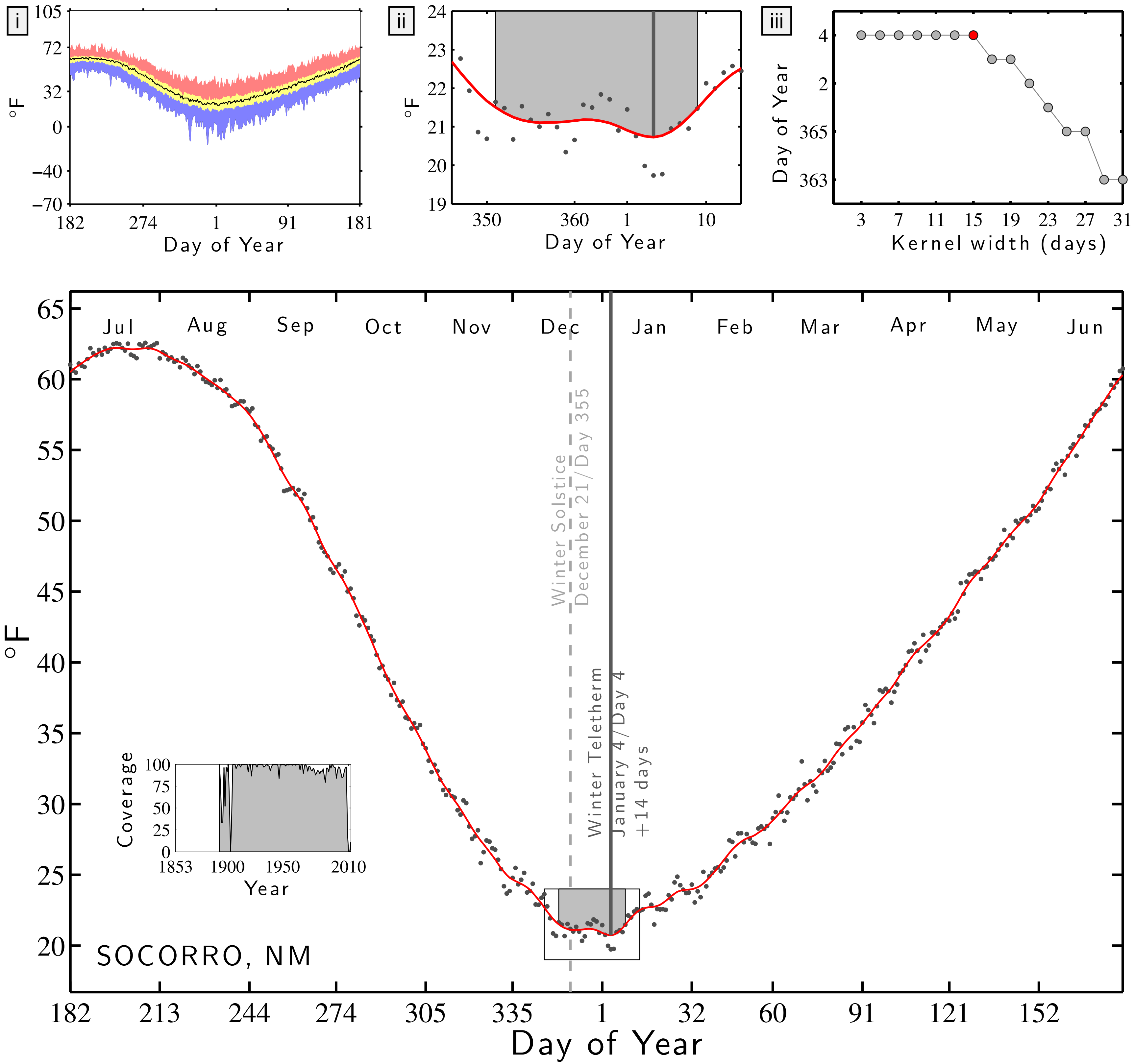

i
105
72
32
0
−40
−70
°F
182
274
1
91
181
Day of Year
ii
24
23
22
21
20
19
°F
350
360
1
10
Day of Year
iii
4
2
365
363
Day of Year
3
7
11
15
19
23
27
31
Kernel width (days)
Jul
Aug
Sep
Oct
Nov
Dec
Jan
Feb
Mar
Apr
May
Jun
65
60
55
50
45
40
35
30
25
20
°F
Winter Solstice
December 21/Day 355
Winter Teletherm
January 4/Day 4
+14 days
Coverage
100
75
50
25
0
1853
1900
1950
2010
Year
SOCORRO, NM
182
213
244
274
305
335
1
32
60
91
121
152
Day of Year

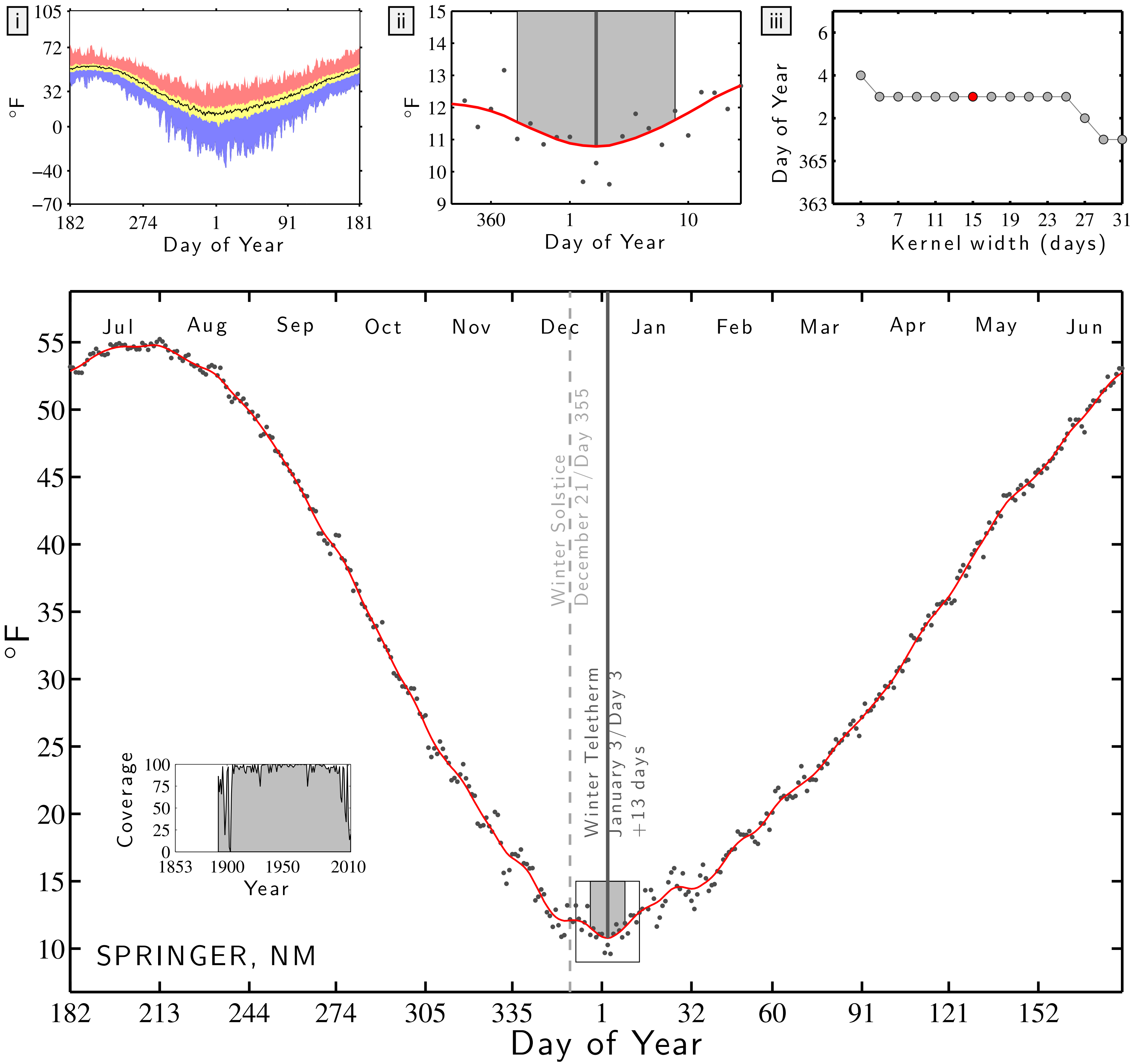

i
105
72
32
0
−40
−70
°F
182
274
1
91
181
Day of Year
ii
15
14
13
12
11
10
9
°F
360
1
10
Day of Year
iii
6
4
2
365
363
Day of Year
3
7
11
15
19
23
27
31
Kernel width (days)
Jul
Aug
Sep
Oct
Nov
Dec
Jan
Feb
Mar
Apr
May
Jun
55
50
45
40
35
30
25
20
15
10
°F
Winter Solstice
December 21/Day 355
Winter Teletherm
January 3/Day 3
+13 days
Coverage
100
75
50
25
0
1853
1900
1950
2010
Year
SPRINGER, NM
182
213
244
274
305
335
1
32
60
91
121
152
Day of Year

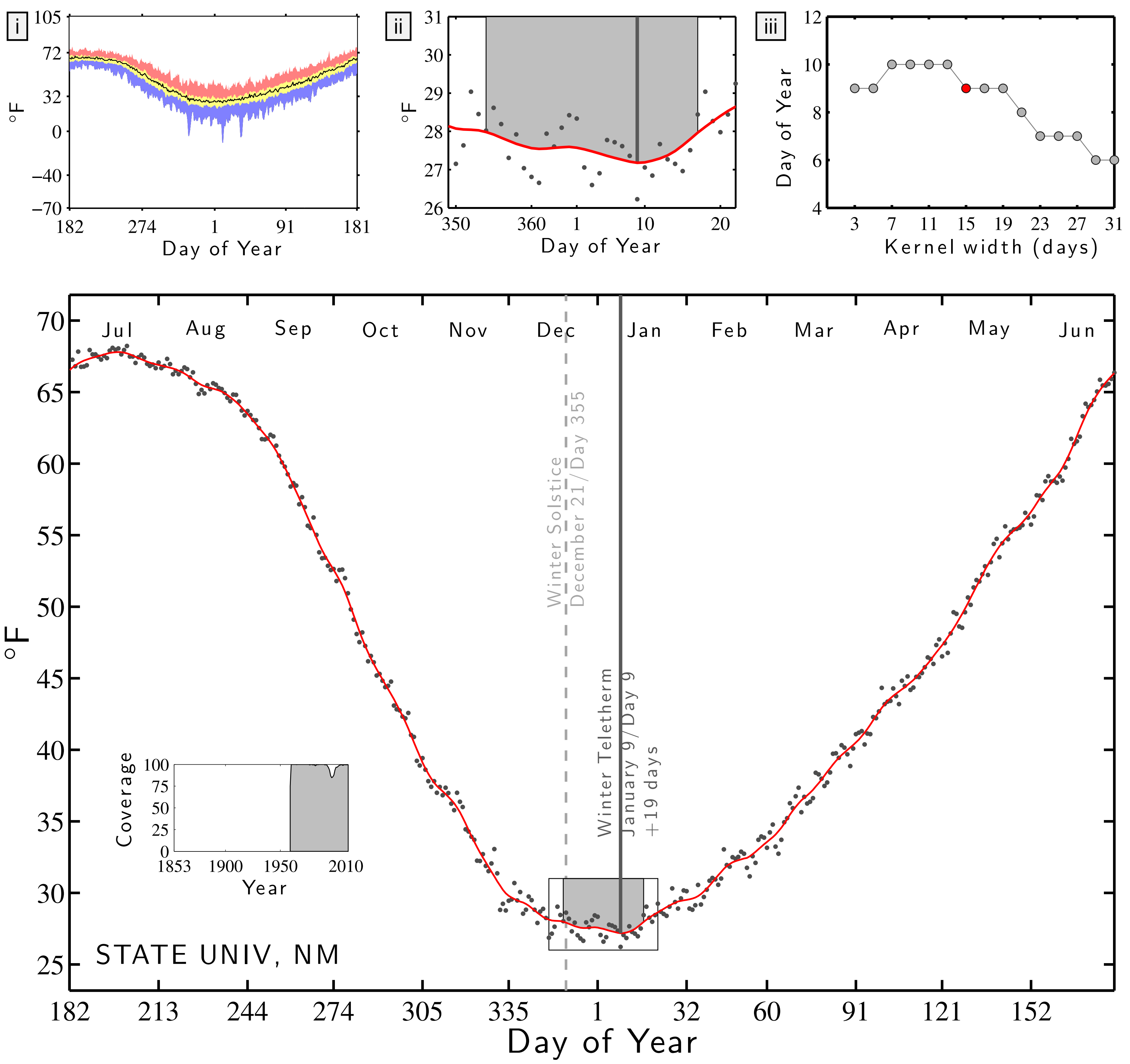

i
°F
105
72
32
0
−40
−70
182
274
1
91
181
Day of Year
ii
°F
31
30
29
28
27
26
350
360
1
10
20
Day of Year
iii
Day of Year
12
10
8
6
4
3
7
11
15
19
23
27
31
Kernel width (days)
70
65
60
55
50
45
40
35
30
25
°F
Jul
Aug
Sep
Oct
Nov
Dec
Jan
Feb
Mar
Apr
May
Jun
Winter Solstice
December 21/Day 355
Winter Teletherm
January 9/Day 9
+19 days
Coverage
100
75
50
25
0
1853
1900
1950
2010
Year
STATE UNIV, NM
182
213
244
274
305
335
1
32
60
91
121
152
Day of Year

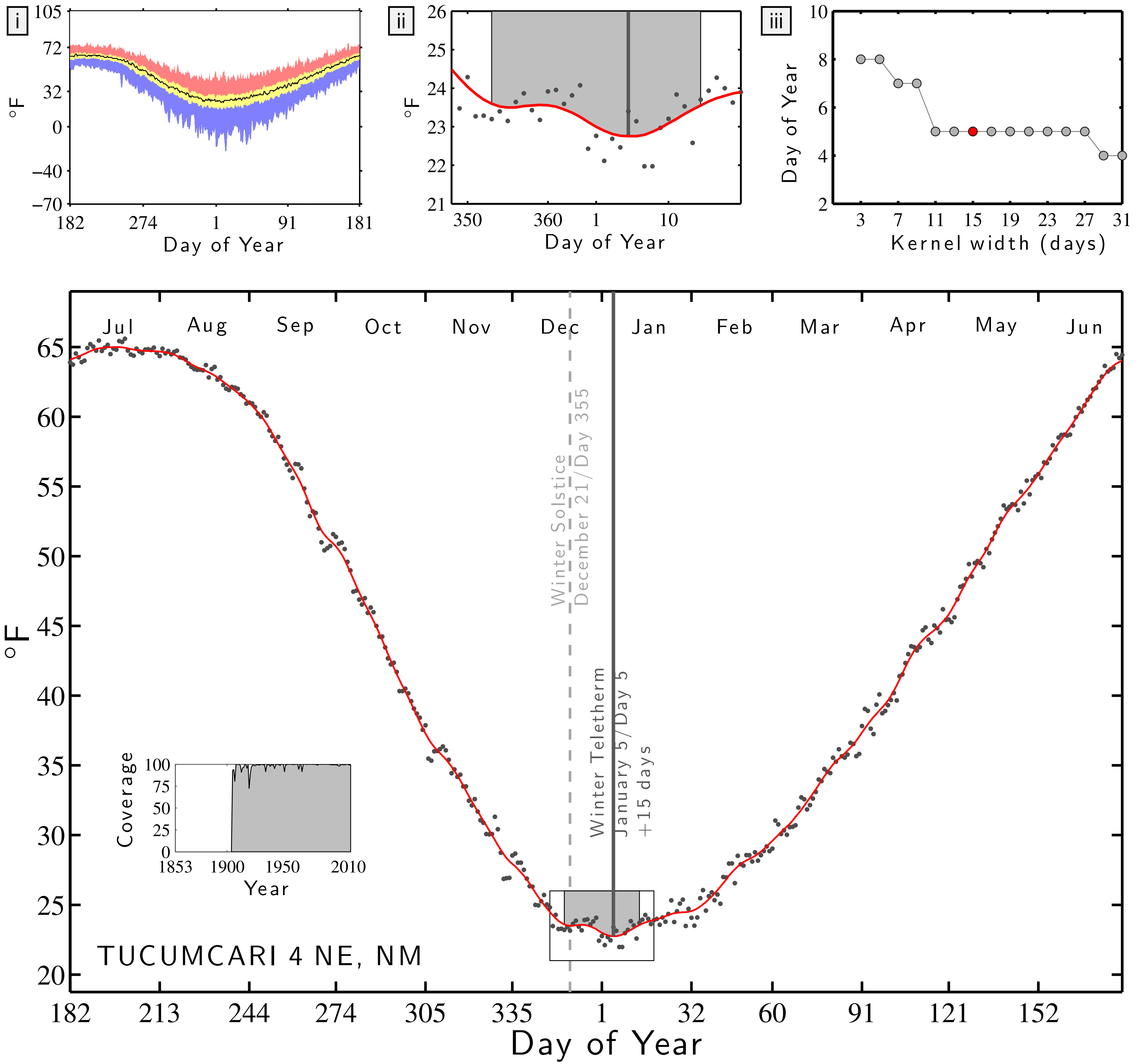

i
°F
Day of Year
ii
°F
Day of Year
iii
Day of Year
Kernel width (days)
Jul
Aug
Sep
Oct
Nov
Dec
Jan
Feb
Mar
Apr
May
Jun
Winter Solstice
December 21/Day 355
Winter Teletherm
January 5/Day 5
+15 days
Coverage
Year
TUCUMCARI 4 NE, NM
°F
Day of Year

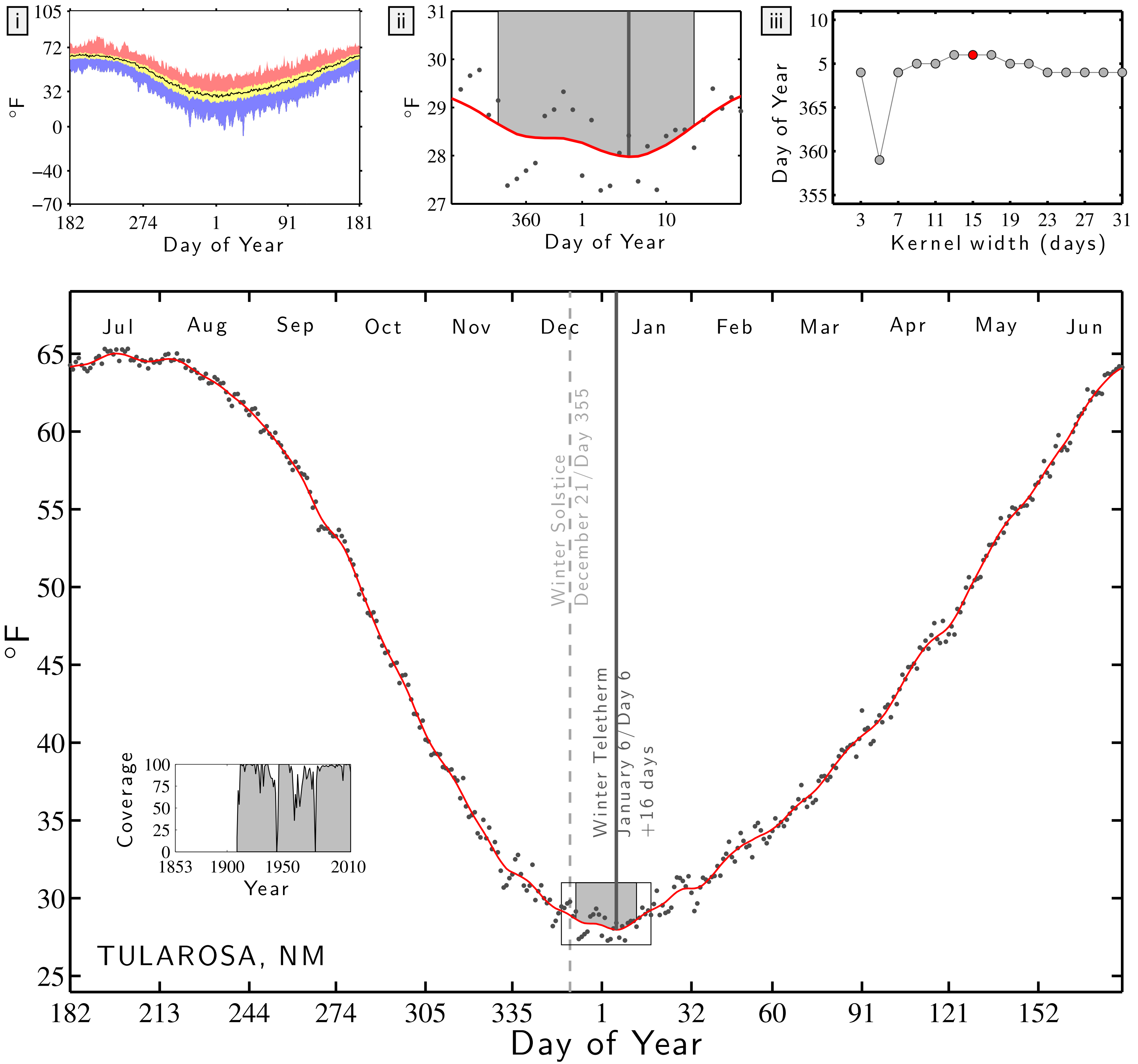

i
°F
Day of Year
ii
°F
Day of Year
iii
Day of Year
Kernel width (days)
Jul
Aug
Sep
Oct
Nov
Dec
Jan
Feb
Mar
Apr
May
Jun
Winter Solstice
December 21/Day 355
Winter Teletherm
January 6/Day 6
+16 days
Coverage
Year
TULAROSA, NM
°F
Day of Year

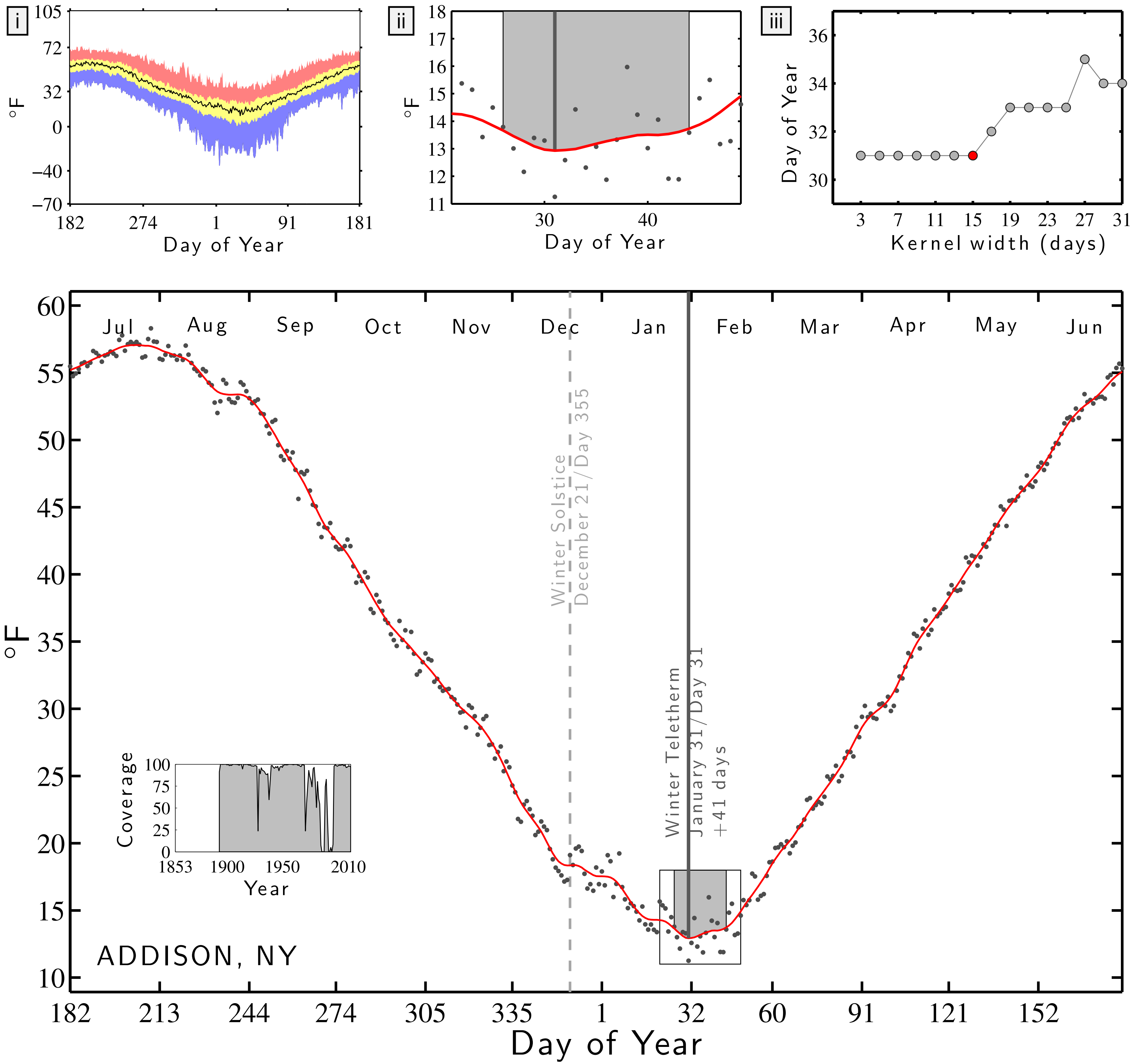

i
ii
iii
°F
Day of Year
Day of Year
Kernel width (days)
Jul
Aug
Sep
Oct
Nov
Dec
Jan
Feb
Mar
Apr
May
Jun
Winter Solstice
December 21/Day 355
Winter Teletherm
January 31/Day 31
+41 days
Coverage
Year
ADDISON, NY

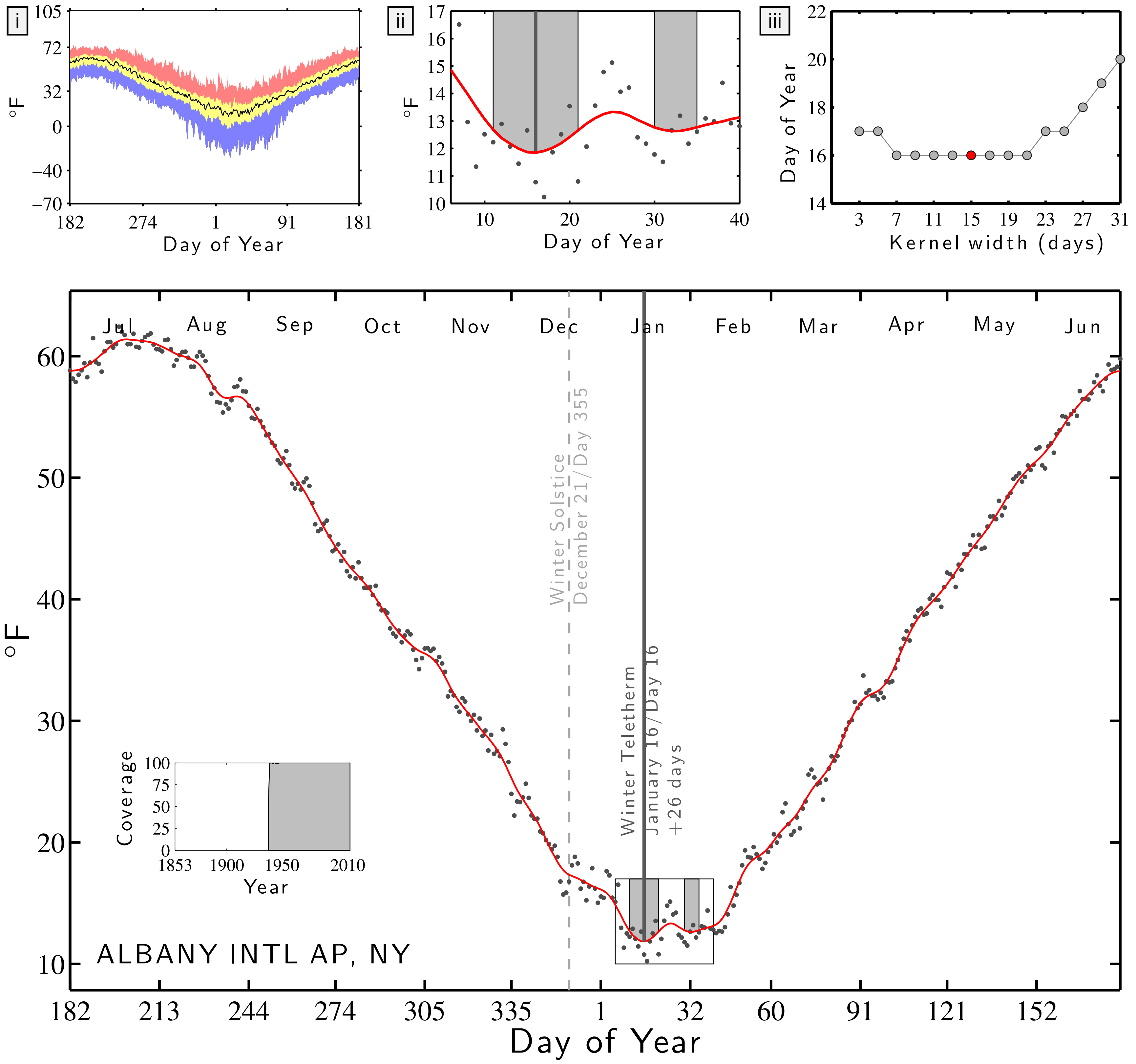

i
°F
Day of Year
ii
°F
Day of Year
iii
Day of Year
Kernel width (days)
Jul
Aug
Sep
Oct
Nov
Dec
Jan
Feb
Mar
Apr
May
Jun
Winter Solstice
December 21/Day 355
Winter Teletherm
January 16/Day 16
+26 days
Coverage
Year
ALBANY INTL AP, NY
°F
Day of Year

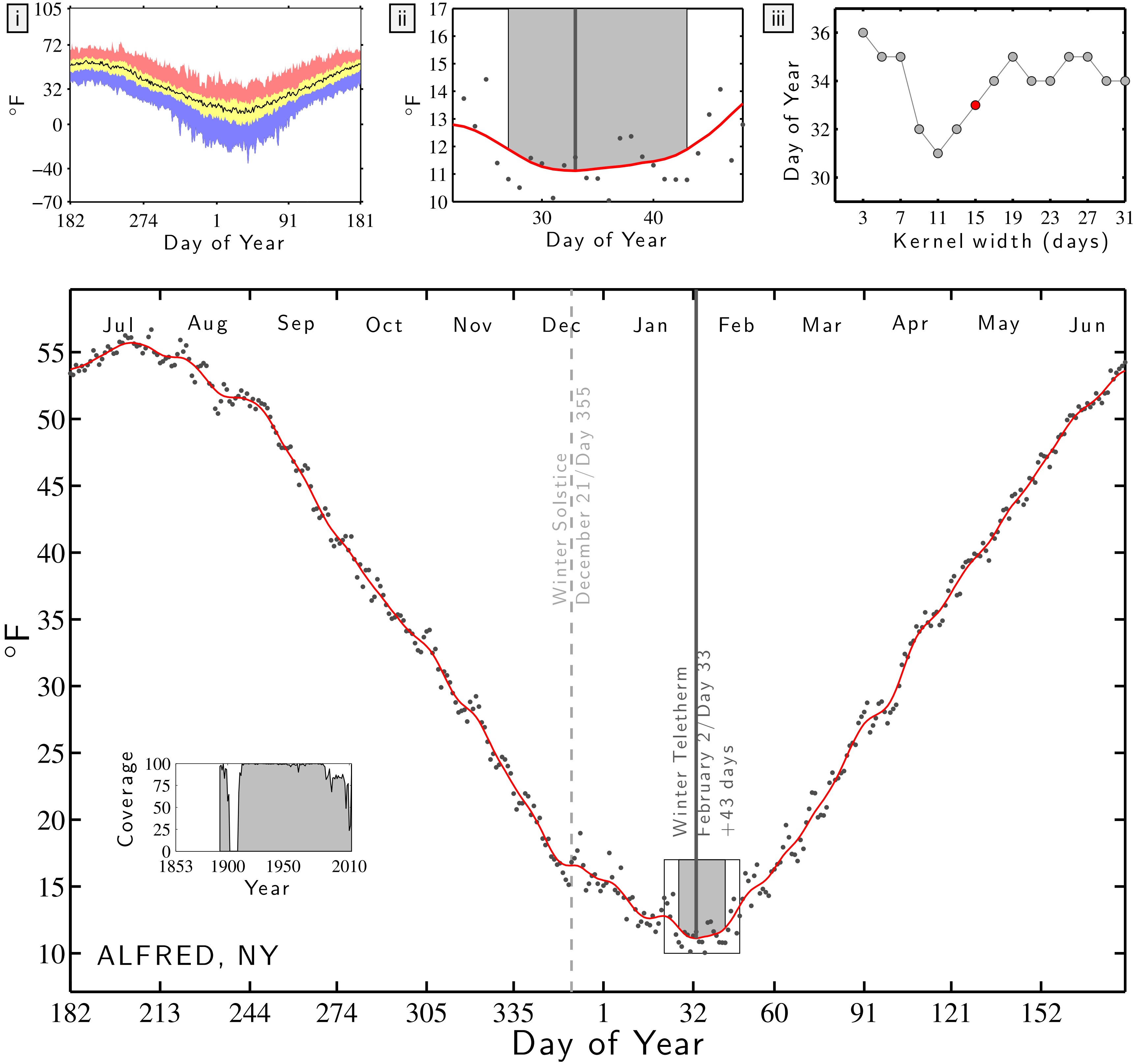

i
ii
iii
Day of Year
Kernel width (days)
°F
Jul Aug Sep Oct Nov Dec Jan Feb Mar Apr May Jun
Winter Solstice
December 21/Day 355
Winter Teletherm
February 2/Day 33
+43 days
Coverage
Year
ALFRED, NY
Day of Year

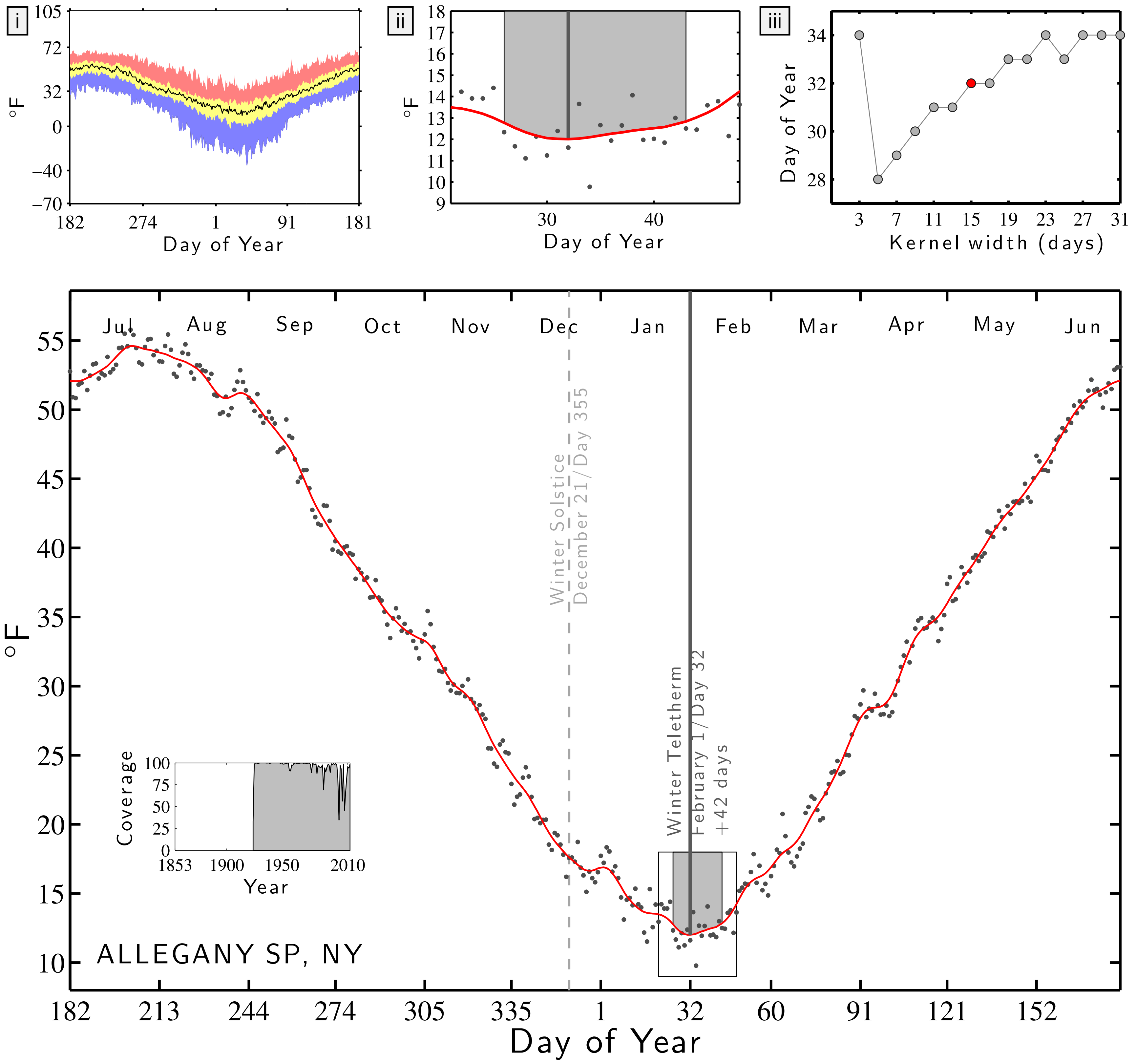

i
ii
iii
°F
Day of Year
Day of Year
Kernel width (days)
Jul
Aug
Sep
Oct
Nov
Dec
Jan
Feb
Mar
Apr
May
Jun
Winter Solstice
December 21/Day 355
Winter Teletherm
February 1/Day 32
+42 days
Coverage
Year
ALLEGANY SP, NY

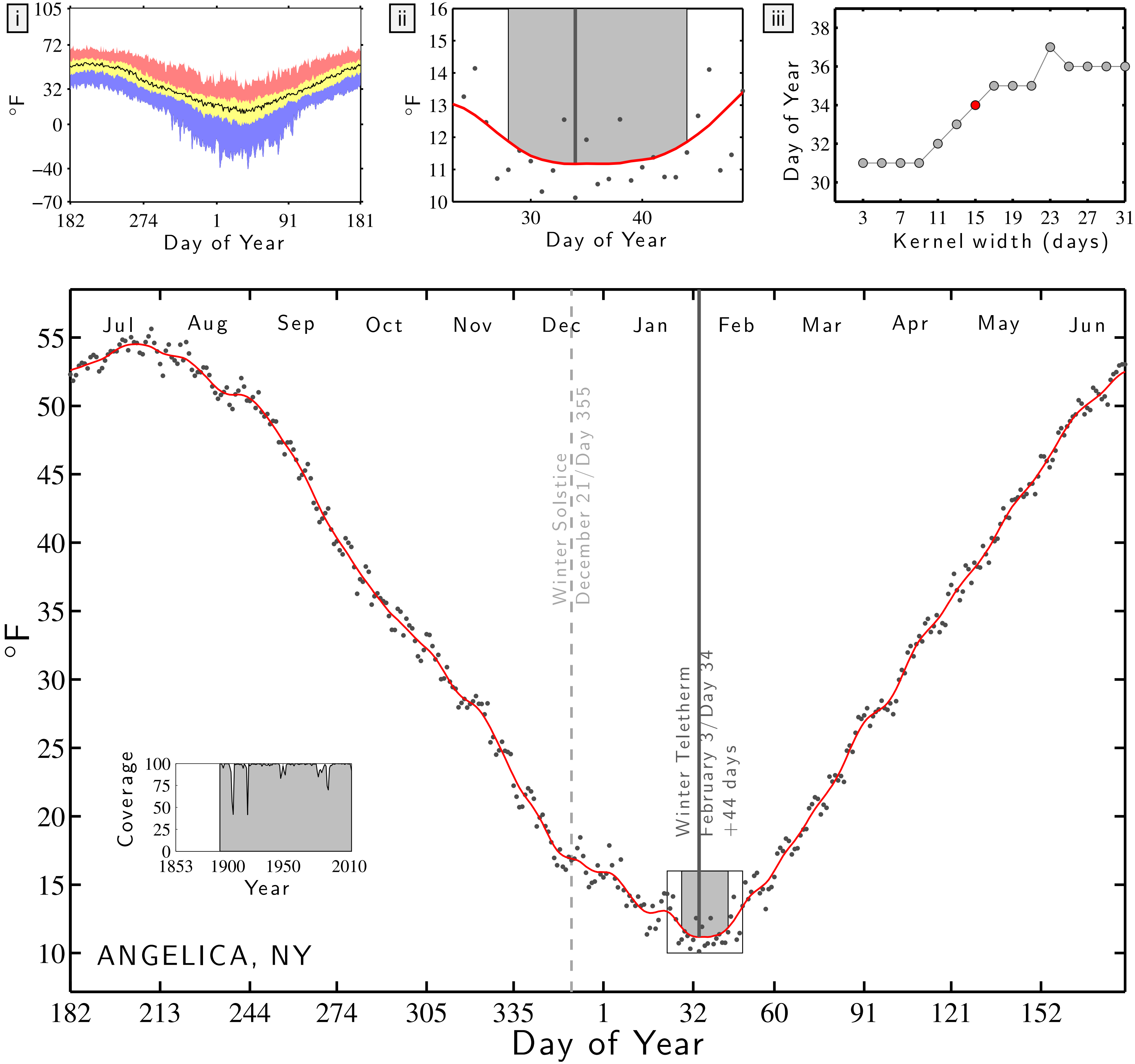
i
105
72
32
0
−40
−70
°F
182
274
1
91
181
Day of Year
ii
16
15
14
13
12
11
10
°F
30
40
Day of Year
iii
38
36
34
32
30
Day of Year
3
7
11
15
19
23
27
31
Kernel width (days)
Jul
Aug
Sep
Oct
Nov
Dec
Jan
Feb
Mar
Apr
May
Jun
Winter Solstice
December 21/Day 355
Winter Teletherm
February 3/Day 34
+44 days
55
50
45
40
35
30
25
20
15
10
°F
Coverage
100
75
50
25
0
1853
1900
1950
2010
Year
ANGELICA, NY
182
213
244
274
305
335
1
32
60
91
121
152
Day of Year

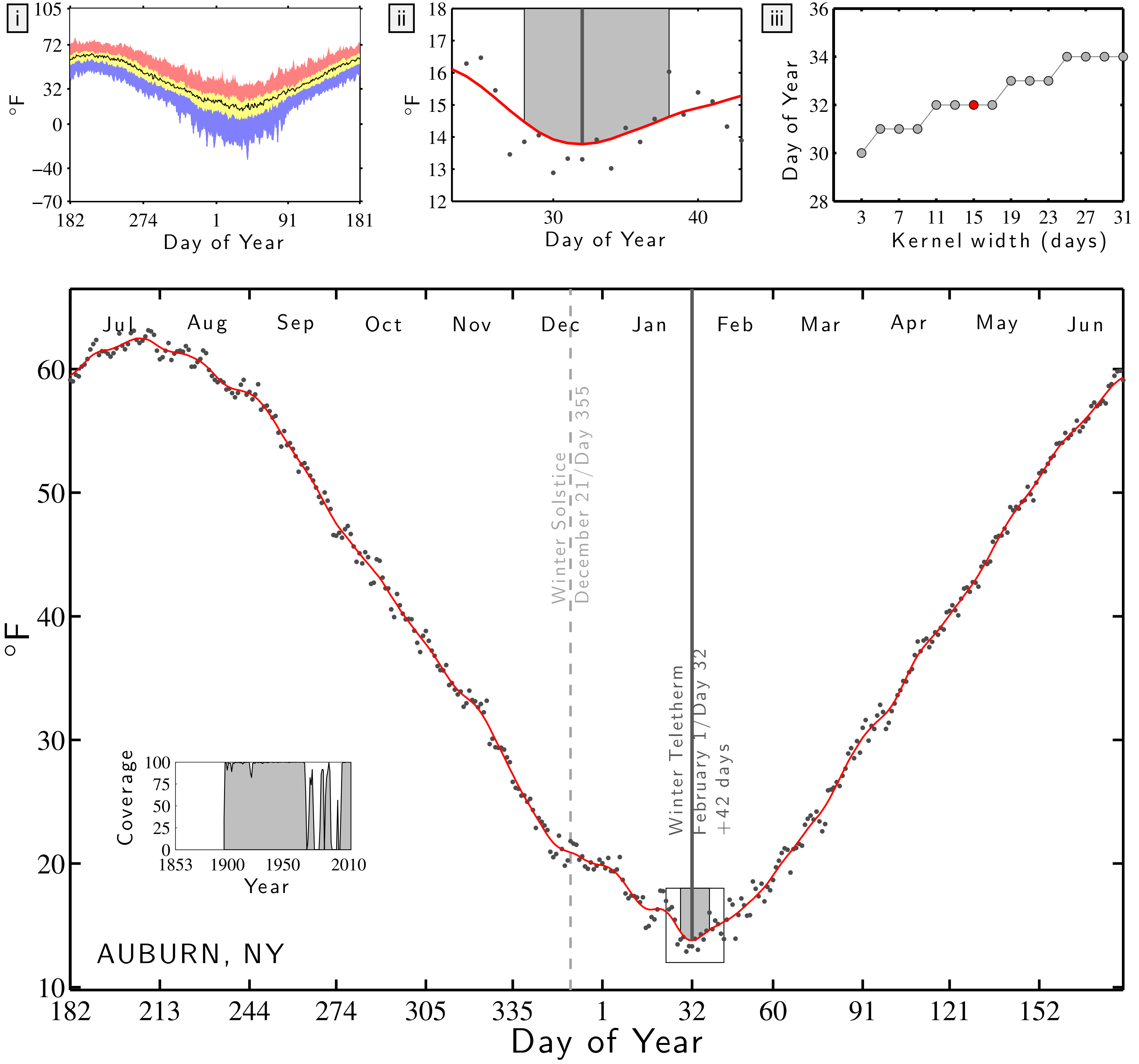
i
°F
Day of Year
ii
°F
Day of Year
iii
Day of Year
Kernel width (days)
Jul
Aug
Sep
Oct
Nov
Dec
Jan
Feb
Mar
Apr
May
Jun
Winter Solstice
December 21/Day 355
Winter Teletherm
February 1/Day 32
+42 days
Coverage
Year
AUBURN, NY
°F
Day of Year

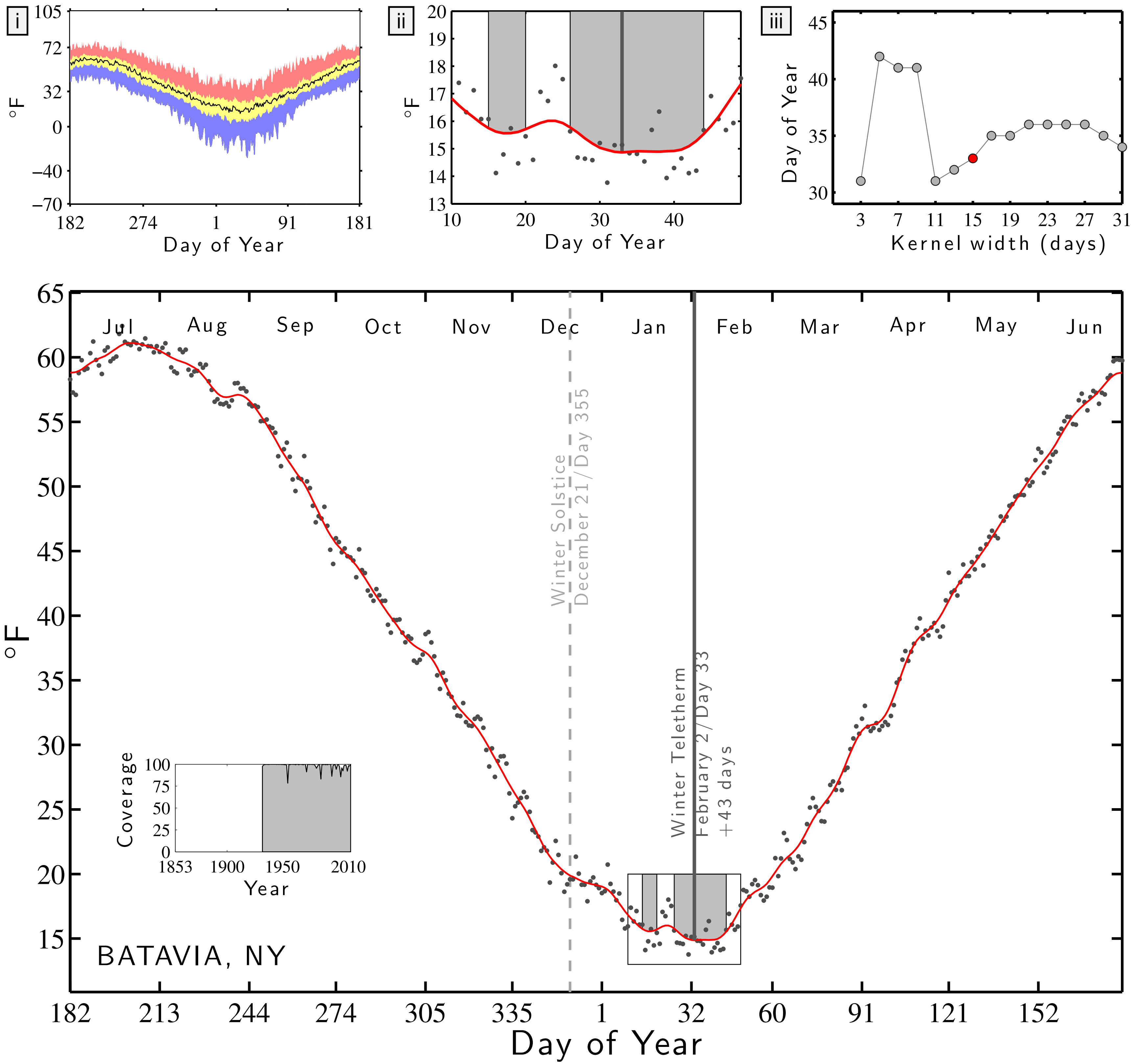

i
°F
Day of Year
ii
°F
Day of Year
iii
Day of Year
Kernel width (days)
Jul
Aug
Sep
Oct
Nov
Dec
Jan
Feb
Mar
Apr
May
Jun
Winter Solstice
December 21/Day 355
Winter Teletherm
February 2/Day 33
+43 days
Coverage
Year
BATAVIA, NY
°F
Day of Year

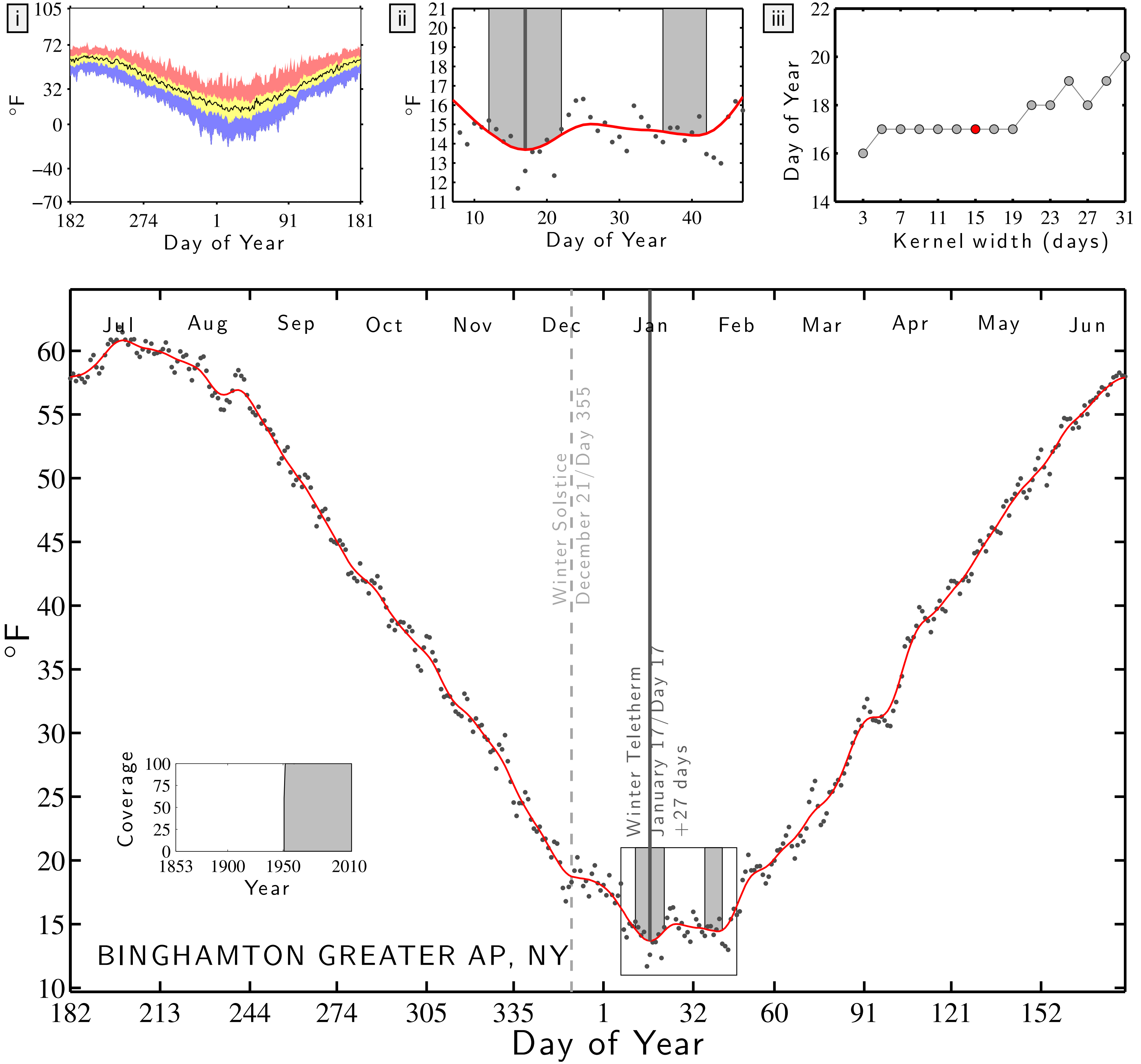

i
ii
iii
°F
Day of Year
Day of Year
Kernel width (days)
Jul
Aug
Sep
Oct
Nov
Dec
Jan
Feb
Mar
Apr
May
Jun
Winter Solstice
December 21/Day 355
Winter Teletherm
January 17/Day 17
+27 days
Coverage
Year
BINGHAMTON GREATER AP, NY

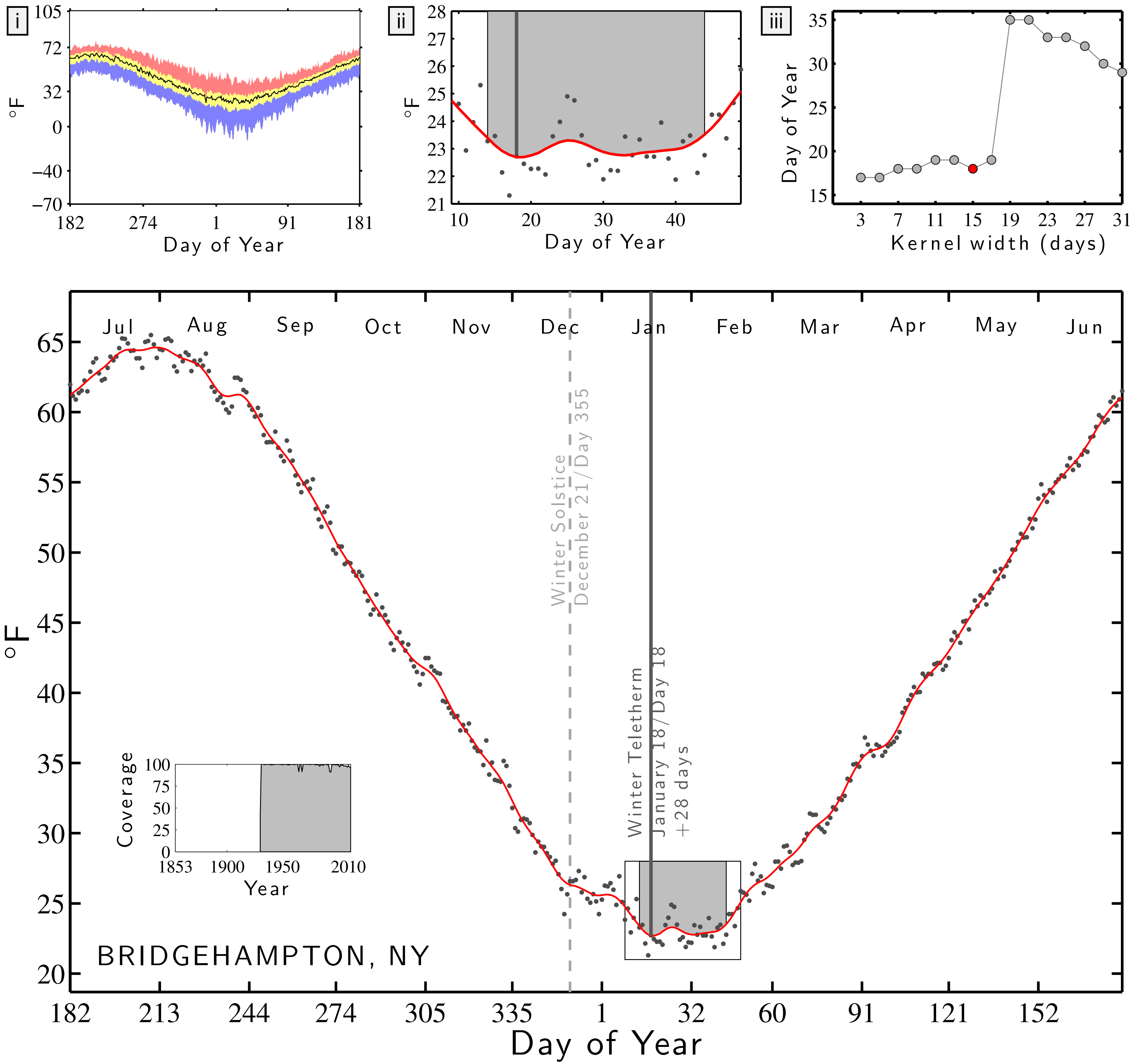

i
ii
iii
°F
Day of Year
Day of Year
Kernel width (days)
Jul
Aug
Sep
Oct
Nov
Dec
Jan
Feb
Mar
Apr
May
Jun
Winter Solstice
December 21/Day 355
Winter Teletherm
January 18/Day 18
+28 days
Coverage
Year
BRIDGEHAMPTON, NY
Day of Year

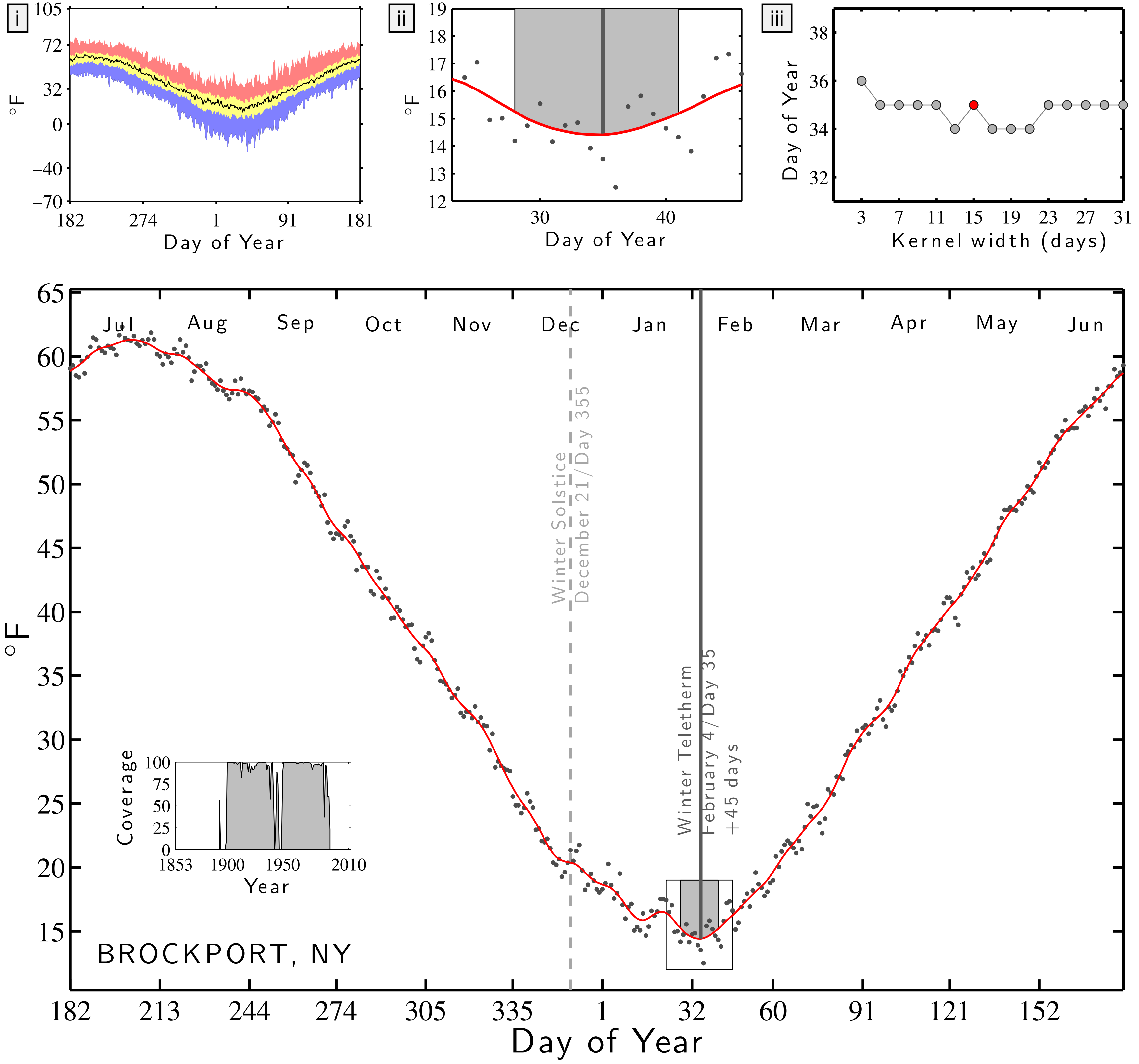

i
°F
Day of Year
ii
°F
Day of Year
iii
Day of Year
Kernel width (days)
Jul
Aug
Sep
Oct
Nov
Dec
Jan
Feb
Mar
Apr
May
Jun
Winter Solstice
December 21/Day 355
Winter Teletherm
February 4/Day 35
+45 days
Coverage
Year
BROCKPORT, NY
°F
Day of Year

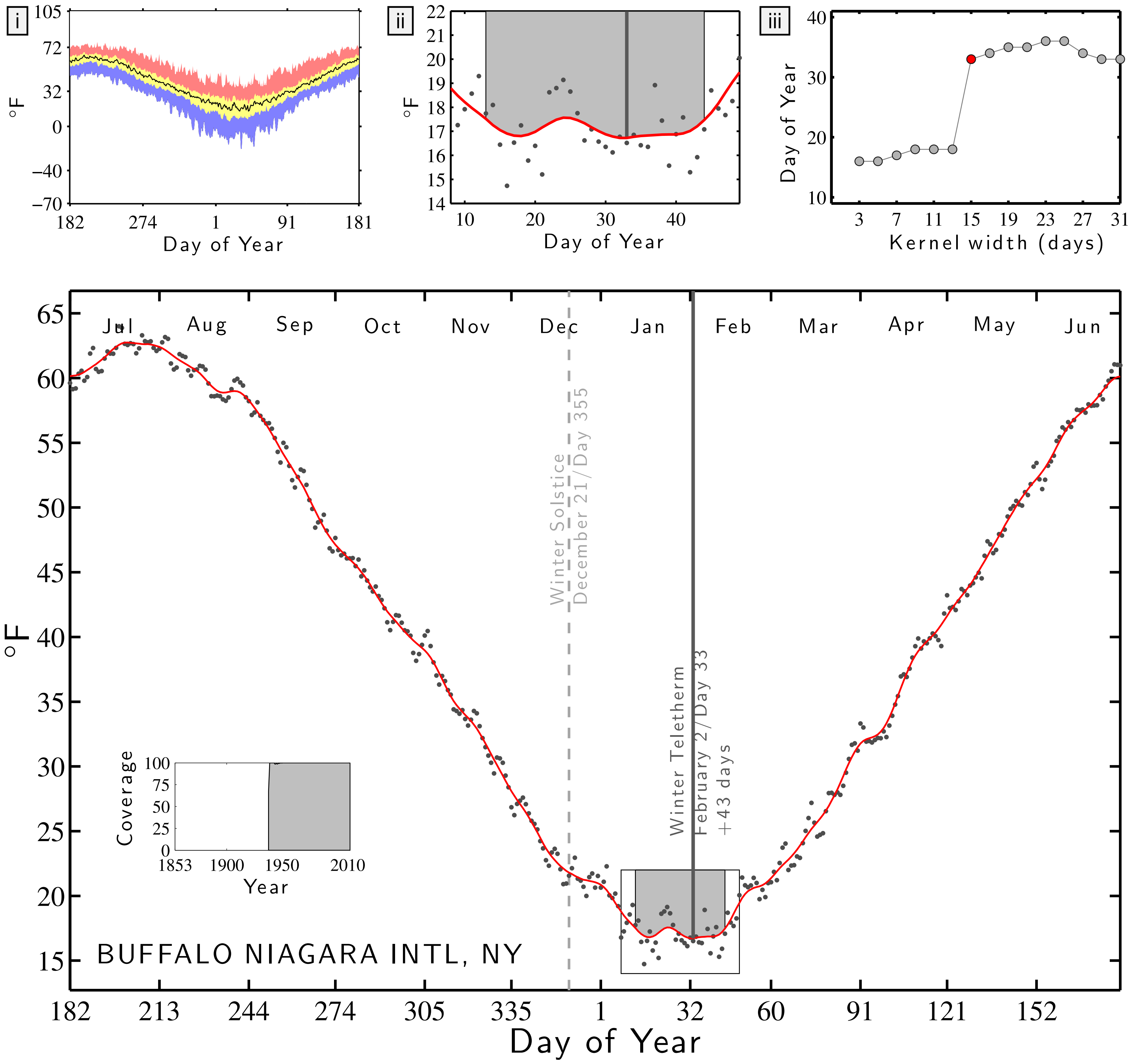

i
°F
Day of Year
ii
°F
Day of Year
iii
Day of Year
Kernel width (days)
Jul
Aug
Sep
Oct
Nov
Dec
Jan
Feb
Mar
Apr
May
Jun
Winter Solstice
December 21/Day 355
Winter Teletherm
February 2/Day 33
+43 days
Coverage
Year
BUFFALO NIAGARA INTL, NY
°F
Day of Year

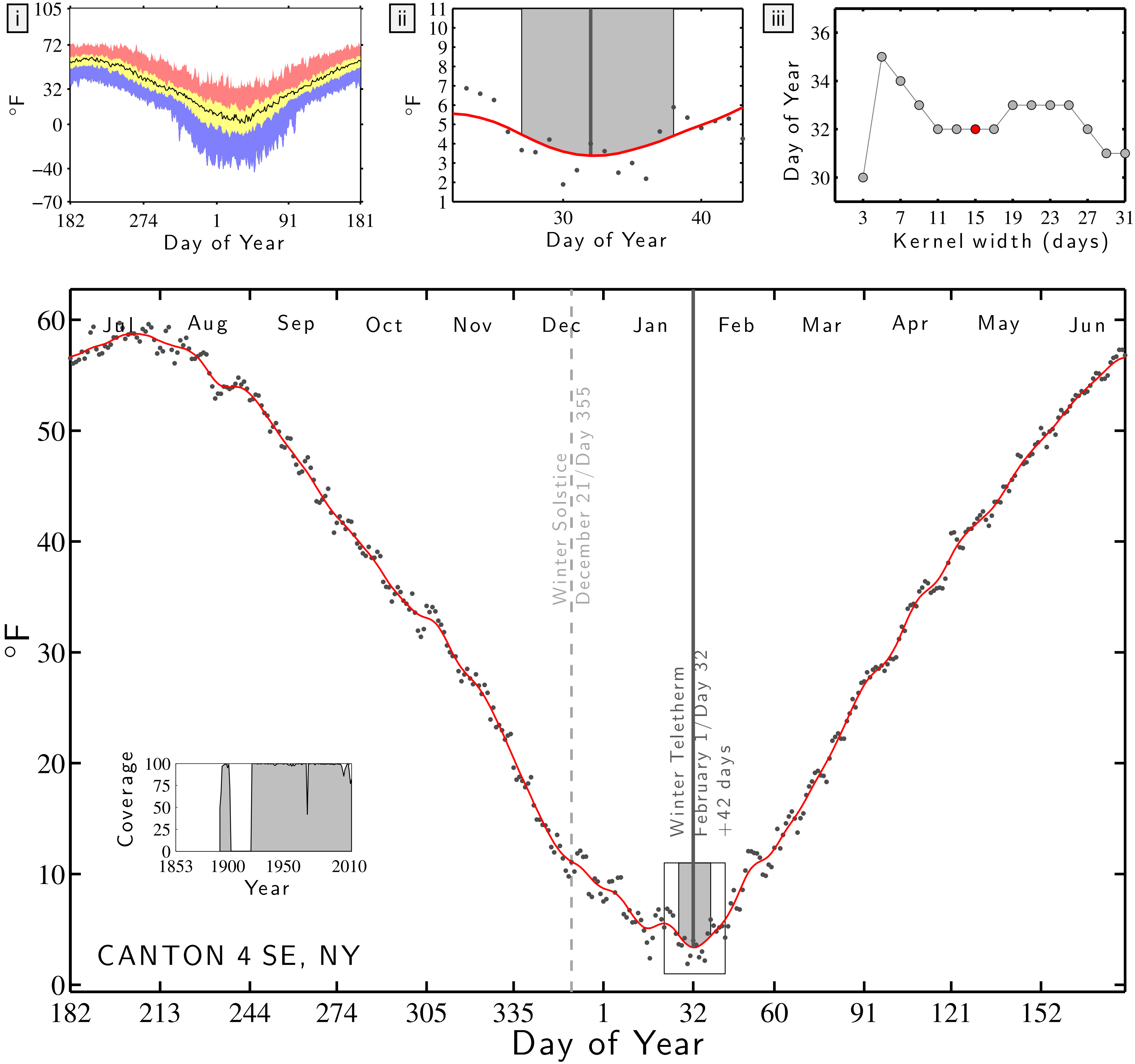

i
ii
iii
°F
Day of Year
Kernel width (days)
Jul
Aug
Sep
Oct
Nov
Dec
Jan
Feb
Mar
Apr
May
Jun
Winter Solstice
December 21/Day 355
Winter Teletherm
February 1/Day 32
+42 days
Coverage
Year
CANTON 4 SE, NY

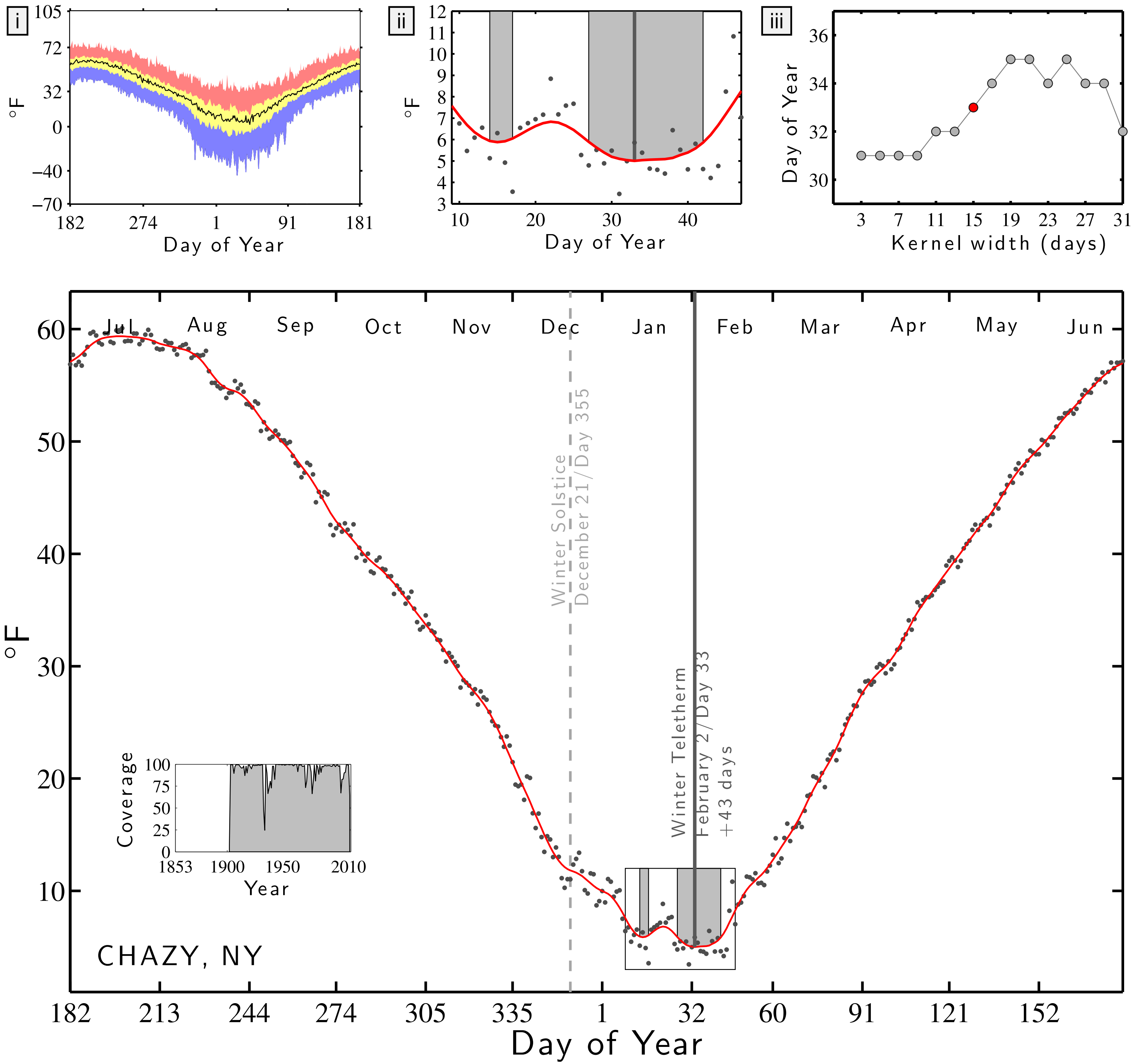

i
105
72
32
0
−40
−70
°F
182
274
1
91
181
Day of Year
ii
12
11
10
9
8
7
6
5
4
3
°F
10
20
30
40
Day of Year
iii
36
34
32
30
Day of Year
3
7
11
15
19
23
27
31
Kernel width (days)
60
50
40
30
20
10
°F
Jul
Aug
Sep
Oct
Nov
Dec
Jan
Feb
Mar
Apr
May
Jun
Winter Solstice
December 21/Day 355
Winter Teletherm
February 2/Day 33
+43 days
Coverage
100
75
50
25
0
1853
1900
1950
2010
Year
CHAZY, NY
182
213
244
274
305
335
1
32
60
91
121
152
Day of Year

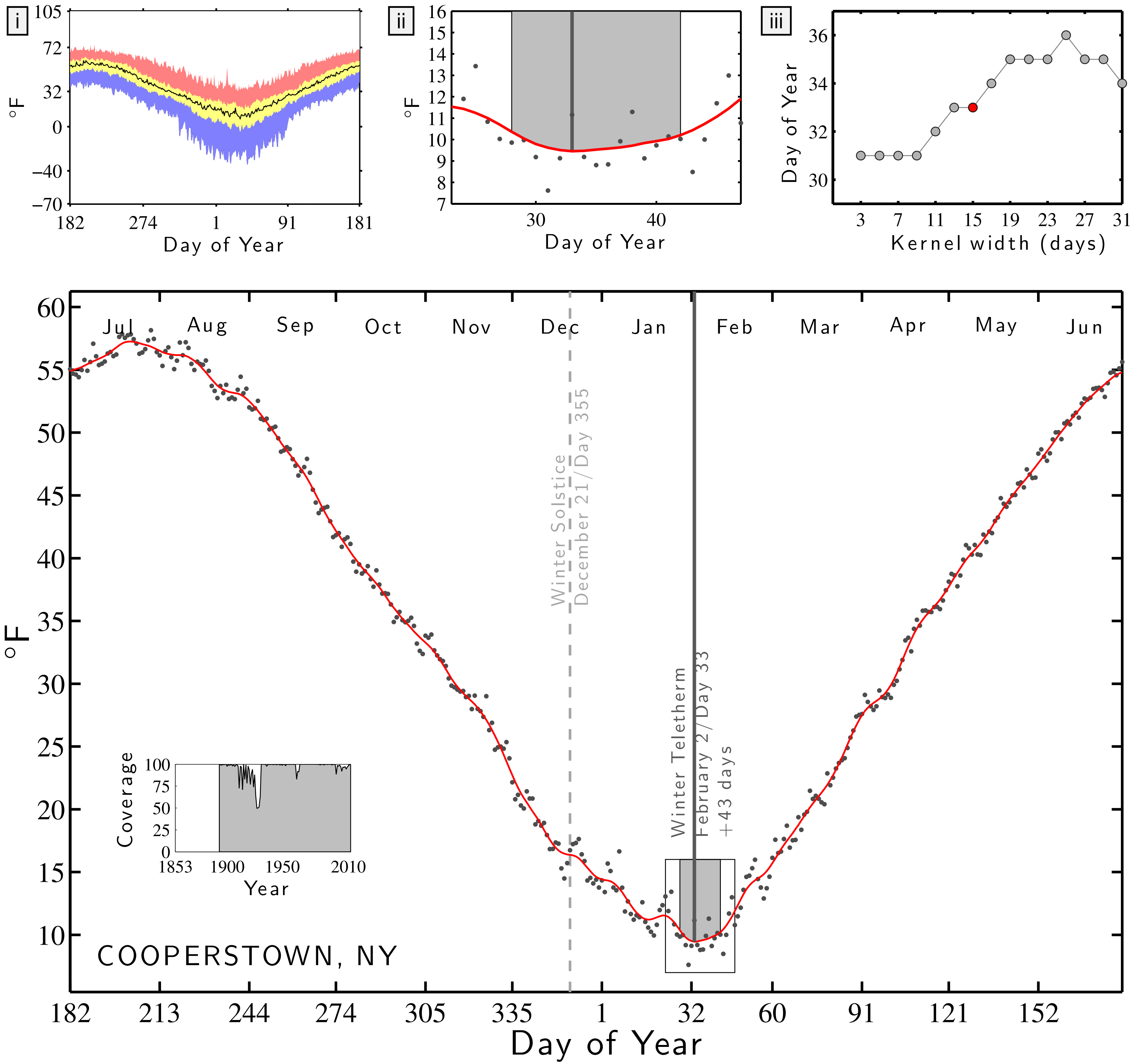

i
°F
Day of Year
ii
iii
Day of Year
Kernel width (days)
Jul
Aug
Sep
Oct
Nov
Dec
Jan
Feb
Mar
Apr
May
Jun
Winter Solstice
December 21/Day 355
Winter Teletherm
February 2/Day 33
+43 days
Coverage
Year
COOPERSTOWN, NY

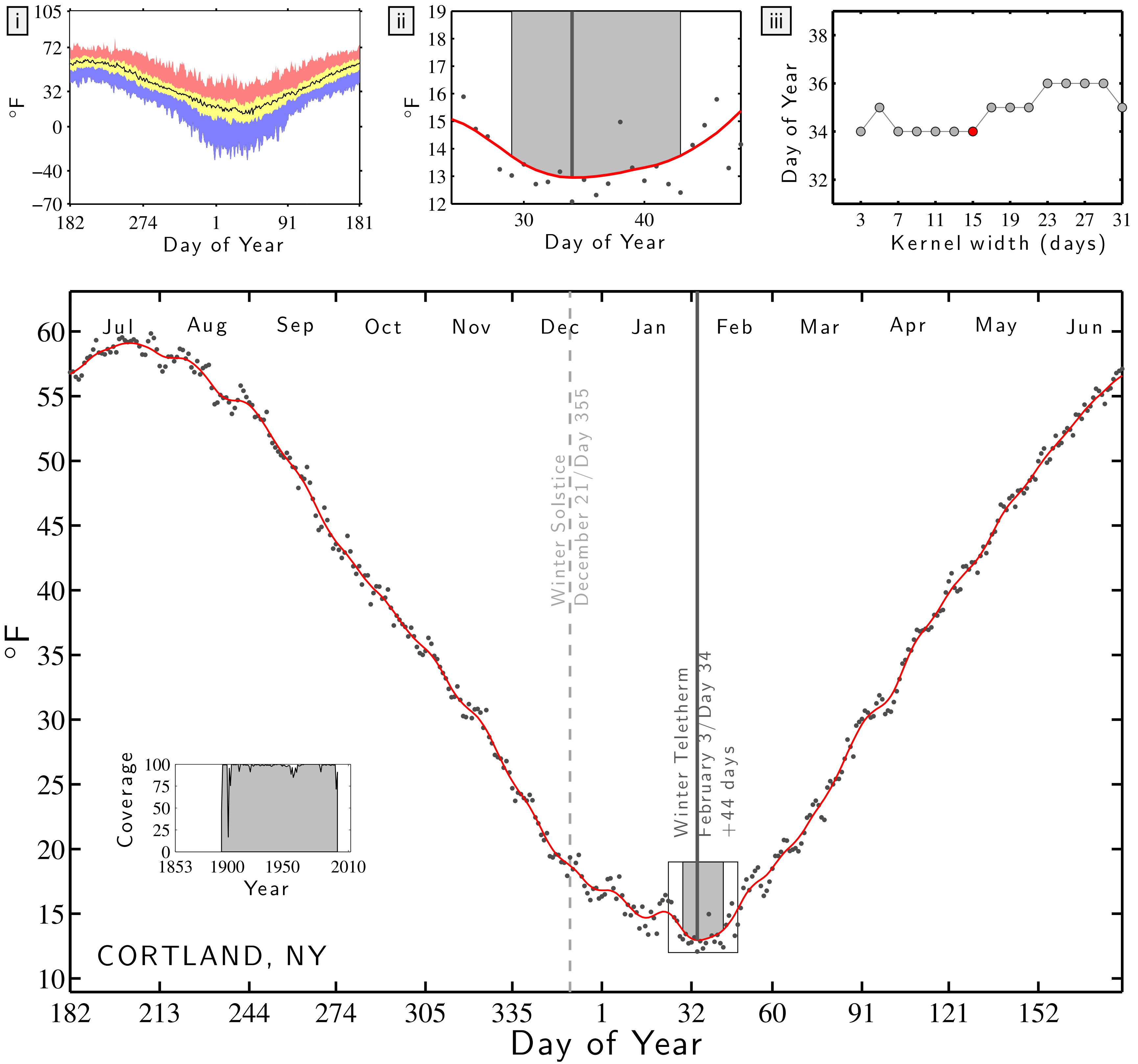

i
°F
Day of Year
ii
°F
Day of Year
iii
Day of Year
Kernel width (days)
Jul
Aug
Sep
Oct
Nov
Dec
Jan
Feb
Mar
Apr
May
Jun
Winter Solstice
December 21/Day 355
Winter Teletherm
February 3/Day 34
+44 days
Coverage
Year
CORTLAND, NY
°F
Day of Year

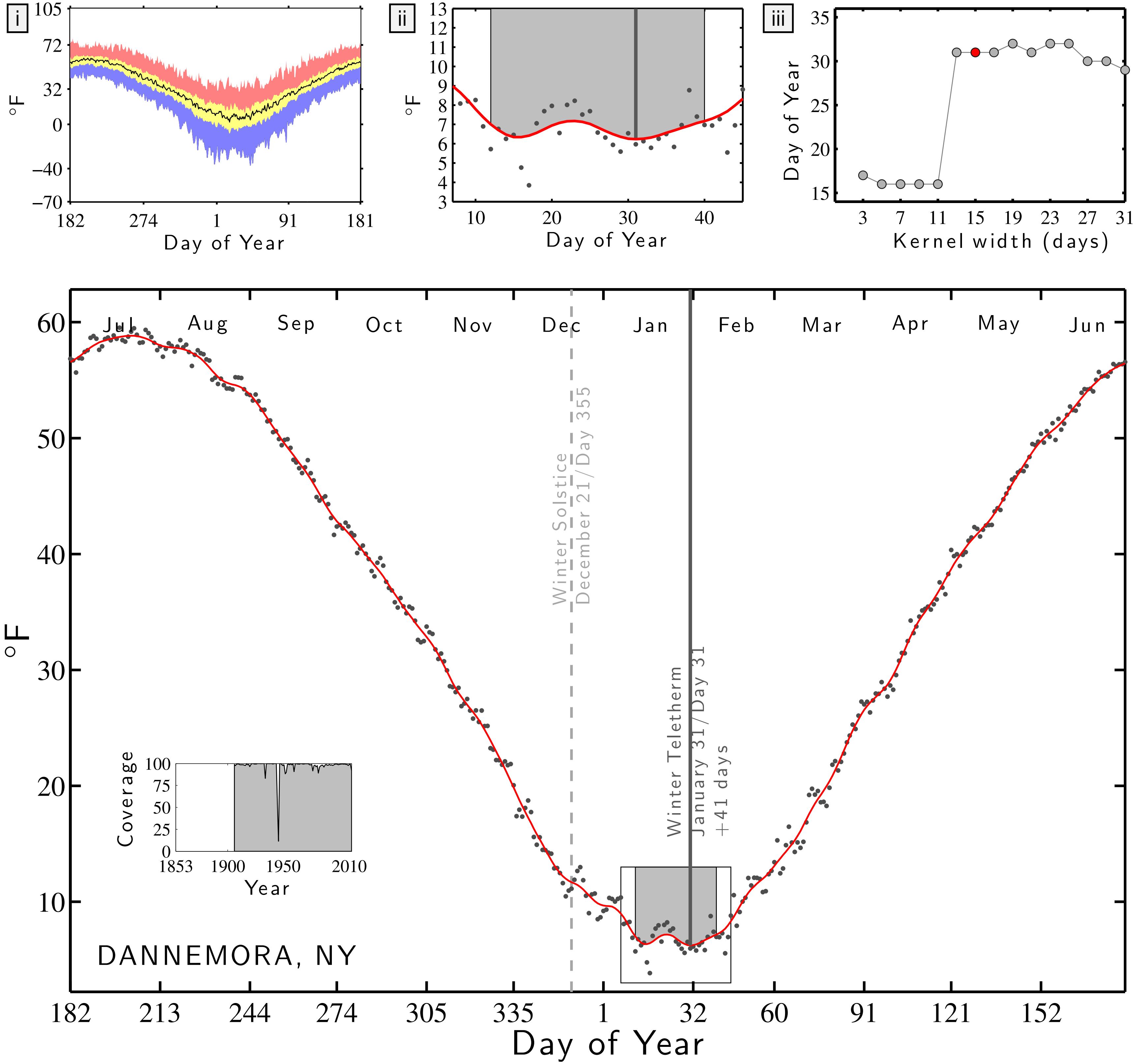

i
°F
Day of Year
ii
°F
Day of Year
iii
Day of Year
Kernel width (days)
Jul
Aug
Sep
Oct
Nov
Dec
Jan
Feb
Mar
Apr
May
Jun
Winter Solstice
December 21/Day 355
Winter Teletherm
January 31/Day 31
+41 days
Coverage
Year
DANNEMORA, NY
°F
Day of Year

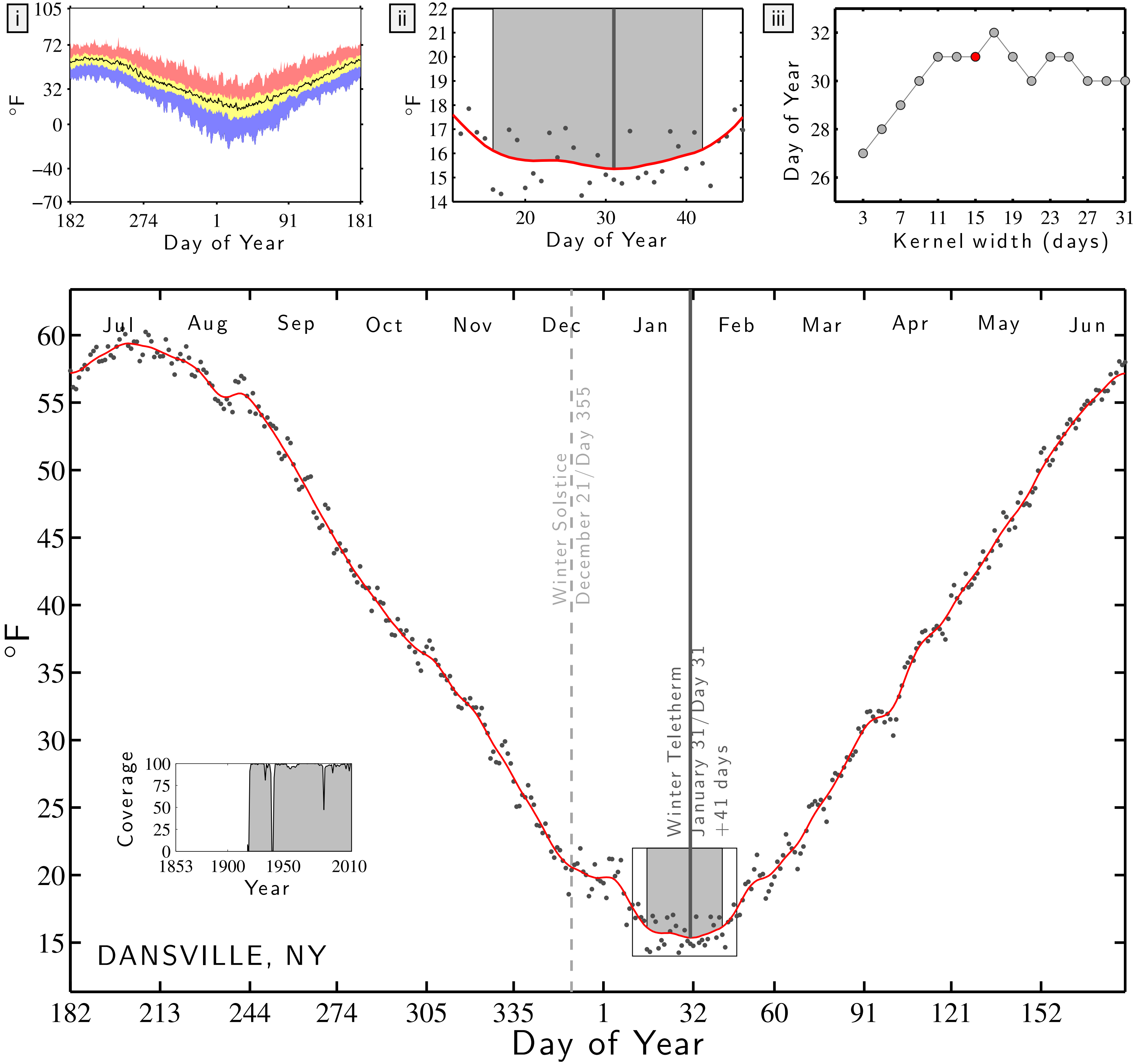

i
°F
Day of Year
ii
iii
Day of Year
Kernel width (days)
Jul
Aug
Sep
Oct
Nov
Dec
Jan
Feb
Mar
Apr
May
Jun
Winter Solstice
December 21/Day 355
Winter Teletherm
January 31/Day 31
+41 days
Coverage
Year
DANSVILLE, NY

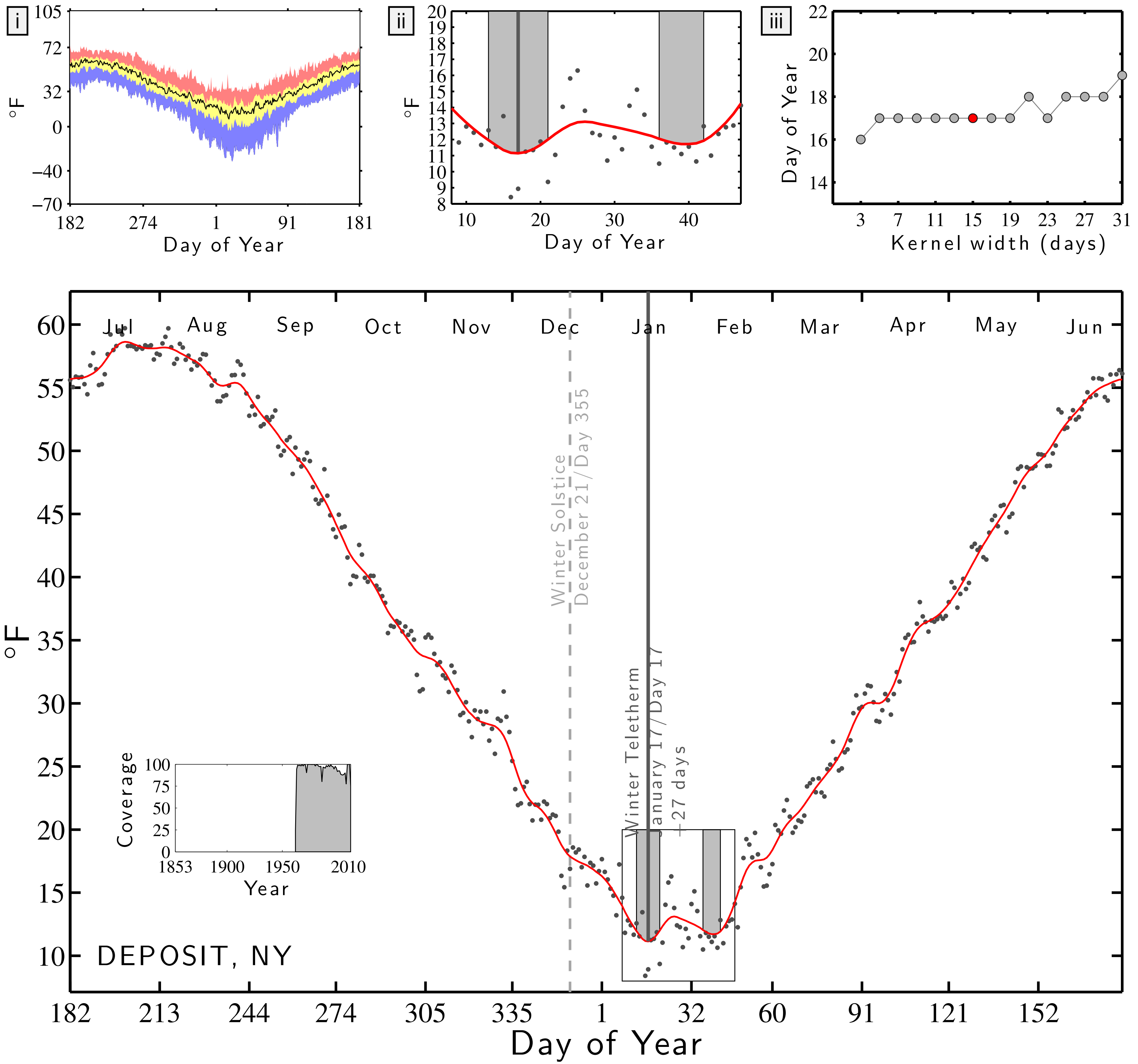

i
ii
iii
Day of Year
°F
Day of Year
Kernel width (days)
Jul
Aug
Sep
Oct
Nov
Dec
Jan
Feb
Mar
Apr
May
Jun
Winter Solstice
December 21/Day 355
Winter Teletherm
January 17/Day 17
±27 days
Coverage
Year
DEPOSIT, NY
Day of Year

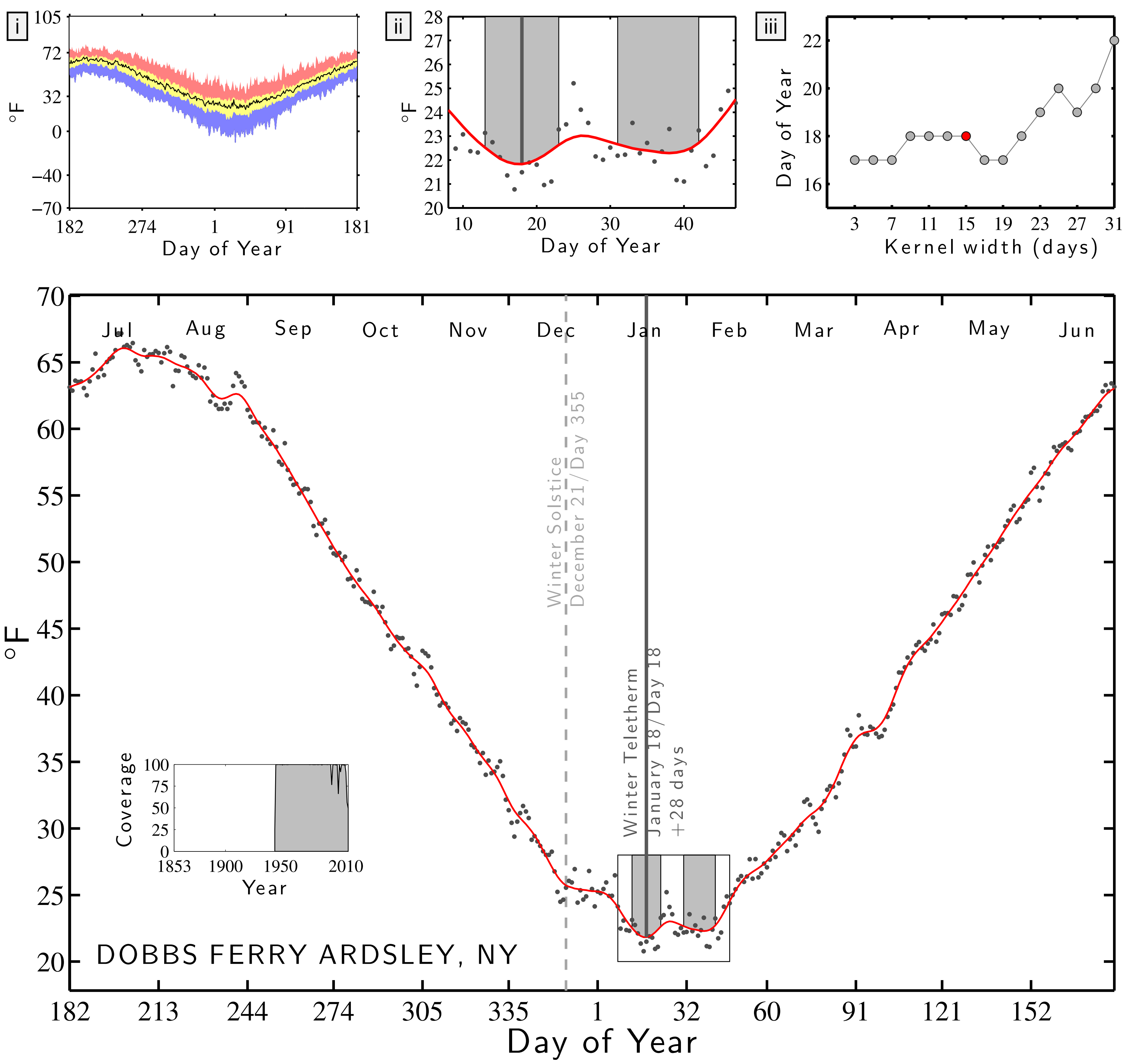
i
ii
iii
Day of Year
°F
Kernel width (days)
Jul
Aug
Sep
Oct
Nov
Dec
Jan
Feb
Mar
Apr
May
Jun
Winter Solstice
December 21/Day 355
Winter Teletherm
January 18/Day 18
+28 days
Coverage
Year
DOBBS FERRY ARDSLEY, NY

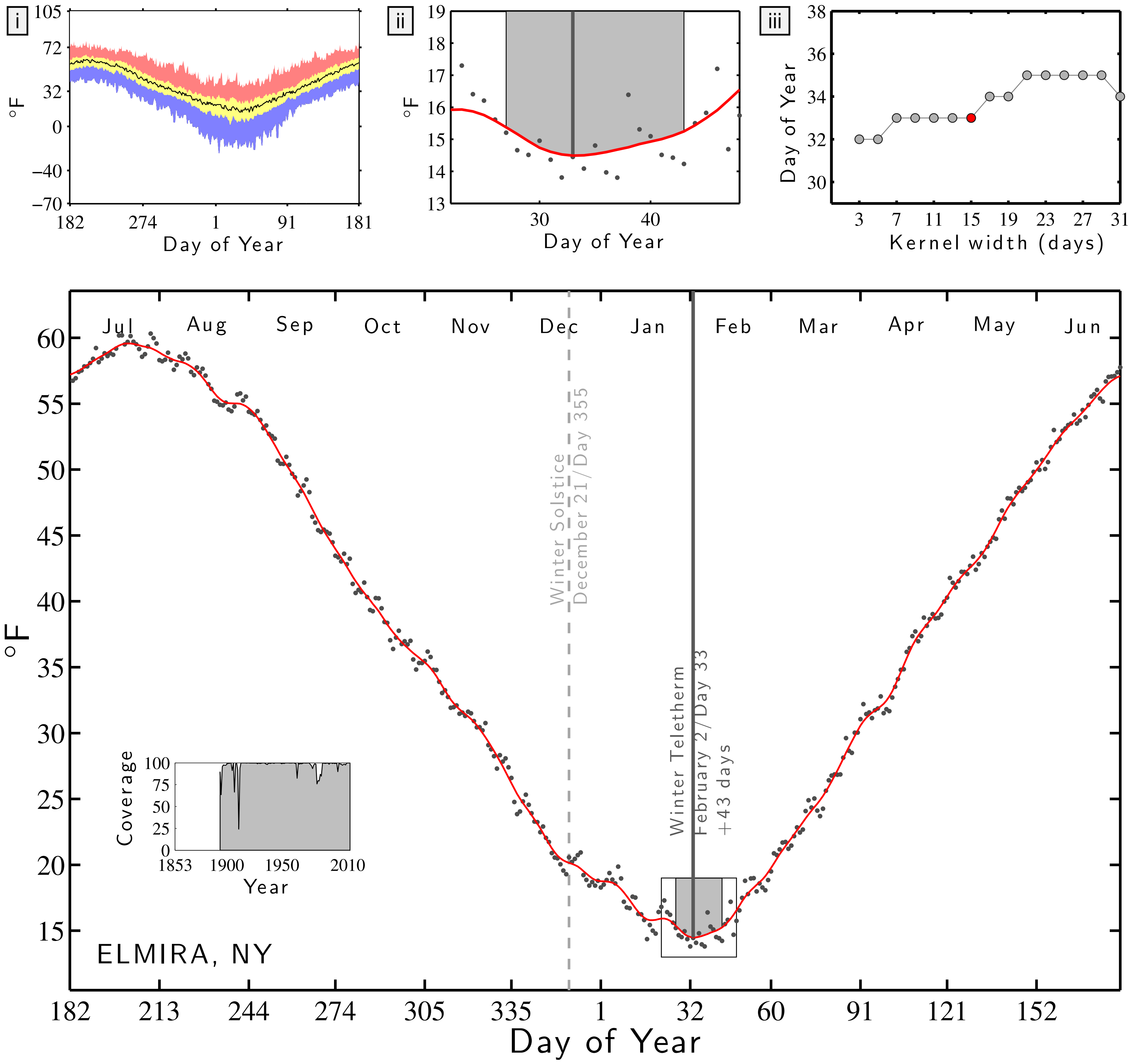

i
Day of Year
°F
ii
Day of Year
°F
iii
Kernel width (days)
Day of Year
Jul
Aug
Sep
Oct
Nov
Dec
Jan
Feb
Mar
Apr
May
Jun
Winter Solstice
December 21/Day 355
Winter Teletherm
February 2/Day 33
+43 days
Coverage
Year
ELMIRA, NY
Day of Year
°F

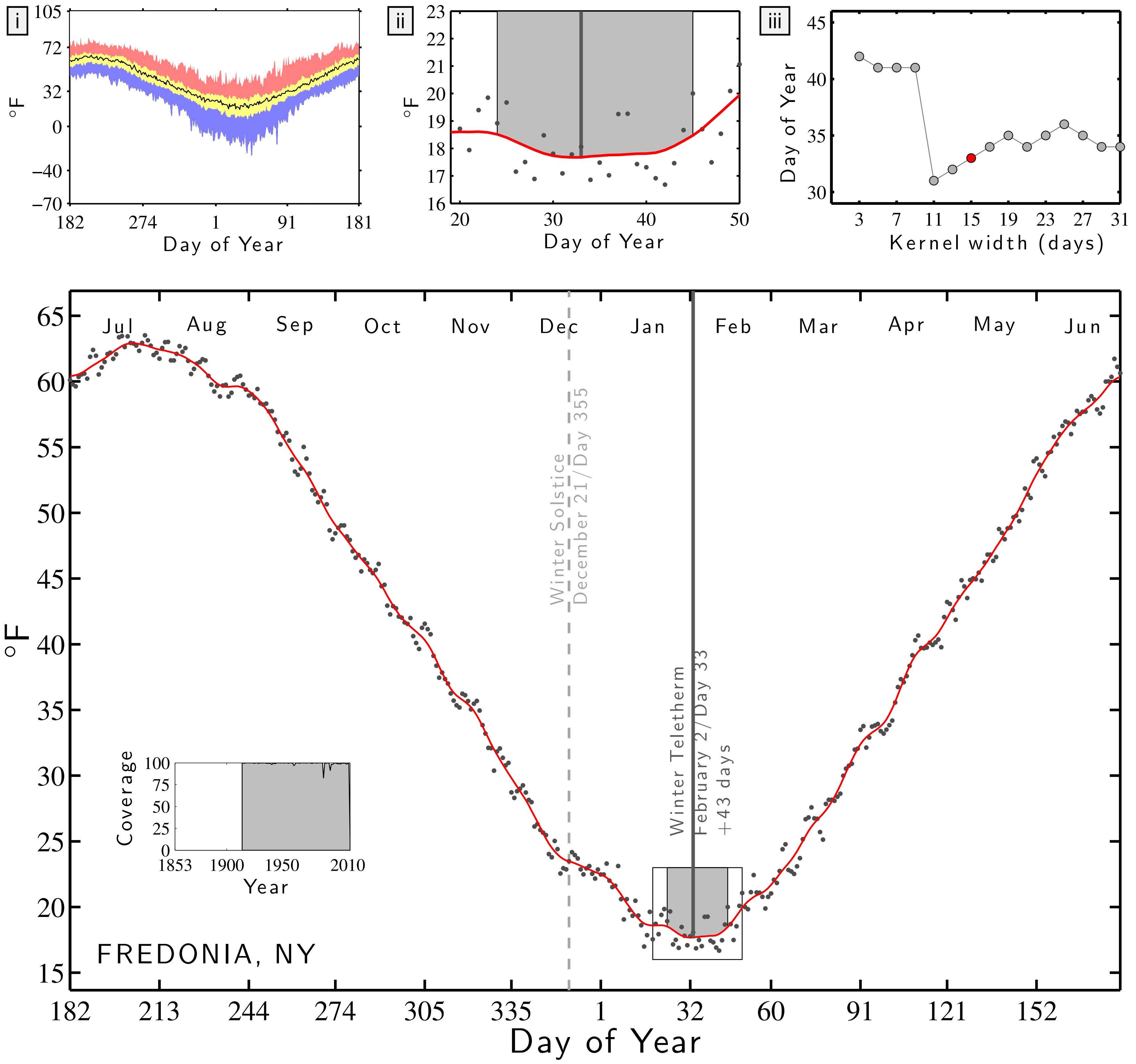

i
ii
iii
°F
Day of Year
Day of Year
Kernel width (days)
Jul
Aug
Sep
Oct
Nov
Dec
Jan
Feb
Mar
Apr
May
Jun
Winter Solstice
December 21/Day 355
Winter Teletherm
February 2/Day 33
+43 days
Coverage
Year
FREDONIA, NY

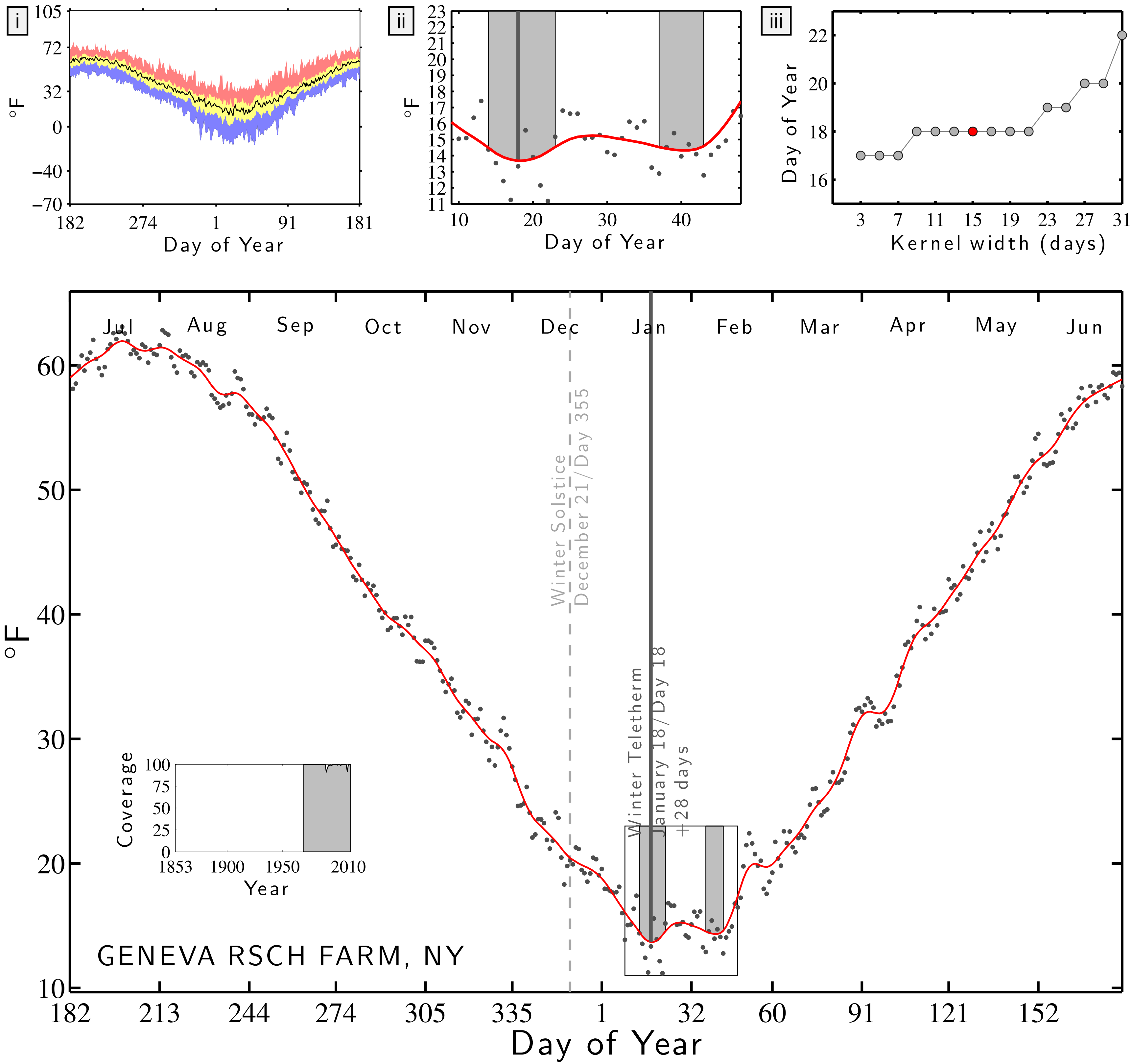
i
ii
iii
°F
Day of Year
Kernel width (days)
Day of Year
Jul
Aug
Sep
Oct
Nov
Dec
Jan
Feb
Mar
Apr
May
Jun
Winter Solstice
December 21/Day 355
Winter Teletherm
January 18/Day 18
±28 days
Coverage
Year
GENEVA RSCH FARM, NY

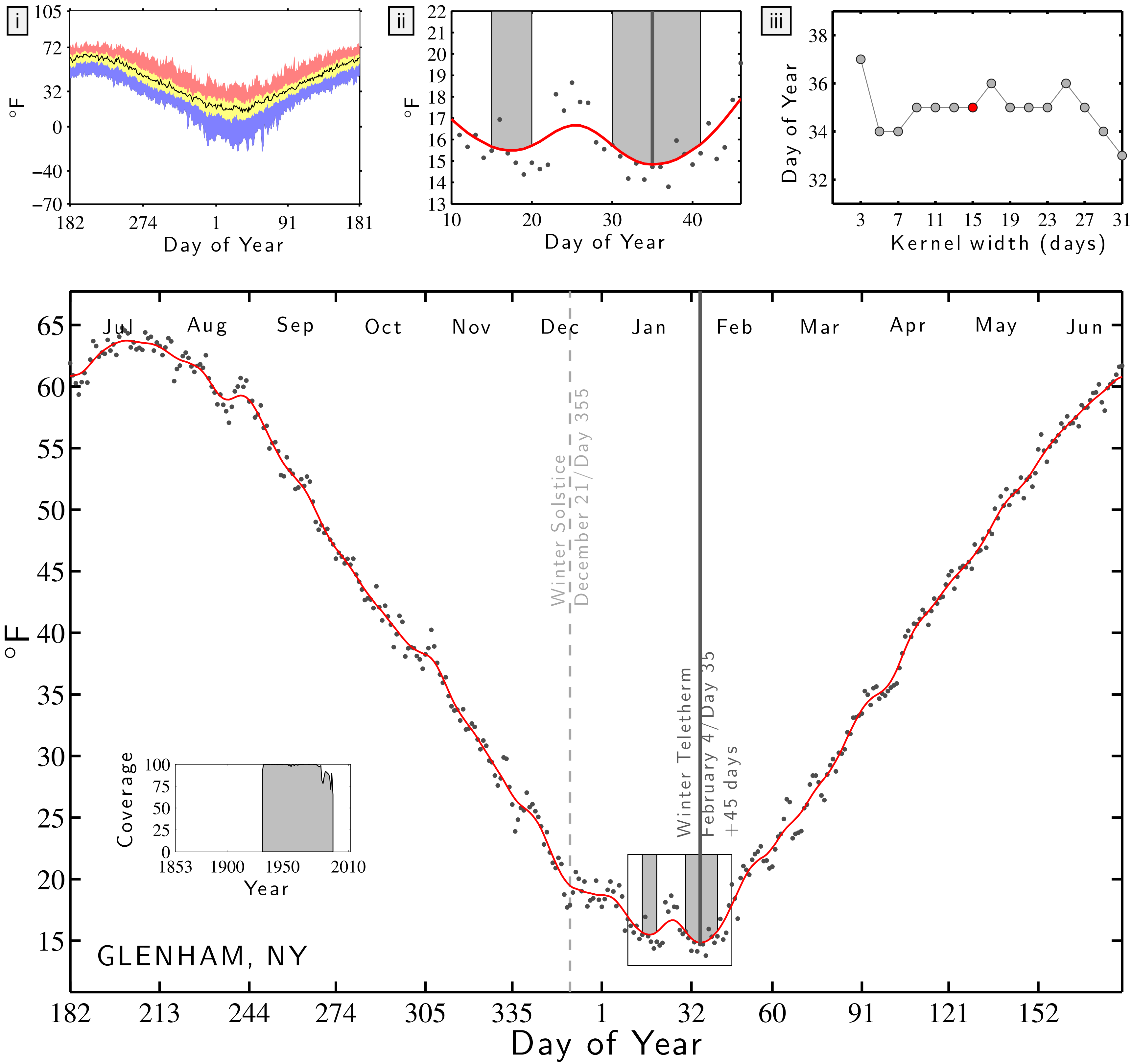

i
°F
Day of Year
ii
°F
Day of Year
iii
Day of Year
Kernel width (days)
Jul
Aug
Sep
Oct
Nov
Dec
Jan
Feb
Mar
Apr
May
Jun
Winter Solstice
December 21/Day 355
Winter Teletherm
February 4/Day 35
+45 days
Coverage
Year
GLENHAM, NY
°F
Day of Year

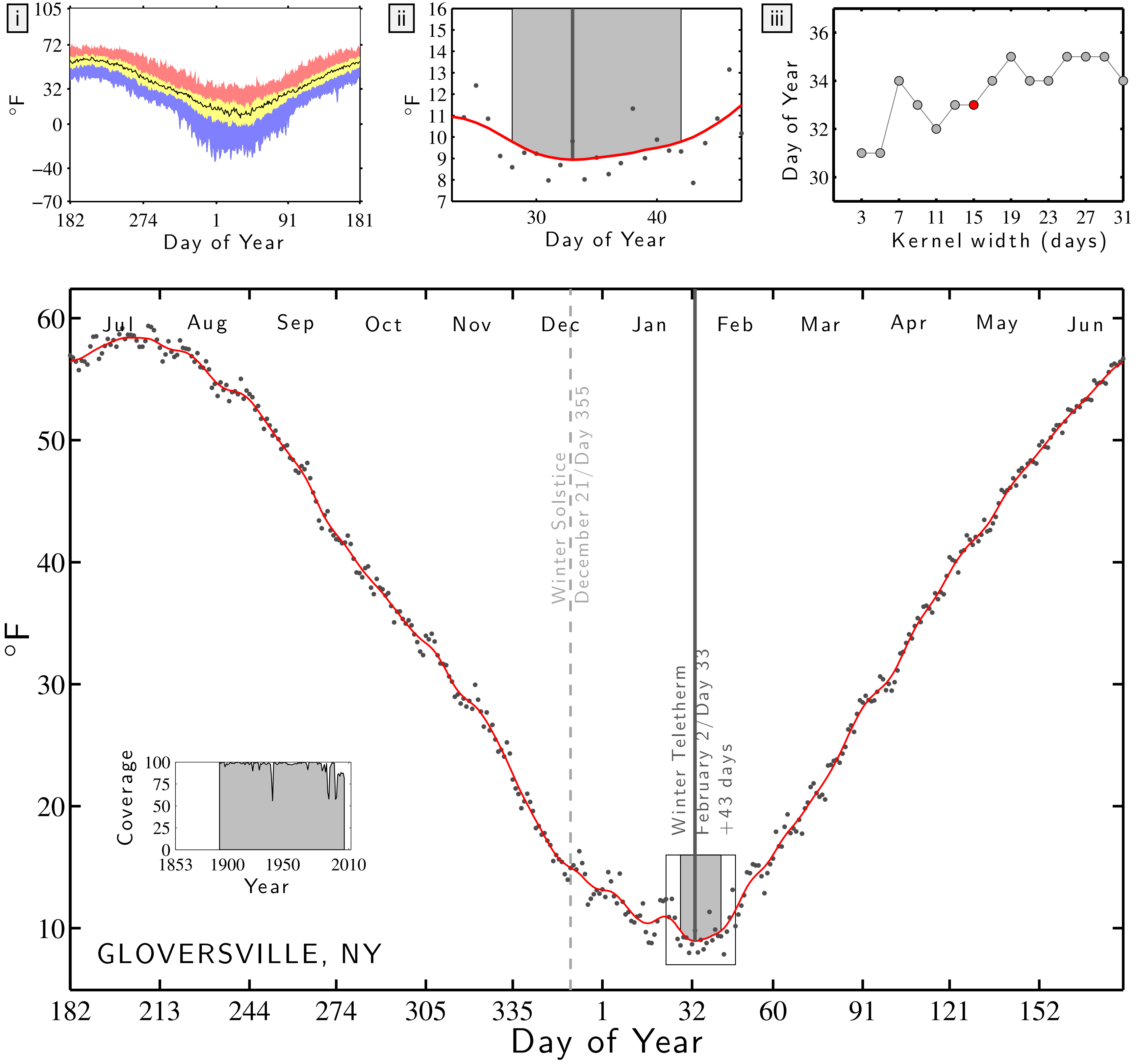

i
105
72
32
0
−40
−70
°F
182
274
1
91
181
Day of Year
ii
°F
30
40
Day of Year
iii
Day of Year
Kernel width (days)
Jul
Aug
Sep
Oct
Nov
Dec
Jan
Feb
Mar
Apr
May
Jun
Winter Solstice
December 21/Day 355
Winter Teletherm
February 2/Day 33
+43 days
Coverage
Year
1853
1900
1950
2010
GLOVERSVILLE, NY
°F
Day of Year
182
213
244
274
305
335
1
32
60
91
121
152

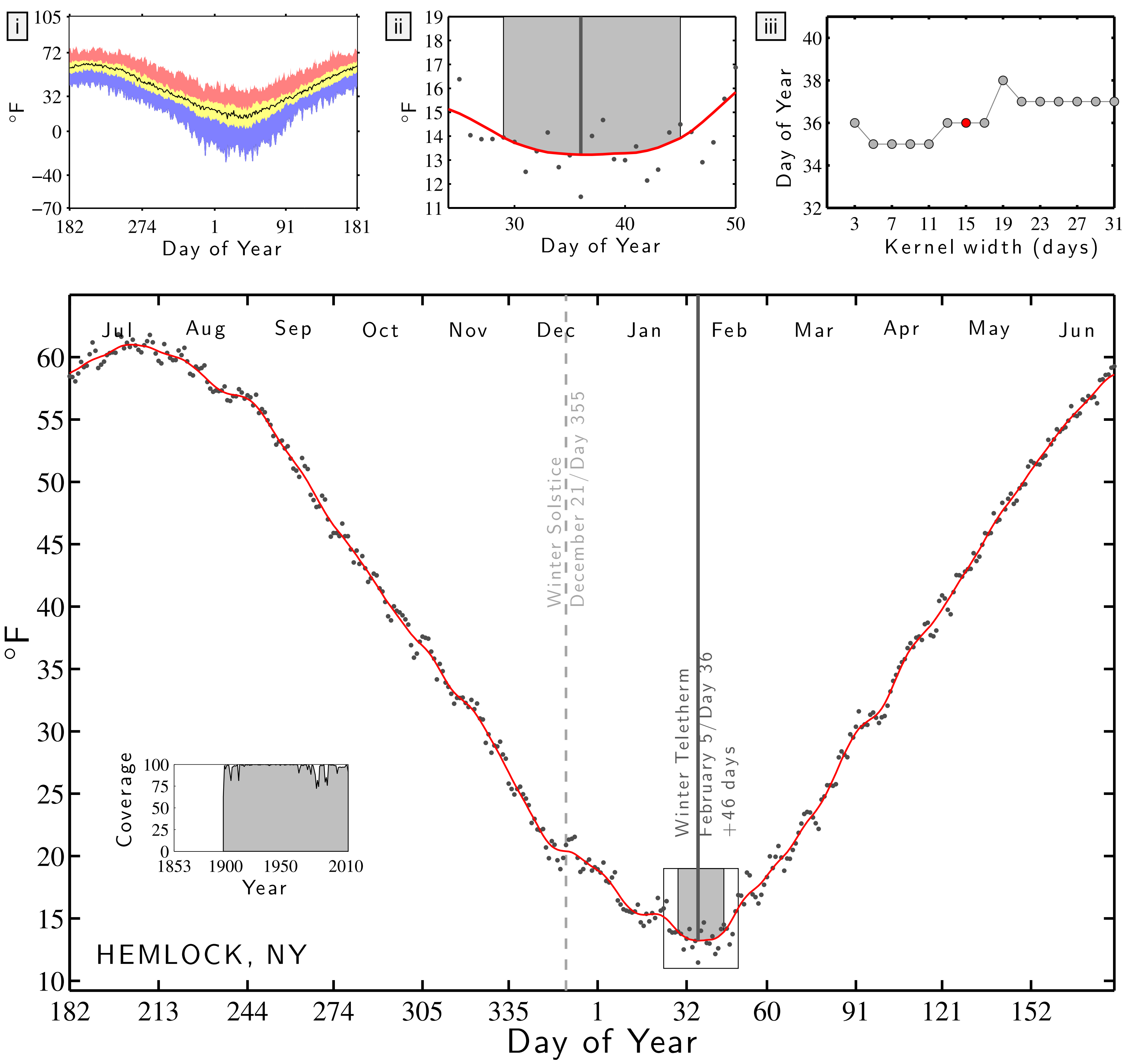

i
°F
Day of Year
ii
°F
Day of Year
iii
Day of Year
Kernel width (days)
Jul
Aug
Sep
Oct
Nov
Dec
Jan
Feb
Mar
Apr
May
Jun
Winter Solstice
December 21/Day 355
Winter Teletherm
February 5/Day 36
+46 days
Coverage
Year
HEMLOCK, NY
°F
Day of Year

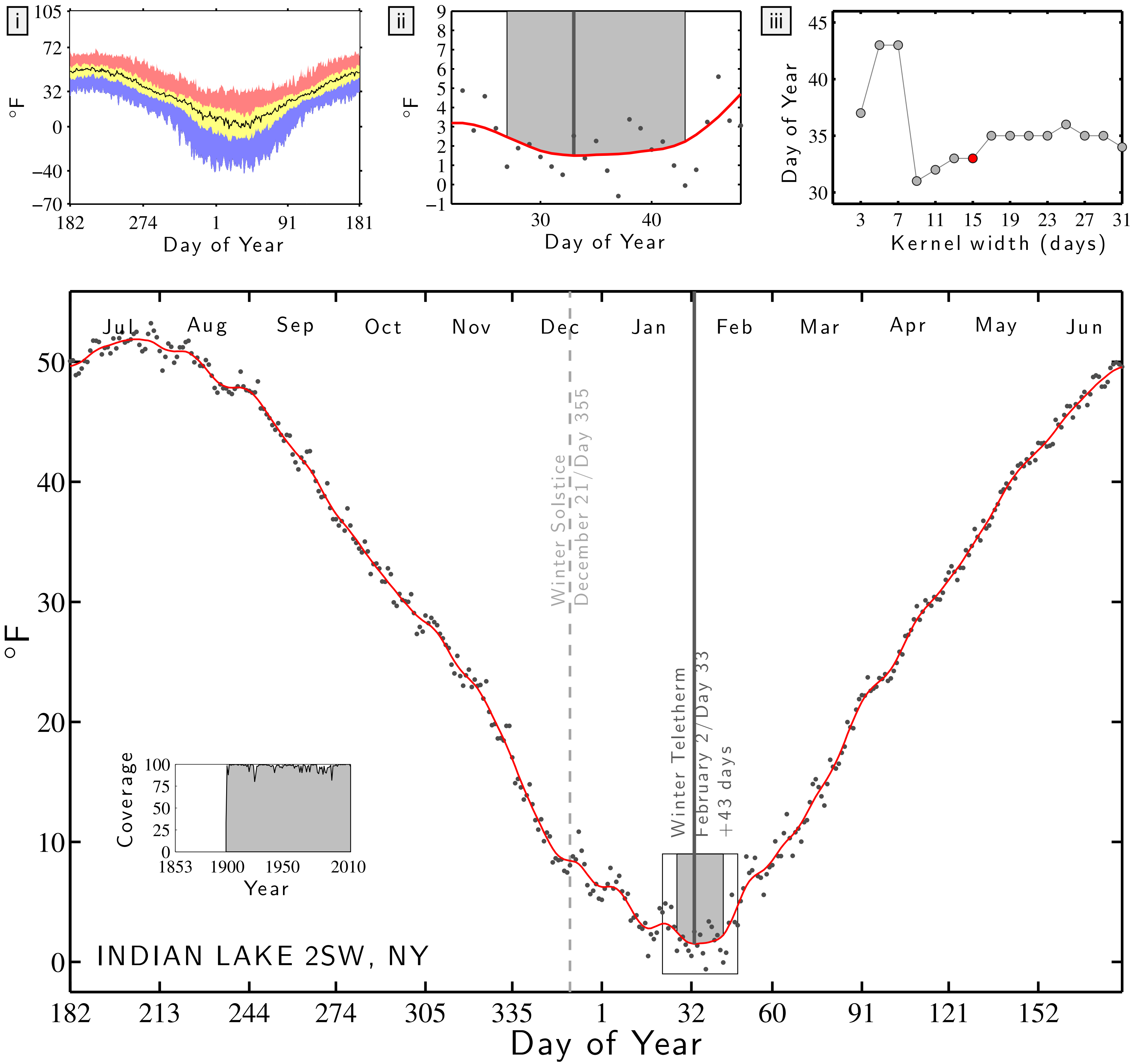

i
ii
iii
°F
Day of Year
Day of Year
Kernel width (days)
Jul
Aug
Sep
Oct
Nov
Dec
Jan
Feb
Mar
Apr
May
Jun
Winter Solstice
December 21/Day 355
Winter Teletherm
February 2/Day 33
+43 days
Coverage
Year
INDIAN LAKE 2SW, NY
Day of Year

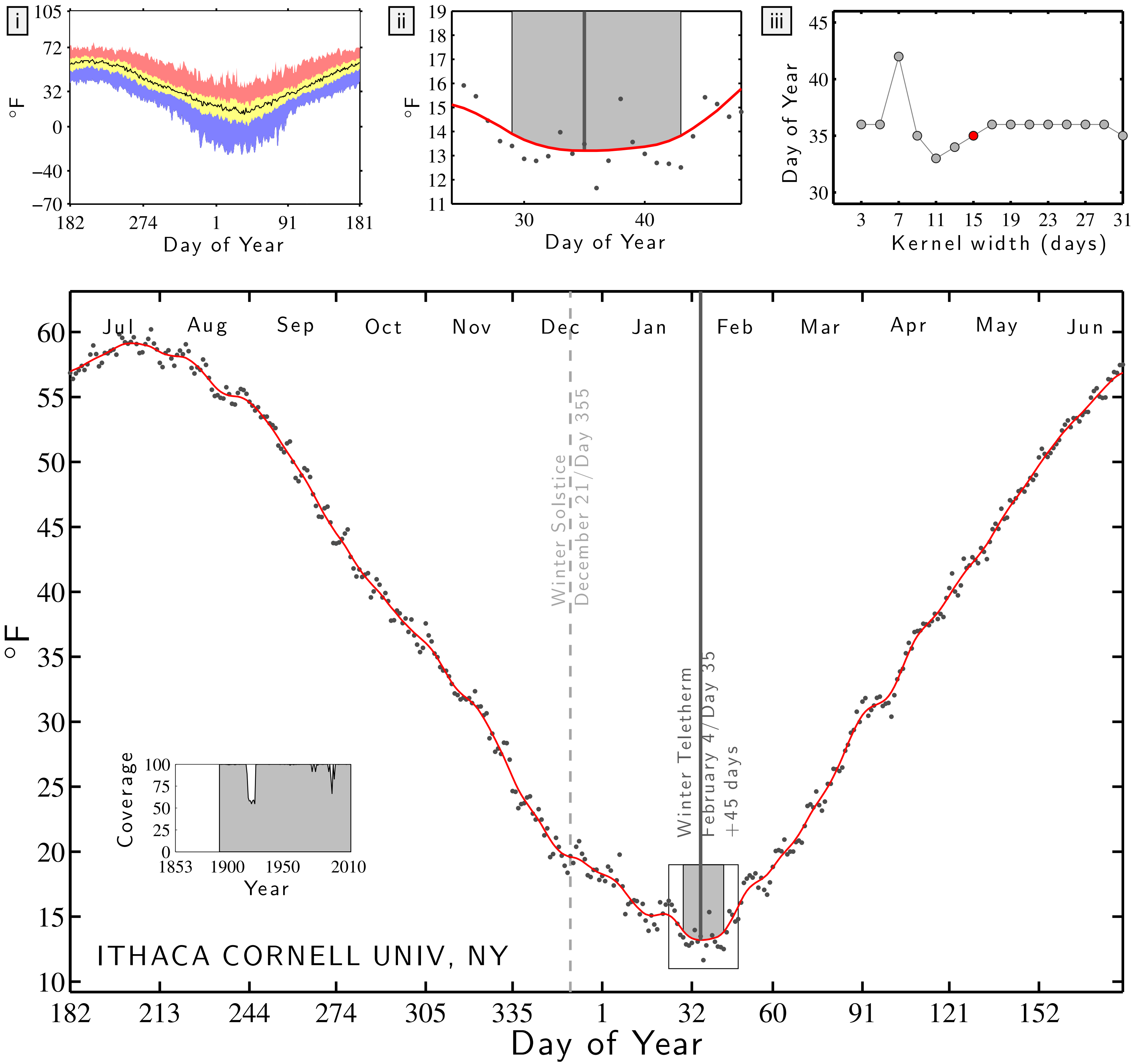

i
°F
Day of Year
ii
°F
Day of Year
iii
Day of Year
Kernel width (days)
Jul
Aug
Sep
Oct
Nov
Dec
Jan
Feb
Mar
Apr
May
Jun
Winter Solstice
December 21/Day 355
Winter Teletherm
February 4/Day 35
+45 days
Coverage
Year
ITHACA CORNELL UNIV, NY
°F
Day of Year

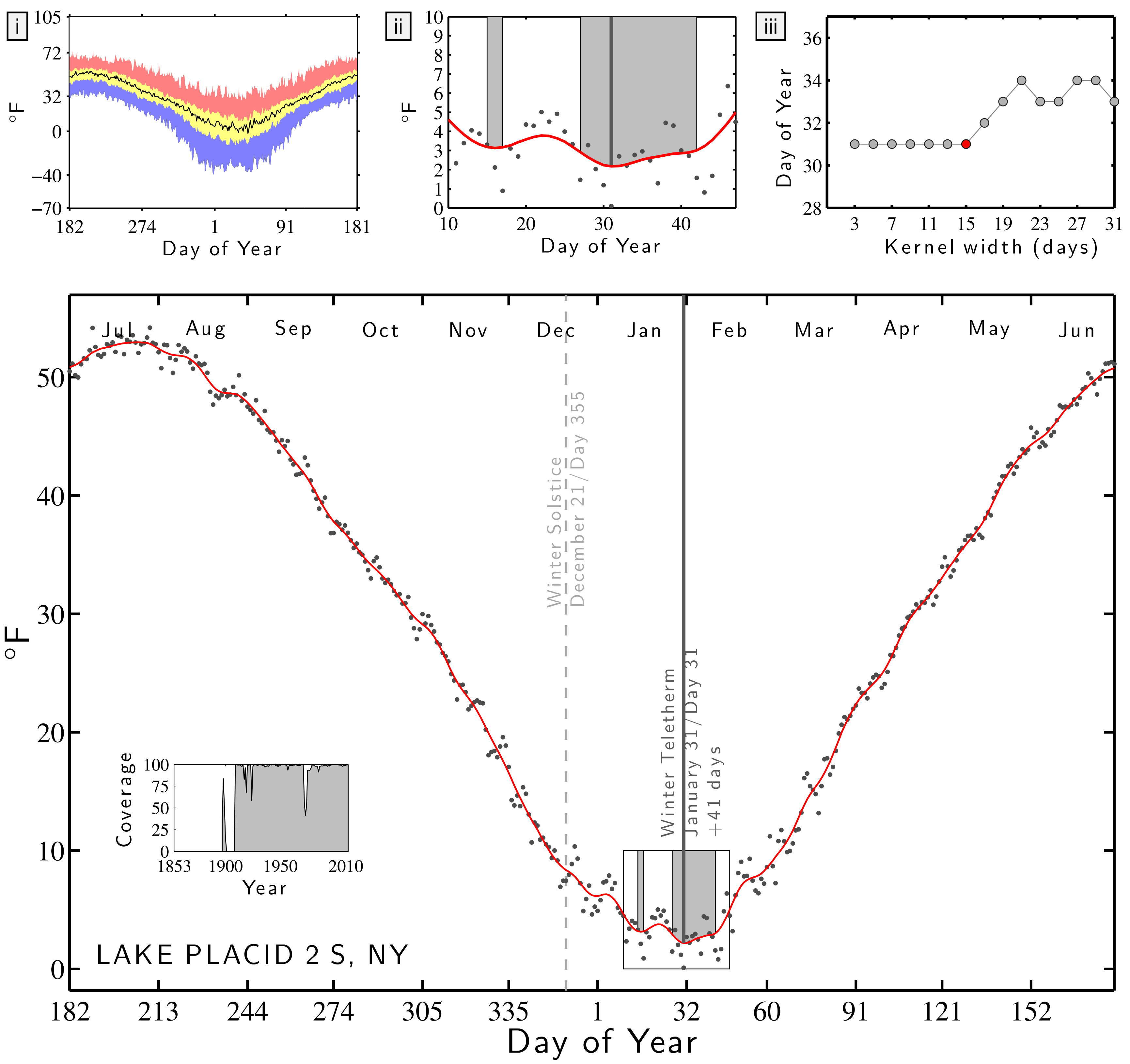

i
°F
Day of Year
ii
iii
Day of Year
Kernel width (days)
Jul
Aug
Sep
Oct
Nov
Dec
Jan
Feb
Mar
Apr
May
Jun
Winter Solstice
December 21/Day 355
Winter Teletherm
January 31/Day 31
+41 days
Coverage
Year
LAKE PLACID 2 S, NY

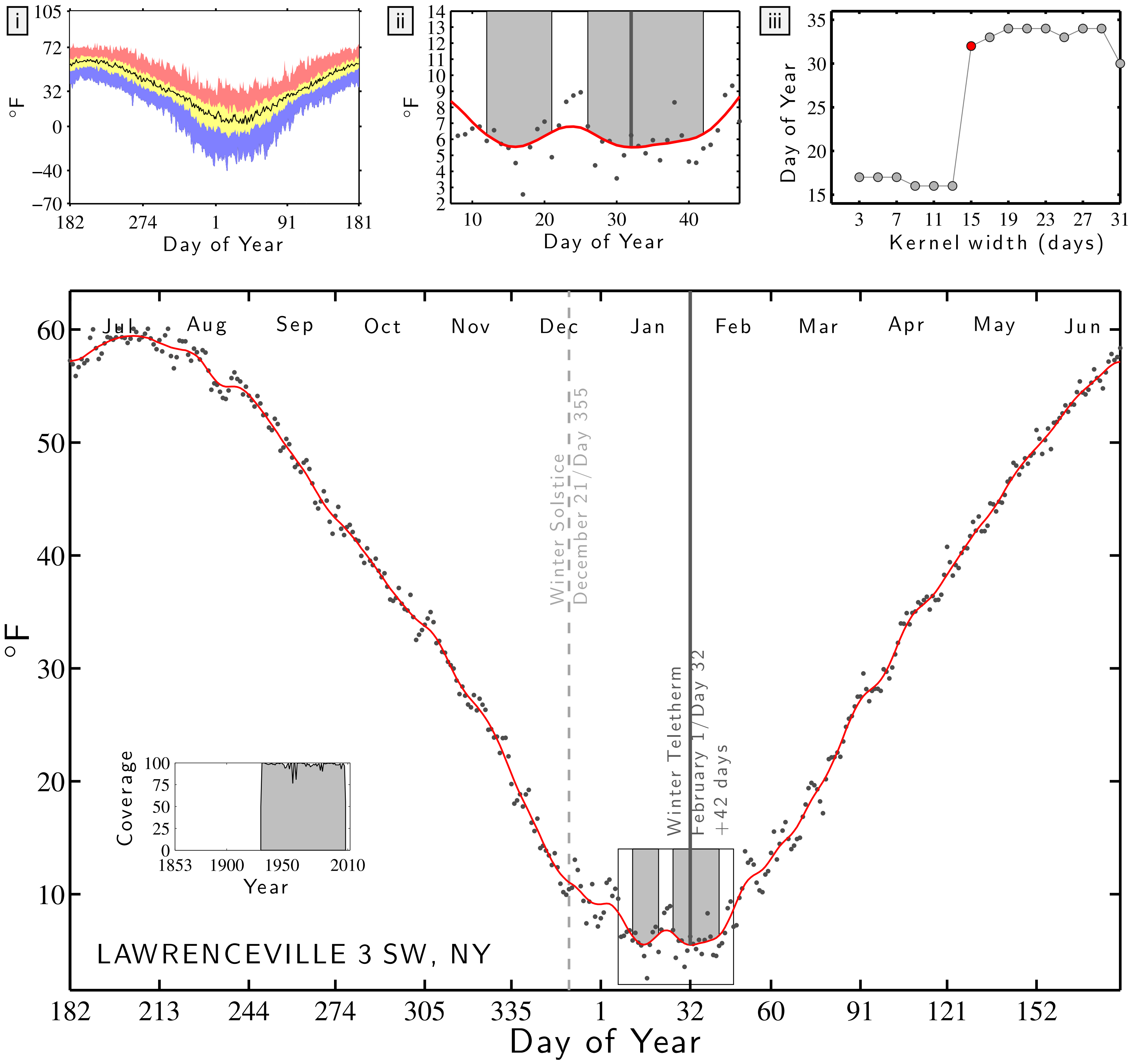

i
ii
iii
°F
Day of Year
Day of Year
Kernel width (days)
Jul
Aug
Sep
Oct
Nov
Dec
Jan
Feb
Mar
Apr
May
Jun
Winter Solstice
December 21/Day 355
Winter Teletherm
February 1/Day 32
+42 days
Coverage
Year
LAWRENCEVILLE 3 SW, NY

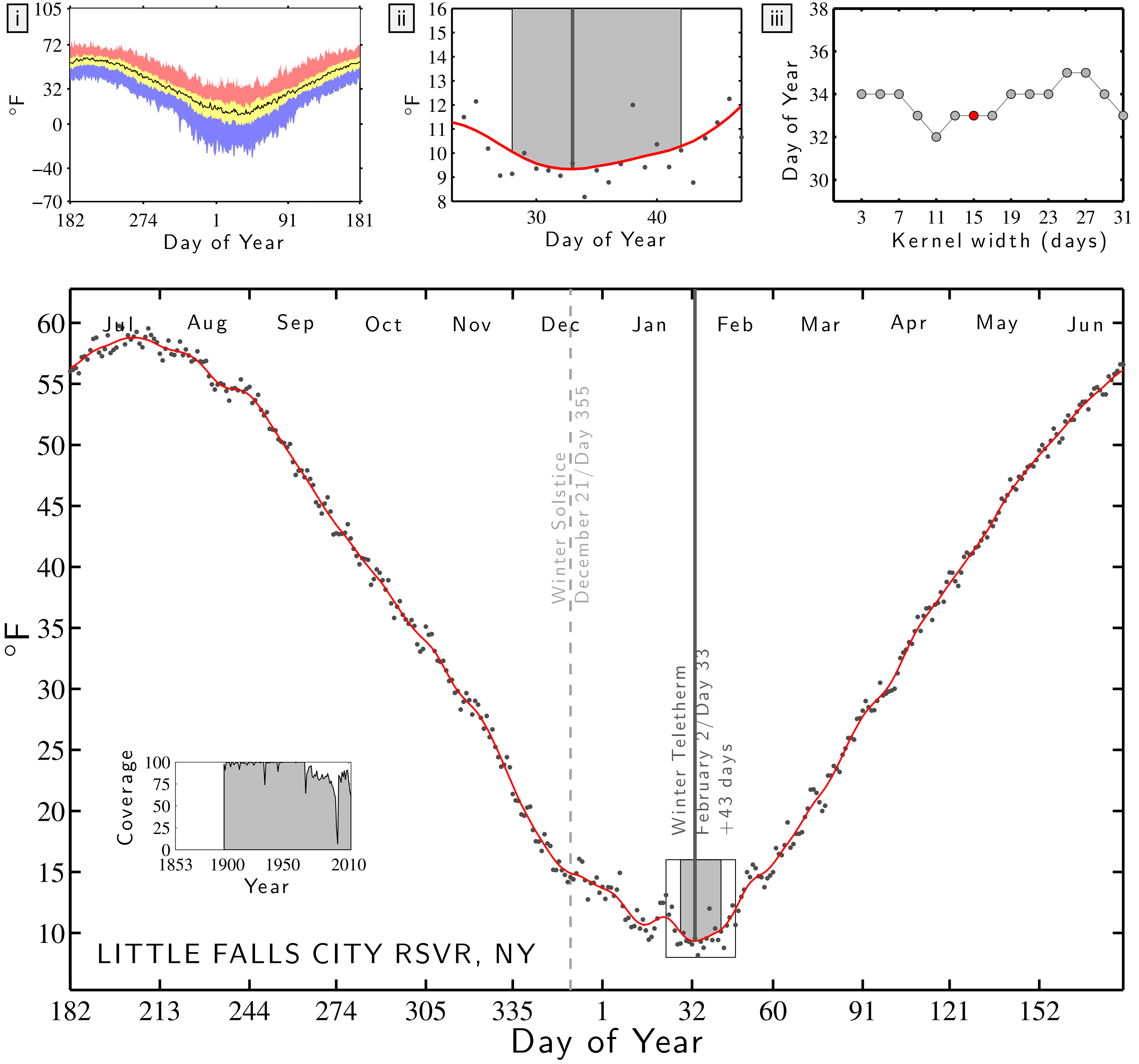

i
°F
Day of Year
ii
°F
Day of Year
iii
Day of Year
Kernel width (days)
Jul
Aug
Sep
Oct
Nov
Dec
Jan
Feb
Mar
Apr
May
Jun
Winter Solstice
December 21/Day 355
Winter Teletherm
February 2/Day 33
+43 days
Coverage
Year
LITTLE FALLS CITY RSVR, NY
°F
Day of Year

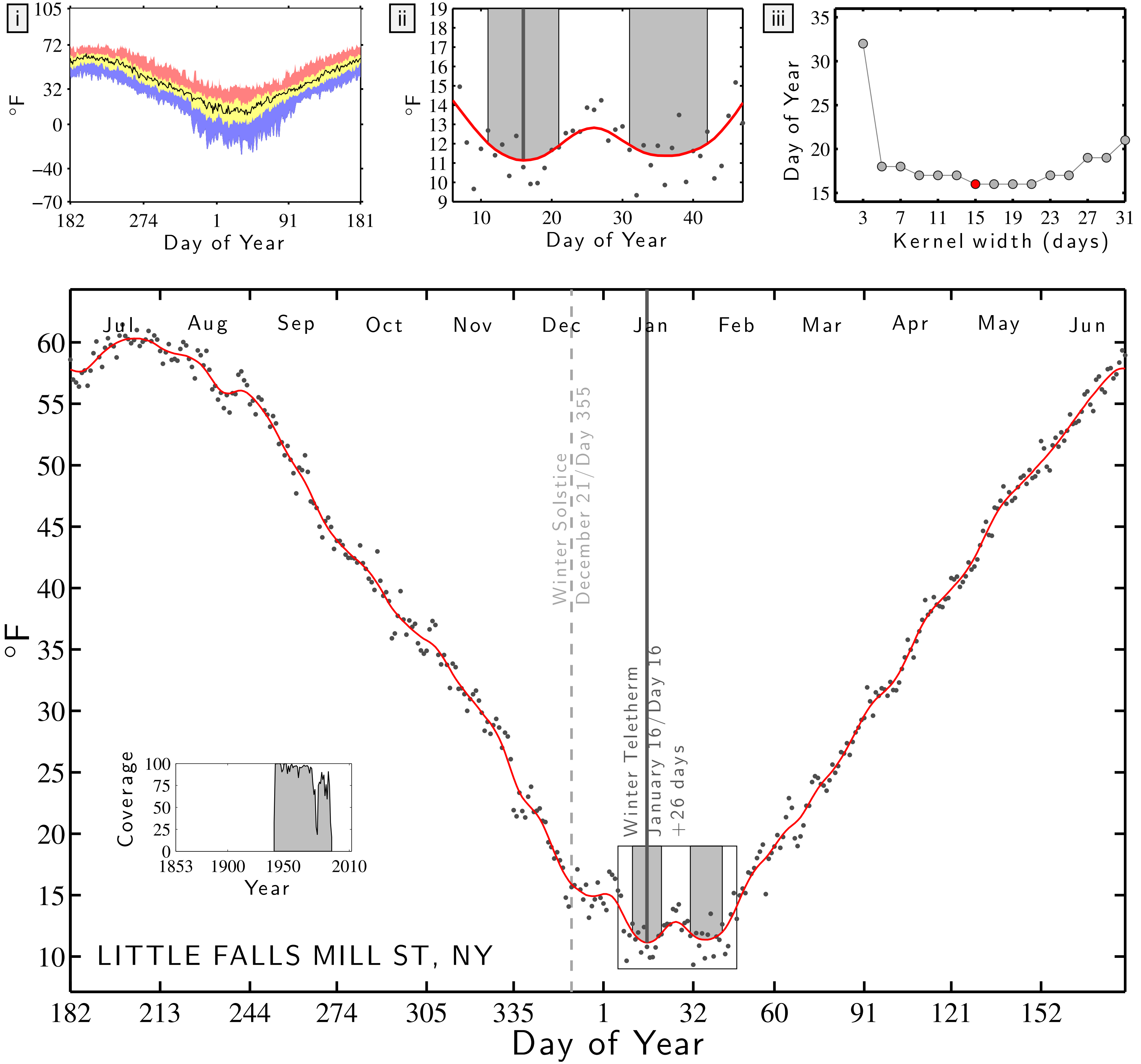

i
°F
Day of Year
ii
°F
Day of Year
iii
Day of Year
Kernel width (days)
Jul
Aug
Sep
Oct
Nov
Dec
Jan
Feb
Mar
Apr
May
Jun
Winter Solstice
December 21/Day 355
Winter Teletherm
January 16/Day 16
+26 days
Coverage
Year
LITTLE FALLS MILL ST, NY
°F
Day of Year

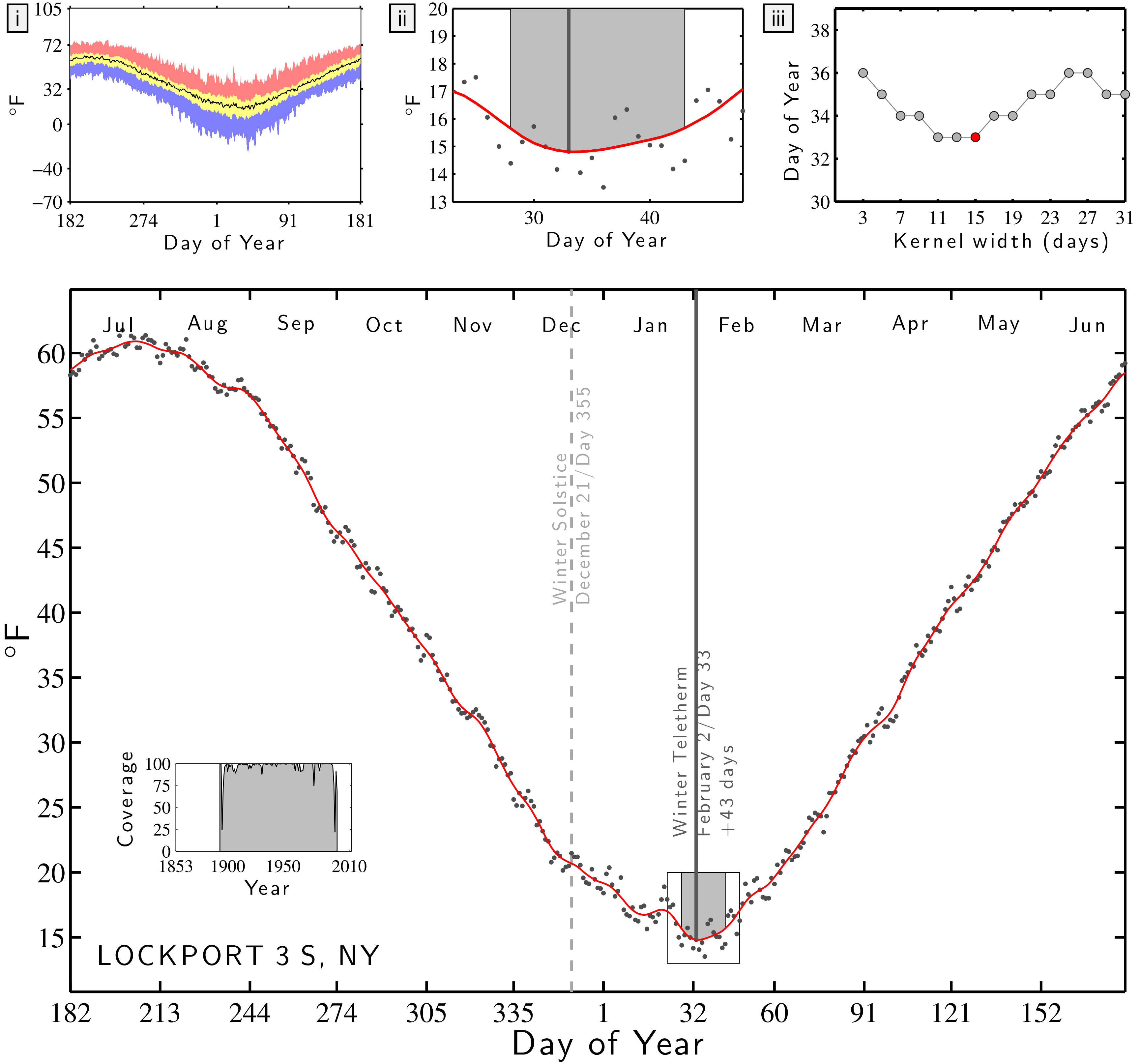

i
ii
iii
°F
Day of Year
Kernel width (days)
Jul
Aug
Sep
Oct
Nov
Dec
Jan
Feb
Mar
Apr
May
Jun
Winter Solstice
December 21/Day 355
Winter Teletherm
February 2/Day 33
+43 days
Coverage
Year
LOCKPORT 3 S, NY

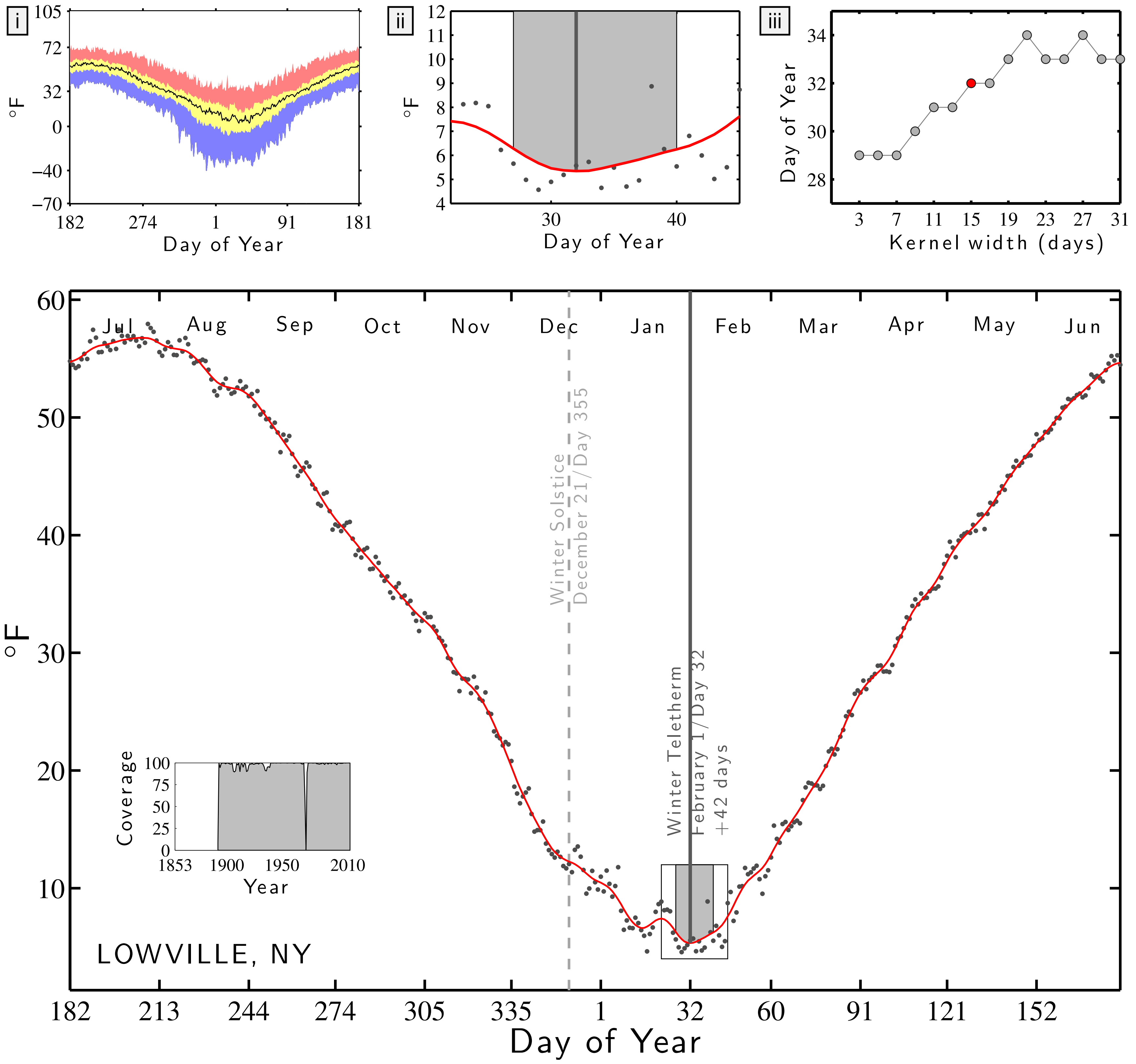

i
°F
Day of Year
ii
Day of Year
iii
Day of Year
Kernel width (days)
Jul
Aug
Sep
Oct
Nov
Dec
Jan
Feb
Mar
Apr
May
Jun
Winter Solstice
December 21/Day 355
Winter Teletherm
February 1/Day 32
+42 days
Coverage
Year
LOWVILLE, NY
Day of Year

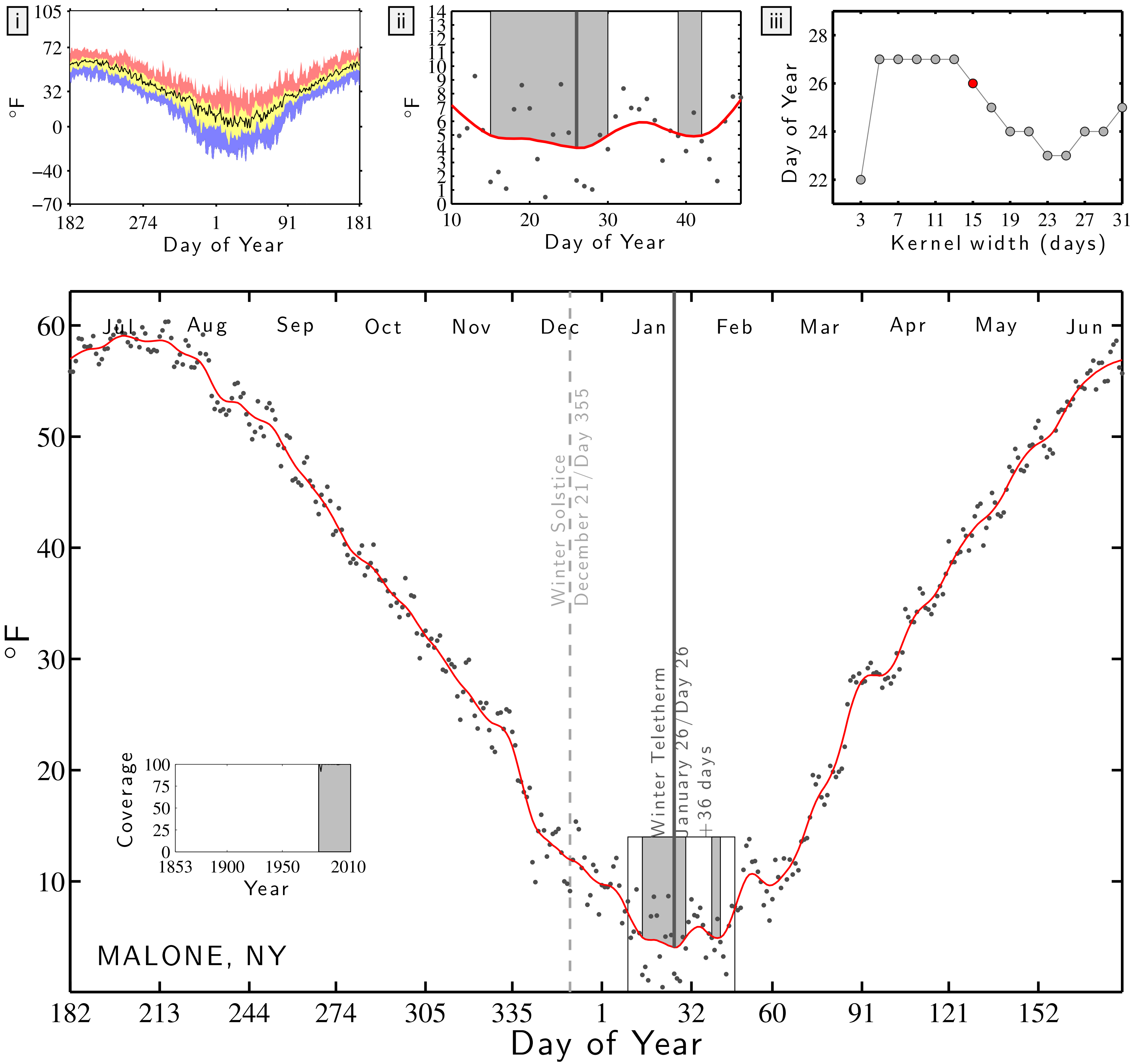

i
°F
Day of Year
ii
°F
Day of Year
iii
Day of Year
Kernel width (days)
Jul
Aug
Sep
Oct
Nov
Dec
Jan
Feb
Mar
Apr
May
Jun
Winter Solstice
December 21/Day 355
Winter Teletherm
January 26/Day 26
+36 days
Coverage
Year
MALONE, NY
°F
Day of Year

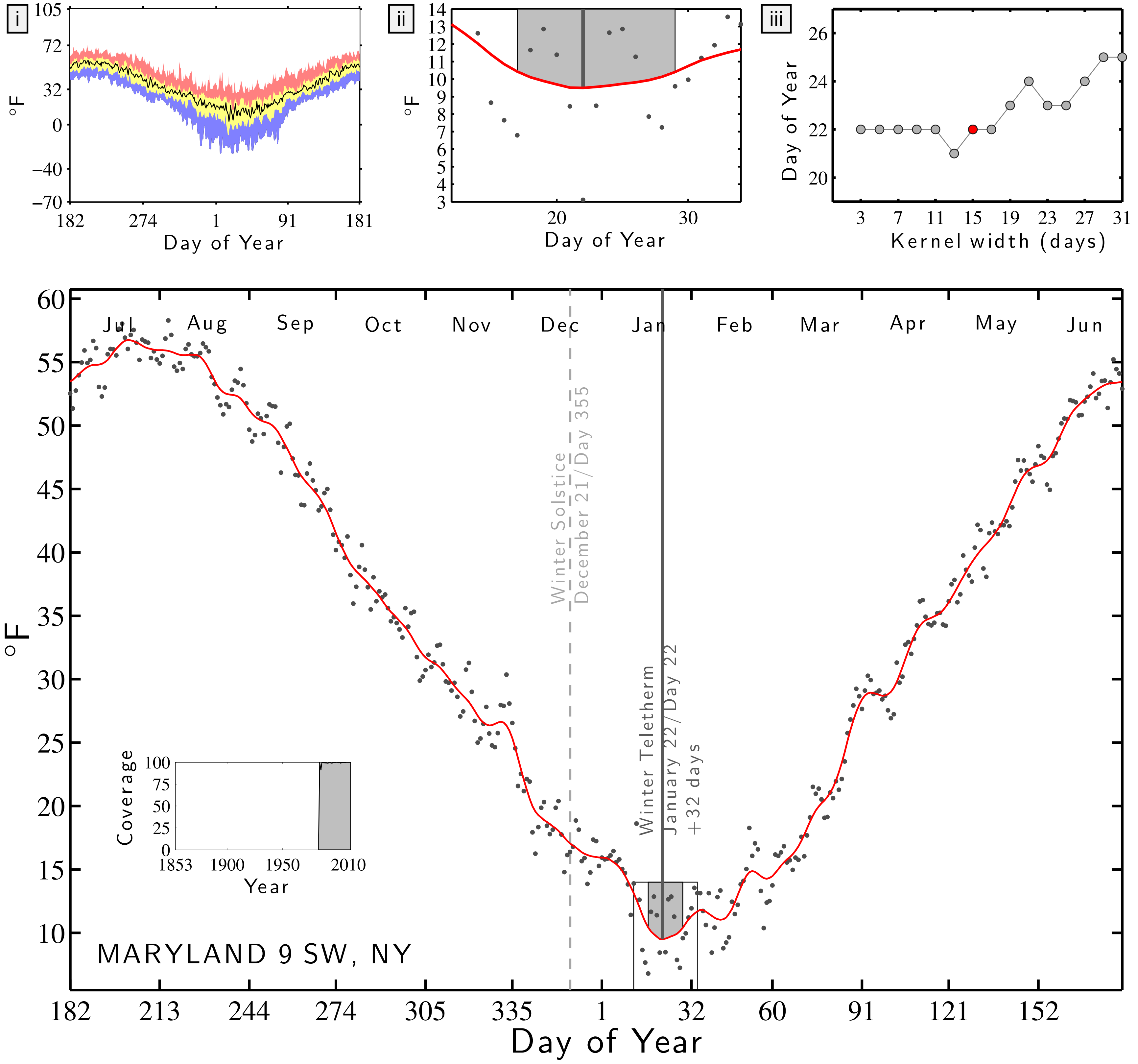

i
°F
Day of Year
ii
Day of Year
iii
Day of Year
Kernel width (days)
Jul
Aug
Sep
Oct
Nov
Dec
Jan
Feb
Mar
Apr
May
Jun
Winter Solstice
December 21/Day 355
Winter Teletherm
January 22/Day 22
+32 days
Coverage
Year
MARYLAND 9 SW, NY
Day of Year

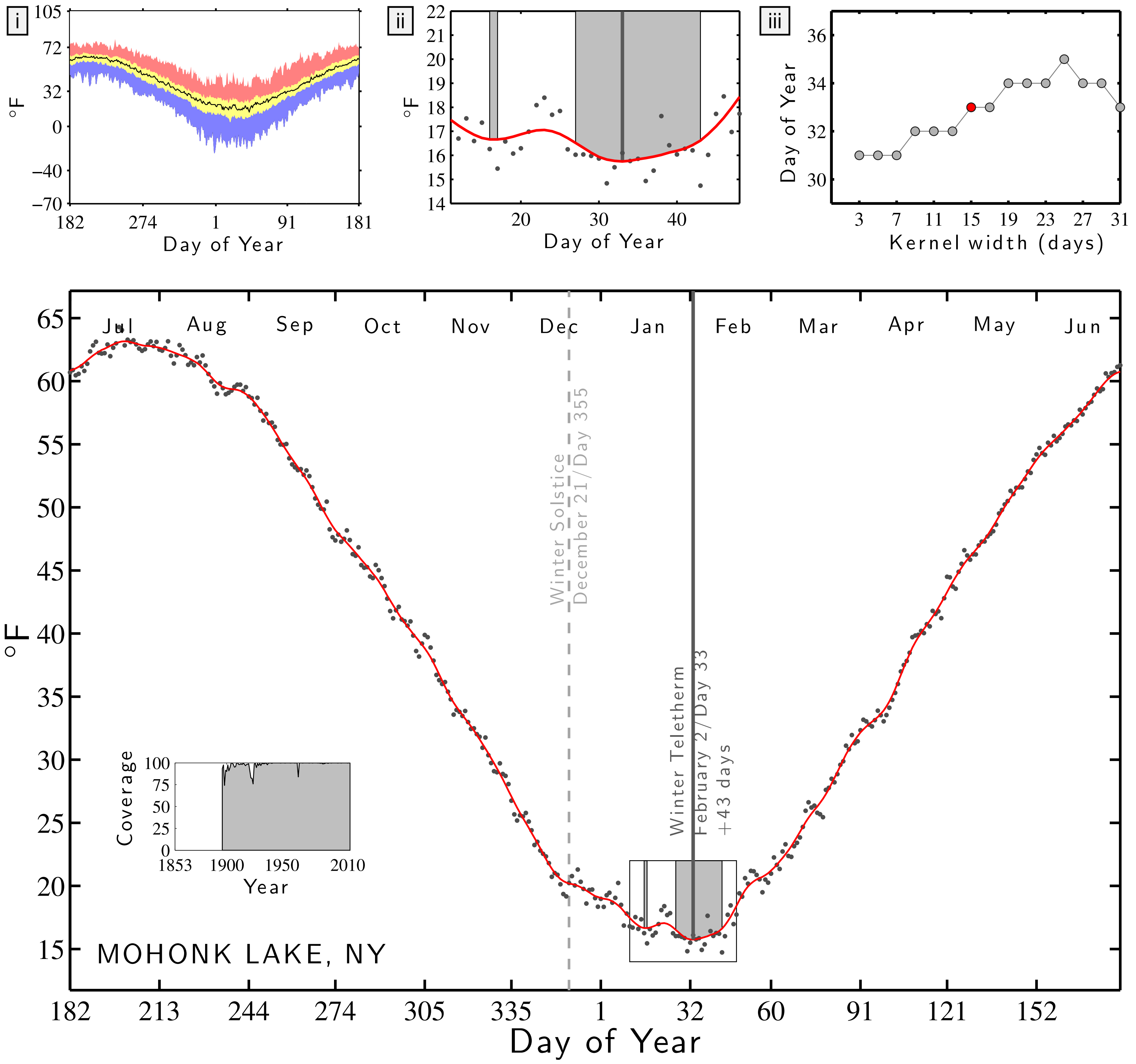

i
°F
Day of Year
ii
°F
Day of Year
iii
Day of Year
Kernel width (days)
Jul
Aug
Sep
Oct
Nov
Dec
Jan
Feb
Mar
Apr
May
Jun
Winter Solstice
December 21/Day 355
Winter Teletherm
February 2/Day 33
+43 days
Coverage
Year
MOHONK LAKE, NY
°F
Day of Year

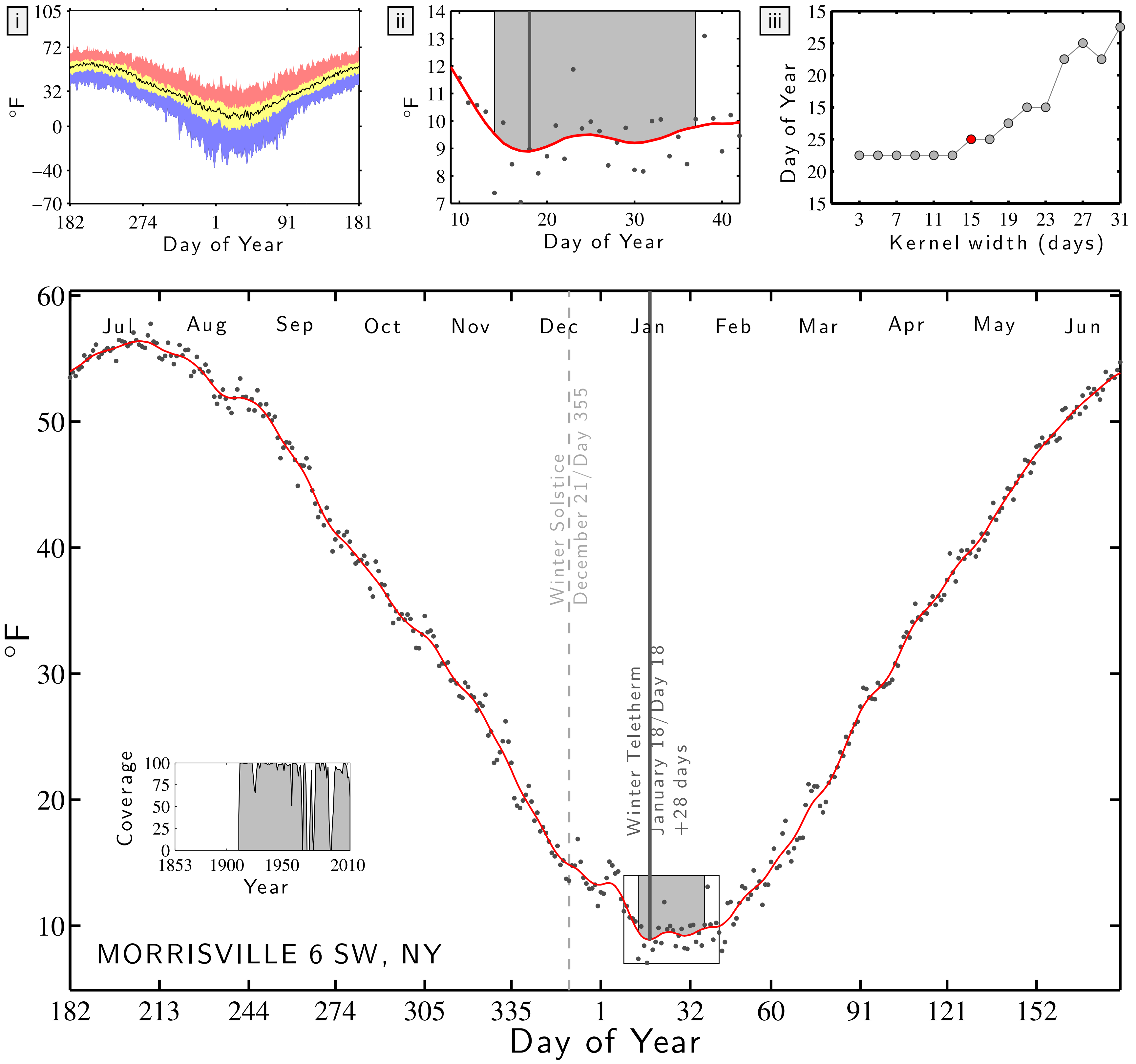

i
°F
Day of Year
ii
°F
Day of Year
iii
Day of Year
Kernel width (days)
Jul
Aug
Sep
Oct
Nov
Dec
Jan
Feb
Mar
Apr
May
Jun
Winter Solstice
December 21/Day 355
Winter Teletherm
January 18/Day 18
+28 days
Coverage
Year
MORRISVILLE 6 SW, NY
°F
Day of Year

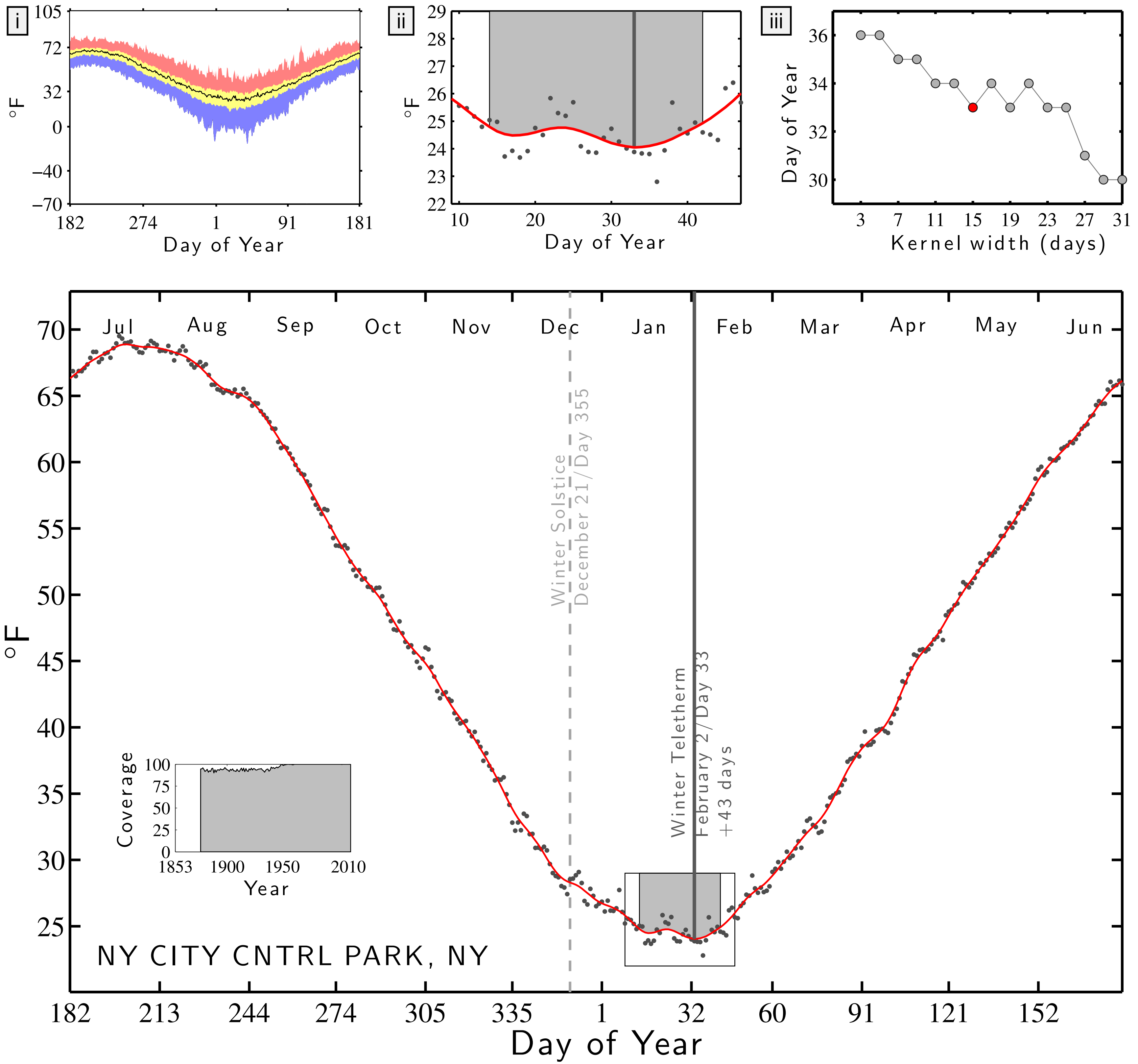

i
ii
iii
°F
Day of Year
Day of Year
Kernel width (days)
Jul
Aug
Sep
Oct
Nov
Dec
Jan
Feb
Mar
Apr
May
Jun
Winter Solstice
December 21/Day 355
Winter Teletherm
February 2/Day 33
+43 days
Coverage
Year
NY CITY CNTRL PARK, NY

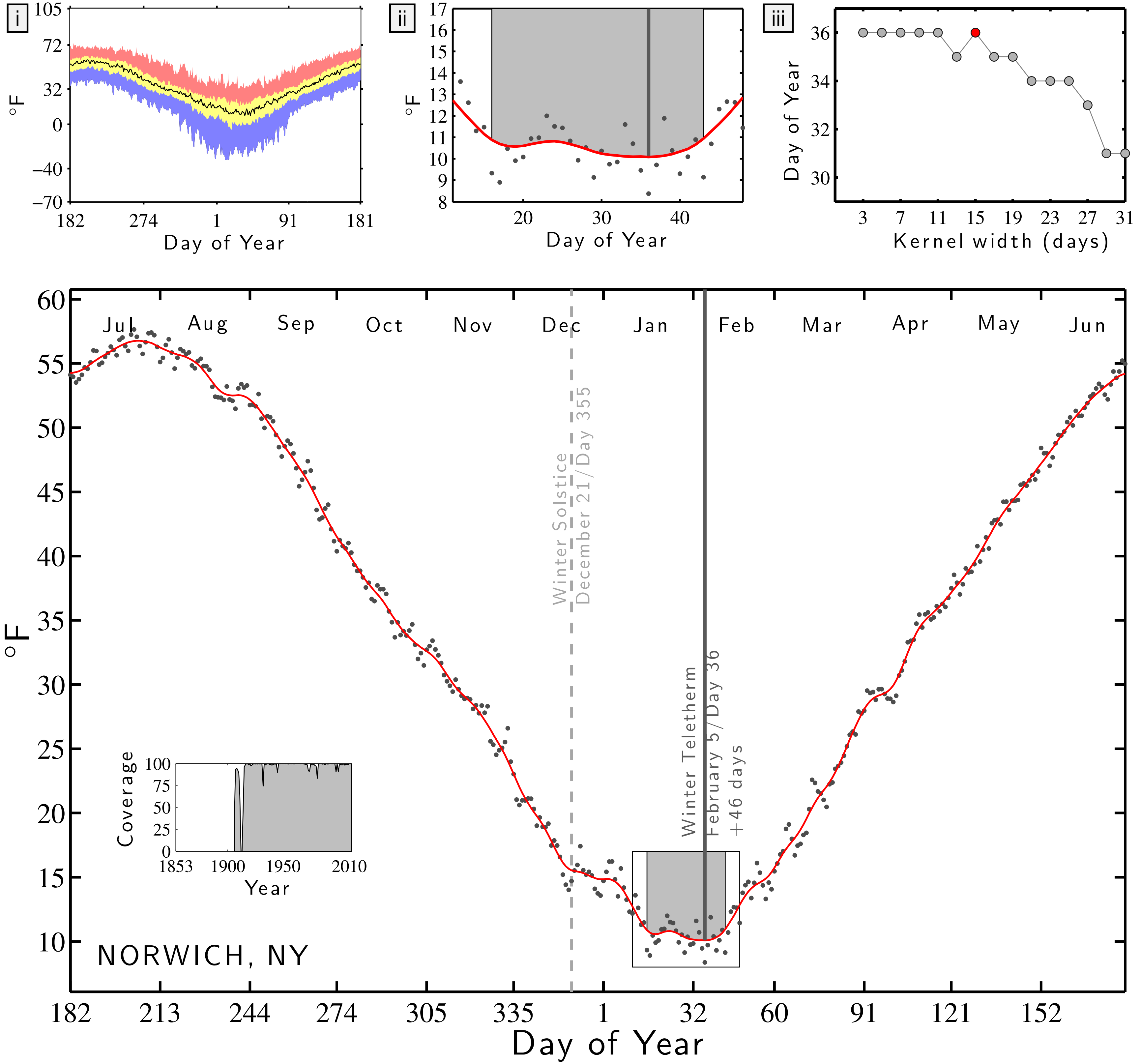

i
ii
iii
°F
Day of Year
Day of Year
Kernel width (days)
Jul
Aug
Sep
Oct
Nov
Dec
Jan
Feb
Mar
Apr
May
Jun
Winter Solstice
December 21/Day 355
Winter Teletherm
February 5/Day 36
+46 days
Coverage
Year
NORWICH, NY

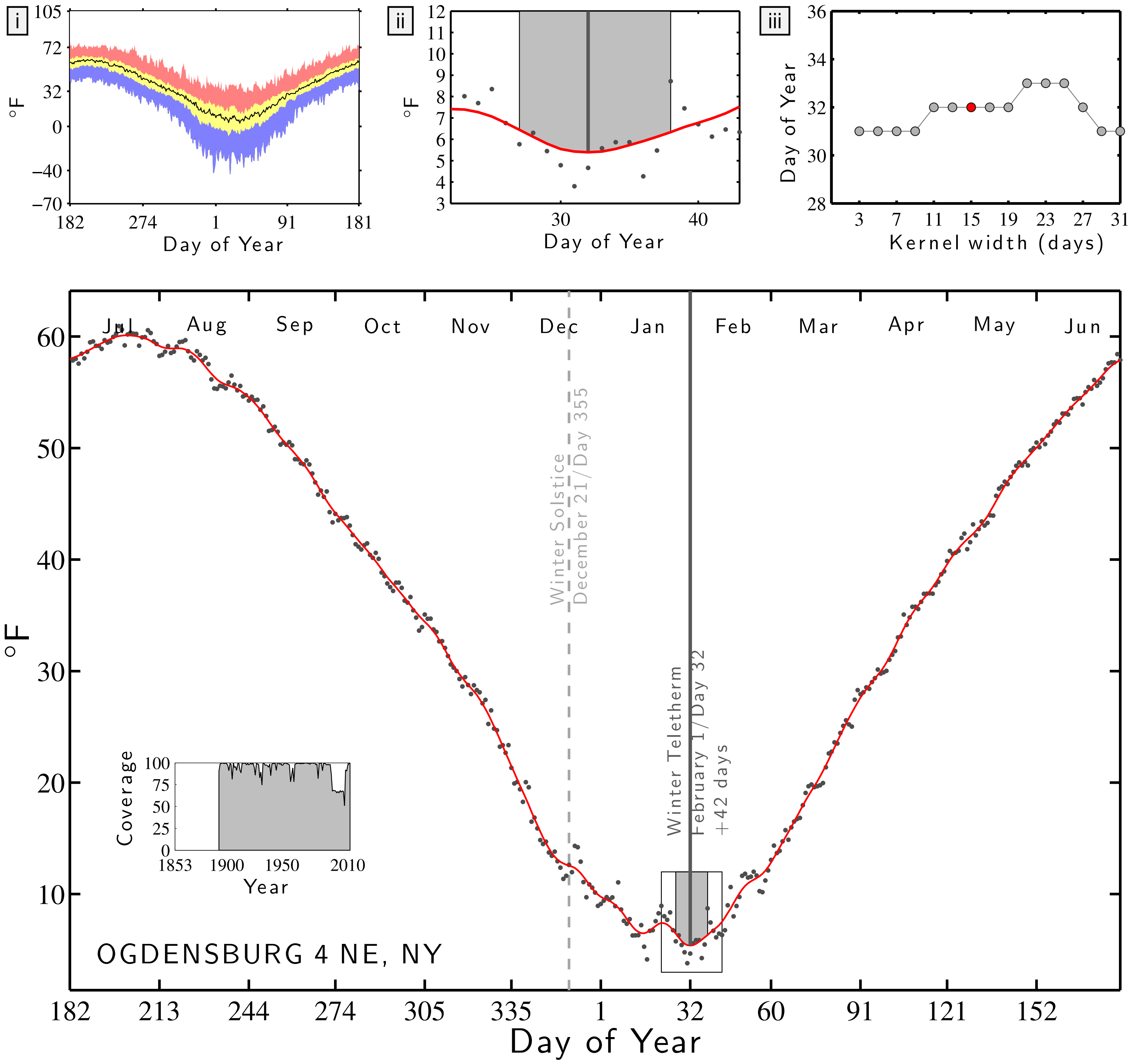

i
°F
Day of Year
ii
°F
Day of Year
iii
Day of Year
Kernel width (days)
Jul
Aug
Sep
Oct
Nov
Dec
Jan
Feb
Mar
Apr
May
Jun
Winter Solstice
December 21/Day 355
Winter Teletherm
February 1/Day 32
+42 days
Coverage
Year
OGDENSBURG 4 NE, NY
°F
Day of Year

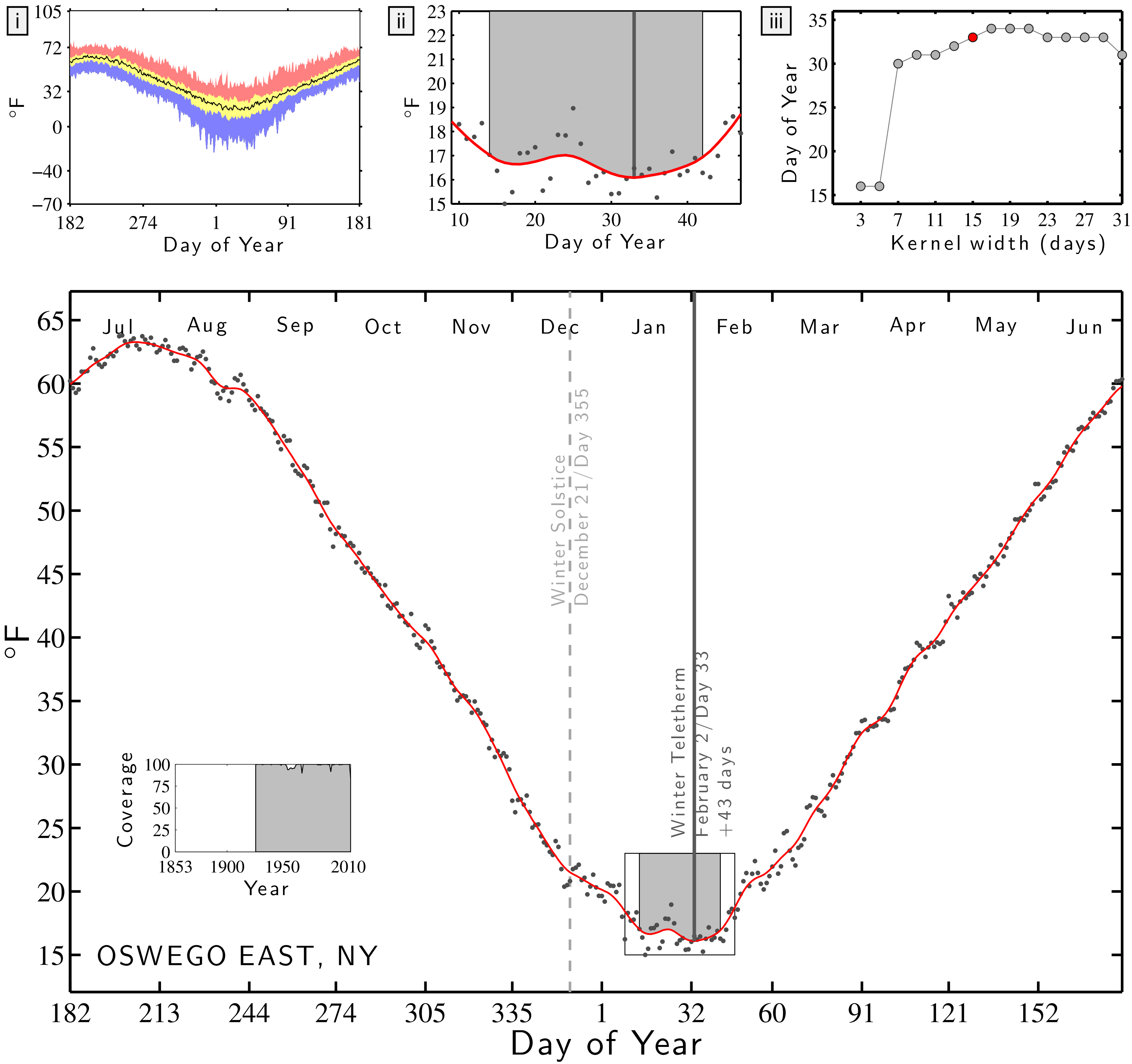

i
°F
105
72
32
0
−40
−70
182
274
1
91
181
Day of Year
ii
°F
23
22
21
20
19
18
17
16
15
10
20
30
40
Day of Year
iii
Day of Year
35
30
25
20
15
3
7
11
15
19
23
27
31
Kernel width (days)
Jul
Aug
Sep
Oct
Nov
Dec
Jan
Feb
Mar
Apr
May
Jun
Winter Solstice
December 21/Day 355
Winter Teletherm
February 2/Day 33
+43 days
Coverage
100
75
50
25
0
1853
1900
1950
2010
Year
OSWEGO EAST, NY
°F
65
60
55
50
45
40
35
30
25
20
15
182
213
244
274
305
335
1
32
60
91
121
152
Day of Year

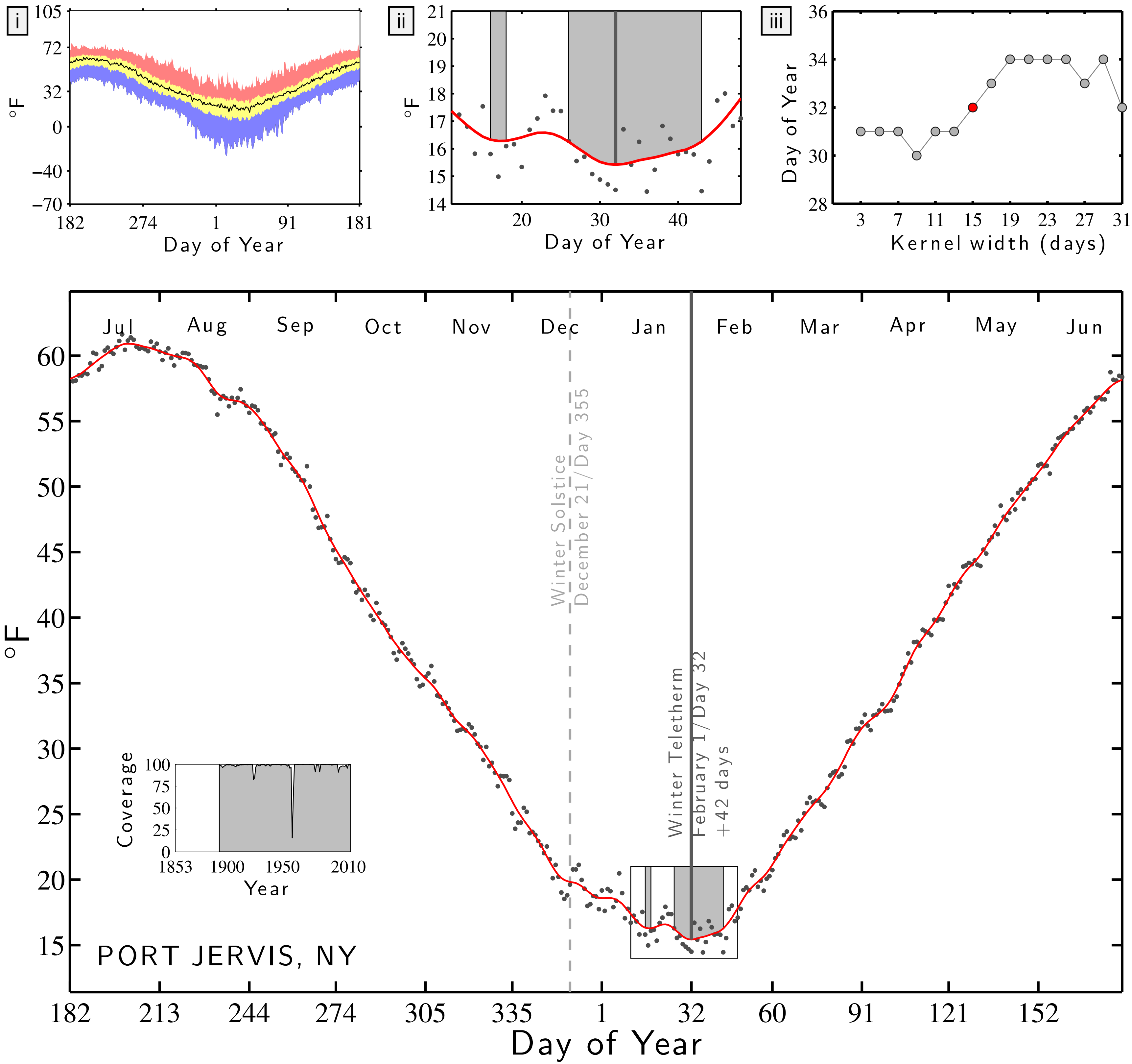

i
°F
Day of Year
ii
Day of Year
iii
Day of Year
Kernel width (days)
Jul
Aug
Sep
Oct
Nov
Dec
Jan
Feb
Mar
Apr
May
Jun
Winter Solstice
December 21/Day 355
Winter Teletherm
February 1/Day 32
+42 days
Coverage
Year
PORT JERVIS, NY
Day of Year

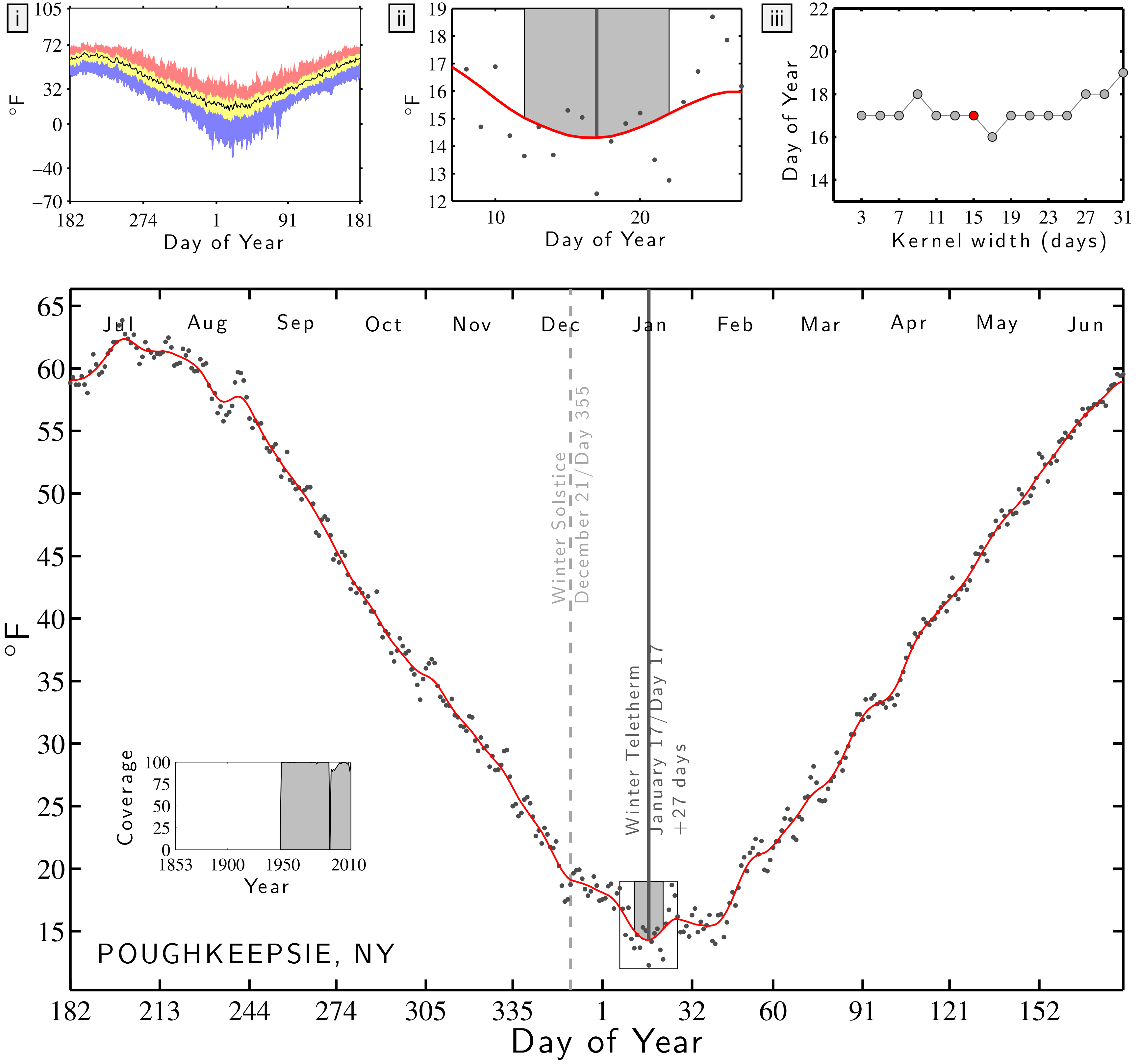

i
ii
iii
°F
Day of Year
Kernel width (days)
Jul
Aug
Sep
Oct
Nov
Dec
Jan
Feb
Mar
Apr
May
Jun
Winter Solstice
December 21/Day 355
Winter Teletherm
January 17/Day 17
+27 days
Coverage
Year
POUGHKEEPSIE, NY

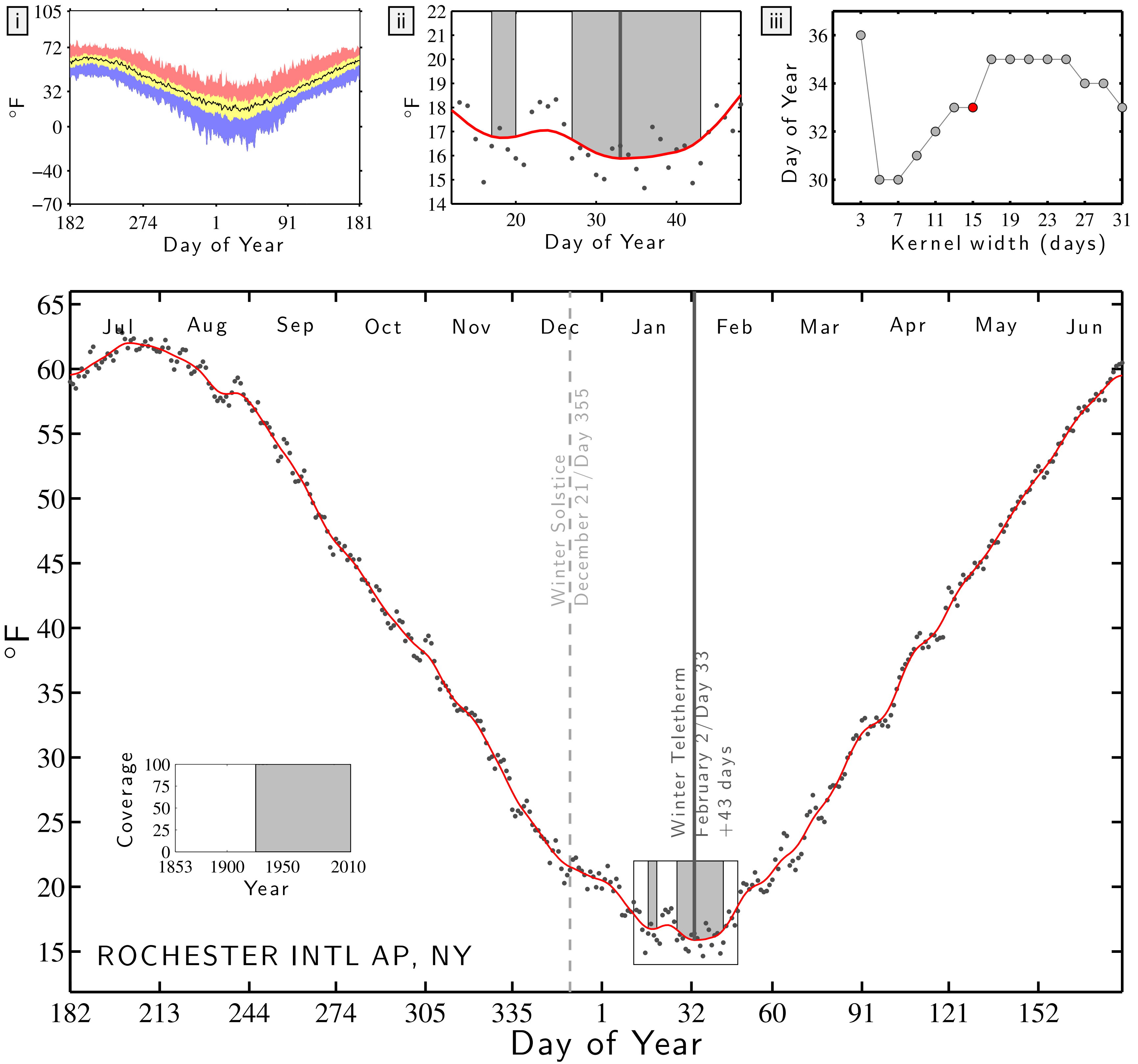

i
ii
iii
Day of Year
Kernel width (days)
°F
Winter Solstice
December 21/Day 355
Winter Teletherm
February 2/Day 33
+43 days
Coverage
Year
ROCHESTER INTL AP, NY
Jul
Aug
Sep
Oct
Nov
Dec
Jan
Feb
Mar
Apr
May
Jun

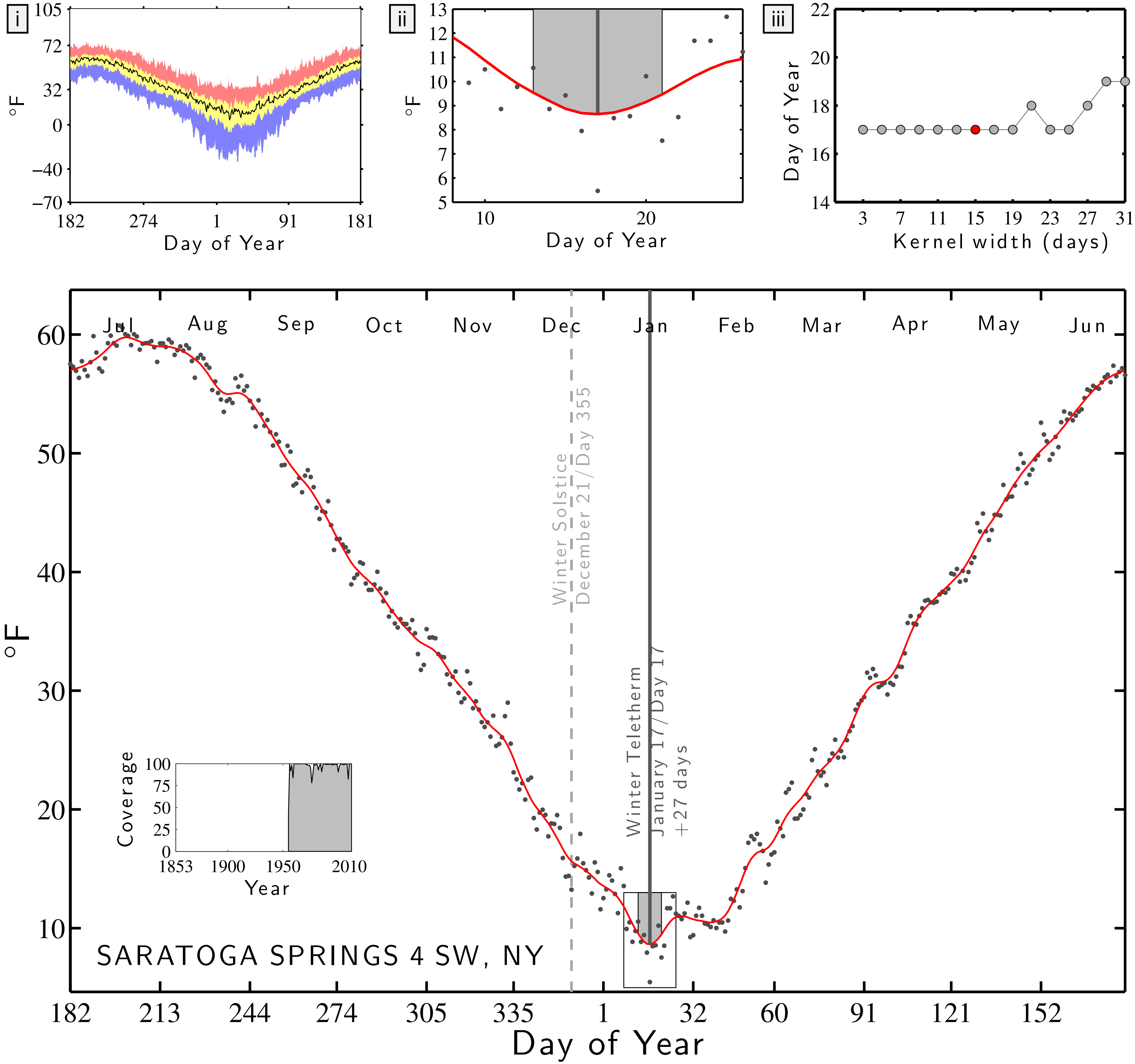

i
ii
iii
°F
Day of Year
Day of Year
Kernel width (days)
Winter Solstice
December 21/Day 355
Winter Teletherm
January 17/Day 17
+27 days
Coverage
Year
SARATOGA SPRINGS 4 SW, NY
Jul
Aug
Sep
Oct
Nov
Dec
Jan
Feb
Mar
Apr
May
Jun

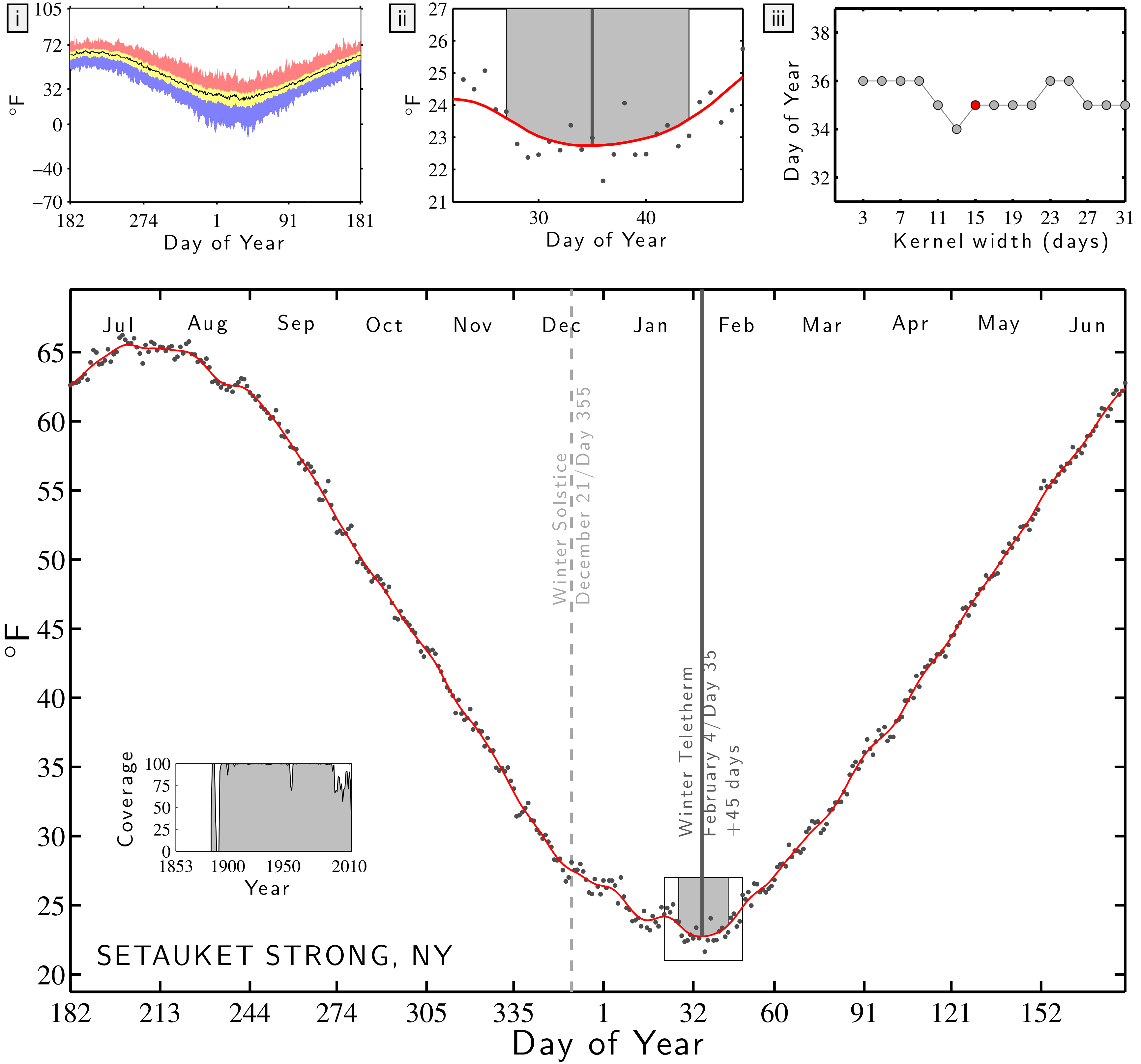

i
°F
Day of Year
ii
°F
Day of Year
iii
Day of Year
Kernel width (days)
Jul
Aug
Sep
Oct
Nov
Dec
Jan
Feb
Mar
Apr
May
Jun
Winter Solstice
December 21/Day 355
Winter Teletherm
February 4/Day 35
+45 days
Coverage
Year
SETAUKET STRONG, NY
°F
Day of Year

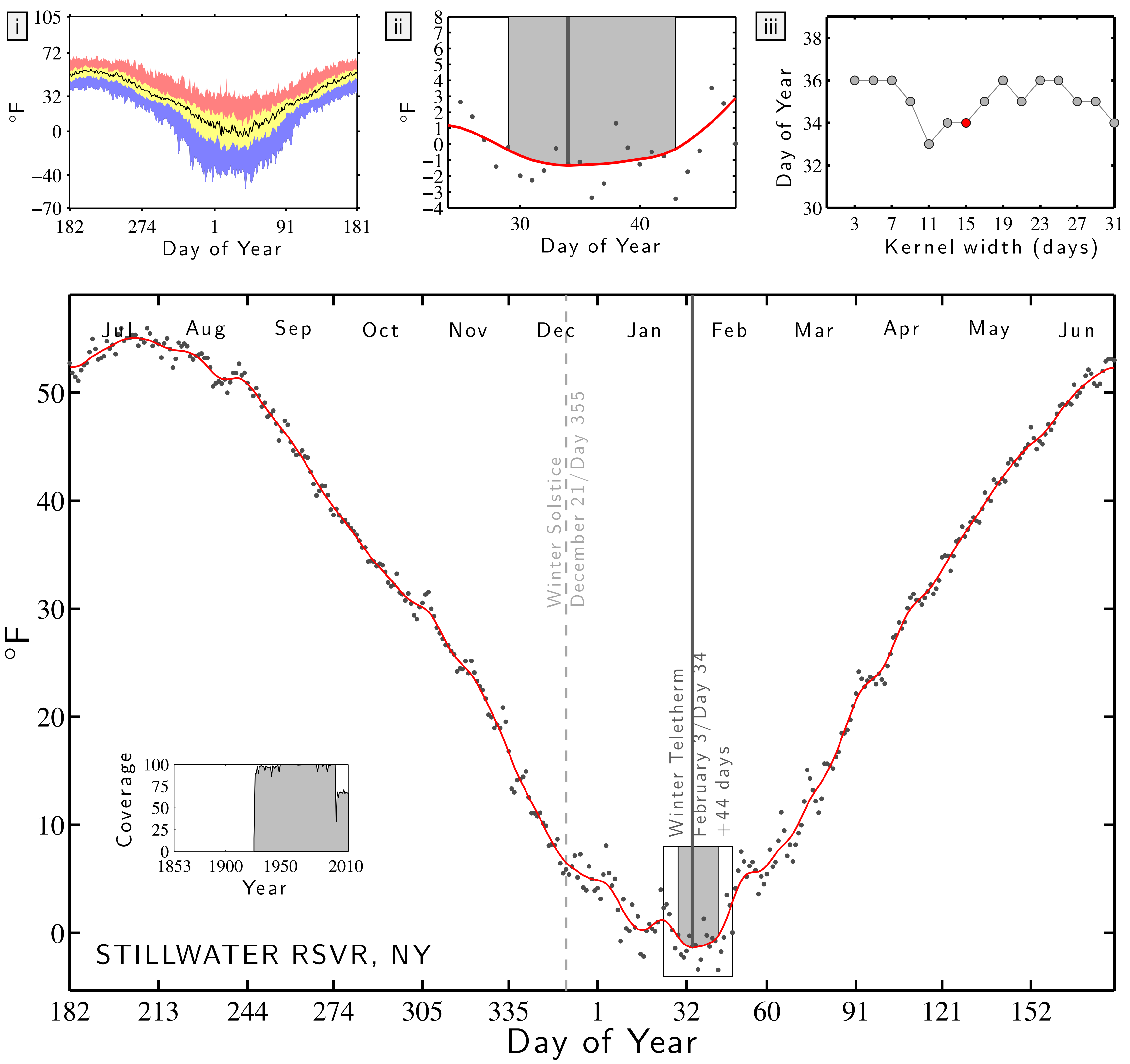

i
°F
Day of Year
ii
°F
Day of Year
iii
Day of Year
Kernel width (days)
Jul
Aug
Sep
Oct
Nov
Dec
Jan
Feb
Mar
Apr
May
Jun
Winter Solstice
December 21/Day 355
Winter Teletherm
February 3/Day 34
+44 days
Coverage
Year
STILLWATER RSVR, NY
°F
Day of Year

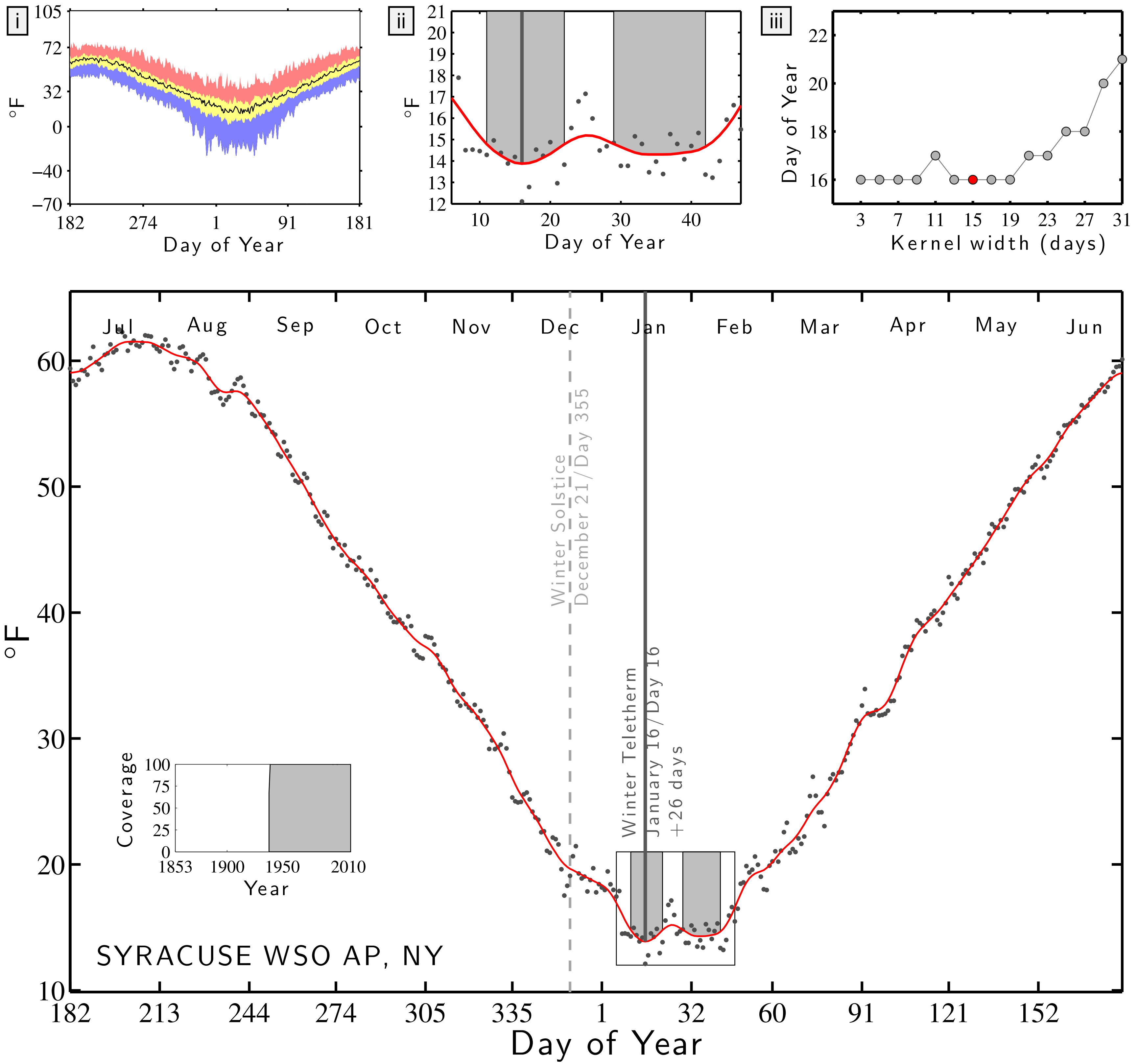
i
ii
iii
°F
Day of Year
Kernel width (days)
Jul
Aug
Sep
Oct
Nov
Dec
Jan
Feb
Mar
Apr
May
Jun
Winter Solstice
December 21/Day 355
Winter Teletherm
January 16/Day 16
+26 days
Coverage
Year
SYRACUSE WSO AP, NY

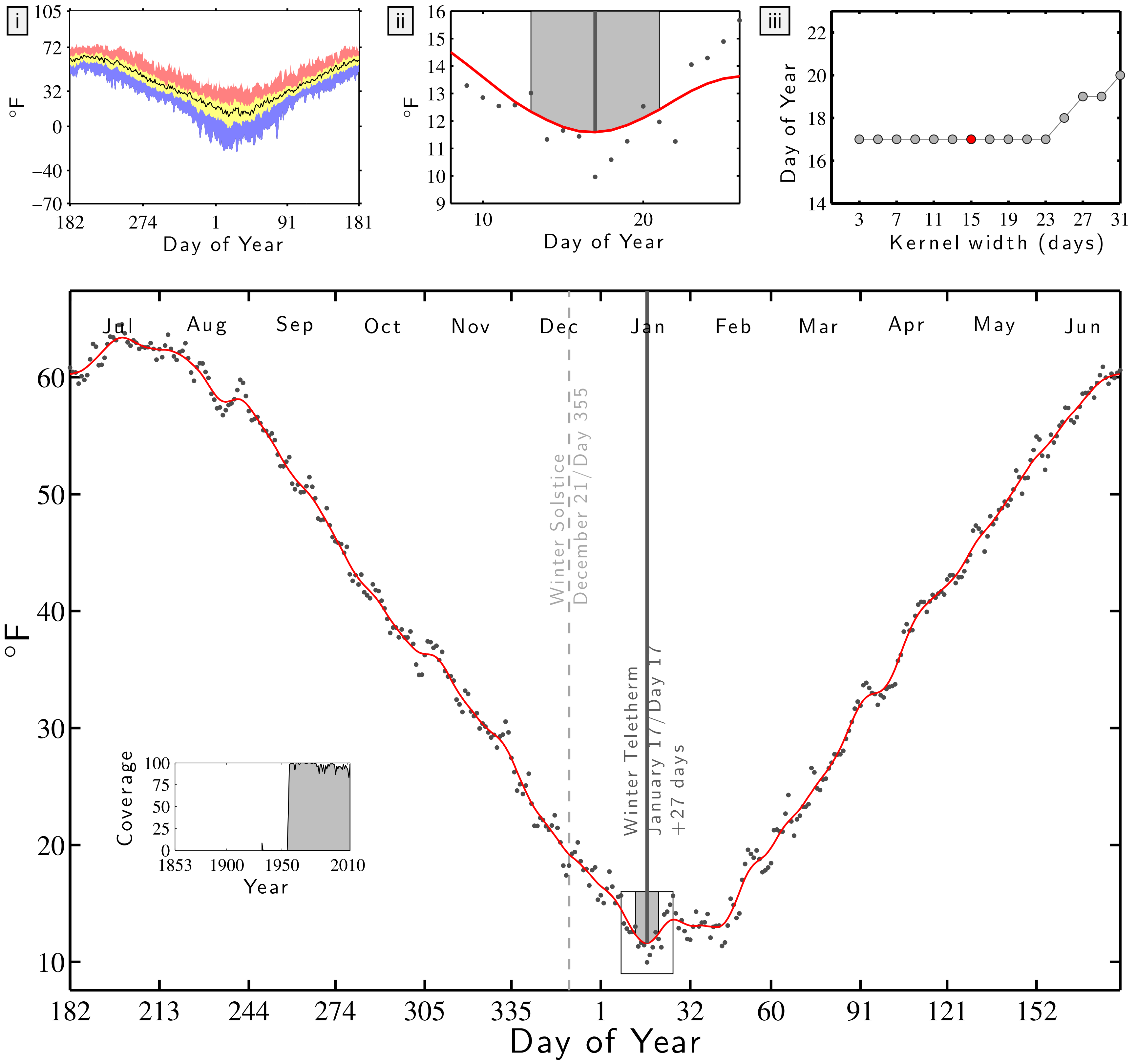

i
ii
iii
°F
Day of Year
Kernel width (days)
Jul
Aug
Sep
Oct
Nov
Dec
Jan
Feb
Mar
Apr
May
Jun
Winter Solstice
December 21/Day 355
Winter Teletherm
January 17/Day 17
+27 days
Coverage
Year

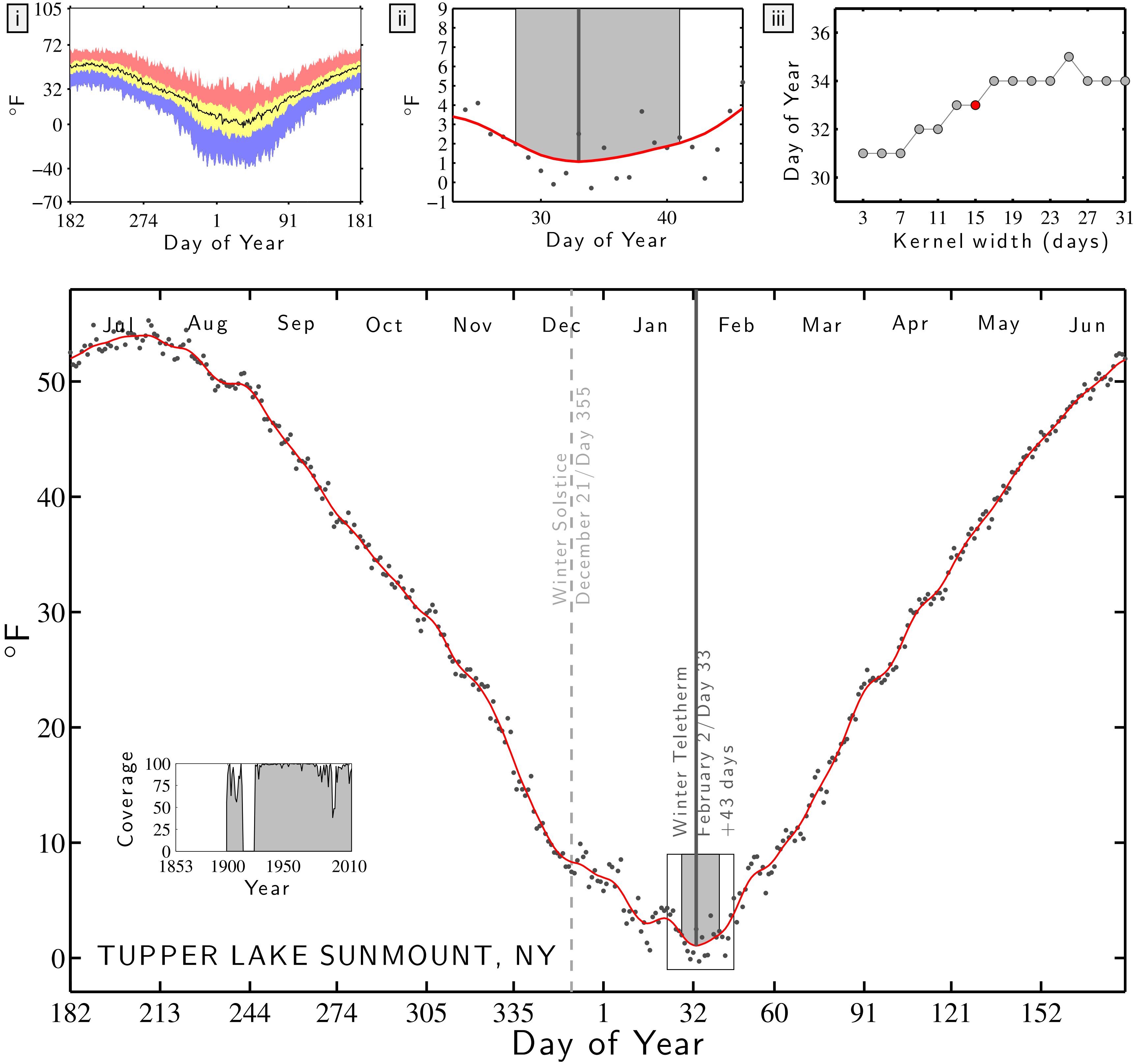

i
°F
Day of Year
ii
°F
Day of Year
iii
Day of Year
Kernel width (days)
Jul
Aug
Sep
Oct
Nov
Dec
Jan
Feb
Mar
Apr
May
Jun
Winter Solstice
December 21/Day 355
Winter Teletherm
February 2/Day 33
+43 days
Coverage
Year
TUPPER LAKE SUNMOUNT, NY
°F
Day of Year

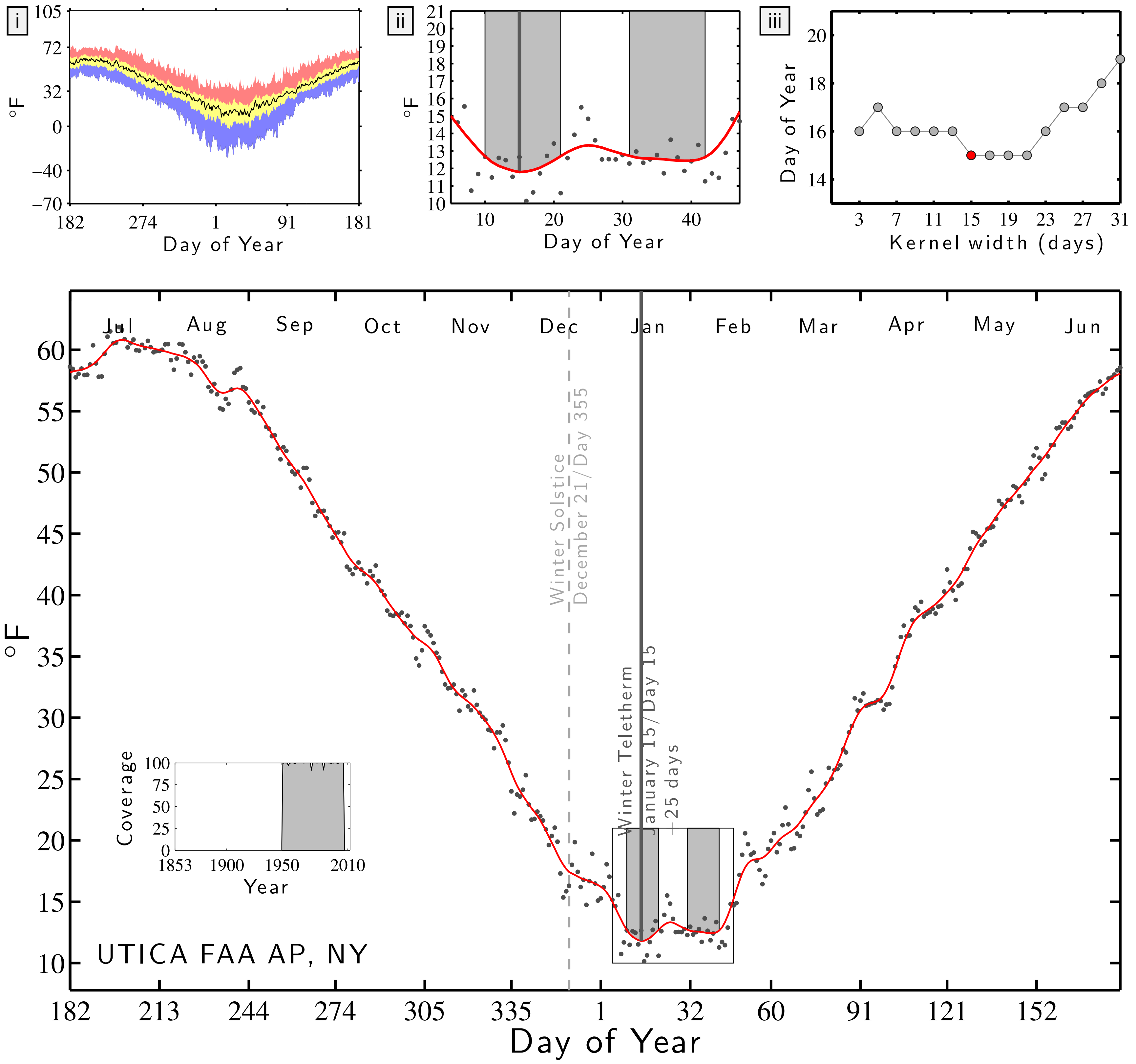

i
°F
Day of Year
ii
°F
Day of Year
iii
Day of Year
Kernel width (days)
Jul
Aug
Sep
Oct
Nov
Dec
Jan
Feb
Mar
Apr
May
Jun
Winter Solstice
December 21/Day 355
Winter Teletherm
January 15/Day 15
±25 days
Coverage
Year
UTICA FAA AP, NY
°F
Day of Year

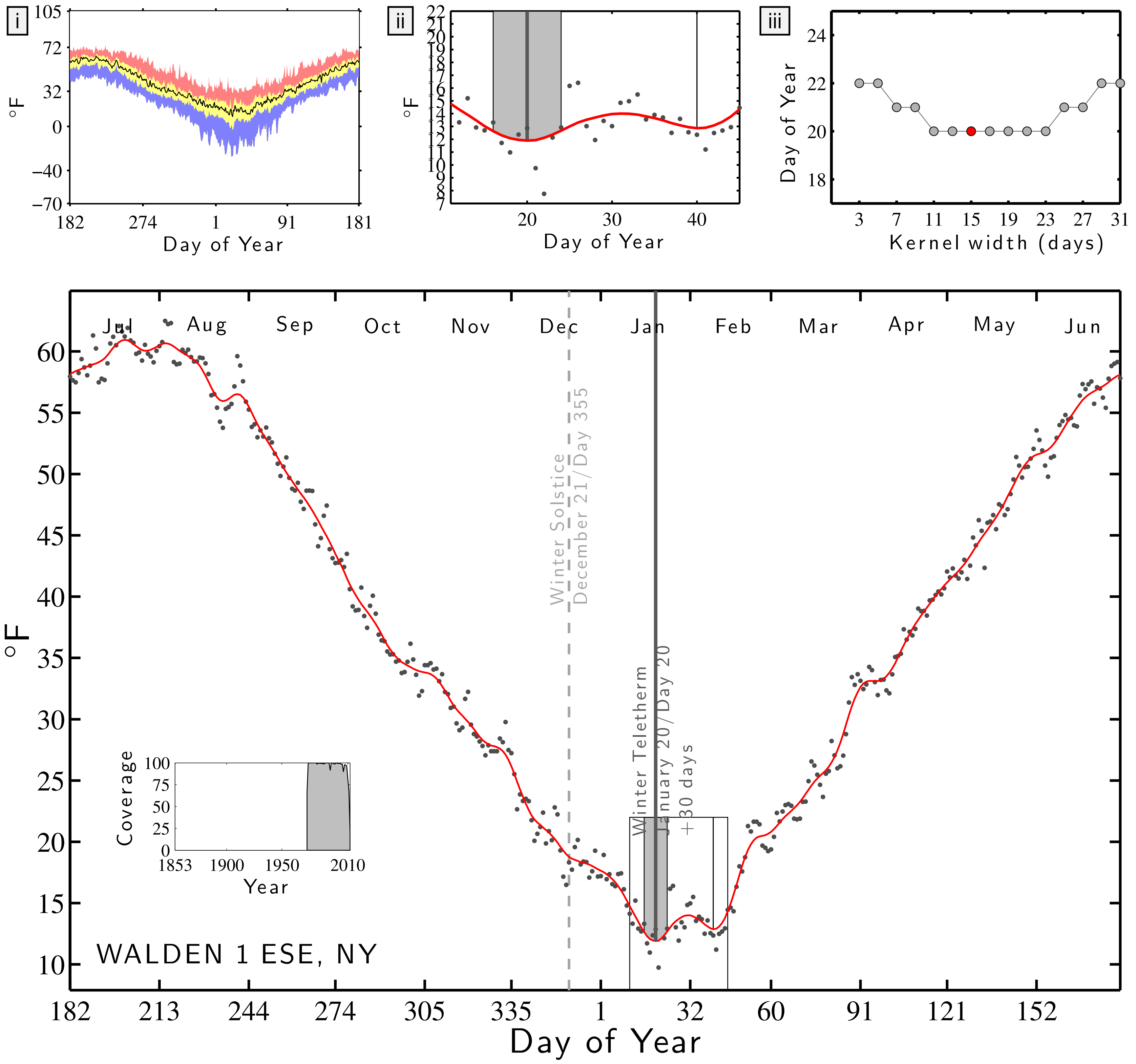

i
°F
Day of Year
ii
°F
Day of Year
iii
Day of Year
Kernel width (days)
Jul
Aug
Sep
Oct
Nov
Dec
Jan
Feb
Mar
Apr
May
Jun
Winter Solstice
December 21/Day 355
Winter Teletherm
January 20/Day 20
±30 days
Coverage
Year
WALDEN 1 ESE, NY
°F
Day of Year

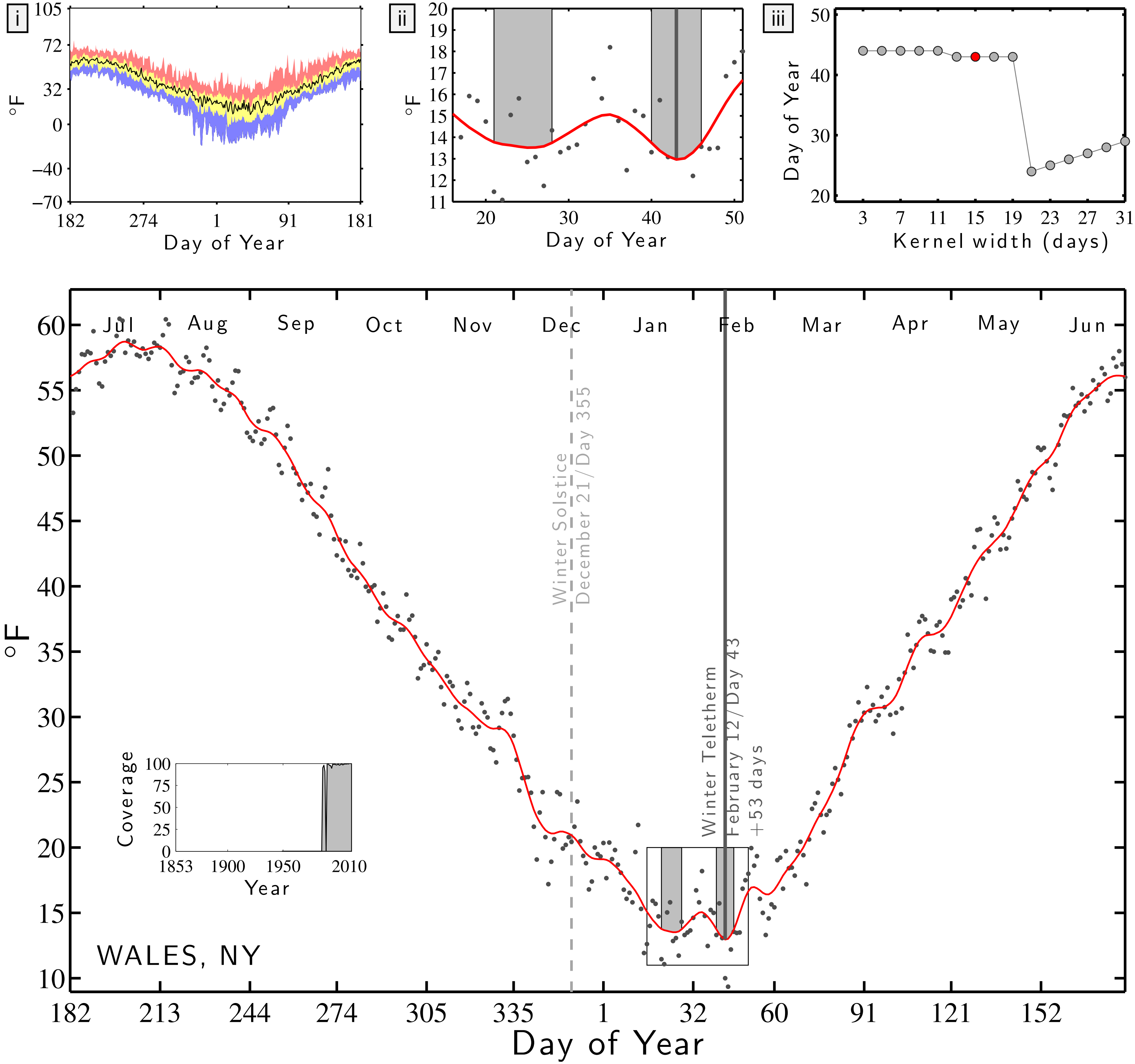
i
°F
Day of Year
ii
°F
Day of Year
iii
Day of Year
Kernel width (days)
Jul
Aug
Sep
Oct
Nov
Dec
Jan
Feb
Mar
Apr
May
Jun
Winter Solstice
December 21/Day 355
Winter Teletherm
February 12/Day 43
+53 days
Coverage
Year
WALES, NY
°F
Day of Year

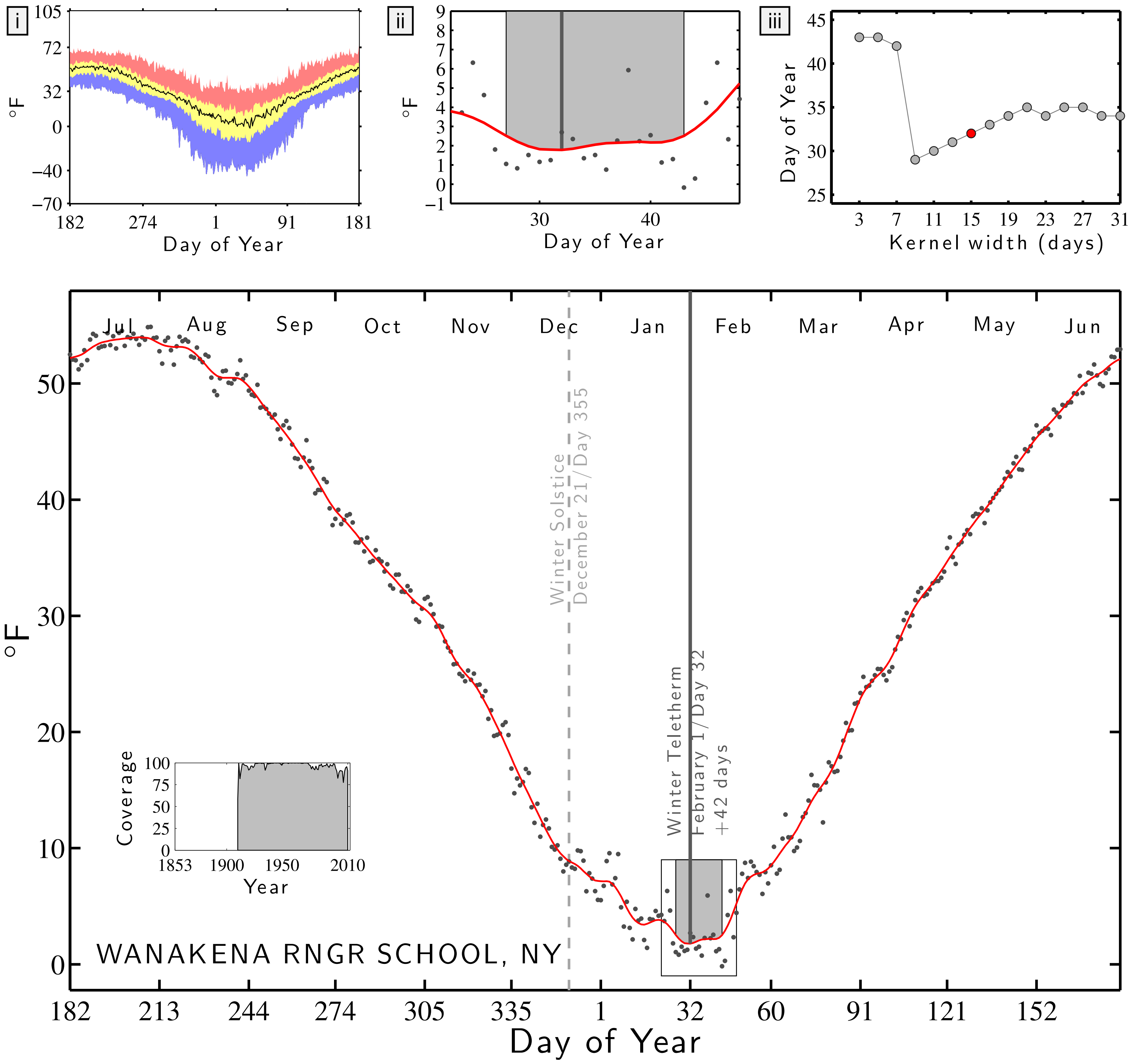

i
ii
iii
°F
Day of Year
Day of Year
Kernel width (days)
Jul
Aug
Sep
Oct
Nov
Dec
Jan
Feb
Mar
Apr
May
Jun
Winter Solstice
December 21/Day 355
Winter Teletherm
February 1/Day 32
+42 days
Coverage
Year
WANAKENA RNGR SCHOOL, NY

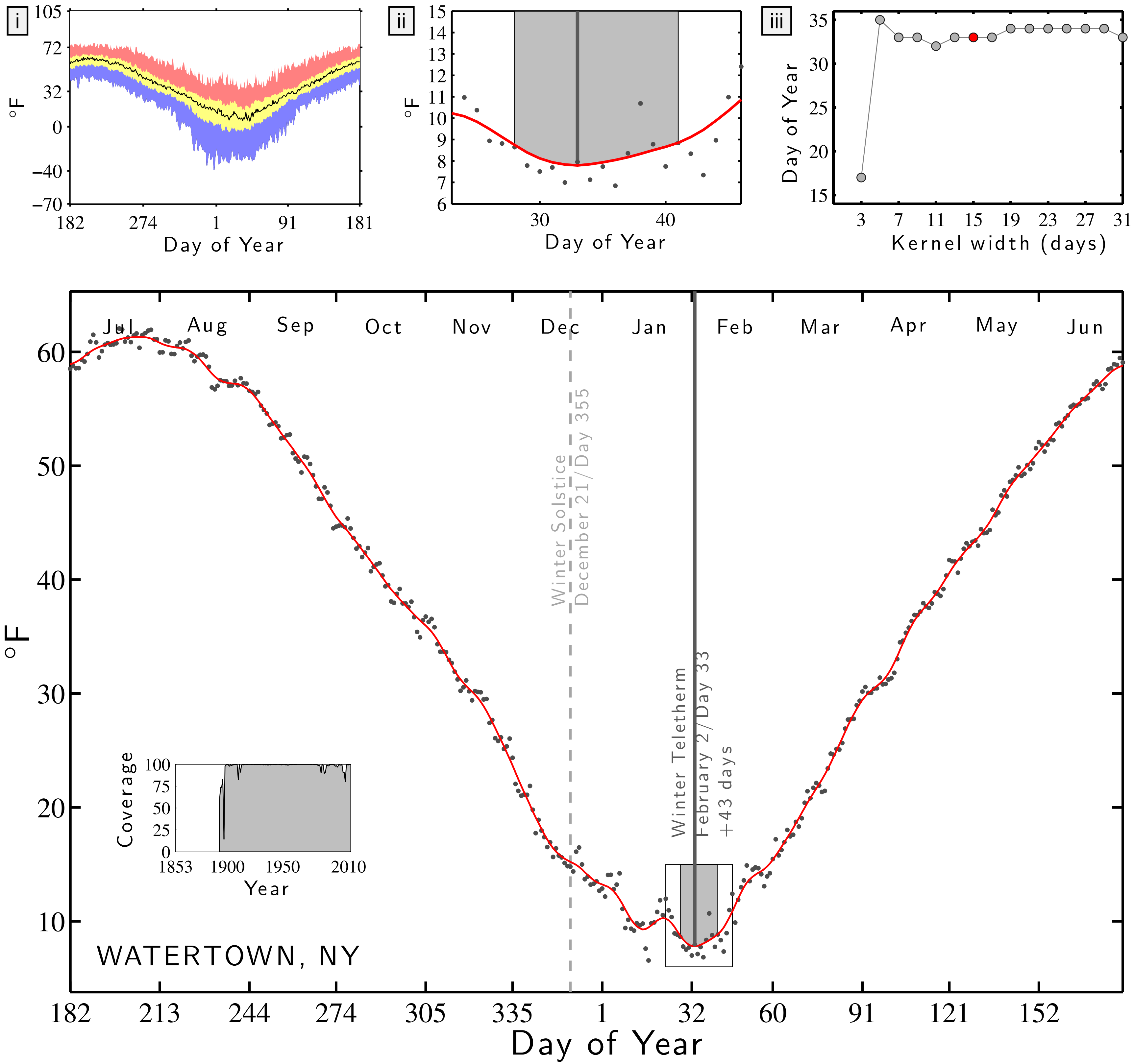

i
°F
105
72
32
0
−40
−70
182
274
1
91
181
Day of Year
ii
°F
15
14
13
12
11
10
9
8
7
6
30
40
Day of Year
iii
Day of Year
35
30
25
20
15
3
7
11
15
19
23
27
31
Kernel width (days)
Jul
Aug
Sep
Oct
Nov
Dec
Jan
Feb
Mar
Apr
May
Jun
Winter Solstice
December 21/Day 355
Winter Teletherm
February 2/Day 33
+43 days
°F
60
50
40
30
20
10
Coverage
100
75
50
25
0
1853
1900
1950
2010
Year
WATERTOWN, NY
182
213
244
274
305
335
1
32
60
91
121
152
Day of Year

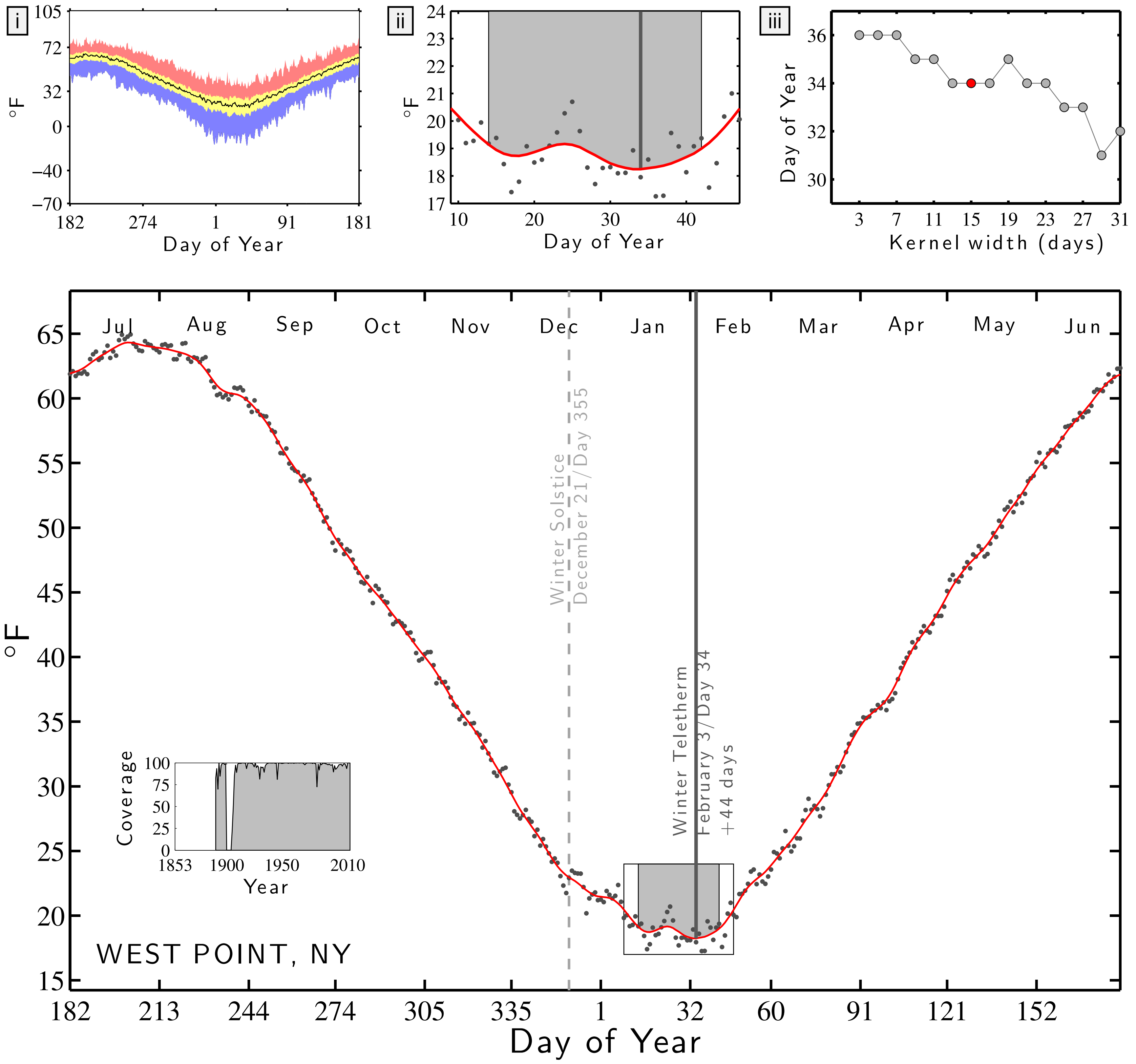

i
ii
iii
°F
Day of Year
Kernel width (days)
Jul
Aug
Sep
Oct
Nov
Dec
Jan
Feb
Mar
Apr
May
Jun
Winter Solstice
December 21/Day 355
Winter Teletherm
February 3/Day 34
+44 days
Coverage
Year
WEST POINT, NY

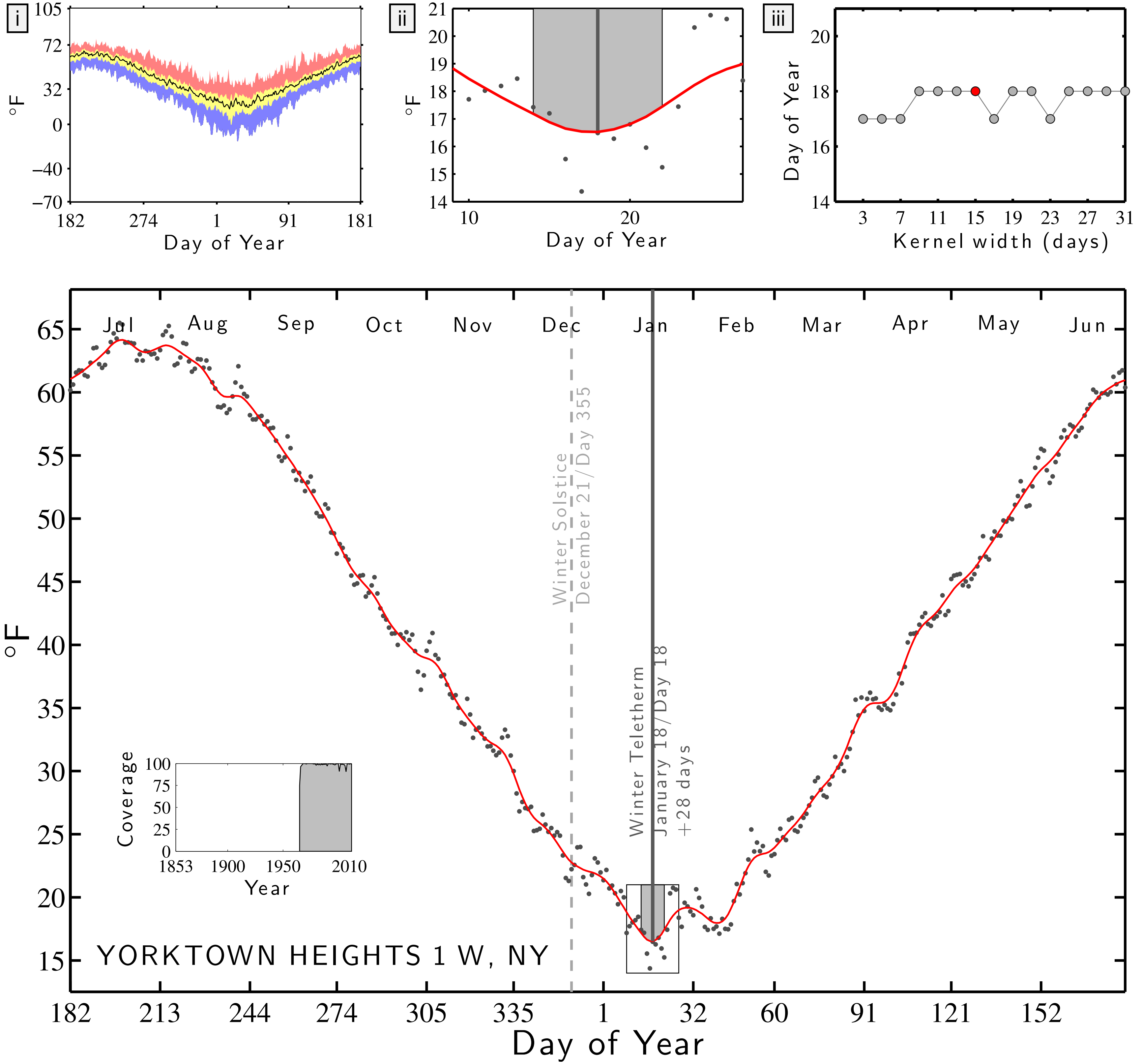

i
ii
iii
°F
Day of Year
Kernel width (days)
Jul
Aug
Sep
Oct
Nov
Dec
Jan
Feb
Mar
Apr
May
Jun
Winter Solstice
December 21/Day 355
Winter Teletherm
January 18/Day 18
+28 days
Coverage
Year
YORKTOWN HEIGHTS 1 W, NY

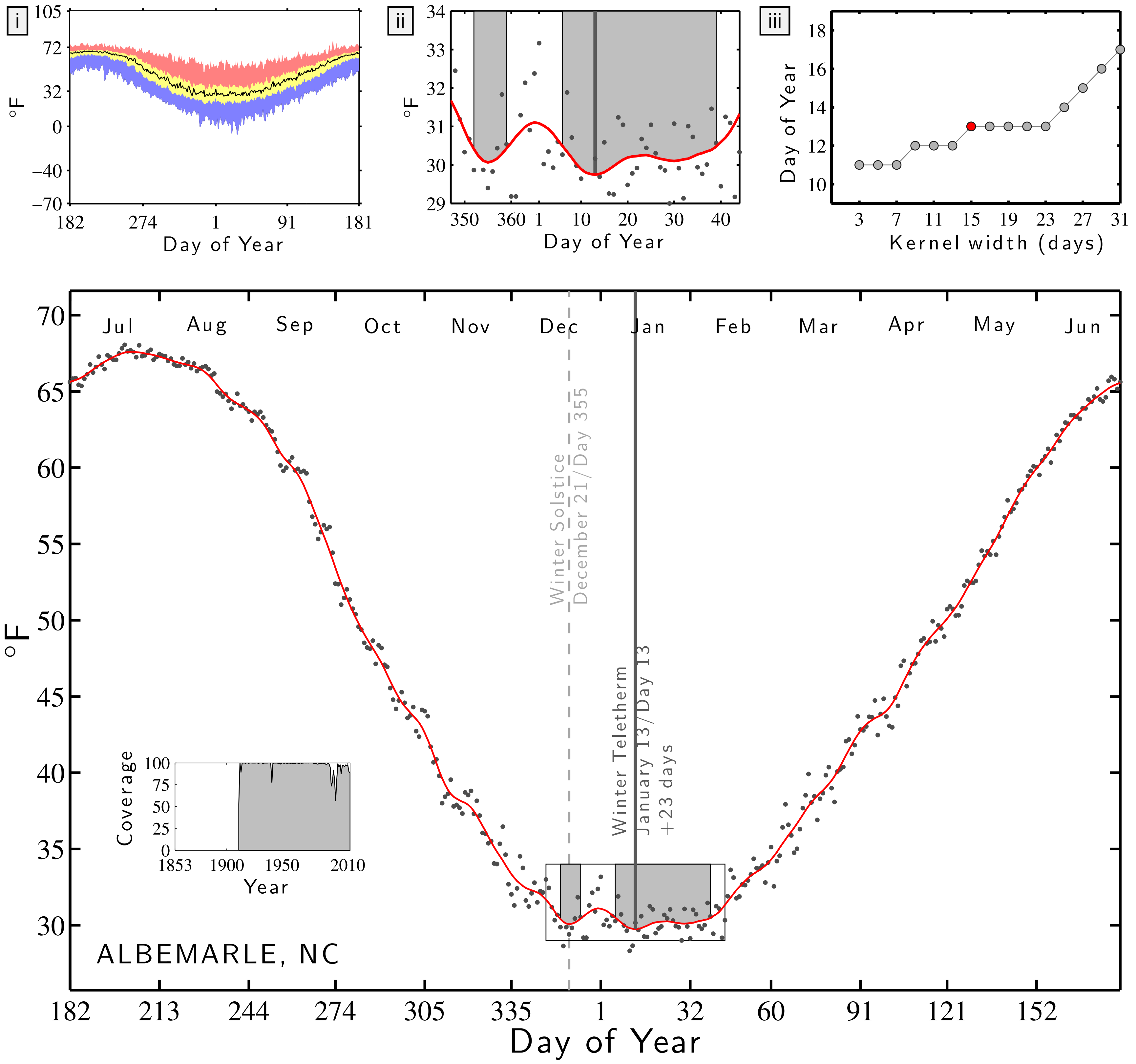

i
°F
Day of Year
ii
°F
Day of Year
iii
Day of Year
Kernel width (days)
Jul
Aug
Sep
Oct
Nov
Dec
Jan
Feb
Mar
Apr
May
Jun
Winter Solstice
December 21/Day 355
Winter Teletherm
January 13/Day 13
+23 days
Coverage
Year
ALBEMARLE, NC
°F
Day of Year

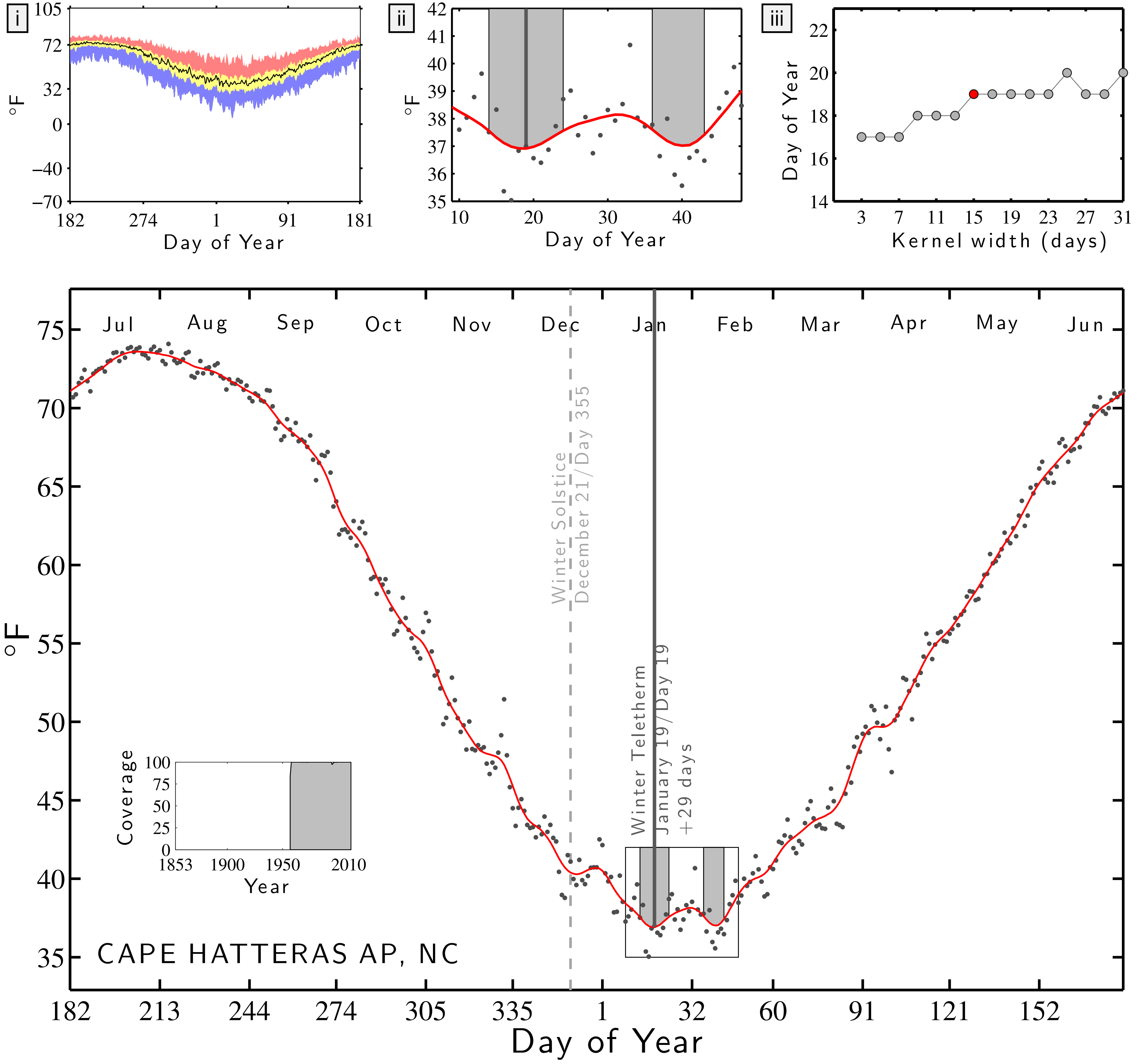

i
Day of Year
°F
ii
Day of Year
°F
iii
Kernel width (days)
Day of Year
Jul
Aug
Sep
Oct
Nov
Dec
Jan
Feb
Mar
Apr
May
Jun
Winter Solstice
December 21/Day 355
Winter Teletherm
January 19/Day 19
+29 days
Coverage
Year
CAPE HATTERAS AP, NC
Day of Year
°F

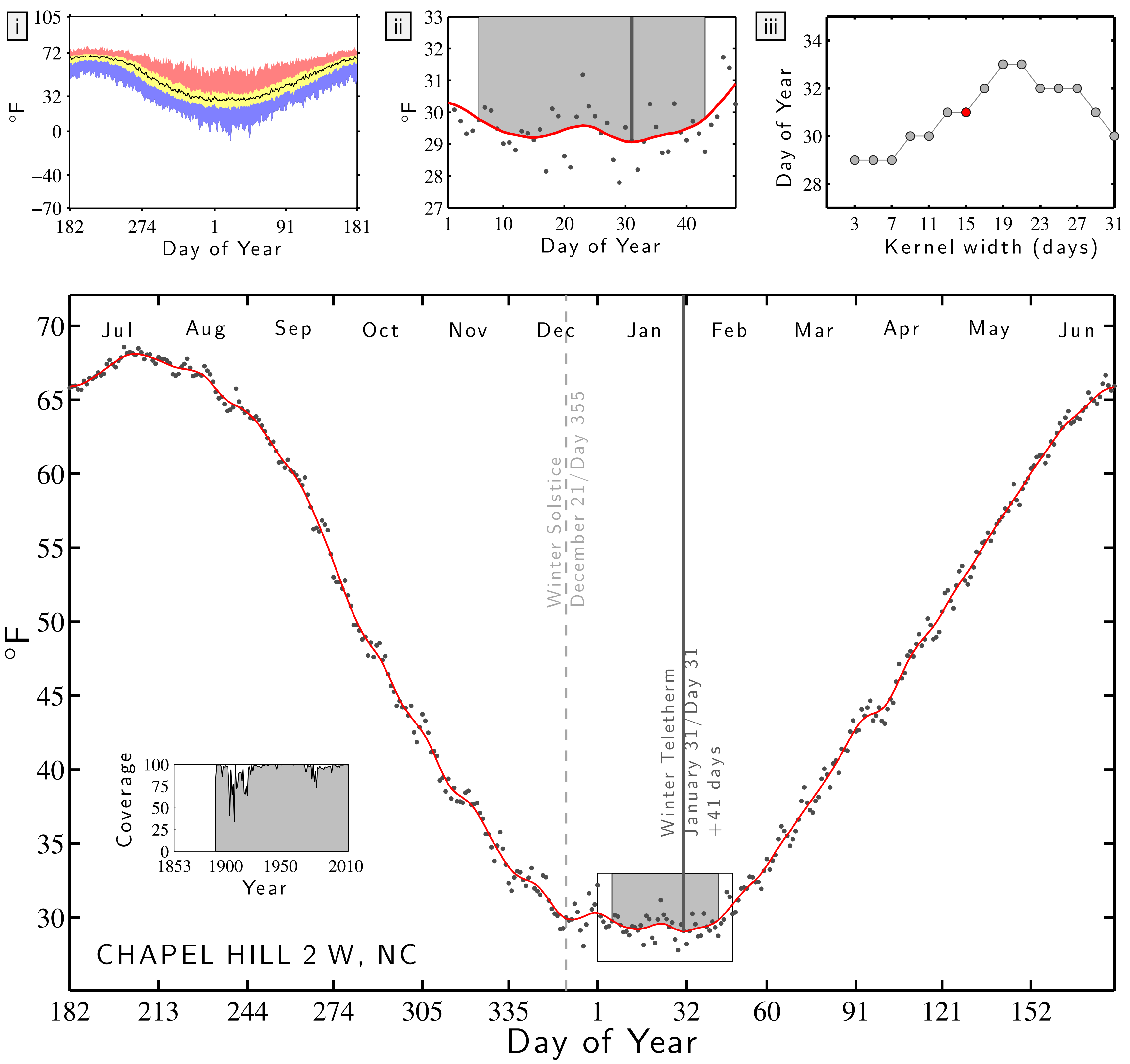

i
°F
Day of Year
ii
°F
Day of Year
iii
Day of Year
Kernel width (days)
Jul Aug Sep Oct Nov Dec Jan Feb Mar Apr May Jun
Winter Solstice
December 21/Day 355
Winter Teletherm
January 31/Day 31
+41 days
Coverage
Year
CHAPEL HILL 2 W, NC
°F
Day of Year

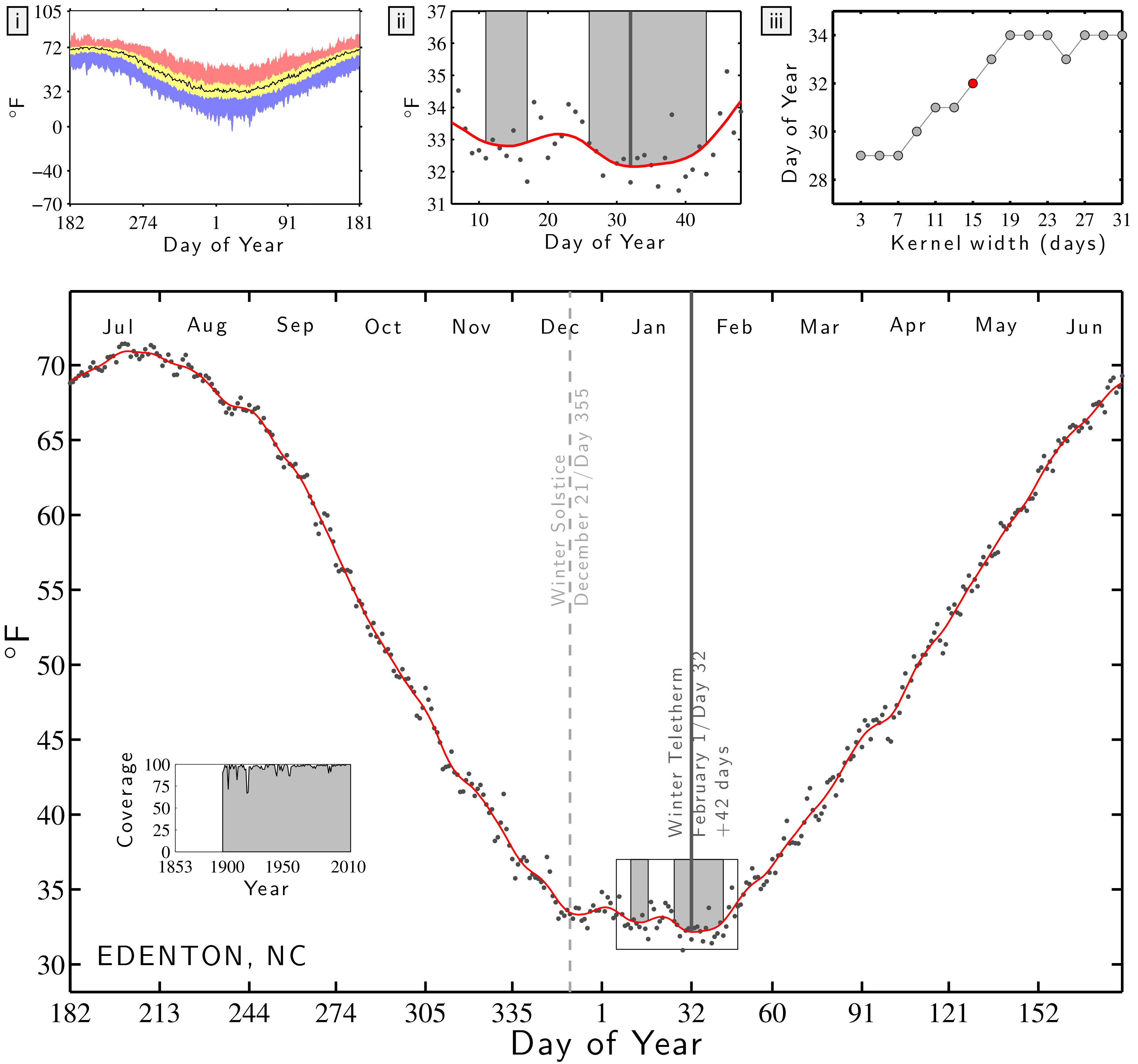

i
ii
iii
°F
Day of Year
Kernel width (days)
Jul
Aug
Sep
Oct
Nov
Dec
Jan
Feb
Mar
Apr
May
Jun
Winter Solstice
December 21/Day 355
Winter Teletherm
February 1/Day 32
+42 days
Coverage
Year
EDENTON, NC

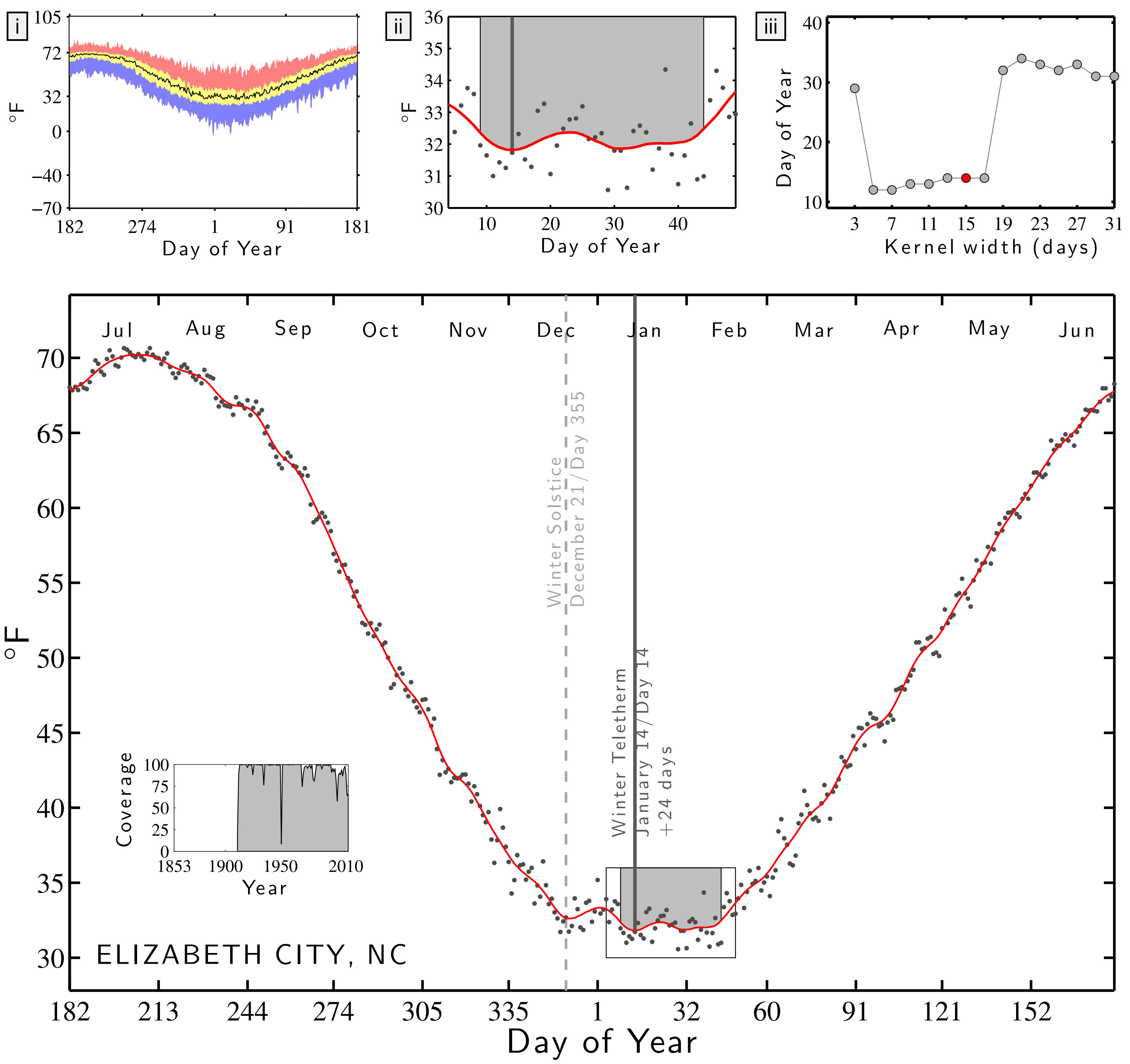

i
ii
iii
°F
Day of Year
Kernel width (days)
Jul
Aug
Sep
Oct
Nov
Dec
Jan
Feb
Mar
Apr
May
Jun
Winter Solstice
December 21/Day 355
Winter Teletherm
January 14/Day 14
+24 days
Coverage
Year
ELIZABETH CITY, NC

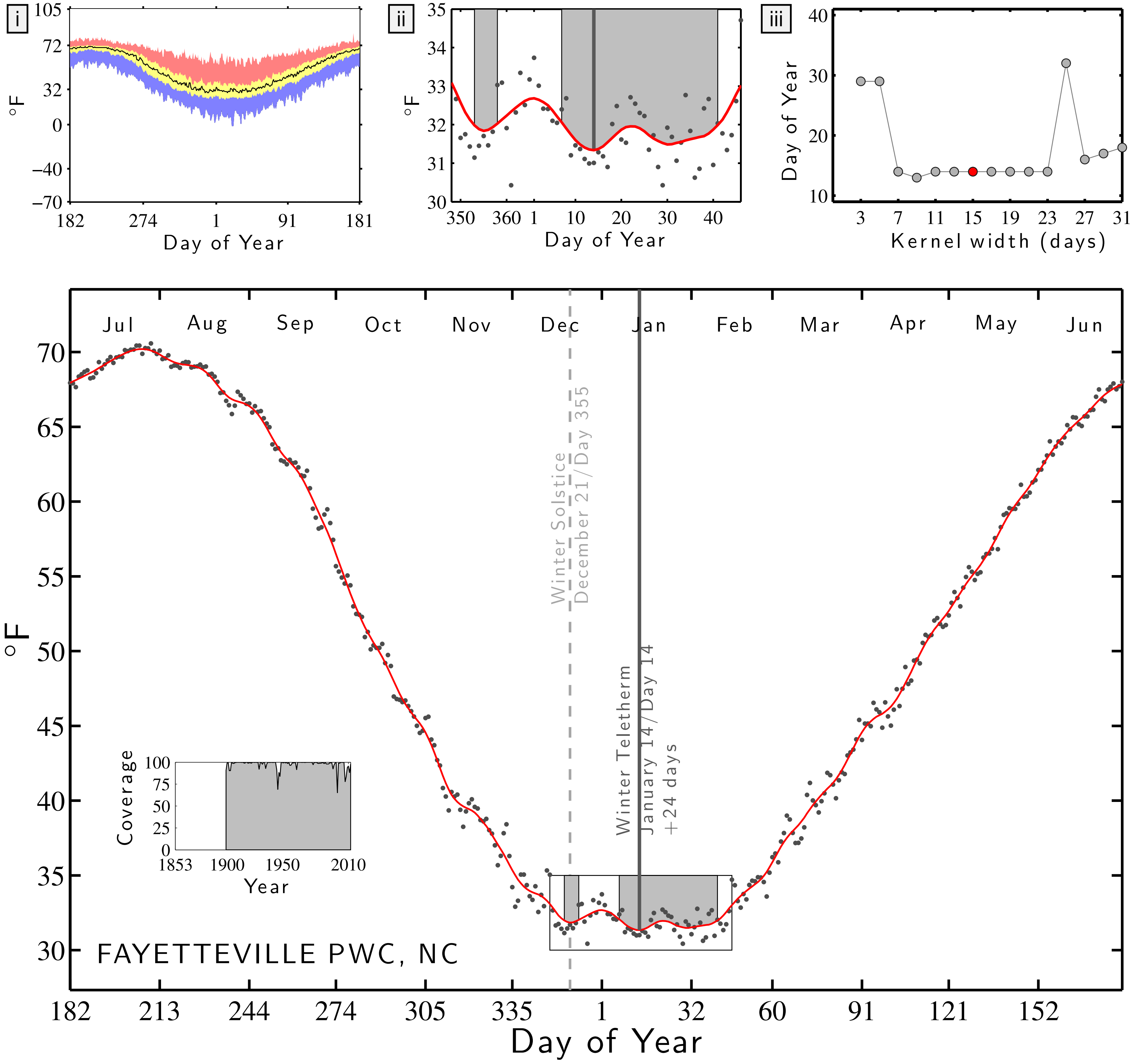

i
°F
Day of Year
ii
°F
Day of Year
iii
Day of Year
Kernel width (days)
Jul
Aug
Sep
Oct
Nov
Dec
Jan
Feb
Mar
Apr
May
Jun
Winter Solstice
December 21/Day 355
Winter Teletherm
January 14/Day 14
+24 days
Coverage
Year
FAYETTEVILLE PWC, NC
°F
Day of Year

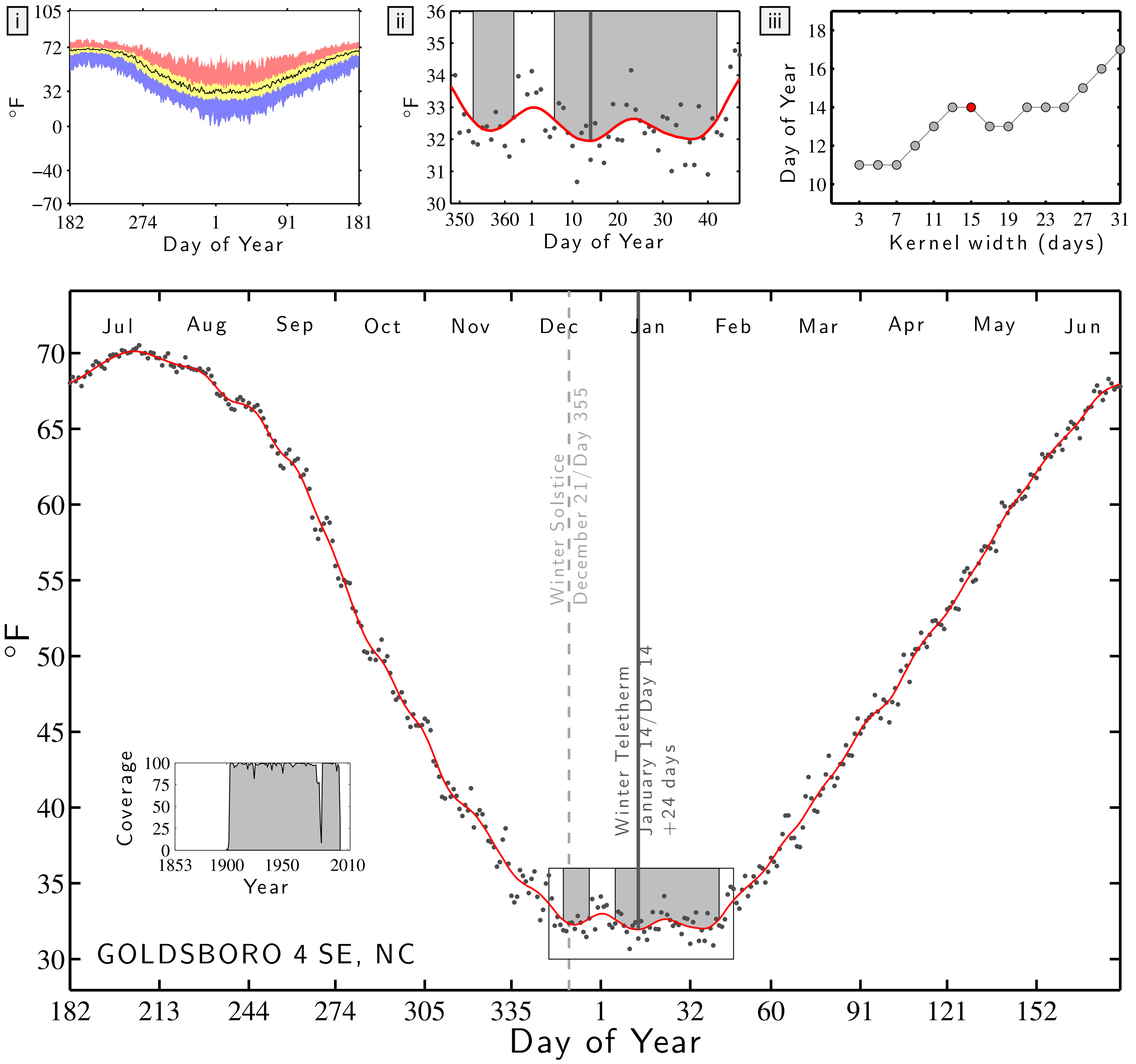

i
°F
Day of Year
ii
°F
Day of Year
iii
Day of Year
Kernel width (days)
Jul
Aug
Sep
Oct
Nov
Dec
Jan
Feb
Mar
Apr
May
Jun
Winter Solstice
December 21/Day 355
Winter Teletherm
January 14/Day 14
+24 days
Coverage
Year
GOLDSBORO 4 SE, NC
°F
Day of Year

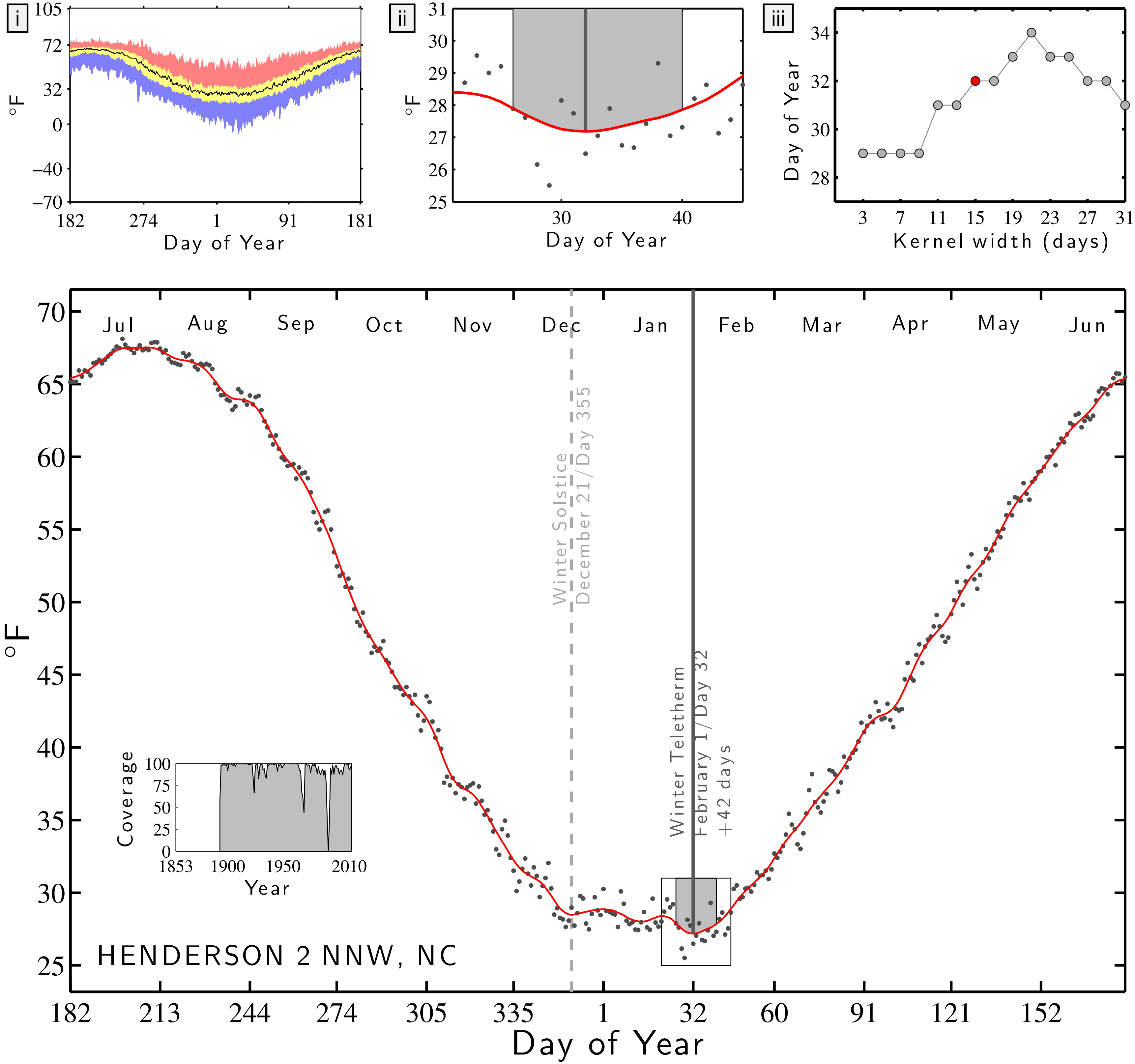

i
°F
Day of Year
ii
°F
Day of Year
iii
Day of Year
Kernel width (days)
Jul
Aug
Sep
Oct
Nov
Dec
Jan
Feb
Mar
Apr
May
Jun
Winter Solstice
December 21/Day 355
Winter Teletherm
February 1/Day 32
+42 days
Coverage
Year
HENDERSON 2 NNW, NC
°F
Day of Year

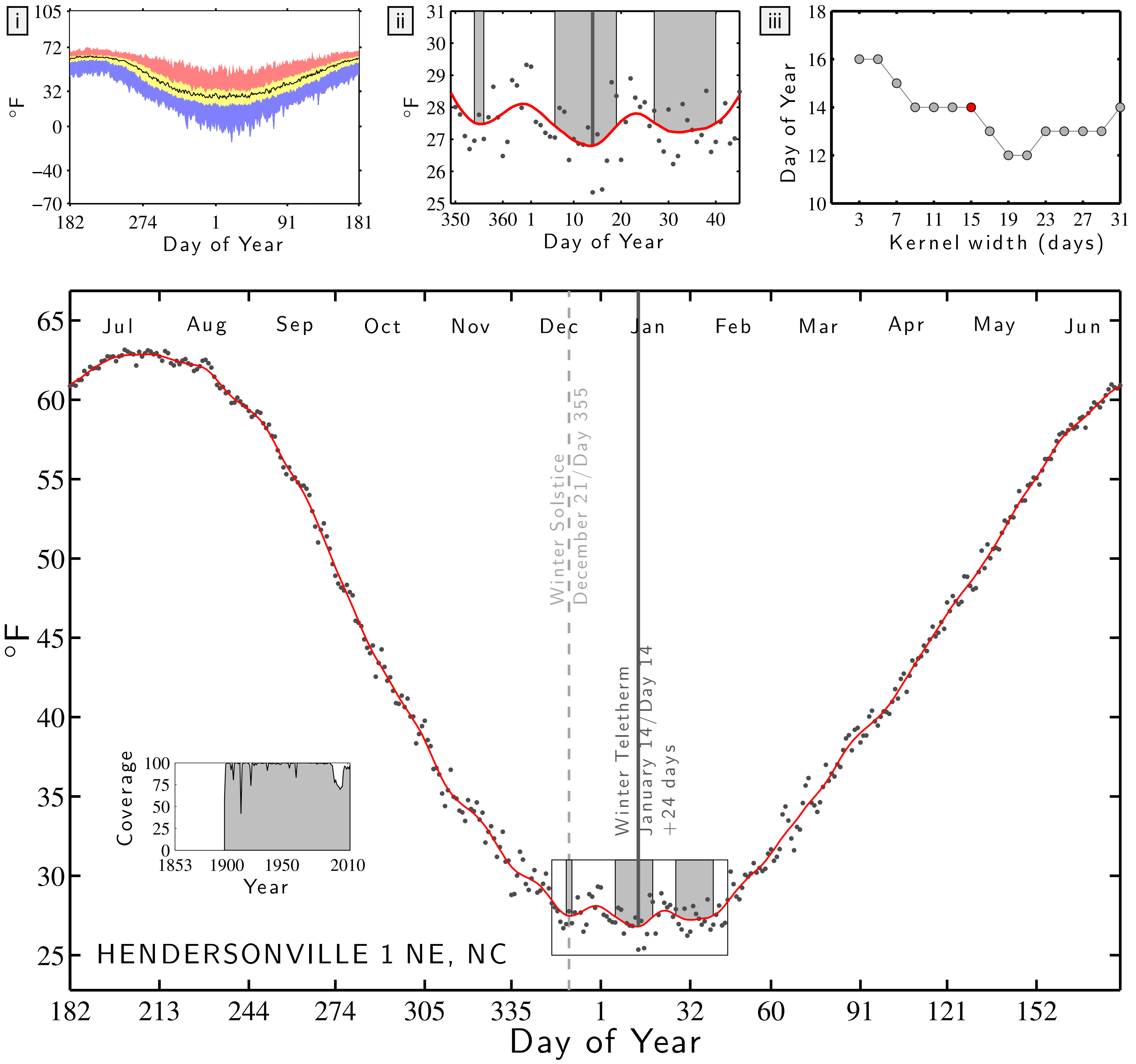

i
°F
Day of Year
ii
Day of Year
iii
Day of Year
Kernel width (days)
Jul
Aug
Sep
Oct
Nov
Dec
Jan
Feb
Mar
Apr
May
Jun
Winter Solstice
December 21/Day 355
Winter Teletherm
January 14/Day 14
+24 days
Coverage
Year
HENDERSONVILLE 1 NE, NC
°F
Day of Year

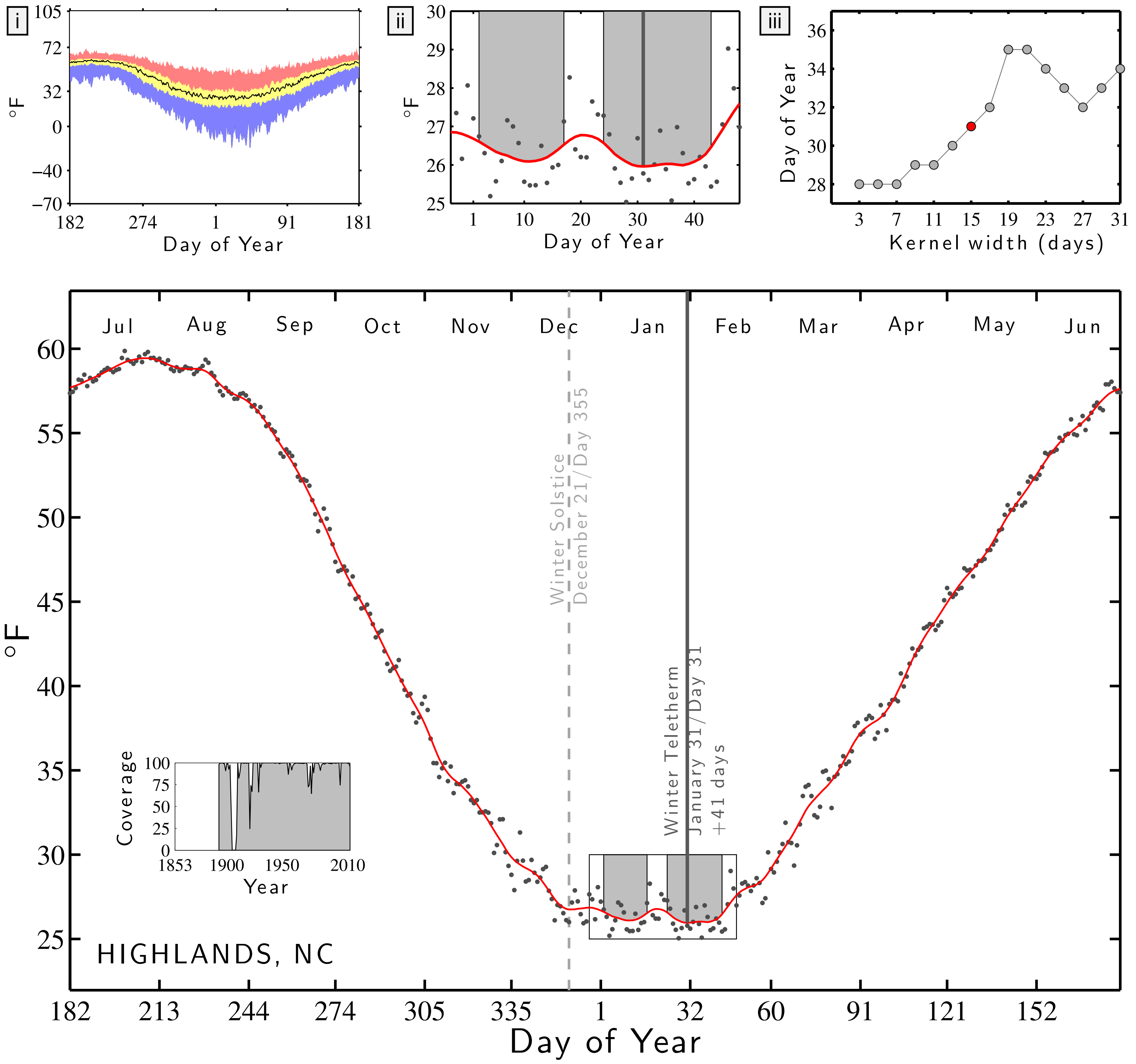

i
ii
iii
°F
Day of Year
Kernel width (days)
Jul
Aug
Sep
Oct
Nov
Dec
Jan
Feb
Mar
Apr
May
Jun
Winter Solstice
December 21/Day 355
Winter Teletherm
January 31/Day 31
+41 days
Coverage
Year
HIGHLANDS, NC

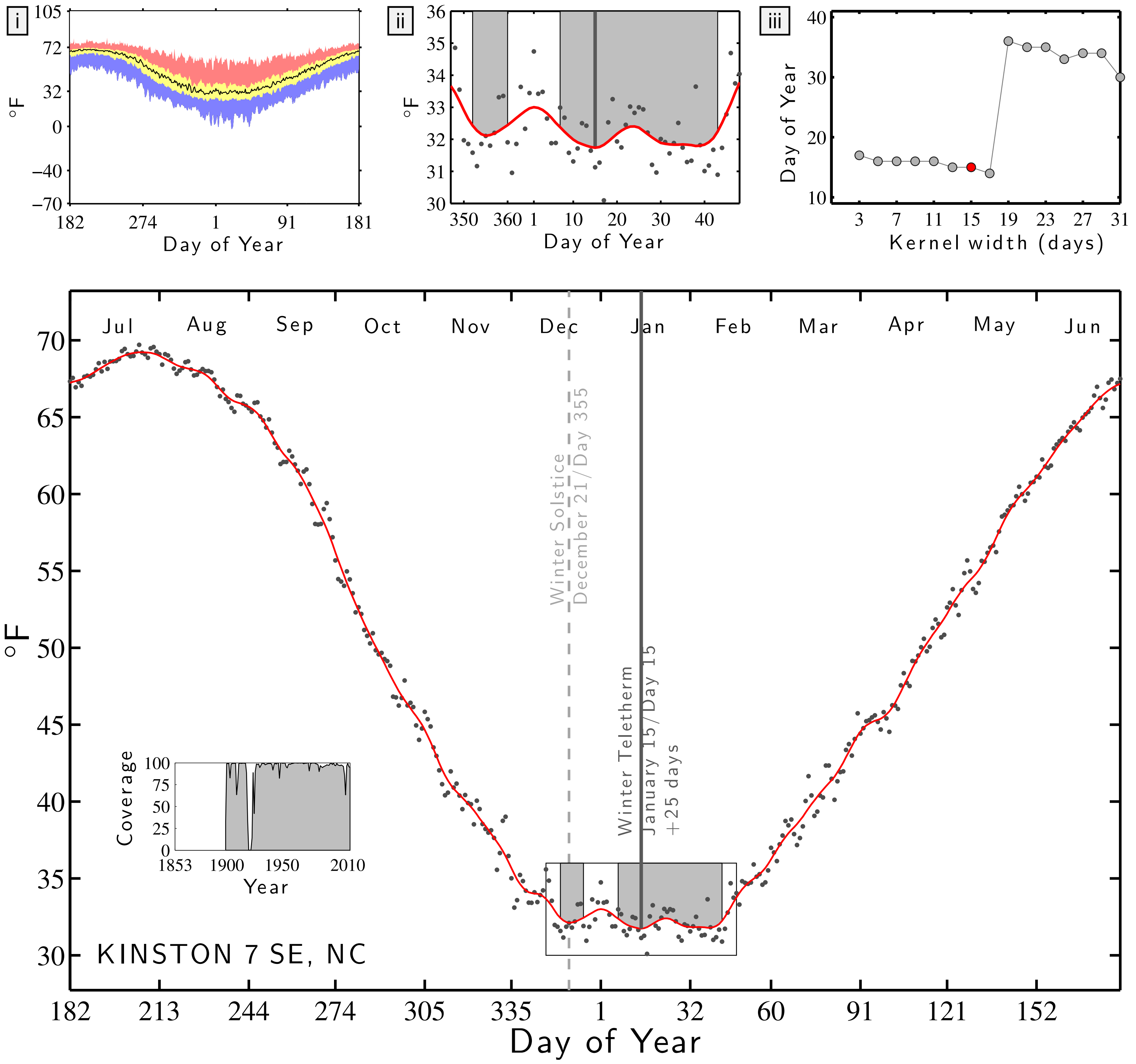

i
105
72
32
0
−40
−70
°F
182
274
1
91
181
Day of Year
ii
36
35
34
33
32
31
30
°F
350
360
1
10
20
30
40
Day of Year
iii
40
30
20
10
Day of Year
3
7
11
15
19
23
27
31
Kernel width (days)
Jul
Aug
Sep
Oct
Nov
Dec
Jan
Feb
Mar
Apr
May
Jun
70
65
60
55
50
45
40
35
30
°F
Winter Solstice
December 21/Day 355
Winter Teletherm
January 15/Day 15
+25 days
Coverage
100
75
50
25
0
1853
1900
1950
2010
Year
KINSTON 7 SE, NC
182
213
244
274
305
335
1
32
60
91
121
152
Day of Year

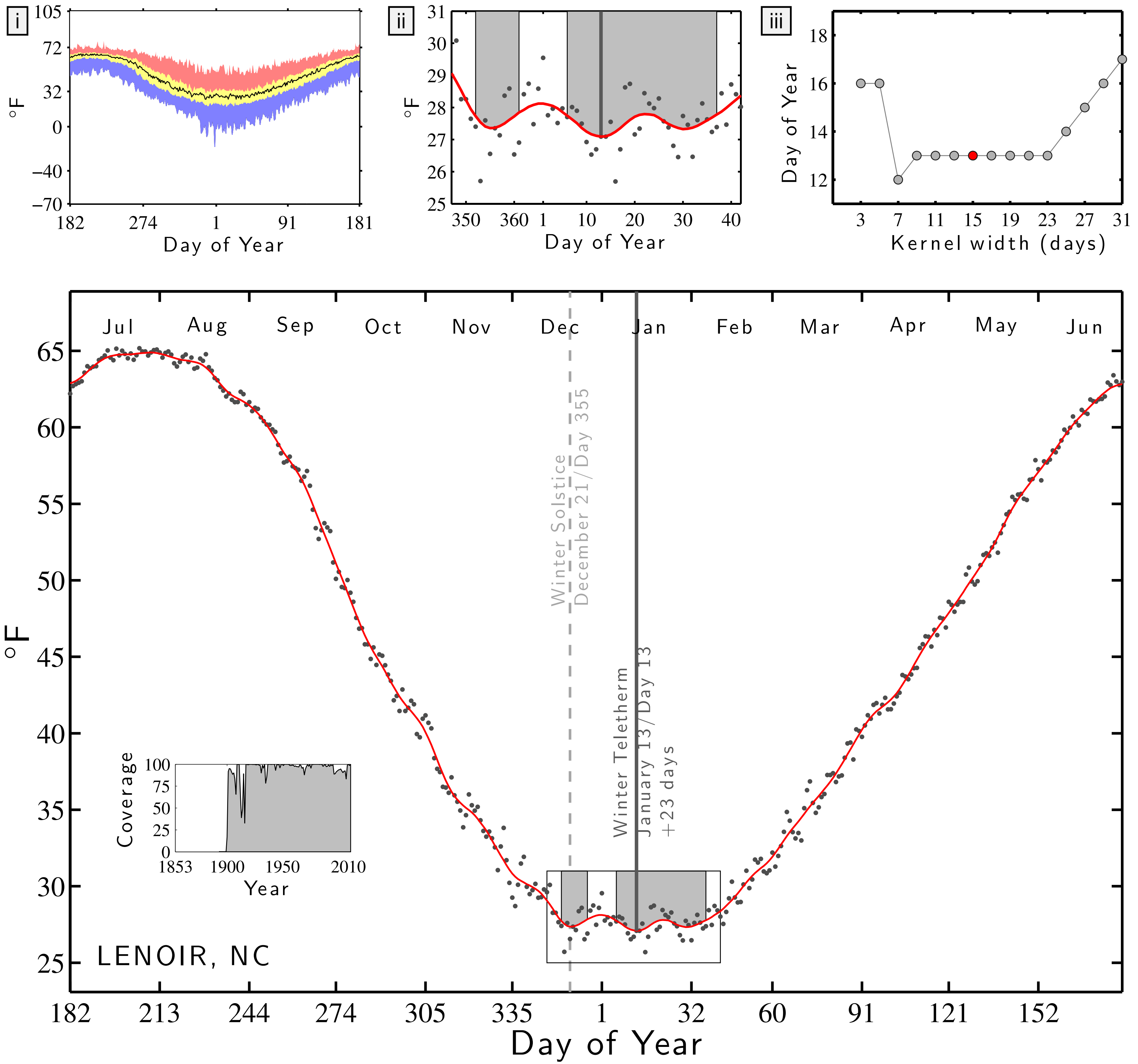

i
°F
Day of Year
ii
°F
Day of Year
iii
Day of Year
Kernel width (days)
Jul
Aug
Sep
Oct
Nov
Dec
Jan
Feb
Mar
Apr
May
Jun
Winter Solstice
December 21/Day 355
Winter Teletherm
January 13/Day 13
+23 days
Coverage
Year
LENOIR, NC
°F
Day of Year

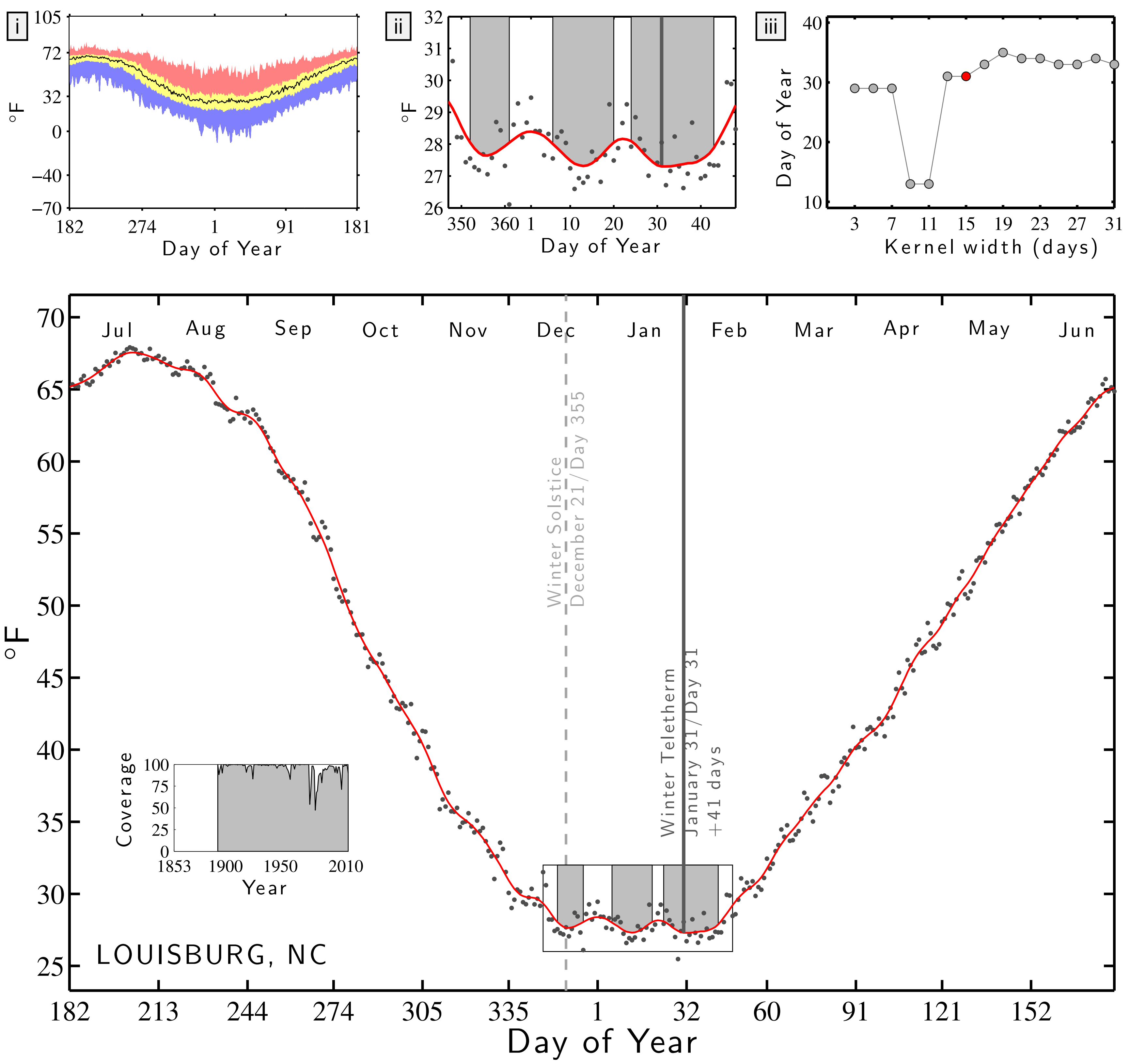
i
ii
iii
°F
Day of Year
Kernel width (days)
Jul
Aug
Sep
Oct
Nov
Dec
Jan
Feb
Mar
Apr
May
Jun
Winter Solstice
December 21/Day 355
Winter Teletherm
January 31/Day 31
+41 days
Coverage
Year
LOUISBURG, NC

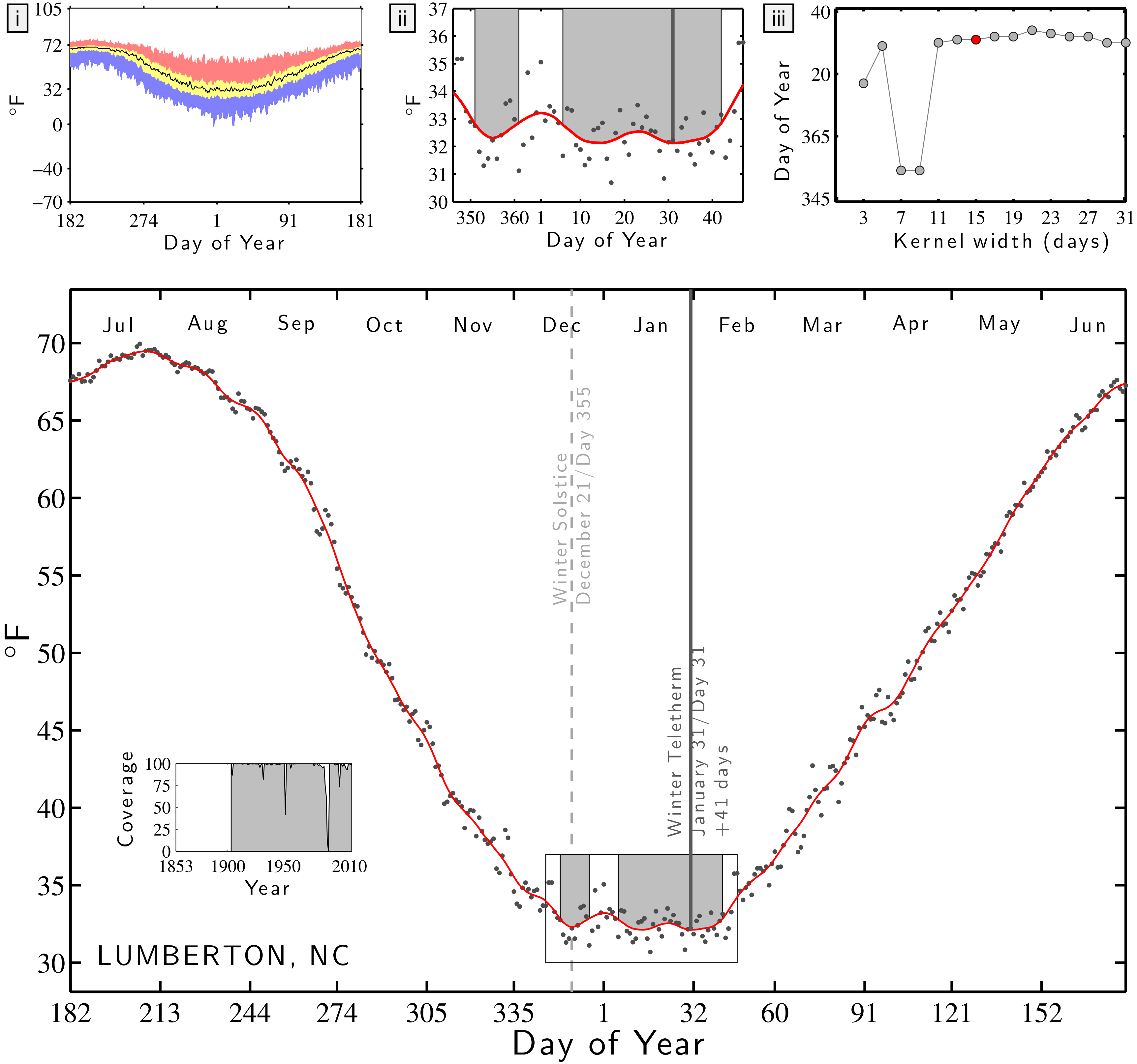
i
°F
Day of Year
ii
°F
Day of Year
iii
Day of Year
Kernel width (days)
Jul
Aug
Sep
Oct
Nov
Dec
Jan
Feb
Mar
Apr
May
Jun
Winter Solstice
December 21/Day 355
Winter Teletherm
January 31/Day 31
+41 days
Coverage
Year
LUMBERTON, NC
°F
Day of Year

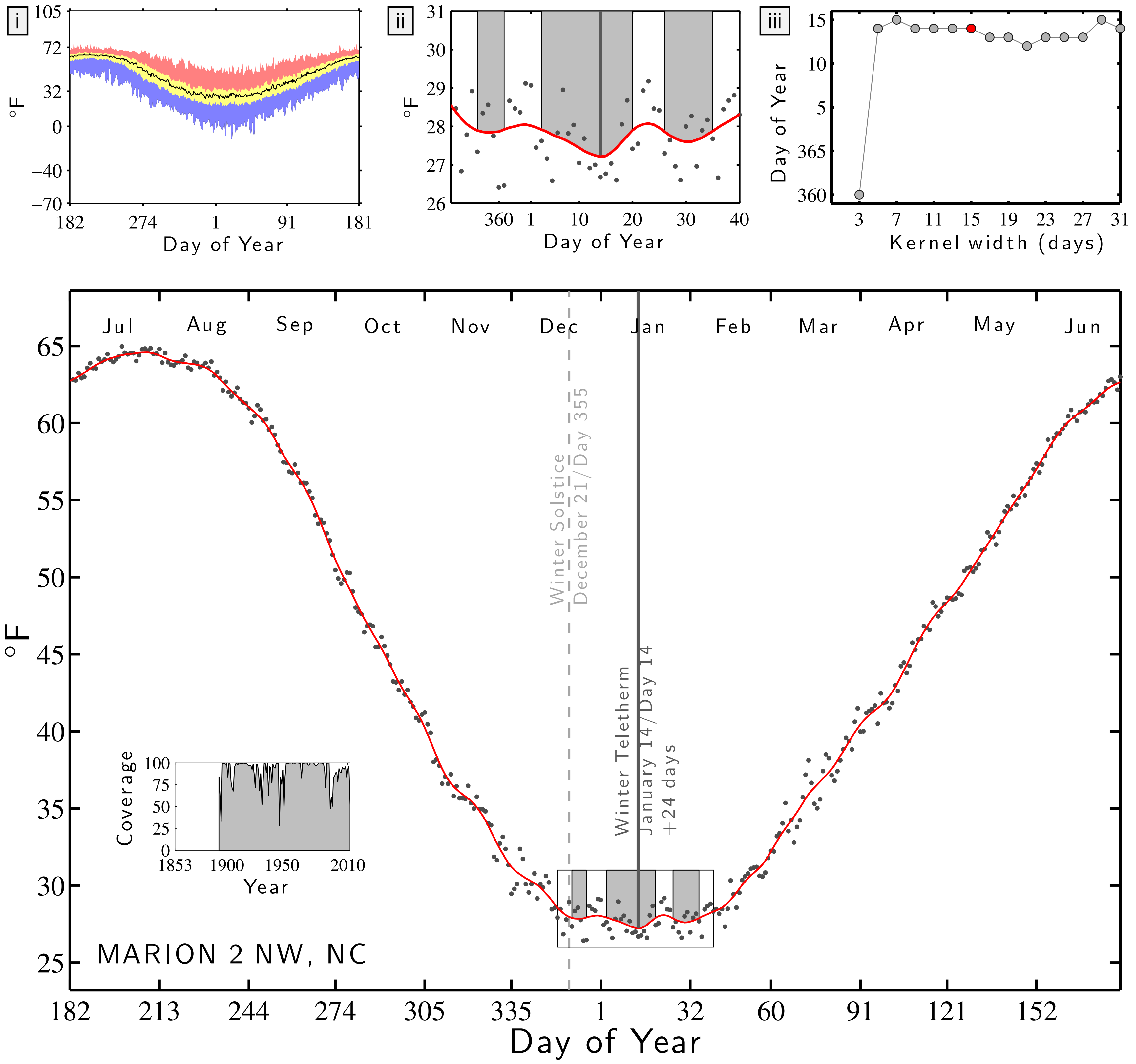

i
°F
Day of Year
ii
°F
Day of Year
iii
Day of Year
Kernel width (days)
Jul Aug Sep Oct Nov Dec Jan Feb Mar Apr May Jun
Winter Solstice
December 21/Day 355
Winter Teletherm
January 14/Day 14
+24 days
Coverage
Year
MARION 2 NW, NC
°F
Day of Year

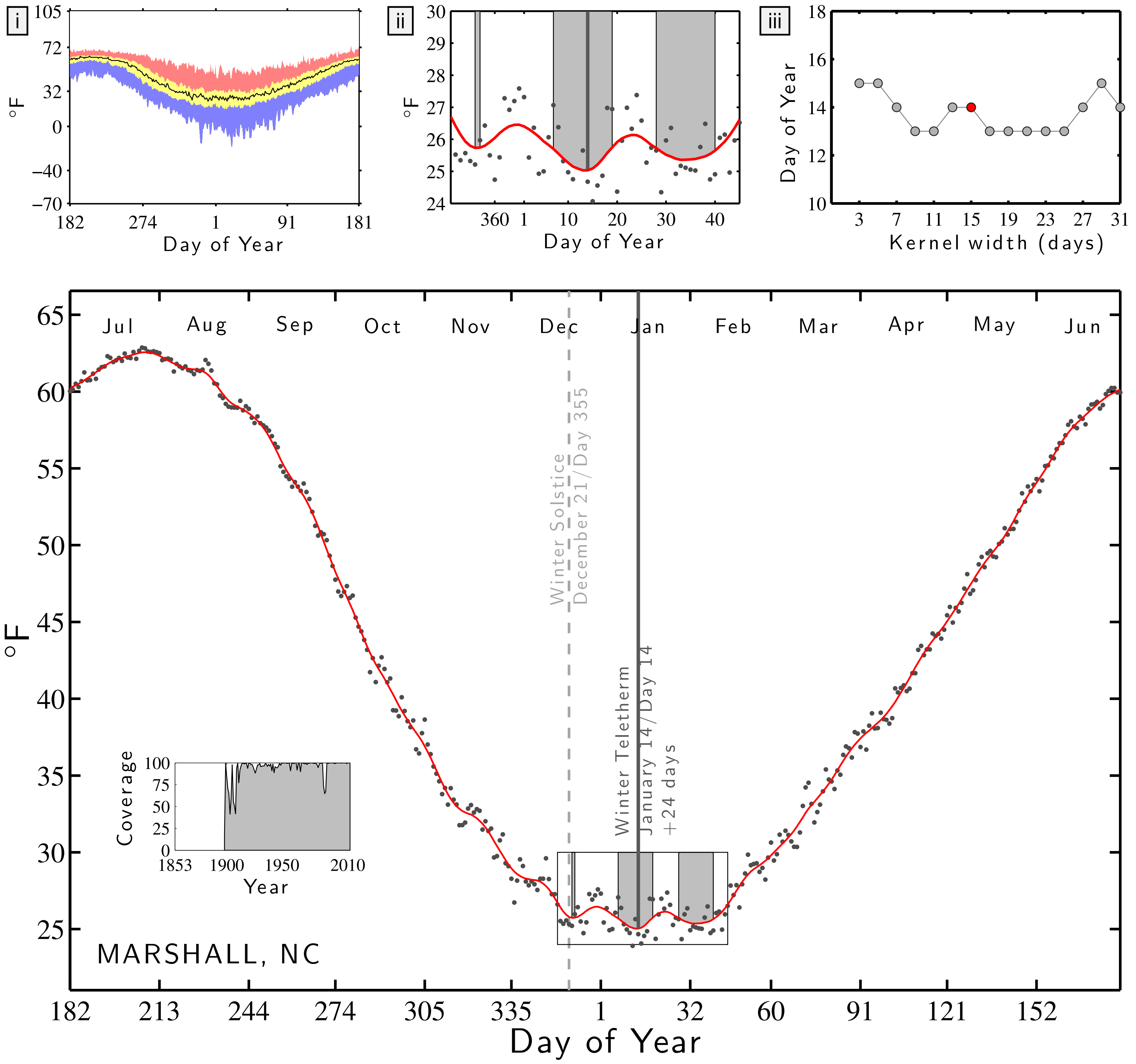

i
Day of Year
°F
ii
Day of Year
°F
iii
Kernel width (days)
Day of Year
Jul
Aug
Sep
Oct
Nov
Dec
Jan
Feb
Mar
Apr
May
Jun
Winter Solstice
December 21/Day 355
Winter Teletherm
January 14/Day 14
+24 days
Coverage
Year
MARSHALL, NC
°F
Day of Year

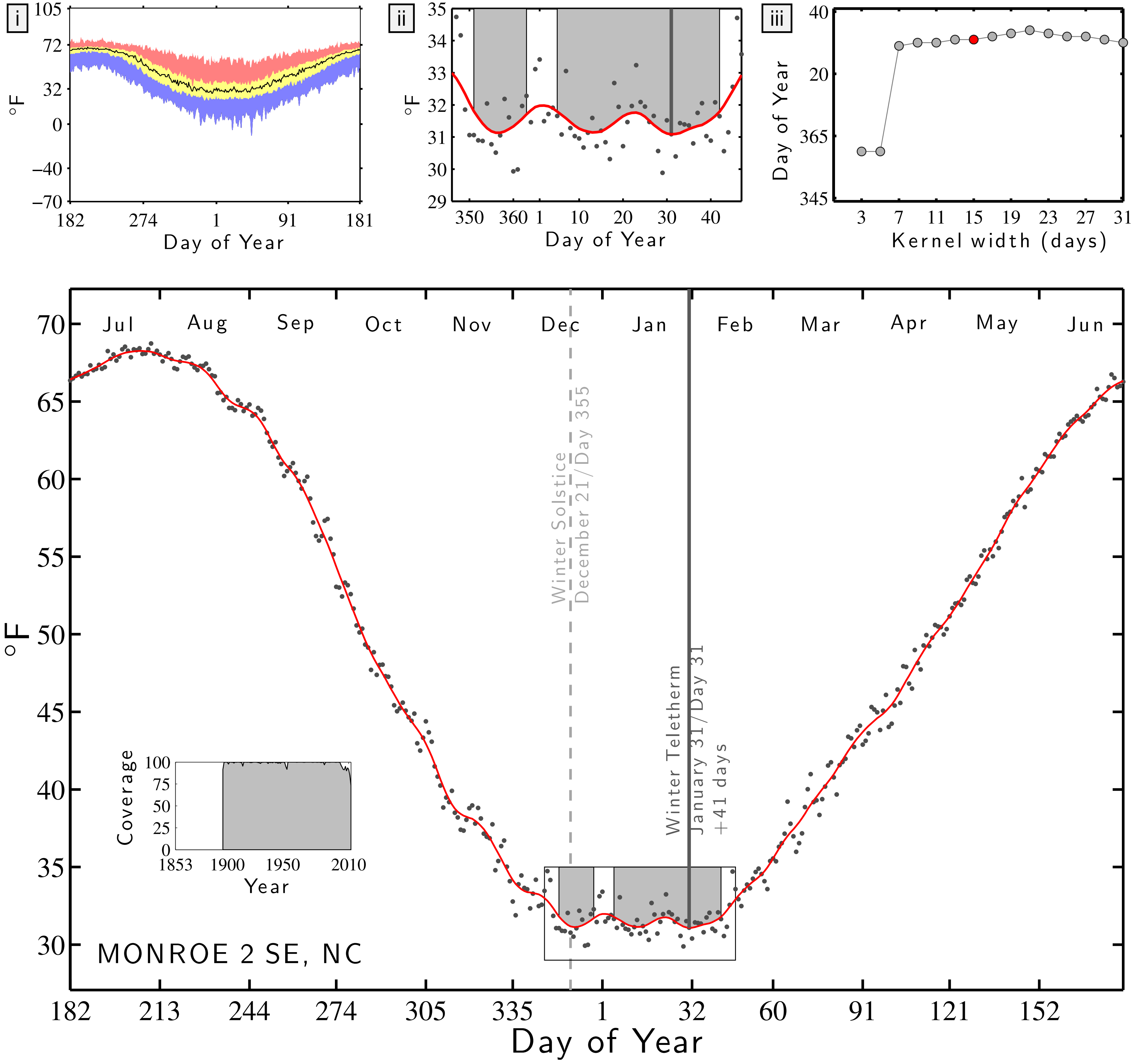
i
°F
Day of Year
ii
°F
Day of Year
iii
Day of Year
Kernel width (days)
Jul
Aug
Sep
Oct
Nov
Dec
Jan
Feb
Mar
Apr
May
Jun
Winter Solstice
December 21/Day 355
Winter Teletherm
January 31/Day 31
+41 days
Coverage
Year
MONROE 2 SE, NC
°F
Day of Year

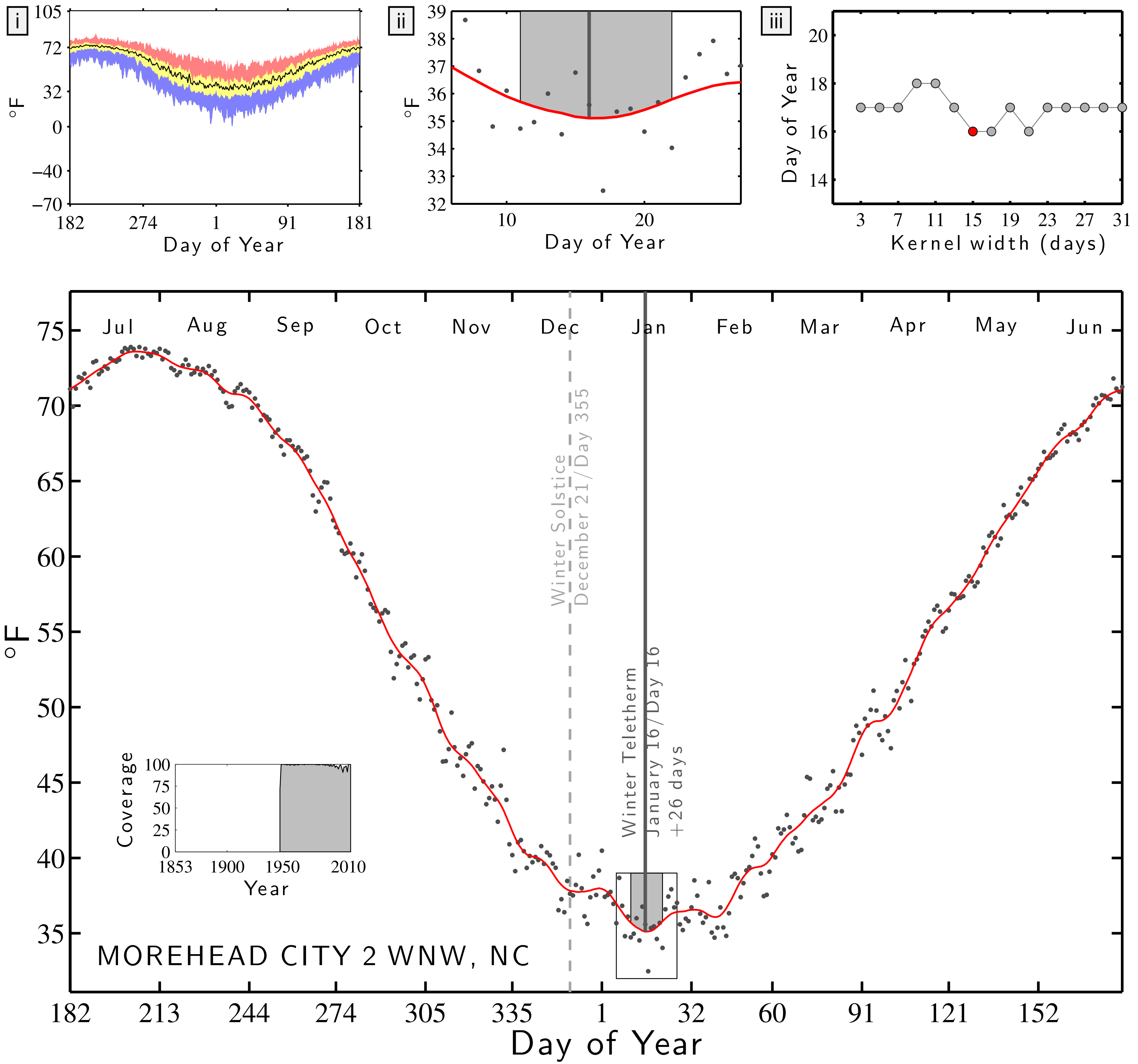

i
105
72
32
0
−40
−70
°F
182
274
1
91
181
Day of Year
ii
39
38
37
36
35
34
33
32
°F
10
20
Day of Year
iii
20
18
16
14
Day of Year
3
7
11
15
19
23
27
31
Kernel width (days)
Jul
Aug
Sep
Oct
Nov
Dec
Jan
Feb
Mar
Apr
May
Jun
75
70
65
60
55
50
45
40
35
°F
Winter Solstice
December 21/Day 355
Winter Teletherm
January 16/Day 16
+26 days
Coverage
100
75
50
25
0
1853
1900
1950
2010
Year
MOREHEAD CITY 2 WNW, NC
182
213
244
274
305
335
1
32
60
91
121
152
Day of Year

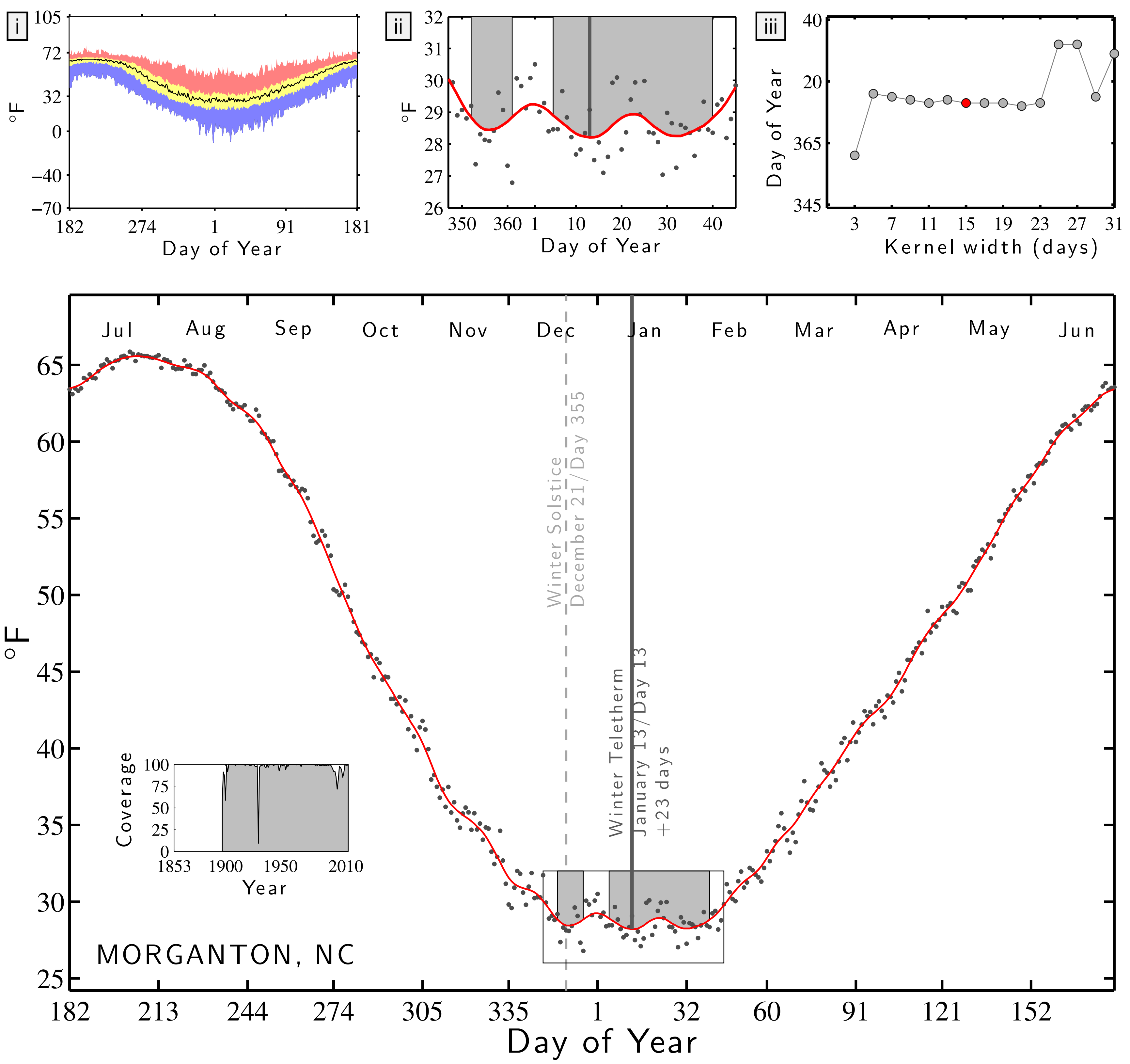
i
ii
iii
Day of Year
Kernel width (days)
Jul
Aug
Sep
Oct
Nov
Dec
Jan
Feb
Mar
Apr
May
Jun
Winter Solstice
December 21/Day 355
Winter Teletherm
January 13/Day 13
+23 days
Coverage
Year
MORGANTON, NC
°F

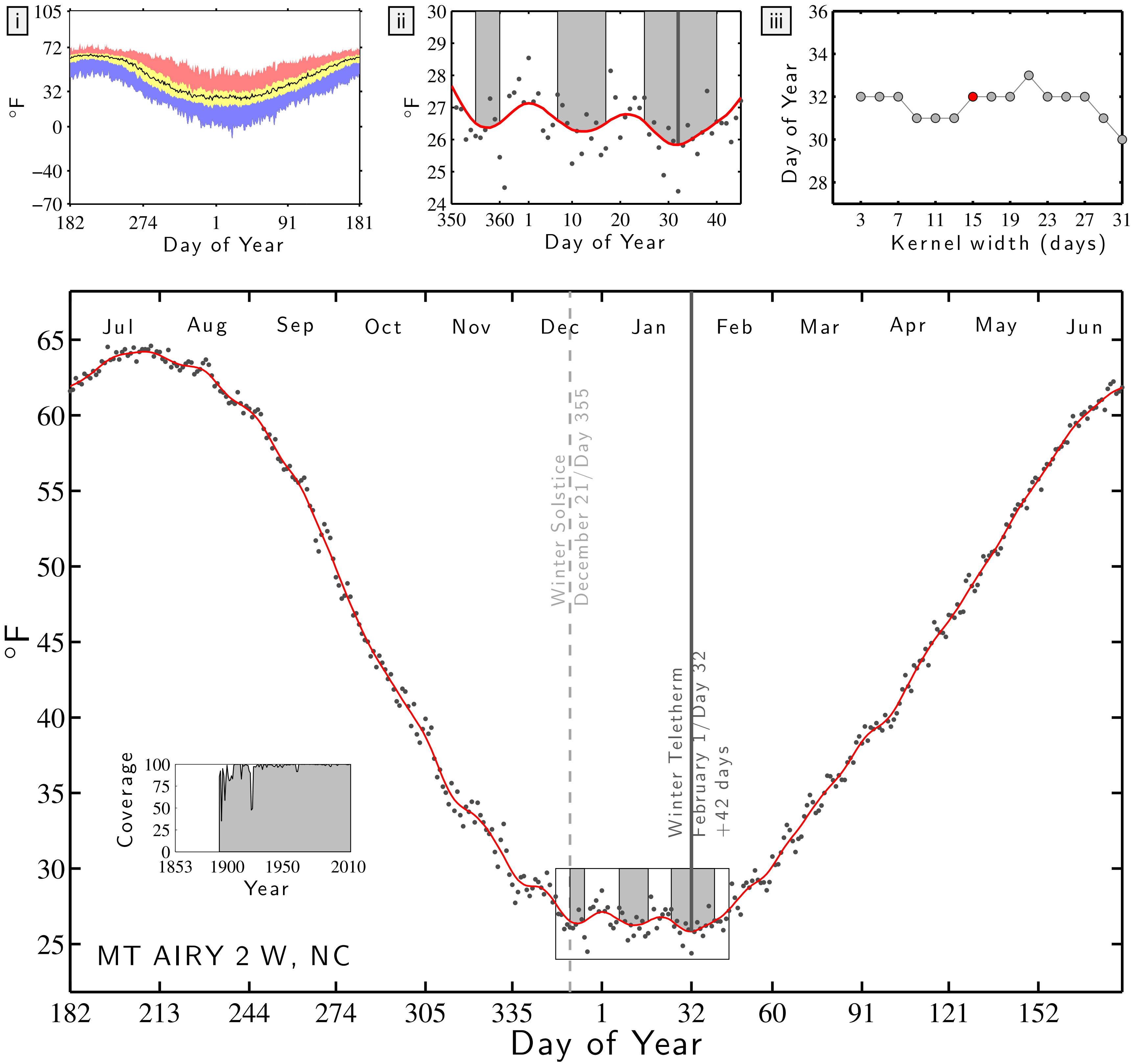

i
°F
Day of Year
ii
°F
Day of Year
iii
Day of Year
Kernel width (days)
Jul
Aug
Sep
Oct
Nov
Dec
Jan
Feb
Mar
Apr
May
Jun
Winter Solstice
December 21/Day 355
Winter Teletherm
February 1/Day 32
+42 days
Coverage
Year
MT AIRY 2 W, NC
°F
Day of Year

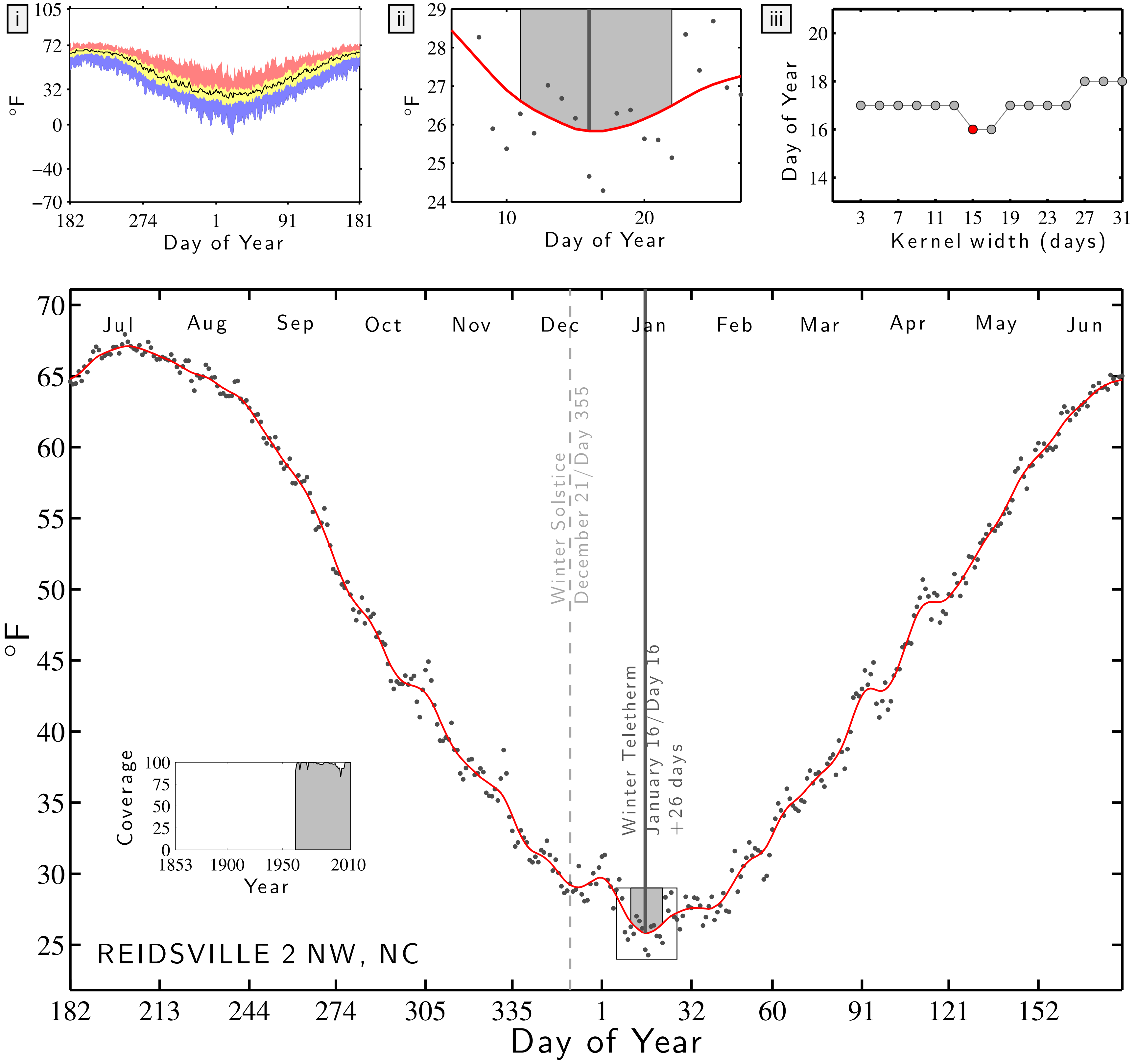

i
°F
Day of Year
ii
Day of Year
iii
Day of Year
Kernel width (days)
Jul
Aug
Sep
Oct
Nov
Dec
Jan
Feb
Mar
Apr
May
Jun
Winter Solstice
December 21/Day 355
Winter Teletherm
January 16/Day 16
+26 days
Coverage
Year
REIDSVILLE 2 NW, NC

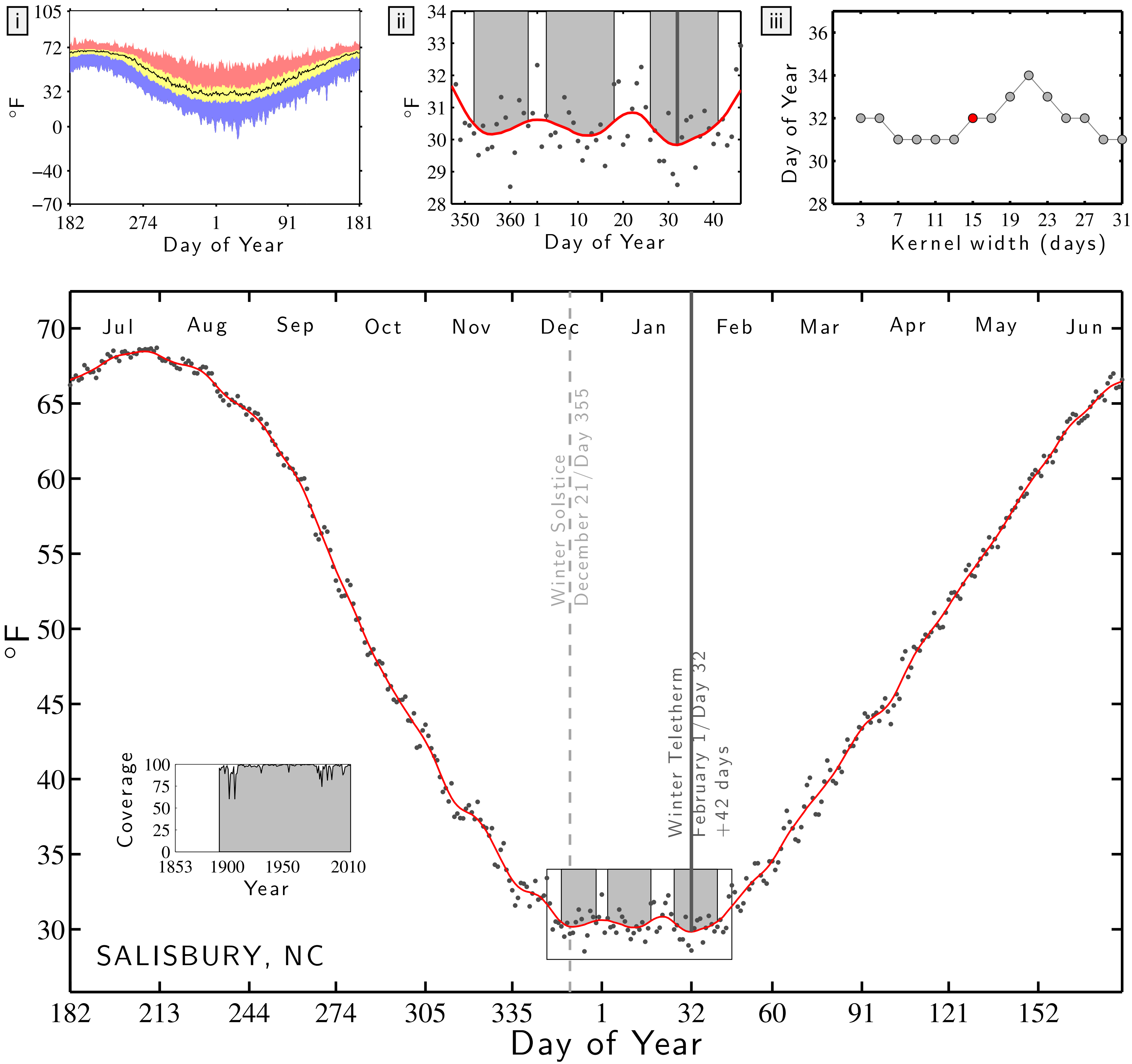

i
105
72
32
0
−40
−70
°F
182
274
1
91
181
Day of Year
ii
34
33
32
31
30
29
28
°F
350
360
1
10
20
30
40
Day of Year
iii
36
34
32
30
28
Day of Year
3
7
11
15
19
23
27
31
Kernel width (days)
Jul
Aug
Sep
Oct
Nov
Dec
Jan
Feb
Mar
Apr
May
Jun
70
65
60
55
50
45
40
35
30
°F
Winter Solstice
December 21/Day 355
Winter Teletherm
February 1/Day 32
+42 days
Coverage
100
75
50
25
0
1853
1900
1950
2010
Year
SALISBURY, NC
182
213
244
274
305
335
1
32
60
91
121
152
Day of Year

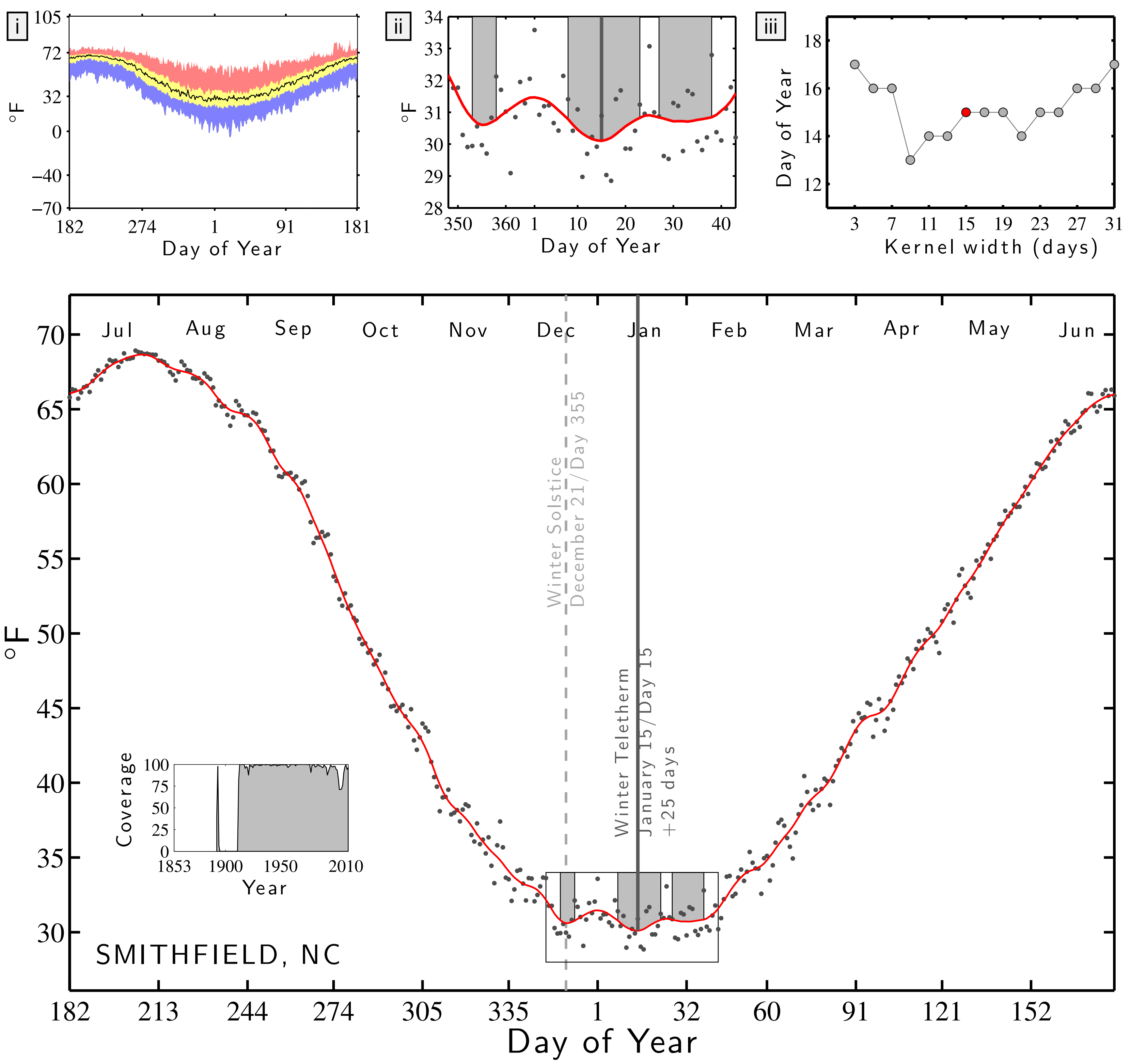

i
°F
Day of Year
ii
°F
Day of Year
iii
Day of Year
Kernel width (days)
Jul
Aug
Sep
Oct
Nov
Dec
Jan
Feb
Mar
Apr
May
Jun
Winter Solstice
December 21/Day 355
Winter Teletherm
January 15/Day 15
+25 days
Coverage
Year
SMITHFIELD, NC
°F
Day of Year

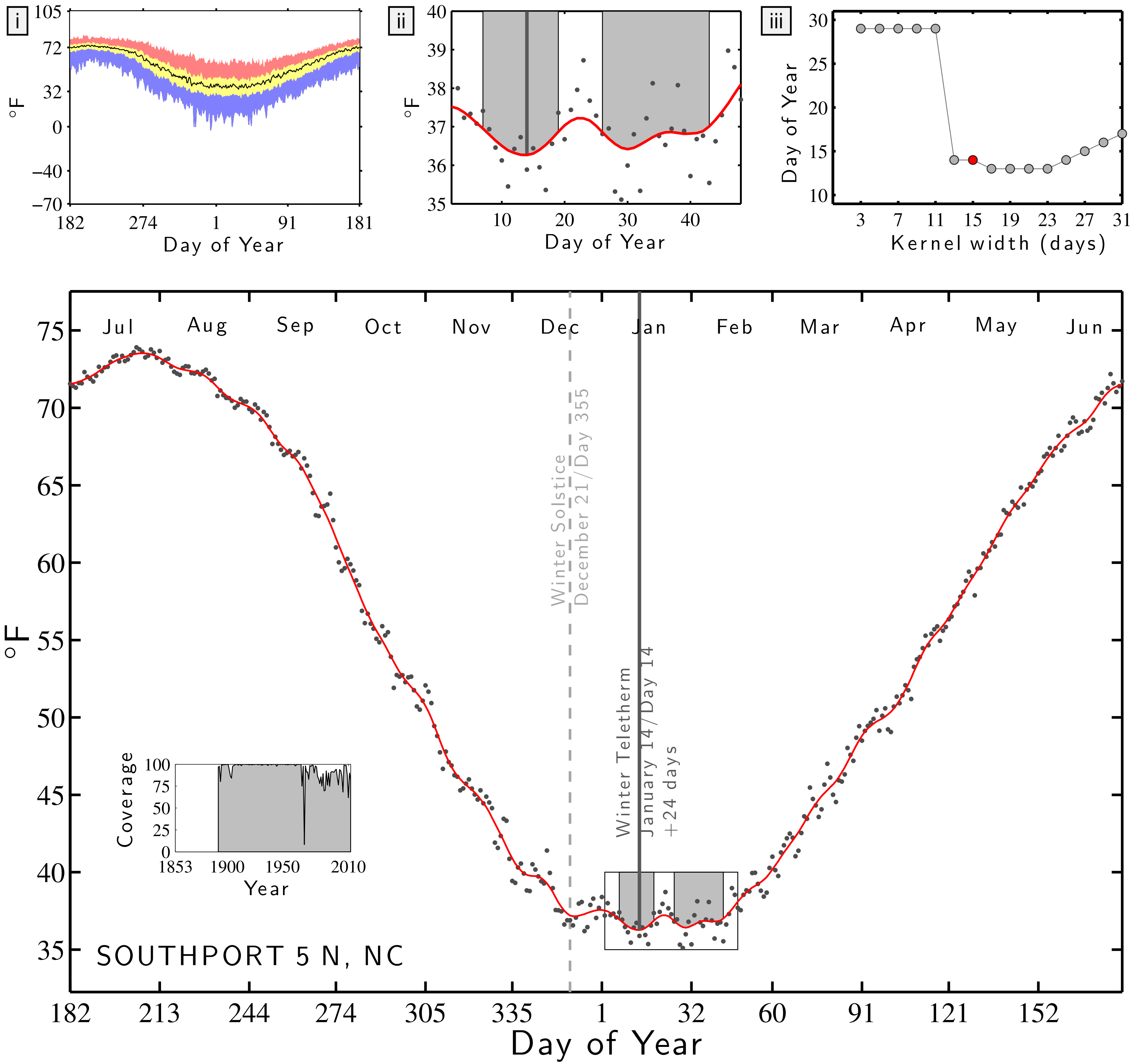

i
°F
Day of Year
ii
°F
Day of Year
iii
Day of Year
Kernel width (days)
Jul
Aug
Sep
Oct
Nov
Dec
Jan
Feb
Mar
Apr
May
Jun
Winter Solstice
December 21/Day 355
Winter Teletherm
January 14/Day 14
+24 days
Coverage
Year
SOUTHPORT 5 N, NC
°F
Day of Year

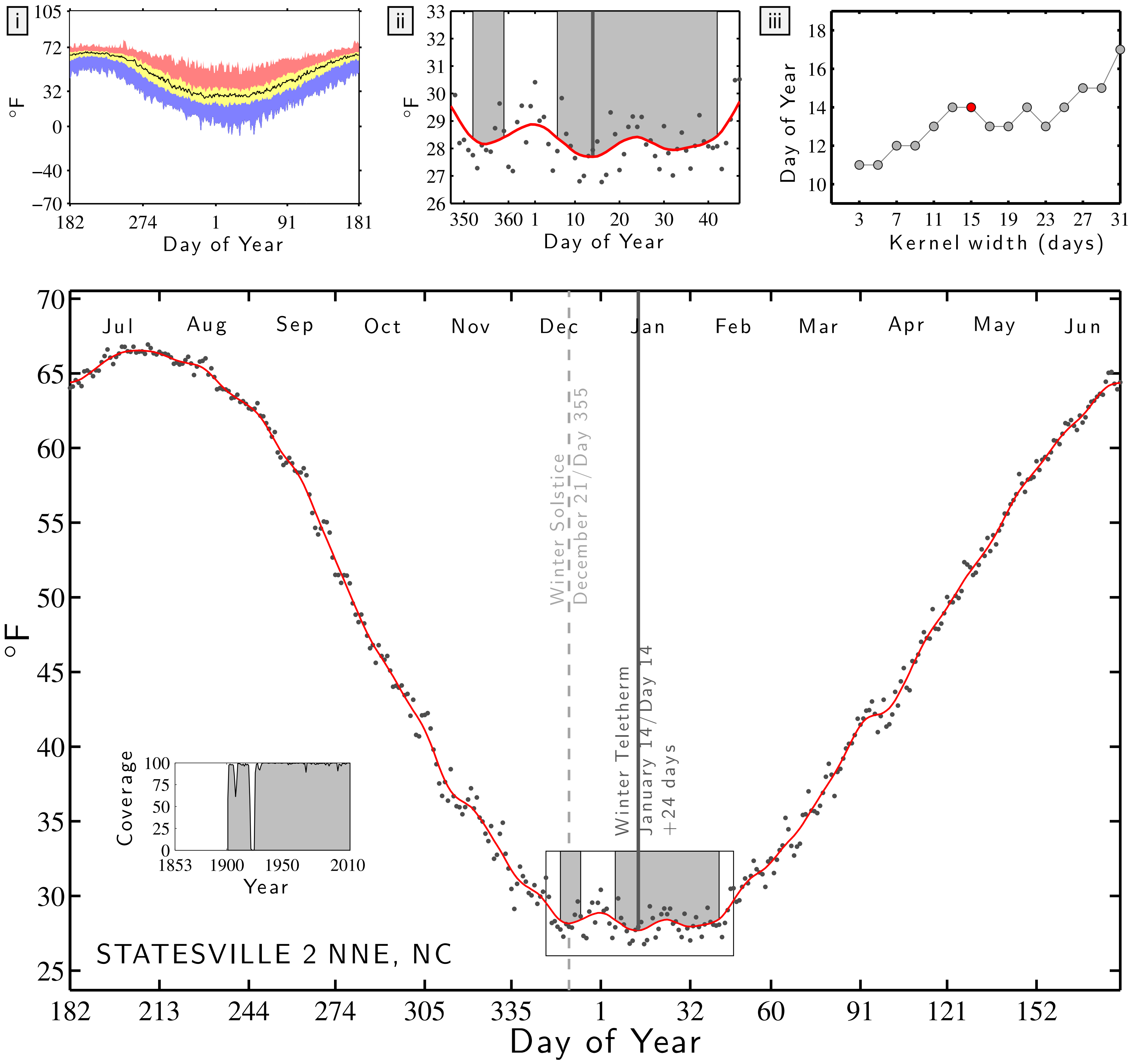

i
°F
Day of Year
ii
°F
Day of Year
iii
Day of Year
Kernel width (days)
Jul
Aug
Sep
Oct
Nov
Dec
Jan
Feb
Mar
Apr
May
Jun
Winter Solstice
December 21/Day 355
Winter Teletherm
January 14/Day 14
+24 days
Coverage
Year
STATESVILLE 2 NNE, NC
°F
Day of Year

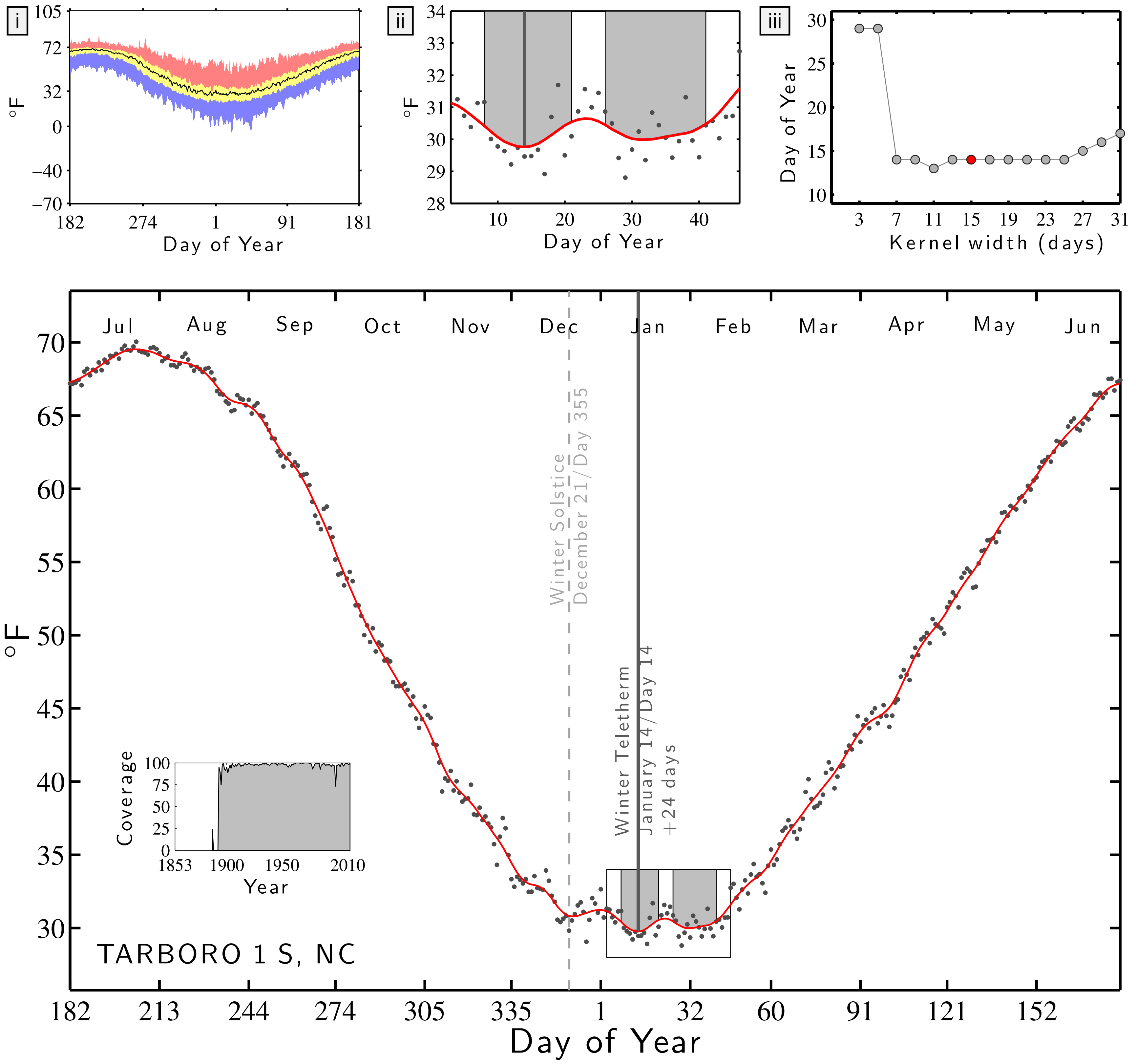

i
ii
iii
°F
Day of Year
Kernel width (days)
Jul
Aug
Sep
Oct
Nov
Dec
Jan
Feb
Mar
Apr
May
Jun
Winter Solstice
December 21/Day 355
Winter Teletherm
January 14/Day 14
+24 days
Coverage
Year
TARBORO 1 S, NC

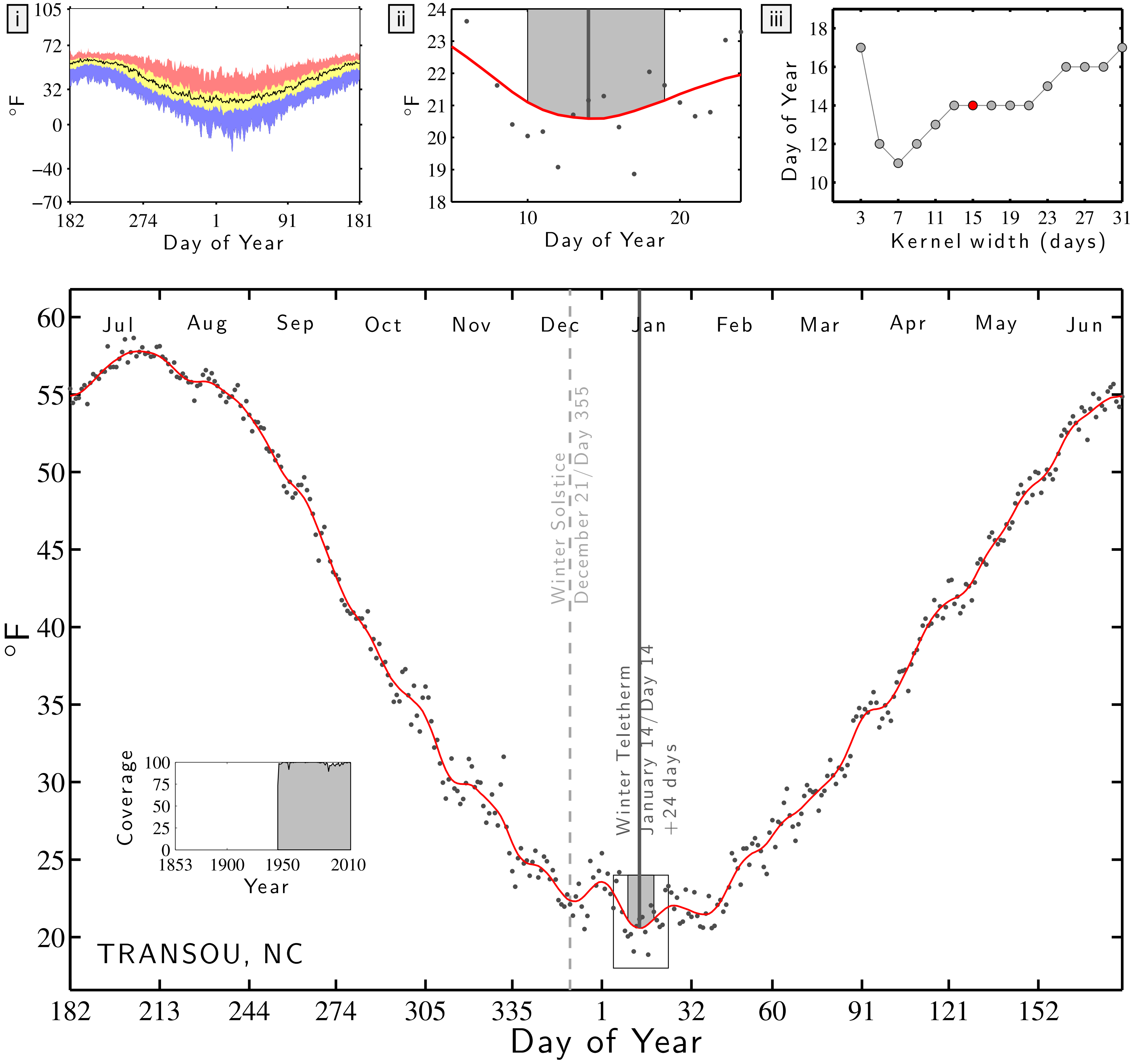

i
ii
iii
Day of Year
Kernel width (days)
°F
Jul
Aug
Sep
Oct
Nov
Dec
Jan
Feb
Mar
Apr
May
Jun
Winter Solstice
December 21/Day 355
Winter Teletherm
January 14/Day 14
+24 days
Coverage
Year
TRANSOU, NC

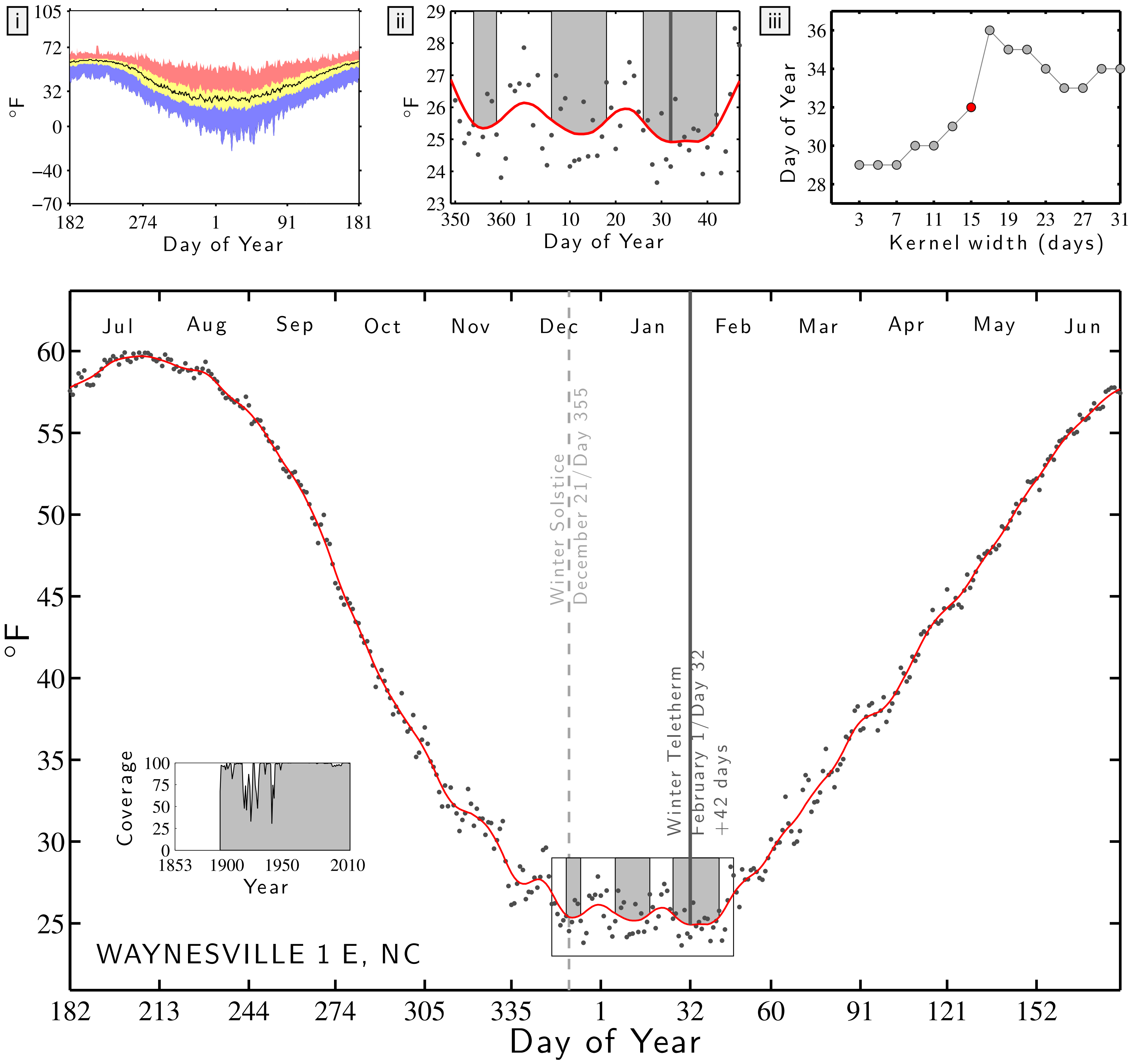

i
°F
Day of Year
ii
°F
Day of Year
iii
Day of Year
Kernel width (days)
Jul
Aug
Sep
Oct
Nov
Dec
Jan
Feb
Mar
Apr
May
Jun
Winter Solstice
December 21/Day 355
Winter Teletherm
February 1/Day 32
+42 days
Coverage
Year
WAYNESVILLE 1 E, NC
°F
Day of Year

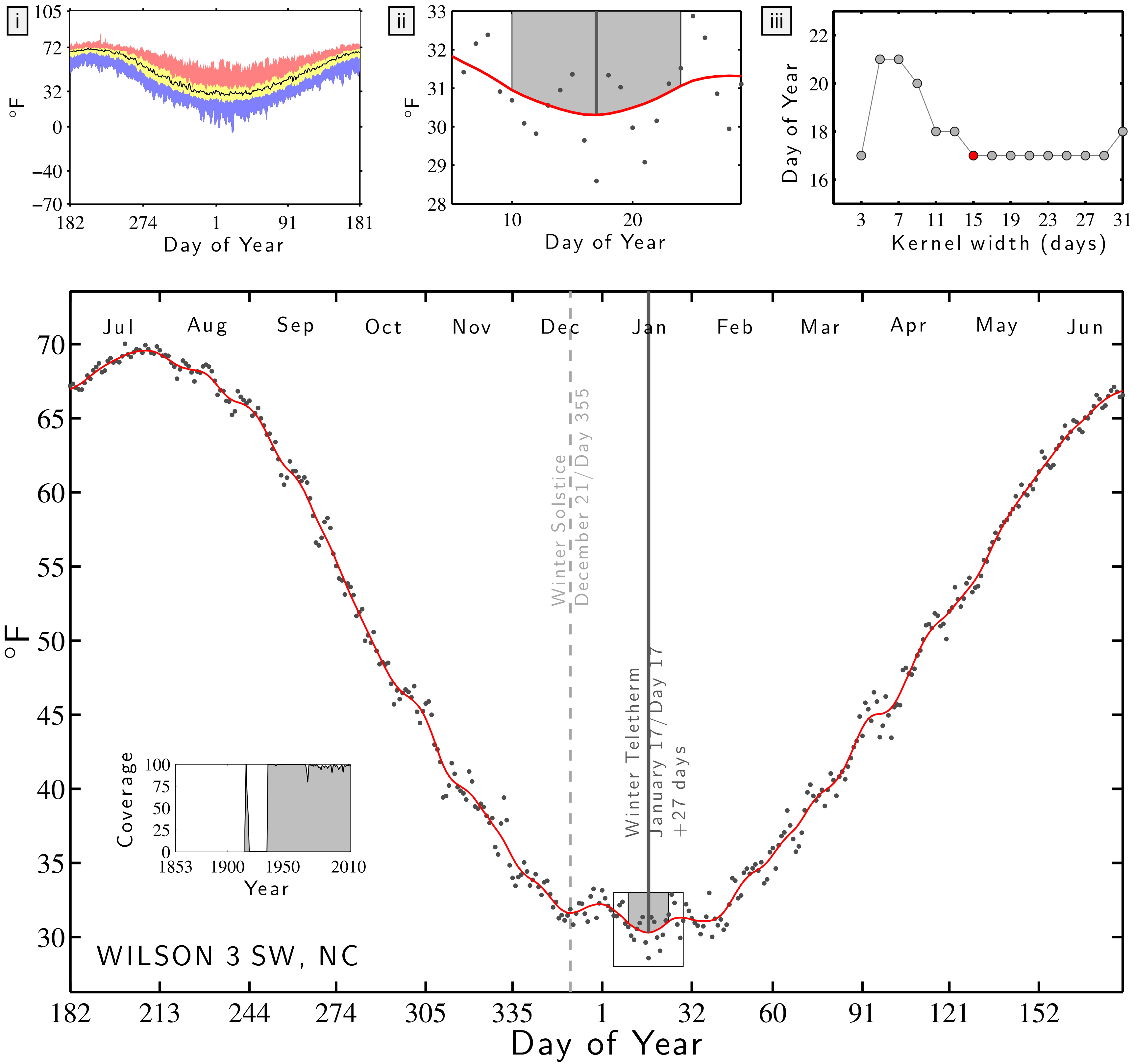

i
°F
Day of Year
ii
°F
Day of Year
iii
Day of Year
Kernel width (days)
Jul
Aug
Sep
Oct
Nov
Dec
Jan
Feb
Mar
Apr
May
Jun
Winter Solstice
December 21/Day 355
Winter Teletherm
January 17/Day 17
+27 days
Coverage
Year
WILSON 3 SW, NC
°F
Day of Year

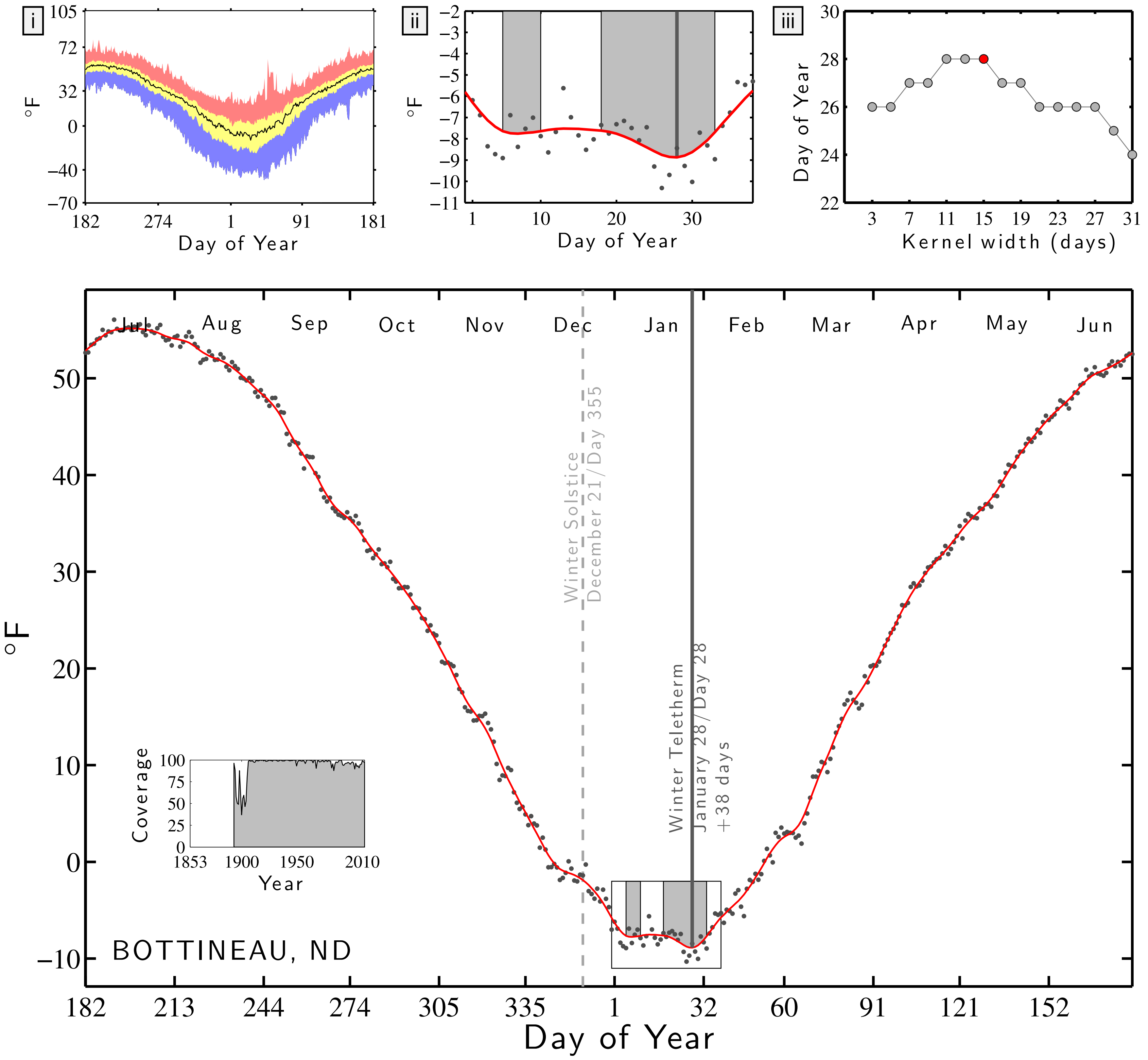

i
°F
Day of Year
ii
°F
Day of Year
iii
Day of Year
Kernel width (days)
Jul
Aug
Sep
Oct
Nov
Dec
Jan
Feb
Mar
Apr
May
Jun
Winter Solstice
December 21/Day 355
Winter Teletherm
January 28/Day 28
+38 days
Coverage
Year
BOTTINEAU, ND
°F
Day of Year

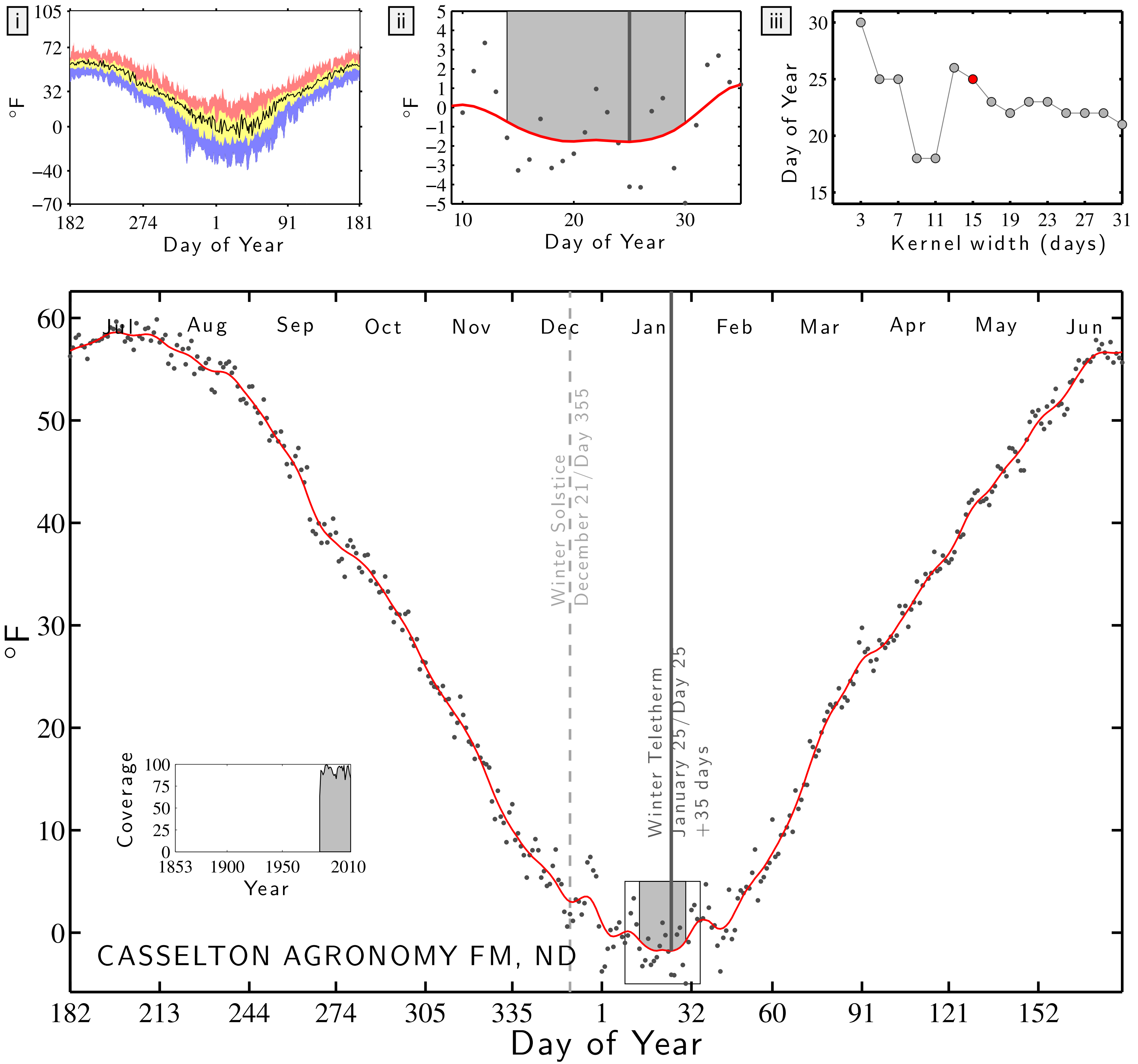

i
ii
iii
°F
Day of Year
Kernel width (days)
Winter Solstice
December 21/Day 355
Winter Teletherm
January 25/Day 25
+35 days
Coverage
Year
CASSELTON AGRONOMY FM, ND
Jul
Aug
Sep
Oct
Nov
Dec
Jan
Feb
Mar
Apr
May
Jun

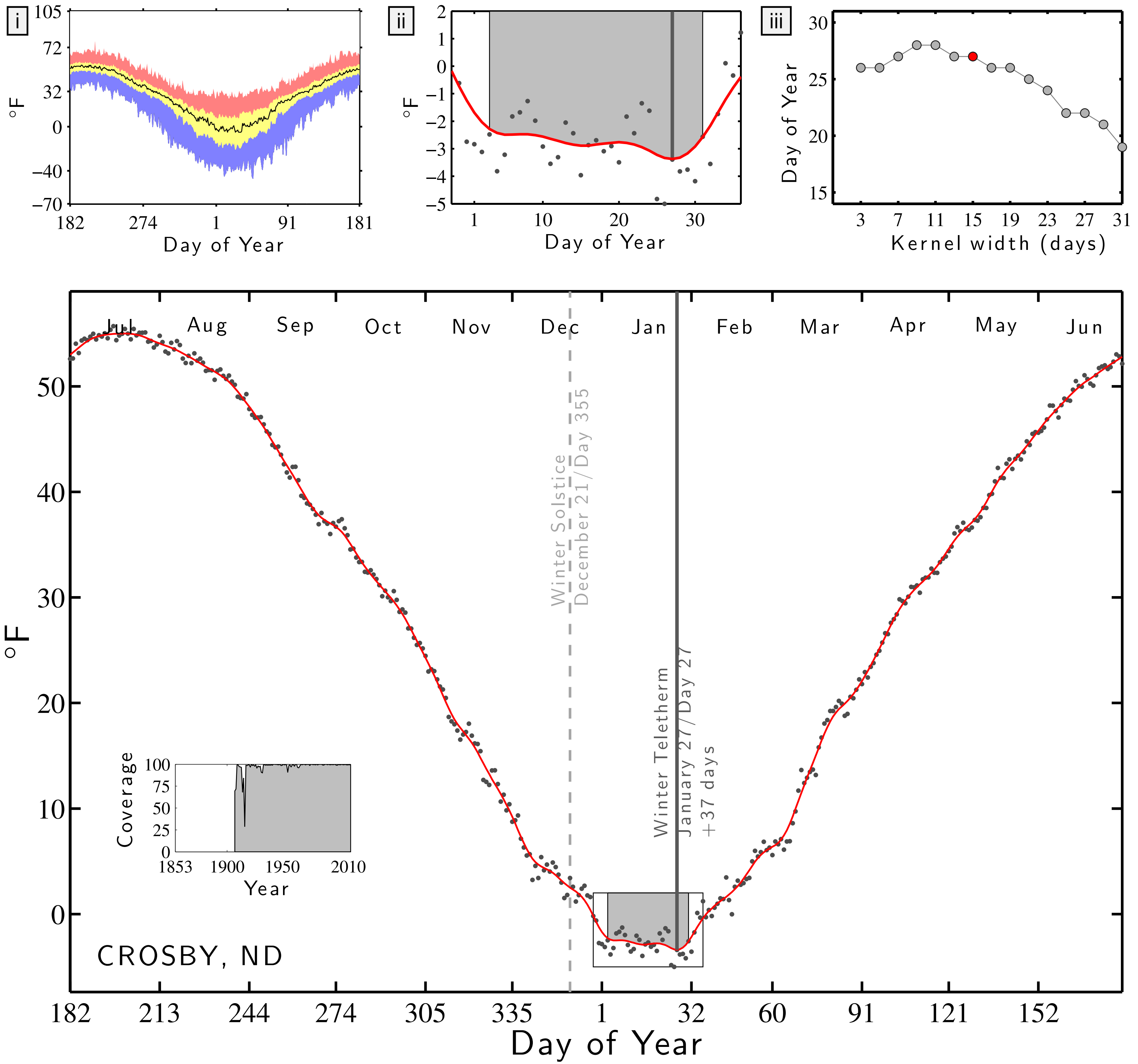

i
ii
iii
°F
Day of Year
Kernel width (days)
Jul
Aug
Sep
Oct
Nov
Dec
Jan
Feb
Mar
Apr
May
Jun
Winter Solstice
December 21/Day 355
Winter Teletherm
January 27/Day 27
+37 days
Coverage
Year
CROSBY, ND

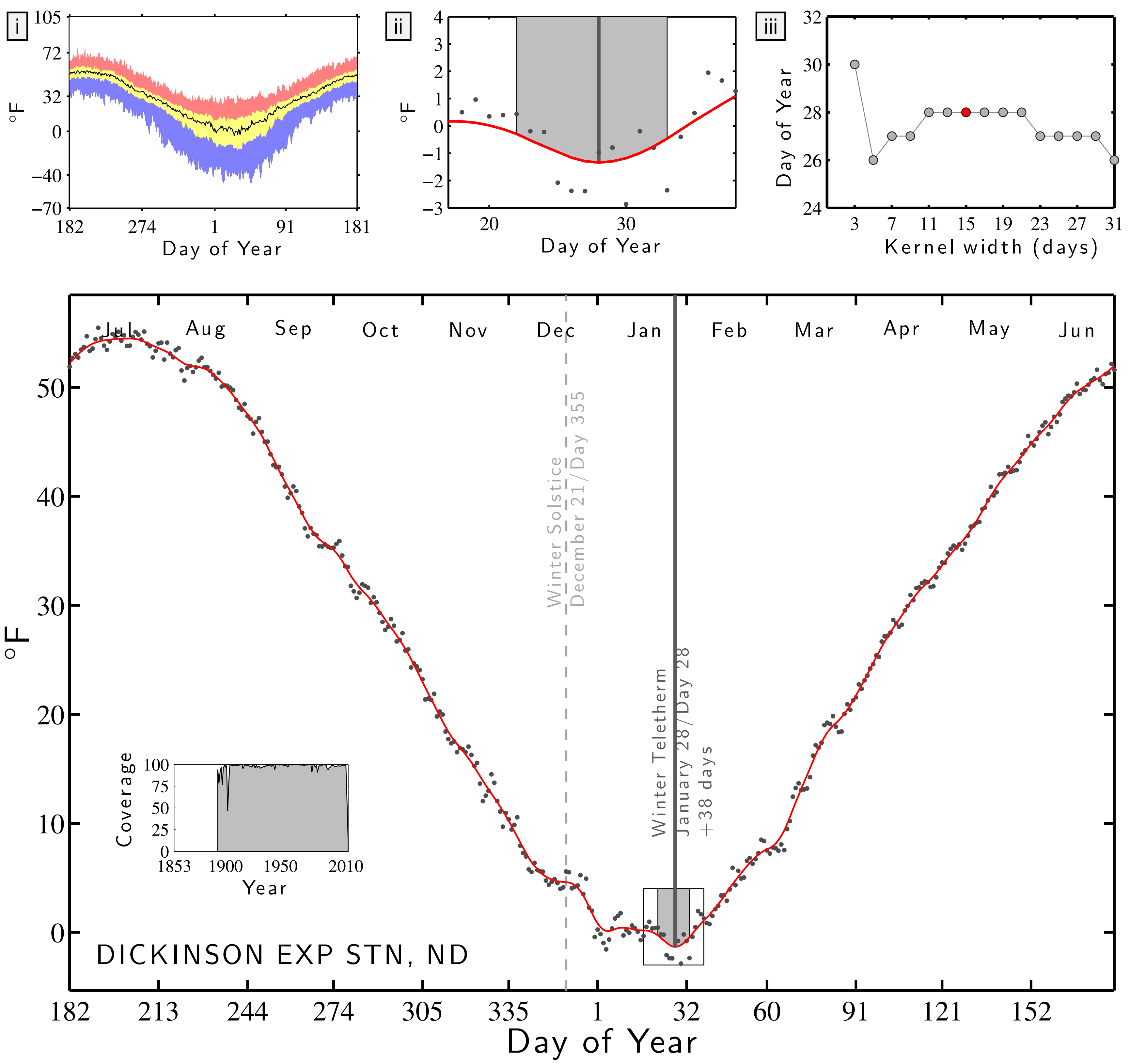

i
°F
Day of Year
ii
°F
Day of Year
iii
Day of Year
Kernel width (days)
Jul
Aug
Sep
Oct
Nov
Dec
Jan
Feb
Mar
Apr
May
Jun
Winter Solstice
December 21/Day 355
Winter Teletherm
January 28/Day 28
+38 days
Coverage
Year
DICKINSON EXP STN, ND
°F
Day of Year

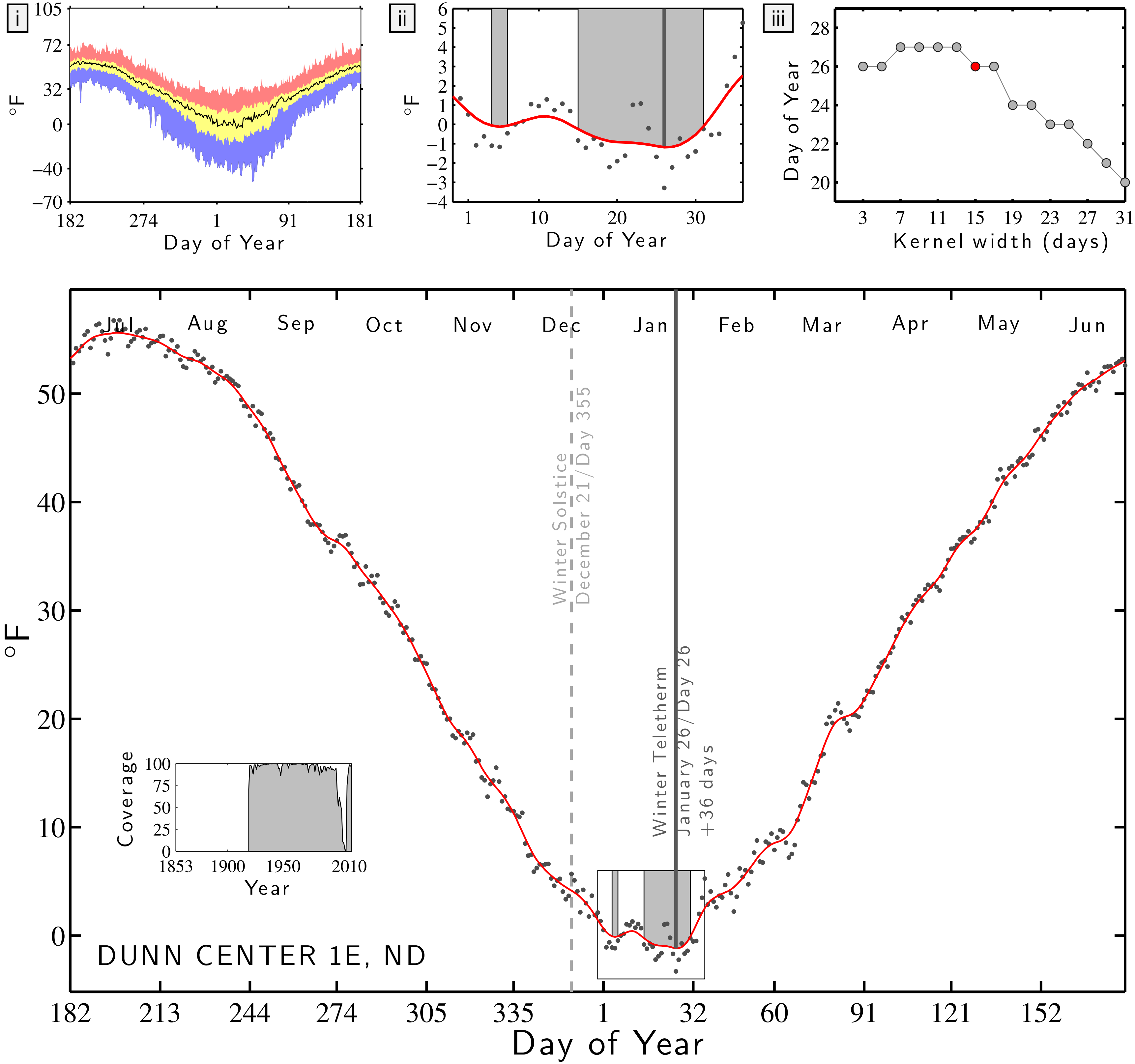

i
ii
iii
°F
Day of Year
Kernel width (days)
Jul
Aug
Sep
Oct
Nov
Dec
Jan
Feb
Mar
Apr
May
Jun
Winter Solstice
December 21/Day 355
Winter Teletherm
January 26/Day 26
+36 days
Coverage
Year
DUNN CENTER 1E, ND

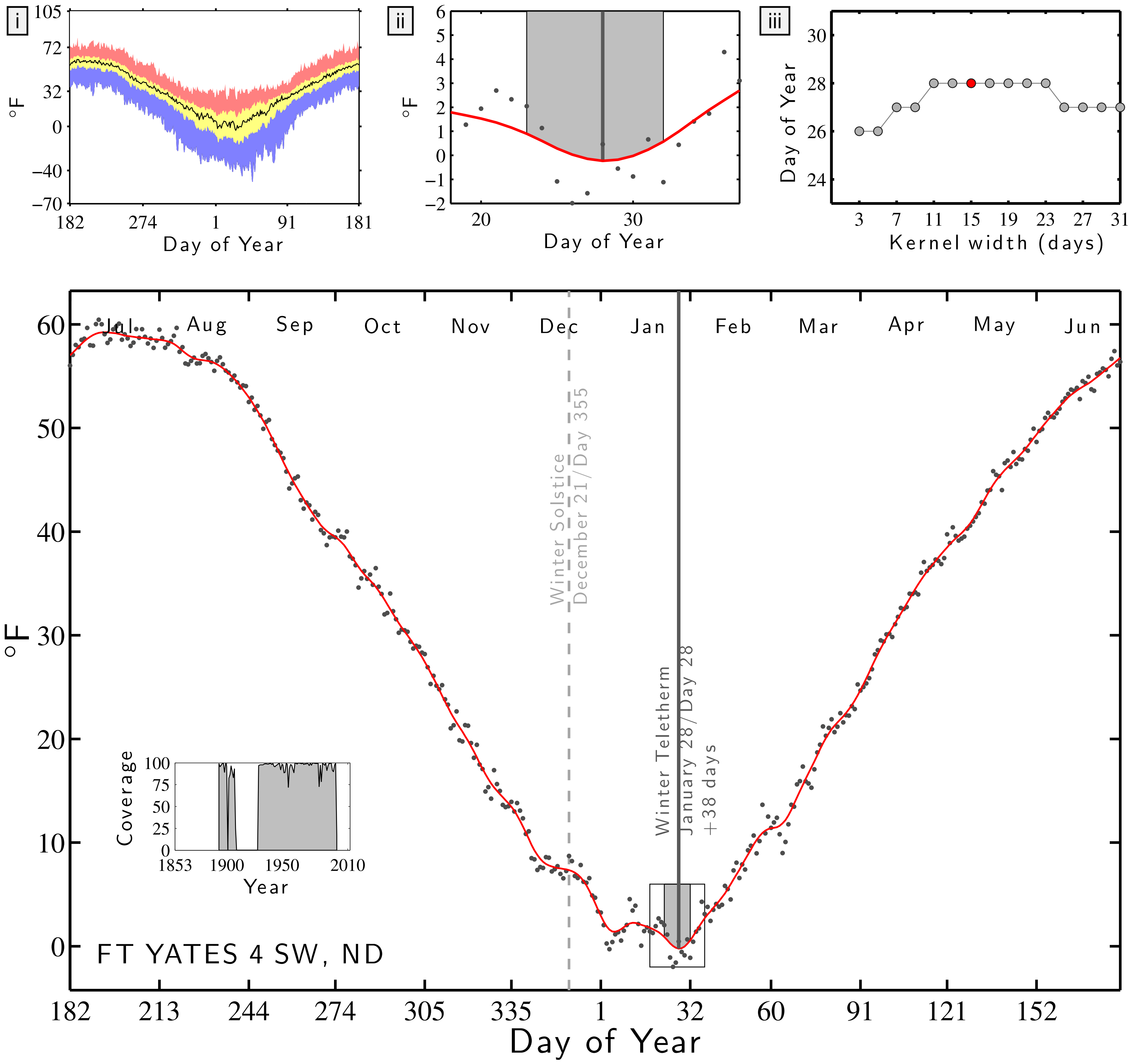

i
ii
iii
°F
Day of Year
Kernel width (days)
Jul
Aug
Sep
Oct
Nov
Dec
Jan
Feb
Mar
Apr
May
Jun
Winter Solstice
December 21/Day 355
Winter Teletherm
January 28/Day 28
+38 days
Coverage
Year
FT YATES 4 SW, ND

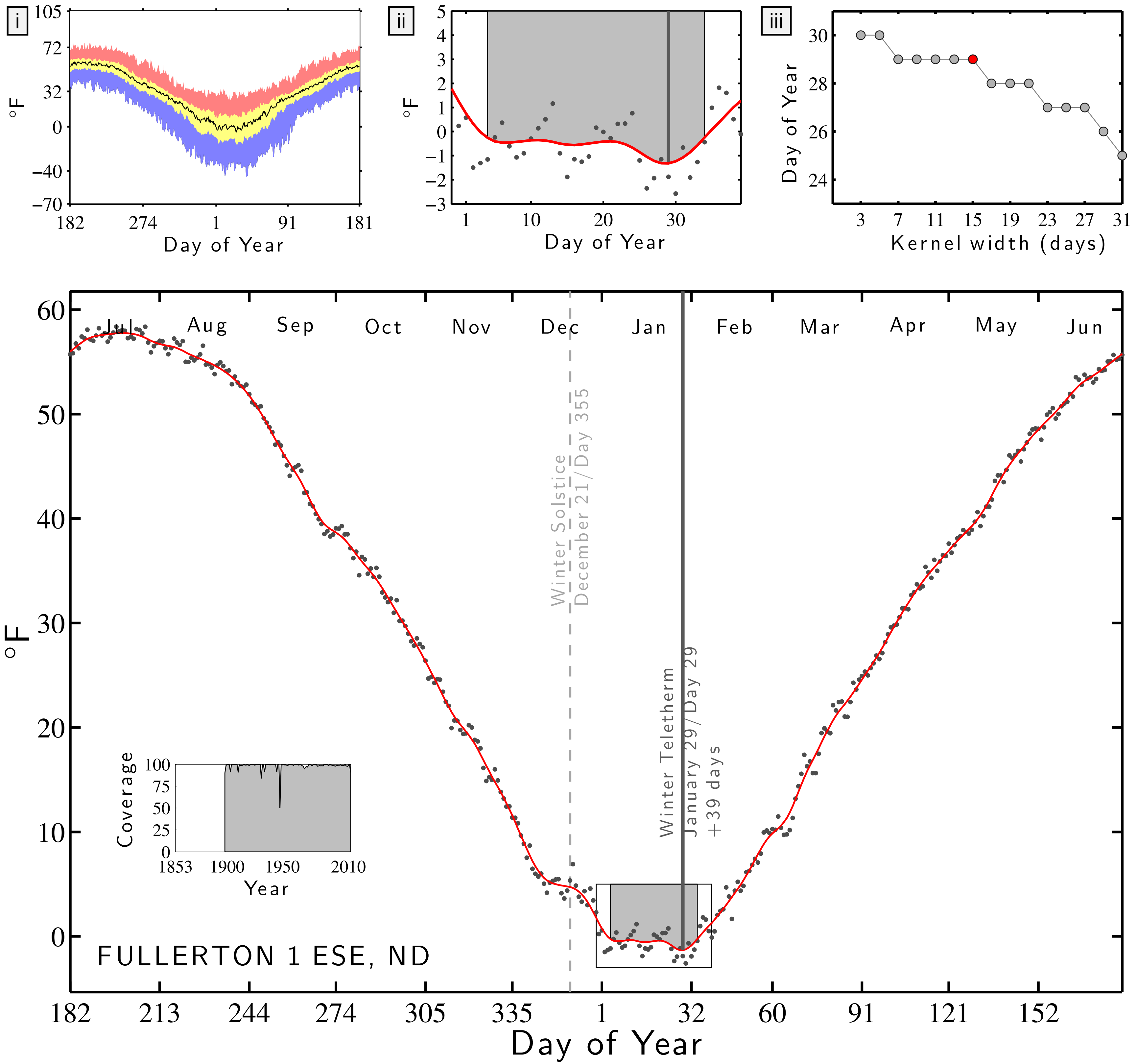
i
°F
Day of Year
ii
°F
Day of Year
iii
Day of Year
Kernel width (days)
Jul
Aug
Sep
Oct
Nov
Dec
Jan
Feb
Mar
Apr
May
Jun
Winter Solstice
December 21/Day 355
Winter Teletherm
January 29/Day 29
+39 days
Coverage
Year
FULLERTON 1 ESE, ND
°F
Day of Year

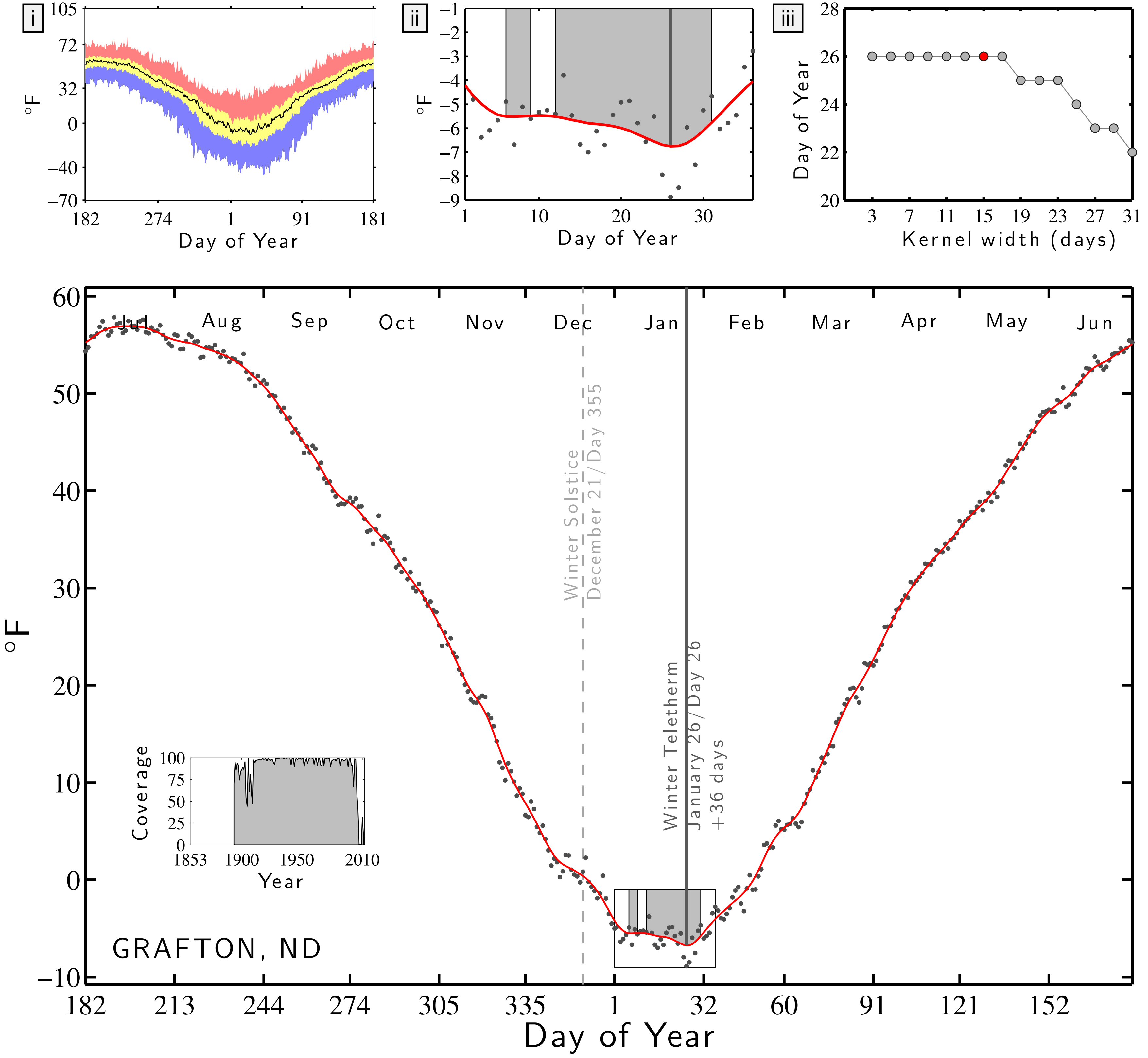

i
°F
Day of Year
ii
Day of Year
iii
Day of Year
Kernel width (days)
Jul
Aug
Sep
Oct
Nov
Dec
Jan
Feb
Mar
Apr
May
Jun
Winter Solstice
December 21/Day 355
Winter Teletherm
January 26/Day 26
+36 days
Coverage
Year
GRAFTON, ND
Day of Year

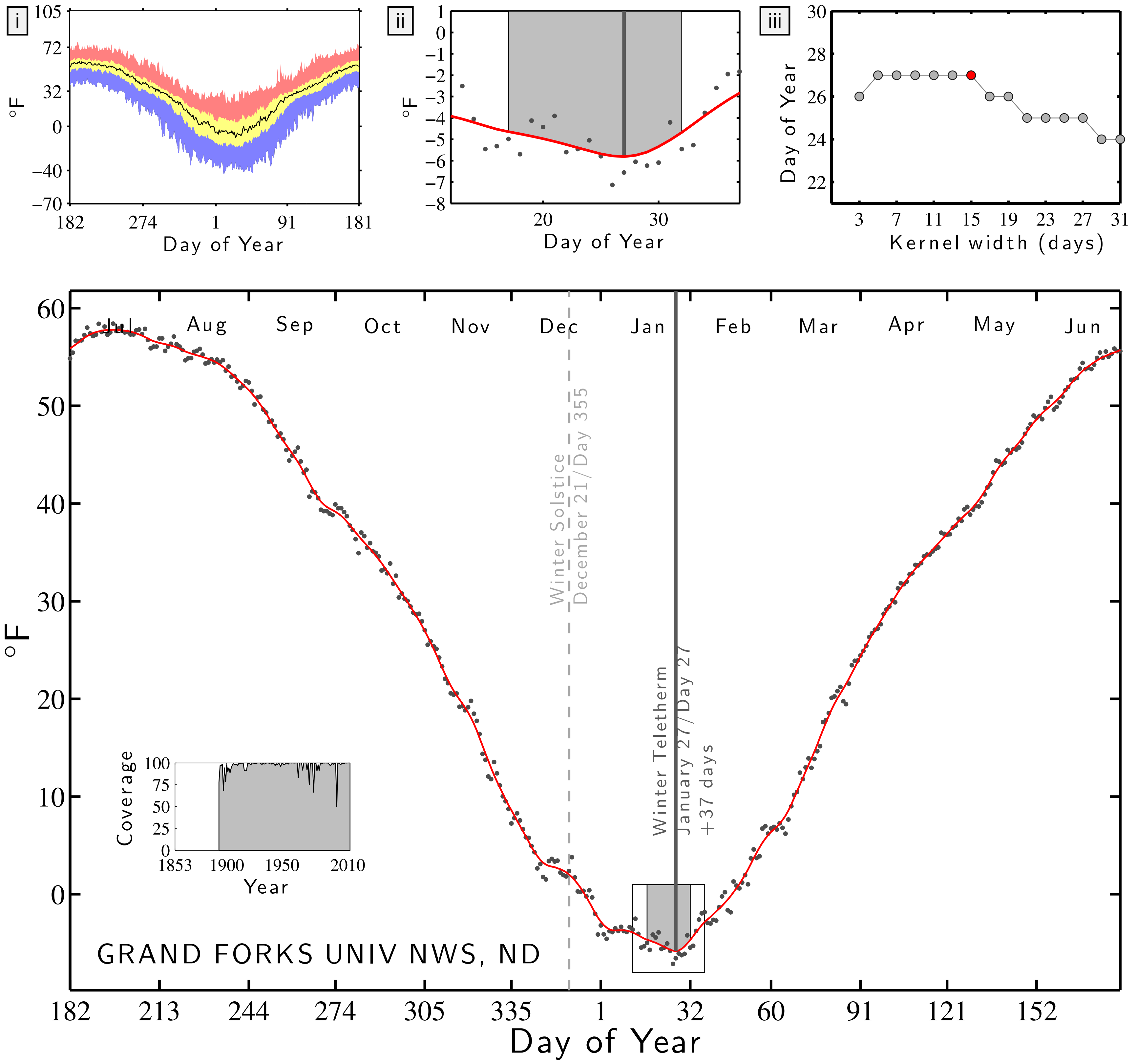

i
°F
Day of Year
ii
°F
Day of Year
iii
Day of Year
Kernel width (days)
Jul
Aug
Sep
Oct
Nov
Dec
Jan
Feb
Mar
Apr
May
Jun
Winter Solstice
December 21/Day 355
Winter Teletherm
January 27/Day 27
+37 days
Coverage
Year
GRAND FORKS UNIV NWS, ND
°F
Day of Year

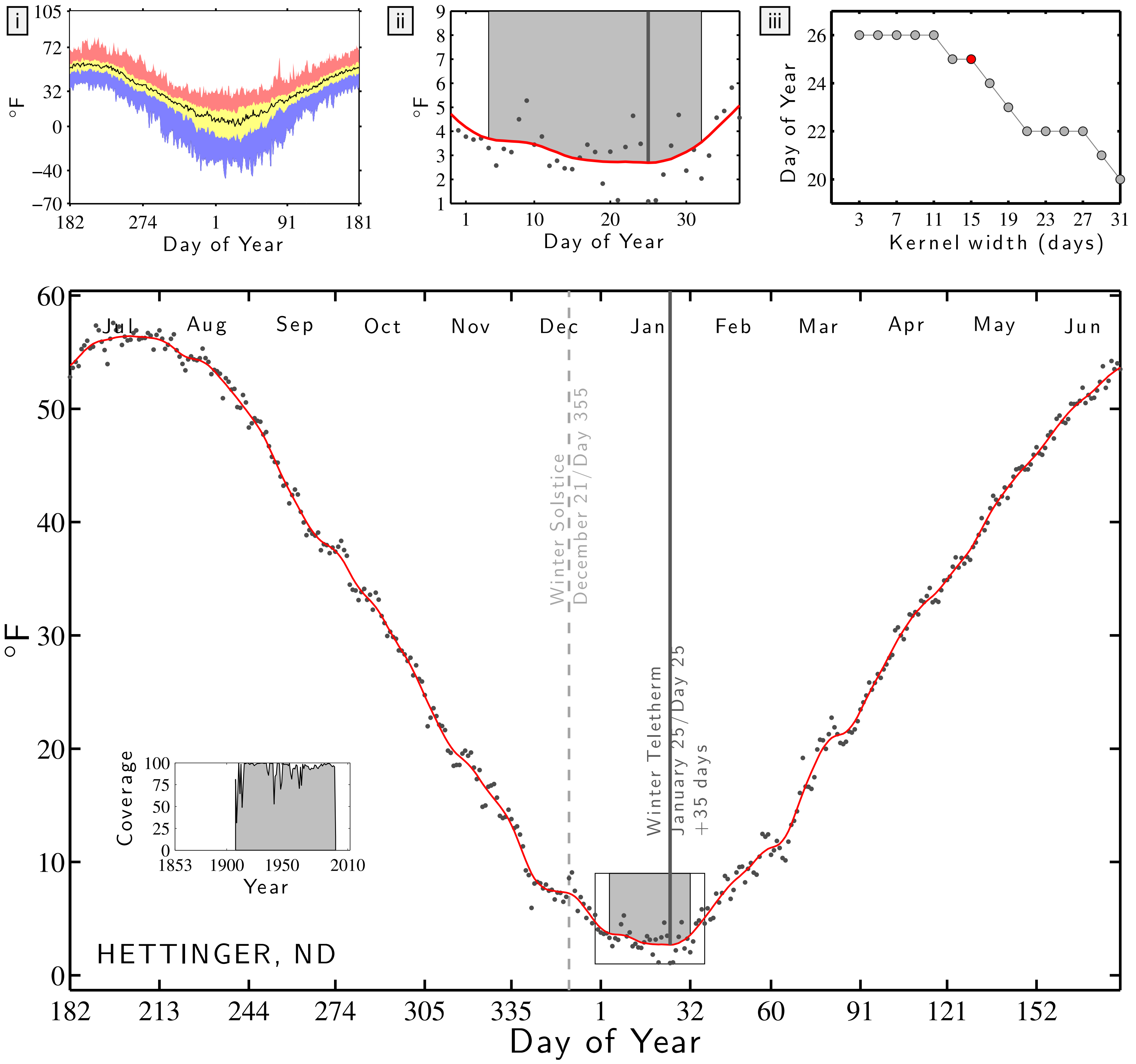

i
°F
Day of Year
ii
°F
Day of Year
iii
Day of Year
Kernel width (days)
°F
Jul
Aug
Sep
Oct
Nov
Dec
Jan
Feb
Mar
Apr
May
Jun
Winter Solstice
December 21/Day 355
Winter Teletherm
January 25/Day 25
+35 days
Coverage
Year
HETTINGER, ND
Day of Year

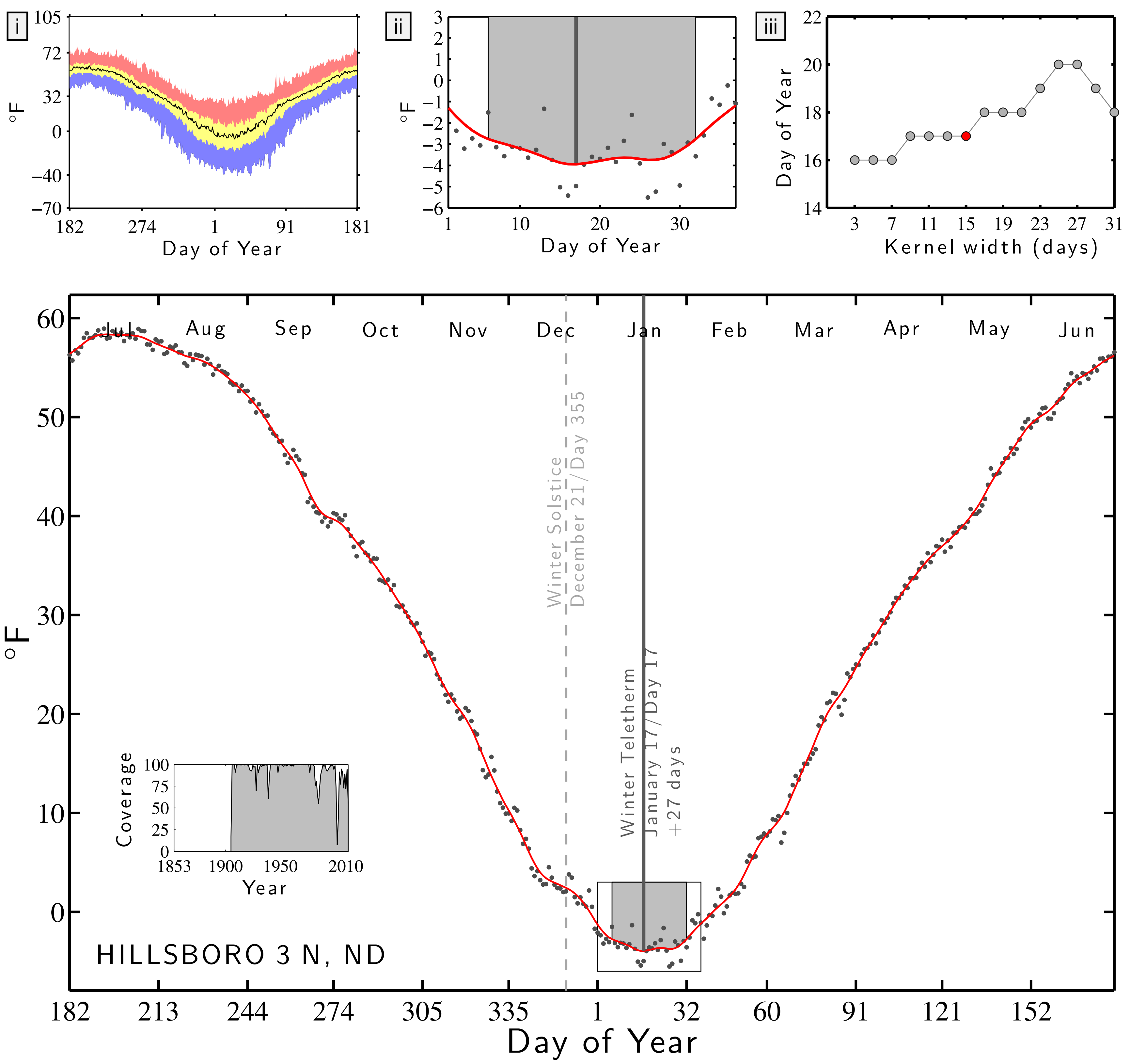

i
ii
iii
Day of Year
Kernel width (days)
°F
Jul
Aug
Sep
Oct
Nov
Dec
Jan
Feb
Mar
Apr
May
Jun
Winter Solstice
December 21/Day 355
Winter Teletherm
January 17/Day 17
+27 days
Coverage
Year
HILLSBORO 3 N, ND

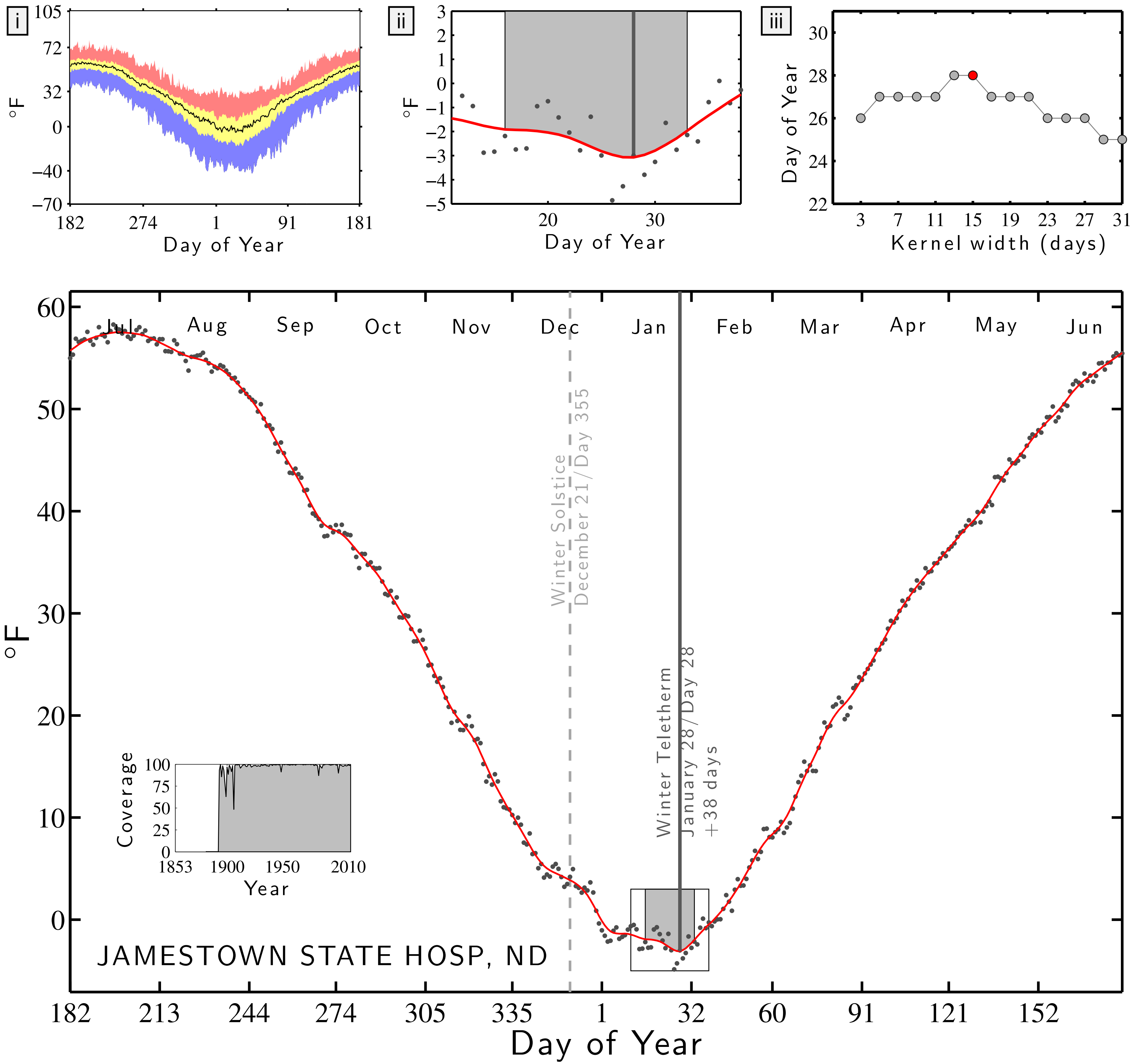

i
°F
Day of Year
ii
iii
Day of Year
Kernel width (days)
Jul
Aug
Sep
Oct
Nov
Dec
Jan
Feb
Mar
Apr
May
Jun
Winter Solstice
December 21/Day 355
Winter Teletherm
January 28/Day 28
+38 days
Coverage
Year
JAMESTOWN STATE HOSP, ND

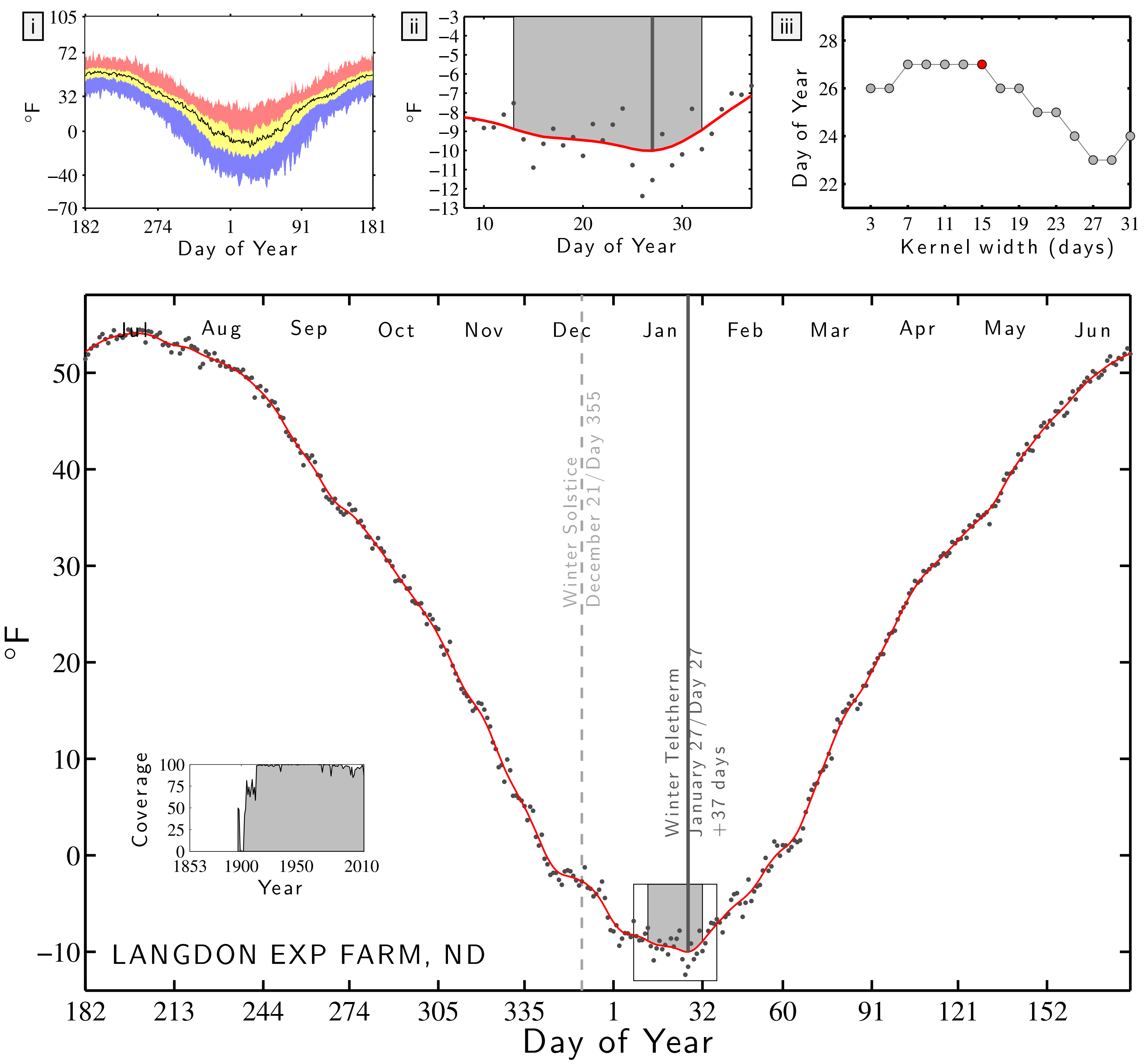

i
ii
iii
°F
Day of Year
Kernel width (days)
Winter Solstice
December 21/Day 355
Winter Teletherm
January 27/Day 27
+37 days
Coverage
Year
LANGDON EXP FARM, ND
Jul
Aug
Sep
Oct
Nov
Dec
Jan
Feb
Mar
Apr
May
Jun

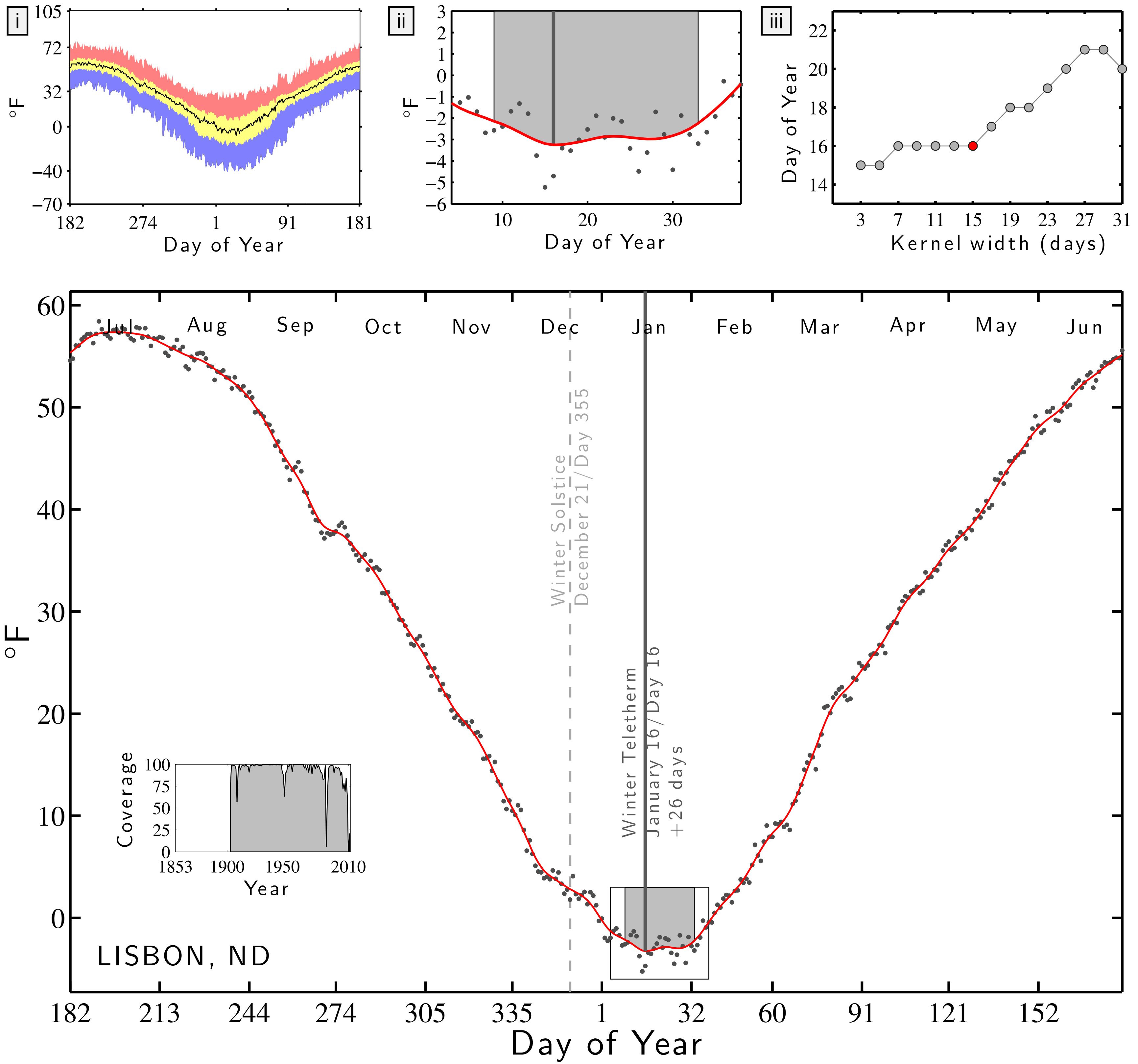

i
°F
Day of Year
ii
°F
Day of Year
iii
Day of Year
Kernel width (days)
Jul
Aug
Sep
Oct
Nov
Dec
Jan
Feb
Mar
Apr
May
Jun
Winter Solstice
December 21/Day 355
Winter Teletherm
January 16/Day 16
+26 days
Coverage
Year
LISBON, ND
°F
Day of Year

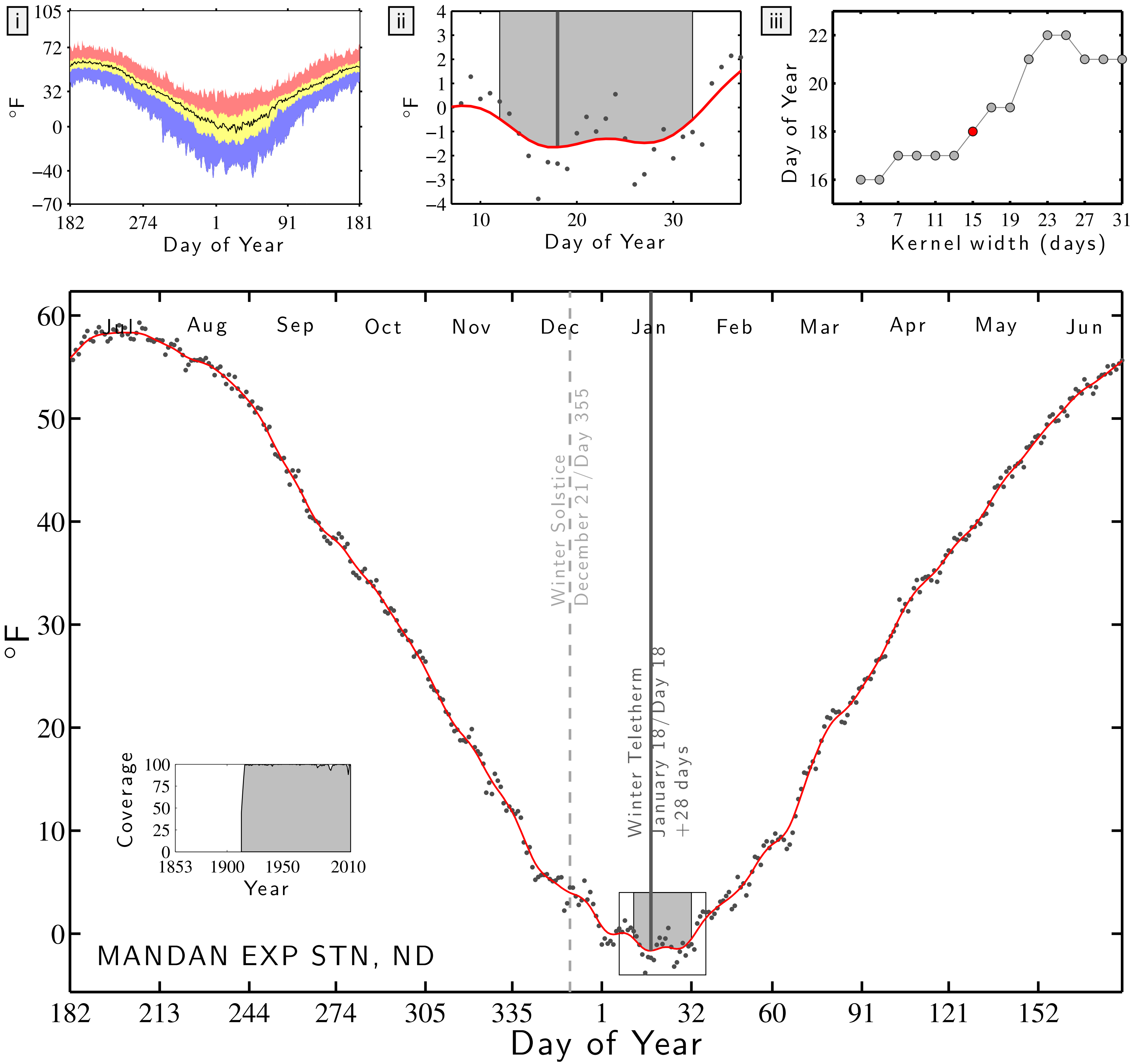

i
105
72
32
0
−40
−70
°F
182
274
1
91
181
Day of Year
ii
4
3
2
1
0
−1
−2
−3
−4
°F
10
20
30
Day of Year
iii
22
20
18
16
Day of Year
3
7
11
15
19
23
27
31
Kernel width (days)
60
50
40
30
20
10
0
°F
Jul
Aug
Sep
Oct
Nov
Dec
Jan
Feb
Mar
Apr
May
Jun
Winter Solstice
December 21/Day 355
Winter Teletherm
January 18/Day 18
+28 days
Coverage
100
75
50
25
0
1853
1900
1950
2010
Year
MANDAN EXP STN, ND
182
213
244
274
305
335
1
32
60
91
121
152
Day of Year

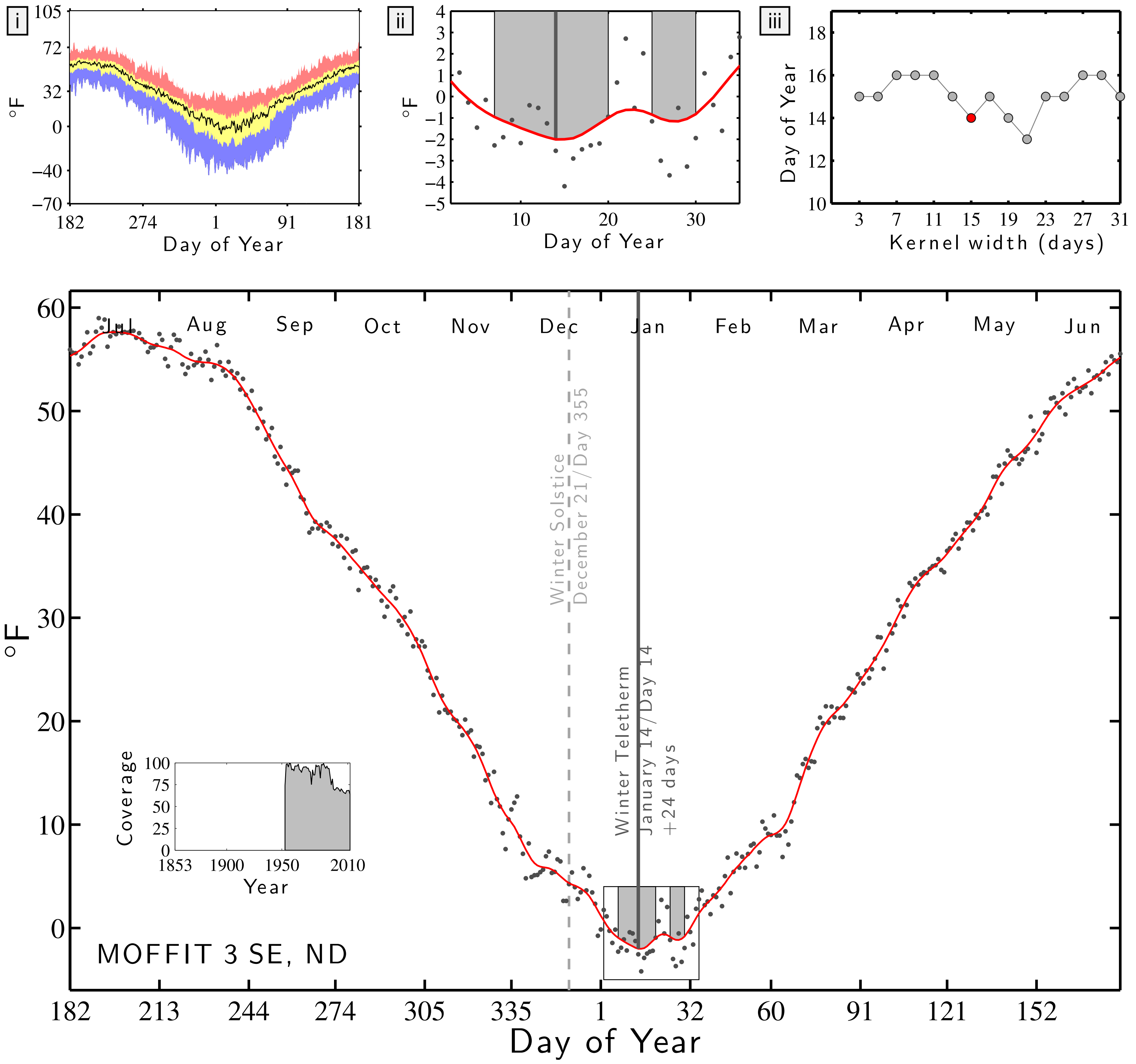
i
ii
iii
°F
Day of Year
Day of Year
Kernel width (days)
Winter Solstice
December 21/Day 355
Winter Teletherm
January 14/Day 14
+24 days
Coverage
Year
MOFFIT 3 SE, ND
Jul
Aug
Sep
Oct
Nov
Dec
Jan
Feb
Mar
Apr
May
Jun
Day of Year

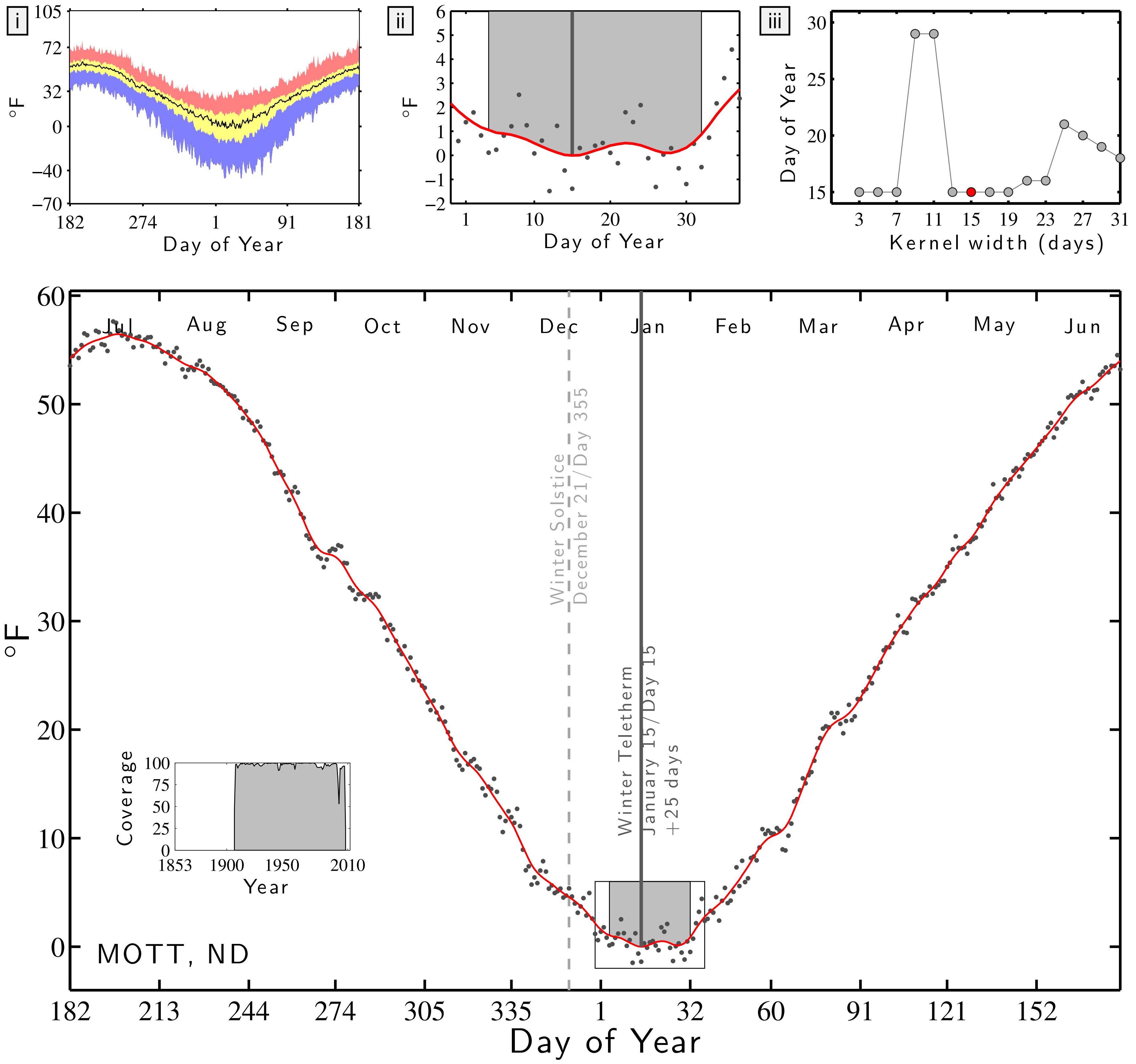

i
°F
Day of Year
ii
°F
Day of Year
iii
Day of Year
Kernel width (days)
Jul
Aug
Sep
Oct
Nov
Dec
Jan
Feb
Mar
Apr
May
Jun
Winter Solstice
December 21/Day 355
Winter Teletherm
January 15/Day 15
+25 days
Coverage
Year
MOTT, ND
°F
Day of Year

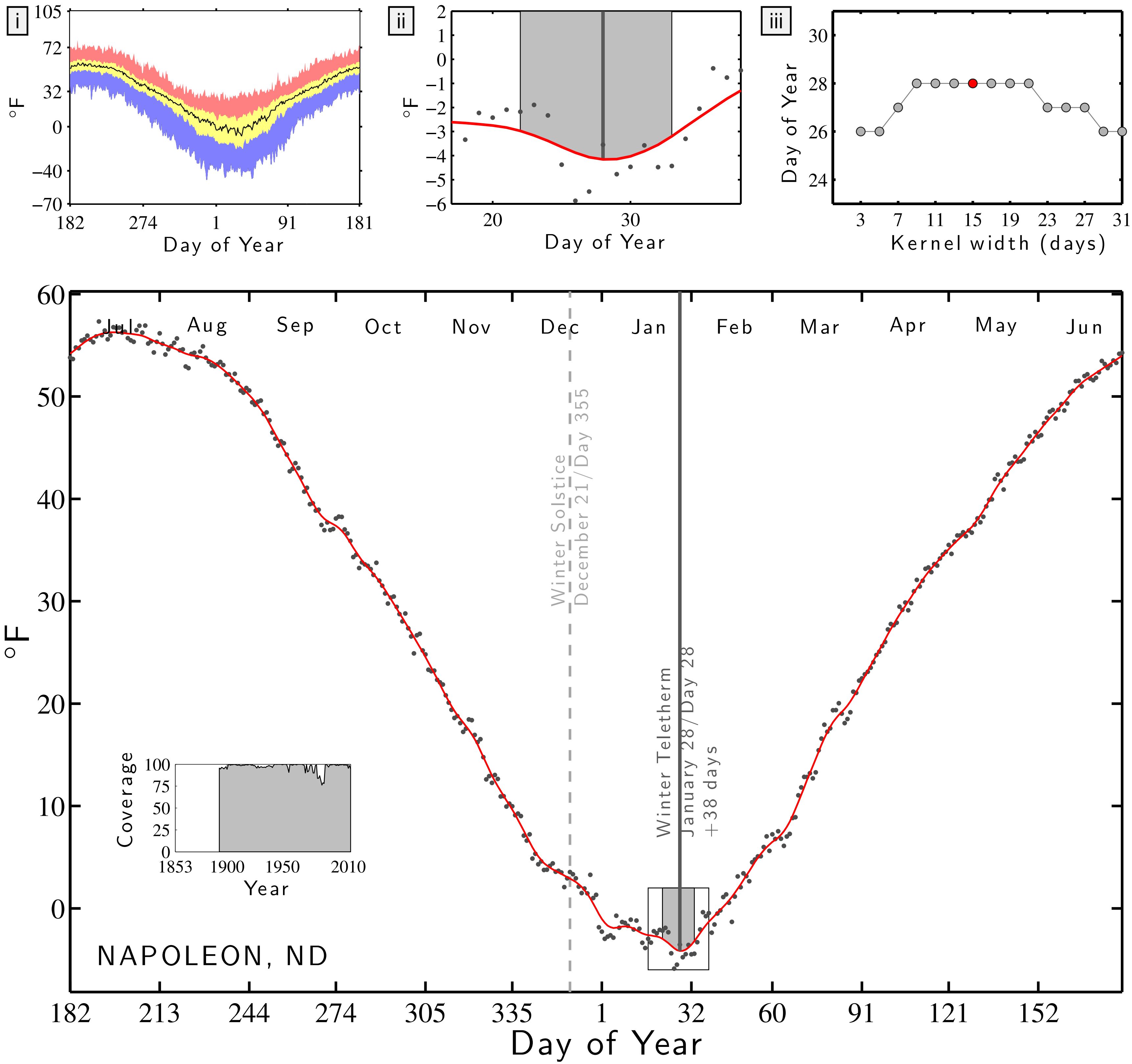

i
°F
105
72
32
0
−40
−70
182
274
1
91
181
Day of Year
ii
°F
2
1
0
−1
−2
−3
−4
−5
−6
20
30
Day of Year
iii
Day of Year
30
28
26
24
3
7
11
15
19
23
27
31
Kernel width (days)
°F
60
50
40
30
20
10
0
Jul
Aug
Sep
Oct
Nov
Dec
Jan
Feb
Mar
Apr
May
Jun
Winter Solstice
December 21/Day 355
Winter Teletherm
January 28/Day 28
+38 days
Coverage
100
75
50
25
0
1853
1900
1950
2010
Year
NAPOLEON, ND
182
213
244
274
305
335
1
32
60
91
121
152
Day of Year

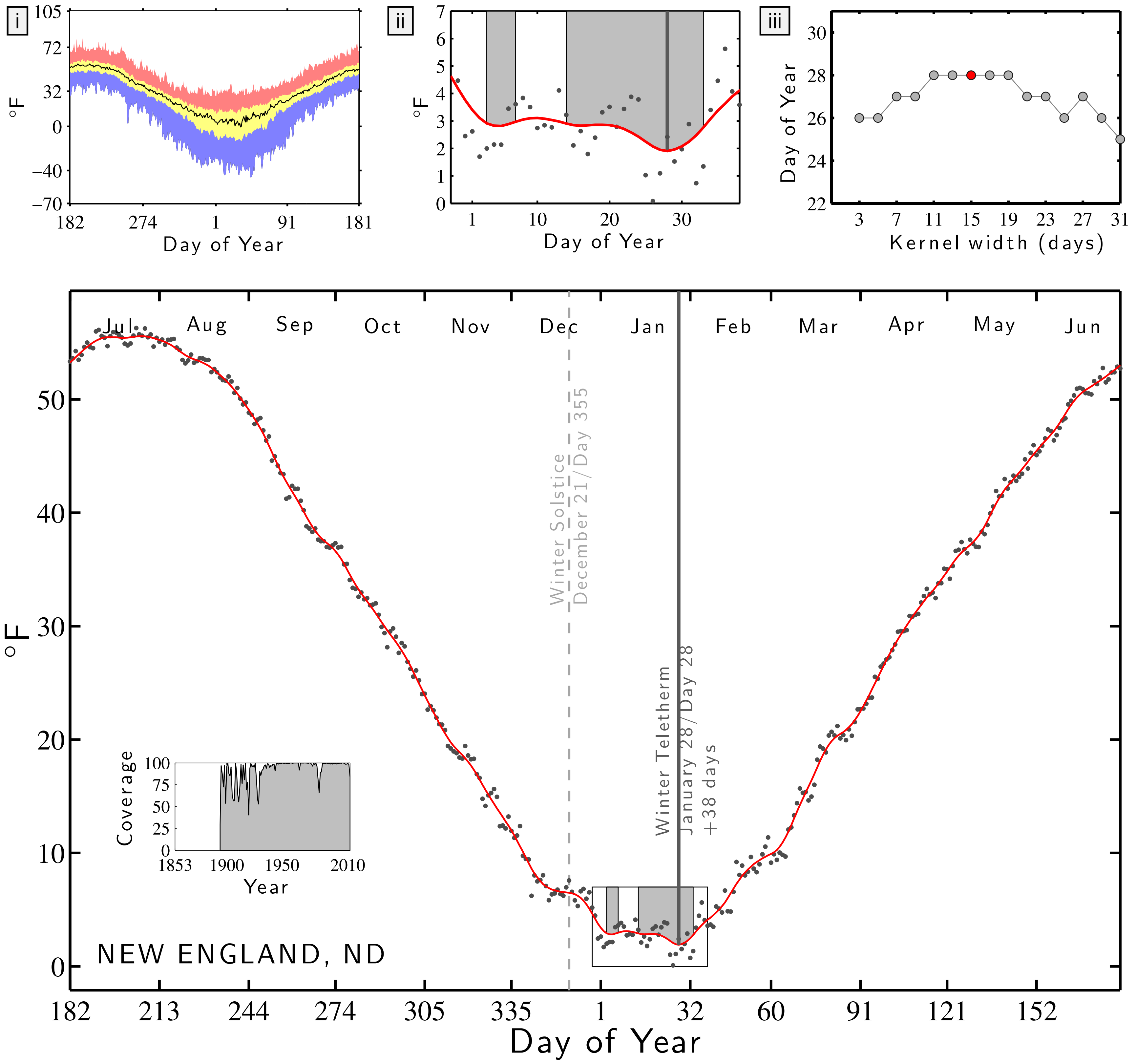

i
ii
iii
°F
Day of Year
Kernel width (days)
Jul
Aug
Sep
Oct
Nov
Dec
Jan
Feb
Mar
Apr
May
Jun
Winter Solstice
December 21/Day 355
Winter Teletherm
January 28/Day 28
+38 days
Coverage
Year
NEW ENGLAND, ND

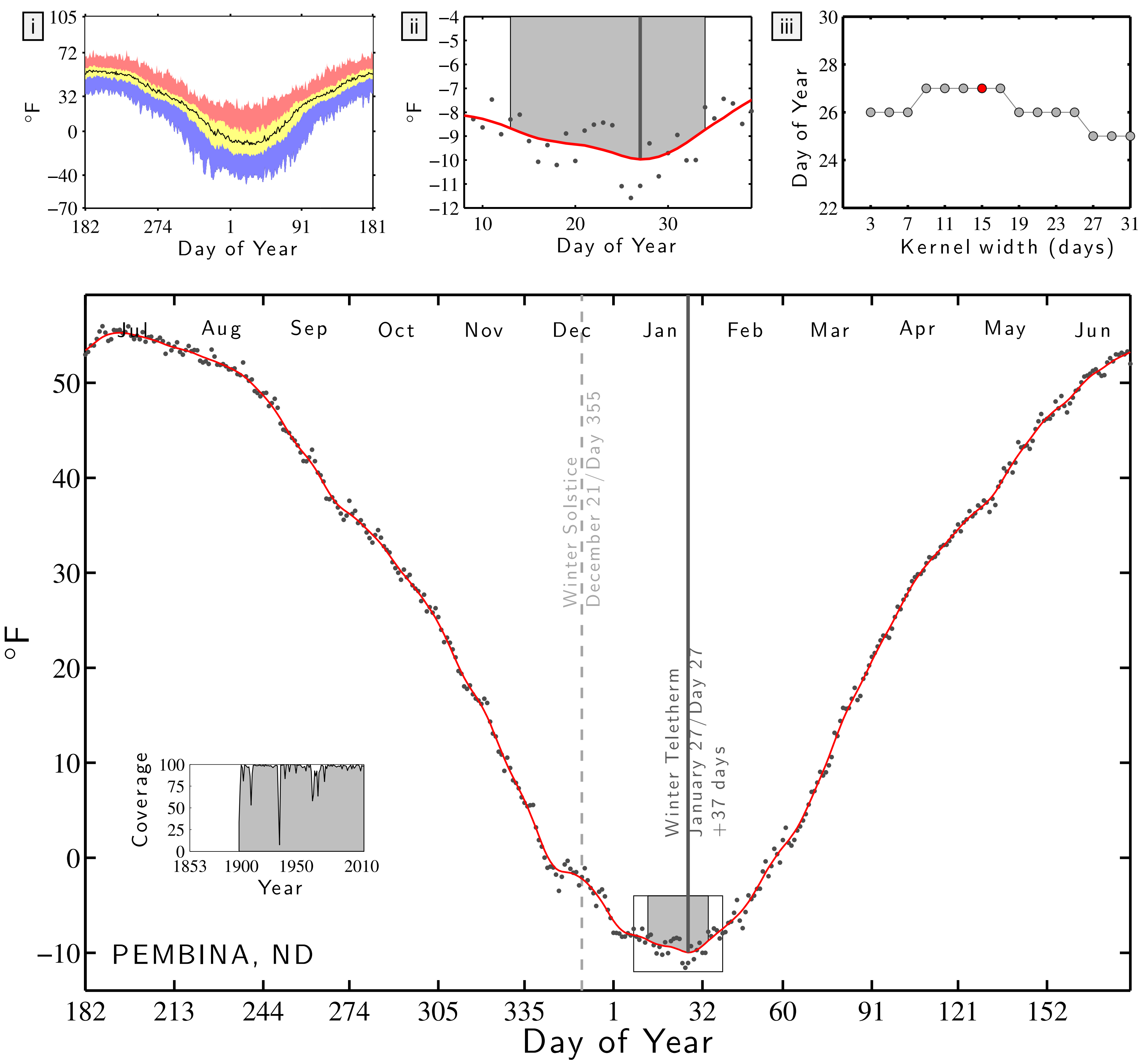

i
ii
iii
°F
Day of Year
Kernel width (days)
Jul
Aug
Sep
Oct
Nov
Dec
Jan
Feb
Mar
Apr
May
Jun
Winter Solstice
December 21/Day 355
Winter Teletherm
January 27/Day 27
+37 days
Coverage
Year
PEMBINA, ND

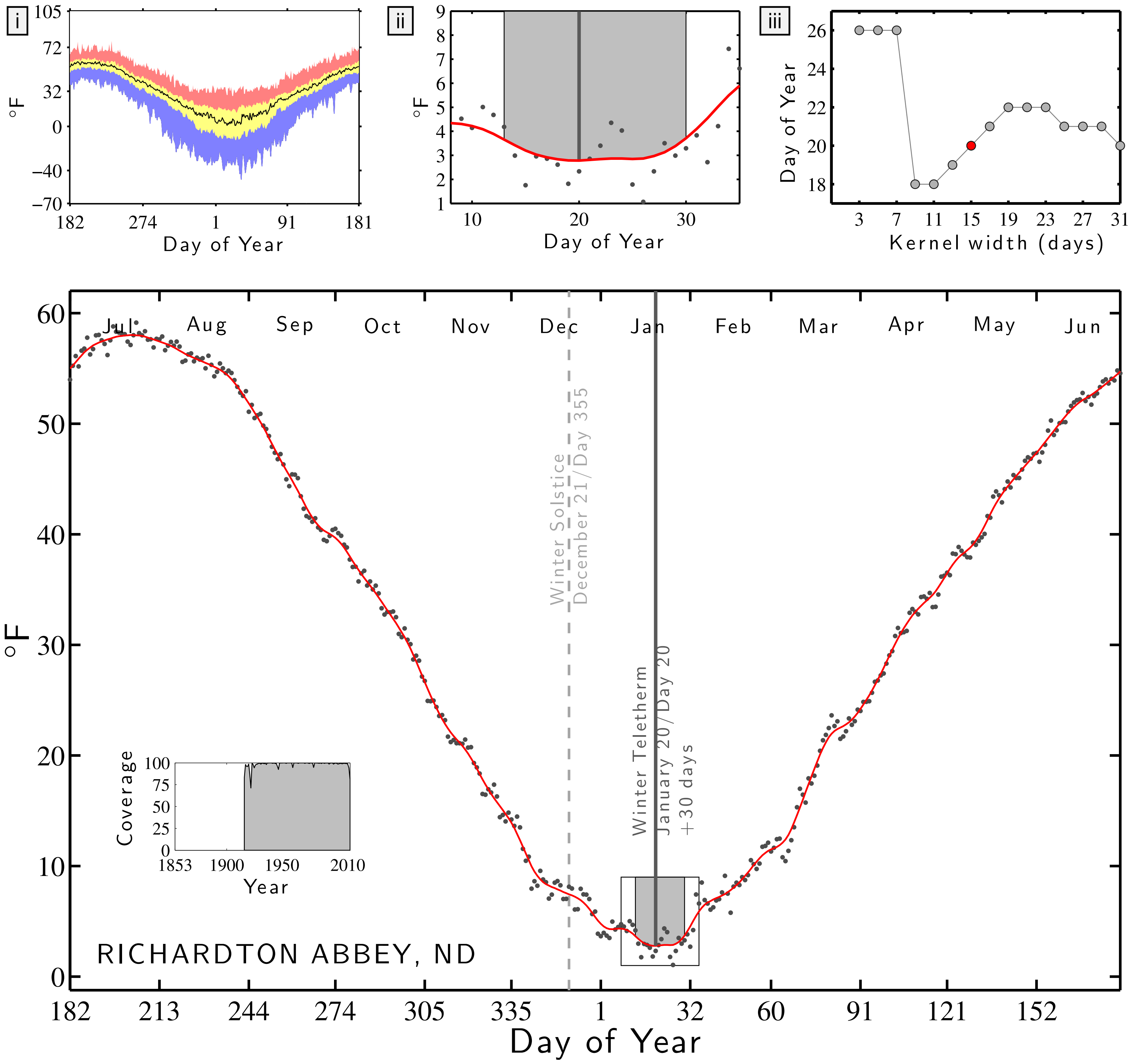

i
°F
105
72
32
0
−40
−70
182
274
1
91
181
Day of Year
ii
°F
9
8
7
6
5
4
3
2
1
10
20
30
Day of Year
iii
Day of Year
26
24
22
20
18
3
7
11
15
19
23
27
31
Kernel width (days)
°F
60
50
40
30
20
10
0
Jul
Aug
Sep
Oct
Nov
Dec
Jan
Feb
Mar
Apr
May
Jun
Winter Solstice
December 21/Day 355
Winter Teletherm
January 20/Day 20
+30 days
Coverage
100
75
50
25
0
1853
1900
1950
2010
Year
RICHARDTON ABBEY, ND
182
213
244
274
305
335
1
32
60
91
121
152
Day of Year

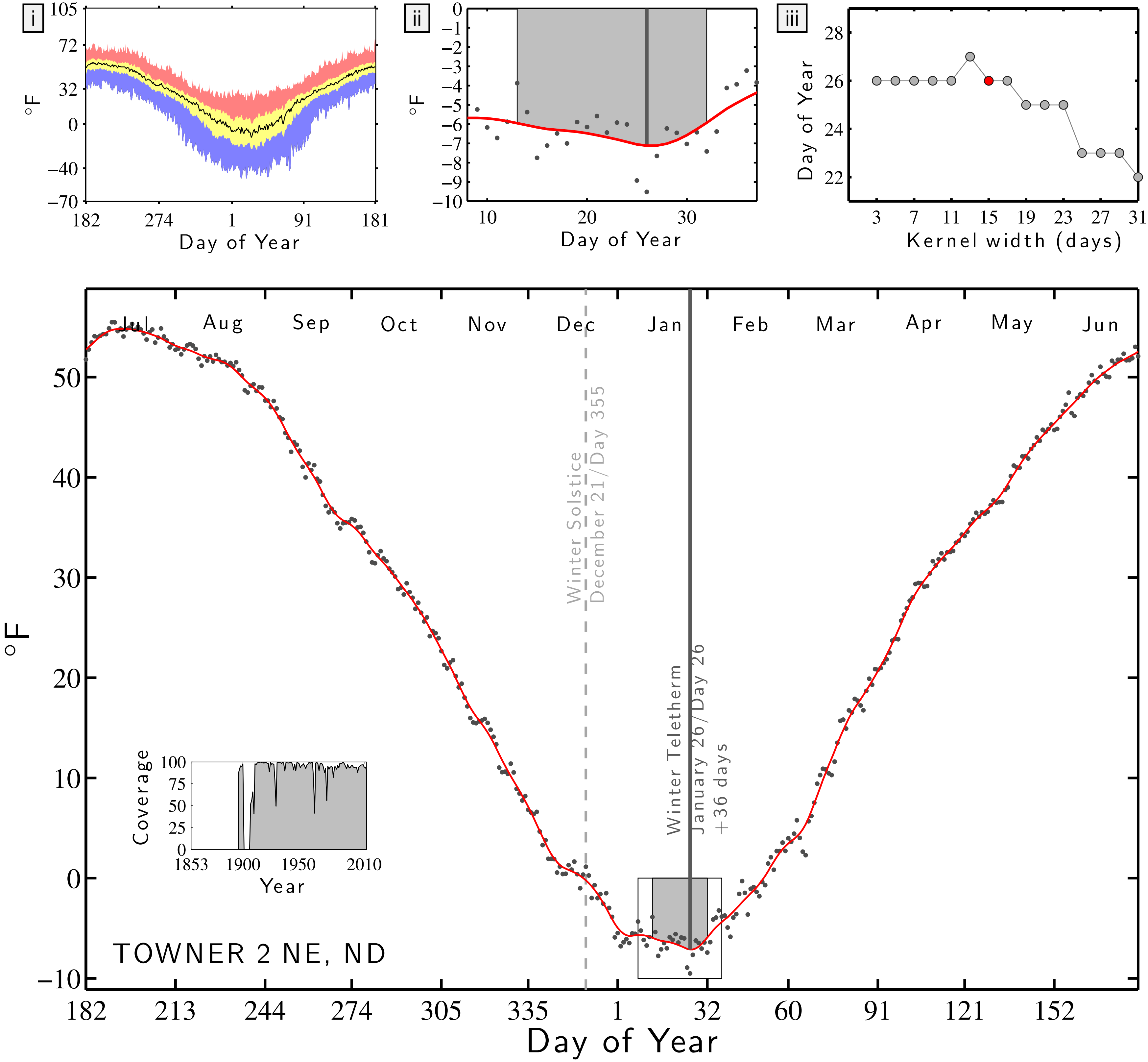

i
ii
iii
°F
Day of Year
Day of Year
Kernel width (days)
Jul
Aug
Sep
Oct
Nov
Dec
Jan
Feb
Mar
Apr
May
Jun
Winter Solstice
December 21/Day 355
Winter Teletherm
January 26/Day 26
+36 days
Coverage
Year
TOWNER 2 NE, ND

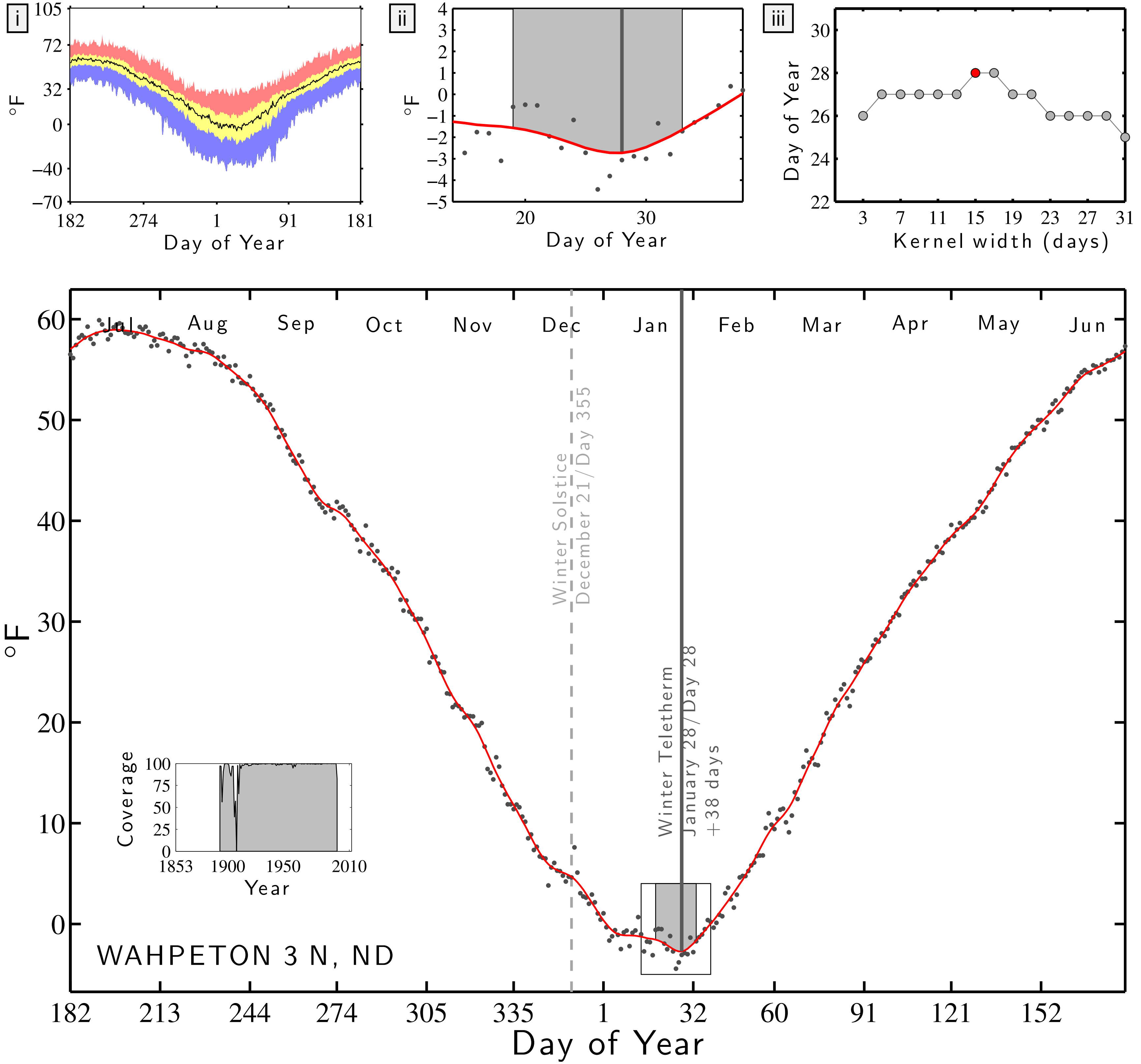

i
°F
105
72
32
0
−40
−70
182
274
1
91
181
Day of Year
ii
°F
Day of Year
iii
Day of Year
Kernel width (days)
Jul
Aug
Sep
Oct
Nov
Dec
Jan
Feb
Mar
Apr
May
Jun
Winter Solstice
December 21/Day 355
Winter Teletherm
January 28/Day 28
+38 days
Coverage
Year
WAHPETON 3 N, ND
°F
Day of Year
182
213
244
274
305
335
1
32
60
91
121
152

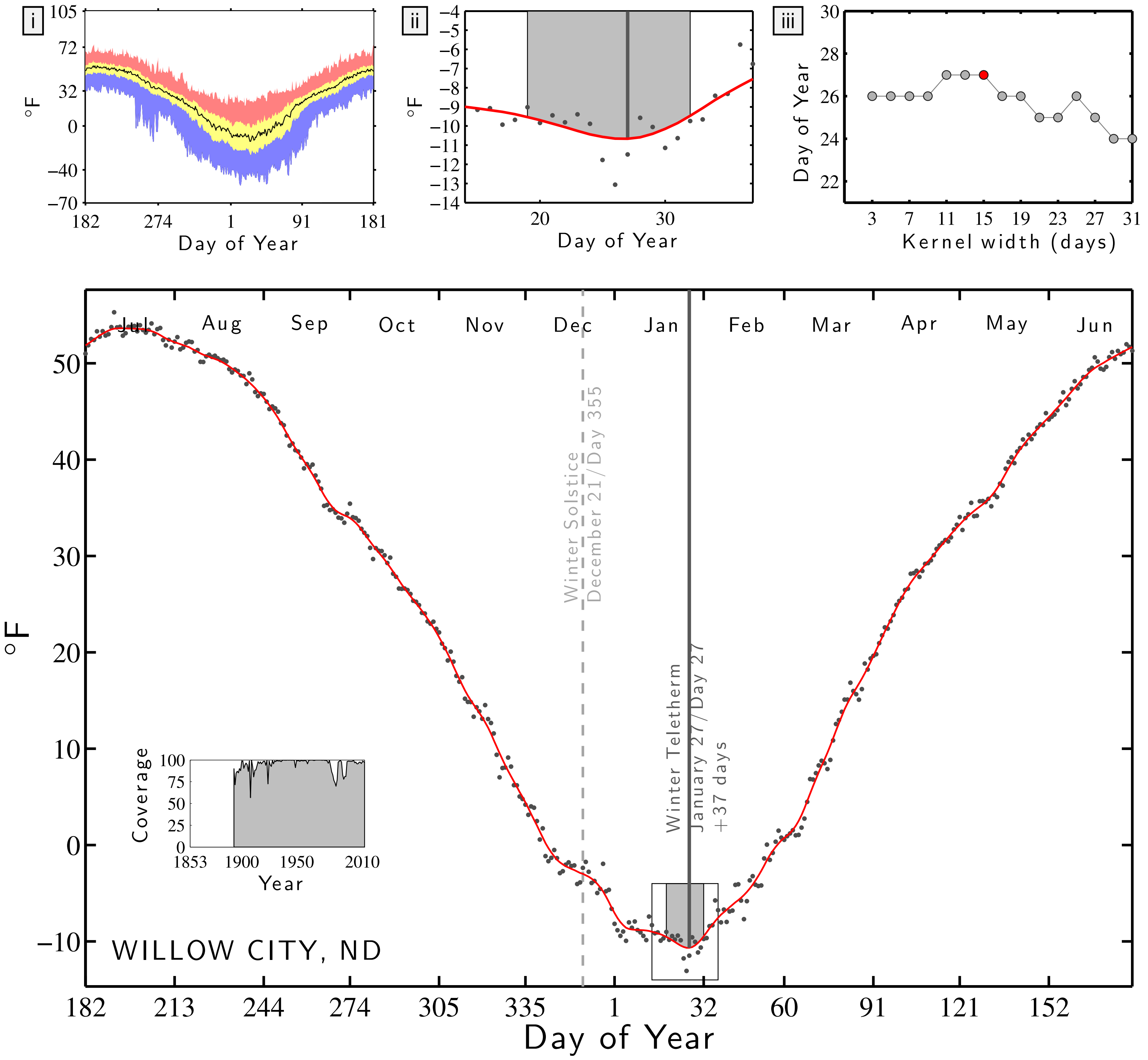

i
°F
Day of Year
ii
°F
Day of Year
iii
Day of Year
Kernel width (days)
Jul
Aug
Sep
Oct
Nov
Dec
Jan
Feb
Mar
Apr
May
Jun
Winter Solstice
December 21/Day 355
Winter Teletherm
January 27/Day 27
+37 days
Coverage
Year
WILLOW CITY, ND
°F
Day of Year

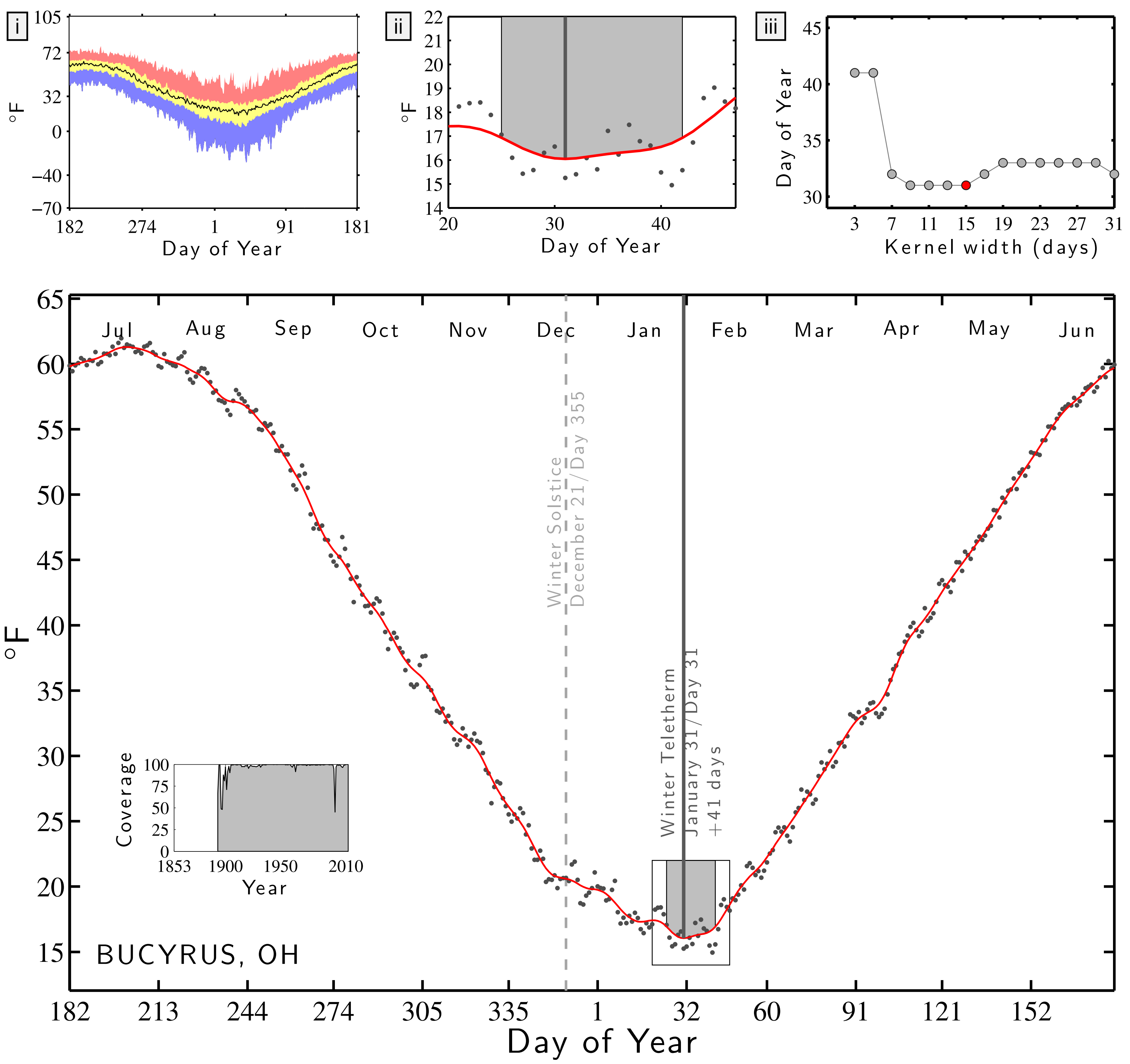

i
ii
iii
°F
Day of Year
Day of Year
Kernel width (days)
Jul
Aug
Sep
Oct
Nov
Dec
Jan
Feb
Mar
Apr
May
Jun
Winter Solstice
December 21/Day 355
Winter Teletherm
January 31/Day 31
+41 days
Coverage
Year
BUCYRUS, OH
Day of Year

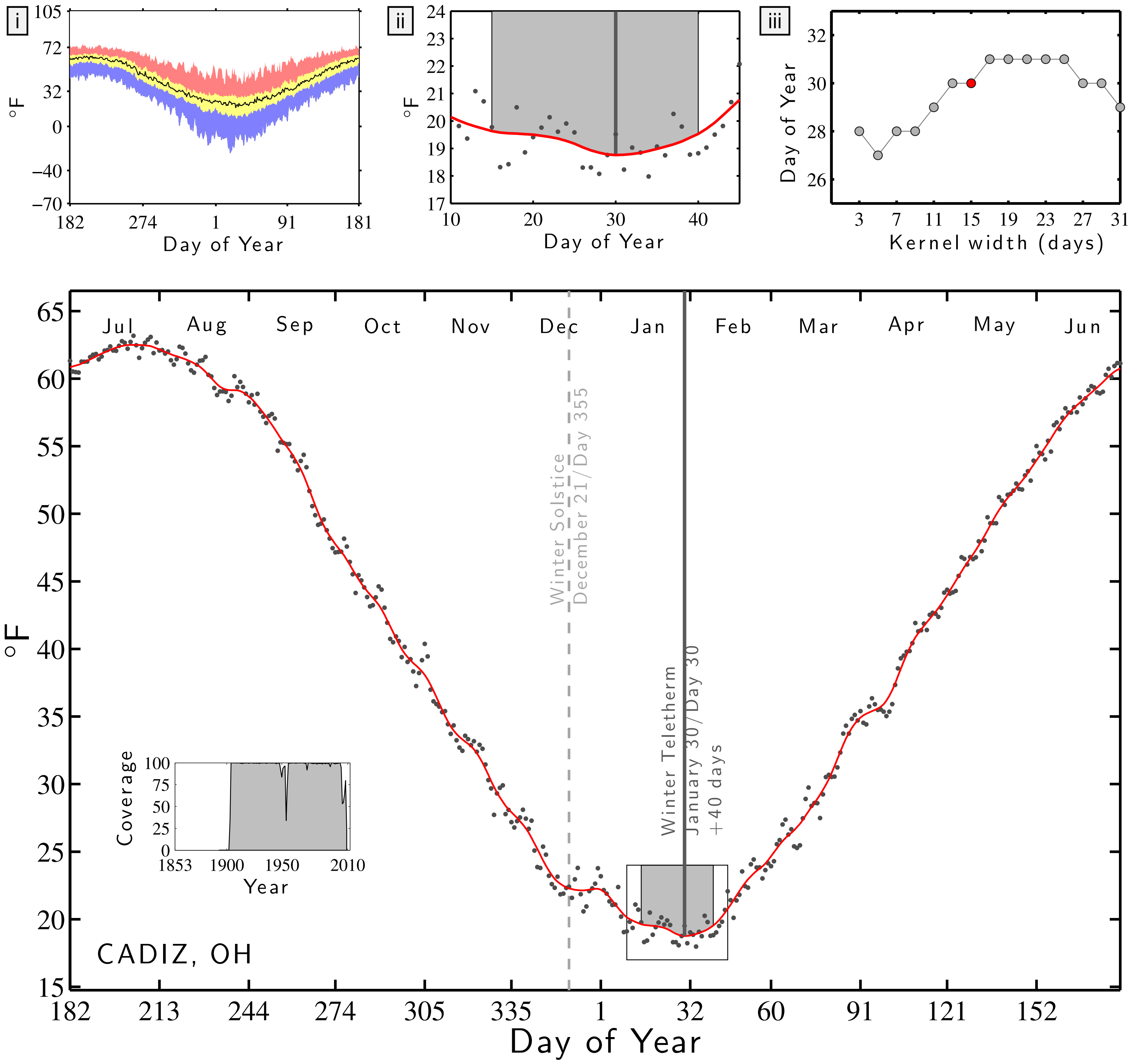

i
°F
Day of Year
ii
°F
Day of Year
iii
Day of Year
Kernel width (days)
Jul
Aug
Sep
Oct
Nov
Dec
Jan
Feb
Mar
Apr
May
Jun
Winter Solstice
December 21/Day 355
Winter Teletherm
January 30/Day 30
+40 days
Coverage
Year
CADIZ, OH
°F
Day of Year

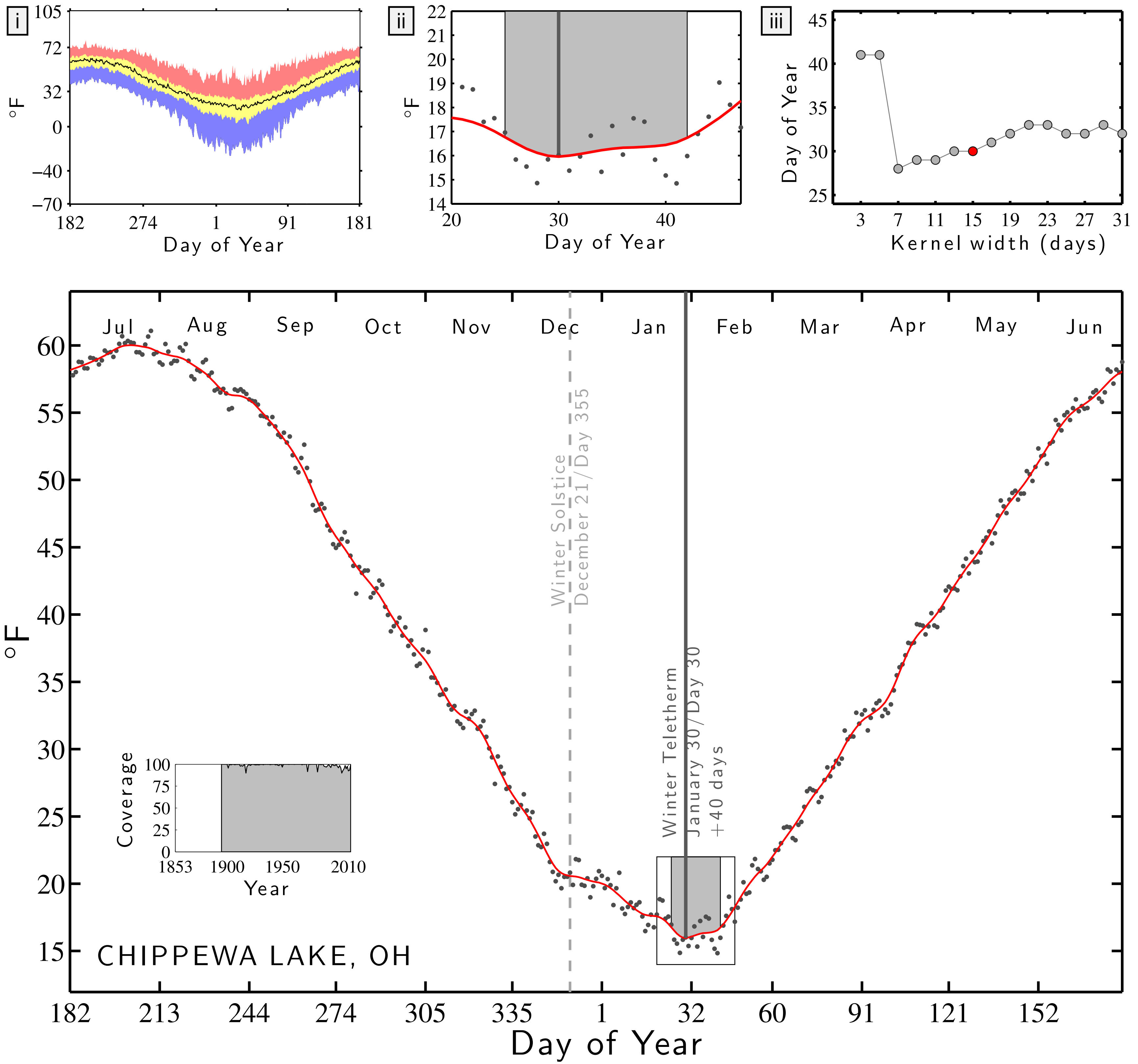

i
ii
iii
°F
Day of Year
Day of Year
Kernel width (days)
CHIPPEWA LAKE, OH
Jul
Aug
Sep
Oct
Nov
Dec
Jan
Feb
Mar
Apr
May
Jun
Winter Solstice
December 21/Day 355
Winter Teletherm
January 30/Day 30
+40 days
Coverage
Year

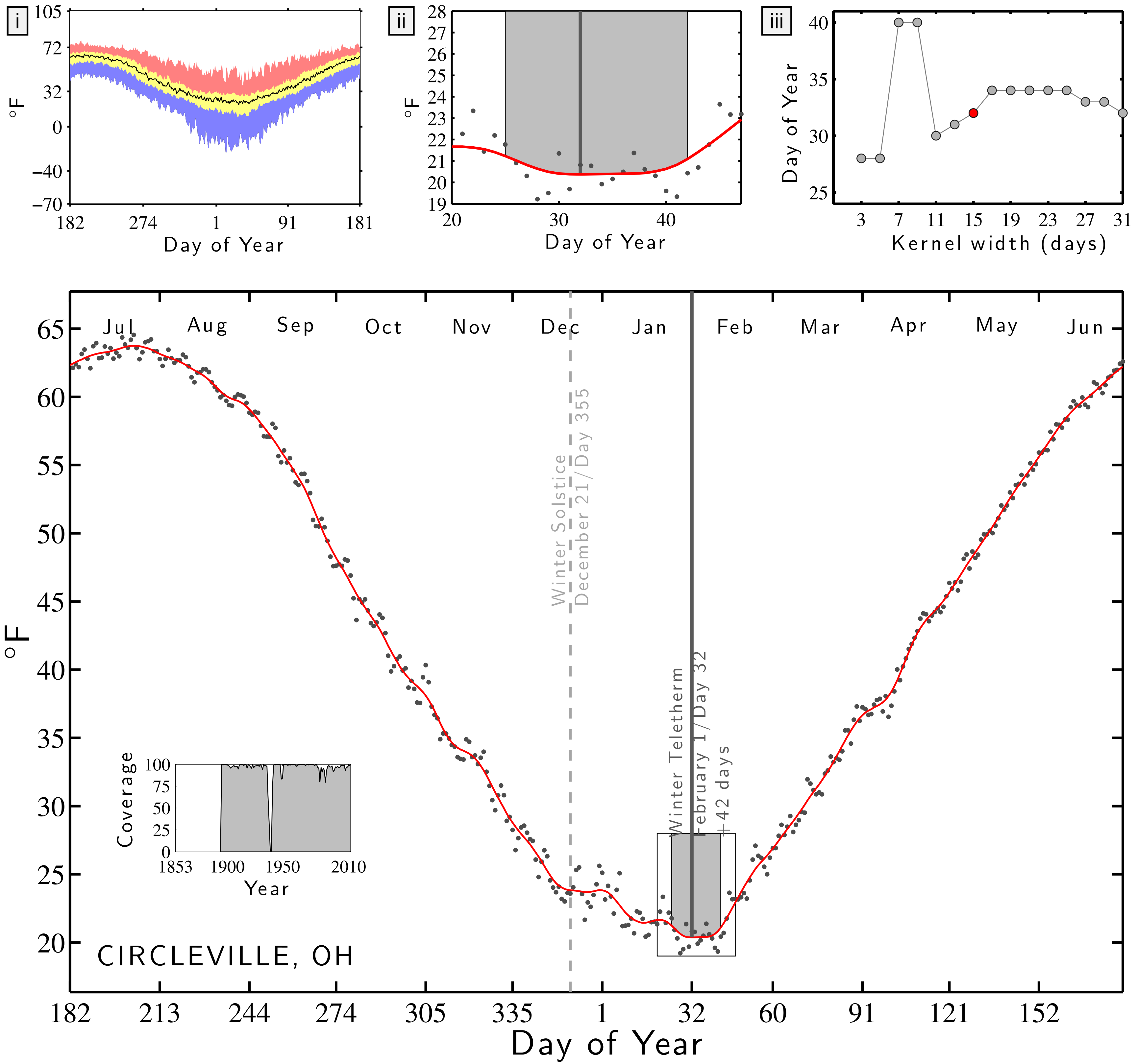

i
°F
Day of Year
ii
°F
Day of Year
iii
Day of Year
Kernel width (days)
Jul
Aug
Sep
Oct
Nov
Dec
Jan
Feb
Mar
Apr
May
Jun
Winter Solstice
December 21/Day 355
Winter Teletherm
February 1/Day 32
±42 days
Coverage
Year
CIRCLEVILLE, OH
°F
Day of Year

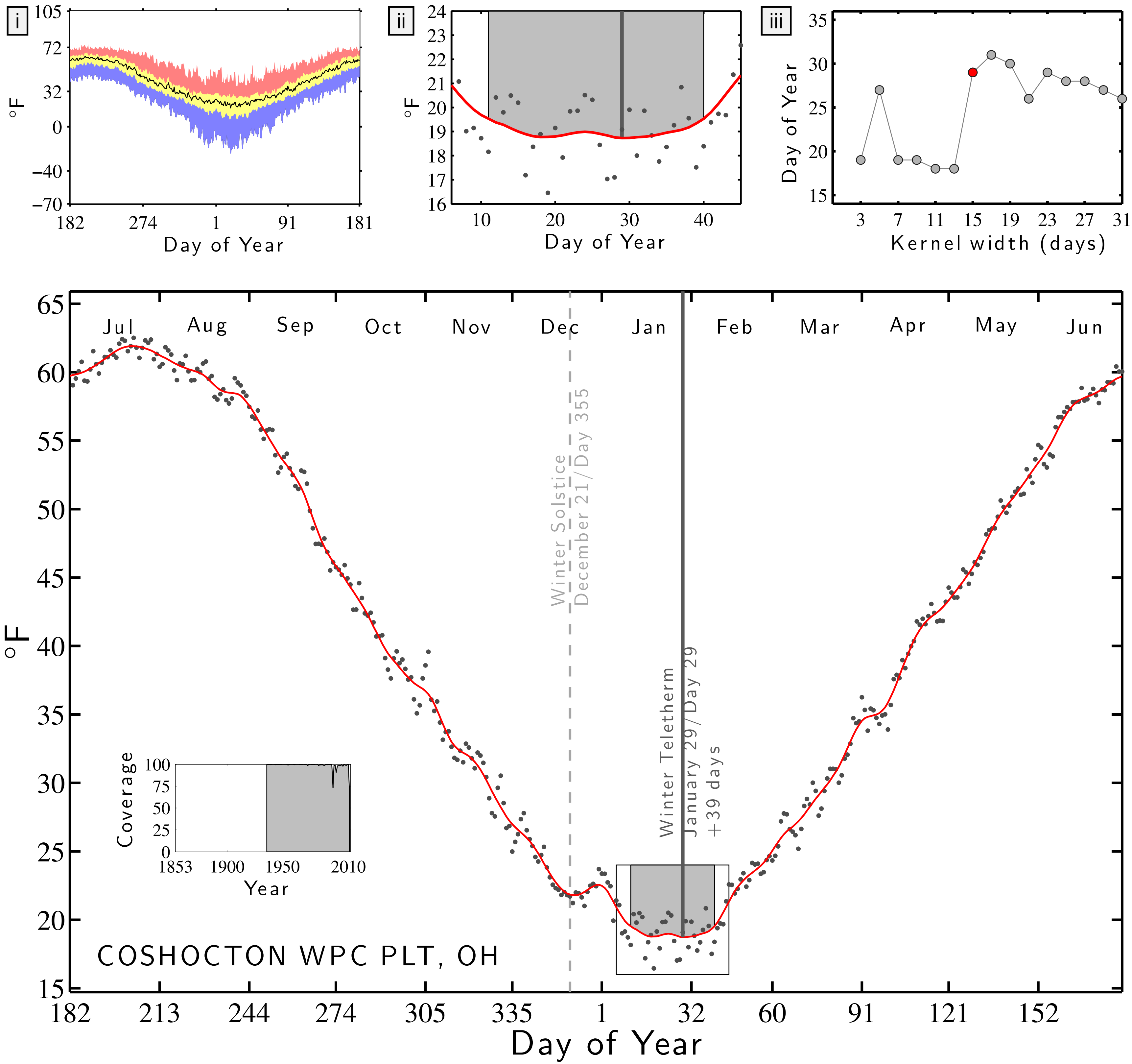

i
ii
iii
°F
Day of Year
Day of Year
Kernel width (days)
Jul
Aug
Sep
Oct
Nov
Dec
Jan
Feb
Mar
Apr
May
Jun
Winter Solstice
December 21/Day 355
Winter Teletherm
January 29/Day 29
+39 days
Coverage
Year
COSHOCTON WPC PLT, OH

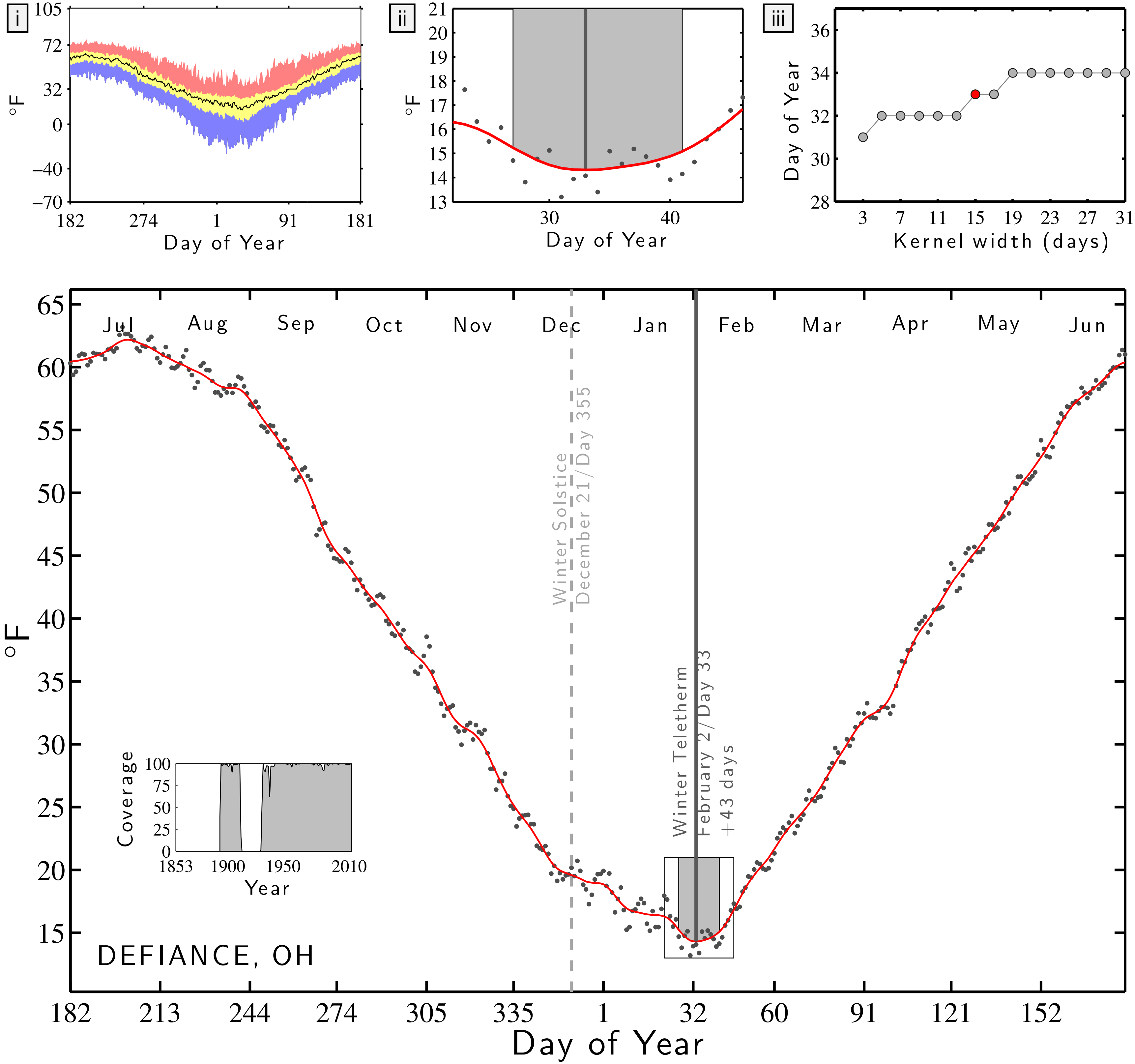

i
°F
Day of Year
ii
°F
Day of Year
iii
Day of Year
Kernel width (days)
Jul
Aug
Sep
Oct
Nov
Dec
Jan
Feb
Mar
Apr
May
Jun
Winter Solstice
December 21/Day 355
Winter Teletherm
February 2/Day 33
+43 days
Coverage
Year
DEFIANCE, OH
°F
Day of Year

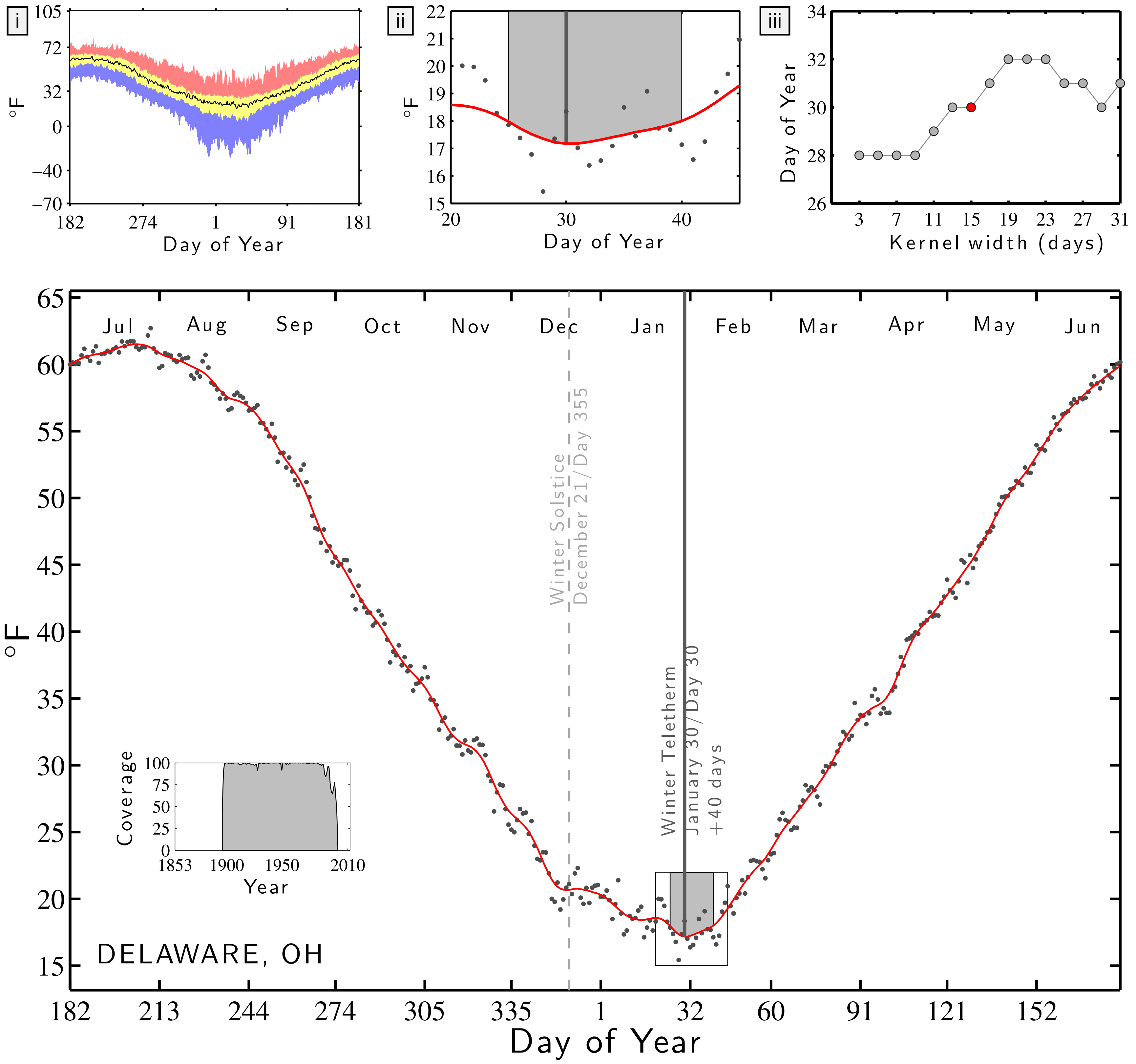

i
ii
iii
Day of Year
°F
Kernel width (days)
Jul
Aug
Sep
Oct
Nov
Dec
Jan
Feb
Mar
Apr
May
Jun
Winter Solstice
December 21/Day 355
Winter Teletherm
January 30/Day 30
+40 days
Coverage
Year
DELAWARE, OH

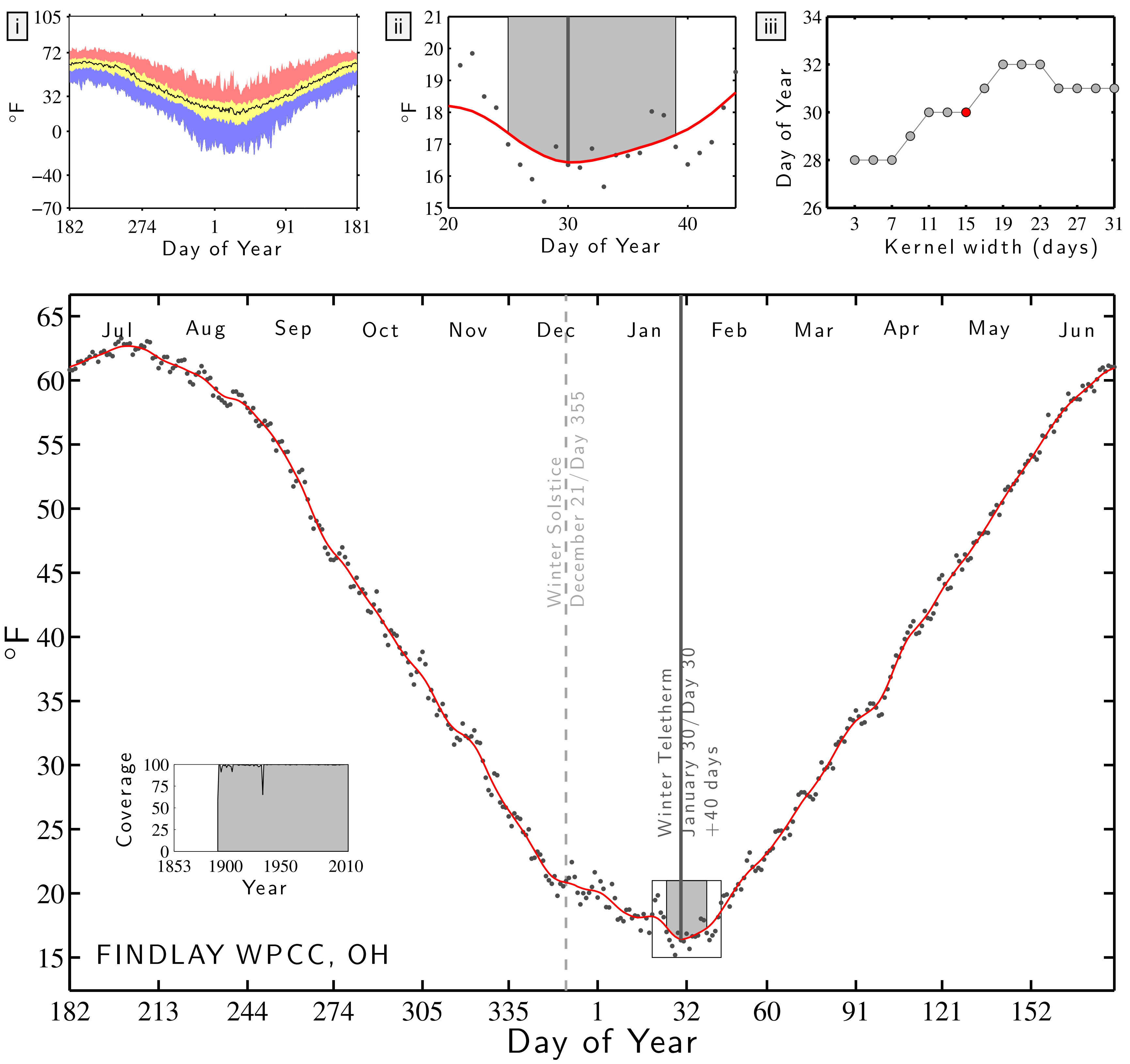

i
ii
iii
°F
Day of Year
Kernel width (days)
Jul
Aug
Sep
Oct
Nov
Dec
Jan
Feb
Mar
Apr
May
Jun
Winter Solstice
December 21/Day 355
Winter Teletherm
January 30/Day 30
+40 days
Coverage
Year
FINDLAY WPCC, OH

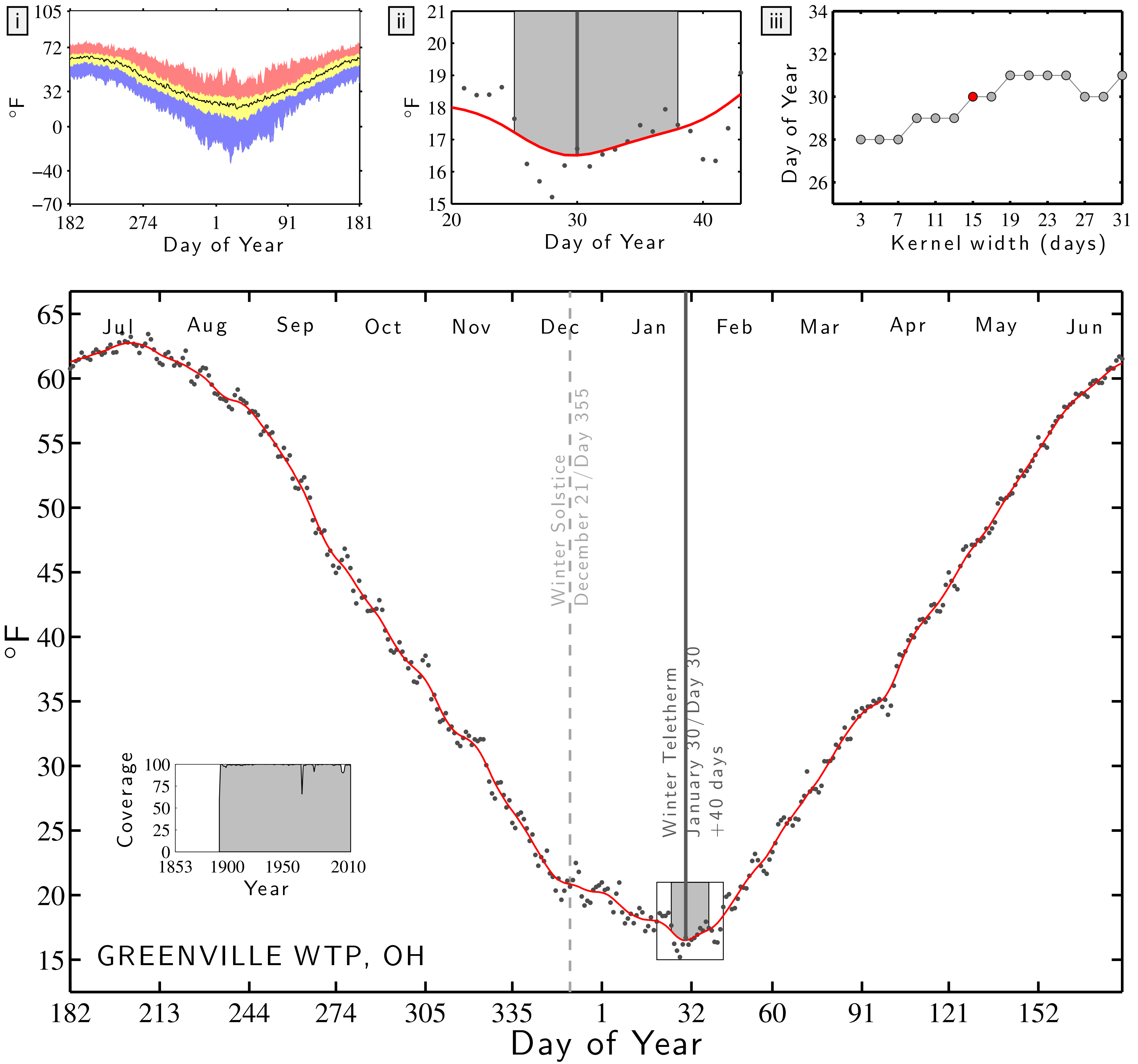

i
ii
iii
°F
Day of Year
Kernel width (days)
Jul
Aug
Sep
Oct
Nov
Dec
Jan
Feb
Mar
Apr
May
Jun
Winter Solstice
December 21/Day 355
Winter Teletherm
January 30/Day 30
+40 days
Coverage
Year
GREENVILLE WTP, OH

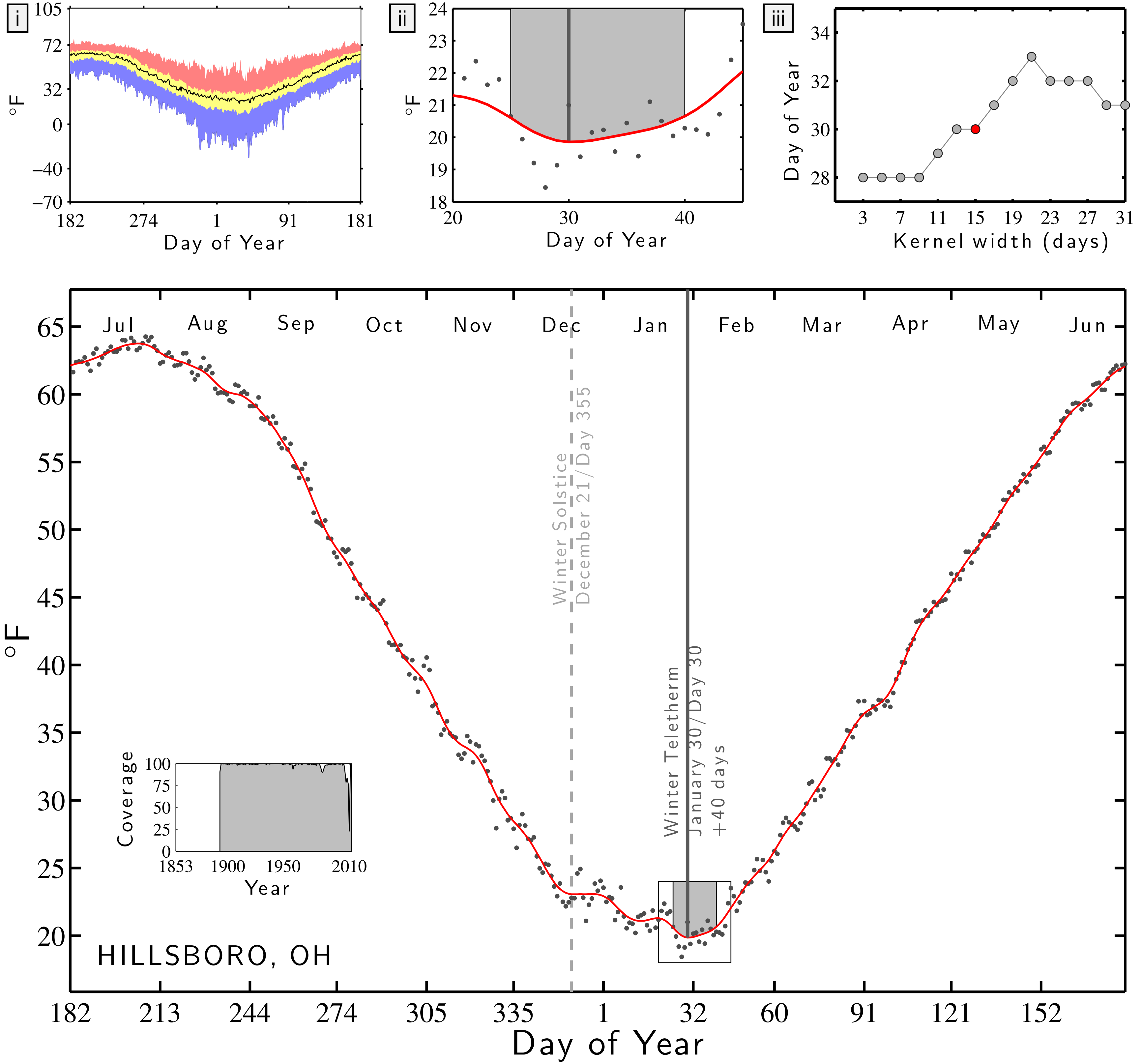
i
ii
iii
°F
Day of Year
Day of Year
Kernel width (days)
Jul
Aug
Sep
Oct
Nov
Dec
Jan
Feb
Mar
Apr
May
Jun
Winter Solstice
December 21/Day 355
Winter Teletherm
January 30/Day 30
+40 days
Coverage
Year
HILLSBORO, OH

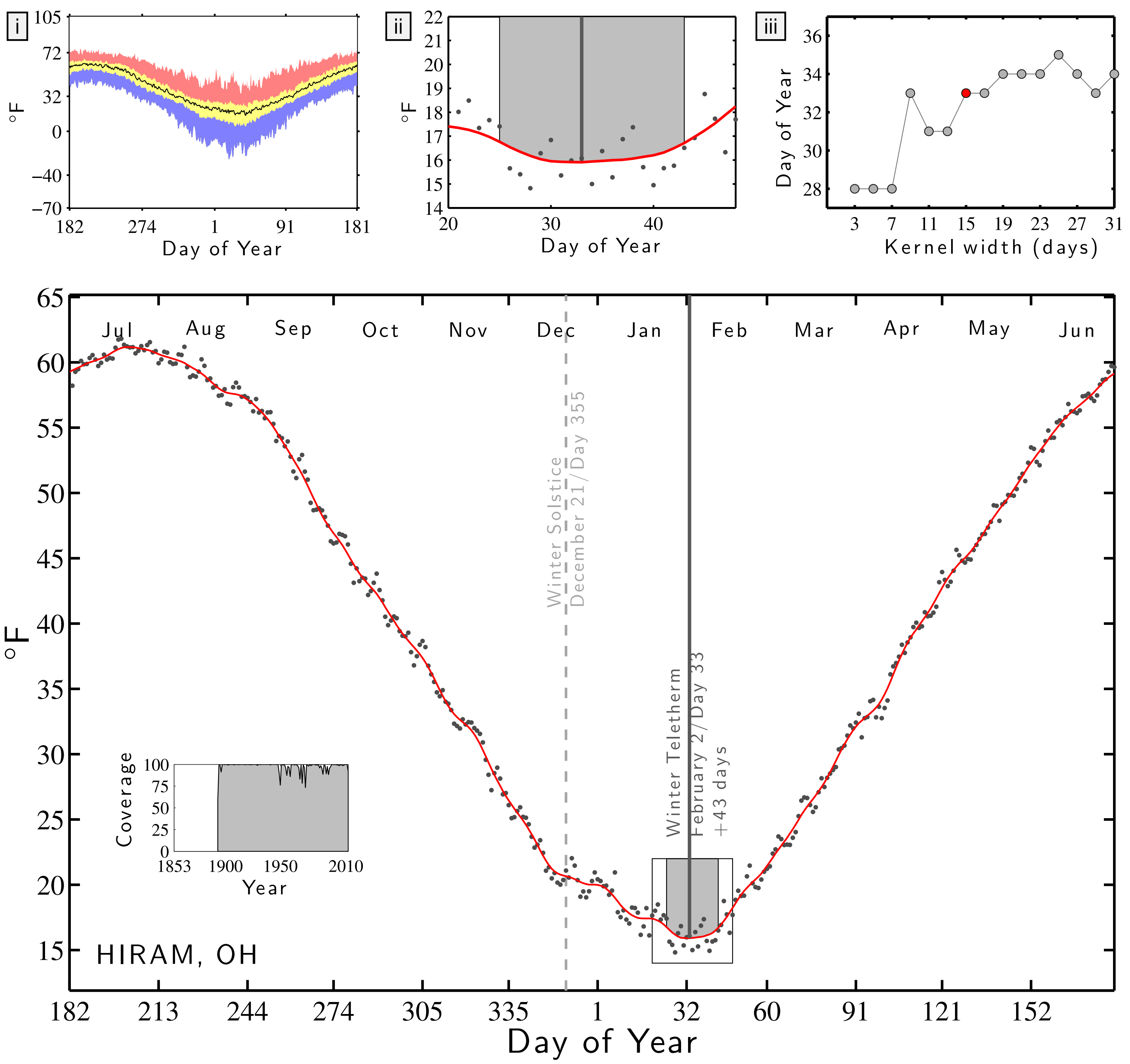

i
ii
iii
Day of Year
°F
Kernel width (days)
Jul
Aug
Sep
Oct
Nov
Dec
Jan
Feb
Mar
Apr
May
Jun
Winter Solstice
December 21/Day 355
Winter Teletherm
February 2/Day 33
+43 days
Coverage
Year
HIRAM, OH

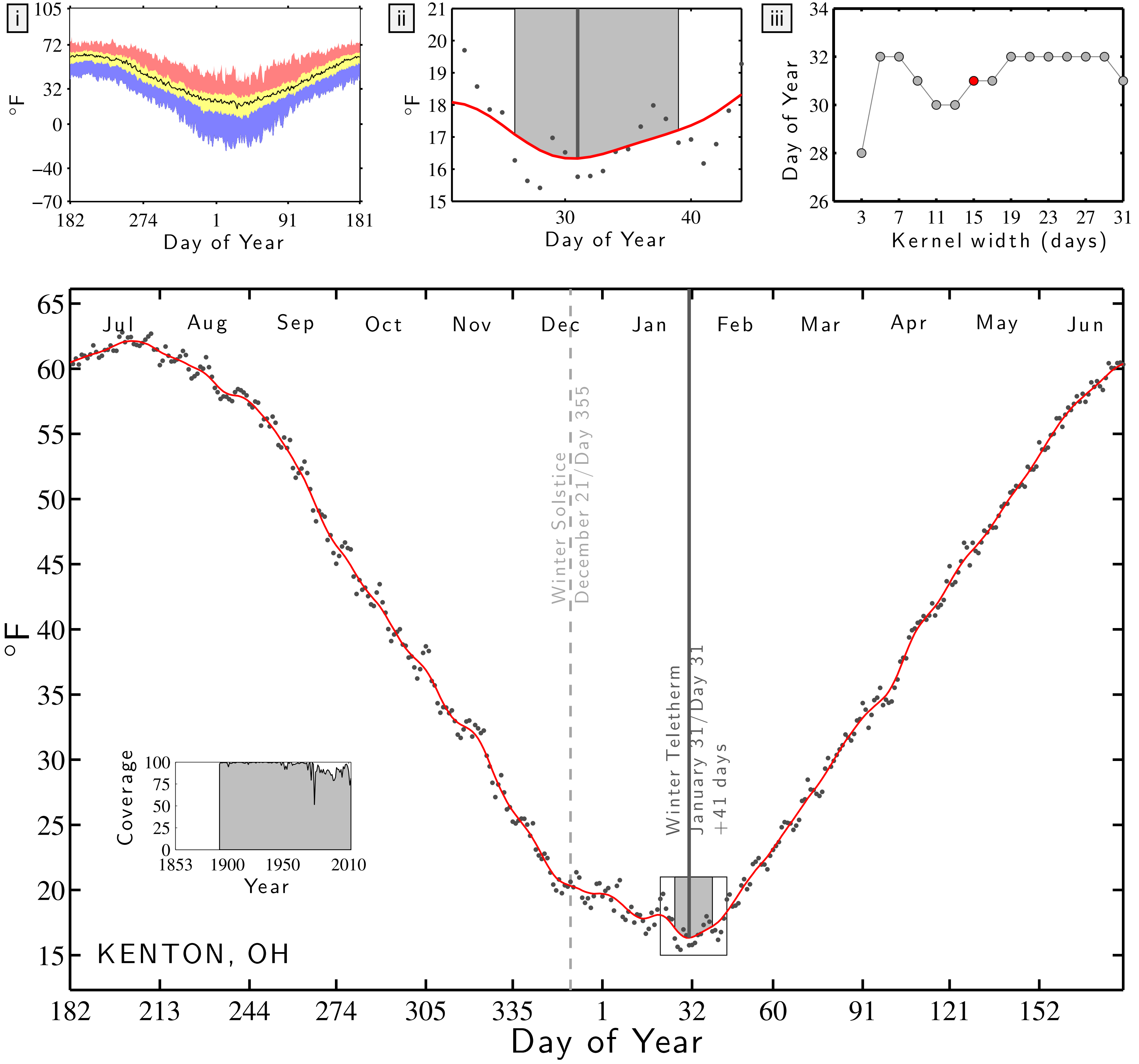

i
ii
iii
°F
Day of Year
Kernel width (days)
Jul
Aug
Sep
Oct
Nov
Dec
Jan
Feb
Mar
Apr
May
Jun
Winter Solstice
December 21/Day 355
Winter Teletherm
January 31/Day 31
+41 days
Coverage
Year
KENTON, OH

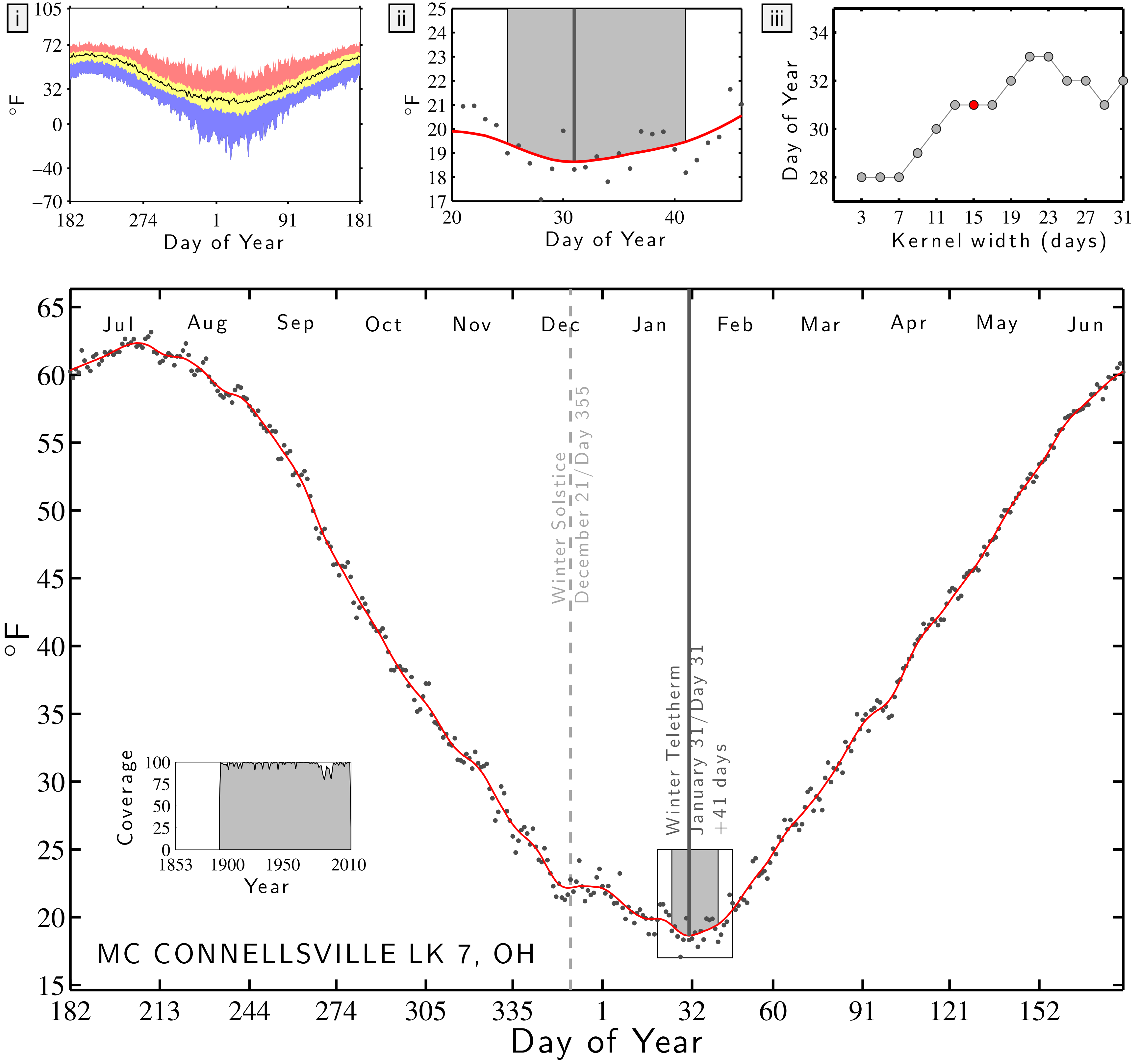

i
°F
Day of Year
ii
Day of Year
iii
Day of Year
Kernel width (days)
Jul
Aug
Sep
Oct
Nov
Dec
Jan
Feb
Mar
Apr
May
Jun
Winter Solstice
December 21/Day 355
Winter Teletherm
January 31/Day 31
+41 days
Coverage
Year
MC CONNELLSVILLE LK 7, OH
Day of Year

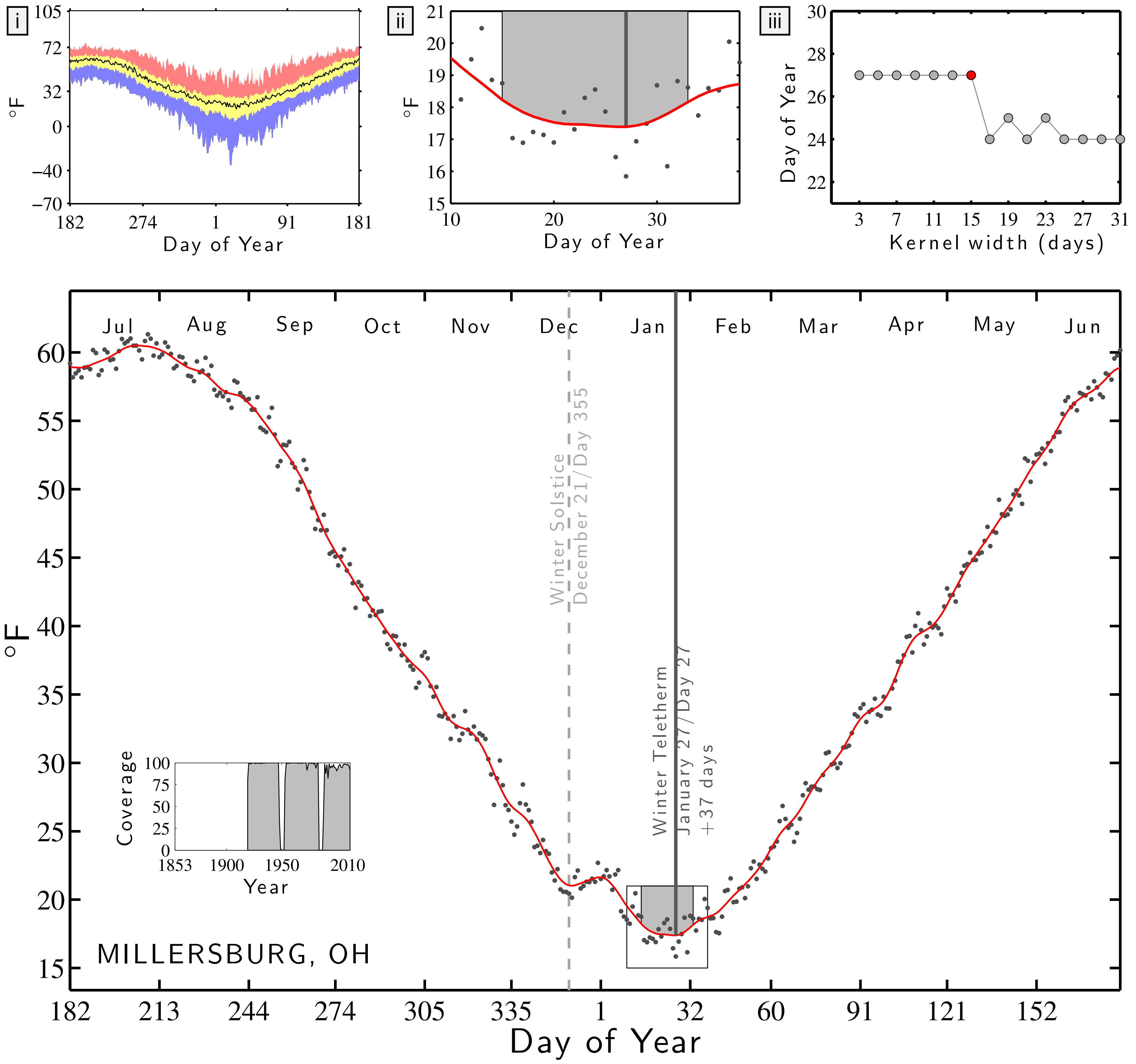
i
105
72
32
0
−40
−70
°F
182
274
1
91
181
Day of Year
ii
21
20
19
18
17
16
15
°F
10
20
30
Day of Year
iii
30
28
26
24
22
Day of Year
3
7
11
15
19
23
27
31
Kernel width (days)
Jul
Aug
Sep
Oct
Nov
Dec
Jan
Feb
Mar
Apr
May
Jun
60
55
50
45
40
35
30
25
20
15
°F
Winter Solstice
December 21/Day 355
Winter Teletherm
January 27/Day 27
+37 days
Coverage
100
75
50
25
0
1853
1900
1950
2010
Year
MILLERSBURG, OH
182
213
244
274
305
335
1
32
60
91
121
152
Day of Year

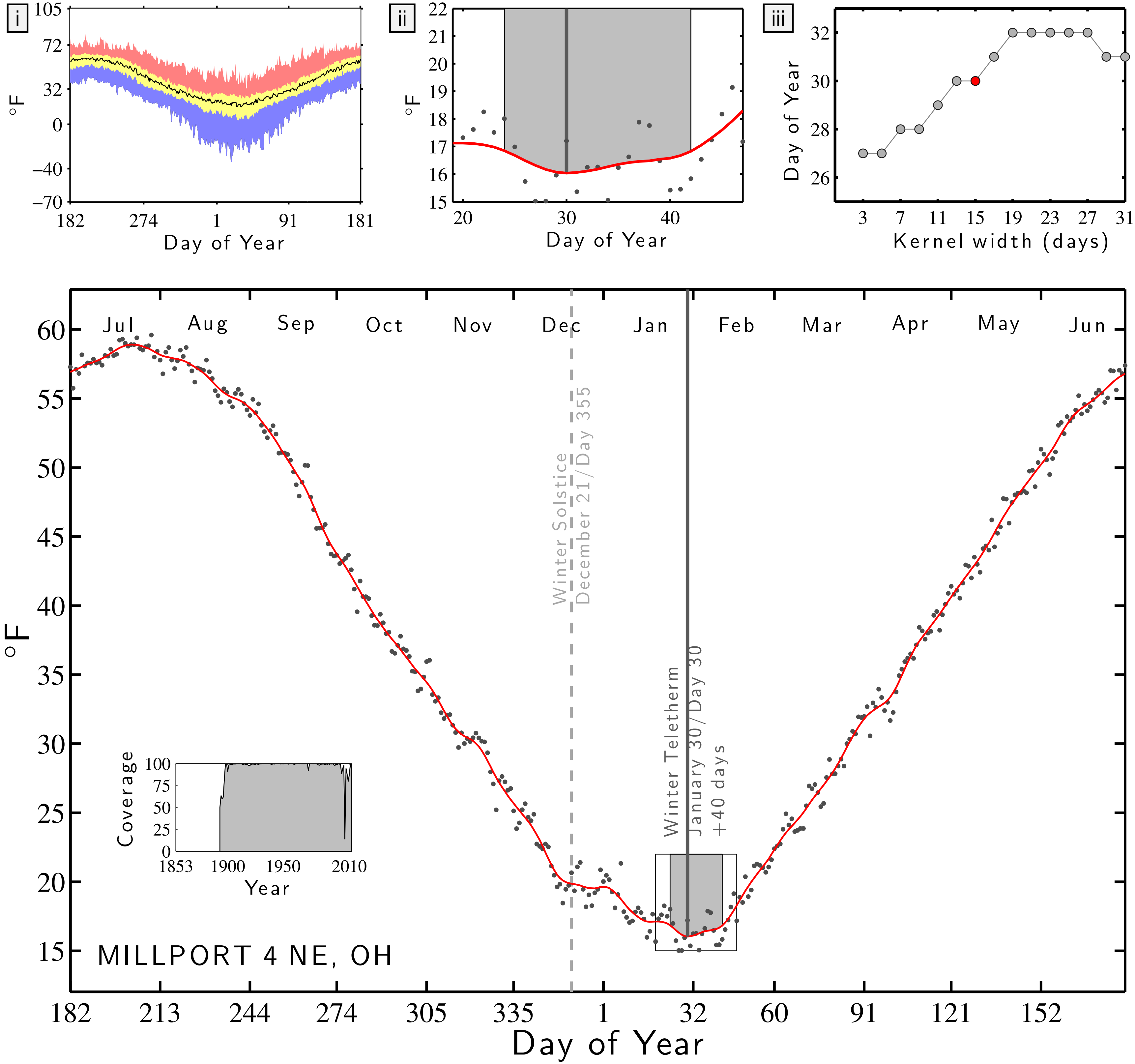

i
ii
iii
°F
Day of Year
Kernel width (days)
Jul
Aug
Sep
Oct
Nov
Dec
Jan
Feb
Mar
Apr
May
Jun
Winter Solstice
December 21/Day 355
Winter Teletherm
January 30/Day 30
+40 days
Coverage
Year
MILLPORT 4 NE, OH

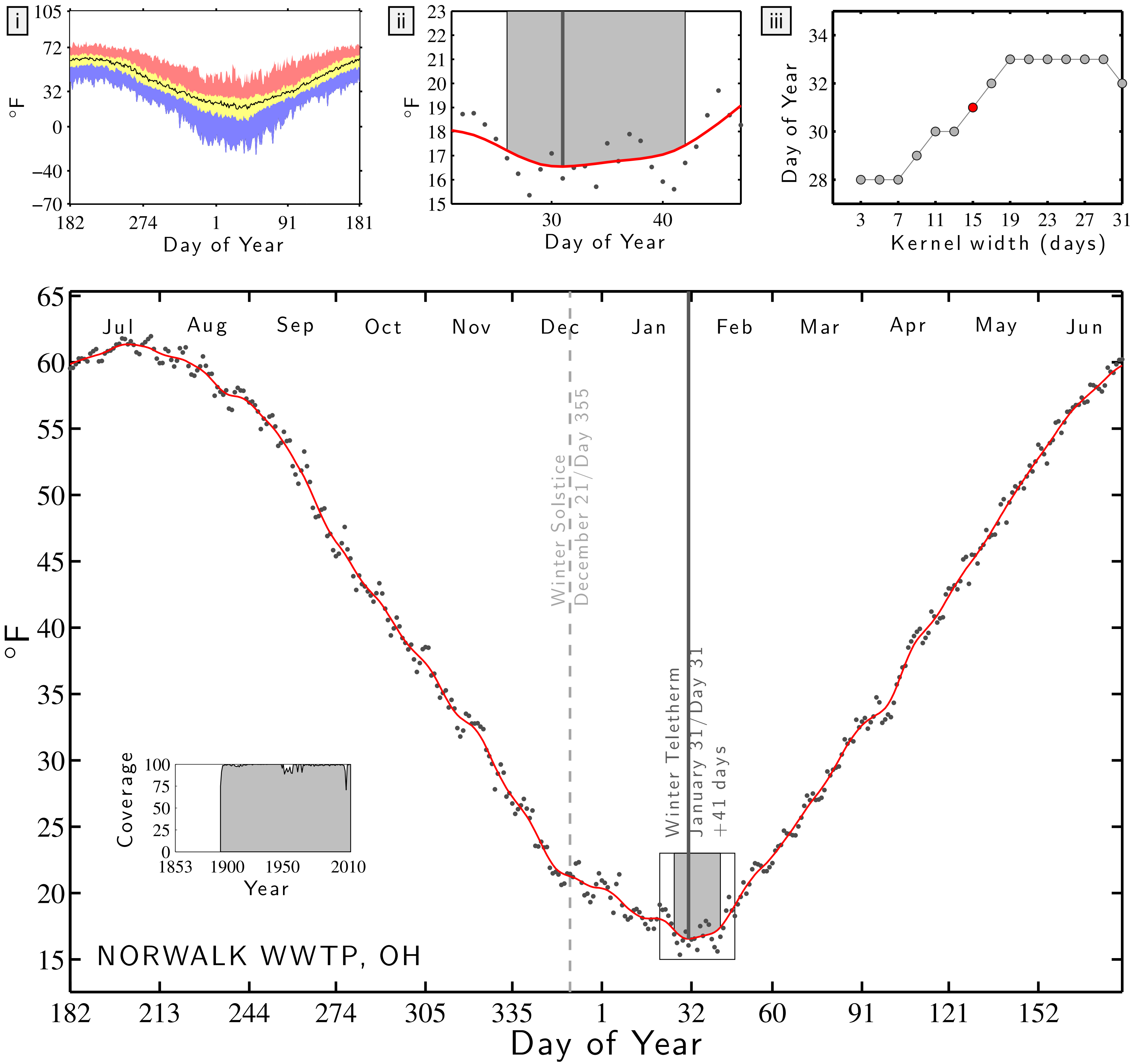

i
°F
105
72
32
0
−40
−70
182
274
1
91
181
Day of Year
ii
23
22
21
20
19
18
17
16
15
30
40
Day of Year
iii
Day of Year
34
32
30
28
3
7
11
15
19
23
27
31
Kernel width (days)
Jul
Aug
Sep
Oct
Nov
Dec
Jan
Feb
Mar
Apr
May
Jun
65
60
55
50
45
40
35
30
25
20
15
Winter Solstice
December 21/Day 355
Winter Teletherm
January 31/Day 31
+41 days
Coverage
100
75
50
25
0
1853
1900
1950
2010
Year
NORWALK WWTP, OH
182
213
244
274
305
335
1
32
60
91
121
152
Day of Year

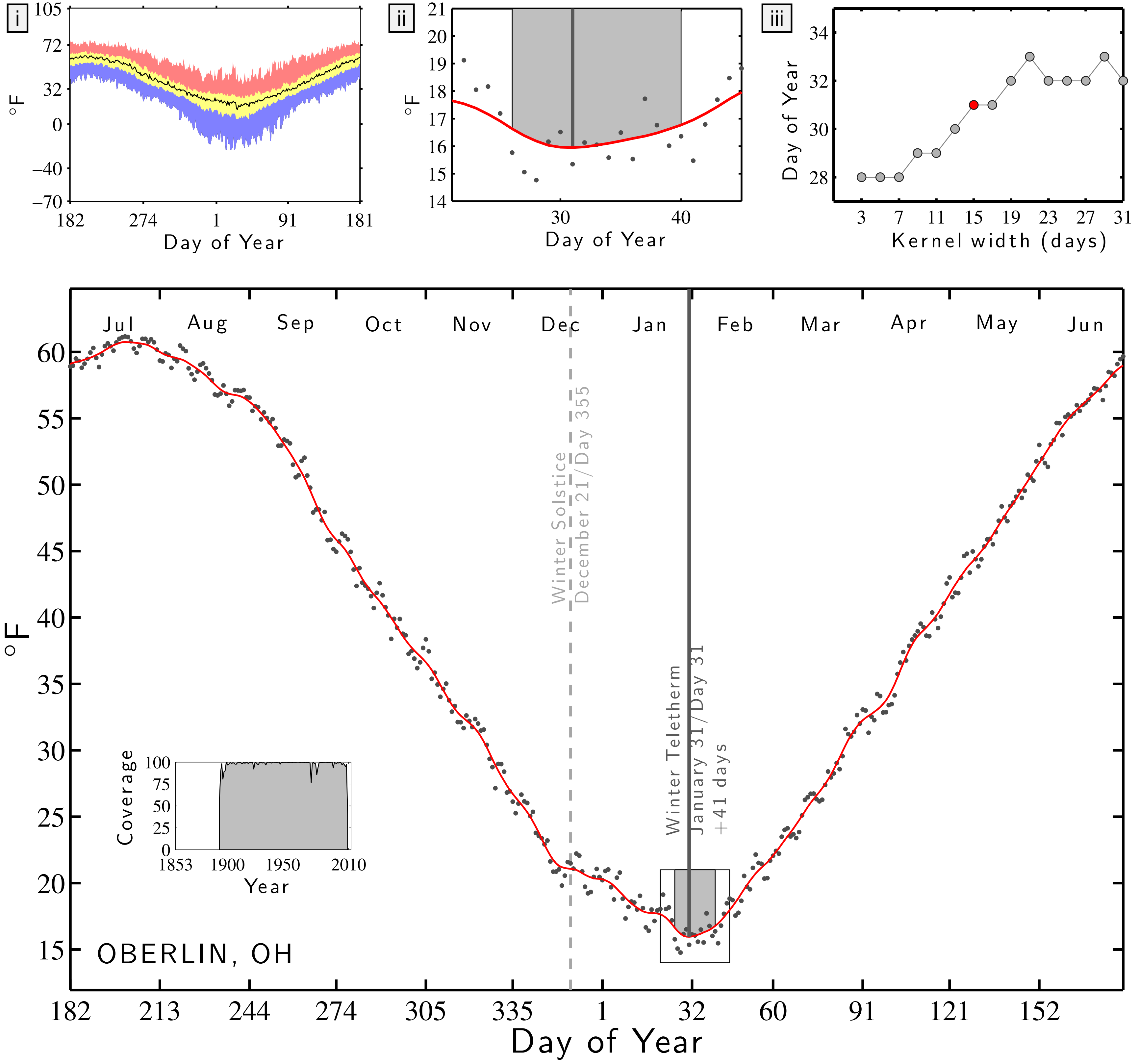

i
ii
iii
°F
Day of Year
Day of Year
Kernel width (days)
Jul
Aug
Sep
Oct
Nov
Dec
Jan
Feb
Mar
Apr
May
Jun
Winter Solstice
December 21/Day 355
Winter Teletherm
January 31/Day 31
+41 days
Coverage
Year
OBERLIN, OH
°F
Day of Year

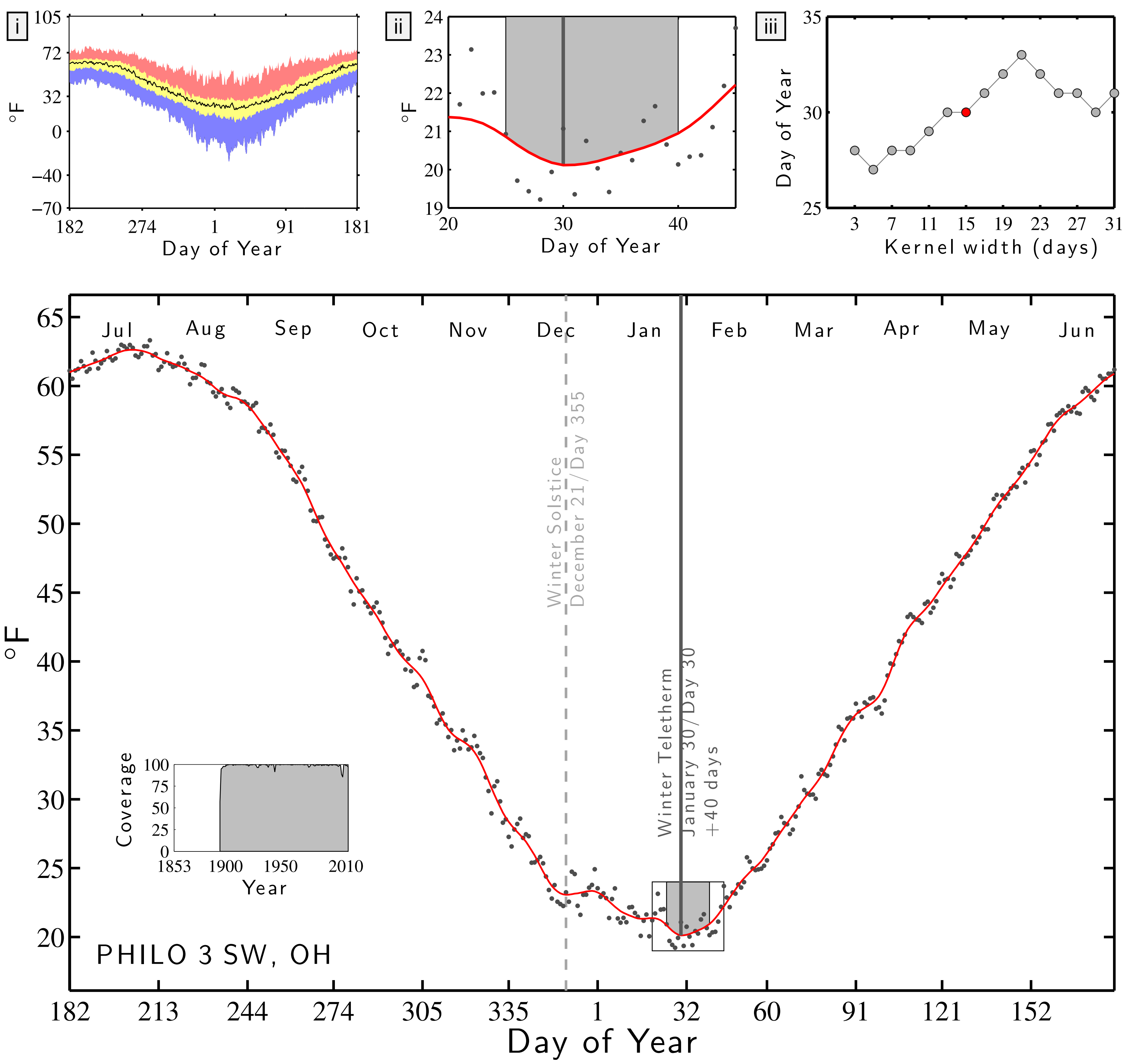

i
°F
Day of Year
ii
°F
Day of Year
iii
Day of Year
Kernel width (days)
Jul
Aug
Sep
Oct
Nov
Dec
Jan
Feb
Mar
Apr
May
Jun
Winter Solstice
December 21/Day 355
Winter Teletherm
January 30/Day 30
+40 days
Coverage
Year
PHILO 3 SW, OH
°F
Day of Year

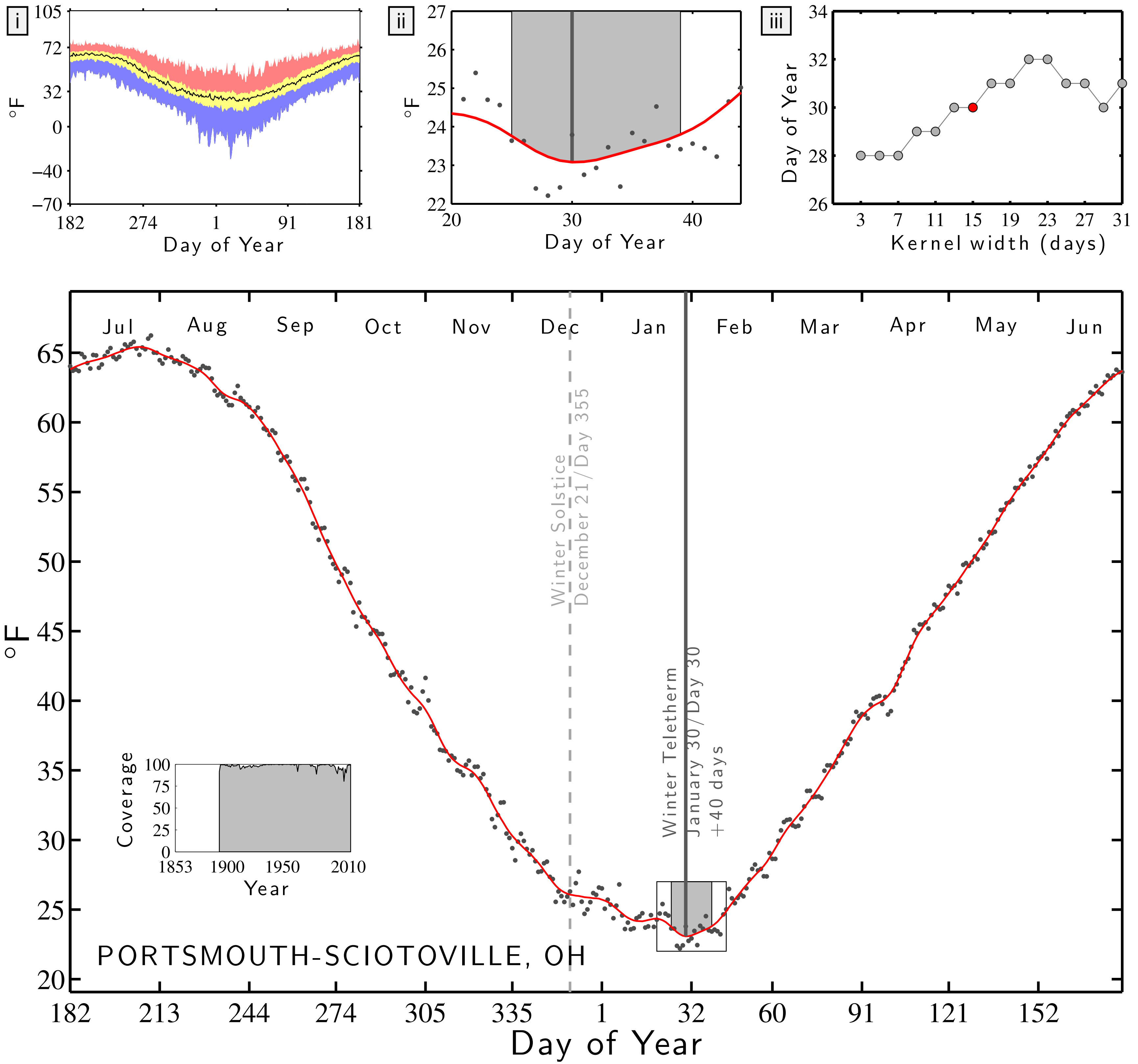
i
ii
iii
°F
Day of Year
Kernel width (days)
Jul
Aug
Sep
Oct
Nov
Dec
Jan
Feb
Mar
Apr
May
Jun
Winter Solstice
December 21/Day 355
Winter Teletherm
January 30/Day 30
+40 days
Coverage
Year
PORTSMOUTH-SCIOTOVILLE, OH

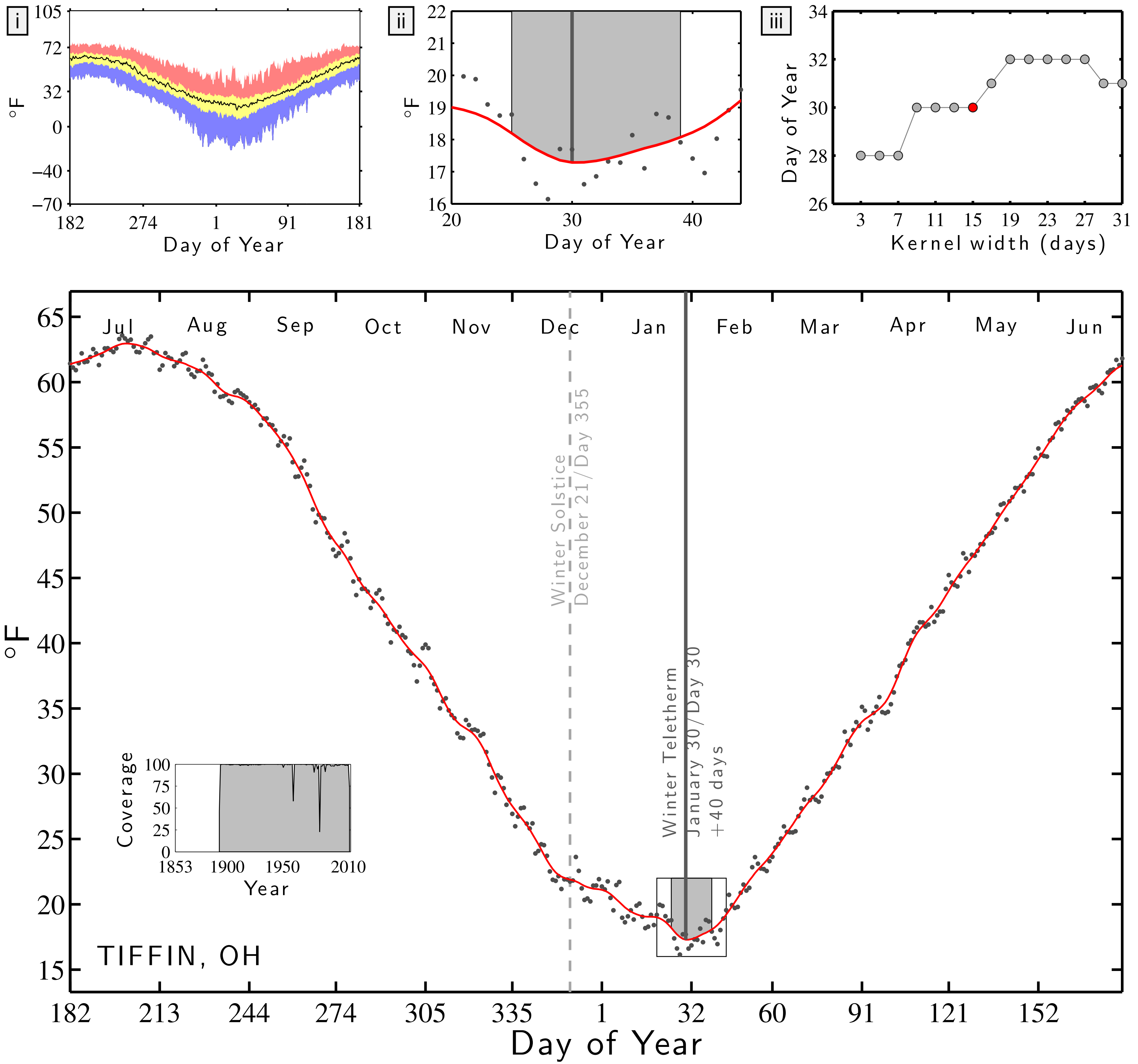

i
ii
iii
°F
Day of Year
Day of Year
Kernel width (days)
Jul
Aug
Sep
Oct
Nov
Dec
Jan
Feb
Mar
Apr
May
Jun
Winter Solstice
December 21/Day 355
Winter Teletherm
January 30/Day 30
+40 days
Coverage
Year
TIFFIN, OH

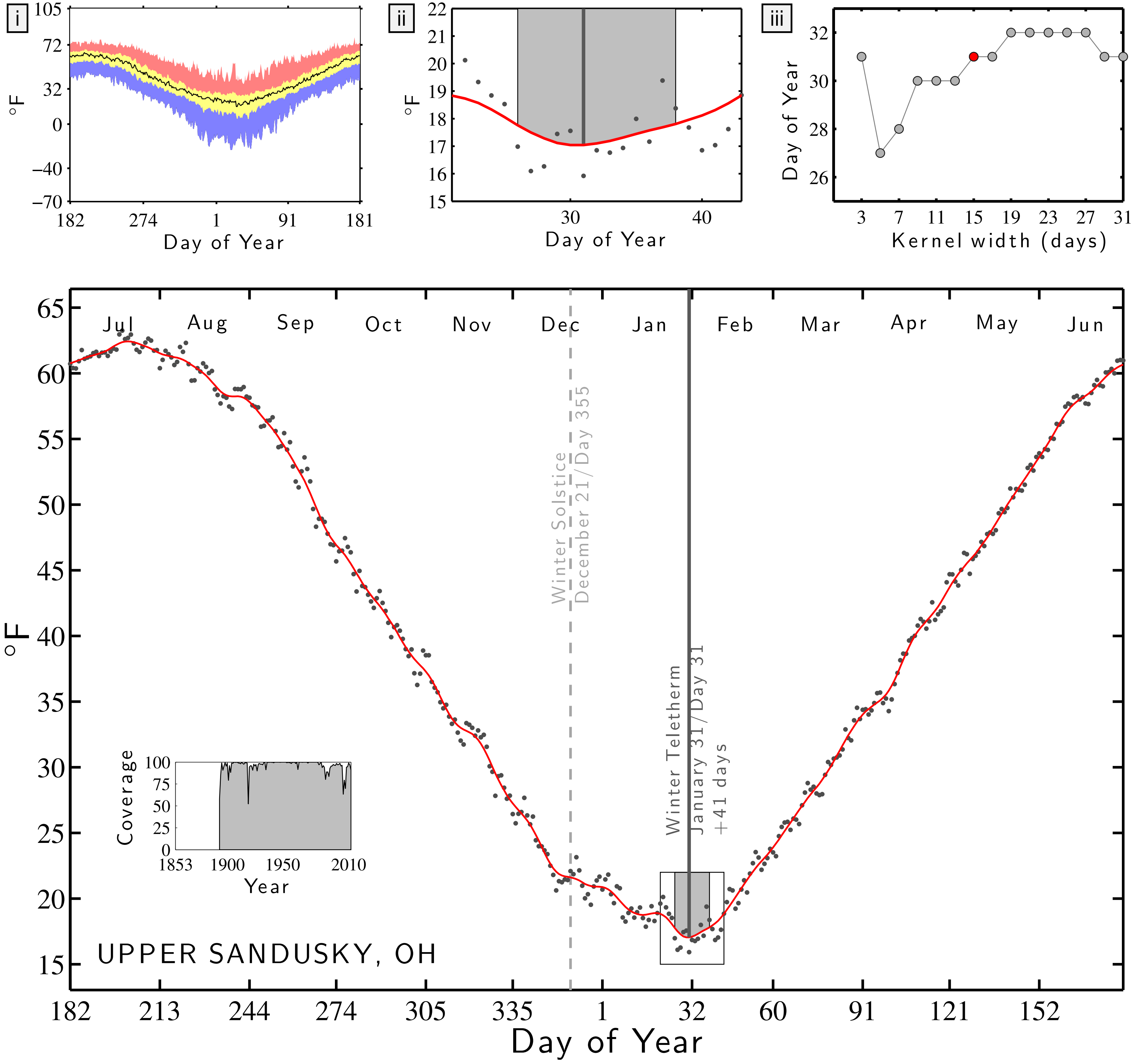

i
ii
iii
°F
Day of Year
Day of Year
Kernel width (days)
Jul
Aug
Sep
Oct
Nov
Dec
Jan
Feb
Mar
Apr
May
Jun
Winter Solstice
December 21/Day 355
Winter Teletherm
January 31/Day 31
+41 days
Coverage
Year
UPPER SANDUSKY, OH

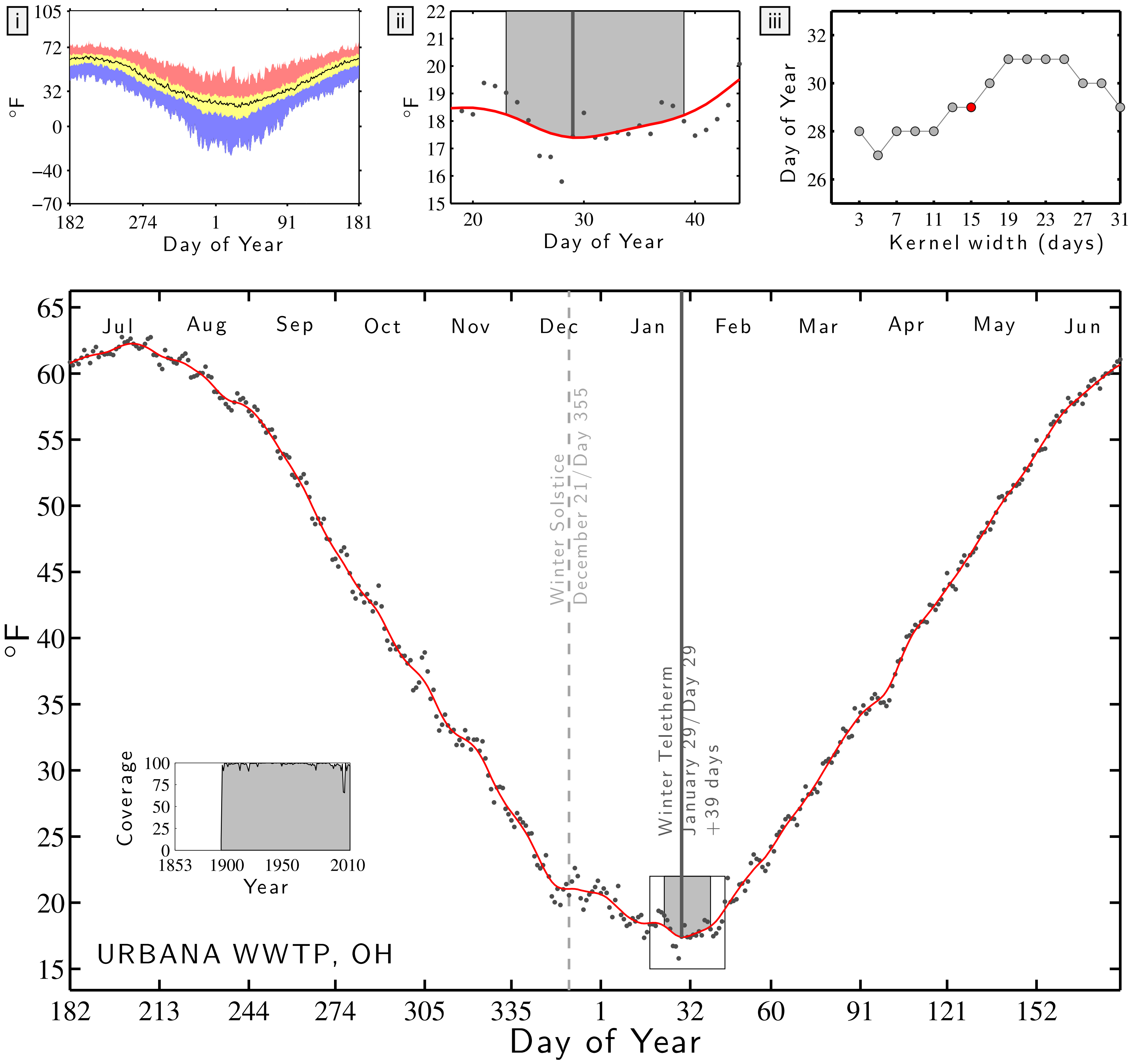

i
ii
iii
°F
Day of Year
Kernel width (days)
Jul
Aug
Sep
Oct
Nov
Dec
Jan
Feb
Mar
Apr
May
Jun
Winter Solstice
December 21/Day 355
Winter Teletherm
January 29/Day 29
+39 days
Coverage
Year
URBANA WWTP, OH

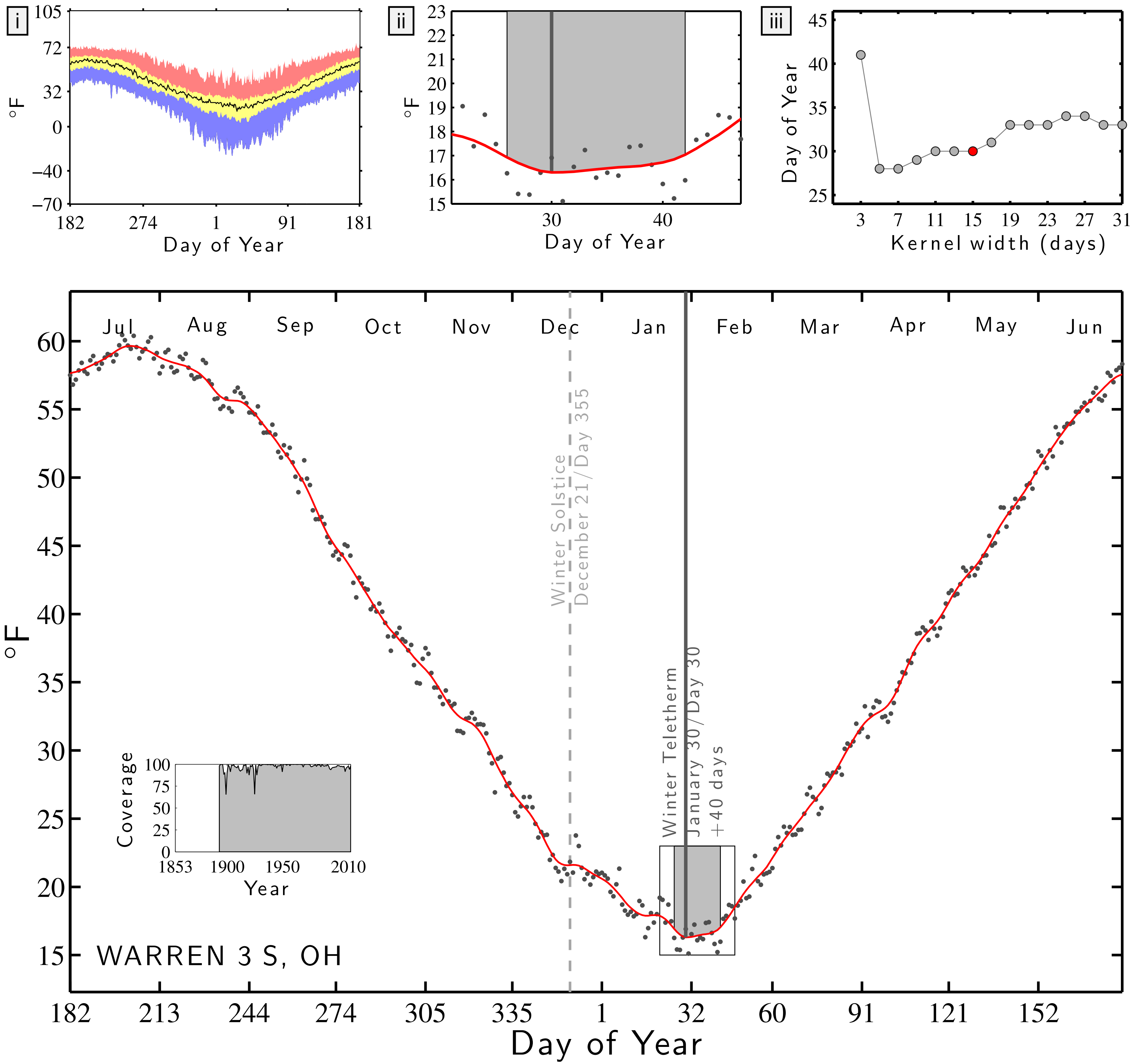

i
ii
iii
Day of Year
°F
Kernel width (days)
Jul
Aug
Sep
Oct
Nov
Dec
Jan
Feb
Mar
Apr
May
Jun
Winter Solstice
December 21/Day 355
Winter Teletherm
January 30/Day 30
+40 days
Coverage
Year
WARREN 3 S, OH

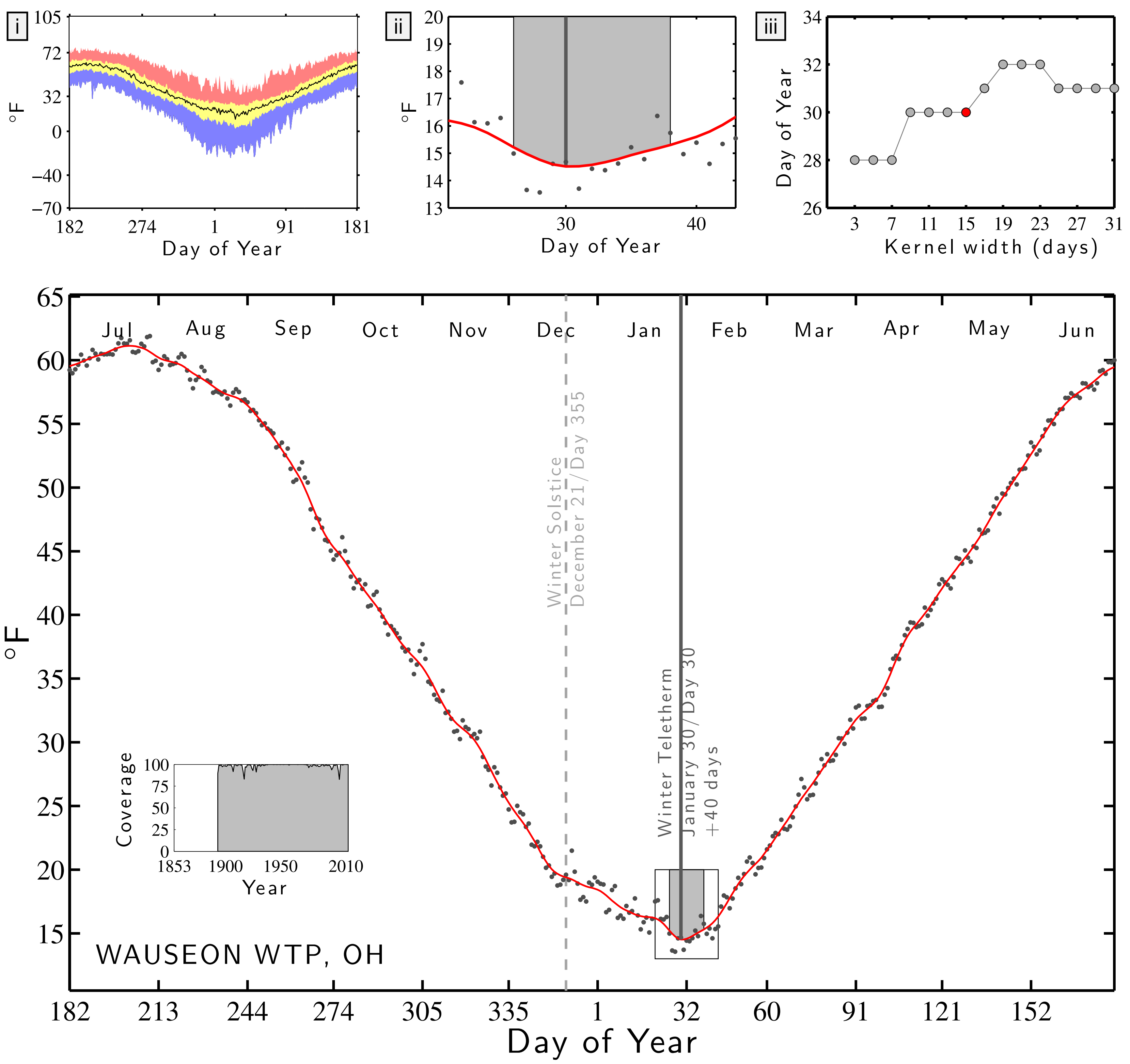
i
°F
Day of Year
ii
°F
Day of Year
iii
Day of Year
Kernel width (days)
Jul
Aug
Sep
Oct
Nov
Dec
Jan
Feb
Mar
Apr
May
Jun
Winter Solstice
December 21/Day 355
Winter Teletherm
January 30/Day 30
+40 days
Coverage
Year
WAUSEON WTP, OH
°F
Day of Year

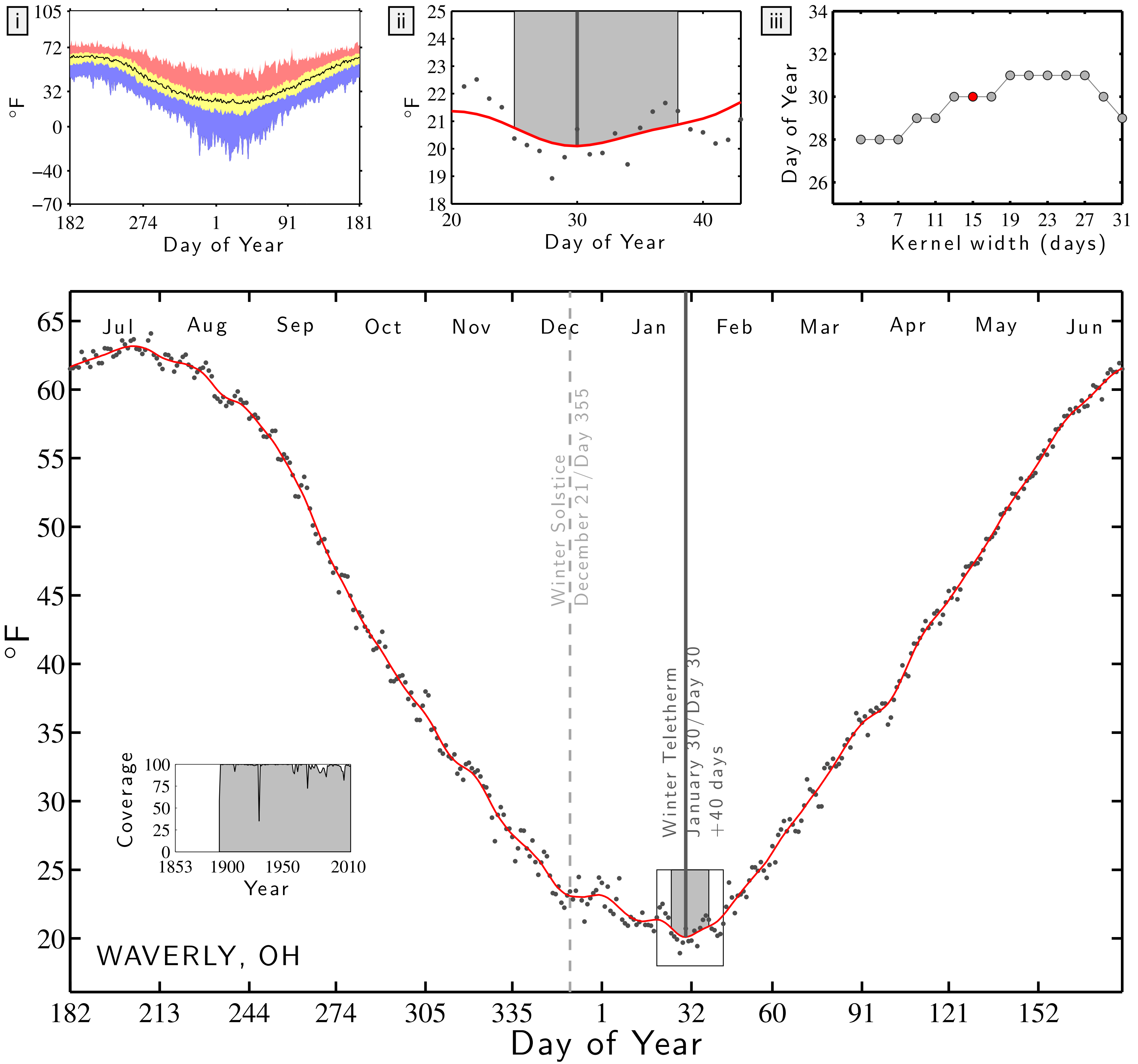

i
°F
Day of Year
ii
°F
Day of Year
iii
Day of Year
Kernel width (days)
Jul
Aug
Sep
Oct
Nov
Dec
Jan
Feb
Mar
Apr
May
Jun
Winter Solstice
December 21/Day 355
Winter Teletherm
January 30/Day 30
+40 days
Coverage
Year
WAVERLY, OH
°F
Day of Year

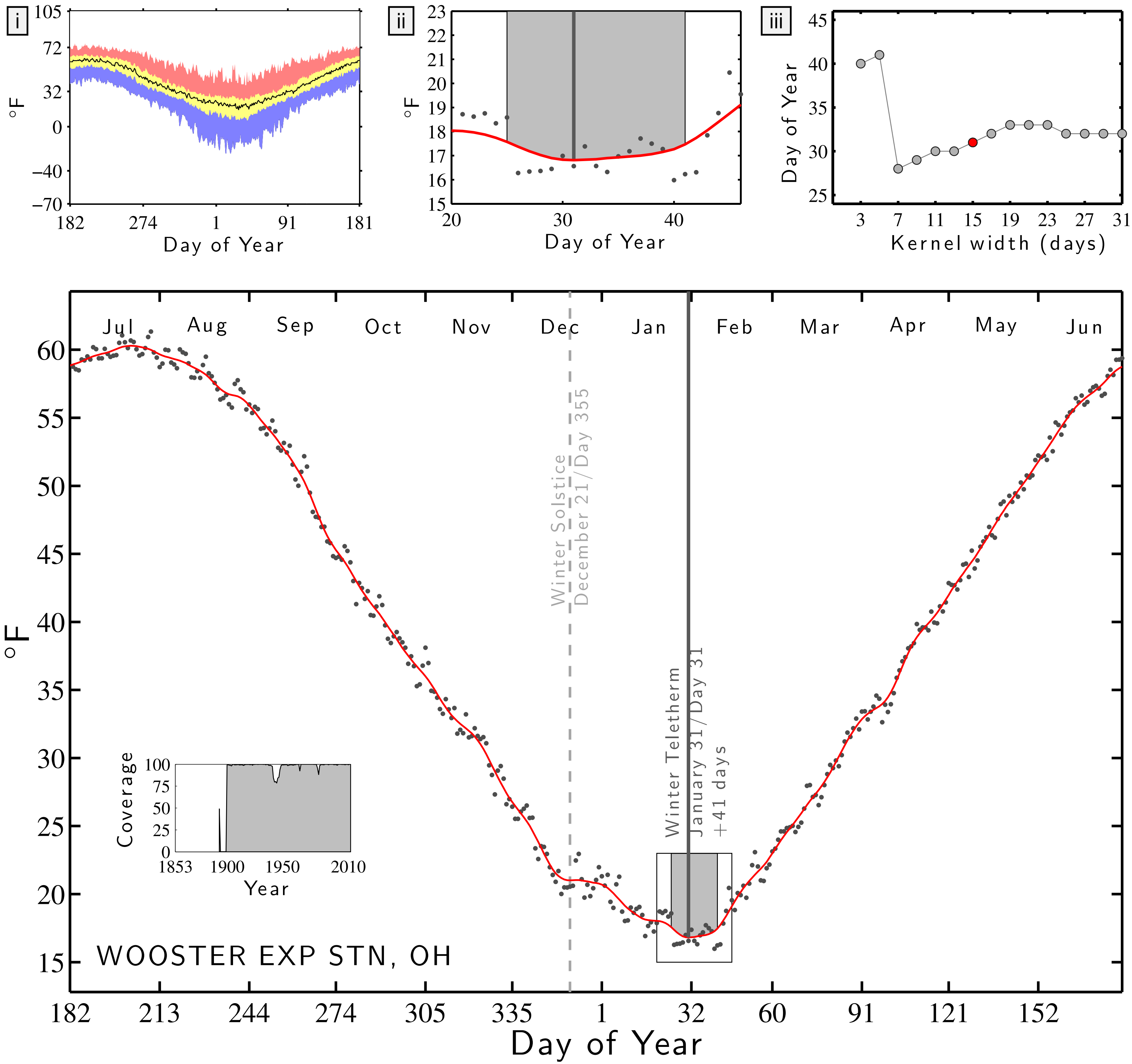

i
ii
iii
°F
Day of Year
Kernel width (days)
Jul
Aug
Sep
Oct
Nov
Dec
Jan
Feb
Mar
Apr
May
Jun
Winter Solstice
December 21/Day 355
Winter Teletherm
January 31/Day 31
+41 days
Coverage
Year
WOOSTER EXP STN, OH

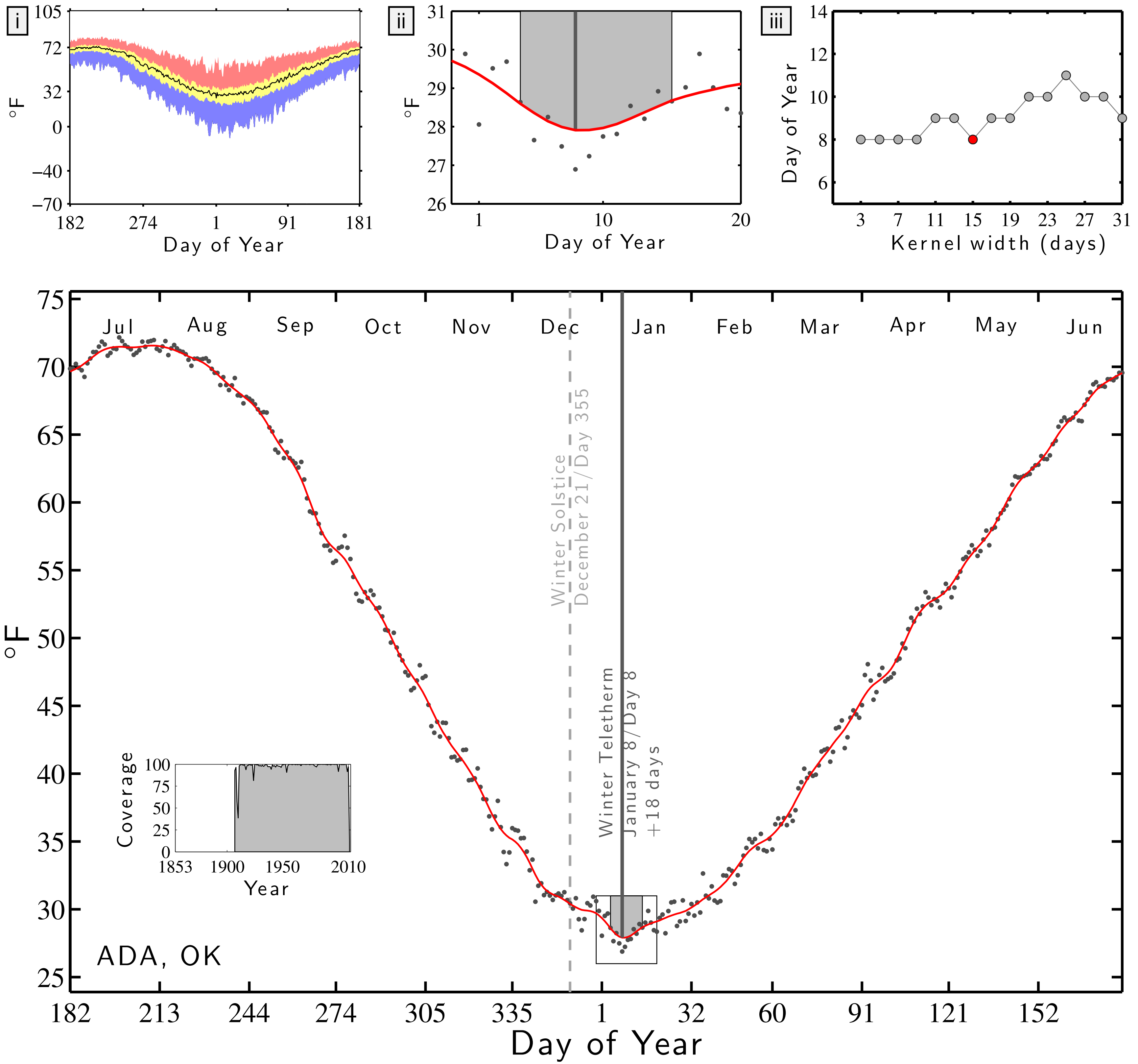

i
°F
Day of Year
ii
°F
Day of Year
iii
Day of Year
Kernel width (days)
Jul
Aug
Sep
Oct
Nov
Dec
Jan
Feb
Mar
Apr
May
Jun
Winter Solstice
December 21/Day 355
Winter Teletherm
January 8/Day 8
+18 days
Coverage
Year
ADA, OK
°F
Day of Year

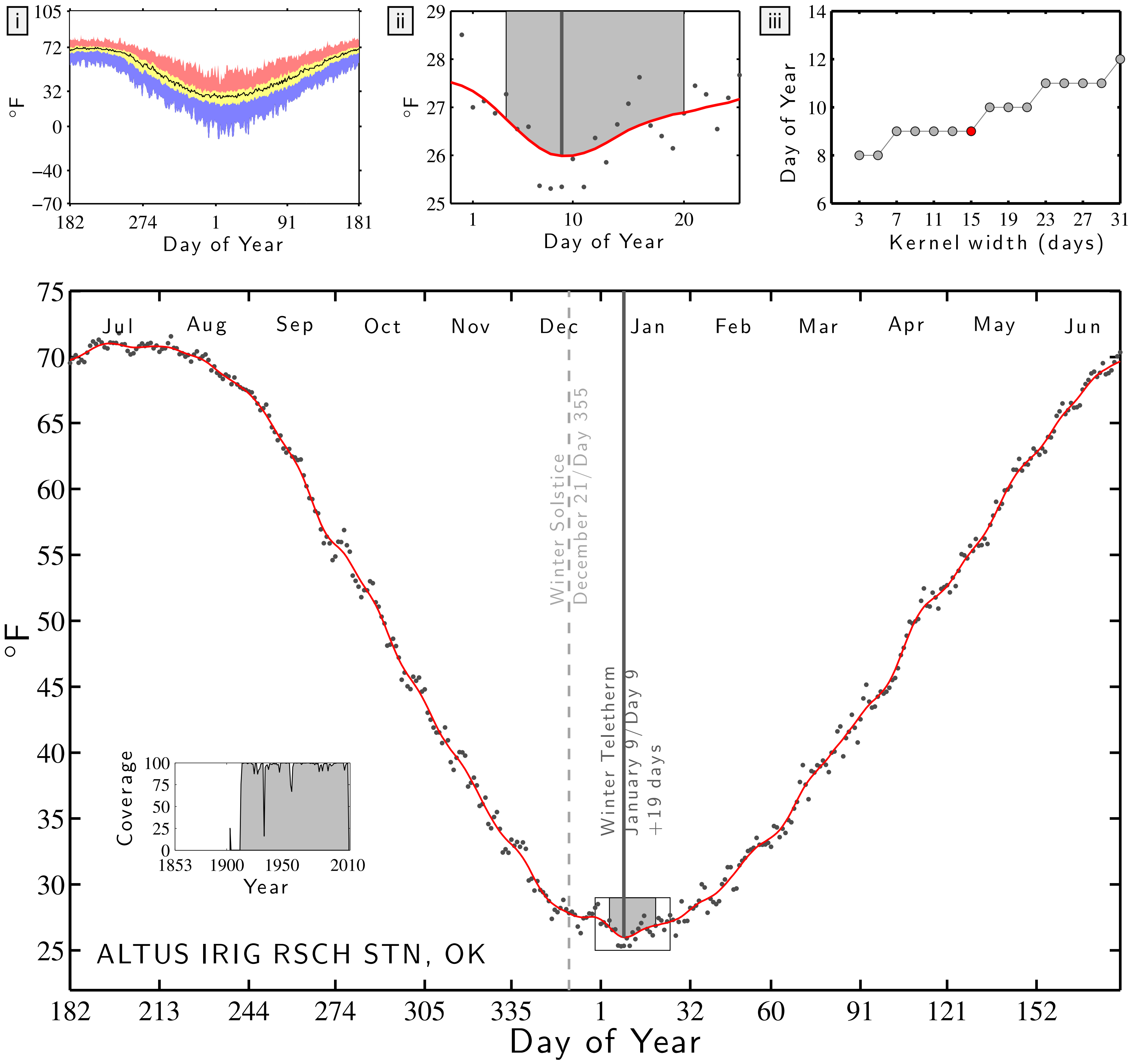

i
ii
iii
°F
Day of Year
Kernel width (days)
Jul
Aug
Sep
Oct
Nov
Dec
Jan
Feb
Mar
Apr
May
Jun
Winter Solstice
December 21/Day 355
Winter Teletherm
January 9/Day 9
+19 days
Coverage
Year
ALTUS IRIG RSCH STN, OK

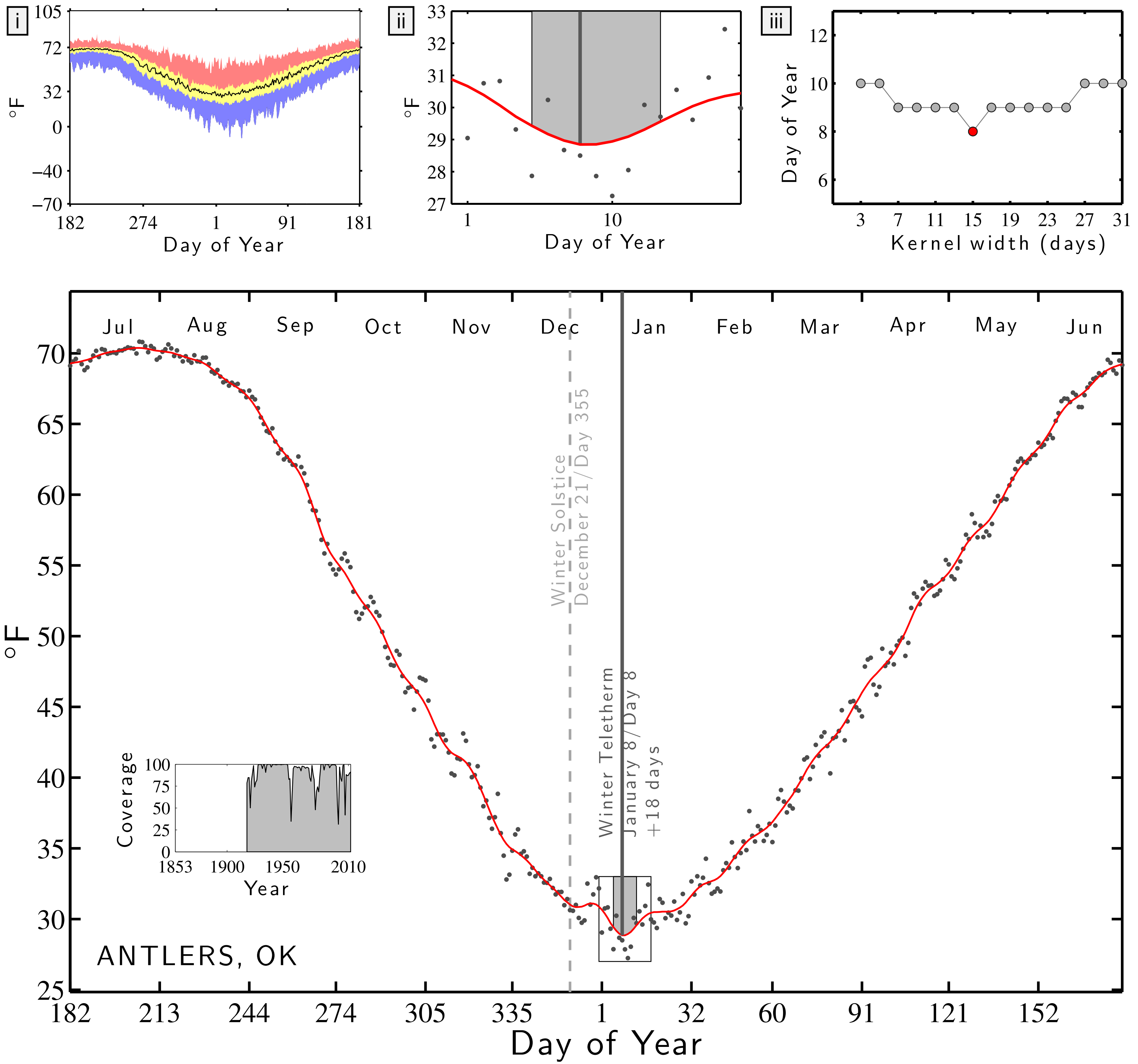

i
°F
Day of Year
ii
°F
Day of Year
iii
Day of Year
Kernel width (days)
Jul
Aug
Sep
Oct
Nov
Dec
Jan
Feb
Mar
Apr
May
Jun
Winter Solstice
December 21/Day 355
Winter Teletherm
January 8/Day 8
+18 days
Coverage
Year
ANTLERS, OK
°F
Day of Year

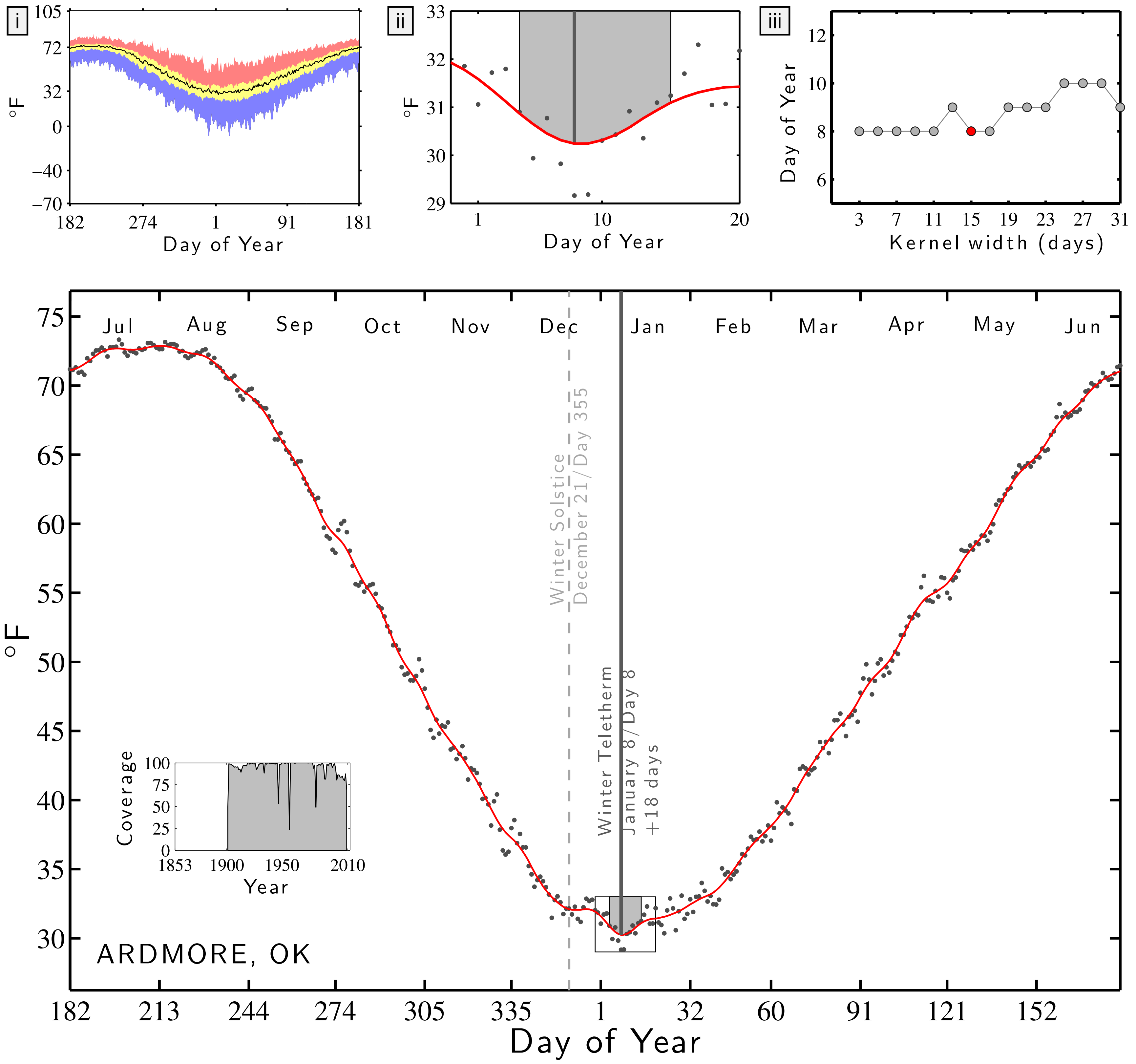
i
ii
iii
°F
Day of Year
Kernel width (days)
Jul
Aug
Sep
Oct
Nov
Dec
Jan
Feb
Mar
Apr
May
Jun
Winter Solstice
December 21/Day 355
Winter Teletherm
January 8/Day 8
+18 days
Coverage
Year
ARDMORE, OK

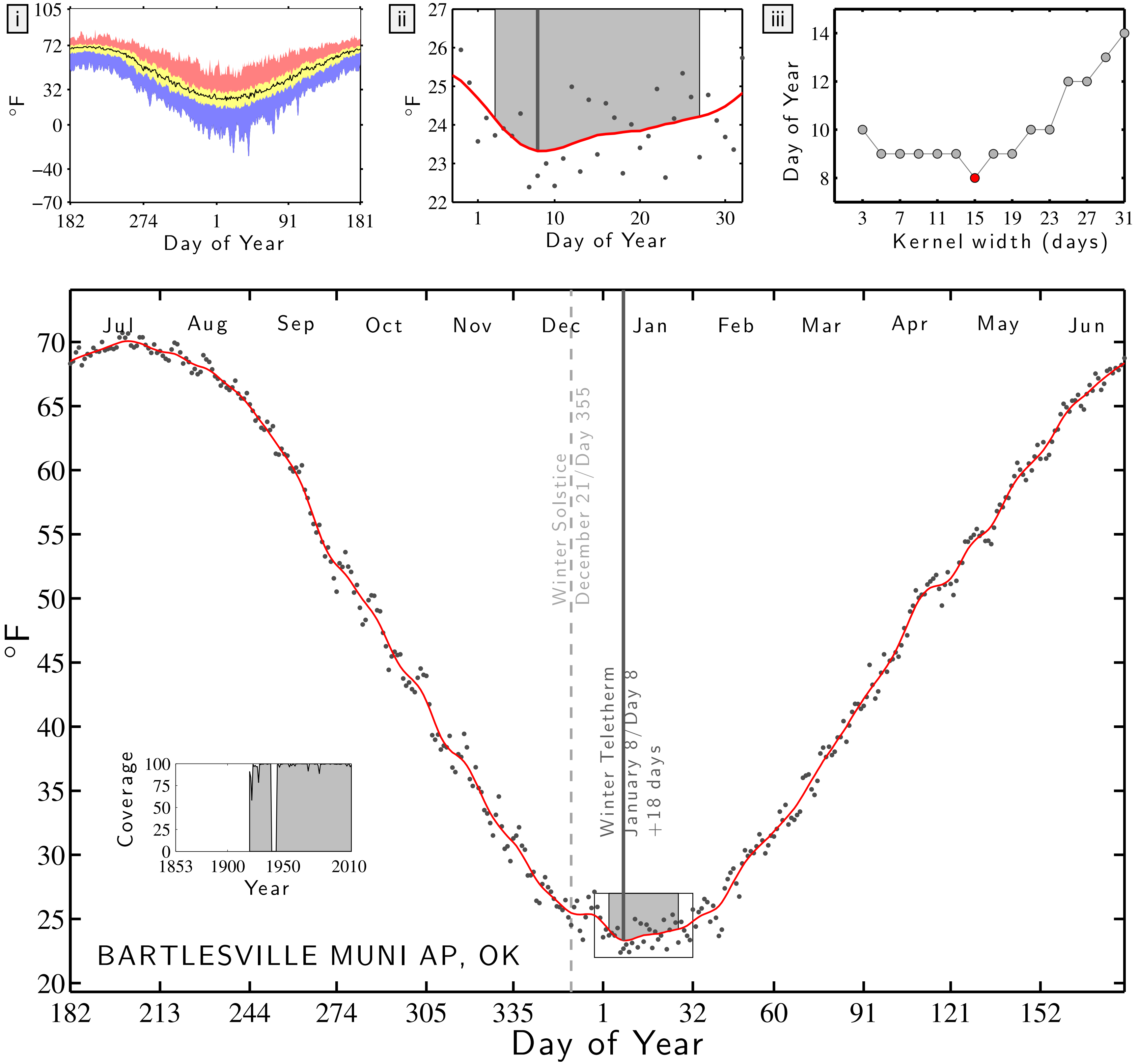

i
°F
Day of Year
ii
°F
Day of Year
iii
Day of Year
Kernel width (days)
Jul
Aug
Sep
Oct
Nov
Dec
Jan
Feb
Mar
Apr
May
Jun
Winter Solstice
December 21/Day 355
Winter Teletherm
January 8/Day 8
+18 days
Coverage
Year
BARTLESVILLE MUNI AP, OK
°F
Day of Year

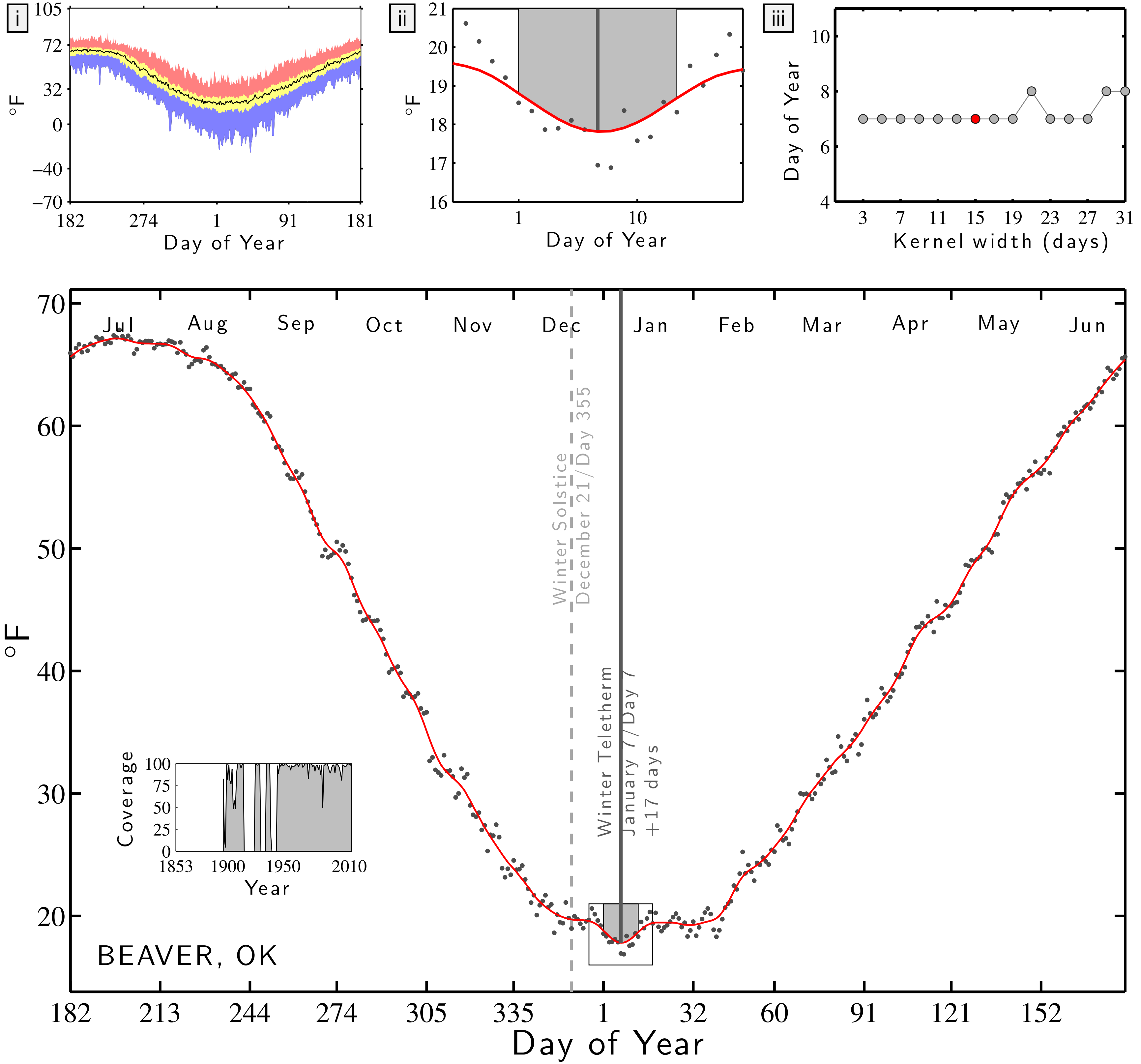

i
°F
105
72
32
0
−40
−70
182
274
1
91
181
Day of Year
ii
21
20
19
18
17
16
1
10
Day of Year
iii
Day of Year
10
8
6
4
3
7
11
15
19
23
27
31
Kernel width (days)
Jul
Aug
Sep
Oct
Nov
Dec
Jan
Feb
Mar
Apr
May
Jun
70
60
50
40
30
20
Winter Solstice
December 21/Day 355
Winter Teletherm
January 7/Day 7
+17 days
Coverage
100
75
50
25
0
1853
1900
1950
2010
Year
BEAVER, OK
182
213
244
274
305
335
1
32
60
91
121
152
Day of Year

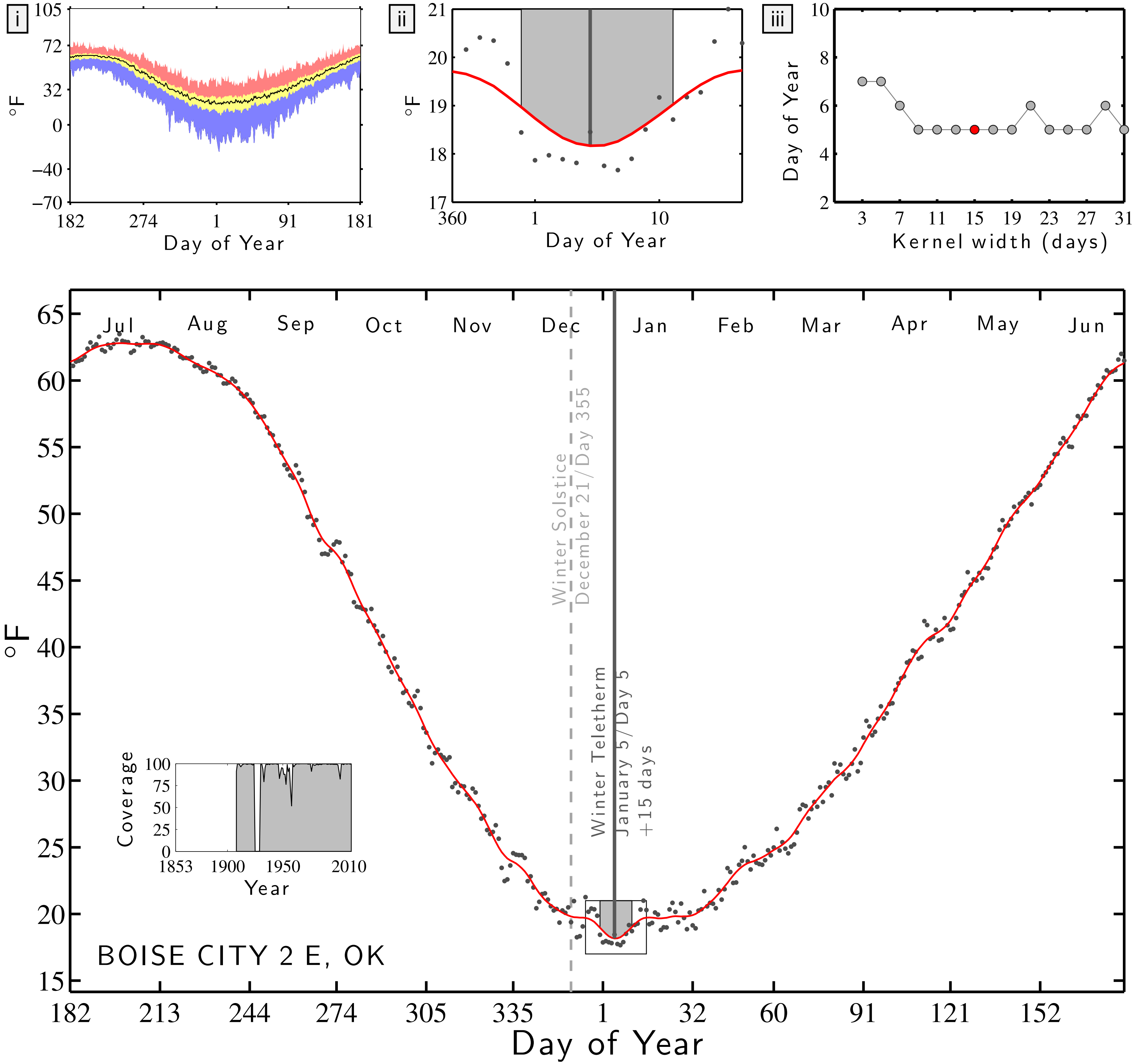

i
ii
iii
°F
Day of Year
Day of Year
Kernel width (days)
Winter Solstice
December 21/Day 355
Winter Teletherm
January 5/Day 5
+15 days
Jul
Aug
Sep
Oct
Nov
Dec
Jan
Feb
Mar
Apr
May
Jun
Coverage
Year
BOISE CITY 2 E, OK
Day of Year

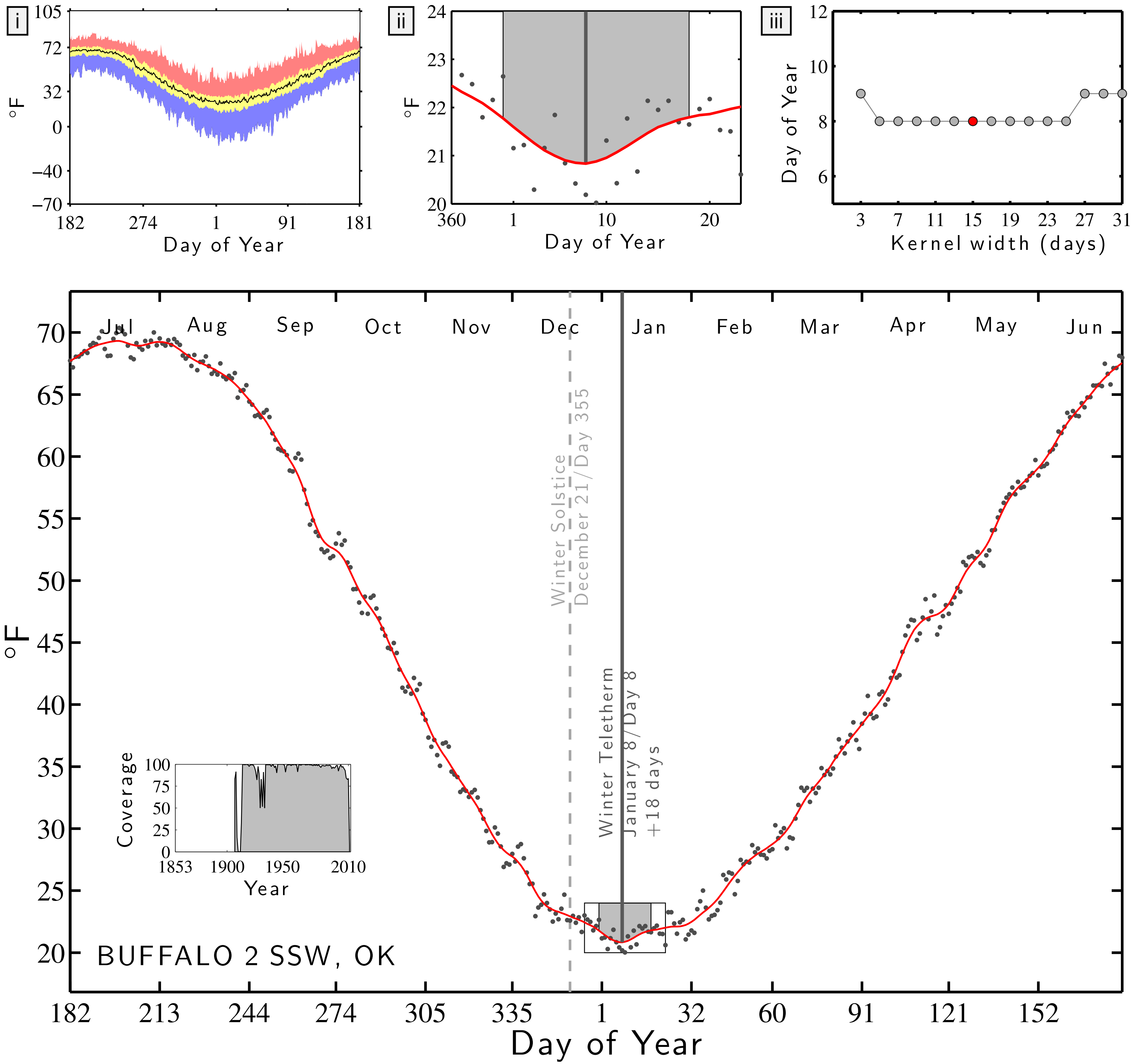

i
ii
iii
°F
Day of Year
Kernel width (days)
Day of Year
Jul
Aug
Sep
Oct
Nov
Dec
Jan
Feb
Mar
Apr
May
Jun
Winter Solstice
December 21/Day 355
Winter Teletherm
January 8/Day 8
+18 days
Coverage
Year
BUFFALO 2 SSW, OK

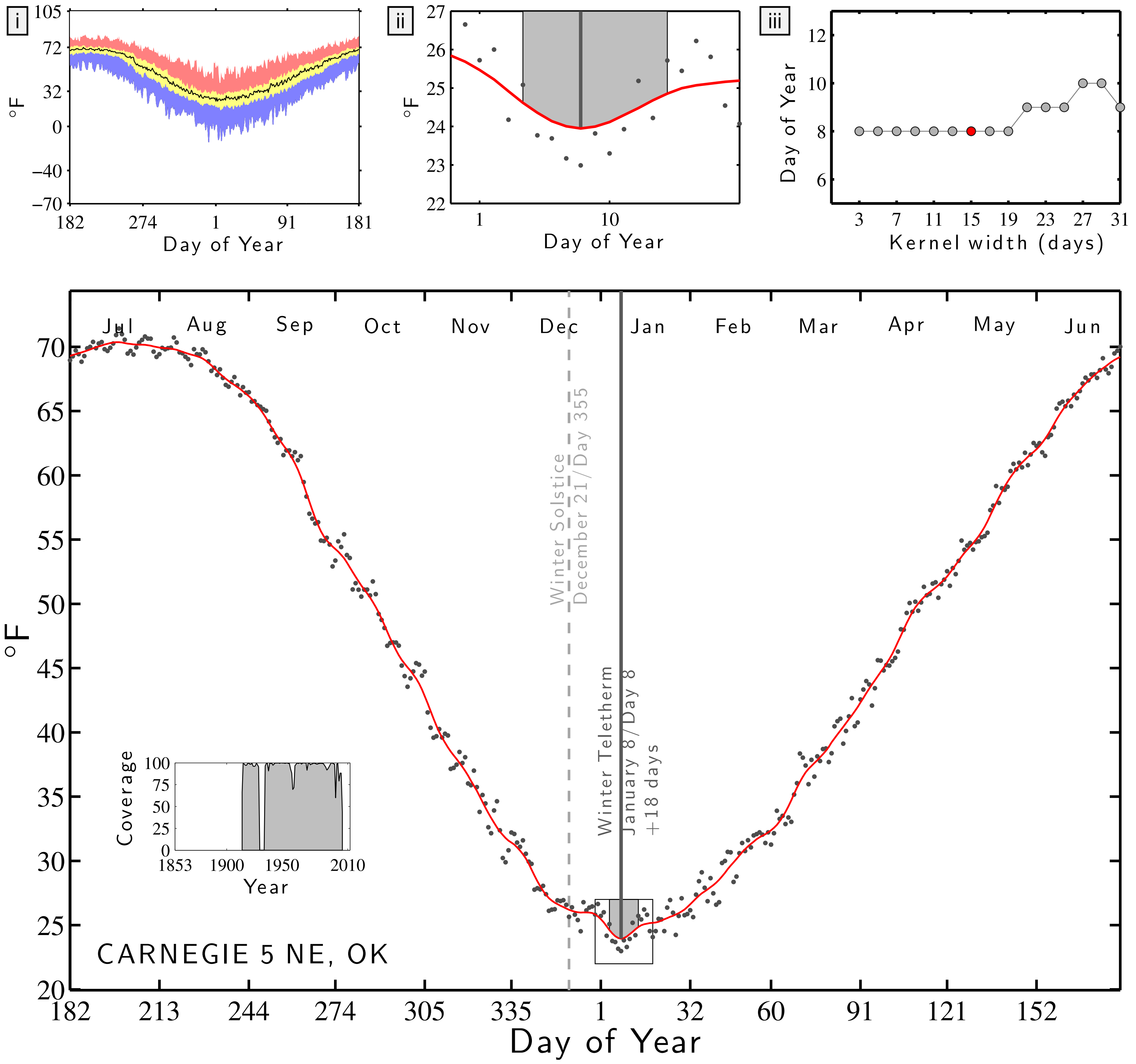

i
ii
iii
Day of Year
Kernel width (days)
Winter Solstice
December 21/Day 355
Winter Teletherm
January 8/Day 8
+18 days
Jul
Aug
Sep
Oct
Nov
Dec
Jan
Feb
Mar
Apr
May
Jun
Coverage
Year
CARNEGIE 5 NE, OK
°F

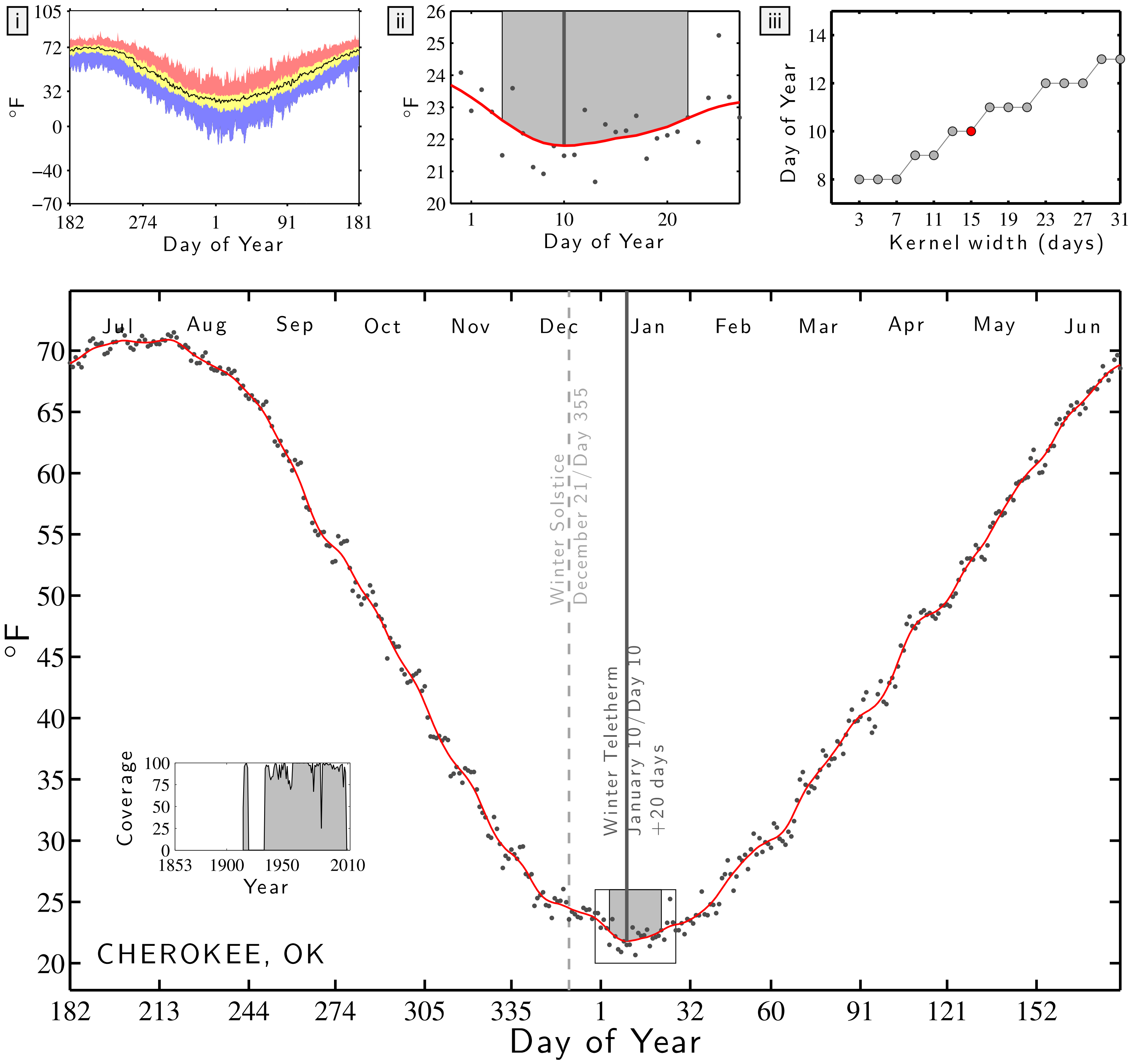
i
105
72
32
0
−40
−70
°F
182
274
1
91
181
Day of Year
ii
26
25
24
23
22
21
20
°F
1
10
20
Day of Year
iii
14
12
10
8
Day of Year
3
7
11
15
19
23
27
31
Kernel width (days)
Jul
Aug
Sep
Oct
Nov
Dec
Jan
Feb
Mar
Apr
May
Jun
70
65
60
55
50
45
40
35
30
25
20
°F
Winter Solstice
December 21/Day 355
Winter Teletherm
January 10/Day 10
+20 days
Coverage
100
75
50
25
0
1853
1900
1950
2010
Year
CHEROKEE, OK
182
213
244
274
305
335
1
32
60
91
121
152
Day of Year

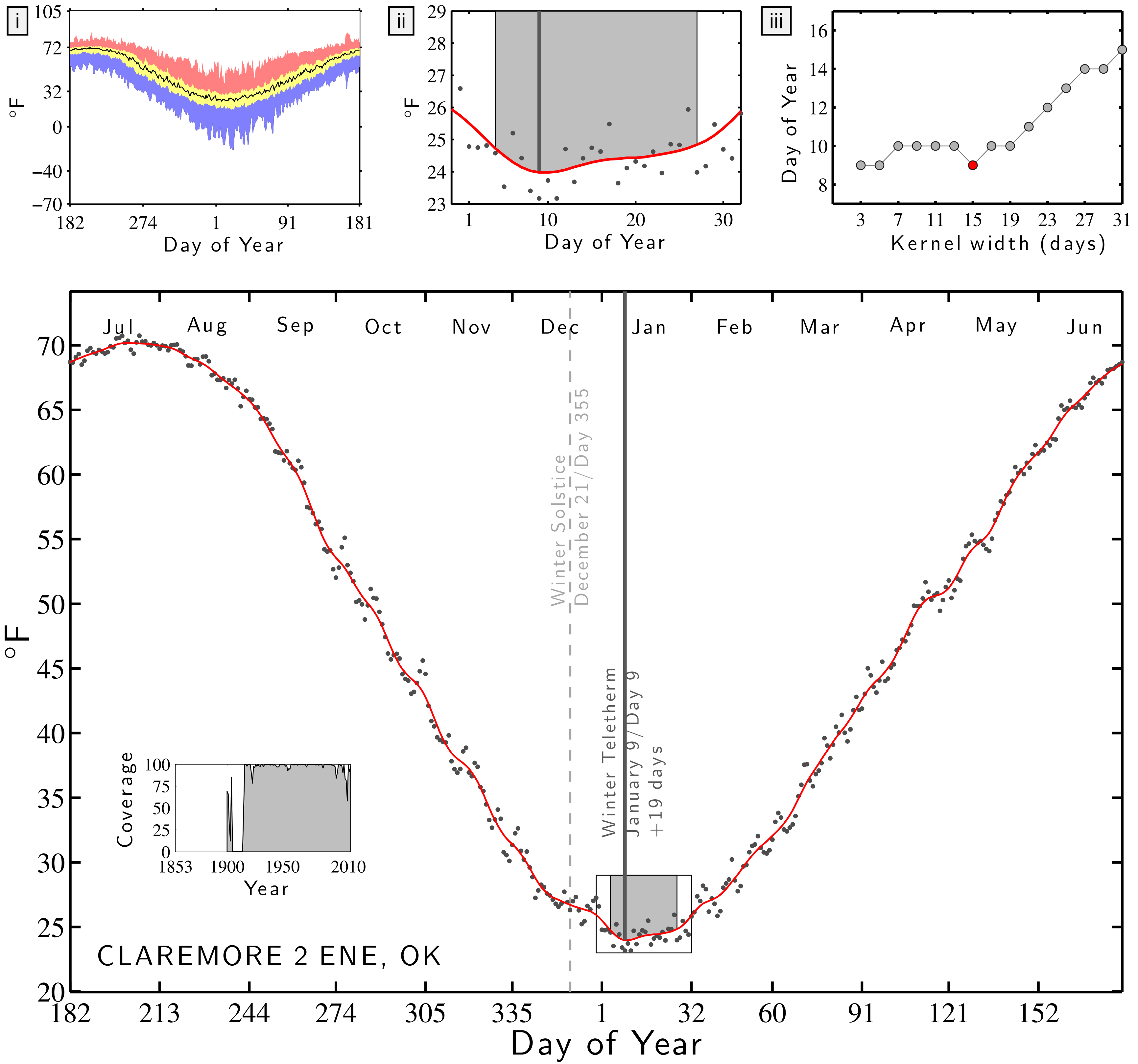

i
°F
Day of Year
ii
°F
Day of Year
iii
Day of Year
Kernel width (days)
Jul
Aug
Sep
Oct
Nov
Dec
Jan
Feb
Mar
Apr
May
Jun
Winter Solstice
December 21/Day 355
Winter Teletherm
January 9/Day 9
+19 days
Coverage
Year
CLAREMORE 2 ENE, OK
°F
Day of Year

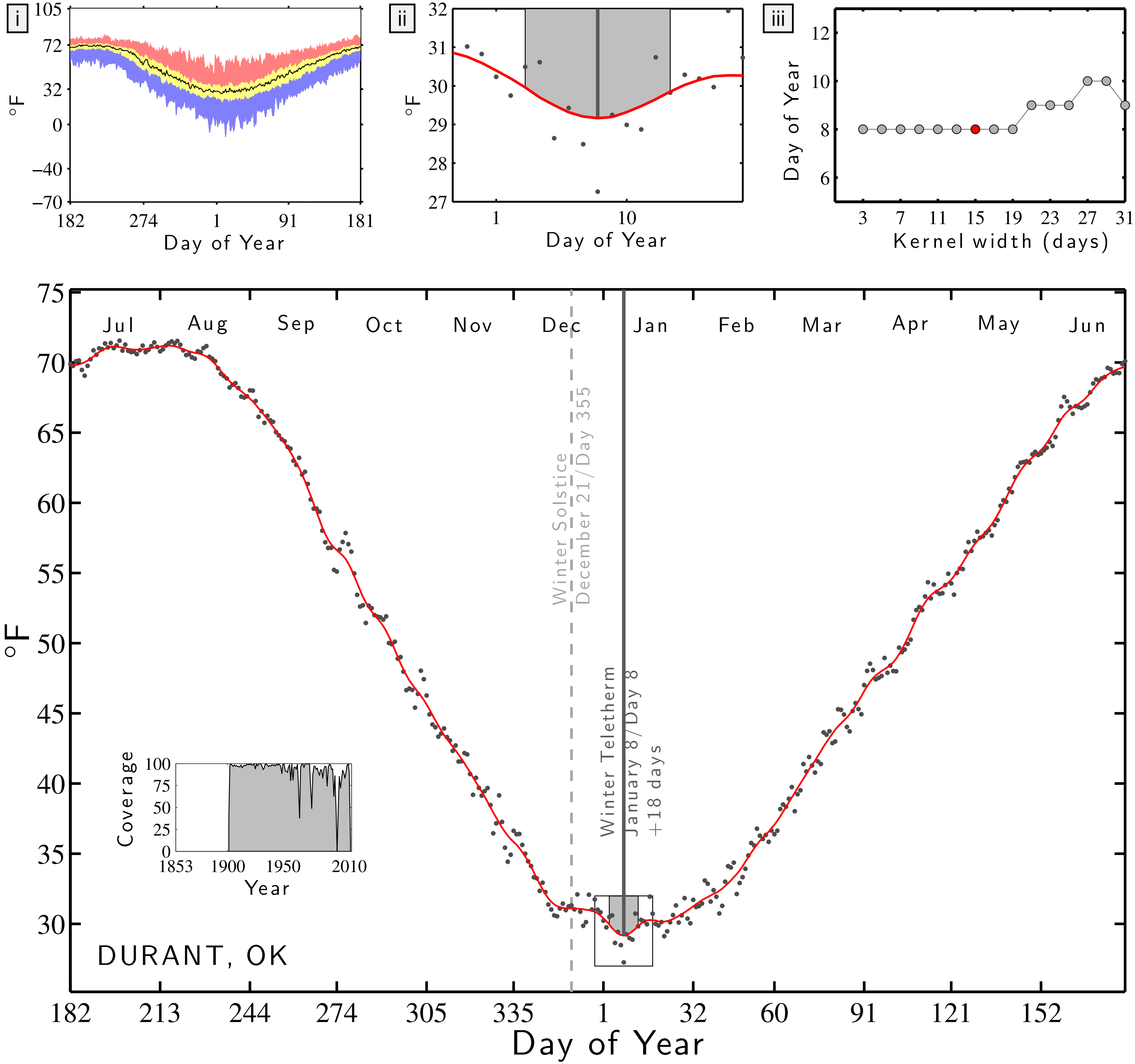

i
°F
Day of Year
ii
Day of Year
iii
Day of Year
Kernel width (days)
Jul
Aug
Sep
Oct
Nov
Dec
Jan
Feb
Mar
Apr
May
Jun
Winter Solstice
December 21/Day 355
Winter Teletherm
January 8/Day 8
+18 days
Coverage
Year
DURANT, OK
Day of Year

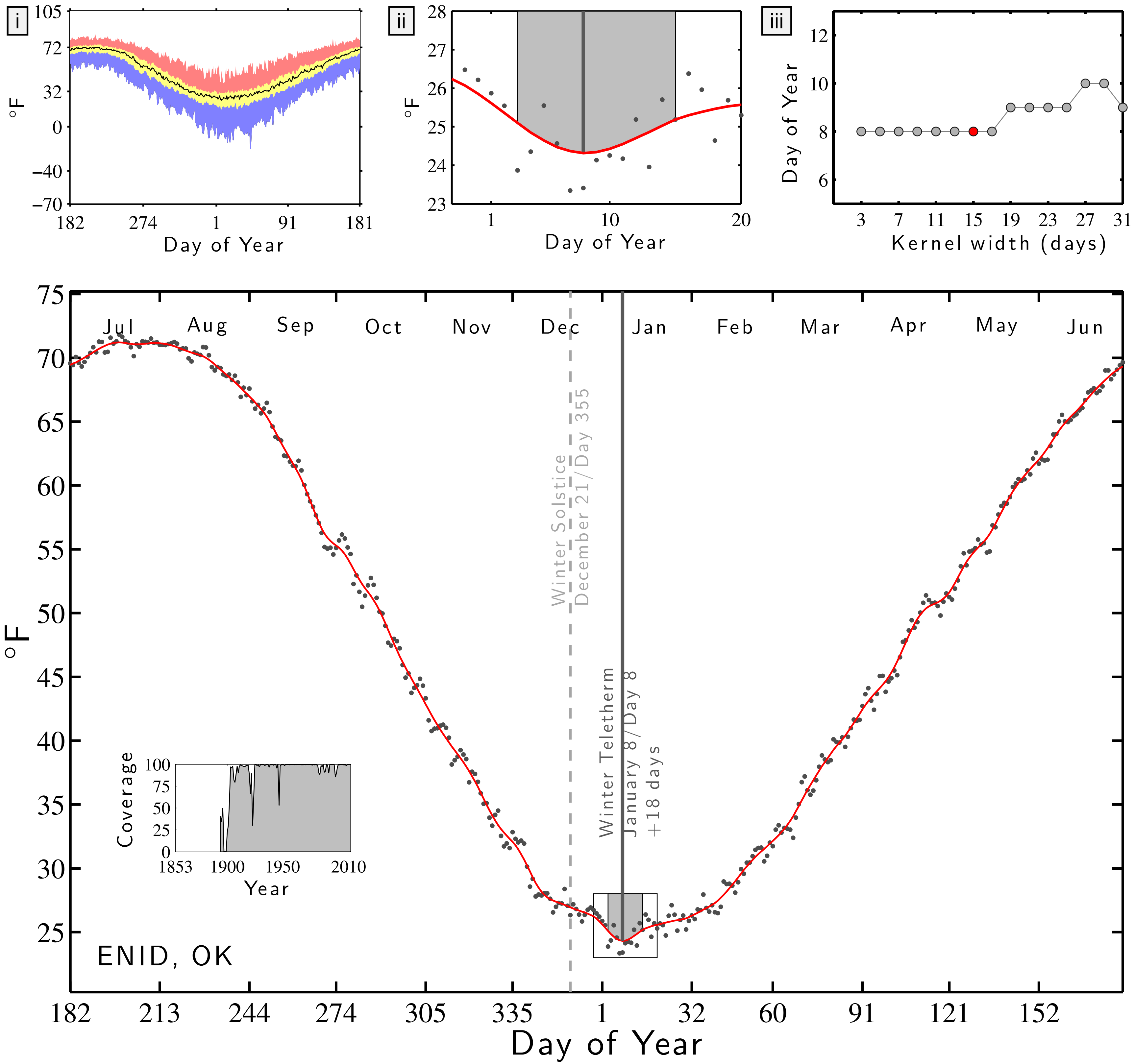

i
°F
105
72
32
0
−40
−70
182
274
1
91
181
Day of Year
ii
°F
28
27
26
25
24
23
1
10
20
Day of Year
iii
Day of Year
12
10
8
6
3
7
11
15
19
23
27
31
Kernel width (days)
Jul
Aug
Sep
Oct
Nov
Dec
Jan
Feb
Mar
Apr
May
Jun
°F
75
70
65
60
55
50
45
40
35
30
25
Winter Solstice
December 21/Day 355
Winter Teletherm
January 8/Day 8
+18 days
Coverage
100
75
50
25
0
1853
1900
1950
2010
Year
ENID, OK
182
213
244
274
305
335
1
32
60
91
121
152
Day of Year

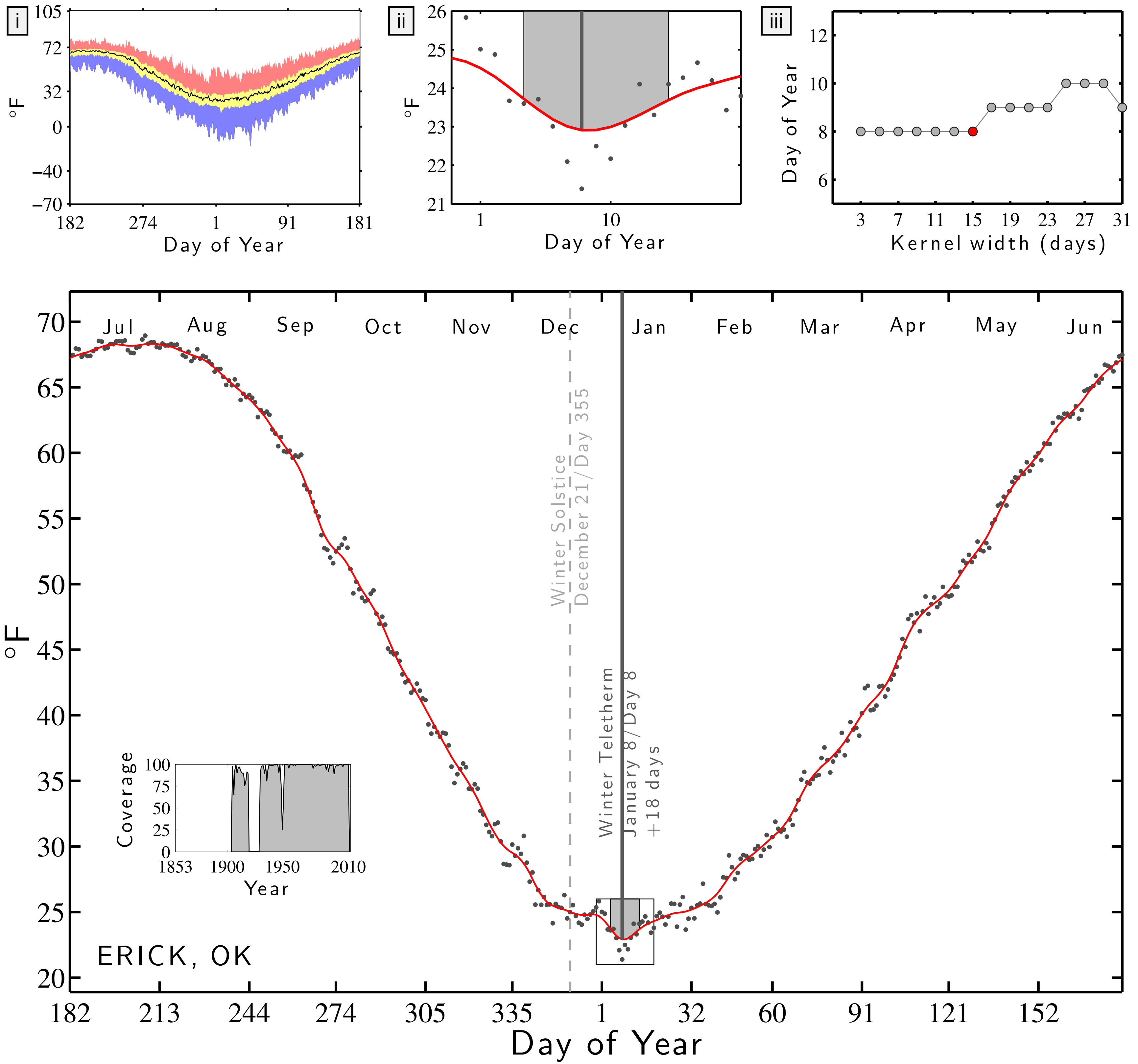

i
ii
iii
°F
Day of Year
Day of Year
Kernel width (days)
Jul
Aug
Sep
Oct
Nov
Dec
Jan
Feb
Mar
Apr
May
Jun
Winter Solstice
December 21/Day 355
Winter Teletherm
January 8/Day 8
+18 days
Coverage
Year
ERICK, OK

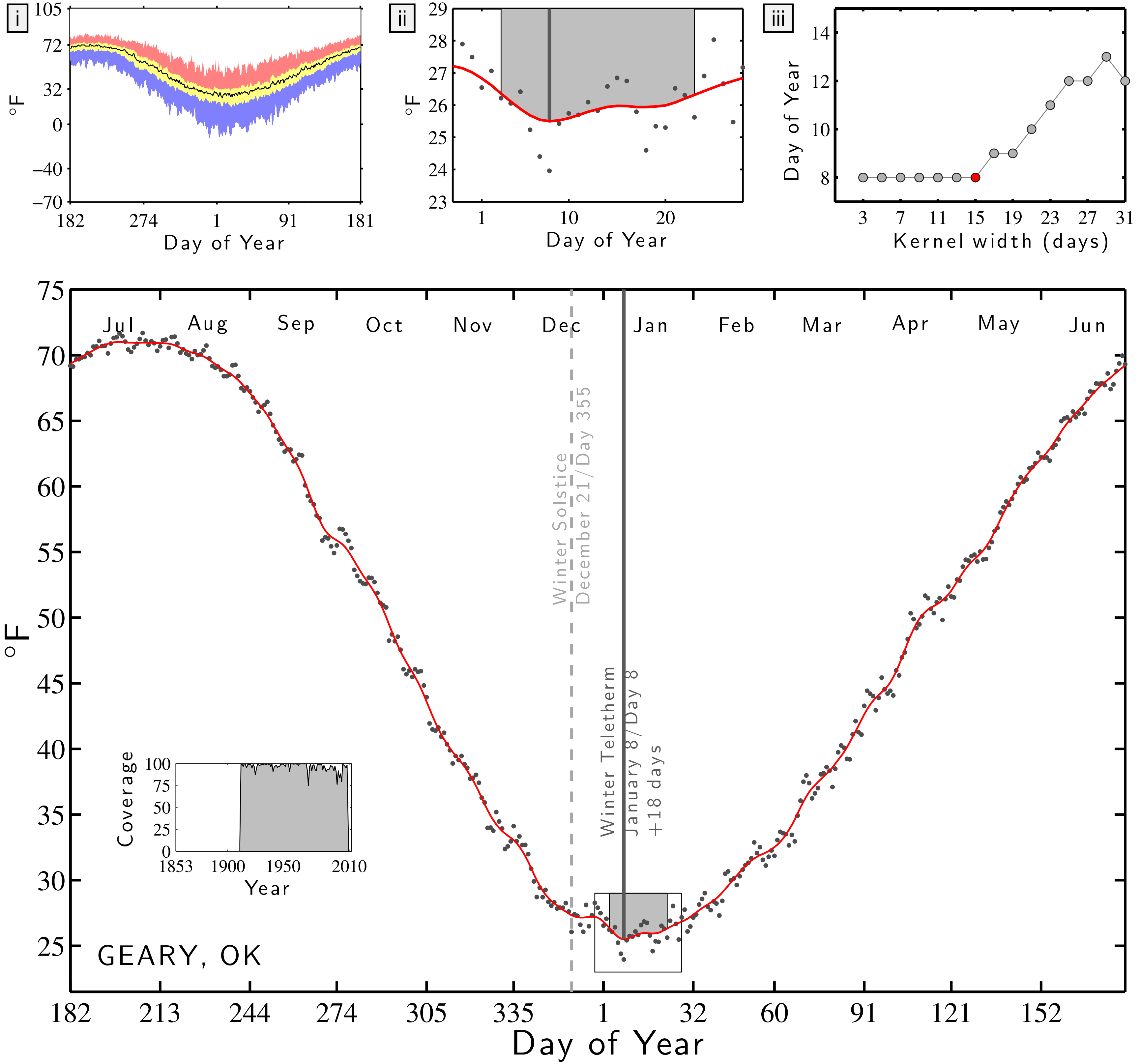

i
ii
iii
°F
Day of Year
Day of Year
Kernel width (days)
Jul
Aug
Sep
Oct
Nov
Dec
Jan
Feb
Mar
Apr
May
Jun
Winter Solstice
December 21/Day 355
Winter Teletherm
January 8/Day 8
+18 days
Coverage
Year
GEARY, OK
°F
Day of Year

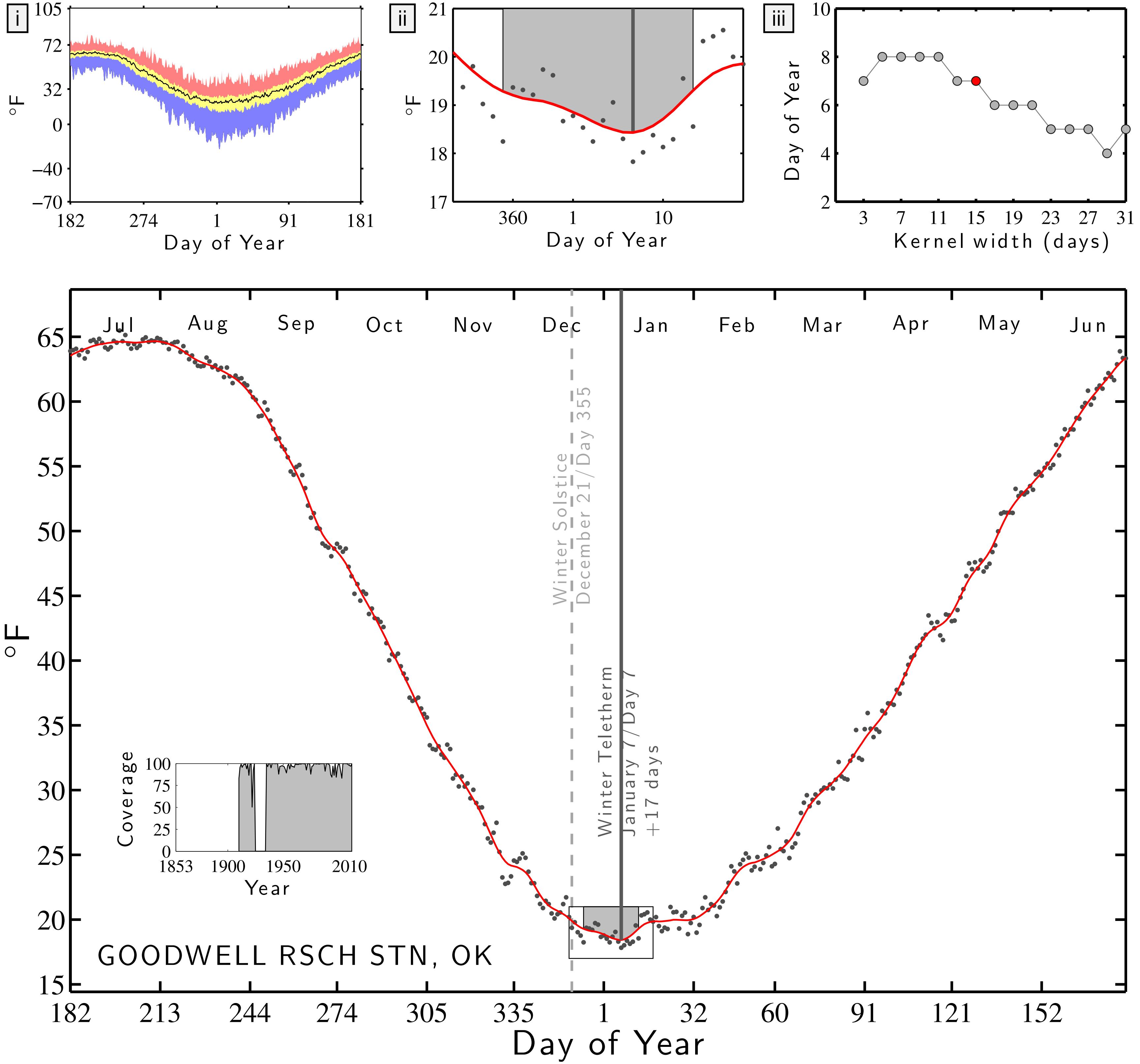

i
°F
Day of Year
ii
°F
Day of Year
iii
Day of Year
Kernel width (days)
Jul
Aug
Sep
Oct
Nov
Dec
Jan
Feb
Mar
Apr
May
Jun
Winter Solstice
December 21/Day 355
Winter Teletherm
January 7/Day 7
+17 days
Coverage
Year
GOODWELL RSCH STN, OK
°F
Day of Year

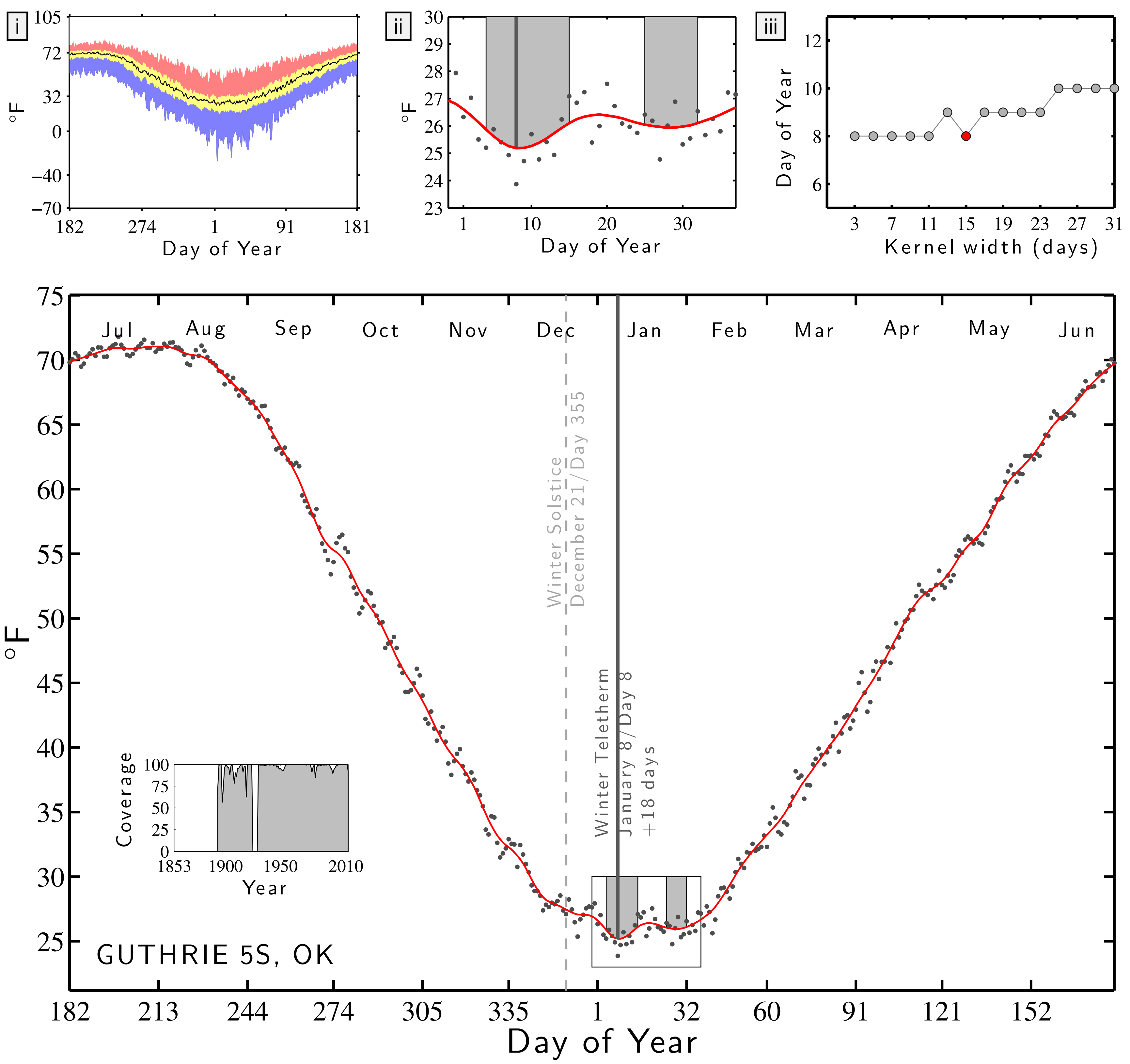

i
°F
Day of Year
ii
Day of Year
iii
Day of Year
Kernel width (days)
Jul
Aug
Sep
Oct
Nov
Dec
Jan
Feb
Mar
Apr
May
Jun
Winter Solstice
December 21/Day 355
Winter Teletherm
January 8/Day 8
+18 days
Coverage
Year
GUTHRIE 5S, OK
Day of Year

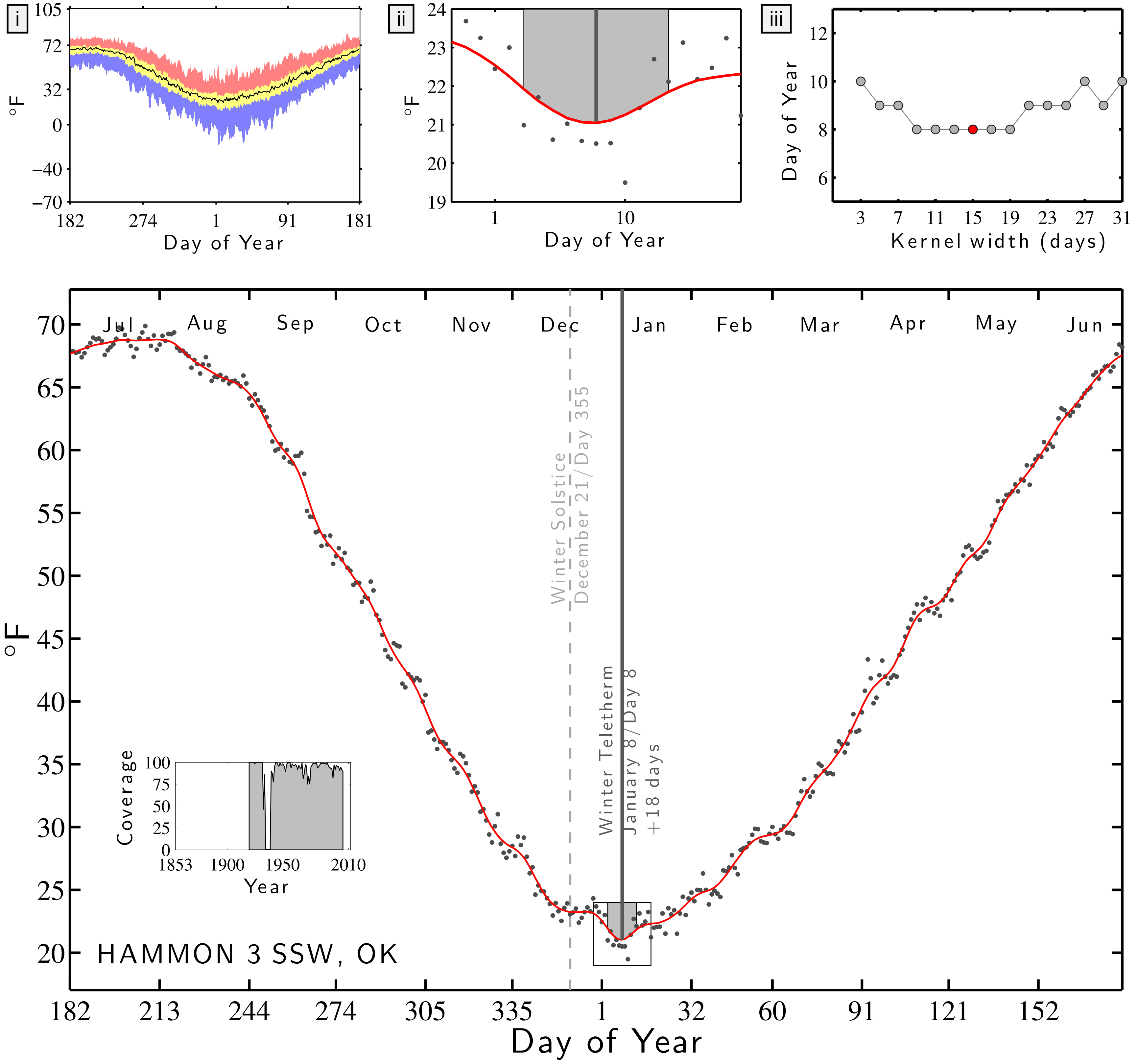

i
°F
Day of Year
ii
Day of Year
iii
Day of Year
Kernel width (days)
Jul
Aug
Sep
Oct
Nov
Dec
Jan
Feb
Mar
Apr
May
Jun
Winter Solstice
December 21/Day 355
Winter Teletherm
January 8/Day 8
+18 days
Coverage
Year
HAMMON 3 SSW, OK

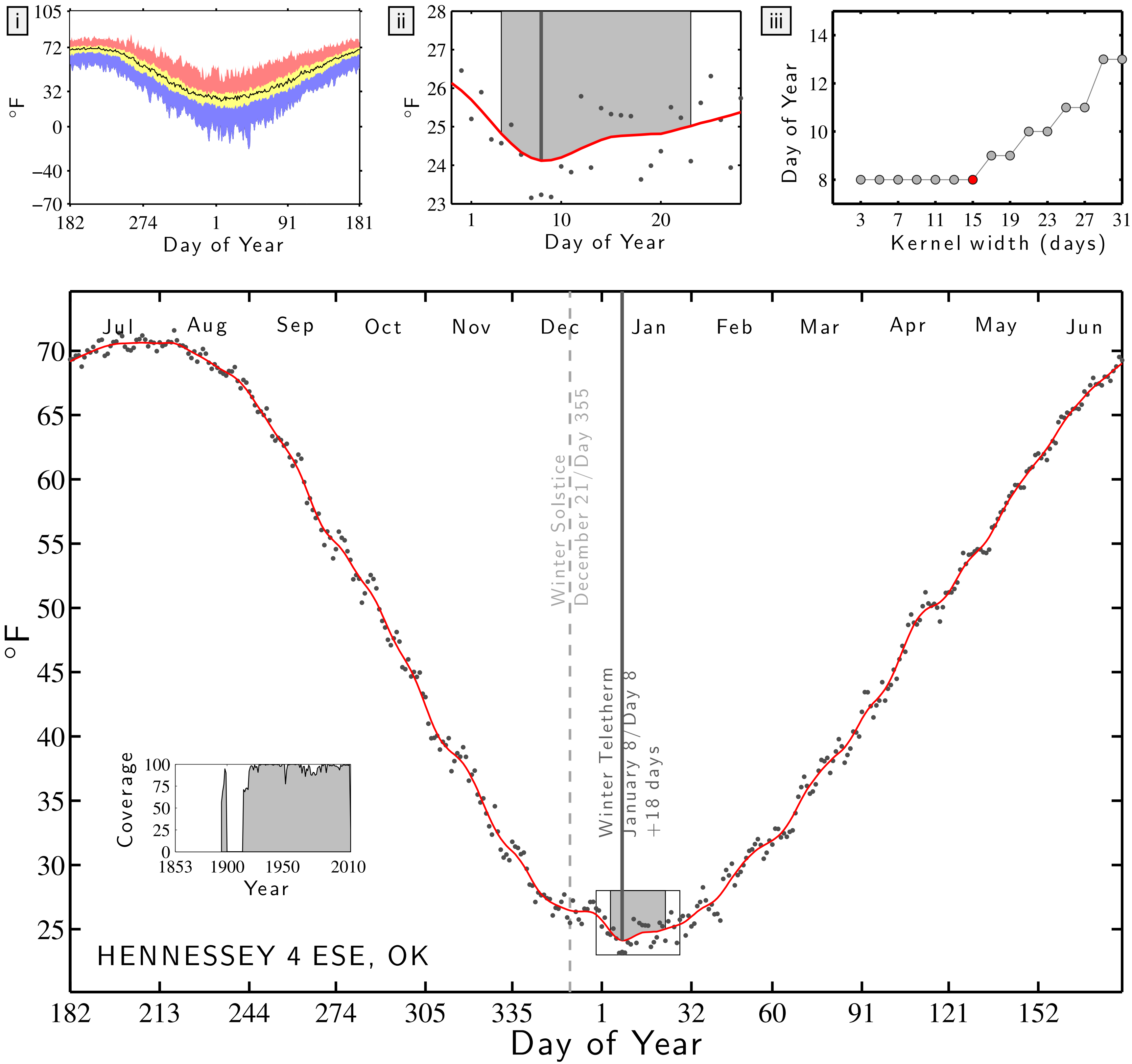

i
ii
iii
°F
Day of Year
Kernel width (days)
Jul
Aug
Sep
Oct
Nov
Dec
Jan
Feb
Mar
Apr
May
Jun
Winter Solstice
December 21/Day 355
Winter Teletherm
January 8/Day 8
+18 days
Coverage
Year
HENNESSEY 4 ESE, OK

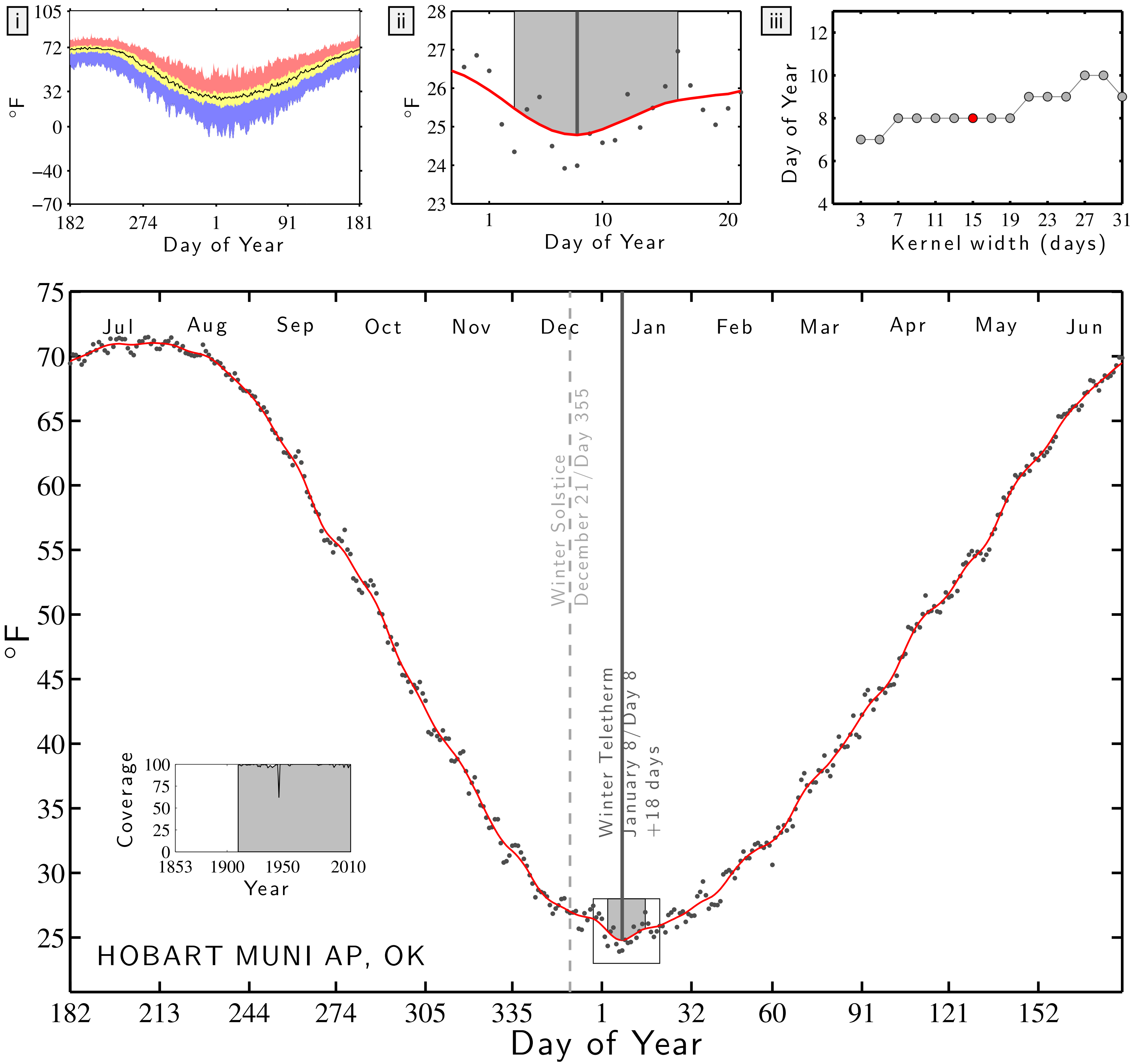

i
ii
iii
°F
Day of Year
Day of Year
Kernel width (days)
Jul
Aug
Sep
Oct
Nov
Dec
Jan
Feb
Mar
Apr
May
Jun
Winter Solstice
December 21/Day 355
Winter Teletherm
January 8/Day 8
+18 days
Coverage
Year
HOBART MUNI AP, OK

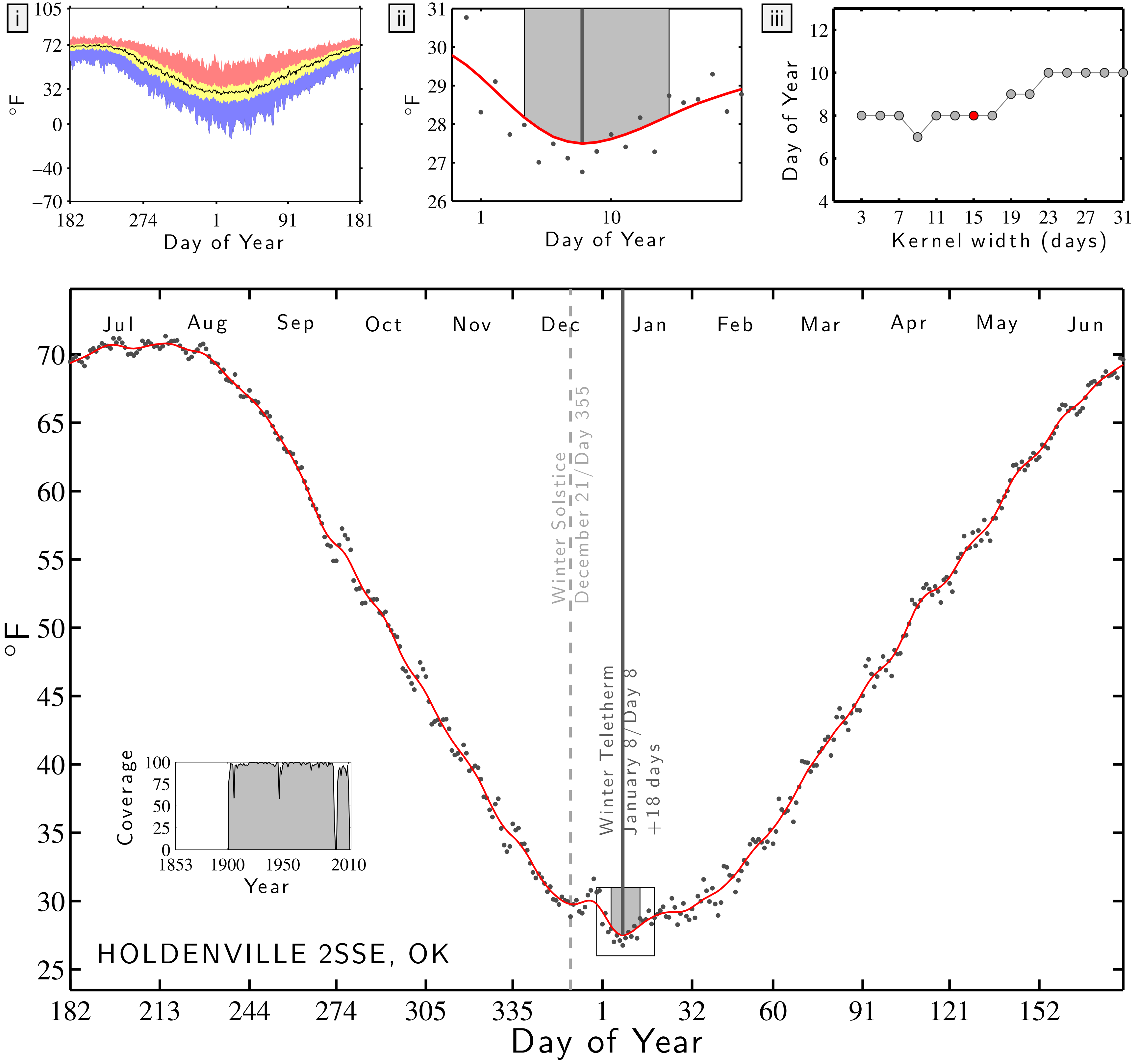

i
°F
Day of Year
ii
°F
Day of Year
iii
Day of Year
Kernel width (days)
Jul
Aug
Sep
Oct
Nov
Dec
Jan
Feb
Mar
Apr
May
Jun
Winter Solstice
December 21/Day 355
Winter Teletherm
January 8/Day 8
+18 days
Coverage
Year
HOLDENVILLE 2SSE, OK
°F
Day of Year

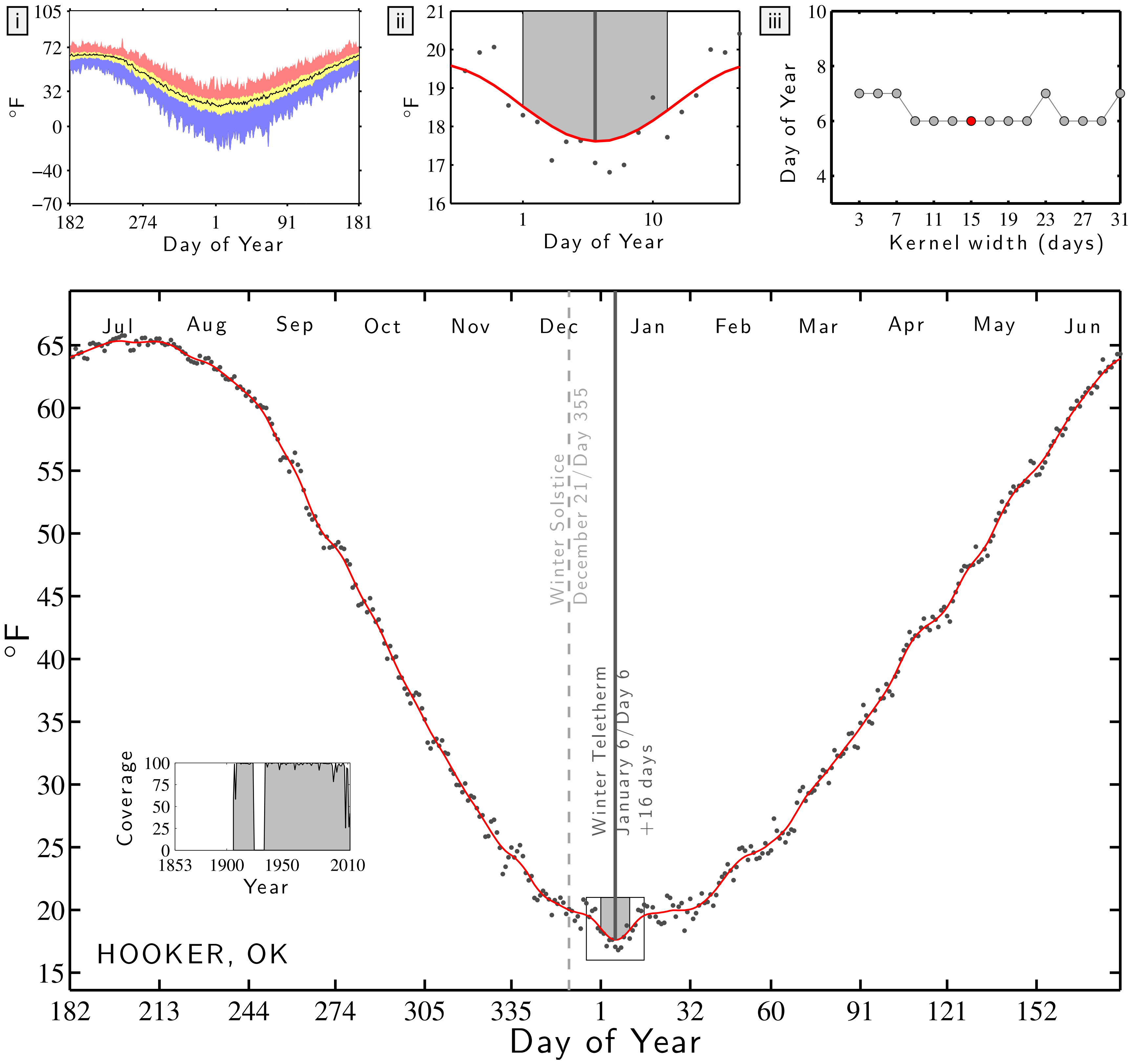

i
ii
iii
°F
Day of Year
Kernel width (days)
Jul
Aug
Sep
Oct
Nov
Dec
Jan
Feb
Mar
Apr
May
Jun
Winter Solstice
December 21/Day 355
Winter Teletherm
January 6/Day 6
+16 days
Coverage
Year
HOOKER, OK

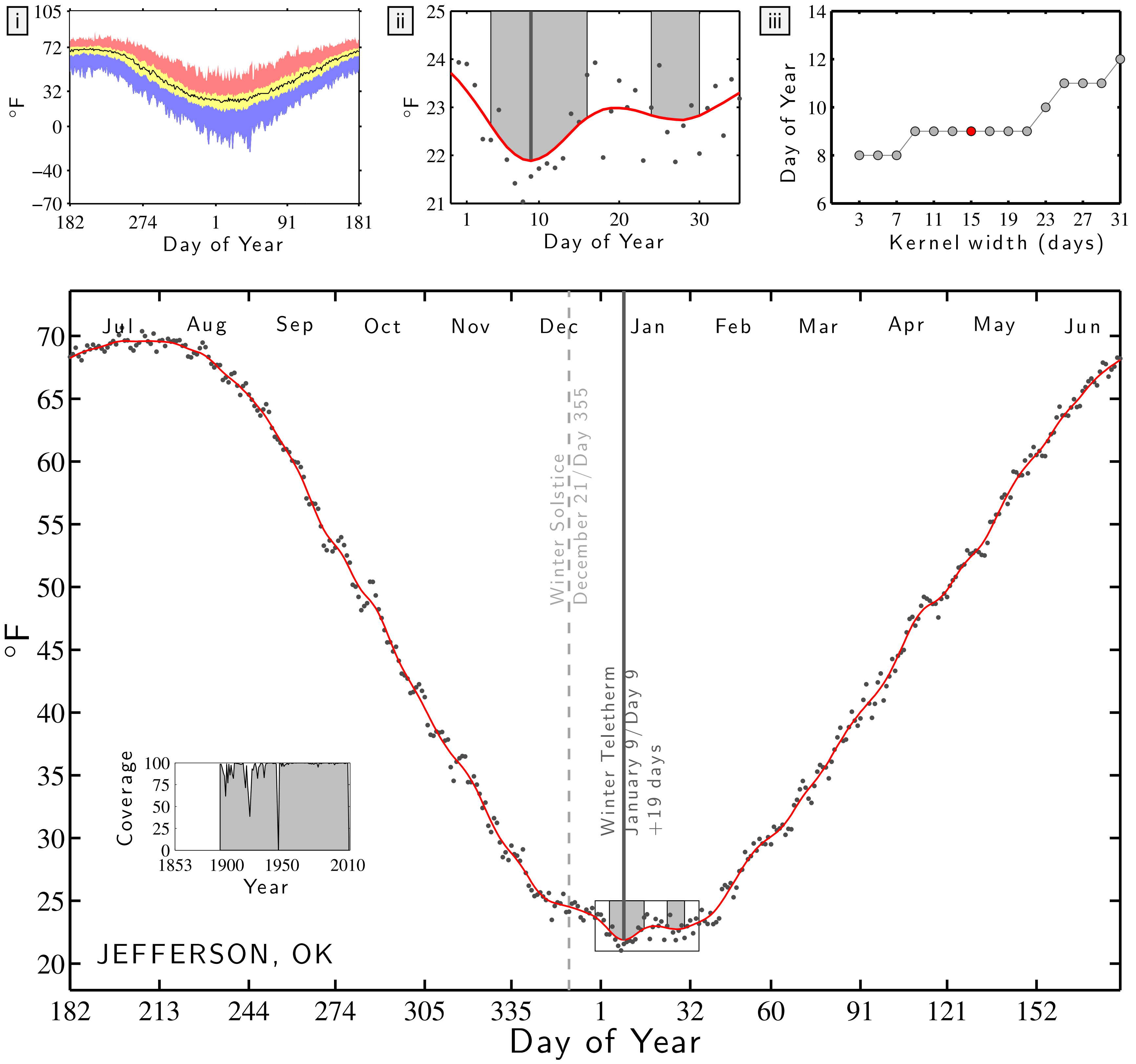

i
ii
iii
°F
Day of Year
Day of Year
Day of Year
Kernel width (days)
Jul
Aug
Sep
Oct
Nov
Dec
Jan
Feb
Mar
Apr
May
Jun
Winter Solstice
December 21/Day 355
Winter Teletherm
January 9/Day 9
+19 days
Coverage
Year
JEFFERSON, OK

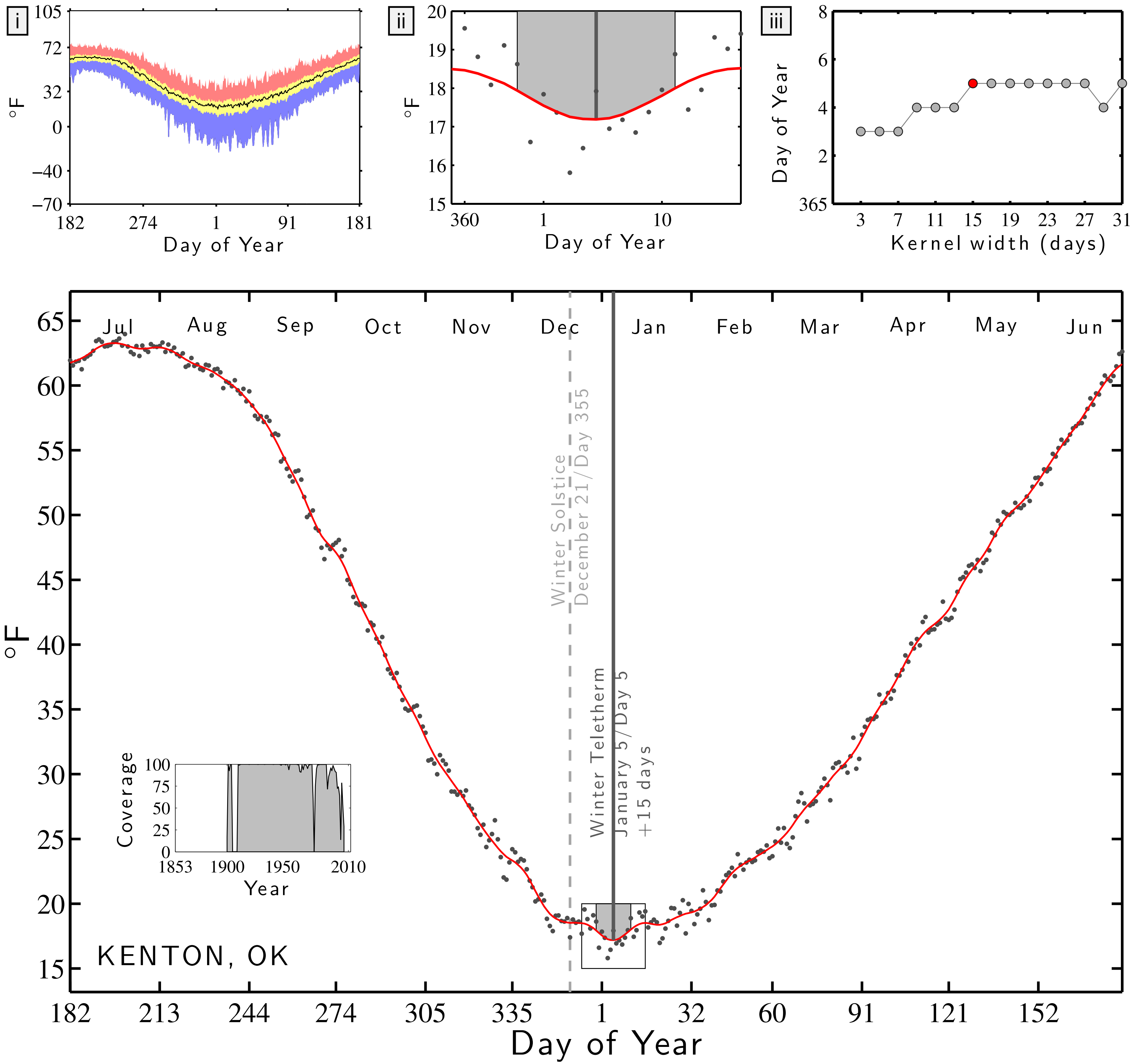

i
ii
iii
°F
Day of Year
Day of Year
Kernel width (days)
Winter Solstice
December 21/Day 355
Winter Teletherm
January 5/Day 5
+15 days
Coverage
Year
KENTON, OK
Jul
Aug
Sep
Oct
Nov
Dec
Jan
Feb
Mar
Apr
May
Jun

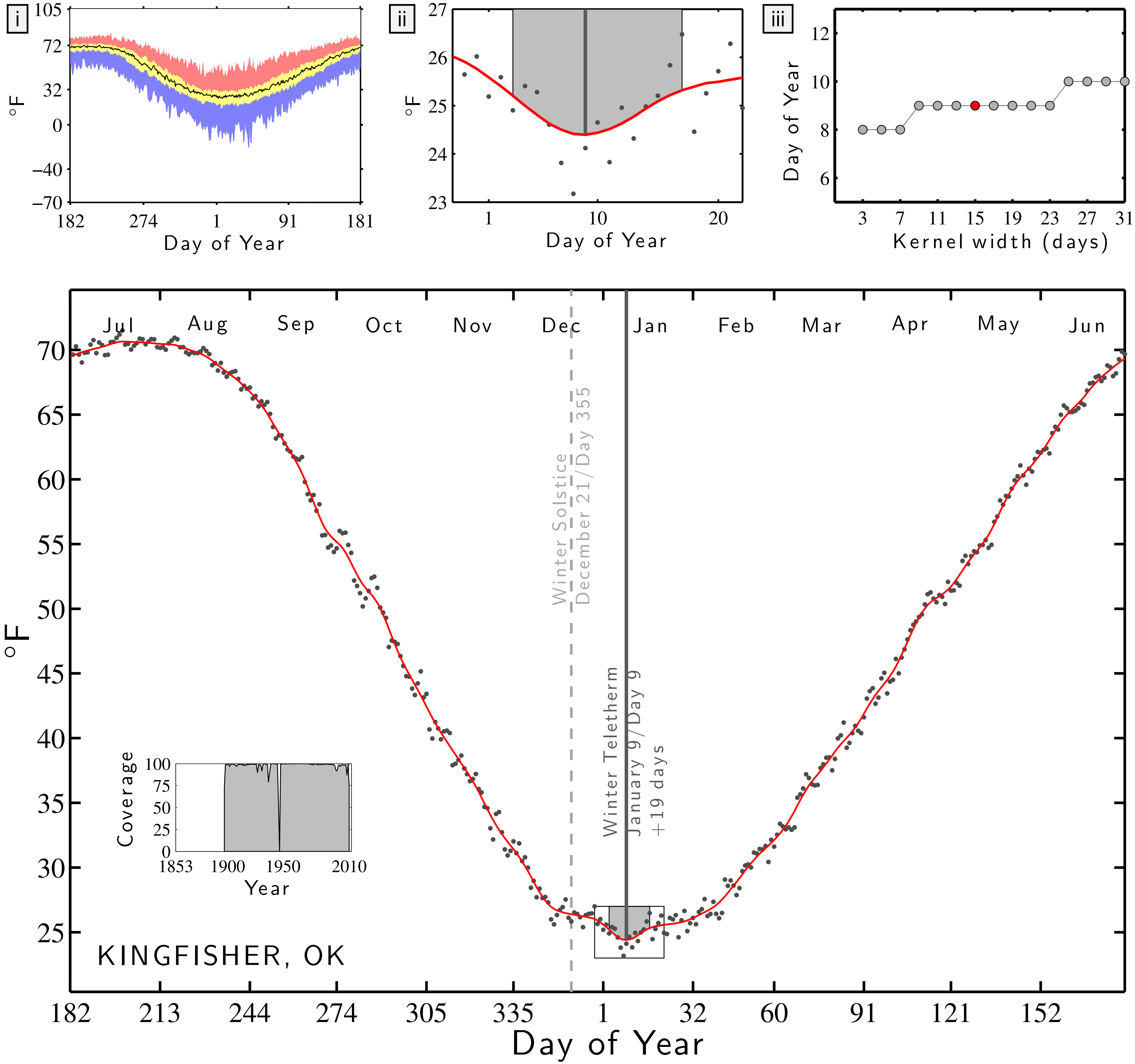

i
°F
Day of Year
182
274
1
91
181
ii
°F
Day of Year
iii
Day of Year
Kernel width (days)
Jul
Aug
Sep
Oct
Nov
Dec
Jan
Feb
Mar
Apr
May
Jun
Winter Solstice
December 21/Day 355
Winter Teletherm
January 9/Day 9
+19 days
Coverage
Year
KINGFISHER, OK
°F
Day of Year

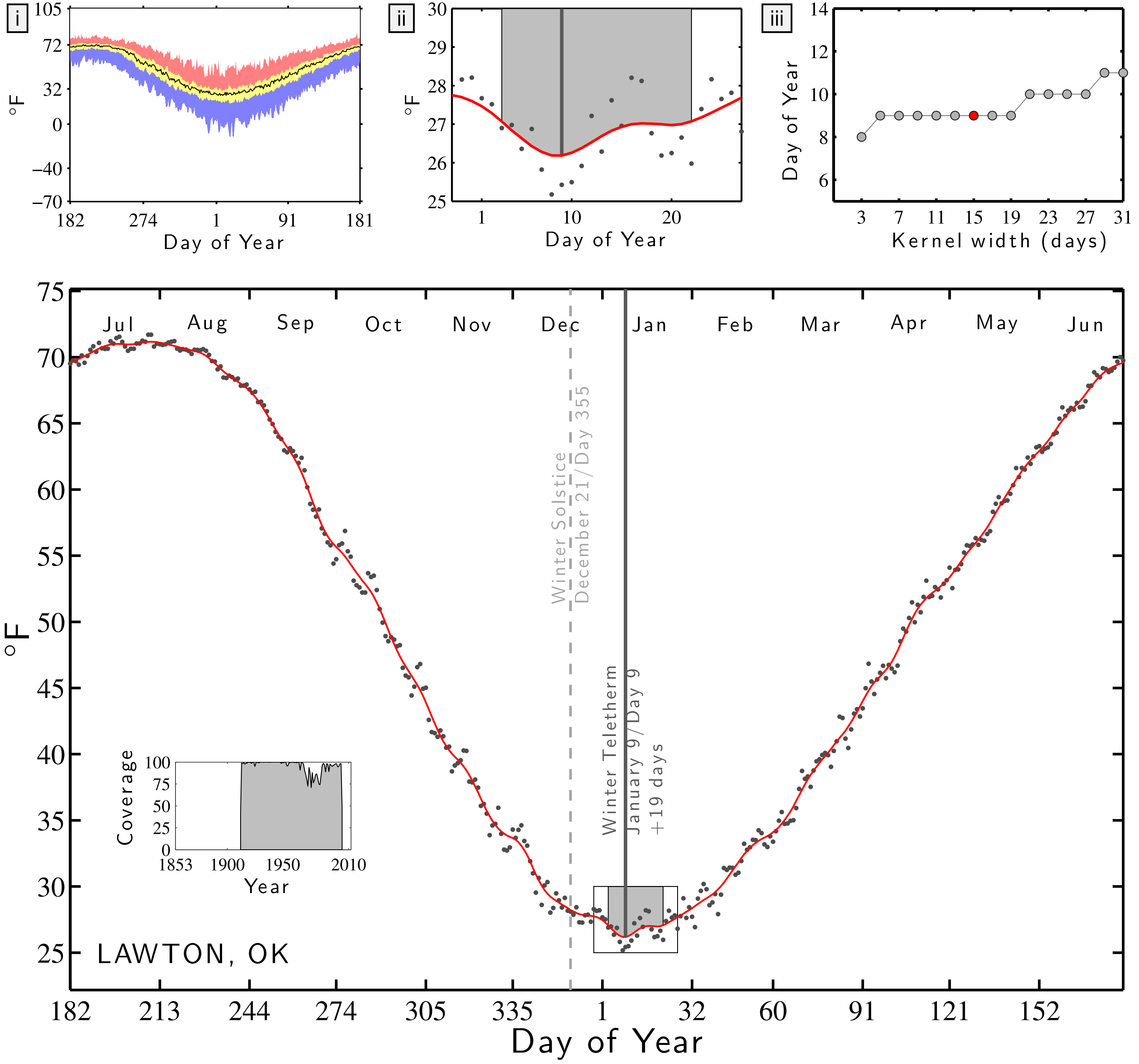

i
°F
105
72
32
0
−40
−70
182
274
1
91
181
Day of Year
ii
30
29
28
27
26
25
1
10
20
Day of Year
iii
Day of Year
14
12
10
8
6
3
7
11
15
19
23
27
31
Kernel width (days)
75
70
65
60
55
50
45
40
35
30
25
Jul
Aug
Sep
Oct
Nov
Dec
Jan
Feb
Mar
Apr
May
Jun
Winter Solstice
December 21/Day 355
Winter Teletherm
January 9/Day 9
+19 days
Coverage
100
75
50
25
0
1853
1900
1950
2010
Year
LAWTON, OK
182
213
244
274
305
335
1
32
60
91
121
152
Day of Year

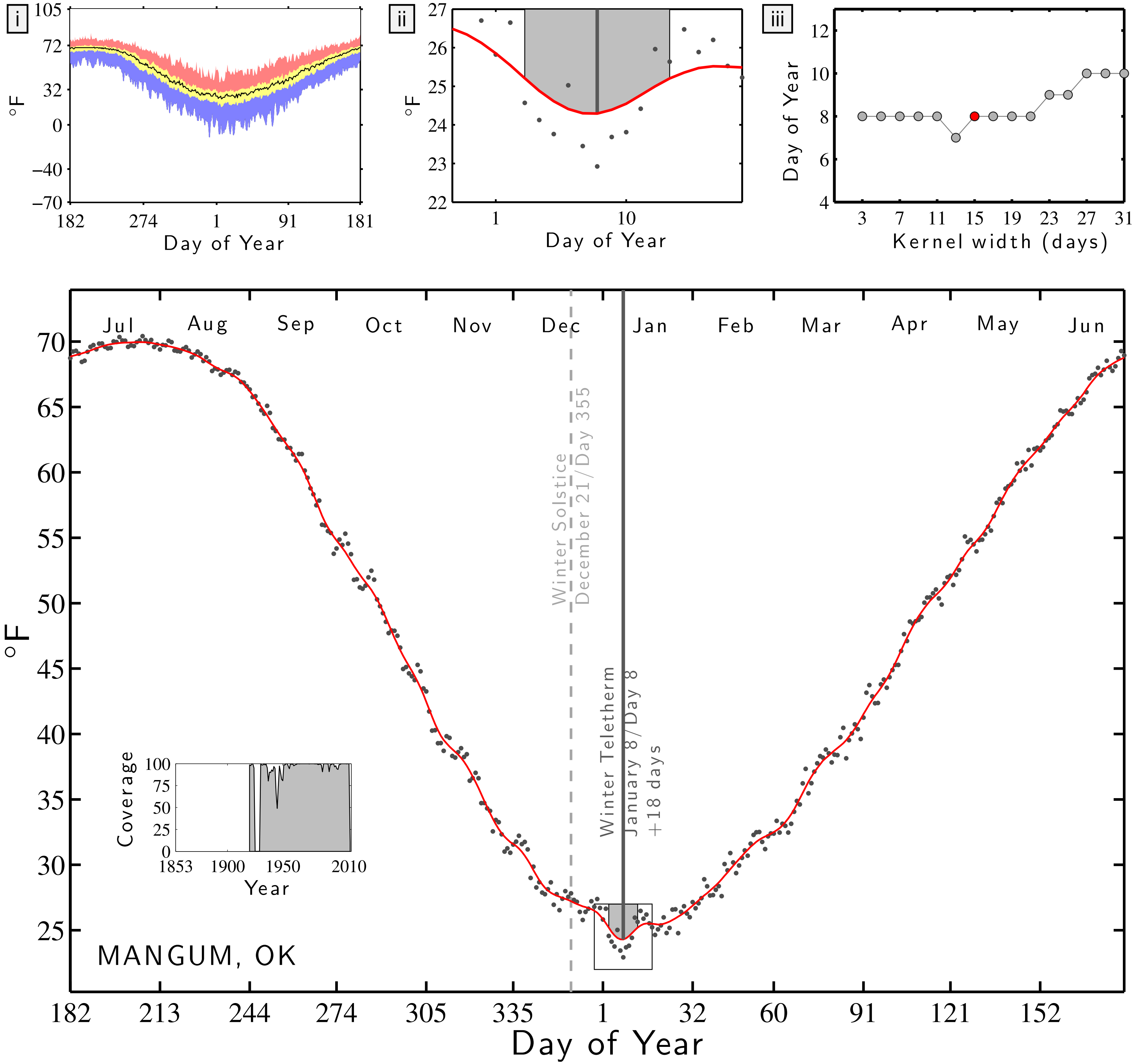

i
°F
Day of Year
ii
°F
Day of Year
iii
Day of Year
Kernel width (days)
Jul
Aug
Sep
Oct
Nov
Dec
Jan
Feb
Mar
Apr
May
Jun
Winter Solstice
December 21/Day 355
Winter Teletherm
January 8/Day 8
+18 days
Coverage
Year
MANGUM, OK
°F
Day of Year

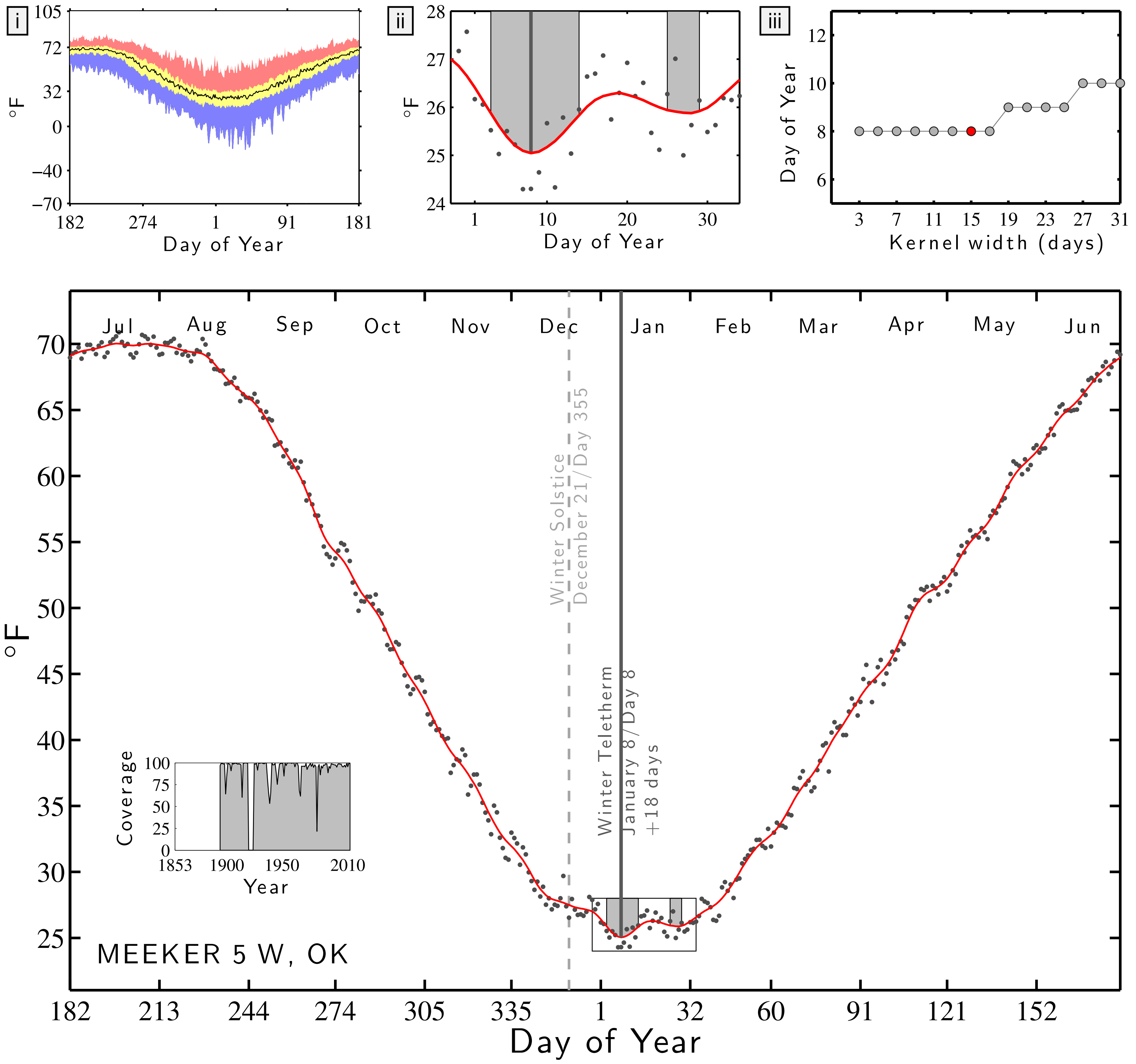

i
ii
iii
°F
Day of Year
Kernel width (days)
Day of Year
Jul
Aug
Sep
Oct
Nov
Dec
Jan
Feb
Mar
Apr
May
Jun
Winter Solstice
December 21/Day 355
Winter Teletherm
January 8/Day 8
+18 days
Coverage
Year
MEEKER 5 W, OK

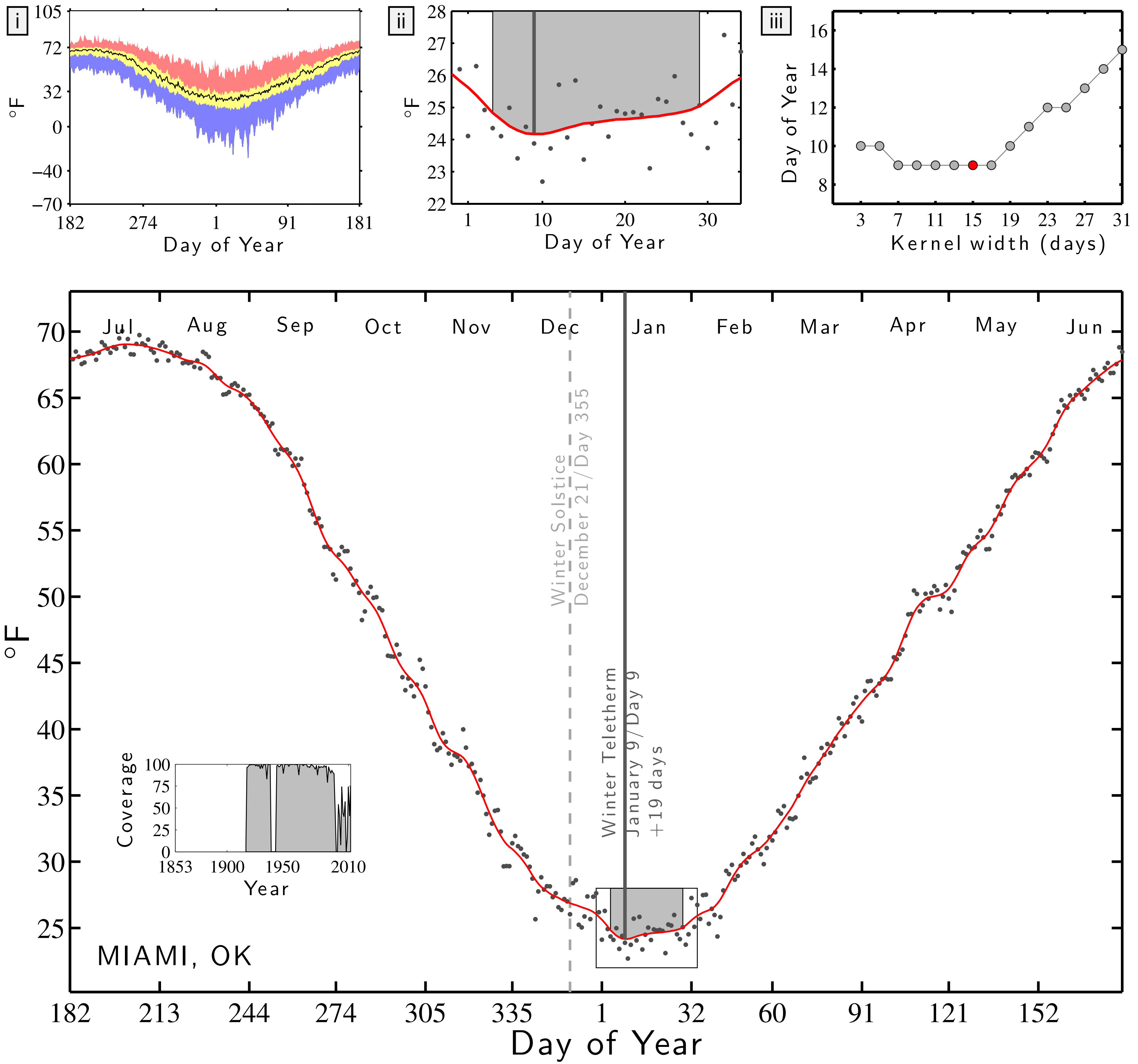

i
°F
Day of Year
ii
°F
Day of Year
iii
Day of Year
Kernel width (days)
Jul
Aug
Sep
Oct
Nov
Dec
Jan
Feb
Mar
Apr
May
Jun
Winter Solstice
December 21/Day 355
Winter Teletherm
January 9/Day 9
+19 days
Coverage
Year
MIAMI, OK
°F
Day of Year

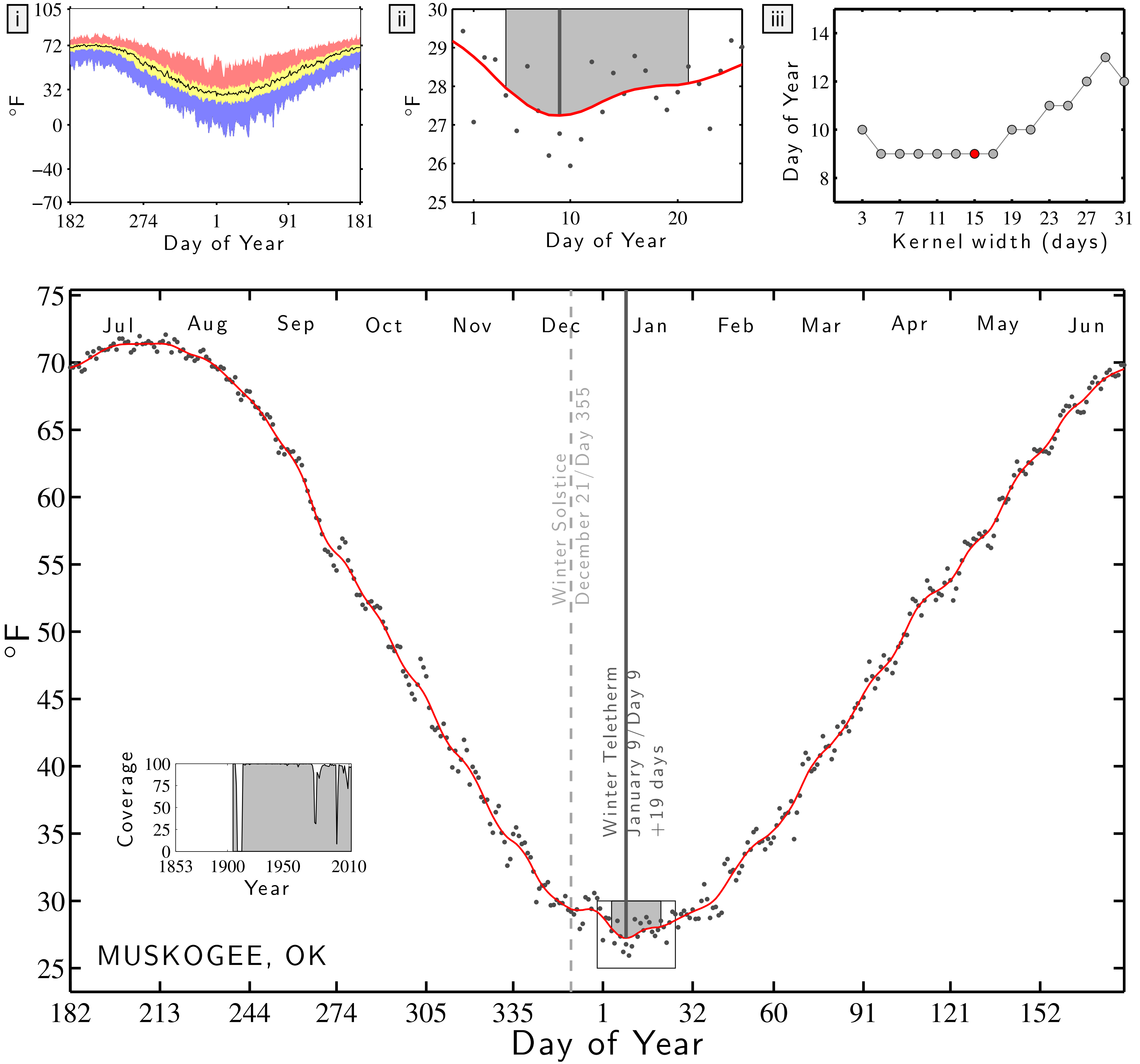

i
°F
Day of Year
ii
°F
Day of Year
iii
Day of Year
Kernel width (days)
Jul
Aug
Sep
Oct
Nov
Dec
Jan
Feb
Mar
Apr
May
Jun
Winter Solstice
December 21/Day 355
Winter Teletherm
January 9/Day 9
+19 days
Coverage
Year
MUSKOGEE, OK
°F
Day of Year

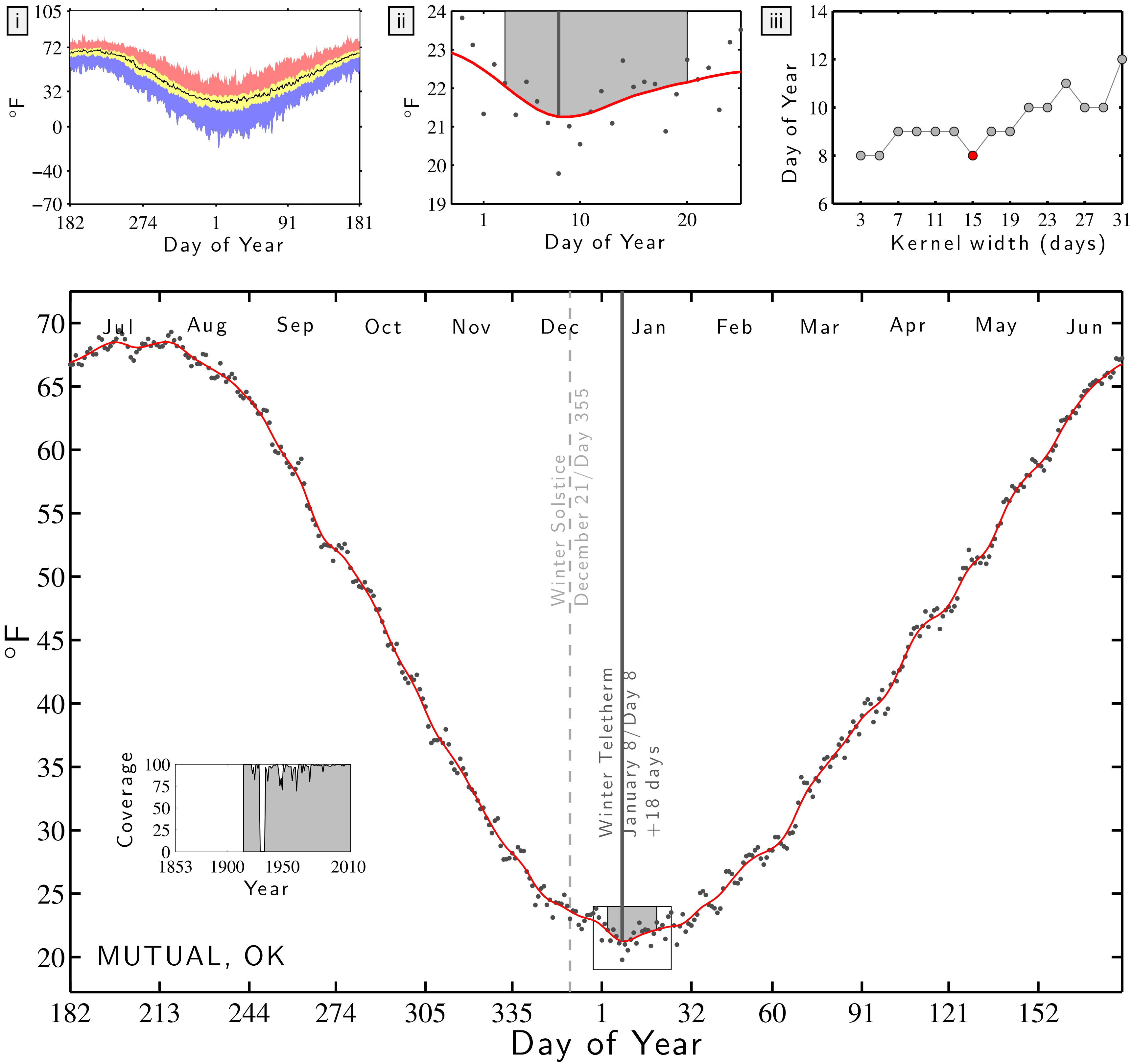

i
°F
Day of Year
ii
°F
Day of Year
iii
Day of Year
Kernel width (days)
Jul
Aug
Sep
Oct
Nov
Dec
Jan
Feb
Mar
Apr
May
Jun
Winter Solstice
December 21/Day 355
Winter Teletherm
January 8/Day 8
+18 days
Coverage
Year
MUTUAL, OK
°F
Day of Year

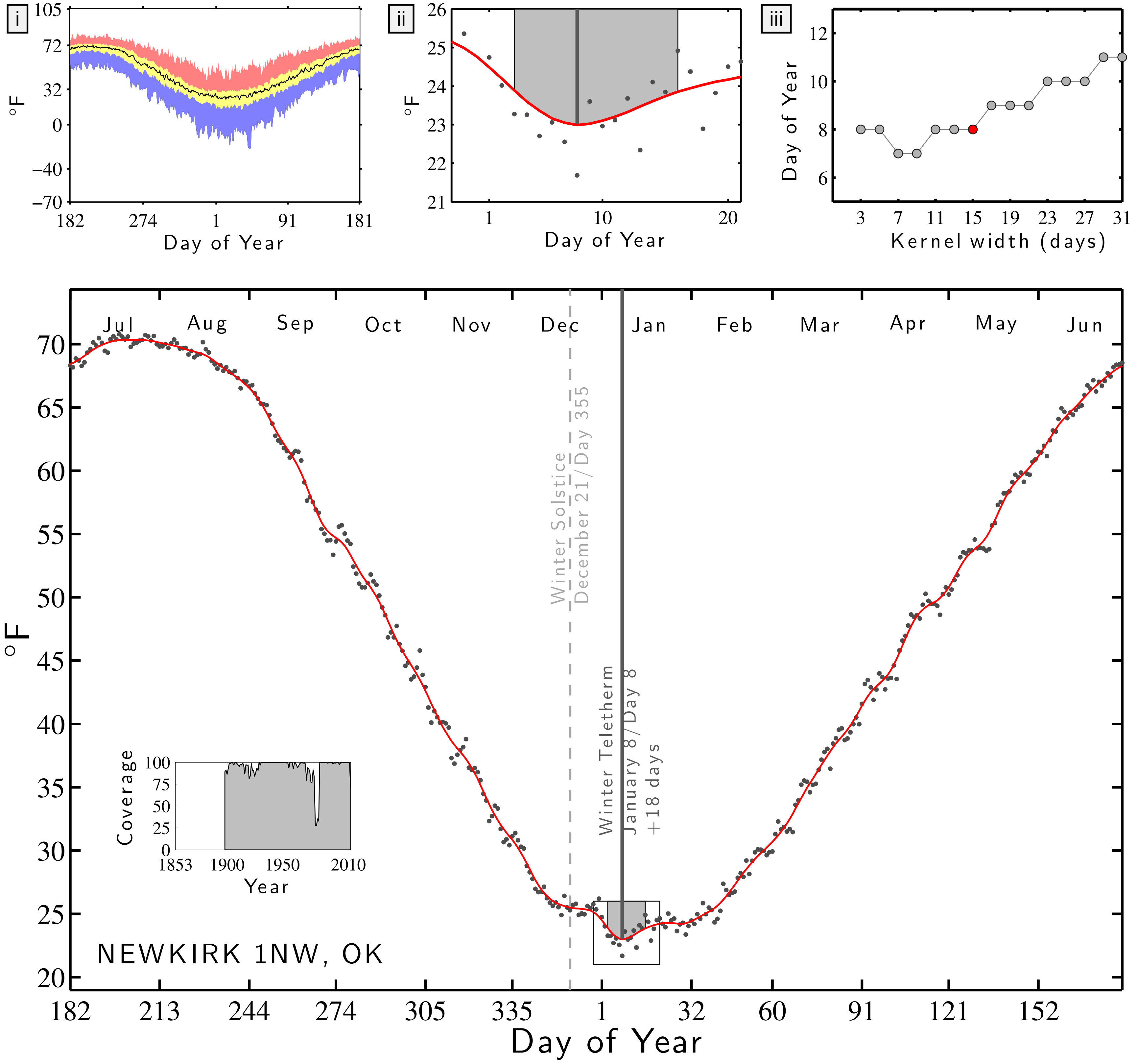

i
°F
Day of Year
ii
°F
Day of Year
iii
Day of Year
Kernel width (days)
Jul
Aug
Sep
Oct
Nov
Dec
Jan
Feb
Mar
Apr
May
Jun
Winter Solstice
December 21/Day 355
Winter Teletherm
January 8/Day 8
+18 days
Coverage
Year
NEWKIRK 1NW, OK
°F
Day of Year

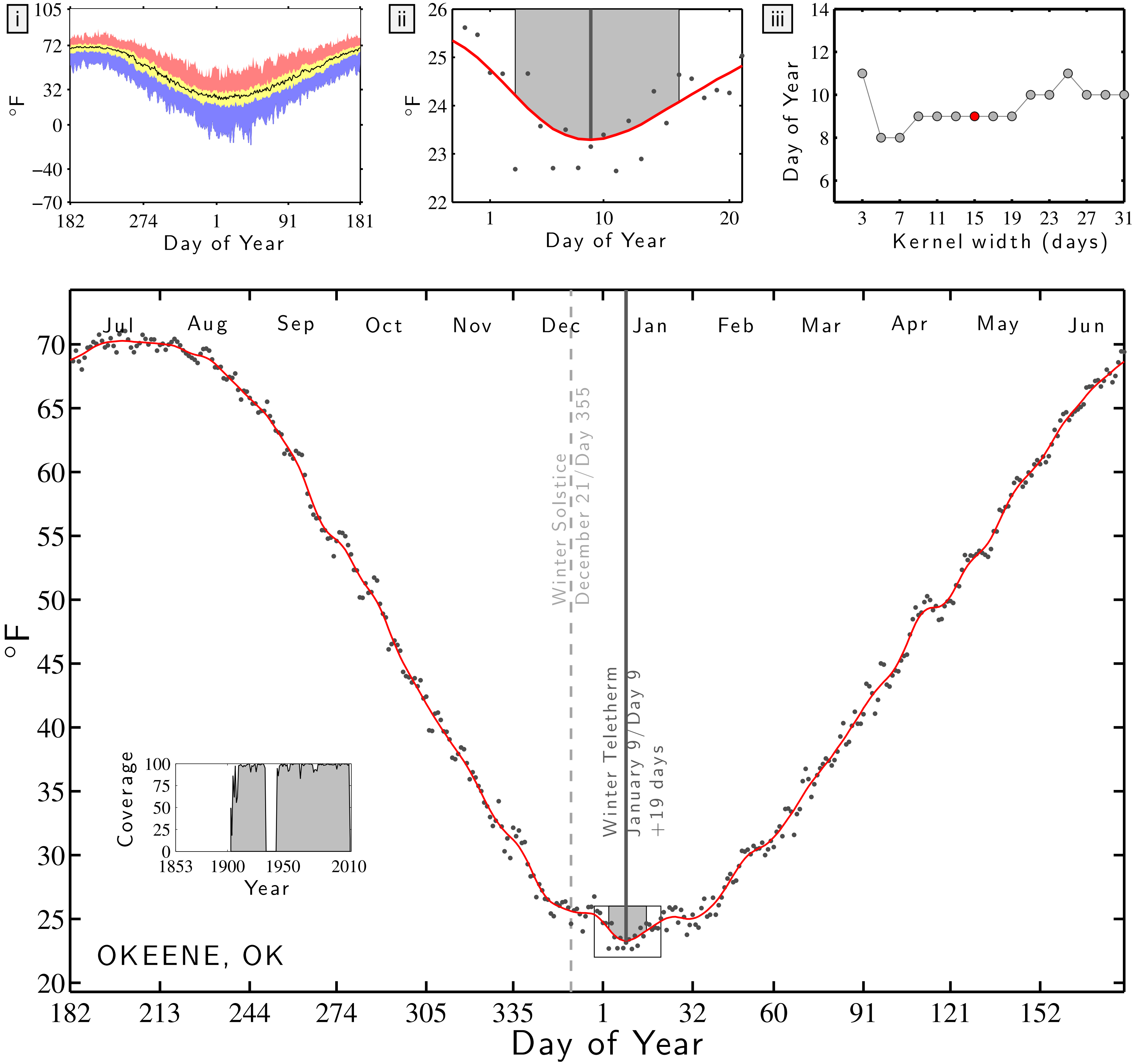

i
°F
105
72
32
0
−40
−70
182
274
1
91
181
Day of Year
ii
°F
26
25
24
23
22
1
10
20
Day of Year
iii
Day of Year
14
12
10
8
6
3
7
11
15
19
23
27
31
Kernel width (days)
°F
70
65
60
55
50
45
40
35
30
25
20
Jul
Aug
Sep
Oct
Nov
Dec
Jan
Feb
Mar
Apr
May
Jun
Winter Solstice
December 21/Day 355
Winter Teletherm
January 9/Day 9
+19 days
Coverage
100
75
50
25
0
1853
1900
1950
2010
Year
OKEENE, OK
182
213
244
274
305
335
1
32
60
91
121
152
Day of Year

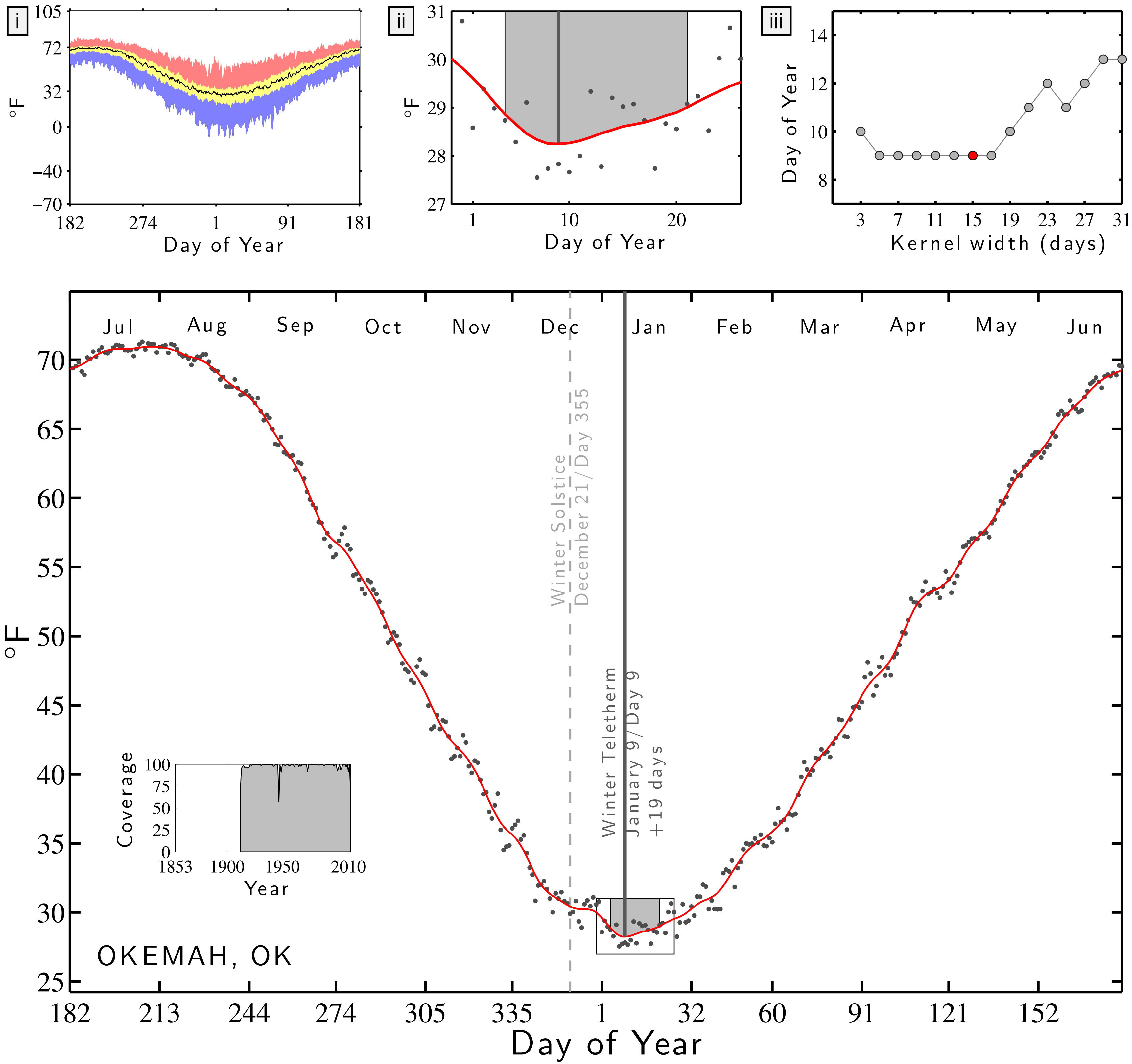

i
°F
Day of Year
ii
Day of Year
iii
Day of Year
Kernel width (days)
Jul
Aug
Sep
Oct
Nov
Dec
Jan
Feb
Mar
Apr
May
Jun
Winter Solstice
December 21/Day 355
Winter Teletherm
January 9/Day 9
+19 days
Coverage
Year
OKEMAH, OK
Day of Year

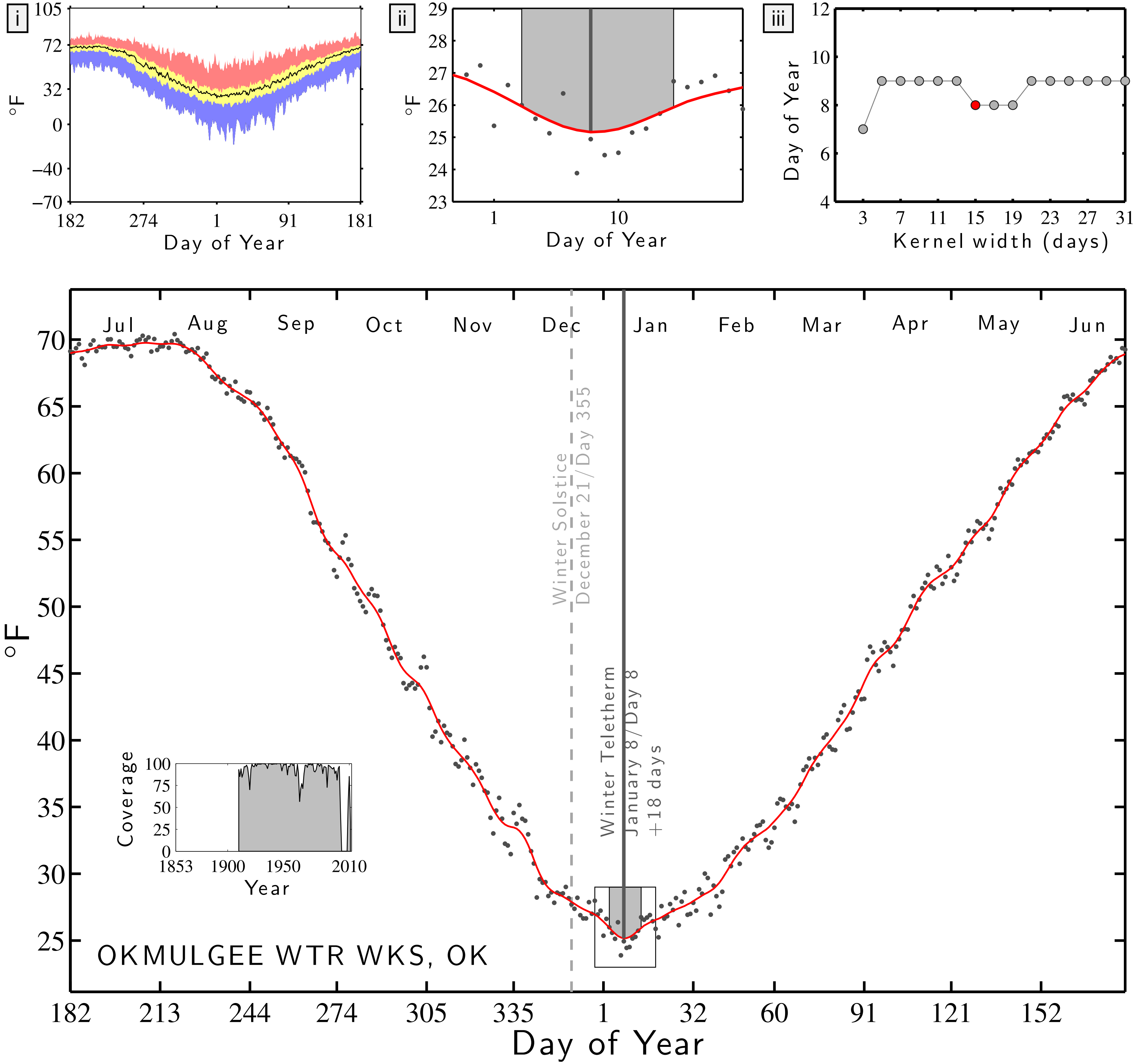

i
°F
Day of Year
ii
Day of Year
iii
Day of Year
Kernel width (days)
Jul
Aug
Sep
Oct
Nov
Dec
Jan
Feb
Mar
Apr
May
Jun
Winter Solstice
December 21/Day 355
Winter Teletherm
January 8/Day 8
+18 days
Coverage
Year
OKMULGEE WTR WKS, OK
Day of Year

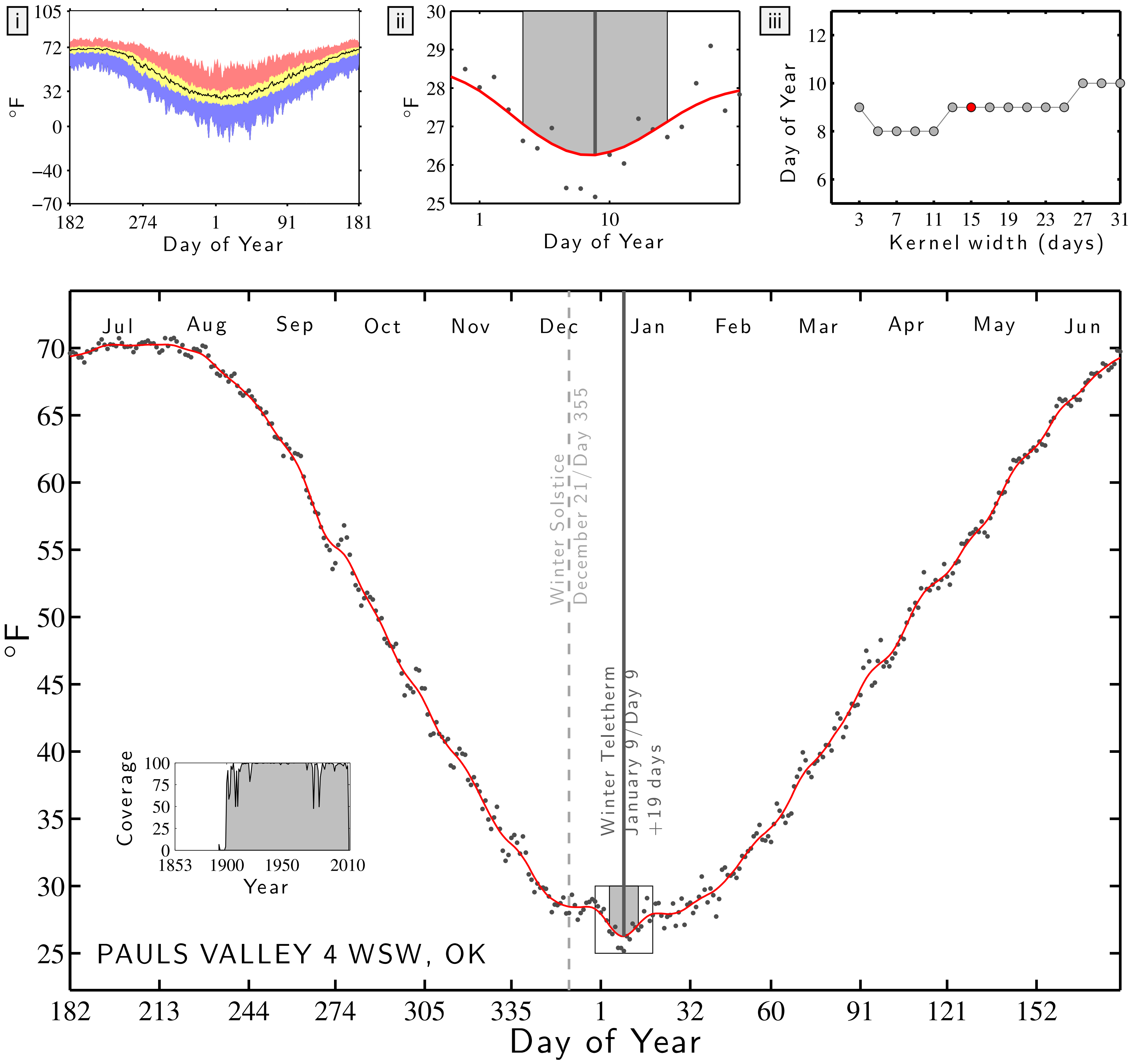

i
°F
Day of Year
ii
°F
Day of Year
iii
Day of Year
Kernel width (days)
Jul
Aug
Sep
Oct
Nov
Dec
Jan
Feb
Mar
Apr
May
Jun
Winter Solstice
December 21/Day 355
Winter Teletherm
January 9/Day 9
+19 days
Coverage
Year
PAULS VALLEY 4 WSW, OK
°F
Day of Year

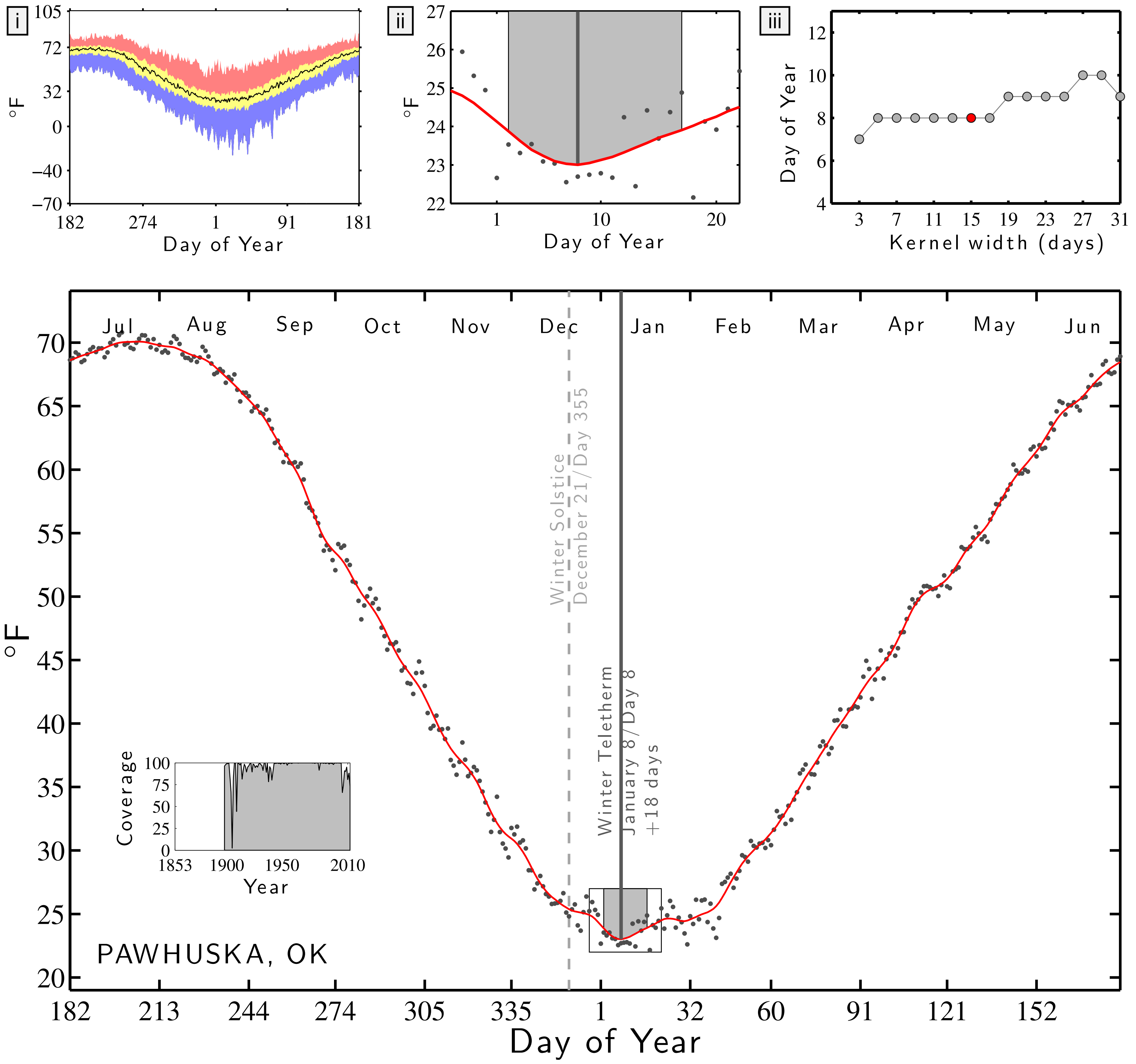

i
°F
Day of Year
ii
°F
Day of Year
iii
Day of Year
Kernel width (days)
Jul
Aug
Sep
Oct
Nov
Dec
Jan
Feb
Mar
Apr
May
Jun
Winter Solstice
December 21/Day 355
Winter Teletherm
January 8/Day 8
+18 days
Coverage
Year
PAWHUSKA, OK
°F
Day of Year

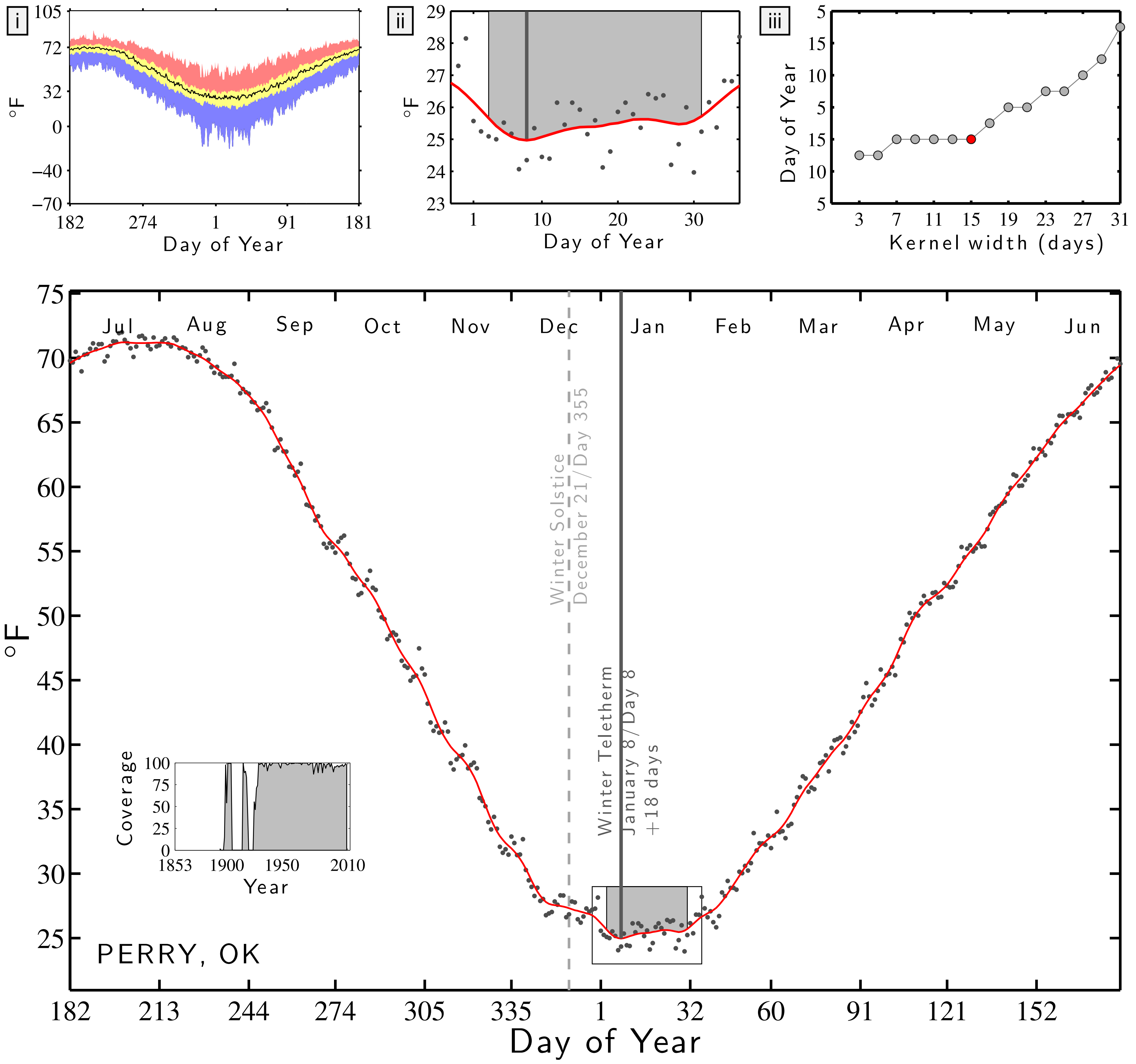

i
°F
Day of Year
ii
°F
Day of Year
iii
Day of Year
Kernel width (days)
Jul
Aug
Sep
Oct
Nov
Dec
Jan
Feb
Mar
Apr
May
Jun
Winter Solstice
December 21/Day 355
Winter Teletherm
January 8/Day 8
+18 days
Coverage
Year
PERRY, OK
°F
Day of Year

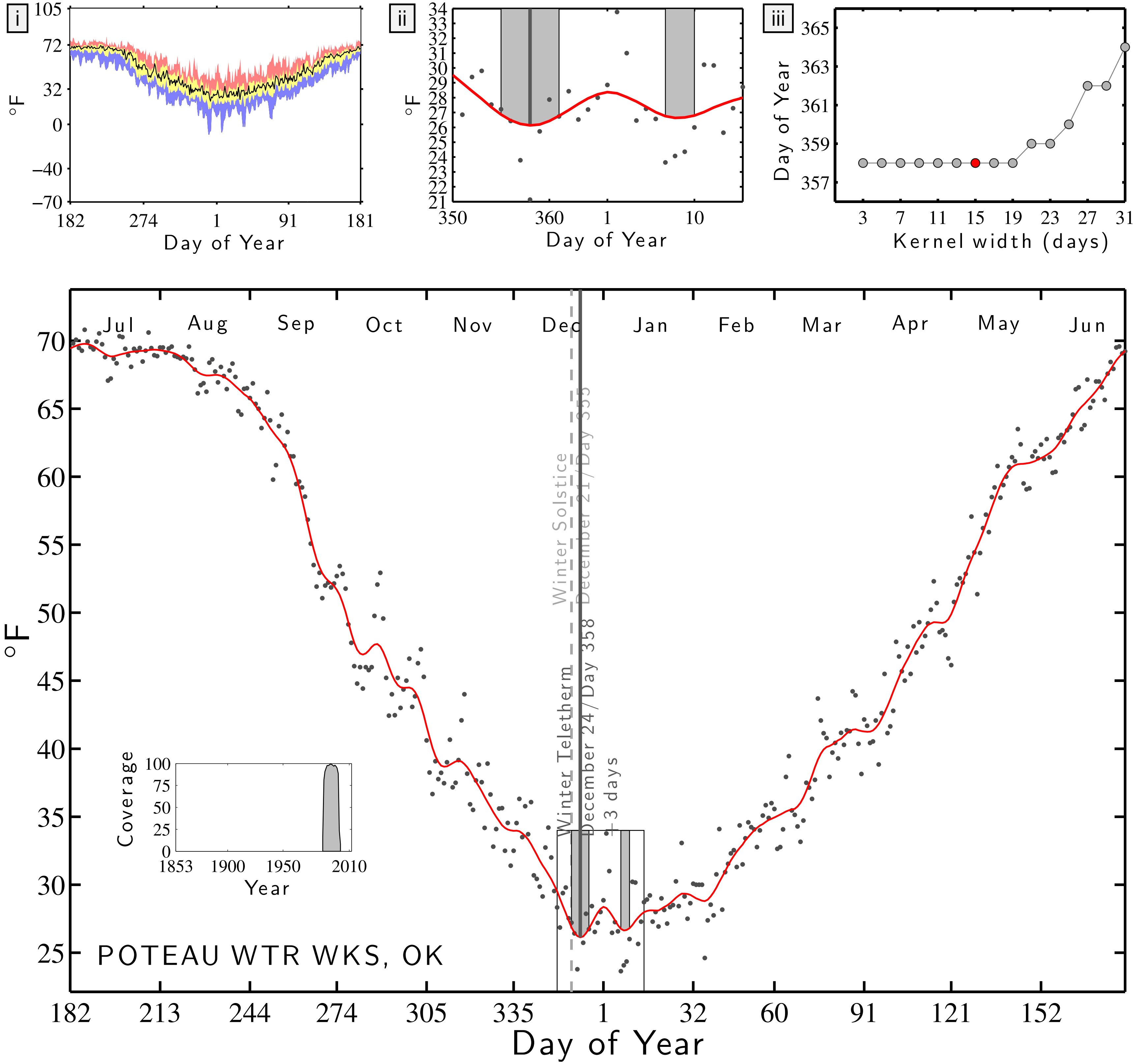

i
ii
iii
°F
Day of Year
Kernel width (days)
Day of Year
Jul
Aug
Sep
Oct
Nov
Dec
Jan
Feb
Mar
Apr
May
Jun
Winter Solstice
December 21/Day 355
Winter Teletherm
December 24/Day 358
± 3 days
Coverage
Year
POTEAU WTR WKS, OK

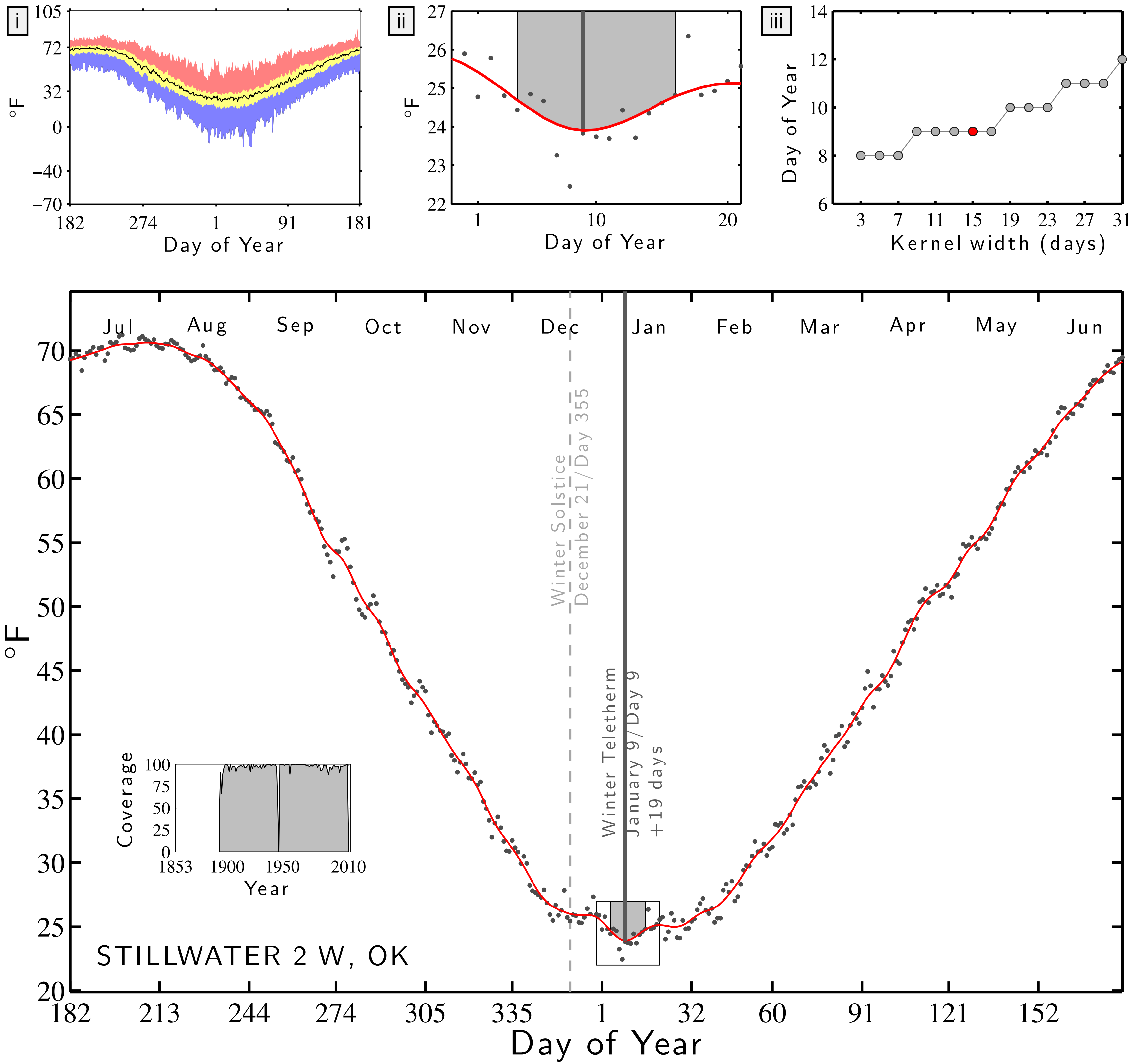

i
105
72
32
0
−40
−70
°F
182
274
1
91
181
Day of Year
ii
27
26
25
24
23
22
°F
1
10
20
Day of Year
iii
14
12
10
8
6
Day of Year
3
7
11
15
19
23
27
31
Kernel width (days)
Jul
Aug
Sep
Oct
Nov
Dec
Jan
Feb
Mar
Apr
May
Jun
Winter Solstice
December 21/Day 355
Winter Teletherm
January 9/Day 9
+19 days
Coverage
100
75
50
25
0
1853
1900
1950
2010
Year
STILLWATER 2 W, OK
70
65
60
55
50
45
40
35
30
25
20
°F
182
213
244
274
305
335
1
32
60
91
121
152
Day of Year

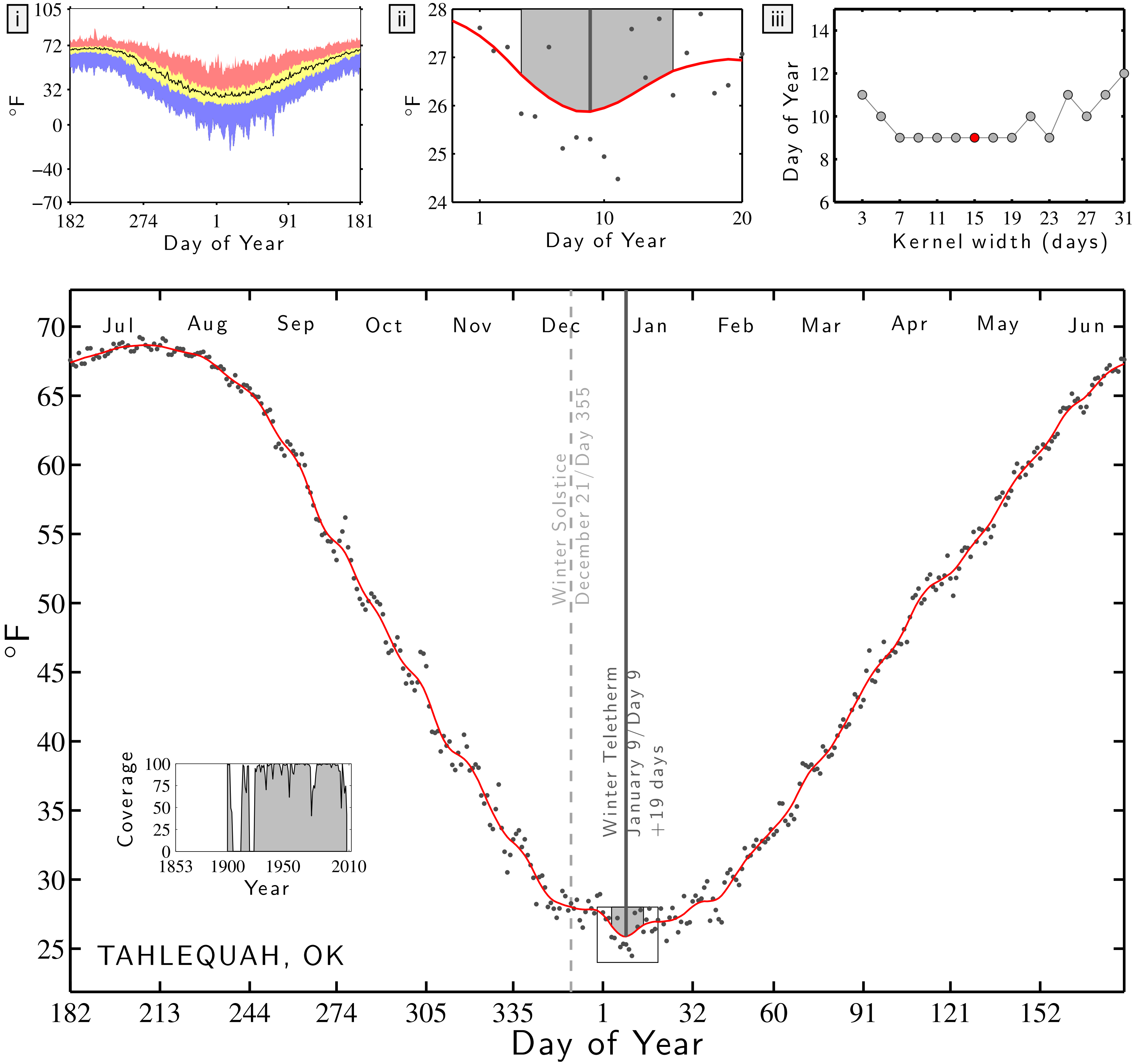

i
ii
iii
°F
Day of Year
Day of Year
Kernel width (days)
Jul
Aug
Sep
Oct
Nov
Dec
Jan
Feb
Mar
Apr
May
Jun
Winter Solstice
December 21/Day 355
Winter Teletherm
January 9/Day 9
+19 days
Coverage
Year
TAHLEQUAH, OK
Day of Year

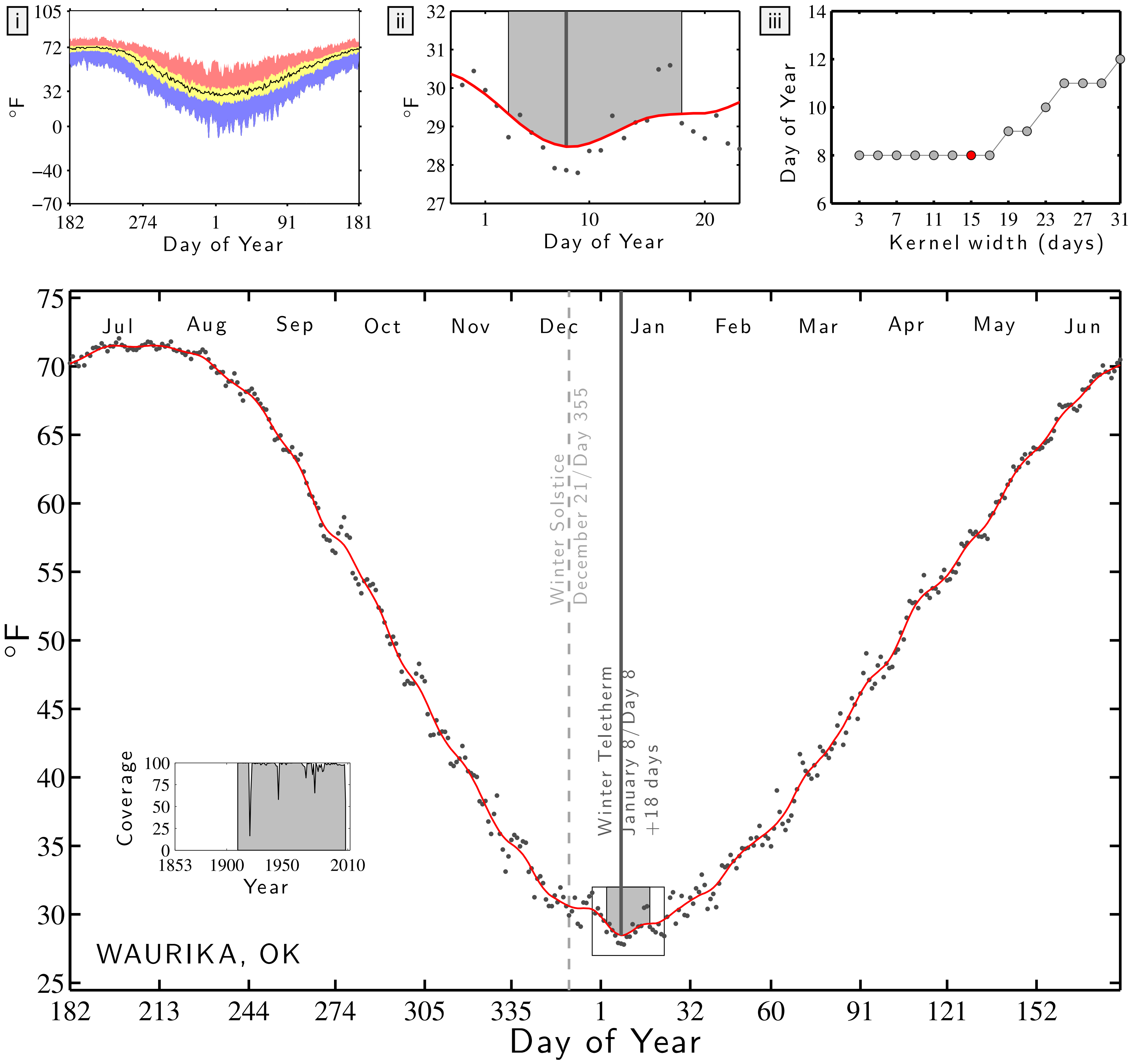
i
ii
iii
°F
Day of Year
Kernel width (days)
Jul
Aug
Sep
Oct
Nov
Dec
Jan
Feb
Mar
Apr
May
Jun
Winter Solstice
December 21/Day 355
Winter Teletherm
January 8/Day 8
+18 days
Coverage
Year
WAURIKA, OK

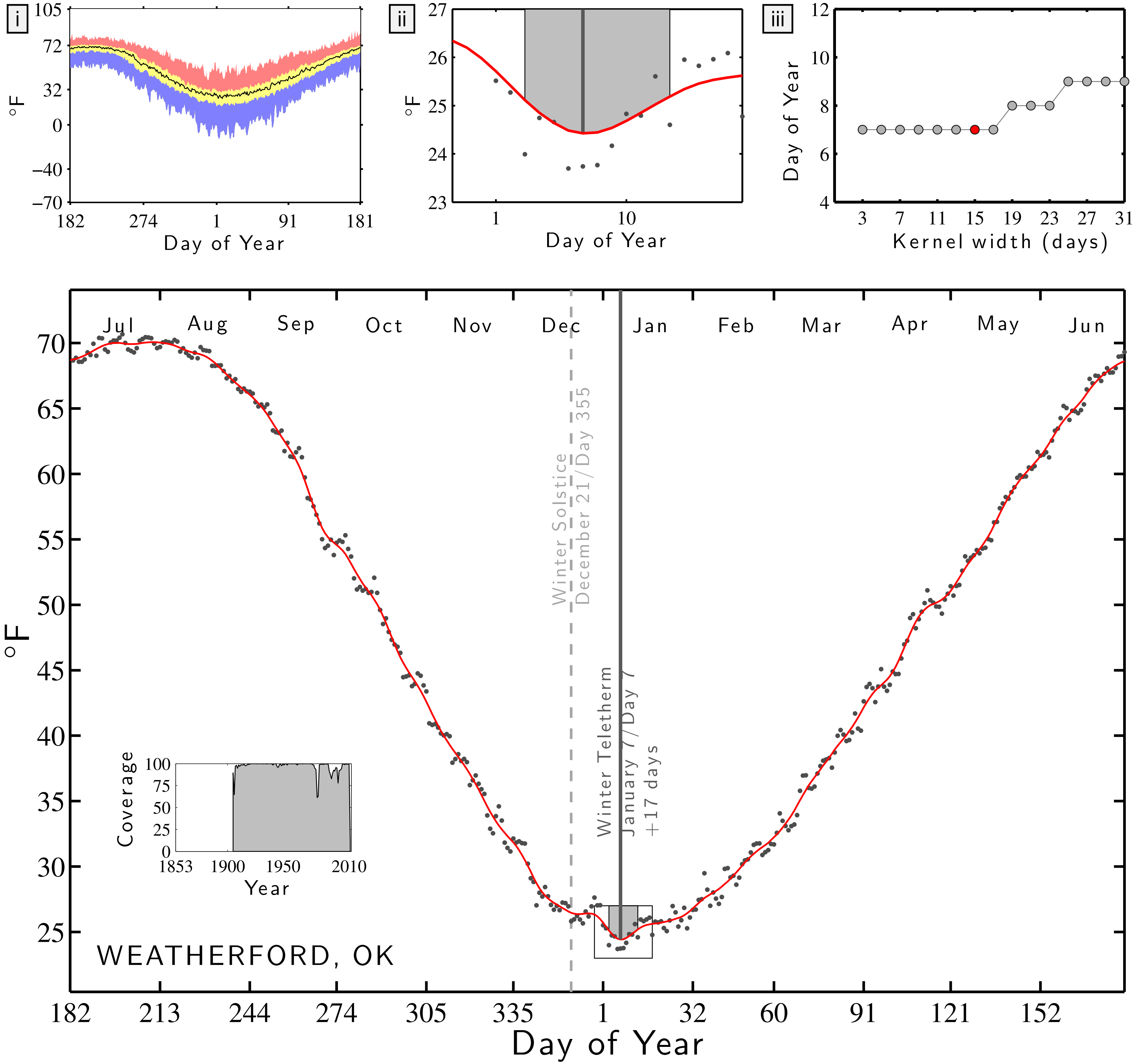

i
°F
Day of Year
ii
°F
Day of Year
iii
Day of Year
Kernel width (days)
Jul
Aug
Sep
Oct
Nov
Dec
Jan
Feb
Mar
Apr
May
Jun
Winter Solstice
December 21/Day 355
Winter Teletherm
January 7/Day 7
+17 days
Coverage
Year
WEATHERFORD, OK
°F
Day of Year

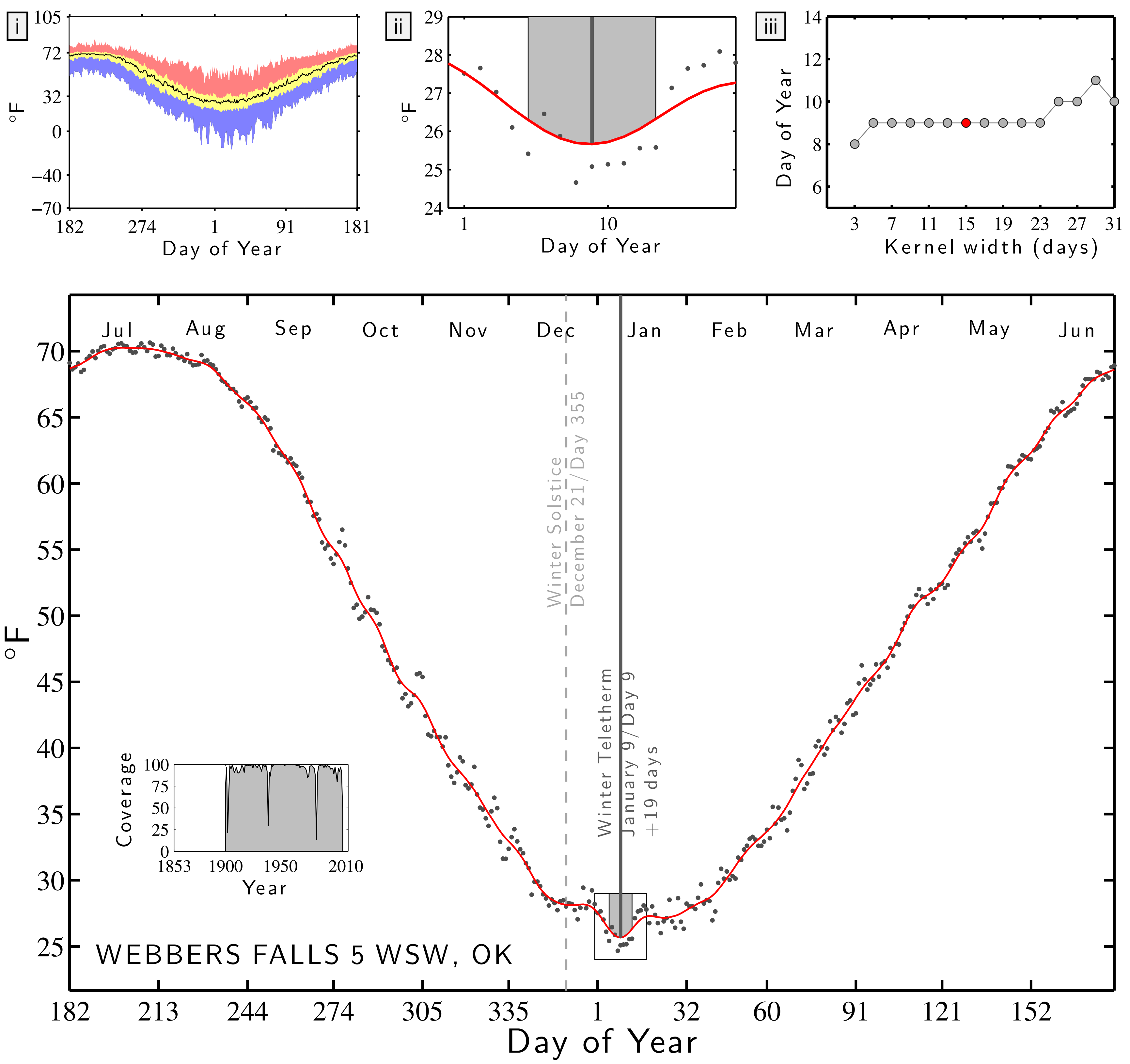

i
°F
105
72
32
0
−40
−70
182
274
1
91
181
Day of Year
ii
°F
29
28
27
26
25
24
1
10
Day of Year
iii
Day of Year
14
12
10
8
6
3
7
11
15
19
23
27
31
Kernel width (days)
Jul
Aug
Sep
Oct
Nov
Dec
Jan
Feb
Mar
Apr
May
Jun
°F
70
65
60
55
50
45
40
35
30
25
Winter Solstice
December 21/Day 355
Winter Teletherm
January 9/Day 9
+19 days
Coverage
100
75
50
25
0
1853
1900
1950
2010
Year
WEBBERS FALLS 5 WSW, OK
182
213
244
274
305
335
1
32
60
91
121
152
Day of Year

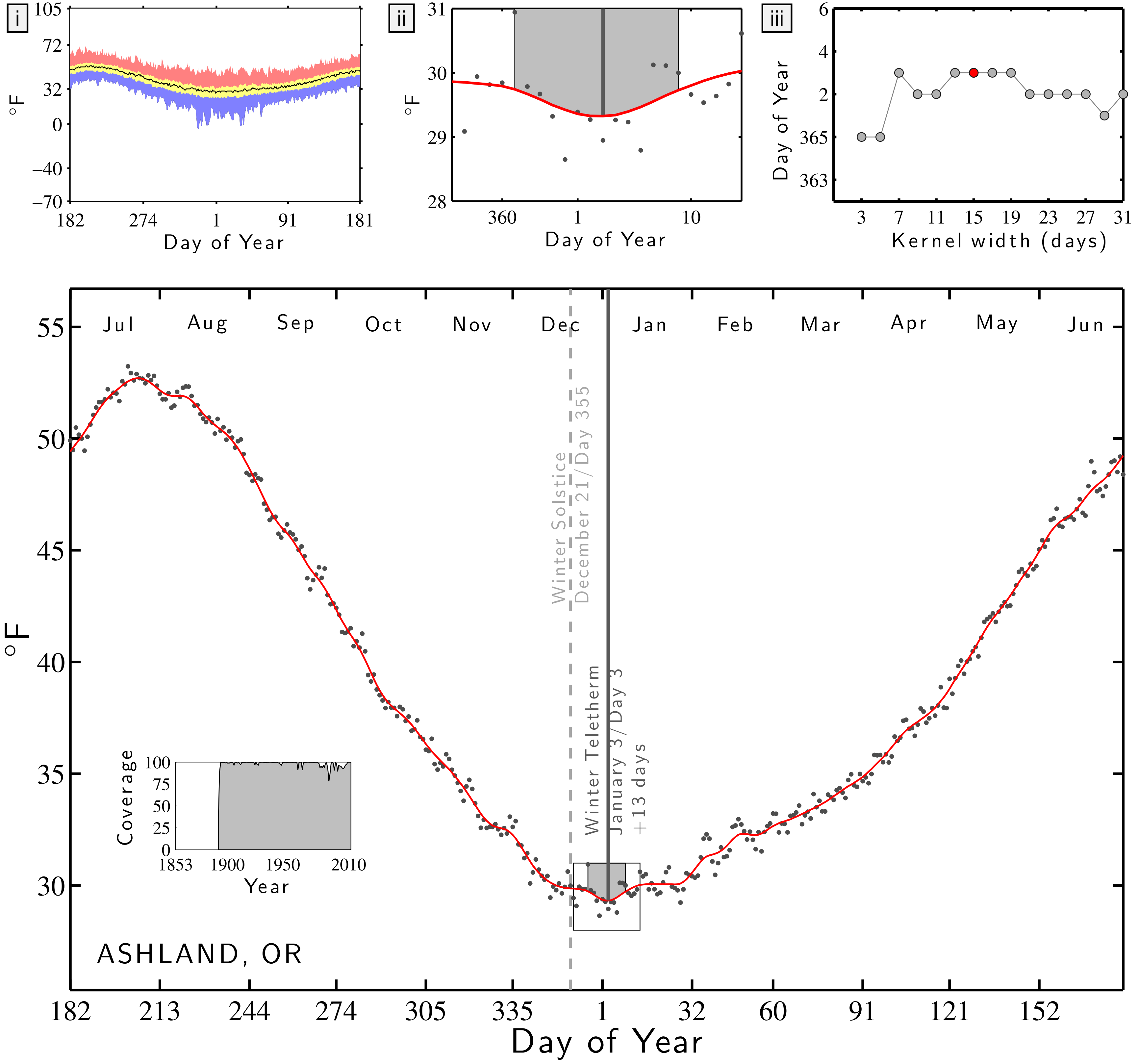

i
ii
iii
°F
Day of Year
Kernel width (days)
Jul
Aug
Sep
Oct
Nov
Dec
Jan
Feb
Mar
Apr
May
Jun
Winter Solstice
December 21/Day 355
Winter Teletherm
January 3/Day 3
+13 days
Coverage
Year
ASHLAND, OR

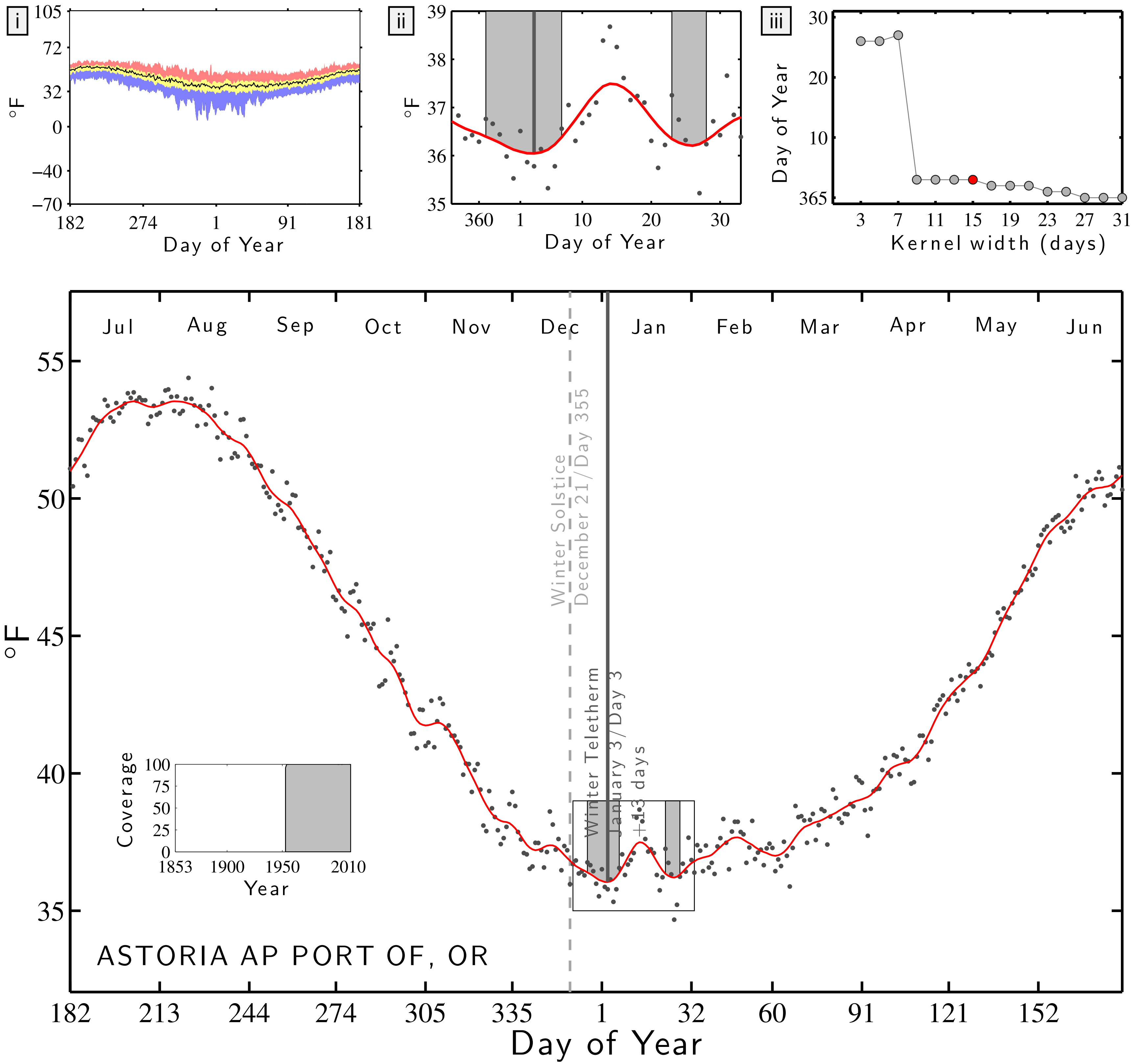

i
°F
105
72
32
0
−40
−70
182
274
1
91
181
Day of Year
ii
39
38
37
36
35
360
1
10
20
30
Day of Year
iii
Day of Year
30
20
10
365
3
7
11
15
19
23
27
31
Kernel width (days)
Jul
Aug
Sep
Oct
Nov
Dec
Jan
Feb
Mar
Apr
May
Jun
55
50
45
40
35
Winter Solstice
December 21/Day 355
Winter Teletherm
January 3/Day 3
+13 days
Coverage
100
75
50
25
0
1853
1900
1950
2010
Year
ASTORIA AP PORT OF, OR
182
213
244
274
305
335
1
32
60
91
121
152
Day of Year

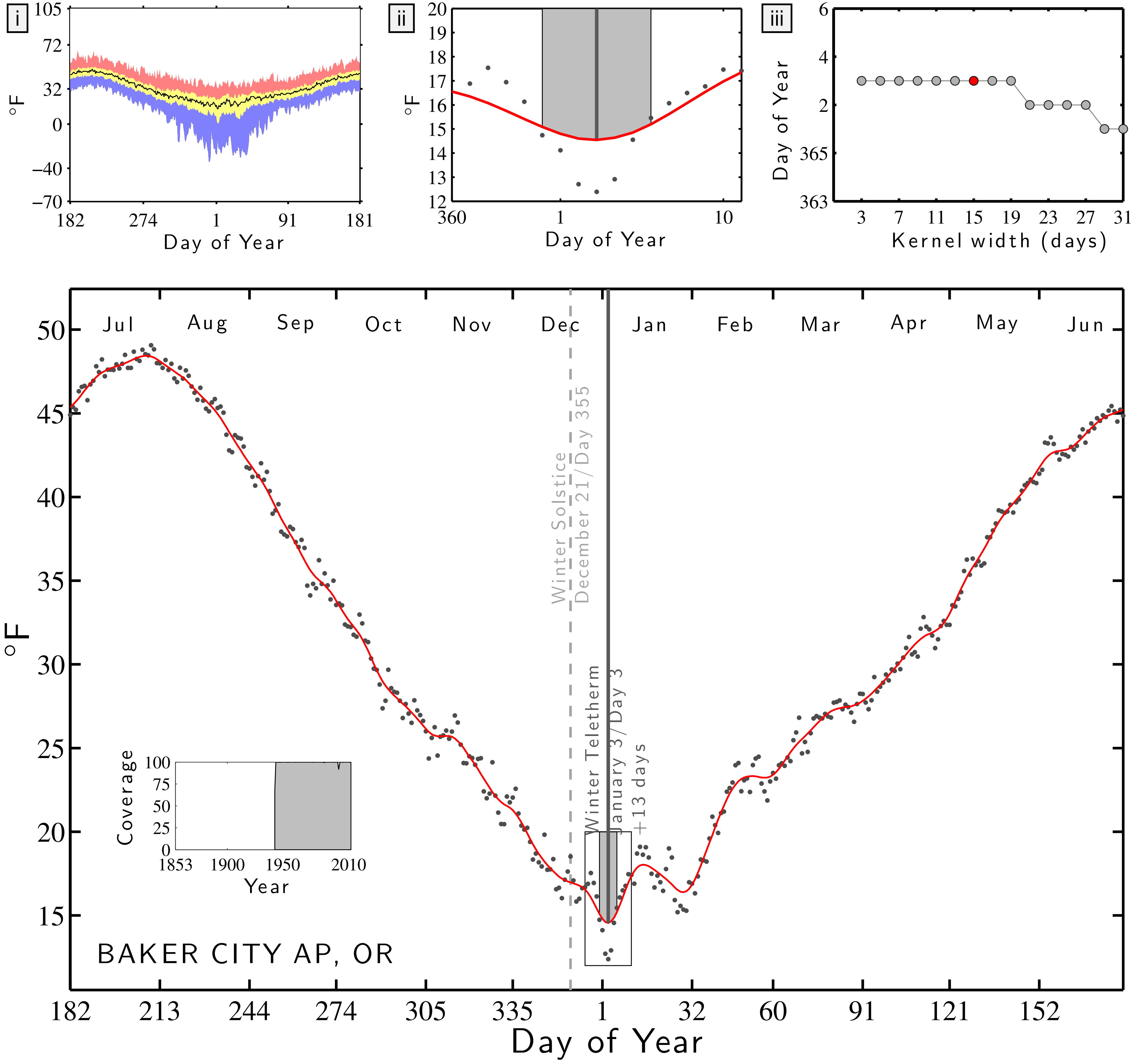

i
ii
iii
°F
Day of Year
Kernel width (days)
Jul
Aug
Sep
Oct
Nov
Dec
Jan
Feb
Mar
Apr
May
Jun
Winter Solstice
December 21/Day 355
Winter Teletherm
January 3/Day 3
+13 days
Coverage
Year
BAKER CITY AP, OR

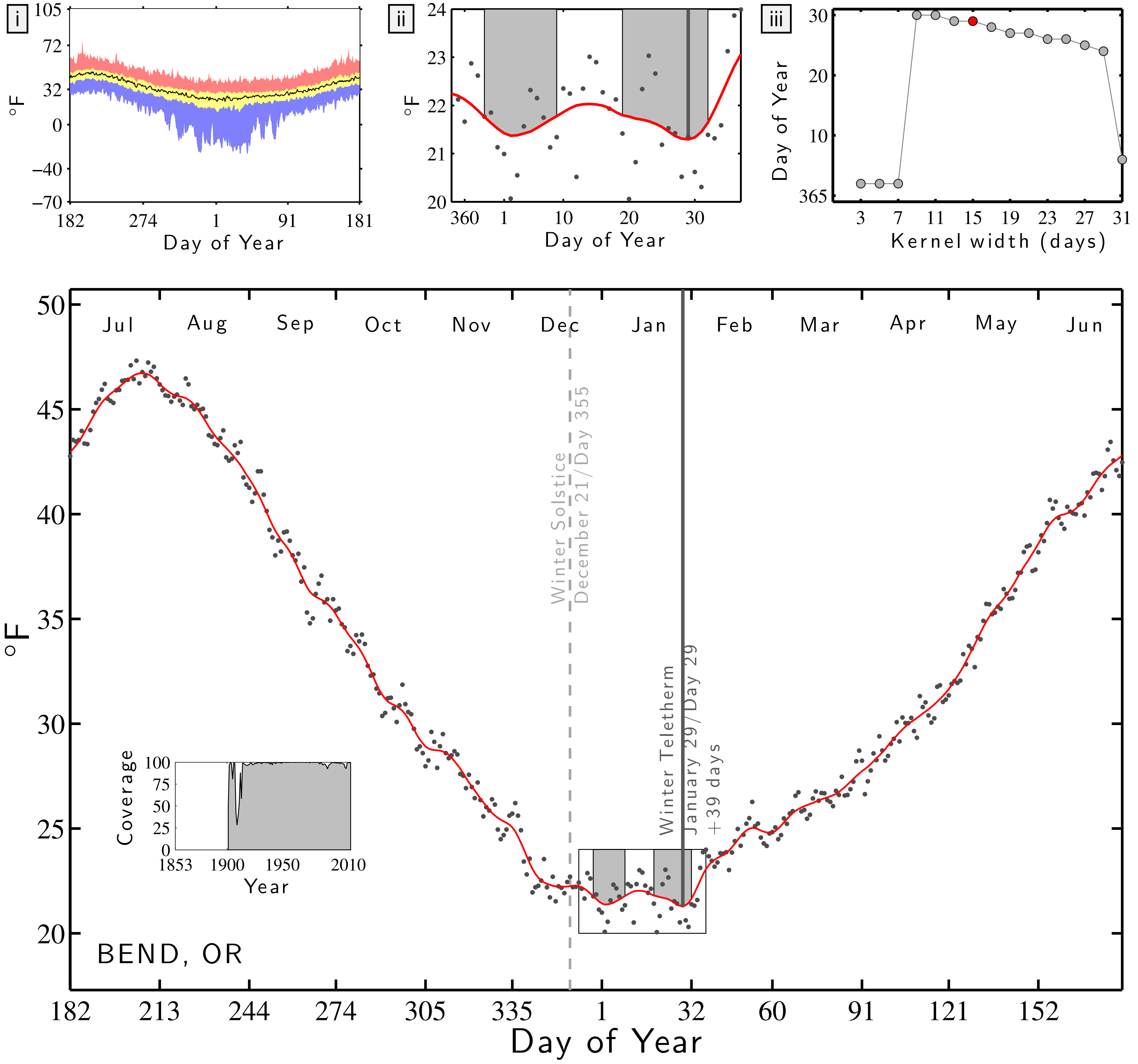

i
°F
105
72
32
0
−40
−70
182
274
1
91
181
Day of Year
ii
°F
24
23
22
21
20
360
1
10
20
30
Day of Year
iii
Day of Year
30
20
10
365
3
7
11
15
19
23
27
31
Kernel width (days)
°F
50
45
40
35
30
25
20
Jul
Aug
Sep
Oct
Nov
Dec
Jan
Feb
Mar
Apr
May
Jun
Winter Solstice
December 21/Day 355
Winter Teletherm
January 29/Day 29
+39 days
Coverage
100
75
50
25
0
1853
1900
1950
2010
Year
BEND, OR
182
213
244
274
305
335
1
32
60
91
121
152
Day of Year

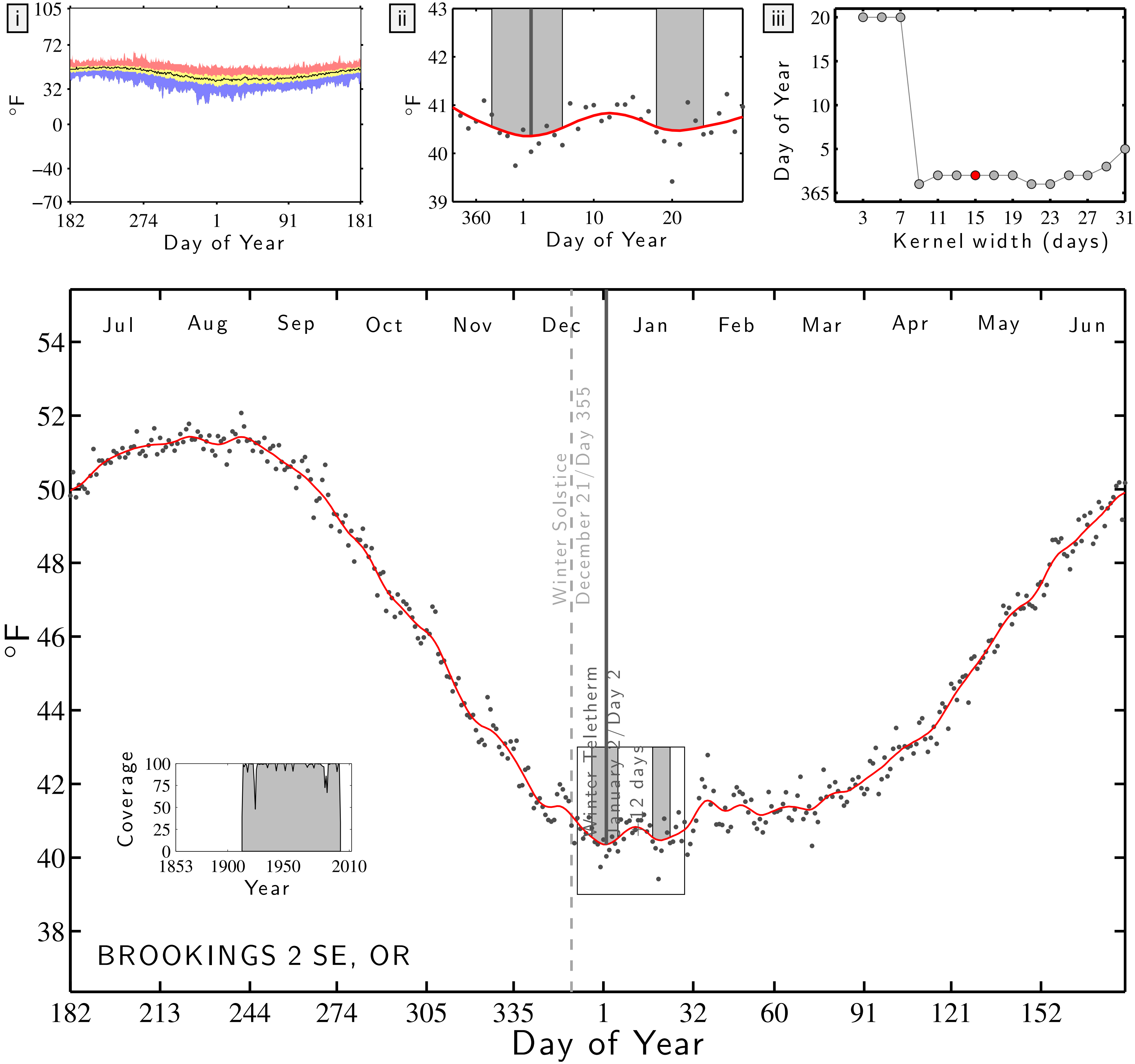

i
ii
iii
Day of Year
Kernel width (days)
°F
Winter Solstice
December 21/Day 355
Winter Teletherm
January 2/Day 2
±12 days
Coverage
Year
BROOKINGS 2 SE, OR
Jul
Aug
Sep
Oct
Nov
Dec
Jan
Feb
Mar
Apr
May
Jun

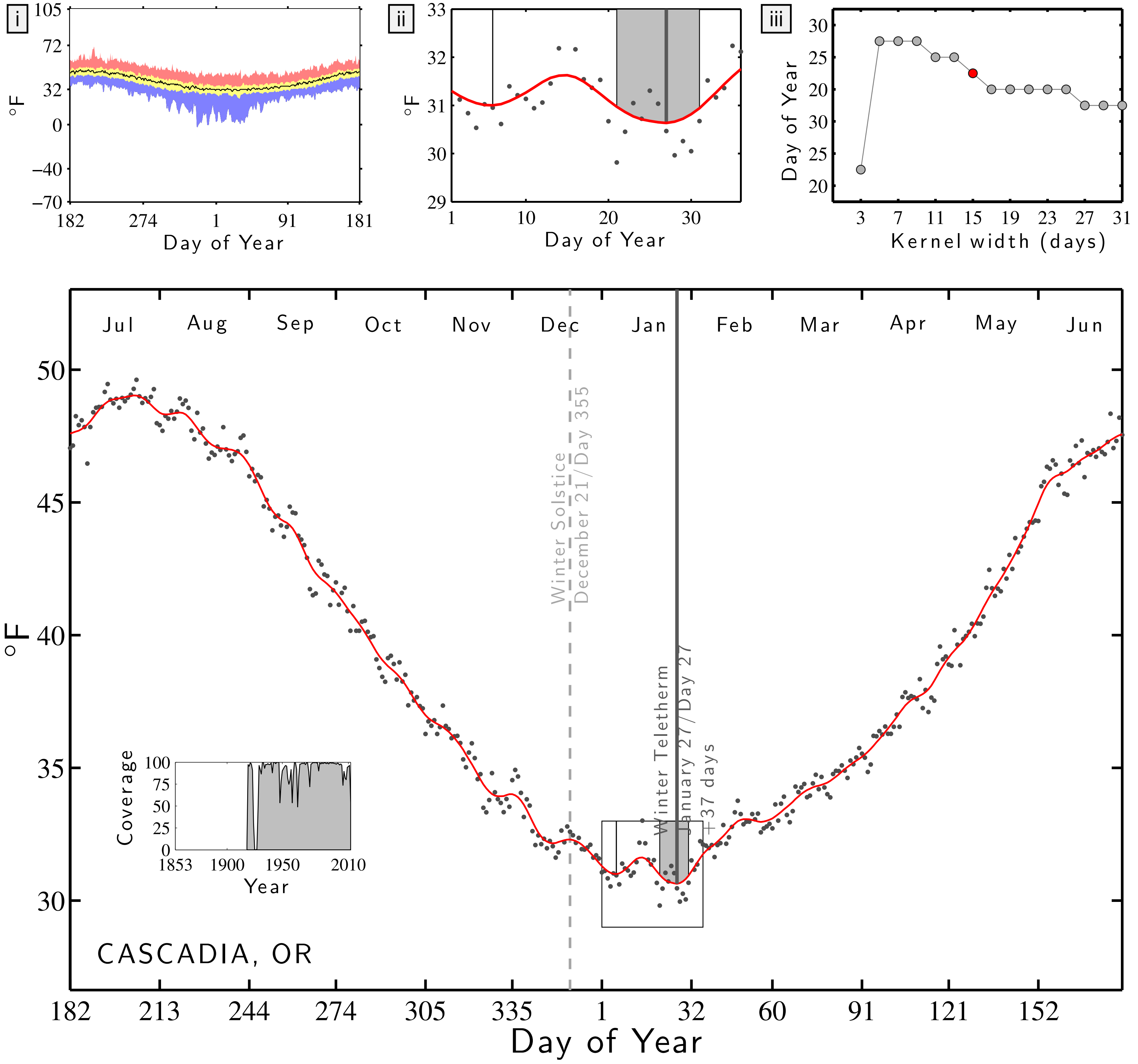

i
ii
iii
Day of Year
°F
Kernel width (days)
Jul
Aug
Sep
Oct
Nov
Dec
Jan
Feb
Mar
Apr
May
Jun
Winter Solstice
December 21/Day 355
Winter Teletherm
January 27/Day 27
+37 days
Coverage
Year
CASCADIA, OR

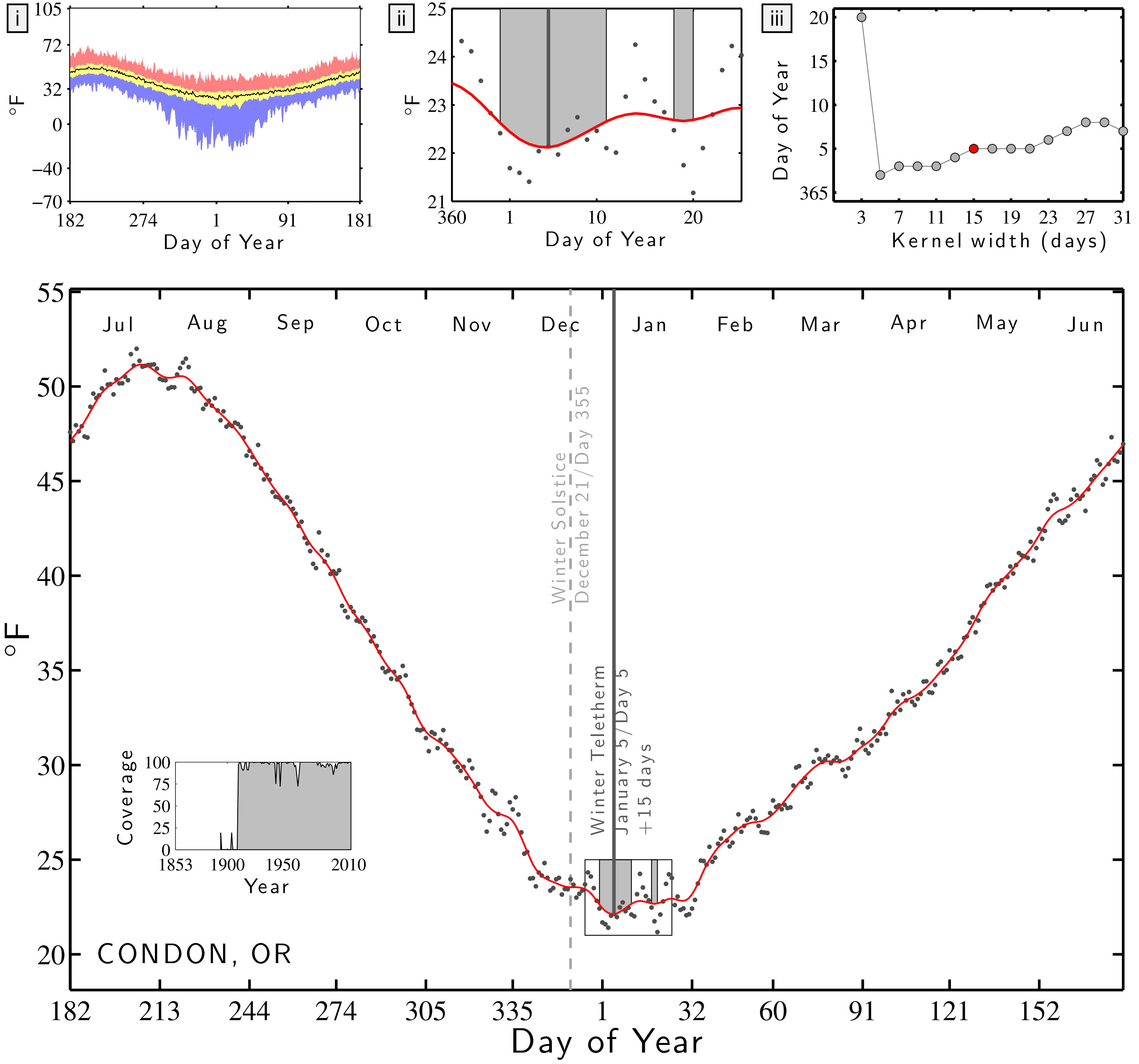

i
ii
iii
°F
Day of Year
Kernel width (days)
Winter Solstice
December 21/Day 355
Winter Teletherm
January 5/Day 5
+15 days
Jul
Aug
Sep
Oct
Nov
Dec
Jan
Feb
Mar
Apr
May
Jun
Coverage
Year
CONDON, OR

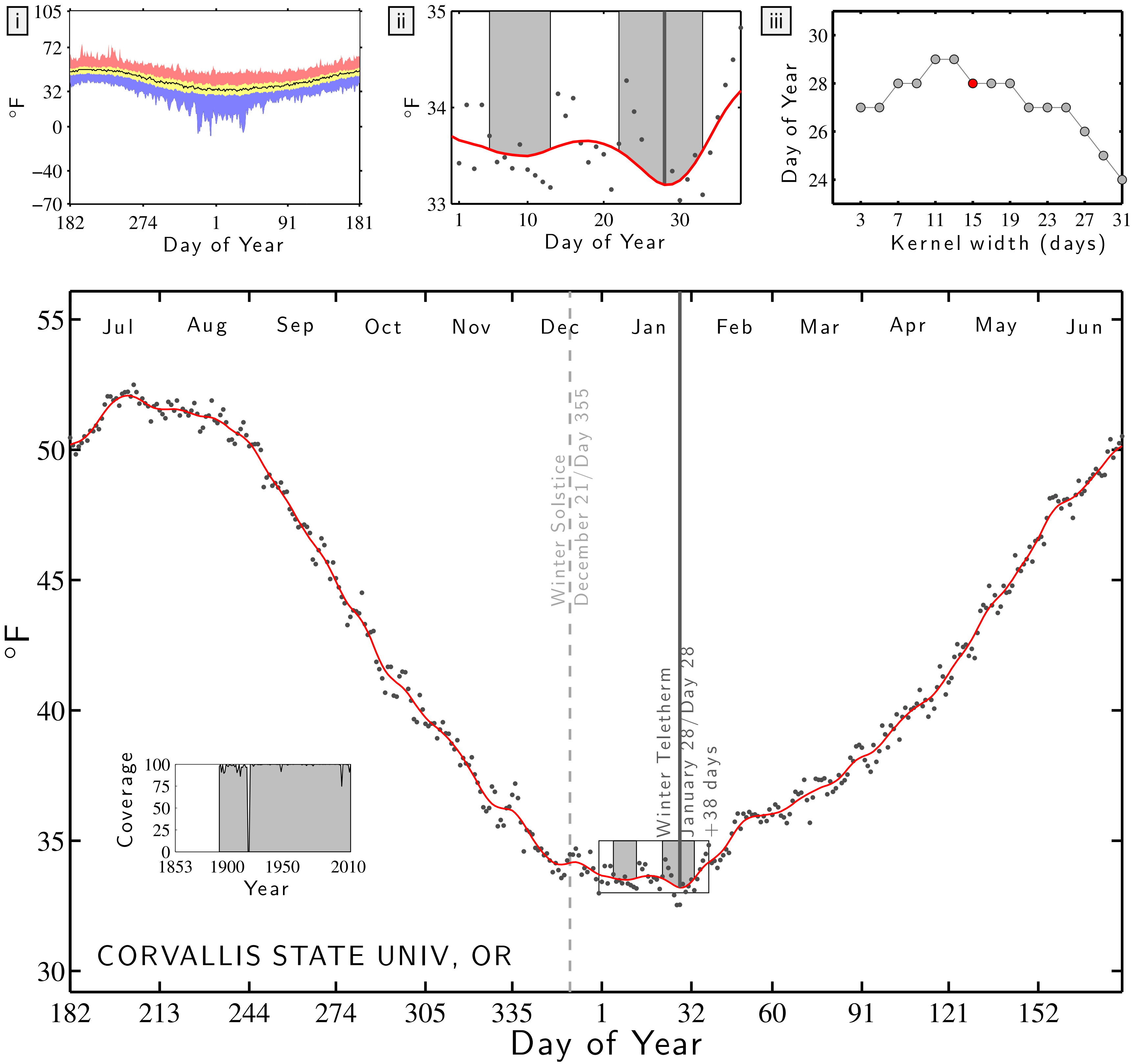
i
°F
Day of Year
ii
°F
Day of Year
iii
Day of Year
Kernel width (days)
Jul
Aug
Sep
Oct
Nov
Dec
Jan
Feb
Mar
Apr
May
Jun
Winter Solstice
December 21/Day 355
Winter Teletherm
January 28/Day 28
+38 days
Coverage
Year
CORVALLIS STATE UNIV, OR
°F
Day of Year

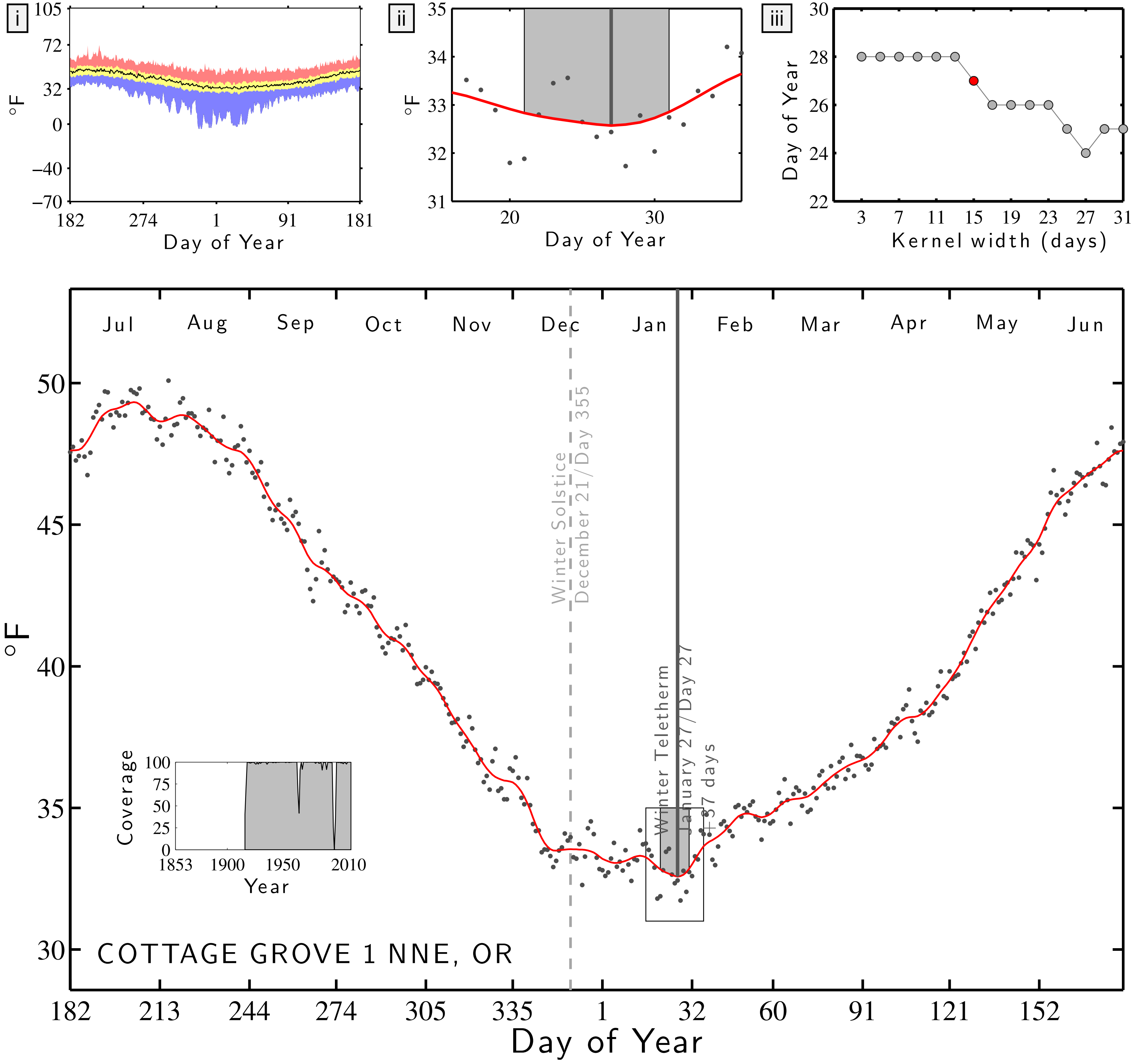

i
ii
iii
°F
Day of Year
Day of Year
Kernel width (days)
Jul
Aug
Sep
Oct
Nov
Dec
Jan
Feb
Mar
Apr
May
Jun
Winter Solstice
December 21/Day 355
Winter Teletherm
January 27/Day 27
+37 days
Coverage
Year
COTTAGE GROVE 1 NNE, OR

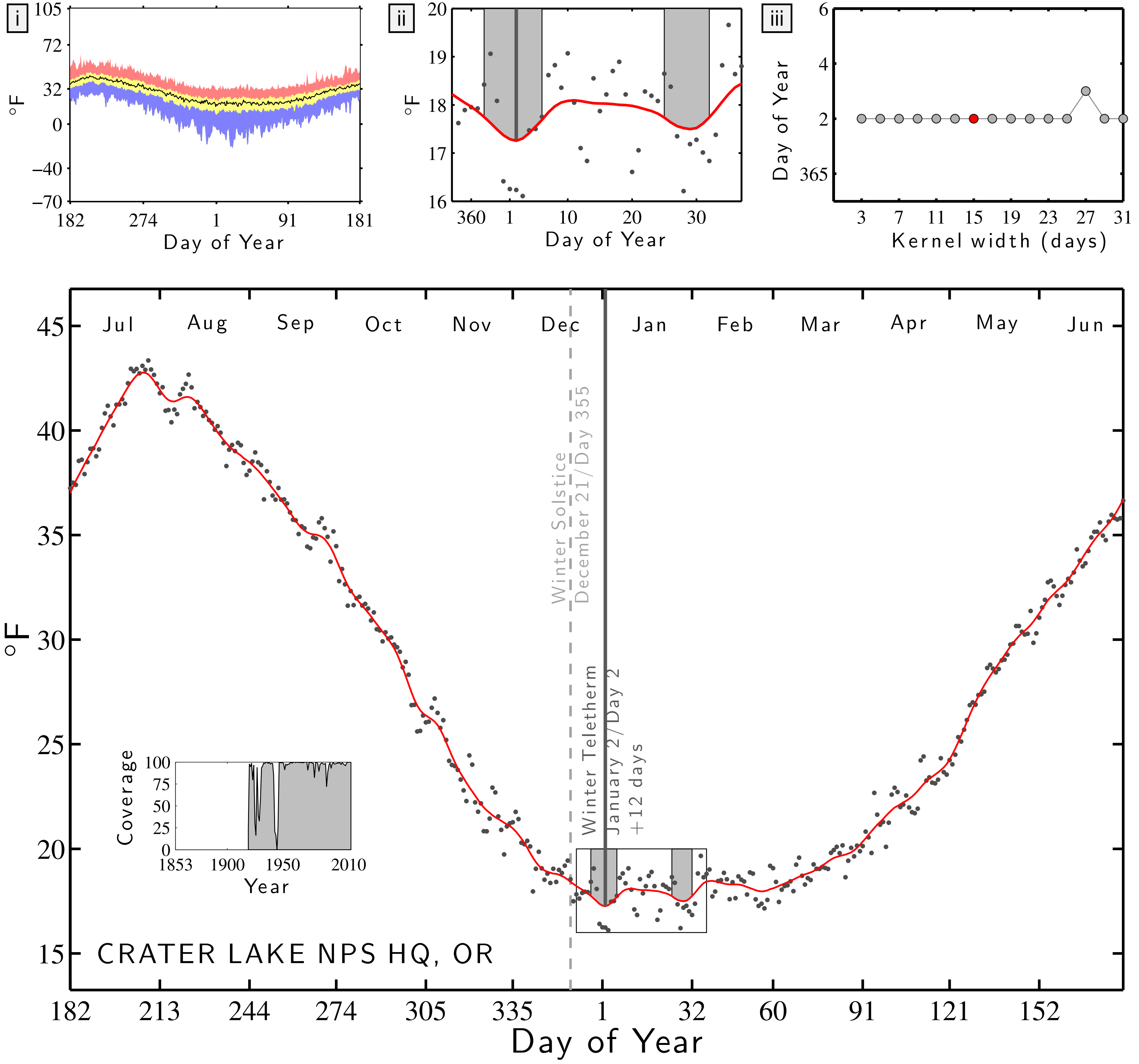

i
ii
iii
°F
Day of Year
Kernel width (days)
Jul
Aug
Sep
Oct
Nov
Dec
Jan
Feb
Mar
Apr
May
Jun
Winter Solstice
December 21/Day 355
Winter Teletherm
January 2/Day 2
+12 days
Coverage
Year
CRATER LAKE NPS HQ, OR

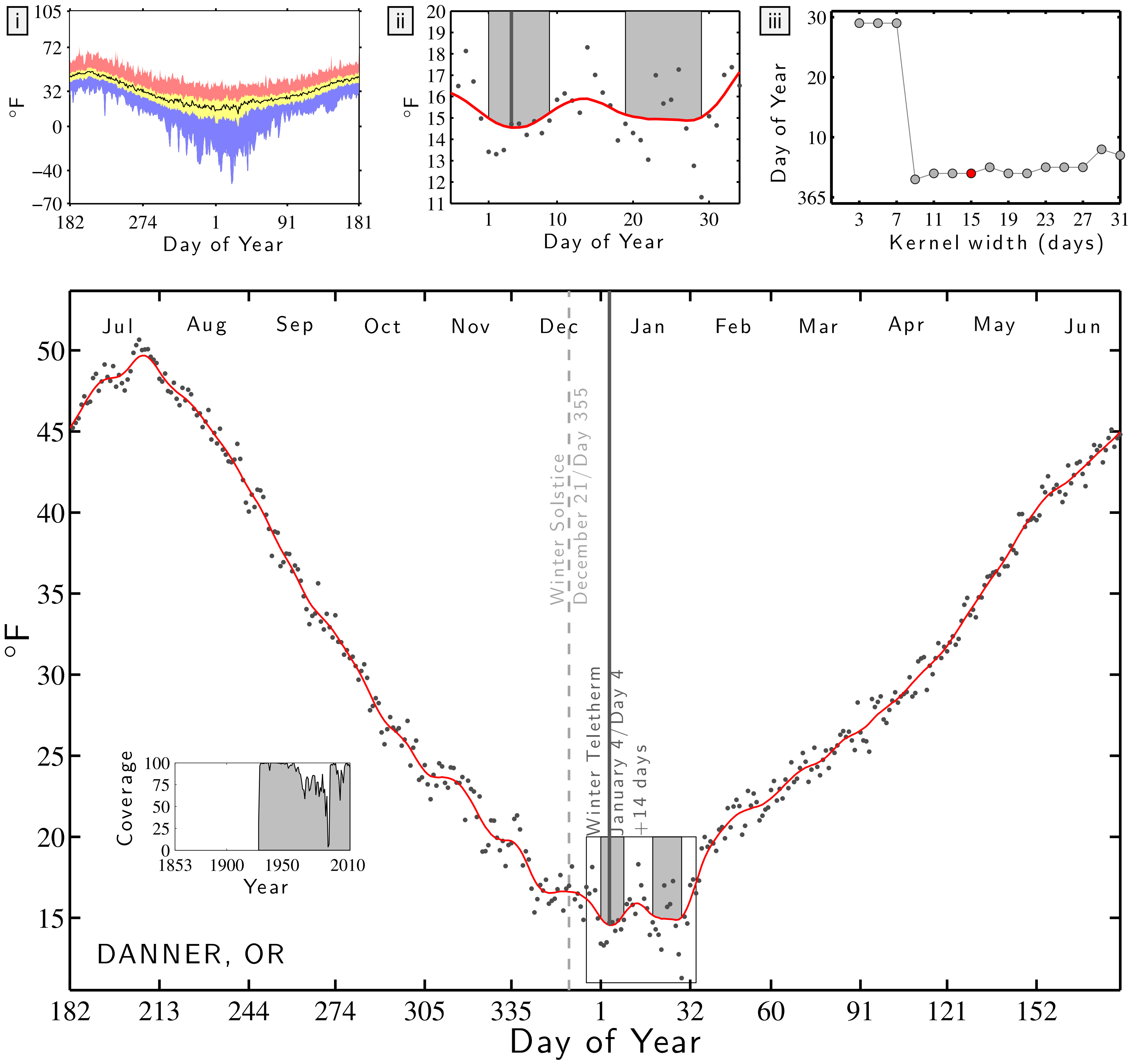

i
ii
iii
°F
Day of Year
Day of Year
Kernel width (days)
Jul
Aug
Sep
Oct
Nov
Dec
Jan
Feb
Mar
Apr
May
Jun
Winter Solstice
December 21/Day 355
Winter Teletherm
January 4/Day 4
+14 days
Coverage
Year
DANNER, OR
Day of Year

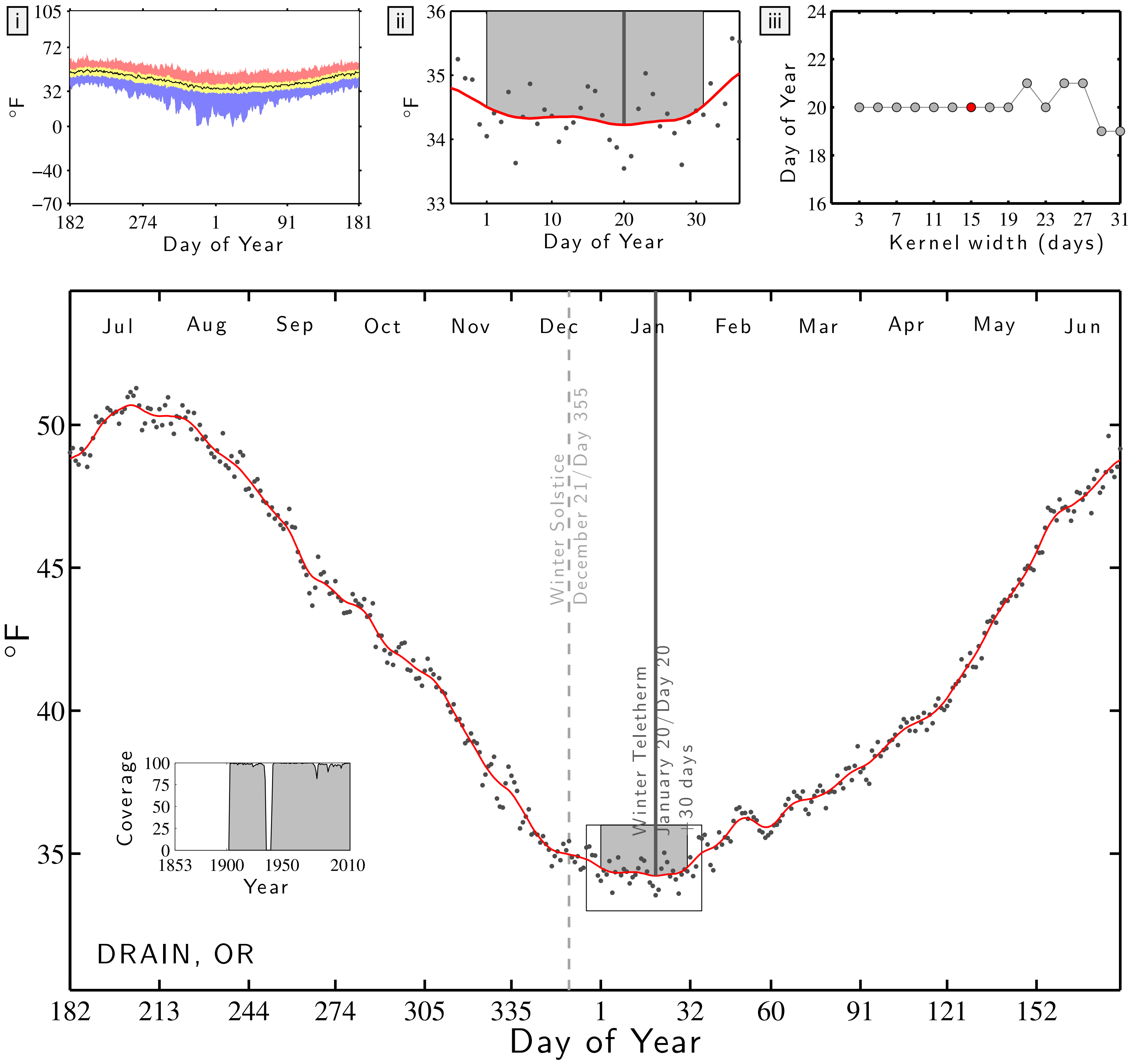

i
ii
iii
°F
Day of Year
Day of Year
Kernel width (days)
Jul
Aug
Sep
Oct
Nov
Dec
Jan
Feb
Mar
Apr
May
Jun
Winter Solstice
December 21/Day 355
Winter Teletherm
January 20/Day 20
±30 days
Coverage
Year
DRAIN, OR

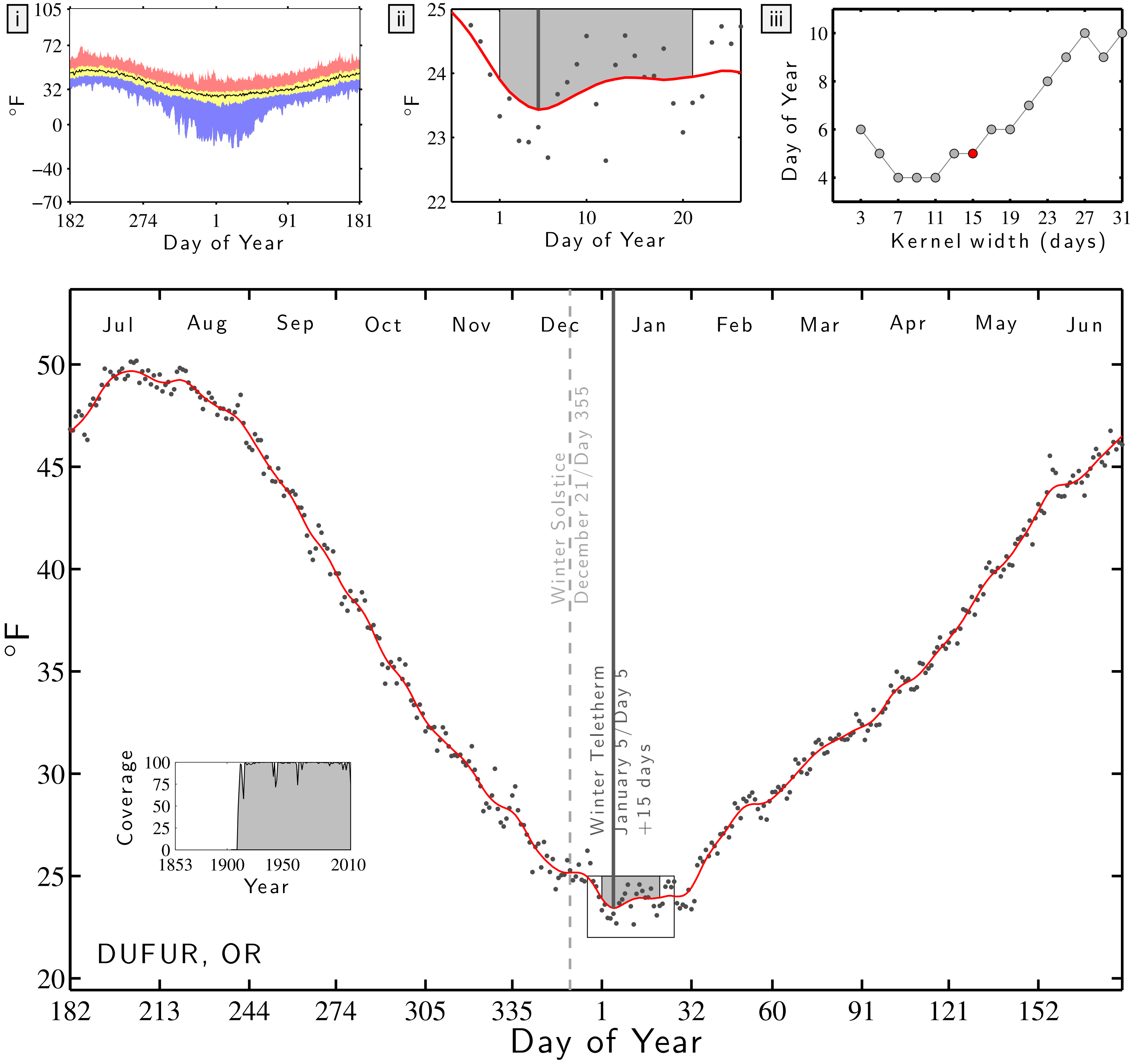

i
ii
iii
°F
Day of Year
Day of Year
Kernel width (days)
Jul
Aug
Sep
Oct
Nov
Dec
Jan
Feb
Mar
Apr
May
Jun
Winter Solstice
December 21/Day 355
Winter Teletherm
January 5/Day 5
+15 days
Coverage
Year
DUFUR, OR
Day of Year

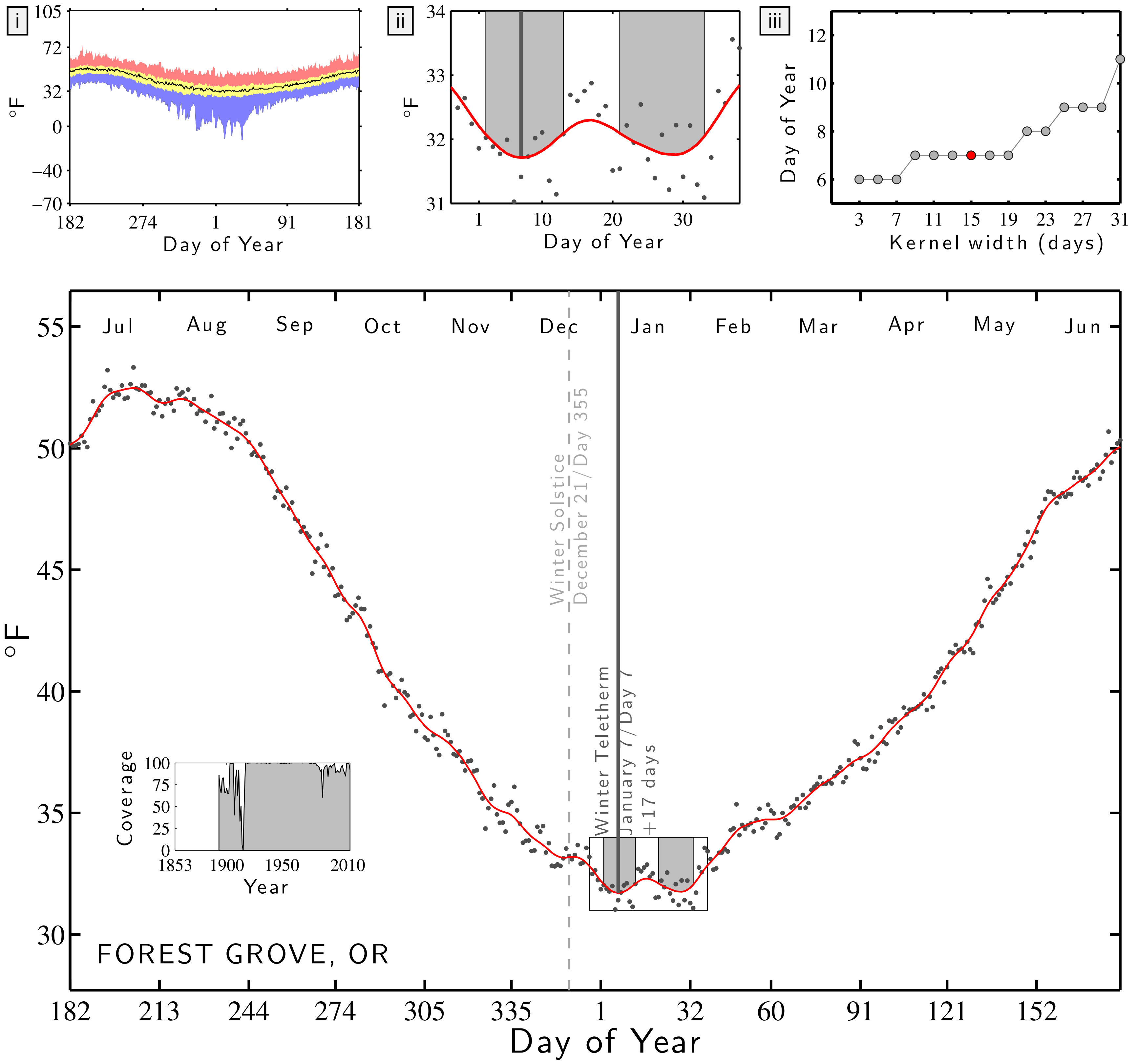

i
°F
Day of Year
ii
°F
Day of Year
iii
Day of Year
Kernel width (days)
Jul Aug Sep Oct Nov Dec Jan Feb Mar Apr May Jun
Winter Solstice
December 21/Day 355
Winter Teletherm
January 7/Day 7
+17 days
Coverage
Year
FOREST GROVE, OR
°F
Day of Year

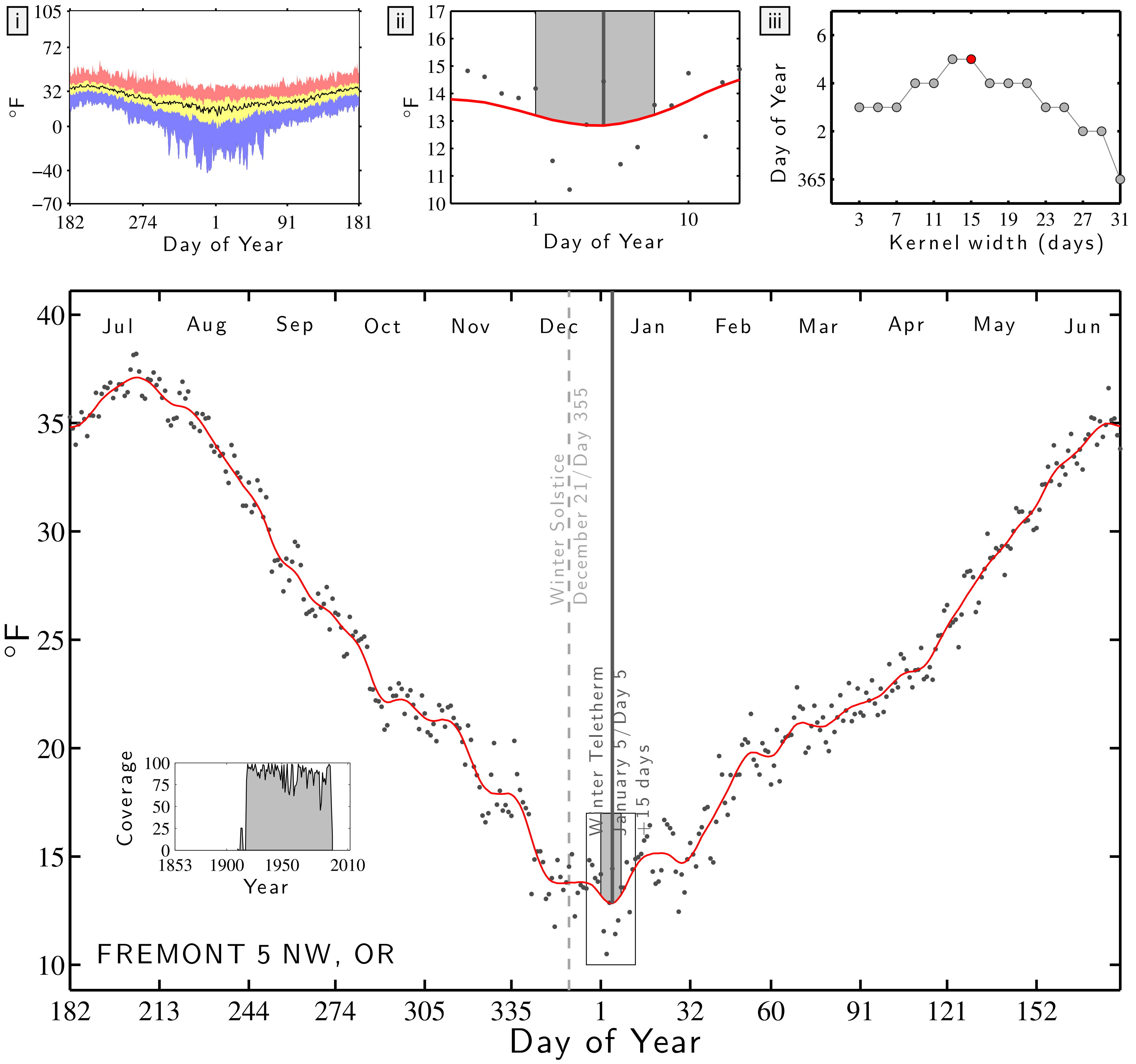

i
°F
Day of Year
ii
°F
Day of Year
iii
Day of Year
Kernel width (days)
Jul Aug Sep Oct Nov Dec Jan Feb Mar Apr May Jun
Winter Solstice
December 21/Day 355
Winter Teletherm
January 5/Day 5
±15 days
Coverage
Year
FREMONT 5 NW, OR
°F
Day of Year

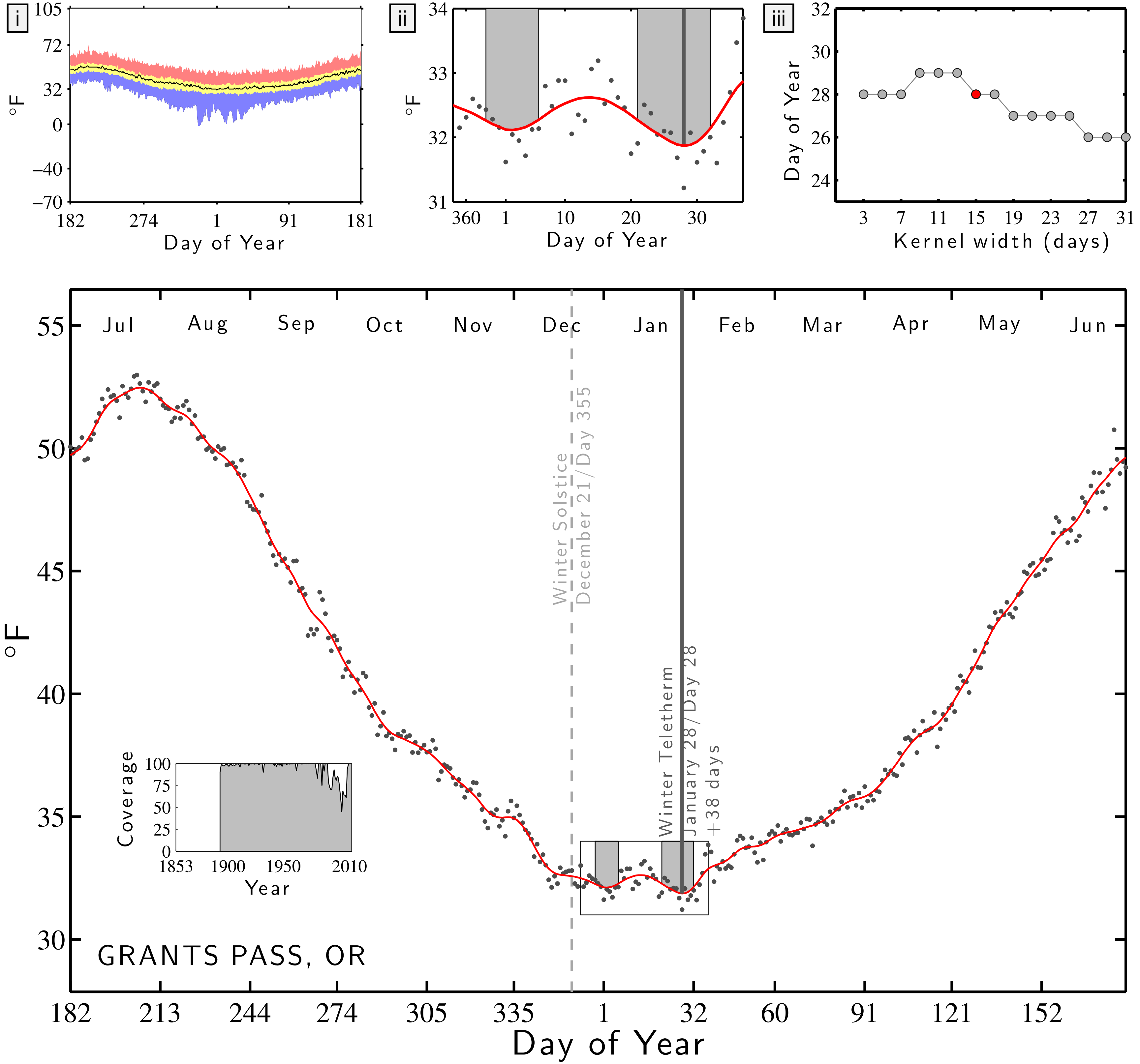

i
°F
Day of Year
ii
°F
Day of Year
iii
Day of Year
Kernel width (days)
Jul
Aug
Sep
Oct
Nov
Dec
Jan
Feb
Mar
Apr
May
Jun
Winter Solstice
December 21/Day 355
Winter Teletherm
January 28/Day 28
+38 days
Coverage
Year
GRANTS PASS, OR
°F
Day of Year

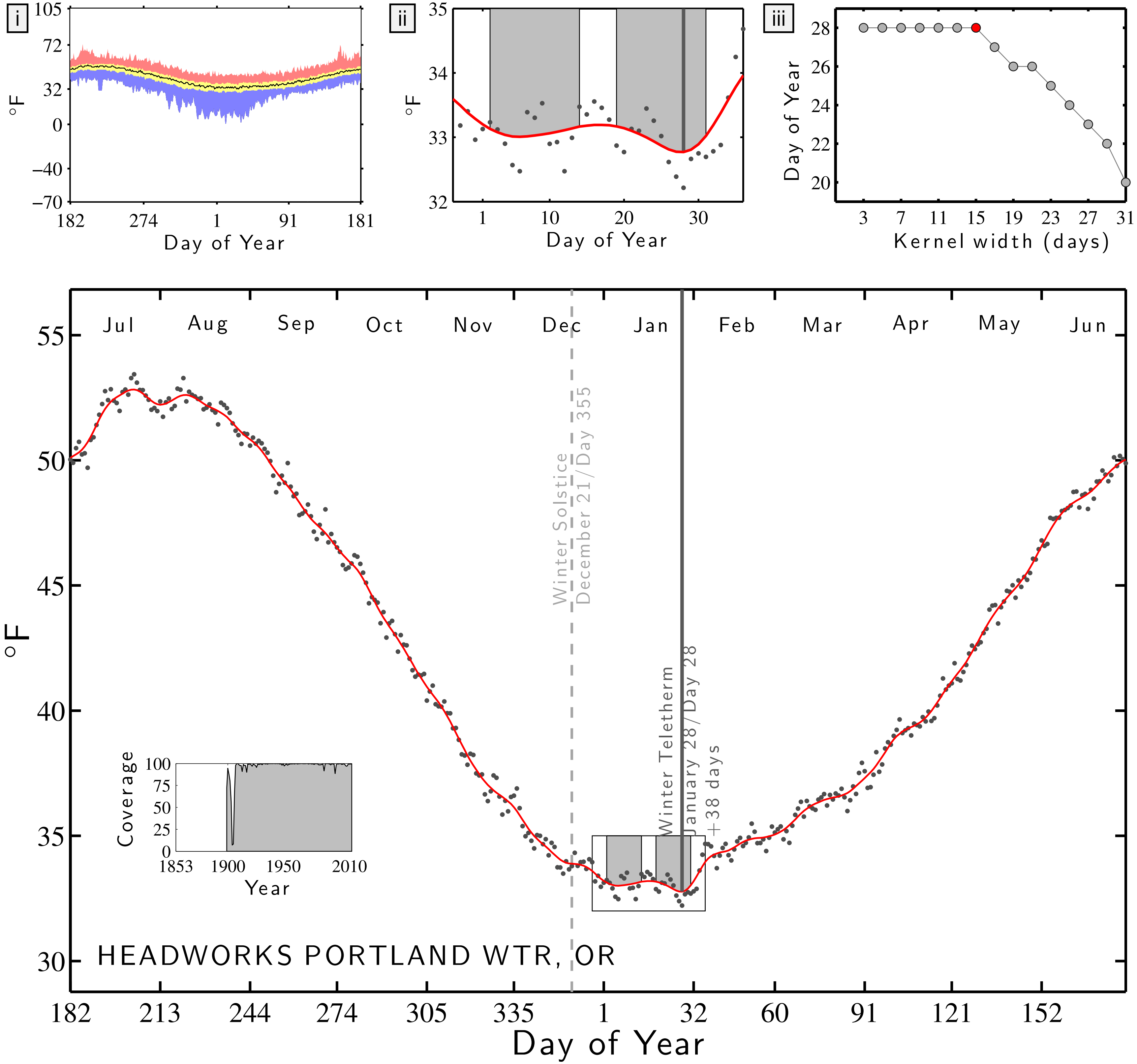

i
ii
iii
°F
Day of Year
Kernel width (days)
Jul
Aug
Sep
Oct
Nov
Dec
Jan
Feb
Mar
Apr
May
Jun
Winter Solstice
December 21/Day 355
Winter Teletherm
January 28/Day 28
+38 days
Coverage
Year
HEADWORKS PORTLAND WTR, OR

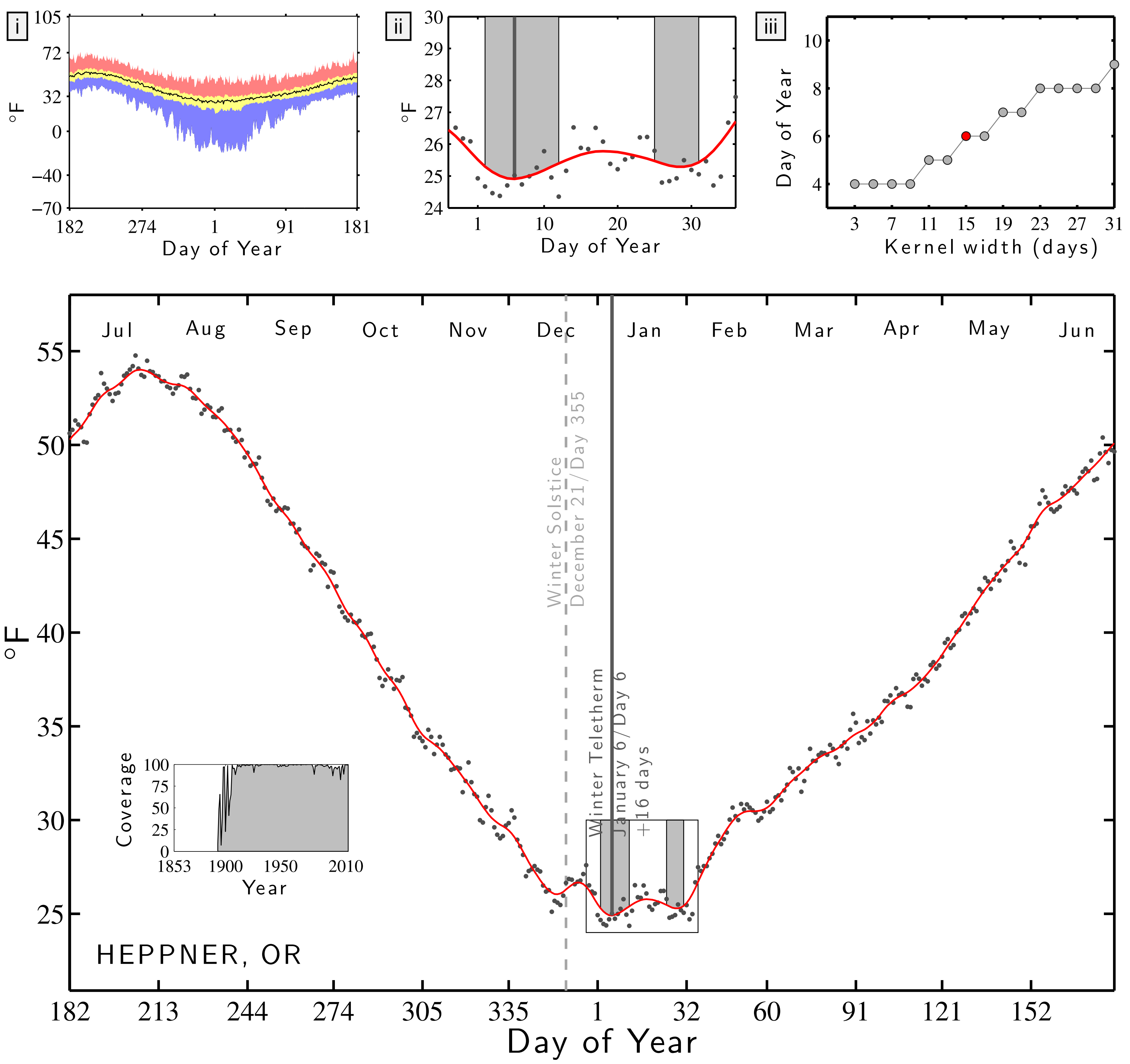

i
ii
iii
°F
Day of Year
Kernel width (days)
Jul
Aug
Sep
Oct
Nov
Dec
Jan
Feb
Mar
Apr
May
Jun
Winter Solstice
December 21/Day 355
Winter Teletherm
January 6/Day 6
+16 days
Coverage
Year
HEPPNER, OR

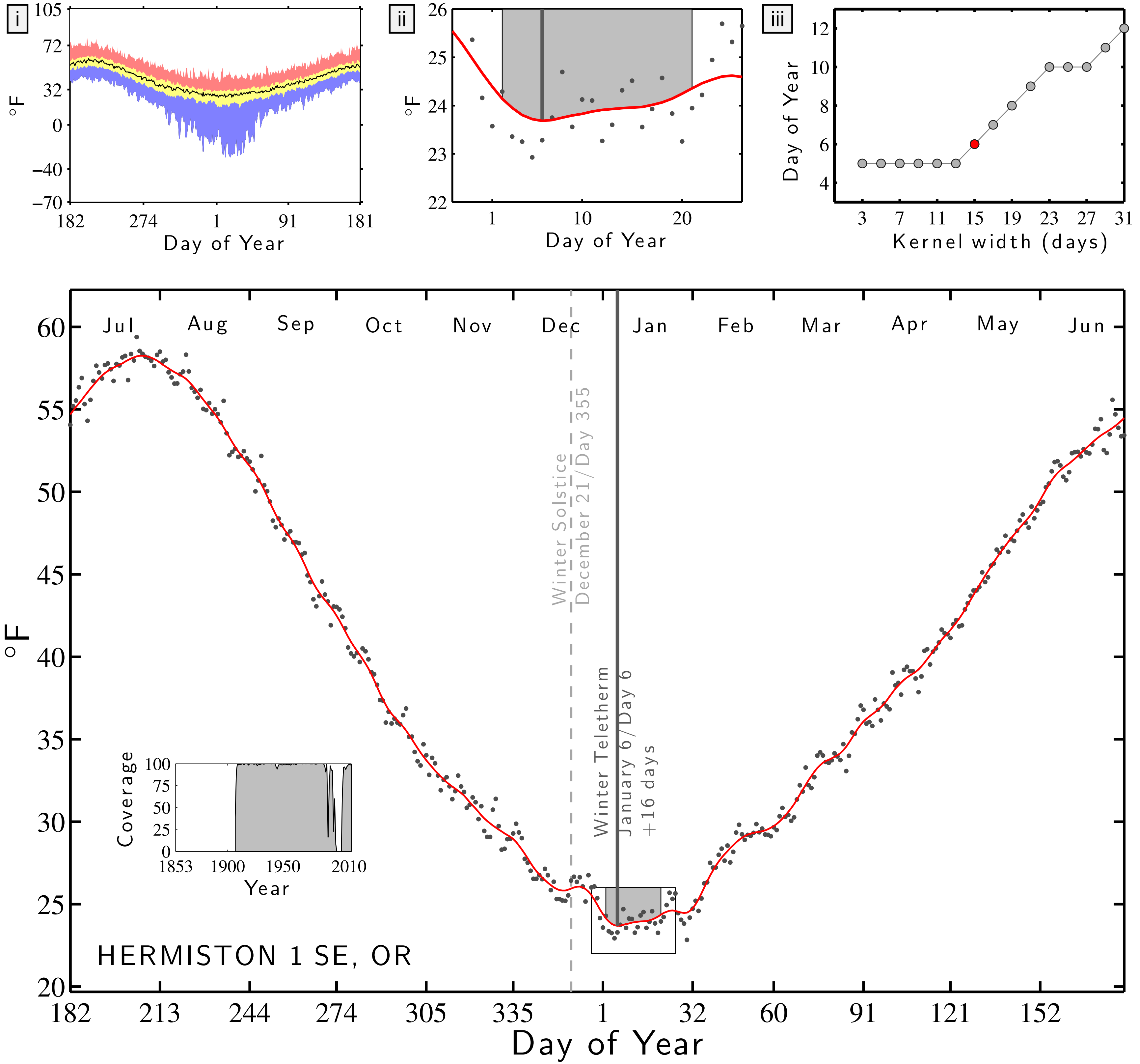

i
105
72
32
0
−40
−70
°F
182
274
1
91
181
Day of Year
ii
26
25
24
23
22
°F
1
10
20
Day of Year
iii
12
10
8
6
4
Day of Year
3
7
11
15
19
23
27
31
Kernel width (days)
60
55
50
45
40
35
30
25
20
°F
Jul
Aug
Sep
Oct
Nov
Dec
Jan
Feb
Mar
Apr
May
Jun
Winter Solstice
December 21/Day 355
Winter Teletherm
January 6/Day 6
+16 days
Coverage
100
75
50
25
0
1853
1900
1950
2010
Year
HERMISTON 1 SE, OR
182
213
244
274
305
335
1
32
60
91
121
152
Day of Year

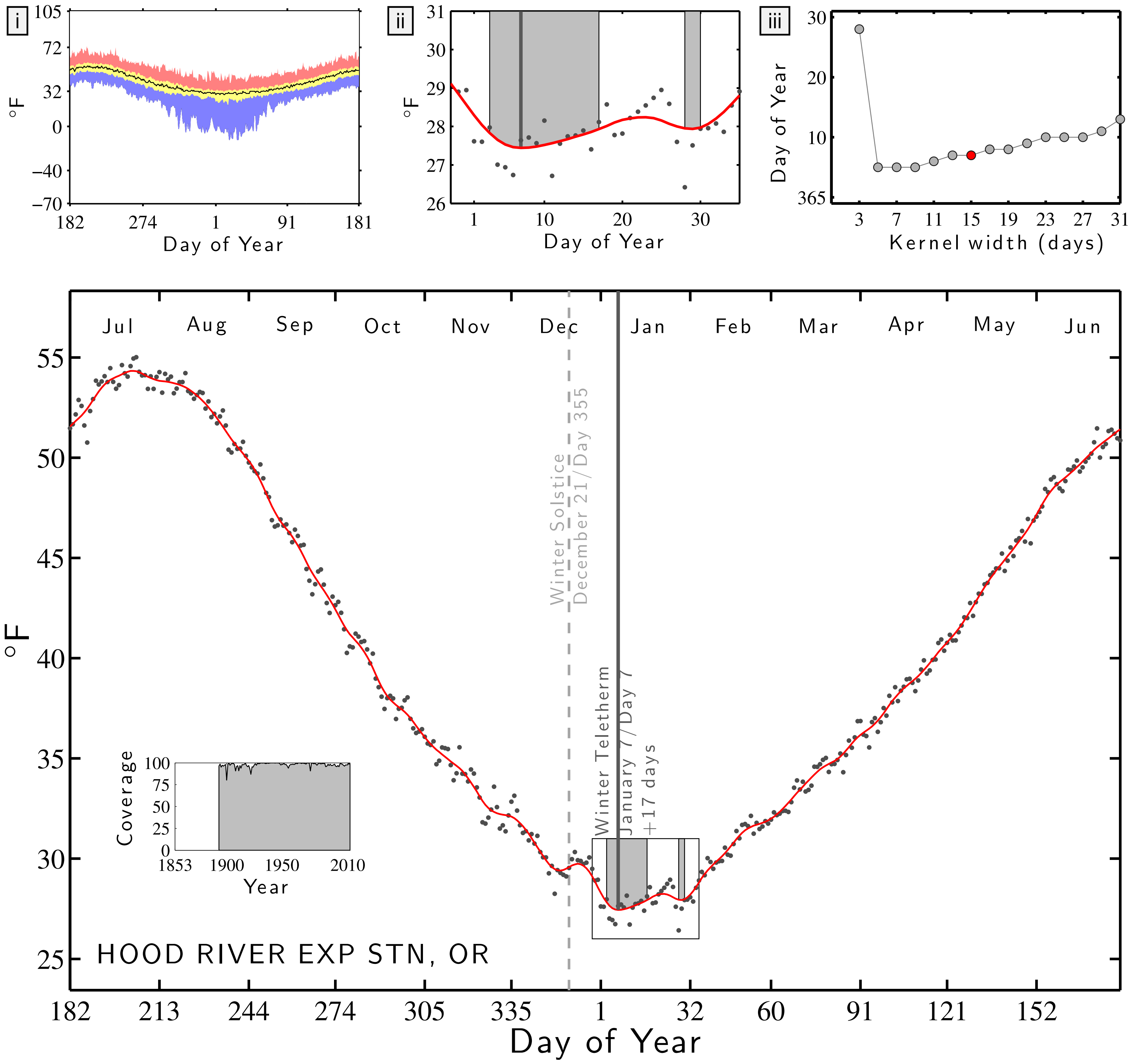

i
°F
Day of Year
ii
°F
Day of Year
iii
Day of Year
Kernel width (days)
Jul
Aug
Sep
Oct
Nov
Dec
Jan
Feb
Mar
Apr
May
Jun
Winter Solstice
December 21/Day 355
Winter Teletherm
January 7/Day 7
+17 days
Coverage
Year
HOOD RIVER EXP STN, OR
°F
Day of Year

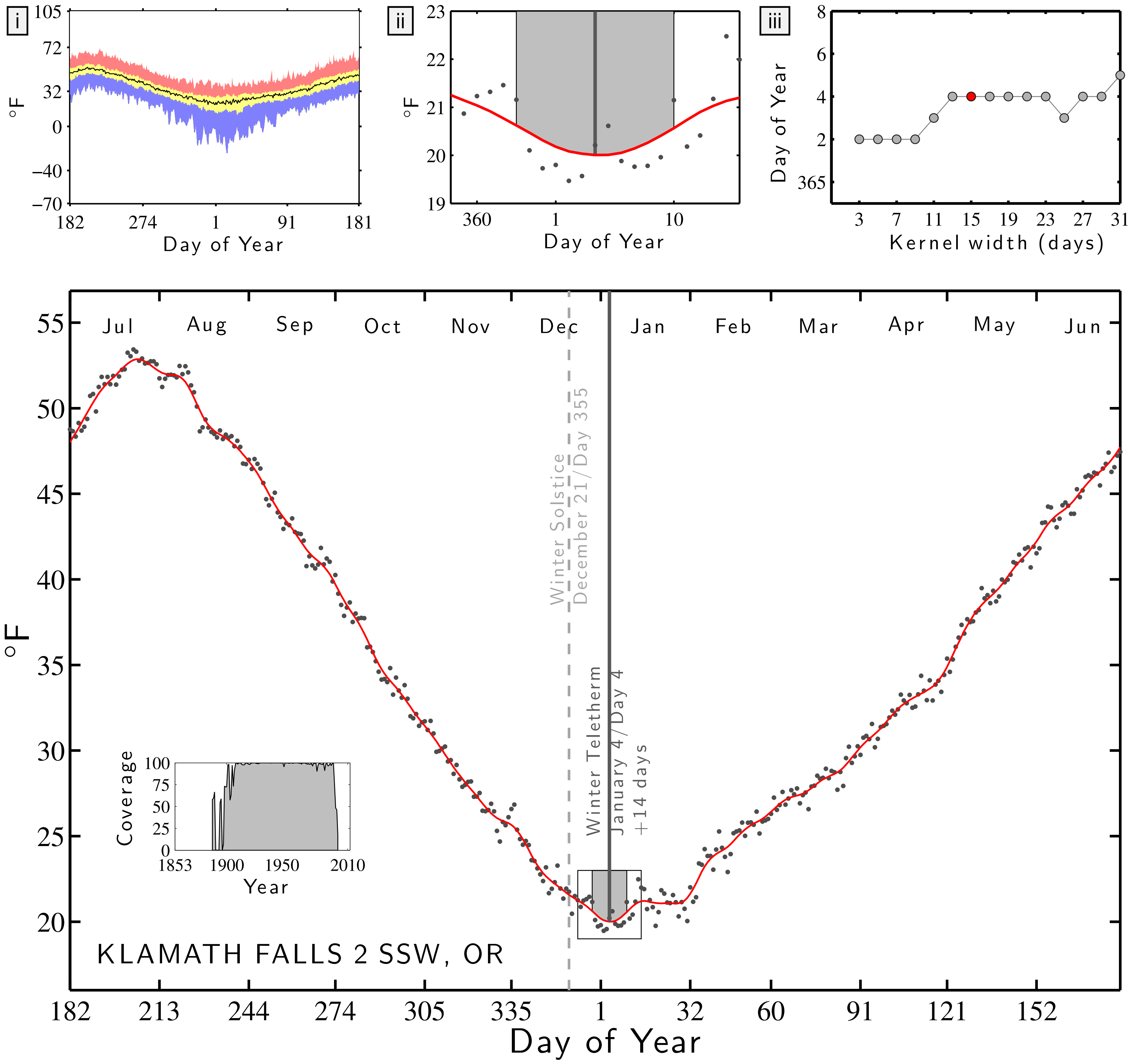
i
°F
Day of Year
ii
Day of Year
iii
Day of Year
Kernel width (days)
Jul
Aug
Sep
Oct
Nov
Dec
Jan
Feb
Mar
Apr
May
Jun
Winter Solstice
December 21/Day 355
Winter Teletherm
January 4/Day 4
+14 days
Coverage
Year
KLAMATH FALLS 2 SSW, OR
Day of Year

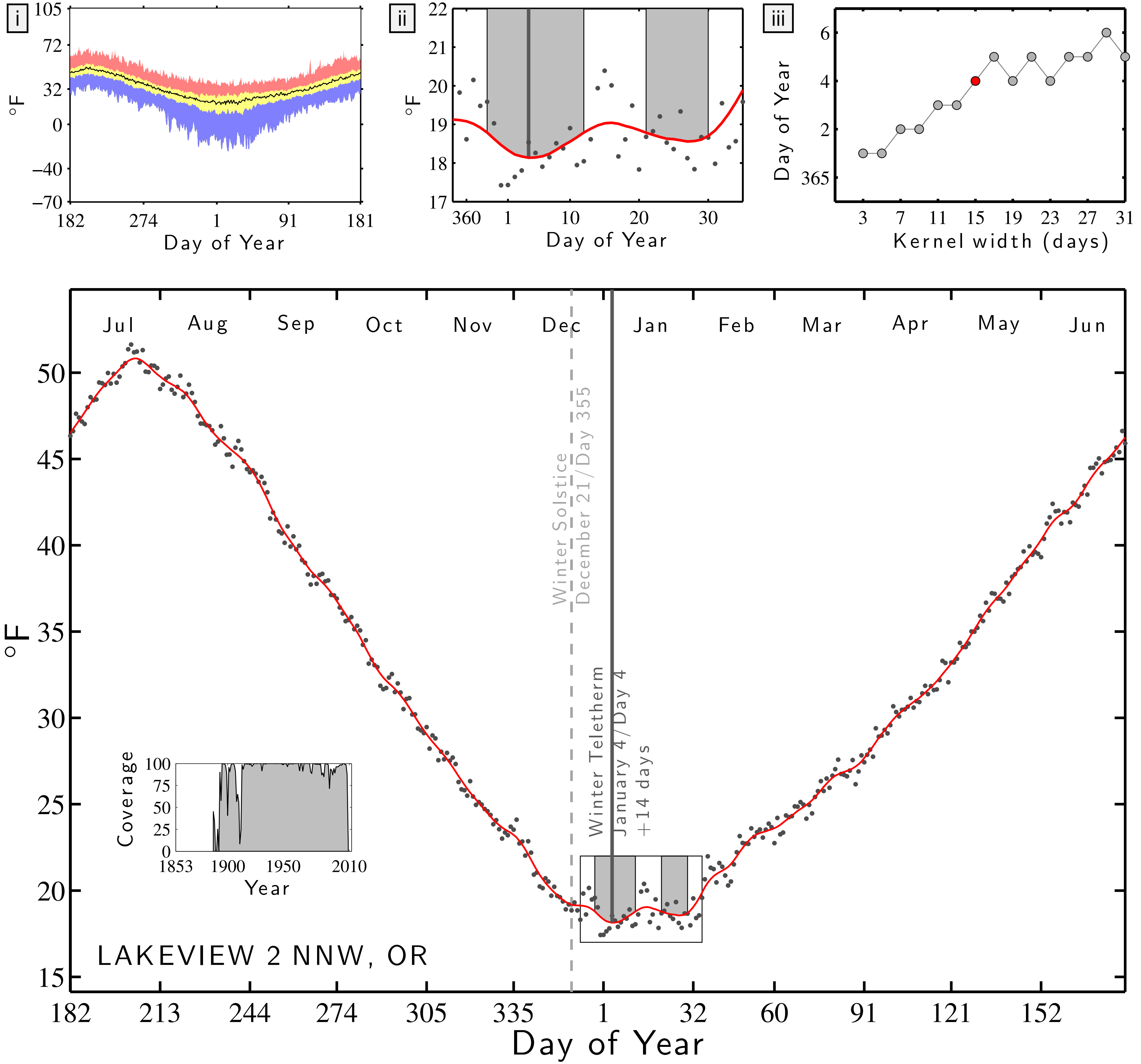

i
105
72
32
0
−40
−70
°F
182
274
1
91
181
Day of Year
ii
22
21
20
19
18
17
°F
360
1
10
20
30
Day of Year
iii
6
4
2
365
Day of Year
3
7
11
15
19
23
27
31
Kernel width (days)
Jul
Aug
Sep
Oct
Nov
Dec
Jan
Feb
Mar
Apr
May
Jun
Winter Solstice
December 21/Day 355
Winter Teletherm
January 4/Day 4
+14 days
50
45
40
35
30
25
20
15
°F
Coverage
100
75
50
25
0
1853
1900
1950
2010
Year
LAKEVIEW 2 NNW, OR
182
213
244
274
305
335
1
32
60
91
121
152
Day of Year

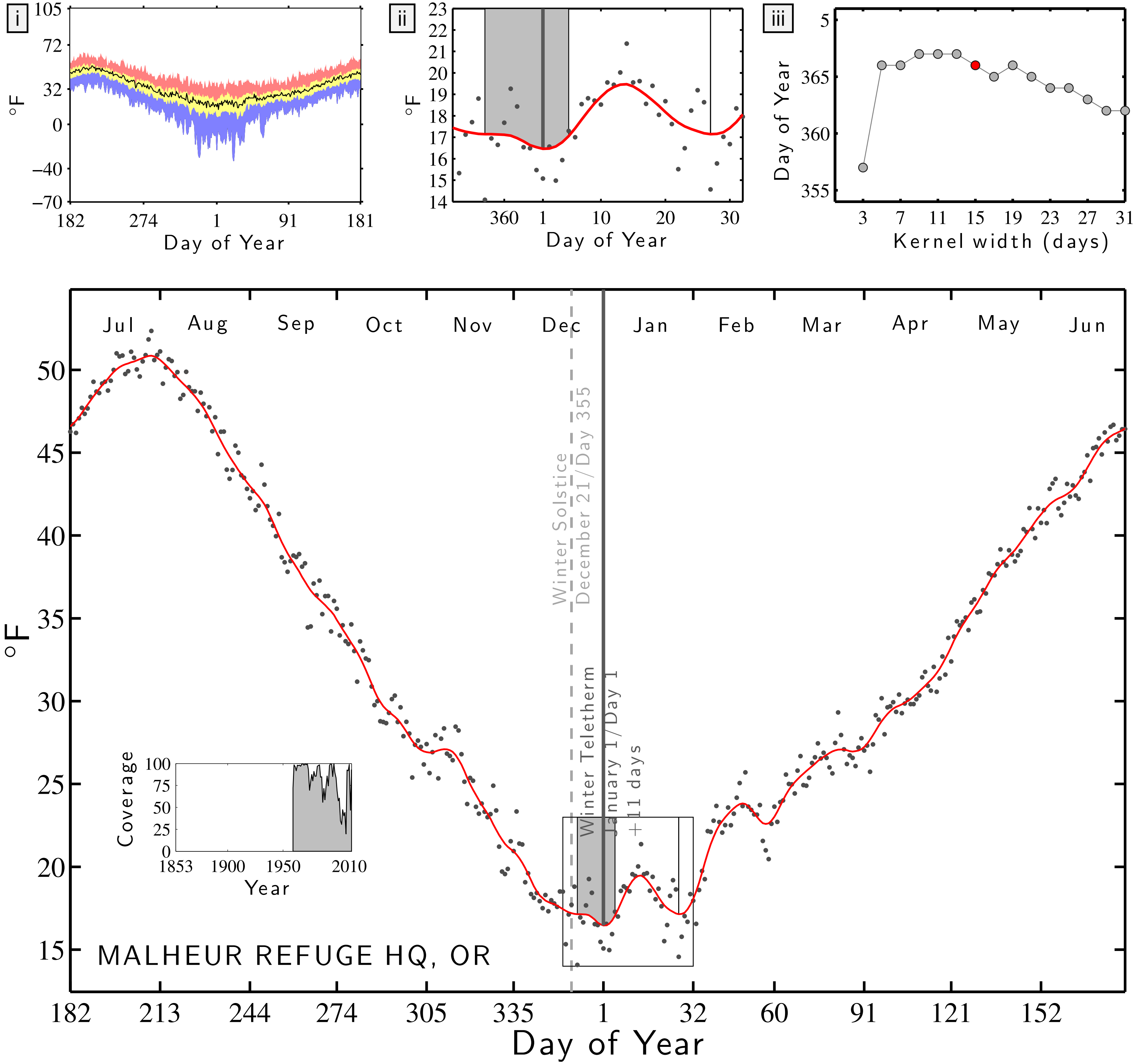

i
ii
iii
°F
Day of Year
Kernel width (days)
Jul
Aug
Sep
Oct
Nov
Dec
Jan
Feb
Mar
Apr
May
Jun
Winter Solstice
December 21/Day 355
Winter Teletherm
January 1/Day 1
+11 days
Coverage
Year
MALHEUR REFUGE HQ, OR

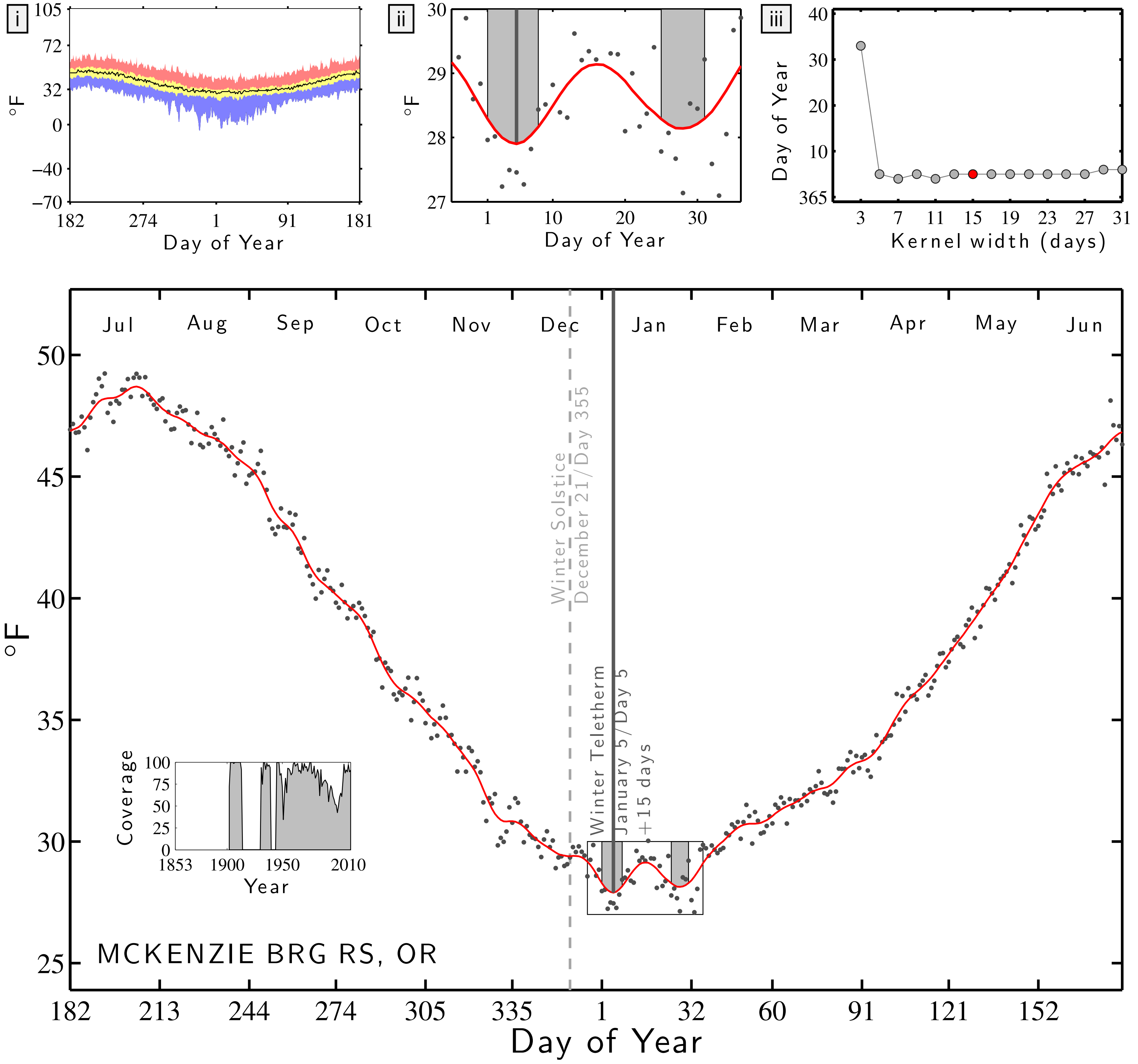

i
°F
Day of Year
ii
°F
Day of Year
iii
Day of Year
Kernel width (days)
Jul
Aug
Sep
Oct
Nov
Dec
Jan
Feb
Mar
Apr
May
Jun
Winter Solstice
December 21/Day 355
Winter Teletherm
January 5/Day 5
+15 days
Coverage
Year
MCKENZIE BRG RS, OR
°F
Day of Year

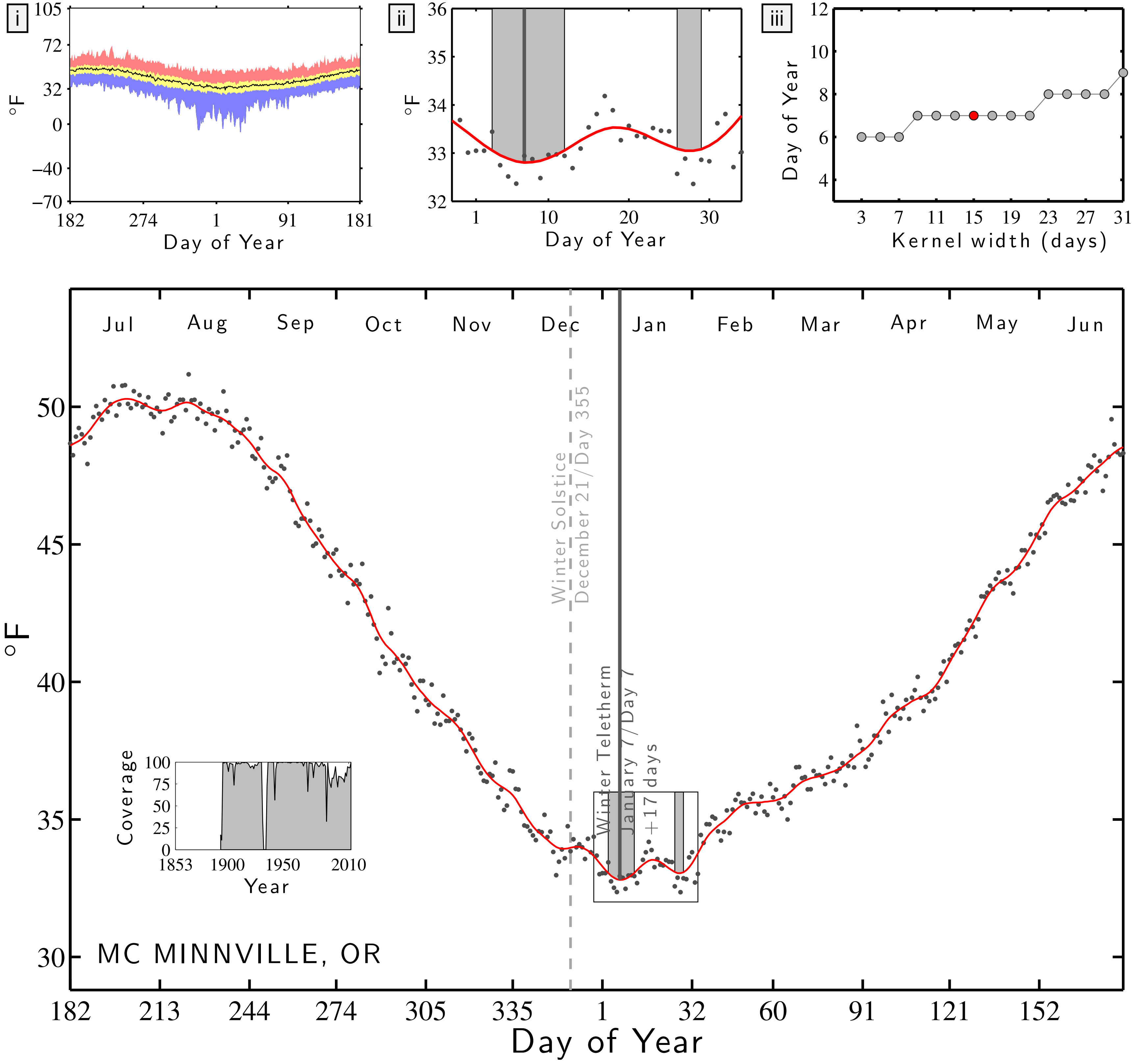

i
ii
iii
°F
Day of Year
Kernel width (days)
Jul
Aug
Sep
Oct
Nov
Dec
Jan
Feb
Mar
Apr
May
Jun
Winter Solstice
December 21/Day 355
Winter Teletherm
January 7/Day 7
+17 days
Coverage
Year
MC MINNVILLE, OR

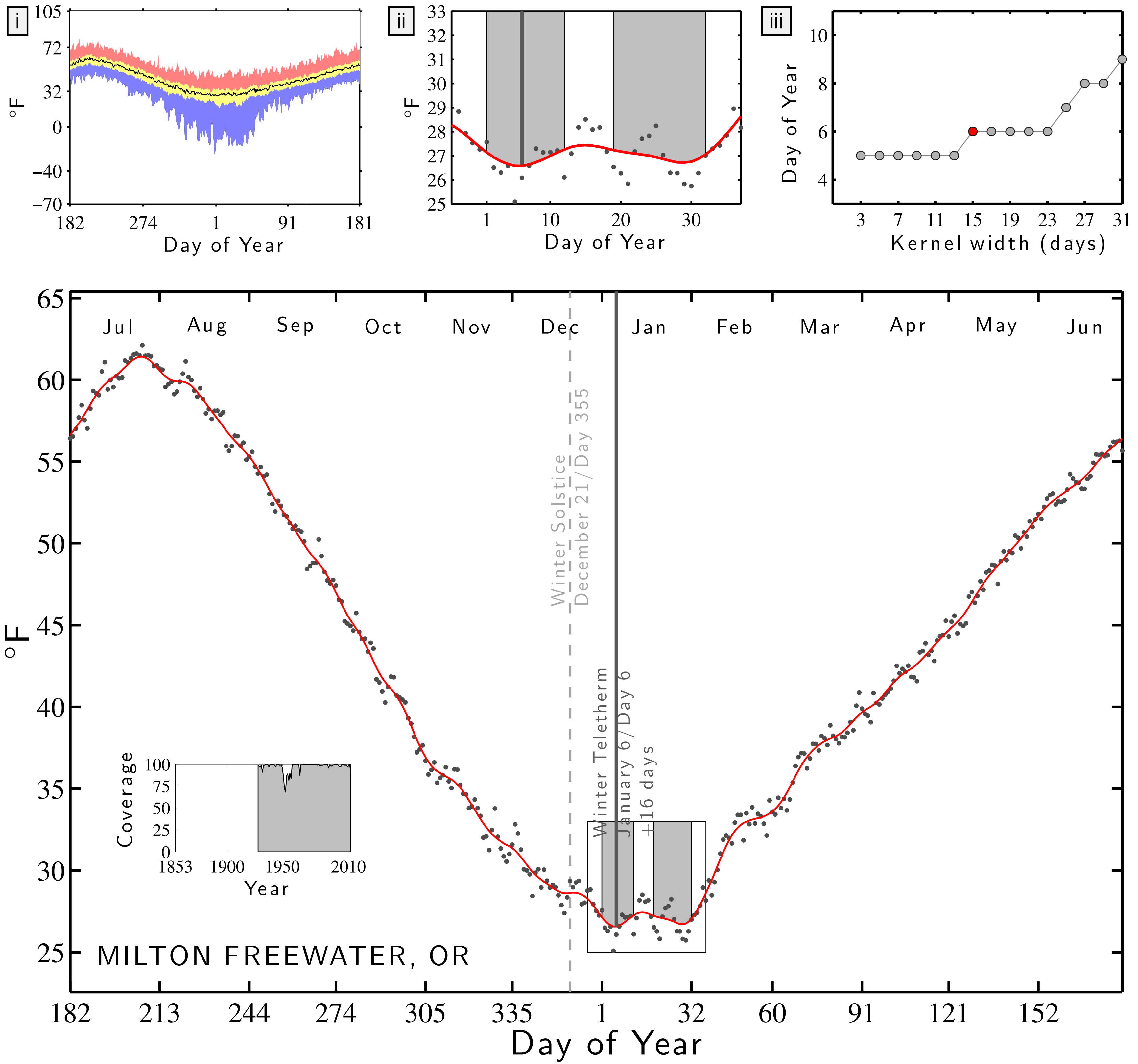

i
ii
iii
°F
Day of Year
Kernel width (days)
Jul
Aug
Sep
Oct
Nov
Dec
Jan
Feb
Mar
Apr
May
Jun
Winter Solstice
December 21/Day 355
Winter Teletherm
January 6/Day 6
±16 days
Coverage
Year
MILTON FREEWATER, OR

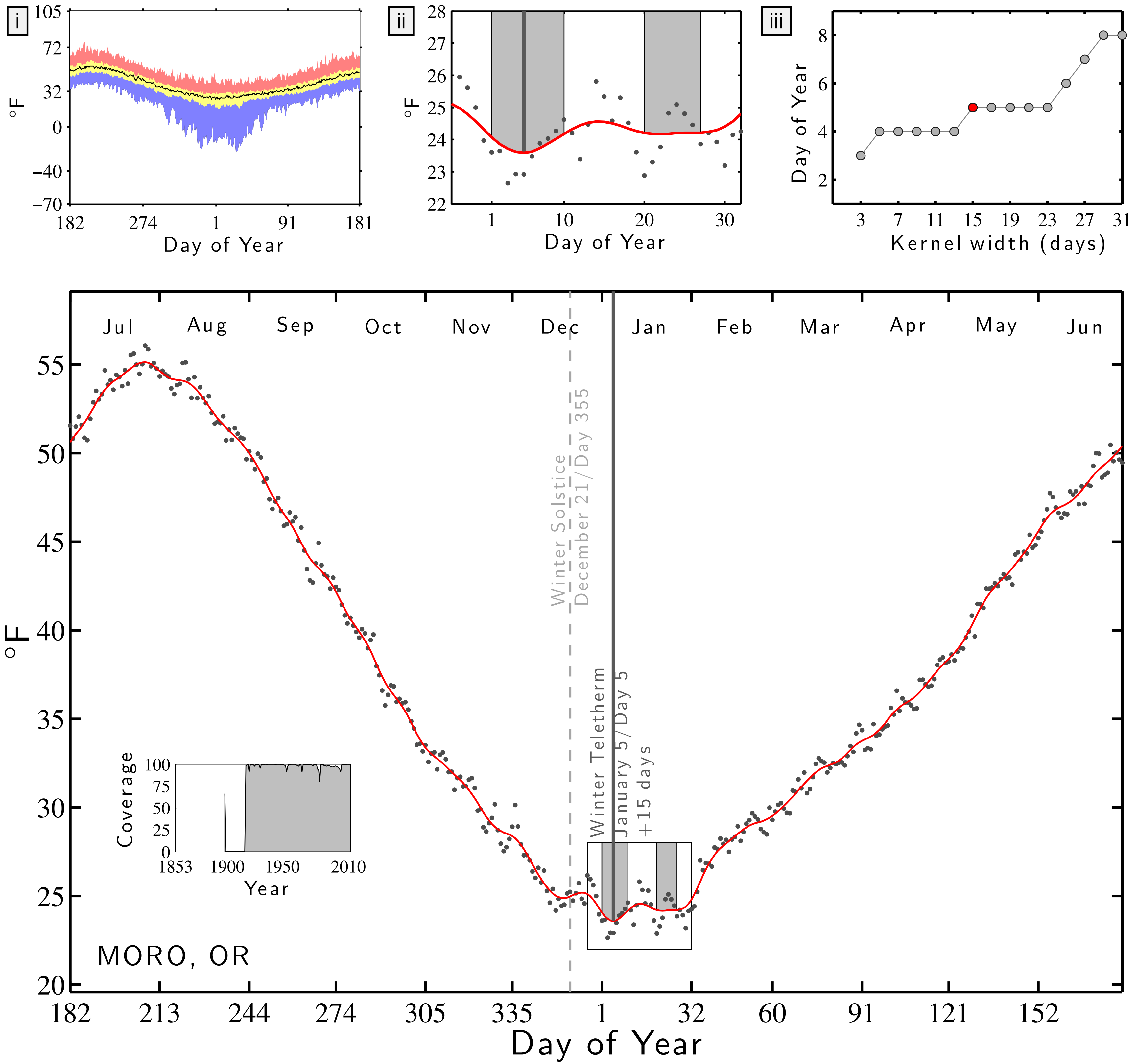

i
ii
iii
Day of Year
°F
Kernel width (days)
Jul
Aug
Sep
Oct
Nov
Dec
Jan
Feb
Mar
Apr
May
Jun
Winter Solstice
December 21/Day 355
Winter Teletherm
January 5/Day 5
+15 days
Coverage
Year
MORO, OR

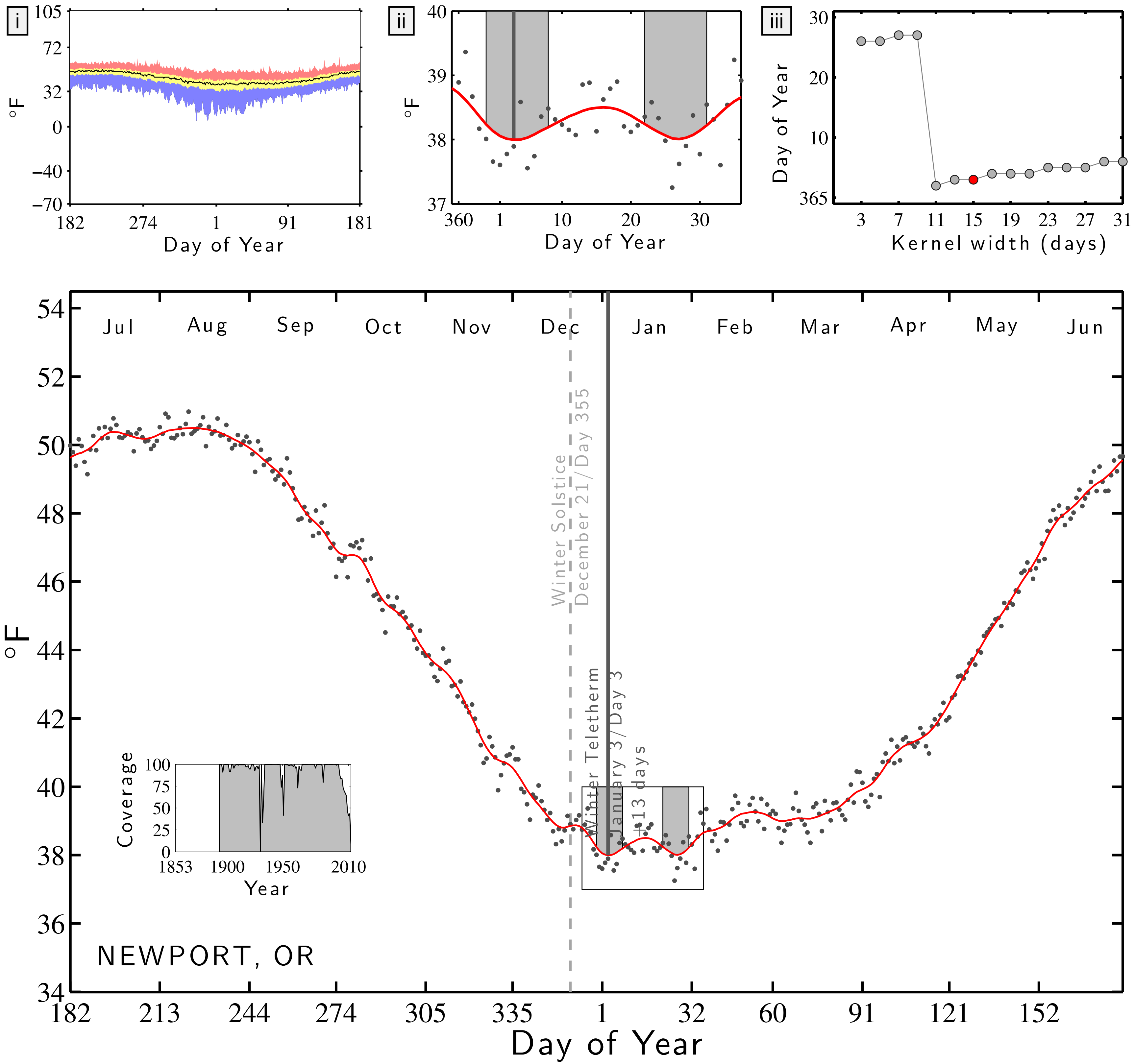

i
ii
iii
°F
Day of Year
Day of Year
Kernel width (days)
Jul
Aug
Sep
Oct
Nov
Dec
Jan
Feb
Mar
Apr
May
Jun
Winter Solstice
December 21/Day 355
Winter Teletherm
January 3/Day 3
+13 days
Coverage
Year
NEWPORT, OR
Day of Year

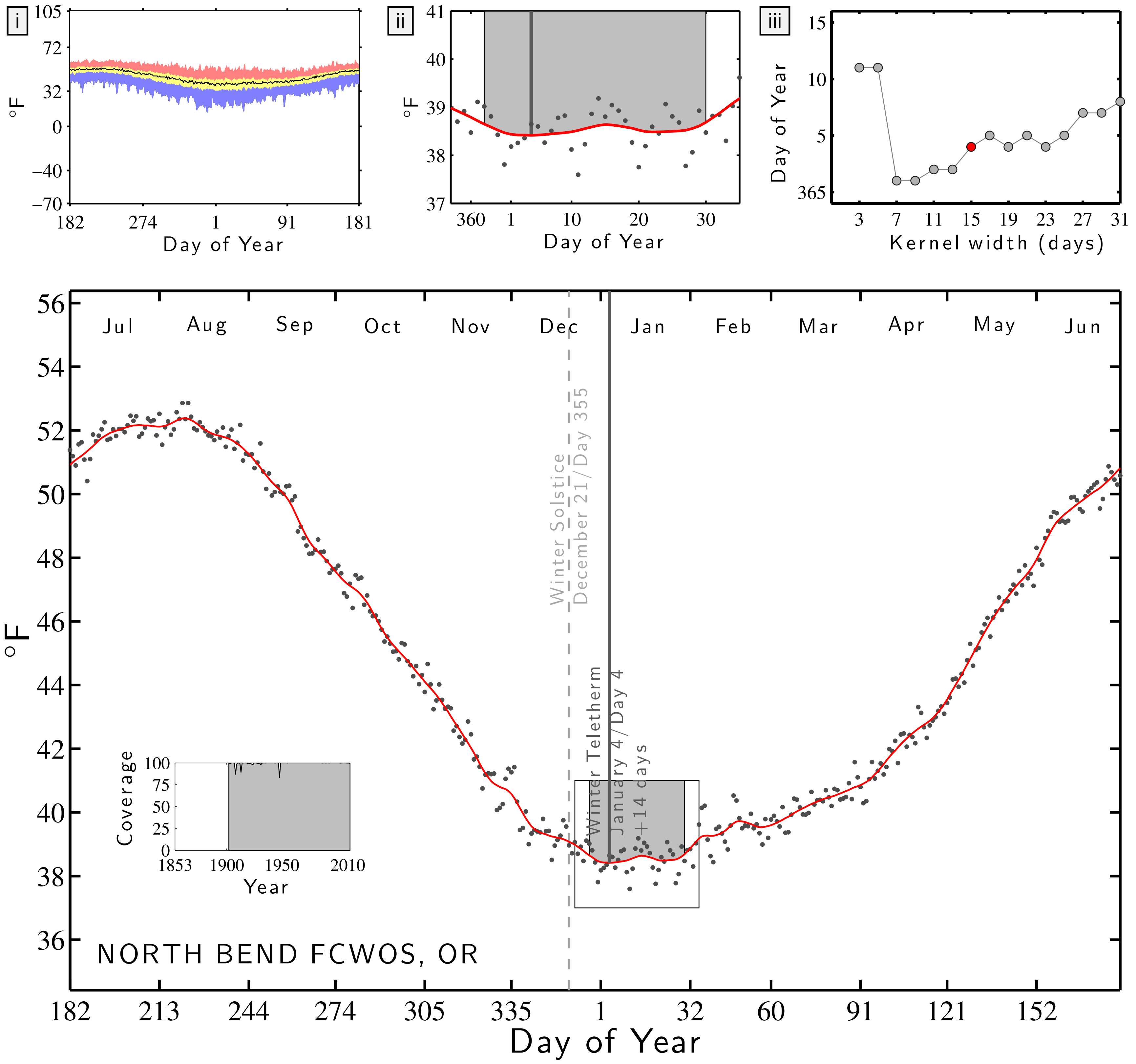

i
°F
Day of Year
ii
°F
Day of Year
iii
Day of Year
Kernel width (days)
Jul
Aug
Sep
Oct
Nov
Dec
Jan
Feb
Mar
Apr
May
Jun
Winter Solstice
December 21/Day 355
Winter Teletherm
January 4/Day 4
+14 days
Coverage
Year
°F
NORTH BEND FCWOS, OR
Day of Year

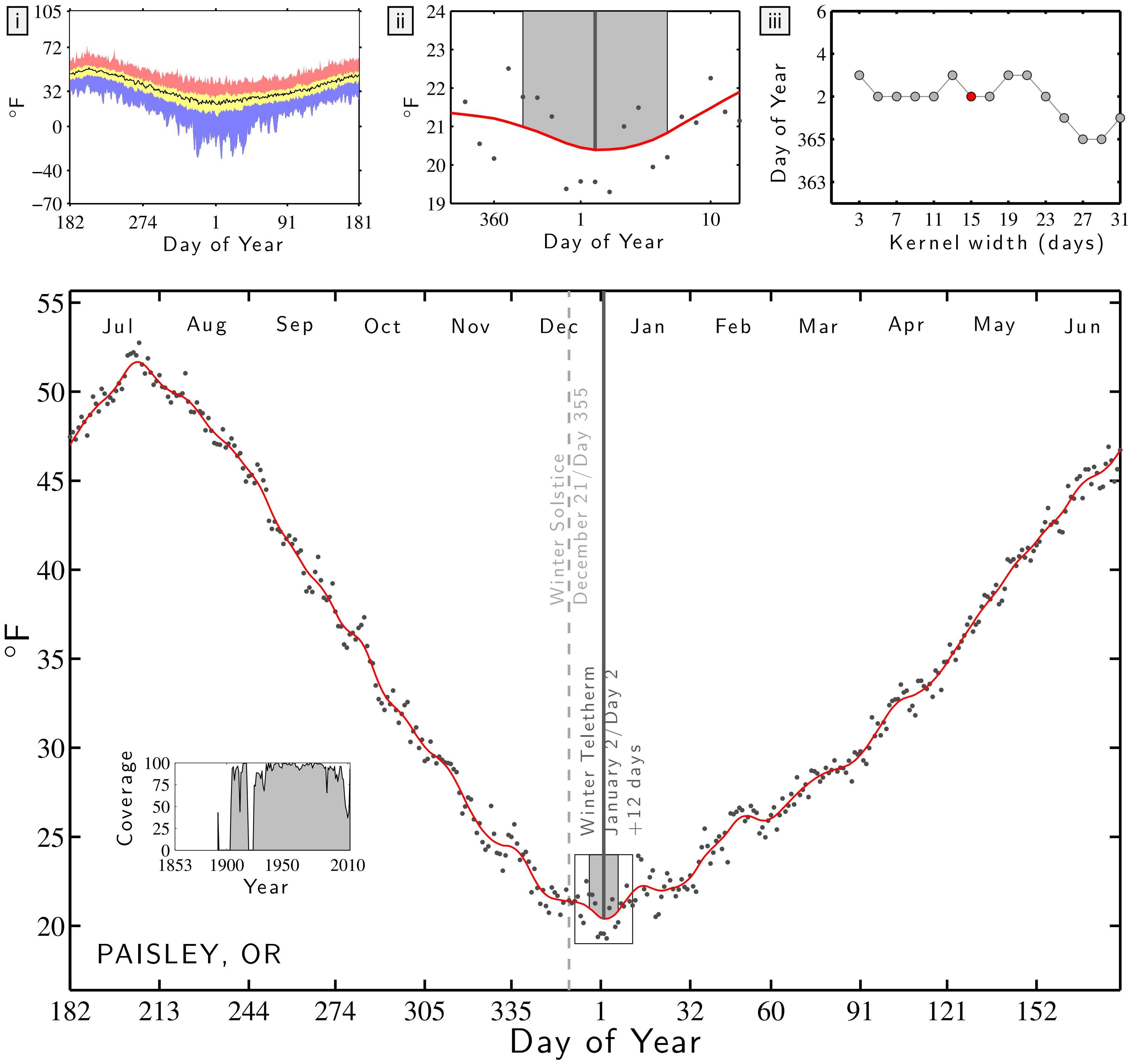

i
°F
Day of Year
ii
°F
Day of Year
iii
Day of Year
Kernel width (days)
Jul
Aug
Sep
Oct
Nov
Dec
Jan
Feb
Mar
Apr
May
Jun
Winter Solstice
December 21/Day 355
Winter Teletherm
January 2/Day 2
+12 days
Coverage
Year
PAISLEY, OR
°F
Day of Year

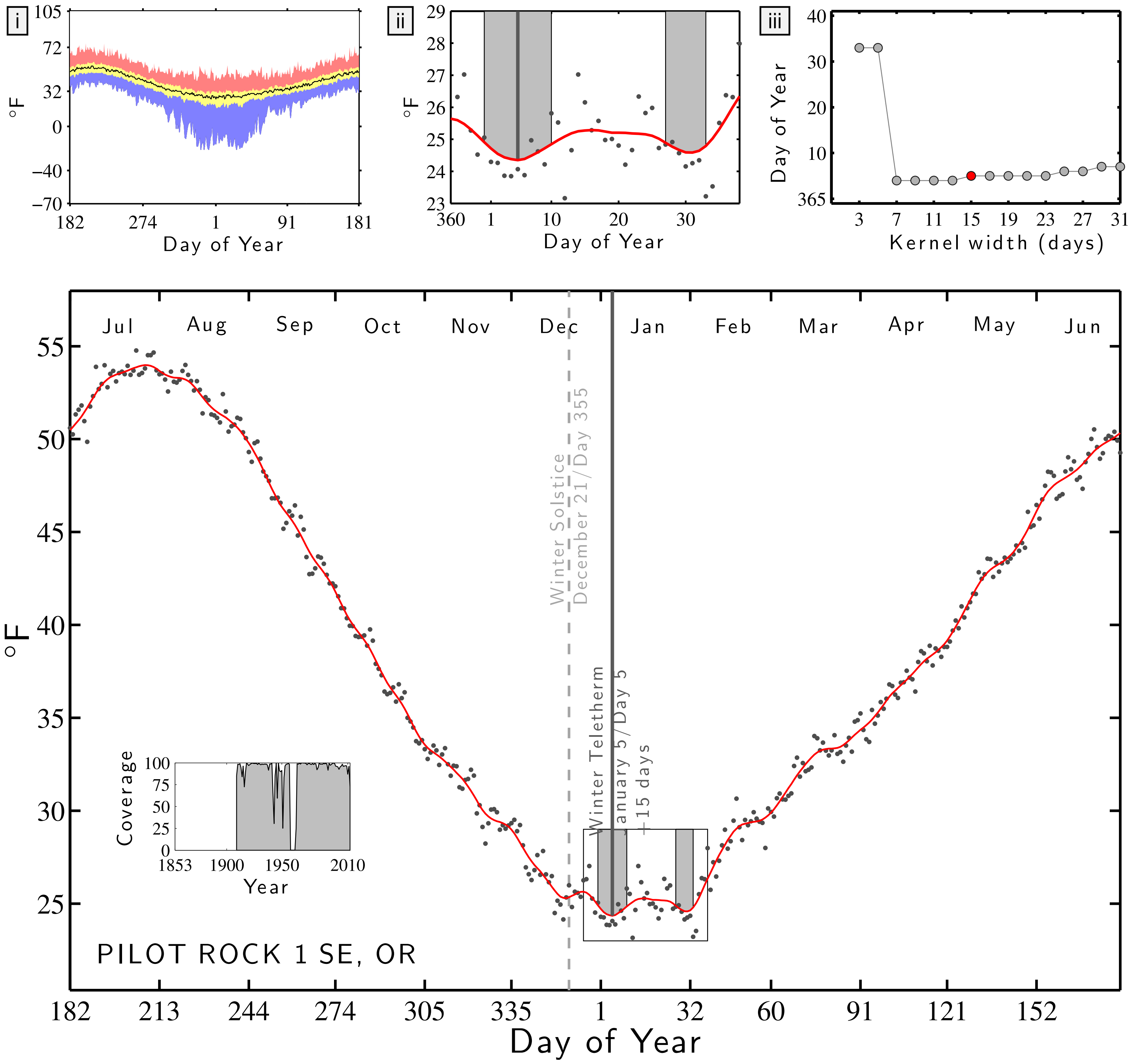

i
°F
Day of Year
ii
°F
Day of Year
iii
Day of Year
Kernel width (days)
Jul Aug Sep Oct Nov Dec Jan Feb Mar Apr May Jun
Winter Solstice
December 21/Day 355
Winter Teletherm
January 5/Day 5
±15 days
Coverage
Year
PILOT ROCK 1 SE, OR
°F
Day of Year

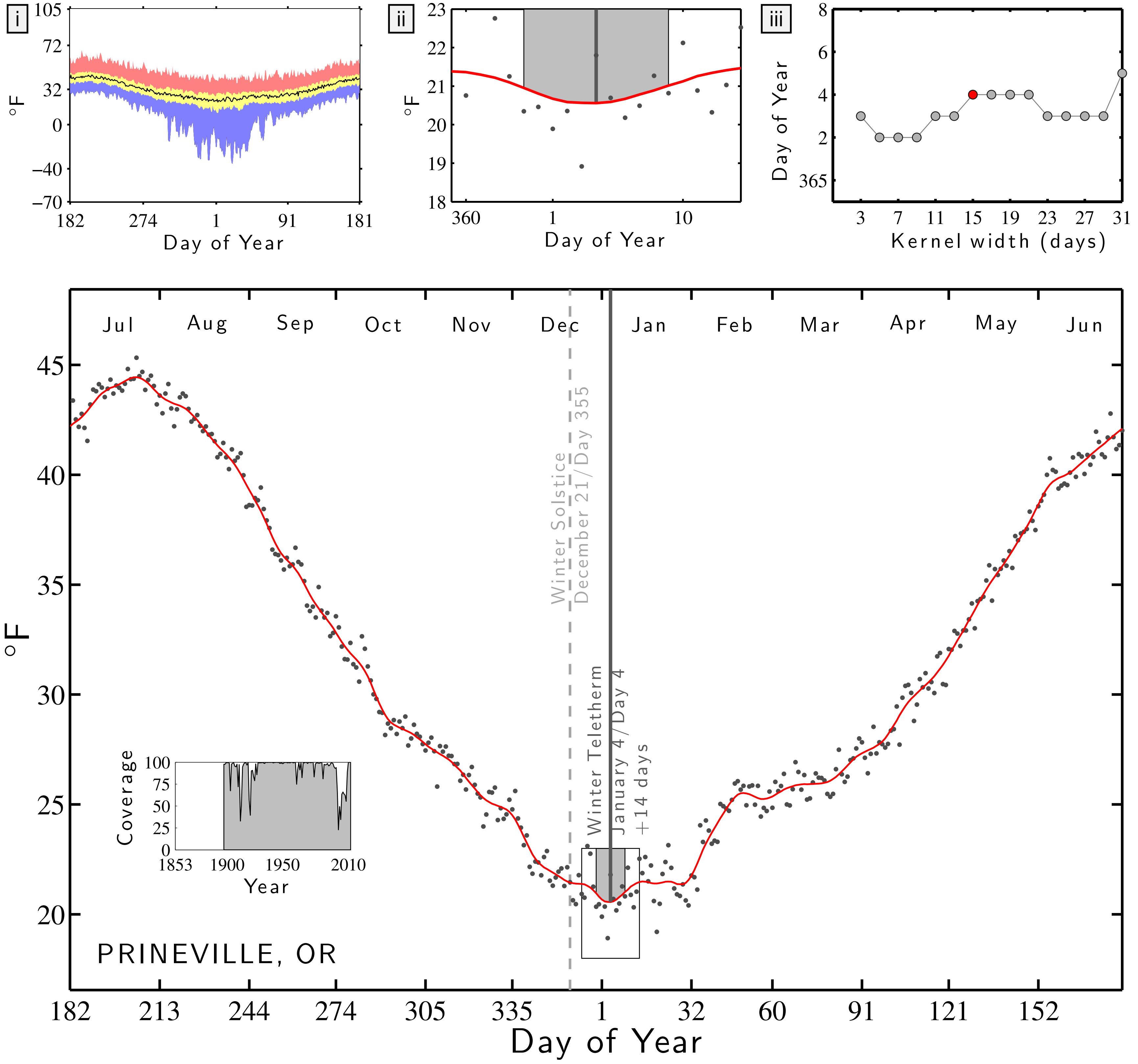

i
ii
iii
°F
Day of Year
Day of Year
Kernel width (days)
Jul
Aug
Sep
Oct
Nov
Dec
Jan
Feb
Mar
Apr
May
Jun
Winter Solstice
December 21/Day 355
Winter Teletherm
January 4/Day 4
+14 days
Coverage
Year
PRINEVILLE, OR
Day of Year

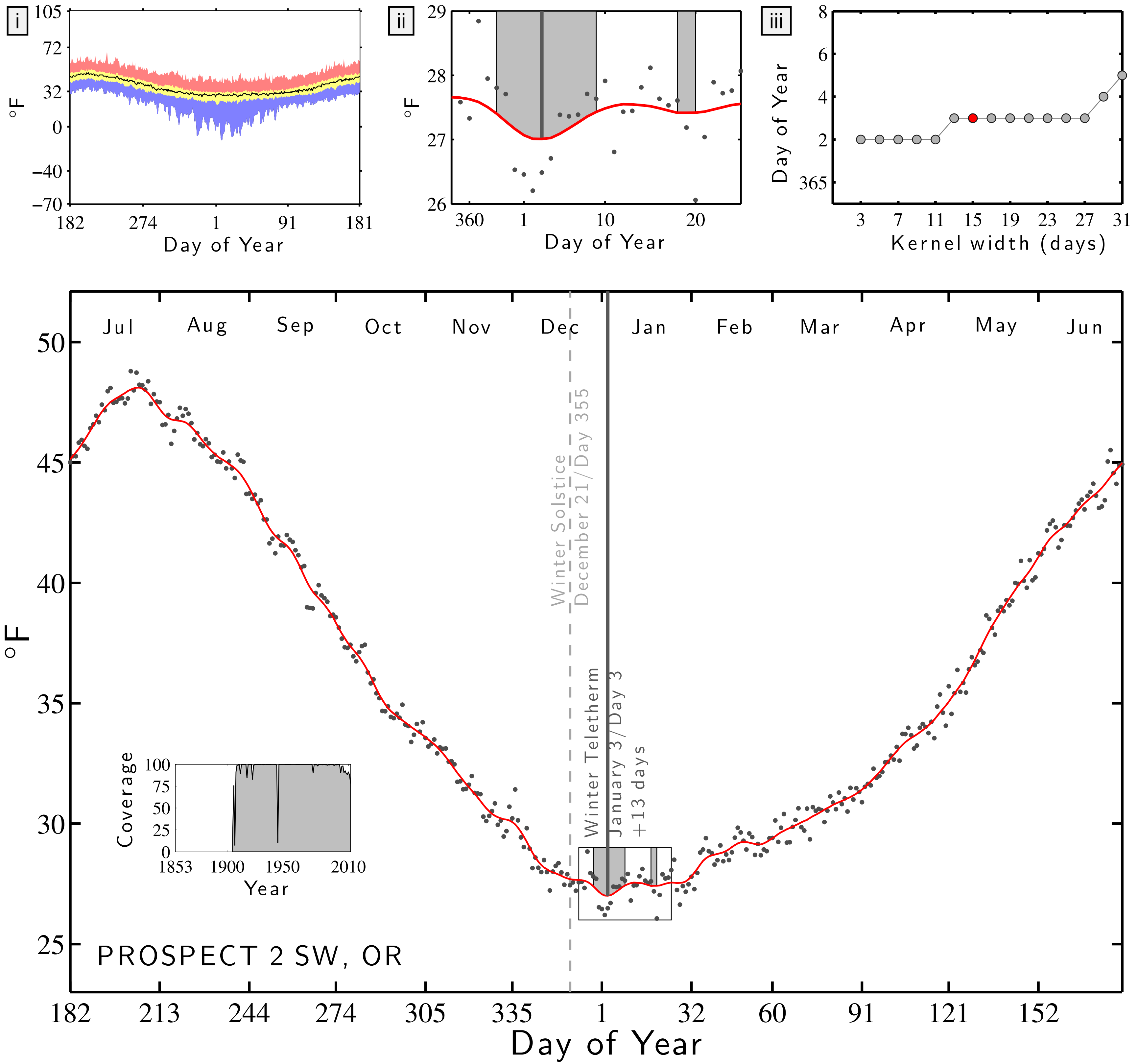

i
ii
iii
°F
Day of Year
Kernel width (days)
Jul
Aug
Sep
Oct
Nov
Dec
Jan
Feb
Mar
Apr
May
Jun
Winter Solstice
December 21/Day 355
Winter Teletherm
January 3/Day 3
+13 days
Coverage
Year
PROSPECT 2 SW, OR

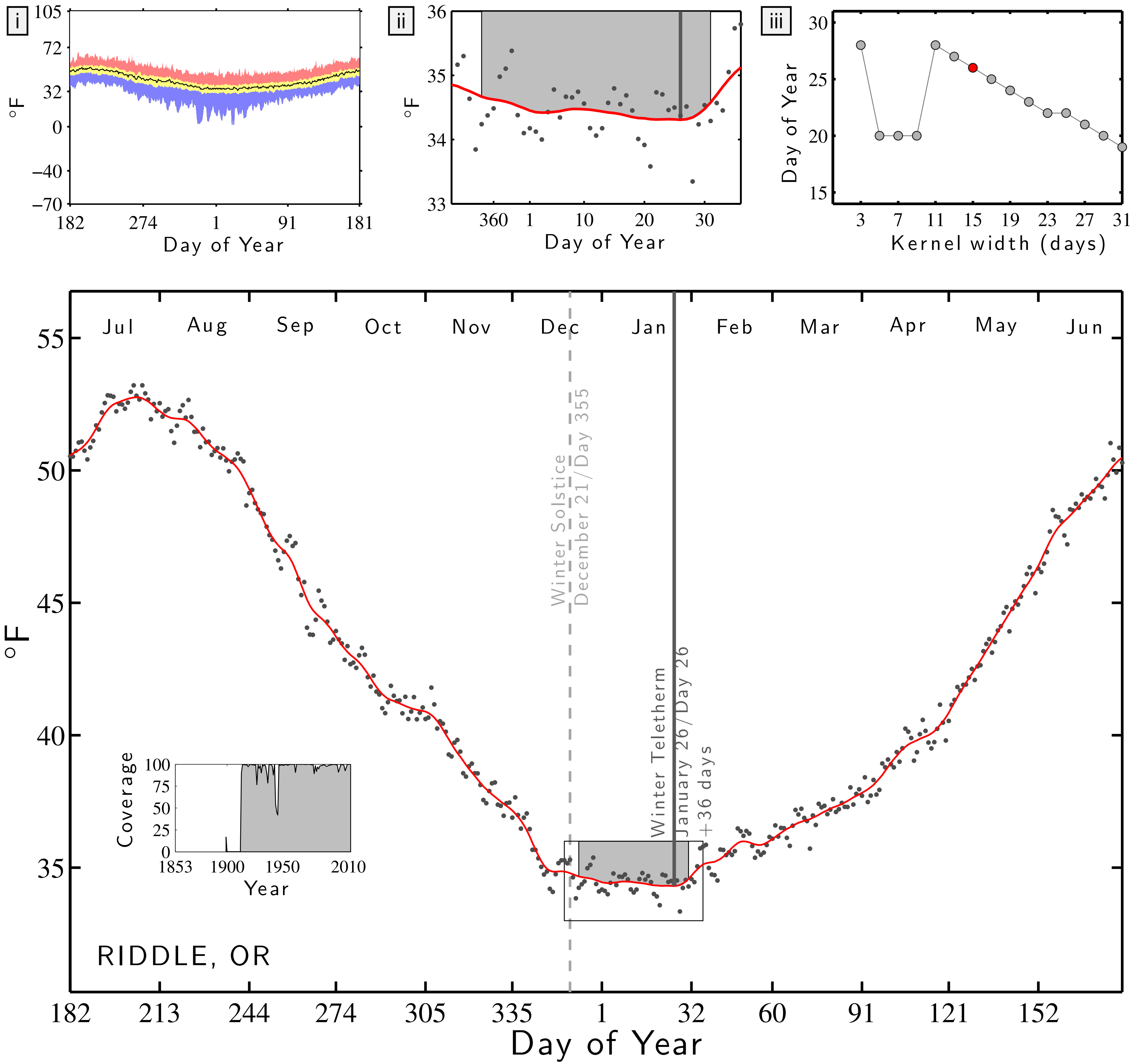

i
105
72
32
0
−40
−70
°F
182
274
1
91
181
Day of Year
ii
36
35
34
33
°F
360
1
10
20
30
Day of Year
iii
30
25
20
15
Day of Year
3
7
11
15
19
23
27
31
Kernel width (days)
Jul
Aug
Sep
Oct
Nov
Dec
Jan
Feb
Mar
Apr
May
Jun
55
50
45
40
35
°F
Winter Solstice
December 21/Day 355
Winter Teletherm
January 26/Day 26
+36 days
Coverage
100
75
50
25
0
1853
1900
1950
2010
Year
RIDDLE, OR
182
213
244
274
305
335
1
32
60
91
121
152
Day of Year

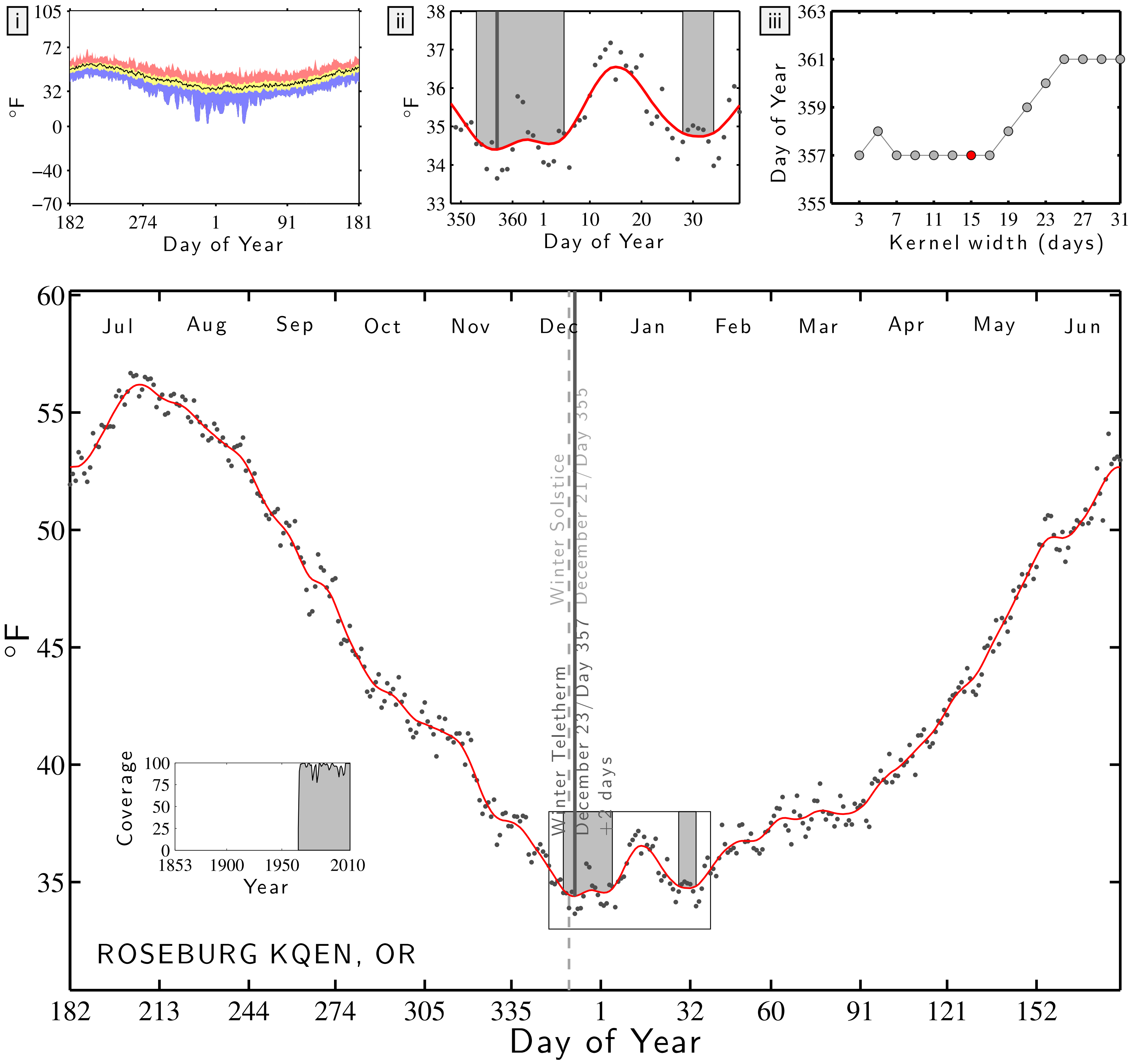

i
°F
Day of Year
ii
°F
Day of Year
iii
Day of Year
Kernel width (days)
Jul
Aug
Sep
Oct
Nov
Dec
Jan
Feb
Mar
Apr
May
Jun
Winter Solstice
December 21/Day 355
Winter Teletherm
December 23/Day 357
+2 days
Coverage
Year
ROSEBURG KQEN, OR
°F
Day of Year

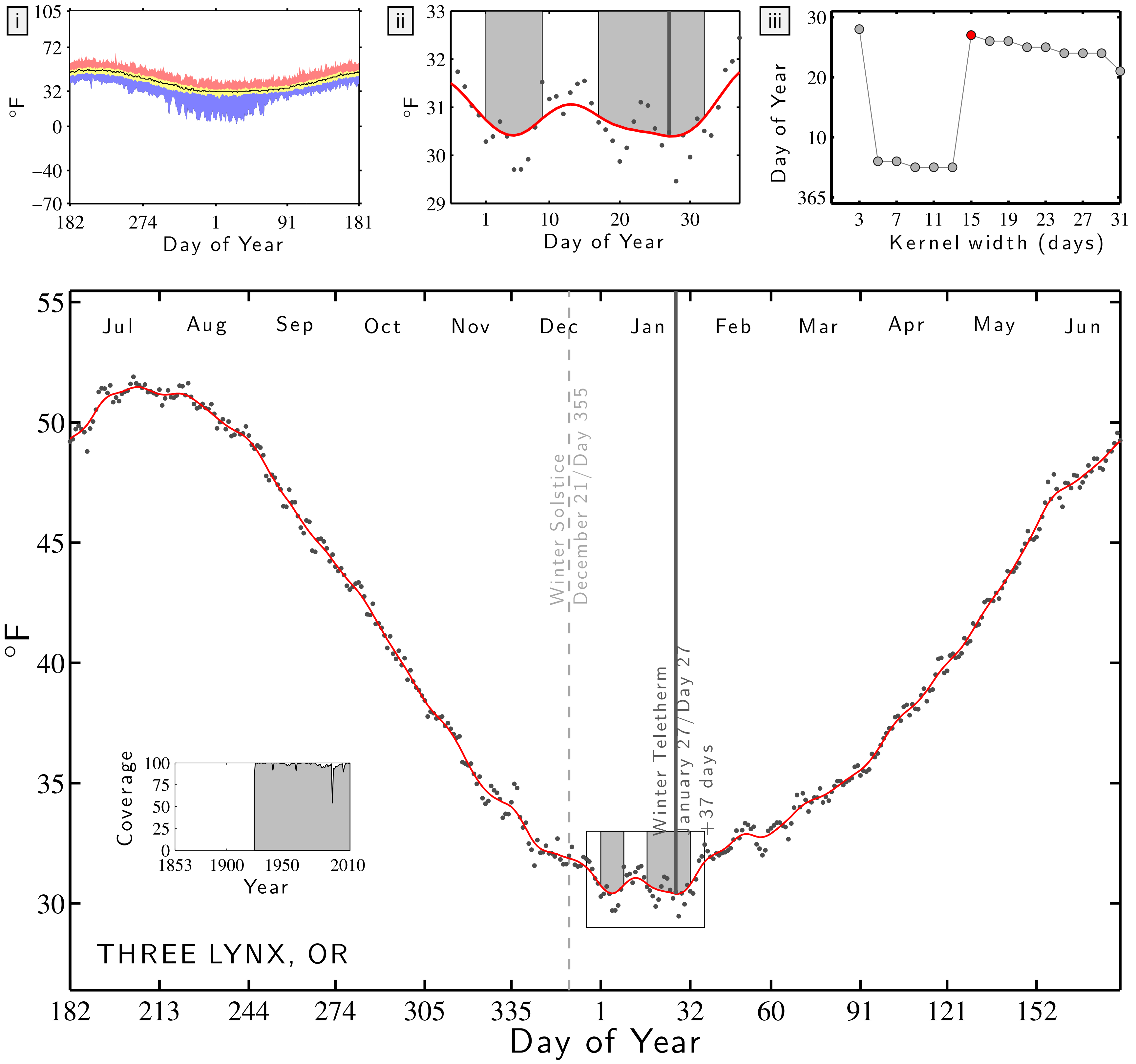

i
Day of Year
°F
ii
Day of Year
°F
iii
Kernel width (days)
Day of Year
Jul
Aug
Sep
Oct
Nov
Dec
Jan
Feb
Mar
Apr
May
Jun
Winter Solstice
December 21/Day 355
Winter Teletherm
January 27/Day 27
±37 days
Coverage
Year
THREE LYNX, OR
Day of Year
°F

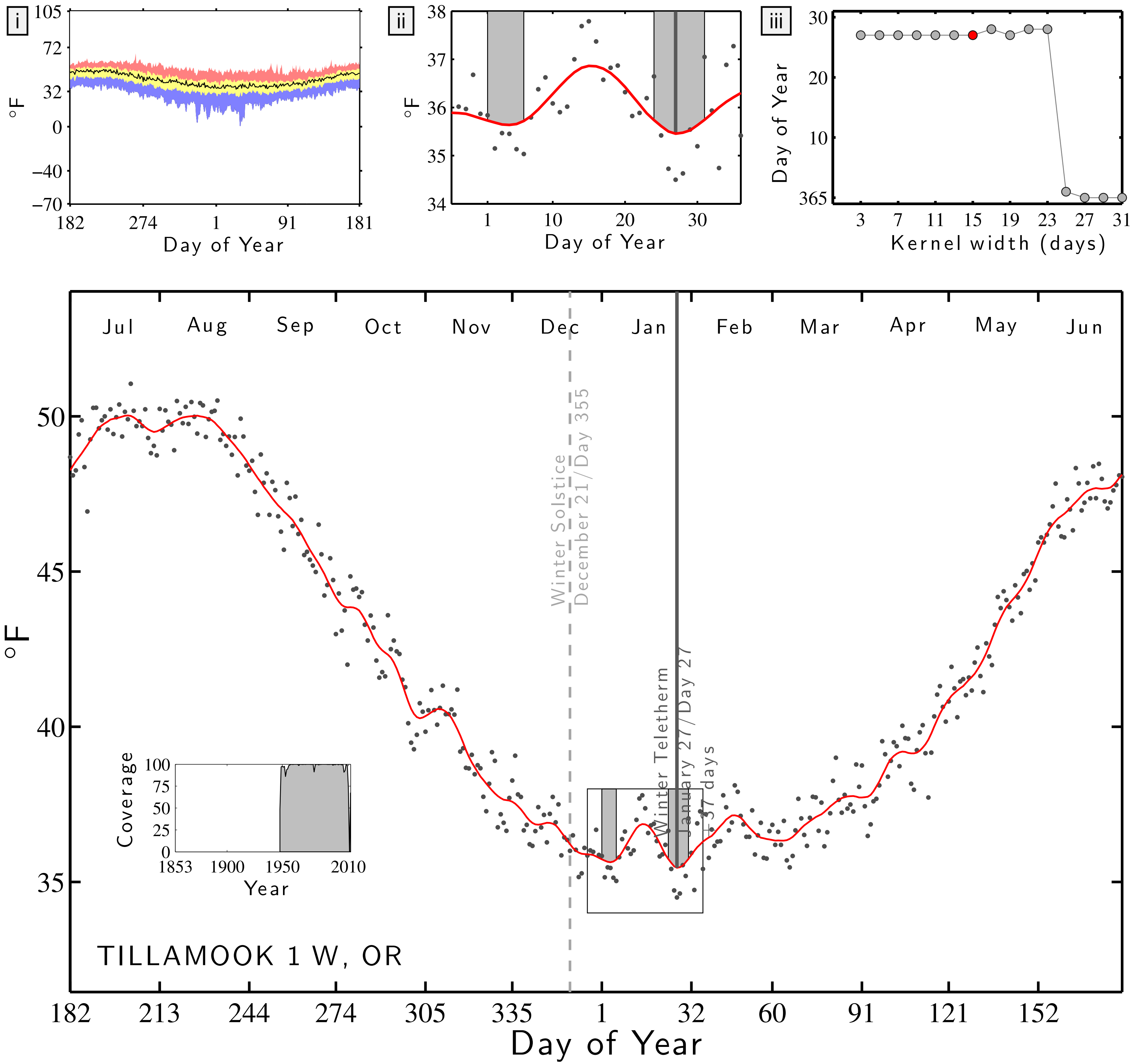

i
ii
iii
Day of Year
°F
Kernel width (days)
Jul
Aug
Sep
Oct
Nov
Dec
Jan
Feb
Mar
Apr
May
Jun
Winter Solstice
December 21/Day 355
Winter Teletherm
January 27/Day 27
±37 days
Coverage
Year
TILLAMOOK 1 W, OR

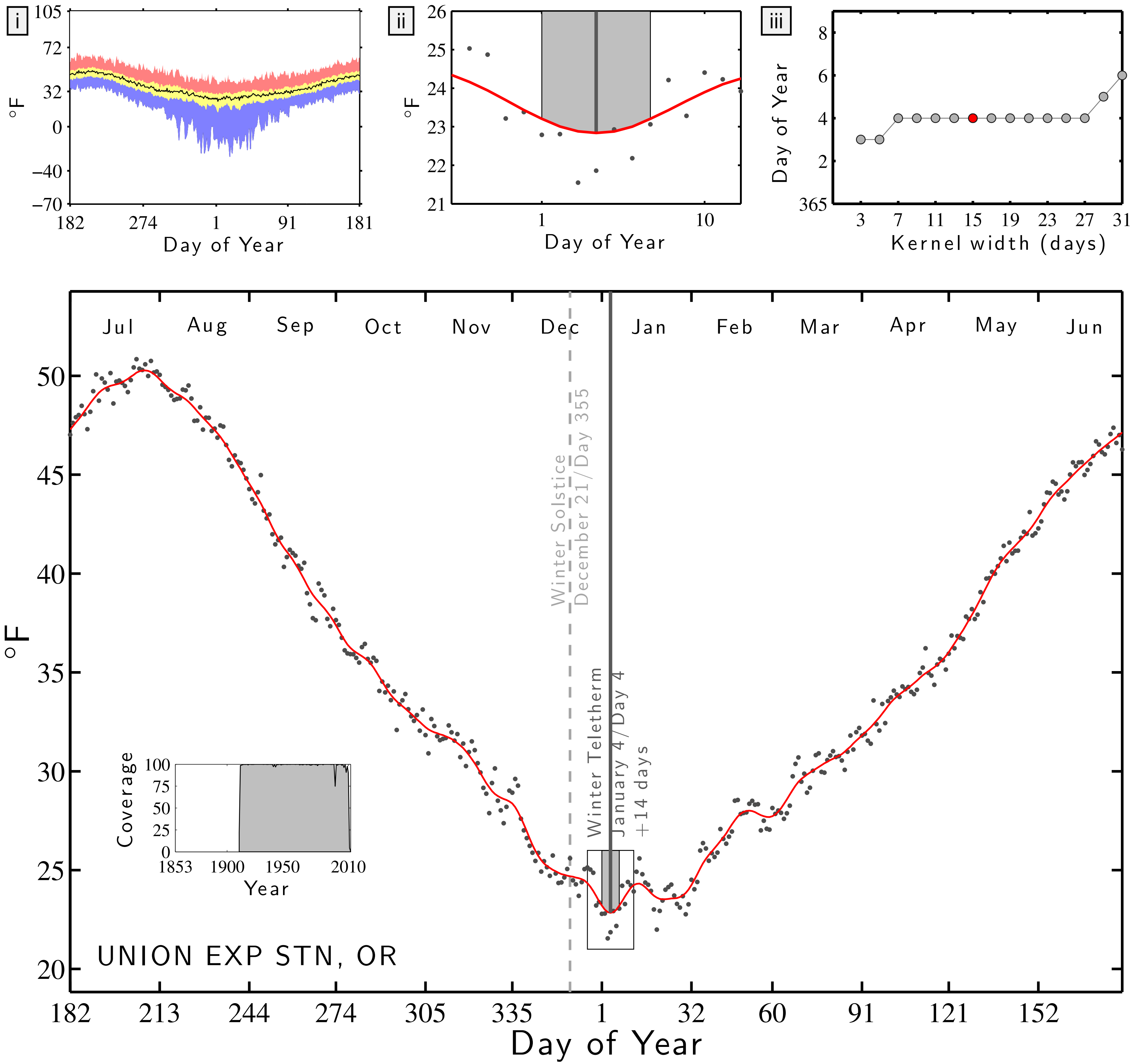

i
°F
105
72
32
0
−40
−70
182
274
1
91
181
Day of Year
ii
26
25
24
23
22
21
1
10
iii
Day of Year
8
6
4
2
365
3
7
11
15
19
23
27
31
Kernel width (days)
Jul
Aug
Sep
Oct
Nov
Dec
Jan
Feb
Mar
Apr
May
Jun
50
45
40
35
30
25
20
Winter Solstice
December 21/Day 355
Winter Teletherm
January 4/Day 4
+14 days
Coverage
100
75
50
25
0
1853
1900
1950
2010
Year
UNION EXP STN, OR
182
213
244
274
305
335
1
32
60
91
121
152
Day of Year

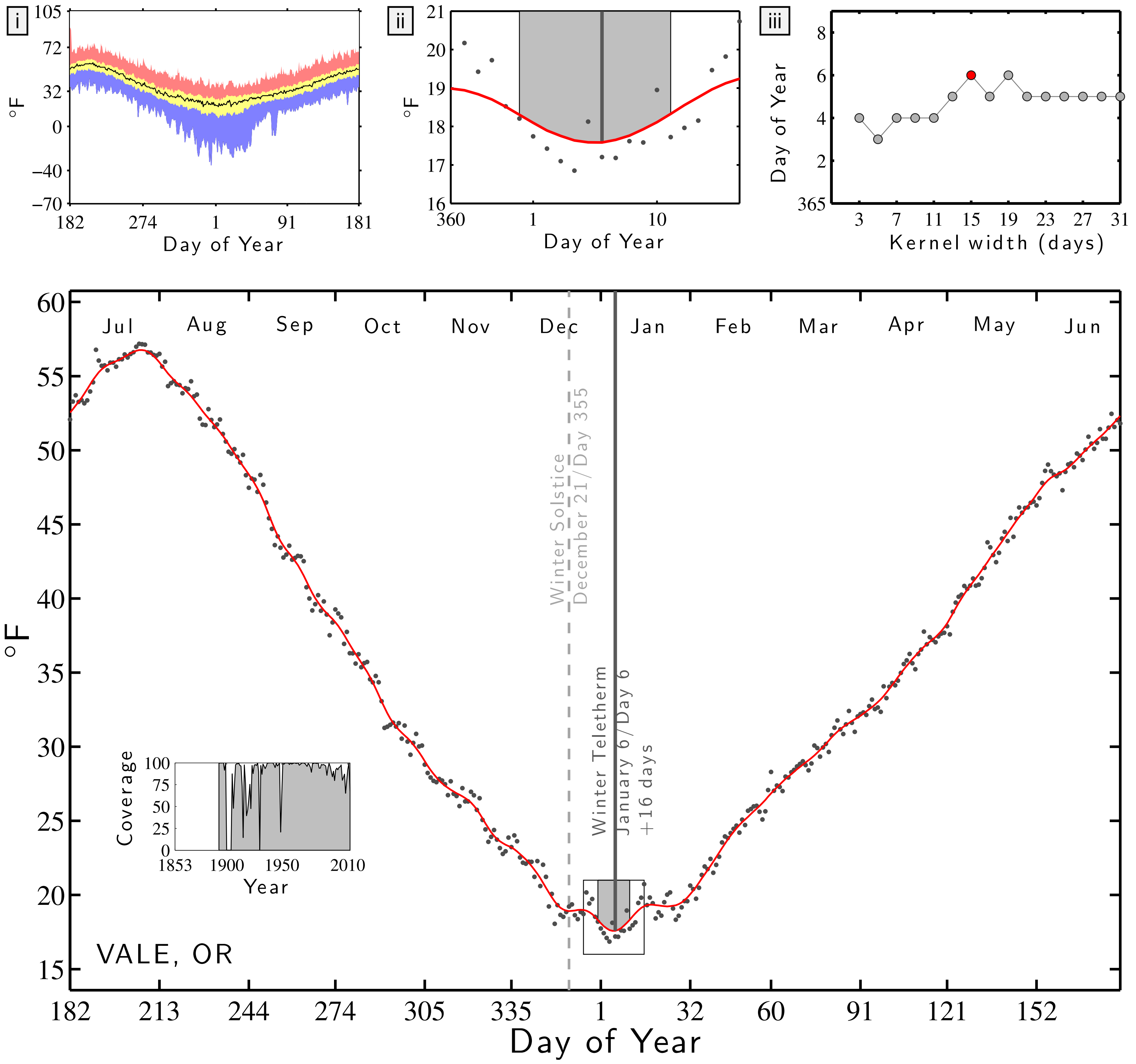
i
°F
Day of Year
ii
°F
Day of Year
iii
Day of Year
Kernel width (days)
Jul
Aug
Sep
Oct
Nov
Dec
Jan
Feb
Mar
Apr
May
Jun
Winter Solstice
December 21/Day 355
Winter Teletherm
January 6/Day 6
+16 days
Coverage
Year
VALE, OR
°F
Day of Year

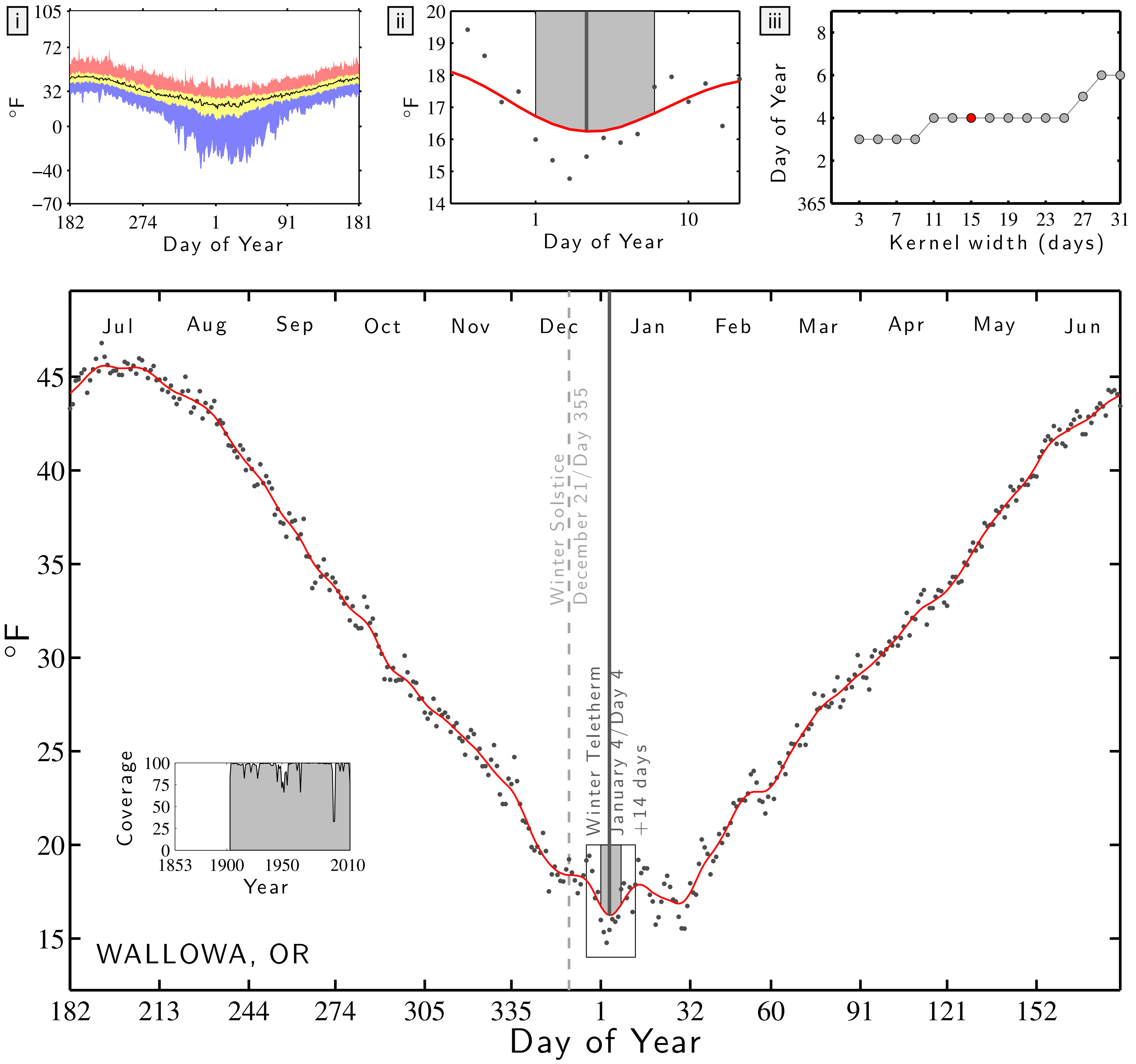

i
°F
Day of Year
ii
°F
Day of Year
iii
Day of Year
Kernel width (days)
Jul
Aug
Sep
Oct
Nov
Dec
Jan
Feb
Mar
Apr
May
Jun
Winter Solstice
December 21/Day 355
Winter Teletherm
January 4/Day 4
+14 days
Coverage
Year
WALLOWA, OR
°F
Day of Year

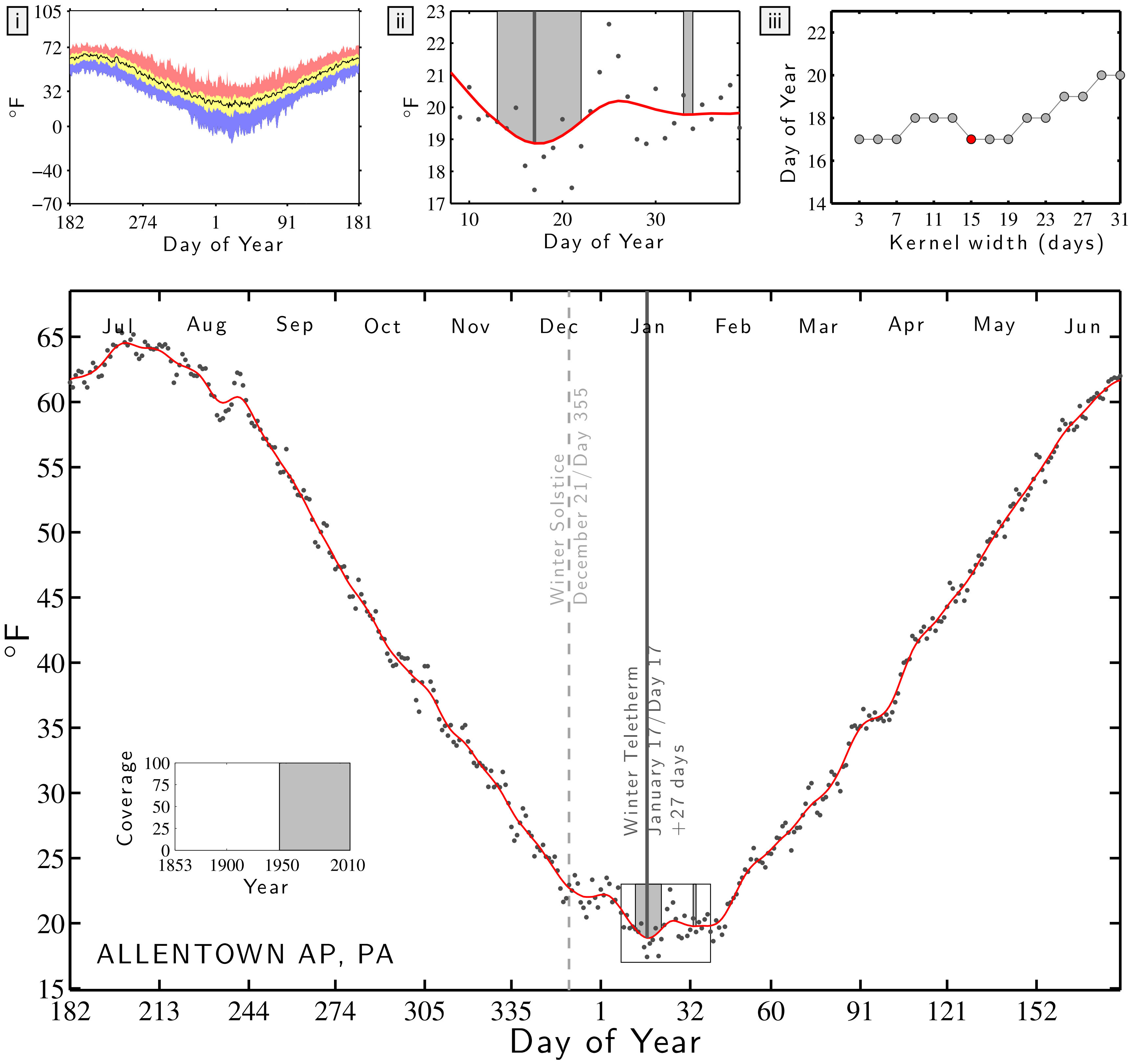

i
ii
iii
°F
Day of Year
Kernel width (days)
Jul
Aug
Sep
Oct
Nov
Dec
Jan
Feb
Mar
Apr
May
Jun
Winter Solstice
December 21/Day 355
Winter Teletherm
January 17/Day 17
+27 days
Coverage
Year
ALLENTOWN AP, PA

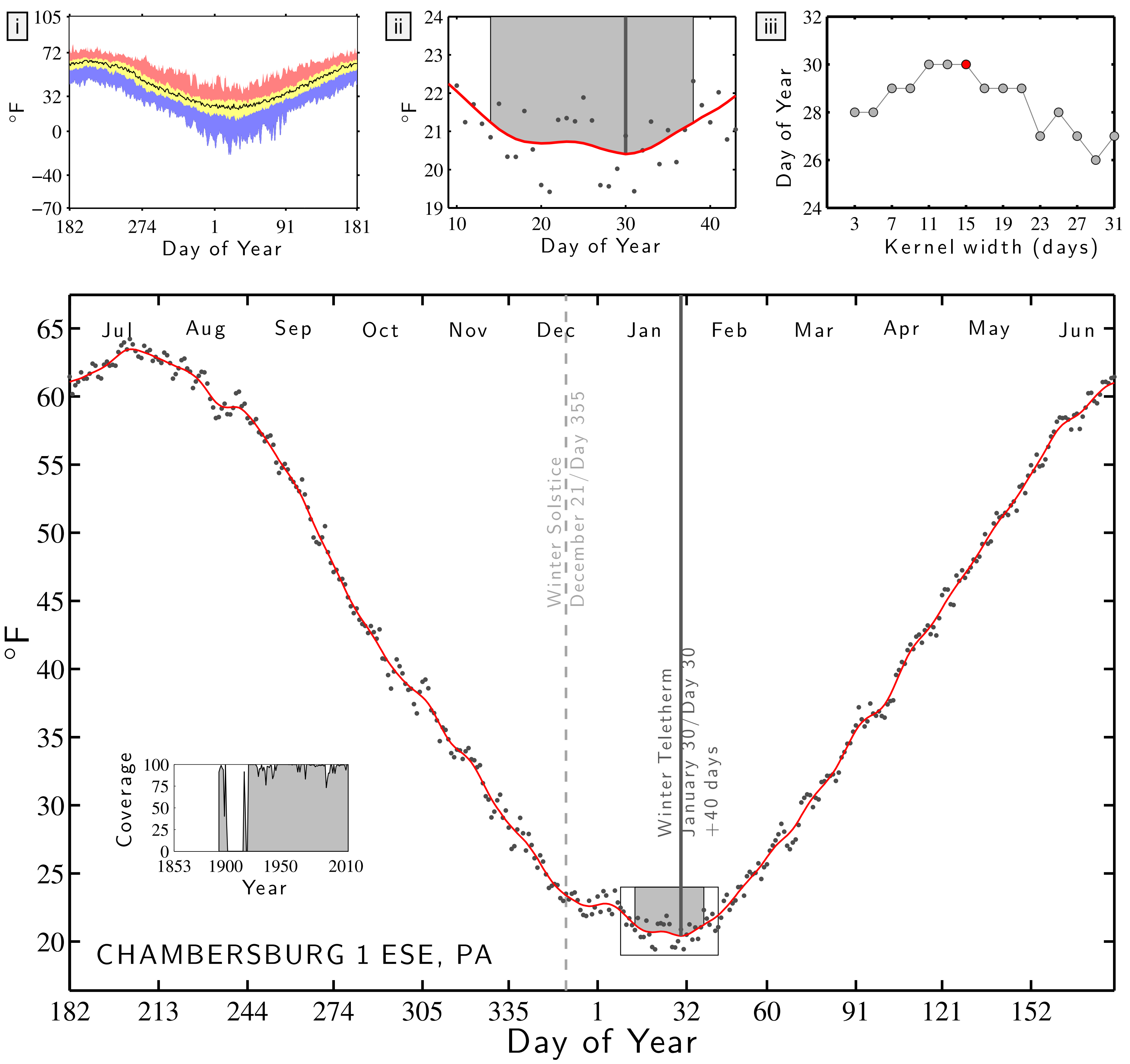

i
°F
105
72
32
0
−40
−70
182
274
1
91
181
Day of Year
ii
24
23
22
21
20
19
10
20
30
40
Day of Year
iii
Day of Year
32
30
28
26
24
3
7
11
15
19
23
27
31
Kernel width (days)
Jul
Aug
Sep
Oct
Nov
Dec
Jan
Feb
Mar
Apr
May
Jun
Winter Solstice
December 21/Day 355
Winter Teletherm
January 30/Day 30
+40 days
Coverage
100
75
50
25
0
1853
1900
1950
2010
Year
CHAMBERSBURG 1 ESE, PA
65
60
55
50
45
40
35
30
25
20
182
213
244
274
305
335
1
32
60
91
121
152
Day of Year

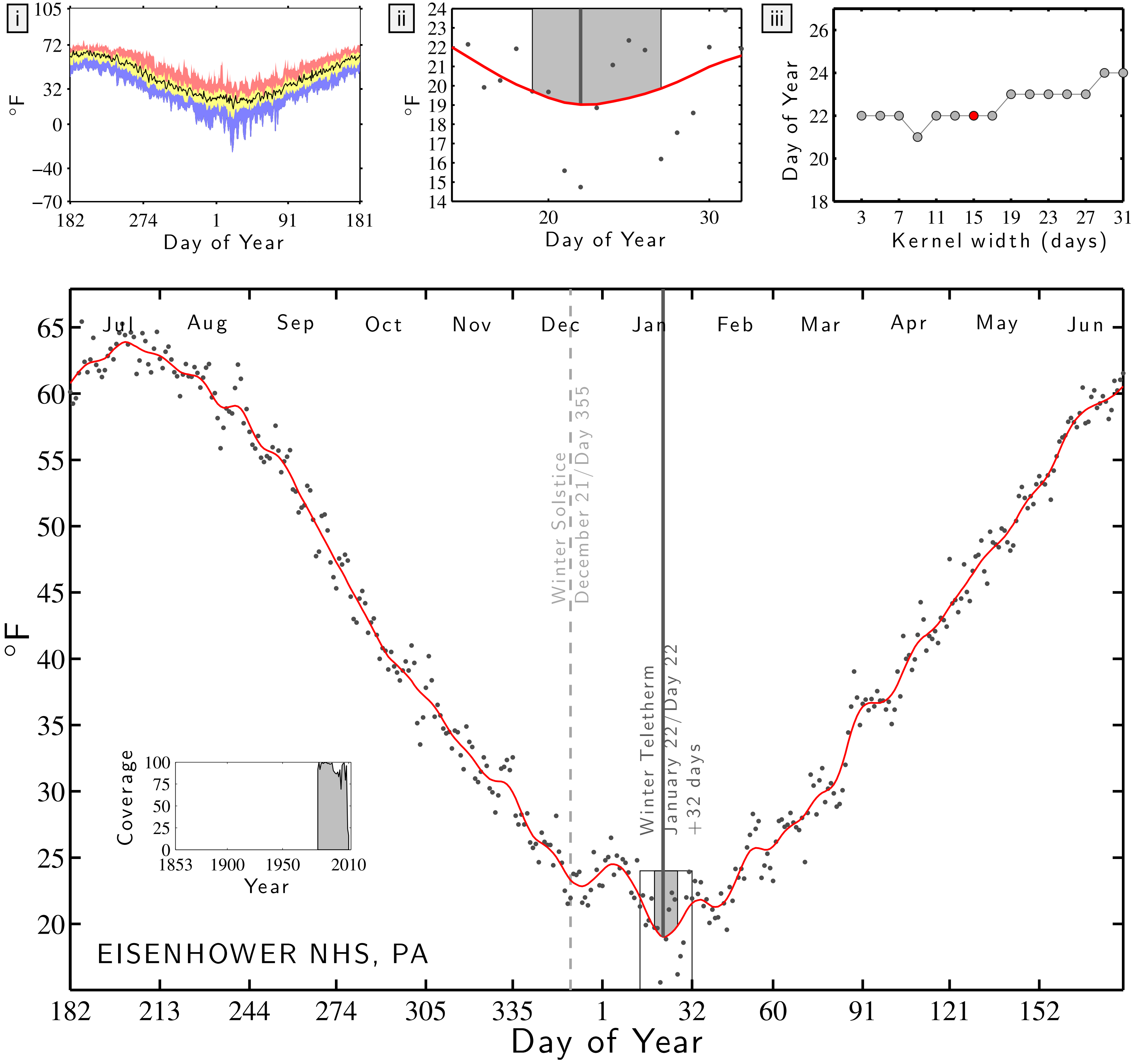
i
ii
iii
°F
Day of Year
Kernel width (days)
Jul
Aug
Sep
Oct
Nov
Dec
Jan
Feb
Mar
Apr
May
Jun
Winter Solstice
December 21/Day 355
Winter Teletherm
January 22/Day 22
+32 days
Coverage
Year
EISENHOWER NHS, PA

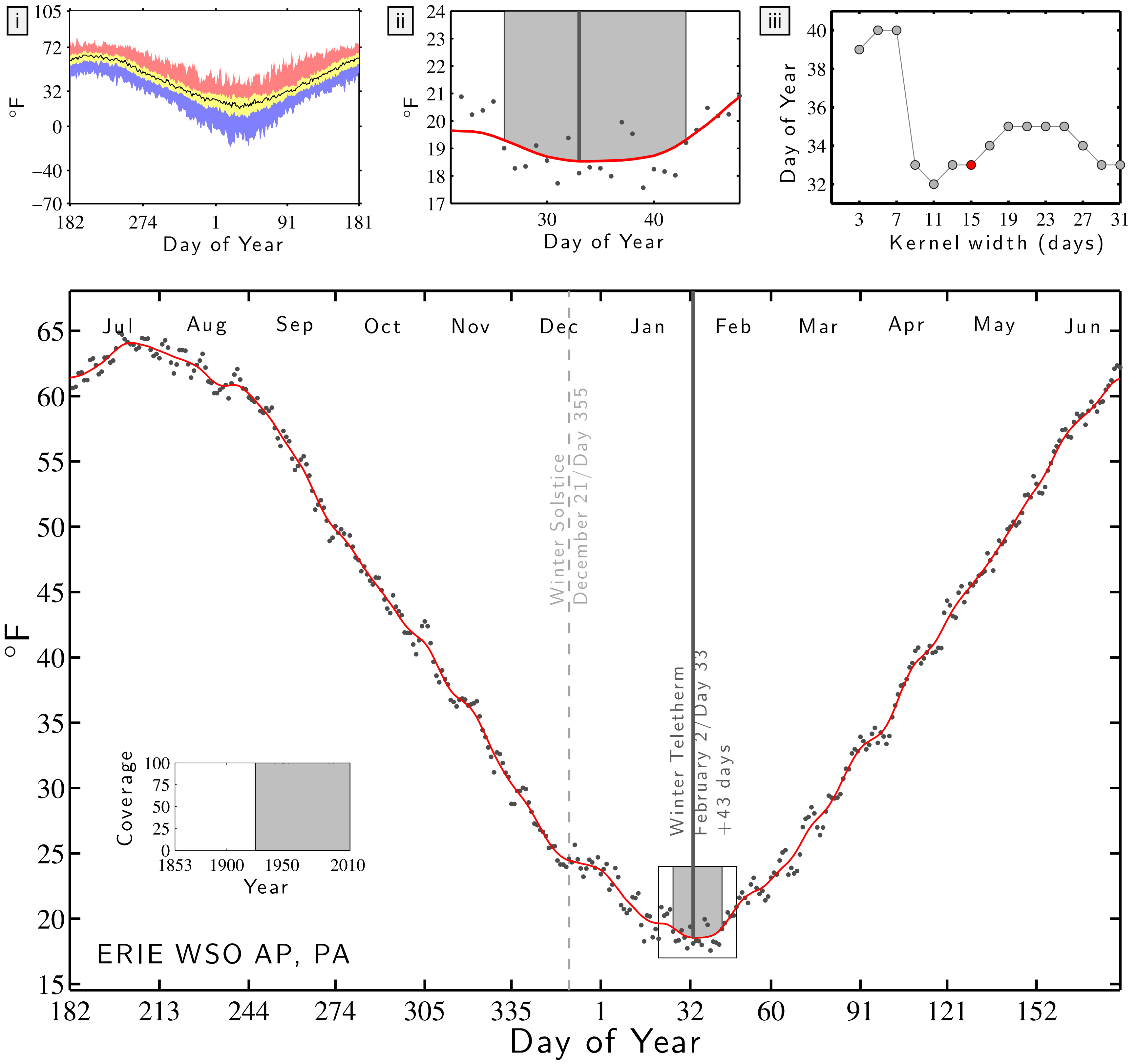

i
°F
Day of Year
ii
Day of Year
iii
Day of Year
Kernel width (days)
Jul
Aug
Sep
Oct
Nov
Dec
Jan
Feb
Mar
Apr
May
Jun
Winter Solstice
December 21/Day 355
Winter Teletherm
February 2/Day 33
+43 days
Coverage
Year
ERIE WSO AP, PA
Day of Year

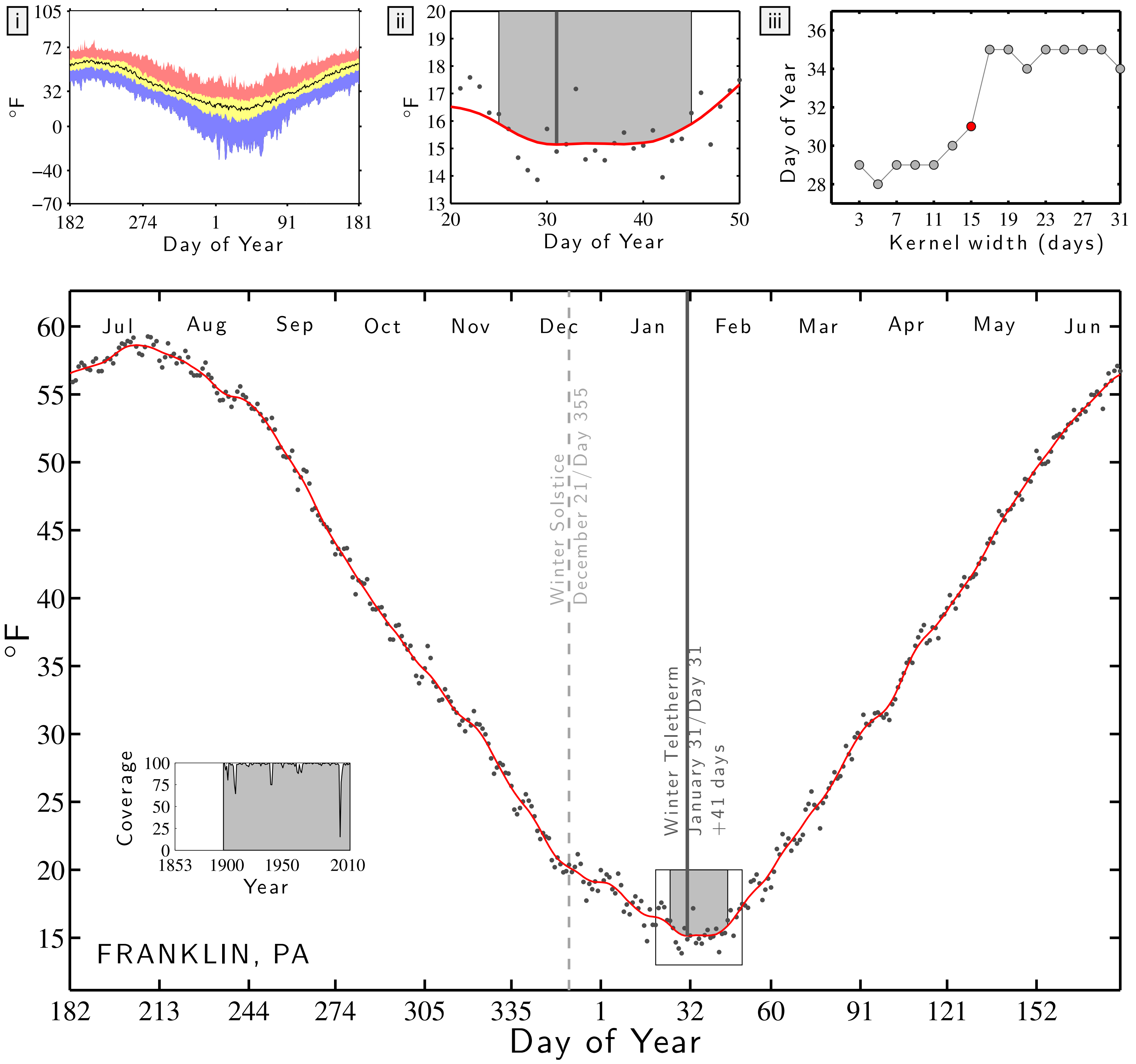

i
ii
iii
Day of Year
°F
Kernel width (days)
Jul
Aug
Sep
Oct
Nov
Dec
Jan
Feb
Mar
Apr
May
Jun
Winter Solstice
December 21/Day 355
Winter Teletherm
January 31/Day 31
+41 days
Coverage
Year
FRANKLIN, PA

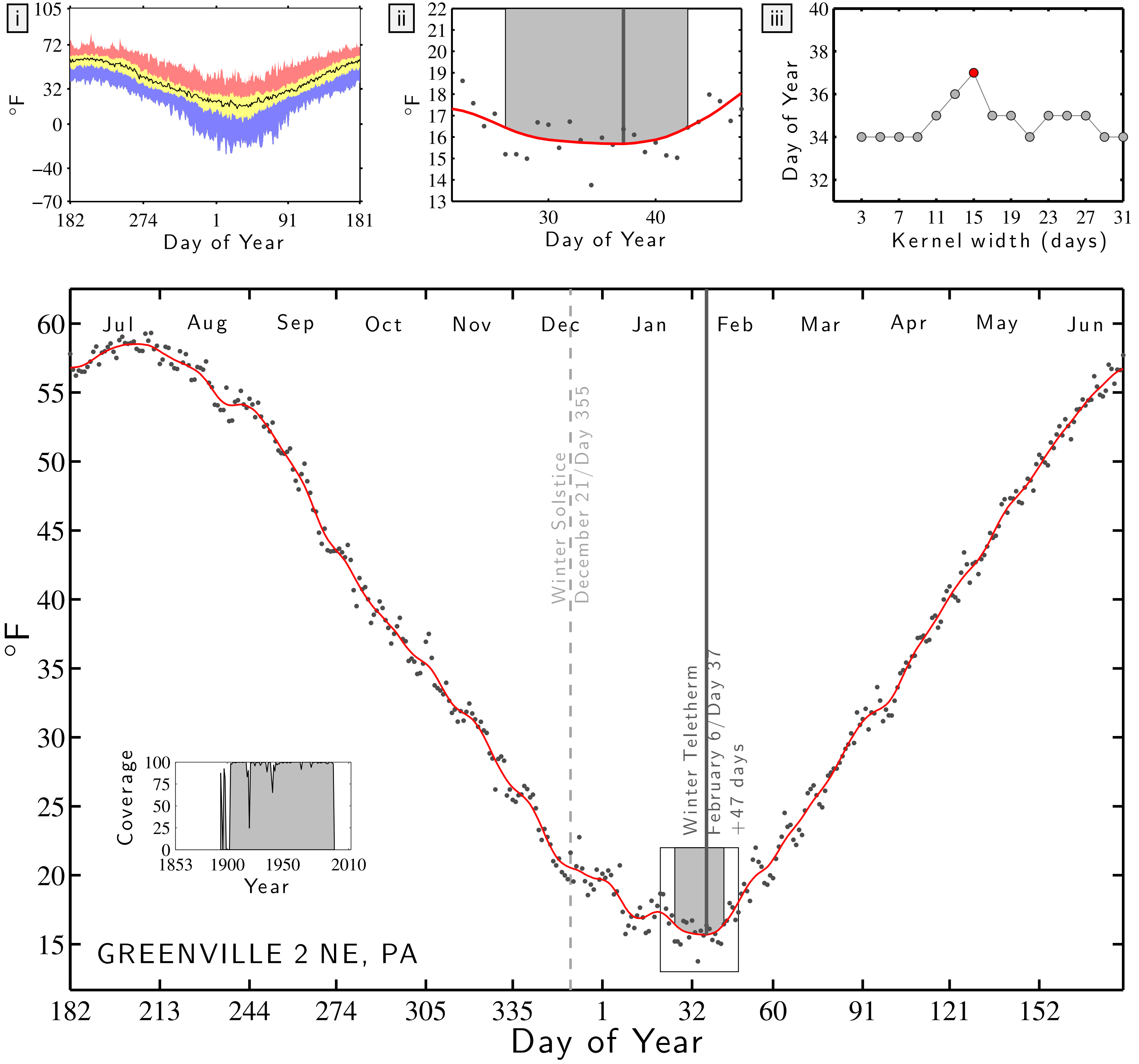

i
ii
iii
°F
Day of Year
Kernel width (days)
Jul
Aug
Sep
Oct
Nov
Dec
Jan
Feb
Mar
Apr
May
Jun
Winter Solstice
December 21/Day 355
Winter Teletherm
February 6/Day 37
+47 days
Coverage
Year
GREENVILLE 2 NE, PA

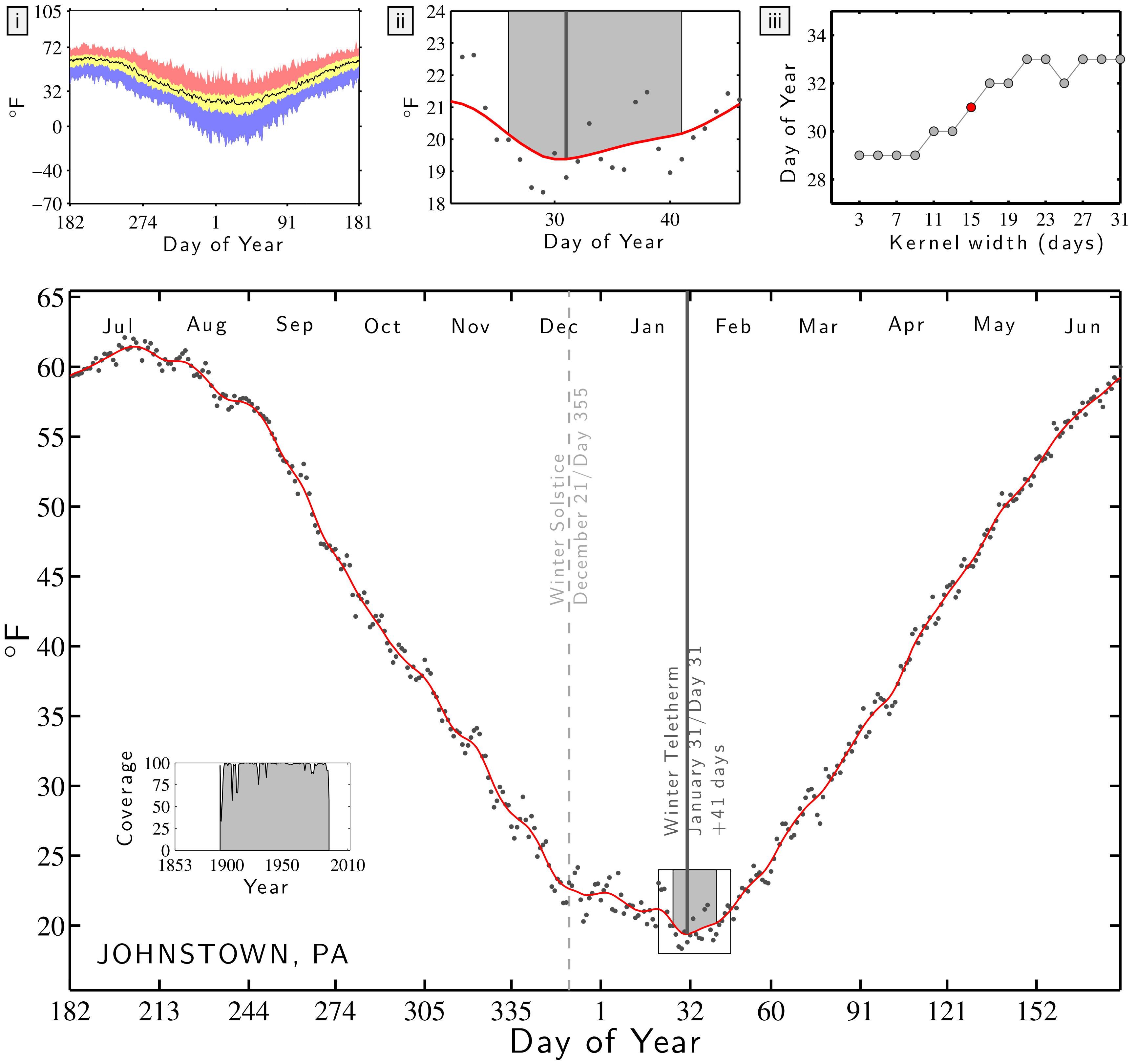

i
ii
iii
°F
Day of Year
Kernel width (days)
Jul
Aug
Sep
Oct
Nov
Dec
Jan
Feb
Mar
Apr
May
Jun
Winter Solstice
December 21/Day 355
Winter Teletherm
January 31/Day 31
+41 days
Coverage
Year
JOHNSTOWN, PA

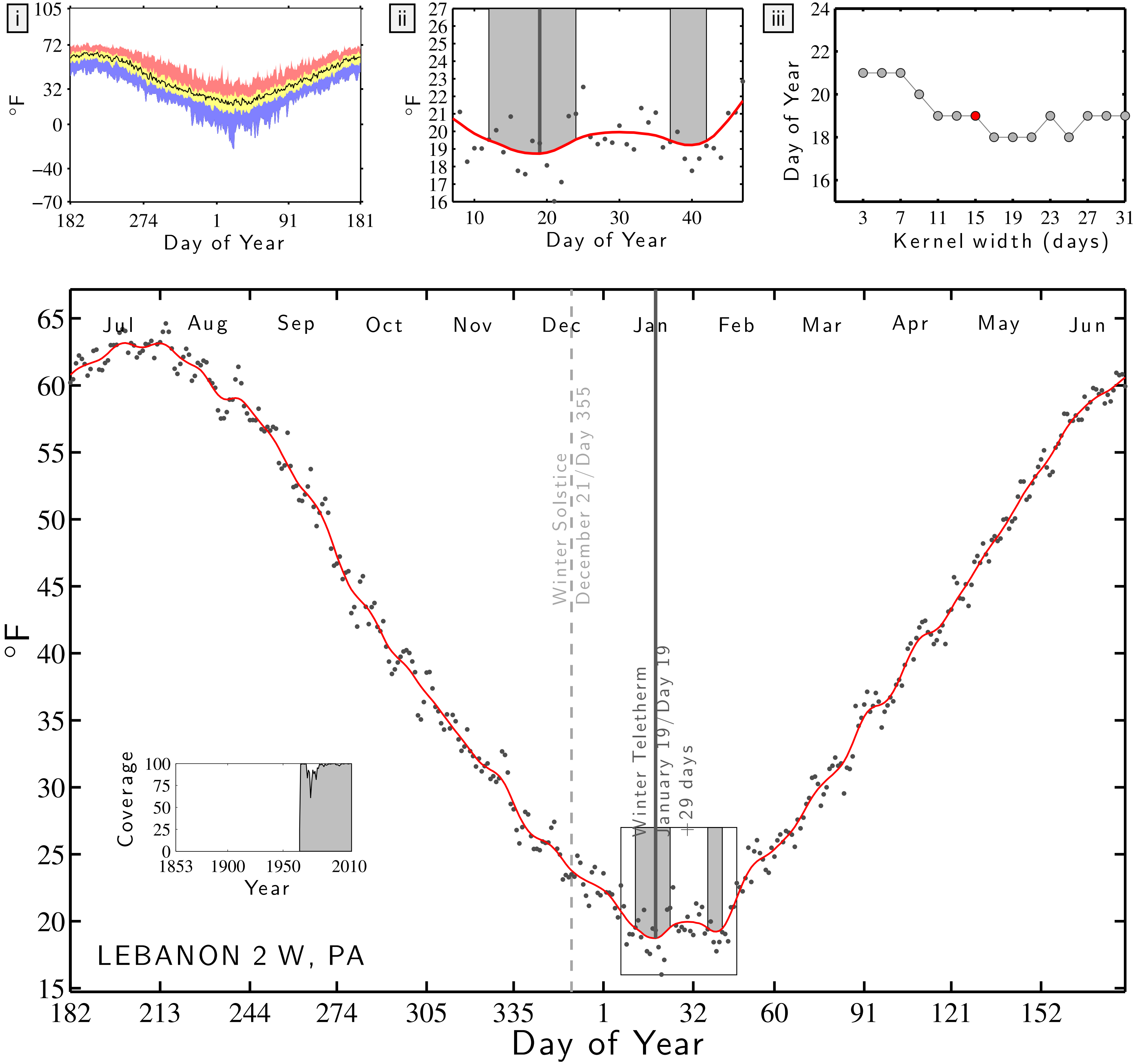

i
°F
Day of Year
ii
°F
Day of Year
iii
Day of Year
Kernel width (days)
Jul
Aug
Sep
Oct
Nov
Dec
Jan
Feb
Mar
Apr
May
Jun
Winter Solstice
December 21/Day 355
Winter Teletherm
January 19/Day 19
+29 days
Coverage
Year
LEBANON 2 W, PA
°F
Day of Year

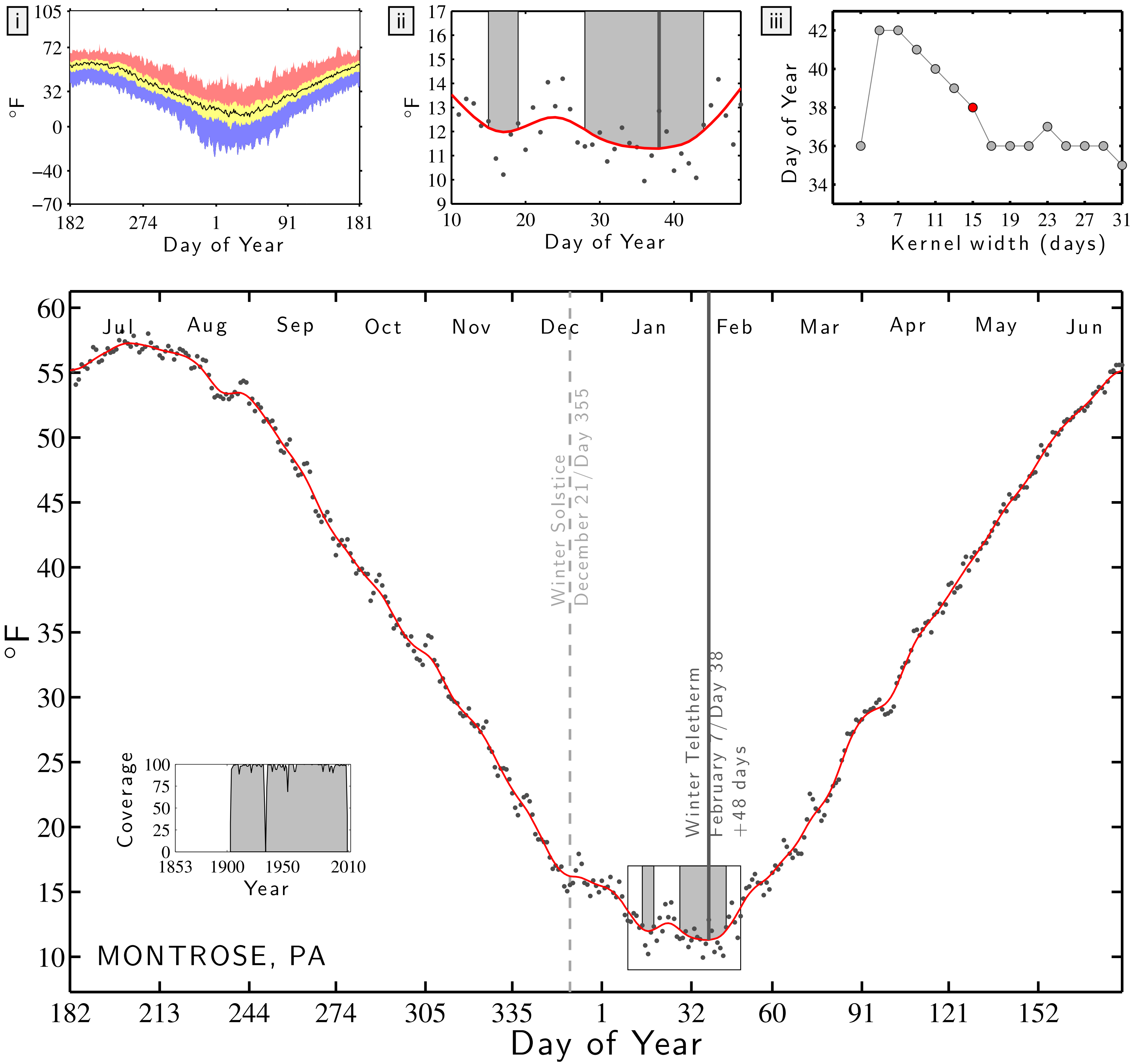

i
ii
iii
°F
Day of Year
Kernel width (days)
Jul Aug Sep Oct Nov Dec Jan Feb Mar Apr May Jun
Winter Solstice
December 21/Day 355
Winter Teletherm
February 7/Day 38
+48 days
Coverage
Year
MONTROSE, PA

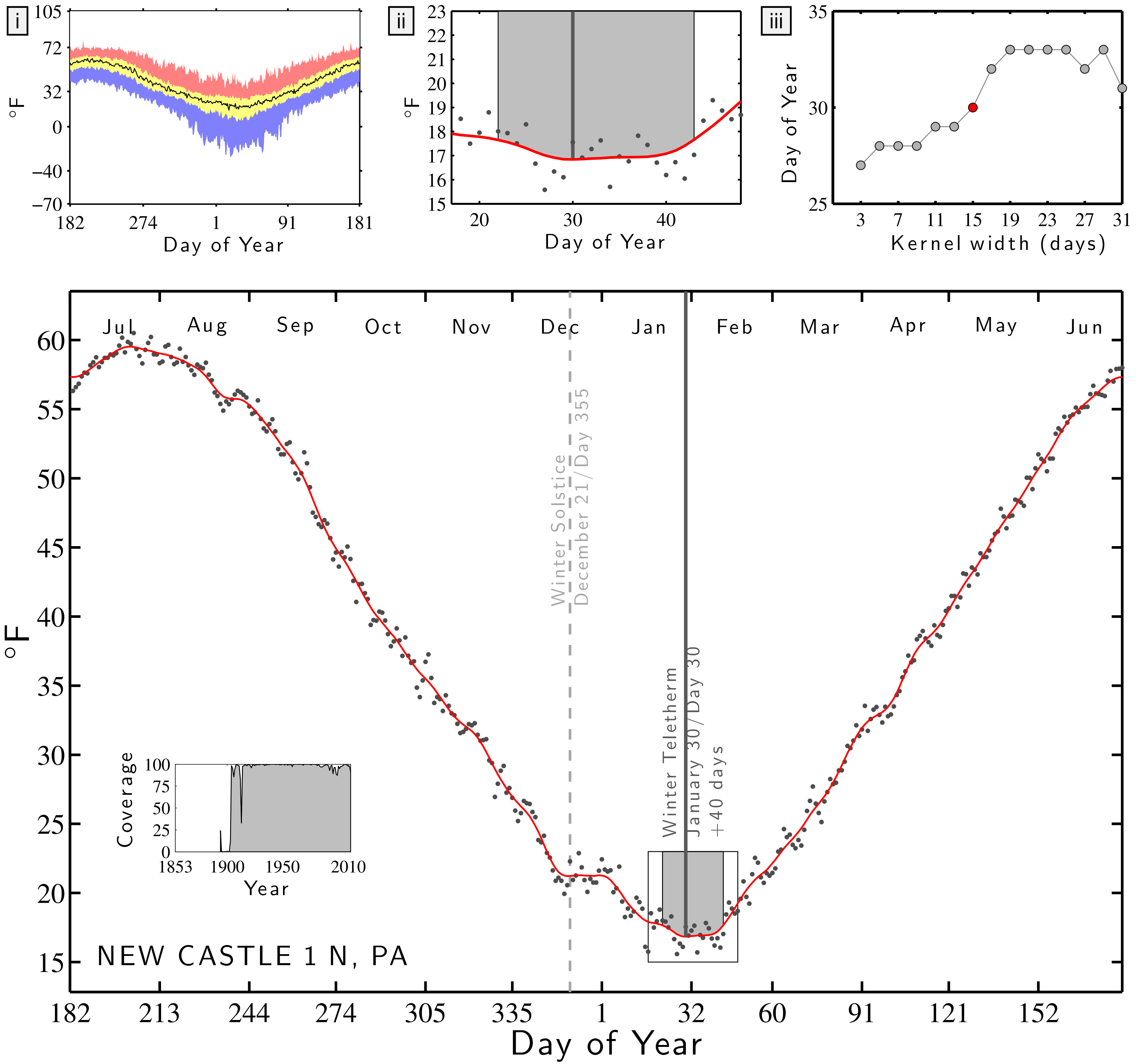

i
°F
Day of Year
ii
°F
Day of Year
iii
Day of Year
Kernel width (days)
Jul
Aug
Sep
Oct
Nov
Dec
Jan
Feb
Mar
Apr
May
Jun
Winter Solstice
December 21/Day 355
Winter Teletherm
January 30/Day 30
+40 days
Coverage
Year
NEW CASTLE 1 N, PA
°F
Day of Year

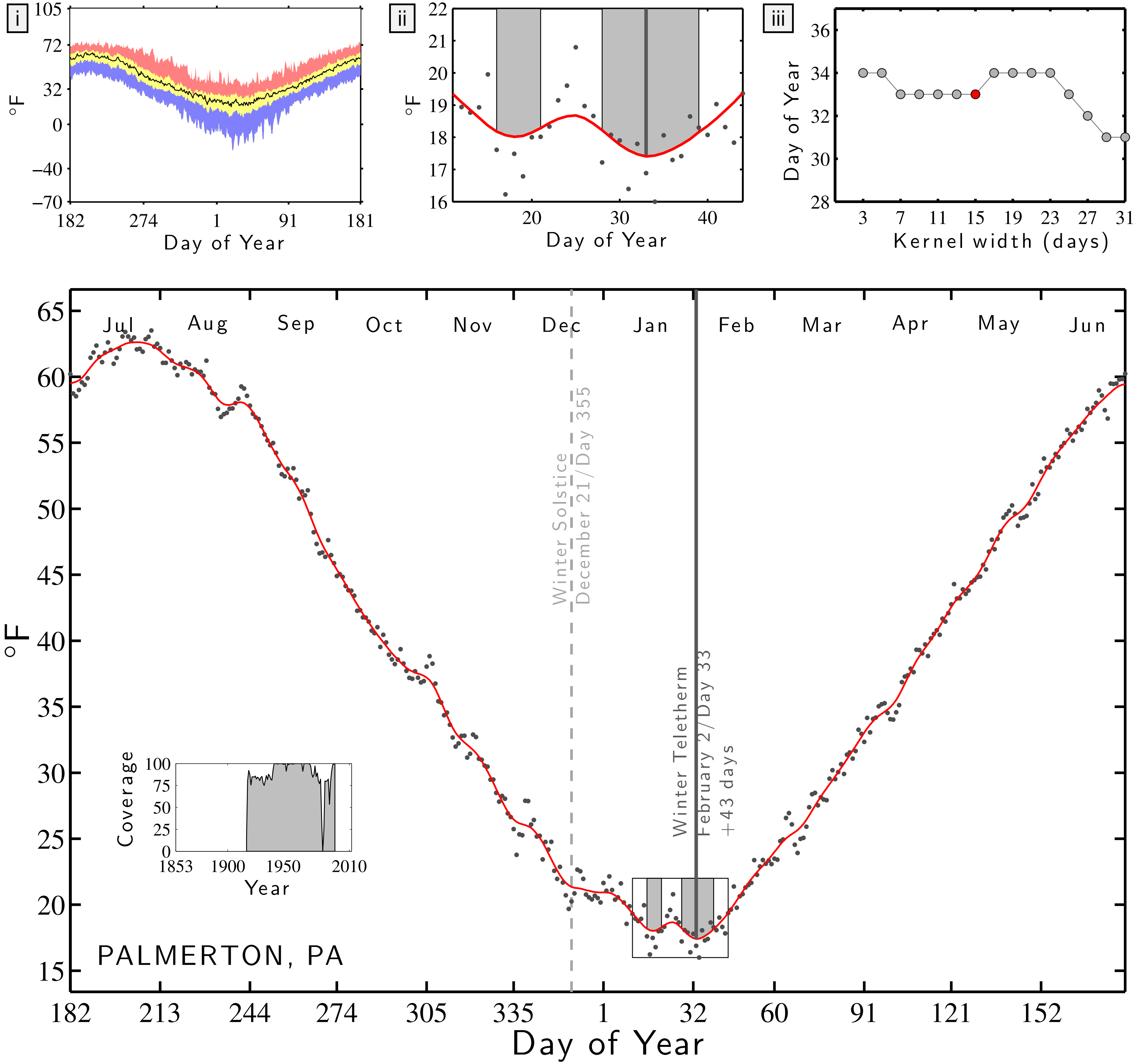

i
ii
iii
°F
Day of Year
Day of Year
Kernel width (days)
Jul
Aug
Sep
Oct
Nov
Dec
Jan
Feb
Mar
Apr
May
Jun
Winter Solstice
December 21/Day 355
Winter Teletherm
February 2/Day 33
+43 days
Coverage
Year
PALMERTON, PA

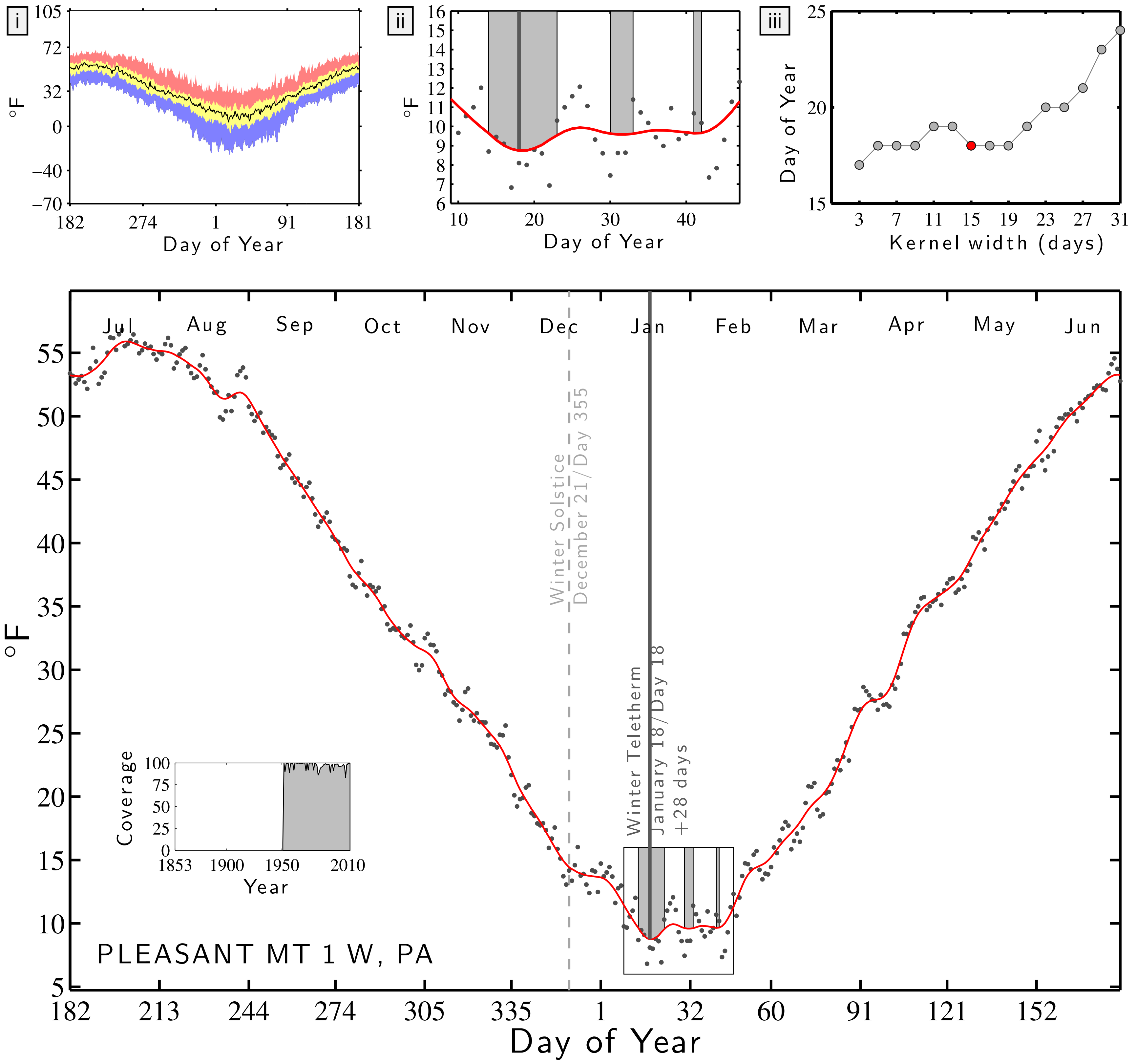

i
°F
Day of Year
ii
°F
Day of Year
iii
Day of Year
Kernel width (days)
Jul
Aug
Sep
Oct
Nov
Dec
Jan
Feb
Mar
Apr
May
Jun
Winter Solstice
December 21/Day 355
Winter Teletherm
January 18/Day 18
+28 days
Coverage
Year
PLEASANT MT 1 W, PA
°F
Day of Year

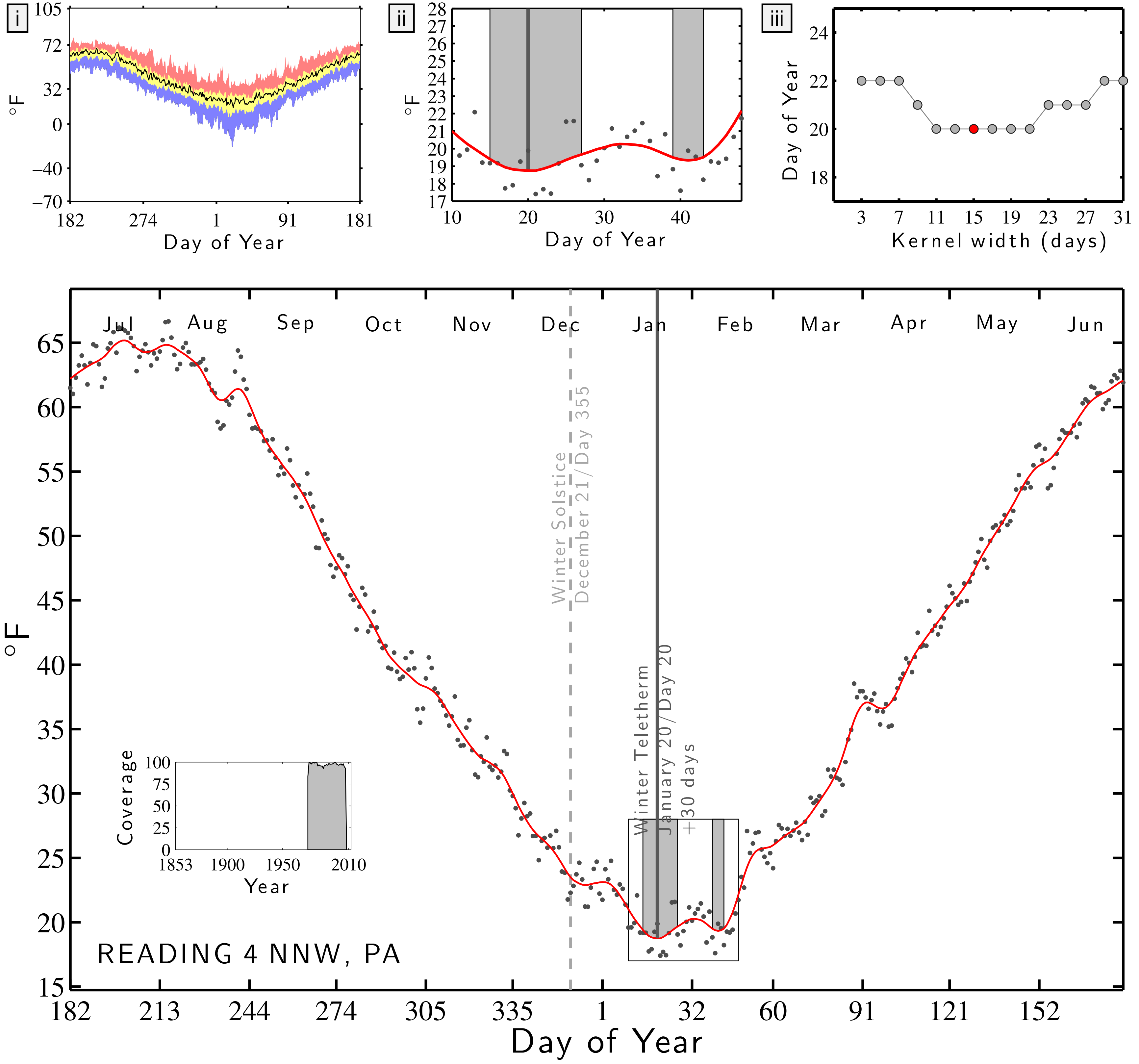
i
ii
iii
Day of Year
Kernel width (days)
Jul Aug Sep Oct Nov Dec Jan Feb Mar Apr May Jun
Winter Solstice
December 21/Day 355
Winter Teletherm
January 20/Day 20
+30 days
Coverage
Year
READING 4 NNW, PA
°F

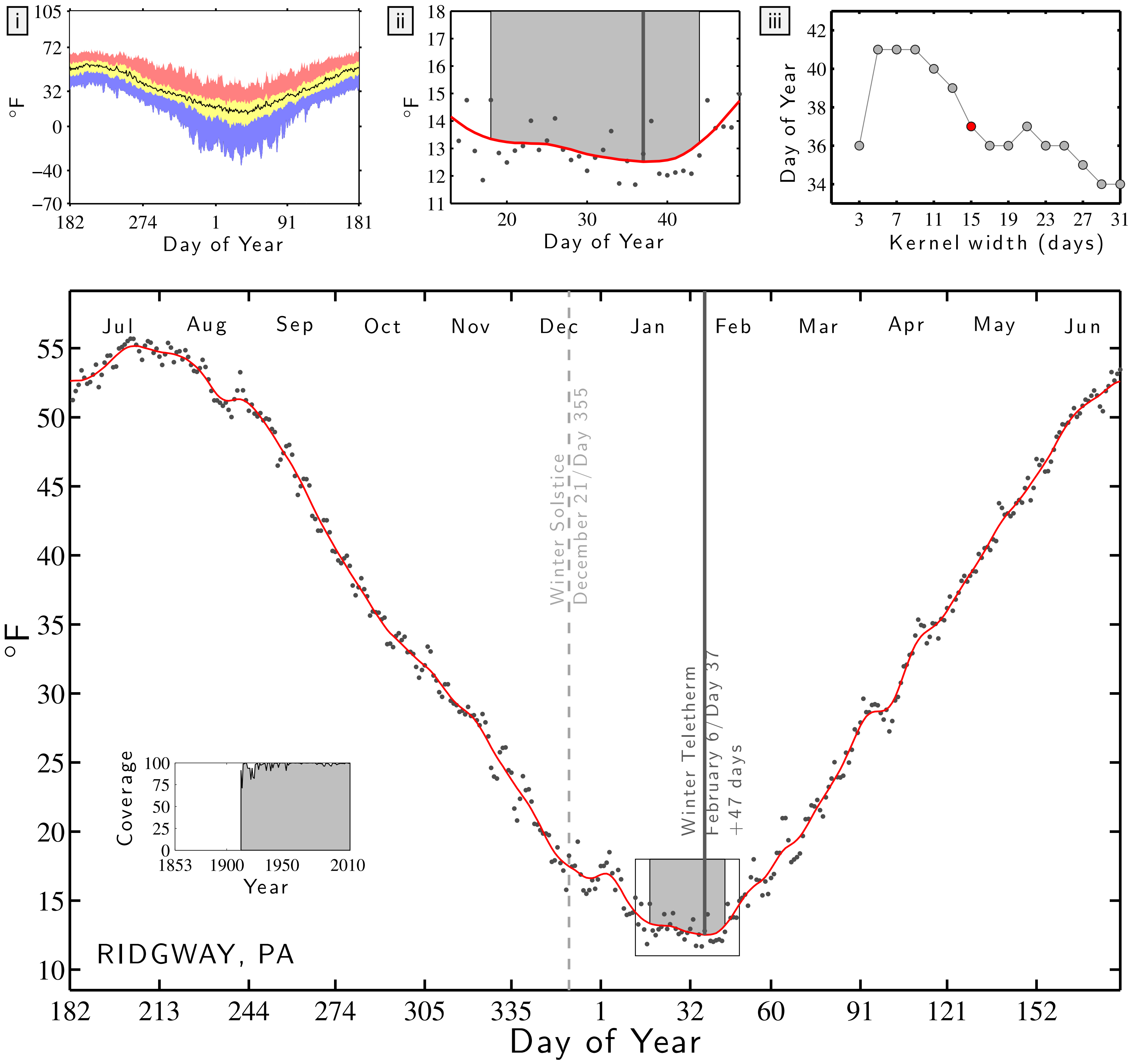

i
ii
iii
°F
Day of Year
Kernel width (days)
Day of Year
Jul
Aug
Sep
Oct
Nov
Dec
Jan
Feb
Mar
Apr
May
Jun
Winter Solstice
December 21/Day 355
Winter Teletherm
February 6/Day 37
+47 days
Coverage
Year
RIDGWAY, PA

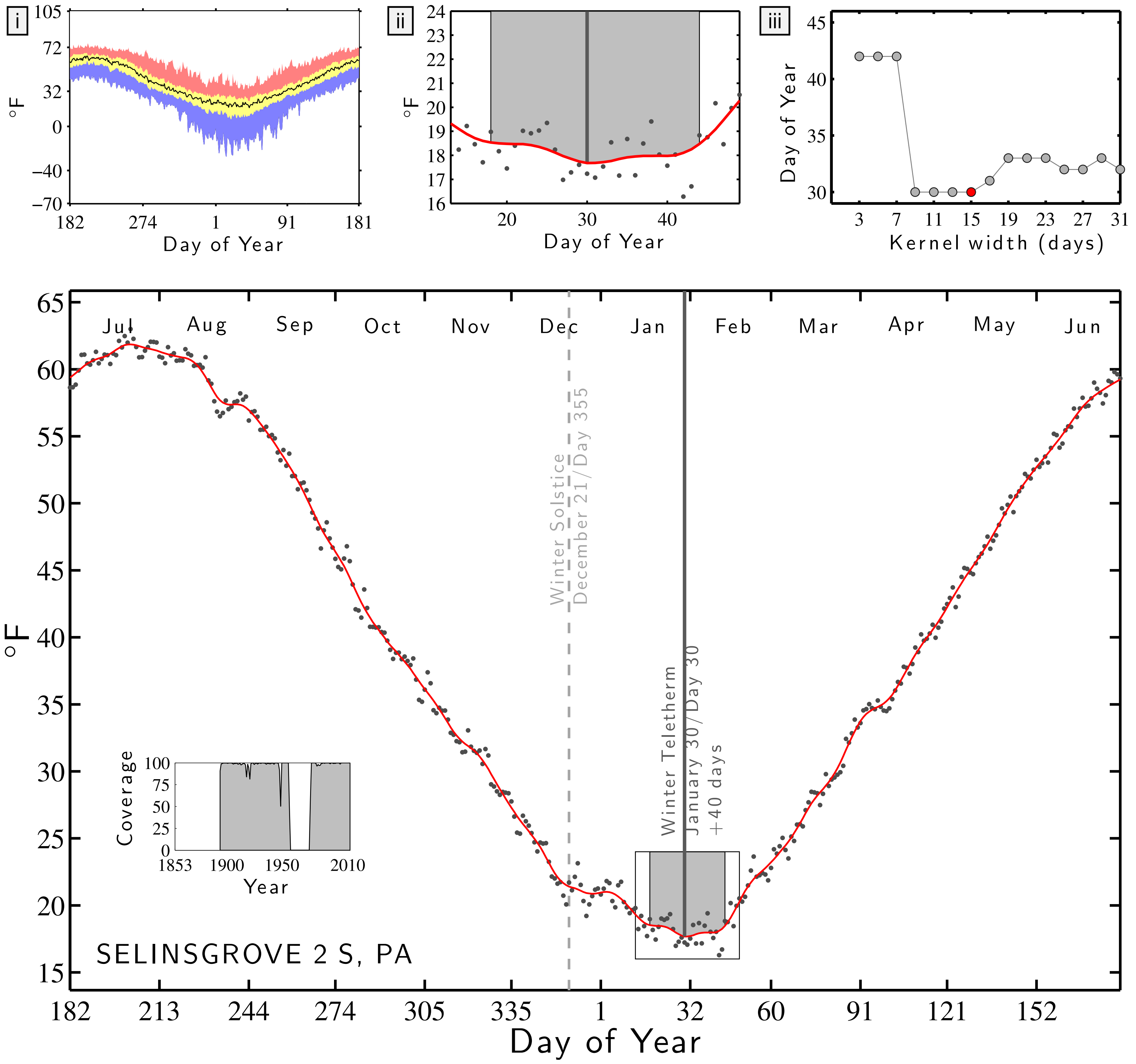

i
°F
Day of Year
ii
°F
Day of Year
iii
Day of Year
Kernel width (days)
Jul
Aug
Sep
Oct
Nov
Dec
Jan
Feb
Mar
Apr
May
Jun
Winter Solstice
December 21/Day 355
Winter Teletherm
January 30/Day 30
+40 days
Coverage
Year
SELINSGROVE 2 S, PA
°F
Day of Year

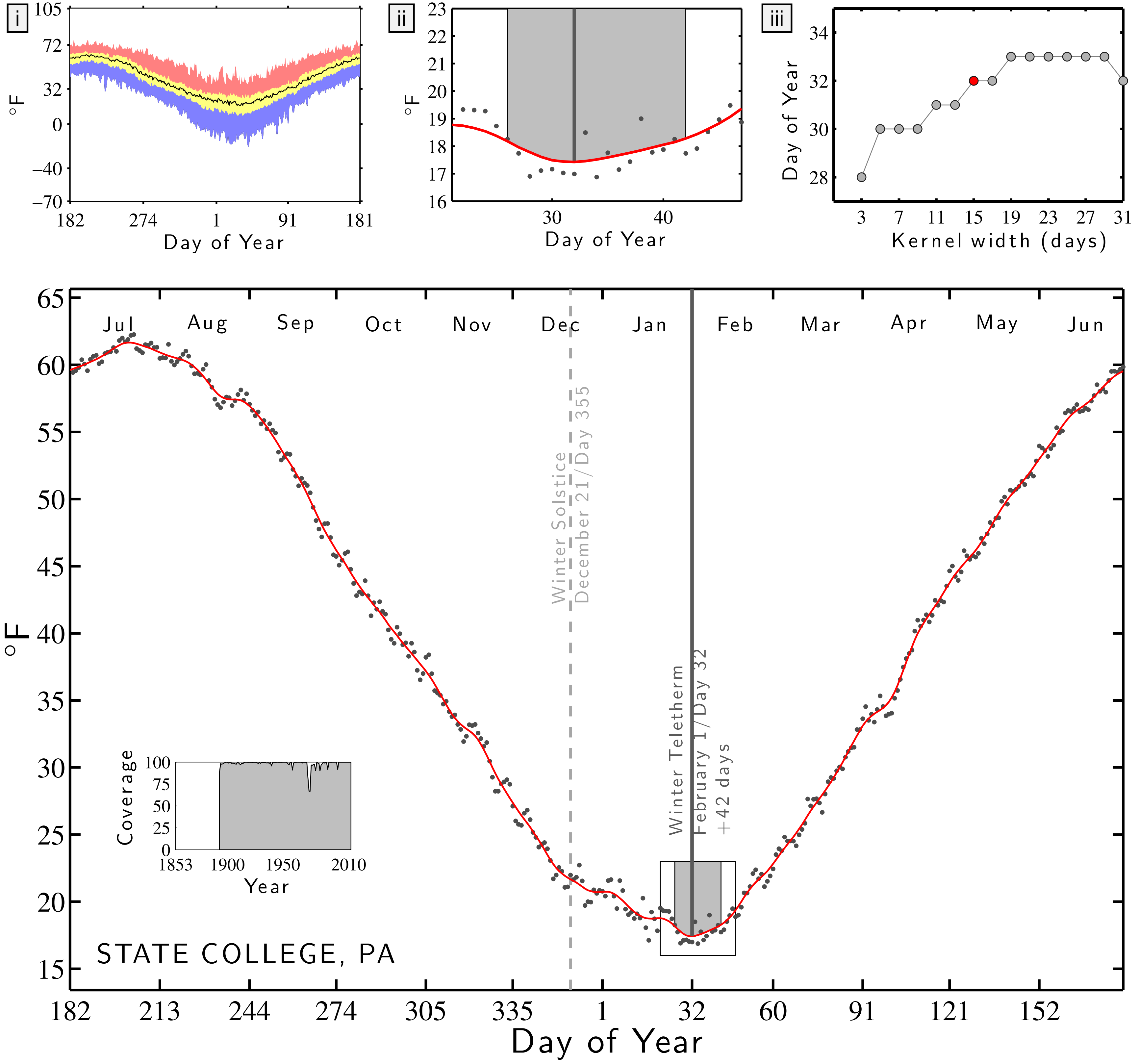

i
°F
Day of Year
ii
°F
Day of Year
iii
Day of Year
Kernel width (days)
Jul
Aug
Sep
Oct
Nov
Dec
Jan
Feb
Mar
Apr
May
Jun
Winter Solstice
December 21/Day 355
Winter Teletherm
February 1/Day 32
+42 days
Coverage
Year
STATE COLLEGE, PA
°F
Day of Year

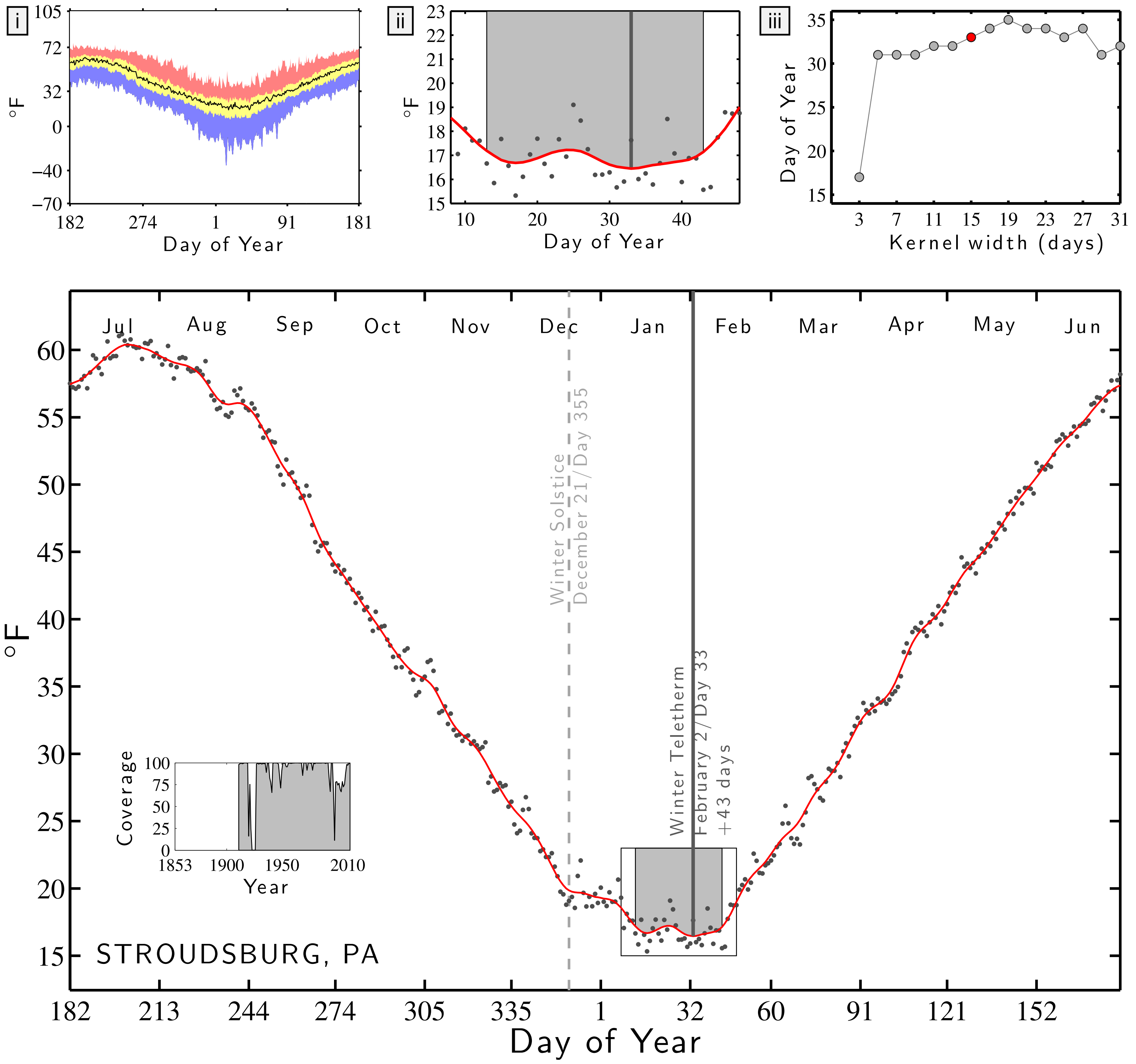

i
ii
iii
Day of Year
Kernel width (days)
Winter Solstice
December 21/Day 355
Winter Teletherm
February 2/Day 33
+43 days
Coverage
Year
STROUDSBURG, PA
Jul
Aug
Sep
Oct
Nov
Dec
Jan
Feb
Mar
Apr
May
Jun
°F
Day of Year

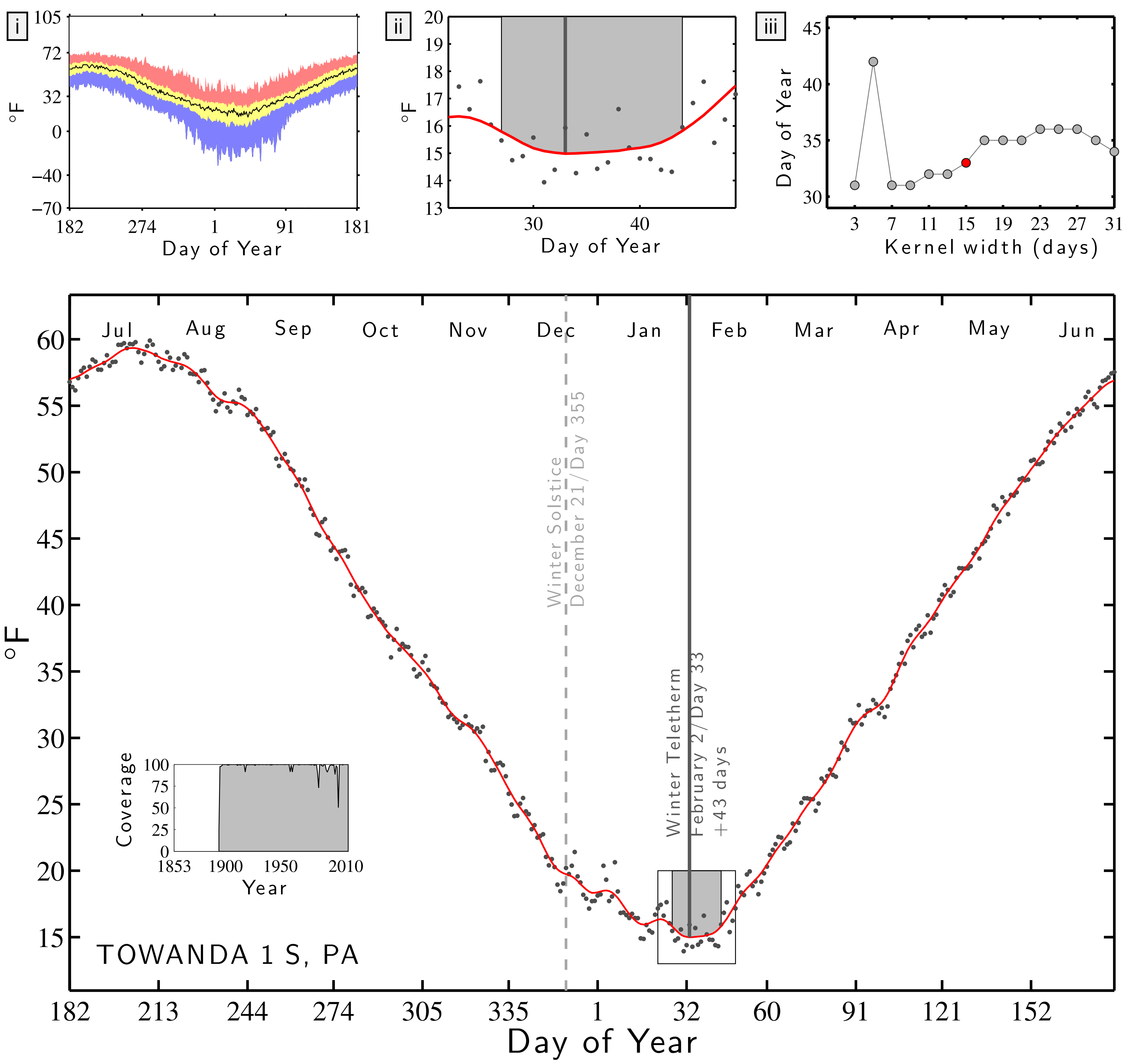

i
°F
Day of Year
ii
°F
Day of Year
iii
Day of Year
Kernel width (days)
Jul
Aug
Sep
Oct
Nov
Dec
Jan
Feb
Mar
Apr
May
Jun
Winter Solstice
December 21/Day 355
Winter Teletherm
February 2/Day 33
+43 days
Coverage
Year
TOWANDA 1 S, PA
°F
Day of Year

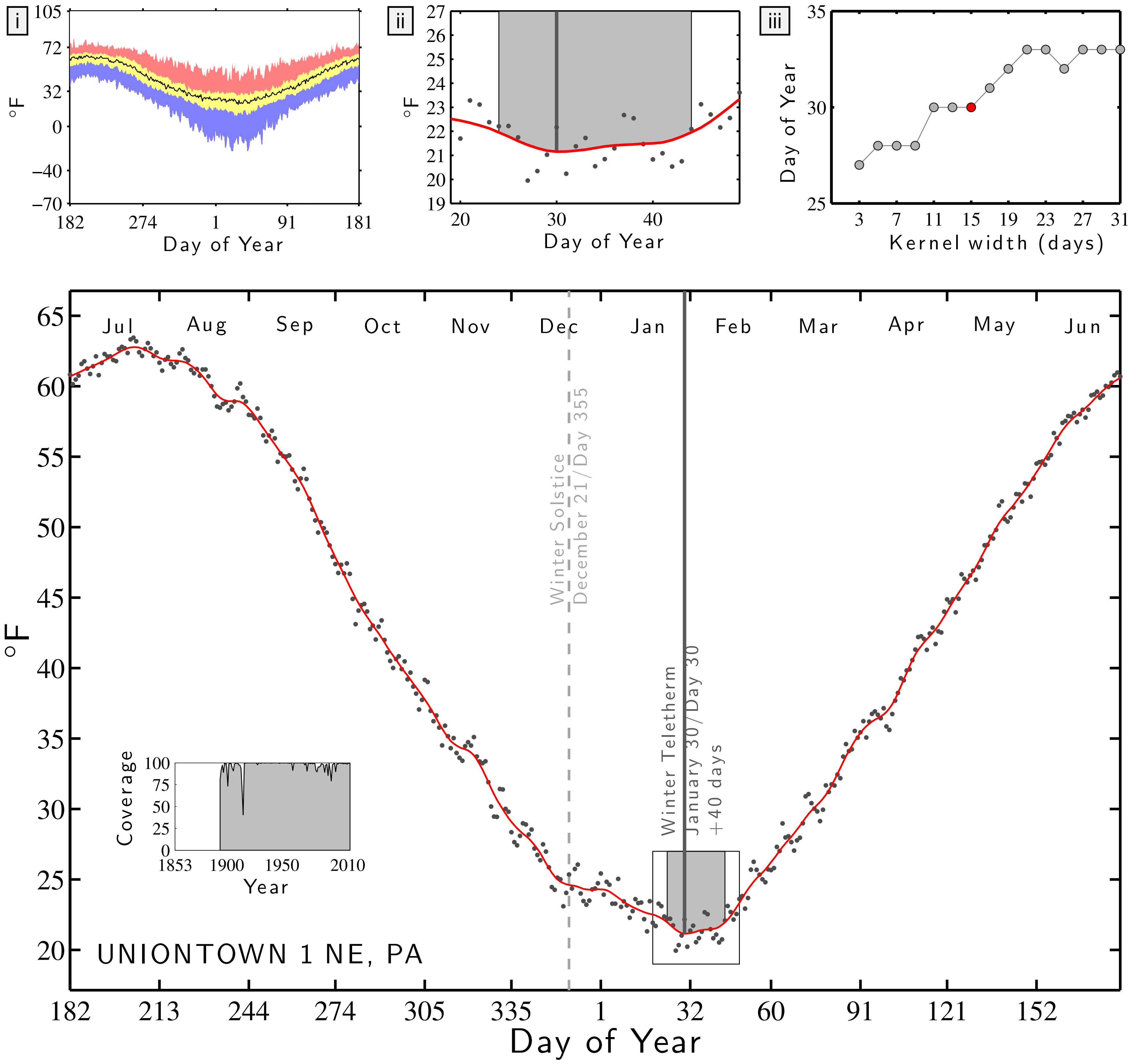

i
°F
Day of Year
ii
°F
Day of Year
iii
Day of Year
Kernel width (days)
Jul
Aug
Sep
Oct
Nov
Dec
Jan
Feb
Mar
Apr
May
Jun
Winter Solstice
December 21/Day 355
Winter Teletherm
January 30/Day 30
+40 days
Coverage
Year
UNIONTOWN 1 NE, PA
°F
Day of Year

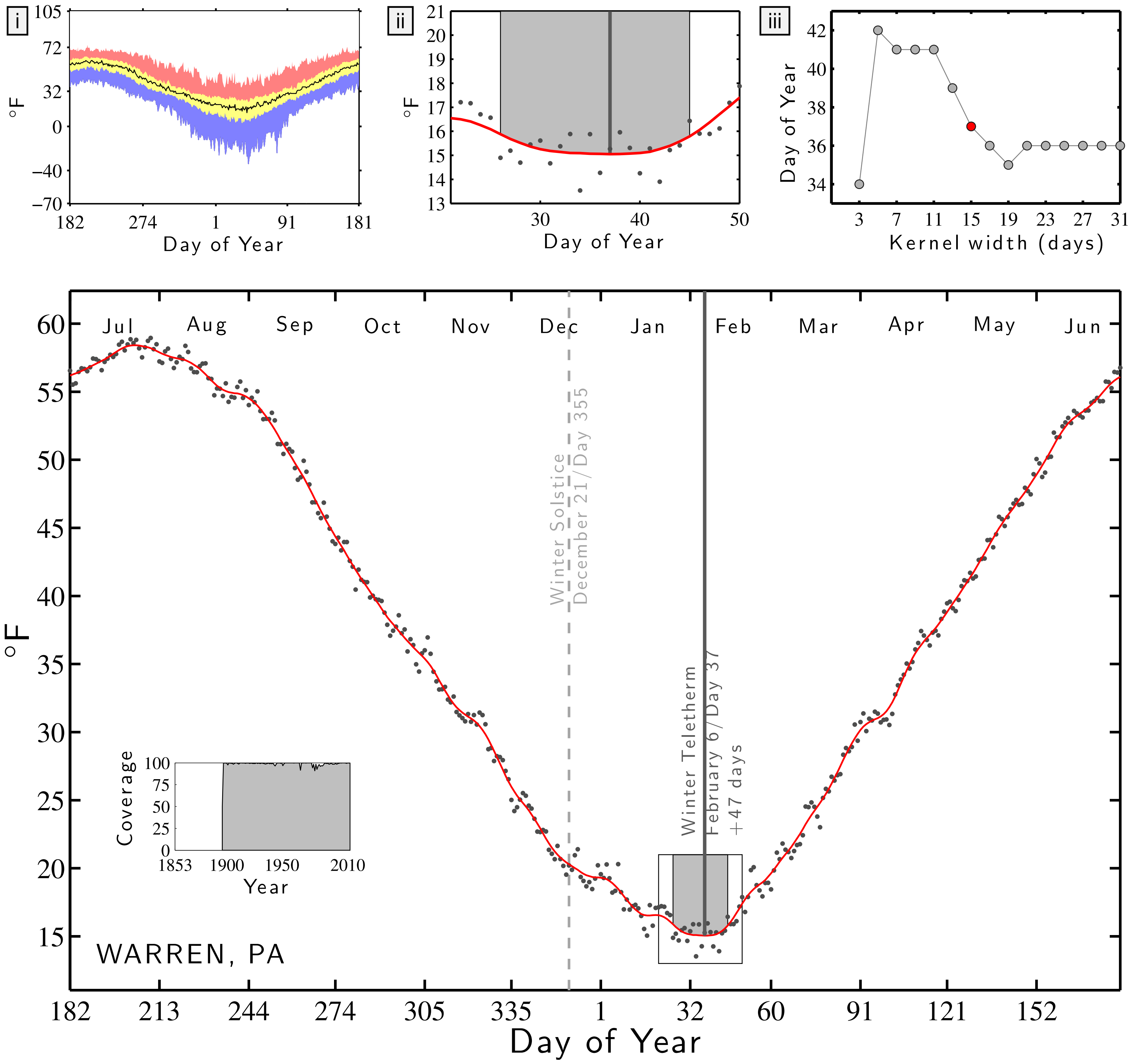

i
ii
iii
°F
Day of Year
Day of Year
Kernel width (days)
Jul
Aug
Sep
Oct
Nov
Dec
Jan
Feb
Mar
Apr
May
Jun
Winter Solstice
December 21/Day 355
Winter Teletherm
February 6/Day 37
+47 days
Coverage
Year
WARREN, PA

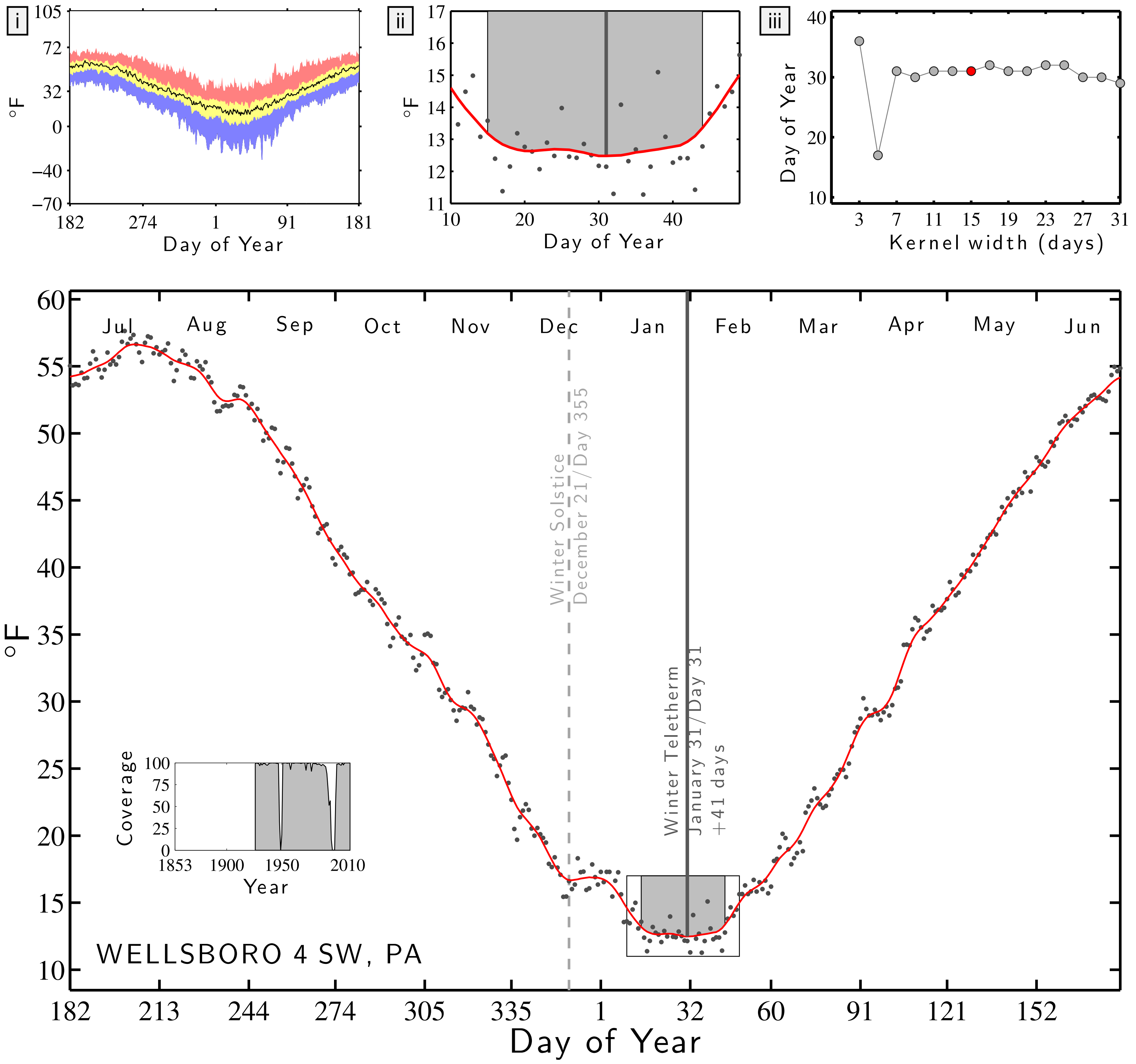

i
°F
Day of Year
ii
°F
Day of Year
iii
Day of Year
Kernel width (days)
Jul
Aug
Sep
Oct
Nov
Dec
Jan
Feb
Mar
Apr
May
Jun
Winter Solstice
December 21/Day 355
Winter Teletherm
January 31/Day 31
+41 days
Coverage
Year
WELLSBORO 4 SW, PA
°F
Day of Year

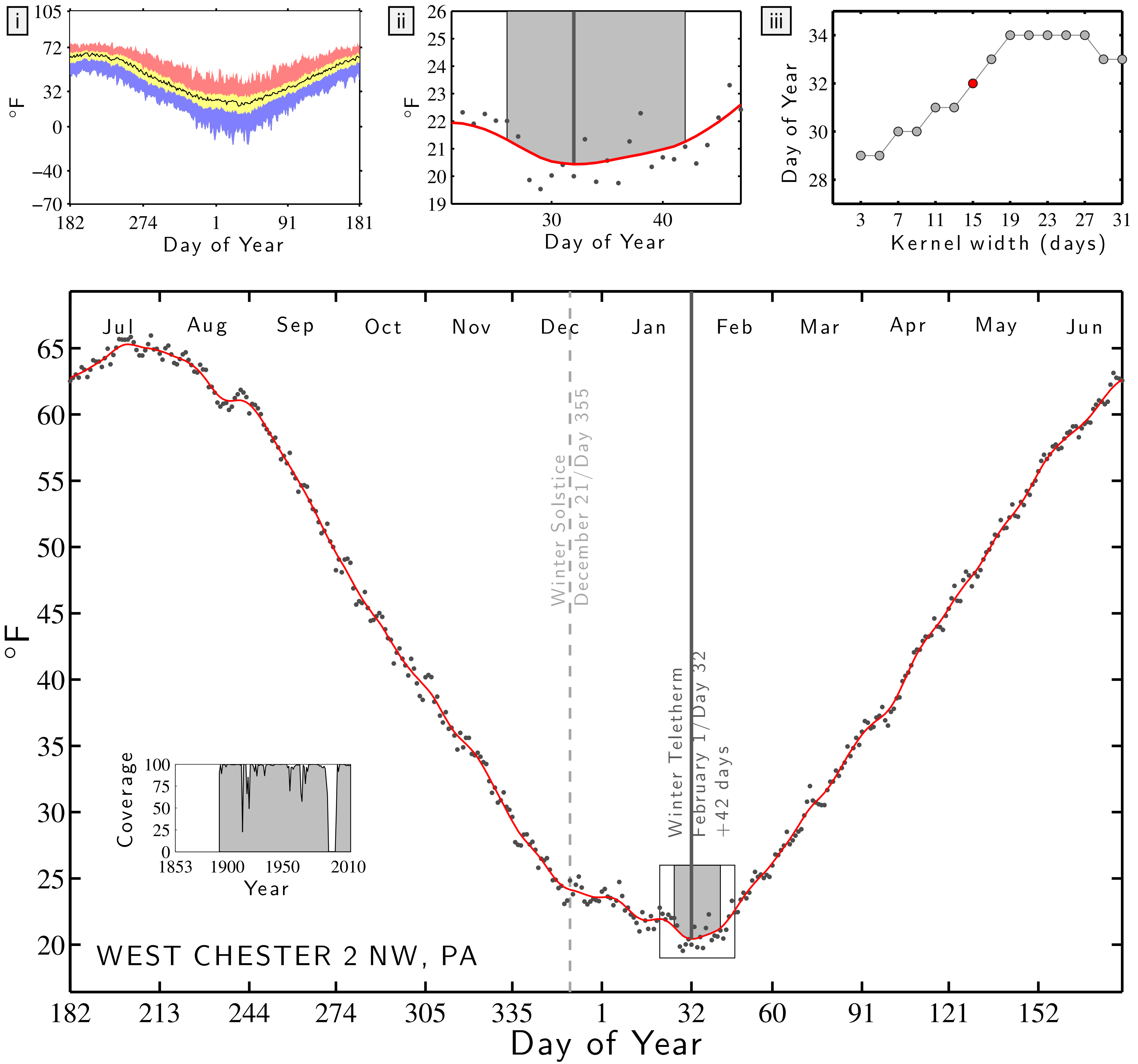

i
°F
Day of Year
ii
°F
Day of Year
iii
Day of Year
Kernel width (days)
Jul
Aug
Sep
Oct
Nov
Dec
Jan
Feb
Mar
Apr
May
Jun
Winter Solstice
December 21/Day 355
Winter Teletherm
February 1/Day 32
+42 days
Coverage
Year
WEST CHESTER 2 NW, PA
°F
Day of Year

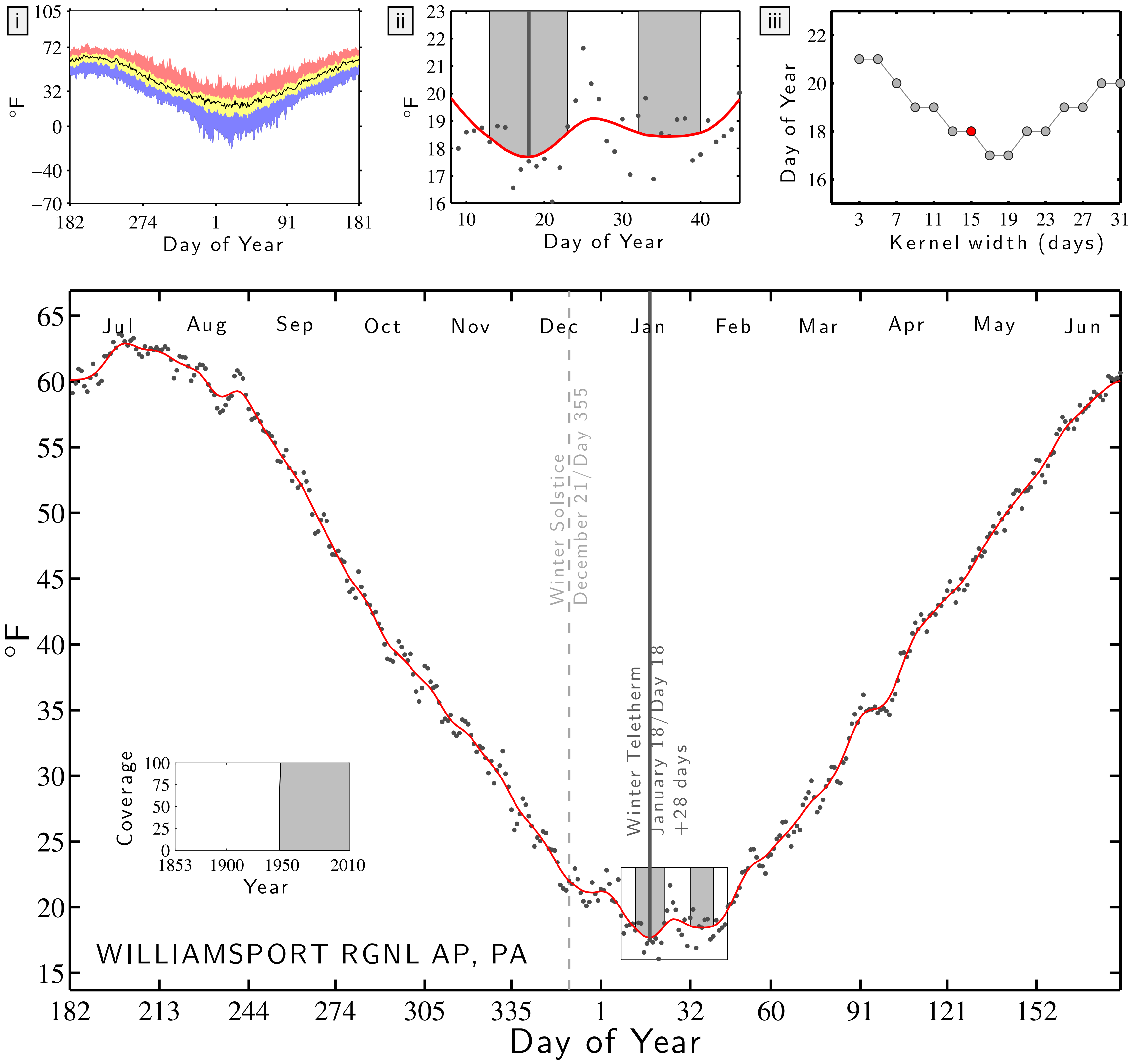

i
°F
Day of Year
ii
°F
Day of Year
iii
Day of Year
Kernel width (days)
Jul
Aug
Sep
Oct
Nov
Dec
Jan
Feb
Mar
Apr
May
Jun
Winter Solstice
December 21/Day 355
Winter Teletherm
January 18/Day 18
+28 days
Coverage
Year
WILLIAMSPORT RGNL AP, PA
°F
Day of Year

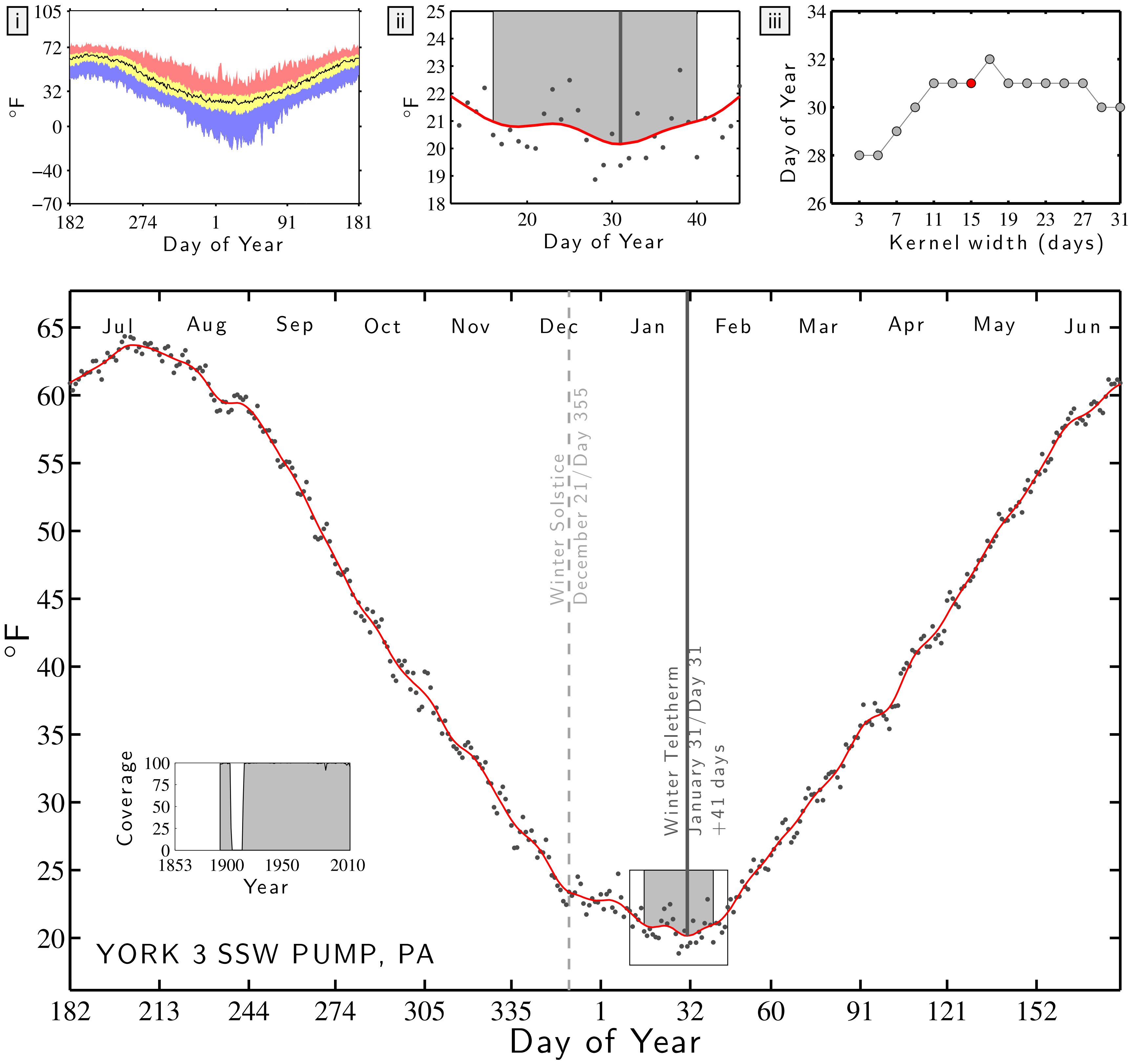

i
105
72
32
0
−40
−70
°F
182
274
1
91
181
Day of Year
ii
25
24
23
22
21
20
19
18
°F
20
30
40
Day of Year
iii
34
32
30
28
26
Day of Year
3
7
11
15
19
23
27
31
Kernel width (days)
Jul
Aug
Sep
Oct
Nov
Dec
Jan
Feb
Mar
Apr
May
Jun
65
60
55
50
45
40
35
30
25
20
°F
Winter Solstice
December 21/Day 355
Winter Teletherm
January 31/Day 31
+41 days
Coverage
100
75
50
25
0
1853
1900
1950
2010
Year
YORK 3 SSW PUMP, PA
182
213
244
274
305
335
1
32
60
91
121
152
Day of Year

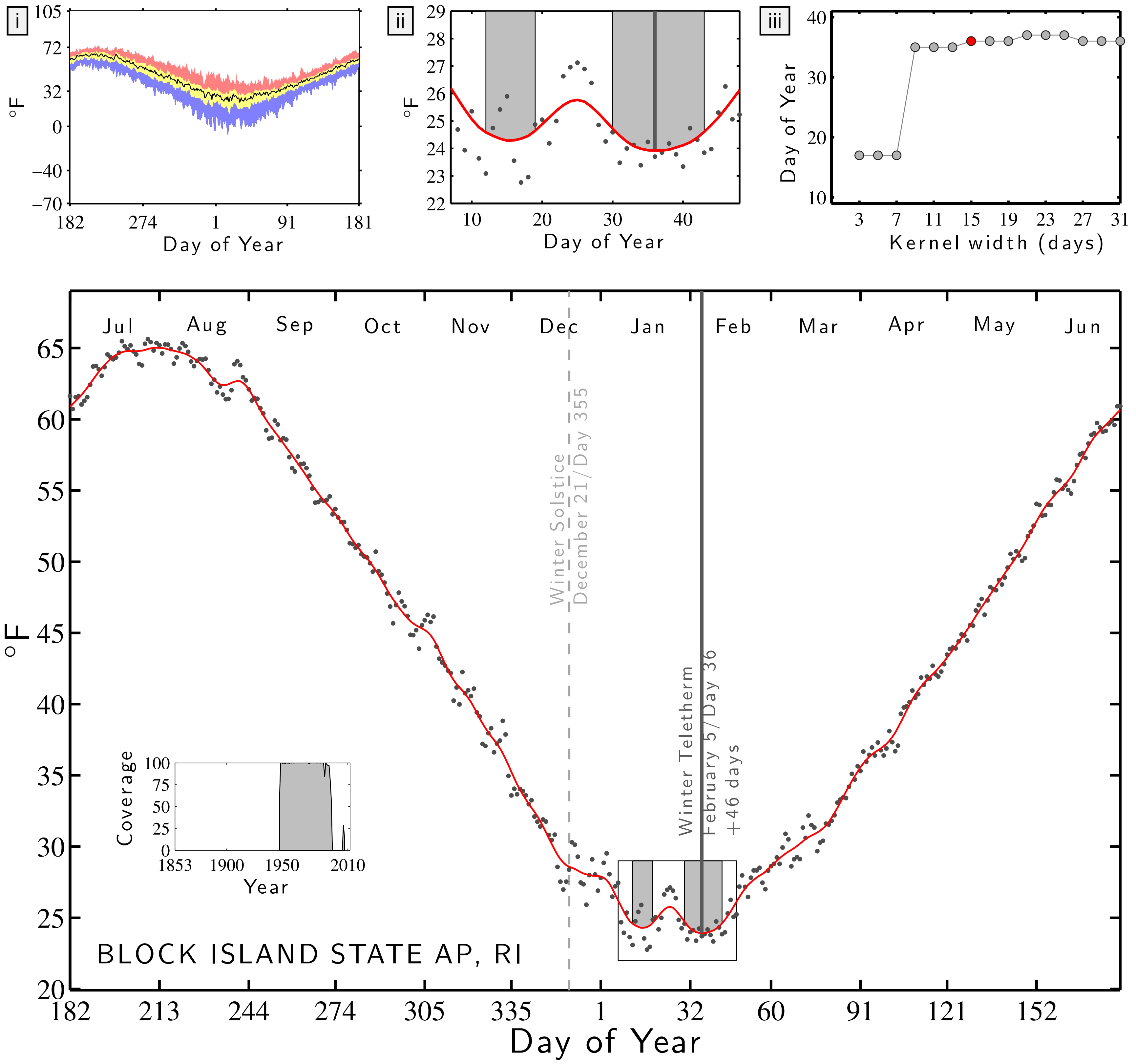

i
ii
iii
Day of Year
°F
Kernel width (days)
Jul
Aug
Sep
Oct
Nov
Dec
Jan
Feb
Mar
Apr
May
Jun
Winter Solstice
December 21/Day 355
Winter Teletherm
February 5/Day 36
+46 days
Coverage
Year
BLOCK ISLAND STATE AP, RI

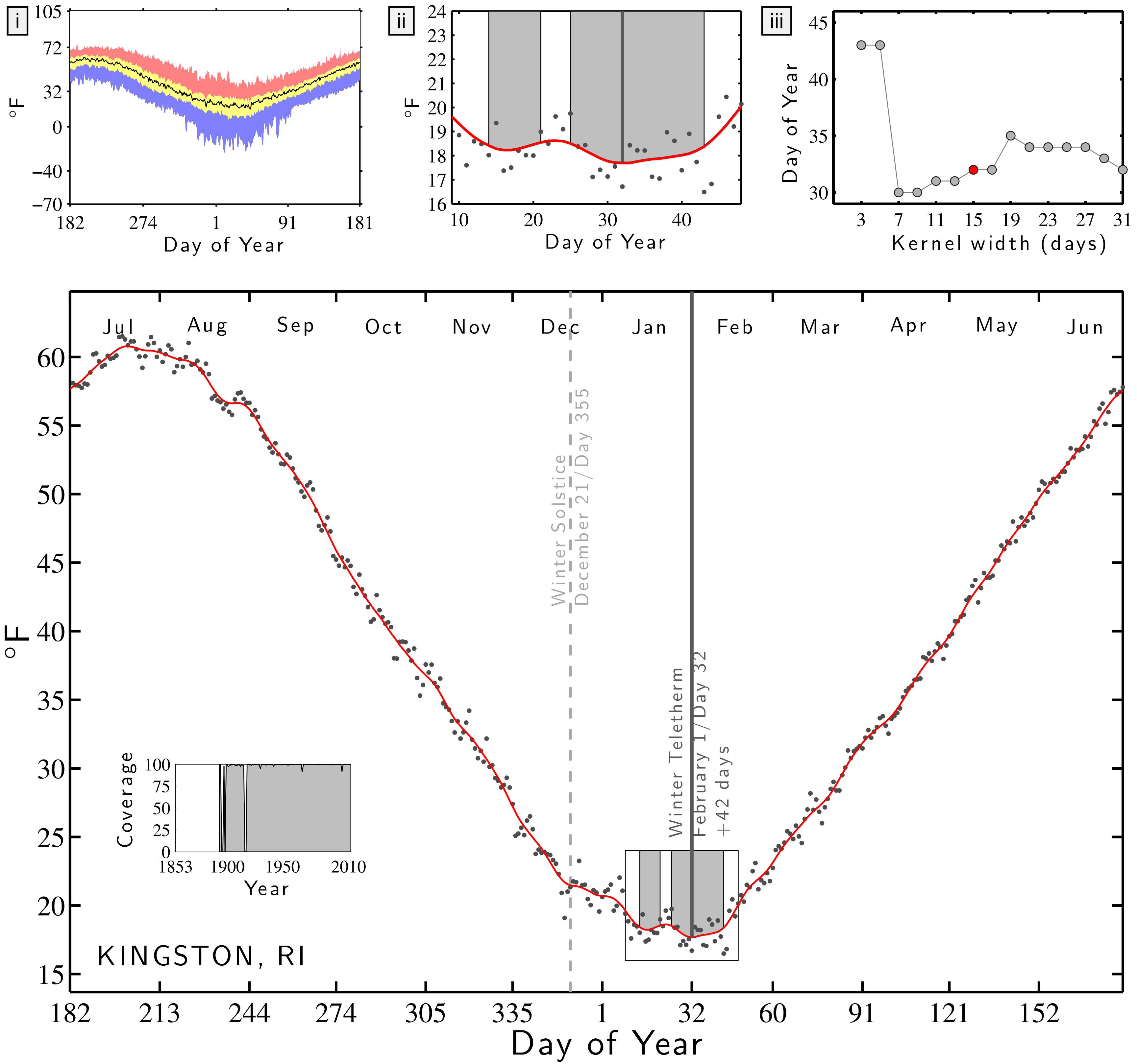

i
°F
Day of Year
ii
°F
Day of Year
iii
Day of Year
Kernel width (days)
Jul
Aug
Sep
Oct
Nov
Dec
Jan
Feb
Mar
Apr
May
Jun
Winter Solstice
December 21/Day 355
Winter Teletherm
February 1/Day 32
+42 days
Coverage
Year
KINGSTON, RI
°F
Day of Year

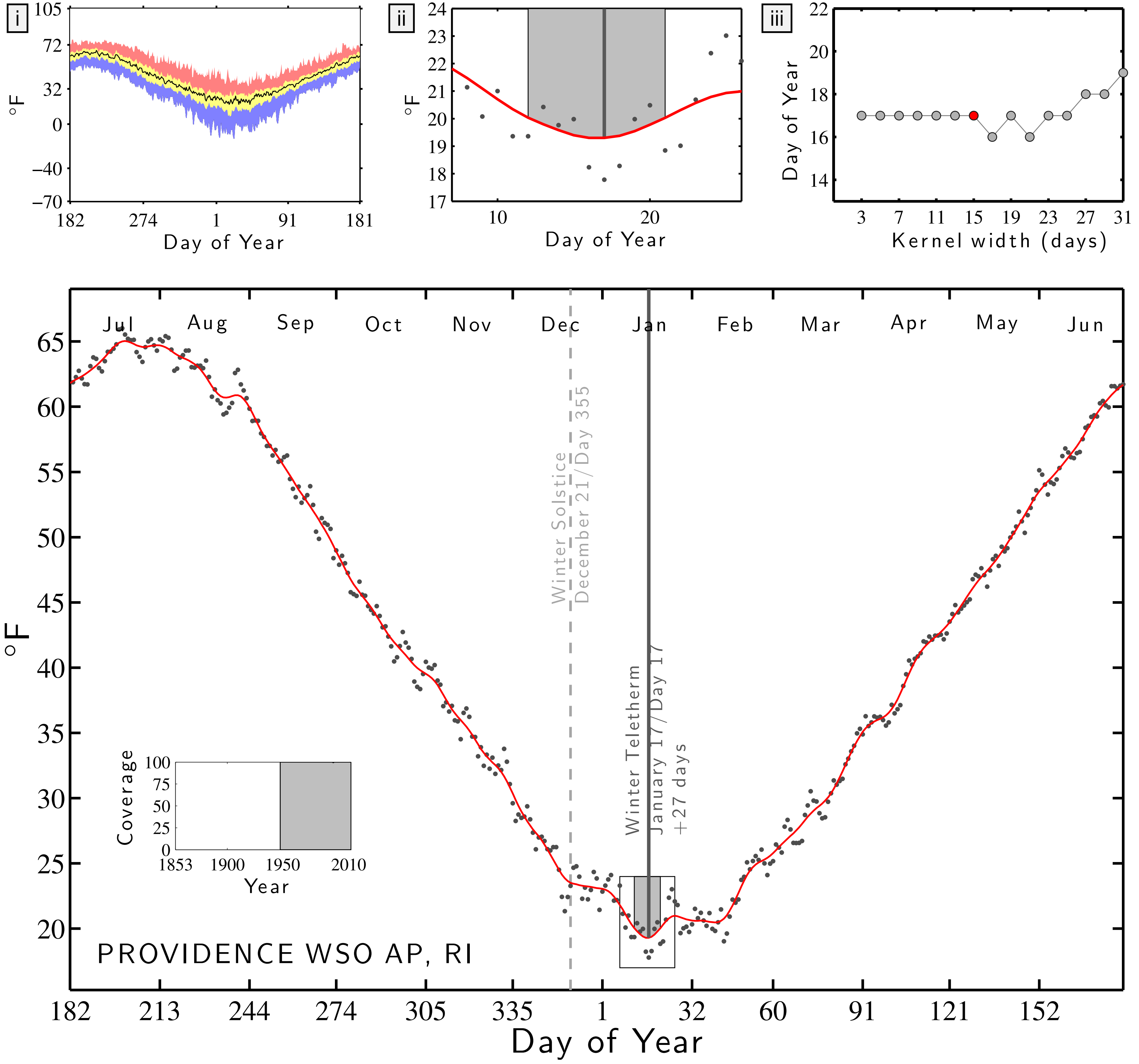

i
ii
iii
°F
Day of Year
Kernel width (days)
Jul
Aug
Sep
Oct
Nov
Dec
Jan
Feb
Mar
Apr
May
Jun
Winter Solstice
December 21/Day 355
Winter Teletherm
January 17/Day 17
+27 days
Coverage
Year
PROVIDENCE WSO AP, RI

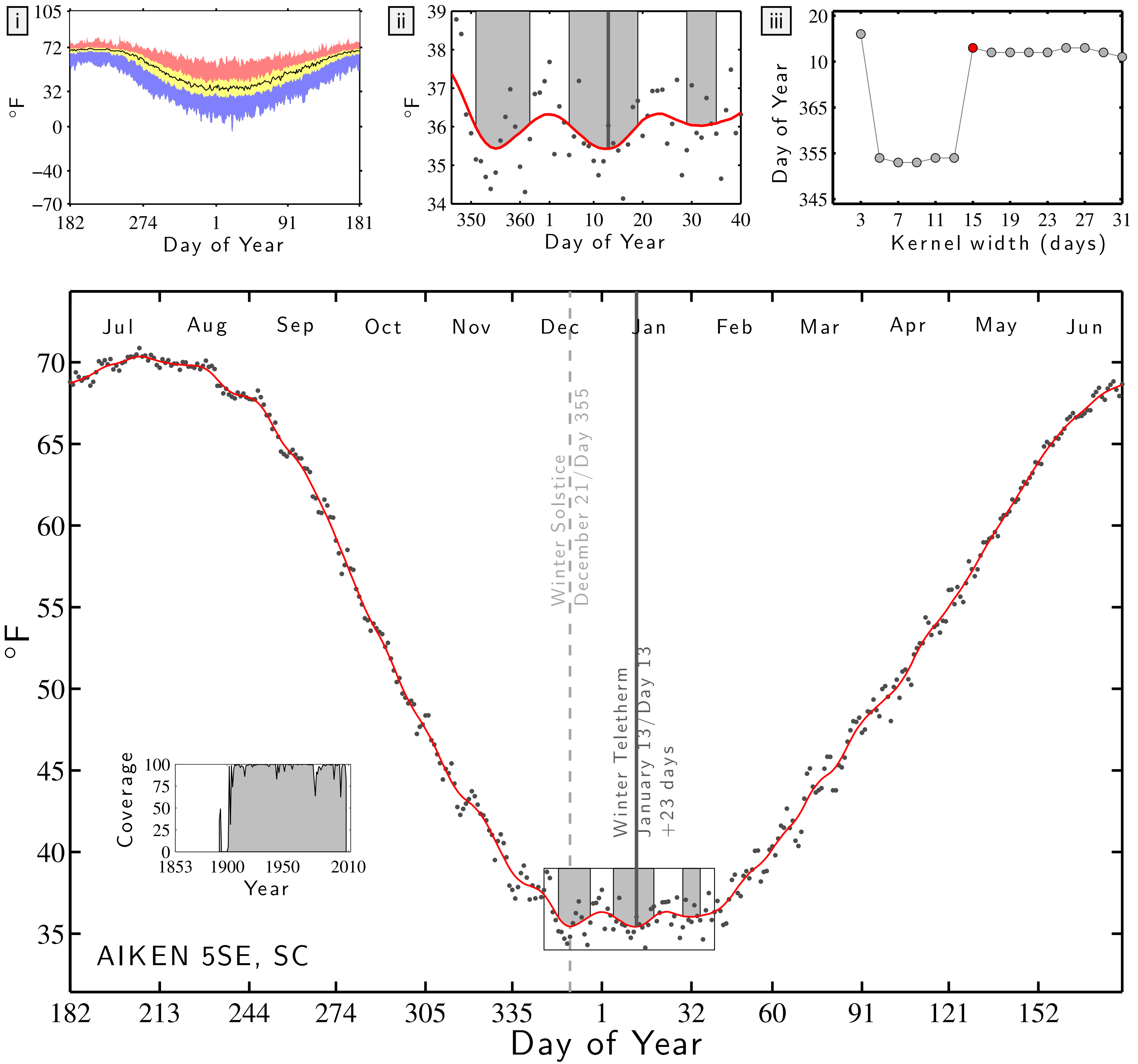

i
°F
Day of Year
ii
°F
Day of Year
iii
Day of Year
Kernel width (days)
Jul
Aug
Sep
Oct
Nov
Dec
Jan
Feb
Mar
Apr
May
Jun
Winter Solstice
December 21/Day 355
Winter Teletherm
January 13/Day 13
+23 days
Coverage
Year
AIKEN 5SE, SC
°F
Day of Year

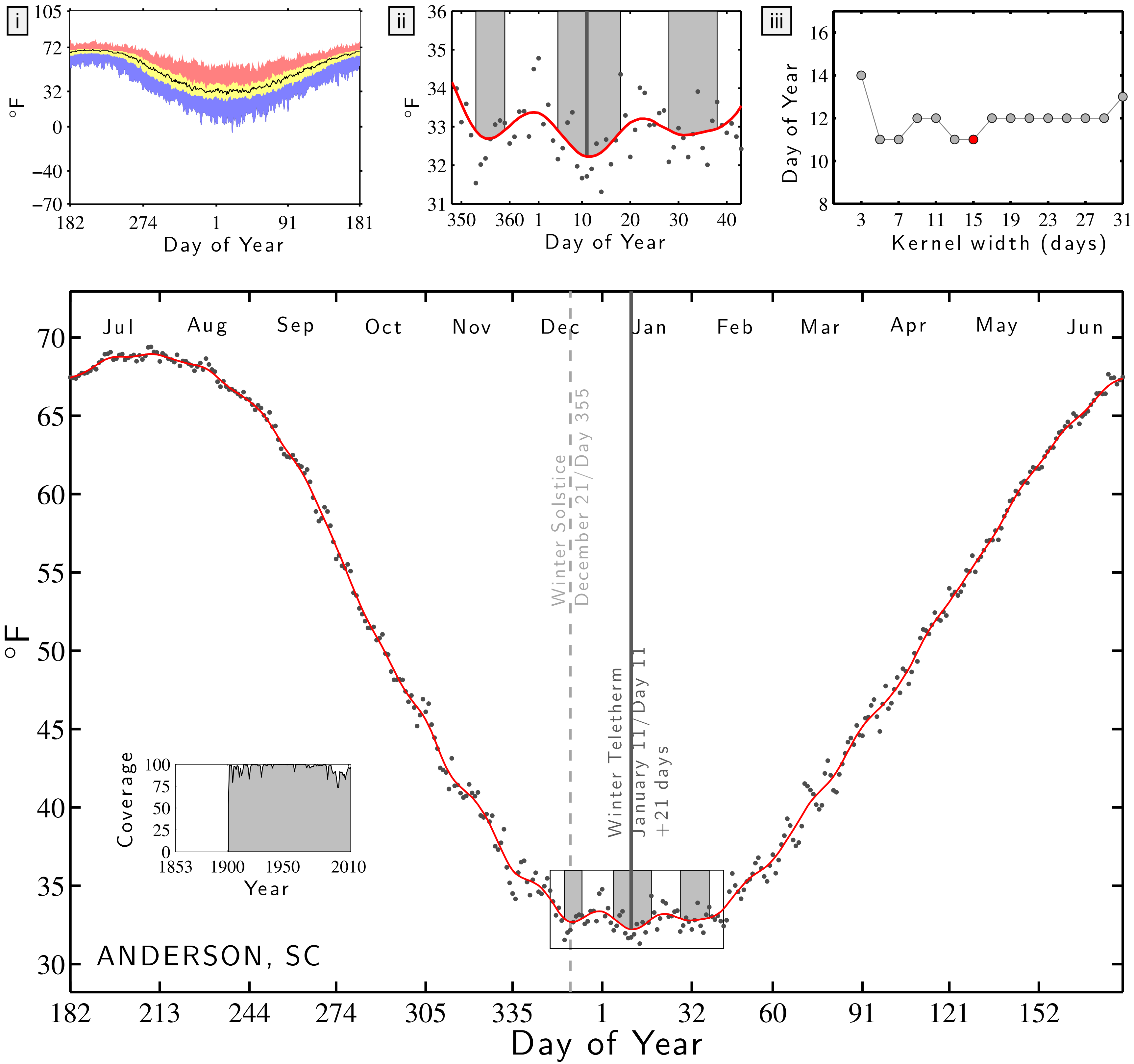

i
ii
iii
°F
Day of Year
Day of Year
Kernel width (days)
Jul
Aug
Sep
Oct
Nov
Dec
Jan
Feb
Mar
Apr
May
Jun
Winter Solstice
December 21/Day 355
Winter Teletherm
January 11/Day 11
+21 days
Coverage
Year
ANDERSON, SC

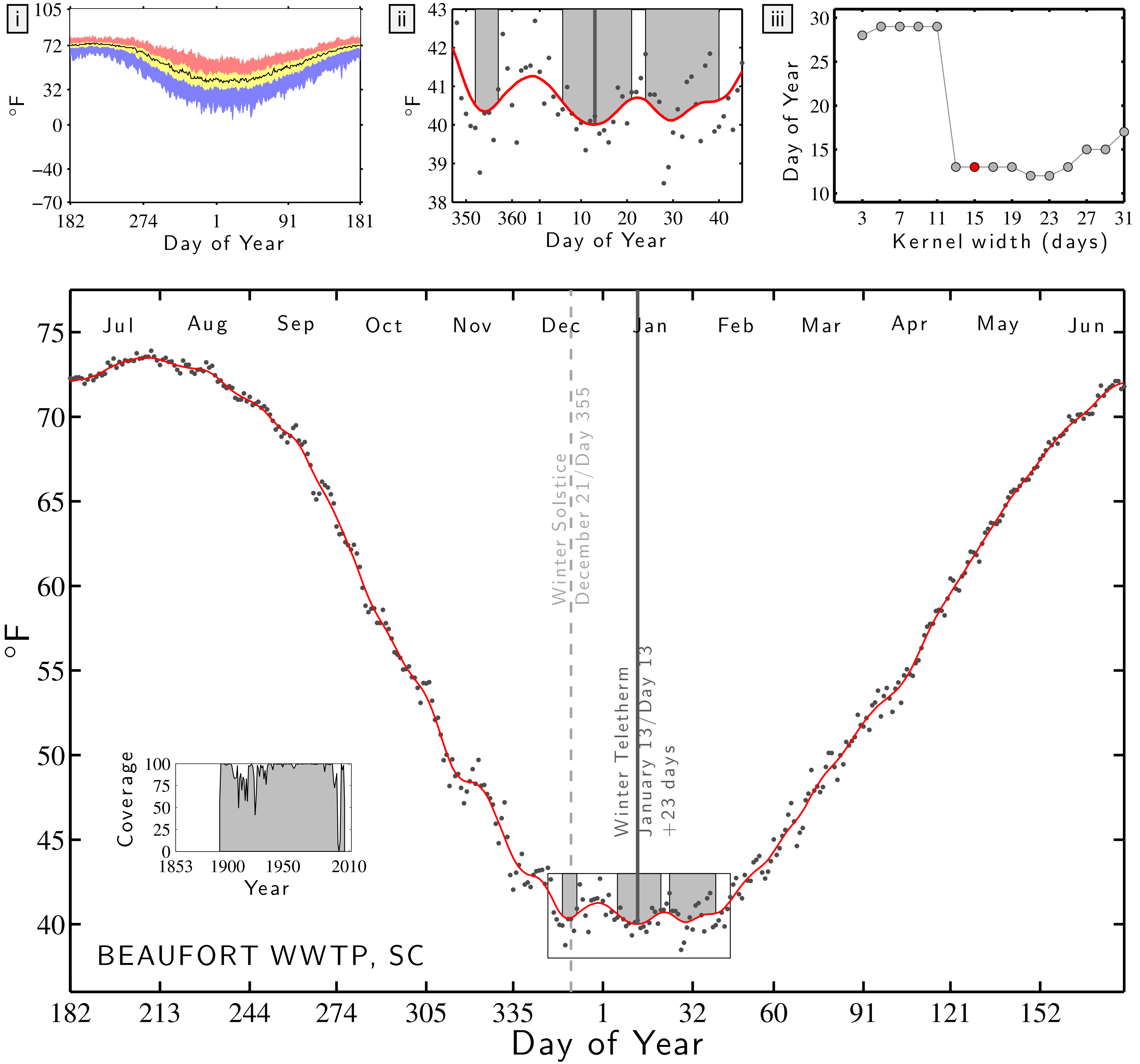

i
°F
Day of Year
ii
°F
Day of Year
iii
Day of Year
Kernel width (days)
Jul
Aug
Sep
Oct
Nov
Dec
Jan
Feb
Mar
Apr
May
Jun
Winter Solstice
December 21/Day 355
Winter Teletherm
January 13/Day 13
+23 days
Coverage
Year
BEAUFORT WWTP, SC
°F
Day of Year

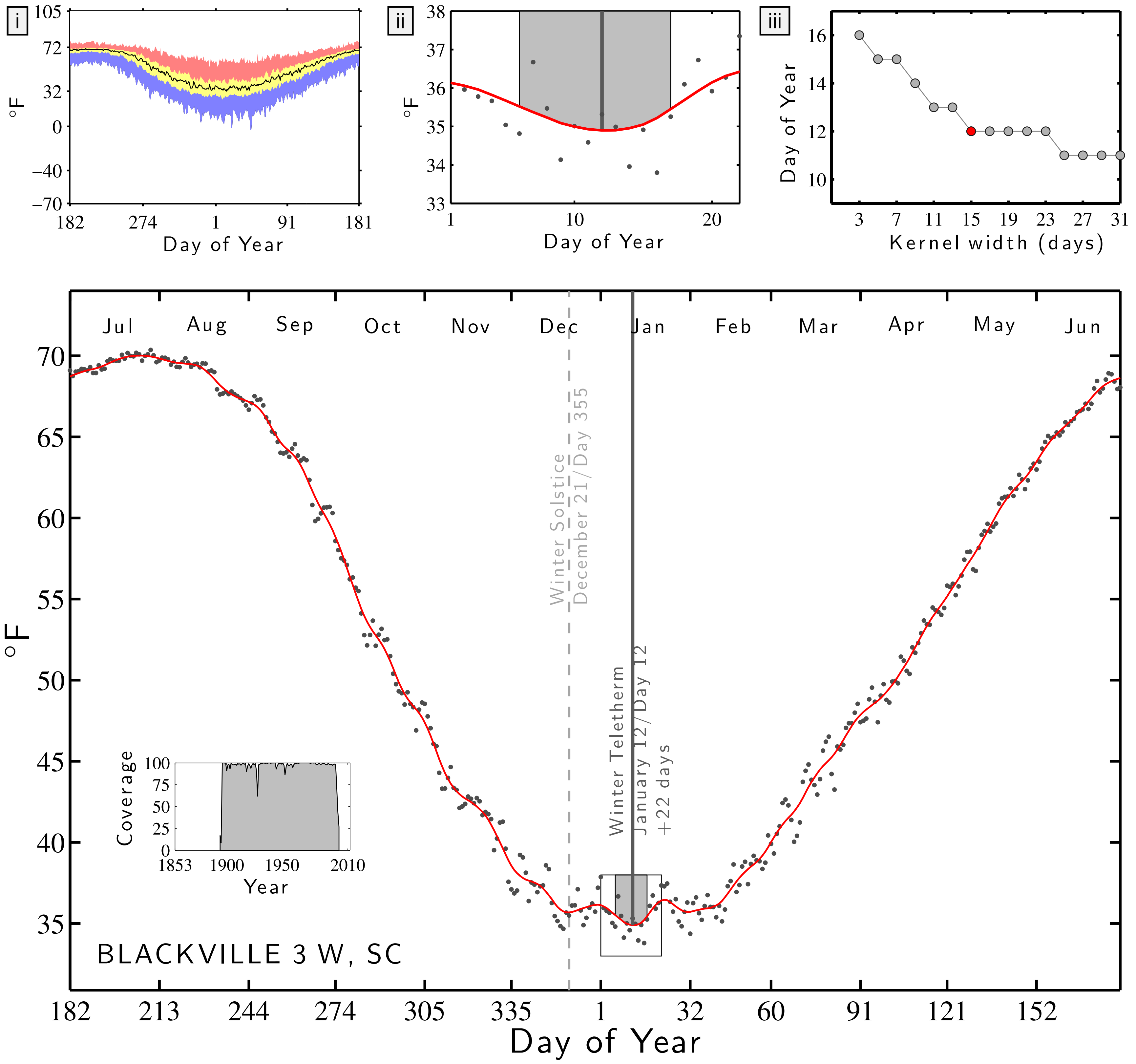

i
105
72
32
0
−40
−70
°F
182
274
1
91
181
Day of Year
ii
38
37
36
35
34
33
°F
1
10
20
Day of Year
iii
16
14
12
10
Day of Year
3
7
11
15
19
23
27
31
Kernel width (days)
Jul
Aug
Sep
Oct
Nov
Dec
Jan
Feb
Mar
Apr
May
Jun
70
65
60
55
50
45
40
35
°F
Winter Solstice
December 21/Day 355
Winter Teletherm
January 12/Day 12
+22 days
Coverage
100
75
50
25
0
1853
1900
1950
2010
Year
BLACKVILLE 3 W, SC
182
213
244
274
305
335
1
32
60
91
121
152
Day of Year

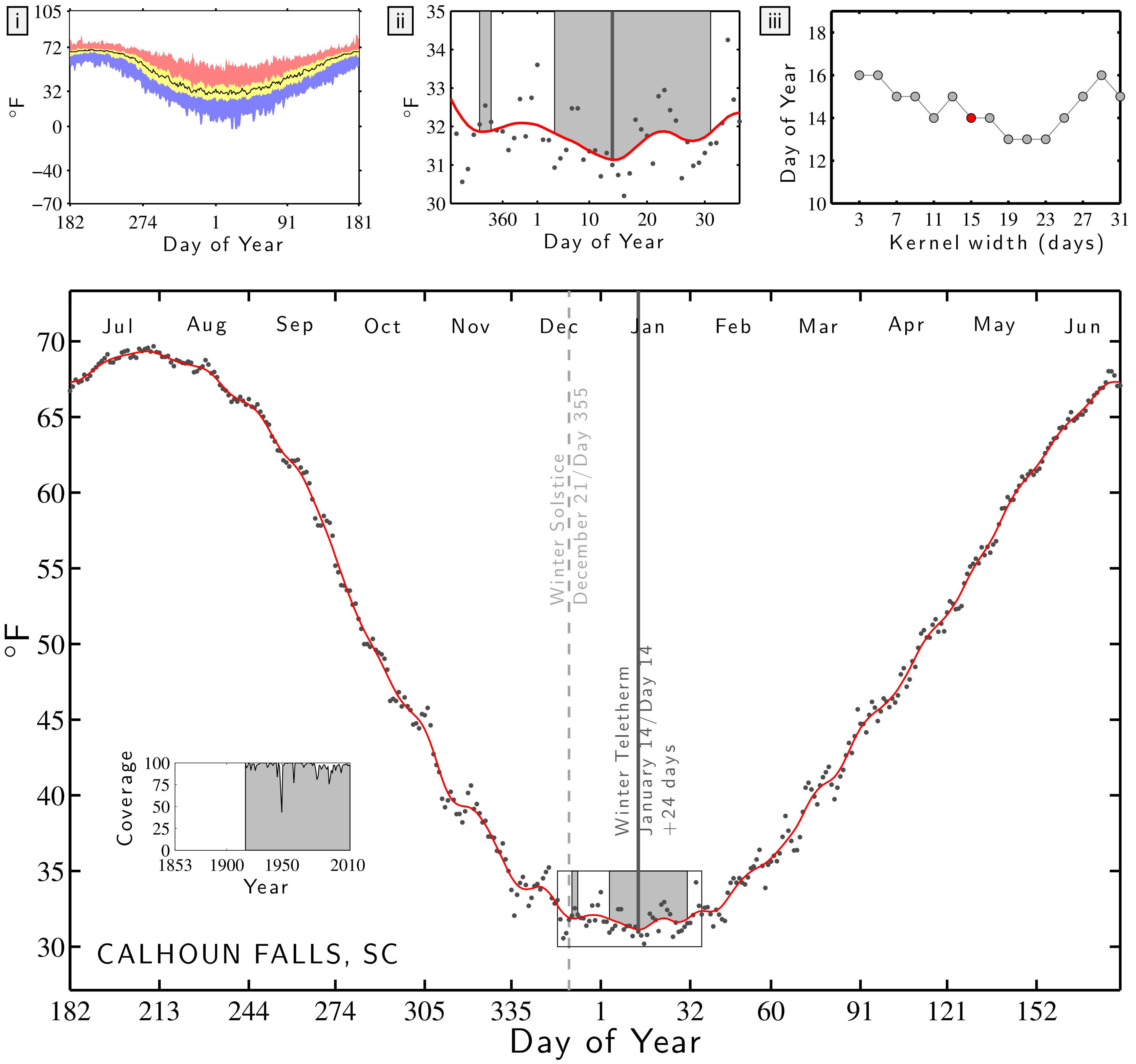

i
°F
Day of Year
ii
°F
Day of Year
iii
Day of Year
Kernel width (days)
Jul
Aug
Sep
Oct
Nov
Dec
Jan
Feb
Mar
Apr
May
Jun
Winter Solstice
December 21/Day 355
Winter Teletherm
January 14/Day 14
+24 days
Coverage
Year
CALHOUN FALLS, SC
°F
Day of Year

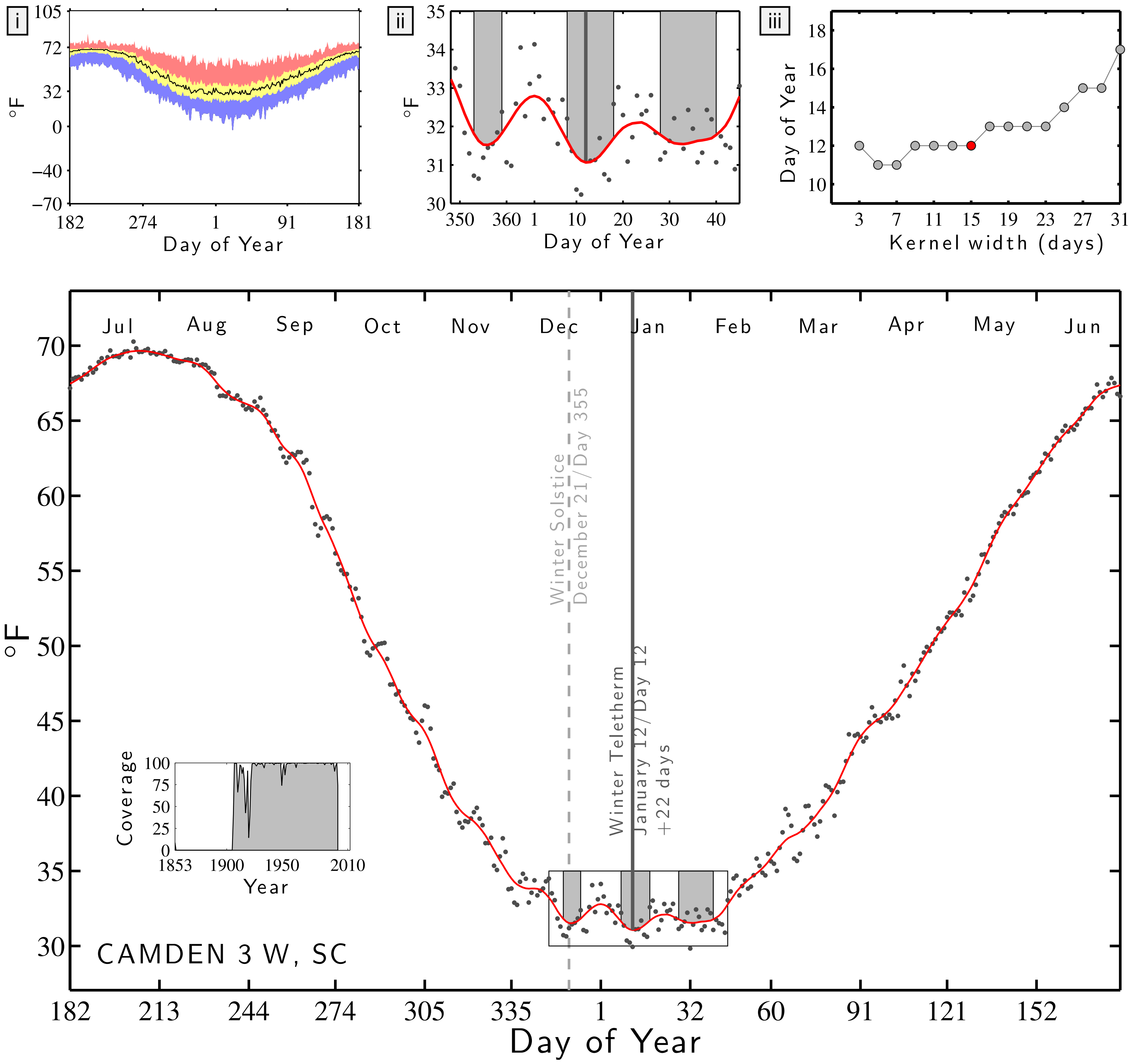

i
°F
105
72
32
0
−40
−70
182
274
1
91
181
Day of Year
ii
35
34
33
32
31
30
350
360
1
10
20
30
40
iii
Day of Year
18
16
14
12
10
3
7
11
15
19
23
27
31
Kernel width (days)
Jul
Aug
Sep
Oct
Nov
Dec
Jan
Feb
Mar
Apr
May
Jun
70
65
60
55
50
45
40
35
30
Winter Solstice
December 21/Day 355
Winter Teletherm
January 12/Day 12
+22 days
Coverage
100
75
50
25
0
1853
1900
1950
2010
Year
CAMDEN 3 W, SC
182
213
244
274
305
335
1
32
60
91
121
152
Day of Year

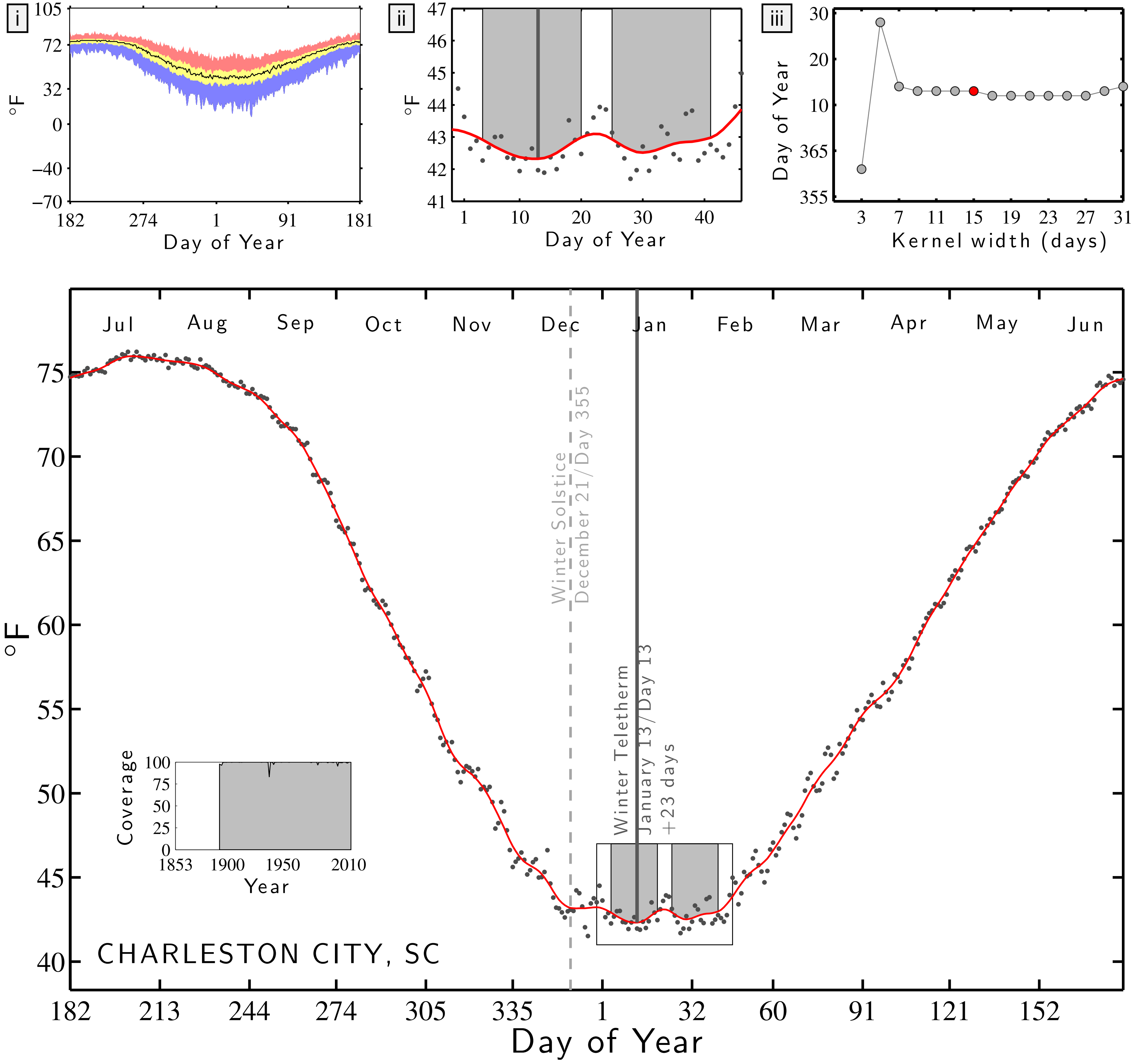

i
ii
iii
°F
Day of Year
Kernel width (days)
Jul
Aug
Sep
Oct
Nov
Dec
Jan
Feb
Mar
Apr
May
Jun
Winter Solstice
December 21/Day 355
Winter Teletherm
January 13/Day 13
+23 days
Coverage
Year
CHARLESTON CITY, SC

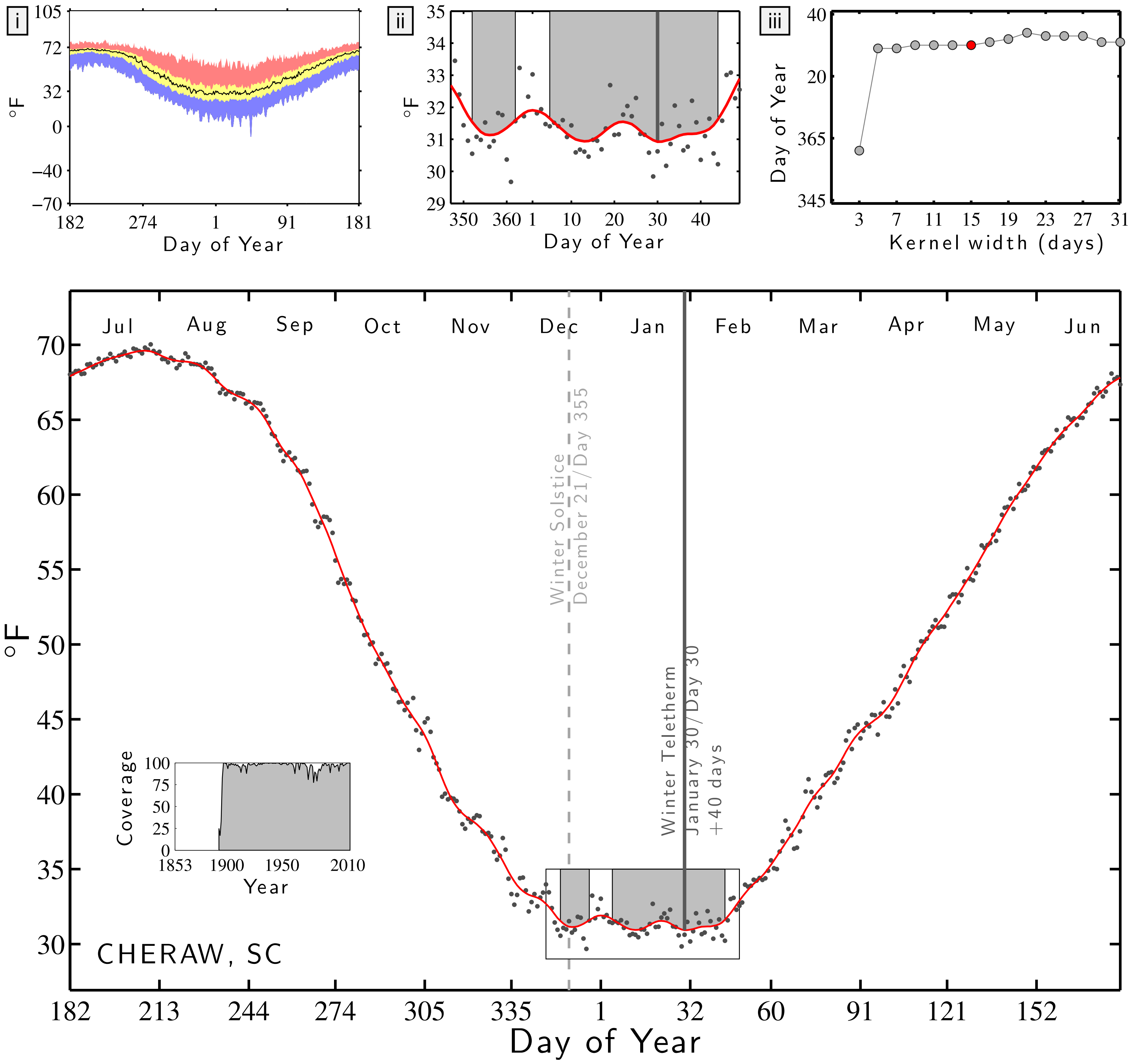

i
ii
iii
°F
Day of Year
Day of Year
Kernel width (days)
Jul
Aug
Sep
Oct
Nov
Dec
Jan
Feb
Mar
Apr
May
Jun
Winter Solstice
December 21/Day 355
Winter Teletherm
January 30/Day 30
+40 days
Coverage
Year
CHERAW, SC
Day of Year

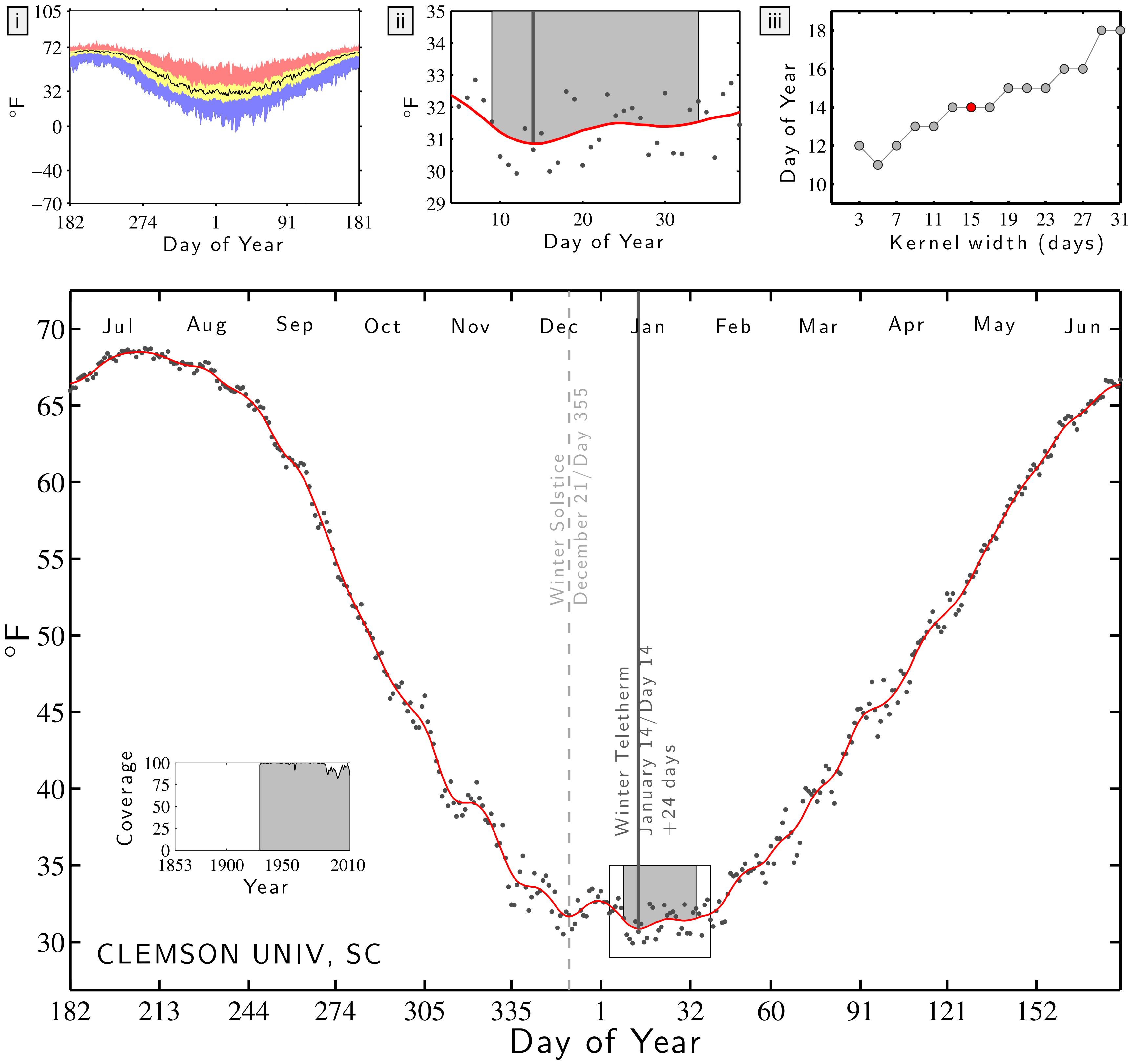
i
ii
iii
°F
Day of Year
Kernel width (days)
Day of Year
Jul
Aug
Sep
Oct
Nov
Dec
Jan
Feb
Mar
Apr
May
Jun
Winter Solstice
December 21/Day 355
Winter Teletherm
January 14/Day 14
+24 days
Coverage
Year
CLEMSON UNIV, SC
Day of Year

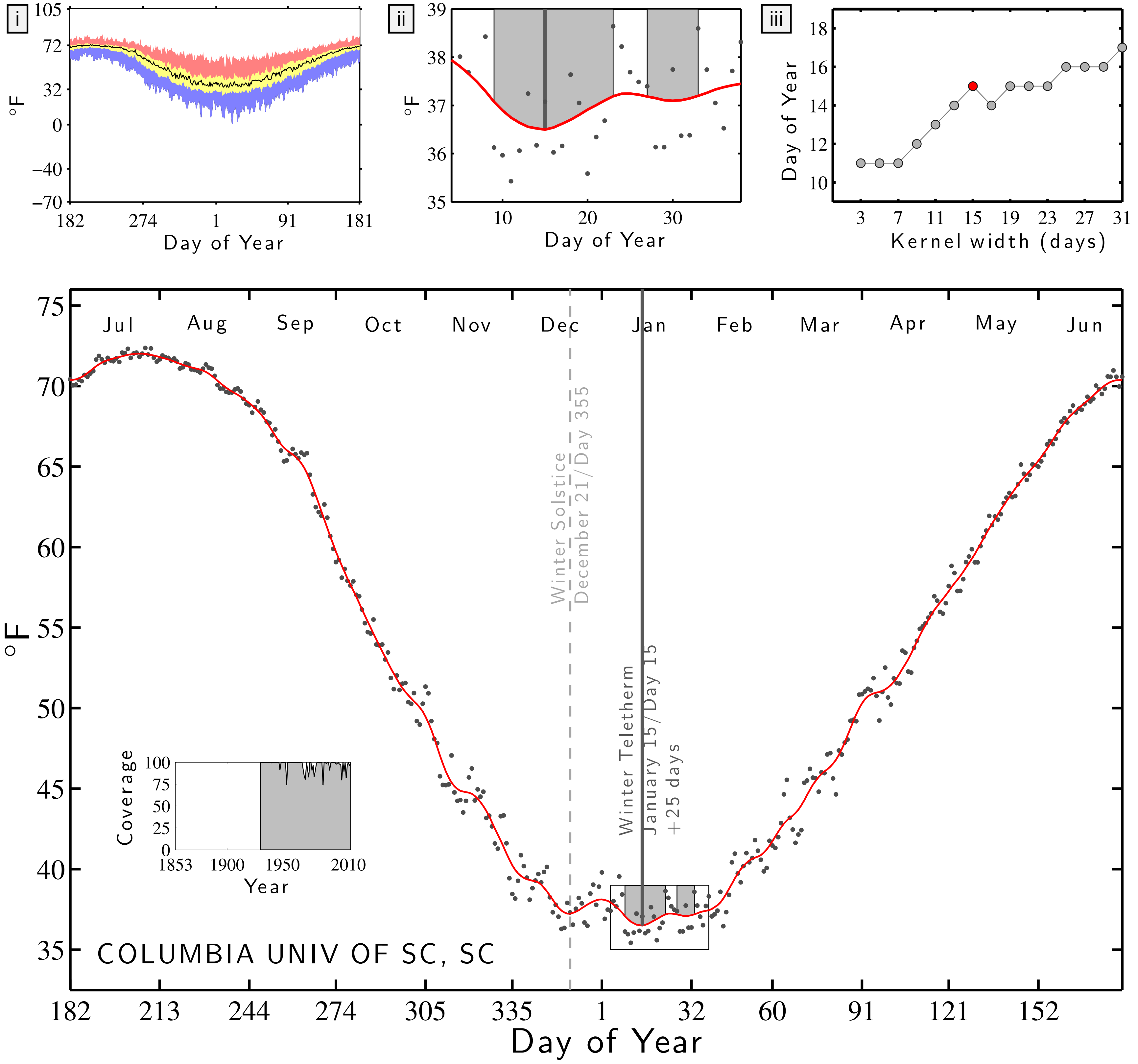

i
ii
iii
Day of Year
Kernel width (days)
°F
Jul
Aug
Sep
Oct
Nov
Dec
Jan
Feb
Mar
Apr
May
Jun
Winter Solstice
December 21/Day 355
Winter Teletherm
January 15/Day 15
+25 days
Coverage
Year
COLUMBIA UNIV OF SC, SC

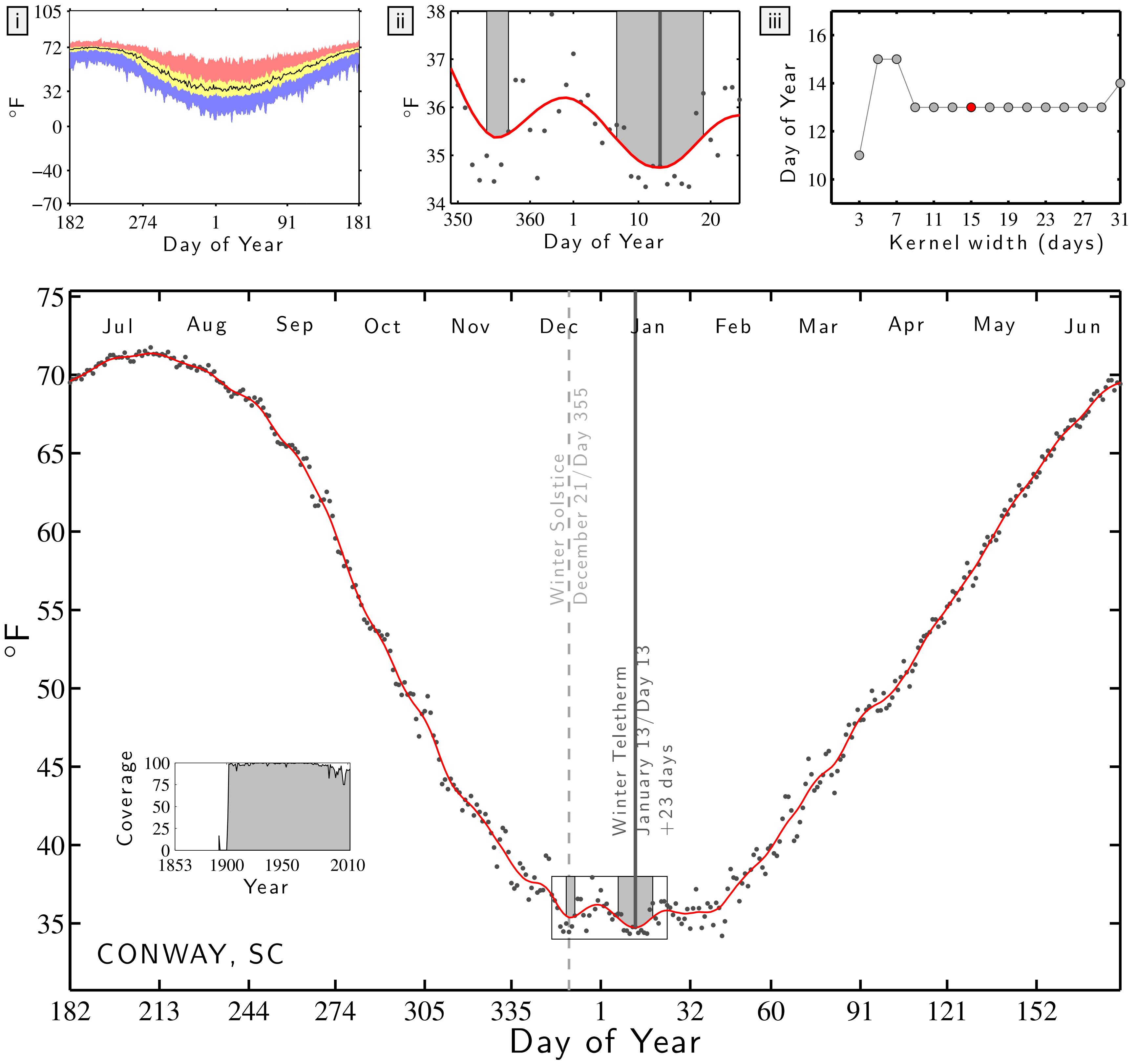

i
°F
Day of Year
ii
°F
Day of Year
iii
Day of Year
Kernel width (days)
Jul
Aug
Sep
Oct
Nov
Dec
Jan
Feb
Mar
Apr
May
Jun
Winter Solstice
December 21/Day 355
Winter Teletherm
January 13/Day 13
+23 days
Coverage
Year
CONWAY, SC
°F
Day of Year

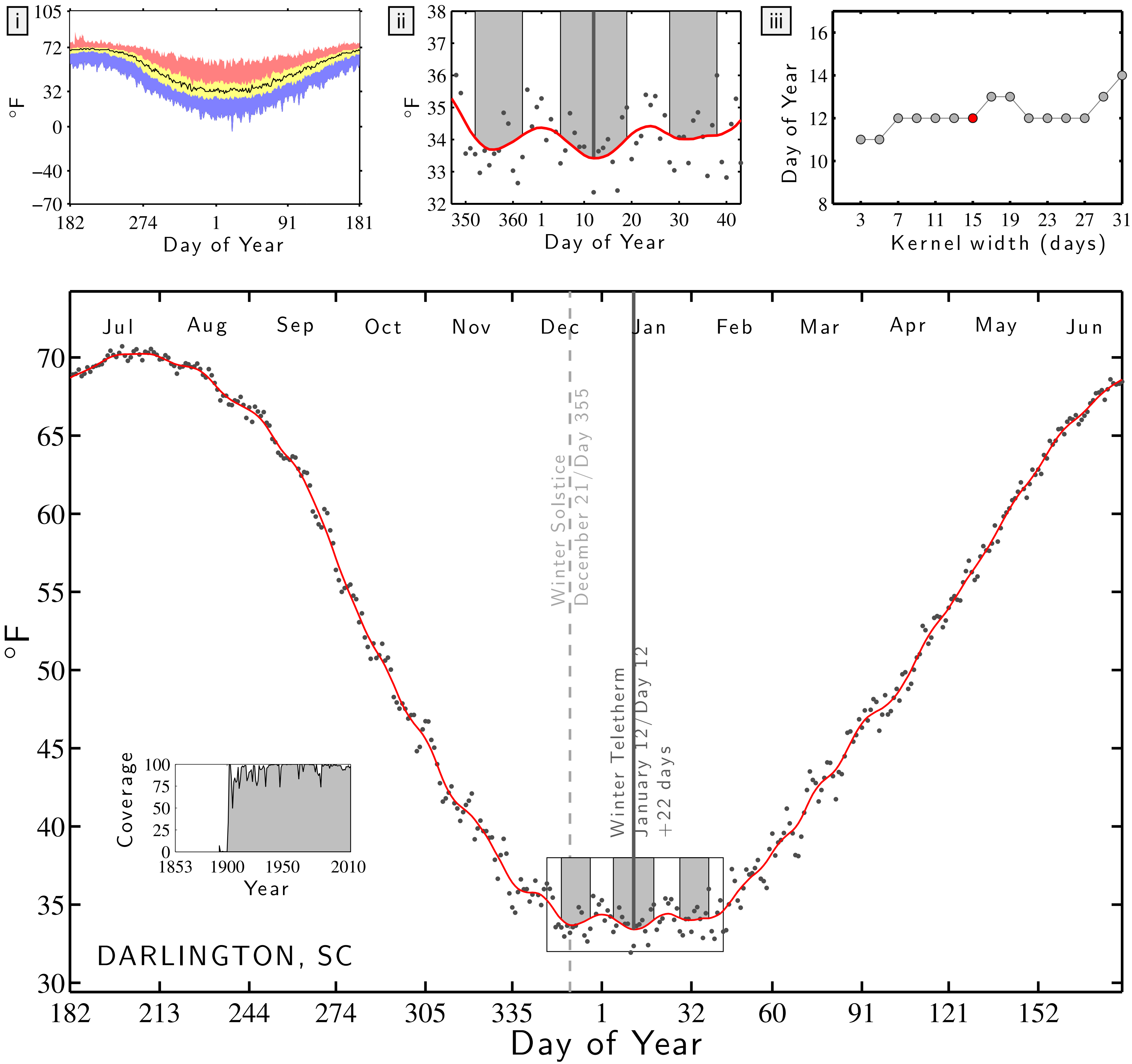

i
°F
Day of Year
ii
°F
Day of Year
iii
Day of Year
Kernel width (days)
Jul
Aug
Sep
Oct
Nov
Dec
Jan
Feb
Mar
Apr
May
Jun
Winter Solstice
December 21/Day 355
Winter Teletherm
January 12/Day 12
+22 days
Coverage
Year
DARLINGTON, SC
°F
Day of Year

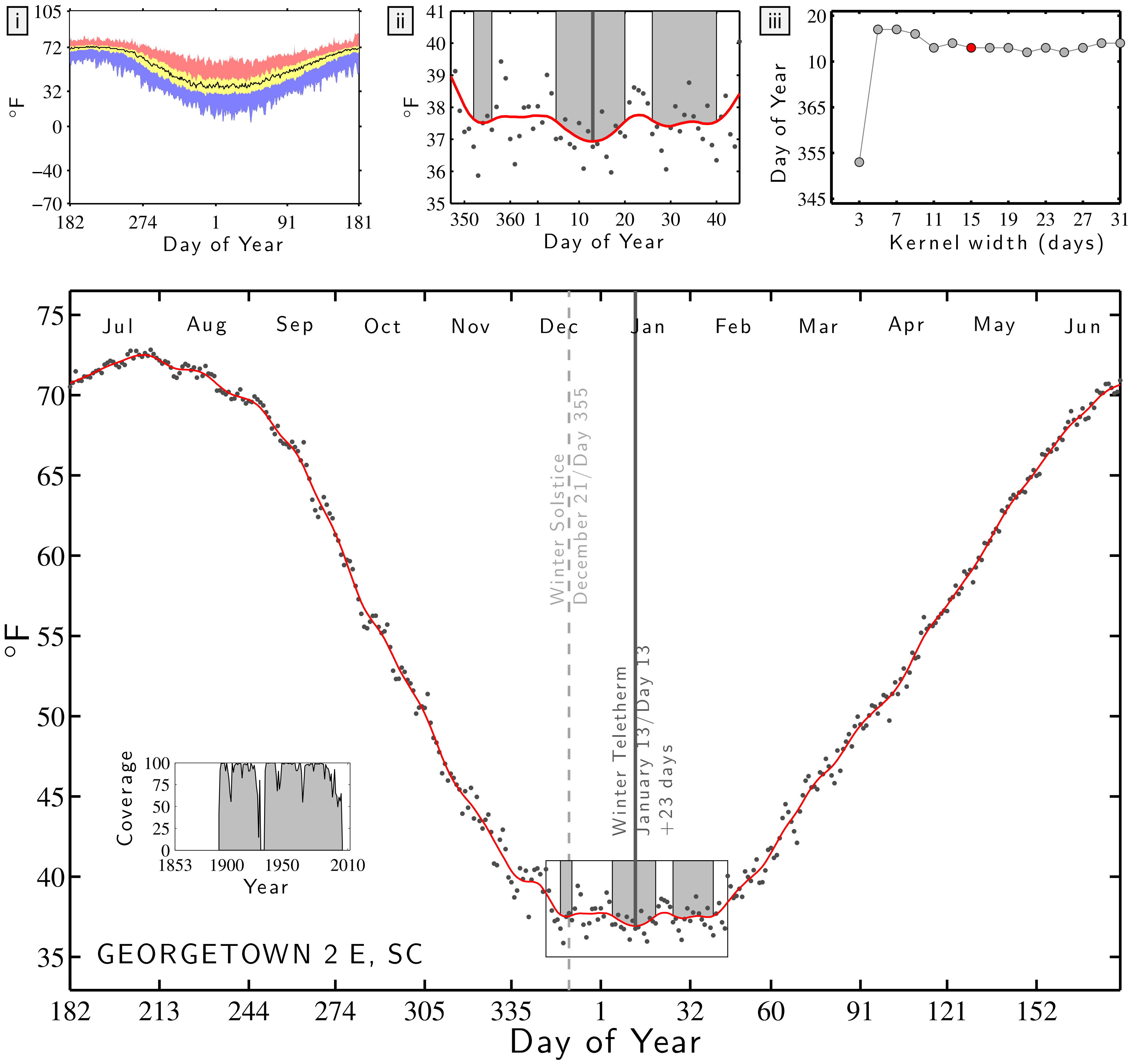

i
°F
Day of Year
ii
°F
Day of Year
iii
Day of Year
Kernel width (days)
Jul
Aug
Sep
Oct
Nov
Dec
Jan
Feb
Mar
Apr
May
Jun
Winter Solstice
December 21/Day 355
Winter Teletherm
January 13/Day 13
+23 days
Coverage
Year
GEORGETOWN 2 E, SC
°F
Day of Year

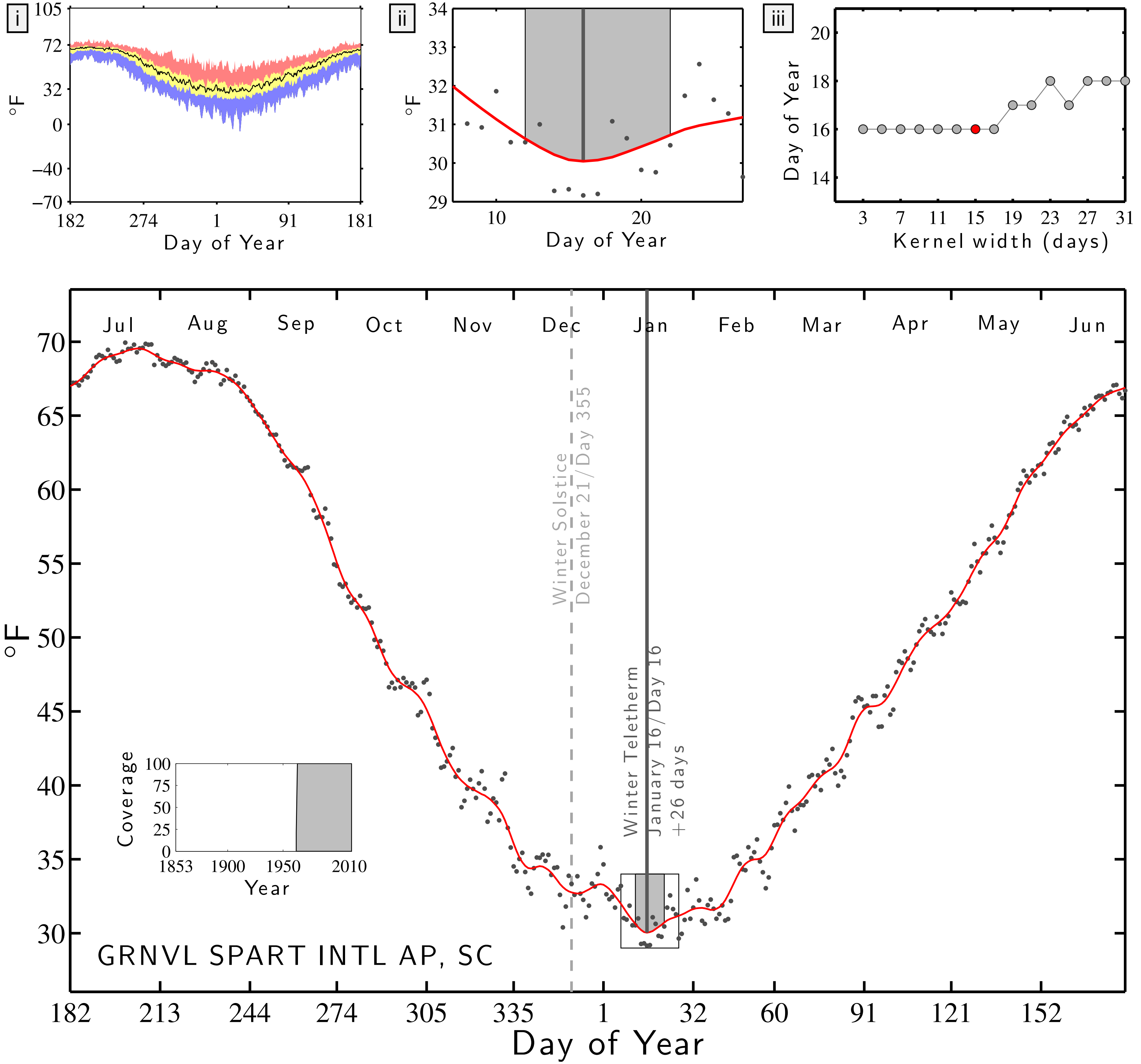

i
ii
iii
Day of Year
Kernel width (days)
°F
Jul
Aug
Sep
Oct
Nov
Dec
Jan
Feb
Mar
Apr
May
Jun
Winter Solstice
December 21/Day 355
Winter Teletherm
January 16/Day 16
+26 days
Coverage
Year
GRNVL SPART INTL AP, SC
Day of Year

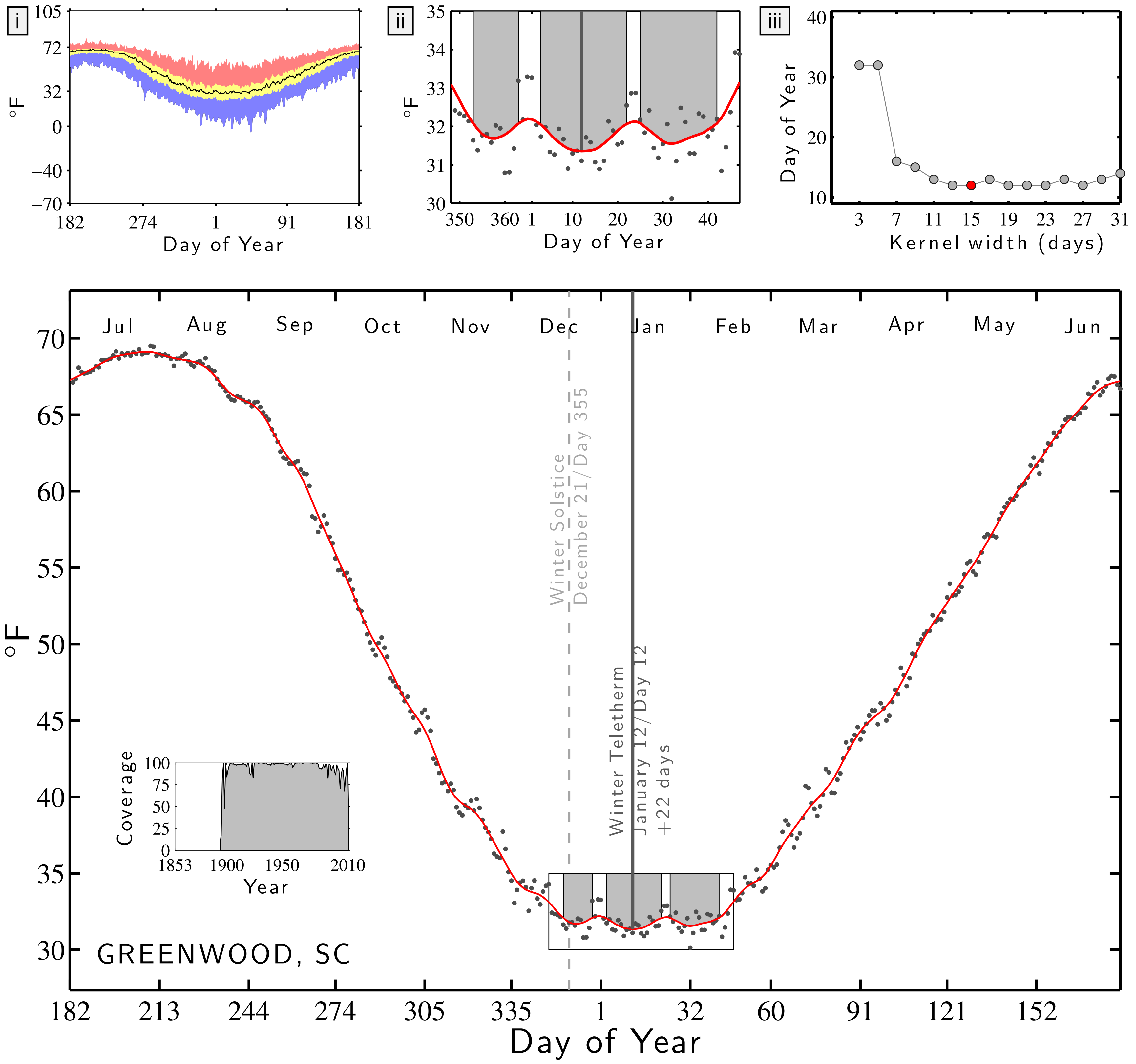
i
ii
iii
°F
Day of Year
Day of Year
Kernel width (days)
Winter Solstice
December 21/Day 355
Winter Teletherm
January 12/Day 12
+22 days
Coverage
Year
GREENWOOD, SC
Jul
Aug
Sep
Oct
Nov
Dec
Jan
Feb
Mar
Apr
May
Jun

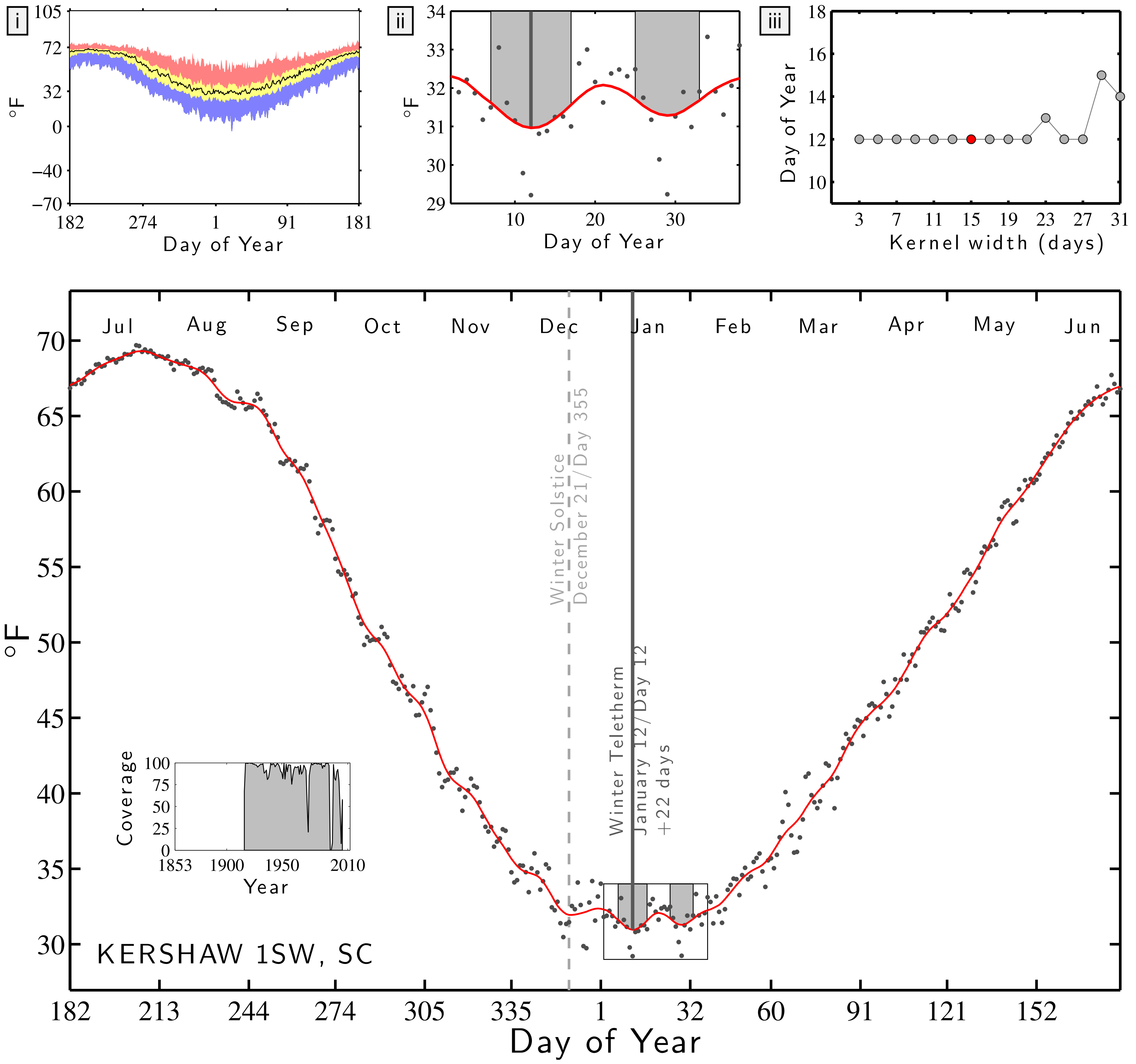

i
ii
iii
Day of Year
Kernel width (days)
°F
Jul
Aug
Sep
Oct
Nov
Dec
Jan
Feb
Mar
Apr
May
Jun
Winter Solstice
December 21/Day 355
Winter Teletherm
January 12/Day 12
+22 days
Coverage
Year
KERSHAW 1SW, SC

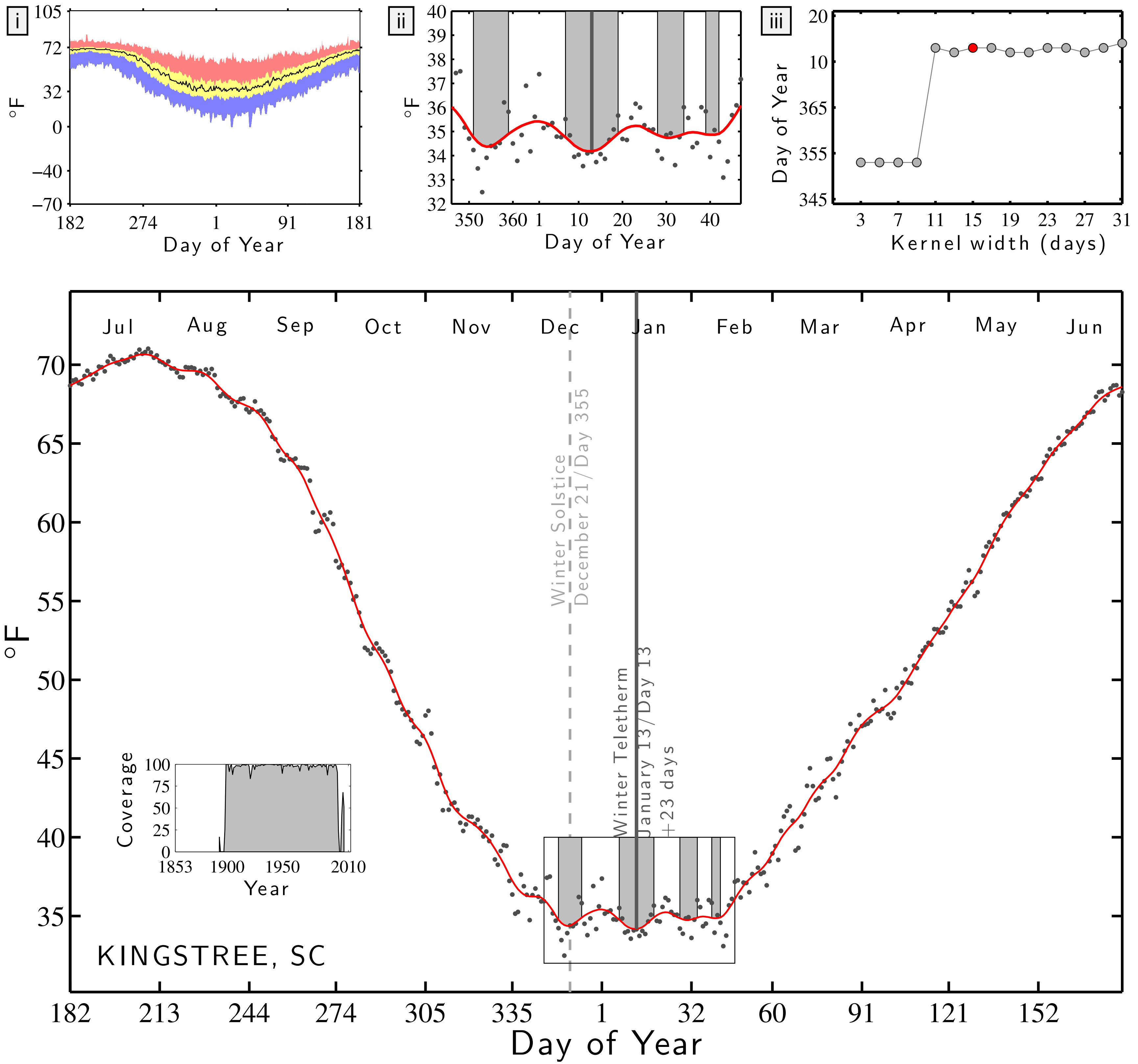

i
°F
Day of Year
ii
iii
Day of Year
Kernel width (days)
Jul
Aug
Sep
Oct
Nov
Dec
Jan
Feb
Mar
Apr
May
Jun
Winter Solstice
December 21/Day 355
Winter Teletherm
January 13/Day 13
+23 days
Coverage
Year
KINGSTREE, SC

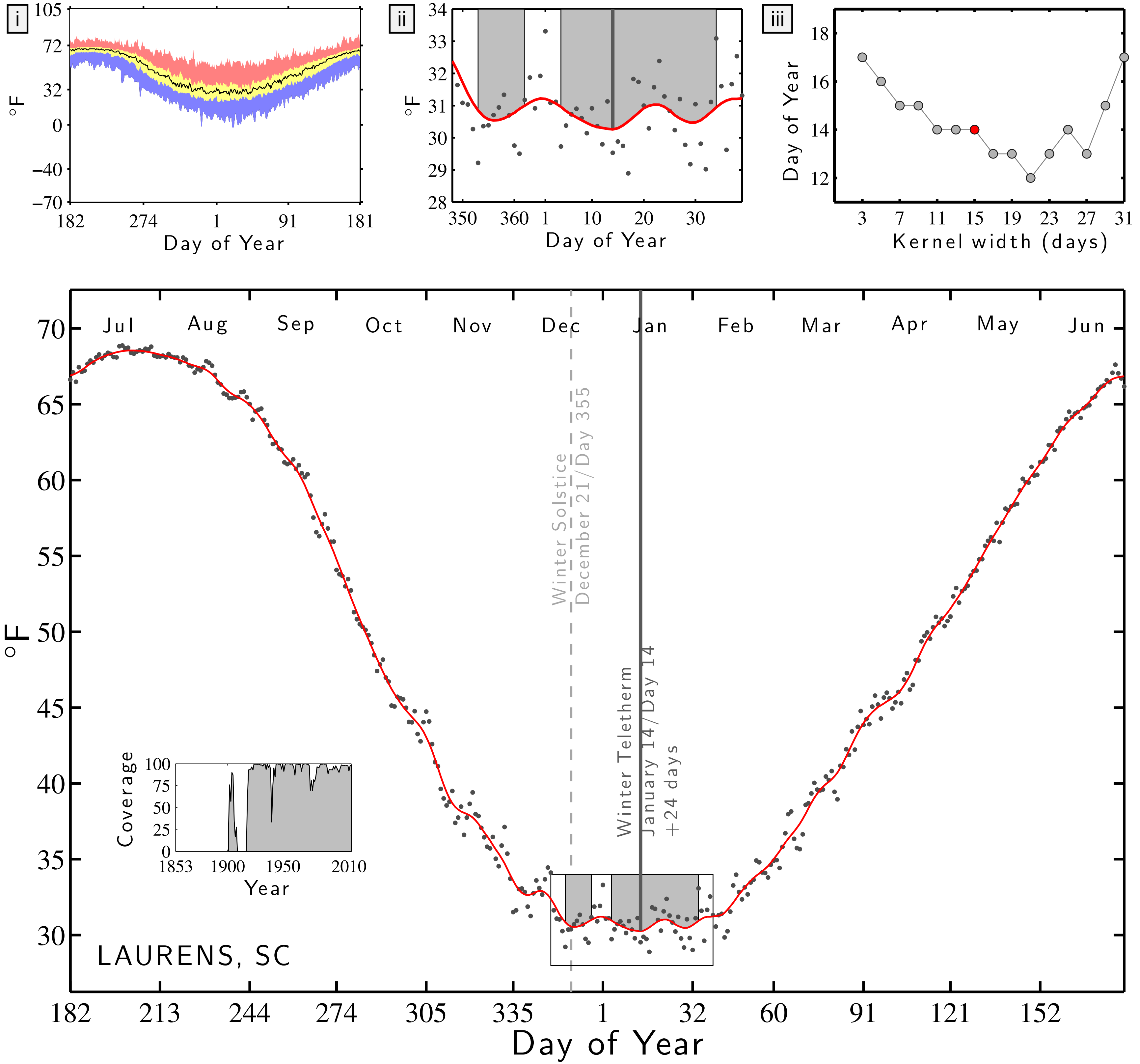

i
ii
iii
°F
Day of Year
Day of Year
Kernel width (days)
Jul
Aug
Sep
Oct
Nov
Dec
Jan
Feb
Mar
Apr
May
Jun
Winter Solstice
December 21/Day 355
Winter Teletherm
January 14/Day 14
+24 days
Coverage
Year
LAURENS, SC

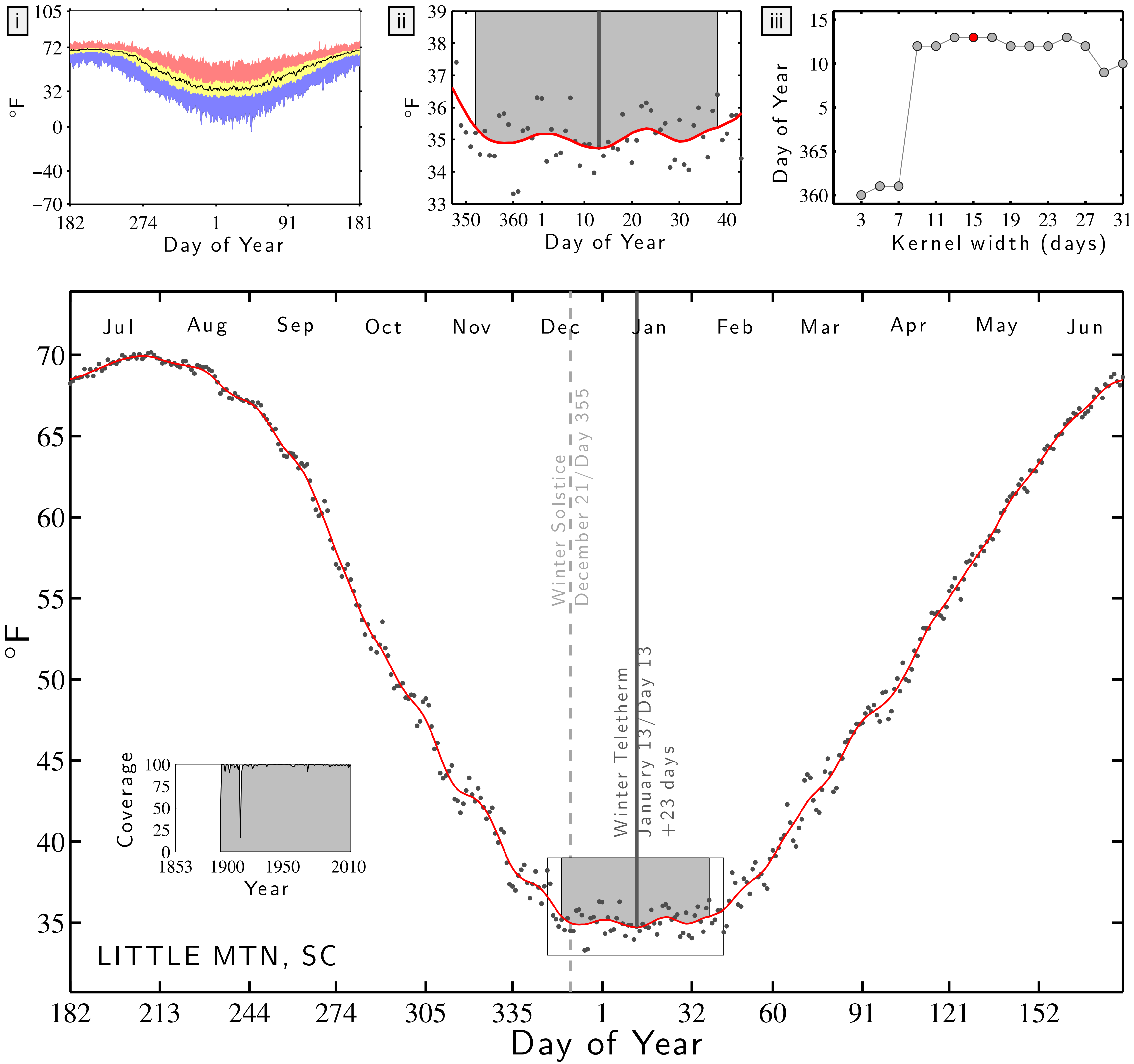

i
ii
iii
°F
Day of Year
Day of Year
Kernel width (days)
Jul
Aug
Sep
Oct
Nov
Dec
Jan
Feb
Mar
Apr
May
Jun
Winter Solstice
December 21/Day 355
Winter Teletherm
January 13/Day 13
+23 days
Coverage
Year
LITTLE MTN, SC

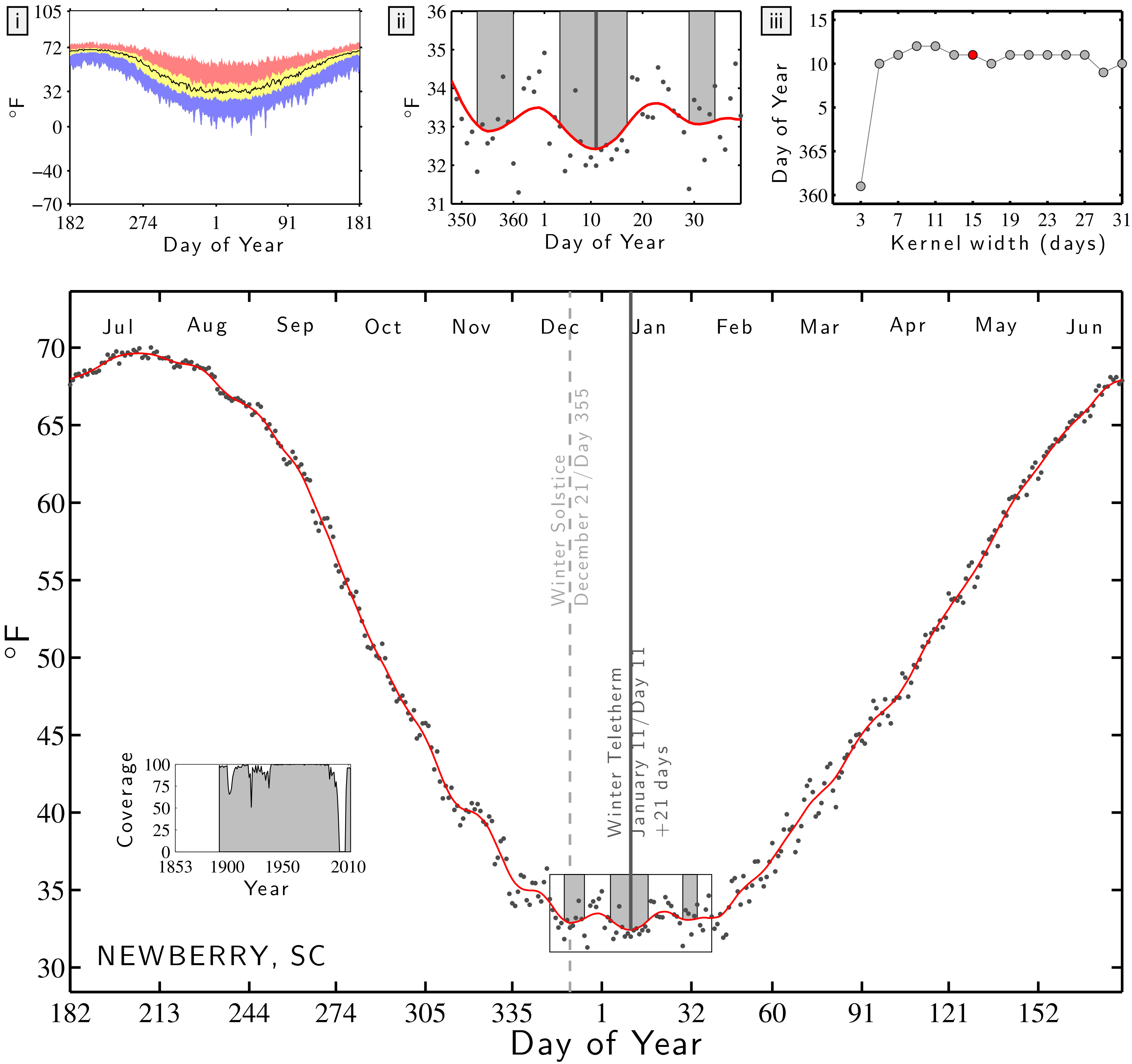

i
105
72
32
0
−40
−70
°F
182
274
1
91
181
Day of Year
ii
36
35
34
33
32
31
°F
350
360
1
10
20
30
Day of Year
iii
15
10
5
365
360
Day of Year
3
7
11
15
19
23
27
31
Kernel width (days)
Jul
Aug
Sep
Oct
Nov
Dec
Jan
Feb
Mar
Apr
May
Jun
70
65
60
55
50
45
40
35
30
°F
Winter Solstice
December 21/Day 355
Winter Teletherm
January 11/Day 11
+21 days
Coverage
100
75
50
25
0
1853
1900
1950
2010
Year
NEWBERRY, SC
182
213
244
274
305
335
1
32
60
91
121
152
Day of Year

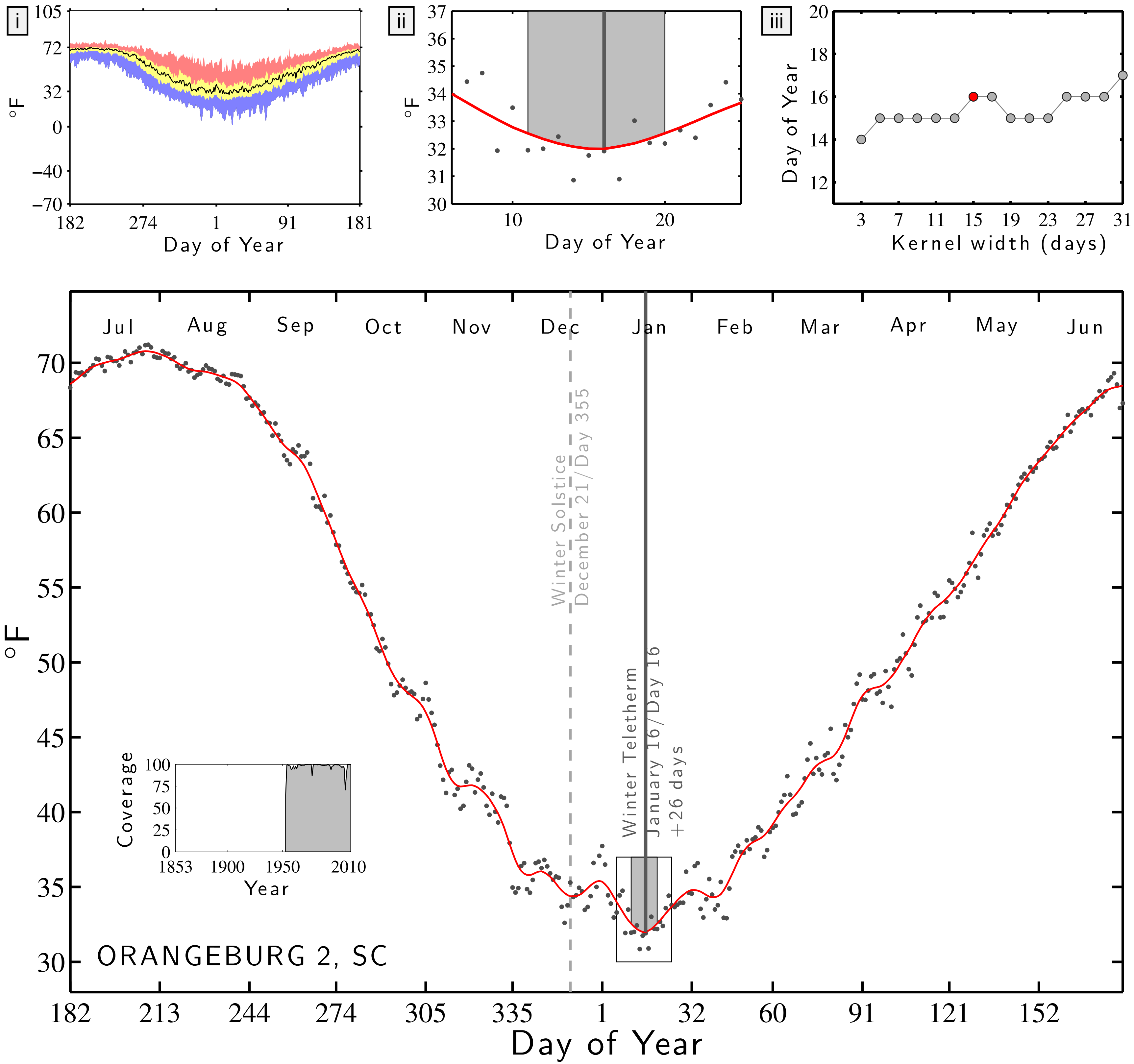

i
°F
105
72
32
0
−40
−70
182
274
1
91
181
Day of Year
ii
37
36
35
34
33
32
31
30
10
20
Day of Year
iii
20
18
16
14
12
Day of Year
3
7
11
15
19
23
27
31
Kernel width (days)
Jul
Aug
Sep
Oct
Nov
Dec
Jan
Feb
Mar
Apr
May
Jun
70
65
60
55
50
45
40
35
30
Winter Solstice
December 21/Day 355
Winter Teletherm
January 16/Day 16
+26 days
Coverage
100
75
50
25
0
1853
1900
1950
2010
Year
ORANGEBURG 2, SC
182
213
244
274
305
335
1
32
60
91
121
152
Day of Year

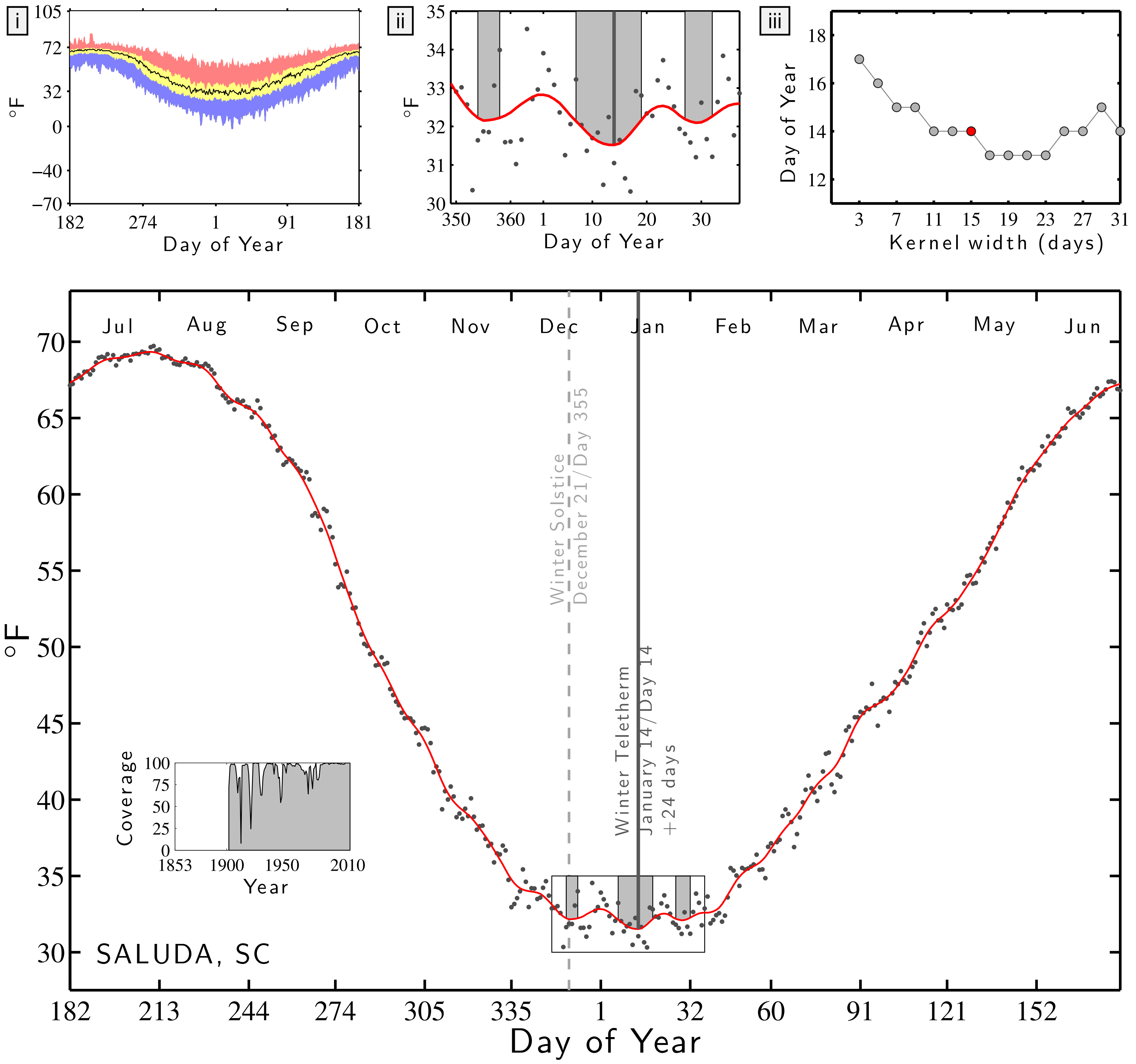

i
ii
iii
°F
Day of Year
Day of Year
Kernel width (days)
Jul
Aug
Sep
Oct
Nov
Dec
Jan
Feb
Mar
Apr
May
Jun
Winter Solstice
December 21/Day 355
Winter Teletherm
January 14/Day 14
+24 days
Coverage
Year
SALUDA, SC
°F
Day of Year

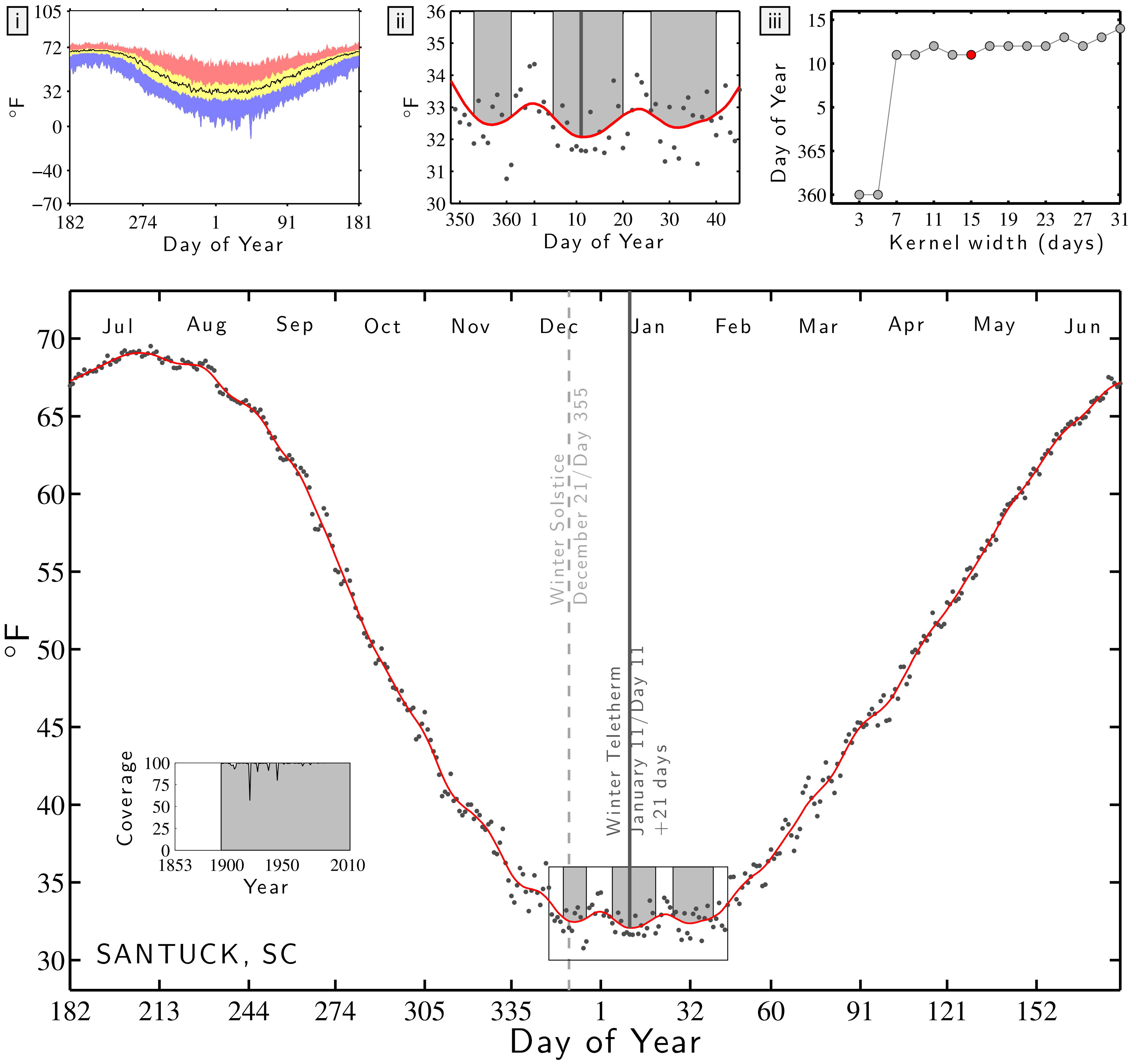

i
105
72
32
0
−40
−70
°F
182
274
1
91
181
Day of Year
ii
36
35
34
33
32
31
30
°F
350
360
1
10
20
30
40
Day of Year
iii
15
10
5
365
360
Day of Year
3
7
11
15
19
23
27
31
Kernel width (days)
Jul
Aug
Sep
Oct
Nov
Dec
Jan
Feb
Mar
Apr
May
Jun
70
65
60
55
50
45
40
35
30
°F
Winter Solstice
December 21/Day 355
Winter Teletherm
January 11/Day 11
+21 days
Coverage
100
75
50
25
0
1853
1900
1950
2010
Year
SANTUCK, SC
182
213
244
274
305
335
1
32
60
91
121
152
Day of Year

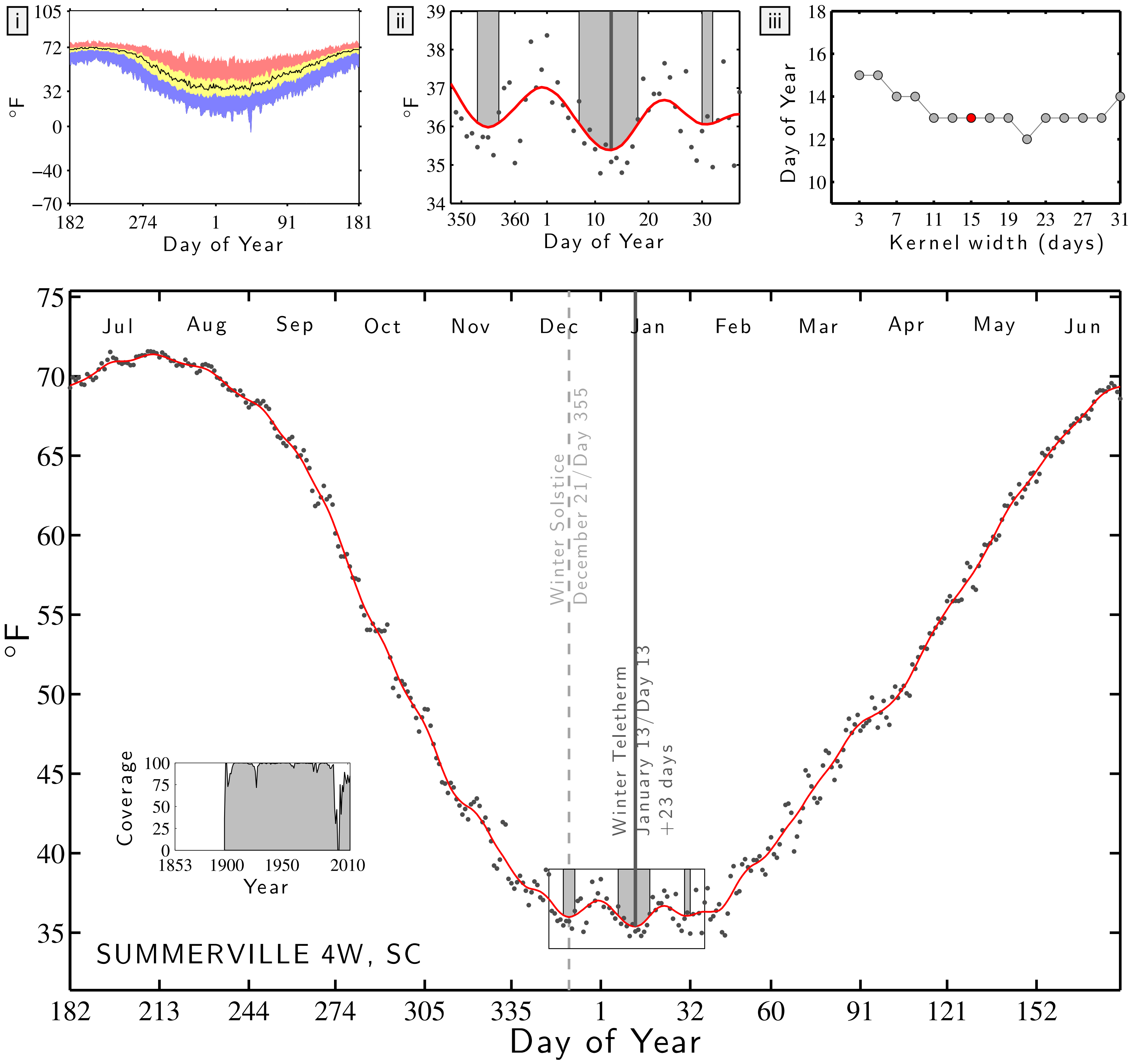

i
ii
iii
°F
Day of Year
Day of Year
Kernel width (days)
Jul
Aug
Sep
Oct
Nov
Dec
Jan
Feb
Mar
Apr
May
Jun
Winter Solstice
December 21/Day 355
Winter Teletherm
January 13/Day 13
+23 days
Coverage
Year
SUMMERVILLE 4W, SC
°F
Day of Year

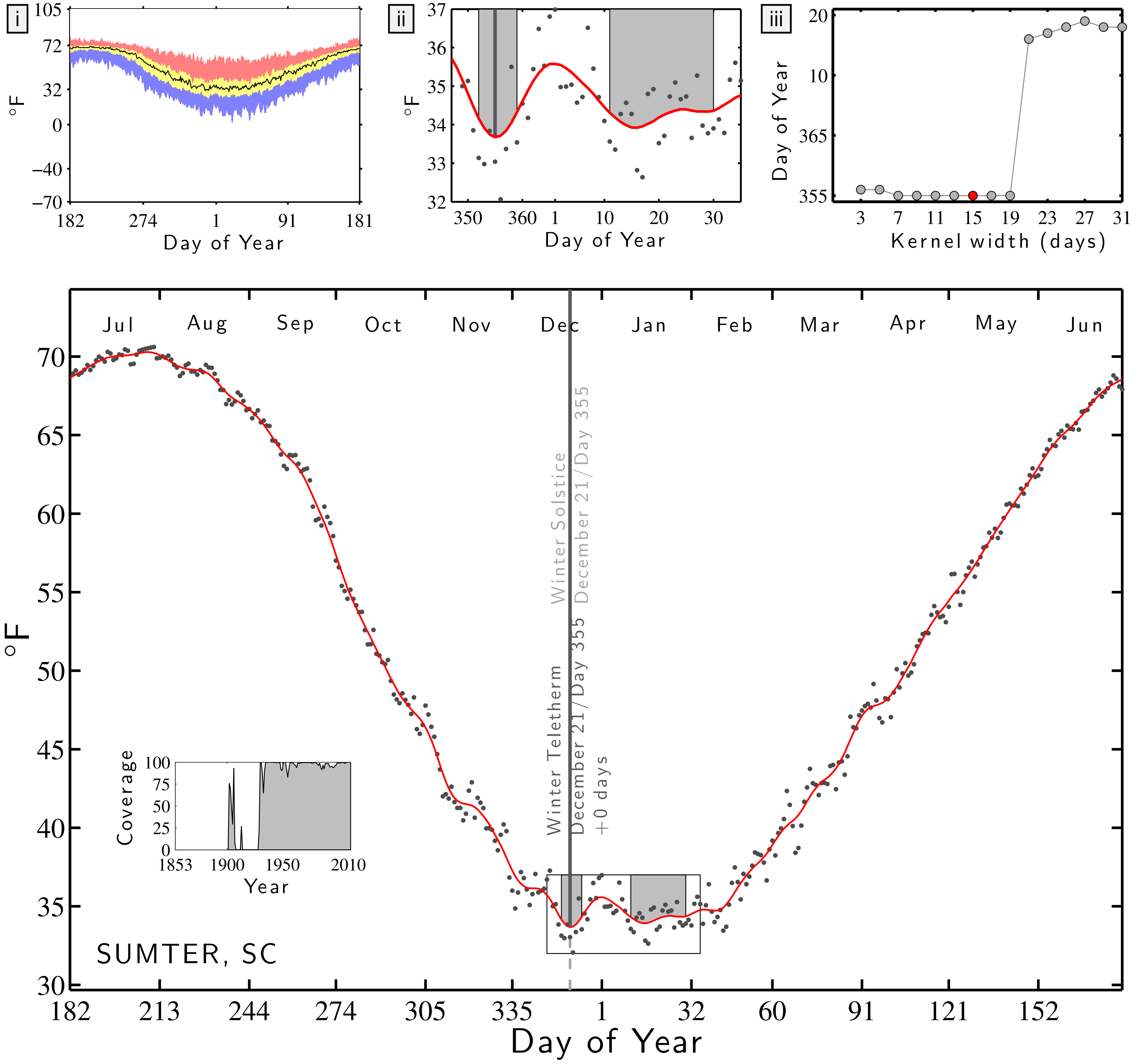

i
ii
iii
°F
Day of Year
Kernel width (days)
Jul
Aug
Sep
Oct
Nov
Dec
Jan
Feb
Mar
Apr
May
Jun
Winter Solstice
December 21 / Day 355
Winter Teletherm
December 21 / Day 355
+0 days
Coverage
Year
SUMTER, SC

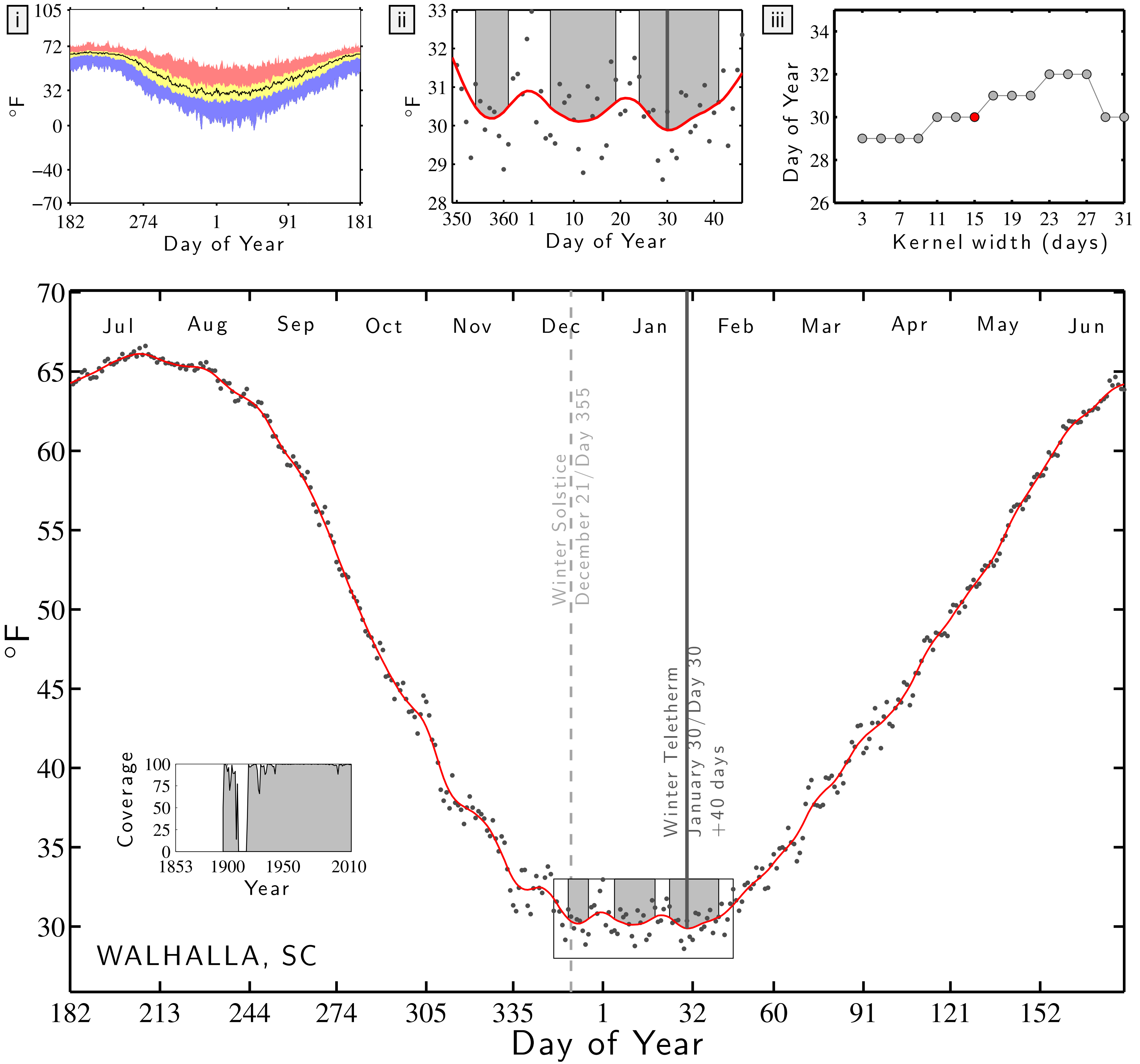

i
°F
105
72
32
0
−40
−70
182
274
1
91
181
Day of Year
ii
°F
33
32
31
30
29
28
350
360
1
10
20
30
40
Day of Year
iii
Day of Year
34
32
30
28
26
3
7
11
15
19
23
27
31
Kernel width (days)
°F
70
65
60
55
50
45
40
35
30
Jul
Aug
Sep
Oct
Nov
Dec
Jan
Feb
Mar
Apr
May
Jun
Winter Solstice
December 21/Day 355
Winter Teletherm
January 30/Day 30
+40 days
Coverage
100
75
50
25
0
1853
1900
1950
2010
Year
WALHALLA, SC
182
213
244
274
305
335
1
32
60
91
121
152
Day of Year

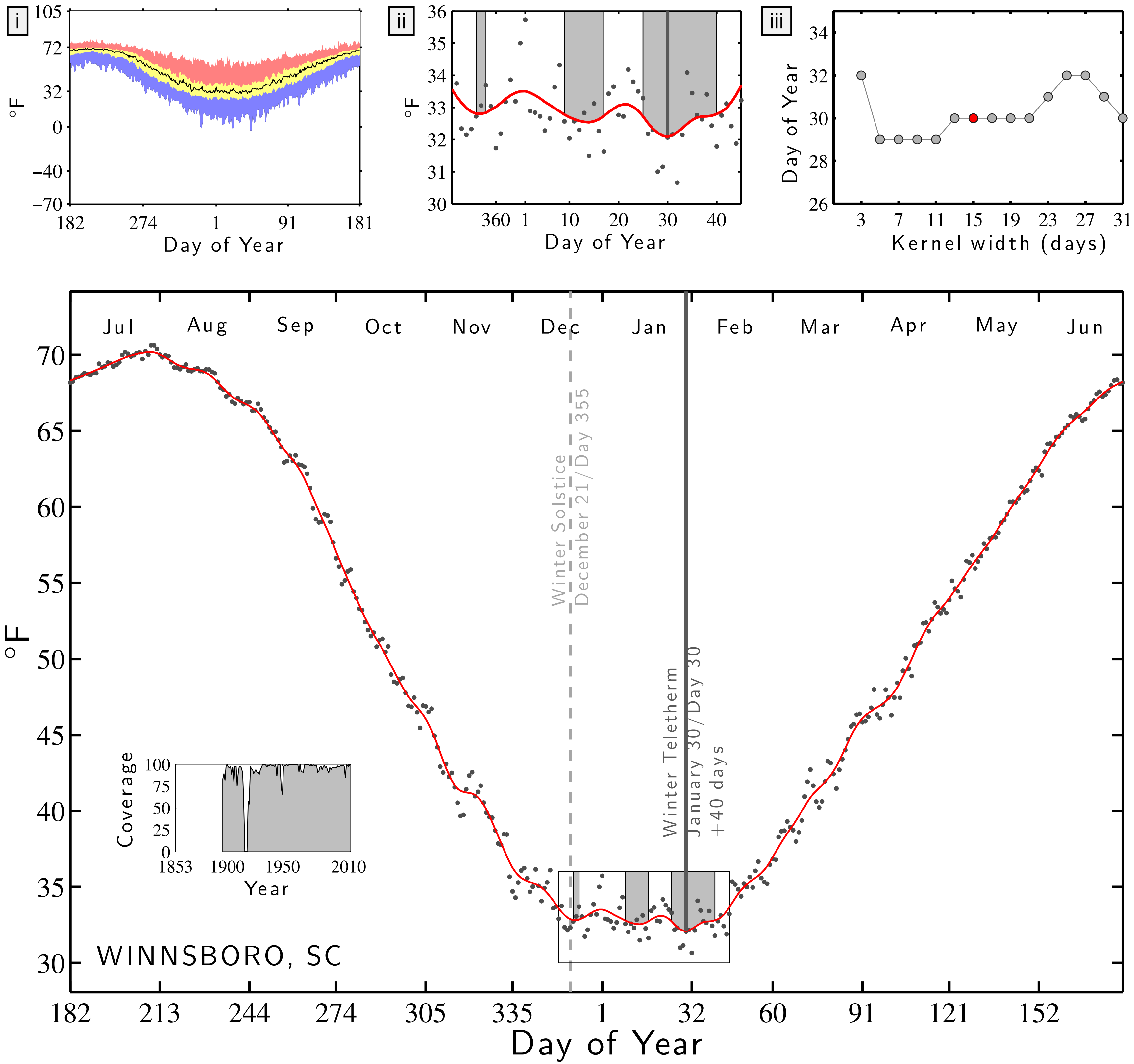

i
ii
iii
°F
Day of Year
Kernel width (days)
Jul
Aug
Sep
Oct
Nov
Dec
Jan
Feb
Mar
Apr
May
Jun
Winter Solstice
December 21/Day 355
Winter Teletherm
January 30/Day 30
+40 days
Coverage
Year
WINNSBORO, SC

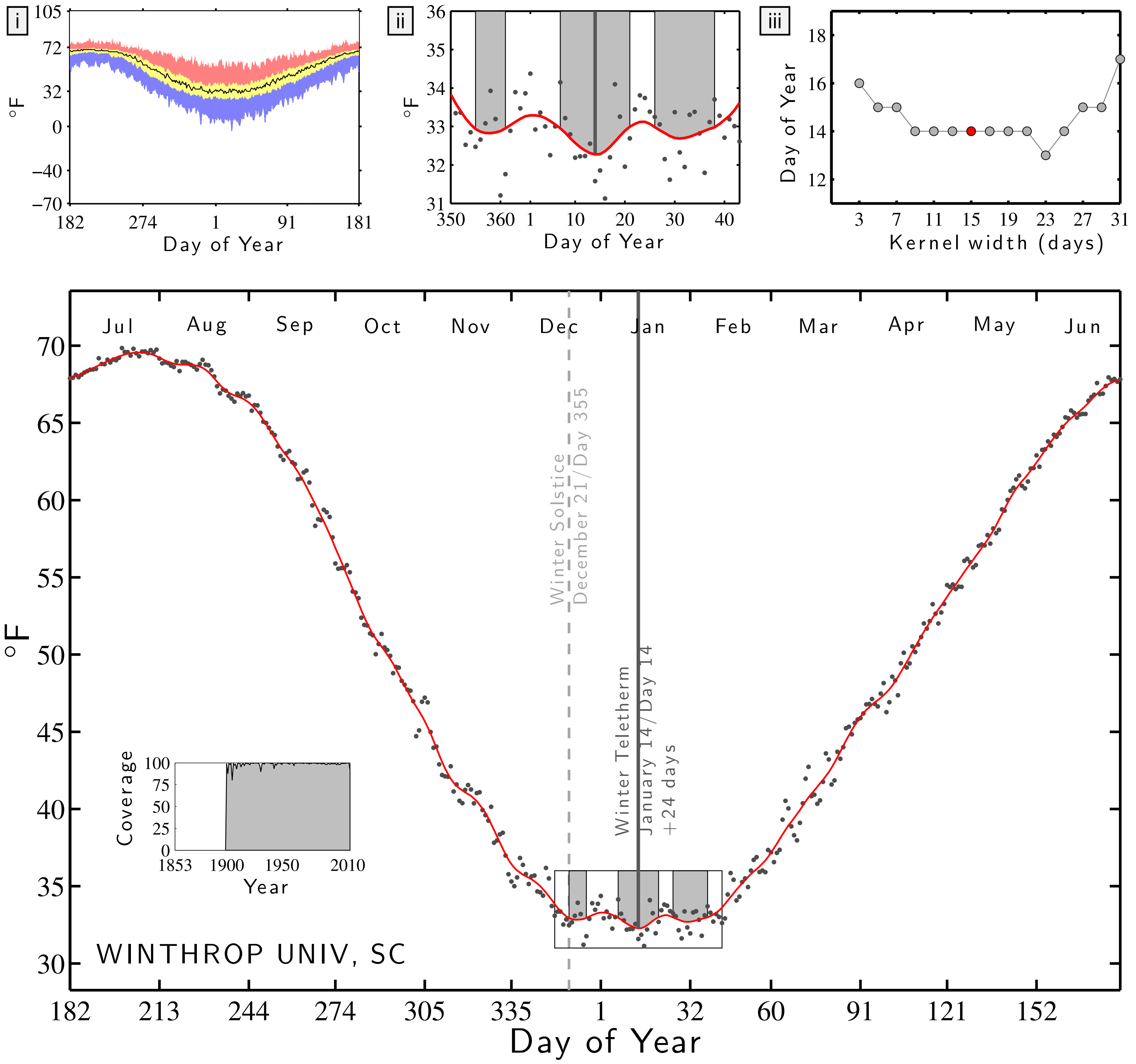

i
ii
iii
°F
Day of Year
Day of Year
Kernel width (days)
Jul
Aug
Sep
Oct
Nov
Dec
Jan
Feb
Mar
Apr
May
Jun
Winter Solstice
December 21/Day 355
Winter Teletherm
January 14/Day 14
+24 days
Coverage
Year
WINTHROP UNIV, SC

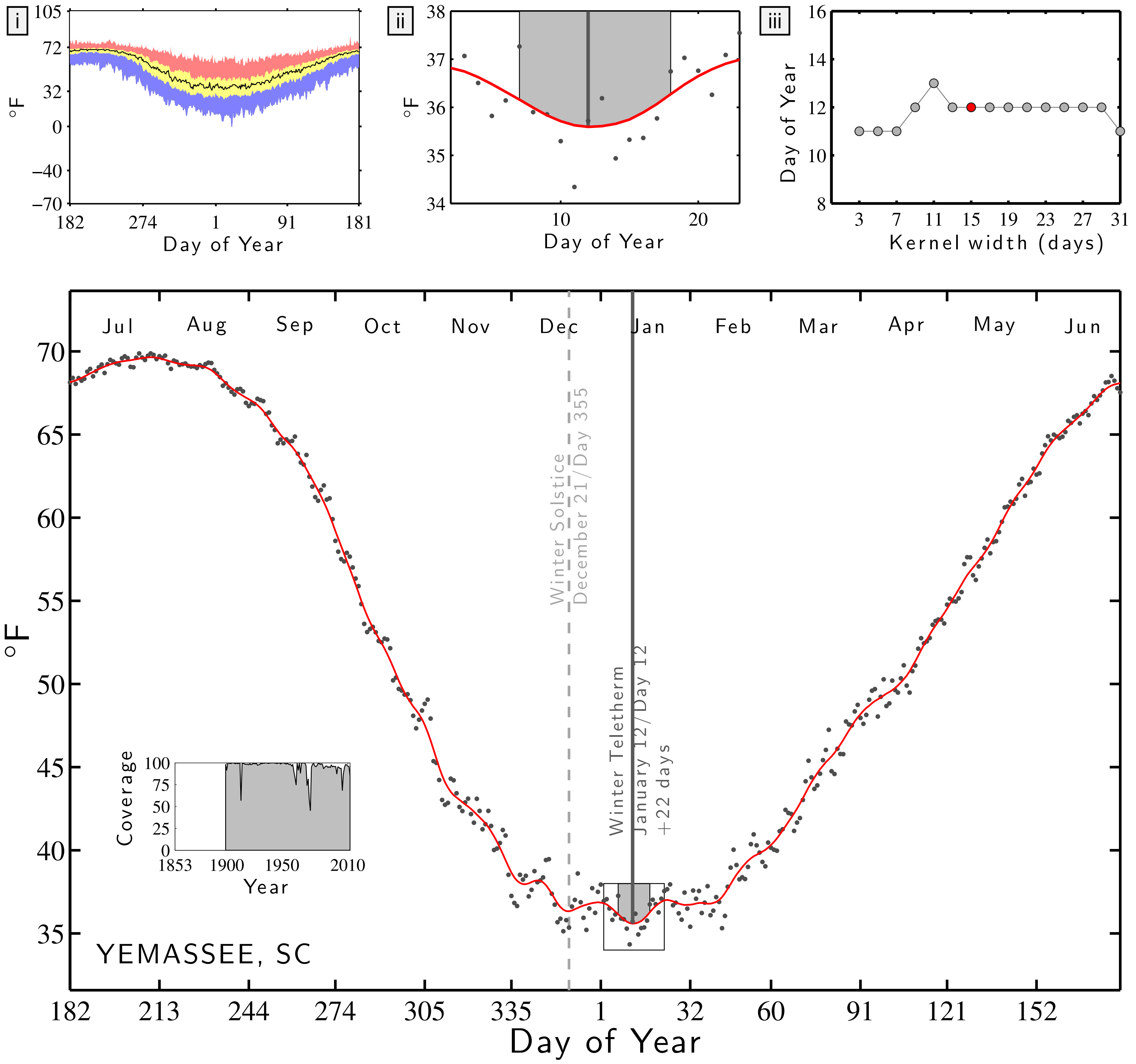

i
°F
Day of Year
ii
°F
Day of Year
iii
Day of Year
Kernel width (days)
Jul
Aug
Sep
Oct
Nov
Dec
Jan
Feb
Mar
Apr
May
Jun
Winter Solstice
December 21/Day 355
Winter Teletherm
January 12/Day 12
+22 days
Coverage
Year
YEMASSEE, SC
°F
Day of Year

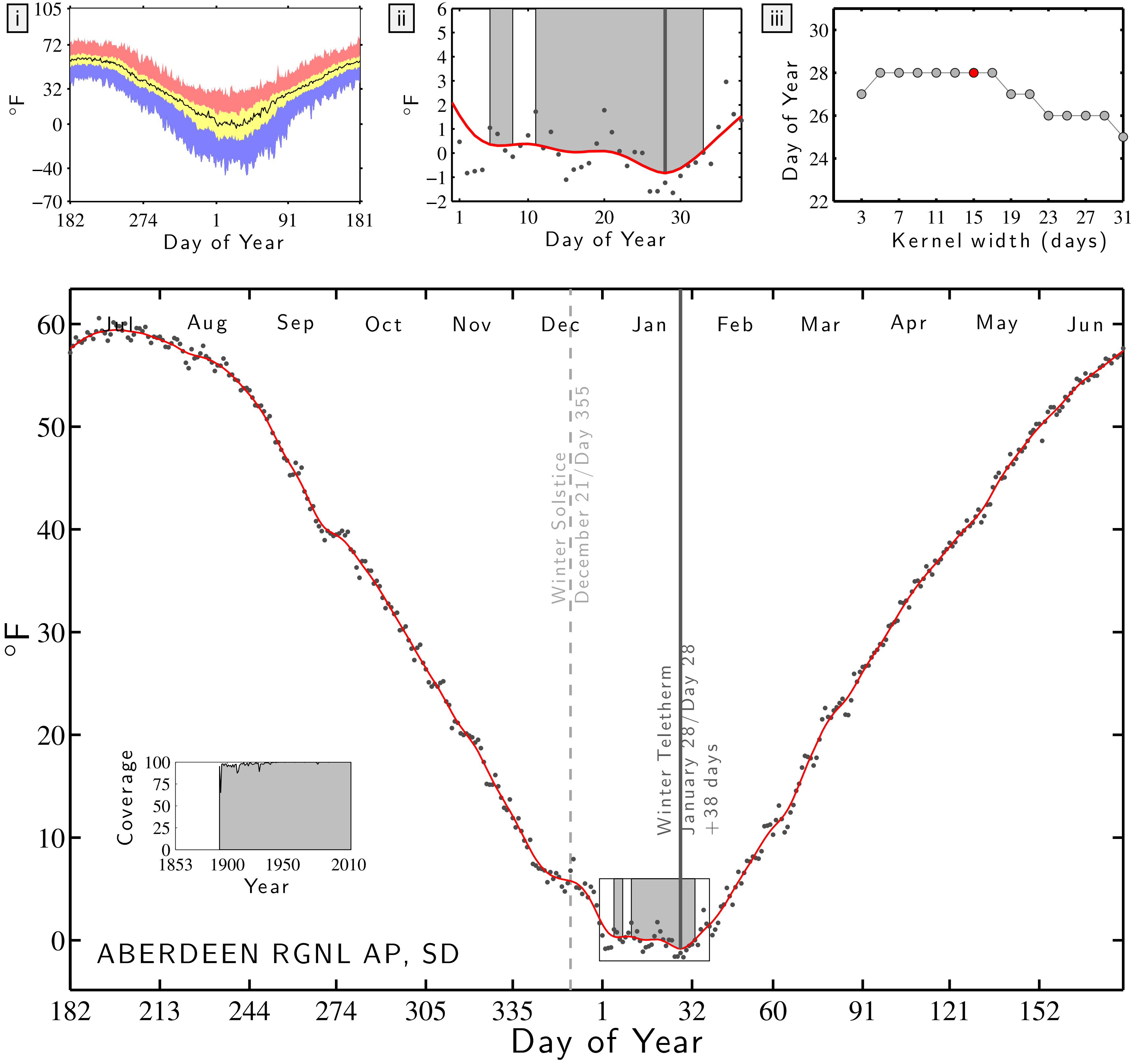

i
ii
iii
°F
Day of Year
Kernel width (days)
Jul
Aug
Sep
Oct
Nov
Dec
Jan
Feb
Mar
Apr
May
Jun
Winter Solstice
December 21/Day 355
Winter Teletherm
January 28/Day 28
+38 days
Coverage
Year
ABERDEEN RGNL AP, SD

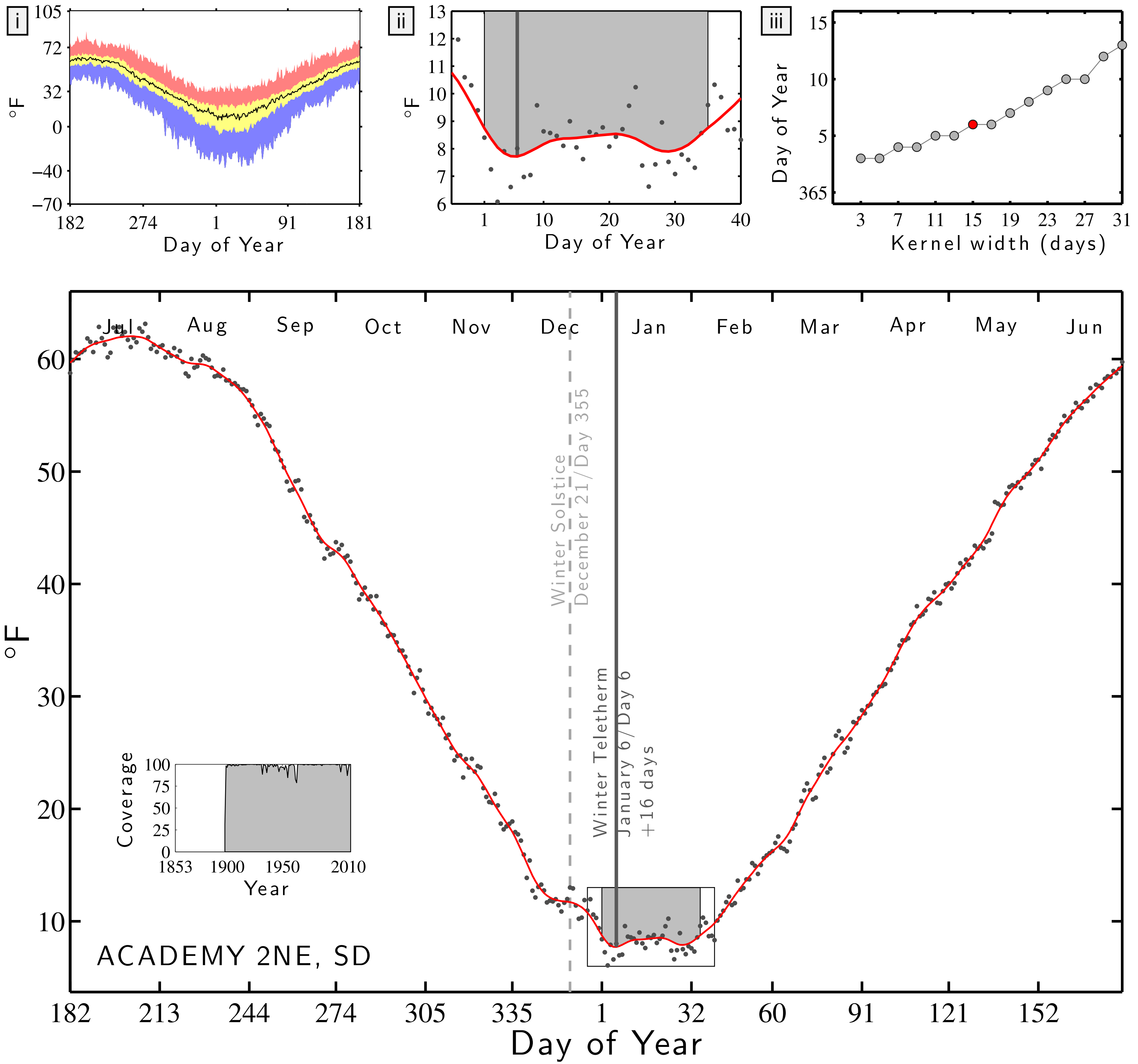

i
ii
iii
°F
Day of Year
Kernel width (days)
Jul
Aug
Sep
Oct
Nov
Dec
Jan
Feb
Mar
Apr
May
Jun
Winter Solstice
December 21/Day 355
Winter Teletherm
January 6/Day 6
+16 days
Coverage
Year
ACADEMY 2NE, SD

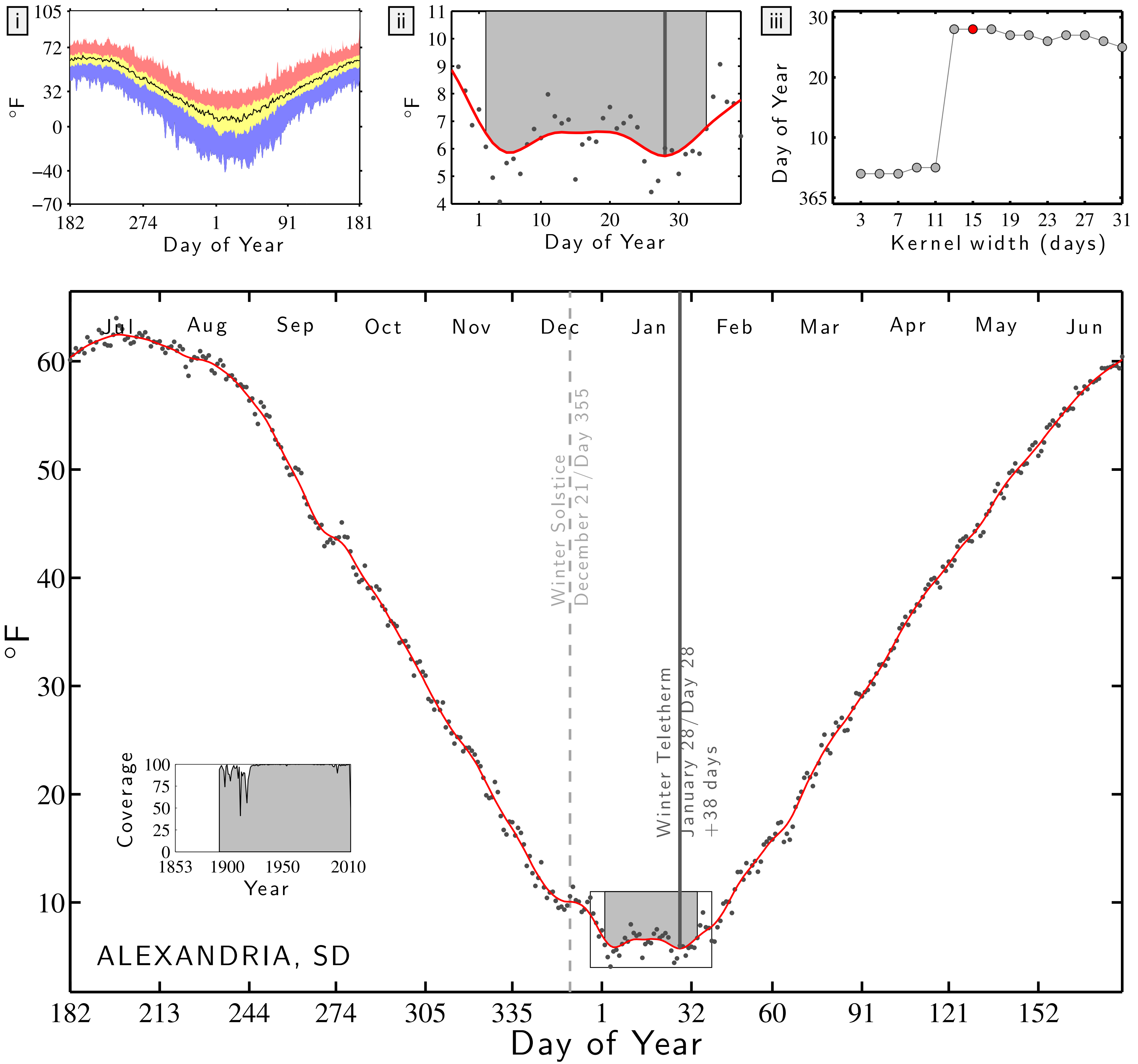

i
°F
105
72
32
0
−40
−70
182
274
1
91
181
Day of Year
ii
°F
11
10
9
8
7
6
5
4
1
10
20
30
Day of Year
iii
Day of Year
30
20
10
365
3
7
11
15
19
23
27
31
Kernel width (days)
Jul
Aug
Sep
Oct
Nov
Dec
Jan
Feb
Mar
Apr
May
Jun
°F
60
50
40
30
20
10
Winter Solstice
December 21/Day 355
Winter Teletherm
January 28/Day 28
+38 days
Coverage
100
75
50
25
0
1853
1900
1950
2010
Year
ALEXANDRIA, SD
182
213
244
274
305
335
1
32
60
91
121
152
Day of Year

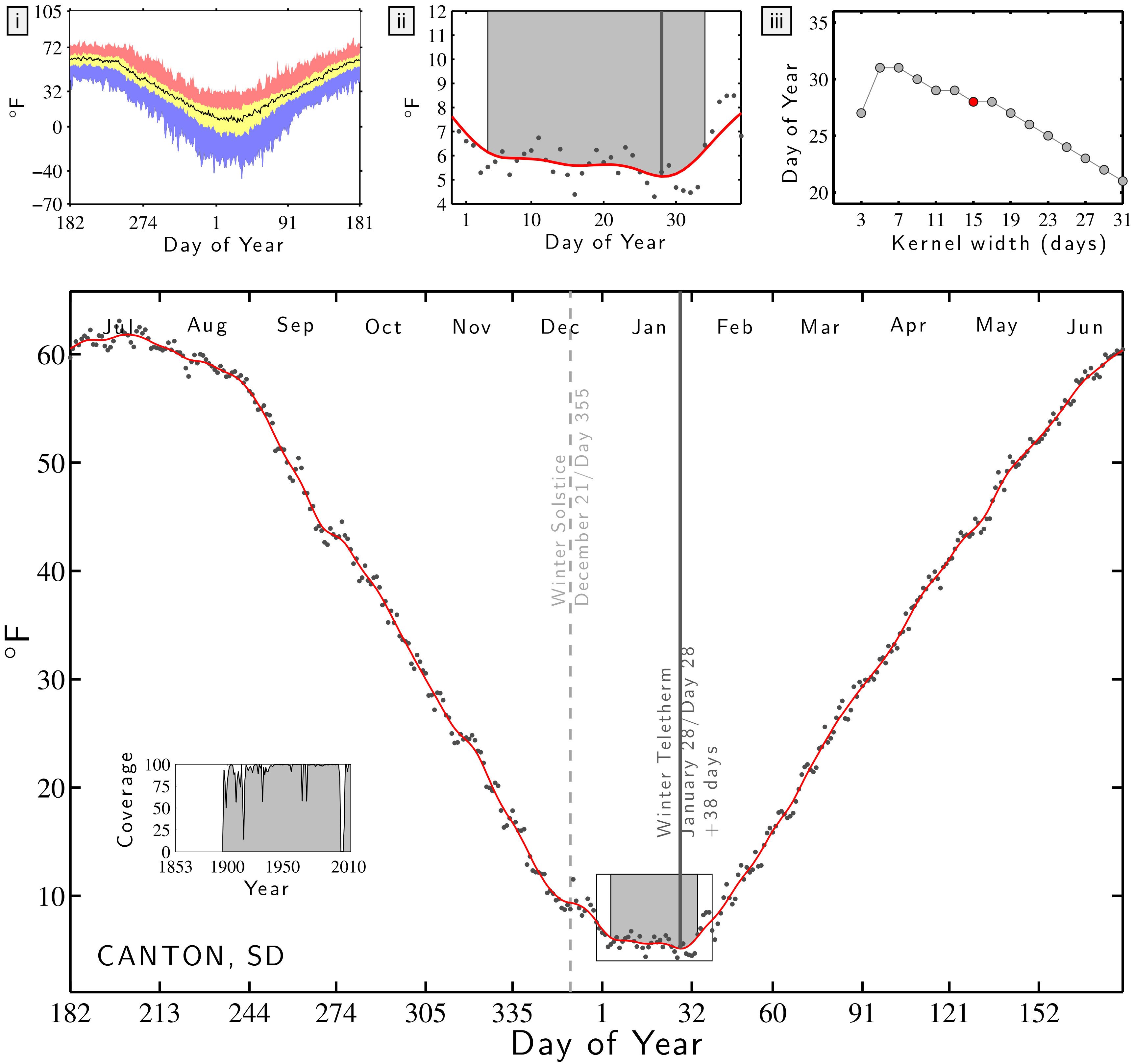

i
°F
Day of Year
ii
°F
Day of Year
iii
Day of Year
Kernel width (days)
Jul
Aug
Sep
Oct
Nov
Dec
Jan
Feb
Mar
Apr
May
Jun
Winter Solstice
December 21/Day 355
Winter Teletherm
January 28/Day 28
+38 days
Coverage
Year
CANTON, SD
°F
Day of Year

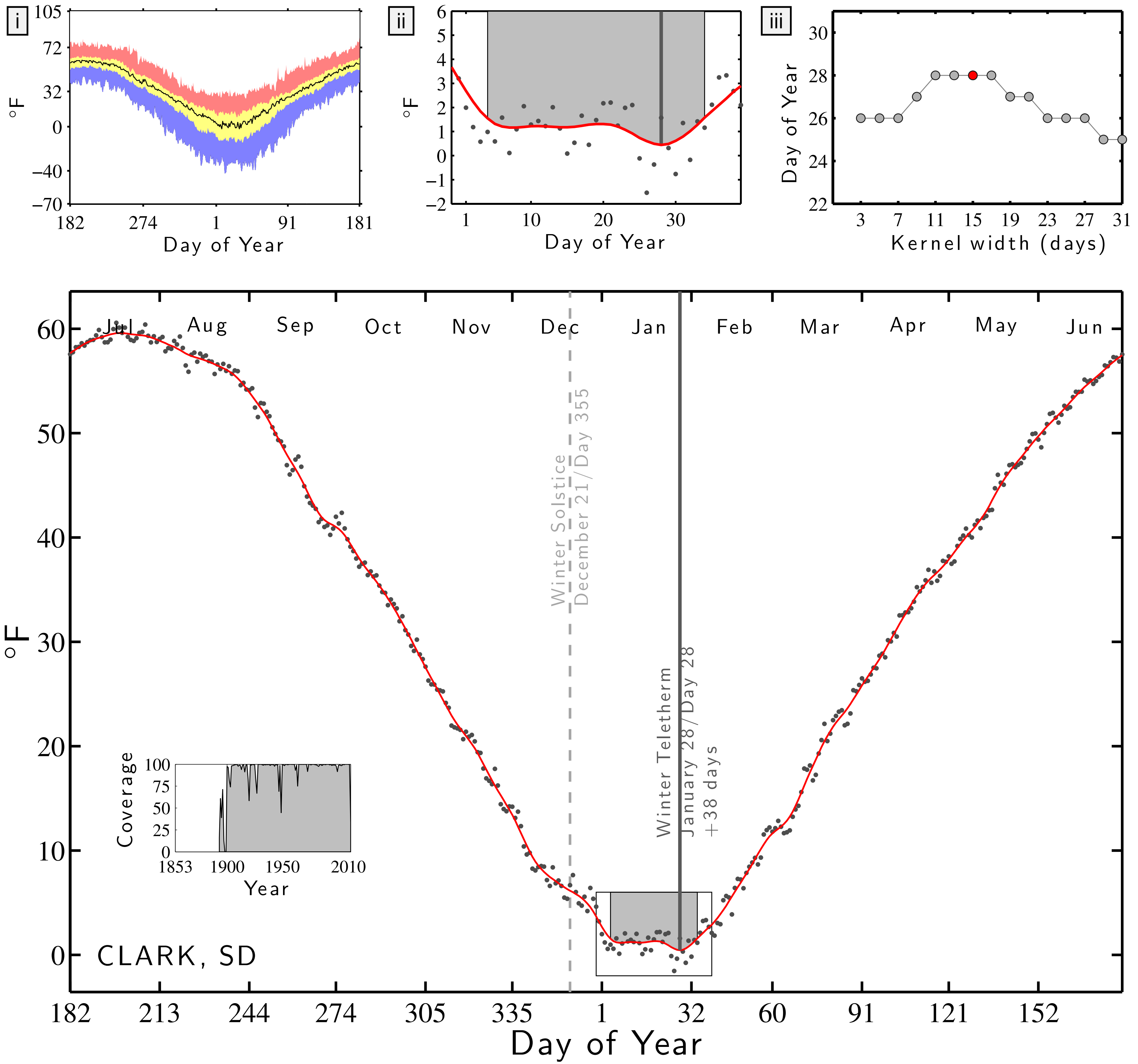

i
°F
Day of Year
ii
Day of Year
iii
Day of Year
Kernel width (days)
Jul
Aug
Sep
Oct
Nov
Dec
Jan
Feb
Mar
Apr
May
Jun
Winter Solstice
December 21/Day 355
Winter Teletherm
January 28/Day 28
+38 days
Coverage
Year
CLARK, SD
Day of Year

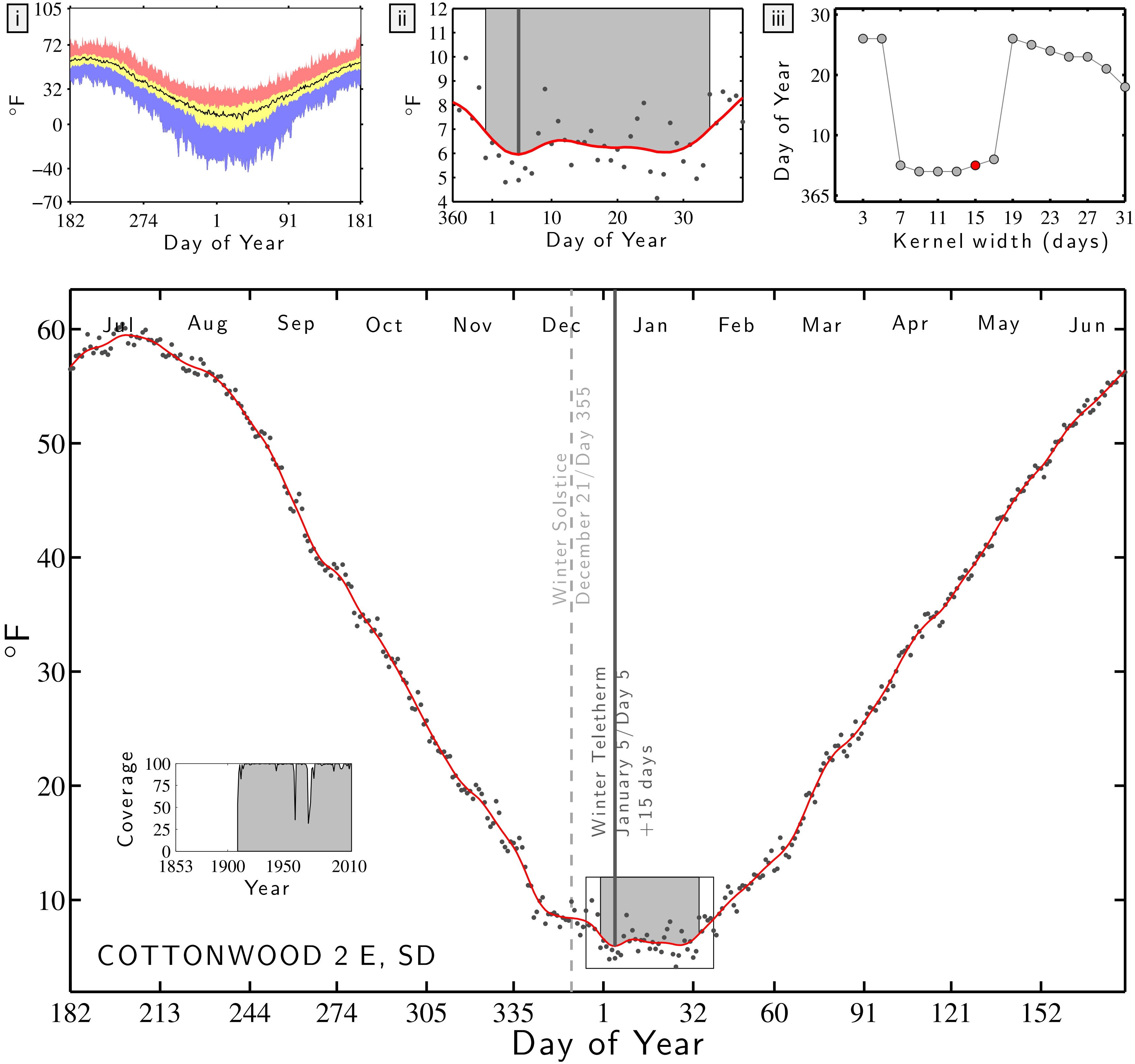

i
°F
105
72
32
0
−40
−70
182
274
1
91
181
Day of Year
ii
°F
360
10
20
30
Day of Year
iii
Day of Year
365
Kernel width (days)
Jul
Aug
Sep
Oct
Nov
Dec
Jan
Feb
Mar
Apr
May
Jun
Winter Solstice
December 21/Day 355
Winter Teletherm
January 5/Day 5
+15 days
Coverage
Year
1853
1900
1950
2010
COTTONWOOD 2 E, SD
°F
Day of Year
182
213
244
274
305
335
1
32
60
91
121
152

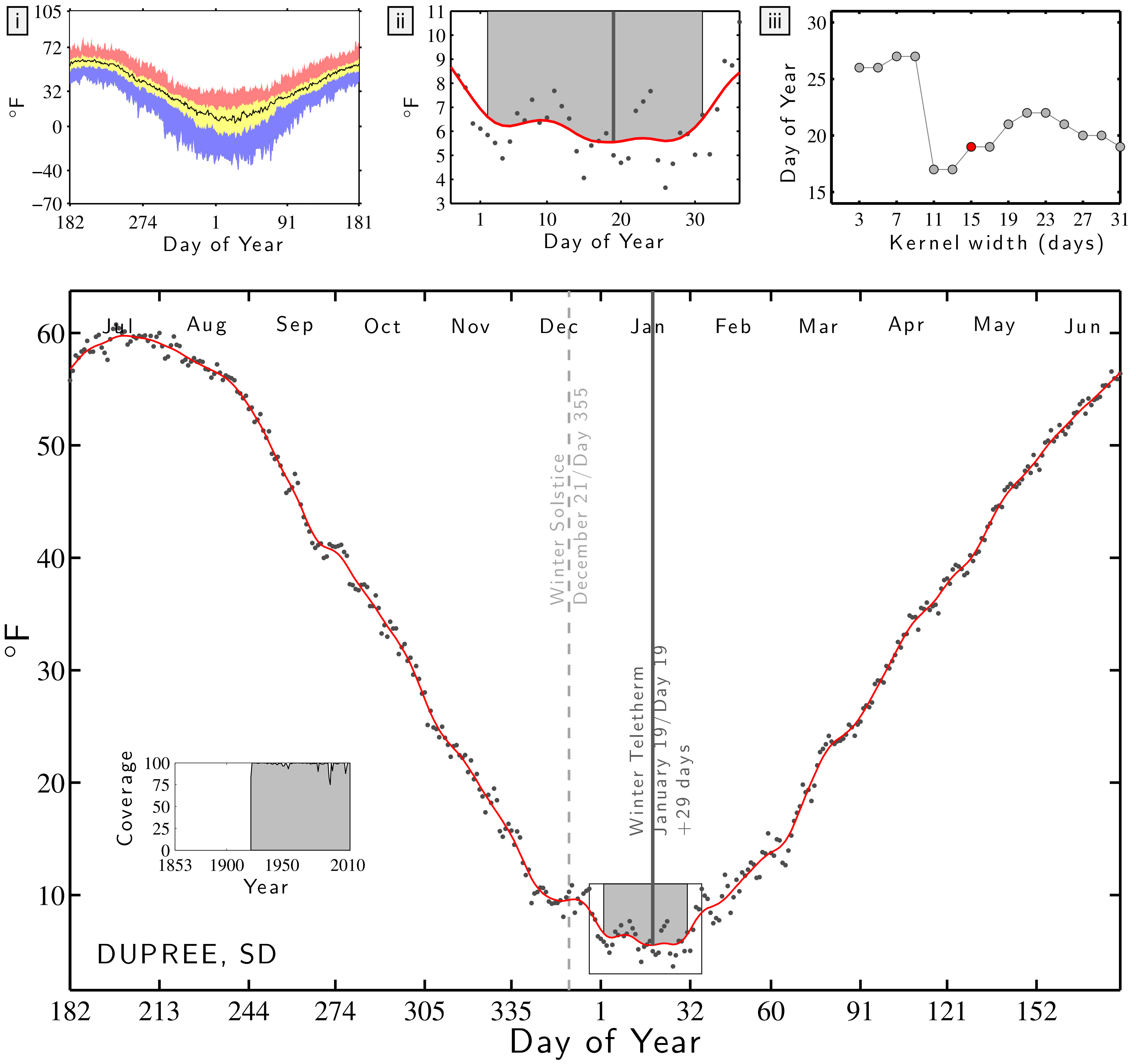
i
ii
iii
°F
Day of Year
Kernel width (days)
Jul
Aug
Sep
Oct
Nov
Dec
Jan
Feb
Mar
Apr
May
Jun
Winter Solstice
December 21/Day 355
Winter Teletherm
January 19/Day 19
+29 days
Coverage
Year
DUPREE, SD

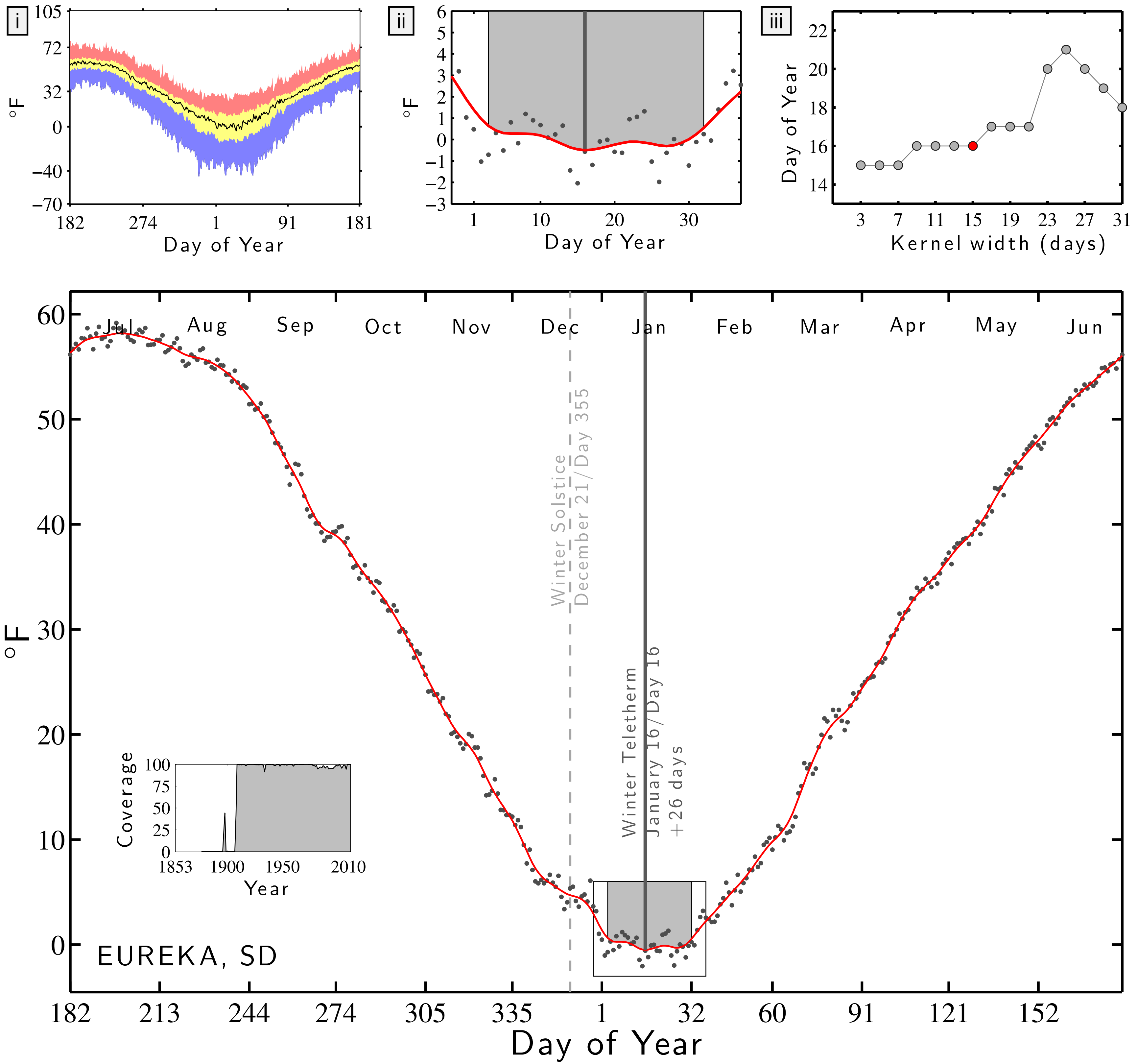

i
°F
Day of Year
ii
°F
Day of Year
iii
Day of Year
Kernel width (days)
Jul
Aug
Sep
Oct
Nov
Dec
Jan
Feb
Mar
Apr
May
Jun
Winter Solstice
December 21/Day 355
Winter Teletherm
January 16/Day 16
+26 days
Coverage
Year
EUREKA, SD
°F
Day of Year

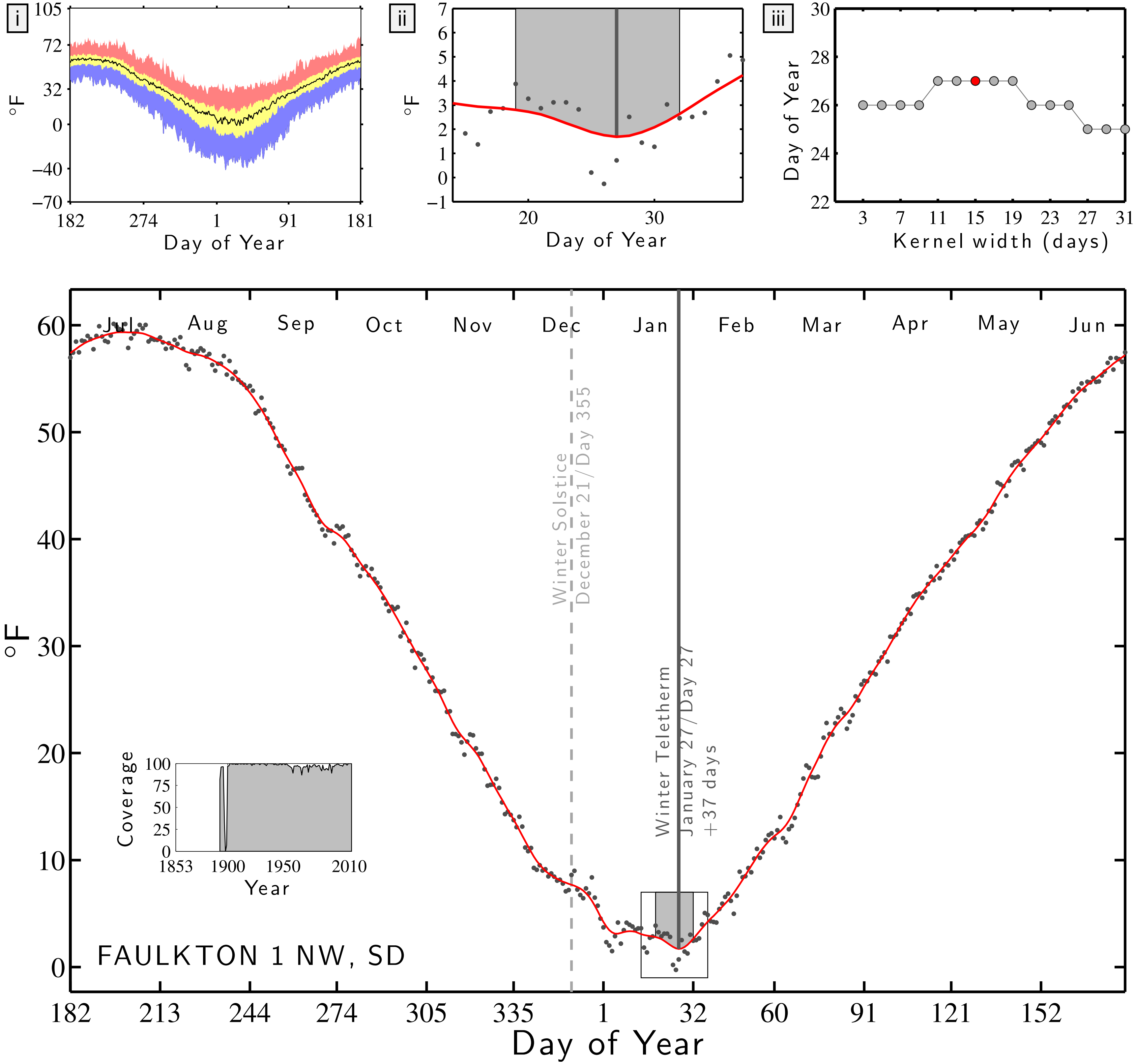

i
ii
iii
°F
Day of Year
Day of Year
Kernel width (days)
Jul
Aug
Sep
Oct
Nov
Dec
Jan
Feb
Mar
Apr
May
Jun
Winter Solstice
December 21/Day 355
Winter Teletherm
January 27/Day 27
+37 days
Coverage
Year
FAULKTON 1 NW, SD

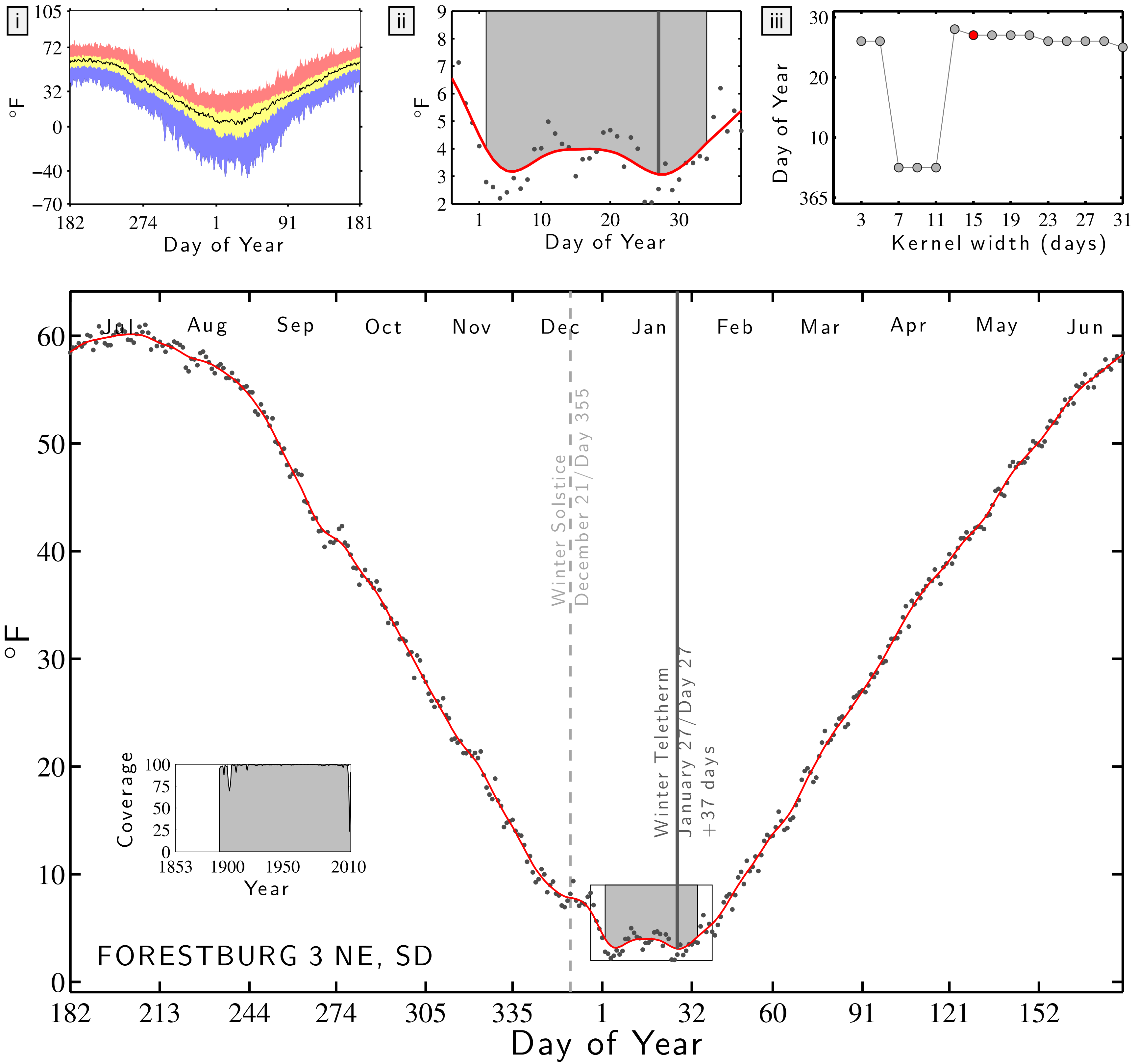

i
°F
Day of Year
ii
°F
Day of Year
iii
Day of Year
Kernel width (days)
Jul
Aug
Sep
Oct
Nov
Dec
Jan
Feb
Mar
Apr
May
Jun
Winter Solstice
December 21/Day 355
Winter Teletherm
January 27/Day 27
+37 days
Coverage
Year
FORESTBURG 3 NE, SD
°F
Day of Year

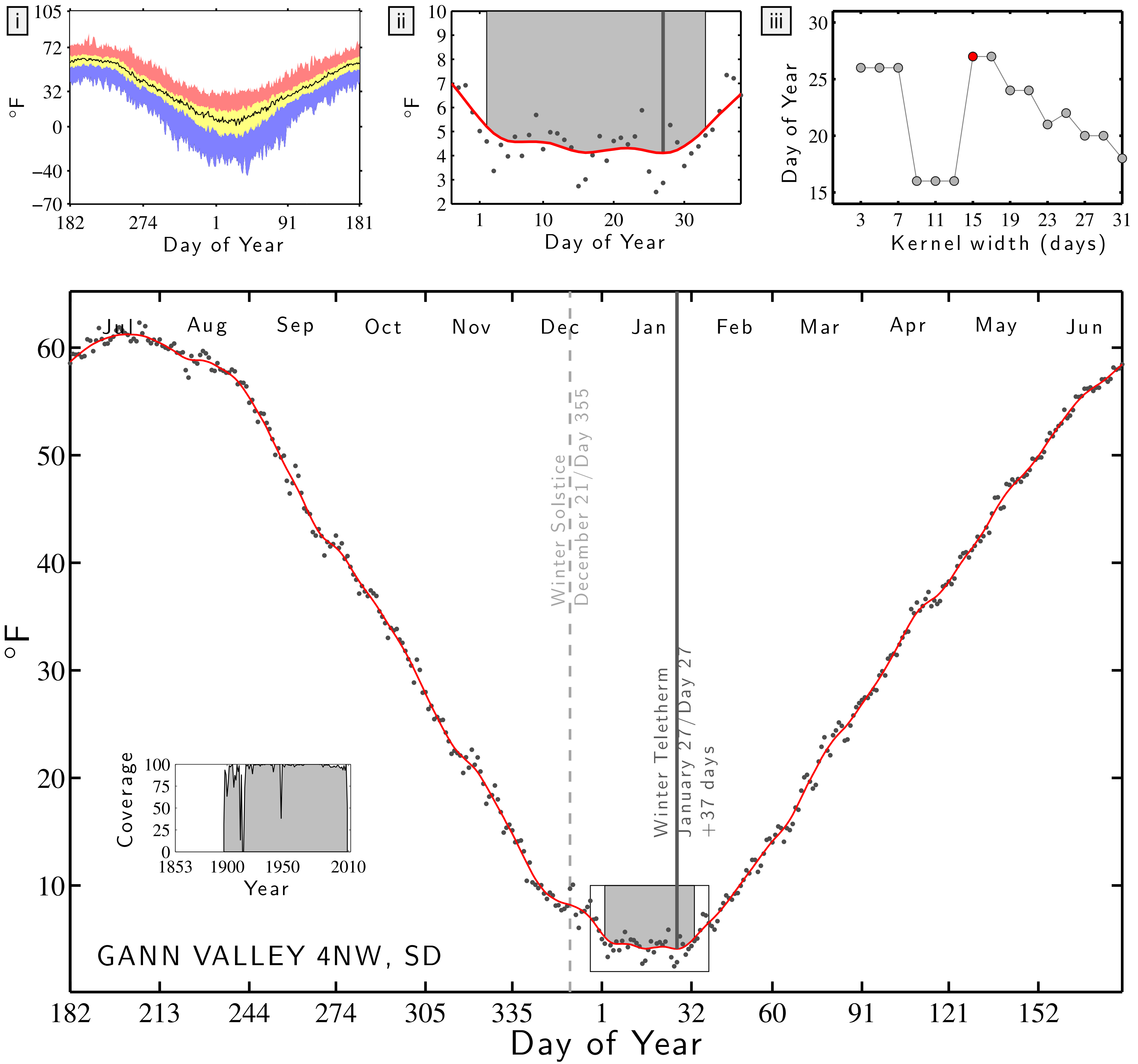

i
105
72
32
0
−40
−70
°F
182
274
1
91
181
Day of Year
ii
10
9
8
7
6
5
4
3
2
°F
1
10
20
30
Day of Year
iii
30
25
20
15
Day of Year
3
7
11
15
19
23
27
31
Kernel width (days)
Jul
Aug
Sep
Oct
Nov
Dec
Jan
Feb
Mar
Apr
May
Jun
60
50
40
30
20
10
°F
Winter Solstice
December 21/Day 355
Winter Teletherm
January 27/Day 27
+37 days
Coverage
100
75
50
25
0
1853
1900
1950
2010
Year
GANN VALLEY 4NW, SD
182
213
244
274
305
335
1
32
60
91
121
152
Day of Year

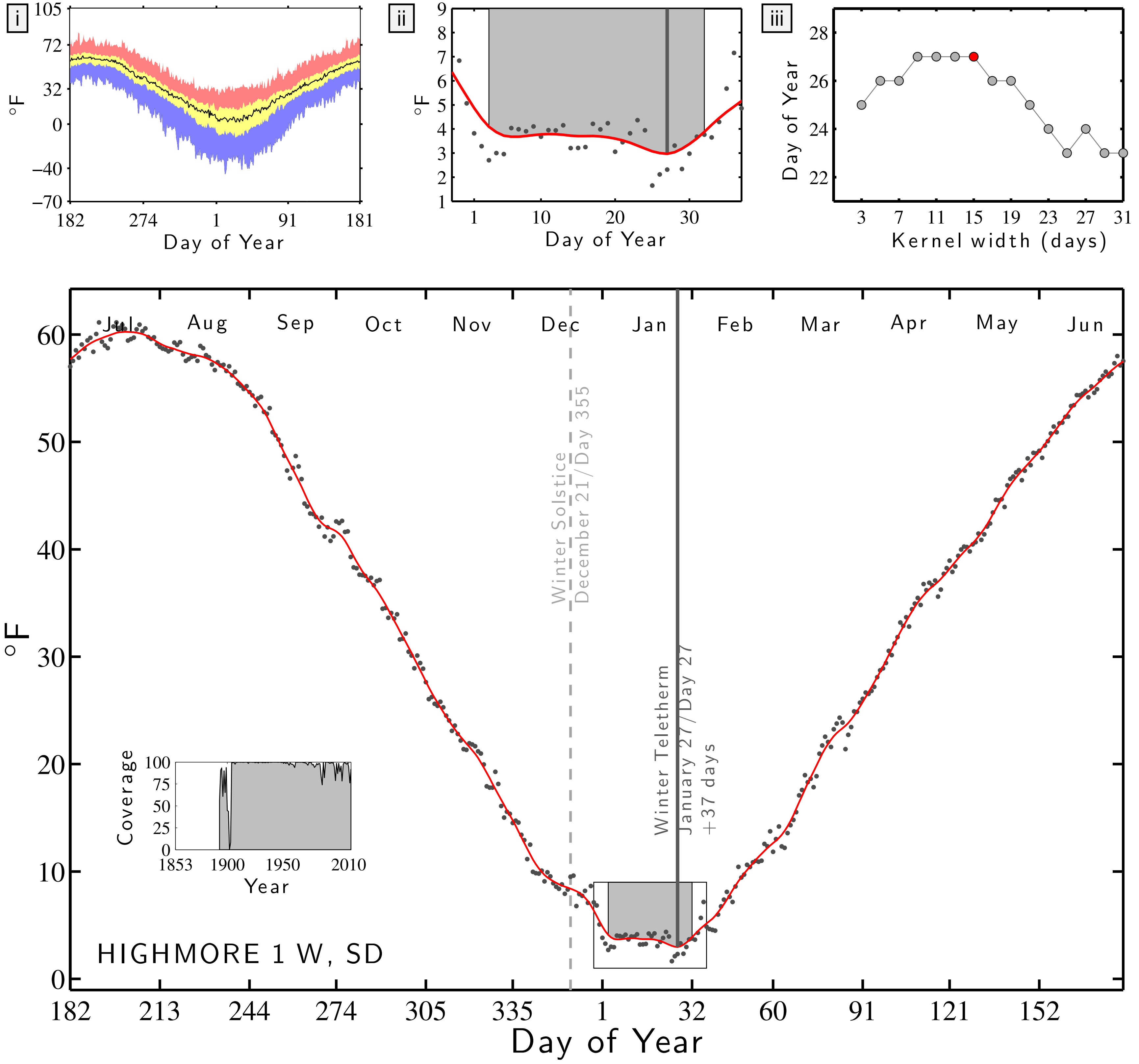

i
ii
iii
°F
Day of Year
Day of Year
Kernel width (days)
Jul
Aug
Sep
Oct
Nov
Dec
Jan
Feb
Mar
Apr
May
Jun
Winter Solstice
December 21/Day 355
Winter Teletherm
January 27/Day 27
+37 days
Coverage
Year
HIGHMORE 1 W, SD

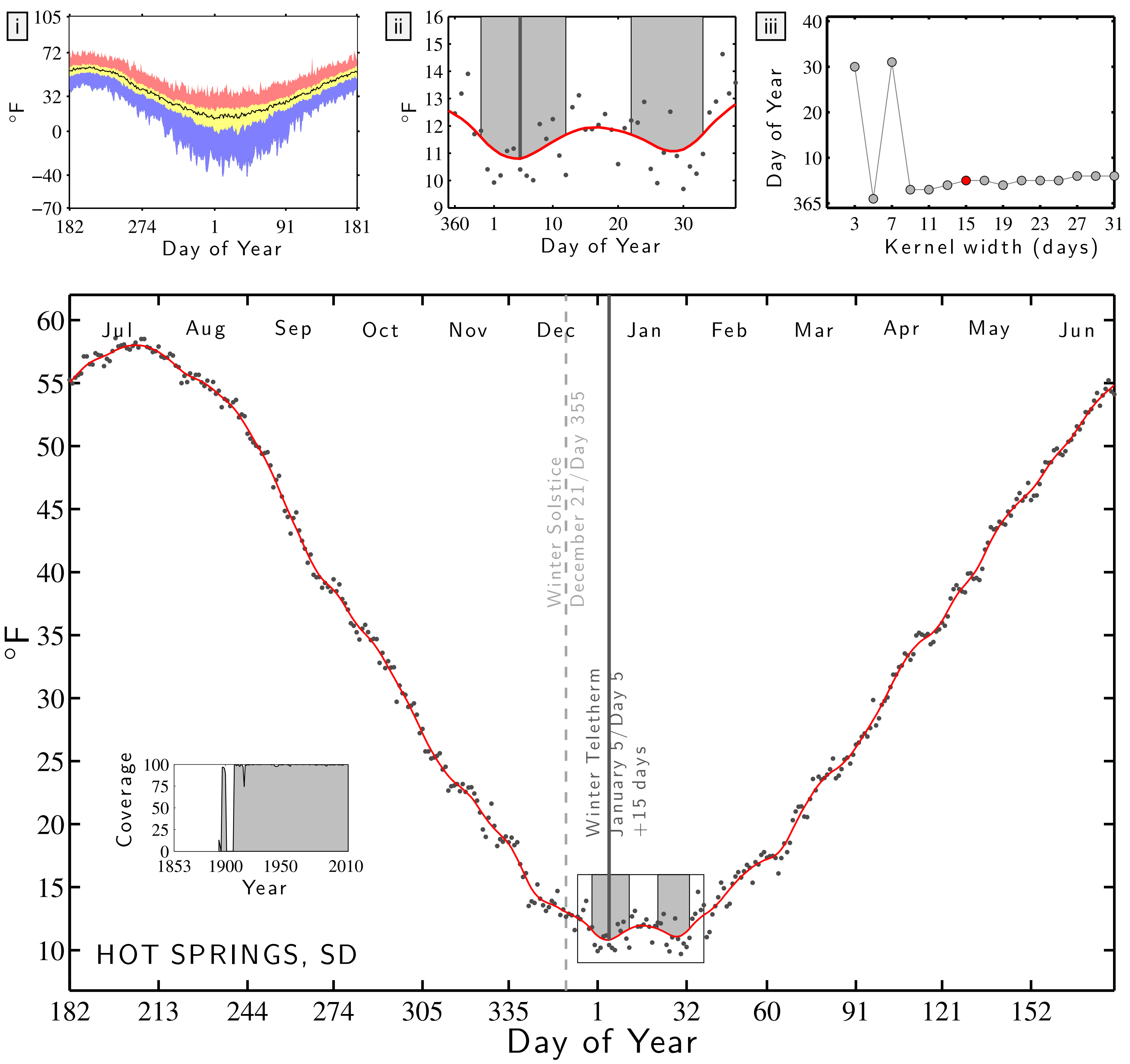

i
°F
Day of Year
ii
Day of Year
iii
Day of Year
Kernel width (days)
Jul
Aug
Sep
Oct
Nov
Dec
Jan
Feb
Mar
Apr
May
Jun
Winter Solstice
December 21/Day 355
Winter Teletherm
January 5/Day 5
+15 days
Coverage
Year
HOT SPRINGS, SD
Day of Year

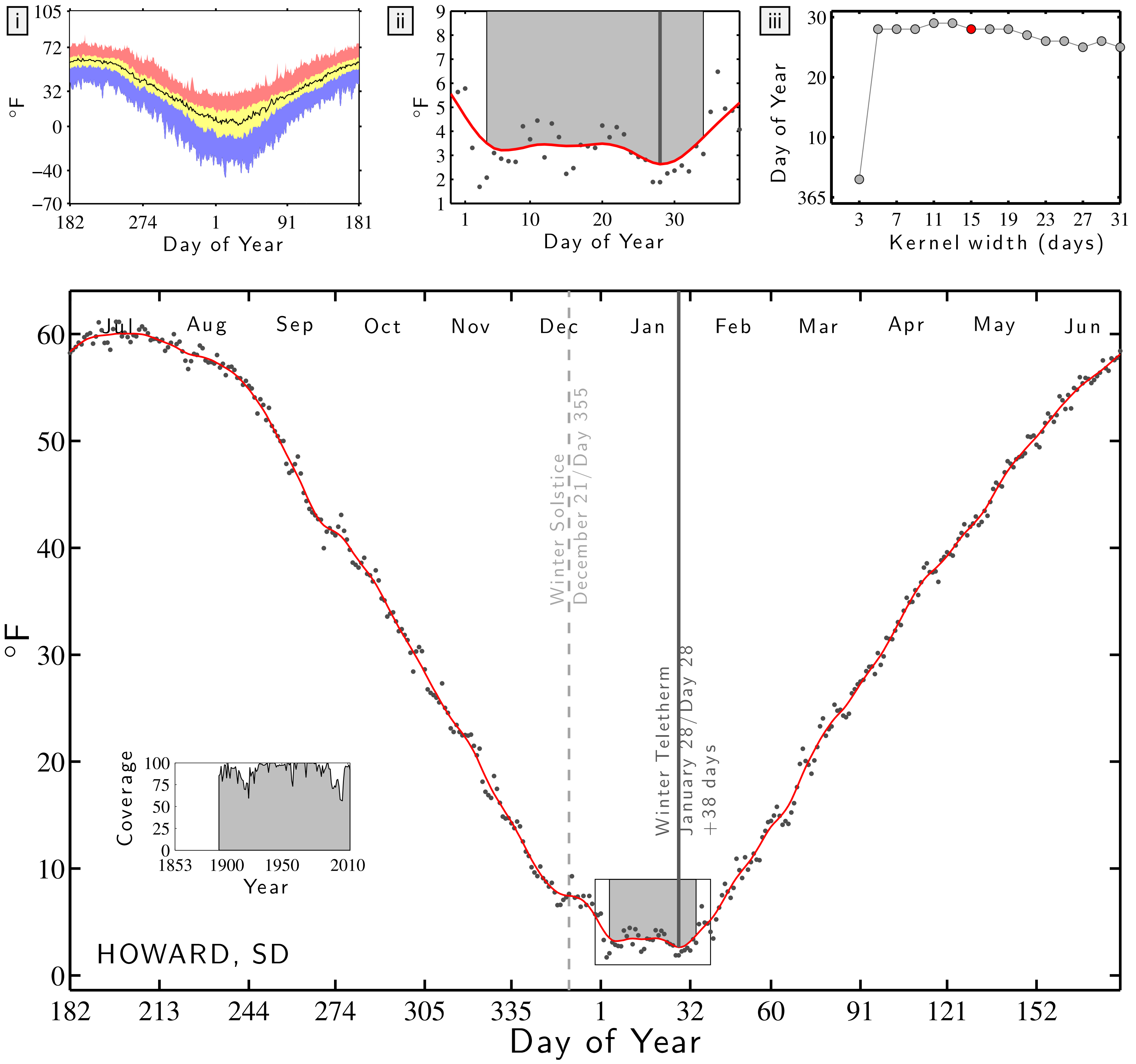

i
ii
iii
°F
Day of Year
Kernel width (days)
Jul
Aug
Sep
Oct
Nov
Dec
Jan
Feb
Mar
Apr
May
Jun
Winter Solstice
December 21/Day 355
Winter Teletherm
January 28/Day 28
+38 days
Coverage
Year
HOWARD, SD

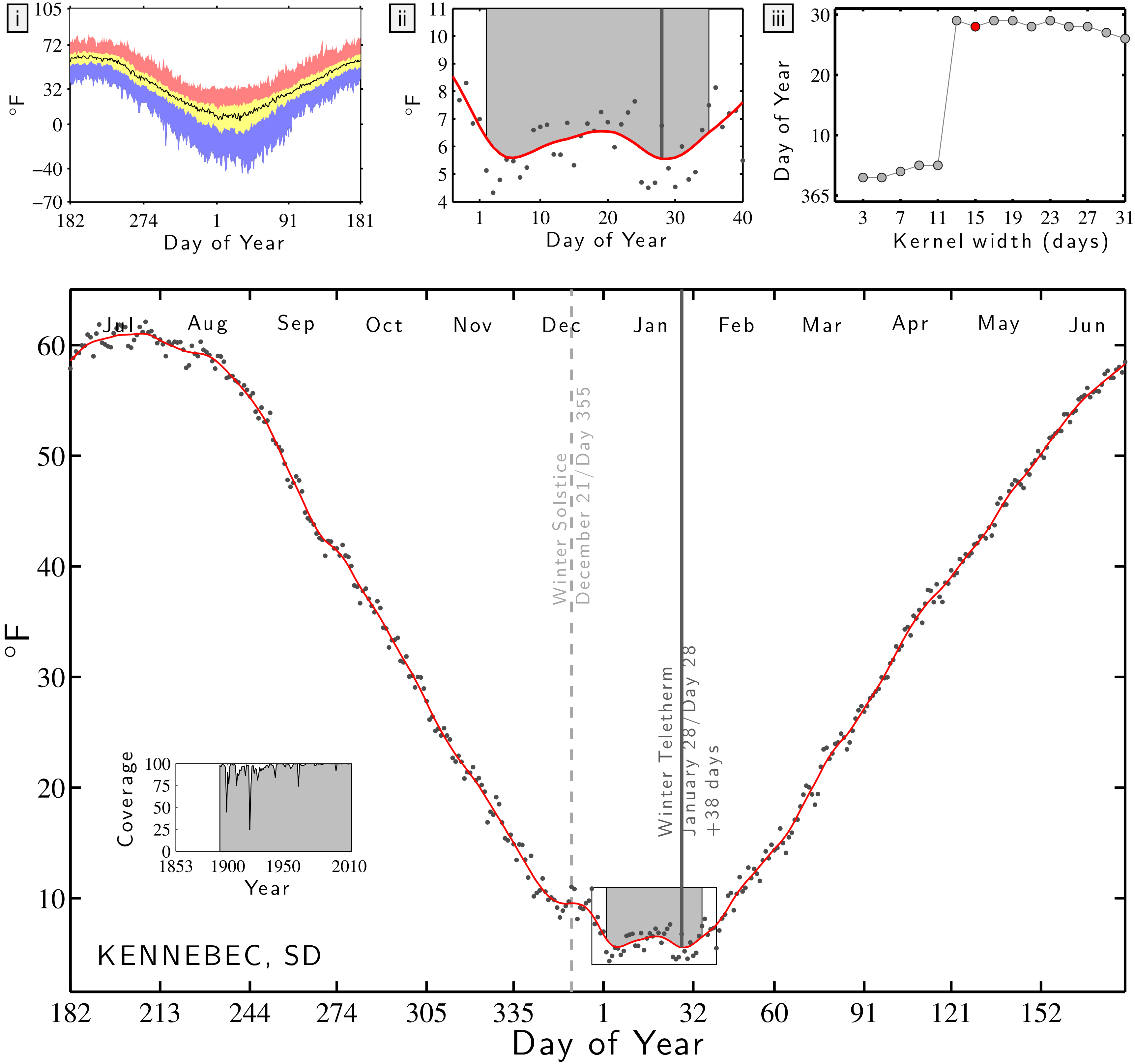

i
°F
Day of Year
ii
°F
Day of Year
iii
Day of Year
Kernel width (days)
Jul
Aug
Sep
Oct
Nov
Dec
Jan
Feb
Mar
Apr
May
Jun
Winter Solstice
December 21/Day 355
Winter Teletherm
January 28/Day 28
+38 days
Coverage
Year
KENNEBEC, SD
°F
Day of Year

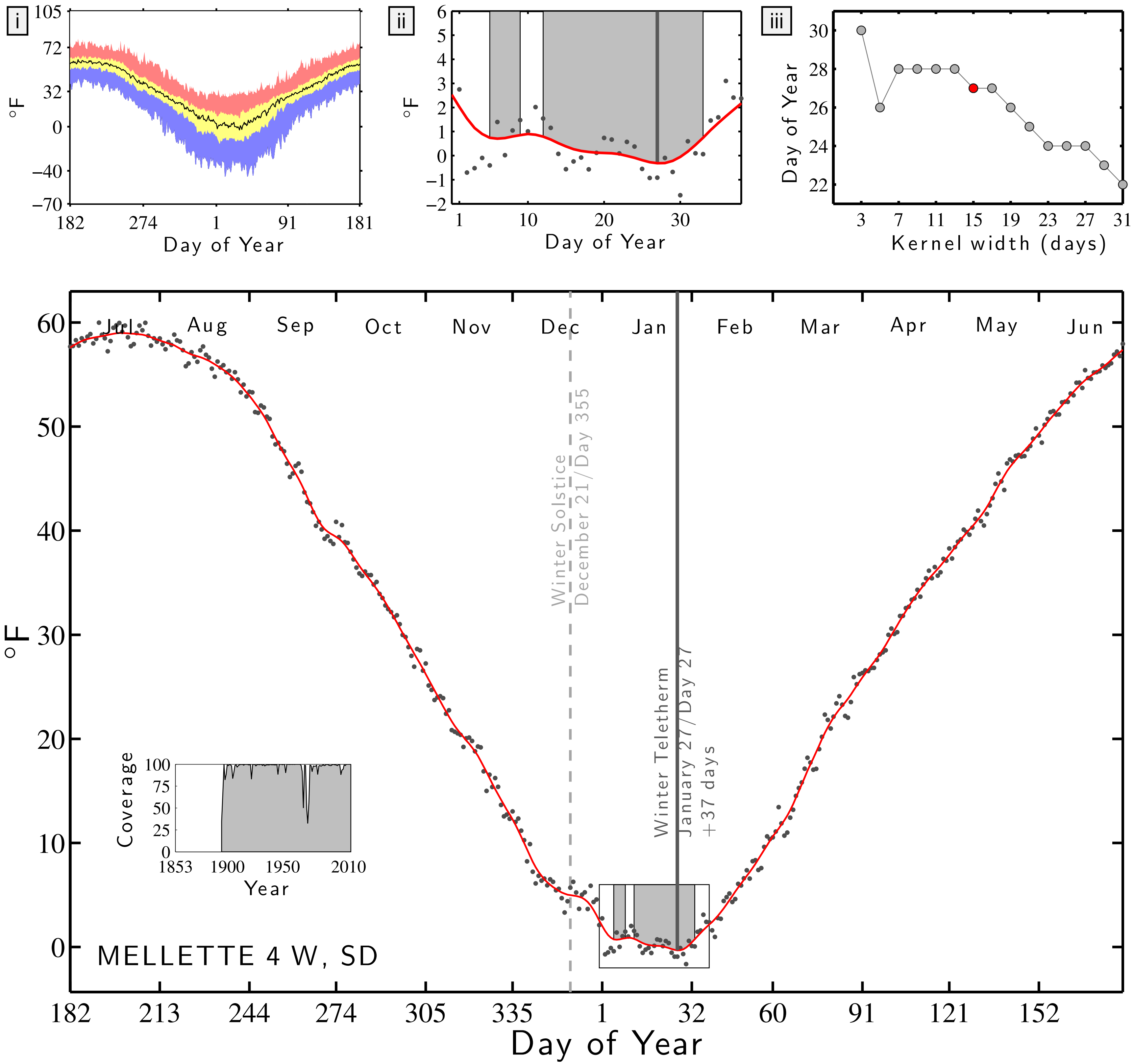

i
ii
iii
°F
Day of Year
Day of Year
Kernel width (days)
Winter Solstice
December 21/Day 355
Winter Teletherm
January 27/Day 27
+37 days
Coverage
Year
MELLETTE 4 W, SD
Jul
Aug
Sep
Oct
Nov
Dec
Jan
Feb
Mar
Apr
May
Jun

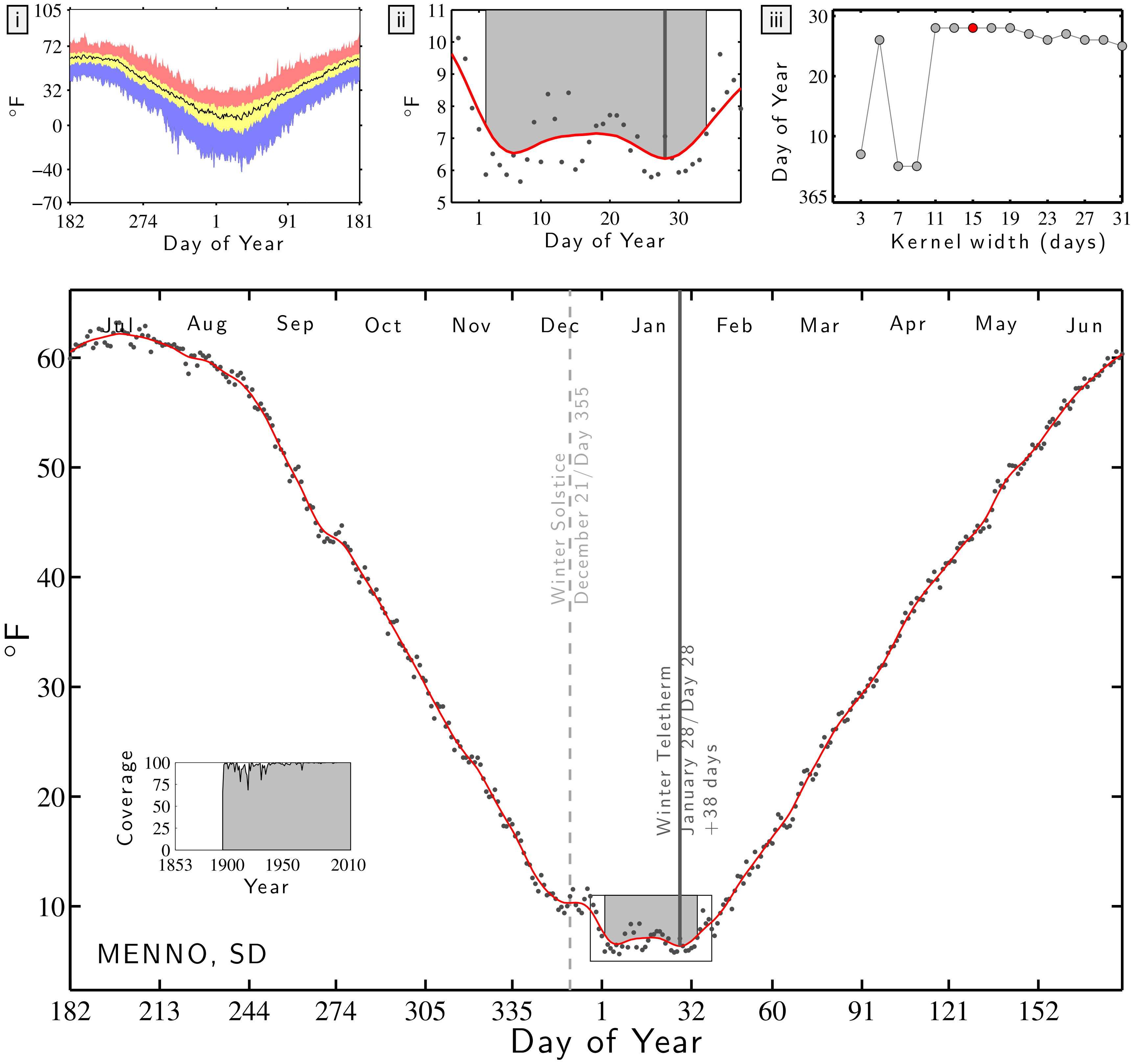

i
°F
105
72
32
0
−40
−70
182
274
1
91
181
Day of Year
ii
°F
11
10
9
8
7
6
5
1
10
20
30
Day of Year
iii
Day of Year
30
20
10
365
3
7
11
15
19
23
27
31
Kernel width (days)
Jul
Aug
Sep
Oct
Nov
Dec
Jan
Feb
Mar
Apr
May
Jun
Winter Solstice
December 21/Day 355
Winter Teletherm
January 28/Day 28
+38 days
°F
60
50
40
30
20
10
Coverage
100
75
50
25
0
1853
1900
1950
2010
Year
MENNO, SD
182
213
244
274
305
335
1
32
60
91
121
152
Day of Year

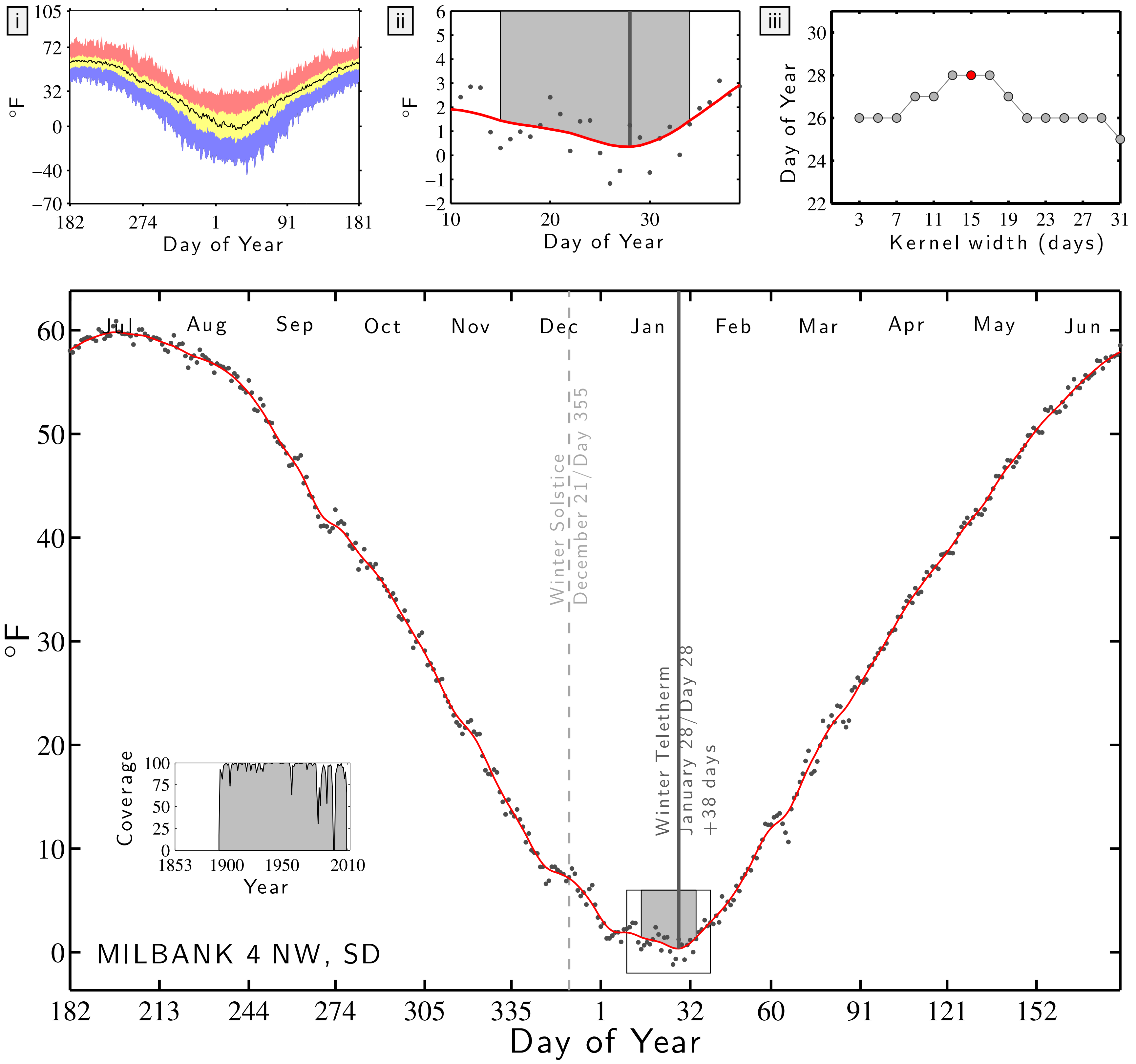

i
°F
Day of Year
ii
°F
Day of Year
iii
Day of Year
Kernel width (days)
Jul
Aug
Sep
Oct
Nov
Dec
Jan
Feb
Mar
Apr
May
Jun
Winter Solstice
December 21/Day 355
Winter Teletherm
January 28/Day 28
+38 days
Coverage
Year
MILBANK 4 NW, SD
°F
Day of Year

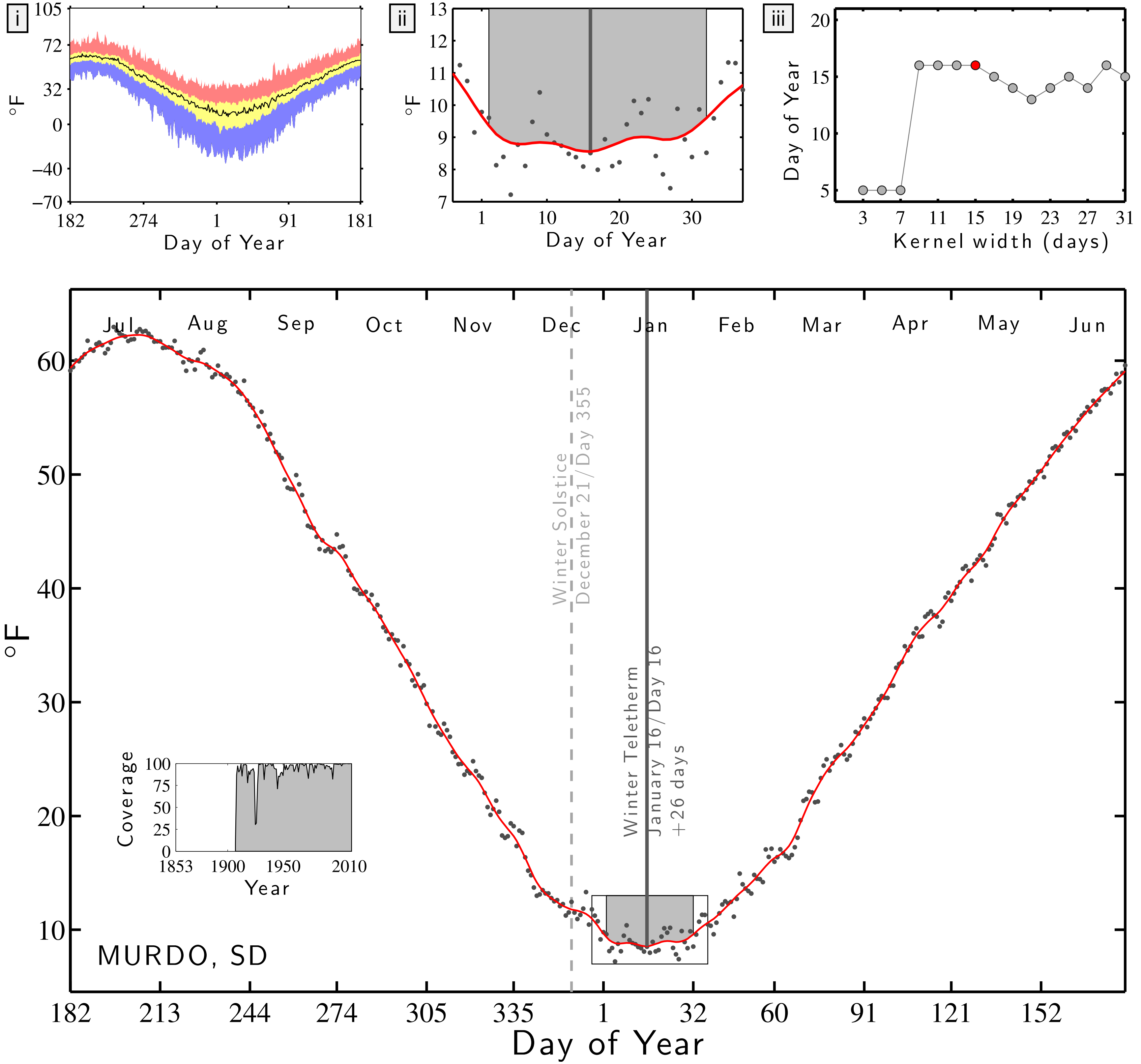

i
ii
iii
°F
Day of Year
Kernel width (days)
Jul
Aug
Sep
Oct
Nov
Dec
Jan
Feb
Mar
Apr
May
Jun
Winter Solstice
December 21/Day 355
Winter Teletherm
January 16/Day 16
+26 days
Coverage
Year
MURDO, SD

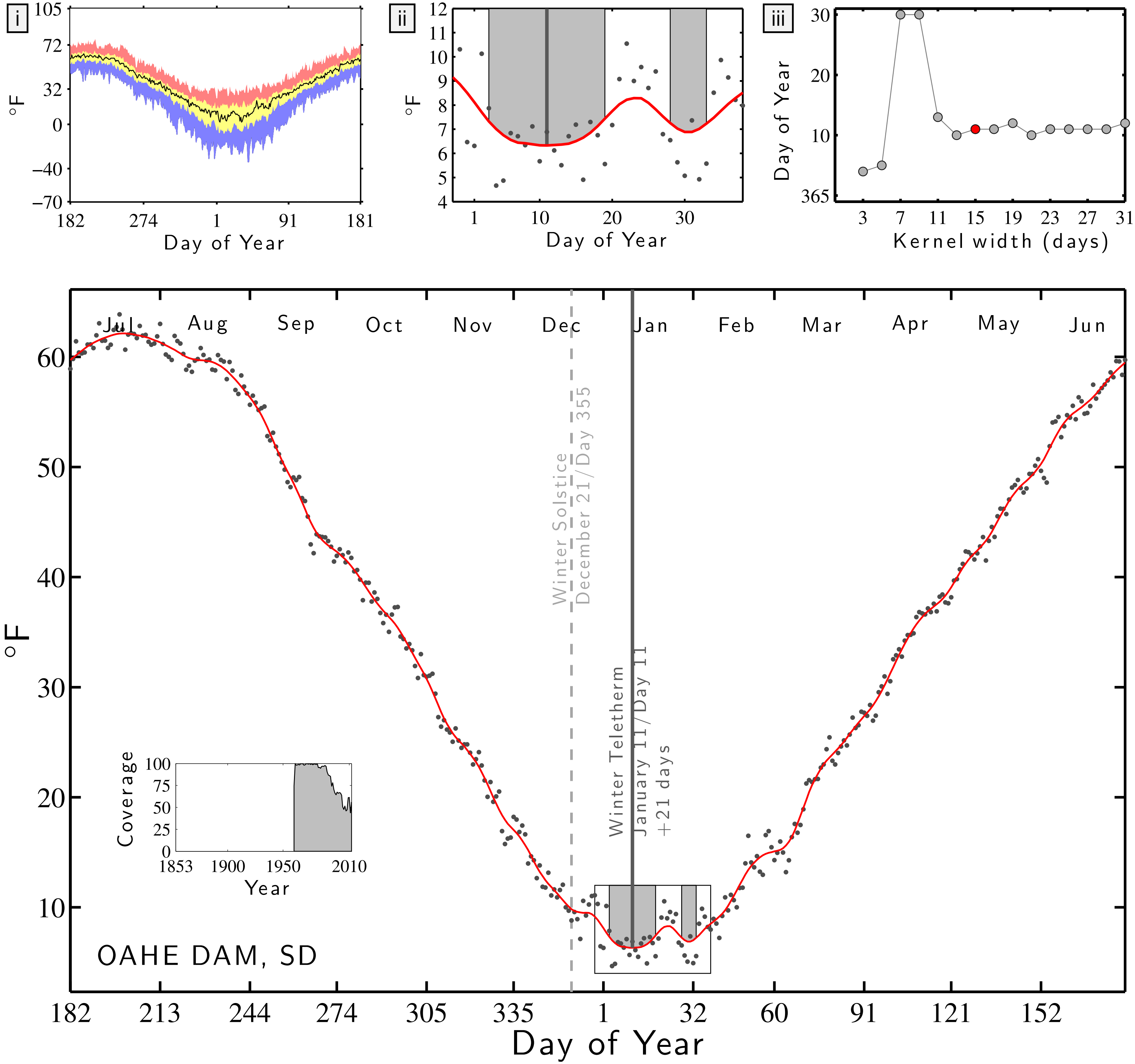

i
°F
Day of Year
ii
°F
Day of Year
iii
Day of Year
Kernel width (days)
Jul
Aug
Sep
Oct
Nov
Dec
Jan
Feb
Mar
Apr
May
Jun
Winter Solstice
December 21/Day 355
Winter Teletherm
January 11/Day 11
+21 days
Coverage
Year
OAHE DAM, SD
°F
Day of Year

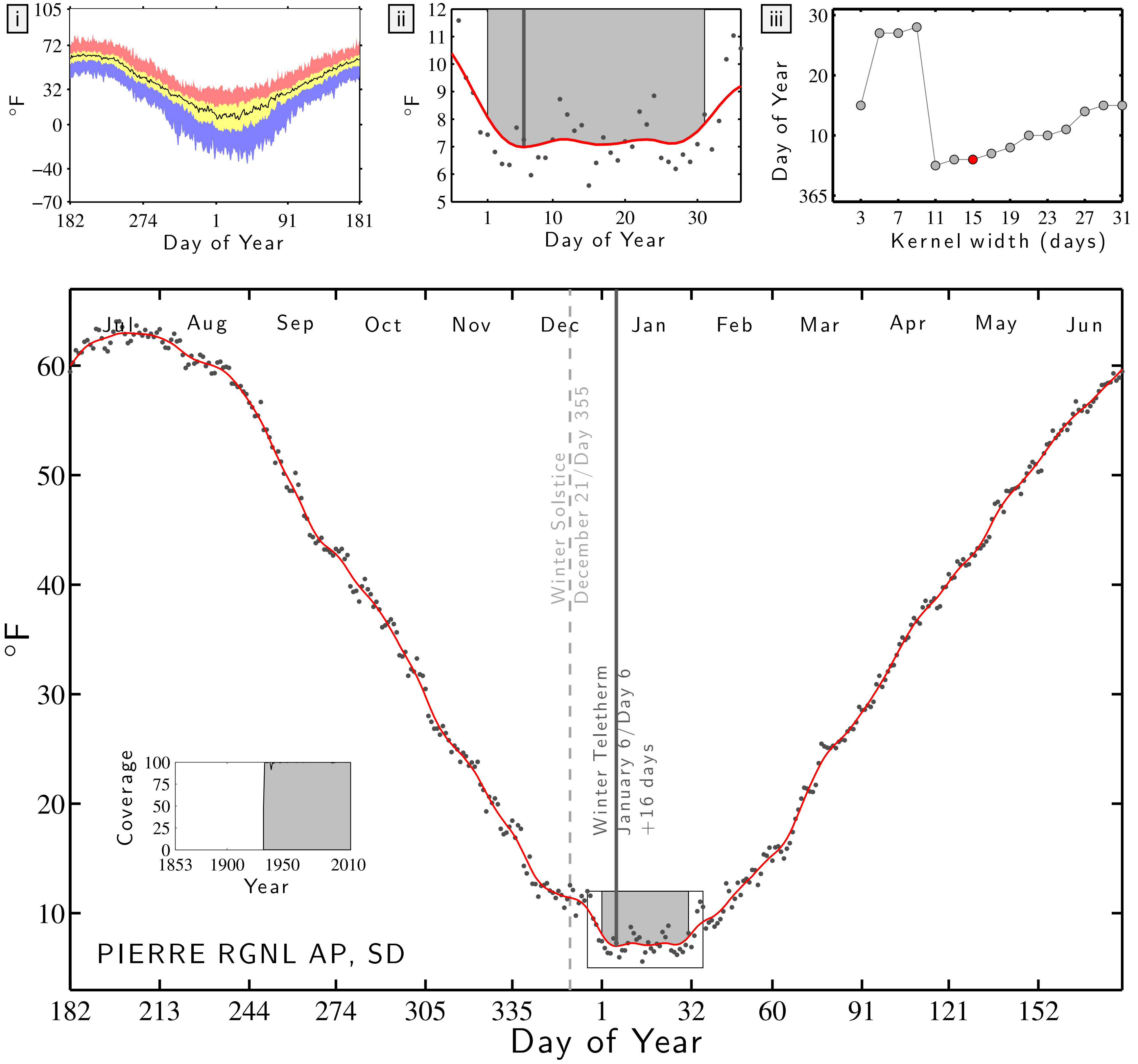

i
ii
iii
°F
Day of Year
Kernel width (days)
Jul
Aug
Sep
Oct
Nov
Dec
Jan
Feb
Mar
Apr
May
Jun
Winter Solstice
December 21/Day 355
Winter Teletherm
January 6/Day 6
+16 days
Coverage
Year
PIERRE RGNL AP, SD

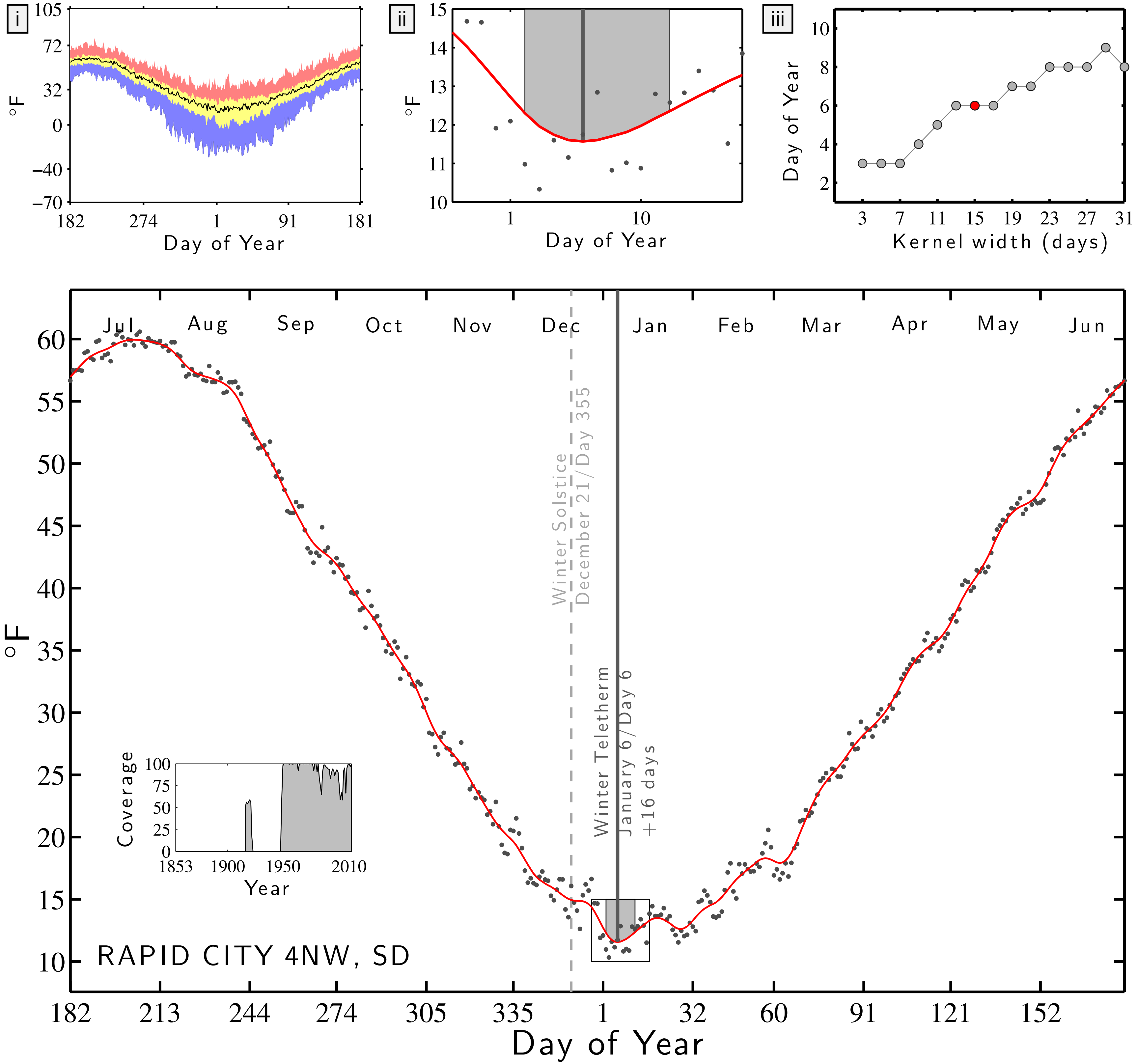

i
ii
iii
Day of Year
Kernel width (days)
°F
Jul
Aug
Sep
Oct
Nov
Dec
Jan
Feb
Mar
Apr
May
Jun
Winter Solstice
December 21/Day 355
Winter Teletherm
January 6/Day 6
+16 days
Coverage
Year
RAPID CITY 4NW, SD

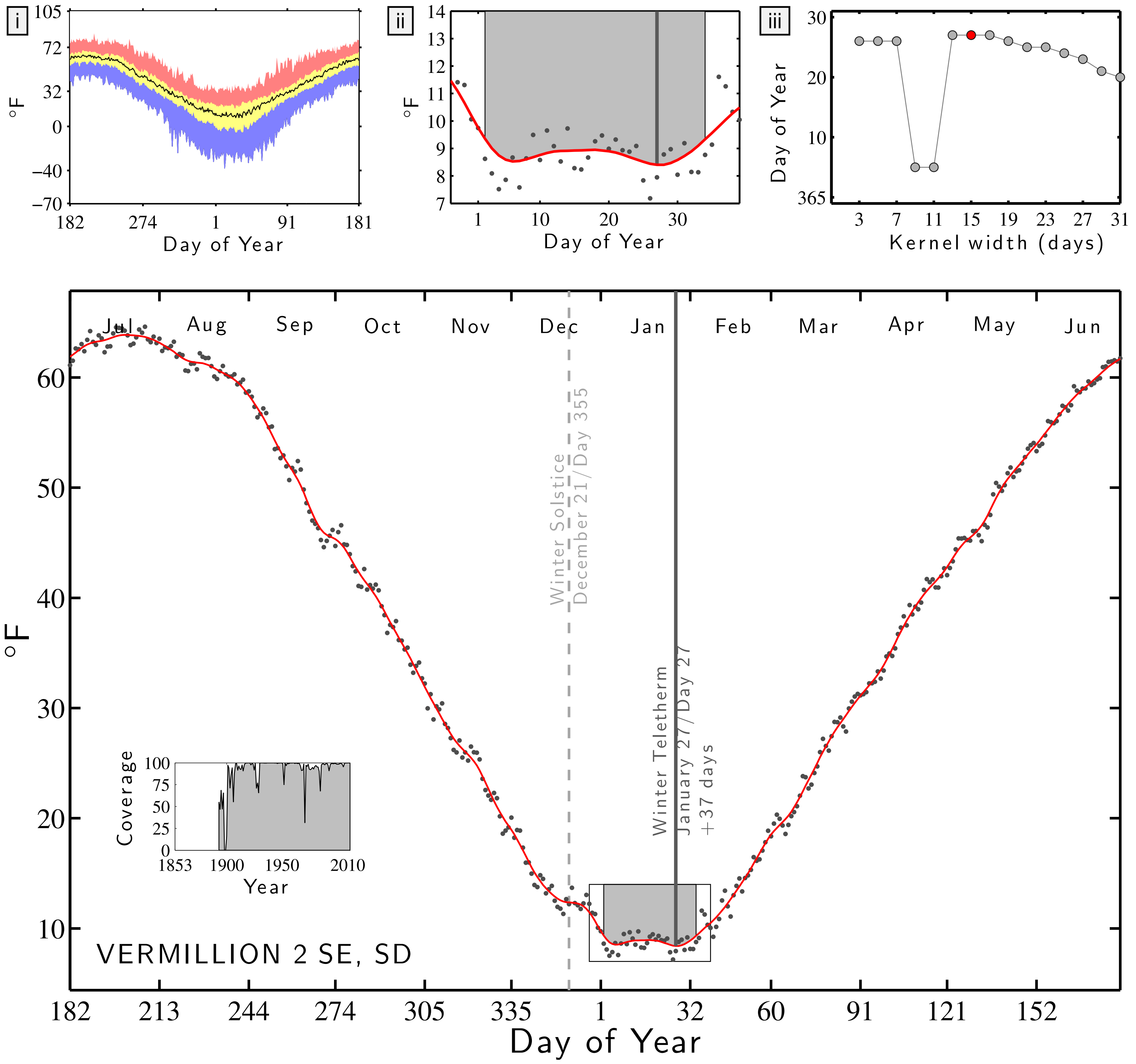

i
ii
iii
°F
Day of Year
Kernel width (days)
Jul
Aug
Sep
Oct
Nov
Dec
Jan
Feb
Mar
Apr
May
Jun
Winter Solstice
December 21/Day 355
Winter Teletherm
January 27/Day 27
+37 days
Coverage
Year
VERMILLION 2 SE, SD

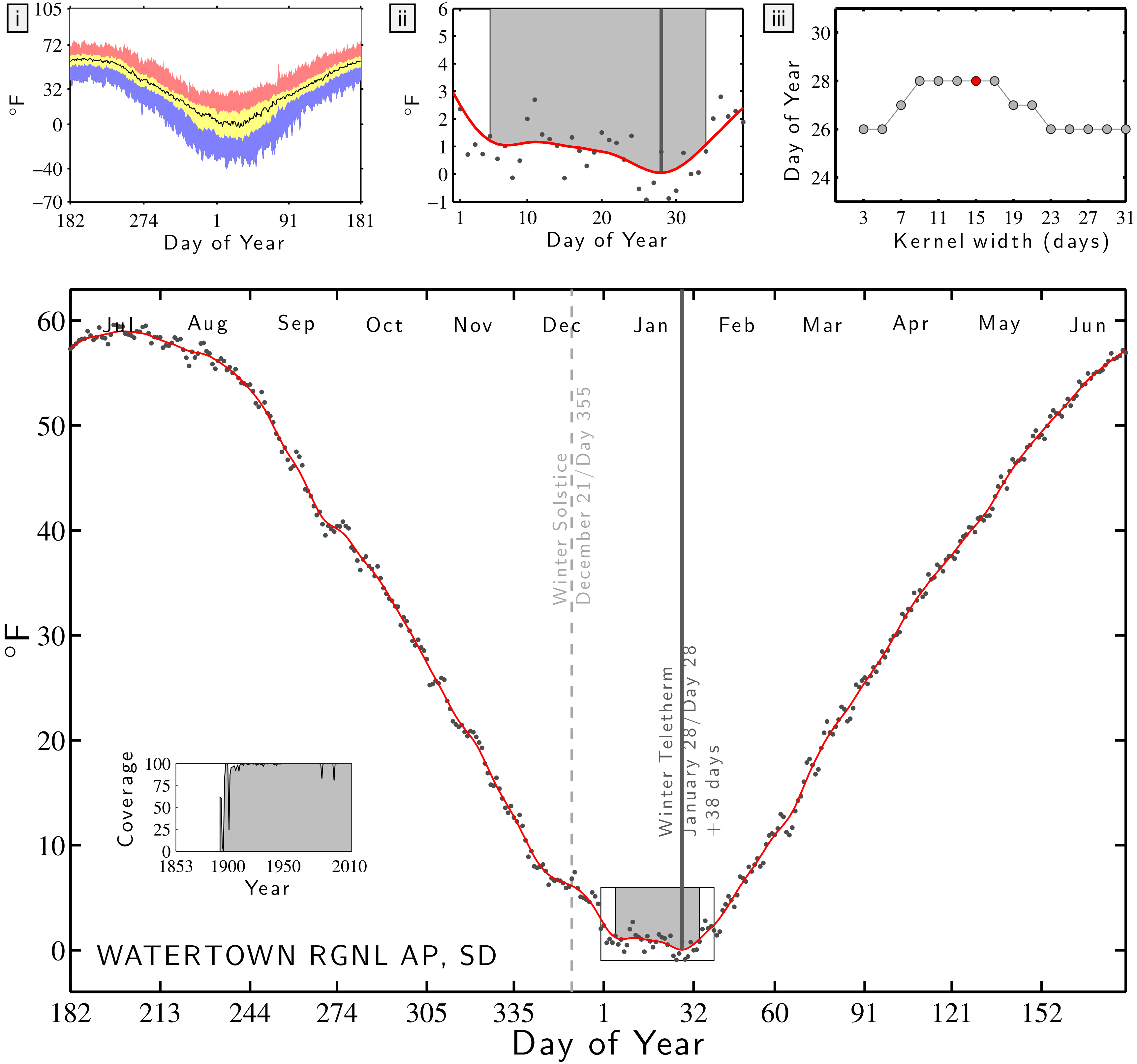

i
ii
iii
°F
Day of Year
Kernel width (days)
Jul
Aug
Sep
Oct
Nov
Dec
Jan
Feb
Mar
Apr
May
Jun
Winter Solstice
December 21/Day 355
Winter Teletherm
January 28/Day 28
+38 days
Coverage
Year
WATERTOWN RGNL AP, SD

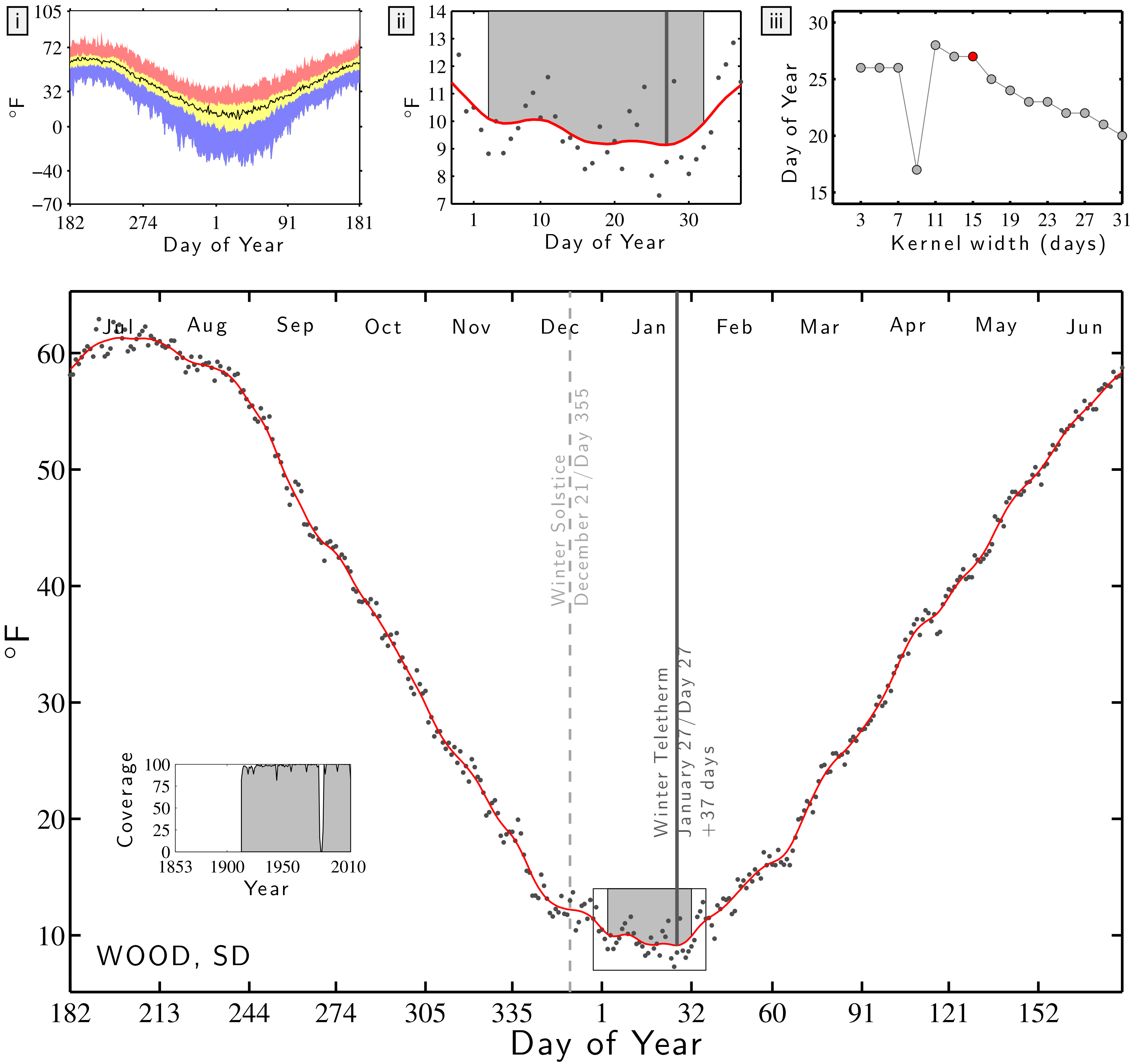

i
ii
iii
°F
Day of Year
Kernel width (days)
Jul
Aug
Sep
Oct
Nov
Dec
Jan
Feb
Mar
Apr
May
Jun
Winter Solstice
December 21/Day 355
Winter Teletherm
January 27/Day 27
+37 days
Coverage
Year
WOOD, SD

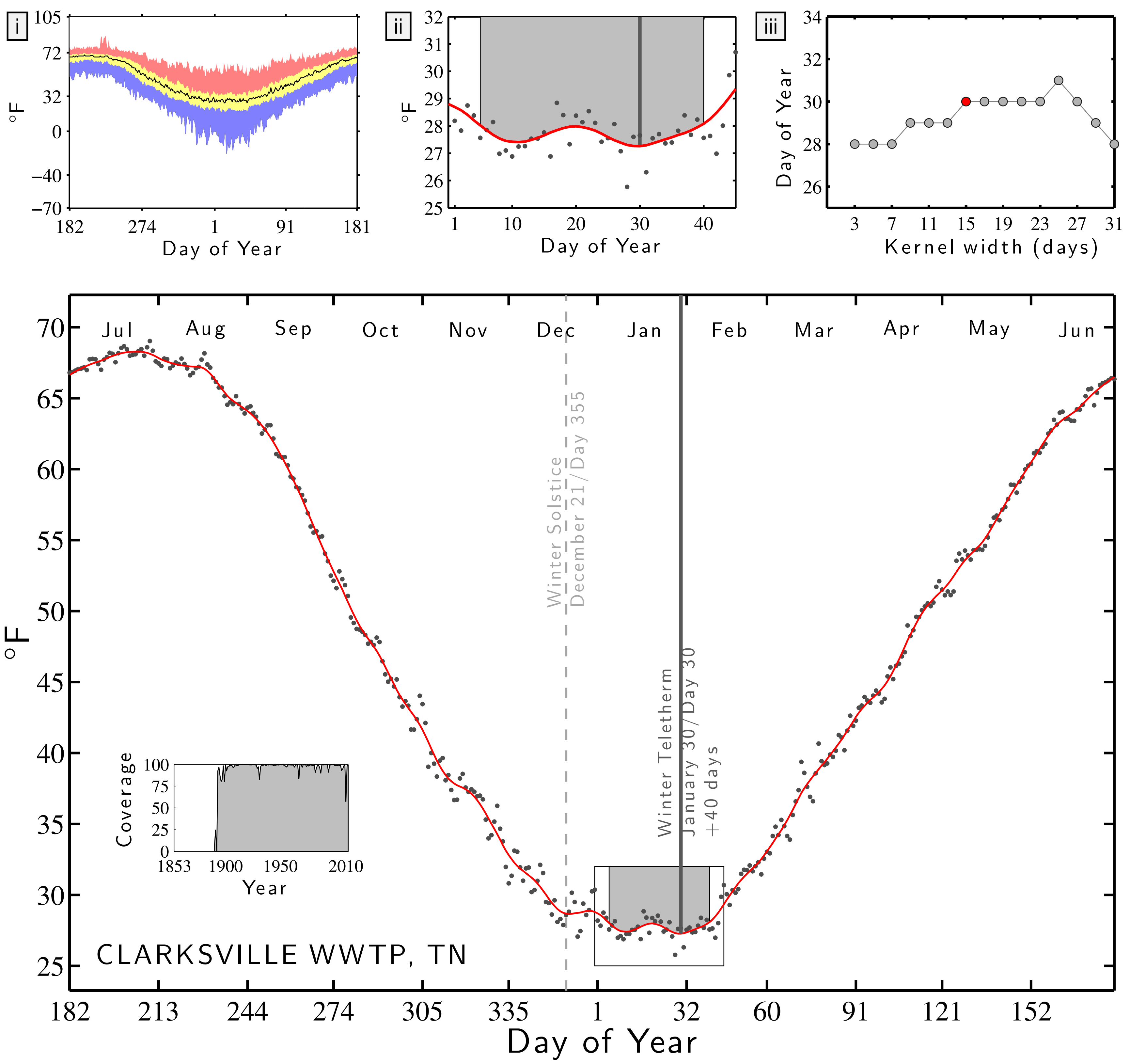

i
ii
iii
°F
Day of Year
Kernel width (days)
Jul
Aug
Sep
Oct
Nov
Dec
Jan
Feb
Mar
Apr
May
Jun
Winter Solstice
December 21/Day 355
Winter Teletherm
January 30/Day 30
+40 days
Coverage
Year
CLARKSVILLE WWTP, TN

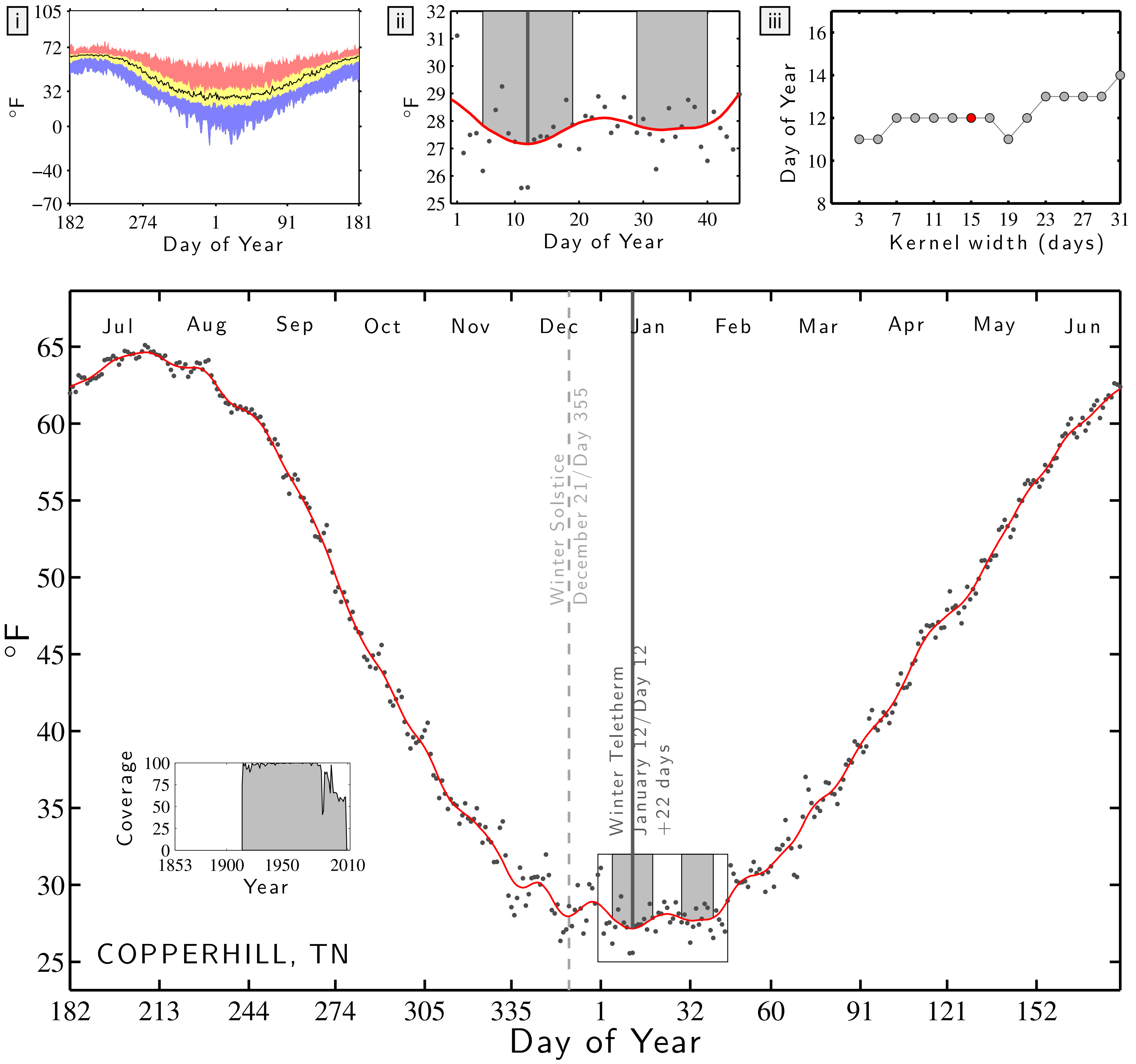

i
°F
105
72
32
0
−40
−70
182
274
1
91
181
Day of Year
ii
°F
32
31
30
29
28
27
26
25
1
10
20
30
40
Day of Year
iii
Day of Year
16
14
12
10
8
3
7
11
15
19
23
27
31
Kernel width (days)
Jul
Aug
Sep
Oct
Nov
Dec
Jan
Feb
Mar
Apr
May
Jun
°F
65
60
55
50
45
40
35
30
25
Winter Solstice
December 21/Day 355
Winter Teletherm
January 12/Day 12
+22 days
Coverage
100
75
50
25
0
1853
1900
1950
2010
Year
COPPERHILL, TN
182
213
244
274
305
335
1
32
60
91
121
152
Day of Year

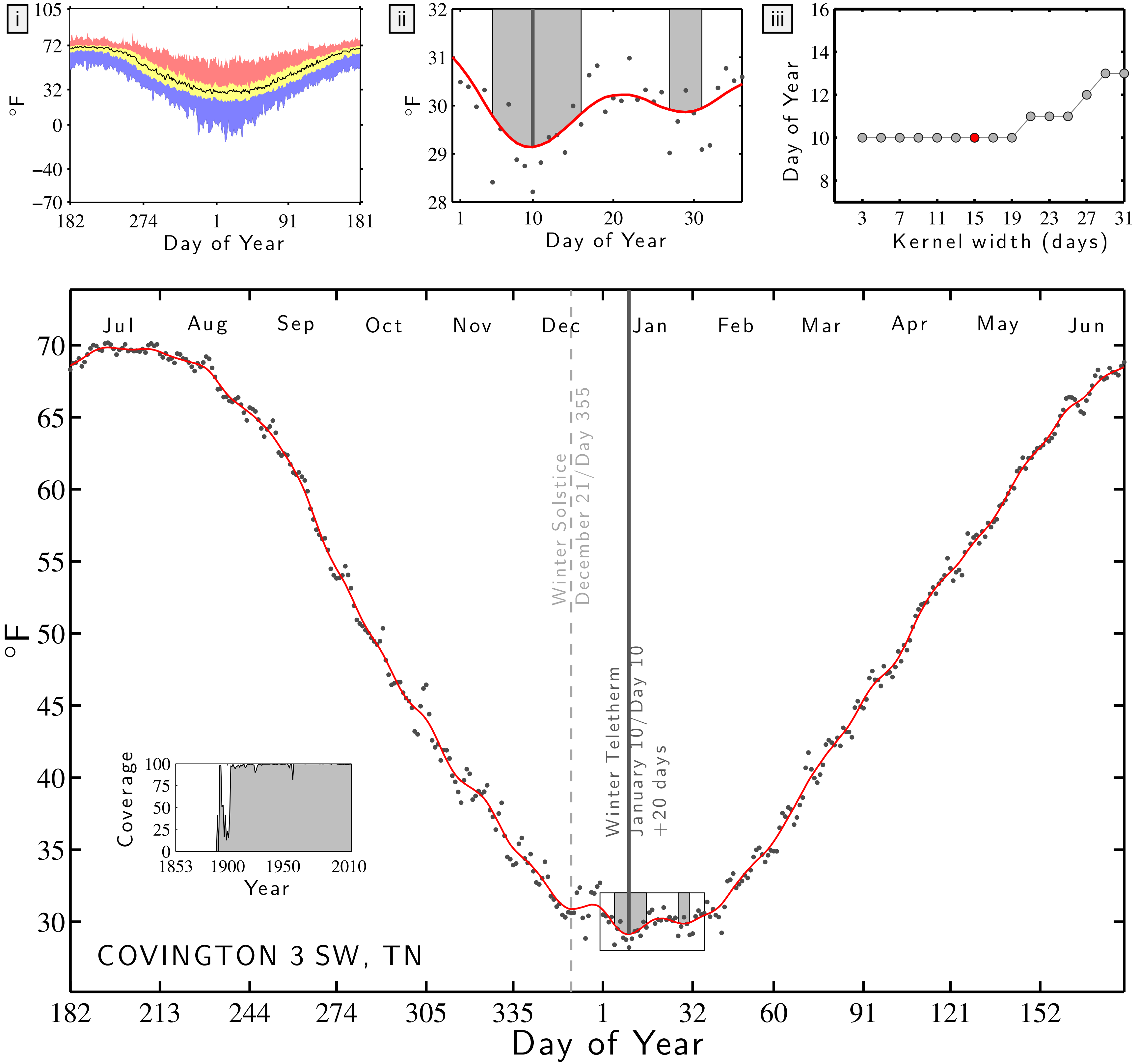

i
ii
iii
°F
Day of Year
Day of Year
Kernel width (days)
Jul
Aug
Sep
Oct
Nov
Dec
Jan
Feb
Mar
Apr
May
Jun
Winter Solstice
December 21/Day 355
Winter Teletherm
January 10/Day 10
+20 days
Coverage
Year
COVINGTON 3 SW, TN
Day of Year

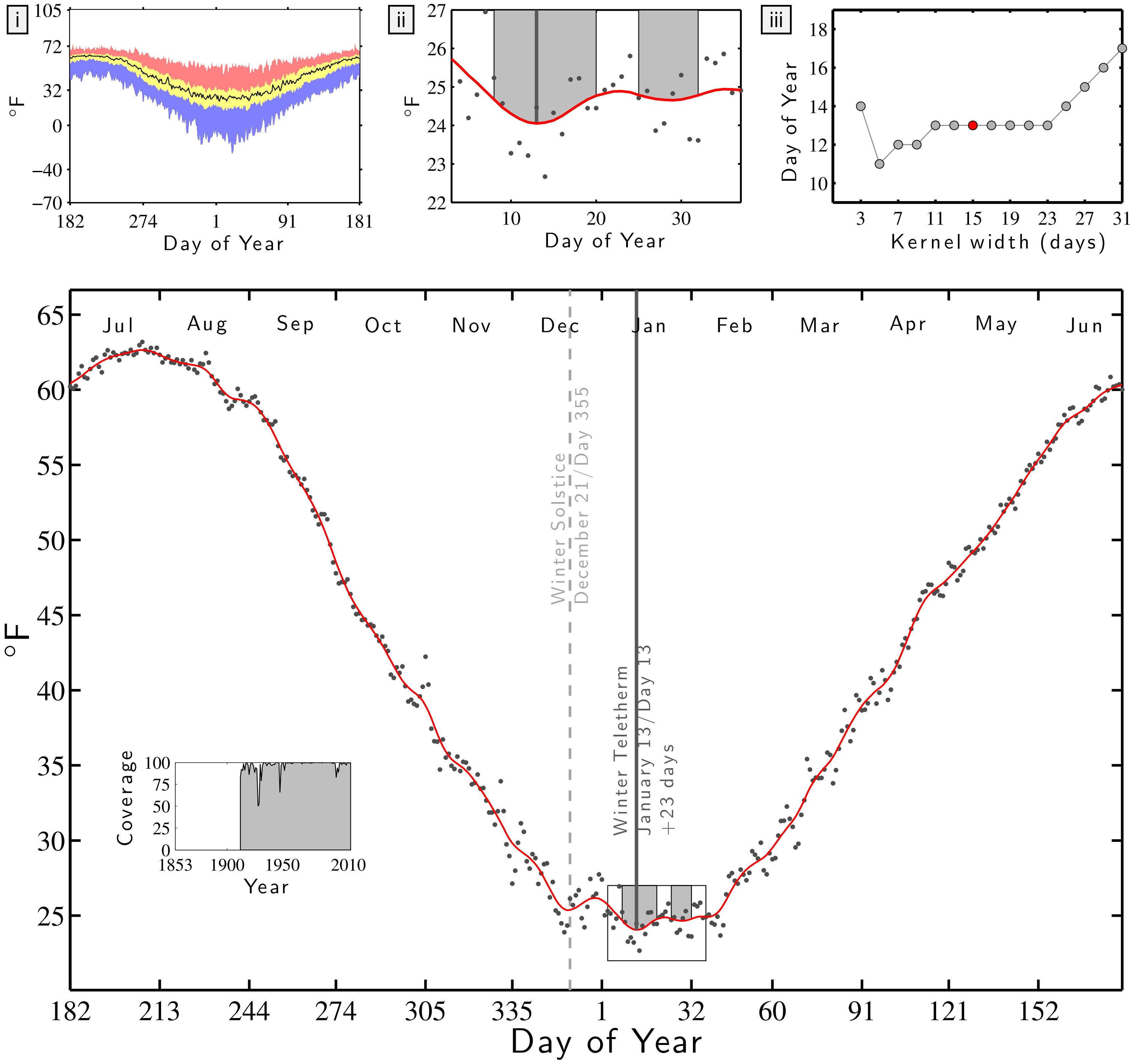

i
ii
iii
°F
Day of Year
Kernel width (days)
Jul
Aug
Sep
Oct
Nov
Dec
Jan
Feb
Mar
Apr
May
Jun
Winter Solstice
December 21/Day 355
Winter Teletherm
January 13/Day 13
+23 days
Coverage
Year

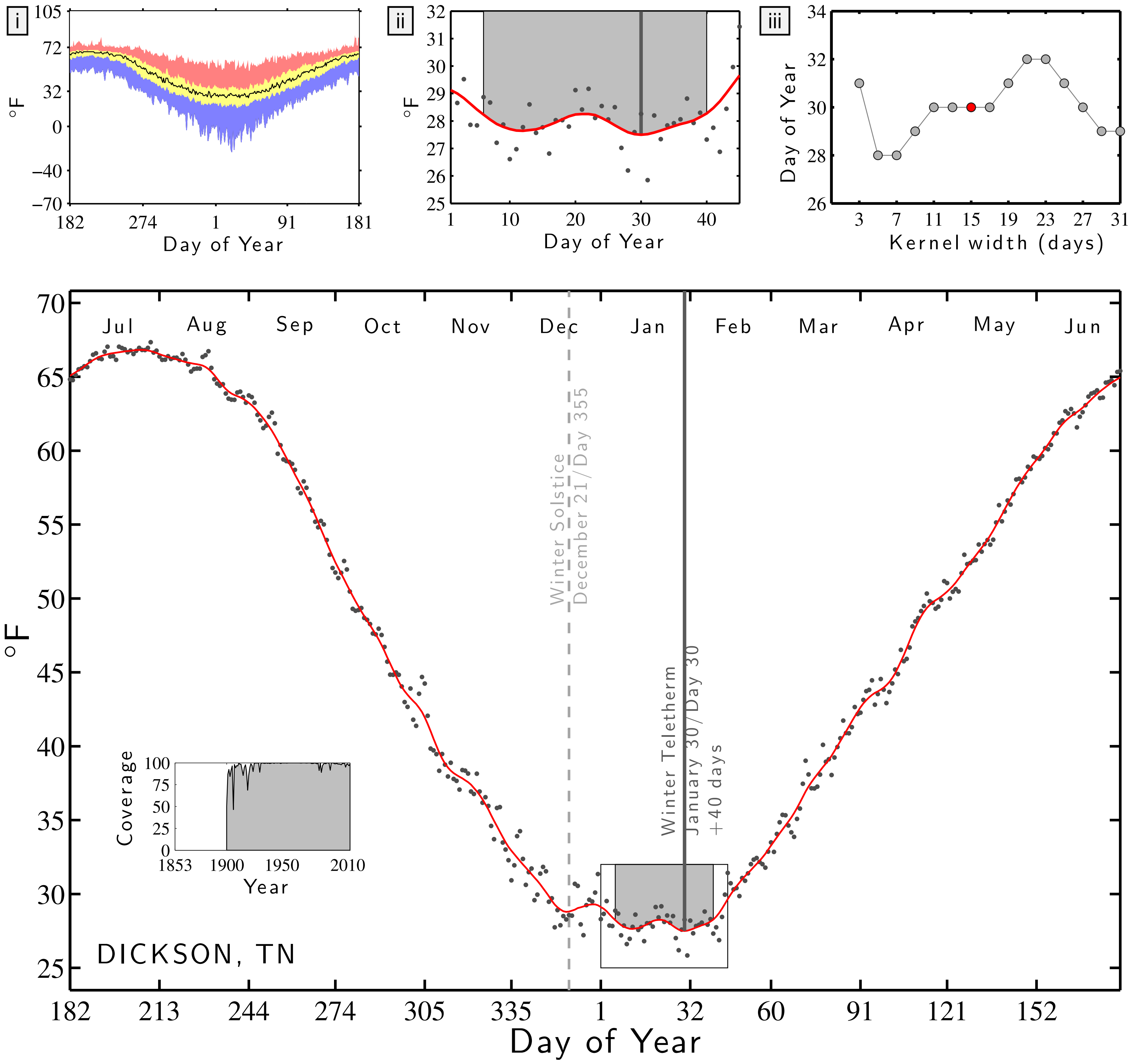

i
ii
iii
°F
Day of Year
Kernel width (days)
Jul
Aug
Sep
Oct
Nov
Dec
Jan
Feb
Mar
Apr
May
Jun
Winter Solstice
December 21/Day 355
Winter Teletherm
January 30/Day 30
+40 days
Coverage
Year
DICKSON, TN

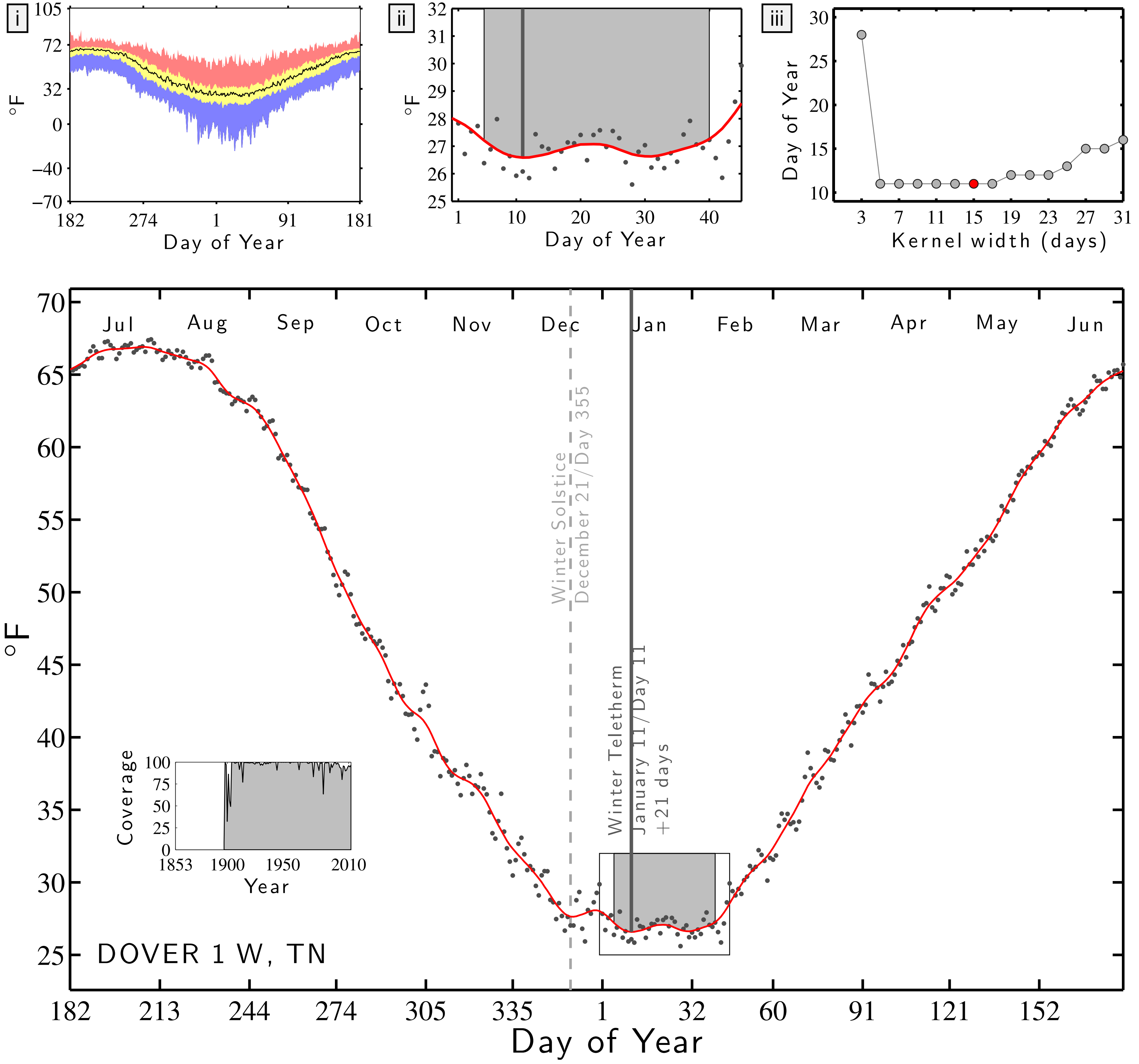

i
°F
Day of Year
ii
°F
Day of Year
iii
Day of Year
Kernel width (days)
Jul
Aug
Sep
Oct
Nov
Dec
Jan
Feb
Mar
Apr
May
Jun
Winter Solstice
December 21/Day 355
Winter Teletherm
January 11/Day 11
+21 days
Coverage
Year
DOVER 1 W, TN
°F
Day of Year

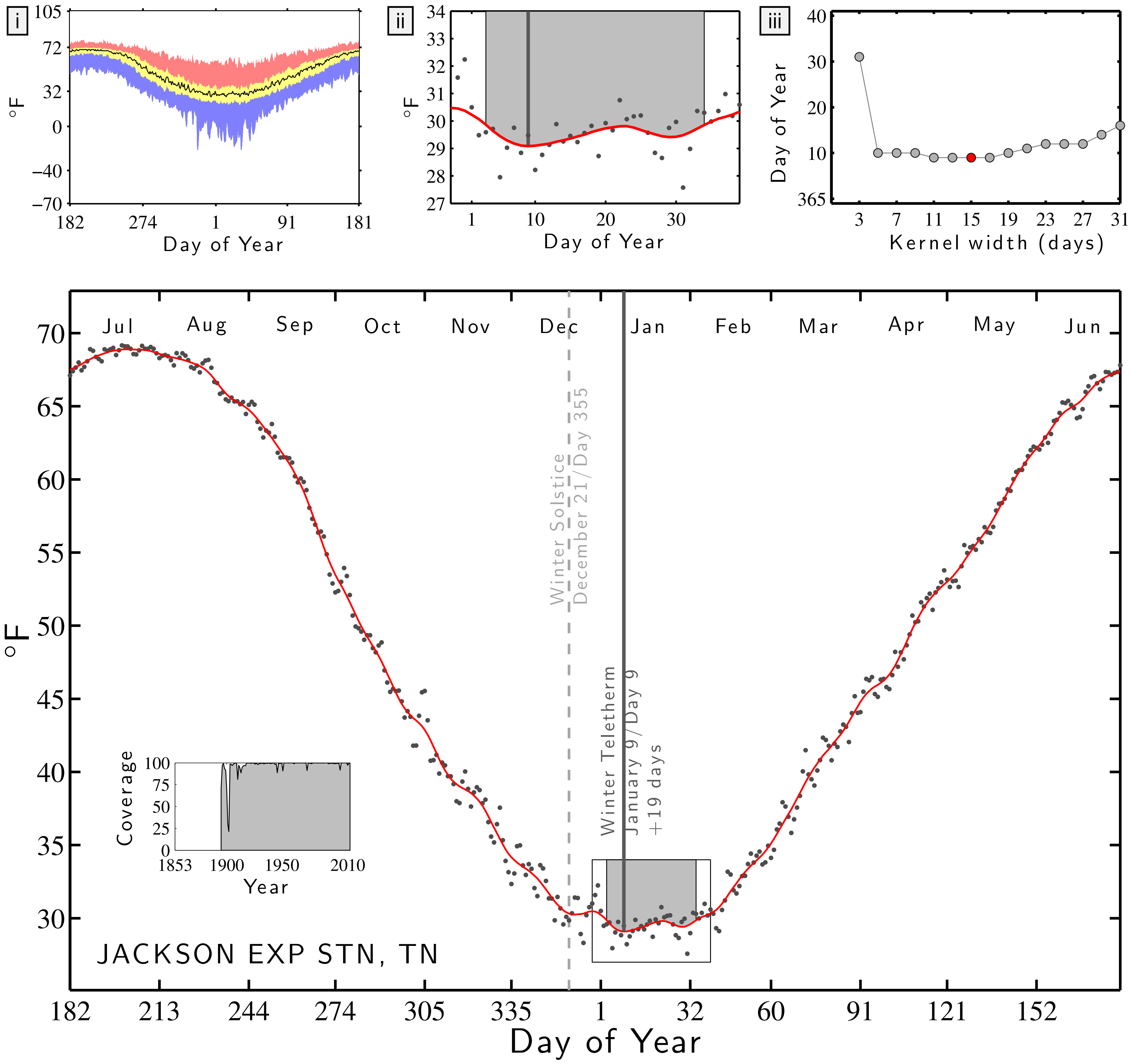

i
°F
Day of Year
ii
Day of Year
iii
Day of Year
Kernel width (days)
Jul Aug Sep Oct Nov Dec Jan Feb Mar Apr May Jun
Winter Solstice
December 21/Day 355
Winter Teletherm
January 9/Day 9
+19 days
Coverage
Year
JACKSON EXP STN, TN
°F
Day of Year

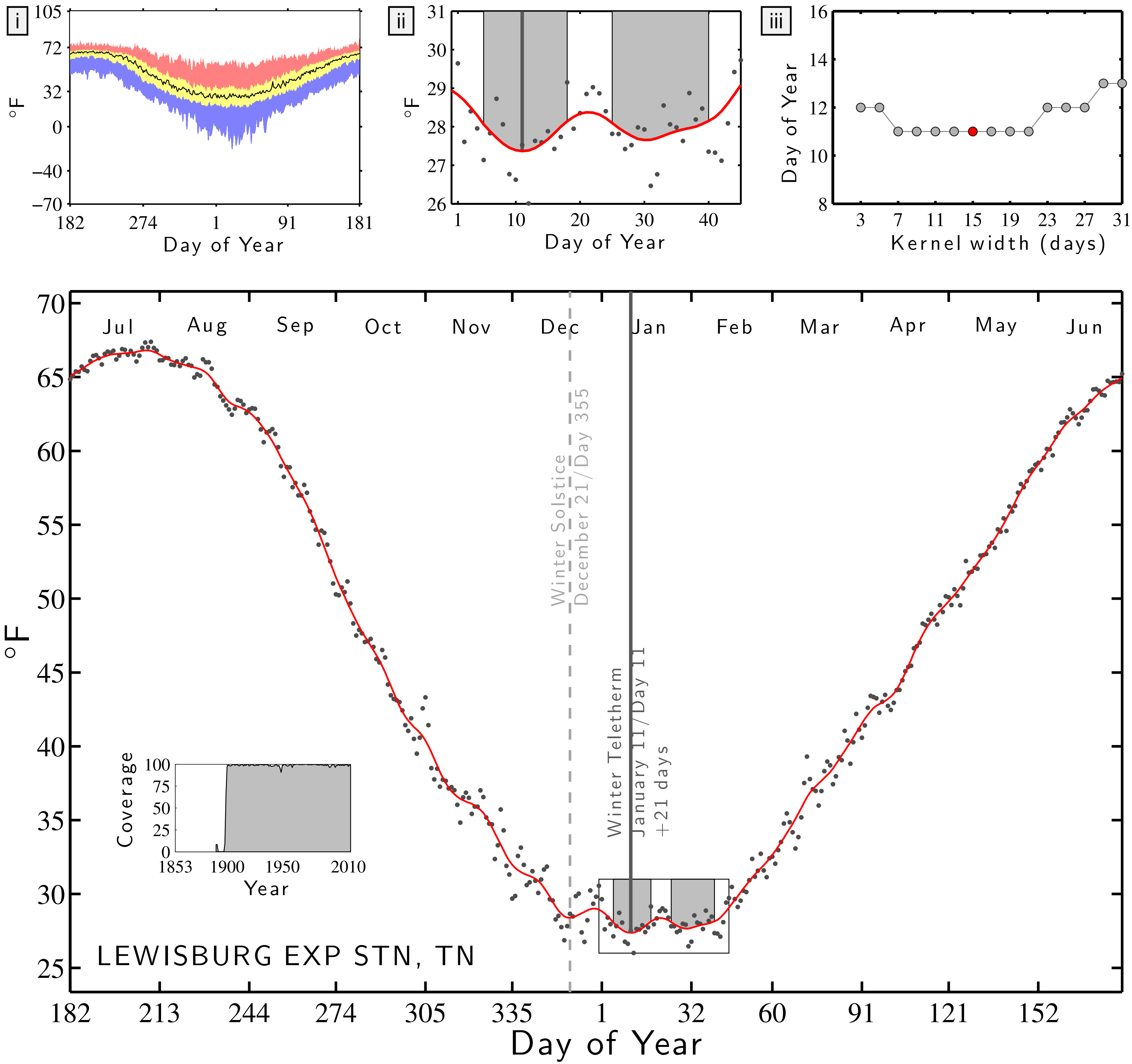

i
ii
iii
°F
Day of Year
Day of Year
Kernel width (days)
Jul
Aug
Sep
Oct
Nov
Dec
Jan
Feb
Mar
Apr
May
Jun
Winter Solstice
December 21/Day 355
Winter Teletherm
January 11/Day 11
+21 days
Coverage
Year
LEWISBURG EXP STN, TN
Day of Year

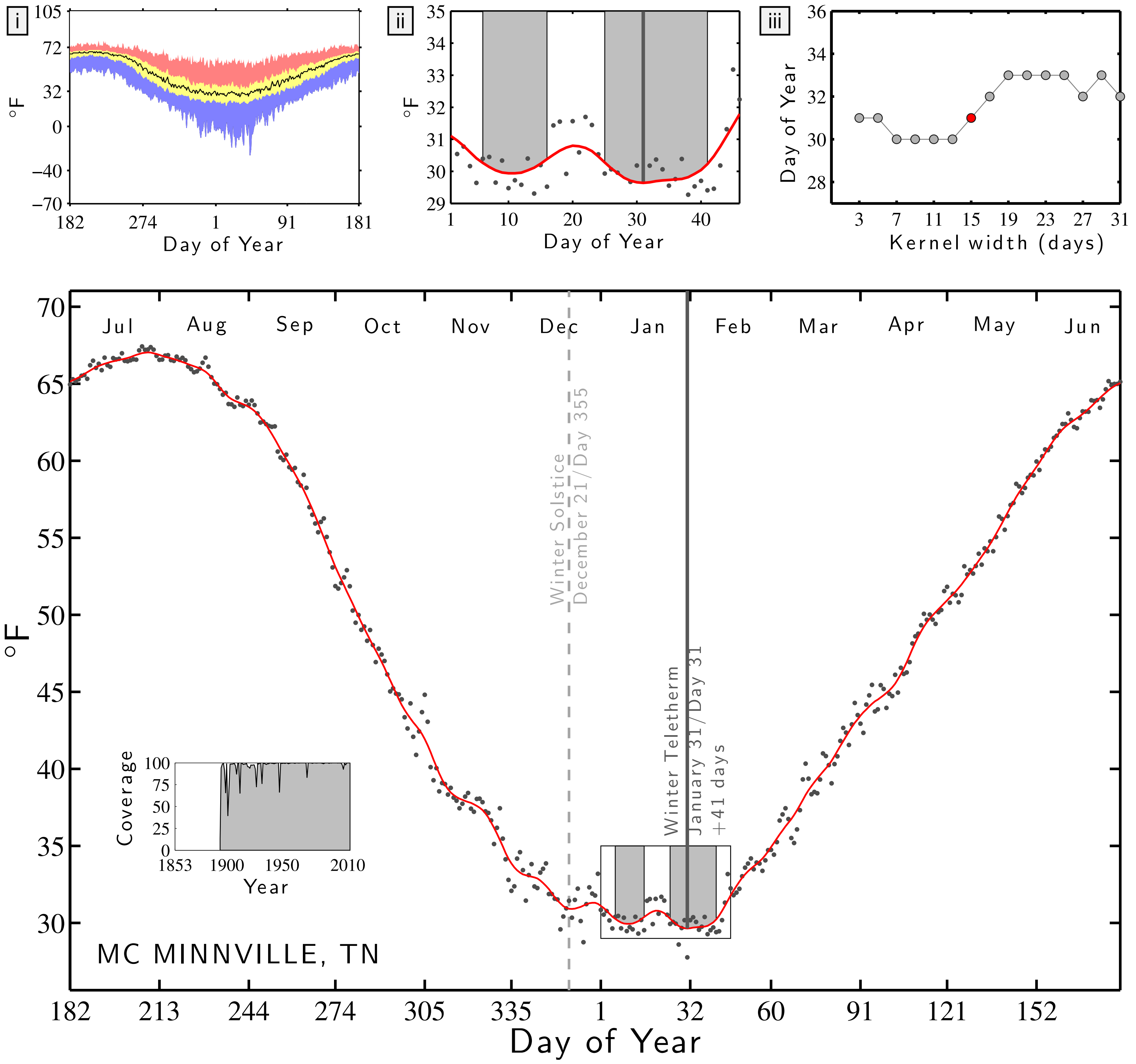

i
°F
Day of Year
ii
Day of Year
iii
Day of Year
Kernel width (days)
Jul
Aug
Sep
Oct
Nov
Dec
Jan
Feb
Mar
Apr
May
Jun
Winter Solstice
December 21/Day 355
Winter Teletherm
January 31/Day 31
+41 days
Coverage
Year
MC MINNVILLE, TN
°F
Day of Year

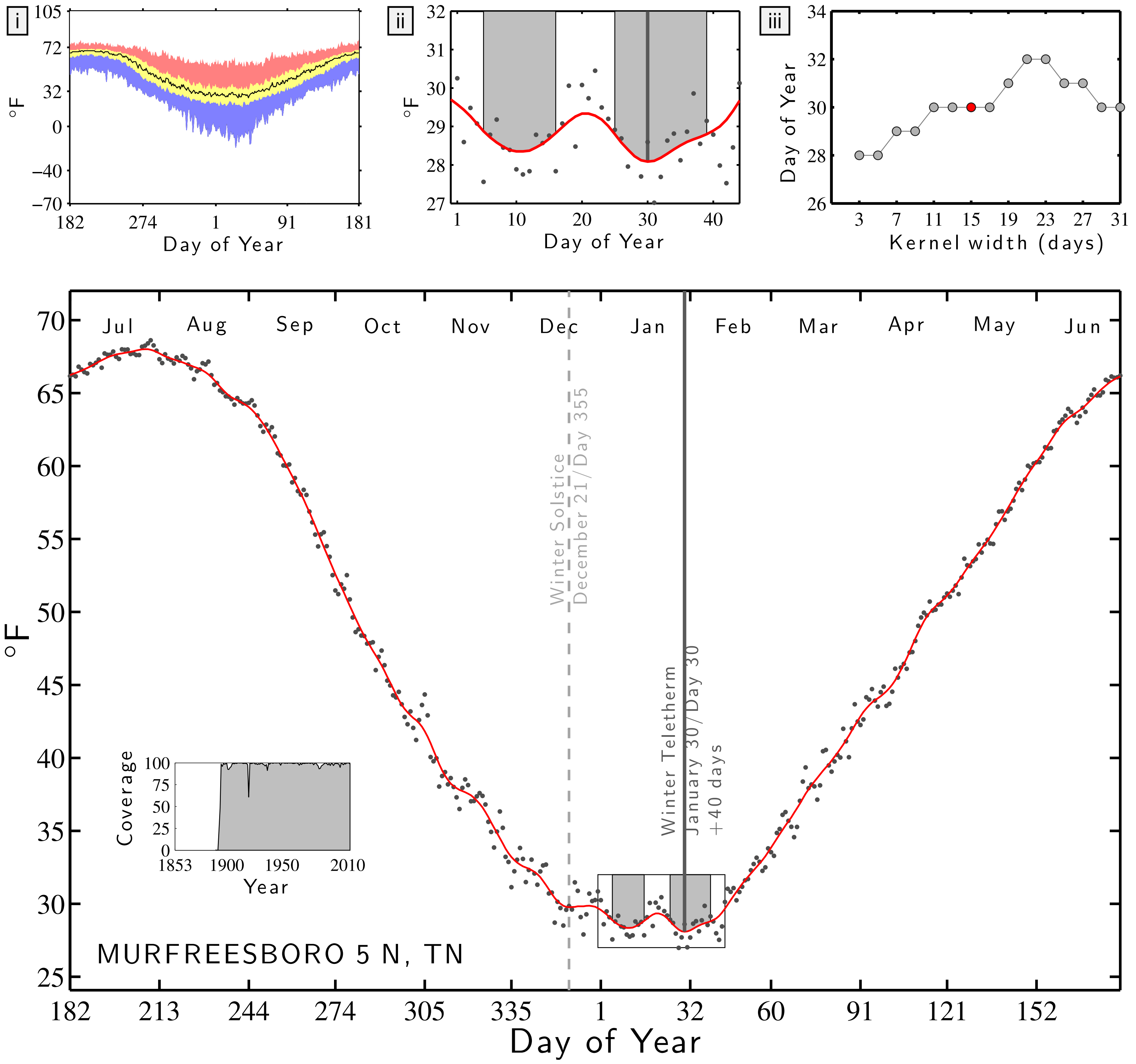

i
ii
iii
Day of Year
°F
Kernel width (days)
Jul
Aug
Sep
Oct
Nov
Dec
Jan
Feb
Mar
Apr
May
Jun
Winter Solstice
December 21/Day 355
Winter Teletherm
January 30/Day 30
+40 days
Coverage
Year
MURFREESBORO 5 N, TN

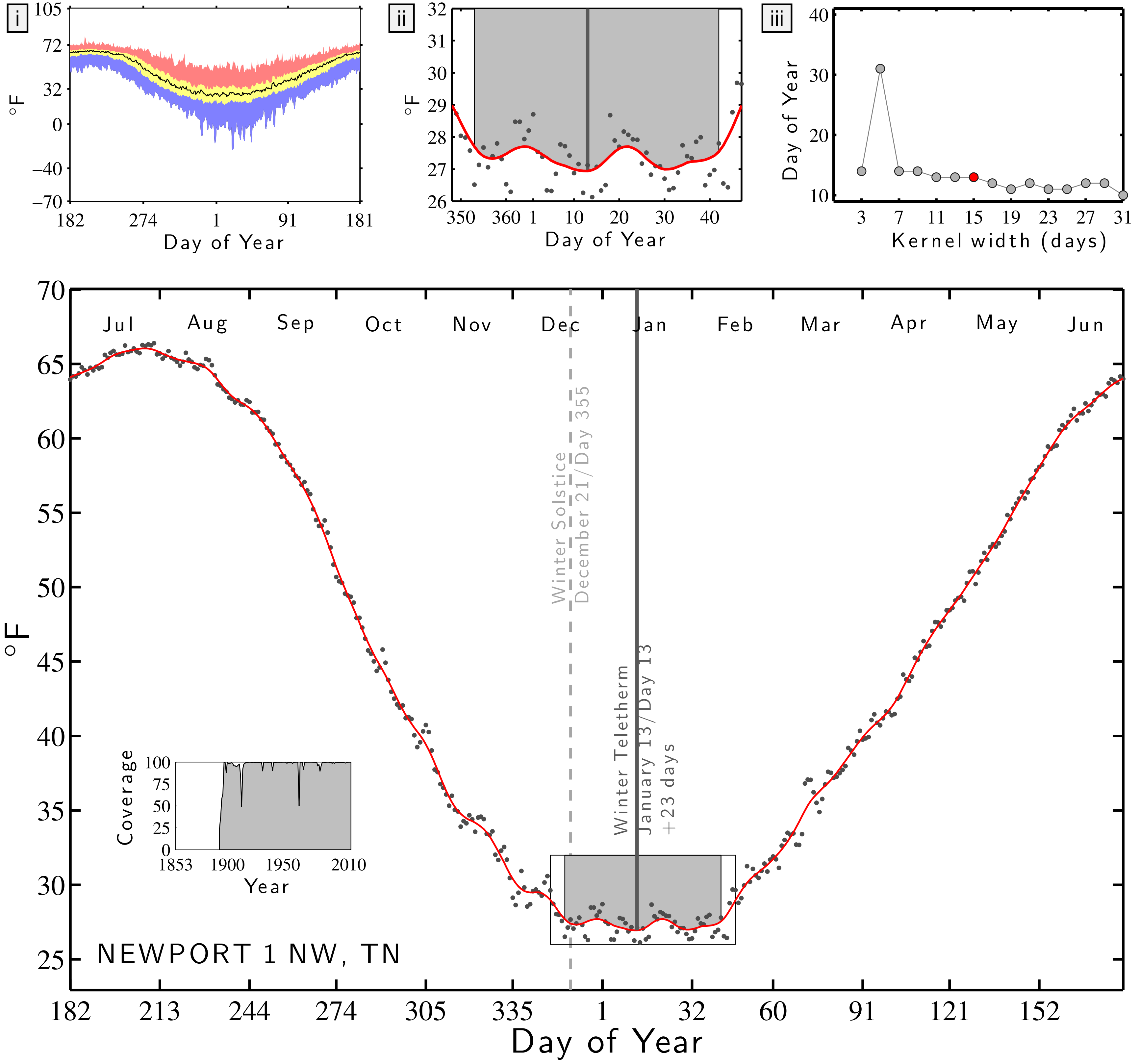

i
°F
Day of Year
ii
°F
Day of Year
iii
Day of Year
Kernel width (days)
Jul
Aug
Sep
Oct
Nov
Dec
Jan
Feb
Mar
Apr
May
Jun
Winter Solstice
December 21/Day 355
Winter Teletherm
January 13/Day 13
+23 days
Coverage
Year
NEWPORT 1 NW, TN
°F
Day of Year

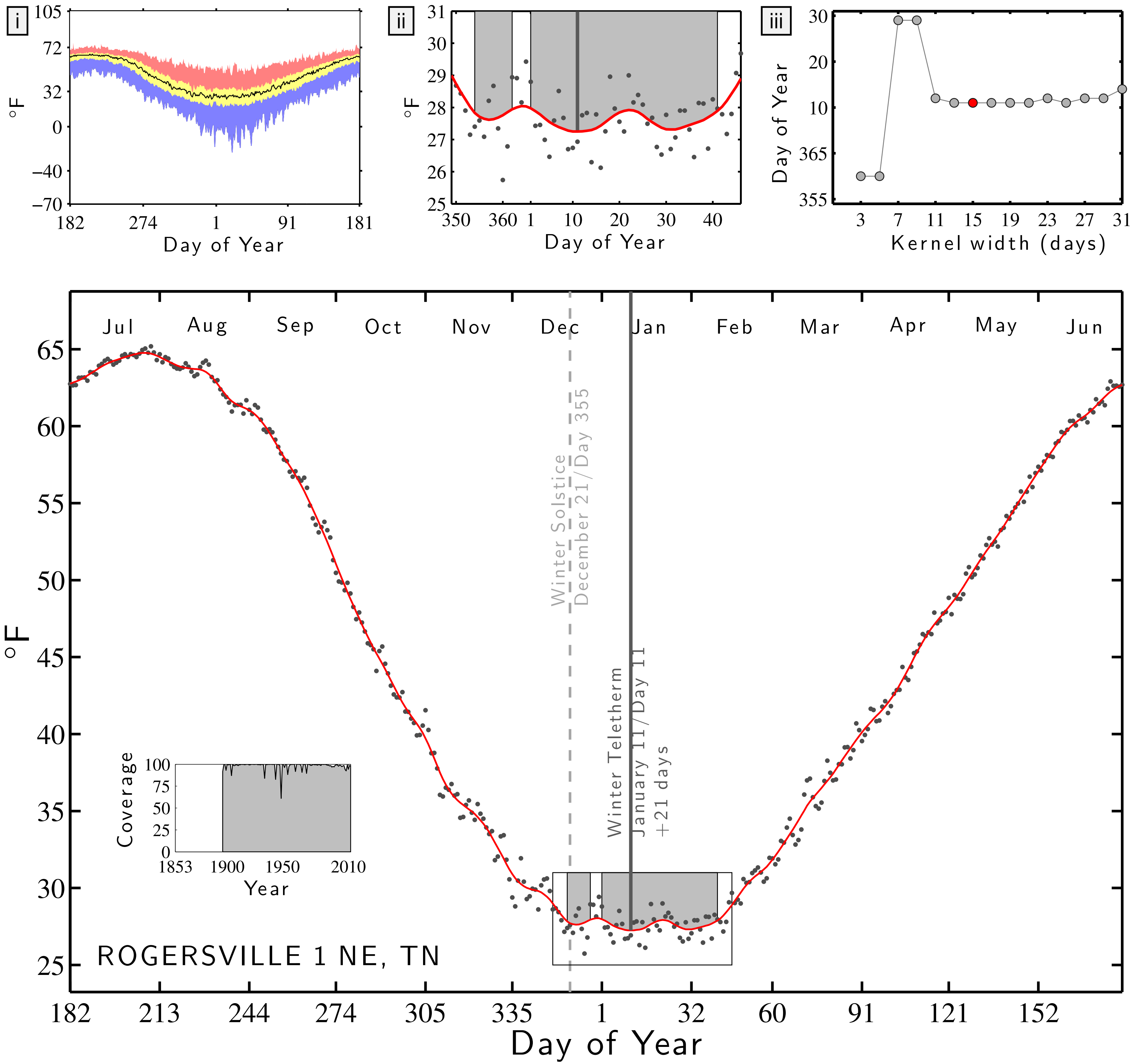

i
°F
Day of Year
ii
iii
Day of Year
Kernel width (days)
Jul Aug Sep Oct Nov Dec Jan Feb Mar Apr May Jun
Winter Solstice
December 21/Day 355
Winter Teletherm
January 11/Day 11
+21 days
Coverage
Year
ROGERSVILLE 1 NE, TN
°F
Day of Year

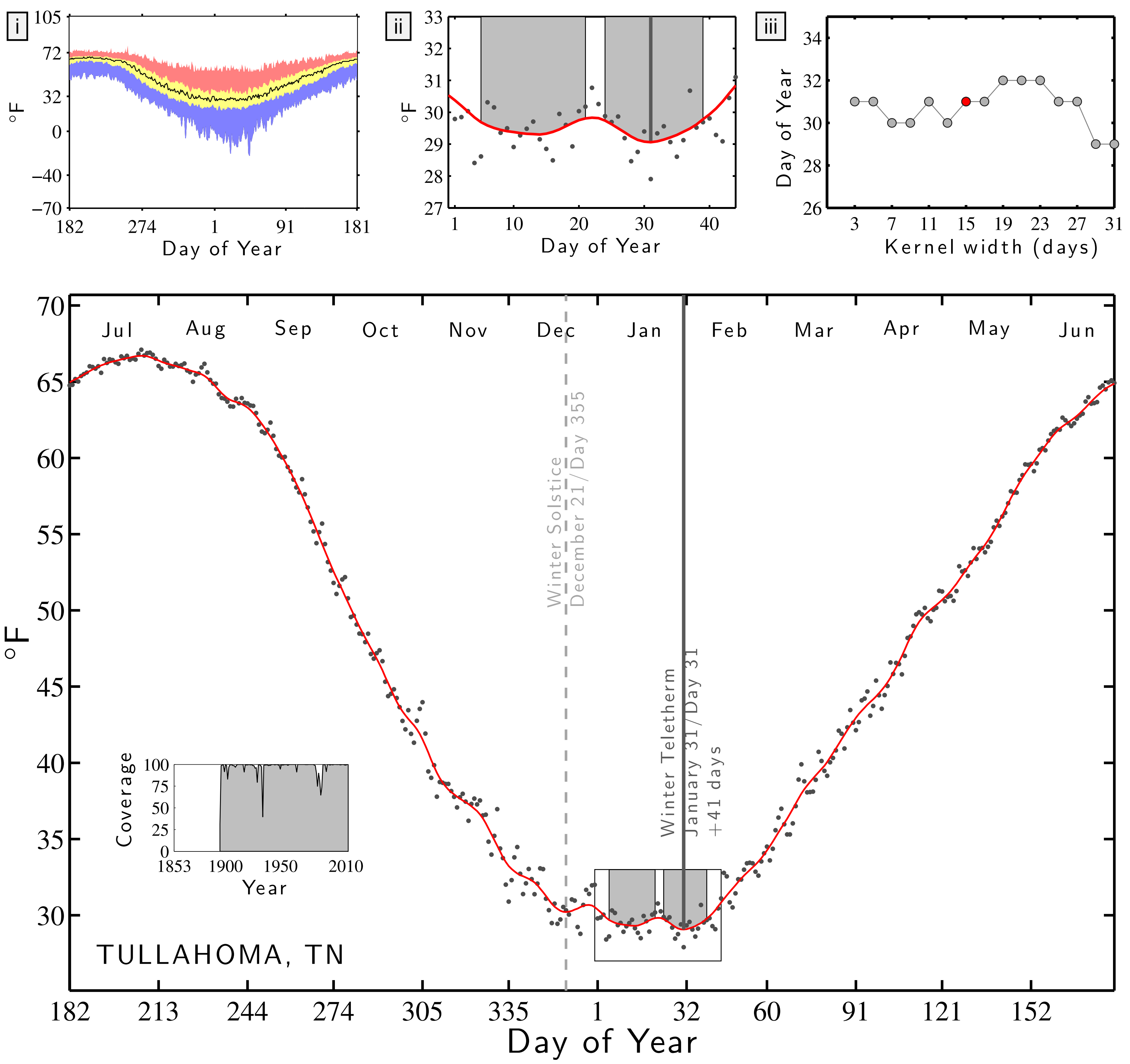

i
ii
iii
Day of Year
°F
Kernel width (days)
Jul
Aug
Sep
Oct
Nov
Dec
Jan
Feb
Mar
Apr
May
Jun
Winter Solstice
December 21/Day 355
Winter Teletherm
January 31/Day 31
+41 days
Coverage
Year
TULLAHOMA, TN

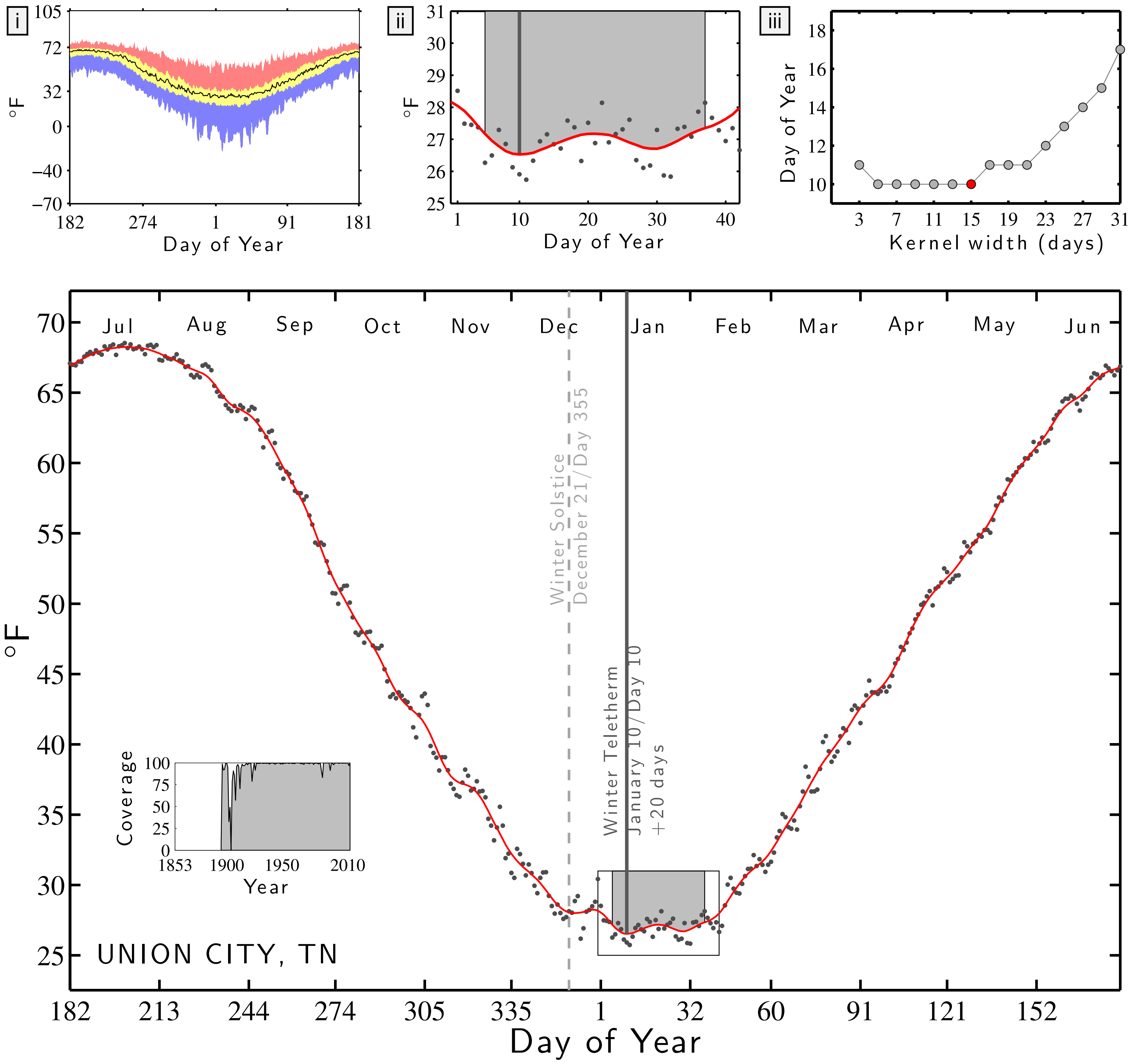

i
°F
Day of Year
ii
°F
Day of Year
iii
Day of Year
Kernel width (days)
Jul
Aug
Sep
Oct
Nov
Dec
Jan
Feb
Mar
Apr
May
Jun
Winter Solstice
December 21/Day 355
Winter Teletherm
January 10/Day 10
+20 days
Coverage
Year
UNION CITY, TN
°F
Day of Year

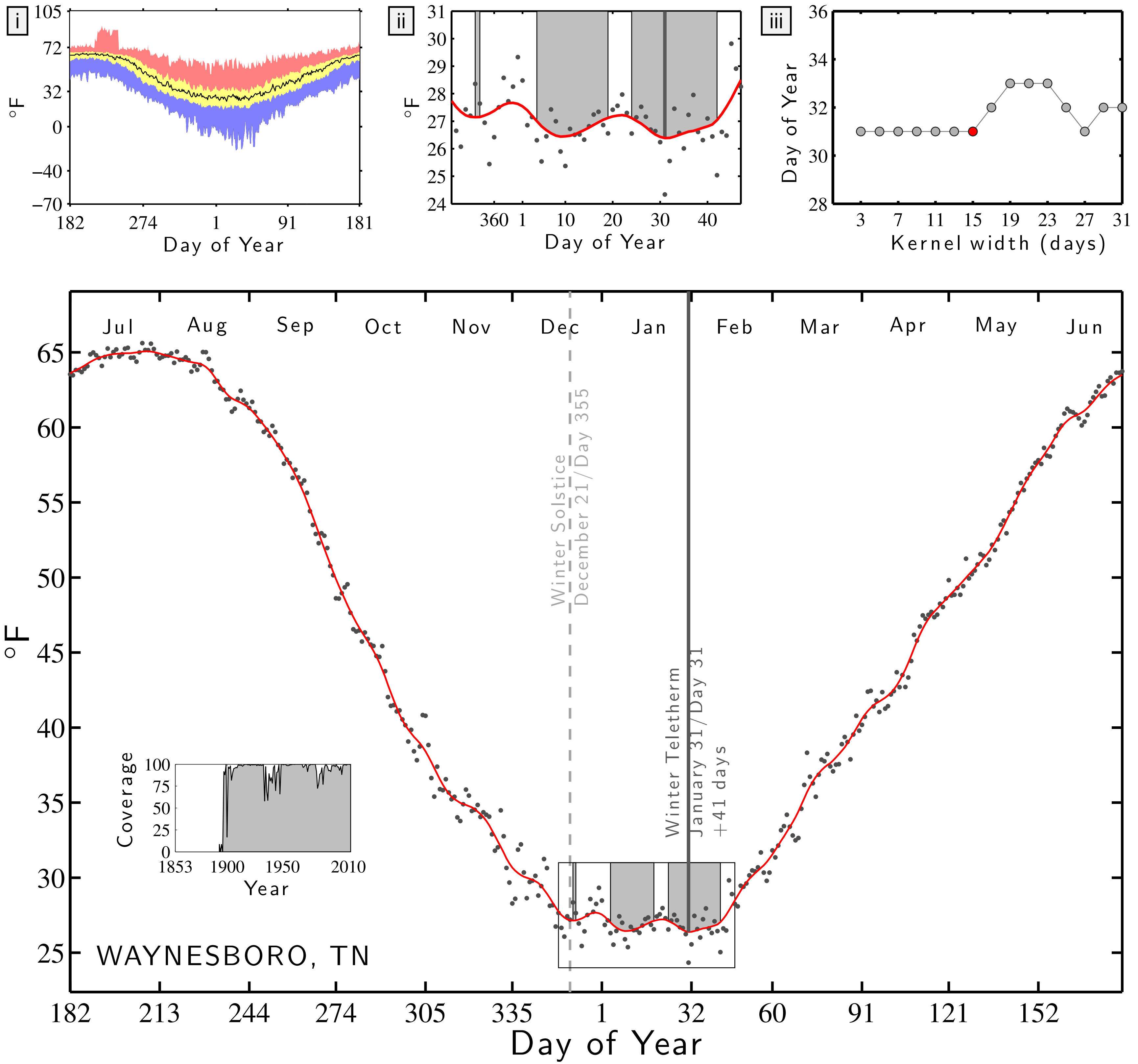

i
°F
Day of Year
ii
°F
Day of Year
iii
Day of Year
Kernel width (days)
Jul
Aug
Sep
Oct
Nov
Dec
Jan
Feb
Mar
Apr
May
Jun
Winter Solstice
December 21/Day 355
Winter Teletherm
January 31/Day 31
+41 days
Coverage
Year
WAYNESBORO, TN
°F
Day of Year

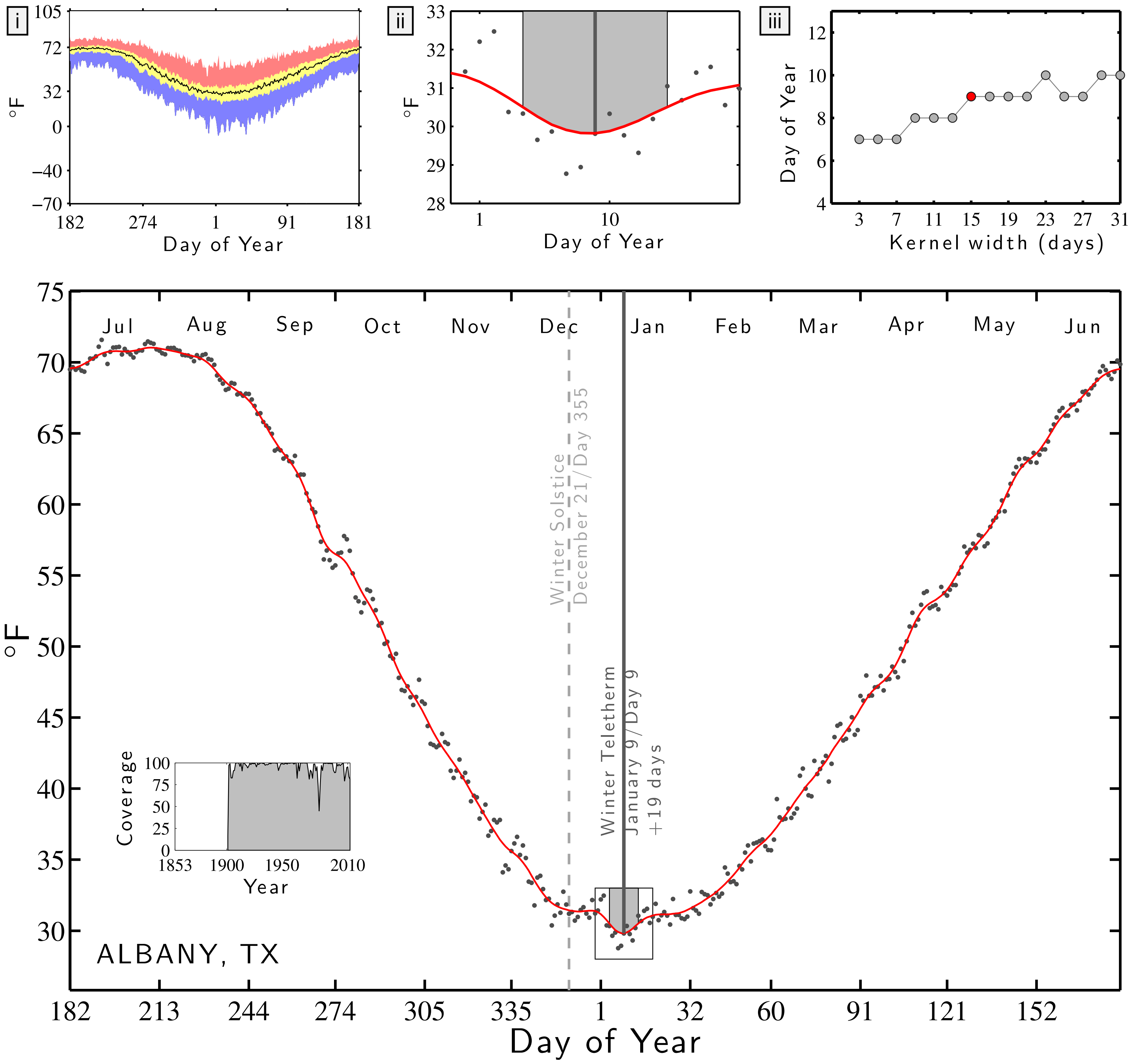

i
°F
Day of Year
182 274 1 91 181
105 72 32 0 −40 −70
ii
°F
Day of Year
1 10
33 32 31 30 29 28
iii
Day of Year
Kernel width (days)
3 7 11 15 19 23 27 31
12 10 8 6 4
Jul Aug Sep Oct Nov Dec Jan Feb Mar Apr May Jun
Winter Solstice
December 21/Day 355
Winter Teletherm
January 9/Day 9
+19 days
Coverage
100 75 50 25 0
1853 1900 1950 2010
Year
ALBANY, TX
°F
75 70 65 60 55 50 45 40 35 30
182 213 244 274 305 335 1 32 60 91 121 152
Day of Year

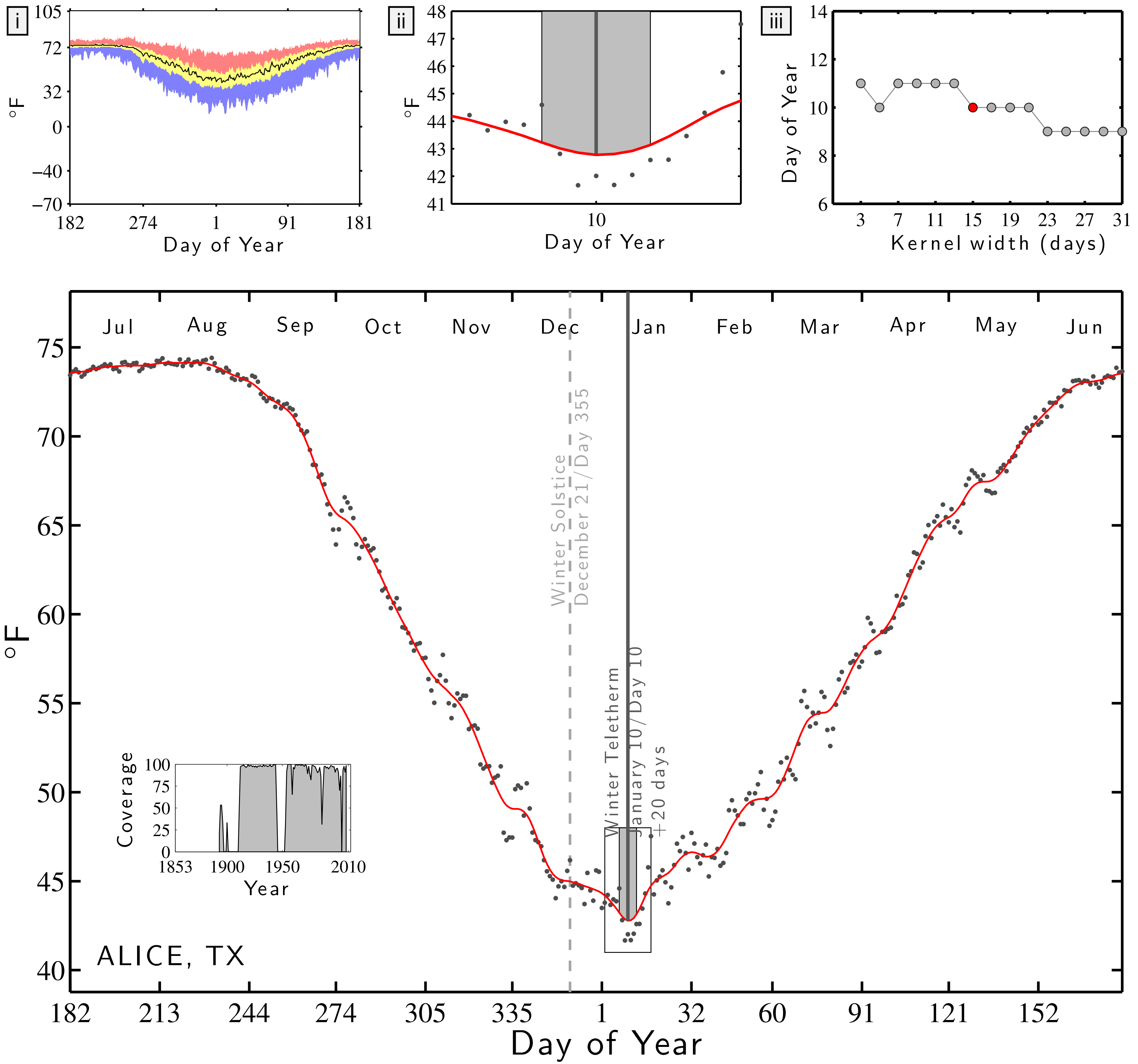

i
105
72
32
0
−40
−70
°F
182
274
1
91
181
Day of Year
ii
48
47
46
45
44
43
42
41
°F
10
Day of Year
iii
14
12
10
8
6
Day of Year
3
7
11
15
19
23
27
31
Kernel width (days)
Jul
Aug
Sep
Oct
Nov
Dec
Jan
Feb
Mar
Apr
May
Jun
75
70
65
60
55
50
45
40
°F
Winter Solstice
December 21/Day 355
Winter Teletherm
January 10/Day 10
+20 days
Coverage
100
75
50
25
0
1853
1900
1950
2010
Year
ALICE, TX
182
213
244
274
305
335
1
32
60
91
121
152
Day of Year

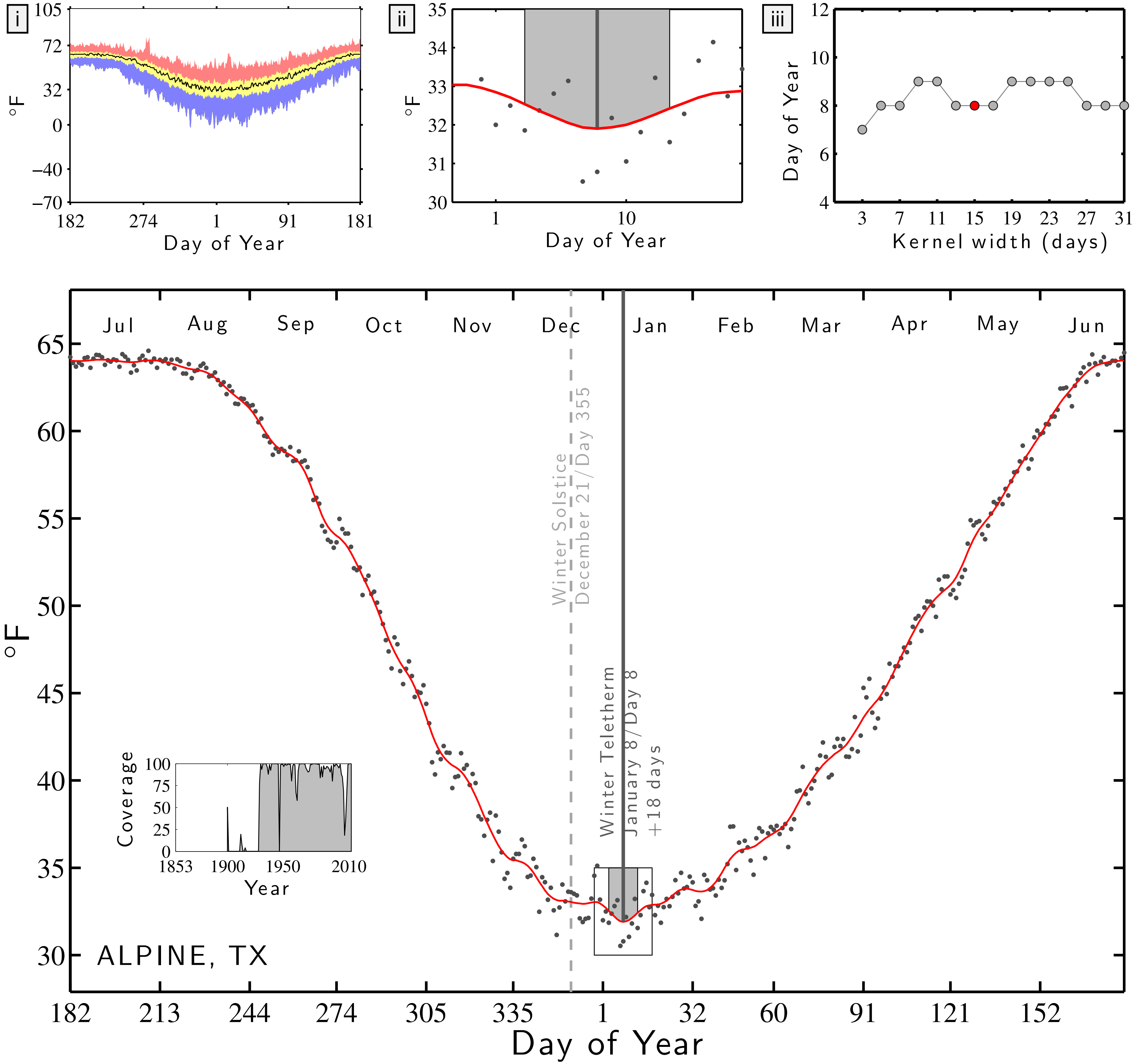

i
ii
iii
°F
Day of Year
Kernel width (days)
Jul
Aug
Sep
Oct
Nov
Dec
Jan
Feb
Mar
Apr
May
Jun
Winter Solstice
December 21/Day 355
Winter Teletherm
January 8/Day 8
+18 days
Coverage
Year
ALPINE, TX

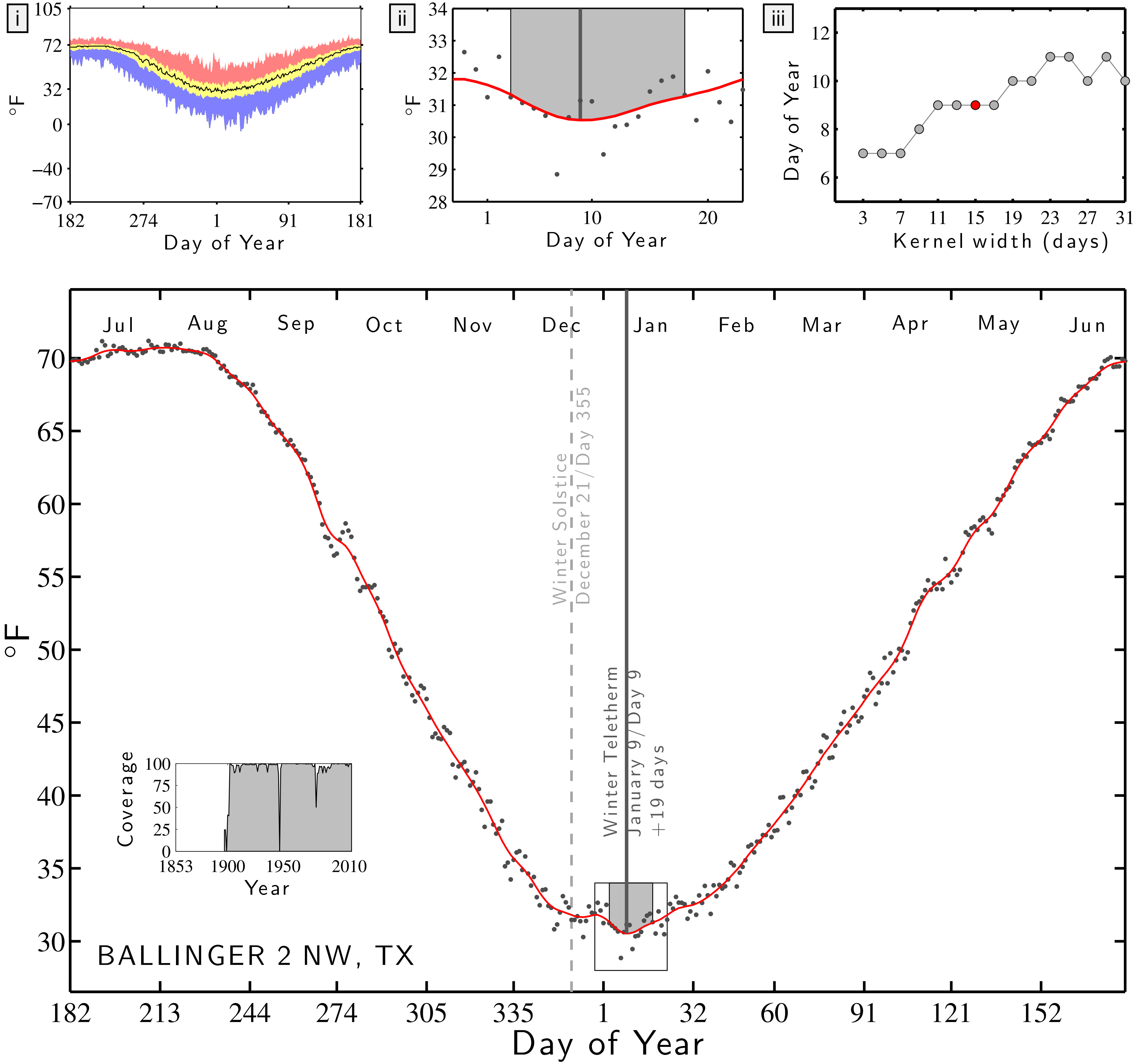

i
°F
Day of Year
ii
°F
Day of Year
iii
Day of Year
Kernel width (days)
Jul
Aug
Sep
Oct
Nov
Dec
Jan
Feb
Mar
Apr
May
Jun
Winter Solstice
December 21/Day 355
Winter Teletherm
January 9/Day 9
+19 days
Coverage
Year
BALLINGER 2 NW, TX
°F
Day of Year

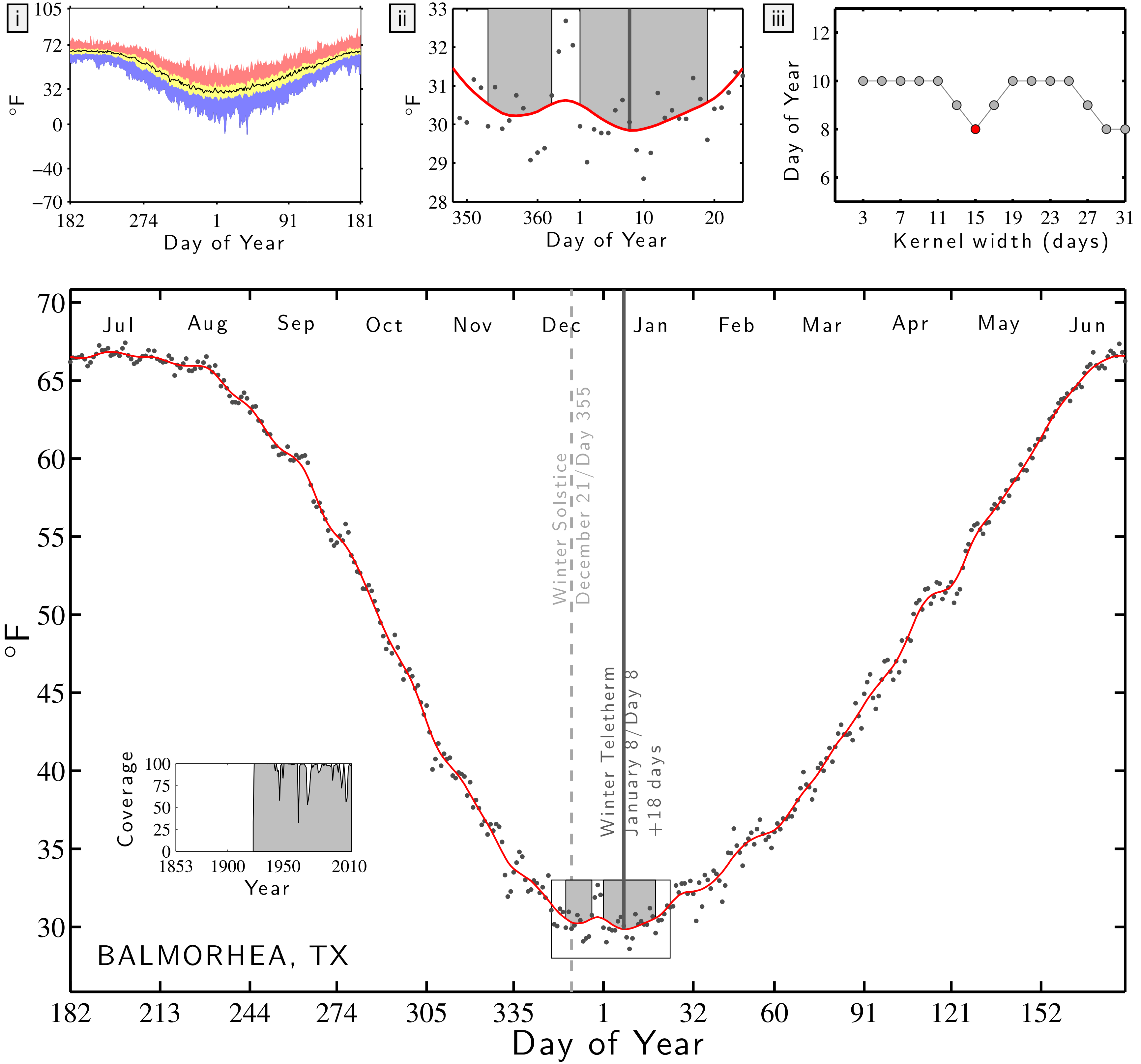

i
Day of Year
°F
ii
Day of Year
°F
iii
Kernel width (days)
Day of Year
Jul
Aug
Sep
Oct
Nov
Dec
Jan
Feb
Mar
Apr
May
Jun
Winter Solstice
December 21/Day 355
Winter Teletherm
January 8/Day 8
+18 days
Coverage
Year
BALMORHEA, TX
Day of Year
°F

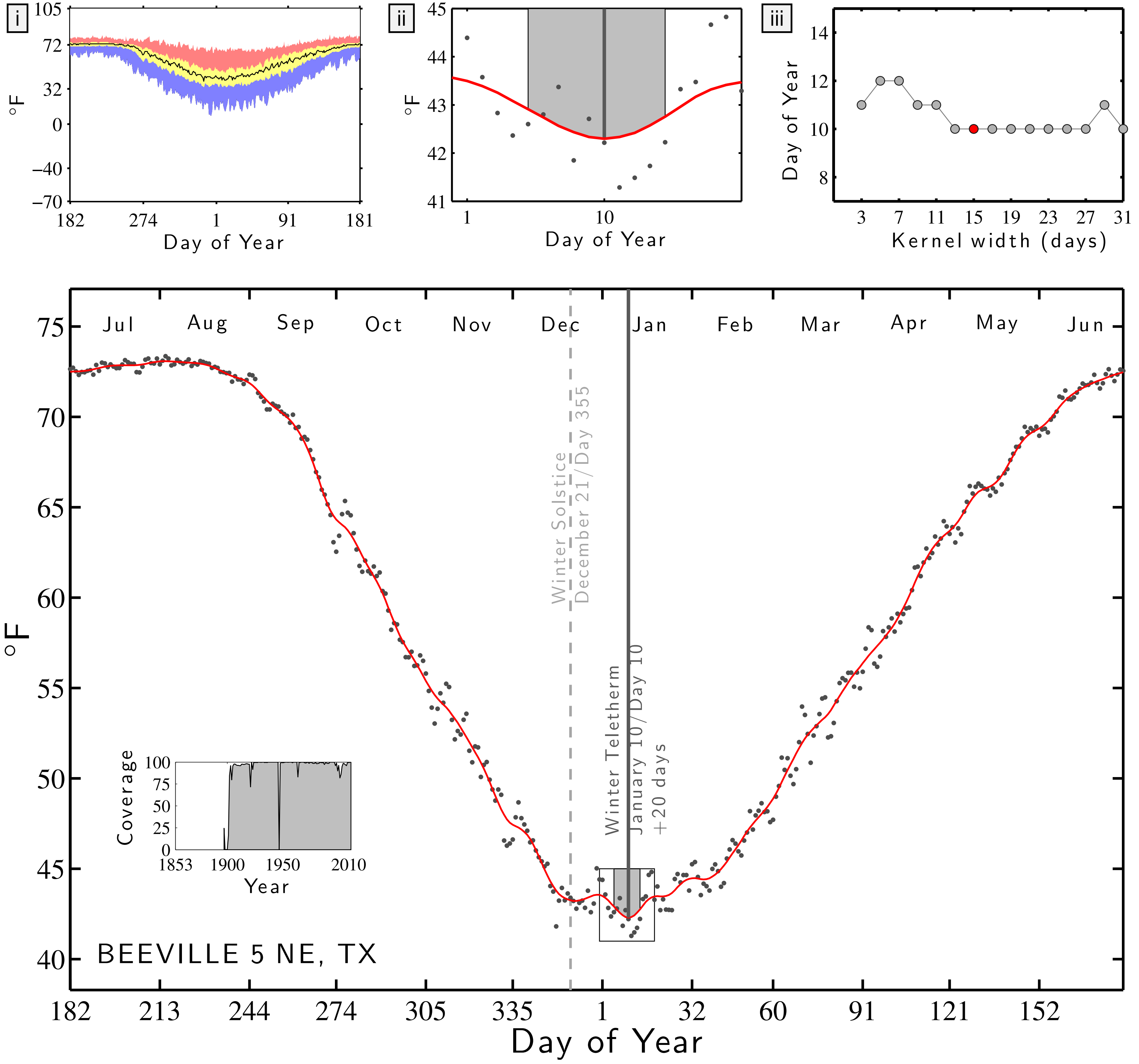

i
ii
iii
°F
Day of Year
Kernel width (days)
Jul
Aug
Sep
Oct
Nov
Dec
Jan
Feb
Mar
Apr
May
Jun
Winter Solstice
December 21/Day 355
Winter Teletherm
January 10/Day 10
+20 days
Coverage
Year
BEEVILLE 5 NE, TX

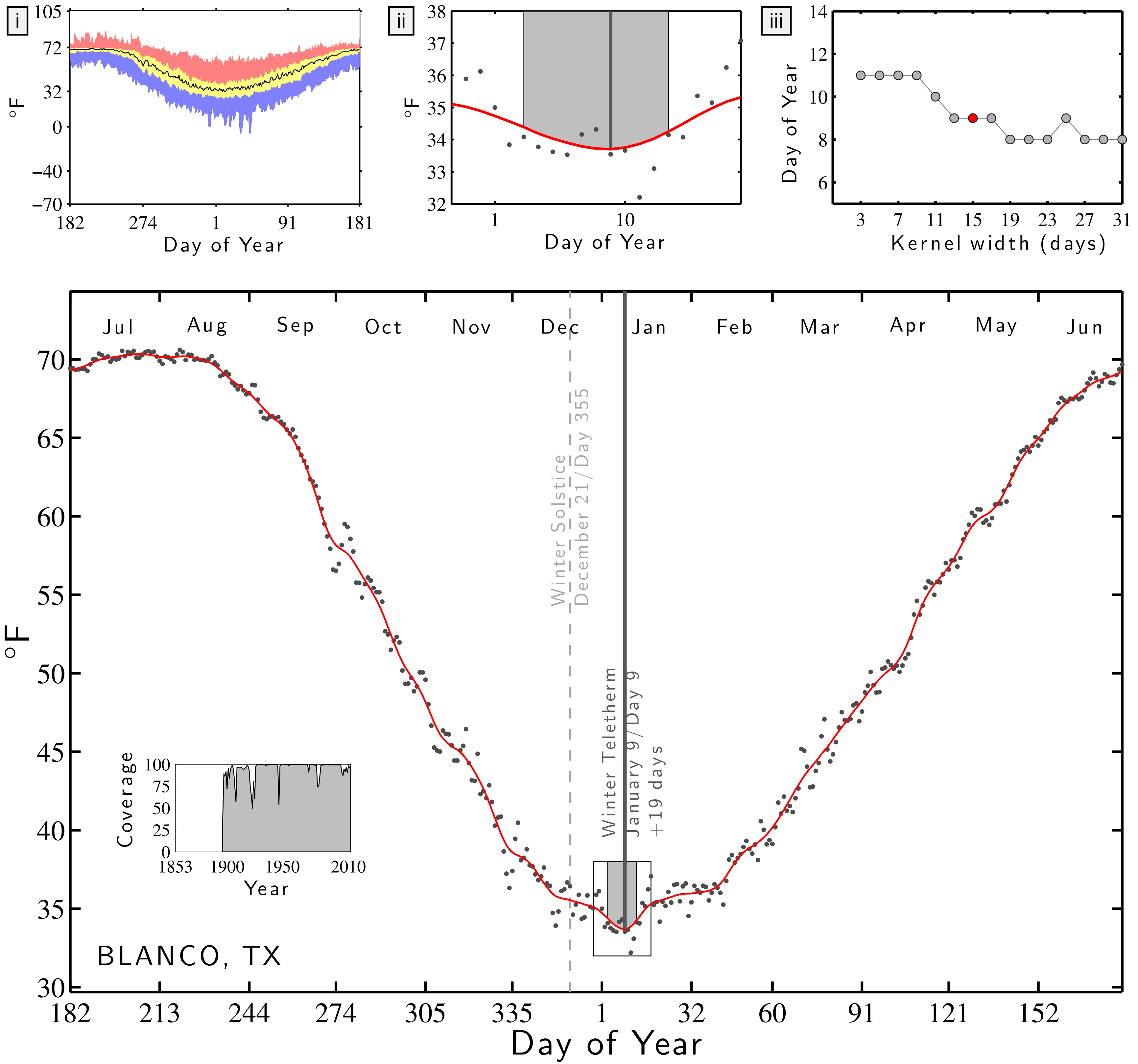

i
ii
iii
°F
Day of Year
Day of Year
Kernel width (days)
Jul
Aug
Sep
Oct
Nov
Dec
Jan
Feb
Mar
Apr
May
Jun
Winter Solstice
December 21/Day 355
Winter Teletherm
January 9/Day 9
+19 days
Coverage
Year
BLANCO, TX
Day of Year

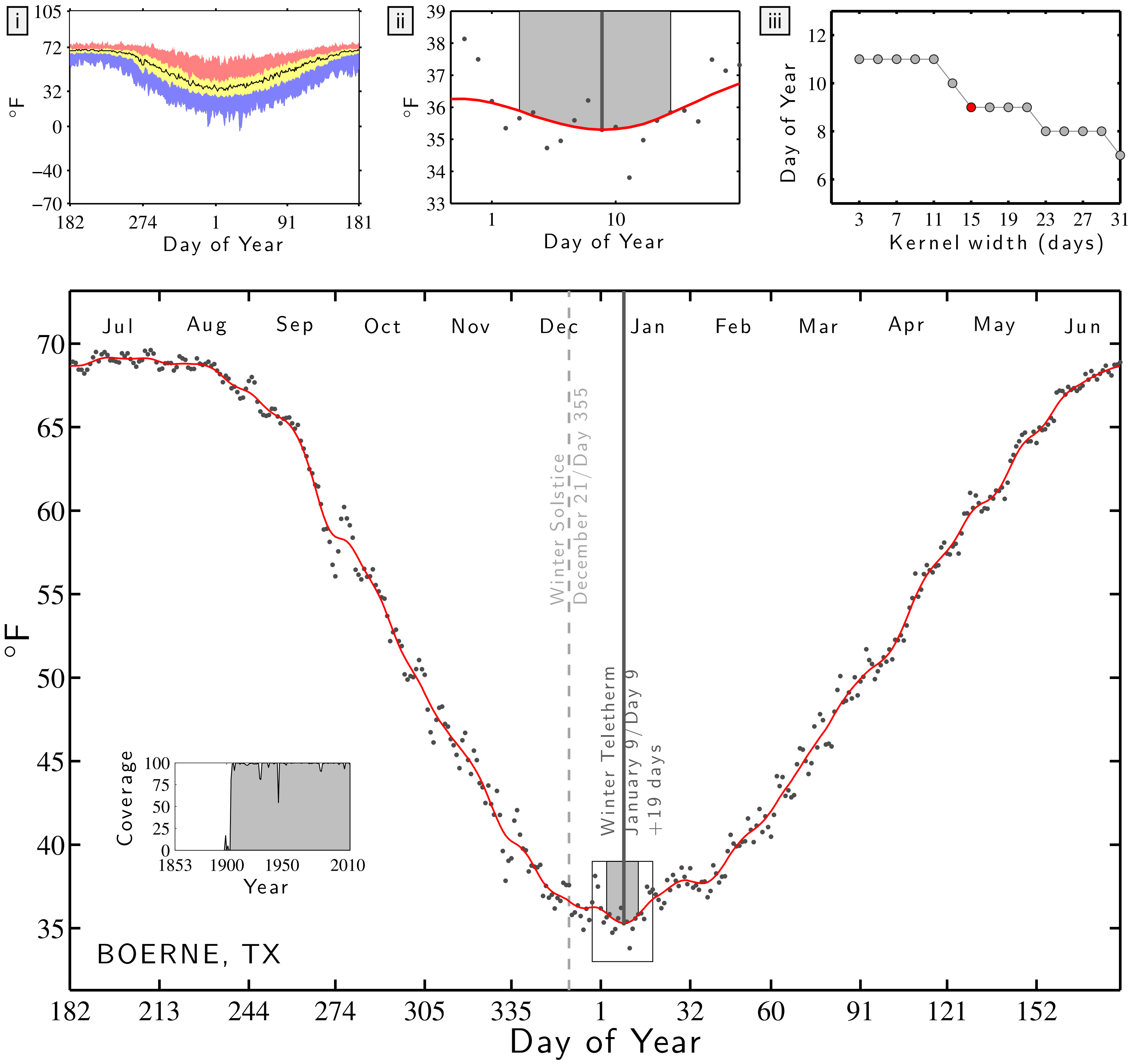

i
ii
iii
°F
Day of Year
Day of Year
Kernel width (days)
Jul
Aug
Sep
Oct
Nov
Dec
Jan
Feb
Mar
Apr
May
Jun
Winter Solstice
December 21/Day 355
Winter Teletherm
January 9/Day 9
+19 days
Coverage
Year
BOERNE, TX

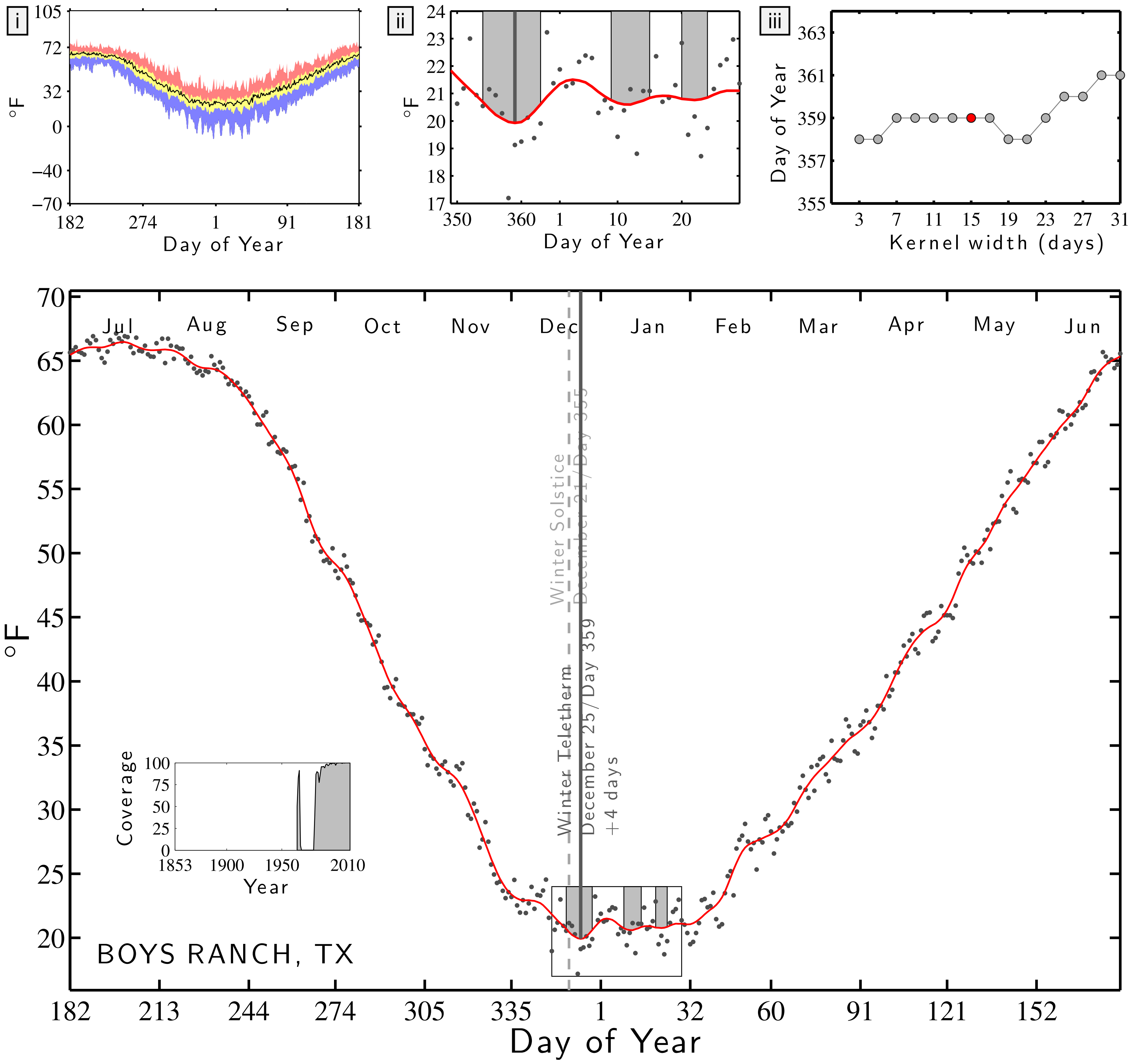

i
ii
iii
°F
Day of Year
Day of Year
Kernel width (days)
Jul
Aug
Sep
Oct
Nov
Dec
Jan
Feb
Mar
Apr
May
Jun
Winter Solstice
December 21/Day 355
Winter Teletherm
December 25/Day 359
+4 days
Coverage
Year
BOYS RANCH, TX
Day of Year

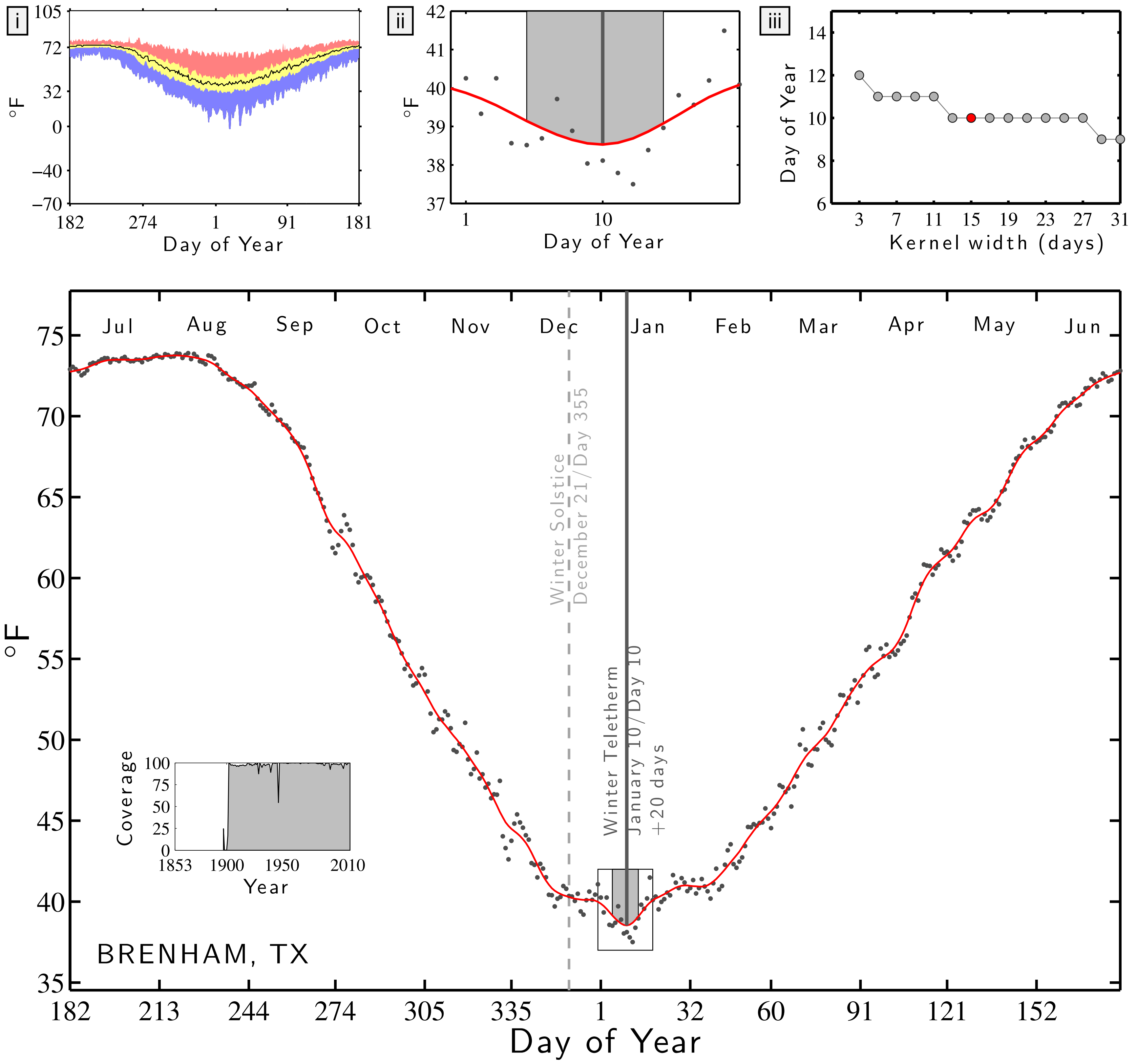

i
ii
iii
°F
Day of Year
Kernel width (days)
Jul
Aug
Sep
Oct
Nov
Dec
Jan
Feb
Mar
Apr
May
Jun
Winter Solstice
December 21/Day 355
Winter Teletherm
January 10/Day 10
+20 days
Coverage
Year
BRENHAM, TX

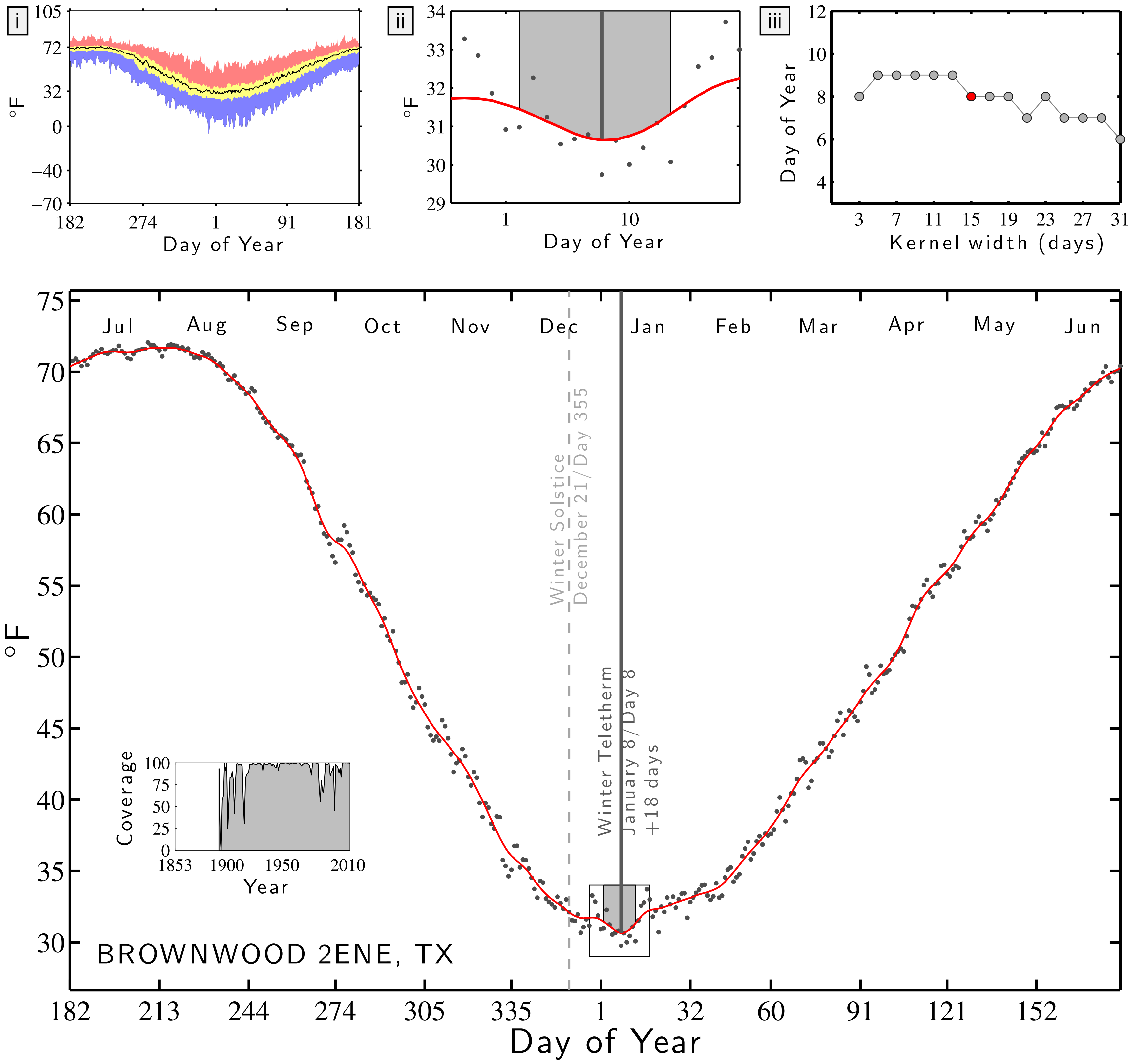

i
ii
iii
°F
Day of Year
Day of Year
Kernel width (days)
Jul
Aug
Sep
Oct
Nov
Dec
Jan
Feb
Mar
Apr
May
Jun
Winter Solstice
December 21/Day 355
Winter Teletherm
January 8/Day 8
+18 days
Coverage
Year
BROWNWOOD 2ENE, TX

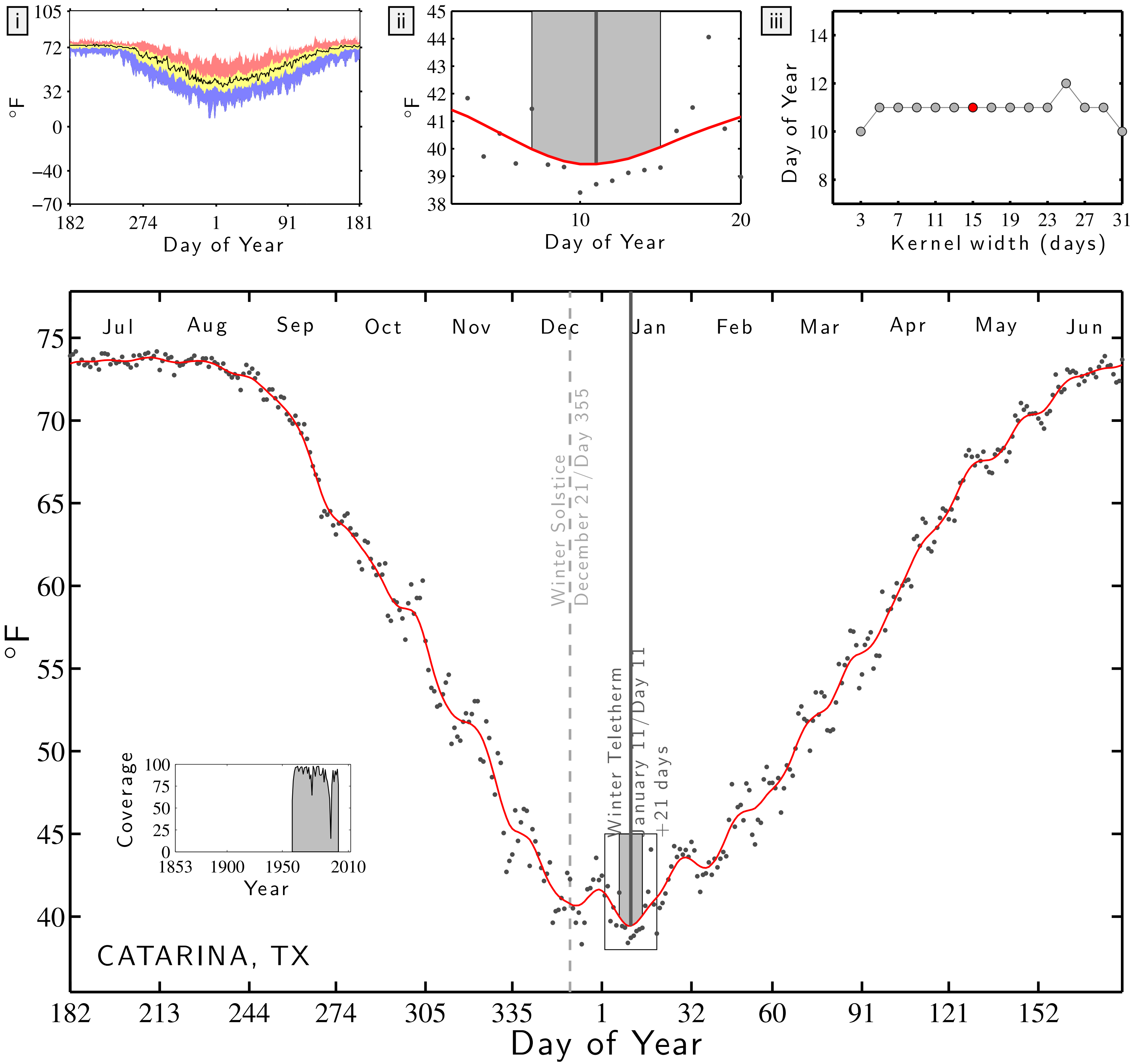

i
ii
iii
°F
Day of Year
Kernel width (days)
Jul
Aug
Sep
Oct
Nov
Dec
Jan
Feb
Mar
Apr
May
Jun
Winter Solstice
December 21/Day 355
Winter Teletherm
January 11/Day 11
±21 days
Coverage
Year
CATARINA, TX

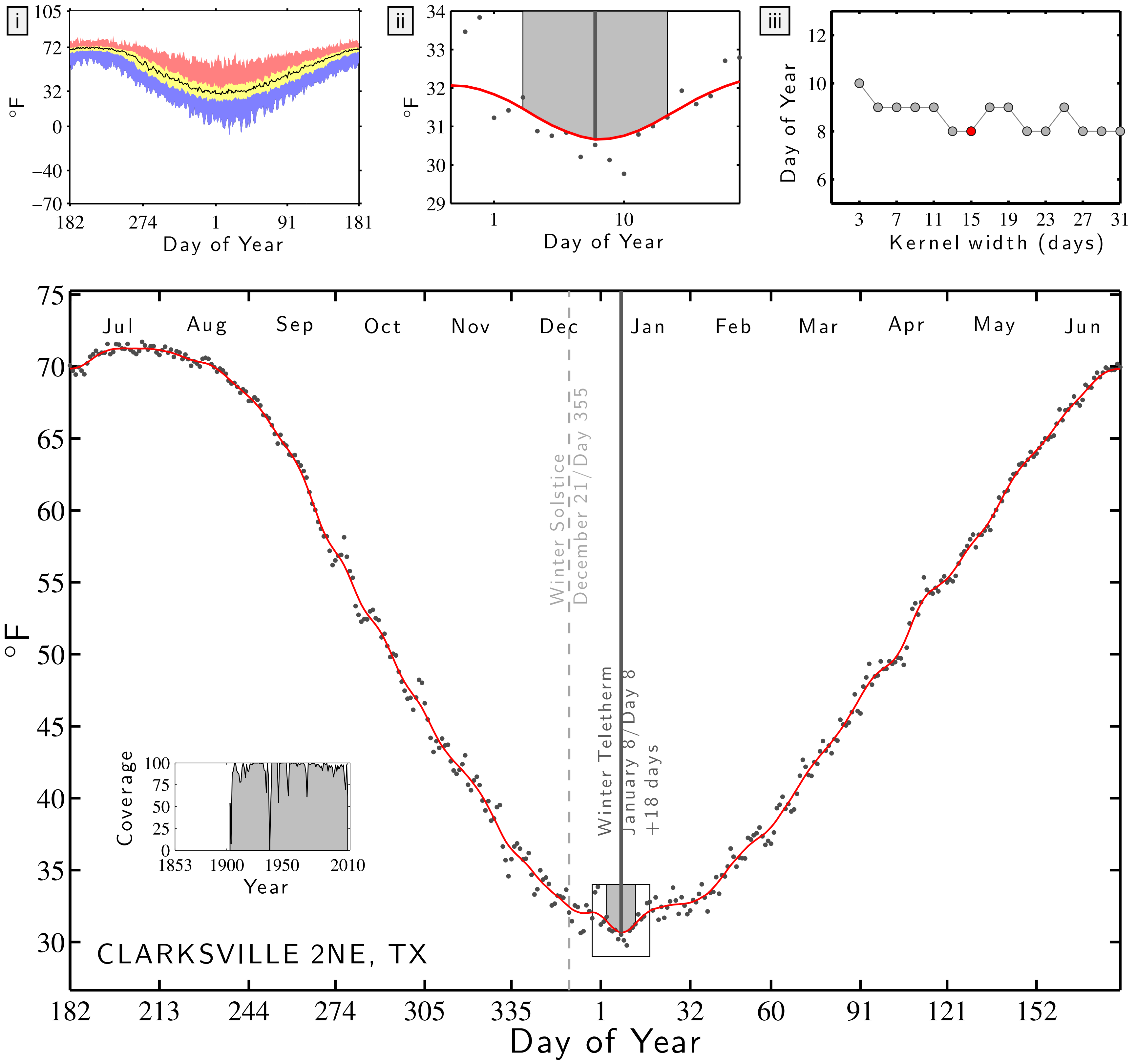

i
105
72
32
0
−40
−70
°F
182
274
1
91
181
Day of Year
ii
34
33
32
31
30
29
°F
1
10
Day of Year
iii
12
10
8
6
Day of Year
3
7
11
15
19
23
27
31
Kernel width (days)
75
70
65
60
55
50
45
40
35
30
°F
Jul
Aug
Sep
Oct
Nov
Dec
Jan
Feb
Mar
Apr
May
Jun
Winter Solstice
December 21/Day 355
Winter Teletherm
January 8/Day 8
+18 days
Coverage
100
75
50
25
0
1853
1900
1950
2010
Year
CLARKSVILLE 2NE, TX
182
213
244
274
305
335
1
32
60
91
121
152
Day of Year

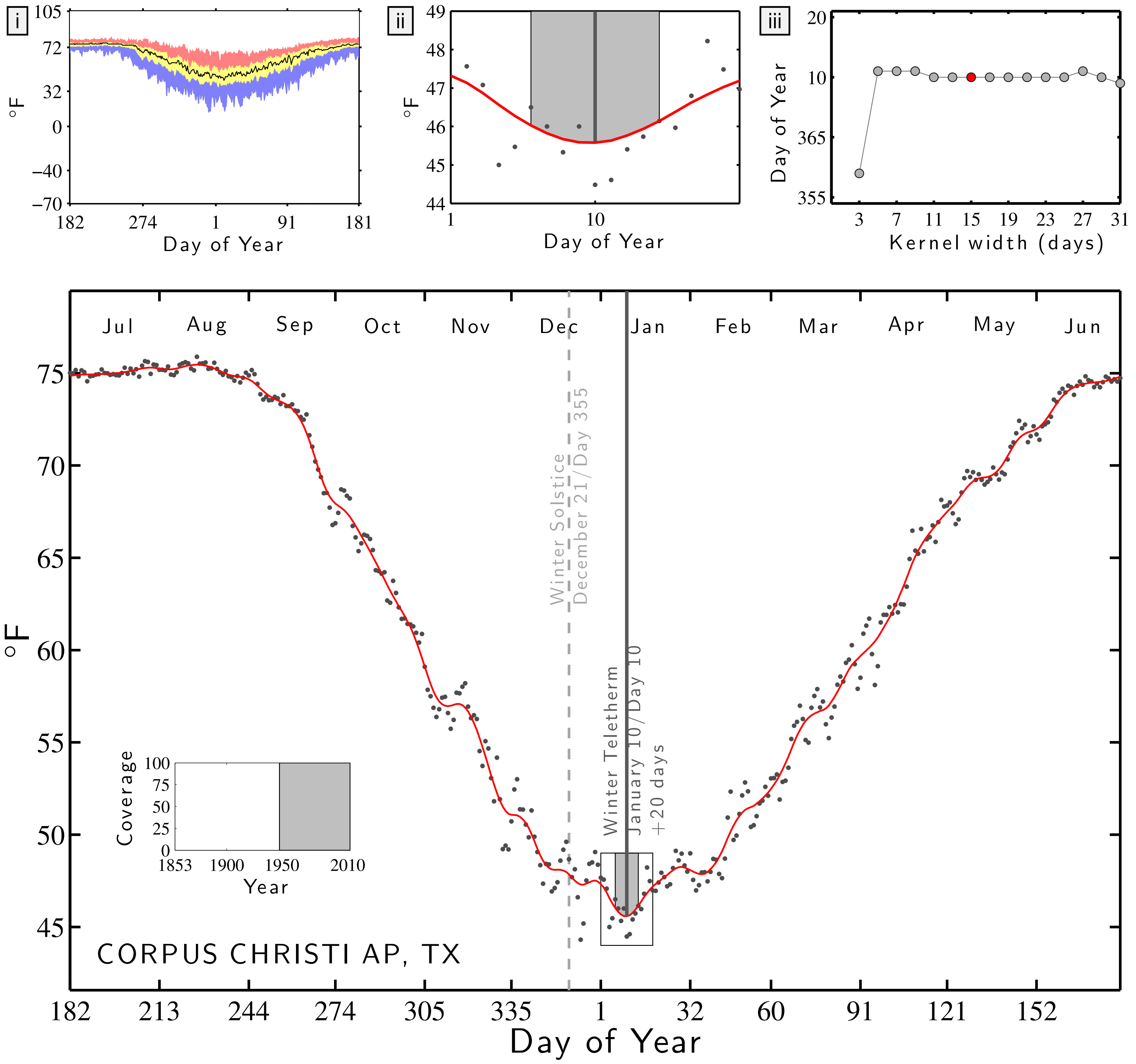

i
°F
Day of Year
ii
°F
Day of Year
iii
Day of Year
Kernel width (days)
Jul
Aug
Sep
Oct
Nov
Dec
Jan
Feb
Mar
Apr
May
Jun
Winter Solstice
December 21/Day 355
Winter Teletherm
January 10/Day 10
+20 days
Coverage
Year
CORPUS CHRISTI AP, TX
°F
Day of Year

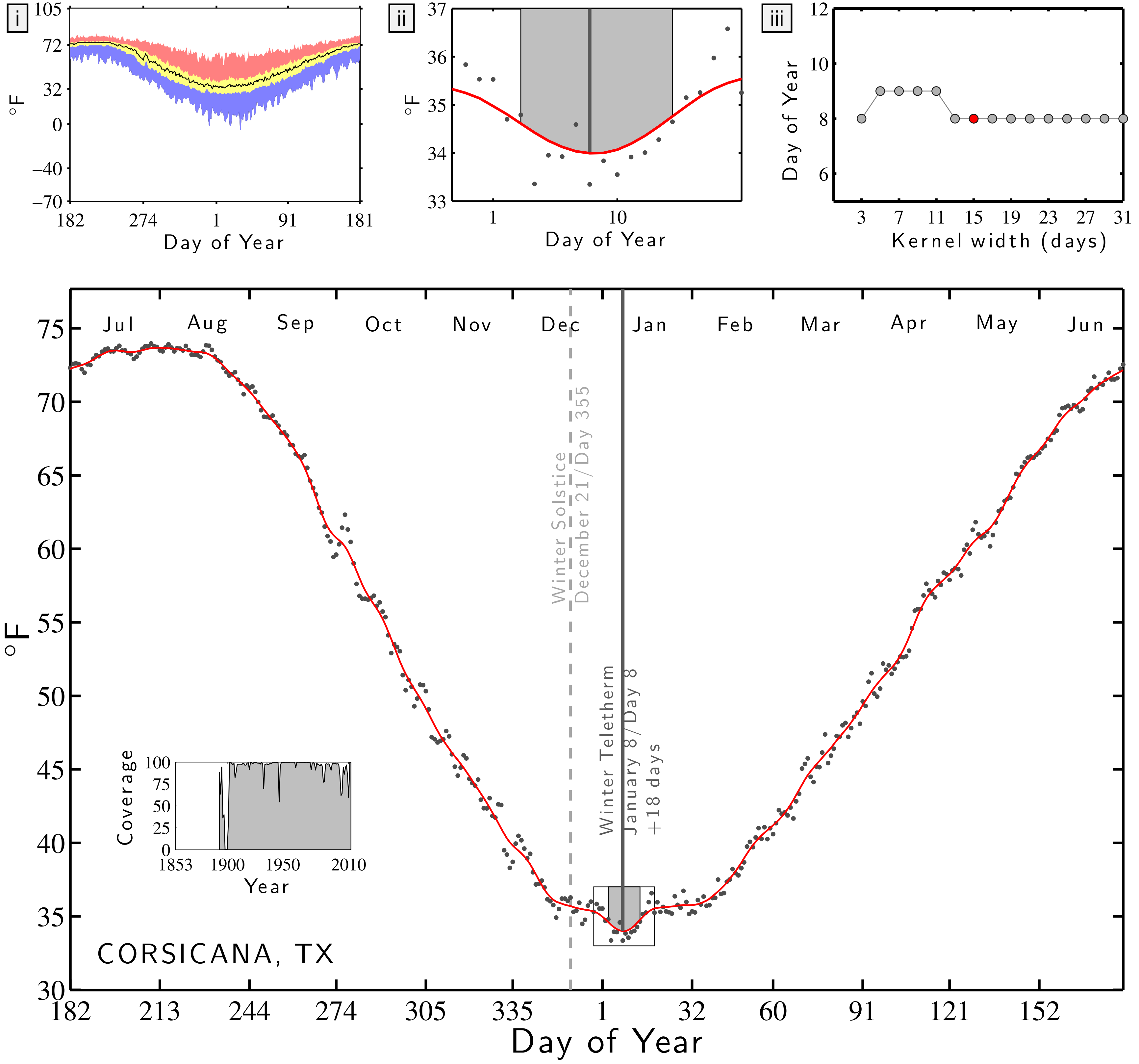

i
ii
iii
°F
Day of Year
Day of Year
Kernel width (days)
Jul
Aug
Sep
Oct
Nov
Dec
Jan
Feb
Mar
Apr
May
Jun
Winter Solstice
December 21/Day 355
Winter Teletherm
January 8/Day 8
+18 days
Coverage
Year
CORSICANA, TX
Day of Year

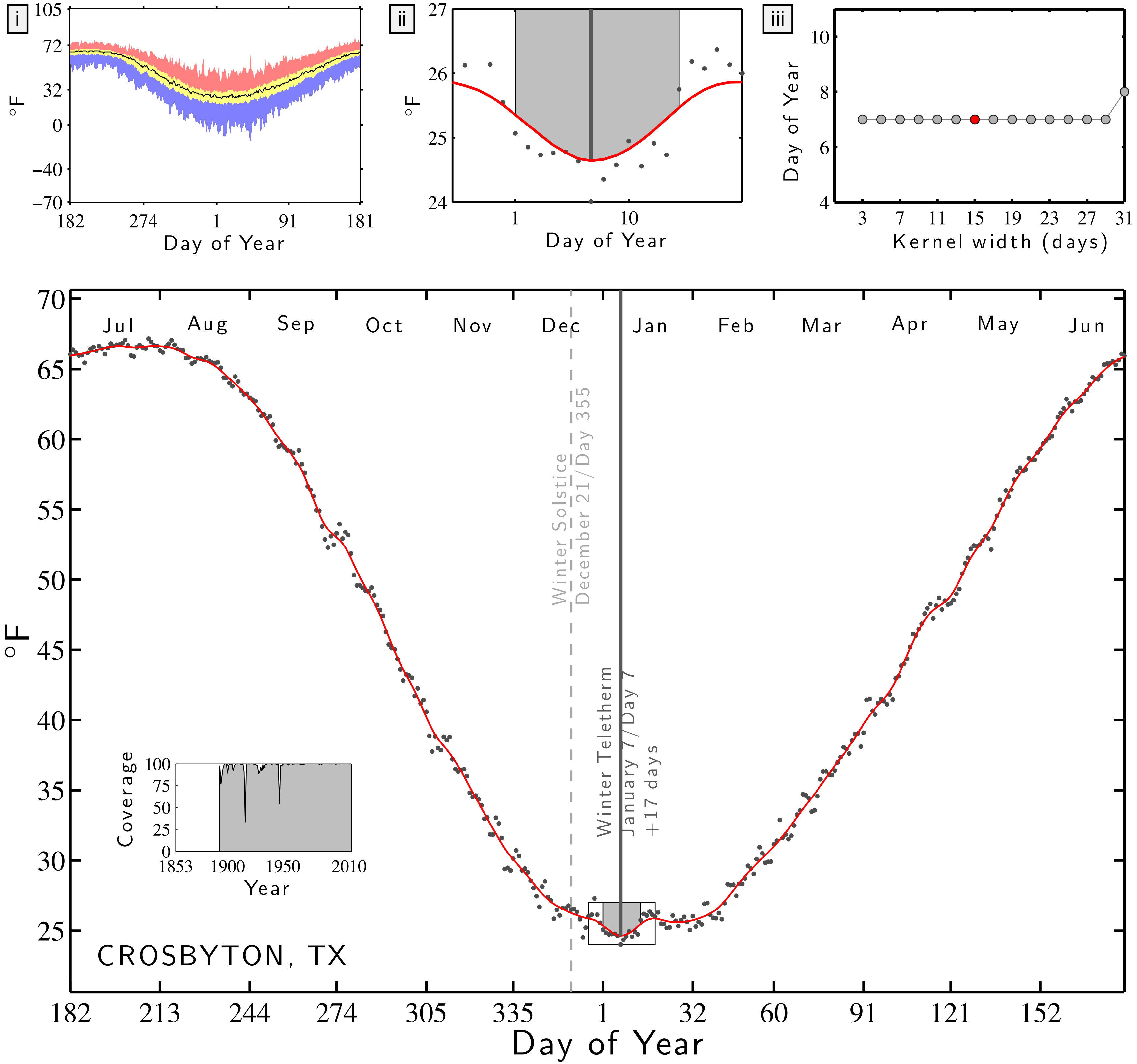

i
°F
105
72
32
0
−40
−70
182
274
1
91
181
Day of Year
ii
°F
27
26
25
24
1
10
Day of Year
iii
Day of Year
10
8
6
4
3
7
11
15
19
23
27
31
Kernel width (days)
°F
70
65
60
55
50
45
40
35
30
25
Jul
Aug
Sep
Oct
Nov
Dec
Jan
Feb
Mar
Apr
May
Jun
Winter Solstice
December 21/Day 355
Winter Teletherm
January 7/Day 7
+17 days
Coverage
100
75
50
25
0
1853
1900
1950
2010
Year
CROSBYTON, TX
182
213
244
274
305
335
1
32
60
91
121
152
Day of Year

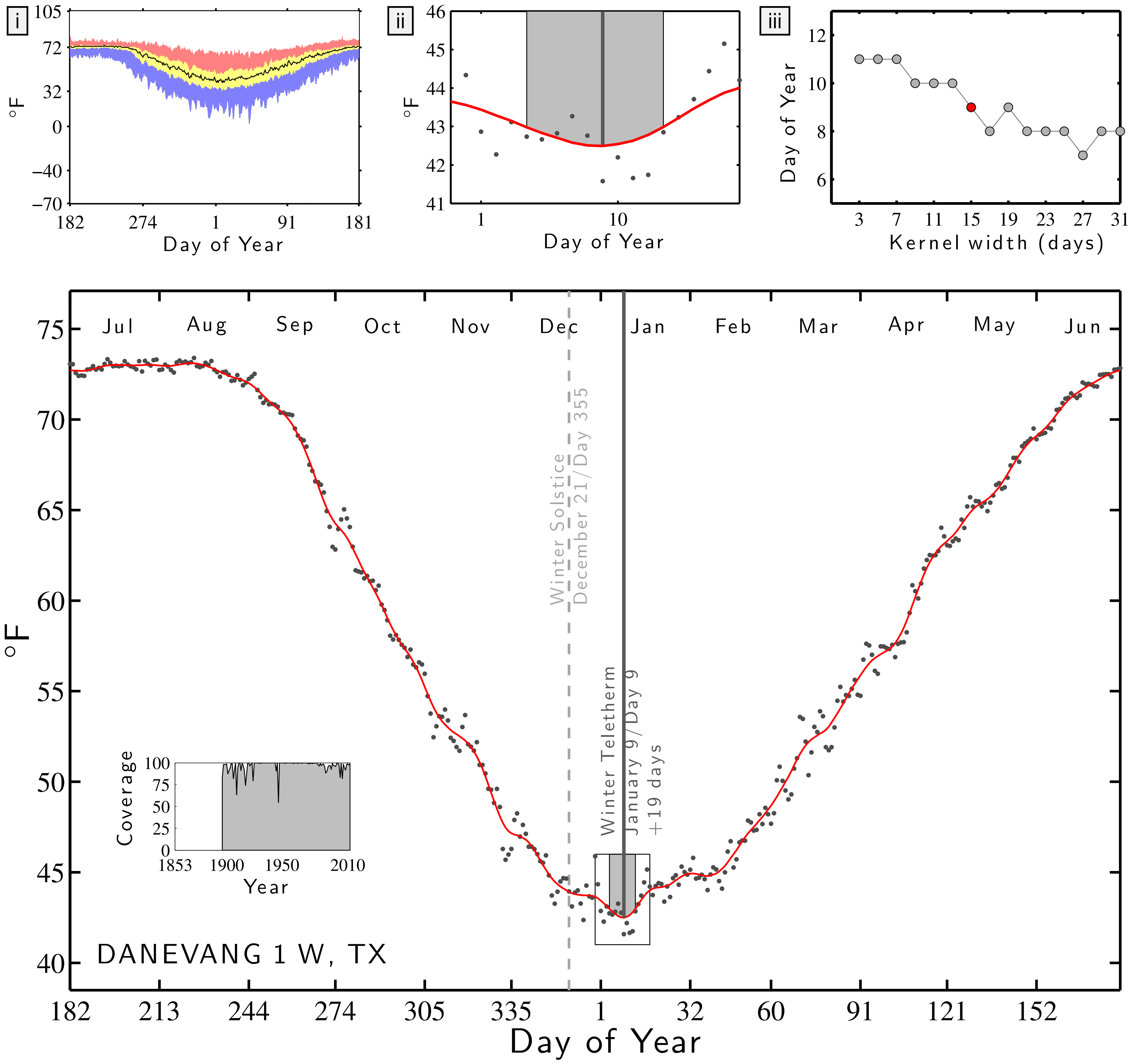

i
ii
iii
Day of Year
Kernel width (days)
°F
Day of Year
Jul
Aug
Sep
Oct
Nov
Dec
Jan
Feb
Mar
Apr
May
Jun
Winter Solstice
December 21/Day 355
Winter Teletherm
January 9/Day 9
+19 days
Coverage
Year
DANEVANG 1 W, TX

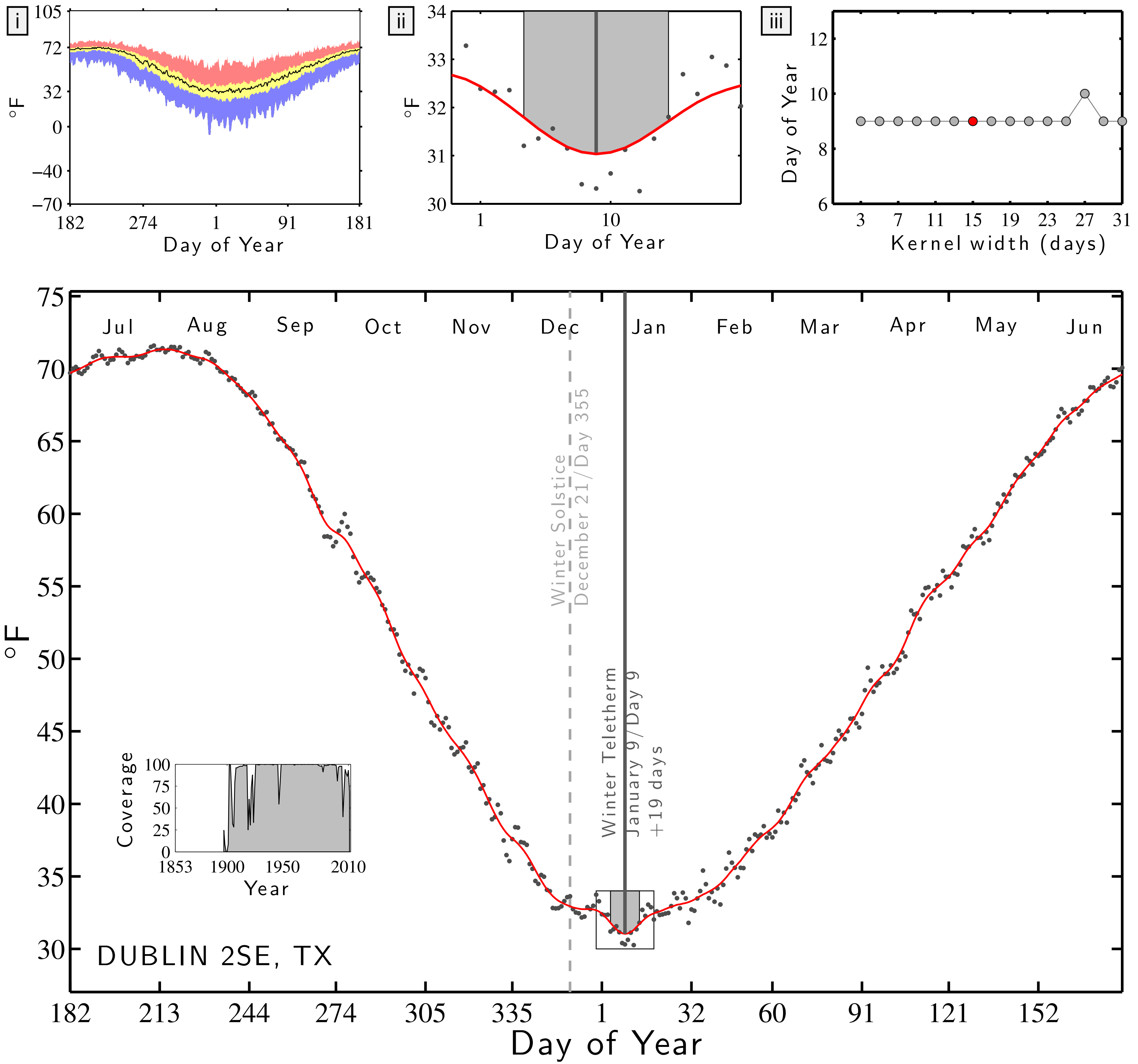
i
ii
iii
°F
Day of Year
Kernel width (days)
Jul
Aug
Sep
Oct
Nov
Dec
Jan
Feb
Mar
Apr
May
Jun
Winter Solstice
December 21/Day 355
Winter Teletherm
January 9/Day 9
+19 days
Coverage
Year
DUBLIN 2SE, TX

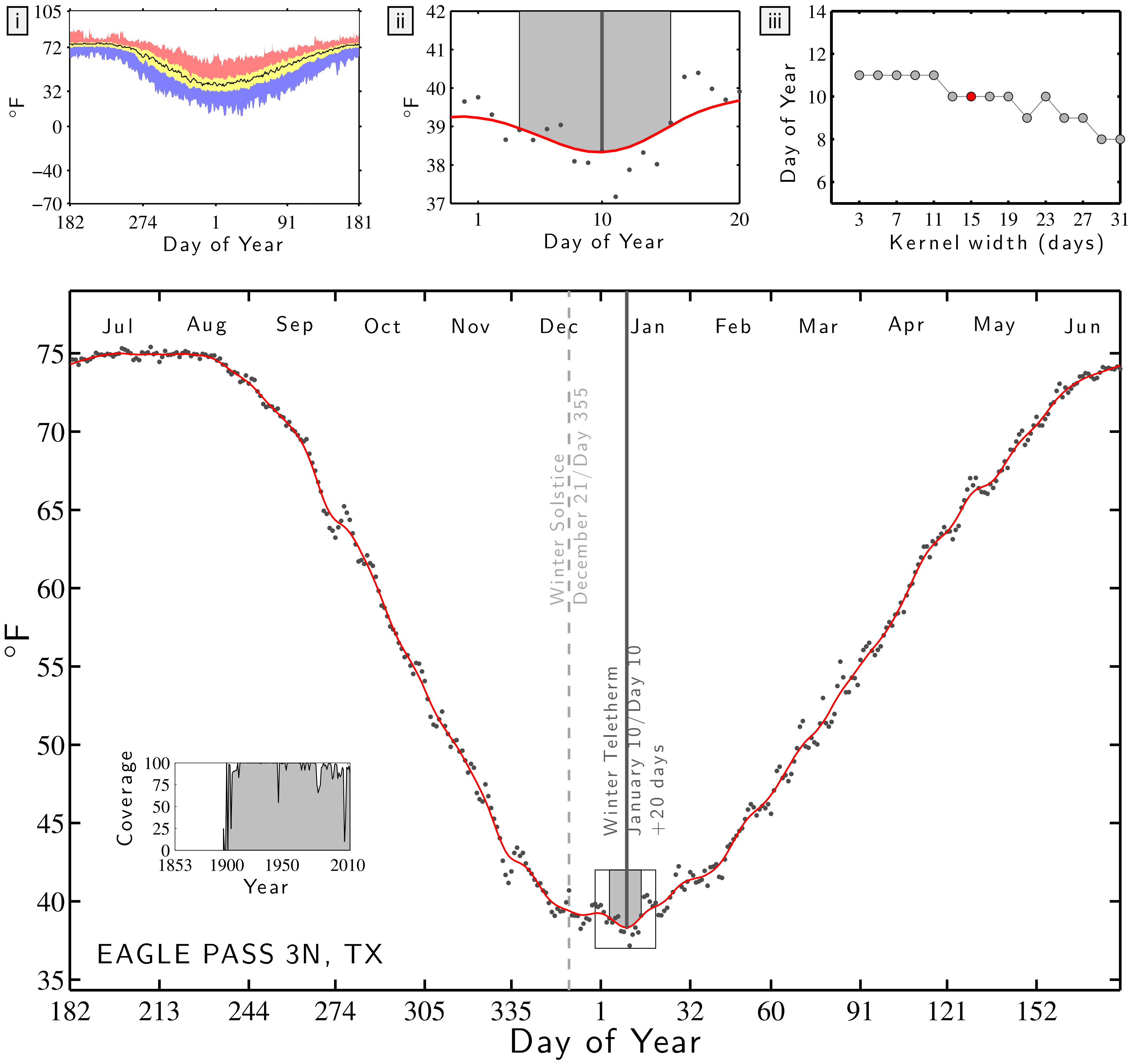

i
ii
iii
°F
Day of Year
Day of Year
Kernel width (days)
Jul
Aug
Sep
Oct
Nov
Dec
Jan
Feb
Mar
Apr
May
Jun
Winter Solstice
December 21/Day 355
Winter Teletherm
January 10/Day 10
+20 days
Coverage
Year
EAGLE PASS 3N, TX
°F
Day of Year

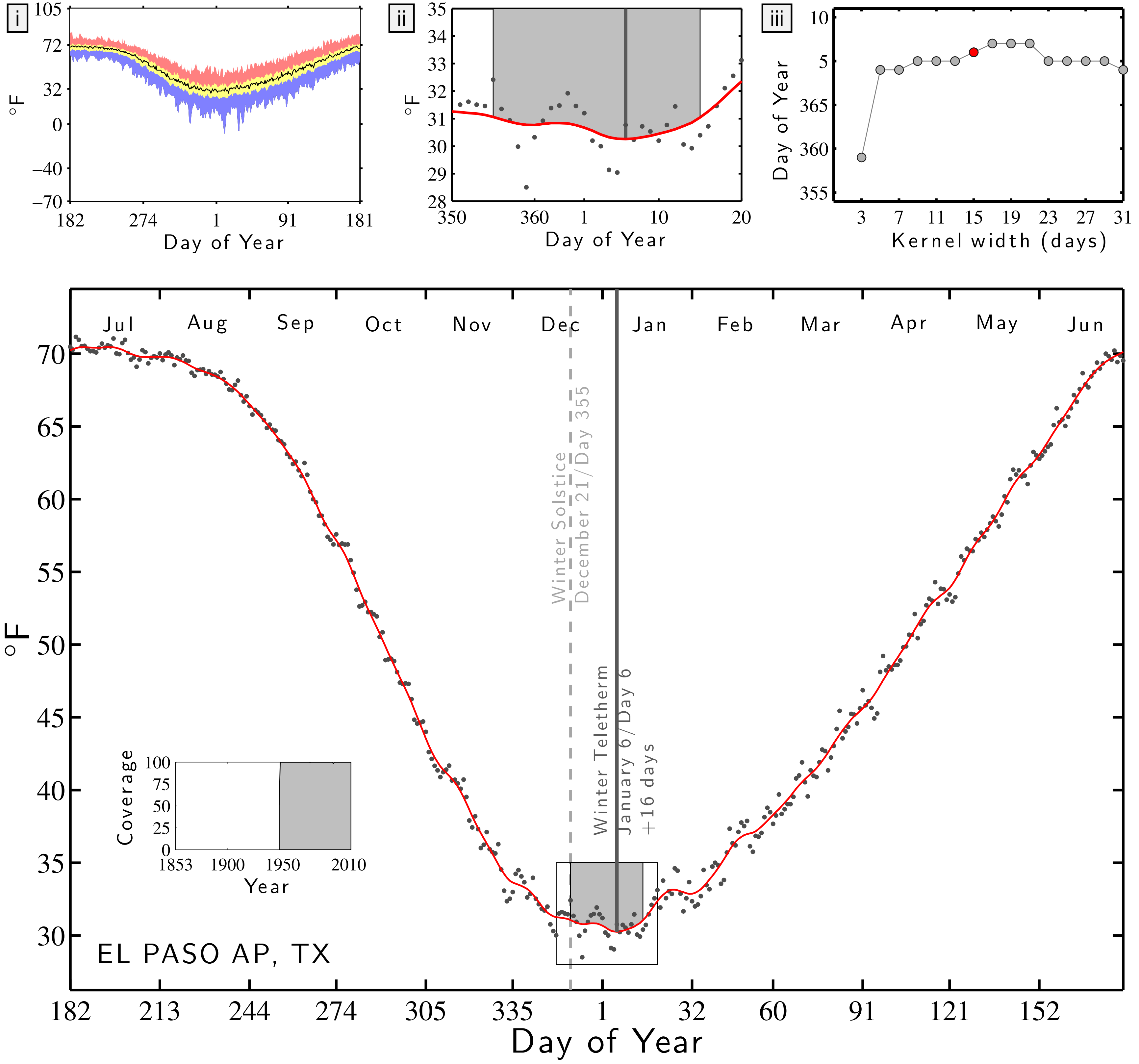

i
ii
iii
°F
Day of Year
Kernel width (days)
Jul
Aug
Sep
Oct
Nov
Dec
Jan
Feb
Mar
Apr
May
Jun
Winter Solstice
December 21/Day 355
Winter Teletherm
January 6/Day 6
+16 days
Coverage
Year
EL PASO AP, TX

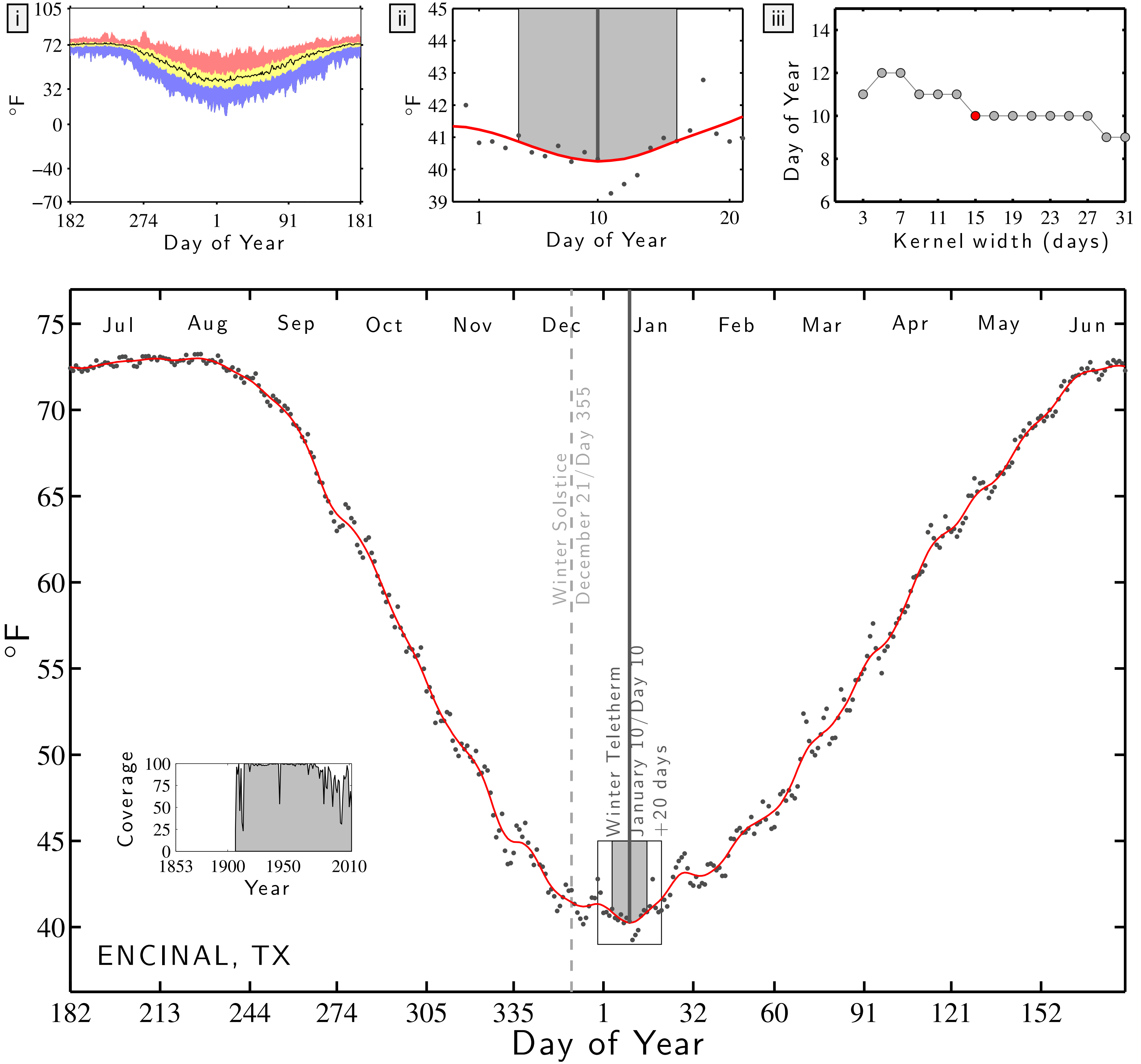

i
°F
Day of Year
ii
°F
Day of Year
iii
Day of Year
Kernel width (days)
Jul
Aug
Sep
Oct
Nov
Dec
Jan
Feb
Mar
Apr
May
Jun
Winter Solstice
December 21/Day 355
Winter Teletherm
January 10/Day 10
+20 days
Coverage
Year
ENCINAL, TX
°F
Day of Year

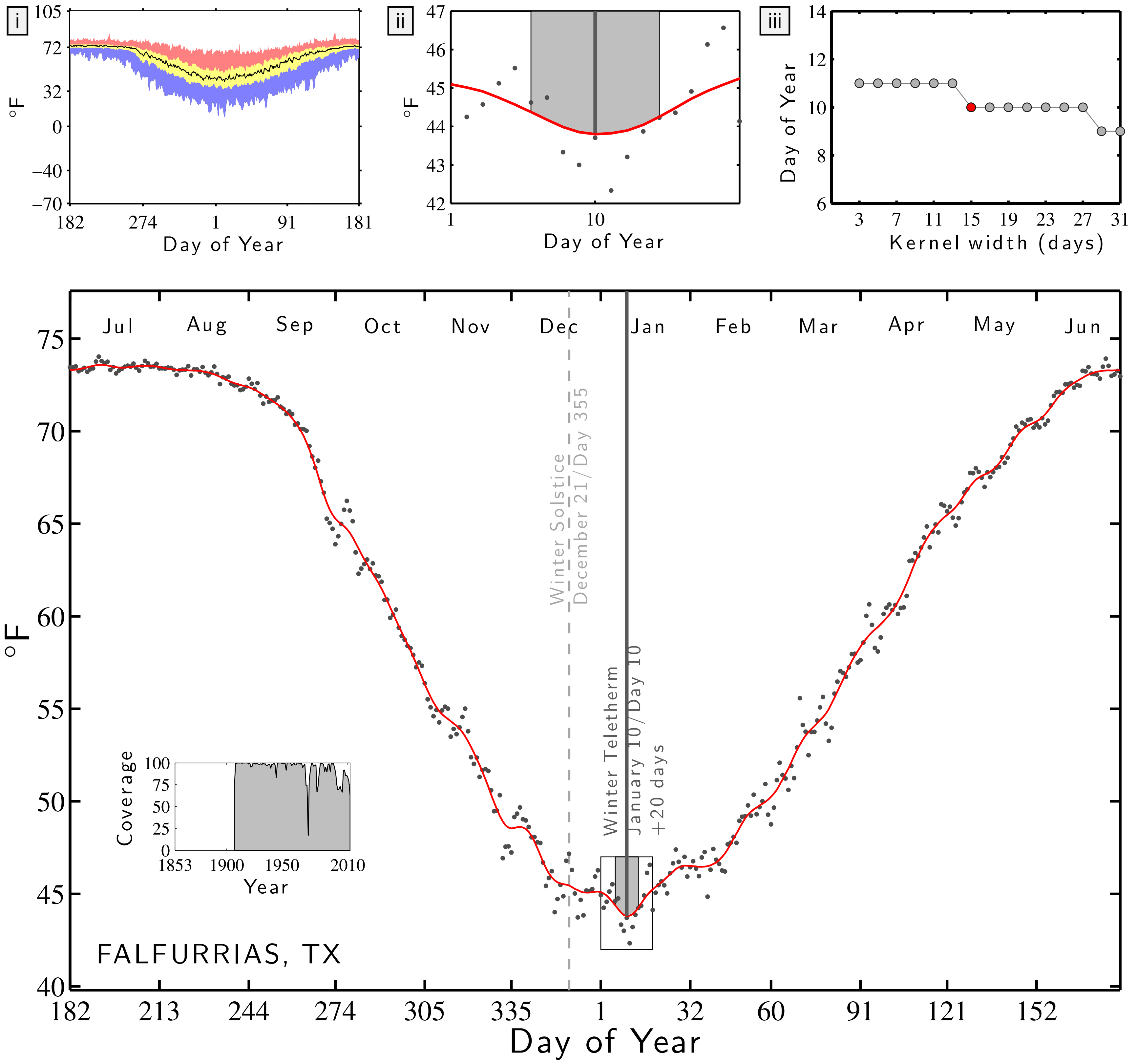

i
°F
Day of Year
ii
°F
Day of Year
iii
Day of Year
Kernel width (days)
Jul
Aug
Sep
Oct
Nov
Dec
Jan
Feb
Mar
Apr
May
Jun
Winter Solstice
December 21/Day 355
Winter Teletherm
January 10/Day 10
+20 days
Coverage
Year
FALFURRIAS, TX
°F
Day of Year

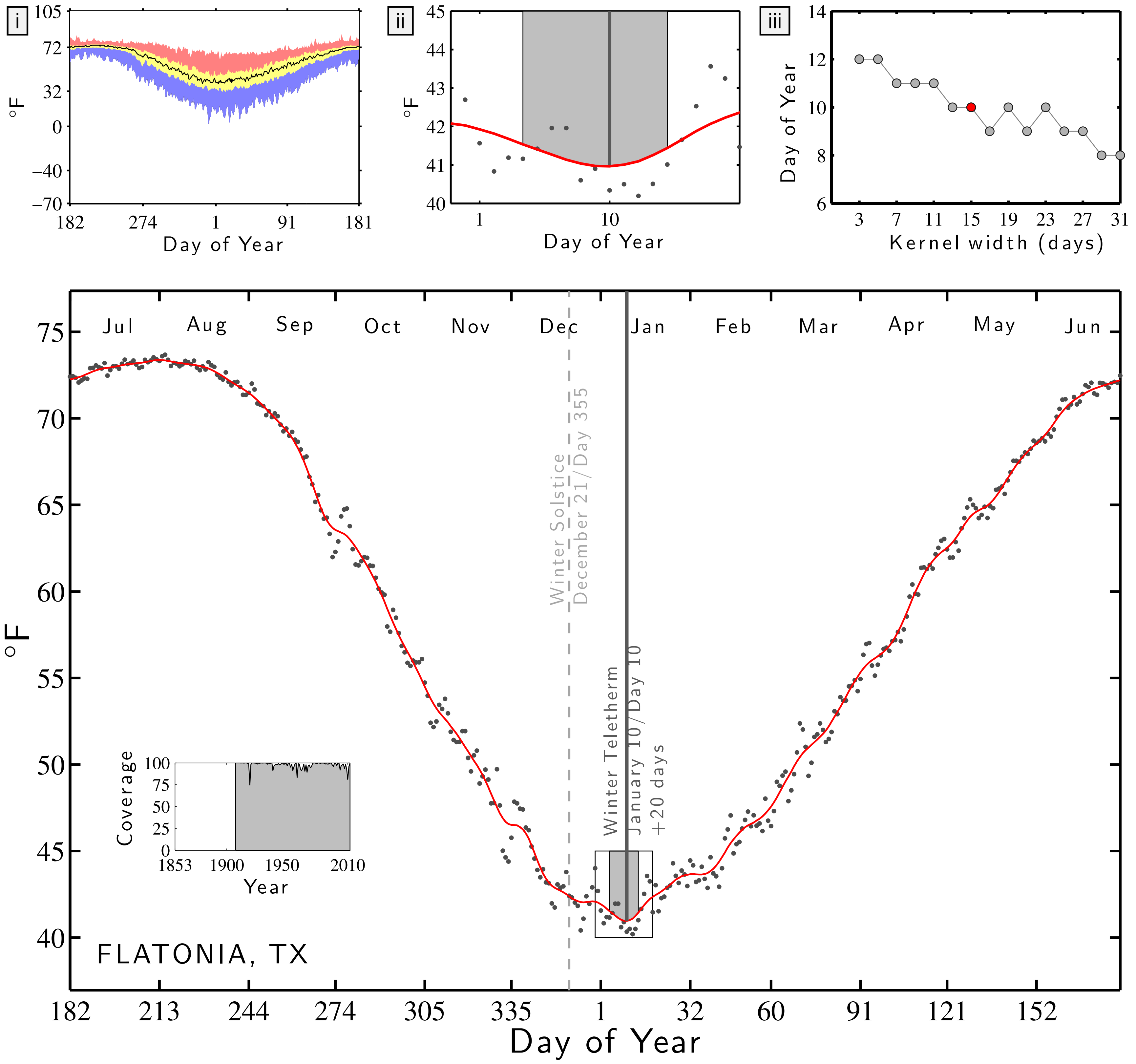

i
°F
Day of Year
ii
°F
Day of Year
iii
Day of Year
Kernel width (days)
Jul
Aug
Sep
Oct
Nov
Dec
Jan
Feb
Mar
Apr
May
Jun
Winter Solstice
December 21/Day 355
Winter Teletherm
January 10/Day 10
+20 days
Coverage
Year
FLATONIA, TX
°F
Day of Year

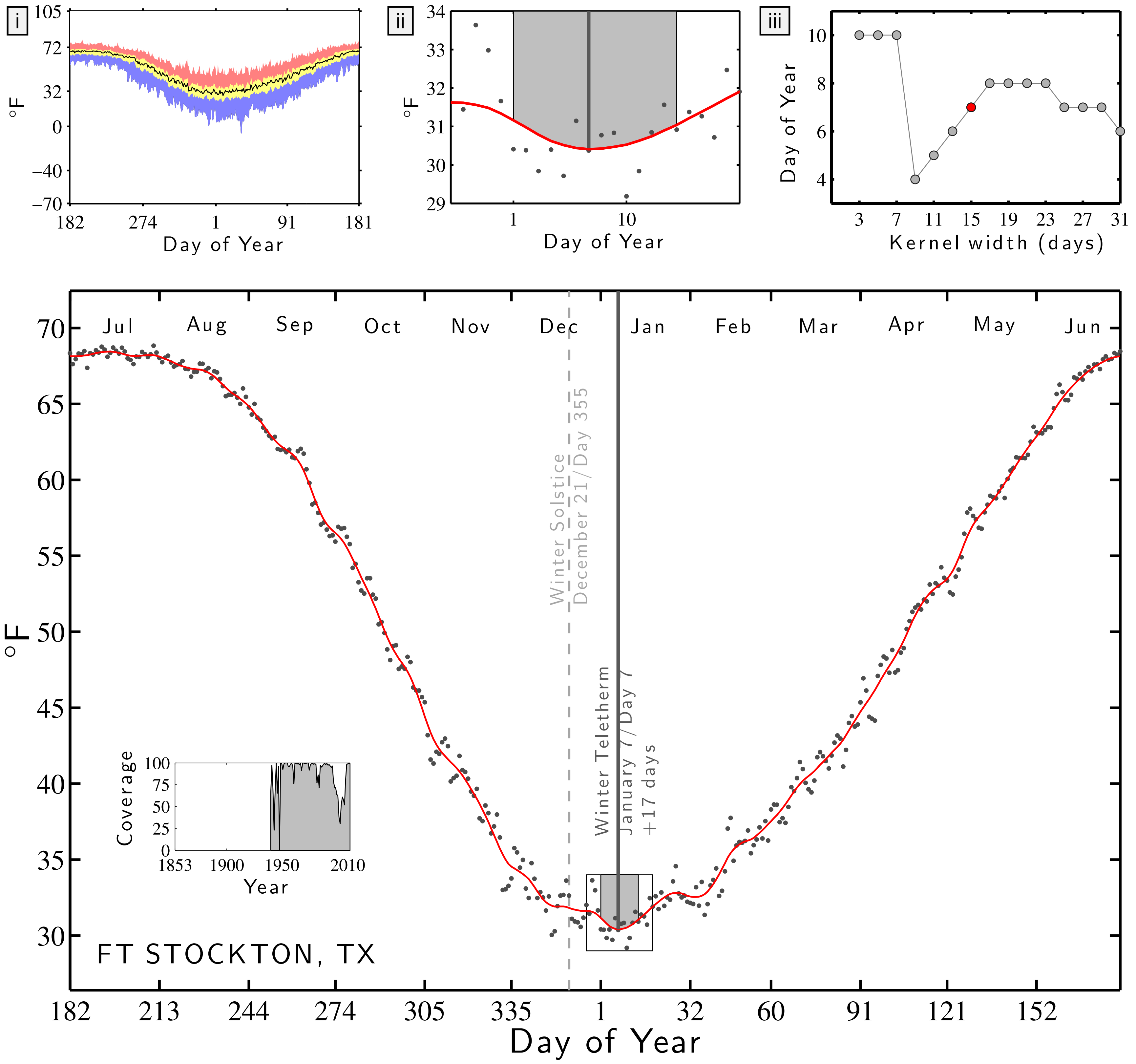

i
ii
iii
°F
Day of Year
Kernel width (days)
Jul
Aug
Sep
Oct
Nov
Dec
Jan
Feb
Mar
Apr
May
Jun
Winter Solstice
December 21/Day 355
Winter Teletherm
January 7/Day 7
+17 days
Coverage
Year
FT STOCKTON, TX

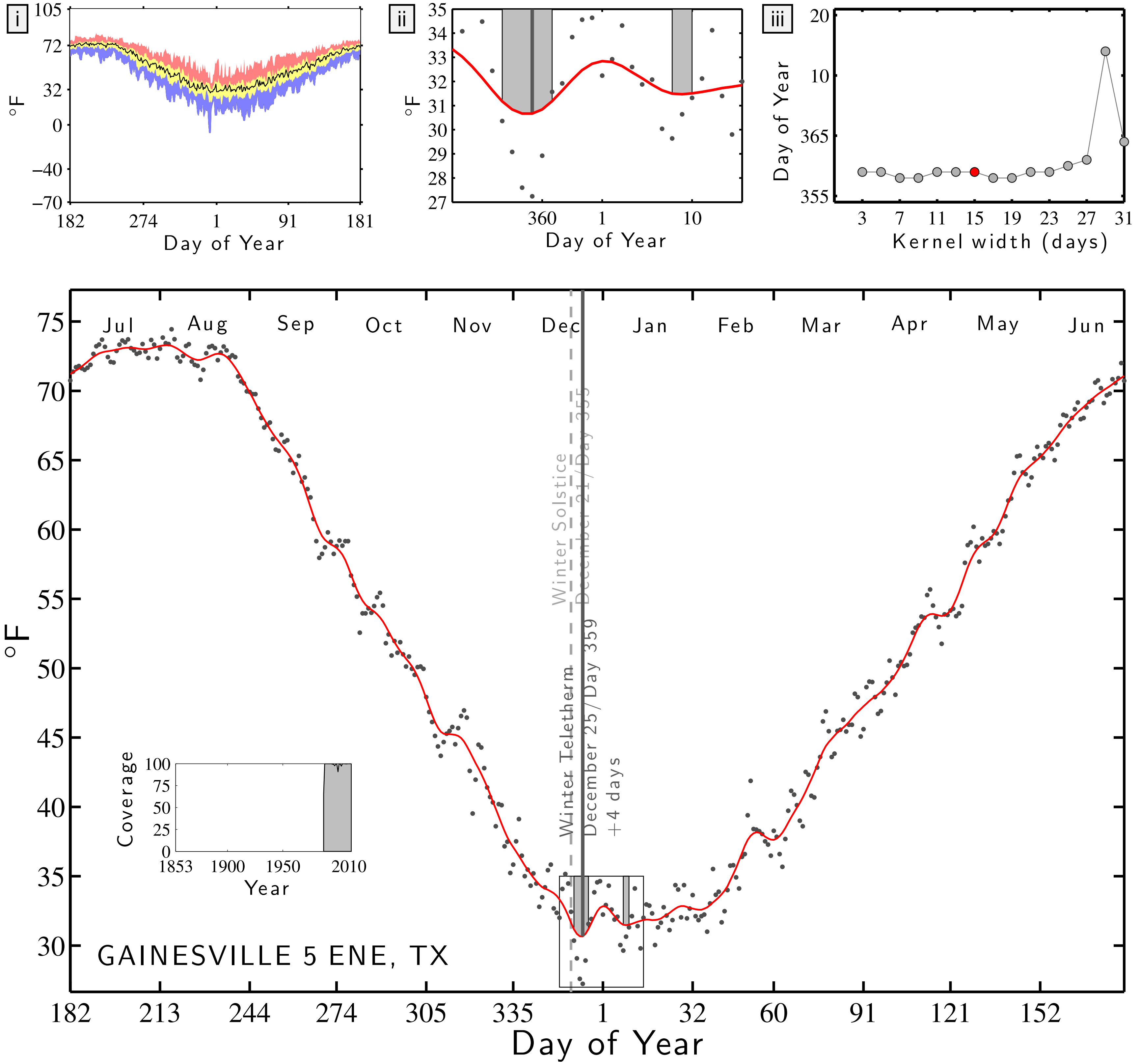

i
ii
iii
°F
Day of Year
Kernel width (days)
Jul
Aug
Sep
Oct
Nov
Dec
Jan
Feb
Mar
Apr
May
Jun
Winter Solstice
December 21/Day 355
Winter Teletherm
December 25/Day 359
+4 days
Coverage
Year
GAINESVILLE 5 ENE, TX

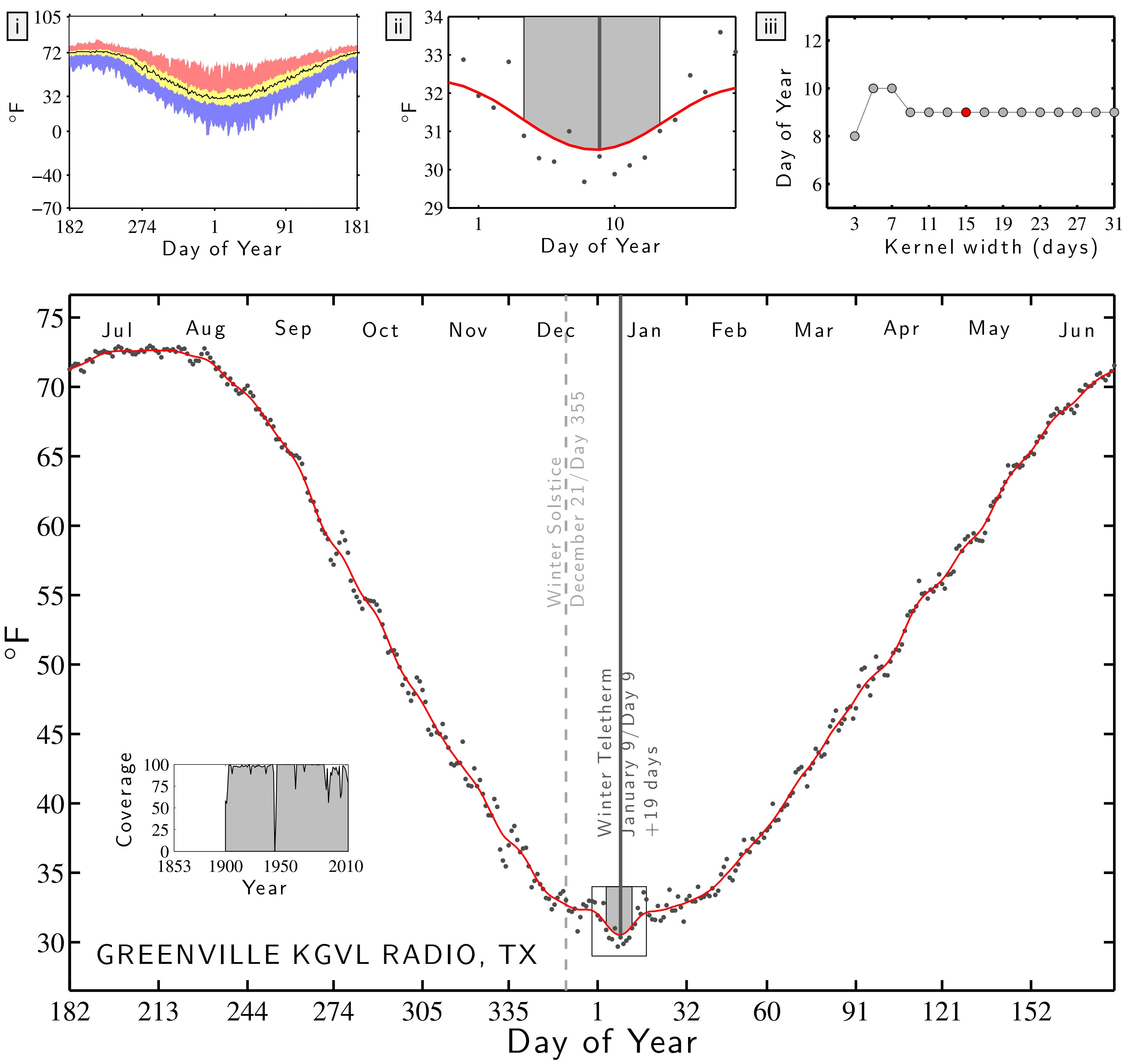

i
ii
iii
°F
Day of Year
Day of Year
Kernel width (days)
Jul
Aug
Sep
Oct
Nov
Dec
Jan
Feb
Mar
Apr
May
Jun
Winter Solstice
December 21/Day 355
Winter Teletherm
January 9/Day 9
+19 days
Coverage
Year
GREENVILLE KGVL RADIO, TX
Day of Year

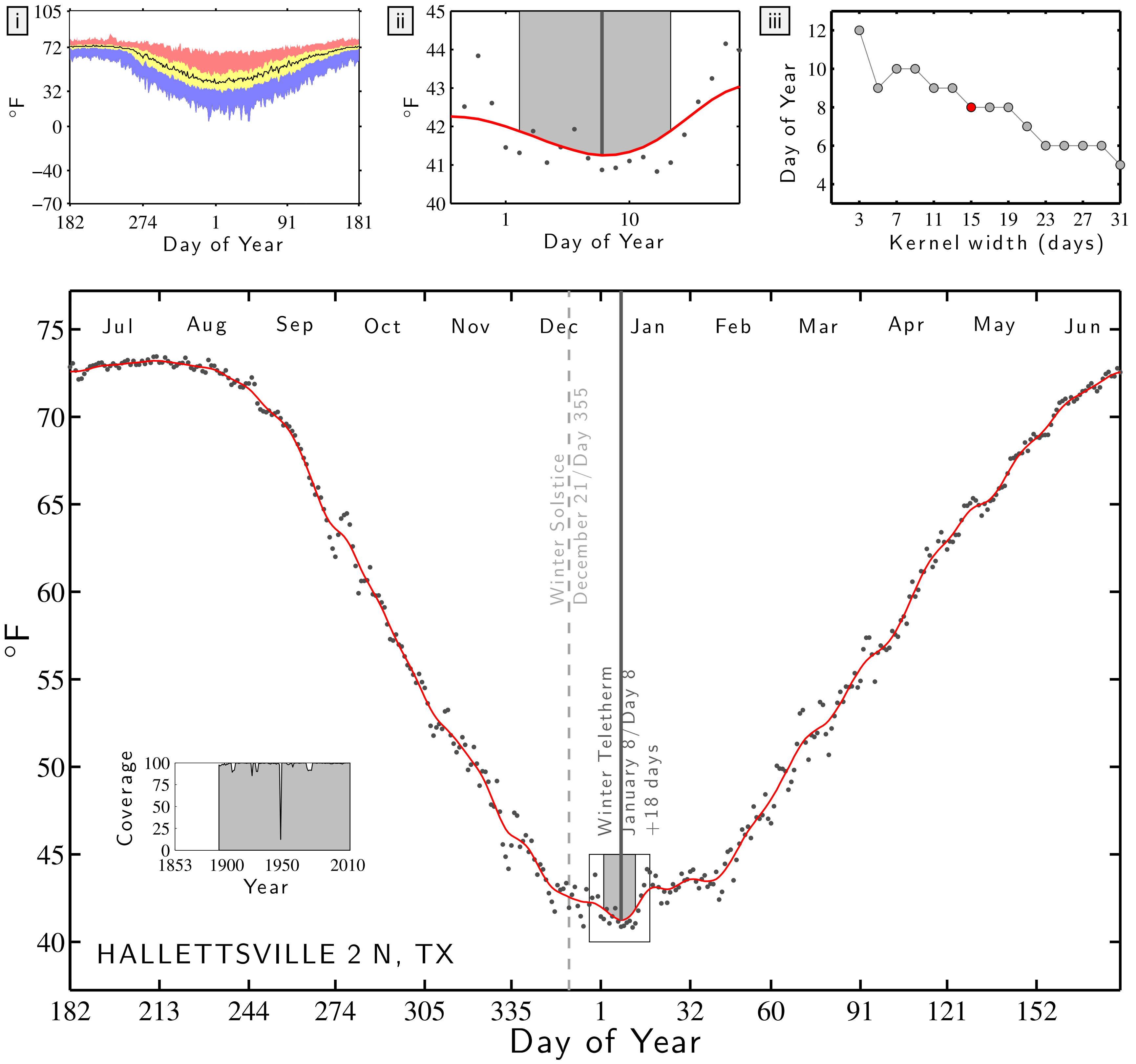

i
ii
iii
°F
Day of Year
Day of Year
Kernel width (days)
Jul
Aug
Sep
Oct
Nov
Dec
Jan
Feb
Mar
Apr
May
Jun
Winter Solstice
December 21/Day 355
Winter Teletherm
January 8/Day 8
+18 days
Coverage
Year
HALLETTSVILLE 2 N, TX
Day of Year

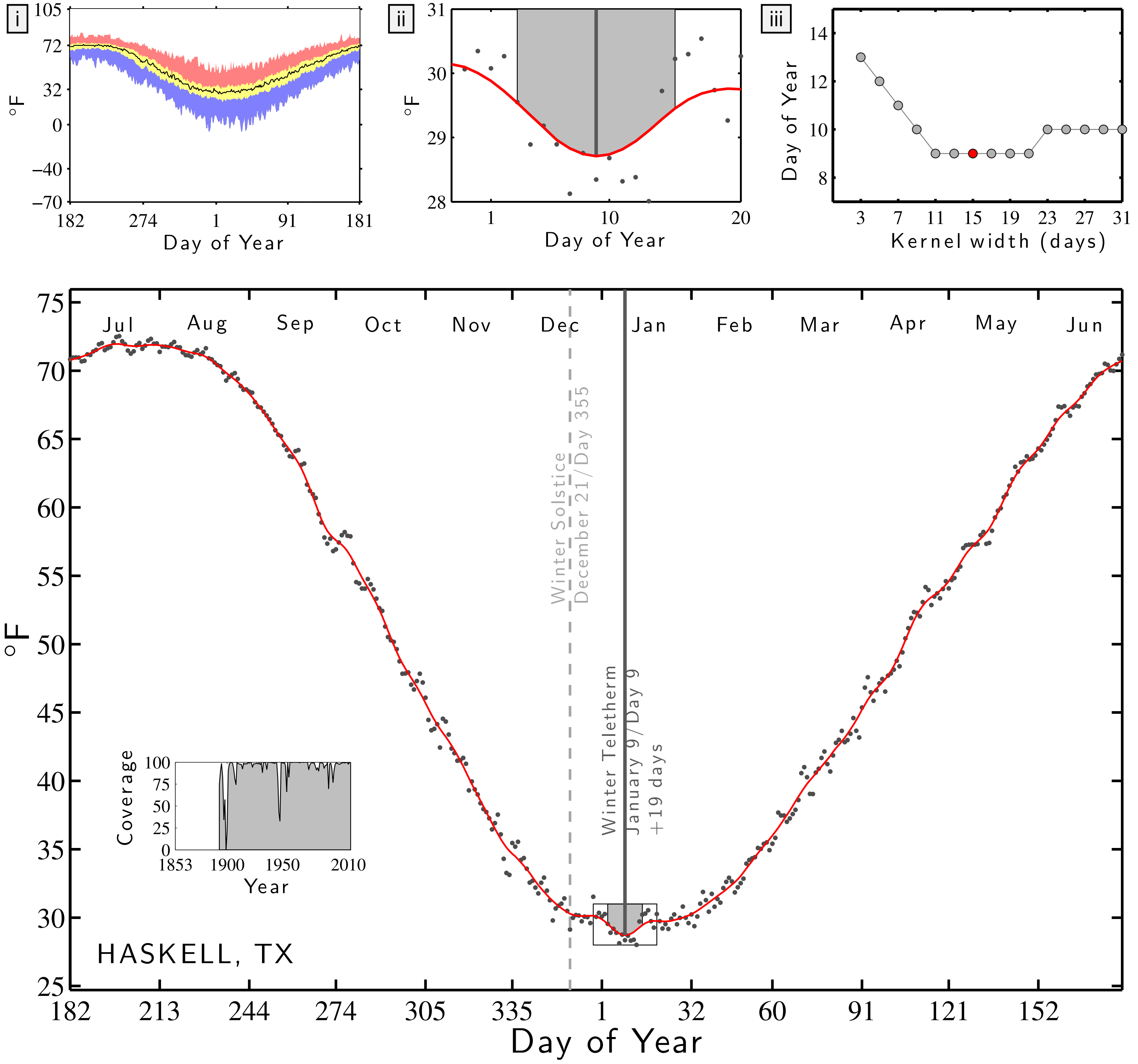

i
°F
Day of Year
ii
°F
Day of Year
iii
Day of Year
Kernel width (days)
Jul
Aug
Sep
Oct
Nov
Dec
Jan
Feb
Mar
Apr
May
Jun
Winter Solstice
December 21/Day 355
Winter Teletherm
January 9/Day 9
+19 days
Coverage
Year
HASKELL, TX
°F
Day of Year

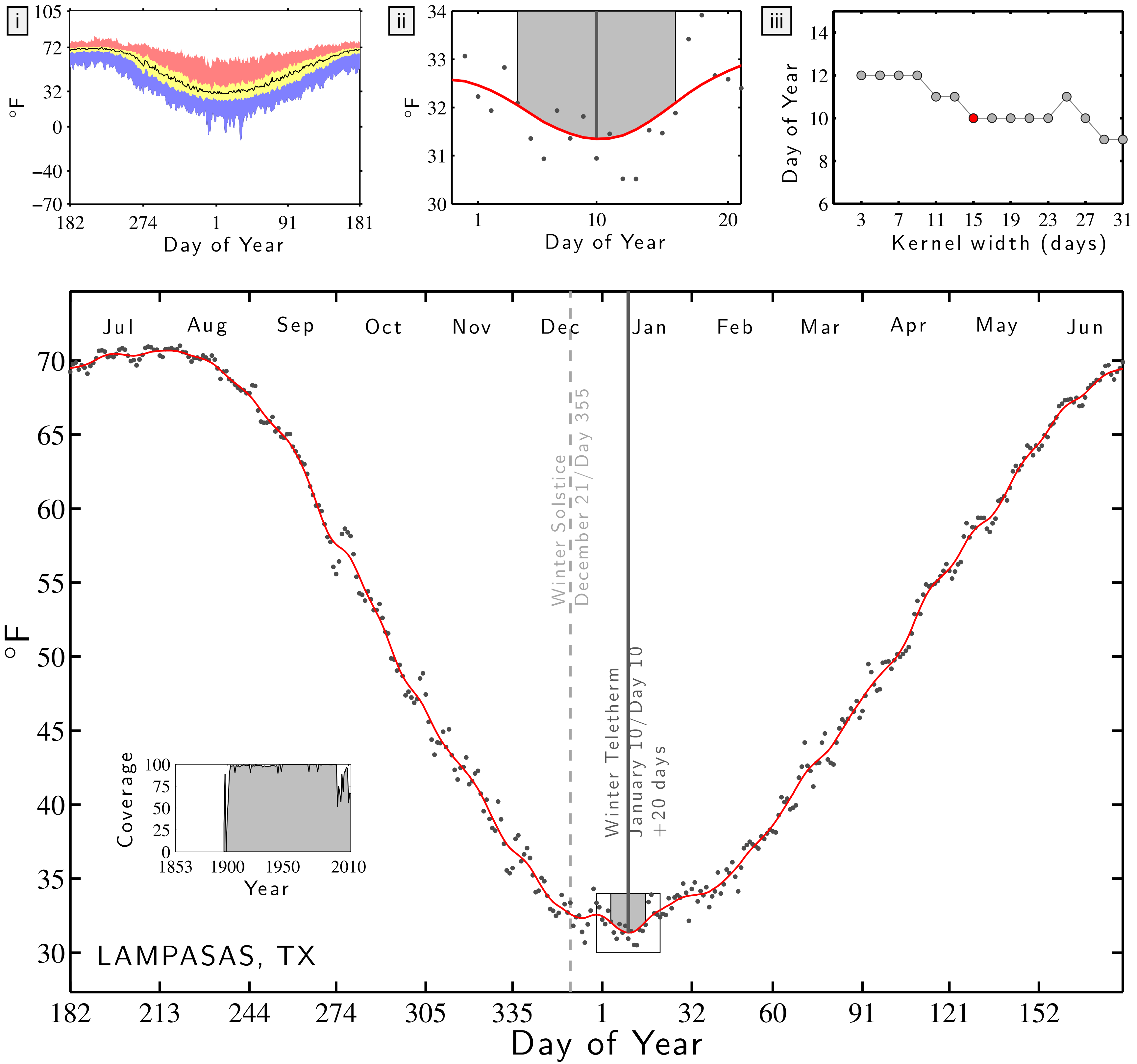

i
ii
iii
°F
Day of Year
Kernel width (days)
Jul
Aug
Sep
Oct
Nov
Dec
Jan
Feb
Mar
Apr
May
Jun
Winter Solstice
December 21/Day 355
Winter Teletherm
January 10/Day 10
+20 days
Coverage
Year
LAMPASAS, TX

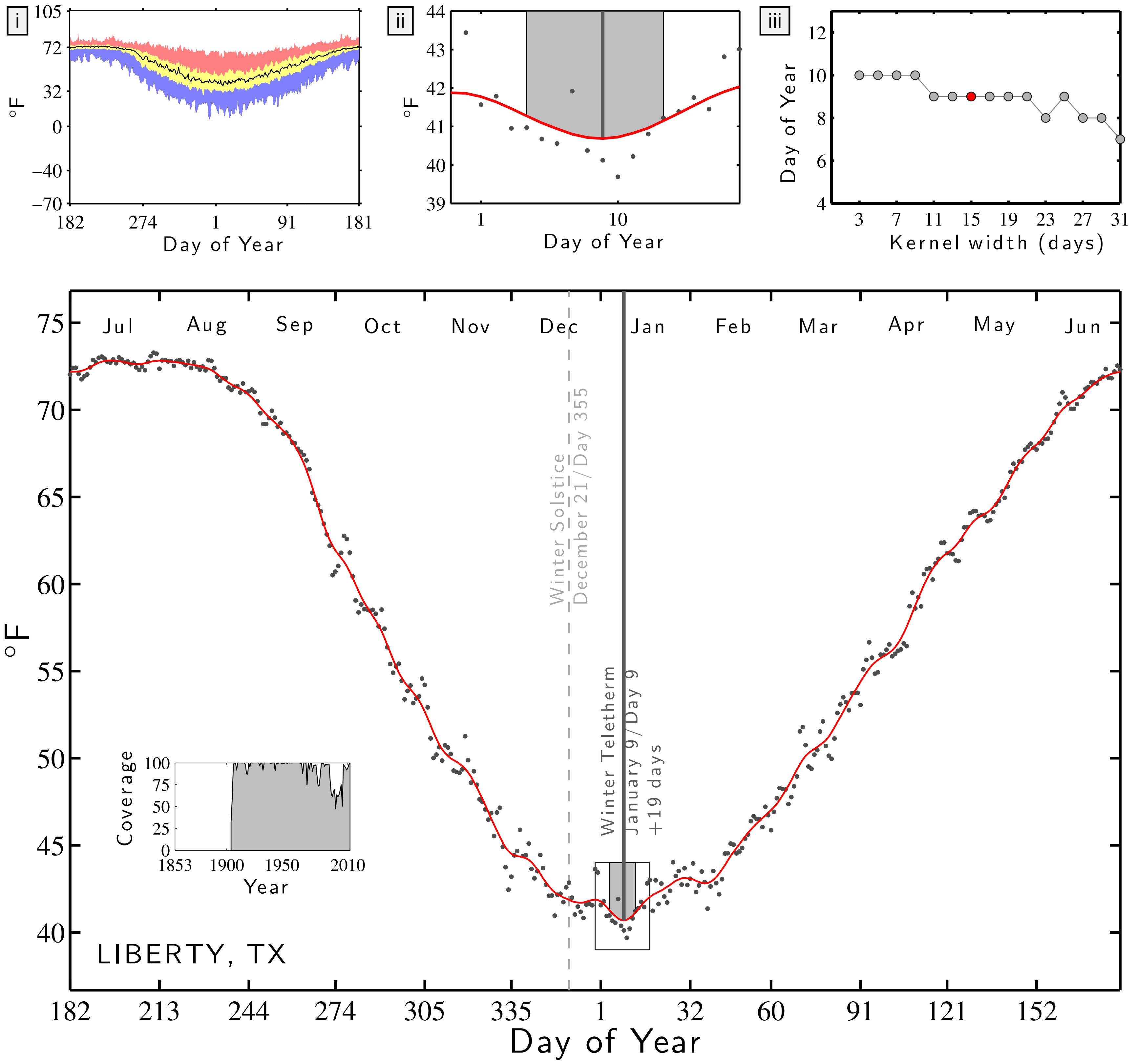

i
ii
iii
°F
Day of Year
Day of Year
Kernel width (days)
Jul
Aug
Sep
Oct
Nov
Dec
Jan
Feb
Mar
Apr
May
Jun
Winter Solstice
December 21/Day 355
Winter Teletherm
January 9/Day 9
+19 days
Coverage
Year
LIBERTY, TX
Day of Year

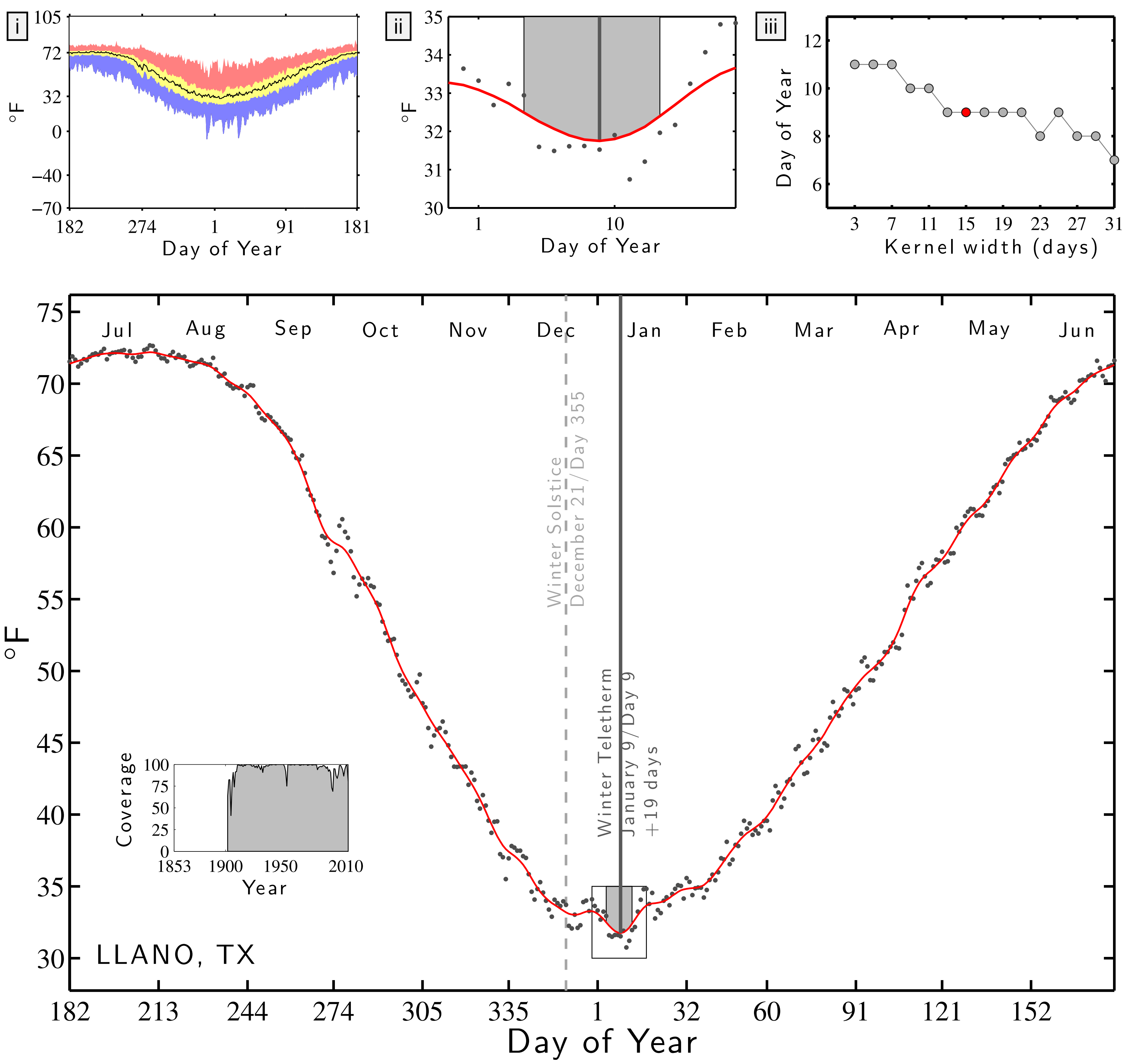

i
°F
Day of Year
ii
°F
Day of Year
iii
Day of Year
Kernel width (days)
Jul
Aug
Sep
Oct
Nov
Dec
Jan
Feb
Mar
Apr
May
Jun
Winter Solstice
December 21/Day 355
Winter Teletherm
January 9/Day 9
+19 days
Coverage
Year
LLANO, TX
°F
Day of Year

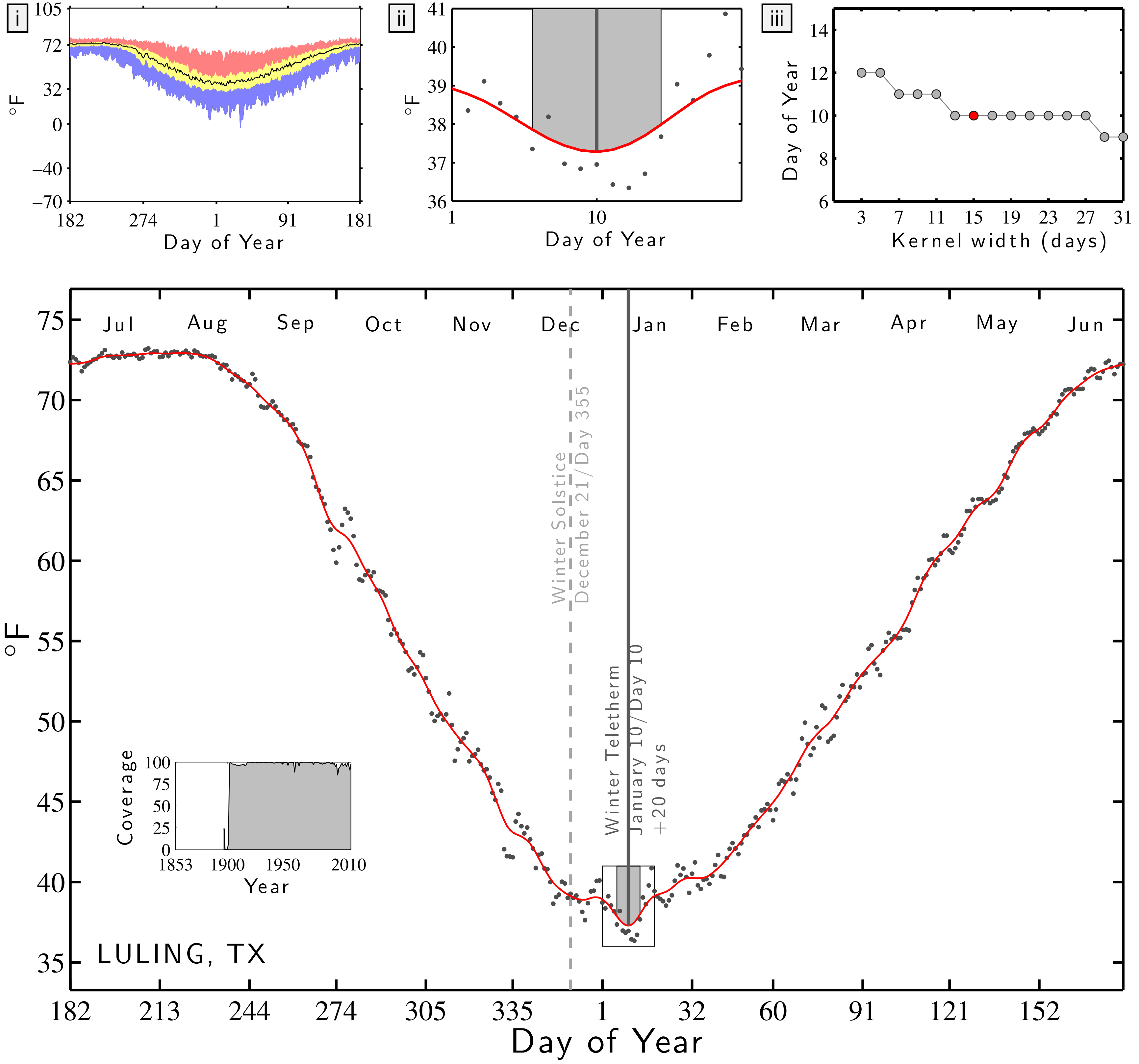
i
°F
Day of Year
ii
°F
Day of Year
iii
Day of Year
Kernel width (days)
Jul
Aug
Sep
Oct
Nov
Dec
Jan
Feb
Mar
Apr
May
Jun
Winter Solstice
December 21/Day 355
Winter Teletherm
January 10/Day 10
+20 days
Coverage
Year
LULING, TX
°F
Day of Year

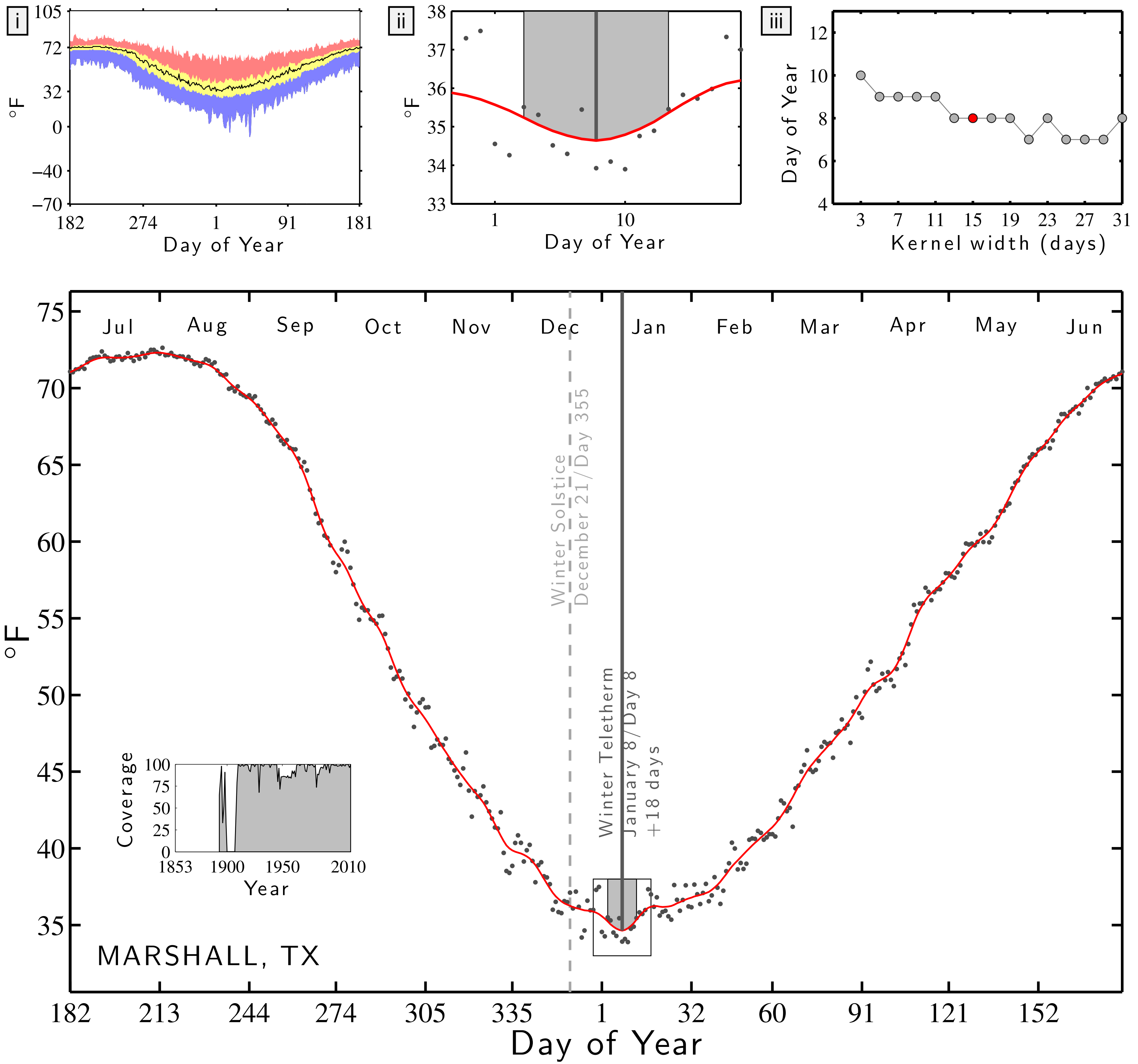

i
°F
Day of Year
ii
°F
Day of Year
iii
Day of Year
Kernel width (days)
Jul
Aug
Sep
Oct
Nov
Dec
Jan
Feb
Mar
Apr
May
Jun
Winter Solstice
December 21/Day 355
Winter Teletherm
January 8/Day 8
+18 days
Coverage
Year
MARSHALL, TX
°F
Day of Year

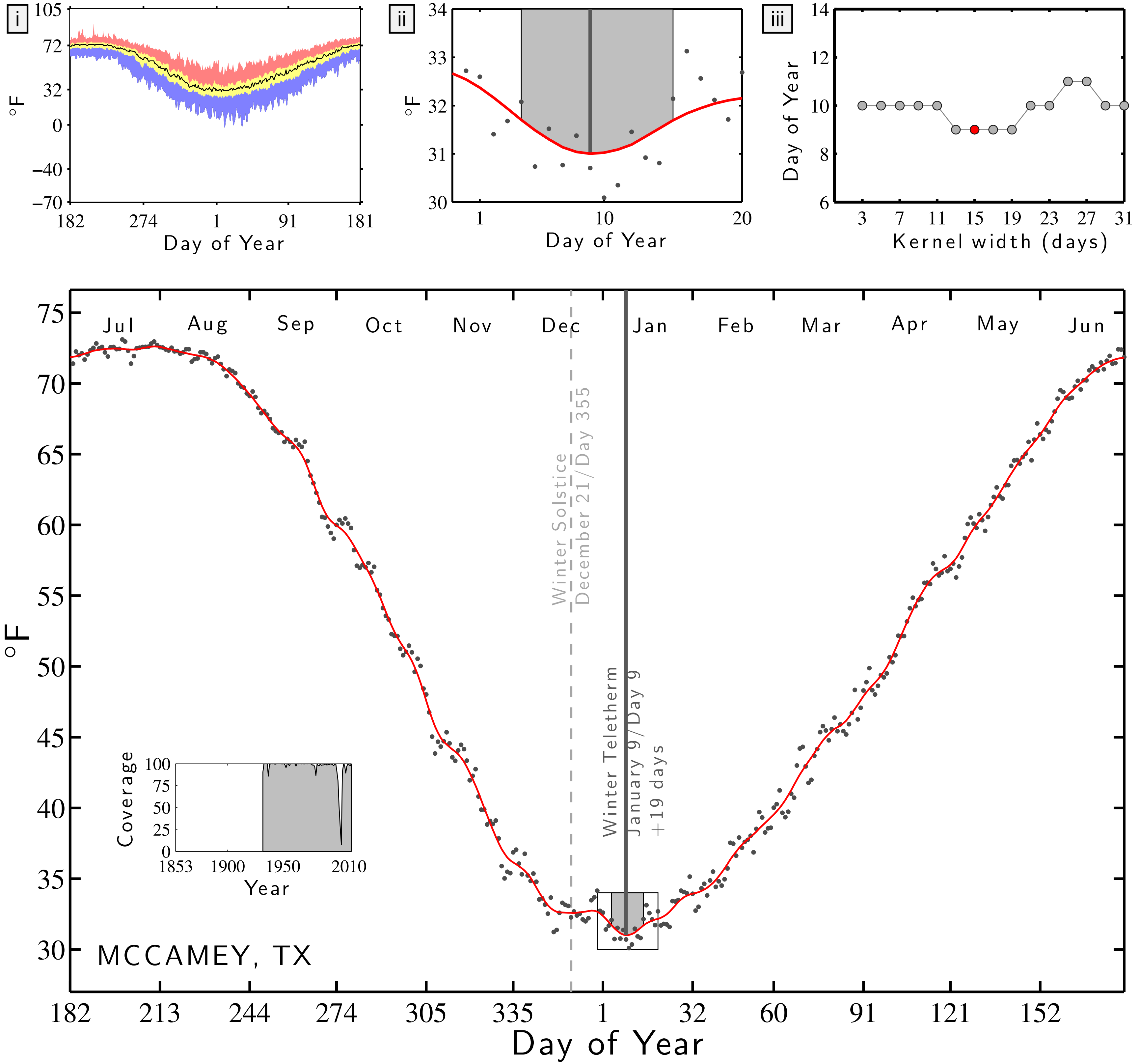

i
ii
iii
°F
Day of Year
Kernel width (days)
Jul
Aug
Sep
Oct
Nov
Dec
Jan
Feb
Mar
Apr
May
Jun
Winter Solstice
December 21/Day 355
Winter Teletherm
January 9/Day 9
+19 days
Coverage
Year
MCCAMEY, TX

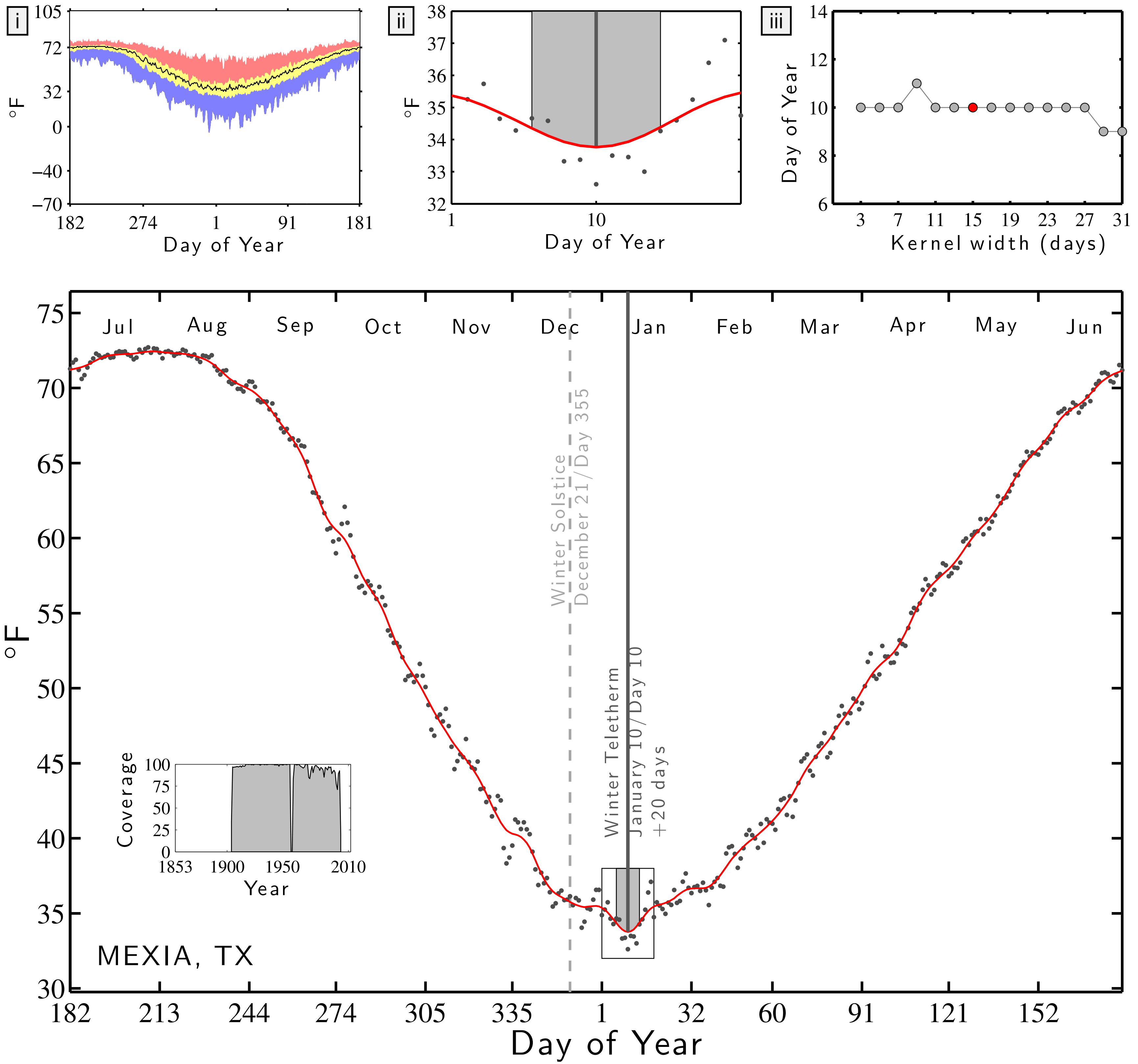
i
°F
Day of Year
ii
Day of Year
iii
Day of Year
Kernel width (days)
Jul
Aug
Sep
Oct
Nov
Dec
Jan
Feb
Mar
Apr
May
Jun
Winter Solstice
December 21/Day 355
Winter Teletherm
January 10/Day 10
+20 days
Coverage
Year
MEXIA, TX
°F
Day of Year

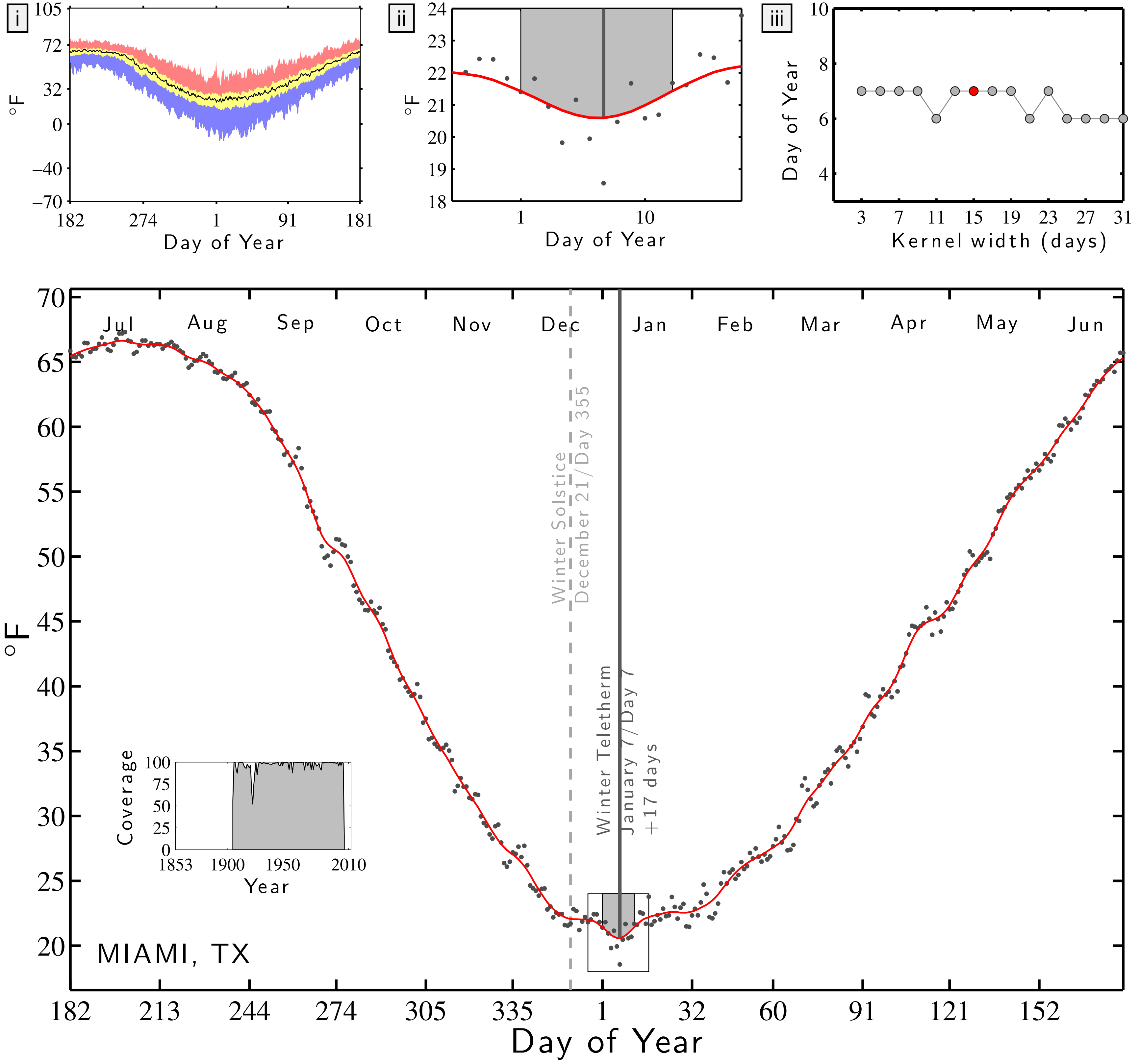

i
Day of Year
°F
ii
Day of Year
iii
Day of Year
Kernel width (days)
Jul
Aug
Sep
Oct
Nov
Dec
Jan
Feb
Mar
Apr
May
Jun
Winter Solstice
December 21/Day 355
Winter Teletherm
January 7/Day 7
+17 days
Coverage
Year
MIAMI, TX

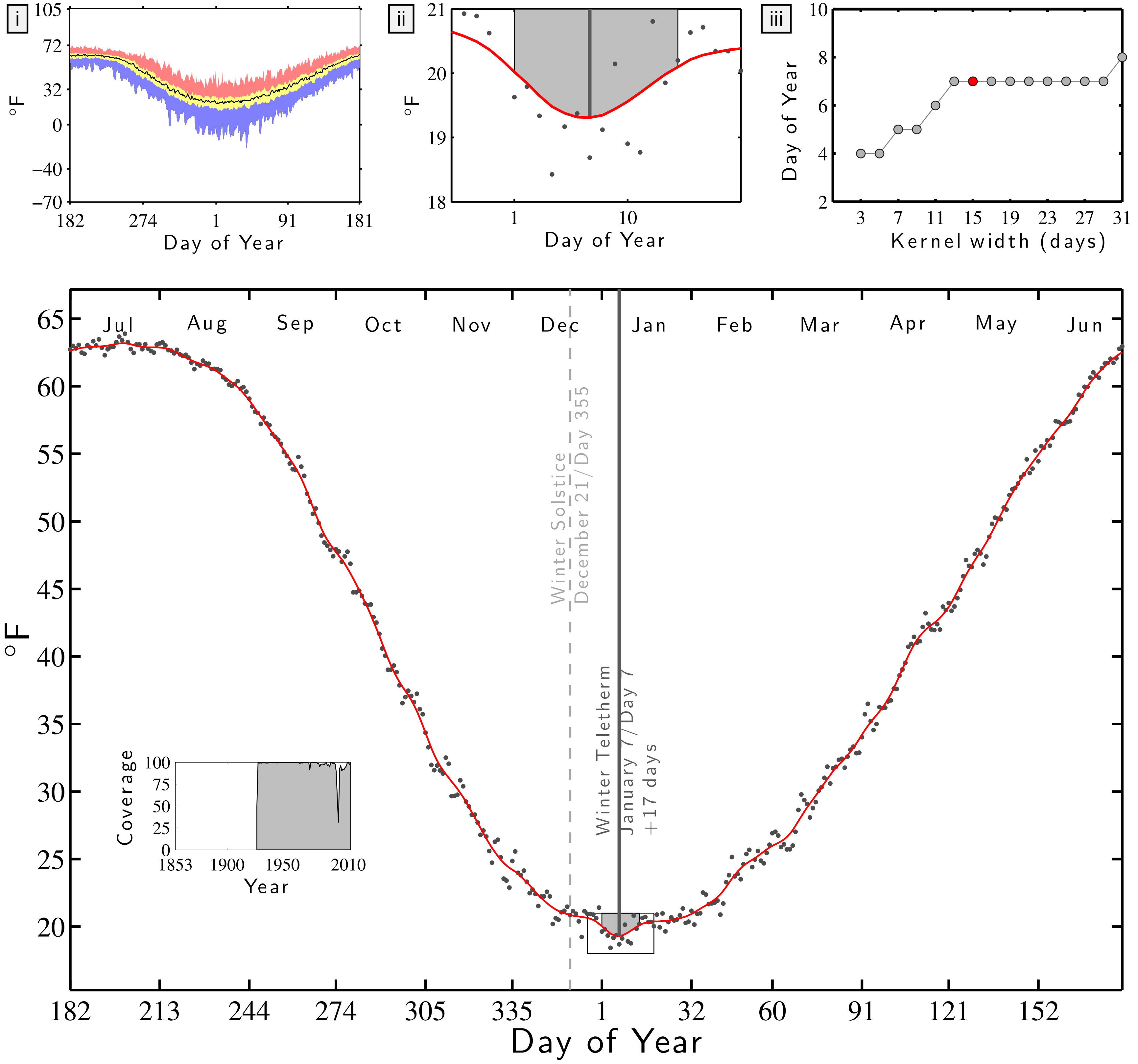

i
°F
Day of Year
ii
Day of Year
iii
Day of Year
Kernel width (days)
Jul
Aug
Sep
Oct
Nov
Dec
Jan
Feb
Mar
Apr
May
Jun
Winter Solstice
December 21/Day 355
Winter Teletherm
January 7/Day 7
+17 days
Coverage
Year
Day of Year

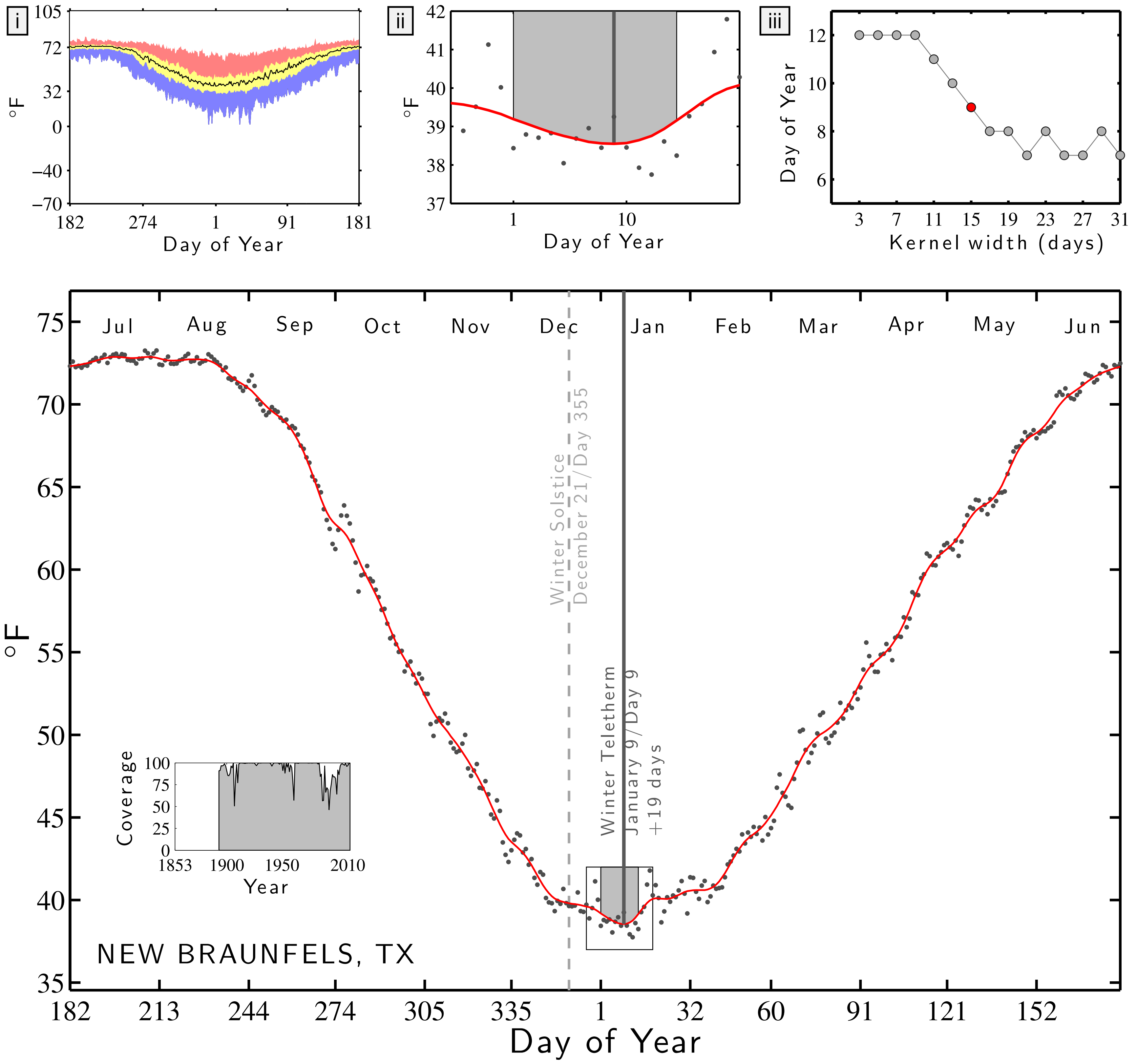

i
°F
Day of Year
ii
°F
Day of Year
iii
Day of Year
Kernel width (days)
Jul
Aug
Sep
Oct
Nov
Dec
Jan
Feb
Mar
Apr
May
Jun
Winter Solstice
December 21/Day 355
Winter Teletherm
January 9/Day 9
+19 days
Coverage
Year
NEW BRAUNFELS, TX
°F
Day of Year

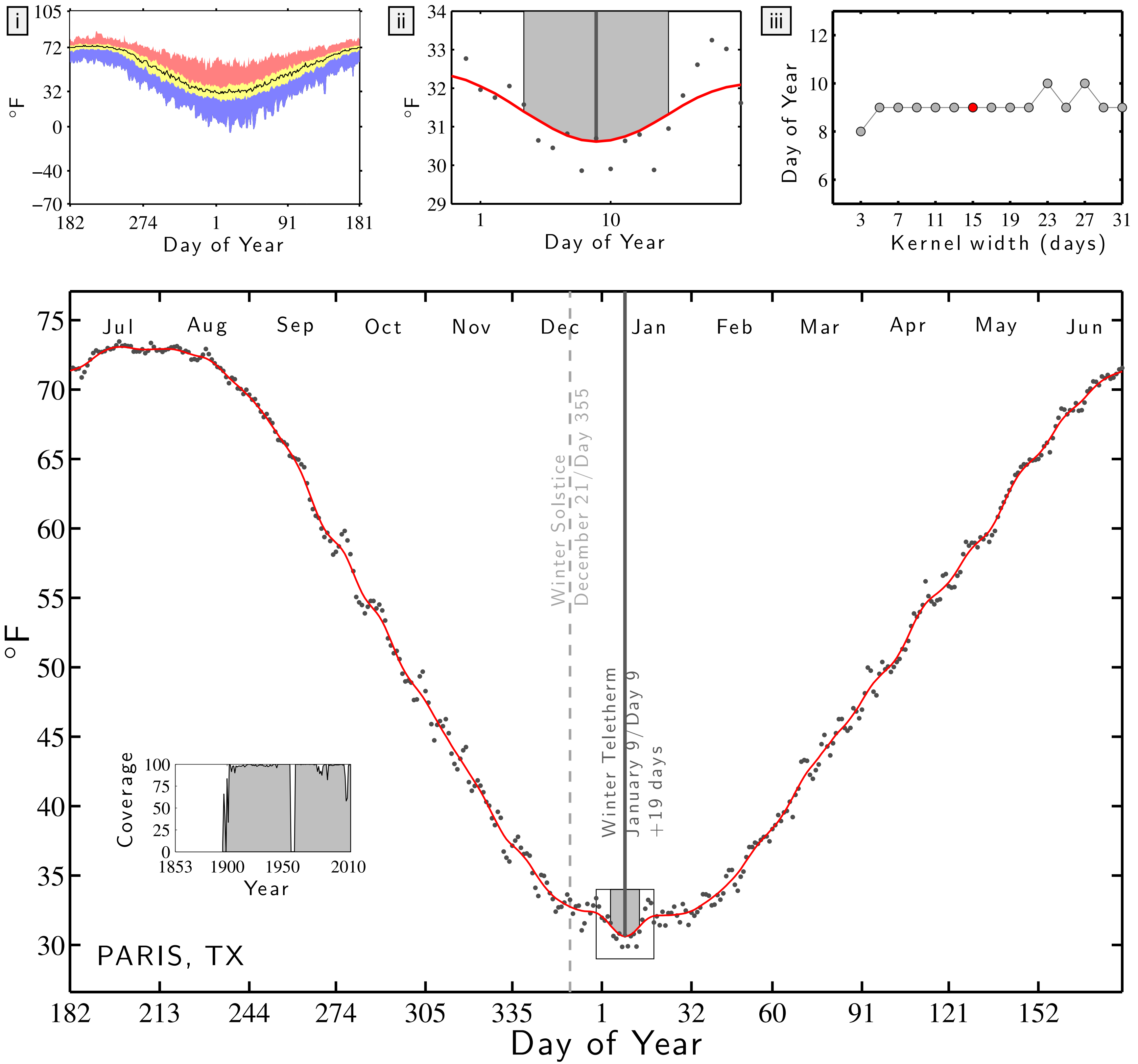

i
ii
iii
°F
Day of Year
Kernel width (days)
Jul
Aug
Sep
Oct
Nov
Dec
Jan
Feb
Mar
Apr
May
Jun
Winter Solstice
December 21/Day 355
Winter Teletherm
January 9/Day 9
+19 days
Coverage
Year
PARIS, TX

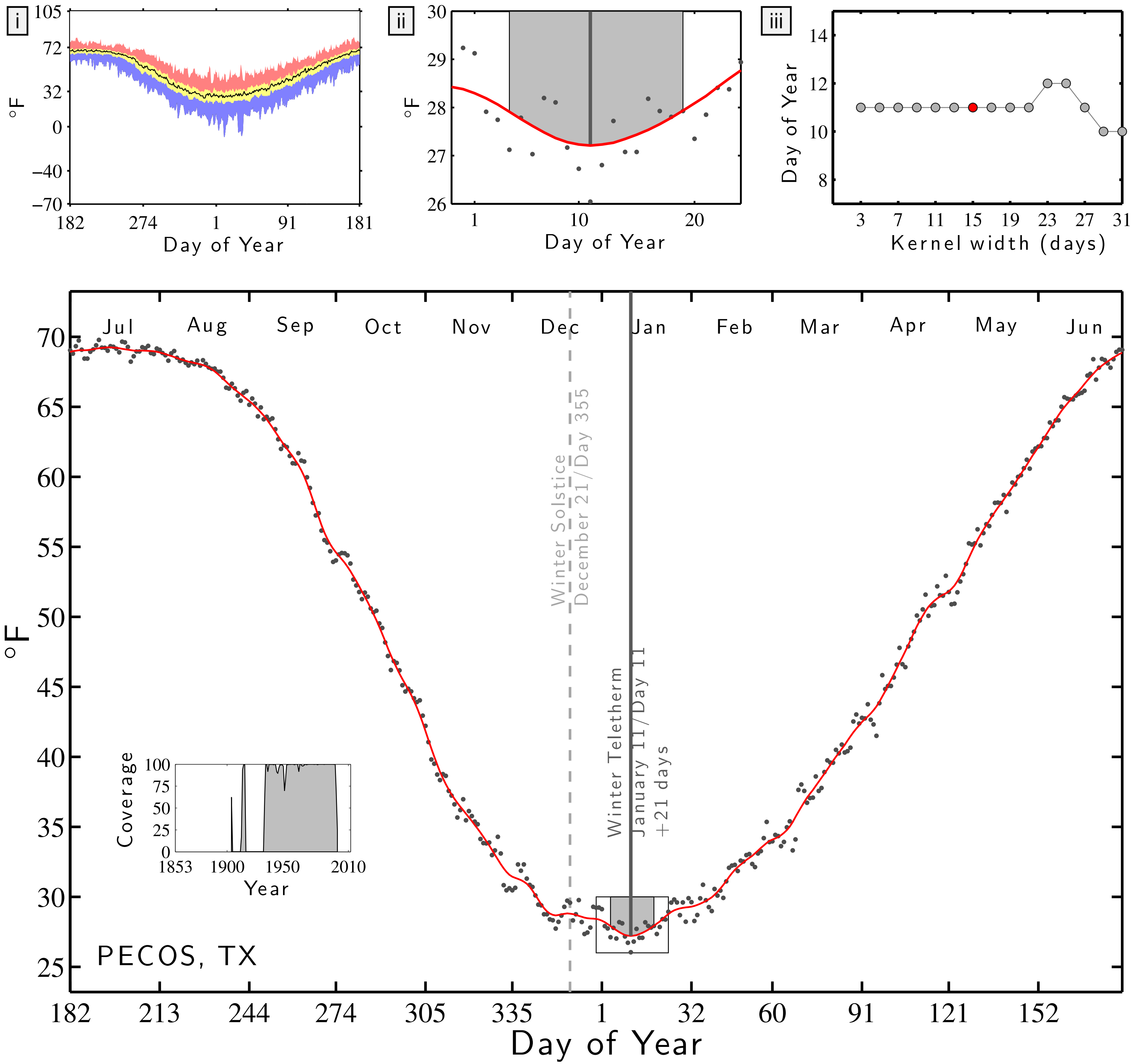

i
105
72
32
0
−40
−70
°F
182
274
1
91
181
Day of Year
ii
30
29
28
27
26
°F
1
10
20
Day of Year
iii
14
12
10
8
Day of Year
3
7
11
15
19
23
27
31
Kernel width (days)
Jul
Aug
Sep
Oct
Nov
Dec
Jan
Feb
Mar
Apr
May
Jun
70
65
60
55
50
45
40
35
30
25
°F
Winter Solstice
December 21/Day 355
Winter Teletherm
January 11/Day 11
+21 days
Coverage
100
75
50
25
0
1853
1900
1950
2010
Year
PECOS, TX
182
213
244
274
305
335
1
32
60
91
121
152
Day of Year

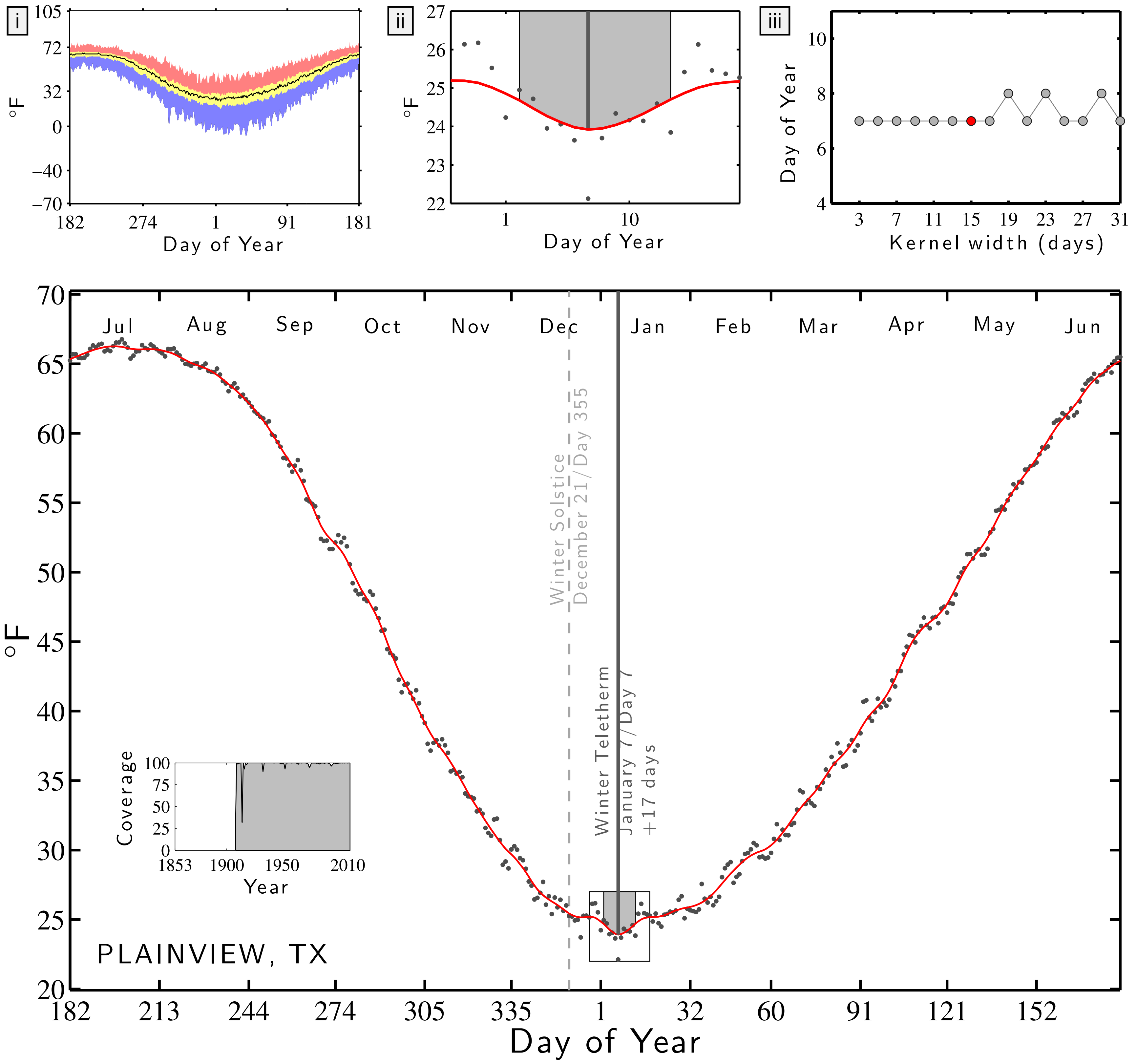

i
ii
iii
°F
Day of Year
Kernel width (days)
Jul
Aug
Sep
Oct
Nov
Dec
Jan
Feb
Mar
Apr
May
Jun
Winter Solstice
December 21/Day 355
Winter Teletherm
January 7/Day 7
+17 days
Coverage
Year
PLAINVIEW, TX

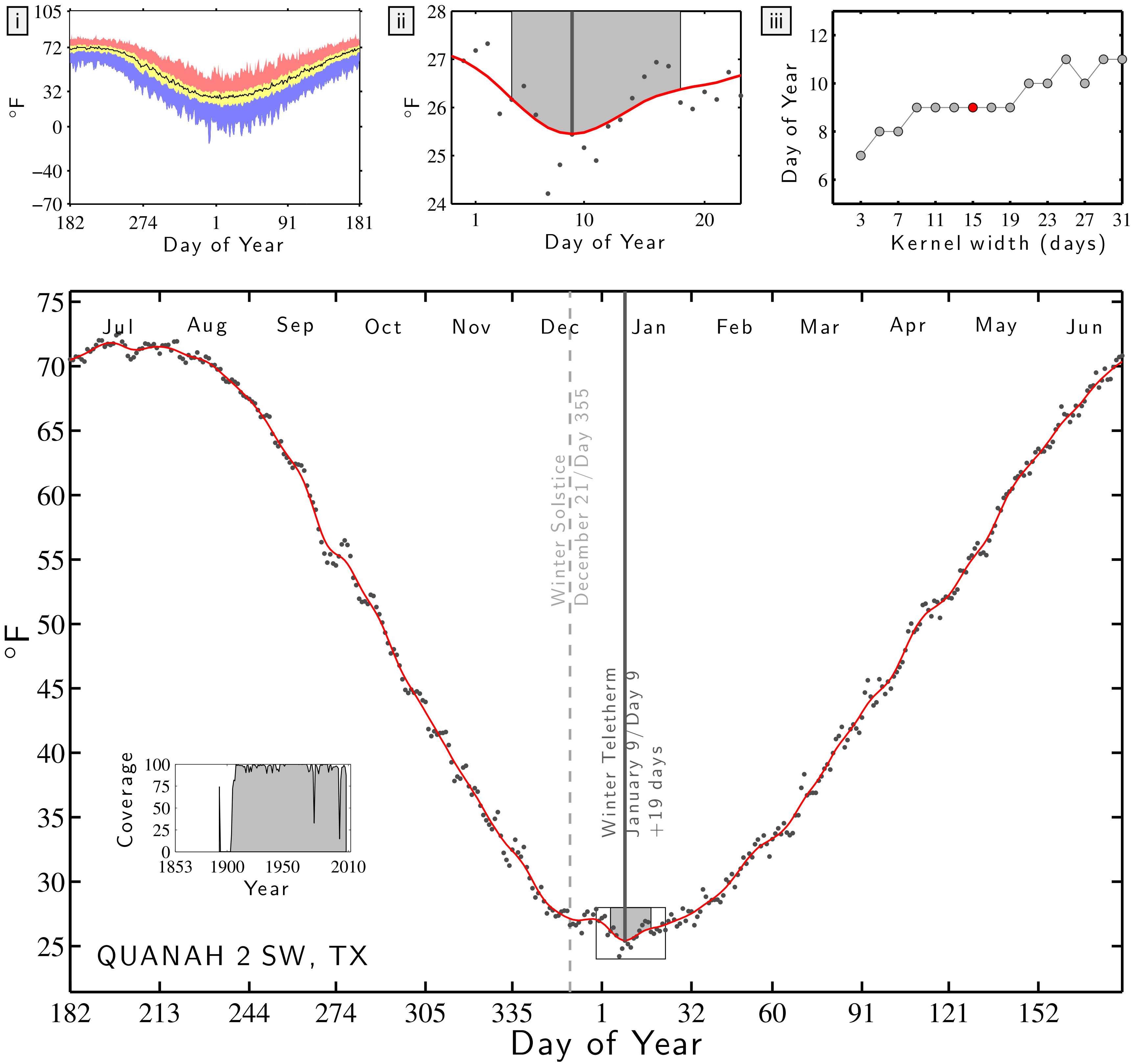

i
ii
iii
°F
Day of Year
Day of Year
Kernel width (days)
Jul
Aug
Sep
Oct
Nov
Dec
Jan
Feb
Mar
Apr
May
Jun
Winter Solstice
December 21/Day 355
Winter Teletherm
January 9/Day 9
+19 days
Coverage
Year
QUANAH 2 SW, TX

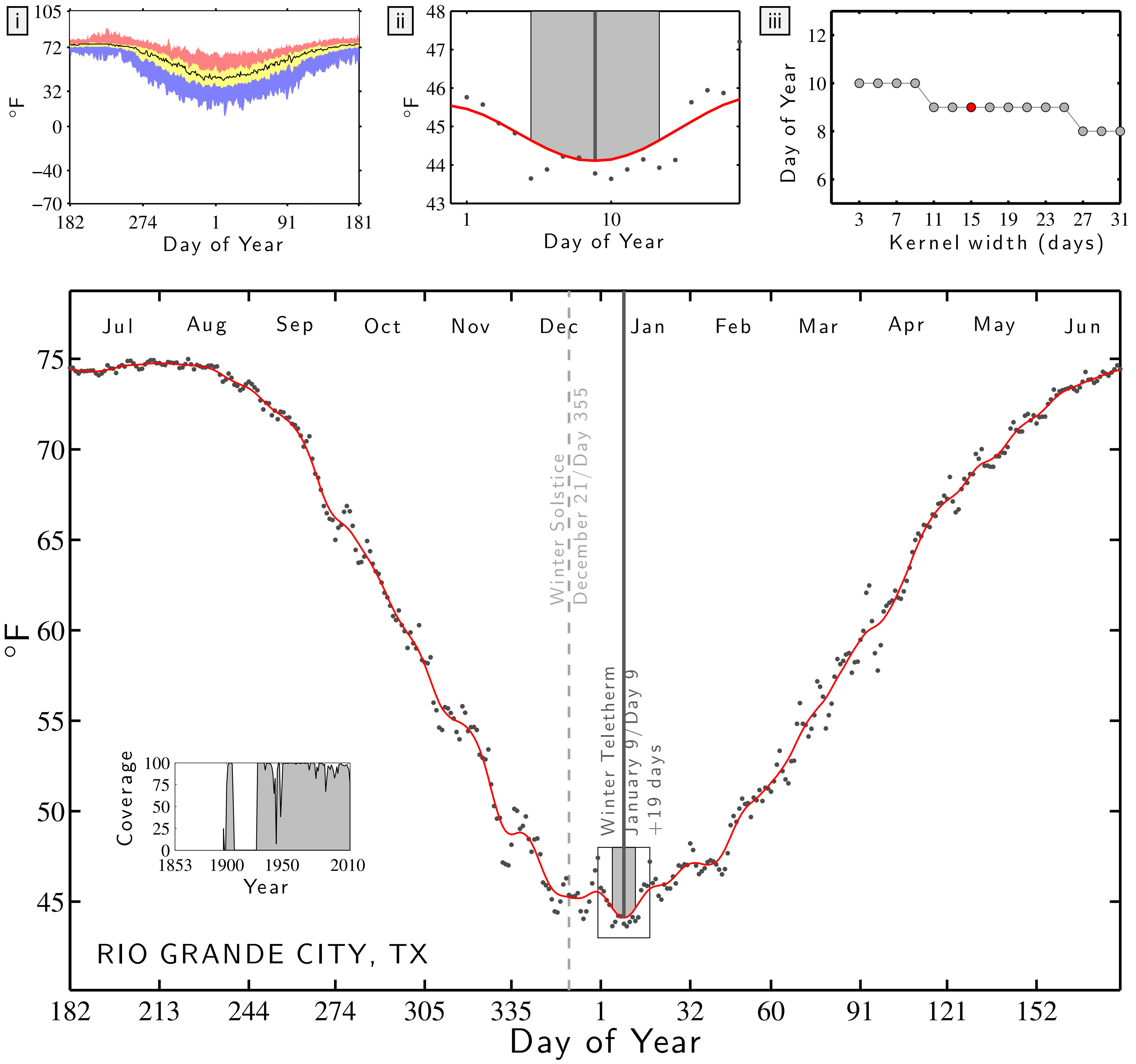

i
ii
iii
Day of Year
Kernel width (days)
°F
Jul
Aug
Sep
Oct
Nov
Dec
Jan
Feb
Mar
Apr
May
Jun
Winter Solstice
December 21/Day 355
Winter Teletherm
January 9/Day 9
+19 days
Coverage
Year
RIO GRANDE CITY, TX
Day of Year

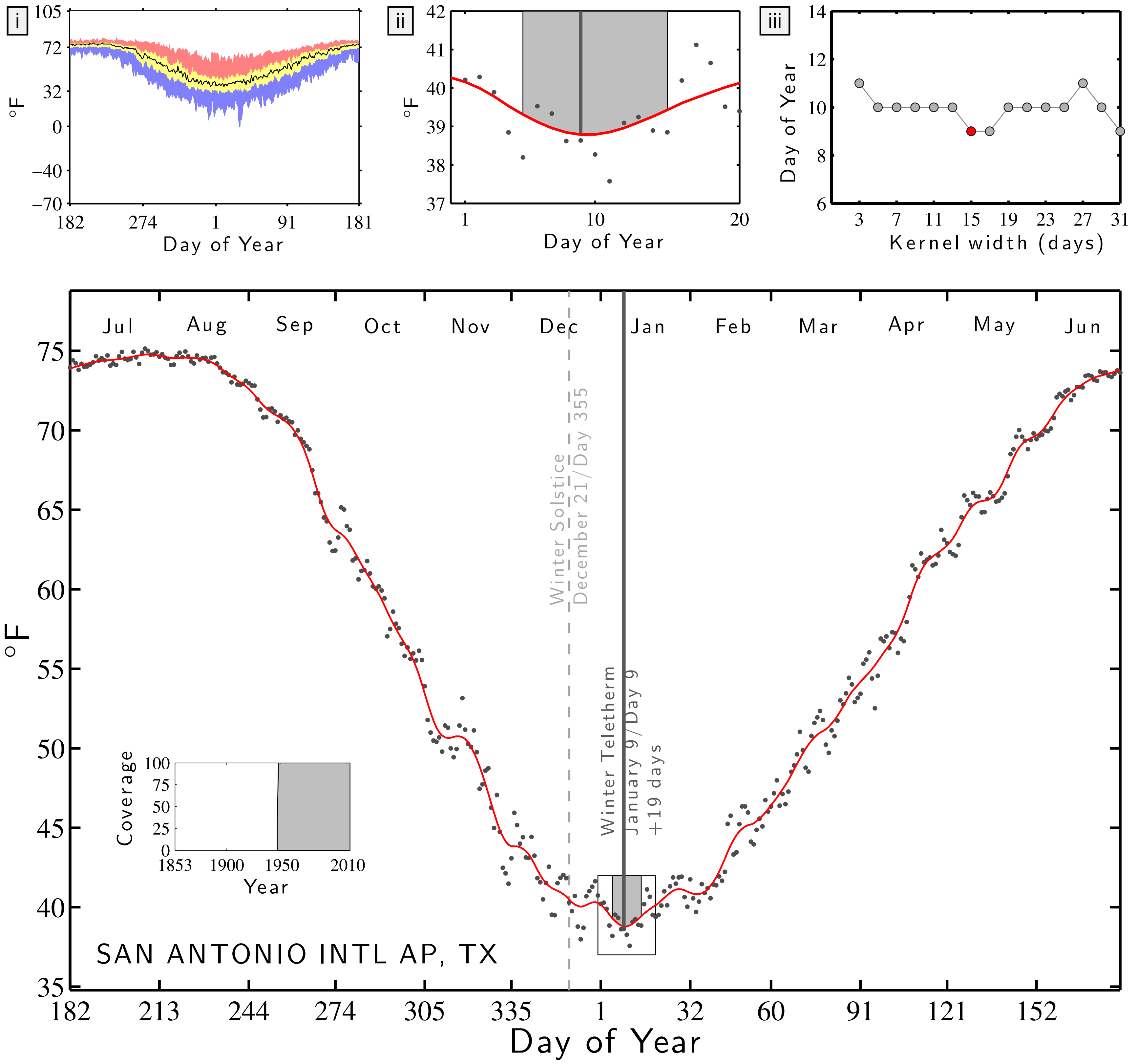
i
ii
iii
°F
Day of Year
Kernel width (days)
Jul
Aug
Sep
Oct
Nov
Dec
Jan
Feb
Mar
Apr
May
Jun
Winter Solstice
December 21/Day 355
Winter Teletherm
January 9/Day 9
+19 days
Coverage
Year
SAN ANTONIO INTL AP, TX

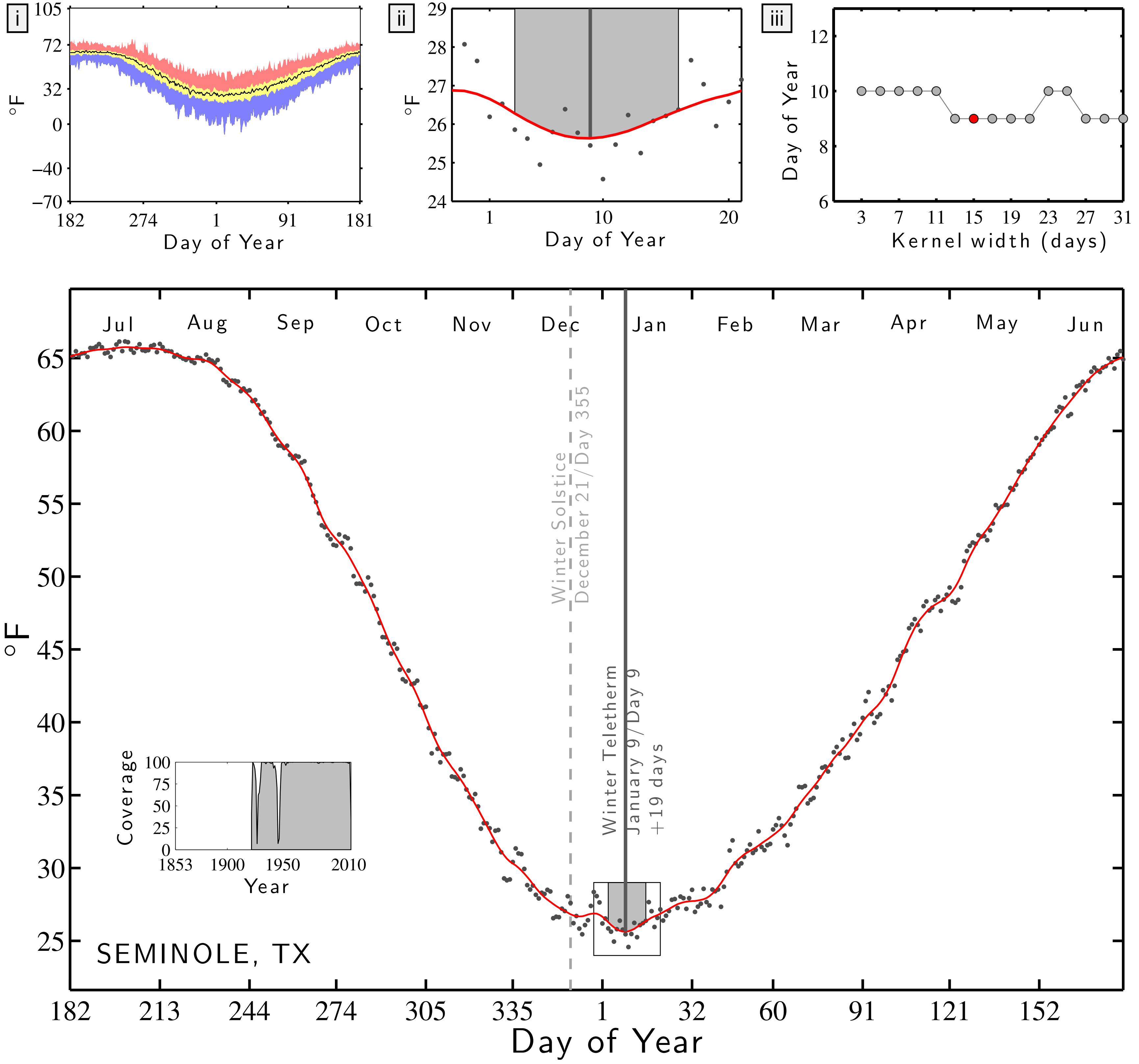

i
ii
iii
°F
Day of Year
Kernel width (days)
Jul
Aug
Sep
Oct
Nov
Dec
Jan
Feb
Mar
Apr
May
Jun
Winter Solstice
December 21/Day 355
Winter Teletherm
January 9/Day 9
+19 days
Coverage
Year
SEMINOLE, TX

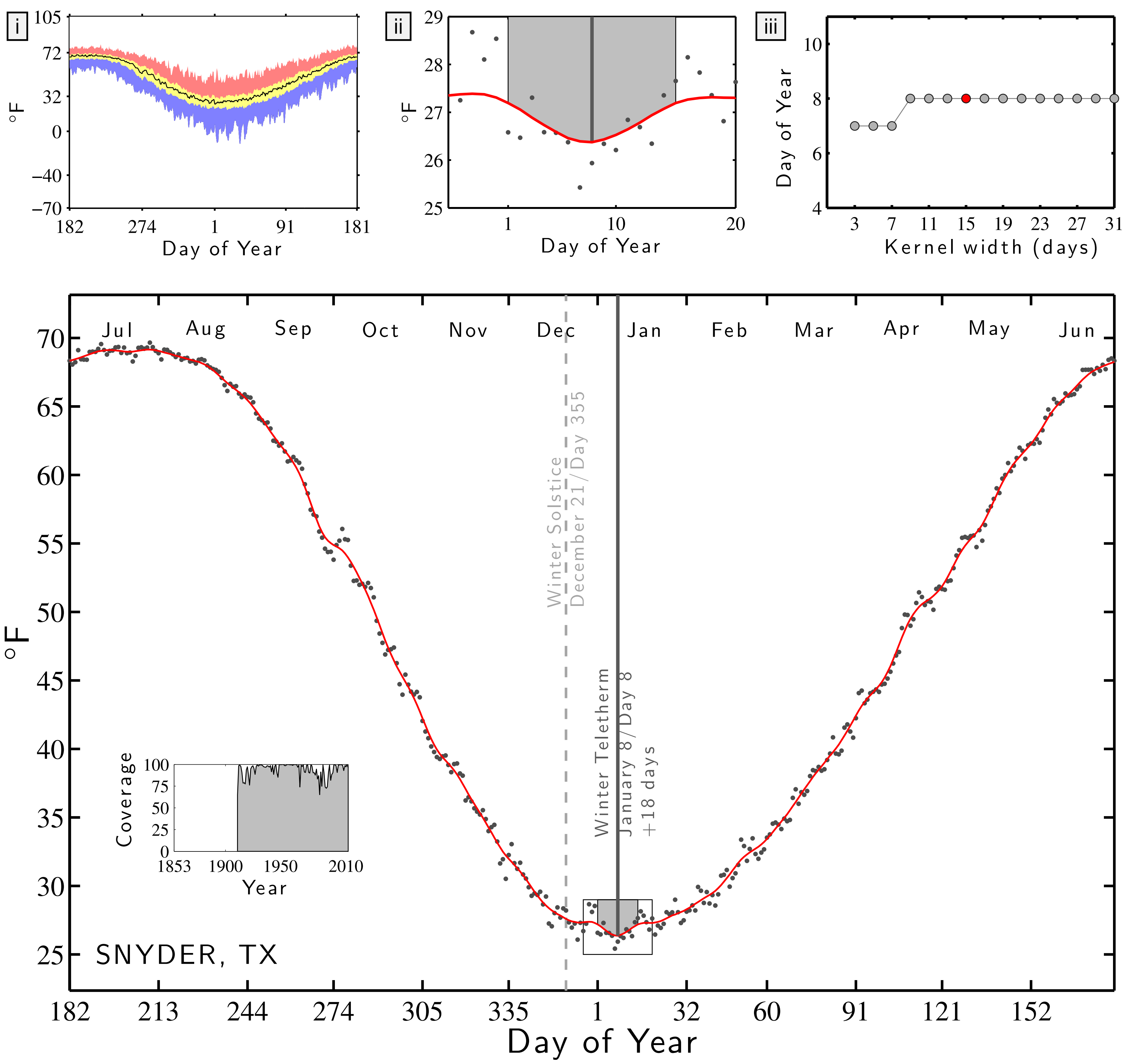

i
°F
Day of Year
ii
°F
Day of Year
iii
Day of Year
Kernel width (days)
Jul
Aug
Sep
Oct
Nov
Dec
Jan
Feb
Mar
Apr
May
Jun
Winter Solstice
December 21/Day 355
Winter Teletherm
January 8/Day 8
+18 days
Coverage
Year
SNYDER, TX
°F
Day of Year

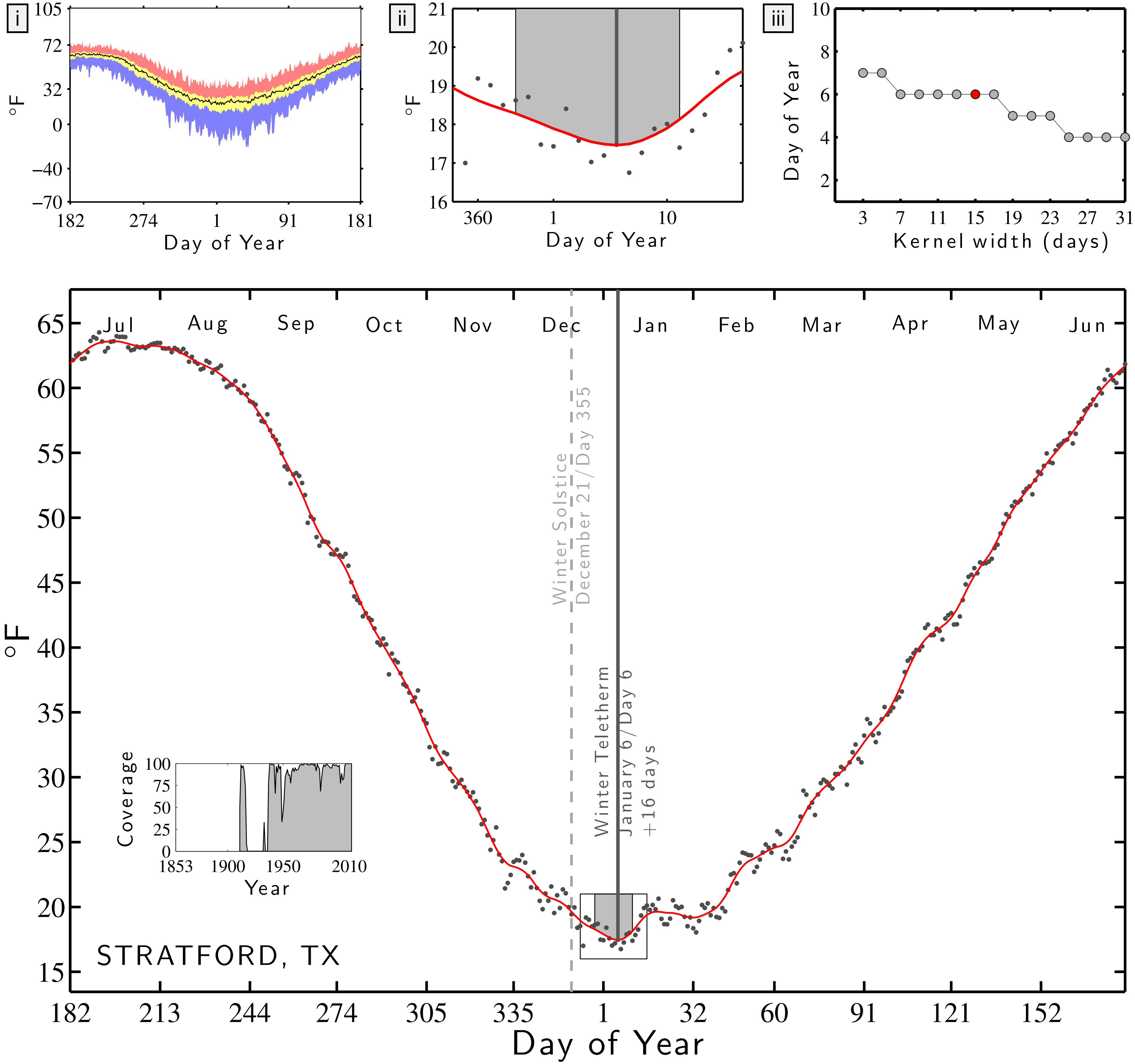

i
°F
Day of Year
ii
°F
Day of Year
iii
Day of Year
Kernel width (days)
Jul
Aug
Sep
Oct
Nov
Dec
Jan
Feb
Mar
Apr
May
Jun
Winter Solstice
December 21/Day 355
Winter Teletherm
January 6/Day 6
+16 days
Coverage
Year
STRATFORD, TX
°F
Day of Year

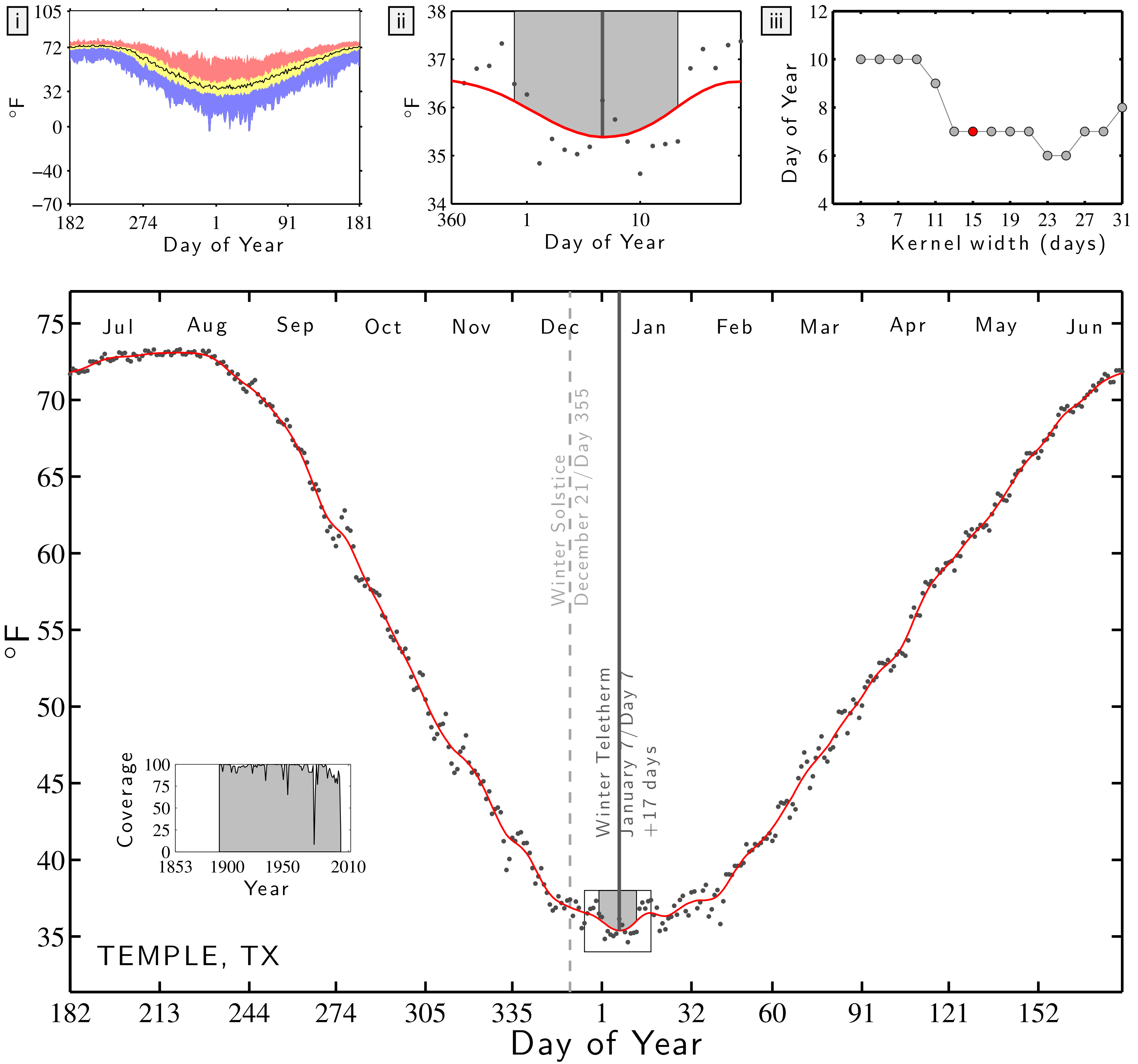
i
°F
Day of Year
ii
Day of Year
iii
Day of Year
Kernel width (days)
Jul
Aug
Sep
Oct
Nov
Dec
Jan
Feb
Mar
Apr
May
Jun
Winter Solstice
December 21/Day 355
Winter Teletherm
January 7/Day 7
+17 days
Coverage
Year
TEMPLE, TX
Day of Year

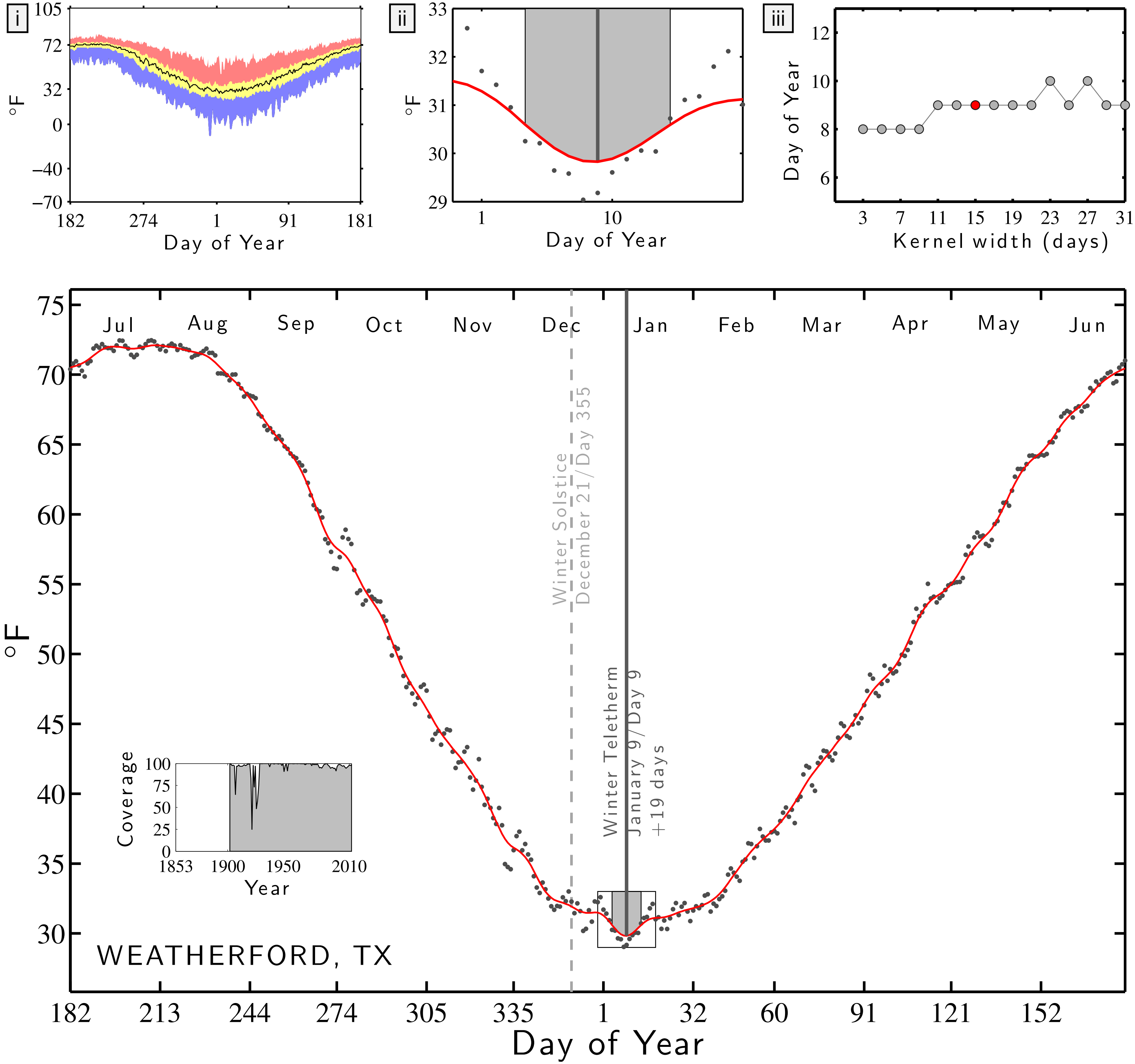

i
°F
105
72
32
0
−40
−70
182
274
1
91
181
Day of Year
ii
°F
33
32
31
30
29
1
10
Day of Year
iii
Day of Year
12
10
8
6
3
7
11
15
19
23
27
31
Kernel width (days)
Jul
Aug
Sep
Oct
Nov
Dec
Jan
Feb
Mar
Apr
May
Jun
Winter Solstice
December 21/Day 355
Winter Teletherm
January 9/Day 9
+19 days
Coverage
100
75
50
25
0
1853
1900
1950
2010
Year
WEATHERFORD, TX
°F
75
70
65
60
55
50
45
40
35
30
182
213
244
274
305
335
1
32
60
91
121
152
Day of Year

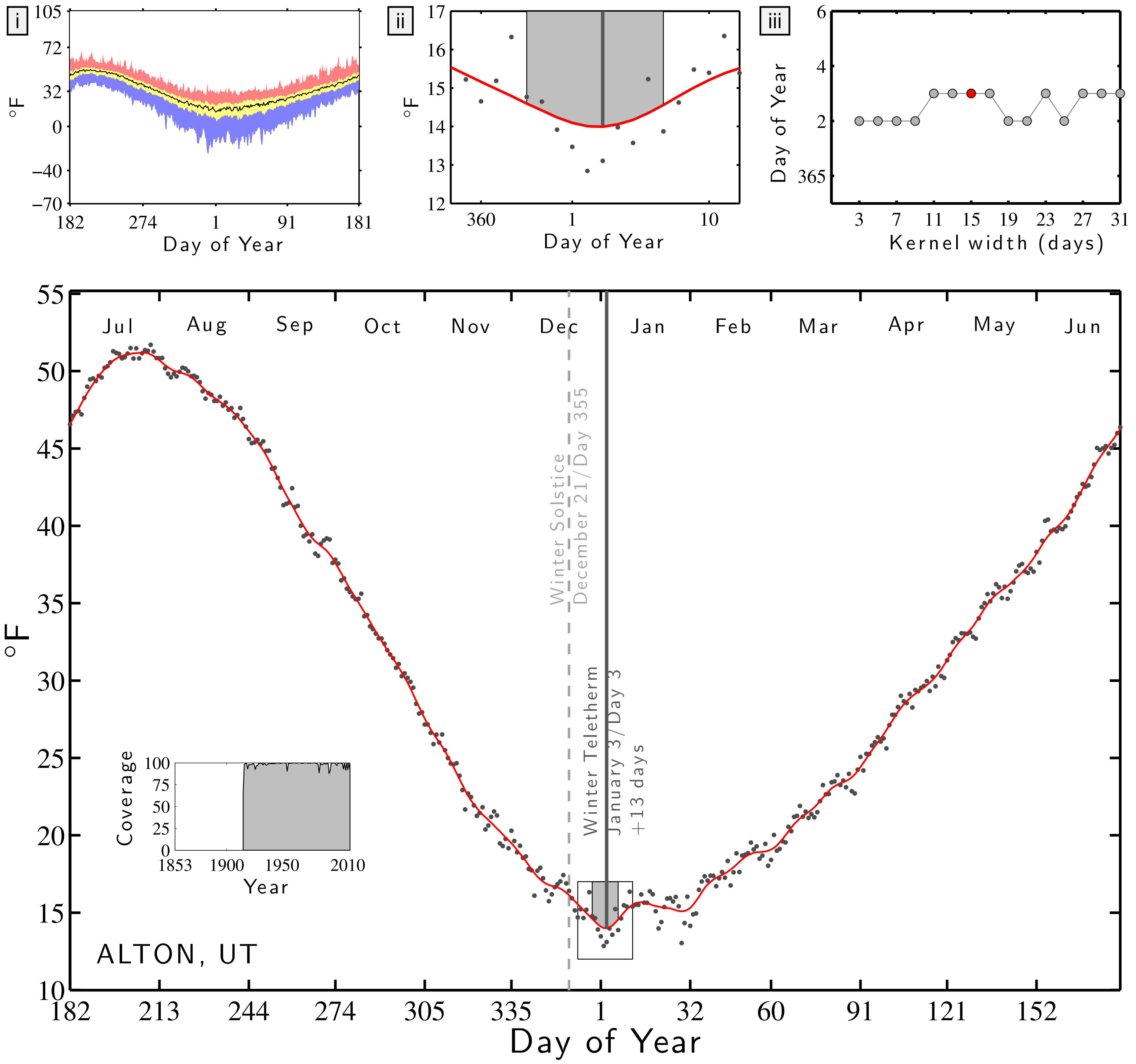

i
°F
Day of Year
ii
°F
Day of Year
iii
Day of Year
Kernel width (days)
Jul
Aug
Sep
Oct
Nov
Dec
Jan
Feb
Mar
Apr
May
Jun
Winter Solstice
December 21/Day 355
Winter Teletherm
January 3/Day 3
+13 days
Coverage
Year
ALTON, UT
°F
Day of Year

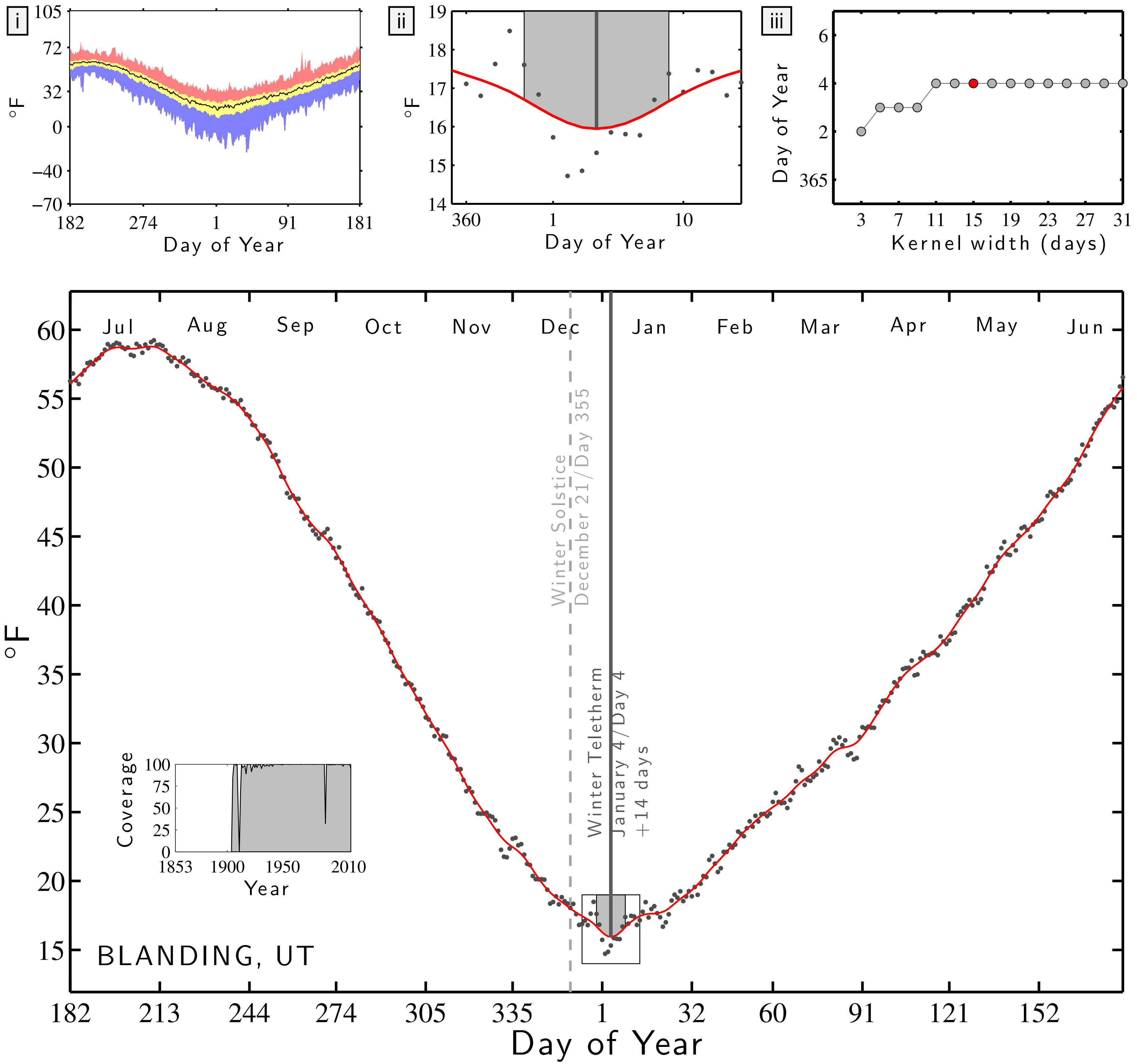

i
105
72
32
0
−40
−70
°F
182
274
1
91
181
Day of Year
ii
19
18
17
16
15
14
°F
360
1
10
Day of Year
iii
6
4
2
365
Day of Year
3
7
11
15
19
23
27
31
Kernel width (days)
Jul
Aug
Sep
Oct
Nov
Dec
Jan
Feb
Mar
Apr
May
Jun
60
55
50
45
40
35
30
25
20
15
°F
Winter Solstice
December 21/Day 355
Winter Teletherm
January 4/Day 4
+14 days
Coverage
100
75
50
25
0
1853
1900
1950
2010
Year
BLANDING, UT
182
213
244
274
305
335
1
32
60
91
121
152
Day of Year

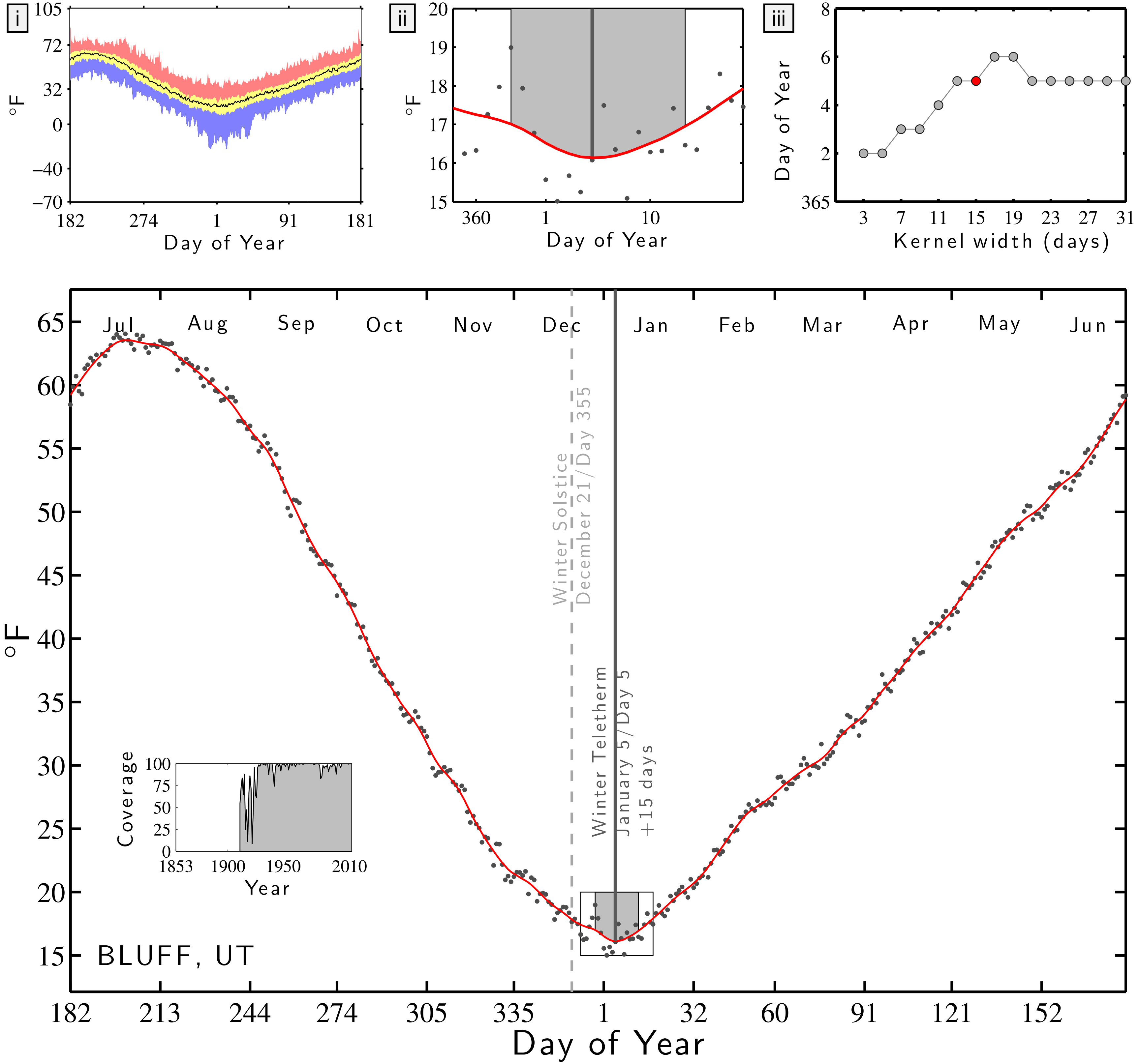

i
°F
Day of Year
ii
°F
Day of Year
iii
Day of Year
Kernel width (days)
Jul
Aug
Sep
Oct
Nov
Dec
Jan
Feb
Mar
Apr
May
Jun
Winter Solstice
December 21/Day 355
Winter Teletherm
January 5/Day 5
+15 days
Coverage
Year
BLUFF, UT
°F
Day of Year

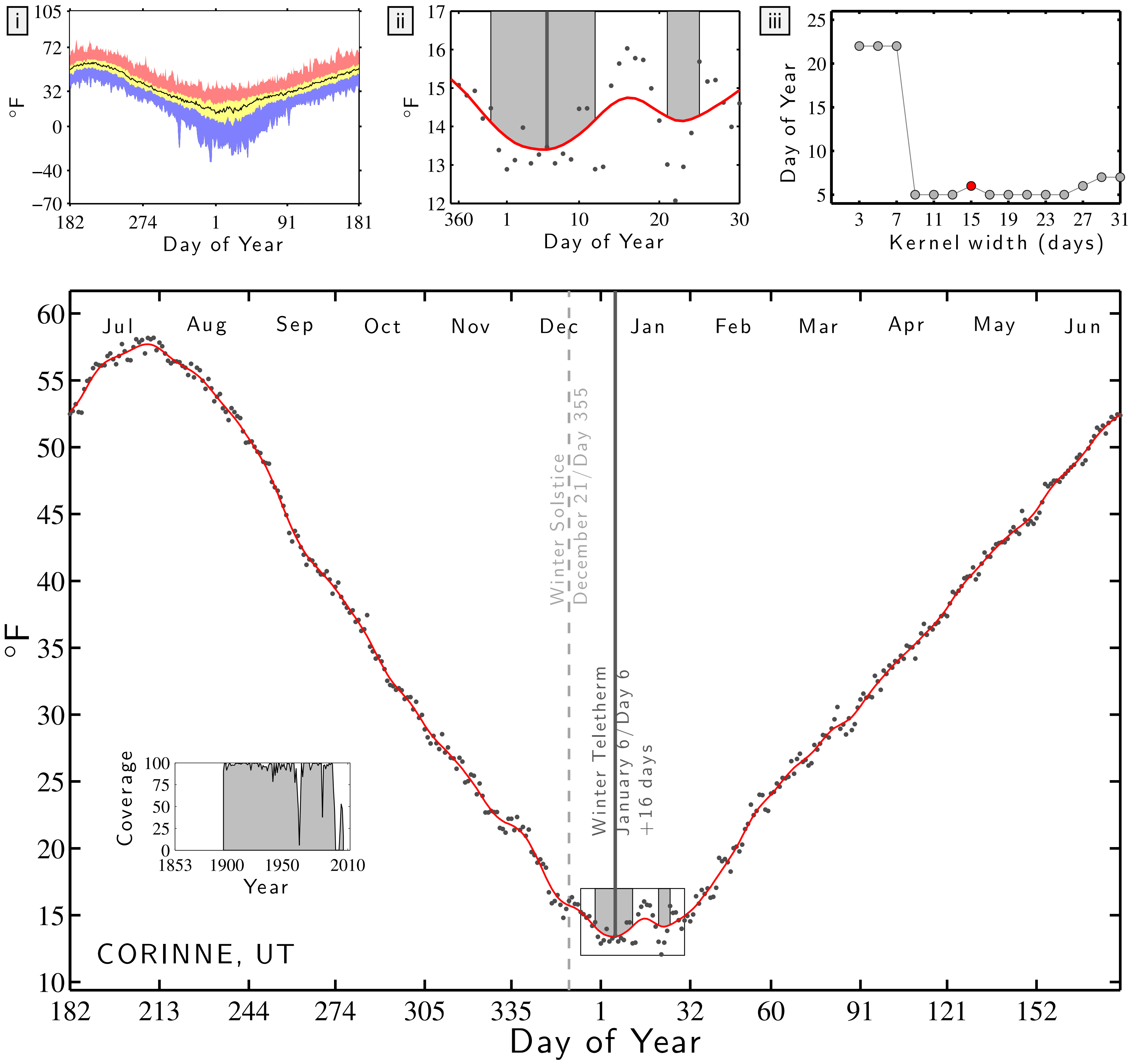

i
ii
iii
Day of Year
°F
Kernel width (days)
Jul
Aug
Sep
Oct
Nov
Dec
Jan
Feb
Mar
Apr
May
Jun
Winter Solstice
December 21/Day 355
Winter Teletherm
January 6/Day 6
+16 days
Coverage
Year
CORINNE, UT

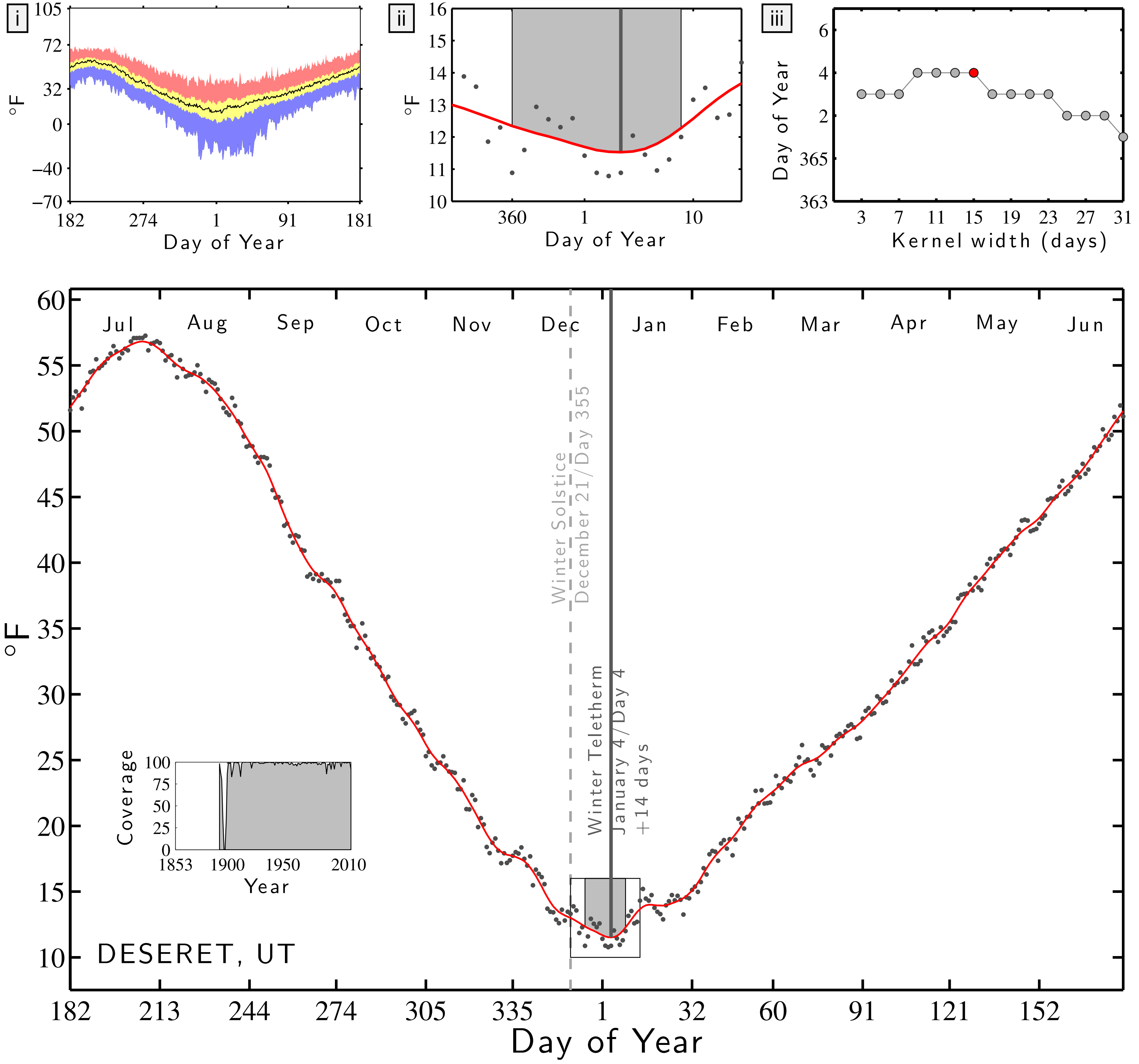

i
°F
Day of Year
ii
°F
Day of Year
iii
Day of Year
Kernel width (days)
Jul
Aug
Sep
Oct
Nov
Dec
Jan
Feb
Mar
Apr
May
Jun
Winter Solstice
December 21/Day 355
Winter Teletherm
January 4/Day 4
+14 days
Coverage
Year
DESERET, UT
°F
Day of Year

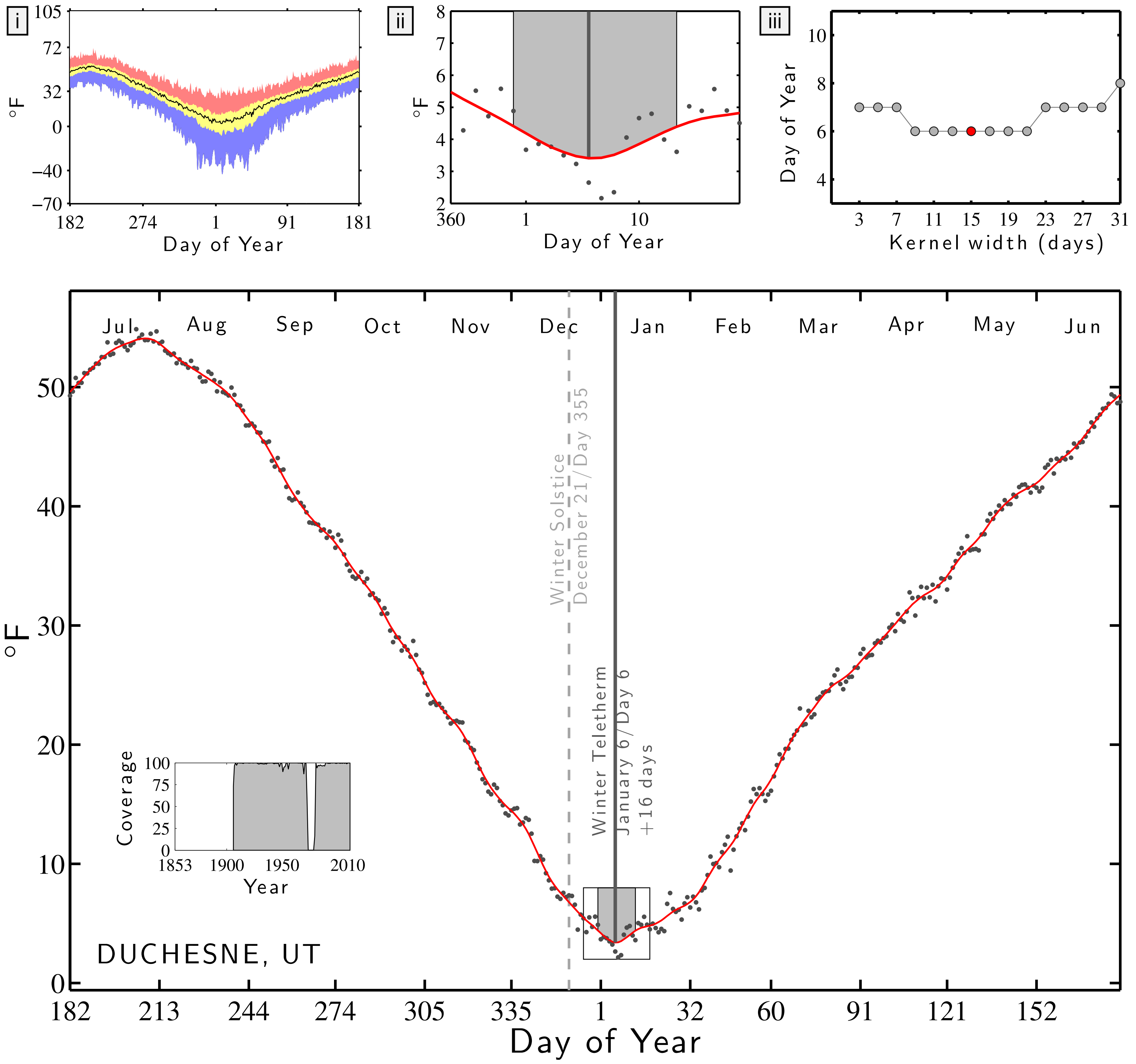
i
°F
Day of Year
ii
°F
Day of Year
iii
Day of Year
Kernel width (days)
Jul
Aug
Sep
Oct
Nov
Dec
Jan
Feb
Mar
Apr
May
Jun
Winter Solstice
December 21/Day 355
Winter Teletherm
January 6/Day 6
+16 days
Coverage
Year
DUCHESNE, UT
°F
Day of Year

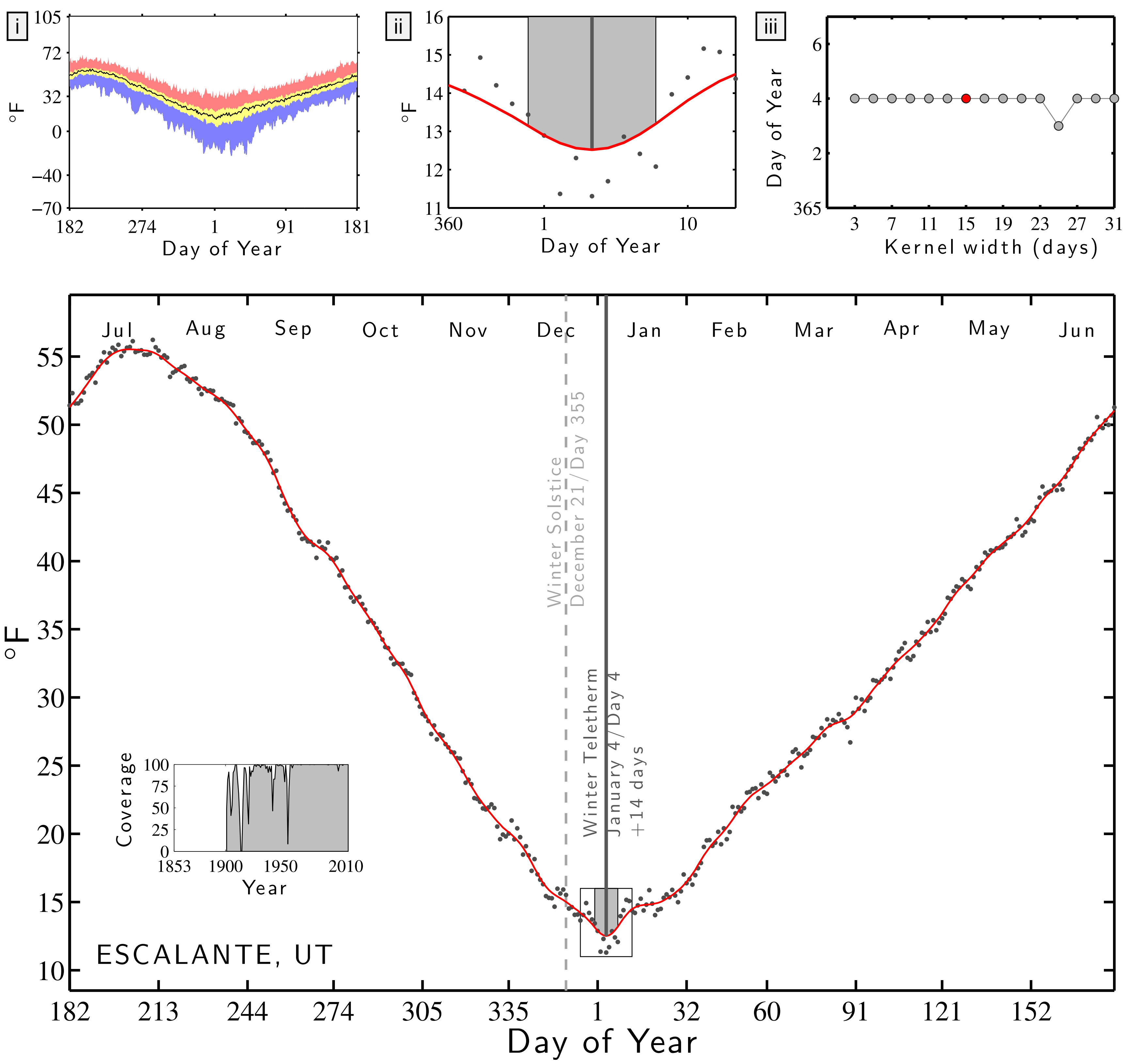

i
105
72
32
0
−40
−70
°F
182
274
1
91
181
Day of Year
ii
16
15
14
13
12
11
°F
360
1
10
Day of Year
iii
6
4
2
365
Day of Year
3
7
11
15
19
23
27
31
Kernel width (days)
Jul
Aug
Sep
Oct
Nov
Dec
Jan
Feb
Mar
Apr
May
Jun
Winter Solstice
December 21/Day 355
Winter Teletherm
January 4/Day 4
+14 days
55
50
45
40
35
30
25
20
15
10
°F
Coverage
100
75
50
25
0
1853
1900
1950
2010
Year
ESCALANTE, UT
182
213
244
274
305
335
1
32
60
91
121
152
Day of Year

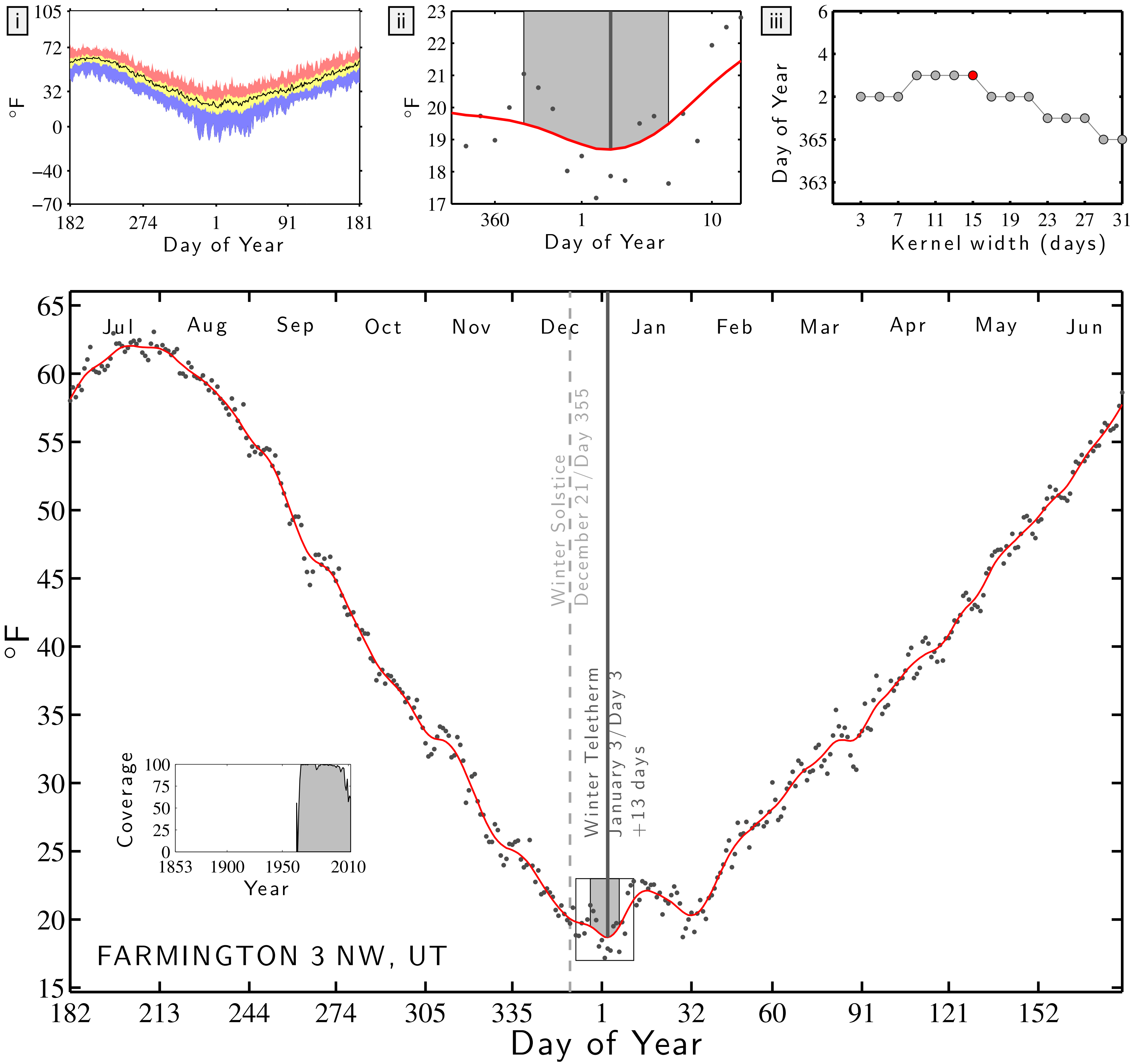

i
ii
iii
°F
Day of Year
Kernel width (days)
Jul
Aug
Sep
Oct
Nov
Dec
Jan
Feb
Mar
Apr
May
Jun
Winter Solstice
December 21/Day 355
Winter Teletherm
January 3/Day 3
+13 days
Coverage
Year
FARMINGTON 3 NW, UT

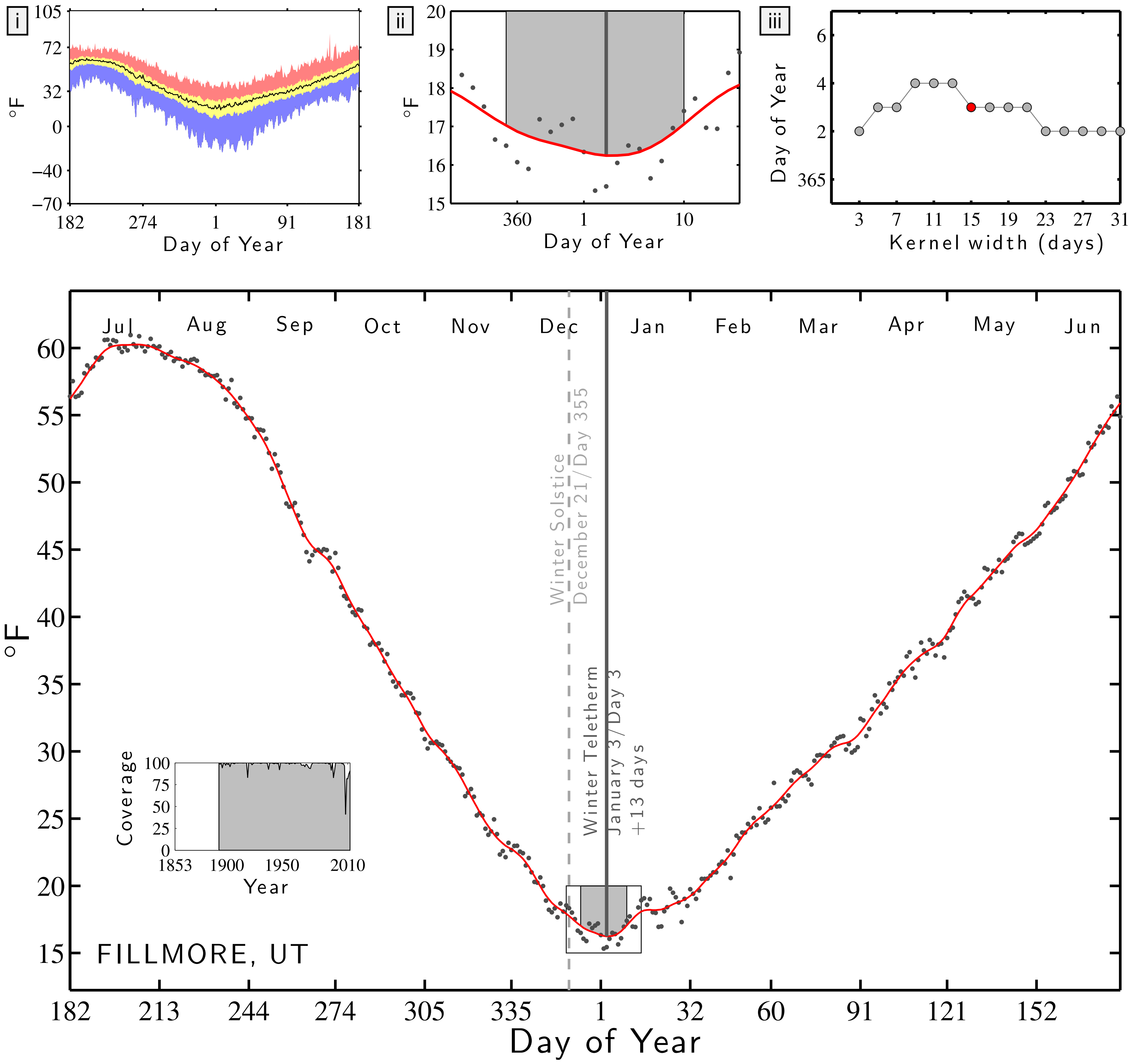

i
ii
iii
°F
Day of Year
Kernel width (days)
Winter Solstice
December 21/Day 355
Winter Teletherm
January 3/Day 3
+13 days
Jul
Aug
Sep
Oct
Nov
Dec
Jan
Feb
Mar
Apr
May
Jun
Coverage
Year
FILLMORE, UT

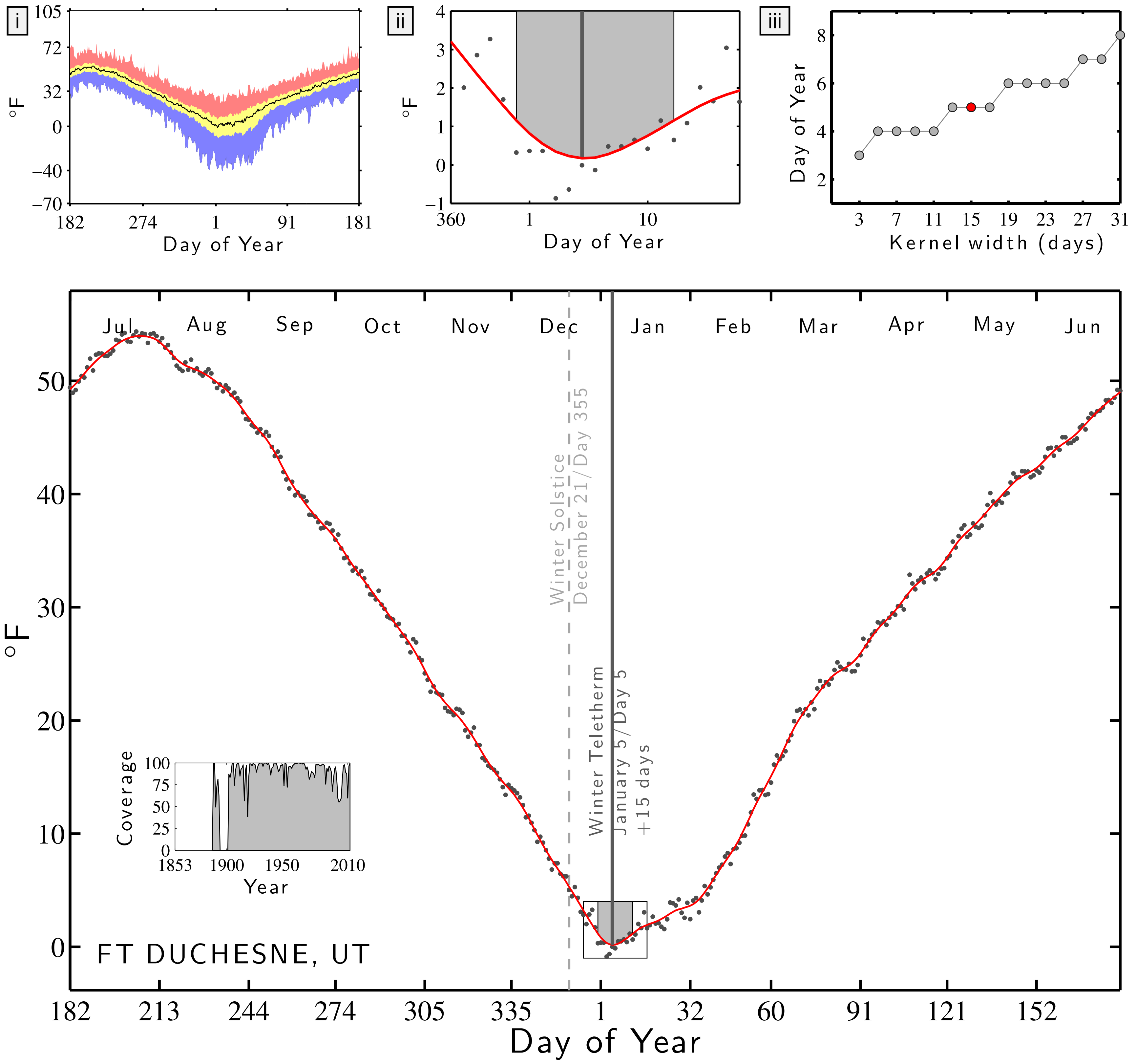

i
°F
Day of Year
ii
°F
Day of Year
iii
Day of Year
Kernel width (days)
Jul
Aug
Sep
Oct
Nov
Dec
Jan
Feb
Mar
Apr
May
Jun
Winter Solstice
December 21/Day 355
Winter Teletherm
January 5/Day 5
+15 days
Coverage
Year
FT DUCHESNE, UT
°F
Day of Year

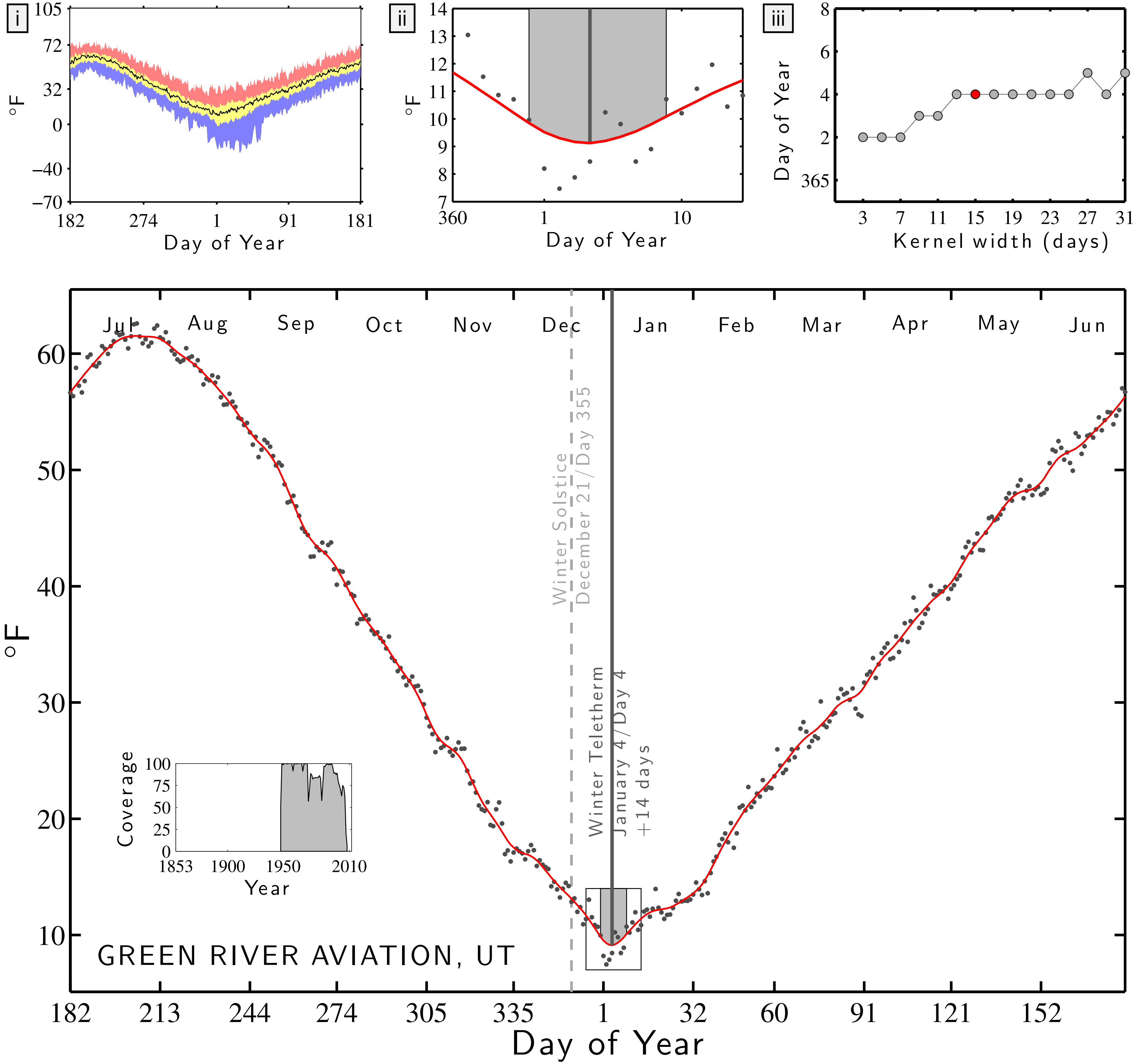

i
ii
iii
°F
Day of Year
Day of Year
Kernel width (days)
Jul
Aug
Sep
Oct
Nov
Dec
Jan
Feb
Mar
Apr
May
Jun
Winter Solstice
December 21/Day 355
Winter Teletherm
January 4/Day 4
+14 days
Coverage
Year
GREEN RIVER AVIATION, UT

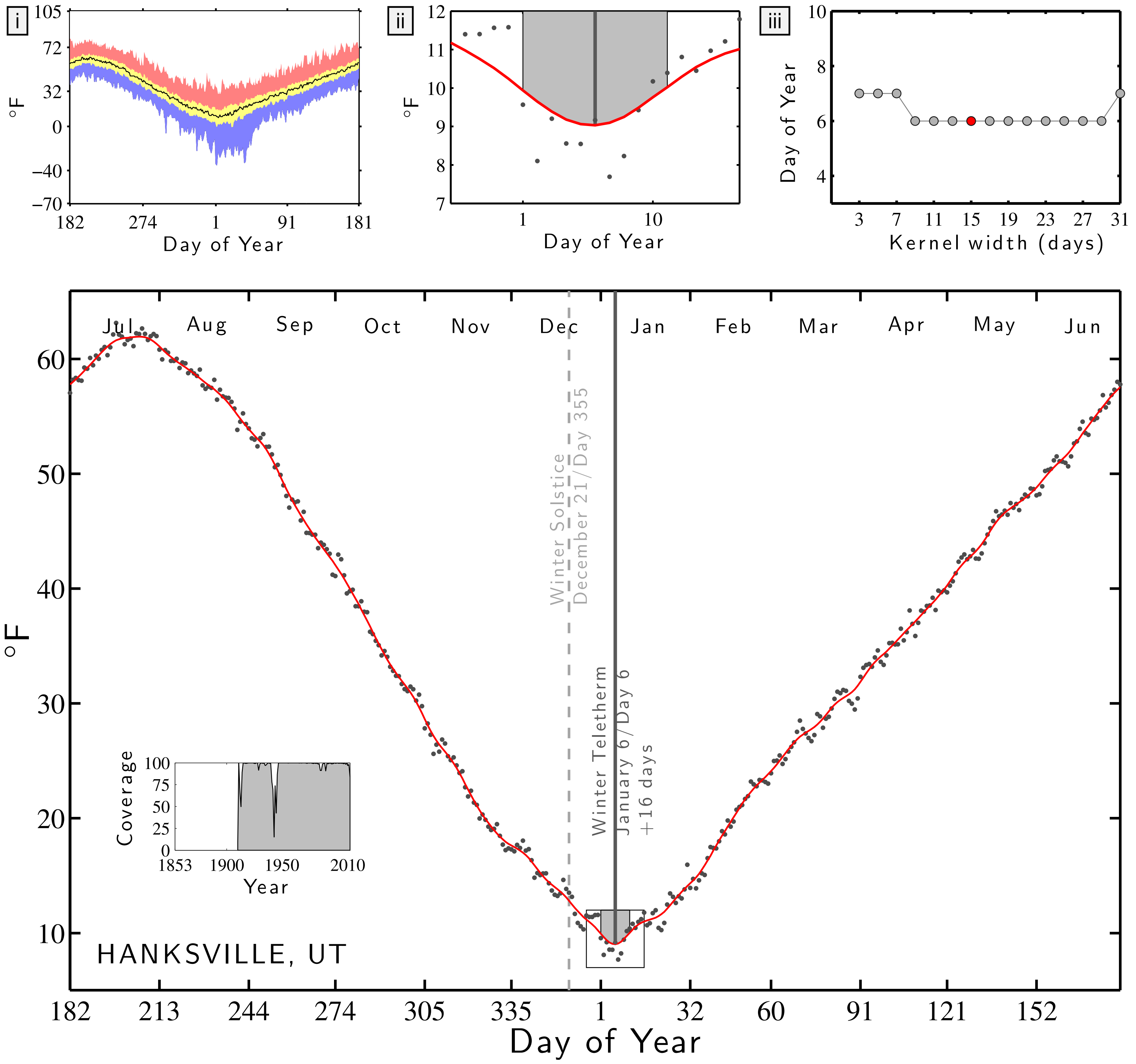

i
°F
Day of Year
ii
°F
Day of Year
iii
Day of Year
Kernel width (days)
Jul
Aug
Sep
Oct
Nov
Dec
Jan
Feb
Mar
Apr
May
Jun
Winter Solstice
December 21/Day 355
Winter Teletherm
January 6/Day 6
+16 days
Coverage
Year
HANKSVILLE, UT
°F
Day of Year

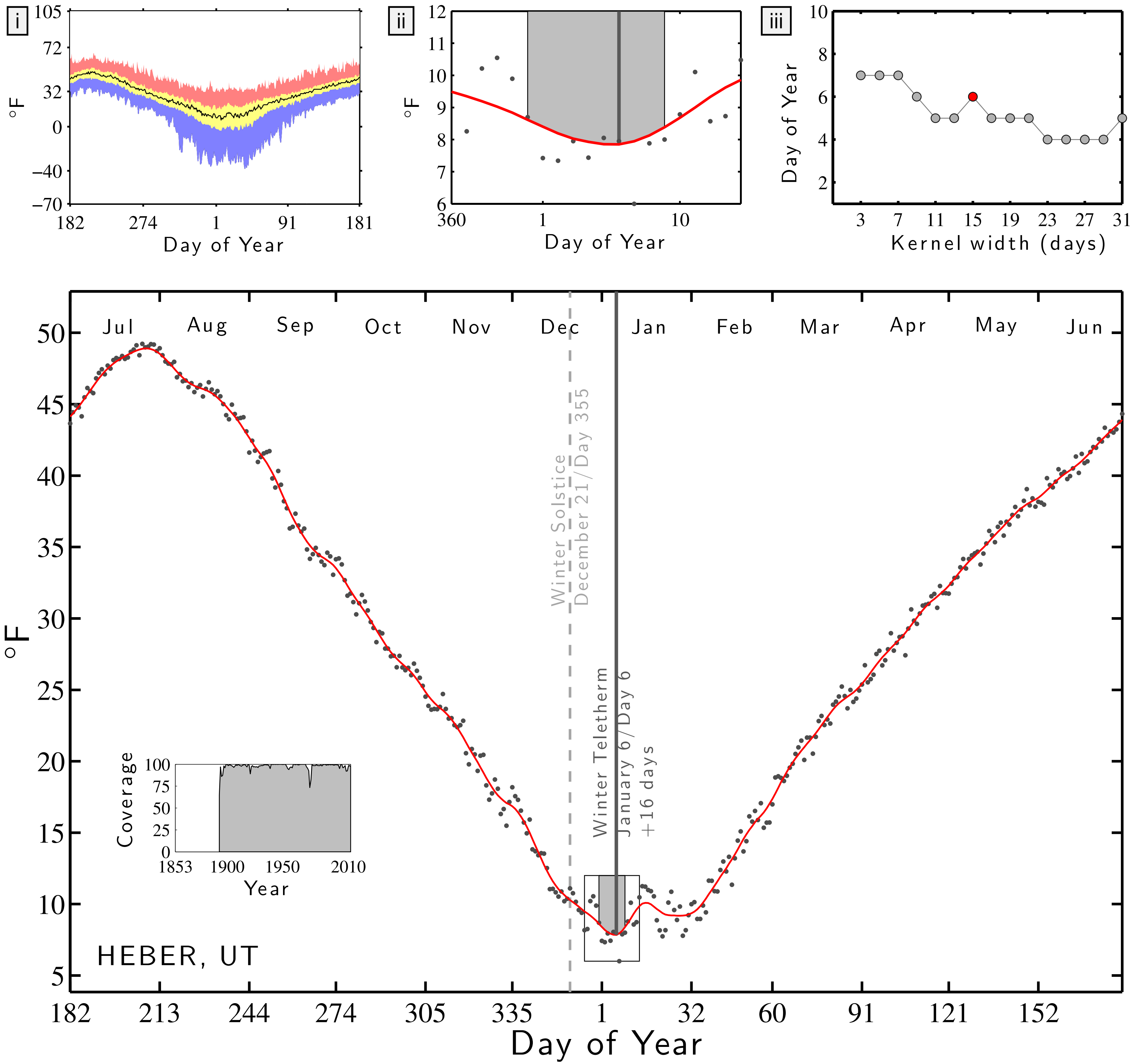

i
°F
Day of Year
182
274
1
91
181
105
72
32
0
−40
−70
ii
Day of Year
360
1
10
iii
Day of Year
Kernel width (days)
Jul
Aug
Sep
Oct
Nov
Dec
Jan
Feb
Mar
Apr
May
Jun
Winter Solstice
December 21/Day 355
Winter Teletherm
January 6/Day 6
+16 days
Coverage
Year
1853
1900
1950
2010
HEBER, UT
°F
Day of Year

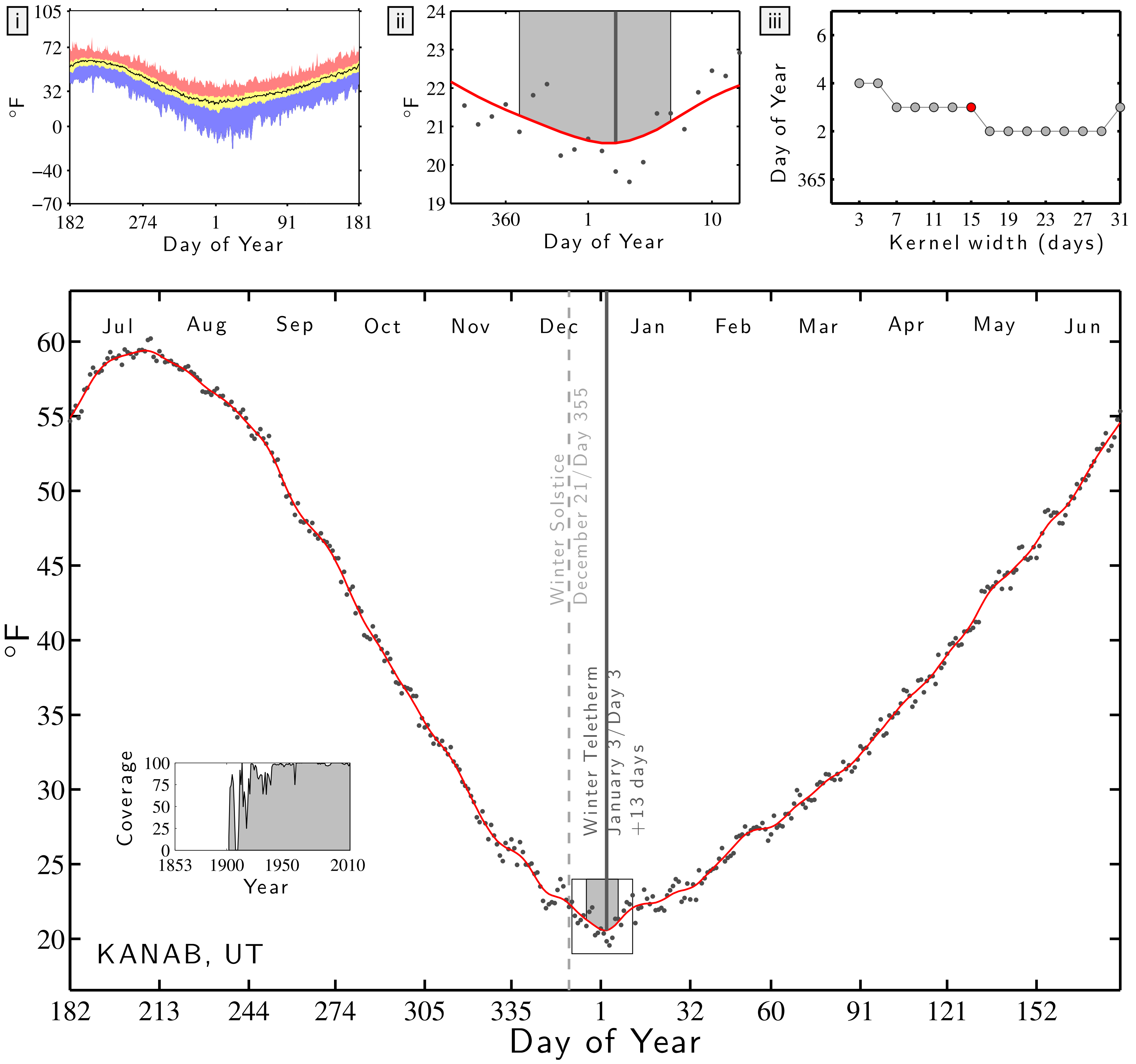

i
ii
iii
°F
Day of Year
Kernel width (days)
Jul
Aug
Sep
Oct
Nov
Dec
Jan
Feb
Mar
Apr
May
Jun
Winter Solstice
December 21/Day 355
Winter Teletherm
January 3/Day 3
+13 days
Coverage
Year
KANAB, UT

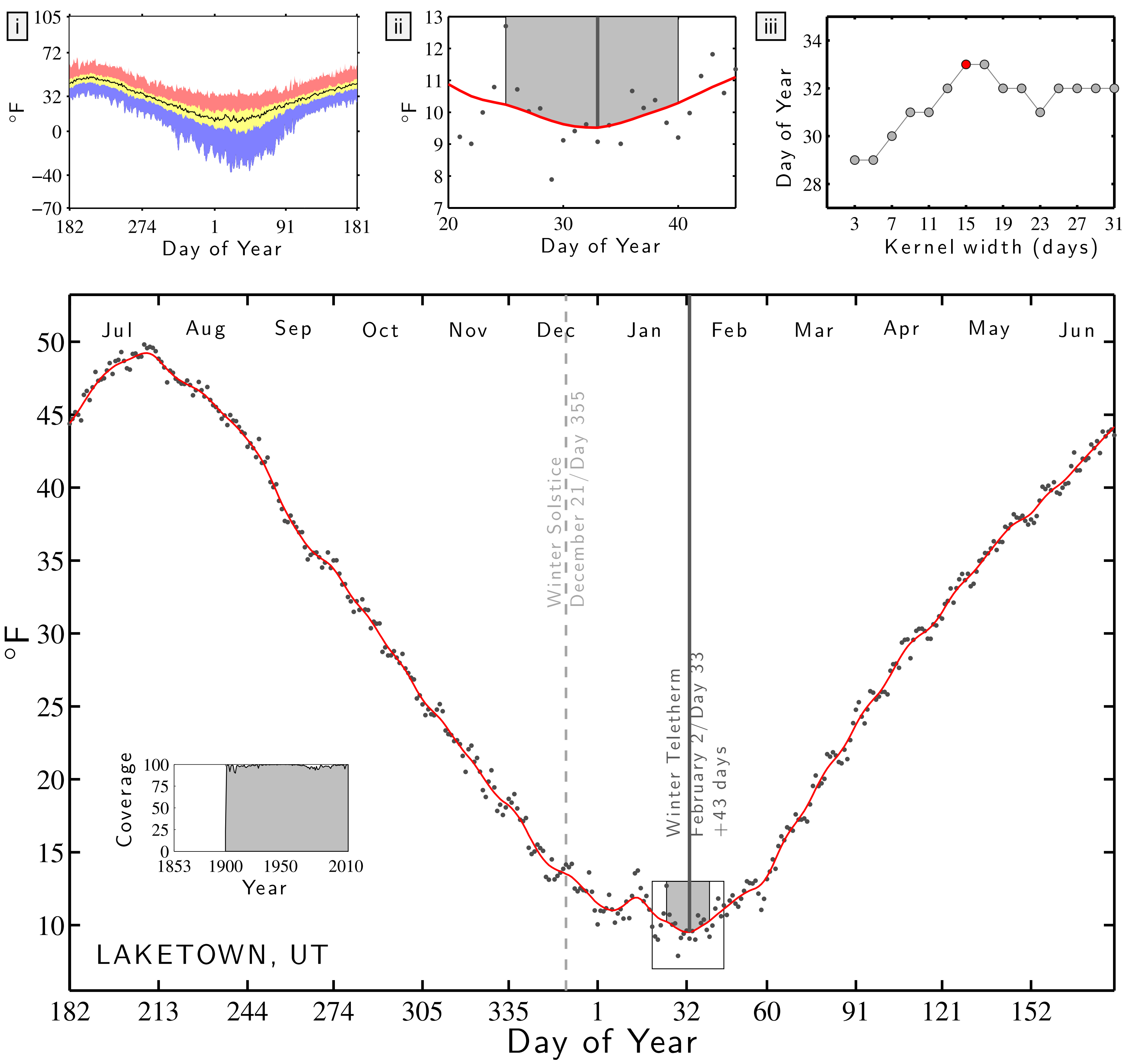

i
°F
Day of Year
ii
°F
Day of Year
iii
Day of Year
Kernel width (days)
Jul
Aug
Sep
Oct
Nov
Dec
Jan
Feb
Mar
Apr
May
Jun
Winter Solstice
December 21/Day 355
Winter Teletherm
February 2/Day 33
+43 days
Coverage
Year
LAKETOWN, UT
°F
Day of Year

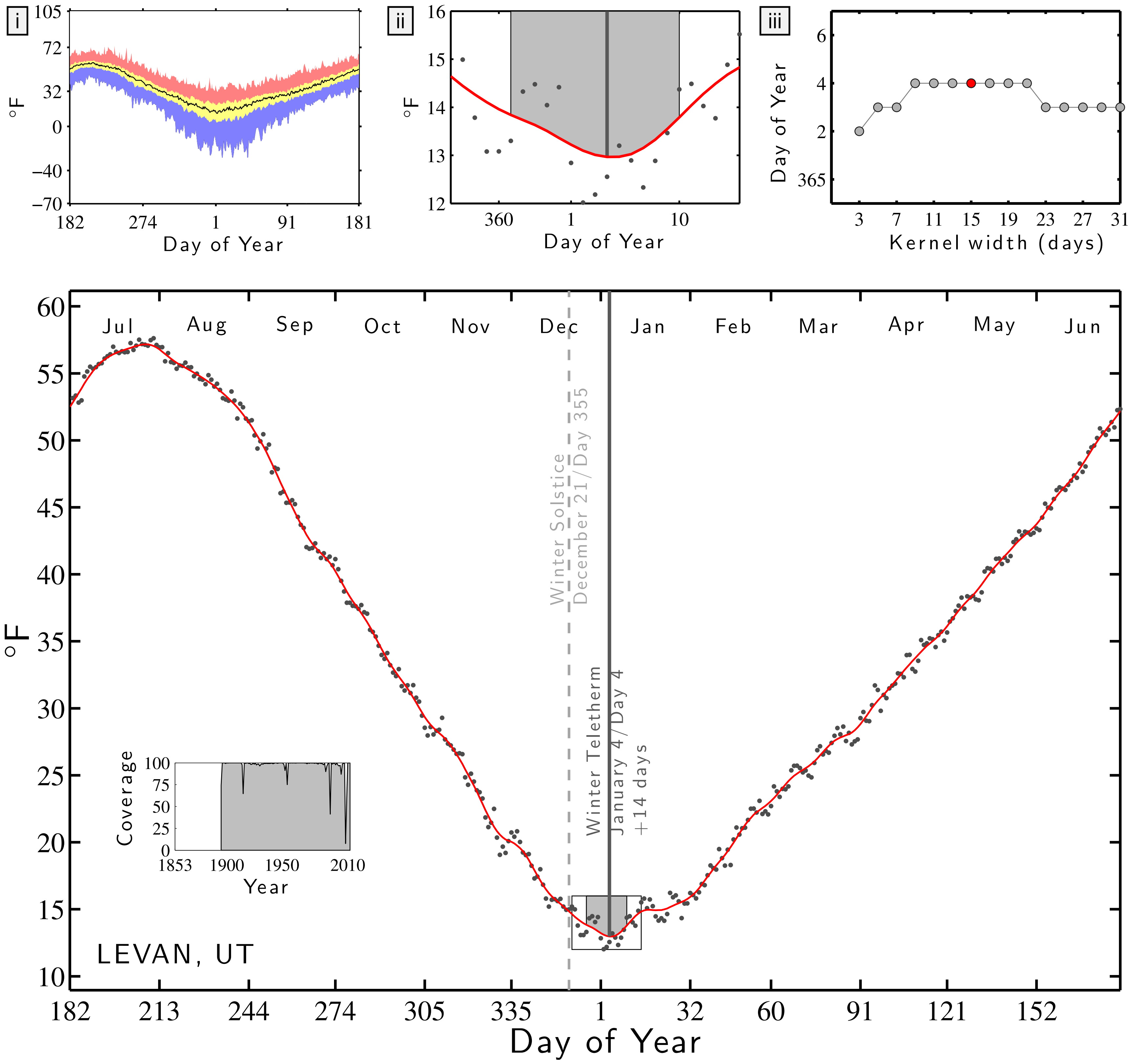

i
°F
Day of Year
ii
Day of Year
iii
Day of Year
Kernel width (days)
Jul
Aug
Sep
Oct
Nov
Dec
Jan
Feb
Mar
Apr
May
Jun
Winter Solstice
December 21/Day 355
Winter Teletherm
January 4/Day 4
+14 days
Coverage
Year
LEVAN, UT
Day of Year

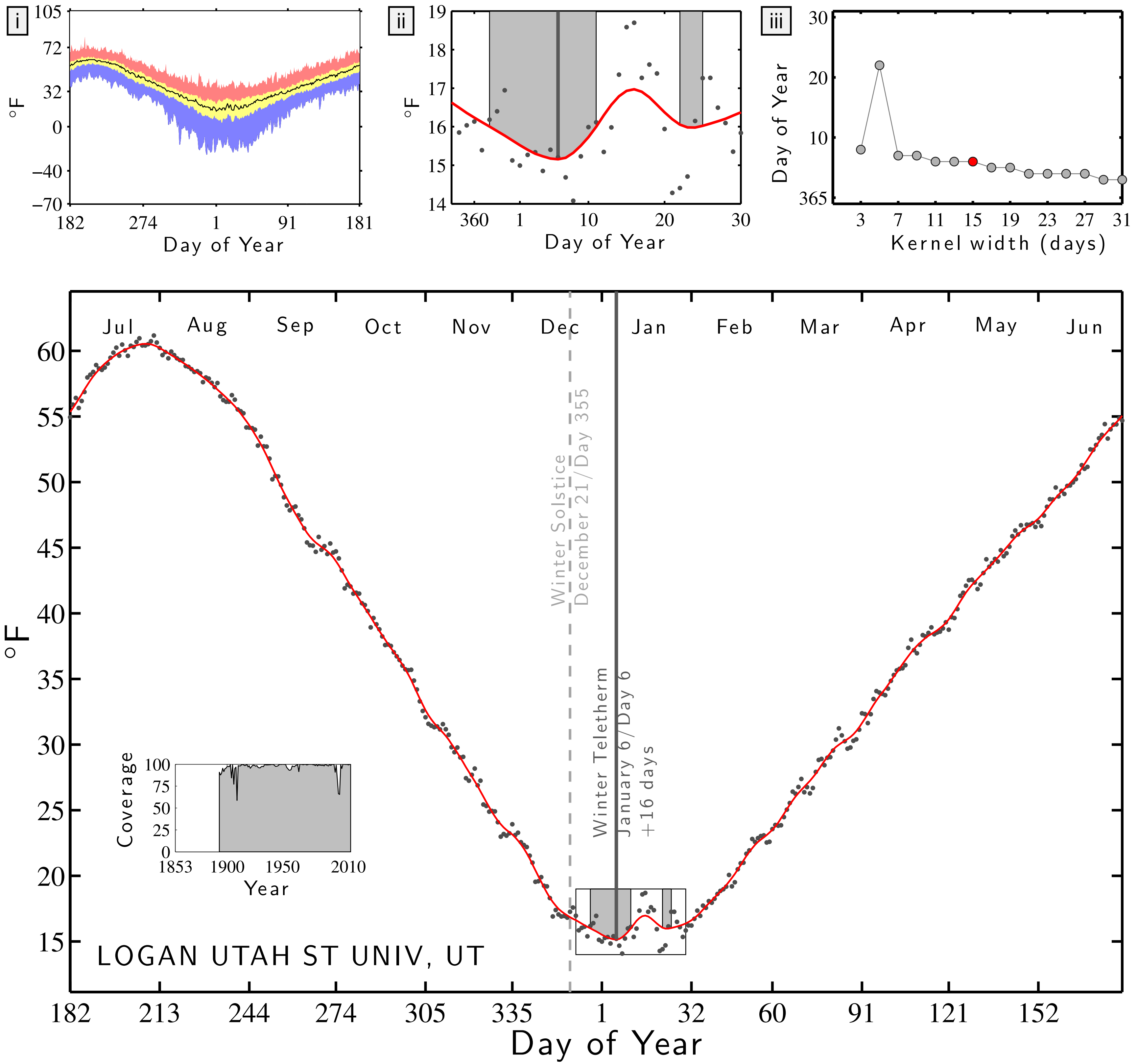

i
°F
Day of Year
ii
Day of Year
iii
Day of Year
Kernel width (days)
Jul
Aug
Sep
Oct
Nov
Dec
Jan
Feb
Mar
Apr
May
Jun
Winter Solstice
December 21/Day 355
Winter Teletherm
January 6/Day 6
+16 days
Coverage
Year
LOGAN UTAH ST UNIV, UT
Day of Year

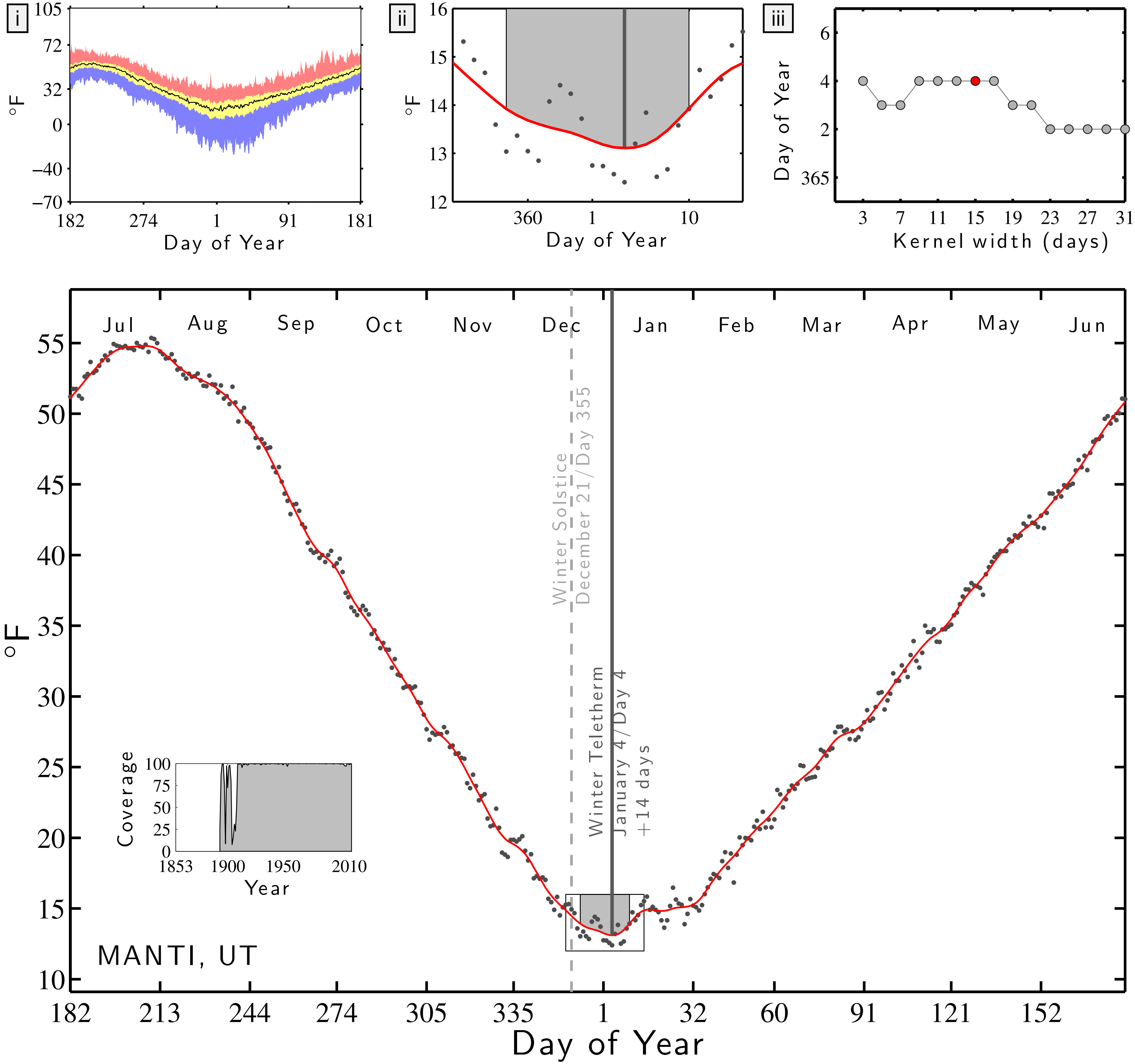

i
105
72
32
0
−40
−70
°F
182
274
1
91
181
Day of Year
ii
16
15
14
13
12
°F
360
1
10
Day of Year
iii
6
4
2
365
Day of Year
3
7
11
15
19
23
27
31
Kernel width (days)
Jul
Aug
Sep
Oct
Nov
Dec
Jan
Feb
Mar
Apr
May
Jun
Winter Solstice
December 21/Day 355
Winter Teletherm
January 4/Day 4
+14 days
Coverage
100
75
50
25
0
1853
1900
1950
2010
Year
MANTI, UT
55
50
45
40
35
30
25
20
15
10
°F
182
213
244
274
305
335
1
32
60
91
121
152
Day of Year

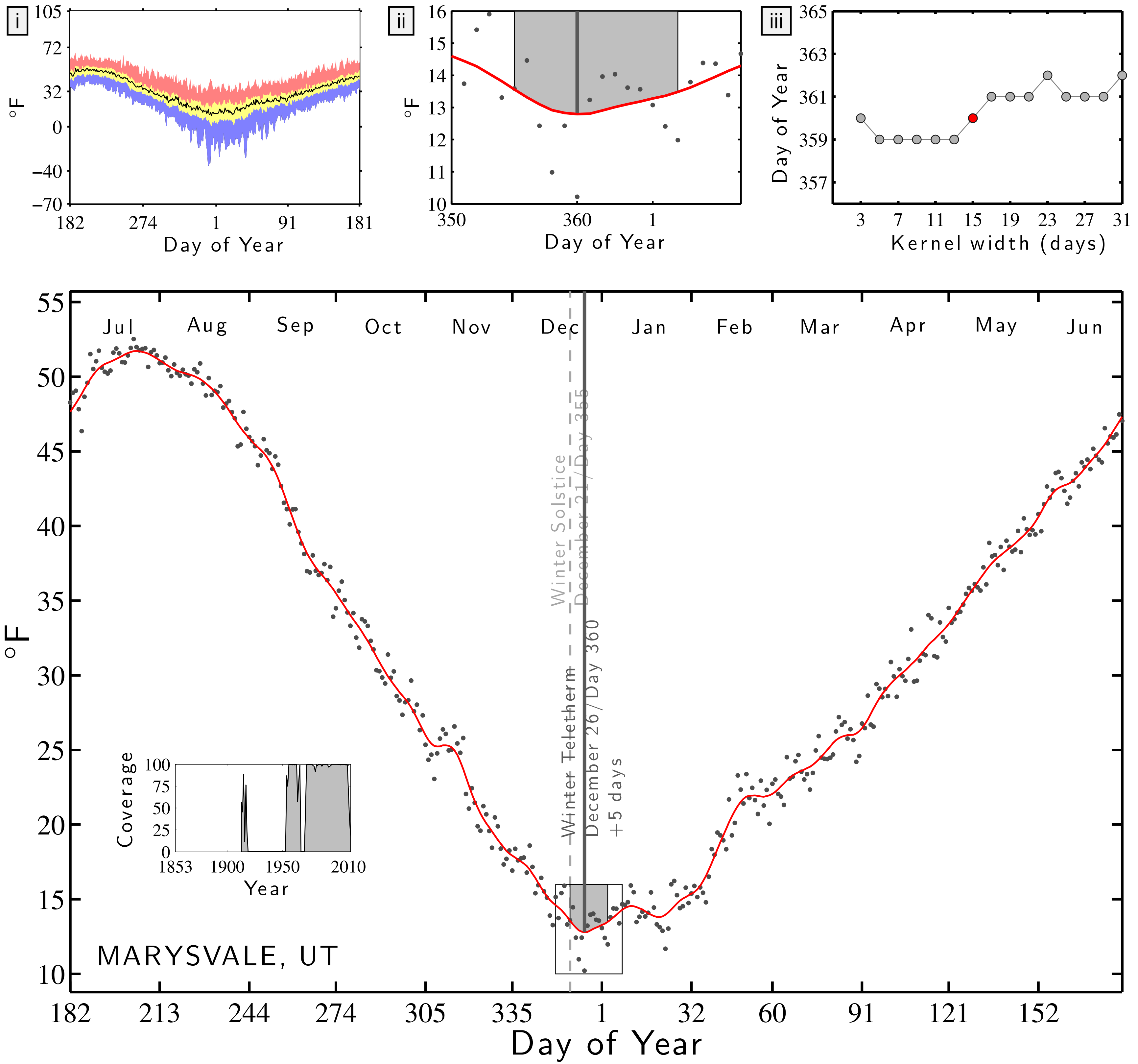

i
°F
Day of Year
ii
°F
Day of Year
iii
Day of Year
Kernel width (days)
Jul
Aug
Sep
Oct
Nov
Dec
Jan
Feb
Mar
Apr
May
Jun
Winter Solstice
December 21/Day 355
Winter Teletherm
December 26/Day 360
+5 days
Coverage
Year
MARYSVALE, UT
°F
Day of Year

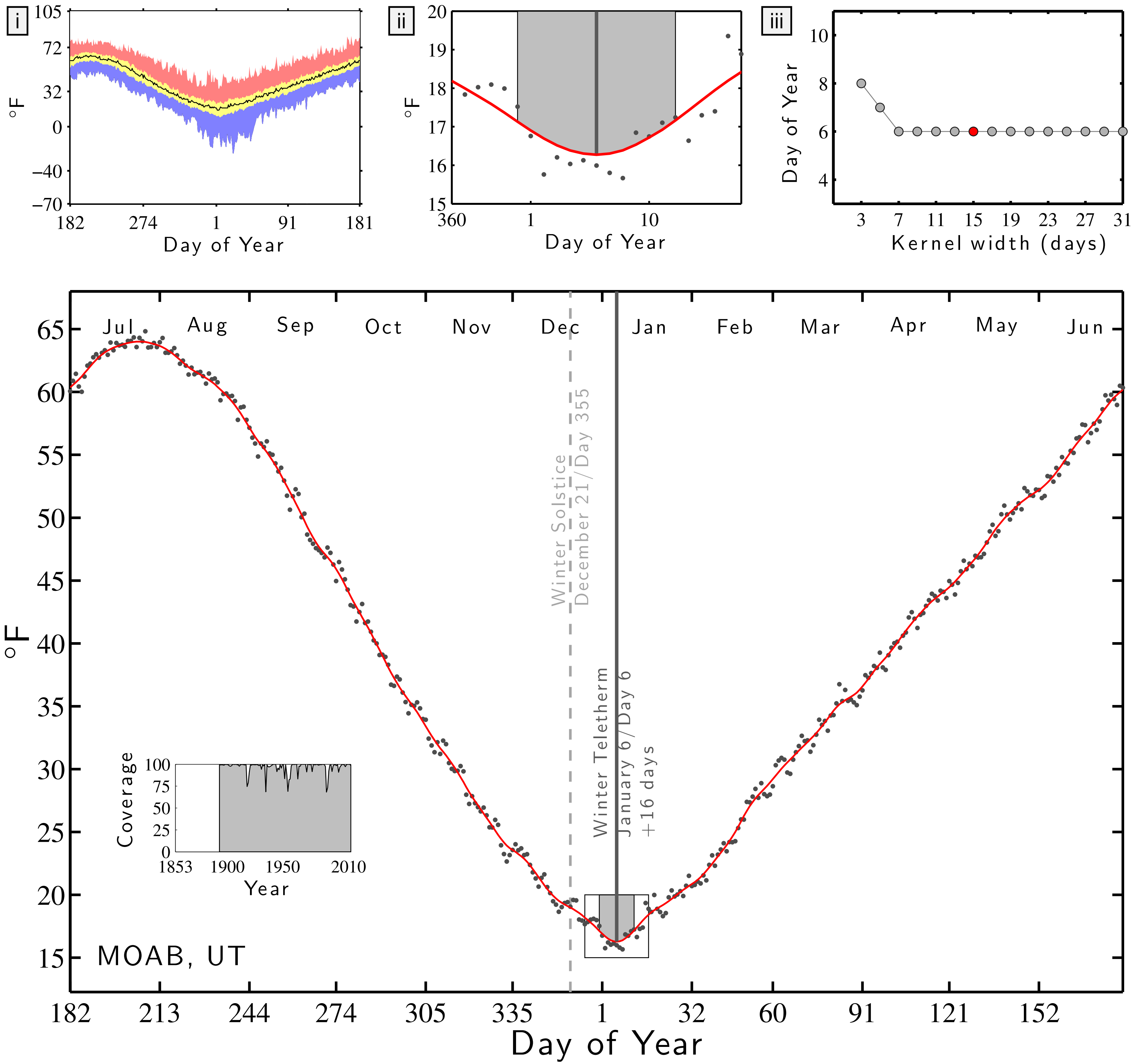

i
°F
Day of Year
ii
°F
Day of Year
iii
Day of Year
Kernel width (days)
Jul
Aug
Sep
Oct
Nov
Dec
Jan
Feb
Mar
Apr
May
Jun
Winter Solstice
December 21/Day 355
Winter Teletherm
January 6/Day 6
+16 days
Coverage
Year
MOAB, UT
°F
Day of Year

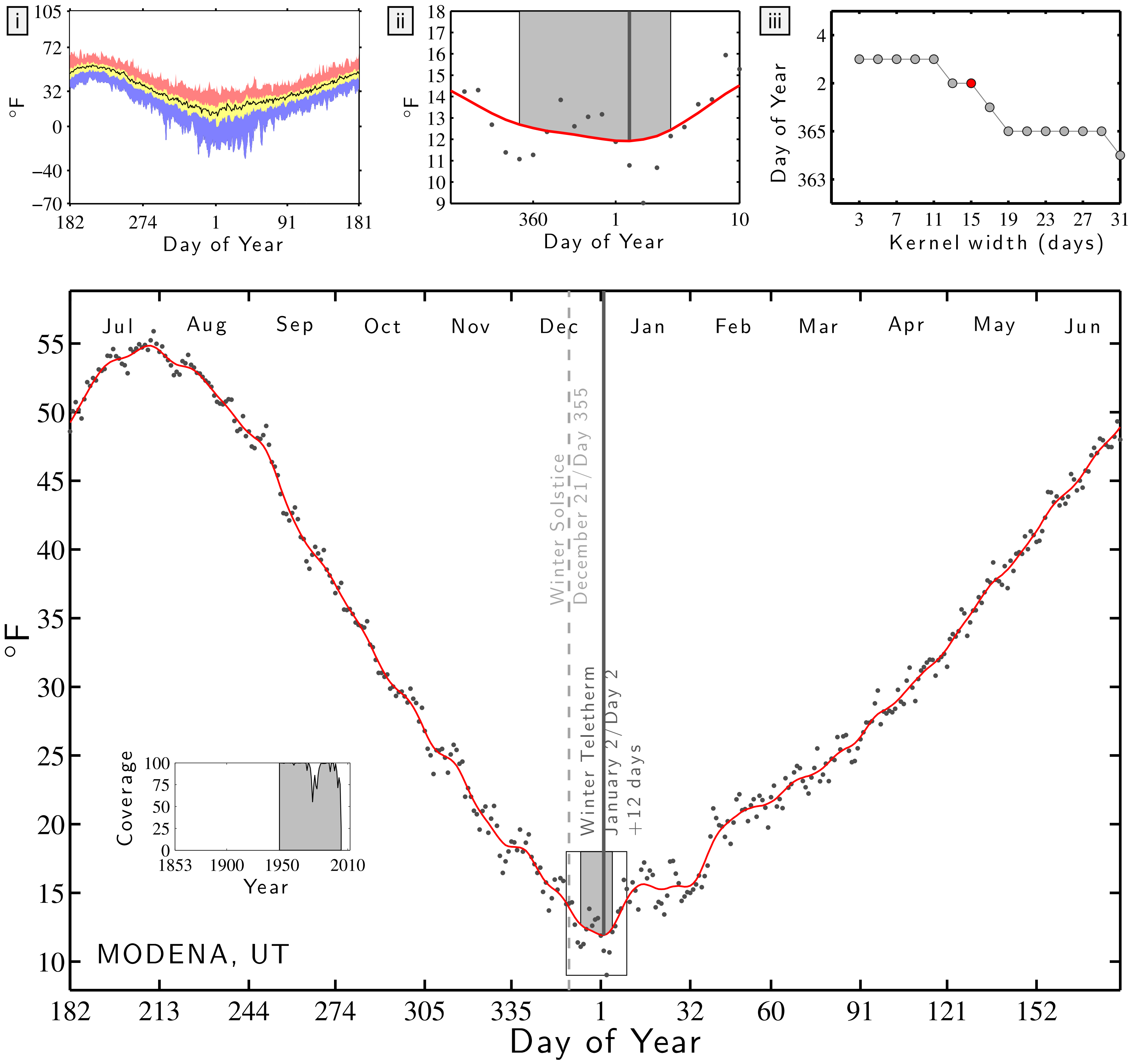

i
°F
105
72
32
0
−40
−70
182
274
1
91
181
Day of Year
ii
°F
18
17
16
15
14
13
12
11
10
9
360
1
10
Day of Year
iii
Day of Year
4
2
365
363
3
7
11
15
19
23
27
31
Kernel width (days)
Jul
Aug
Sep
Oct
Nov
Dec
Jan
Feb
Mar
Apr
May
Jun
Winter Solstice
December 21/Day 355
Winter Teletherm
January 2/Day 2
+12 days
°F
55
50
45
40
35
30
25
20
15
10
Coverage
100
75
50
25
0
1853
1900
1950
2010
Year
MODENA, UT
182
213
244
274
305
335
1
32
60
91
121
152
Day of Year

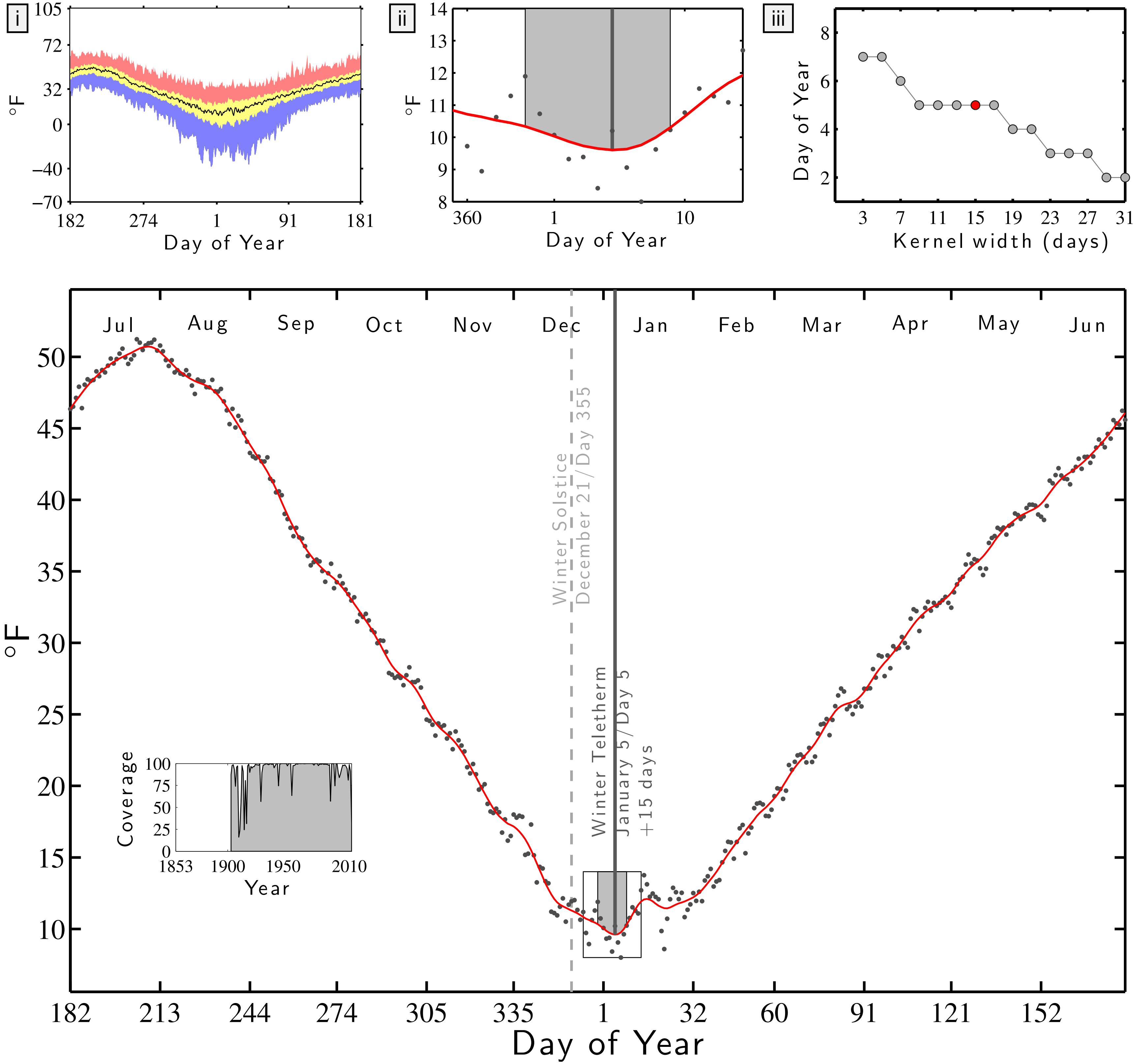
i
ii
iii
°F
Day of Year
Kernel width (days)
Jul
Aug
Sep
Oct
Nov
Dec
Jan
Feb
Mar
Apr
May
Jun
Winter Solstice
December 21/Day 355
Winter Teletherm
January 5/Day 5
+15 days
Coverage
Year

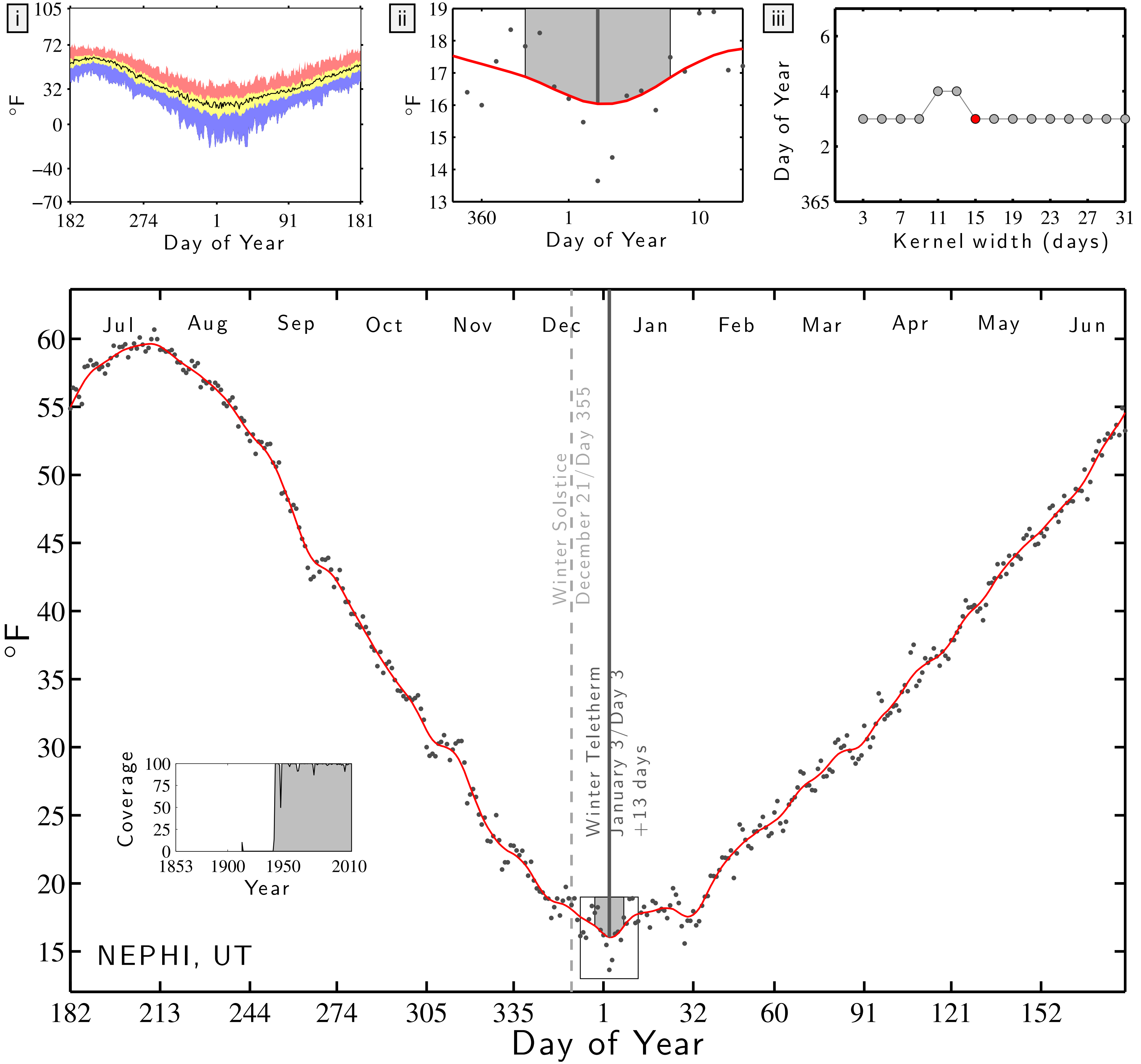

i
ii
iii
°F
Day of Year
Day of Year
Kernel width (days)
Jul
Aug
Sep
Oct
Nov
Dec
Jan
Feb
Mar
Apr
May
Jun
Winter Solstice
December 21/Day 355
Winter Teletherm
January 3/Day 3
+13 days
Coverage
Year
NEPHI, UT

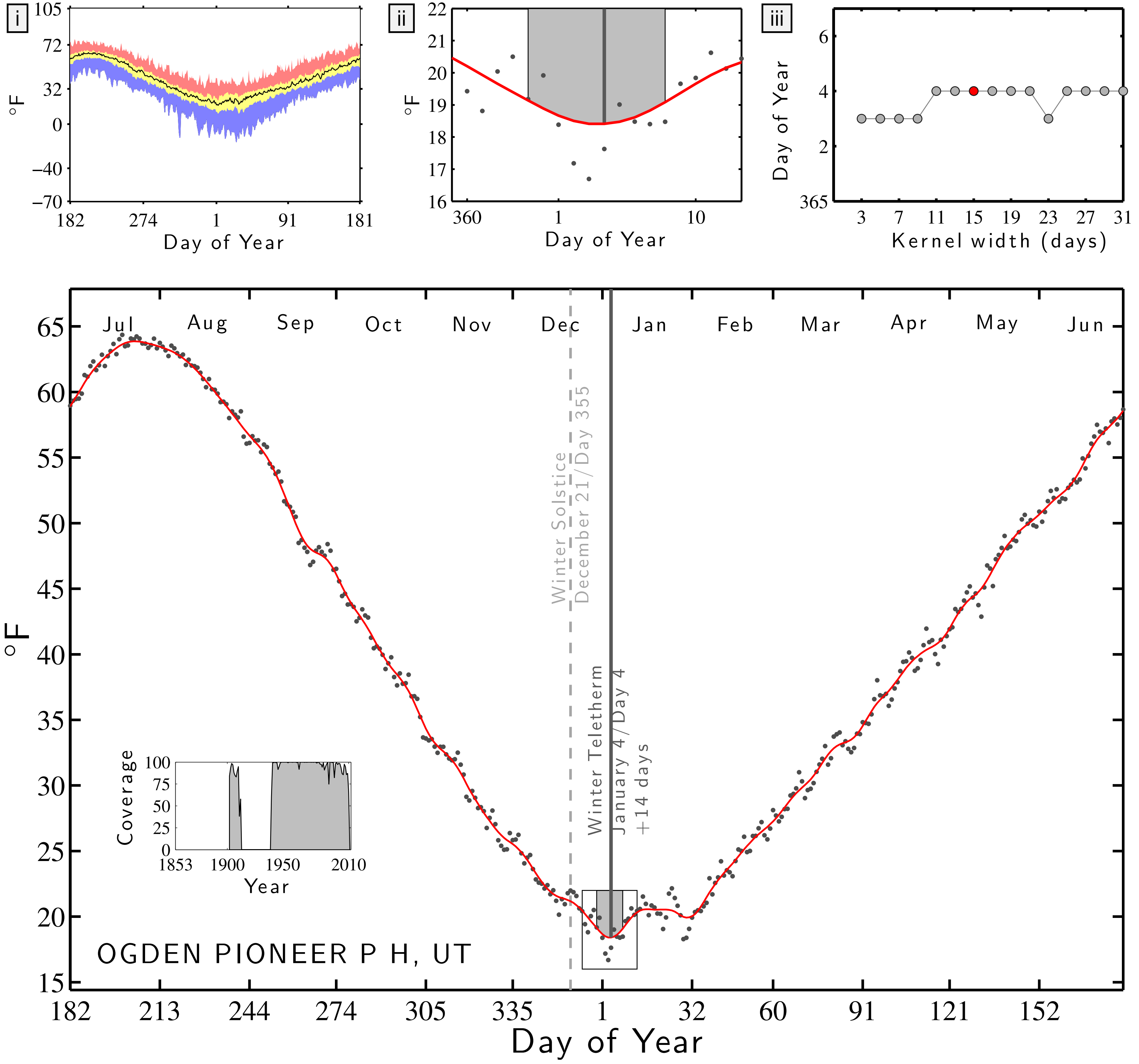

i
°F
Day of Year
ii
°F
Day of Year
iii
Day of Year
Kernel width (days)
Jul
Aug
Sep
Oct
Nov
Dec
Jan
Feb
Mar
Apr
May
Jun
Winter Solstice
December 21/Day 355
Winter Teletherm
January 4/Day 4
+14 days
Coverage
Year
OGDEN PIONEER P H, UT
°F
Day of Year

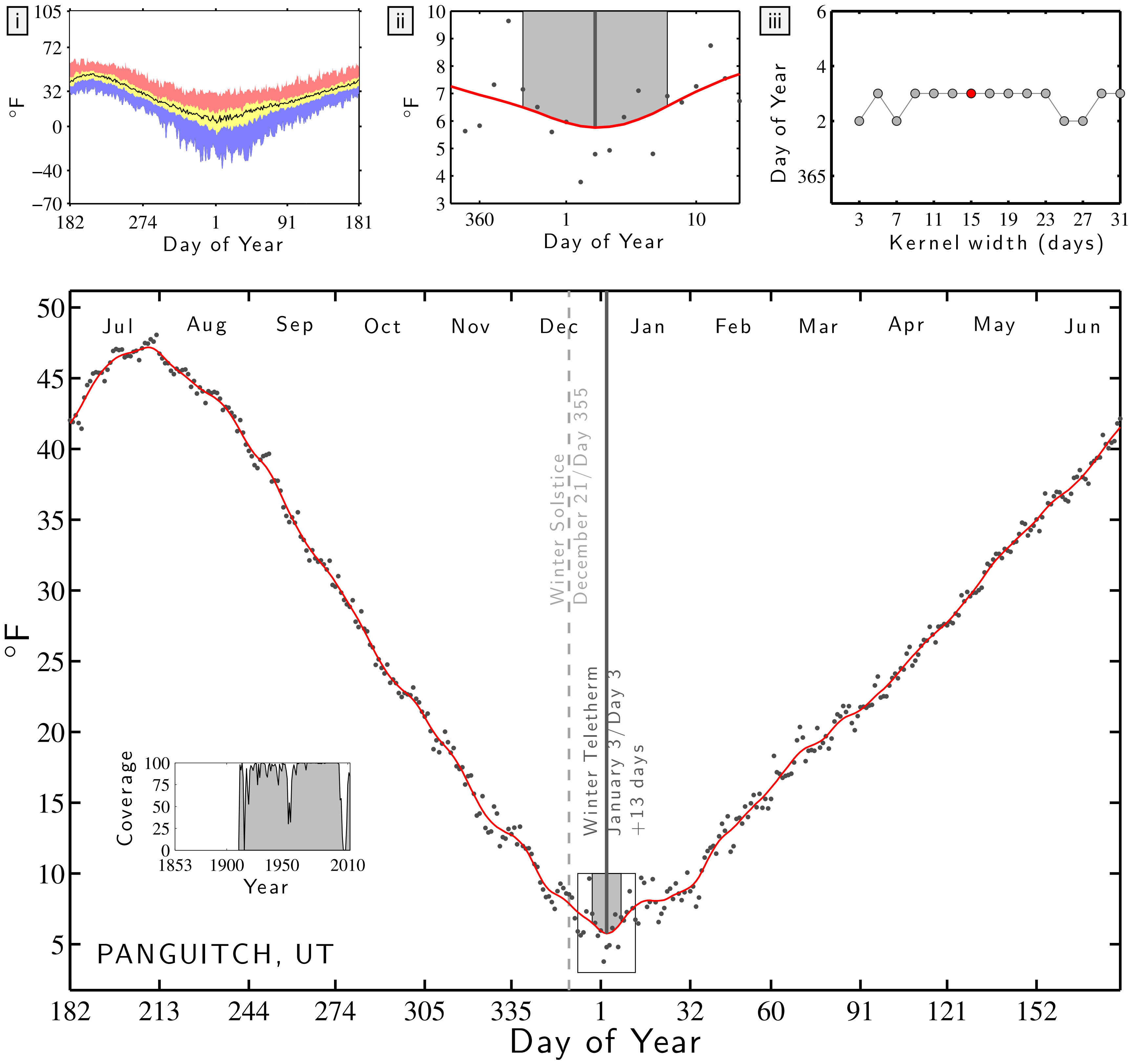

i
°F
Day of Year
ii
Day of Year
iii
Day of Year
Kernel width (days)
Jul
Aug
Sep
Oct
Nov
Dec
Jan
Feb
Mar
Apr
May
Jun
Winter Solstice
December 21/Day 355
Winter Teletherm
January 3/Day 3
+13 days
Coverage
Year
PANGUITCH, UT
Day of Year

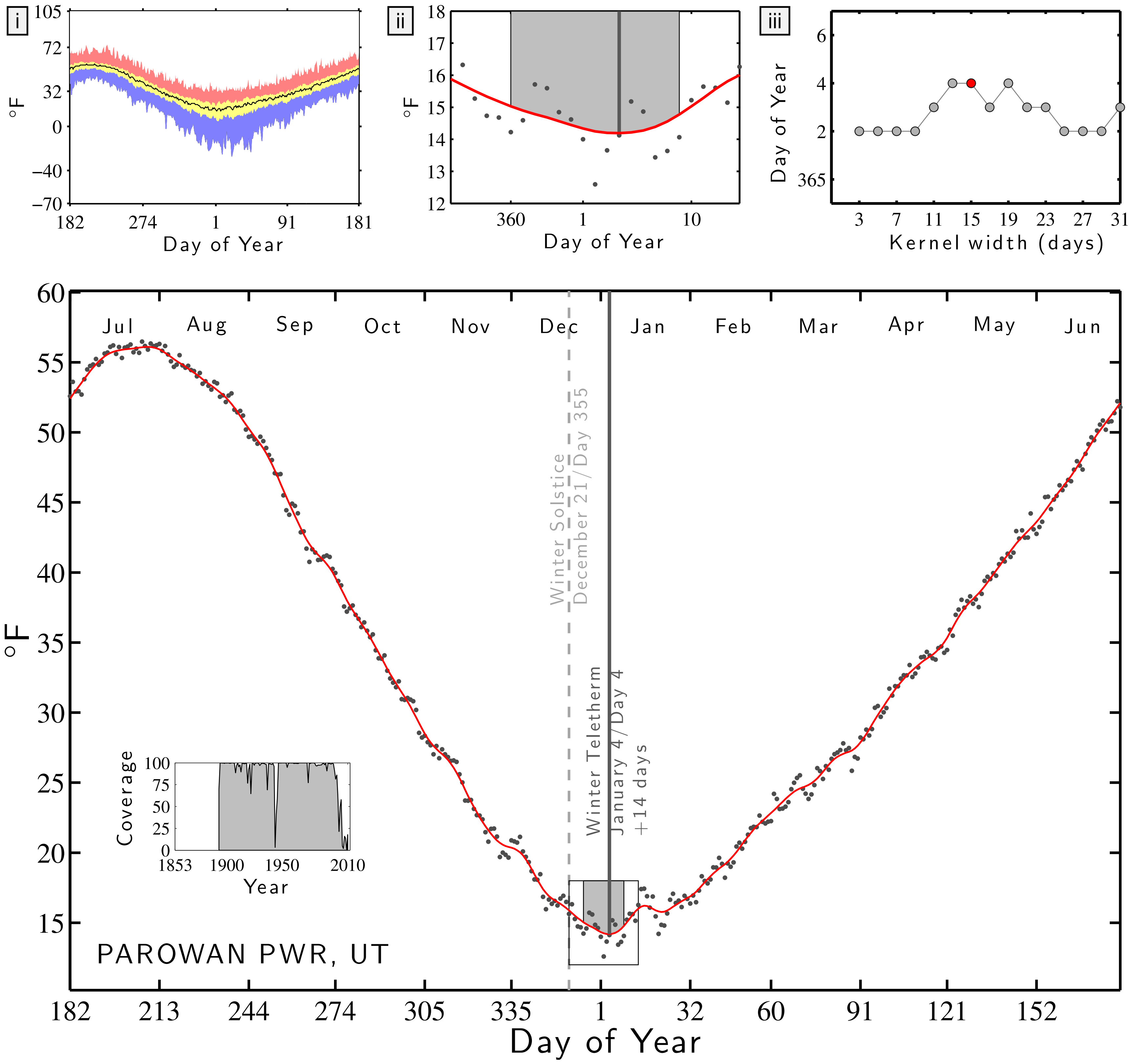

i
ii
iii
°F
Day of Year
Kernel width (days)
Jul
Aug
Sep
Oct
Nov
Dec
Jan
Feb
Mar
Apr
May
Jun
Winter Solstice
December 21/Day 355
Winter Teletherm
January 4/Day 4
+14 days
Coverage
Year
PAROWAN PWR, UT

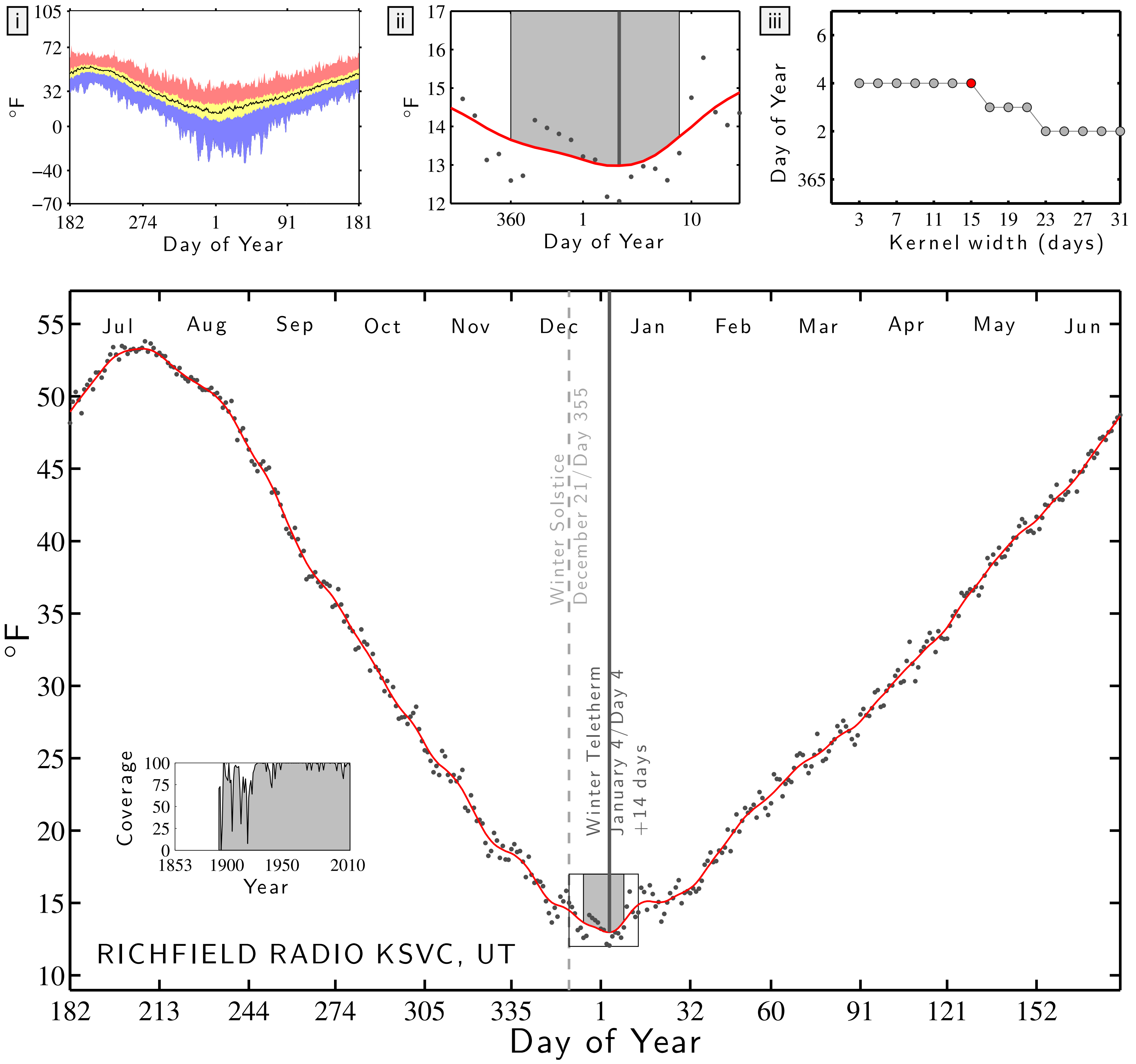

i
°F
Day of Year
ii
iii
Day of Year
Kernel width (days)
Jul
Aug
Sep
Oct
Nov
Dec
Jan
Feb
Mar
Apr
May
Jun
Winter Solstice
December 21/Day 355
Winter Teletherm
January 4/Day 4
+14 days
Coverage
Year
RICHFIELD RADIO KSVC, UT

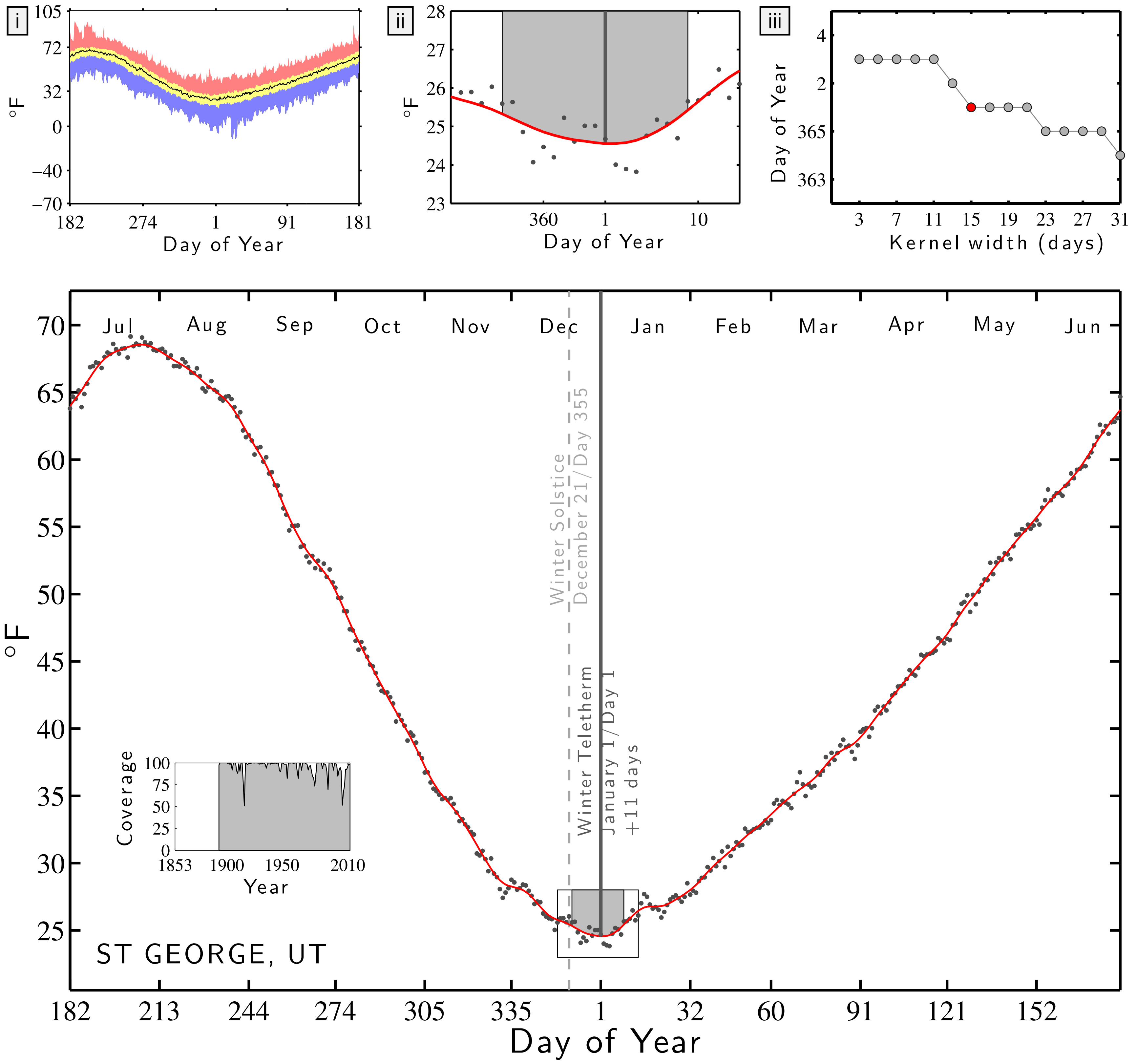

i
ii
iii
°F
Day of Year
Day of Year
Kernel width (days)
Jul
Aug
Sep
Oct
Nov
Dec
Jan
Feb
Mar
Apr
May
Jun
Winter Solstice
December 21/Day 355
Winter Teletherm
January 1/Day 1
+11 days
Coverage
Year
ST GEORGE, UT

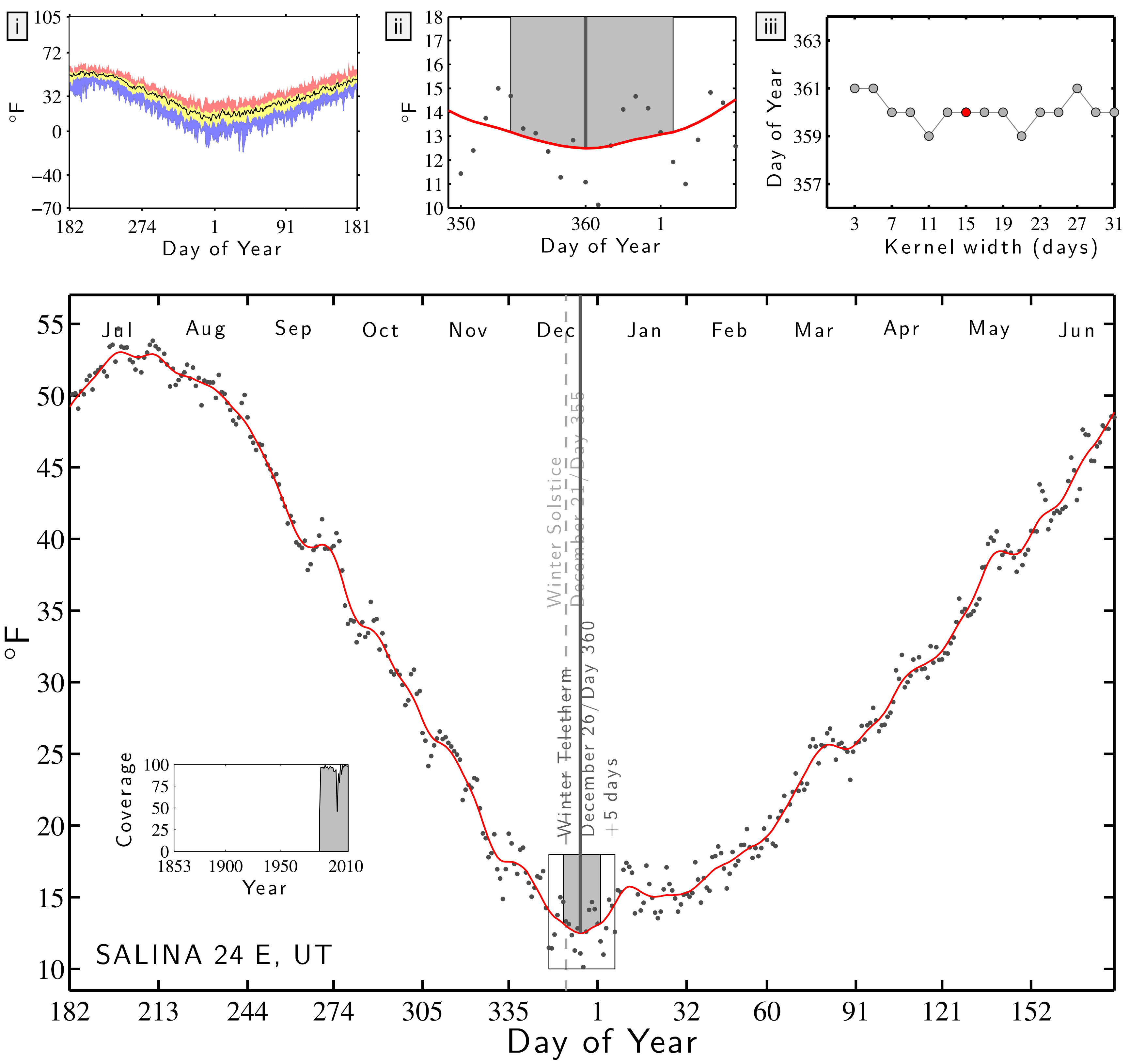

i
ii
iii
Day of Year
Kernel width (days)
°F
Jul
Aug
Sep
Oct
Nov
Dec
Jan
Feb
Mar
Apr
May
Jun
Winter Solstice
December 21/Day 355
Winter Teletherm
December 26/Day 360
+5 days
Coverage
Year
SALINA 24 E, UT

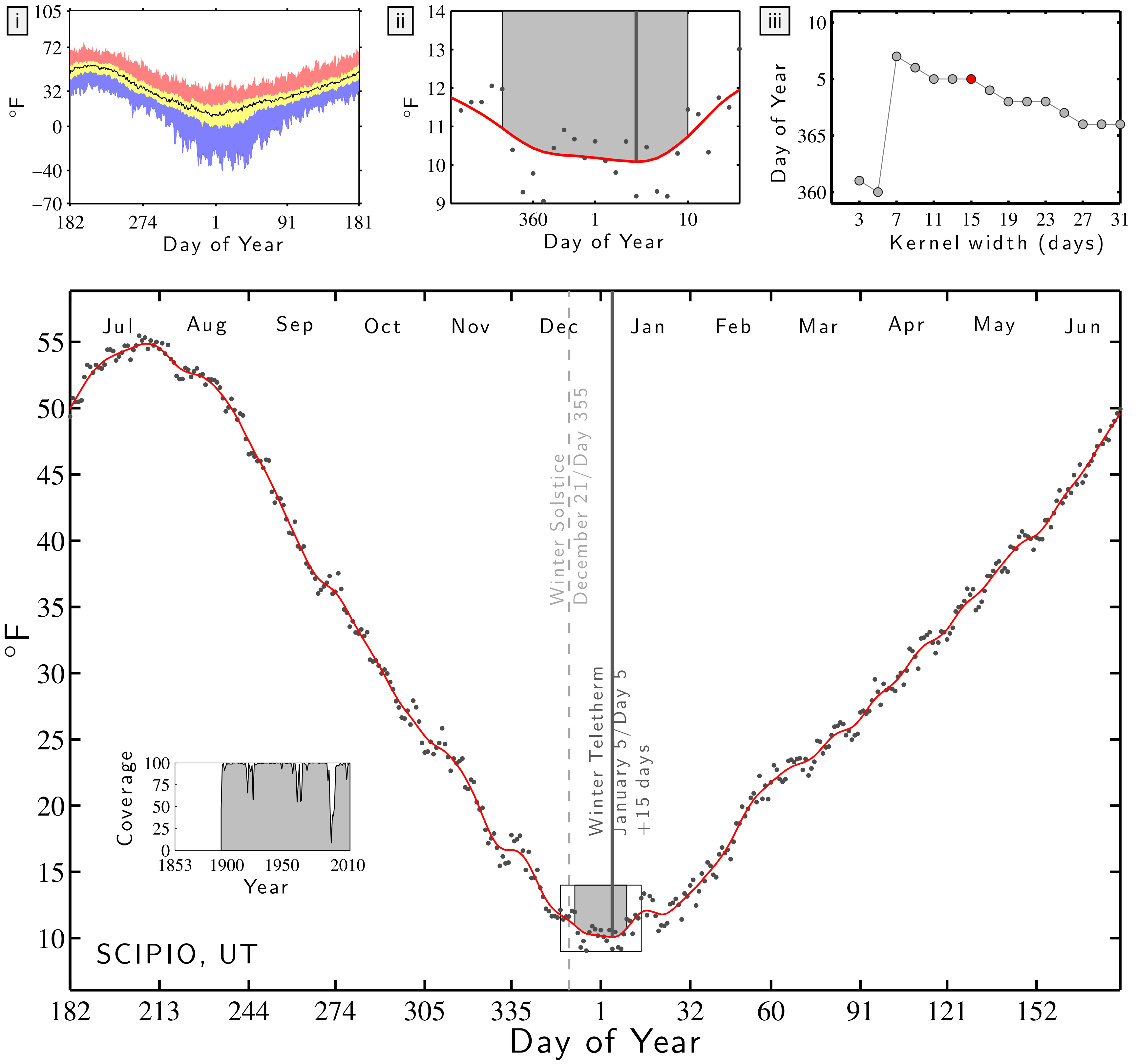

i
°F
Day of Year
ii
°F
Day of Year
iii
Day of Year
Kernel width (days)
Jul
Aug
Sep
Oct
Nov
Dec
Jan
Feb
Mar
Apr
May
Jun
Winter Solstice
December 21/Day 355
Winter Teletherm
January 5/Day 5
+15 days
Coverage
Year
SCIPIO, UT
°F
Day of Year

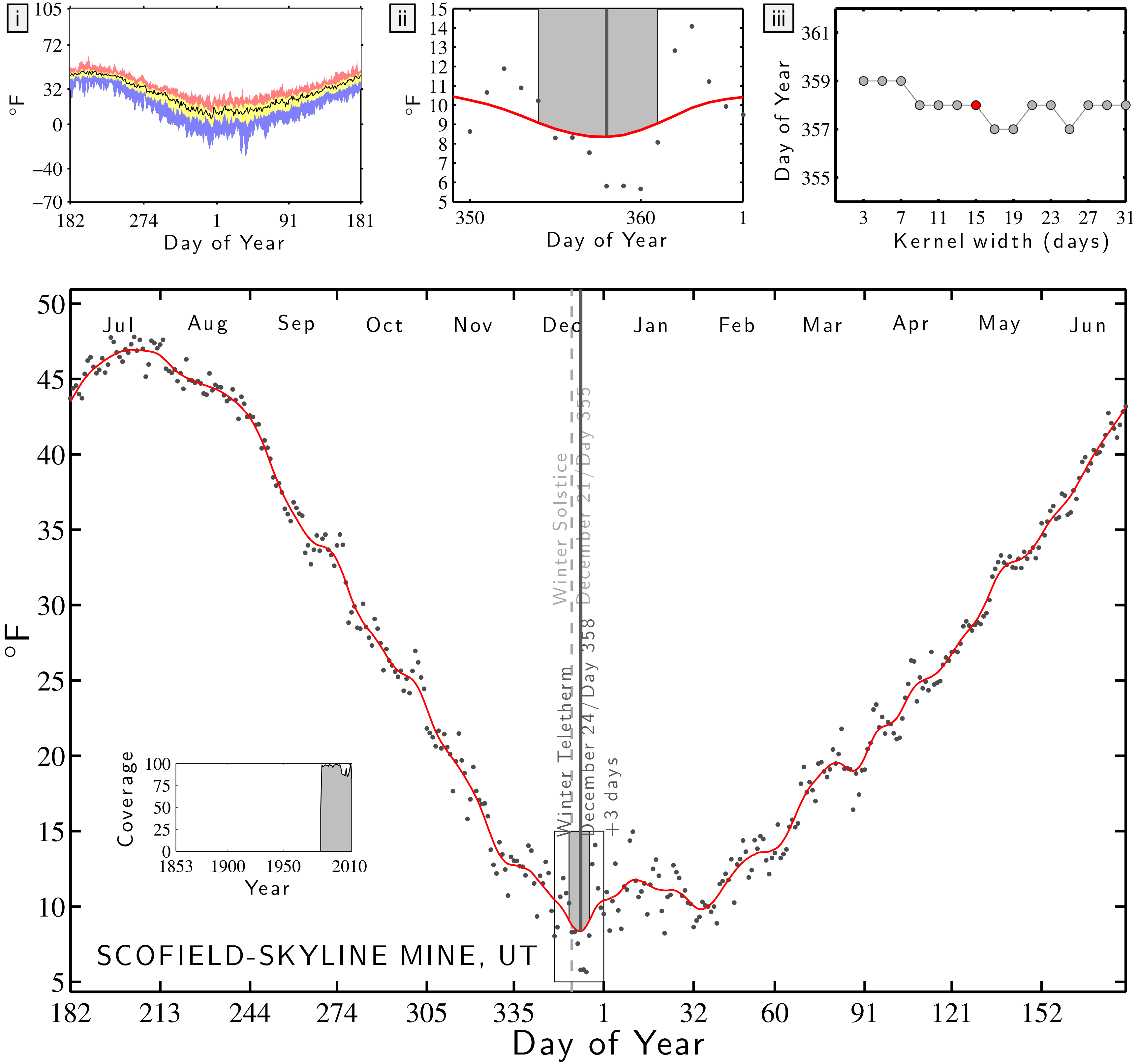

i
°F
Day of Year
ii
°F
Day of Year
iii
Day of Year
Kernel width (days)
Jul
Aug
Sep
Oct
Nov
Dec
Jan
Feb
Mar
Apr
May
Jun
Winter Solstice
December 21/Day 355
Winter Teletherm
December 24/Day 358
+3 days
Coverage
Year
SCOFIELD-SKYLINE MINE, UT
°F
Day of Year

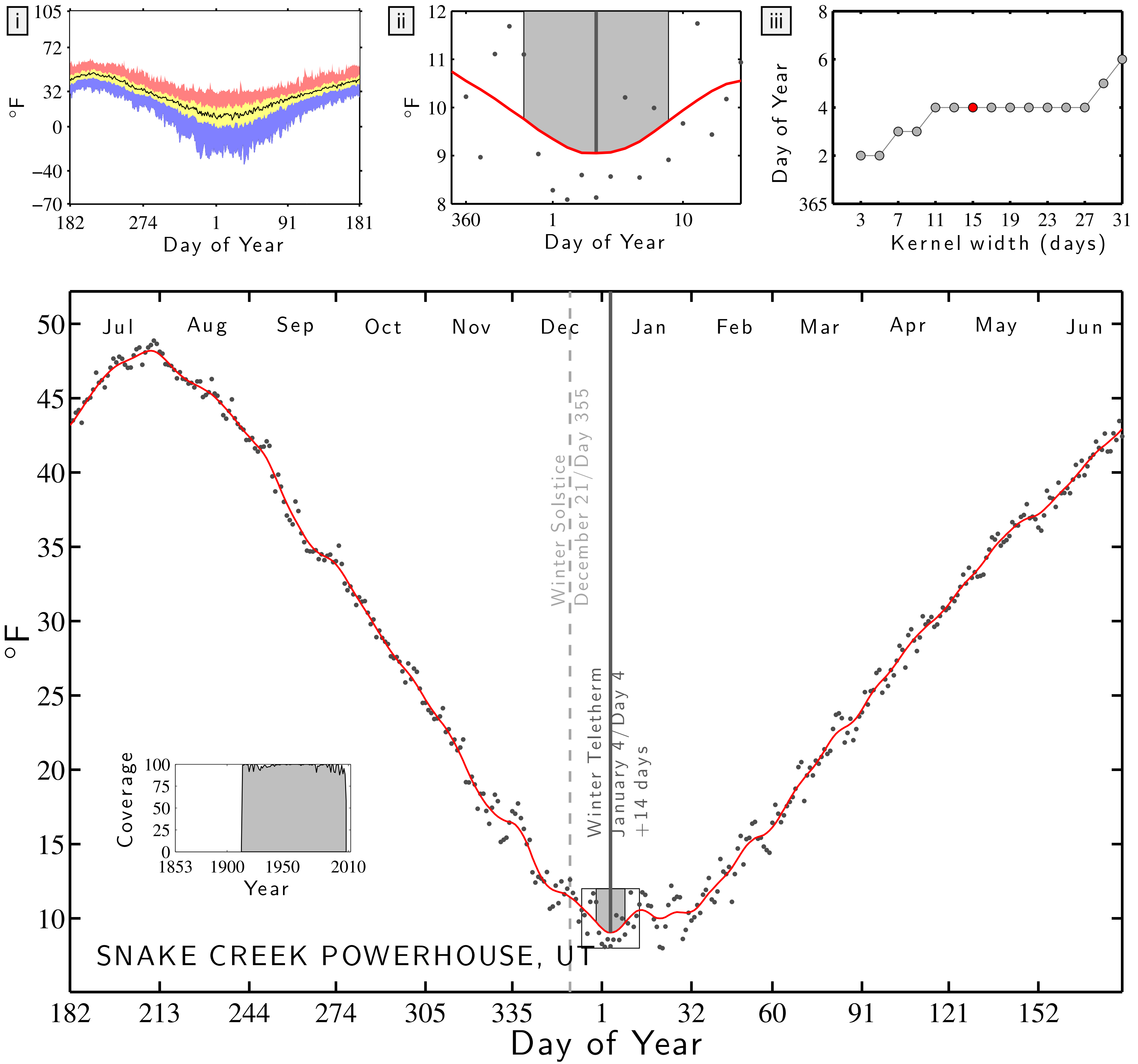

i
°F
Day of Year
ii
°F
Day of Year
iii
Day of Year
Kernel width (days)
Jul
Aug
Sep
Oct
Nov
Dec
Jan
Feb
Mar
Apr
May
Jun
Winter Solstice
December 21/Day 355
Winter Teletherm
January 4/Day 4
+14 days
Coverage
Year
SNAKE CREEK POWERHOUSE, UT
°F
Day of Year

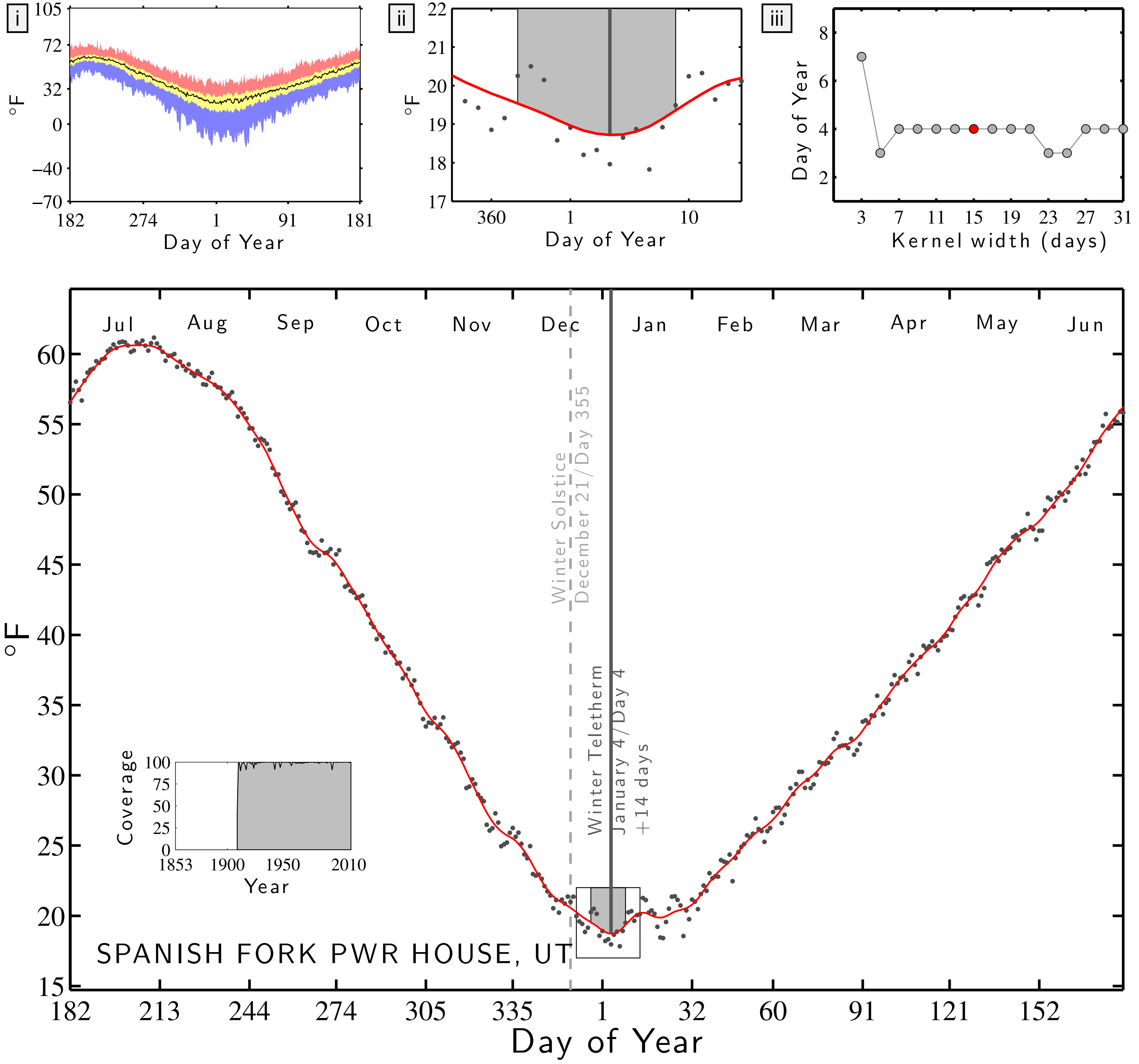

i
°F
Day of Year
ii
Day of Year
iii
Day of Year
Kernel width (days)
Jul
Aug
Sep
Oct
Nov
Dec
Jan
Feb
Mar
Apr
May
Jun
Winter Solstice
December 21/Day 355
Winter Teletherm
January 4/Day 4
+14 days
Coverage
Year
SPANISH FORK PWR HOUSE, UT
Day of Year

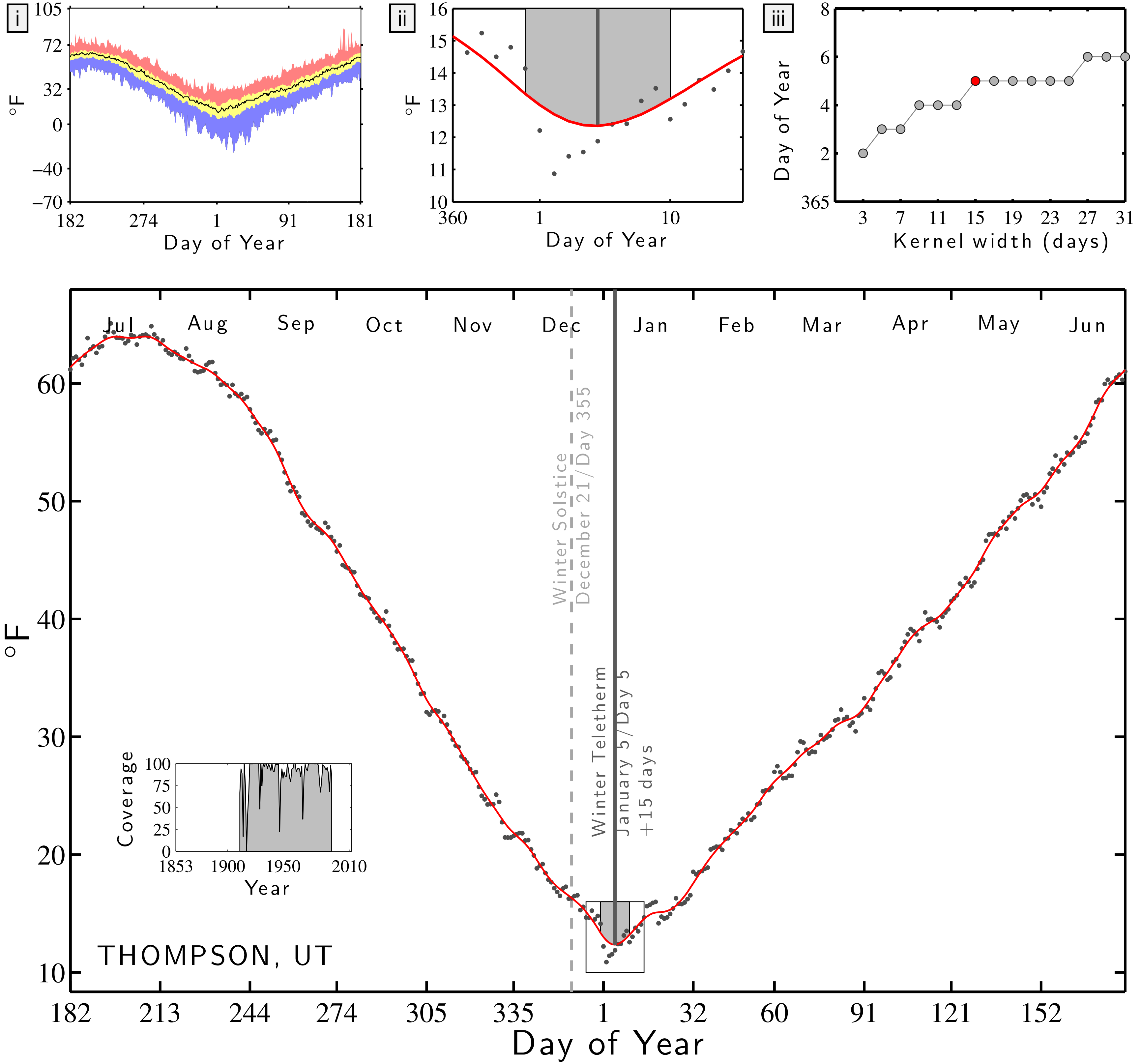

i
°F
Day of Year
ii
°F
Day of Year
iii
Day of Year
Kernel width (days)
Jul
Aug
Sep
Oct
Nov
Dec
Jan
Feb
Mar
Apr
May
Jun
Winter Solstice
December 21/Day 355
Winter Teletherm
January 5/Day 5
+15 days
Coverage
Year
THOMPSON, UT
°F
Day of Year

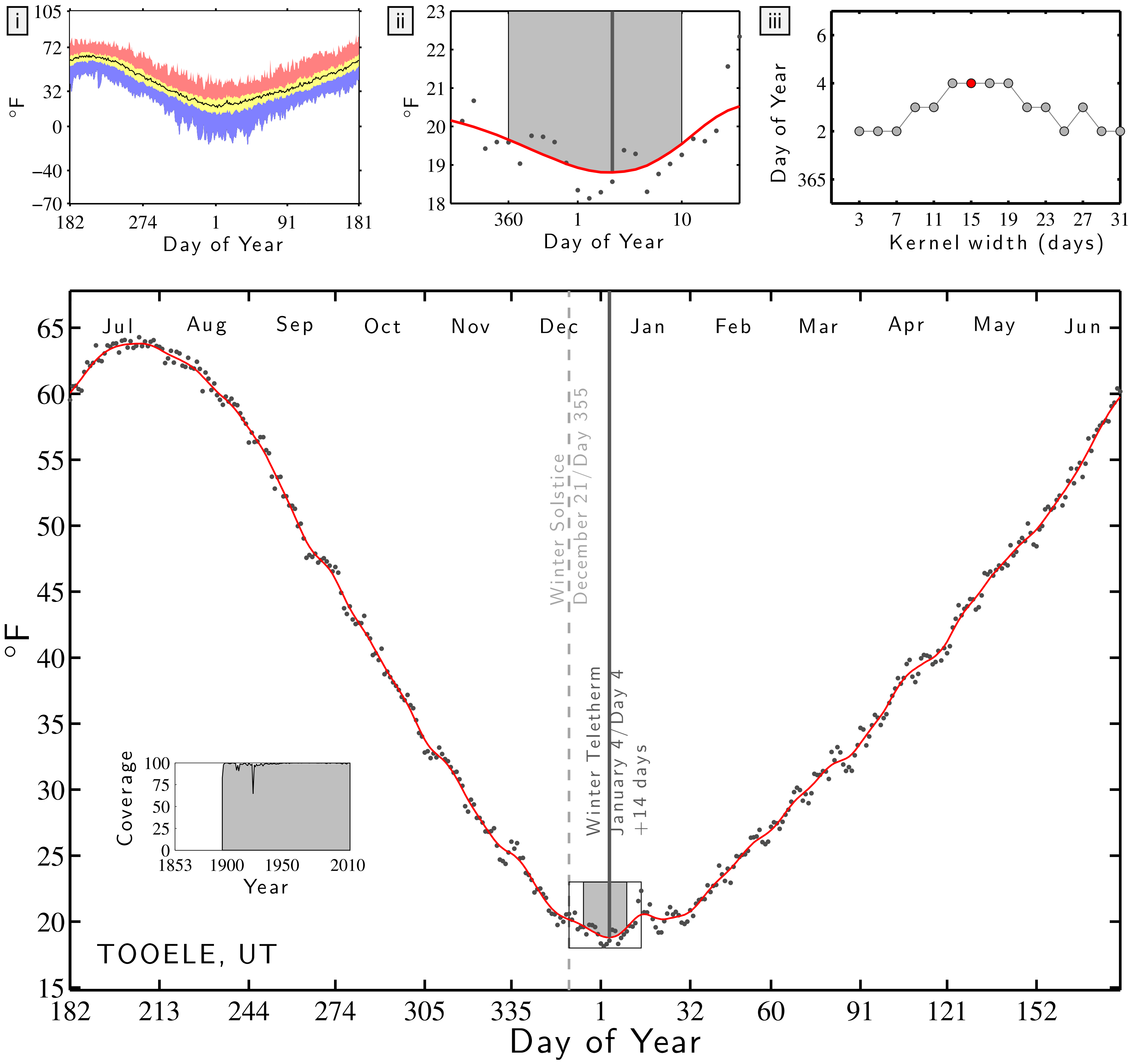

i
°F
Day of Year
ii
°F
Day of Year
iii
Day of Year
Kernel width (days)
Jul
Aug
Sep
Oct
Nov
Dec
Jan
Feb
Mar
Apr
May
Jun
Winter Solstice
December 21/Day 355
Winter Teletherm
January 4/Day 4
+14 days
Coverage
Year
TOOELE, UT
°F
Day of Year

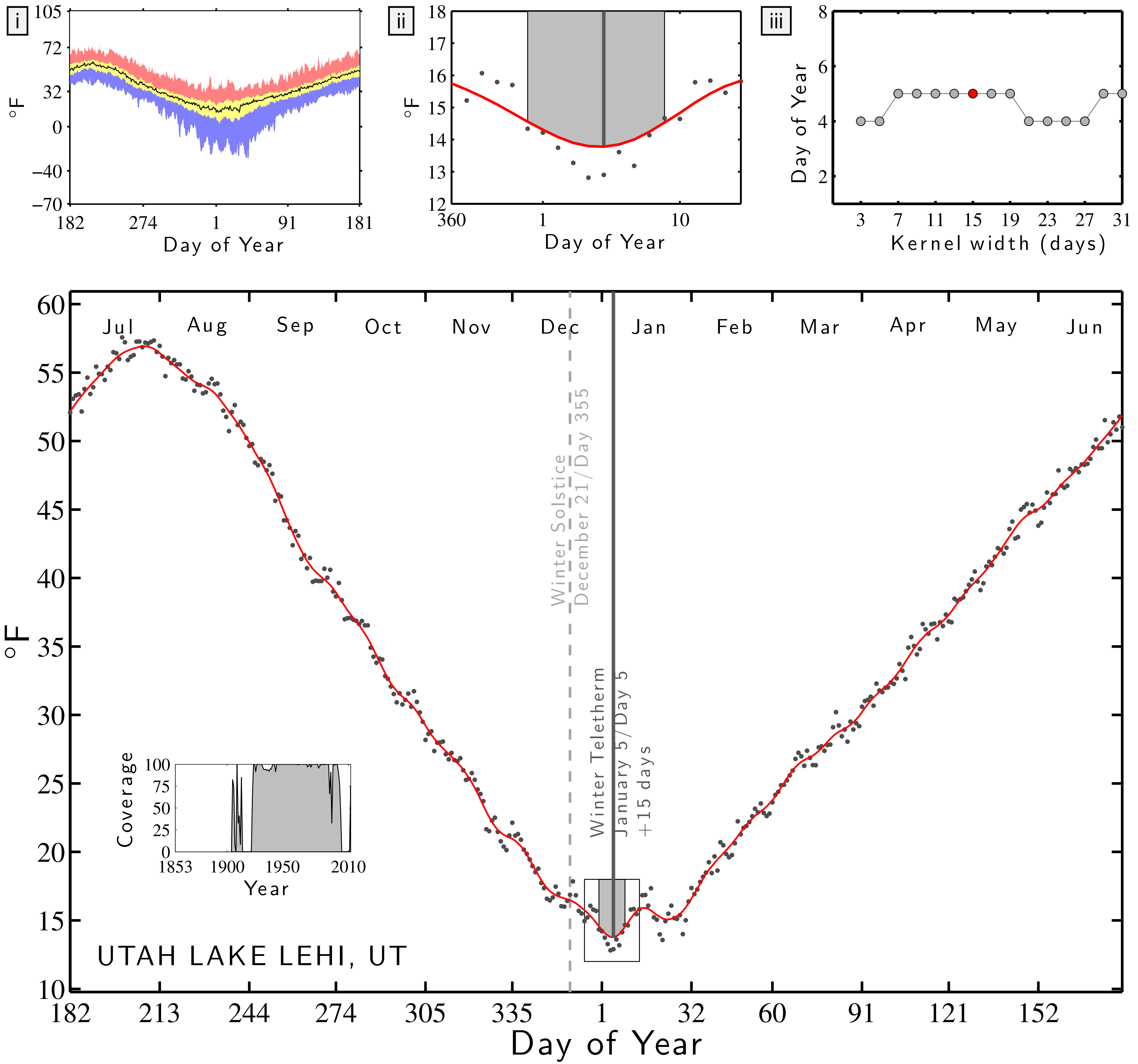

i
°F
Day of Year
ii
°F
Day of Year
iii
Day of Year
Kernel width (days)
Jul
Aug
Sep
Oct
Nov
Dec
Jan
Feb
Mar
Apr
May
Jun
Winter Solstice
December 21/Day 355
Winter Teletherm
January 5/Day 5
+15 days
Coverage
Year
UTAH LAKE LEHI, UT
°F
Day of Year

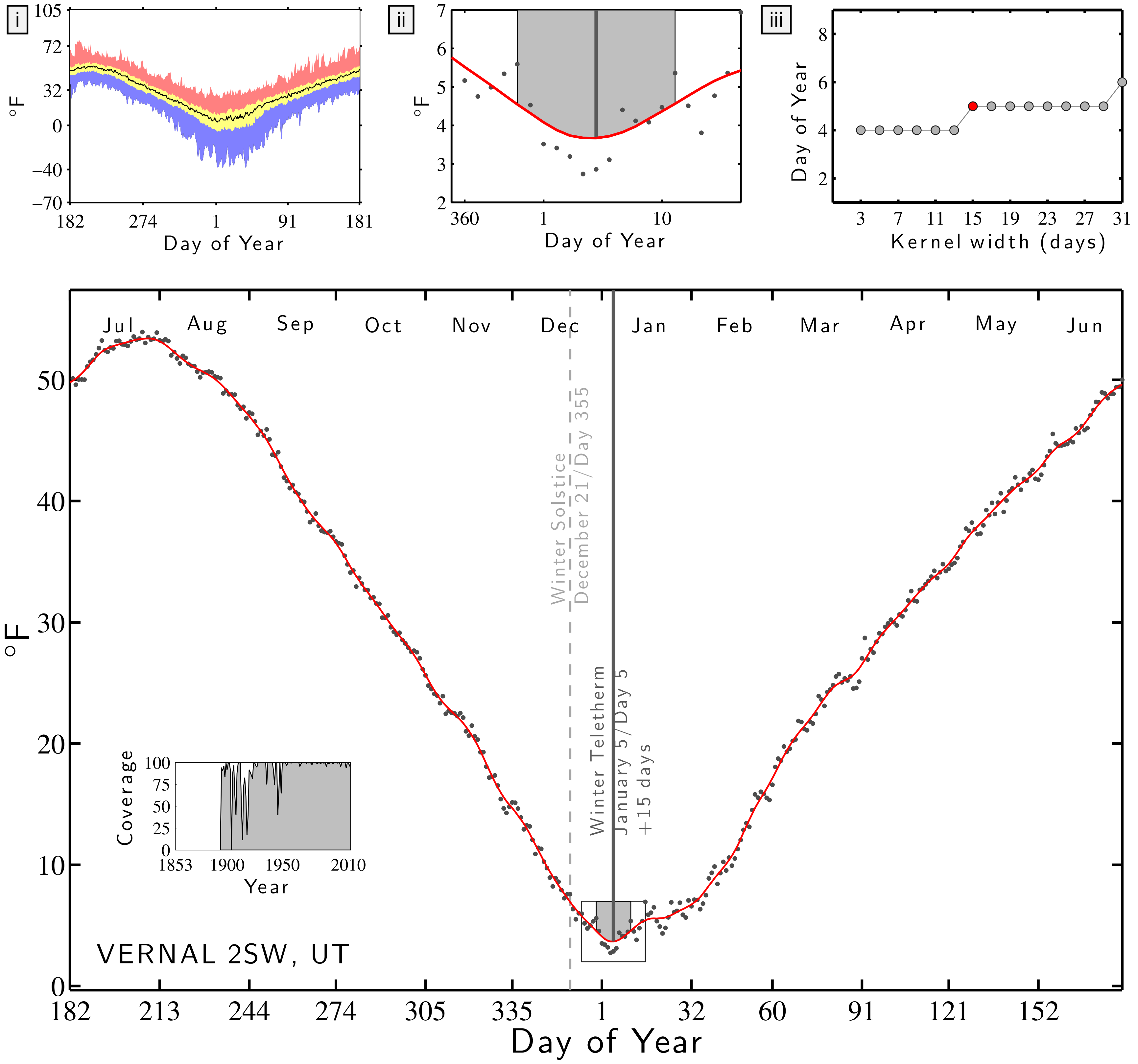

i
°F
Day of Year
ii
°F
Day of Year
iii
Day of Year
Kernel width (days)
Jul
Aug
Sep
Oct
Nov
Dec
Jan
Feb
Mar
Apr
May
Jun
Winter Solstice
December 21/Day 355
Winter Teletherm
January 5/Day 5
+15 days
Coverage
Year
VERNAL 2SW, UT
°F
Day of Year

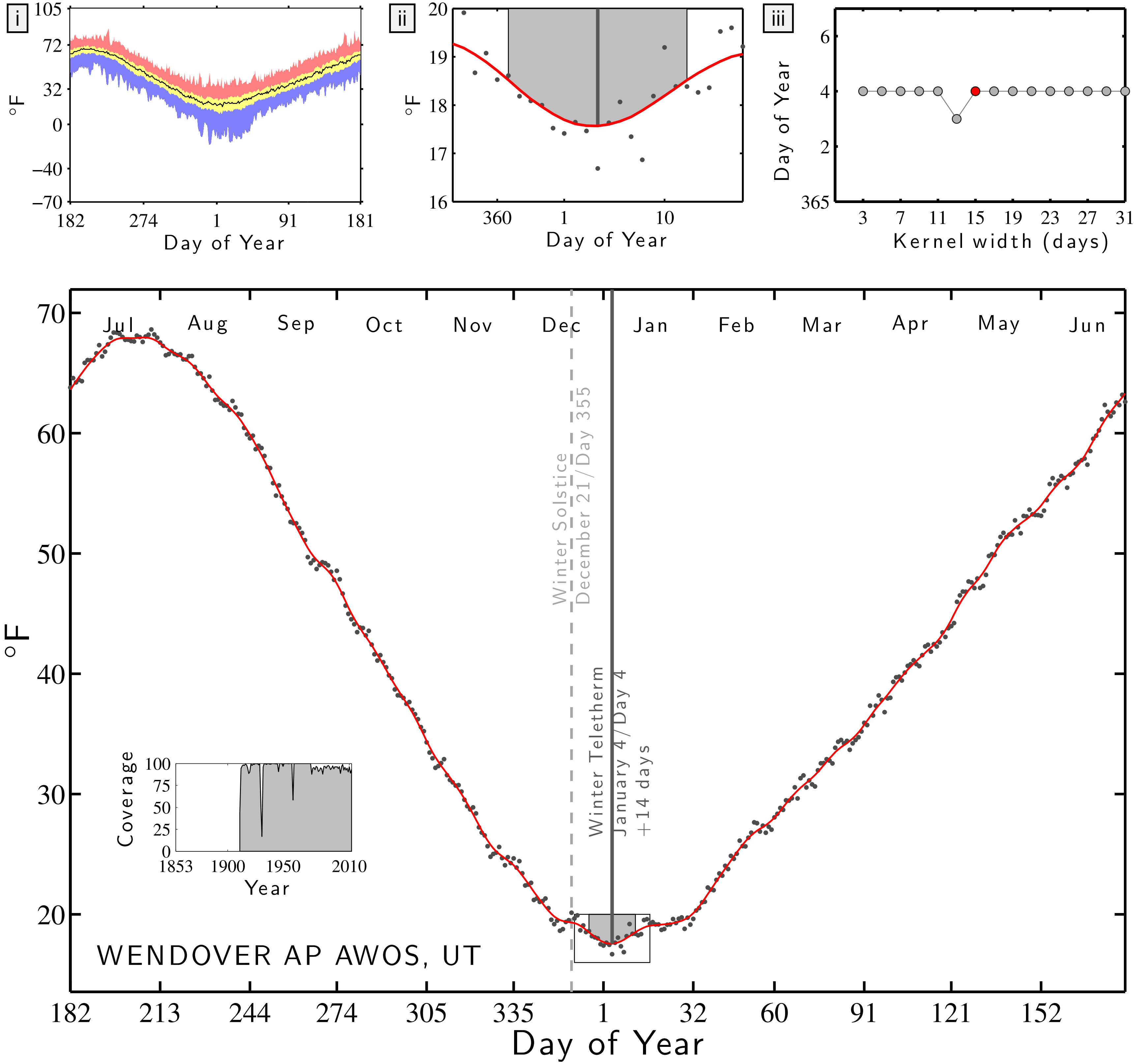

i
105
72
32
0
−40
−70
°F
182
274
1
91
181
Day of Year
ii
20
19
18
17
16
°F
360
1
10
Day of Year
iii
6
4
2
365
Day of Year
3
7
11
15
19
23
27
31
Kernel width (days)
70
60
50
40
30
20
°F
Jul
Aug
Sep
Oct
Nov
Dec
Jan
Feb
Mar
Apr
May
Jun
Winter Solstice
December 21/Day 355
Winter Teletherm
January 4/Day 4
+14 days
Coverage
100
75
50
25
0
1853
1900
1950
2010
Year
WENDOVER AP AWOS, UT
182
213
244
274
305
335
1
32
60
91
121
152
Day of Year

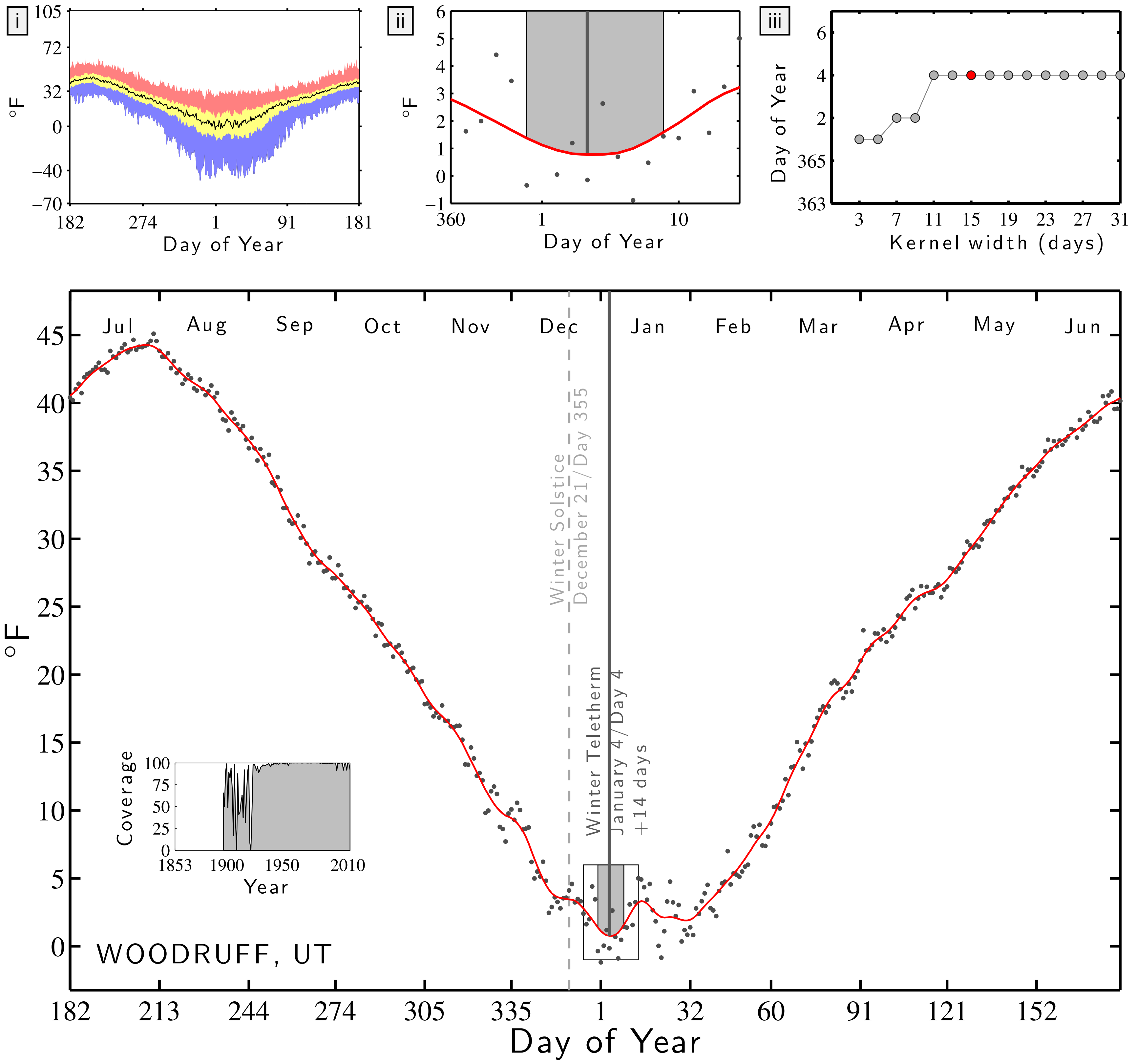

i
ii
iii
°F
Day of Year
Kernel width (days)
Jul
Aug
Sep
Oct
Nov
Dec
Jan
Feb
Mar
Apr
May
Jun
Winter Solstice
December 21/Day 355
Winter Teletherm
January 4/Day 4
+14 days
Coverage
Year
WOODRUFF, UT

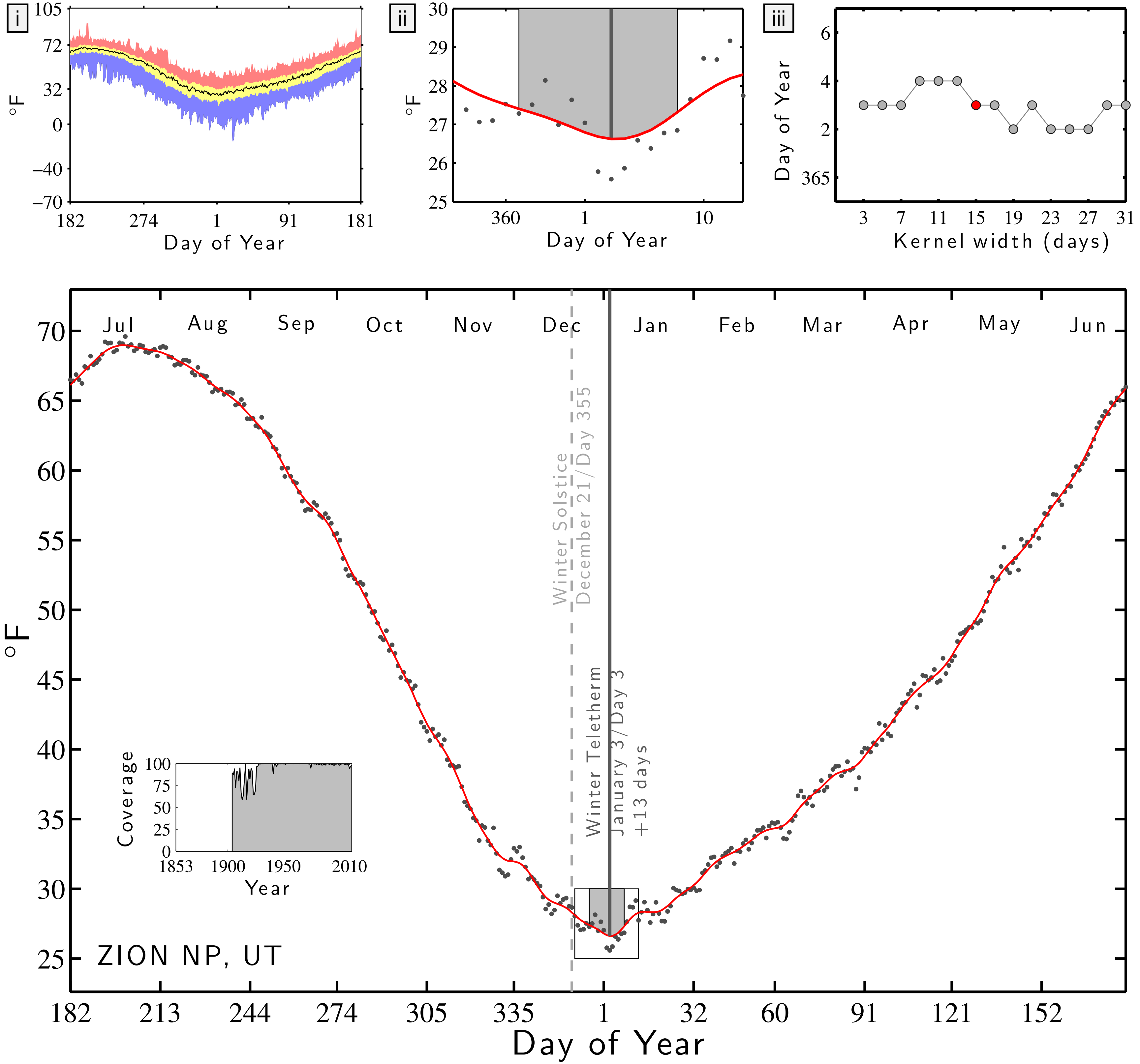

i
°F
Day of Year
ii
°F
Day of Year
iii
Day of Year
Kernel width (days)
Jul
Aug
Sep
Oct
Nov
Dec
Jan
Feb
Mar
Apr
May
Jun
Winter Solstice
December 21/Day 355
Winter Teletherm
January 3/Day 3
+13 days
Coverage
Year
ZION NP, UT
°F
Day of Year

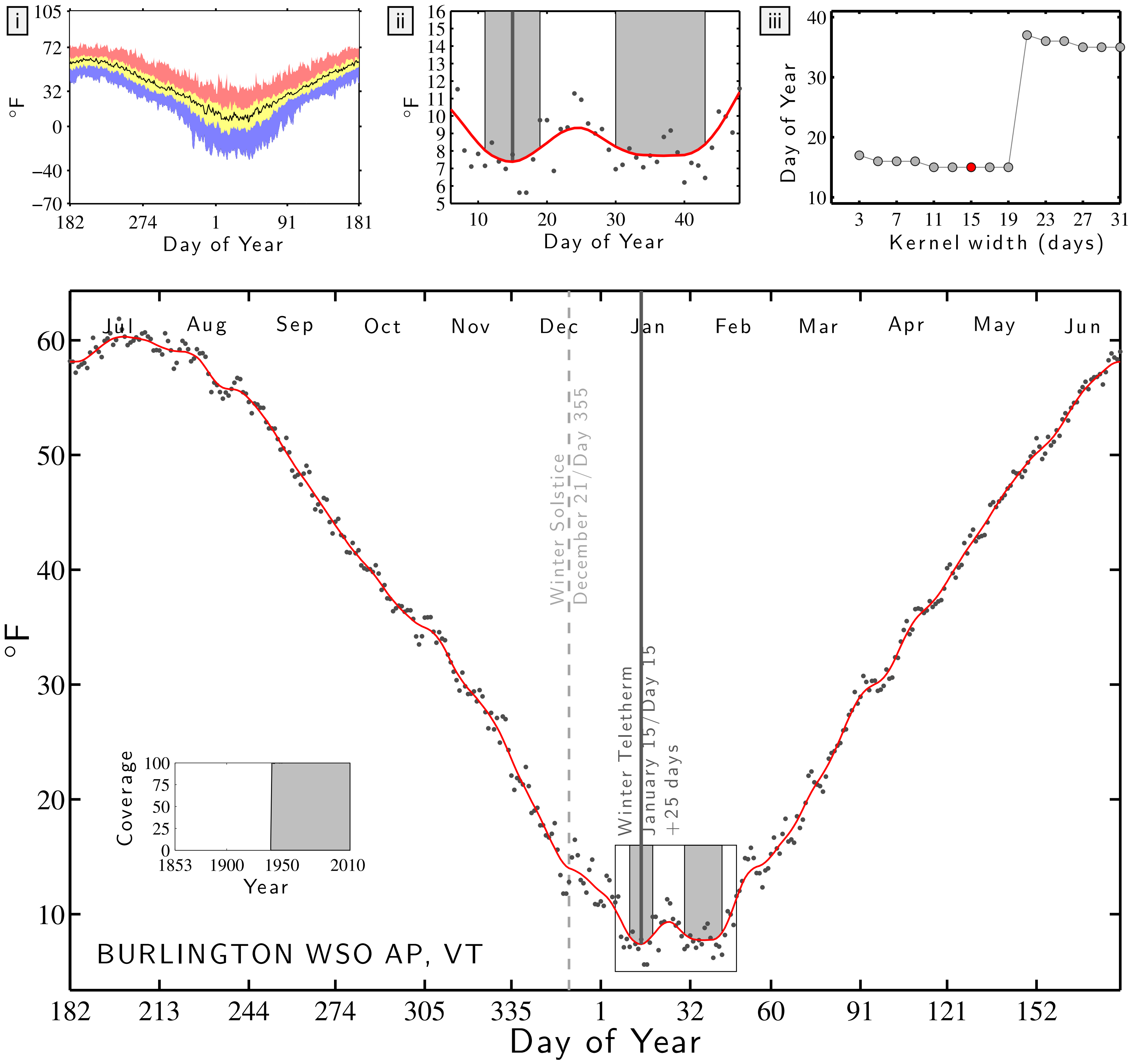

i
°F
Day of Year
ii
°F
Day of Year
iii
Day of Year
Kernel width (days)
Jul
Aug
Sep
Oct
Nov
Dec
Jan
Feb
Mar
Apr
May
Jun
Winter Solstice
December 21/Day 355
Winter Teletherm
January 15/Day 15
+25 days
Coverage
Year
BURLINGTON WSO AP, VT
°F
Day of Year

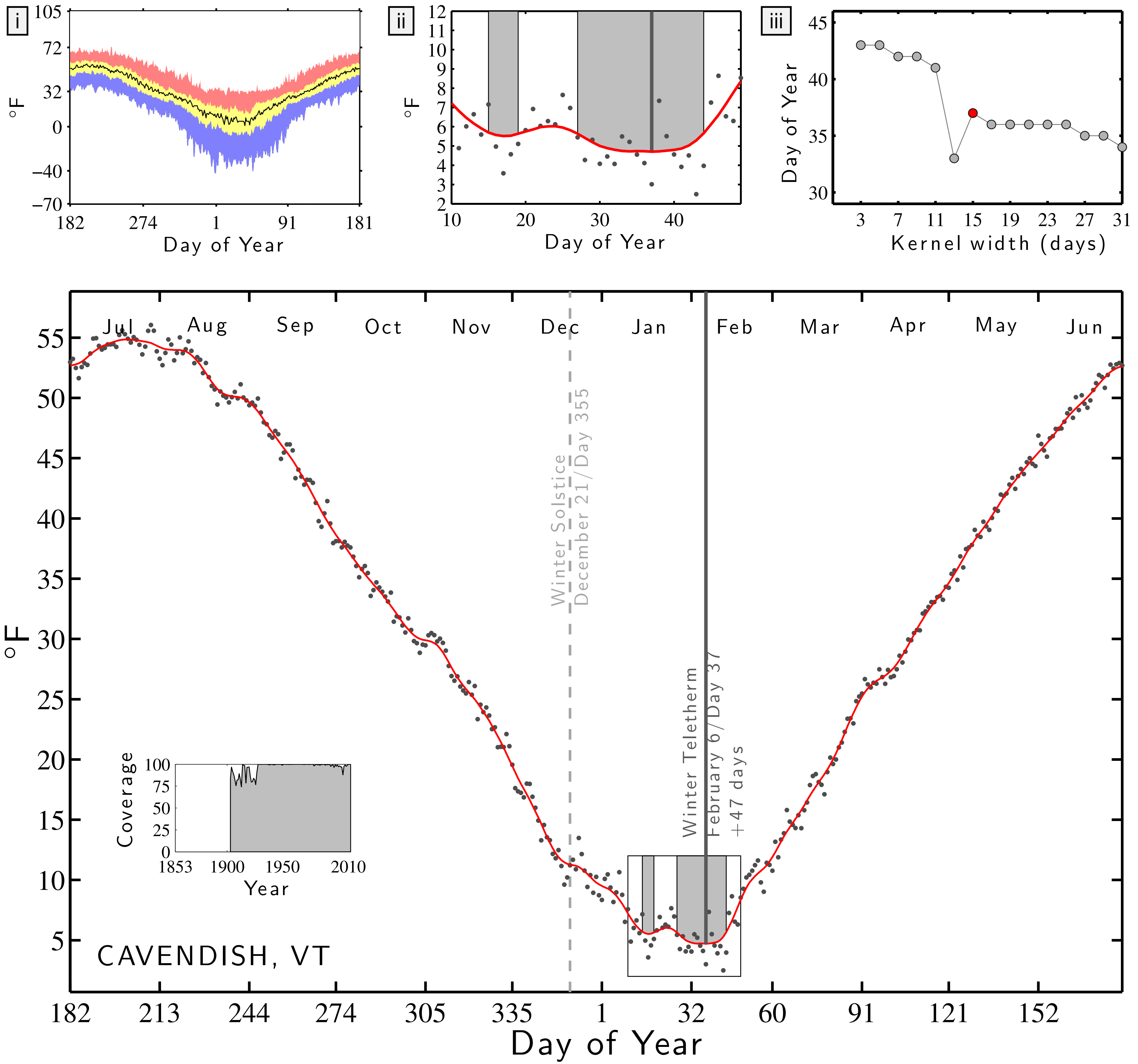

i
ii
iii
°F
Day of Year
Kernel width (days)
Jul
Aug
Sep
Oct
Nov
Dec
Jan
Feb
Mar
Apr
May
Jun
Winter Solstice
December 21/Day 355
Winter Teletherm
February 6/Day 37
+47 days
Coverage
Year
CAVENDISH, VT

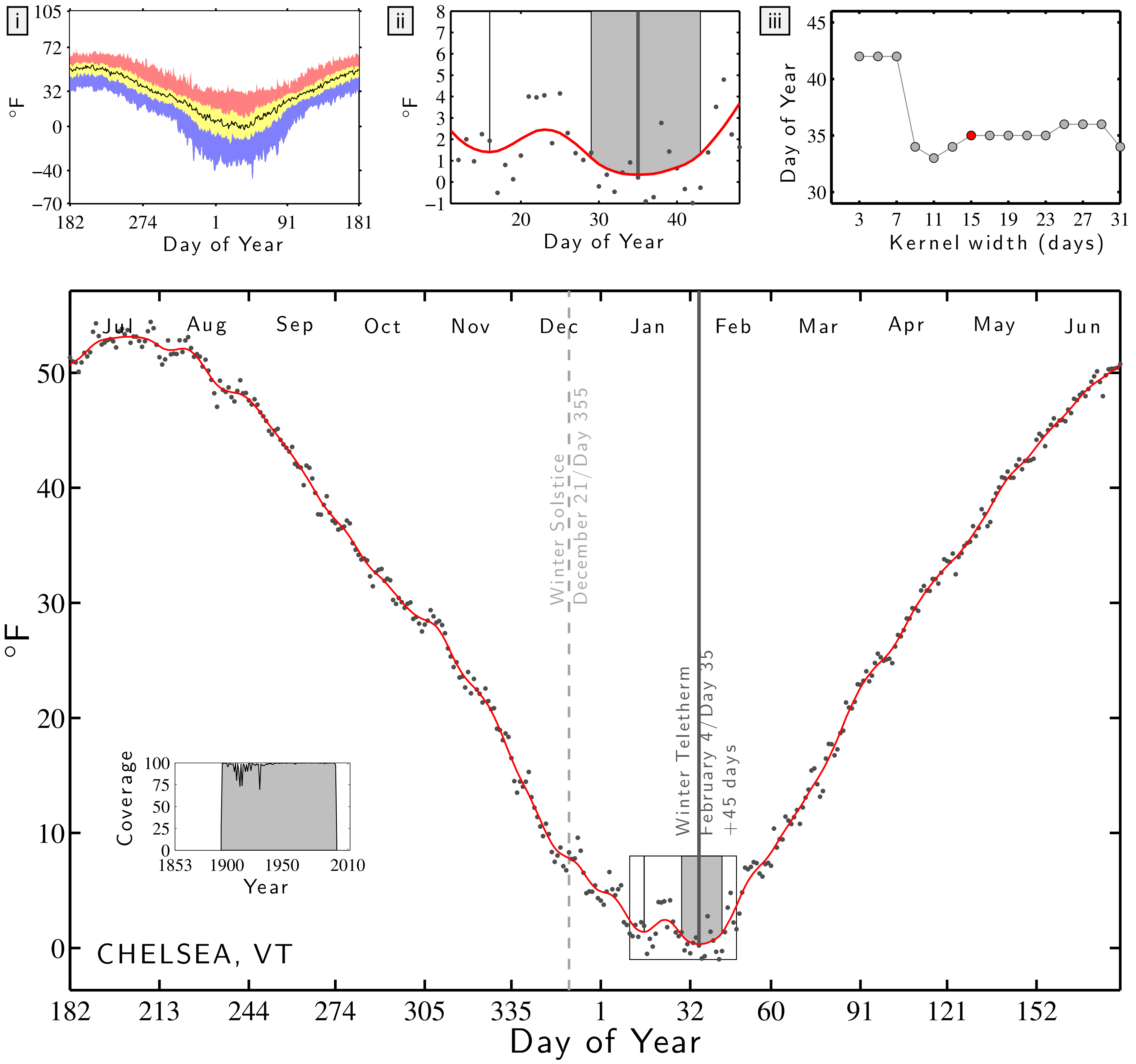

i
°F
Day of Year
ii
°F
Day of Year
iii
Day of Year
Kernel width (days)
Jul
Aug
Sep
Oct
Nov
Dec
Jan
Feb
Mar
Apr
May
Jun
Winter Solstice
December 21/Day 355
Winter Teletherm
February 4/Day 35
+45 days
Coverage
Year
CHELSEA, VT
°F
Day of Year

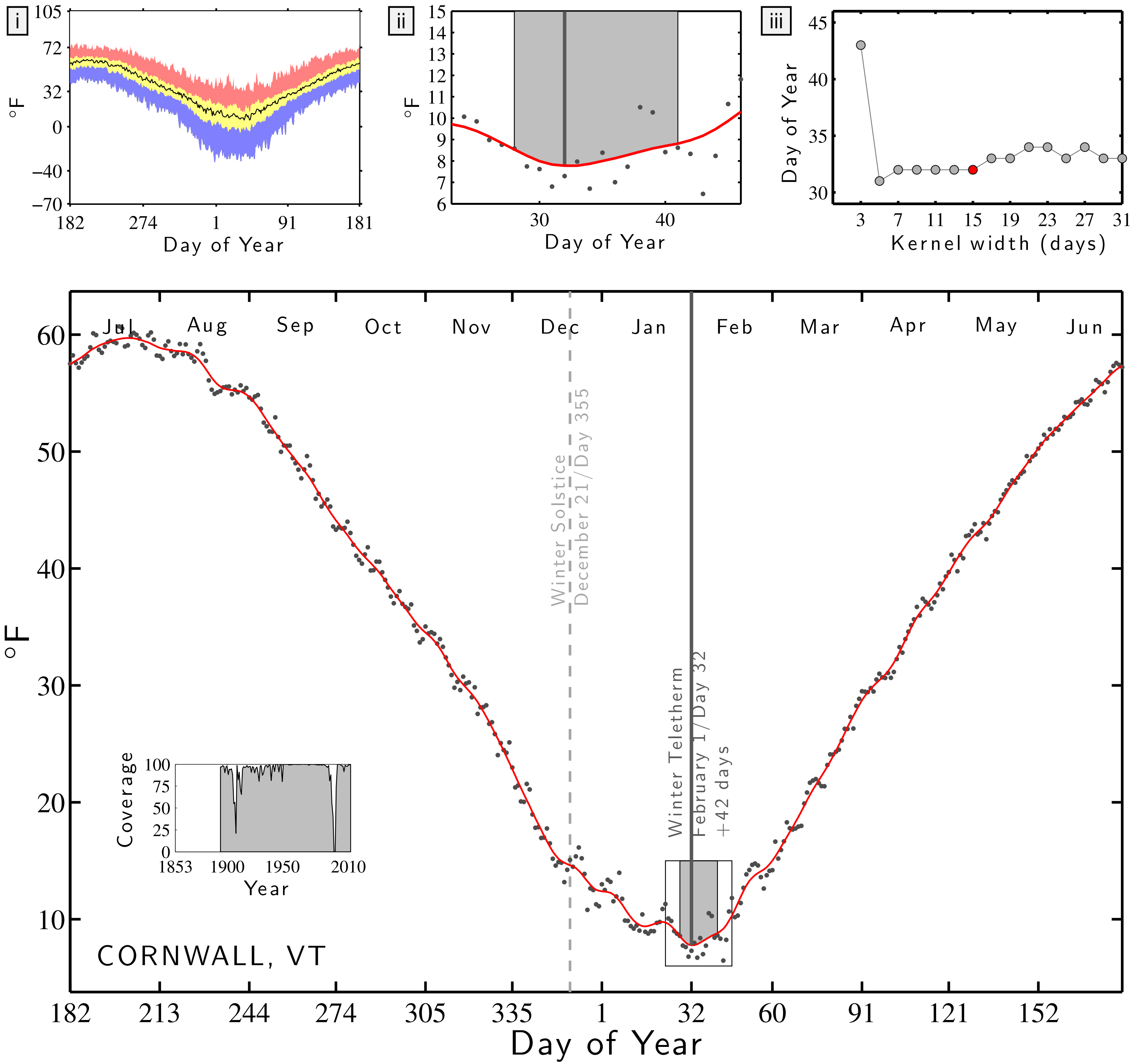
i
°F
Day of Year
ii
°F
Day of Year
iii
Day of Year
Kernel width (days)
Jul
Aug
Sep
Oct
Nov
Dec
Jan
Feb
Mar
Apr
May
Jun
Winter Solstice
December 21/Day 355
Winter Teletherm
February 1/Day 32
+42 days
Coverage
Year
CORNWALL, VT
°F
Day of Year

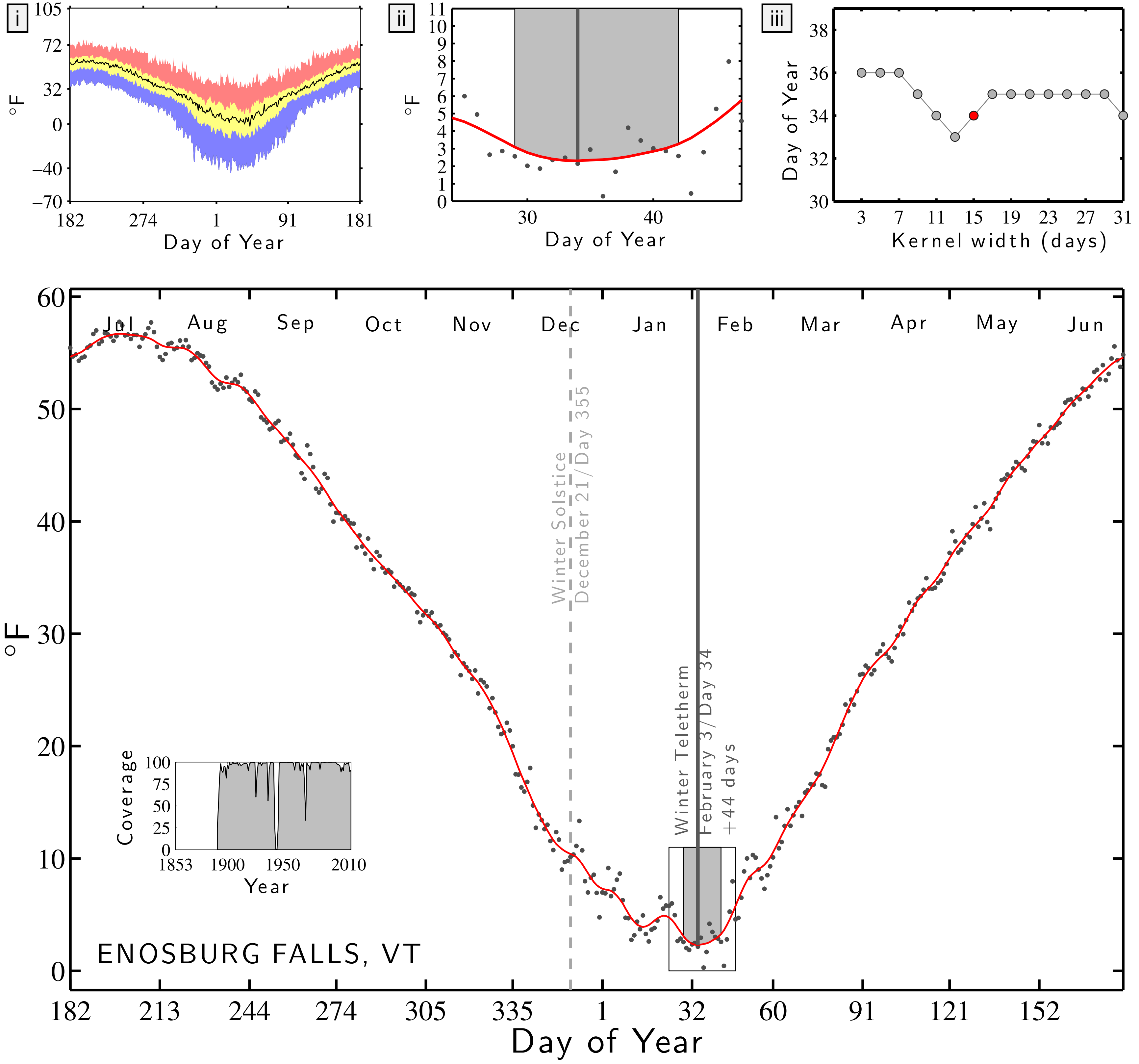

i
°F
Day of Year
ii
°F
Day of Year
iii
Day of Year
Kernel width (days)
Jul
Aug
Sep
Oct
Nov
Dec
Jan
Feb
Mar
Apr
May
Jun
Winter Solstice
December 21/Day 355
Winter Teletherm
February 3/Day 34
+44 days
Coverage
Year
ENOSBURG FALLS, VT
°F
Day of Year

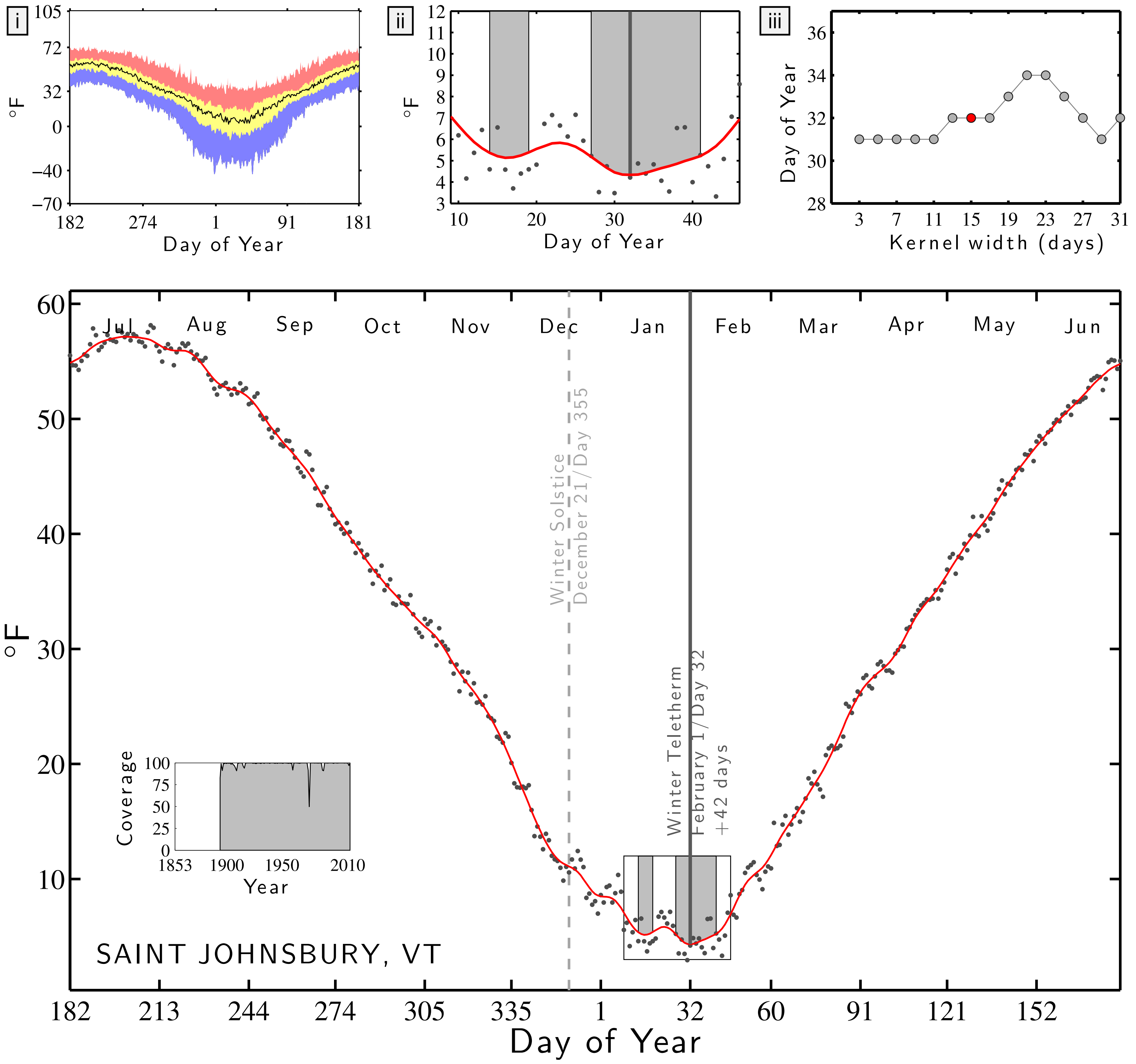
i
°F
Day of Year
ii
°F
Day of Year
iii
Day of Year
Kernel width (days)
Jul
Aug
Sep
Oct
Nov
Dec
Jan
Feb
Mar
Apr
May
Jun
Winter Solstice
December 21/Day 355
Winter Teletherm
February 1/Day 32
+42 days
Coverage
Year
SAINT JOHNSBURY, VT
°F
Day of Year

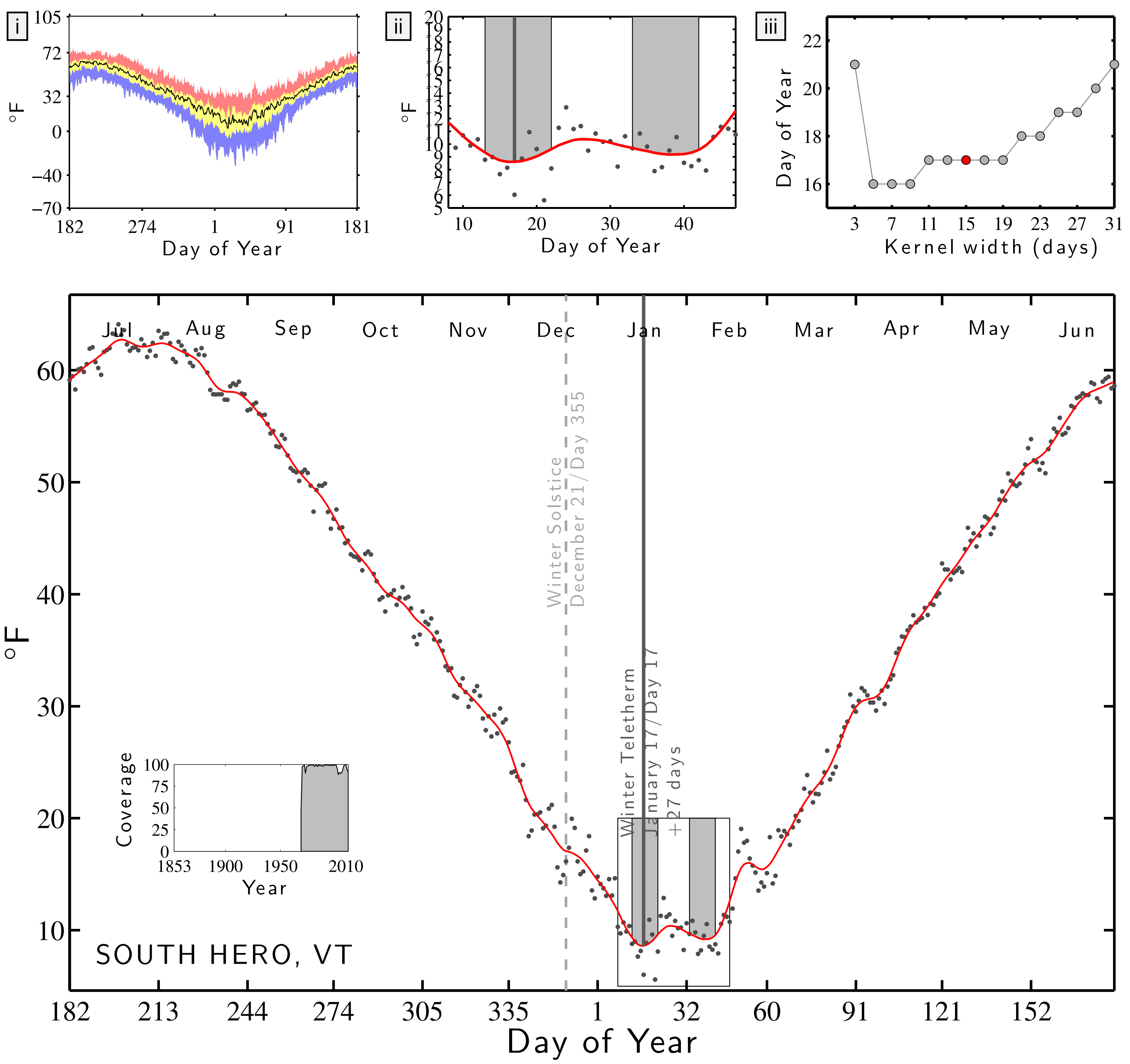

i
°F
Day of Year
ii
°F
Day of Year
iii
Day of Year
Kernel width (days)
Jul
Aug
Sep
Oct
Nov
Dec
Jan
Feb
Mar
Apr
May
Jun
Winter Solstice
December 21/Day 355
Winter Teletherm
January 17/Day 17
±27 days
Coverage
Year
SOUTH HERO, VT
°F
Day of Year

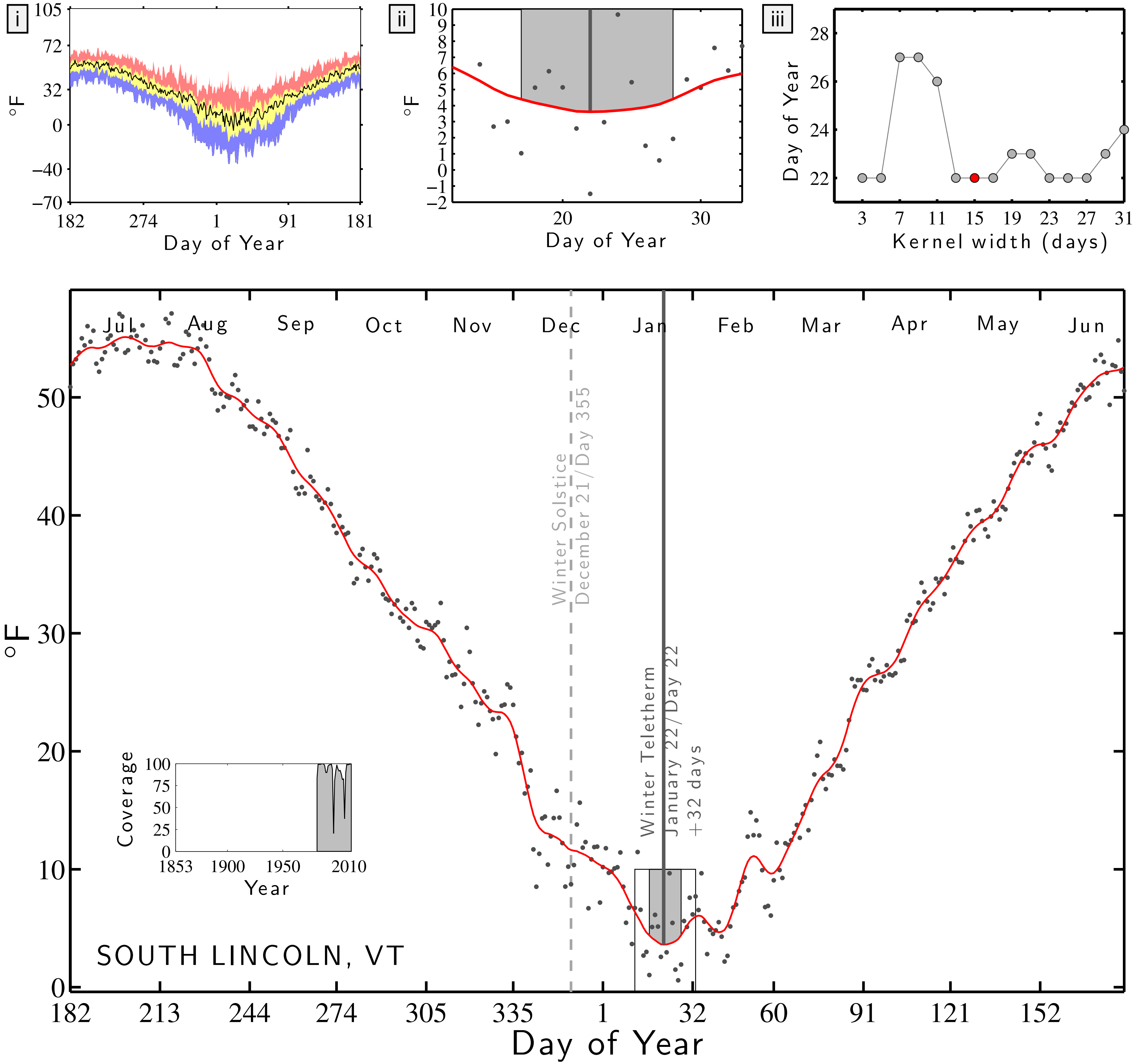

i
°F
Day of Year
ii
°F
Day of Year
iii
Day of Year
Kernel width (days)
Jul
Aug
Sep
Oct
Nov
Dec
Jan
Feb
Mar
Apr
May
Jun
Winter Solstice
December 21/Day 355
Winter Teletherm
January 22/Day 22
+32 days
Coverage
Year
SOUTH LINCOLN, VT
°F
Day of Year

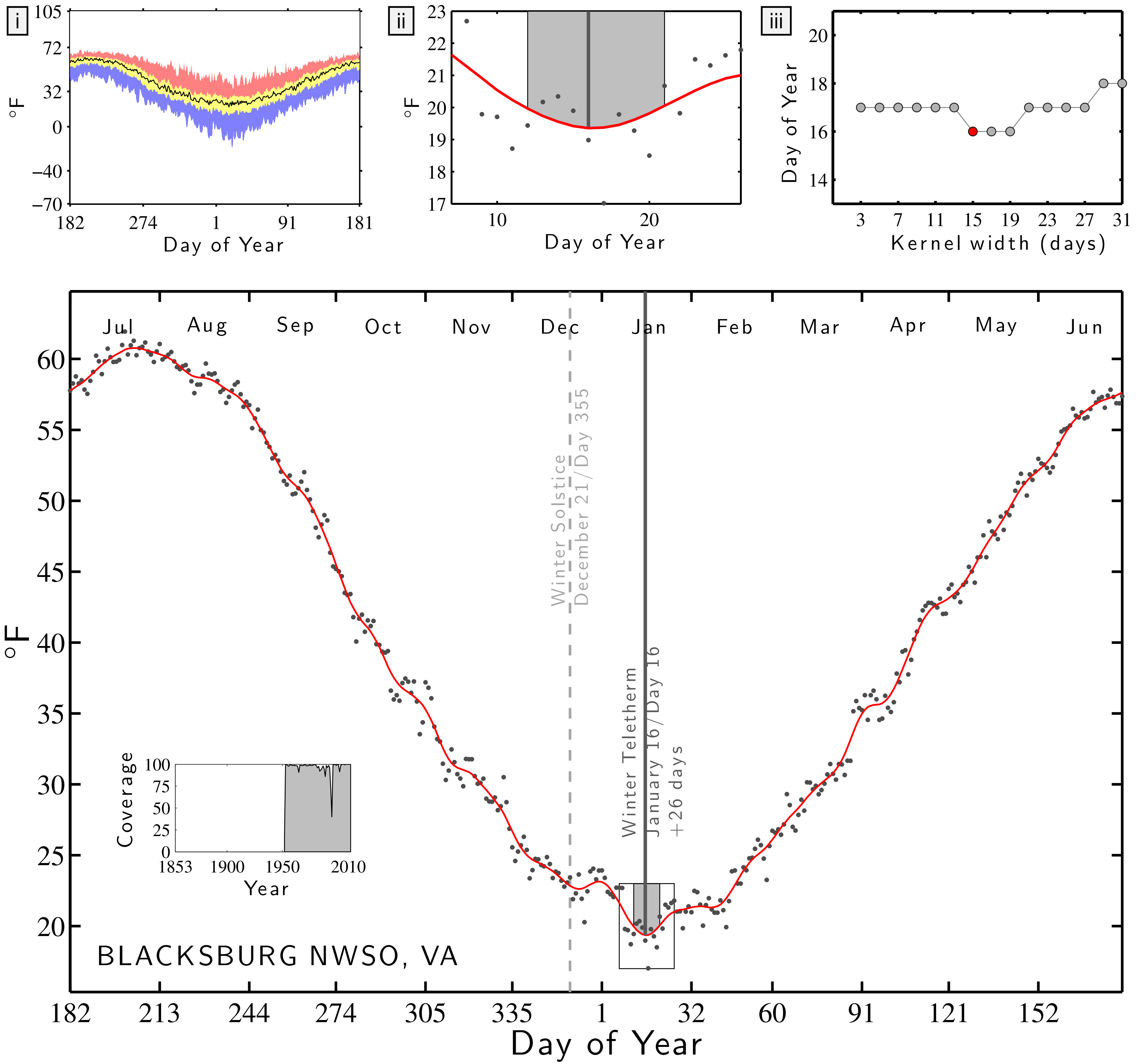
i
°F
Day of Year
ii
°F
Day of Year
iii
Day of Year
Kernel width (days)
Jul
Aug
Sep
Oct
Nov
Dec
Jan
Feb
Mar
Apr
May
Jun
Winter Solstice
December 21/Day 355
Winter Teletherm
January 16/Day 16
+26 days
Coverage
Year
BLACKSBURG NWSO, VA
°F
Day of Year

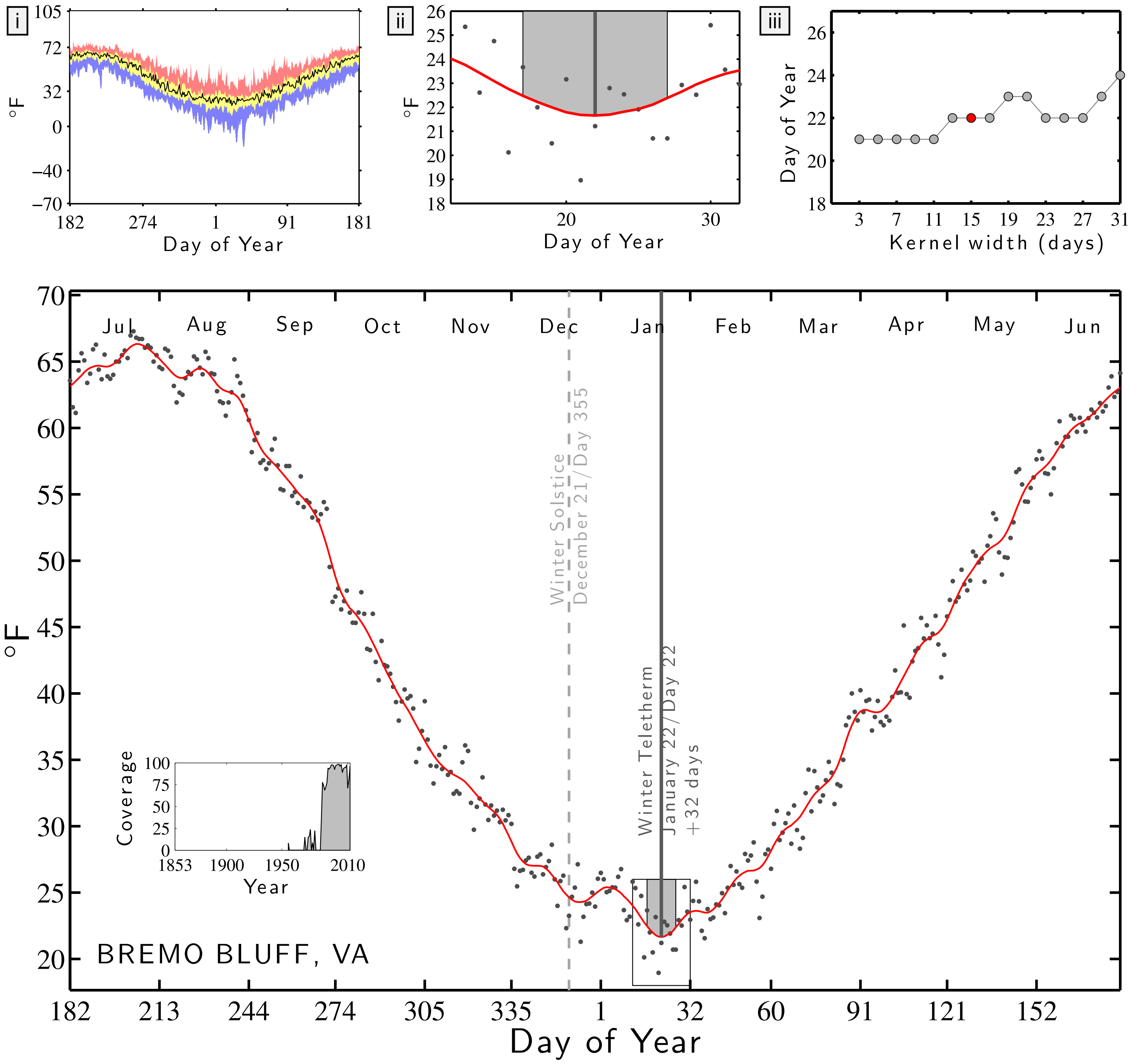

i
ii
iii
°F
Day of Year
Day of Year
Kernel width (days)
Jul
Aug
Sep
Oct
Nov
Dec
Jan
Feb
Mar
Apr
May
Jun
Winter Solstice
December 21/Day 355
Winter Teletherm
January 22/Day 22
+32 days
Coverage
Year
BREMO BLUFF, VA

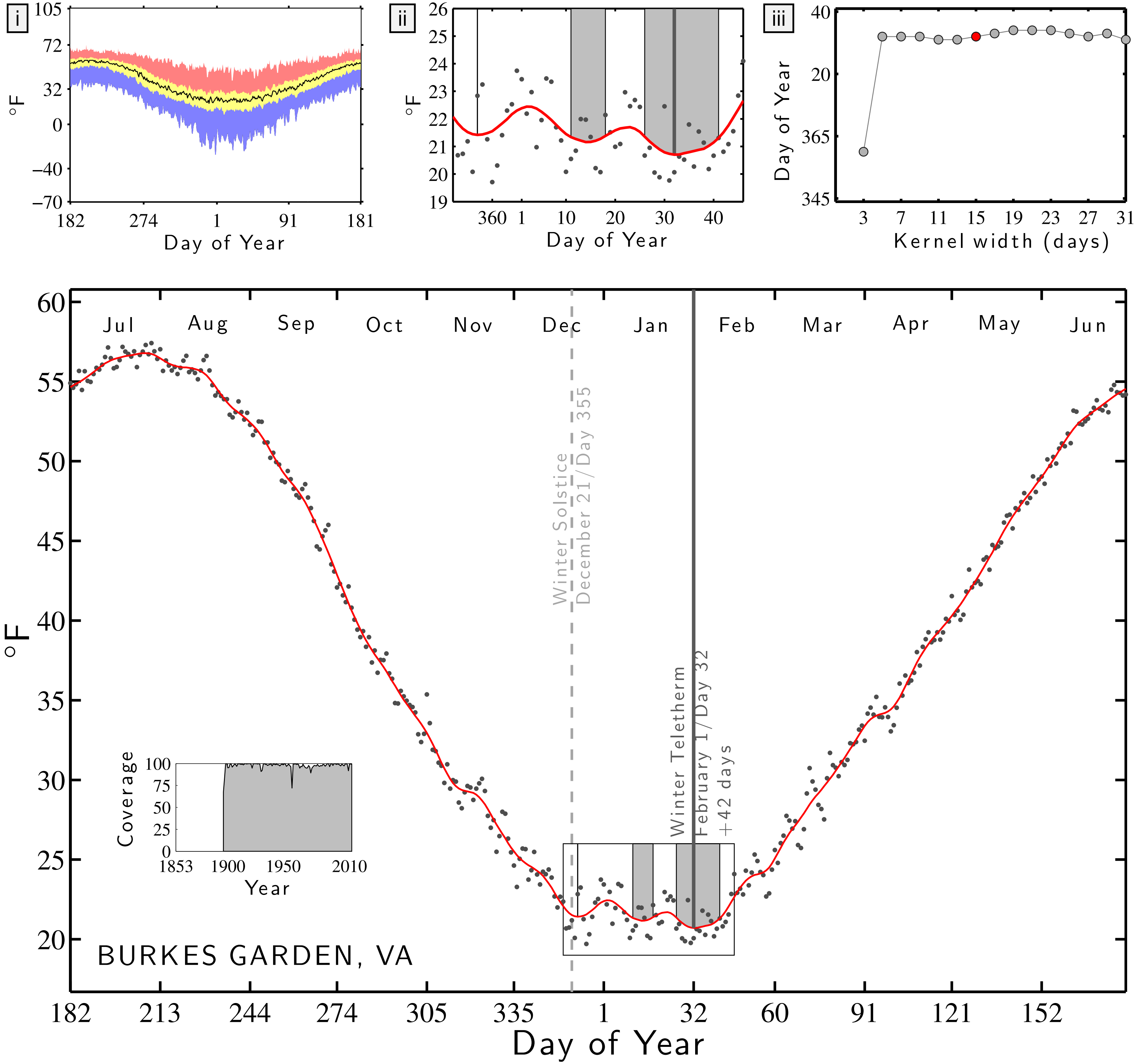

i
°F
Day of Year
ii
°F
Day of Year
iii
Day of Year
Kernel width (days)
Jul
Aug
Sep
Oct
Nov
Dec
Jan
Feb
Mar
Apr
May
Jun
Winter Solstice
December 21/Day 355
Winter Teletherm
February 1/Day 32
+42 days
Coverage
Year
BURKES GARDEN, VA
°F
Day of Year

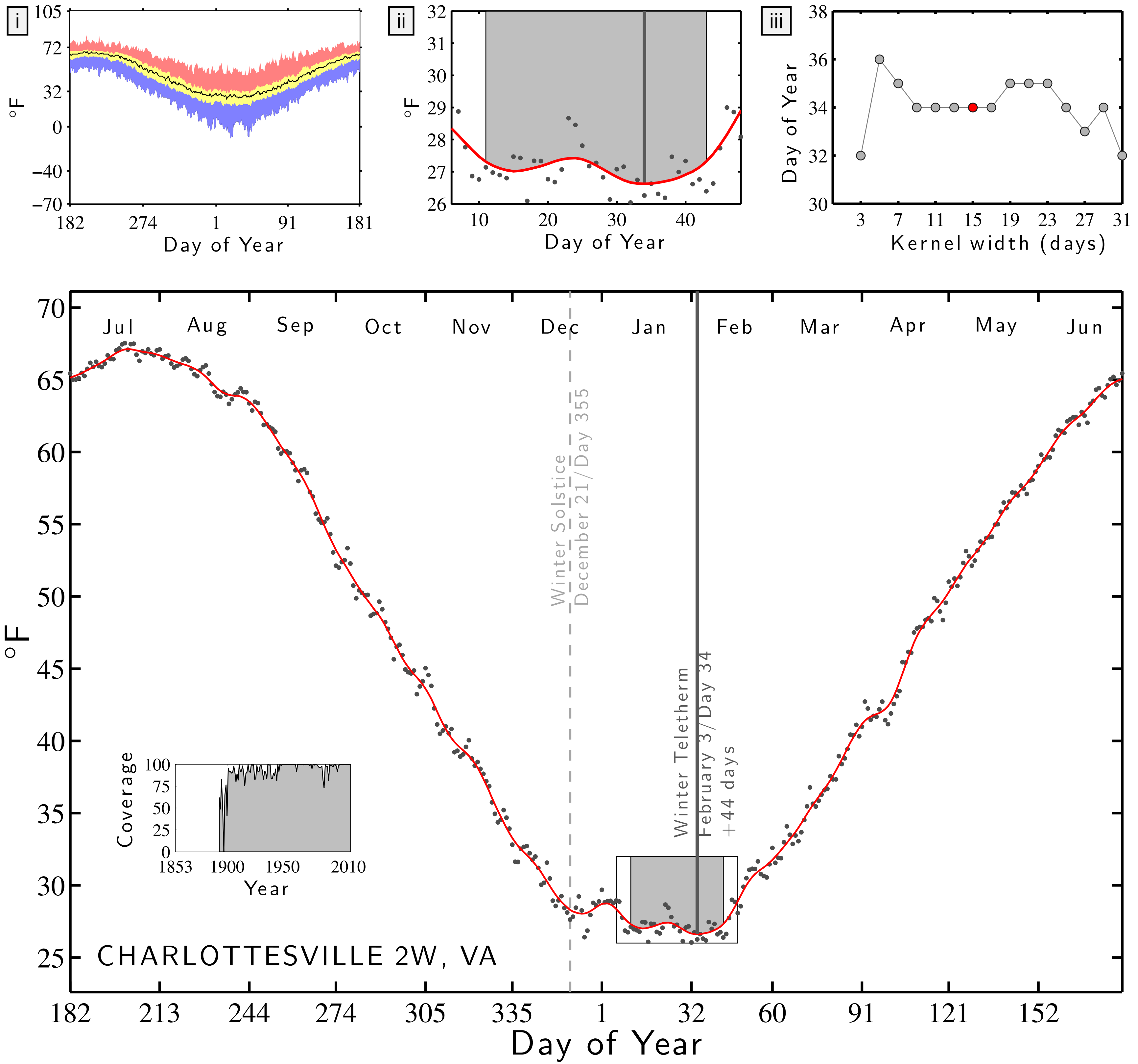

i
ii
iii
Day of Year
°F
Day of Year
Kernel width (days)
Jul
Aug
Sep
Oct
Nov
Dec
Jan
Feb
Mar
Apr
May
Jun
Winter Solstice
December 21/Day 355
Winter Teletherm
February 3/Day 34
+44 days
Coverage
Year
CHARLOTTESVILLE 2W, VA
Day of Year

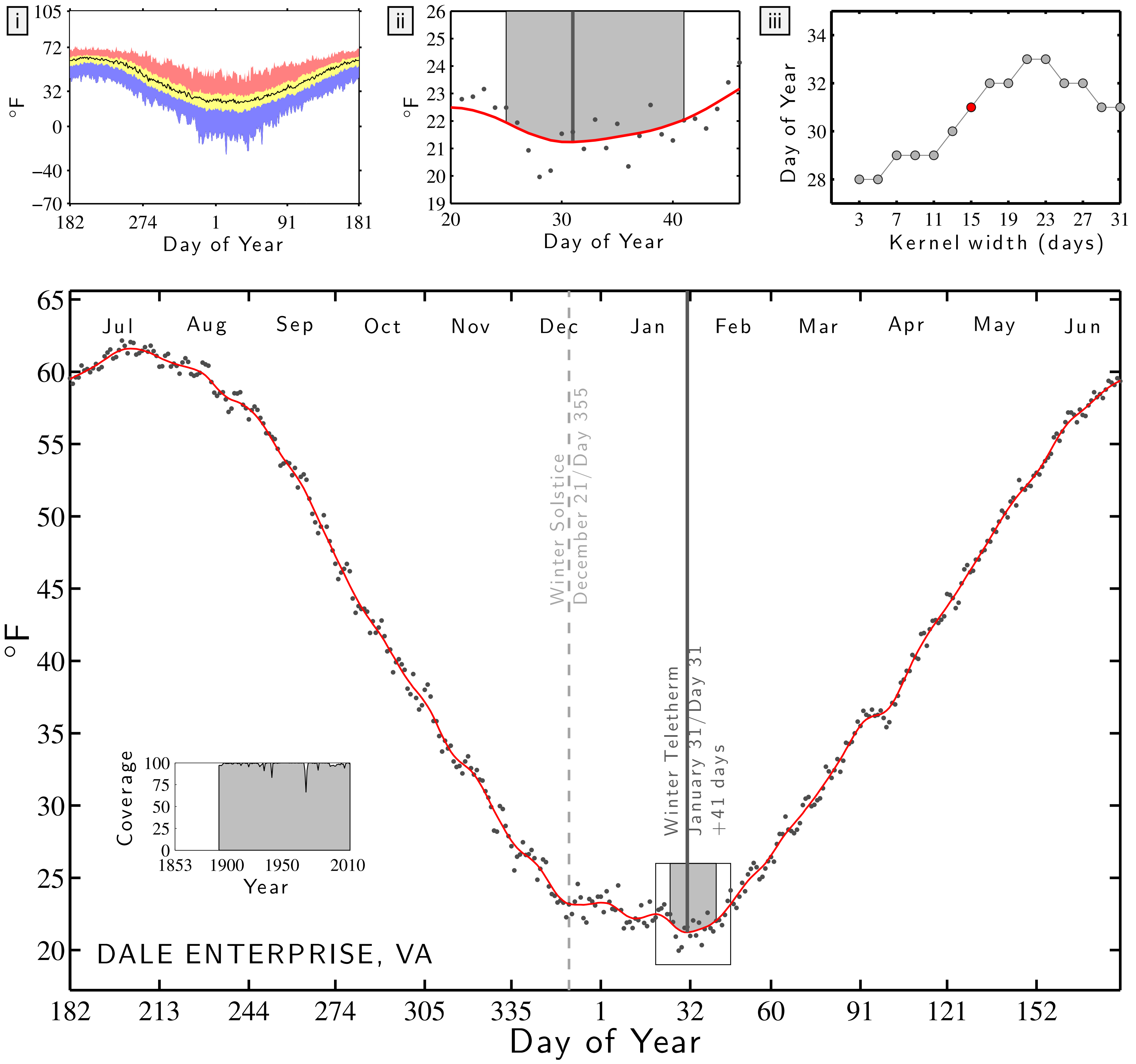

i
ii
iii
°F
Day of Year
Kernel width (days)
Day of Year
Jul
Aug
Sep
Oct
Nov
Dec
Jan
Feb
Mar
Apr
May
Jun
Winter Solstice
December 21/Day 355
Winter Teletherm
January 31/Day 31
+41 days
Coverage
Year
DALE ENTERPRISE, VA

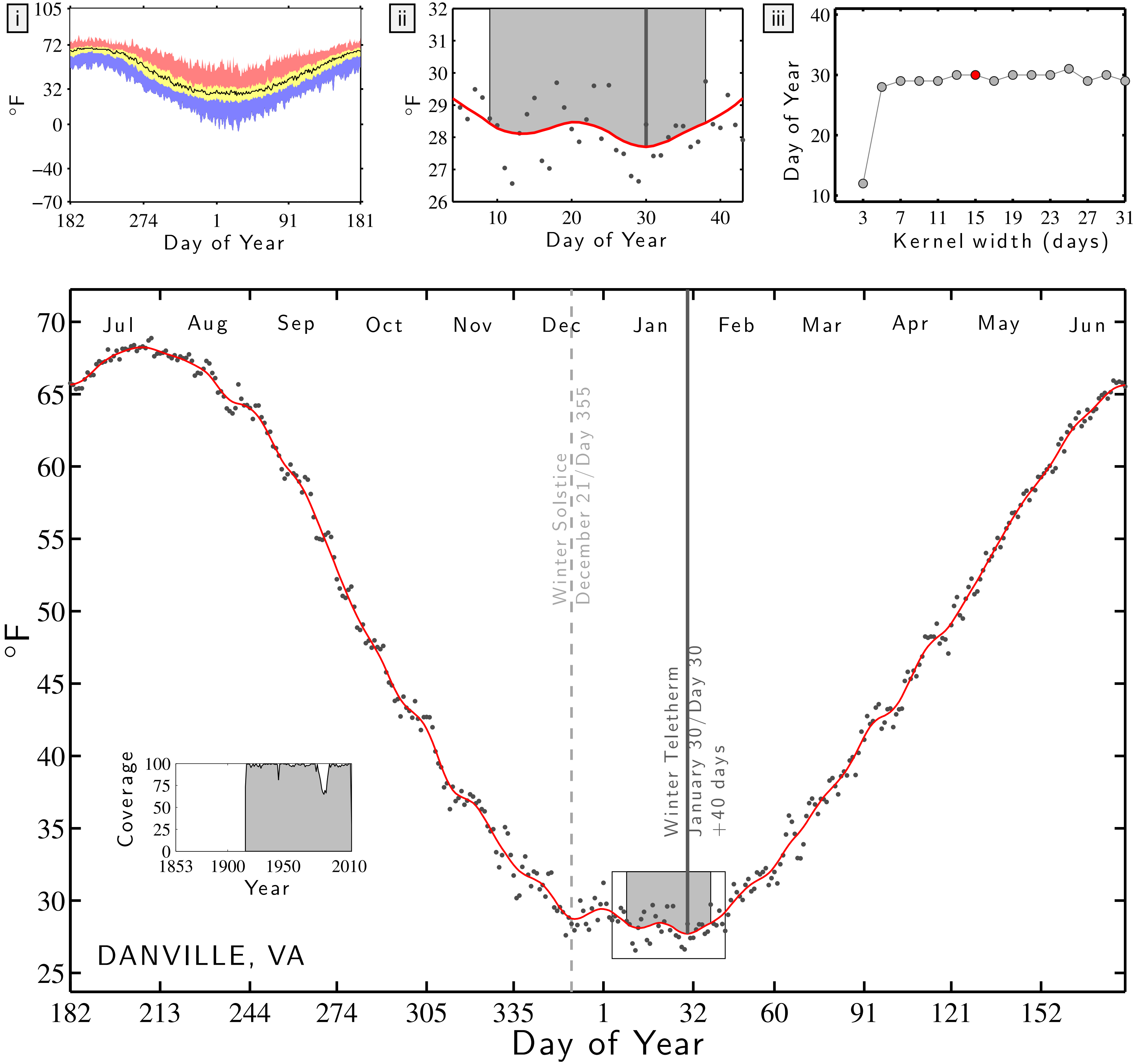

i
ii
iii
°F
Day of Year
Day of Year
Kernel width (days)
Jul
Aug
Sep
Oct
Nov
Dec
Jan
Feb
Mar
Apr
May
Jun
Winter Solstice
December 21/Day 355
Winter Teletherm
January 30/Day 30
+40 days
Coverage
Year
DANVILLE, VA

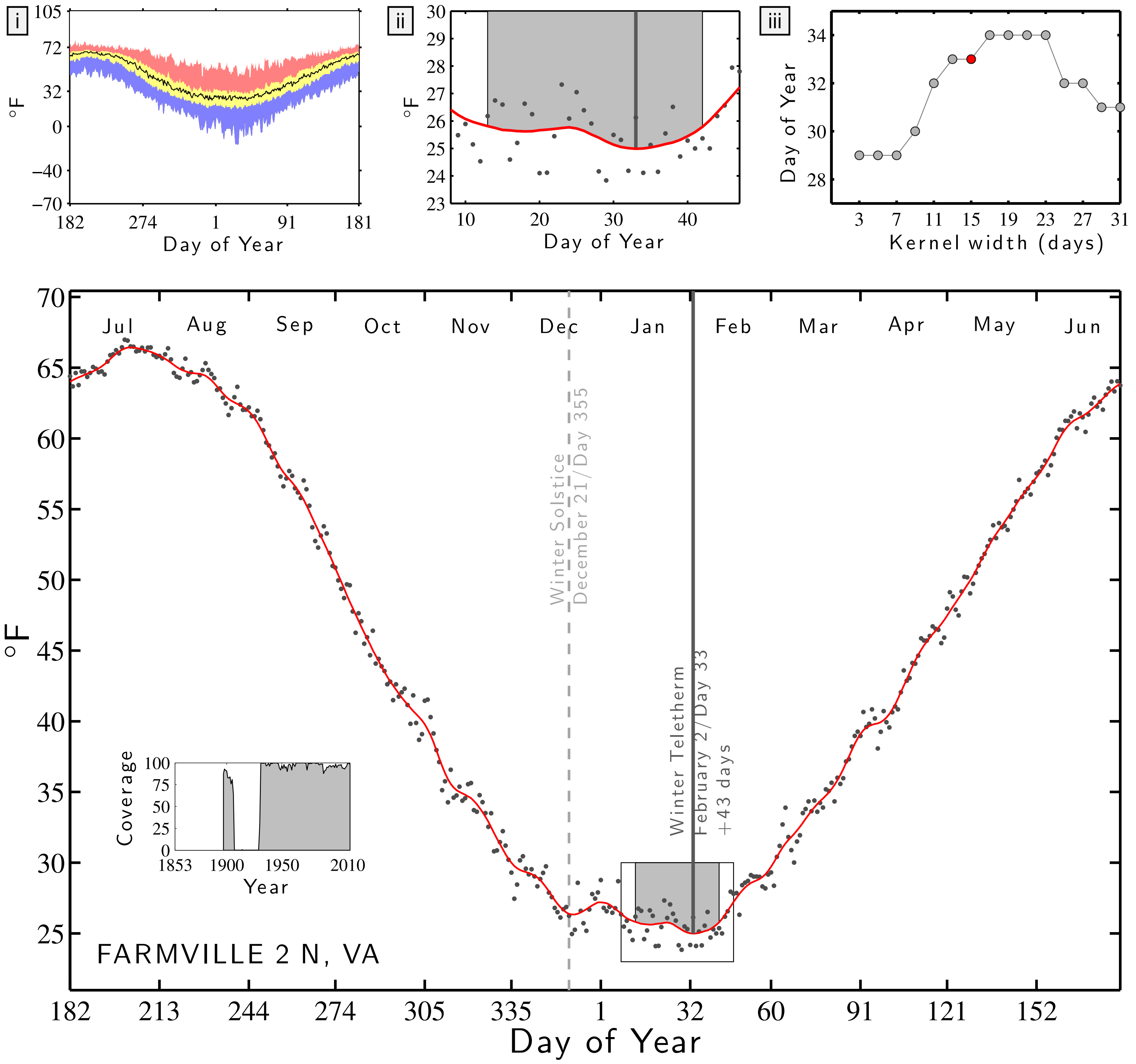

i
ii
iii
°F
Day of Year
Kernel width (days)
Jul
Aug
Sep
Oct
Nov
Dec
Jan
Feb
Mar
Apr
May
Jun
Winter Solstice
December 21/Day 355
Winter Teletherm
February 2/Day 33
+43 days
Coverage
Year
FARMVILLE 2 N, VA

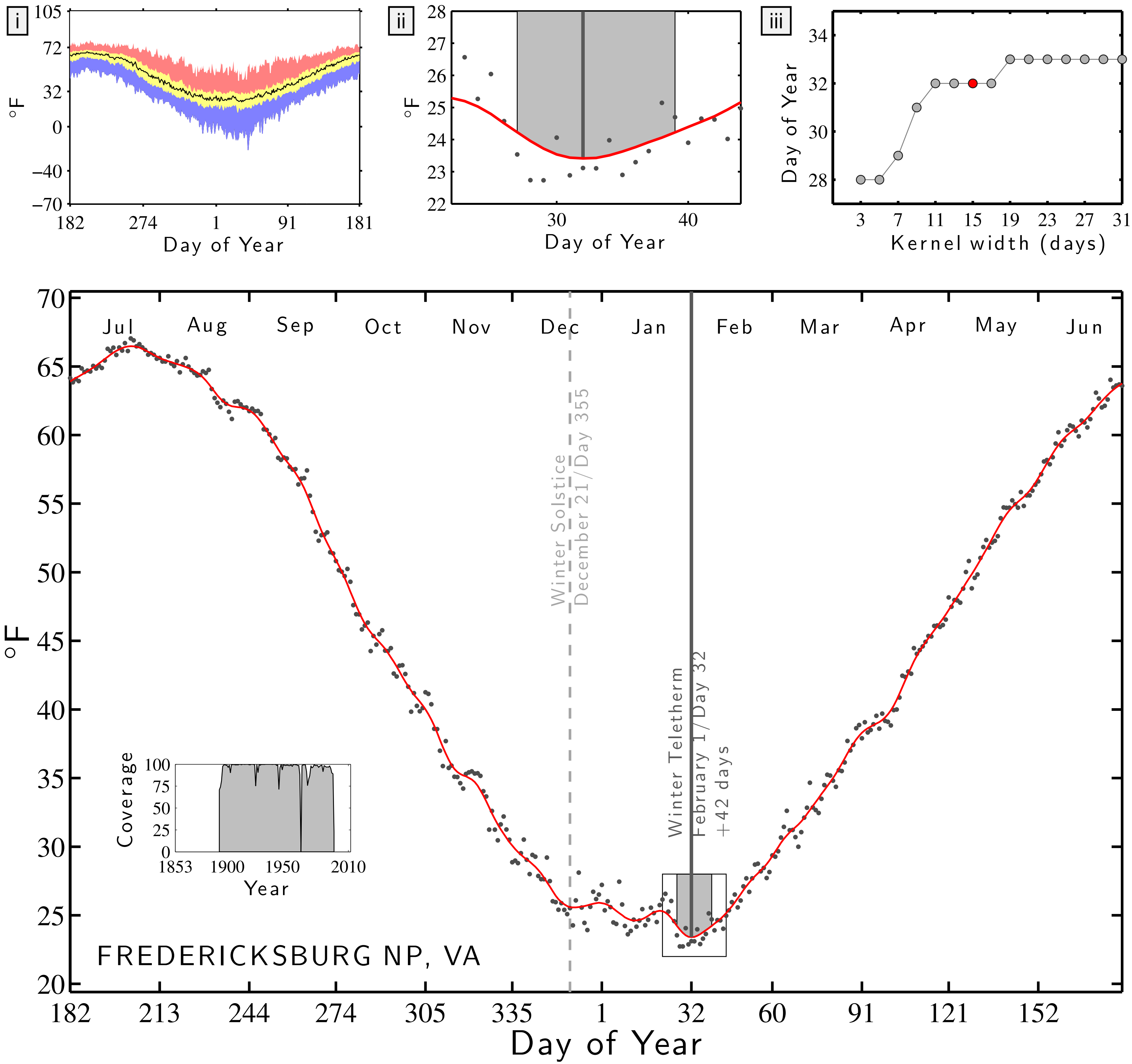

i
°F
Day of Year
ii
°F
Day of Year
iii
Day of Year
Kernel width (days)
Jul
Aug
Sep
Oct
Nov
Dec
Jan
Feb
Mar
Apr
May
Jun
Winter Solstice
December 21/Day 355
Winter Teletherm
February 1/Day 32
+42 days
Coverage
Year
FREDERICKSBURG NP, VA
°F
Day of Year

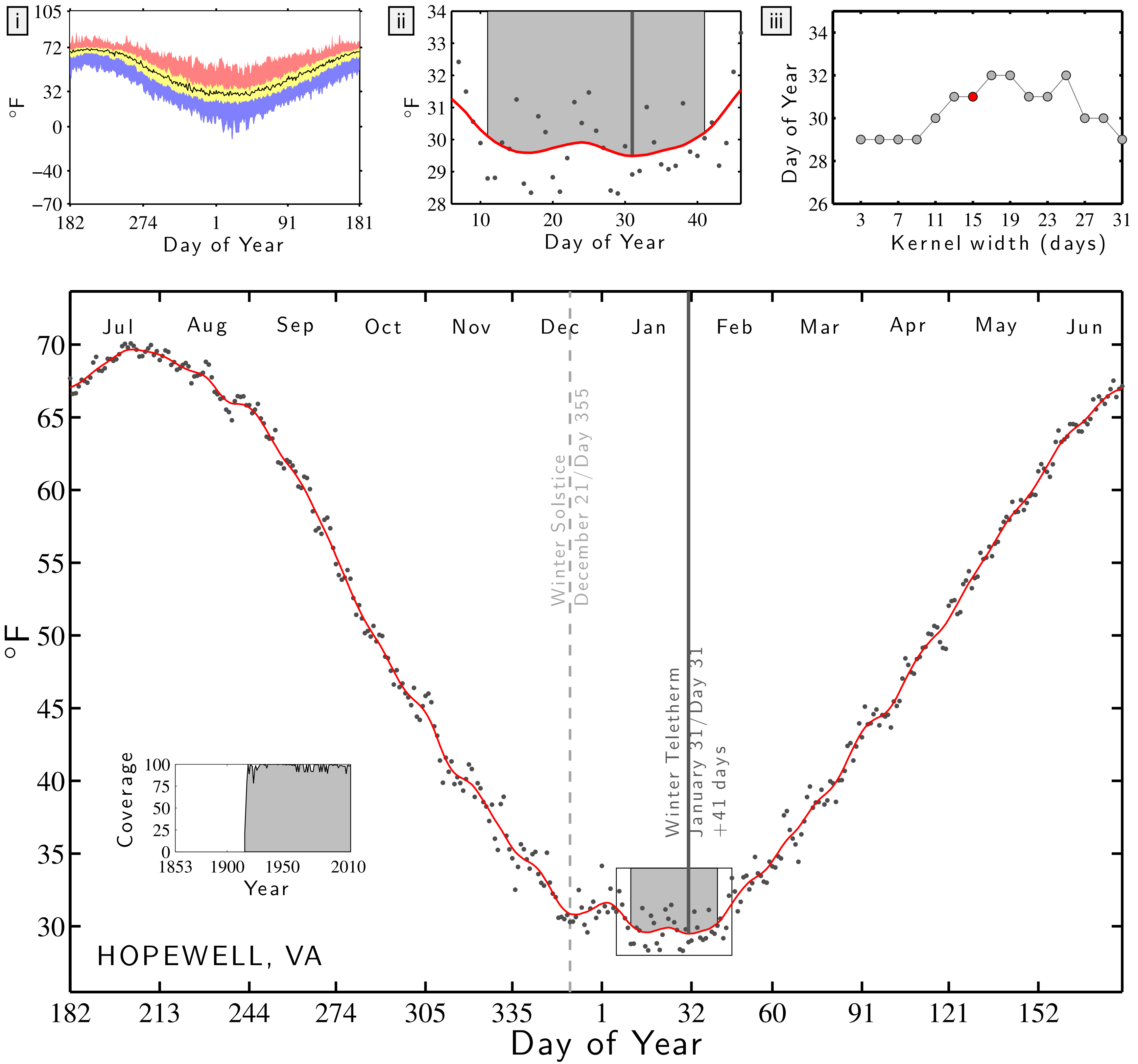

i
Day of Year
°F
ii
Day of Year
°F
iii
Kernel width (days)
Day of Year
Jul
Aug
Sep
Oct
Nov
Dec
Jan
Feb
Mar
Apr
May
Jun
Winter Solstice
December 21/Day 355
Winter Teletherm
January 31/Day 31
+41 days
Coverage
Year
HOPEWELL, VA
Day of Year
°F

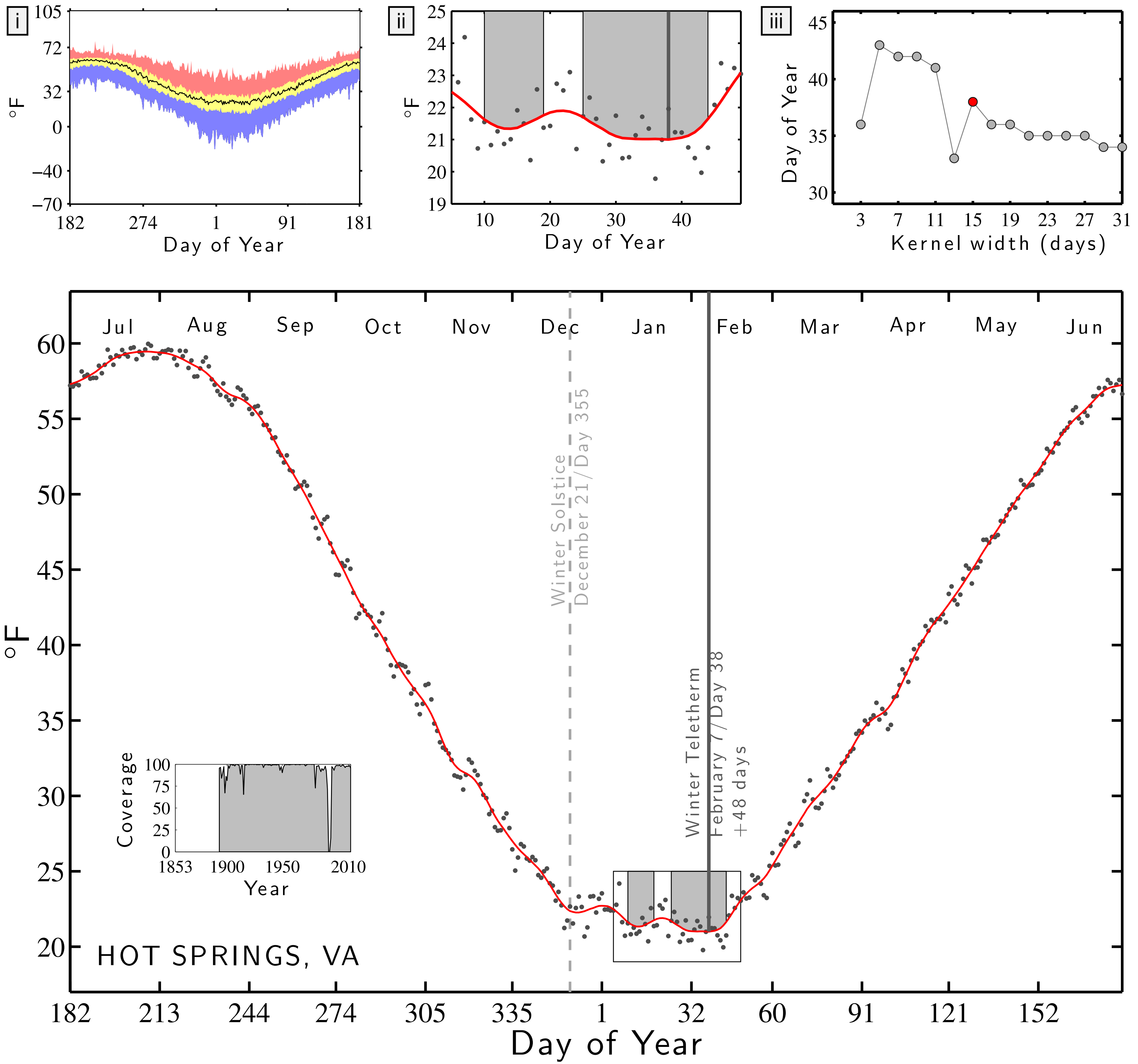

i
°F
105
72
32
0
−40
−70
182
274
1
91
181
Day of Year
ii
25
24
23
22
21
20
19
10
20
30
40
Day of Year
iii
45
40
35
30
Day of Year
3
7
11
15
19
23
27
31
Kernel width (days)
Jul
Aug
Sep
Oct
Nov
Dec
Jan
Feb
Mar
Apr
May
Jun
60
55
50
45
40
35
30
25
20
Winter Solstice
December 21/Day 355
Winter Teletherm
February 7/Day 38
+48 days
Coverage
100
75
50
25
0
1853
1900
1950
2010
Year
HOT SPRINGS, VA
182
213
244
274
305
335
1
32
60
91
121
152
Day of Year

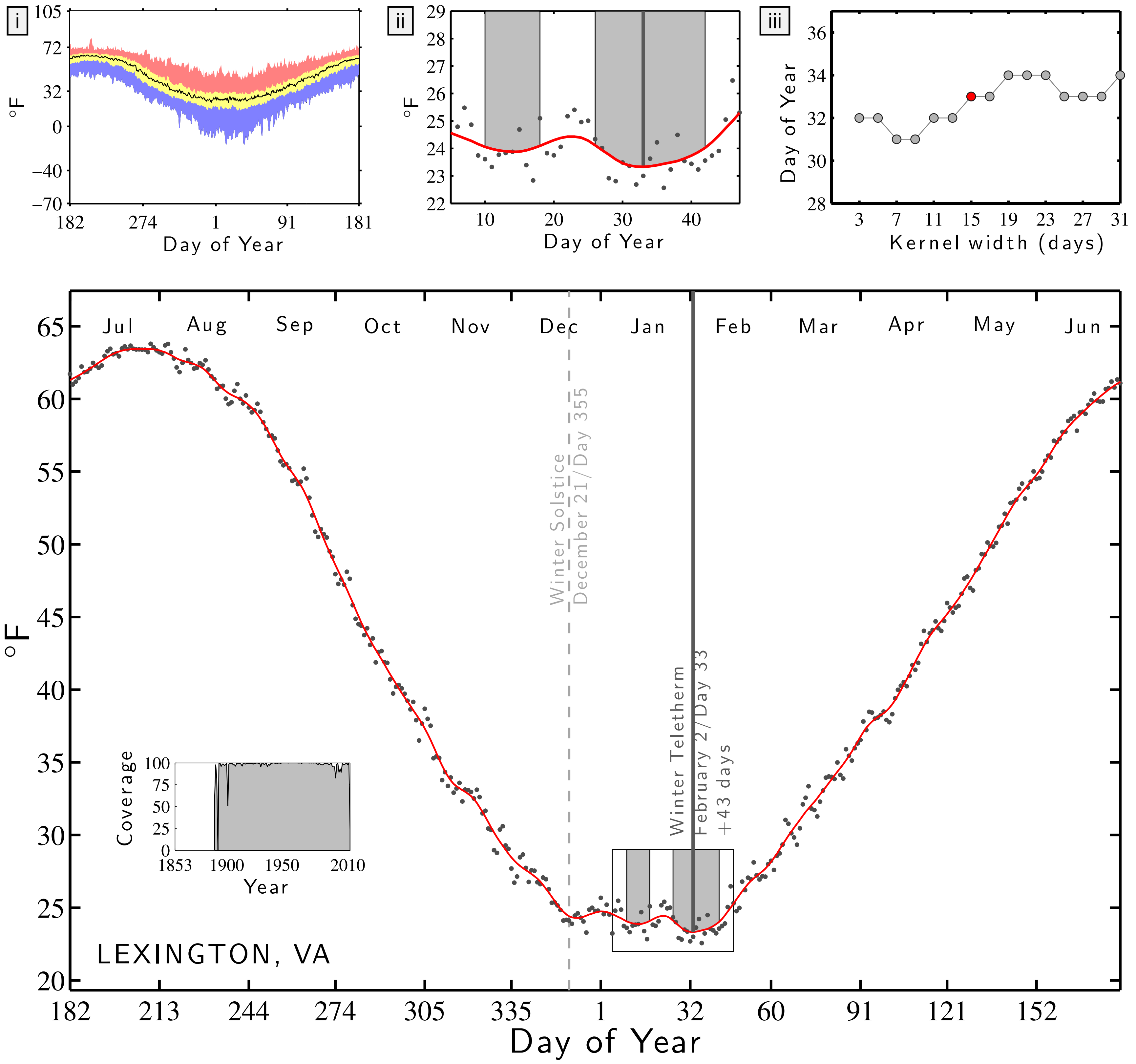

i
°F
Day of Year
ii
°F
Day of Year
iii
Day of Year
Kernel width (days)
Jul
Aug
Sep
Oct
Nov
Dec
Jan
Feb
Mar
Apr
May
Jun
Winter Solstice
December 21/Day 355
Winter Teletherm
February 2/Day 33
+43 days
Coverage
Year
LEXINGTON, VA
°F
Day of Year

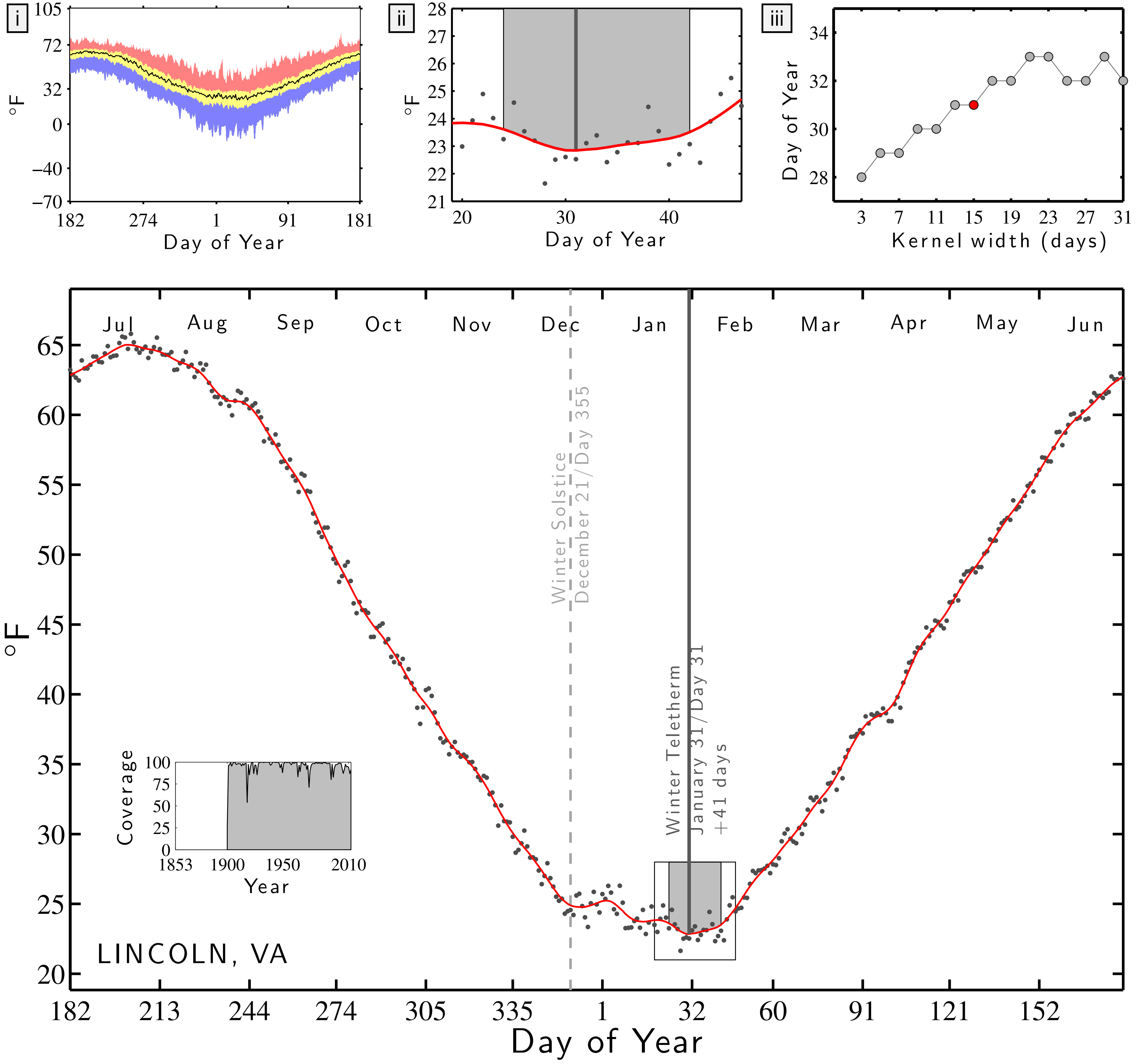

i
ii
iii
°F
Day of Year
Day of Year
Kernel width (days)
Jul
Aug
Sep
Oct
Nov
Dec
Jan
Feb
Mar
Apr
May
Jun
Winter Solstice
December 21/Day 355
Winter Teletherm
January 31/Day 31
+41 days
Coverage
Year
LINCOLN, VA
Day of Year

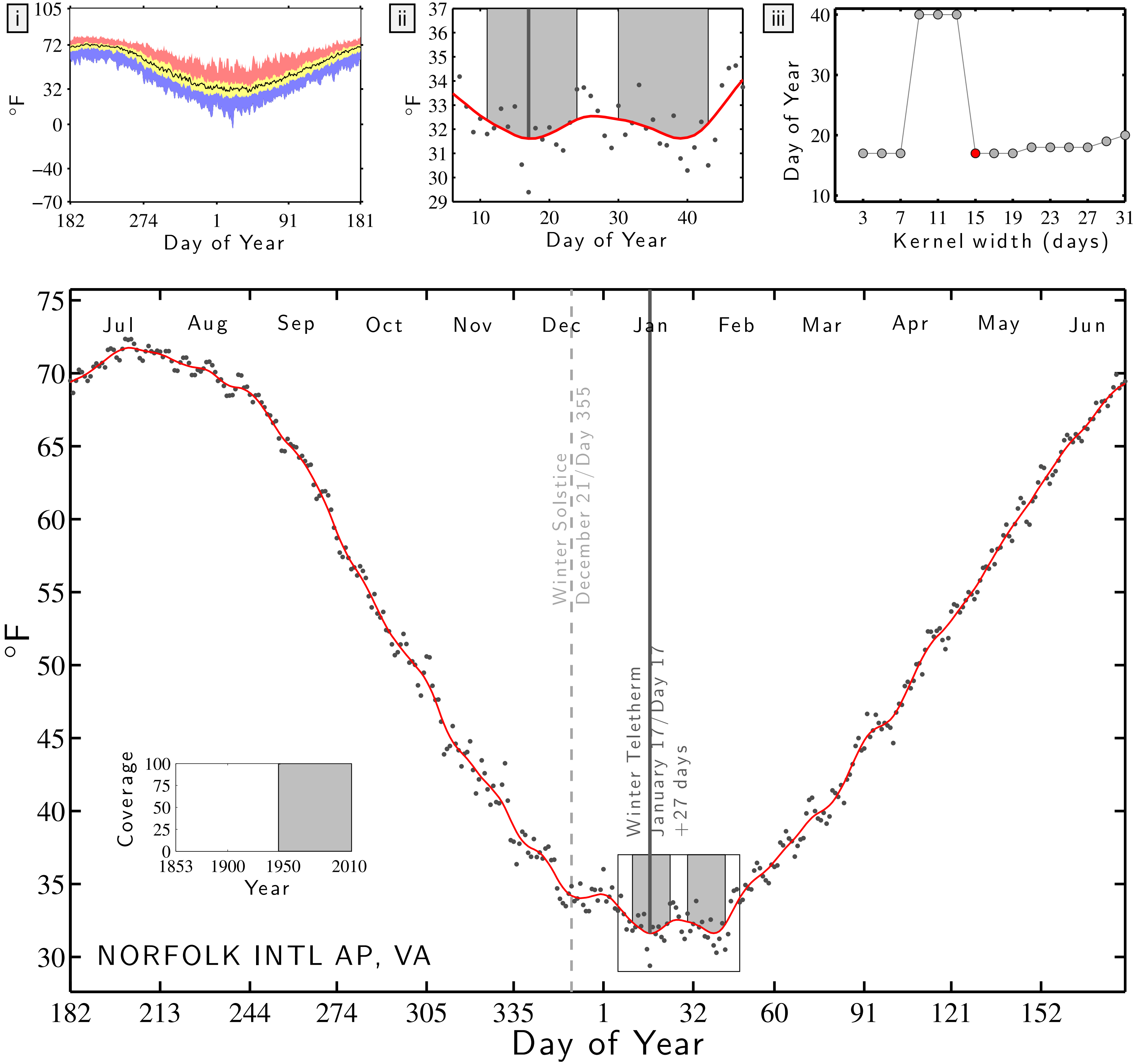

i
ii
iii
°F
Day of Year
Kernel width (days)
Jul
Aug
Sep
Oct
Nov
Dec
Jan
Feb
Mar
Apr
May
Jun
Winter Solstice
December 21/Day 355
Winter Teletherm
January 17/Day 17
+27 days
Coverage
Year
NORFOLK INTL AP, VA

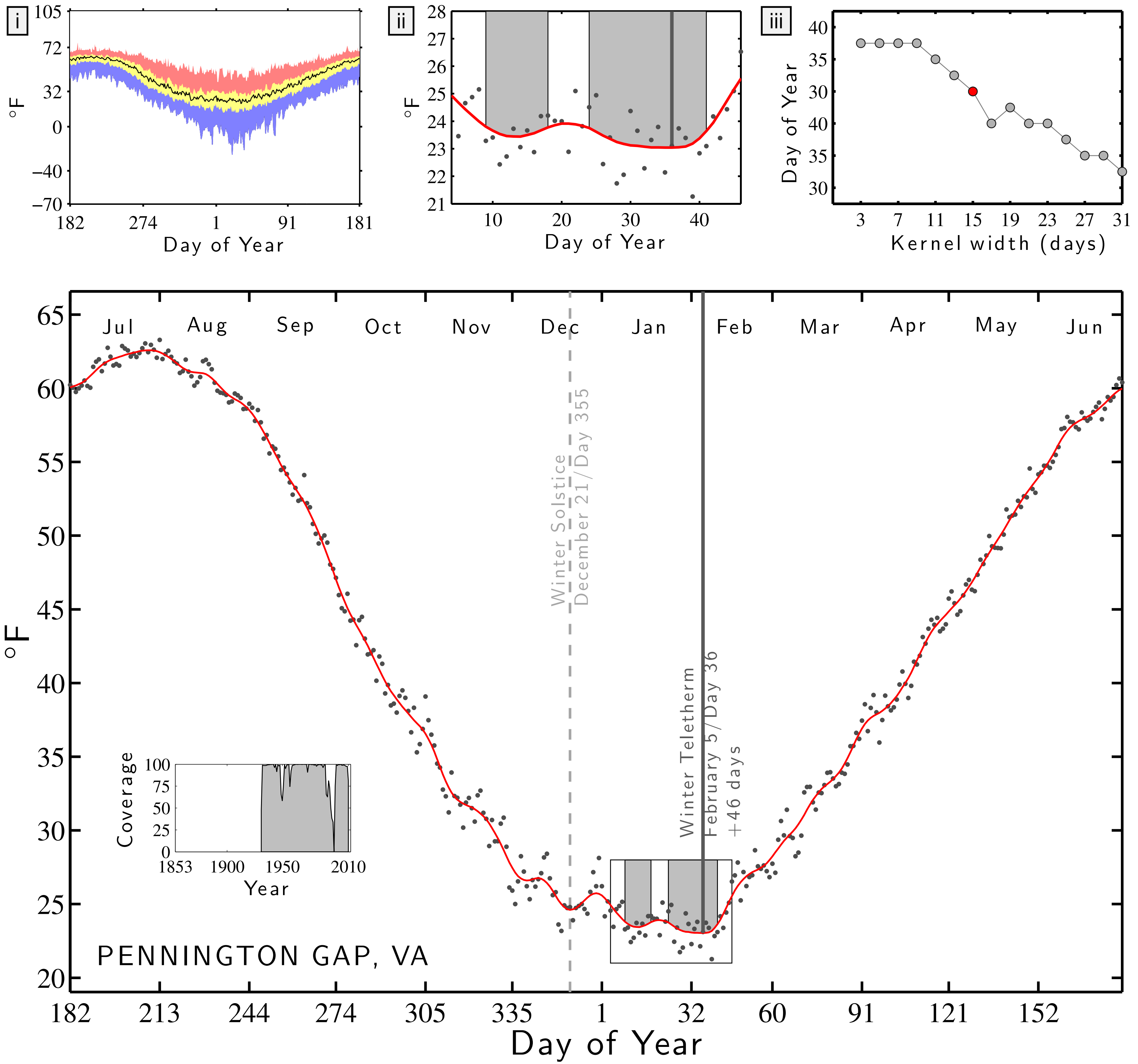

i
°F
Day of Year
ii
°F
Day of Year
iii
Day of Year
Kernel width (days)
Jul
Aug
Sep
Oct
Nov
Dec
Jan
Feb
Mar
Apr
May
Jun
Winter Solstice
December 21/Day 355
Winter Teletherm
February 5/Day 36
+46 days
Coverage
Year
PENNINGTON GAP, VA
°F
Day of Year

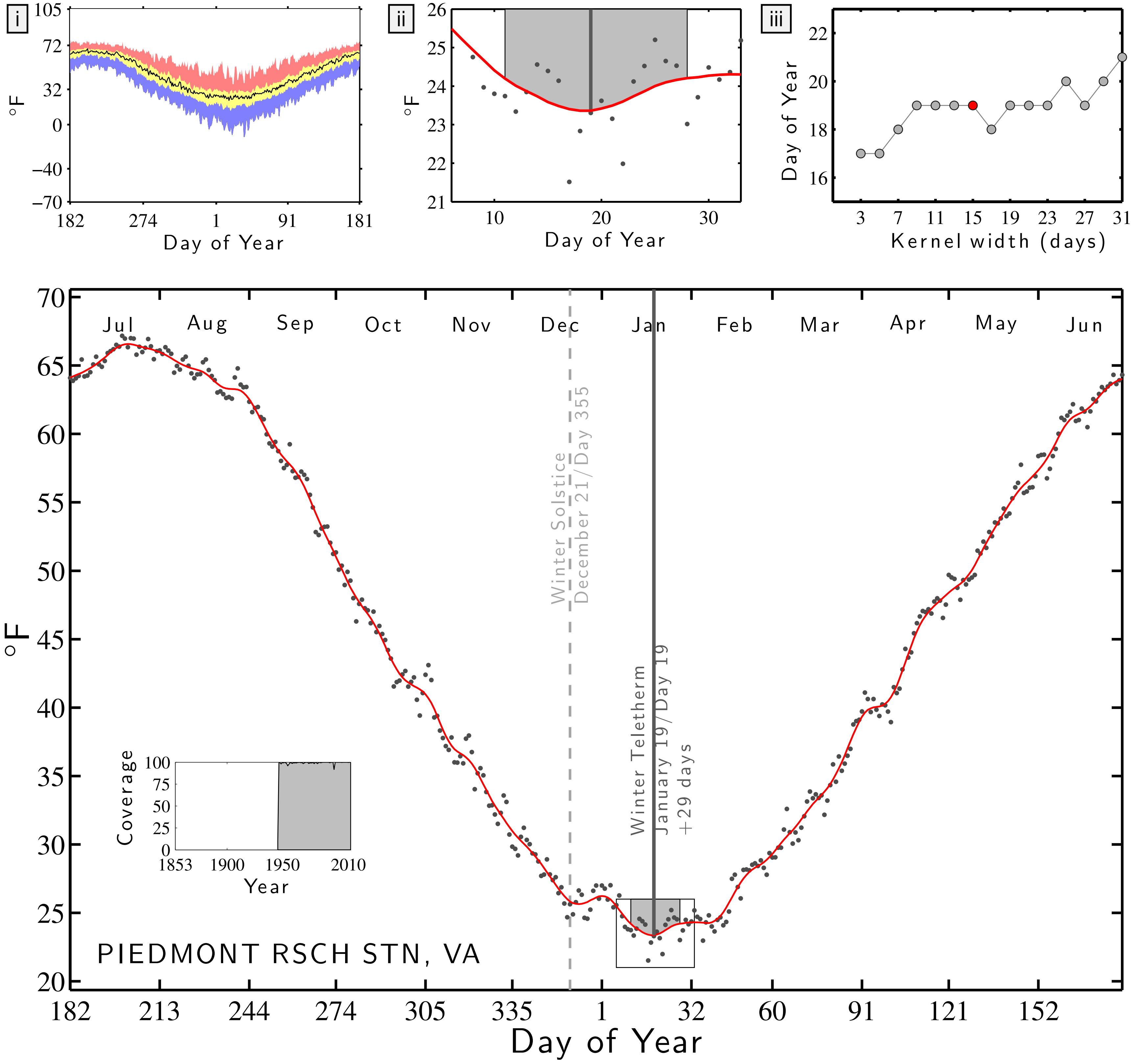

i
ii
iii
Day of Year
Kernel width (days)
Winter Solstice
December 21/Day 355
Winter Teletherm
January 19/Day 19
+29 days
Coverage
Year
PIEDMONT RSCH STN, VA
Jul
Aug
Sep
Oct
Nov
Dec
Jan
Feb
Mar
Apr
May
Jun
°F

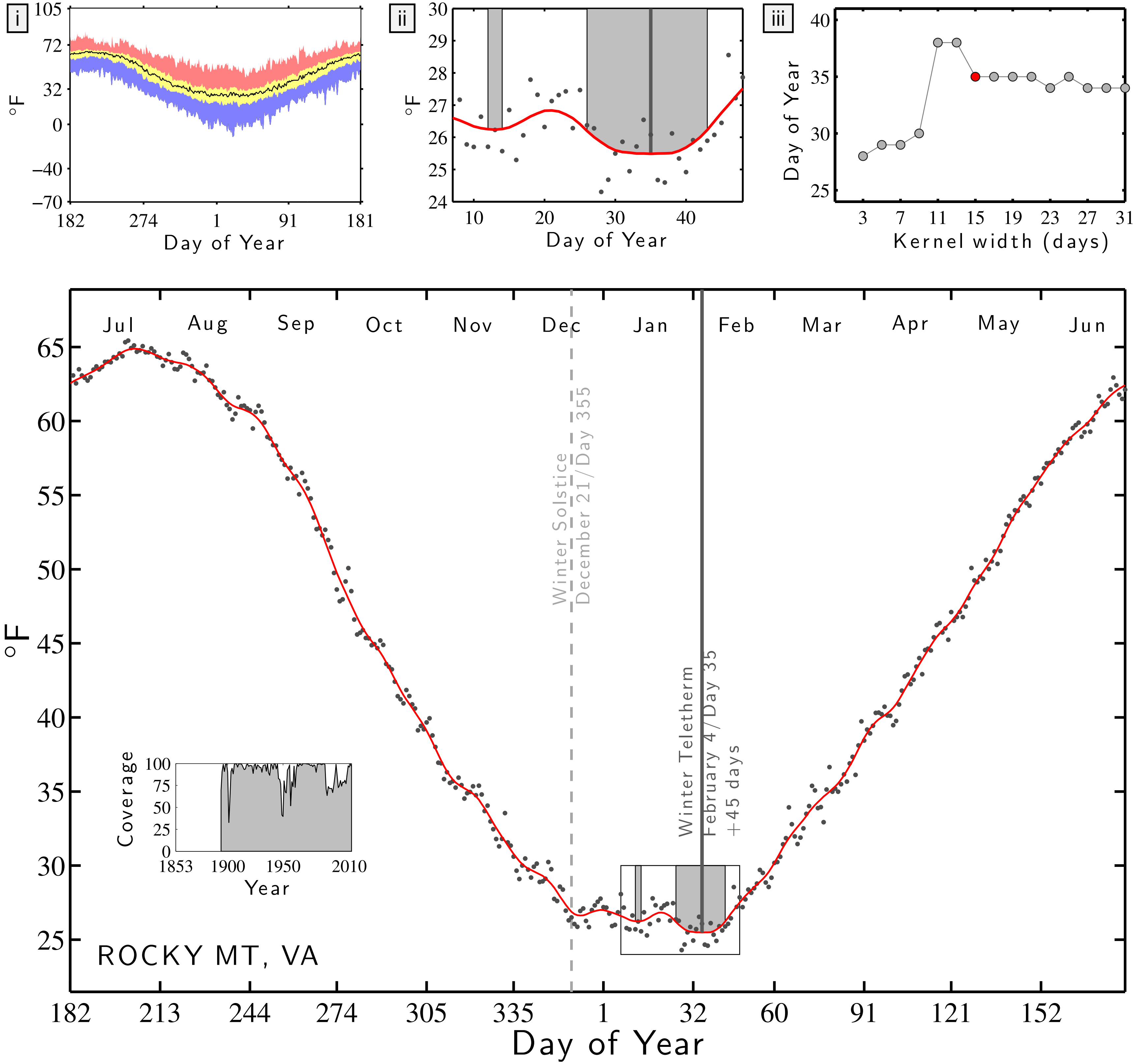

i
°F
105
72
32
0
−40
−70
182
274
1
91
181
Day of Year
ii
30
29
28
27
26
25
24
10
20
30
40
iii
Day of Year
40
35
30
25
3
7
11
15
19
23
27
31
Kernel width (days)
Jul
Aug
Sep
Oct
Nov
Dec
Jan
Feb
Mar
Apr
May
Jun
65
60
55
50
45
40
35
30
25
Winter Solstice
December 21/Day 355
Winter Teletherm
February 4/Day 35
+45 days
Coverage
100
75
50
25
0
1853
1900
1950
2010
Year
ROCKY MT, VA
182
213
244
274
305
335
1
32
60
91
121
152
Day of Year

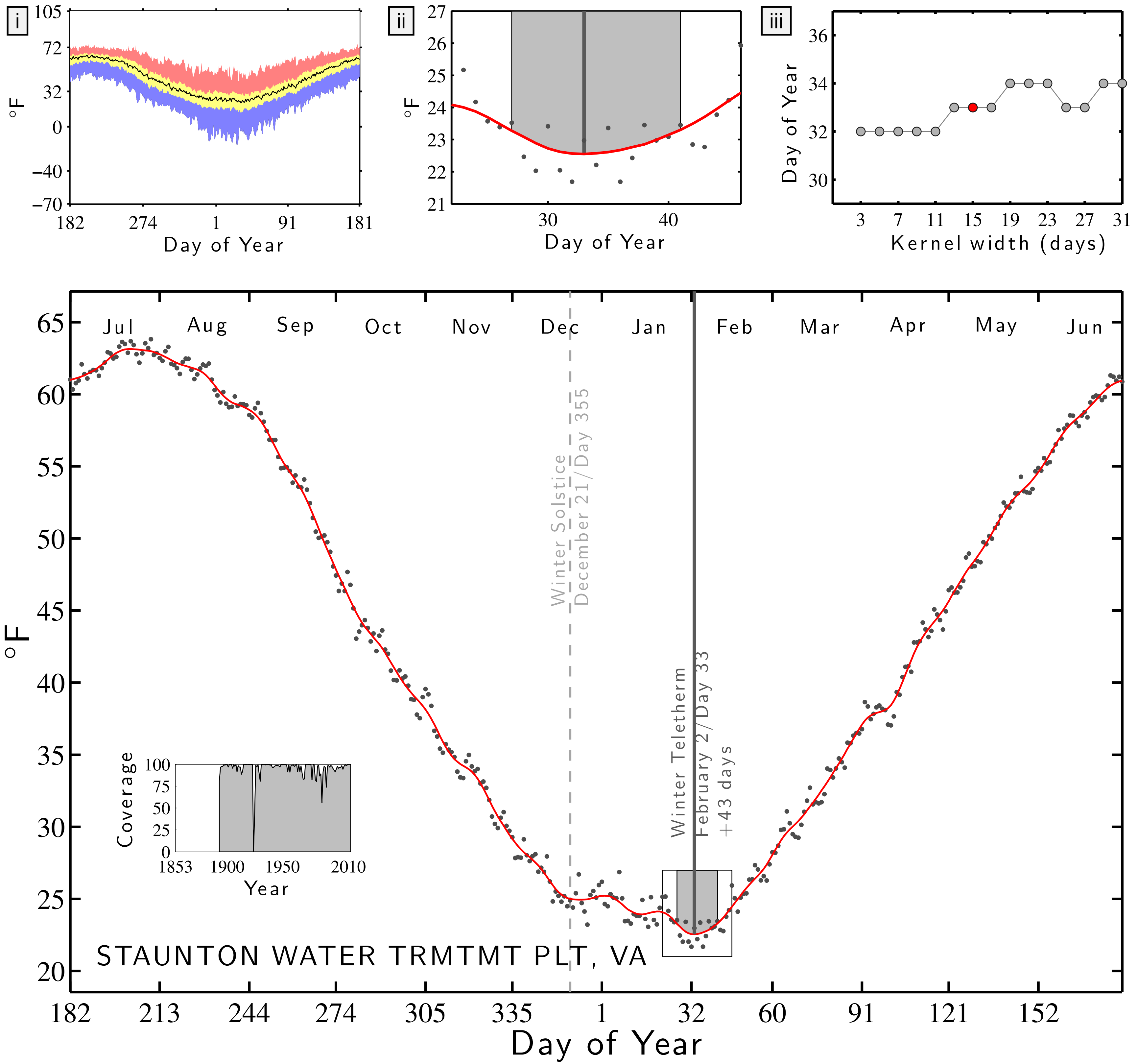

i
°F
Day of Year
ii
°F
Day of Year
iii
Day of Year
Kernel width (days)
Jul
Aug
Sep
Oct
Nov
Dec
Jan
Feb
Mar
Apr
May
Jun
Winter Solstice
December 21/Day 355
Winter Teletherm
February 2/Day 33
+43 days
°F
Coverage
Year
STAUNTON WATER TRMTMT PLT, VA
Day of Year

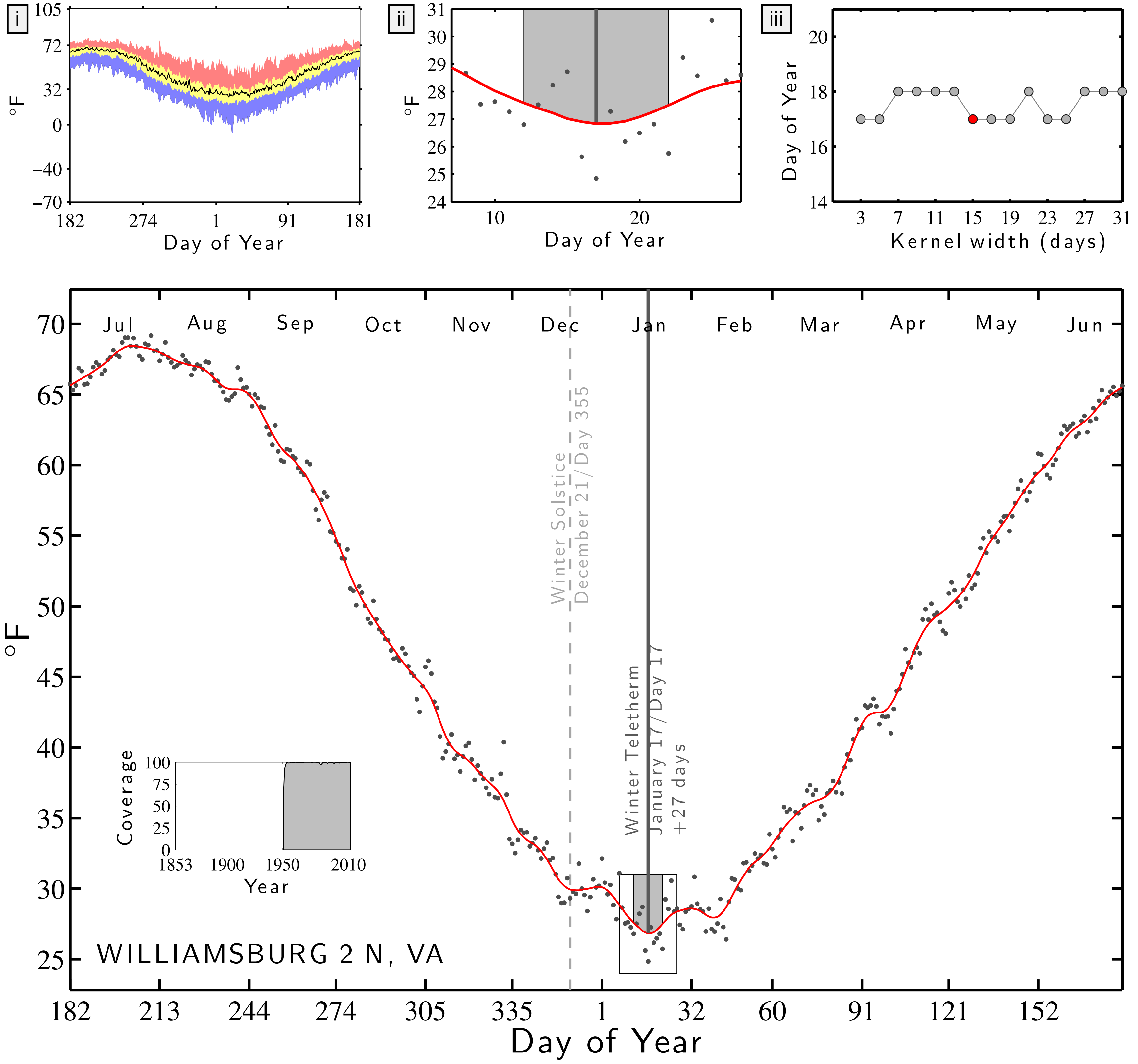

i
°F
Day of Year
ii
°F
Day of Year
iii
Day of Year
Kernel width (days)
Jul
Aug
Sep
Oct
Nov
Dec
Jan
Feb
Mar
Apr
May
Jun
Winter Solstice
December 21/Day 355
Winter Teletherm
January 17/Day 17
+27 days
Coverage
Year
WILLIAMSBURG 2 N, VA
°F
Day of Year

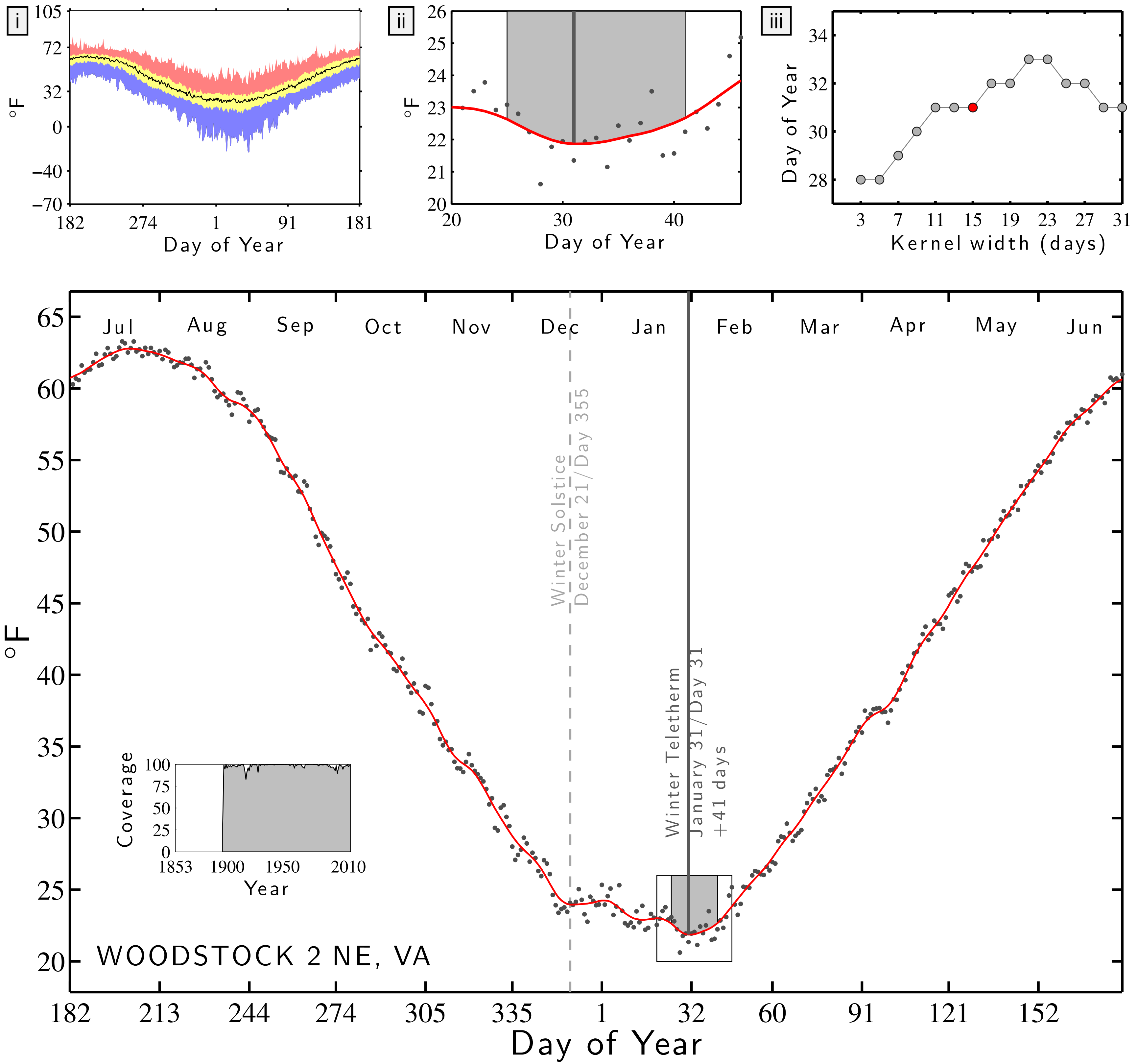

i
°F
Day of Year
ii
°F
Day of Year
iii
Day of Year
Kernel width (days)
Jul
Aug
Sep
Oct
Nov
Dec
Jan
Feb
Mar
Apr
May
Jun
Winter Solstice
December 21/Day 355
Winter Teletherm
January 31/Day 31
+41 days
Coverage
Year
WOODSTOCK 2 NE, VA
°F
Day of Year

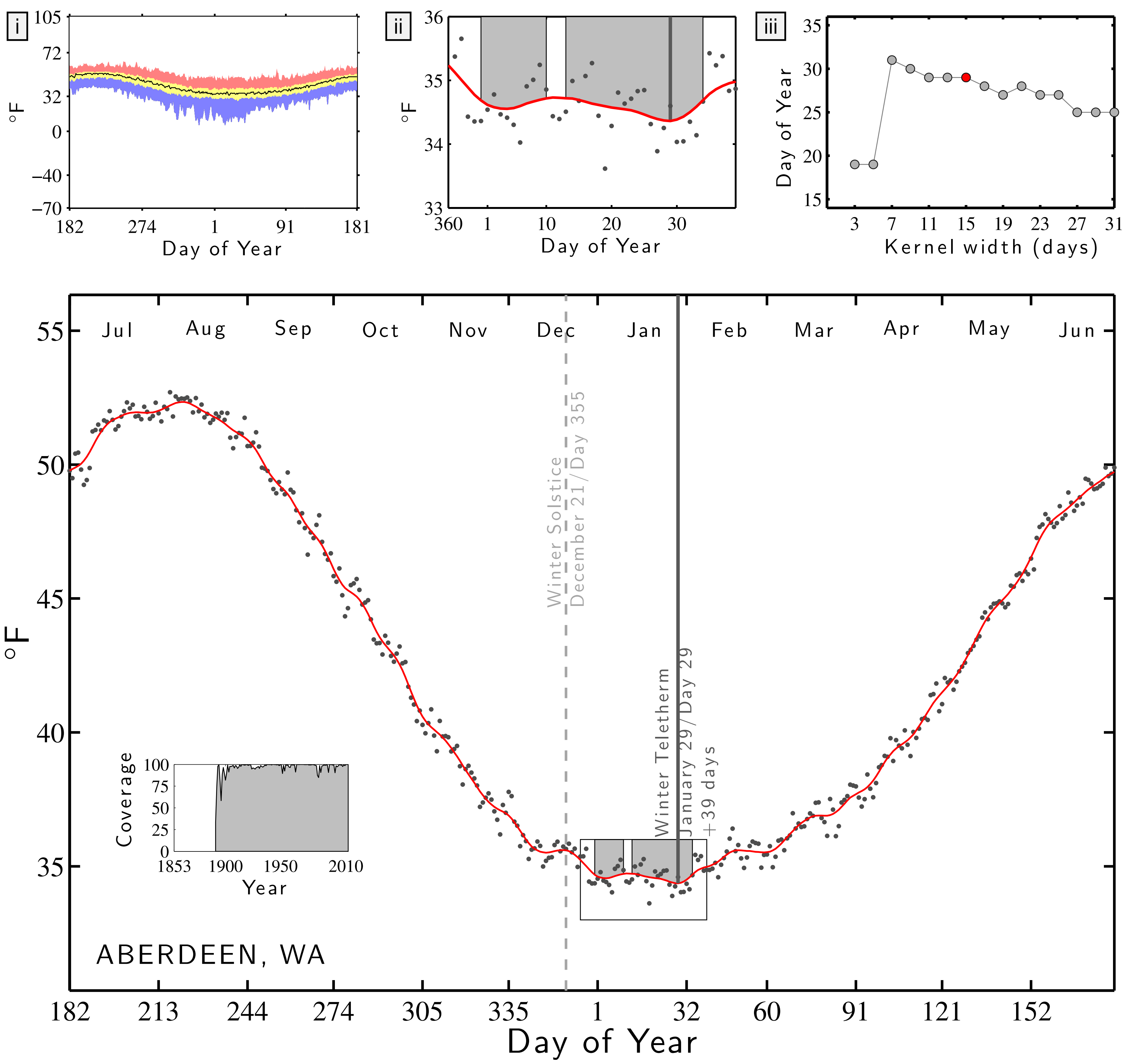
i
ii
iii
°F
Day of Year
Kernel width (days)
Jul
Aug
Sep
Oct
Nov
Dec
Jan
Feb
Mar
Apr
May
Jun
Winter Solstice
December 21/Day 355
Winter Teletherm
January 29/Day 29
+39 days
Coverage
Year
ABERDEEN, WA

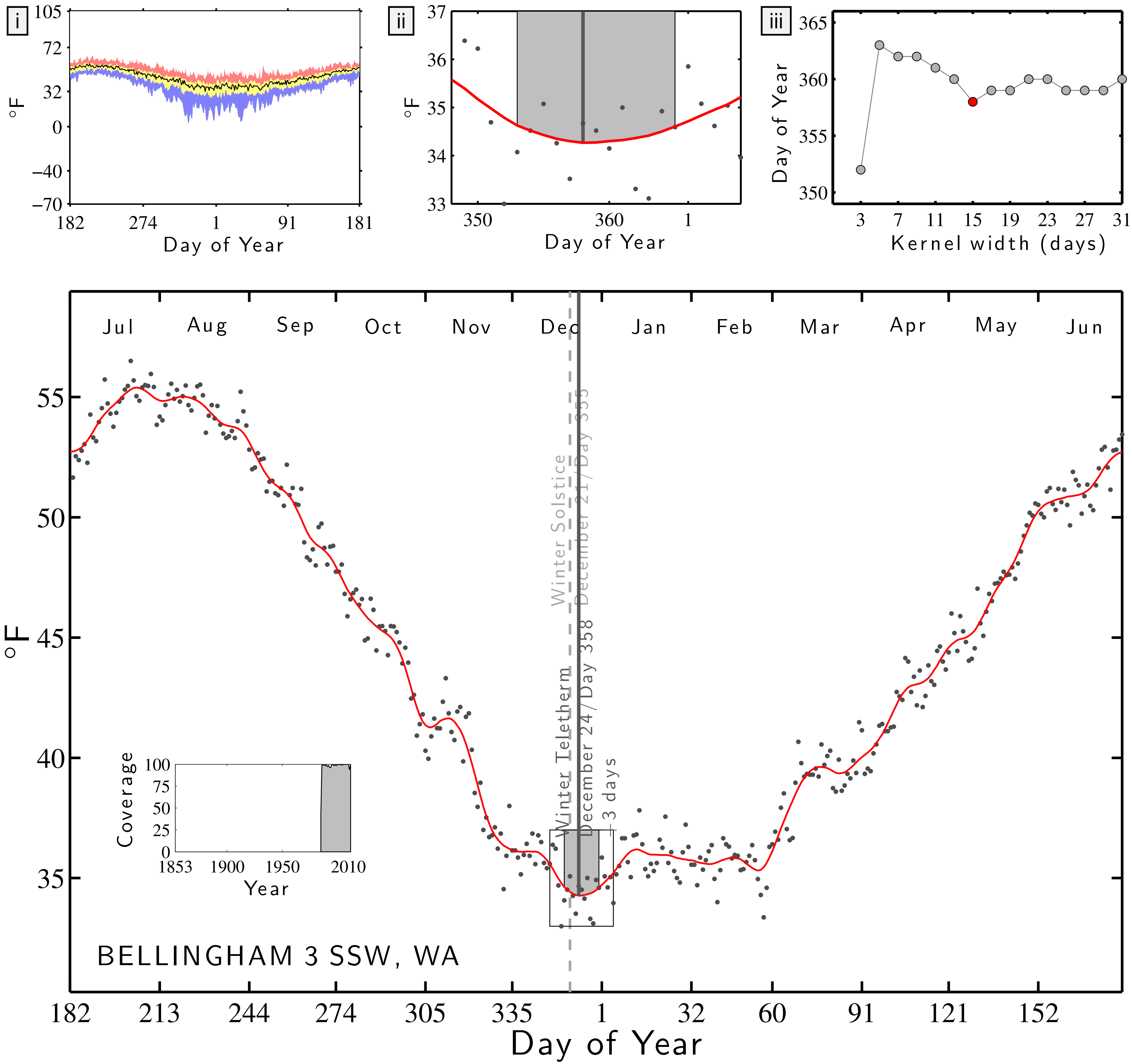

i
105
72
32
0
−40
−70
°F
182
274
1
91
181
Day of Year
ii
37
36
35
34
33
°F
350
360
1
Day of Year
iii
365
360
355
350
Day of Year
3
7
11
15
19
23
27
31
Kernel width (days)
Jul
Aug
Sep
Oct
Nov
Dec
Jan
Feb
Mar
Apr
May
Jun
55
50
45
40
35
°F
Winter Solstice
December 21/Day 355
Winter Teletherm
December 24/Day 358
3 days
Coverage
100
75
50
25
0
1853
1900
1950
2010
Year
BELLINGHAM 3 SSW, WA
182
213
244
274
305
335
1
32
60
91
121
152
Day of Year

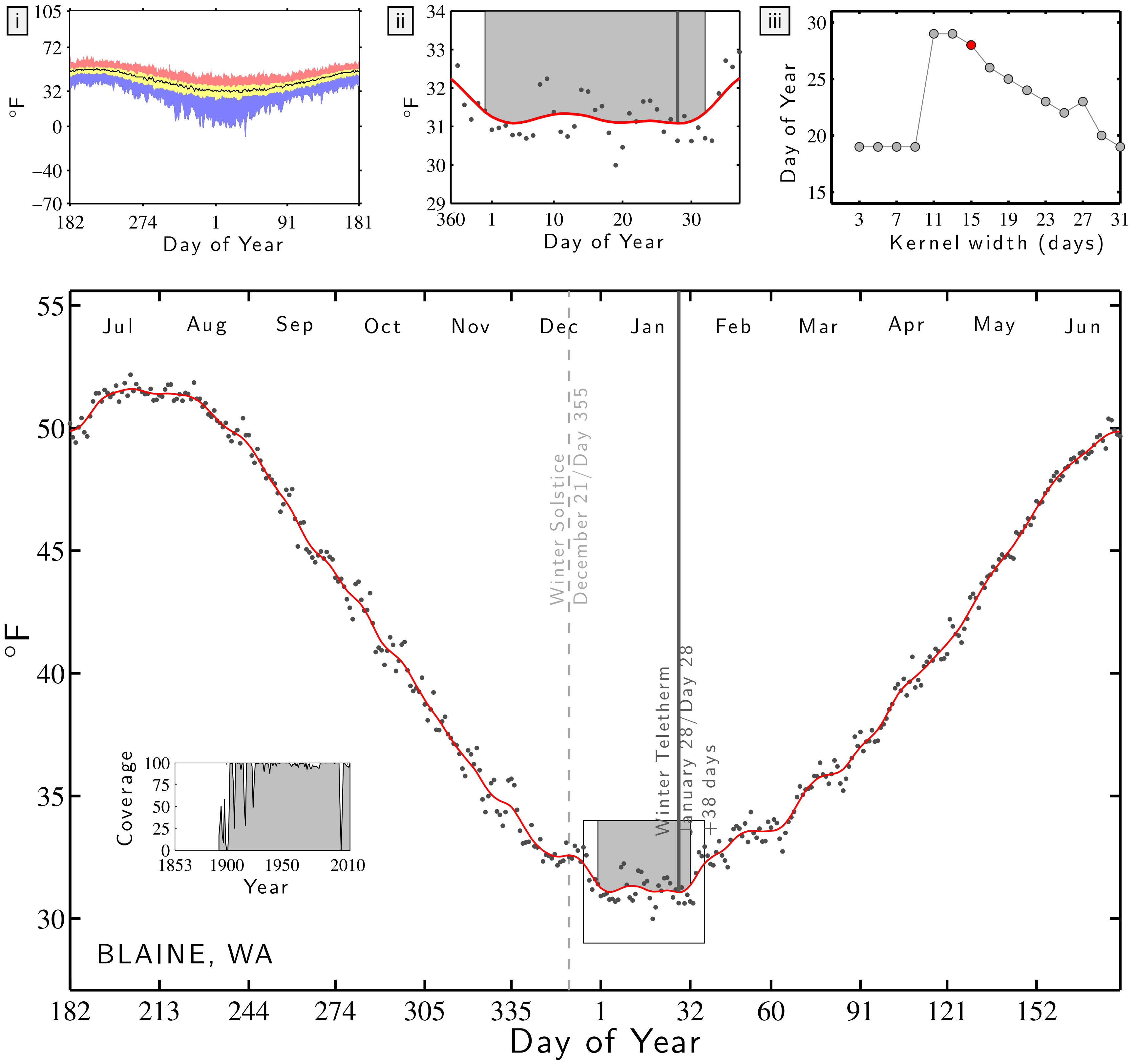

i
ii
iii
°F
Day of Year
Day of Year
Day of Year
Kernel width (days)
Jul
Aug
Sep
Oct
Nov
Dec
Jan
Feb
Mar
Apr
May
Jun
Winter Solstice
December 21/Day 355
Winter Teletherm
January 28/Day 28
±38 days
Coverage
Year
BLAINE, WA

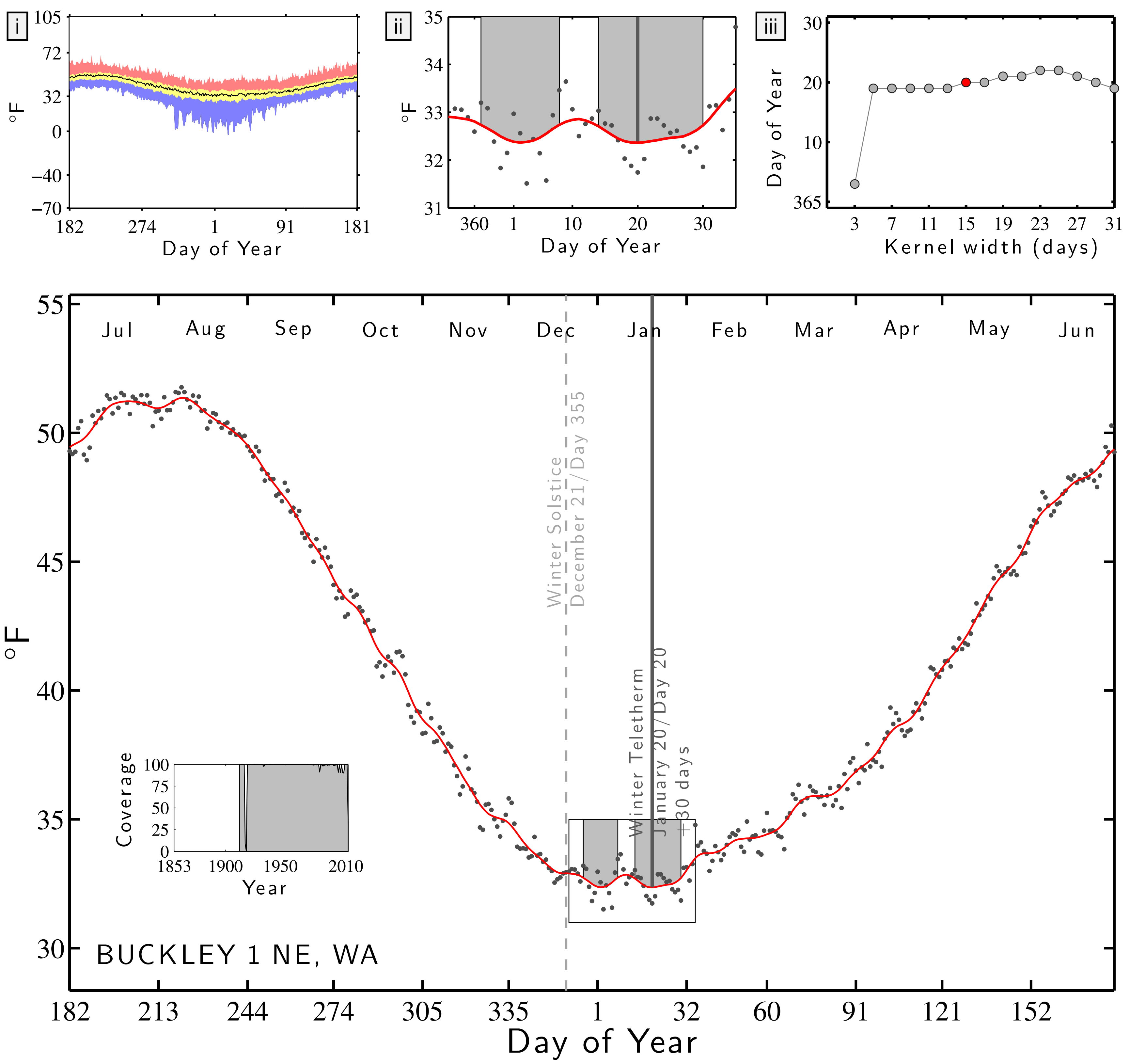

i
ii
iii
°F
Day of Year
Day of Year
Kernel width (days)
Winter Solstice
December 21/Day 355
Winter Teletherm
January 20/Day 20
±30 days
Jul
Aug
Sep
Oct
Nov
Dec
Jan
Feb
Mar
Apr
May
Jun
Coverage
Year
BUCKLEY 1 NE, WA

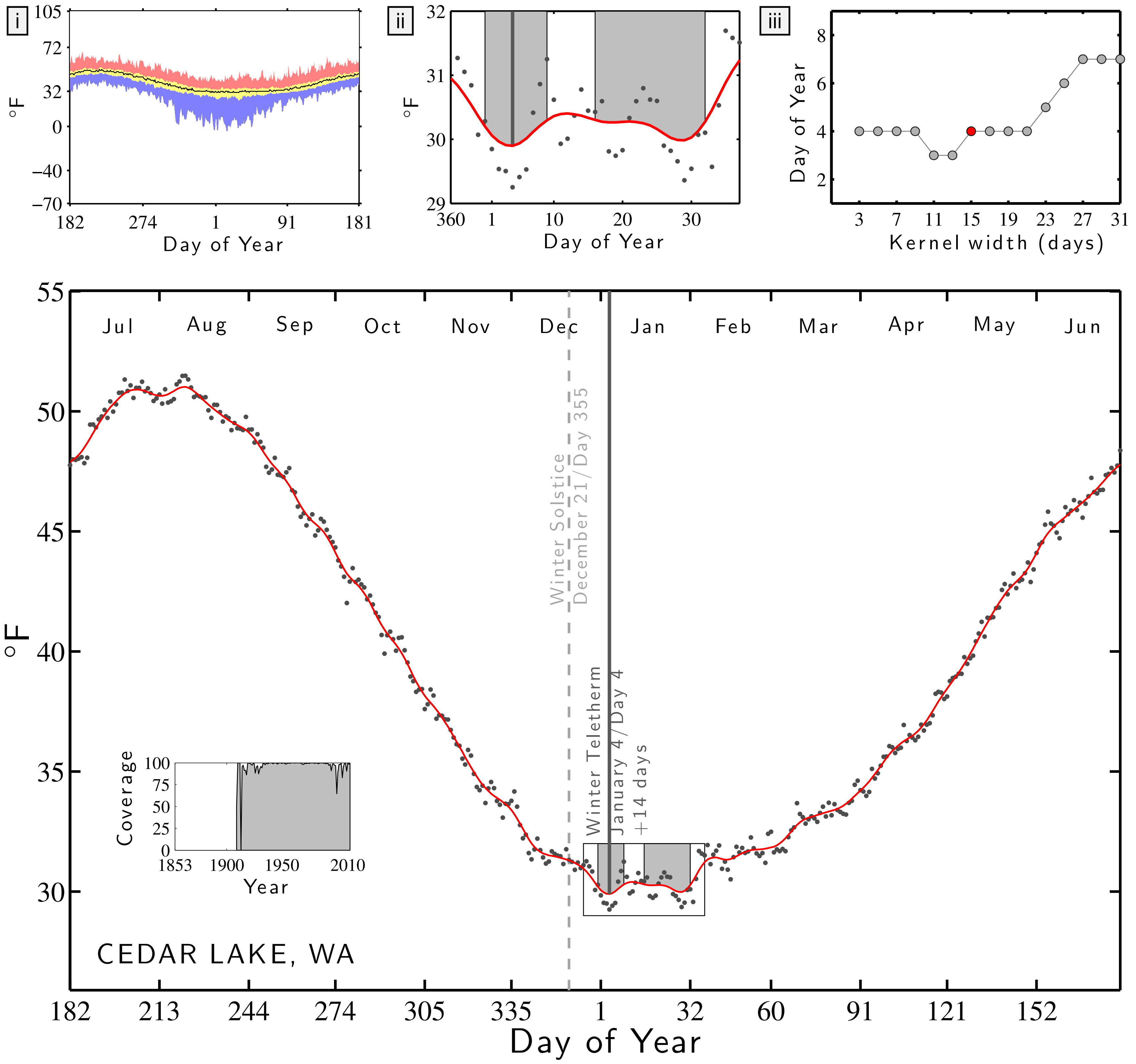

i
105
72
32
0
−40
−70
°F
182
274
1
91
181
Day of Year
ii
32
31
30
29
°F
360
1
10
20
30
Day of Year
iii
8
6
4
2
Day of Year
3
7
11
15
19
23
27
31
Kernel width (days)
55
50
45
40
35
30
°F
Jul
Aug
Sep
Oct
Nov
Dec
Jan
Feb
Mar
Apr
May
Jun
Winter Solstice
December 21/Day 355
Winter Teletherm
January 4/Day 4
+14 days
Coverage
100
75
50
25
0
1853
1900
1950
2010
Year
CEDAR LAKE, WA
182
213
244
274
305
335
1
32
60
91
121
152
Day of Year

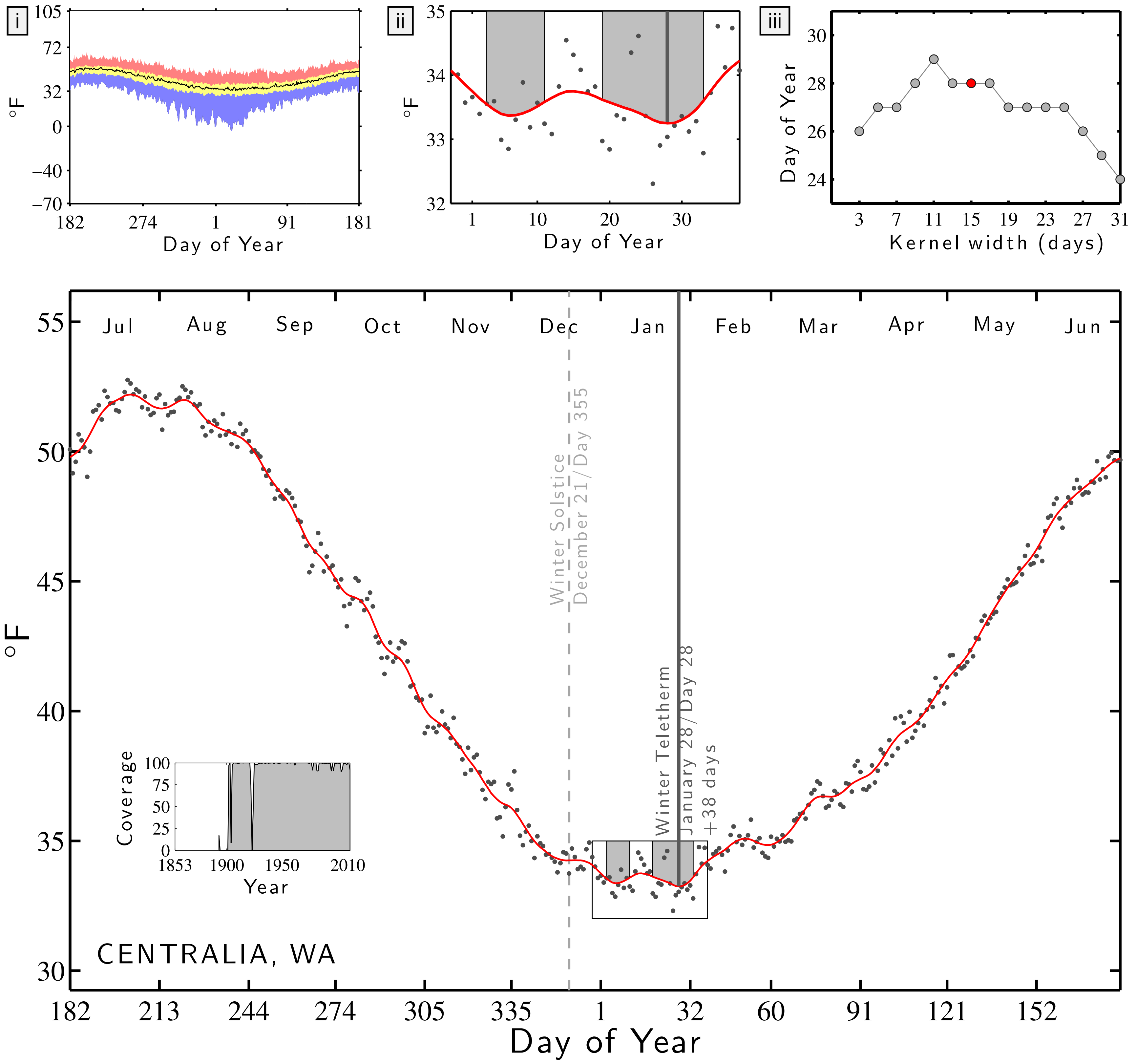

i
ii
iii
°F
Day of Year
Day of Year
Kernel width (days)
Jul
Aug
Sep
Oct
Nov
Dec
Jan
Feb
Mar
Apr
May
Jun
Winter Solstice
December 21/Day 355
Winter Teletherm
January 28/Day 28
+38 days
Coverage
Year
CENTRALIA, WA

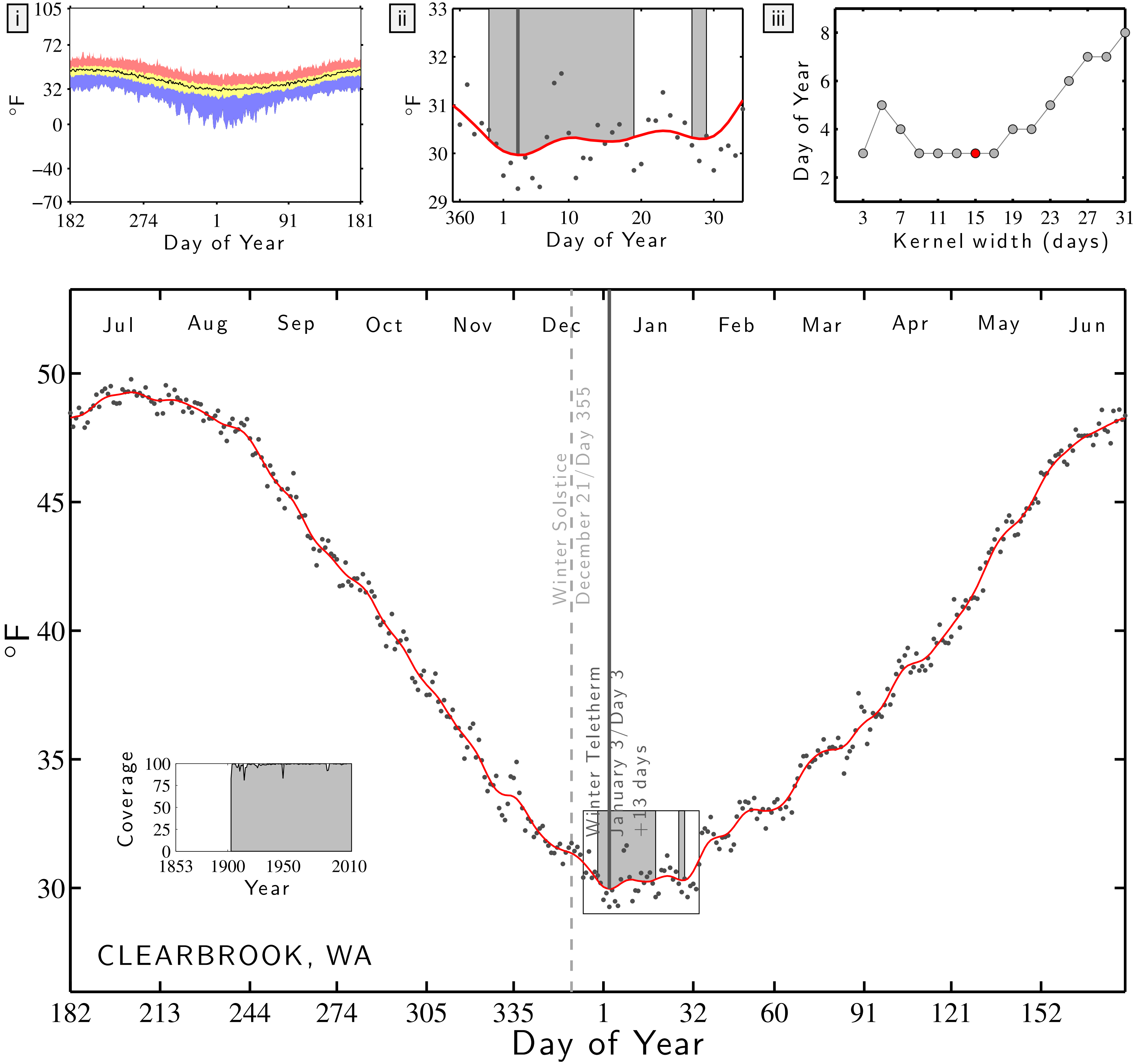

i
ii
iii
°F
Day of Year
Kernel width (days)
Jul
Aug
Sep
Oct
Nov
Dec
Jan
Feb
Mar
Apr
May
Jun
Winter Solstice
December 21/Day 355
Winter Teletherm
January 3/Day 3
+13 days
Coverage
Year
CLEARBROOK, WA

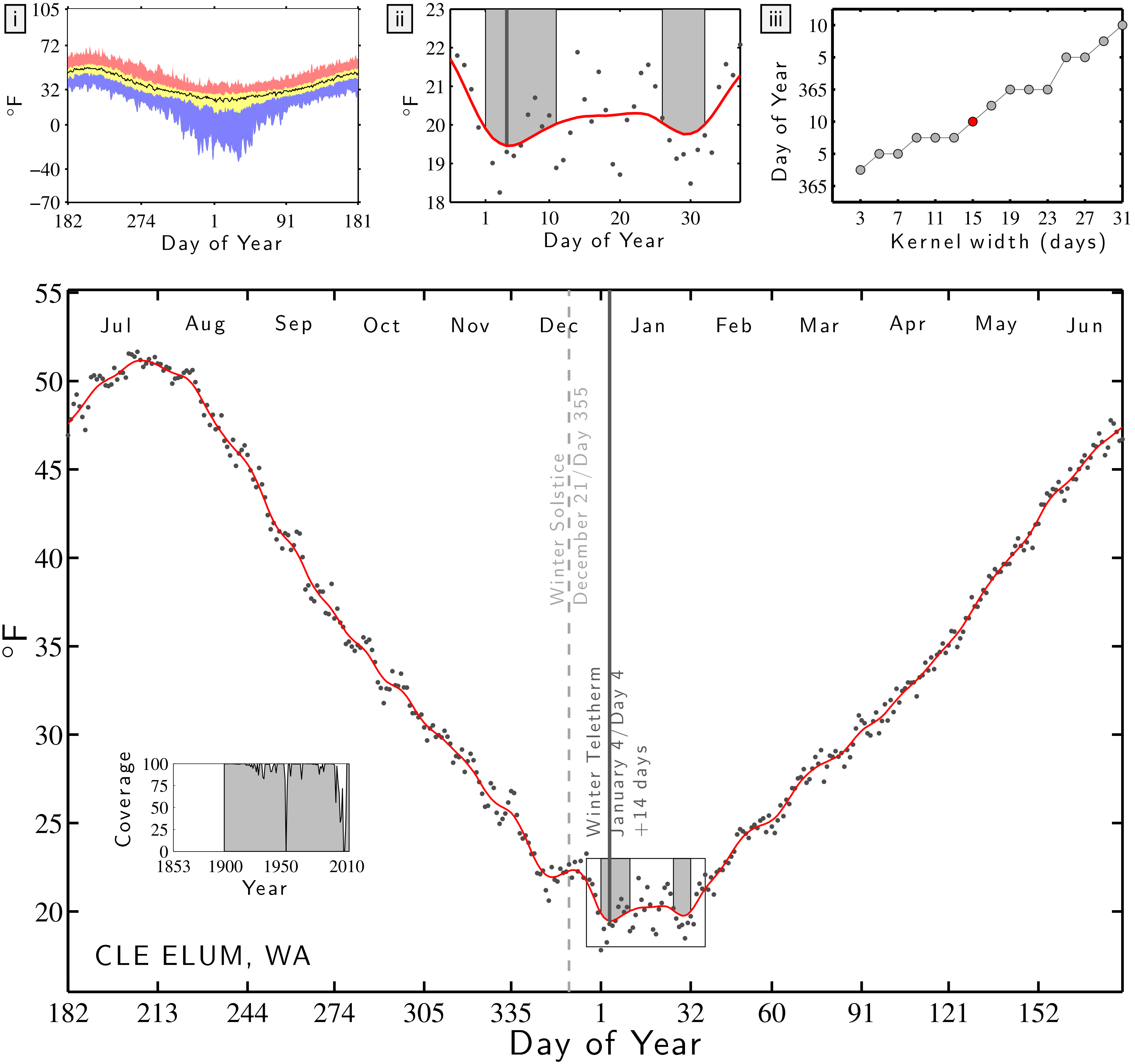

i
°F
Day of Year
ii
°F
Day of Year
iii
Day of Year
Kernel width (days)
Jul
Aug
Sep
Oct
Nov
Dec
Jan
Feb
Mar
Apr
May
Jun
Winter Solstice
December 21/Day 355
Winter Teletherm
January 4/Day 4
+14 days
Coverage
Year
CLE ELUM, WA
°F
Day of Year

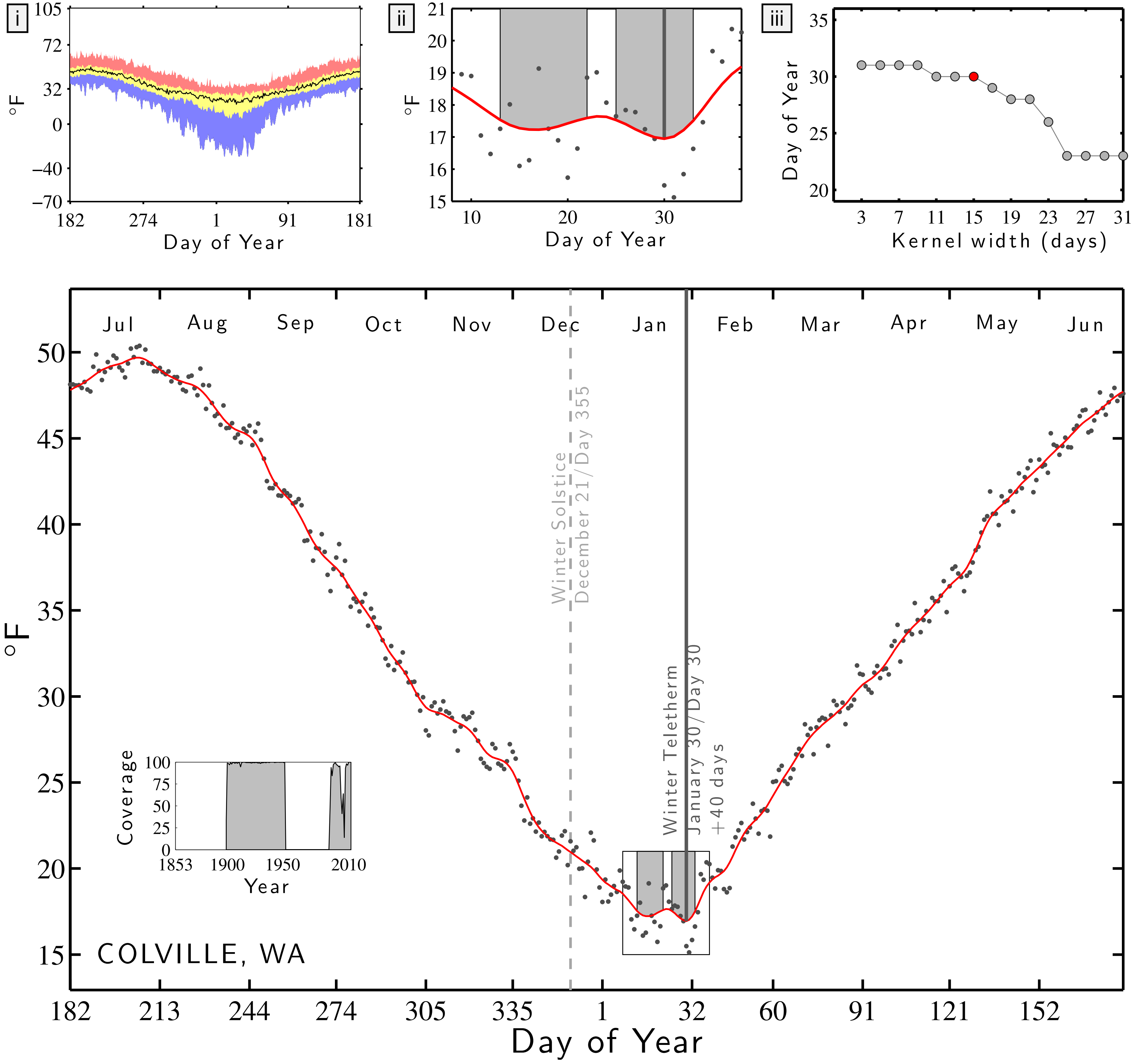

i
°F
Day of Year
ii
°F
Day of Year
iii
Day of Year
Kernel width (days)
Jul
Aug
Sep
Oct
Nov
Dec
Jan
Feb
Mar
Apr
May
Jun
Winter Solstice
December 21/Day 355
Winter Teletherm
January 30/Day 30
+40 days
°F
Coverage
Year
COLVILLE, WA
Day of Year

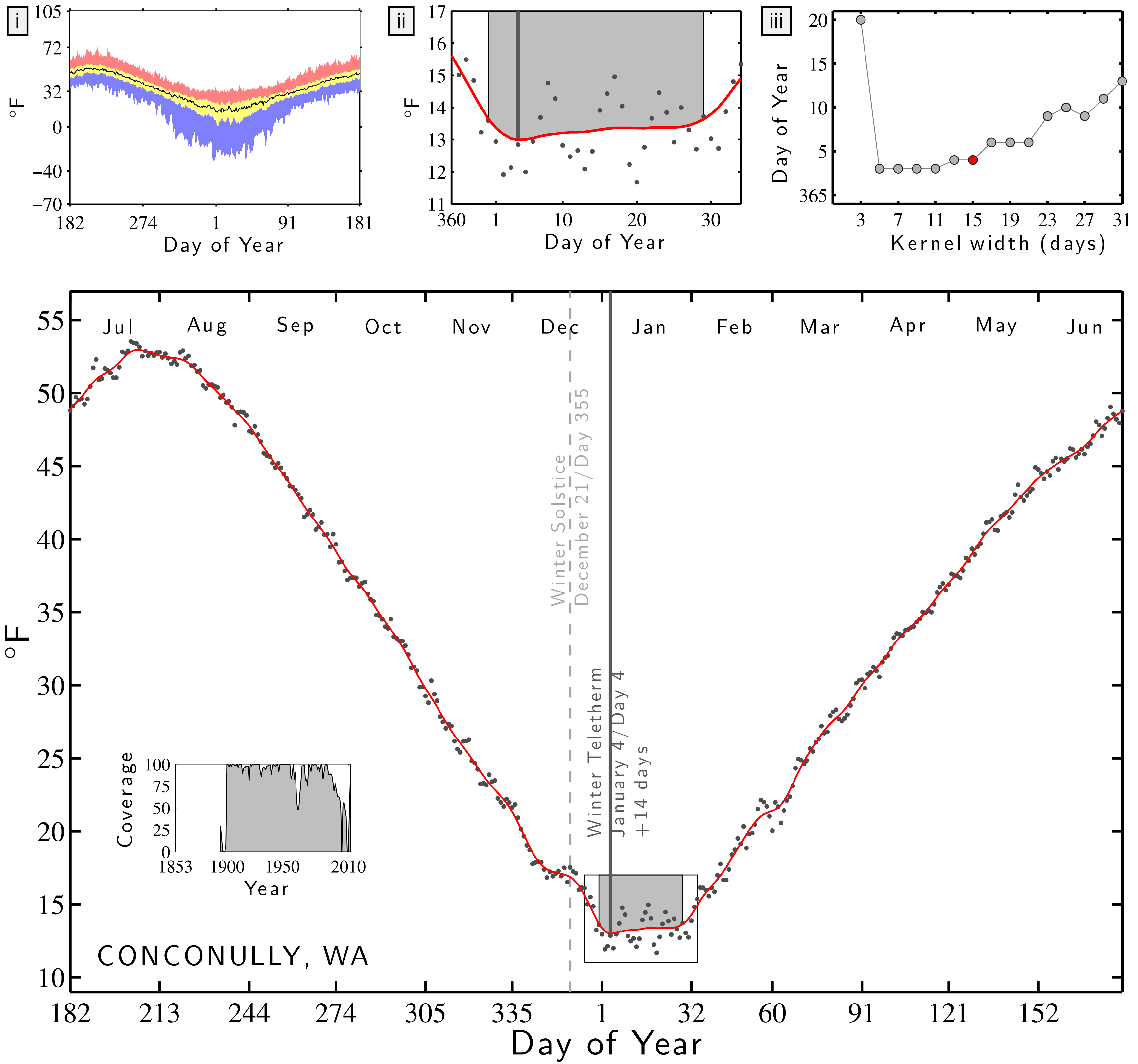

i
ii
iii
Day of Year
°F
Kernel width (days)
Jul
Aug
Sep
Oct
Nov
Dec
Jan
Feb
Mar
Apr
May
Jun
Winter Solstice
December 21/Day 355
Winter Teletherm
January 4/Day 4
+14 days
Coverage
Year
CONCONULLY, WA

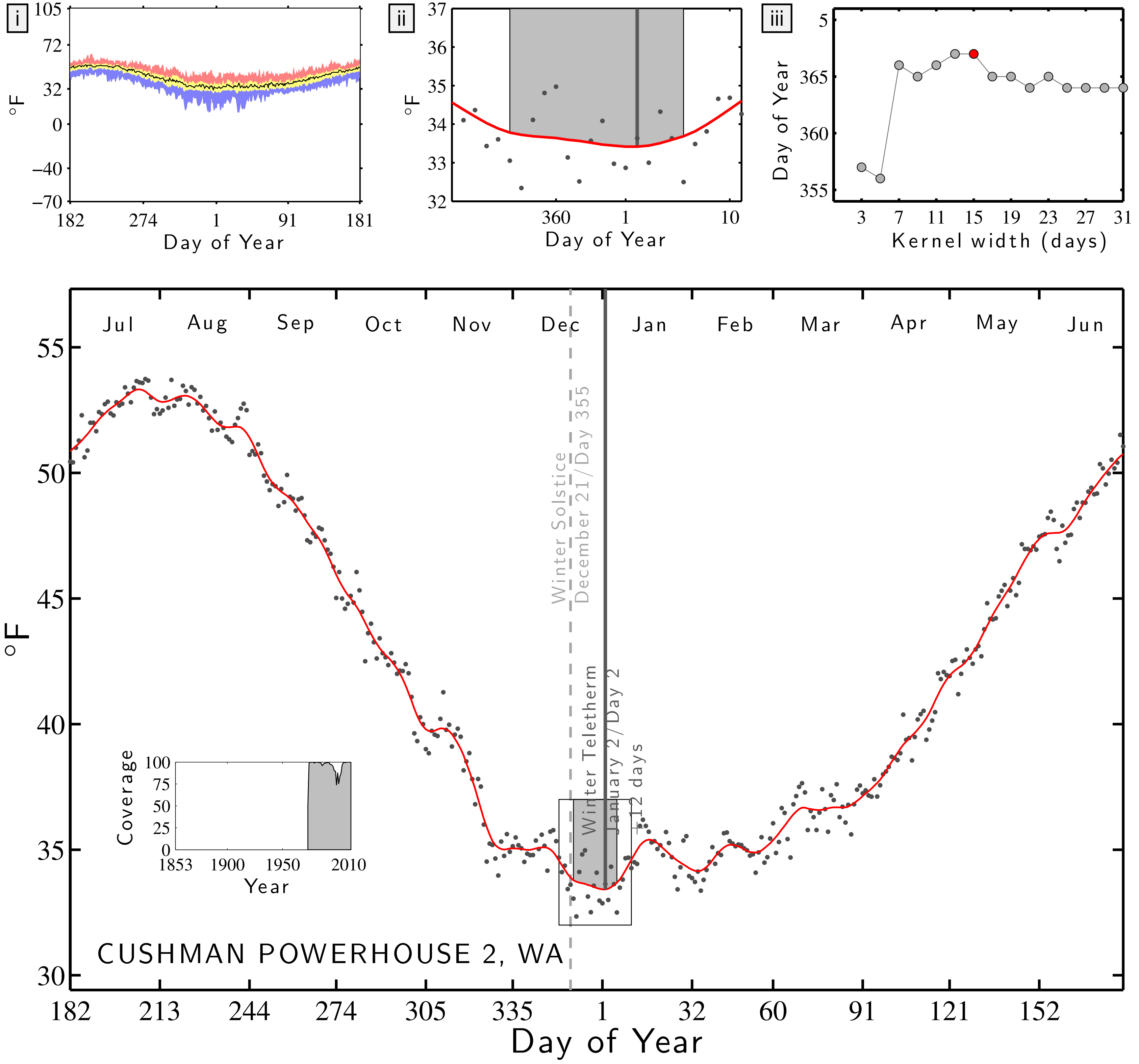
i
ii
iii
°F
Day of Year
Kernel width (days)
Jul
Aug
Sep
Oct
Nov
Dec
Jan
Feb
Mar
Apr
May
Jun
Winter Solstice
December 21/Day 355
Winter Teletherm
January 2/Day 2
±12 days
Coverage
Year
CUSHMAN POWERHOUSE 2, WA

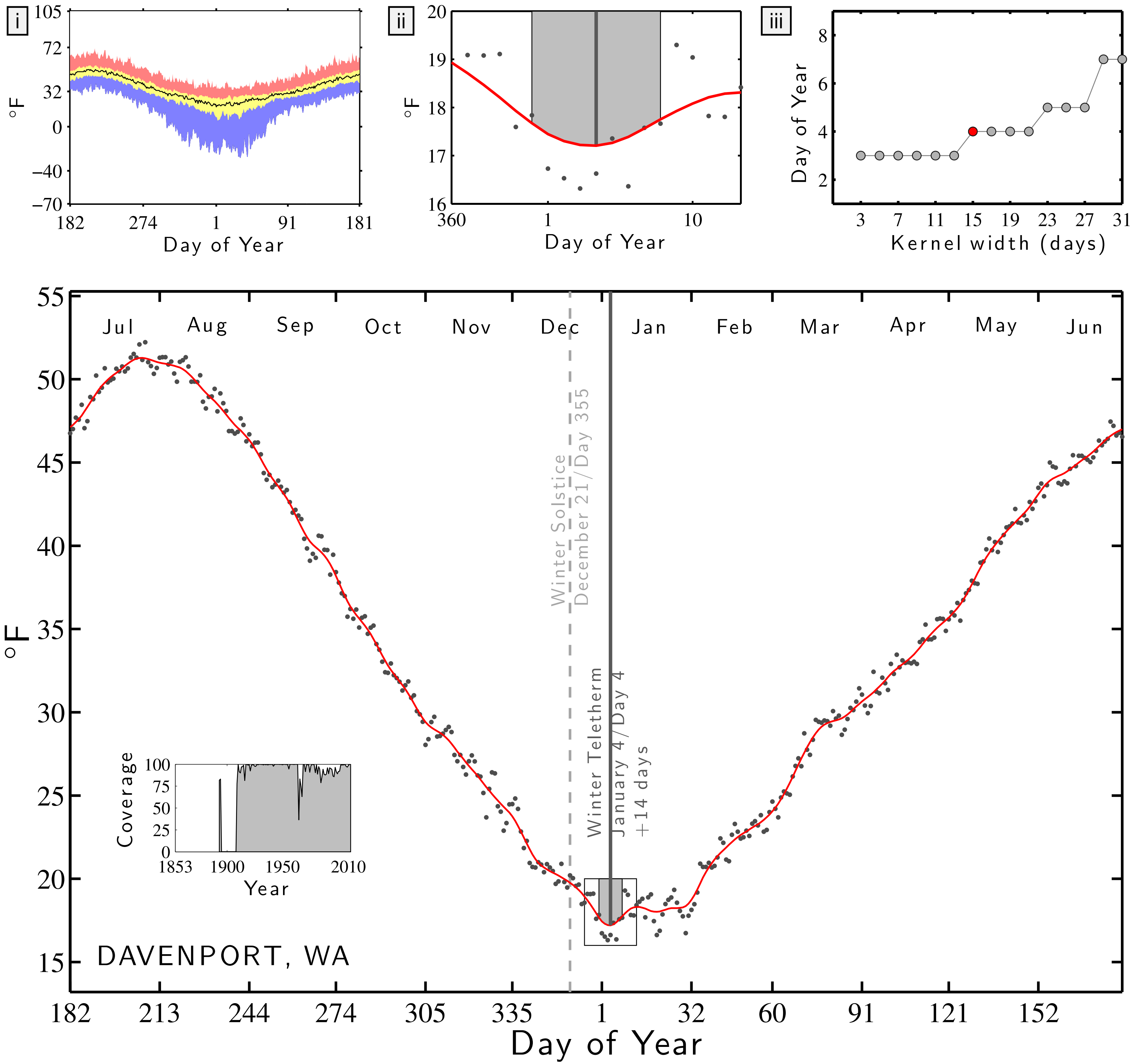

i
105
72
32
0
−40
−70
°F
182
274
1
91
181
Day of Year
ii
20
19
18
17
16
°F
360
1
10
Day of Year
iii
8
6
4
2
Day of Year
3
7
11
15
19
23
27
31
Kernel width (days)
Jul
Aug
Sep
Oct
Nov
Dec
Jan
Feb
Mar
Apr
May
Jun
Winter Solstice
December 21/Day 355
Winter Teletherm
January 4/Day 4
+14 days
Coverage
100
75
50
25
0
1853
1900
1950
2010
Year
DAVENPORT, WA
55
50
45
40
35
30
25
20
15
°F
182
213
244
274
305
335
1
32
60
91
121
152
Day of Year

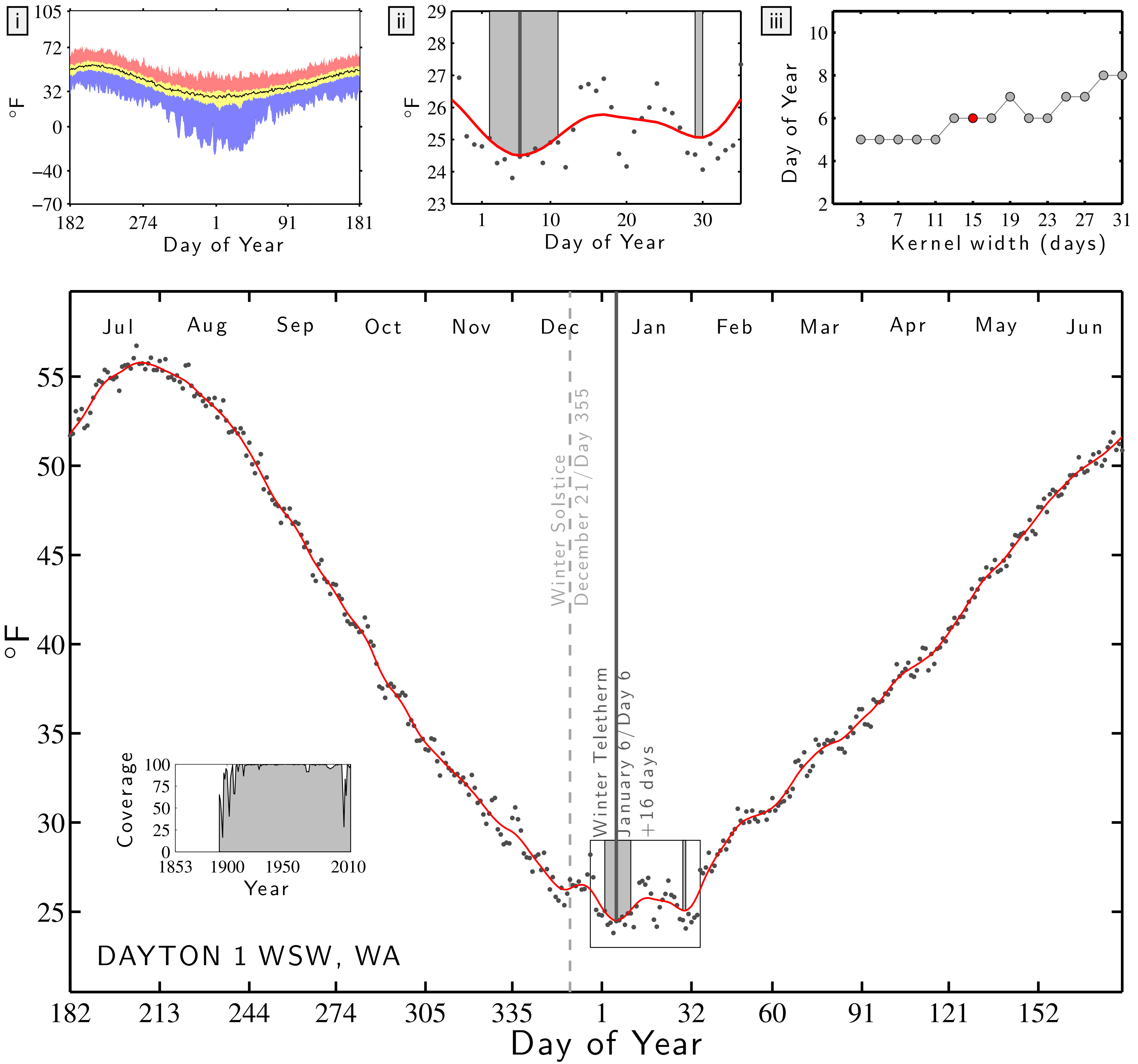

i
ii
iii
°F
Day of Year
Kernel width (days)
Jul
Aug
Sep
Oct
Nov
Dec
Jan
Feb
Mar
Apr
May
Jun
Winter Solstice
December 21/Day 355
Winter Teletherm
January 6/Day 6
+16 days
Coverage
Year
DAYTON 1 WSW, WA

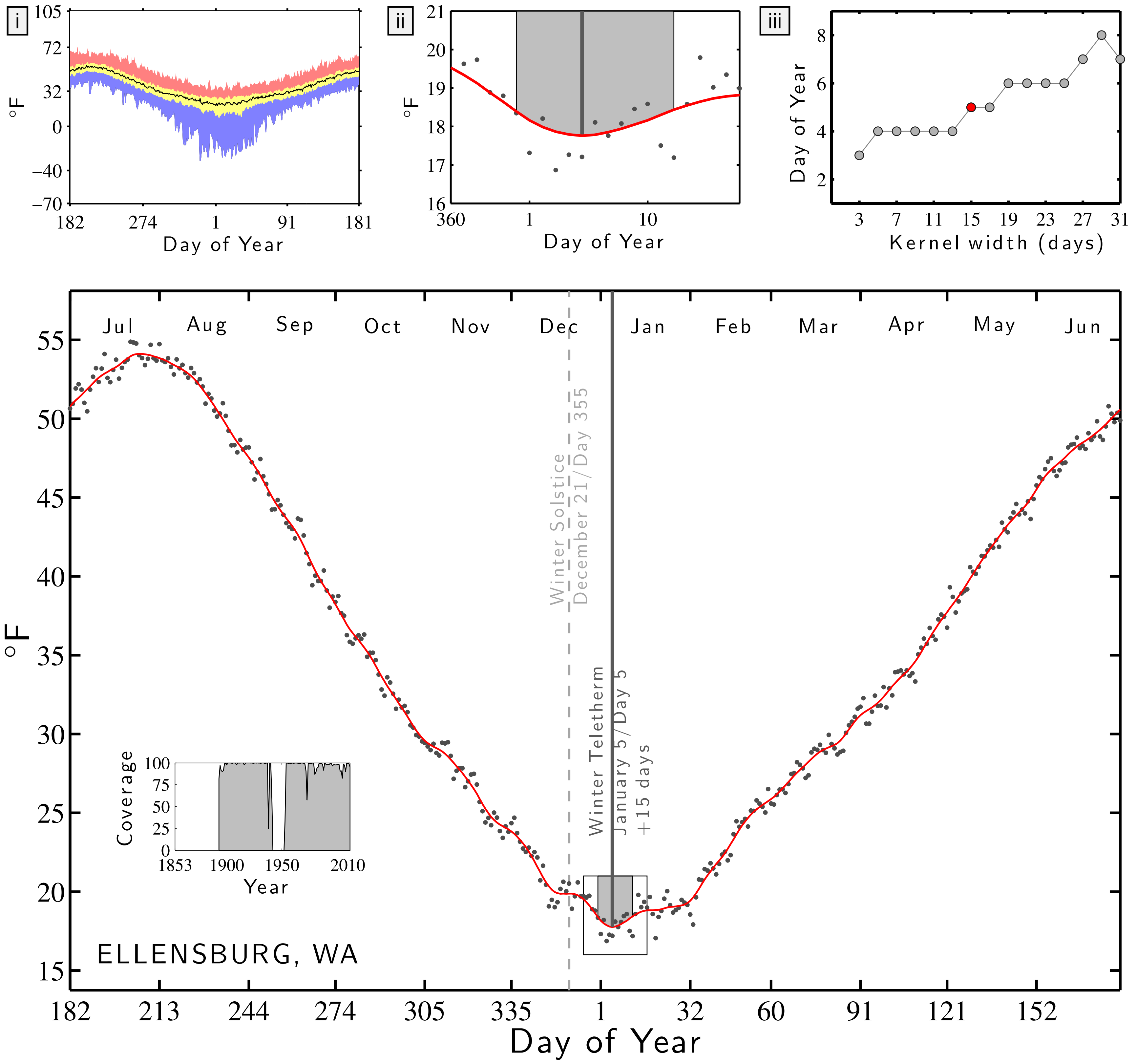

i
ii
iii
Day of Year
°F
Kernel width (days)
Jul
Aug
Sep
Oct
Nov
Dec
Jan
Feb
Mar
Apr
May
Jun
Winter Solstice
December 21/Day 355
Winter Teletherm
January 5/Day 5
+15 days
Coverage
Year
ELLENSBURG, WA

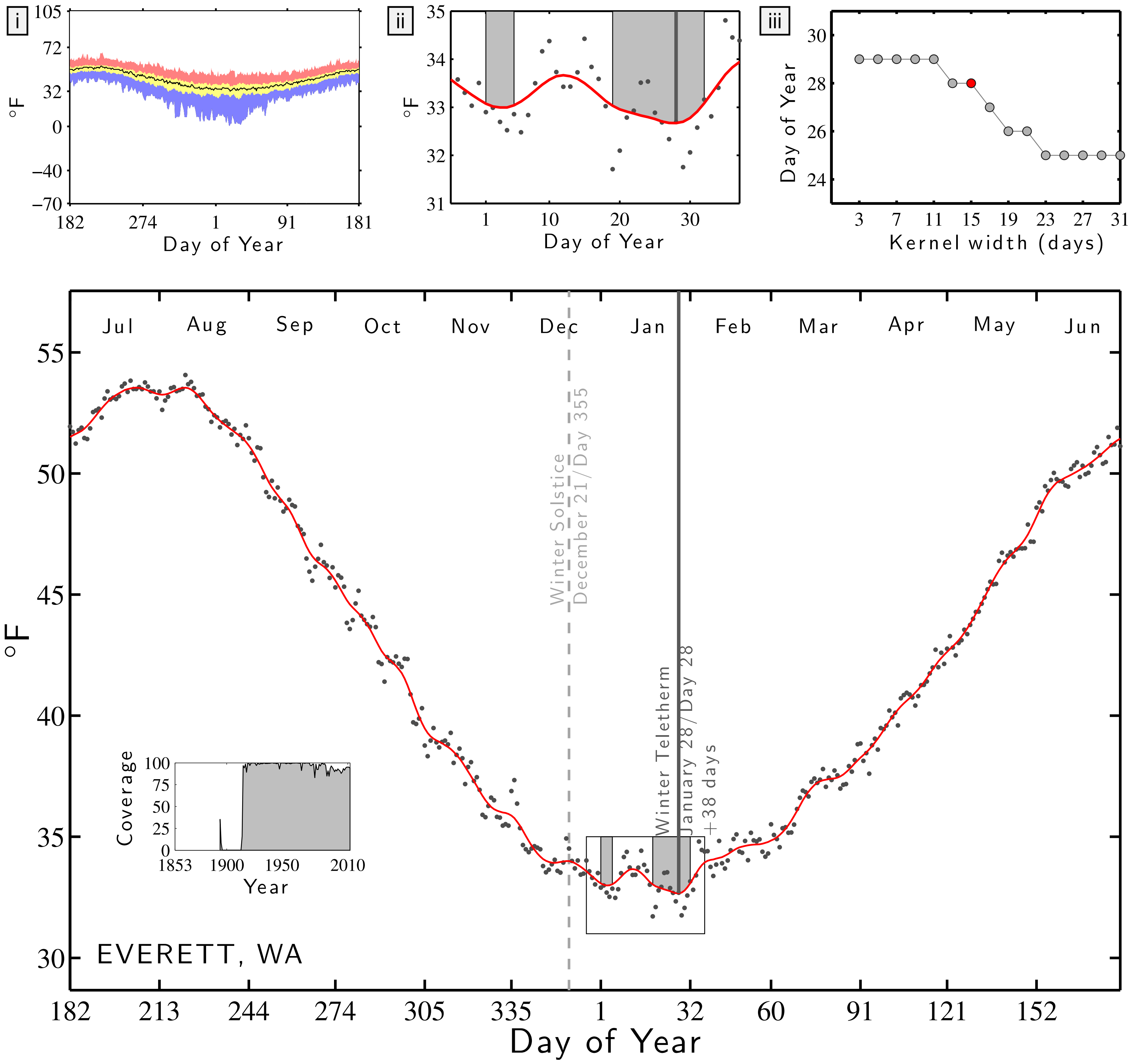

i
ii
iii
°F
Day of Year
Kernel width (days)
Jul
Aug
Sep
Oct
Nov
Dec
Jan
Feb
Mar
Apr
May
Jun
Winter Solstice
December 21/Day 355
Winter Teletherm
January 28/Day 28
+38 days
Coverage
Year
EVERETT, WA

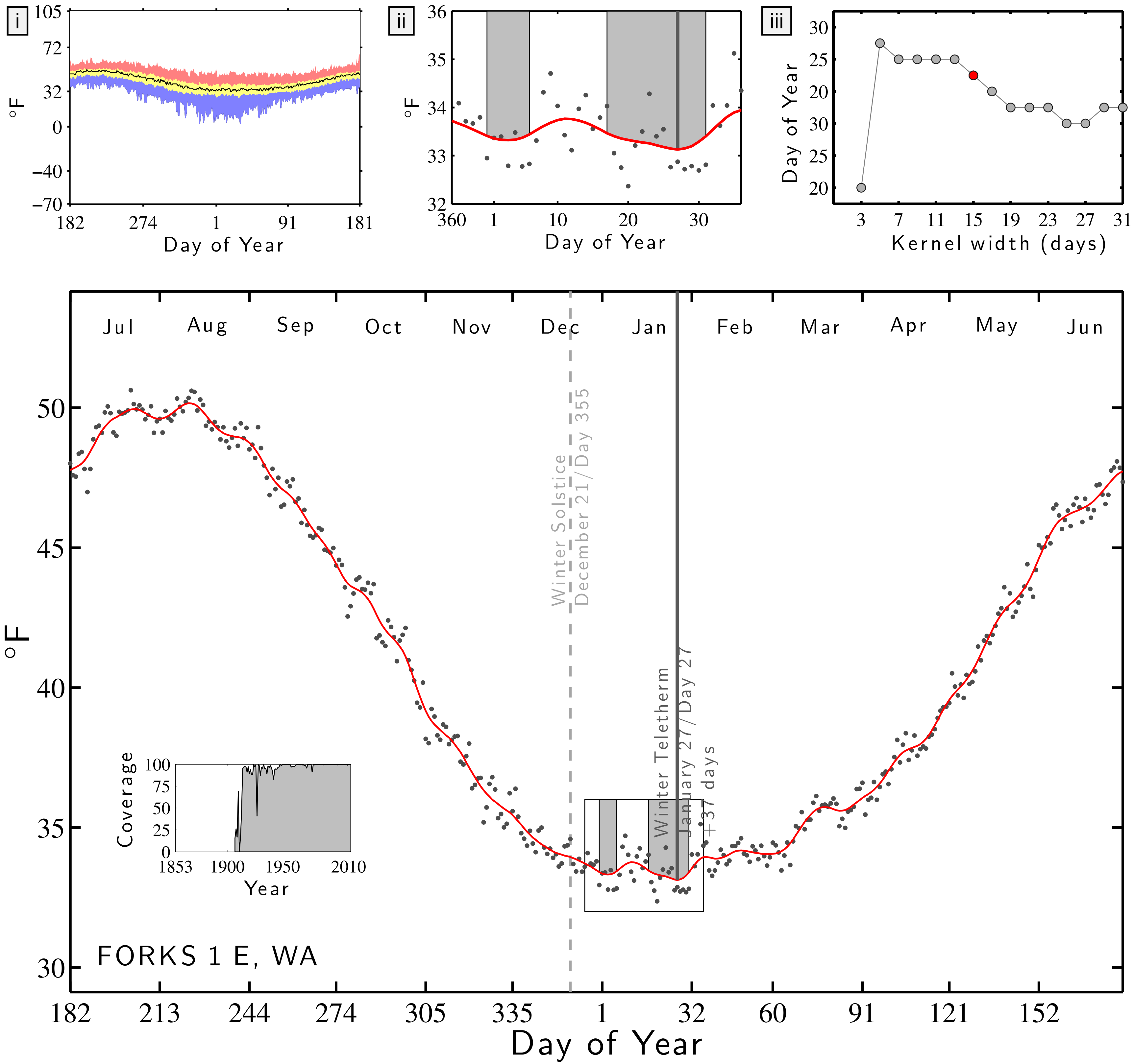

i
105
72
32
0
−40
−70
°F
182
274
1
91
181
Day of Year
ii
36
35
34
33
32
°F
360
1
10
20
30
Day of Year
iii
30
25
20
30
25
20
Day of Year
3
7
11
15
19
23
27
31
Kernel width (days)
Jul
Aug
Sep
Oct
Nov
Dec
Jan
Feb
Mar
Apr
May
Jun
50
45
40
35
30
°F
Winter Solstice
December 21/Day 355
Winter Teletherm
January 27/Day 27
+37 days
Coverage
100
75
50
25
0
1853
1900
1950
2010
Year
FORKS 1 E, WA
182
213
244
274
305
335
1
32
60
91
121
152
Day of Year

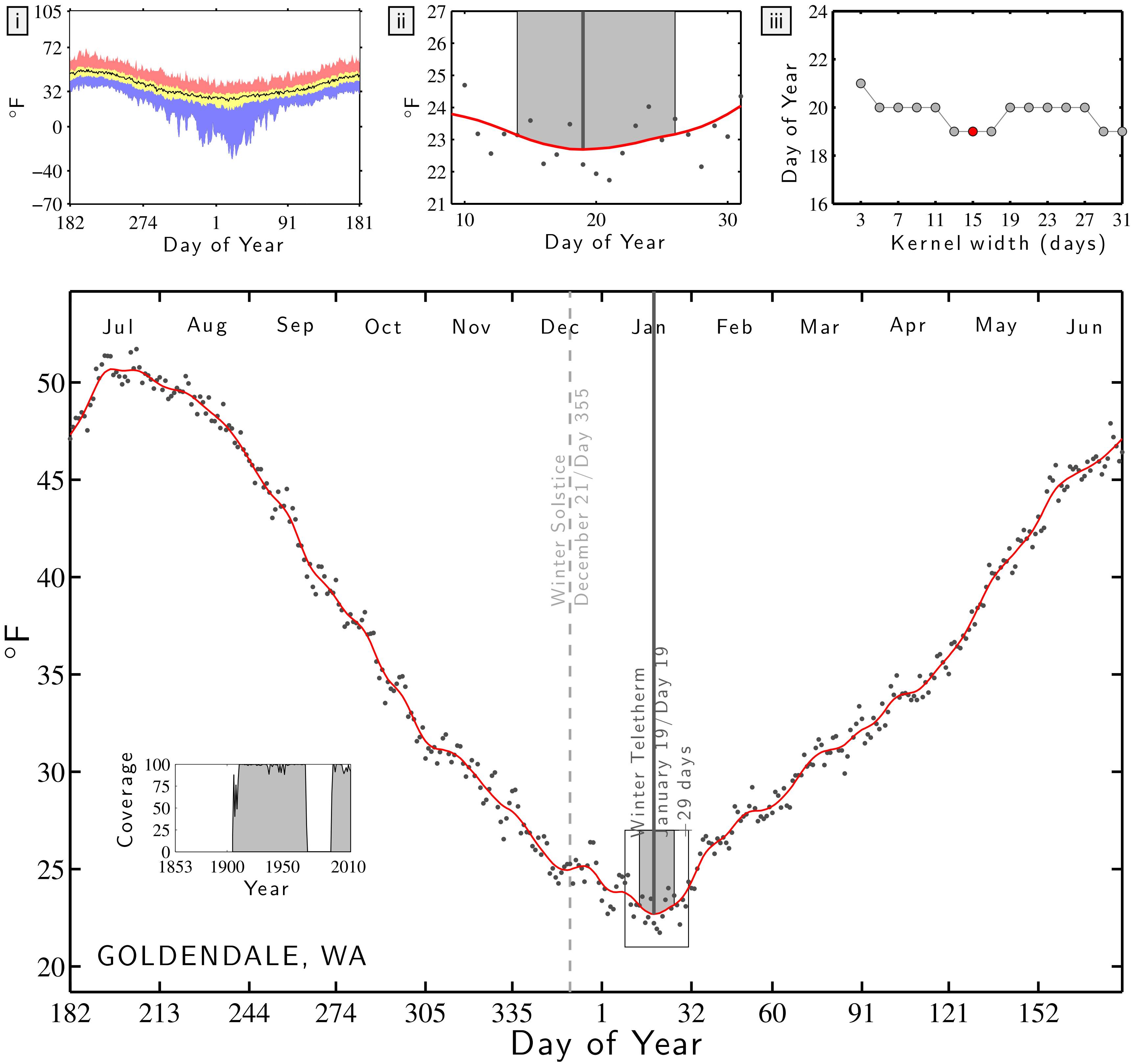

i
°F
105
72
32
0
−40
−70
182
274
1
91
181
Day of Year
ii
°F
27
26
25
24
23
22
21
10
20
30
Day of Year
iii
Day of Year
24
22
20
18
16
3
7
11
15
19
23
27
31
Kernel width (days)
Jul
Aug
Sep
Oct
Nov
Dec
Jan
Feb
Mar
Apr
May
Jun
°F
50
45
40
35
30
25
20
Winter Solstice
December 21/Day 355
Winter Teletherm
January 19/Day 19
± 29 days
Coverage
100
75
50
25
0
1853
1900
1950
2010
Year
GOLDENDALE, WA
182
213
244
274
305
335
1
32
60
91
121
152
Day of Year

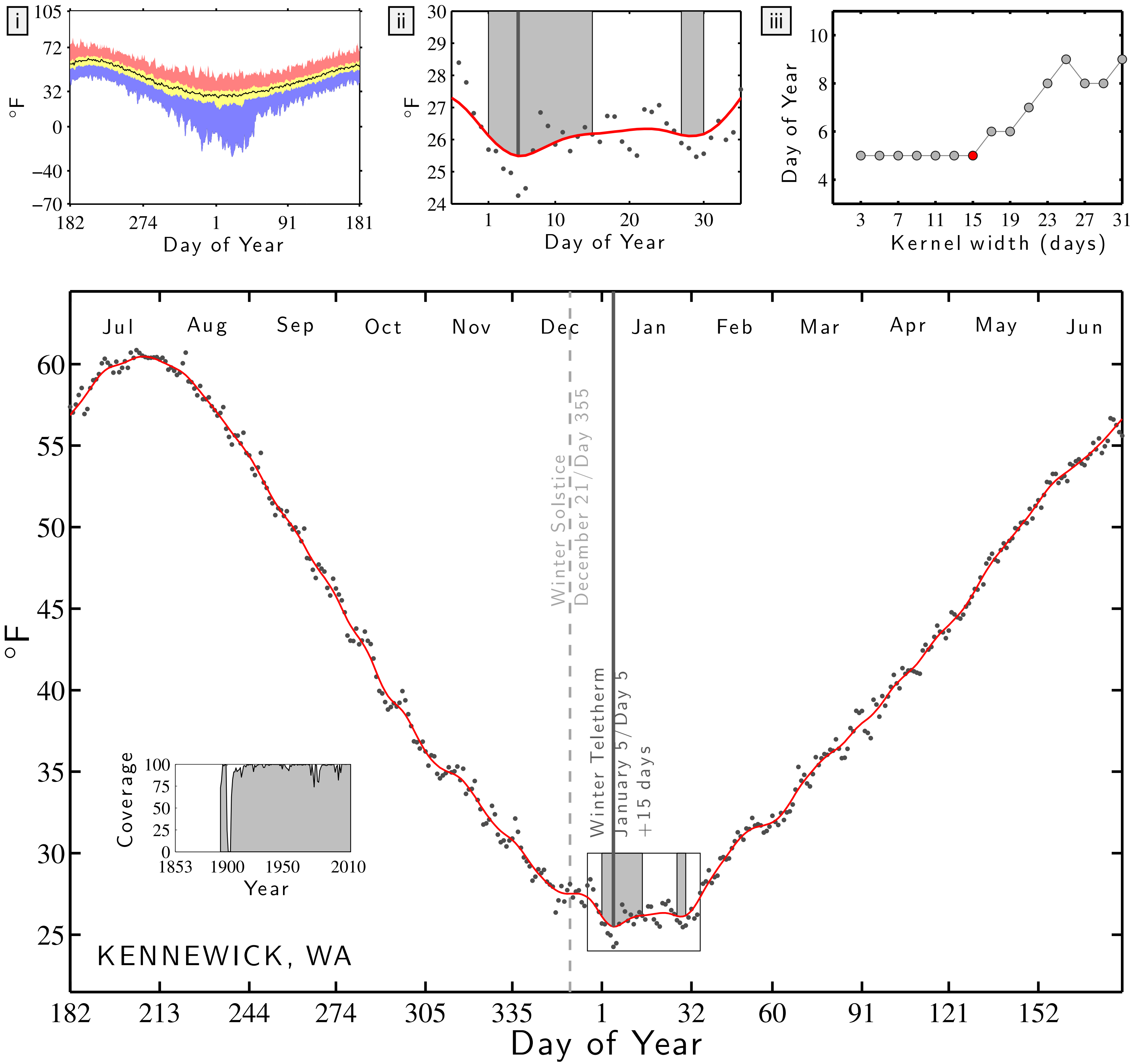

i
105
72
32
0
−40
−70
°F
182
274
1
91
181
Day of Year
ii
30
29
28
27
26
25
24
°F
1
10
20
30
Day of Year
iii
10
8
6
4
Day of Year
3
7
11
15
19
23
27
31
Kernel width (days)
Jul
Aug
Sep
Oct
Nov
Dec
Jan
Feb
Mar
Apr
May
Jun
60
55
50
45
40
35
30
25
°F
Winter Solstice
December 21/Day 355
Winter Teletherm
January 5/Day 5
+15 days
Coverage
100
75
50
25
0
1853
1900
1950
2010
Year
KENNEWICK, WA
182
213
244
274
305
335
1
32
60
91
121
152
Day of Year

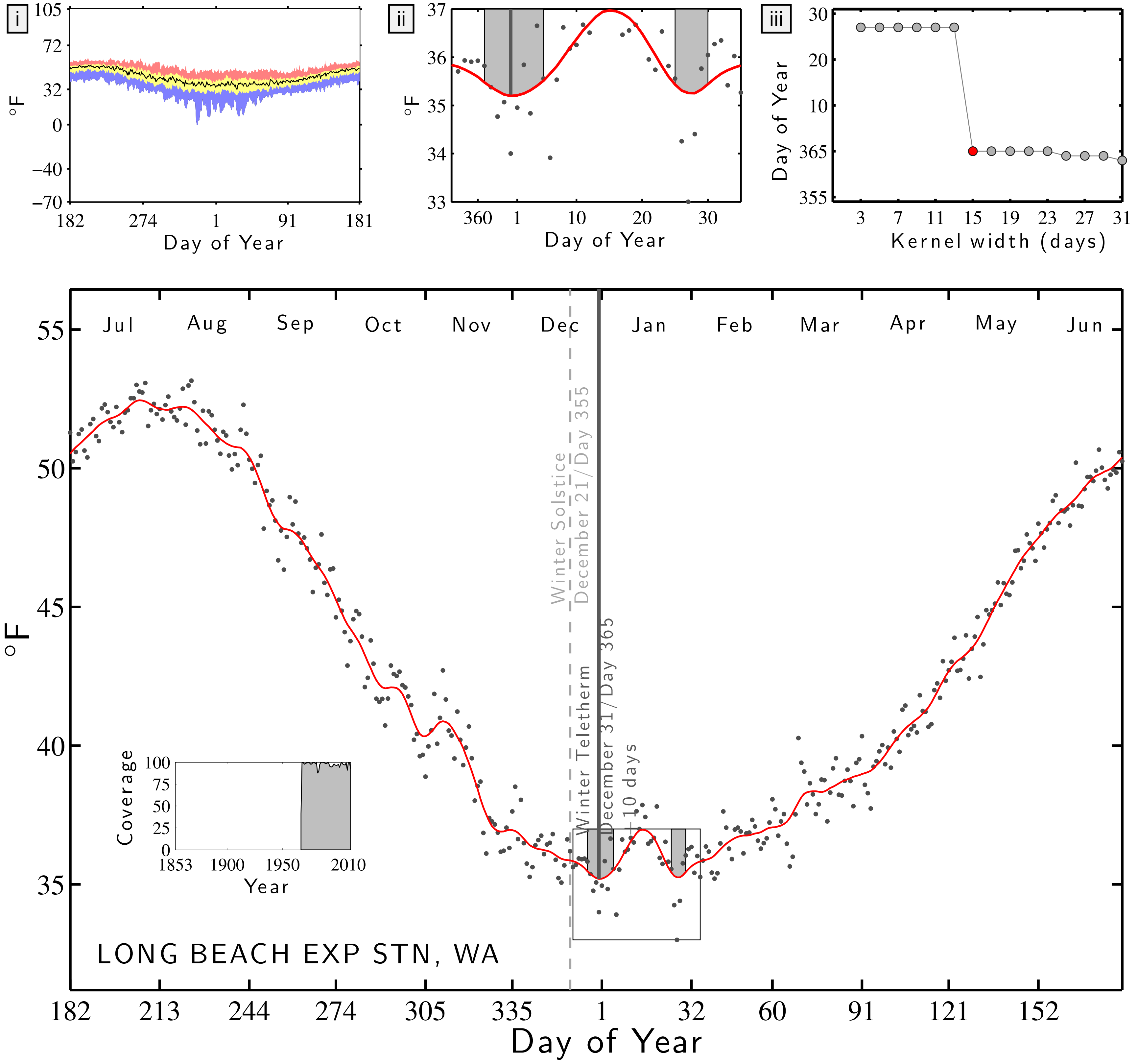

i
ii
iii
°F
Day of Year
Kernel width (days)
Jul
Aug
Sep
Oct
Nov
Dec
Jan
Feb
Mar
Apr
May
Jun
Winter Solstice
December 21/Day 355
Winter Teletherm
December 31/Day 365
±10 days
Coverage
Year
LONG BEACH EXP STN, WA

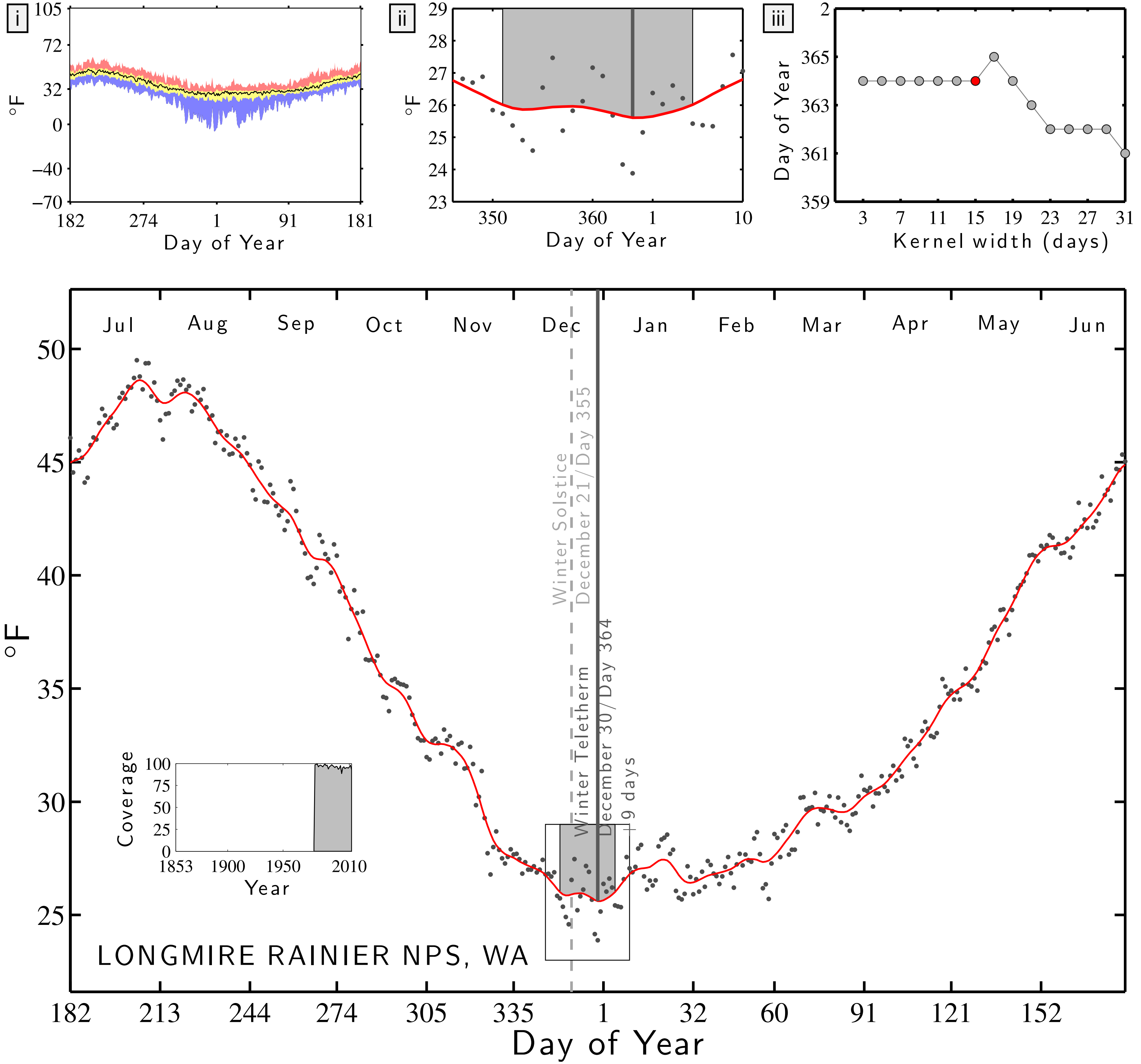

i
°F
Day of Year
ii
°F
Day of Year
iii
Day of Year
Kernel width (days)
Jul
Aug
Sep
Oct
Nov
Dec
Jan
Feb
Mar
Apr
May
Jun
Winter Solstice
December 21/Day 355
Winter Teletherm
December 30/Day 364
9 days
Coverage
Year
LONGMIRE RAINIER NPS, WA
°F
Day of Year

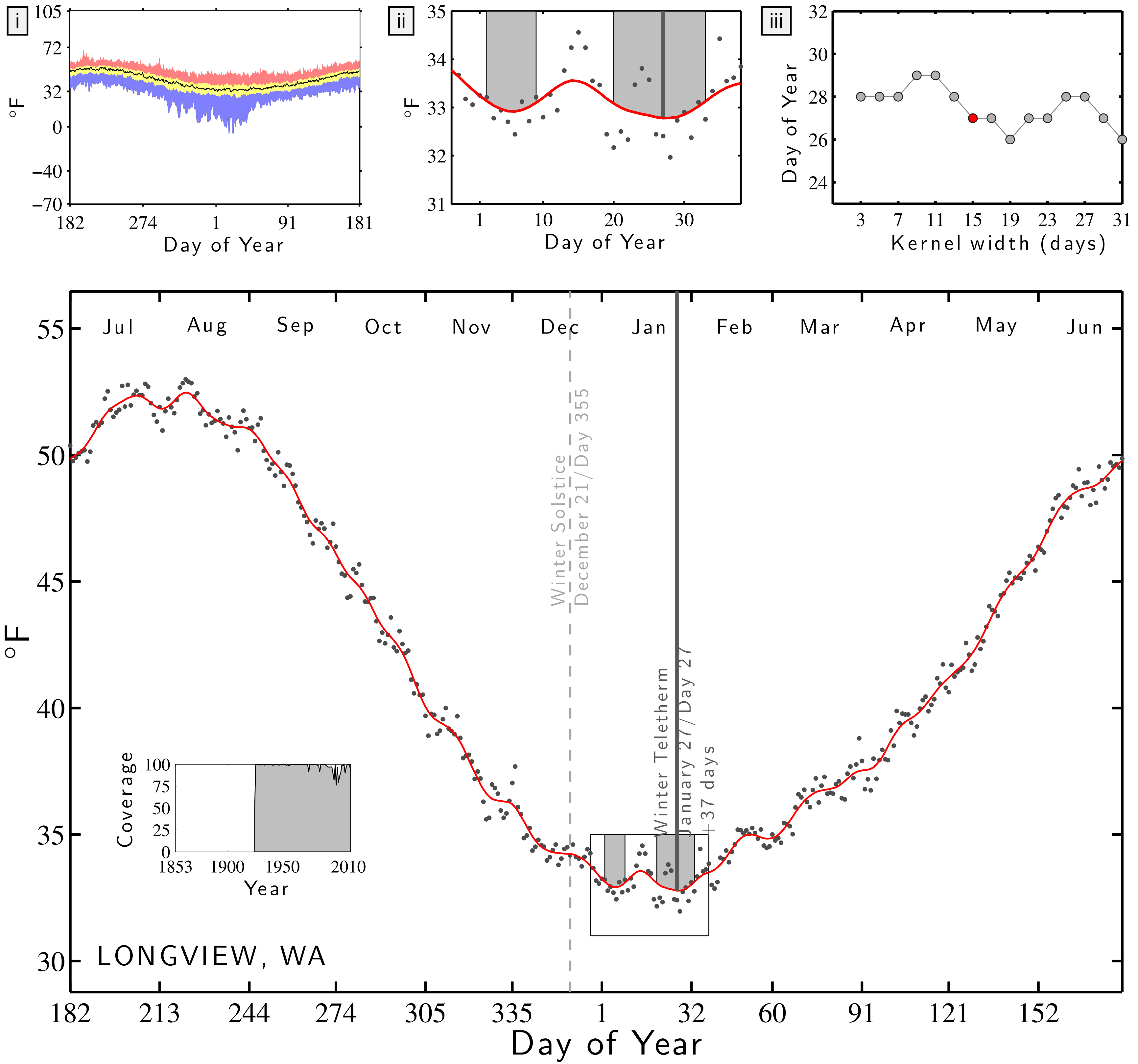

i
105
72
32
0
−40
−70
°F
182
274
1
91
181
Day of Year
ii
35
34
33
32
31
°F
1
10
20
30
Day of Year
iii
32
30
28
26
24
Day of Year
3
7
11
15
19
23
27
31
Kernel width (days)
Jul
Aug
Sep
Oct
Nov
Dec
Jan
Feb
Mar
Apr
May
Jun
55
50
45
40
35
30
°F
Winter Solstice
December 21/Day 355
Winter Teletherm
January 27/Day 27
±37 days
Coverage
100
75
50
25
0
1853
1900
1950
2010
Year
LONGVIEW, WA
182
213
244
274
305
335
1
32
60
91
121
152
Day of Year

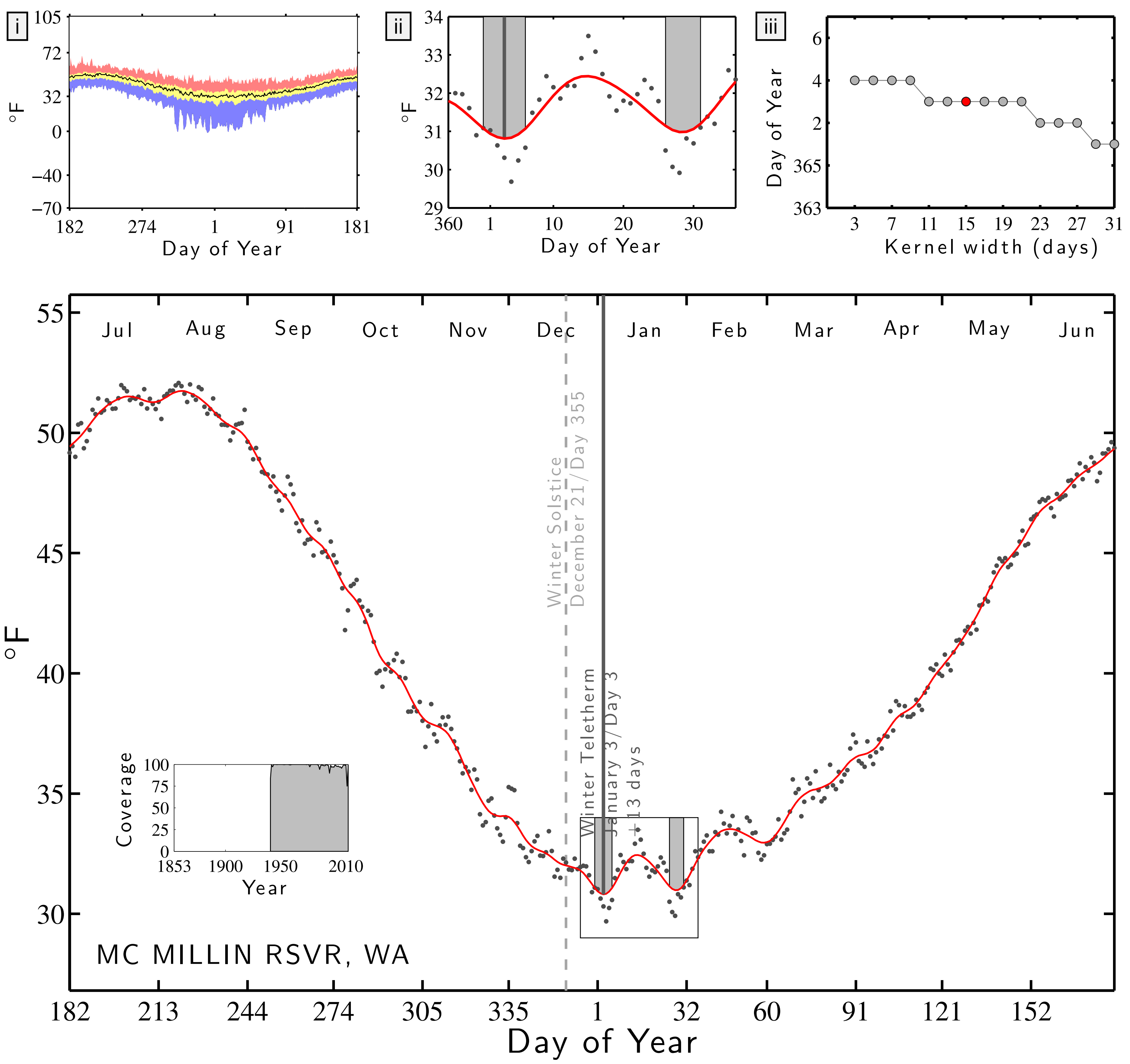

i
°F
Day of Year
ii
°F
Day of Year
iii
Day of Year
Kernel width (days)
Jul
Aug
Sep
Oct
Nov
Dec
Jan
Feb
Mar
Apr
May
Jun
Winter Solstice
December 21/Day 355
Winter Teletherm
January 3/Day 3
± 13 days
Coverage
Year
MC MILLIN RSVR, WA
°F
Day of Year

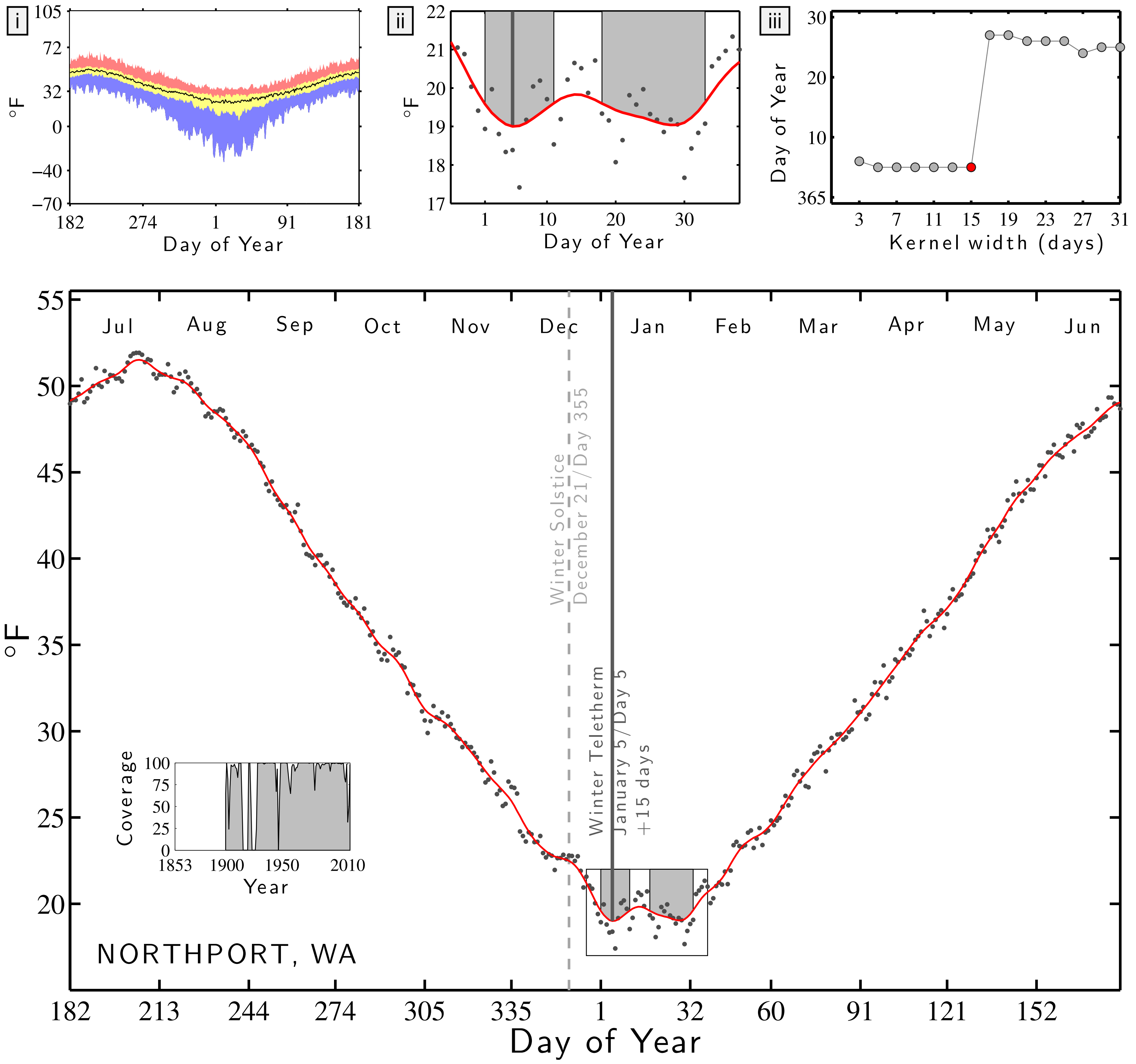

i
°F
105
72
32
0
−40
−70
182
274
1
91
181
Day of Year
ii
22
21
20
19
18
17
1
10
20
30
iii
30
20
10
365
3
7
11
15
19
23
27
31
Day of Year
Kernel width (days)
Jul
Aug
Sep
Oct
Nov
Dec
Jan
Feb
Mar
Apr
May
Jun
55
50
45
40
35
30
25
20
Winter Solstice
December 21/Day 355
Winter Teletherm
January 5/Day 5
+15 days
Coverage
100
75
50
25
0
1853
1900
1950
2010
Year
NORTHPORT, WA
182
213
244
274
305
335
1
32
60
91
121
152
Day of Year

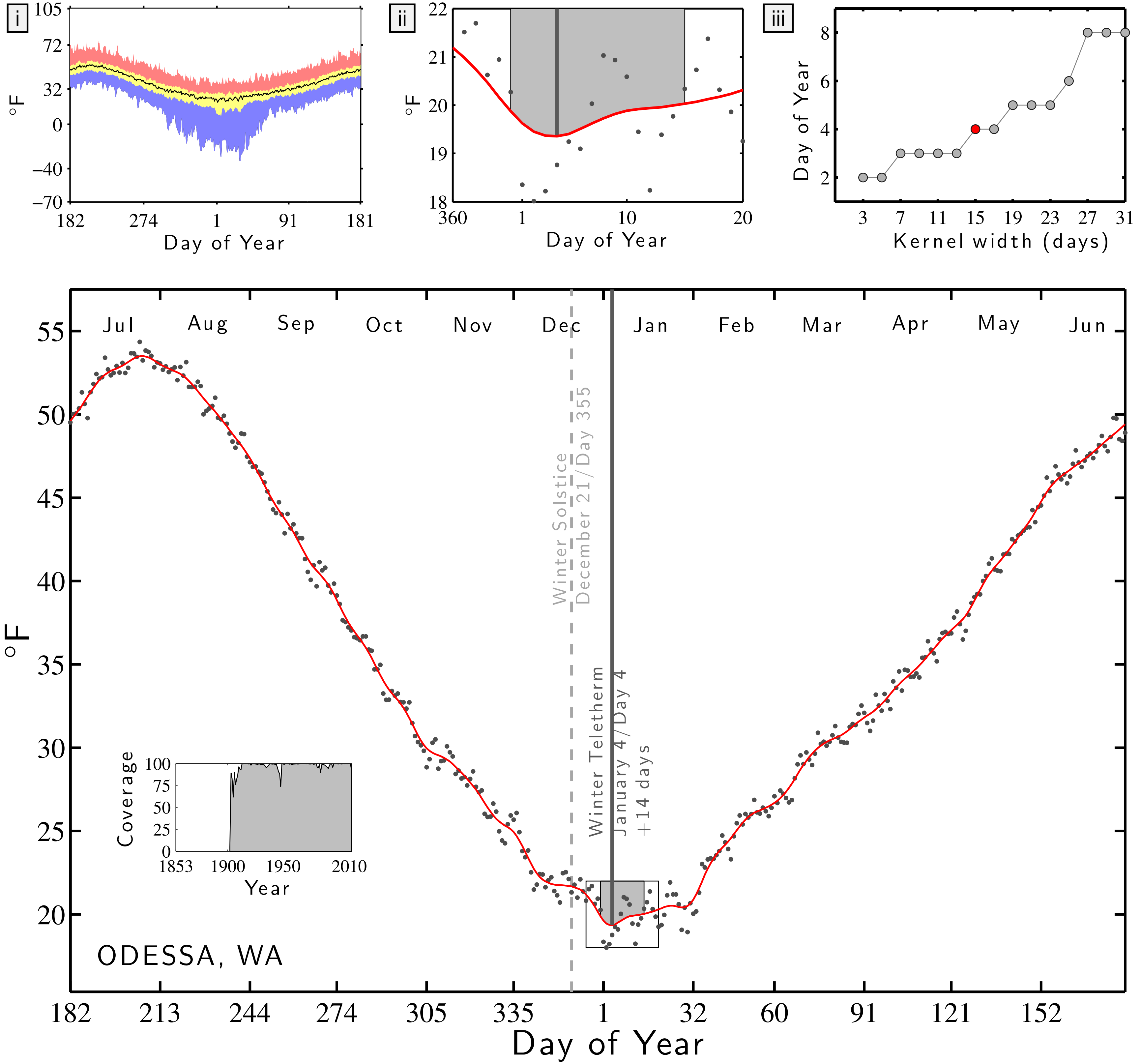

i
105
72
32
0
−40
−70
°F
182
274
1
91
181
Day of Year
ii
22
21
20
19
18
°F
360
1
10
20
Day of Year
iii
8
6
4
2
Day of Year
3
7
11
15
19
23
27
31
Kernel width (days)
Jul
Aug
Sep
Oct
Nov
Dec
Jan
Feb
Mar
Apr
May
Jun
55
50
45
40
35
30
25
20
°F
Winter Solstice
December 21/Day 355
Winter Teletherm
January 4/Day 4
+14 days
Coverage
100
75
50
25
0
1853
1900
1950
2010
Year
ODESSA, WA
182
213
244
274
305
335
1
32
60
91
121
152
Day of Year

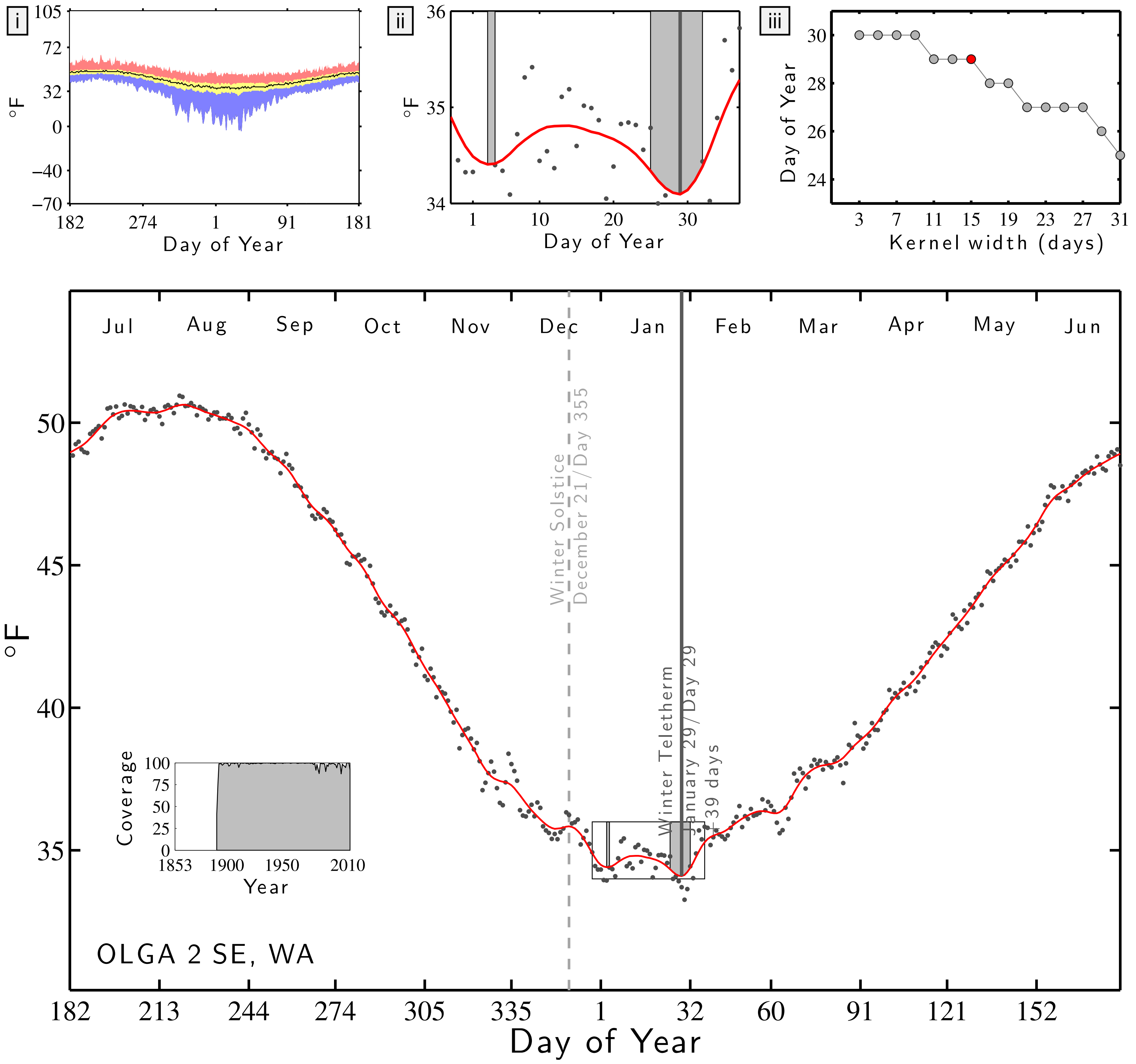

i
ii
iii
°F
Day of Year
Day of Year
Kernel width (days)
Jul
Aug
Sep
Oct
Nov
Dec
Jan
Feb
Mar
Apr
May
Jun
Winter Solstice
December 21/Day 355
Winter Teletherm
January 29/Day 29
±39 days
Coverage
Year
OLGA 2 SE, WA
Day of Year

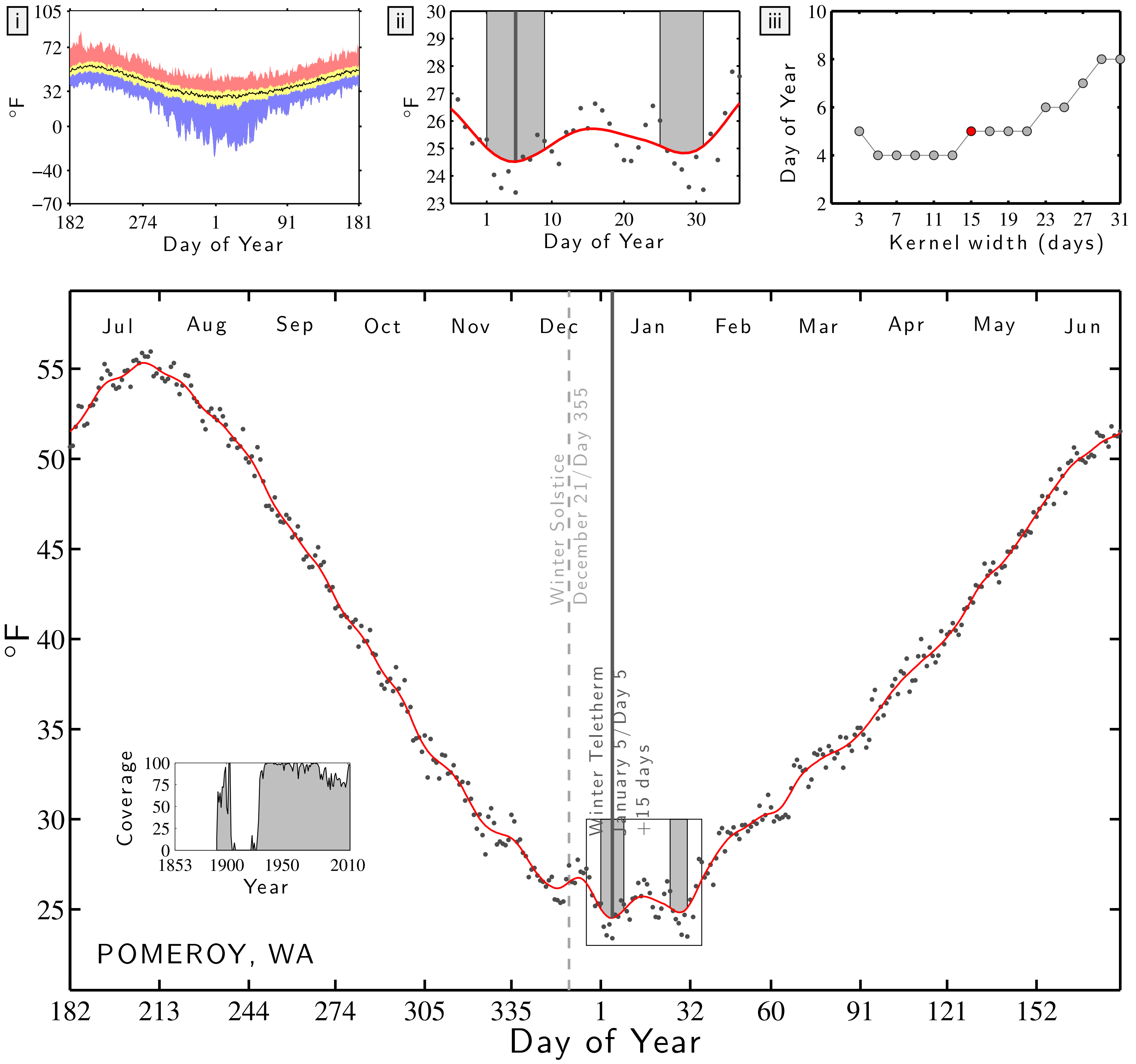

i
°F
Day of Year
ii
°F
Day of Year
iii
Day of Year
Kernel width (days)
Jul
Aug
Sep
Oct
Nov
Dec
Jan
Feb
Mar
Apr
May
Jun
Winter Solstice
December 21/Day 355
Winter Teletherm
January 5/Day 5
±15 days
Coverage
Year
POMEROY, WA
°F
Day of Year

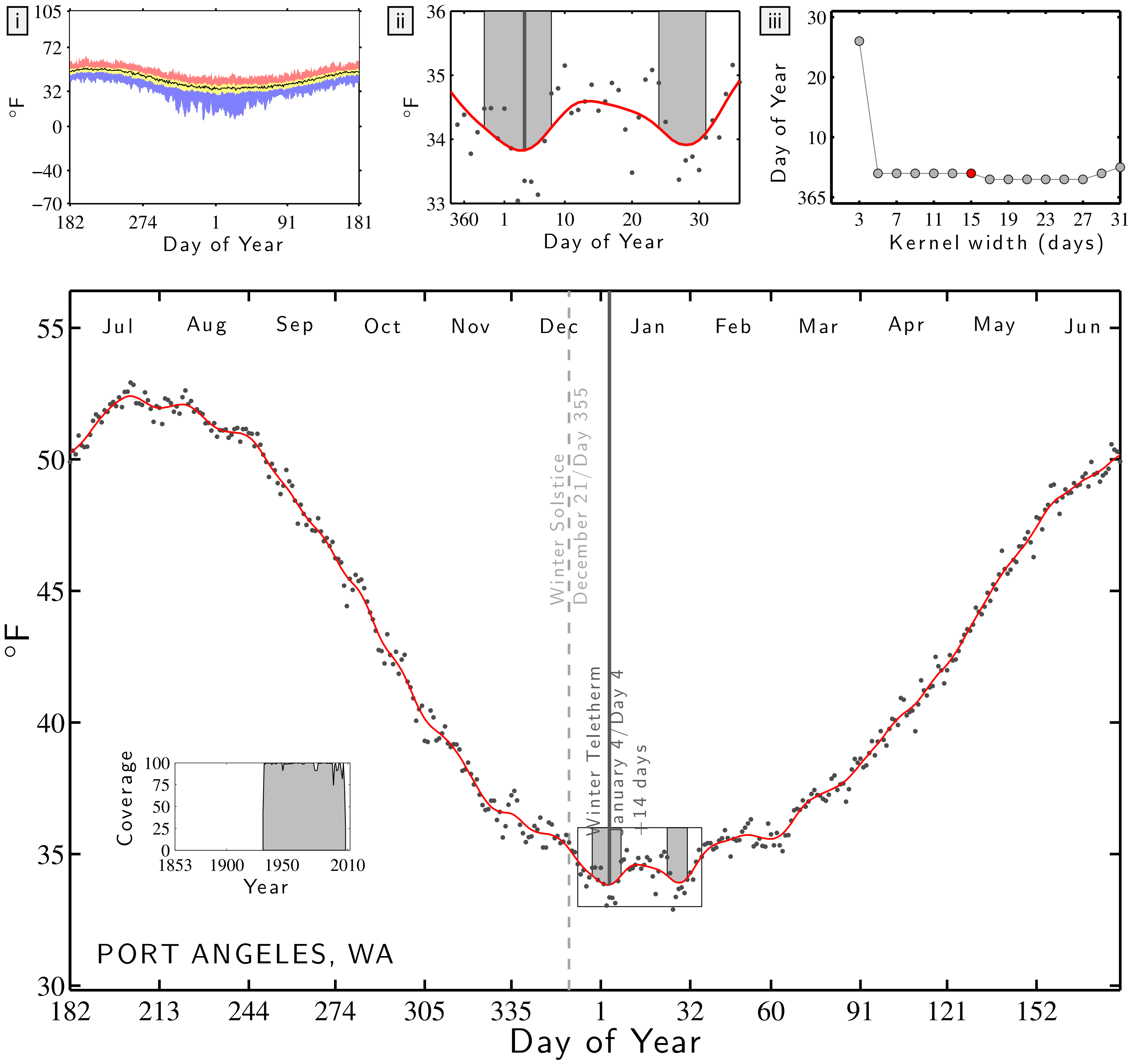

i
ii
iii
Day of Year
Kernel width (days)
°F
Jul
Aug
Sep
Oct
Nov
Dec
Jan
Feb
Mar
Apr
May
Jun
Winter Solstice
December 21/Day 355
Winter Teletherm
January 4/Day 4
±14 days
Coverage
Year
PORT ANGELES, WA

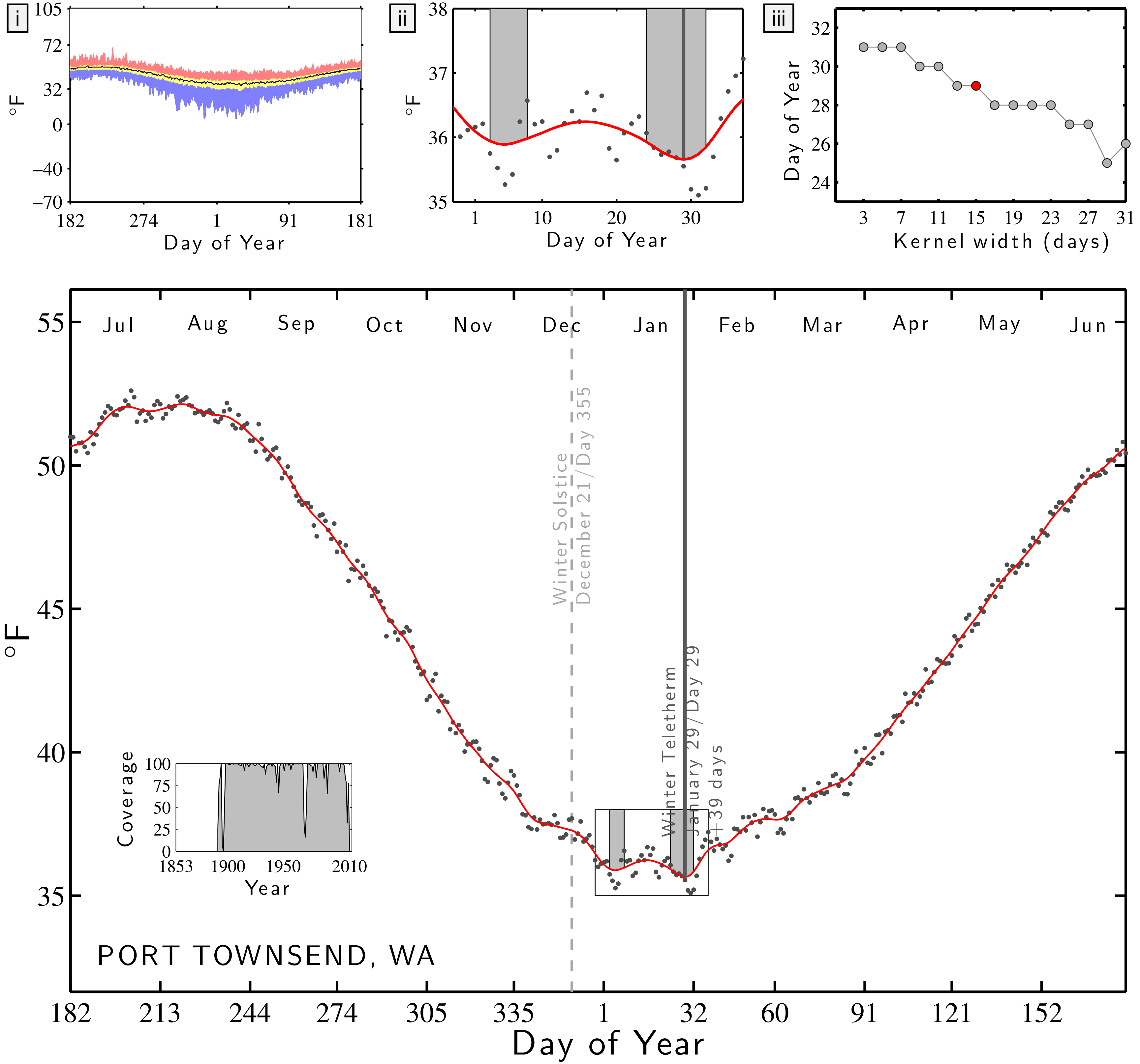

i
ii
iii
°F
Day of Year
Kernel width (days)
Day of Year
Jul
Aug
Sep
Oct
Nov
Dec
Jan
Feb
Mar
Apr
May
Jun
Winter Solstice
December 21/Day 355
Winter Teletherm
January 29/Day 29
+39 days
Coverage
Year
PORT TOWNSEND, WA

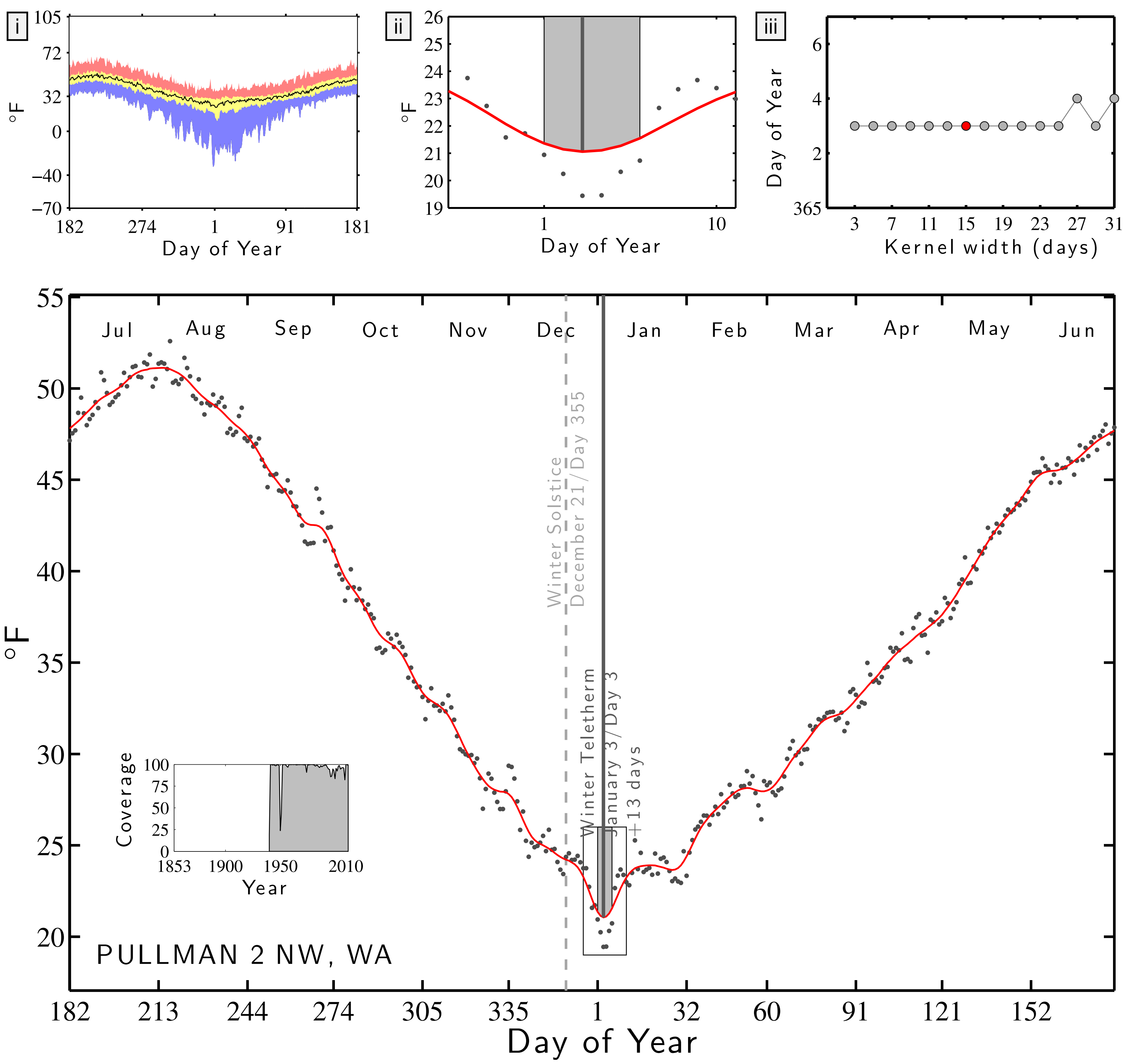

i
°F
Day of Year
ii
Day of Year
iii
Day of Year
Kernel width (days)
Jul
Aug
Sep
Oct
Nov
Dec
Jan
Feb
Mar
Apr
May
Jun
Winter Solstice
December 21/Day 355
Winter Teletherm
January 3/Day 3
+13 days
Coverage
Year
PULLMAN 2 NW, WA
°F
Day of Year

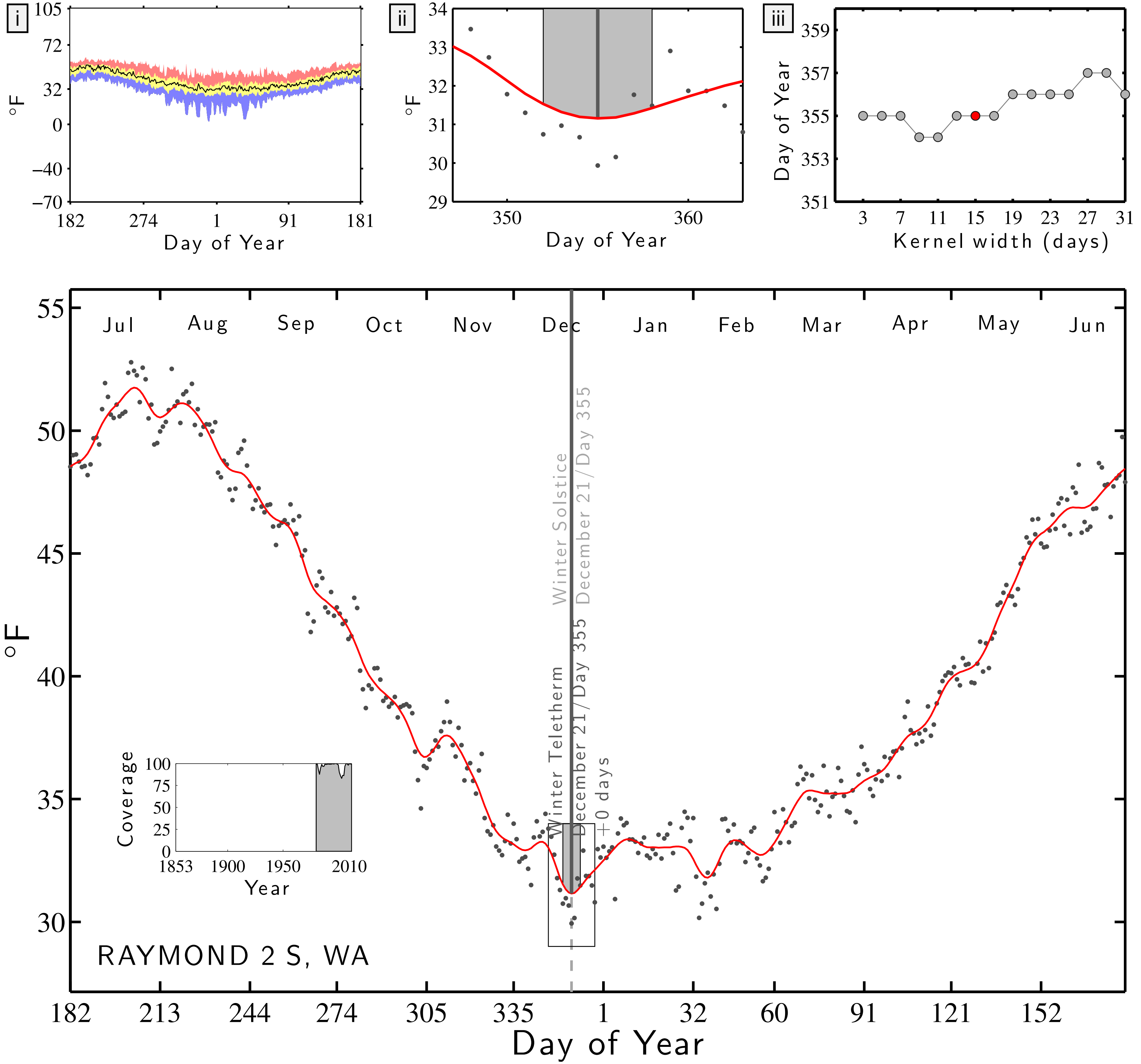

i
105
72
32
0
−40
−70
°F
182
274
1
91
181
Day of Year
ii
34
33
32
31
30
29
°F
350
360
Day of Year
iii
359
357
355
353
351
Day of Year
3
7
11
15
19
23
27
31
Kernel width (days)
Jul
Aug
Sep
Oct
Nov
Dec
Jan
Feb
Mar
Apr
May
Jun
55
50
45
40
35
30
°F
Winter Solstice
December 21/Day 355
Winter Teletherm
December 21/Day 355
+0 days
Coverage
100
75
50
25
0
1853
1900
1950
2010
Year
RAYMOND 2 S, WA
182
213
244
274
305
335
1
32
60
91
121
152
Day of Year

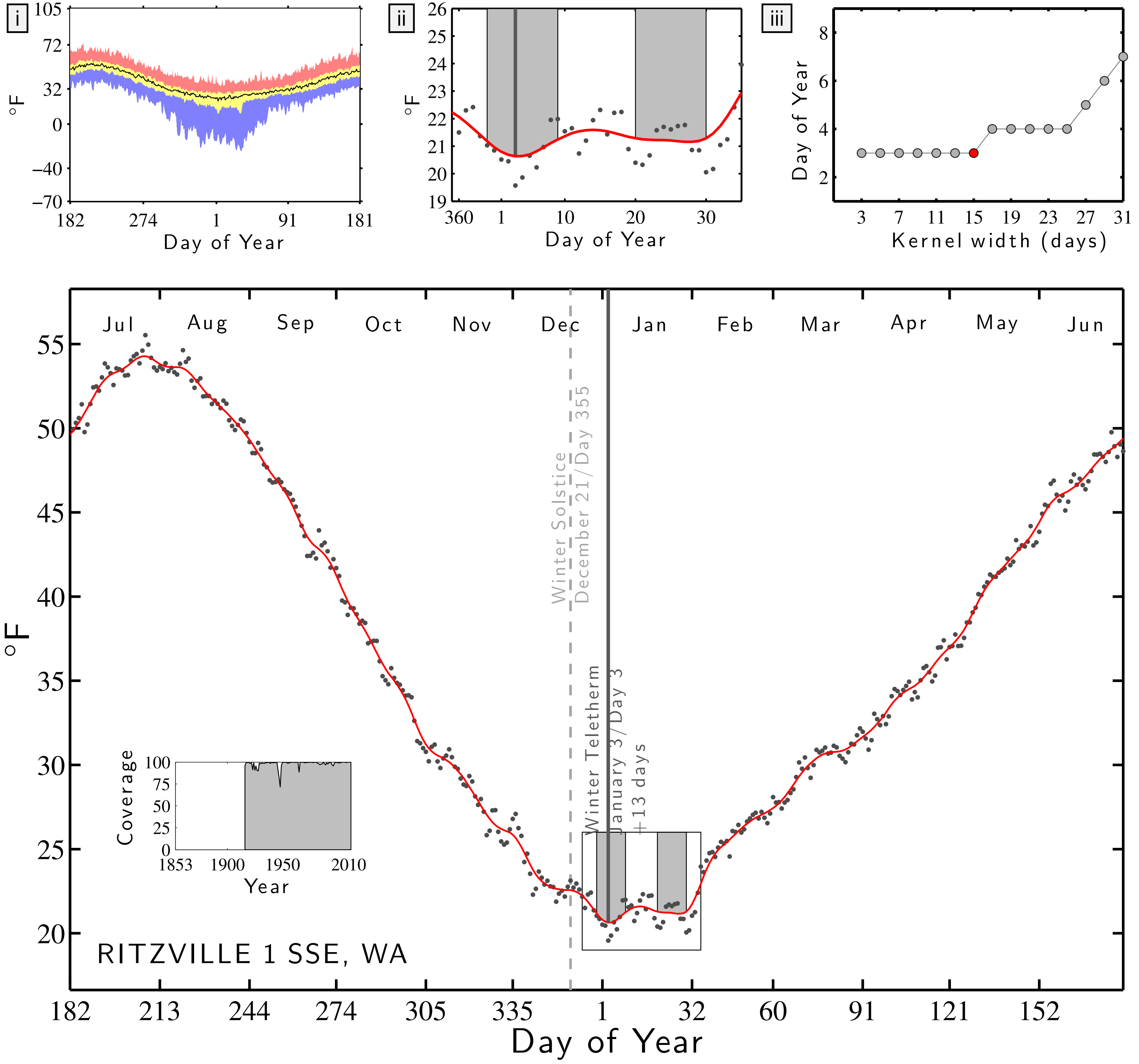
i
ii
iii
°F
Day of Year
Kernel width (days)
Jul
Aug
Sep
Oct
Nov
Dec
Jan
Feb
Mar
Apr
May
Jun
Winter Solstice
December 21/Day 355
Winter Teletherm
January 3/Day 3
+13 days
Coverage
Year
RITZVILLE 1 SSE, WA

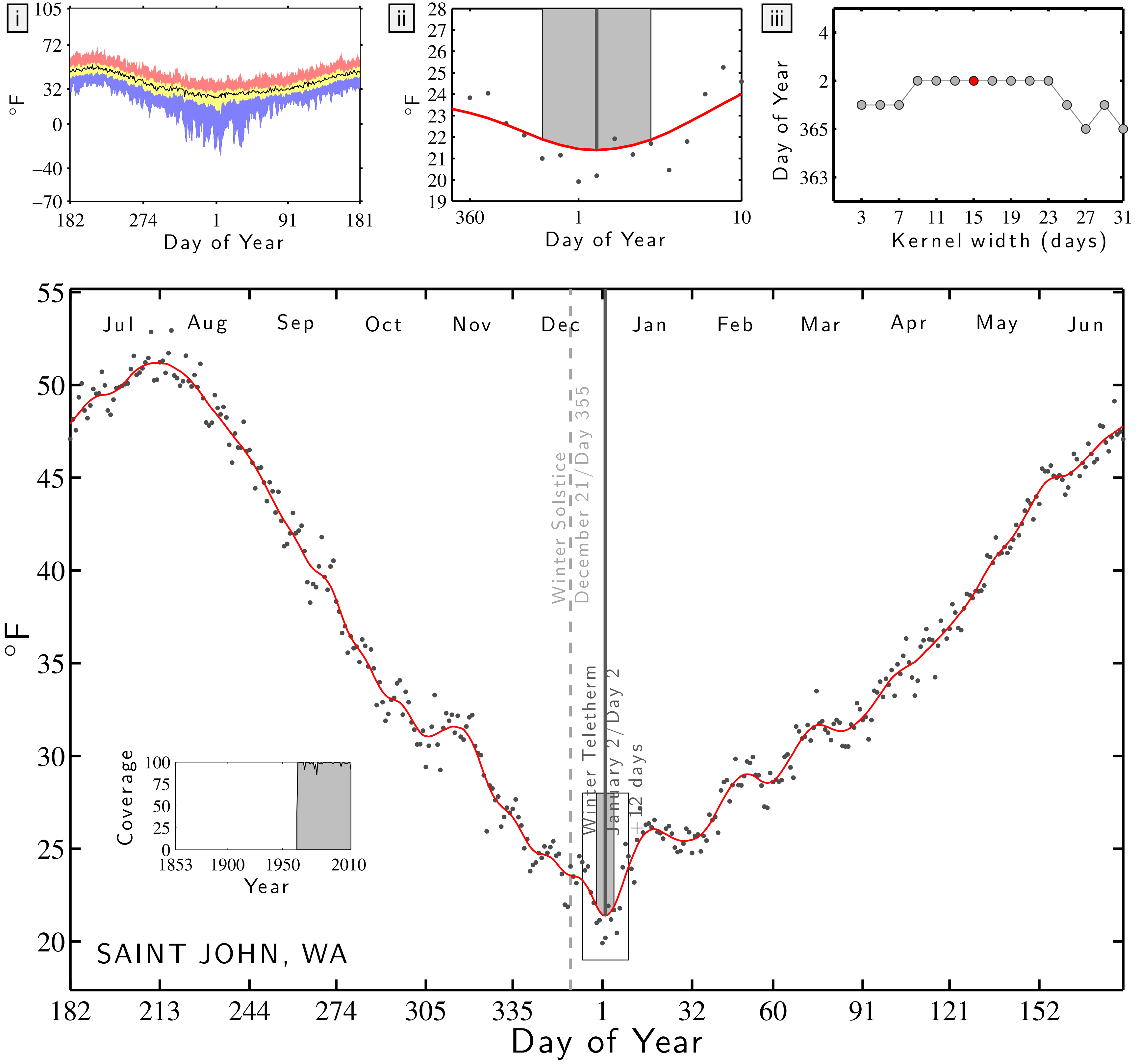

i
°F
Day of Year
ii
Day of Year
iii
Day of Year
Kernel width (days)
Jul
Aug
Sep
Oct
Nov
Dec
Jan
Feb
Mar
Apr
May
Jun
Winter Solstice
December 21/Day 355
Winter Teletherm
January 2/Day 2
+12 days
Coverage
Year
SAINT JOHN, WA
Day of Year

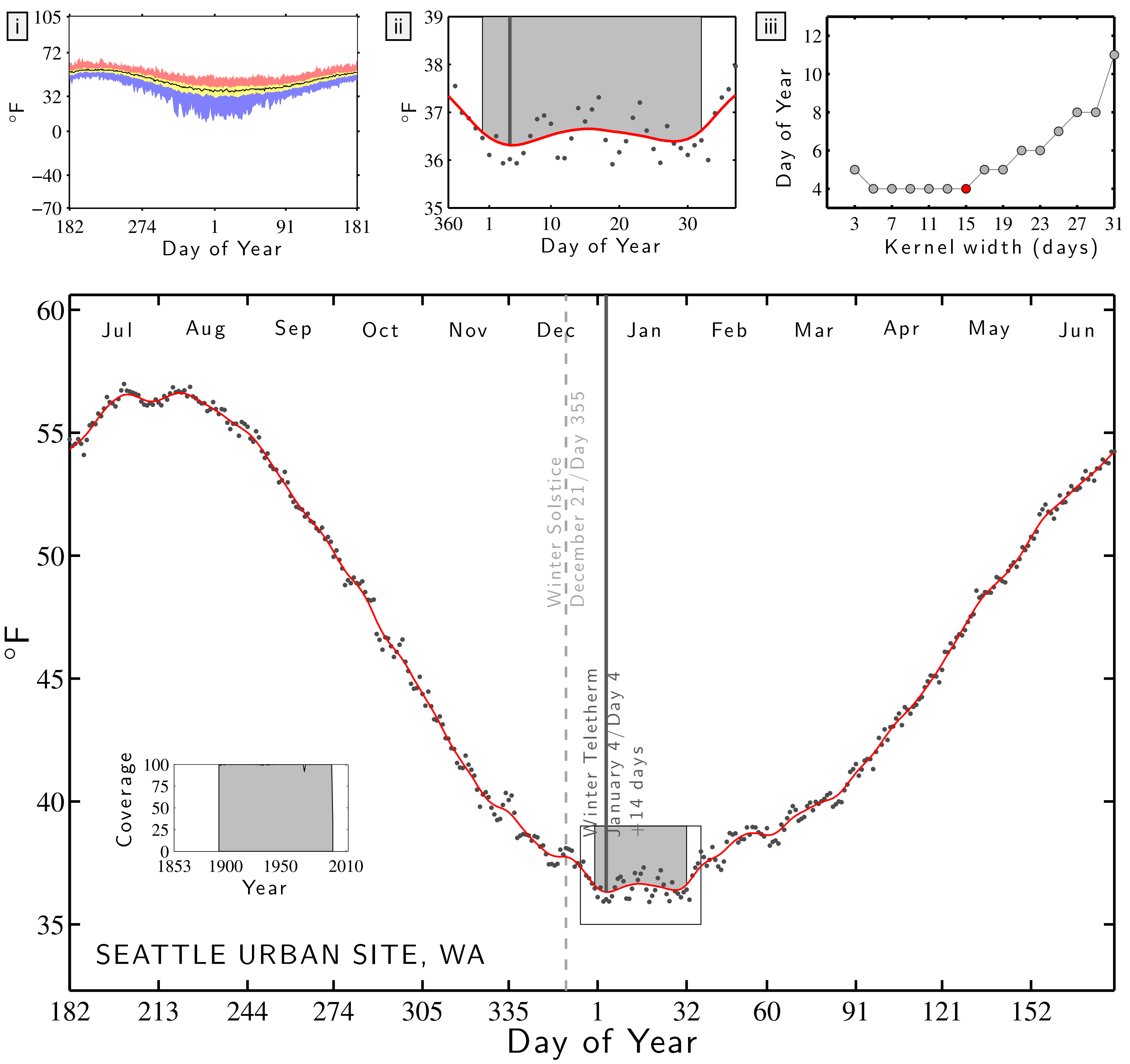

i
ii
iii
Day of Year
Kernel width (days)
Jul
Aug
Sep
Oct
Nov
Dec
Jan
Feb
Mar
Apr
May
Jun
Winter Solstice
December 21/Day 355
Winter Teletherm
January 4/Day 4
+14 days
Coverage
Year
SEATTLE URBAN SITE, WA
Day of Year

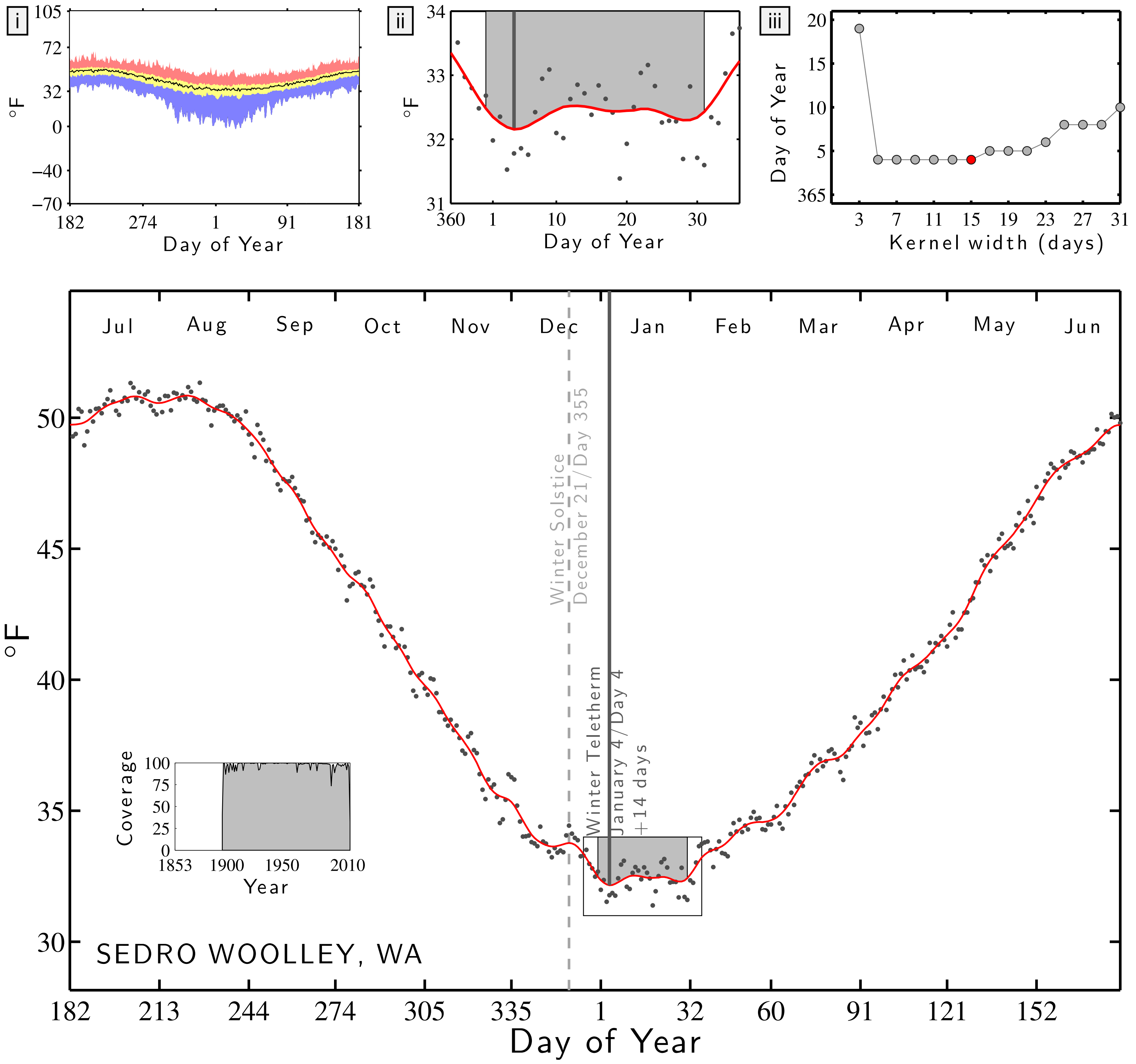

i
°F
Day of Year
ii
°F
Day of Year
iii
Day of Year
Kernel width (days)
Jul
Aug
Sep
Oct
Nov
Dec
Jan
Feb
Mar
Apr
May
Jun
Winter Solstice
December 21/Day 355
Winter Teletherm
January 4/Day 4
+14 days
Coverage
Year
SEDRO WOOLLEY, WA
°F
Day of Year

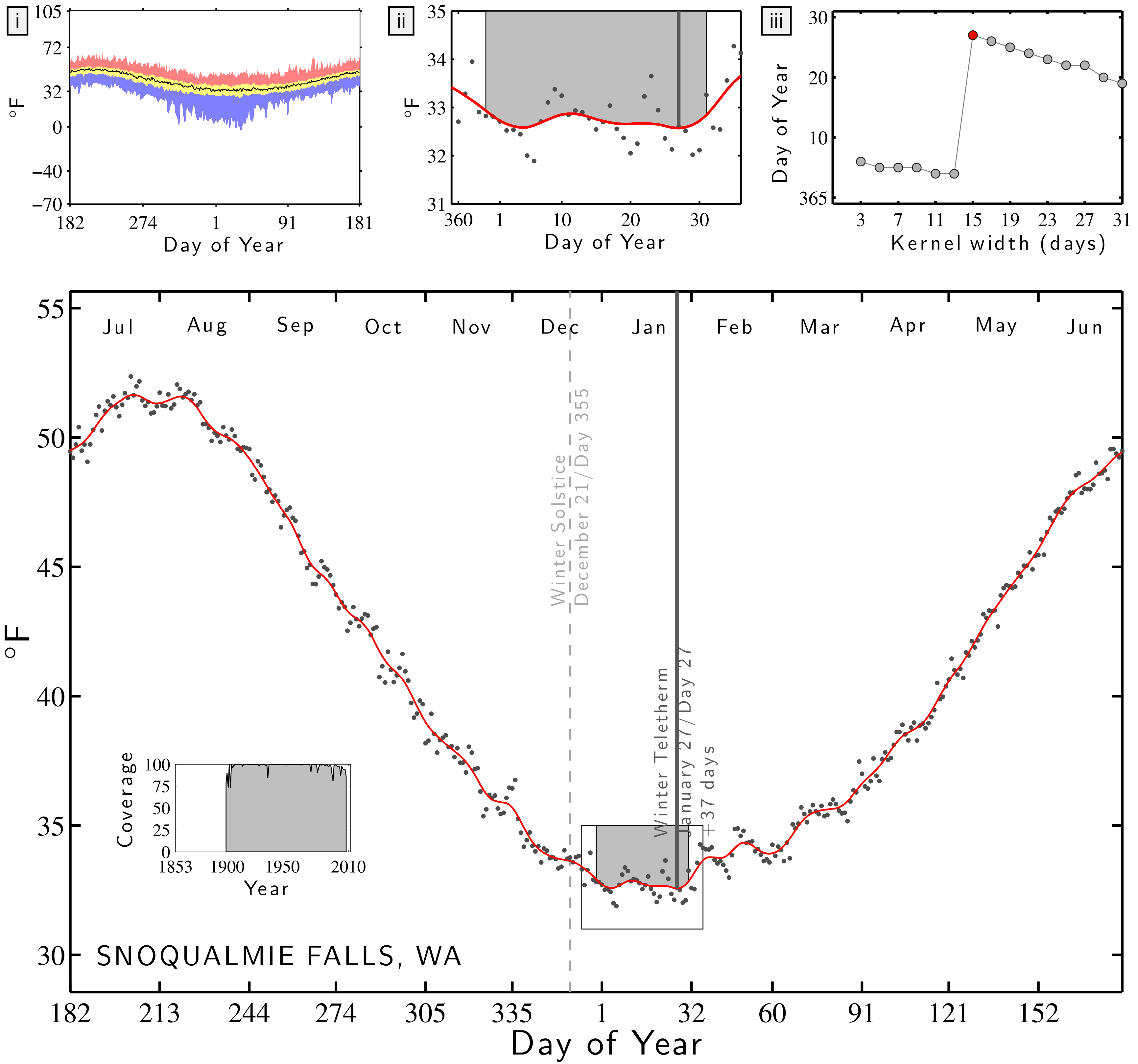

i
°F
Day of Year
ii
°F
Day of Year
iii
Day of Year
Kernel width (days)
Jul
Aug
Sep
Oct
Nov
Dec
Jan
Feb
Mar
Apr
May
Jun
Winter Solstice
December 21/Day 355
Winter Teletherm
January 27/Day 27
+37 days
Coverage
Year
SNOQUALMIE FALLS, WA
°F
Day of Year

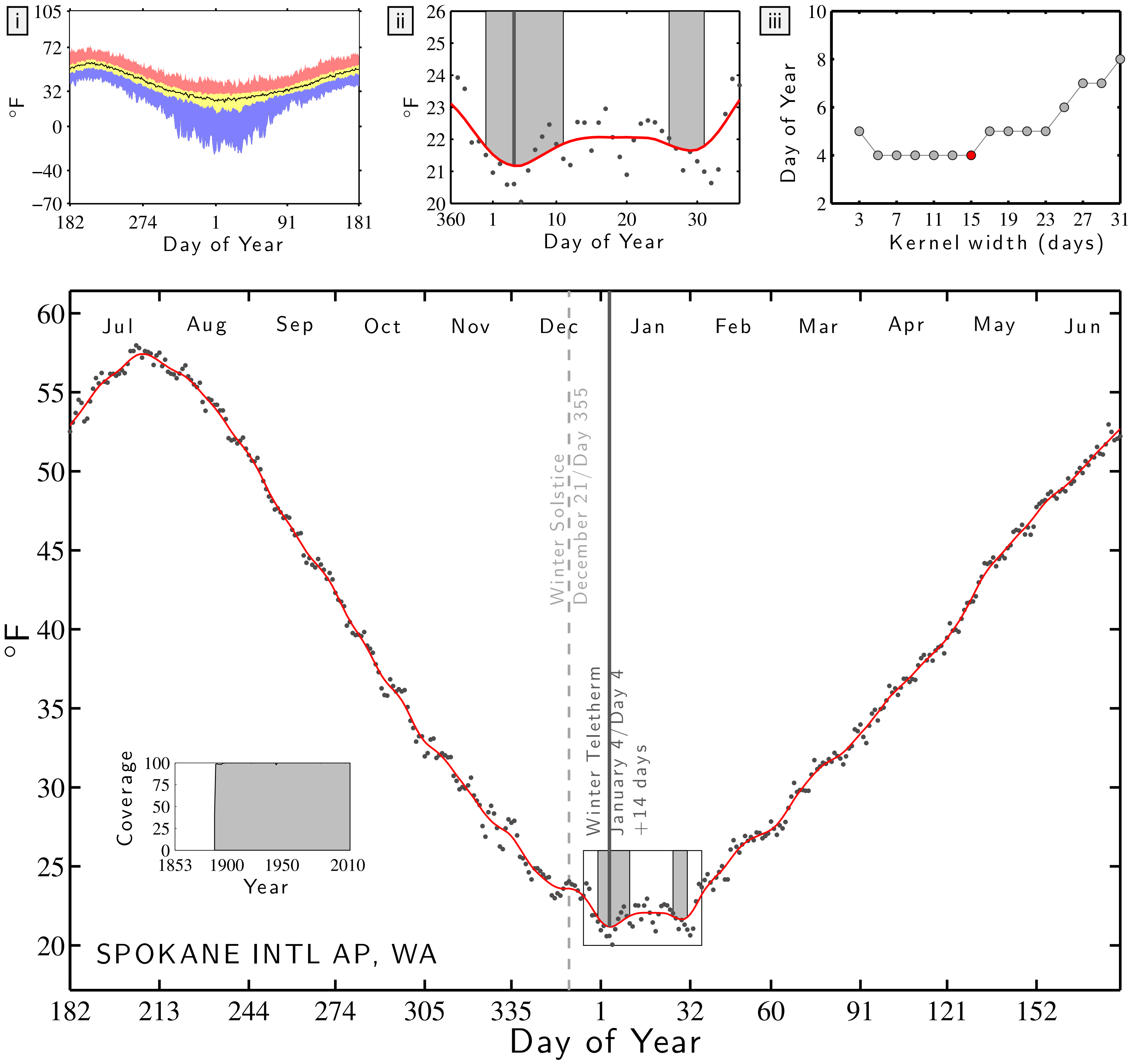

i
ii
iii
°F
Day of Year
Kernel width (days)
Jul
Aug
Sep
Oct
Nov
Dec
Jan
Feb
Mar
Apr
May
Jun
Winter Solstice
December 21/Day 355
Winter Teletherm
January 4/Day 4
+14 days
Coverage
Year
SPOKANE INTL AP, WA

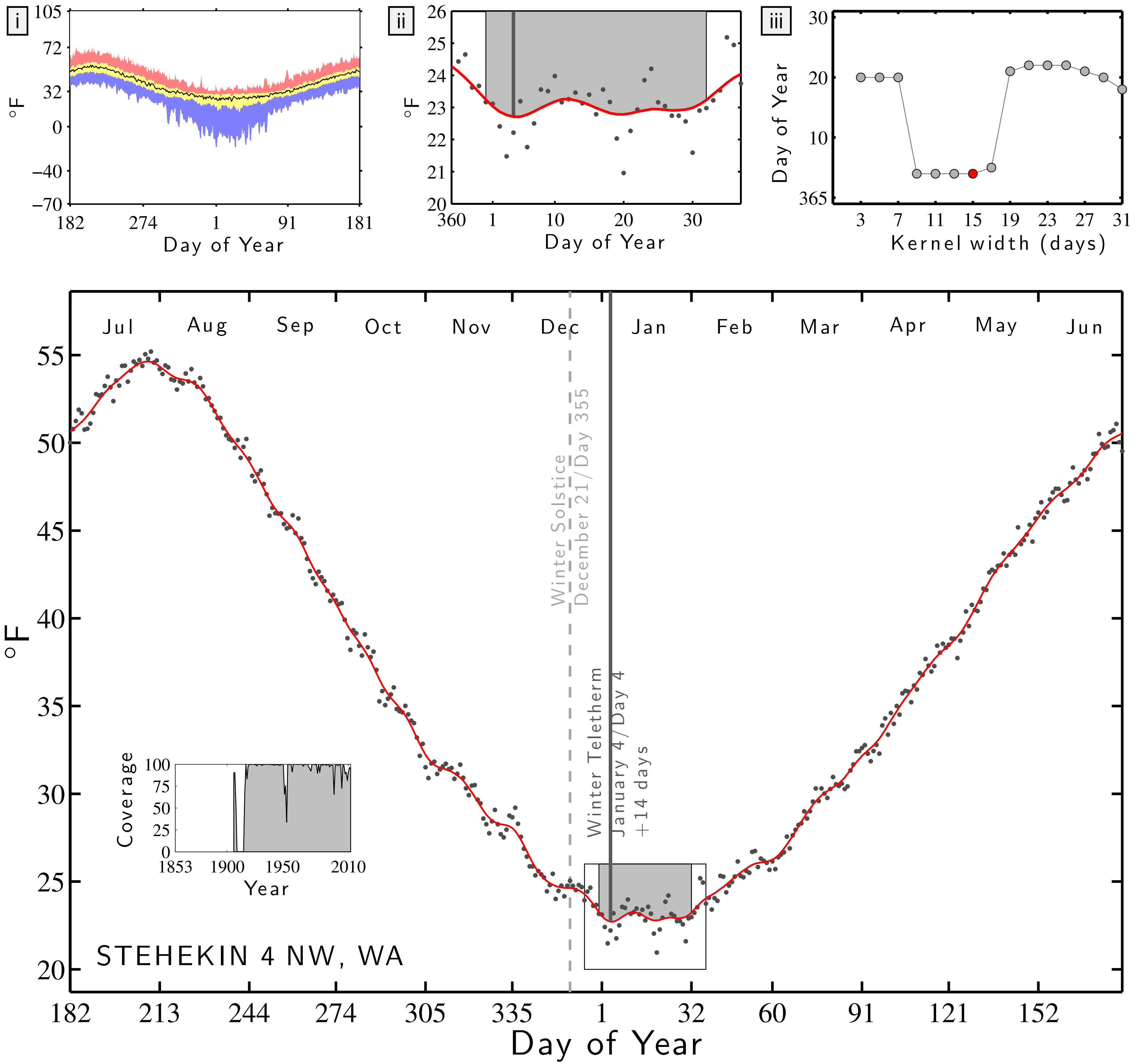

i
105
72
32
0
−40
−70
°F
182
274
1
91
181
Day of Year
ii
26
25
24
23
22
21
20
°F
360
1
10
20
30
Day of Year
iii
30
20
10
365
Day of Year
3
7
11
15
19
23
27
31
Kernel width (days)
Jul
Aug
Sep
Oct
Nov
Dec
Jan
Feb
Mar
Apr
May
Jun
55
50
45
40
35
30
25
20
°F
Winter Solstice
December 21/Day 355
Winter Teletherm
January 4/Day 4
+14 days
Coverage
100
75
50
25
0
1853
1900
1950
2010
Year
STEHEKIN 4 NW, WA
182
213
244
274
305
335
1
32
60
91
121
152
Day of Year

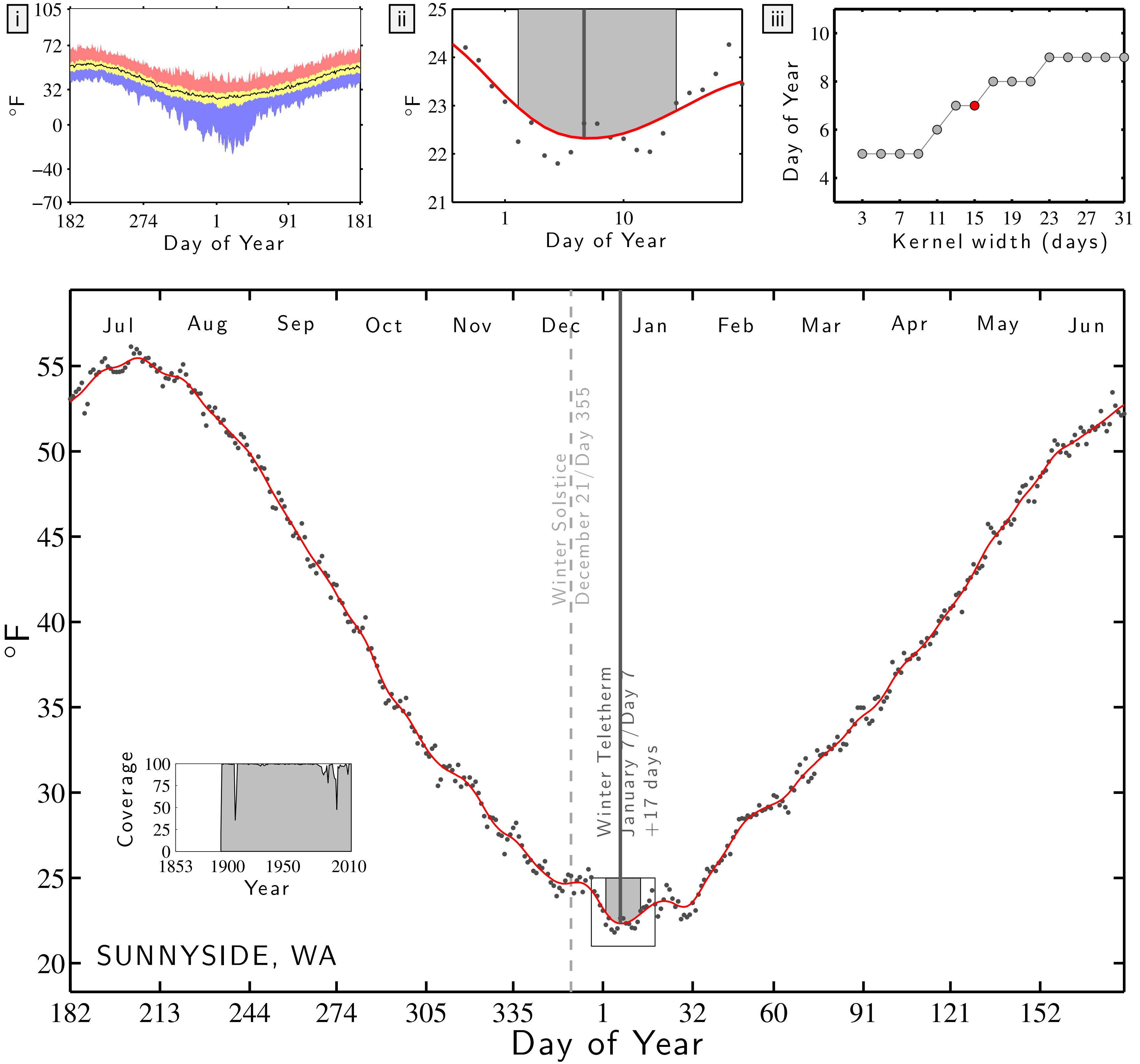

i
ii
iii
Day of Year
°F
Kernel width (days)
Jul
Aug
Sep
Oct
Nov
Dec
Jan
Feb
Mar
Apr
May
Jun
Winter Solstice
December 21/Day 355
Winter Teletherm
January 7/Day 7
+17 days
Coverage
Year
SUNNYSIDE, WA

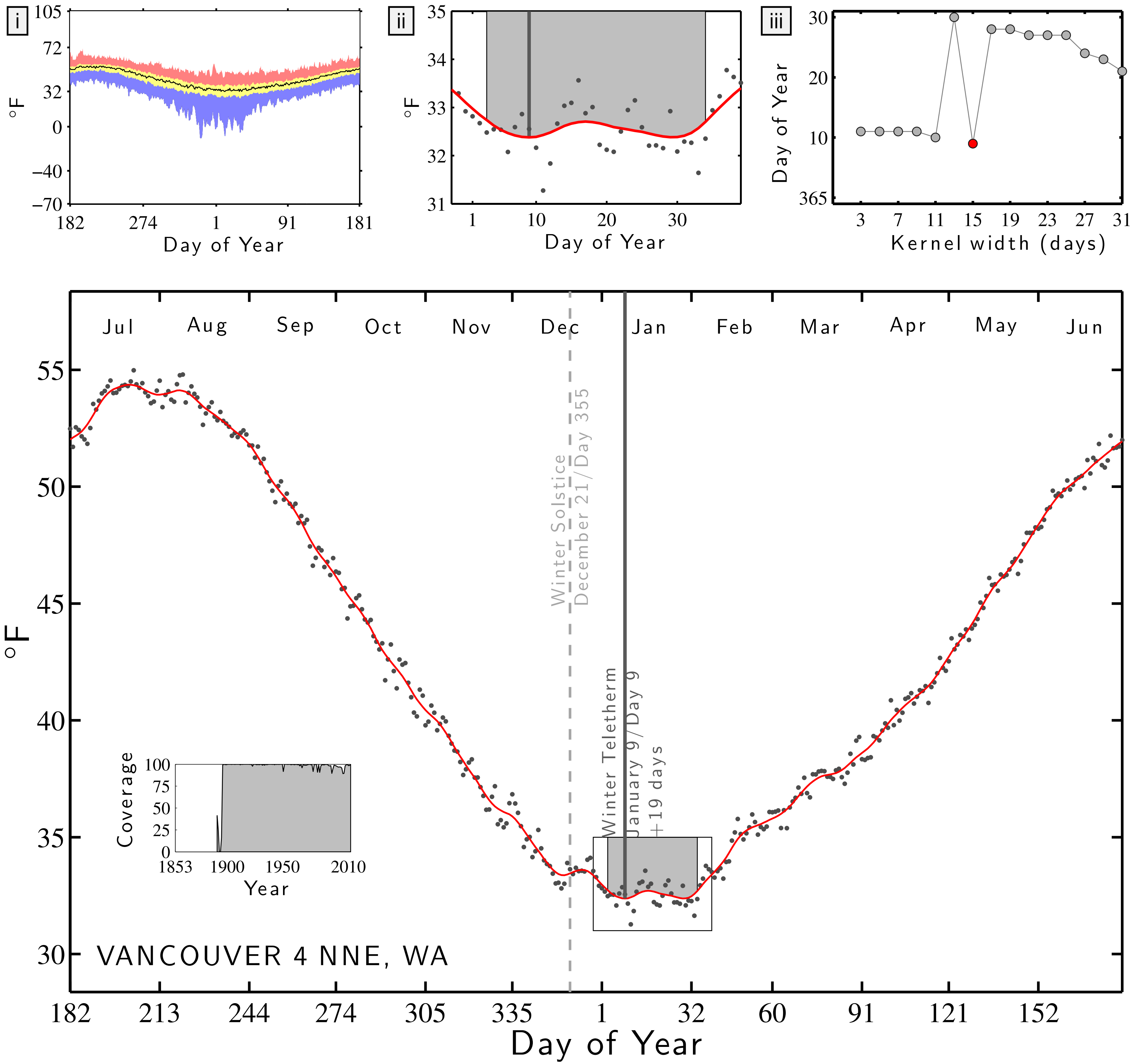

i
°F
Day of Year
ii
°F
Day of Year
iii
Day of Year
Kernel width (days)
Jul
Aug
Sep
Oct
Nov
Dec
Jan
Feb
Mar
Apr
May
Jun
Winter Solstice
December 21/Day 355
Winter Teletherm
January 9/Day 9
±19 days
Coverage
Year
VANCOUVER 4 NNE, WA
°F
Day of Year

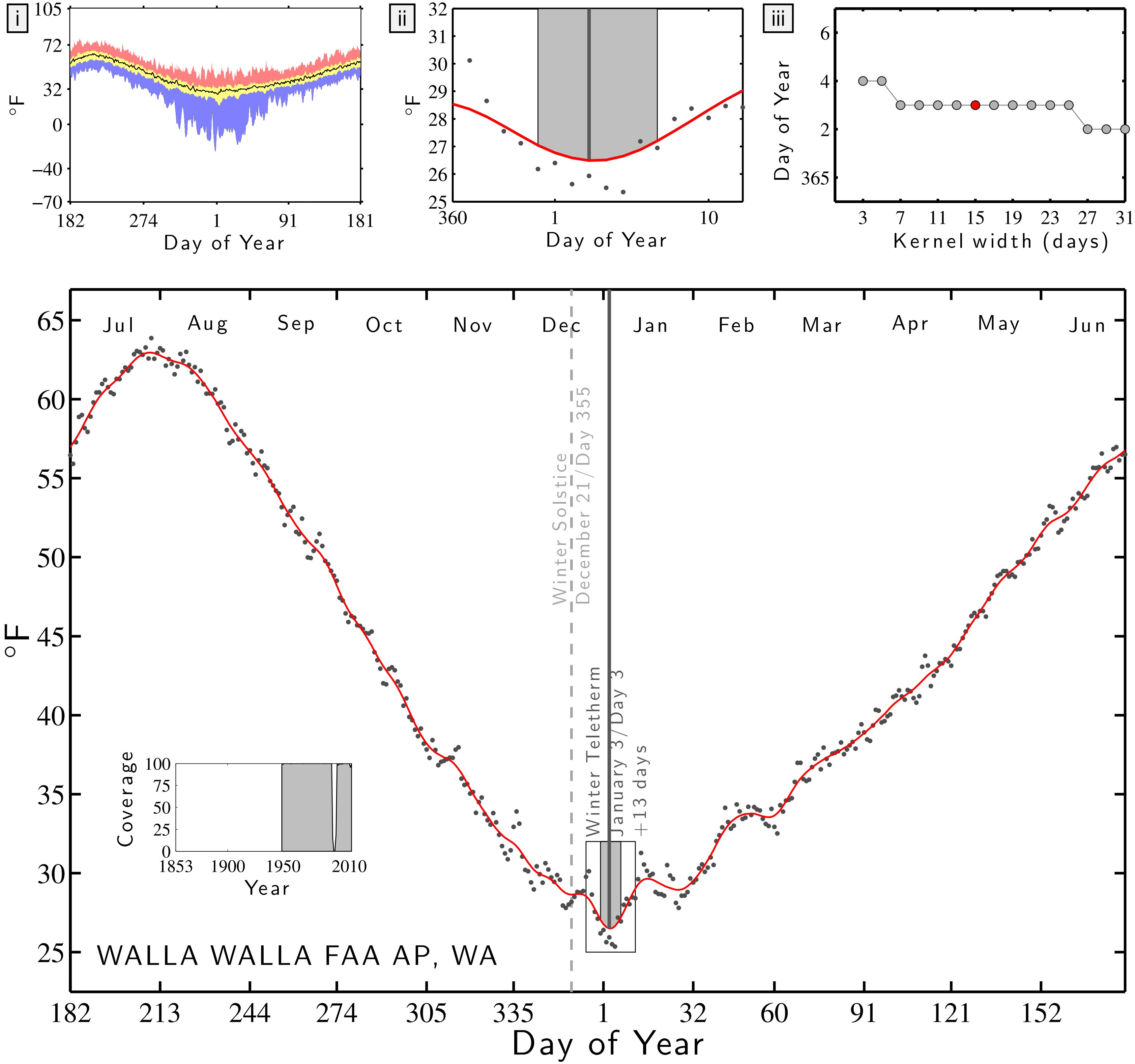

i
ii
iii
°F
Day of Year
Kernel width (days)
Jul
Aug
Sep
Oct
Nov
Dec
Jan
Feb
Mar
Apr
May
Jun
Winter Solstice
December 21/Day 355
Winter Teletherm
January 3/Day 3
+13 days
Coverage
Year
WALLA WALLA FAA AP, WA

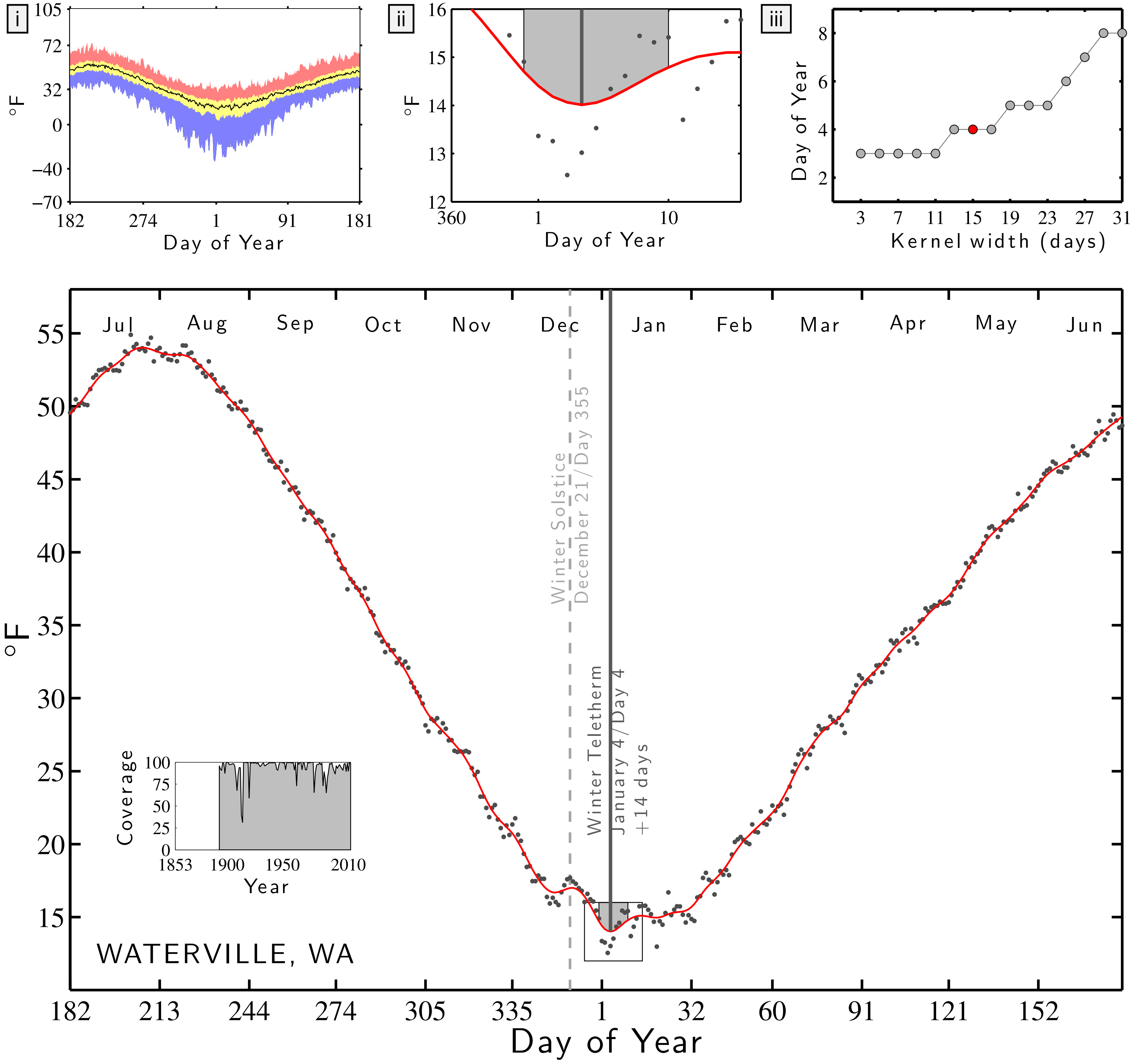

i
Day of Year
°F
ii
Day of Year
°F
iii
Kernel width (days)
Day of Year
Jul
Aug
Sep
Oct
Nov
Dec
Jan
Feb
Mar
Apr
May
Jun
Winter Solstice
December 21/Day 355
Winter Teletherm
January 4/Day 4
+14 days
Coverage
Year
WATERVILLE, WA
Day of Year
°F

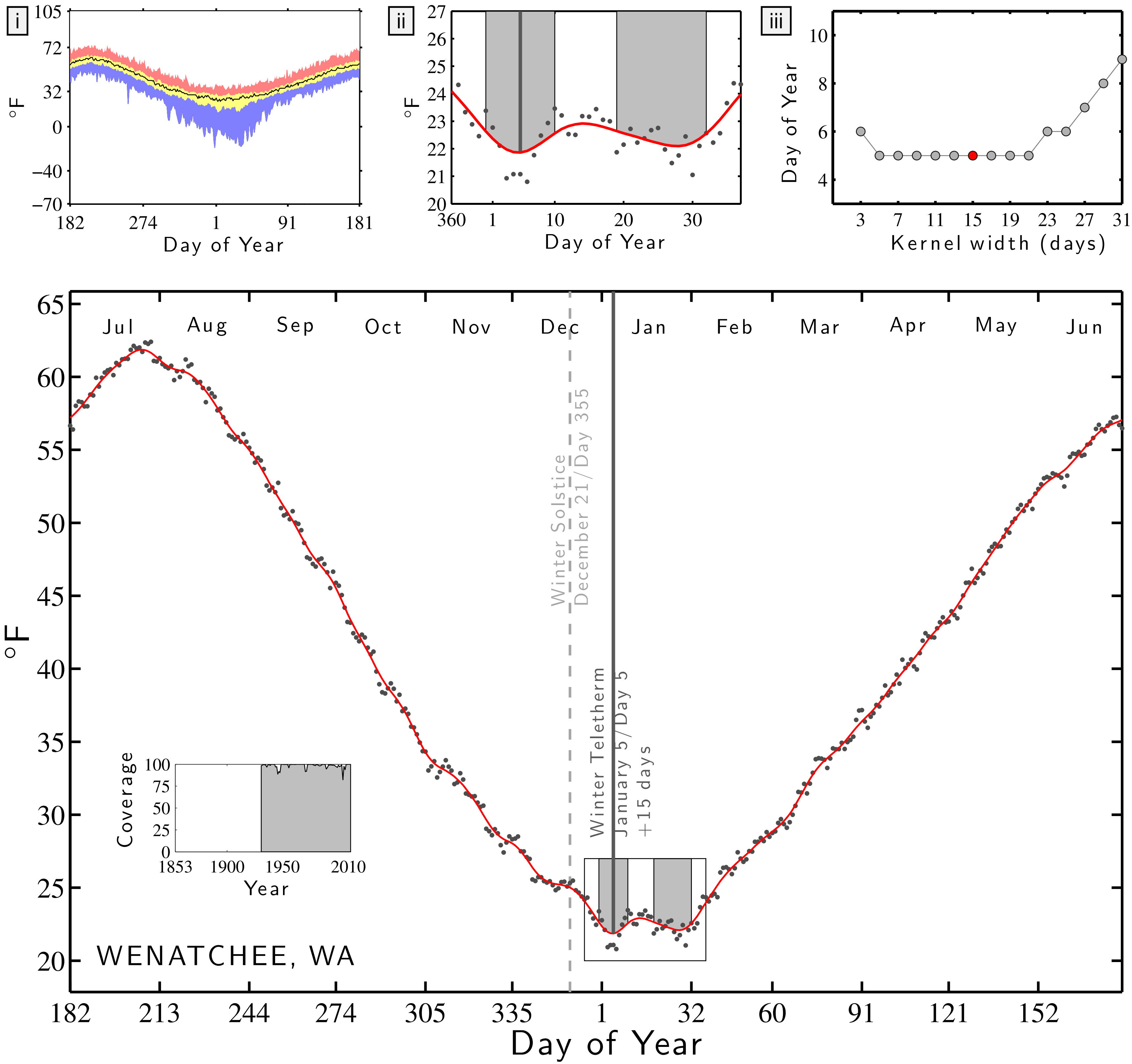

i
105
72
32
0
−40
−70
°F
182
274
1
91
181
Day of Year
ii
27
26
25
24
23
22
21
20
°F
360
1
10
20
30
Day of Year
iii
10
8
6
4
Day of Year
3
7
11
15
19
23
27
31
Kernel width (days)
65
60
55
50
45
40
35
30
25
20
°F
Jul
Aug
Sep
Oct
Nov
Dec
Jan
Feb
Mar
Apr
May
Jun
Winter Solstice
December 21/Day 355
Winter Teletherm
January 5/Day 5
+15 days
Coverage
100
75
50
25
0
1853
1900
1950
2010
Year
WENATCHEE, WA
182
213
244
274
305
335
1
32
60
91
121
152
Day of Year

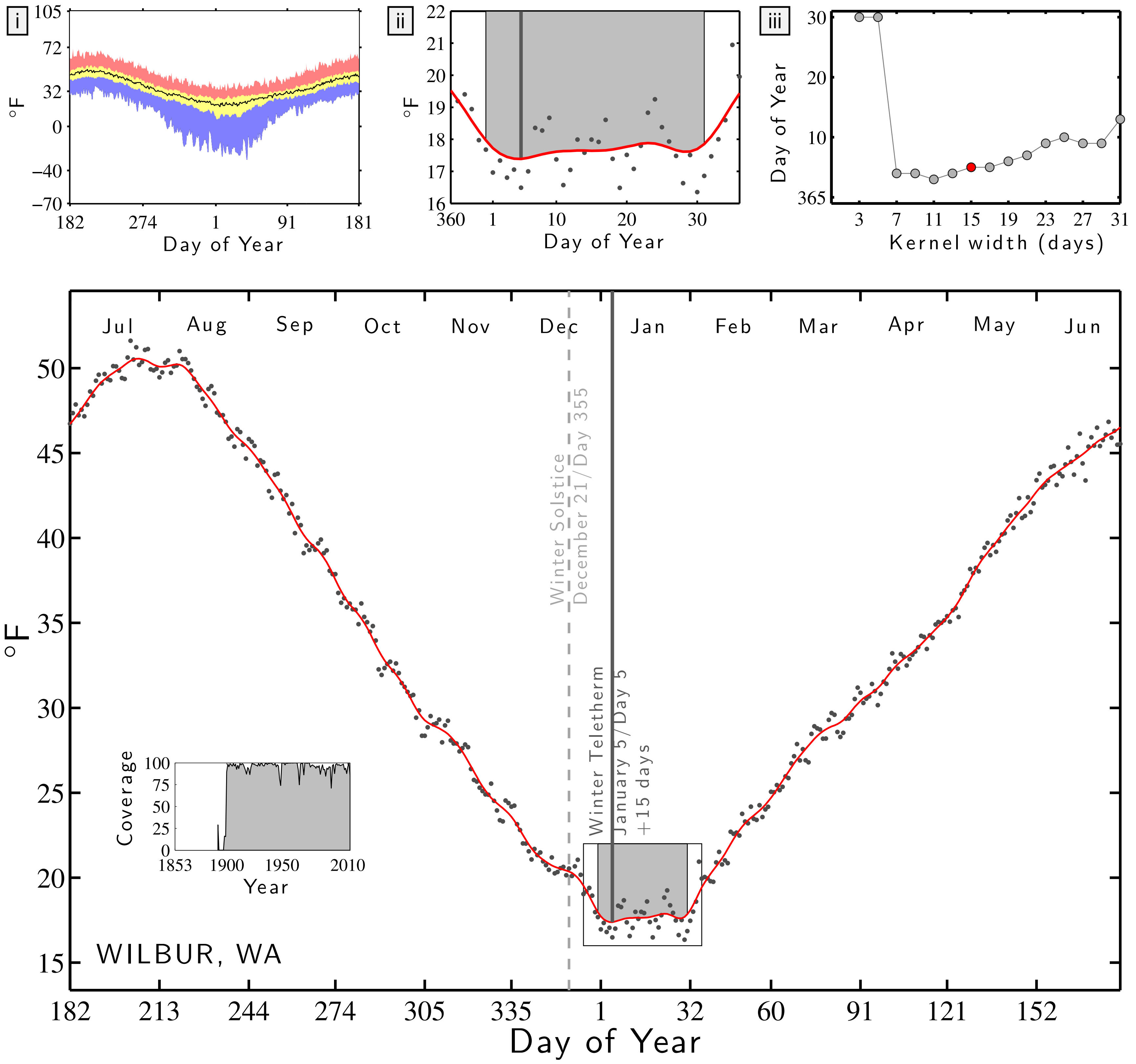

i
ii
iii
°F
Day of Year
Kernel width (days)
Jul
Aug
Sep
Oct
Nov
Dec
Jan
Feb
Mar
Apr
May
Jun
Winter Solstice
December 21/Day 355
Winter Teletherm
January 5/Day 5
+15 days
Coverage
Year
WILBUR, WA

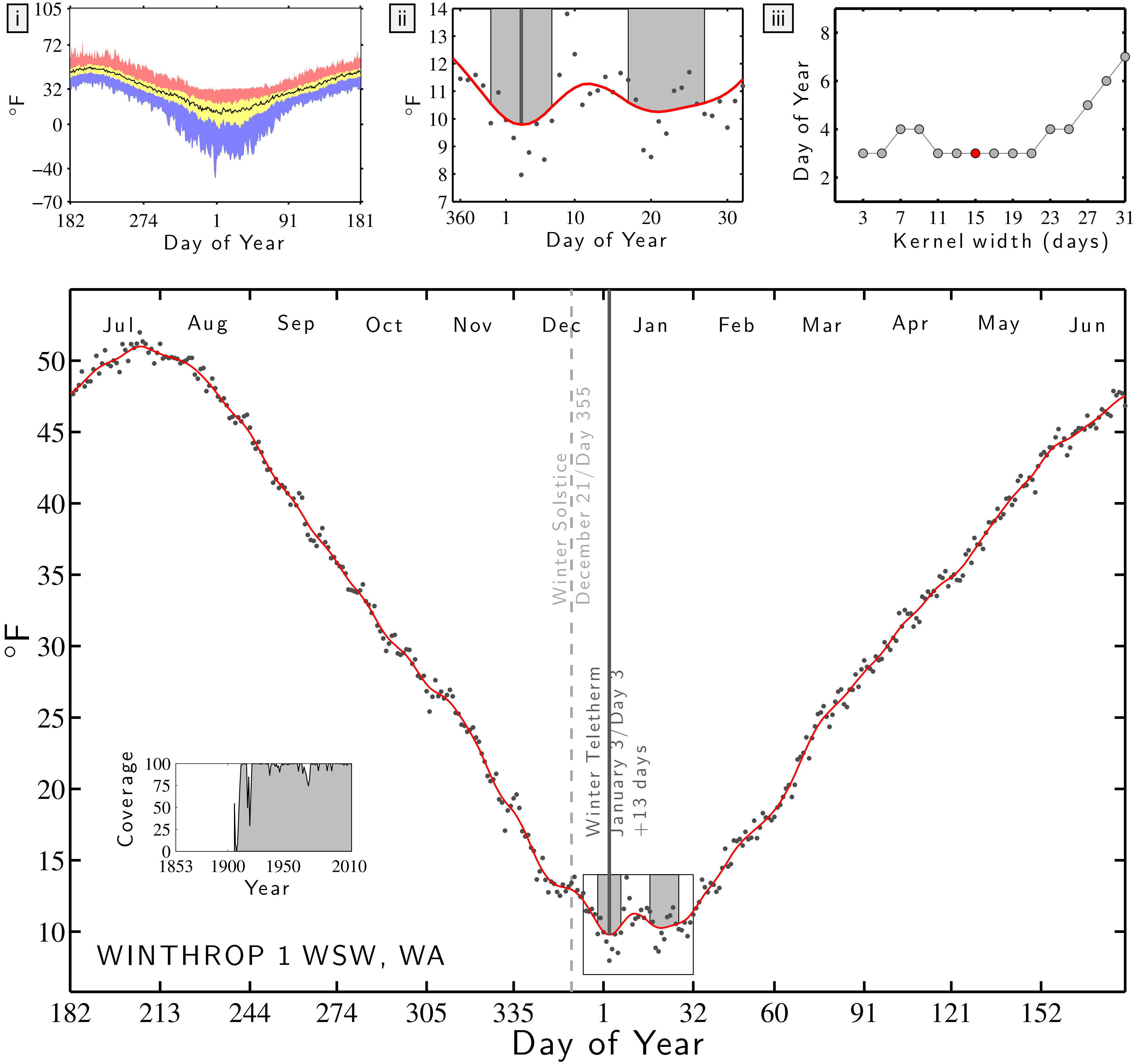

i
°F
Day of Year
ii
°F
Day of Year
iii
Day of Year
Kernel width (days)
Jul
Aug
Sep
Oct
Nov
Dec
Jan
Feb
Mar
Apr
May
Jun
Winter Solstice
December 21/Day 355
Winter Teletherm
January 3/Day 3
+13 days
Coverage
Year
WINTHROP 1 WSW, WA
°F
Day of Year

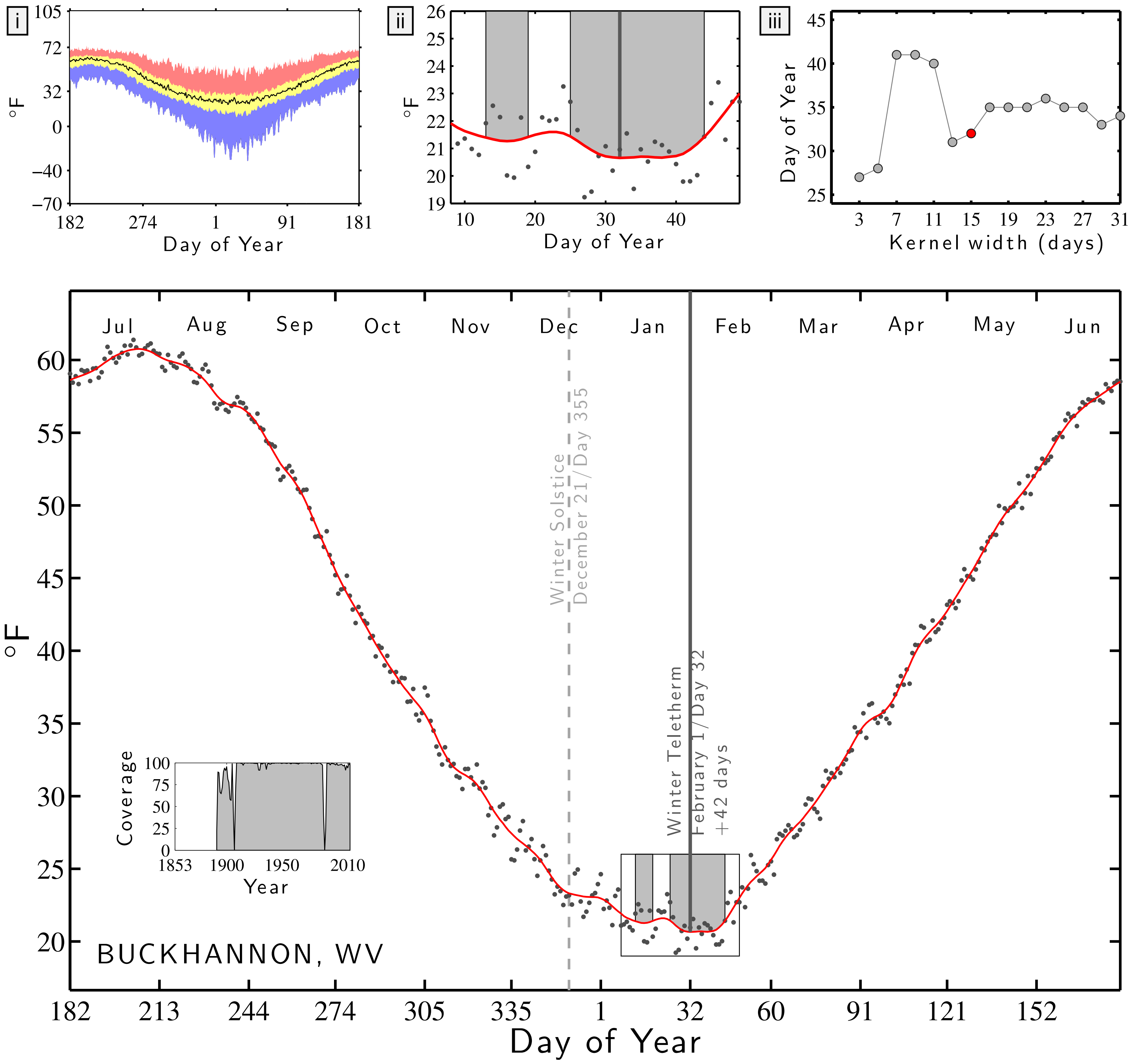
i
ii
iii
°F
Day of Year
Kernel width (days)
Jul
Aug
Sep
Oct
Nov
Dec
Jan
Feb
Mar
Apr
May
Jun
Winter Solstice
December 21/Day 355
Winter Teletherm
February 1/Day 32
+42 days
Coverage
Year
BUCKHANNON, WV

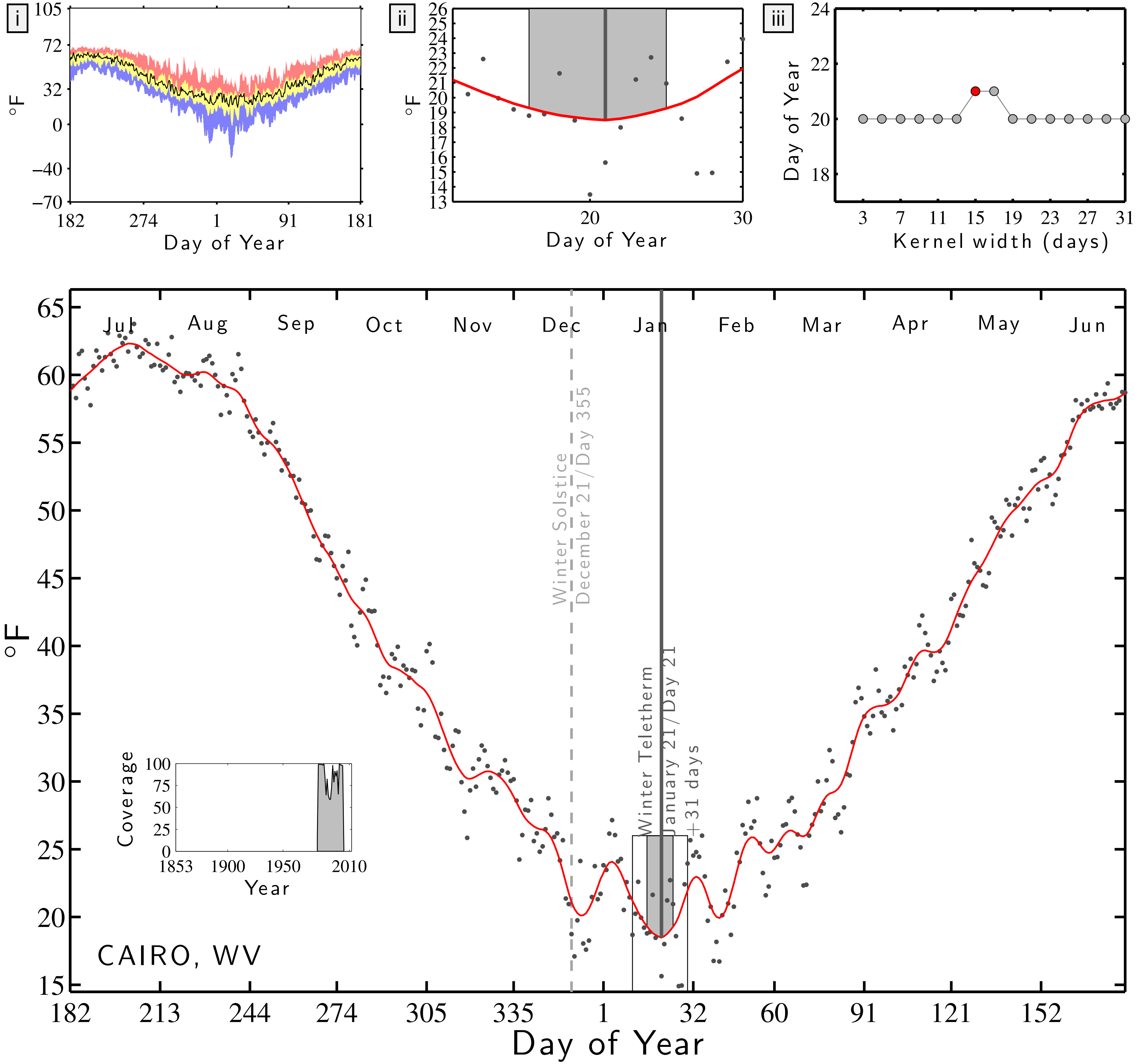

i
°F
Day of Year
ii
°F
Day of Year
iii
Day of Year
Kernel width (days)
Jul
Aug
Sep
Oct
Nov
Dec
Jan
Feb
Mar
Apr
May
Jun
Winter Solstice
December 21/Day 355
Winter Teletherm
January 21/Day 21
±31 days
Coverage
Year
CAIRO, WV
°F
Day of Year

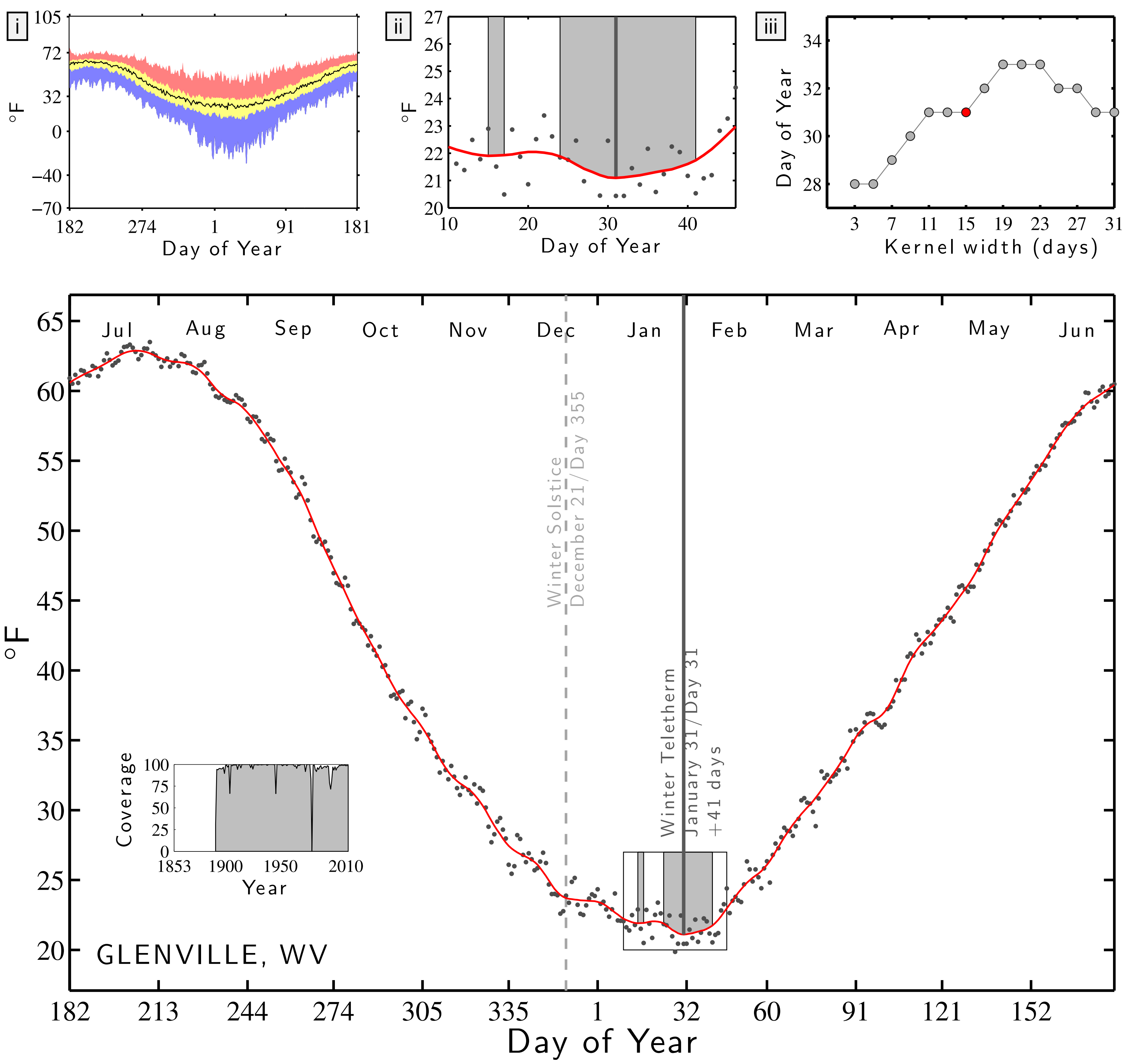

i
ii
iii
Day of Year
°F
Kernel width (days)
Jul
Aug
Sep
Oct
Nov
Dec
Jan
Feb
Mar
Apr
May
Jun
Winter Solstice
December 21/Day 355
Winter Teletherm
January 31/Day 31
+41 days
Coverage
Year
GLENVILLE, WV

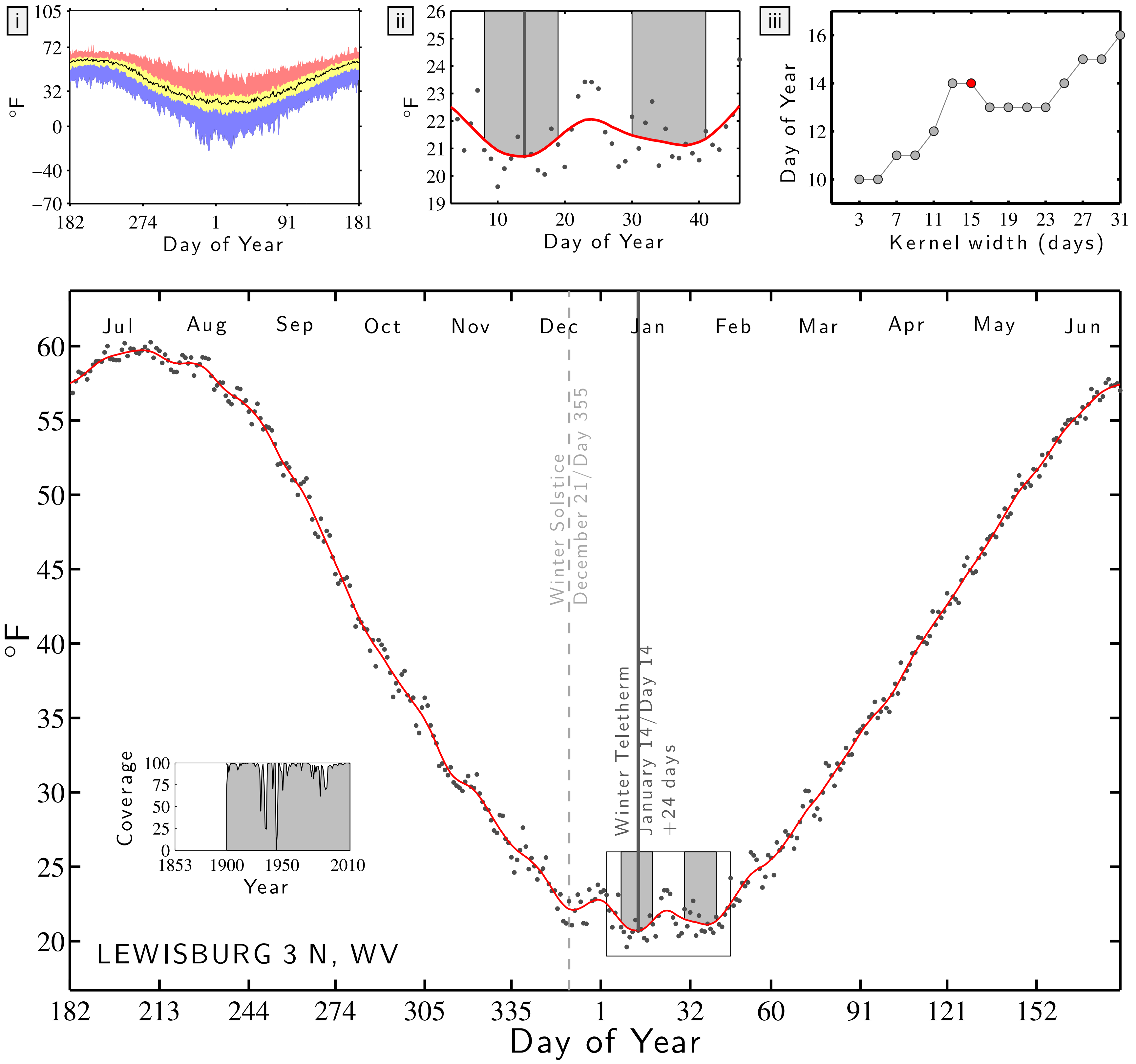

i
ii
iii
°F
Day of Year
Kernel width (days)
Jul
Aug
Sep
Oct
Nov
Dec
Jan
Feb
Mar
Apr
May
Jun
Winter Solstice
December 21/Day 355
Winter Teletherm
January 14/Day 14
+24 days
Coverage
Year
LEWISBURG 3 N, WV

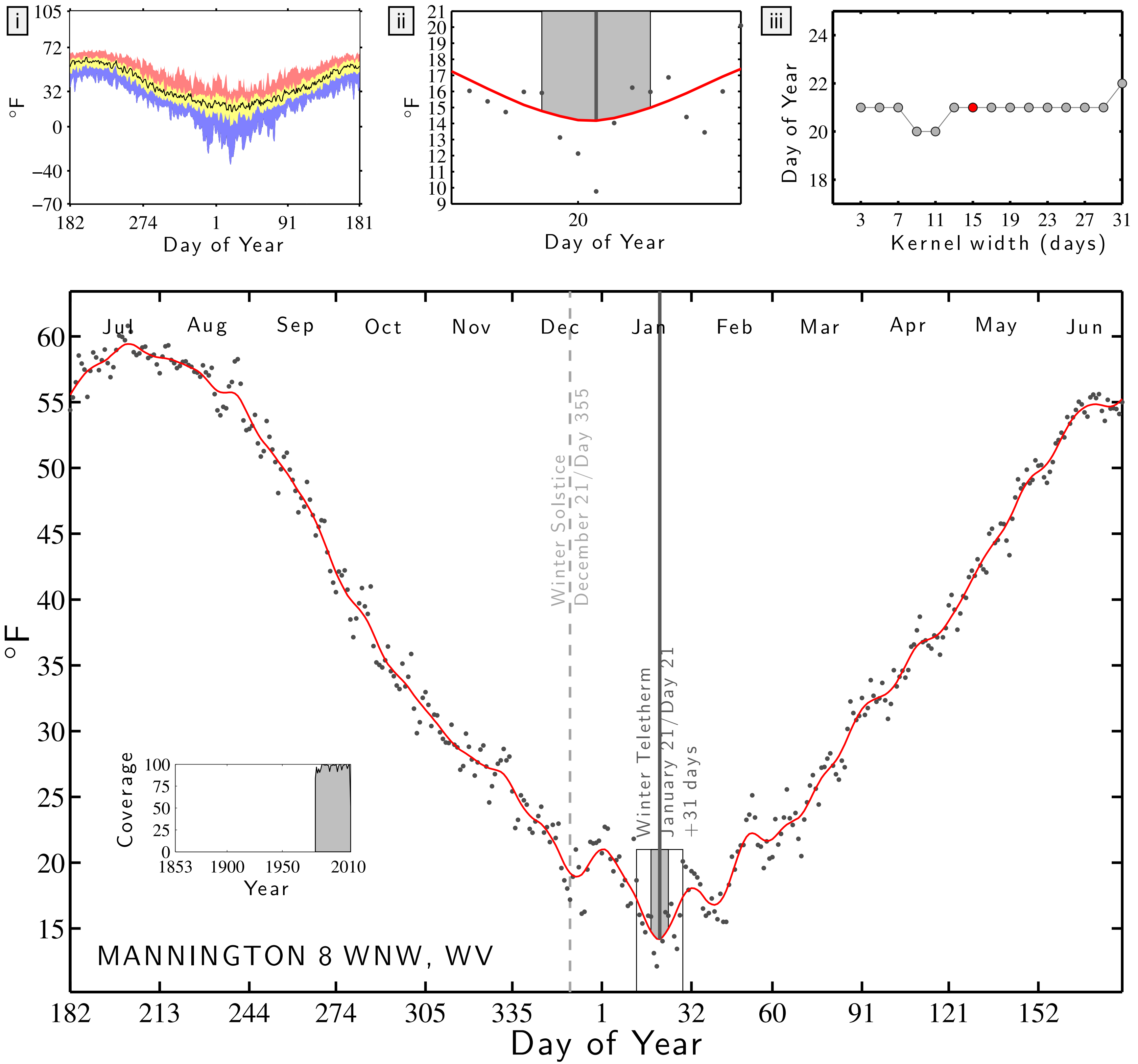

i
ii
iii
°F
Day of Year
Day of Year
Kernel width (days)
Jul
Aug
Sep
Oct
Nov
Dec
Jan
Feb
Mar
Apr
May
Jun
Winter Solstice
December 21/Day 355
Winter Teletherm
January 21/Day 21
+31 days
Coverage
Year
MANNINGTON 8 WNW, WV
°F
Day of Year

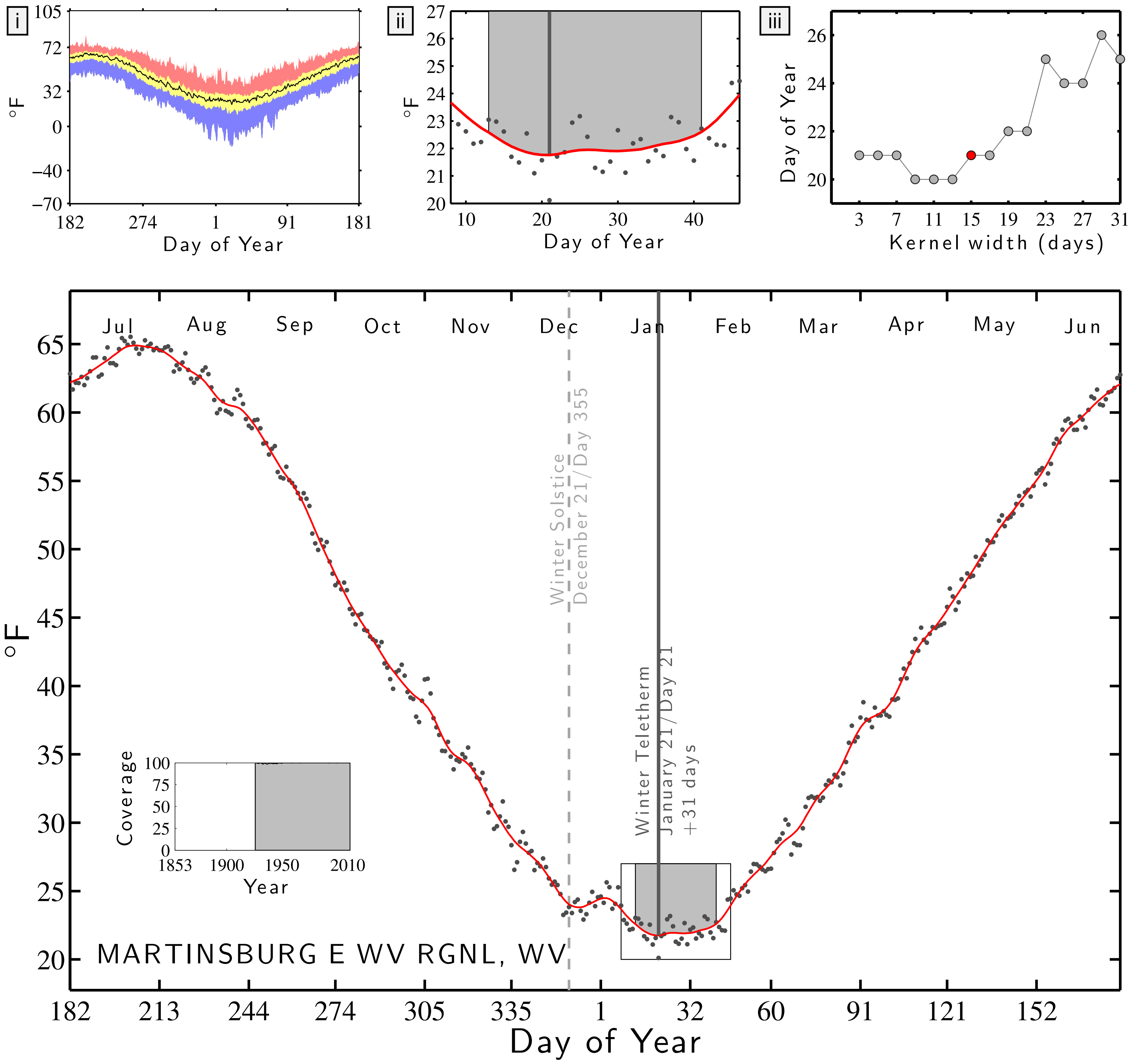

i
ii
iii
°F
Day of Year
Kernel width (days)
Jul
Aug
Sep
Oct
Nov
Dec
Jan
Feb
Mar
Apr
May
Jun
Winter Solstice
December 21/Day 355
Winter Teletherm
January 21/Day 21
+31 days
Coverage
Year
MARTINSBURG E WV RGNL, WV

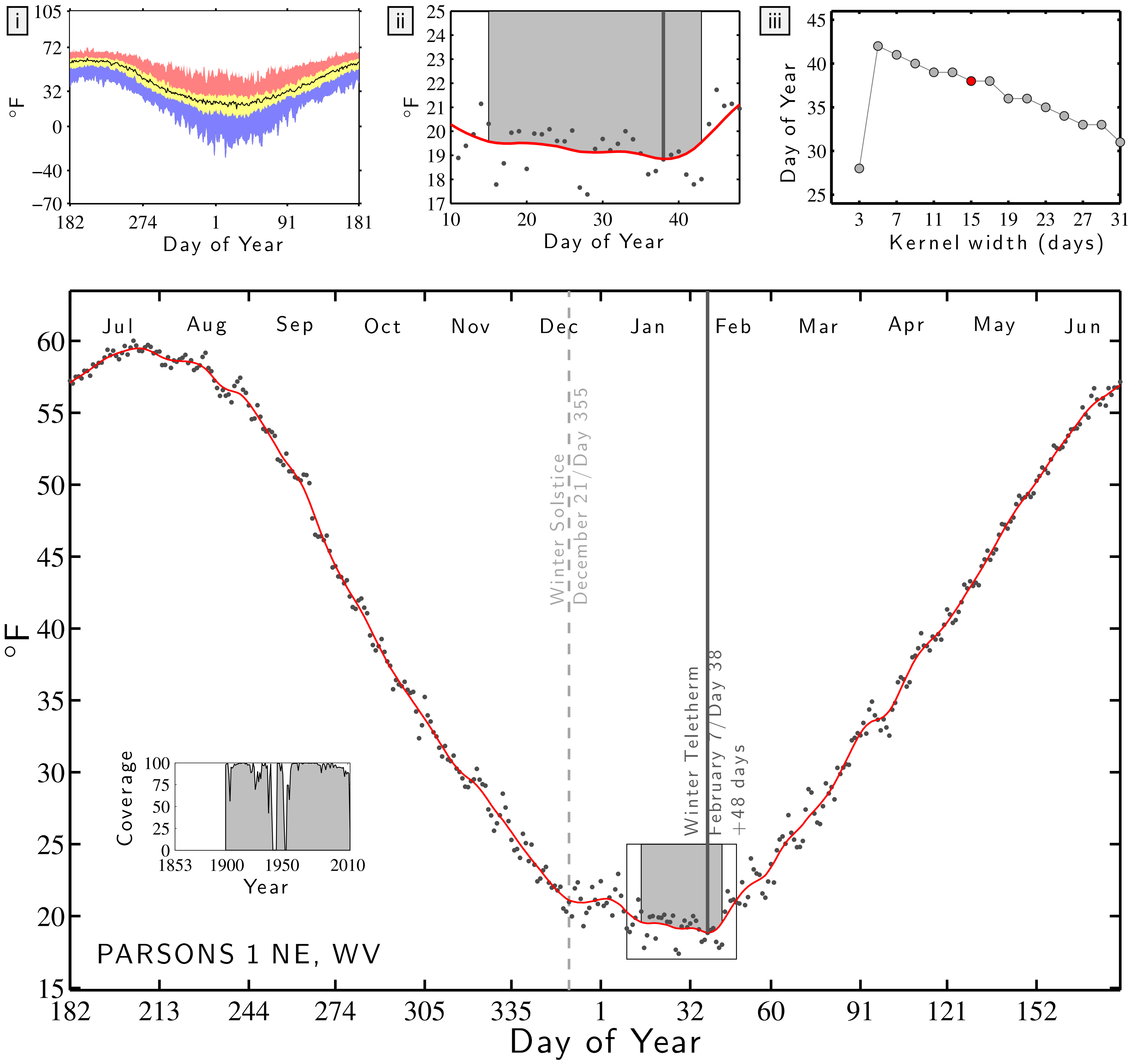

i
°F
Day of Year
ii
°F
Day of Year
iii
Day of Year
Kernel width (days)
Jul
Aug
Sep
Oct
Nov
Dec
Jan
Feb
Mar
Apr
May
Jun
Winter Solstice
December 21/Day 355
Winter Teletherm
February 7/Day 38
+48 days
Coverage
Year
PARSONS 1 NE, WV
°F
Day of Year

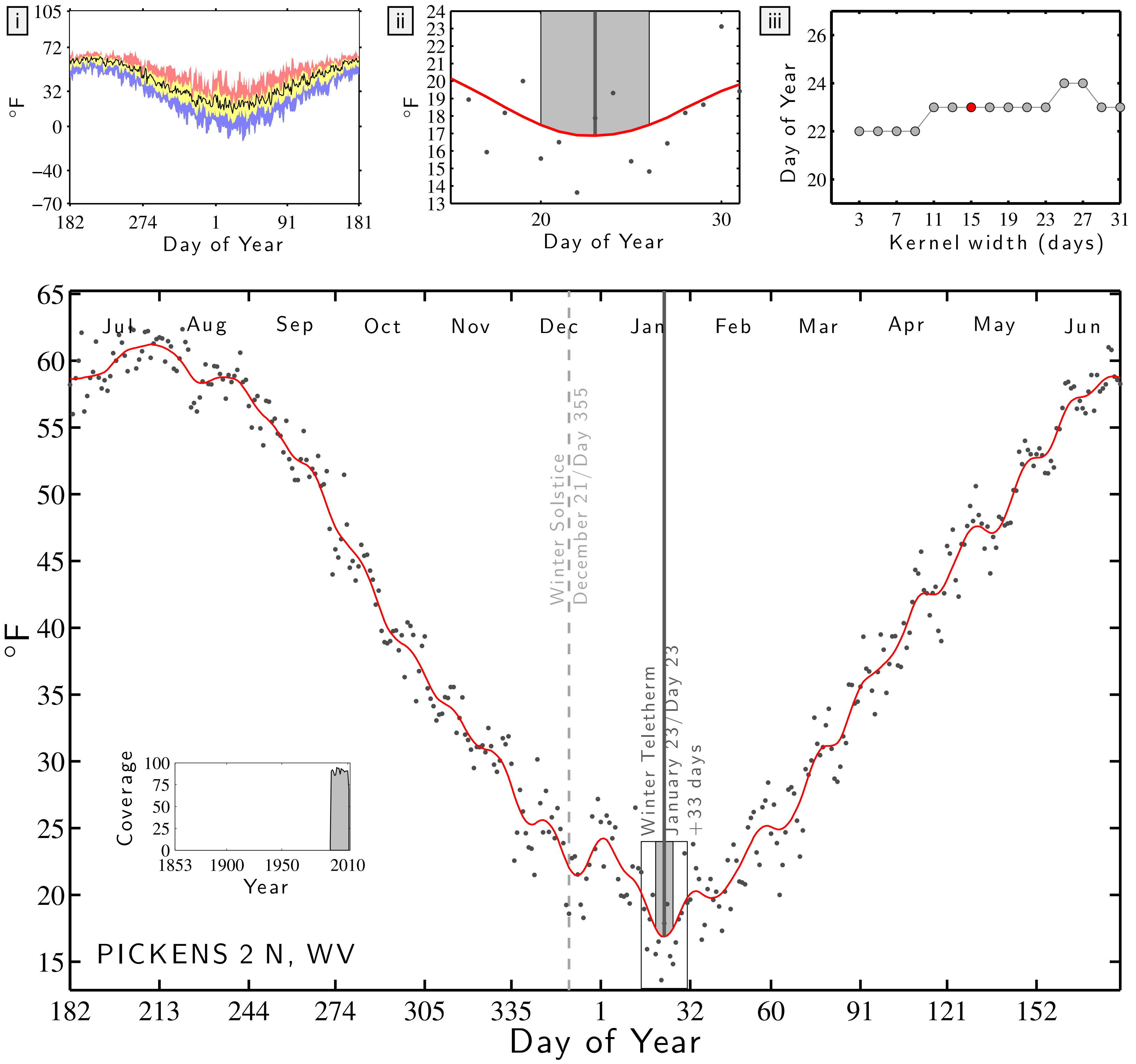

i
°F
Day of Year
ii
°F
Day of Year
iii
Day of Year
Kernel width (days)
Jul
Aug
Sep
Oct
Nov
Dec
Jan
Feb
Mar
Apr
May
Jun
Winter Solstice
December 21/Day 355
Winter Teletherm
January 23/Day 23
+33 days
Coverage
Year
PICKENS 2 N, WV
°F
Day of Year

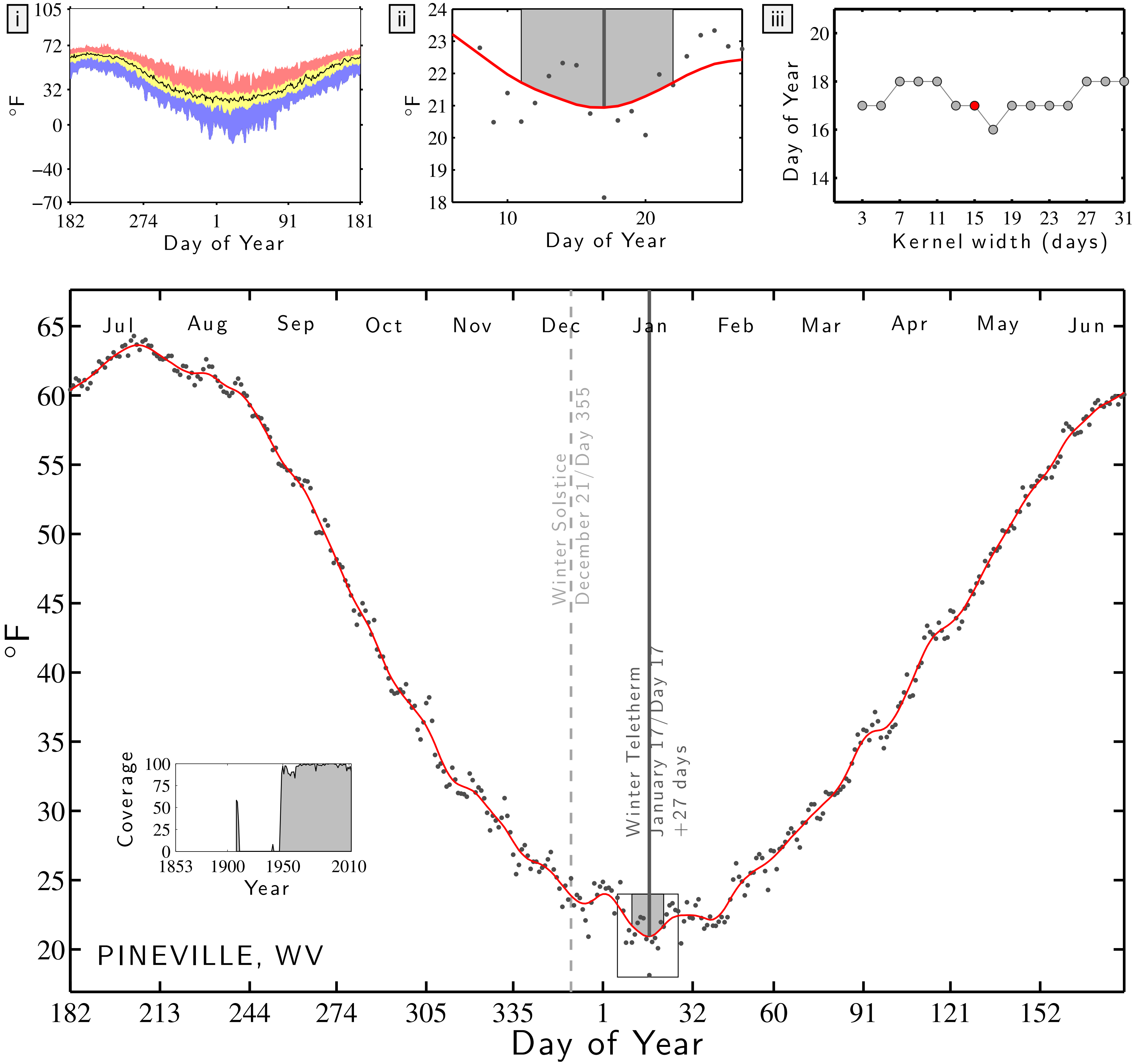

i
ii
iii
°F
Day of Year
Kernel width (days)
Winter Solstice
December 21/Day 355
Winter Teletherm
January 17/Day 17
+27 days
Coverage
Year
PINEVILLE, WV
Jul
Aug
Sep
Oct
Nov
Dec
Jan
Feb
Mar
Apr
May
Jun

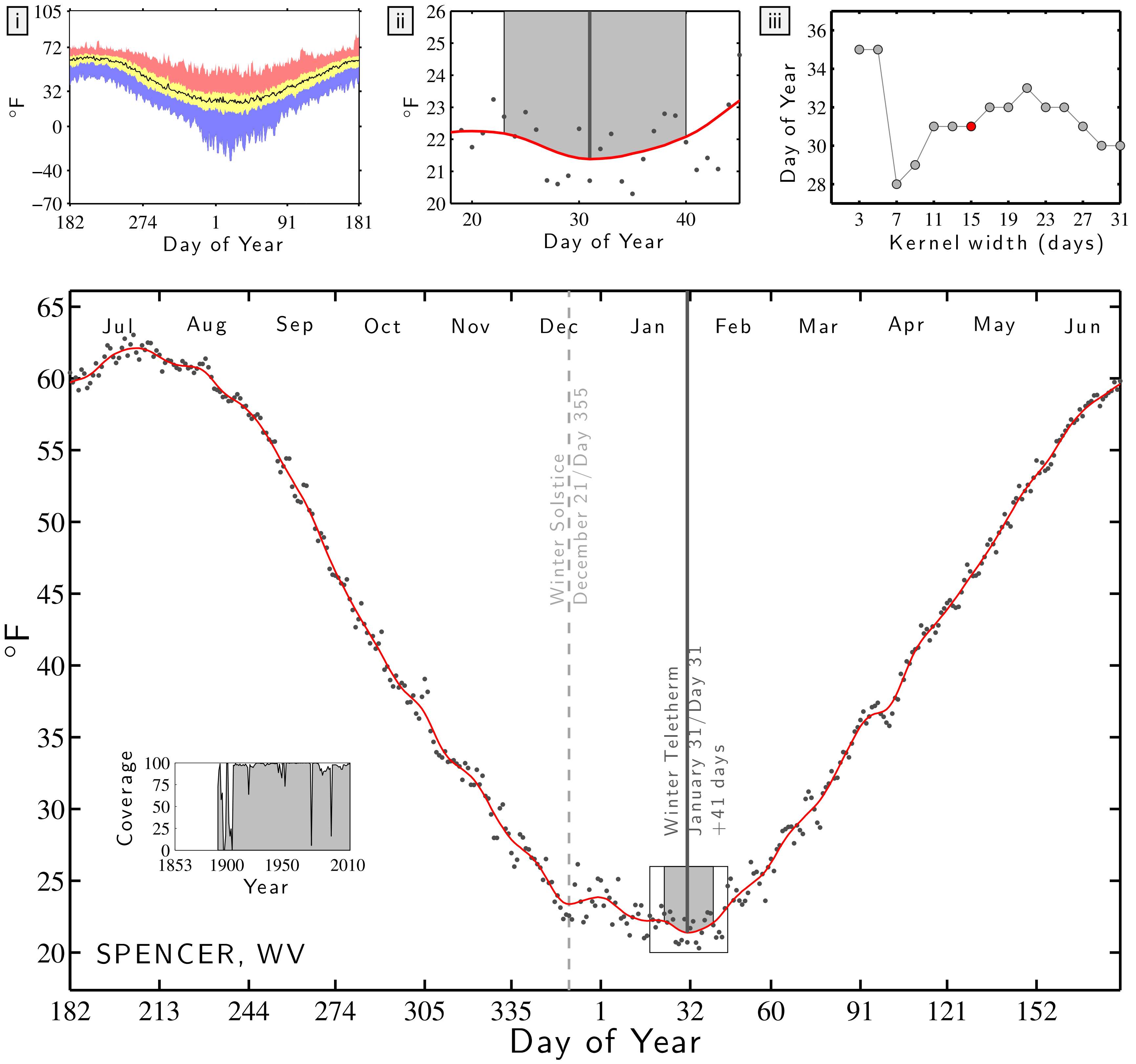

i
°F
Day of Year
ii
°F
Day of Year
iii
Day of Year
Kernel width (days)
Jul
Aug
Sep
Oct
Nov
Dec
Jan
Feb
Mar
Apr
May
Jun
Winter Solstice
December 21/Day 355
Winter Teletherm
January 31/Day 31
+41 days
Coverage
Year
SPENCER, WV
°F
Day of Year

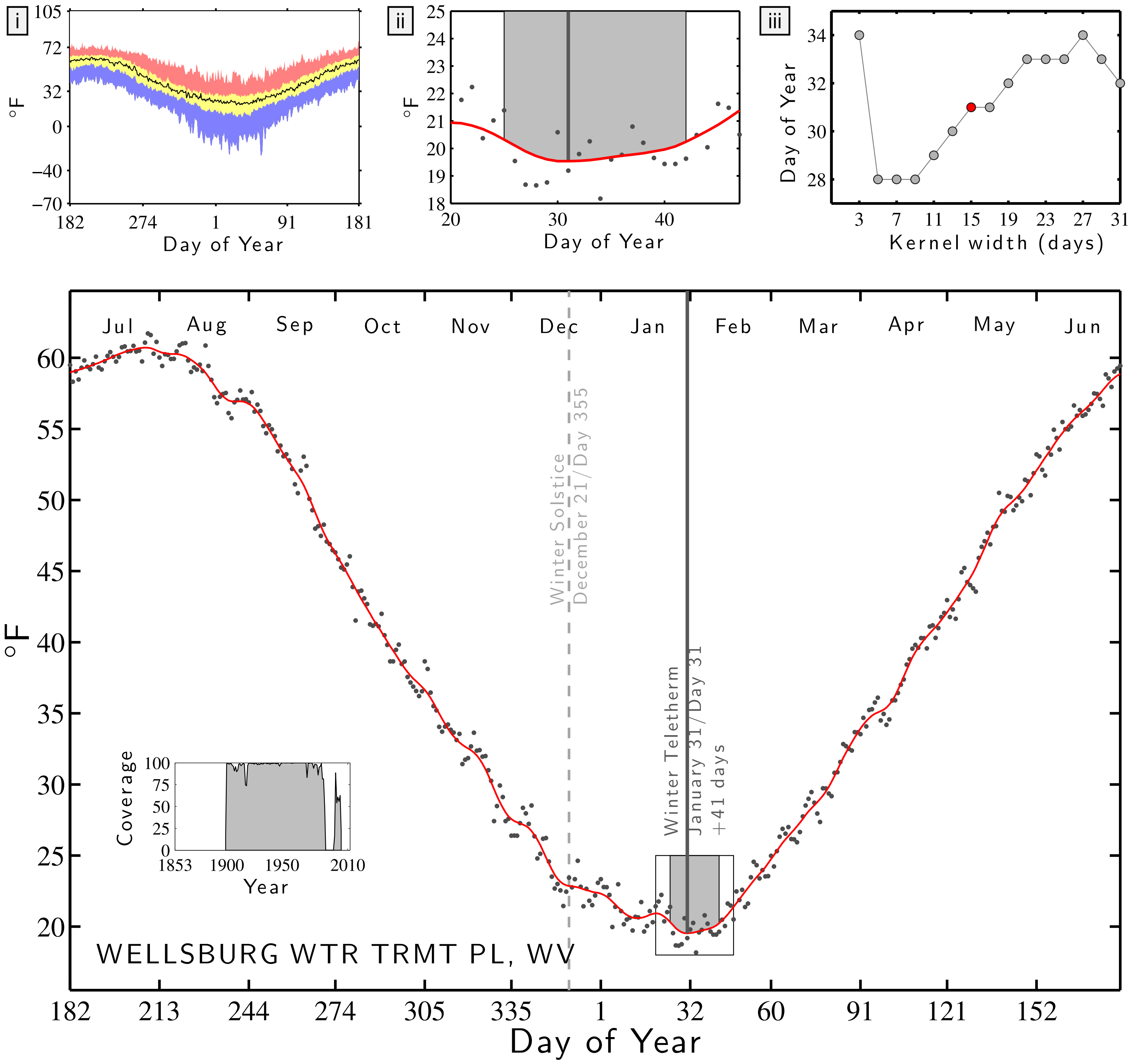

i
ii
iii
°F
Day of Year
Kernel width (days)
Jul
Aug
Sep
Oct
Nov
Dec
Jan
Feb
Mar
Apr
May
Jun
Winter Solstice
December 21/Day 355
Winter Teletherm
January 31/Day 31
+41 days
Coverage
Year
WELLSBURG WTR TRMT PL, WV

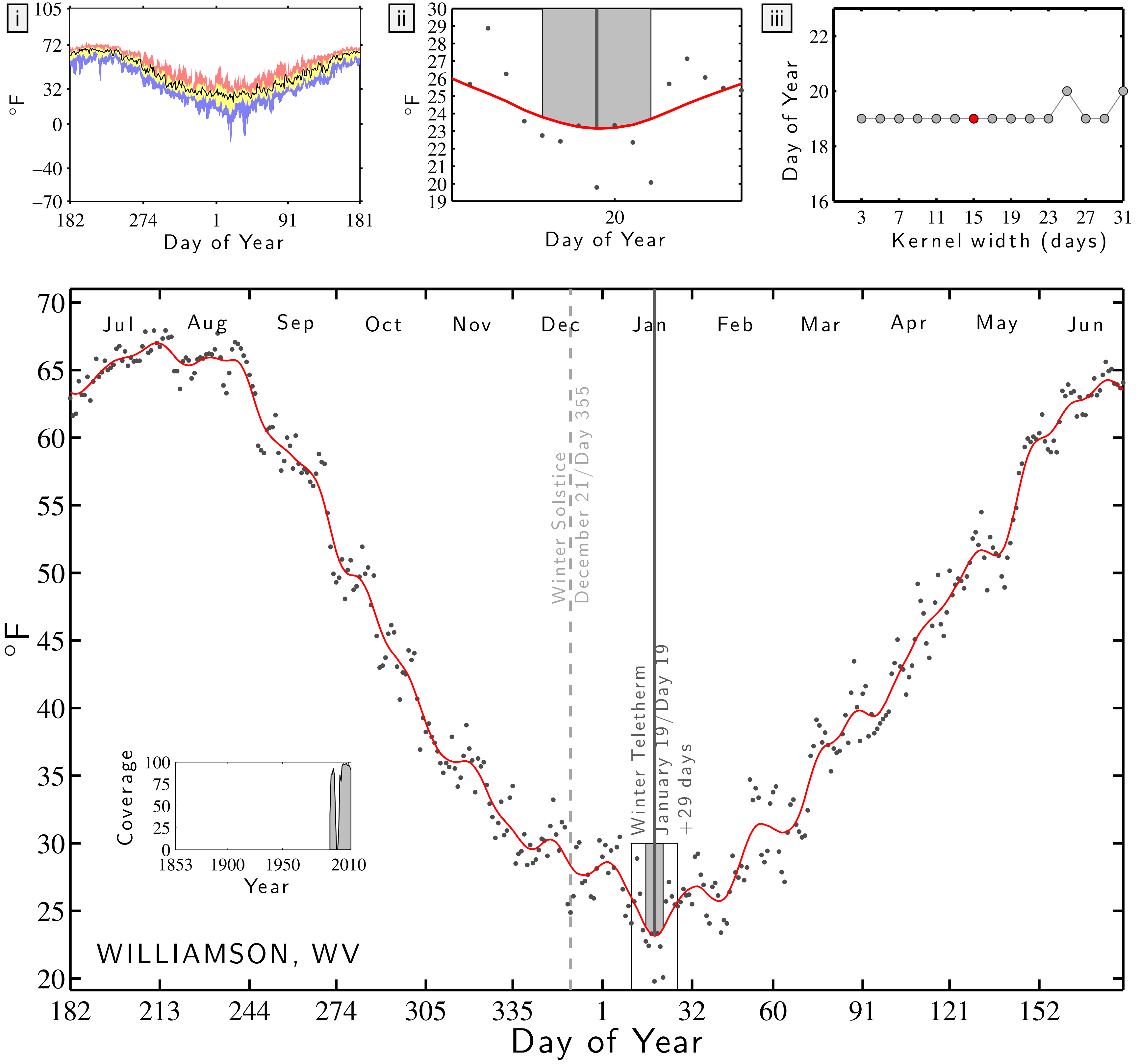

i
ii
iii
Day of Year
°F
Kernel width (days)
Jul
Aug
Sep
Oct
Nov
Dec
Jan
Feb
Mar
Apr
May
Jun
Winter Solstice
December 21/Day 355
Winter Teletherm
January 19/Day 19
+29 days
Coverage
Year
WILLIAMSON, WV

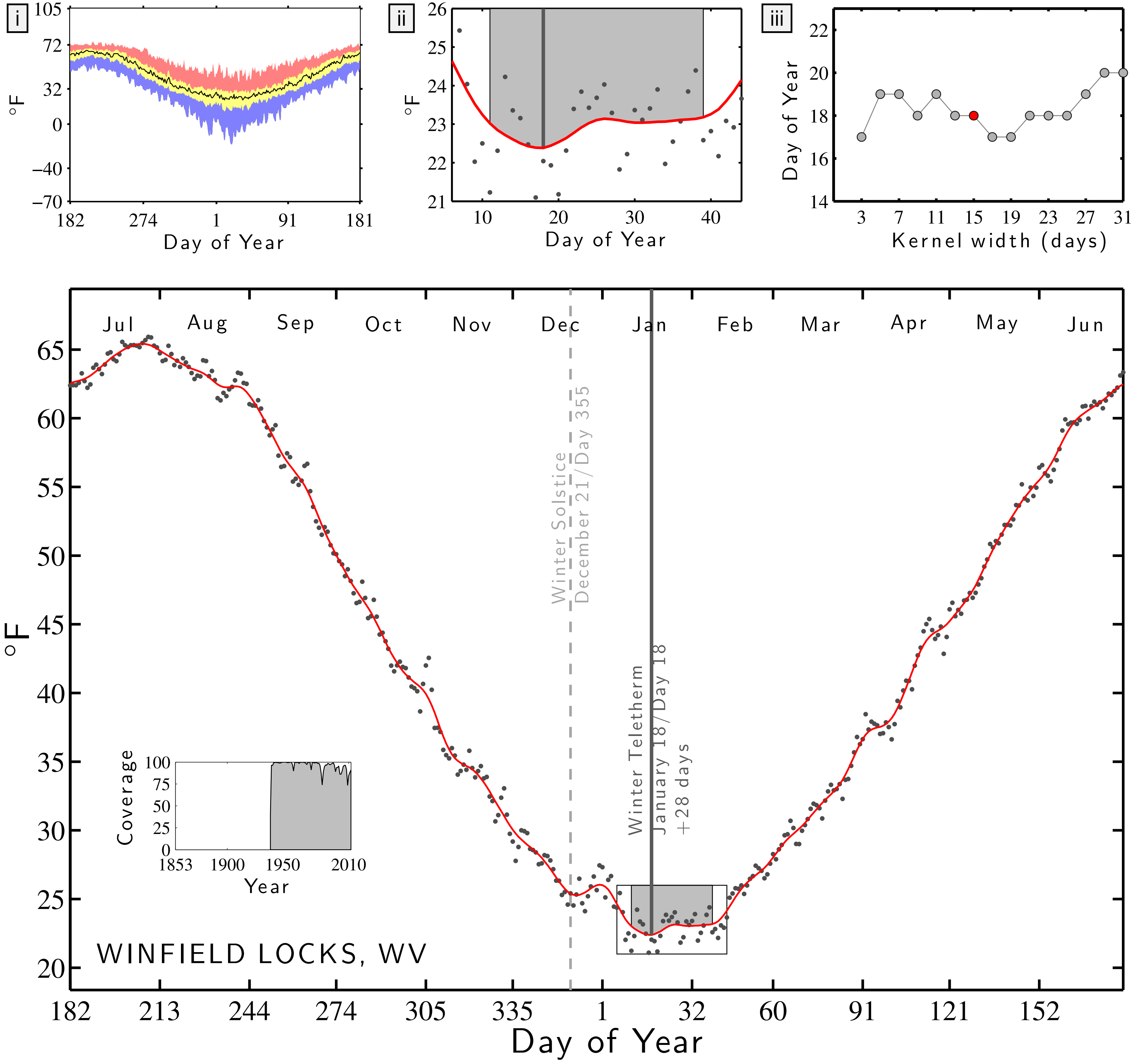

i
°F
Day of Year
ii
°F
Day of Year
iii
Day of Year
Kernel width (days)
Jul
Aug
Sep
Oct
Nov
Dec
Jan
Feb
Mar
Apr
May
Jun
Winter Solstice
December 21/Day 355
Winter Teletherm
January 18/Day 18
+28 days
Coverage
Year
WINFIELD LOCKS, WV
°F
Day of Year

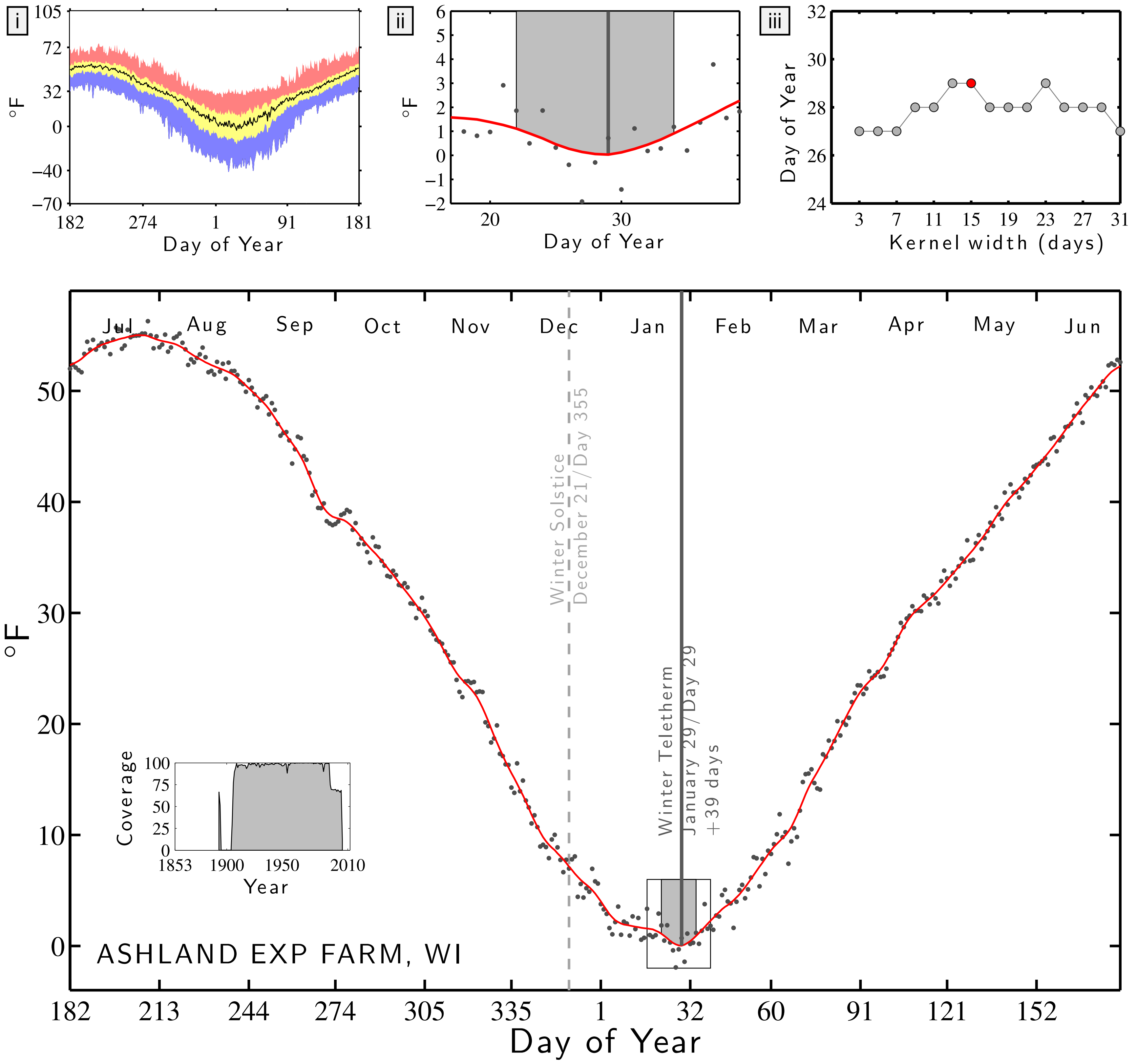

i
ii
iii
°F
Day of Year
Day of Year
Kernel width (days)
Jul
Aug
Sep
Oct
Nov
Dec
Jan
Feb
Mar
Apr
May
Jun
Winter Solstice
December 21/Day 355
Winter Teletherm
January 29/Day 29
+39 days
Coverage
Year
ASHLAND EXP FARM, WI

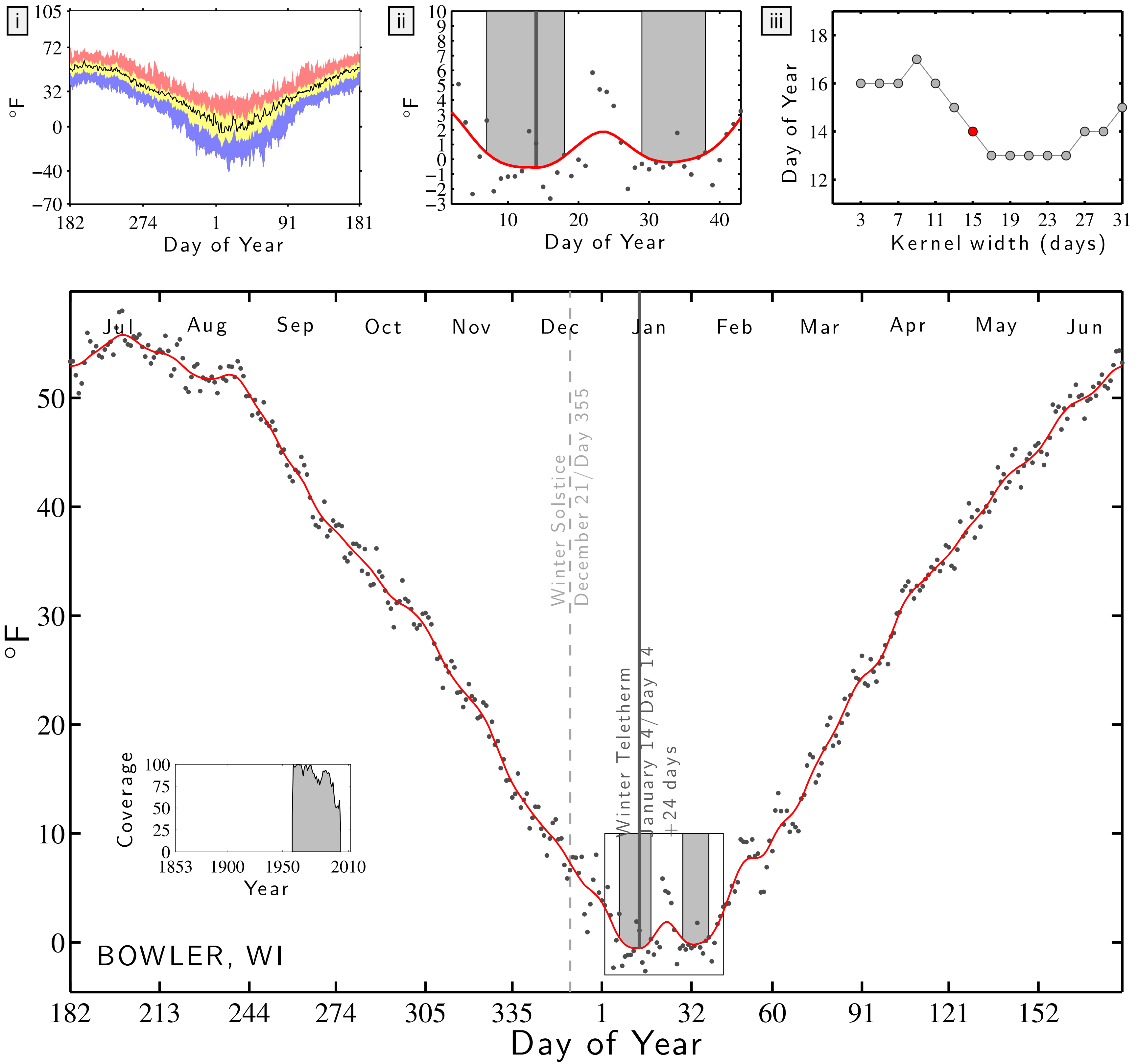

i
ii
iii
°F
Day of Year
Kernel width (days)
Jul
Aug
Sep
Oct
Nov
Dec
Jan
Feb
Mar
Apr
May
Jun
Winter Solstice
December 21/Day 355
Winter Teletherm
January 14/Day 14
+24 days
Coverage
Year
BOWLER, WI

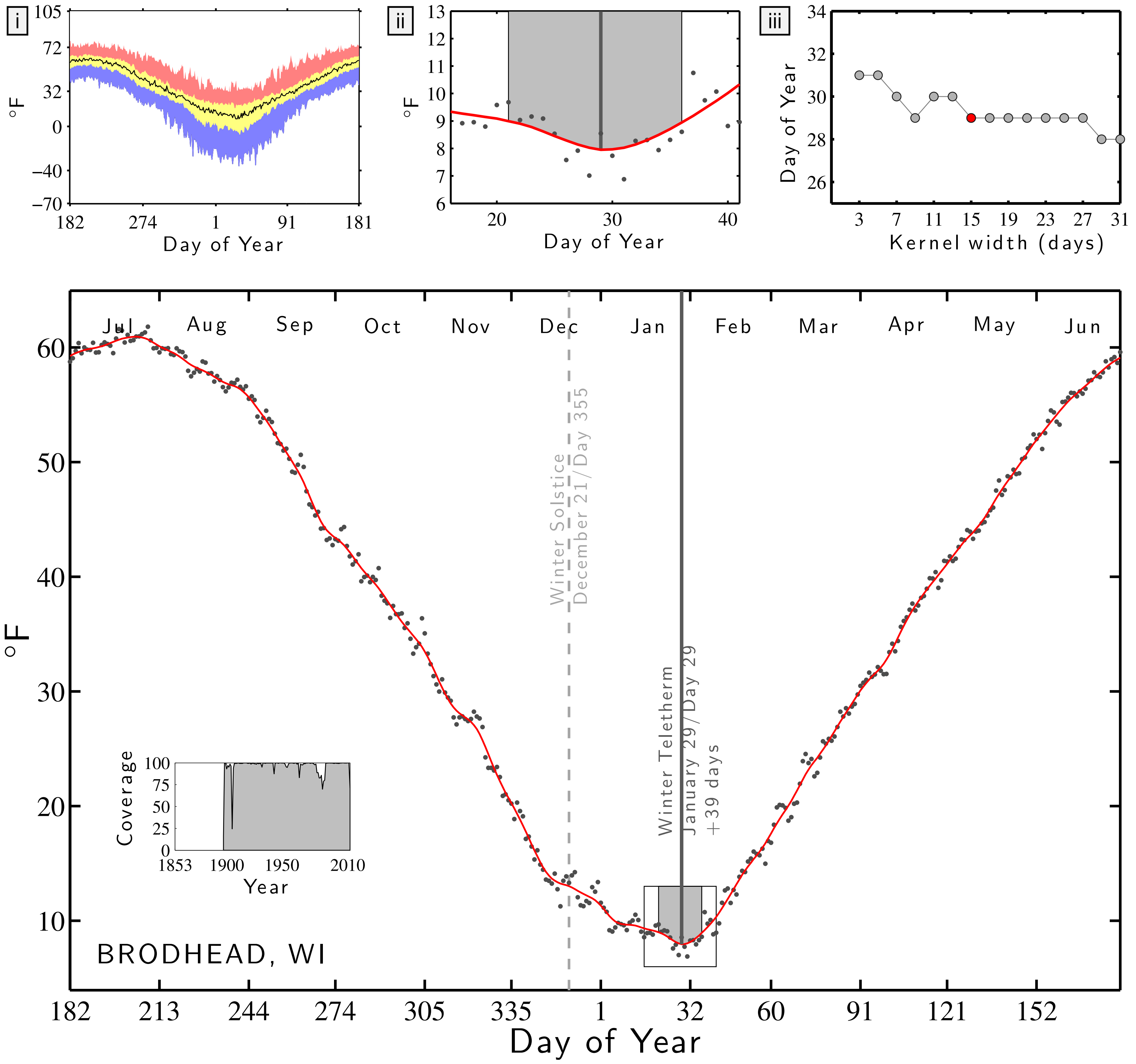

i
°F
Day of Year
ii
iii
Day of Year
Kernel width (days)
Jul
Aug
Sep
Oct
Nov
Dec
Jan
Feb
Mar
Apr
May
Jun
Winter Solstice
December 21/Day 355
Winter Teletherm
January 29/Day 29
+39 days
Coverage
Year
BRODHEAD, WI

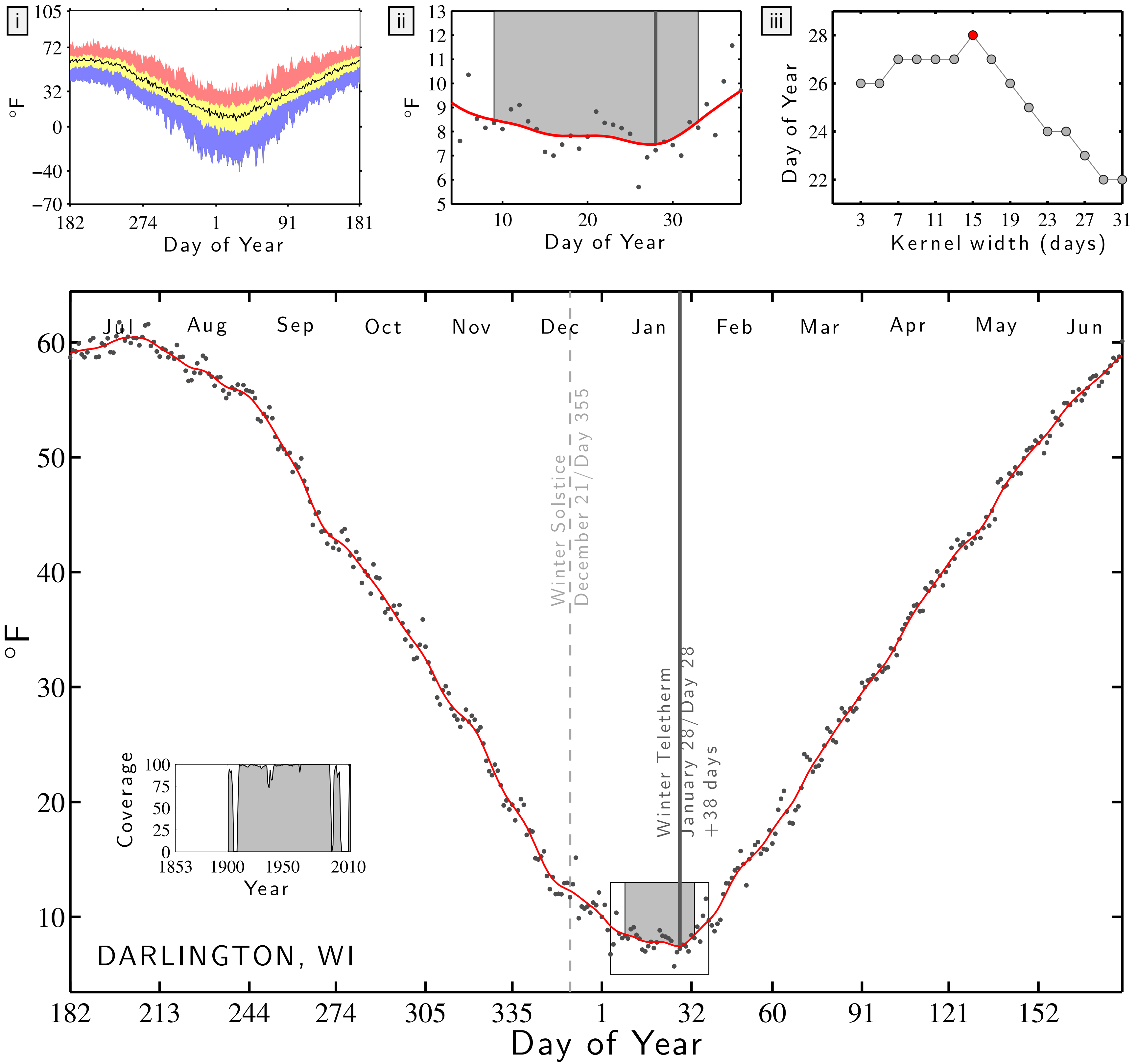

i
ii
iii
°F
Day of Year
Day of Year
Kernel width (days)
Jul
Aug
Sep
Oct
Nov
Dec
Jan
Feb
Mar
Apr
May
Jun
Winter Solstice
December 21/Day 355
Winter Teletherm
January 28/Day 28
+38 days
Coverage
Year
DARLINGTON, WI
Day of Year

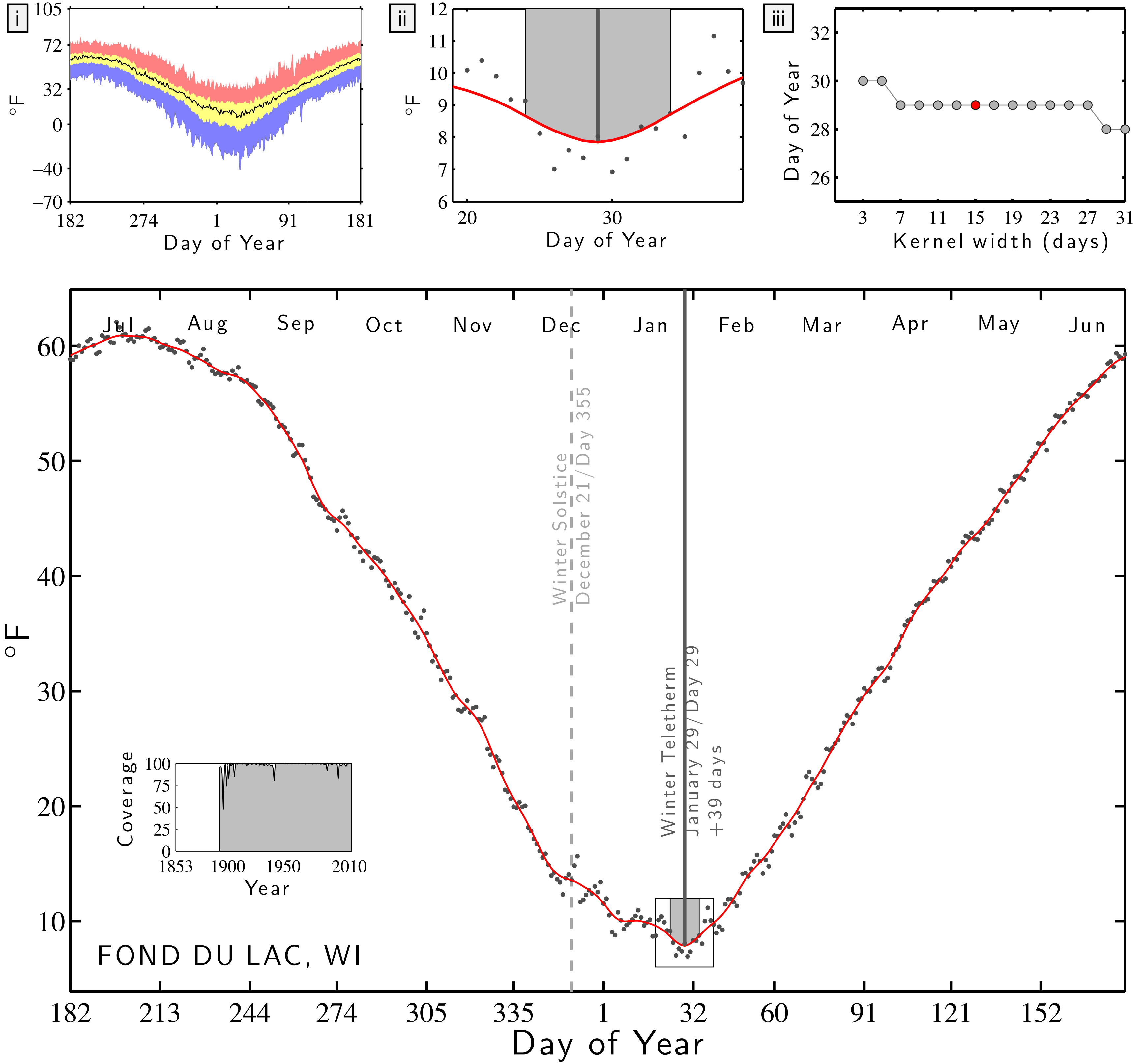
i
°F
Day of Year
ii
°F
Day of Year
iii
Day of Year
Kernel width (days)
Jul
Aug
Sep
Oct
Nov
Dec
Jan
Feb
Mar
Apr
May
Jun
Winter Solstice
December 21/Day 355
Winter Teletherm
January 29/Day 29
+39 days
Coverage
Year
FOND DU LAC, WI
°F
Day of Year

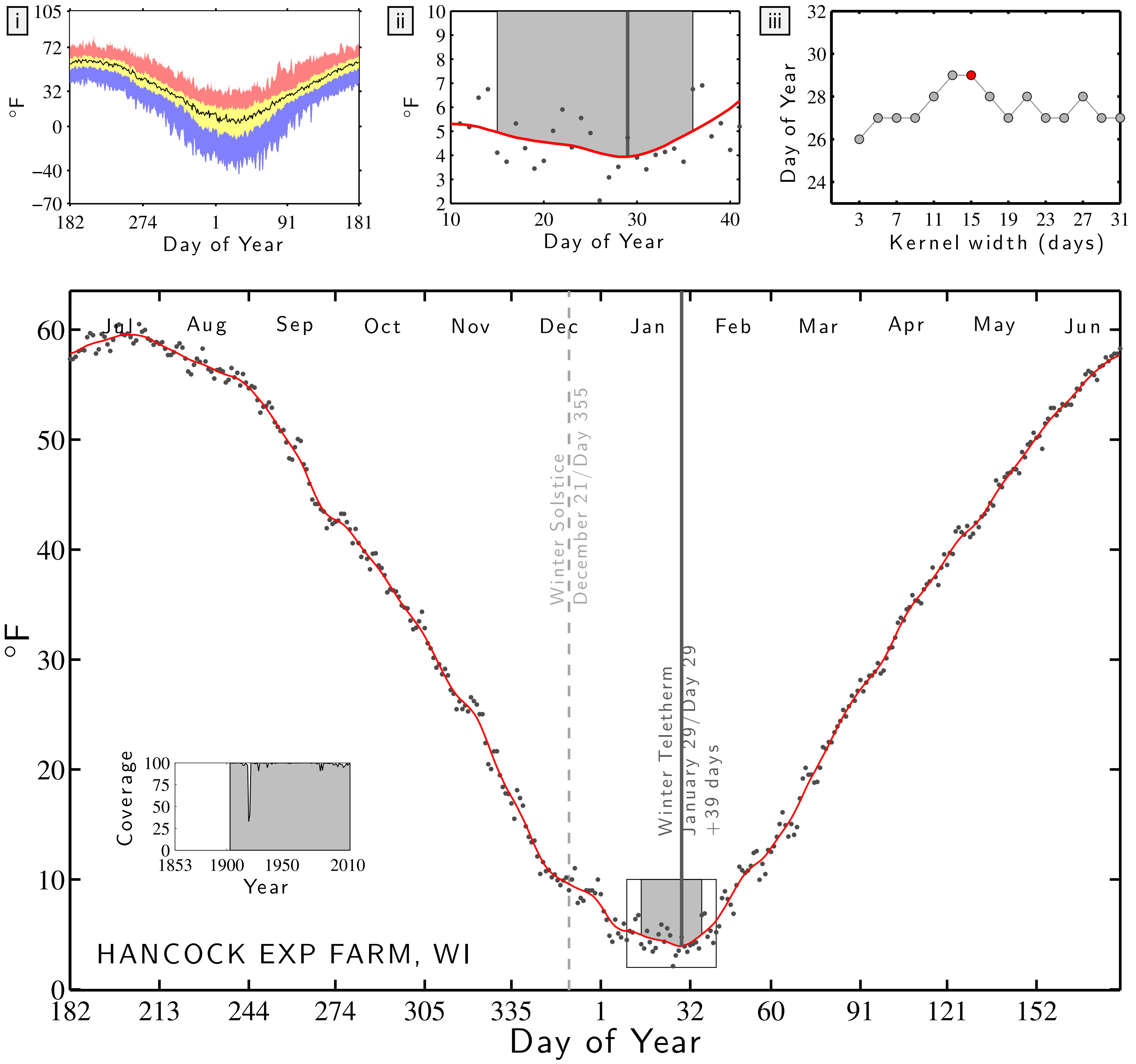

i
ii
iii
°F
Day of Year
Kernel width (days)
Jul
Aug
Sep
Oct
Nov
Dec
Jan
Feb
Mar
Apr
May
Jun
Winter Solstice
December 21/Day 355
Winter Teletherm
January 29/Day 29
+39 days
Coverage
Year
HANCOCK EXP FARM, WI

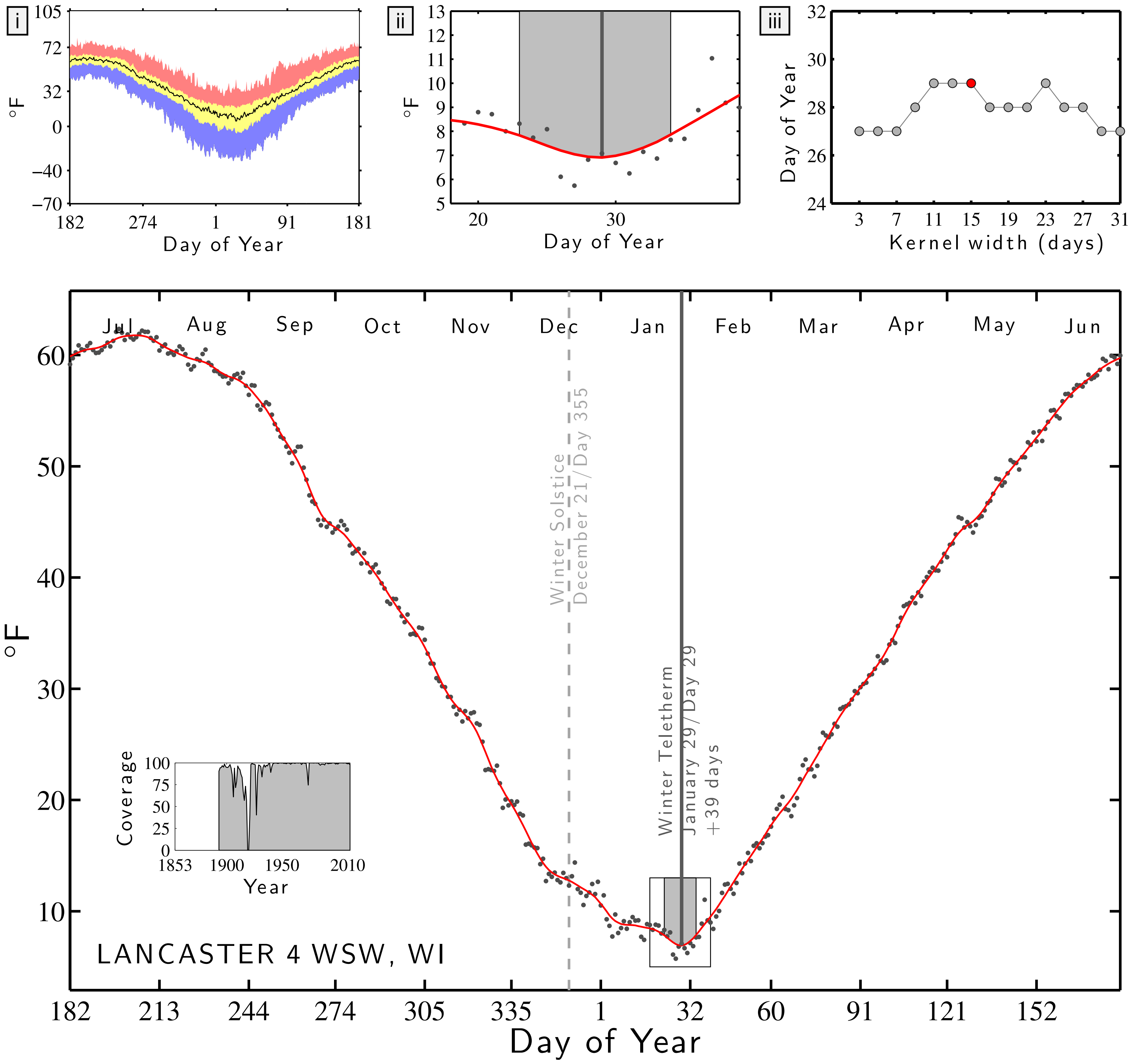

i
°F
105
72
32
0
−40
−70
182
274
1
91
181
Day of Year
ii
13
12
11
10
9
8
7
6
5
20
30
Day of Year
iii
32
30
28
26
24
Day of Year
3
7
11
15
19
23
27
31
Kernel width (days)
Jul
Aug
Sep
Oct
Nov
Dec
Jan
Feb
Mar
Apr
May
Jun
60
50
40
30
20
10
Winter Solstice
December 21/Day 355
Winter Teletherm
January 29/Day 29
+39 days
Coverage
100
75
50
25
0
1853
1900
1950
2010
Year
LANCASTER 4 WSW, WI
182
213
244
274
305
335
1
32
60
91
121
152
Day of Year

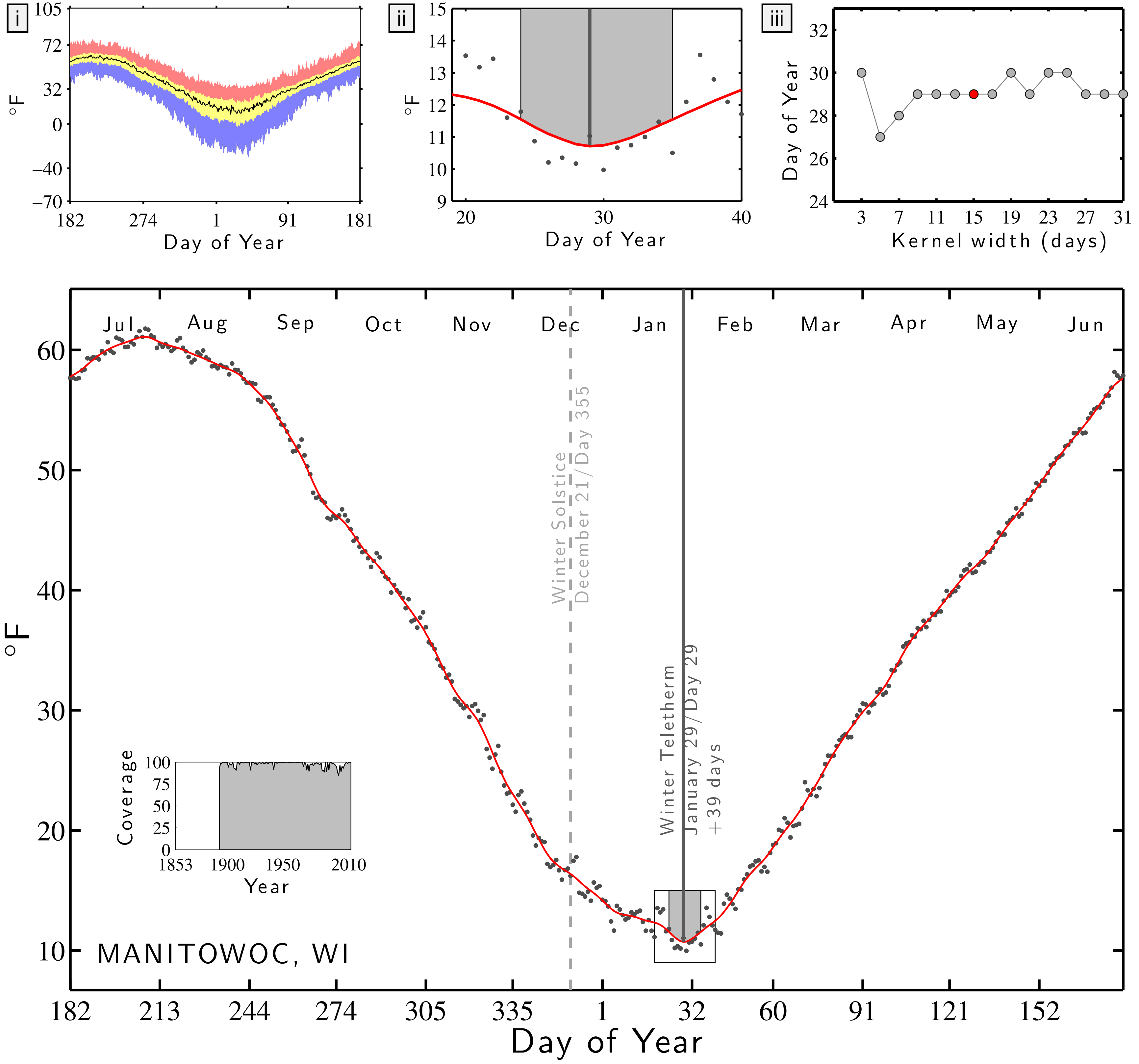

i
ii
iii
°F
Day of Year
Kernel width (days)
Jul
Aug
Sep
Oct
Nov
Dec
Jan
Feb
Mar
Apr
May
Jun
Winter Solstice
December 21/Day 355
Winter Teletherm
January 29/Day 29
+39 days
Coverage
Year
MANITOWOC, WI

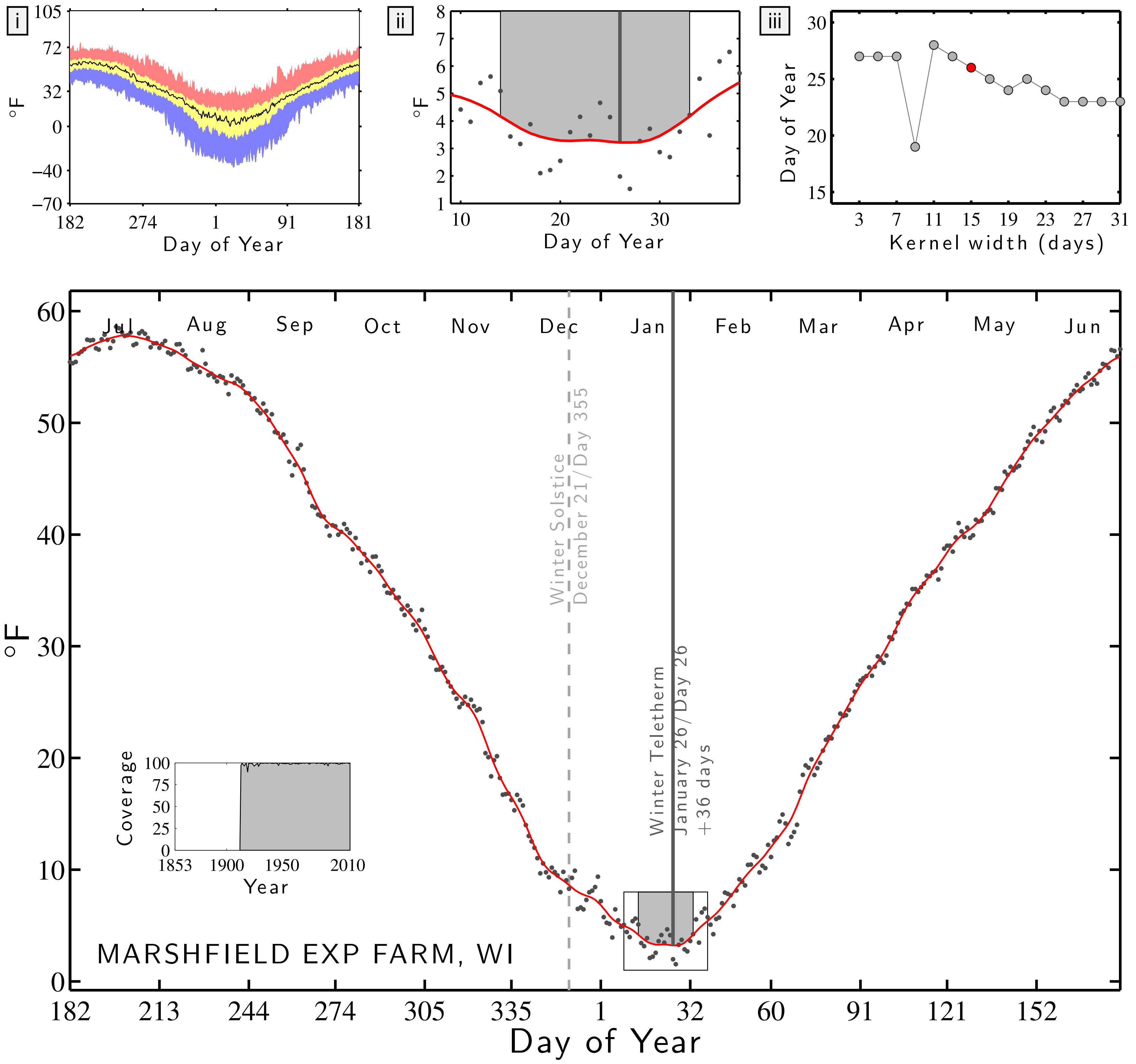

i
ii
iii
°F
Day of Year
Kernel width (days)
Jul
Aug
Sep
Oct
Nov
Dec
Jan
Feb
Mar
Apr
May
Jun
Winter Solstice
December 21/Day 355
Winter Teletherm
January 26/Day 26
+36 days
Coverage
Year
MARSHFIELD EXP FARM, WI

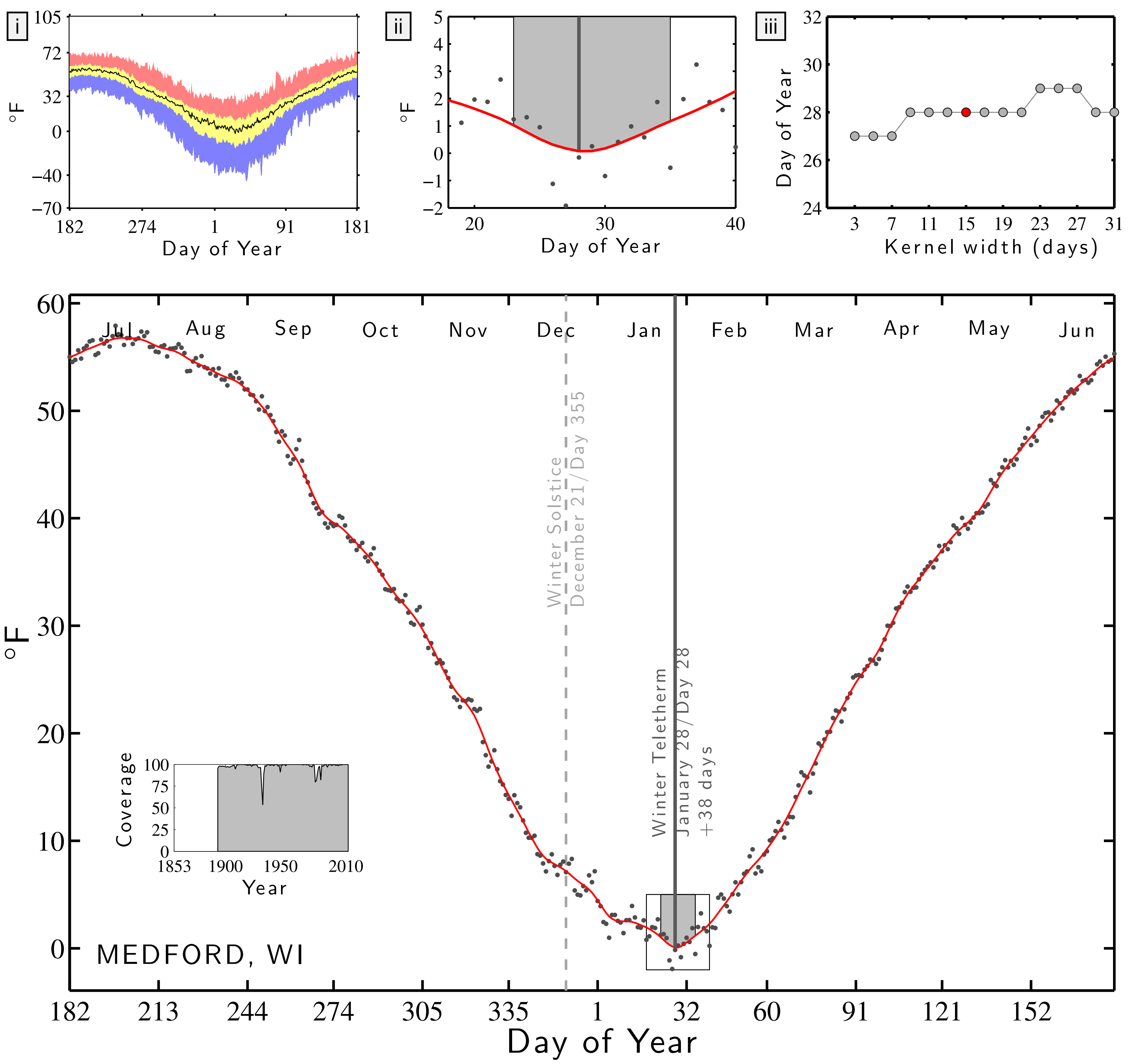

i
ii
iii
°F
Day of Year
Day of Year
Kernel width (days)
Jul
Aug
Sep
Oct
Nov
Dec
Jan
Feb
Mar
Apr
May
Jun
Winter Solstice
December 21/Day 355
Winter Teletherm
January 28/Day 28
+38 days
Coverage
Year
MEDFORD, WI

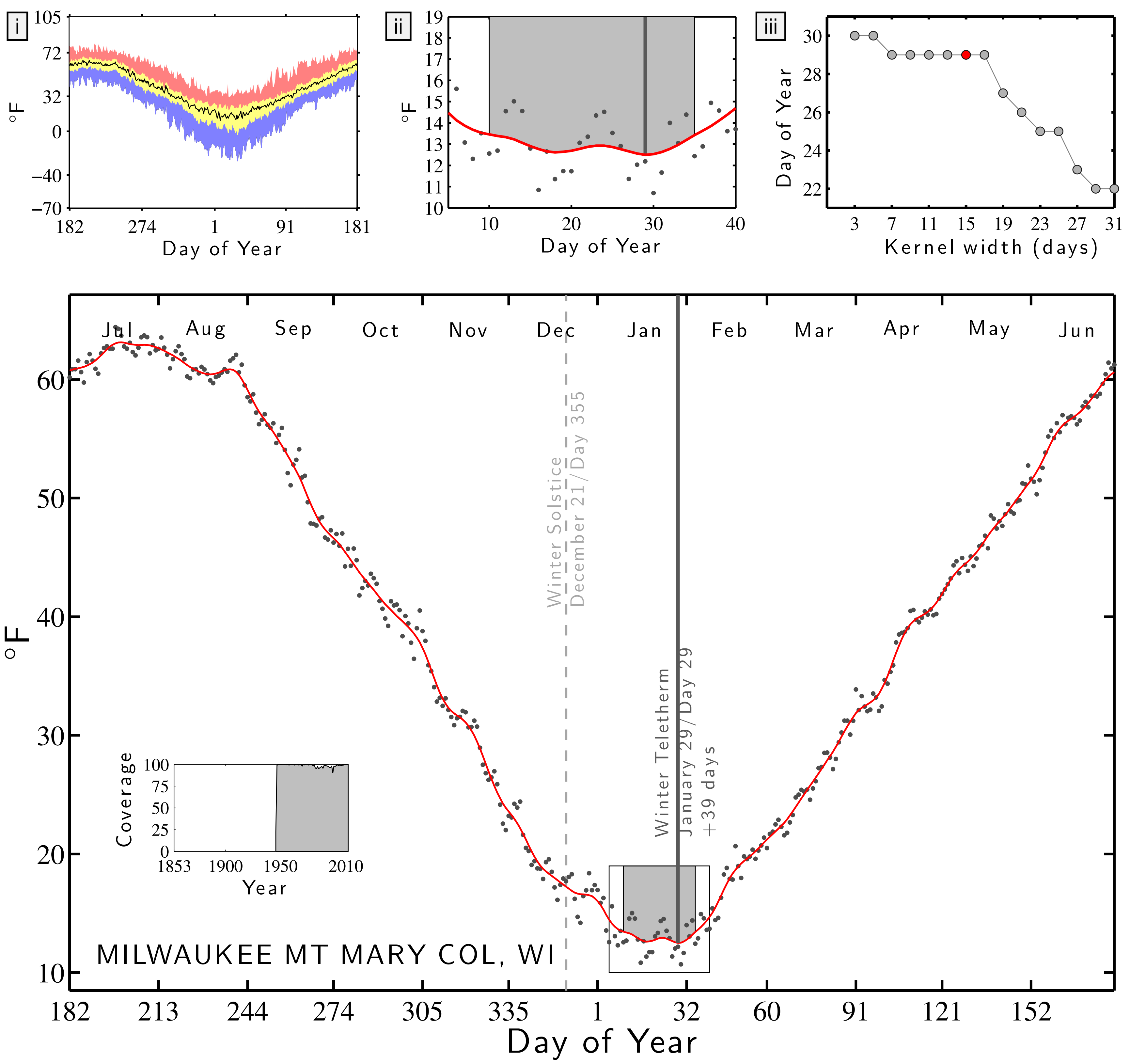

i
°F
Day of Year
ii
°F
Day of Year
iii
Day of Year
Kernel width (days)
Jul
Aug
Sep
Oct
Nov
Dec
Jan
Feb
Mar
Apr
May
Jun
Winter Solstice
December 21/Day 355
Winter Teletherm
January 29/Day 29
+39 days
Coverage
Year
MILWAUKEE MT MARY COL, WI
°F
Day of Year

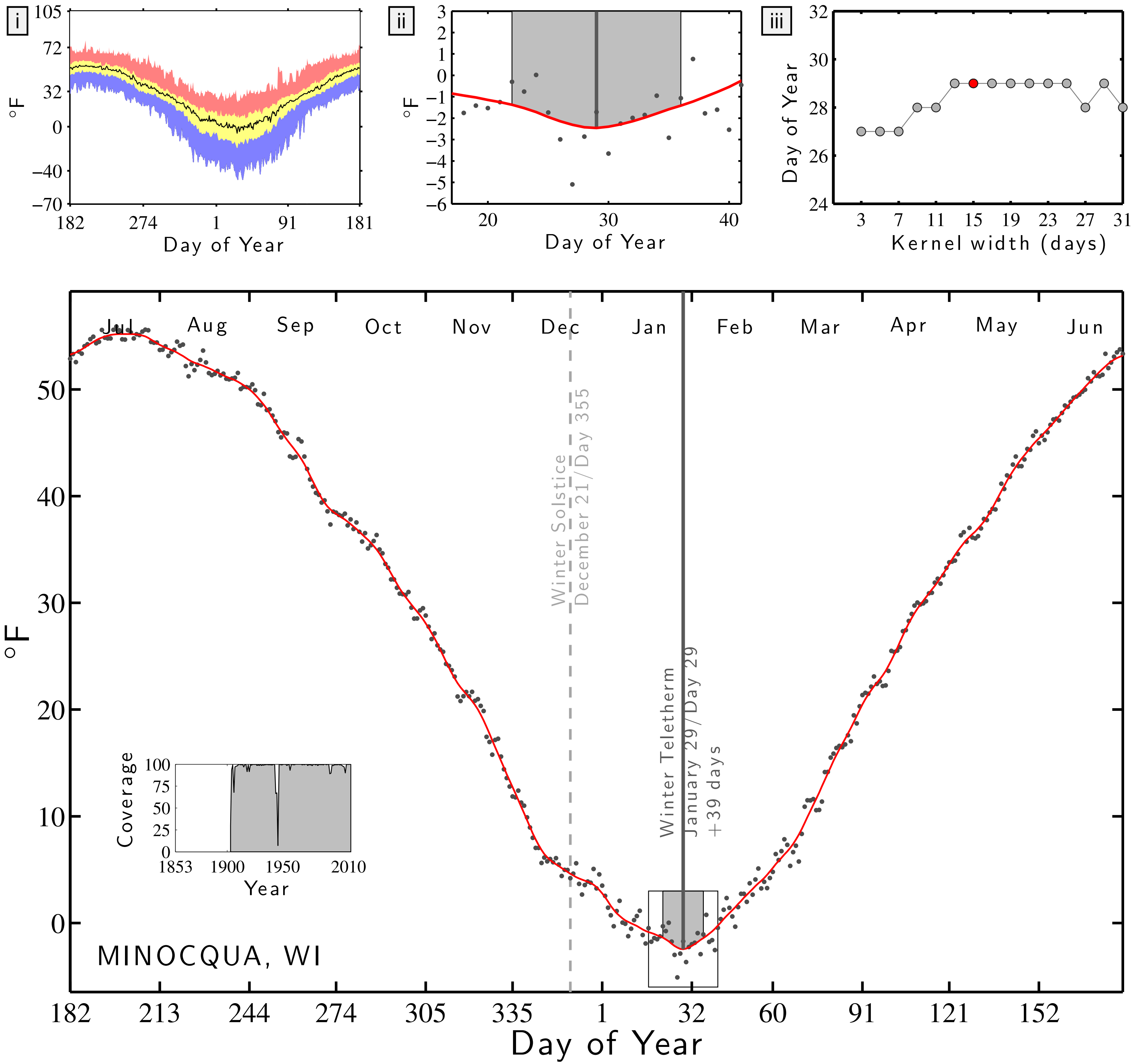

i
°F
Day of Year
ii
°F
Day of Year
iii
Day of Year
Kernel width (days)
Jul
Aug
Sep
Oct
Nov
Dec
Jan
Feb
Mar
Apr
May
Jun
Winter Solstice
December 21/Day 355
Winter Teletherm
January 29/Day 29
+39 days
Coverage
Year
MINOCQUA, WI
°F
Day of Year

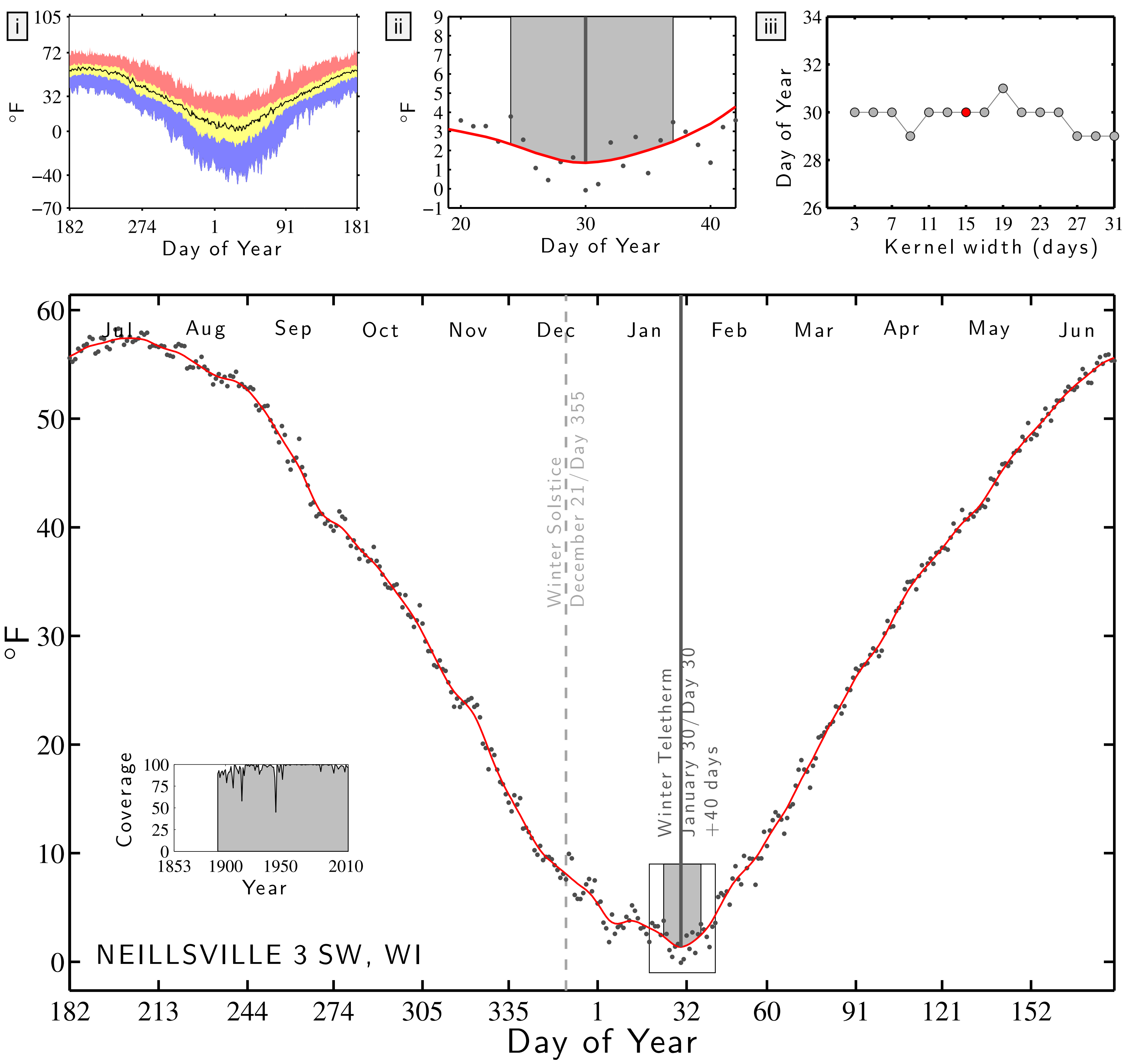

i
ii
iii
°F
Day of Year
Kernel width (days)
Day of Year
Winter Solstice
December 21/Day 355
Winter Teletherm
January 30/Day 30
+40 days
Jul
Aug
Sep
Oct
Nov
Dec
Jan
Feb
Mar
Apr
May
Jun
Coverage
Year
NEILLSVILLE 3 SW, WI

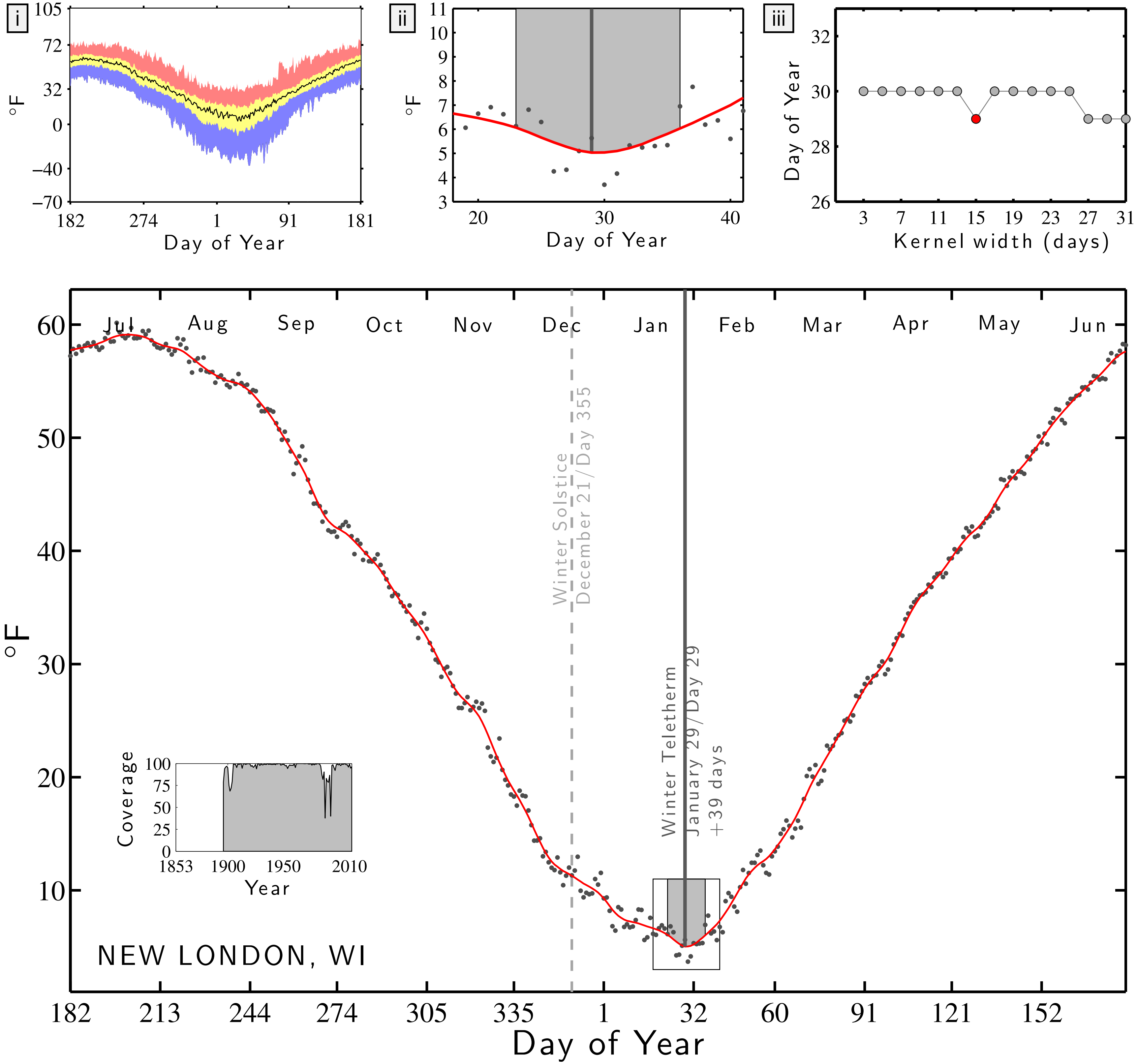
i
°F
105
72
32
0
−40
−70
182
274
1
91
181
Day of Year
ii
11
10
9
8
7
6
5
4
3
20
30
40
Day of Year
iii
Day of Year
32
30
28
26
3
7
11
15
19
23
27
31
Kernel width (days)
Jul
Aug
Sep
Oct
Nov
Dec
Jan
Feb
Mar
Apr
May
Jun
Winter Solstice
December 21/Day 355
Winter Teletherm
January 29/Day 29
+39 days
Coverage
100
75
50
25
0
1853
1900
1950
2010
Year
NEW LONDON, WI
60
50
40
30
20
10
182
213
244
274
305
335
1
32
60
91
121
152
Day of Year

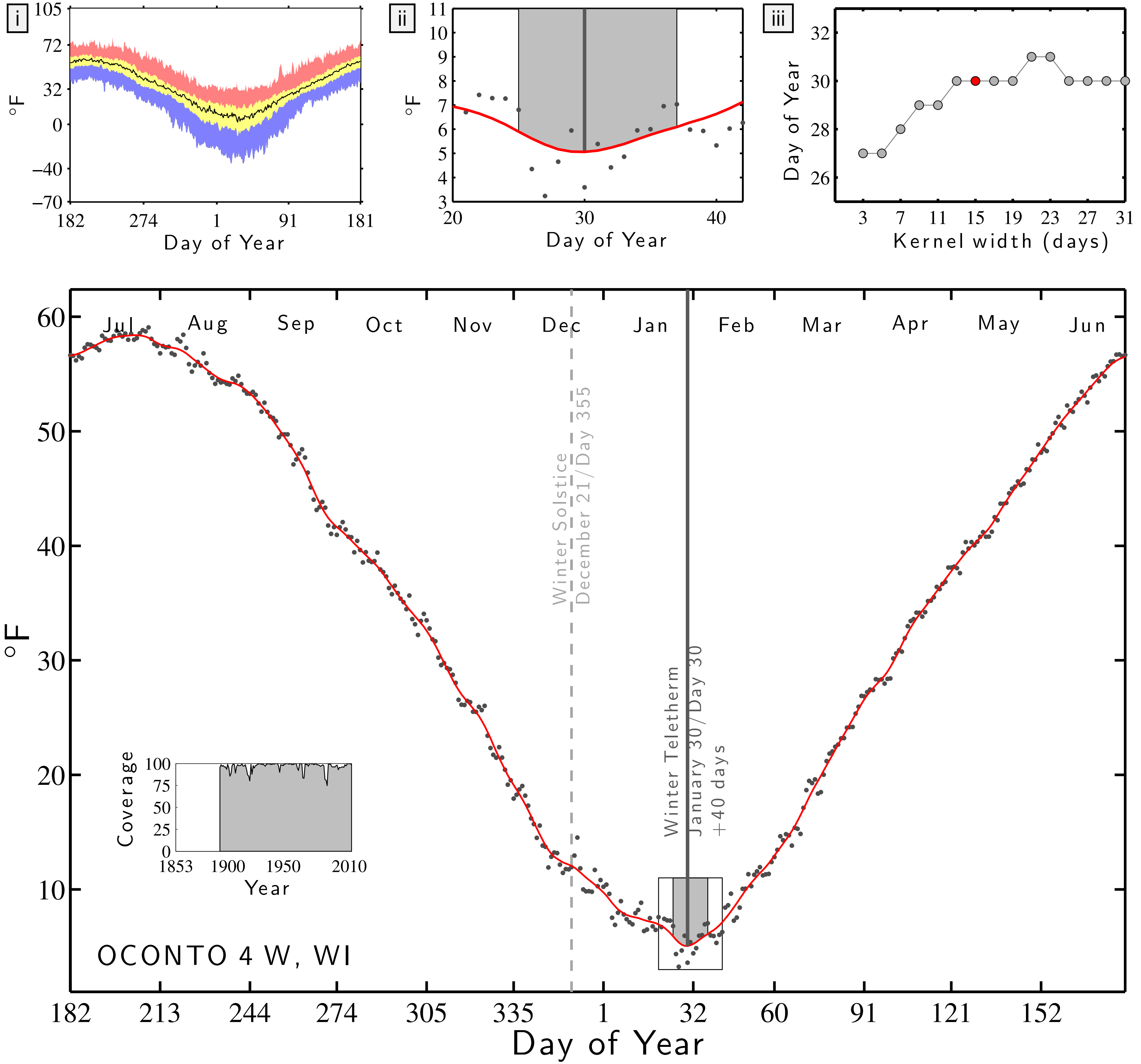

i
ii
iii
°F
Day of Year
Day of Year
Kernel width (days)
Jul
Aug
Sep
Oct
Nov
Dec
Jan
Feb
Mar
Apr
May
Jun
Winter Solstice
December 21/Day 355
Winter Teletherm
January 30/Day 30
+40 days
Coverage
Year
OCONTO 4 W, WI

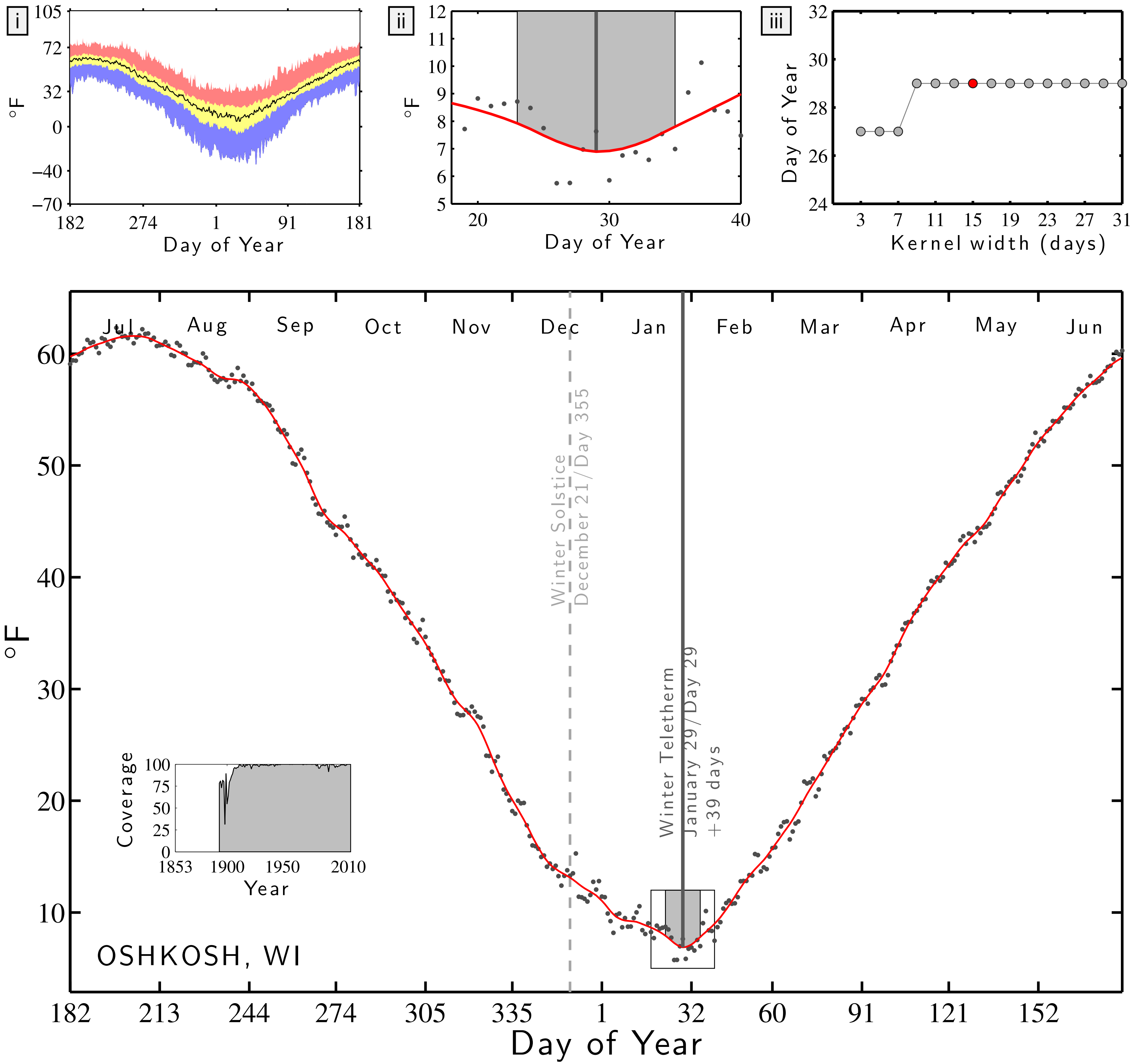

i
°F
Day of Year
ii
°F
Day of Year
iii
Day of Year
Kernel width (days)
Jul
Aug
Sep
Oct
Nov
Dec
Jan
Feb
Mar
Apr
May
Jun
Winter Solstice
December 21/Day 355
Winter Teletherm
January 29/Day 29
+39 days
Coverage
Year
OSHKOSH, WI
°F
Day of Year

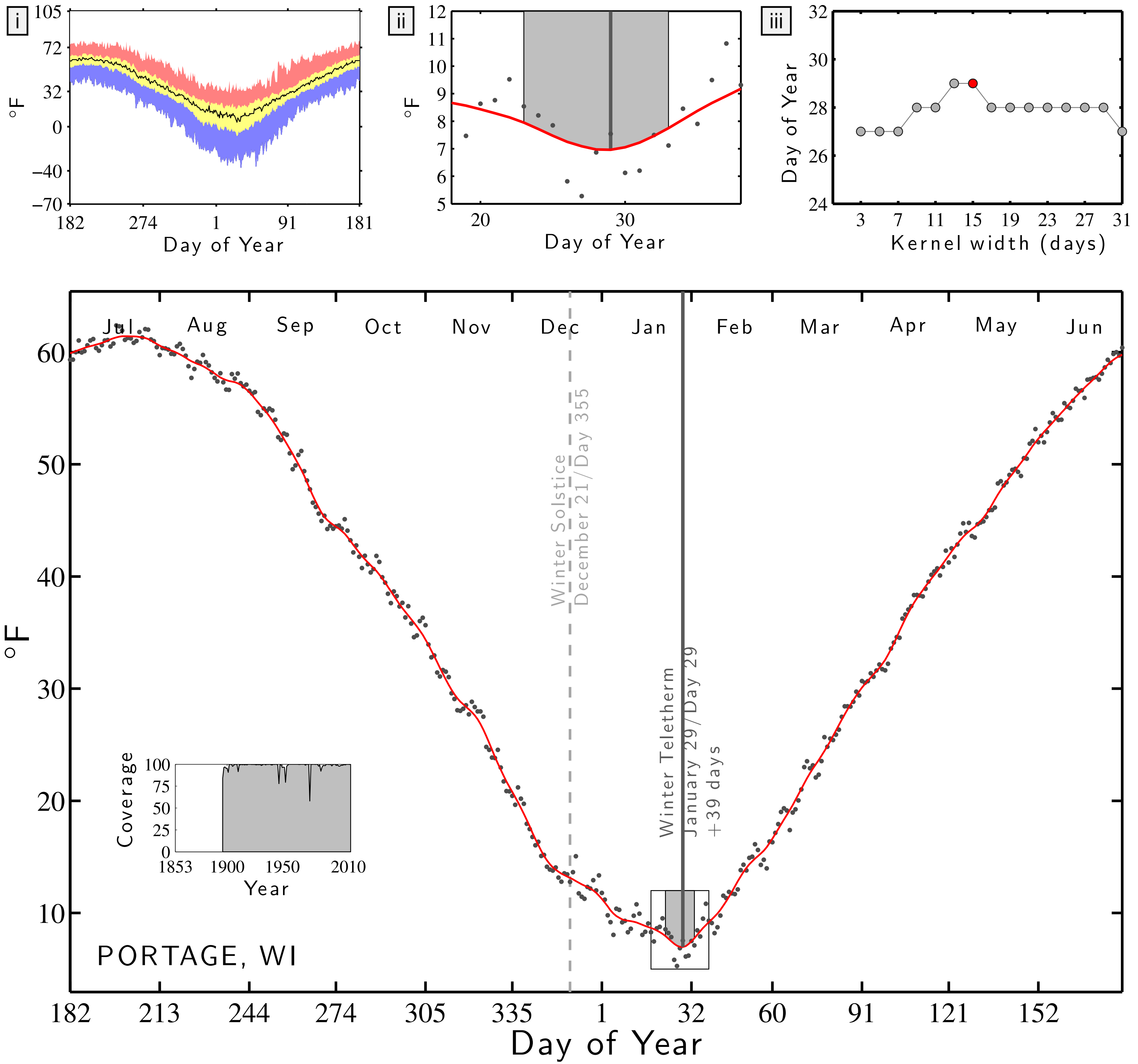

i
ii
iii
°F
Day of Year
Kernel width (days)
Jul
Aug
Sep
Oct
Nov
Dec
Jan
Feb
Mar
Apr
May
Jun
Winter Solstice
December 21/Day 355
Winter Teletherm
January 29/Day 29
+39 days
Coverage
Year
PORTAGE, WI

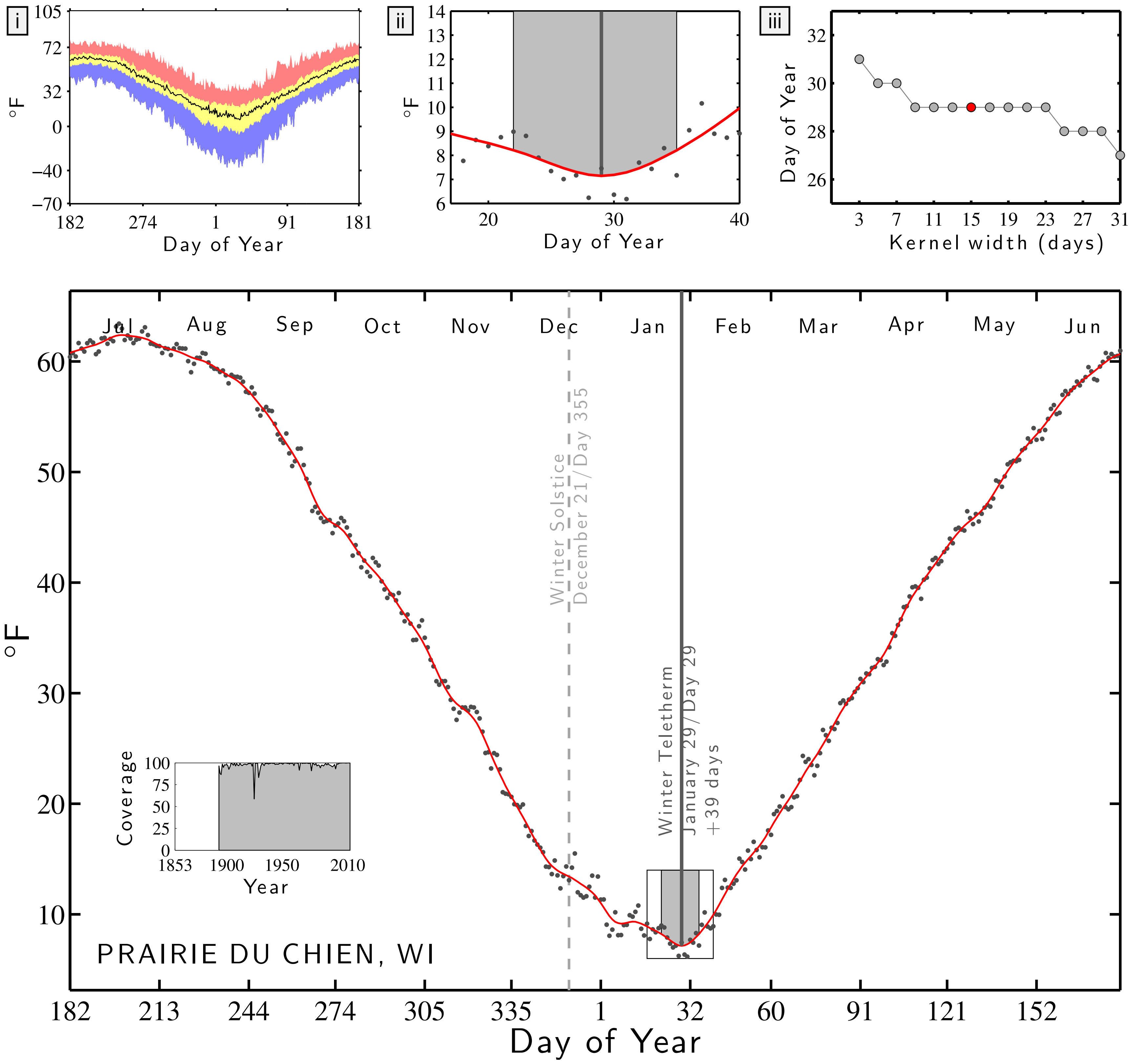
i
°F
Day of Year
ii
Day of Year
iii
Day of Year
Kernel width (days)
Jul
Aug
Sep
Oct
Nov
Dec
Jan
Feb
Mar
Apr
May
Jun
Winter Solstice
December 21/Day 355
Winter Teletherm
January 29/Day 29
+39 days
Coverage
Year
PRAIRIE DU CHIEN, WI
Day of Year

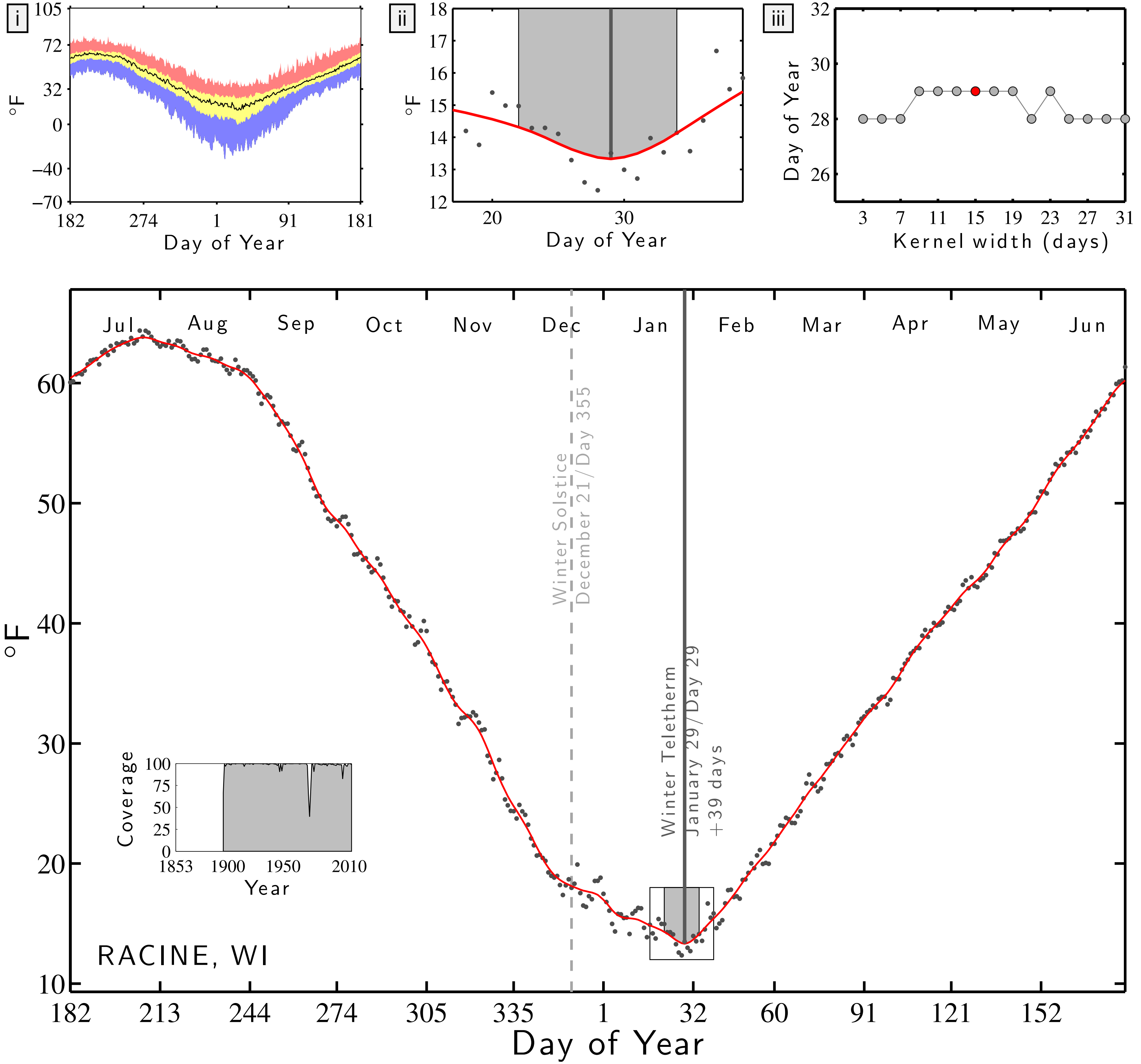

i
ii
iii
°F
Day of Year
Day of Year
Kernel width (days)
Jul
Aug
Sep
Oct
Nov
Dec
Jan
Feb
Mar
Apr
May
Jun
Winter Solstice
December 21/Day 355
Winter Teletherm
January 29/Day 29
+39 days
Coverage
Year
RACINE, WI

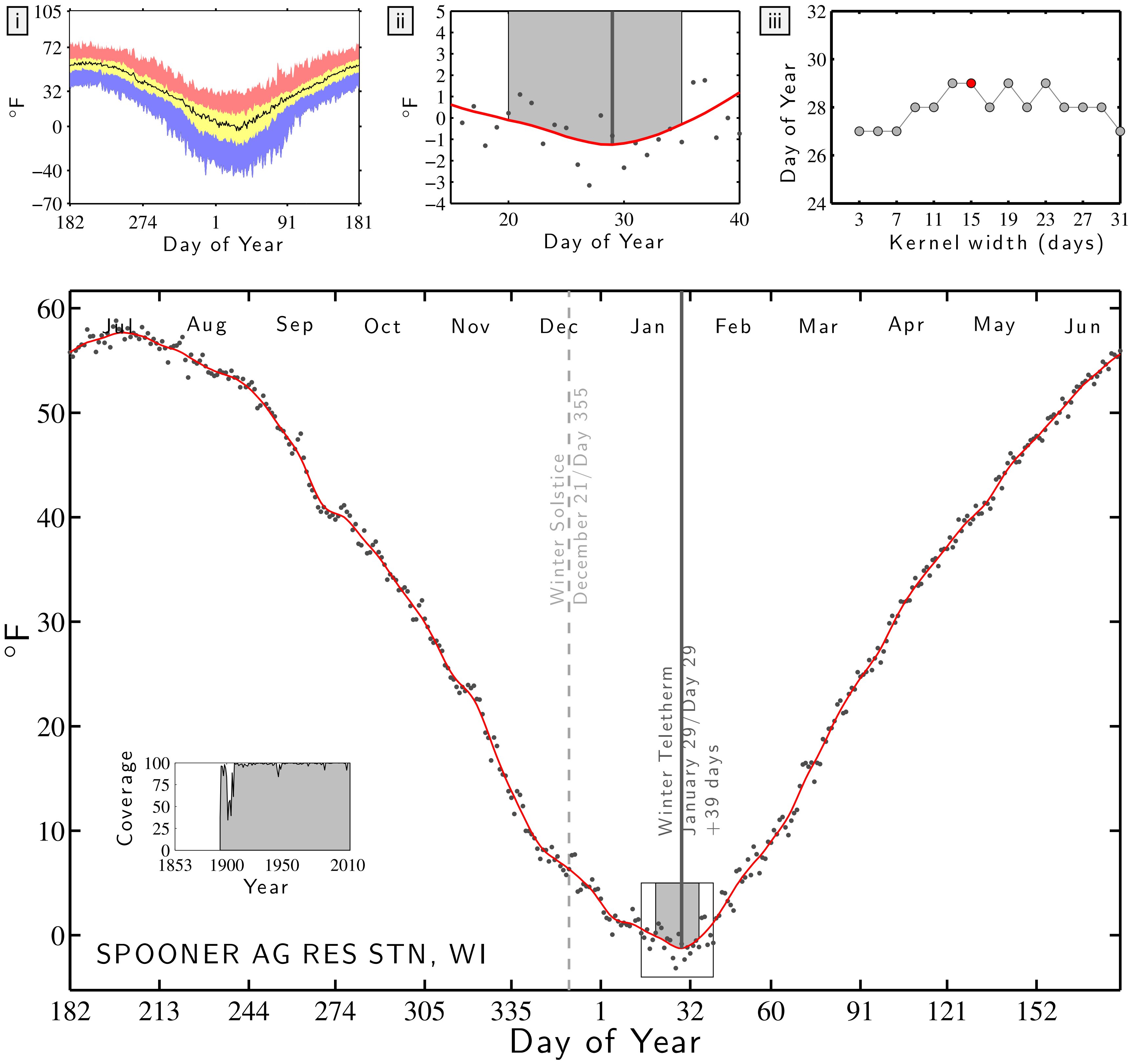
i
ii
iii
Day of Year
Kernel width (days)
°F
Day of Year
Jul
Aug
Sep
Oct
Nov
Dec
Jan
Feb
Mar
Apr
May
Jun
Winter Solstice
December 21/Day 355
Winter Teletherm
January 29/Day 29
+39 days
Coverage
Year
SPOONER AG RES STN, WI

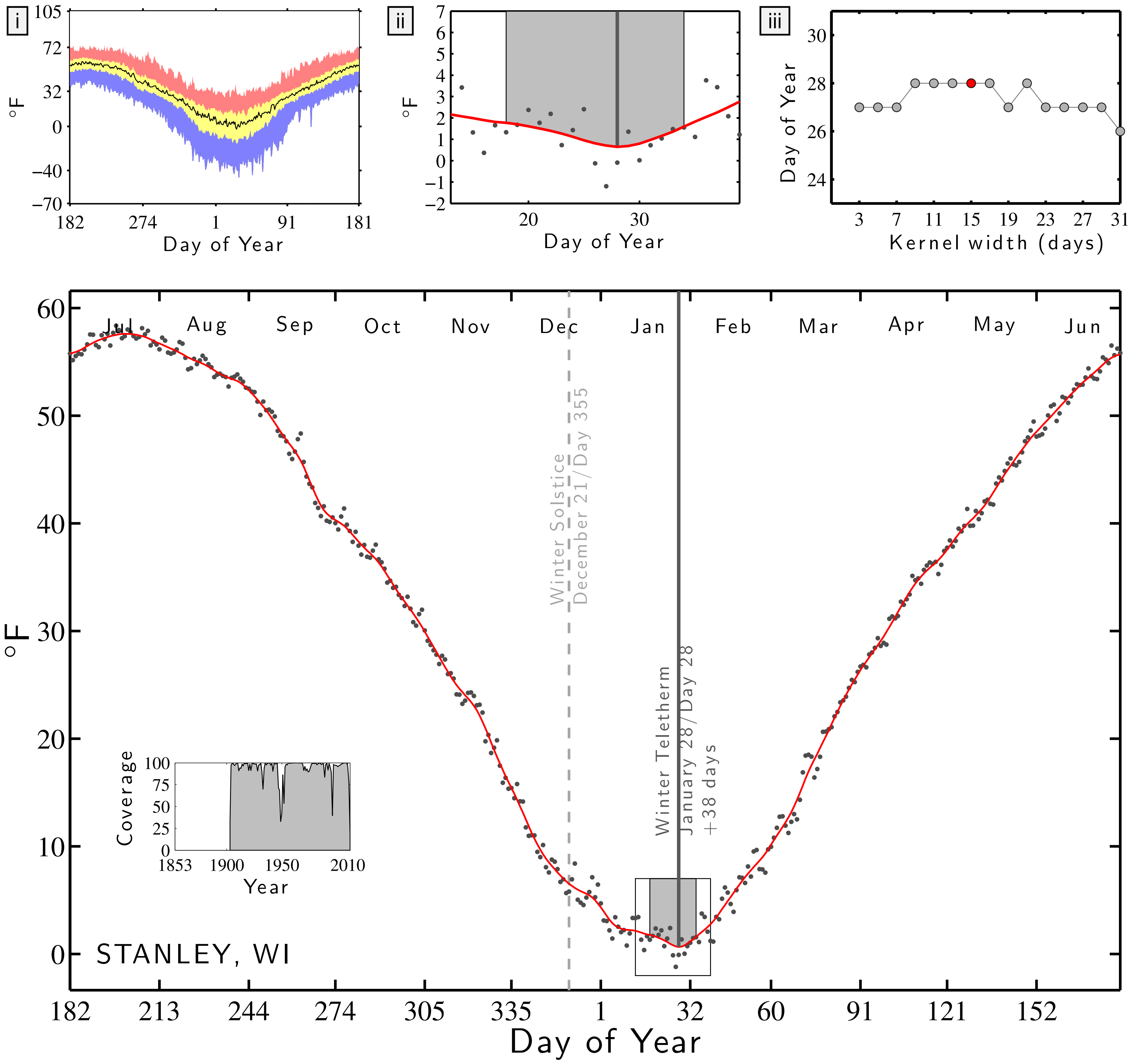
i
°F
Day of Year
ii
°F
Day of Year
iii
Day of Year
Kernel width (days)
Jul
Aug
Sep
Oct
Nov
Dec
Jan
Feb
Mar
Apr
May
Jun
Winter Solstice
December 21/Day 355
Winter Teletherm
January 28/Day 28
+38 days
Coverage
Year
STANLEY, WI
°F
Day of Year

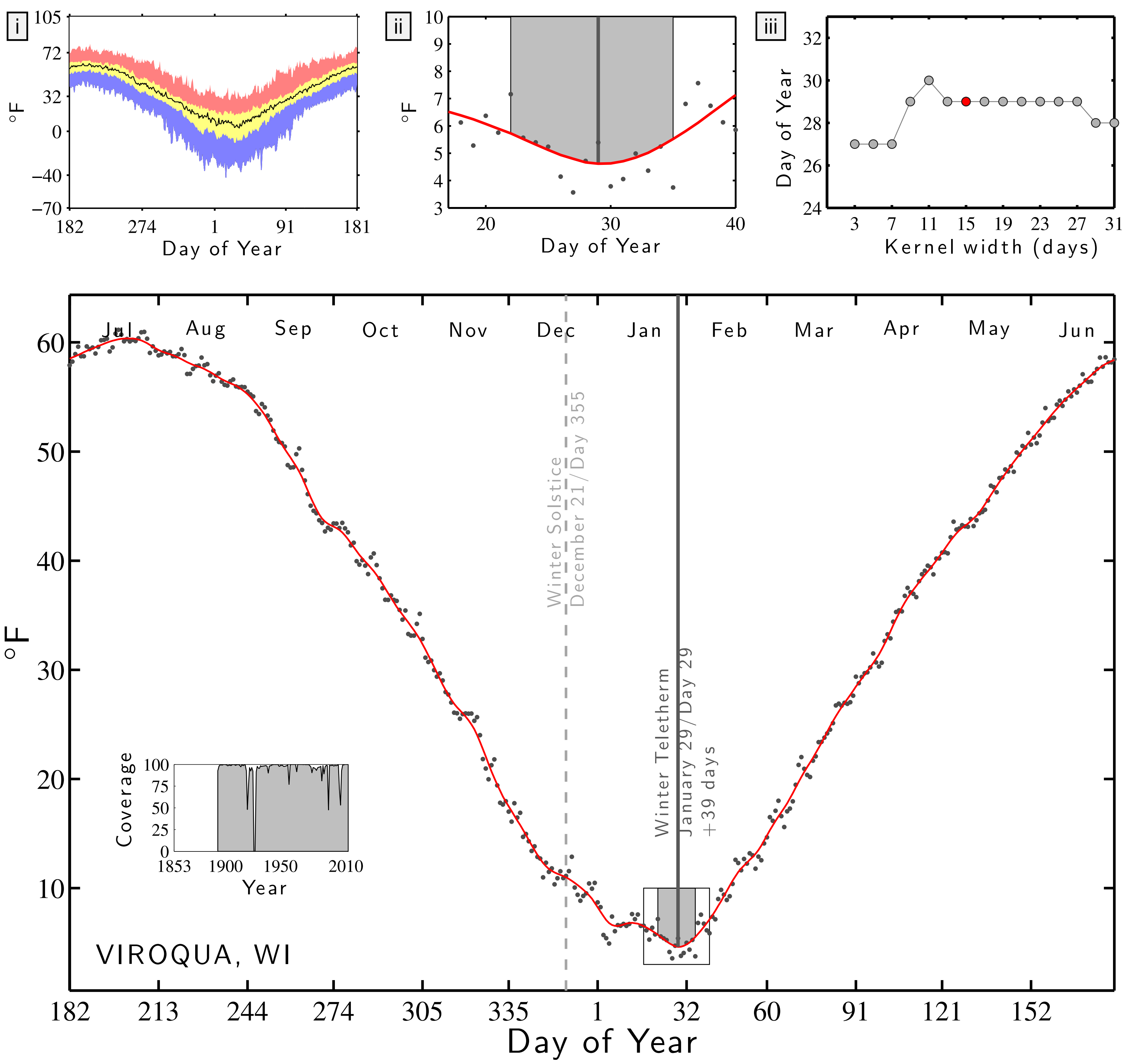

i
105
72
32
0
−40
−70
°F
182
274
1
91
181
Day of Year
ii
10
9
8
7
6
5
4
3
°F
20
30
40
Day of Year
iii
32
30
28
26
24
Day of Year
3
7
11
15
19
23
27
31
Kernel width (days)
Jul
Aug
Sep
Oct
Nov
Dec
Jan
Feb
Mar
Apr
May
Jun
60
50
40
30
20
10
°F
Winter Solstice
December 21/Day 355
Winter Teletherm
January 29/Day 29
+39 days
Coverage
100
75
50
25
0
1853
1900
1950
2010
Year
VIROQUA, WI
182
213
244
274
305
335
1
32
60
91
121
152
Day of Year

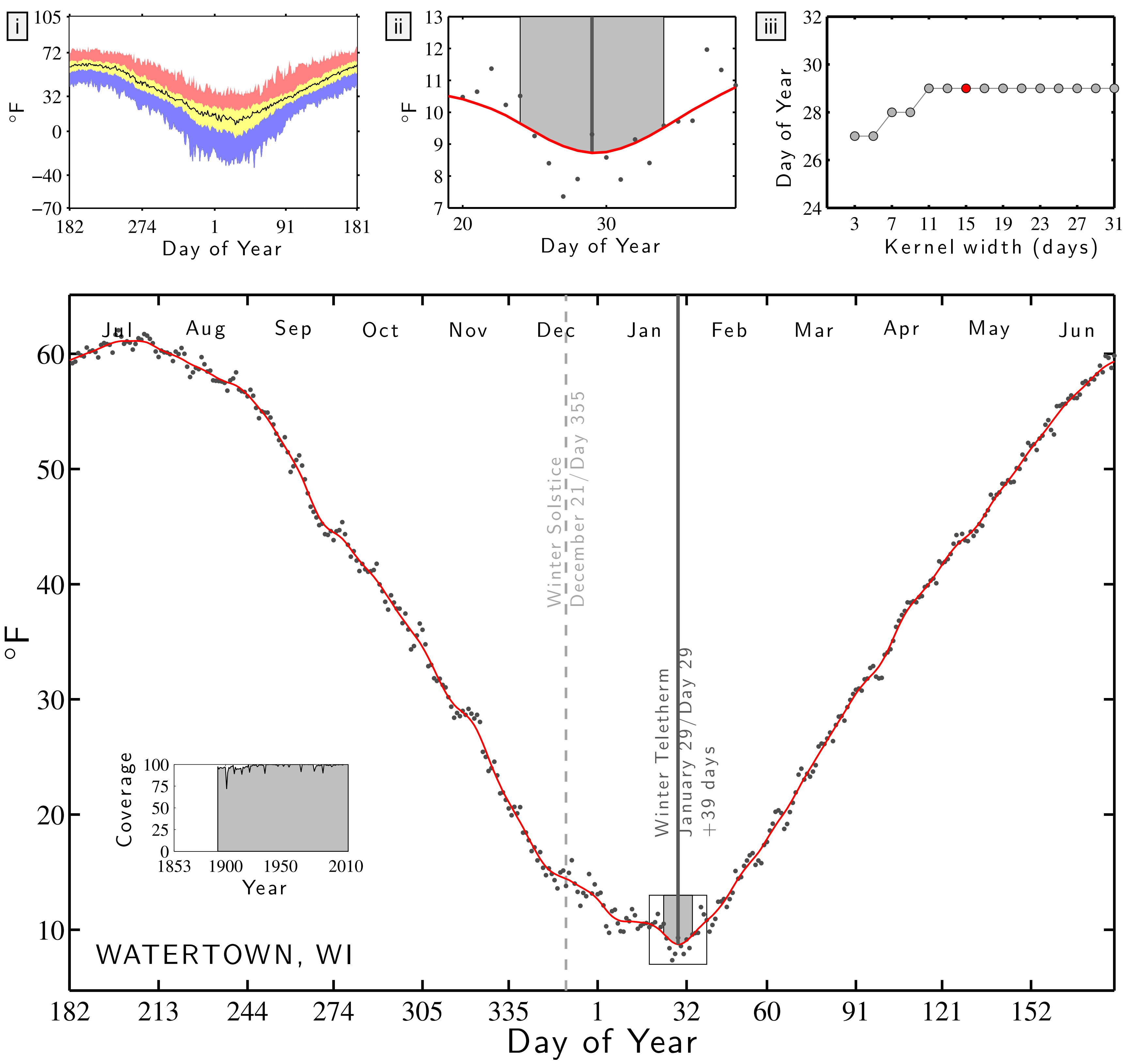
i
ii
iii
°F
Day of Year
Kernel width (days)
Day of Year
Jul
Aug
Sep
Oct
Nov
Dec
Jan
Feb
Mar
Apr
May
Jun
Winter Solstice
December 21/Day 355
Winter Teletherm
January 29/Day 29
+39 days
Coverage
Year
WATERTOWN, WI

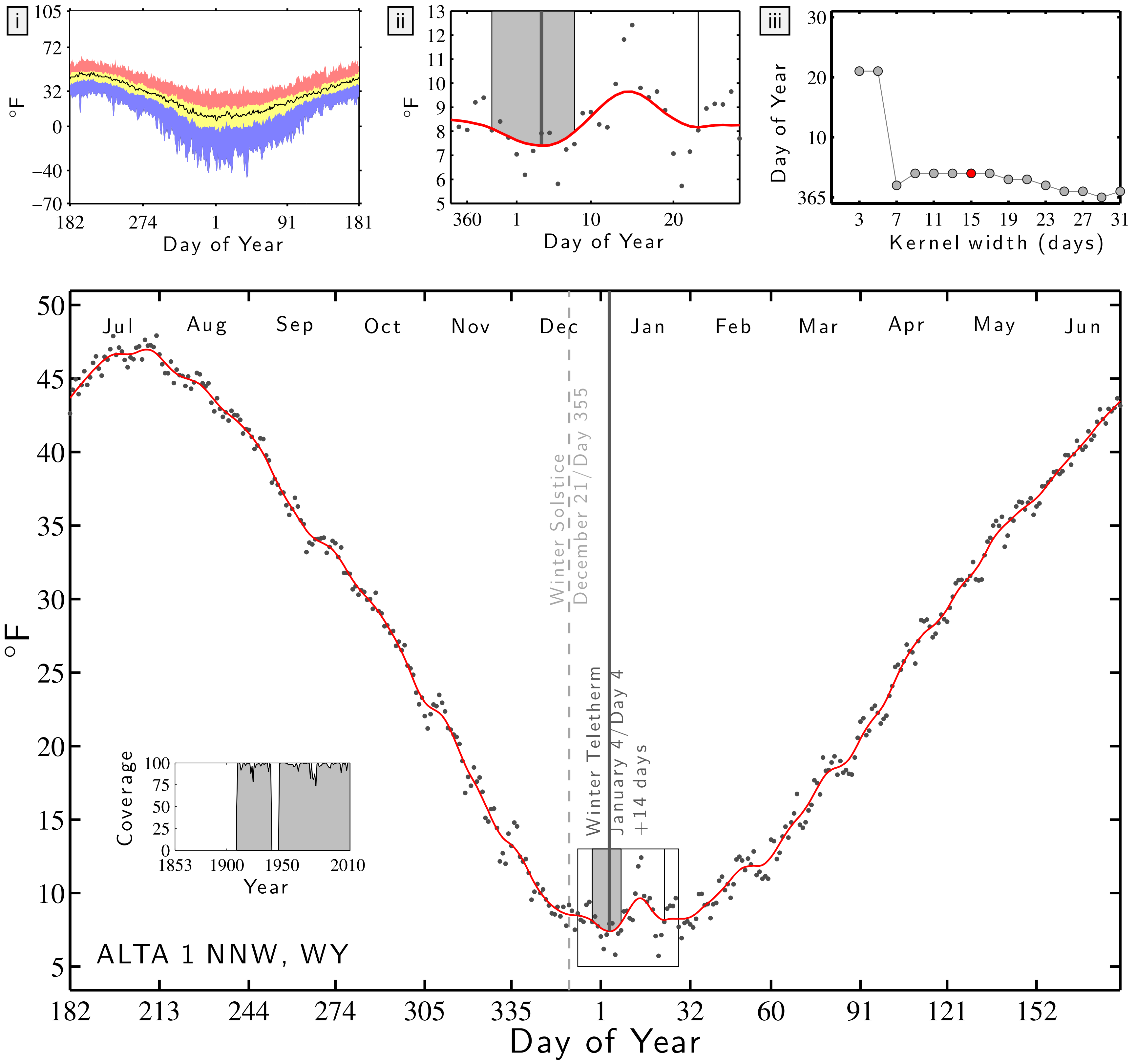

i
°F
Day of Year
ii
°F
Day of Year
iii
Day of Year
Kernel width (days)
Jul
Aug
Sep
Oct
Nov
Dec
Jan
Feb
Mar
Apr
May
Jun
Winter Solstice
December 21/Day 355
Winter Teletherm
January 4/Day 4
+14 days
Coverage
Year
ALTA 1 NNW, WY
°F
Day of Year

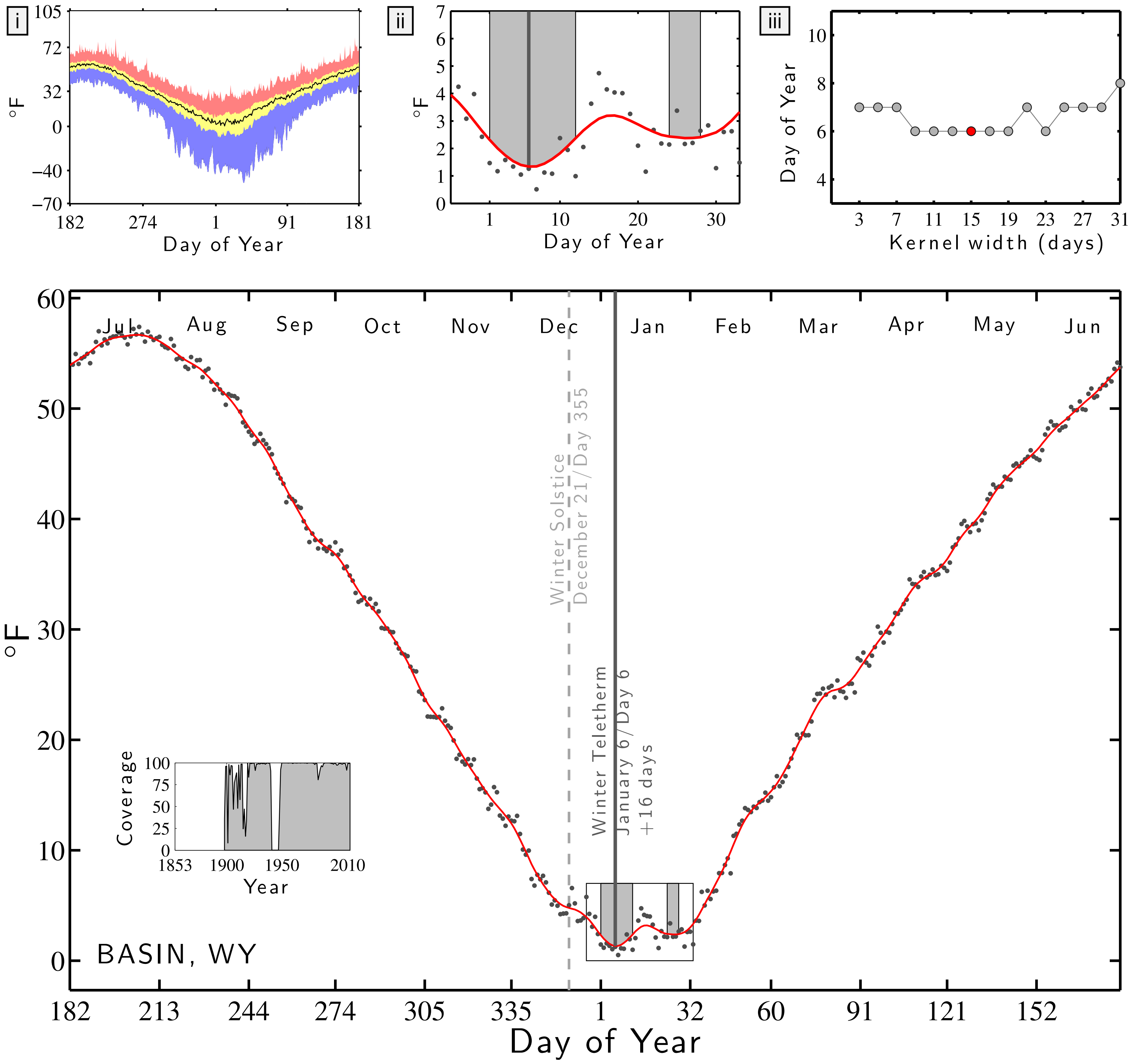

i
ii
iii
°F
Day of Year
Kernel width (days)
Jul
Aug
Sep
Oct
Nov
Dec
Jan
Feb
Mar
Apr
May
Jun
Winter Solstice
December 21/Day 355
Winter Teletherm
January 6/Day 6
+16 days
Coverage
Year
BASIN, WY

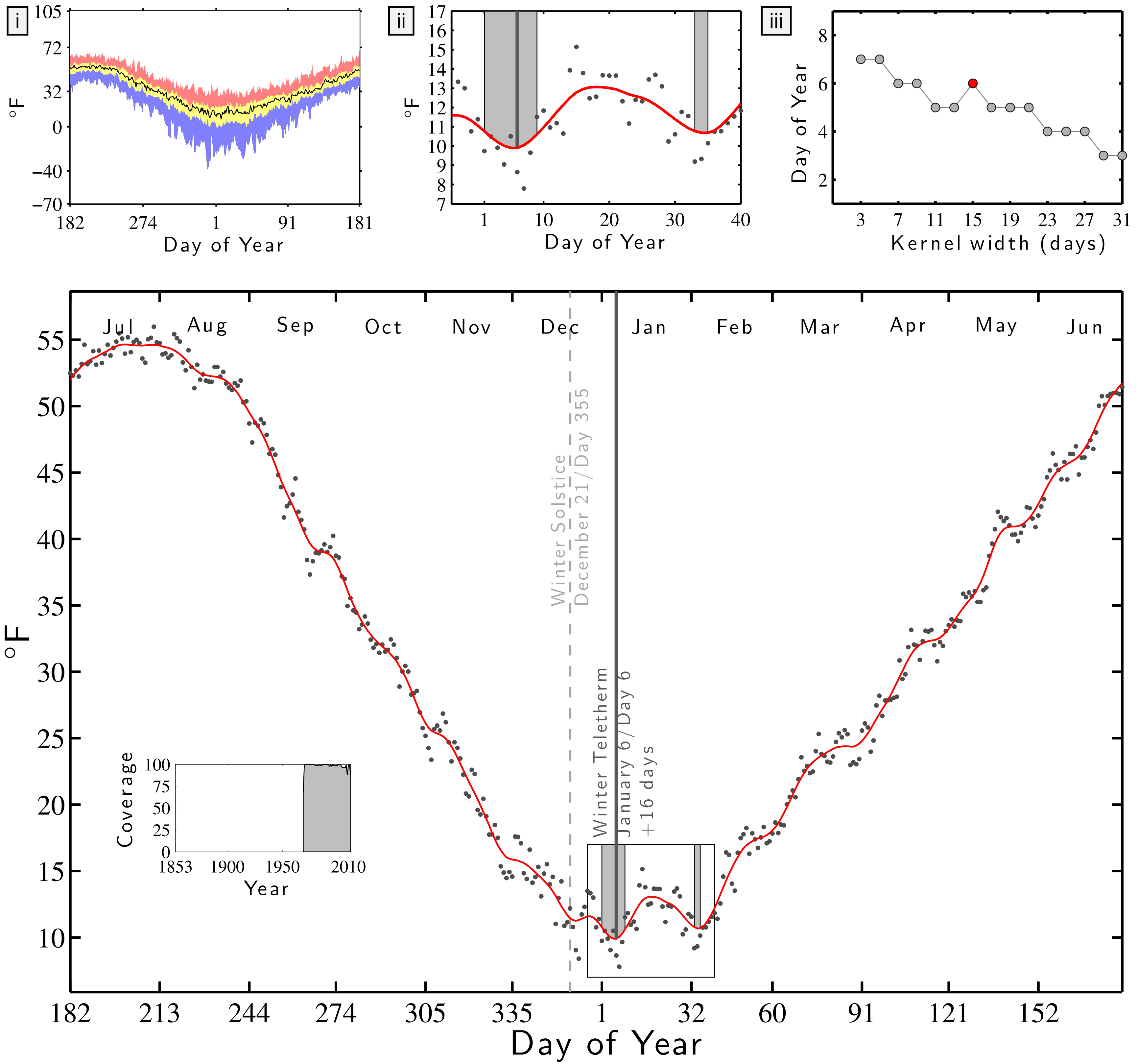

i
°F
Day of Year
ii
°F
Day of Year
iii
Day of Year
Kernel width (days)
Jul
Aug
Sep
Oct
Nov
Dec
Jan
Feb
Mar
Apr
May
Jun
Winter Solstice
December 21/Day 355
Winter Teletherm
January 6/Day 6
+16 days
Coverage
Year
°F
Day of Year

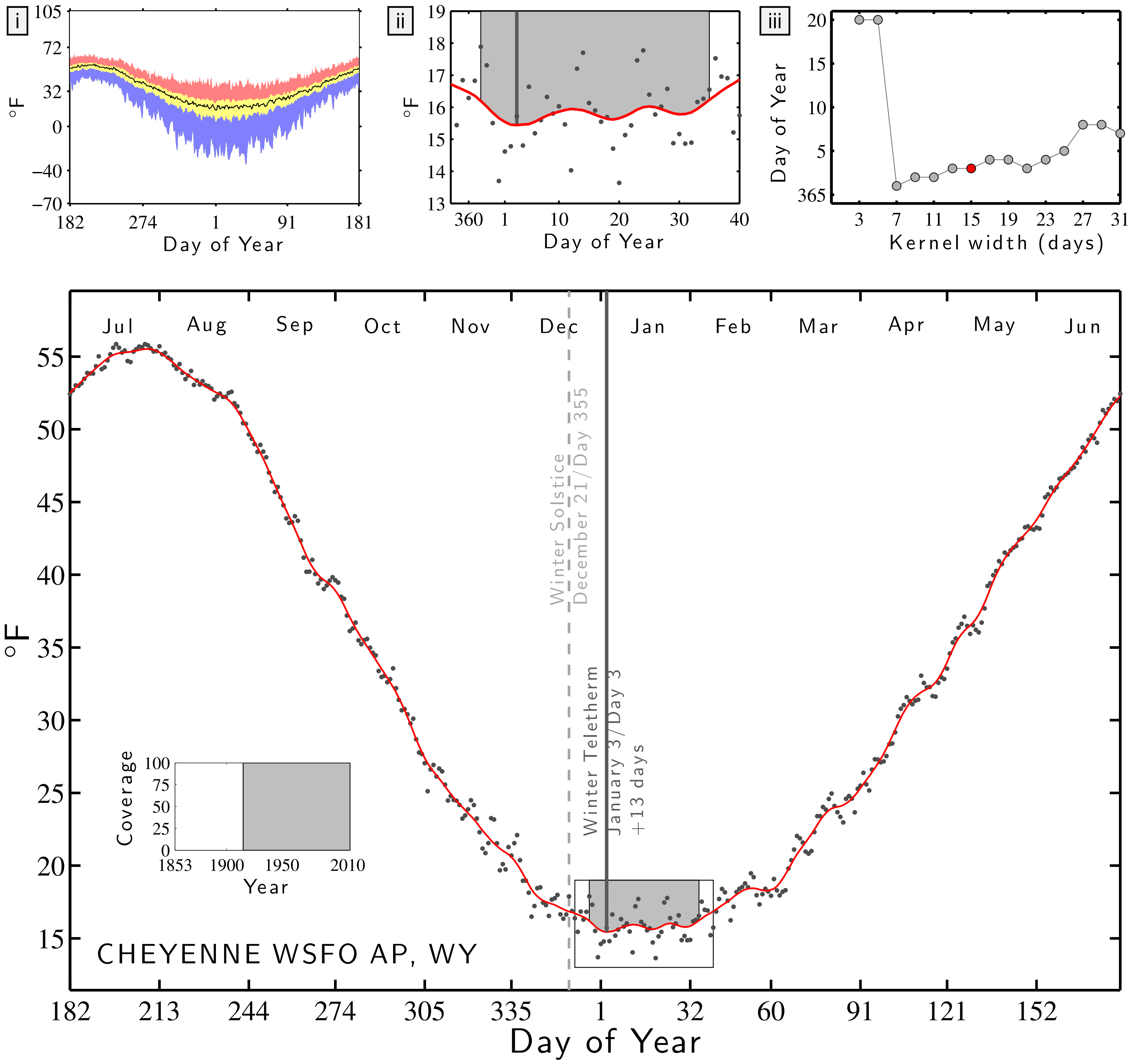

i
°F
Day of Year
ii
°F
Day of Year
iii
Day of Year
Kernel width (days)
Jul
Aug
Sep
Oct
Nov
Dec
Jan
Feb
Mar
Apr
May
Jun
Winter Solstice
December 21/Day 355
Winter Teletherm
January 3/Day 3
+13 days
Coverage
Year
CHEYENNE WSFO AP, WY
°F
Day of Year

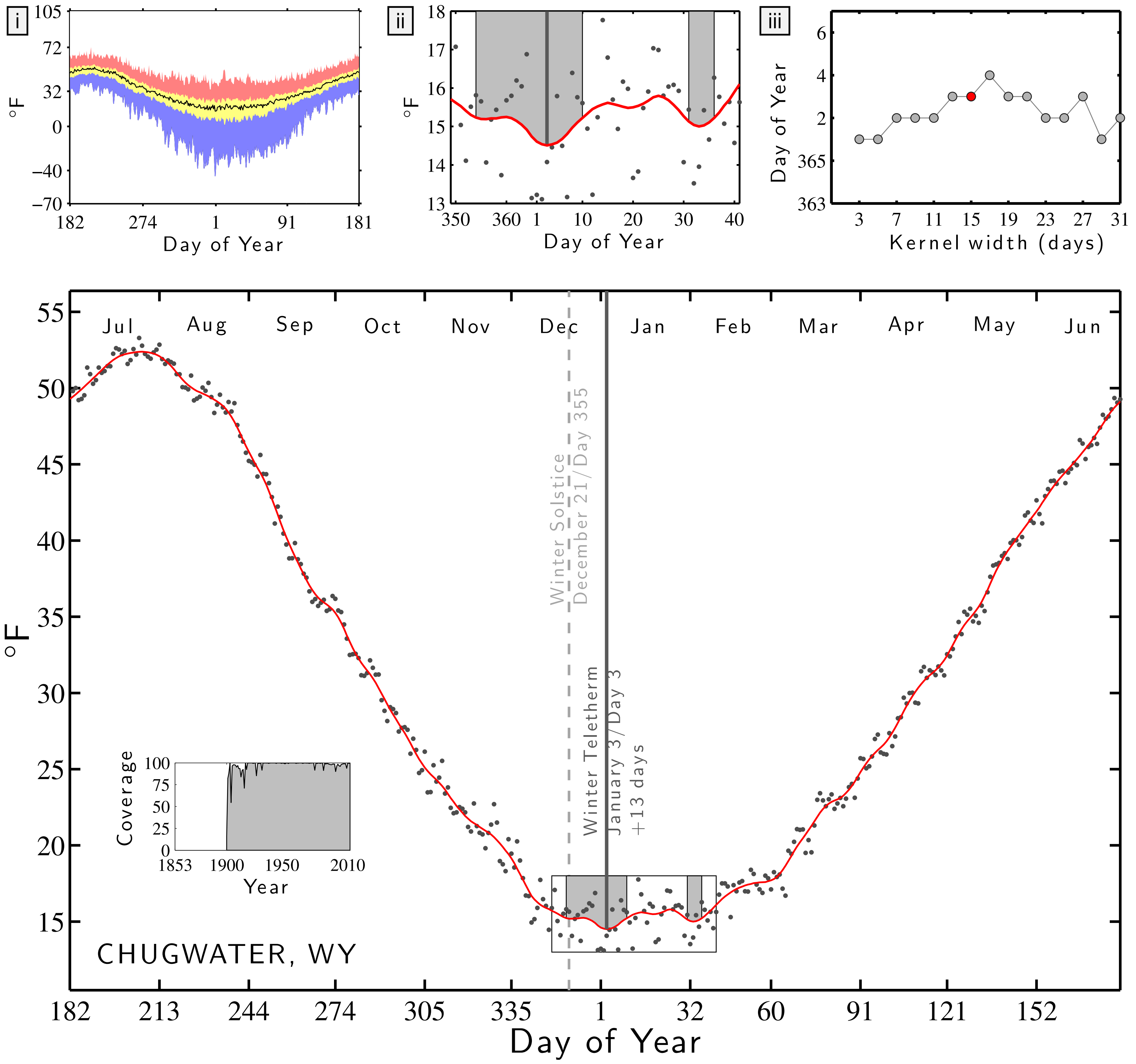

i
ii
iii
°F
Day of Year
Day of Year
Kernel width (days)
Jul
Aug
Sep
Oct
Nov
Dec
Jan
Feb
Mar
Apr
May
Jun
Winter Solstice
December 21/Day 355
Winter Teletherm
January 3/Day 3
+13 days
Coverage
Year
CHUGWATER, WY

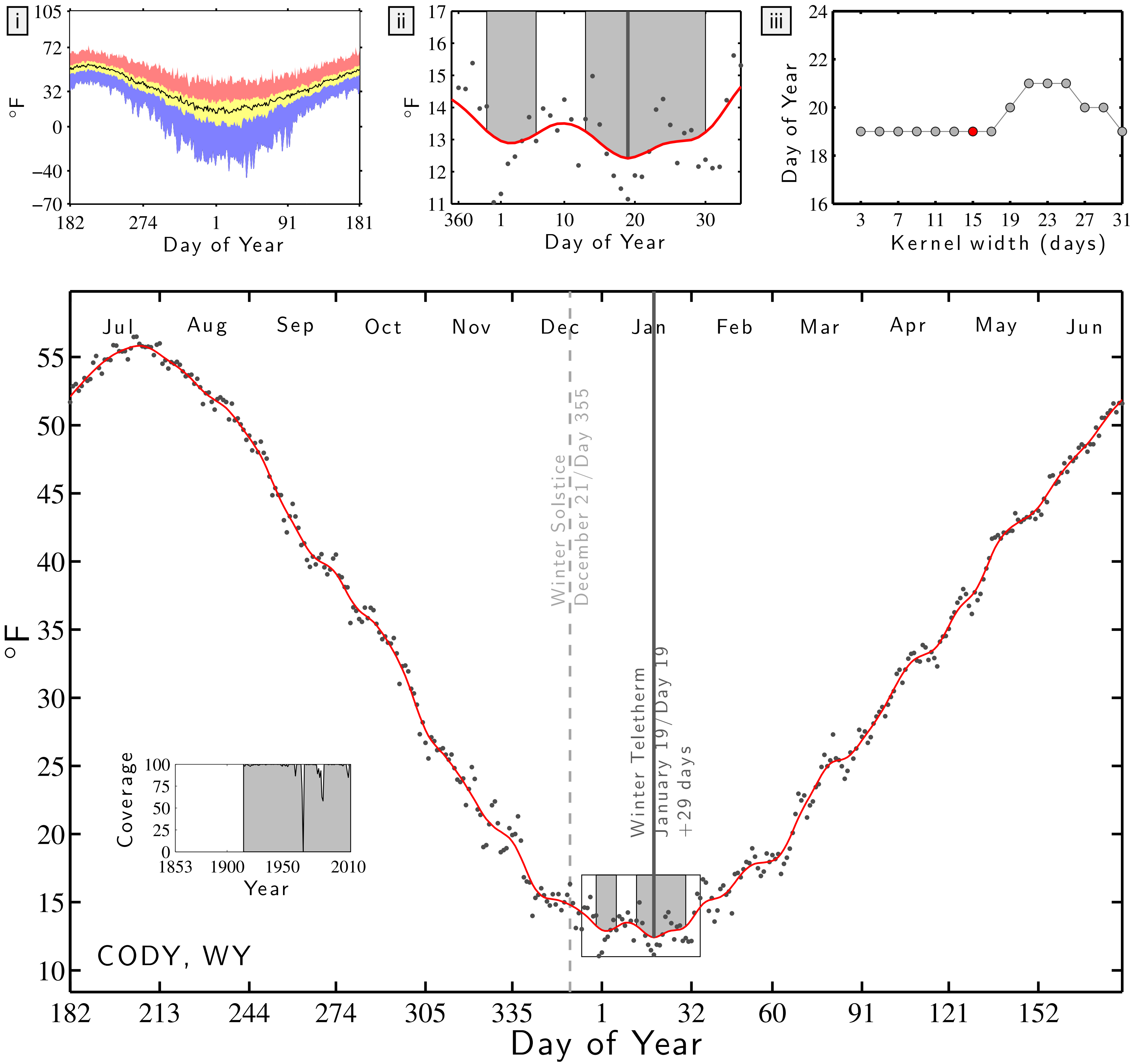

i
ii
iii
°F
Day of Year
Day of Year
Kernel width (days)
Jul
Aug
Sep
Oct
Nov
Dec
Jan
Feb
Mar
Apr
May
Jun
Winter Solstice
December 21/Day 355
Winter Teletherm
January 19/Day 19
+29 days
Coverage
Year
CODY, WY
Day of Year

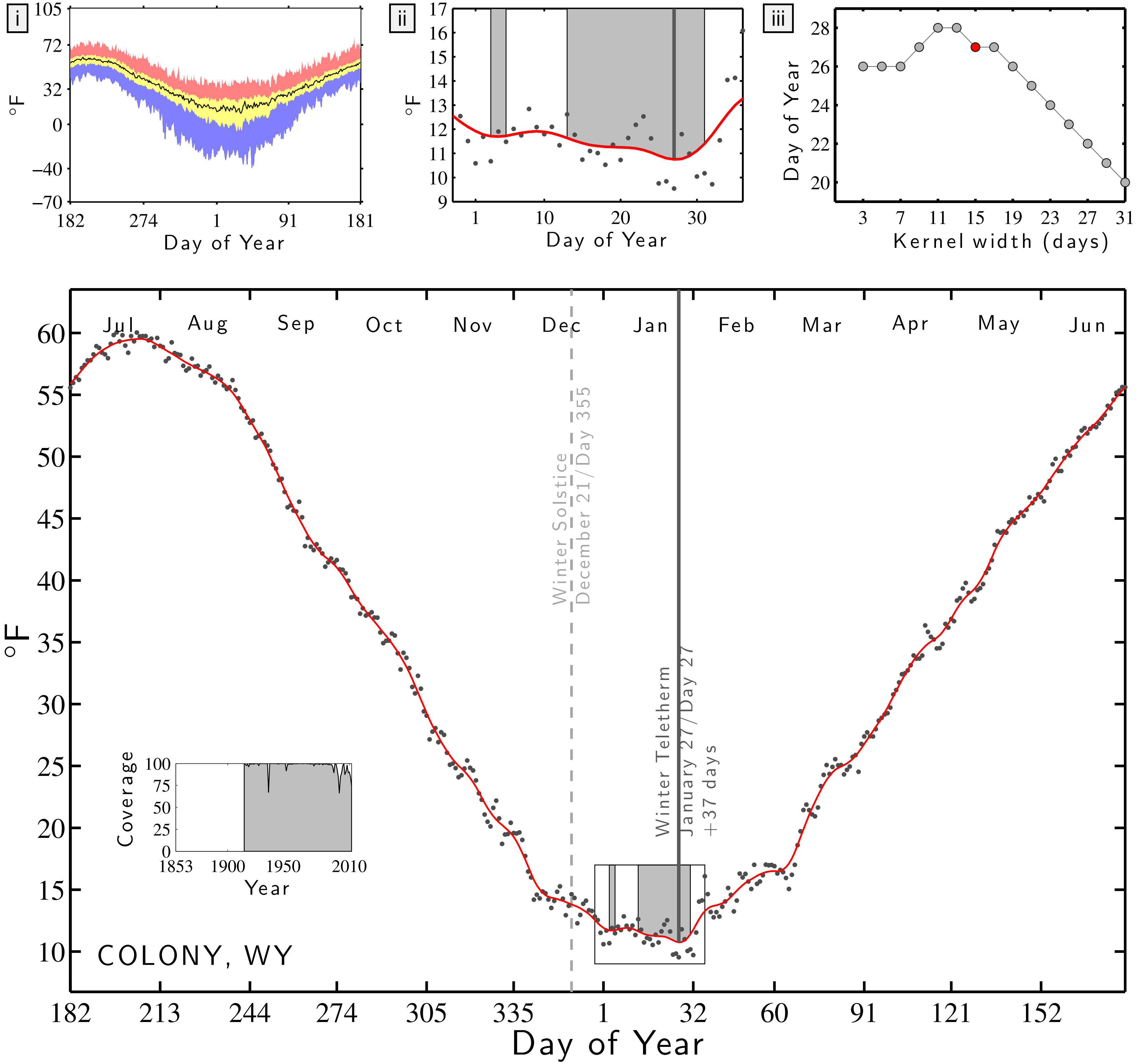

i
ii
iii
°F
Day of Year
Day of Year
Kernel width (days)
Jul
Aug
Sep
Oct
Nov
Dec
Jan
Feb
Mar
Apr
May
Jun
Winter Solstice
December 21/Day 355
Winter Teletherm
January 27/Day 27
+37 days
Coverage
Year
COLONY, WY
Day of Year

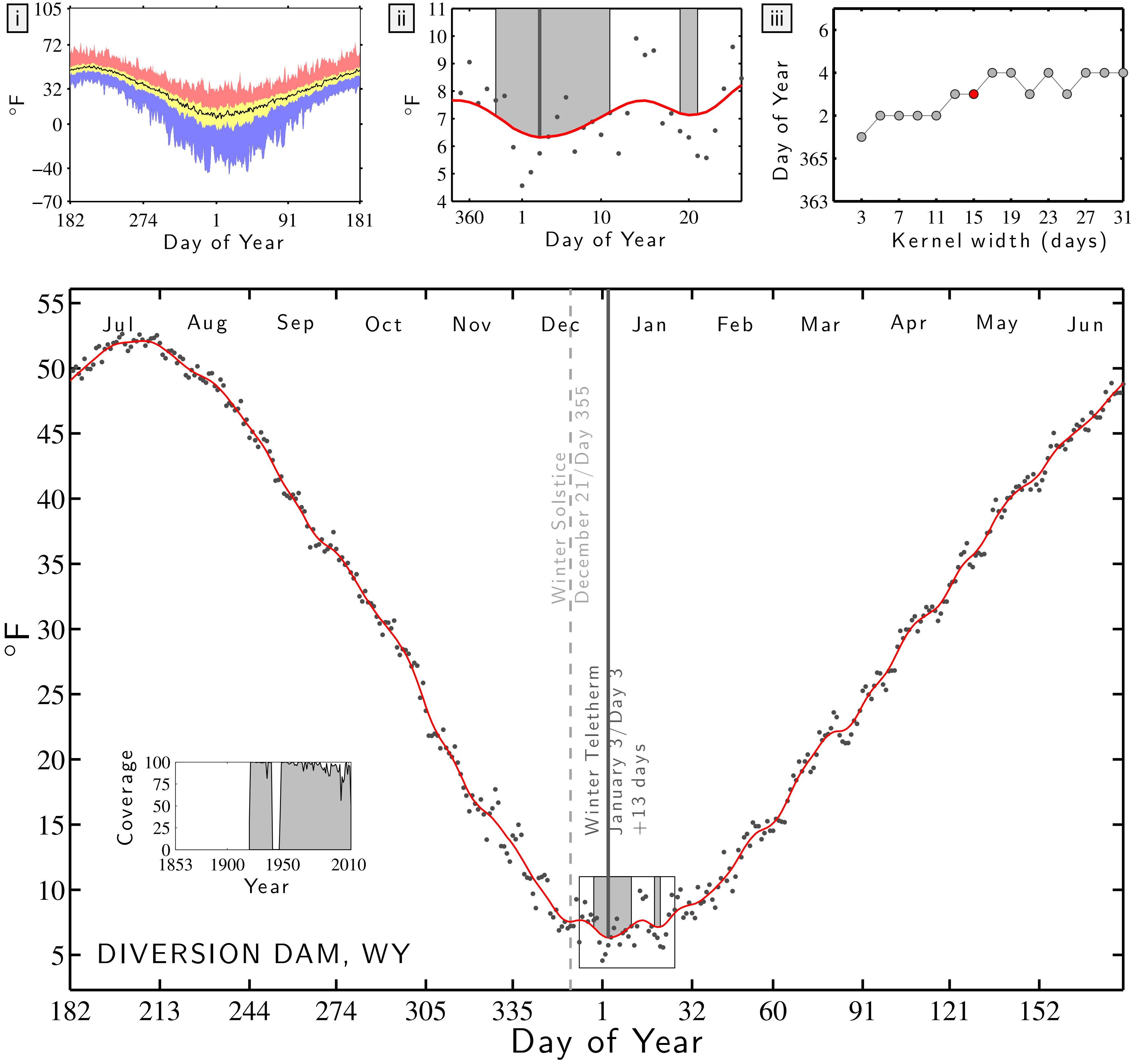

i
ii
iii
°F
Day of Year
Kernel width (days)
Winter Solstice
December 21/Day 355
Winter Teletherm
January 3/Day 3
+13 days
Coverage
Year
DIVERSION DAM, WY
Jul
Aug
Sep
Oct
Nov
Dec
Jan
Feb
Mar
Apr
May
Jun

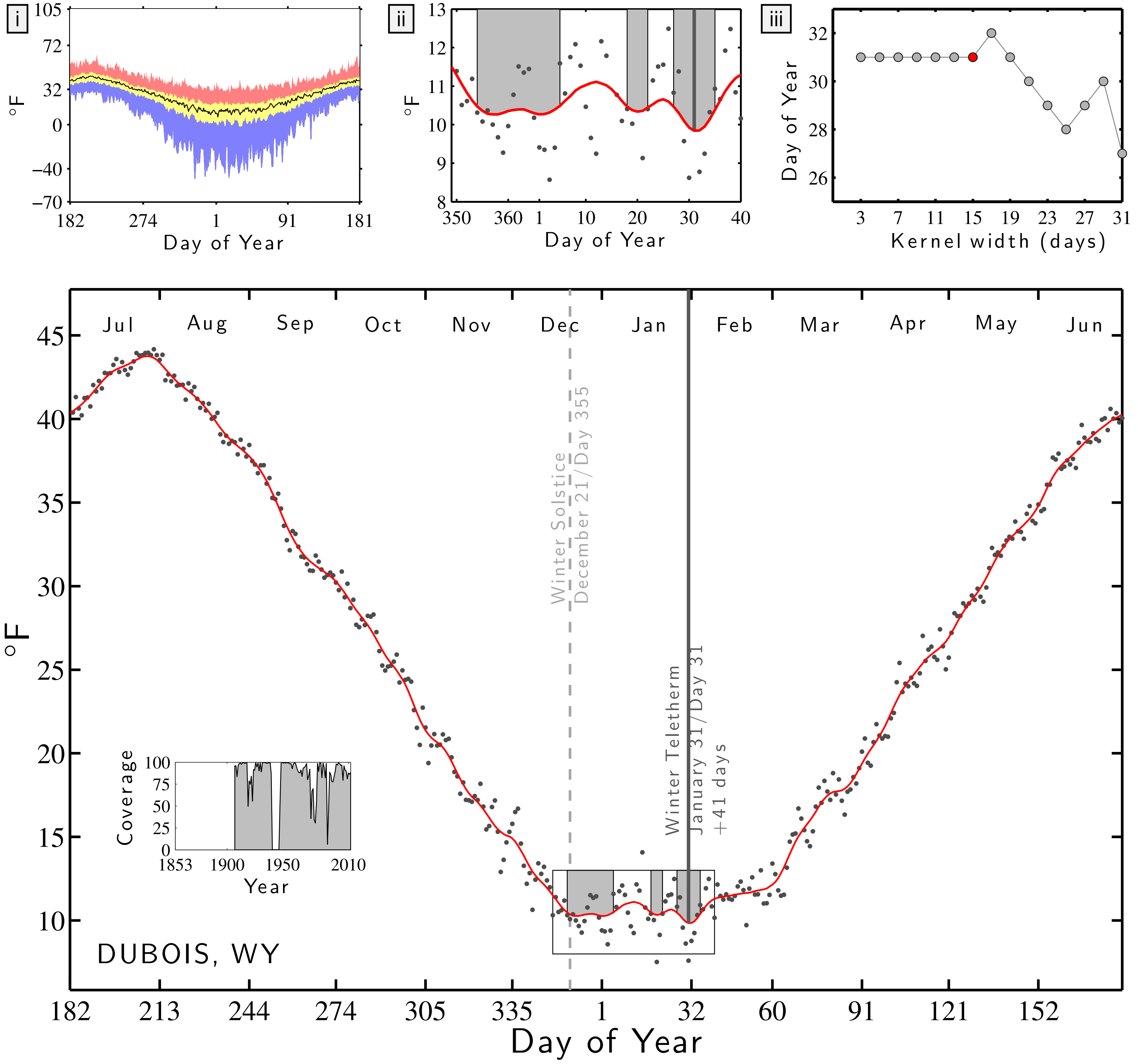

i
ii
iii
°F
Day of Year
Kernel width (days)
Day of Year
Jul
Aug
Sep
Oct
Nov
Dec
Jan
Feb
Mar
Apr
May
Jun
Winter Solstice
December 21/Day 355
Winter Teletherm
January 31/Day 31
+41 days
Coverage
Year
DUBOIS, WY

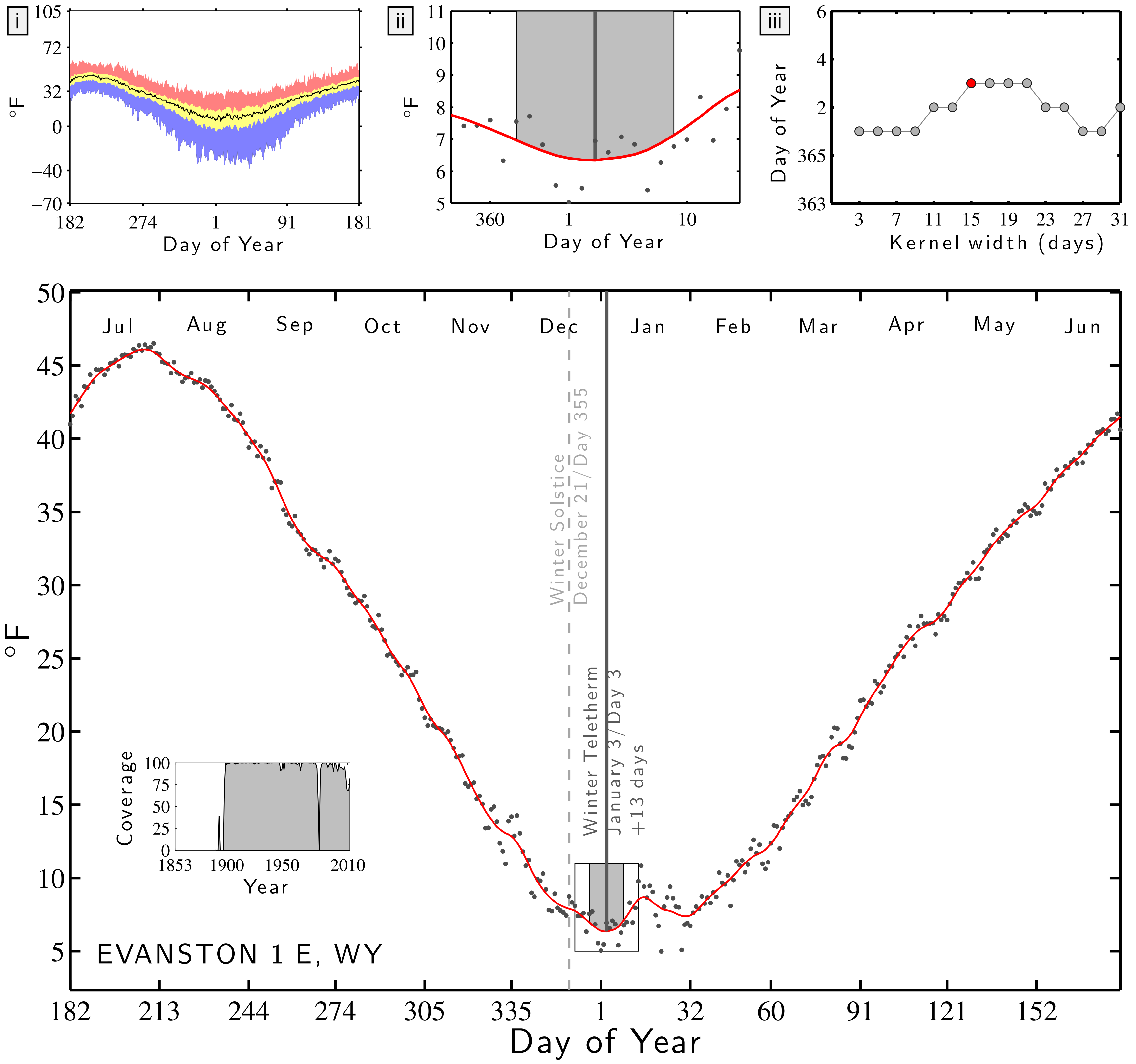

i
°F
Day of Year
ii
°F
Day of Year
iii
Day of Year
Kernel width (days)
Jul
Aug
Sep
Oct
Nov
Dec
Jan
Feb
Mar
Apr
May
Jun
Winter Solstice
December 21/Day 355
Winter Teletherm
January 3/Day 3
+13 days
Coverage
Year
EVANSTON 1 E, WY
°F
Day of Year

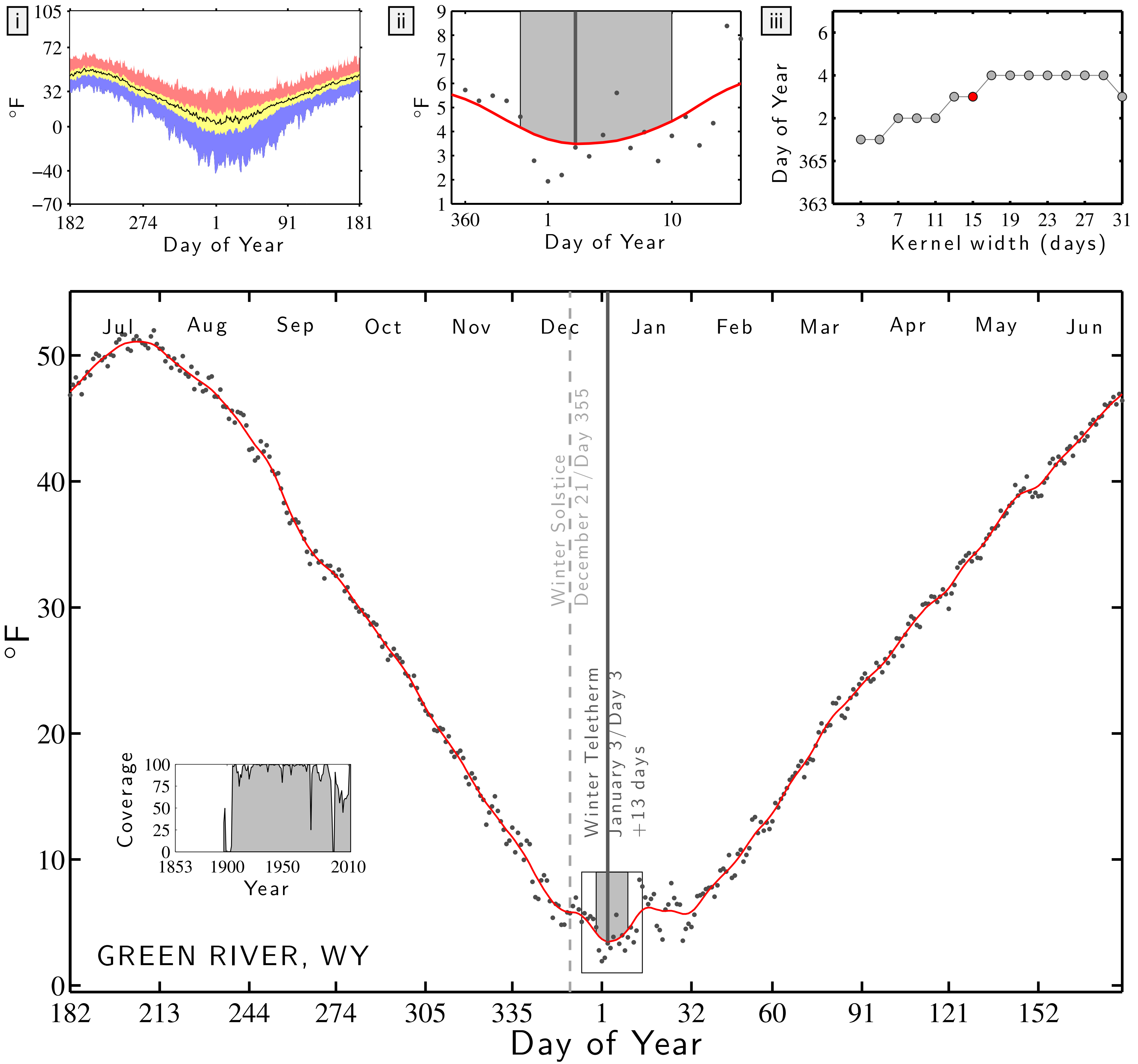

i
105
72
32
0
−40
−70
°F
182
274
1
91
181
Day of Year
ii
°F
360
1
10
Day of Year
iii
Day of Year
363
365
2
4
6
3
7
11
15
19
23
27
31
Kernel width (days)
Jul
Aug
Sep
Oct
Nov
Dec
Jan
Feb
Mar
Apr
May
Jun
Winter Solstice
December 21/Day 355
Winter Teletherm
January 3/Day 3
+13 days
Coverage
Year
1853
1900
1950
2010
GREEN RIVER, WY
°F
Day of Year
182
213
244
274
305
335
1
32
60
91
121
152

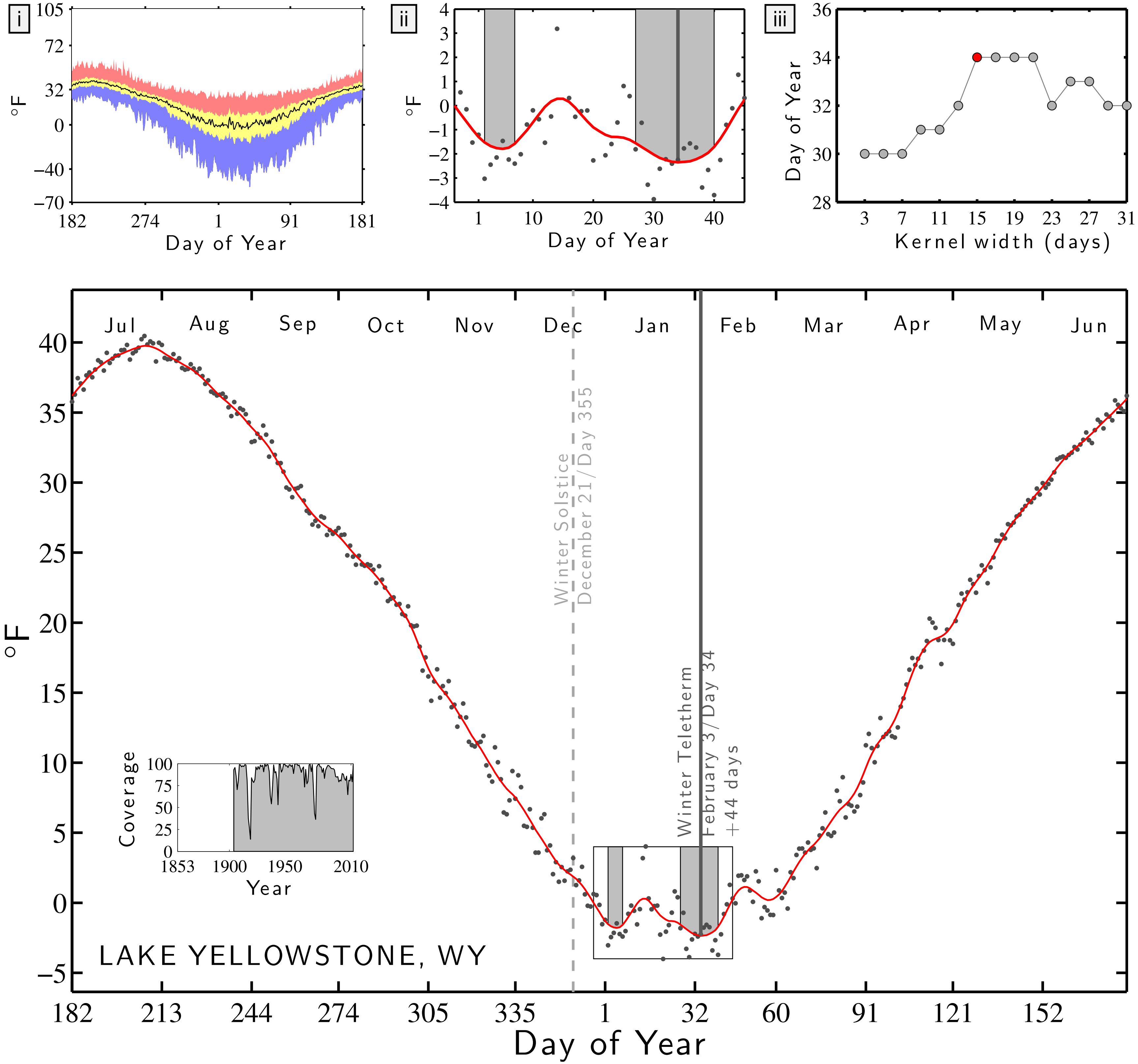

i
°F
Day of Year
ii
°F
Day of Year
iii
Day of Year
Kernel width (days)
Jul
Aug
Sep
Oct
Nov
Dec
Jan
Feb
Mar
Apr
May
Jun
Winter Solstice
December 21/Day 355
Winter Teletherm
February 3/Day 34
+44 days
Coverage
Year
LAKE YELLOWSTONE, WY
°F
Day of Year

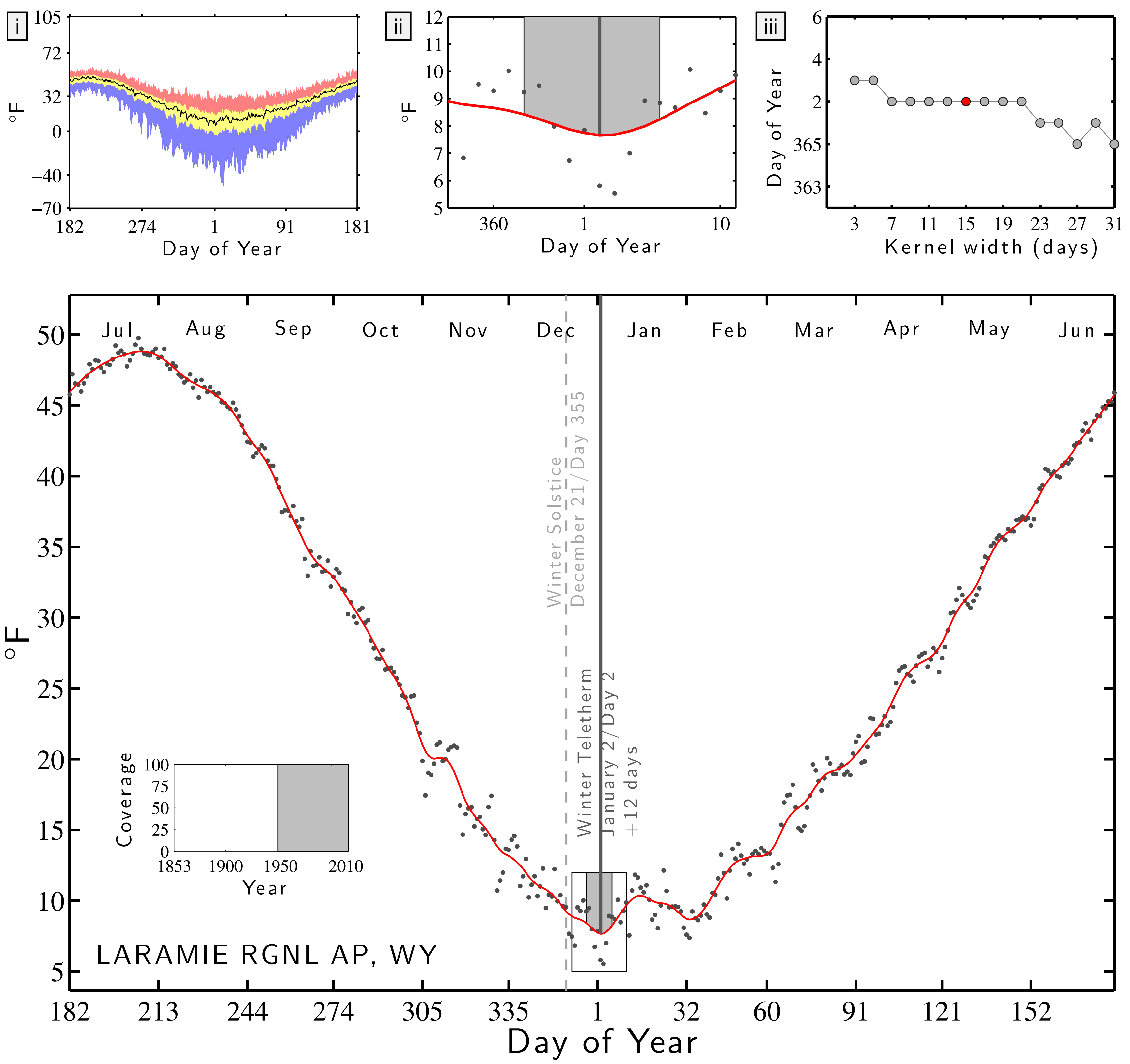

i
ii
iii
°F
Day of Year
Day of Year
Kernel width (days)
Winter Solstice
December 21/Day 355
Winter Teletherm
January 2/Day 2
+12 days
Jul
Aug
Sep
Oct
Nov
Dec
Jan
Feb
Mar
Apr
May
Jun
Coverage
Year
LARAMIE RGNL AP, WY

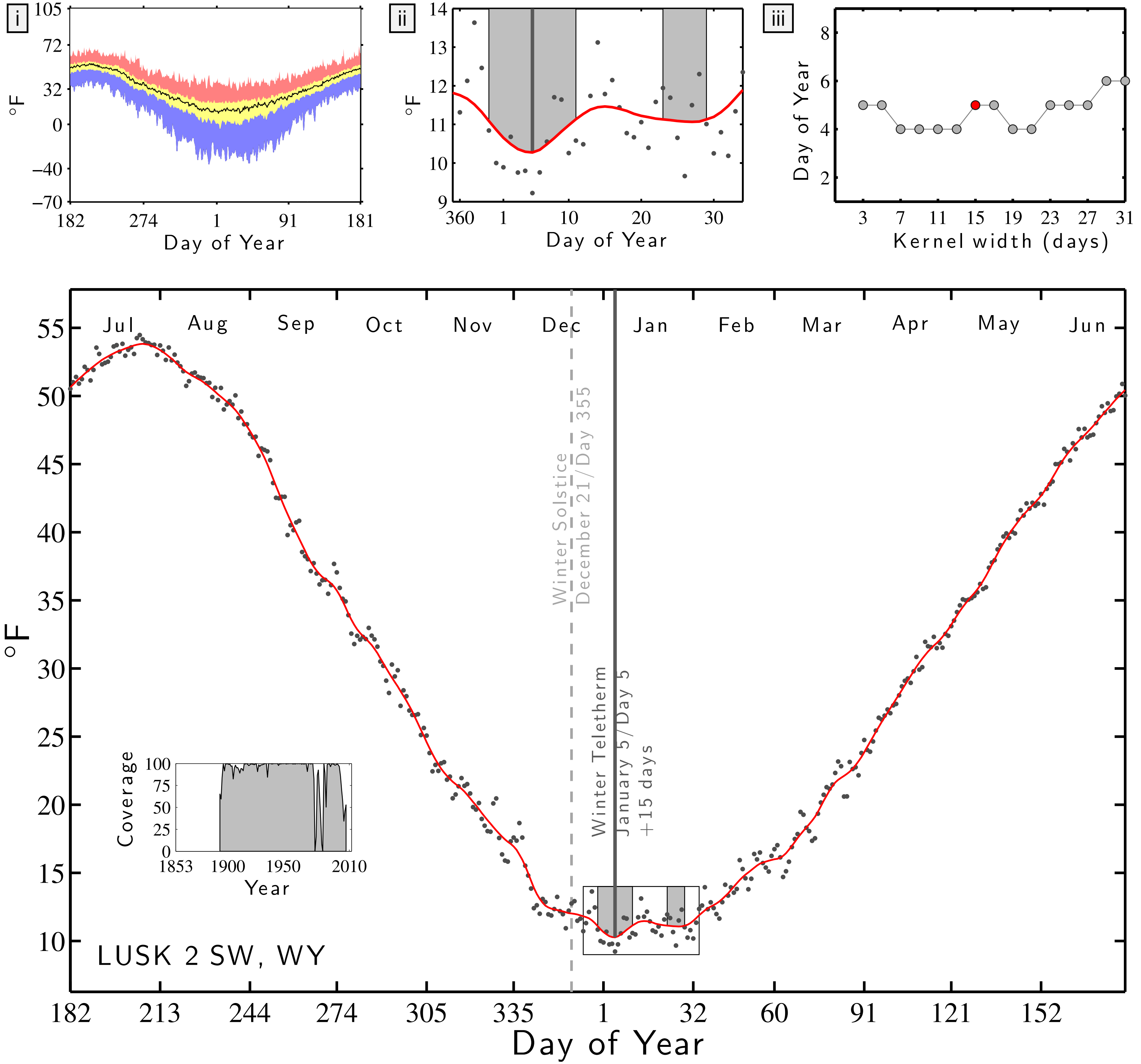

i
°F
Day of Year
ii
°F
Day of Year
iii
Day of Year
Kernel width (days)
Jul
Aug
Sep
Oct
Nov
Dec
Jan
Feb
Mar
Apr
May
Jun
Winter Solstice
December 21/Day 355
Winter Teletherm
January 5/Day 5
+15 days
Coverage
Year
LUSK 2 SW, WY
°F
Day of Year

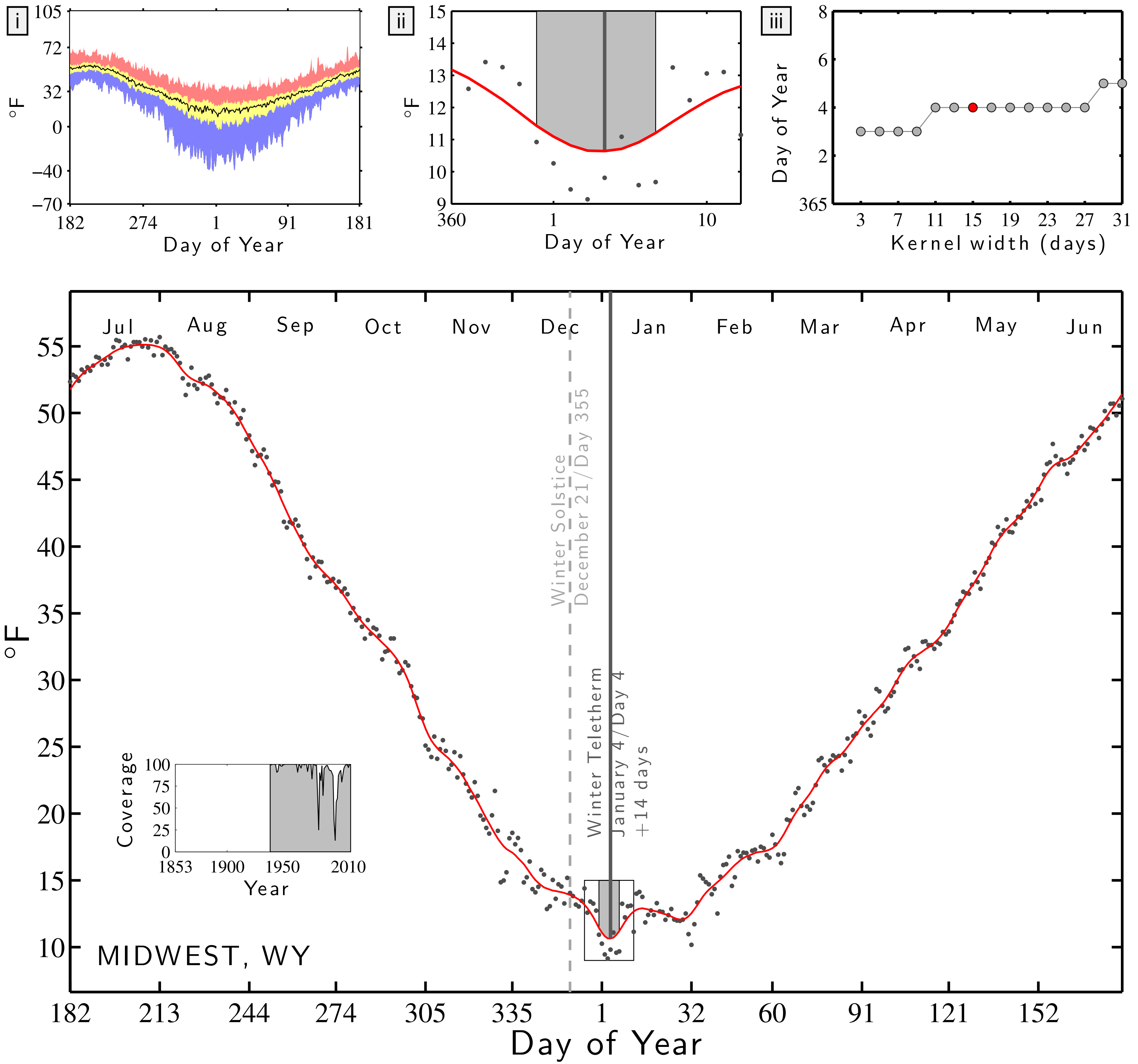

i
°F
Day of Year
ii
°F
Day of Year
iii
Day of Year
Kernel width (days)
Jul
Aug
Sep
Oct
Nov
Dec
Jan
Feb
Mar
Apr
May
Jun
Winter Solstice
December 21/Day 355
Winter Teletherm
January 4/Day 4
+14 days
Coverage
Year
MIDWEST, WY
°F
Day of Year

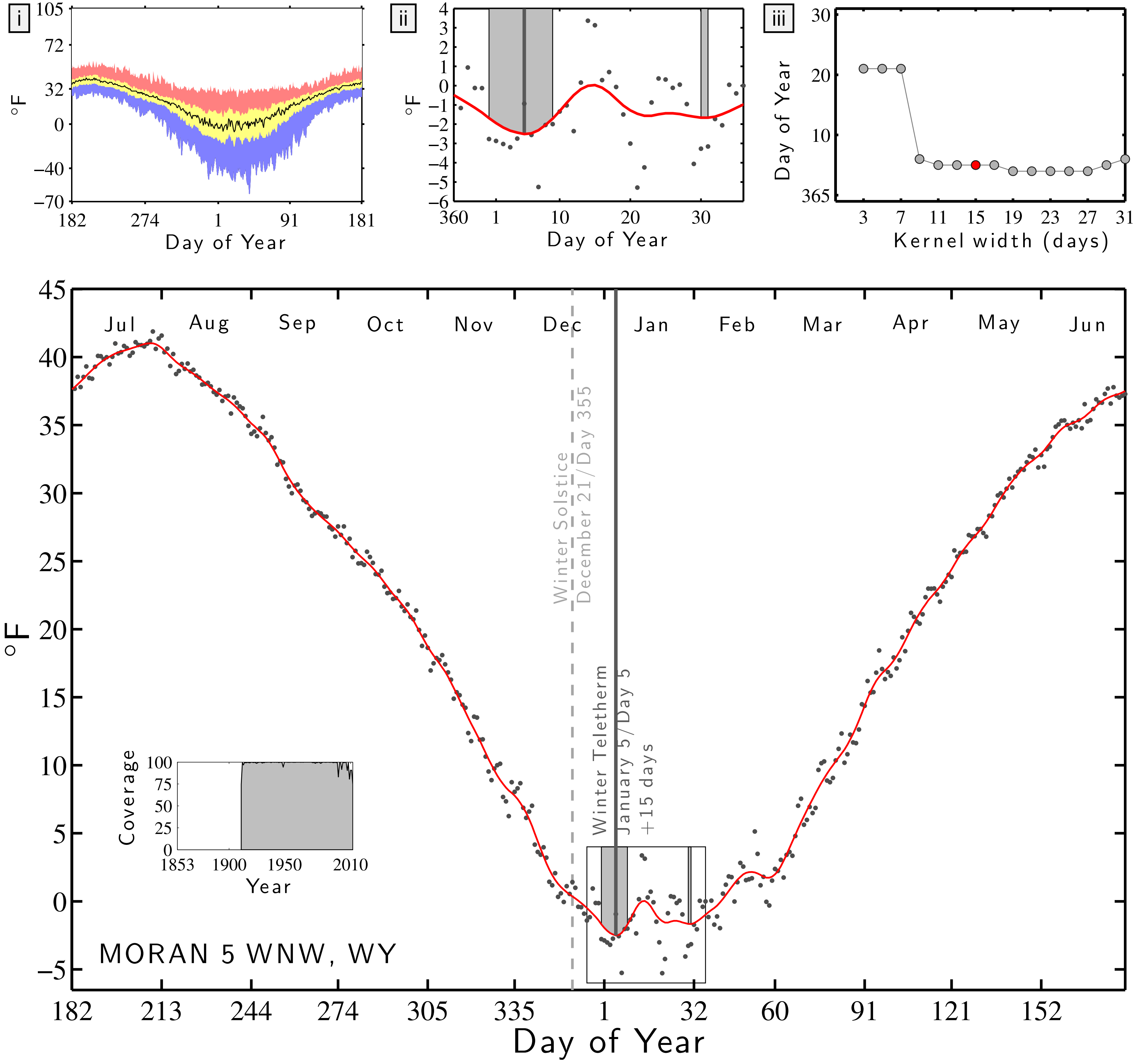

i
°F
Day of Year
ii
°F
Day of Year
iii
Day of Year
Kernel width (days)
Jul
Aug
Sep
Oct
Nov
Dec
Jan
Feb
Mar
Apr
May
Jun
Winter Solstice
December 21/Day 355
Winter Teletherm
January 5/Day 5
+15 days
Coverage
Year
MORAN 5 WNW, WY
°F
Day of Year

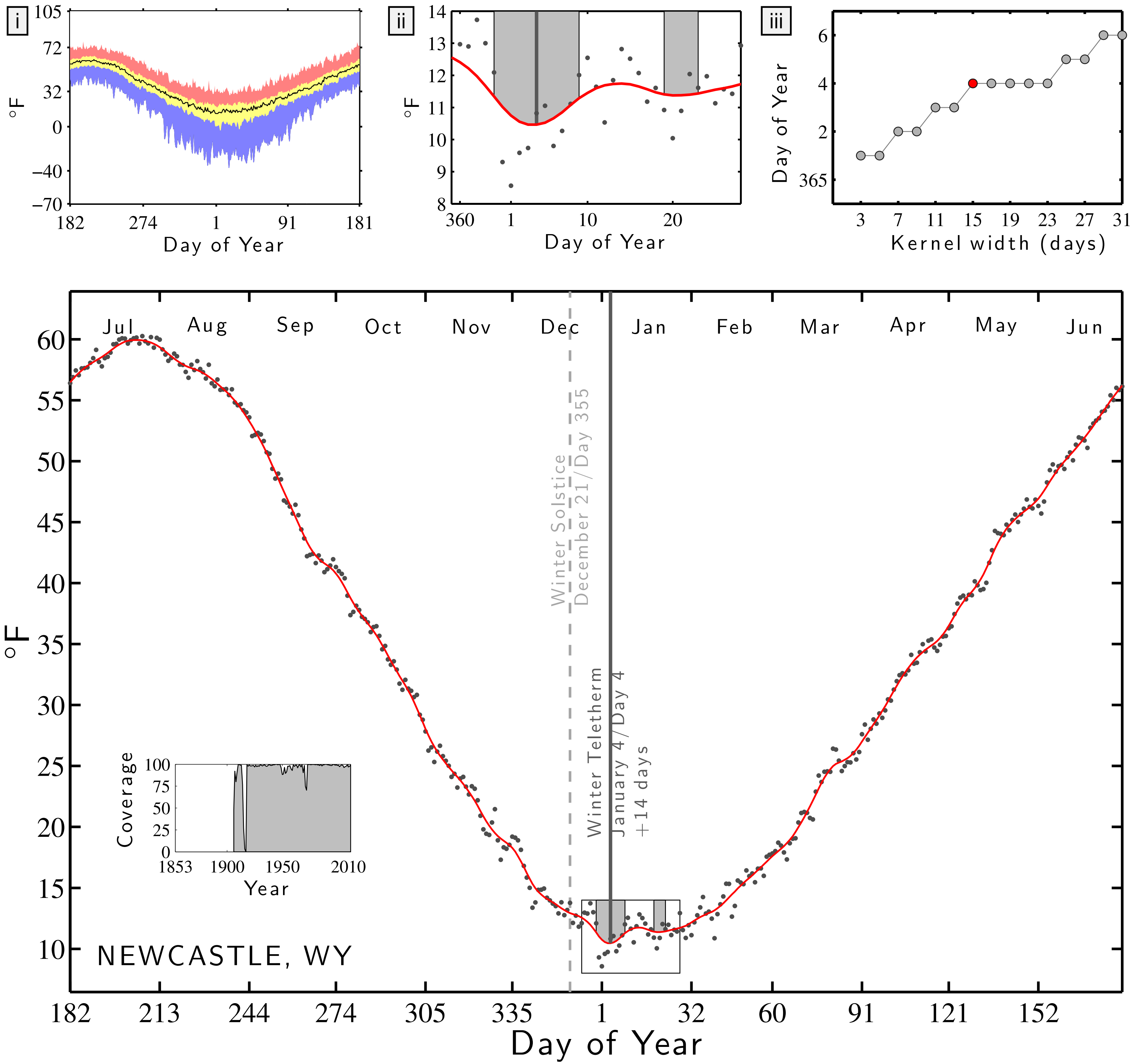

i
°F
Day of Year
ii
°F
Day of Year
iii
Day of Year
Kernel width (days)
Jul
Aug
Sep
Oct
Nov
Dec
Jan
Feb
Mar
Apr
May
Jun
Winter Solstice
December 21/Day 355
Winter Teletherm
January 4/Day 4
+14 days
Coverage
Year
NEWCASTLE, WY
°F
Day of Year

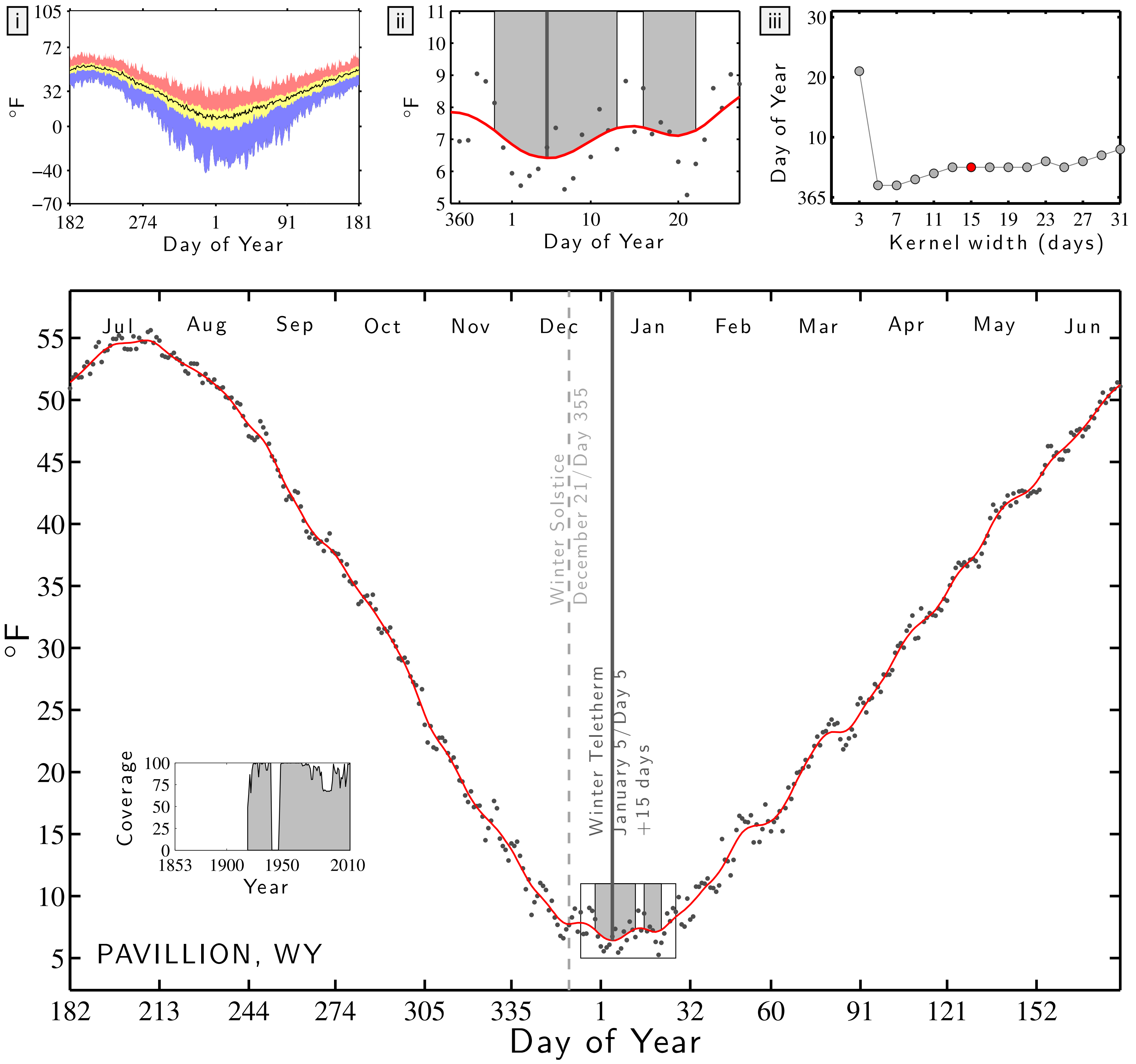

i
°F
Day of Year
ii
°F
Day of Year
iii
Day of Year
Kernel width (days)
Jul
Aug
Sep
Oct
Nov
Dec
Jan
Feb
Mar
Apr
May
Jun
Winter Solstice
December 21/Day 355
Winter Teletherm
January 5/Day 5
+15 days
Coverage
Year
PAVILLION, WY
°F
Day of Year

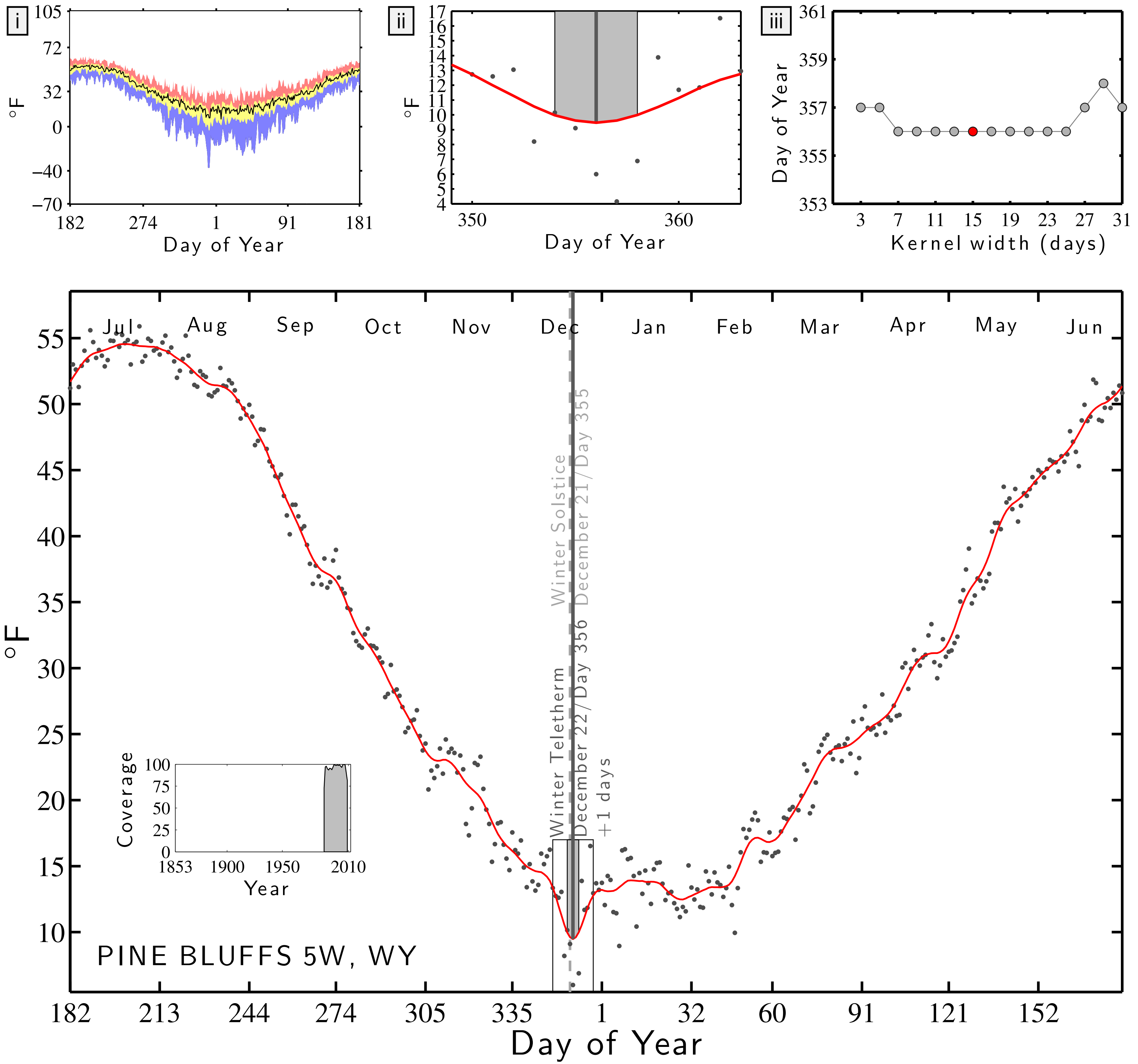

i
°F
105
72
32
0
−40
−70
182
274
1
91
181
Day of Year
ii
°F
350
360
Day of Year
iii
Day of Year
361
359
357
355
353
3
7
11
15
19
23
27
31
Kernel width (days)
Jul
Aug
Sep
Oct
Nov
Dec
Jan
Feb
Mar
Apr
May
Jun
°F
Winter Solstice December 21/Day 355
Winter Teletherm December 22/Day 356
+1 days
Coverage
100
75
50
25
0
1853
1900
1950
2010
Year
PINE BLUFFS 5W, WY
182
213
244
274
305
335
1
32
60
91
121
152
Day of Year

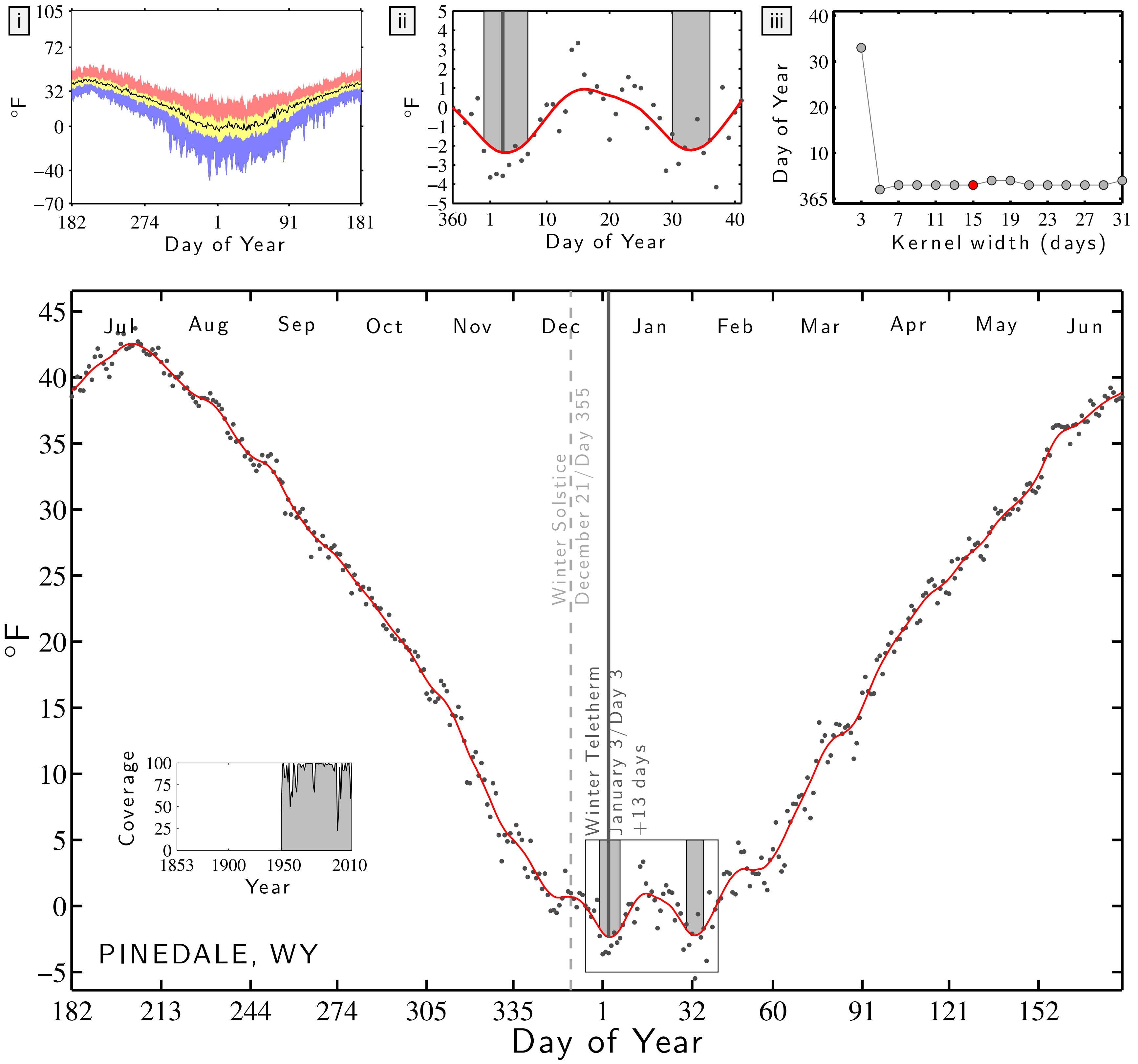
i
°F
Day of Year
ii
°F
Day of Year
iii
Day of Year
Kernel width (days)
Jul
Aug
Sep
Oct
Nov
Dec
Jan
Feb
Mar
Apr
May
Jun
Winter Solstice
December 21/Day 355
Winter Teletherm
January 3/Day 3
+13 days
Coverage
Year
PINEDALE, WY
°F
Day of Year

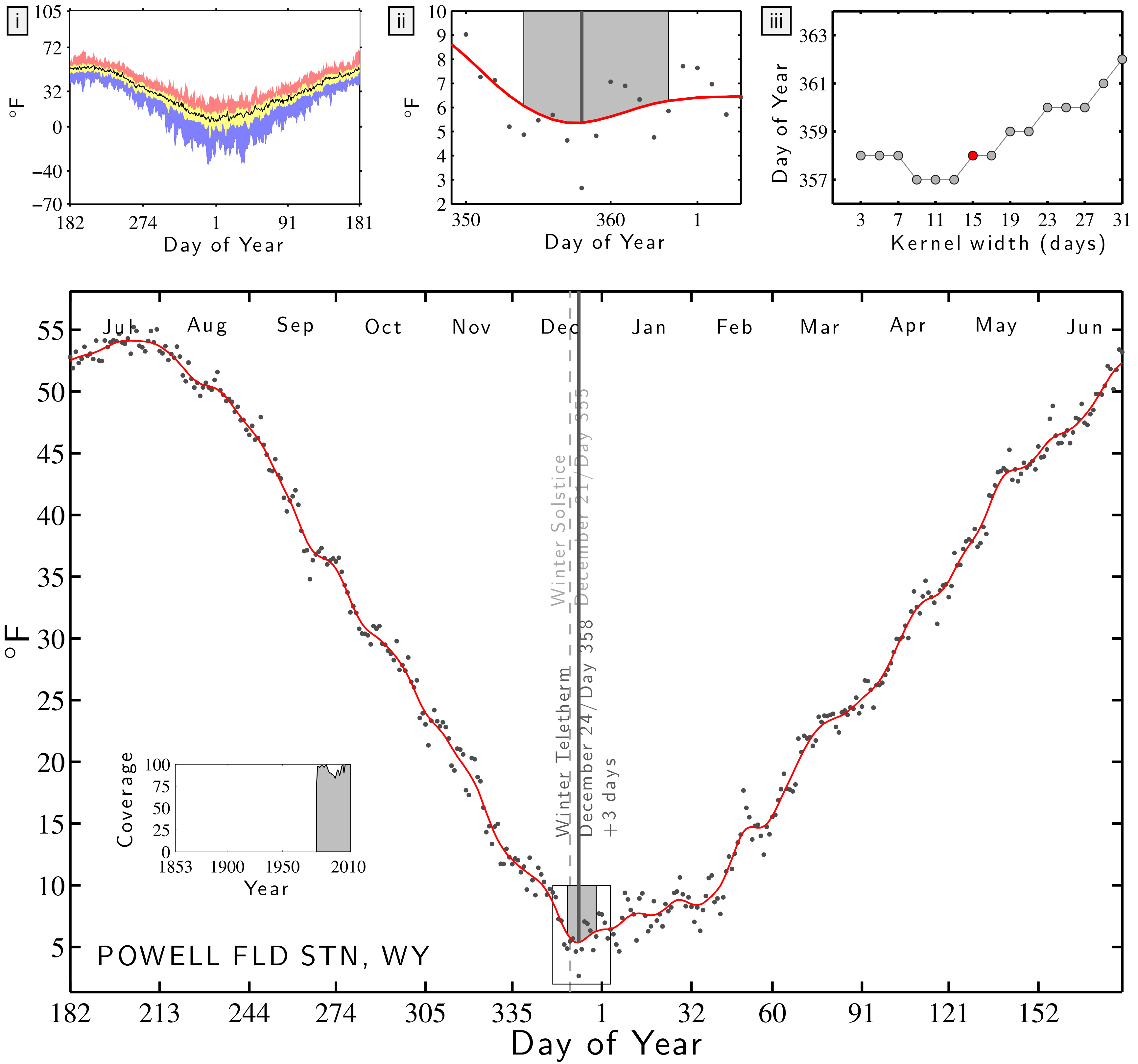

i
°F
Day of Year
ii
°F
Day of Year
iii
Day of Year
Kernel width (days)
Jul
Aug
Sep
Oct
Nov
Dec
Jan
Feb
Mar
Apr
May
Jun
Winter Solstice
December 21/Day 355
Winter Teletherm
December 24/Day 358
+3 days
Coverage
Year
POWELL FLD STN, WY
°F
Day of Year

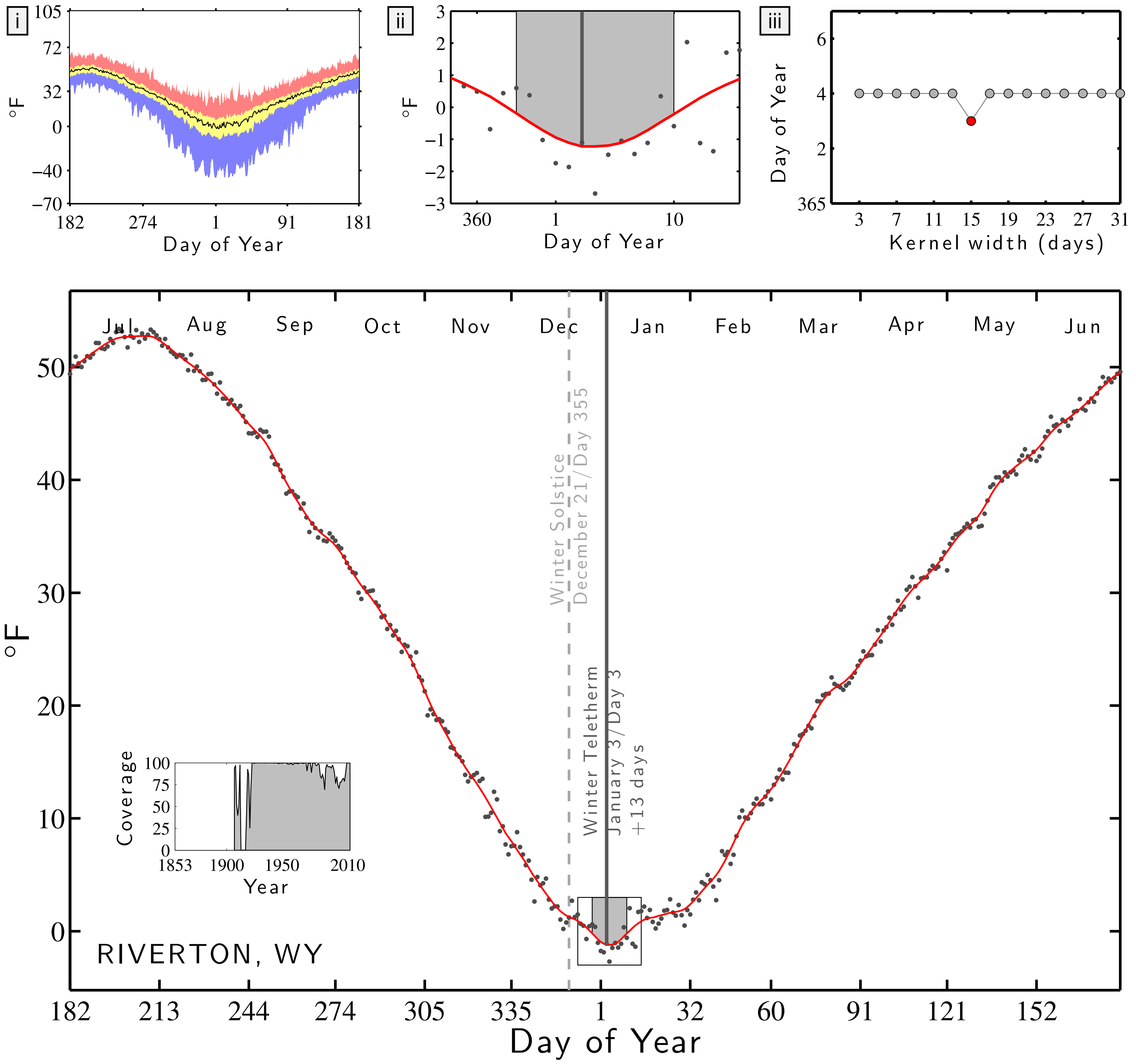

i
°F
Day of Year
ii
°F
Day of Year
iii
Day of Year
Kernel width (days)
Jul
Aug
Sep
Oct
Nov
Dec
Jan
Feb
Mar
Apr
May
Jun
Winter Solstice
December 21/Day 355
Winter Teletherm
January 3/Day 3
+13 days
Coverage
Year
RIVERTON, WY
°F
Day of Year

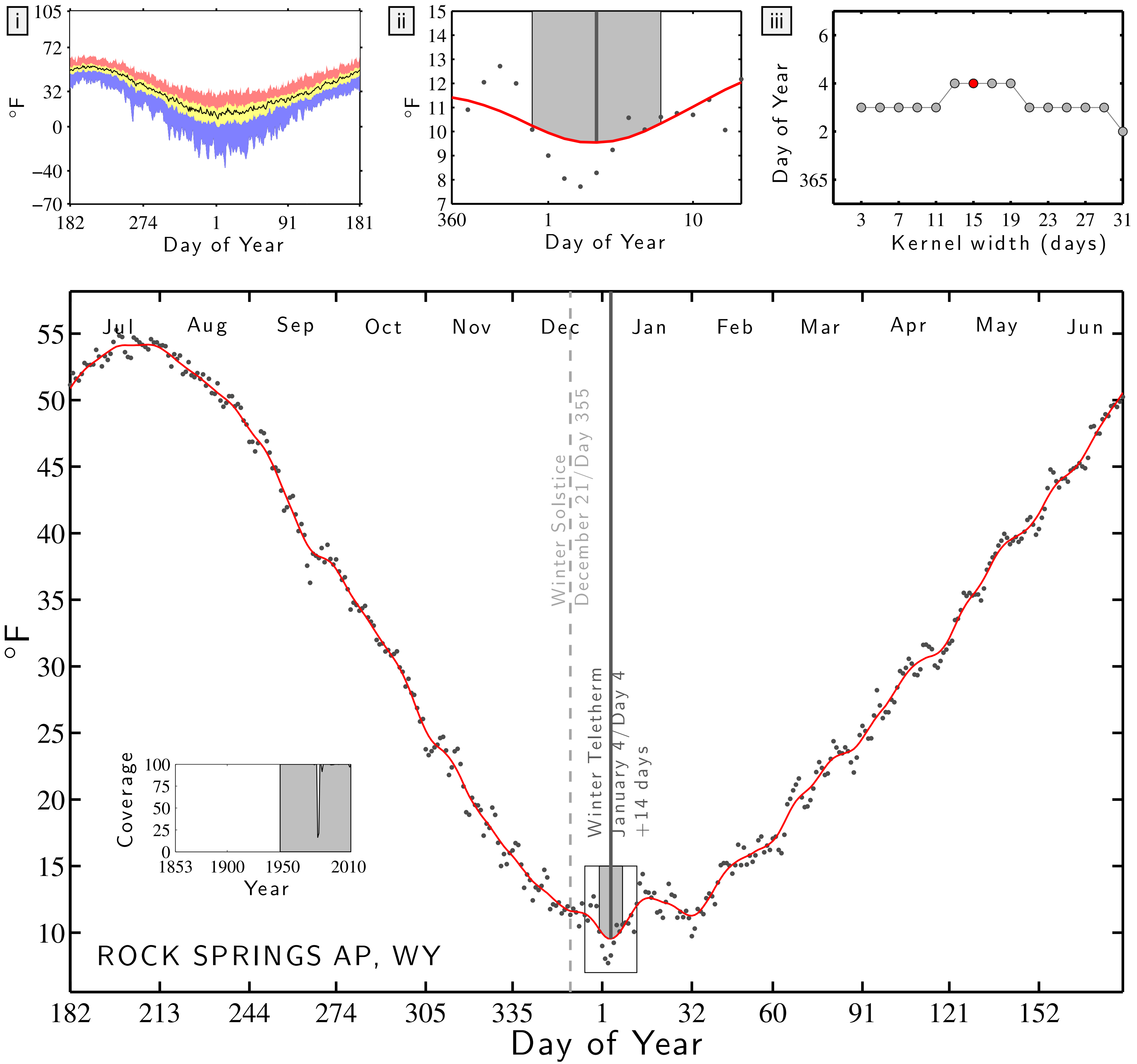

i
°F
Day of Year
ii
Day of Year
iii
Day of Year
Kernel width (days)
Jul
Aug
Sep
Oct
Nov
Dec
Jan
Feb
Mar
Apr
May
Jun
Winter Solstice
December 21/Day 355
Winter Teletherm
January 4/Day 4
+14 days
Coverage
Year
ROCK SPRINGS AP, WY
Day of Year

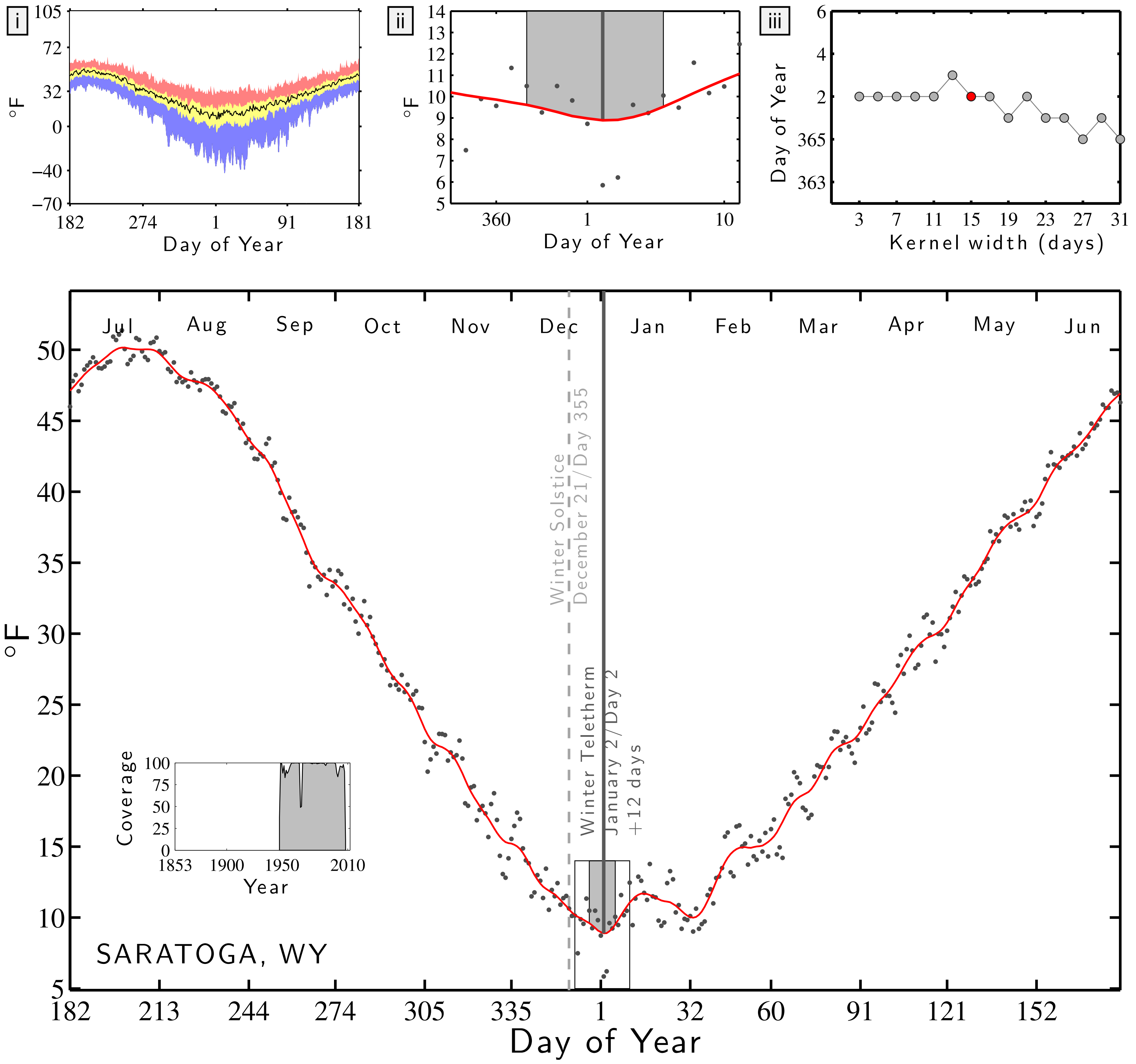

i
ii
iii
Day of Year
°F
Kernel width (days)
Jul
Aug
Sep
Oct
Nov
Dec
Jan
Feb
Mar
Apr
May
Jun
Winter Solstice
December 21/Day 355
Winter Teletherm
January 2/Day 2
+12 days
Coverage
Year
SARATOGA, WY

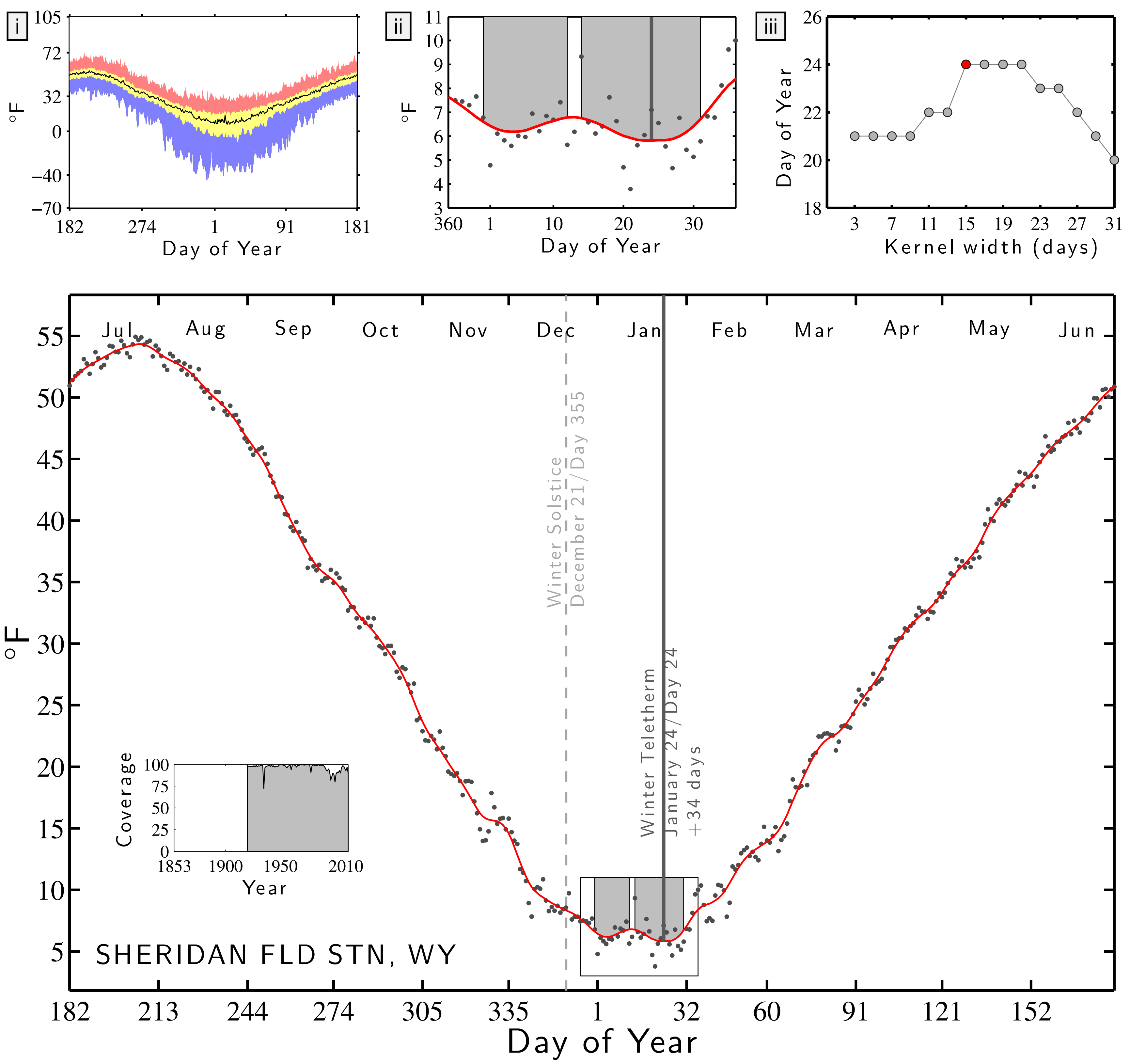

i
°F
Day of Year
ii
°F
Day of Year
iii
Day of Year
Kernel width (days)
Jul
Aug
Sep
Oct
Nov
Dec
Jan
Feb
Mar
Apr
May
Jun
Winter Solstice
December 21/Day 355
Winter Teletherm
January 24/Day 24
+34 days
Coverage
Year
SHERIDAN FLD STN, WY
°F
Day of Year

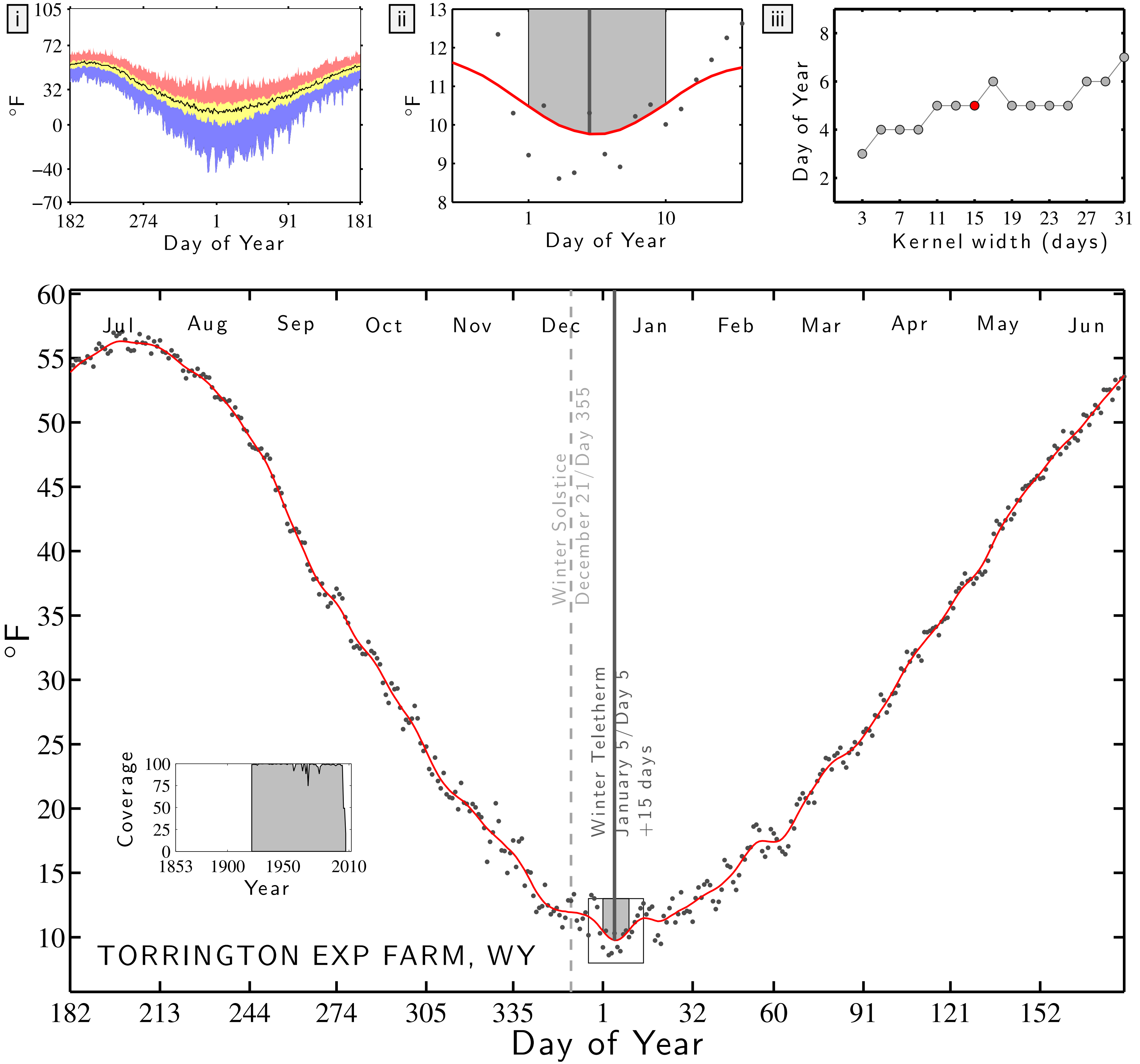

i
ii
iii
°F
Day of Year
Kernel width (days)
Jul
Aug
Sep
Oct
Nov
Dec
Jan
Feb
Mar
Apr
May
Jun
Winter Solstice
December 21/Day 355
Winter Teletherm
January 5/Day 5
+15 days
Coverage
Year
TORRINGTON EXP FARM, WY

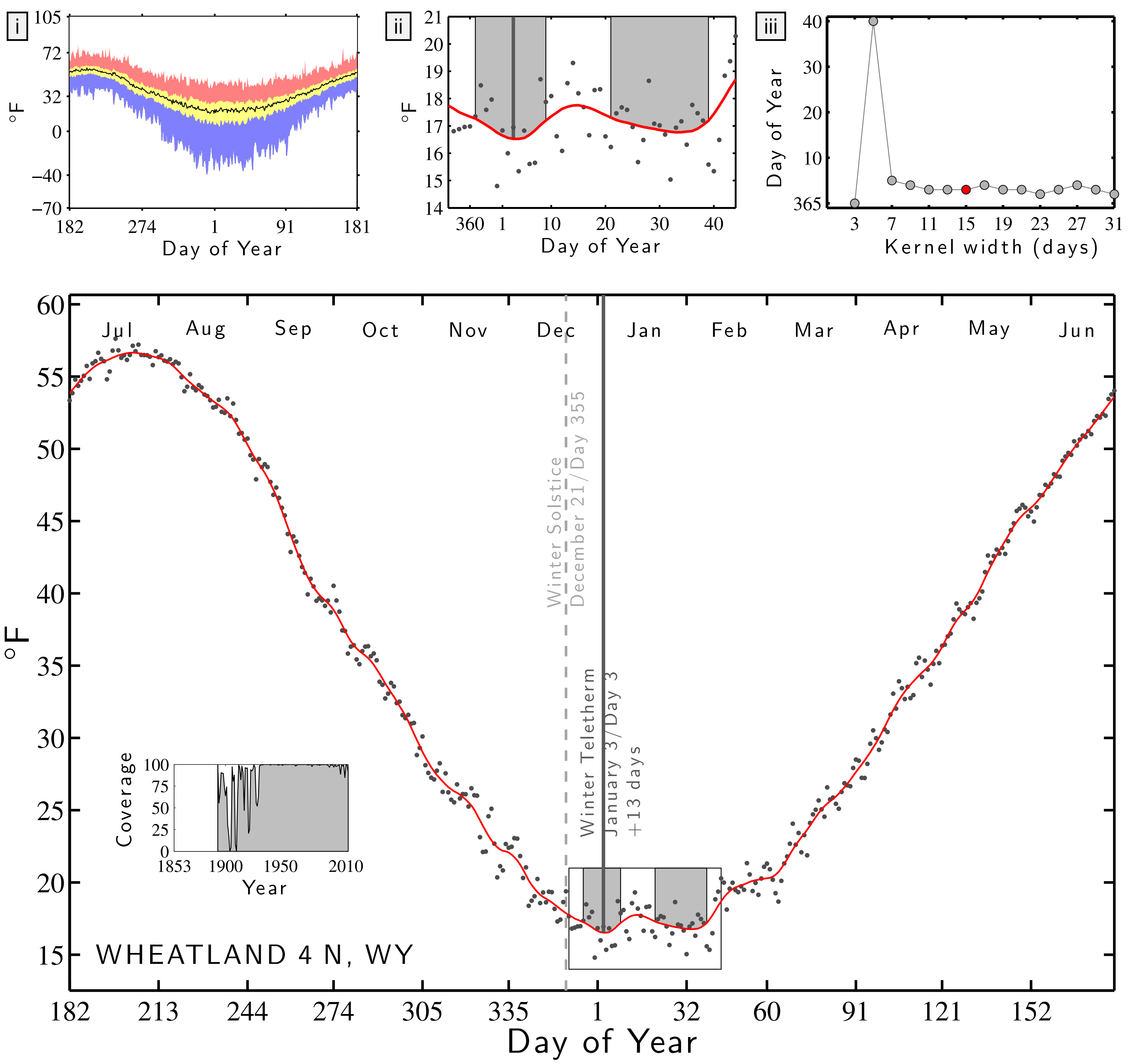

i
°F
Day of Year
ii
Day of Year
iii
Day of Year
Kernel width (days)
Jul
Aug
Sep
Oct
Nov
Dec
Jan
Feb
Mar
Apr
May
Jun
Winter Solstice
December 21/Day 355
Winter Teletherm
January 3/Day 3
+13 days
Coverage
Year
WHEATLAND 4 N, WY
Day of Year

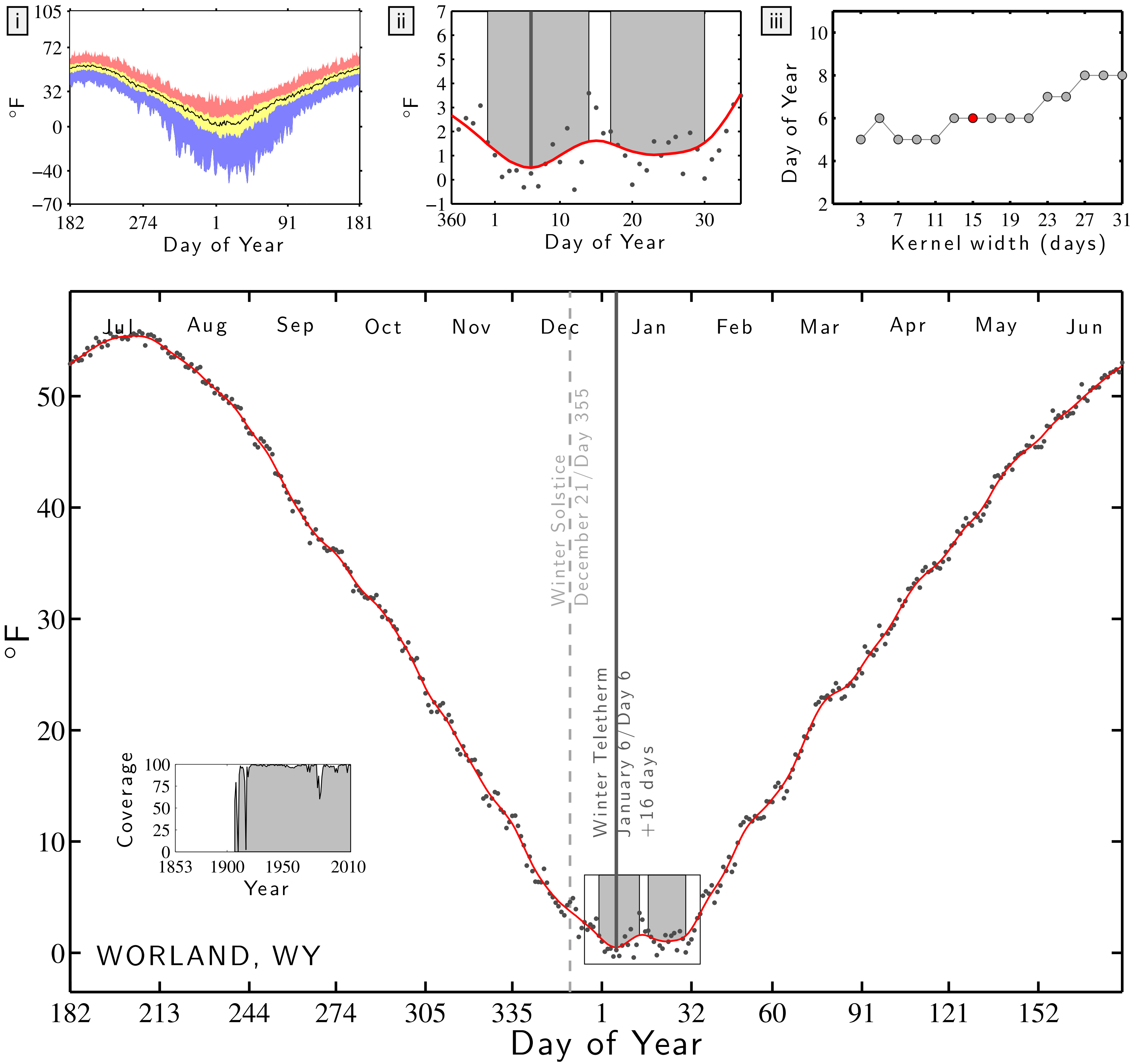

i
°F
Day of Year
ii
°F
Day of Year
iii
Day of Year
Kernel width (days)
Jul
Aug
Sep
Oct
Nov
Dec
Jan
Feb
Mar
Apr
May
Jun
Winter Solstice
December 21/Day 355
Winter Teletherm
January 6/Day 6
+16 days
Coverage
Year
WORLAND, WY
°F
Day of Year

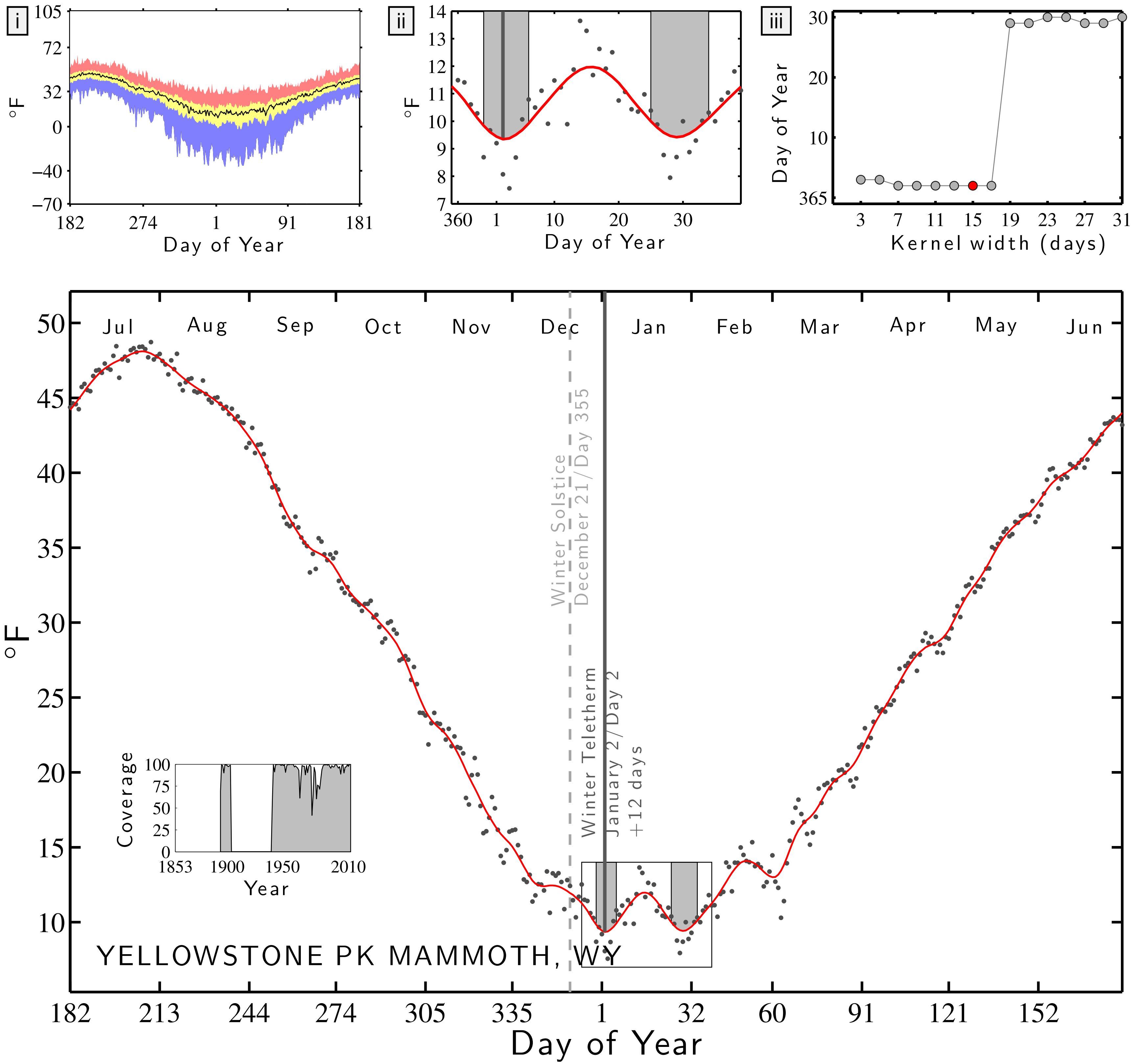

i
105
72
32
0
−40
−70
°F
182
274
1
91
181
Day of Year
ii
14
13
12
11
10
9
8
7
°F
360
1
10
20
30
Day of Year
iii
30
20
10
365
Day of Year
3
7
11
15
19
23
27
31
Kernel width (days)
Jul
Aug
Sep
Oct
Nov
Dec
Jan
Feb
Mar
Apr
May
Jun
50
45
40
35
30
25
20
15
10
°F
Winter Solstice
December 21/Day 355
Winter Teletherm
January 2/Day 2
+12 days
Coverage
100
75
50
25
0
1853
1900
1950
2010
Year
YELLOWSTONE PK MAMMOTH, WY
182
213
244
274
305
335
1
32
60
91
121
152
Day of Year
