# Supplementary material for: Tracking Climate Change through the Spatiotemporal Dynamics of the Teletherms, the Statistically Hottest and Coldest Days of the Year
Source: PLoS One. 2016 May 11;11(5):e0154184. doi: 10.1371/journal.pone.0154184 (PMC4864332; doi:10.1371/journal.pone.0154184)
Supplement: S3 File — (PDF) [file pone.0154184.s024.pdf]

50 year Summer Teletherm dynamics for BREWTON 3 SSE, AL:

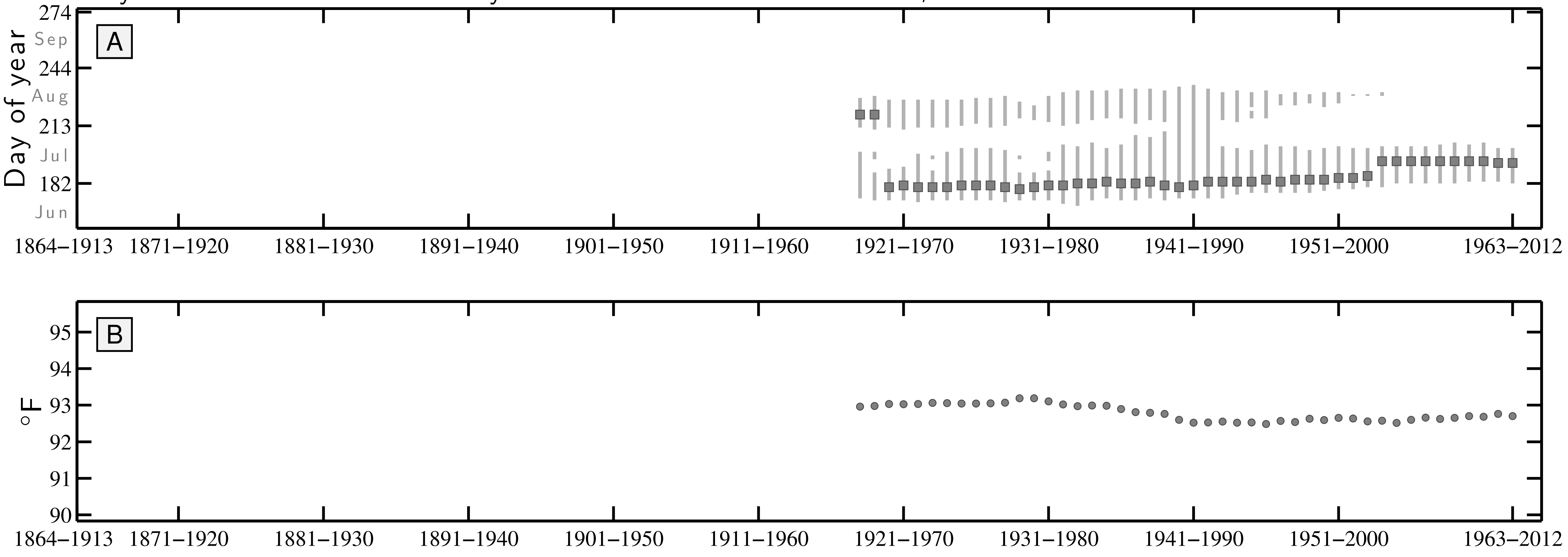

50 year Winter Teletherm dynamics for BREWTON 3 SSE, AL:

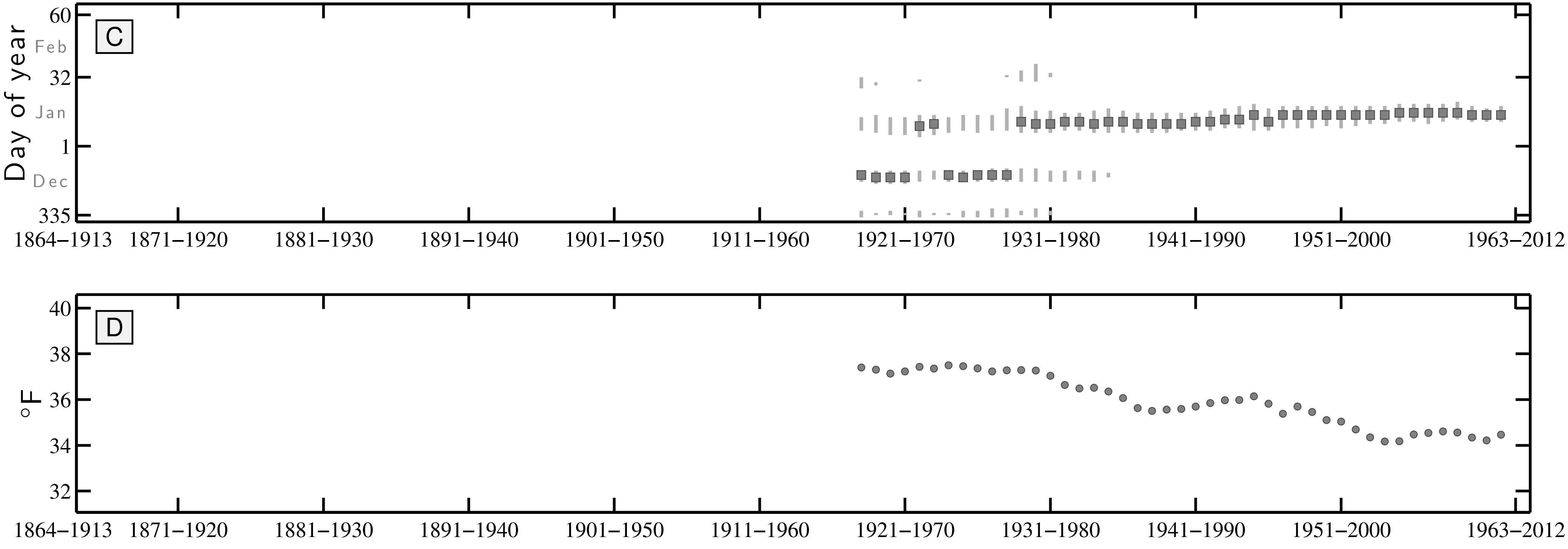

50 year Summer Teletherm dynamics for FAIRHOPE 2 NE, AL:

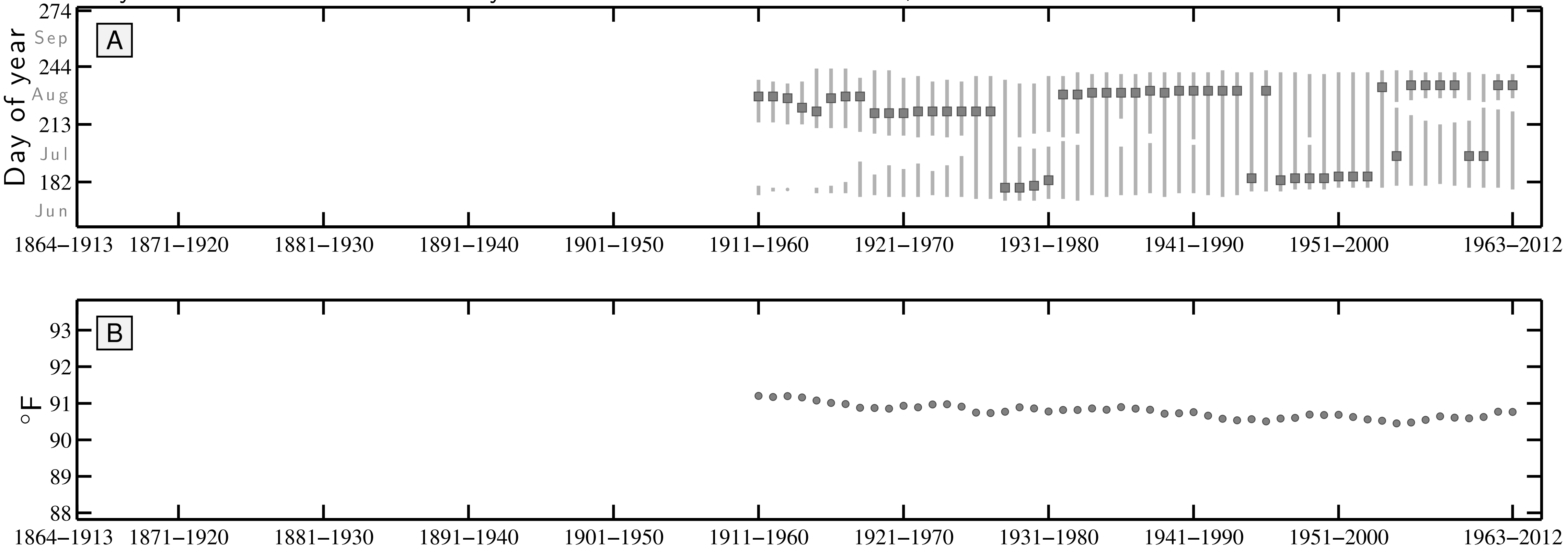

50 year Winter Teletherm dynamics for FAIRHOPE 2 NE, AL:

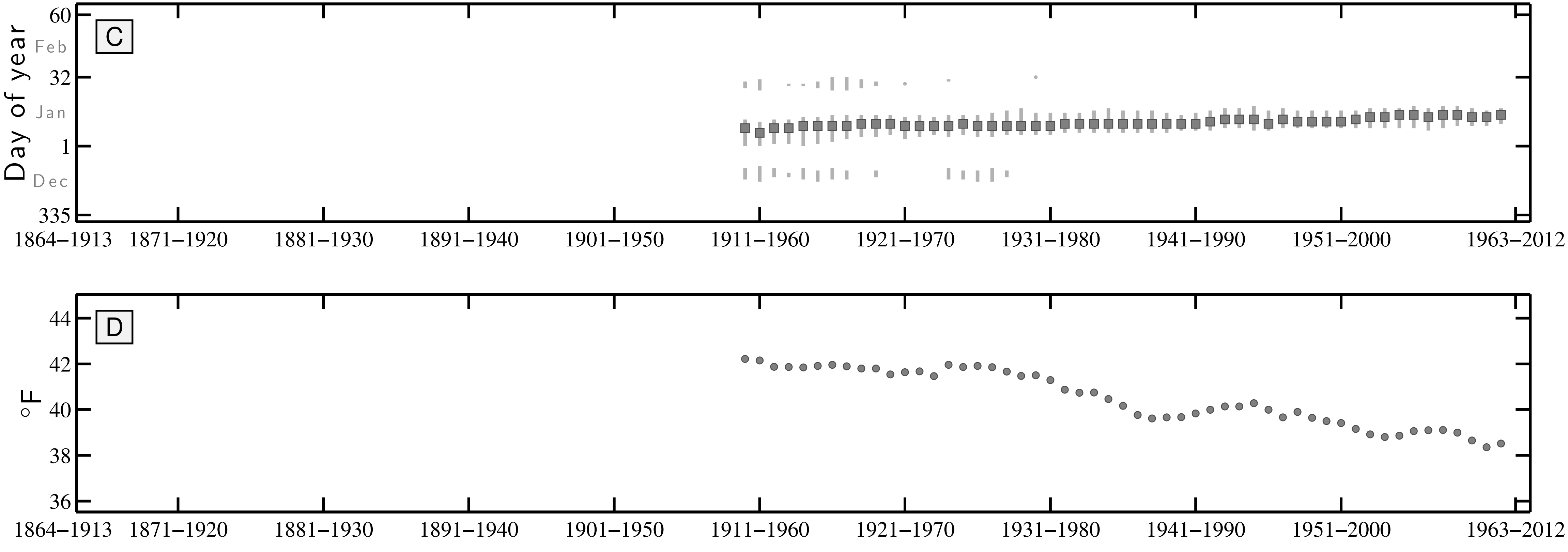

50 year Summer Teletherm dynamics for GAINESVILLE LOCK, AL:

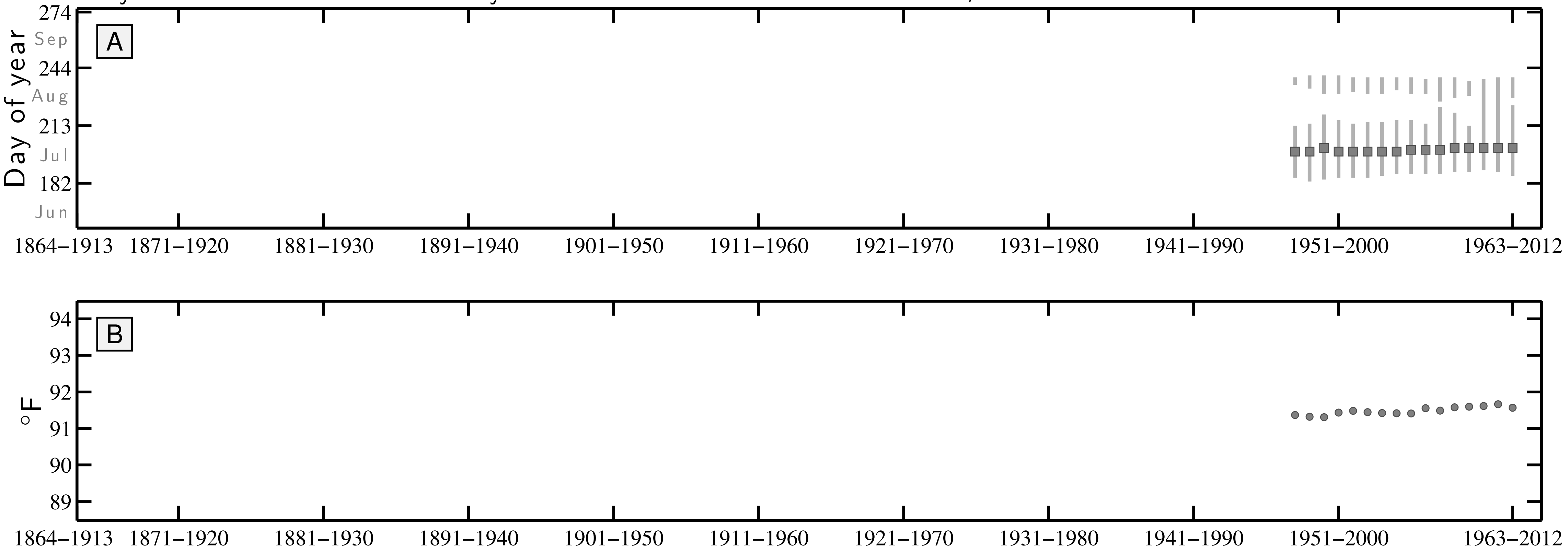

50 year Winter Teletherm dynamics for GAINESVILLE LOCK, AL:

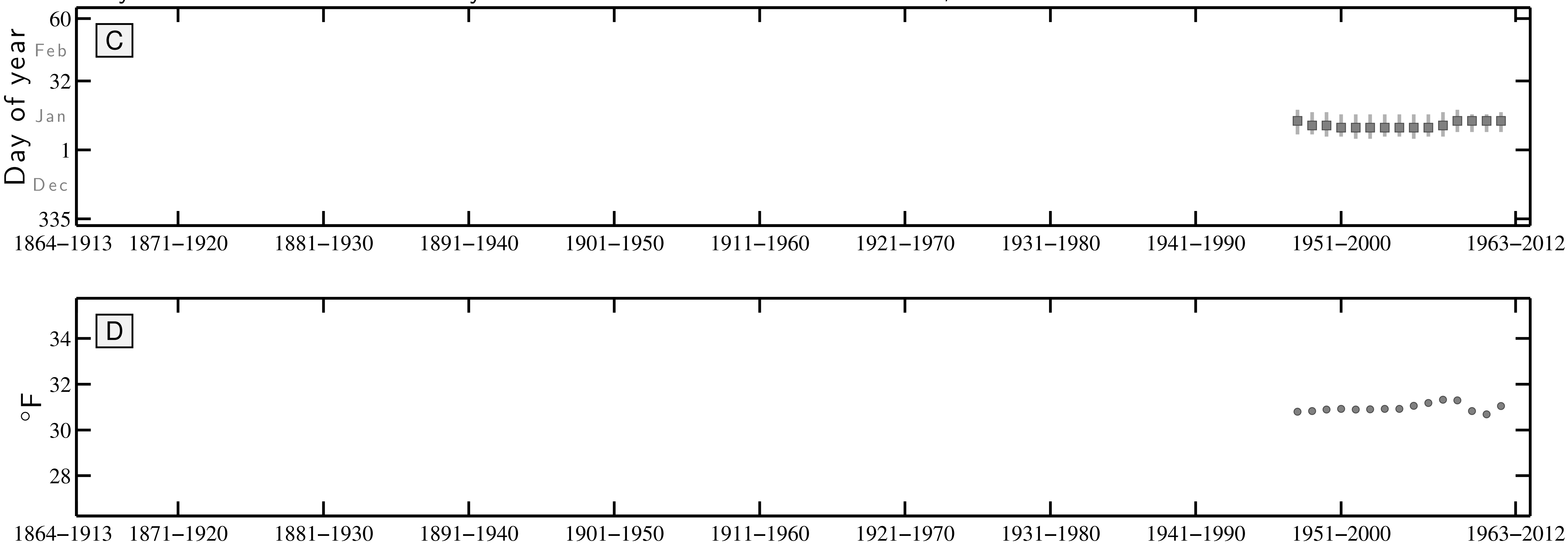

50 year Summer Teletherm dynamics for GREENSBORO, AL:

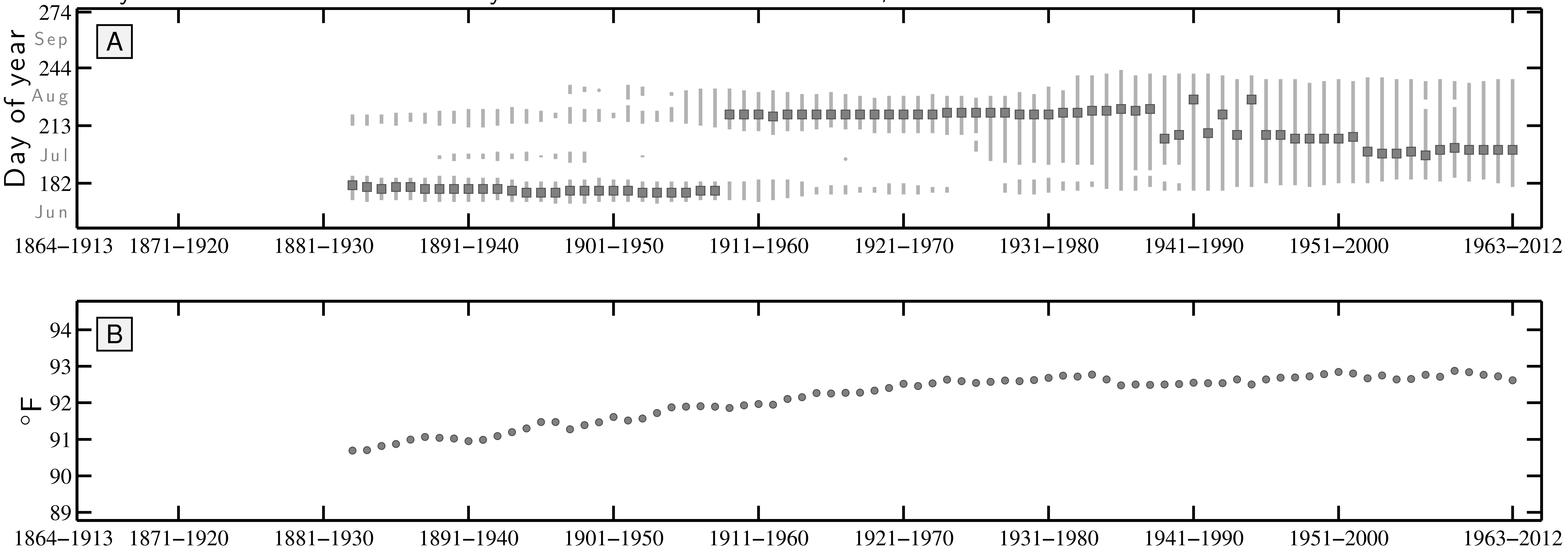

50 year Winter Teletherm dynamics for GREENSBORO, AL:

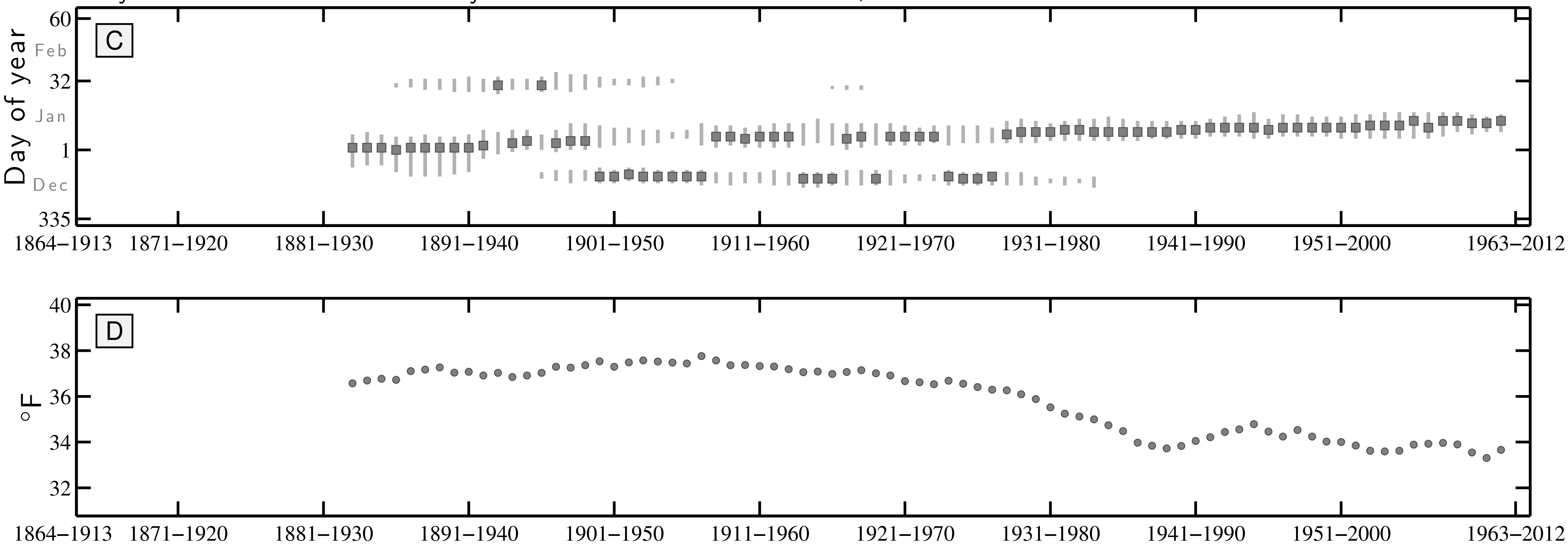

50 year Summer Teletherm dynamics for HIGHLAND HOME, AL:

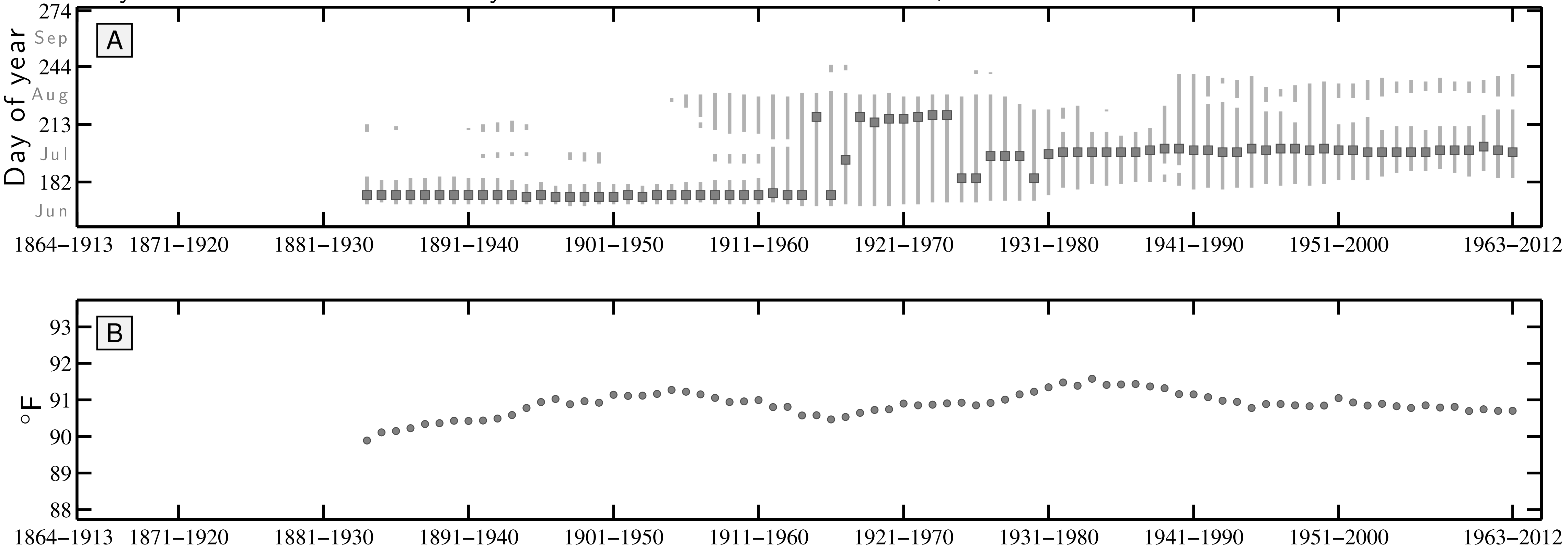

50 year Winter Teletherm dynamics for HIGHLAND HOME, AL:

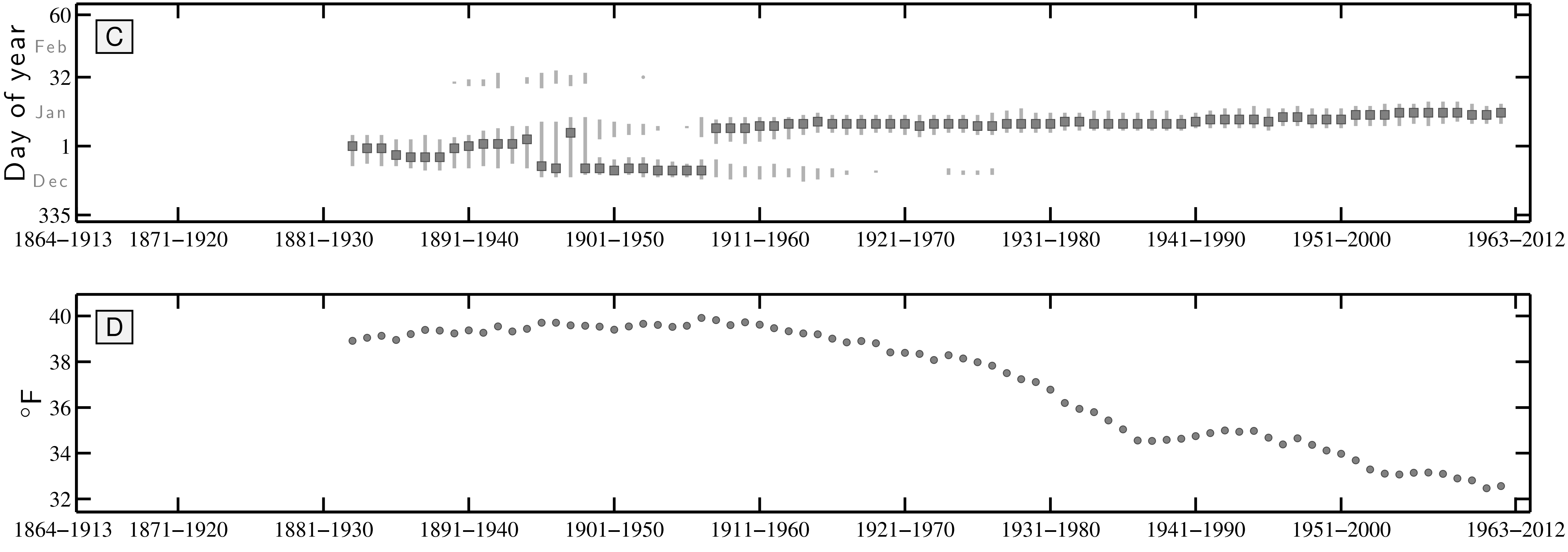

50 year Summer Teletherm dynamics for MUSCLE SHOALS AP, AL:

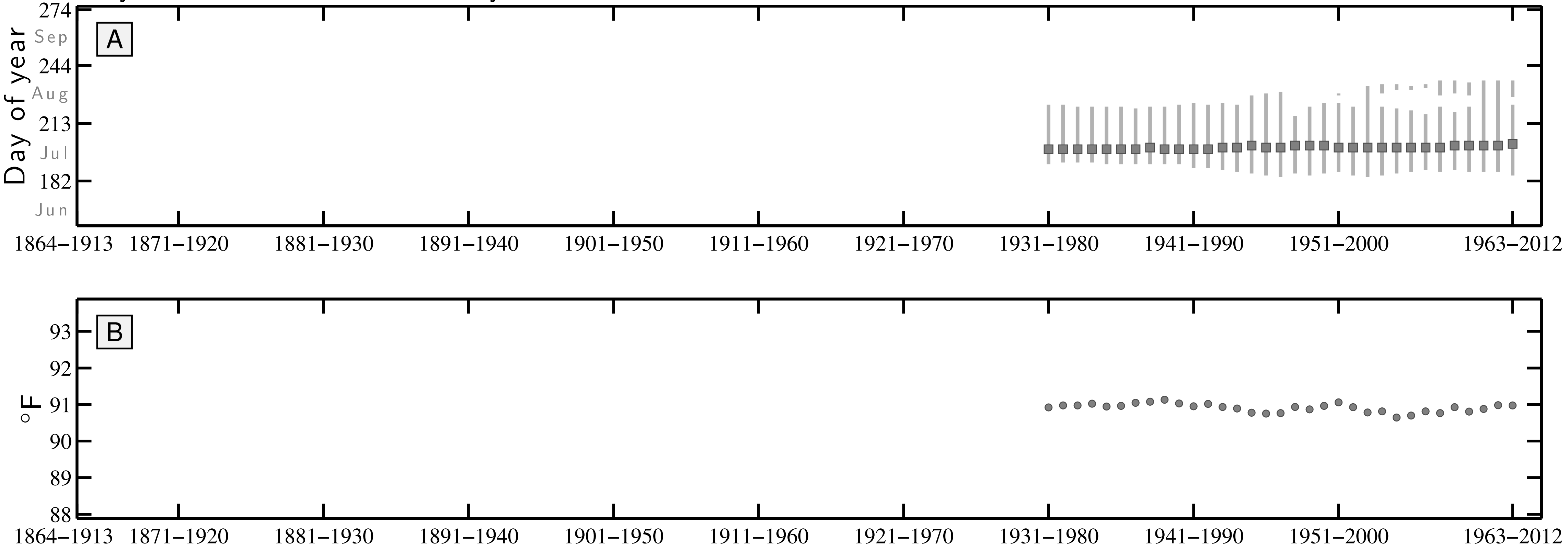

50 year Winter Teletherm dynamics for MUSCLE SHOALS AP, AL:

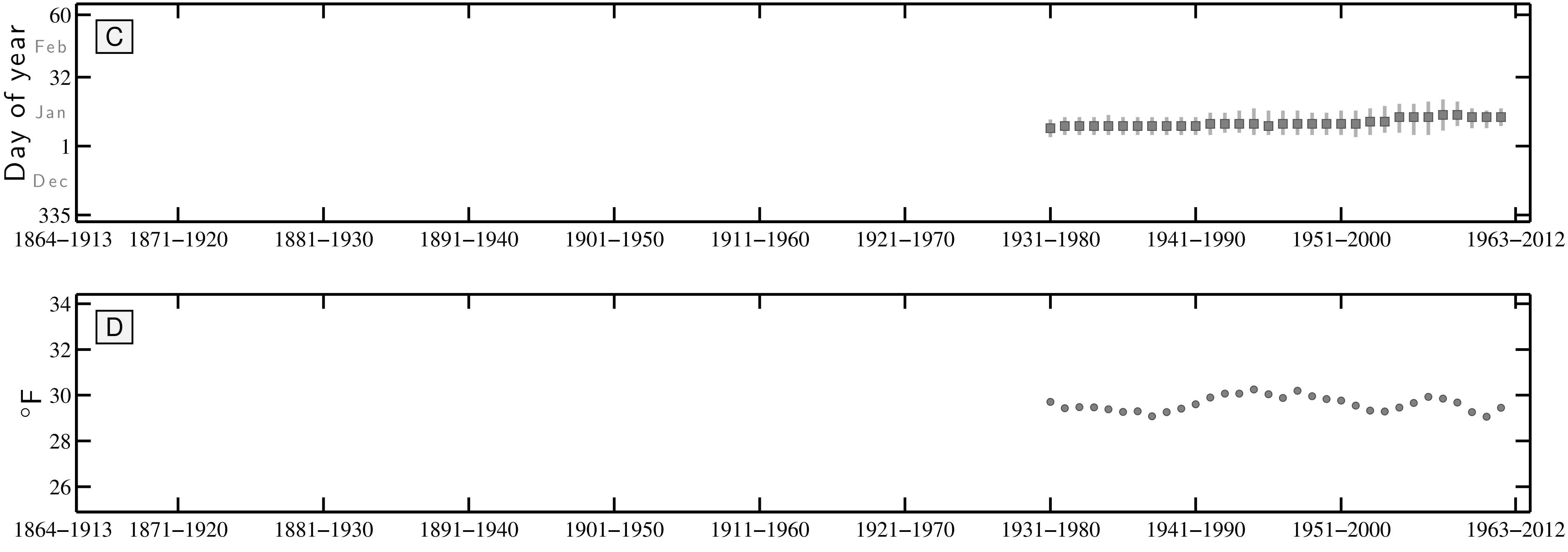

50 year Summer Teletherm dynamics for SAINT BERNARD, AL:

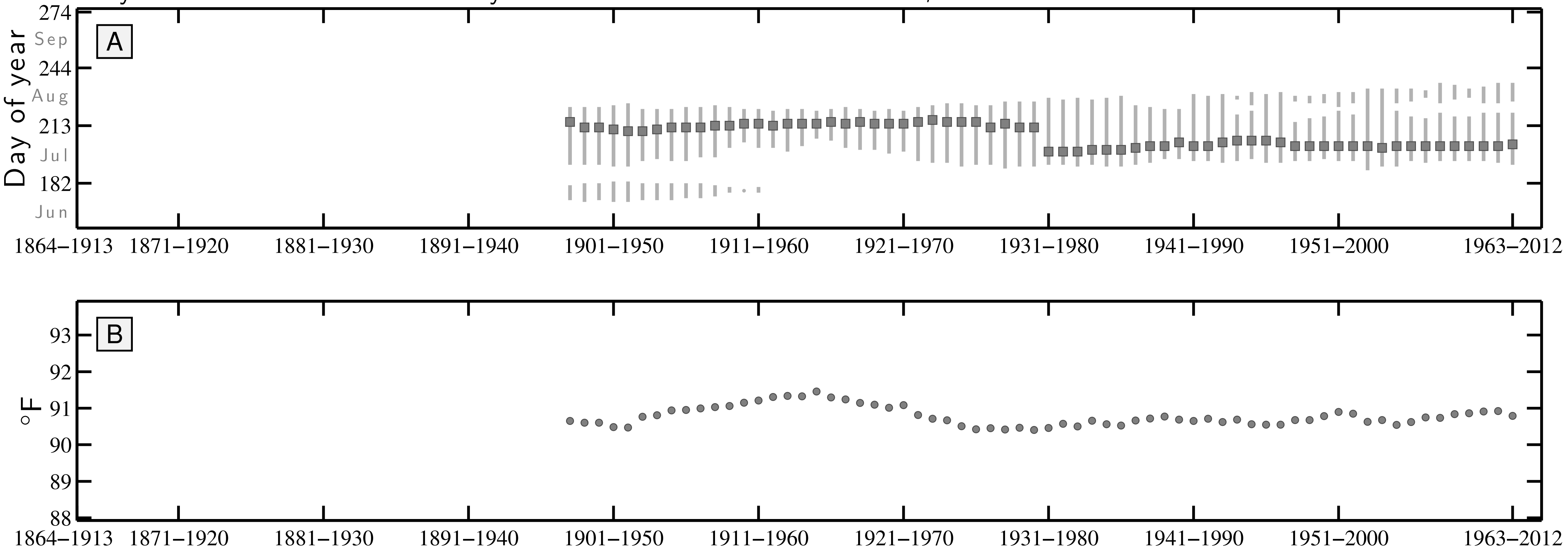

50 year Winter Teletherm dynamics for SAINT BERNARD, AL:

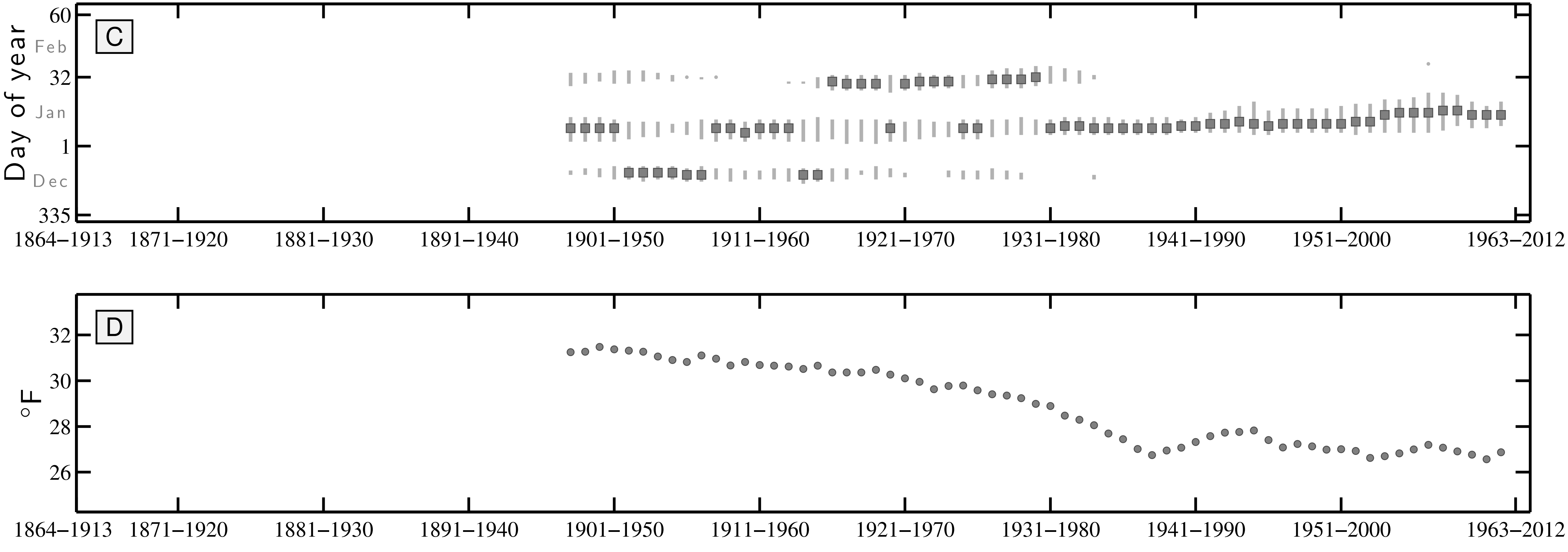

50 year Summer Teletherm dynamics for SCOTTSBORO, AL:

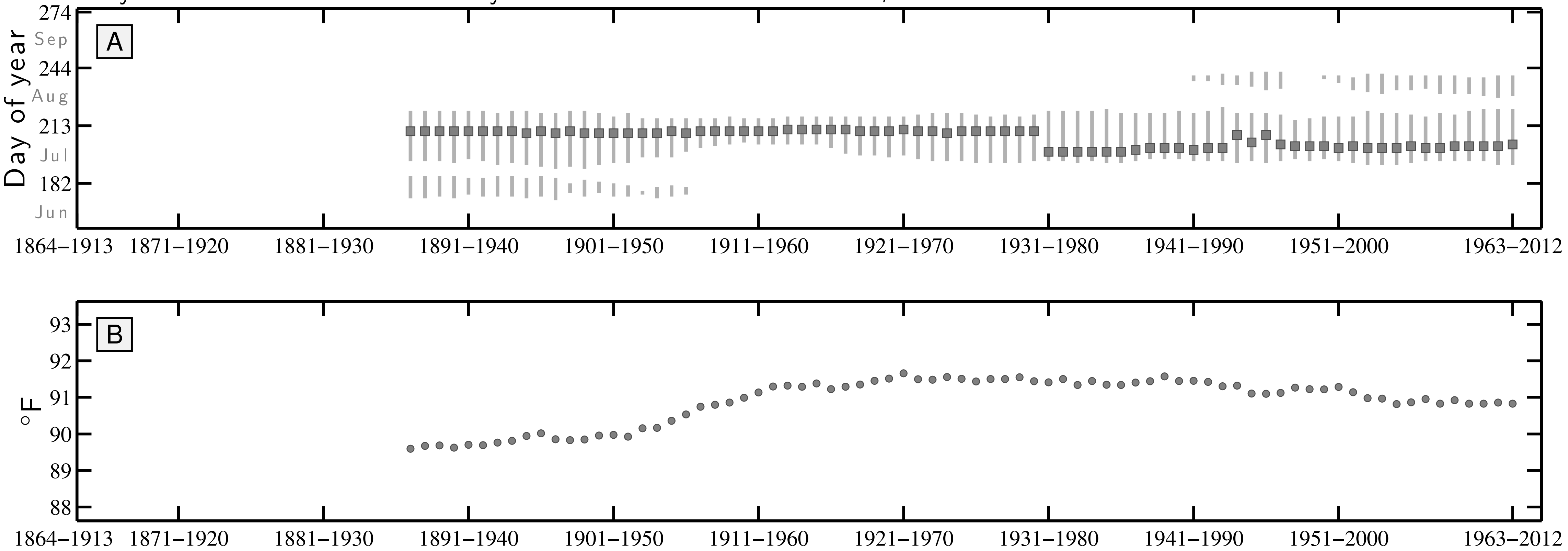

50 year Winter Teletherm dynamics for SCOTTSBORO, AL:

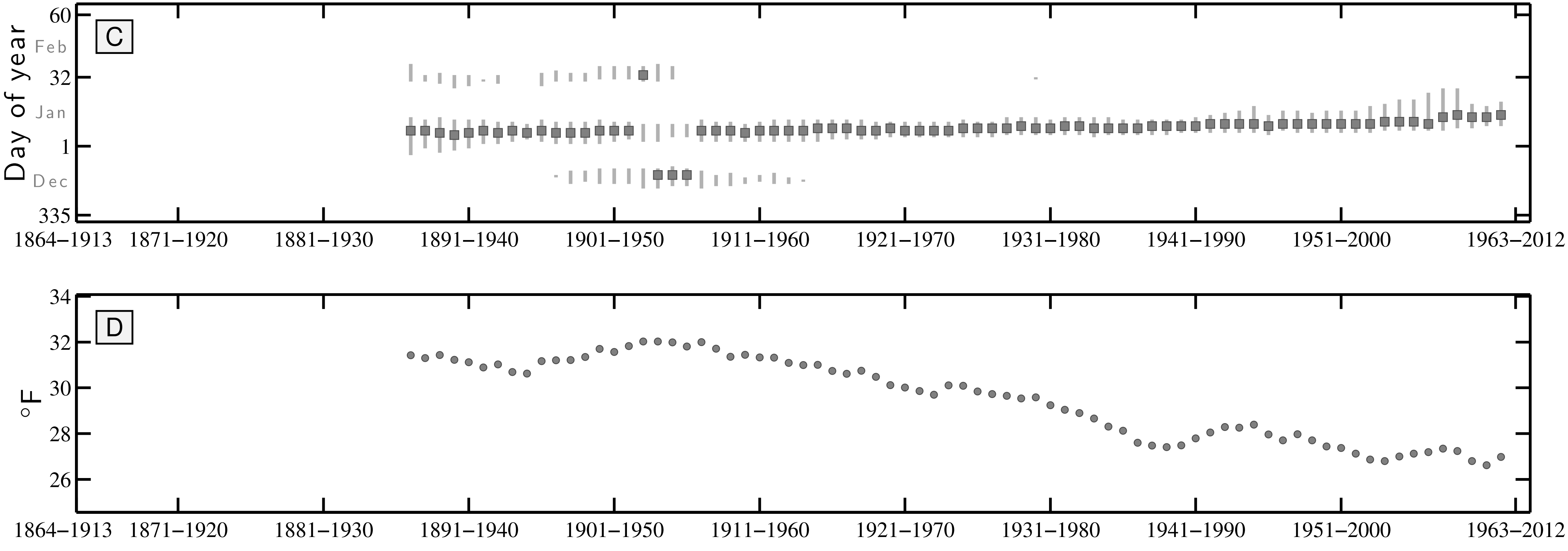

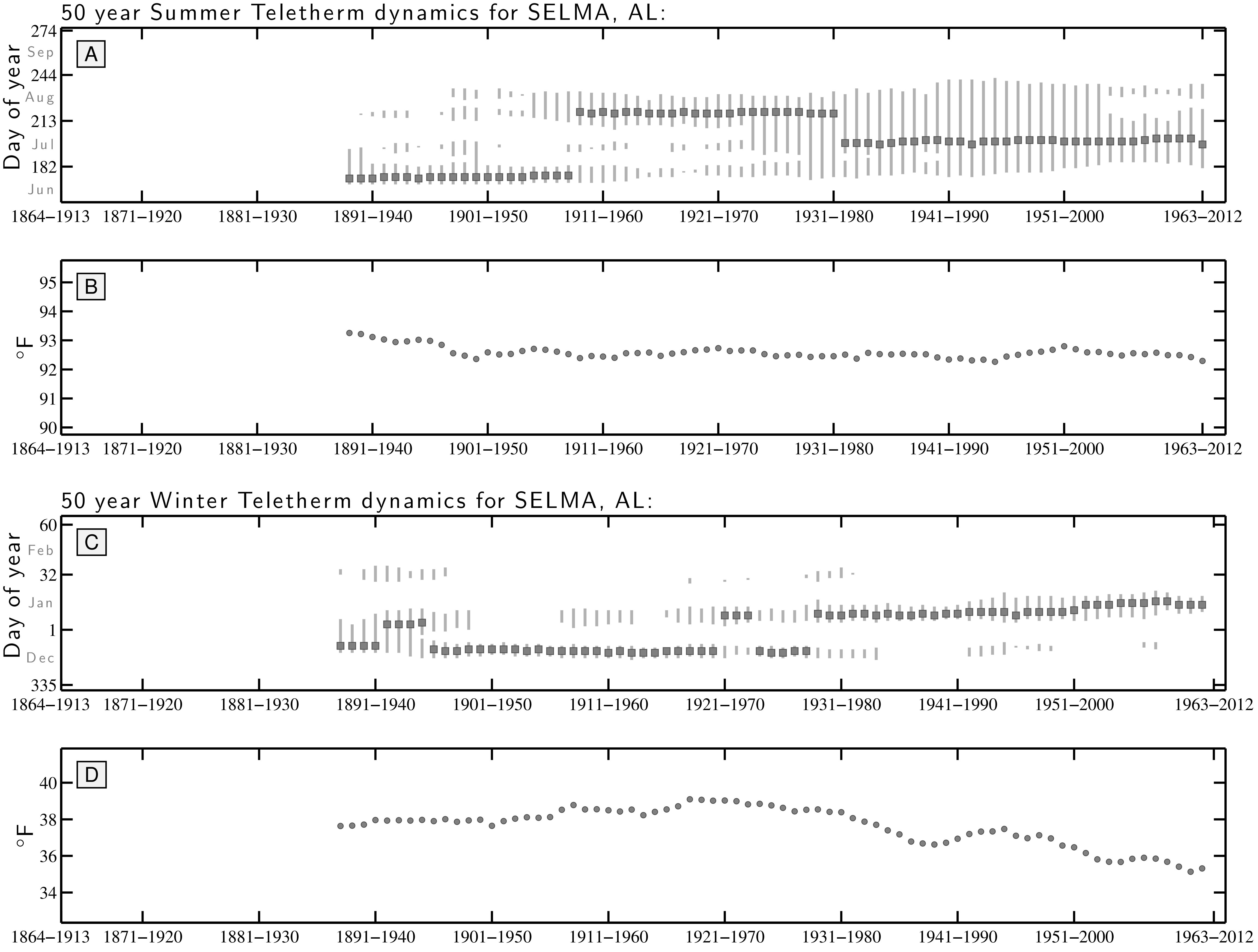
50 year Summer Teletherm dynamics for SELMA, AL:
A
Day of year
Sep
Aug
Jul
Jun
274
244
213
182
B
°F
95
94
93
92
91
90
50 year Winter Teletherm dynamics for SELMA, AL:
C
Day of year
Feb
Jan
Dec
60
32
1
335
D
°F
40
38
36
34
1864–1913
1871–1920
1881–1930
1891–1940
1901–1950
1911–1960
1921–1970
1931–1980
1941–1990
1951–2000
1963–2012

50 year Summer Teletherm dynamics for TALLADEGA, AL:

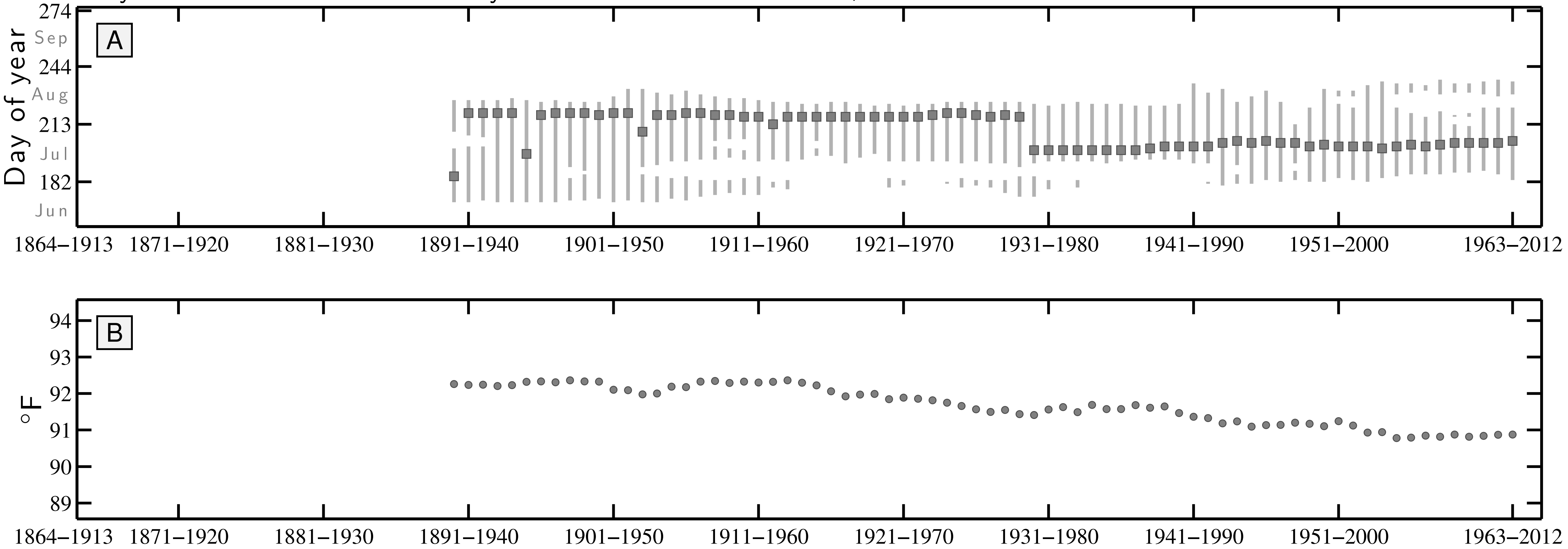

50 year Winter Teletherm dynamics for TALLADEGA, AL:

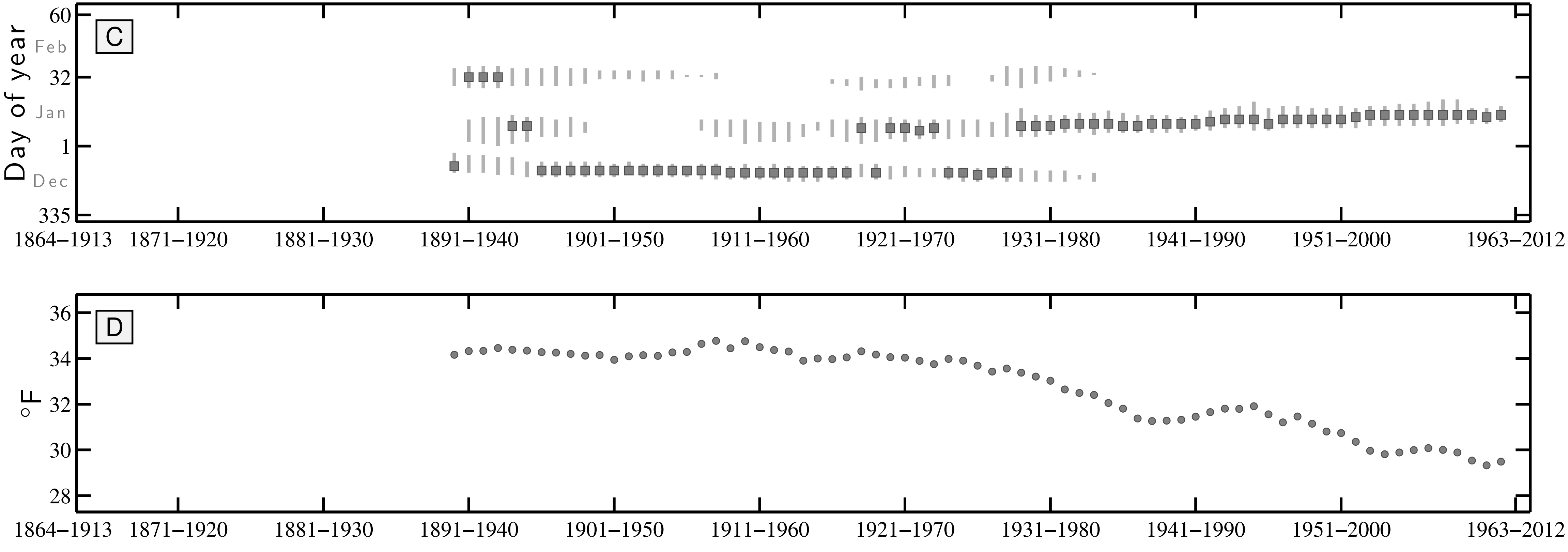

50 year Summer Teletherm dynamics for THOMASVILLE, AL:

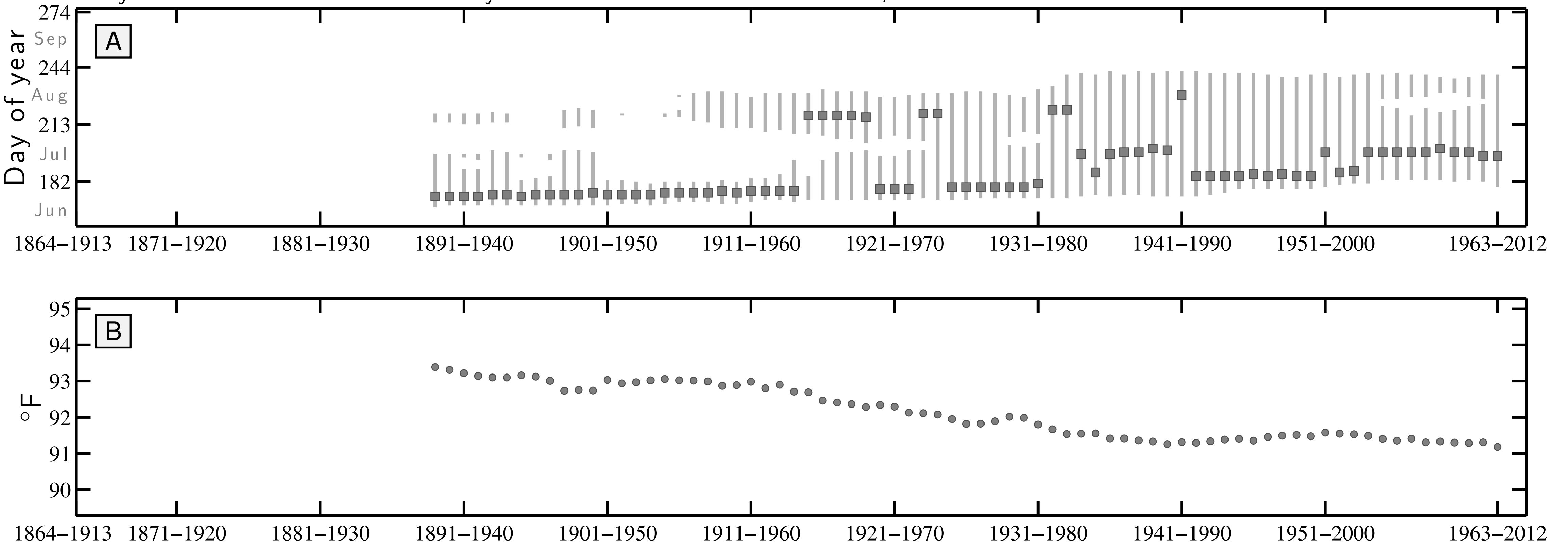

50 year Winter Teletherm dynamics for THOMASVILLE, AL:

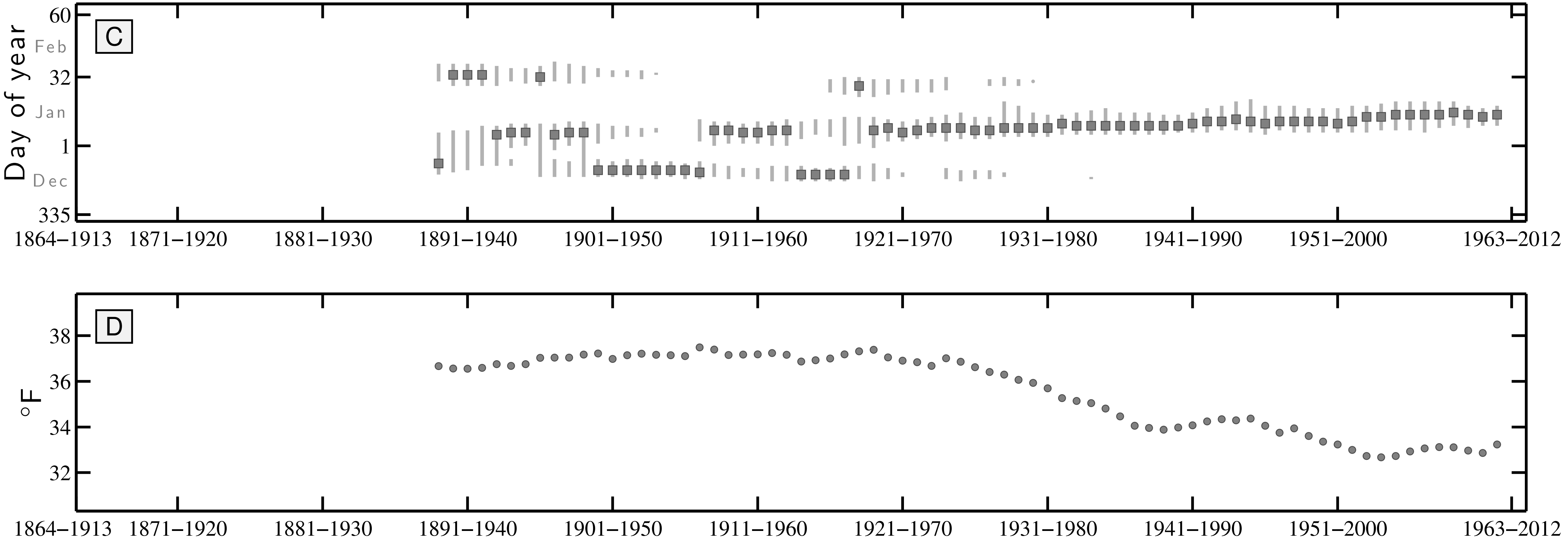

50 year Summer Teletherm dynamics for TROY, AL:

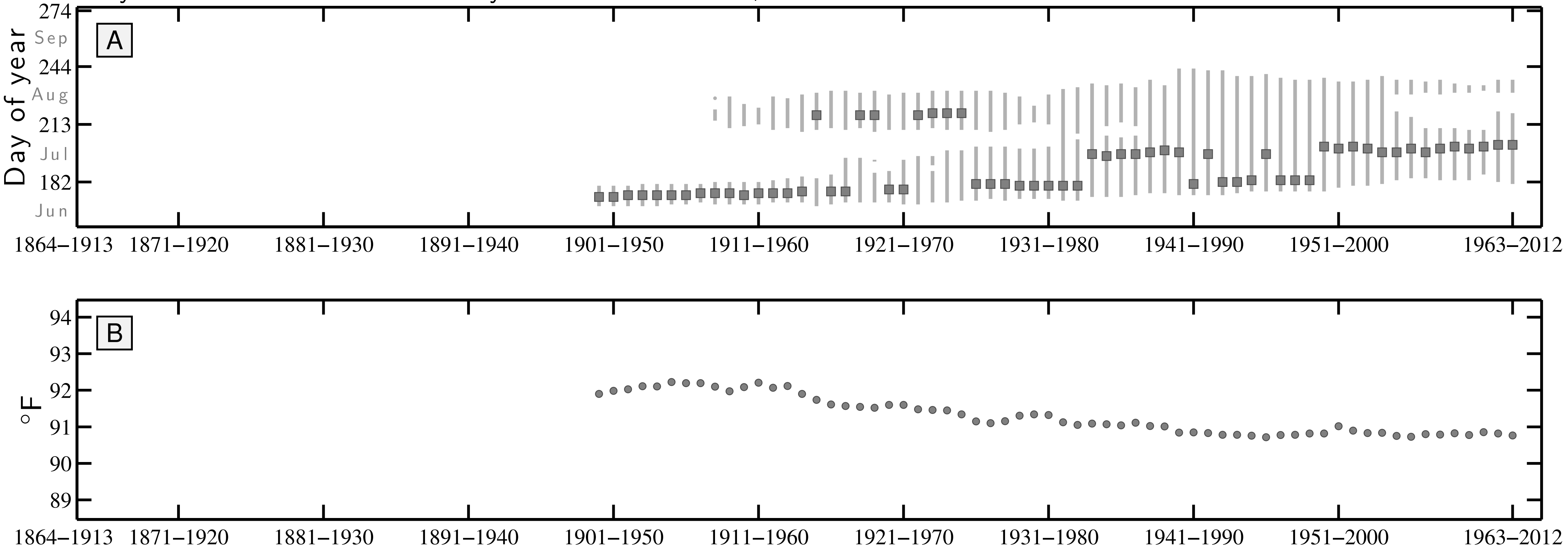

50 year Winter Teletherm dynamics for TROY, AL:

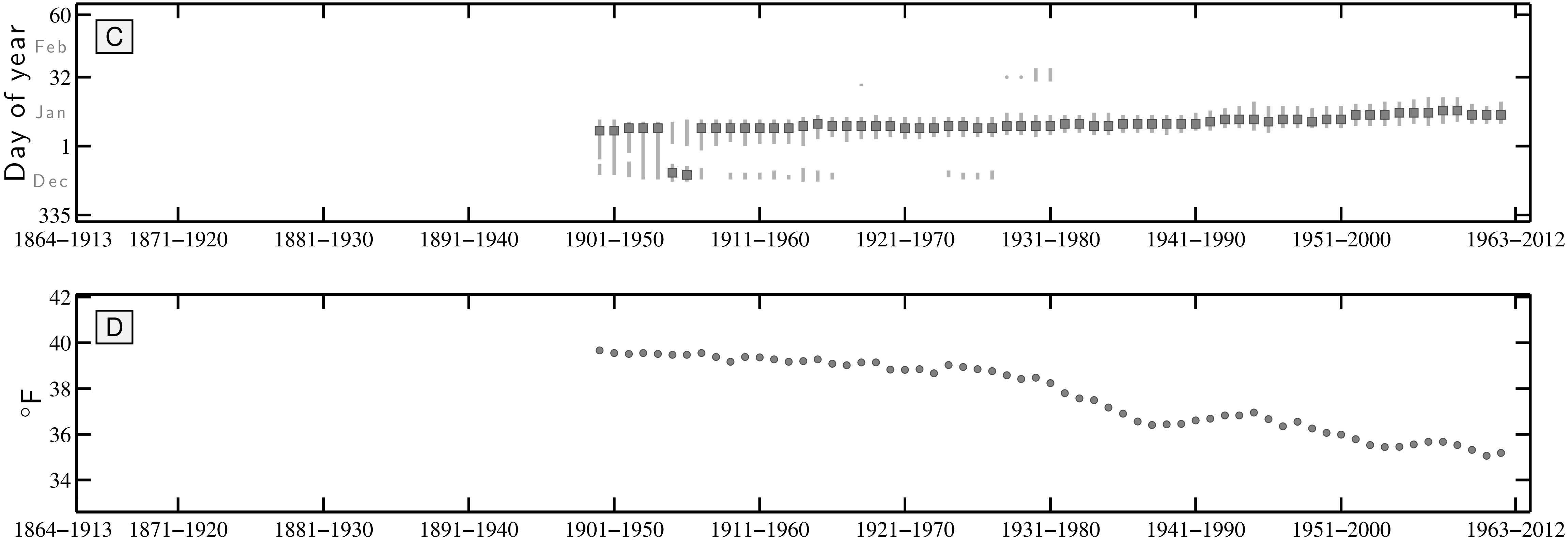

50 year Summer Teletherm dynamics for TUSCALOOSA ACFD, AL:

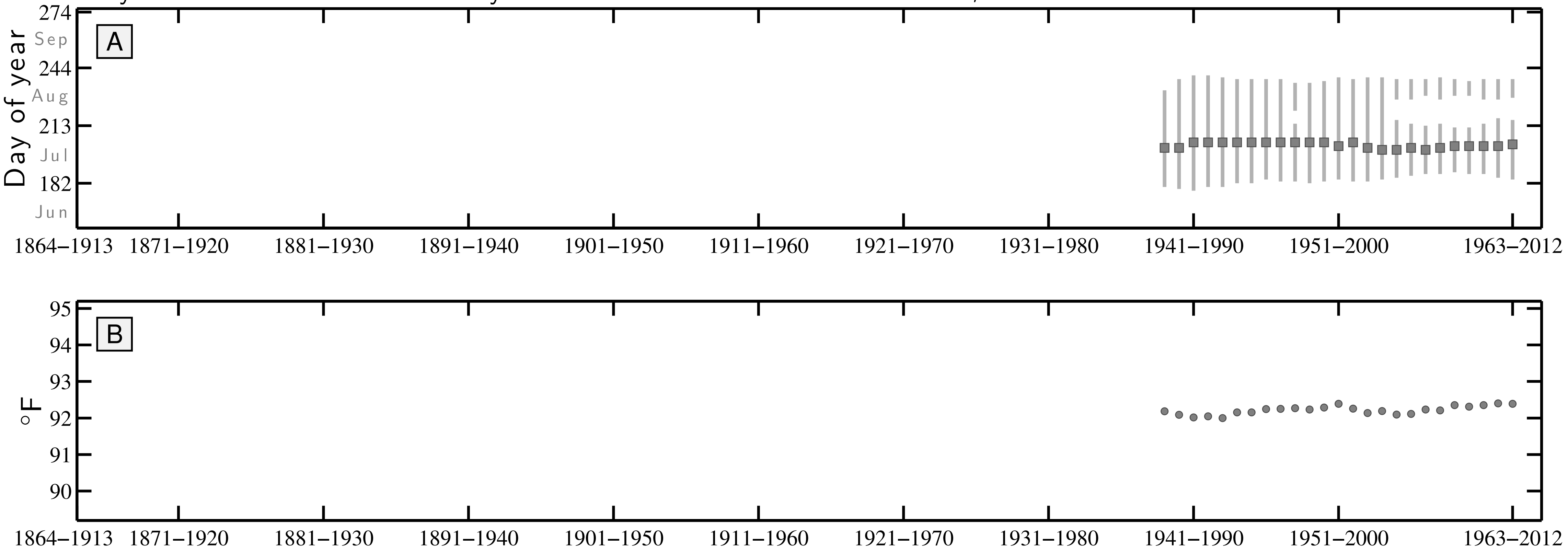

50 year Winter Teletherm dynamics for TUSCALOOSA ACFD, AL:

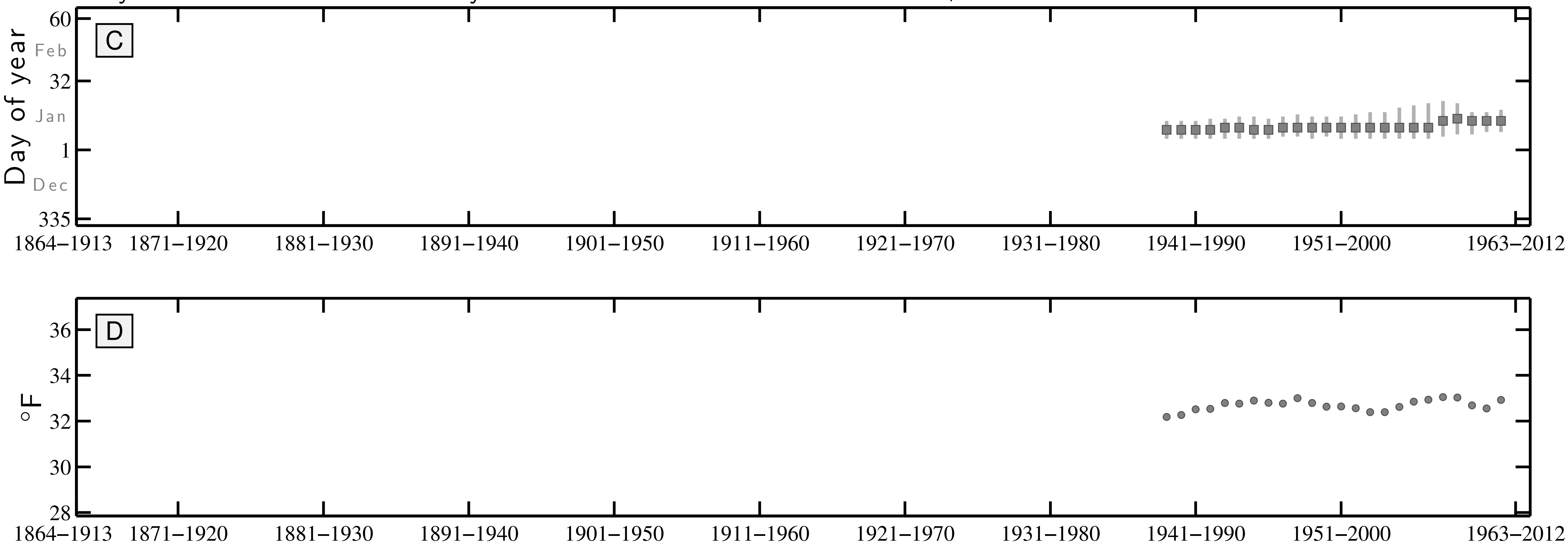

50 year Summer Teletherm dynamics for UNION SPRINGS 9 S, AL:

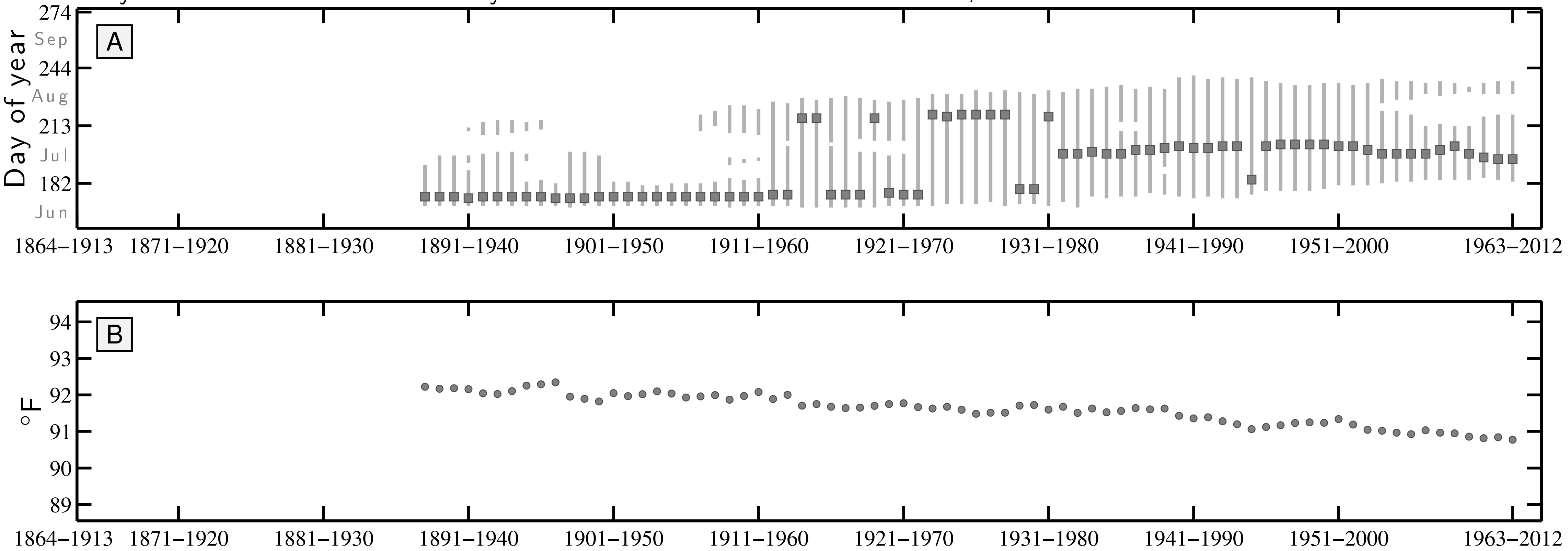

50 year Winter Teletherm dynamics for UNION SPRINGS 9 S, AL:

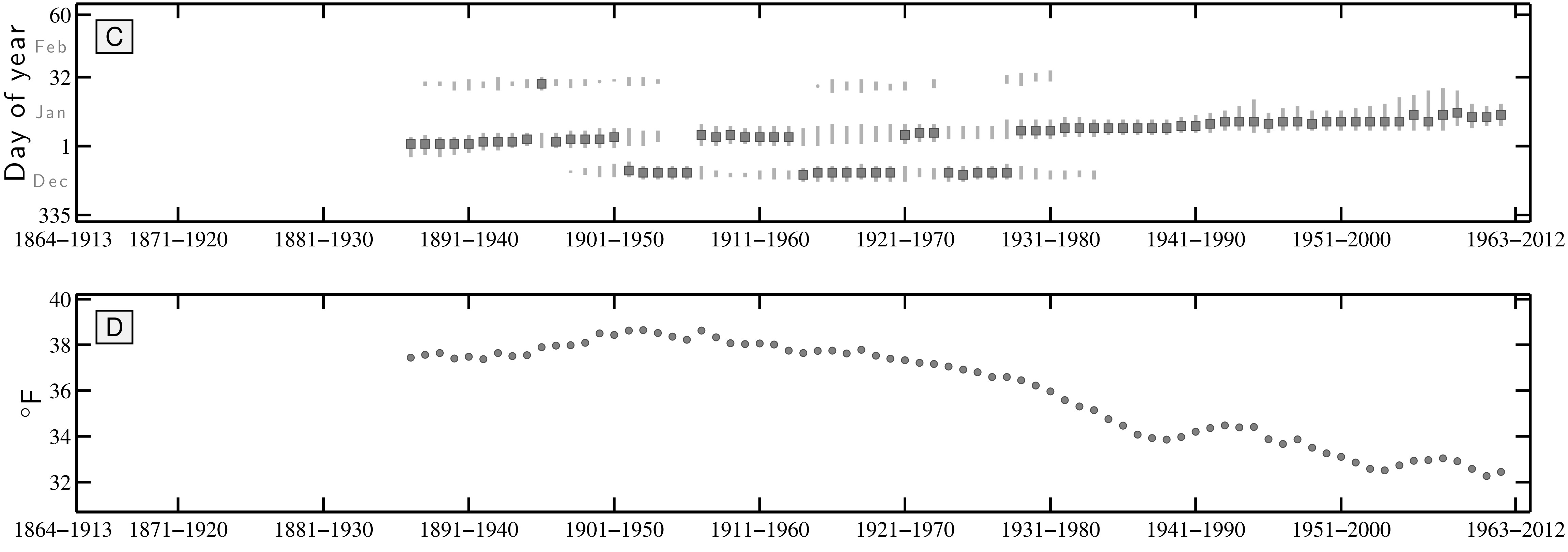

50 year Summer Teletherm dynamics for VALLEY HEAD, AL:

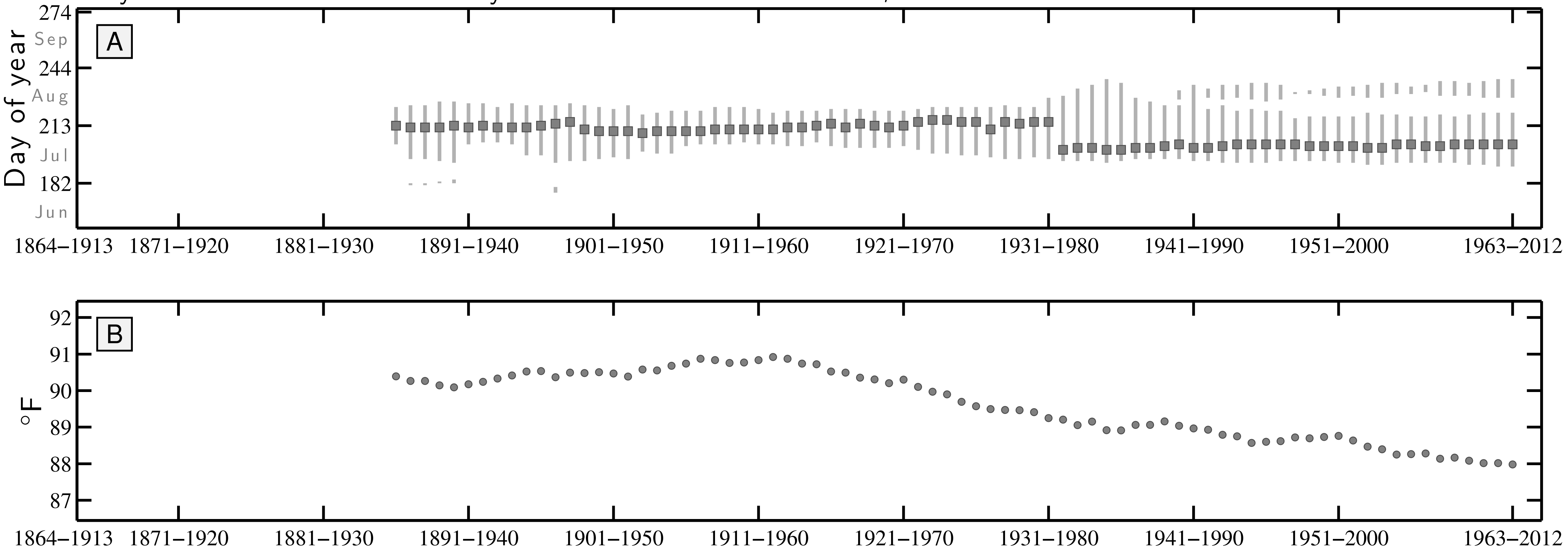

50 year Winter Teletherm dynamics for VALLEY HEAD, AL:

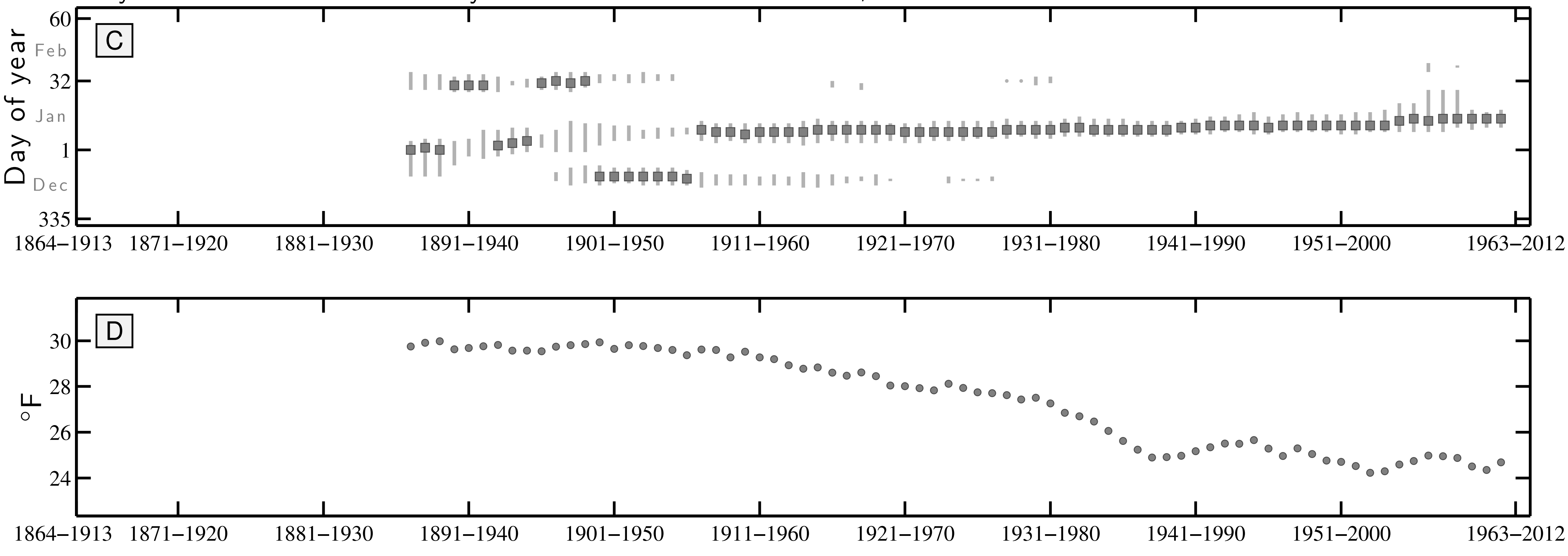

50 year Summer Teletherm dynamics for AJO, AZ:

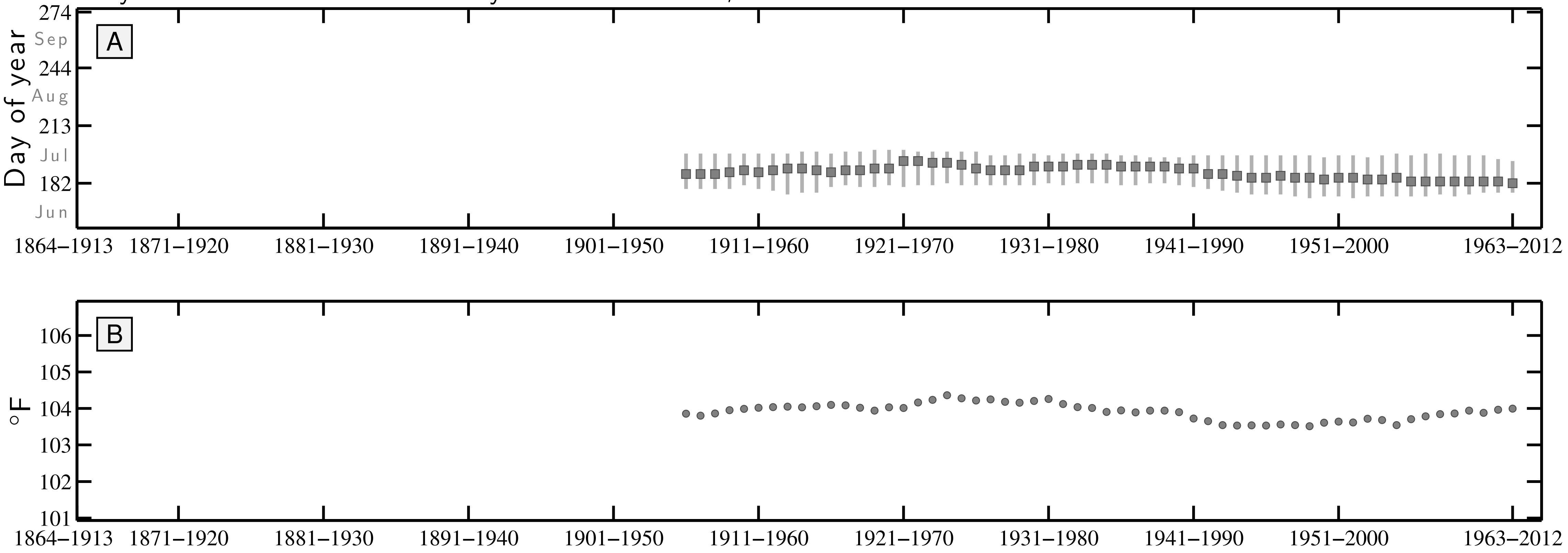

50 year Winter Teletherm dynamics for AJO, AZ:

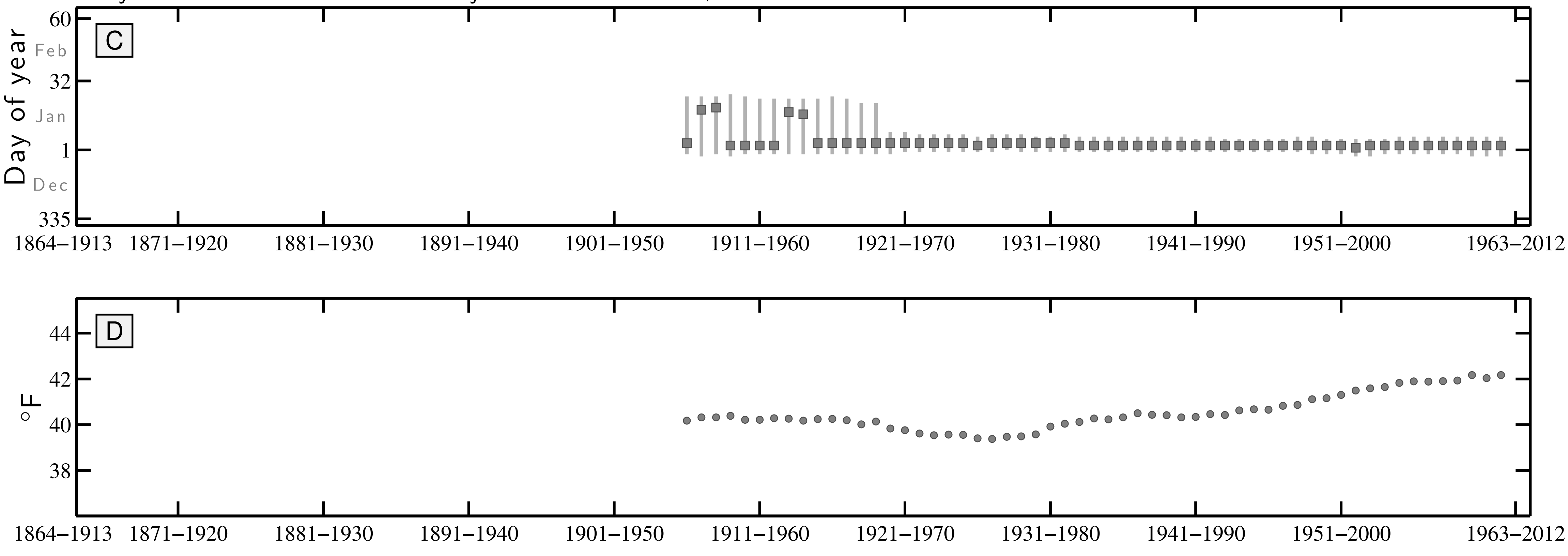

50 year Summer Teletherm dynamics for BUCKEYE, AZ:

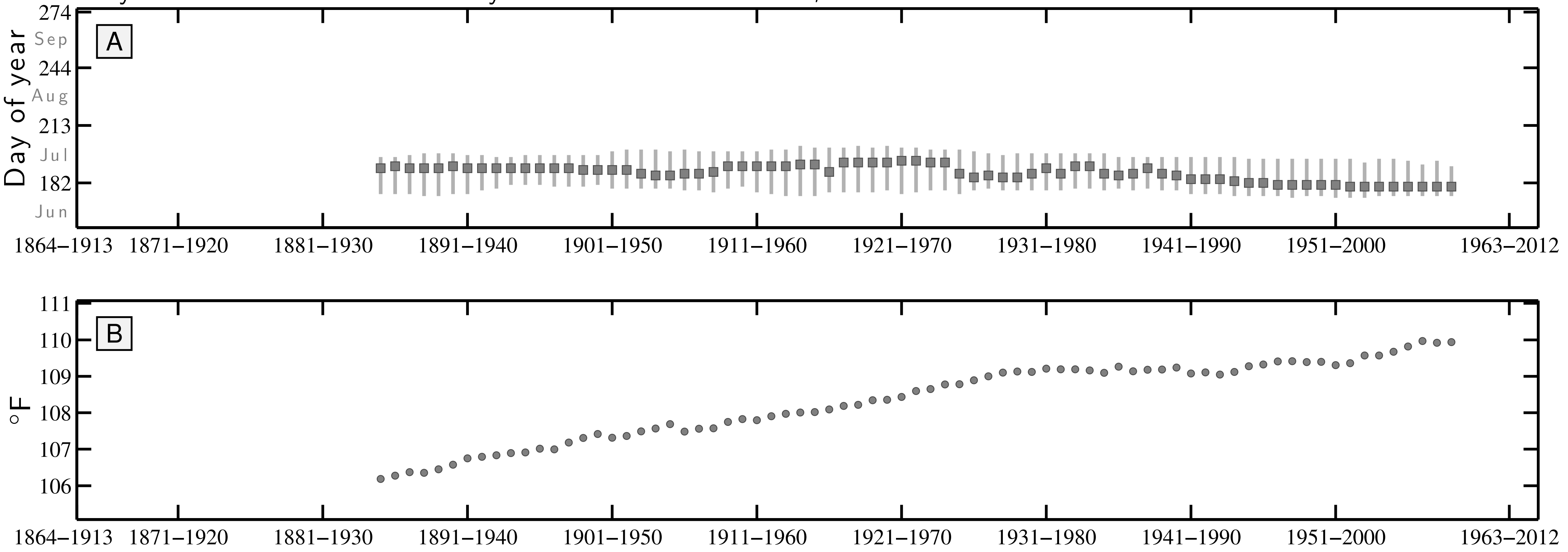

50 year Winter Teletherm dynamics for BUCKEYE, AZ:

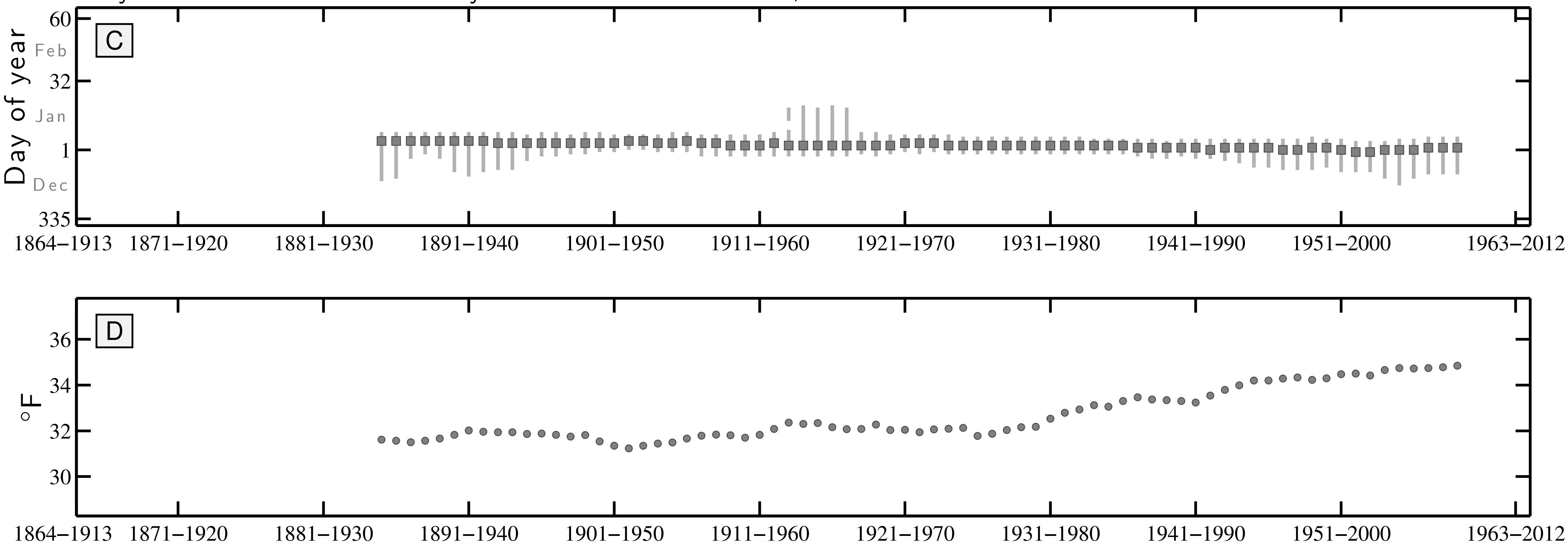

50 year Summer Teletherm dynamics for CHANDLER HEIGHTS, AZ:

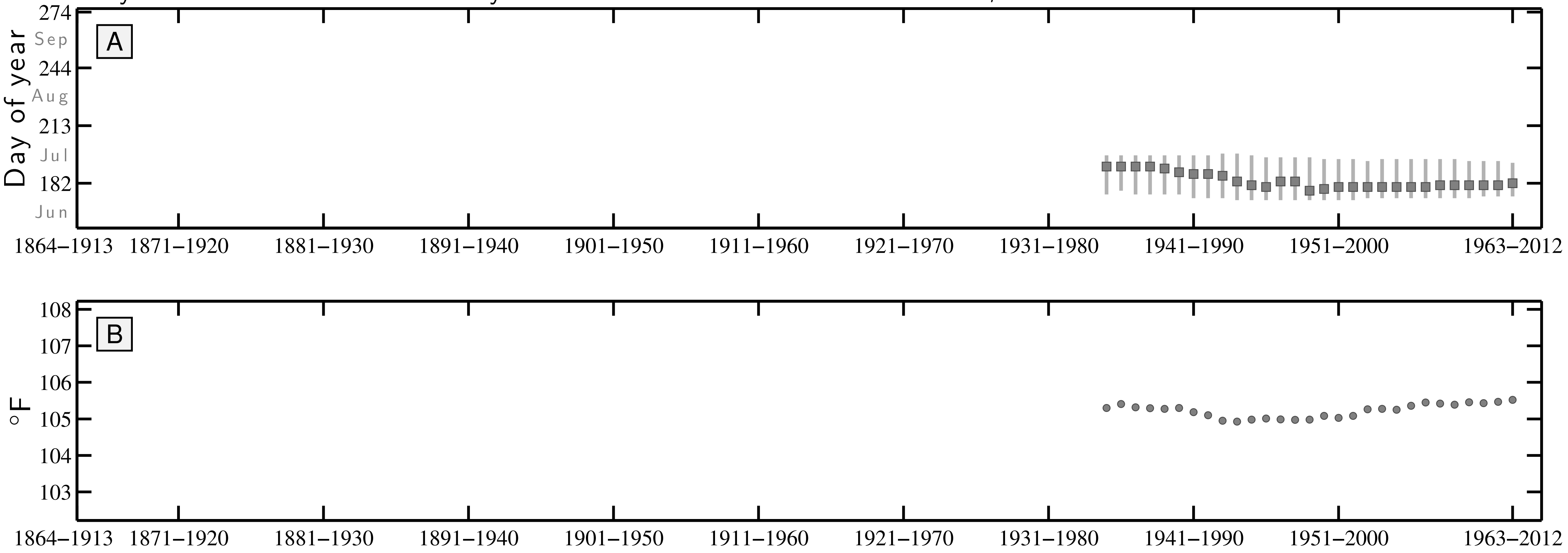

50 year Winter Teletherm dynamics for CHANDLER HEIGHTS, AZ:

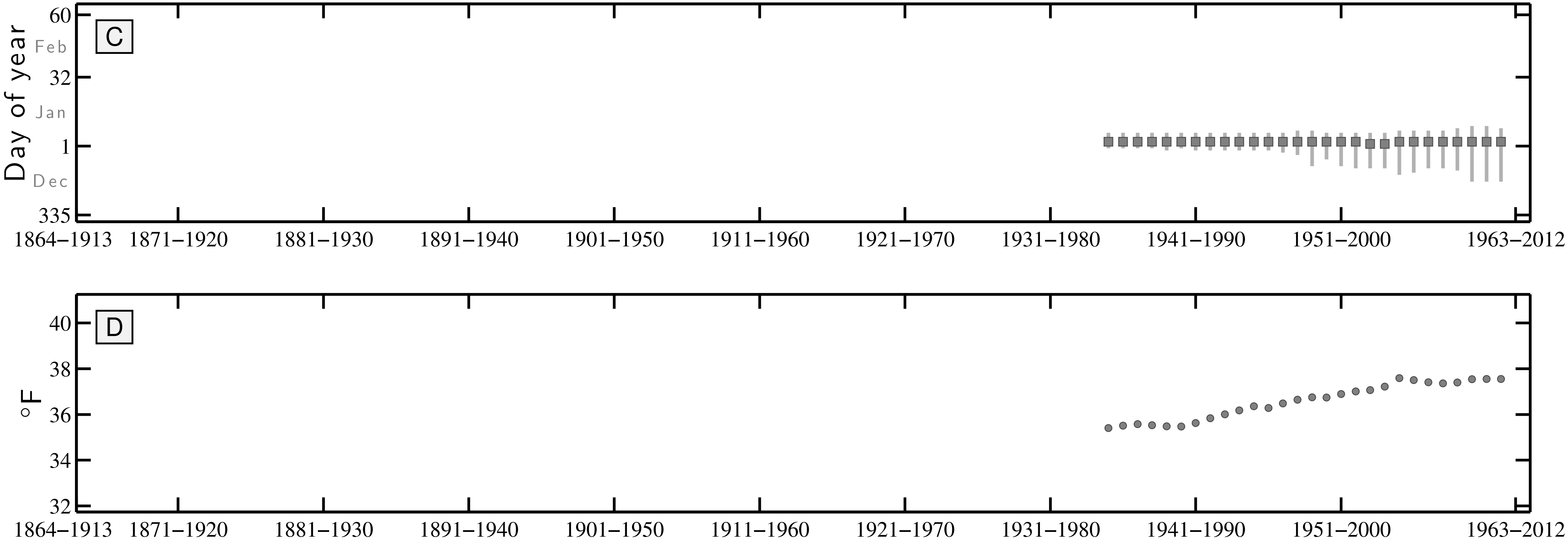

50 year Summer Teletherm dynamics for CHILDS, AZ:

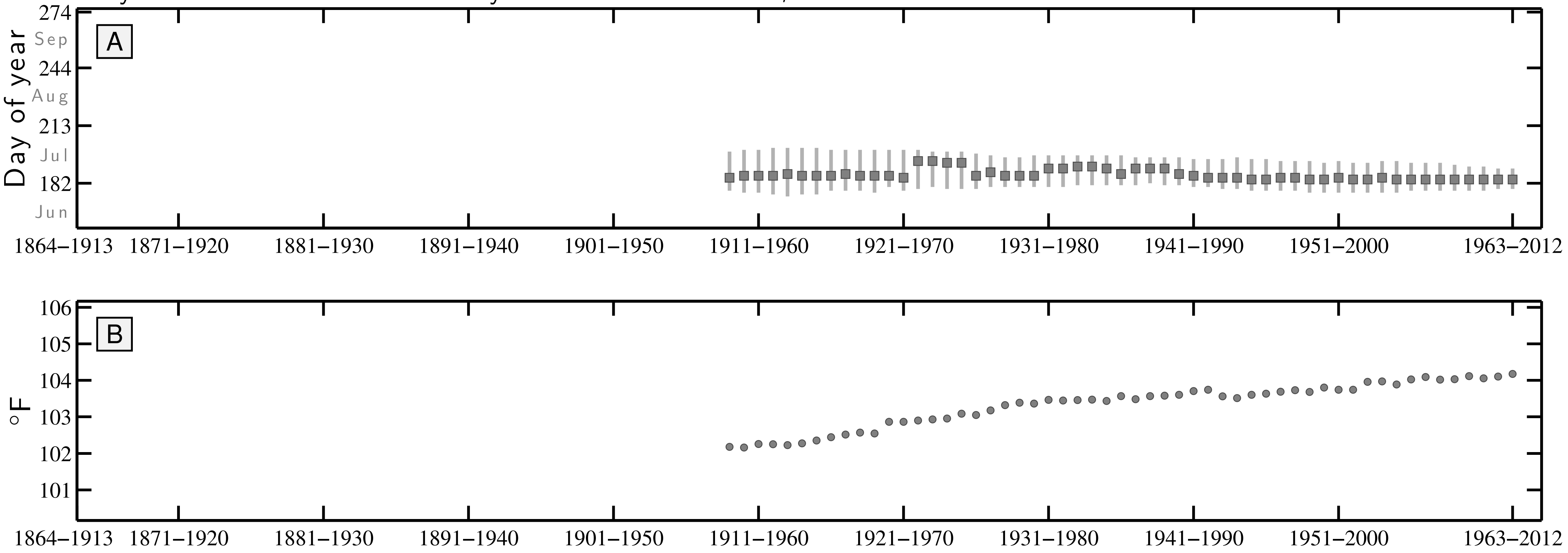

50 year Winter Teletherm dynamics for CHILDS, AZ:

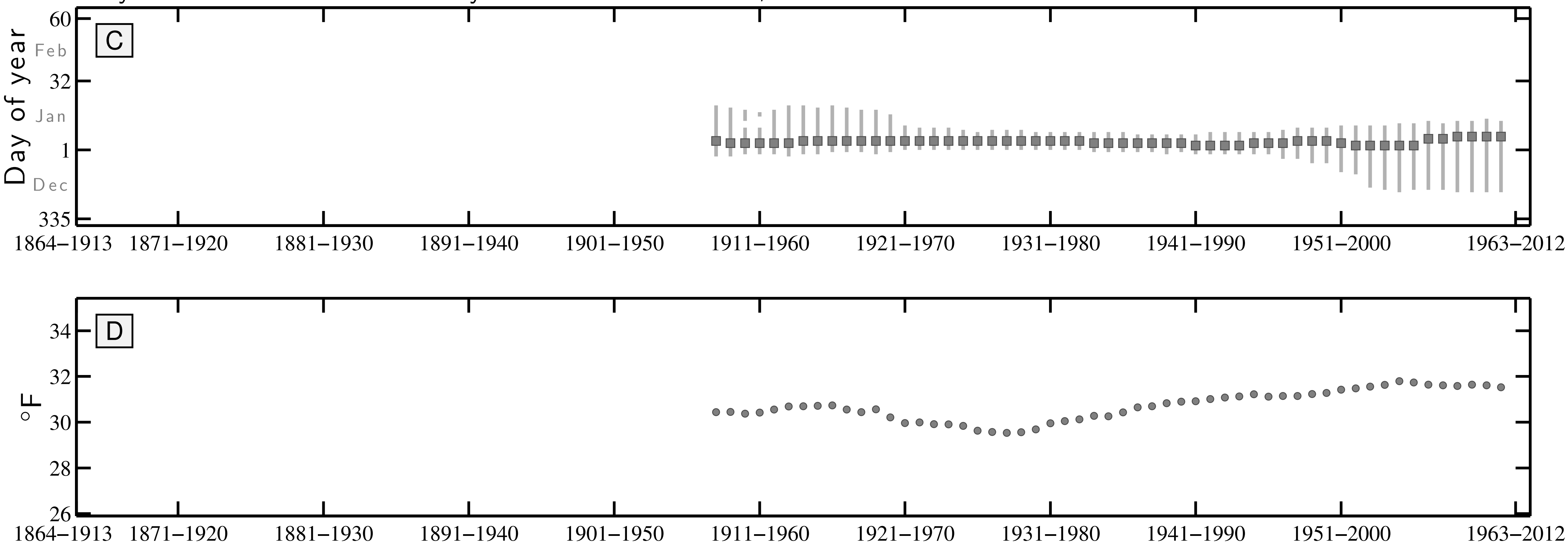

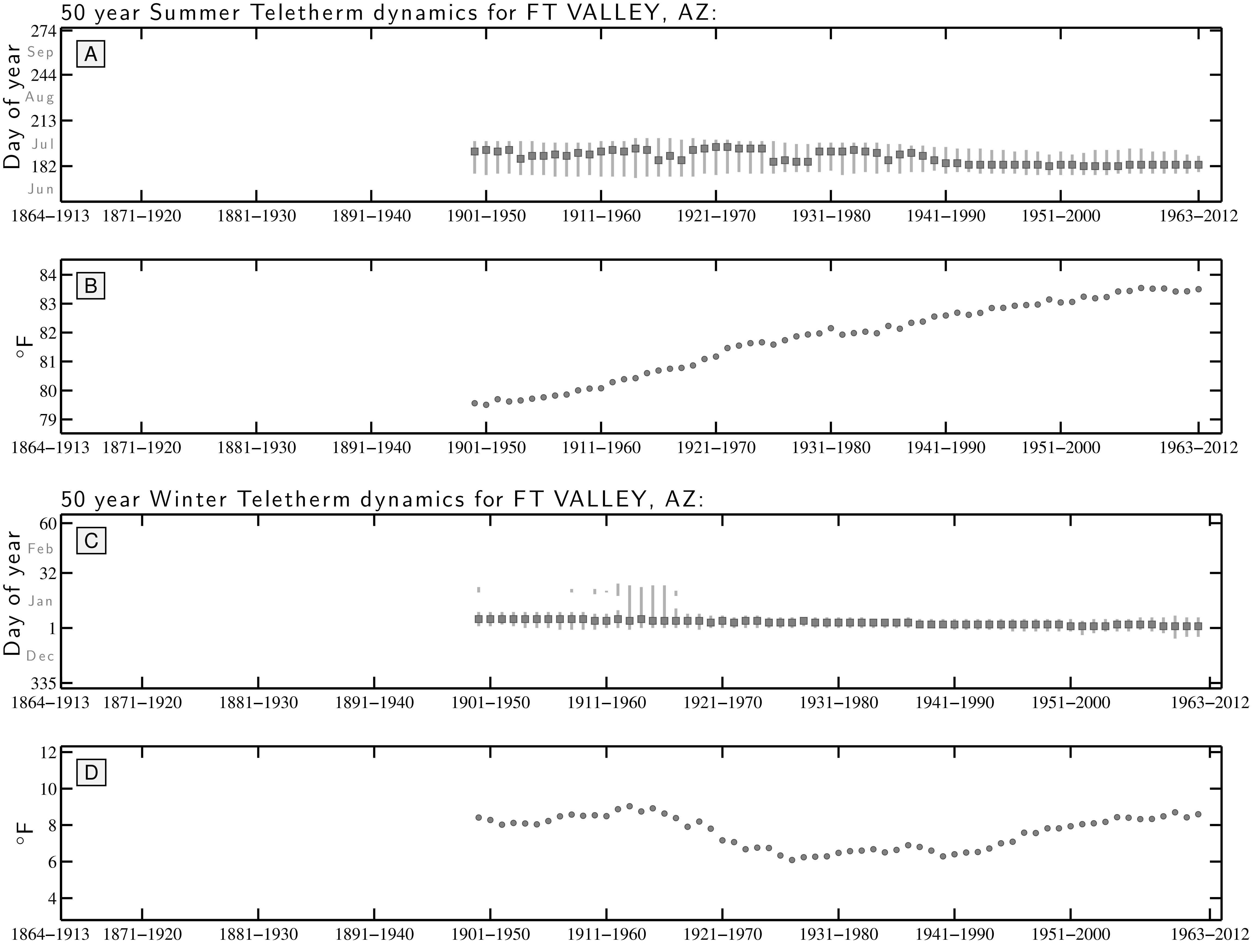
50 year Summer Teletherm dynamics for FT VALLEY, AZ:
A
B
Day of year
°F
Sep
Aug
Jul
Jun
274
244
213
182
84
83
82
81
80
79
1864–1913
1871–1920
1881–1930
1891–1940
1901–1950
1911–1960
1921–1970
1931–1980
1941–1990
1951–2000
1963–2012
50 year Winter Teletherm dynamics for FT VALLEY, AZ:
C
D
Feb
Jan
Dec
60
32
1
335
12
10
8
6
4

50 year Summer Teletherm dynamics for HOLBROOK, AZ:

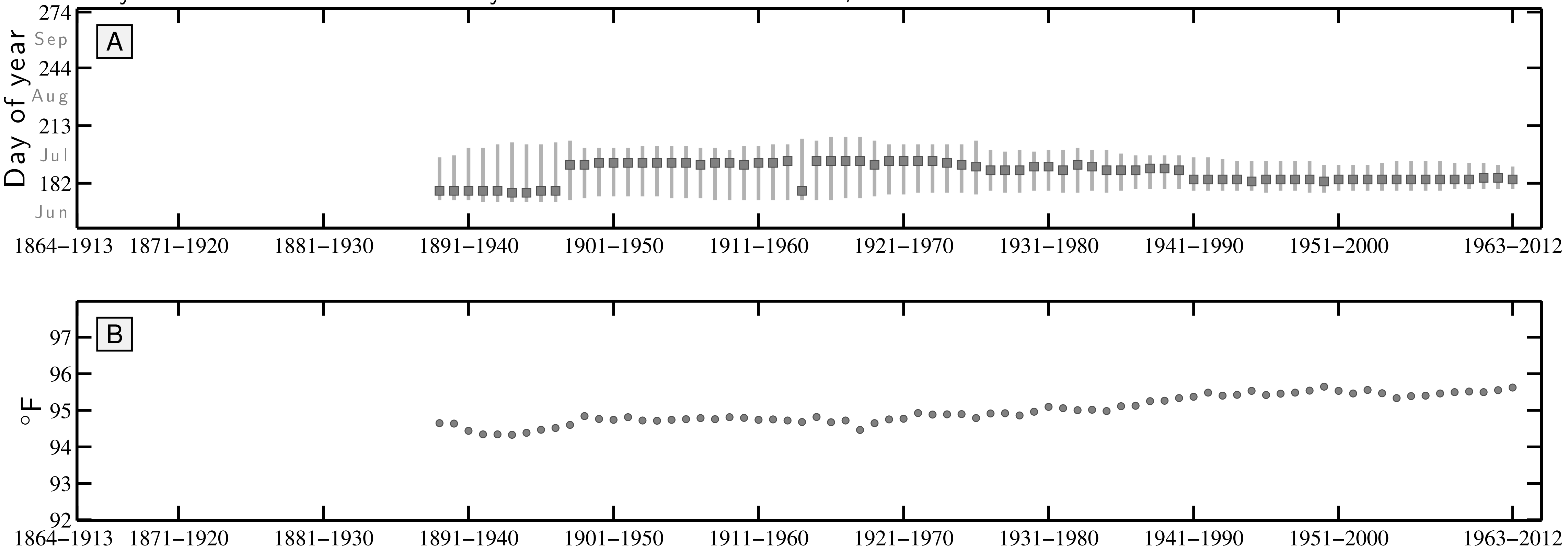

50 year Winter Teletherm dynamics for HOLBROOK, AZ:

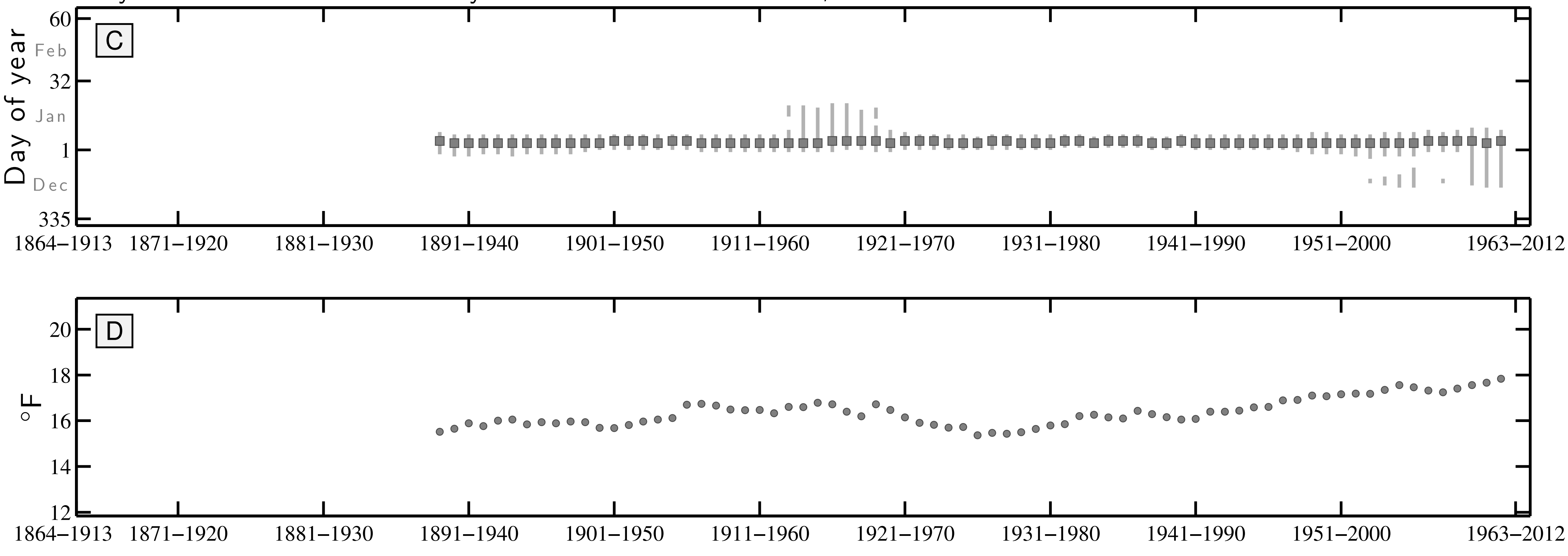

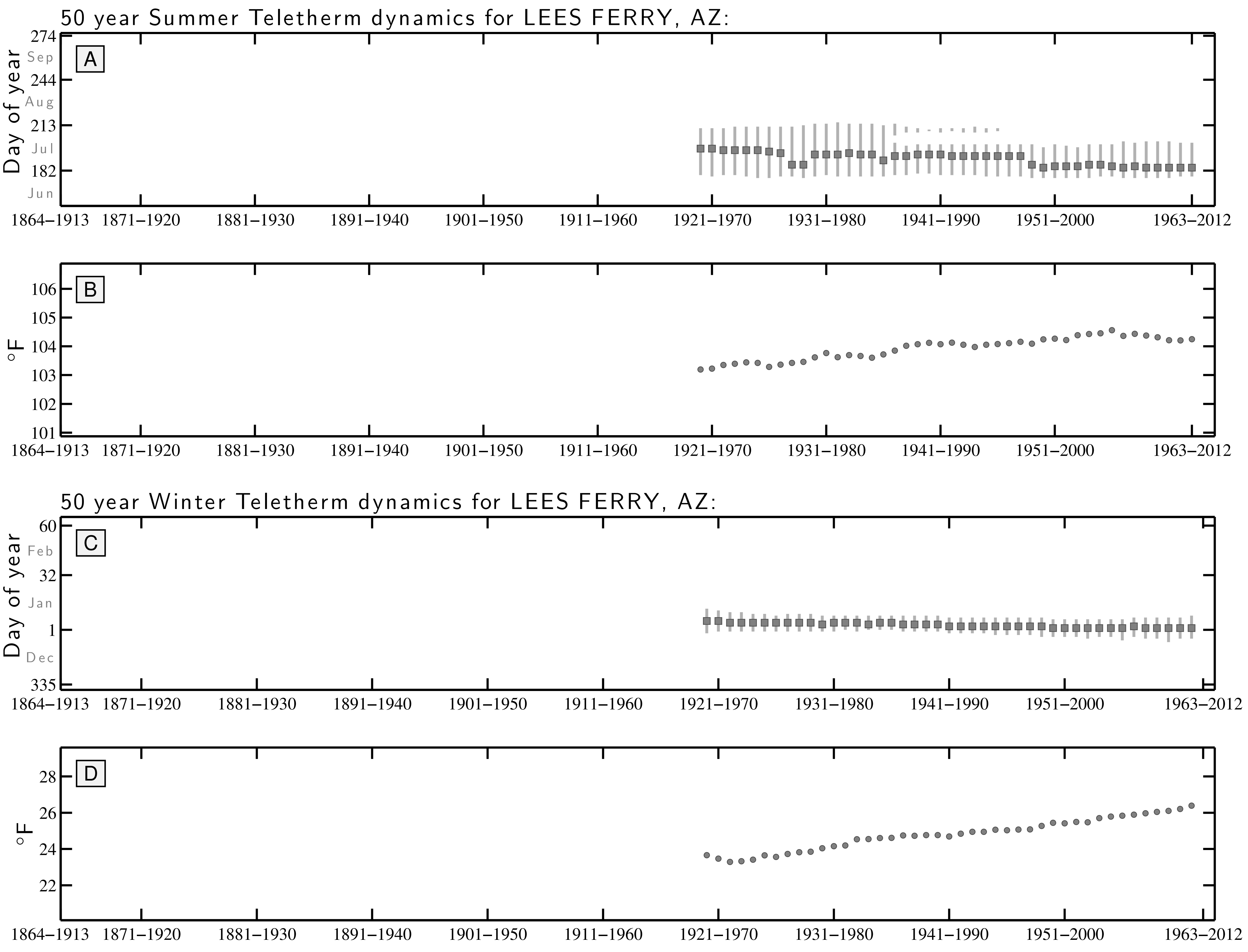
50 year Summer Teletherm dynamics for LEES FERRY, AZ:
A
B
50 year Winter Teletherm dynamics for LEES FERRY, AZ:
C
D
Day of year
°F
274
Sep
244
Aug
213
Jul
182
Jun
106
105
104
103
102
101
60
Feb
32
Jan
1
Dec
335
28
26
24
22
1864–1913
1871–1920
1881–1930
1891–1940
1901–1950
1911–1960
1921–1970
1931–1980
1941–1990
1951–2000
1963–2012

50 year Summer Teletherm dynamics for MIAMI, AZ:

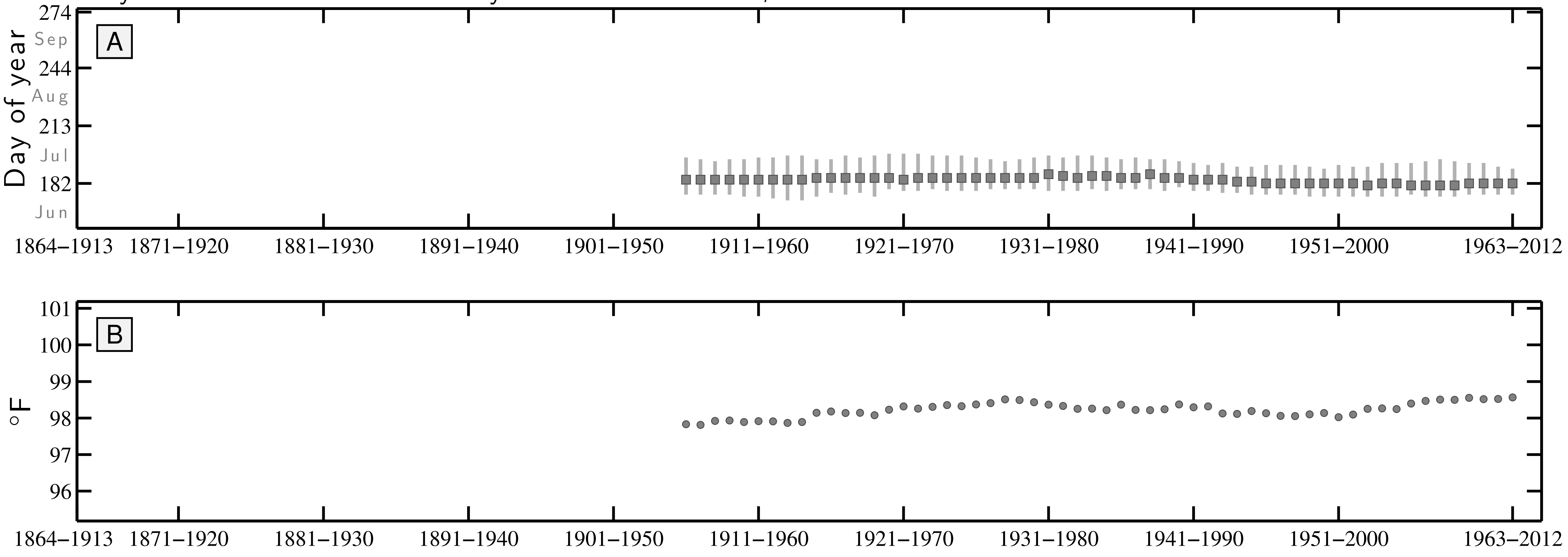

50 year Winter Teletherm dynamics for MIAMI, AZ:

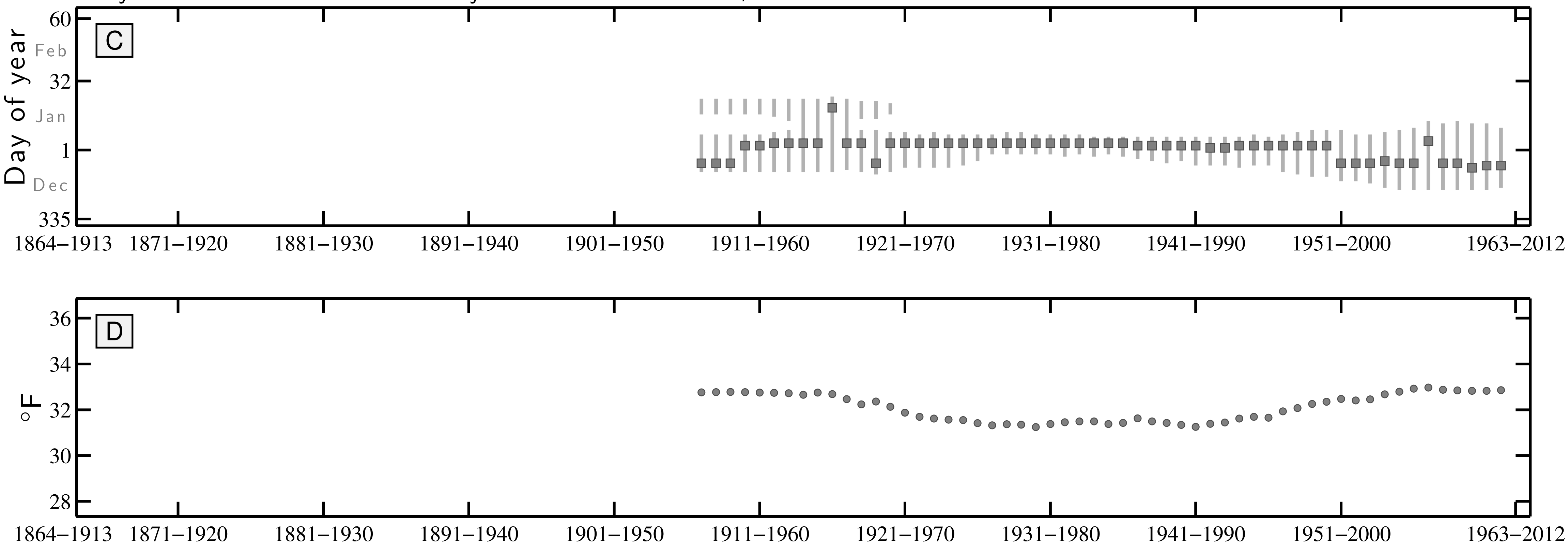

50 year Summer Teletherm dynamics for PARKER, AZ:

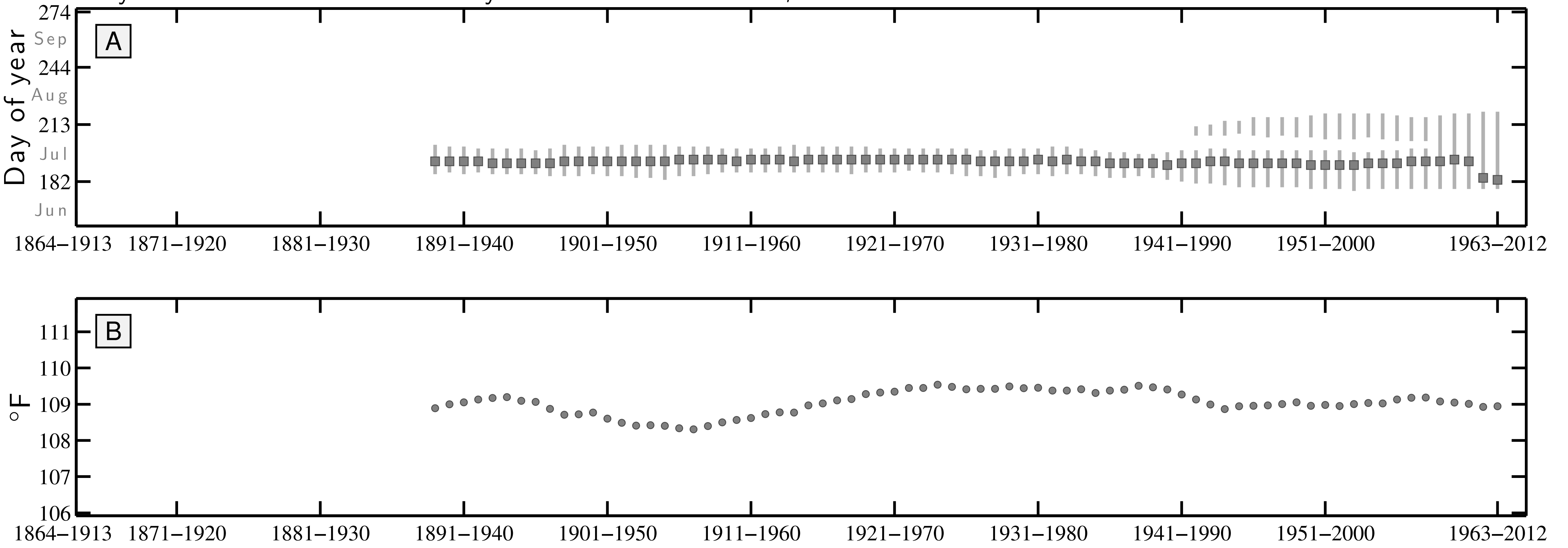

50 year Winter Teletherm dynamics for PARKER, AZ:

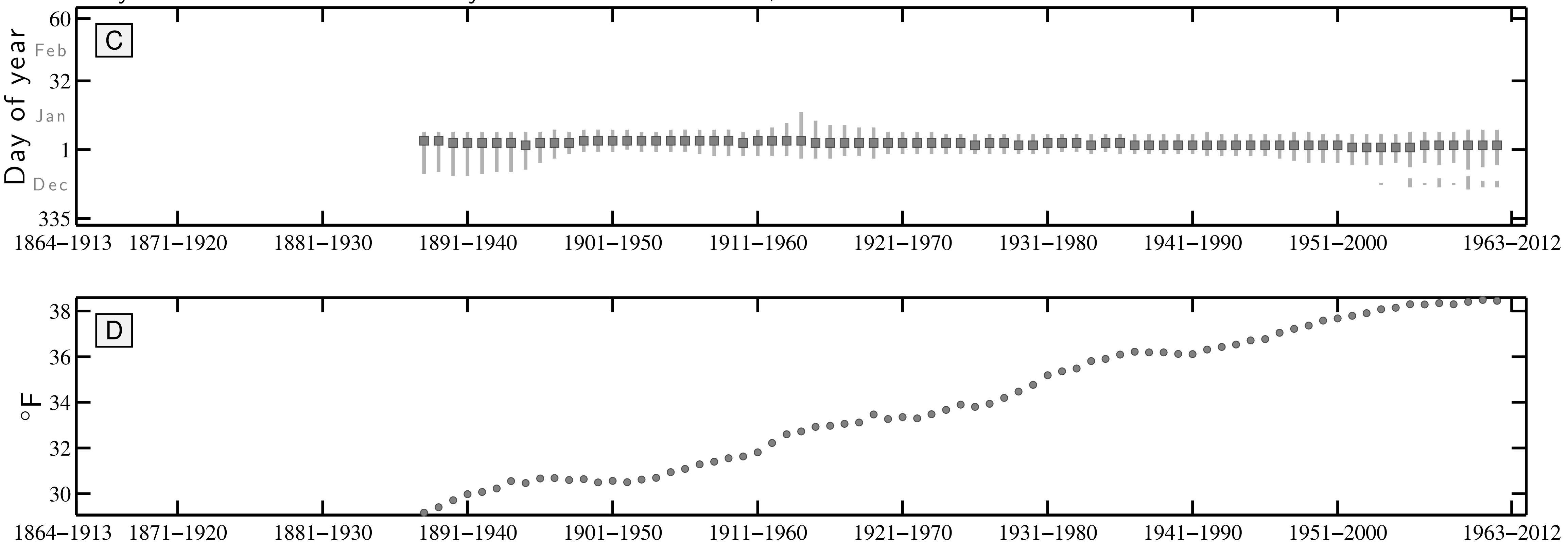

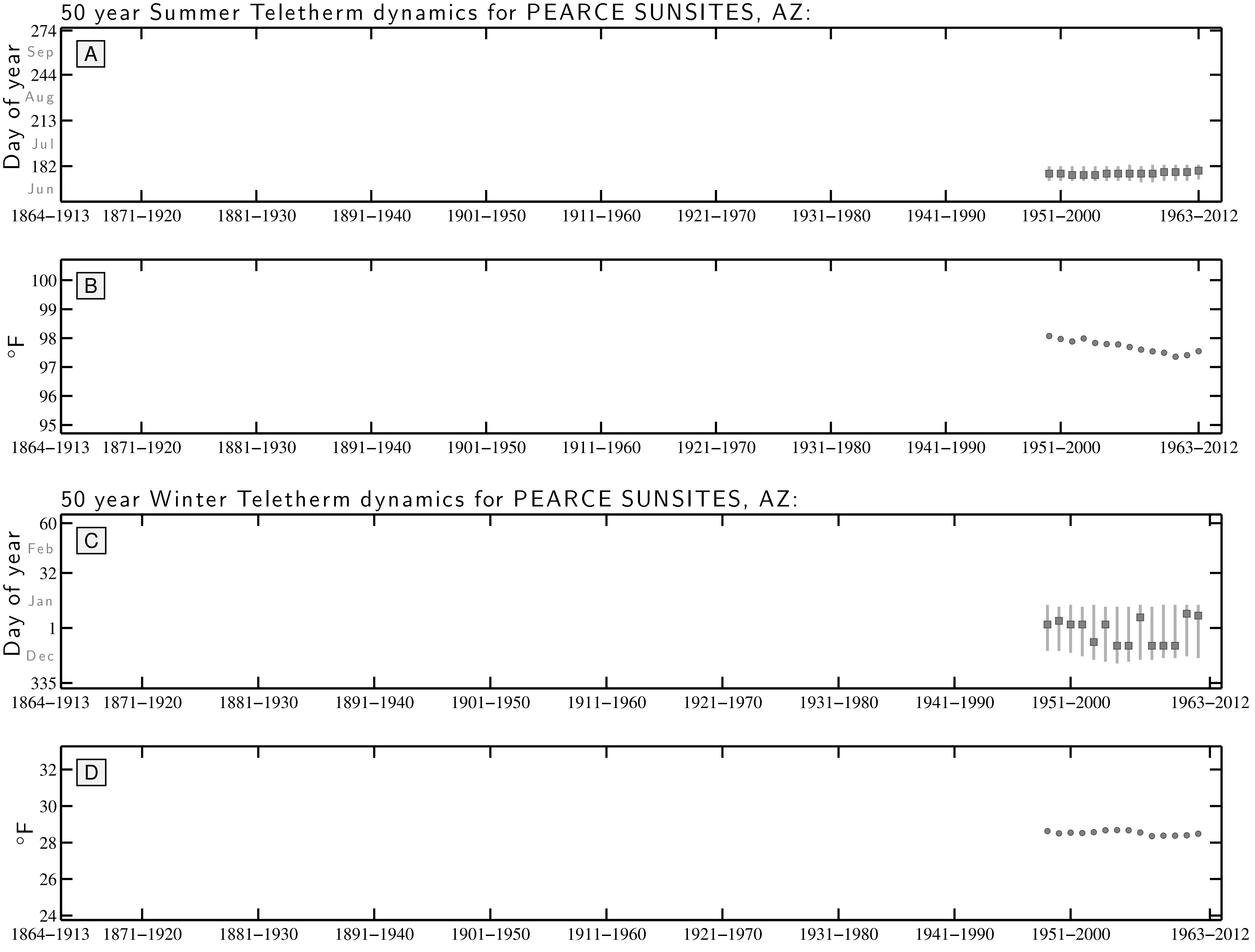
50 year Summer Teletherm dynamics for PEARCE SUNSITES, AZ:
A
B
Day of year
°F
274
Sep
244
Aug
213
Jul
182
Jun
100
99
98
97
96
95
1864–1913
1871–1920
1881–1930
1891–1940
1901–1950
1911–1960
1921–1970
1931–1980
1941–1990
1951–2000
1963–2012
50 year Winter Teletherm dynamics for PEARCE SUNSITES, AZ:
C
D
60
Feb
32
Jan
1
Dec
335
32
30
28
26
24

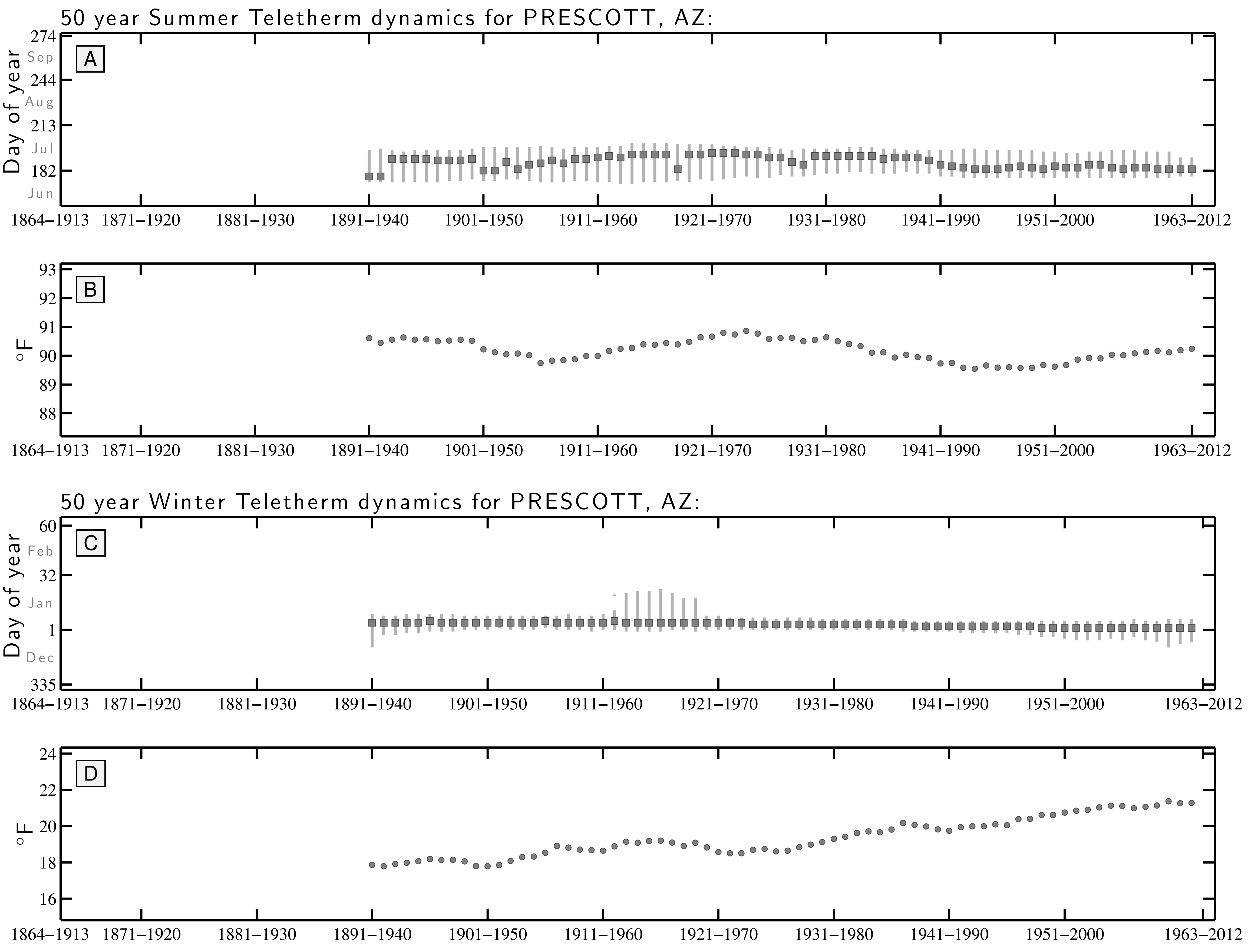
50 year Summer Teletherm dynamics for PRESCOTT, AZ:
A
Day of year
274
Sep
244
Aug
213
Jul
182
Jun
1864–1913
1871–1920
1881–1930
1891–1940
1901–1950
1911–1960
1921–1970
1931–1980
1941–1990
1951–2000
1963–2012
B
°F
93
92
91
90
89
88
50 year Winter Teletherm dynamics for PRESCOTT, AZ:
C
60
Feb
32
Jan
1
Dec
335
D
24
22
20
18
16

50 year Summer Teletherm dynamics for ROOSEVELT 1 WNW, AZ:

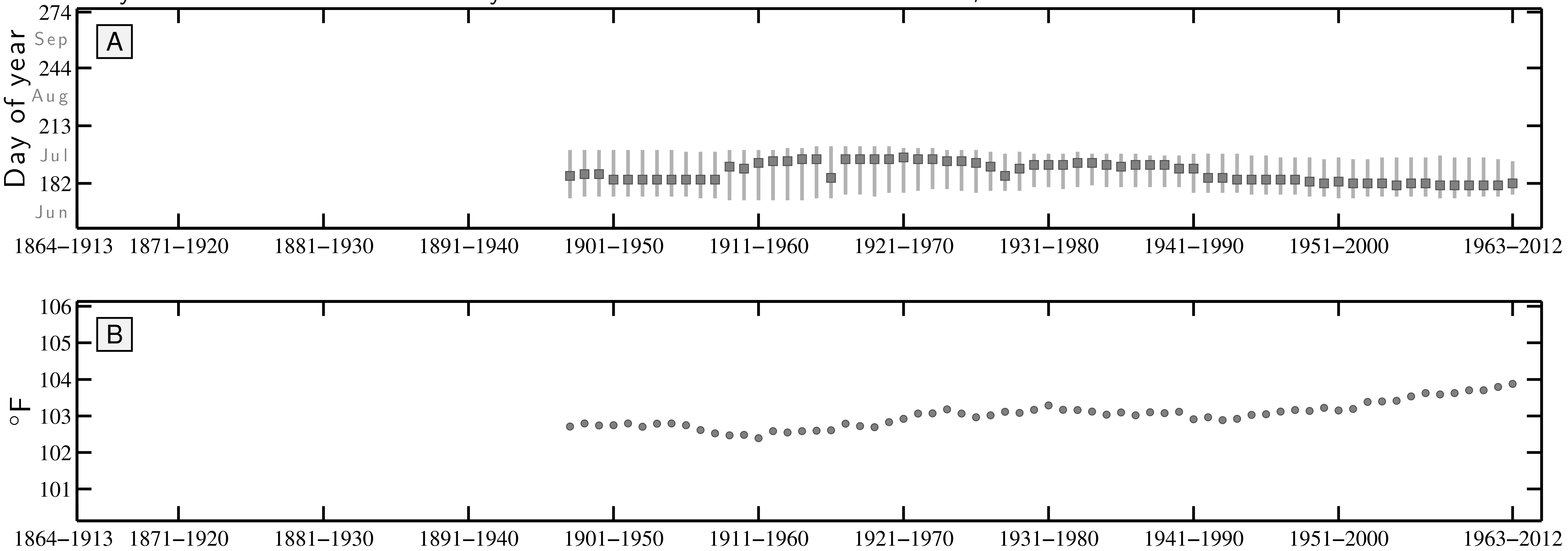

50 year Winter Teletherm dynamics for ROOSEVELT 1 WNW, AZ:

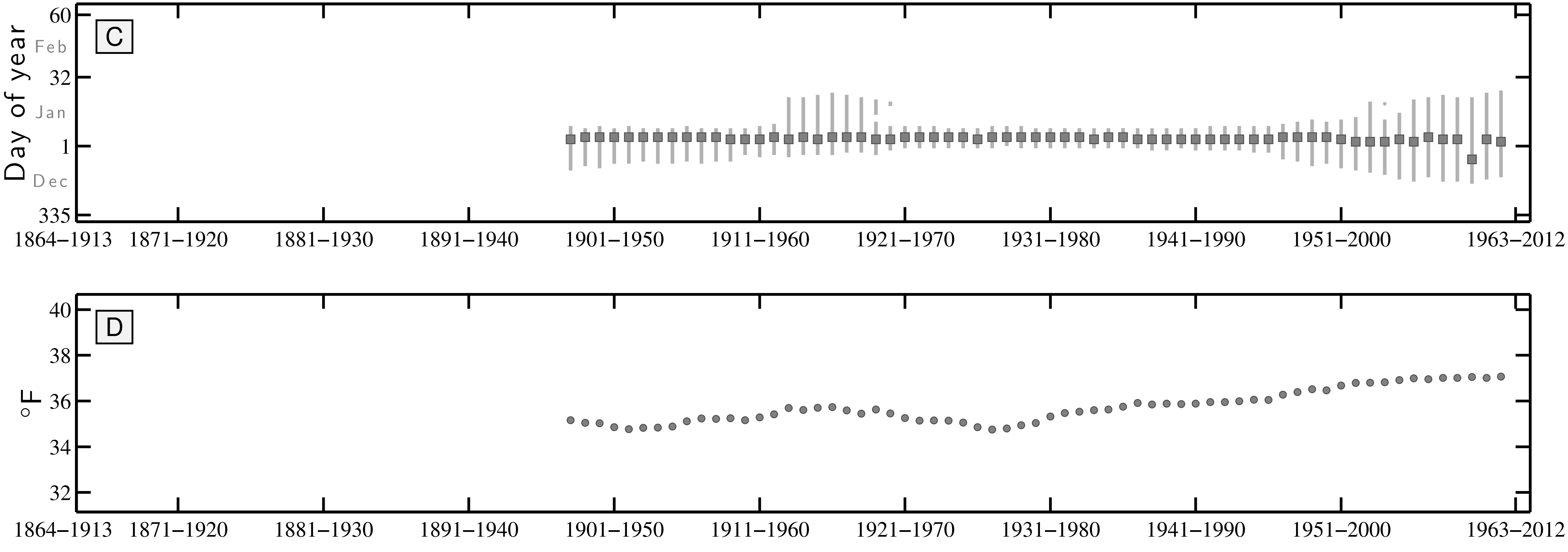

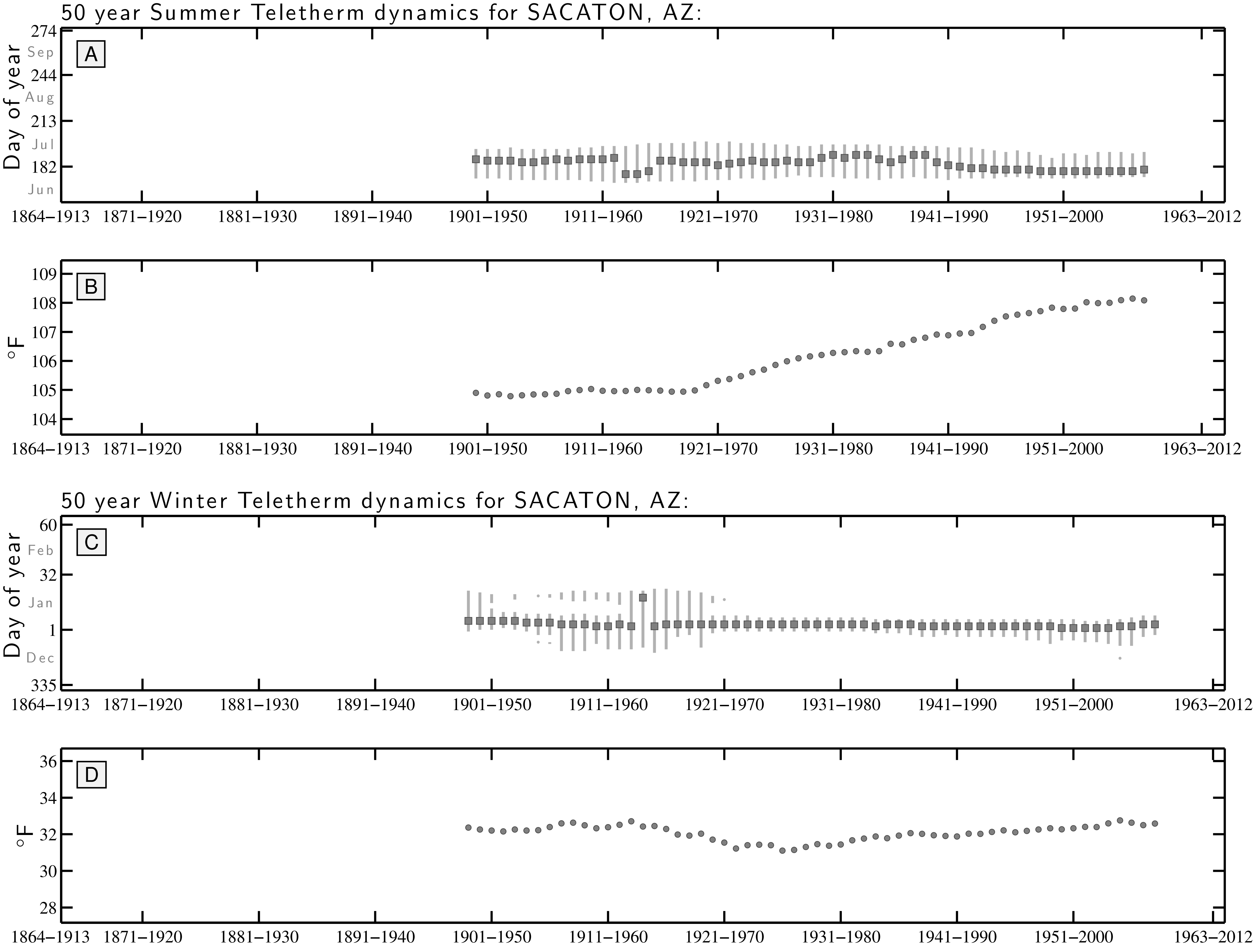

50 year Summer Teletherm dynamics for SACATON, AZ:
A
Day of year
274
Sep
244
Aug
213
Jul
182
Jun
1864–1913
1871–1920
1881–1930
1891–1940
1901–1950
1911–1960
1921–1970
1931–1980
1941–1990
1951–2000
1963–2012
B
°F
109
108
107
106
105
104
50 year Winter Teletherm dynamics for SACATON, AZ:
C
60
Feb
32
Jan
1
Dec
335
D
36
34
32
30
28

50 year Summer Teletherm dynamics for SAFFORD AGRICULTRL CTR, AZ:

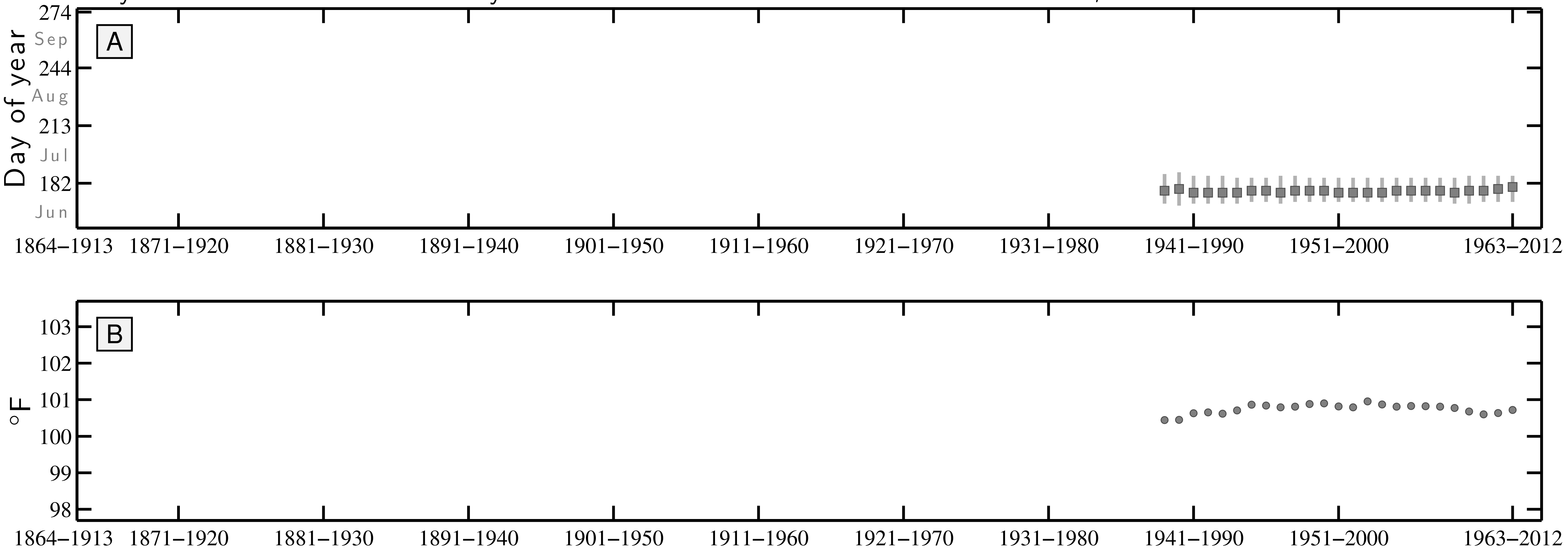

50 year Winter Teletherm dynamics for SAFFORD AGRICULTRL CTR, AZ:

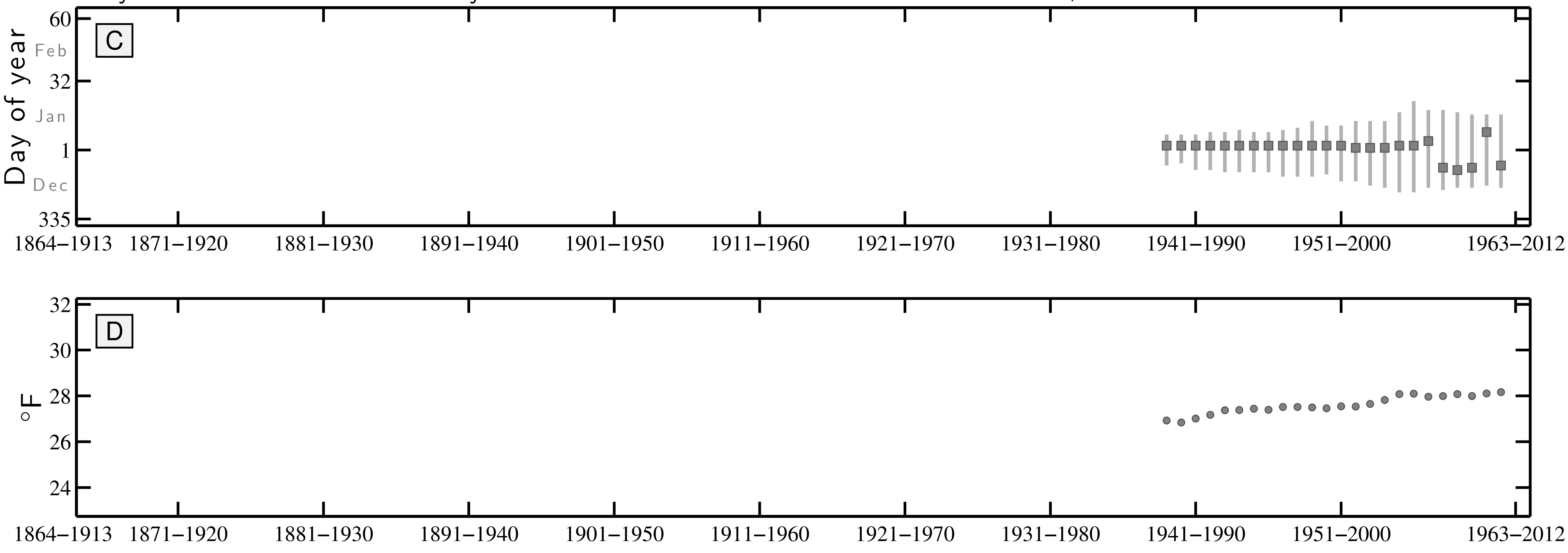

50 year Summer Teletherm dynamics for SAINT JOHNS, AZ:

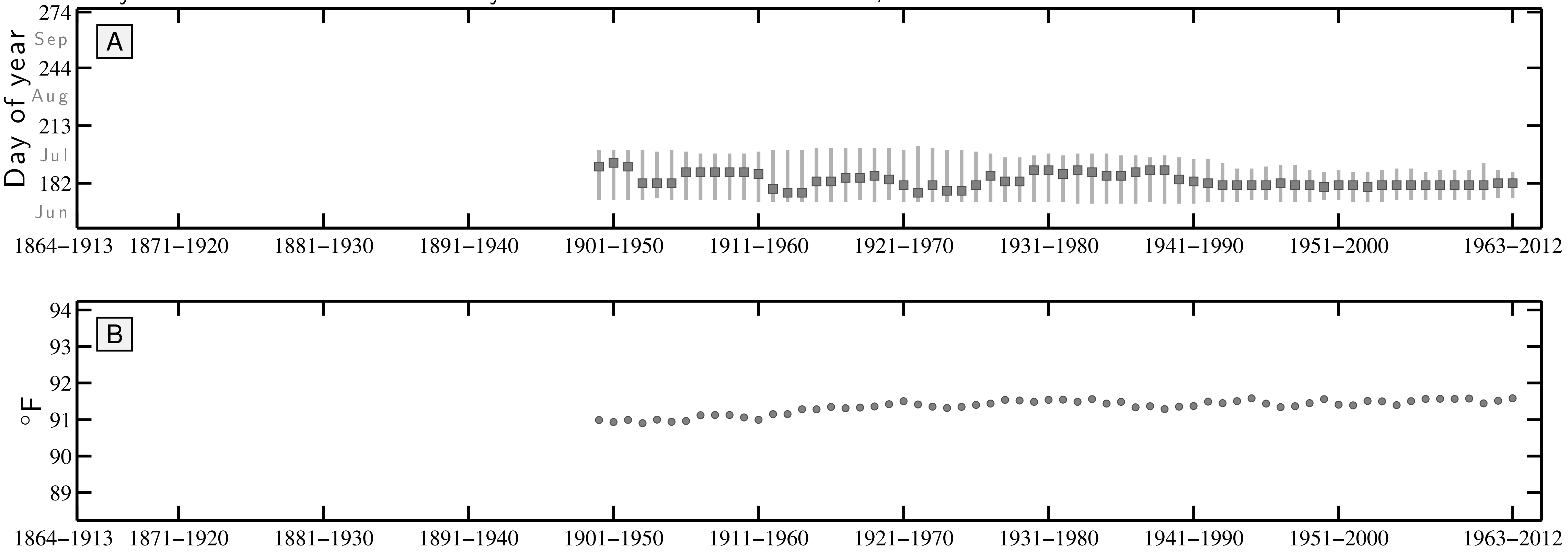

50 year Winter Teletherm dynamics for SAINT JOHNS, AZ:

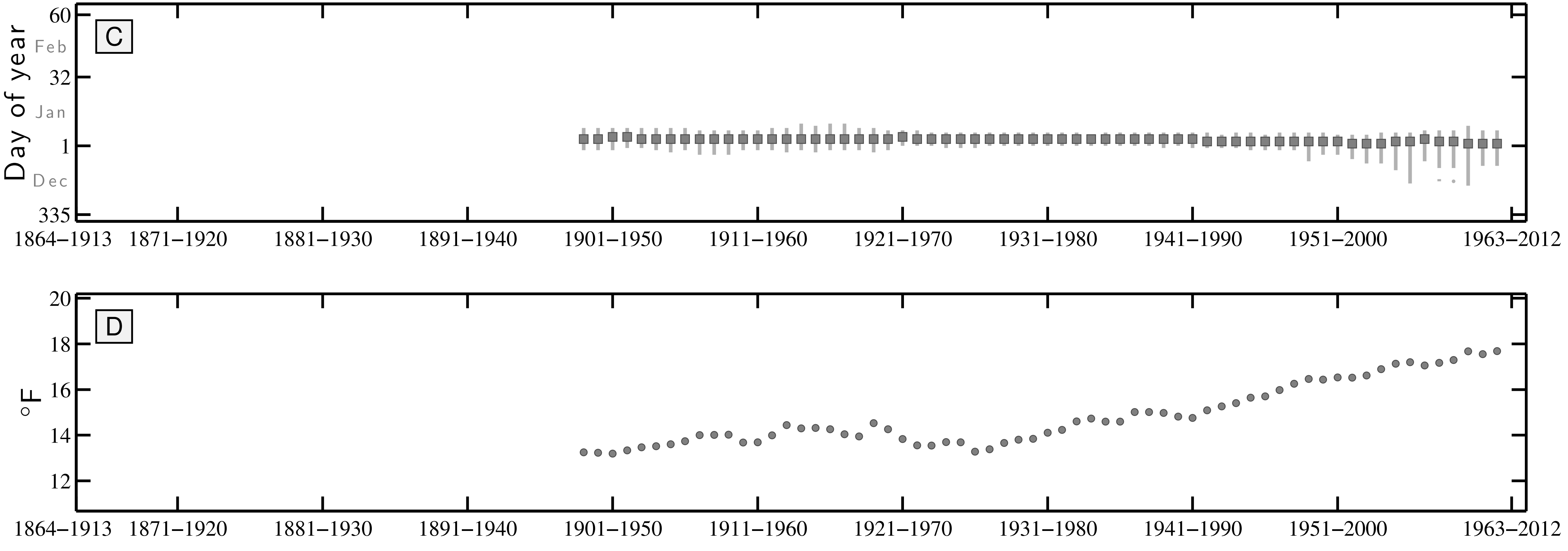

50 year Summer Teletherm dynamics for SELIGMAN, AZ:

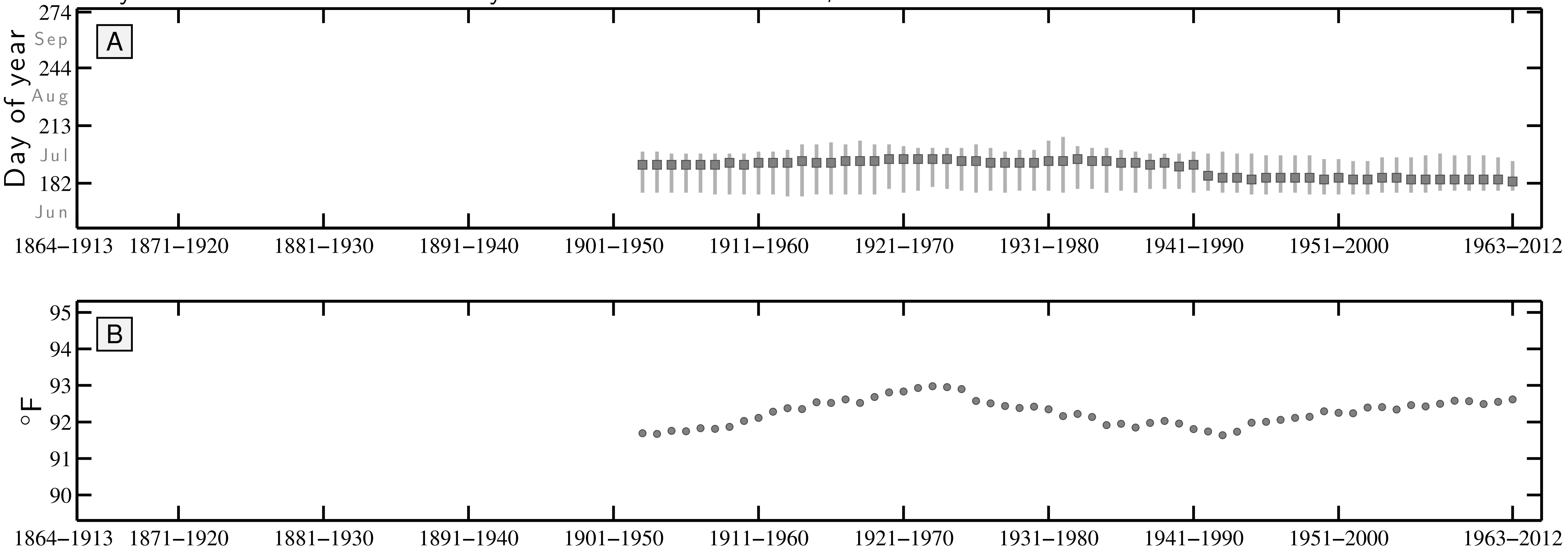

50 year Winter Teletherm dynamics for SELIGMAN, AZ:

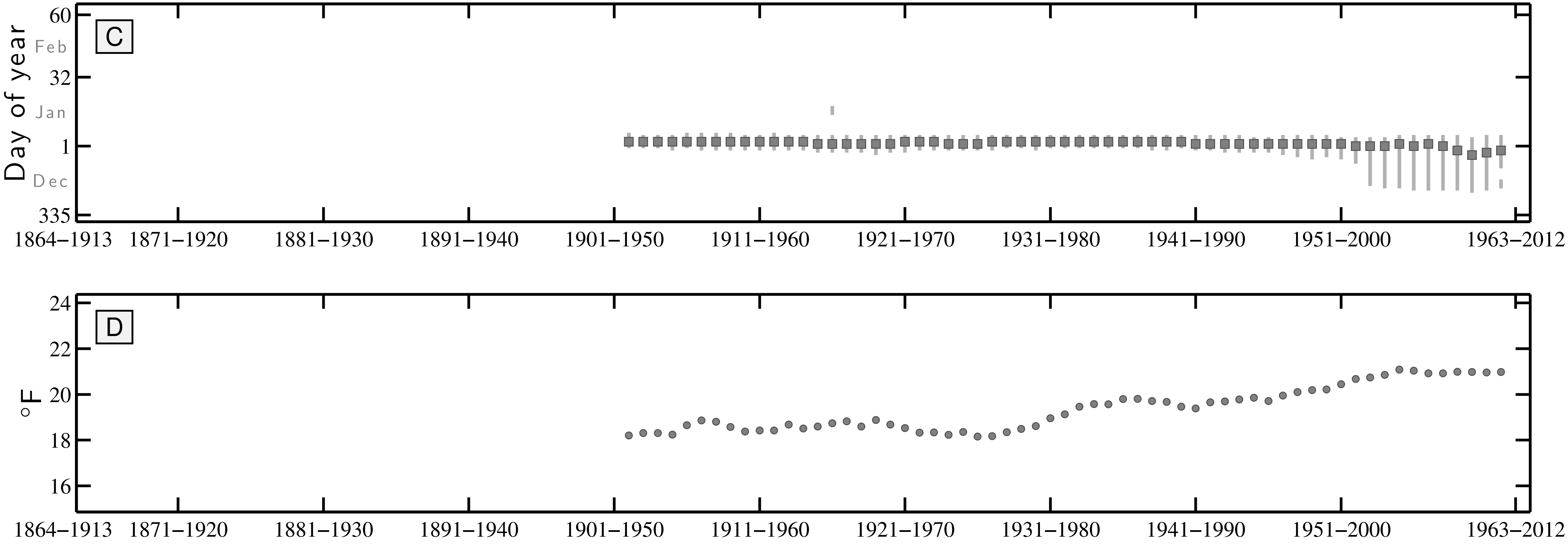

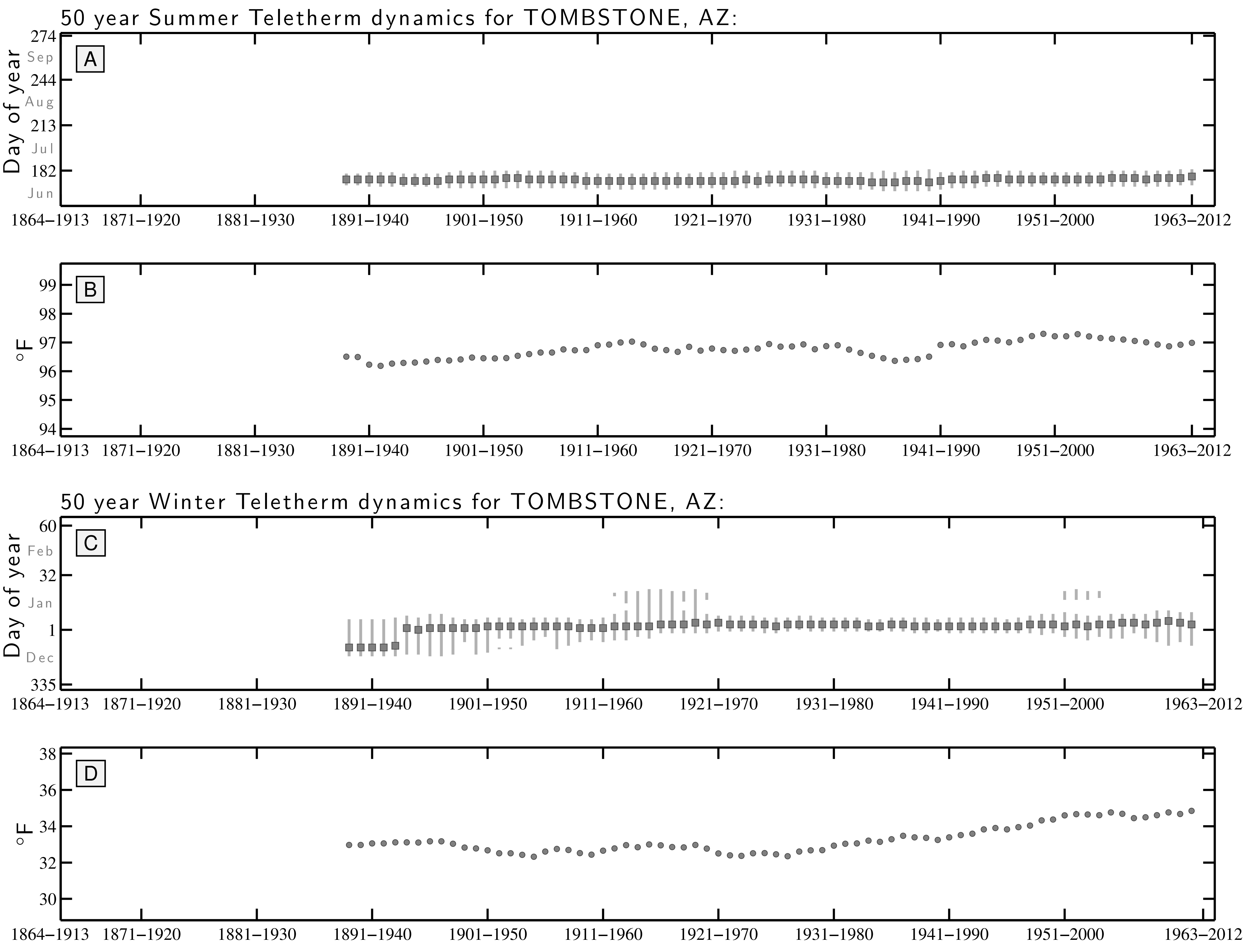
50 year Summer Teletherm dynamics for TOMBSTONE, AZ:
A
Day of year
274
Sep
244
Aug
213
Jul
182
Jun
1864–1913
1871–1920
1881–1930
1891–1940
1901–1950
1911–1960
1921–1970
1931–1980
1941–1990
1951–2000
1963–2012
B
°F
99
98
97
96
95
94
50 year Winter Teletherm dynamics for TOMBSTONE, AZ:
C
60
Feb
32
Jan
1
Dec
335
D
38
36
34
32
30

50 year Summer Teletherm dynamics for TUCSON WFO, AZ:

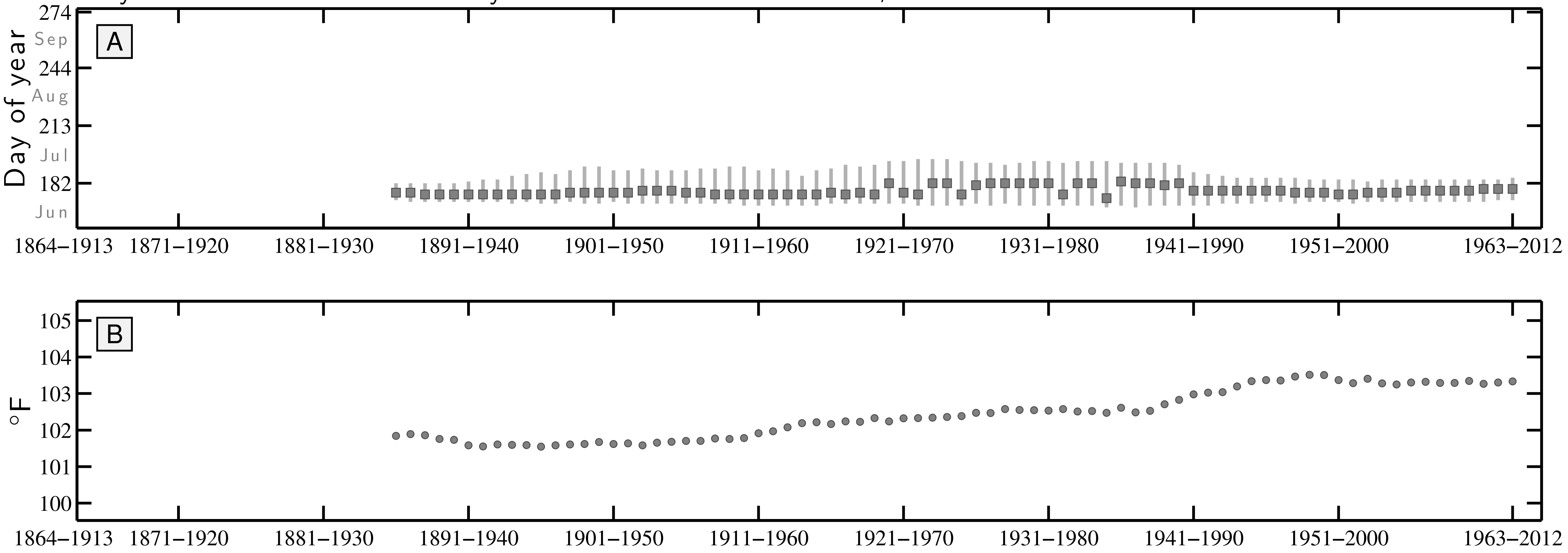

50 year Winter Teletherm dynamics for TUCSON WFO, AZ:

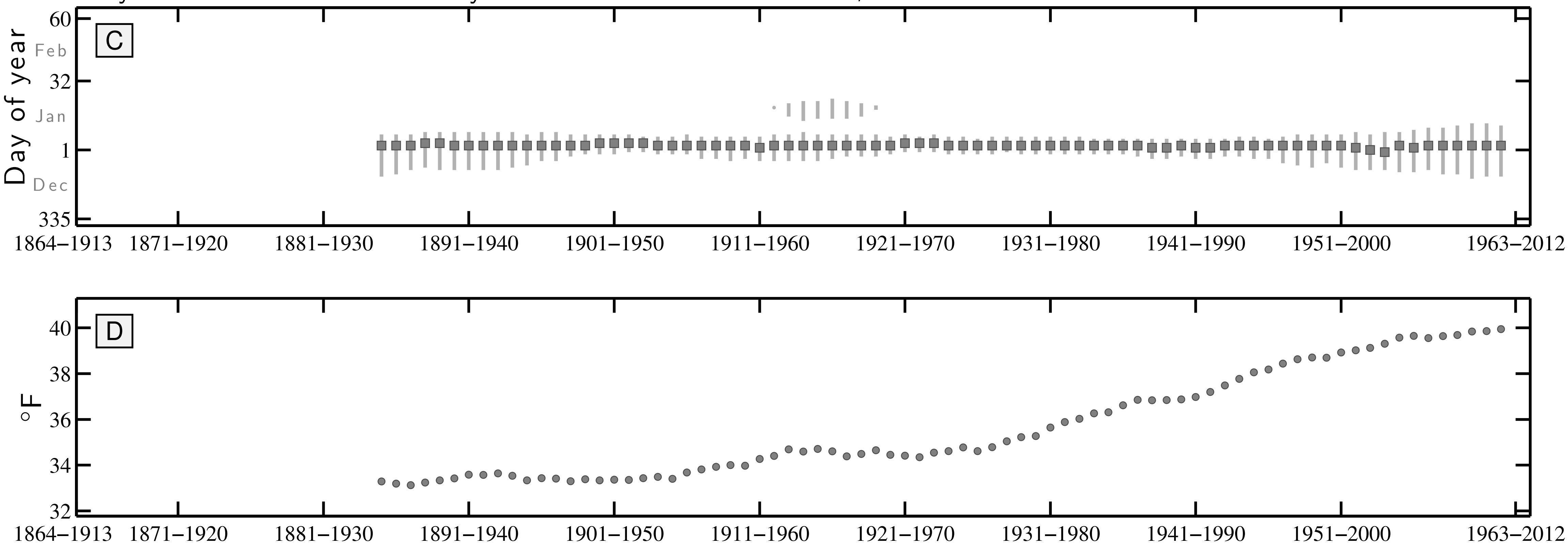

50 year Summer Teletherm dynamics for WHITERIVER 1 SW, AZ:

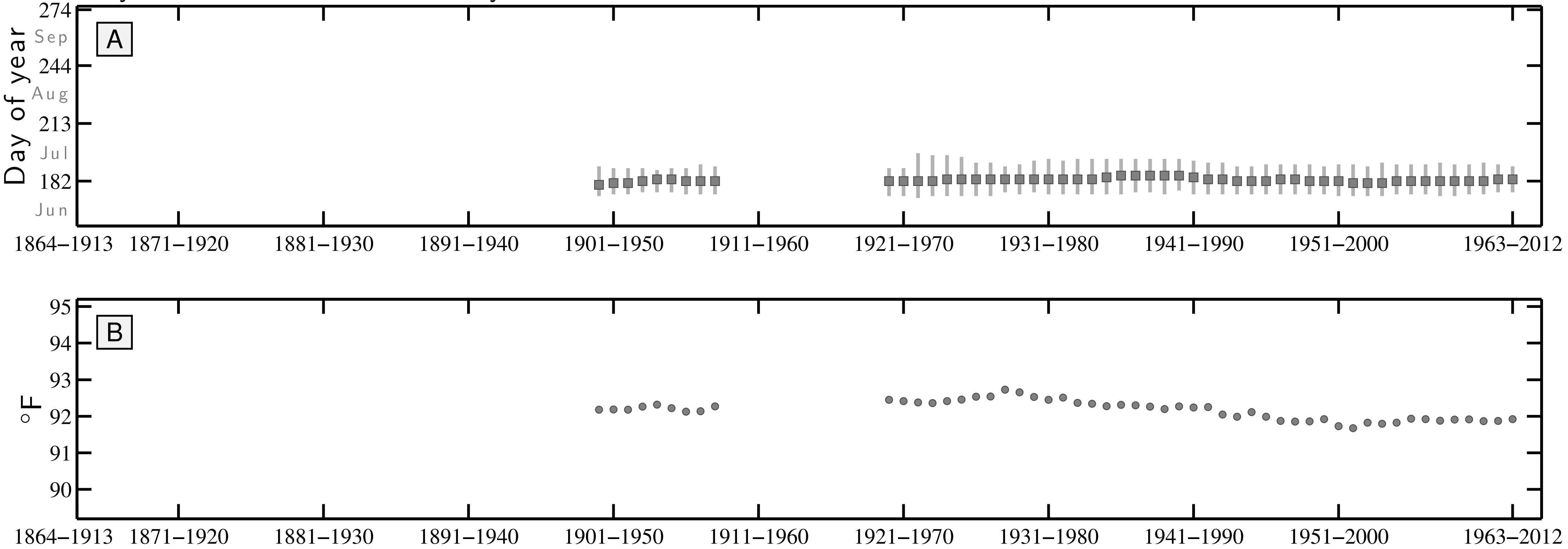

50 year Winter Teletherm dynamics for WHITERIVER 1 SW, AZ:

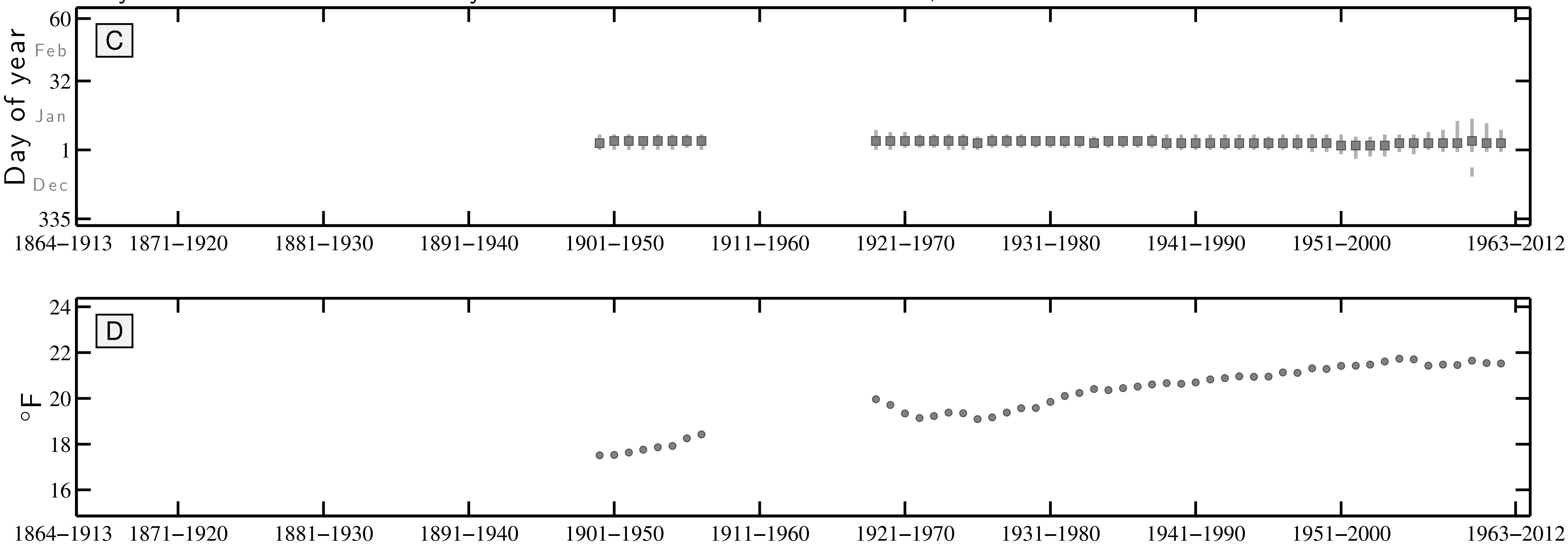

50 year Summer Teletherm dynamics for WICKENBURG, AZ:

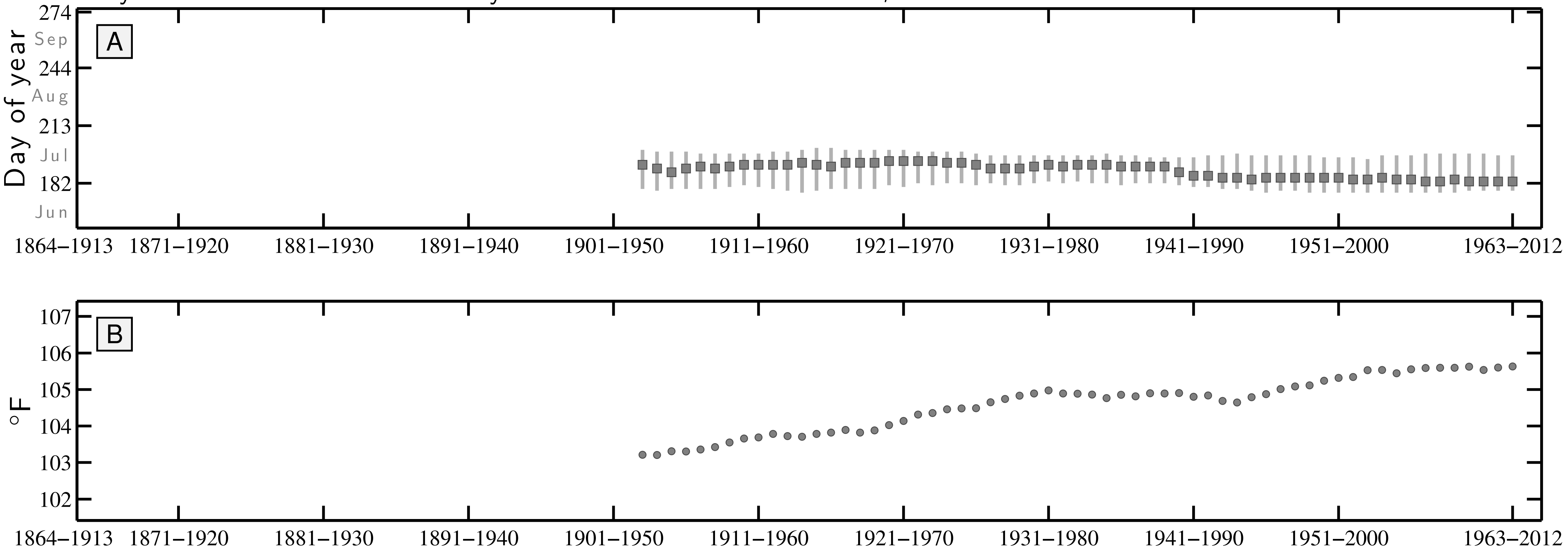

50 year Winter Teletherm dynamics for WICKENBURG, AZ:

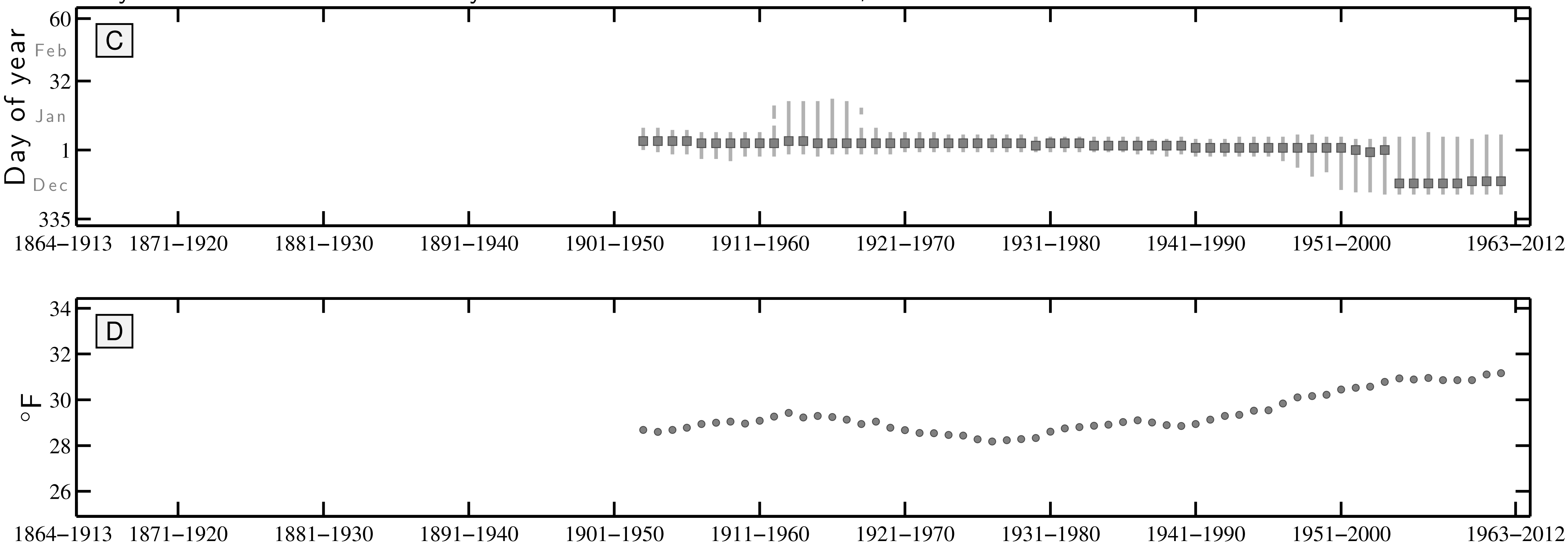

50 year Summer Teletherm dynamics for WILLIAMS, AZ:

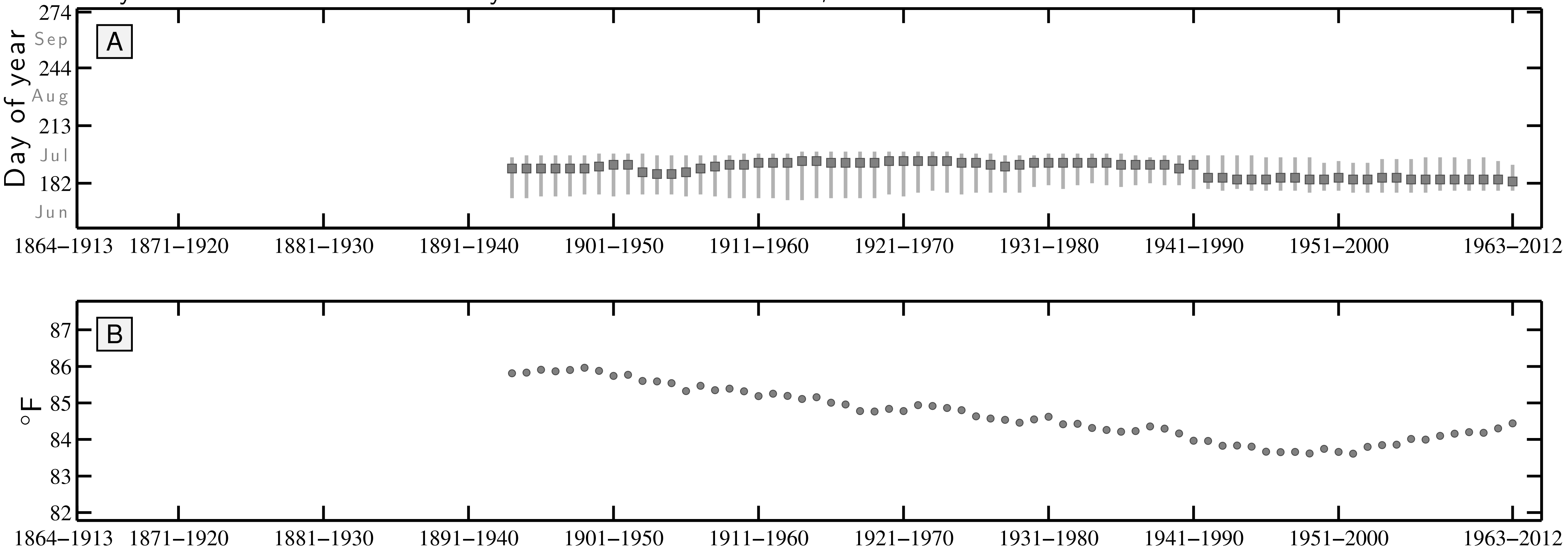

50 year Winter Teletherm dynamics for WILLIAMS, AZ:

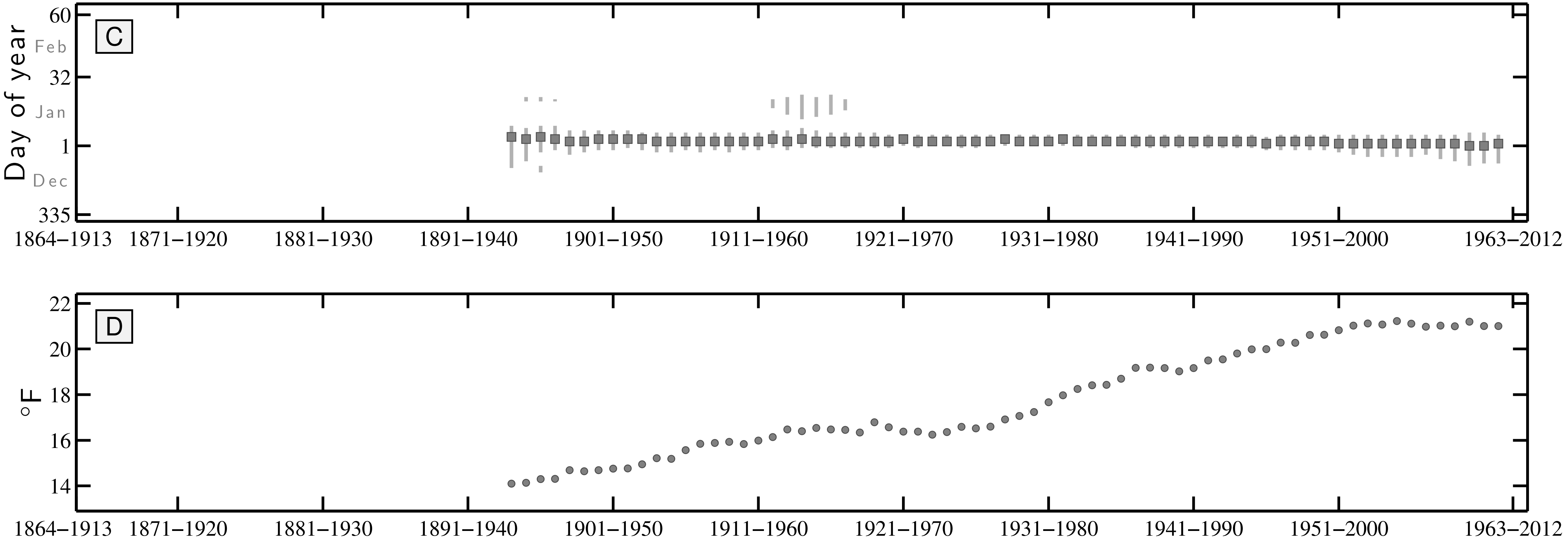

50 year Summer Teletherm dynamics for YUMA CITRUS STN, AZ:

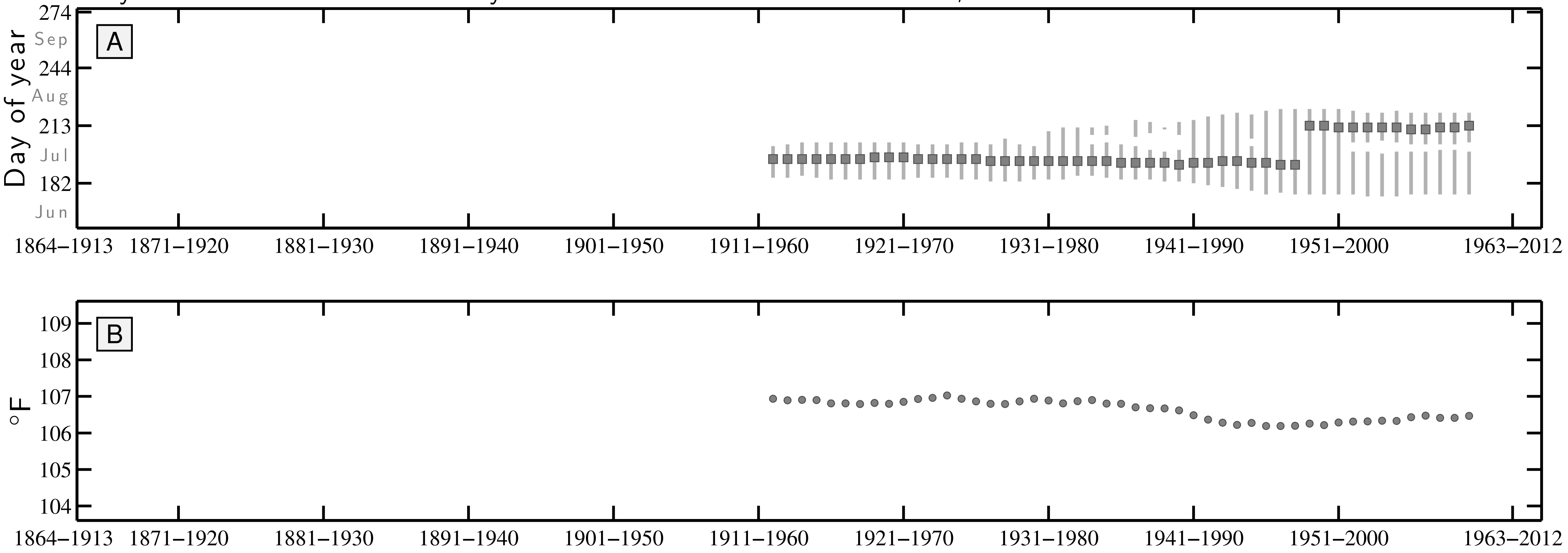

50 year Winter Teletherm dynamics for YUMA CITRUS STN, AZ:

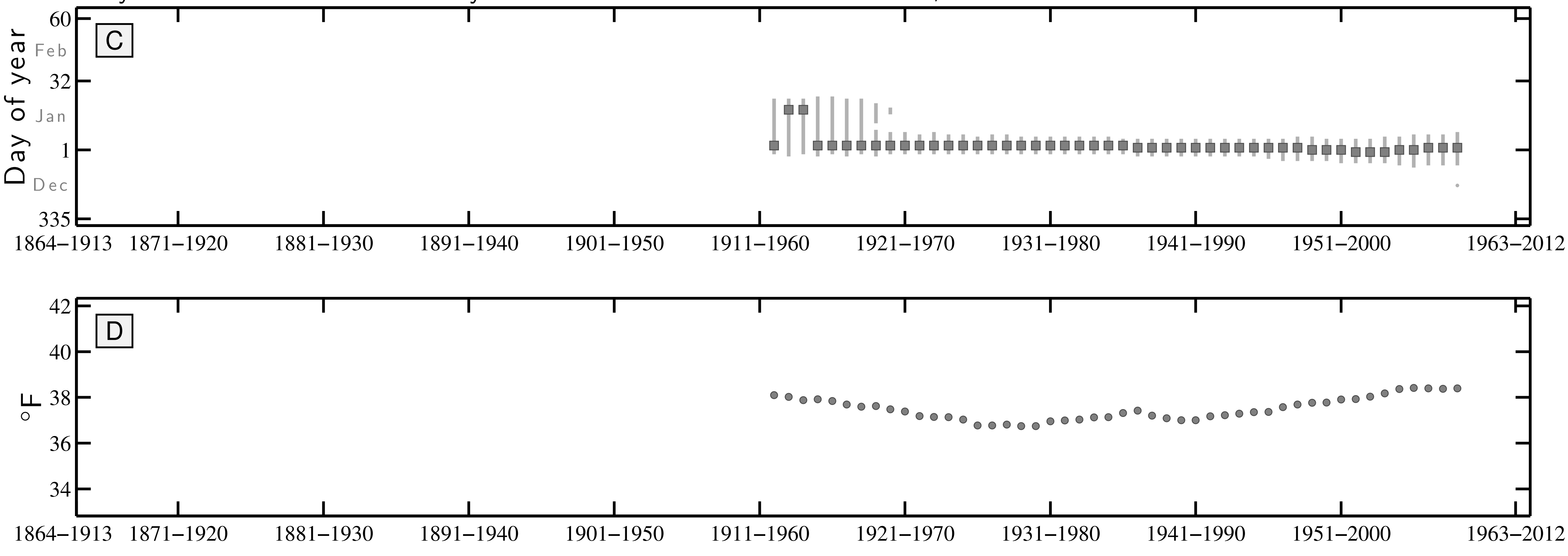

50 year Summer Teletherm dynamics for BRINKLEY, AR:

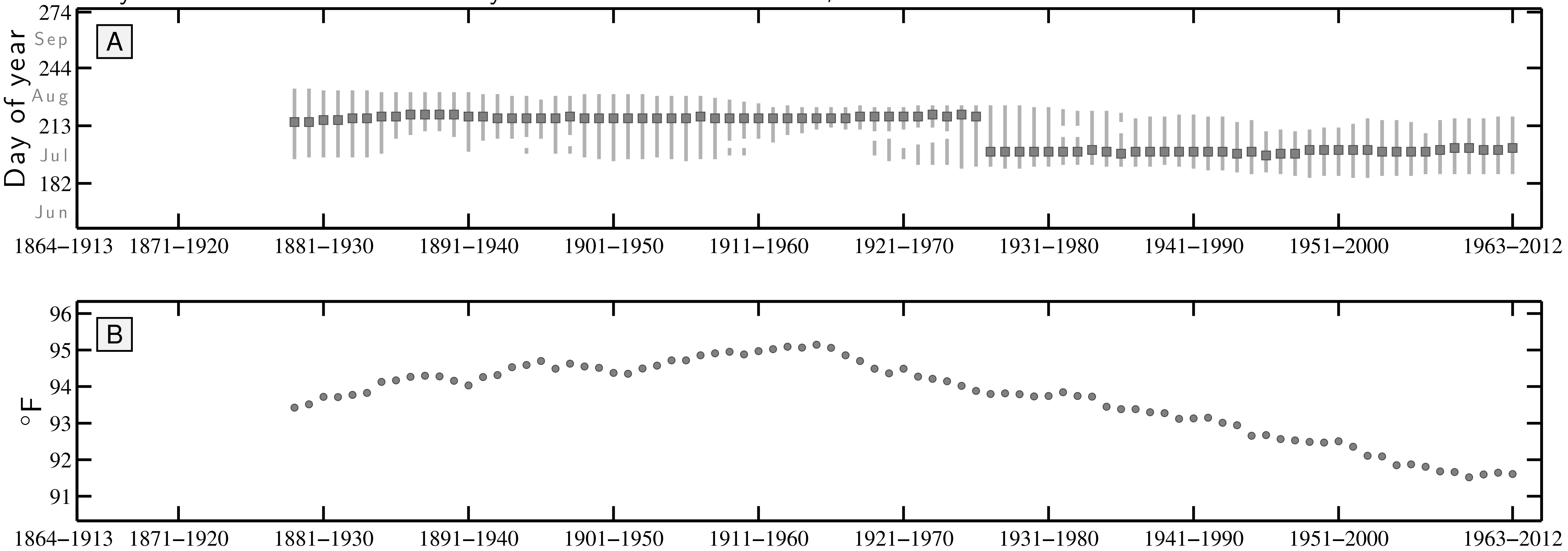

50 year Winter Teletherm dynamics for BRINKLEY, AR:

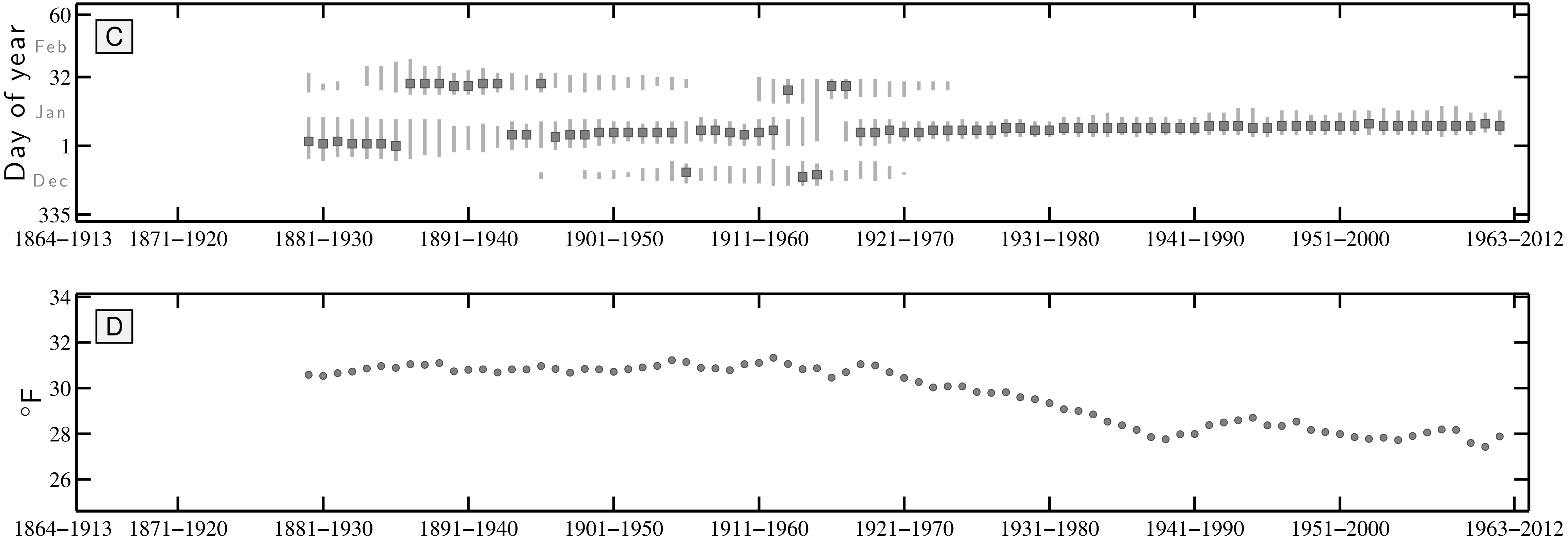

50 year Summer Teletherm dynamics for CONWAY, AR:

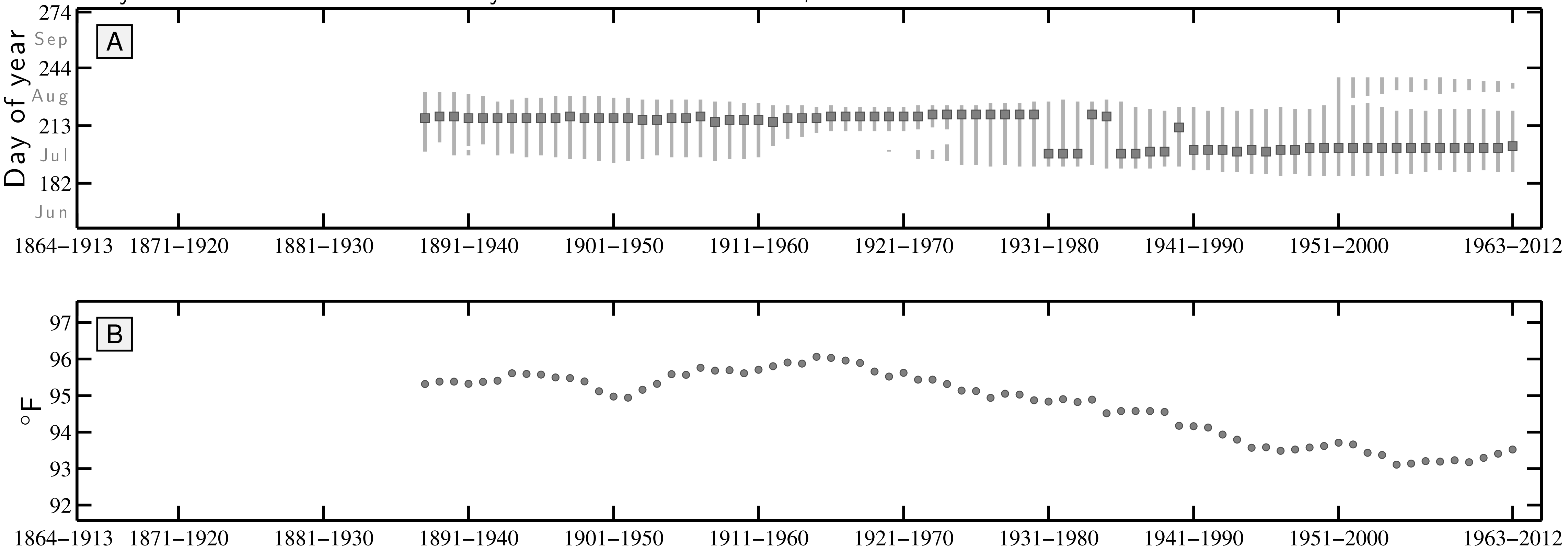

50 year Winter Teletherm dynamics for CONWAY, AR:

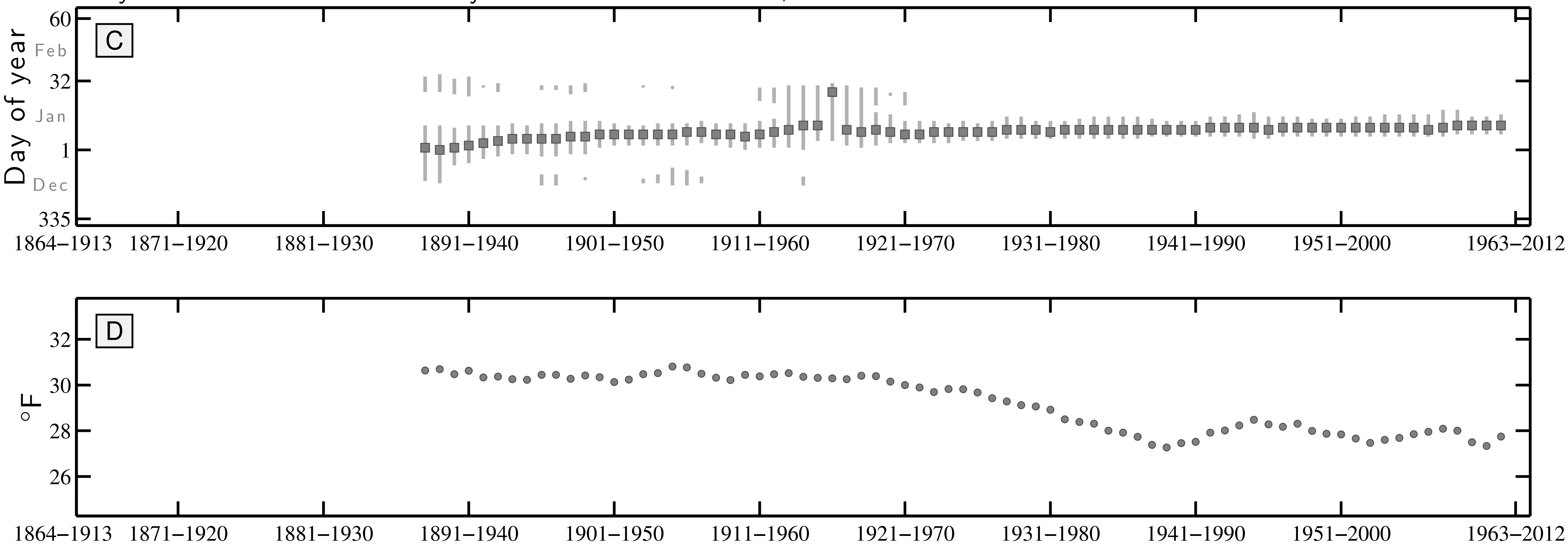

50 year Summer Teletherm dynamics for CORNING, AR:

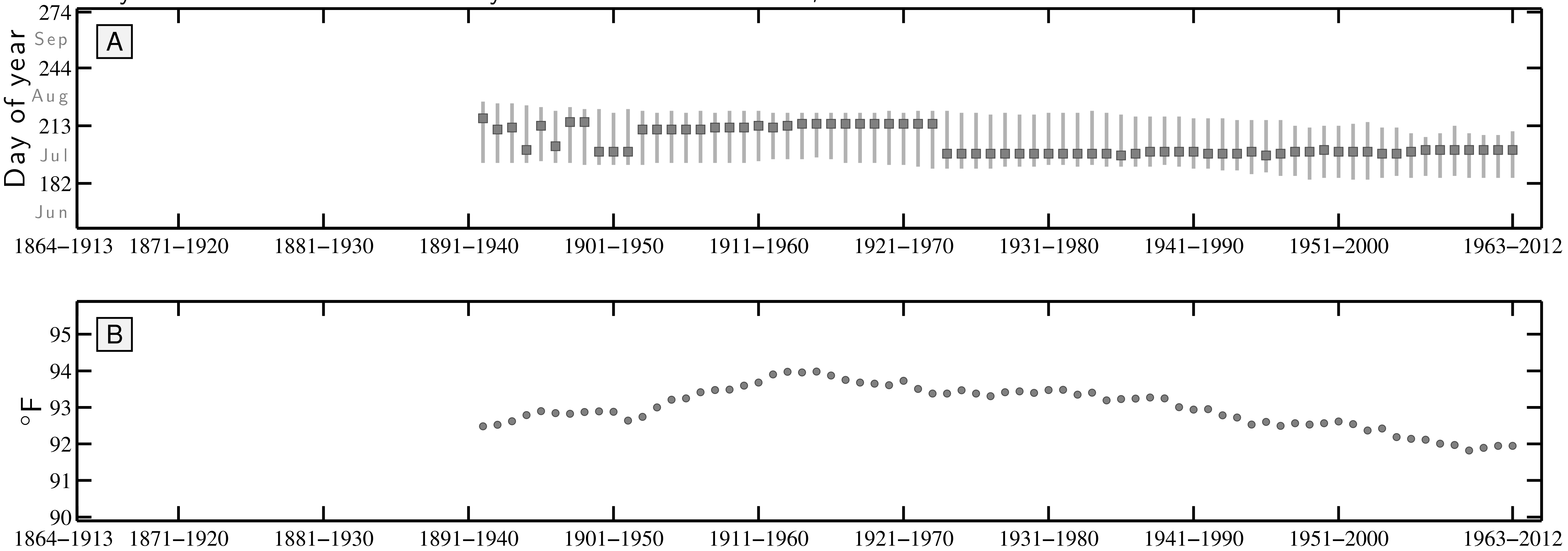

50 year Winter Teletherm dynamics for CORNING, AR:

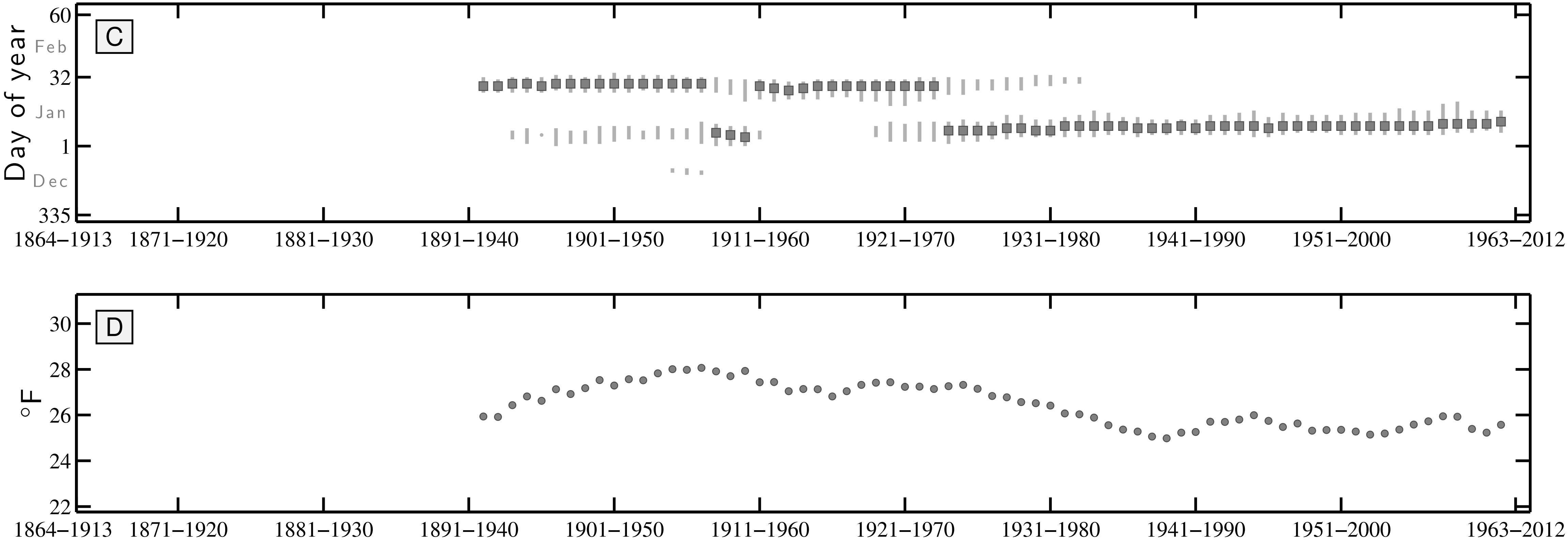

50 year Summer Teletherm dynamics for EUREKA SPRINGS 3 WNW, AR:

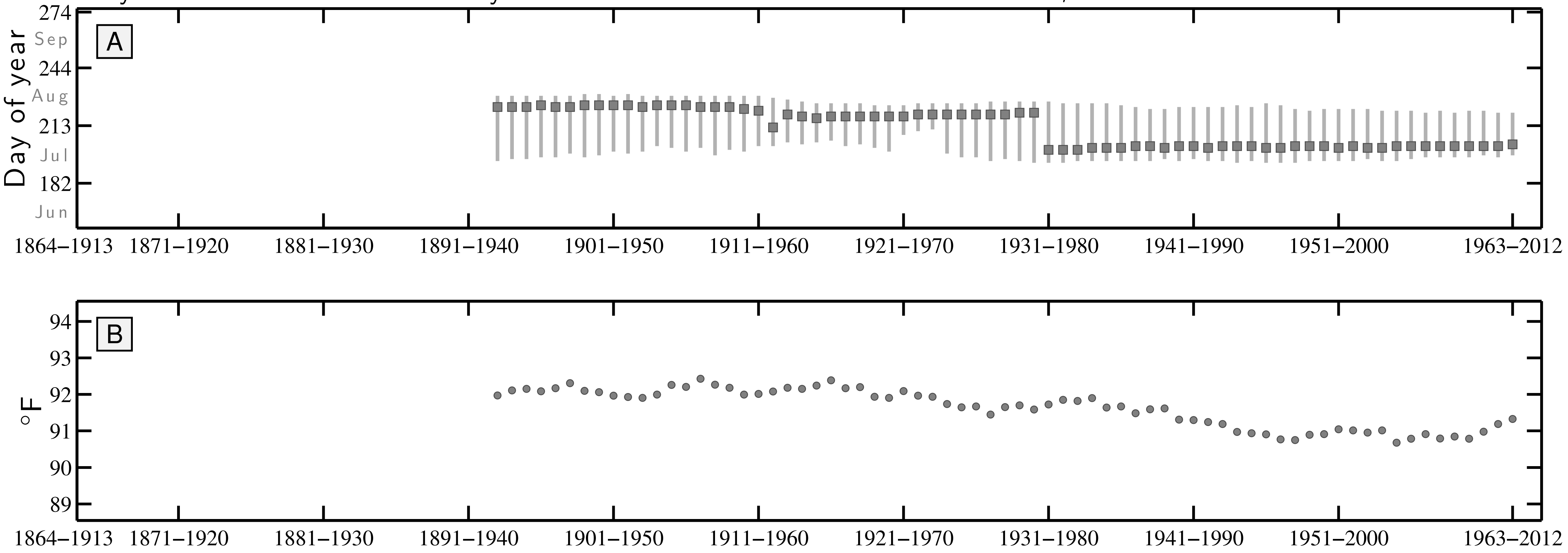

50 year Winter Teletherm dynamics for EUREKA SPRINGS 3 WNW, AR:

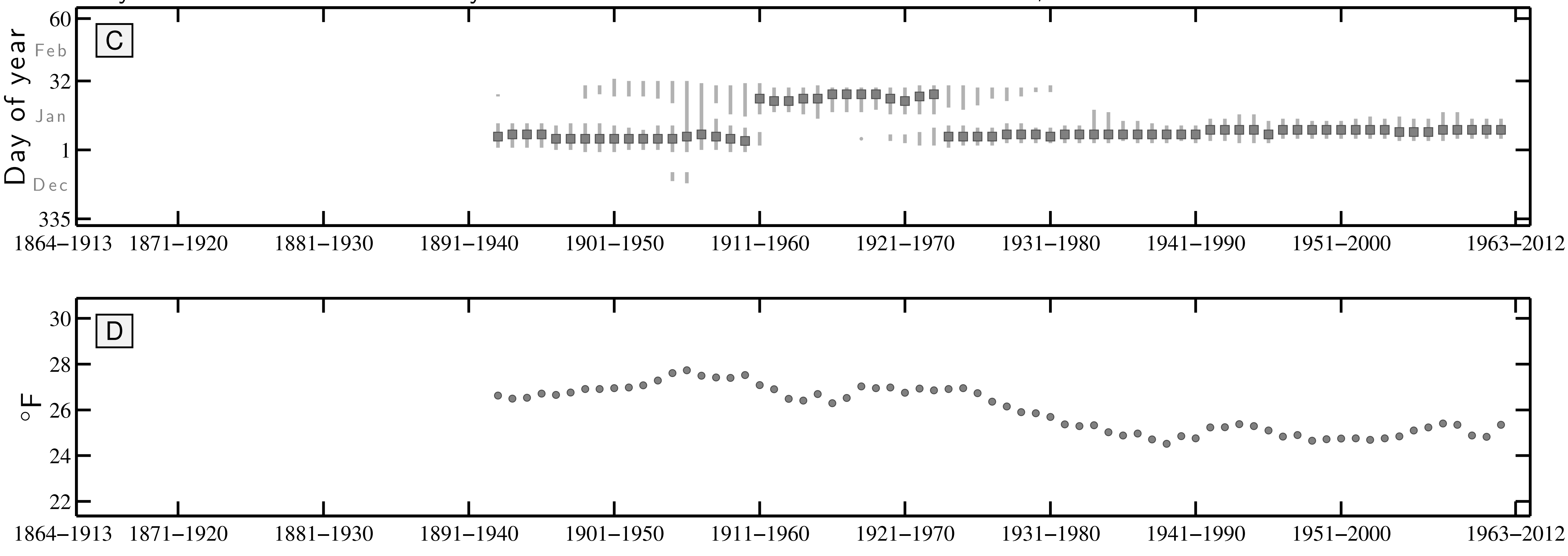

50 year Summer Teletherm dynamics for FAYETTEVILLE EXP STN, AR:

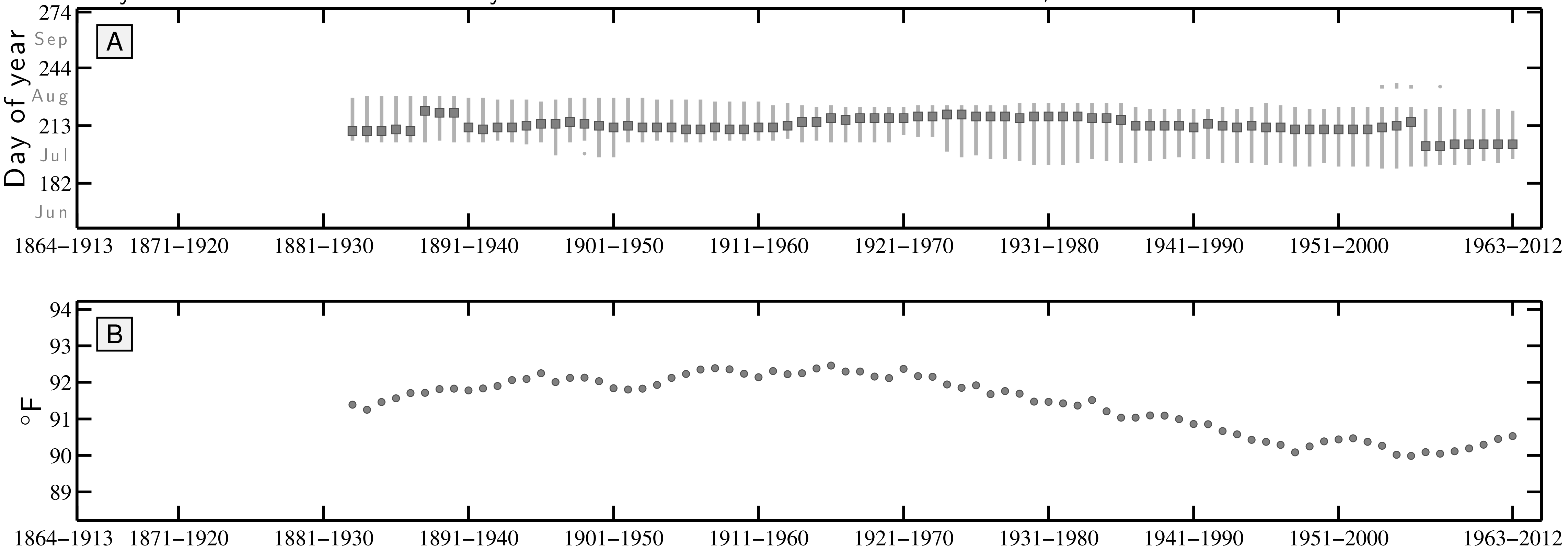

50 year Winter Teletherm dynamics for FAYETTEVILLE EXP STN, AR:

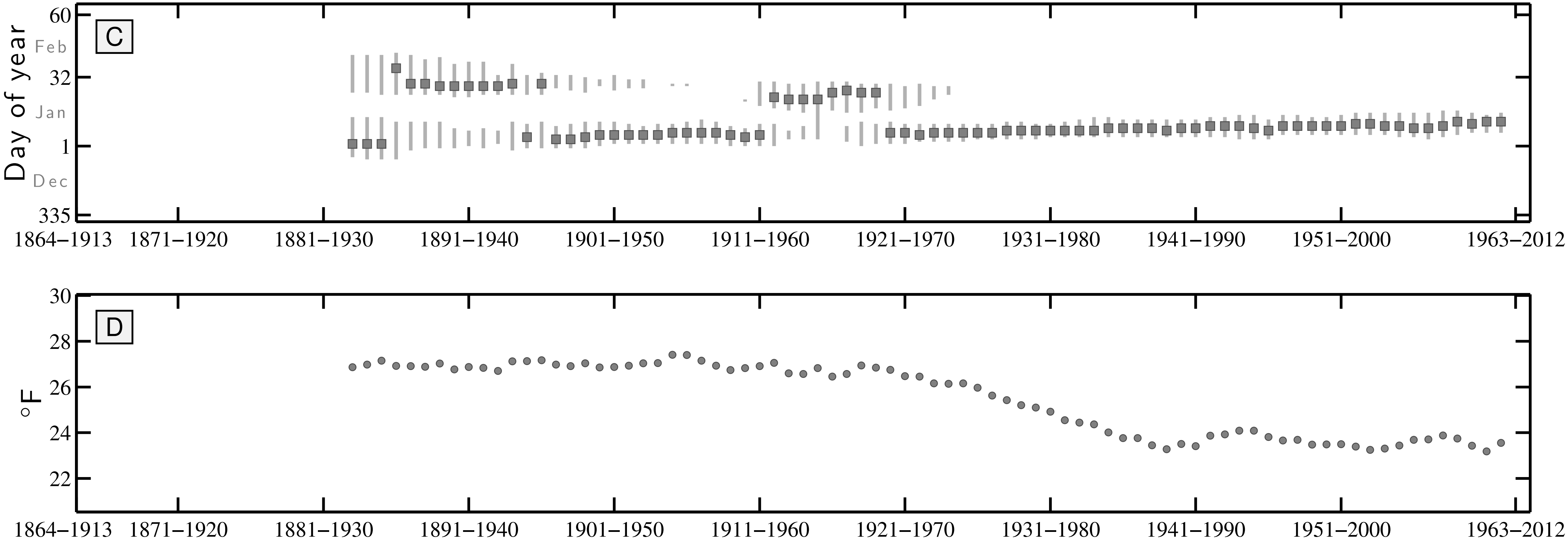

50 year Summer Teletherm dynamics for GRAVETTE, AR:

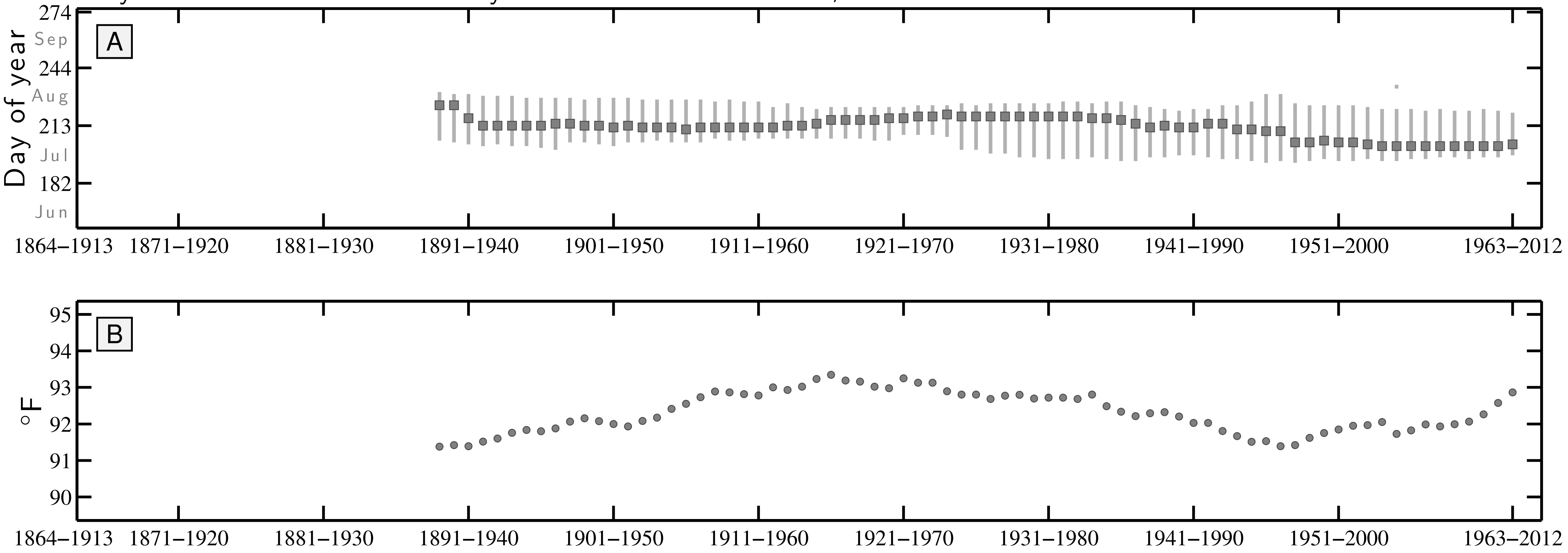

50 year Winter Teletherm dynamics for GRAVETTE, AR:

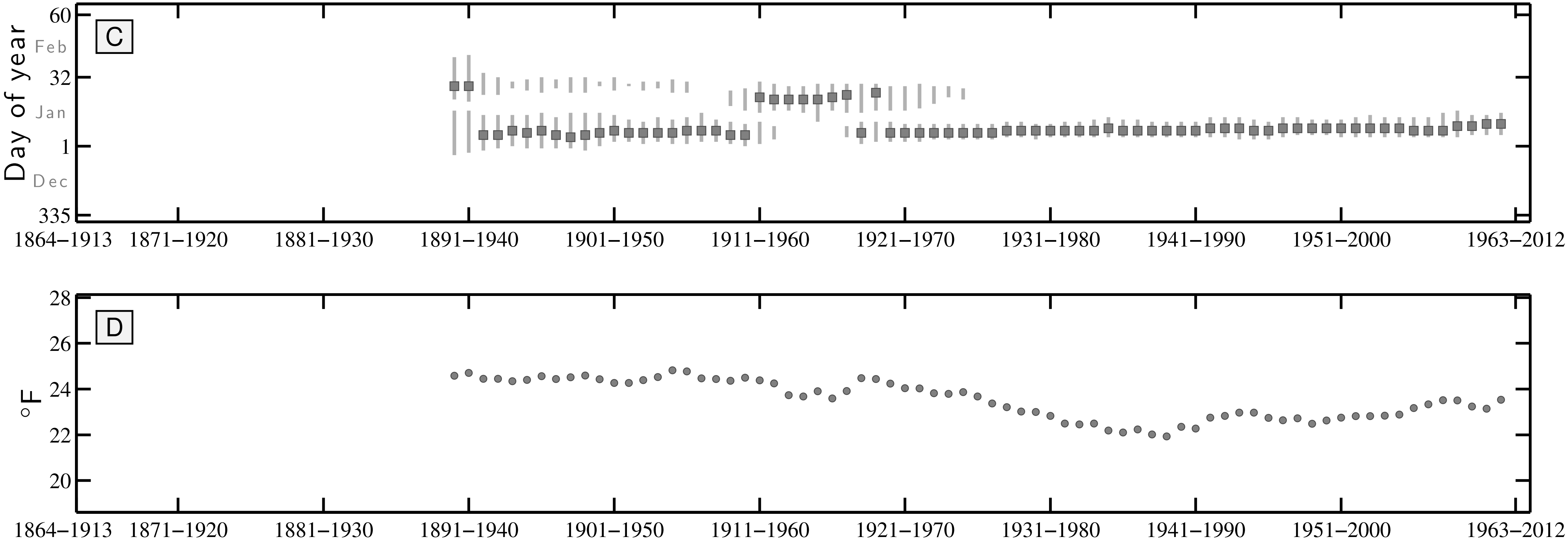

50 year Summer Teletherm dynamics for MAMMOTH SPRING, AR:

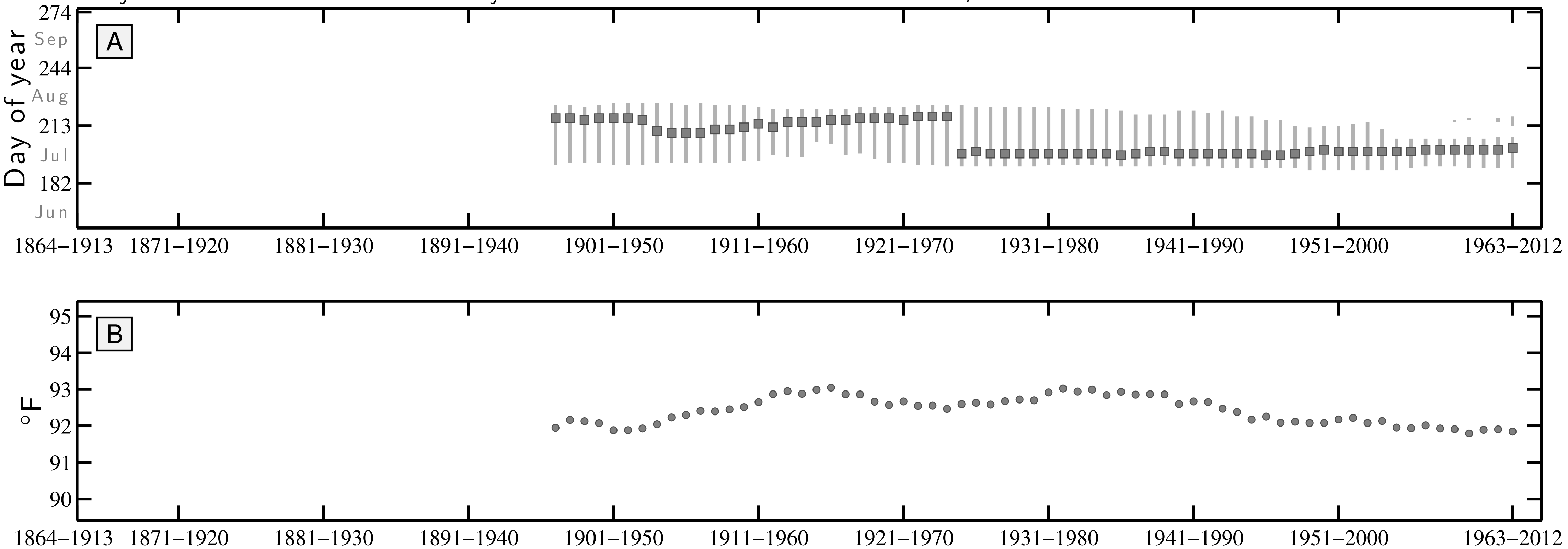

50 year Winter Teletherm dynamics for MAMMOTH SPRING, AR:

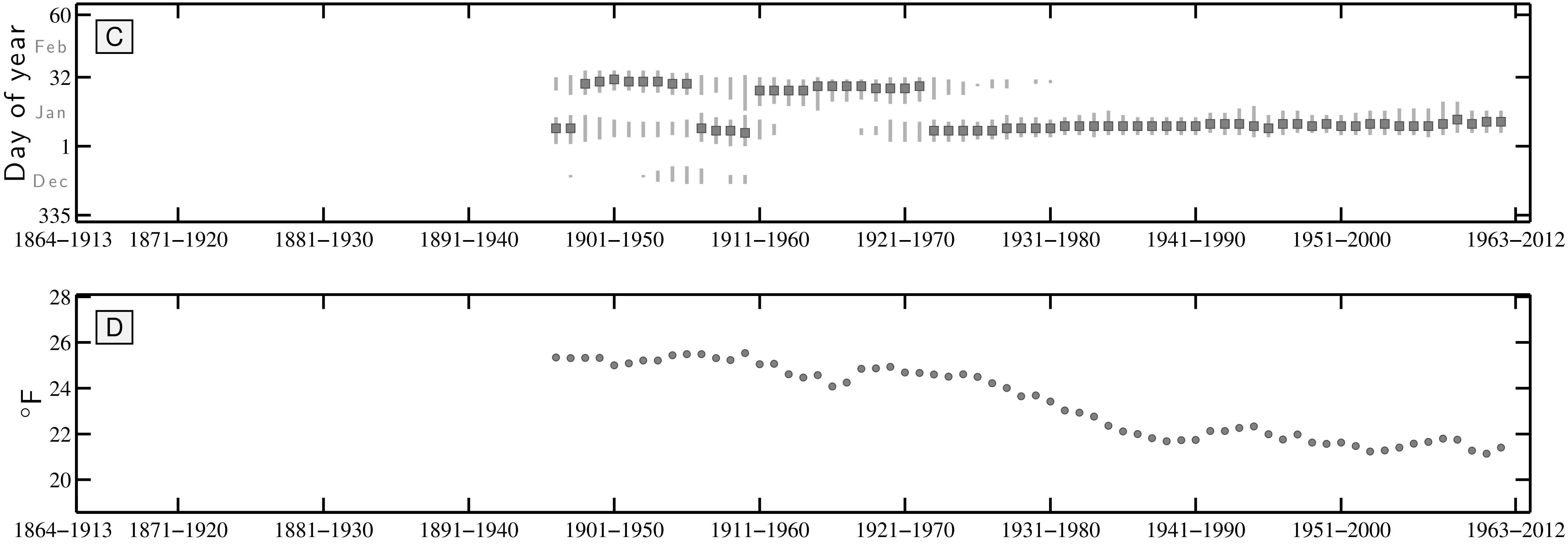

50 year Summer Teletherm dynamics for MENA, AR:

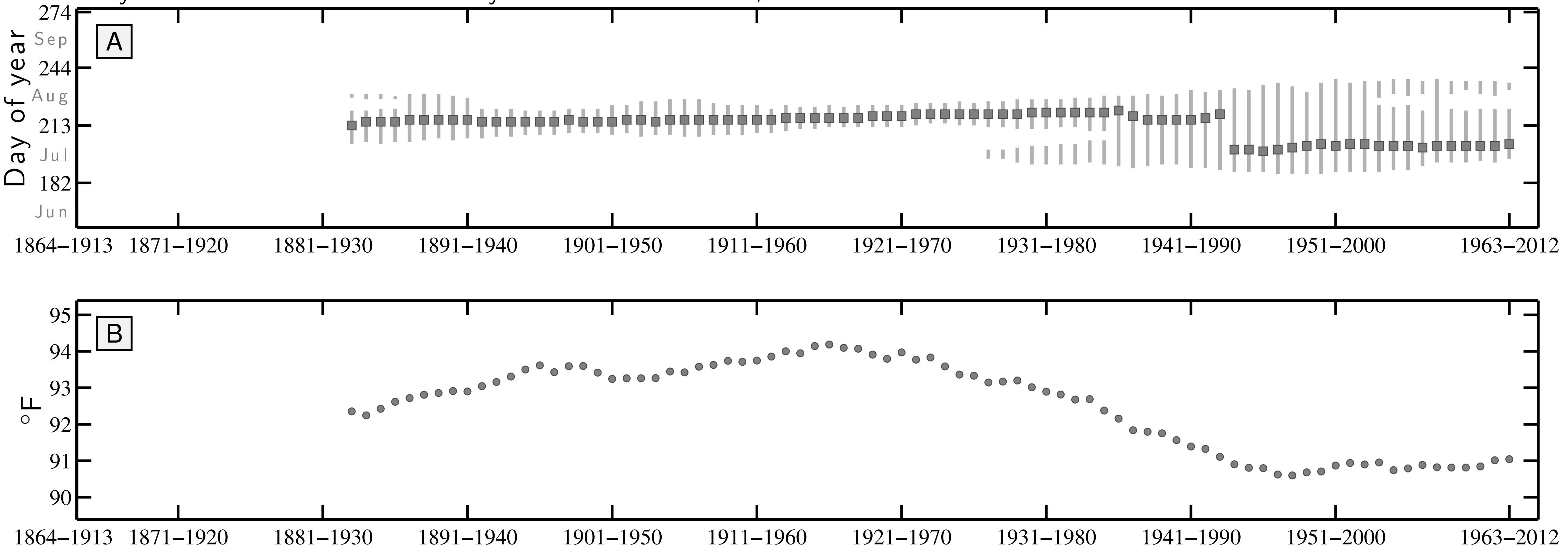

50 year Winter Teletherm dynamics for MENA, AR:

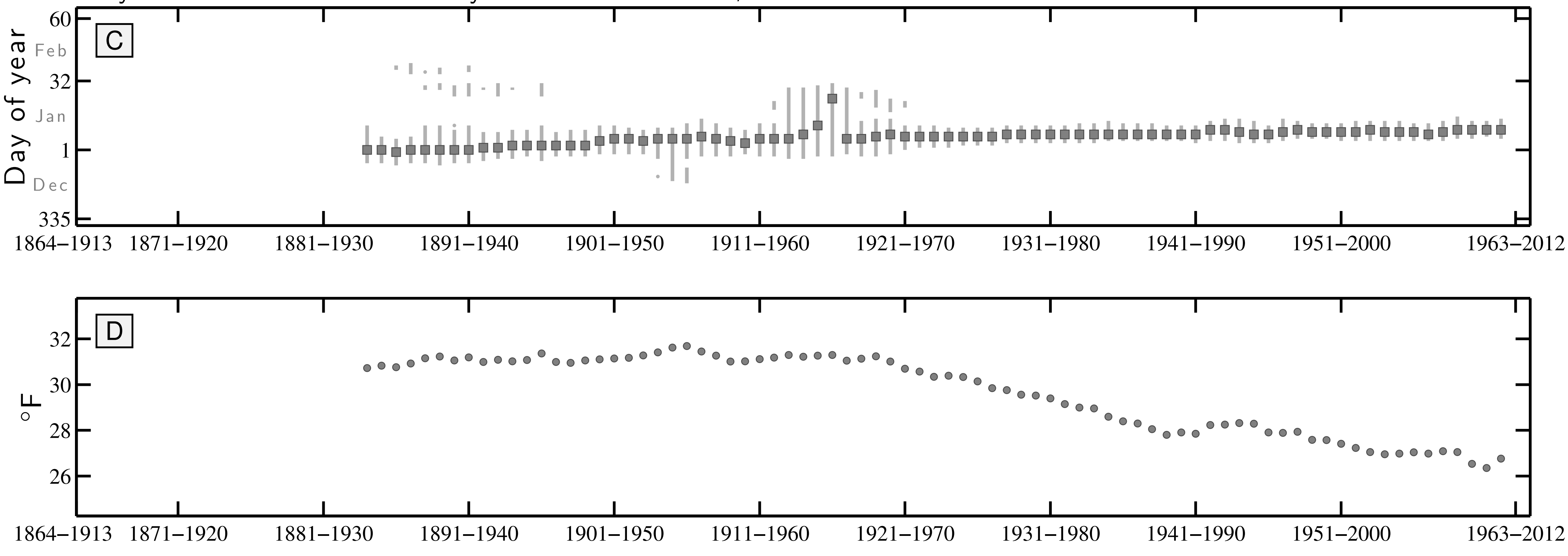

50 year Summer Teletherm dynamics for NEWPORT, AR:

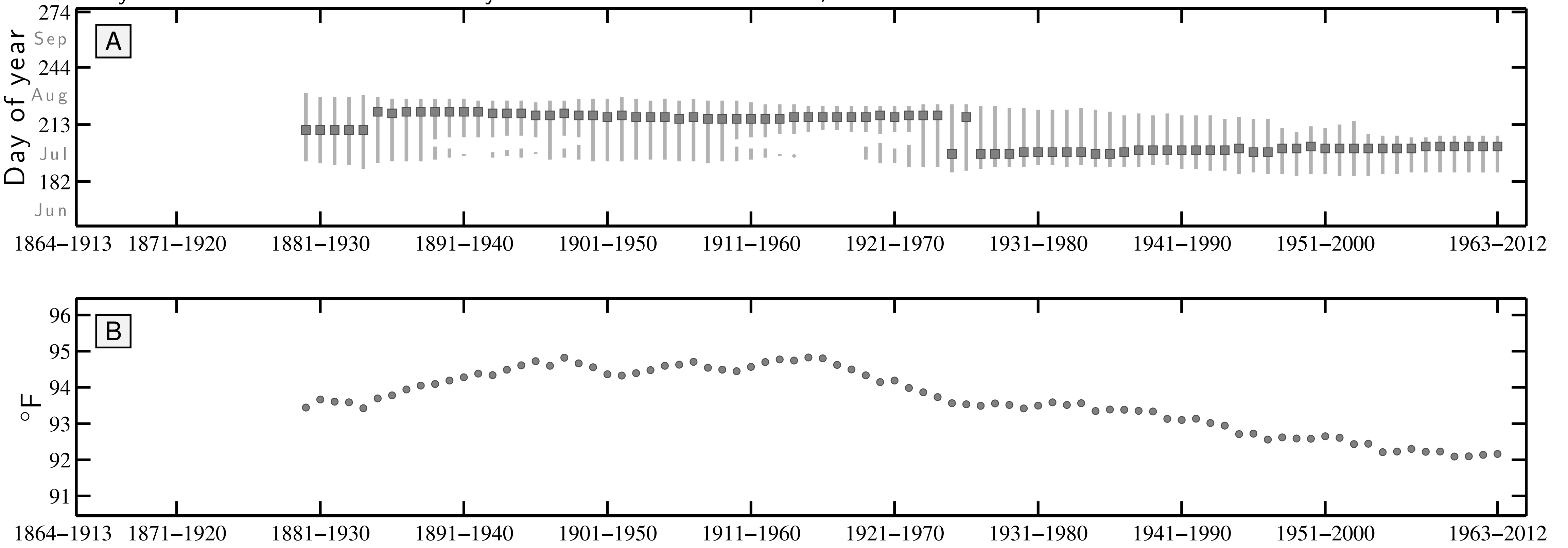

50 year Winter Teletherm dynamics for NEWPORT, AR:

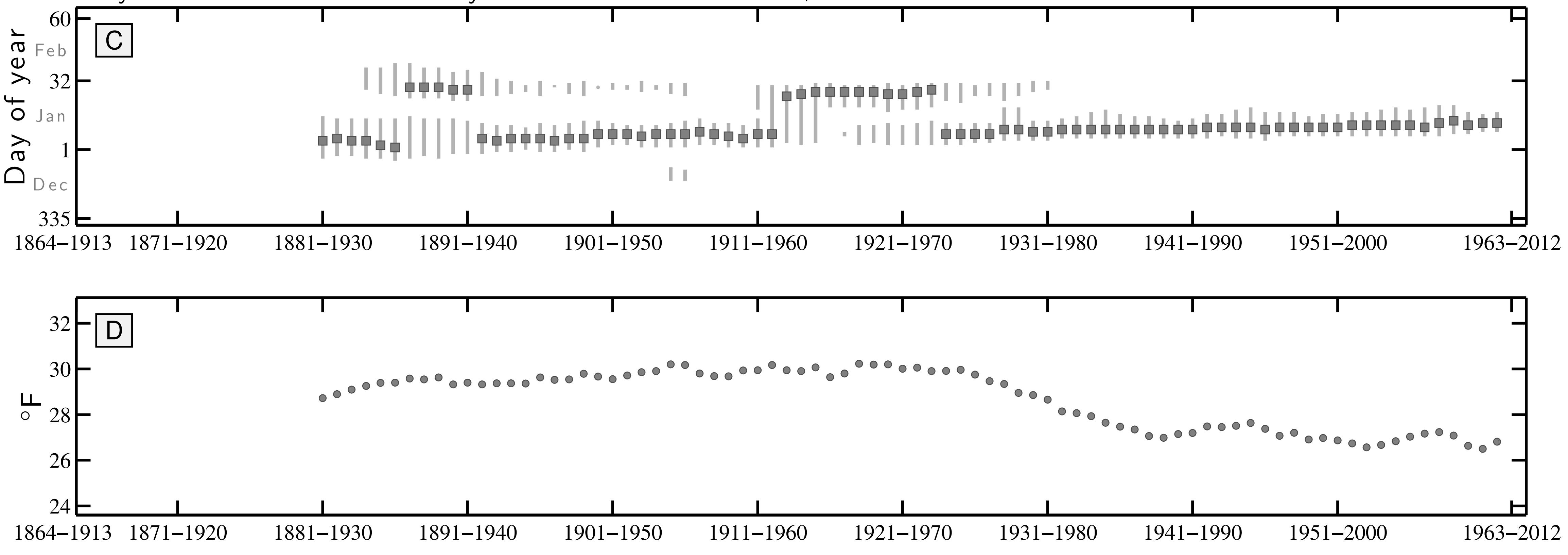

50 year Summer Teletherm dynamics for PINE BLUFF, AR:

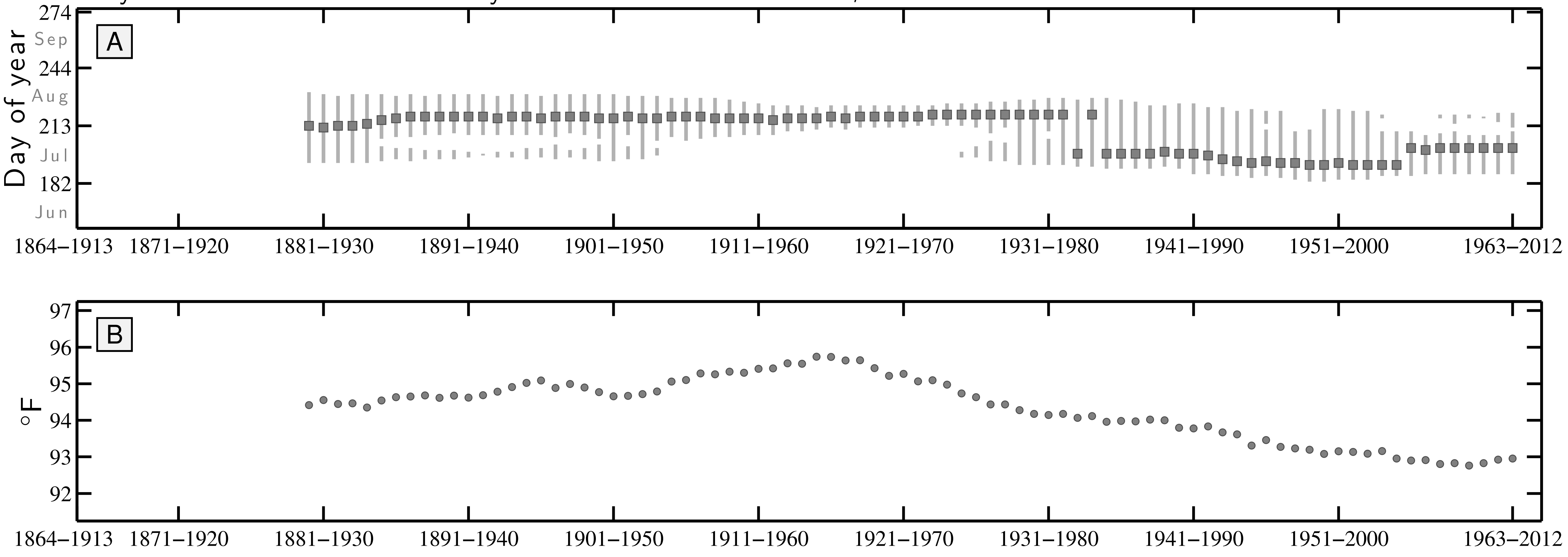

50 year Winter Teletherm dynamics for PINE BLUFF, AR:

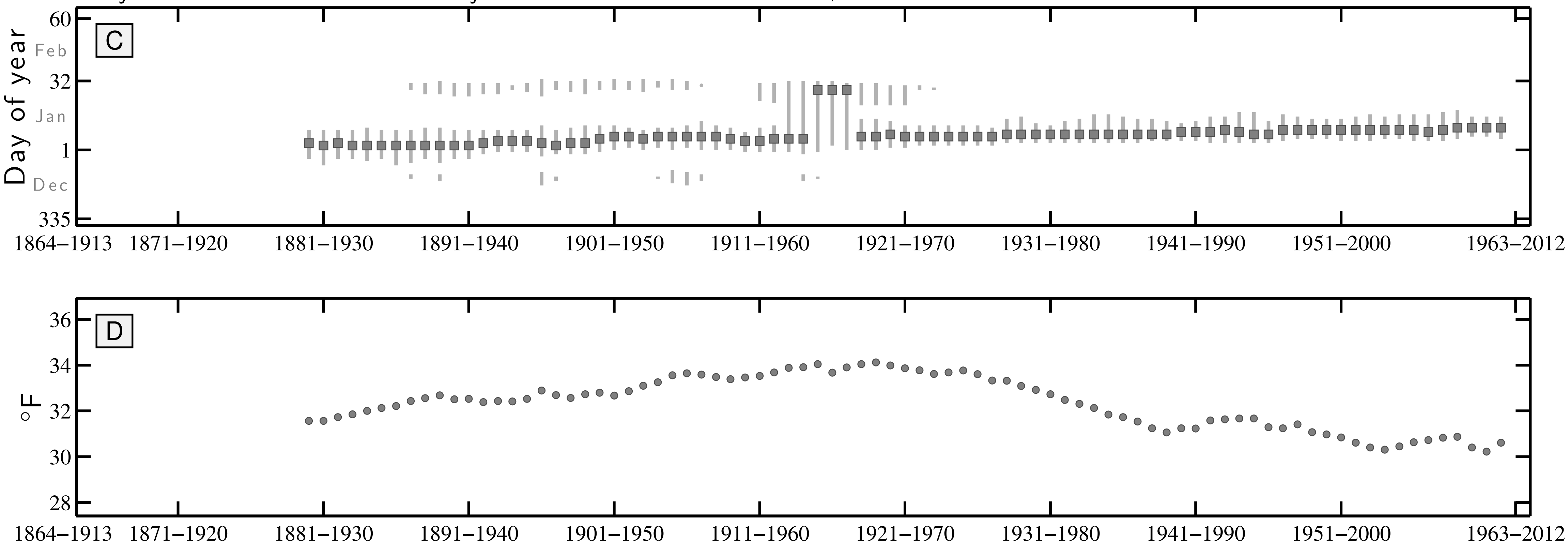

50 year Summer Teletherm dynamics for POCAHONTAS 1, AR:

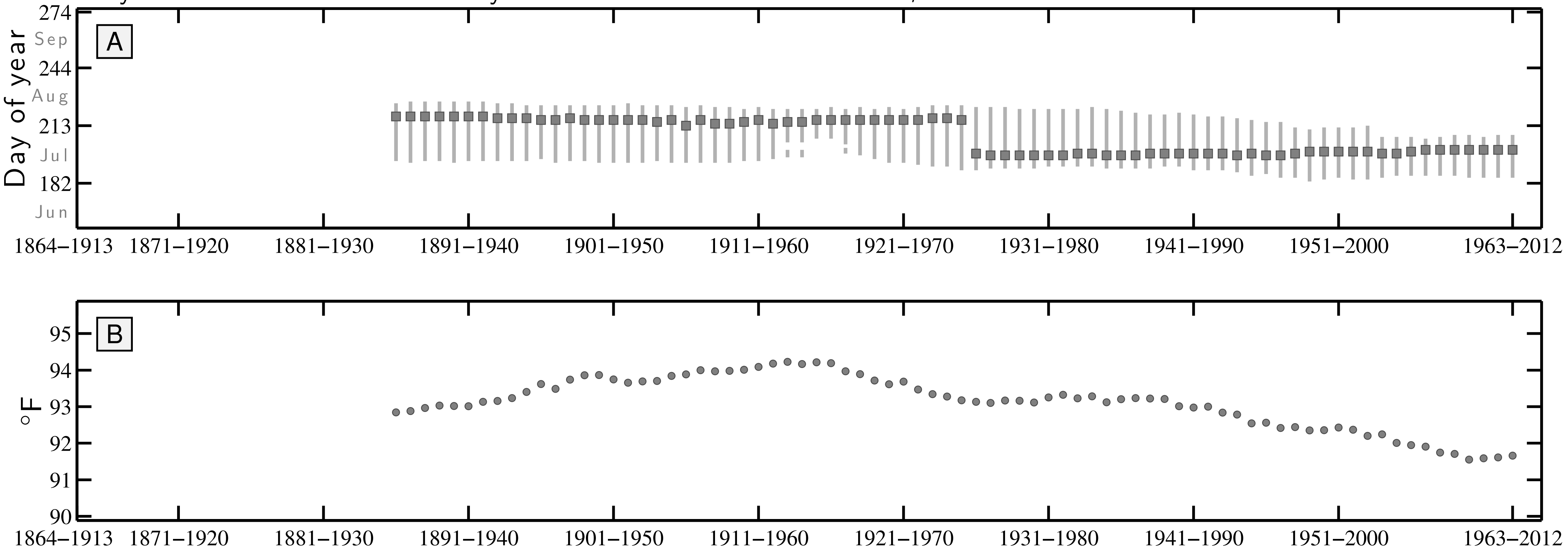

50 year Winter Teletherm dynamics for POCAHONTAS 1, AR:

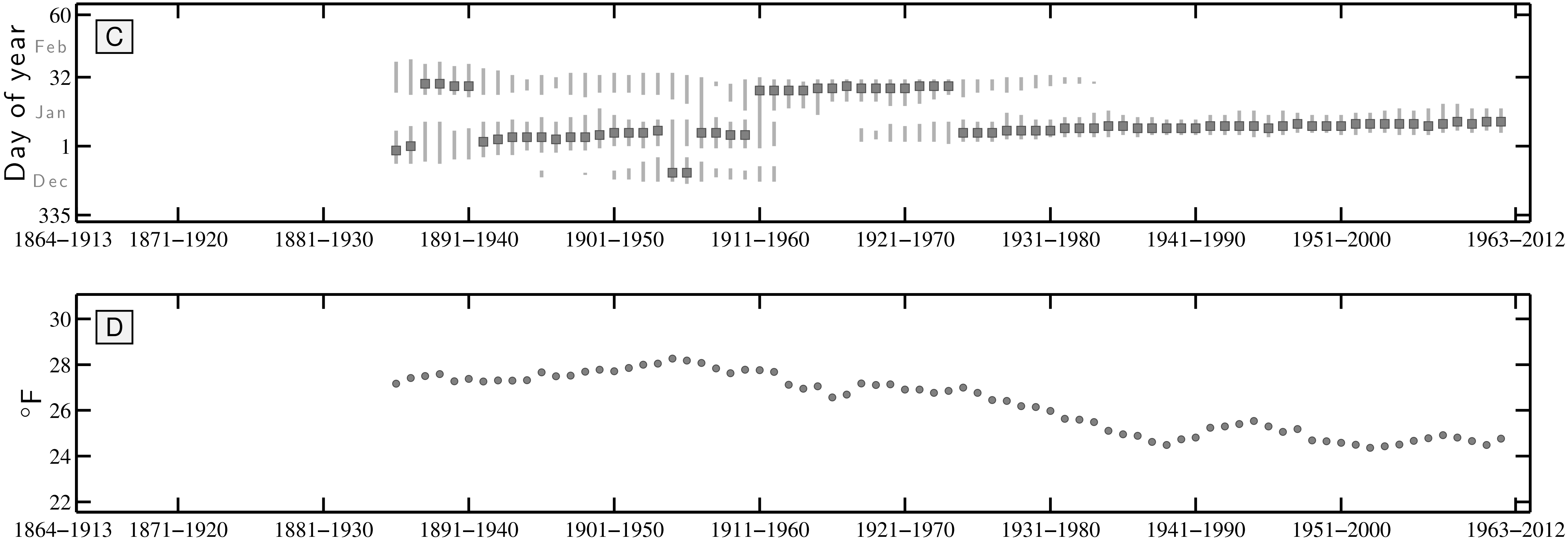

50 year Summer Teletherm dynamics for PRESCOTT 2 NNW, AR:

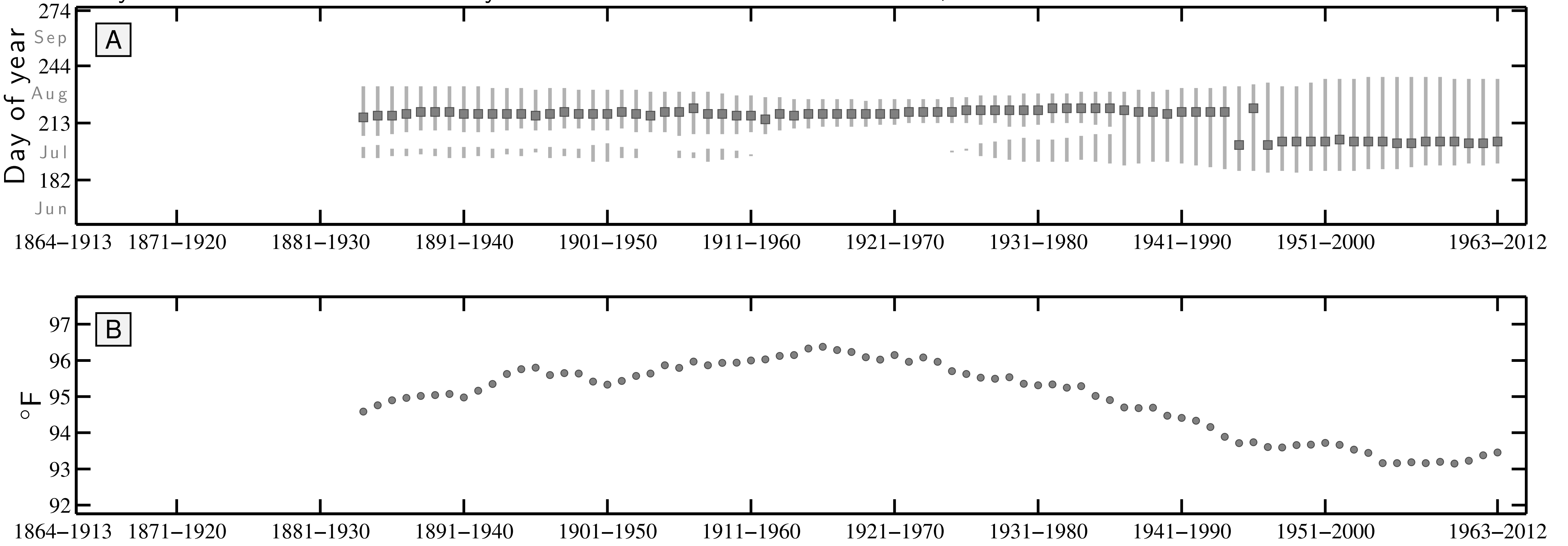

50 year Winter Teletherm dynamics for PRESCOTT 2 NNW, AR:

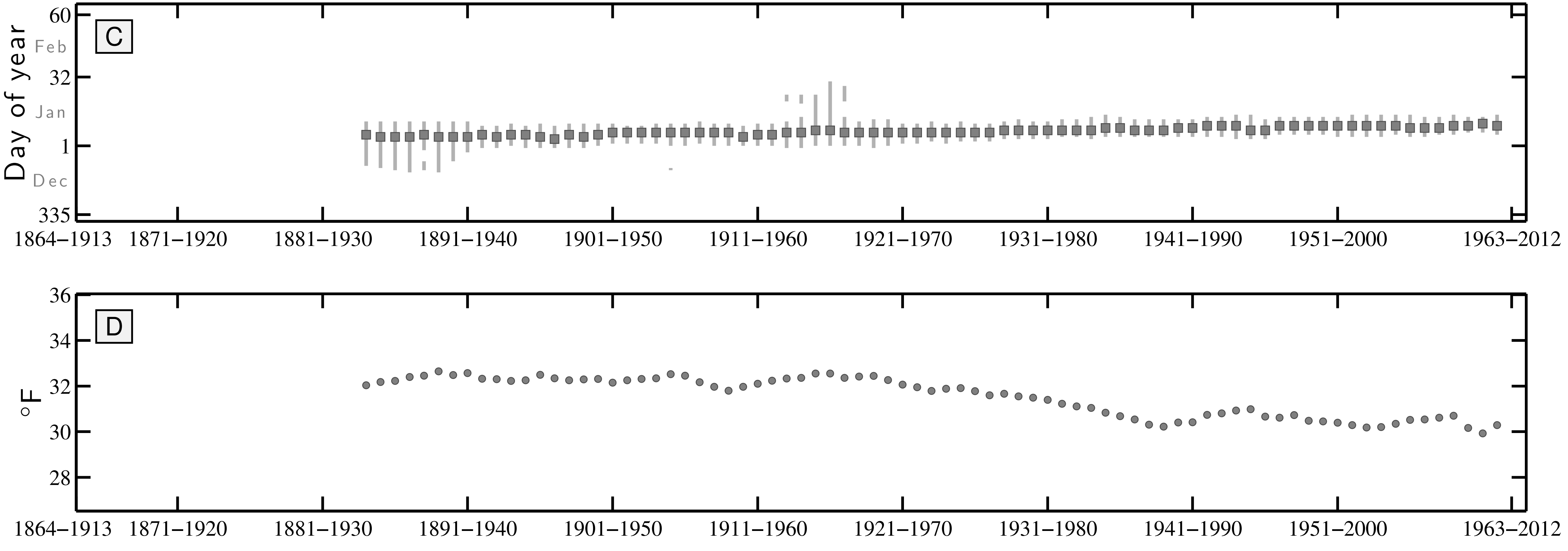

50 year Summer Teletherm dynamics for ROHWER 2 NNE, AR:

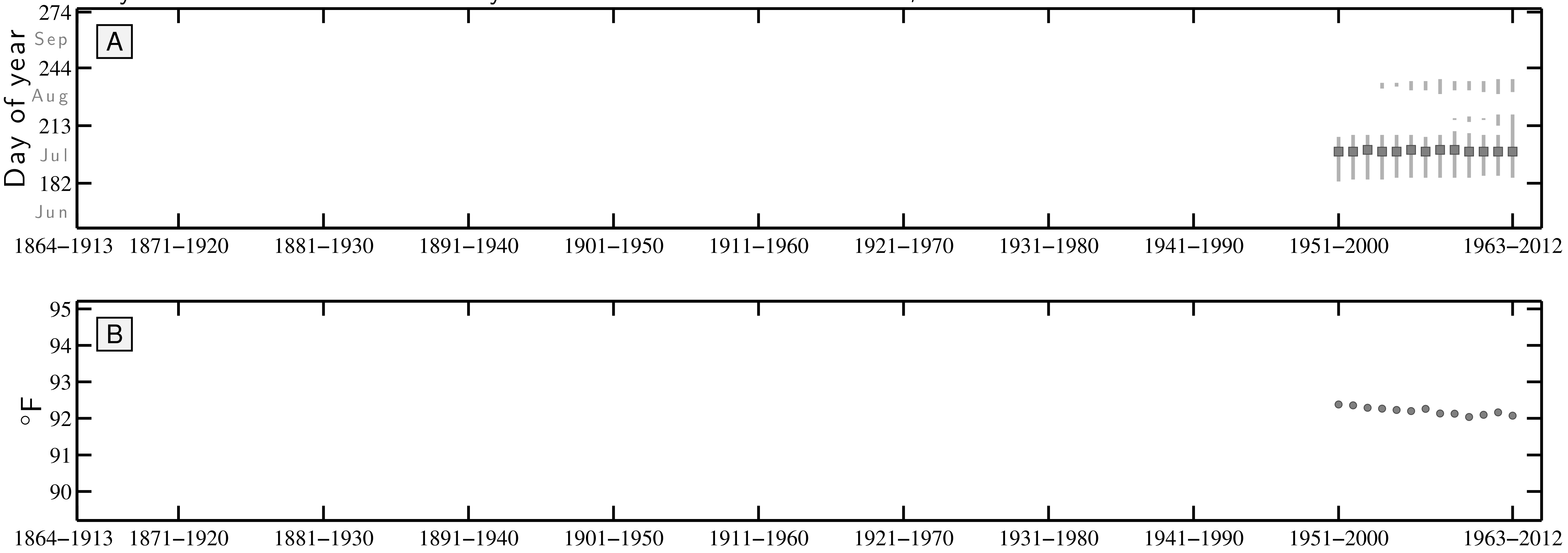

50 year Winter Teletherm dynamics for ROHWER 2 NNE, AR:

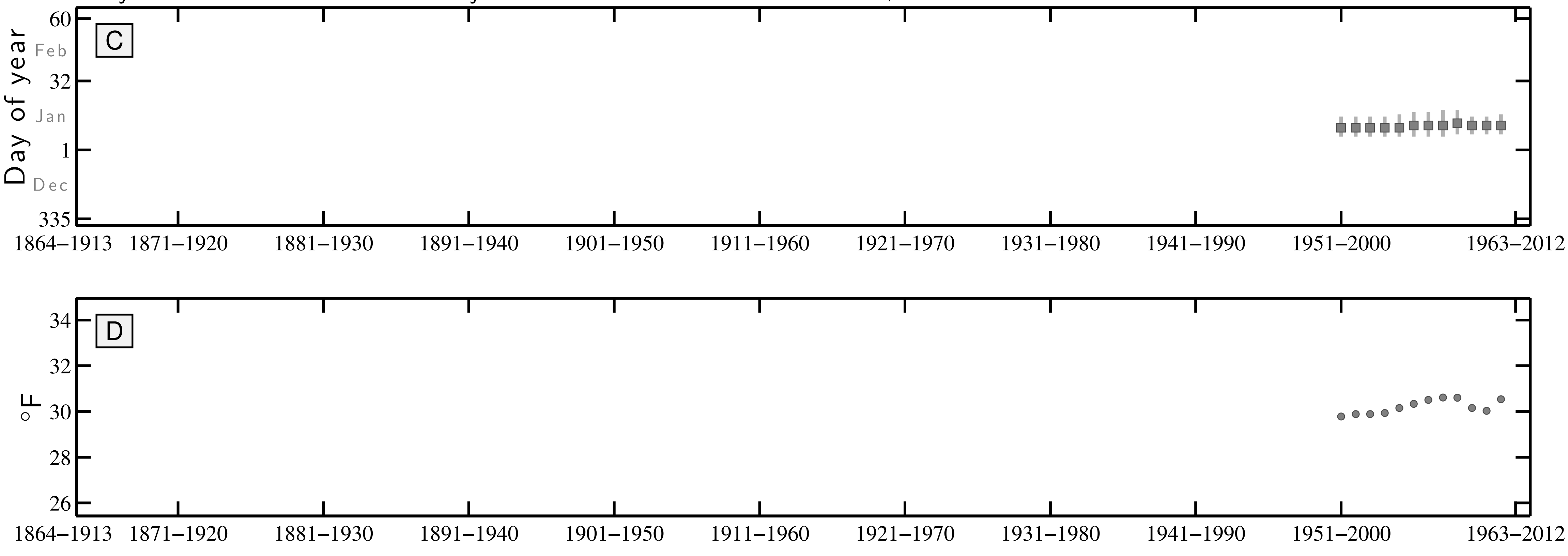

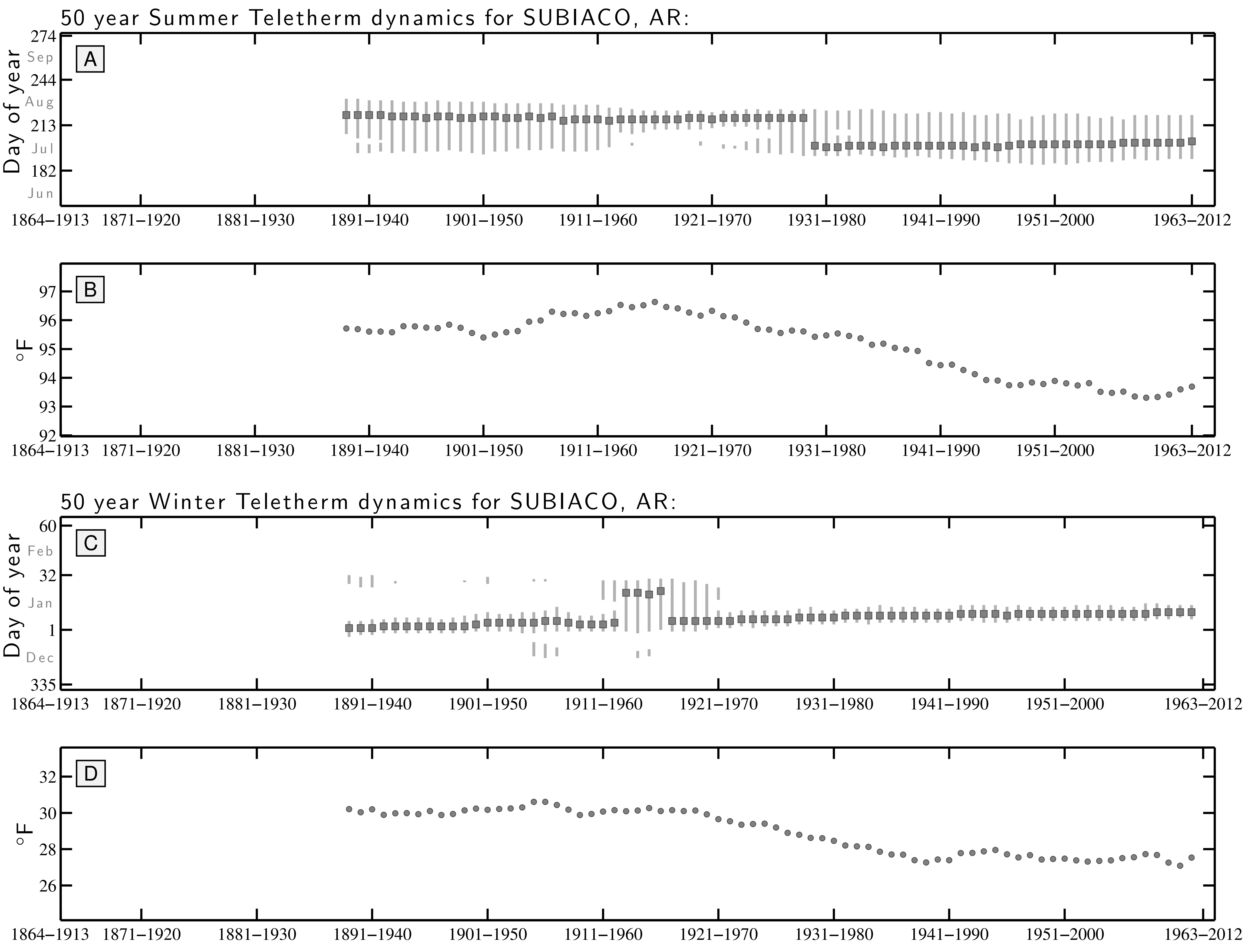
50 year Summer Teletherm dynamics for SUBIACO, AR:
A
Day of year
274
Sep
244
Aug
213
Jul
182
Jun
B
°F
97
96
95
94
93
92
50 year Winter Teletherm dynamics for SUBIACO, AR:
C
Day of year
60
Feb
32
Jan
1
Dec
335
D
°F
32
30
28
26
1864–1913
1871–1920
1881–1930
1891–1940
1901–1950
1911–1960
1921–1970
1931–1980
1941–1990
1951–2000
1963–2012

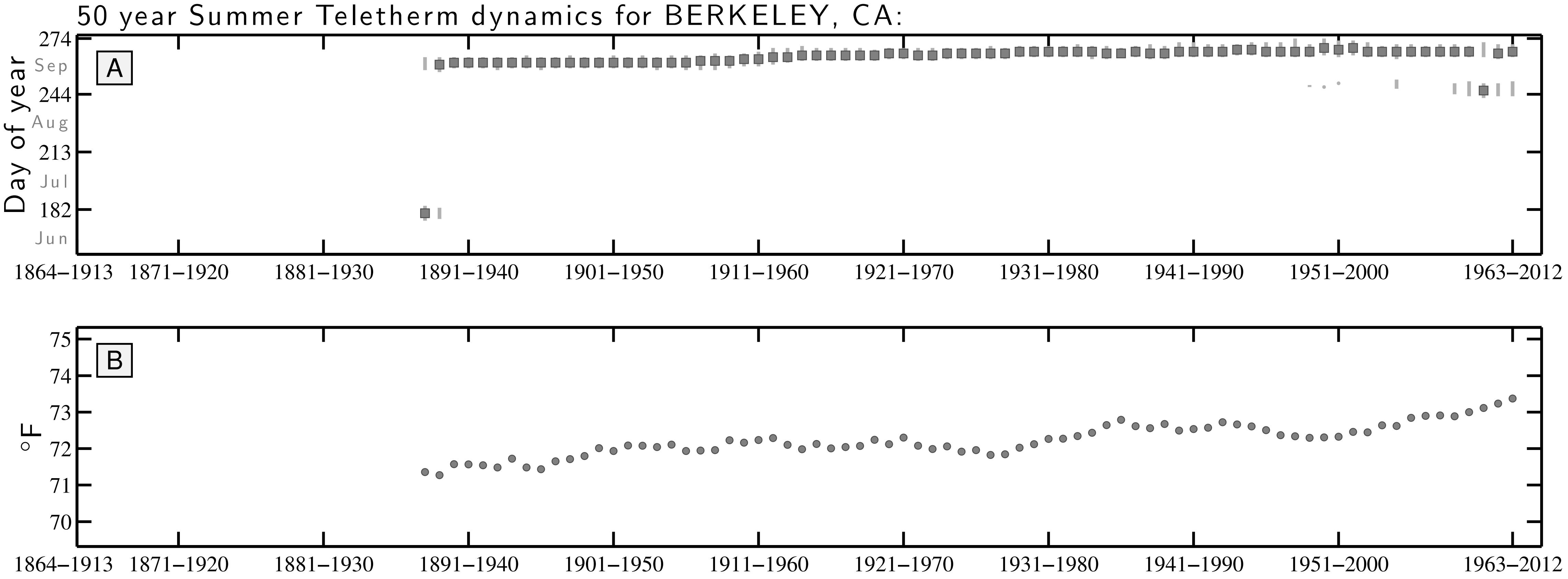
50 year Summer Teletherm dynamics for BERKELEY, CA:
A
B
Day of year
°F
274
244
213
182
Sep
Aug
Jul
Jun
75
74
73
72
71
70
1864–1913
1871–1920
1881–1930
1891–1940
1901–1950
1911–1960
1921–1970
1931–1980
1941–1990
1951–2000
1963–2012

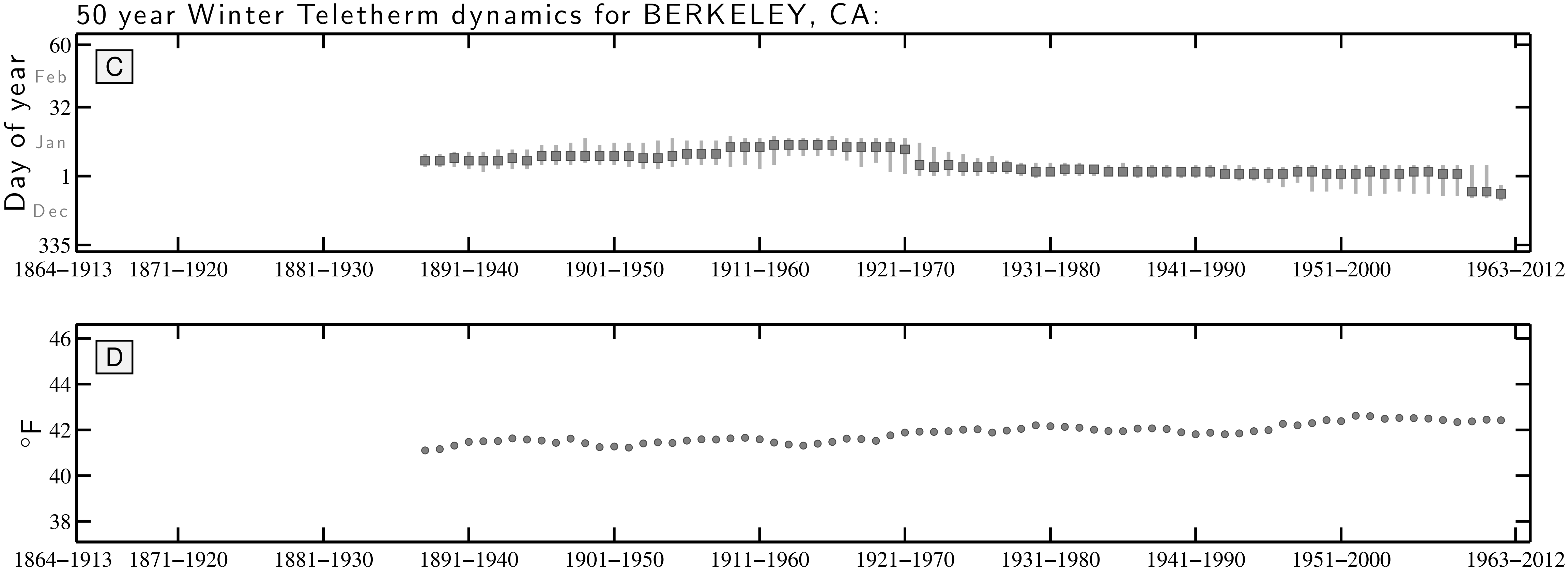
50 year Winter Teletherm dynamics for BERKELEY, CA:
C
D
Day of year
°F
60
32
1
335
Feb
Jan
Dec
46
44
42
40
38
1864–1913
1871–1920
1881–1930
1891–1940
1901–1950
1911–1960
1921–1970
1931–1980
1941–1990
1951–2000
1963–2012

50 year Summer Teletherm dynamics for BLYTHE, CA:

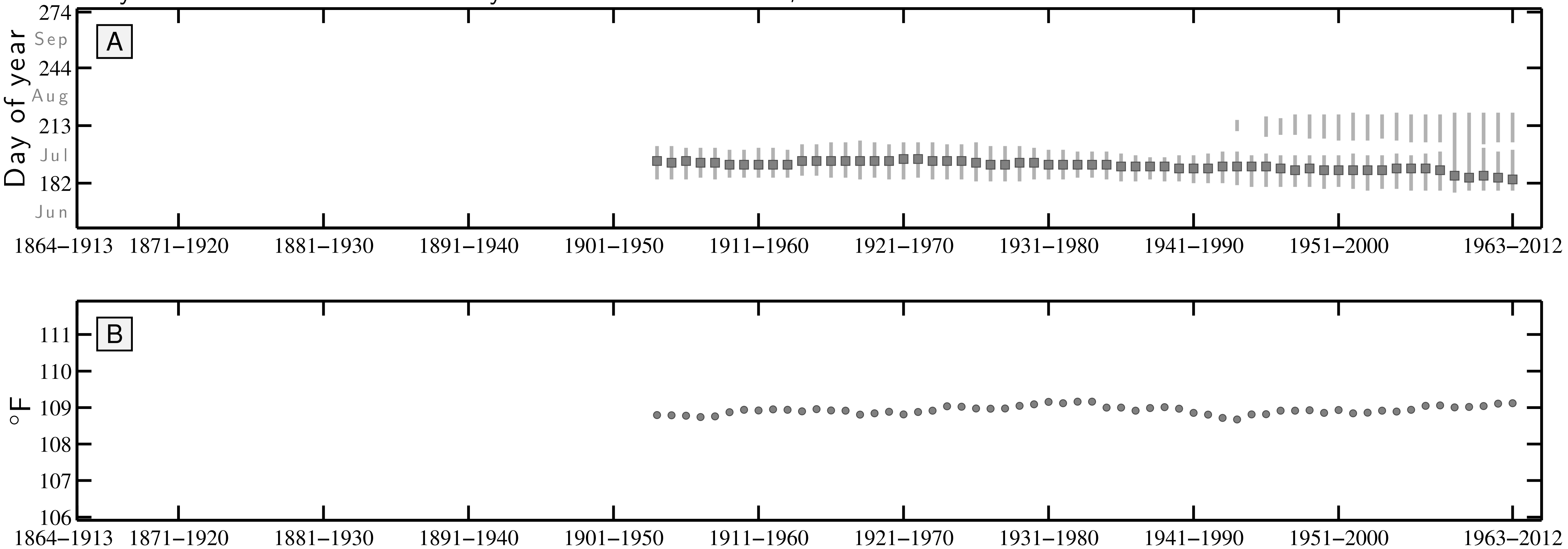

50 year Winter Teletherm dynamics for BLYTHE, CA:

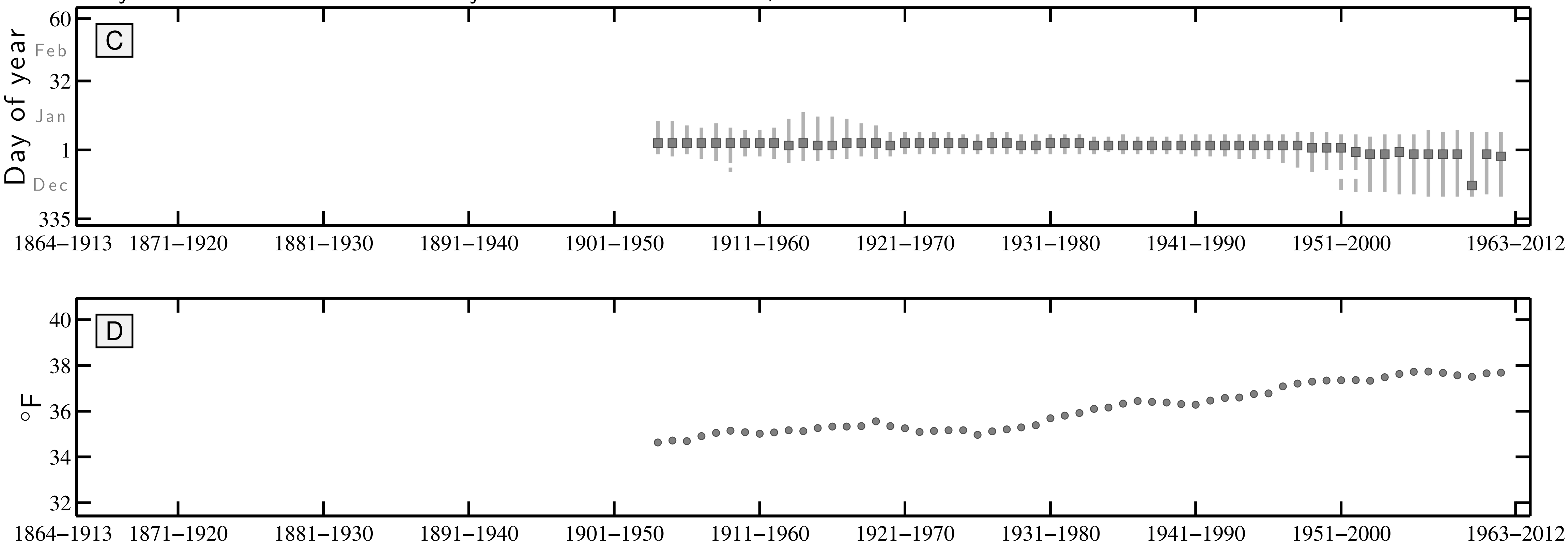

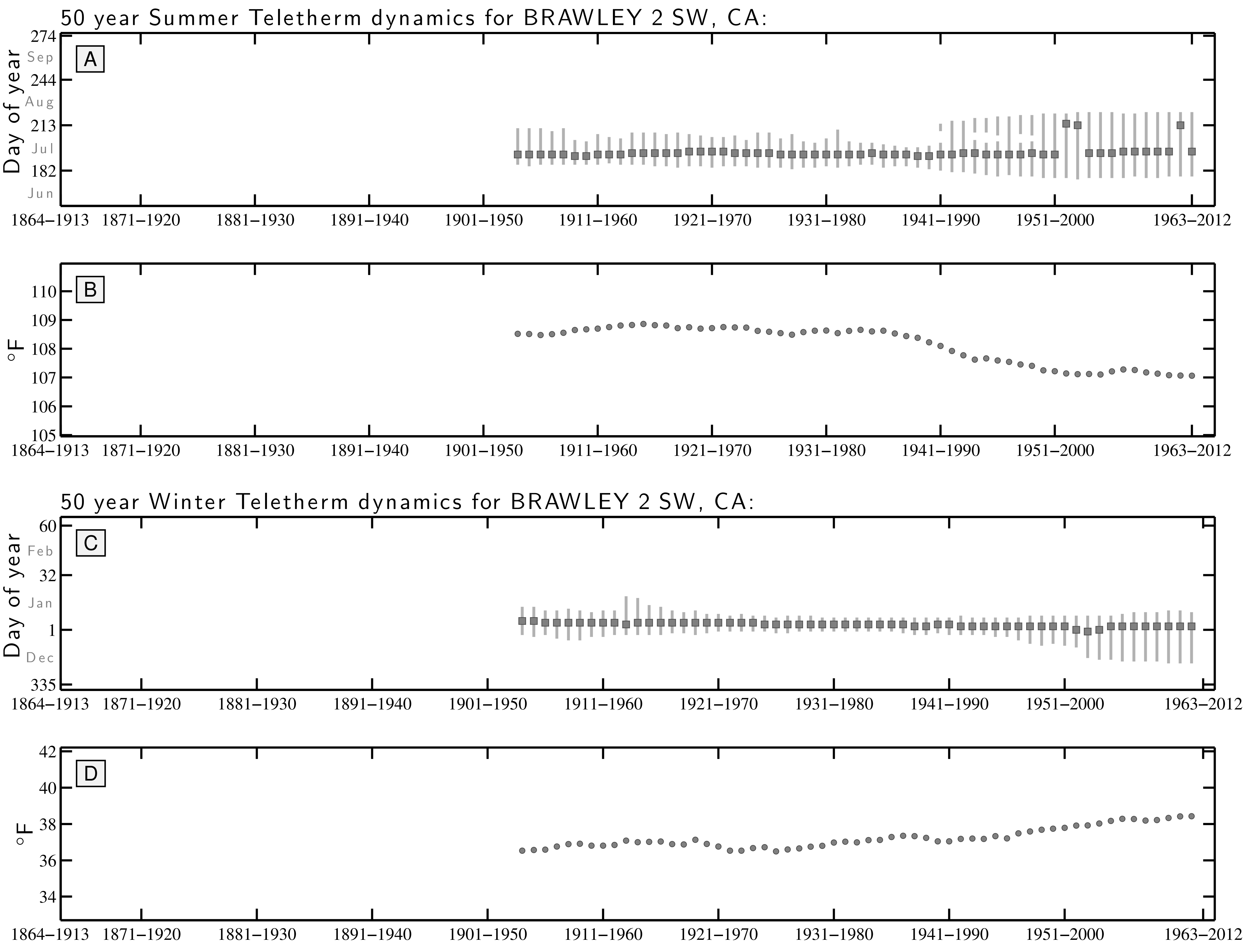

50 year Summer Teletherm dynamics for BRAWLEY 2 SW, CA:
A
B
Day of year
°F
274
Sep
244
Aug
213
Jul
182
Jun
110
109
108
107
106
105
50 year Winter Teletherm dynamics for BRAWLEY 2 SW, CA:
C
D
60
Feb
32
Jan
1
Dec
335
42
40
38
36
34
1864–1913
1871–1920
1881–1930
1891–1940
1901–1950
1911–1960
1921–1970
1931–1980
1941–1990
1951–2000
1963–2012

50 year Summer Teletherm dynamics for CEDARVILLE, CA:

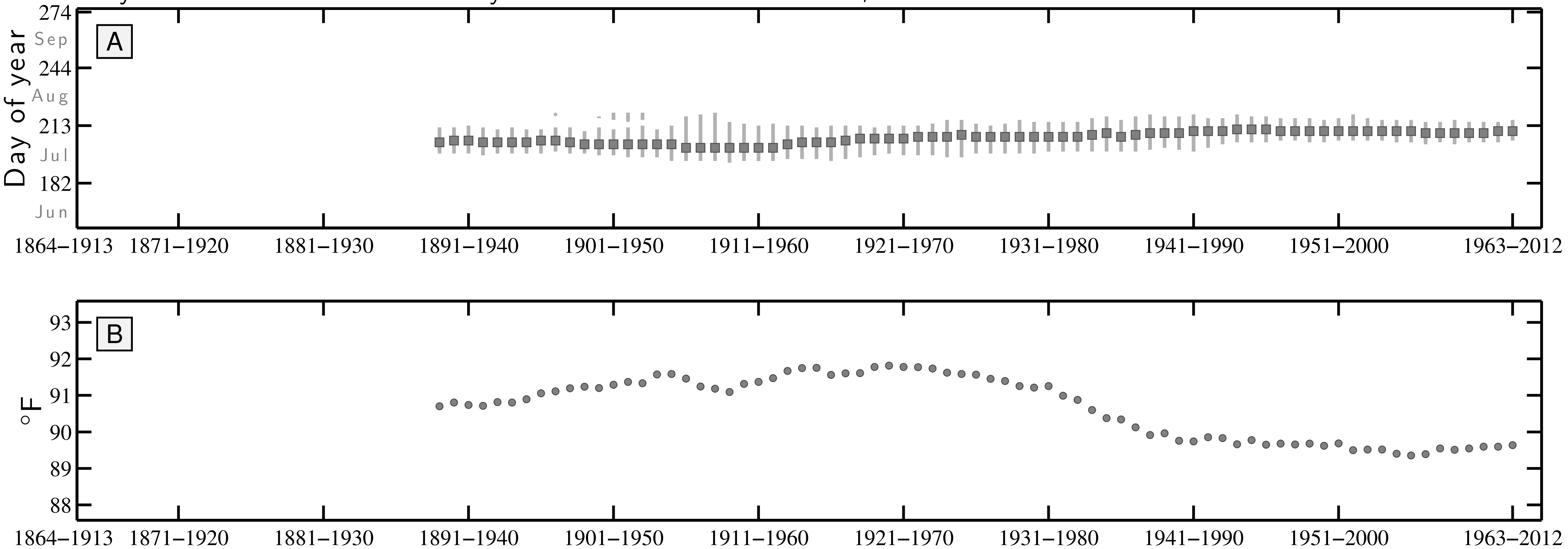

50 year Winter Teletherm dynamics for CEDARVILLE, CA:

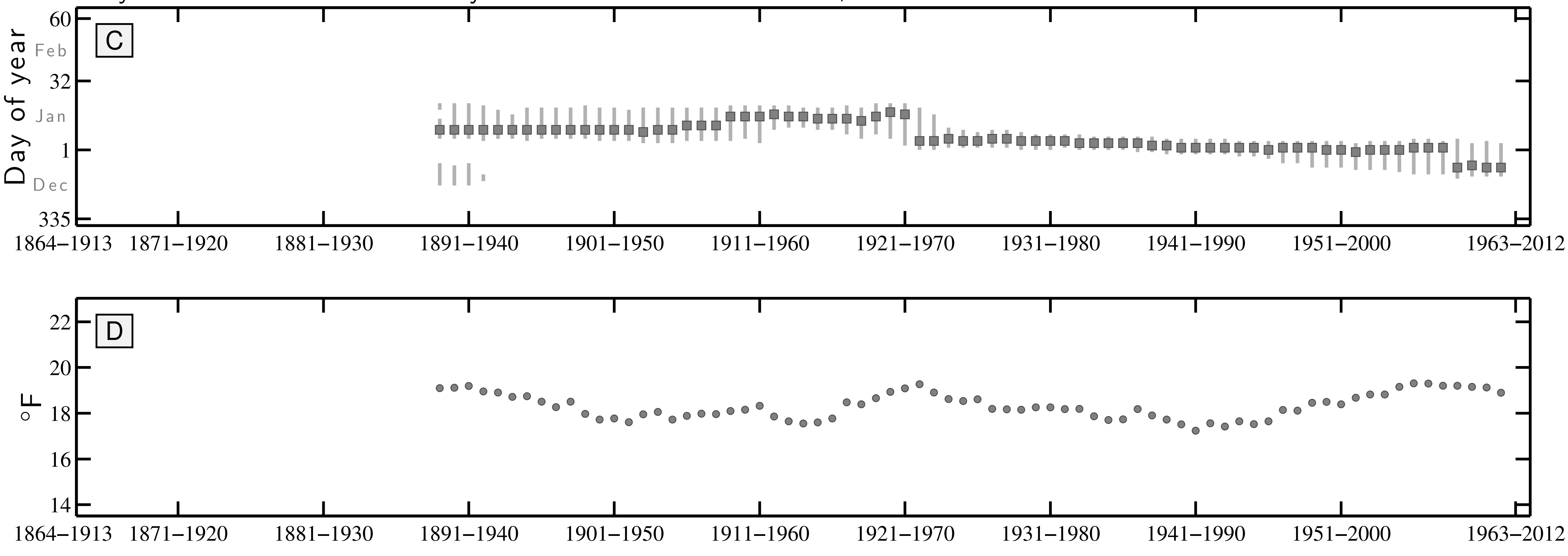

50 year Summer Teletherm dynamics for CHICO UNIV FARM, CA:

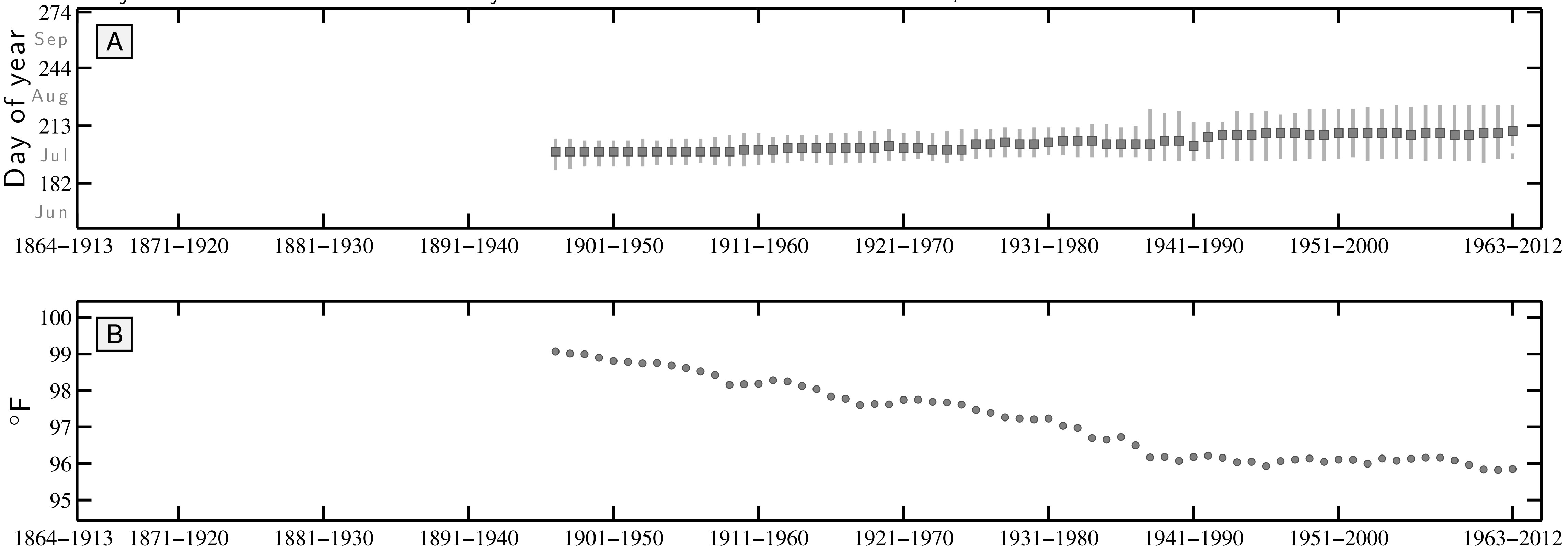

50 year Winter Teletherm dynamics for CHICO UNIV FARM, CA:

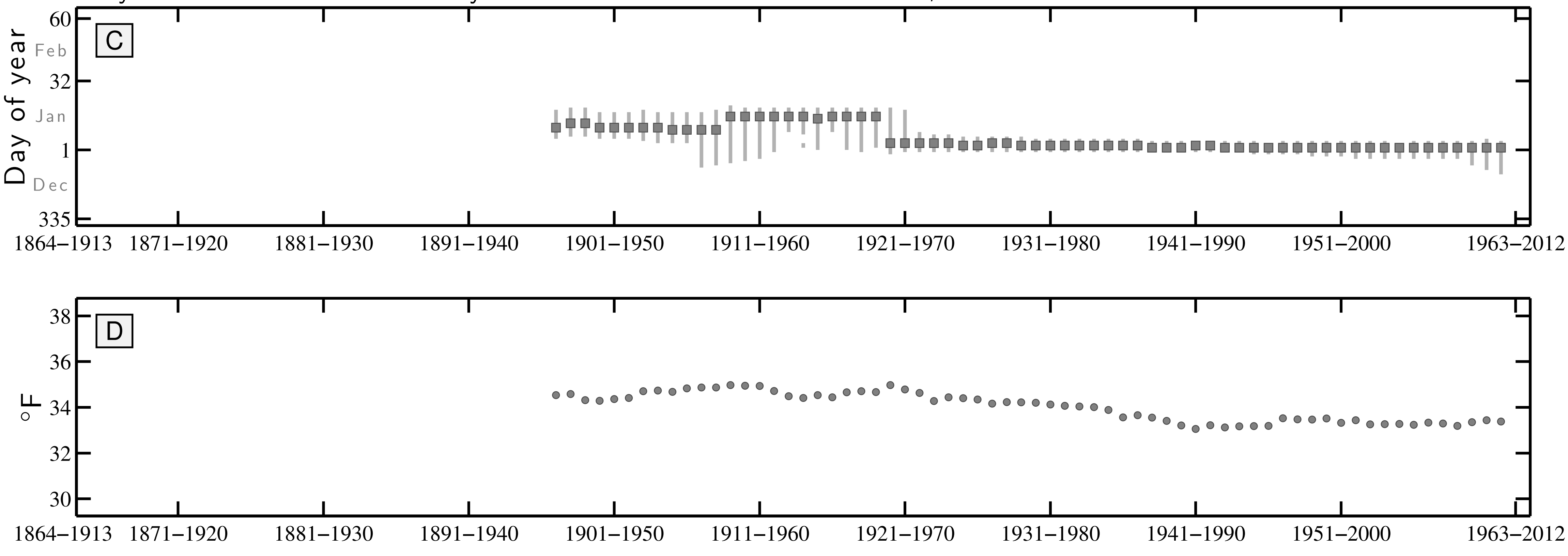

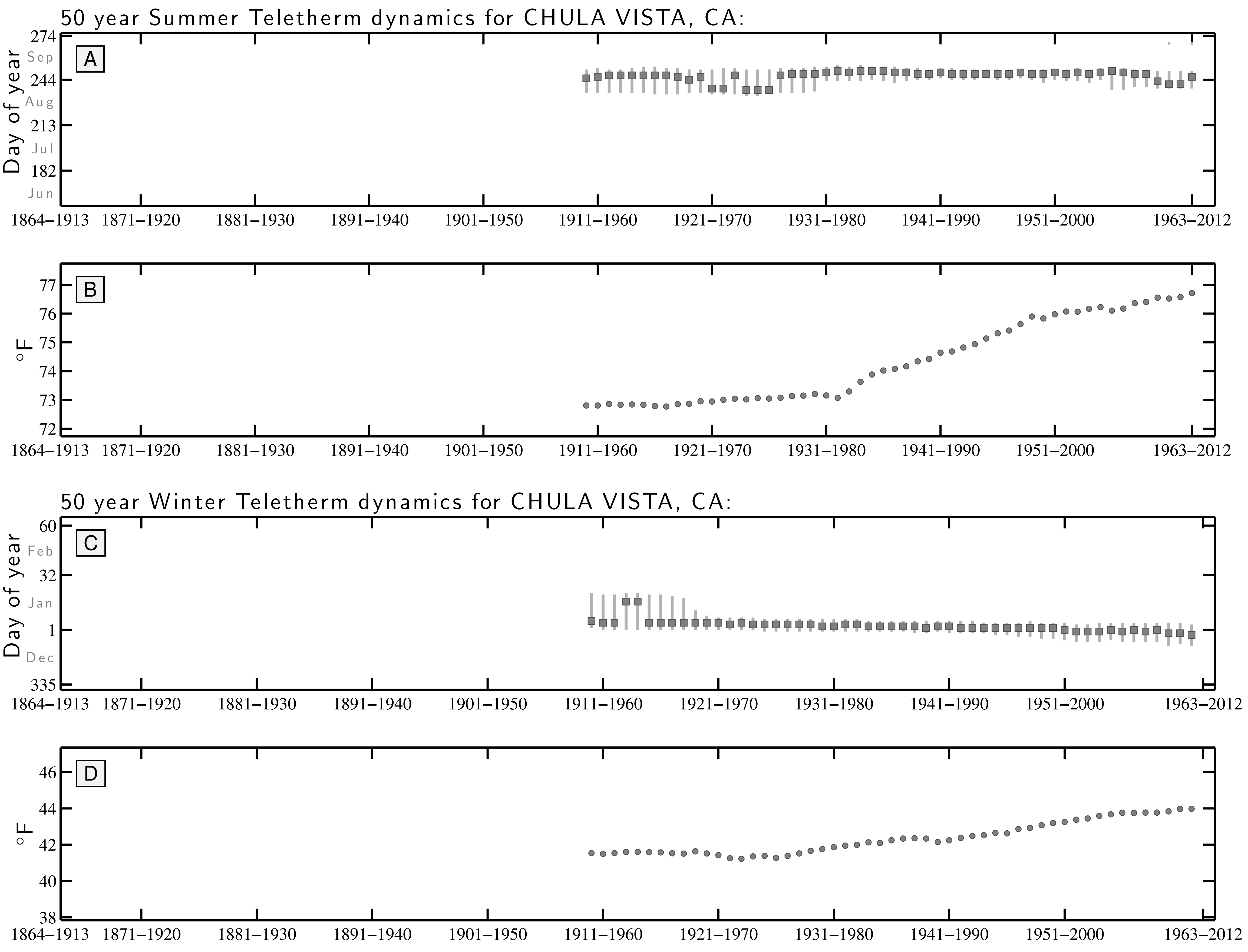

50 year Summer Teletherm dynamics for CHULA VISTA, CA:
A
Day of year
274
Sep
244
Aug
213
Jul
182
Jun
B
°F
77
76
75
74
73
72
50 year Winter Teletherm dynamics for CHULA VISTA, CA:
C
Day of year
60
Feb
32
Jan
1
Dec
335
D
°F
46
44
42
40
38
1864–1913
1871–1920
1881–1930
1891–1940
1901–1950
1911–1960
1921–1970
1931–1980
1941–1990
1951–2000
1963–2012

50 year Summer Teletherm dynamics for COLFAX, CA:

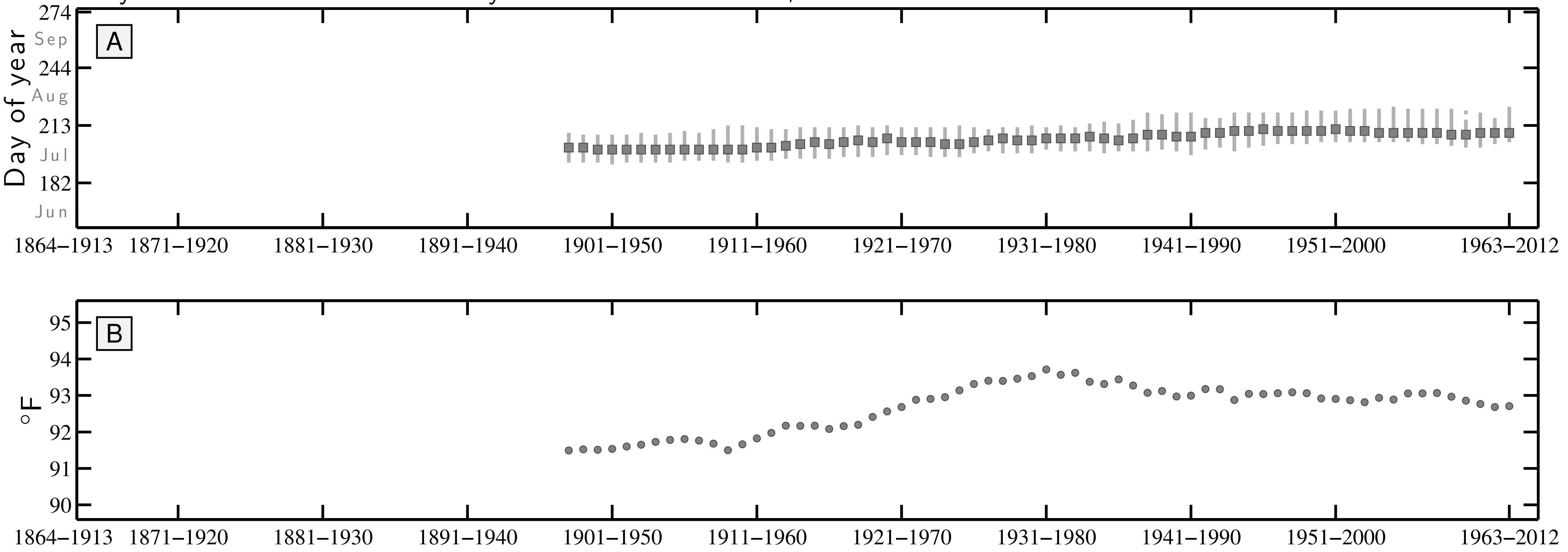

50 year Winter Teletherm dynamics for COLFAX, CA:

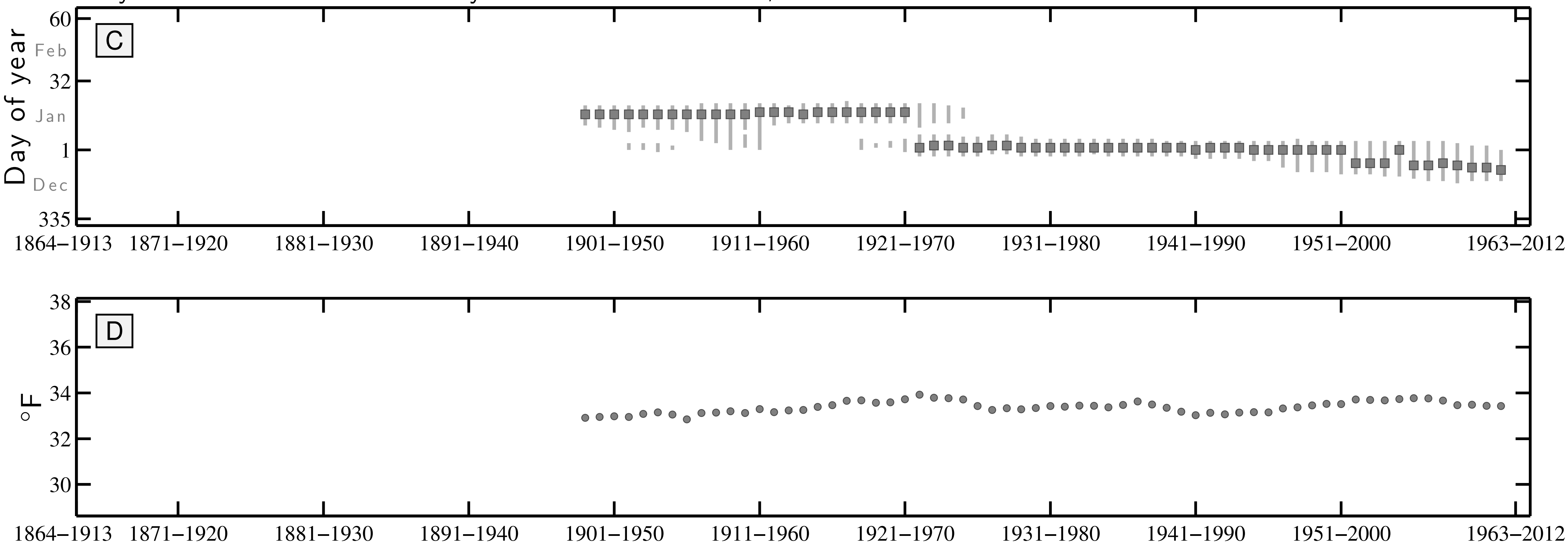

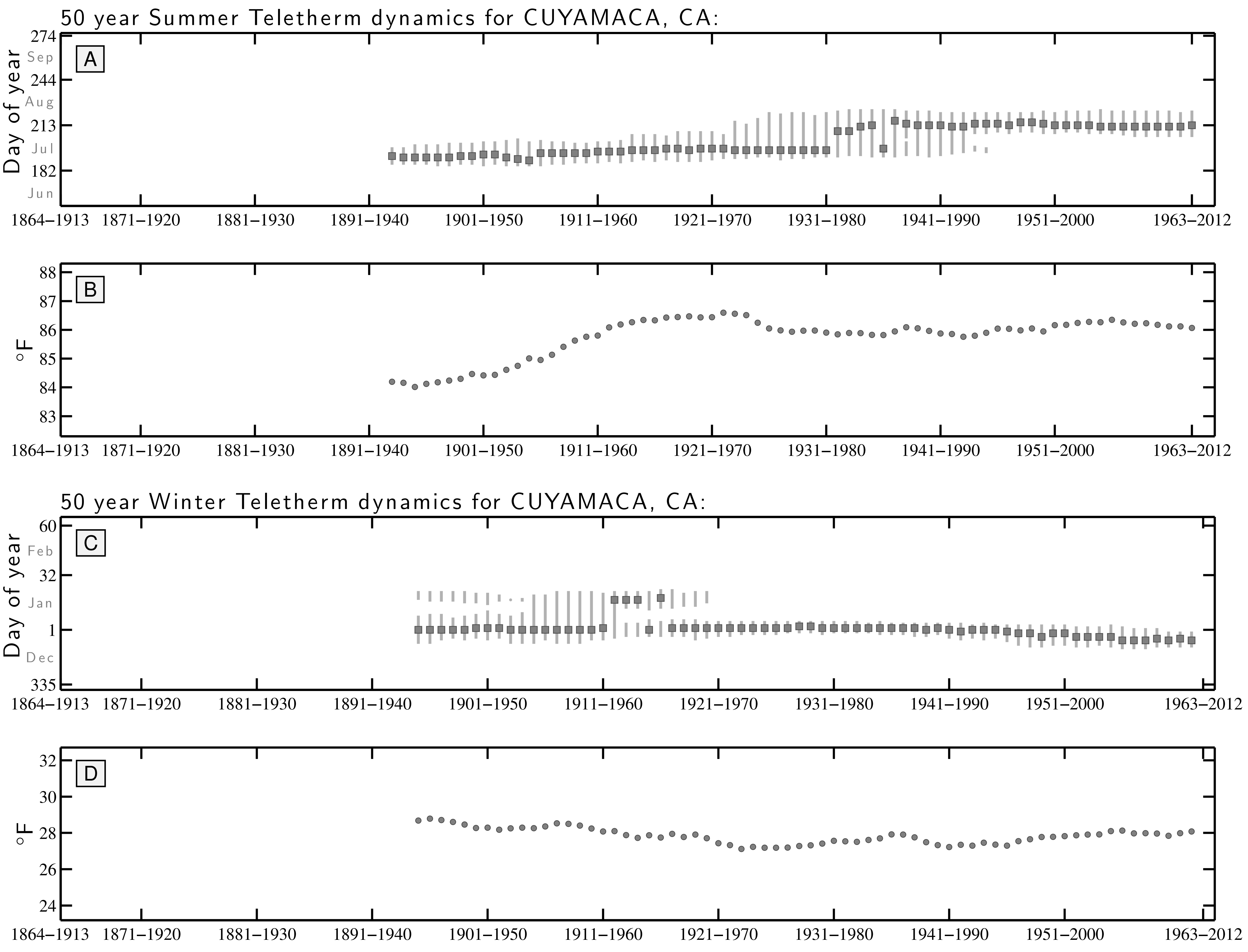
50 year Summer Teletherm dynamics for CUYAMACA, CA:
A
B
50 year Winter Teletherm dynamics for CUYAMACA, CA:
C
D
Day of year
°F
Jun
Jul
Aug
Sep
Dec
Jan
Feb
182
213
244
274
335
1
32
60
83
84
85
86
87
88
24
26
28
30
32
1864–1913
1871–1920
1881–1930
1891–1940
1901–1950
1911–1960
1921–1970
1931–1980
1941–1990
1951–2000
1963–2012

50 year Summer Teletherm dynamics for DAVIS 2 WSW EXP, CA:

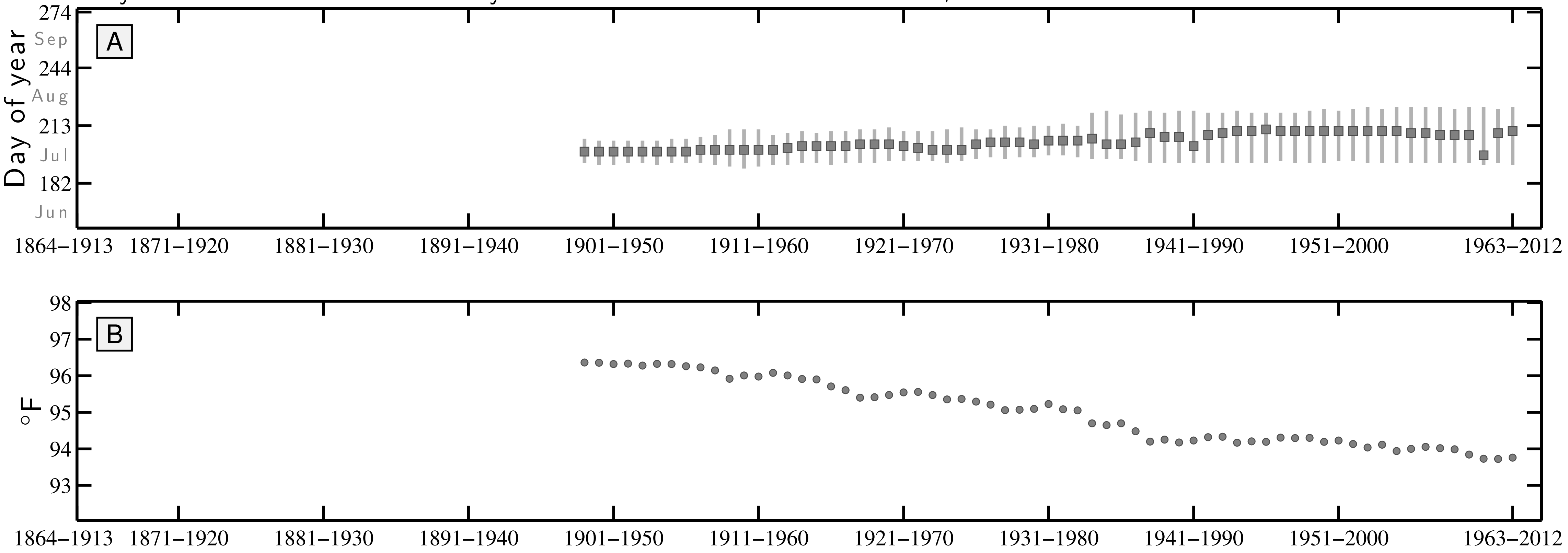

50 year Winter Teletherm dynamics for DAVIS 2 WSW EXP, CA:

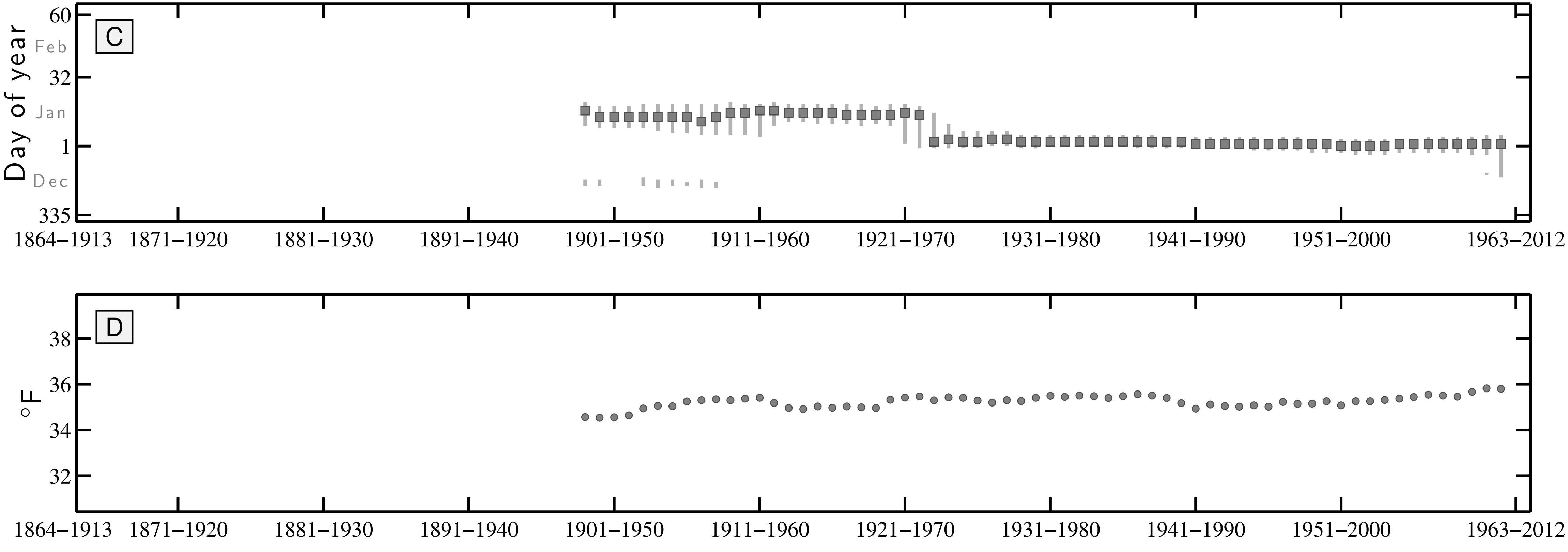

50 year Summer Teletherm dynamics for DEATH VALLEY, CA:

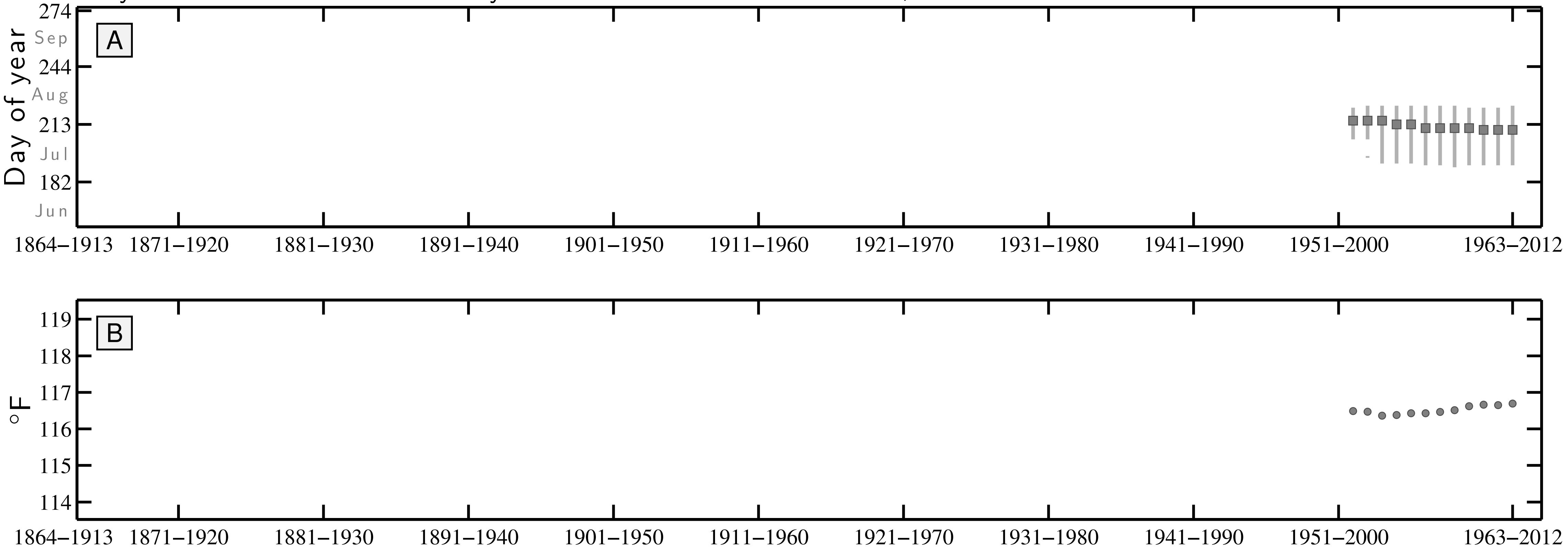

50 year Winter Teletherm dynamics for DEATH VALLEY, CA:

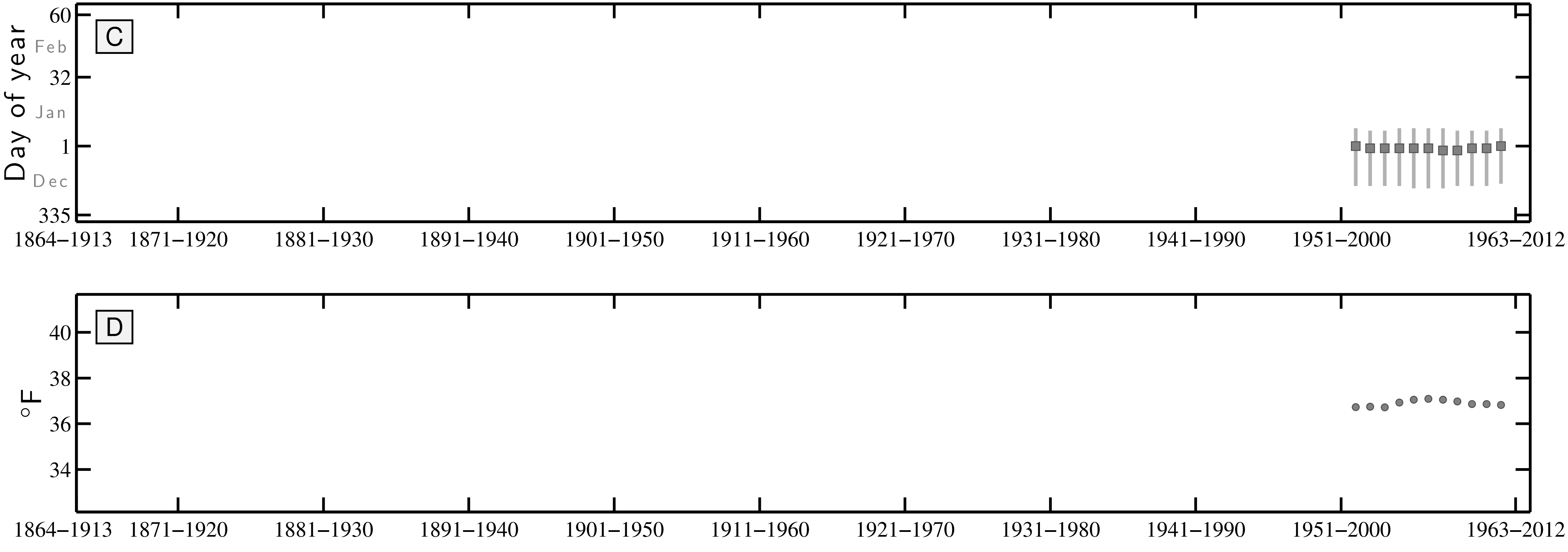

50 year Summer Teletherm dynamics for ELECTRA P H, CA:

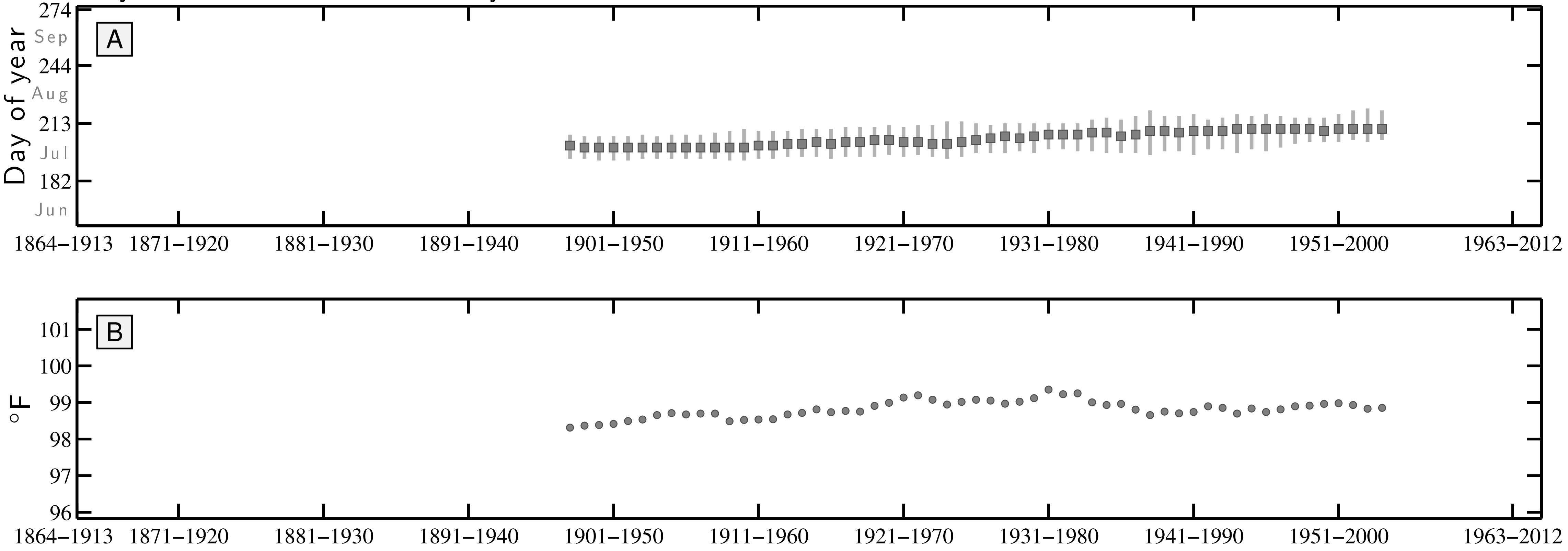

50 year Winter Teletherm dynamics for ELECTRA P H, CA:

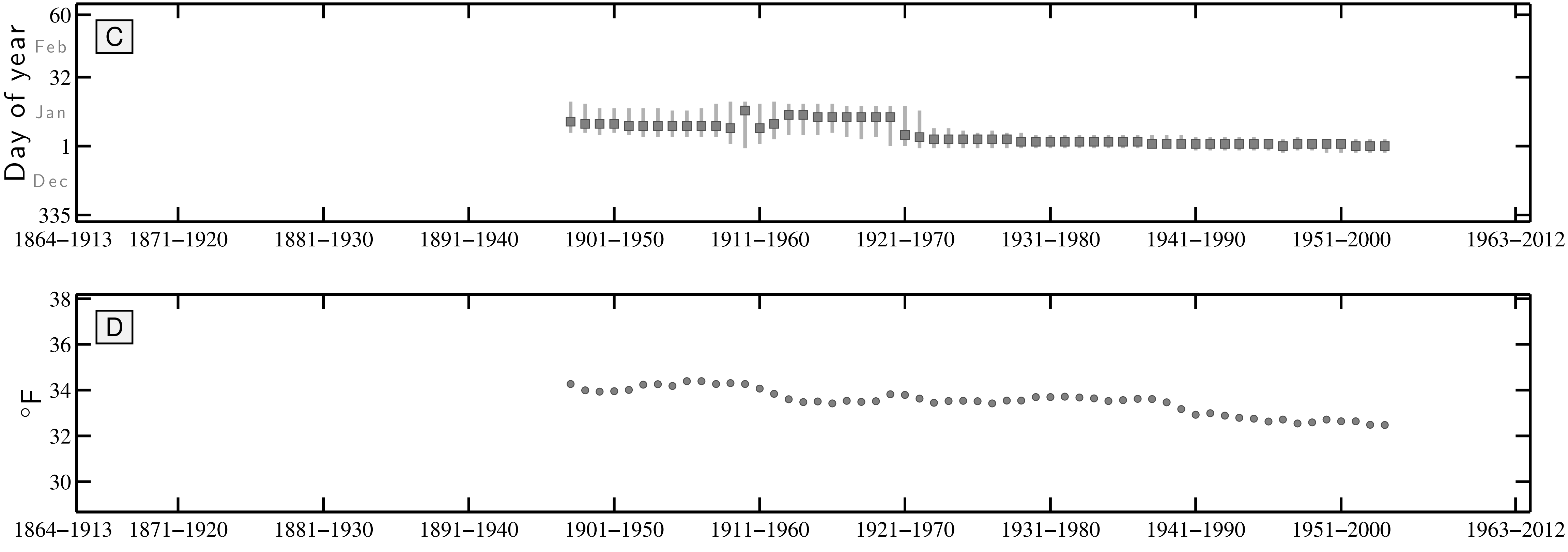

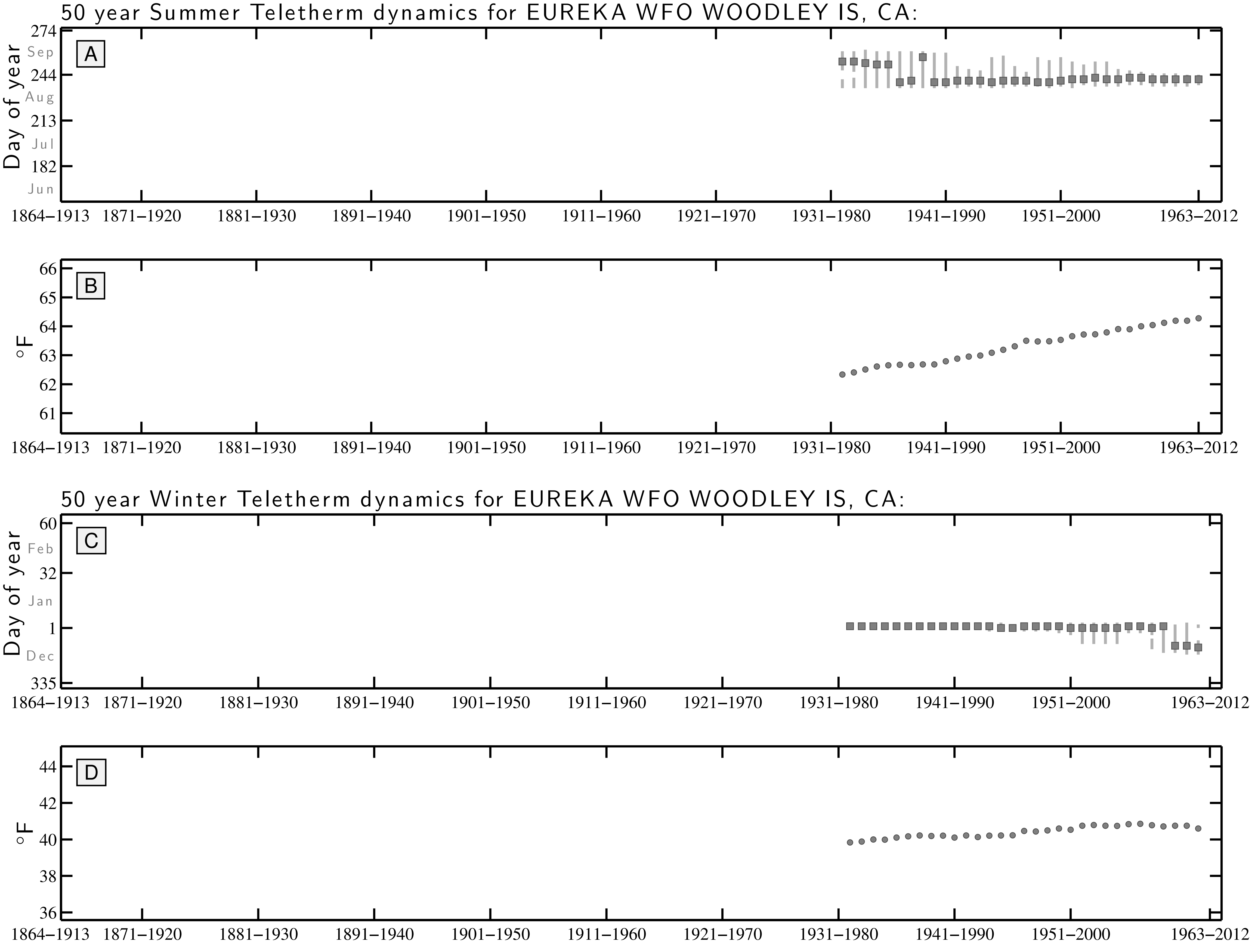
50 year Summer Teletherm dynamics for EUREKA WFO WOODLEY IS, CA:
A
B
Day of year
°F
274
244
213
182
Sep
Aug
Jul
Jun
66
65
64
63
62
61
50 year Winter Teletherm dynamics for EUREKA WFO WOODLEY IS, CA:
C
D
60
32
1
335
Feb
Jan
Dec
44
42
40
38
36
1864–1913
1871–1920
1881–1930
1891–1940
1901–1950
1911–1960
1921–1970
1931–1980
1941–1990
1951–2000
1963–2012

50 year Summer Teletherm dynamics for FAIRMONT, CA:

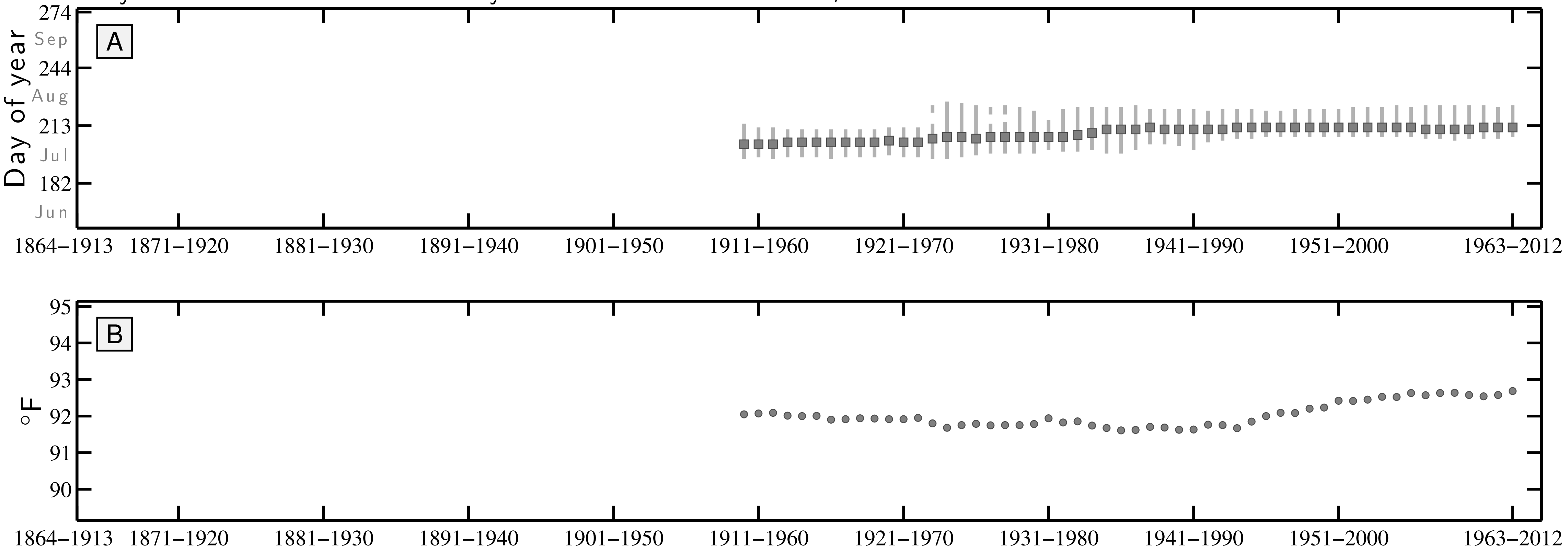

50 year Winter Teletherm dynamics for FAIRMONT, CA:

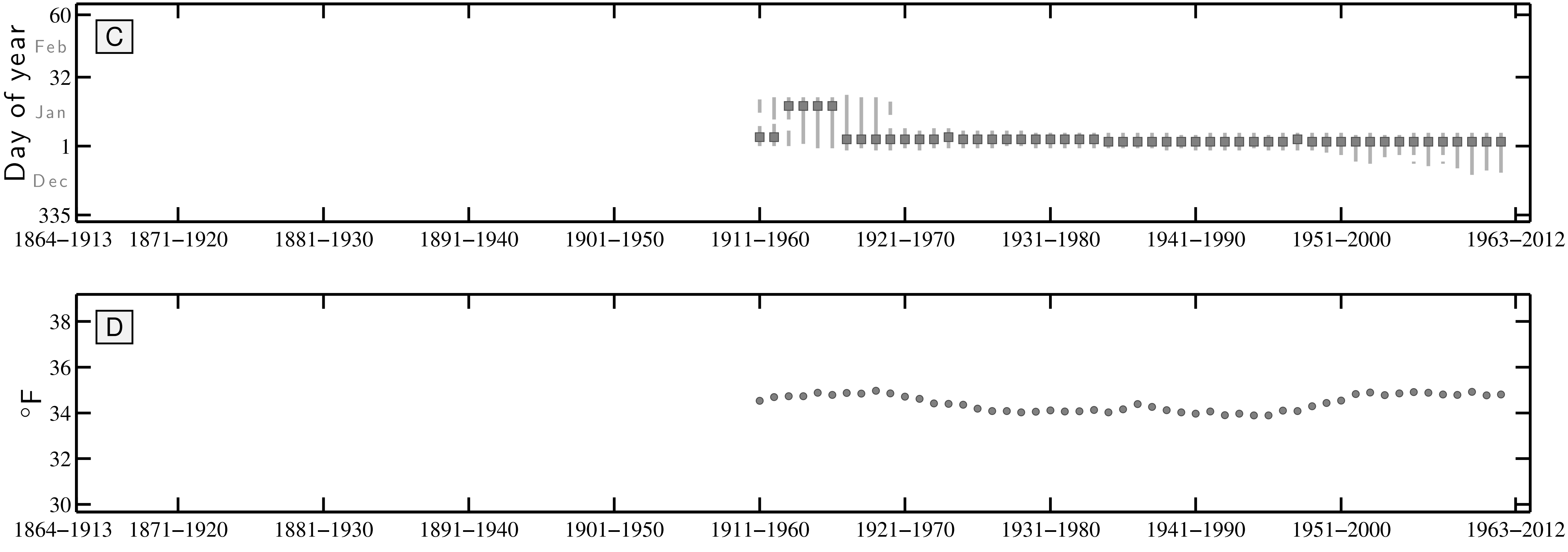

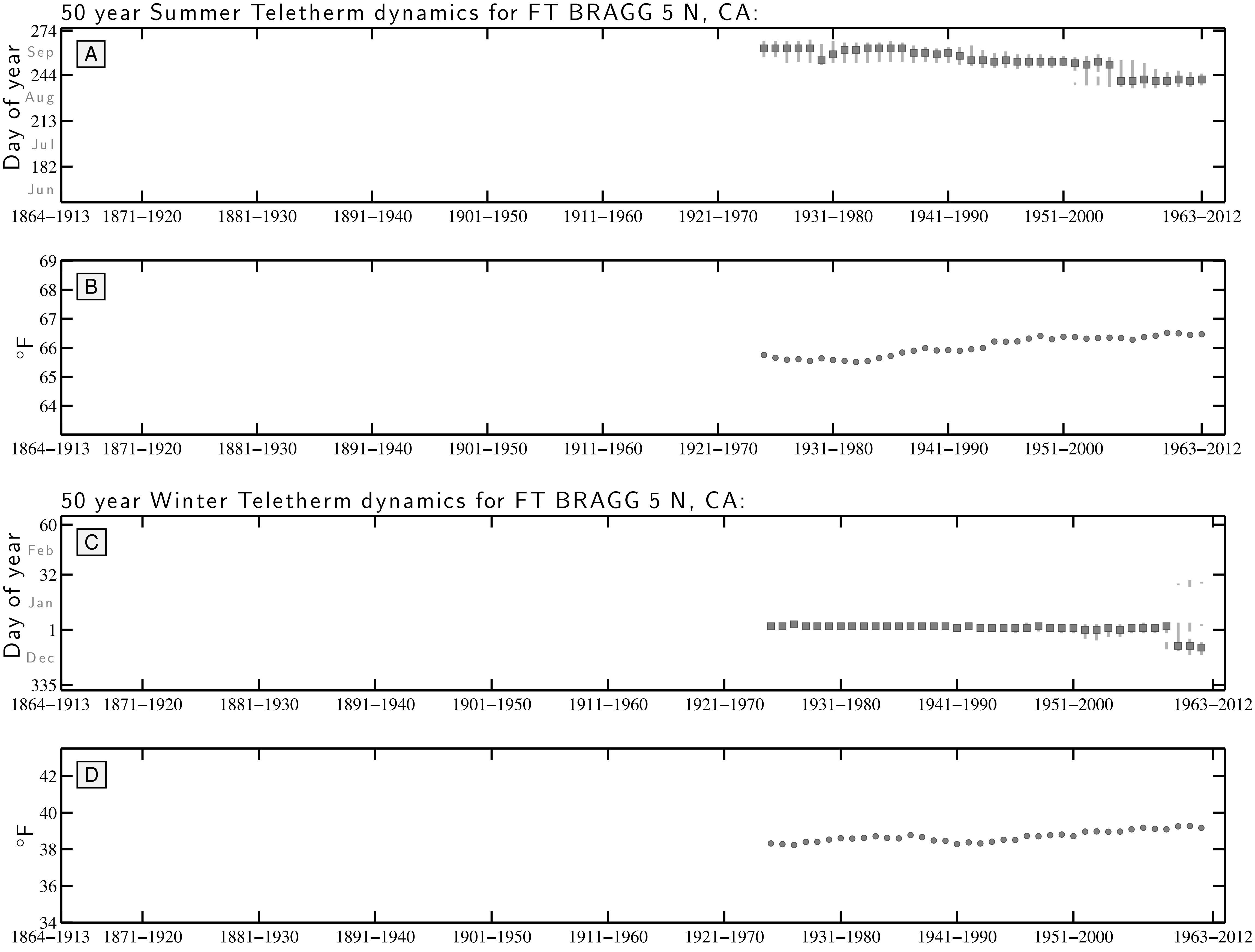

50 year Summer Teletherm dynamics for FT BRAGG 5 N, CA:
A
B
Day of year
°F
Sep
Aug
Jul
Jun
274
244
213
182
69
68
67
66
65
64
50 year Winter Teletherm dynamics for FT BRAGG 5 N, CA:
C
D
Feb
Jan
Dec
60
32
1
335
42
40
38
36
34
1864–1913
1871–1920
1881–1930
1891–1940
1901–1950
1911–1960
1921–1970
1931–1980
1941–1990
1951–2000
1963–2012

50 year Summer Teletherm dynamics for FRESNO YOSEMITE AP, CA:

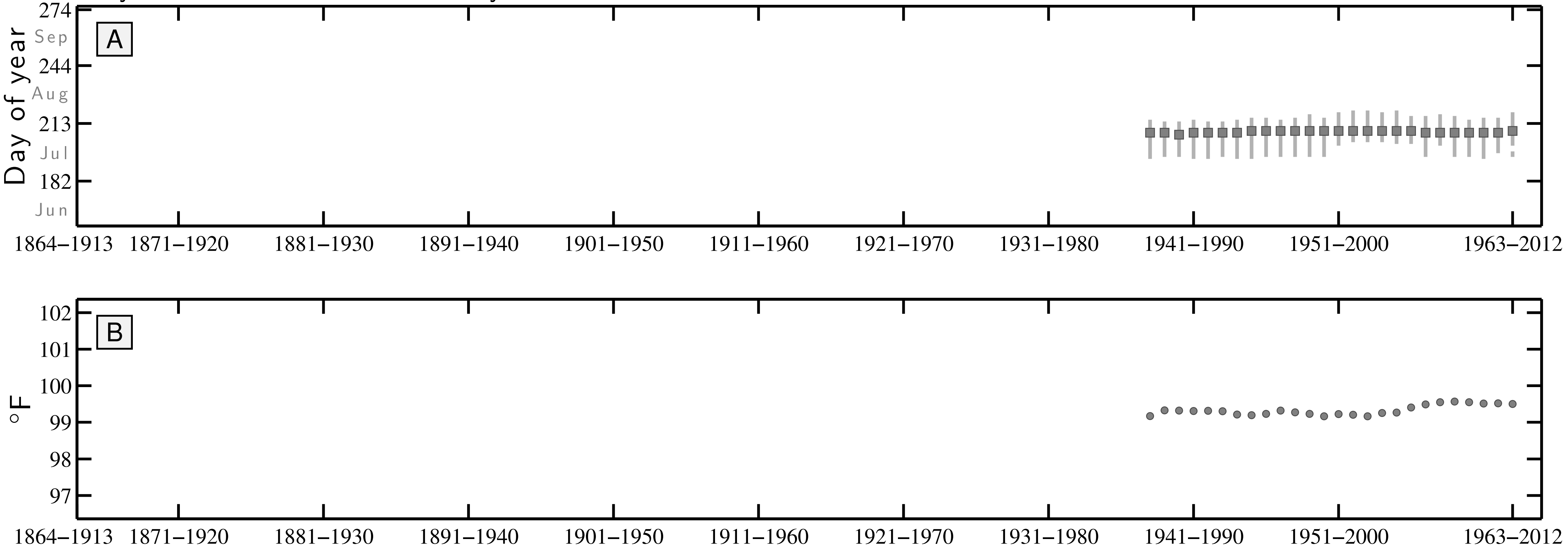

50 year Winter Teletherm dynamics for FRESNO YOSEMITE AP, CA:

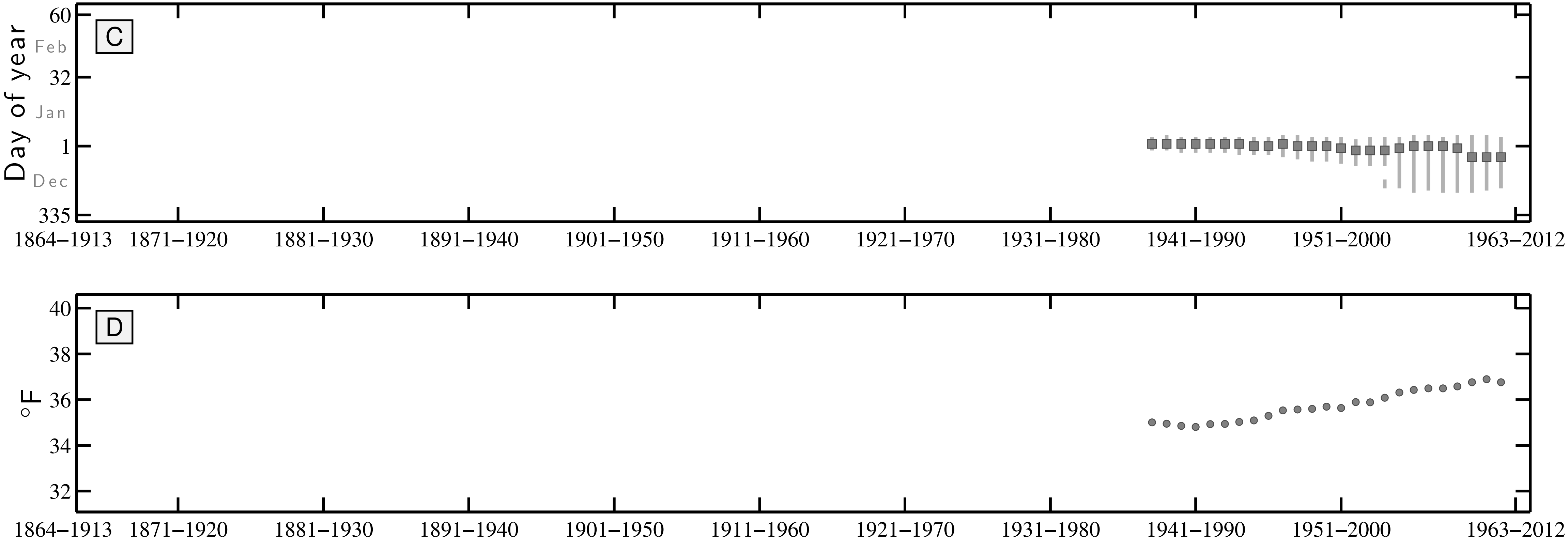

50 year Summer Teletherm dynamics for HANFORD 1 S, CA:

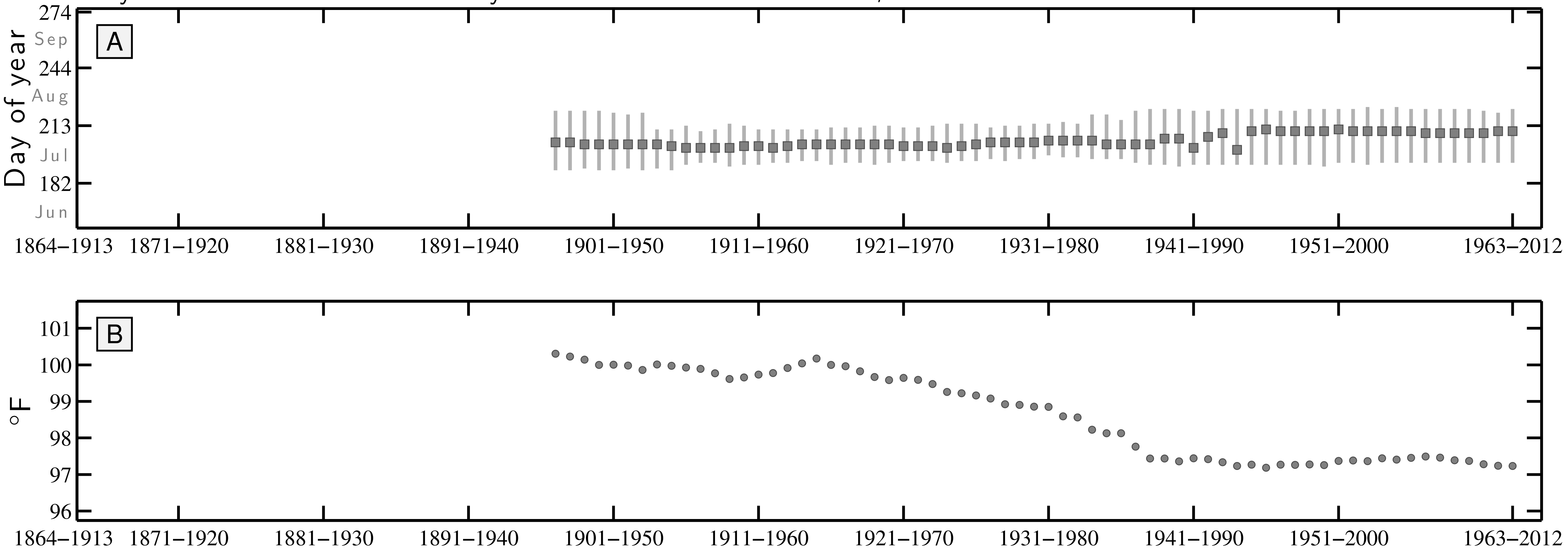

50 year Winter Teletherm dynamics for HANFORD 1 S, CA:

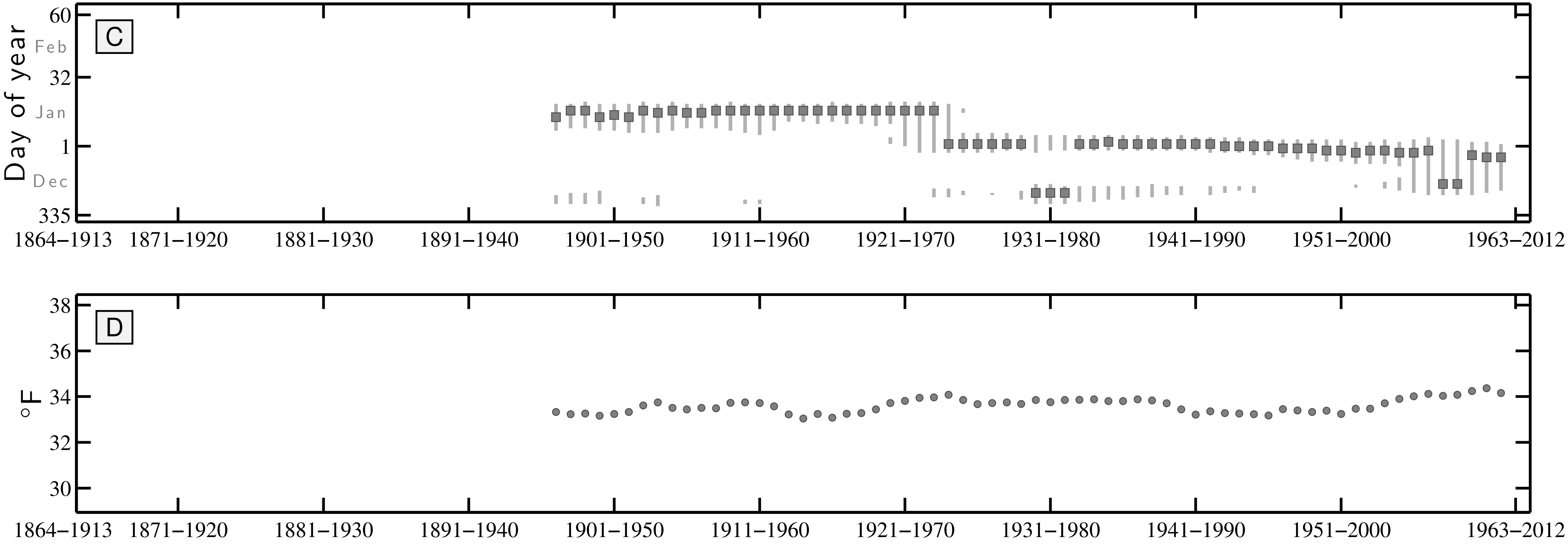

50 year Summer Teletherm dynamics for HAPPY CAMP RS, CA:

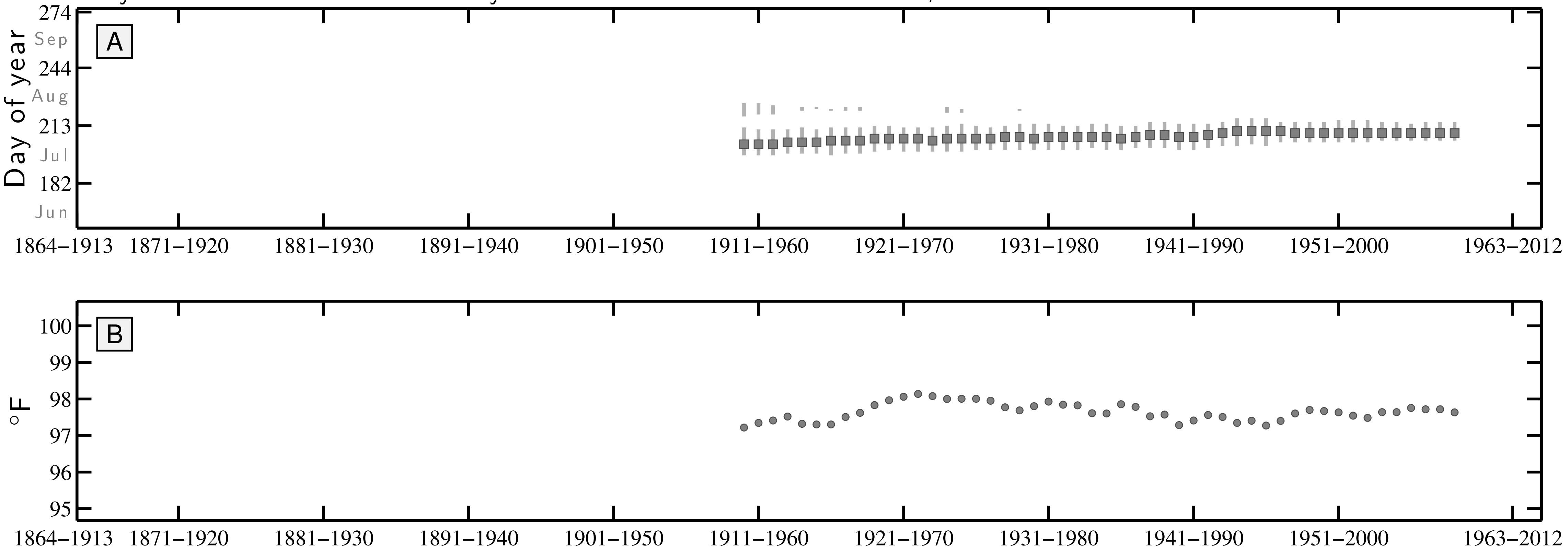

50 year Winter Teletherm dynamics for HAPPY CAMP RS, CA:

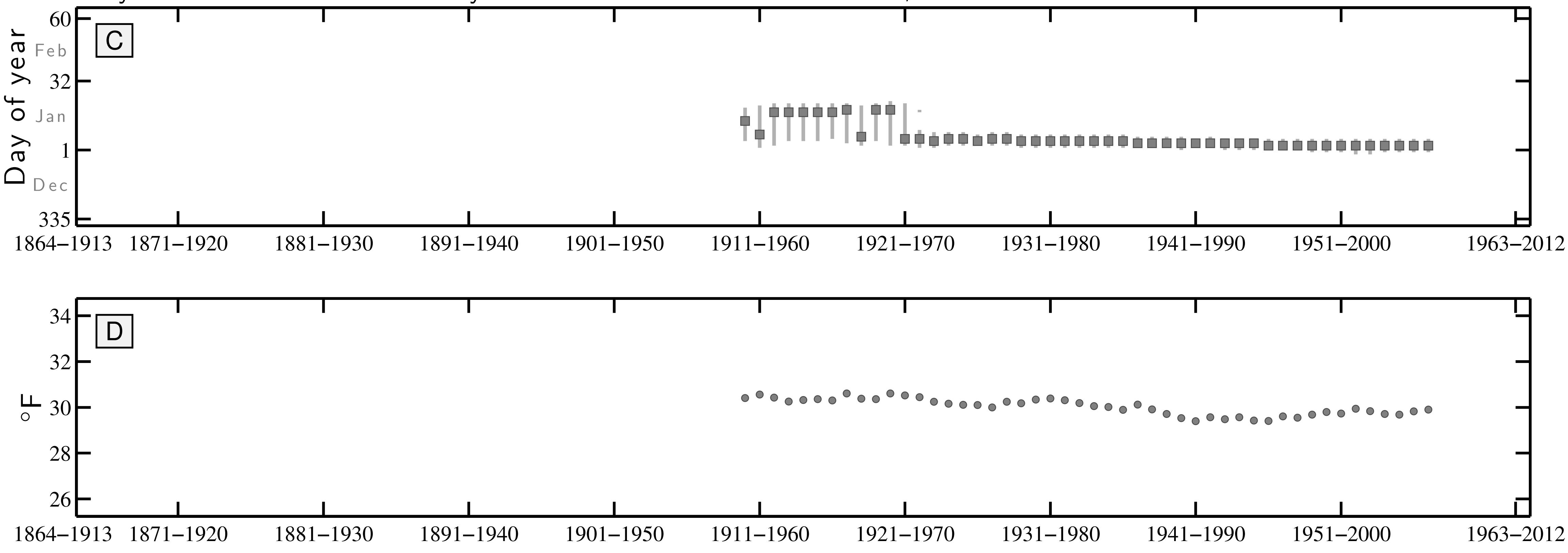

50 year Summer Teletherm dynamics for HEALDSBURG, CA:

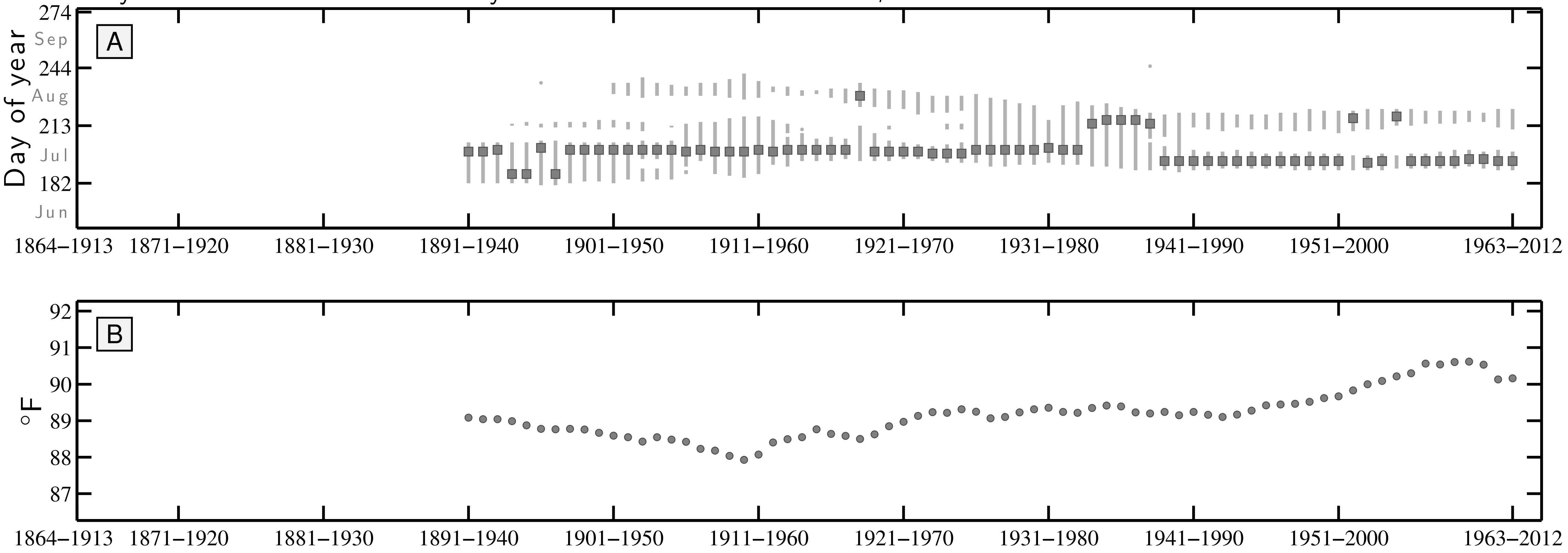

50 year Winter Teletherm dynamics for HEALDSBURG, CA:

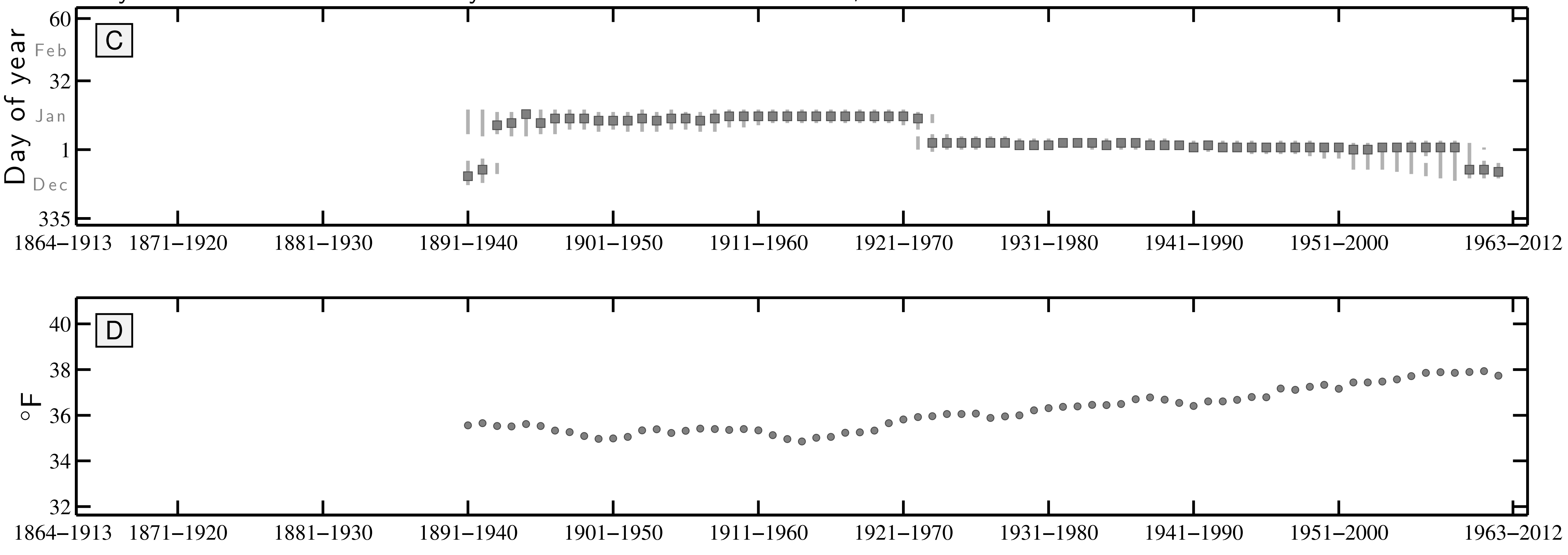

50 year Summer Teletherm dynamics for INDEPENDENCE, CA:

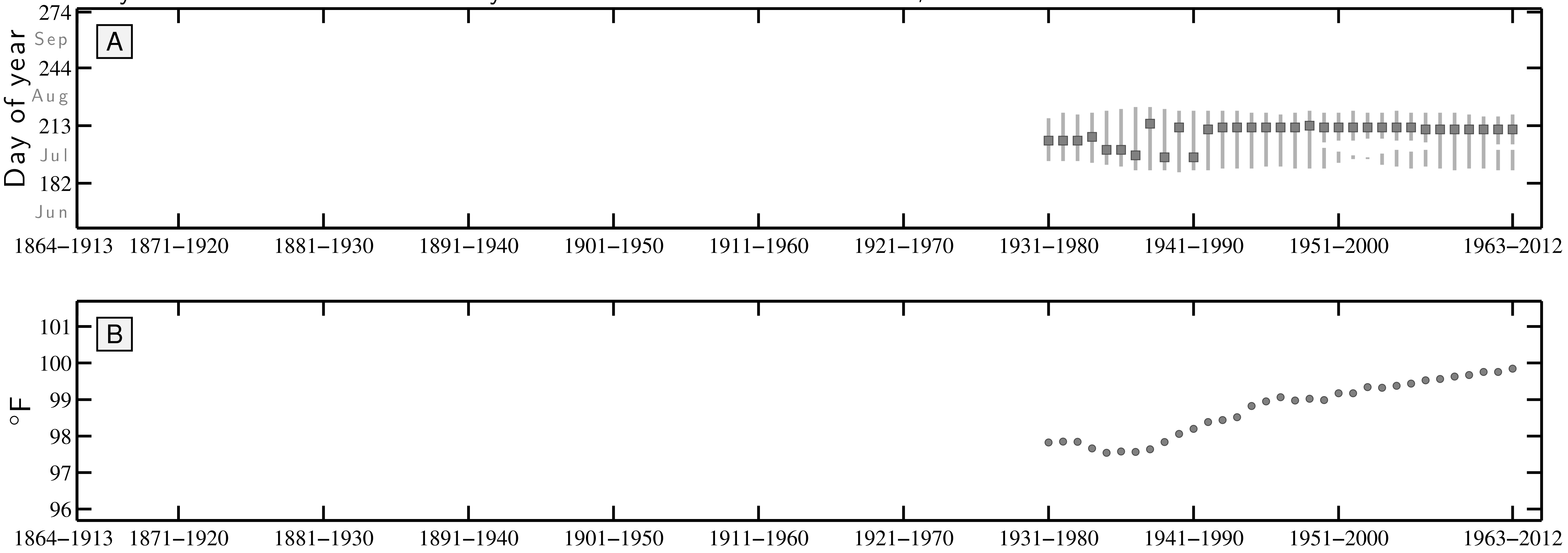

50 year Winter Teletherm dynamics for INDEPENDENCE, CA:

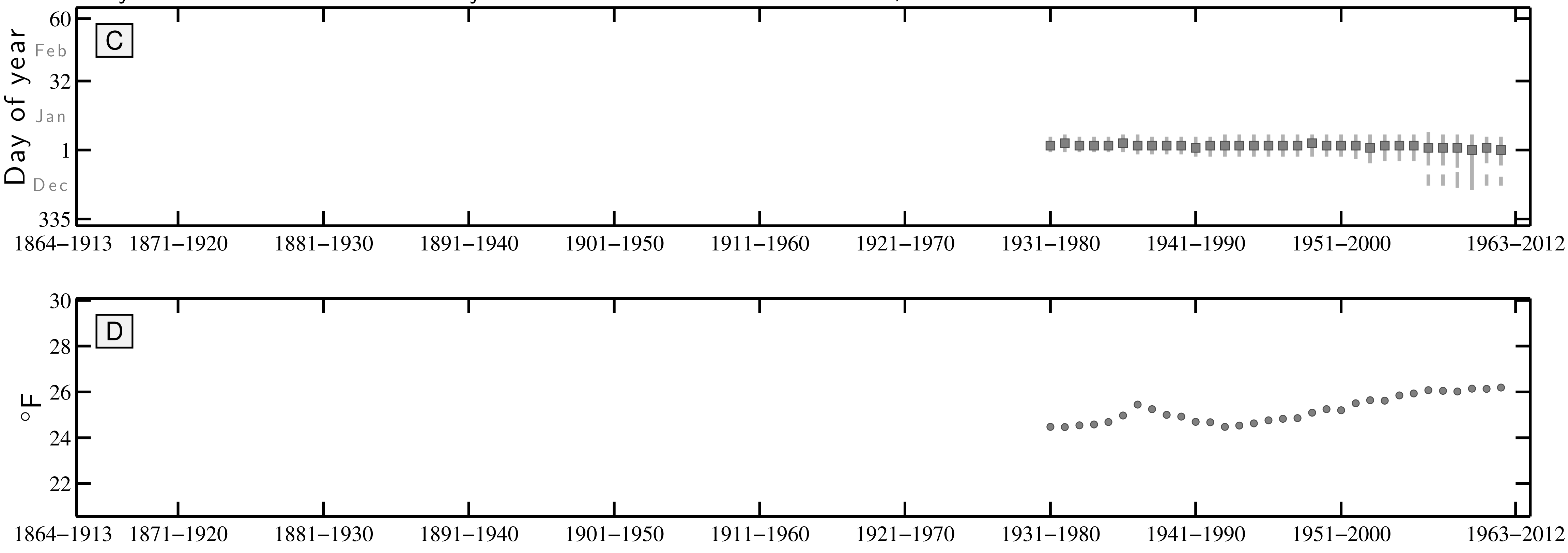

50 year Summer Teletherm dynamics for INDIO FIRE STN, CA:

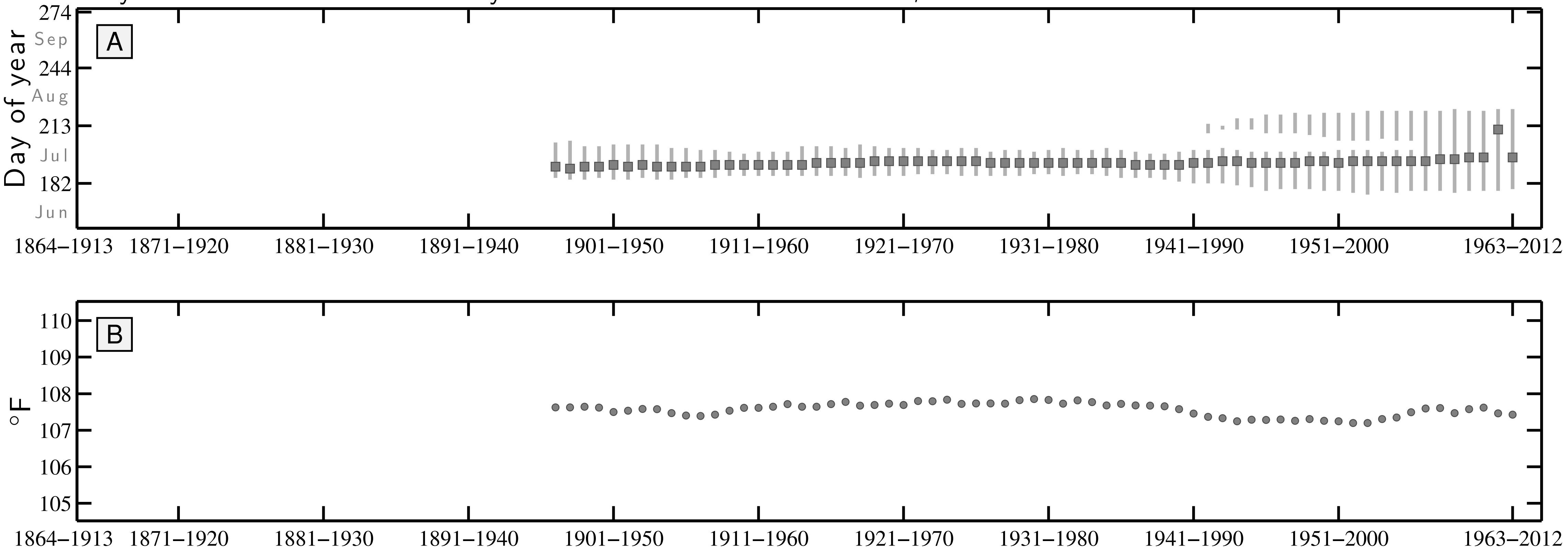

50 year Winter Teletherm dynamics for INDIO FIRE STN, CA:

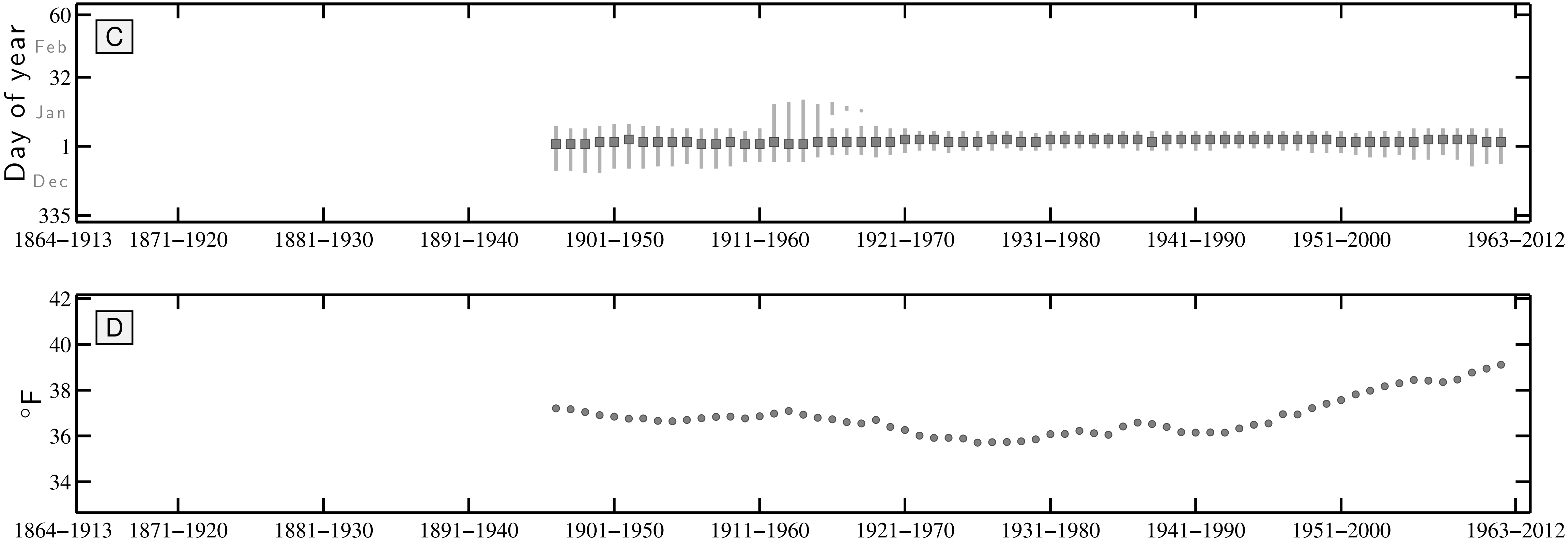

50 year Summer Teletherm dynamics for LAKE SPAULDING, CA:

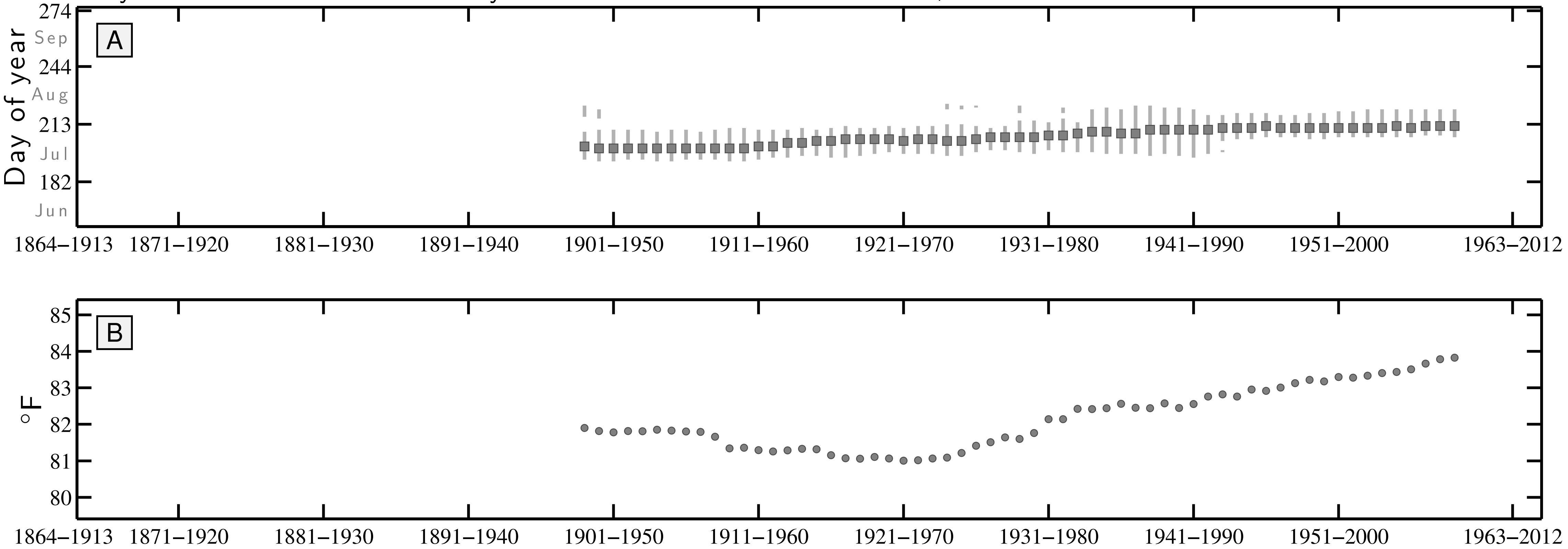

50 year Winter Teletherm dynamics for LAKE SPAULDING, CA:

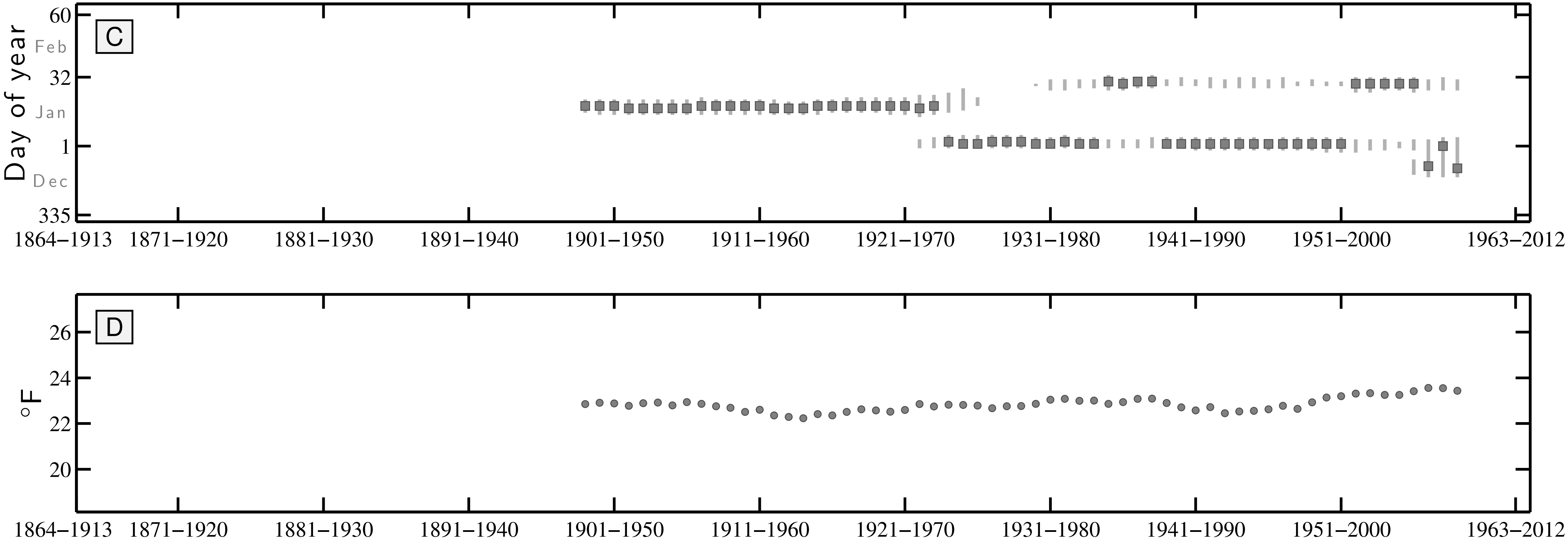

50 year Summer Teletherm dynamics for LEMON COVE, CA:

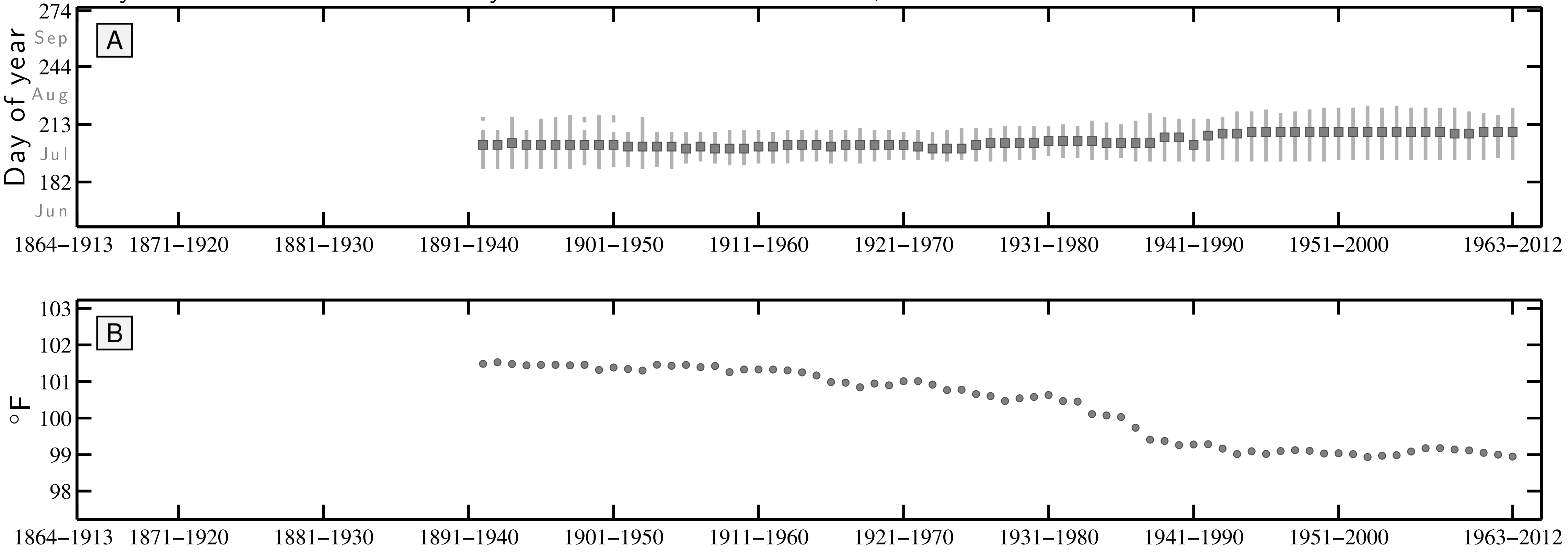

50 year Winter Teletherm dynamics for LEMON COVE, CA:

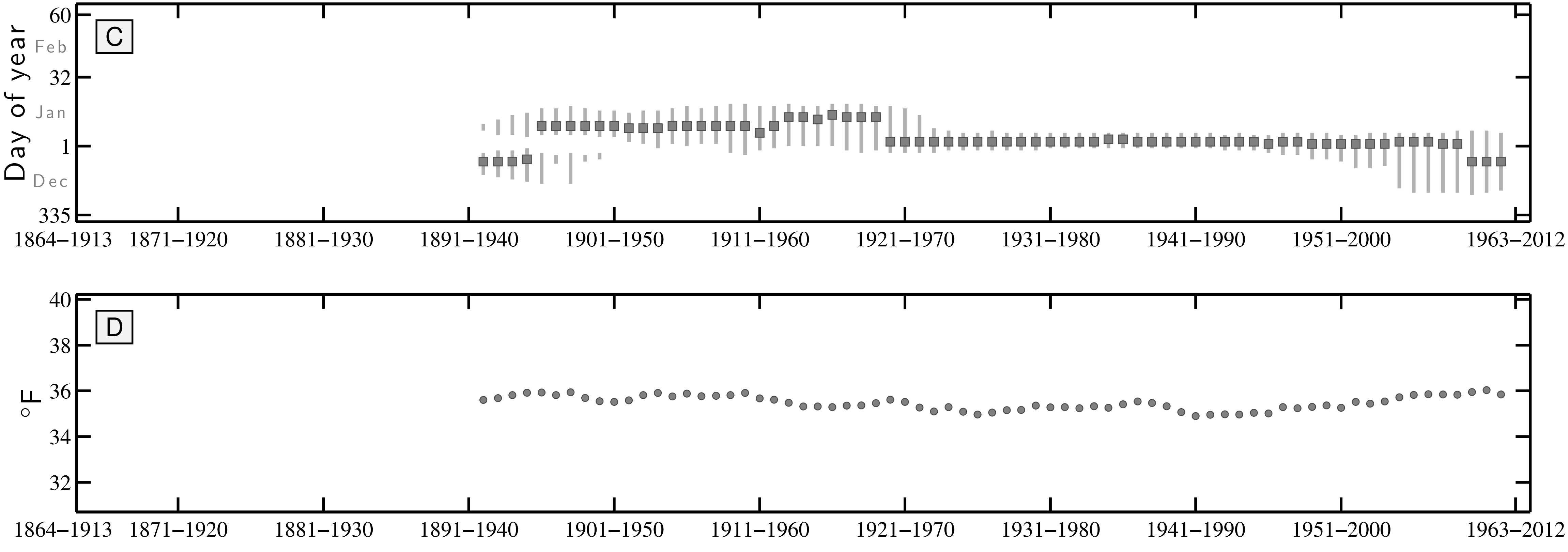

50 year Summer Teletherm dynamics for LIVERMORE, CA:

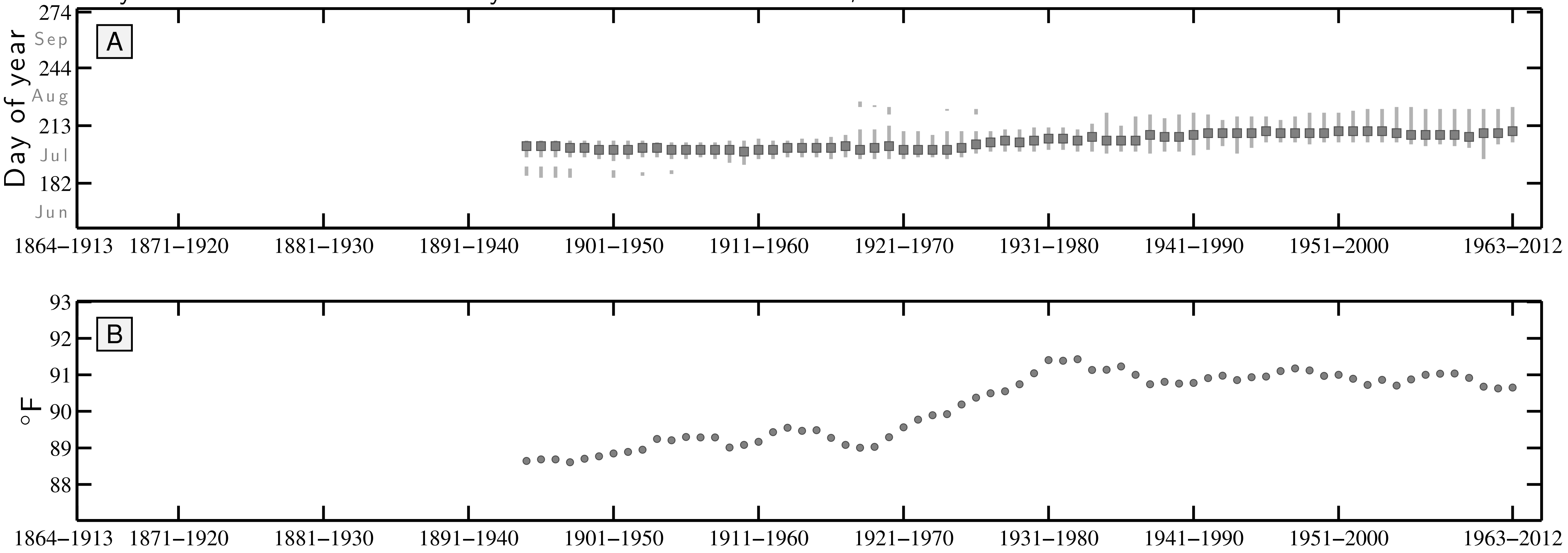

50 year Winter Teletherm dynamics for LIVERMORE, CA:

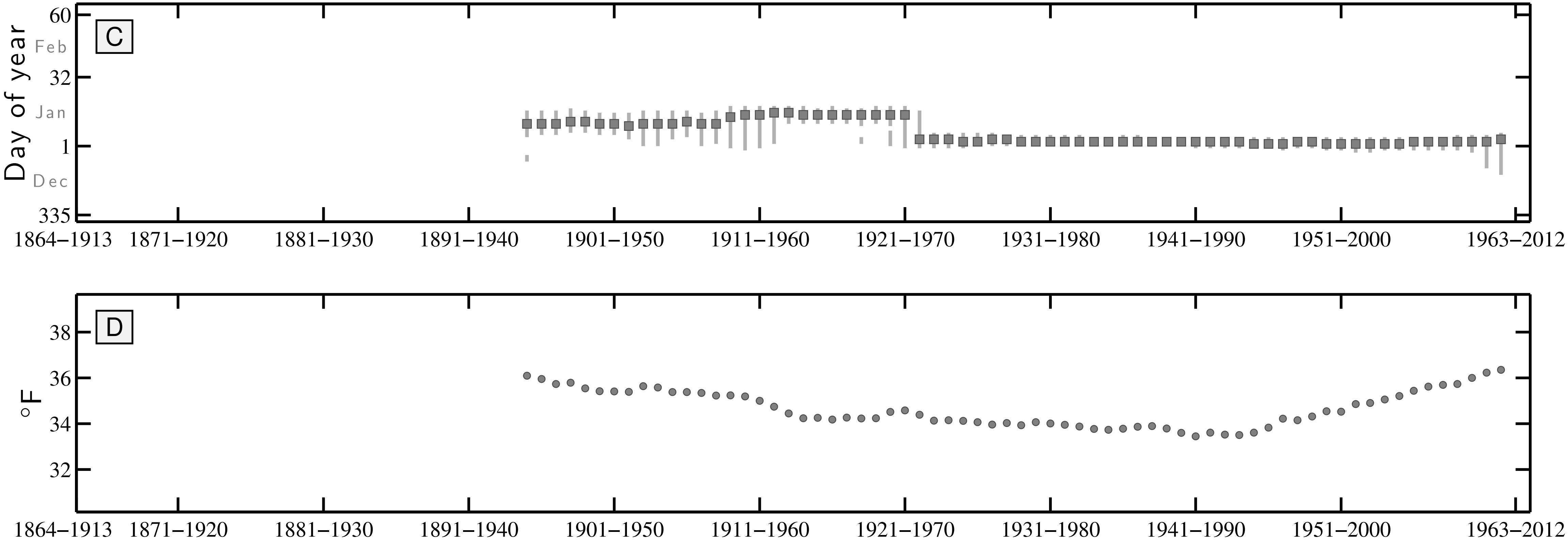

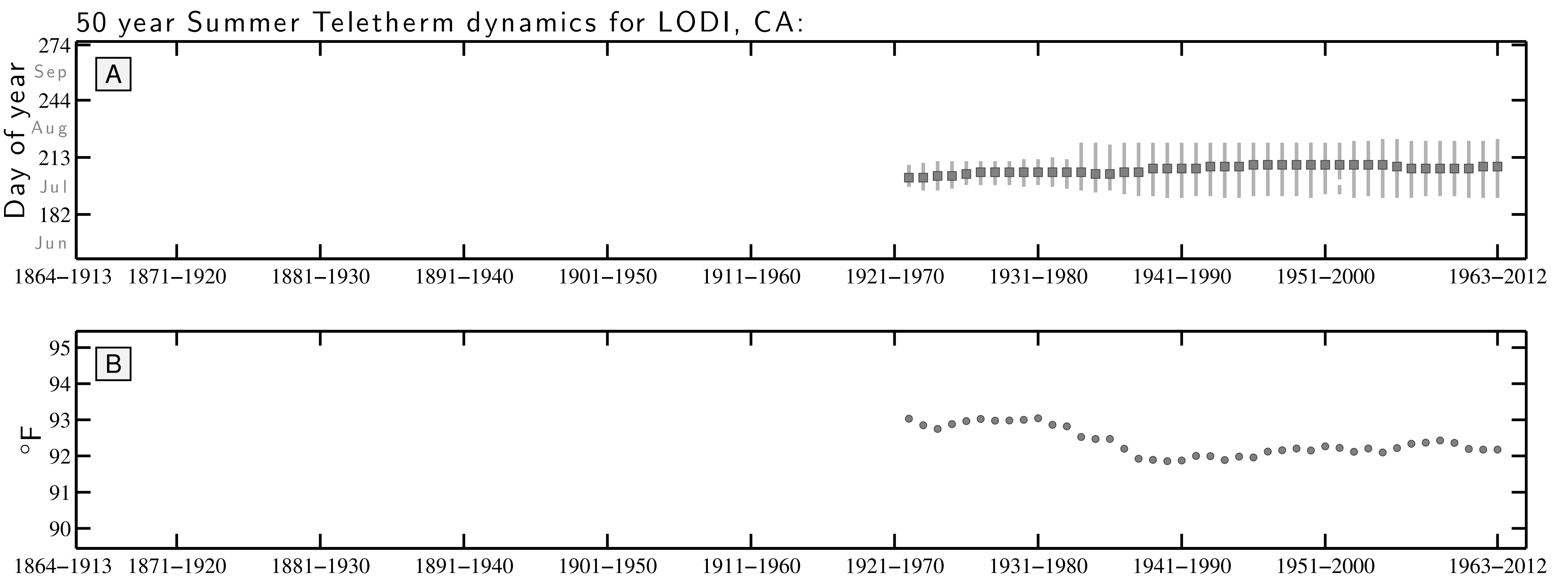
50 year Summer Teletherm dynamics for LODI, CA:
A
B
Day of year
°F
274
Sep
244
Aug
213
Jul
182
Jun
95
94
93
92
91
90
1864–1913
1871–1920
1881–1930
1891–1940
1901–1950
1911–1960
1921–1970
1931–1980
1941–1990
1951–2000
1963–2012

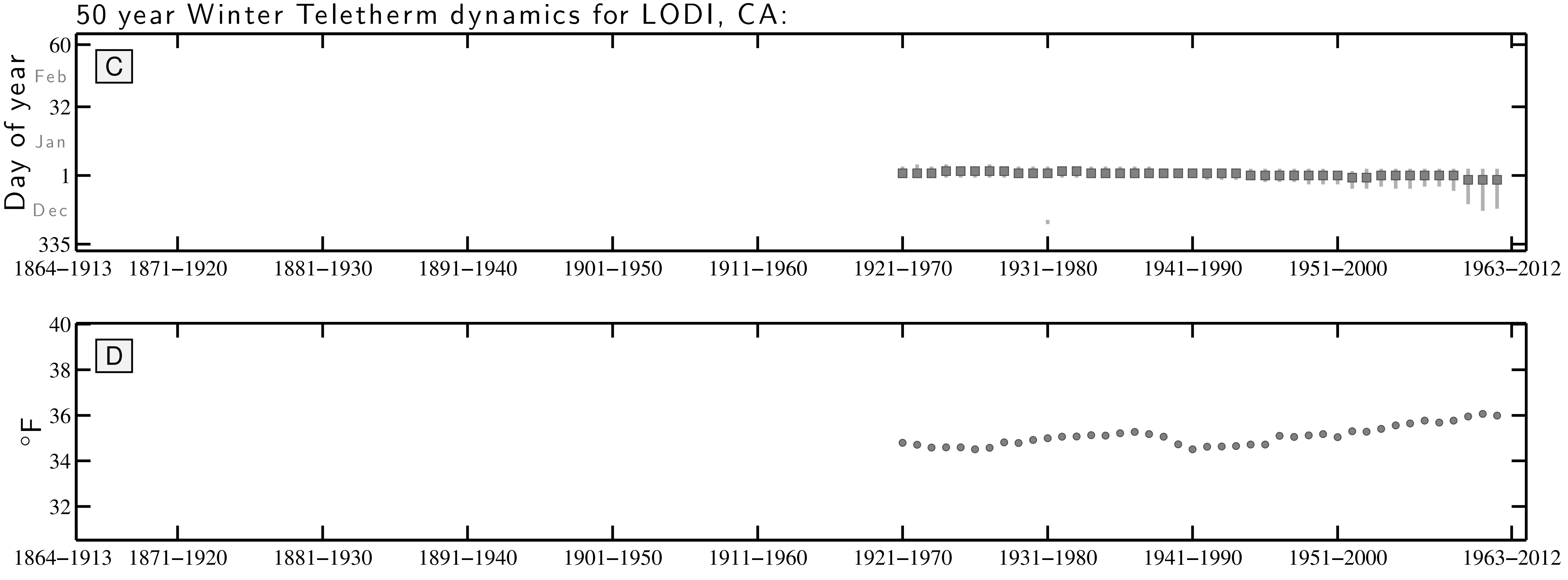
50 year Winter Teletherm dynamics for LODI, CA:
C
D
Day of year
°F
60
Feb
32
Jan
1
Dec
335
40
38
36
34
32
1864–1913
1871–1920
1881–1930
1891–1940
1901–1950
1911–1960
1921–1970
1931–1980
1941–1990
1951–2000
1963–2012

50 year Summer Teletherm dynamics for MARYSVILLE, CA:

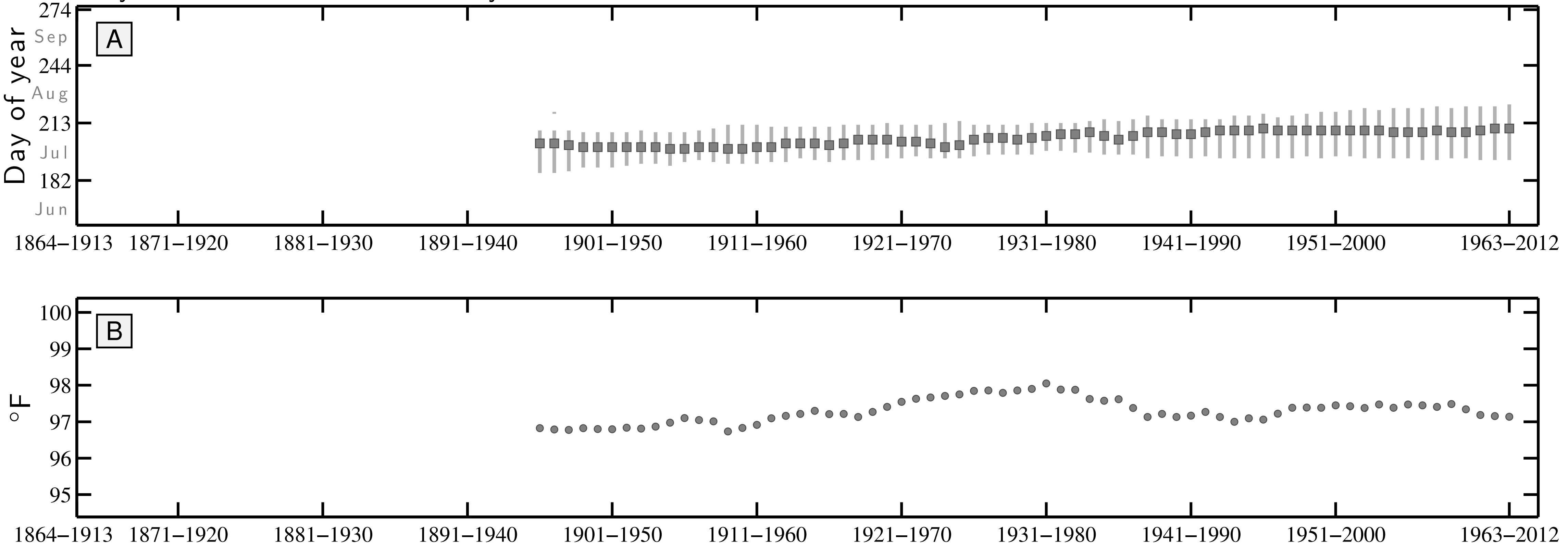

50 year Winter Teletherm dynamics for MARYSVILLE, CA:

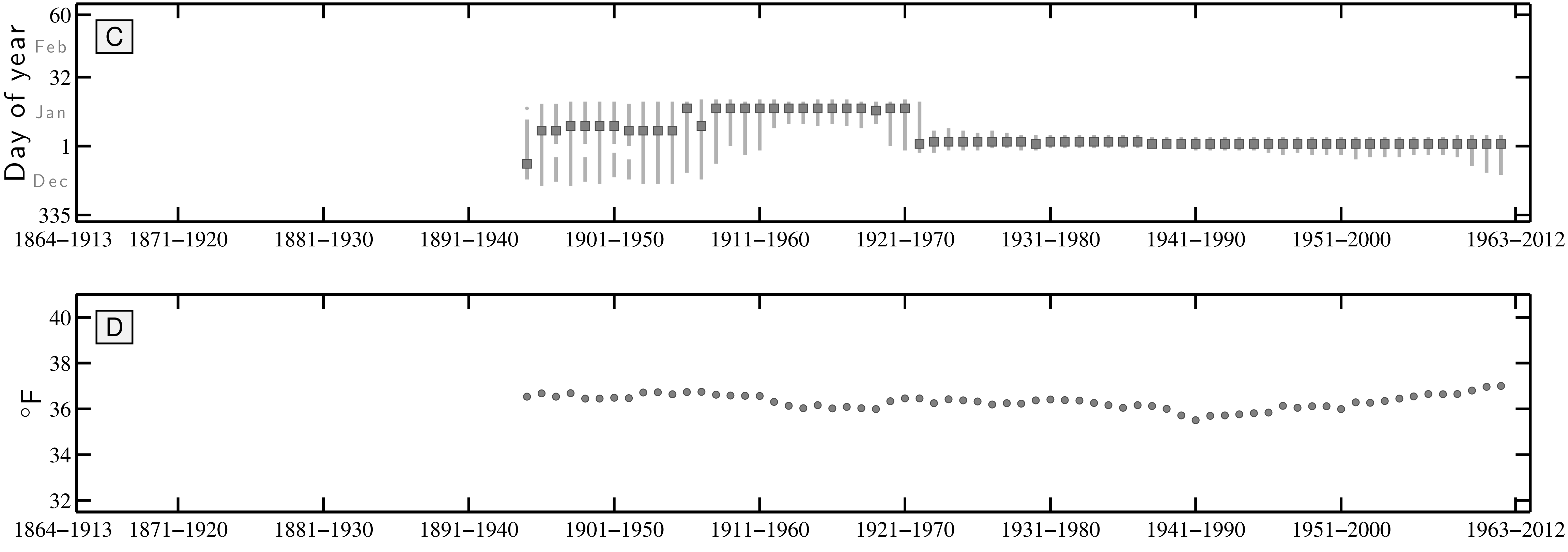

50 year Summer Teletherm dynamics for MERCED, CA:

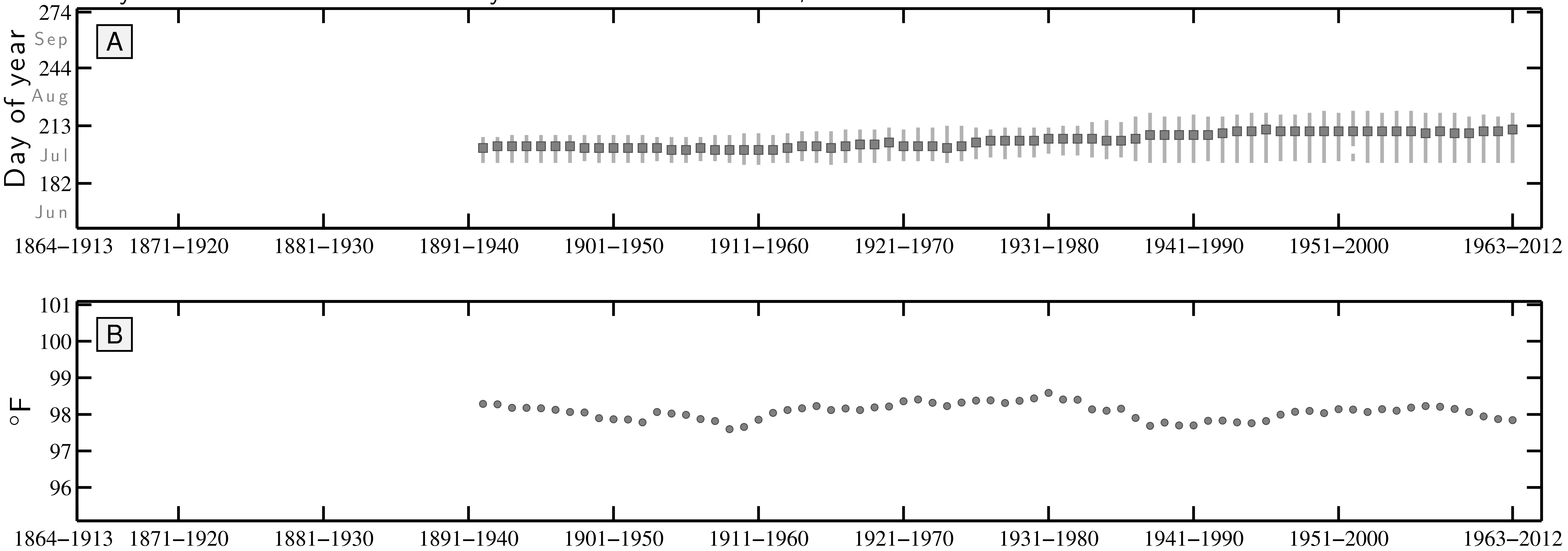

50 year Winter Teletherm dynamics for MERCED, CA:

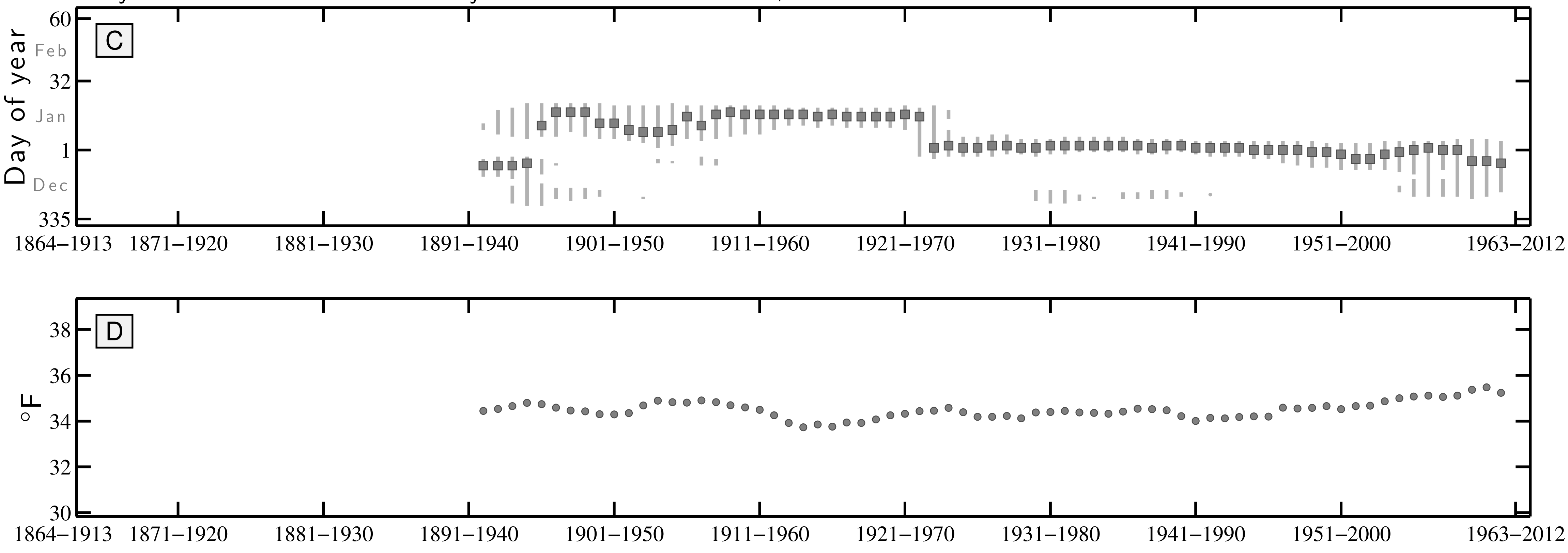

50 year Summer Teletherm dynamics for MT SHASTA, CA:

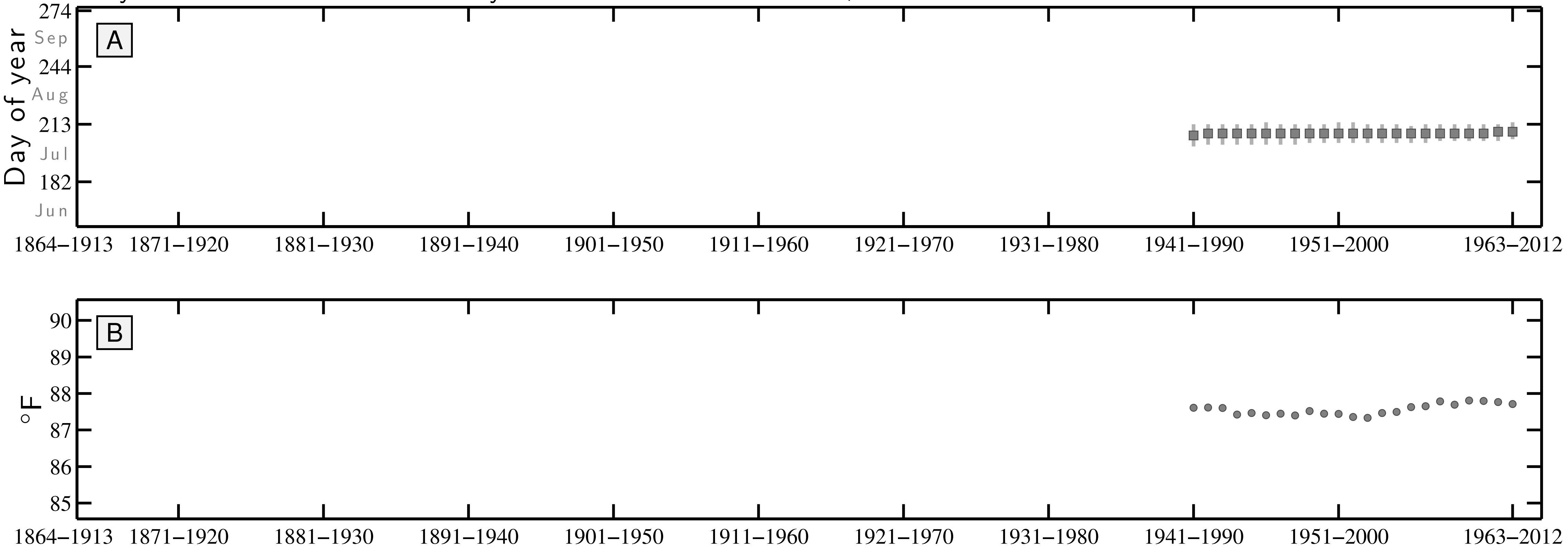

50 year Winter Teletherm dynamics for MT SHASTA, CA:

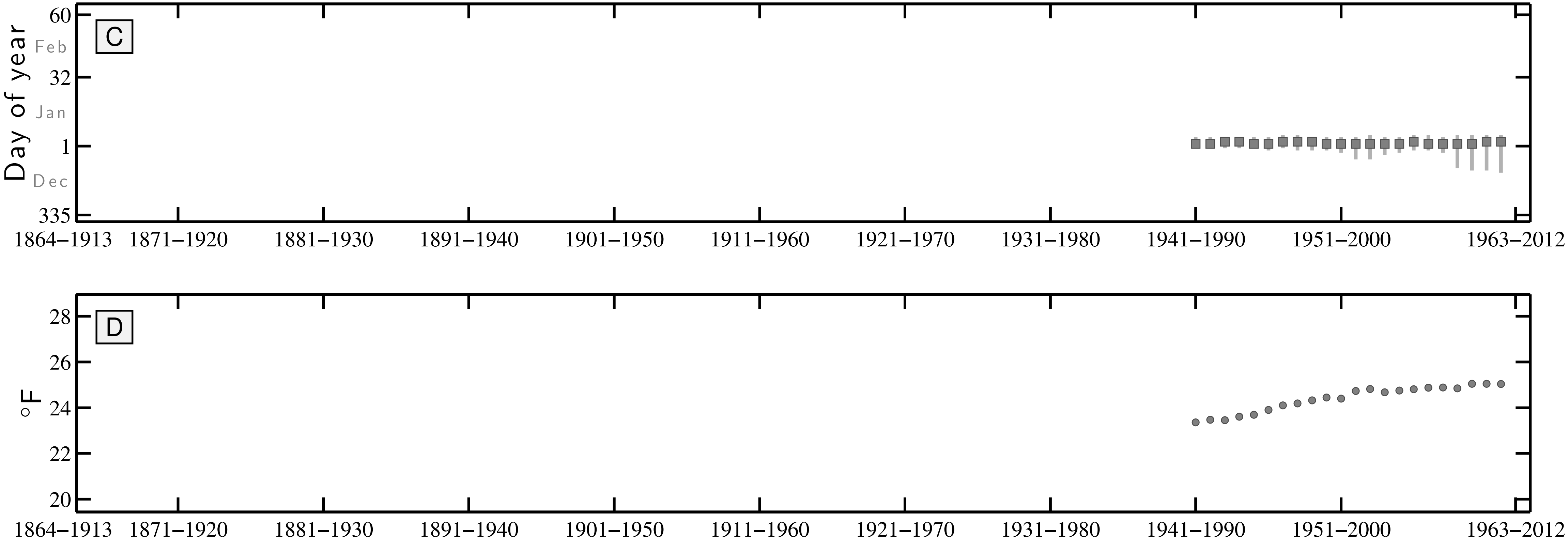

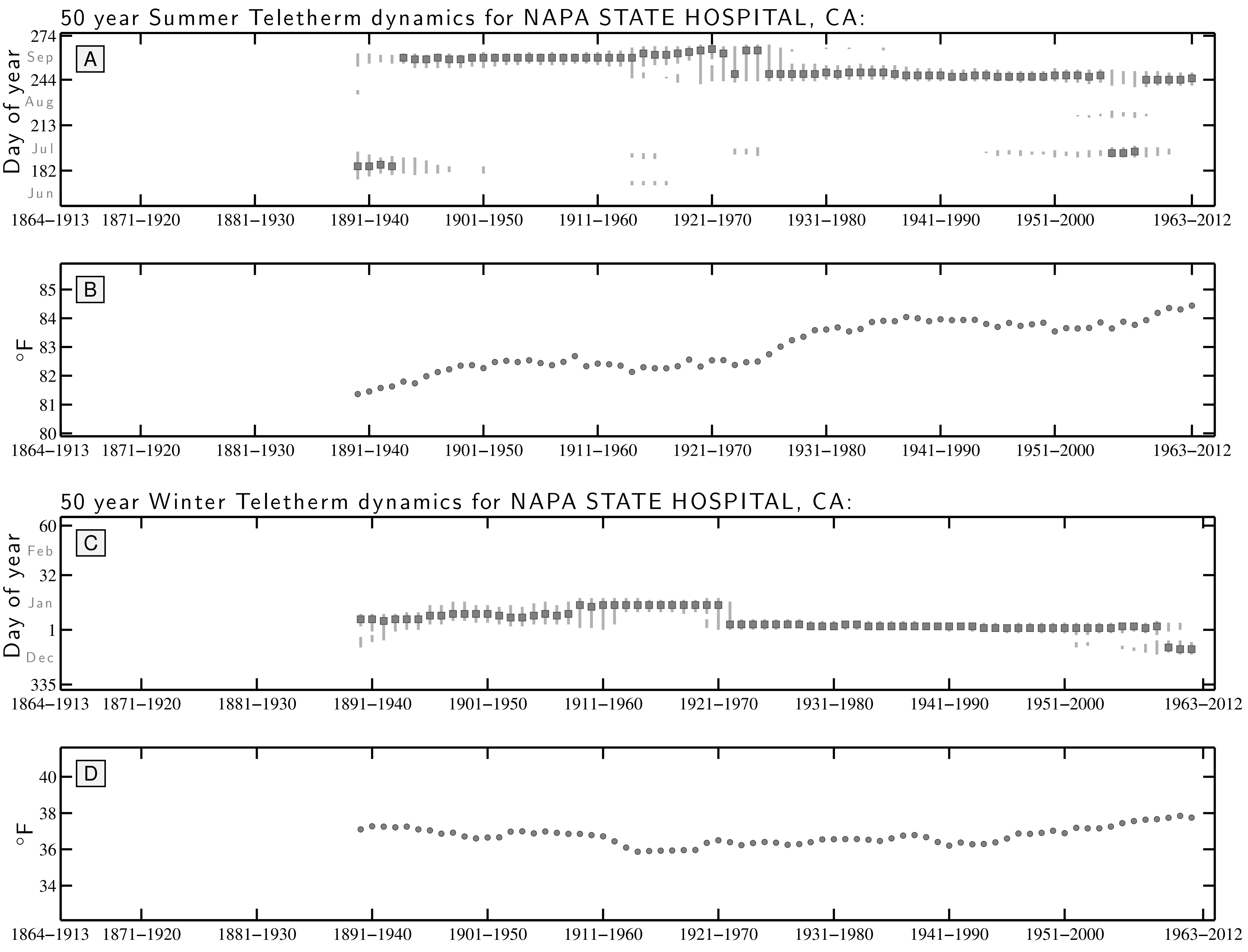

50 year Summer Teletherm dynamics for NAPA STATE HOSPITAL, CA:
A
Day of year
274
Sep
244
Aug
213
Jul
182
Jun
1864–1913
1871–1920
1881–1930
1891–1940
1901–1950
1911–1960
1921–1970
1931–1980
1941–1990
1951–2000
1963–2012
B
°F
85
84
83
82
81
80
50 year Winter Teletherm dynamics for NAPA STATE HOSPITAL, CA:
C
60
Feb
32
Jan
1
Dec
335
D
40
38
36
34

50 year Summer Teletherm dynamics for NEEDLES AP, CA:

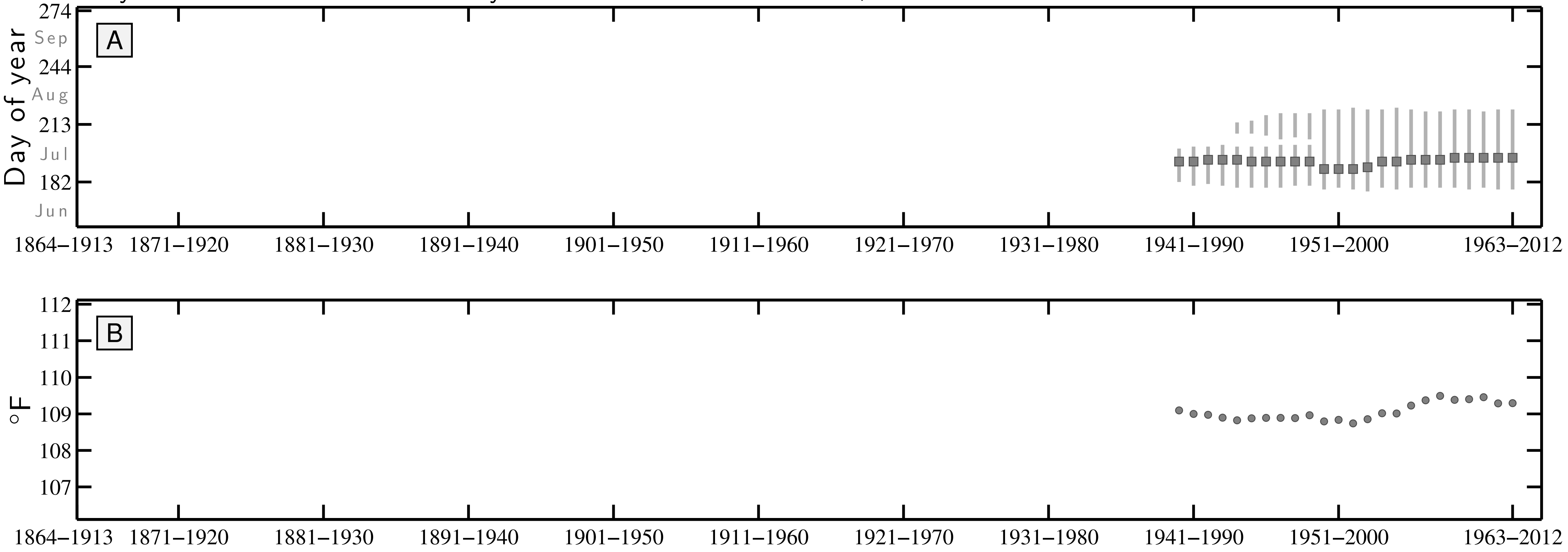

50 year Winter Teletherm dynamics for NEEDLES AP, CA:

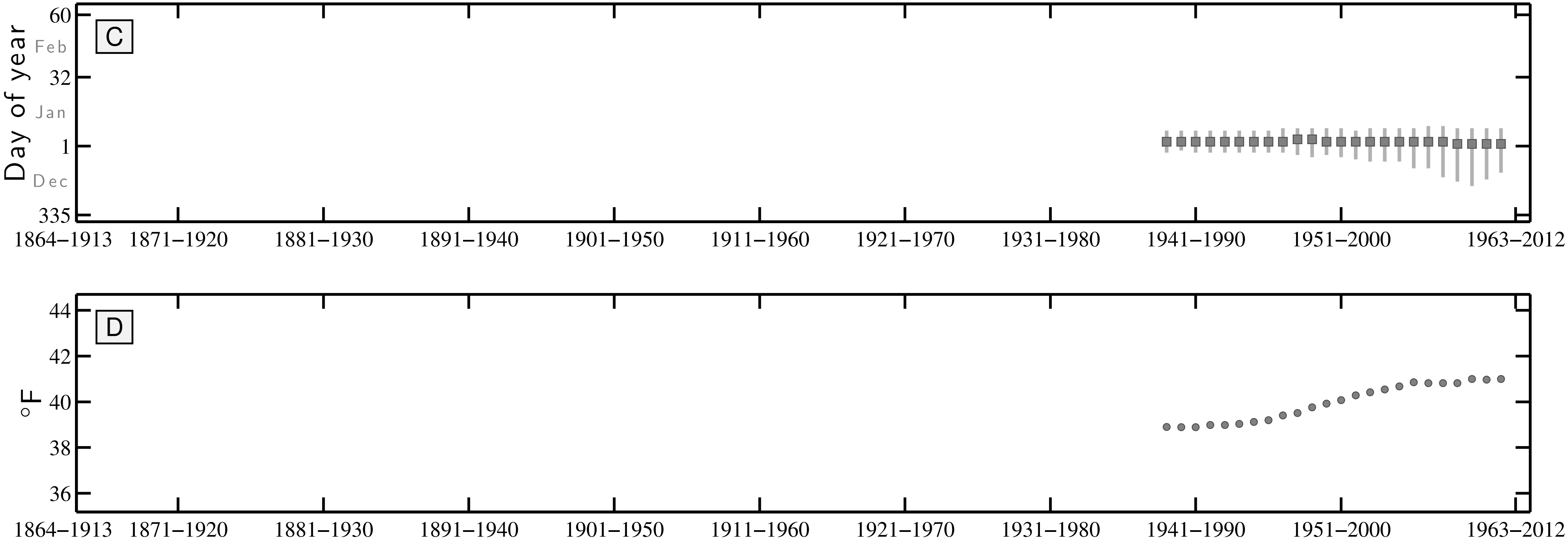

50 year Summer Teletherm dynamics for NEWPORT BEACH HARBOR, CA:

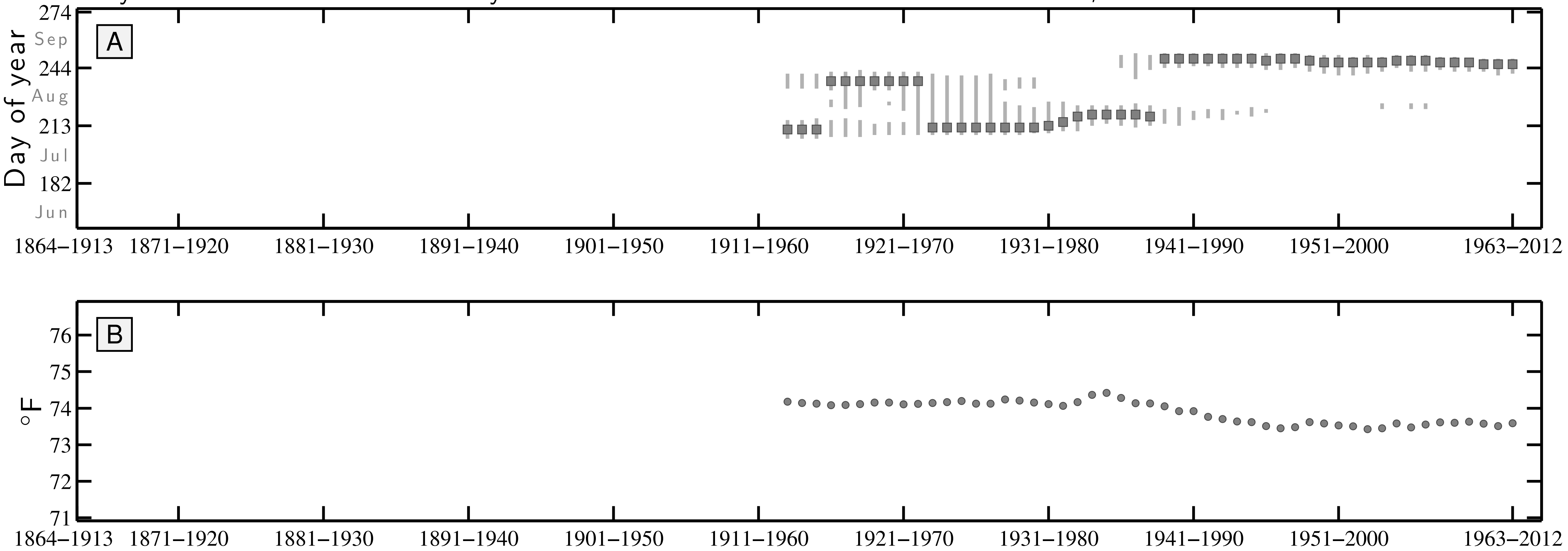

50 year Winter Teletherm dynamics for NEWPORT BEACH HARBOR, CA:

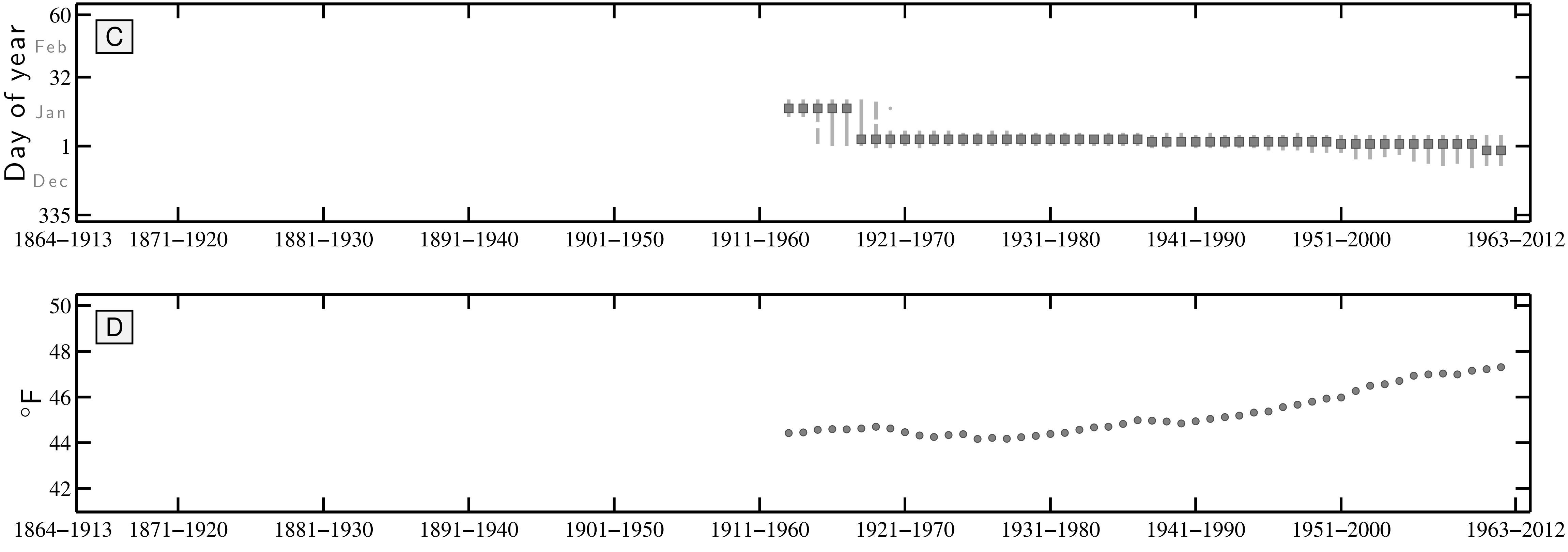

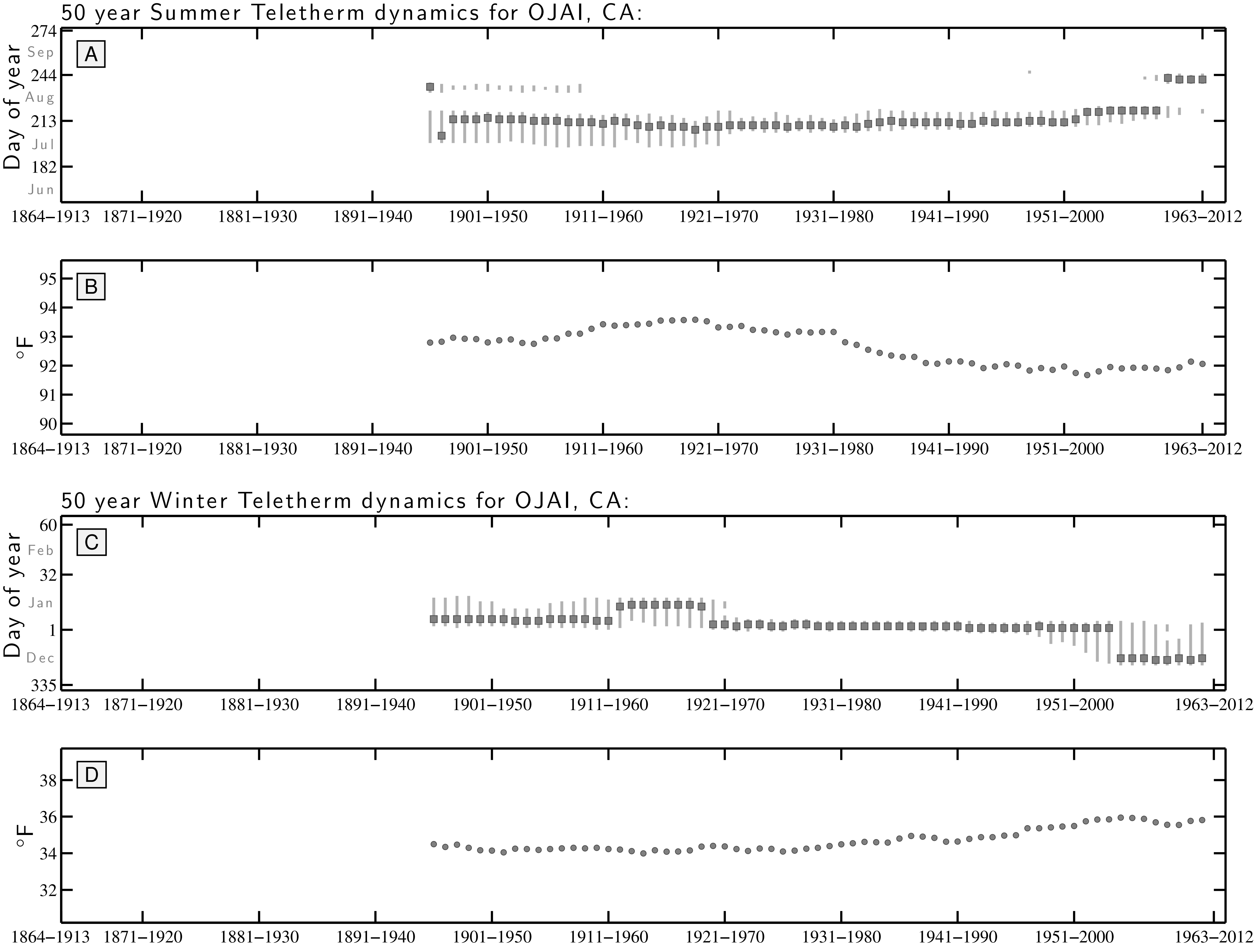
50 year Summer Teletherm dynamics for OJAI, CA:
A
Day of year
274
Sep
244
Aug
213
Jul
182
Jun
1864–1913
1871–1920
1881–1930
1891–1940
1901–1950
1911–1960
1921–1970
1931–1980
1941–1990
1951–2000
1963–2012
B
°F
95
94
93
92
91
90
50 year Winter Teletherm dynamics for OJAI, CA:
C
Day of year
60
Feb
32
Jan
1
Dec
335
D
°F
38
36
34
32

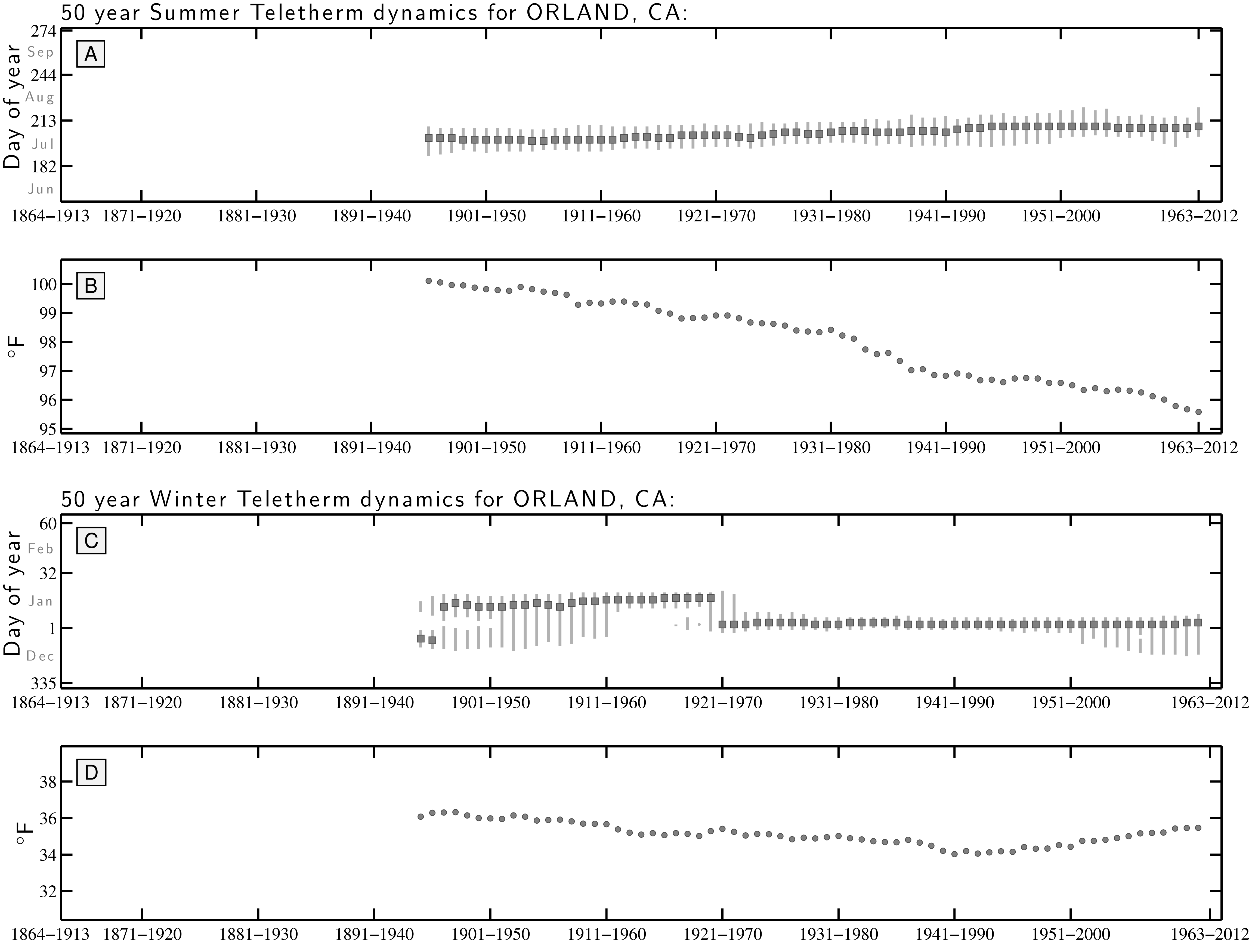
50 year Summer Teletherm dynamics for ORLAND, CA:
A
Day of year
274
Sep
244
Aug
213
Jul
182
Jun
1864–1913
1871–1920
1881–1930
1891–1940
1901–1950
1911–1960
1921–1970
1931–1980
1941–1990
1951–2000
1963–2012
B
°F
100
99
98
97
96
95
50 year Winter Teletherm dynamics for ORLAND, CA:
C
Day of year
60
Feb
32
Jan
1
Dec
335
D
°F
38
36
34
32

50 year Summer Teletherm dynamics for ORLEANS, CA:

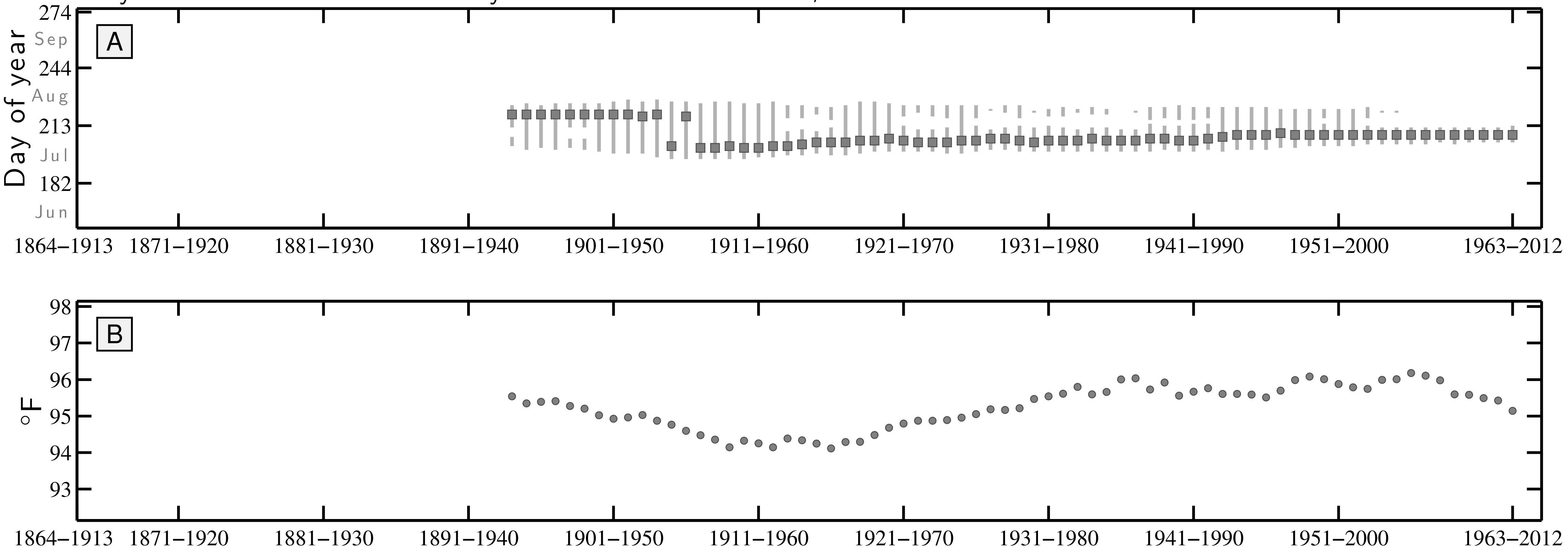

50 year Winter Teletherm dynamics for ORLEANS, CA:

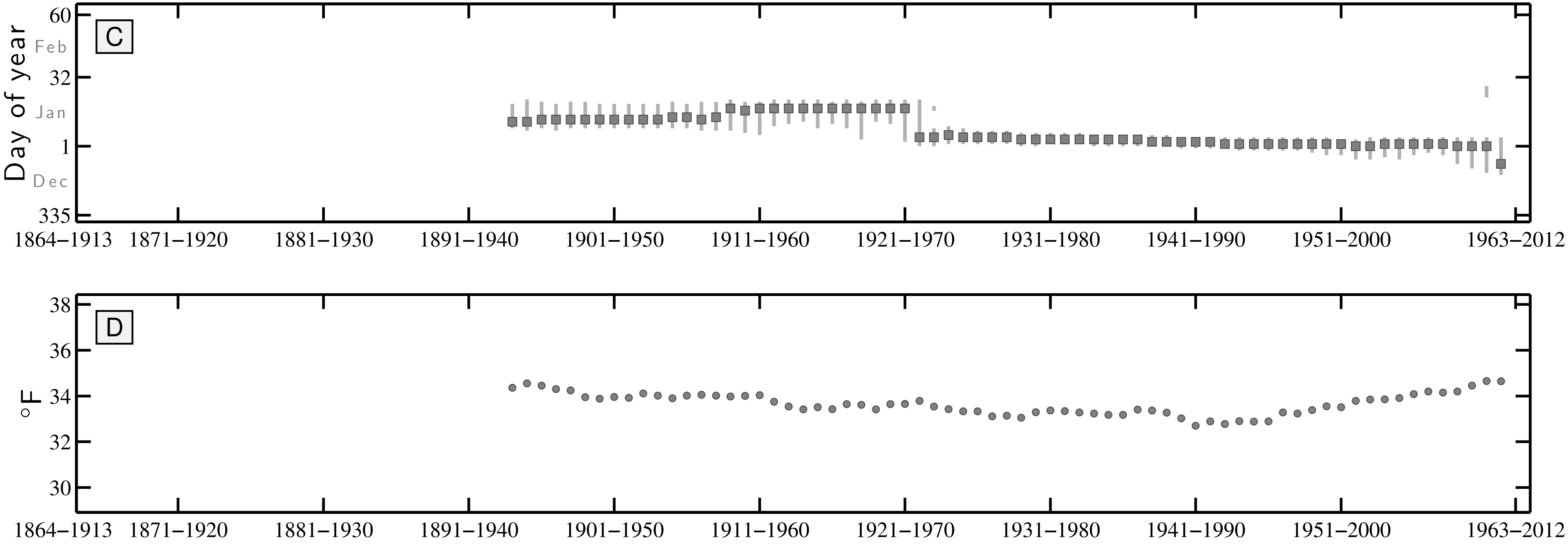

50 year Summer Teletherm dynamics for PASADENA, CA:

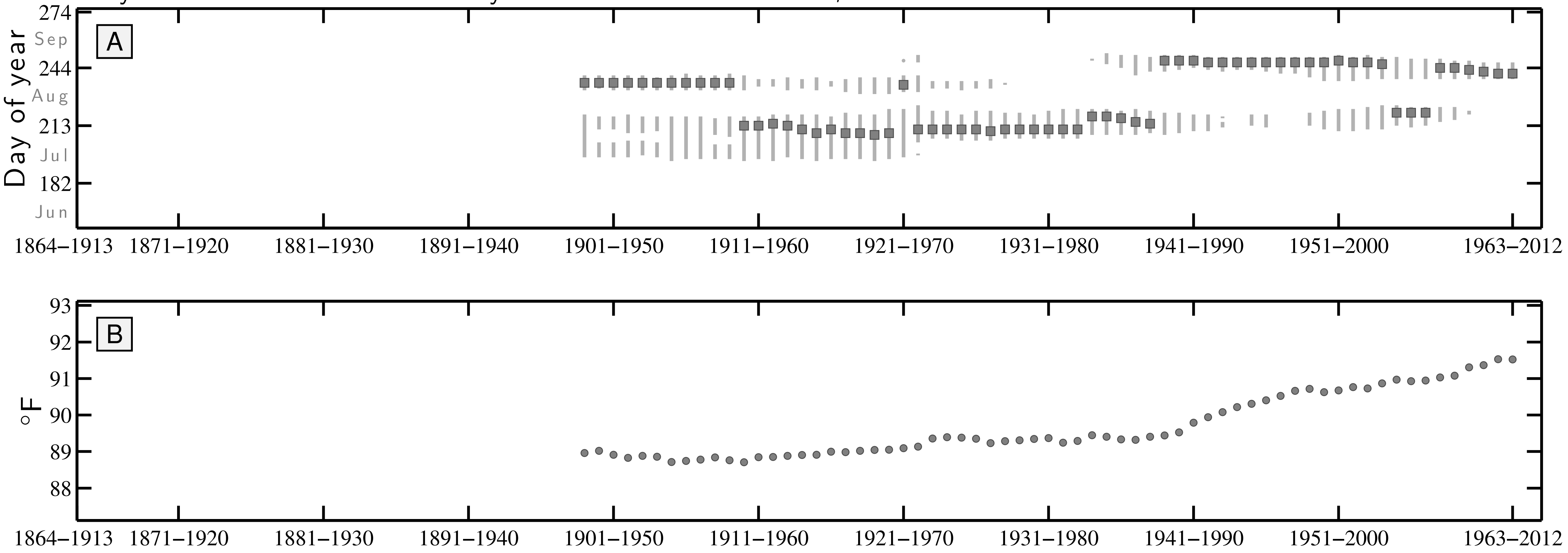

50 year Winter Teletherm dynamics for PASADENA, CA:

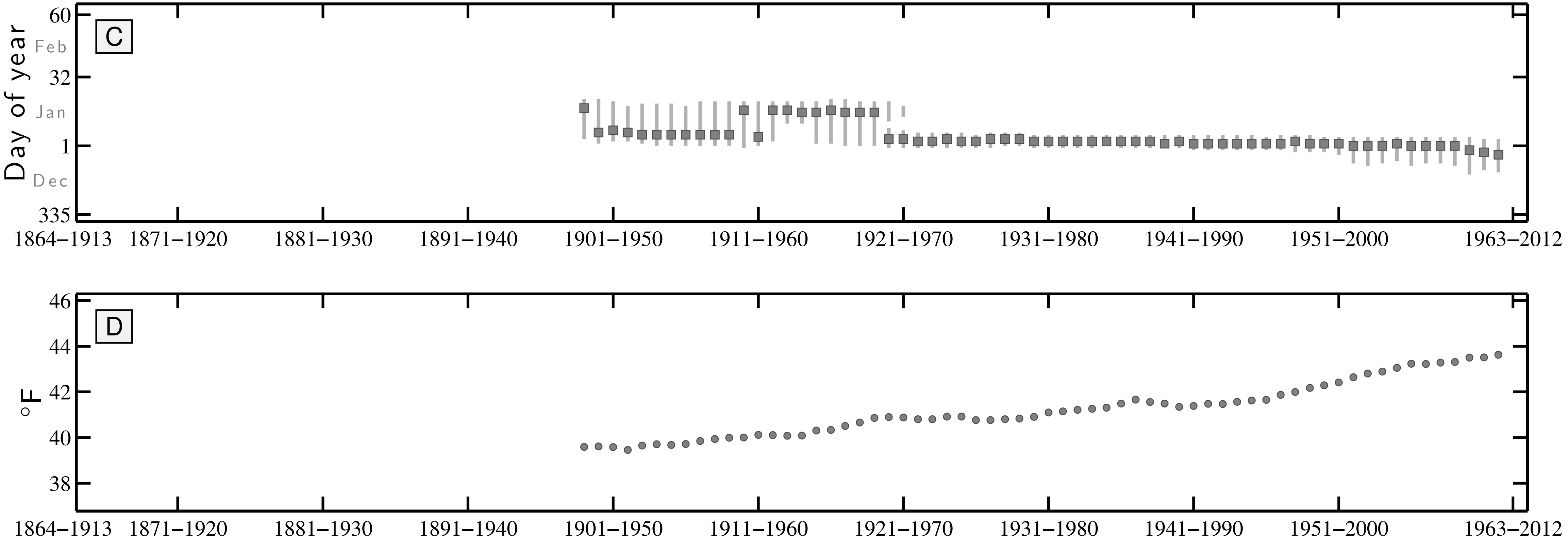

50 year Summer Teletherm dynamics for PASO ROBLES, CA:

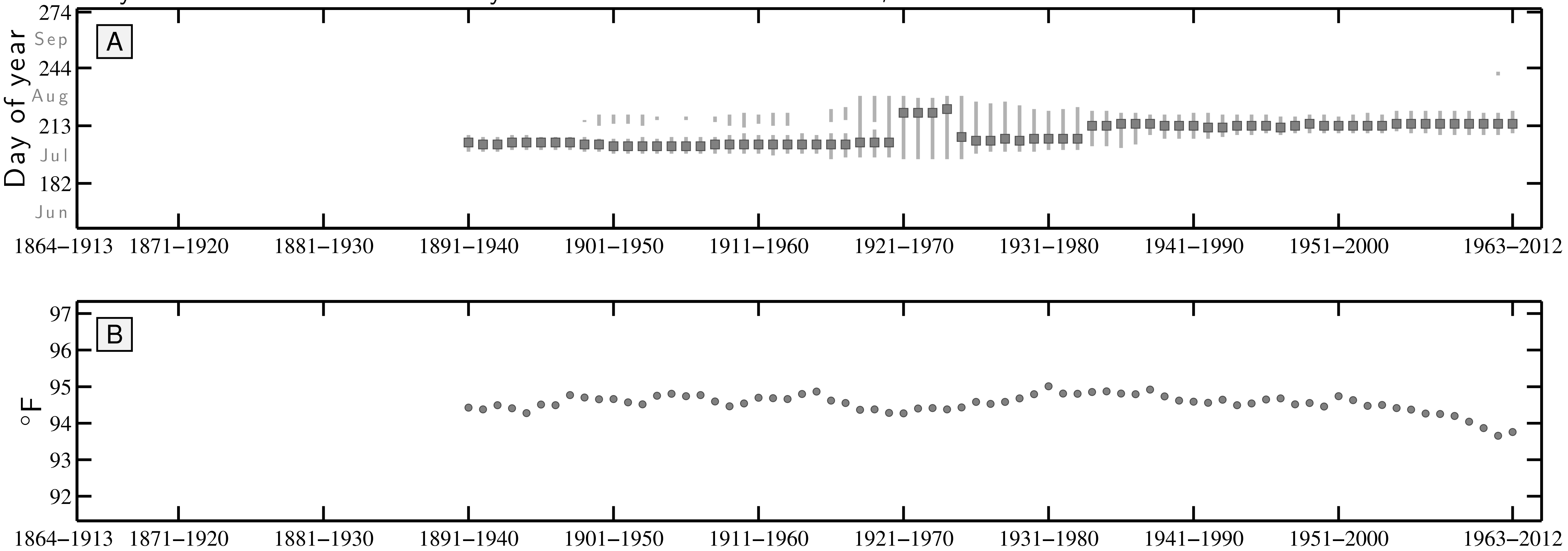

50 year Winter Teletherm dynamics for PASO ROBLES, CA:

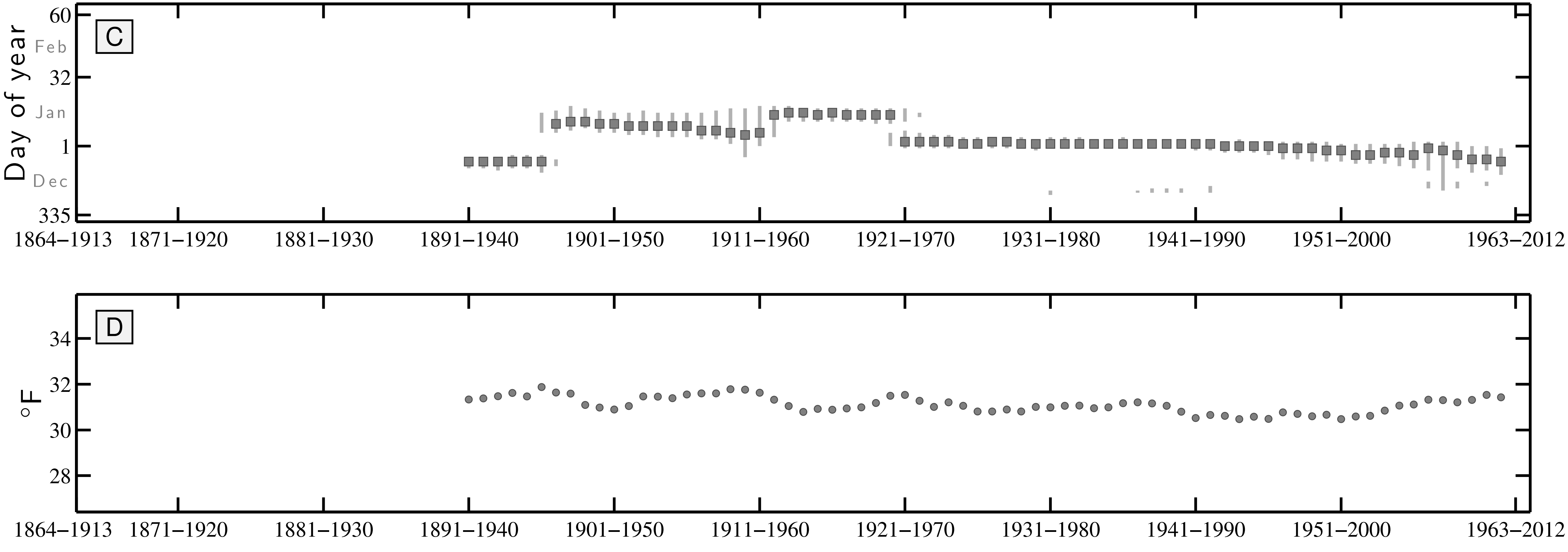

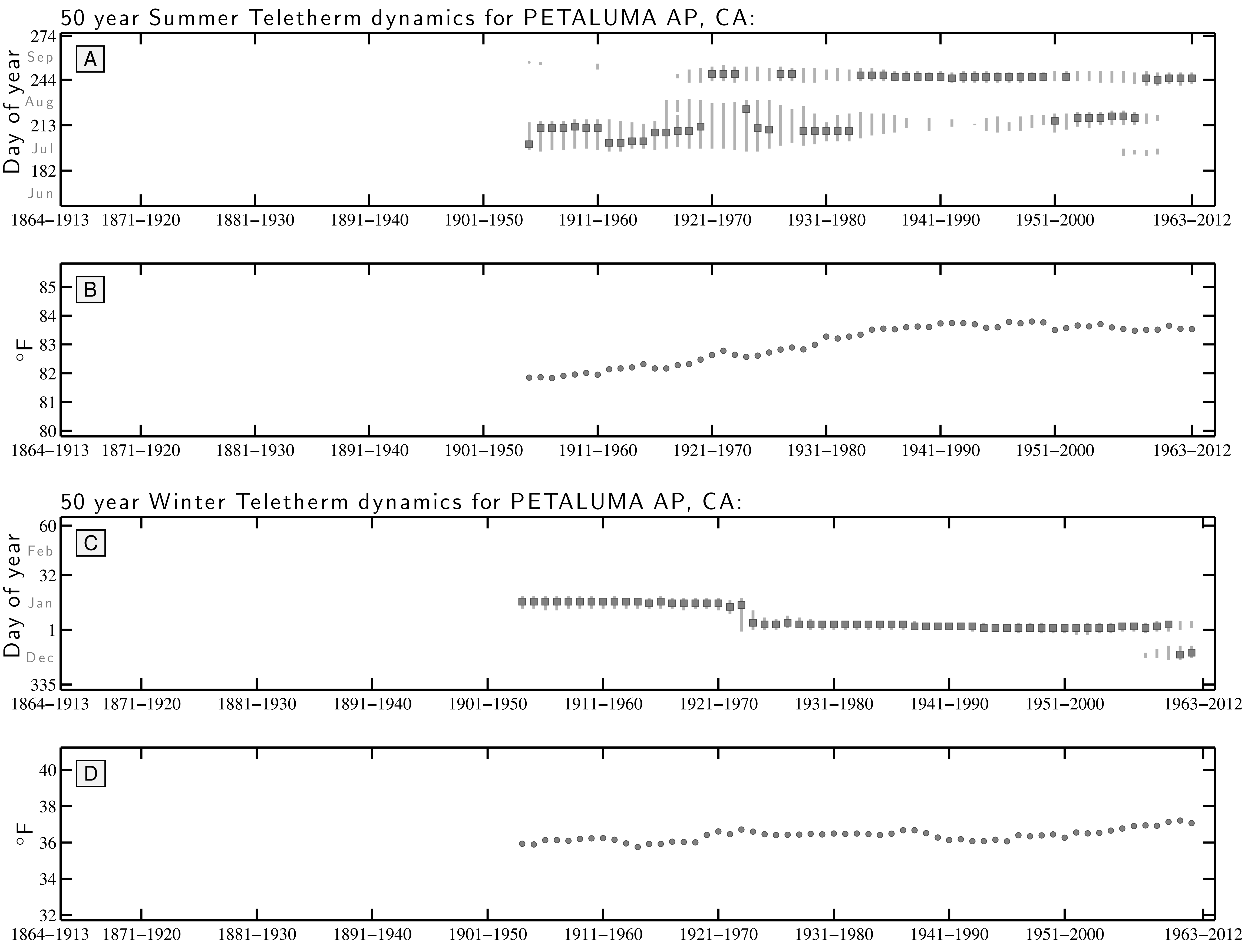
50 year Summer Teletherm dynamics for PETALUMA AP, CA:
A
Day of year
274
Sep
244
Aug
213
Jul
182
Jun
1864–1913
1871–1920
1881–1930
1891–1940
1901–1950
1911–1960
1921–1970
1931–1980
1941–1990
1951–2000
1963–2012
B
°F
85
84
83
82
81
80
50 year Winter Teletherm dynamics for PETALUMA AP, CA:
C
60
Feb
32
Jan
1
Dec
335
D
40
38
36
34
32

50 year Summer Teletherm dynamics for QUINCY, CA:

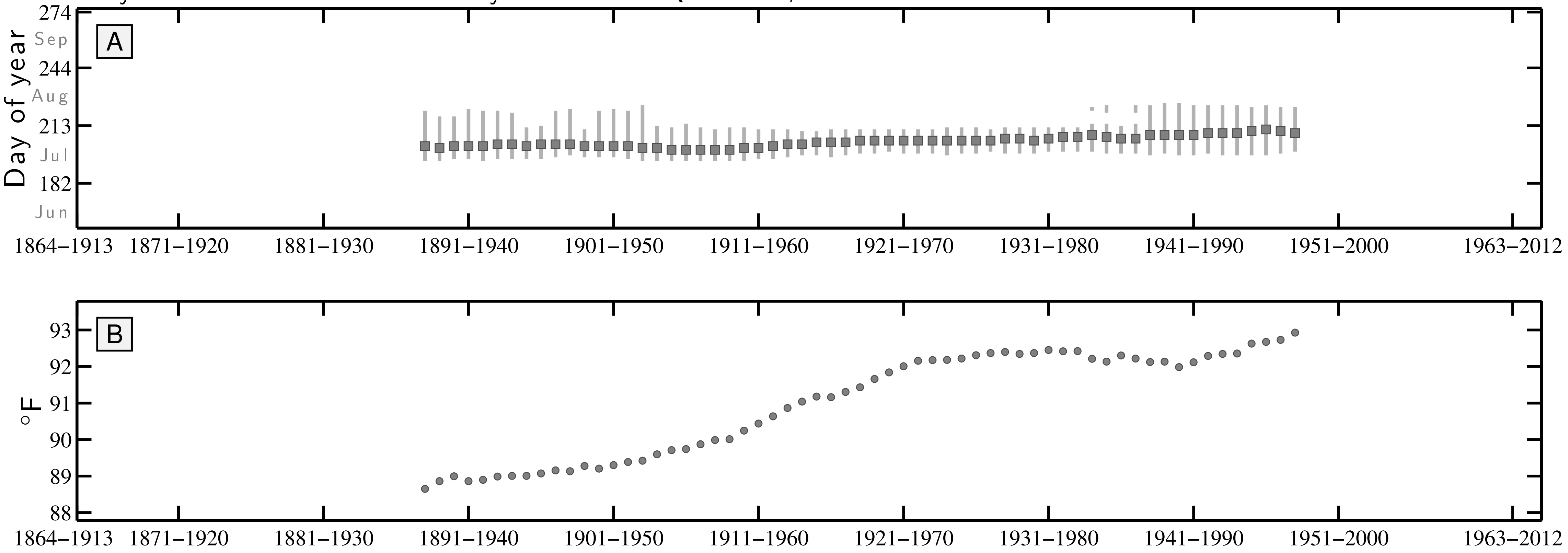

50 year Winter Teletherm dynamics for QUINCY, CA:

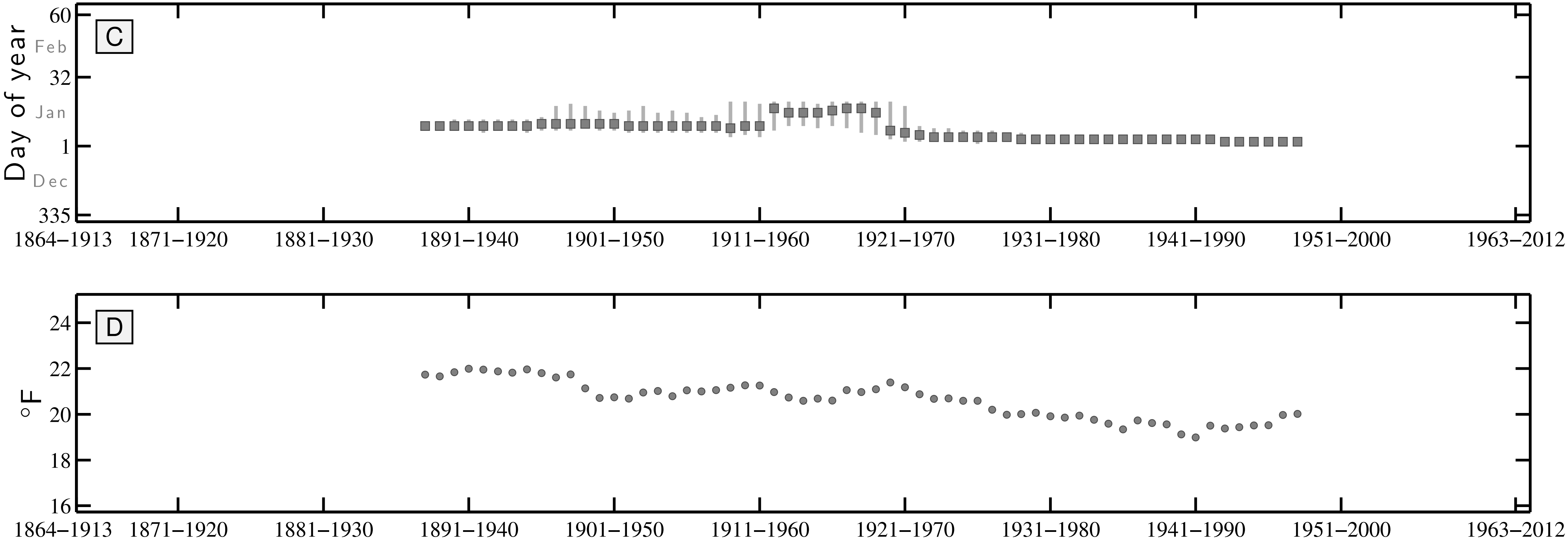

50 year Summer Teletherm dynamics for REDLANDS, CA:

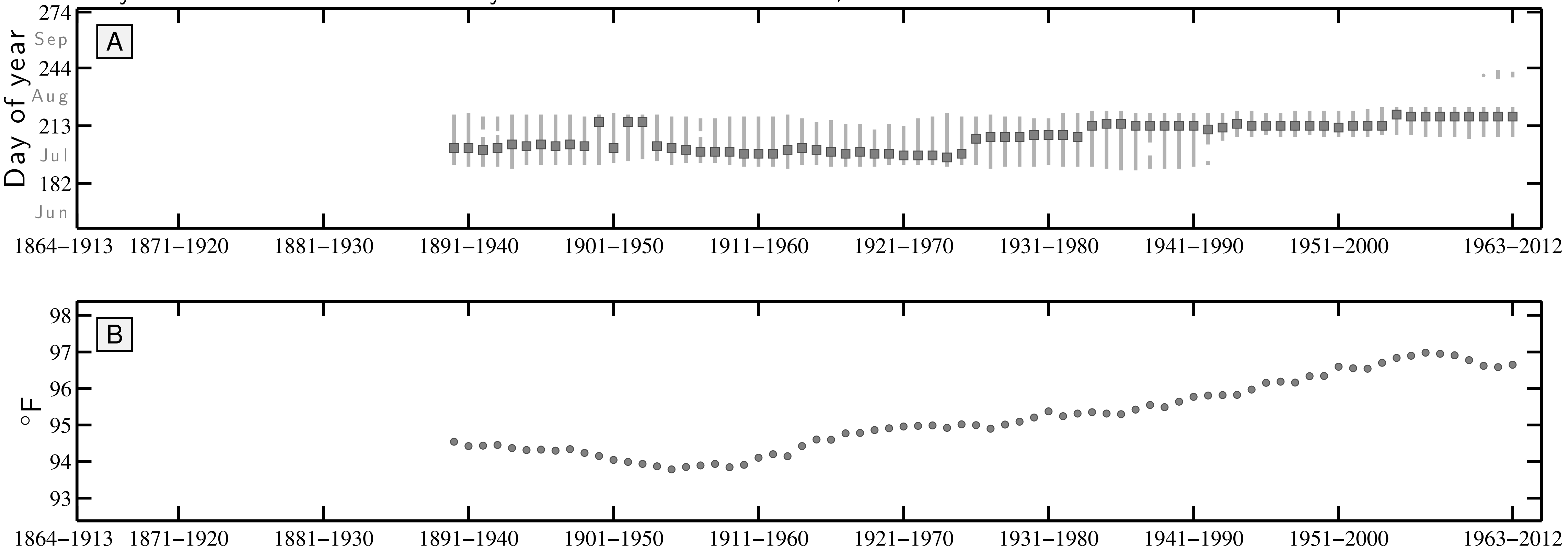

50 year Winter Teletherm dynamics for REDLANDS, CA:

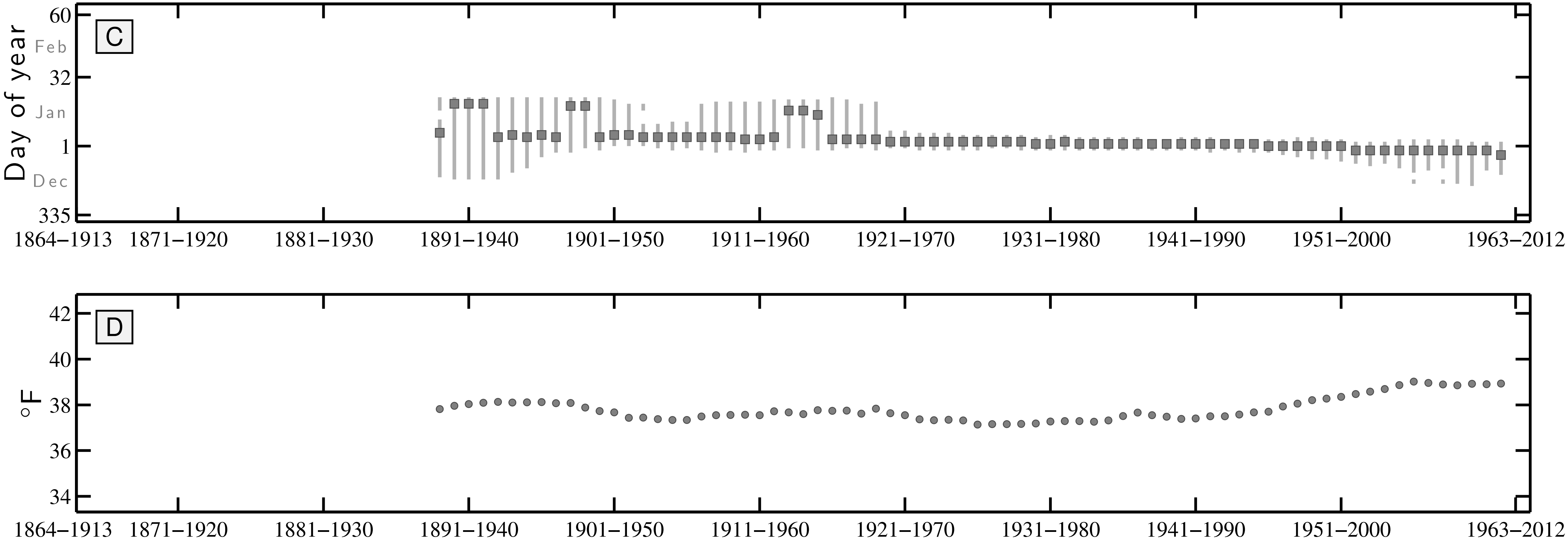

50 year Summer Teletherm dynamics for SAN LUIS OBISPO POLY, CA:

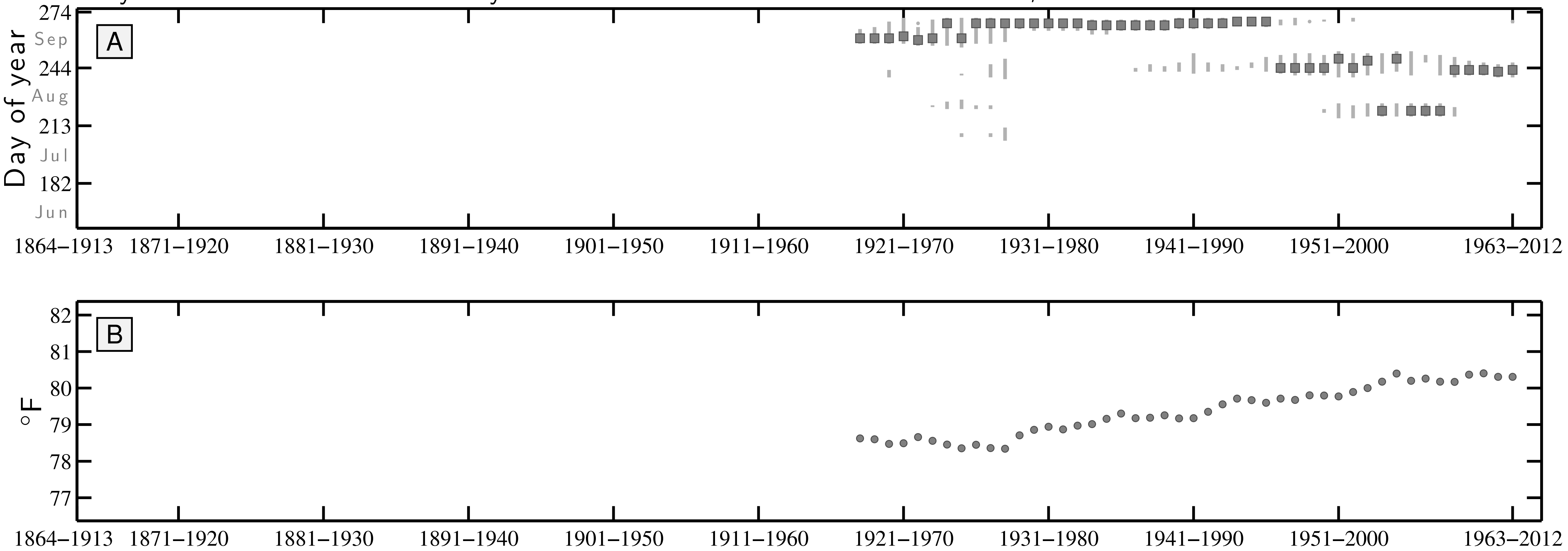

50 year Winter Teletherm dynamics for SAN LUIS OBISPO POLY, CA:

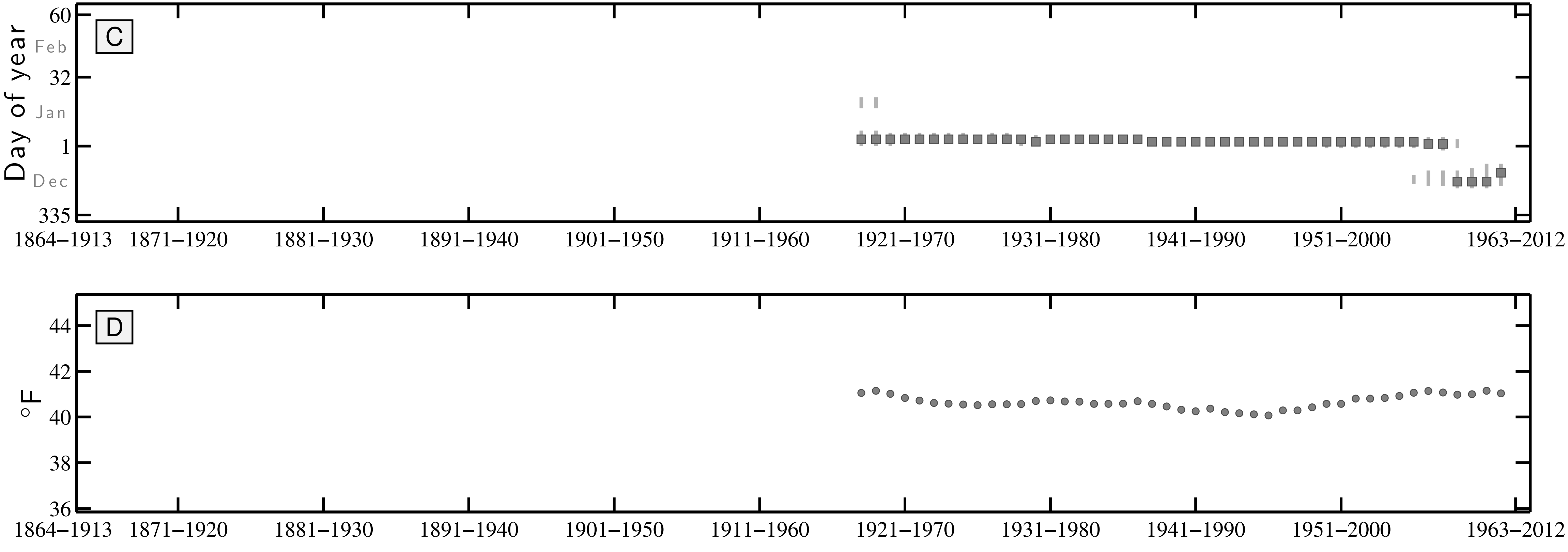

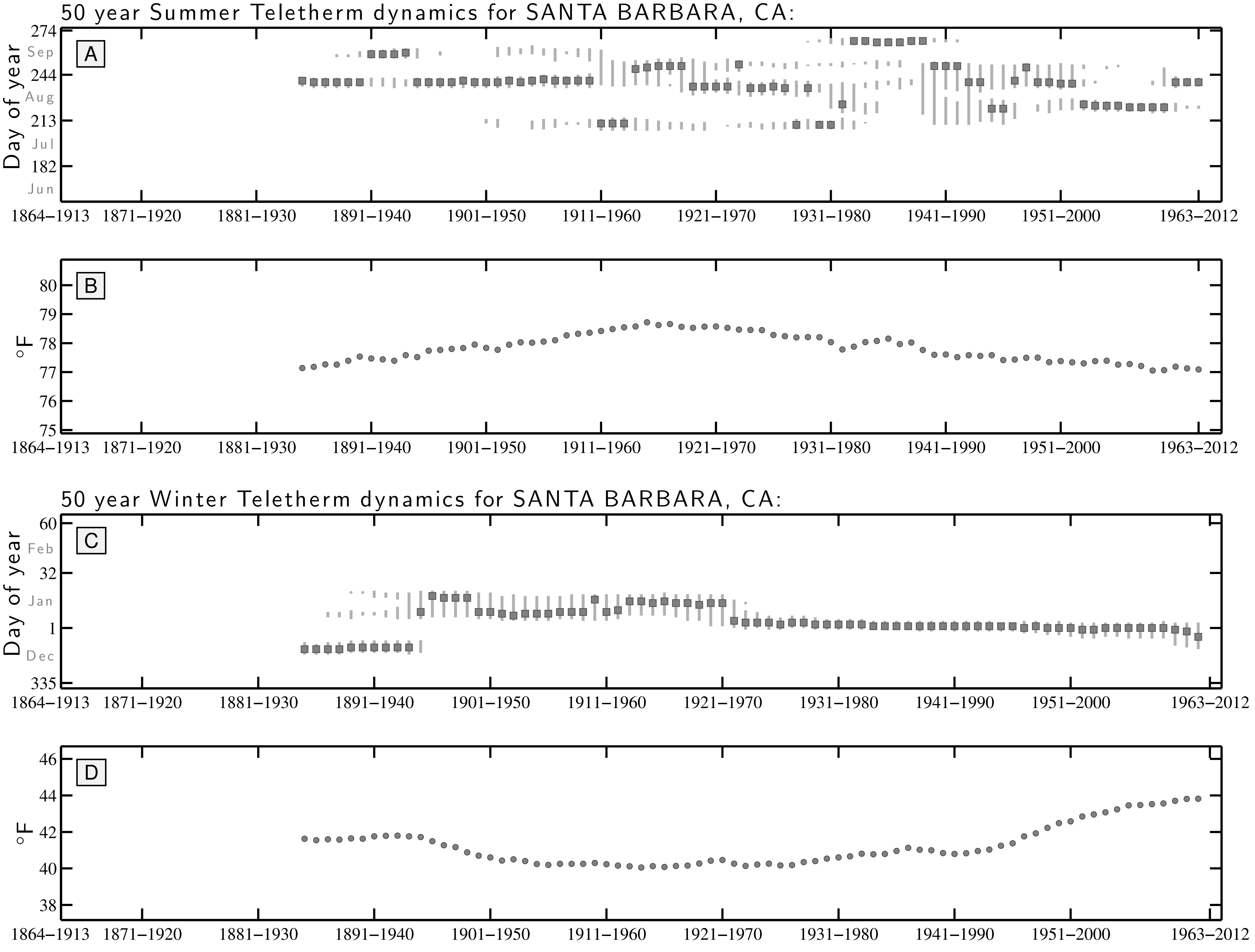
50 year Summer Teletherm dynamics for SANTA BARBARA, CA:
A
Day of year
274
Sep
244
Aug
213
Jul
182
Jun
1864–1913
1871–1920
1881–1930
1891–1940
1901–1950
1911–1960
1921–1970
1931–1980
1941–1990
1951–2000
1963–2012
B
°F
80
79
78
77
76
75
50 year Winter Teletherm dynamics for SANTA BARBARA, CA:
C
Day of year
60
Feb
32
Jan
1
Dec
335
D
°F
46
44
42
40
38

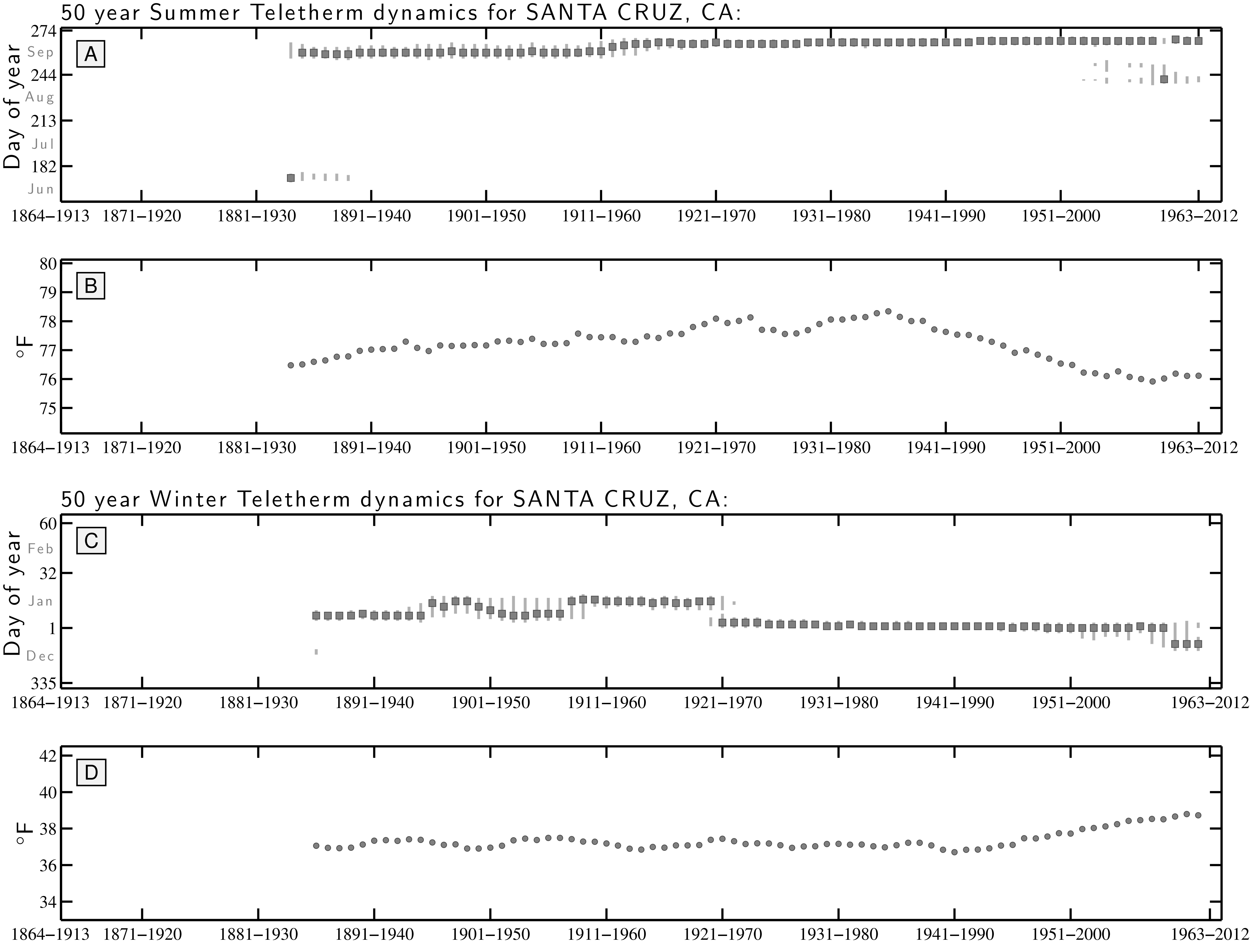
50 year Summer Teletherm dynamics for SANTA CRUZ, CA:
A
Day of year
274
Sep
244
Aug
213
Jul
182
Jun
B
°F
80
79
78
77
76
75
50 year Winter Teletherm dynamics for SANTA CRUZ, CA:
C
Day of year
60
Feb
32
Jan
1
Dec
335
D
°F
42
40
38
36
34
1864–1913
1871–1920
1881–1930
1891–1940
1901–1950
1911–1960
1921–1970
1931–1980
1941–1990
1951–2000
1963–2012

50 year Summer Teletherm dynamics for SANTA ROSA, CA:

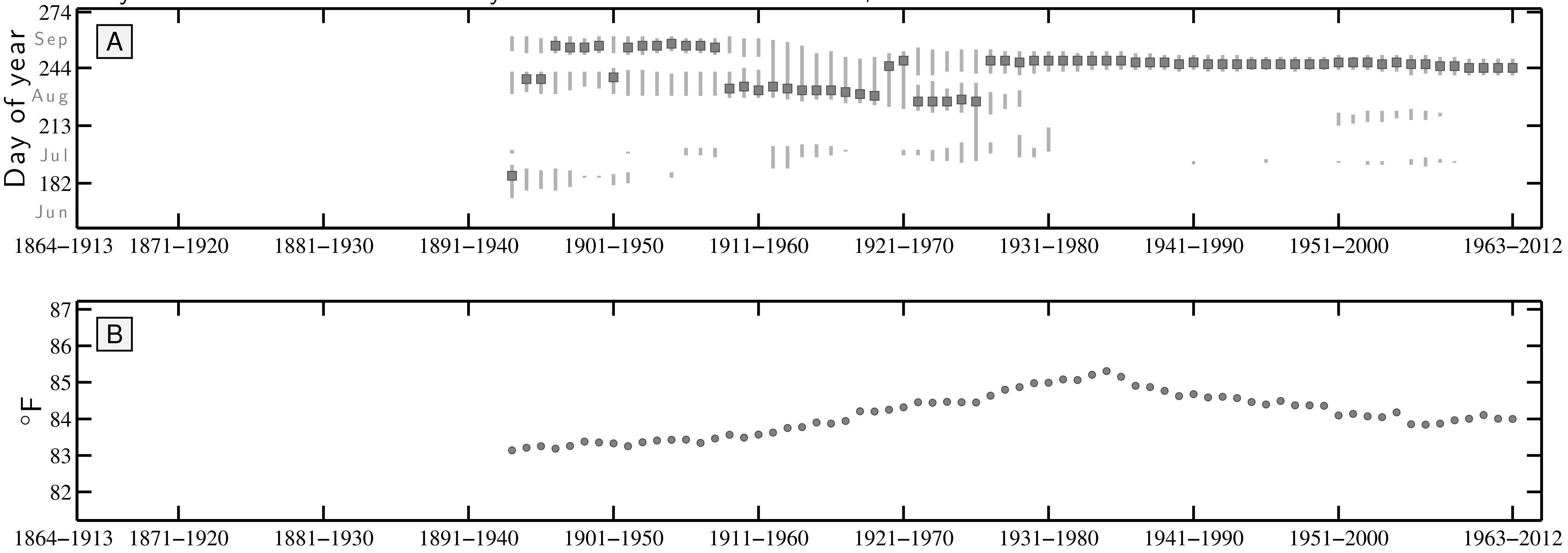

50 year Winter Teletherm dynamics for SANTA ROSA, CA:

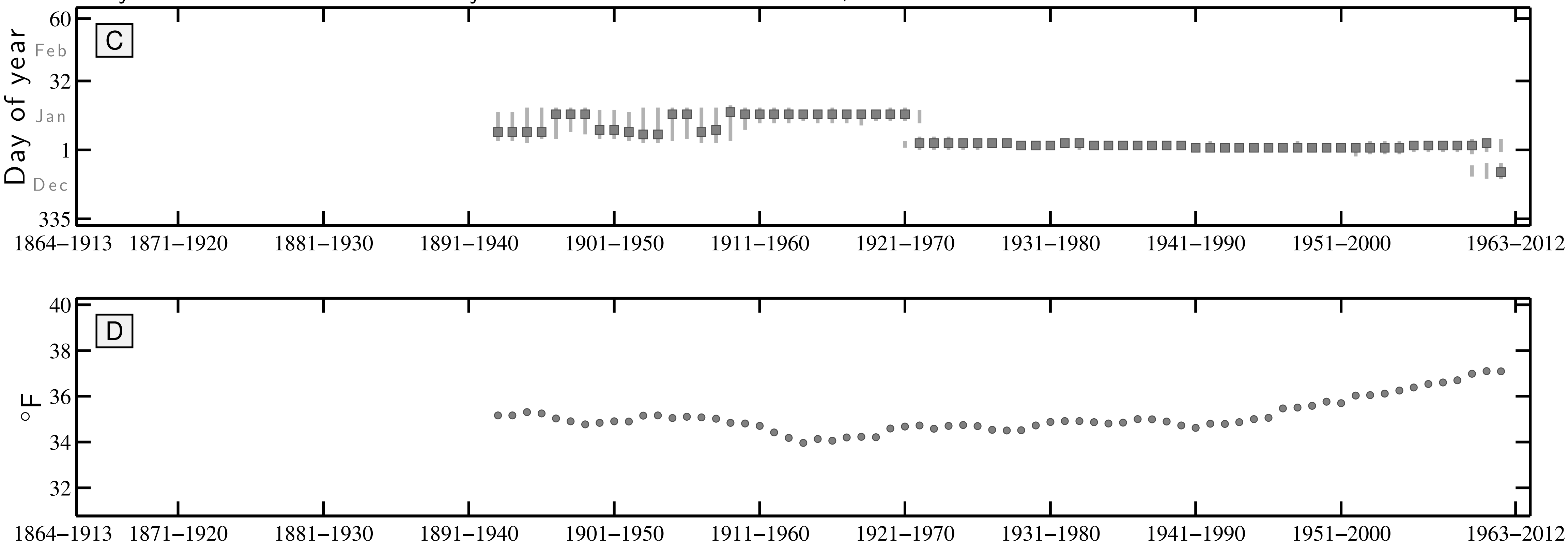

50 year Summer Teletherm dynamics for SUSANVILLE 2SW, CA:

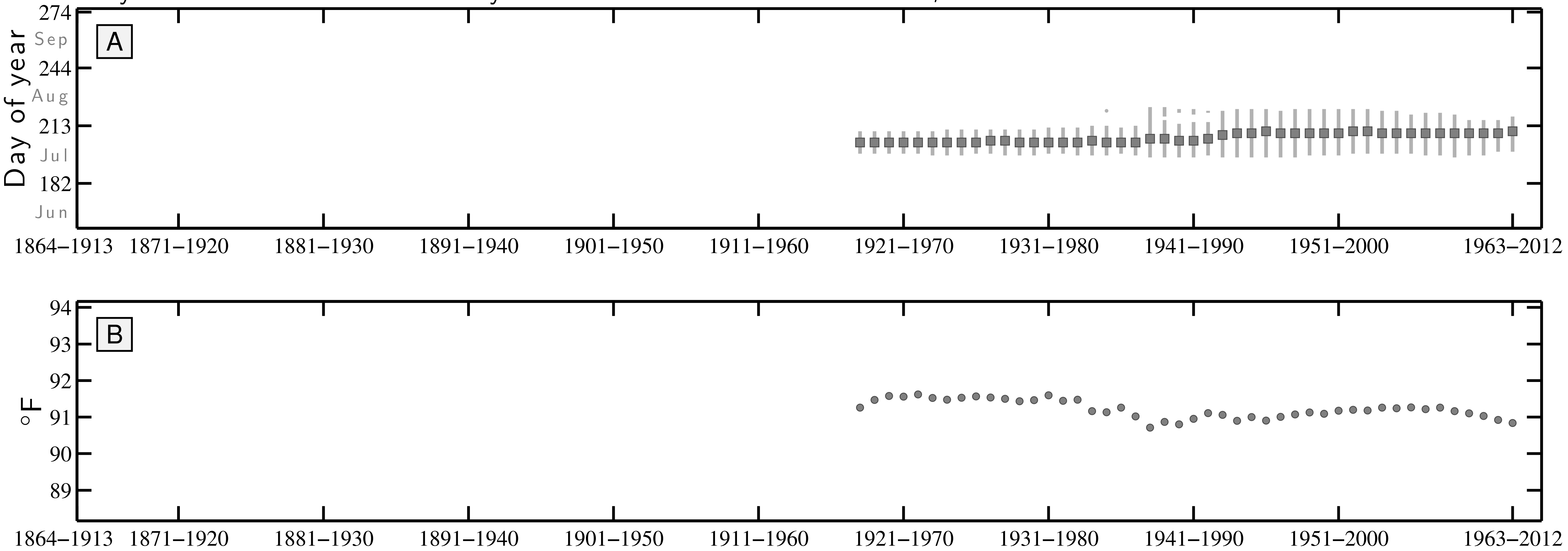

50 year Winter Teletherm dynamics for SUSANVILLE 2SW, CA:

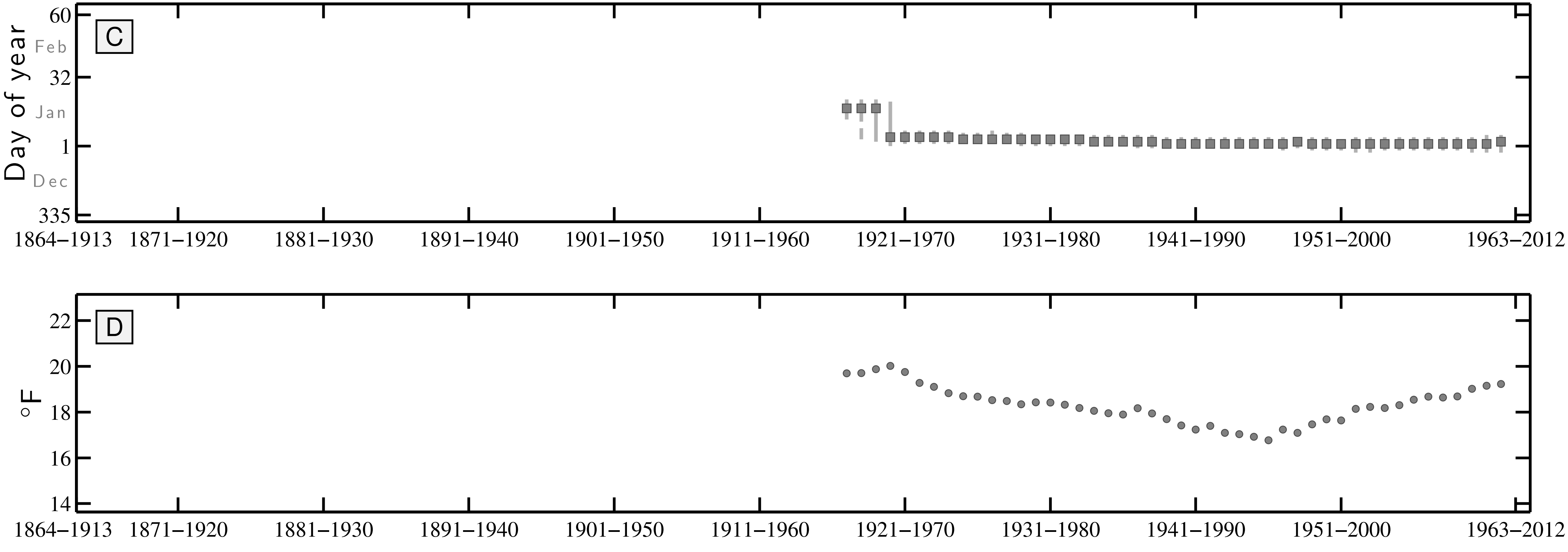

50 year Summer Teletherm dynamics for TAHOE CITY, CA:

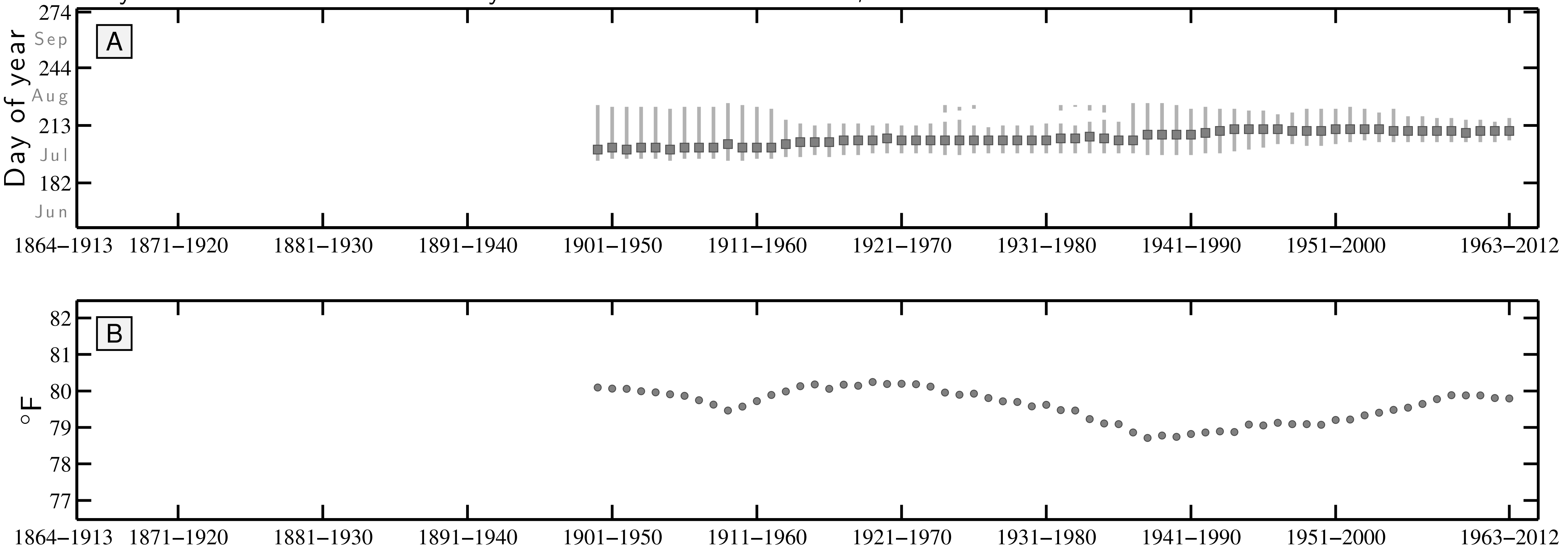

50 year Winter Teletherm dynamics for TAHOE CITY, CA:

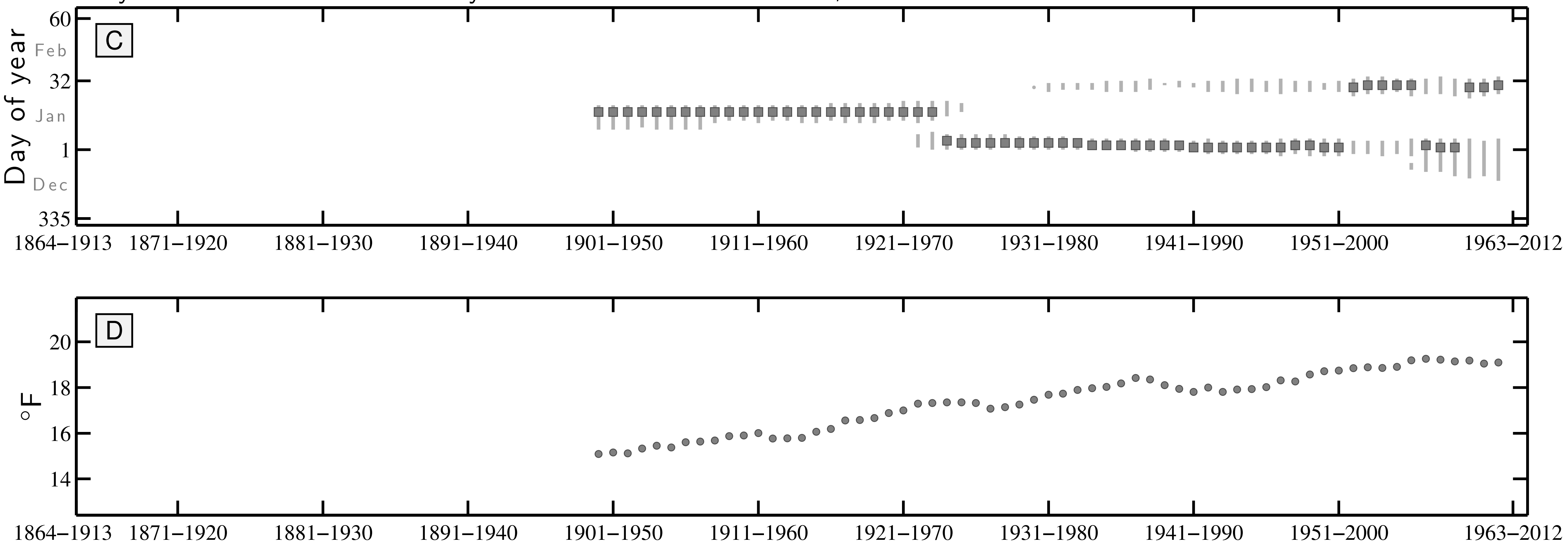

50 year Summer Teletherm dynamics for TEJON RANCHO, CA:

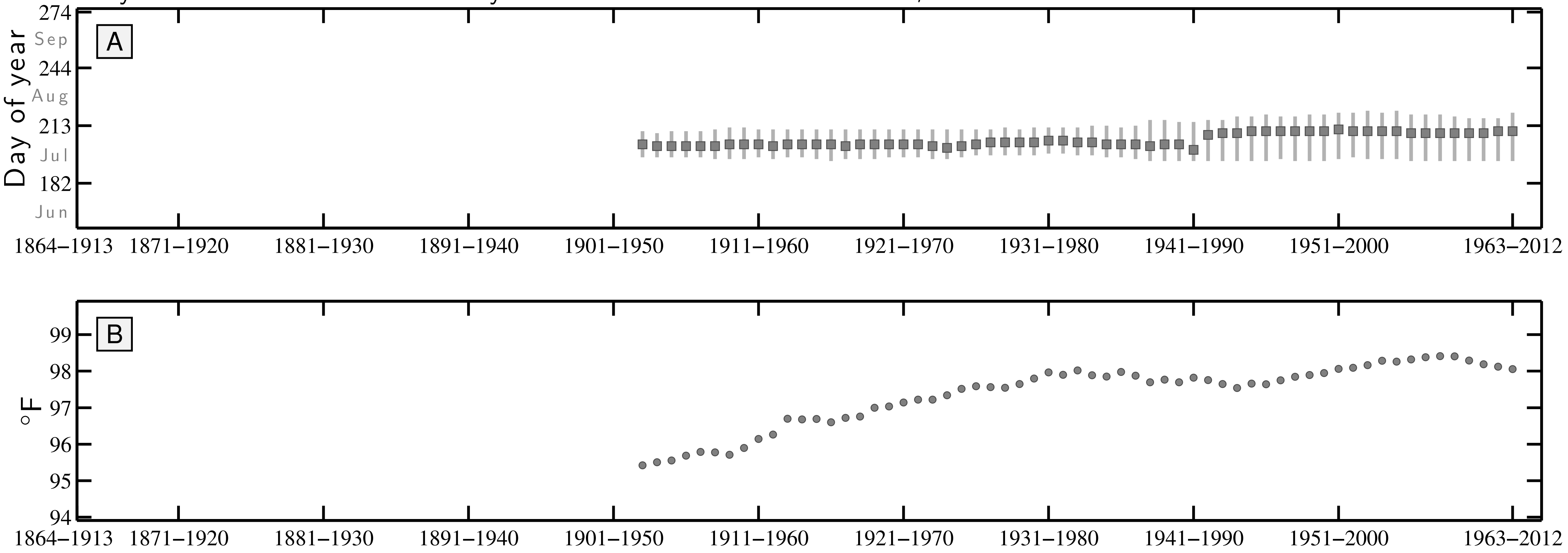

50 year Winter Teletherm dynamics for TEJON RANCHO, CA:

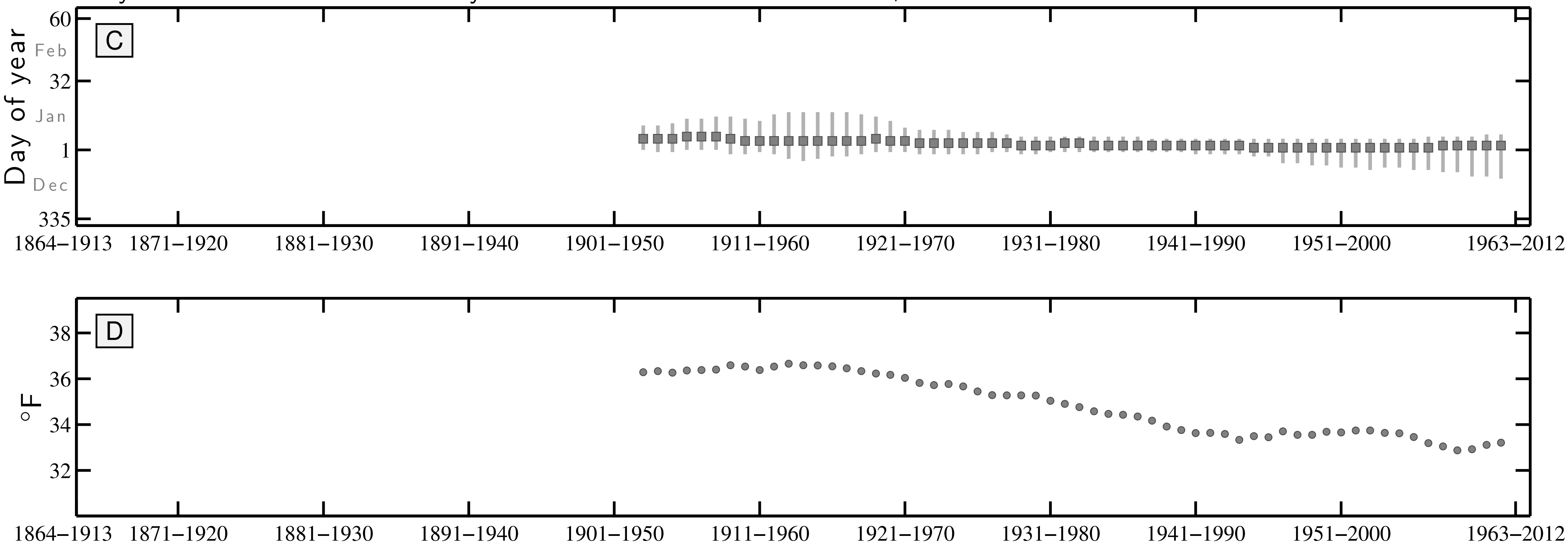

50 year Summer Teletherm dynamics for TUSTIN IRVINE RCH, CA:

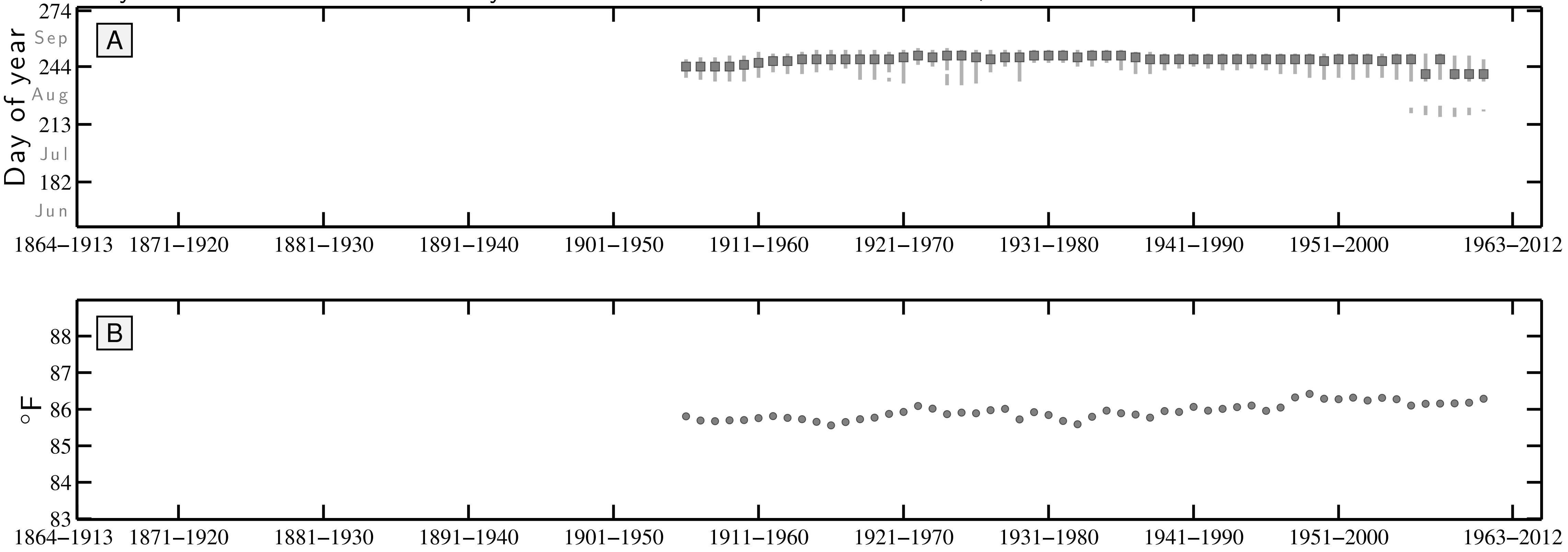

50 year Winter Teletherm dynamics for TUSTIN IRVINE RCH, CA:

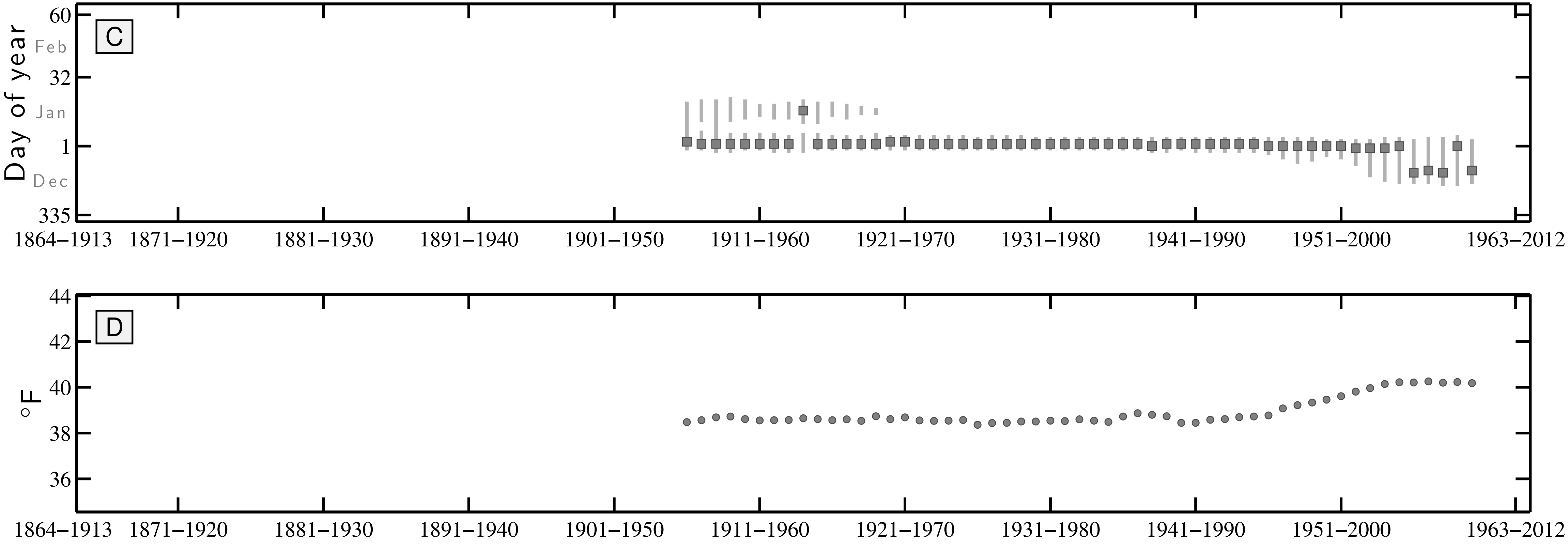

50 year Summer Teletherm dynamics for UKIAH, CA:

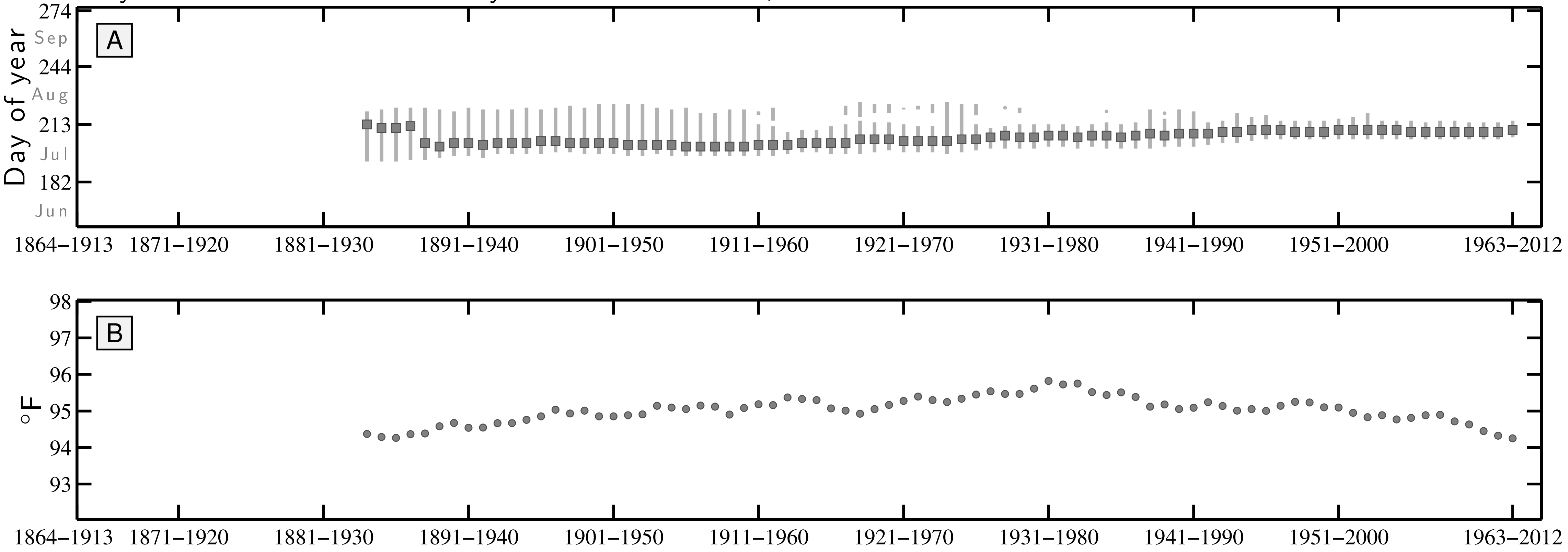

50 year Winter Teletherm dynamics for UKIAH, CA:

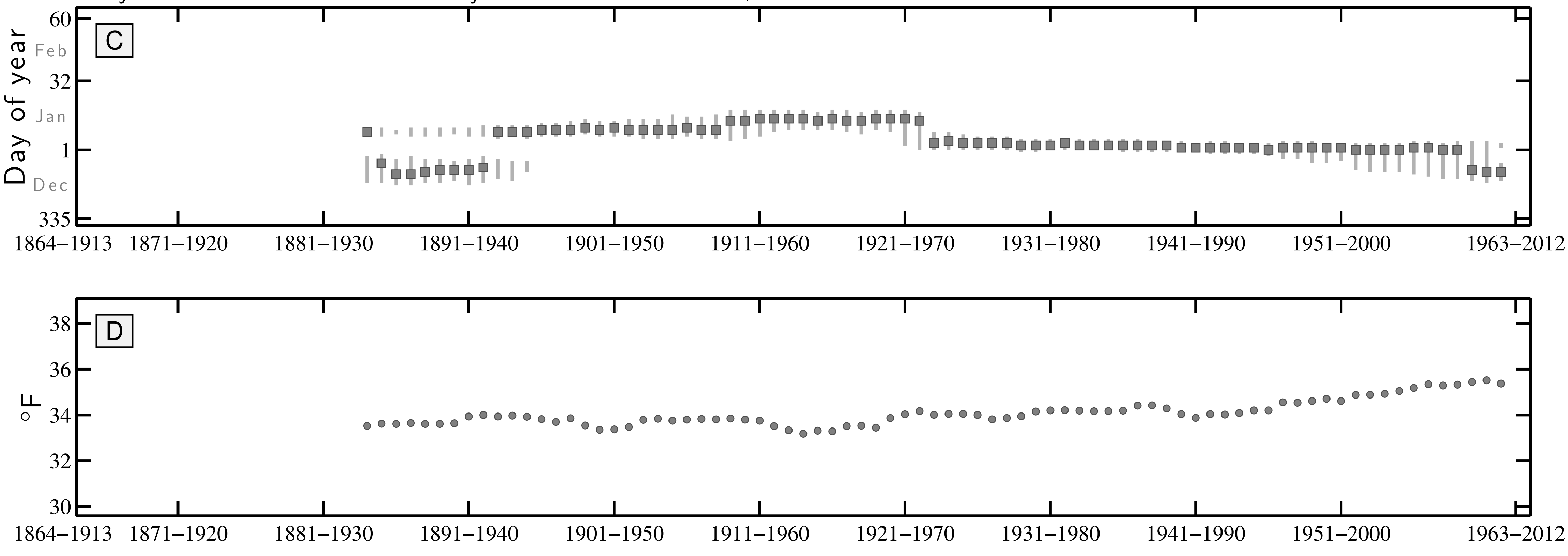

50 year Summer Teletherm dynamics for VACAVILLE, CA:

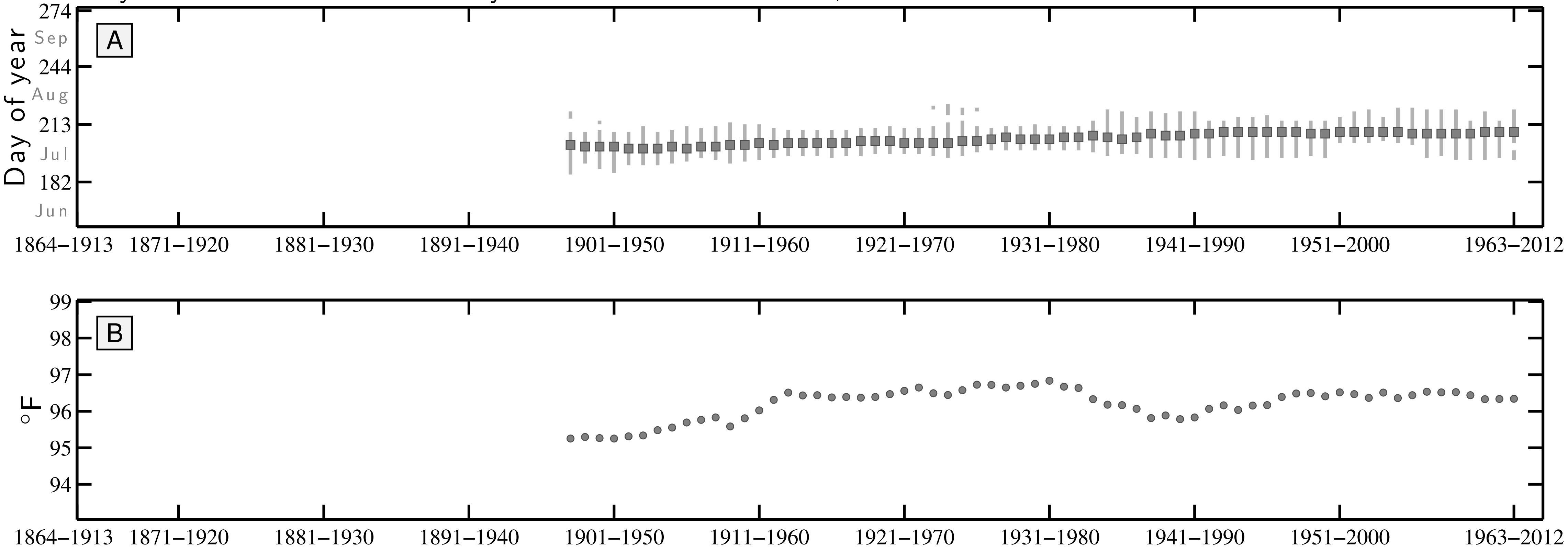

50 year Winter Teletherm dynamics for VACAVILLE, CA:

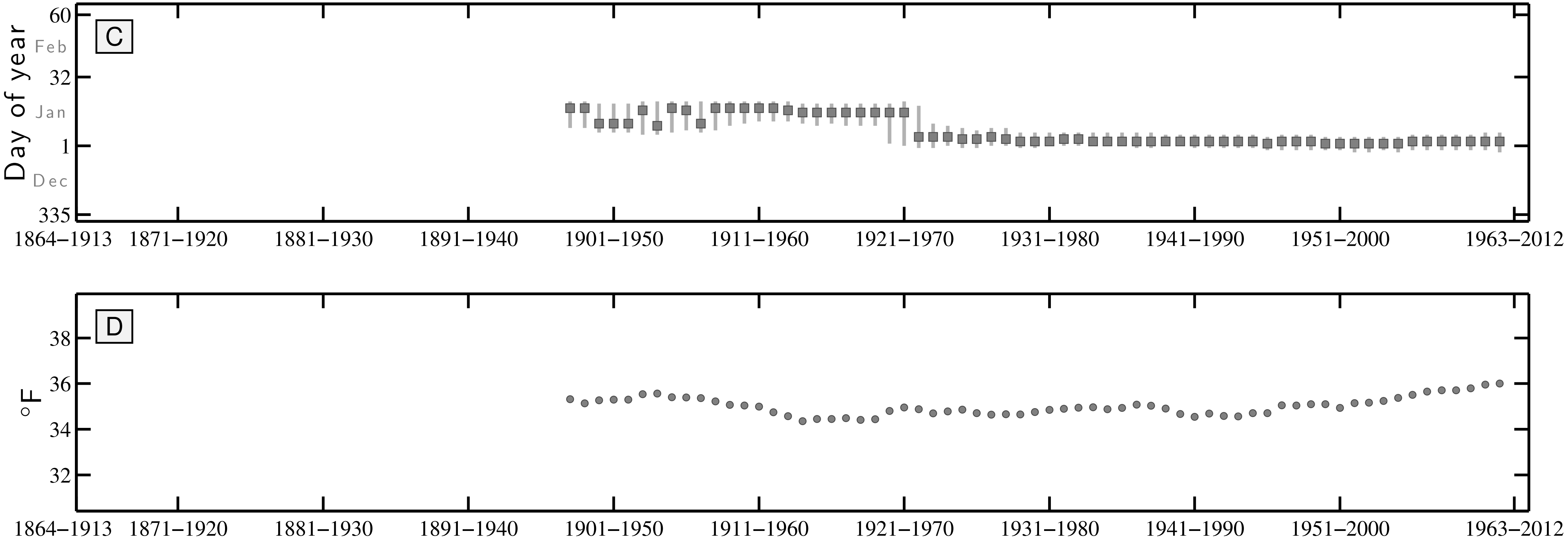

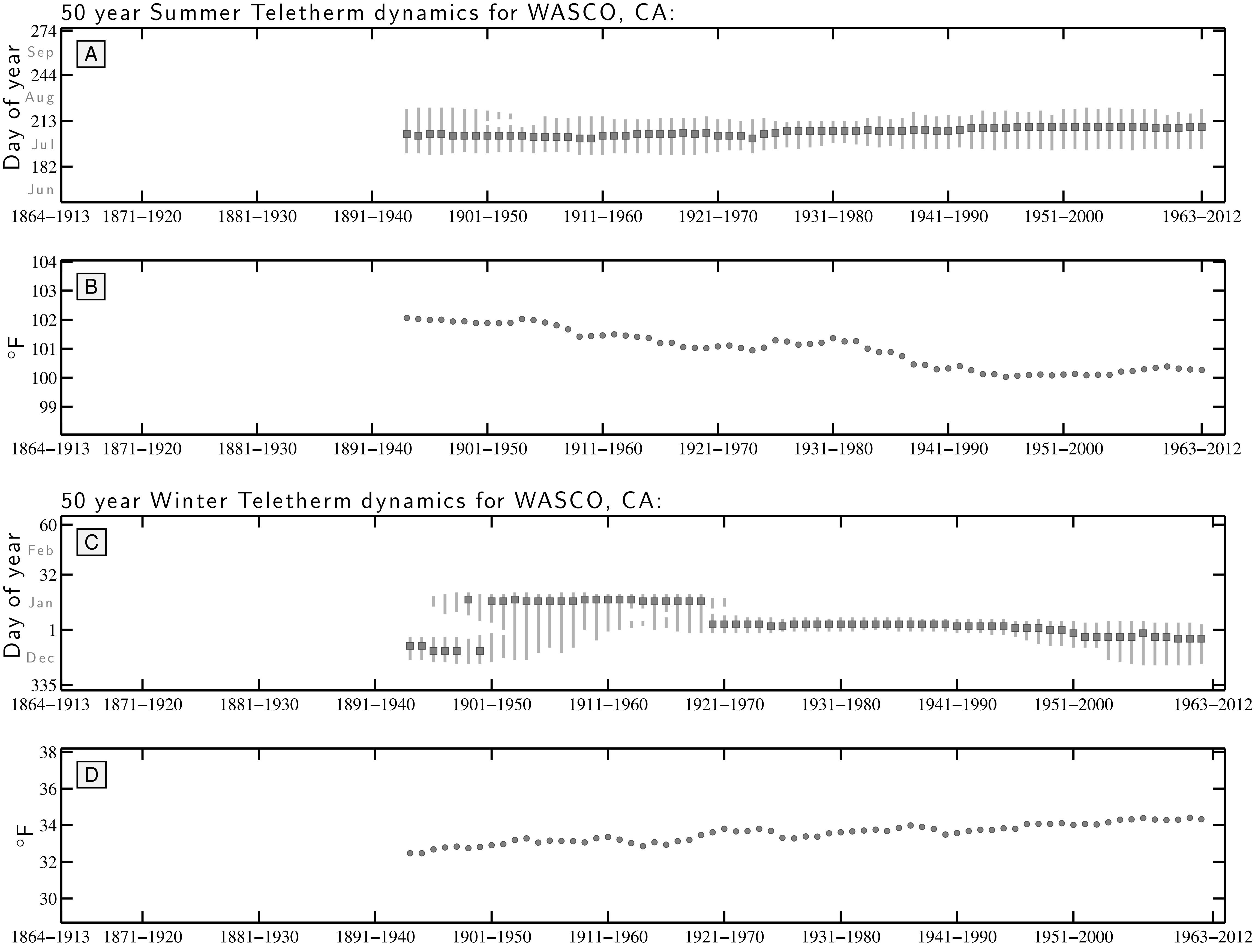
50 year Summer Teletherm dynamics for WASCO, CA:
A
Day of year
274
Sep
244
Aug
213
Jul
182
Jun
1864–1913
1871–1920
1881–1930
1891–1940
1901–1950
1911–1960
1921–1970
1931–1980
1941–1990
1951–2000
1963–2012
B
°F
104
103
102
101
100
99
50 year Winter Teletherm dynamics for WASCO, CA:
C
Day of year
60
Feb
32
Jan
1
Dec
335
D
°F
38
36
34
32
30

50 year Summer Teletherm dynamics for WEAVERVILLE, CA:

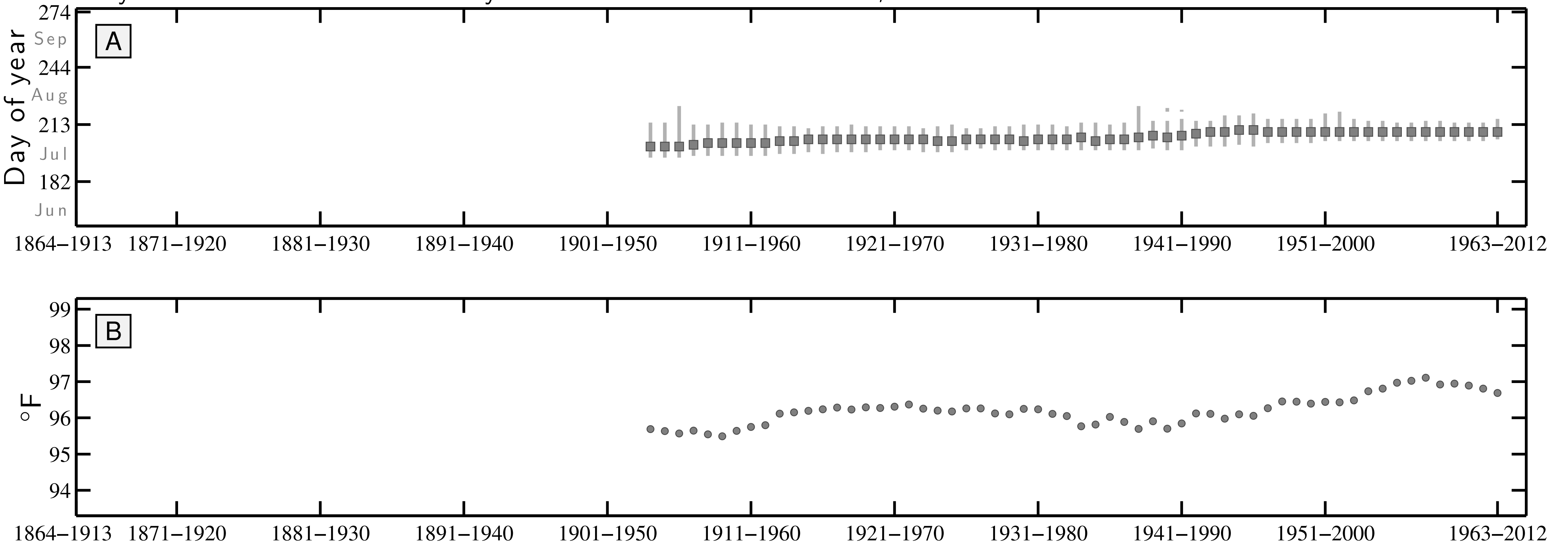

50 year Winter Teletherm dynamics for WEAVERVILLE, CA:

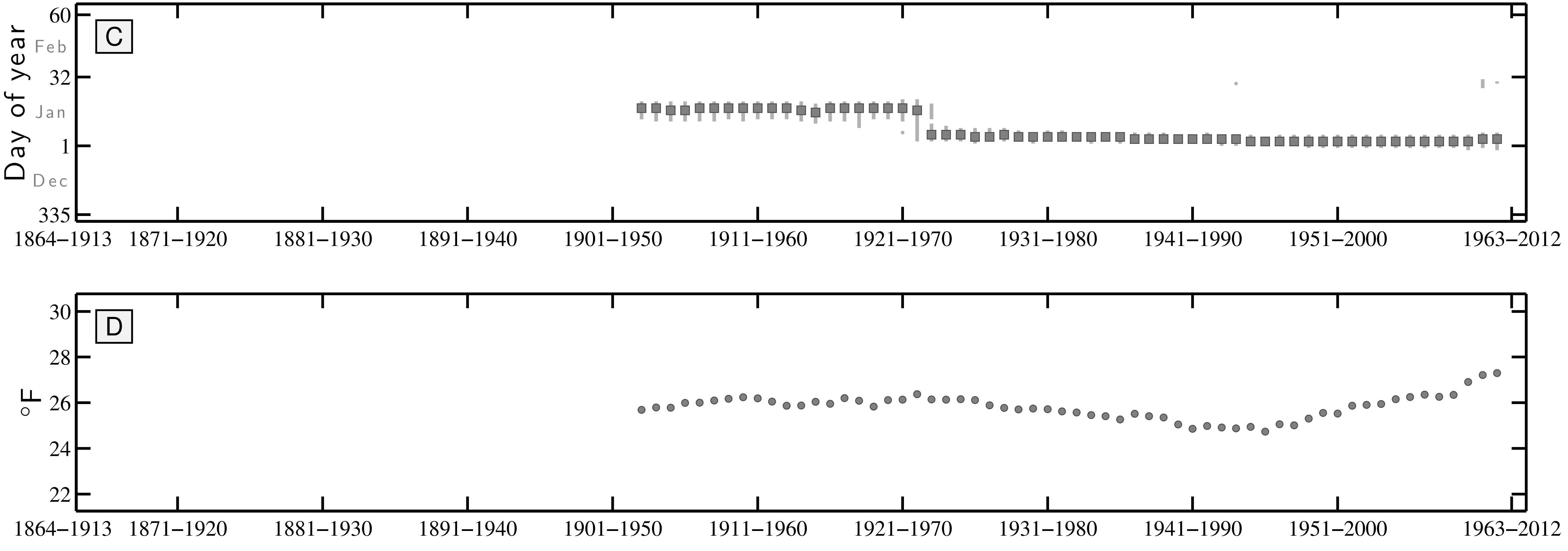

50 year Summer Teletherm dynamics for WILLOWS 6 W, CA:

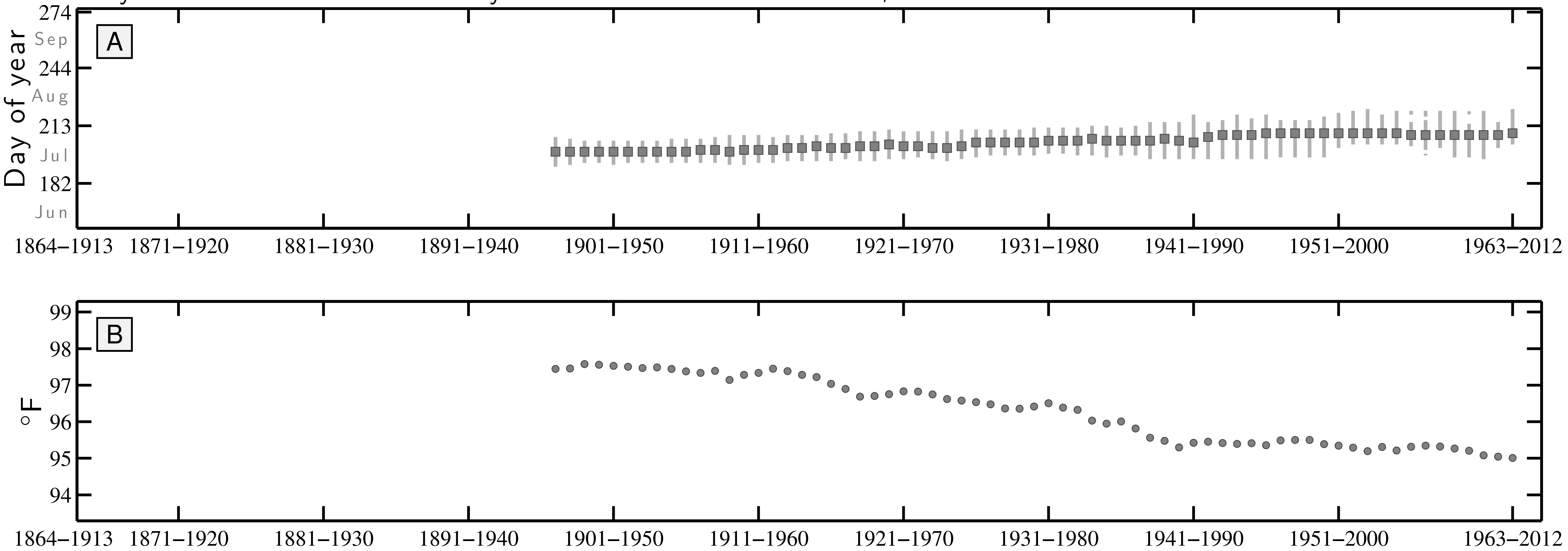

50 year Winter Teletherm dynamics for WILLOWS 6 W, CA:

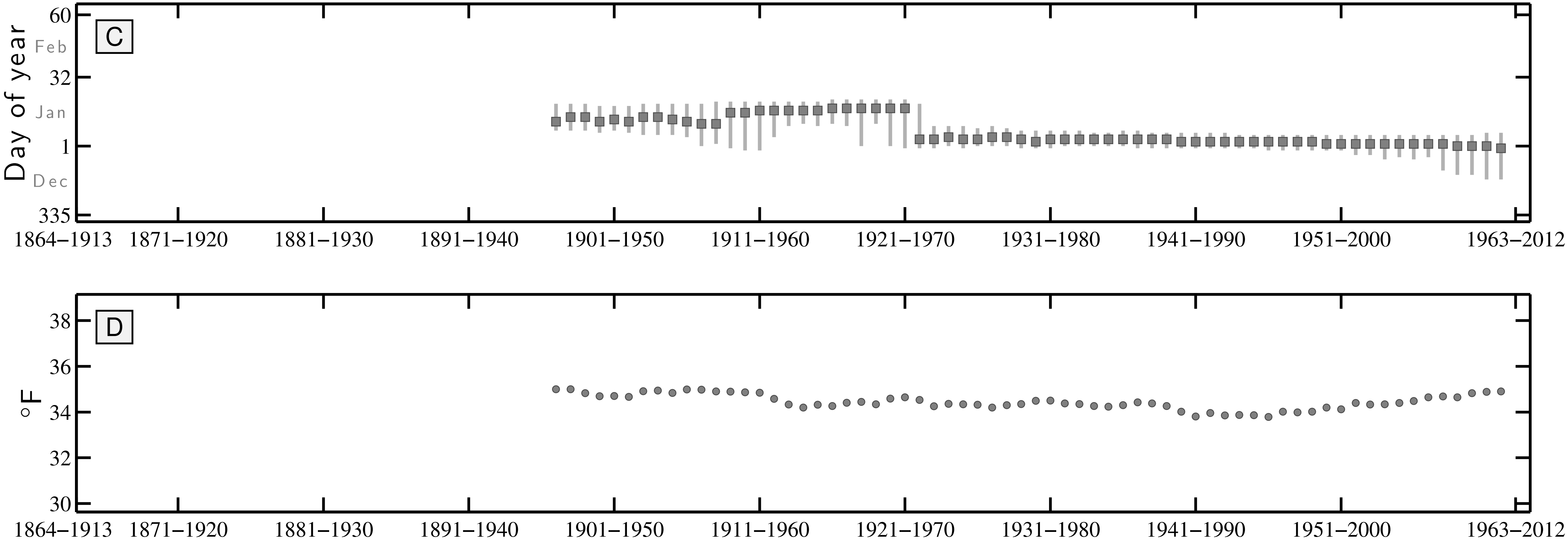

50 year Summer Teletherm dynamics for YOSEMITE PARK HQ, CA:

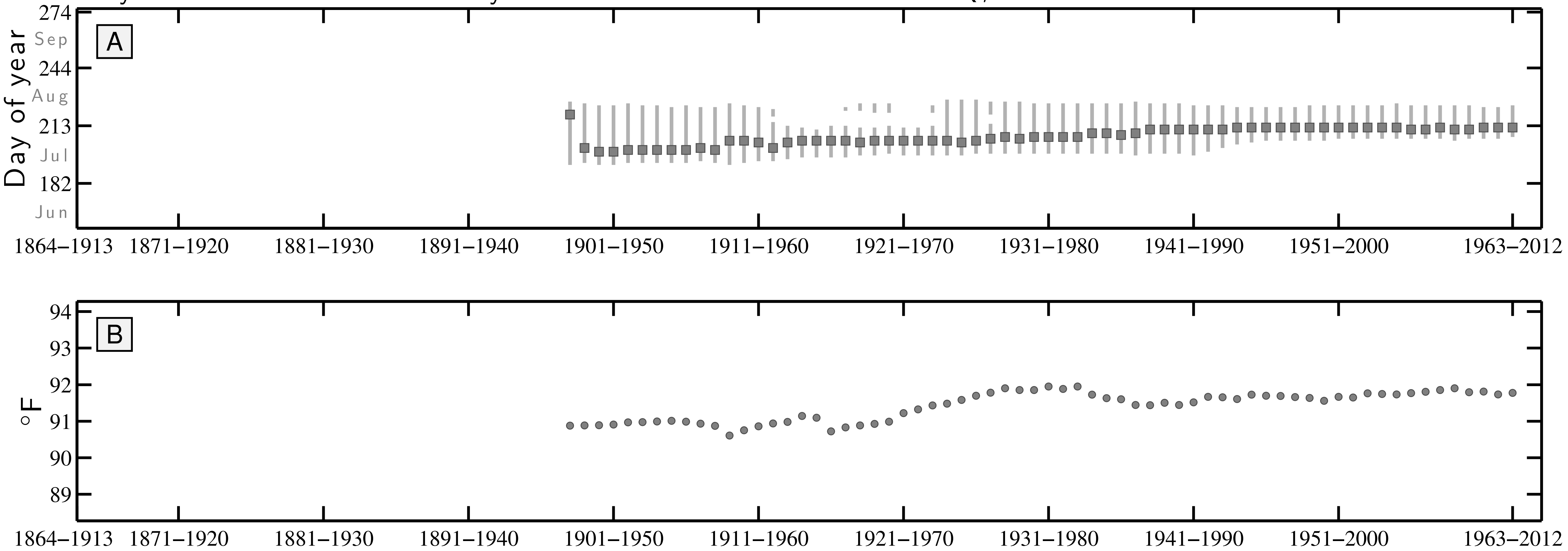

50 year Winter Teletherm dynamics for YOSEMITE PARK HQ, CA:

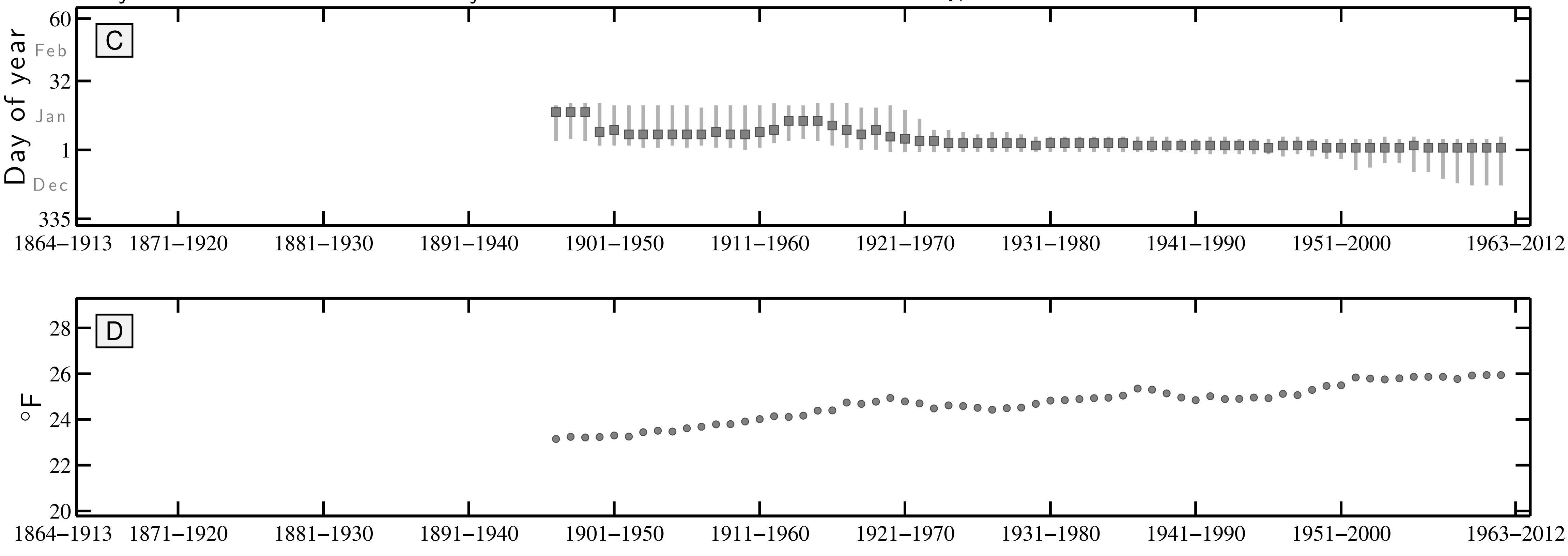

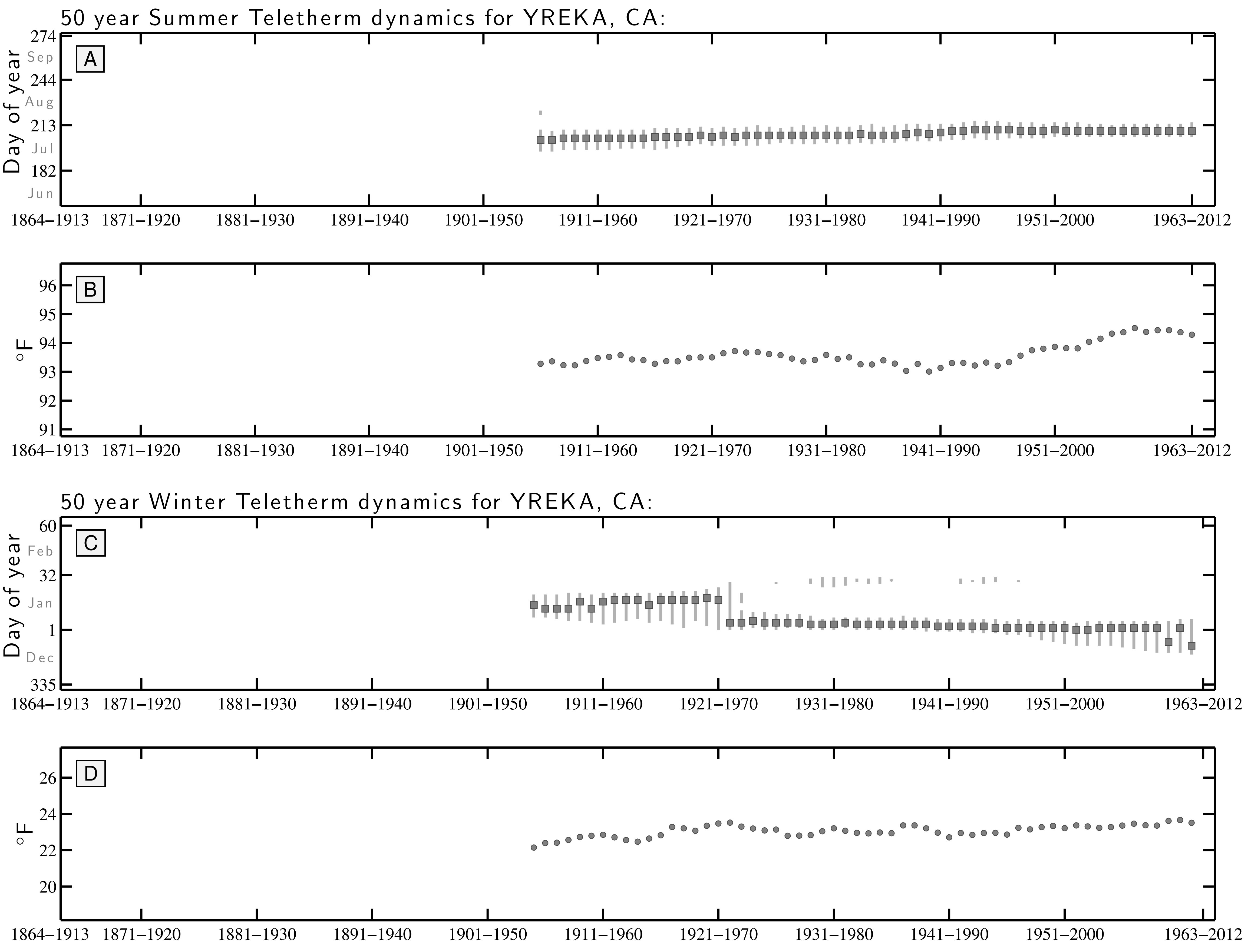

50 year Summer Teletherm dynamics for YREKA, CA:
A
Day of year
274
Sep
244
Aug
213
Jul
182
Jun
1864–1913
1871–1920
1881–1930
1891–1940
1901–1950
1911–1960
1921–1970
1931–1980
1941–1990
1951–2000
1963–2012
B
°F
96
95
94
93
92
91
50 year Winter Teletherm dynamics for YREKA, CA:
C
Day of year
60
Feb
32
Jan
1
Dec
335
D
°F
26
24
22
20

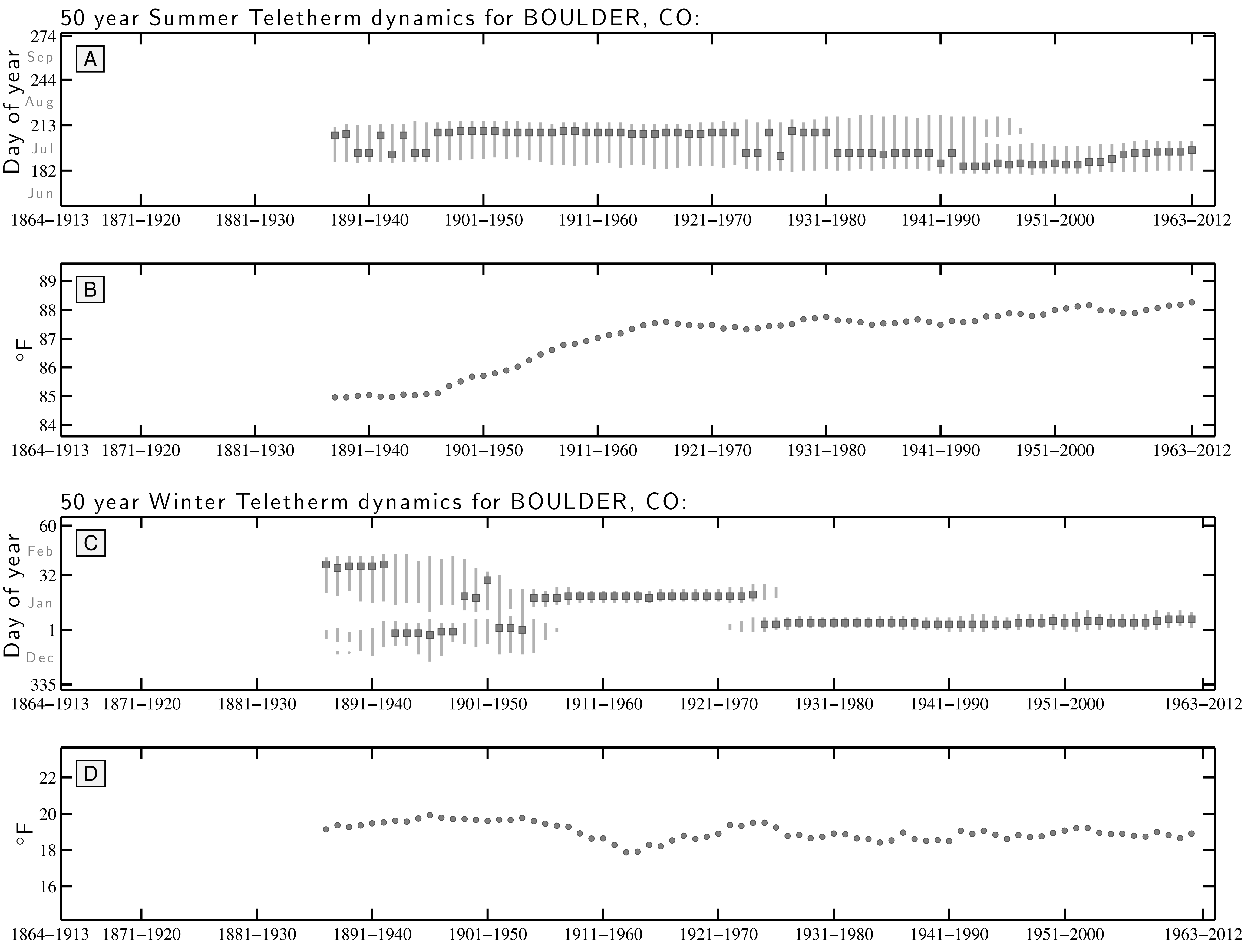

50 year Summer Teletherm dynamics for BOULDER, CO:
A
B
Day of year
°F
Sep
Aug
Jul
Jun
274
244
213
182
89
88
87
86
85
84
1864–1913
1871–1920
1881–1930
1891–1940
1901–1950
1911–1960
1921–1970
1931–1980
1941–1990
1951–2000
1963–2012
50 year Winter Teletherm dynamics for BOULDER, CO:
C
D
Feb
Jan
Dec
60
32
1
335
22
20
18
16

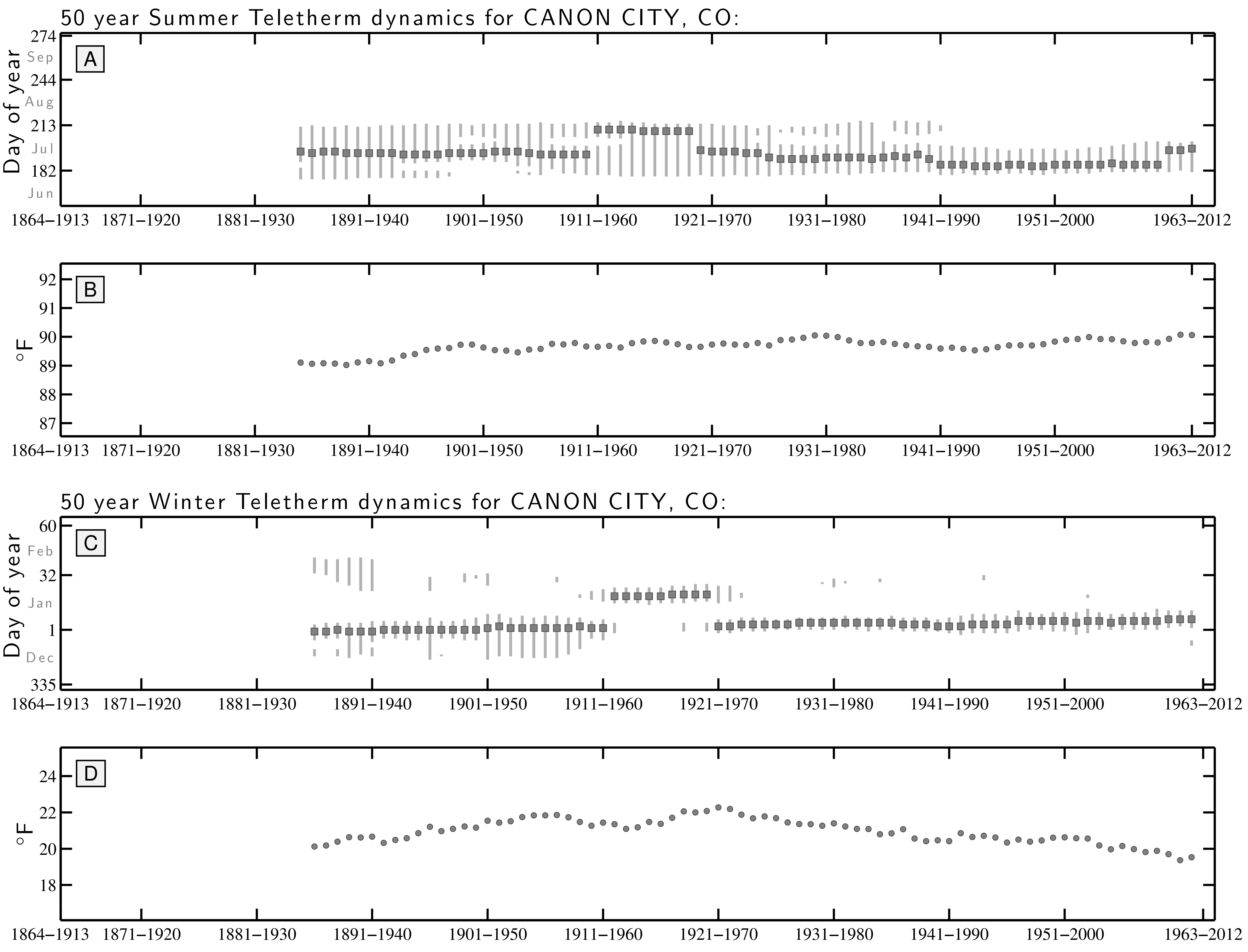

50 year Summer Teletherm dynamics for CANON CITY, CO:
A
Day of year
274
Sep
244
Aug
213
Jul
182
Jun
1864–1913
1871–1920
1881–1930
1891–1940
1901–1950
1911–1960
1921–1970
1931–1980
1941–1990
1951–2000
1963–2012
B
°F
92
91
90
89
88
87
50 year Winter Teletherm dynamics for CANON CITY, CO:
C
Day of year
60
Feb
32
Jan
1
Dec
335
D
°F
24
22
20
18

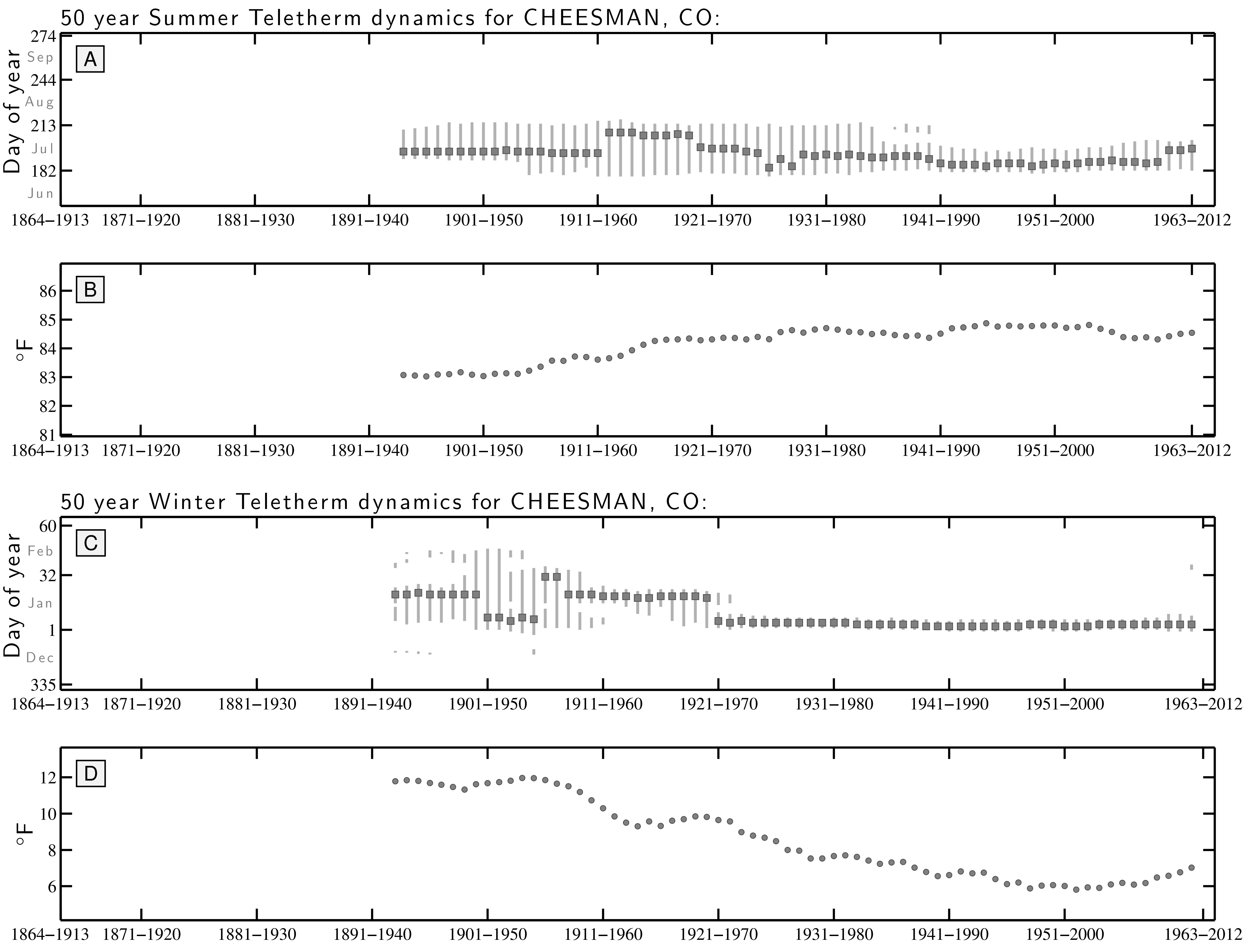
50 year Summer Teletherm dynamics for CHEESMAN, CO:
A
Day of year
274
Sep
244
Aug
213
Jul
182
Jun
B
°F
86
85
84
83
82
81
50 year Winter Teletherm dynamics for CHEESMAN, CO:
C
Day of year
60
Feb
32
Jan
1
Dec
335
D
°F
12
10
8
6
1864–1913
1871–1920
1881–1930
1891–1940
1901–1950
1911–1960
1921–1970
1931–1980
1941–1990
1951–2000
1963–2012

50 year Summer Teletherm dynamics for CHEYENNE WELLS, CO:

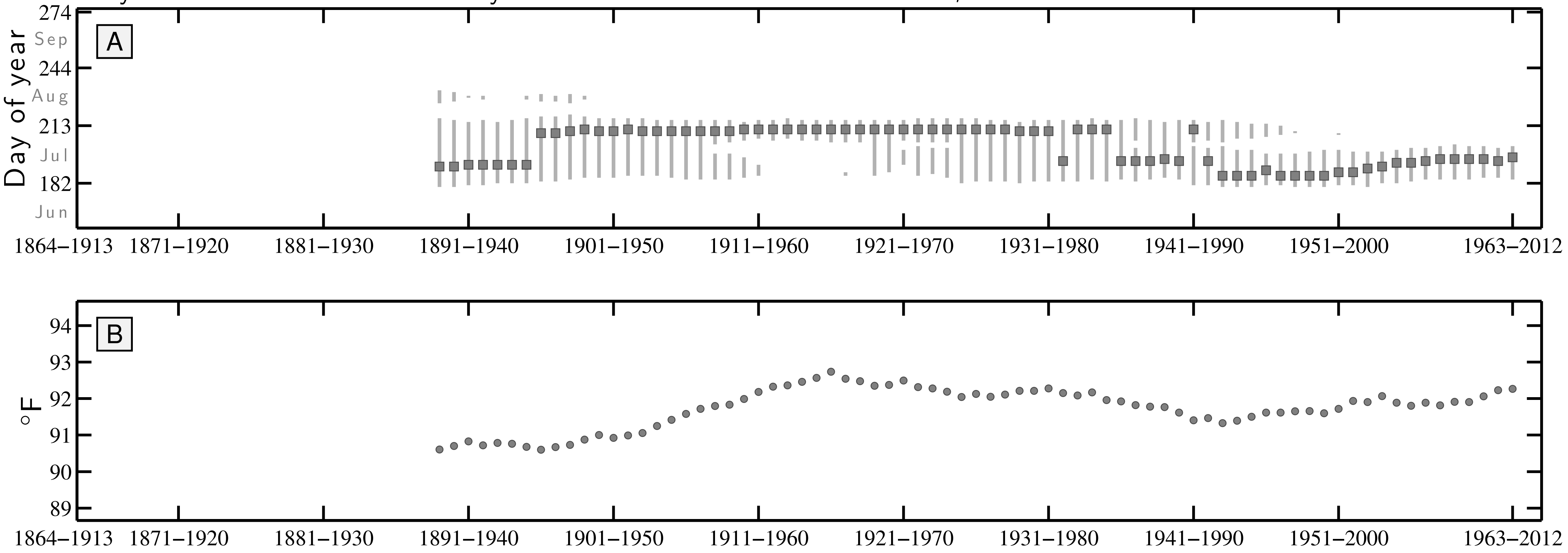

50 year Winter Teletherm dynamics for CHEYENNE WELLS, CO:

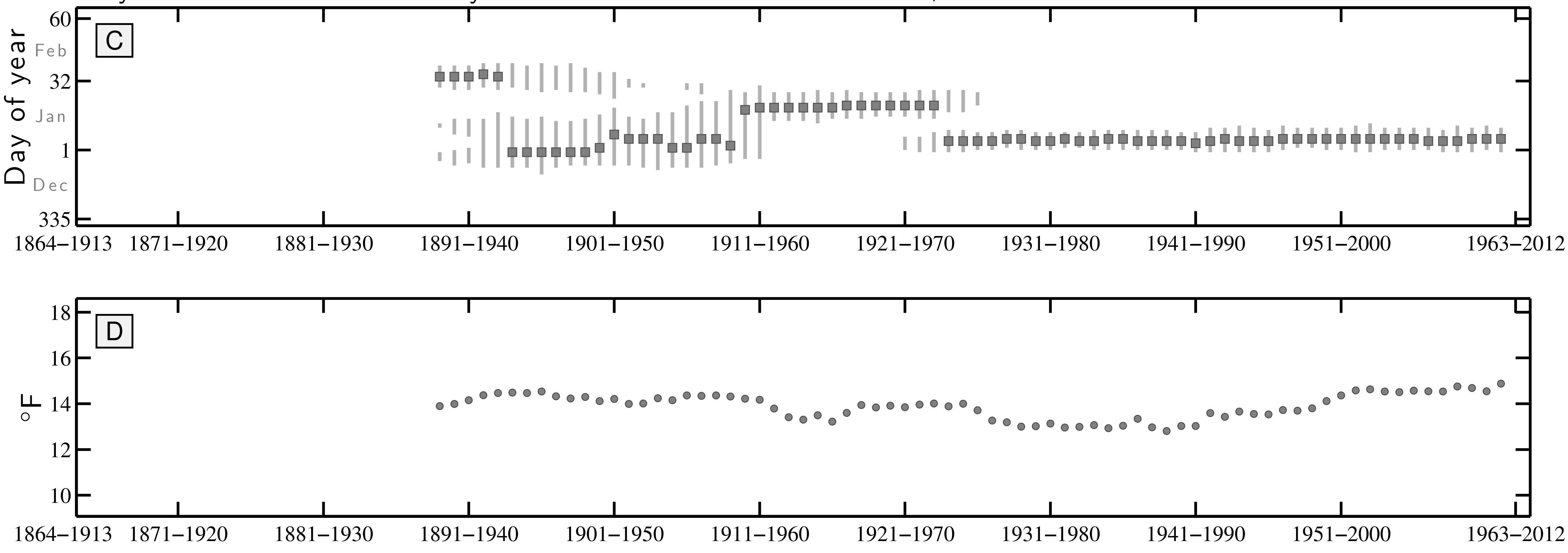

50 year Summer Teletherm dynamics for COLLBRAN, CO:

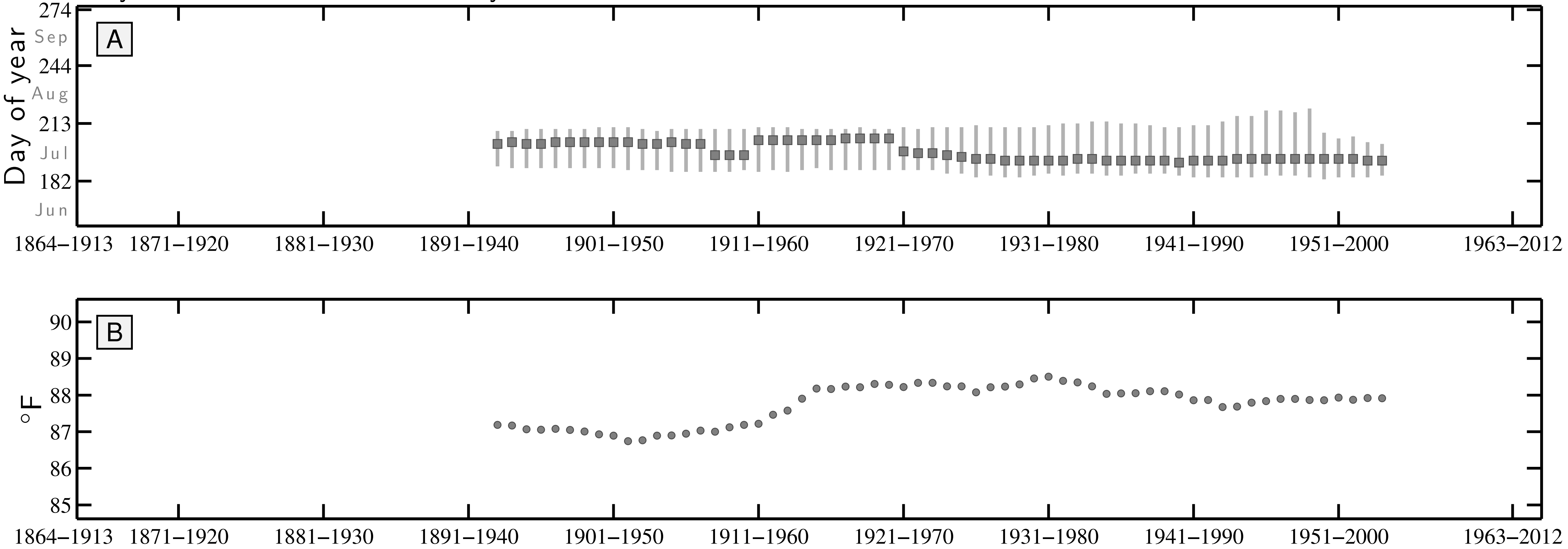

50 year Winter Teletherm dynamics for COLLBRAN, CO:

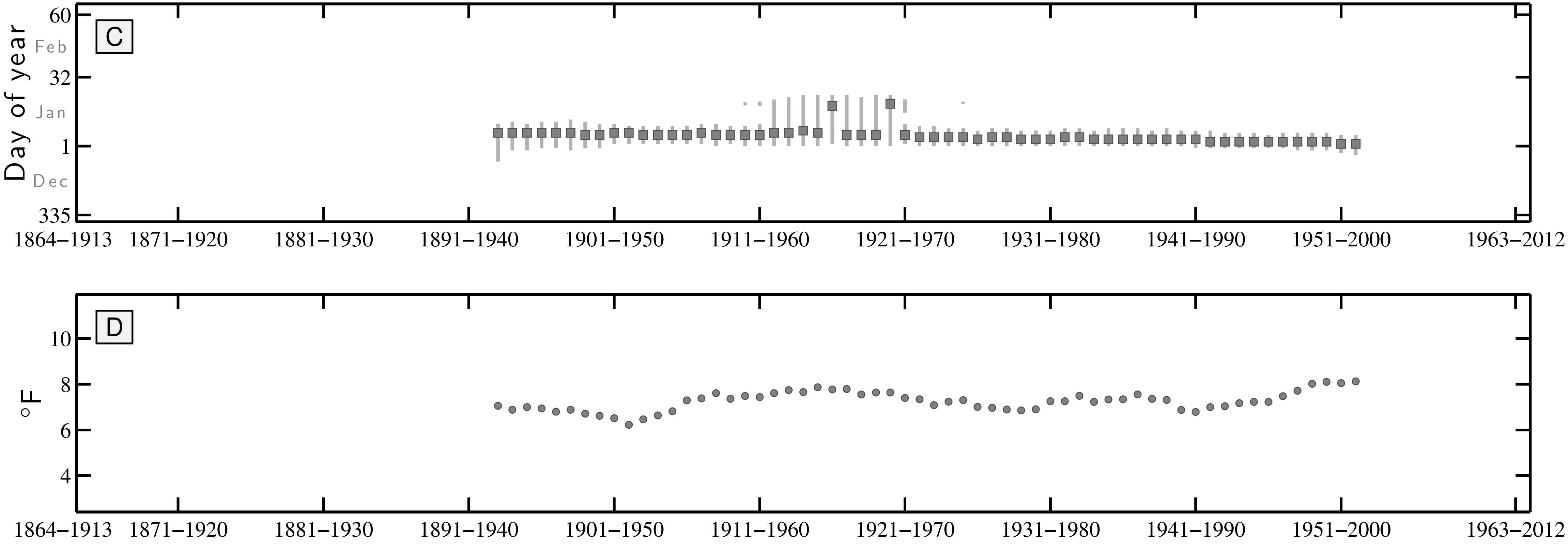

50 year Summer Teletherm dynamics for DEL NORTE 2E, CO:

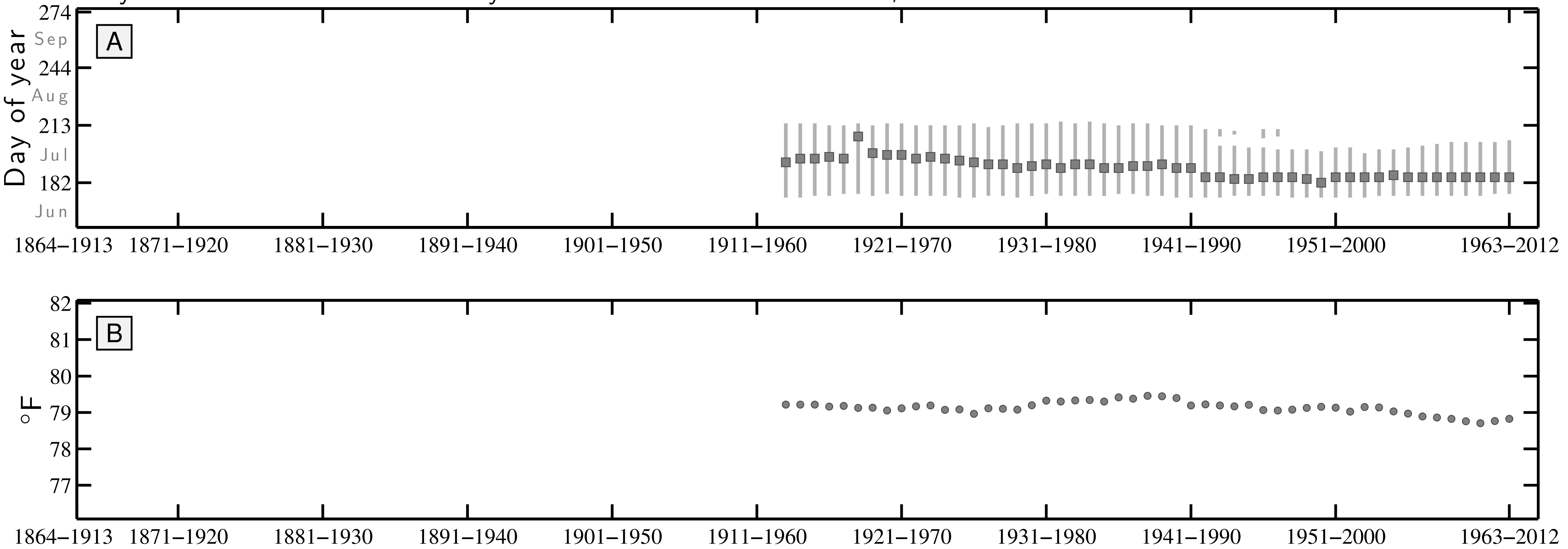

50 year Winter Teletherm dynamics for DEL NORTE 2E, CO:

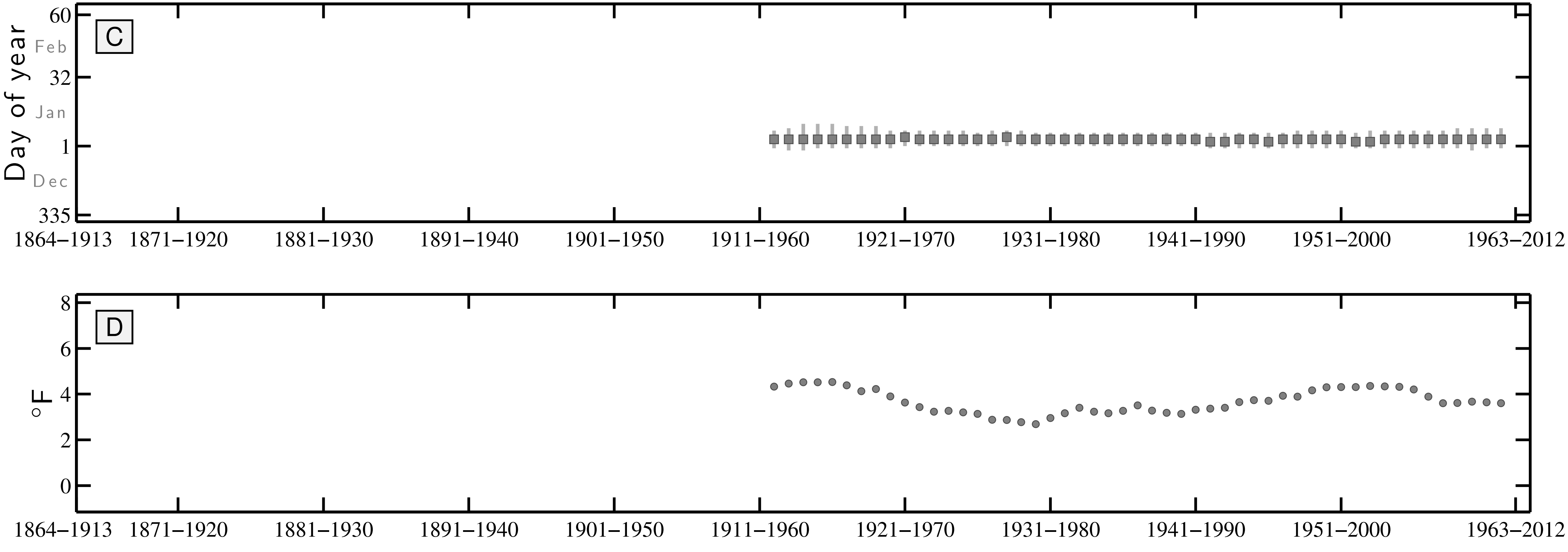

50 year Summer Teletherm dynamics for DILLON 1 E, CO:

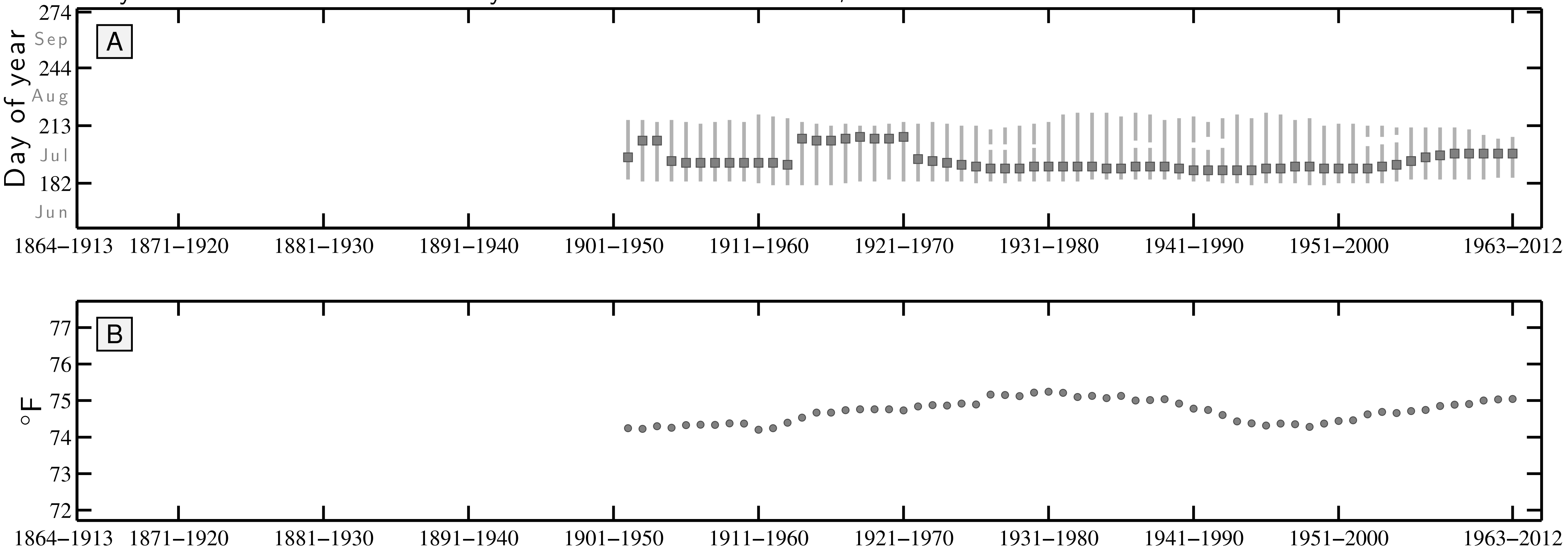

50 year Winter Teletherm dynamics for DILLON 1 E, CO:

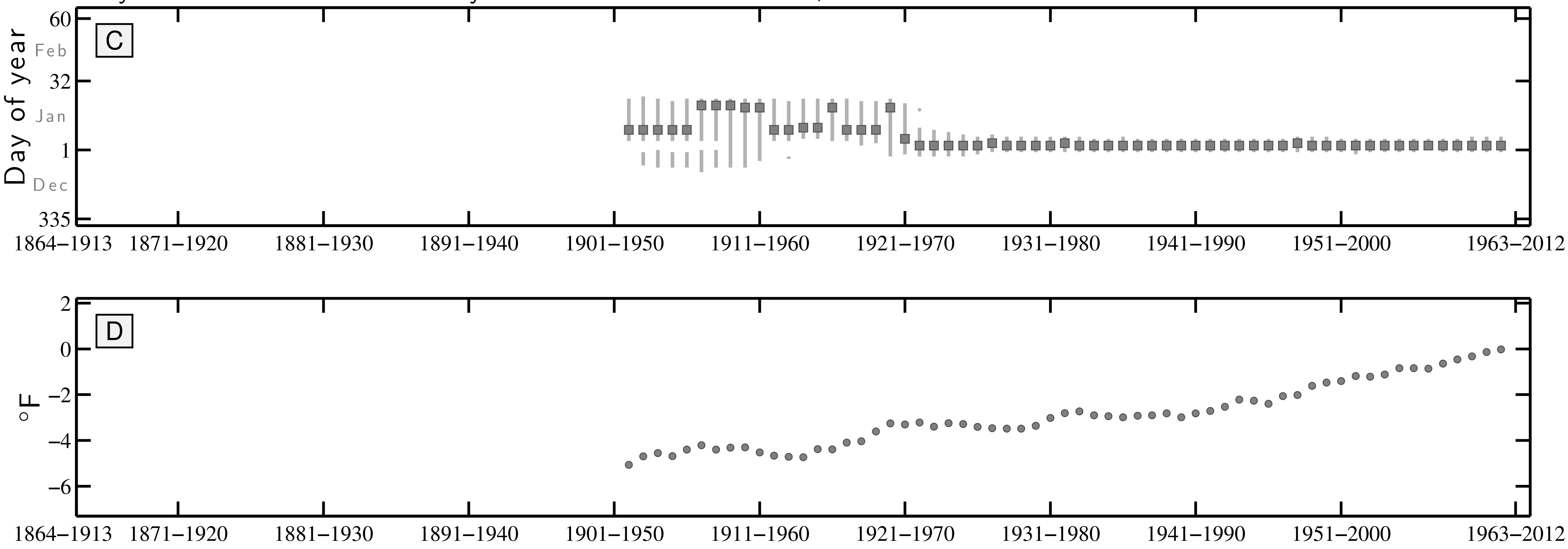

50 year Summer Teletherm dynamics for EADS, CO:

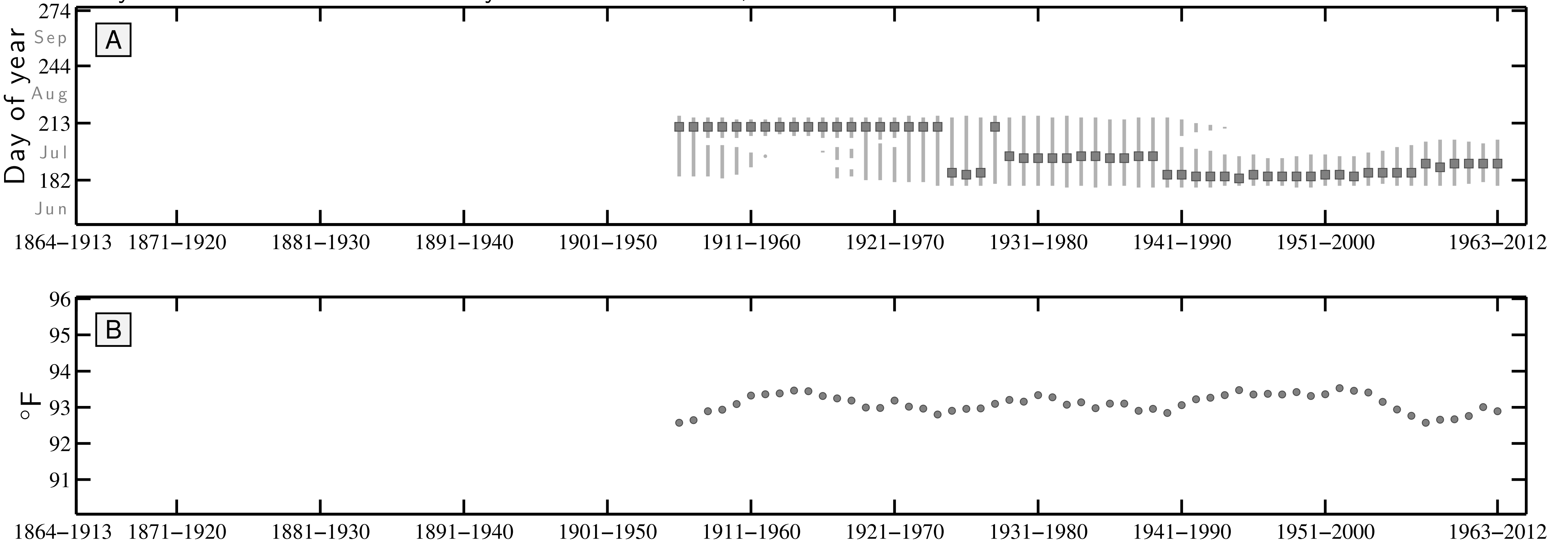

50 year Winter Teletherm dynamics for EADS, CO:

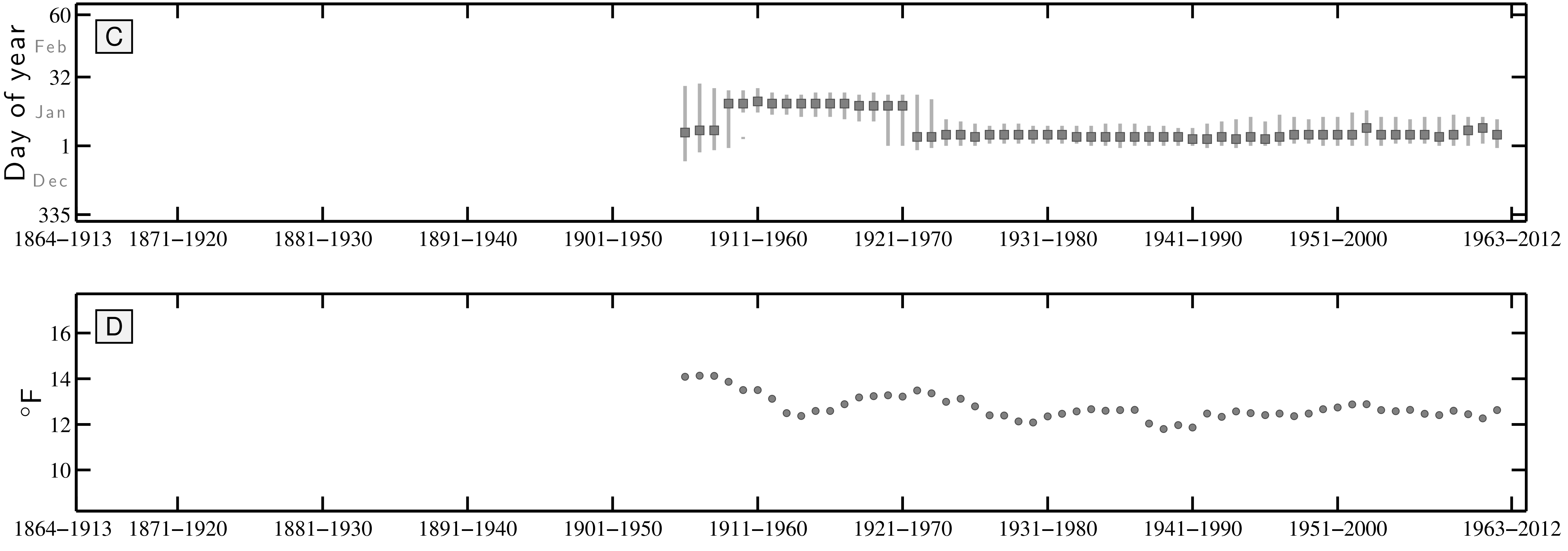

50 year Summer Teletherm dynamics for FT COLLINS, CO:

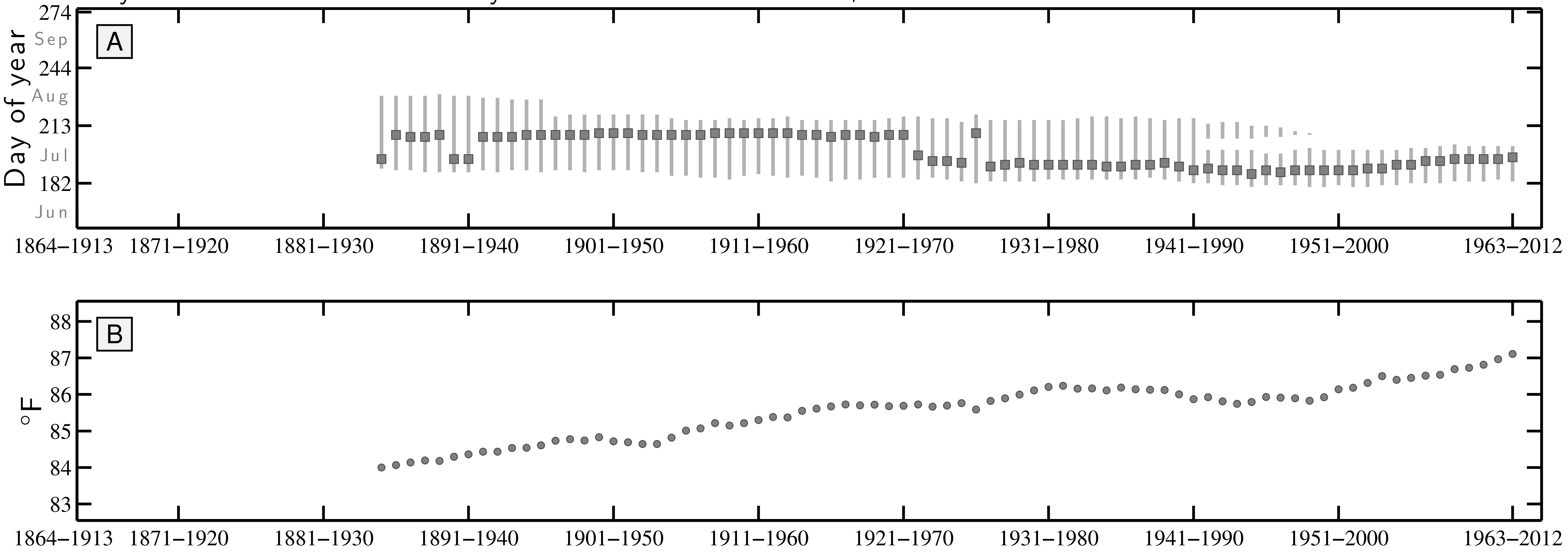

50 year Winter Teletherm dynamics for FT COLLINS, CO:

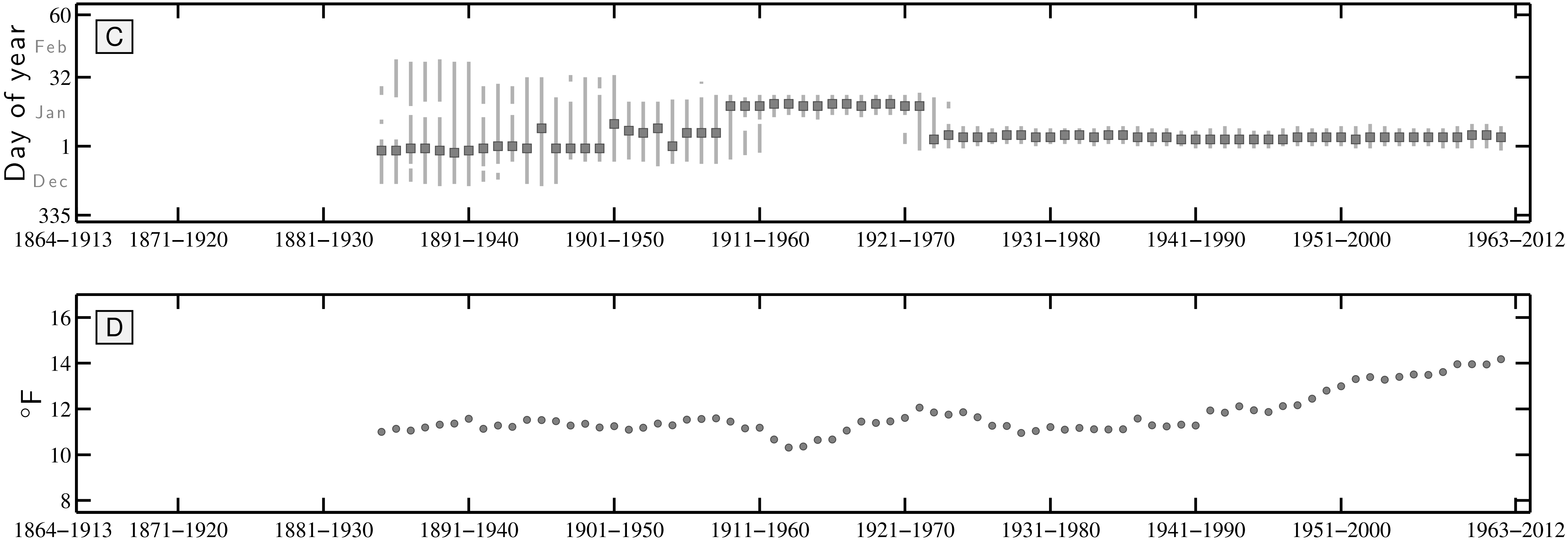

50 year Summer Teletherm dynamics for FT MORGAN, CO:

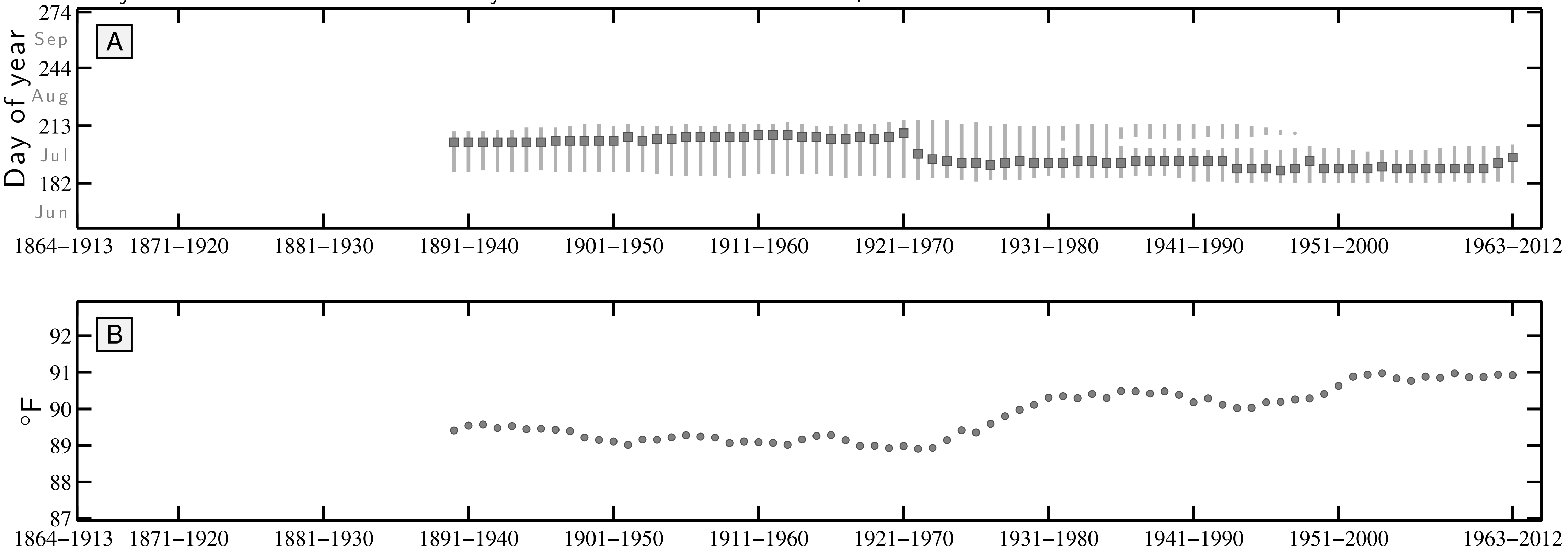

50 year Winter Teletherm dynamics for FT MORGAN, CO:

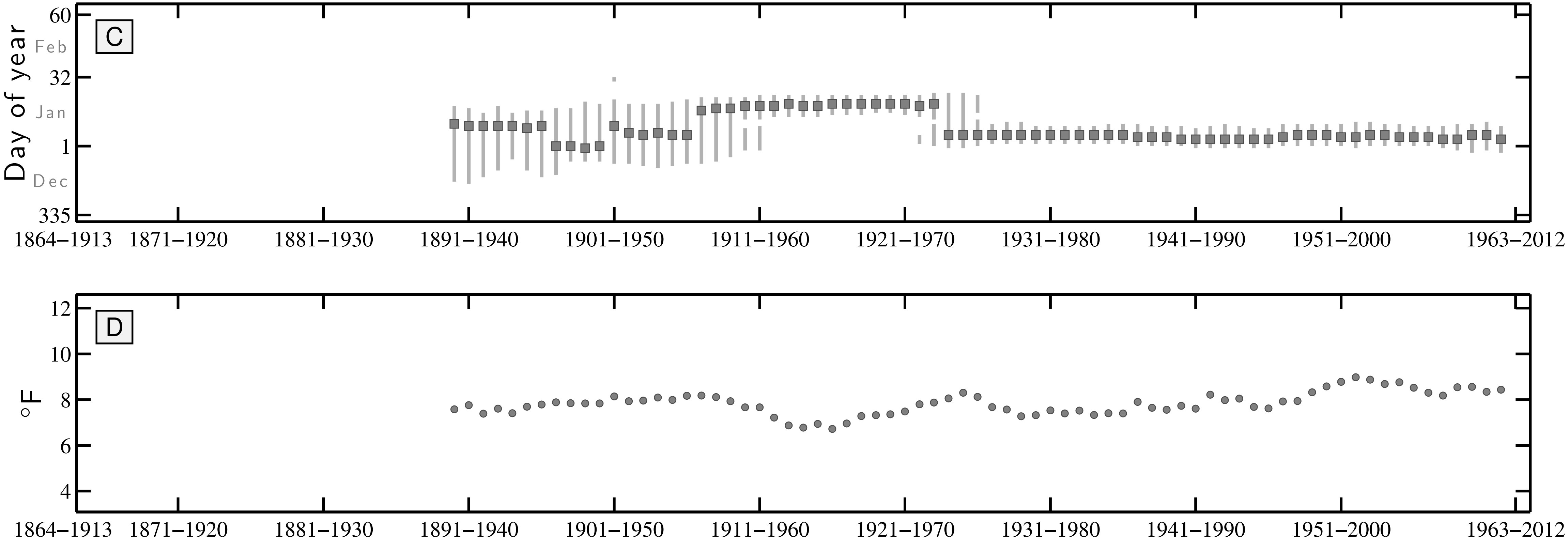

50 year Summer Teletherm dynamics for FRUITA, CO:

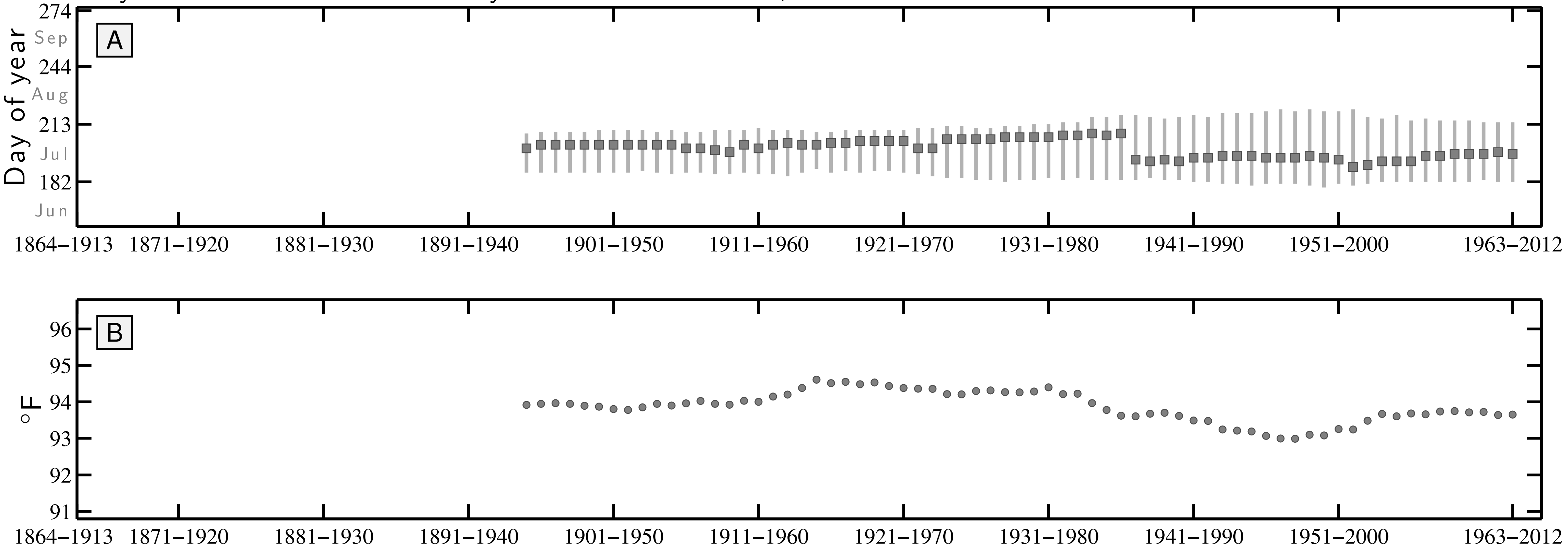

50 year Winter Teletherm dynamics for FRUITA, CO:

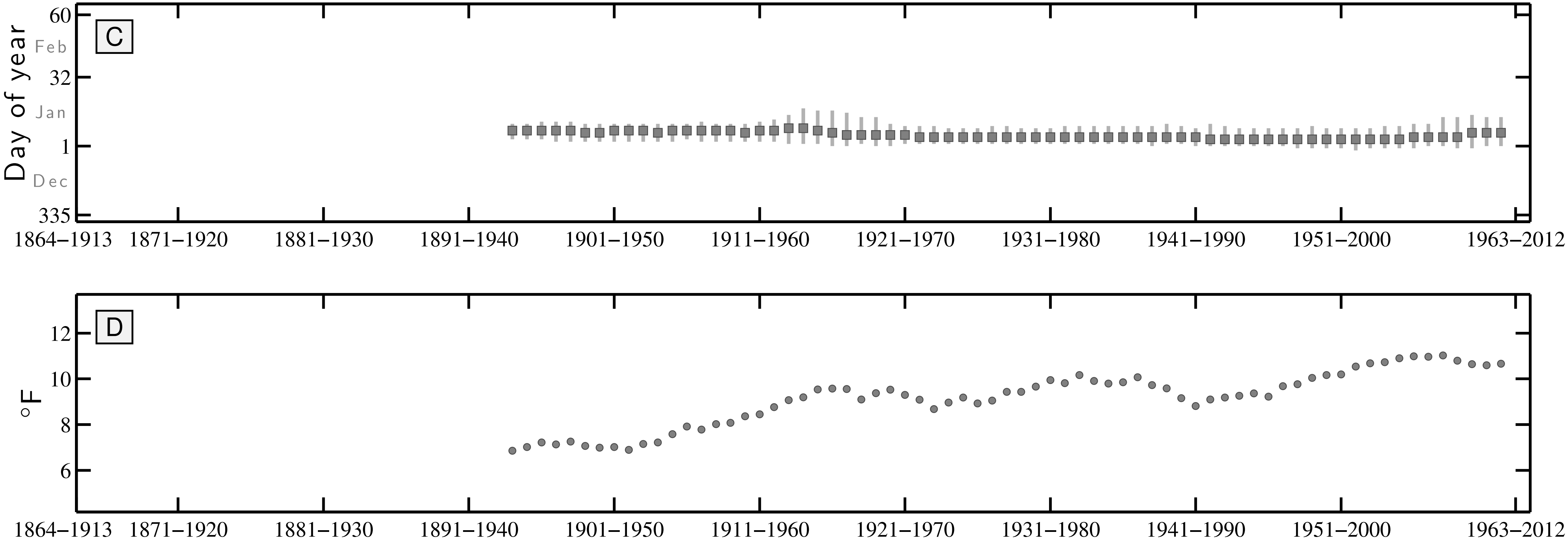

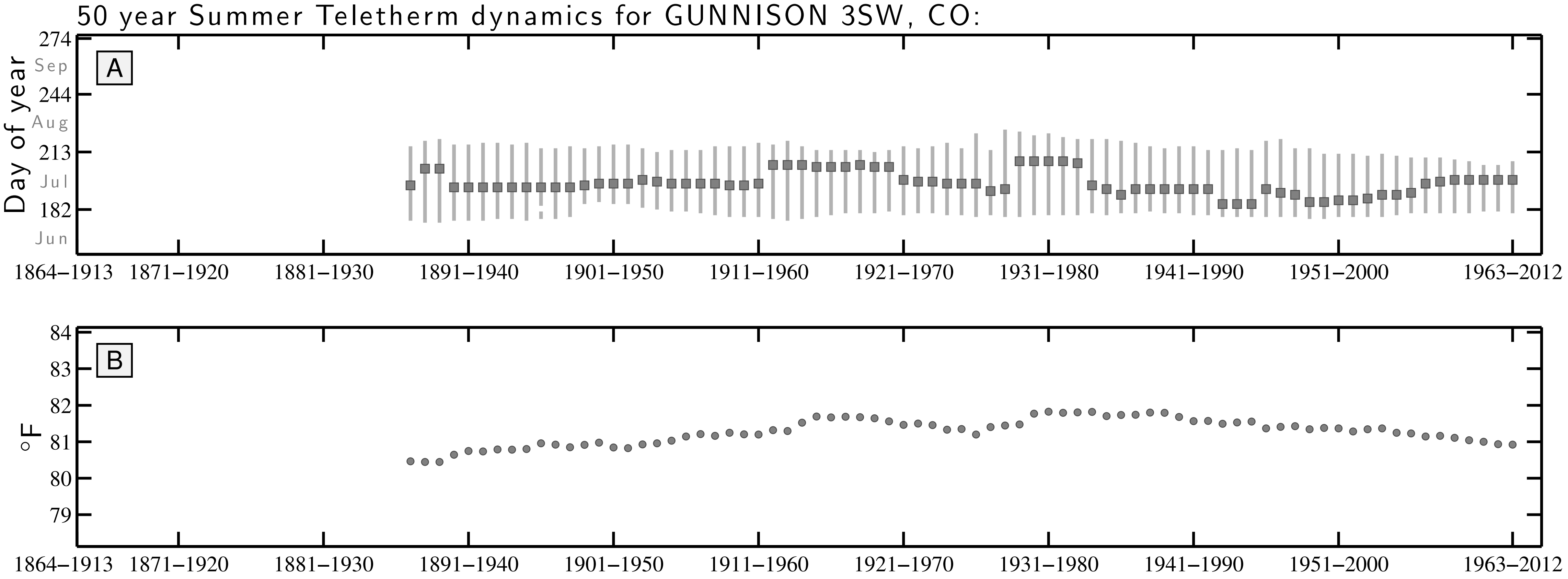
50 year Summer Teletherm dynamics for GUNNISON 3SW, CO:
A
B
Day of year
°F
Jun
Jul
Aug
Sep
182
213
244
274
79
80
81
82
83
84
1864–1913
1871–1920
1881–1930
1891–1940
1901–1950
1911–1960
1921–1970
1931–1980
1941–1990
1951–2000
1963–2012

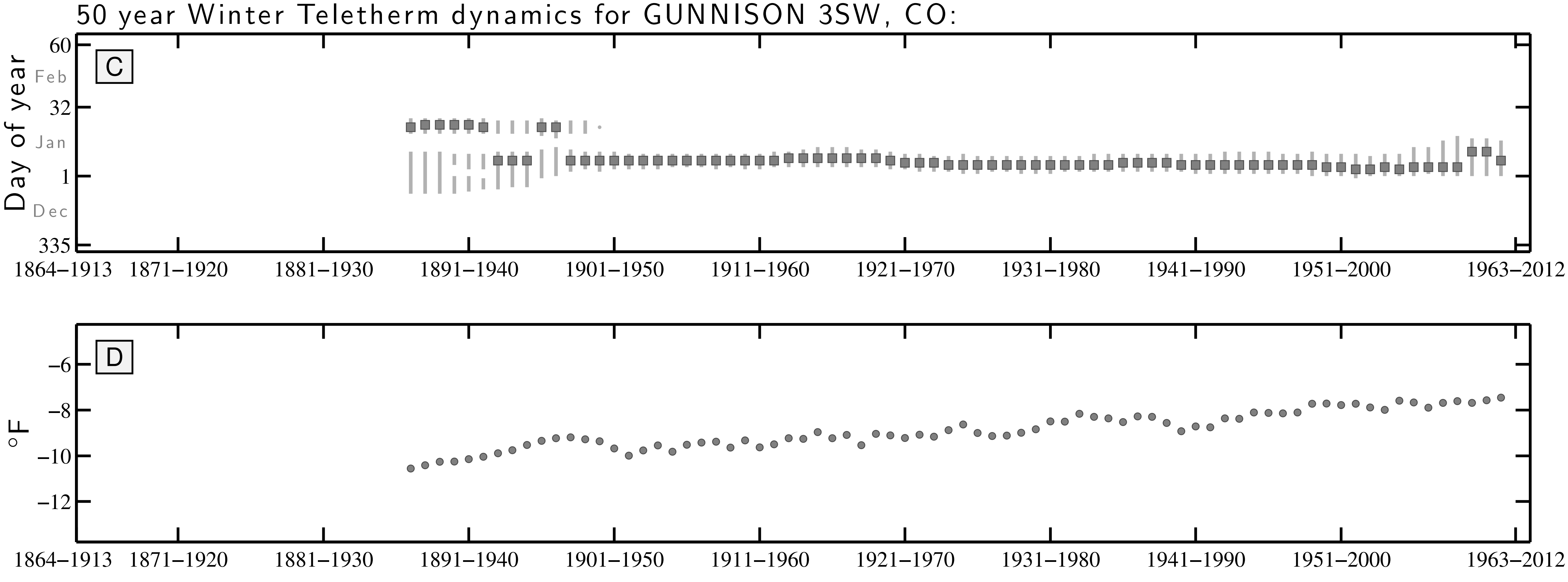
50 year Winter Teletherm dynamics for GUNNISON 3SW, CO:
C
D
Day of year
°F
Dec
Jan
Feb
335
1
32
60
−12
−10
−8
−6
1864–1913
1871–1920
1881–1930
1891–1940
1901–1950
1911–1960
1921–1970
1931–1980
1941–1990
1951–2000
1963–2012

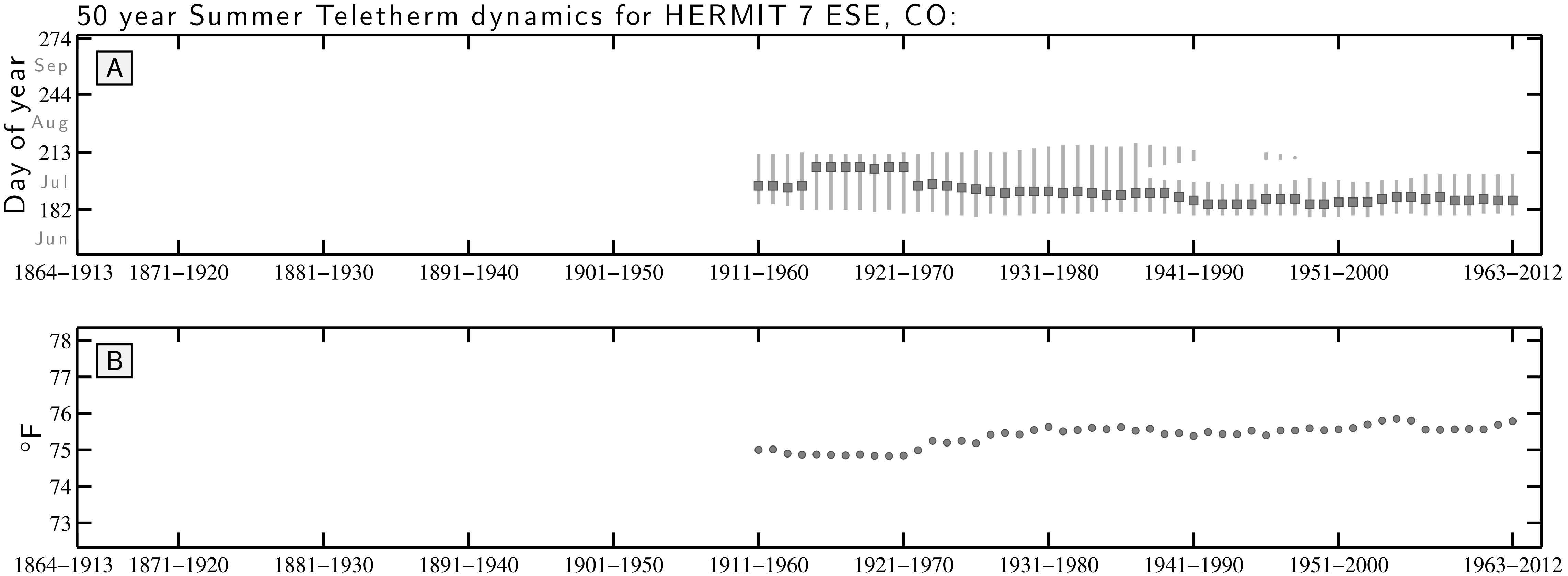
50 year Summer Teletherm dynamics for HERMIT 7 ESE, CO:
A
Day of year
274
Sep
244
Aug
213
Jul
182
Jun
1864–1913
1871–1920
1881–1930
1891–1940
1901–1950
1911–1960
1921–1970
1931–1980
1941–1990
1951–2000
1963–2012
B
°F
78
77
76
75
74
73

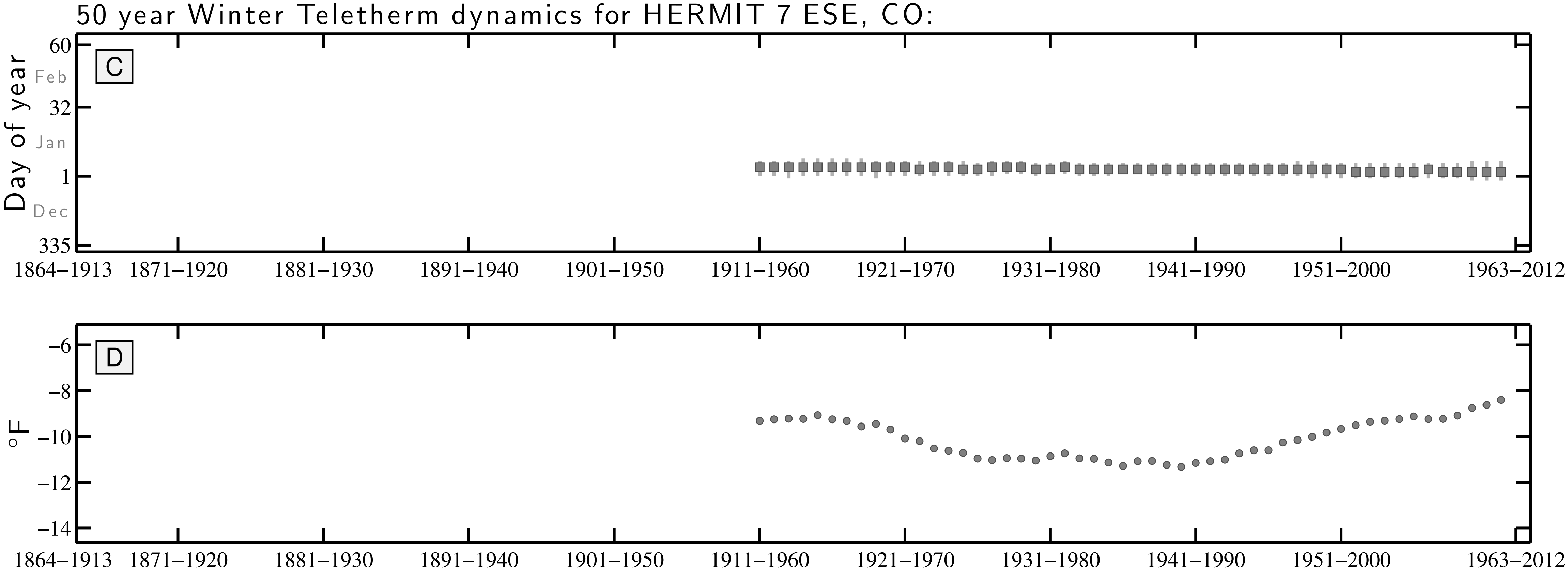
50 year Winter Teletherm dynamics for HERMIT 7 ESE, CO:
C
Day of year
60
Feb
32
Jan
1
Dec
335
1864–1913
1871–1920
1881–1930
1891–1940
1901–1950
1911–1960
1921–1970
1931–1980
1941–1990
1951–2000
1963–2012
D
°F
−6
−8
−10
−12
−14

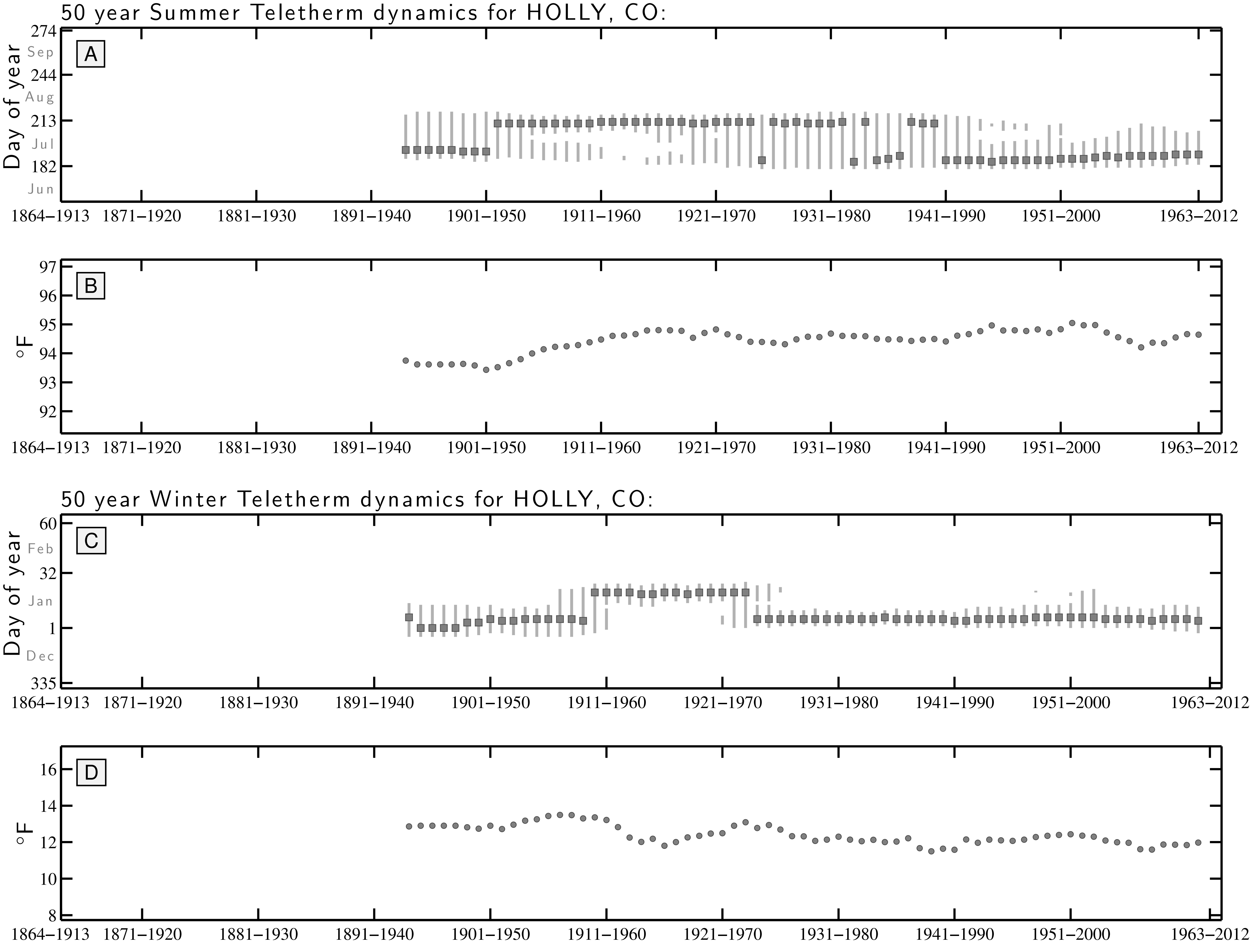

50 year Summer Teletherm dynamics for HOLLY, CO:
A
Day of year
274
Sep
244
Aug
213
Jul
182
Jun
1864–1913
1871–1920
1881–1930
1891–1940
1901–1950
1911–1960
1921–1970
1931–1980
1941–1990
1951–2000
1963–2012
B
°F
97
96
95
94
93
92
50 year Winter Teletherm dynamics for HOLLY, CO:
C
Day of year
60
Feb
32
Jan
1
Dec
335
D
°F
16
14
12
10
8

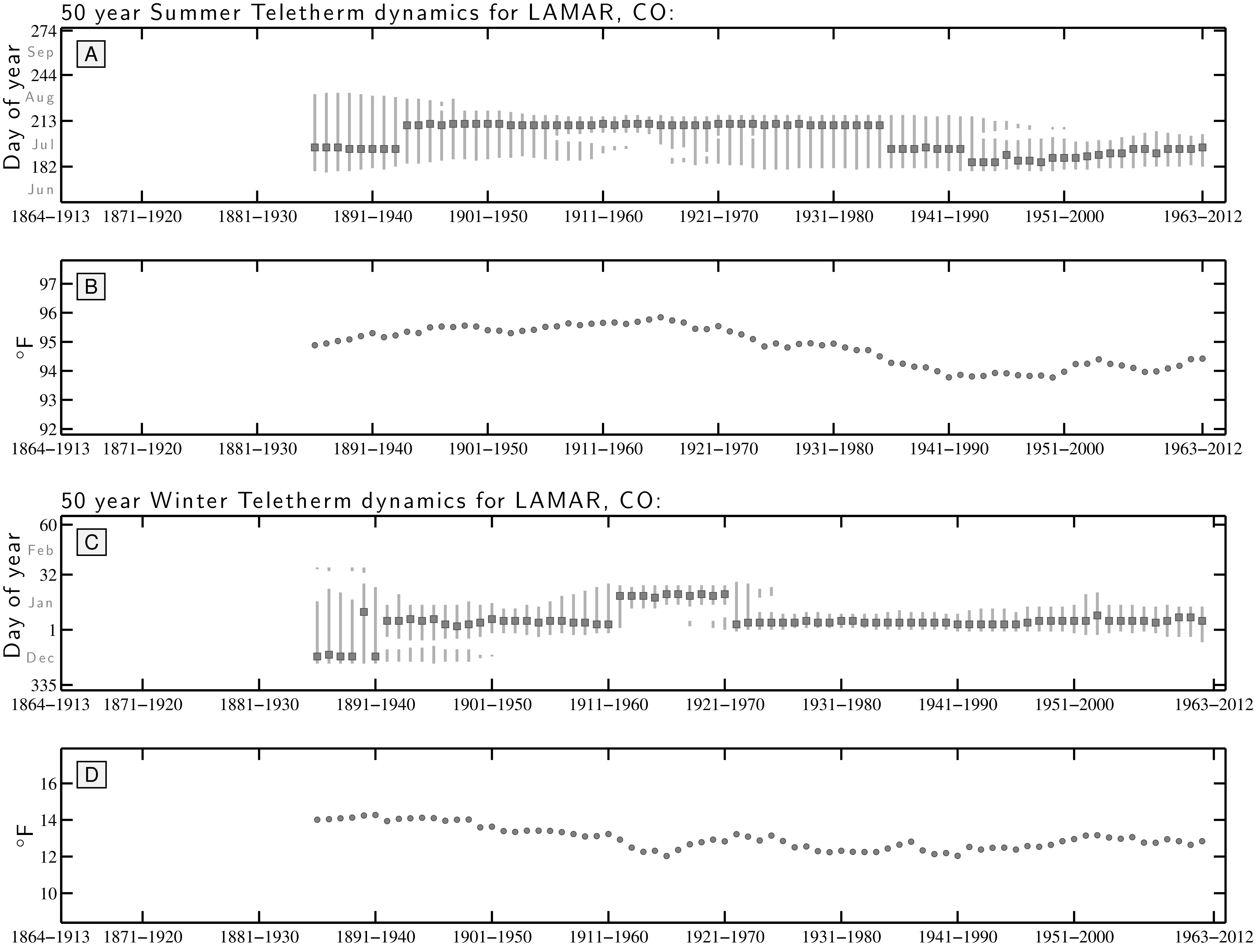

50 year Summer Teletherm dynamics for LAMAR, CO:
A
B
50 year Winter Teletherm dynamics for LAMAR, CO:
C
D
Day of year
°F
274
Sep
244
Aug
213
Jul
182
Jun
97
96
95
94
93
92
60
Feb
32
Jan
1
Dec
335
16
14
12
10
1864–1913
1871–1920
1881–1930
1891–1940
1901–1950
1911–1960
1921–1970
1931–1980
1941–1990
1951–2000
1963–2012

50 year Summer Teletherm dynamics for LAS ANIMAS, CO:

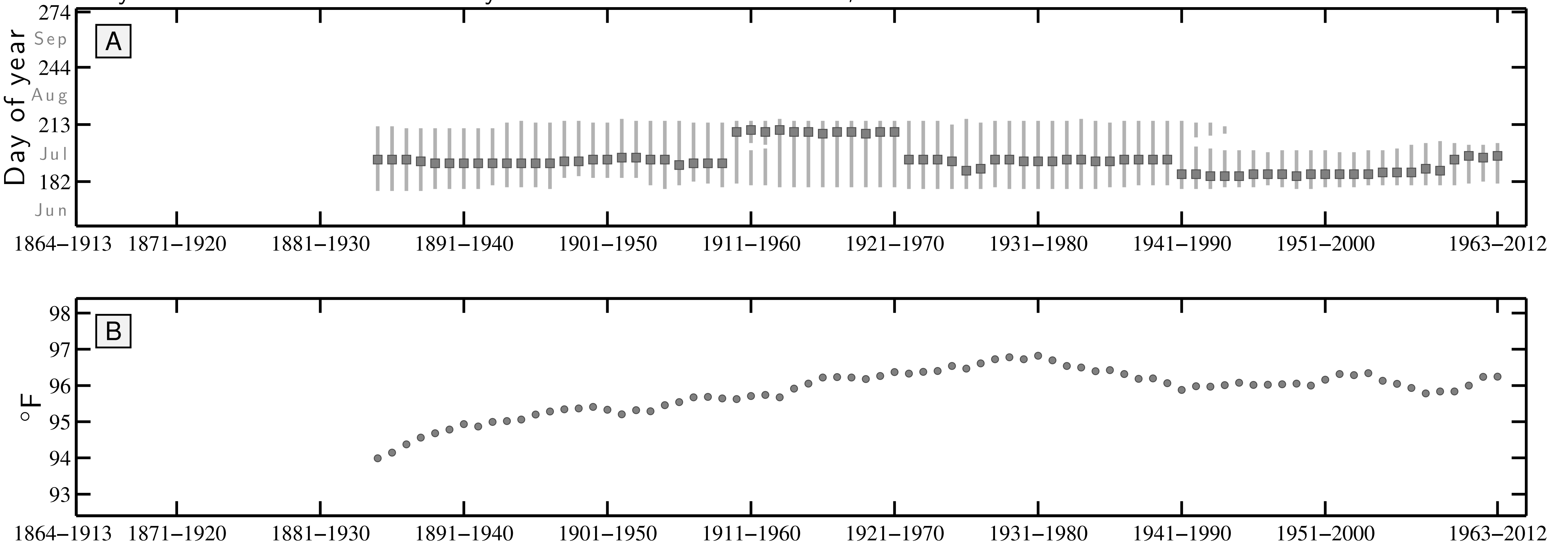

50 year Winter Teletherm dynamics for LAS ANIMAS, CO:

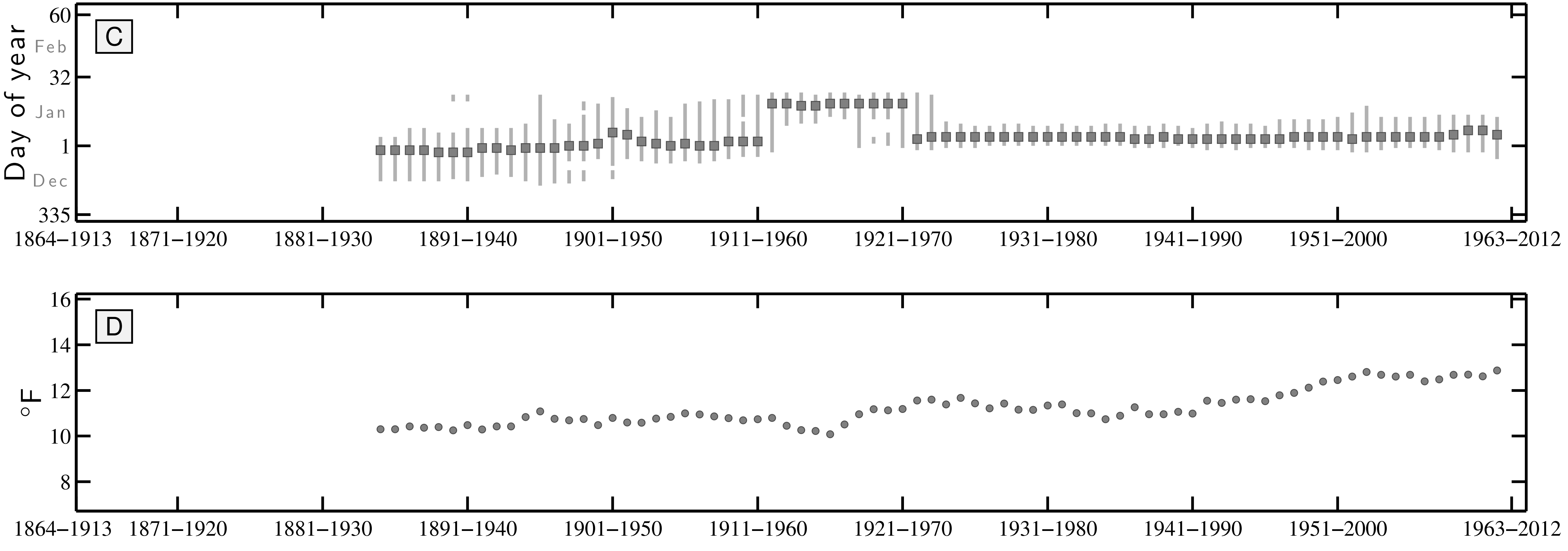

50 year Summer Teletherm dynamics for MANASSA, CO:

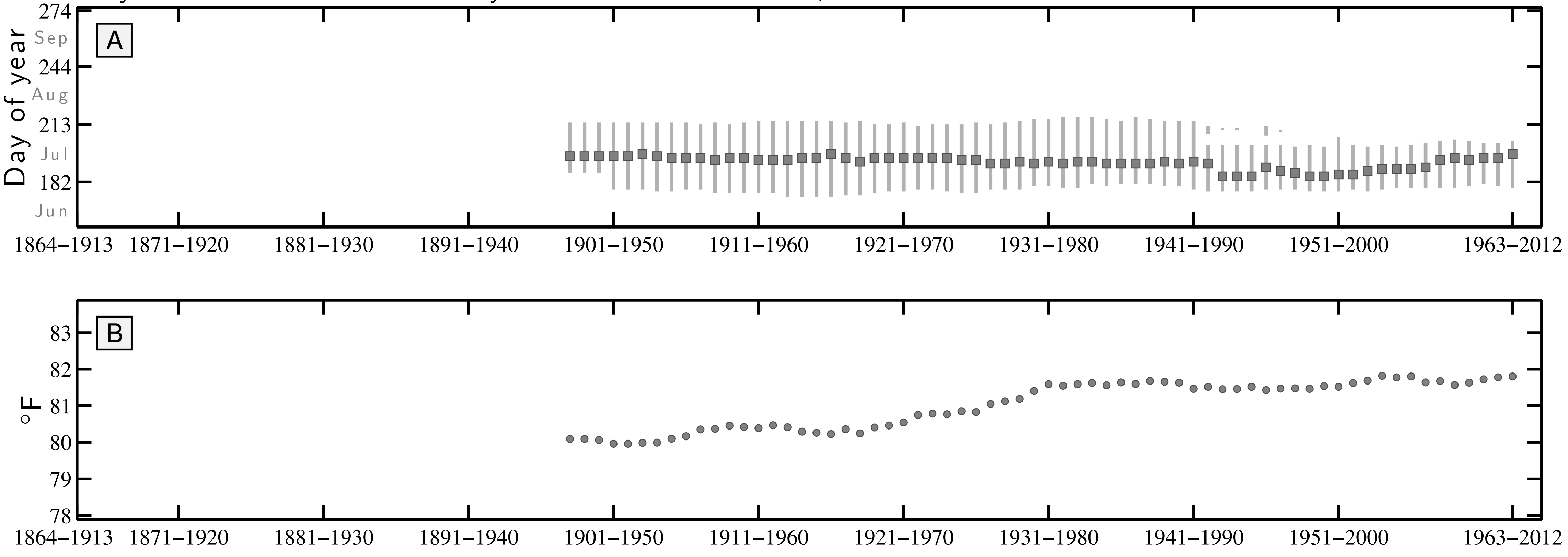

50 year Winter Teletherm dynamics for MANASSA, CO:

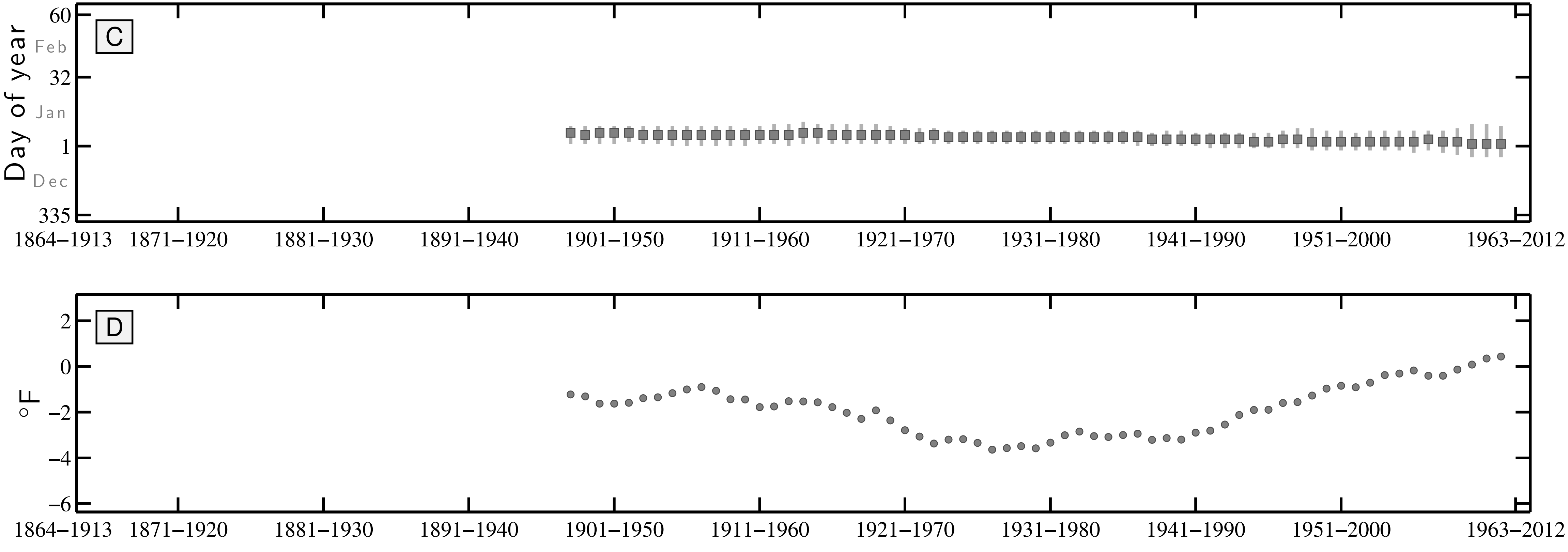

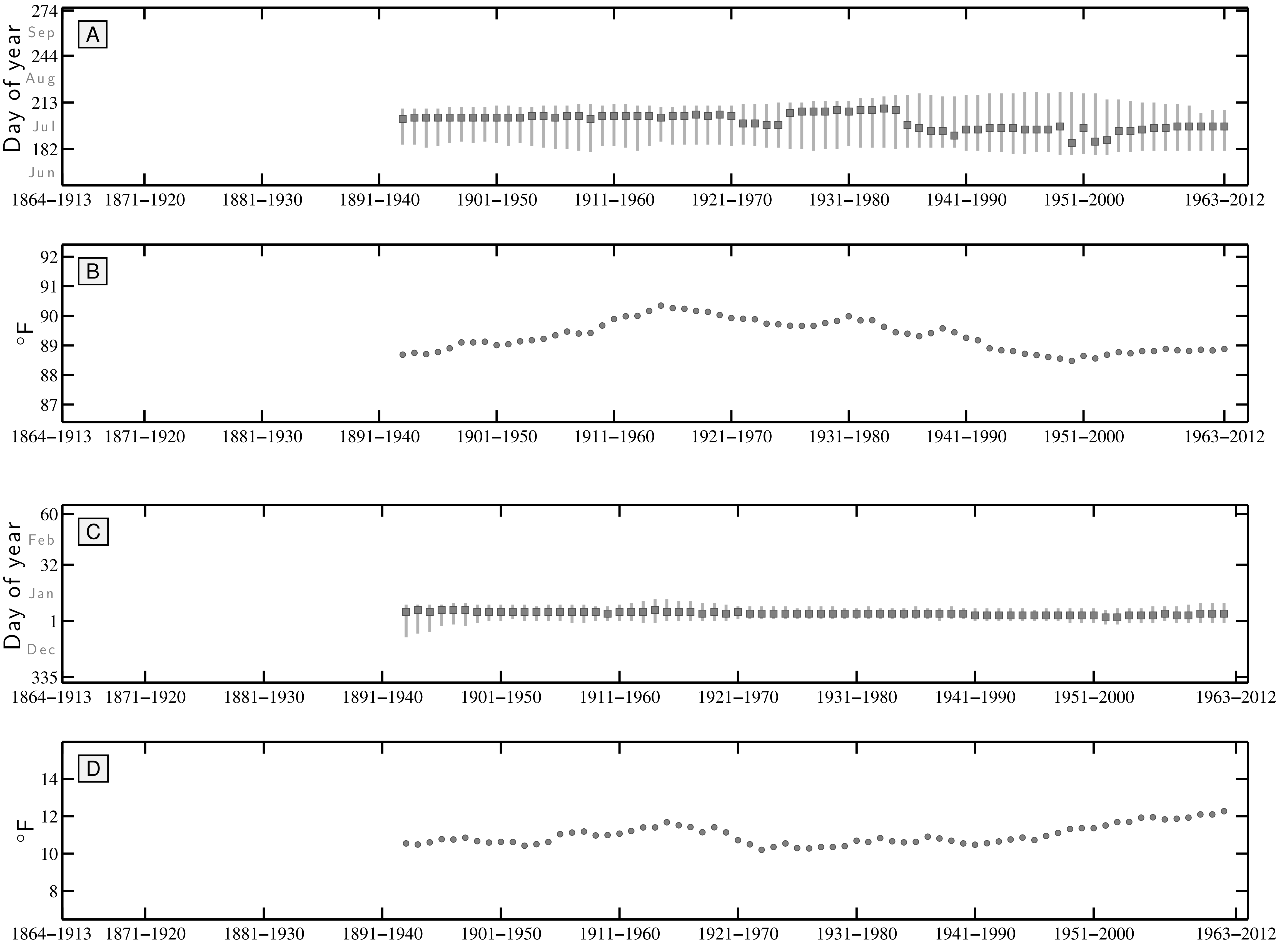
A
Day of year
274
Sep
244
Aug
213
Jul
182
Jun
1864–1913
1871–1920
1881–1930
1891–1940
1901–1950
1911–1960
1921–1970
1931–1980
1941–1990
1951–2000
1963–2012
B
°F
92
91
90
89
88
87
C
60
Feb
32
Jan
1
Dec
335
D
14
12
10
8

50 year Summer Teletherm dynamics for ROCKY FORD 2 SE, CO:

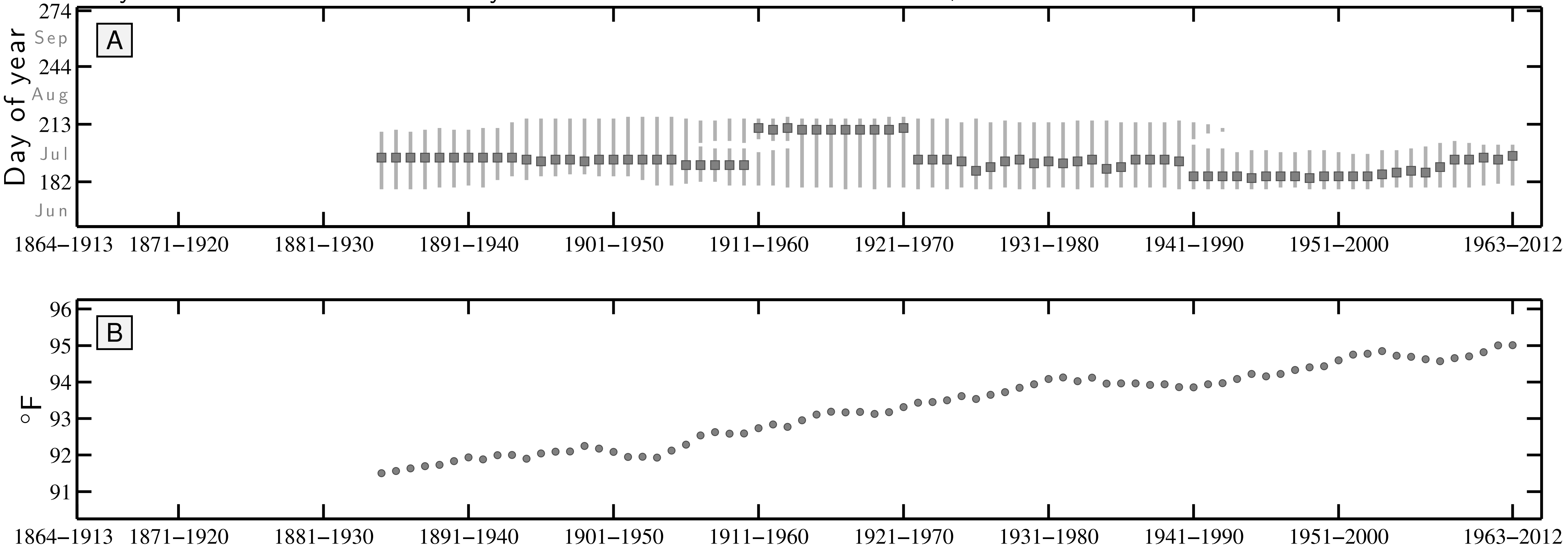

50 year Winter Teletherm dynamics for ROCKY FORD 2 SE, CO:

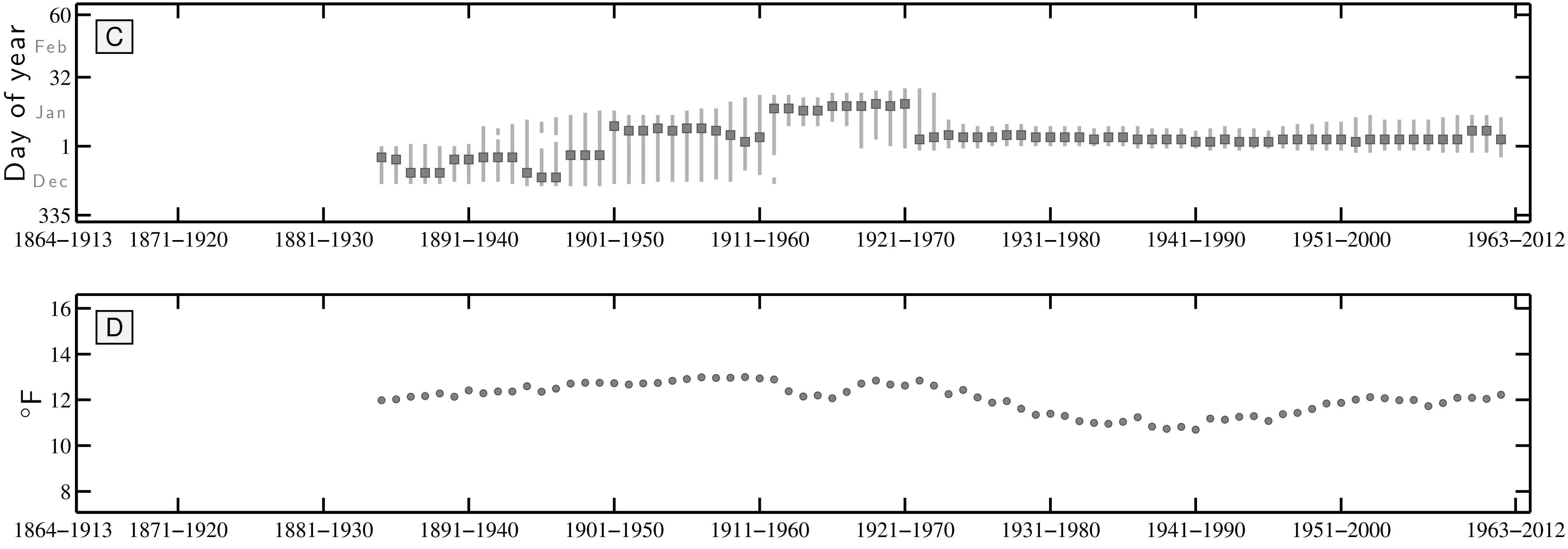

50 year Summer Teletherm dynamics for SAGUACHE, CO:

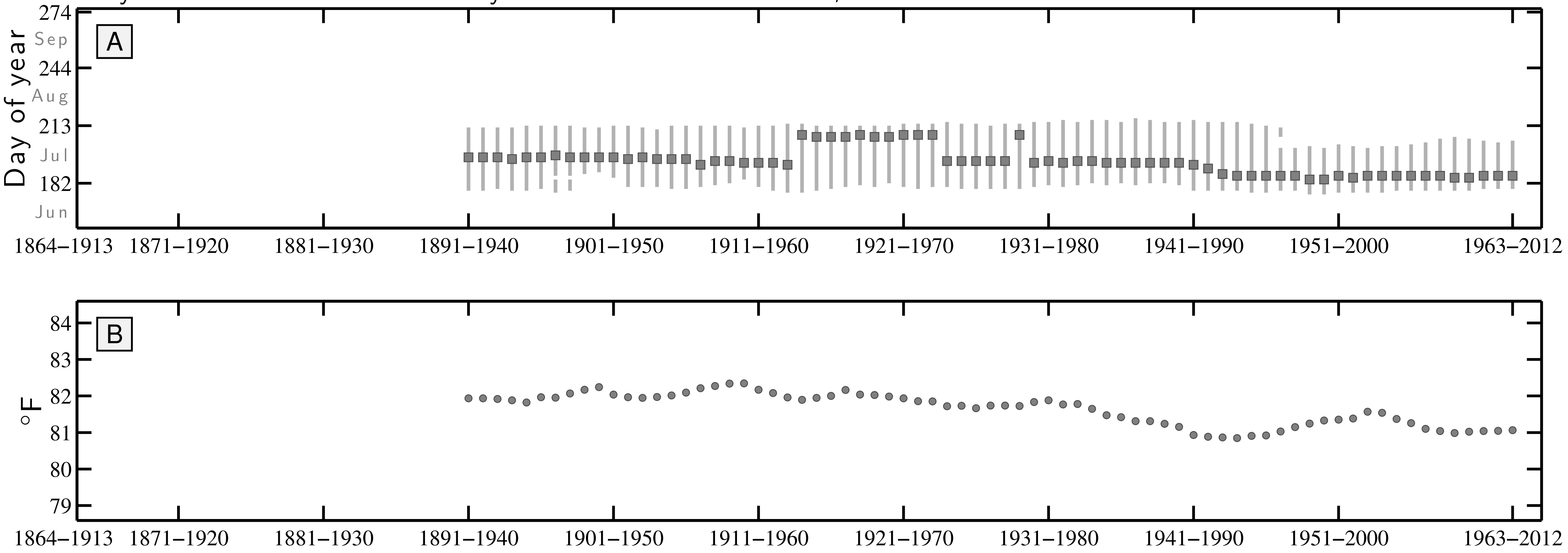

50 year Winter Teletherm dynamics for SAGUACHE, CO:

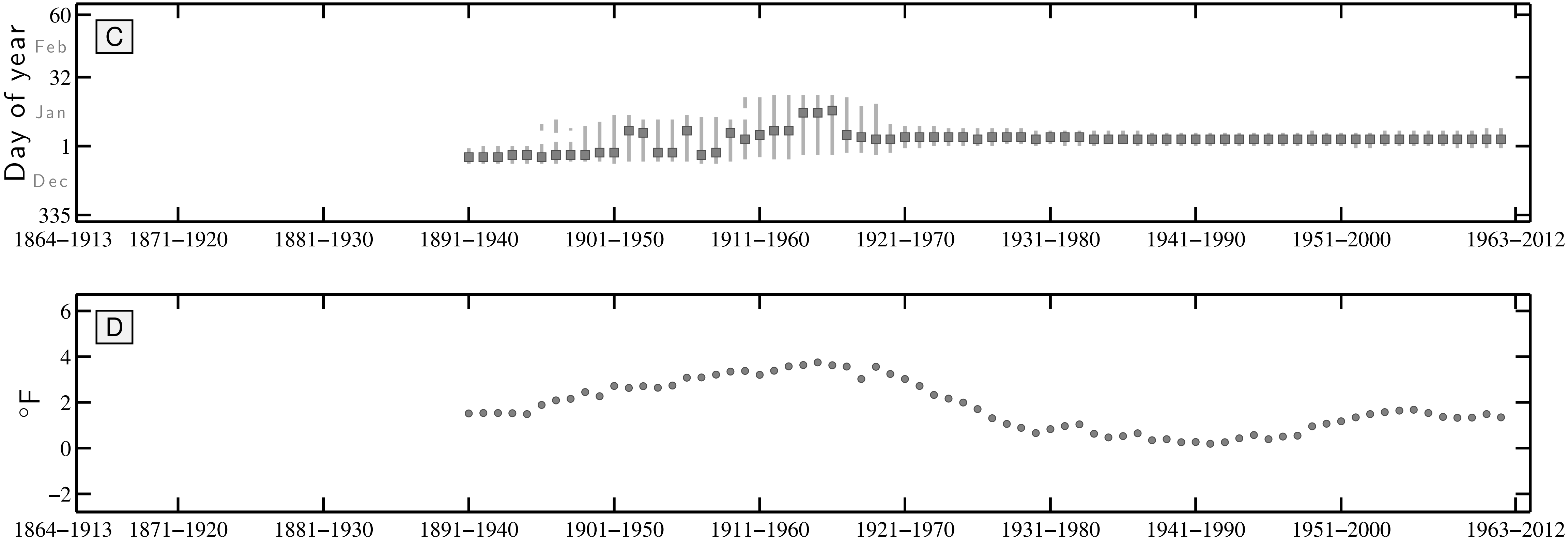

50 year Summer Teletherm dynamics for STEAMBOAT SPRINGS, CO:

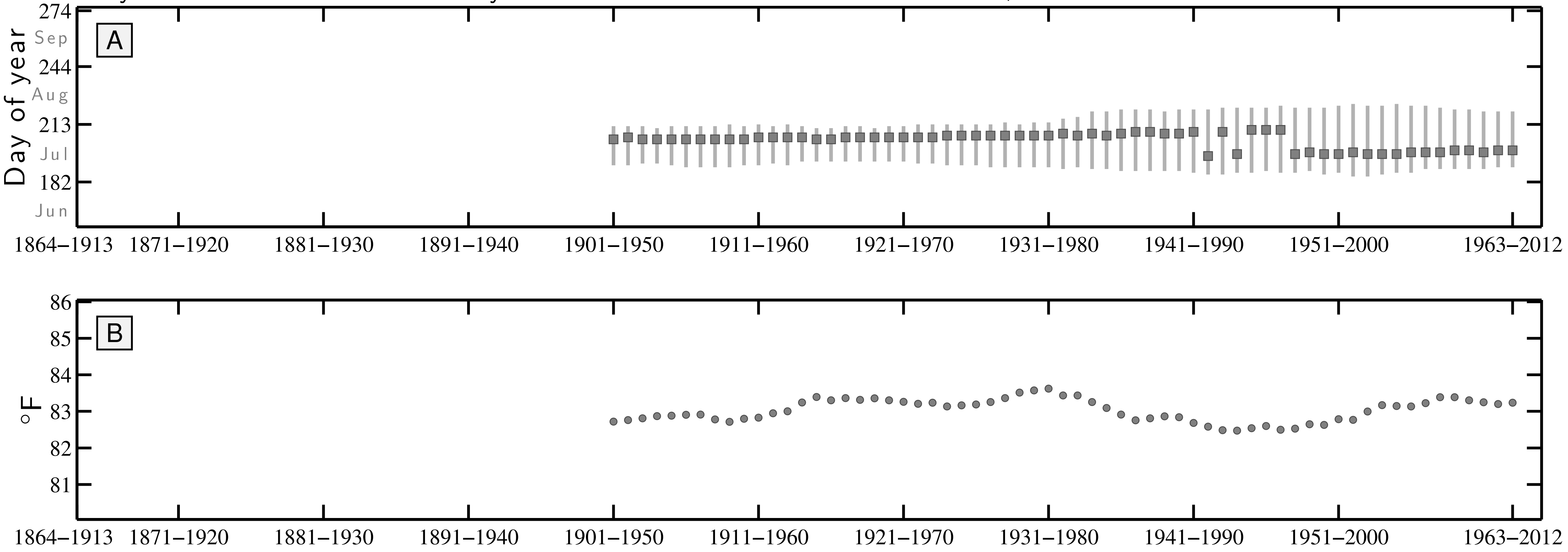

50 year Winter Teletherm dynamics for STEAMBOAT SPRINGS, CO:

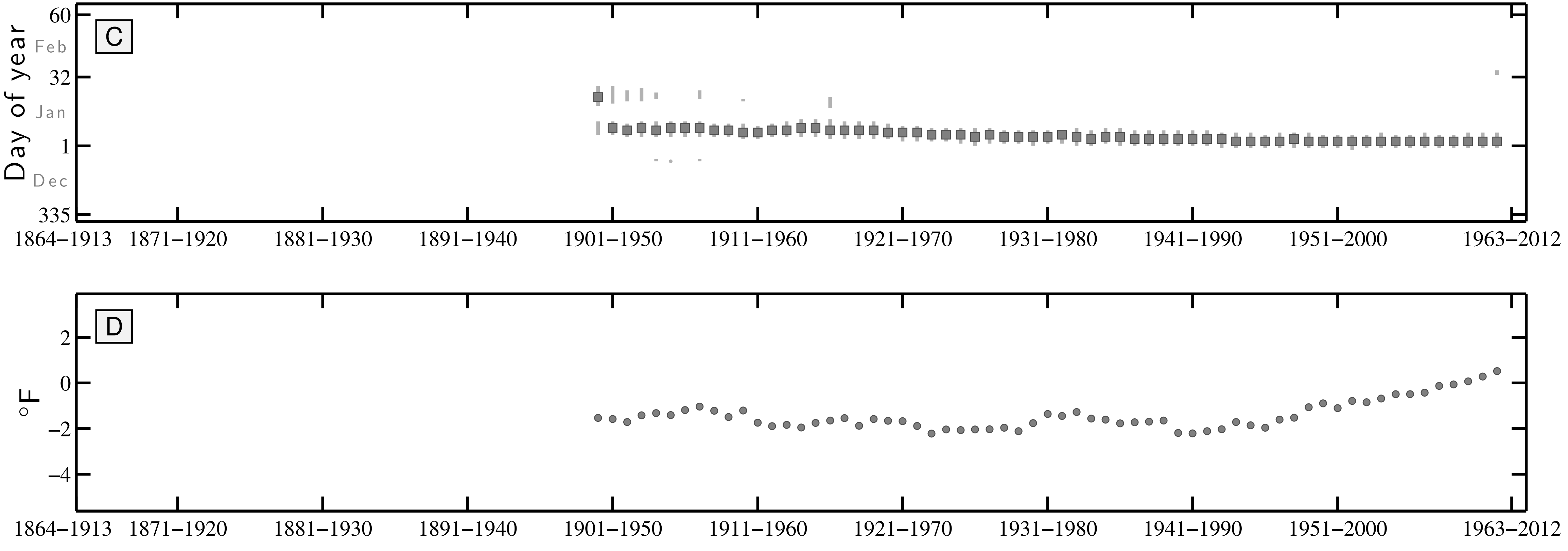

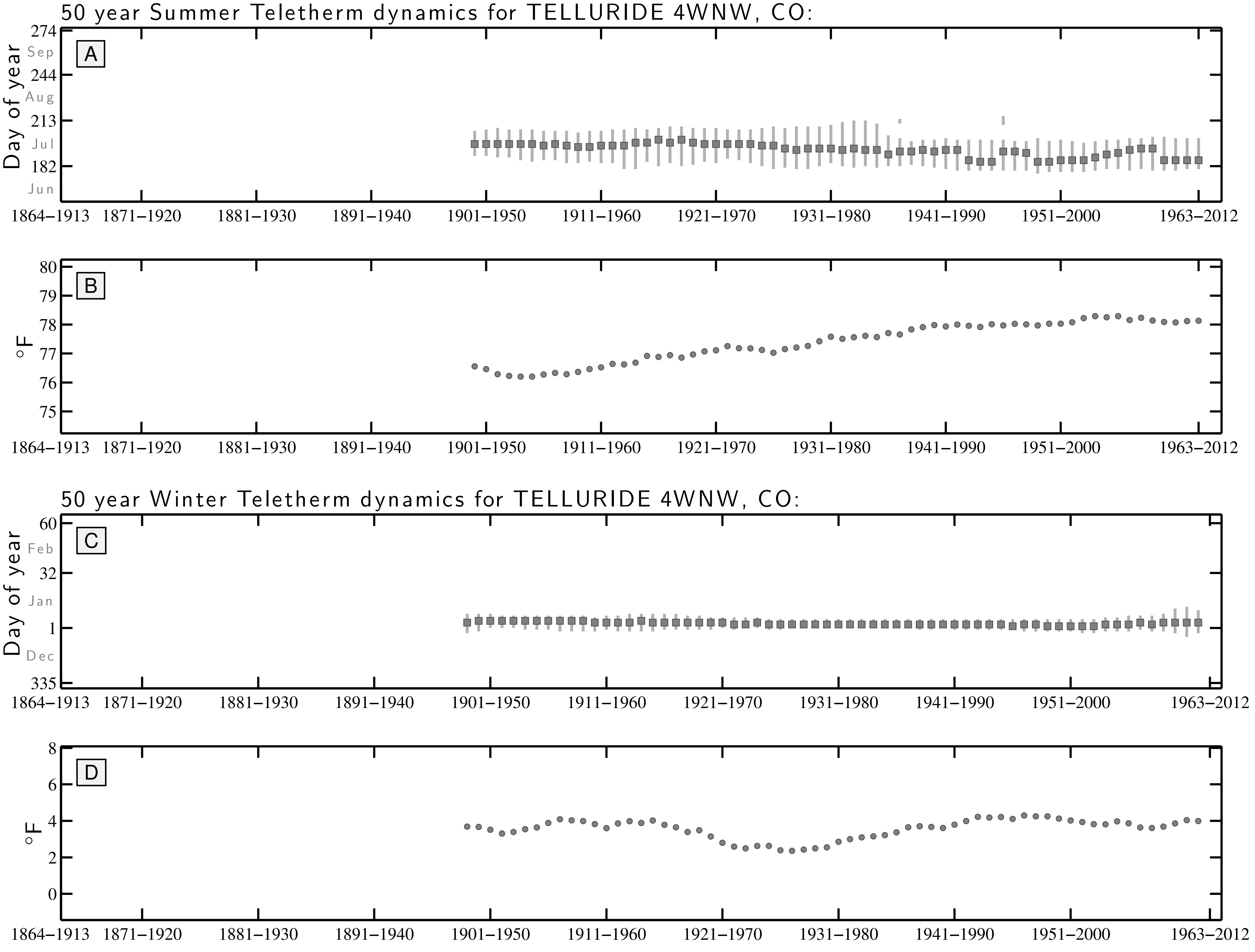
50 year Summer Teletherm dynamics for TELLURIDE 4WNW, CO:
A
B
Day of year
°F
Sep
Aug
Jul
Jun
274
244
213
182
80
79
78
77
76
75
50 year Winter Teletherm dynamics for TELLURIDE 4WNW, CO:
C
D
Feb
Jan
Dec
60
32
1
335
8
6
4
2
0
1864–1913
1871–1920
1881–1930
1891–1940
1901–1950
1911–1960
1921–1970
1931–1980
1941–1990
1951–2000
1963–2012

50 year Summer Teletherm dynamics for TRINIDAD, CO:

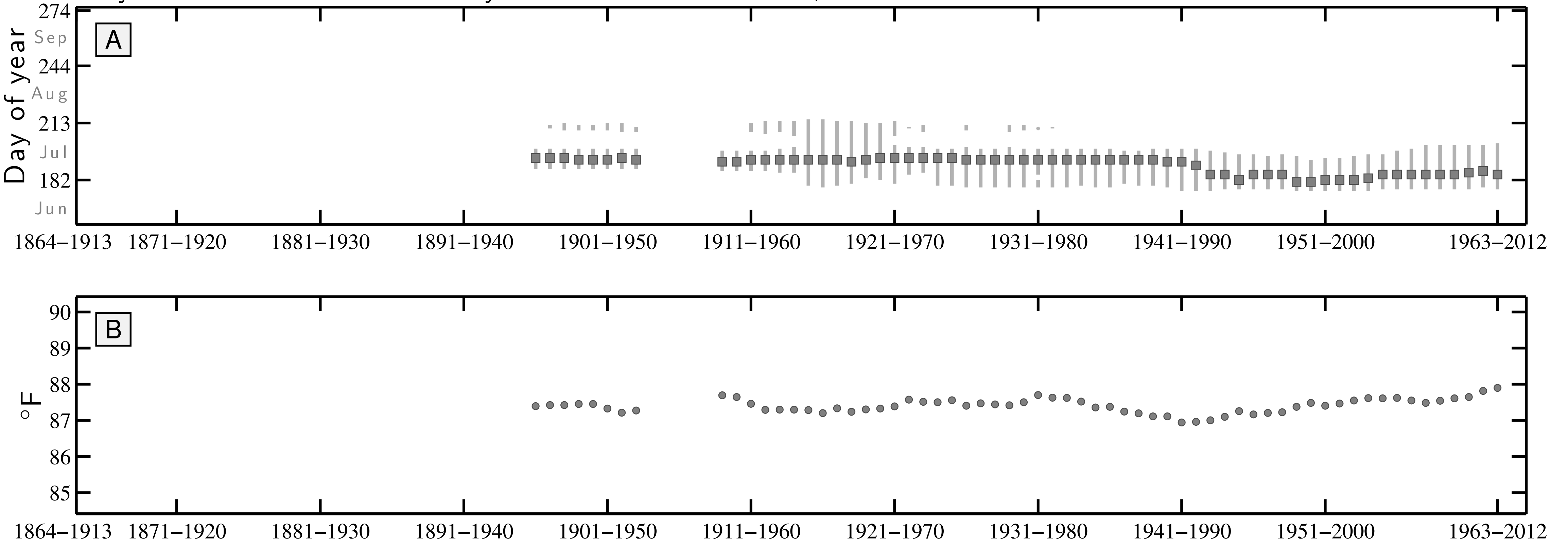

50 year Winter Teletherm dynamics for TRINIDAD, CO:

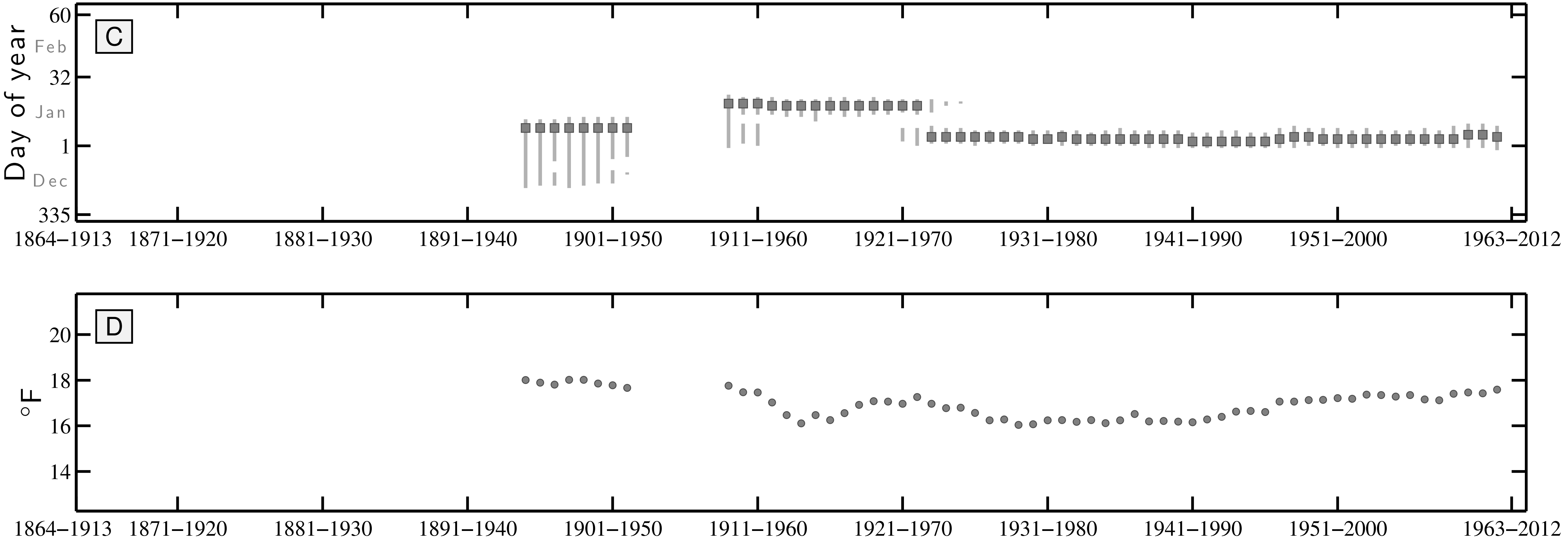

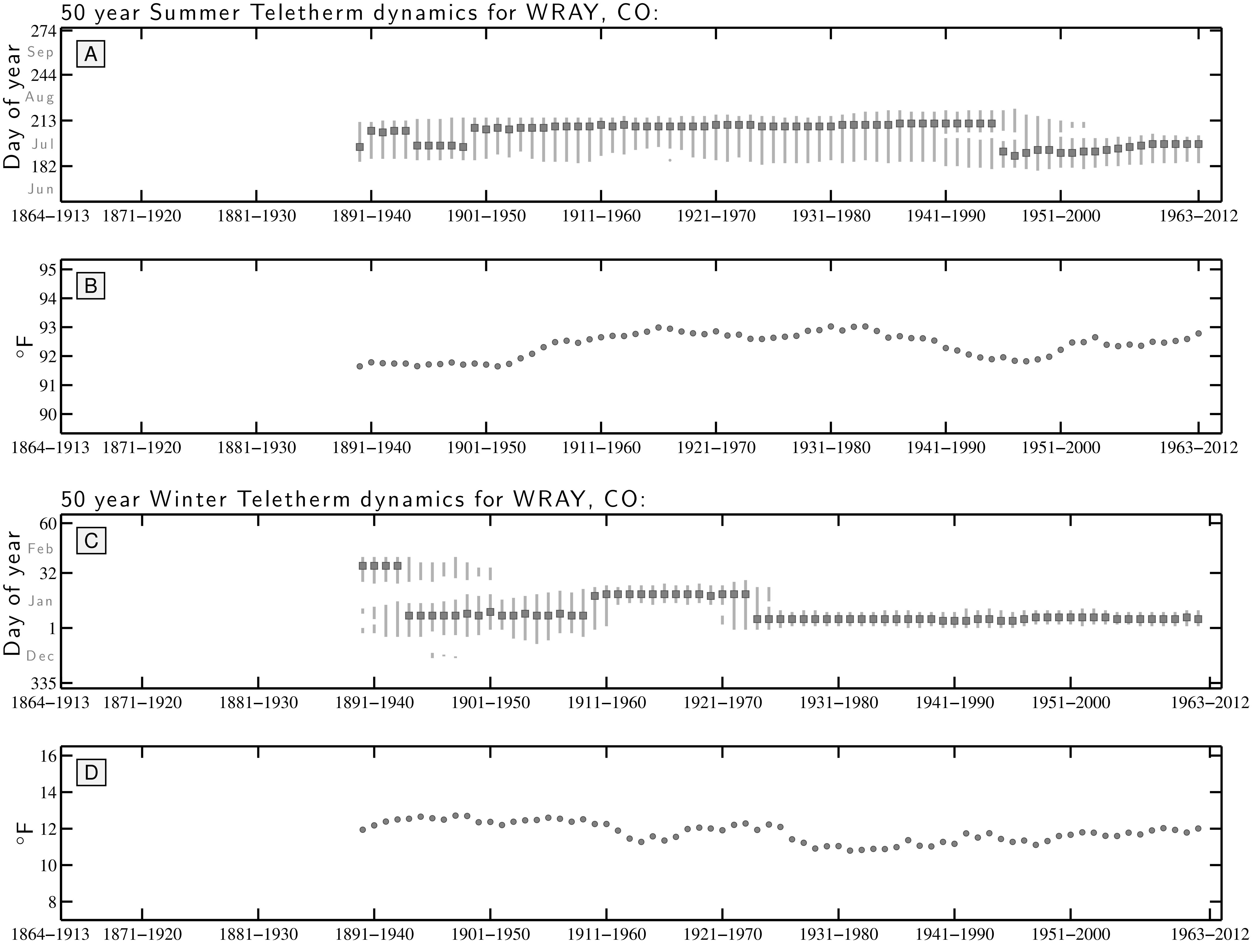
50 year Summer Teletherm dynamics for WRAY, CO:
A
Day of year
274
Sep
244
Aug
213
Jul
182
Jun
1864–1913
1871–1920
1881–1930
1891–1940
1901–1950
1911–1960
1921–1970
1931–1980
1941–1990
1951–2000
1963–2012
B
°F
95
94
93
92
91
90
50 year Winter Teletherm dynamics for WRAY, CO:
C
Day of year
60
Feb
32
Jan
1
Dec
335
D
°F
16
14
12
10
8

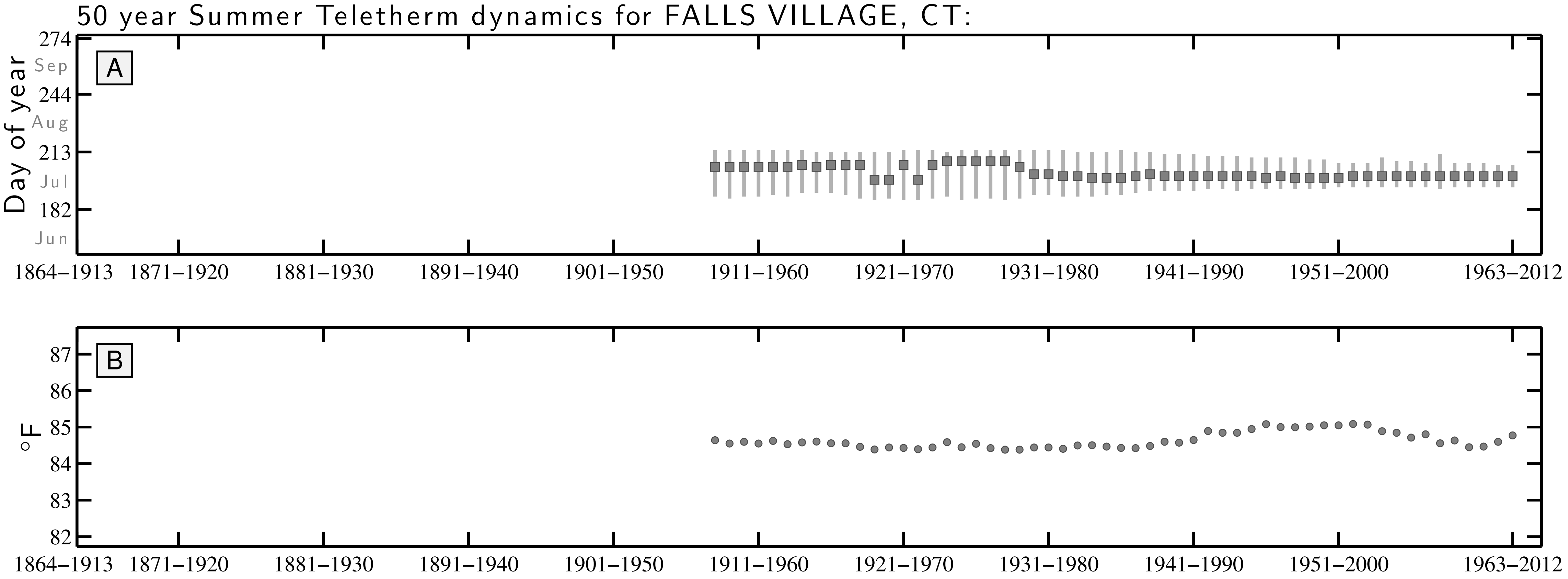
50 year Summer Teletherm dynamics for FALLS VILLAGE, CT:
A
Day of year
274
Sep
244
Aug
213
Jul
182
Jun
B
°F
87
86
85
84
83
82
1864–1913
1871–1920
1881–1930
1891–1940
1901–1950
1911–1960
1921–1970
1931–1980
1941–1990
1951–2000
1963–2012

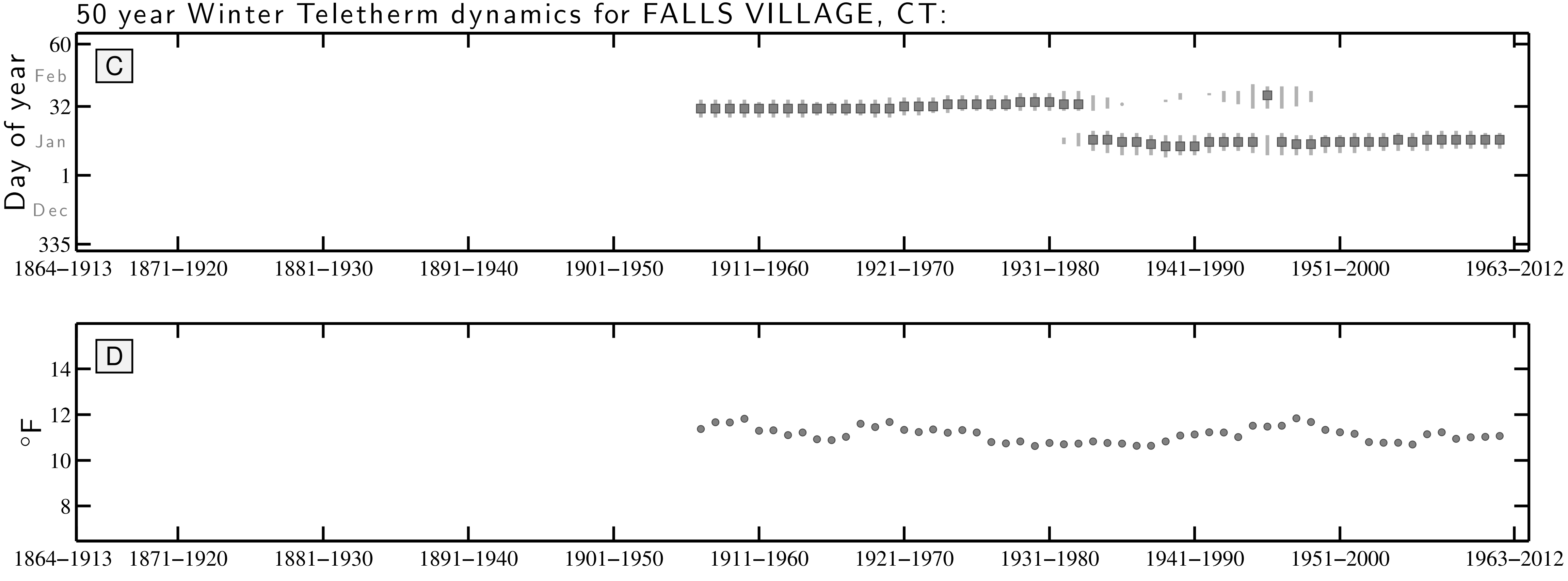
50 year Winter Teletherm dynamics for FALLS VILLAGE, CT:
C
Day of year
60
Feb
32
Jan
1
Dec
335
D
°F
14
12
10
8
1864–1913
1871–1920
1881–1930
1891–1940
1901–1950
1911–1960
1921–1970
1931–1980
1941–1990
1951–2000
1963–2012

50 year Summer Teletherm dynamics for GROTON, CT:

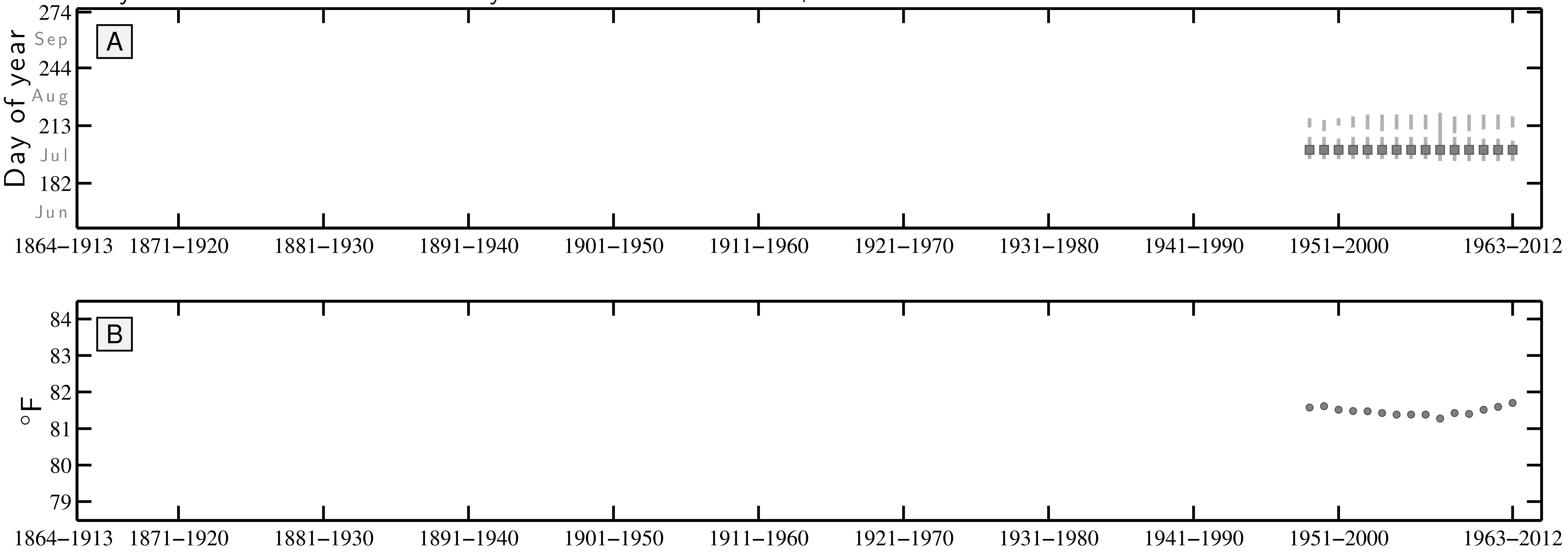

50 year Winter Teletherm dynamics for GROTON, CT:

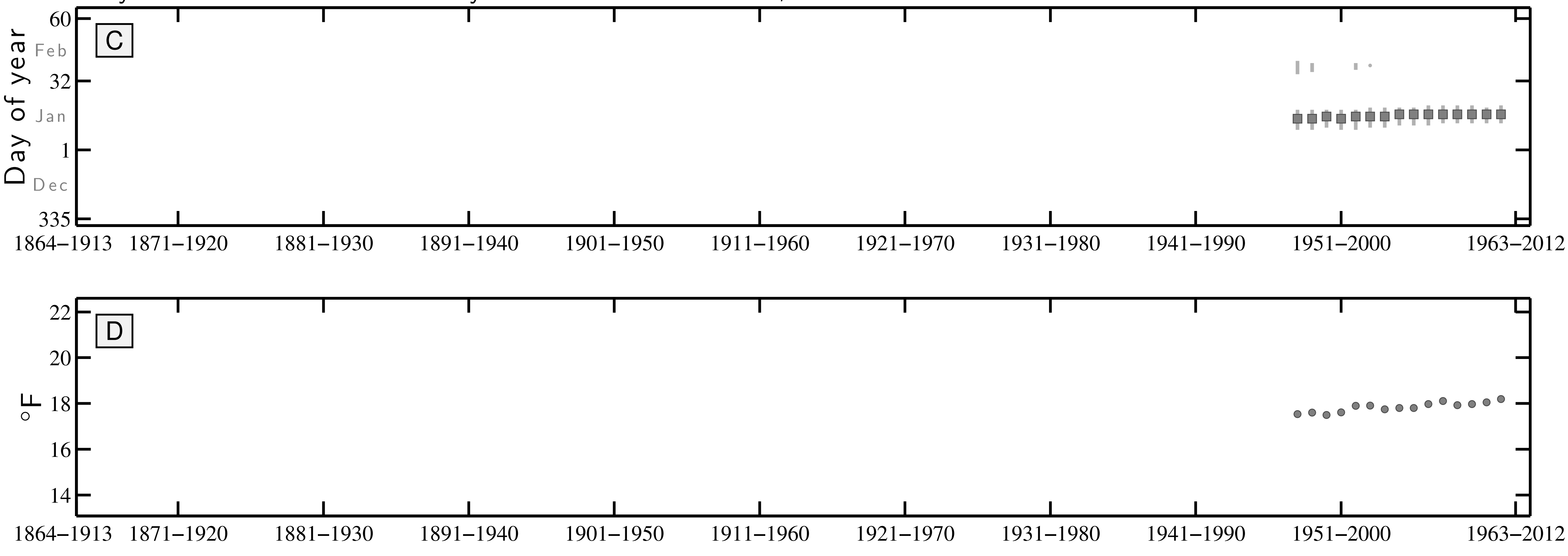

50 year Summer Teletherm dynamics for STAMFORD 5 N, CT:

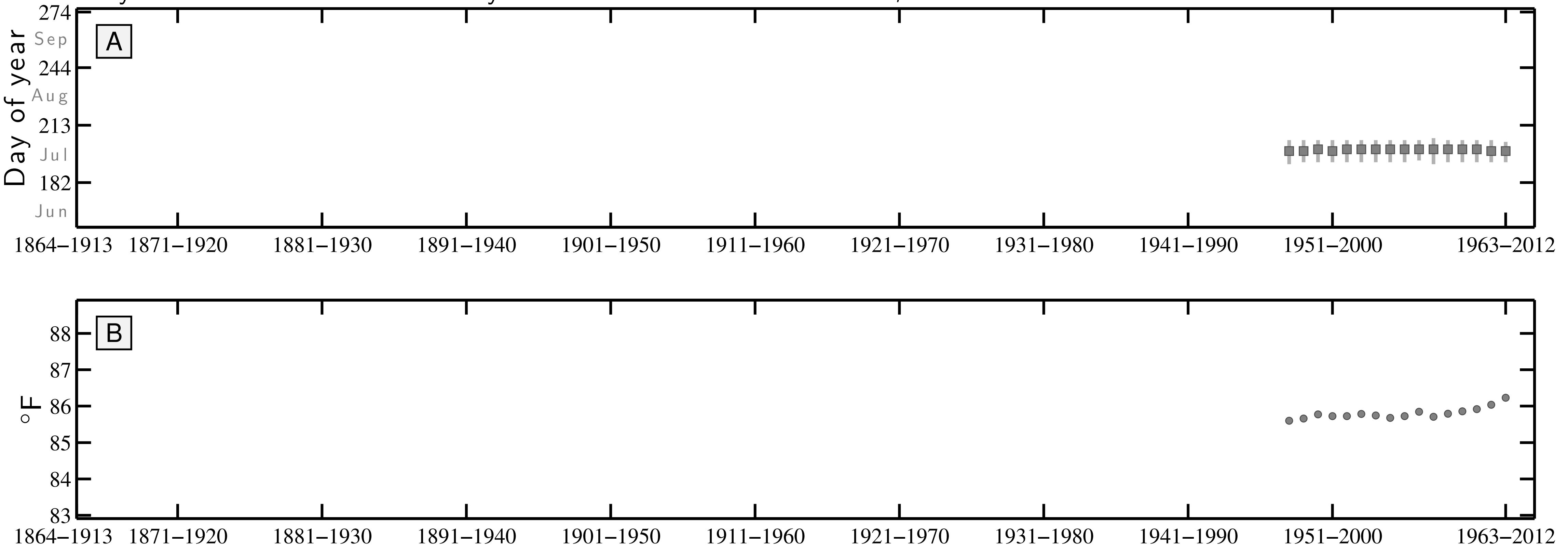

50 year Winter Teletherm dynamics for STAMFORD 5 N, CT:

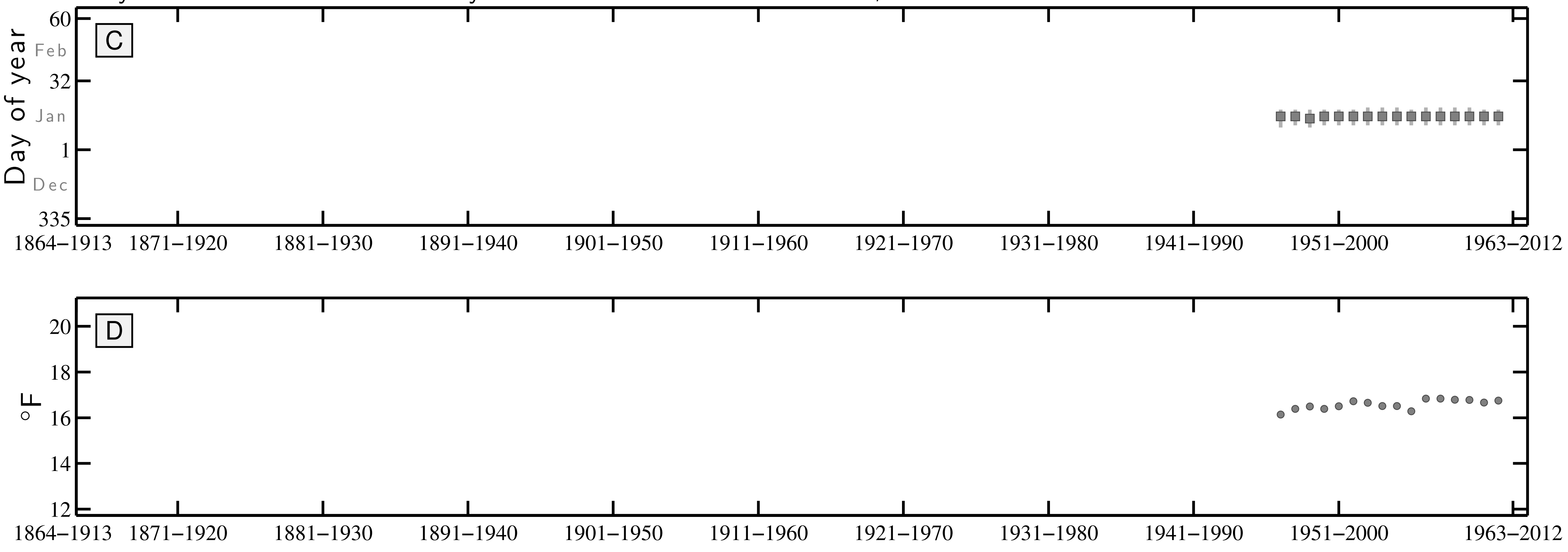

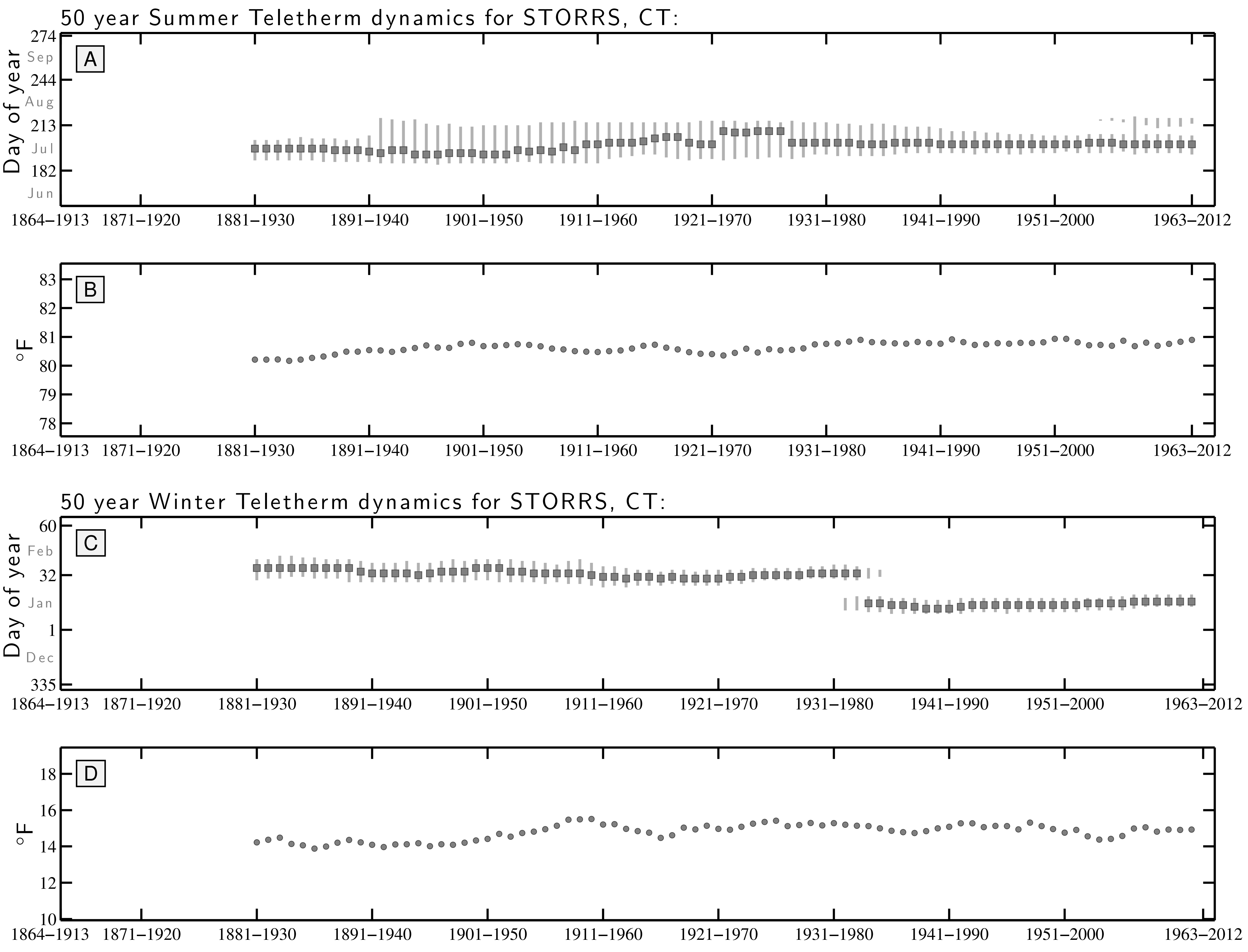
50 year Summer Teletherm dynamics for STORRS, CT:
A
Day of year
274
Sep
244
Aug
213
Jul
182
Jun
1864–1913
1871–1920
1881–1930
1891–1940
1901–1950
1911–1960
1921–1970
1931–1980
1941–1990
1951–2000
1963–2012
B
°F
83
82
81
80
79
78
50 year Winter Teletherm dynamics for STORRS, CT:
C
60
Feb
32
Jan
1
Dec
335
D
18
16
14
12
10

50 year Summer Teletherm dynamics for DOVER, DE:

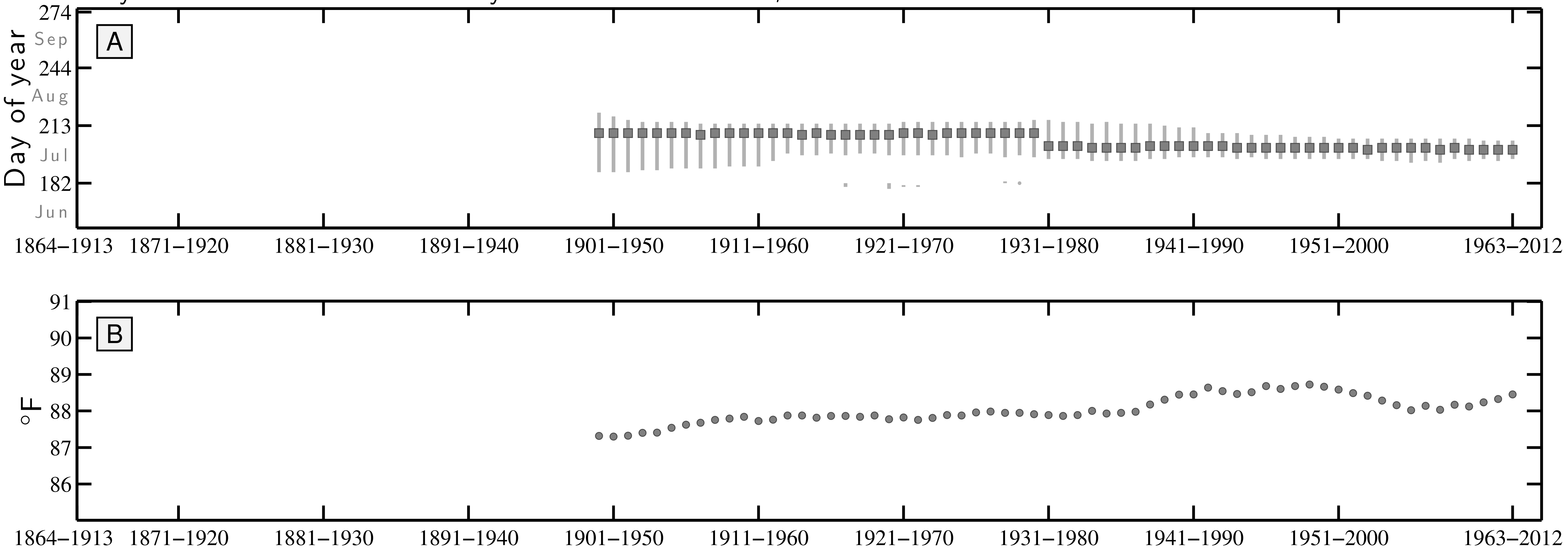

50 year Winter Teletherm dynamics for DOVER, DE:

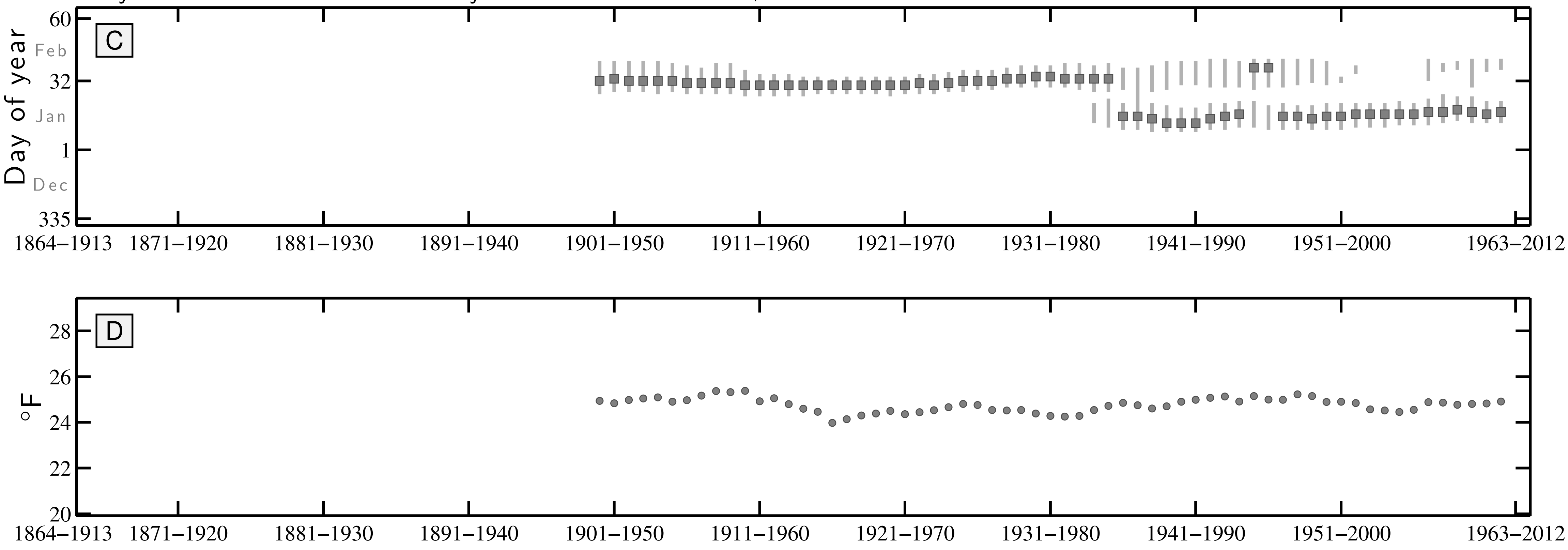

50 year Summer Teletherm dynamics for MILFORD 2 SE, DE:

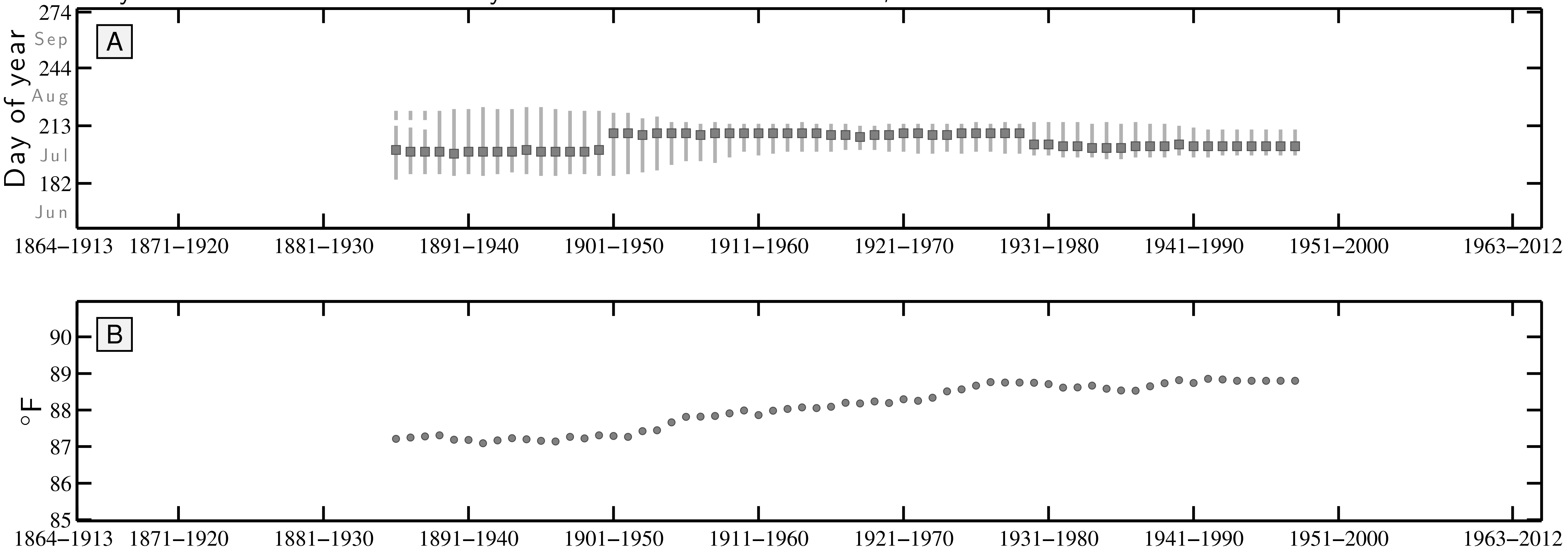

50 year Winter Teletherm dynamics for MILFORD 2 SE, DE:

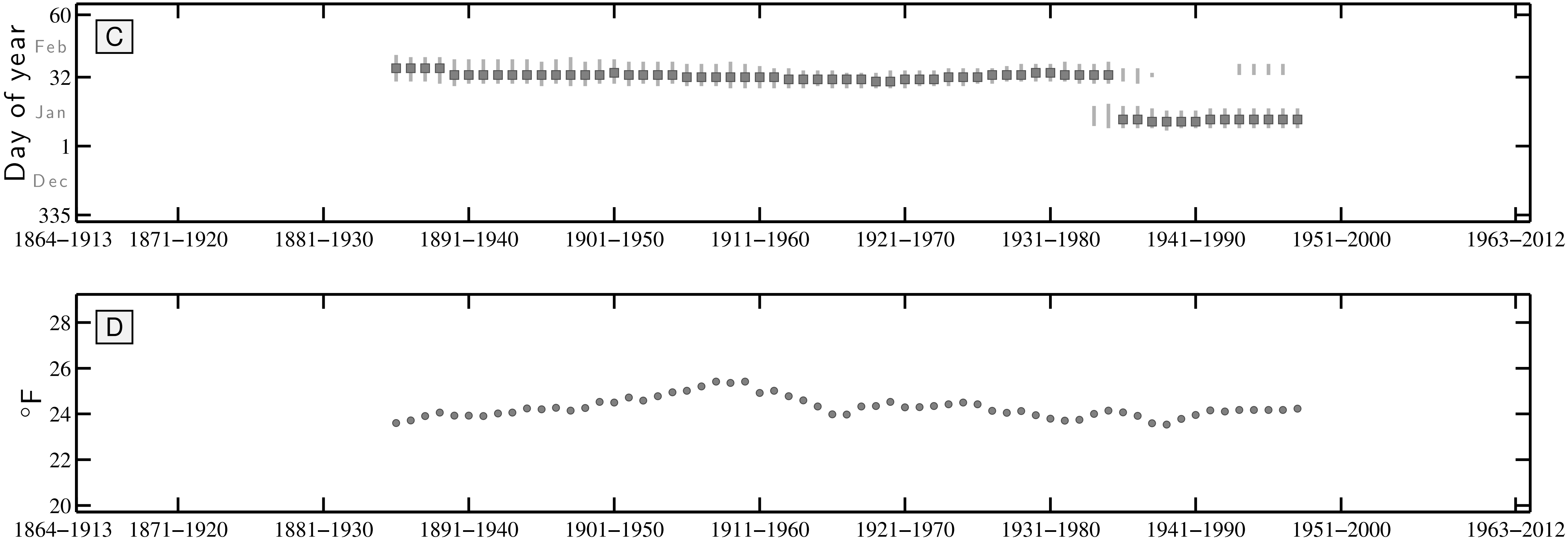

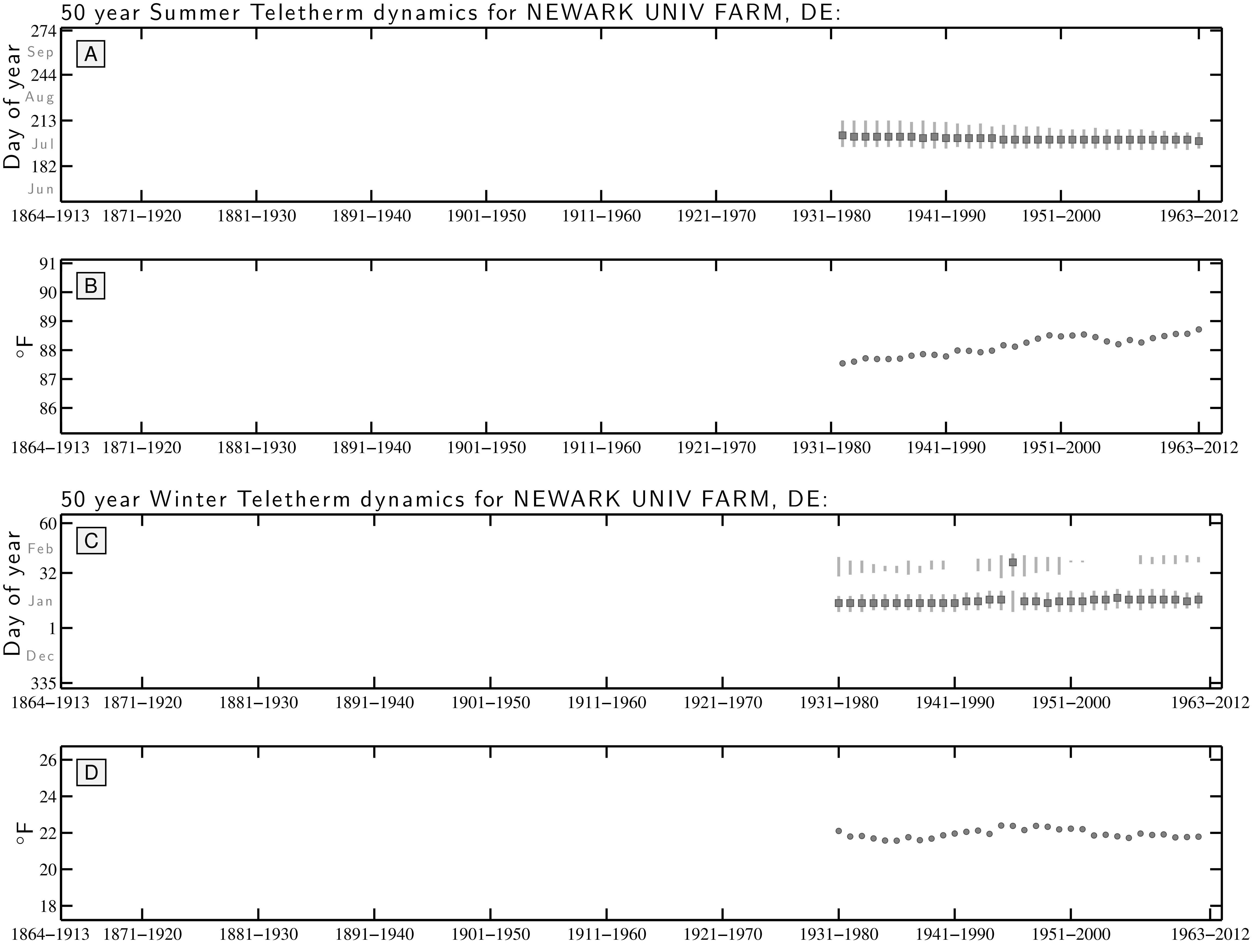
50 year Summer Teletherm dynamics for NEWARK UNIV FARM, DE:
A
B
Day of year
°F
274
244
213
182
Sep
Aug
Jul
Jun
91
90
89
88
87
86
50 year Winter Teletherm dynamics for NEWARK UNIV FARM, DE:
C
D
60
32
1
335
Feb
Jan
Dec
26
24
22
20
18
1864–1913
1871–1920
1881–1930
1891–1940
1901–1950
1911–1960
1921–1970
1931–1980
1941–1990
1951–2000
1963–2012

50 year Summer Teletherm dynamics for WILMINGTON PORTER RES, DE:

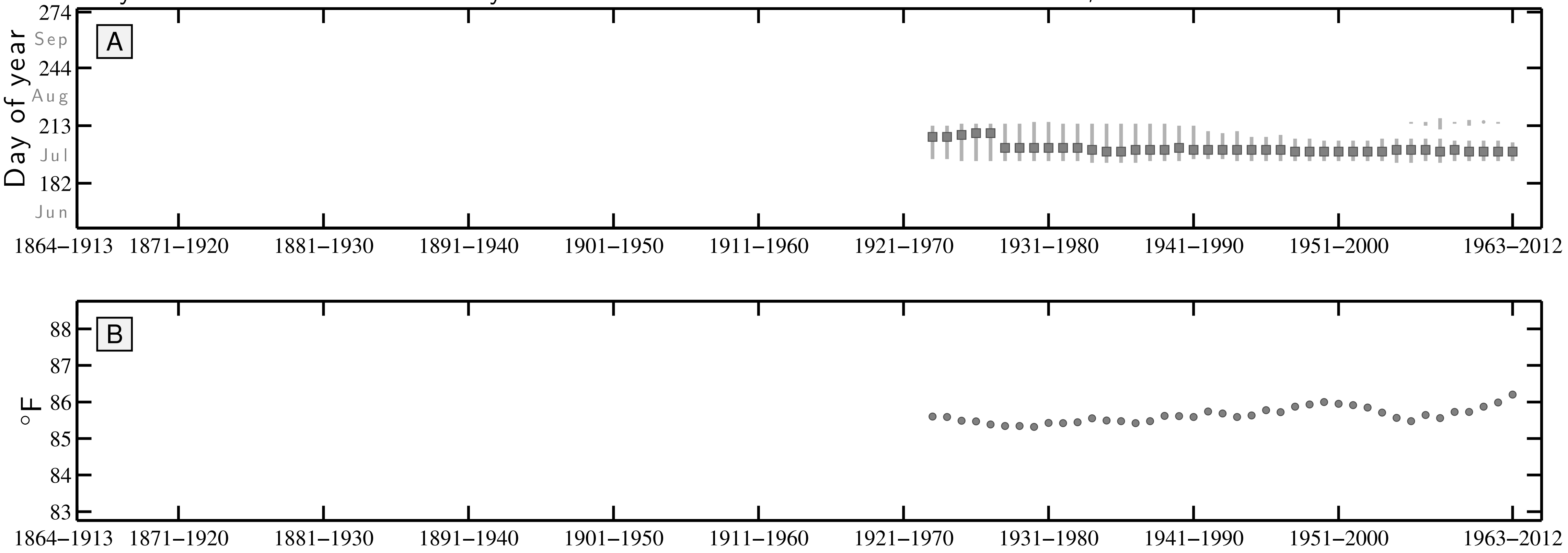

50 year Winter Teletherm dynamics for WILMINGTON PORTER RES, DE:

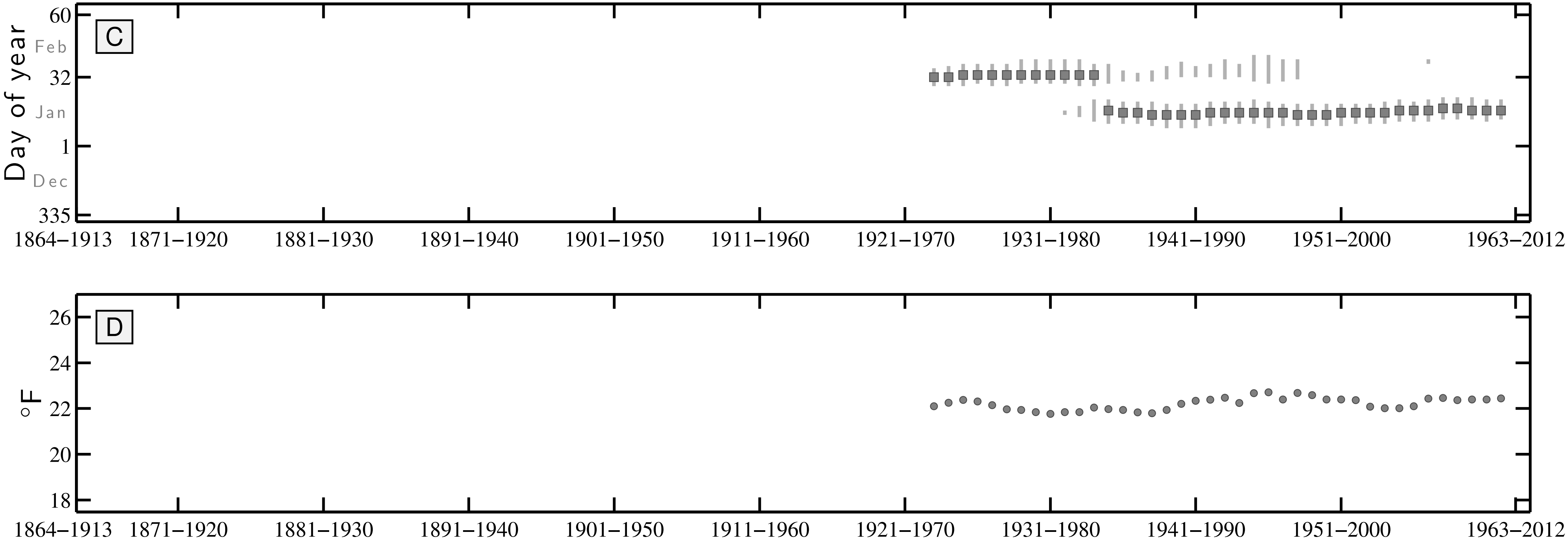

50 year Summer Teletherm dynamics for APALACHICOLA AP, FL:

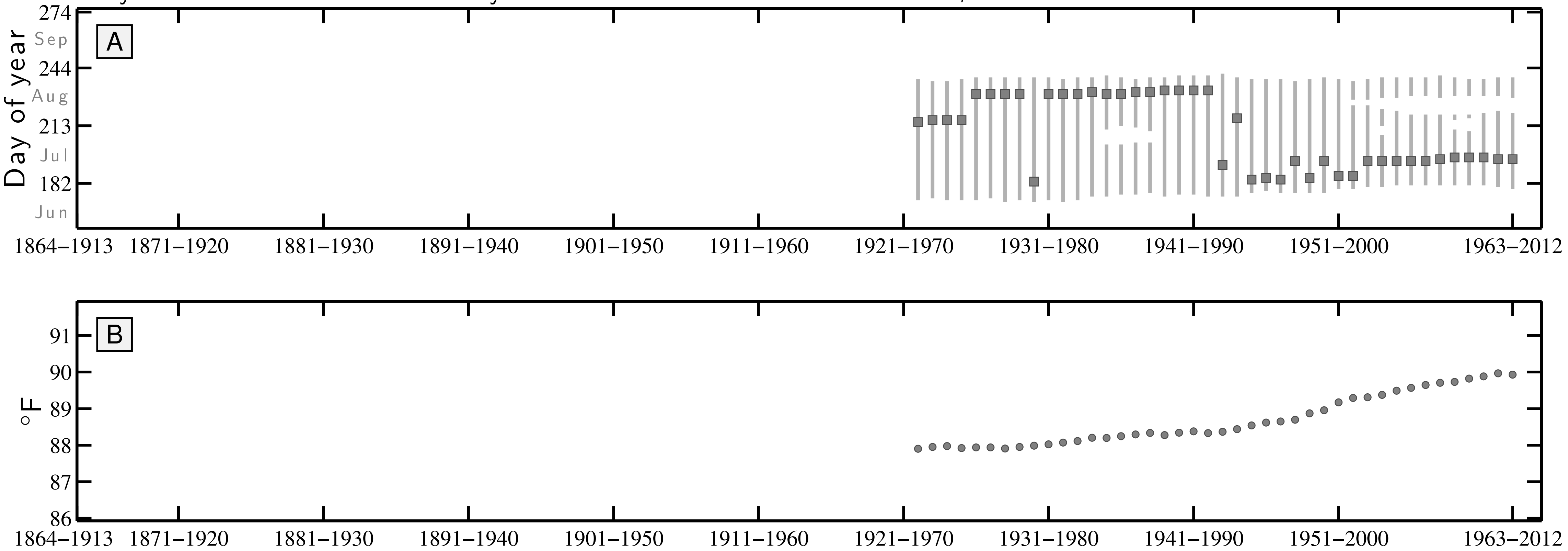

50 year Winter Teletherm dynamics for APALACHICOLA AP, FL:

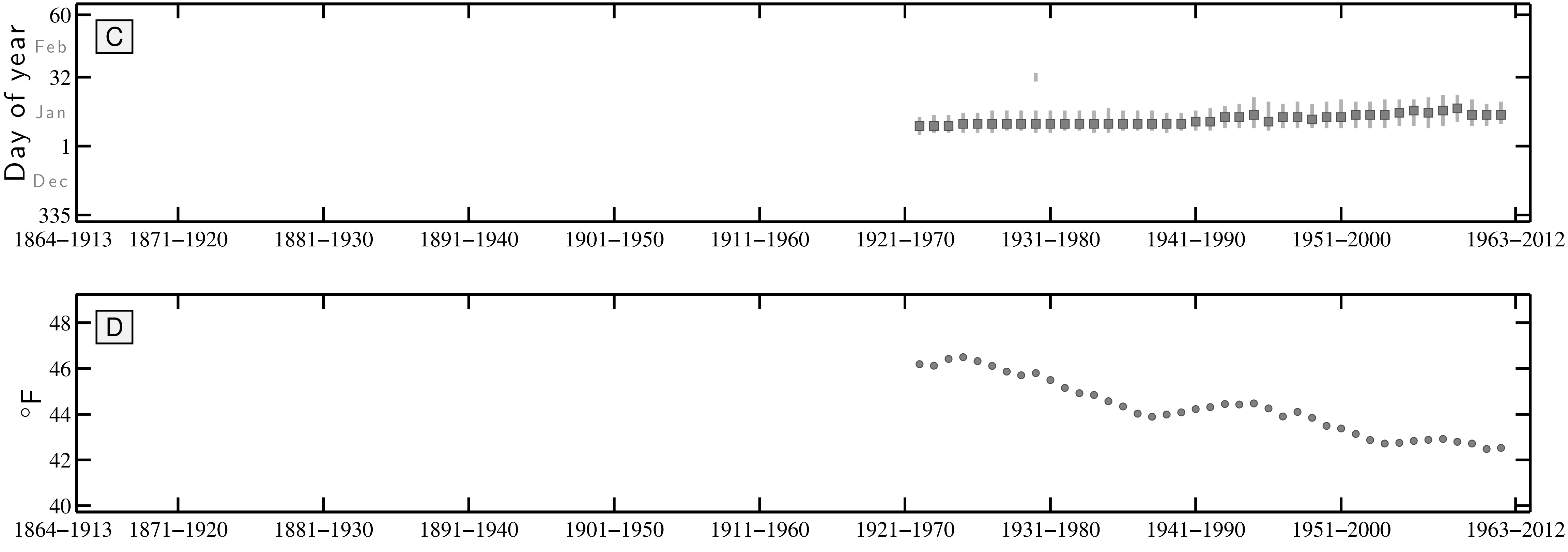

50 year Summer Teletherm dynamics for ARCADIA, FL:

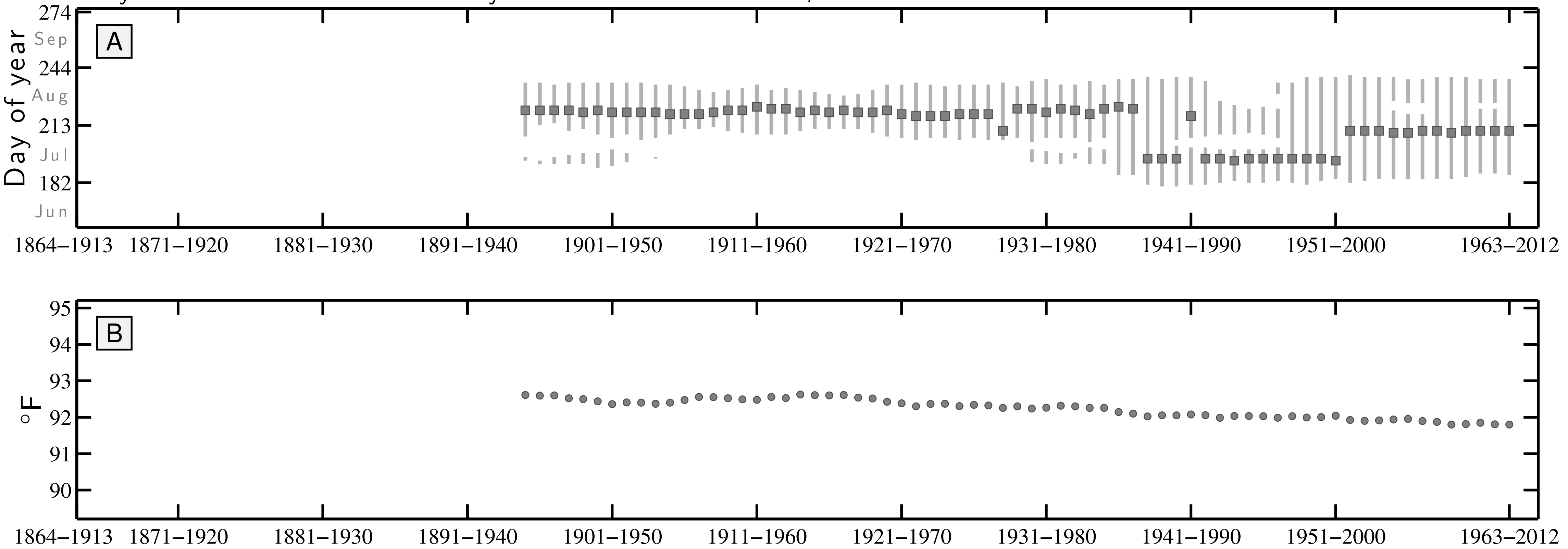

50 year Winter Teletherm dynamics for ARCADIA, FL:

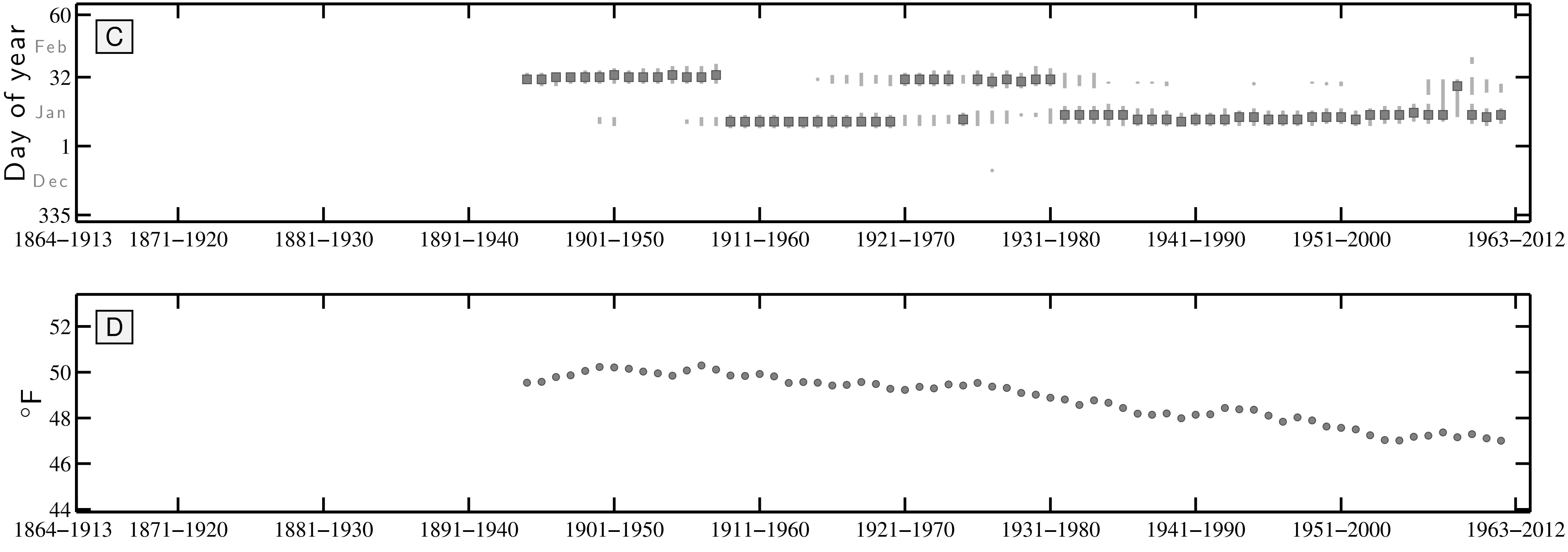

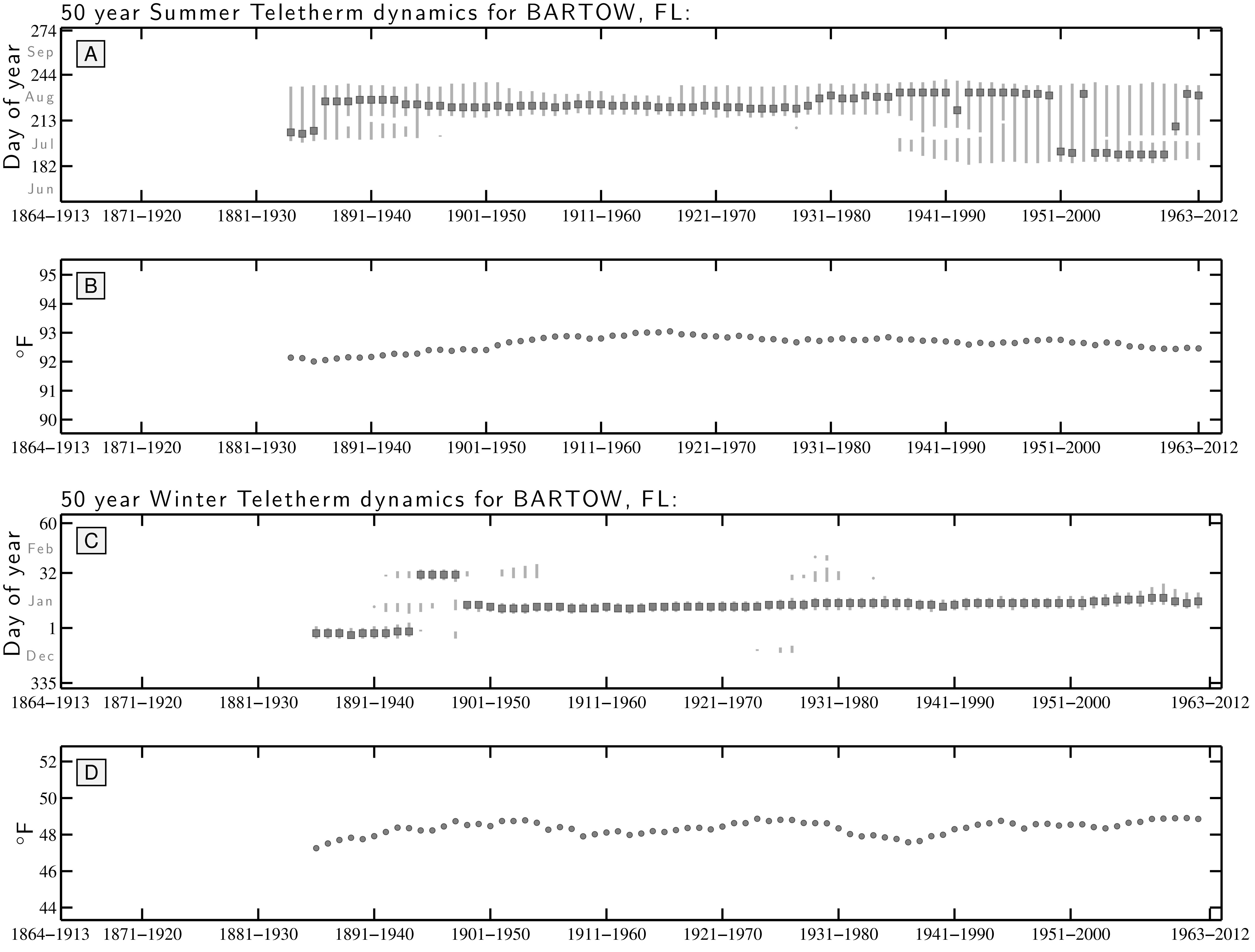
50 year Summer Teletherm dynamics for BARTOW, FL:
A
Day of year
274
Sep
244
Aug
213
Jul
182
Jun
1864–1913
1871–1920
1881–1930
1891–1940
1901–1950
1911–1960
1921–1970
1931–1980
1941–1990
1951–2000
1963–2012
B
°F
95
94
93
92
91
90
50 year Winter Teletherm dynamics for BARTOW, FL:
C
Day of year
60
Feb
32
Jan
1
Dec
335
D
°F
52
50
48
46
44

50 year Summer Teletherm dynamics for BELLE GLADE, FL:

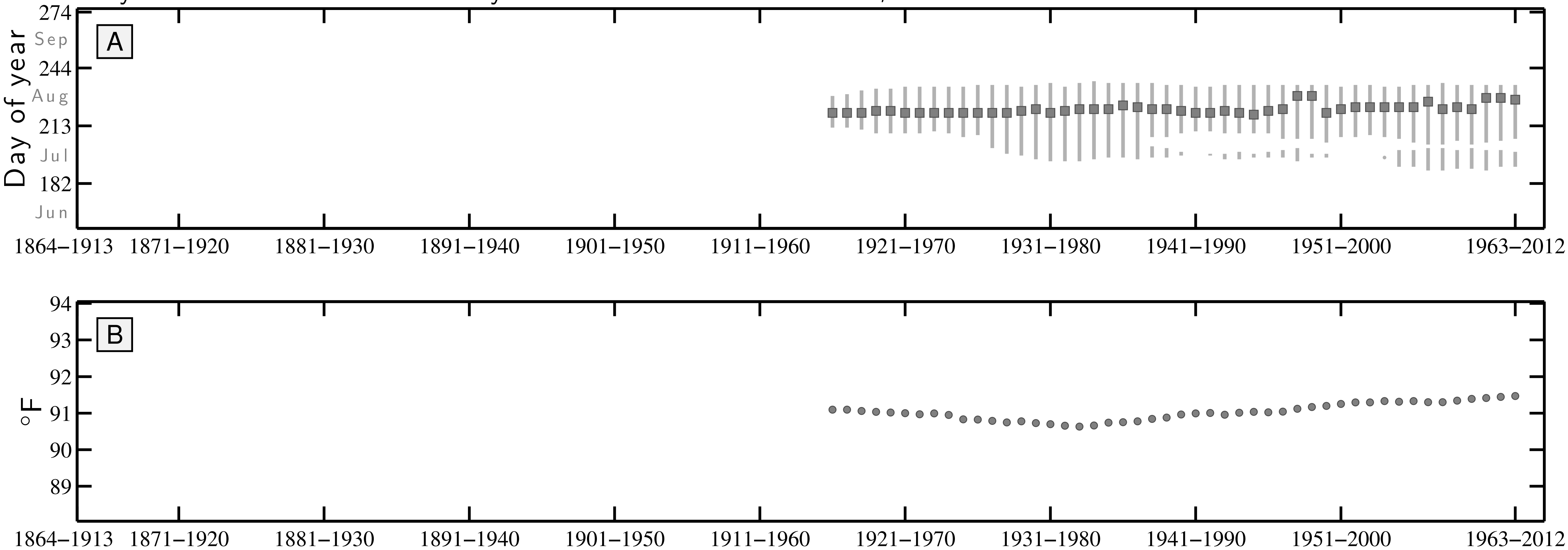

50 year Winter Teletherm dynamics for BELLE GLADE, FL:

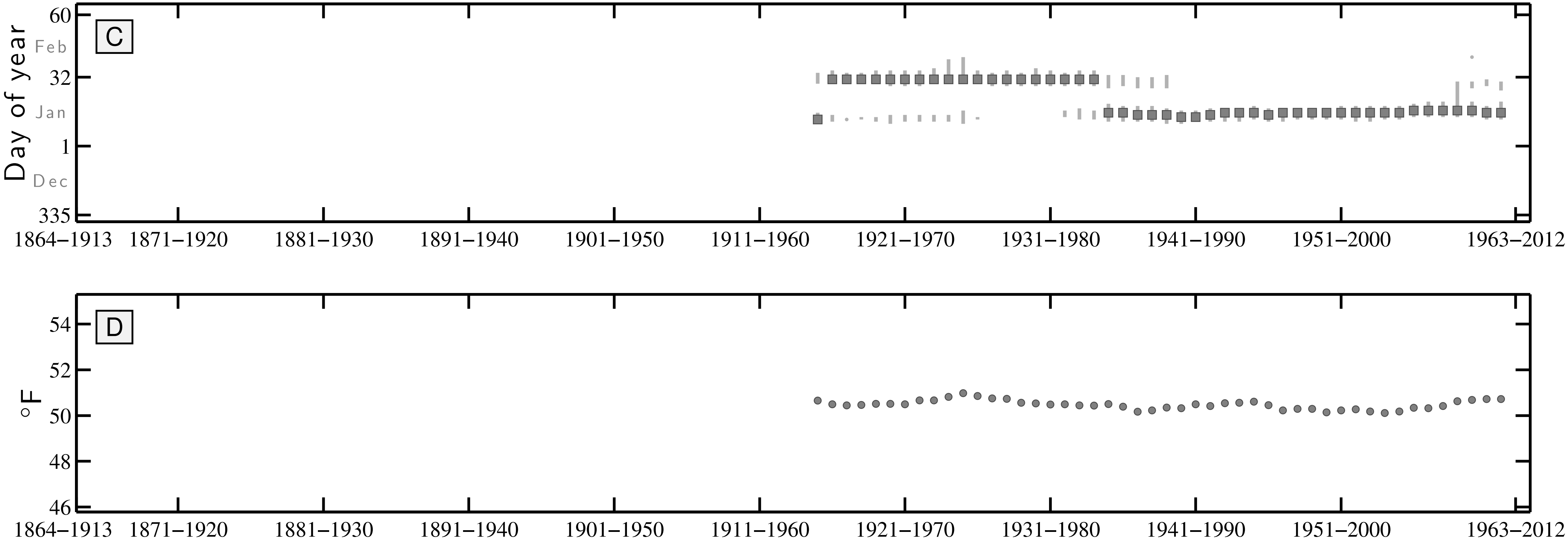

50 year Summer Teletherm dynamics for DE FUNIAK SPRINGS 1, FL:

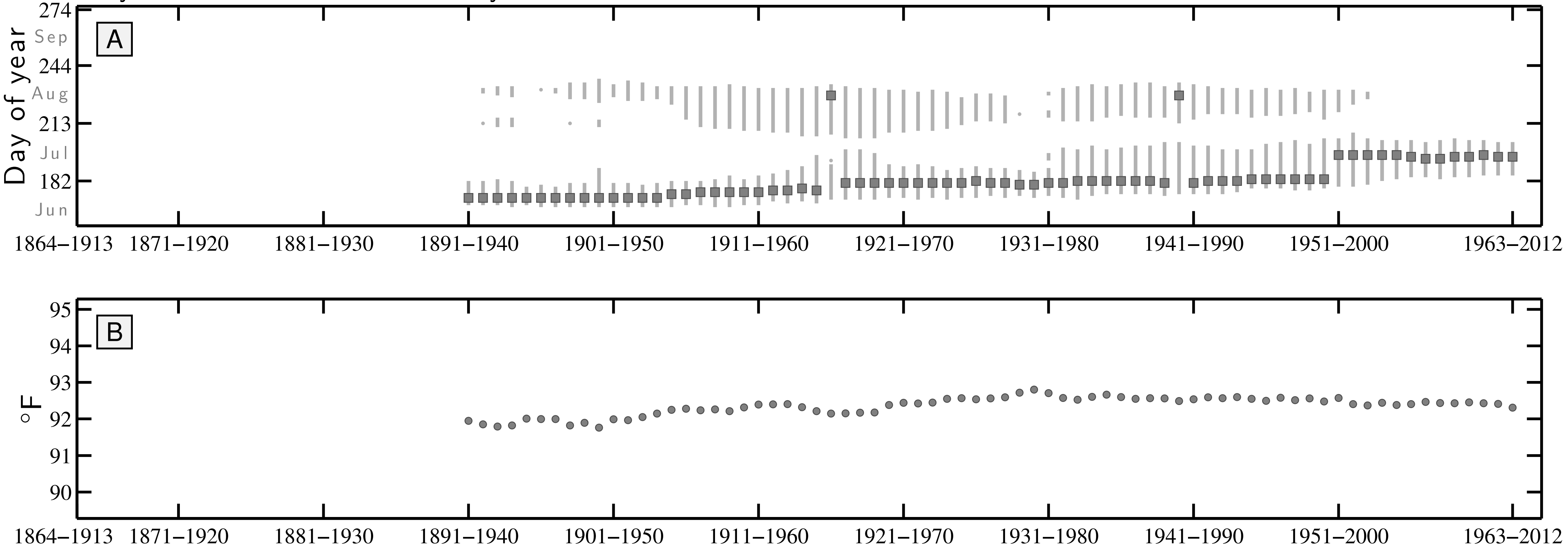

50 year Winter Teletherm dynamics for DE FUNIAK SPRINGS 1, FL:

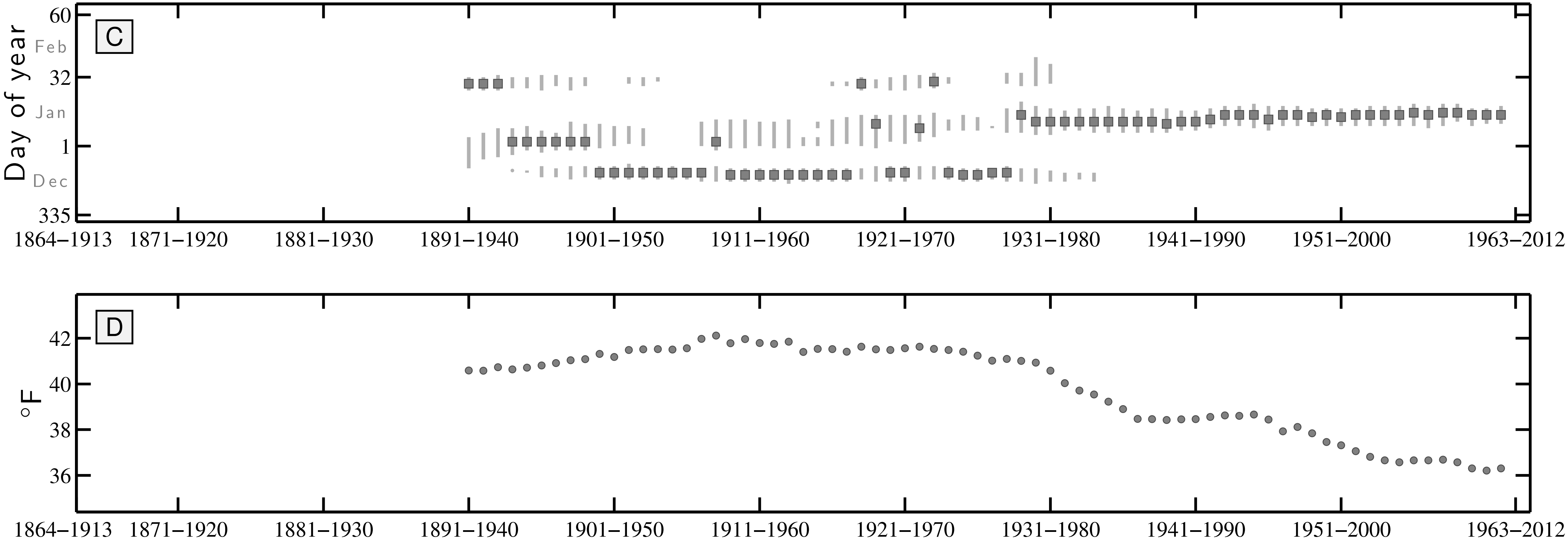

50 year Summer Teletherm dynamics for EVERGLADES, FL:

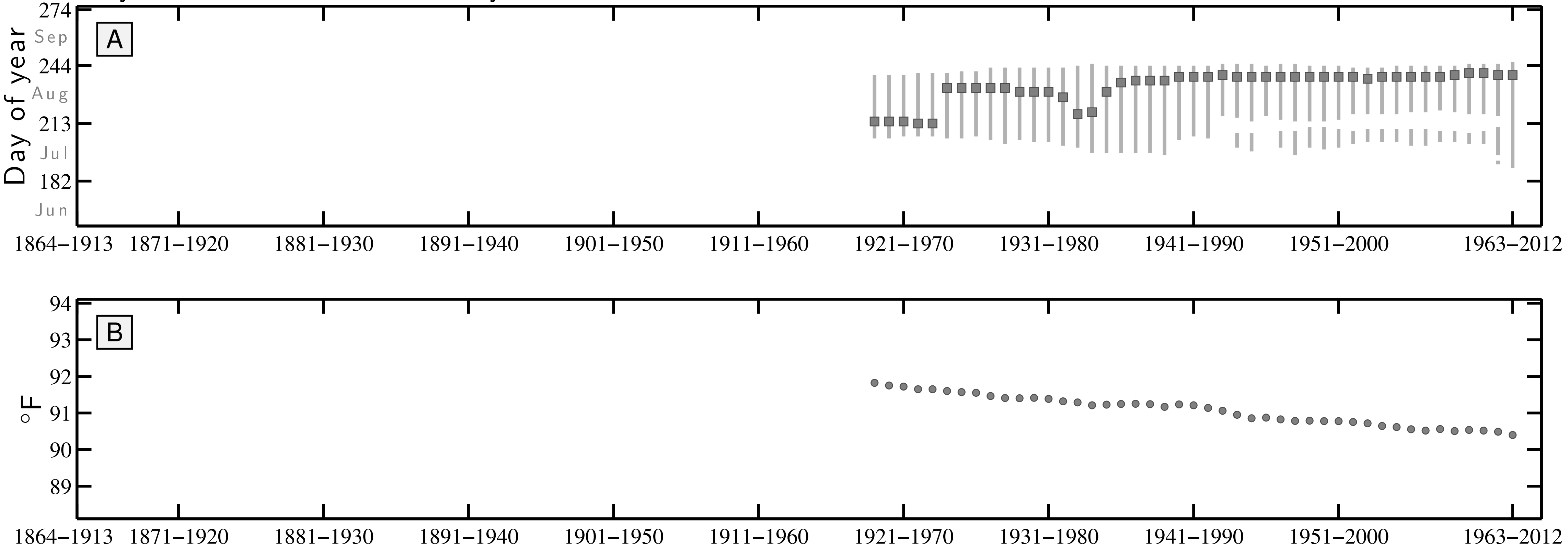

50 year Winter Teletherm dynamics for EVERGLADES, FL:

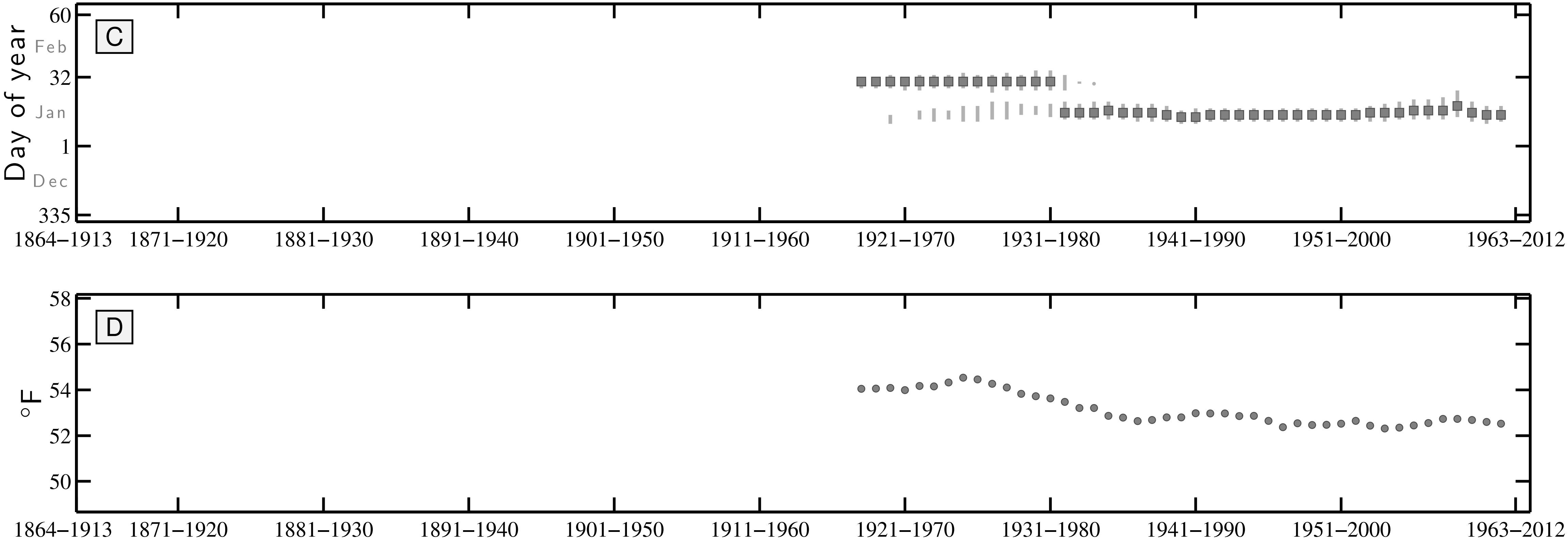

50 year Summer Teletherm dynamics for FEDERAL POINT, FL:

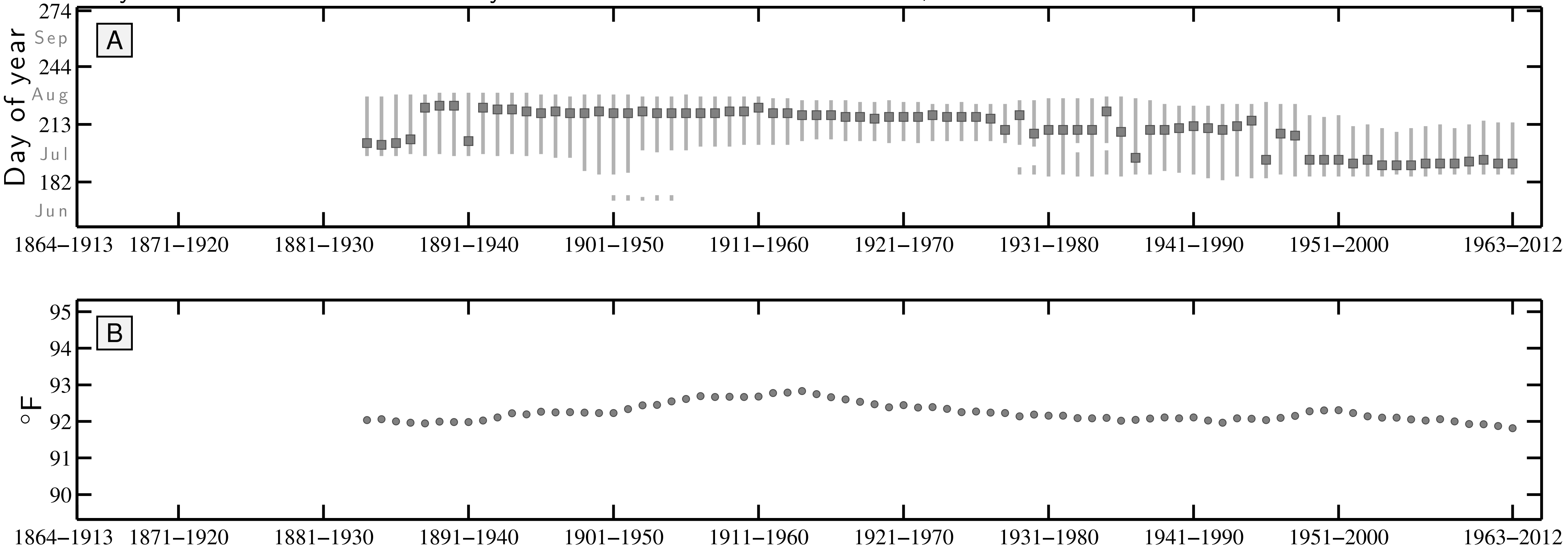

50 year Winter Teletherm dynamics for FEDERAL POINT, FL:

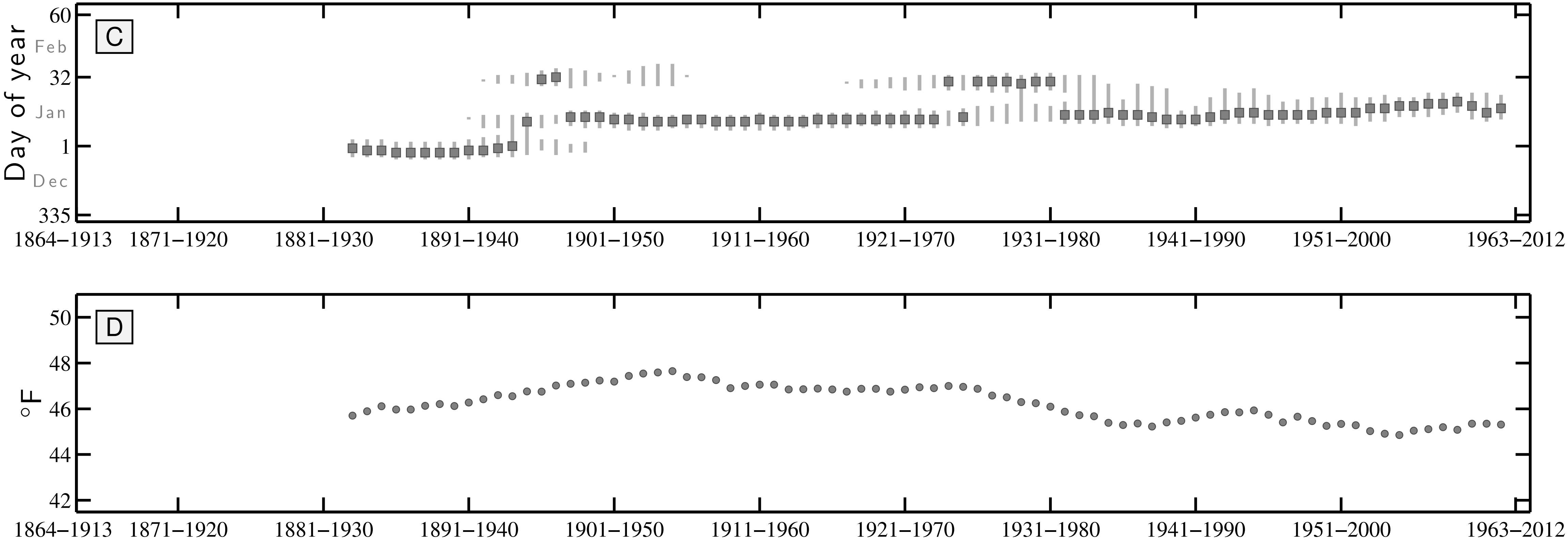

50 year Summer Teletherm dynamics for FERNANDINA BEACH, FL:

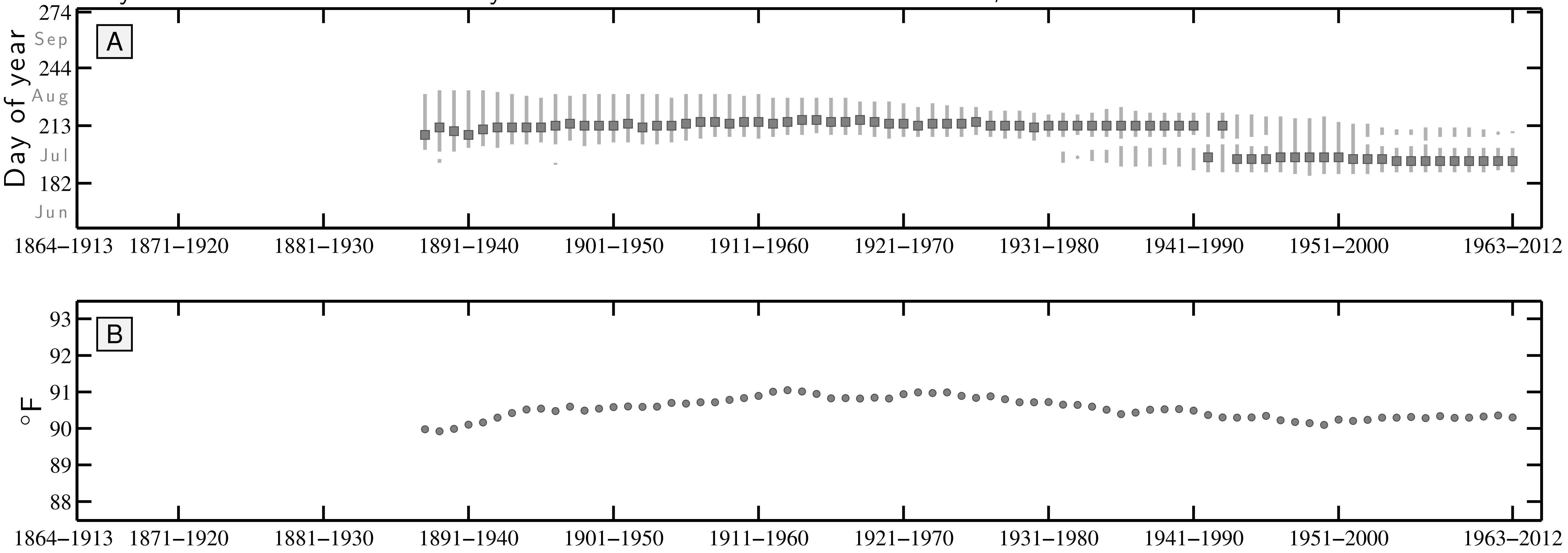

50 year Winter Teletherm dynamics for FERNANDINA BEACH, FL:

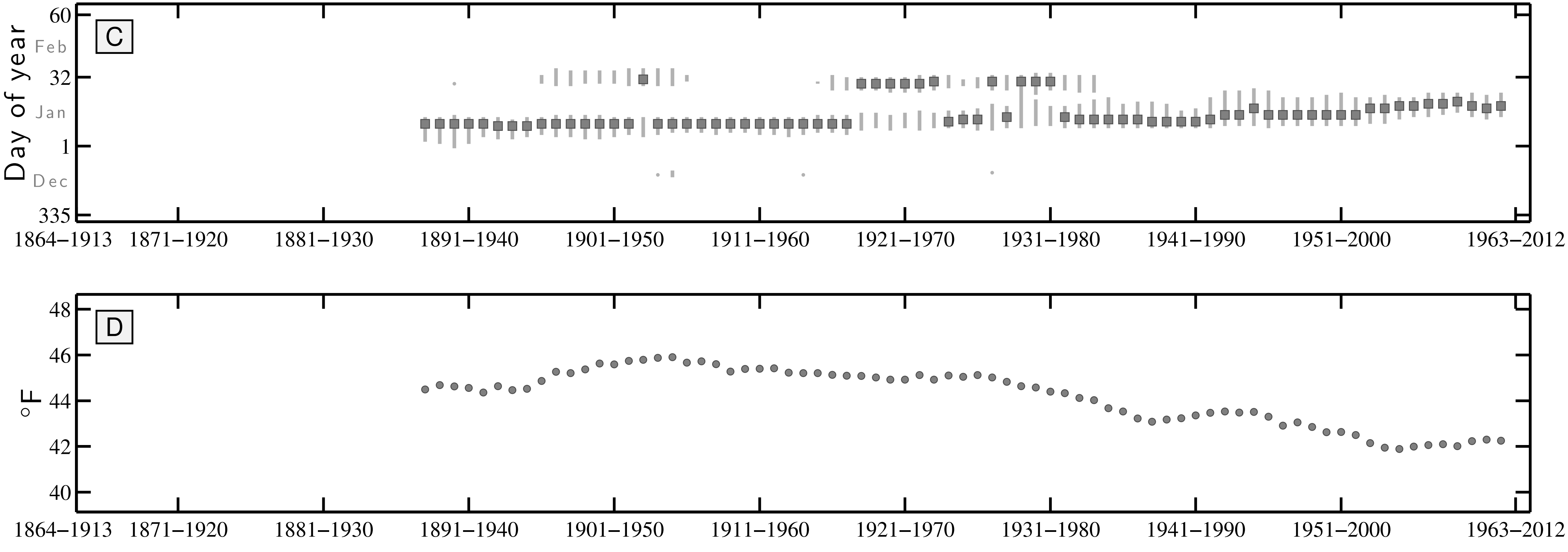

50 year Summer Teletherm dynamics for FT LAUDERDALE, FL:

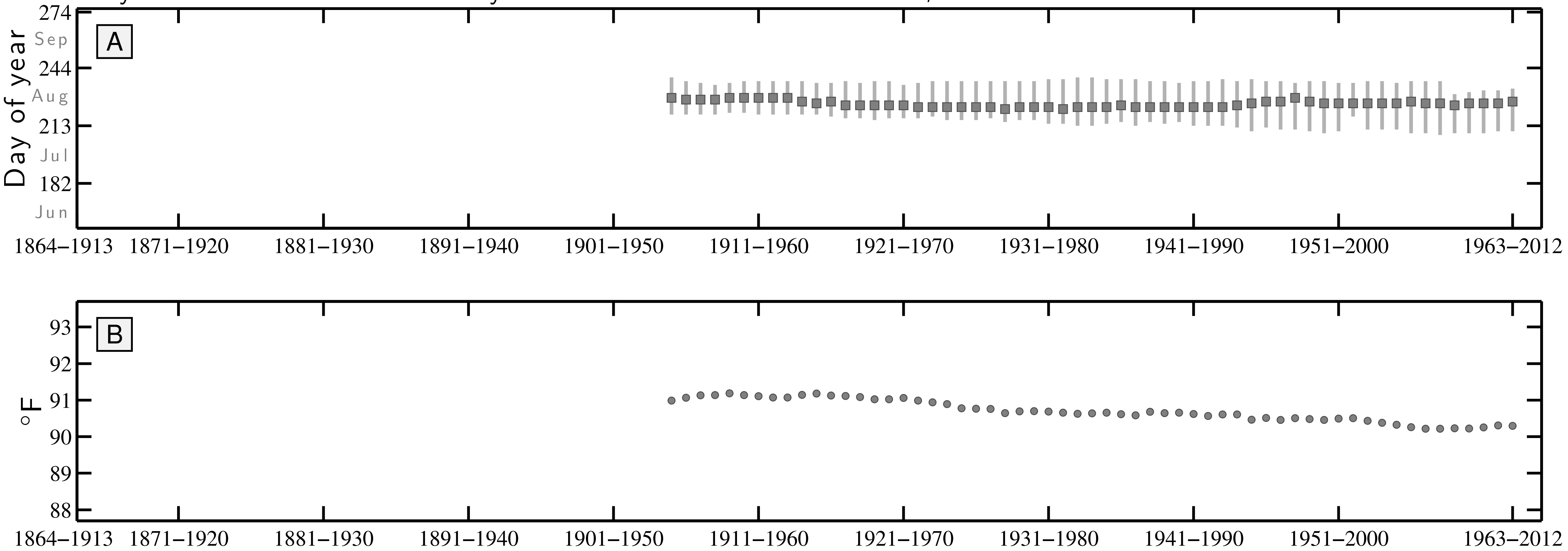

50 year Winter Teletherm dynamics for FT LAUDERDALE, FL:

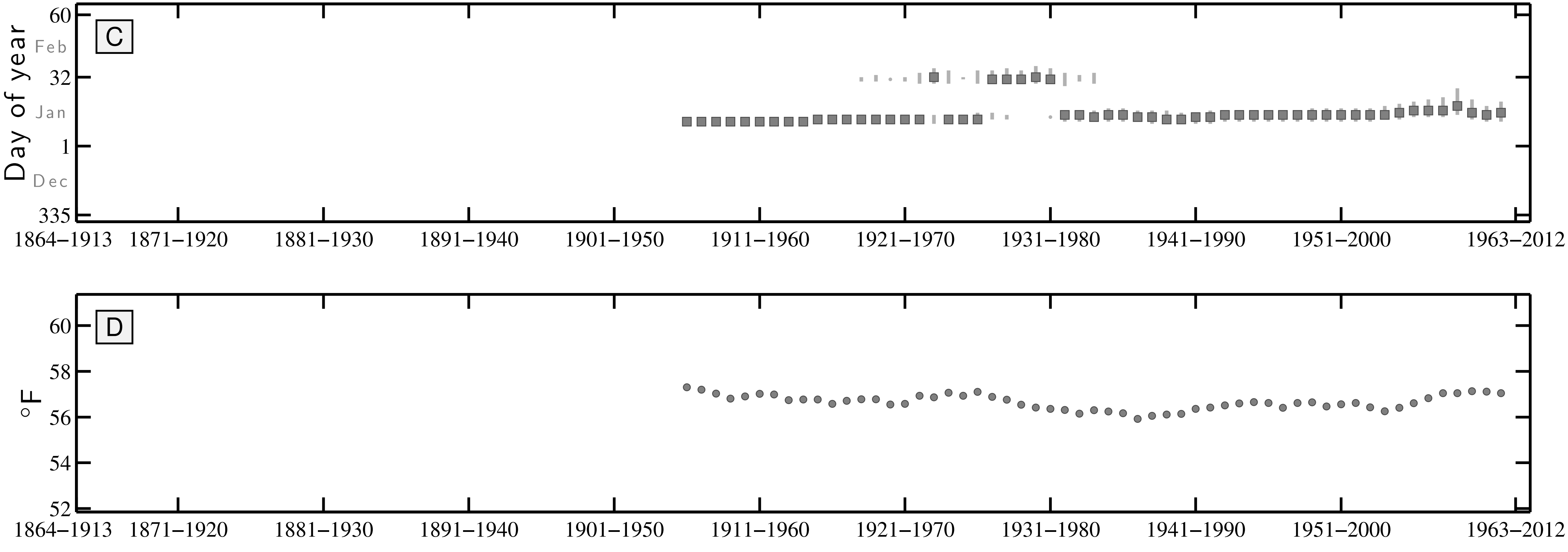

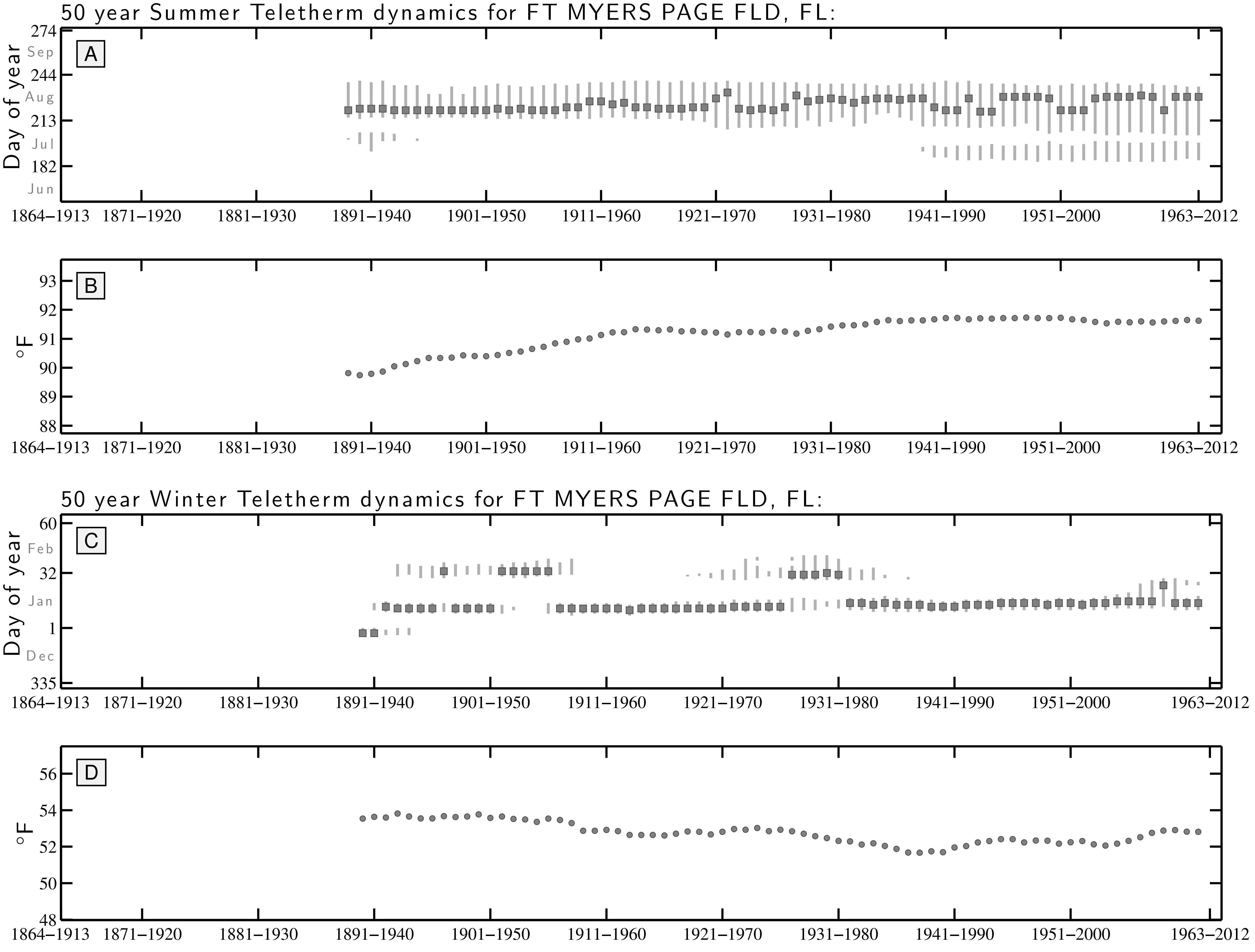
50 year Summer Teletherm dynamics for FT MYERS PAGE FLD, FL:
A
B
Day of year
274
Sep
244
Aug
213
Jul
182
Jun
°F
93
92
91
90
89
88
50 year Winter Teletherm dynamics for FT MYERS PAGE FLD, FL:
C
D
60
Feb
32
Jan
1
Dec
335
56
54
52
50
48
1864–1913
1871–1920
1881–1930
1891–1940
1901–1950
1911–1960
1921–1970
1931–1980
1941–1990
1951–2000
1963–2012

50 year Summer Teletherm dynamics for FT PIERCE, FL:

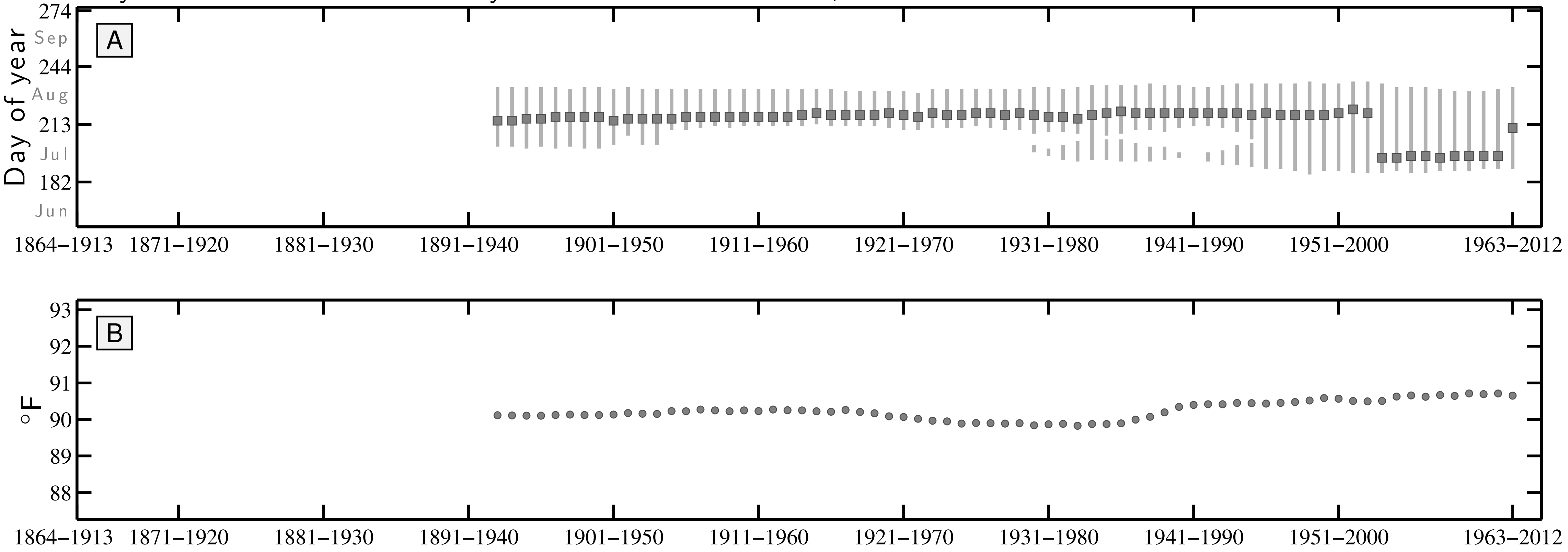

50 year Winter Teletherm dynamics for FT PIERCE, FL:

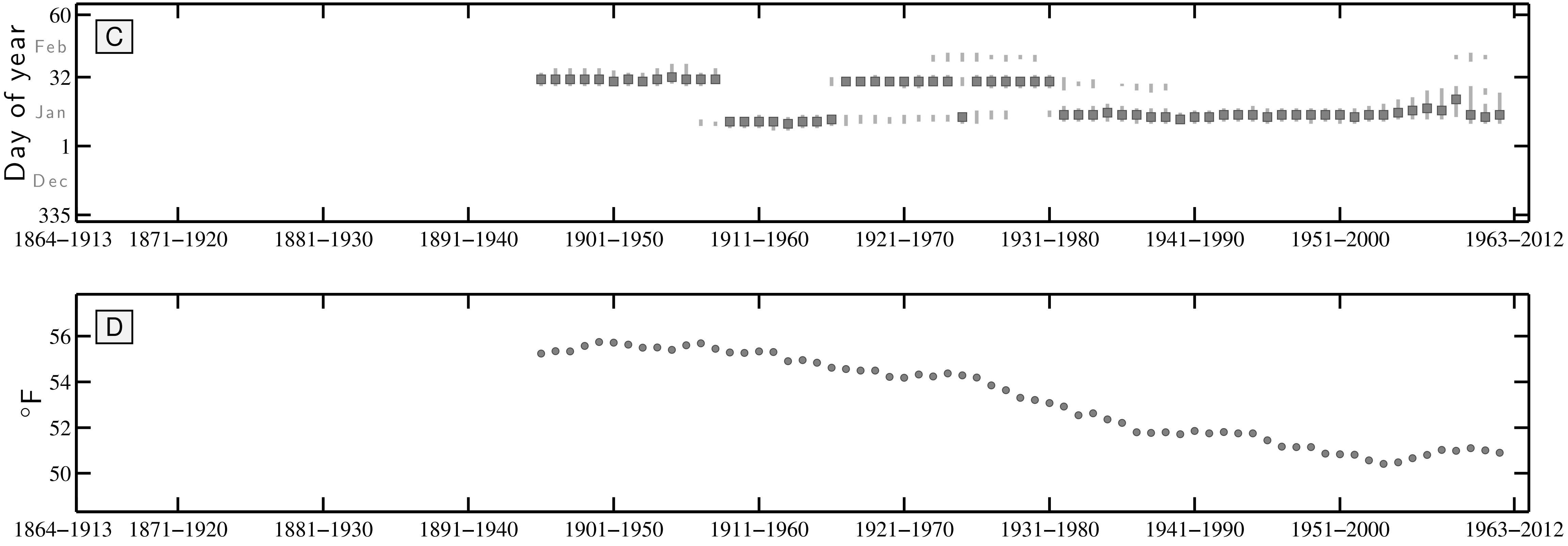

50 year Summer Teletherm dynamics for INVERNESS 3 SE, FL:

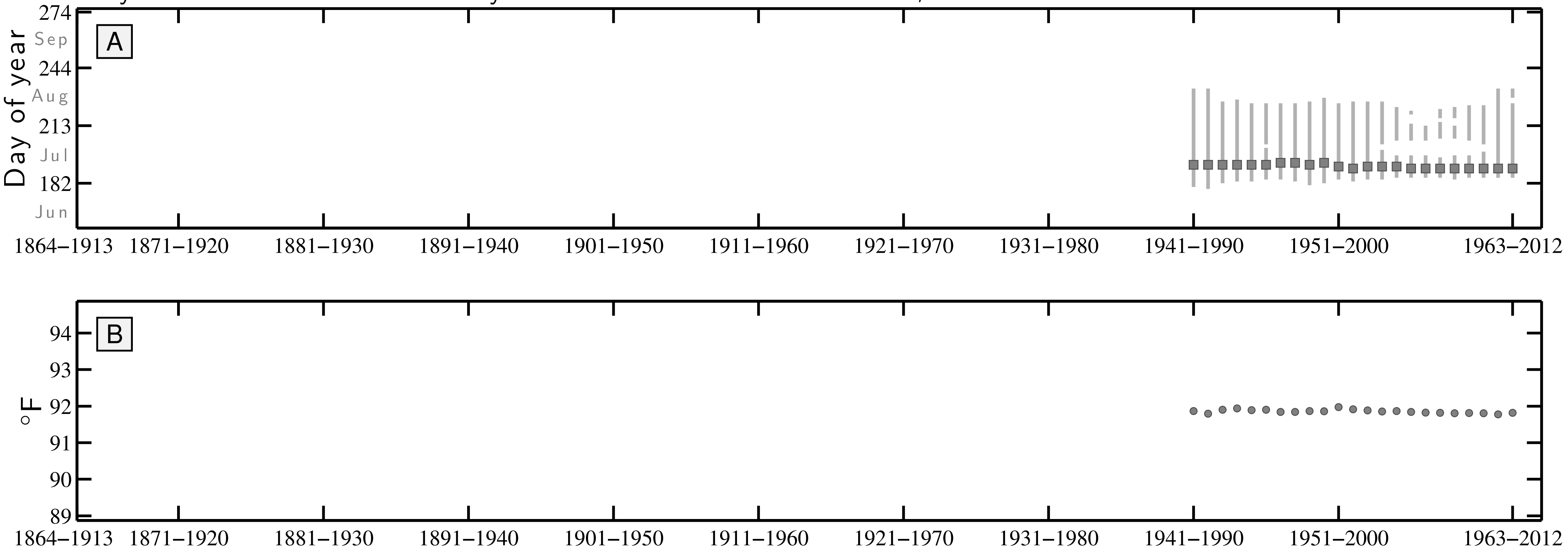

50 year Winter Teletherm dynamics for INVERNESS 3 SE, FL:

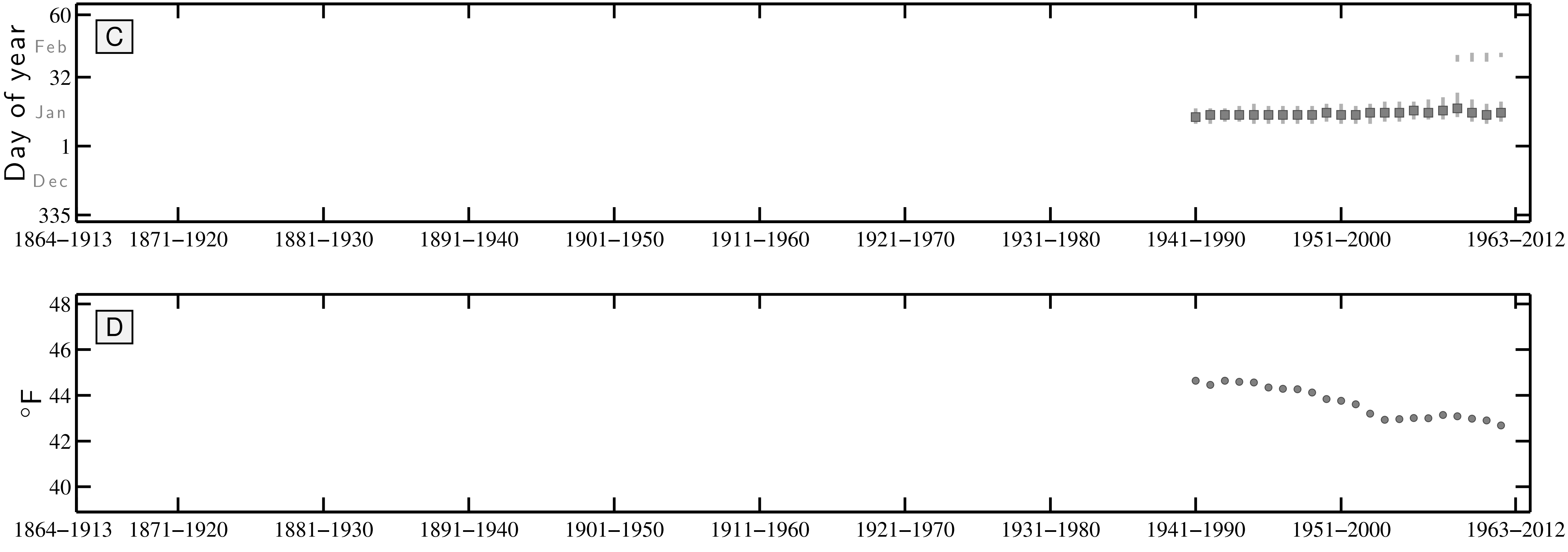

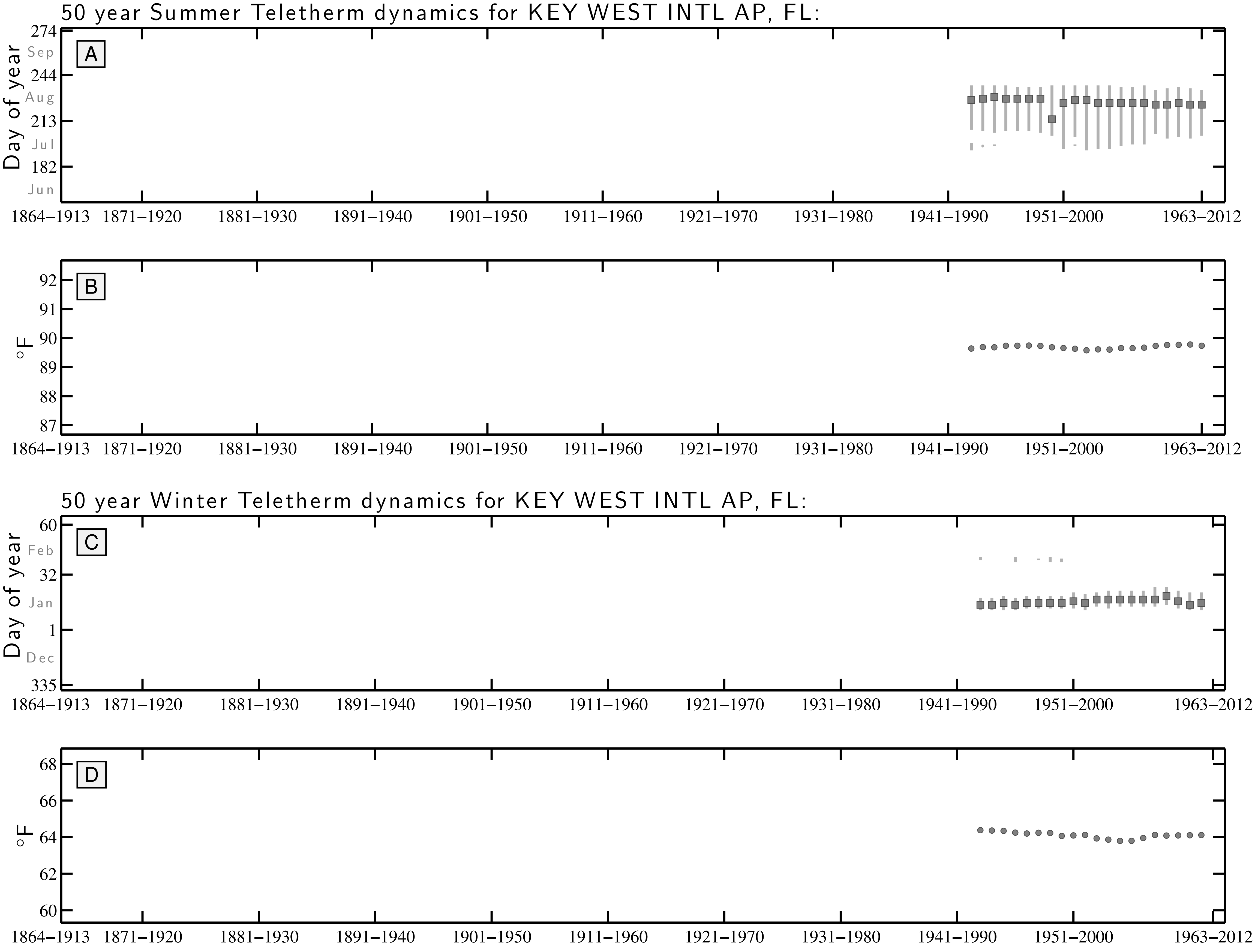

50 year Summer Teletherm dynamics for KEY WEST INTL AP, FL:
A
Day of year
274
Sep
244
Aug
213
Jul
182
Jun
B
°F
92
91
90
89
88
87
50 year Winter Teletherm dynamics for KEY WEST INTL AP, FL:
C
Day of year
60
Feb
32
Jan
1
Dec
335
D
°F
68
66
64
62
60
1864–1913
1871–1920
1881–1930
1891–1940
1901–1950
1911–1960
1921–1970
1931–1980
1941–1990
1951–2000
1963–2012

50 year Summer Teletherm dynamics for LAKE CITY 2 E, FL:

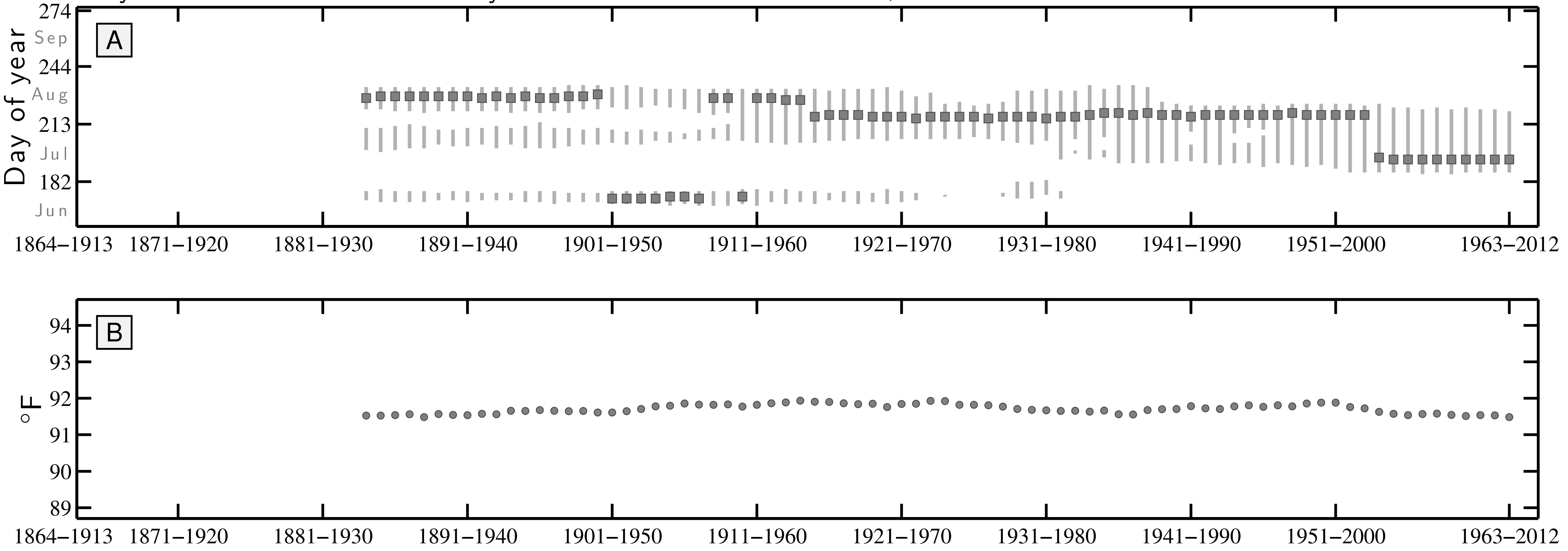

50 year Winter Teletherm dynamics for LAKE CITY 2 E, FL:

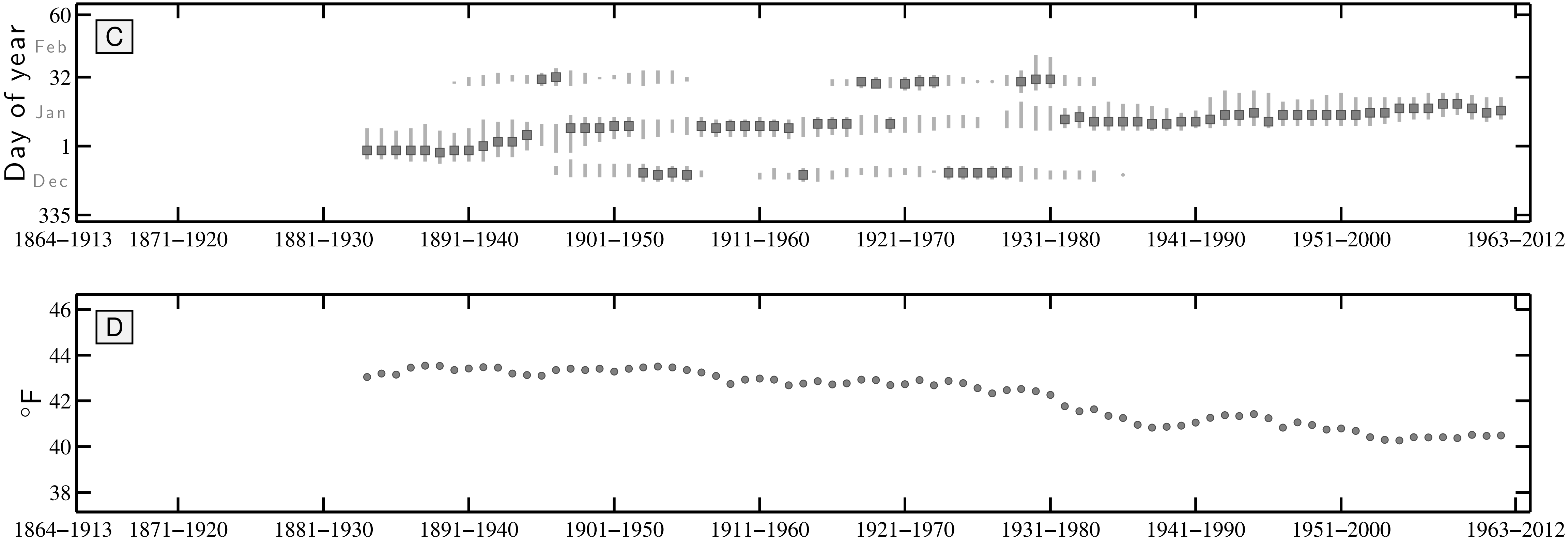

50 year Summer Teletherm dynamics for MADISON, FL:

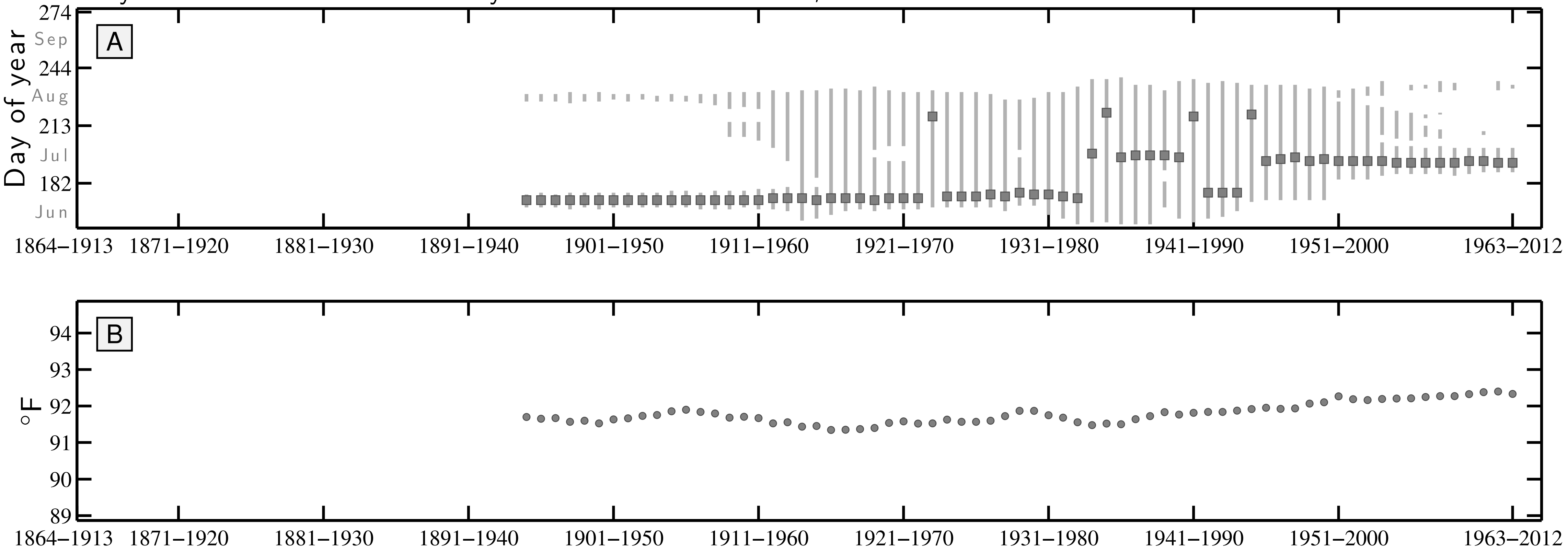

50 year Winter Teletherm dynamics for MADISON, FL:

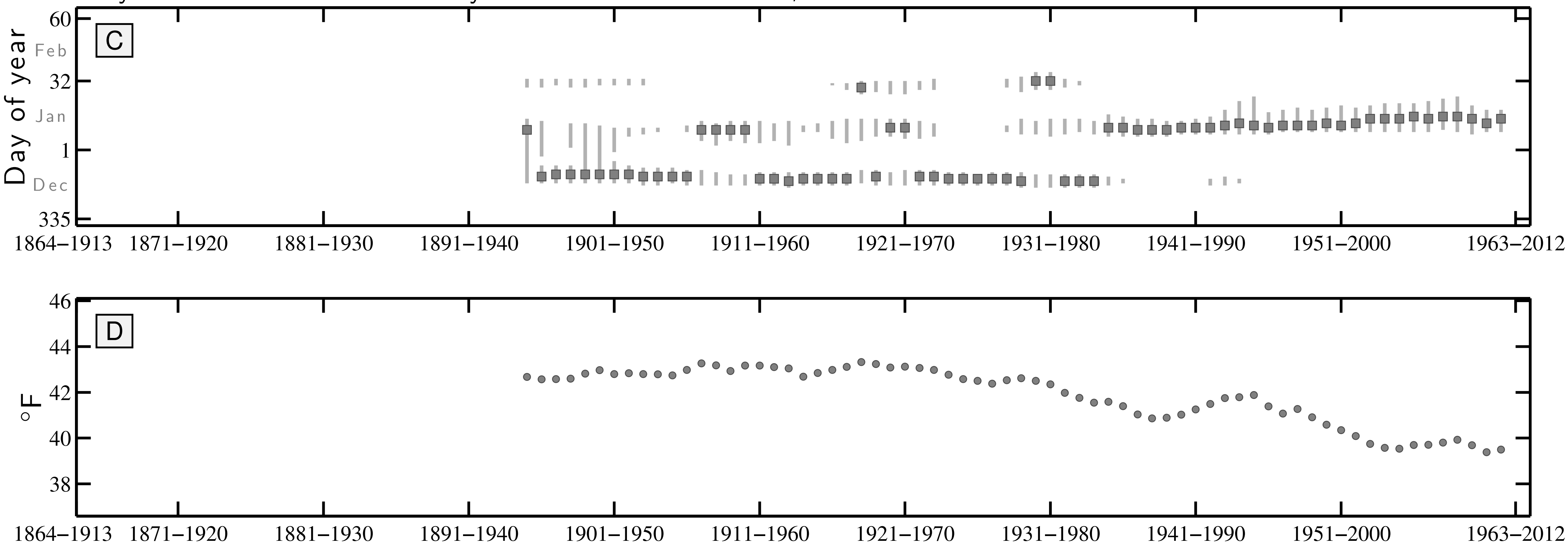

50 year Summer Teletherm dynamics for OCALA, FL:

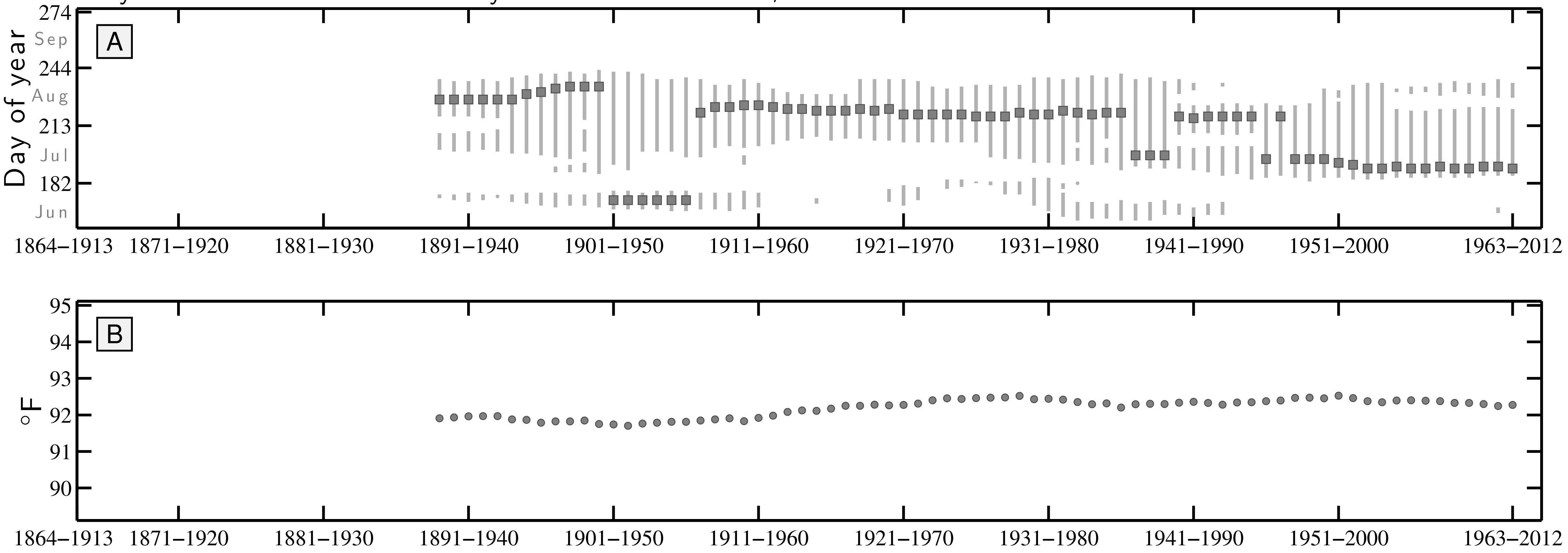

50 year Winter Teletherm dynamics for OCALA, FL:

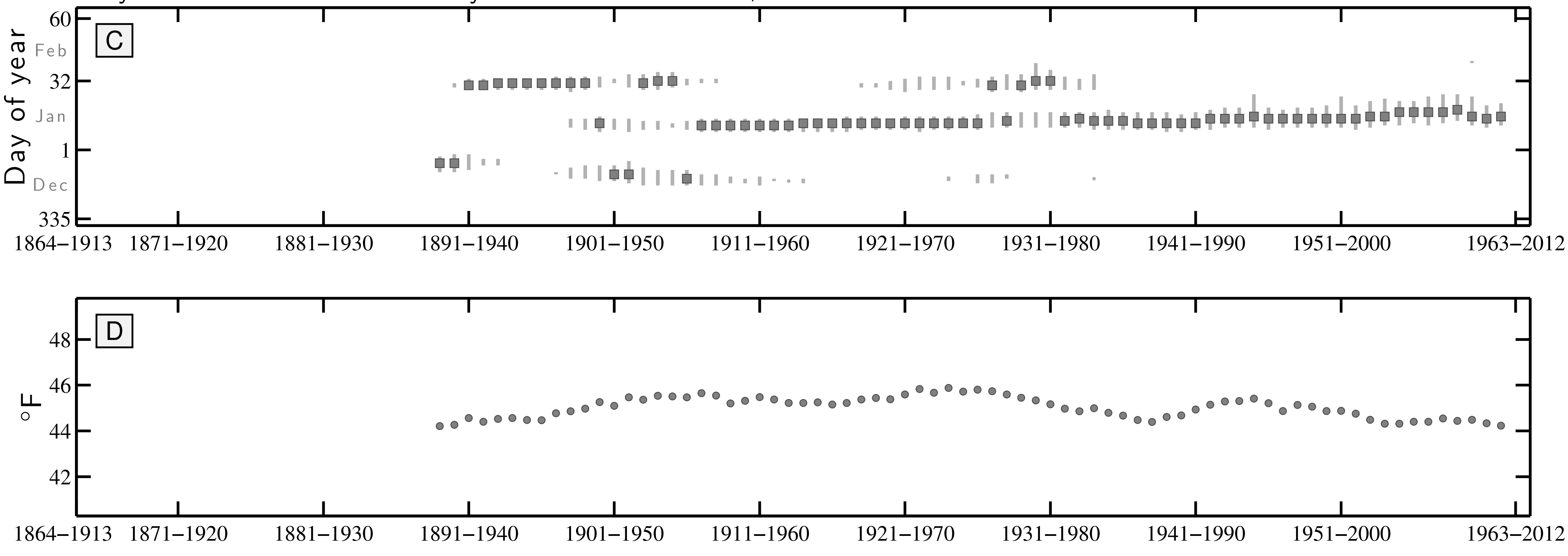

50 year Summer Teletherm dynamics for PENSACOLA RGNL AP, FL:

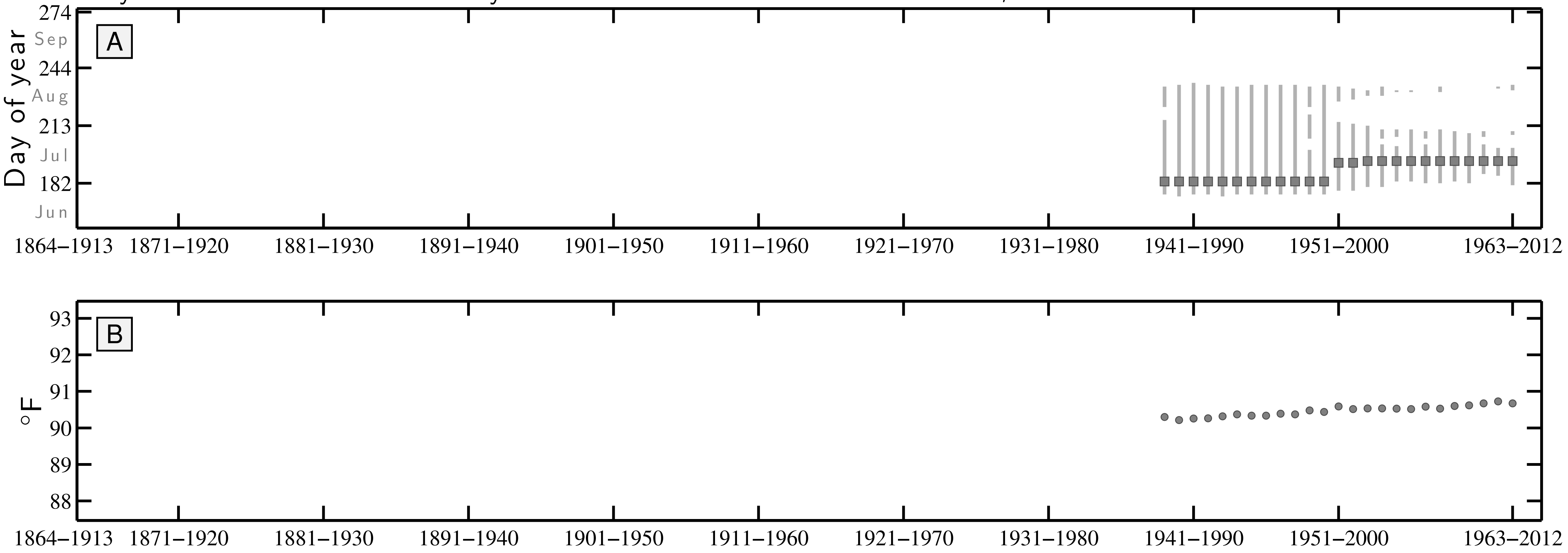

50 year Winter Teletherm dynamics for PENSACOLA RGNL AP, FL:

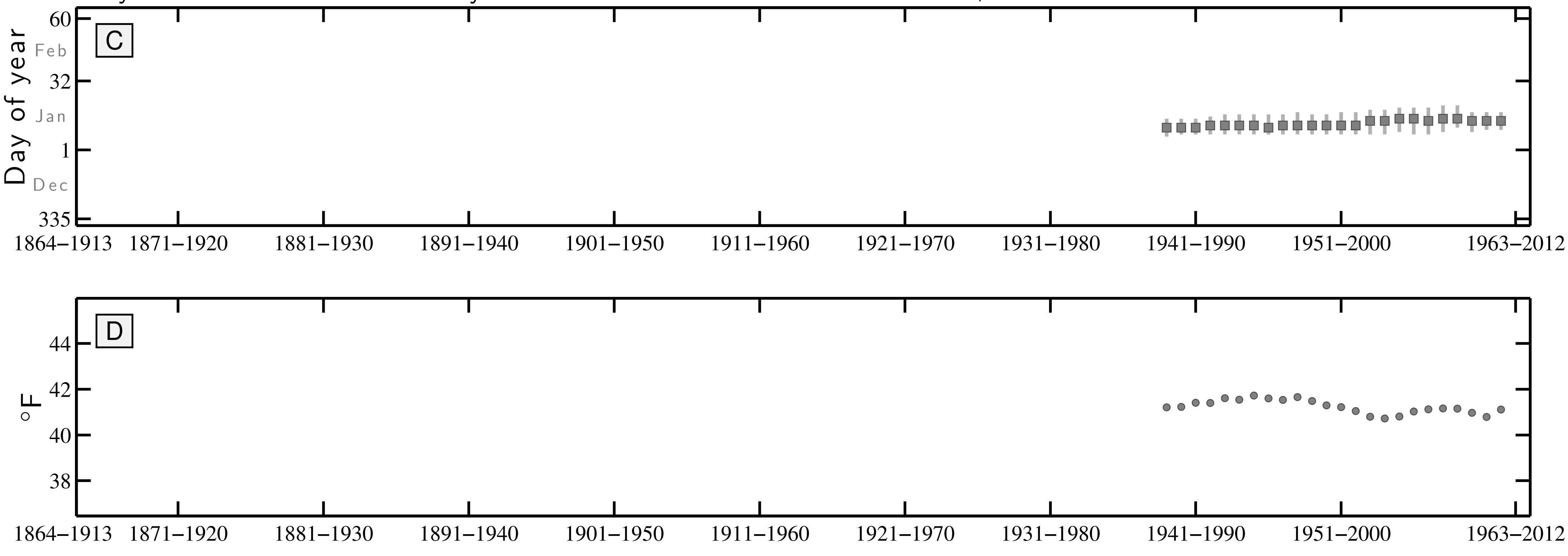

50 year Summer Teletherm dynamics for SAINT LEO, FL:

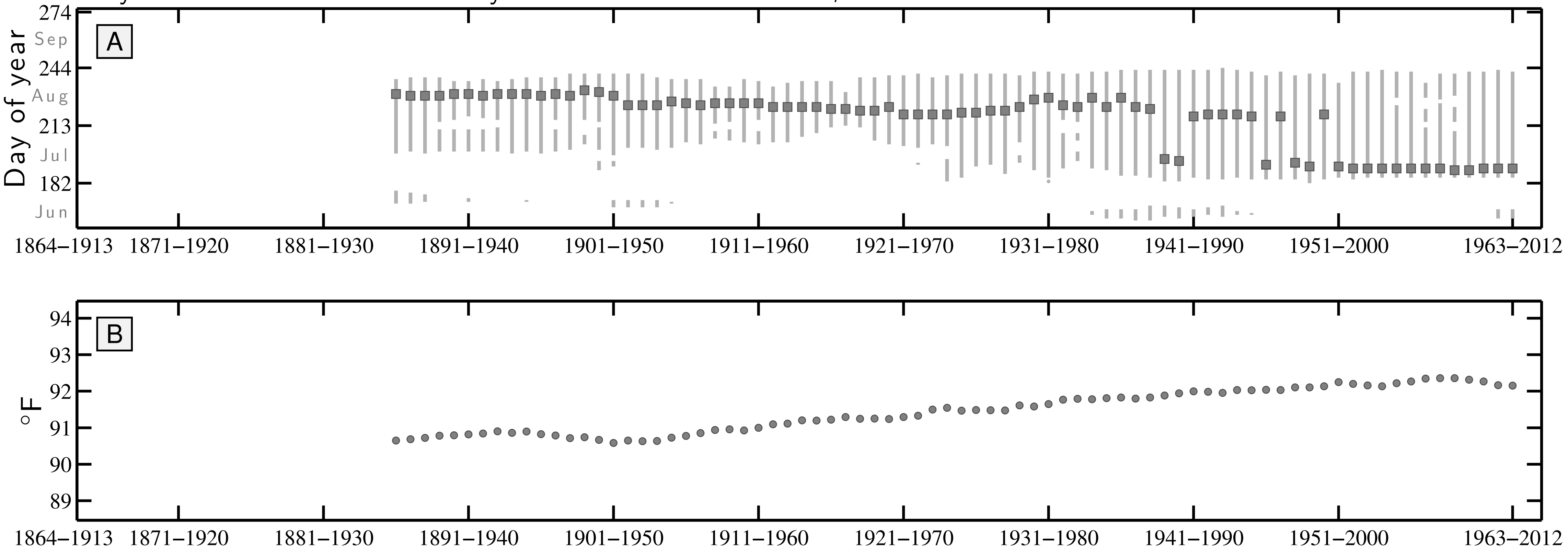

50 year Winter Teletherm dynamics for SAINT LEO, FL:

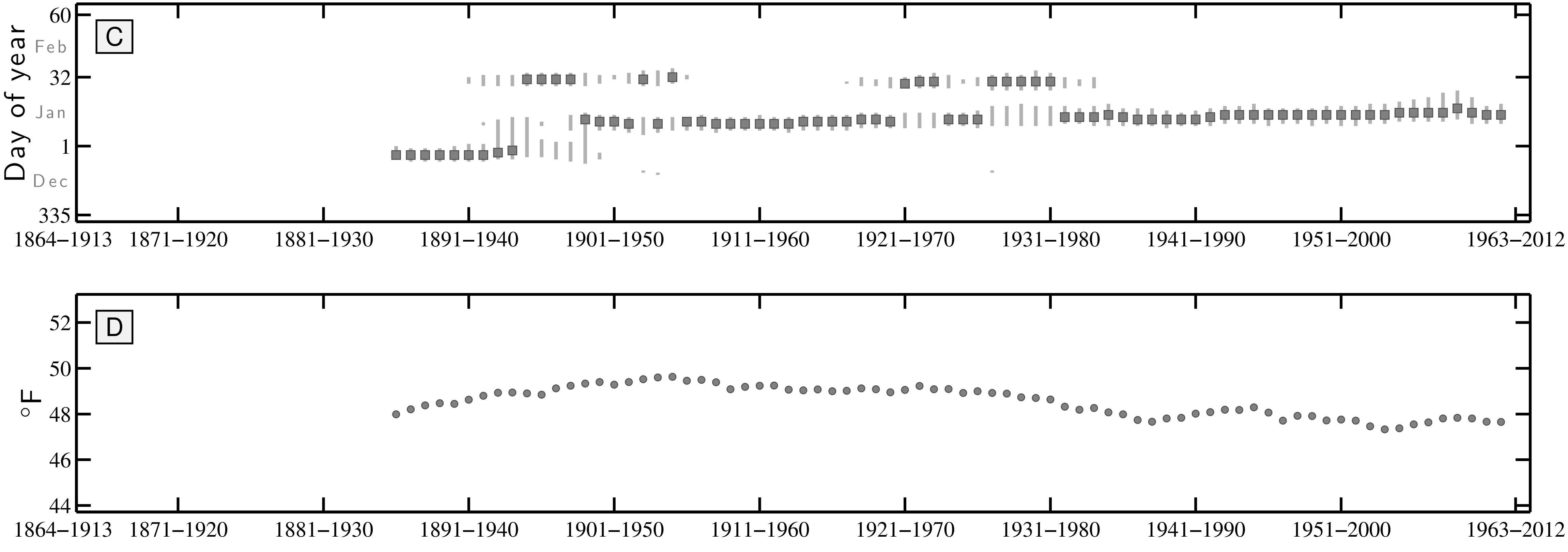

50 year Summer Teletherm dynamics for TALLAHASSEE WSO AP, FL:

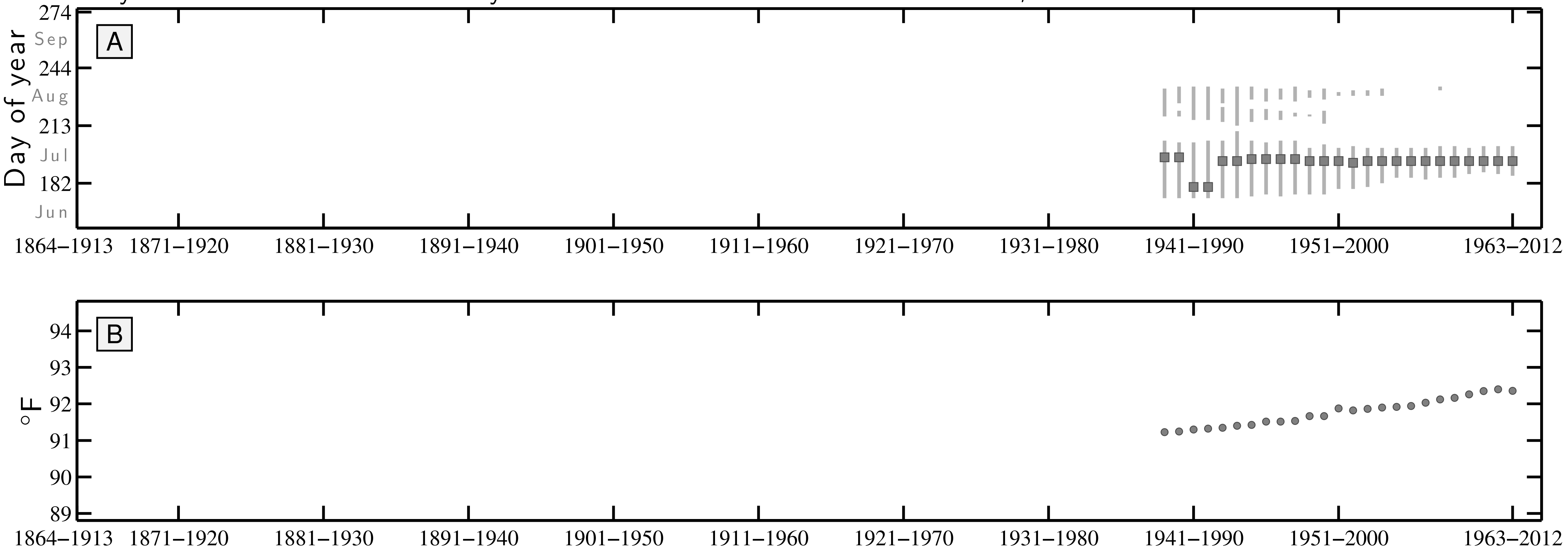

50 year Winter Teletherm dynamics for TALLAHASSEE WSO AP, FL:

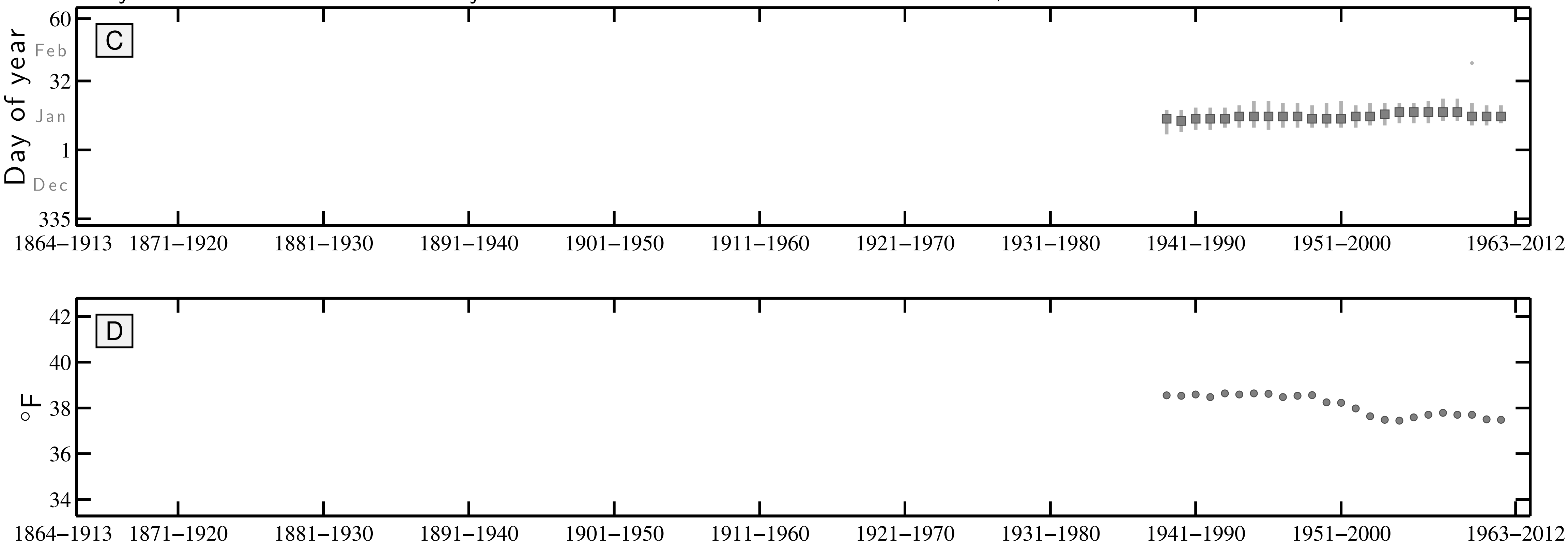

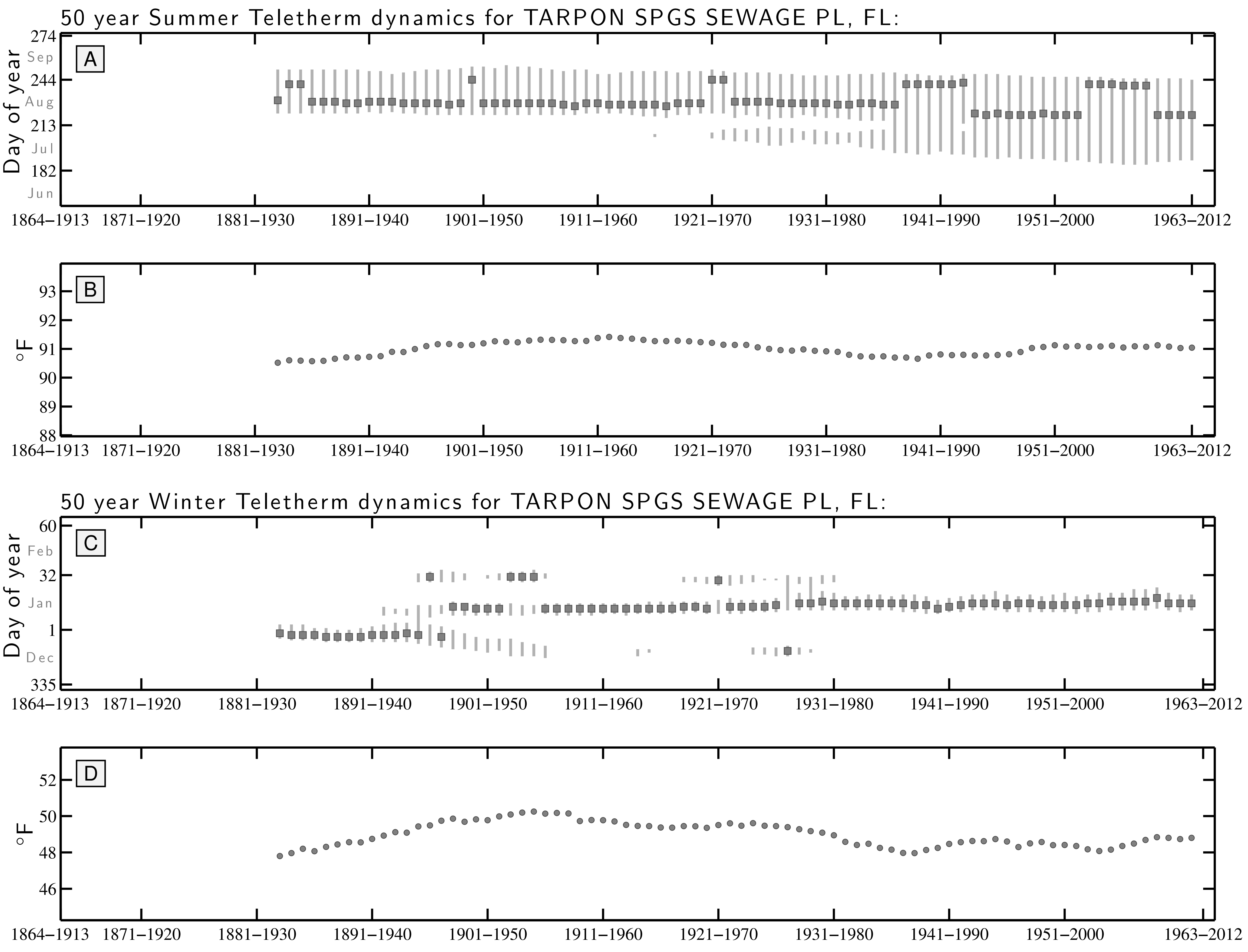

50 year Summer Teletherm dynamics for TARPON SPGS SEWAGE PL, FL:
A
Day of year
274
Sep
244
Aug
213
Jul
182
Jun
1864–1913
1871–1920
1881–1930
1891–1940
1901–1950
1911–1960
1921–1970
1931–1980
1941–1990
1951–2000
1963–2012
B
°F
93
92
91
90
89
88
50 year Winter Teletherm dynamics for TARPON SPGS SEWAGE PL, FL:
C
60
Feb
32
Jan
1
Dec
335
D
52
50
48
46

50 year Summer Teletherm dynamics for TITUSVILLE, FL:

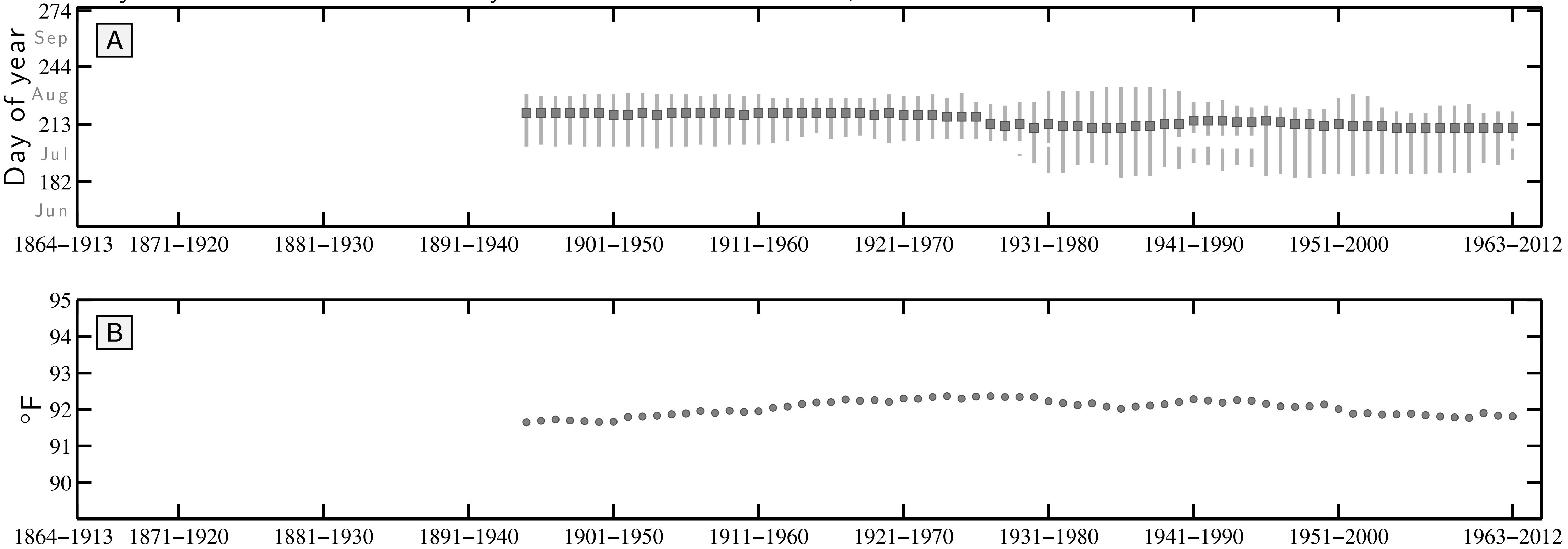

50 year Winter Teletherm dynamics for TITUSVILLE, FL:

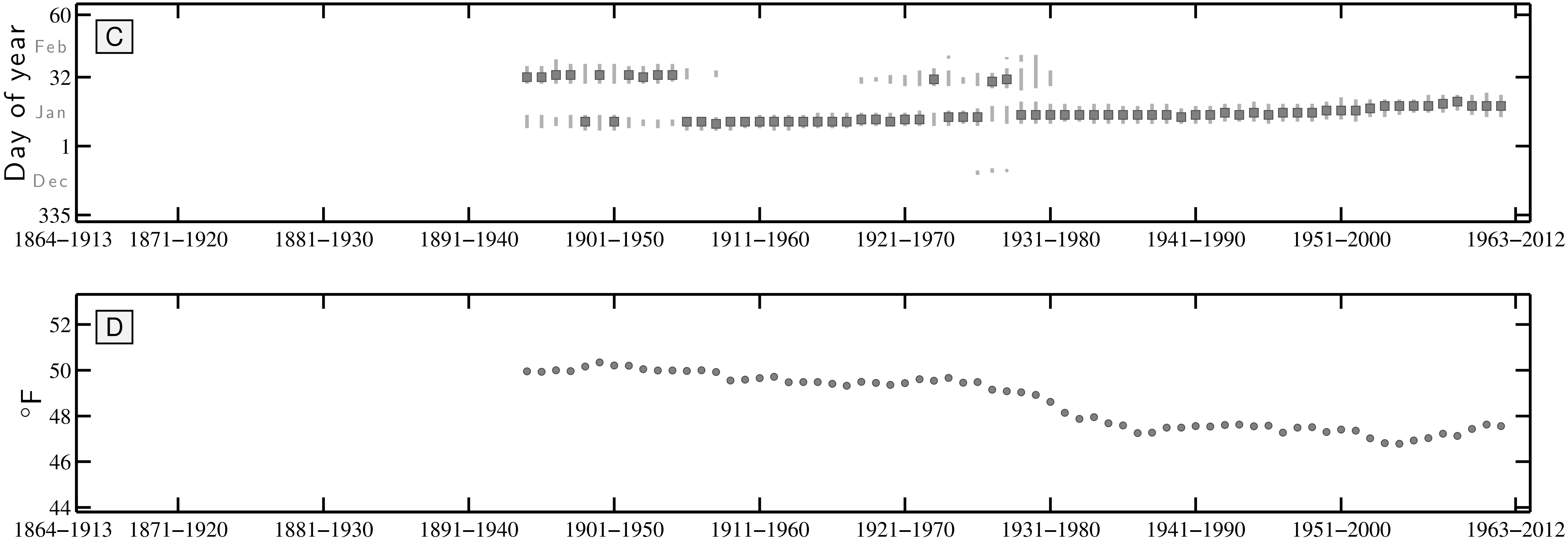

50 year Summer Teletherm dynamics for ALBANY 3 SE, GA:

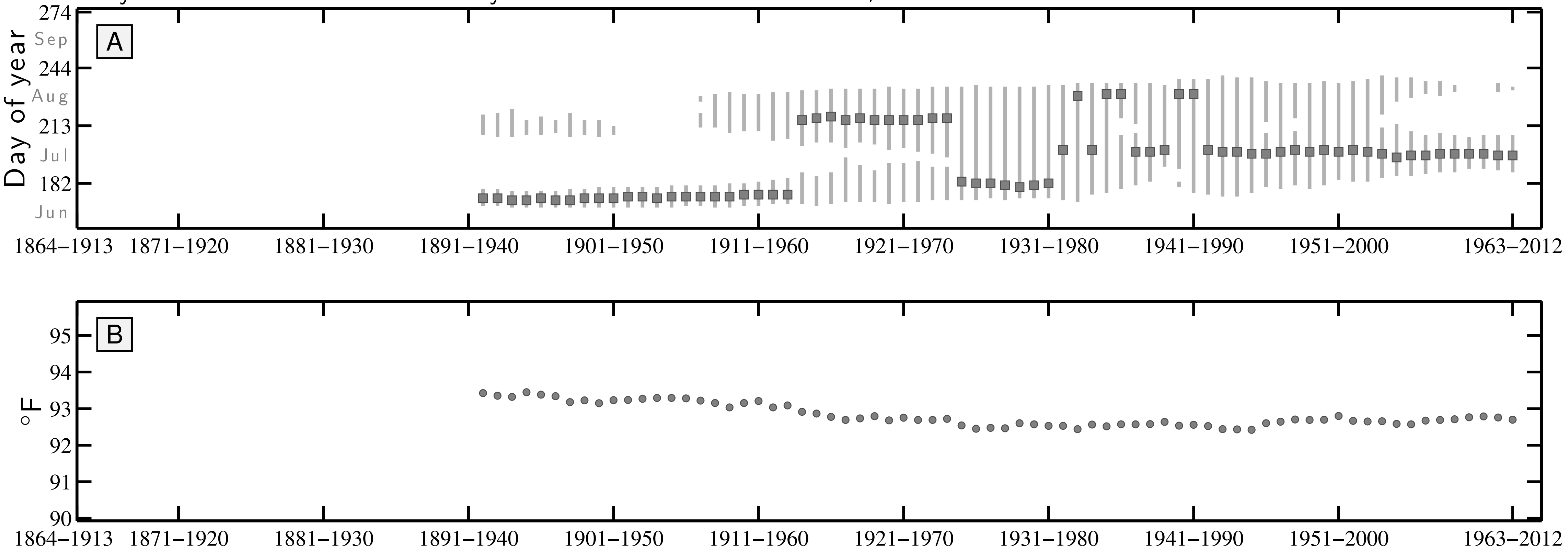

50 year Winter Teletherm dynamics for ALBANY 3 SE, GA:

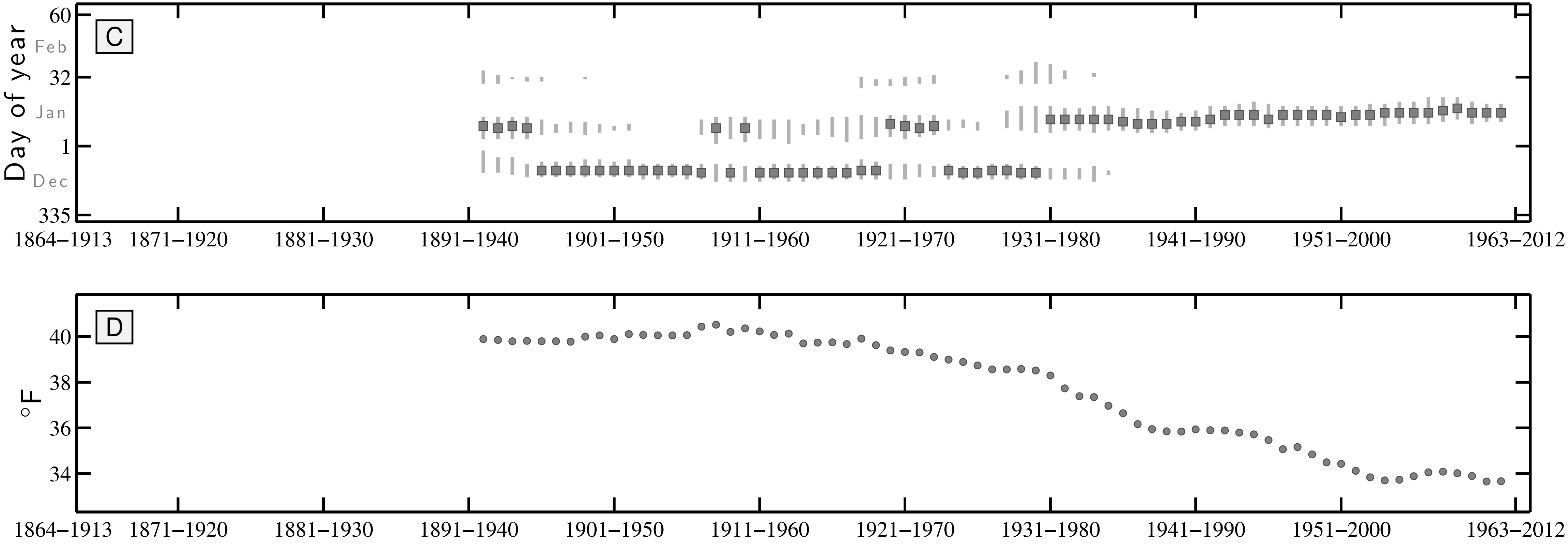

50 year Summer Teletherm dynamics for BRUNSWICK, GA:

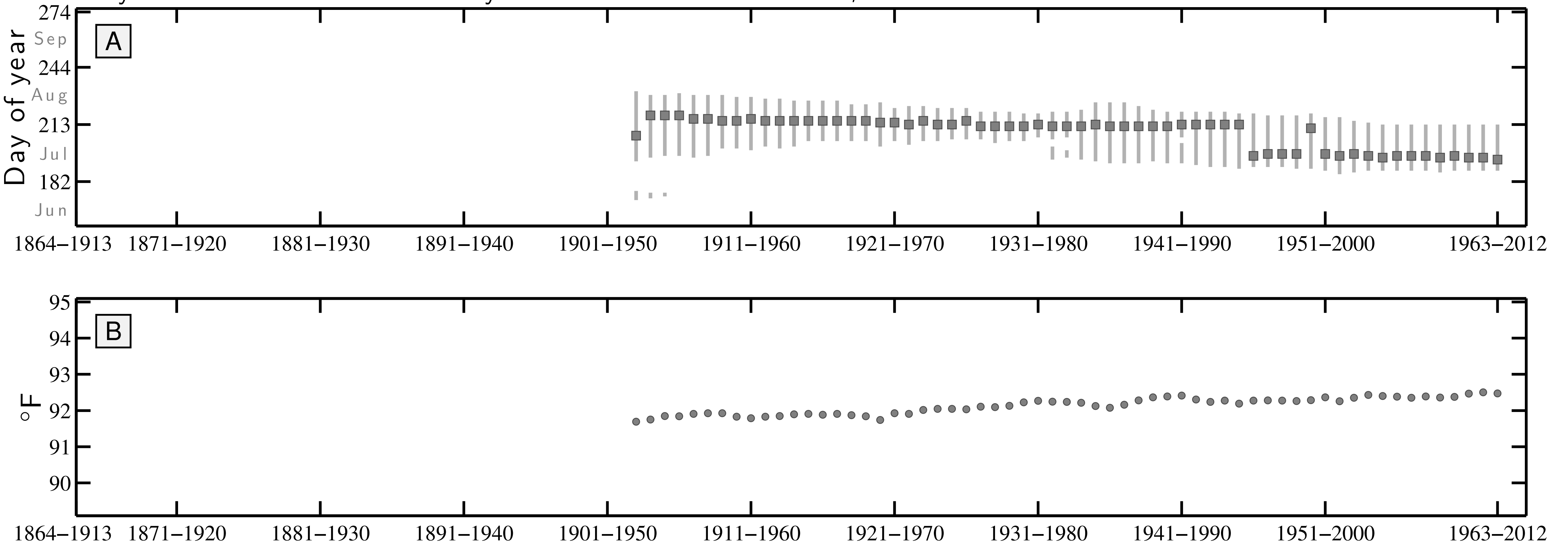

50 year Winter Teletherm dynamics for BRUNSWICK, GA:

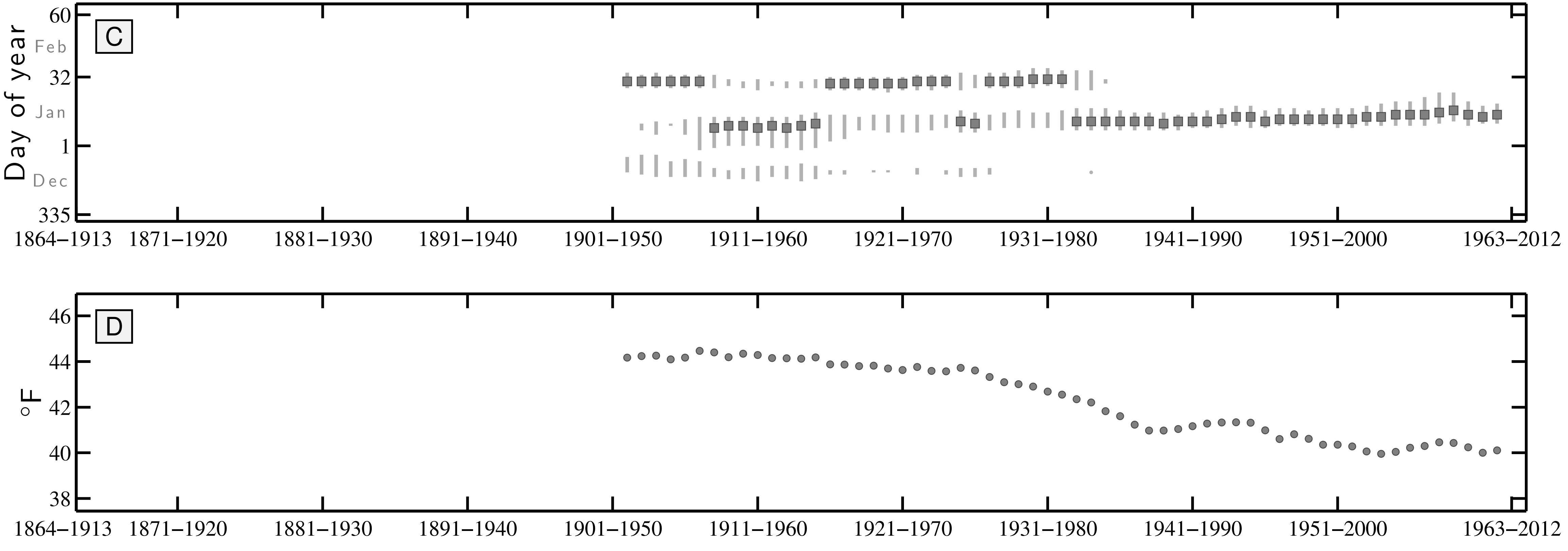

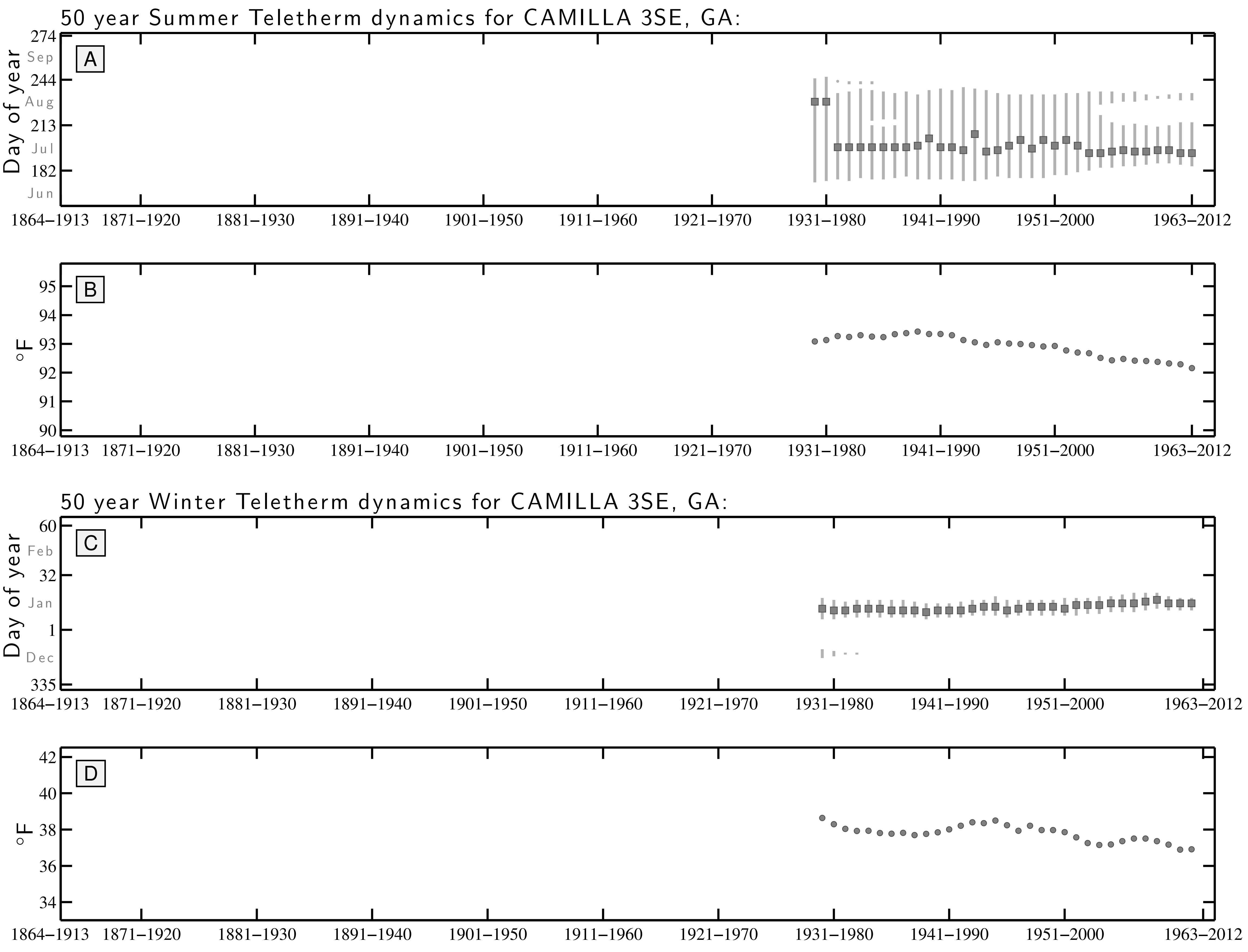
50 year Summer Teletherm dynamics for CAMILLA 3SE, GA:
A
Day of year
274
Sep
244
Aug
213
Jul
182
Jun
B
°F
95
94
93
92
91
90
50 year Winter Teletherm dynamics for CAMILLA 3SE, GA:
C
Day of year
60
Feb
32
Jan
1
Dec
335
D
°F
42
40
38
36
34
1864–1913
1871–1920
1881–1930
1891–1940
1901–1950
1911–1960
1921–1970
1931–1980
1941–1990
1951–2000
1963–2012

50 year Summer Teletherm dynamics for COVINGTON, GA:

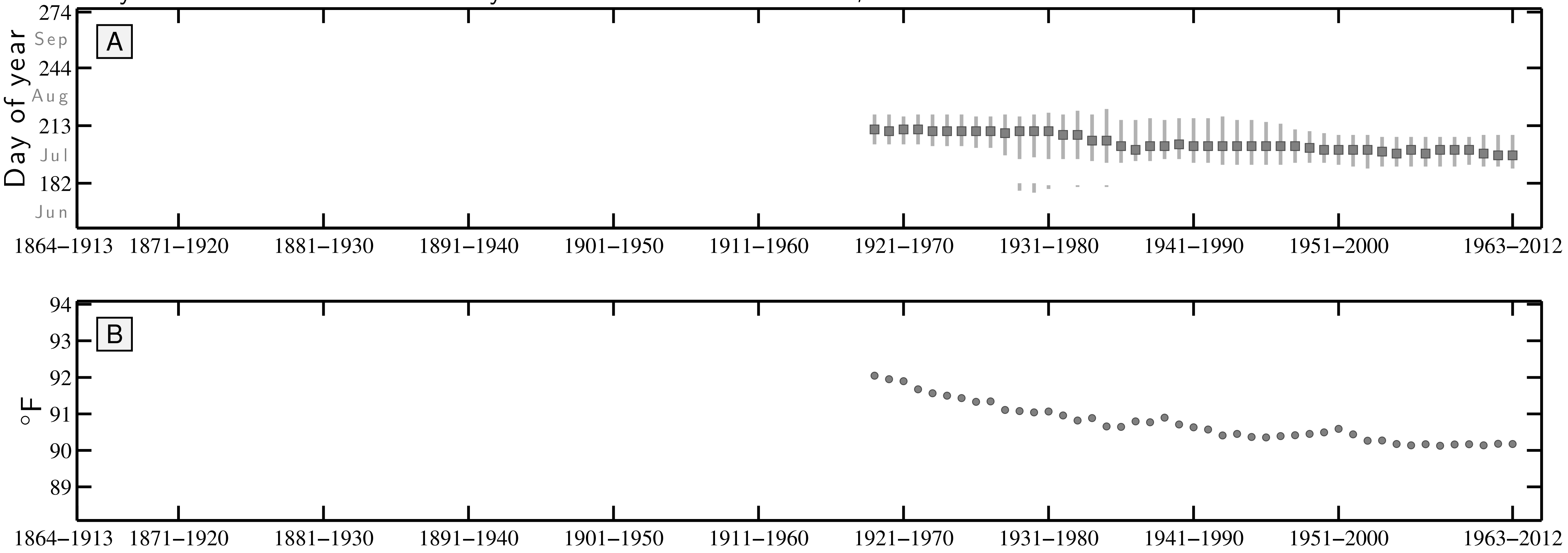

50 year Winter Teletherm dynamics for COVINGTON, GA:

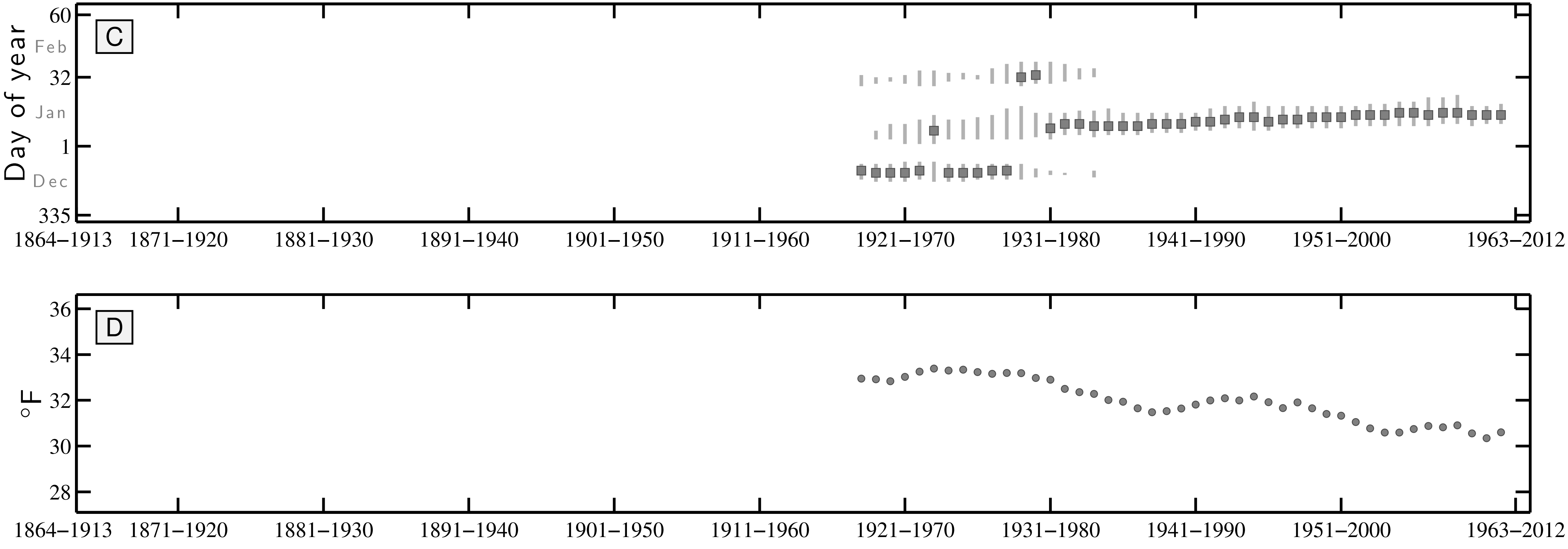

50 year Summer Teletherm dynamics for DAHLONEGA, GA:

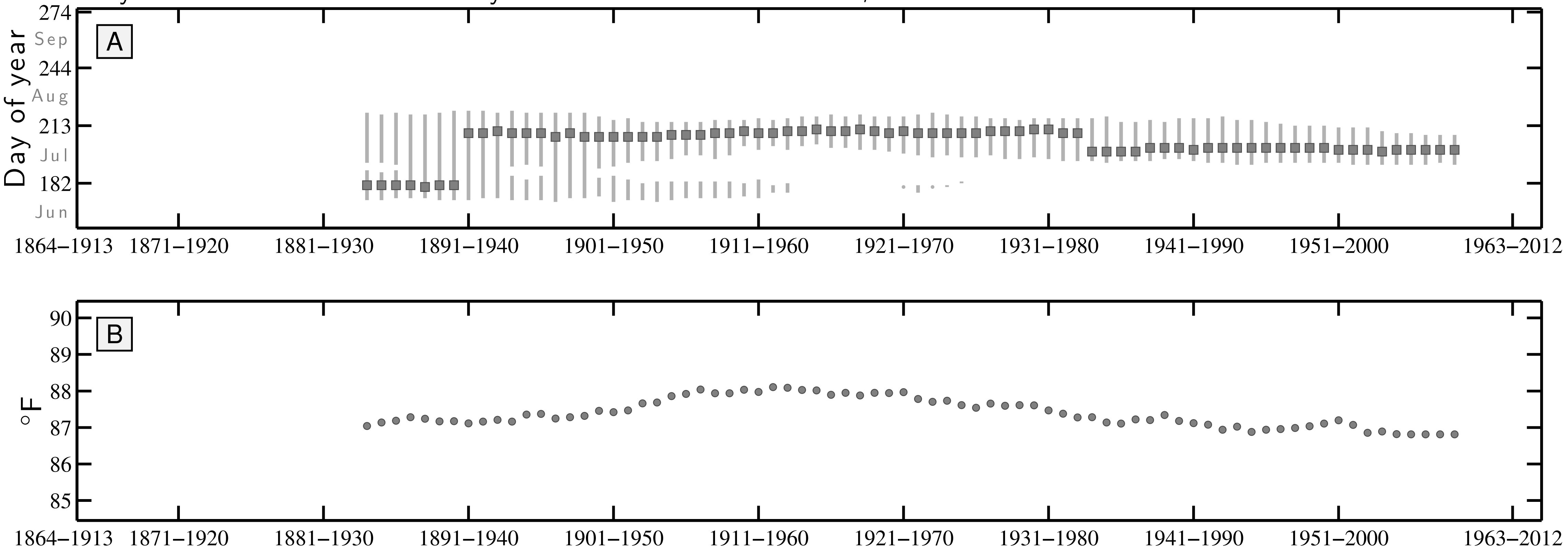

50 year Winter Teletherm dynamics for DAHLONEGA, GA:

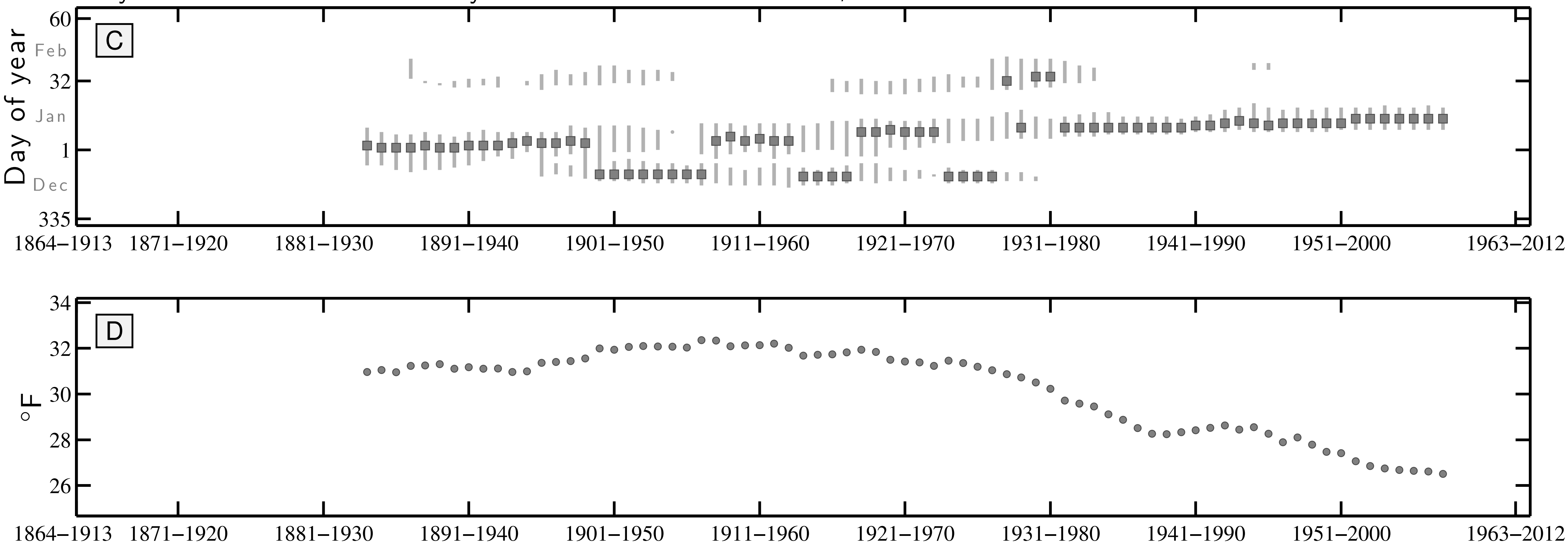

50 year Summer Teletherm dynamics for EASTMAN 1 W, GA:

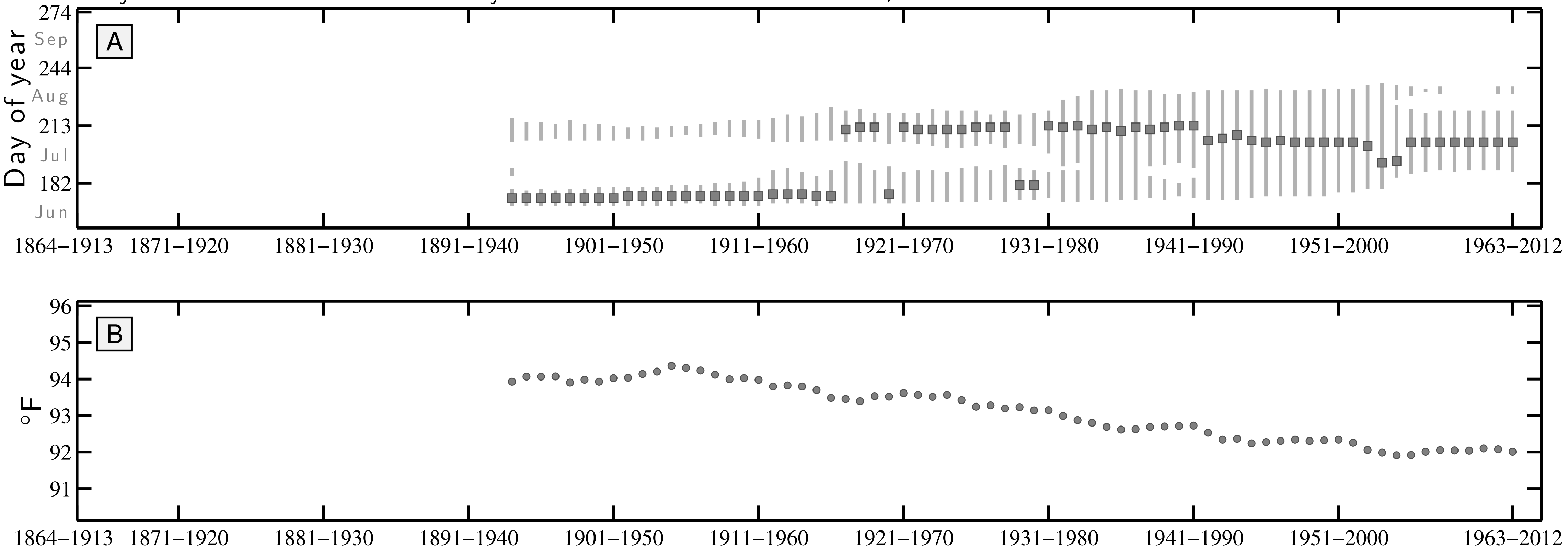

50 year Winter Teletherm dynamics for EASTMAN 1 W, GA:

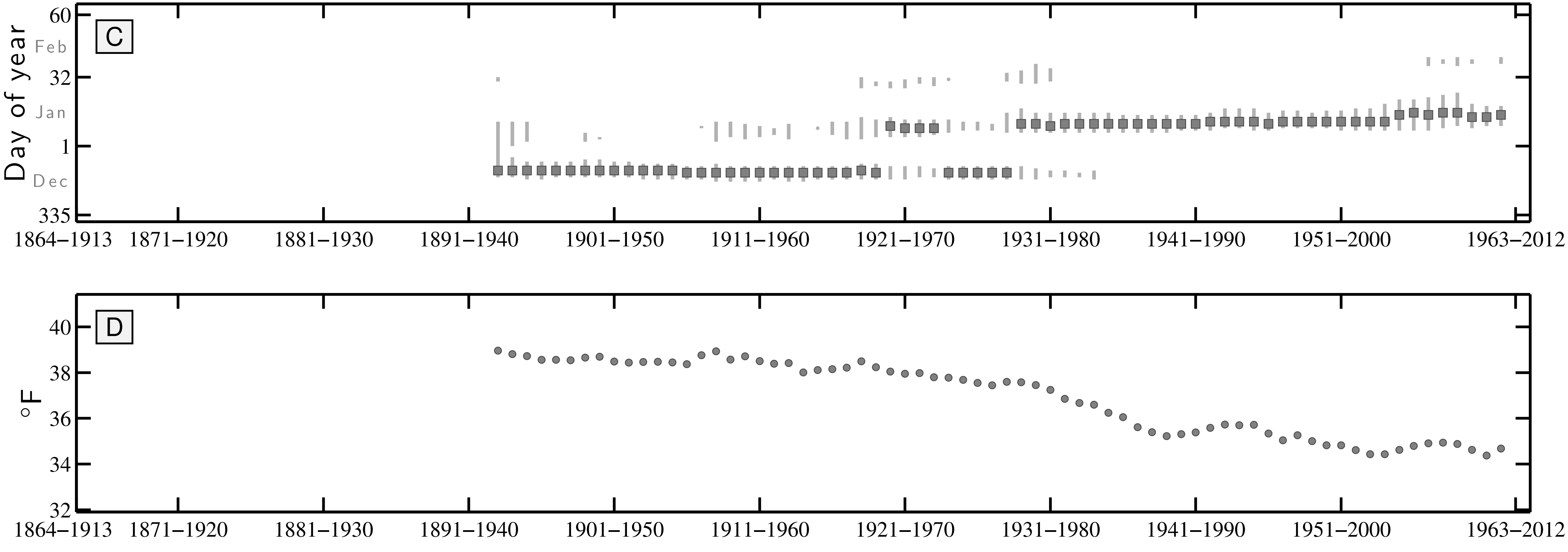

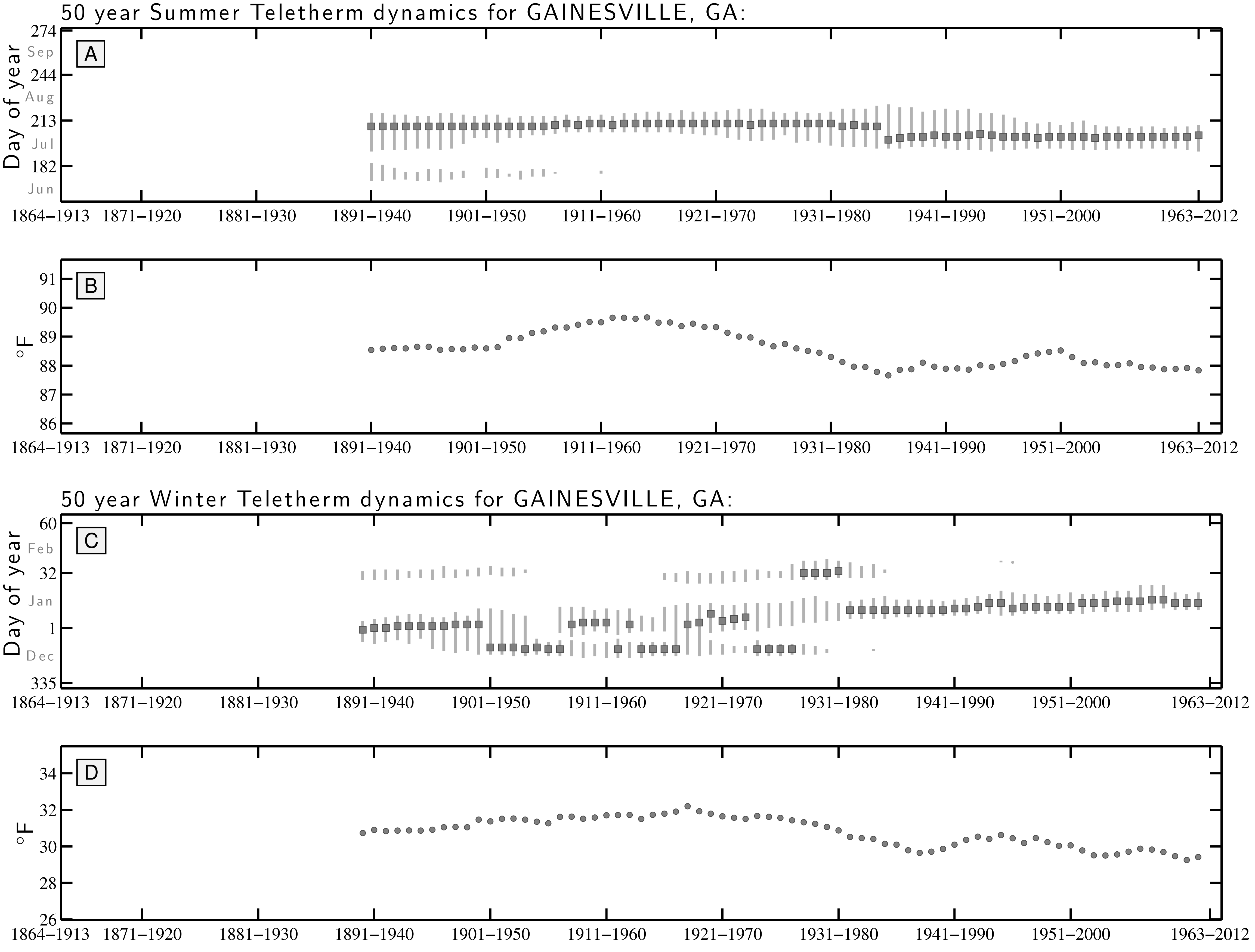
50 year Summer Teletherm dynamics for GAINESVILLE, GA:
A
Day of year
274
Sep
244
Aug
213
Jul
182
Jun
1864–1913
1871–1920
1881–1930
1891–1940
1901–1950
1911–1960
1921–1970
1931–1980
1941–1990
1951–2000
1963–2012
B
°F
91
90
89
88
87
86
50 year Winter Teletherm dynamics for GAINESVILLE, GA:
C
Day of year
60
Feb
32
Jan
1
Dec
335
D
°F
34
32
30
28
26

50 year Summer Teletherm dynamics for GLENNVILLE 3NW, GA:

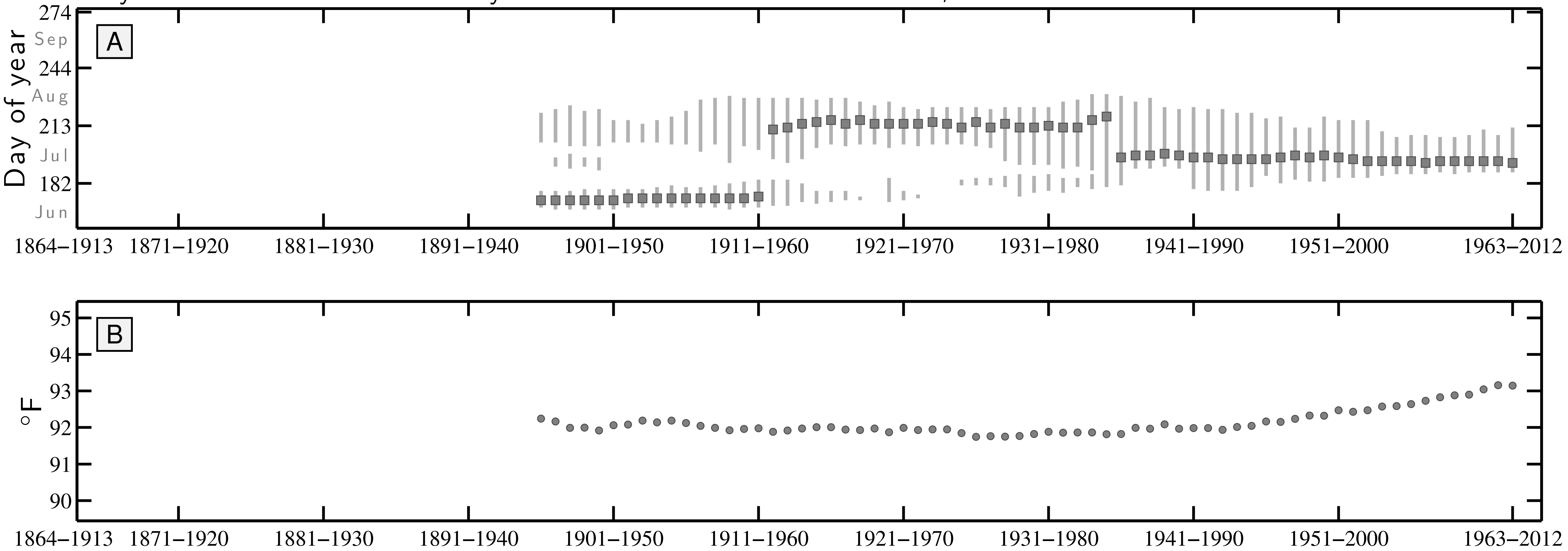

50 year Winter Teletherm dynamics for GLENNVILLE 3NW, GA:

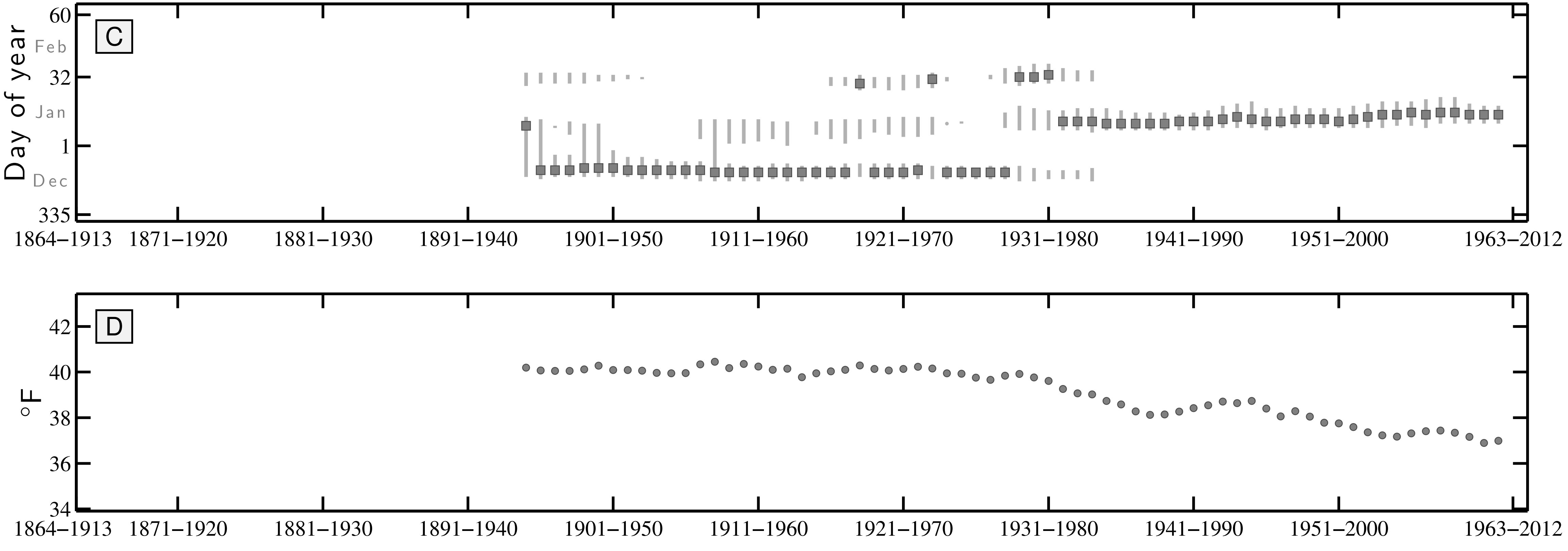

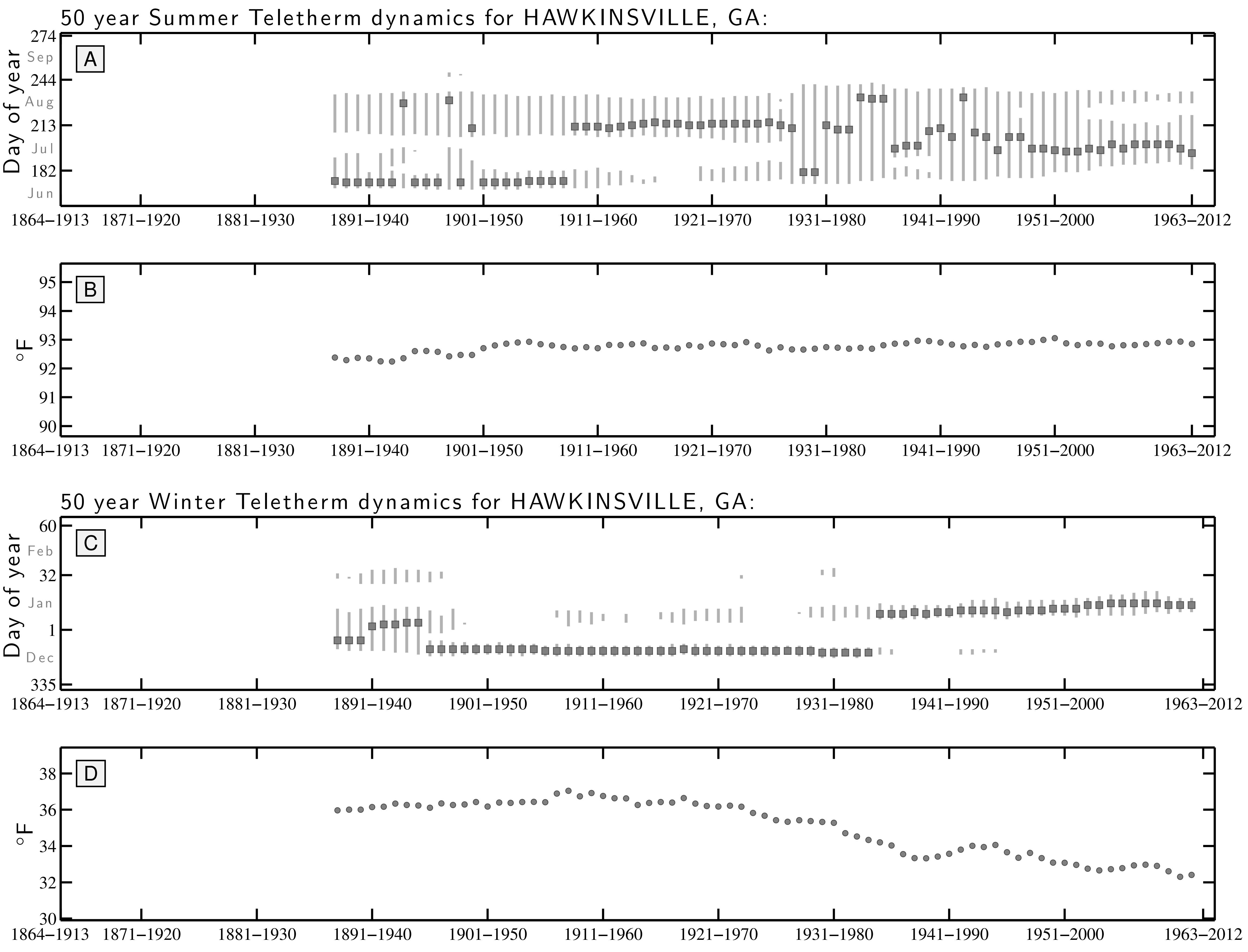
50 year Summer Teletherm dynamics for HAWKINSVILLE, GA:
A
B
Day of year
°F
274
Sep
244
Aug
213
Jul
182
Jun
95
94
93
92
91
90
1864–1913
1871–1920
1881–1930
1891–1940
1901–1950
1911–1960
1921–1970
1931–1980
1941–1990
1951–2000
1963–2012
50 year Winter Teletherm dynamics for HAWKINSVILLE, GA:
C
D
60
Feb
32
Jan
1
Dec
335
38
36
34
32
30

50 year Summer Teletherm dynamics for MILLEDGEVILLE, GA:

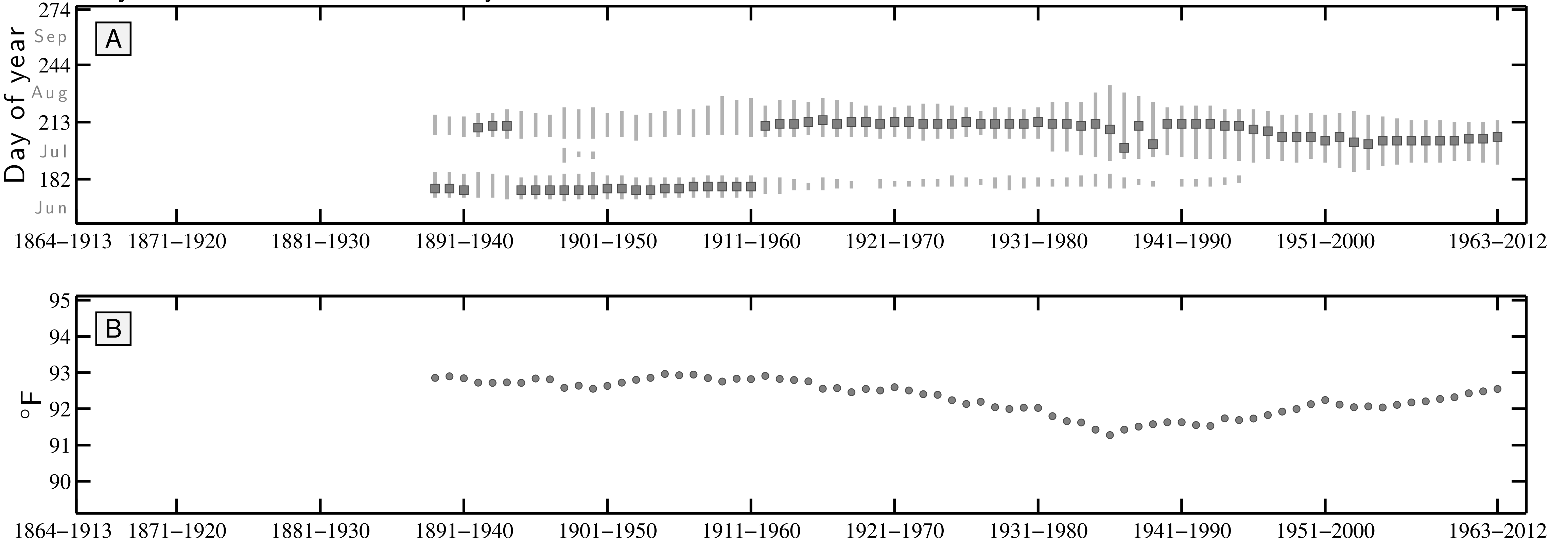

50 year Winter Teletherm dynamics for MILLEDGEVILLE, GA:

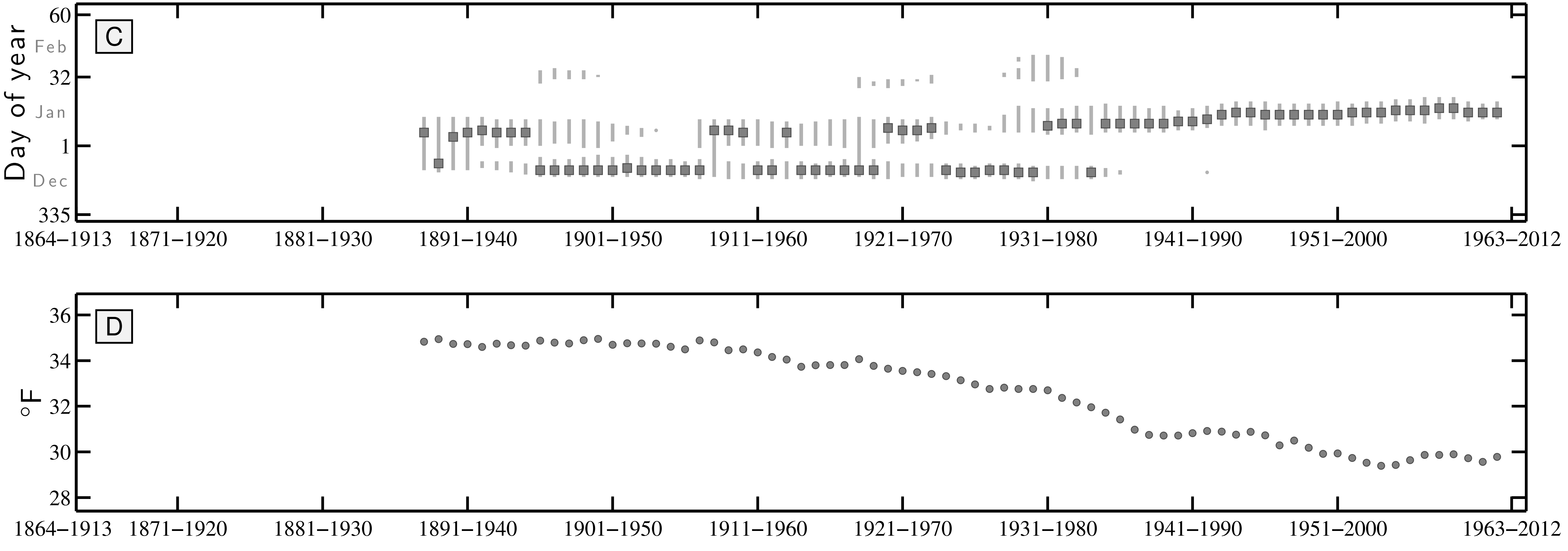

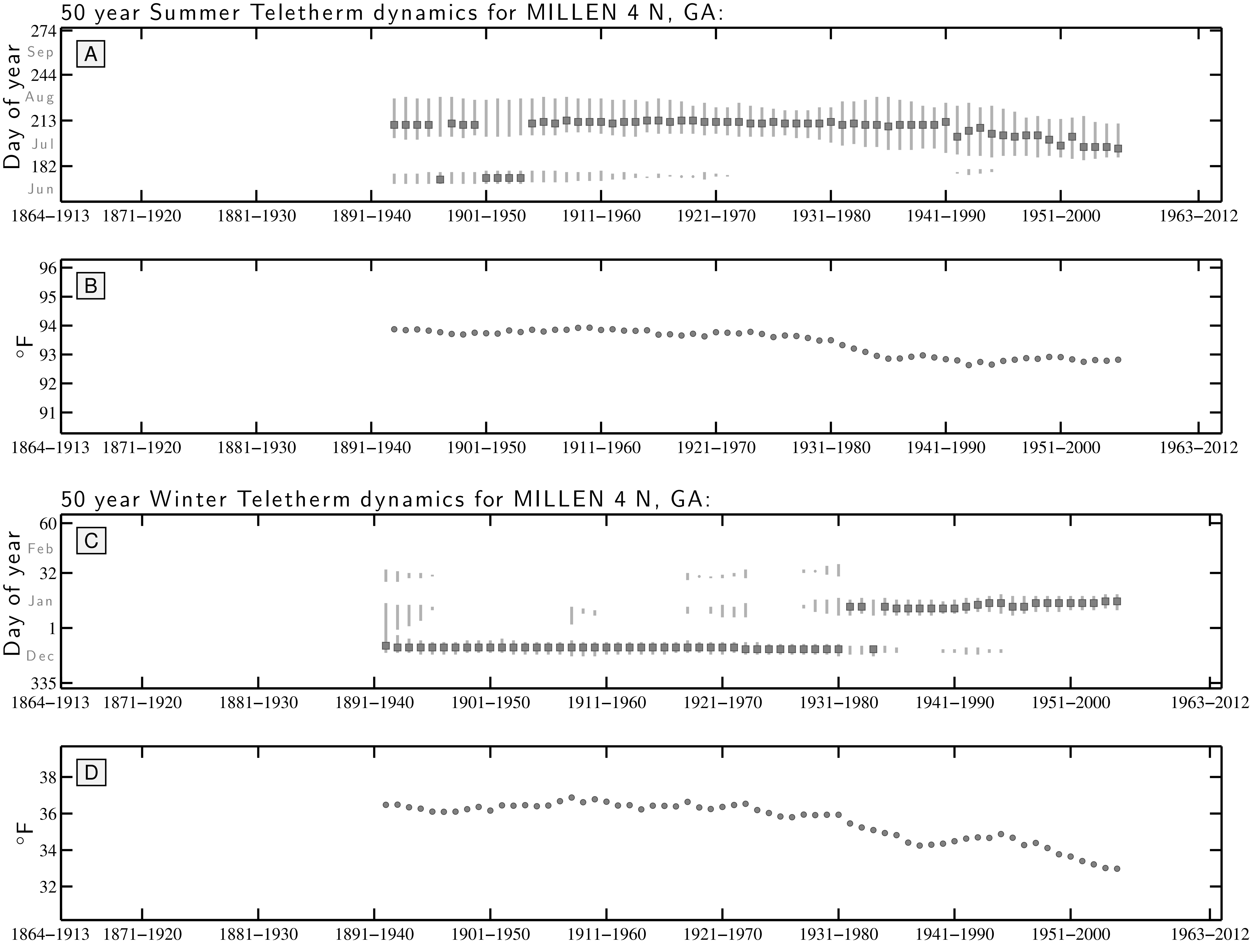

50 year Summer Teletherm dynamics for MILLEN 4 N, GA:
A
B
Day of year
°F
Sep
Aug
Jul
Jun
274
244
213
182
96
95
94
93
92
91
1864–1913
1871–1920
1881–1930
1891–1940
1901–1950
1911–1960
1921–1970
1931–1980
1941–1990
1951–2000
1963–2012
50 year Winter Teletherm dynamics for MILLEN 4 N, GA:
C
D
Feb
Jan
Dec
60
32
1
335
38
36
34
32

50 year Summer Teletherm dynamics for NEWNAN 5N, GA:

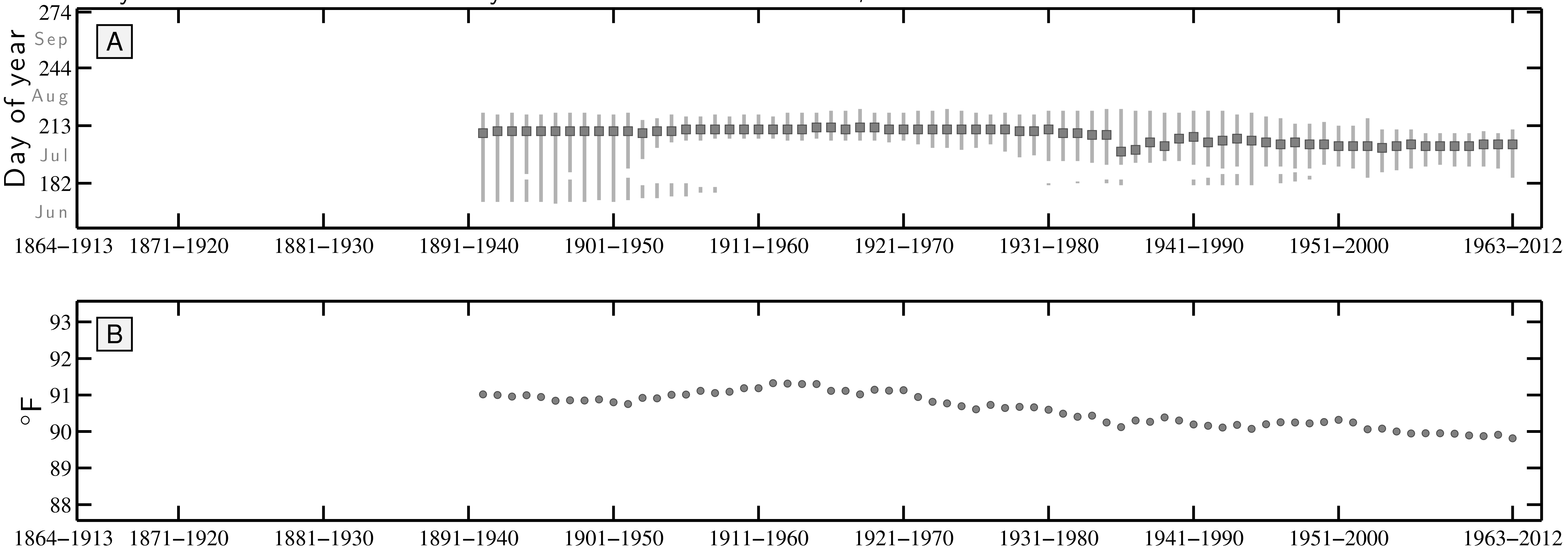

50 year Winter Teletherm dynamics for NEWNAN 5N, GA:

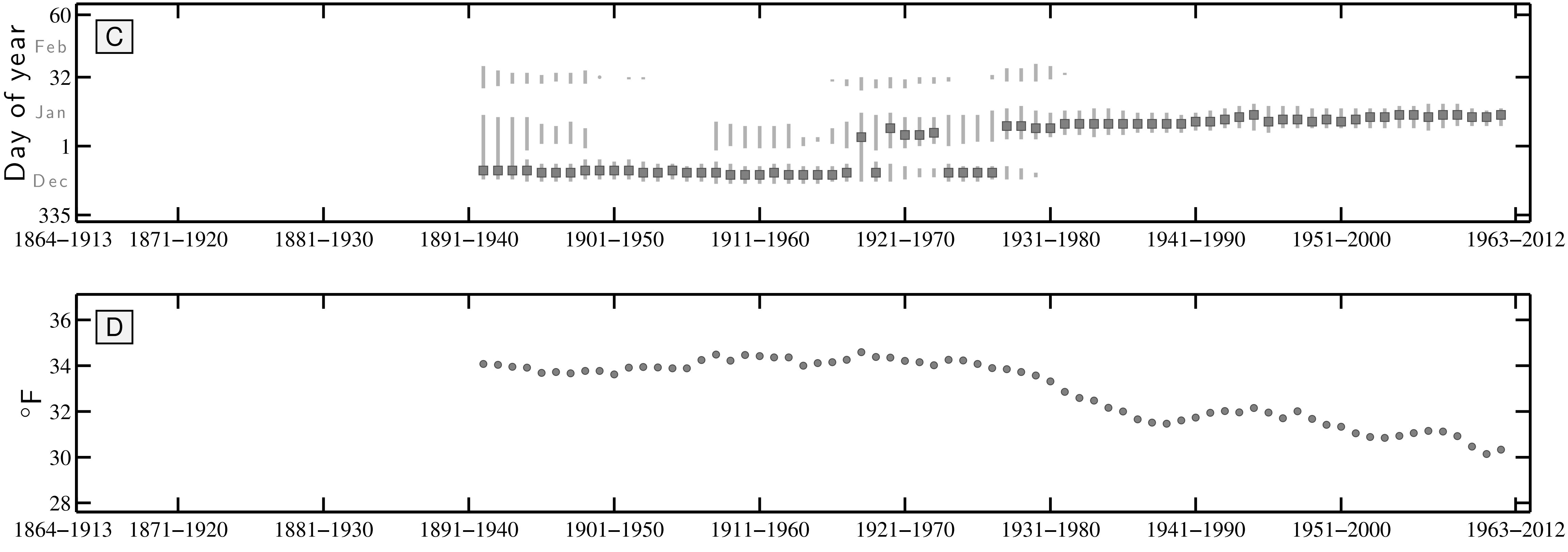

50 year Summer Teletherm dynamics for QUITMAN 2 NW, GA:

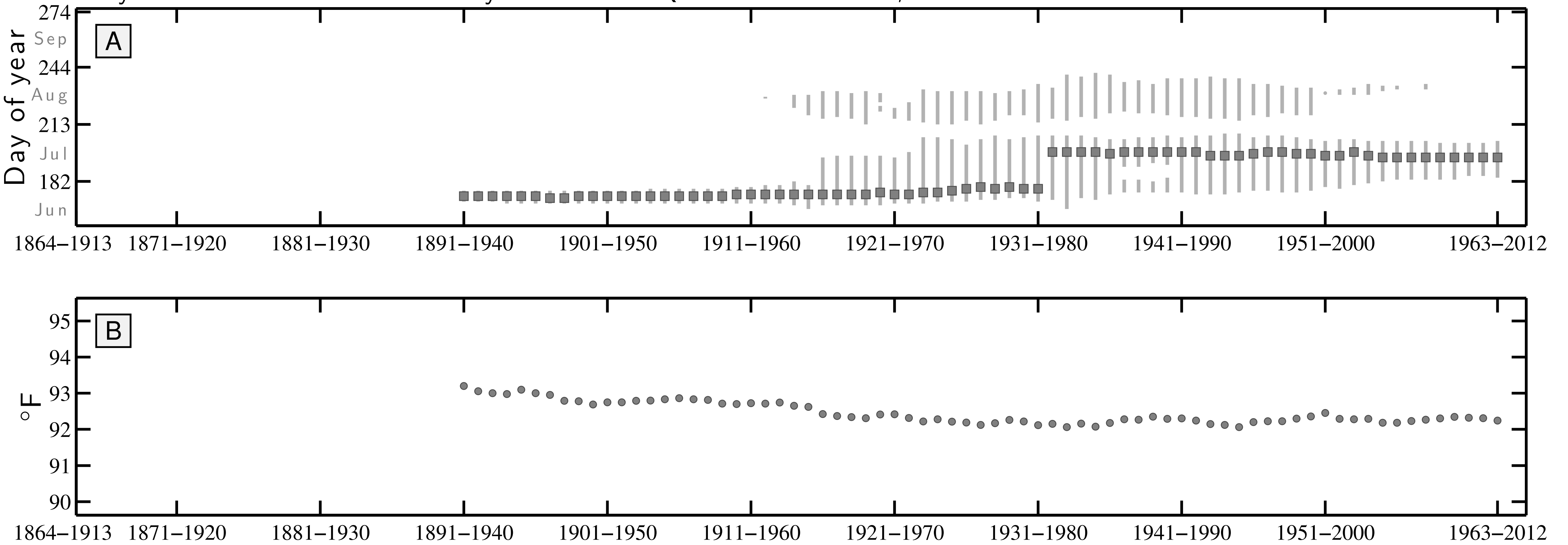

50 year Winter Teletherm dynamics for QUITMAN 2 NW, GA:

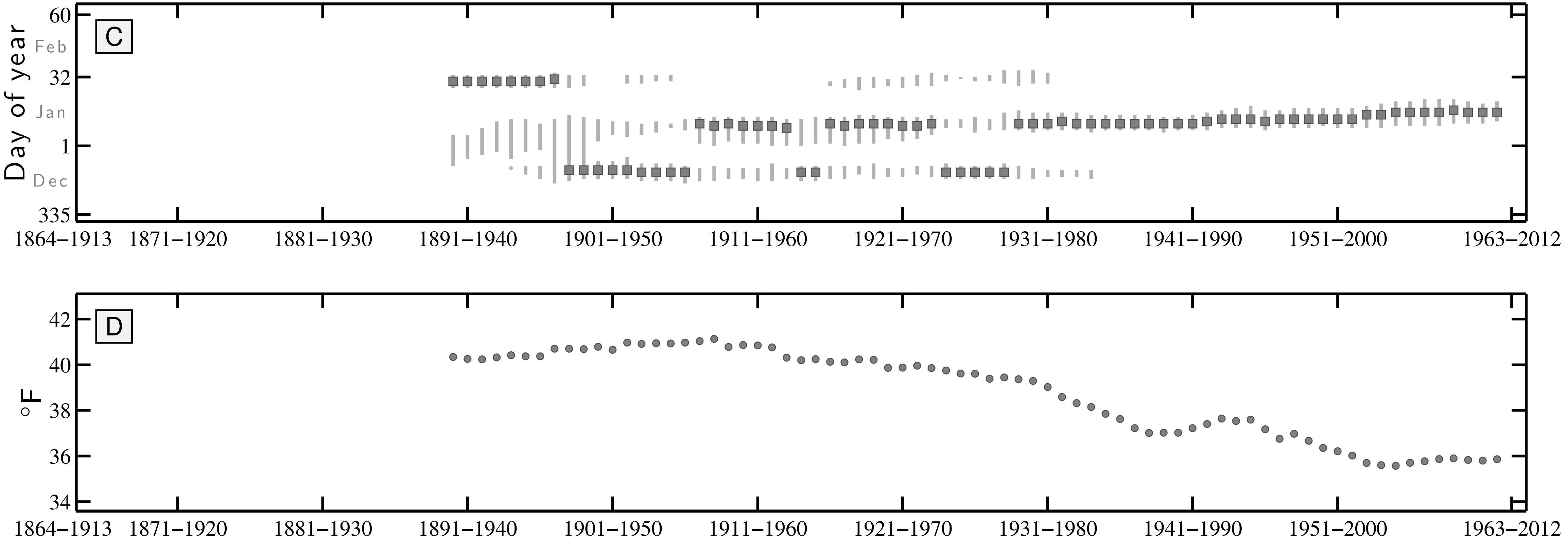

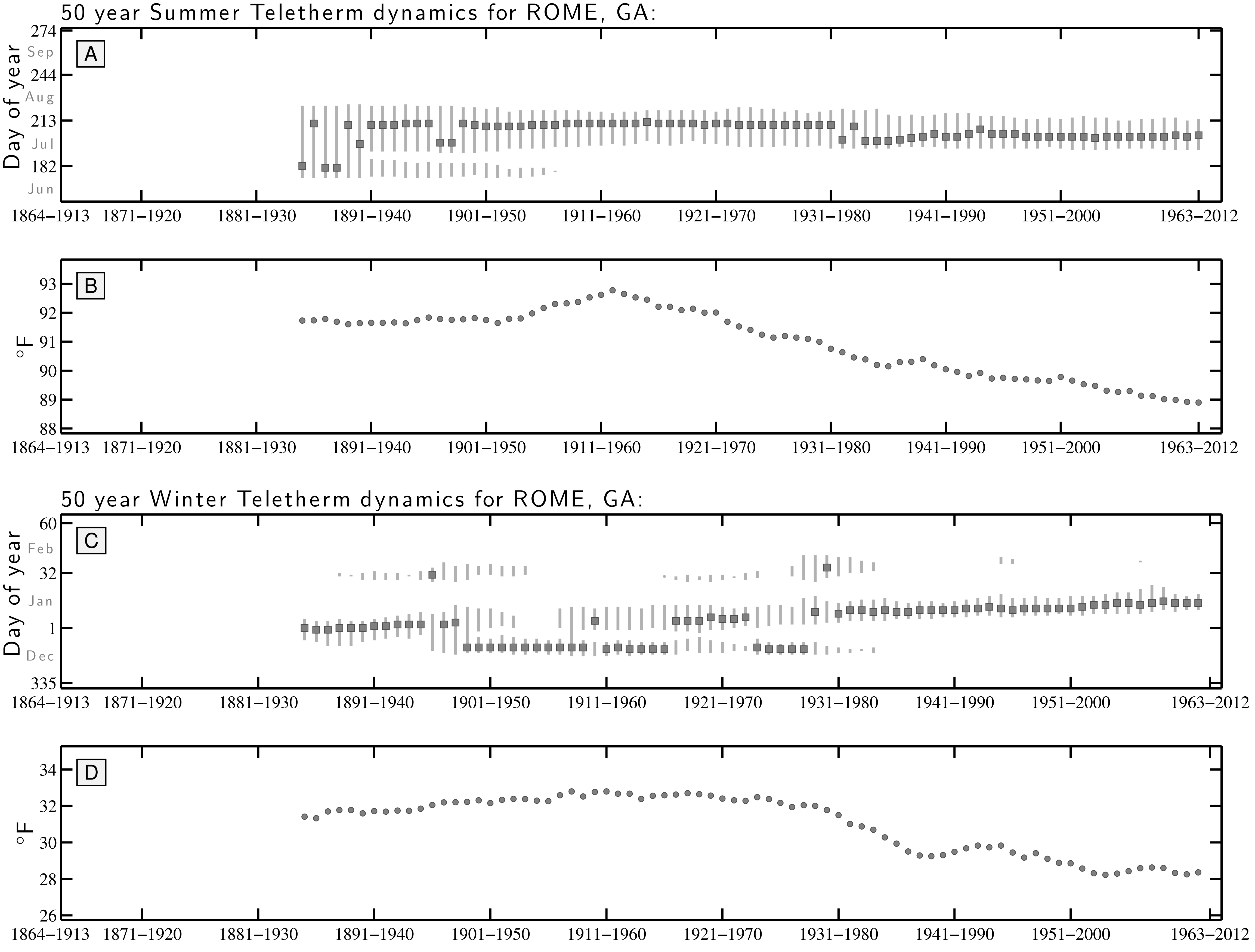

50 year Summer Teletherm dynamics for ROME, GA:
A
Day of year
274
Sep
244
Aug
213
Jul
182
Jun
1864–1913
1871–1920
1881–1930
1891–1940
1901–1950
1911–1960
1921–1970
1931–1980
1941–1990
1951–2000
1963–2012
B
°F
93
92
91
90
89
88
50 year Winter Teletherm dynamics for ROME, GA:
C
Day of year
60
Feb
32
Jan
1
Dec
335
D
°F
34
32
30
28
26

50 year Summer Teletherm dynamics for SAVANNAH INTL AP, GA:

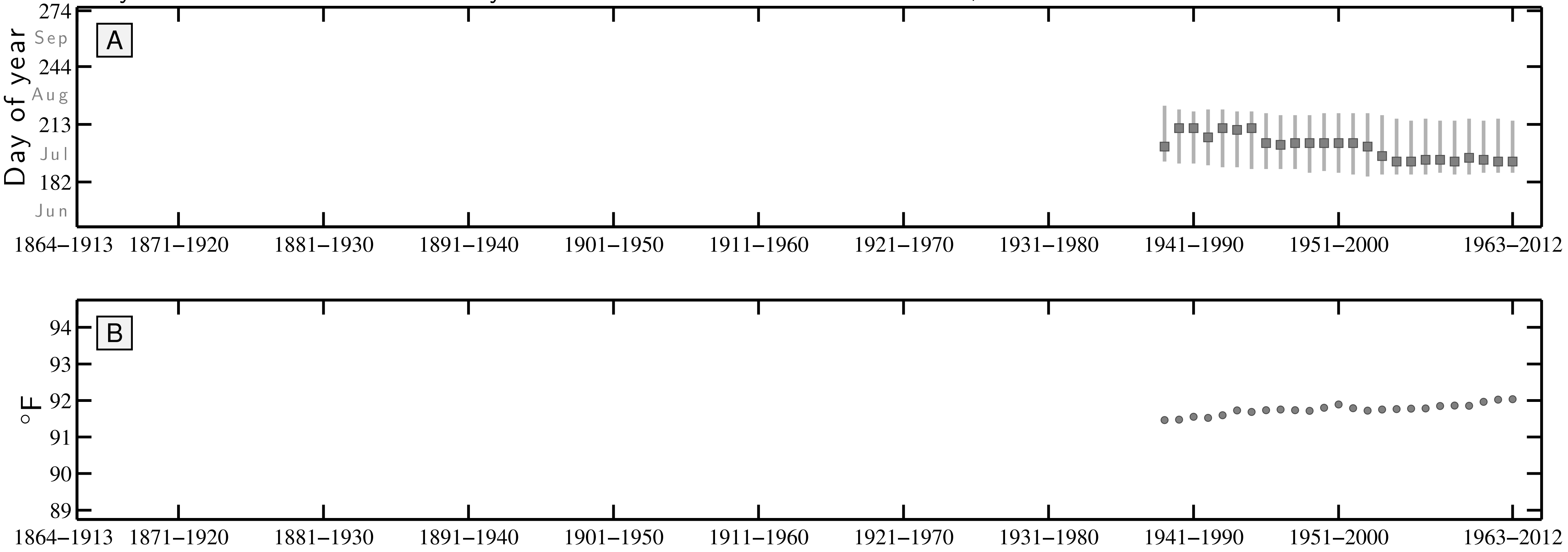

50 year Winter Teletherm dynamics for SAVANNAH INTL AP, GA:

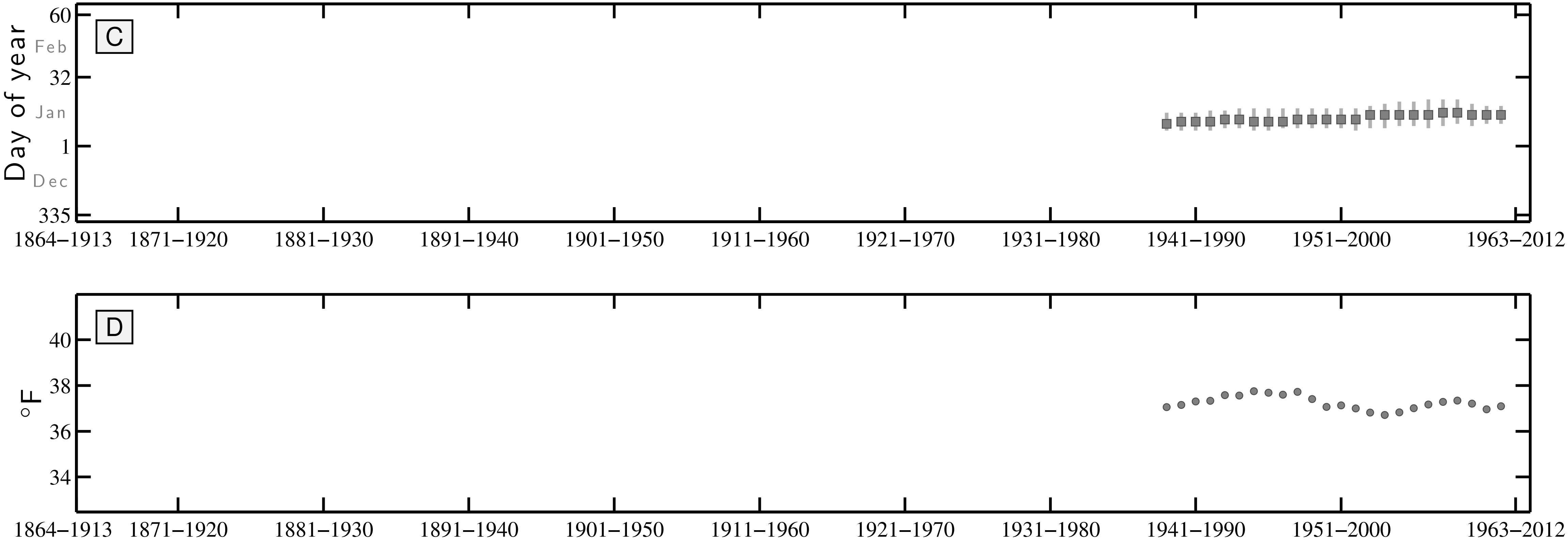

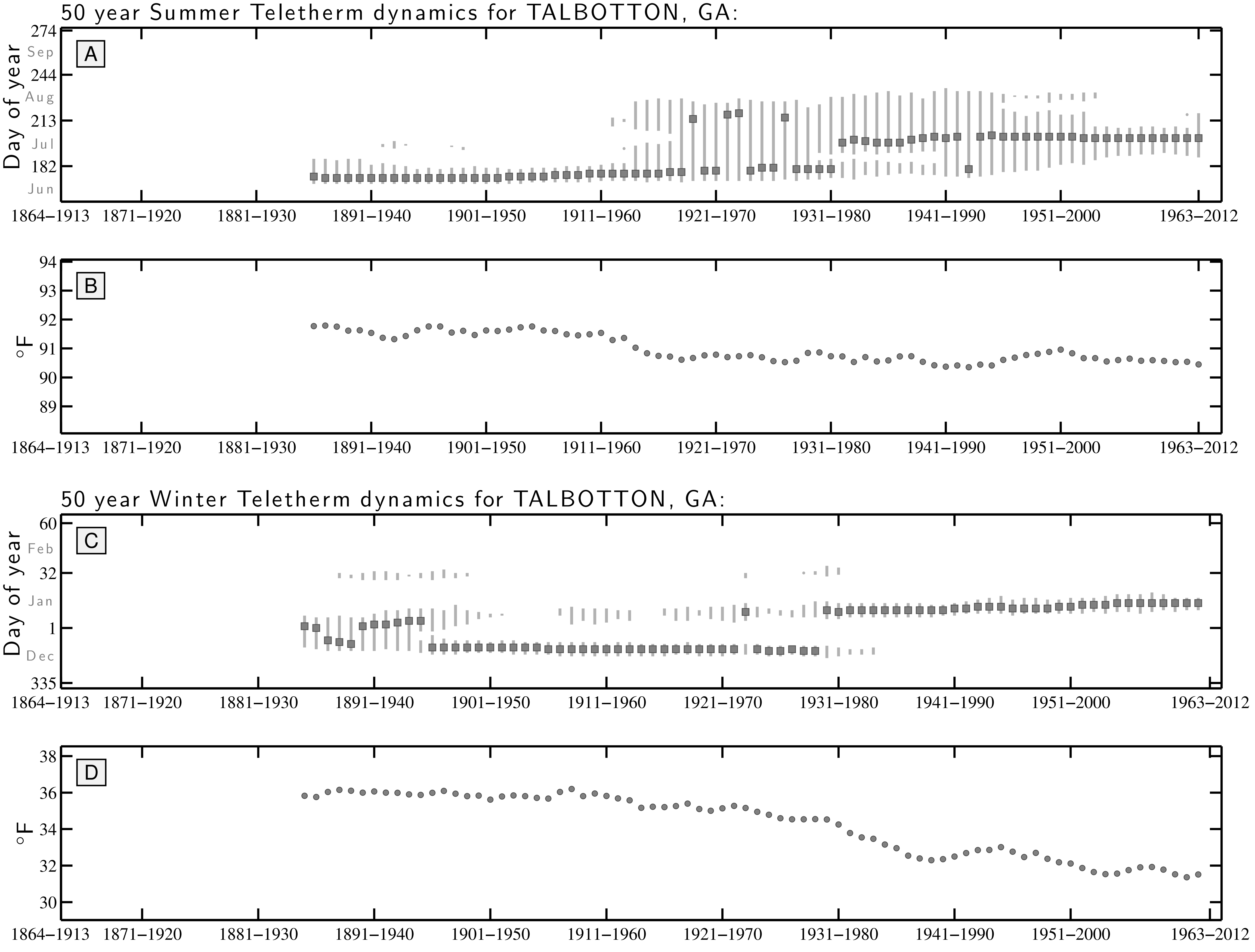

50 year Summer Teletherm dynamics for TALBOTTON, GA:
A
Day of year
274
Sep
244
Aug
213
Jul
182
Jun
1864–1913
1871–1920
1881–1930
1891–1940
1901–1950
1911–1960
1921–1970
1931–1980
1941–1990
1951–2000
1963–2012
B
°F
94
93
92
91
90
89
50 year Winter Teletherm dynamics for TALBOTTON, GA:
C
60
Feb
32
Jan
1
Dec
335
D
38
36
34
32
30

50 year Summer Teletherm dynamics for TIFTON, GA:

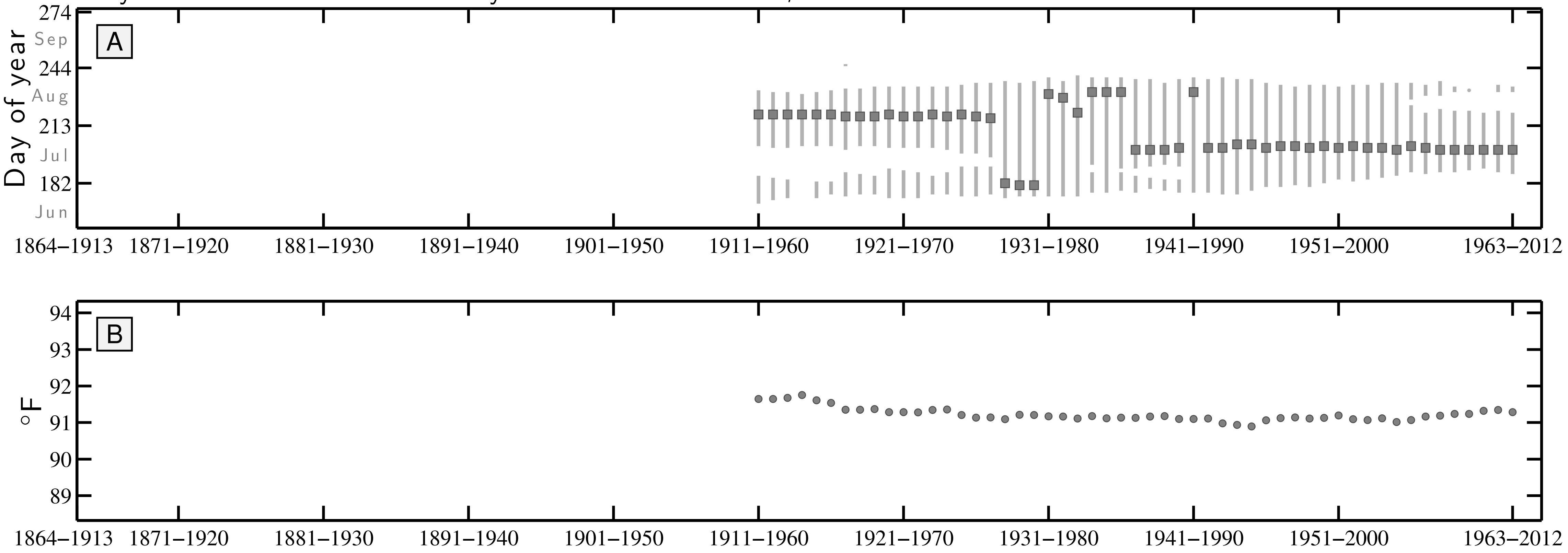

50 year Winter Teletherm dynamics for TIFTON, GA:

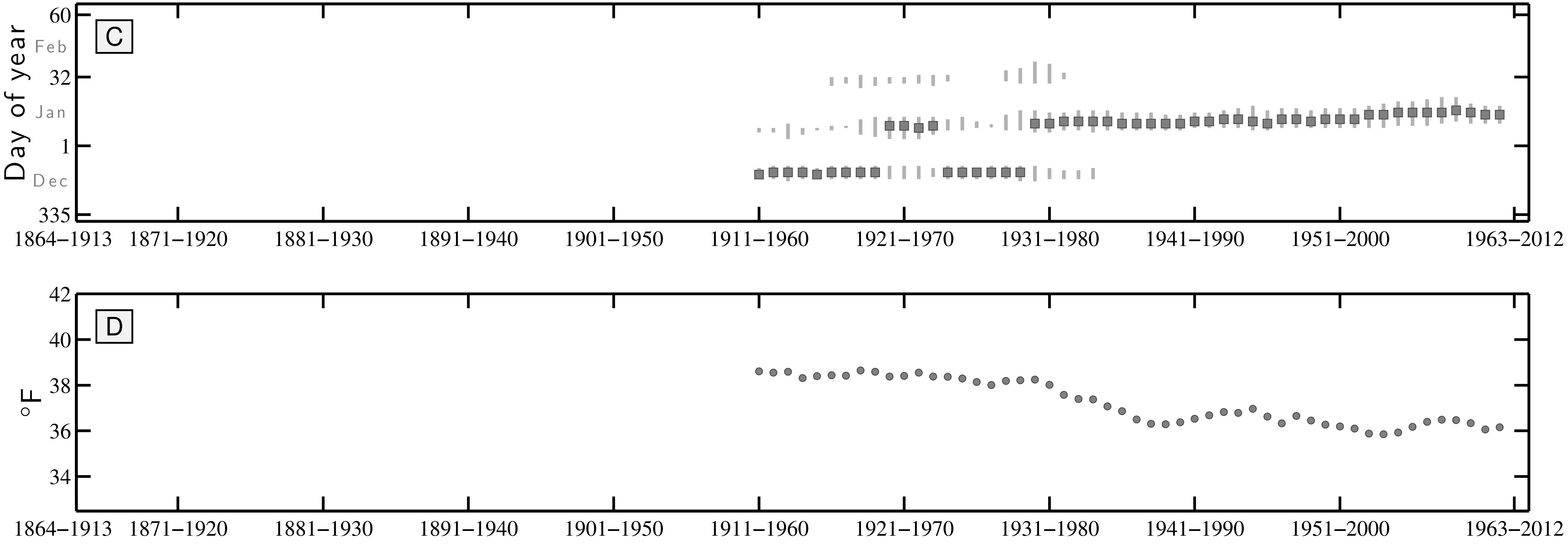

50 year Summer Teletherm dynamics for TOCCOA, GA:

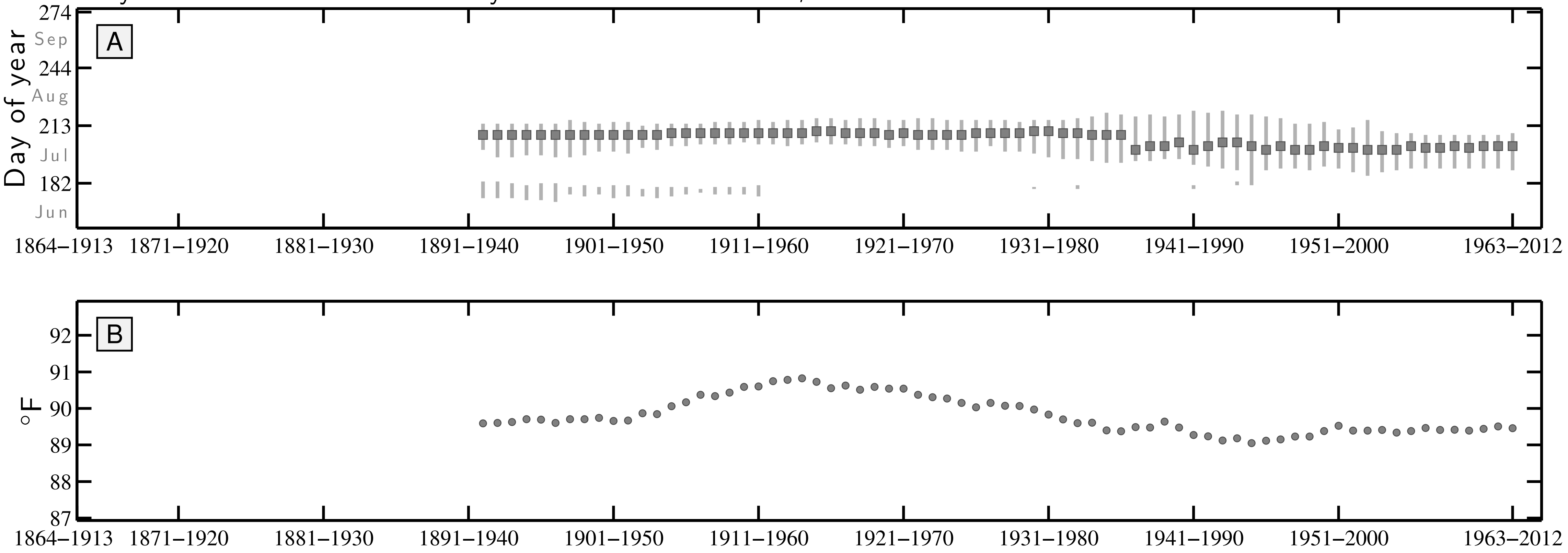

50 year Winter Teletherm dynamics for TOCCOA, GA:

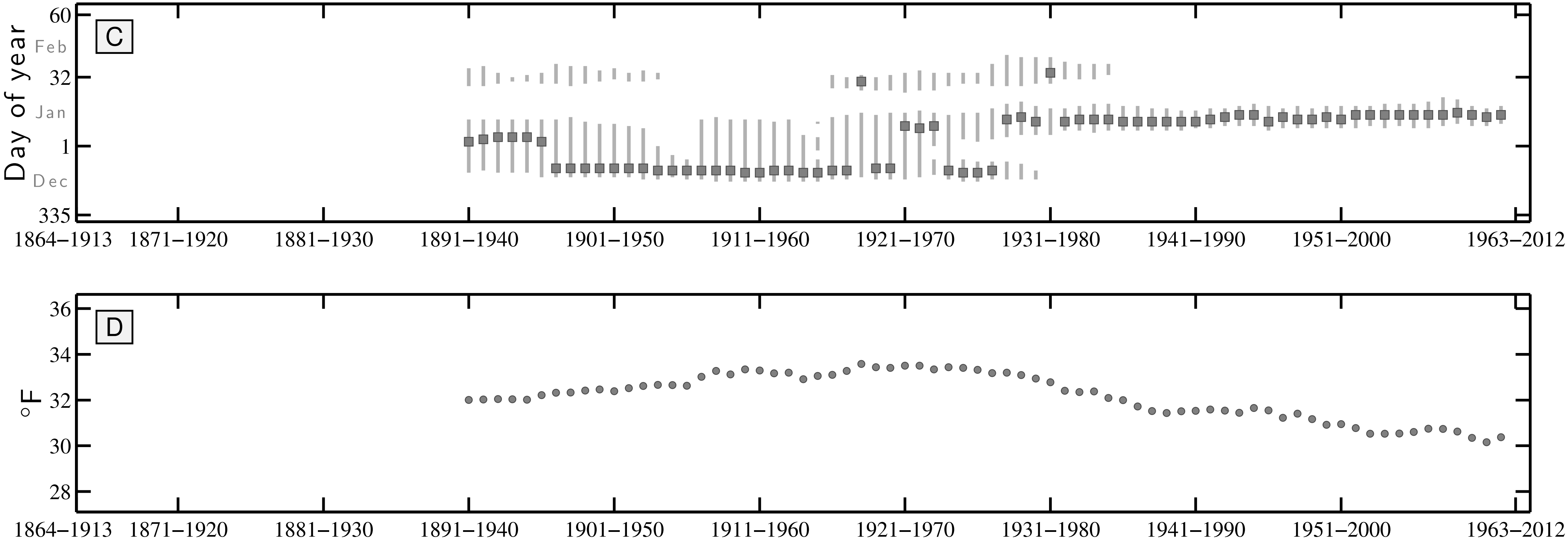

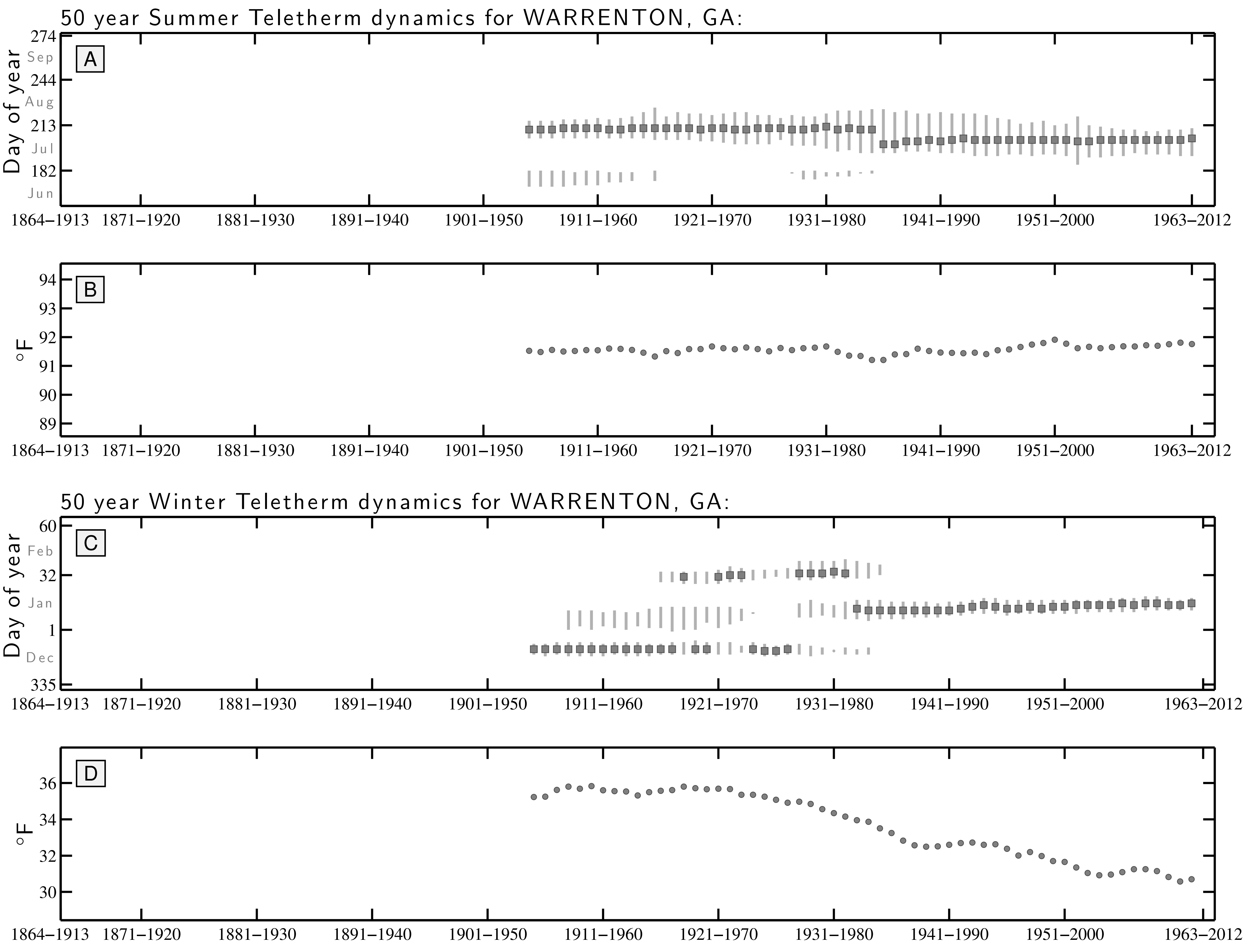
50 year Summer Teletherm dynamics for WARRENTON, GA:
A
B
Day of year
274
244
213
182
Sep
Aug
Jul
Jun
°F
94
93
92
91
90
89
50 year Winter Teletherm dynamics for WARRENTON, GA:
C
D
60
32
1
335
Feb
Jan
Dec
36
34
32
30
1864–1913
1871–1920
1881–1930
1891–1940
1901–1950
1911–1960
1921–1970
1931–1980
1941–1990
1951–2000
1963–2012

50 year Summer Teletherm dynamics for WASHINGTON 2 ESE, GA:

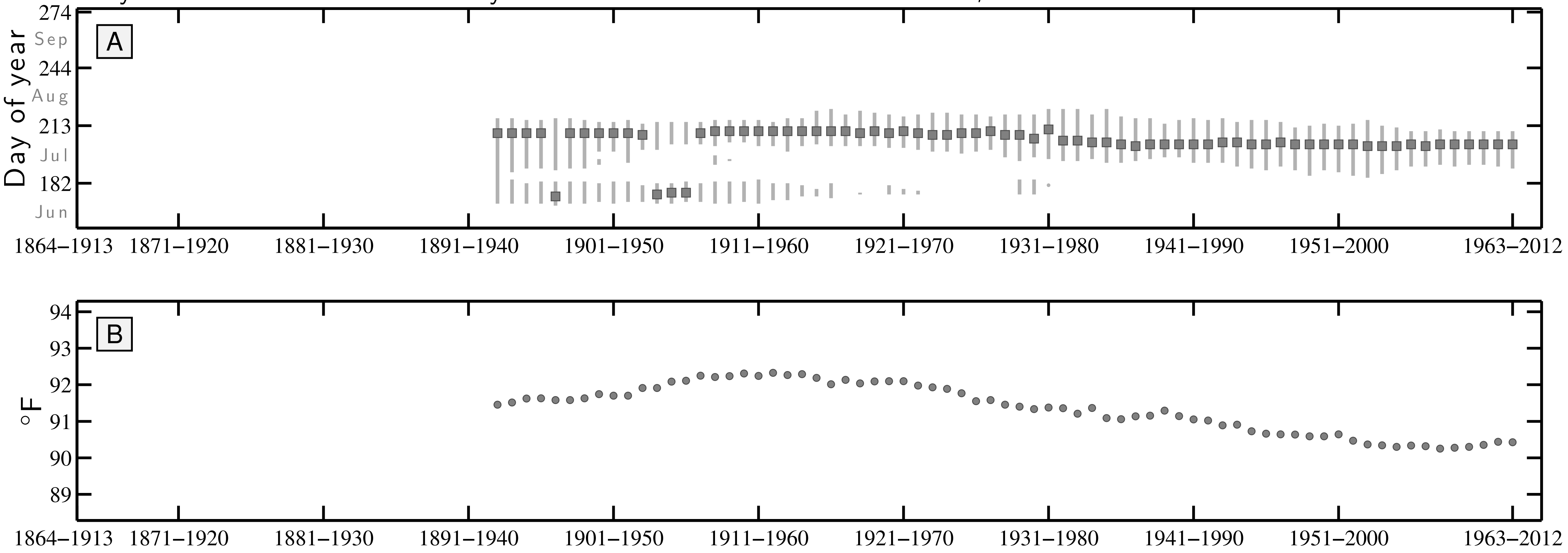

50 year Winter Teletherm dynamics for WASHINGTON 2 ESE, GA:

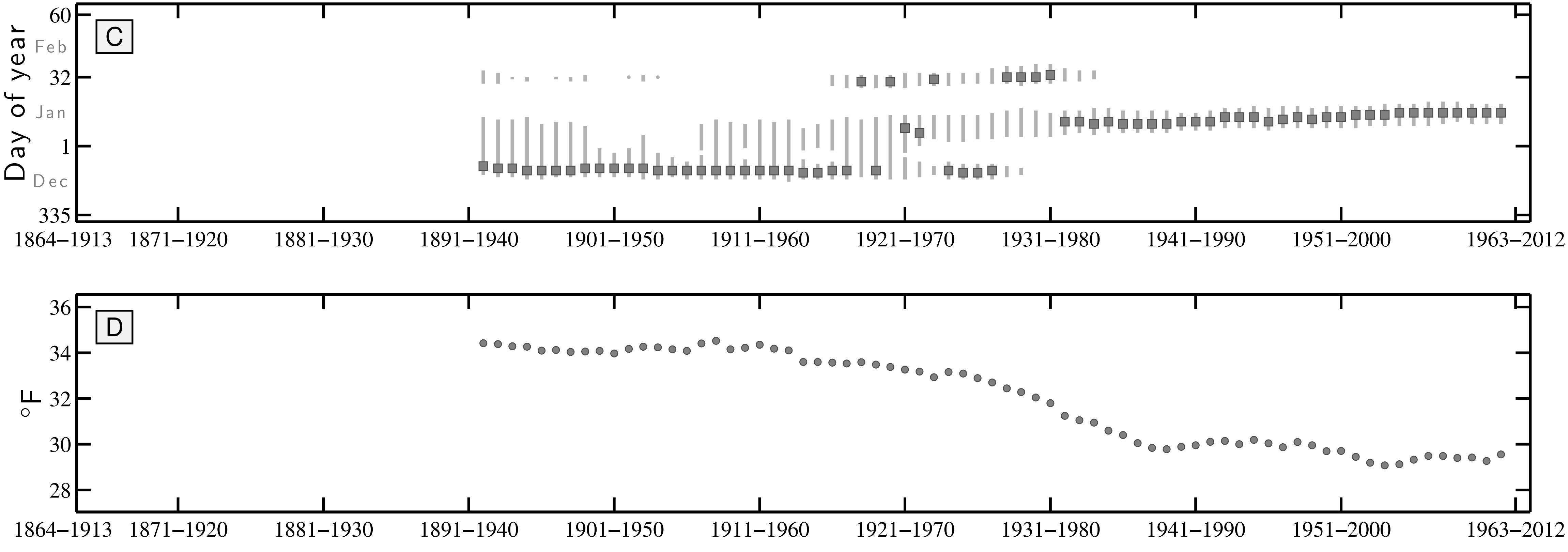

50 year Summer Teletherm dynamics for WAYCROSS 4 NE, GA:

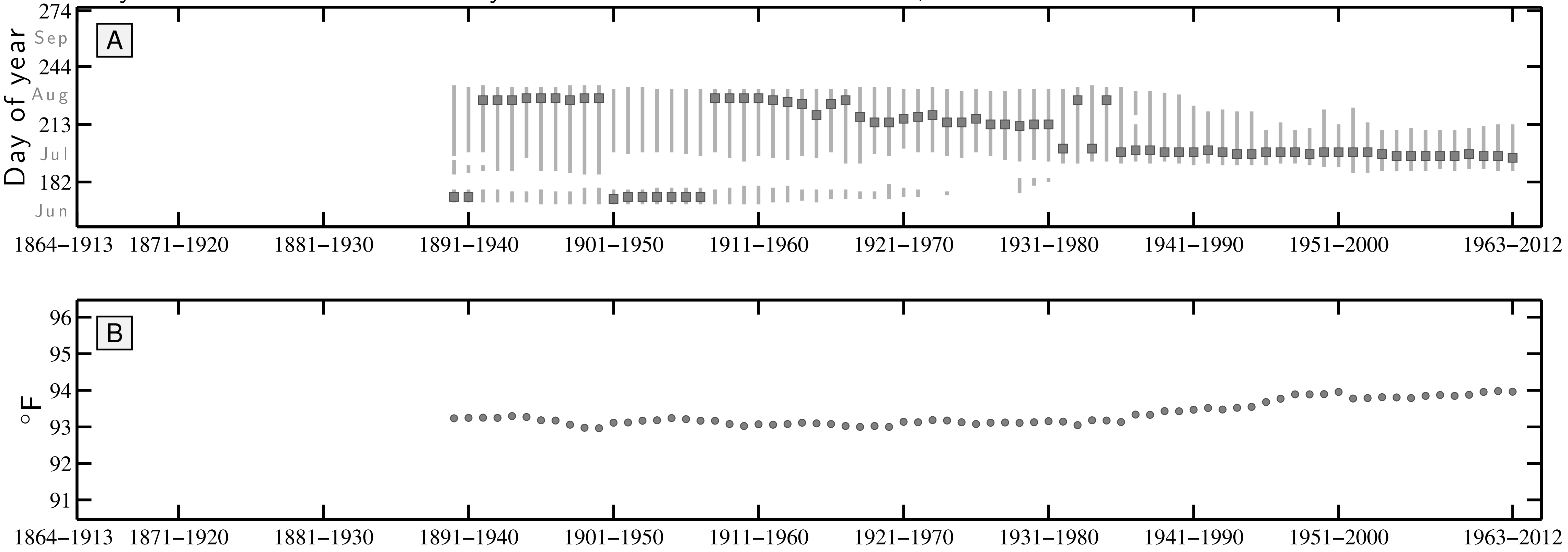

50 year Winter Teletherm dynamics for WAYCROSS 4 NE, GA:

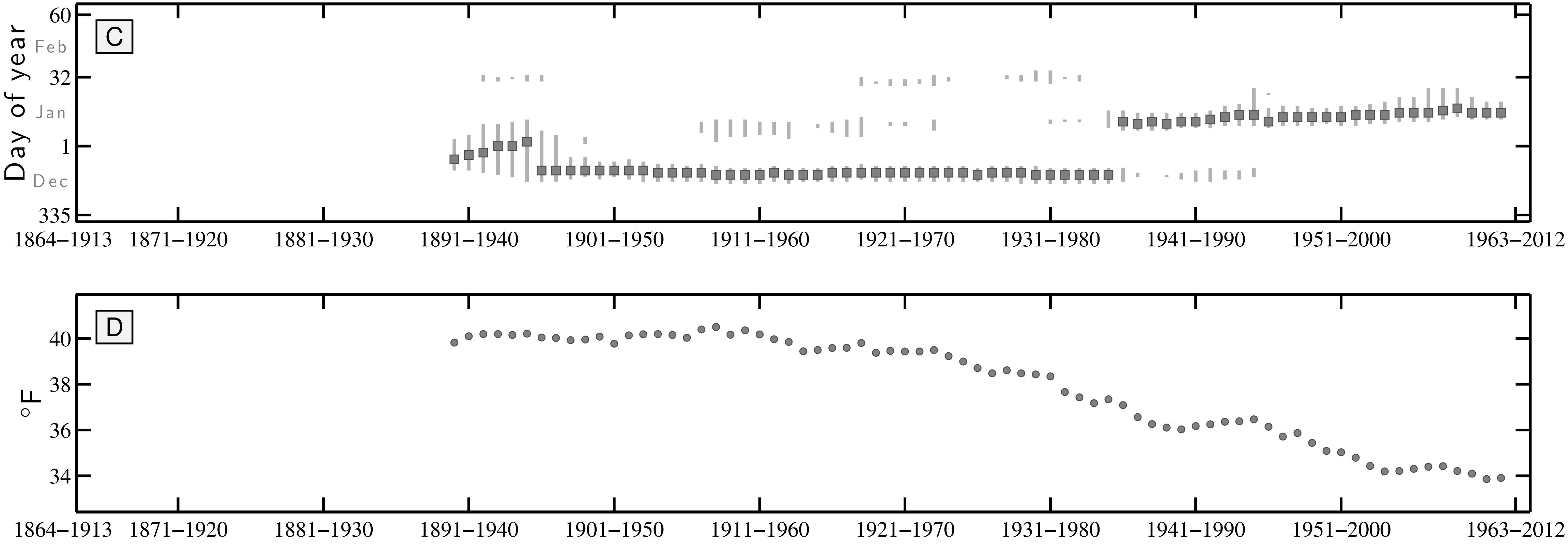

50 year Summer Teletherm dynamics for WEST POINT, GA:

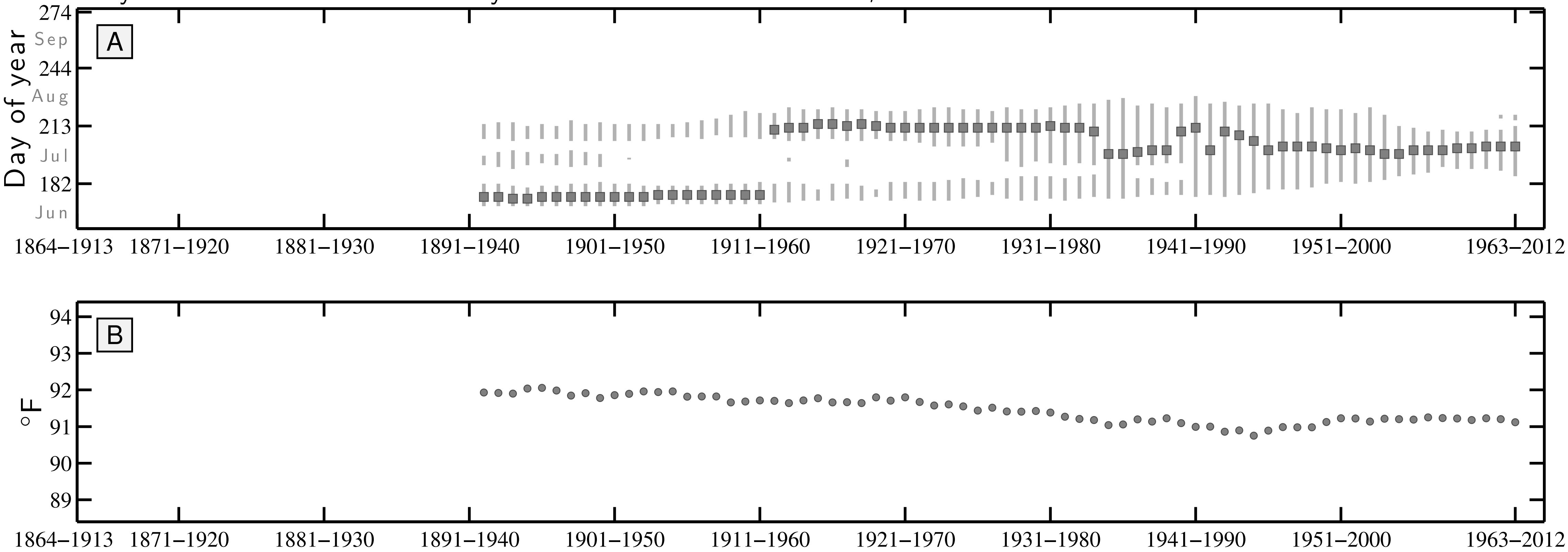

50 year Winter Teletherm dynamics for WEST POINT, GA:

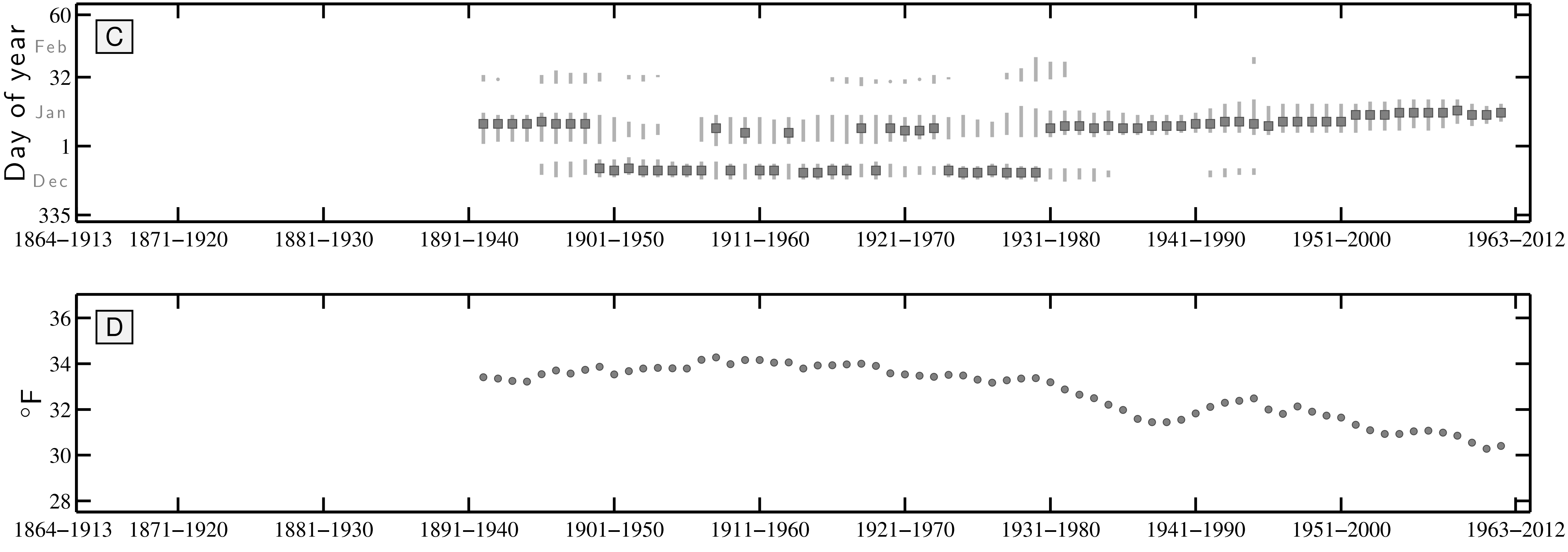

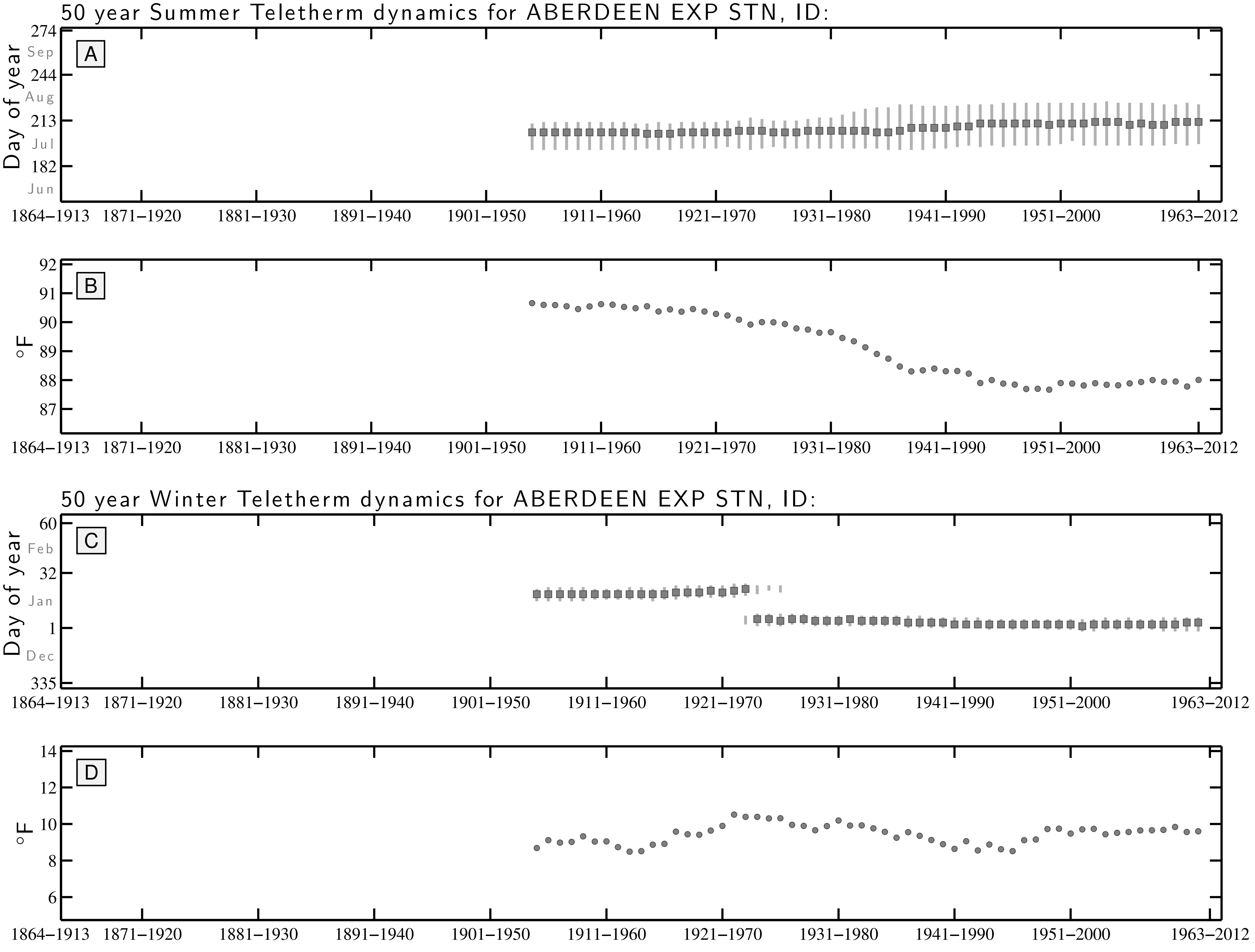
50 year Summer Teletherm dynamics for ABERDEEN EXP STN, ID:
A
Day of year
274
Sep
244
Aug
213
Jul
182
Jun
B
92
91
90
89
88
87
°F
50 year Winter Teletherm dynamics for ABERDEEN EXP STN, ID:
C
Day of year
60
Feb
32
Jan
1
Dec
335
D
14
12
10
8
6
°F
1864–1913
1871–1920
1881–1930
1891–1940
1901–1950
1911–1960
1921–1970
1931–1980
1941–1990
1951–2000
1963–2012

50 year Summer Teletherm dynamics for ARROWROCK DAM, ID:

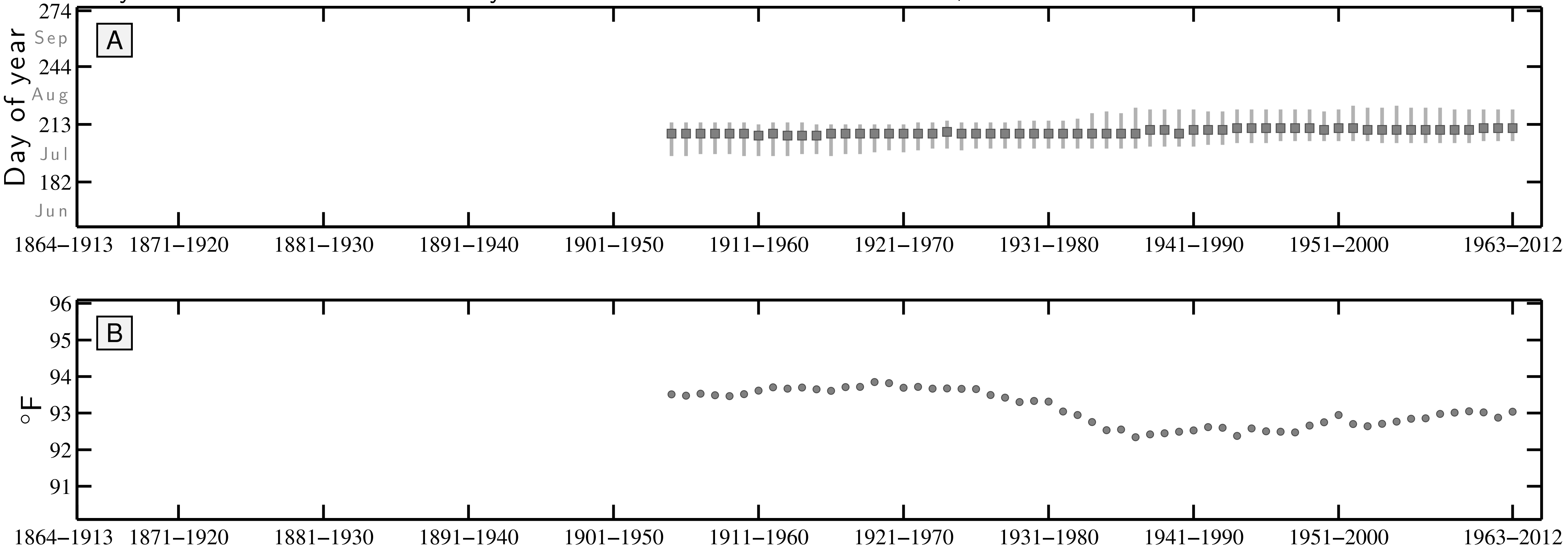

50 year Winter Teletherm dynamics for ARROWROCK DAM, ID:

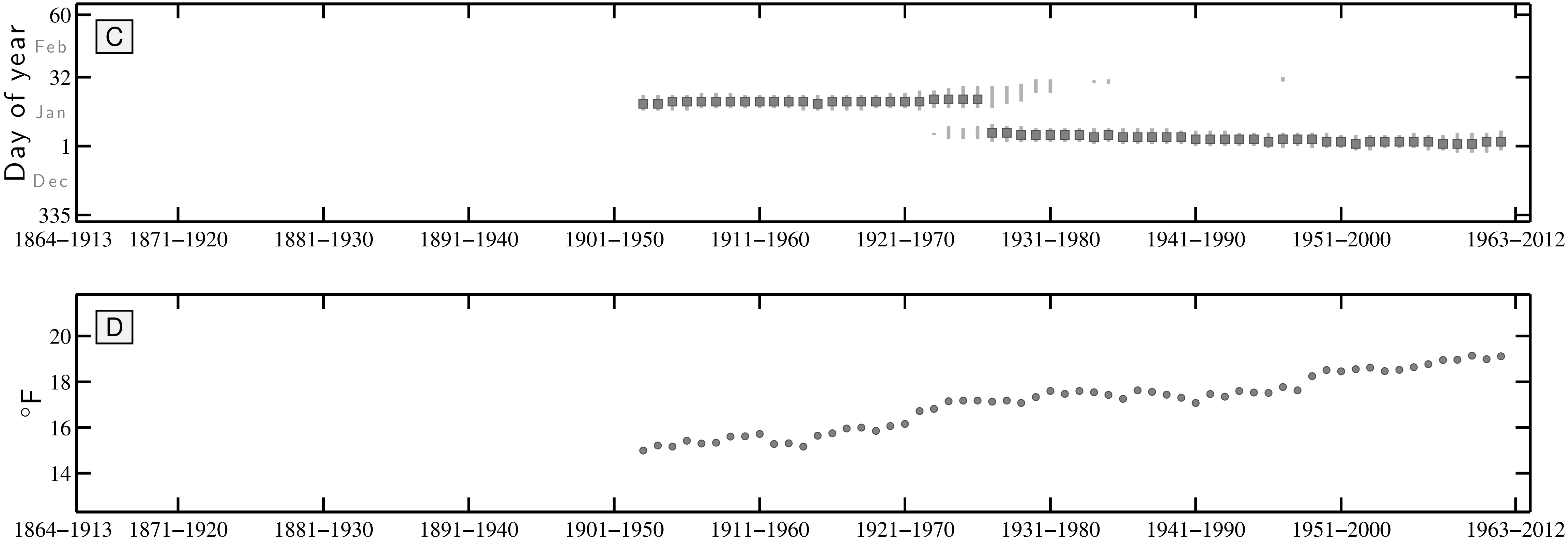

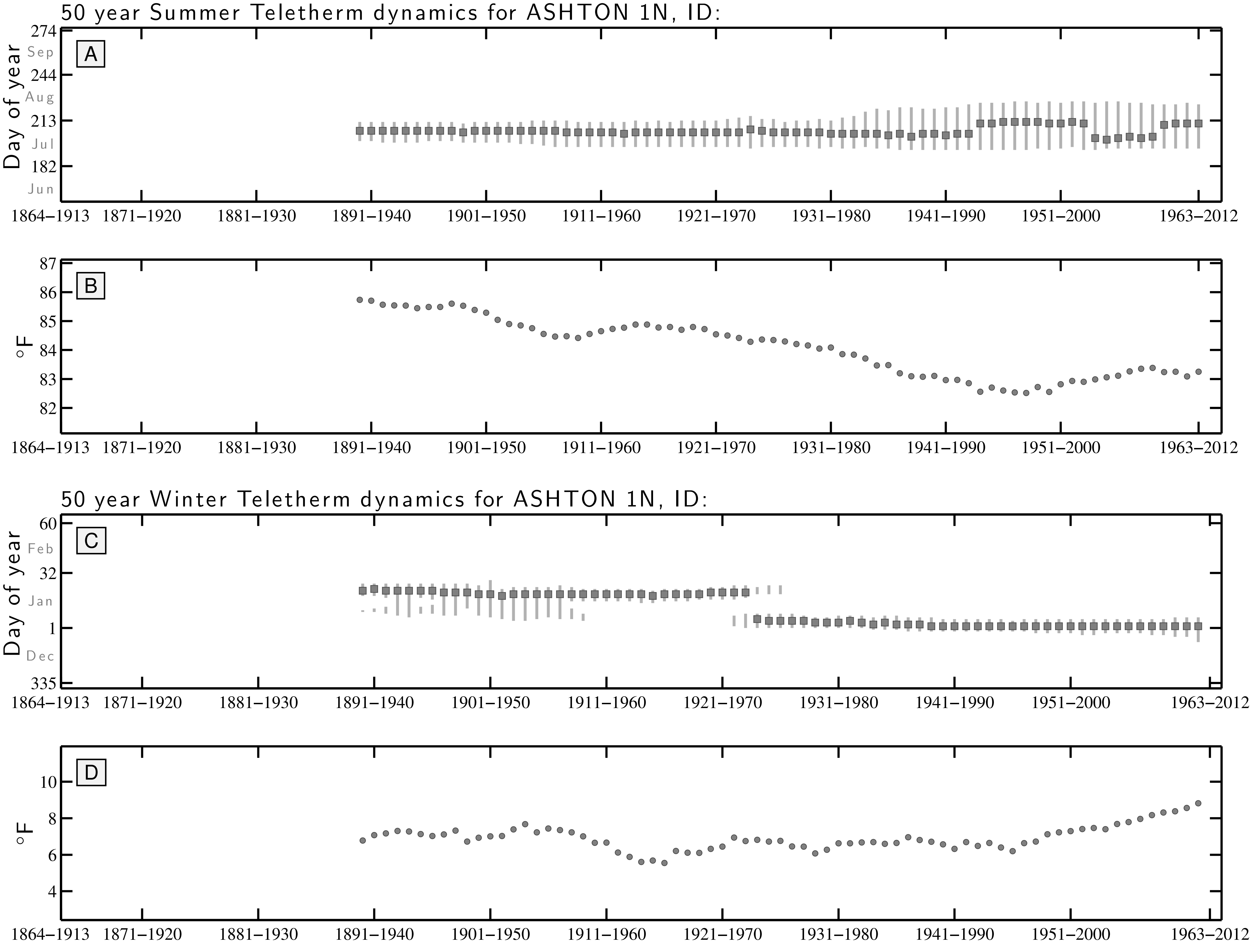
50 year Summer Teletherm dynamics for ASHTON 1N, ID:
A
B
50 year Winter Teletherm dynamics for ASHTON 1N, ID:
C
D
Day of year
°F
274
244
213
182
Sep
Aug
Jul
Jun
87
86
85
84
83
82
60
32
1
335
Feb
Jan
Dec
10
8
6
4
1864–1913
1871–1920
1881–1930
1891–1940
1901–1950
1911–1960
1921–1970
1931–1980
1941–1990
1951–2000
1963–2012

50 year Summer Teletherm dynamics for CAMBRIDGE, ID:

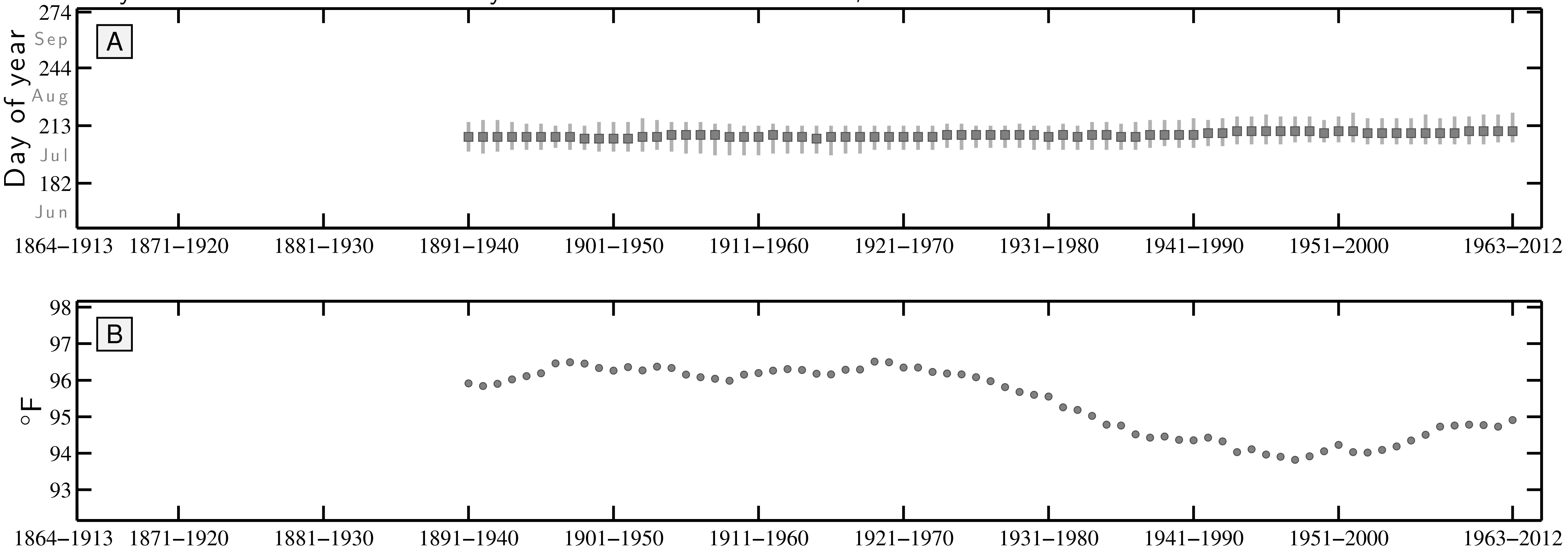

50 year Winter Teletherm dynamics for CAMBRIDGE, ID:

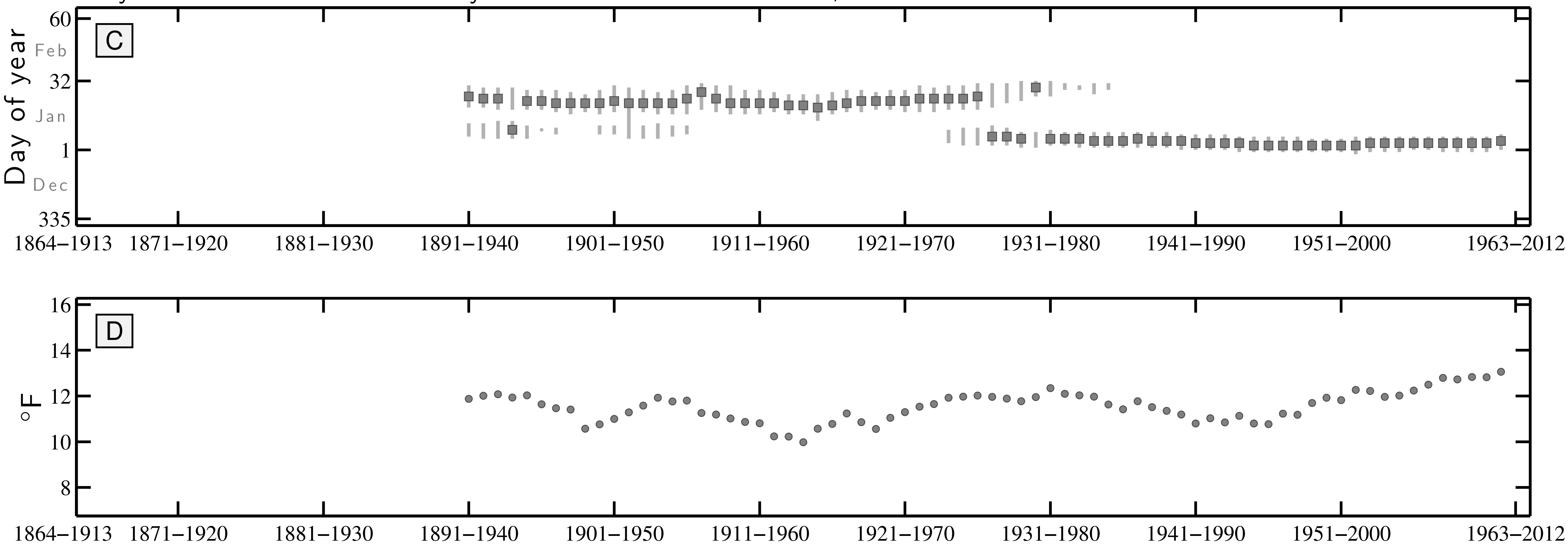

50 year Summer Teletherm dynamics for COEUR D'ALENE, ID:

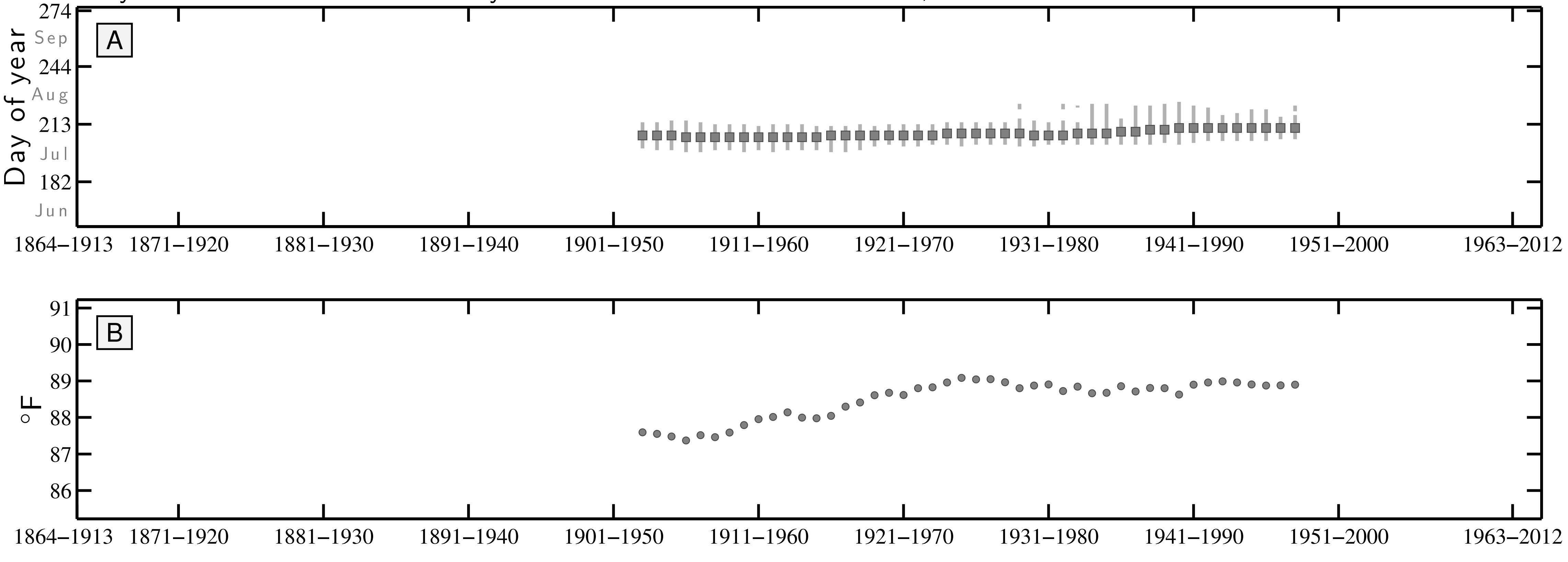

50 year Winter Teletherm dynamics for COEUR D'ALENE, ID:

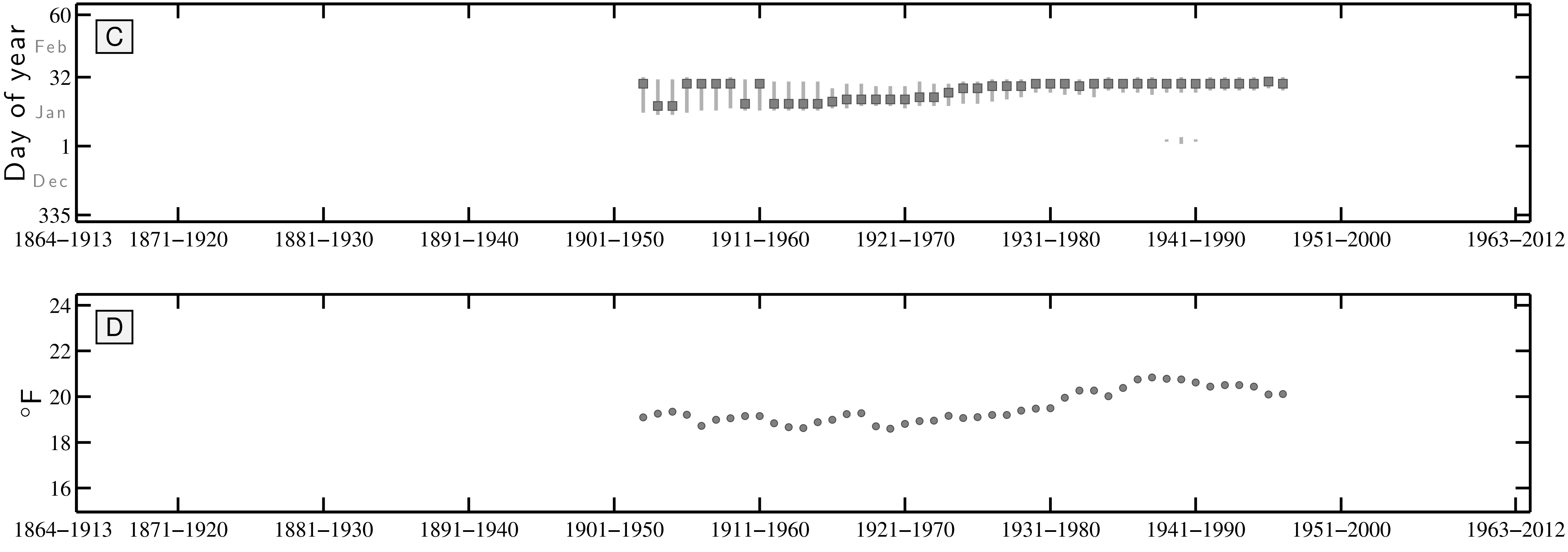

50 year Summer Teletherm dynamics for DWORSHAK FISH HATCHERY, ID:

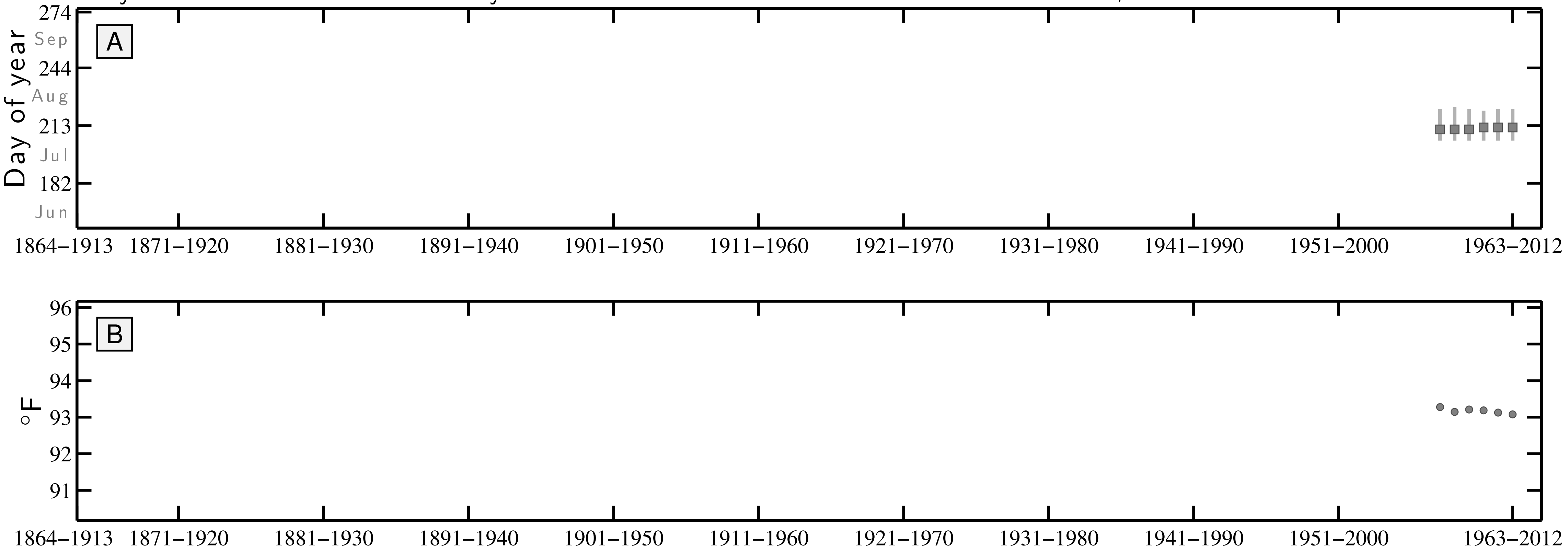

50 year Winter Teletherm dynamics for DWORSHAK FISH HATCHERY, ID:

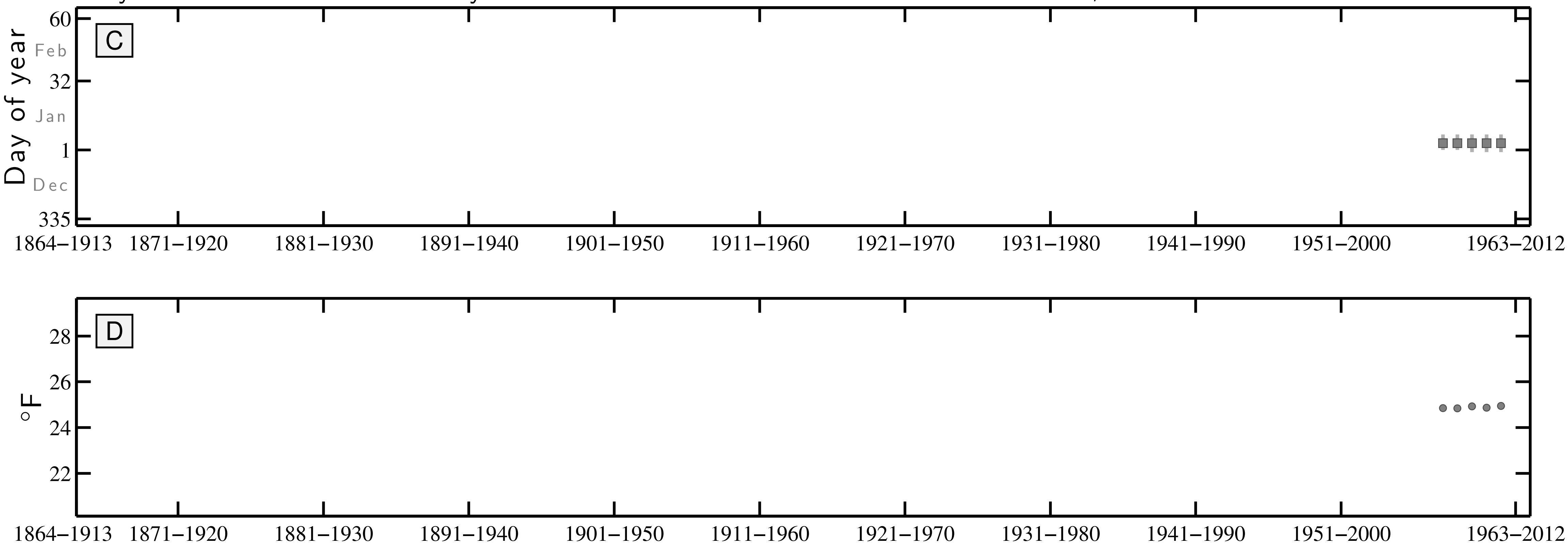

50 year Summer Teletherm dynamics for FENN RS, ID:

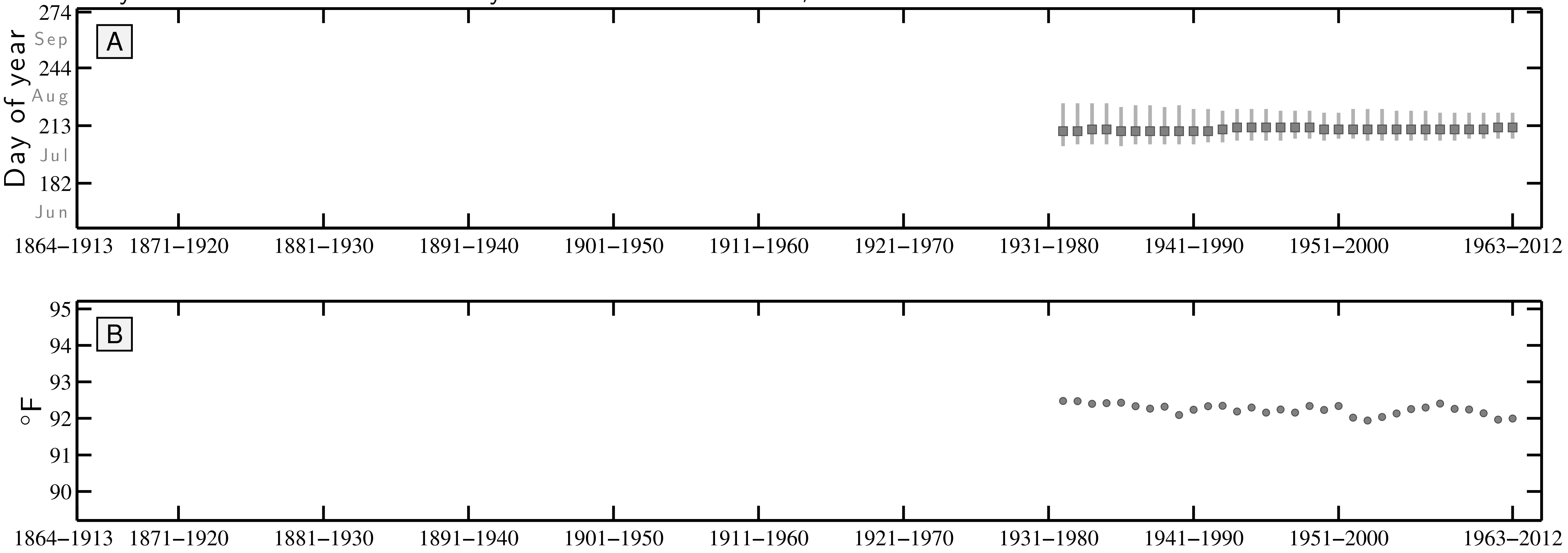

50 year Winter Teletherm dynamics for FENN RS, ID:

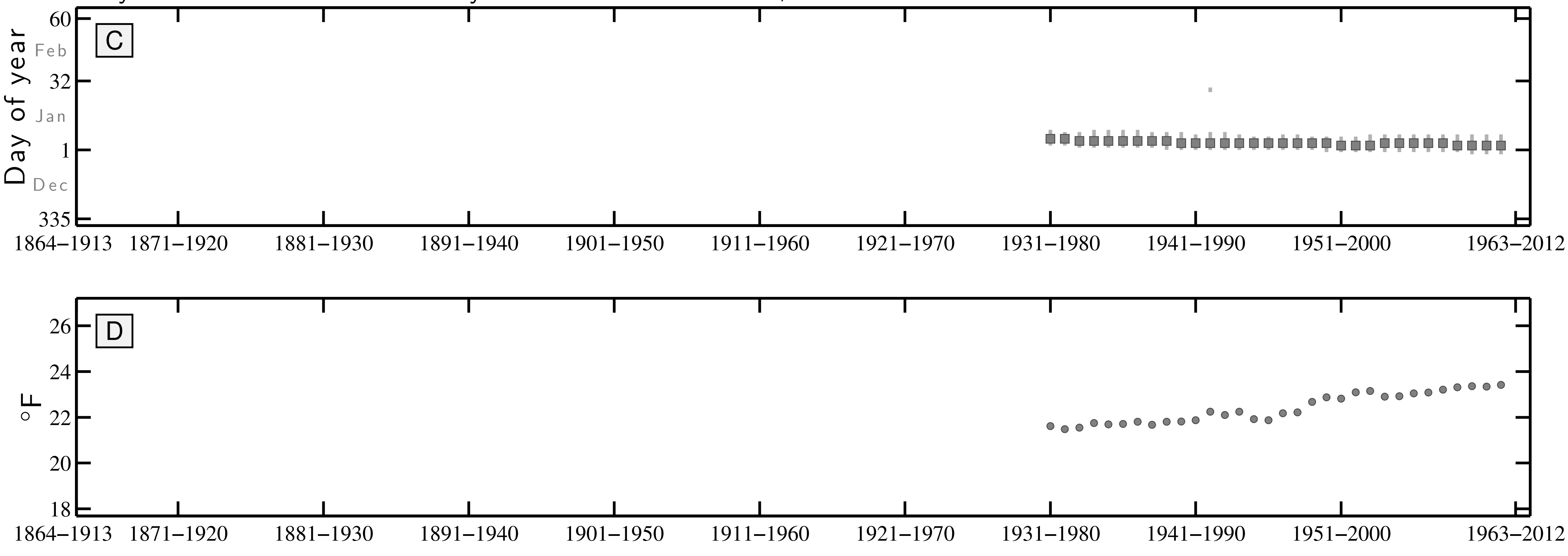

50 year Summer Teletherm dynamics for GLENNS FERRY, ID:

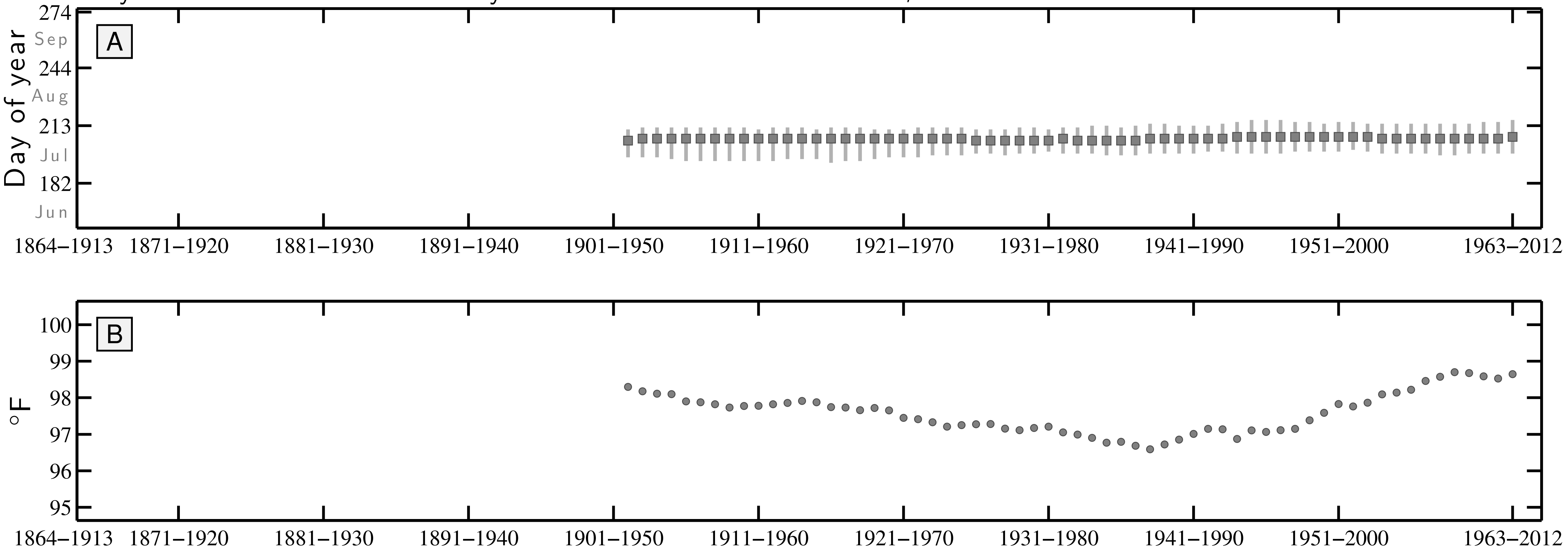

50 year Winter Teletherm dynamics for GLENNS FERRY, ID:

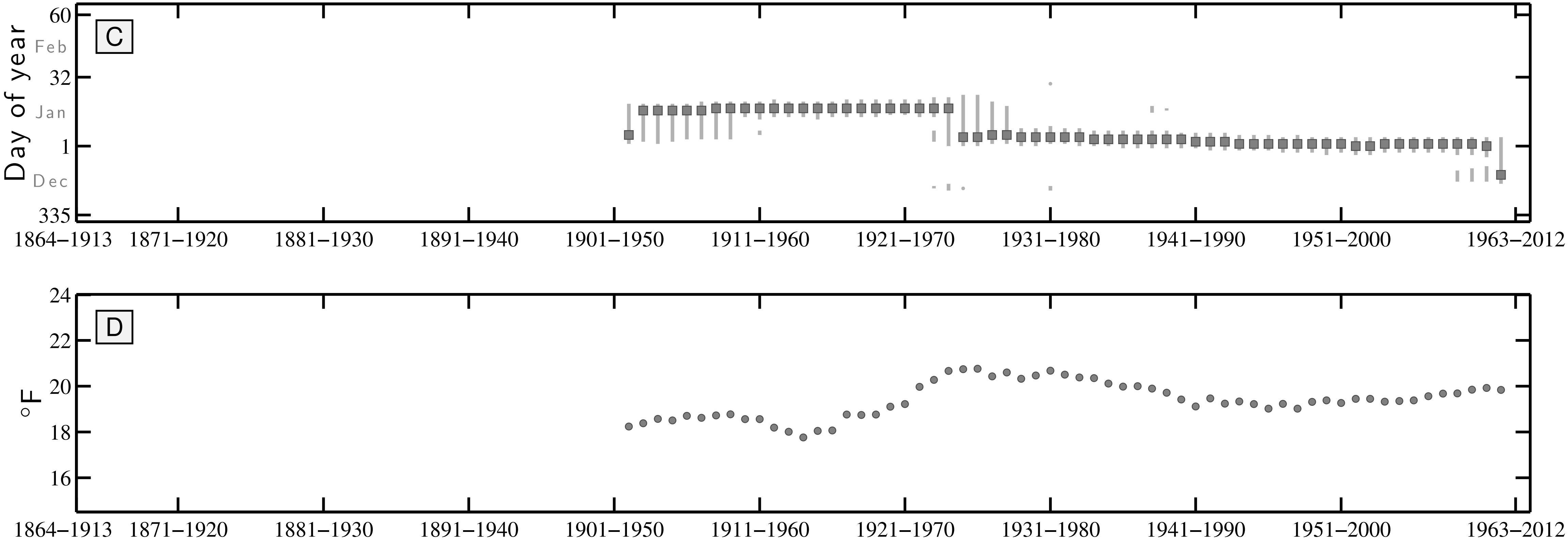

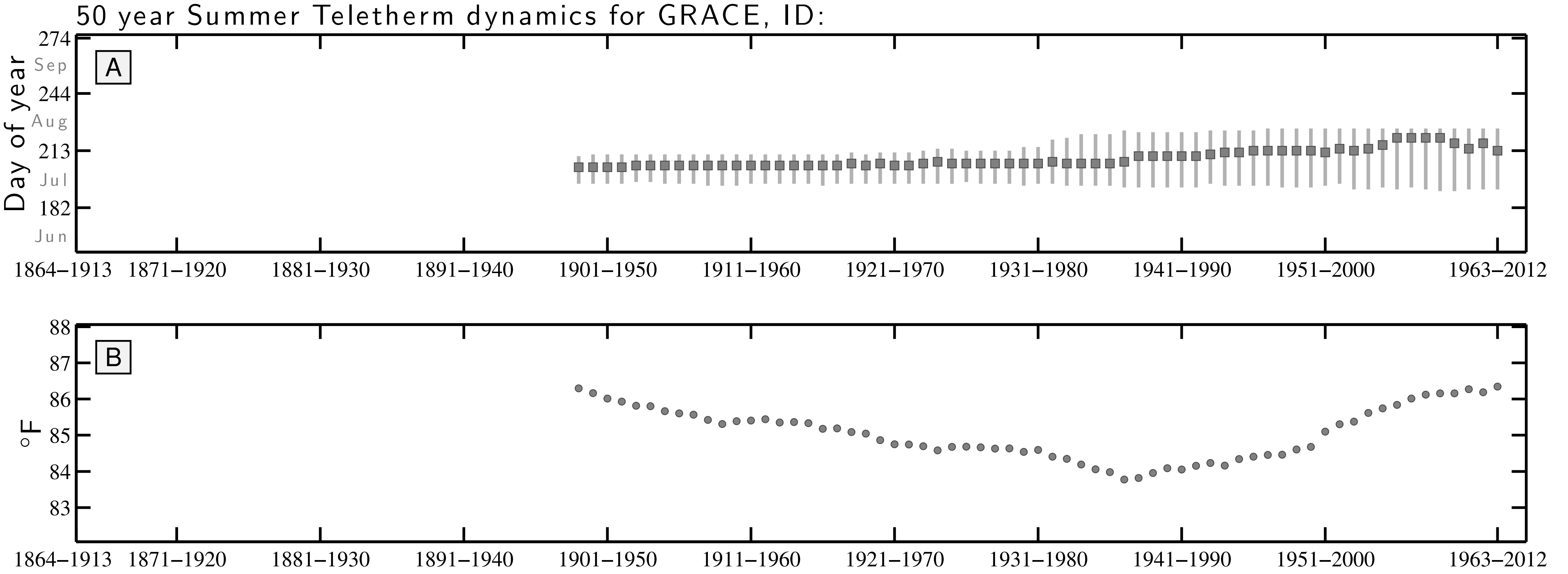
50 year Summer Teletherm dynamics for GRACE, ID:
A
B
Day of year
Sep
Aug
Jul
Jun
274
244
213
182
°F
88
87
86
85
84
83
1864–1913
1871–1920
1881–1930
1891–1940
1901–1950
1911–1960
1921–1970
1931–1980
1941–1990
1951–2000
1963–2012

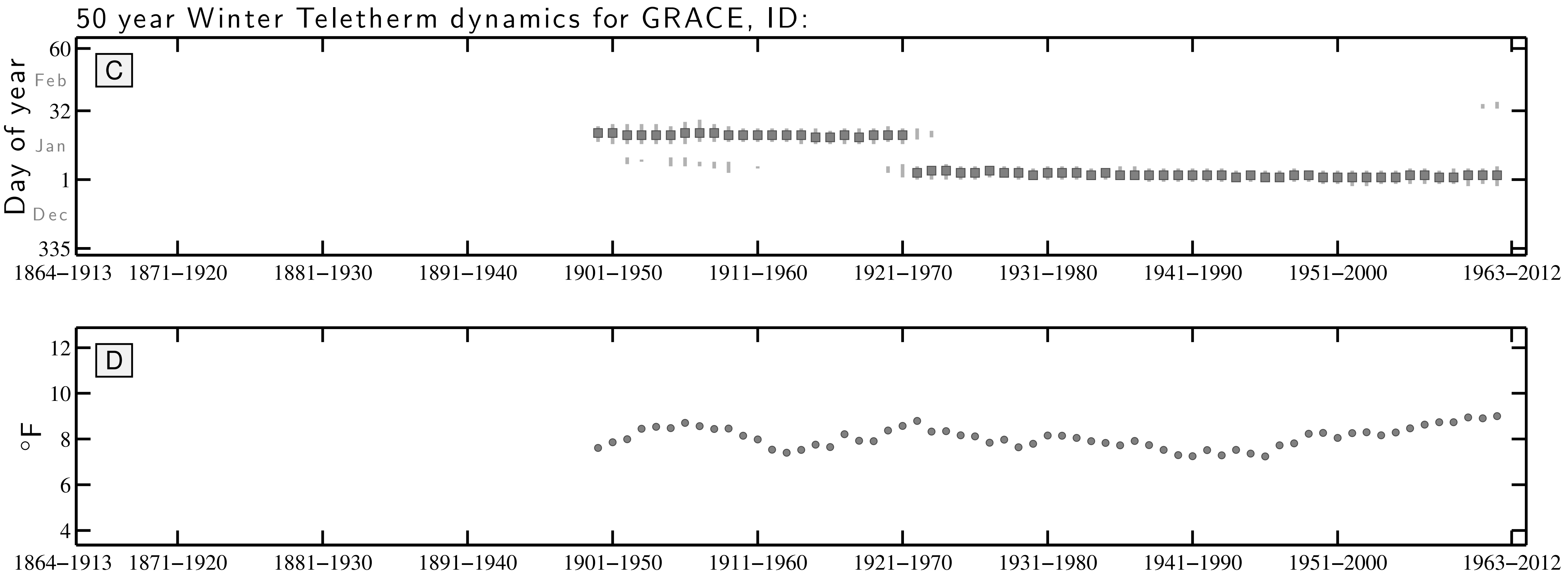
50 year Winter Teletherm dynamics for GRACE, ID:
C
D
Day of year
Feb
Jan
Dec
60
32
1
335
°F
12
10
8
6
4
1864–1913
1871–1920
1881–1930
1891–1940
1901–1950
1911–1960
1921–1970
1931–1980
1941–1990
1951–2000
1963–2012

50 year Summer Teletherm dynamics for HAZELTON, ID:

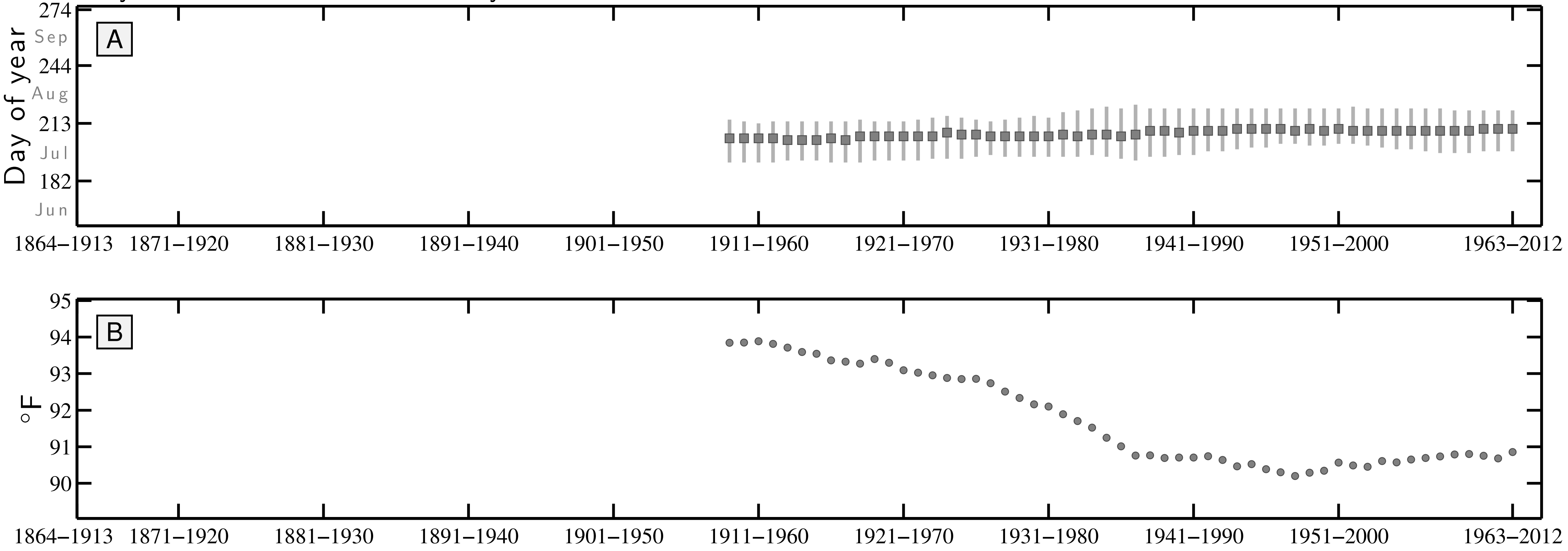

50 year Winter Teletherm dynamics for HAZELTON, ID:

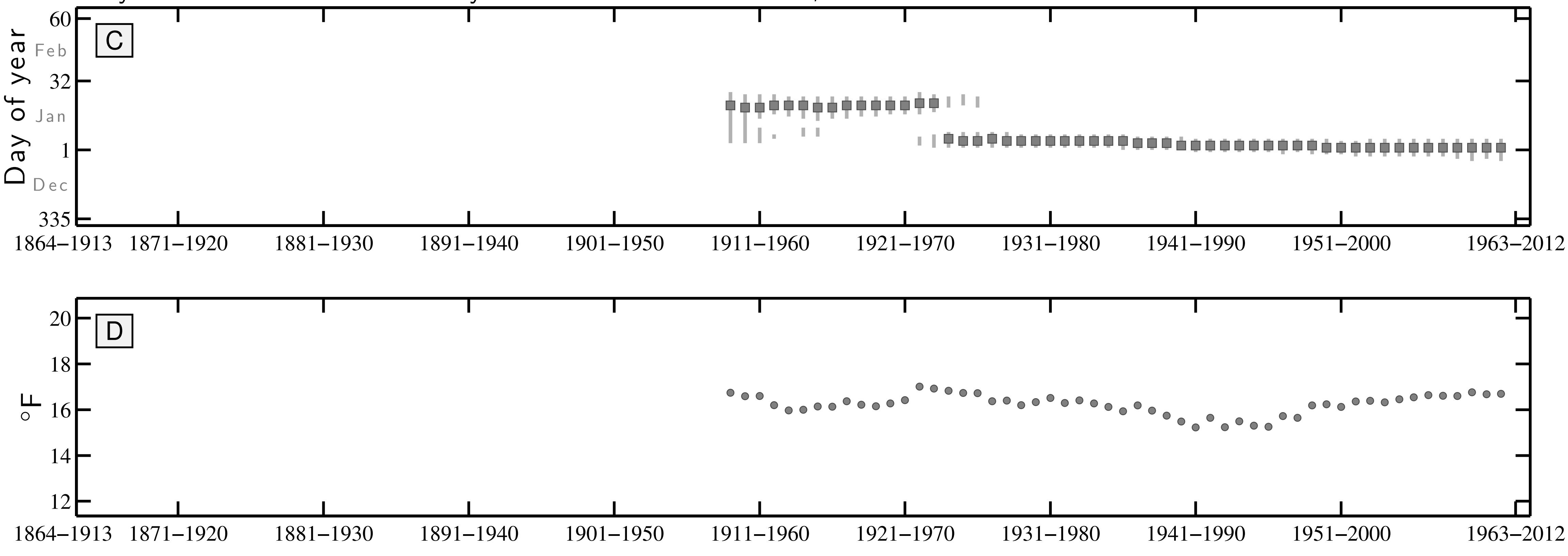

50 year Summer Teletherm dynamics for HOLLISTER, ID:

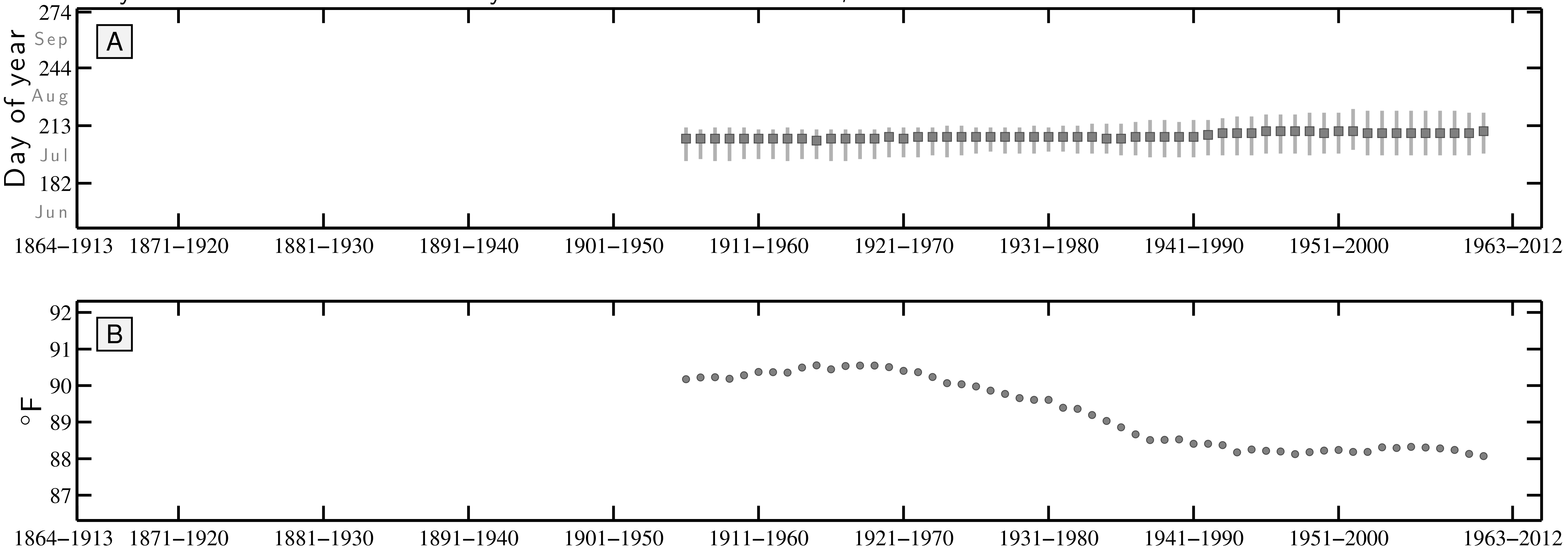

50 year Winter Teletherm dynamics for HOLLISTER, ID:

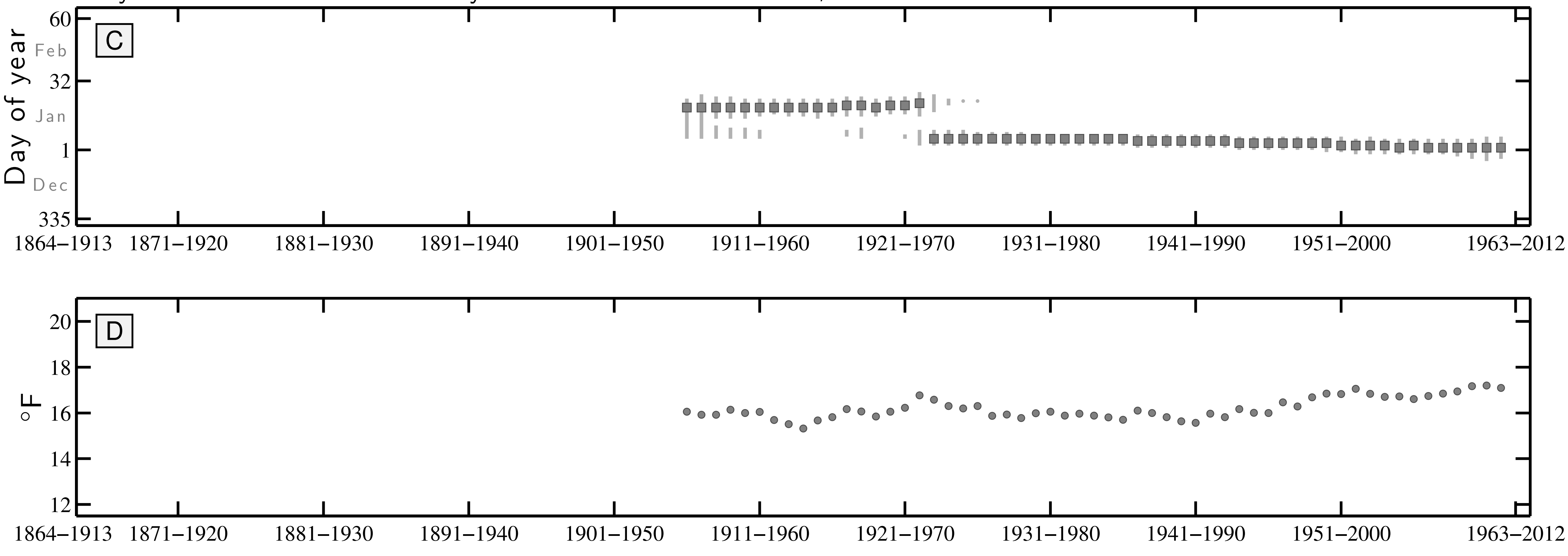

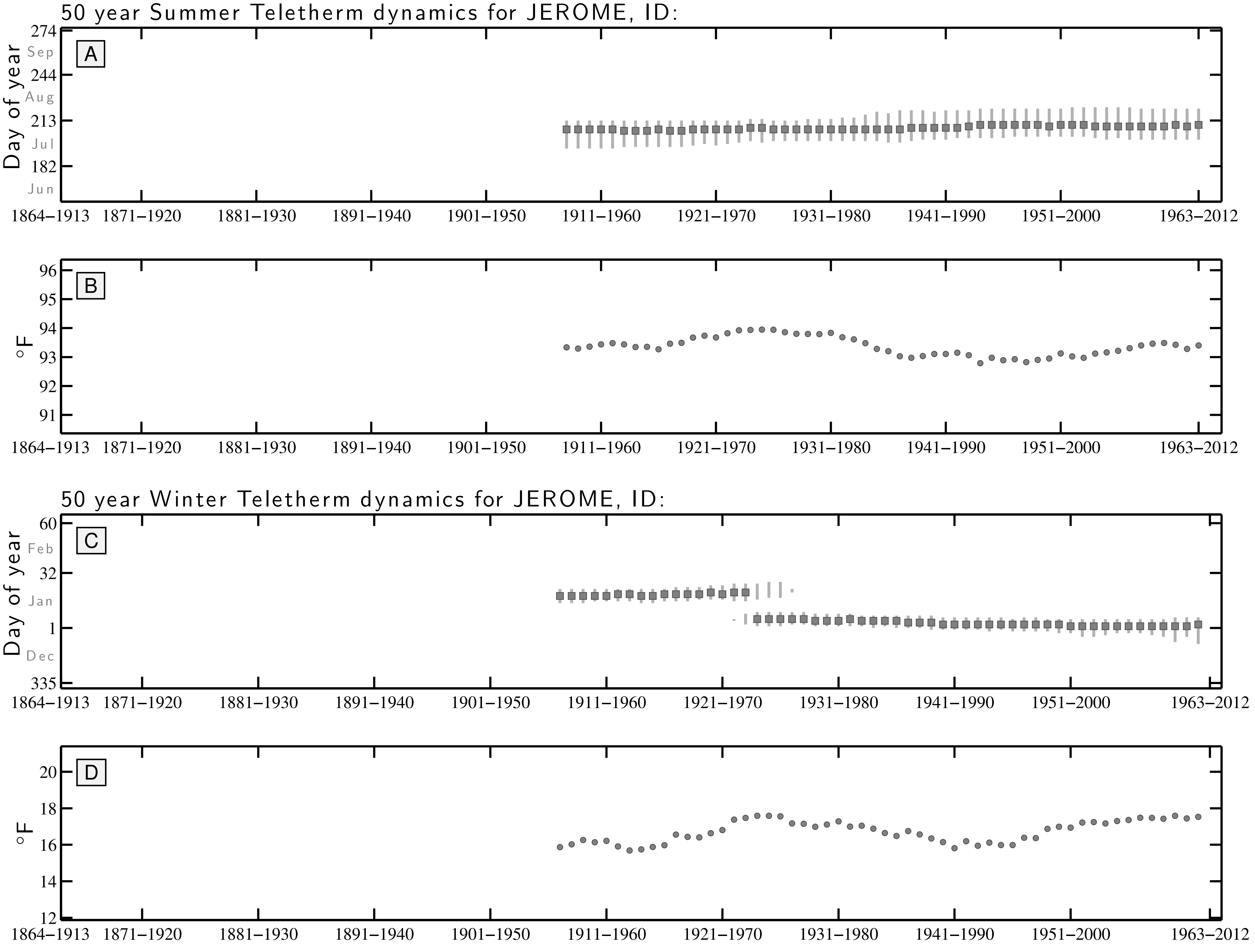
50 year Summer Teletherm dynamics for JEROME, ID:
A
Day of year
274
Sep
244
Aug
213
Jul
182
Jun
1864–1913
1871–1920
1881–1930
1891–1940
1901–1950
1911–1960
1921–1970
1931–1980
1941–1990
1951–2000
1963–2012
B
°F
96
95
94
93
92
91
50 year Winter Teletherm dynamics for JEROME, ID:
C
Day of year
60
Feb
32
Jan
1
Dec
335
D
°F
20
18
16
14
12

50 year Summer Teletherm dynamics for KELLOGG, ID:

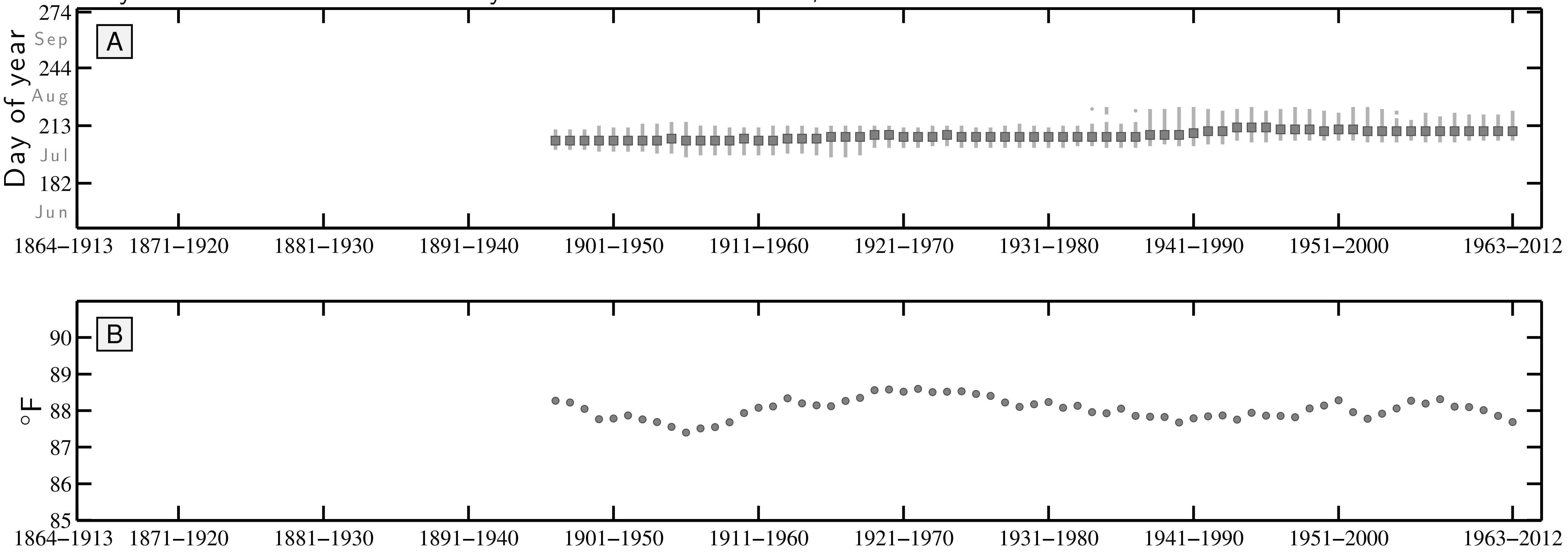

50 year Winter Teletherm dynamics for KELLOGG, ID:

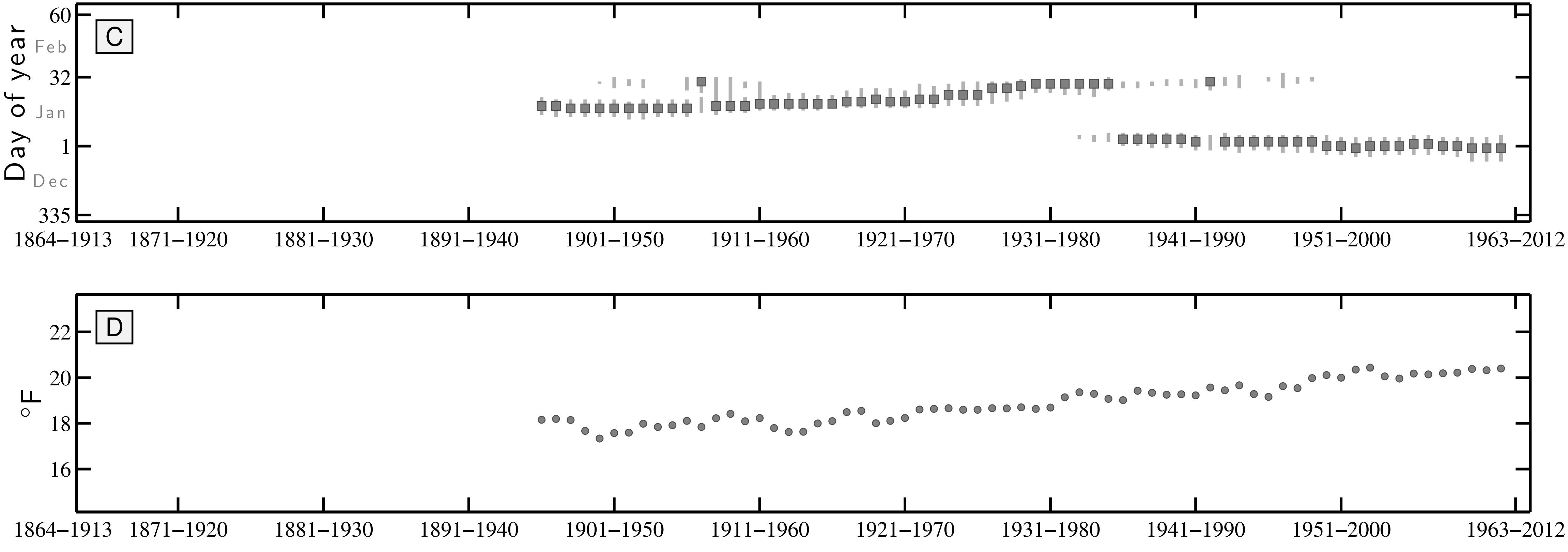

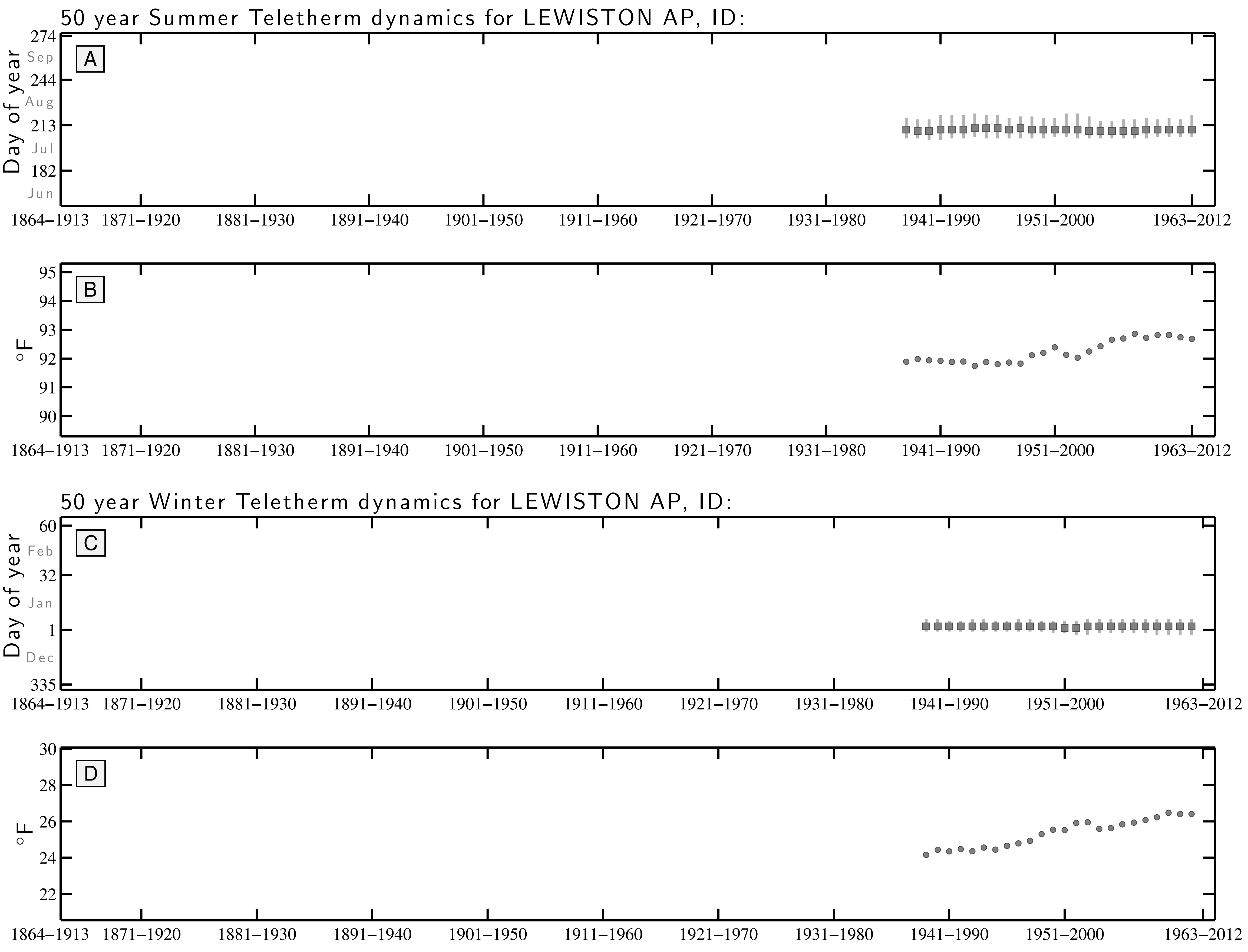

50 year Summer Teletherm dynamics for LEWISTON AP, ID:
A
B
Day of year
°F
274
Sep
244
Aug
213
Jul
182
Jun
95
94
93
92
91
90
1864–1913
1871–1920
1881–1930
1891–1940
1901–1950
1911–1960
1921–1970
1931–1980
1941–1990
1951–2000
1963–2012
50 year Winter Teletherm dynamics for LEWISTON AP, ID:
C
D
60
Feb
32
Jan
1
Dec
335
30
28
26
24
22

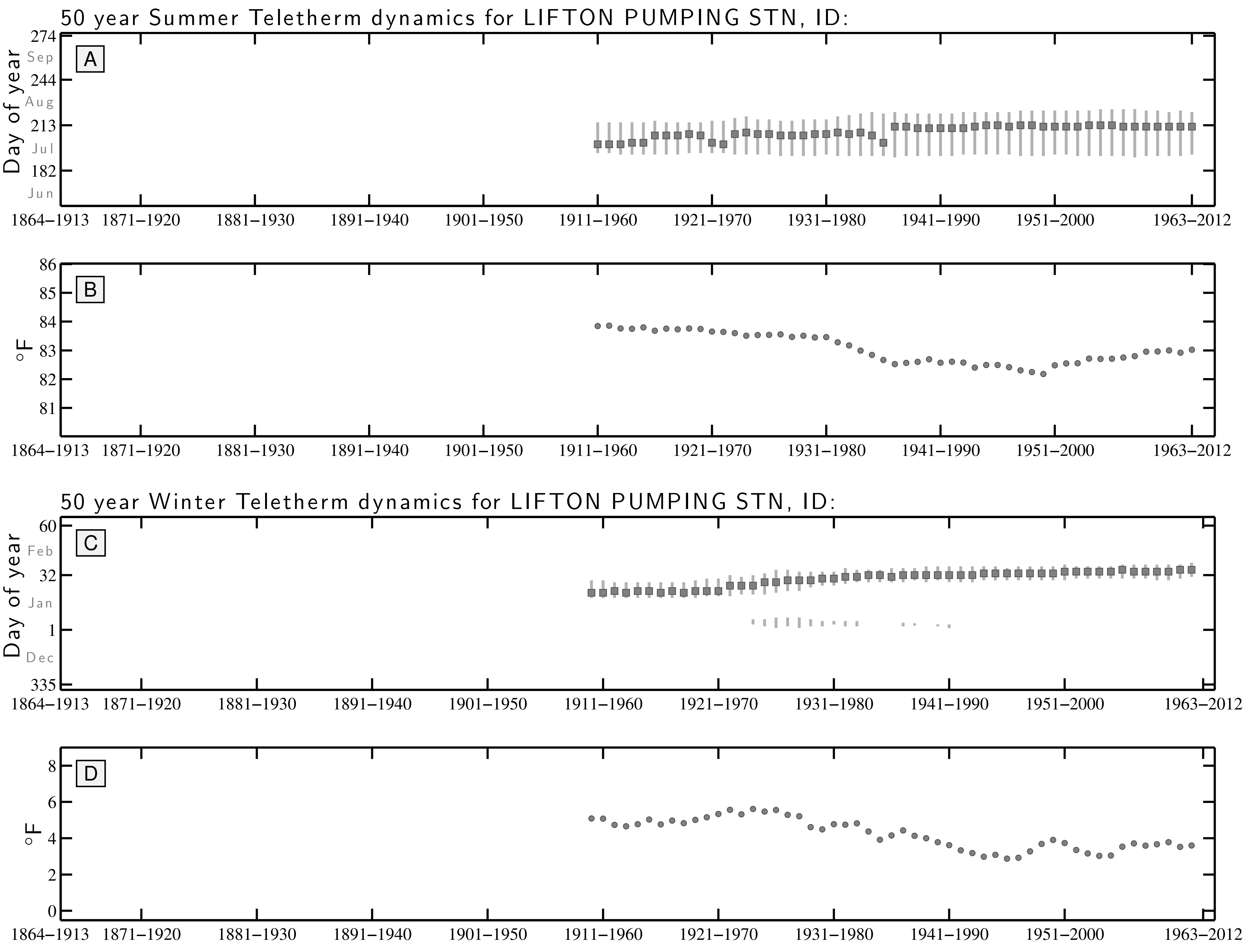

50 year Summer Teletherm dynamics for LIFTON PUMPING STN, ID:
A
Day of year
274
Sep
244
Aug
213
Jul
182
Jun
1864–1913
1871–1920
1881–1930
1891–1940
1901–1950
1911–1960
1921–1970
1931–1980
1941–1990
1951–2000
1963–2012
B
°F
86
85
84
83
82
81
50 year Winter Teletherm dynamics for LIFTON PUMPING STN, ID:
C
60
Feb
32
Jan
1
Dec
335
D
8
6
4
2
0

50 year Summer Teletherm dynamics for MACKAY LOST RIVER RS, ID:

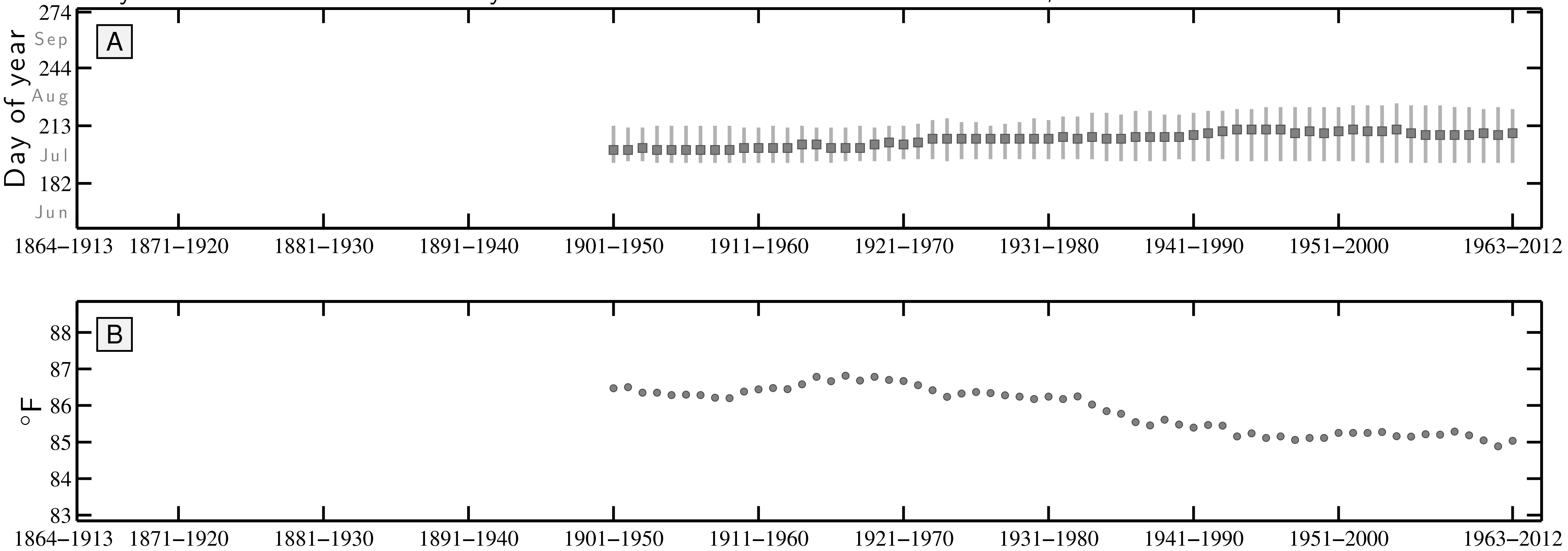

50 year Winter Teletherm dynamics for MACKAY LOST RIVER RS, ID:

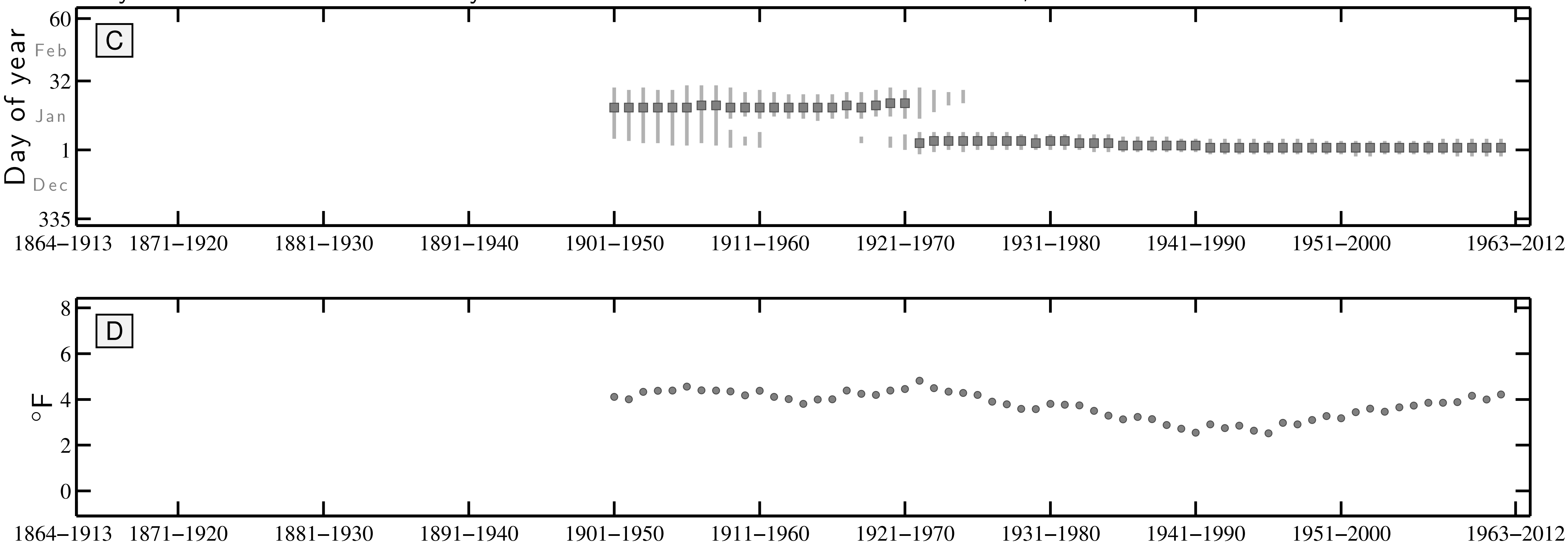

50 year Summer Teletherm dynamics for MALAD CITY, ID:

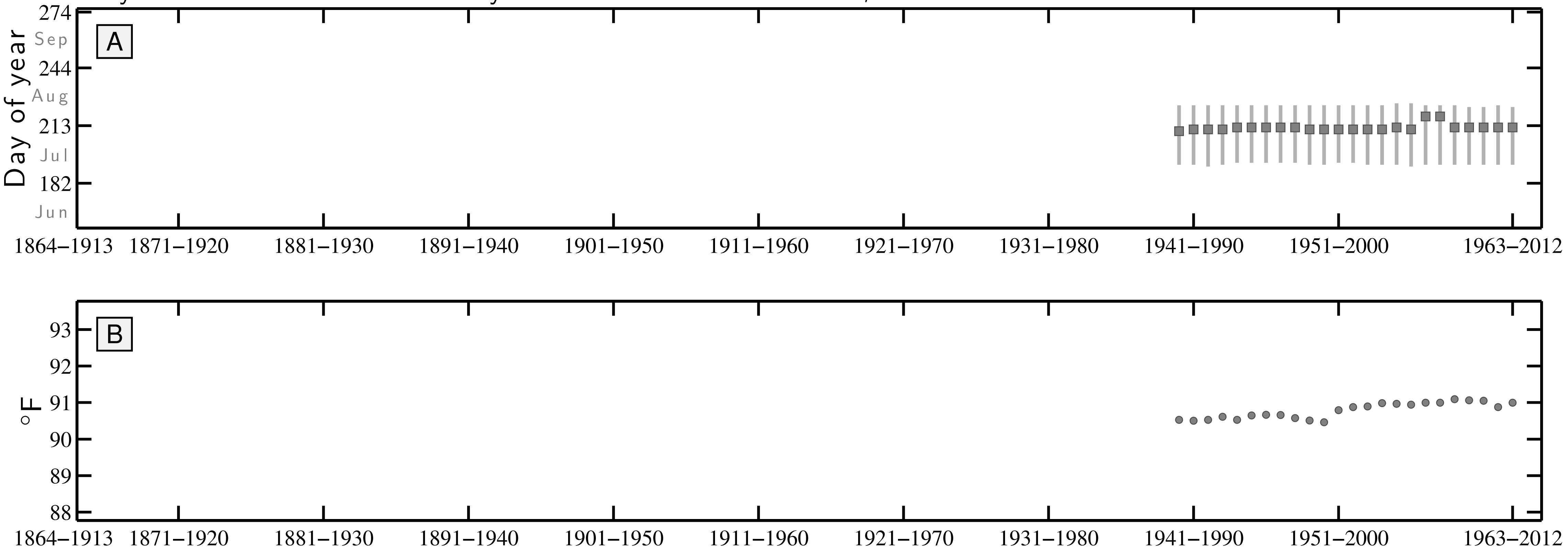

50 year Winter Teletherm dynamics for MALAD CITY, ID:

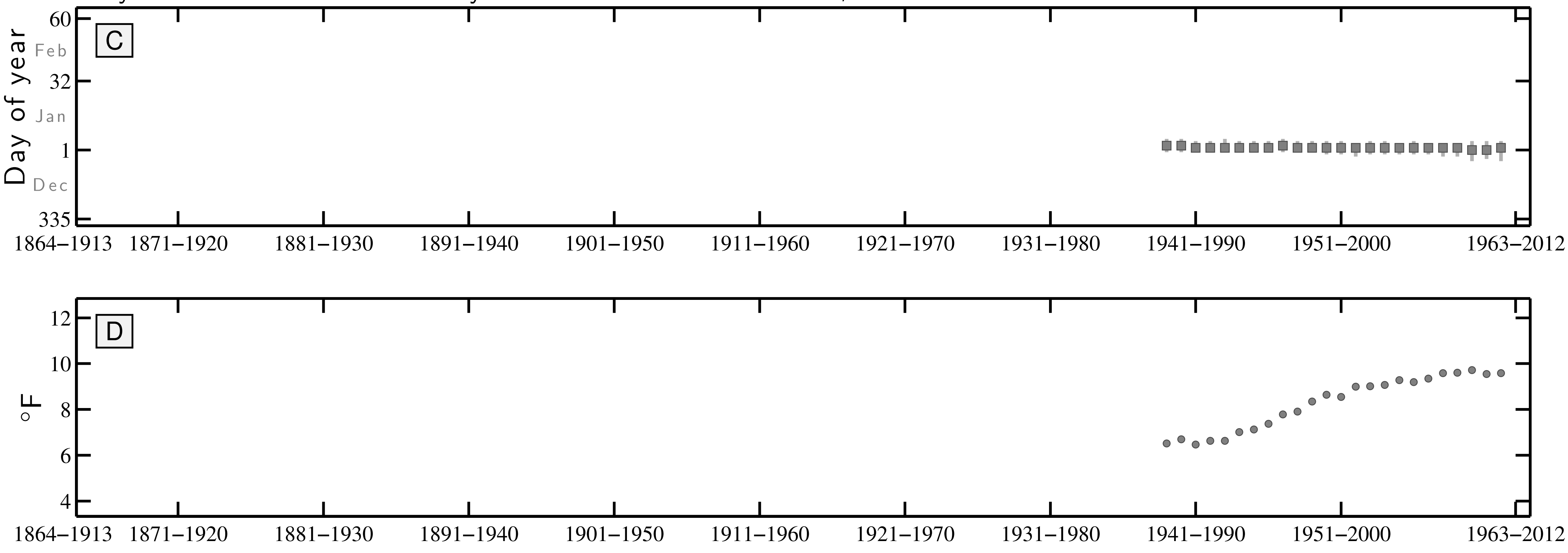

50 year Summer Teletherm dynamics for MAY 2SSE, ID:

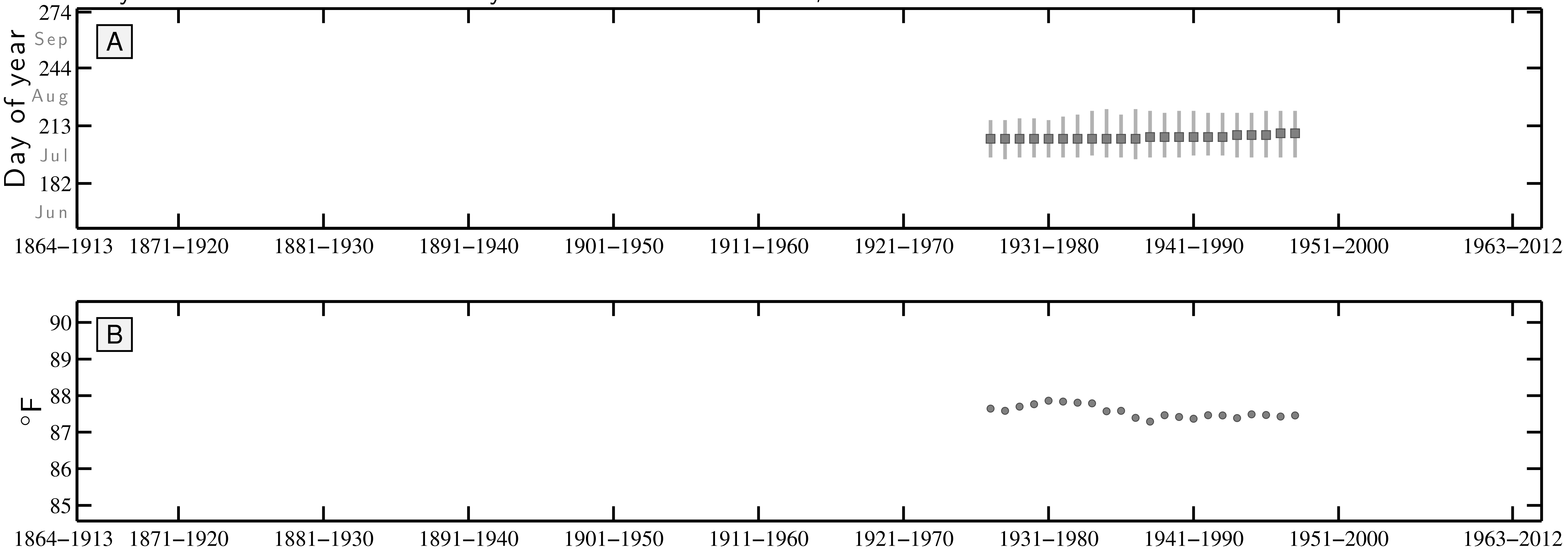

50 year Winter Teletherm dynamics for MAY 2SSE, ID:

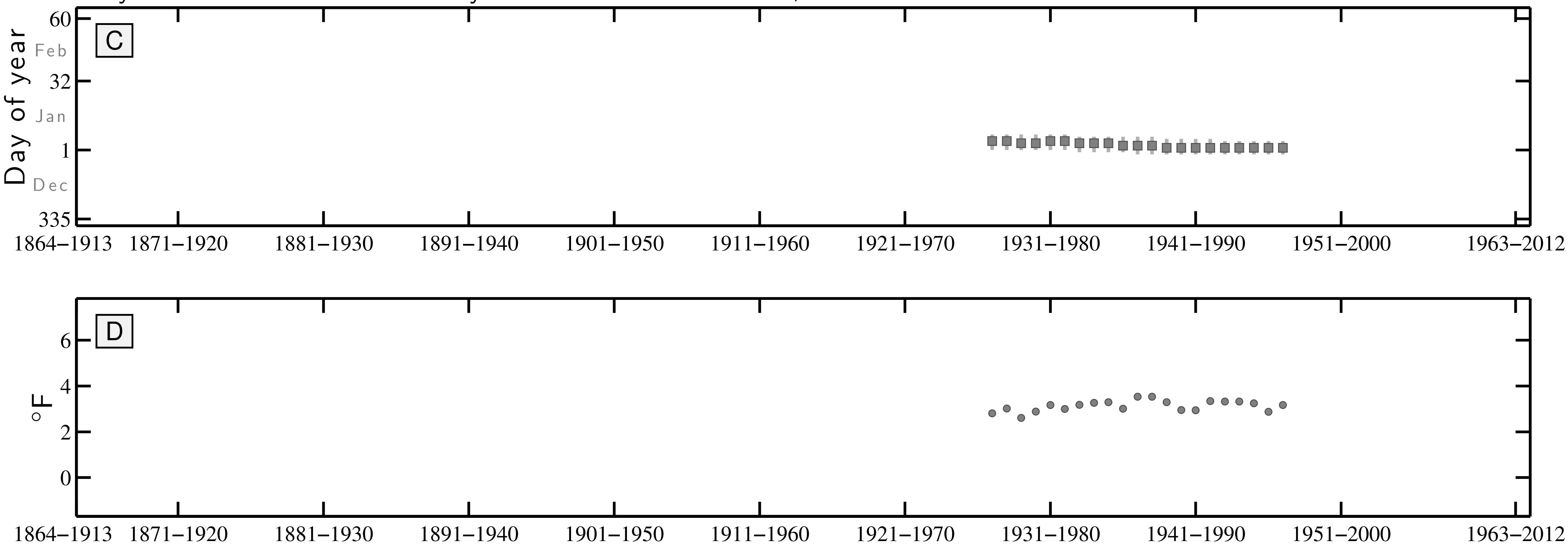

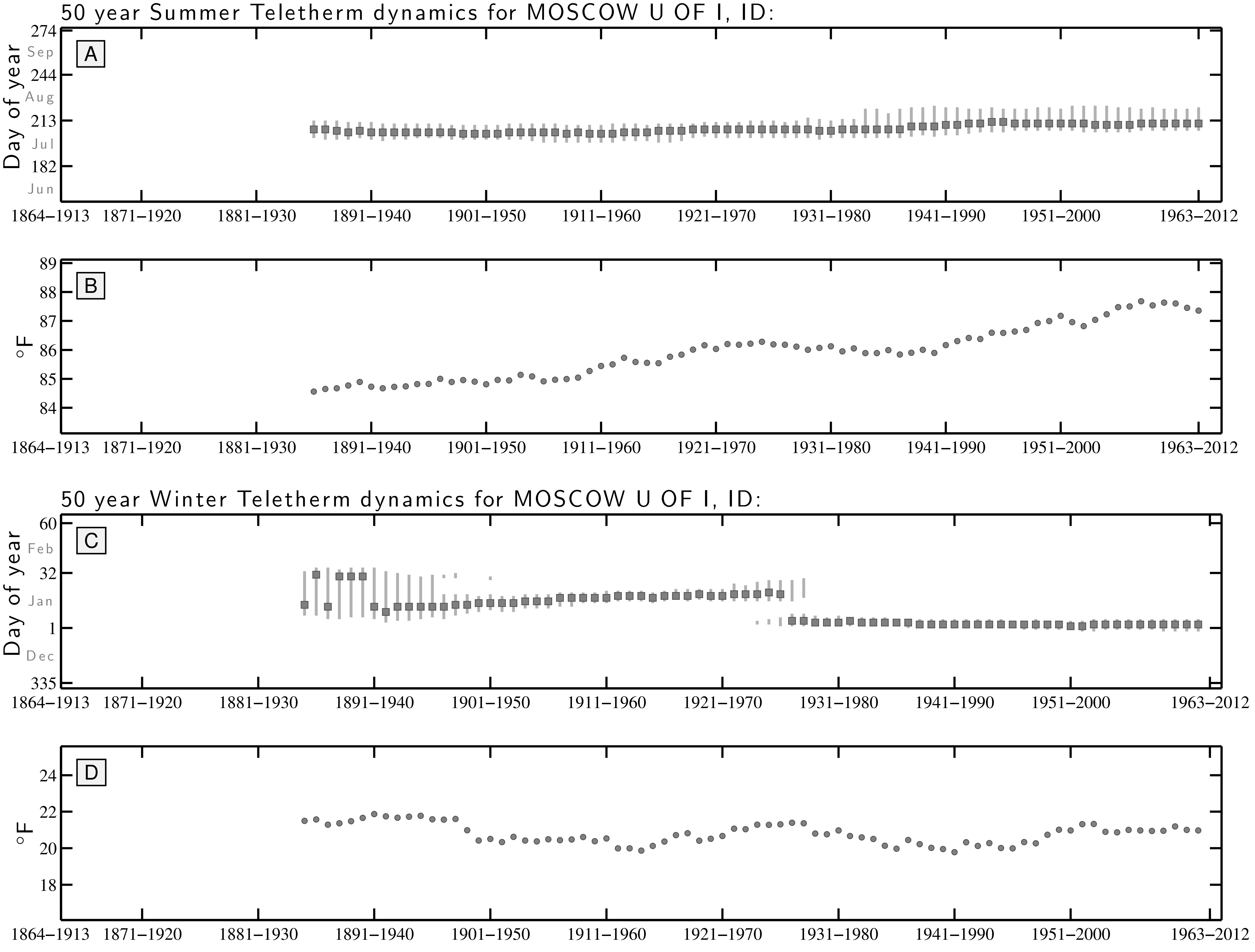

50 year Summer Teletherm dynamics for MOSCOW U OF I, ID:
A
Day of year
274
Sep
244
Aug
213
Jul
182
Jun
B
°F
89
88
87
86
85
84
50 year Winter Teletherm dynamics for MOSCOW U OF I, ID:
C
Day of year
60
Feb
32
Jan
1
Dec
335
D
°F
24
22
20
18
1864–1913
1871–1920
1881–1930
1891–1940
1901–1950
1911–1960
1921–1970
1931–1980
1941–1990
1951–2000
1963–2012

50 year Summer Teletherm dynamics for NEW MEADOWS RS, ID:

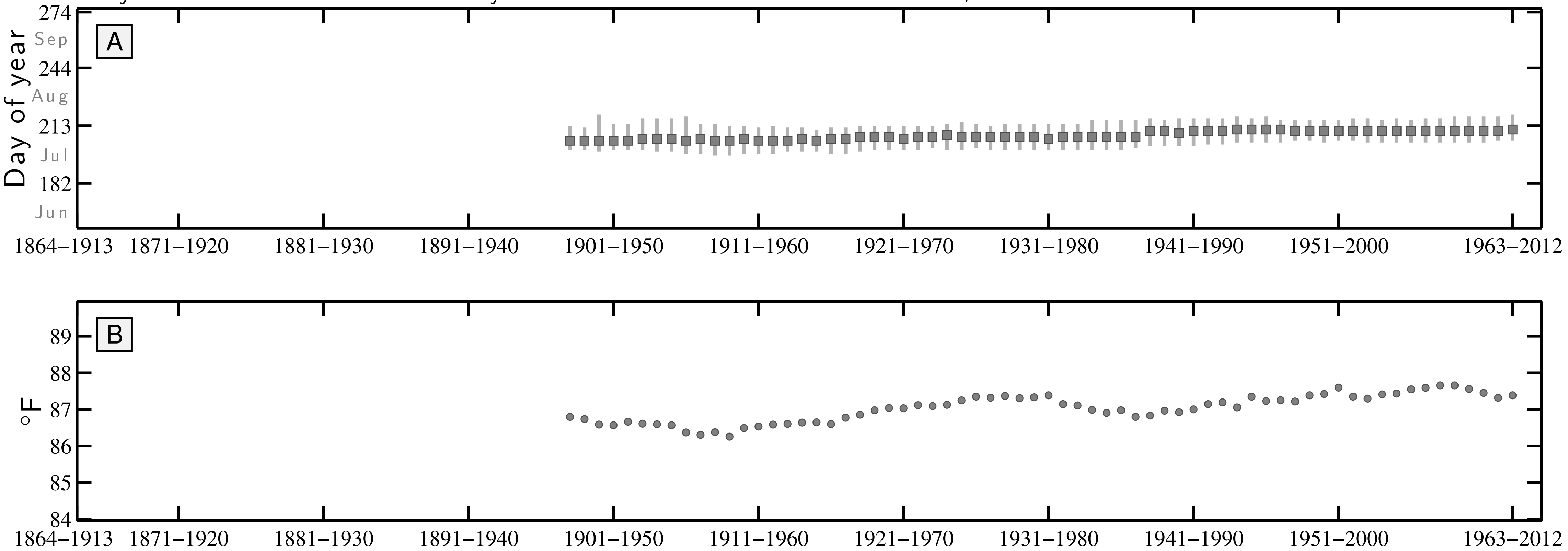

50 year Winter Teletherm dynamics for NEW MEADOWS RS, ID:

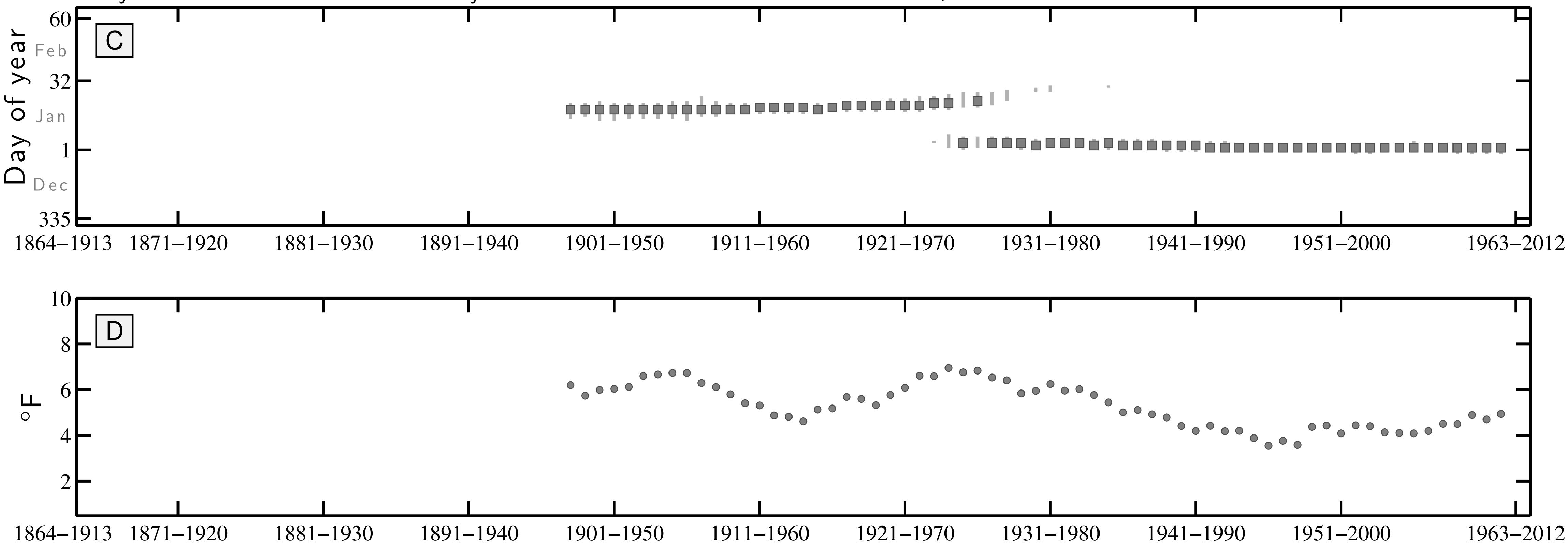

50 year Summer Teletherm dynamics for OAKLEY, ID:

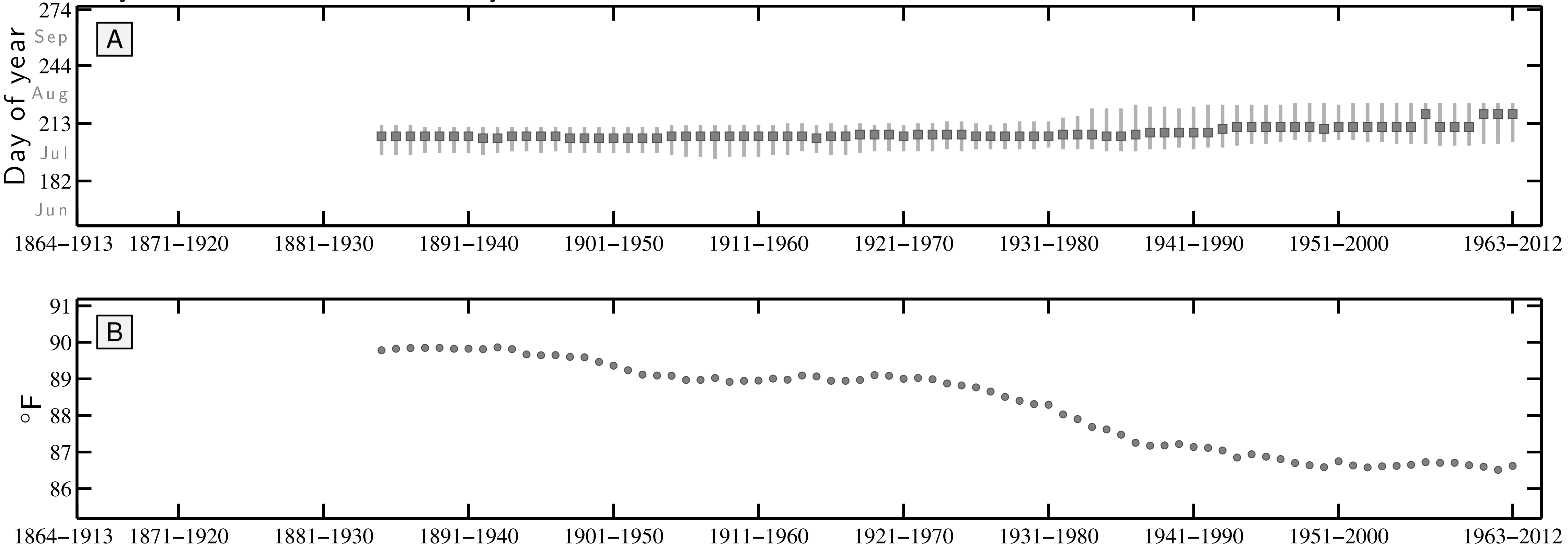

50 year Winter Teletherm dynamics for OAKLEY, ID:

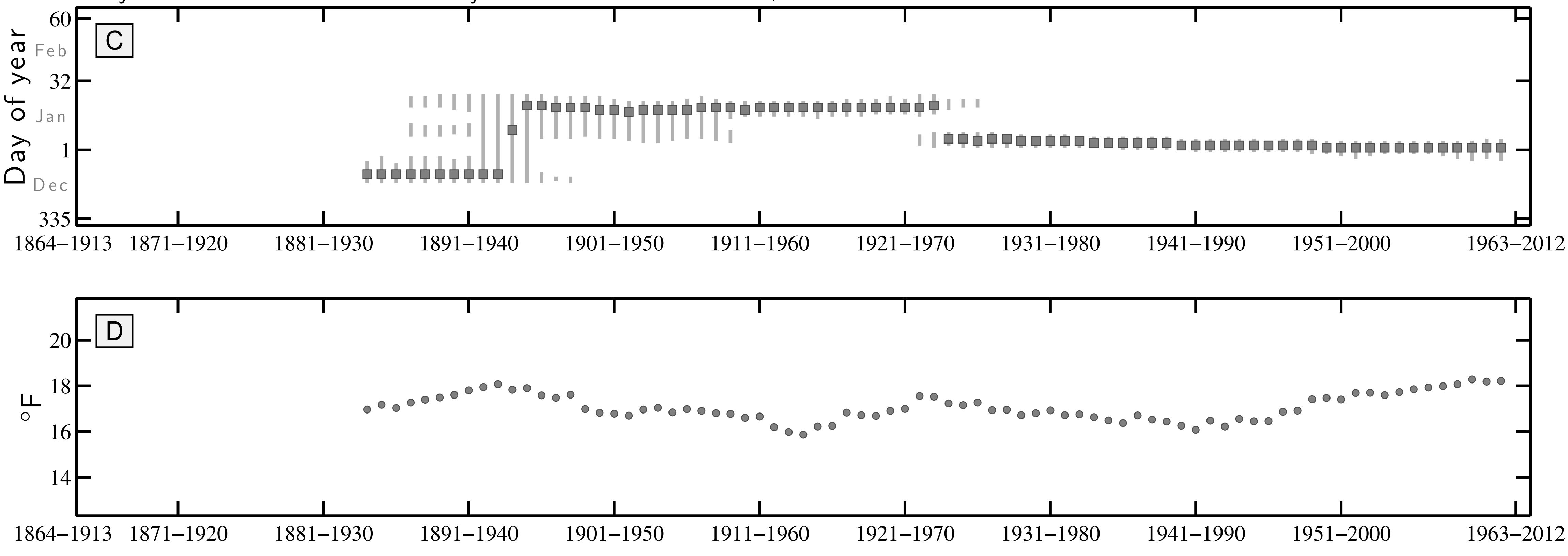

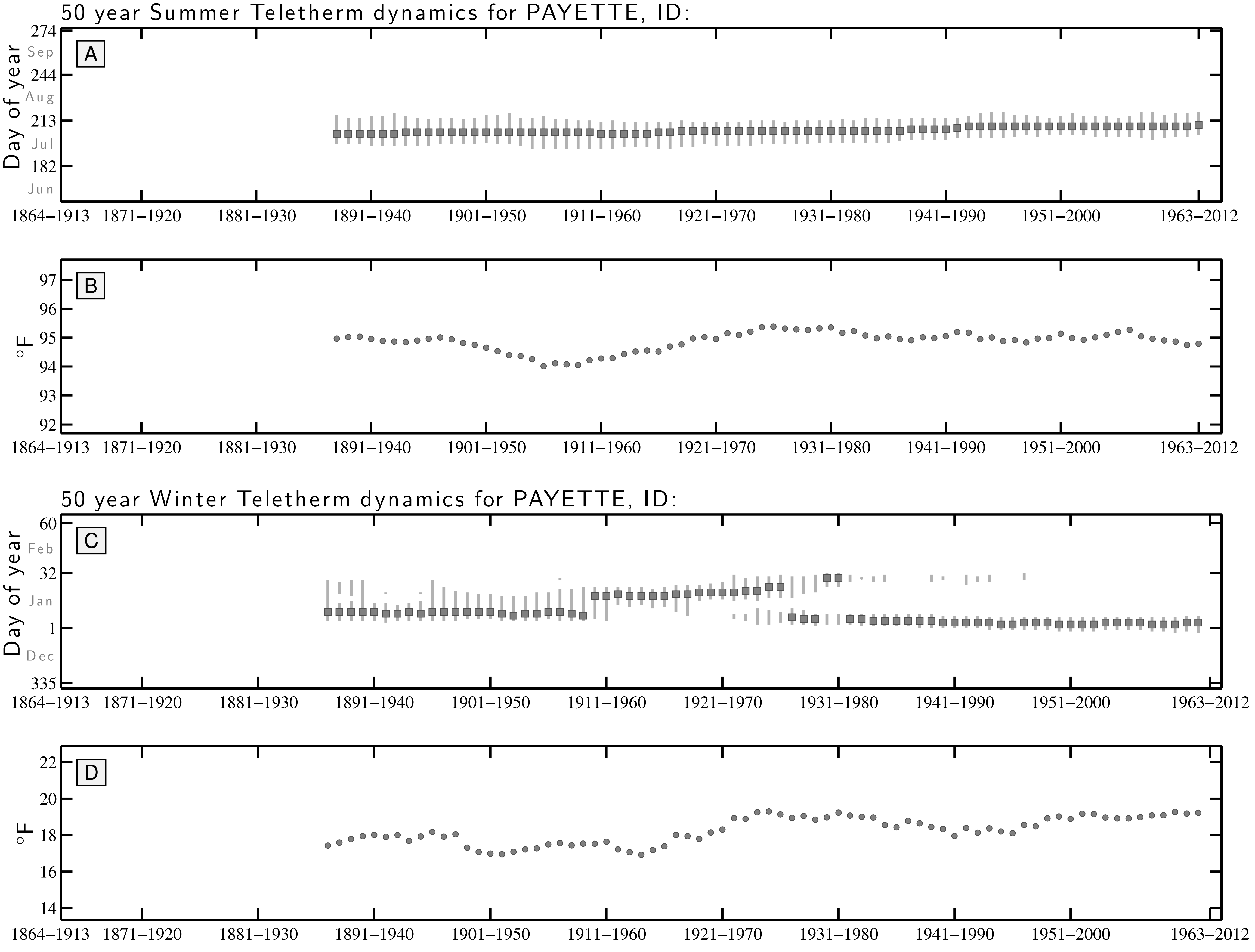
50 year Summer Teletherm dynamics for PAYETTE, ID:
A
Day of year
274
Sep
244
Aug
213
Jul
182
Jun
1864–1913
1871–1920
1881–1930
1891–1940
1901–1950
1911–1960
1921–1970
1931–1980
1941–1990
1951–2000
1963–2012
B
°F
97
96
95
94
93
92
50 year Winter Teletherm dynamics for PAYETTE, ID:
C
Day of year
60
Feb
32
Jan
1
Dec
335
D
°F
22
20
18
16
14

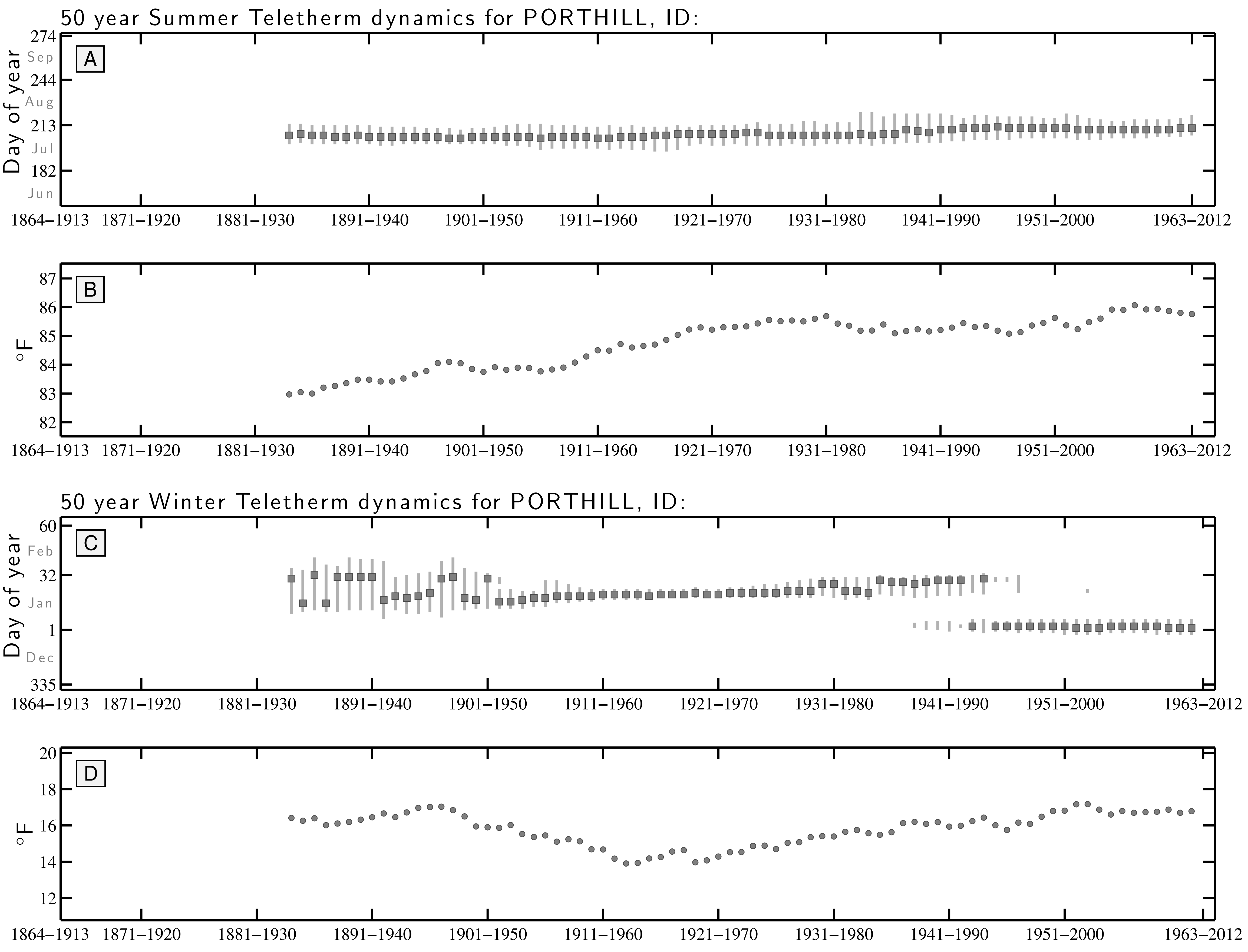
50 year Summer Teletherm dynamics for PORTHILL, ID:
A
B
Day of year
°F
274
Sep
244
Aug
213
Jul
182
Jun
87
86
85
84
83
82
1864–1913
1871–1920
1881–1930
1891–1940
1901–1950
1911–1960
1921–1970
1931–1980
1941–1990
1951–2000
1963–2012
50 year Winter Teletherm dynamics for PORTHILL, ID:
C
D
60
Feb
32
Jan
1
Dec
335
20
18
16
14
12

50 year Summer Teletherm dynamics for PRIEST RIVER EXP STN, ID:

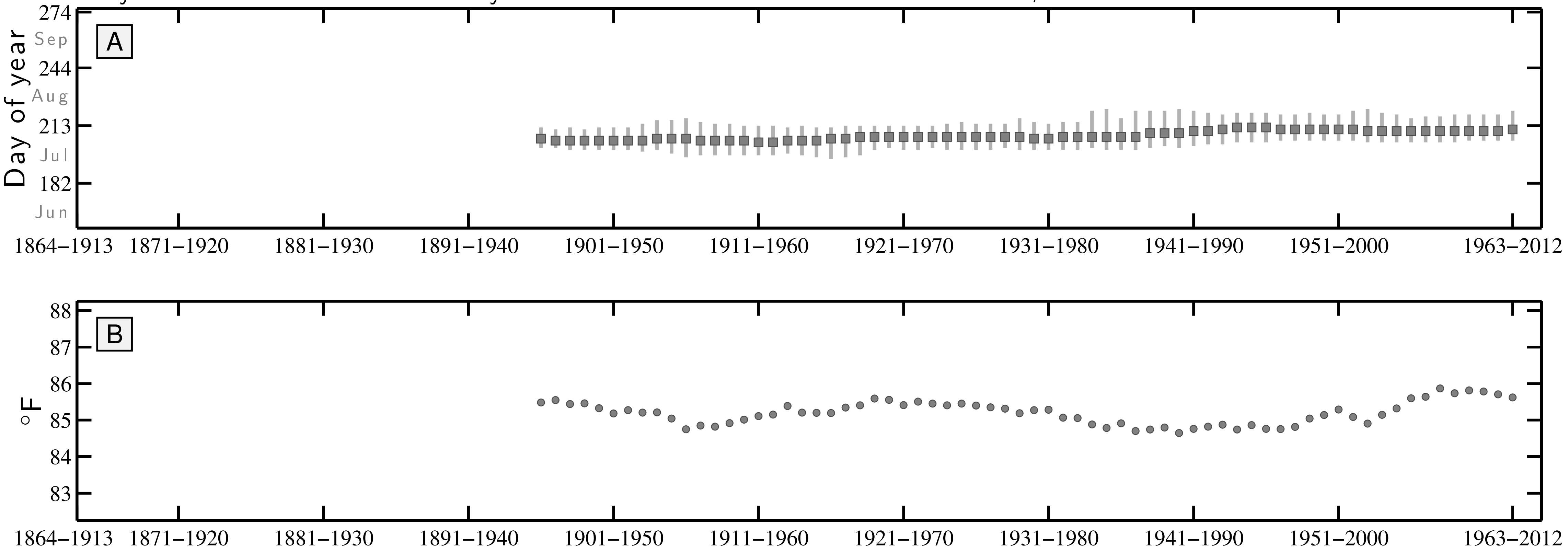

50 year Winter Teletherm dynamics for PRIEST RIVER EXP STN, ID:

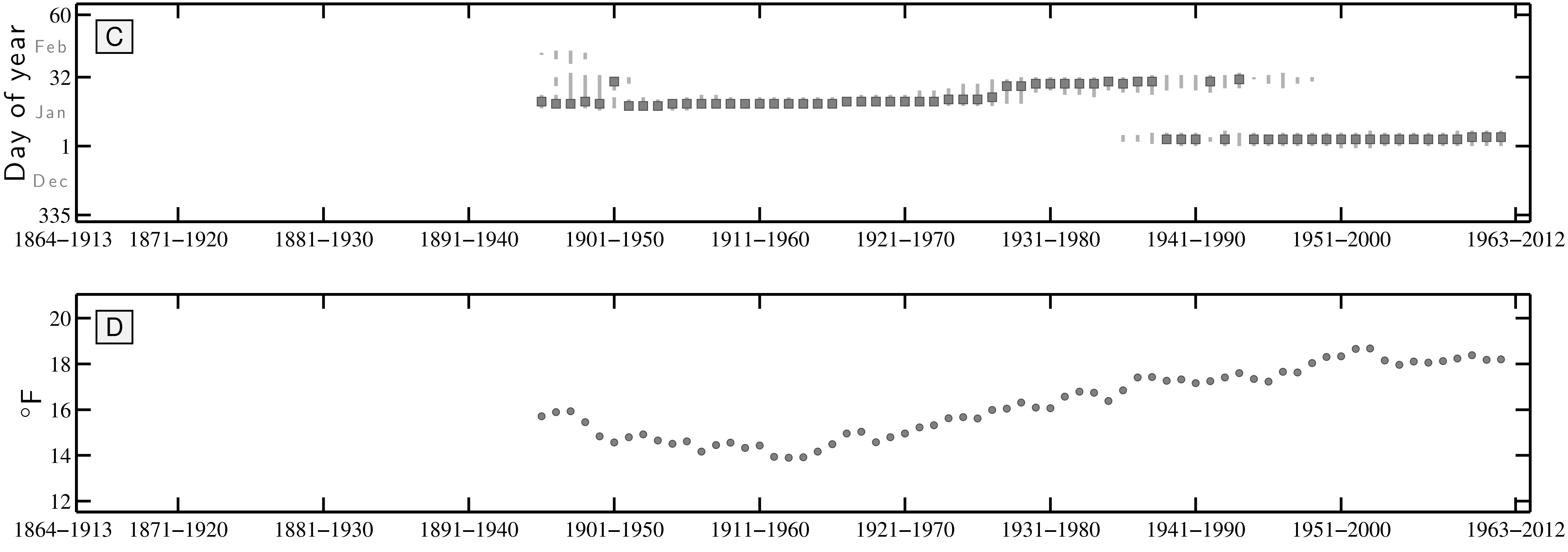

50 year Summer Teletherm dynamics for SALMON-KSRA, ID:

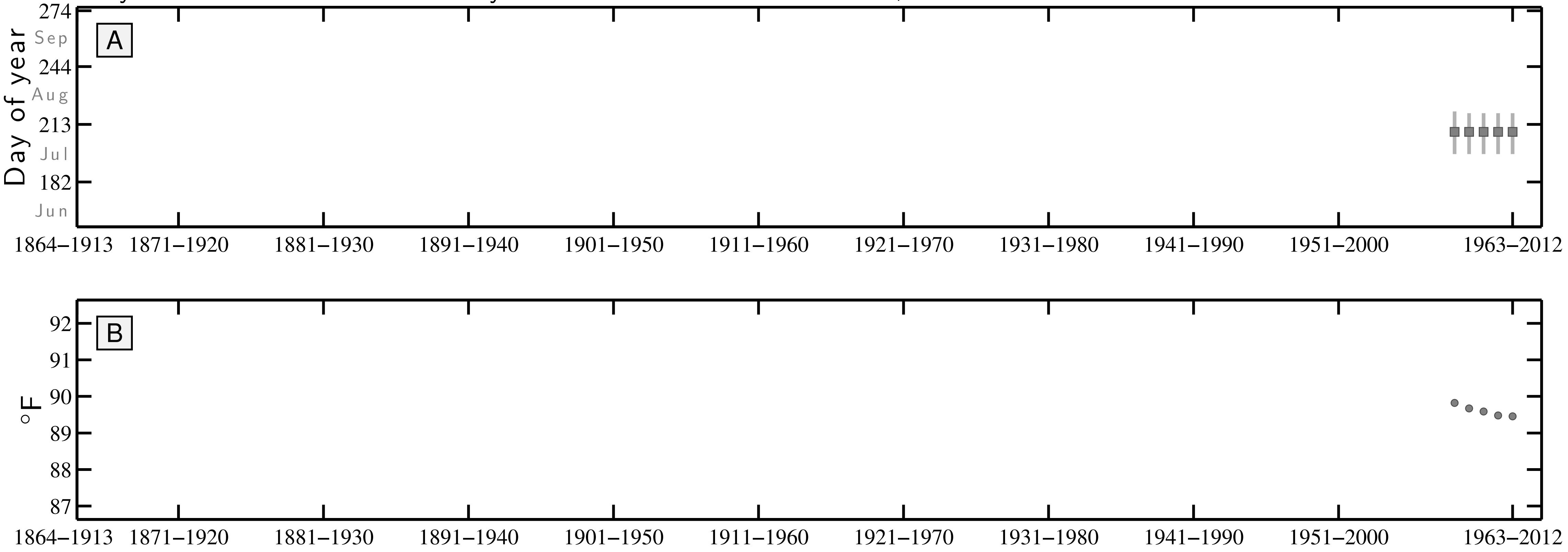

50 year Winter Teletherm dynamics for SALMON-KSRA, ID:

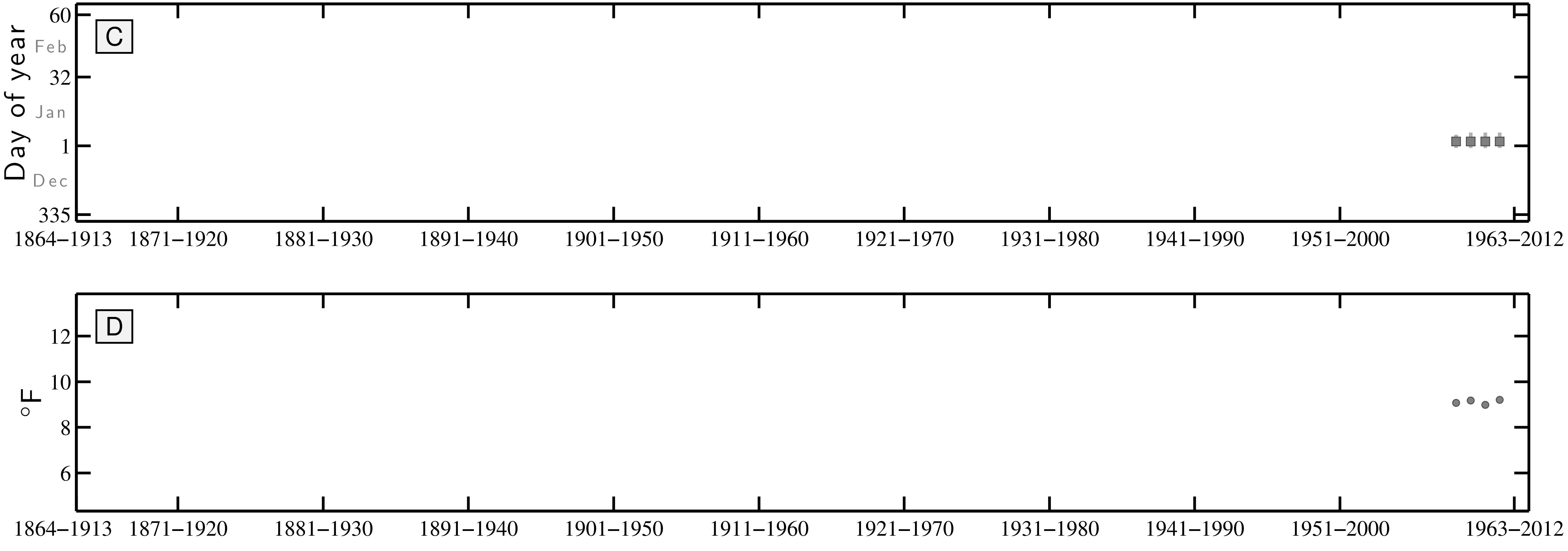

50 year Summer Teletherm dynamics for SANDPOINT EXP STN, ID:

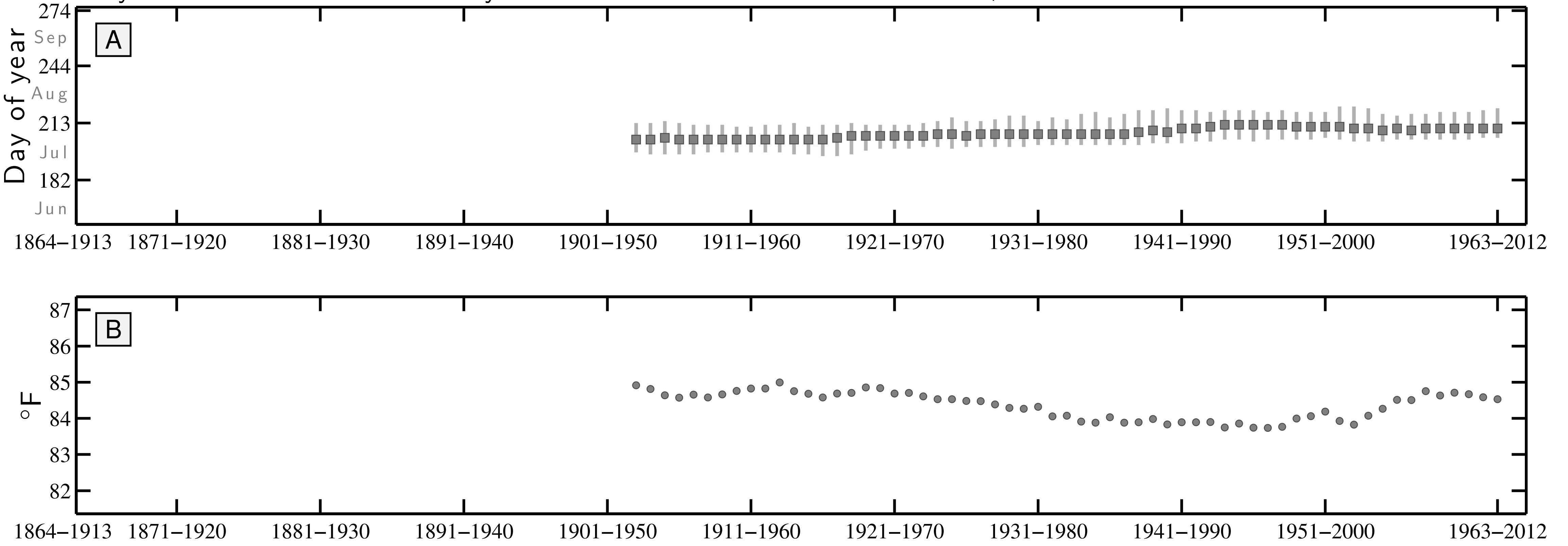

50 year Winter Teletherm dynamics for SANDPOINT EXP STN, ID:

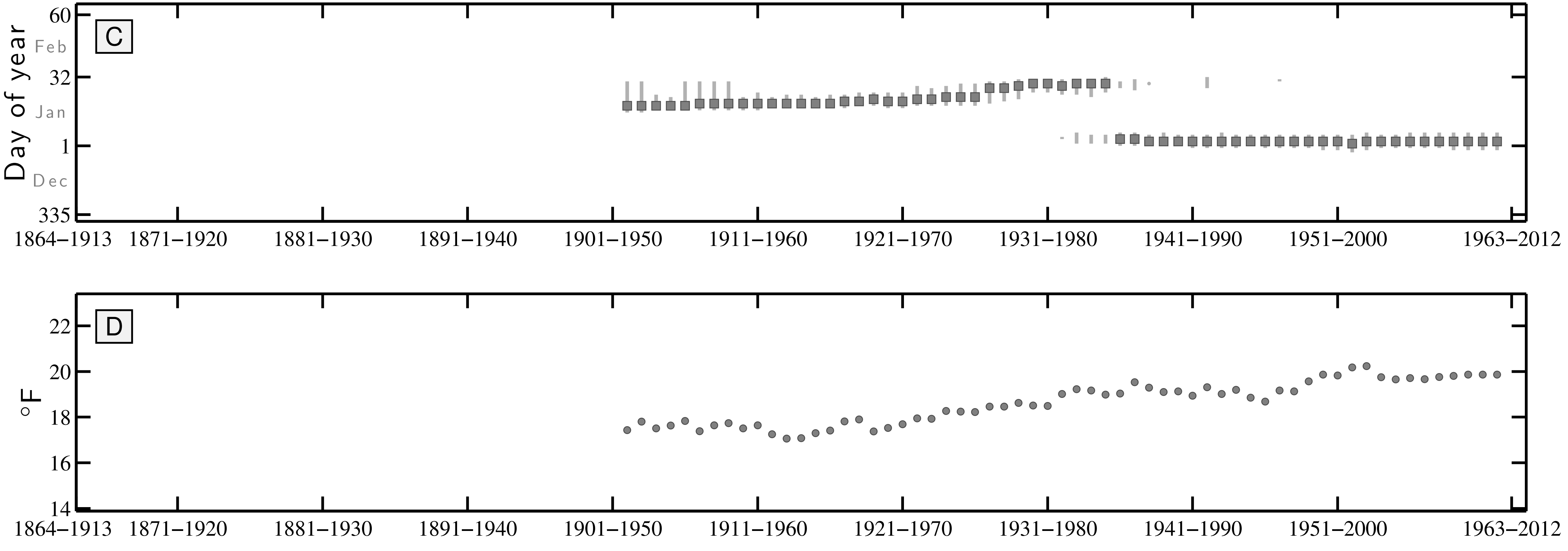

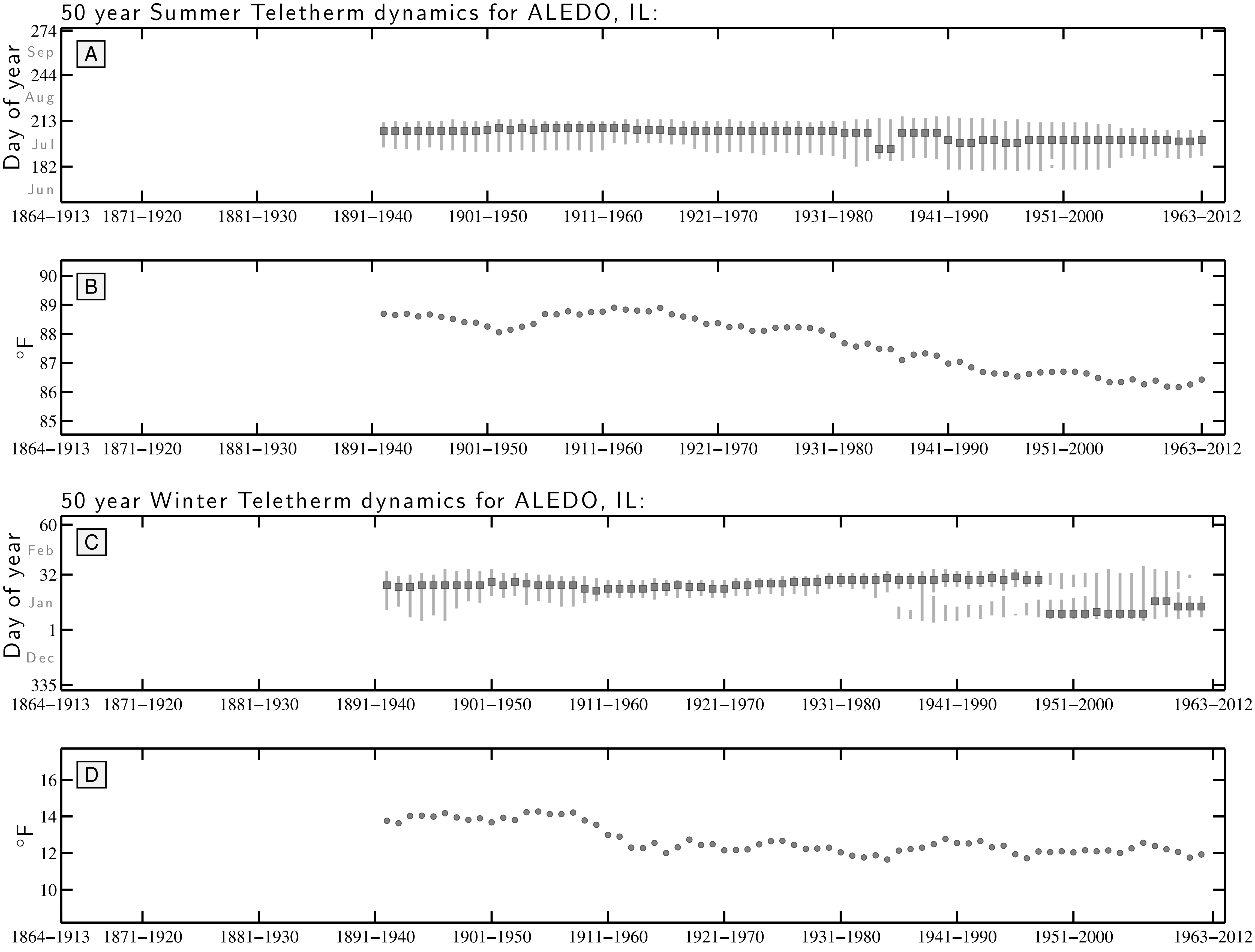

50 year Summer Teletherm dynamics for ALEDO, IL:
A
Day of year
274
Sep
244
Aug
213
Jul
182
Jun
1864–1913
1871–1920
1881–1930
1891–1940
1901–1950
1911–1960
1921–1970
1931–1980
1941–1990
1951–2000
1963–2012
B
°F
90
89
88
87
86
85
50 year Winter Teletherm dynamics for ALEDO, IL:
C
60
Feb
32
Jan
1
Dec
335
D
16
14
12
10

50 year Summer Teletherm dynamics for ANNA 2 NNE, IL:

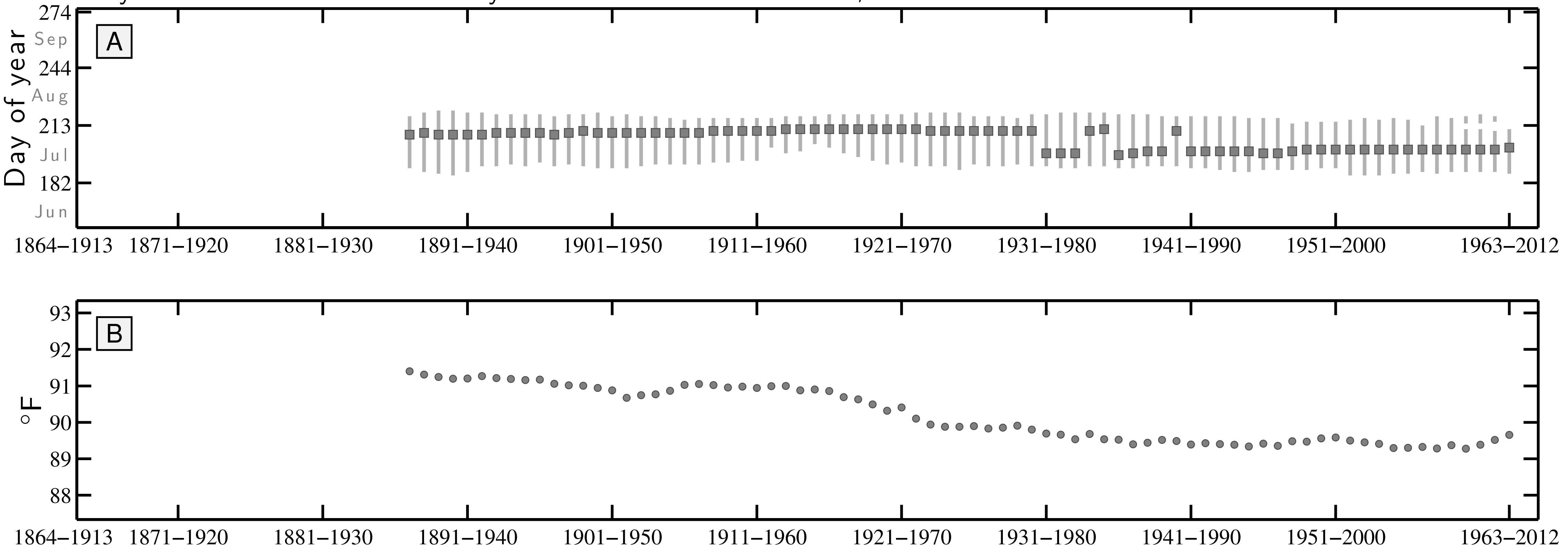

50 year Winter Teletherm dynamics for ANNA 2 NNE, IL:

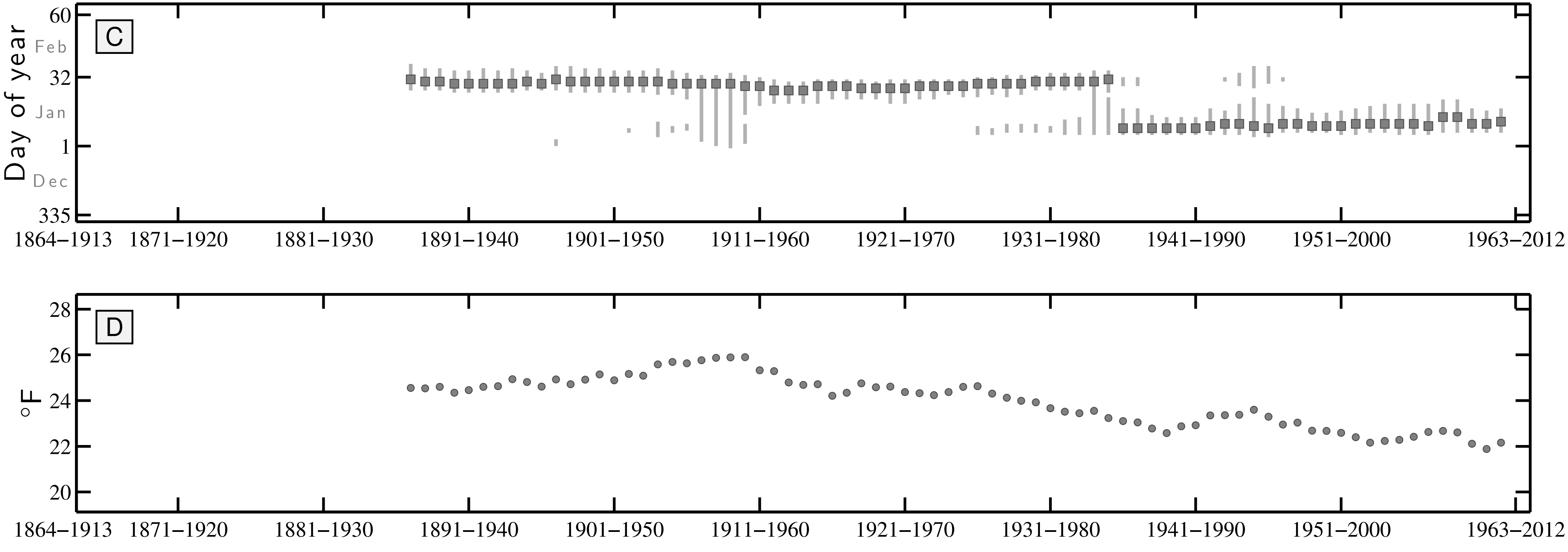

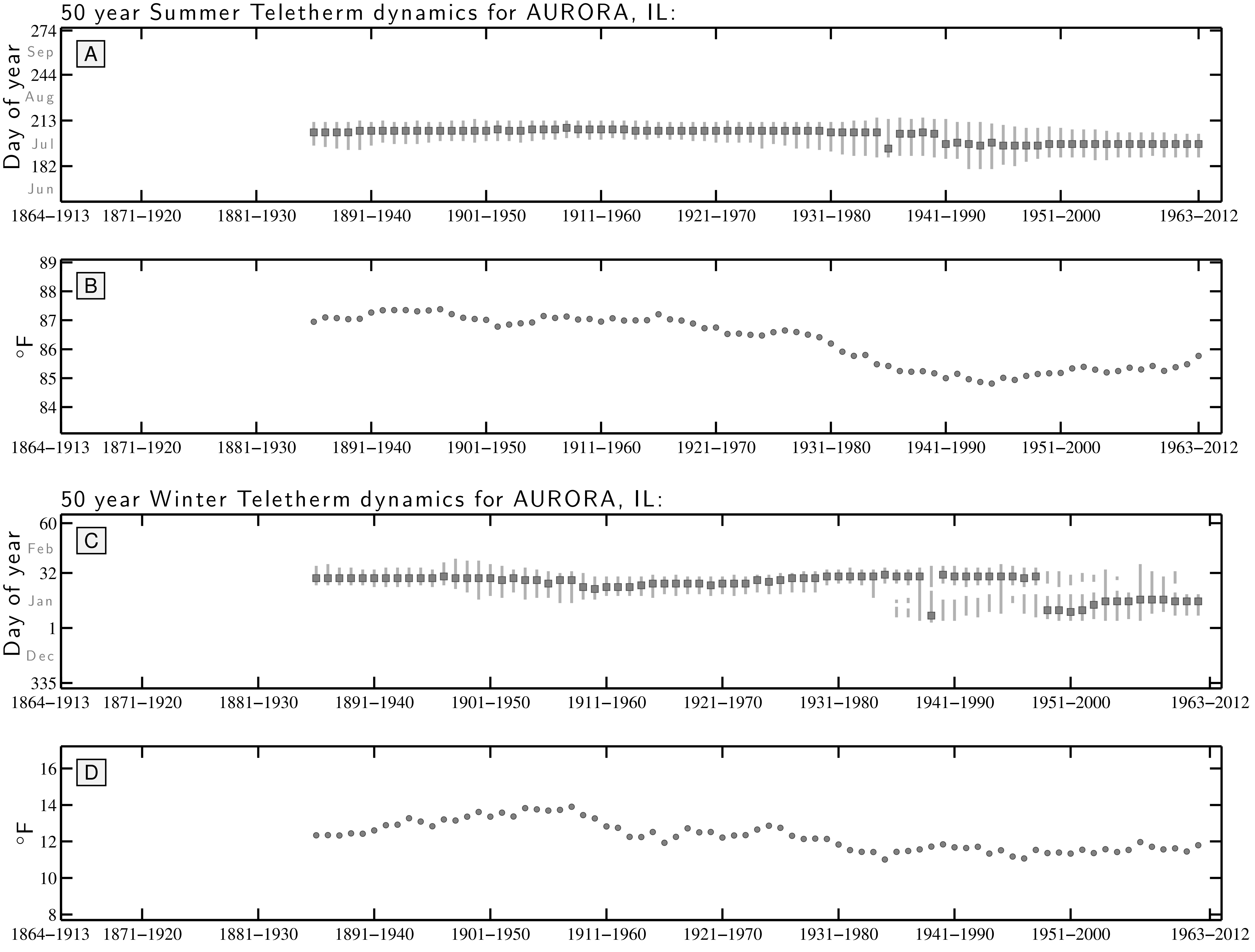
50 year Summer Teletherm dynamics for AURORA, IL:
A
B
50 year Winter Teletherm dynamics for AURORA, IL:
C
D
Day of year
°F
Jun
Jul
Aug
Sep
Dec
Jan
Feb
182
213
244
274
335
1
32
60
84
85
86
87
88
89
8
10
12
14
16
1864–1913
1871–1920
1881–1930
1891–1940
1901–1950
1911–1960
1921–1970
1931–1980
1941–1990
1951–2000
1963–2012

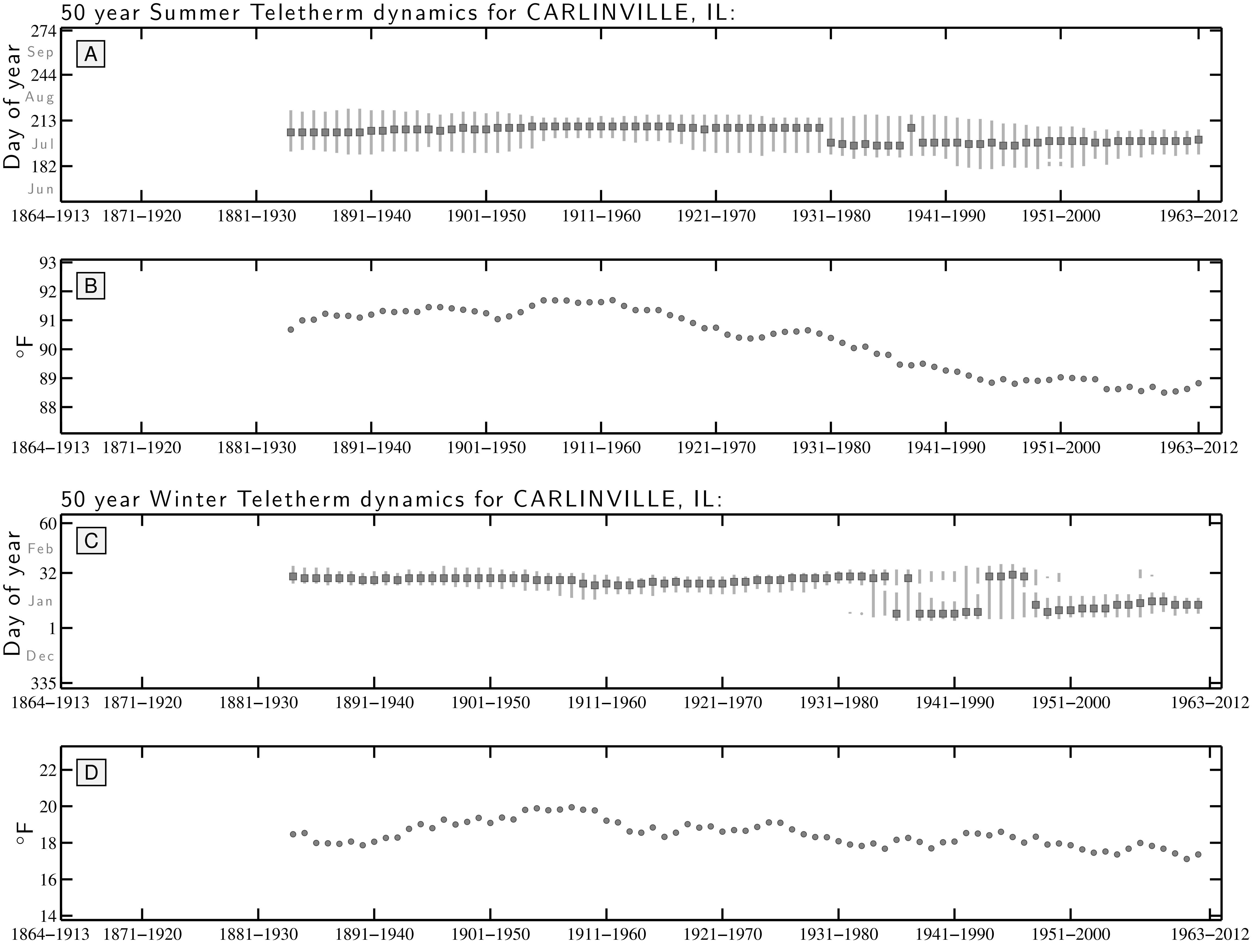

50 year Summer Teletherm dynamics for CARLINVILLE, IL:
A
B
Day of year
°F
274
244
213
182
Sep
Aug
Jul
Jun
93
92
91
90
89
88
1864–1913
1871–1920
1881–1930
1891–1940
1901–1950
1911–1960
1921–1970
1931–1980
1941–1990
1951–2000
1963–2012
50 year Winter Teletherm dynamics for CARLINVILLE, IL:
C
D
60
32
1
335
Feb
Jan
Dec
22
20
18
16
14

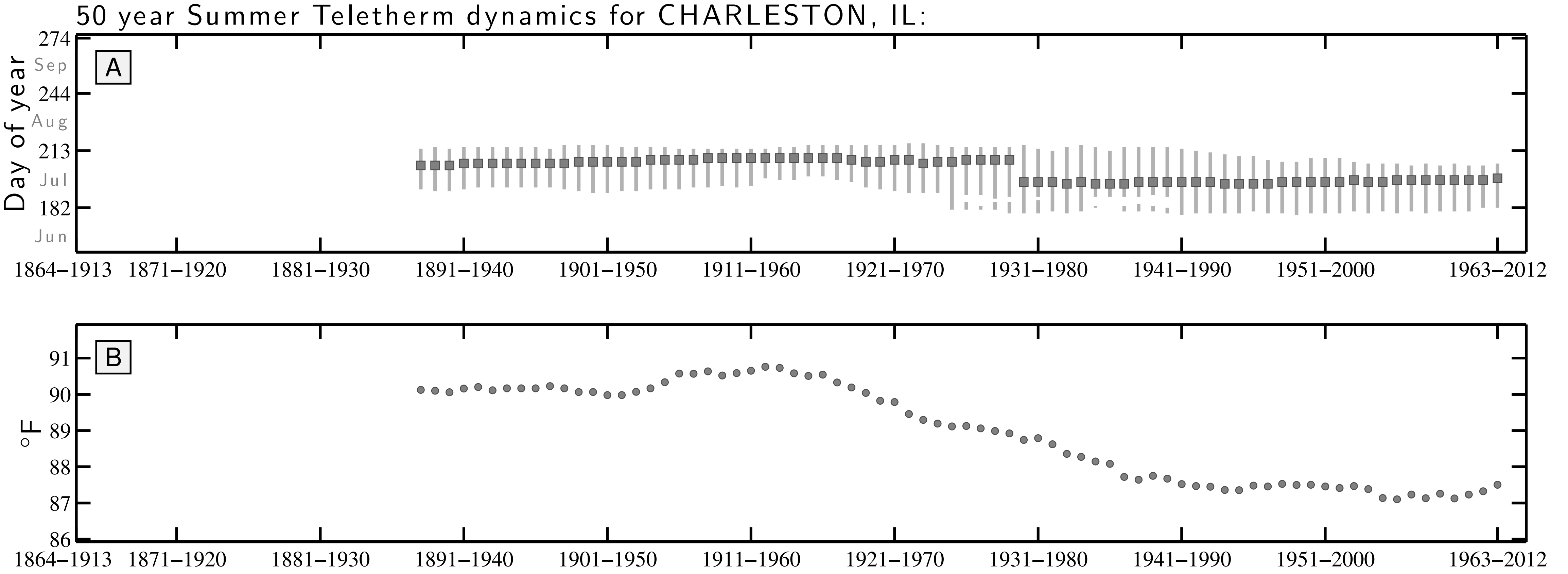
50 year Summer Teletherm dynamics for CHARLESTON, IL:
A
B
Day of year
°F
274
244
213
182
Sep
Aug
Jul
Jun
91
90
89
88
87
86
1864–1913
1871–1920
1881–1930
1891–1940
1901–1950
1911–1960
1921–1970
1931–1980
1941–1990
1951–2000
1963–2012

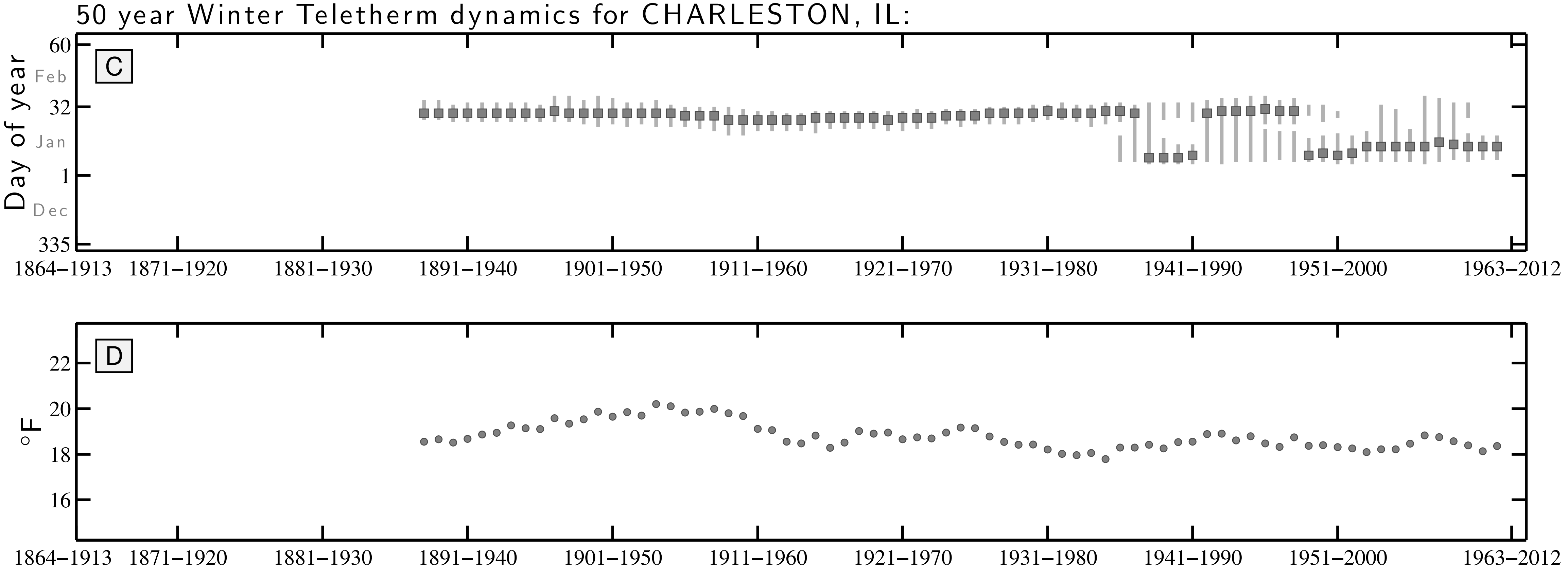
50 year Winter Teletherm dynamics for CHARLESTON, IL:
C
D
Day of year
°F
60
32
1
335
Feb
Jan
Dec
22
20
18
16
1864–1913
1871–1920
1881–1930
1891–1940
1901–1950
1911–1960
1921–1970
1931–1980
1941–1990
1951–2000
1963–2012

50 year Summer Teletherm dynamics for DANVILLE, IL:

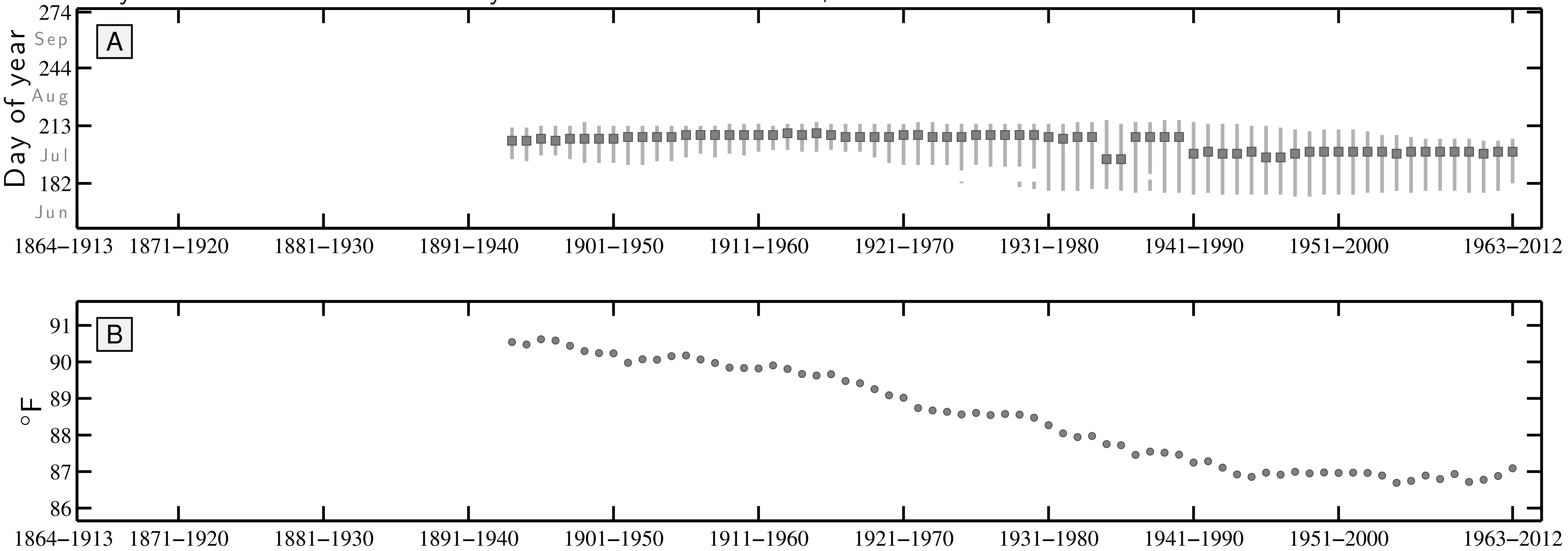

50 year Winter Teletherm dynamics for DANVILLE, IL:

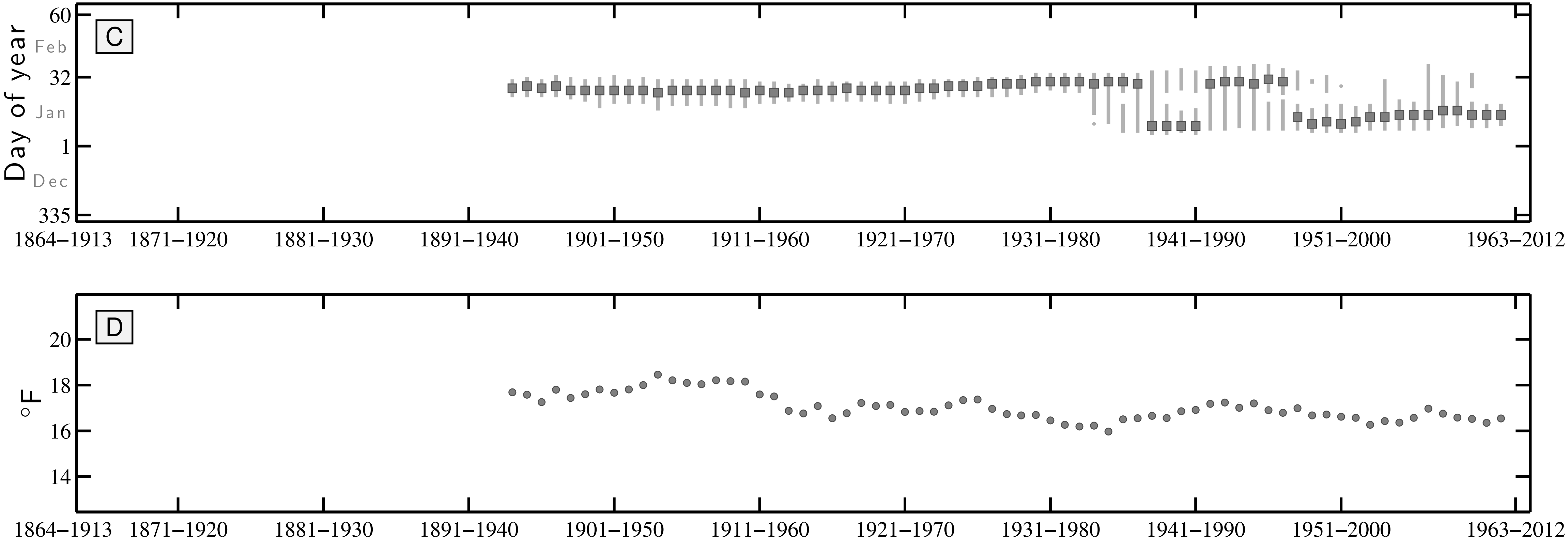

50 year Summer Teletherm dynamics for DECATUR WTP, IL:

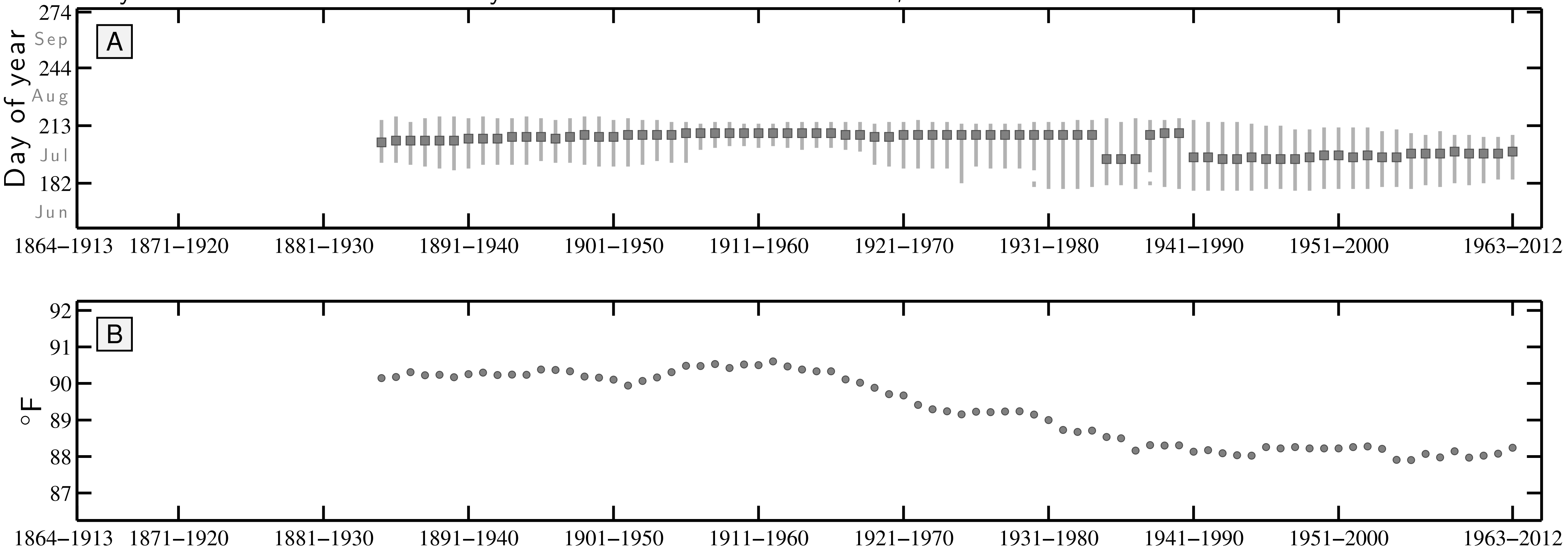

50 year Winter Teletherm dynamics for DECATUR WTP, IL:

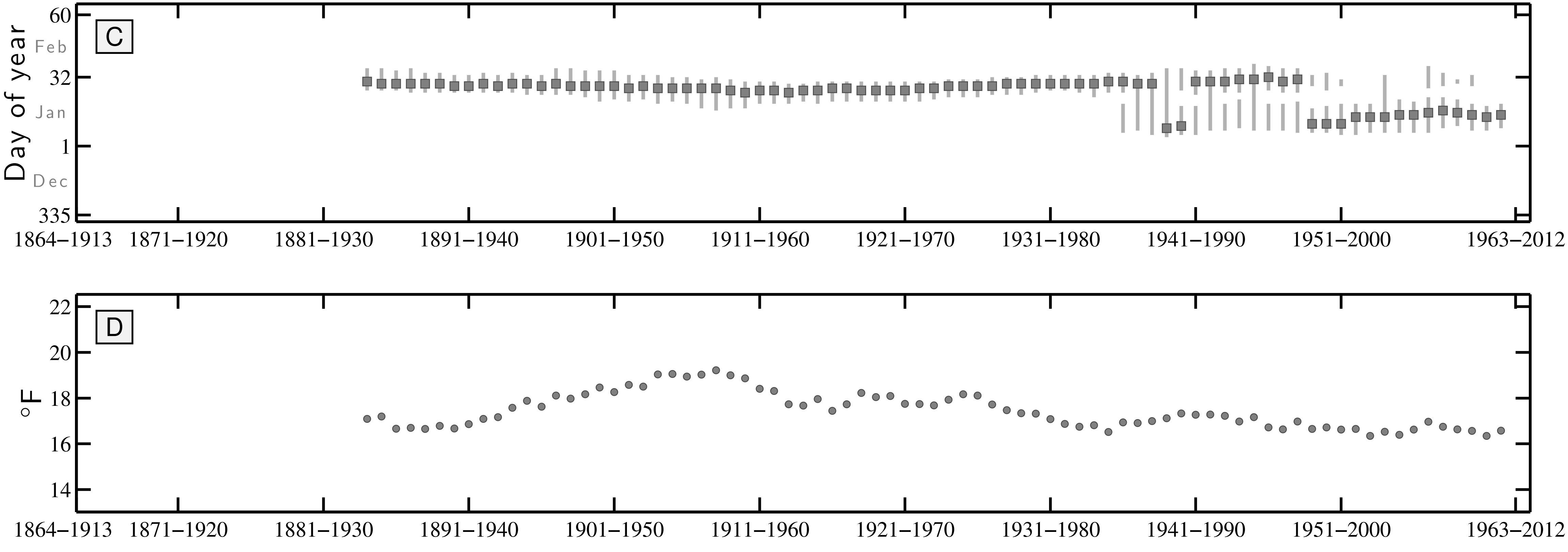

50 year Summer Teletherm dynamics for DIXON 1 NW, IL:

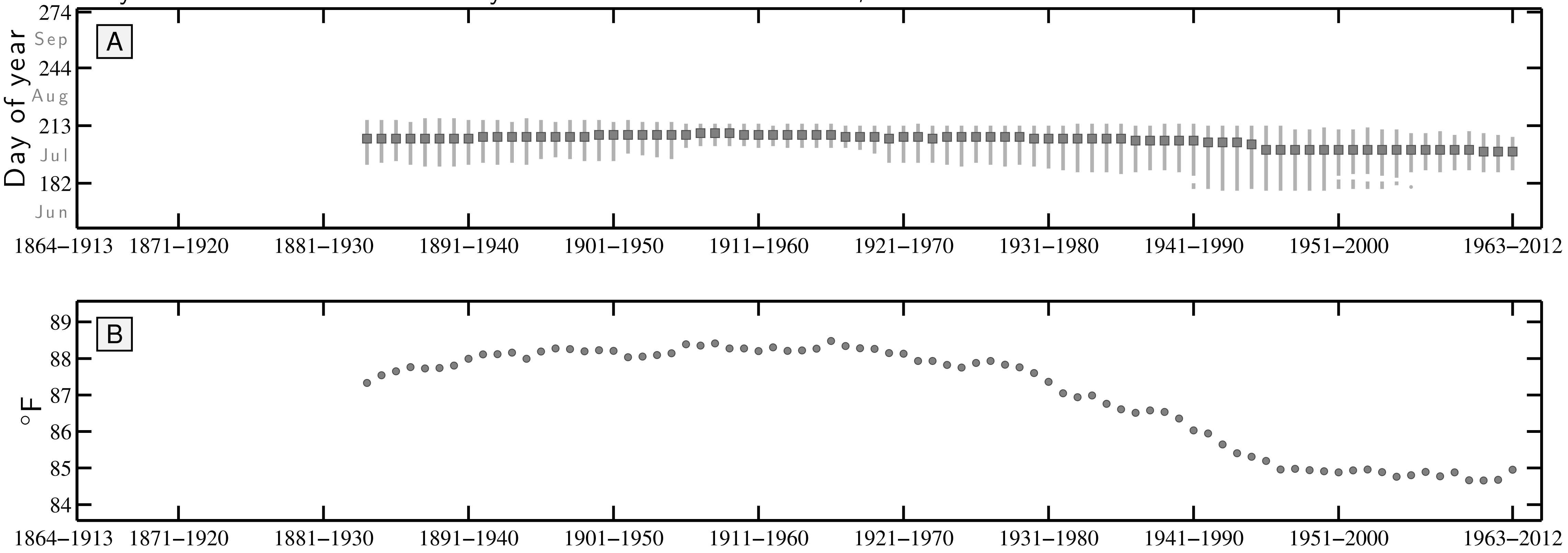

50 year Winter Teletherm dynamics for DIXON 1 NW, IL:

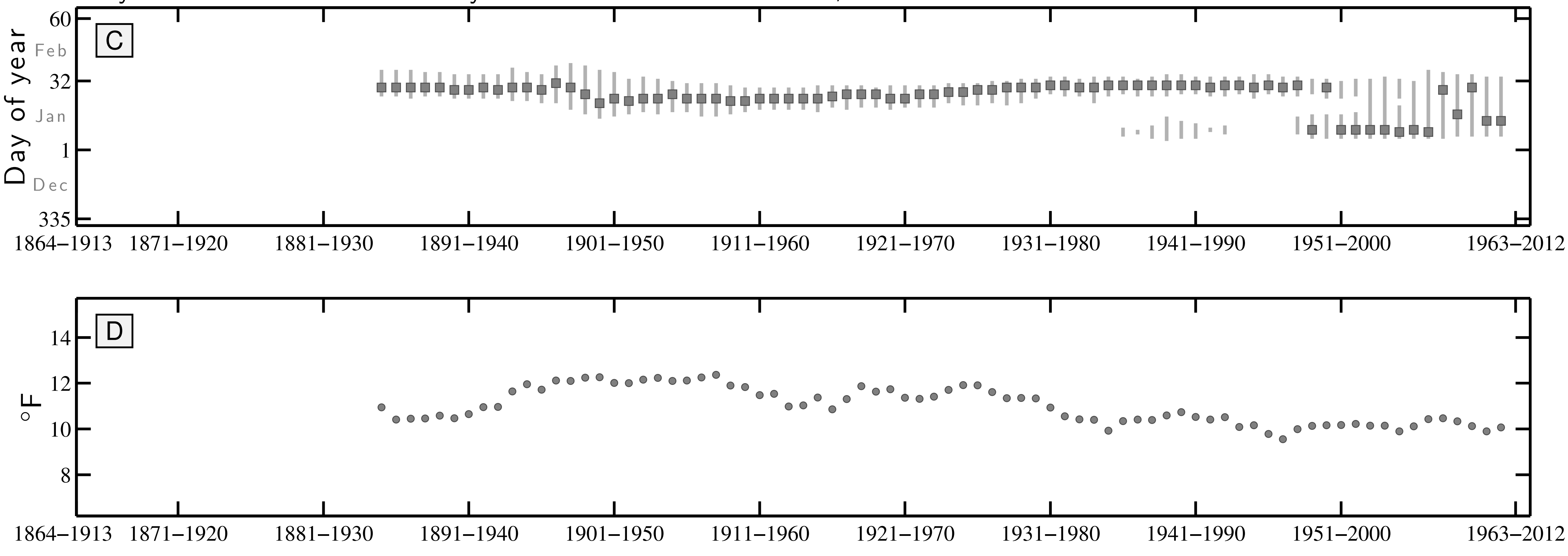

50 year Summer Teletherm dynamics for DU QUOIN 4 SE, IL:

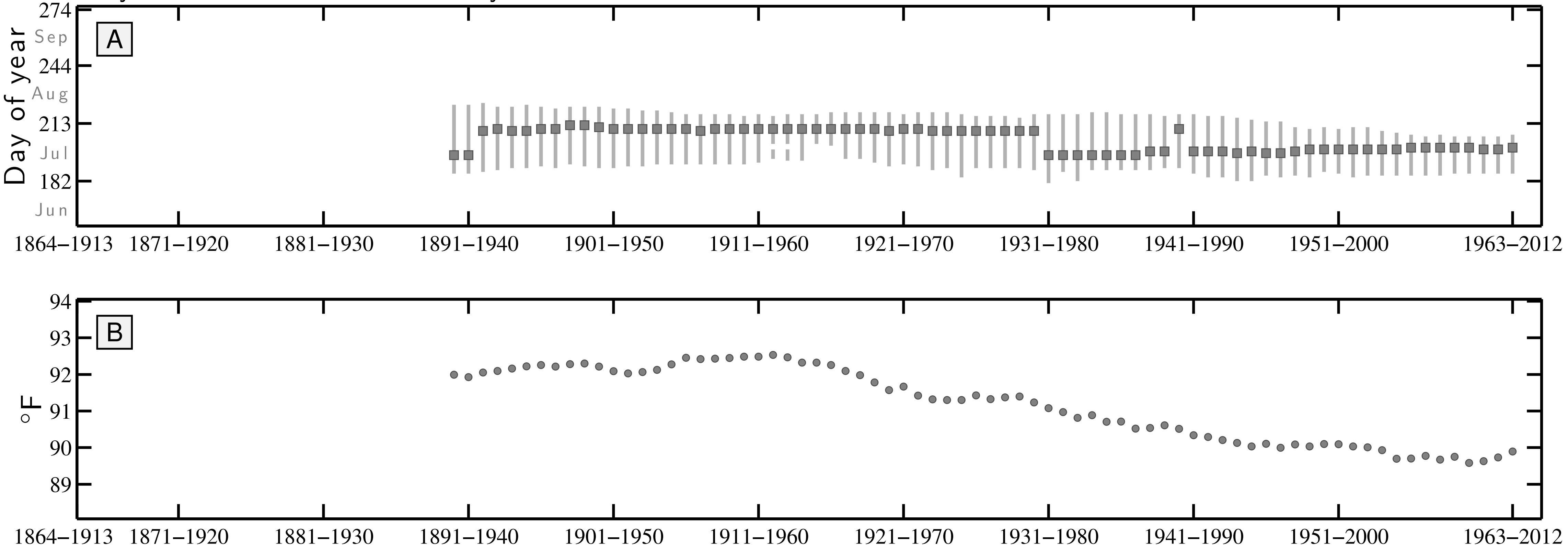

50 year Winter Teletherm dynamics for DU QUOIN 4 SE, IL:

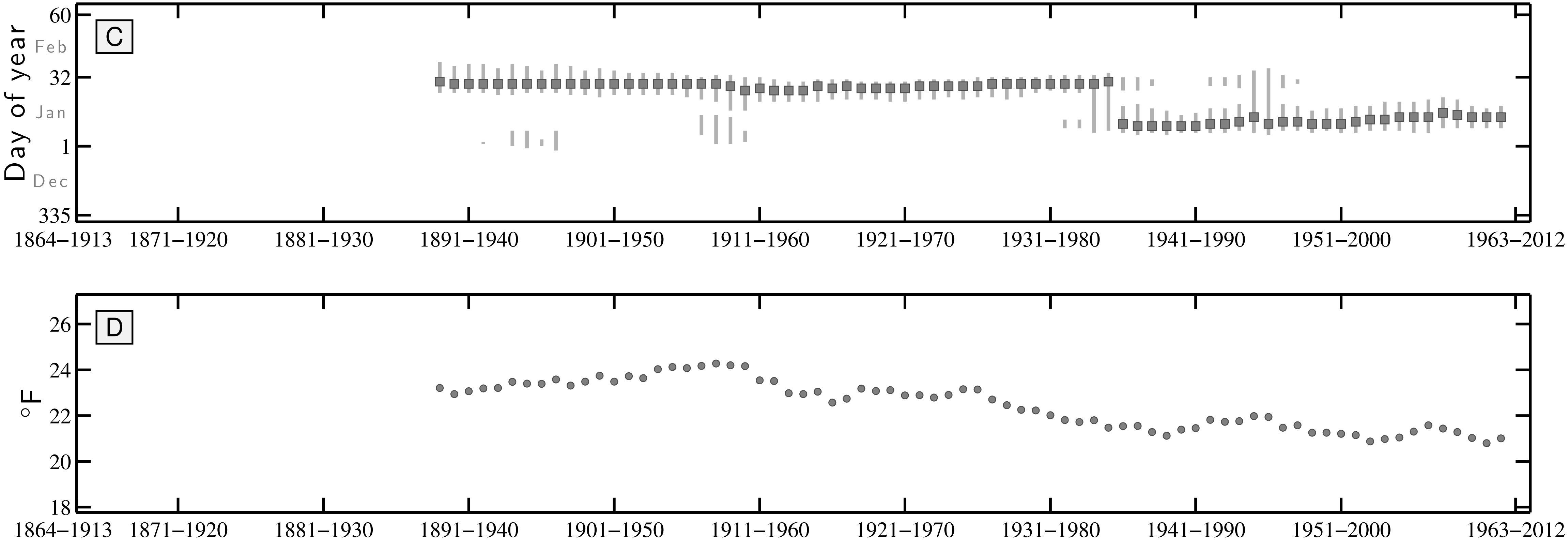

50 year Summer Teletherm dynamics for GALVA, IL:

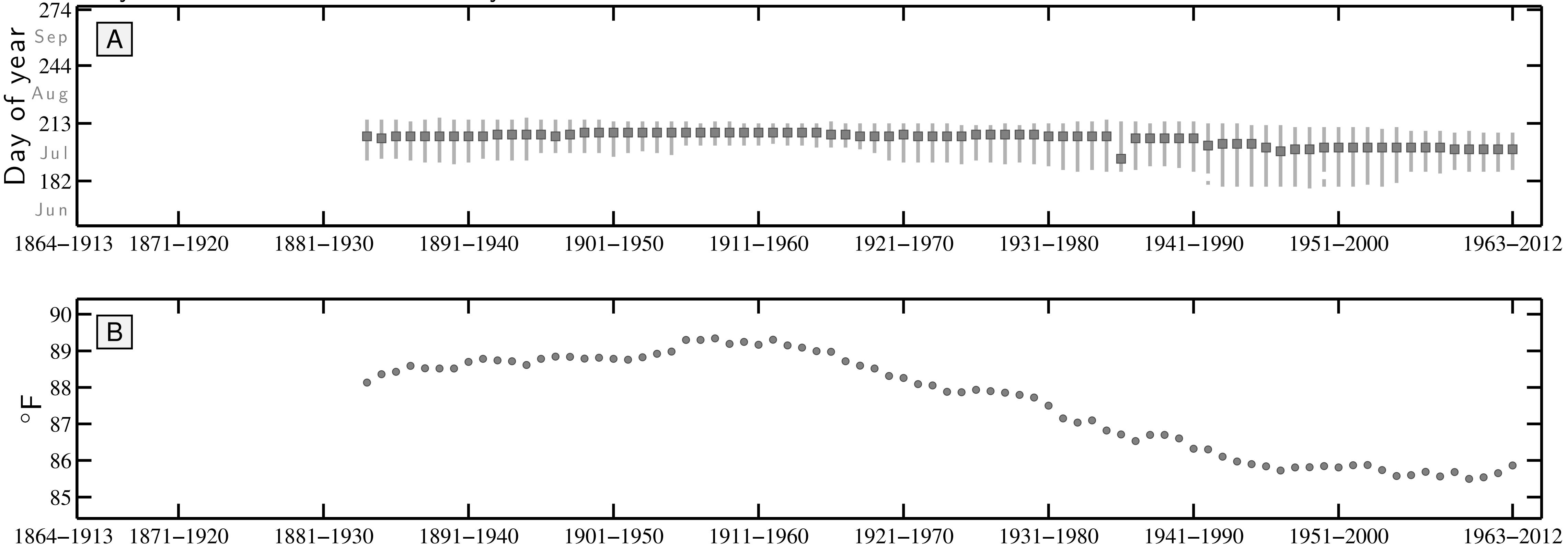

50 year Winter Teletherm dynamics for GALVA, IL:

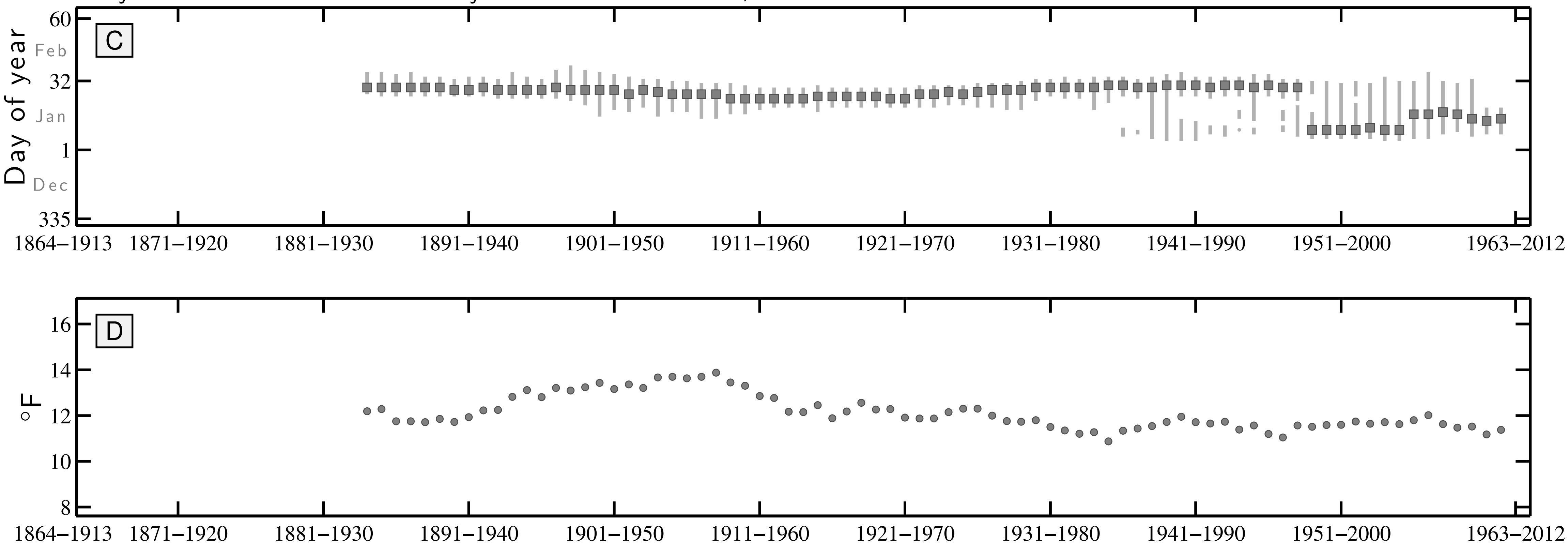

50 year Summer Teletherm dynamics for HARRISBURG, IL:

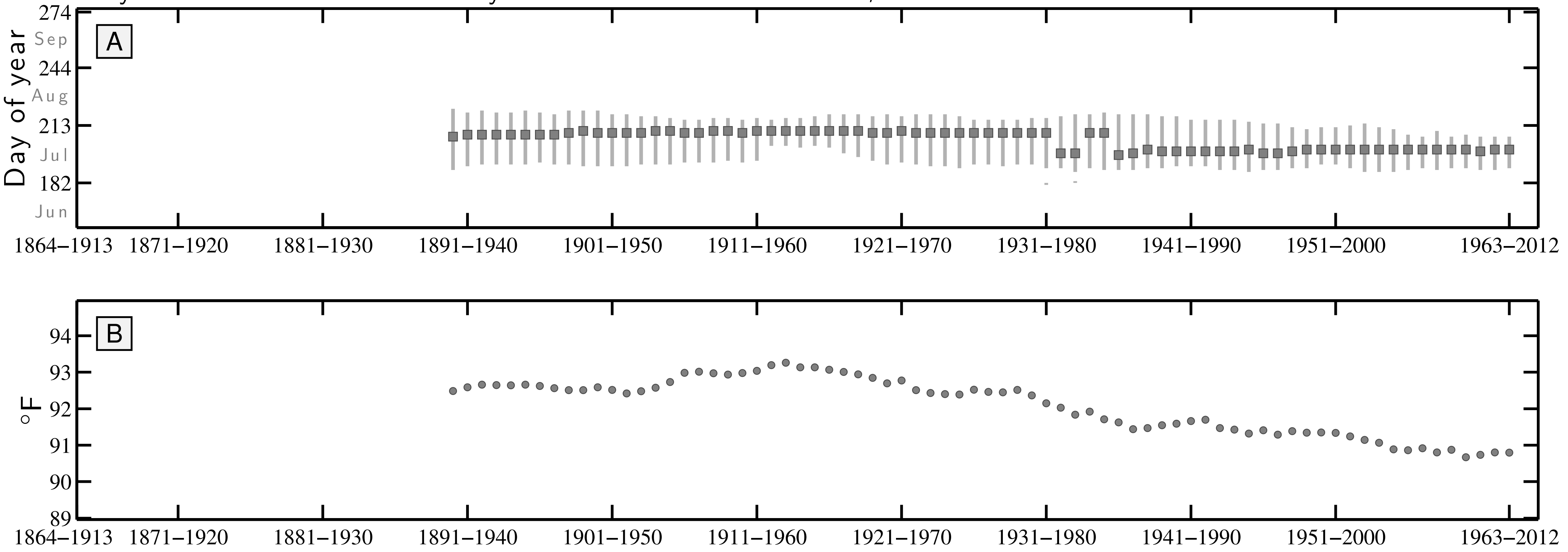

50 year Winter Teletherm dynamics for HARRISBURG, IL:

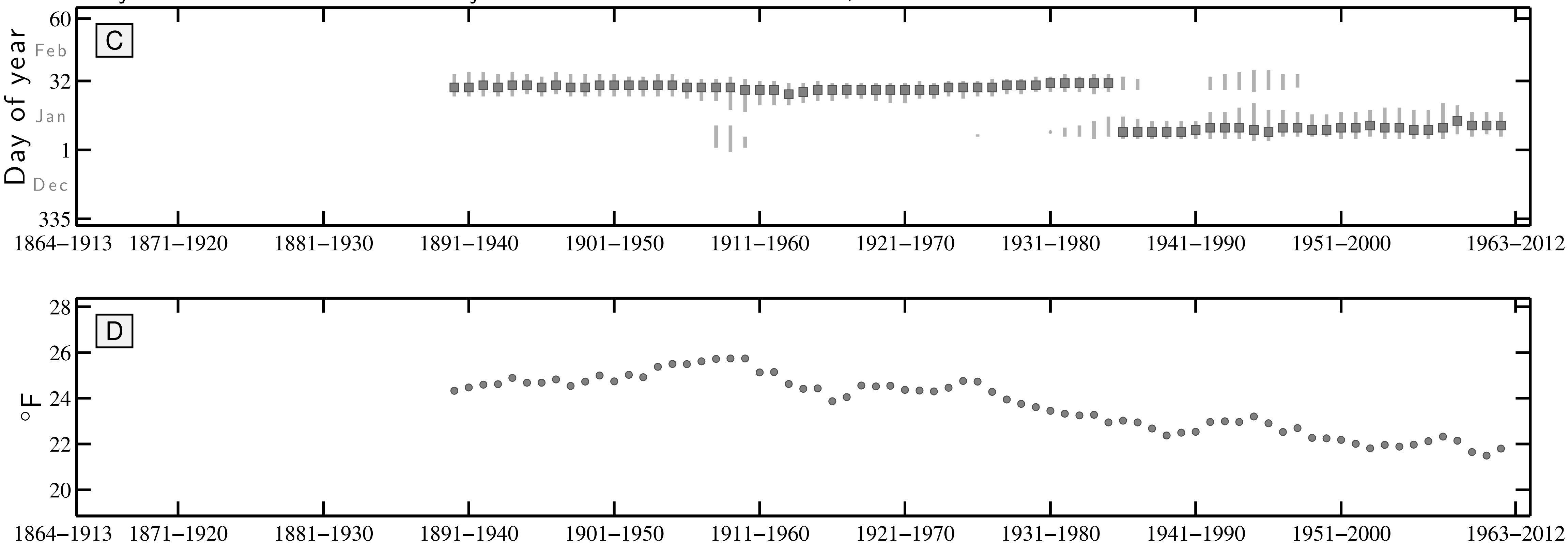

50 year Summer Teletherm dynamics for HILLSBORO, IL:

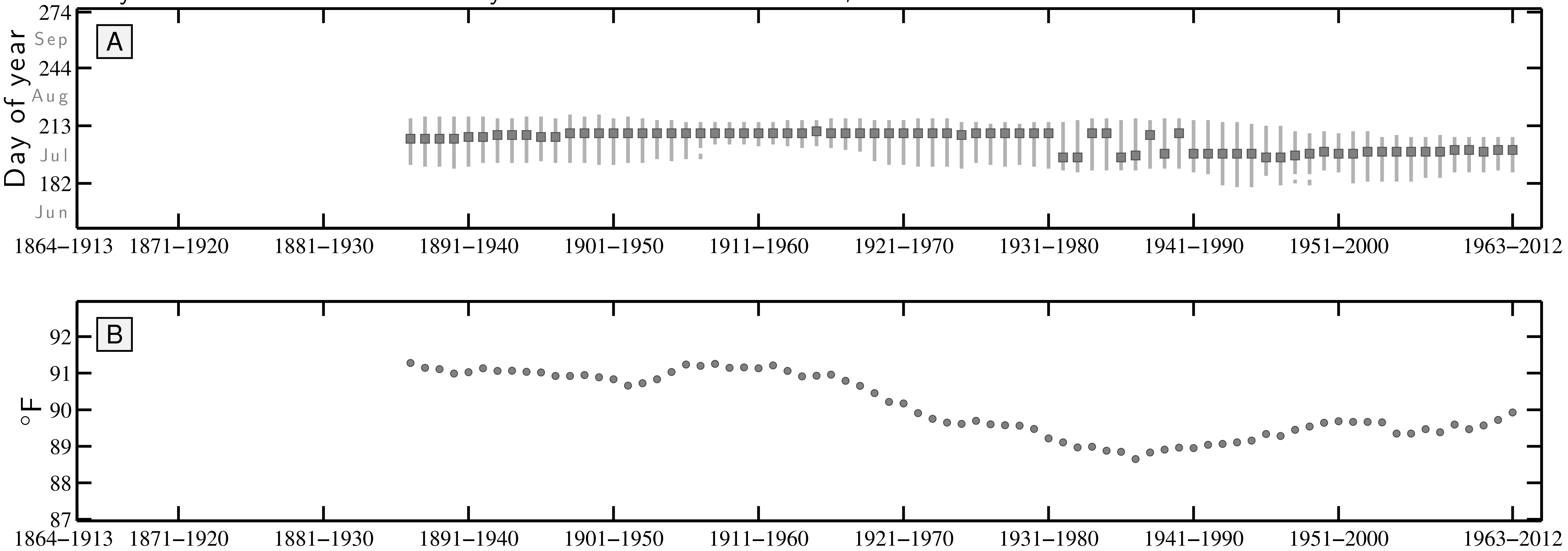

50 year Winter Teletherm dynamics for HILLSBORO, IL:

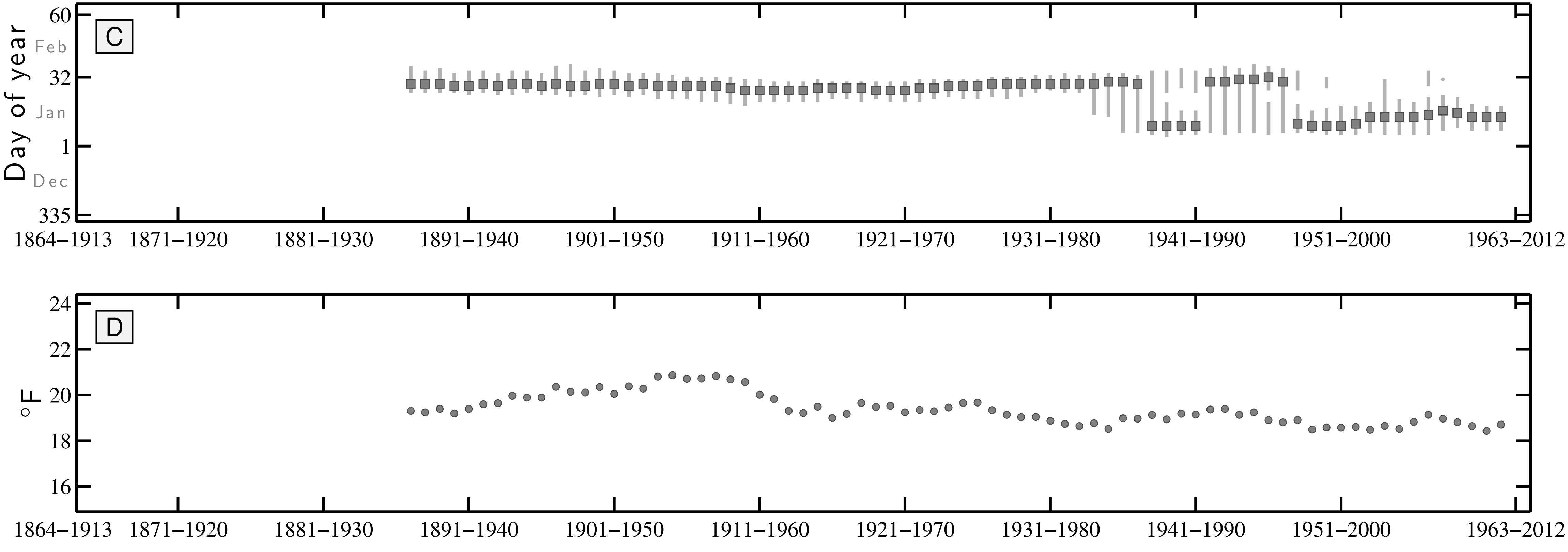

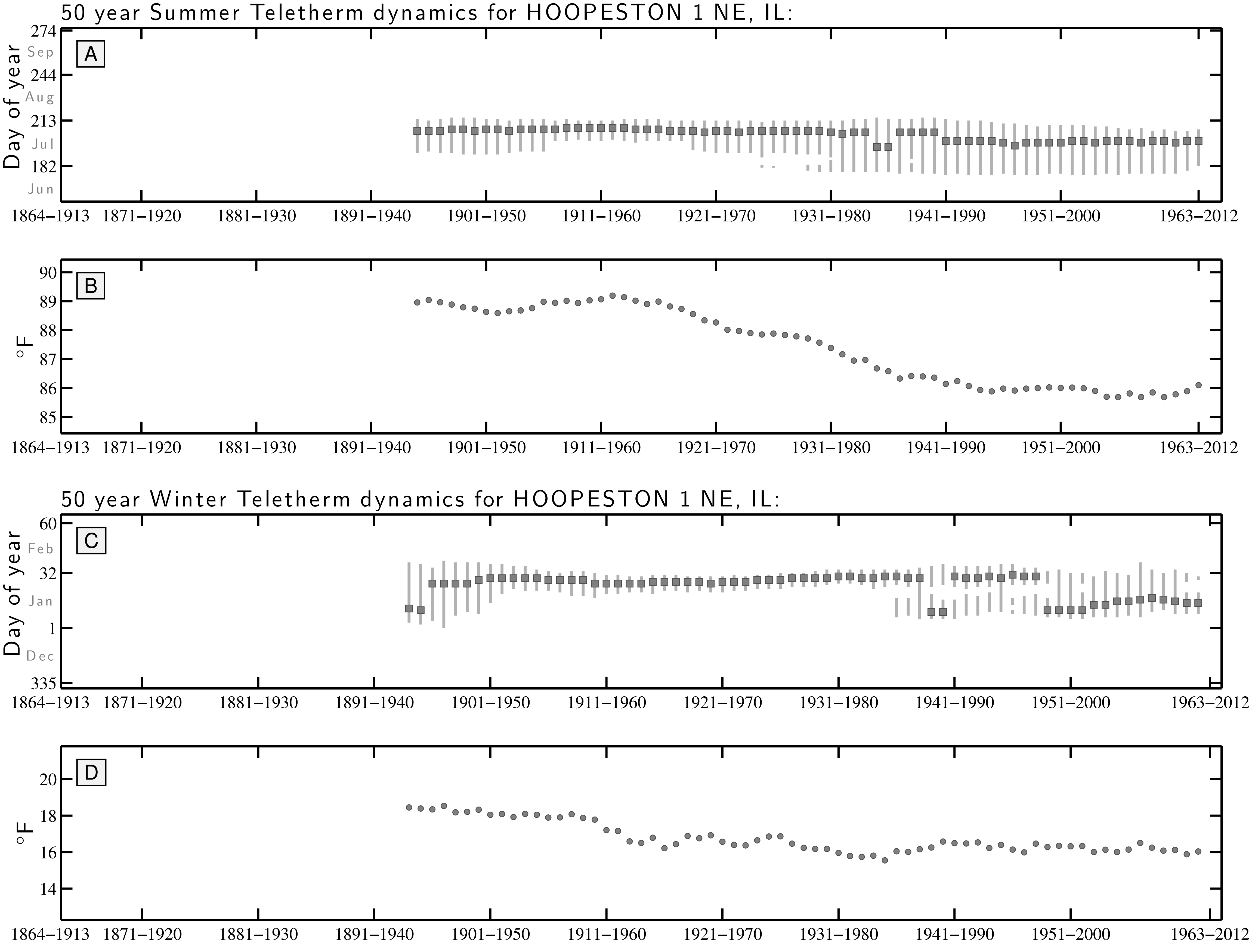

50 year Summer Teletherm dynamics for HOOPESTON 1 NE, IL:
A
Day of year
274
Sep
244
Aug
213
Jul
182
Jun
1864–1913
1871–1920
1881–1930
1891–1940
1901–1950
1911–1960
1921–1970
1931–1980
1941–1990
1951–2000
1963–2012
B
°F
90
89
88
87
86
85
50 year Winter Teletherm dynamics for HOOPESTON 1 NE, IL:
C
60
Feb
32
Jan
1
Dec
335
D
20
18
16
14

50 year Summer Teletherm dynamics for JACKSONVILLE 2E, IL:

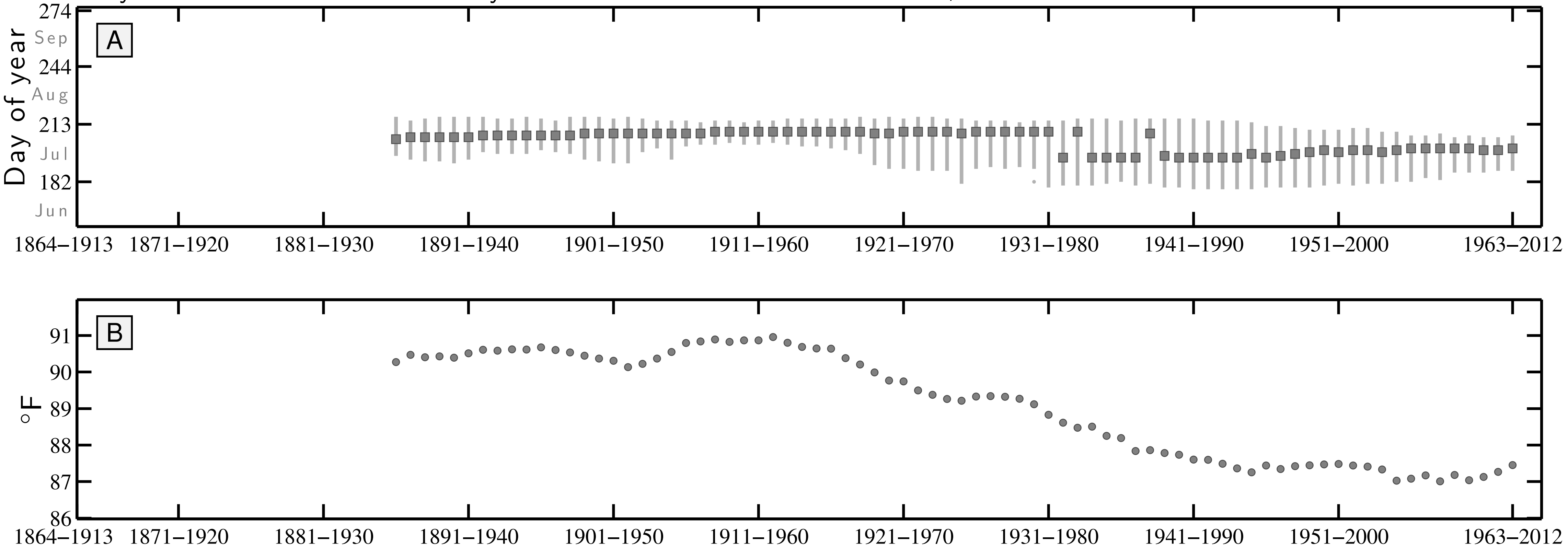

50 year Winter Teletherm dynamics for JACKSONVILLE 2E, IL:

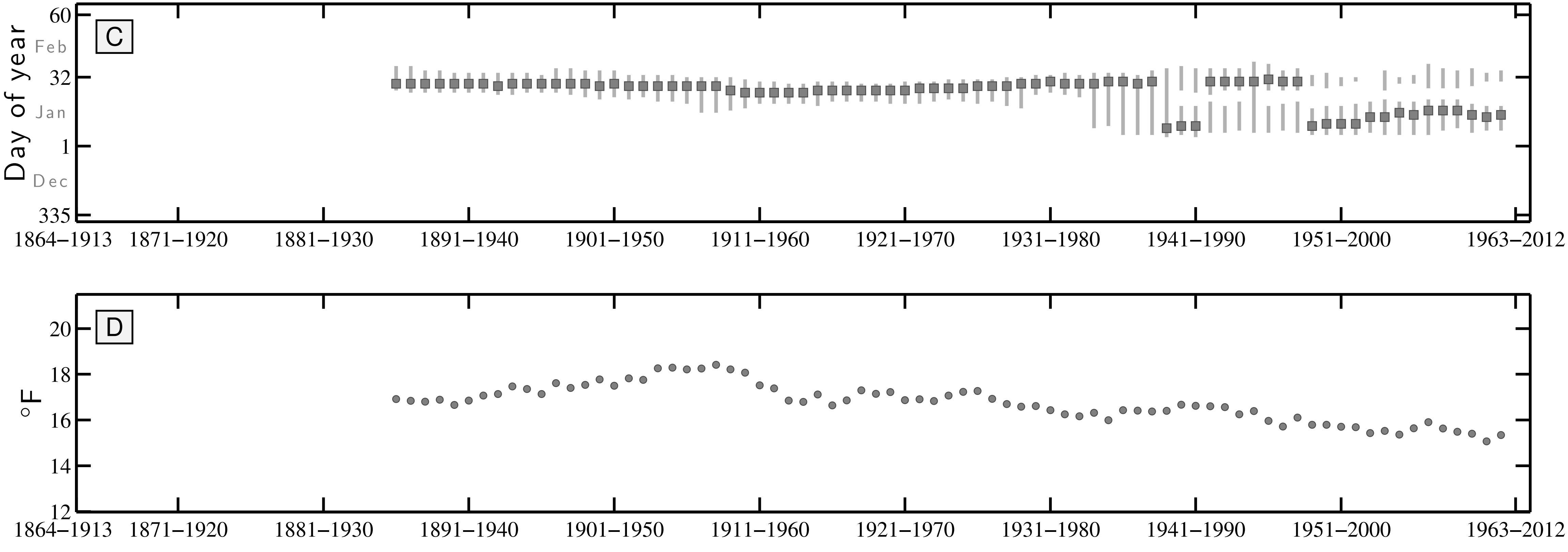

50 year Summer Teletherm dynamics for LA HARPE, IL:

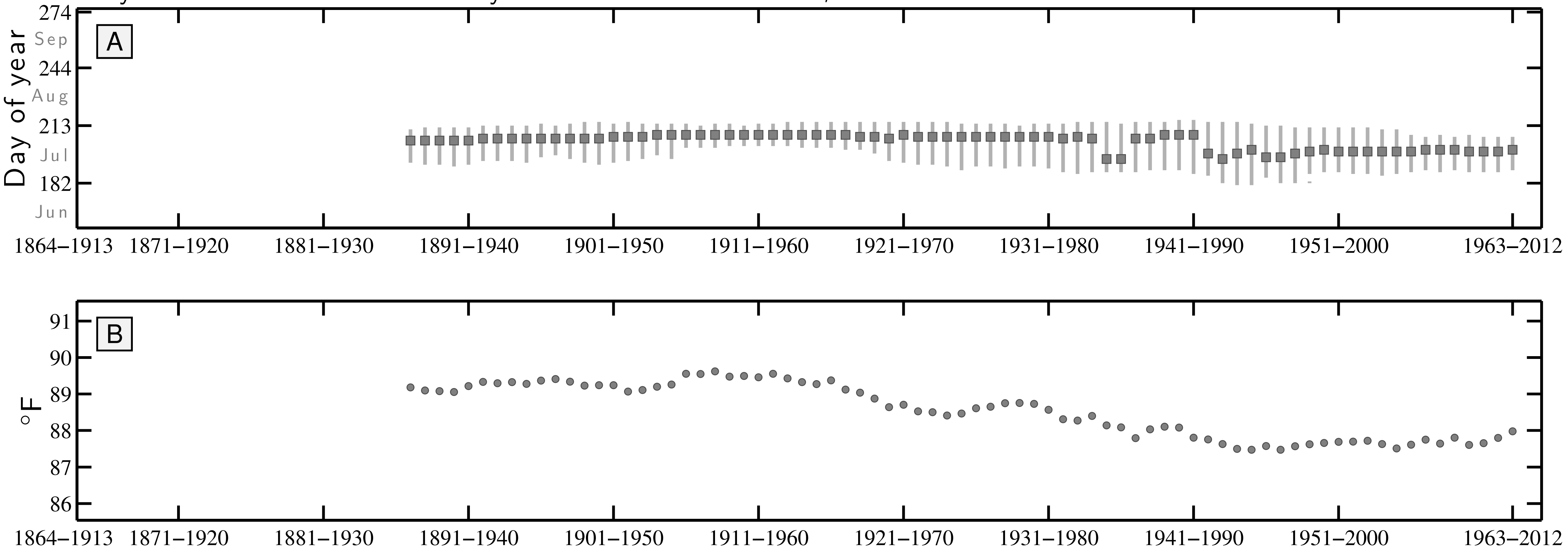

50 year Winter Teletherm dynamics for LA HARPE, IL:

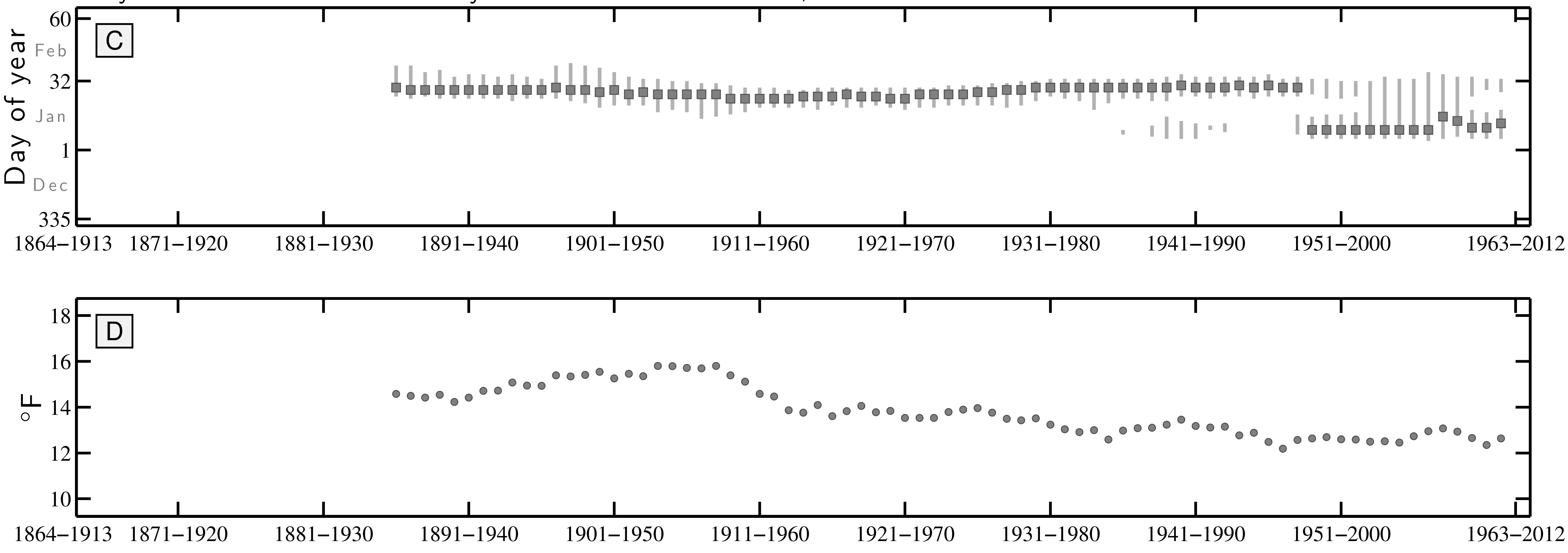

50 year Summer Teletherm dynamics for LINCOLN, IL:

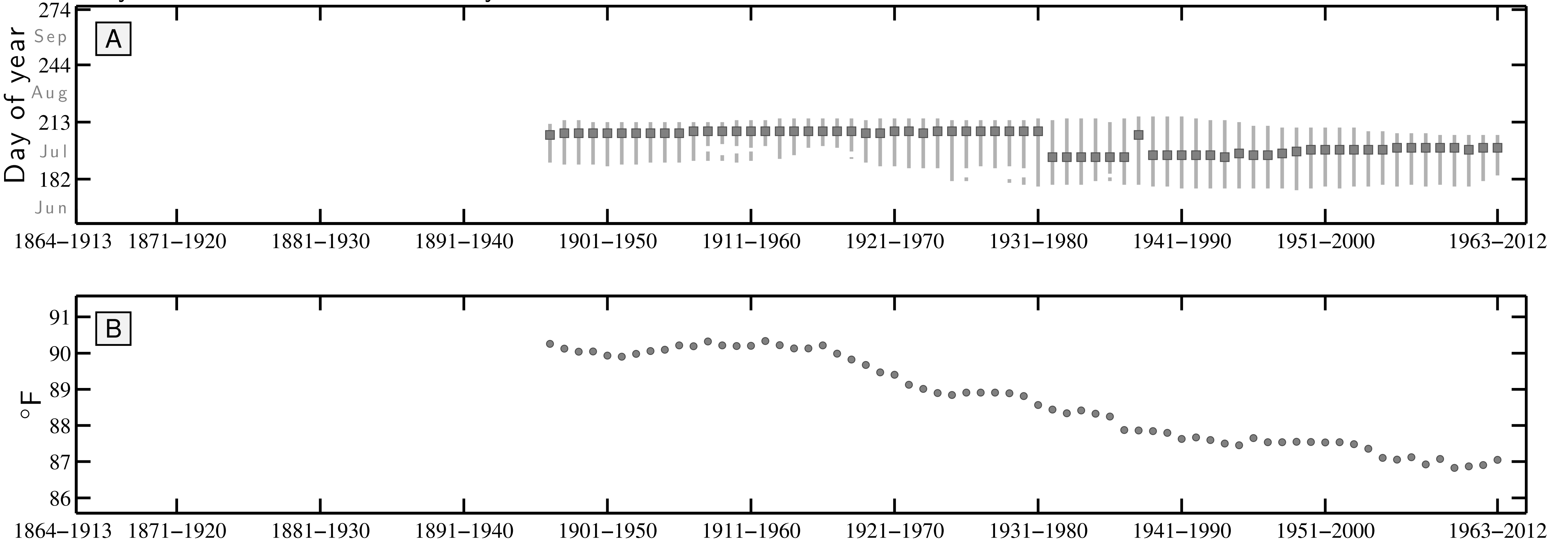

50 year Winter Teletherm dynamics for LINCOLN, IL:

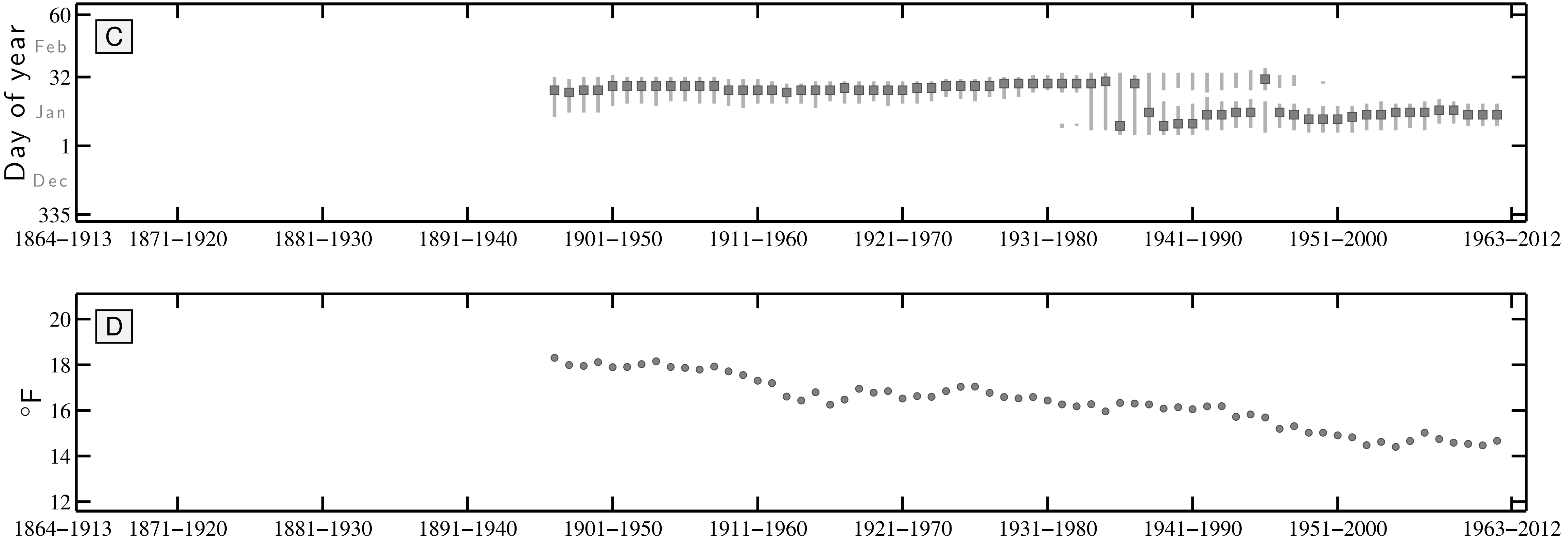

50 year Summer Teletherm dynamics for MARENGO, IL:

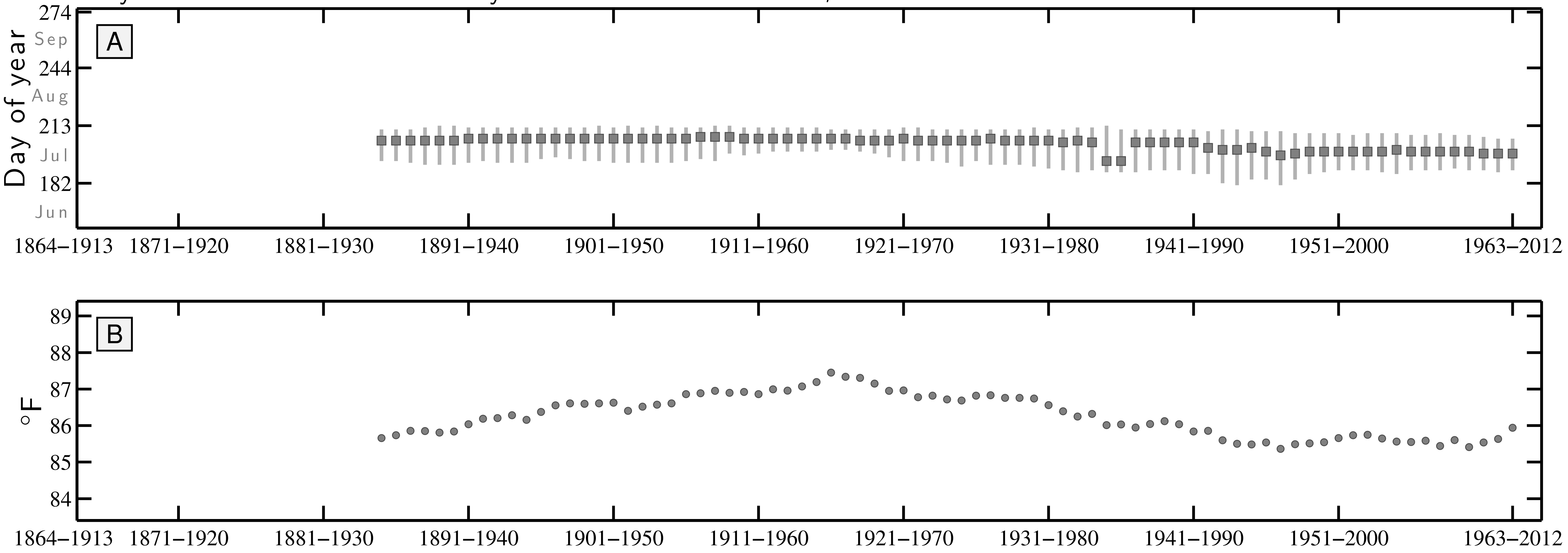

50 year Winter Teletherm dynamics for MARENGO, IL:

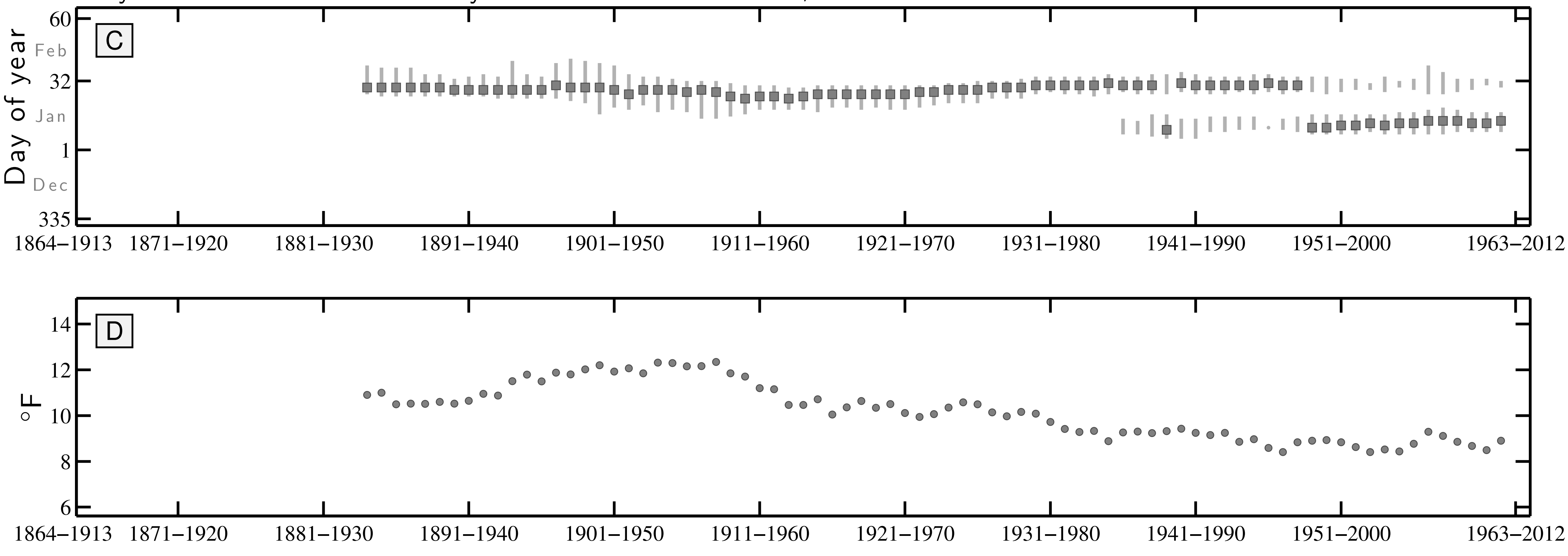

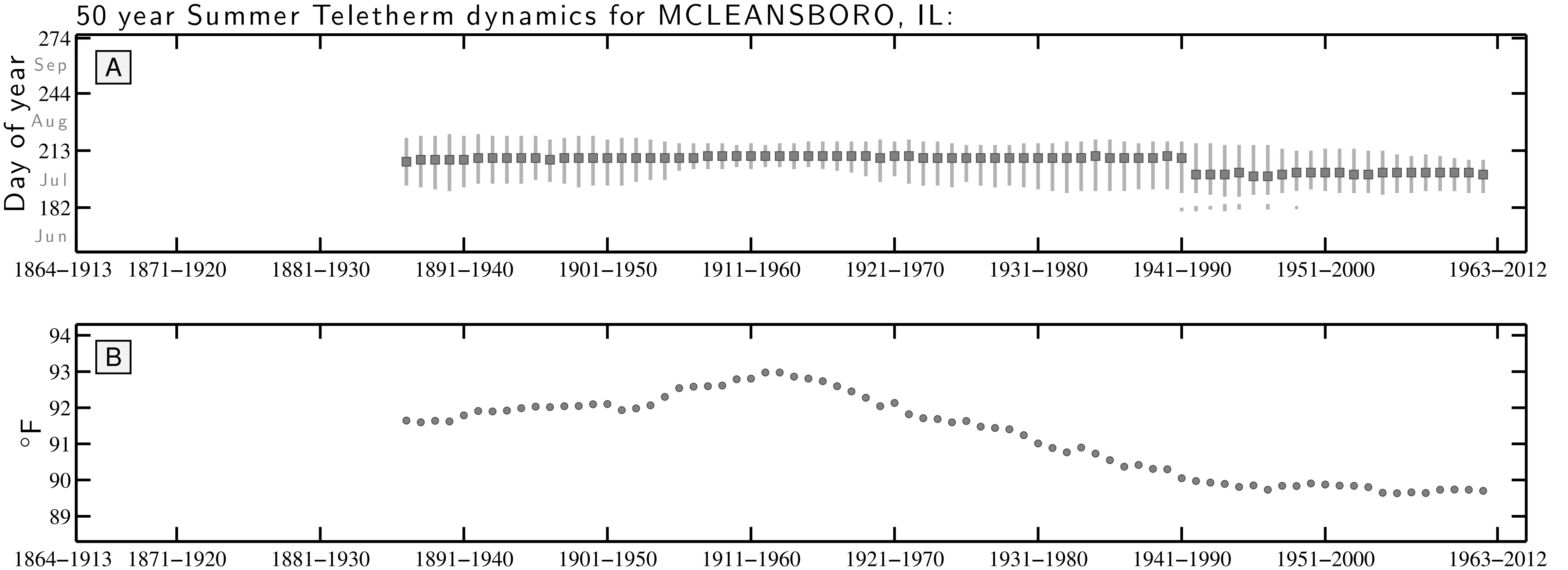
50 year Summer Teletherm dynamics for MCLEANSBORO, IL:
A
B
Day of year
°F
274
244
213
182
Sep
Aug
Jul
Jun
94
93
92
91
90
89
1864–1913
1871–1920
1881–1930
1891–1940
1901–1950
1911–1960
1921–1970
1931–1980
1941–1990
1951–2000
1963–2012

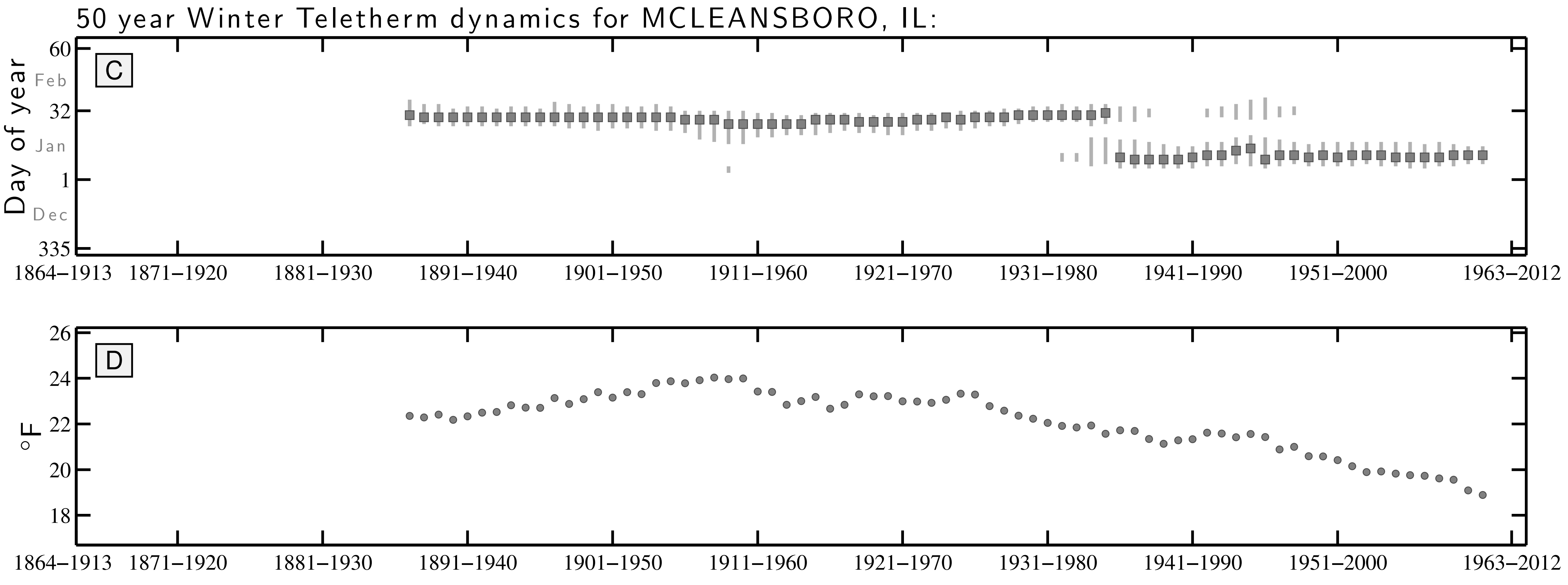
50 year Winter Teletherm dynamics for MCLEANSBORO, IL:
C
D
Day of year
°F
60
32
1
335
Feb
Jan
Dec
26
24
22
20
18
1864–1913
1871–1920
1881–1930
1891–1940
1901–1950
1911–1960
1921–1970
1931–1980
1941–1990
1951–2000
1963–2012

50 year Summer Teletherm dynamics for MINONK, IL:

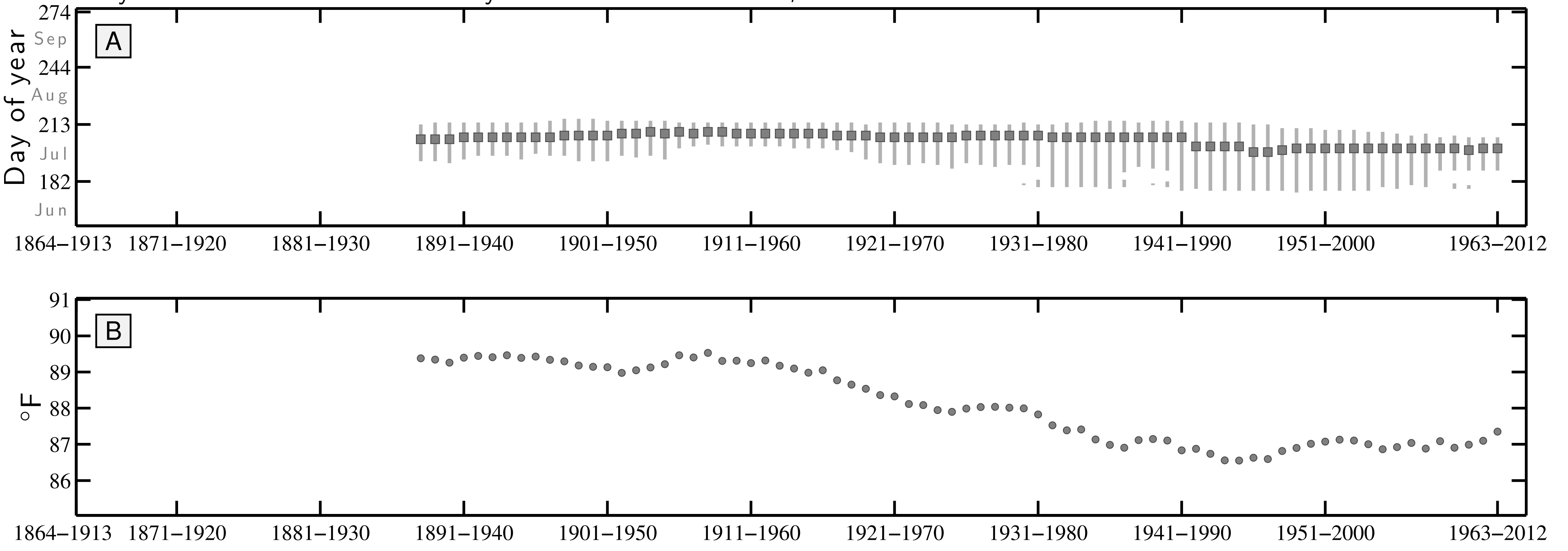

50 year Winter Teletherm dynamics for MINONK, IL:

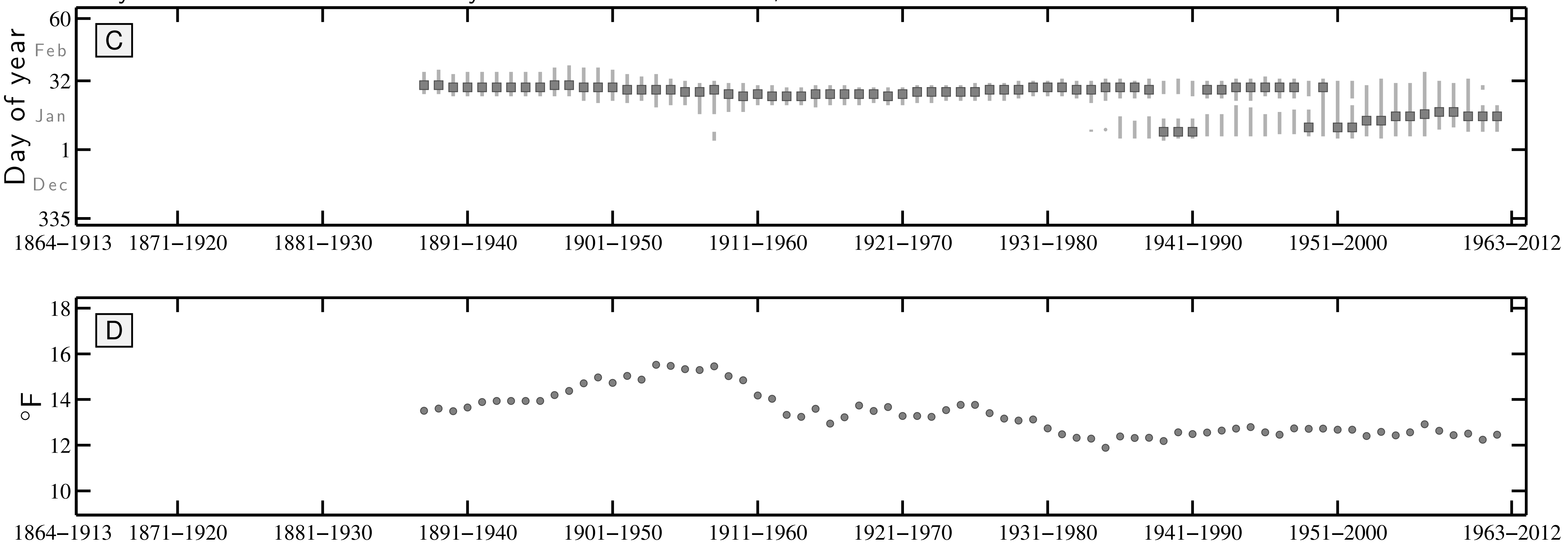

50 year Summer Teletherm dynamics for MONMOUTH, IL:

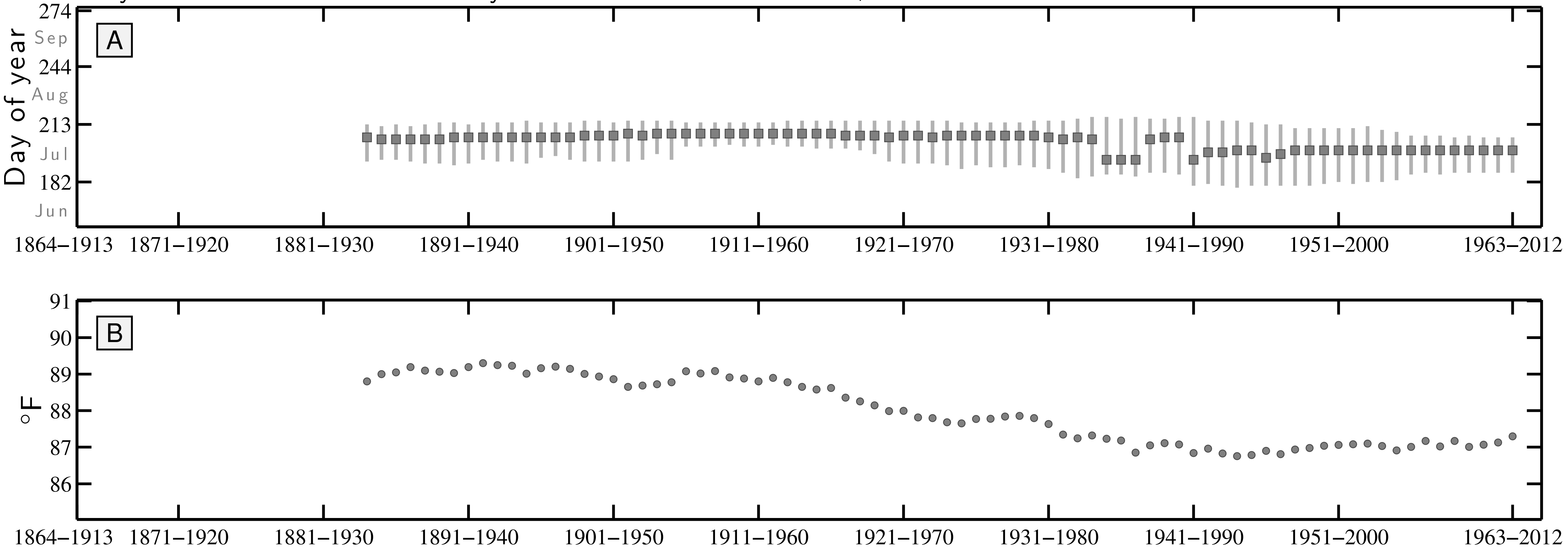

50 year Winter Teletherm dynamics for MONMOUTH, IL:

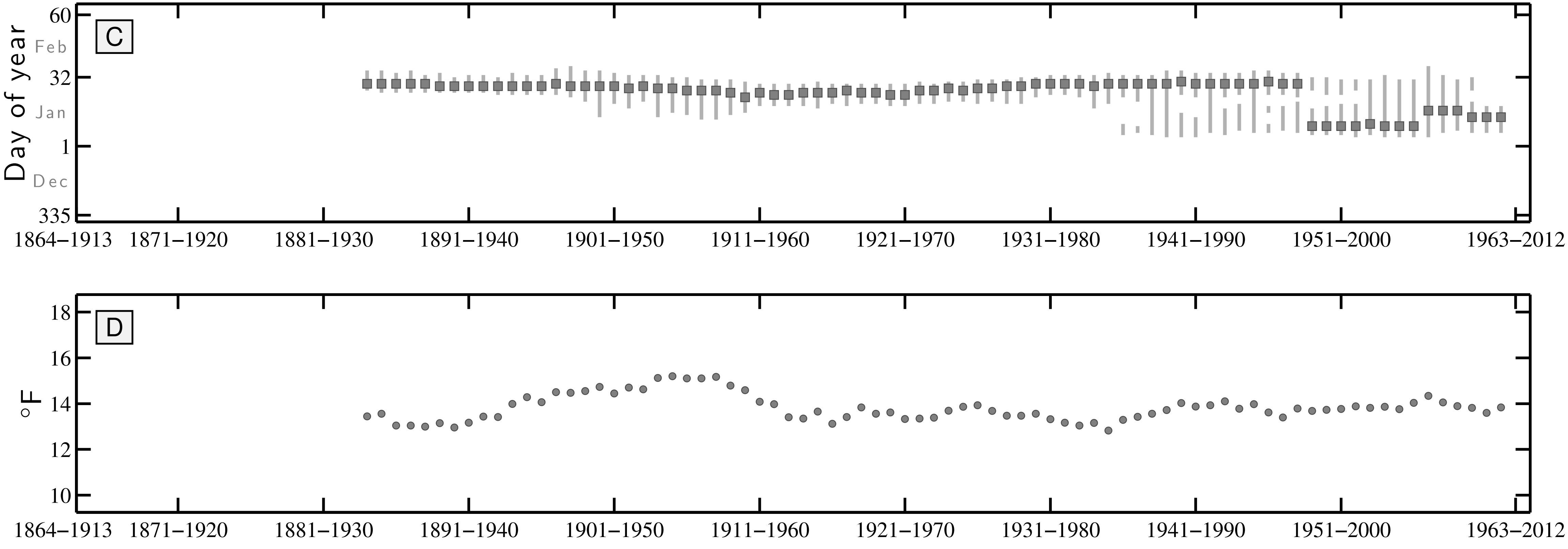

50 year Summer Teletherm dynamics for MORRISON, IL:

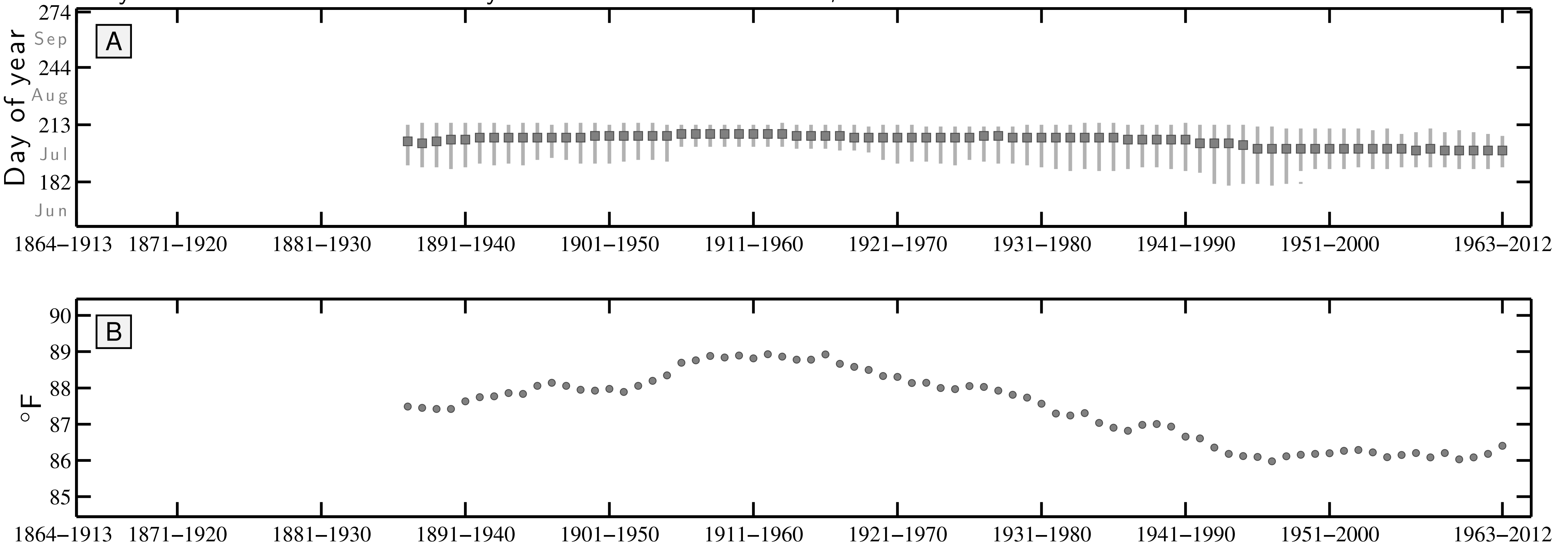

50 year Winter Teletherm dynamics for MORRISON, IL:

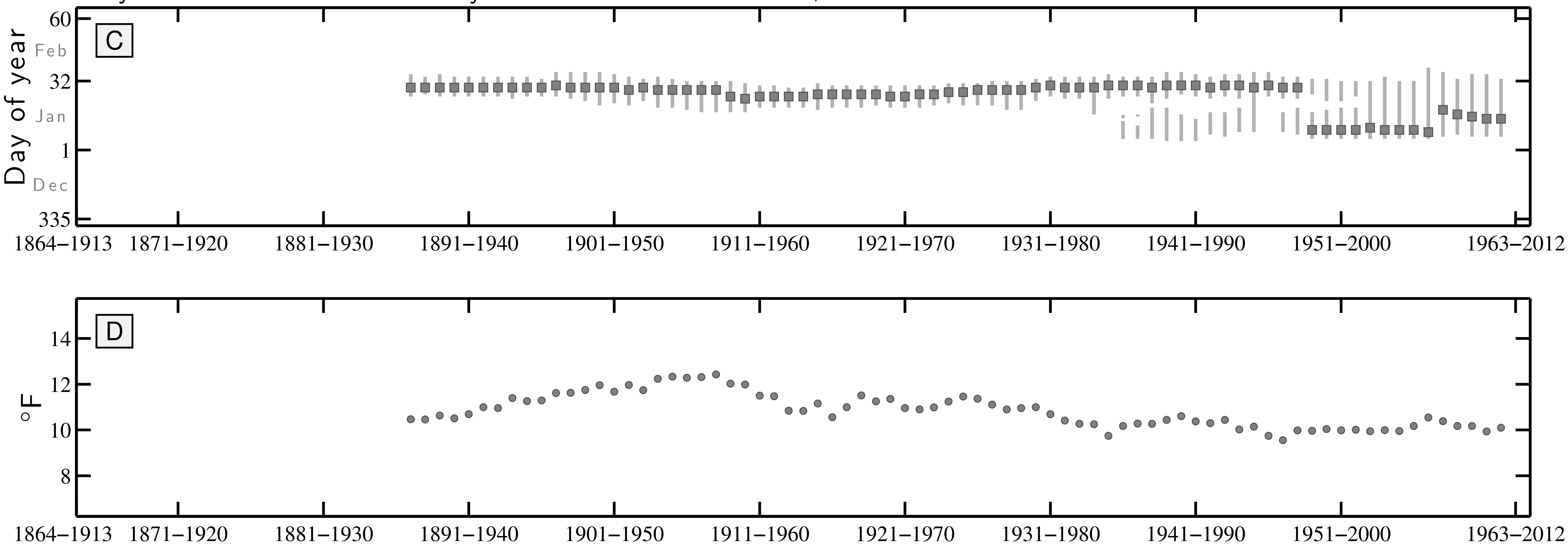

50 year Summer Teletherm dynamics for MT CARROLL, IL:

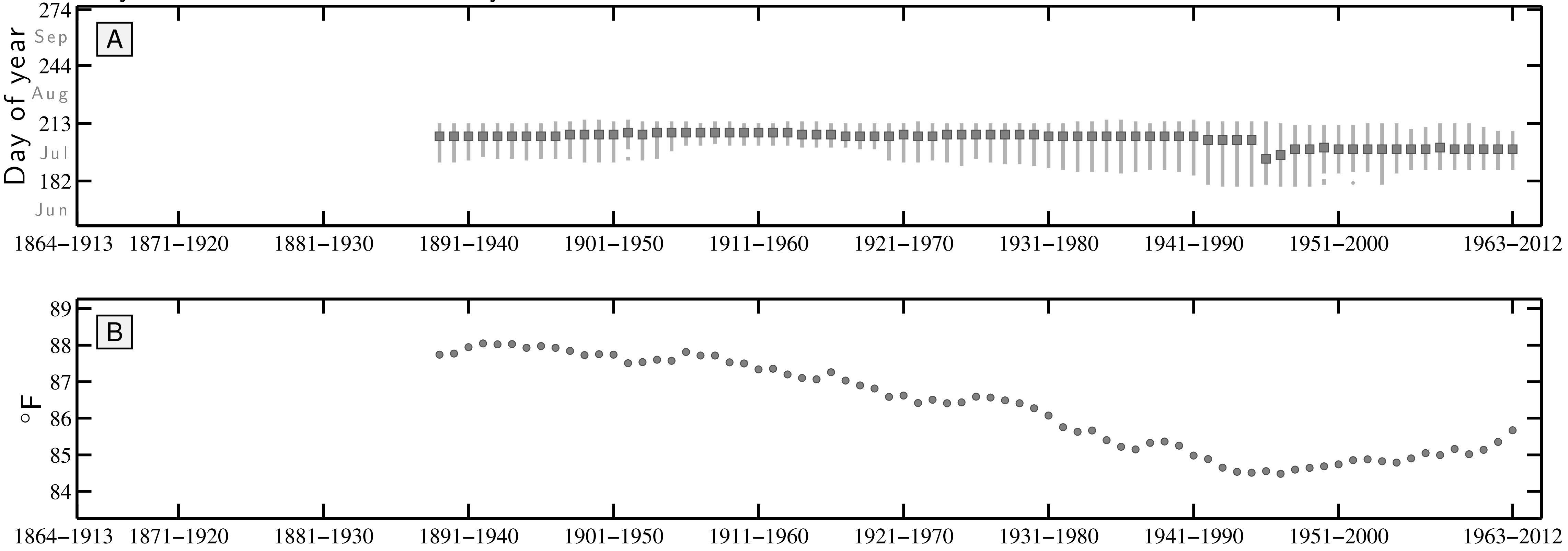

50 year Winter Teletherm dynamics for MT CARROLL, IL:

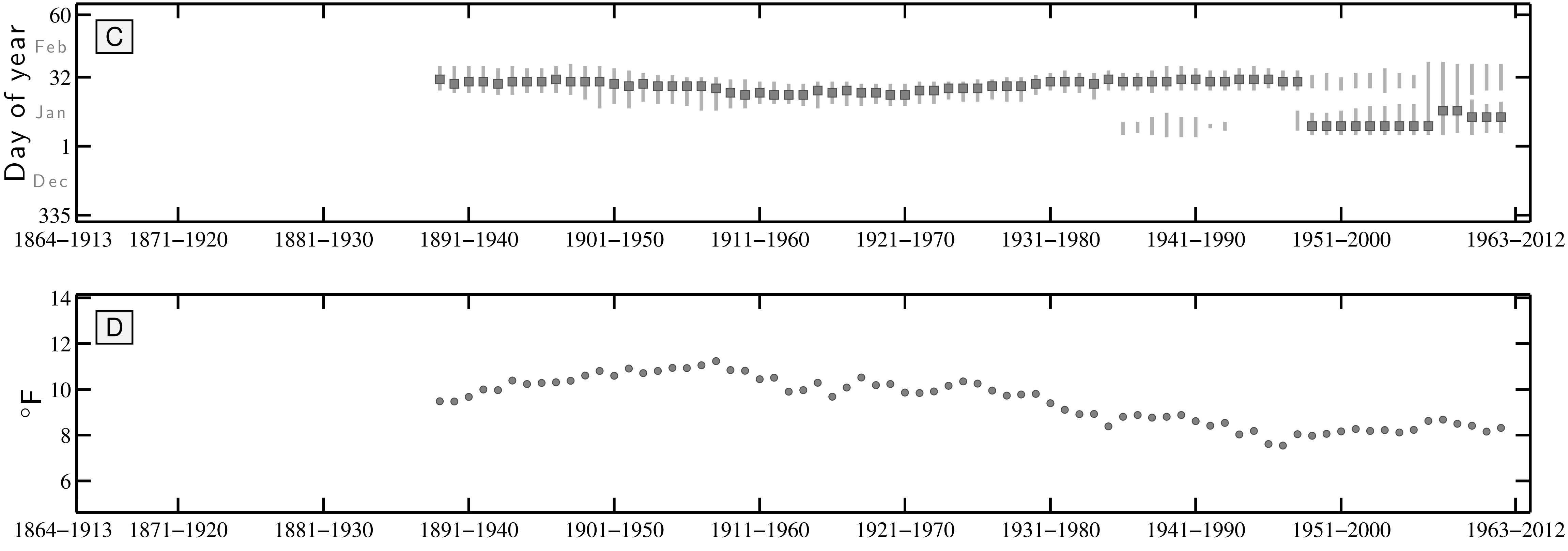

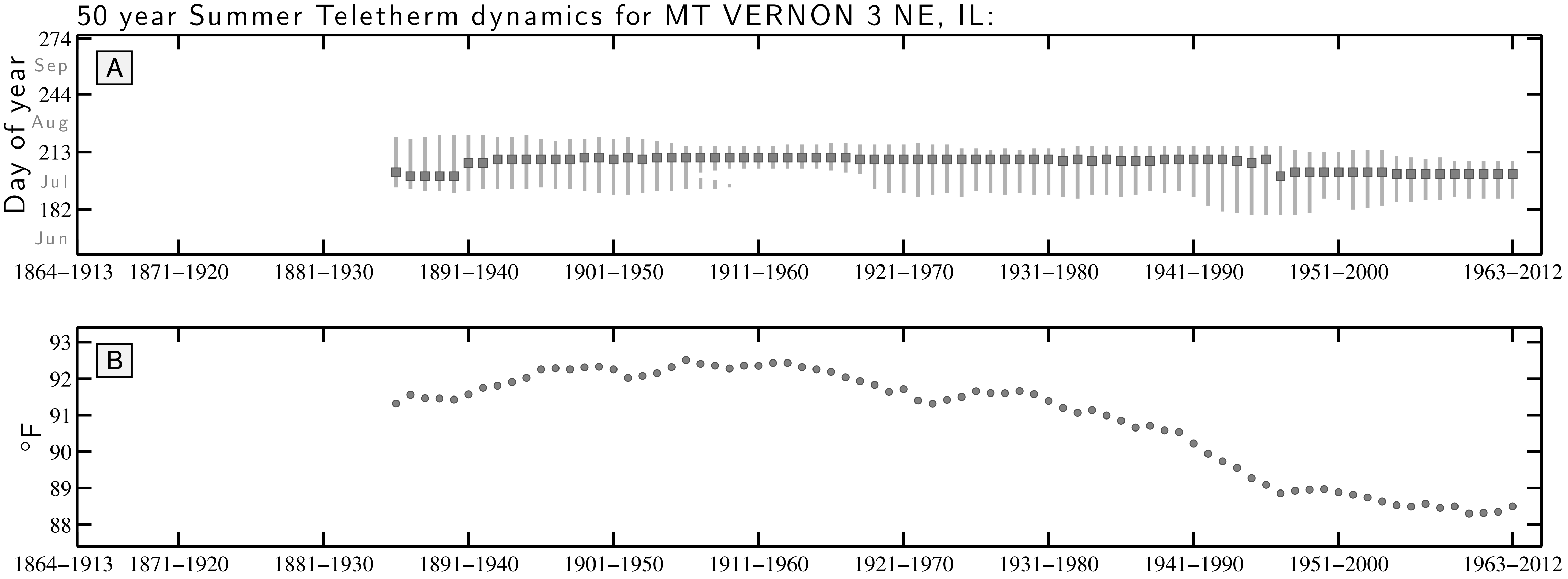

50 year Summer Teletherm dynamics for MT VERNON 3 NE, IL:
A
B
Day of year
°F
274
244
213
182
Sep
Aug
Jul
Jun
93
92
91
90
89
88
1864–1913
1871–1920
1881–1930
1891–1940
1901–1950
1911–1960
1921–1970
1931–1980
1941–1990
1951–2000
1963–2012

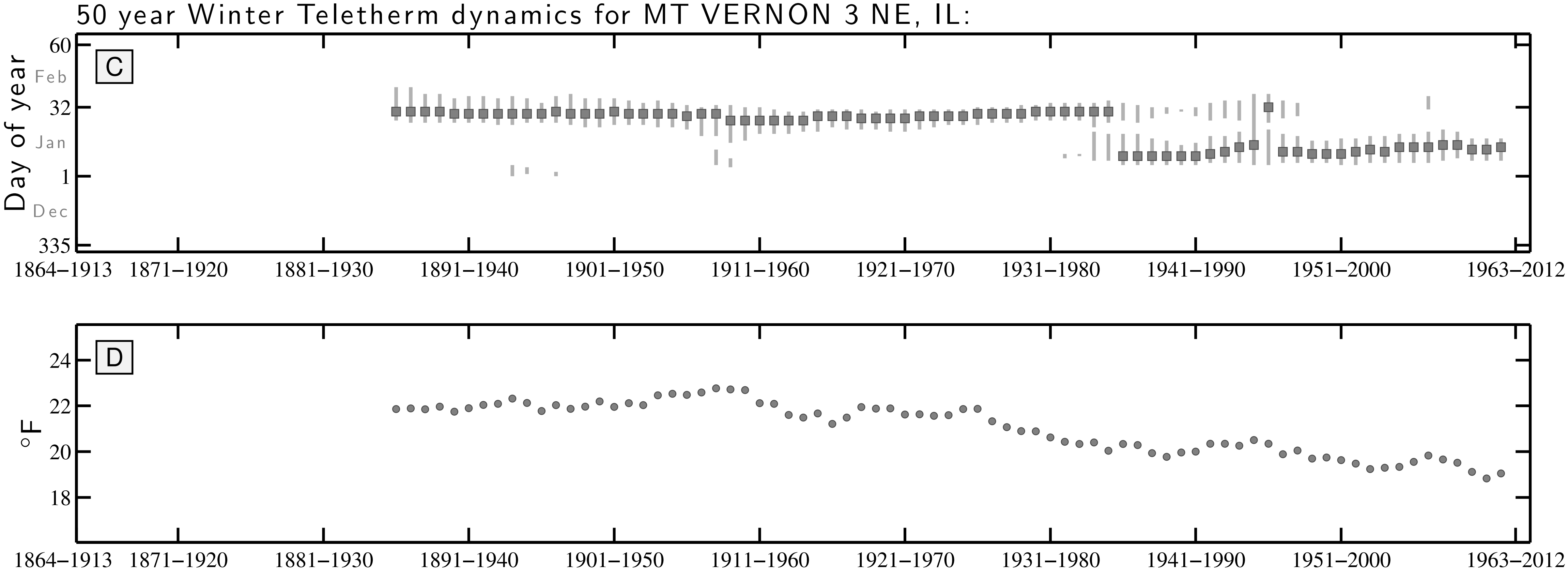

50 year Winter Teletherm dynamics for MT VERNON 3 NE, IL:
C
D
Day of year
°F
60
32
1
335
Feb
Jan
Dec
24
22
20
18
1864–1913
1871–1920
1881–1930
1891–1940
1901–1950
1911–1960
1921–1970
1931–1980
1941–1990
1951–2000
1963–2012

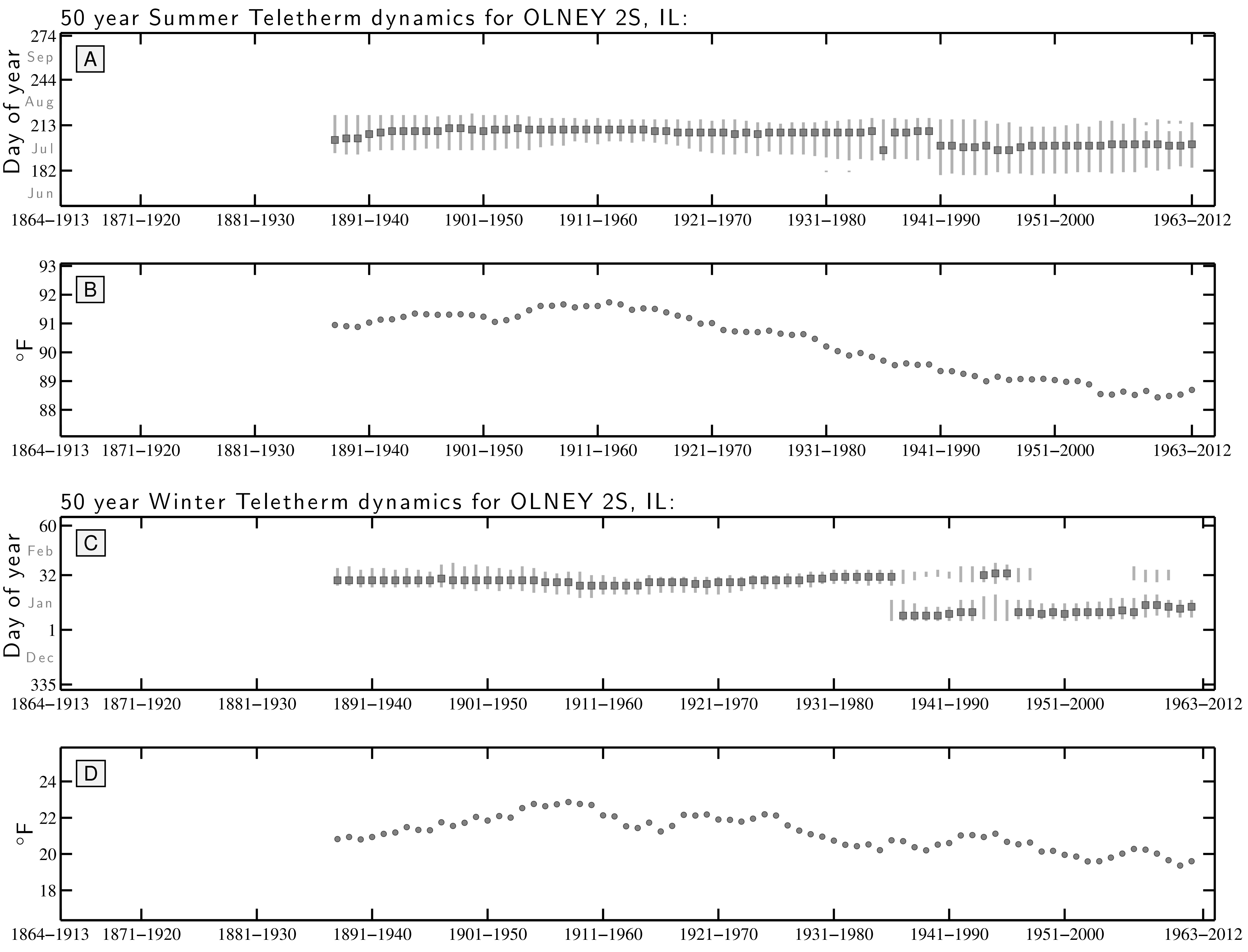
50 year Summer Teletherm dynamics for OLNEY 2S, IL:
A
B
Day of year
°F
Sep
Aug
Jul
Jun
274
244
213
182
93
92
91
90
89
88
1864–1913
1871–1920
1881–1930
1891–1940
1901–1950
1911–1960
1921–1970
1931–1980
1941–1990
1951–2000
1963–2012
50 year Winter Teletherm dynamics for OLNEY 2S, IL:
C
D
Feb
Jan
Dec
60
32
1
335
24
22
20
18

50 year Summer Teletherm dynamics for OTTAWA 5SW, IL:

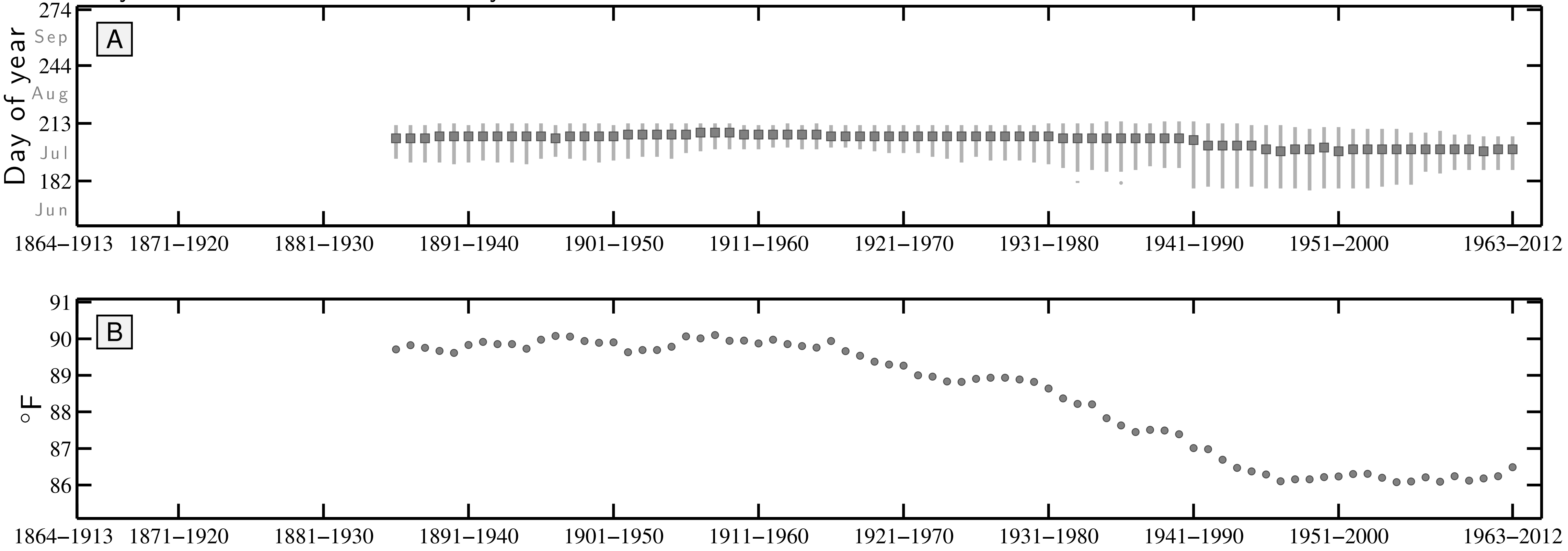

50 year Winter Teletherm dynamics for OTTAWA 5SW, IL:

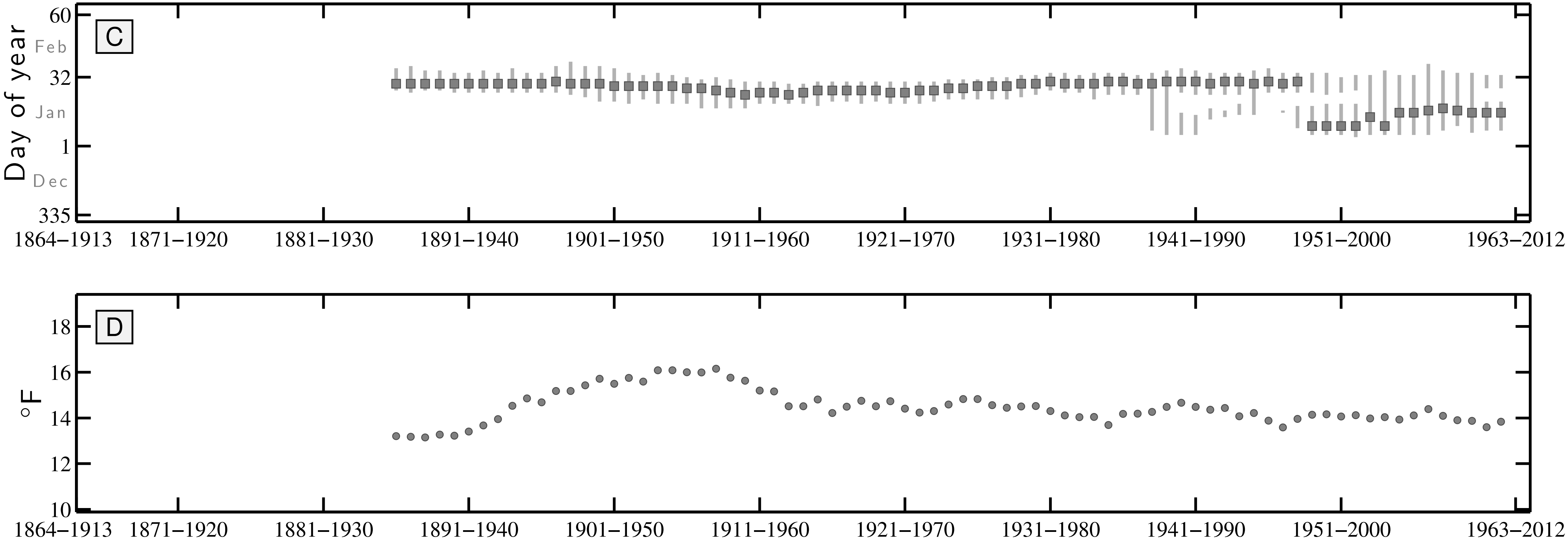

50 year Summer Teletherm dynamics for PALESTINE, IL:

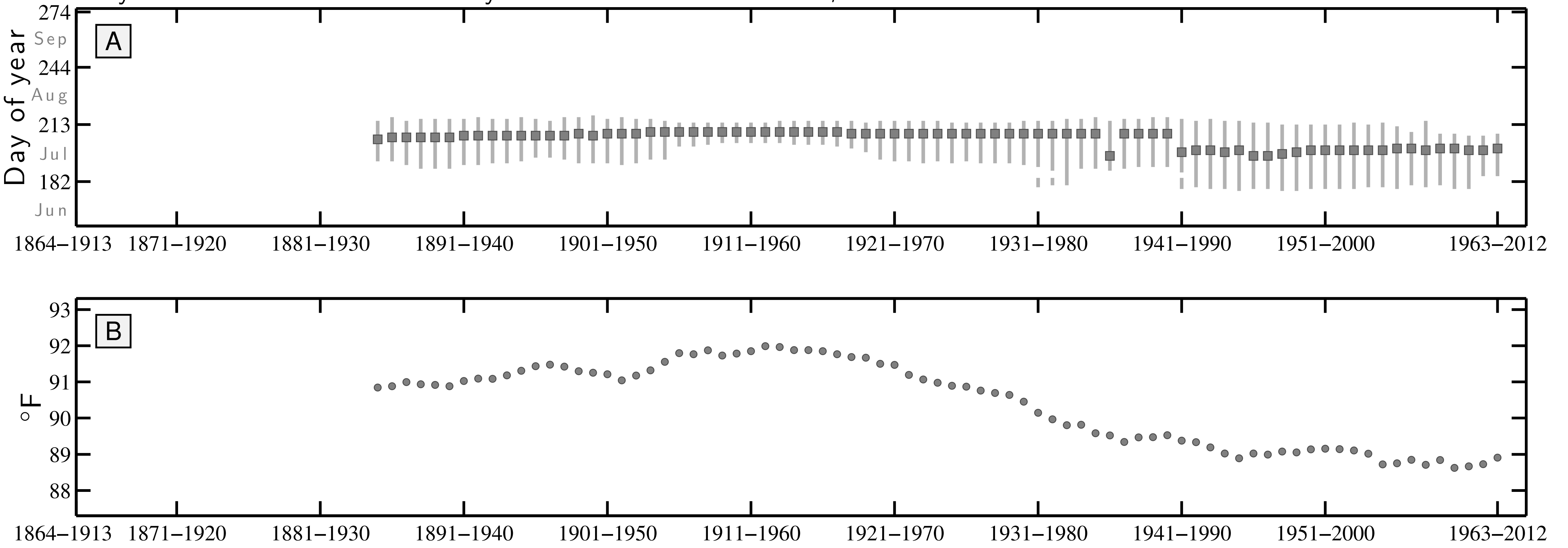

50 year Winter Teletherm dynamics for PALESTINE, IL:

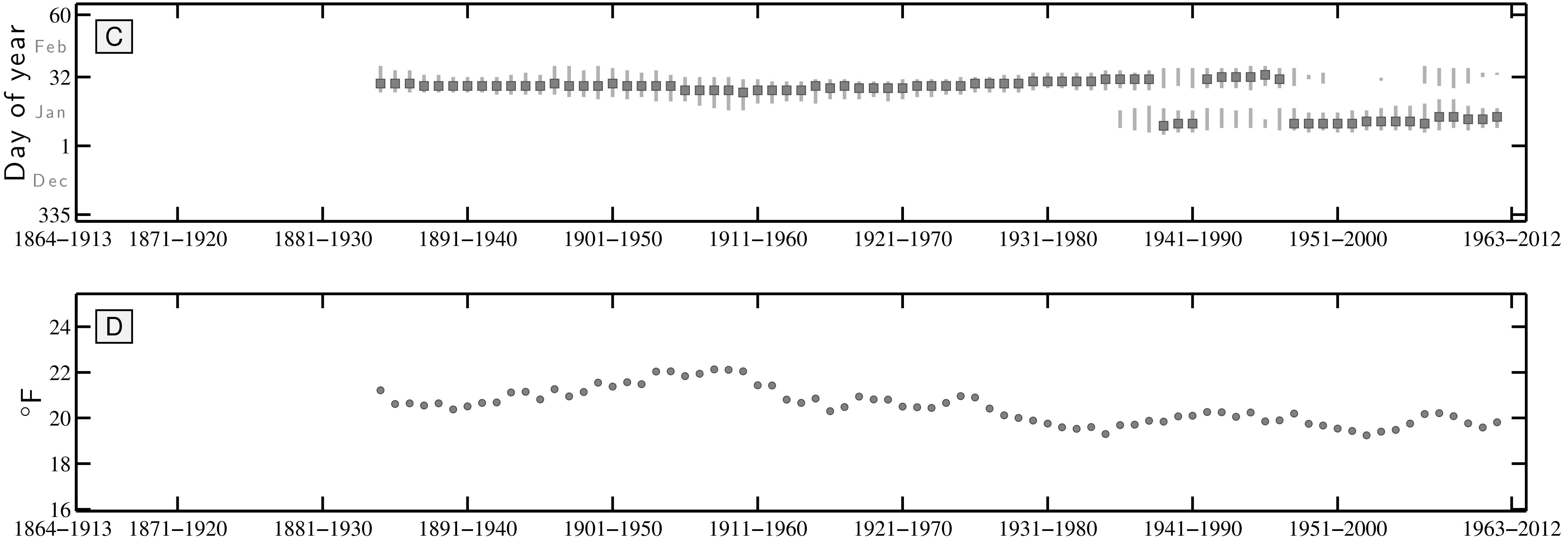

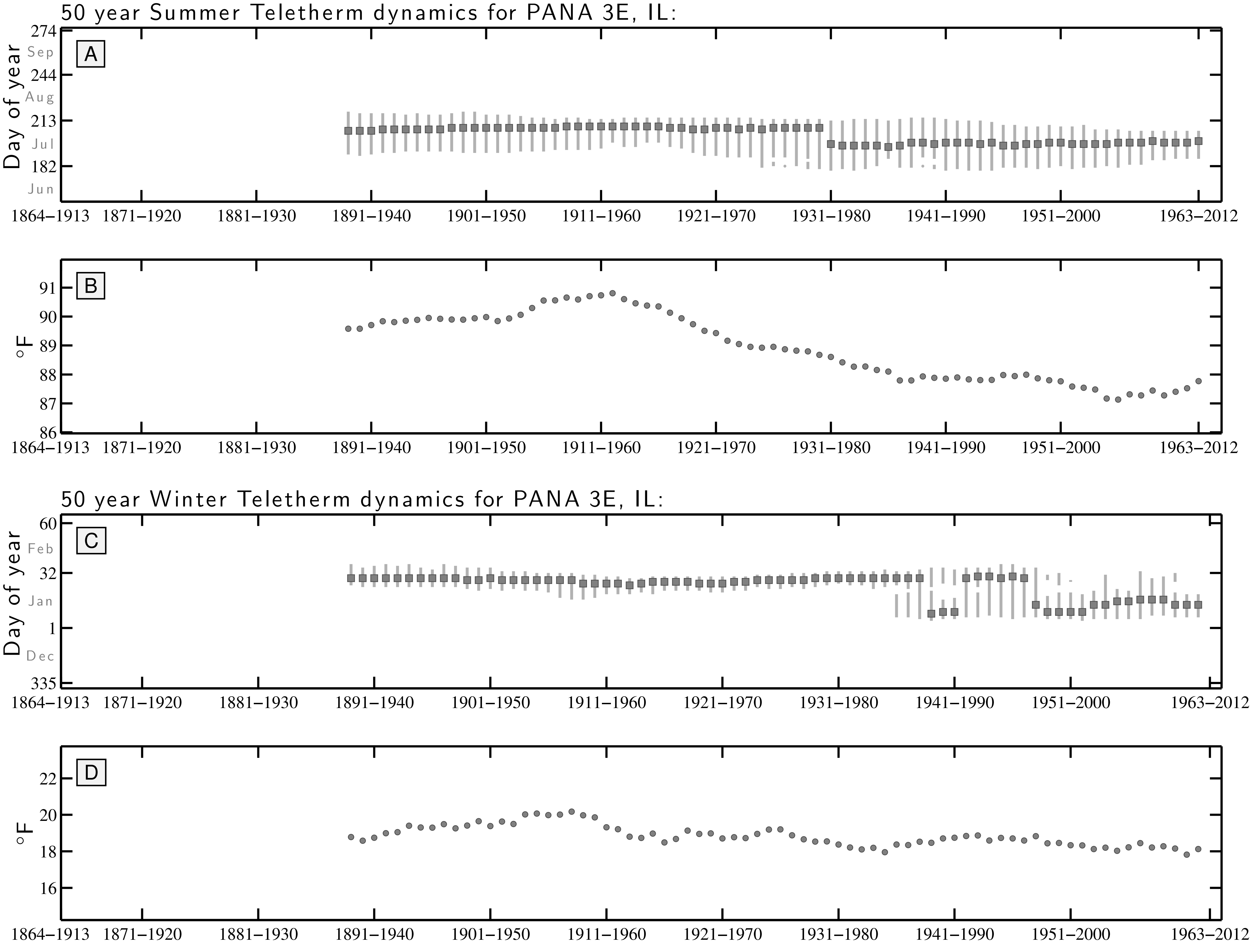
50 year Summer Teletherm dynamics for PANA 3E, IL:
A
Day of year
274
Sep
244
Aug
213
Jul
182
Jun
1864–1913
1871–1920
1881–1930
1891–1940
1901–1950
1911–1960
1921–1970
1931–1980
1941–1990
1951–2000
1963–2012
B
°F
91
90
89
88
87
86
50 year Winter Teletherm dynamics for PANA 3E, IL:
C
60
Feb
32
Jan
1
Dec
335
D
22
20
18
16

50 year Summer Teletherm dynamics for PARIS WTR WKS, IL:

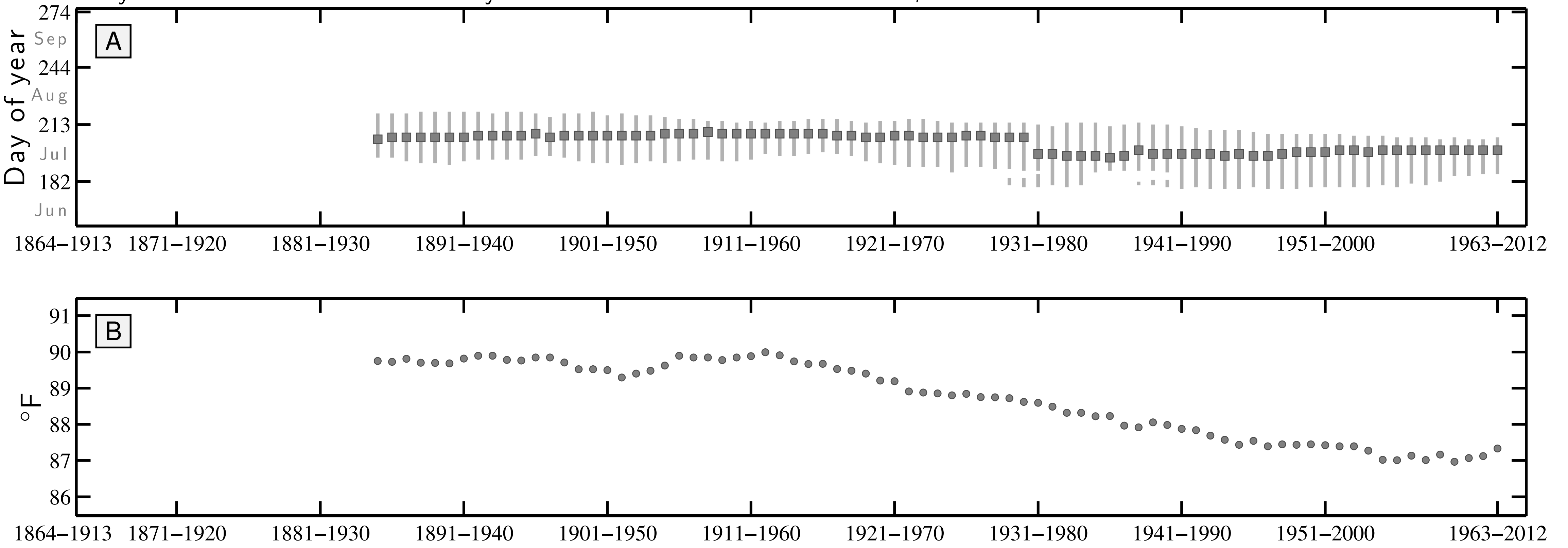

50 year Winter Teletherm dynamics for PARIS WTR WKS, IL:

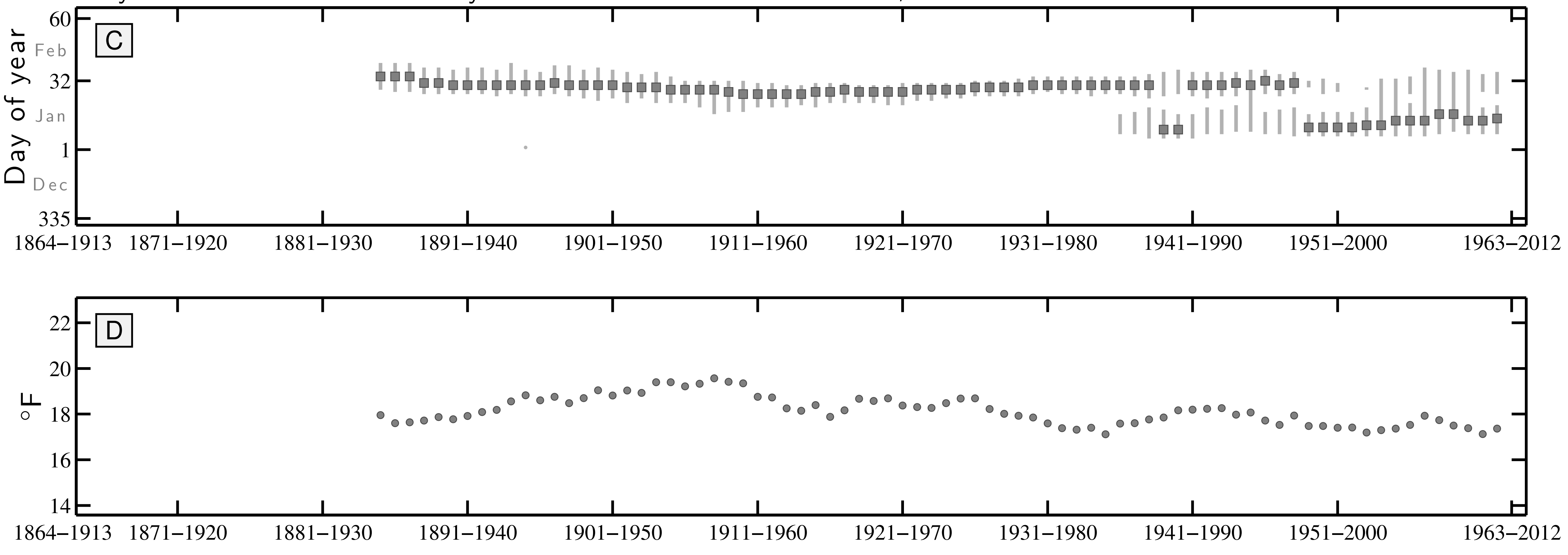

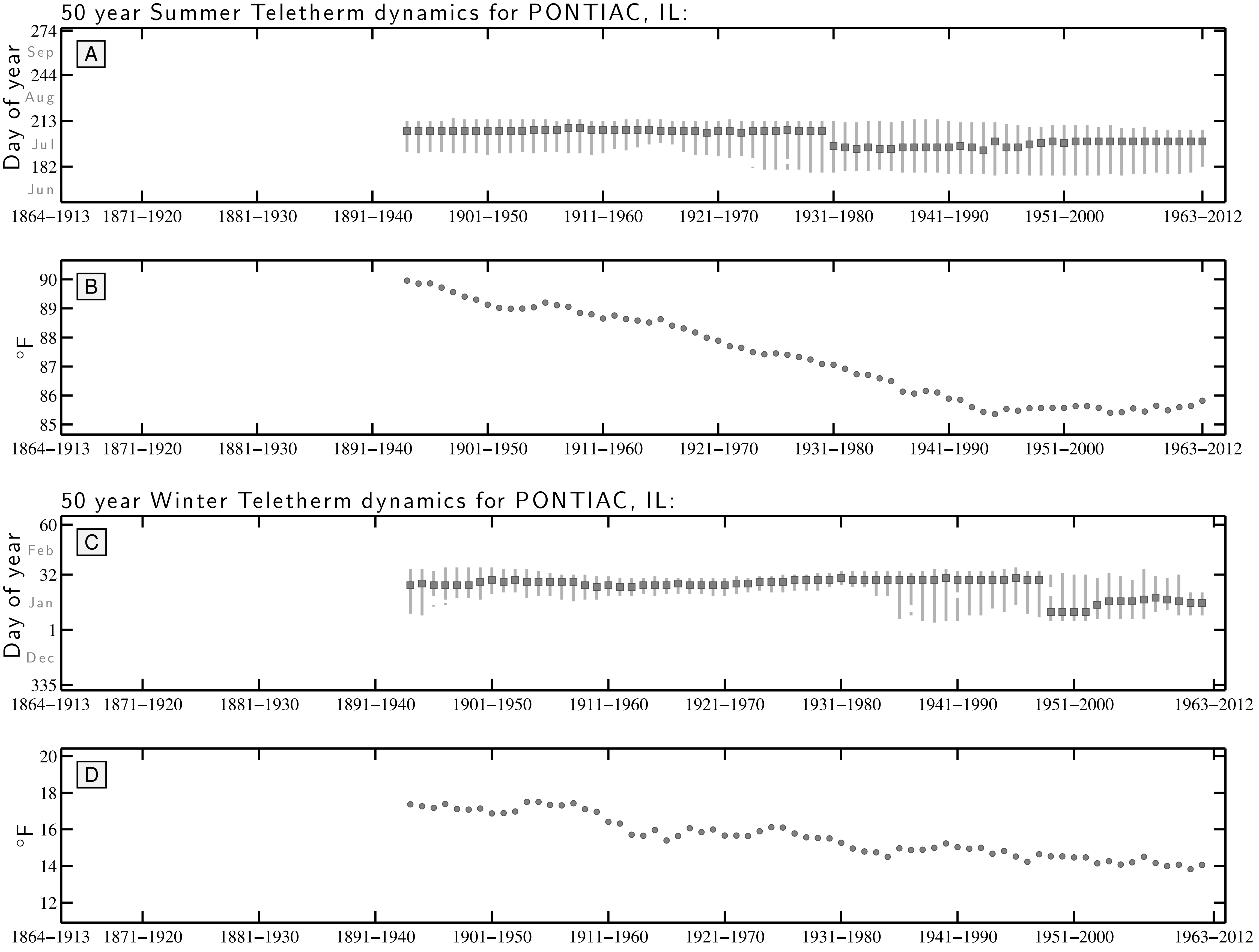
50 year Summer Teletherm dynamics for PONTIAC, IL:
A
Day of year
274
Sep
244
Aug
213
Jul
182
Jun
1864–1913
1871–1920
1881–1930
1891–1940
1901–1950
1911–1960
1921–1970
1931–1980
1941–1990
1951–2000
1963–2012
B
°F
90
89
88
87
86
85
50 year Winter Teletherm dynamics for PONTIAC, IL:
C
Day of year
60
Feb
32
Jan
1
Dec
335
D
°F
20
18
16
14
12

50 year Summer Teletherm dynamics for RUSHVILLE, IL:

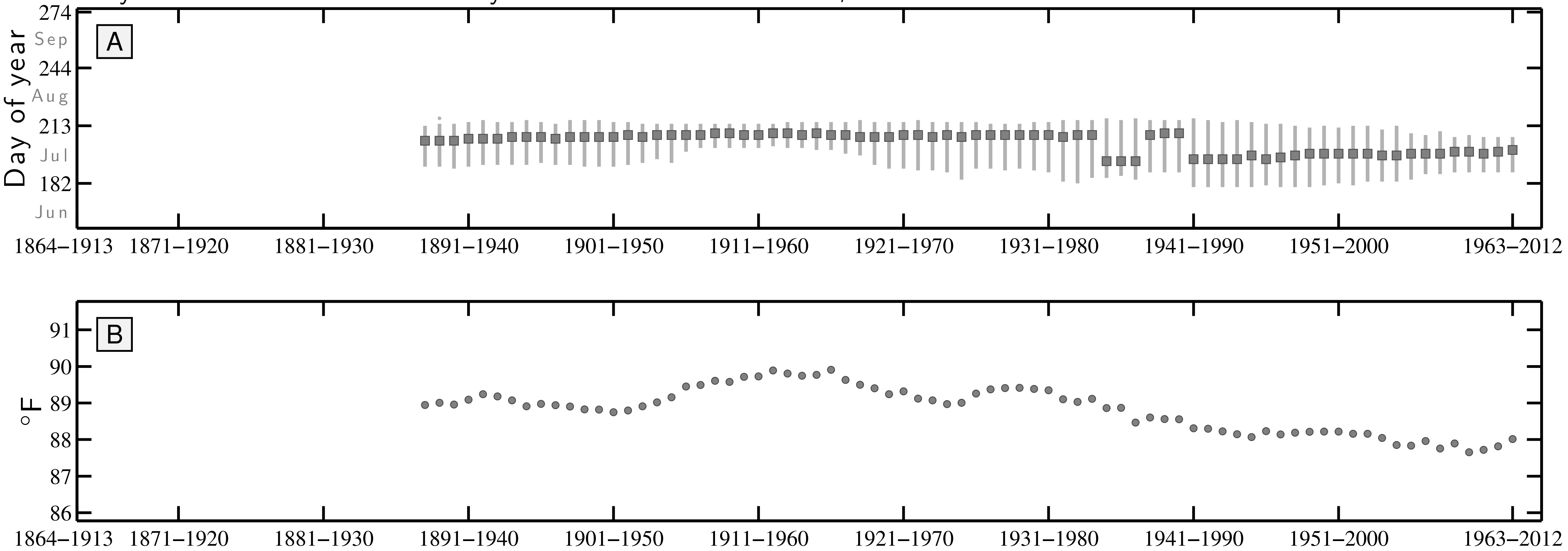

50 year Winter Teletherm dynamics for RUSHVILLE, IL:

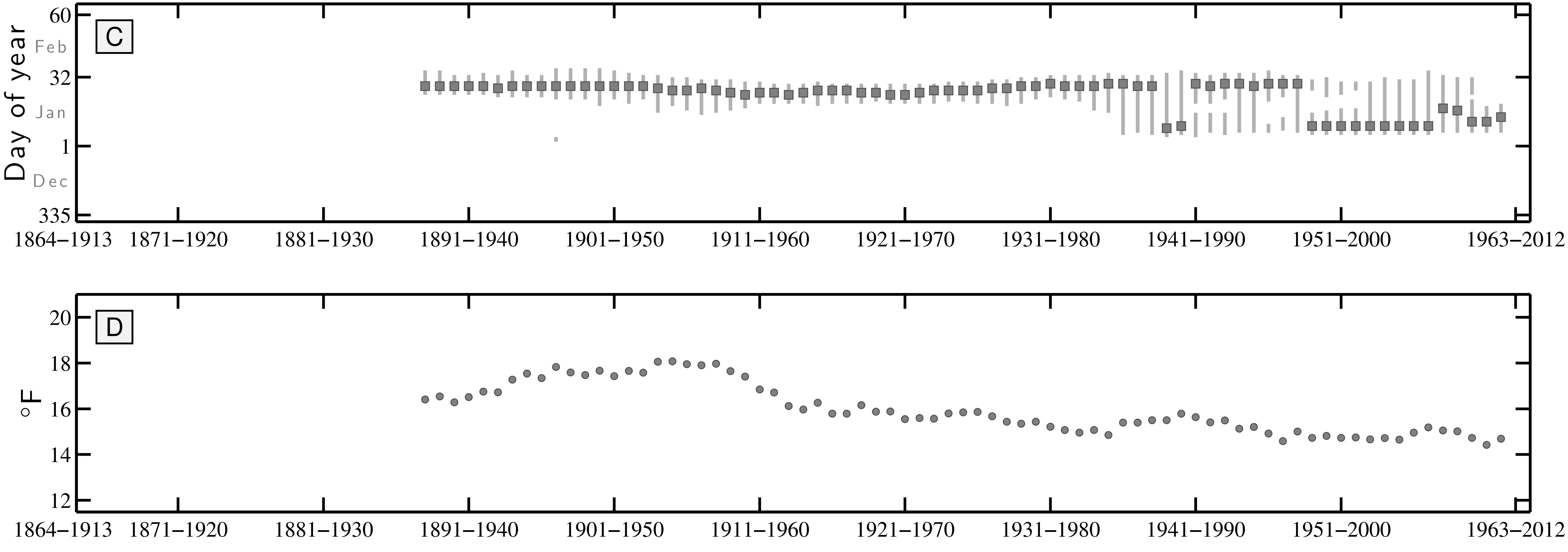

50 year Summer Teletherm dynamics for SPARTA 1 W, IL:

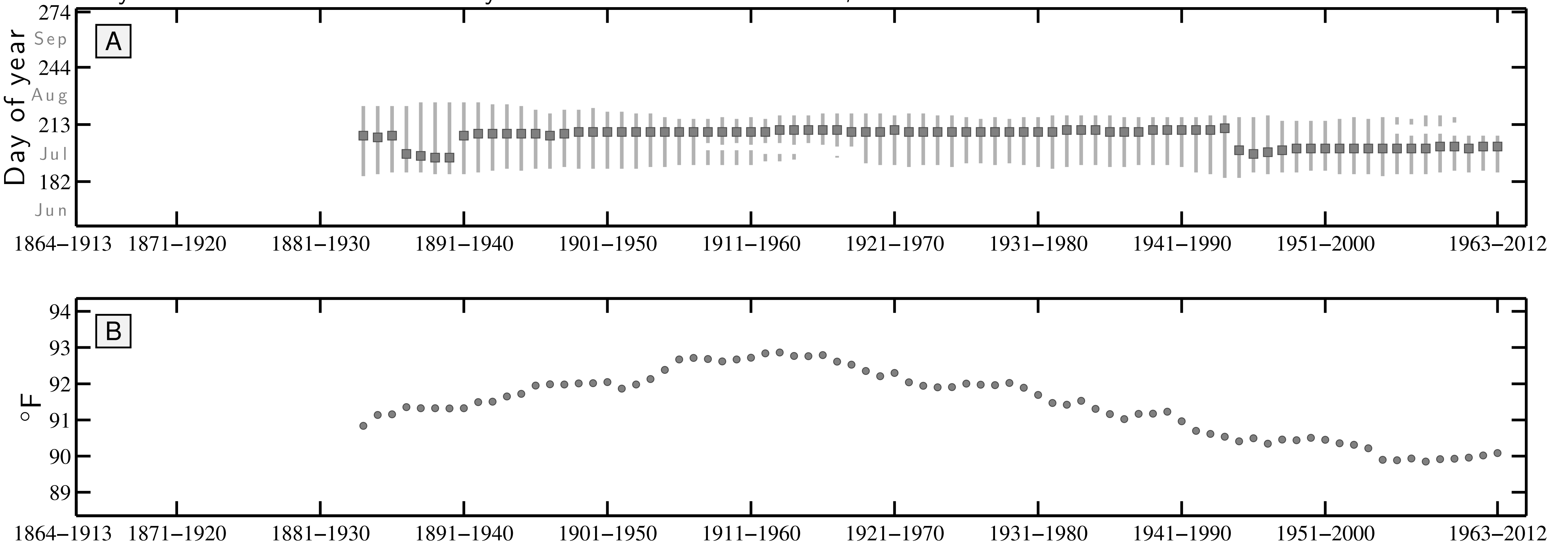

50 year Winter Teletherm dynamics for SPARTA 1 W, IL:

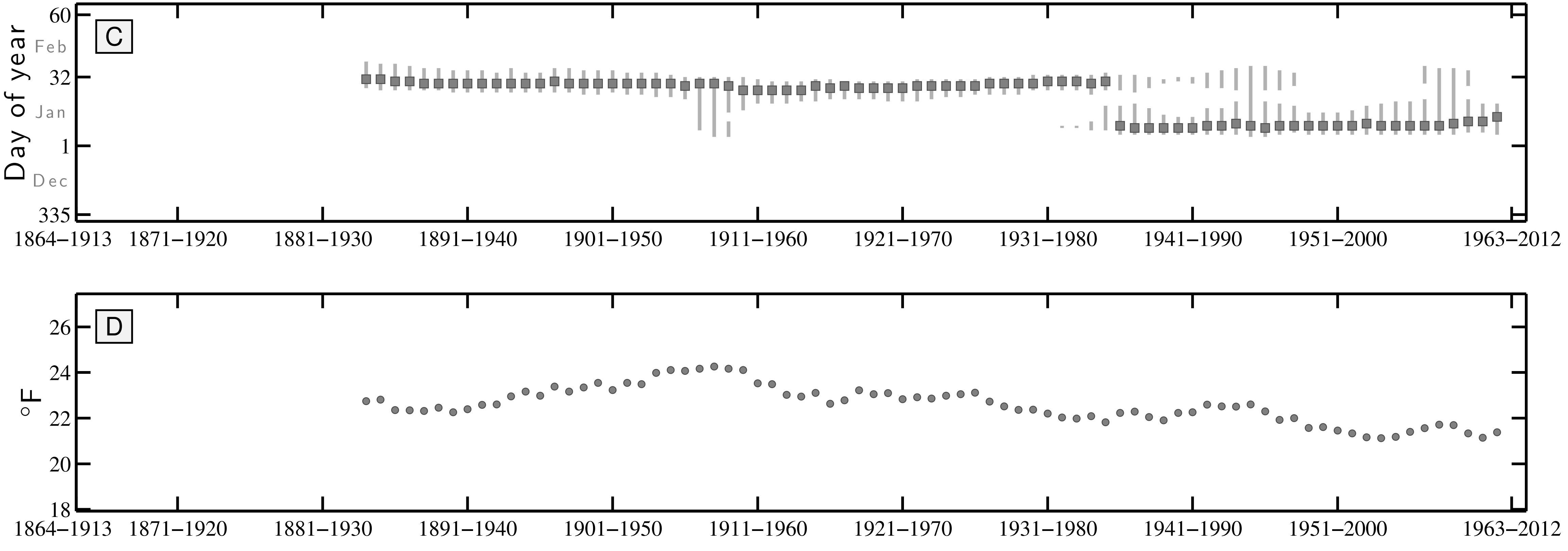

50 year Summer Teletherm dynamics for URBANA, IL:

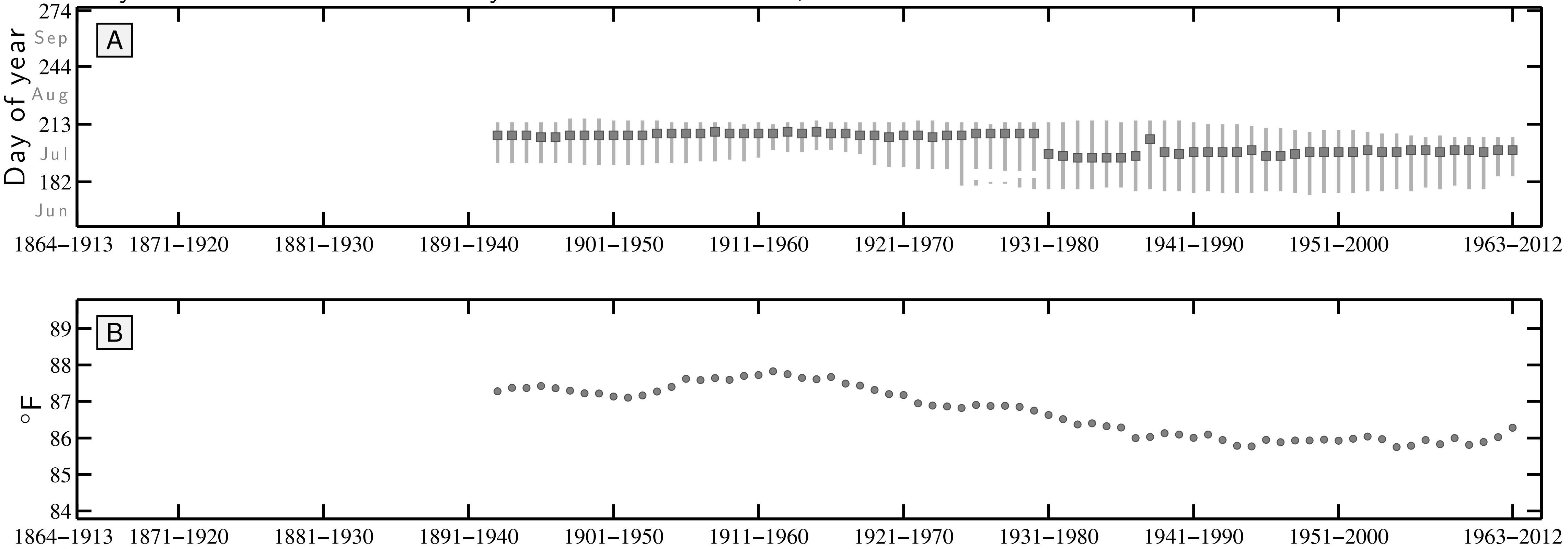

50 year Winter Teletherm dynamics for URBANA, IL:

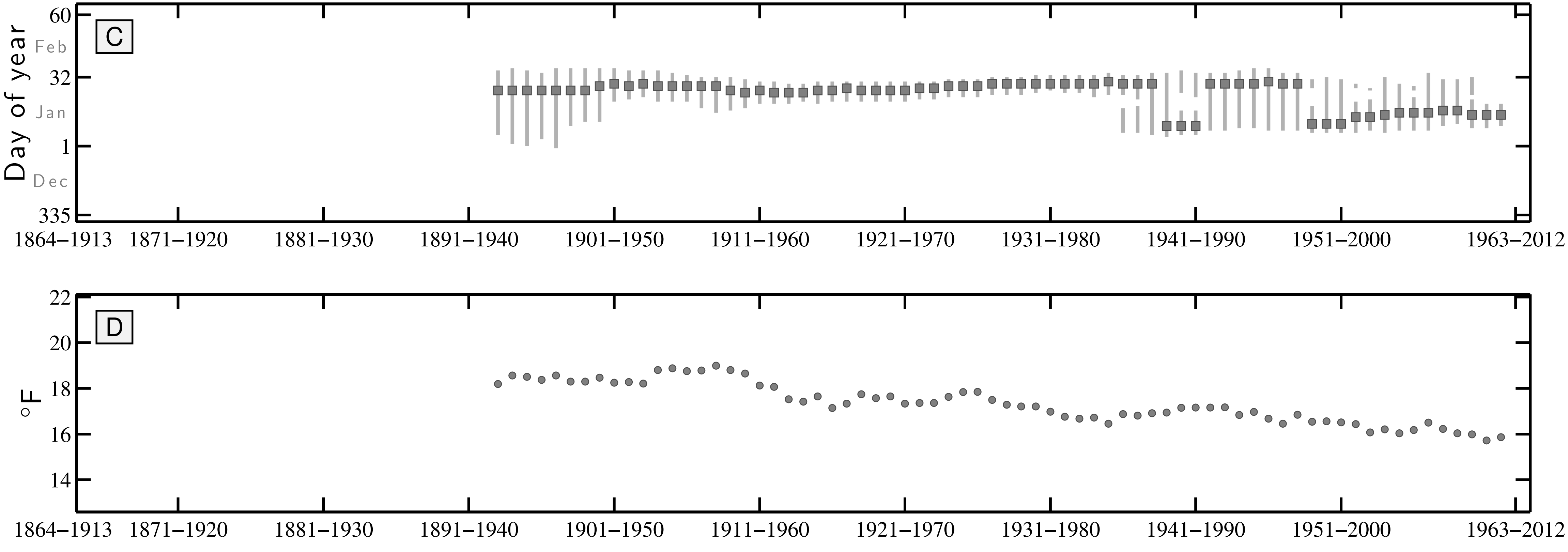

50 year Summer Teletherm dynamics for WALNUT, IL:

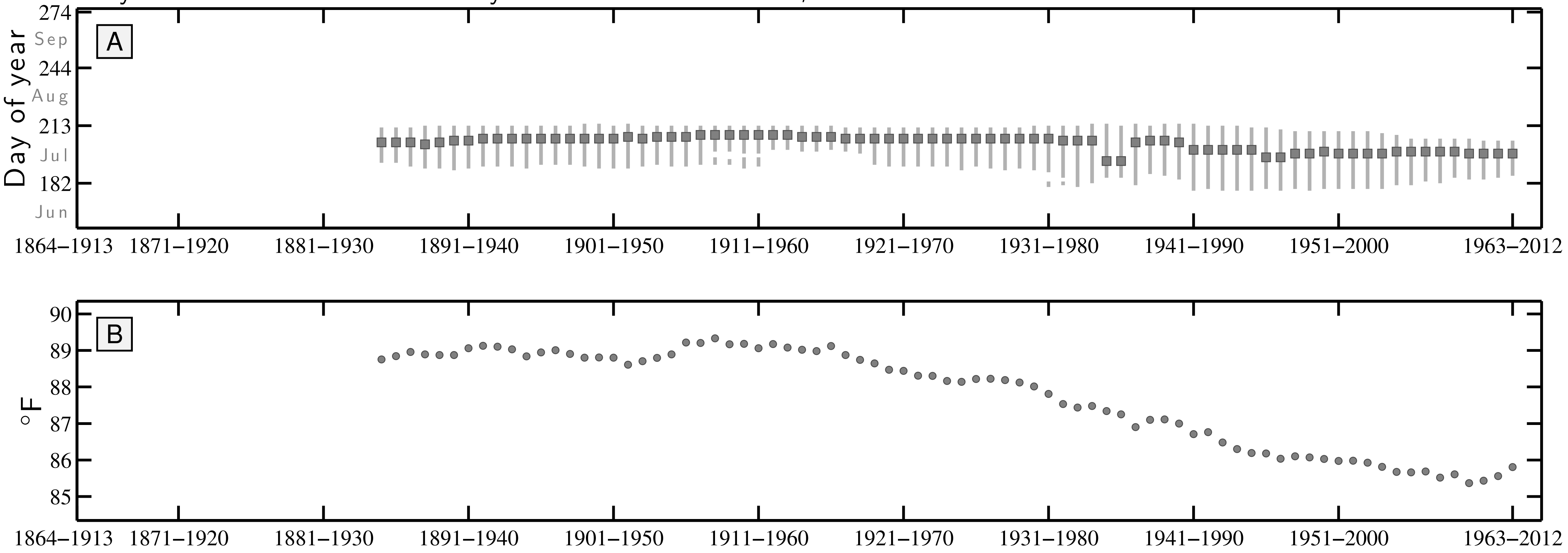

50 year Winter Teletherm dynamics for WALNUT, IL:

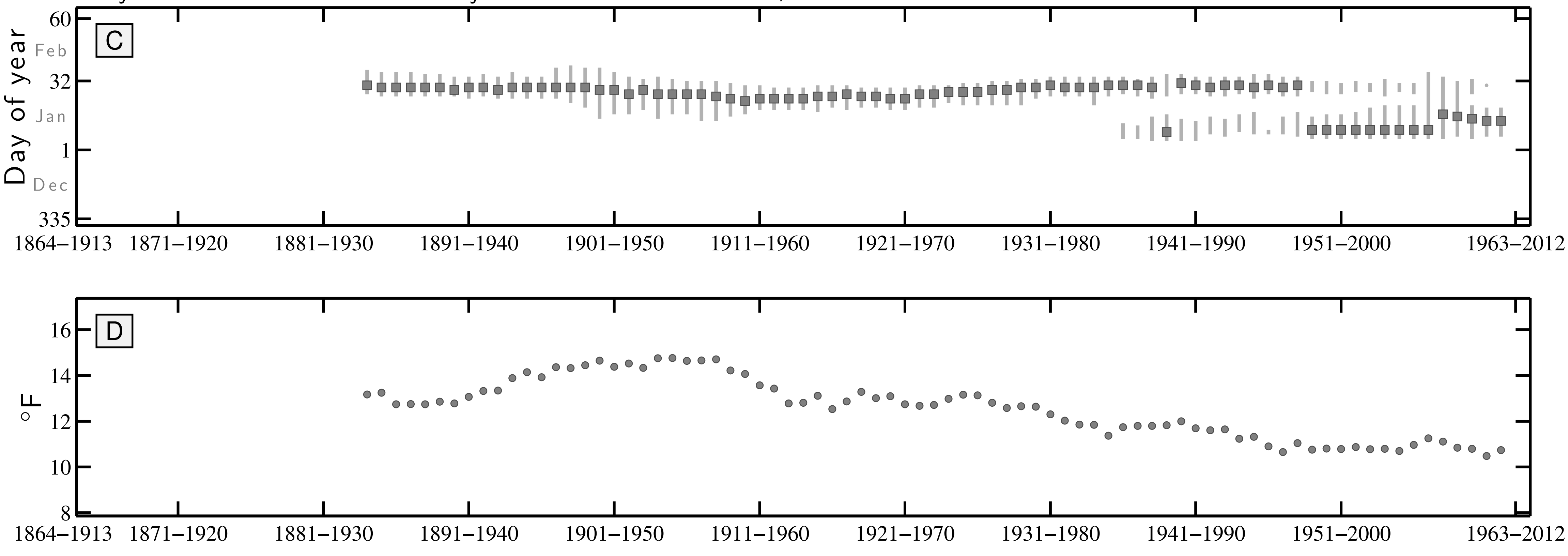

50 year Summer Teletherm dynamics for WHITE HALL 1 E, IL:

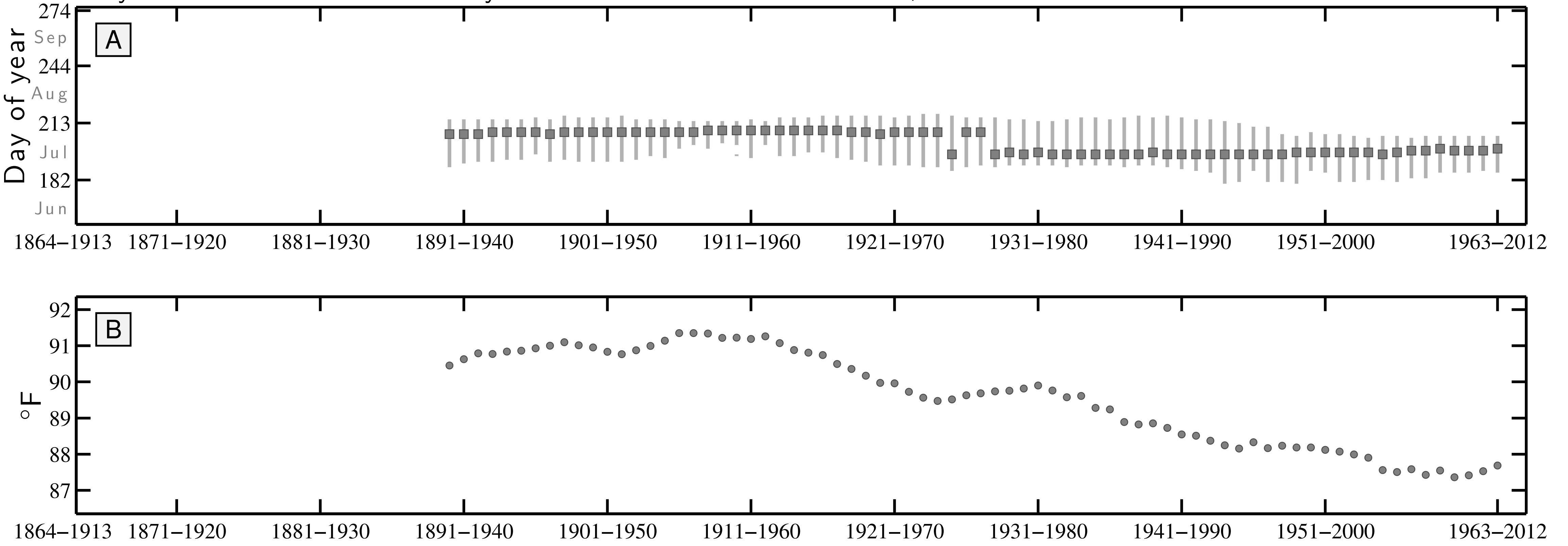

50 year Winter Teletherm dynamics for WHITE HALL 1 E, IL:

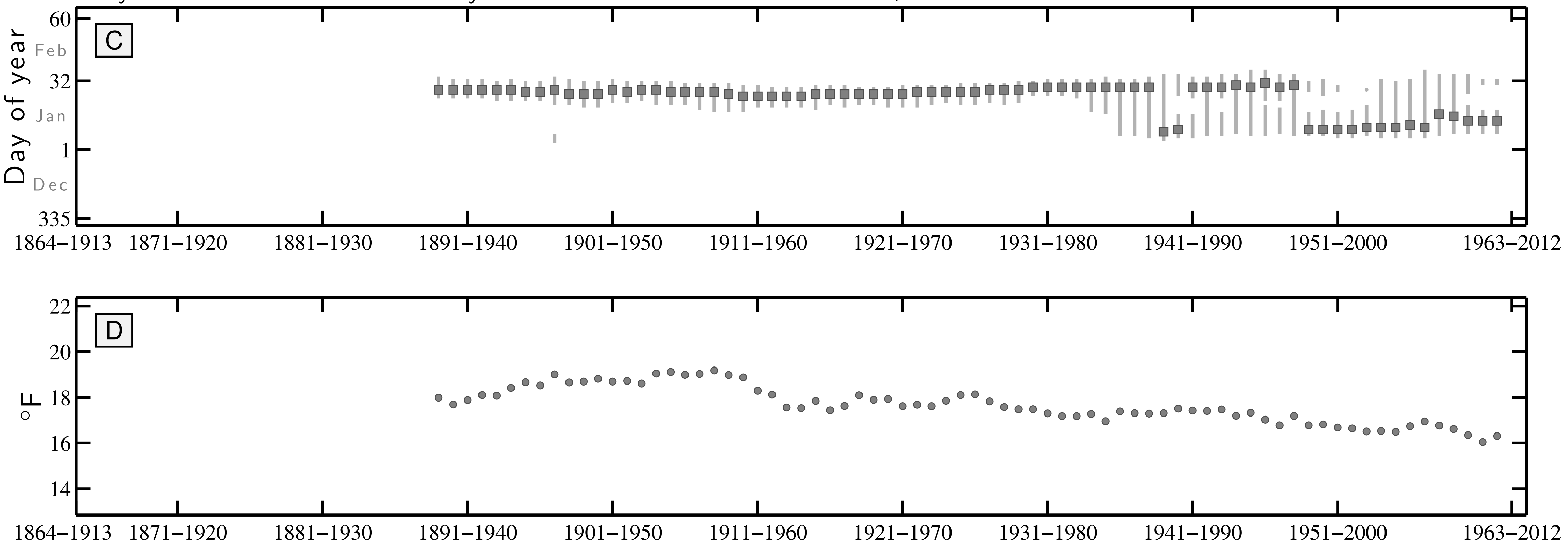

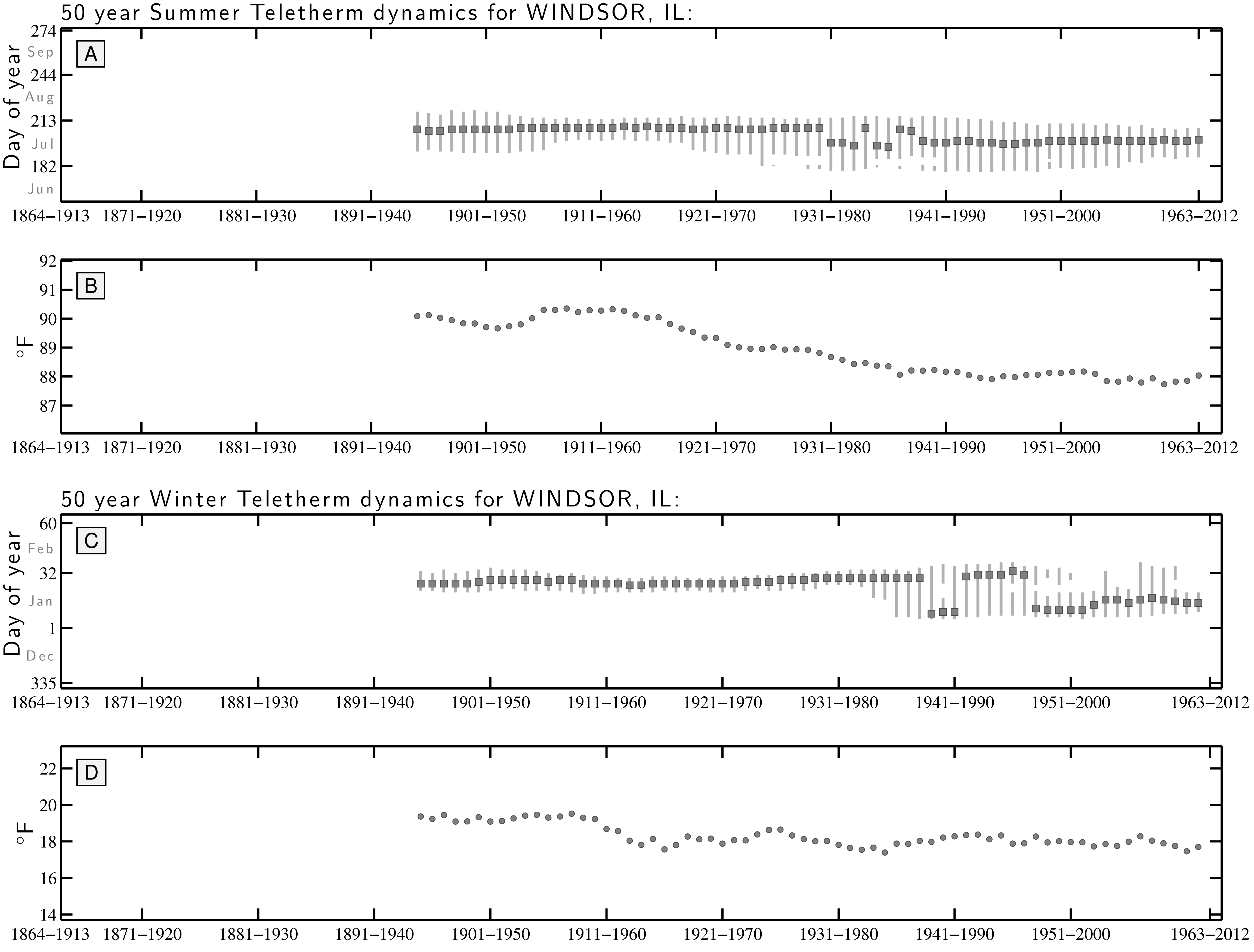
50 year Summer Teletherm dynamics for WINDSOR, IL:
A
B
50 year Winter Teletherm dynamics for WINDSOR, IL:
C
D
Day of year
°F
274
244
213
182
Sep
Aug
Jul
Jun
92
91
90
89
88
87
60
32
1
335
Feb
Jan
Dec
22
20
18
16
14
1864–1913
1871–1920
1881–1930
1891–1940
1901–1950
1911–1960
1921–1970
1931–1980
1941–1990
1951–2000
1963–2012

50 year Summer Teletherm dynamics for ANDERSON SEWAGE PLT, IN:

50 year Winter Teletherm dynamics for ANDERSON SEWAGE PLT, IN:

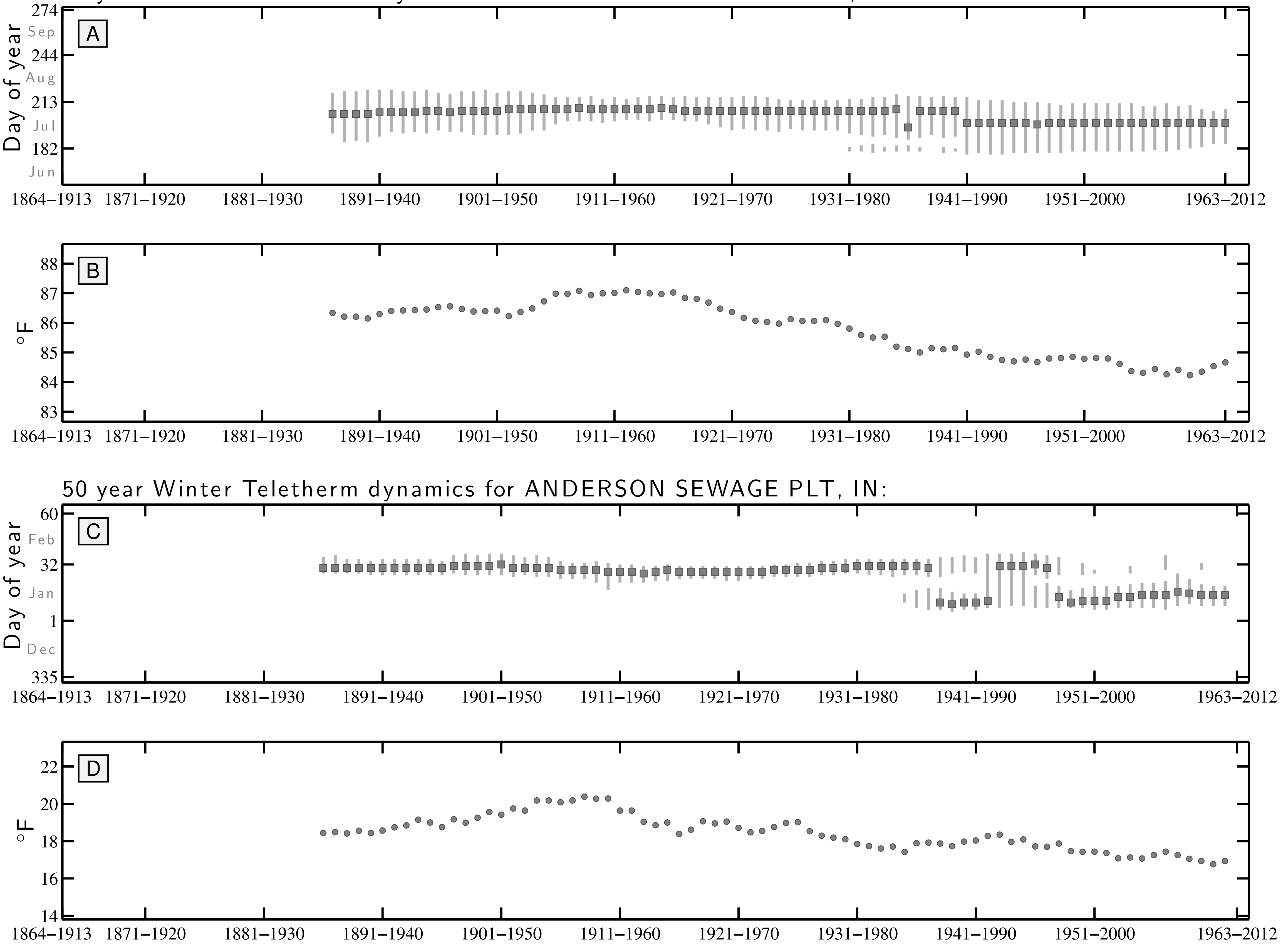

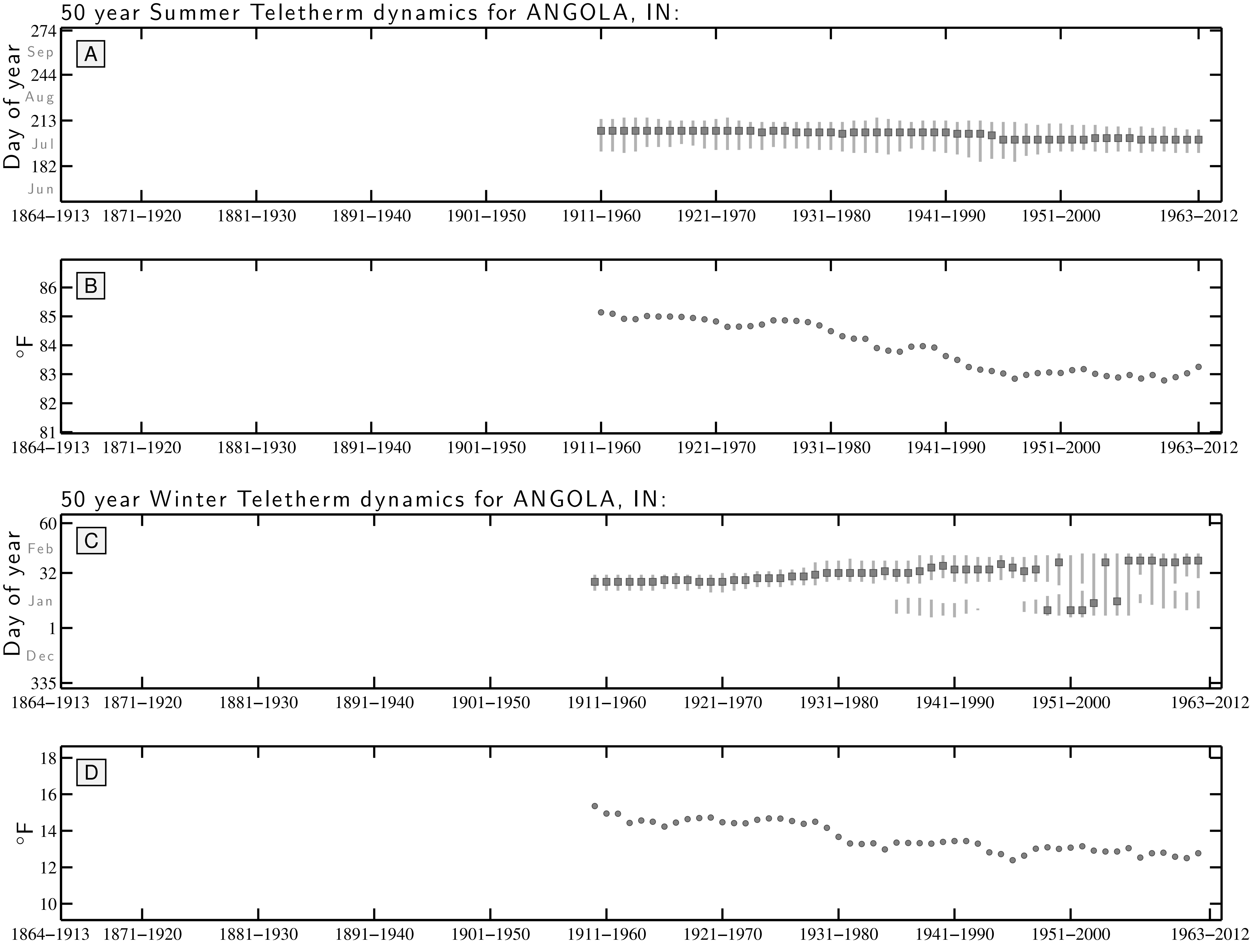
50 year Summer Teletherm dynamics for ANGOLA, IN:
A
B
Day of year
°F
274
244
213
182
Sep
Aug
Jul
Jun
86
85
84
83
82
81
1864–1913
1871–1920
1881–1930
1891–1940
1901–1950
1911–1960
1921–1970
1931–1980
1941–1990
1951–2000
1963–2012
50 year Winter Teletherm dynamics for ANGOLA, IN:
C
D
60
32
1
335
Feb
Jan
Dec
18
16
14
12
10

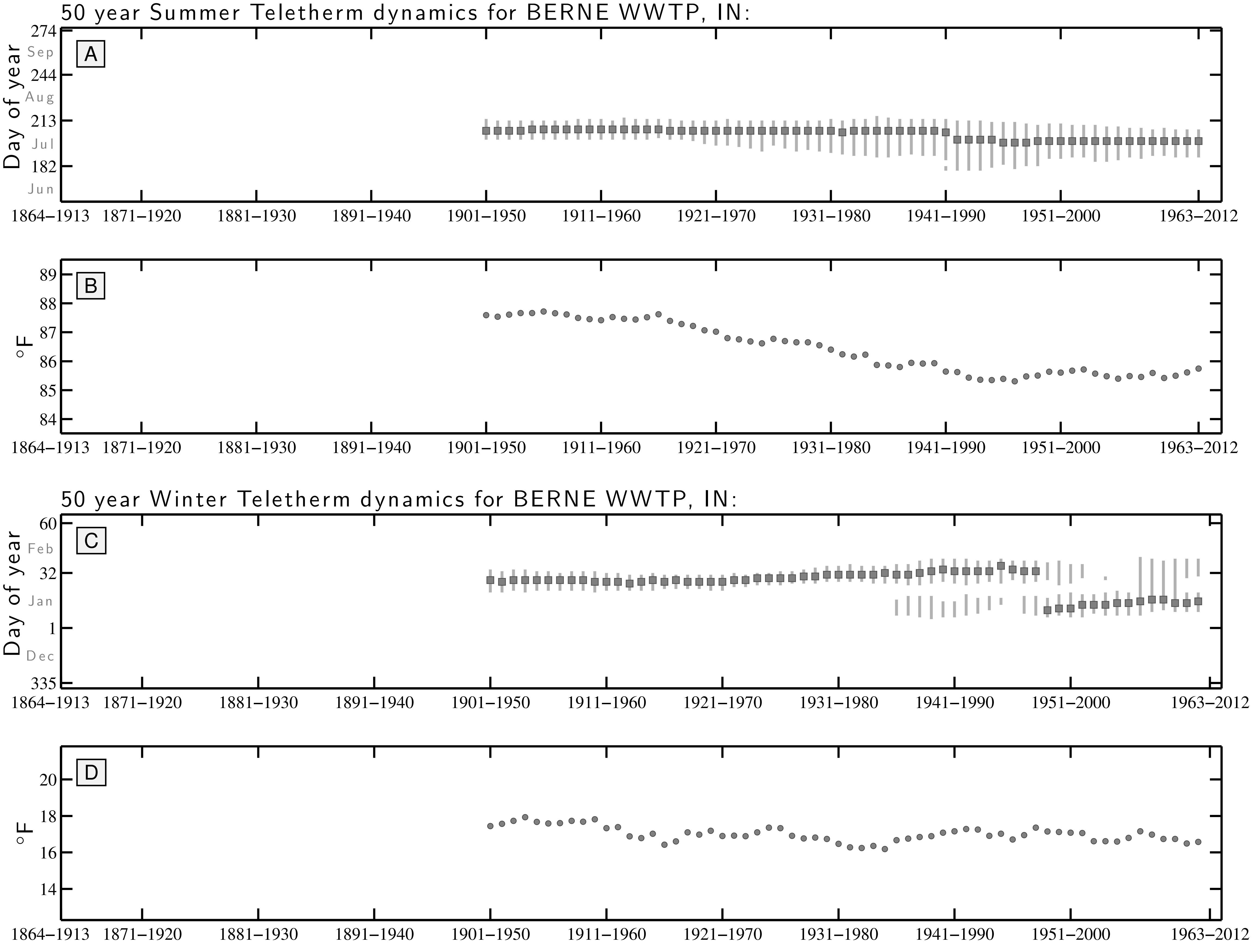
50 year Summer Teletherm dynamics for BERNE WWTP, IN:
A
Day of year
274
Sep
244
Aug
213
Jul
182
Jun
1864–1913
1871–1920
1881–1930
1891–1940
1901–1950
1911–1960
1921–1970
1931–1980
1941–1990
1951–2000
1963–2012
B
°F
89
88
87
86
85
84
50 year Winter Teletherm dynamics for BERNE WWTP, IN:
C
Day of year
60
Feb
32
Jan
1
Dec
335
D
°F
20
18
16
14

50 year Summer Teletherm dynamics for BLOOMINGTON IN UNIV, IN:

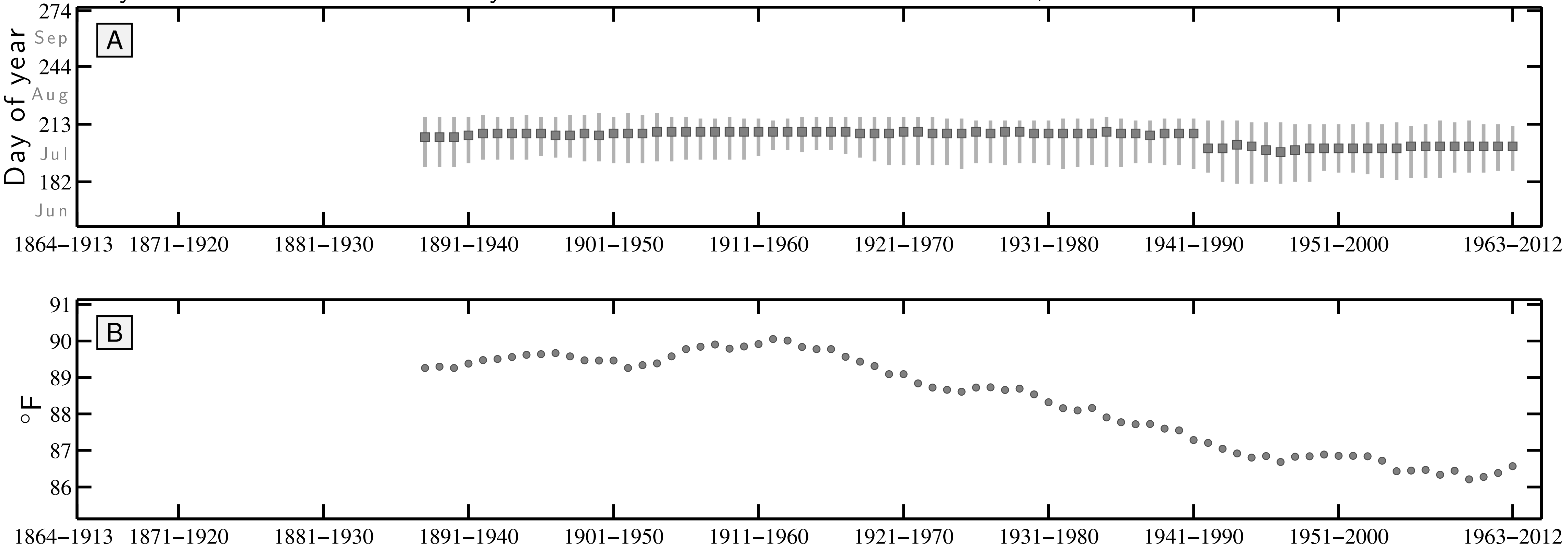

50 year Winter Teletherm dynamics for BLOOMINGTON IN UNIV, IN:

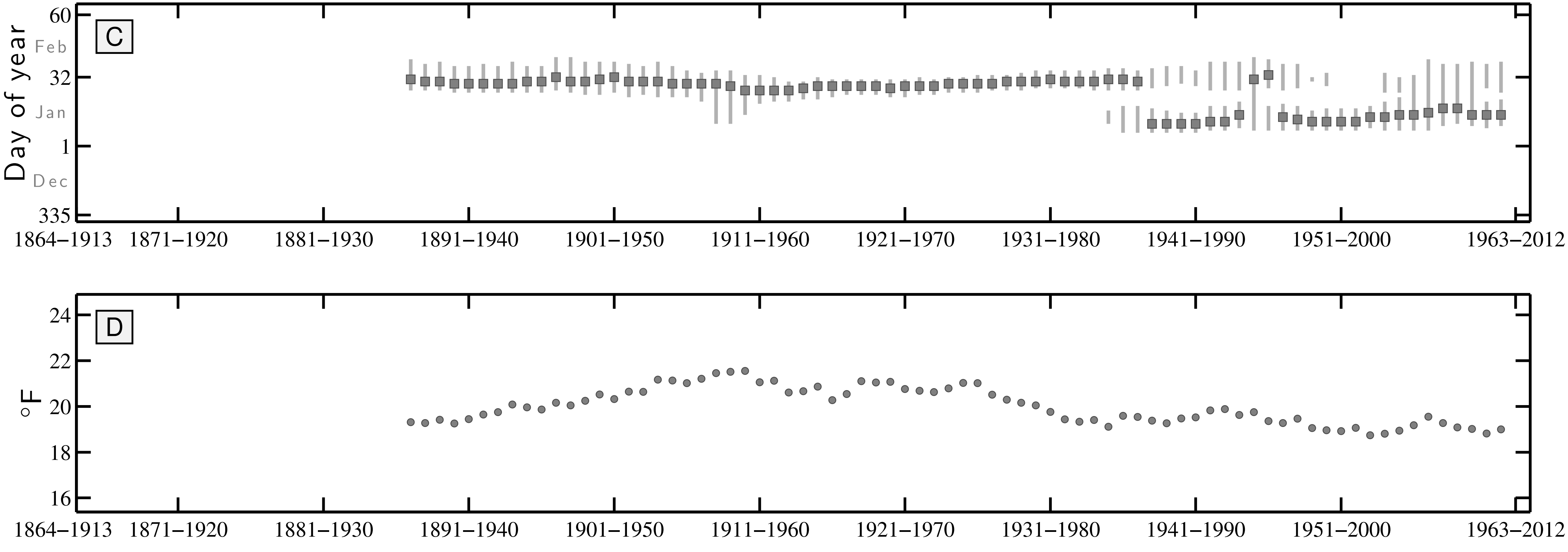

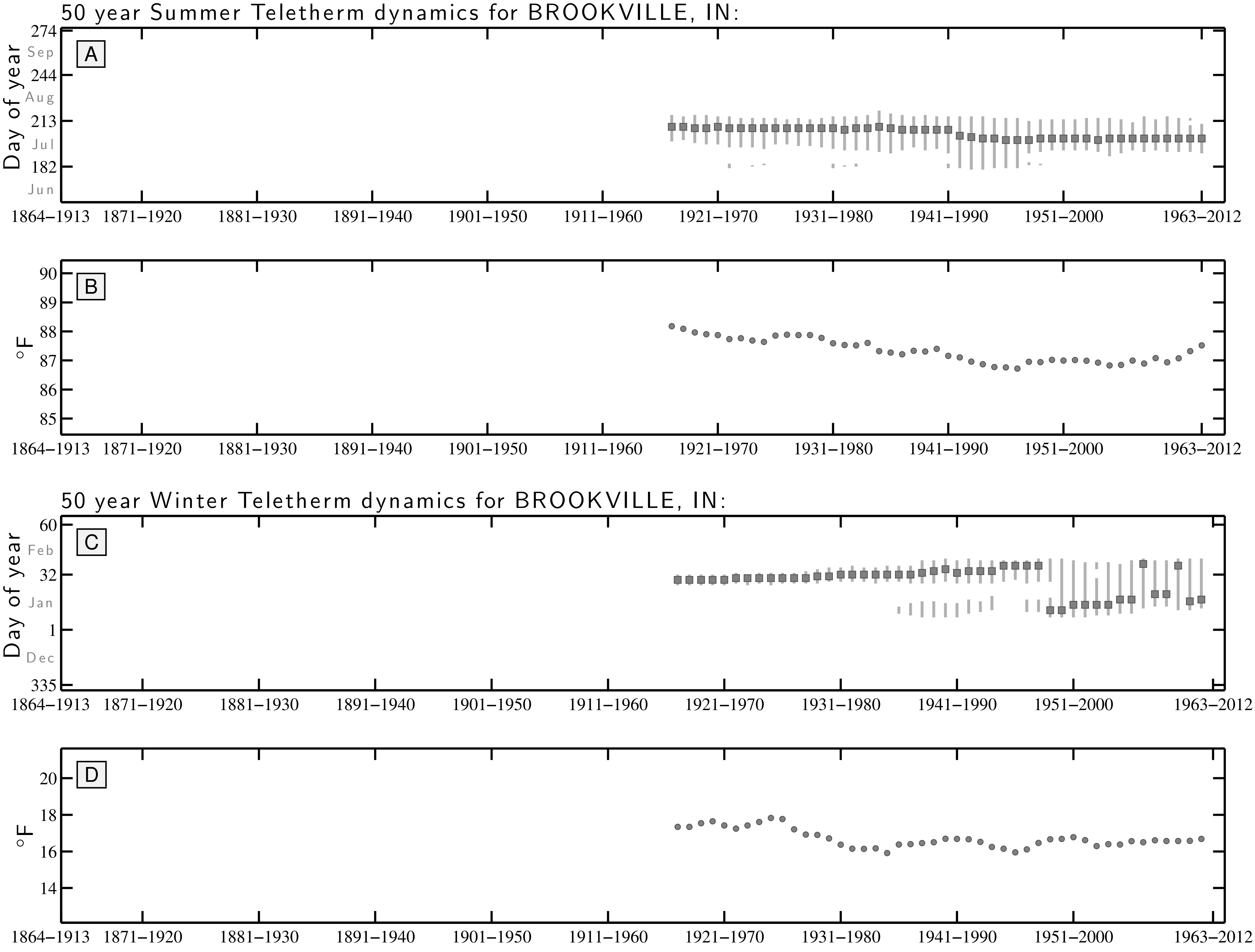
50 year Summer Teletherm dynamics for BROOKVILLE, IN:
A
Day of year
274
Sep
244
Aug
213
Jul
182
Jun
1864–1913
1871–1920
1881–1930
1891–1940
1901–1950
1911–1960
1921–1970
1931–1980
1941–1990
1951–2000
1963–2012
B
°F
90
89
88
87
86
85
50 year Winter Teletherm dynamics for BROOKVILLE, IN:
C
Day of year
60
Feb
32
Jan
1
Dec
335
D
°F
20
18
16
14

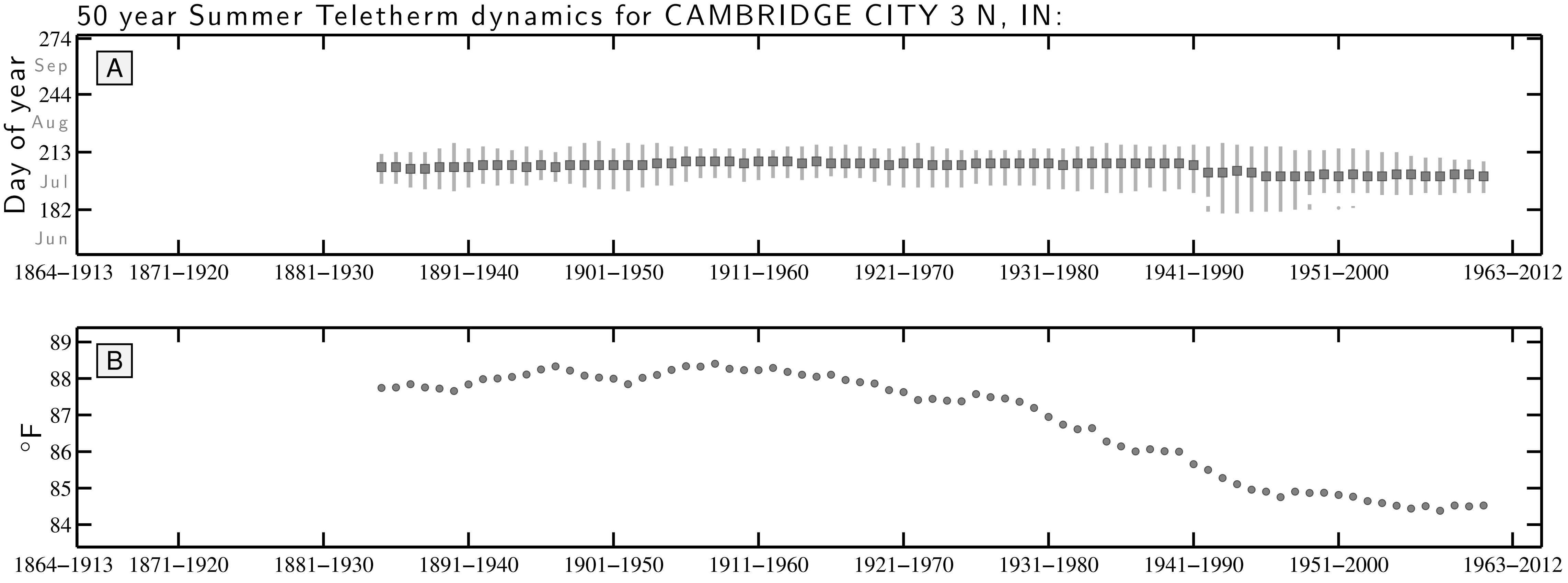
50 year Summer Teletherm dynamics for CAMBRIDGE CITY 3 N, IN:
A
B
Day of year
°F
274
244
213
182
Sep
Aug
Jul
Jun
89
88
87
86
85
84
1864–1913
1871–1920
1881–1930
1891–1940
1901–1950
1911–1960
1921–1970
1931–1980
1941–1990
1951–2000
1963–2012

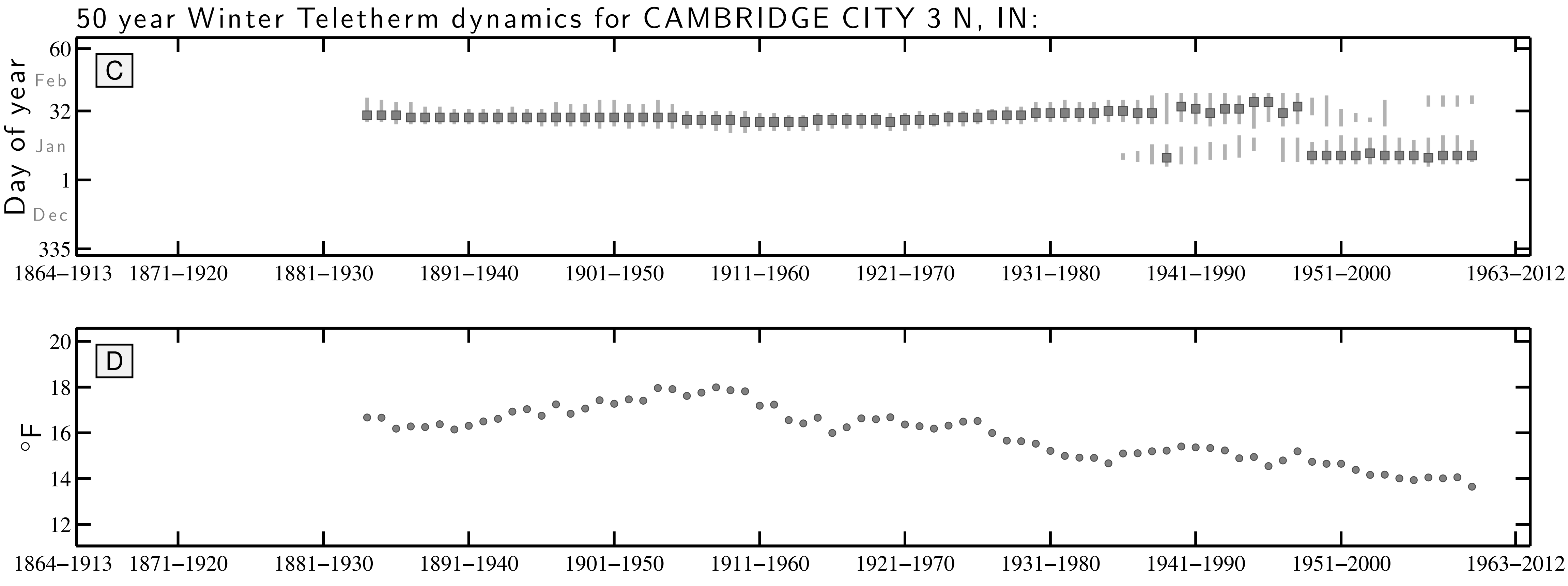
50 year Winter Teletherm dynamics for CAMBRIDGE CITY 3 N, IN:
C
D
Day of year
°F
60
32
1
335
Feb
Jan
Dec
20
18
16
14
12
1864–1913
1871–1920
1881–1930
1891–1940
1901–1950
1911–1960
1921–1970
1931–1980
1941–1990
1951–2000
1963–2012

50 year Summer Teletherm dynamics for COLUMBUS, IN:

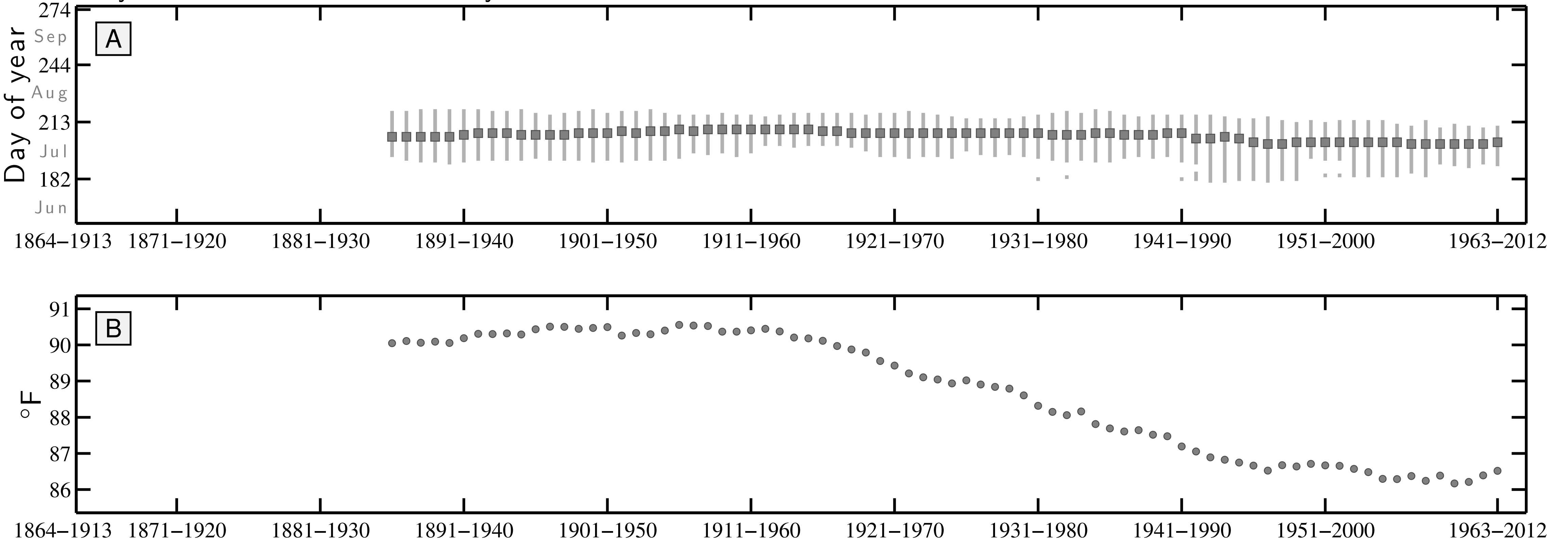

50 year Winter Teletherm dynamics for COLUMBUS, IN:

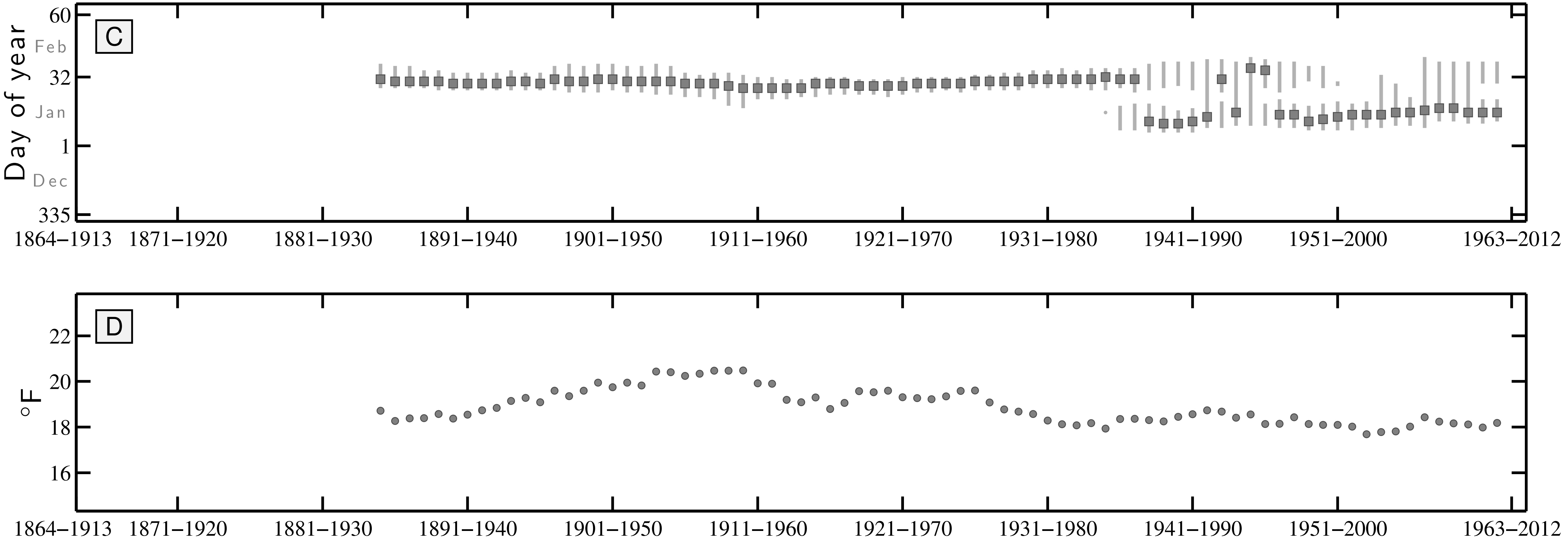

50 year Summer Teletherm dynamics for DELPHI 2 N, IN:

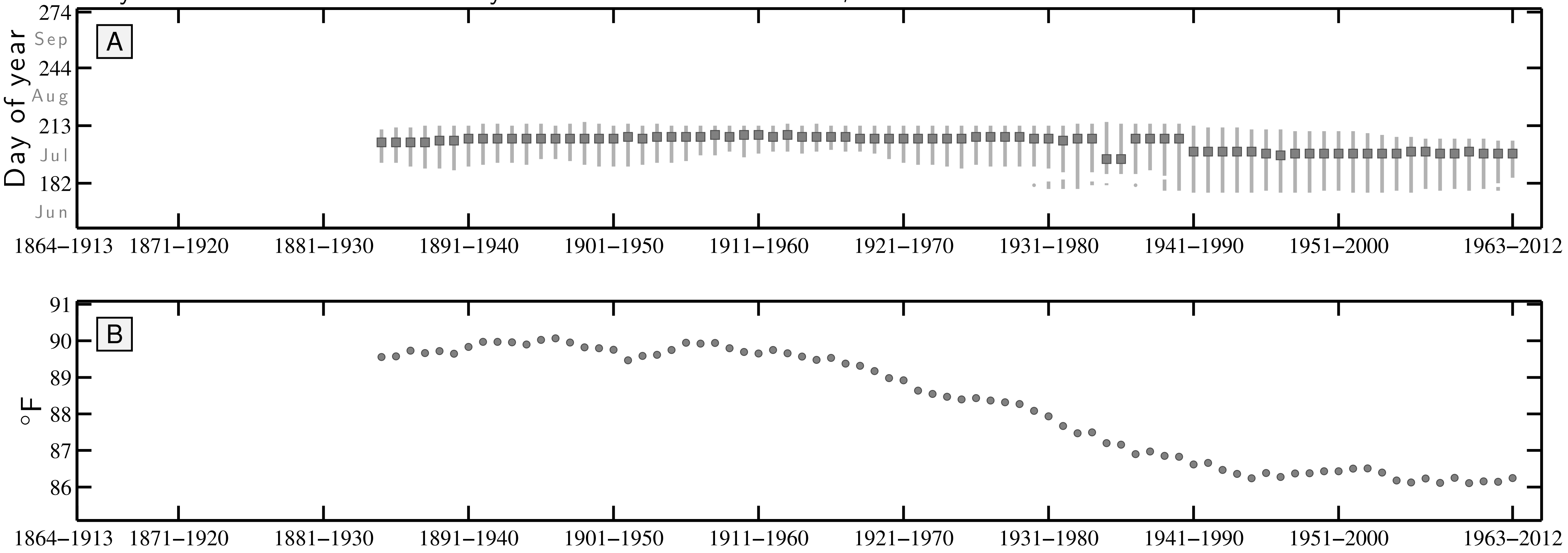

50 year Winter Teletherm dynamics for DELPHI 2 N, IN:

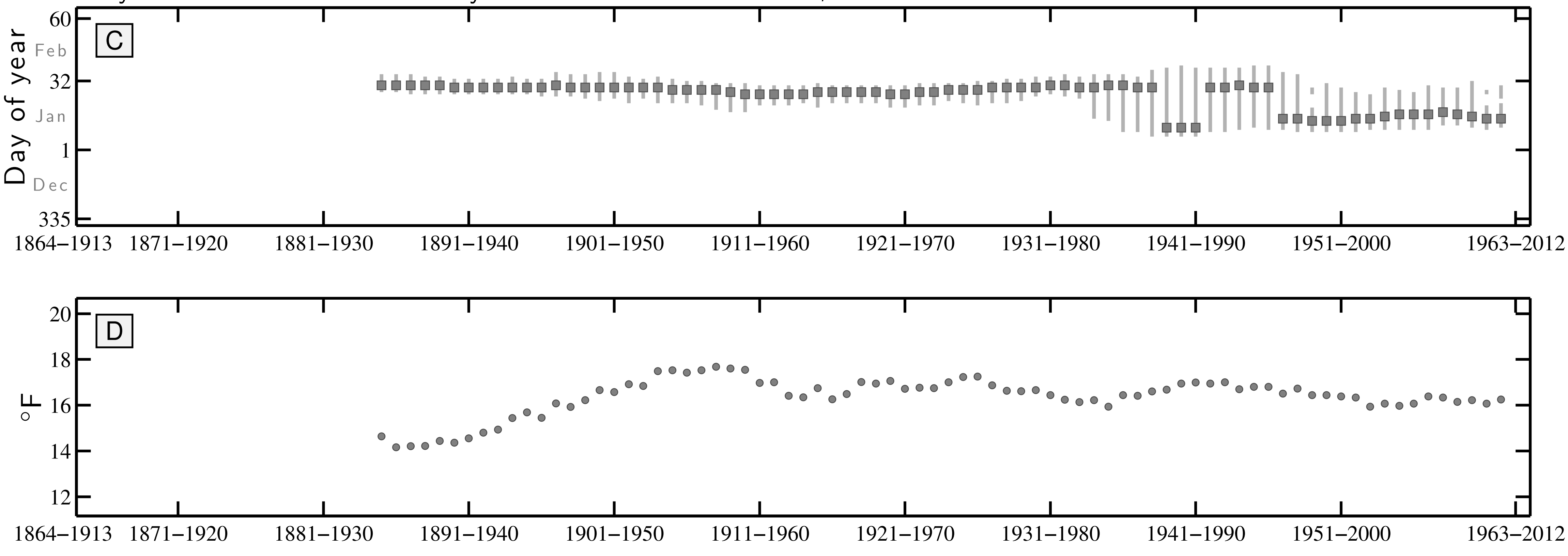

50 year Summer Teletherm dynamics for GOSHEN 3SW, IN:

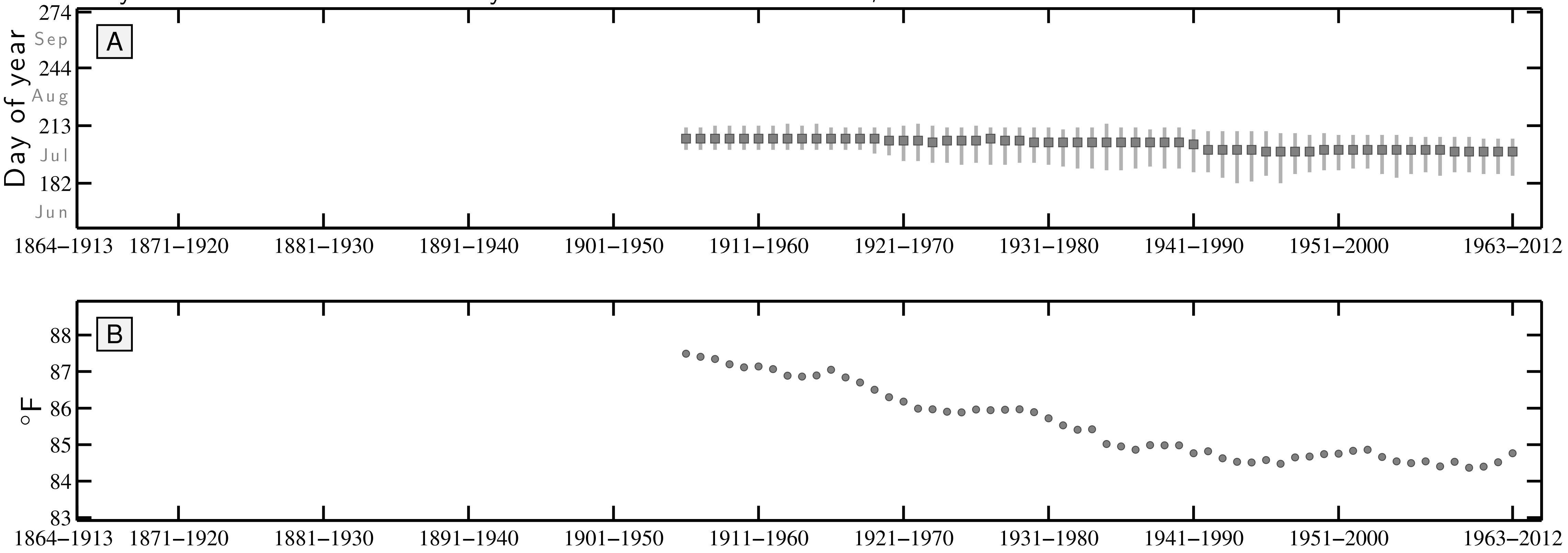

50 year Winter Teletherm dynamics for GOSHEN 3SW, IN:

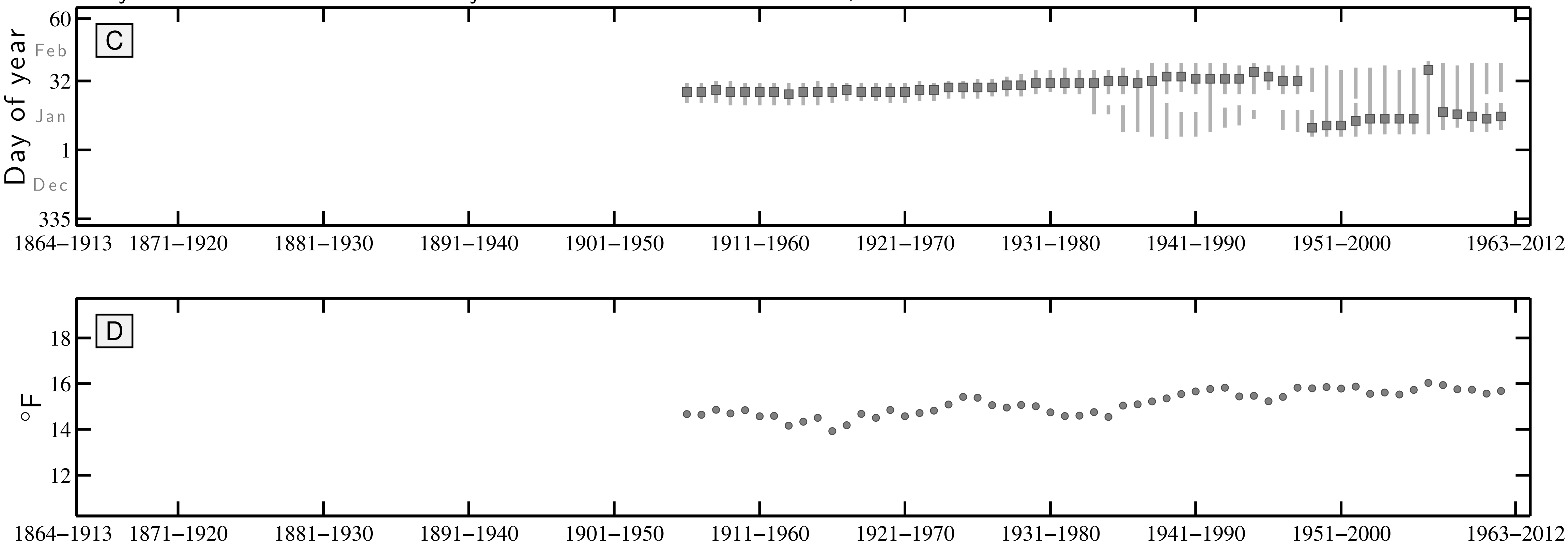

50 year Summer Teletherm dynamics for GREENCASTLE 1 W, IN:

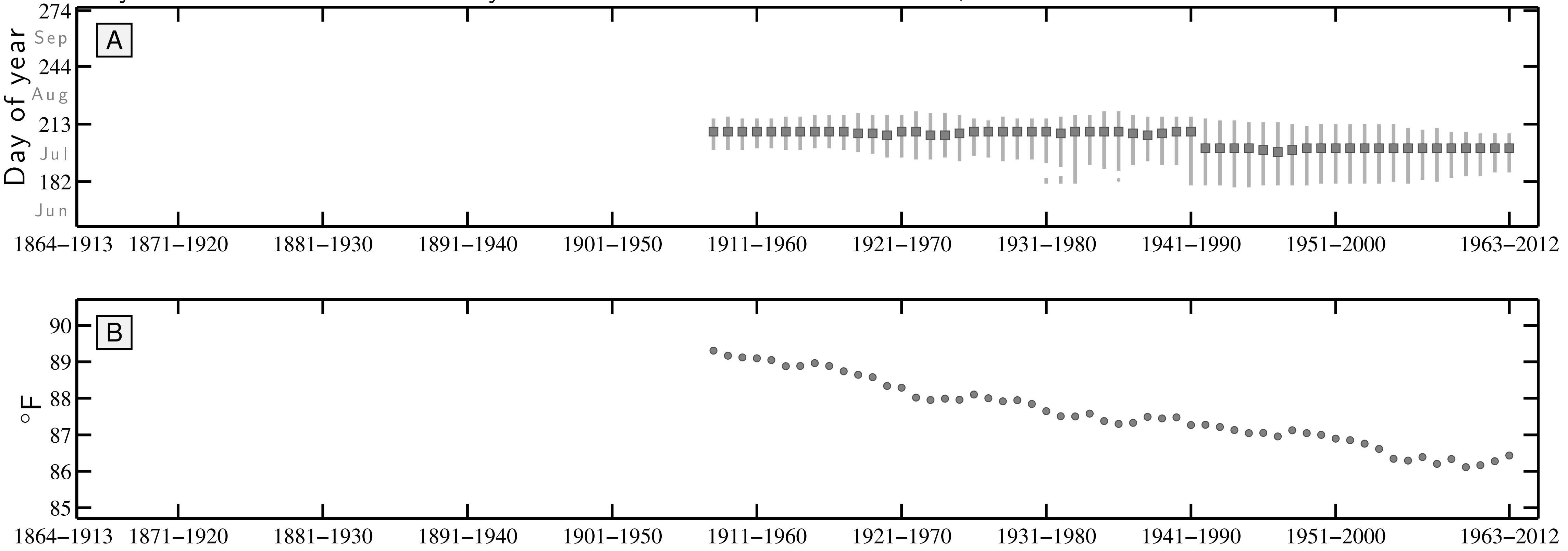

50 year Winter Teletherm dynamics for GREENCASTLE 1 W, IN:

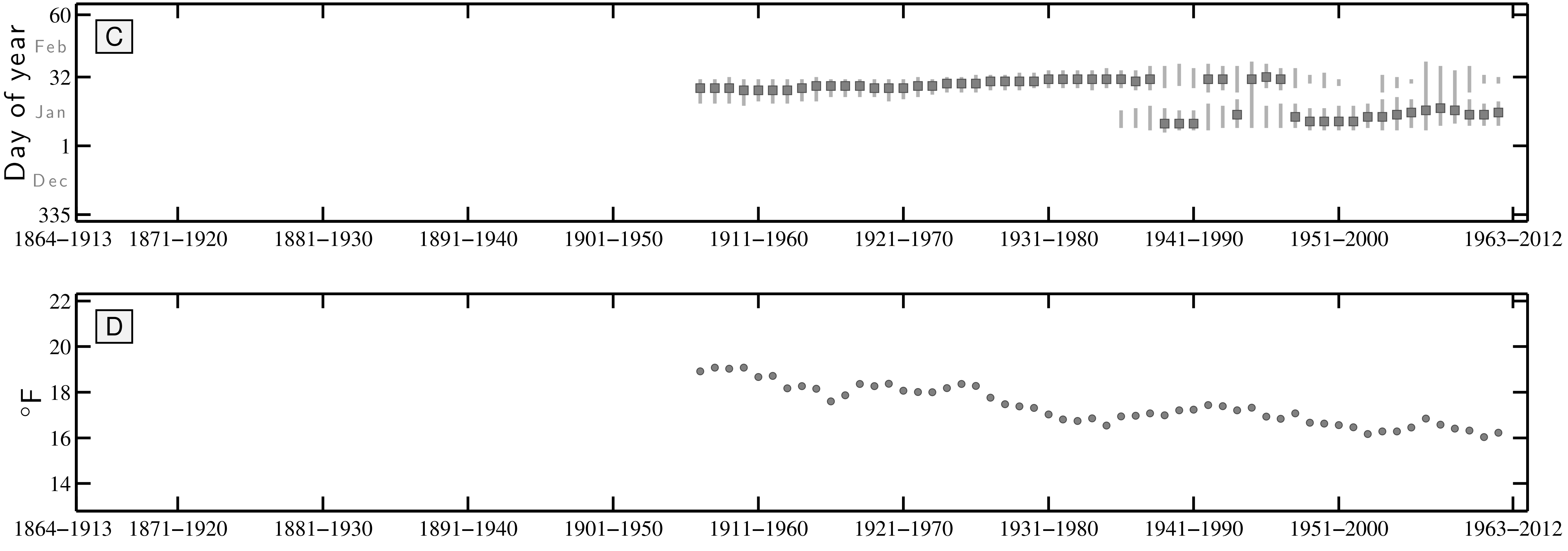

50 year Summer Teletherm dynamics for GREENFIELD, IN:

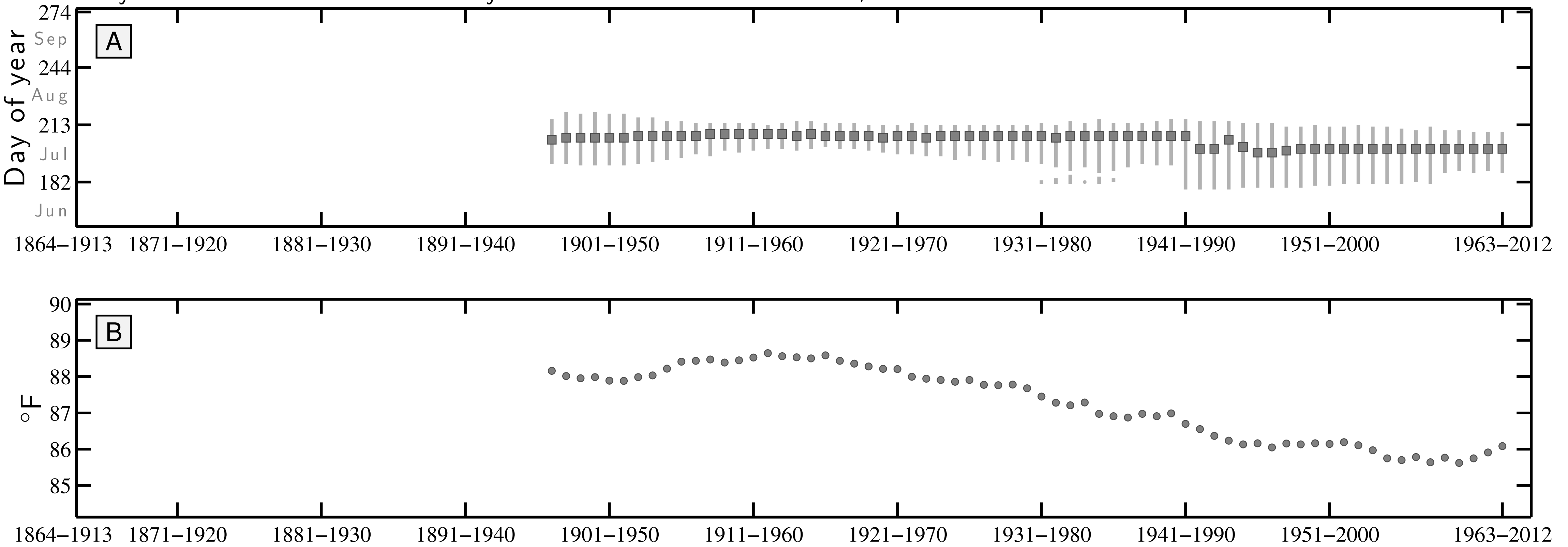

50 year Winter Teletherm dynamics for GREENFIELD, IN:

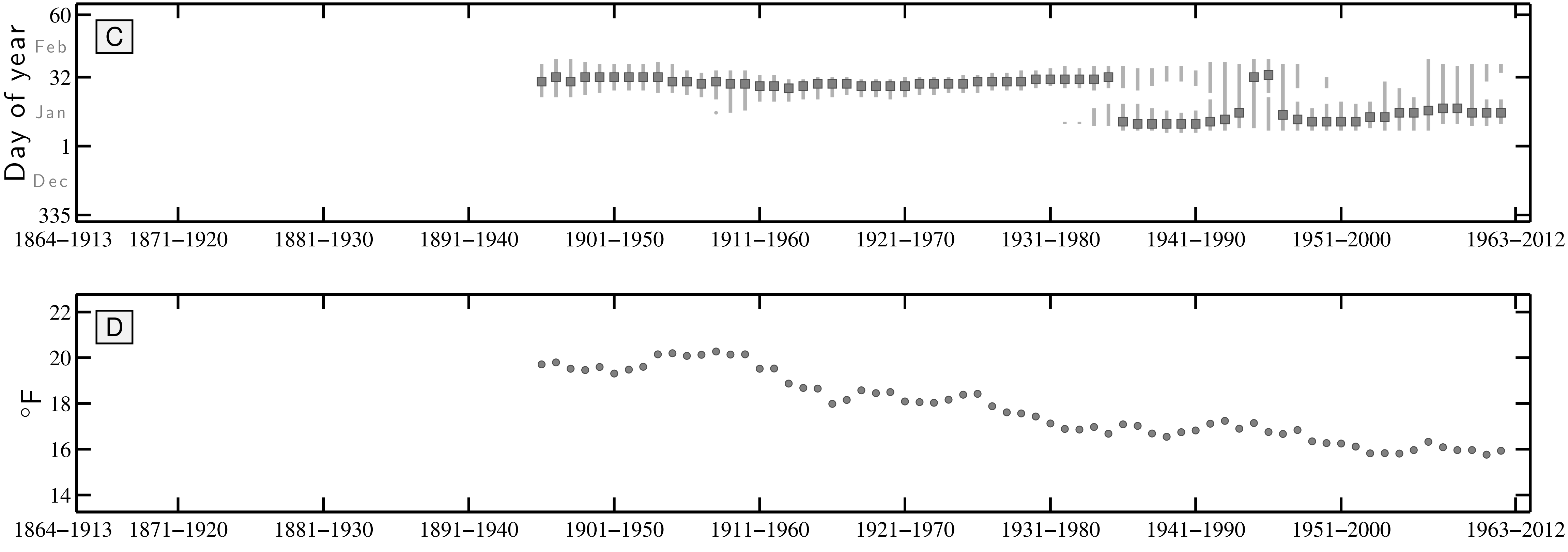

50 year Summer Teletherm dynamics for HOBART 2 WNW, IN:

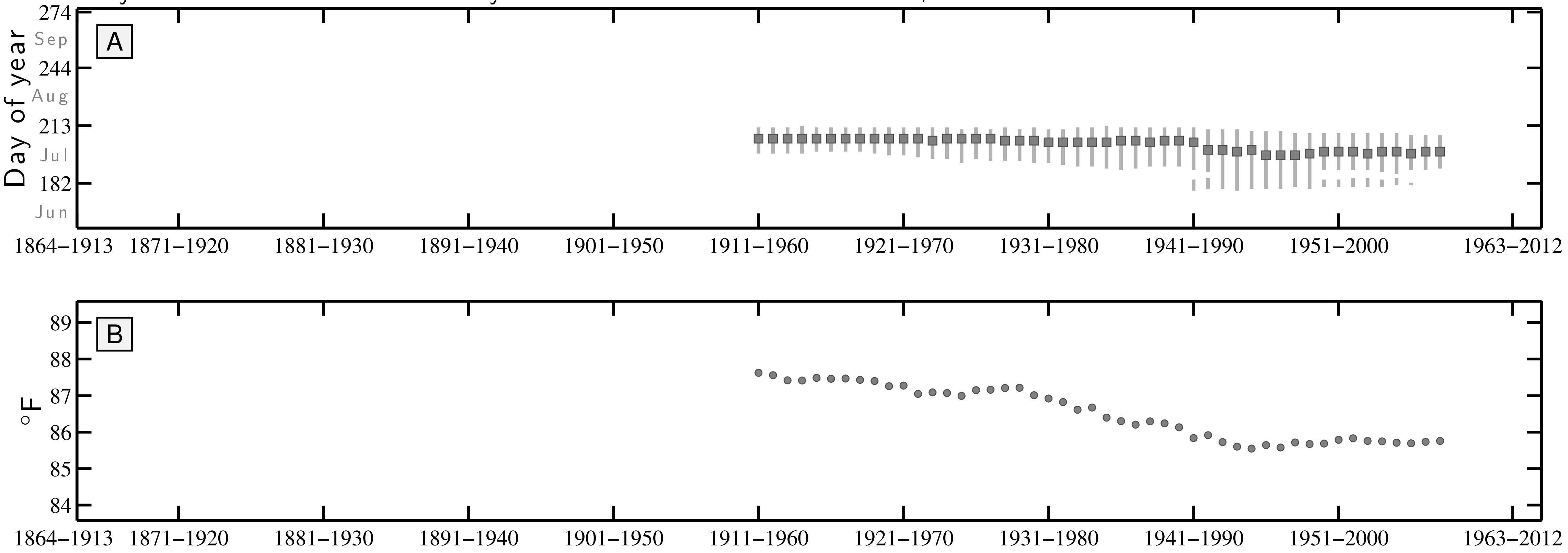

50 year Winter Teletherm dynamics for HOBART 2 WNW, IN:

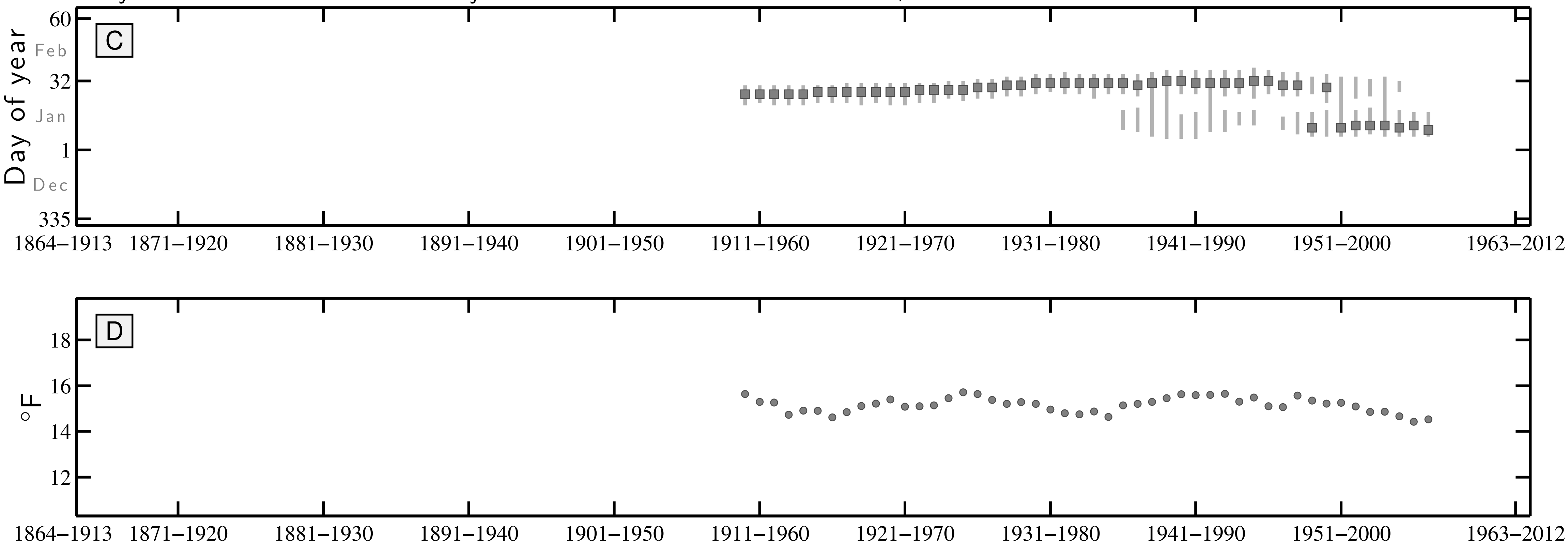

50 year Summer Teletherm dynamics for LAPORTE, IN:

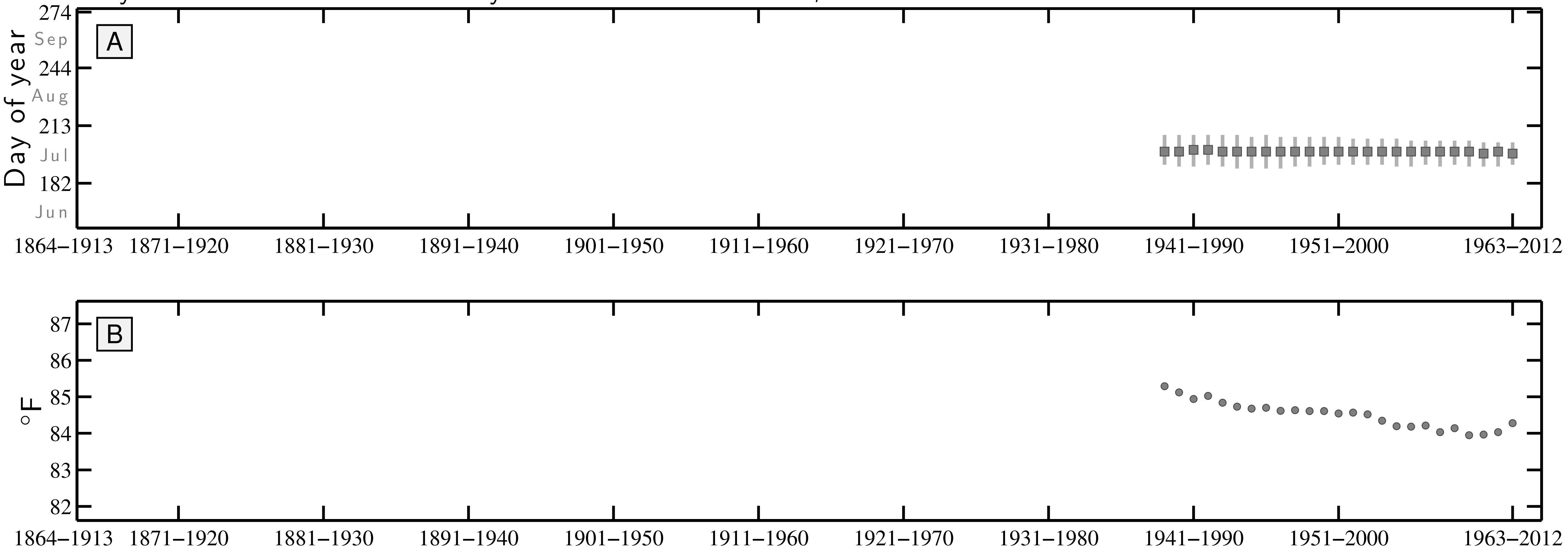

50 year Winter Teletherm dynamics for LAPORTE, IN:

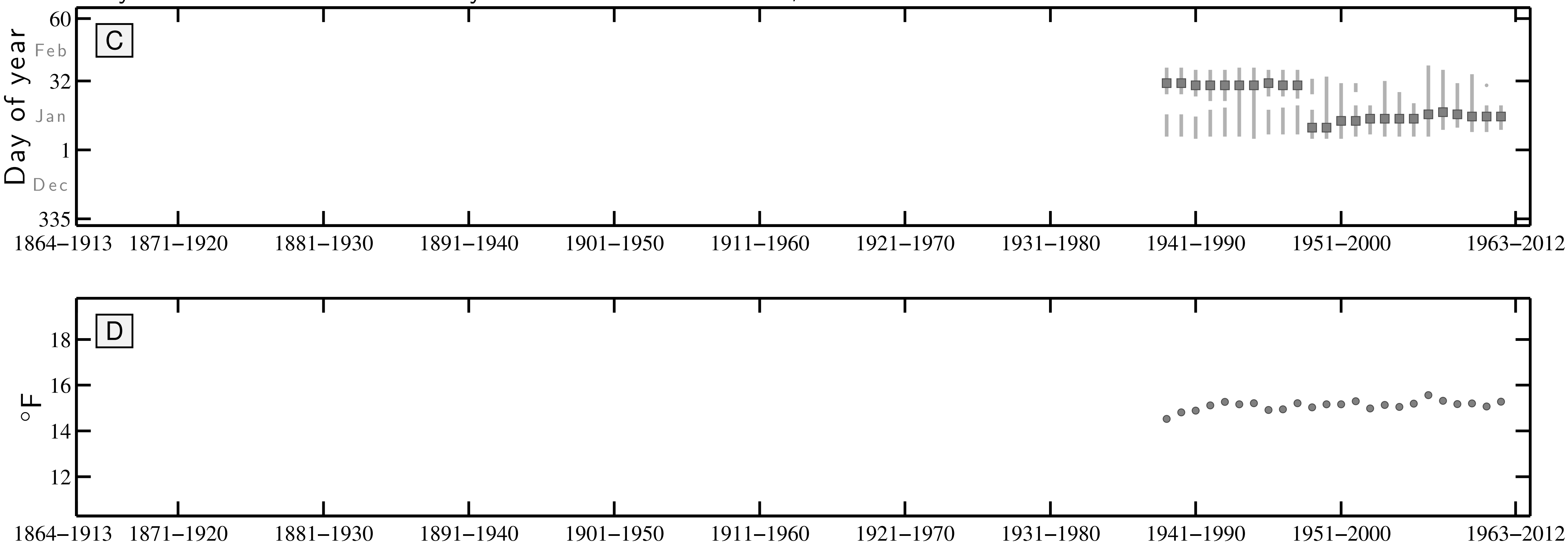

50 year Summer Teletherm dynamics for MADISON SEWAGE PLT, IN:

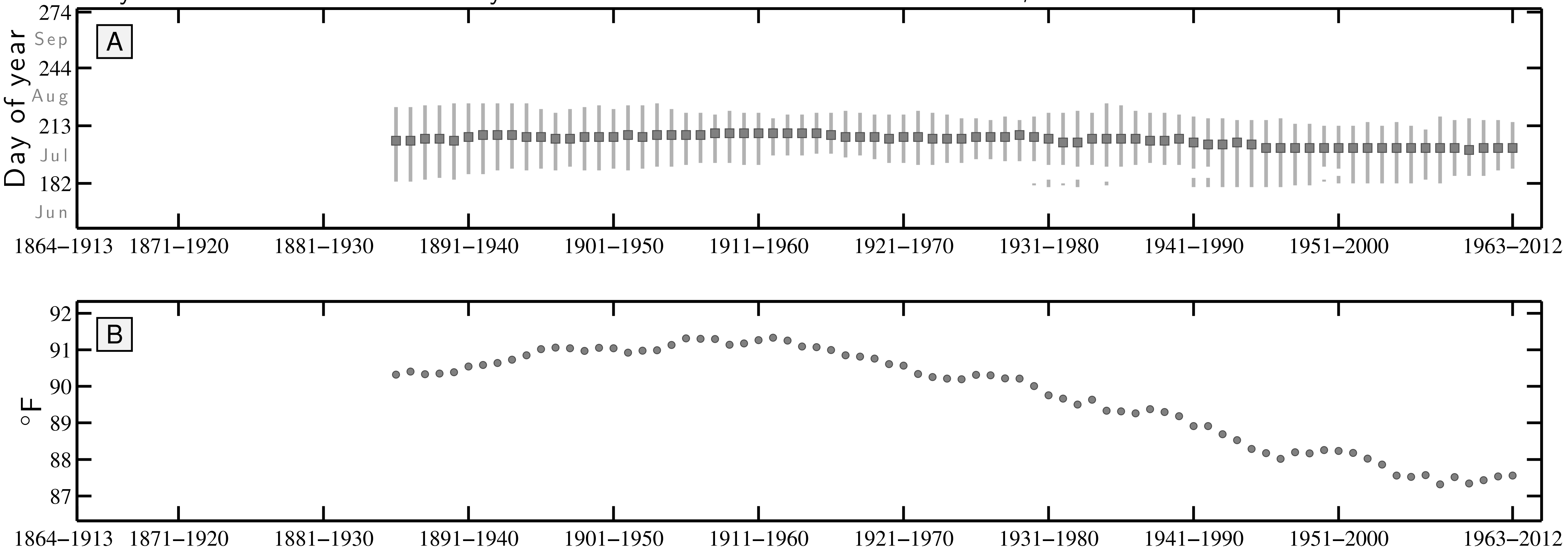

50 year Winter Teletherm dynamics for MADISON SEWAGE PLT, IN:

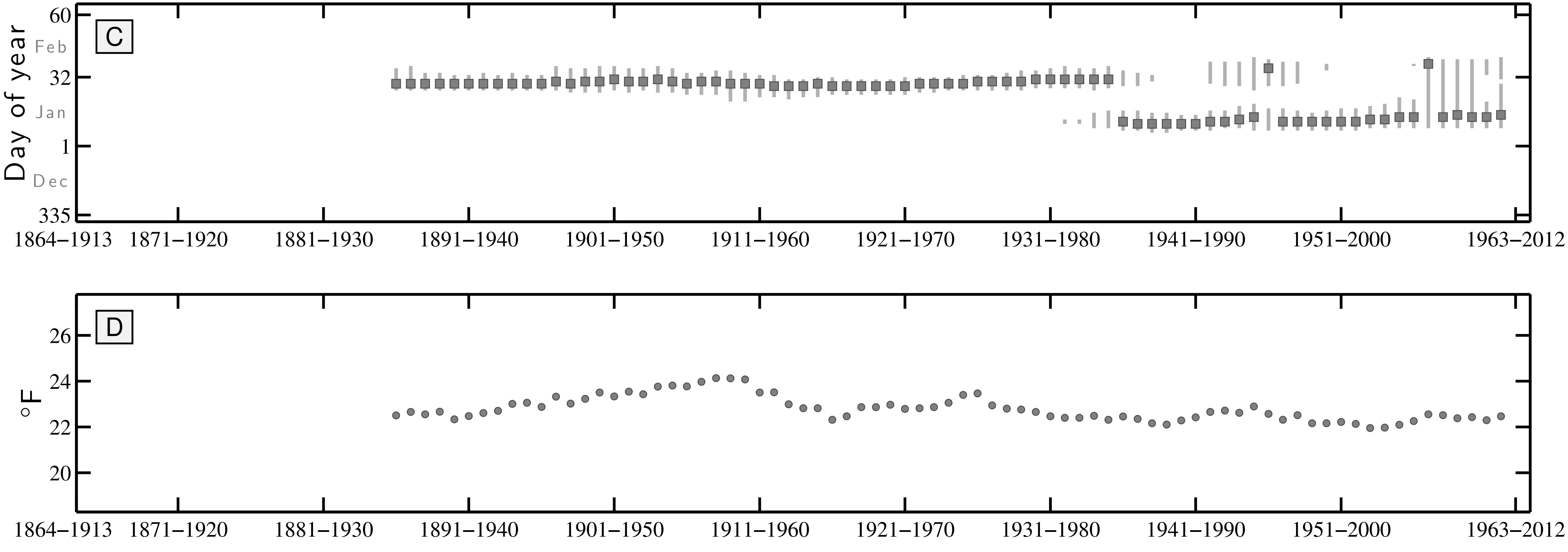

50 year Summer Teletherm dynamics for MARION 2 N, IN:

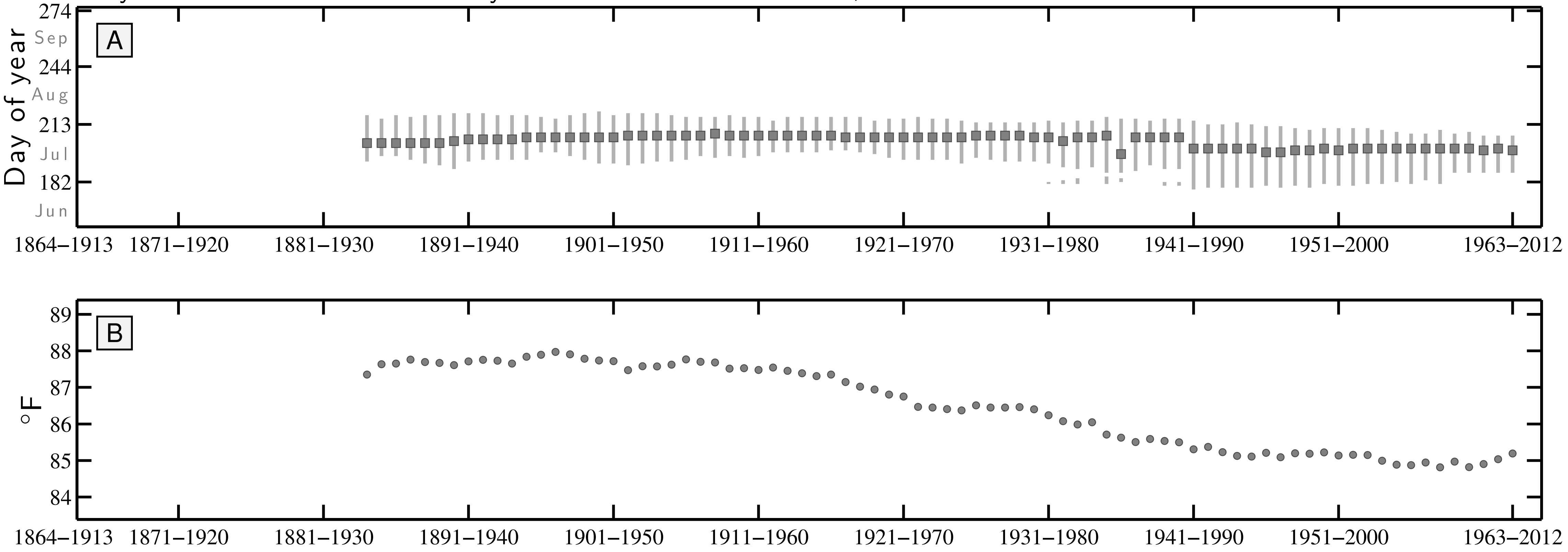

50 year Winter Teletherm dynamics for MARION 2 N, IN:

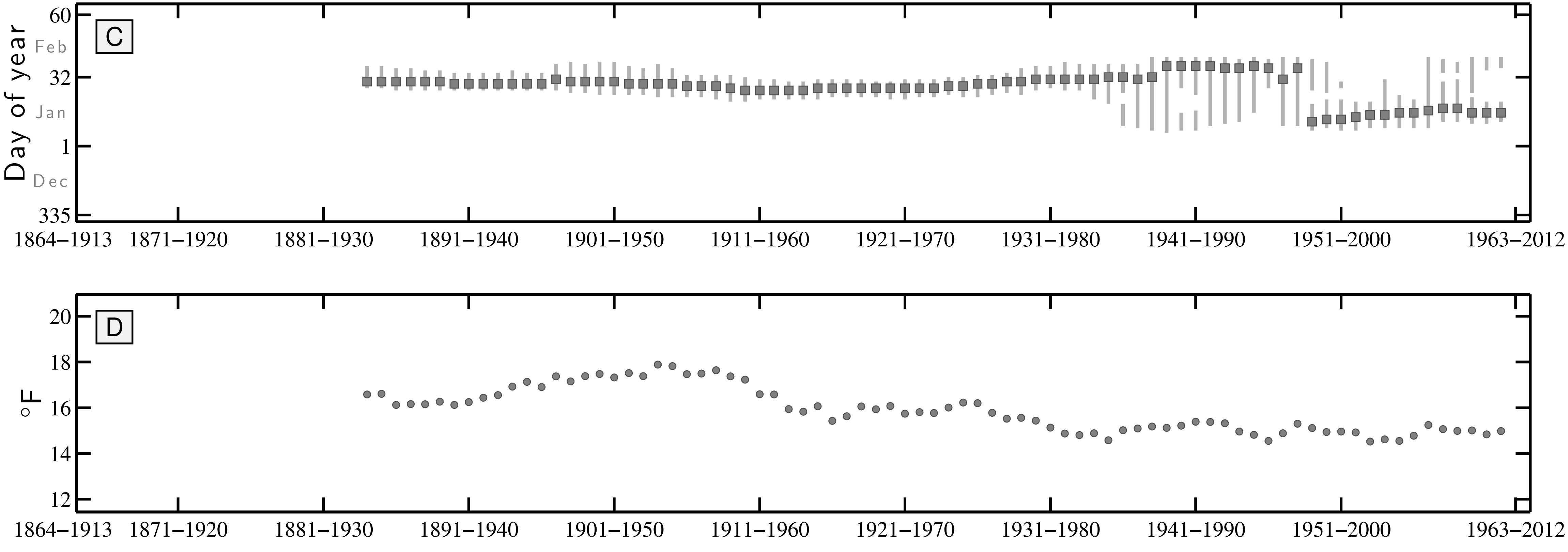

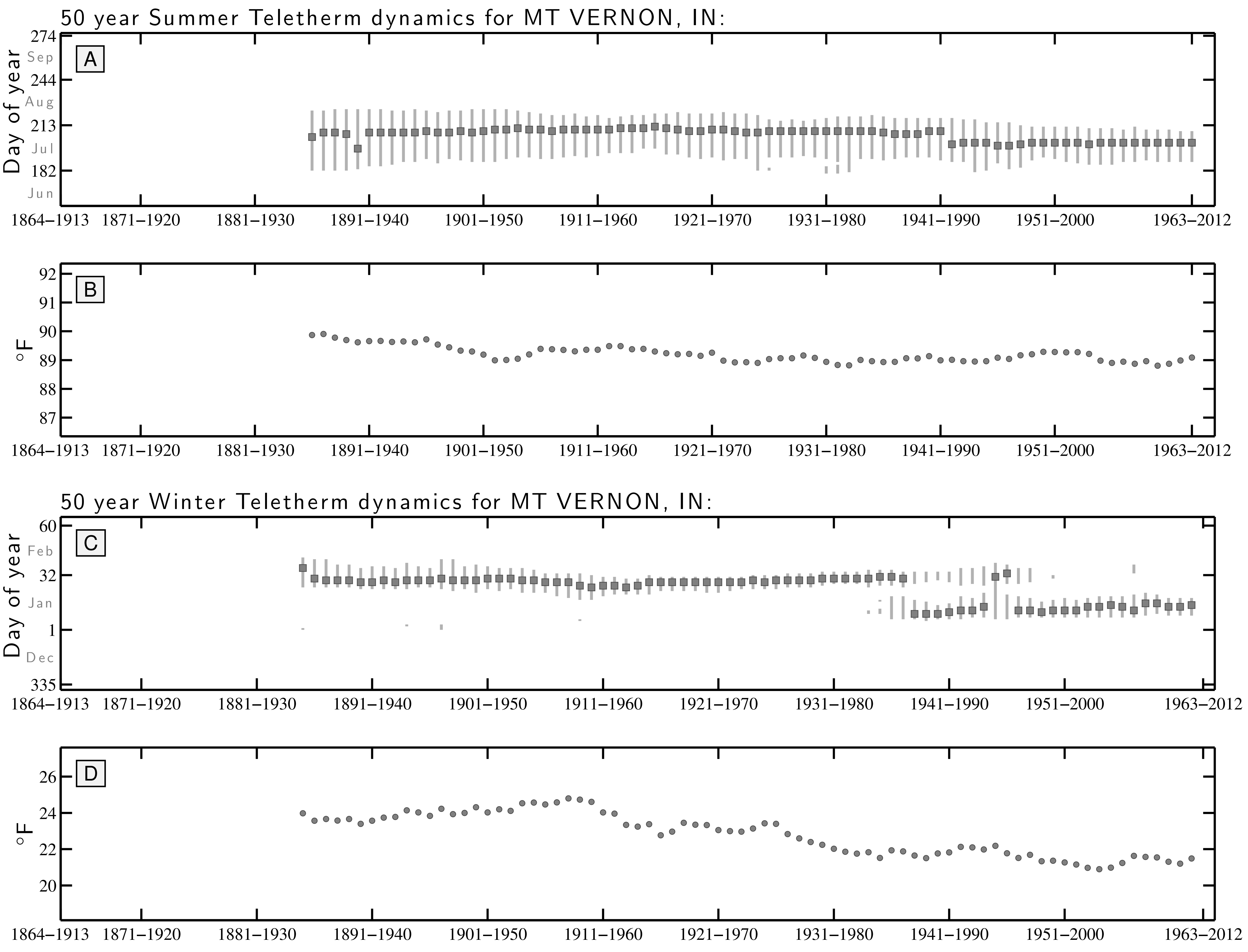

50 year Summer Teletherm dynamics for MT VERNON, IN:
A
B
Day of year
°F
Sep
Aug
Jul
Jun
274
244
213
182
92
91
90
89
88
87
50 year Winter Teletherm dynamics for MT VERNON, IN:
C
D
Feb
Jan
Dec
60
32
1
335
26
24
22
20
1864–1913
1871–1920
1881–1930
1891–1940
1901–1950
1911–1960
1921–1970
1931–1980
1941–1990
1951–2000
1963–2012

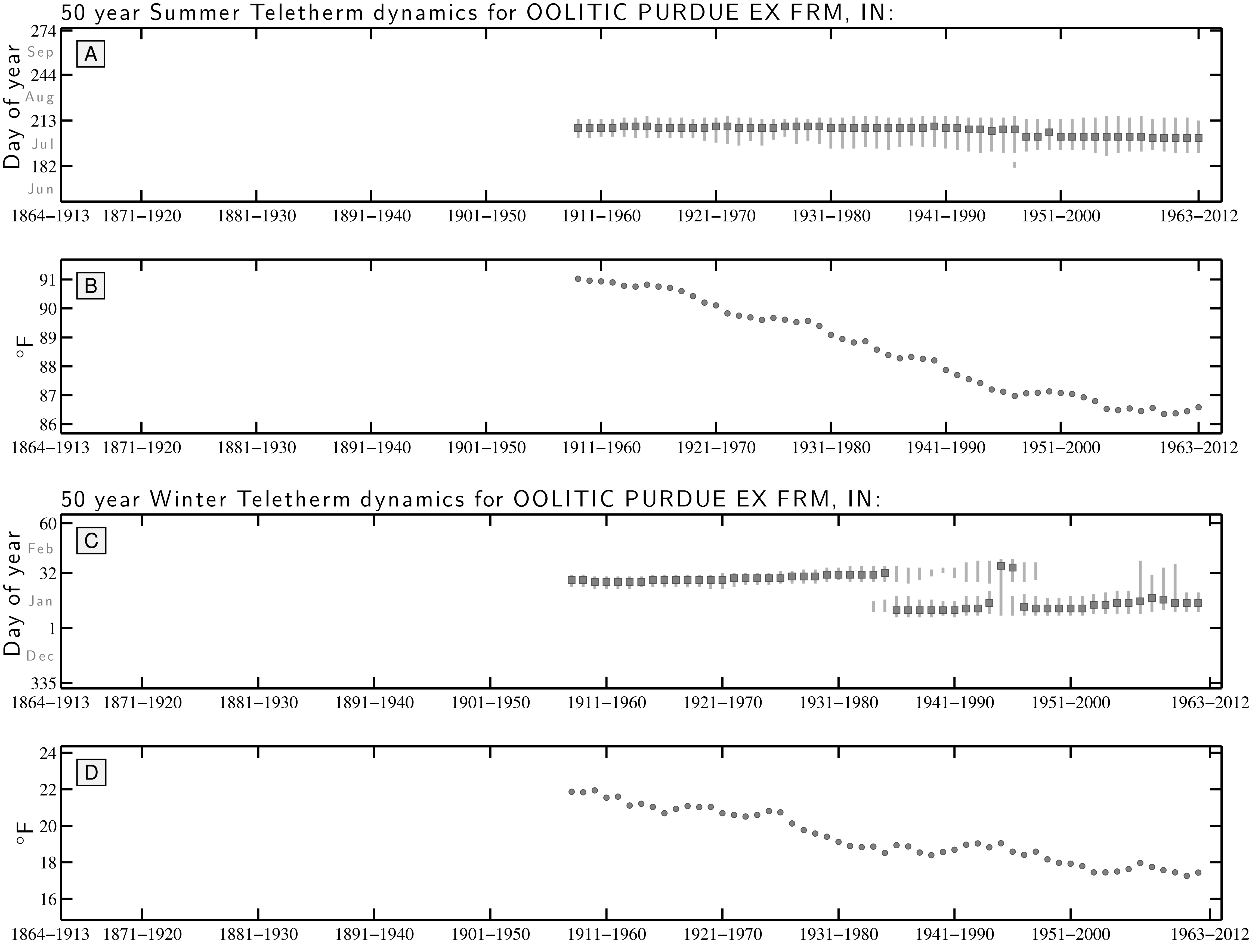
50 year Summer Teletherm dynamics for OOLITIC PURDUE EX FRM, IN:
A
Day of year
274
Sep
244
Aug
213
Jul
182
Jun
B
°F
91
90
89
88
87
86
50 year Winter Teletherm dynamics for OOLITIC PURDUE EX FRM, IN:
C
Day of year
60
Feb
32
Jan
1
Dec
335
D
°F
24
22
20
18
16
1864–1913
1871–1920
1881–1930
1891–1940
1901–1950
1911–1960
1921–1970
1931–1980
1941–1990
1951–2000
1963–2012

50 year Summer Teletherm dynamics for PAOLI, IN:

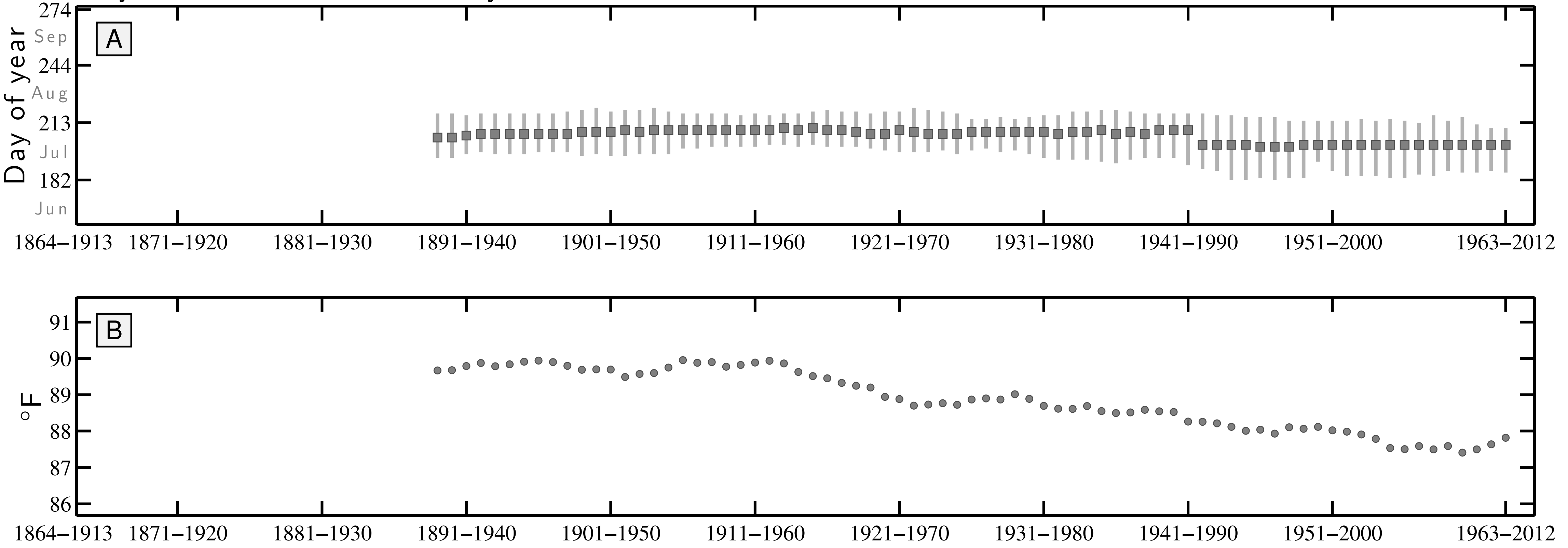

50 year Winter Teletherm dynamics for PAOLI, IN:

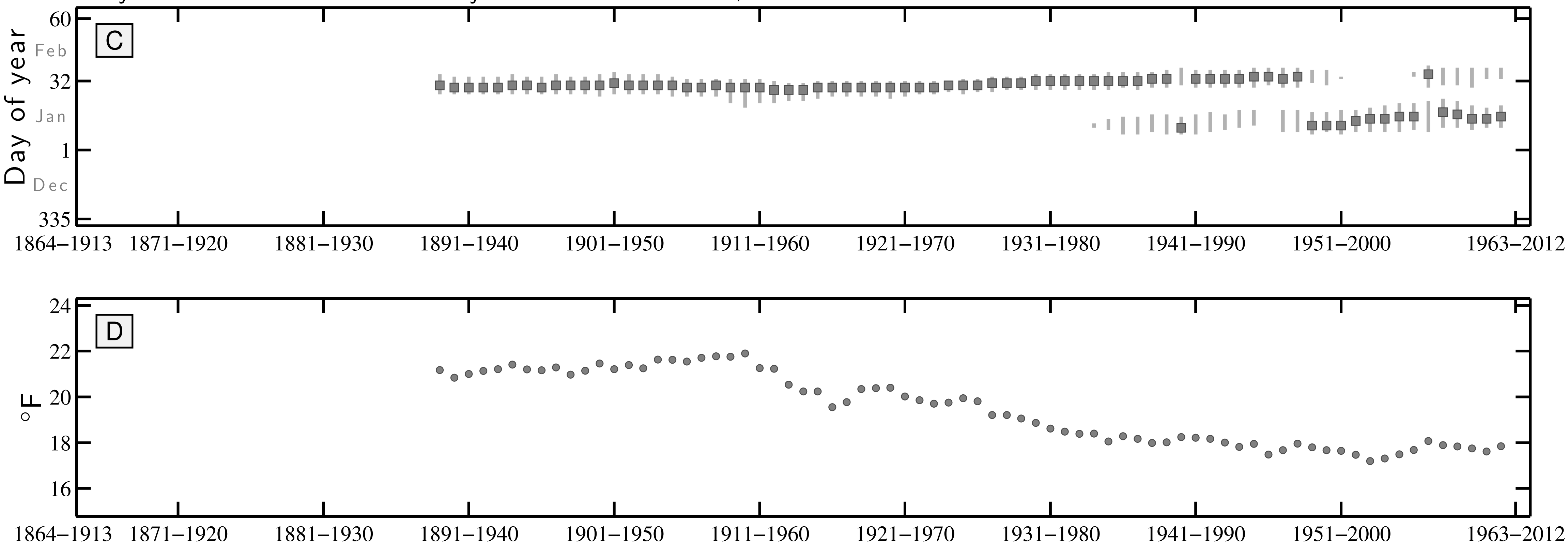

50 year Summer Teletherm dynamics for PRINCETON 1 W, IN:

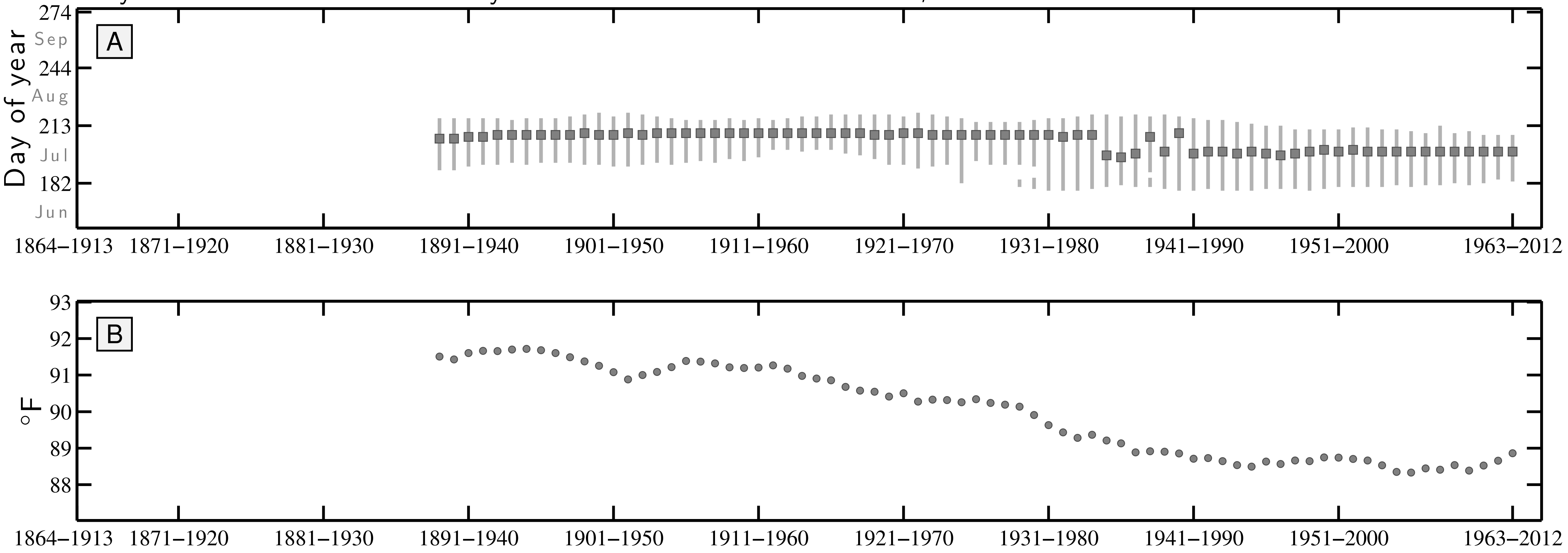

50 year Winter Teletherm dynamics for PRINCETON 1 W, IN:

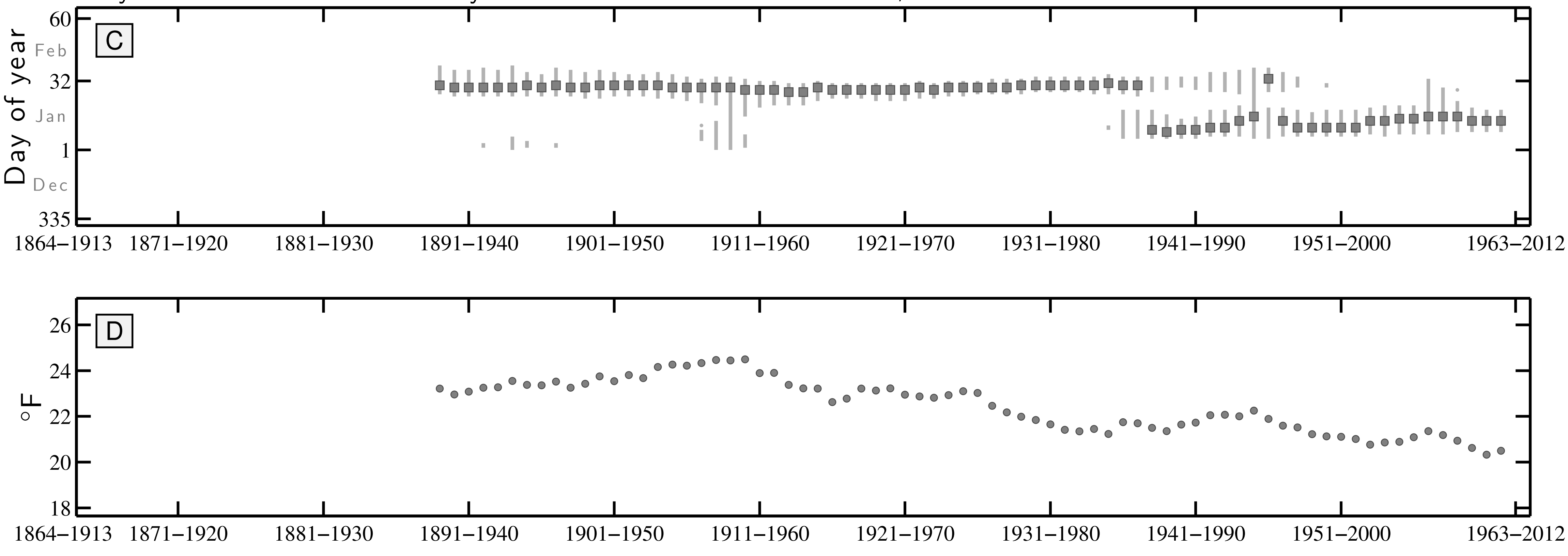

50 year Summer Teletherm dynamics for RENSSELAER, IN:

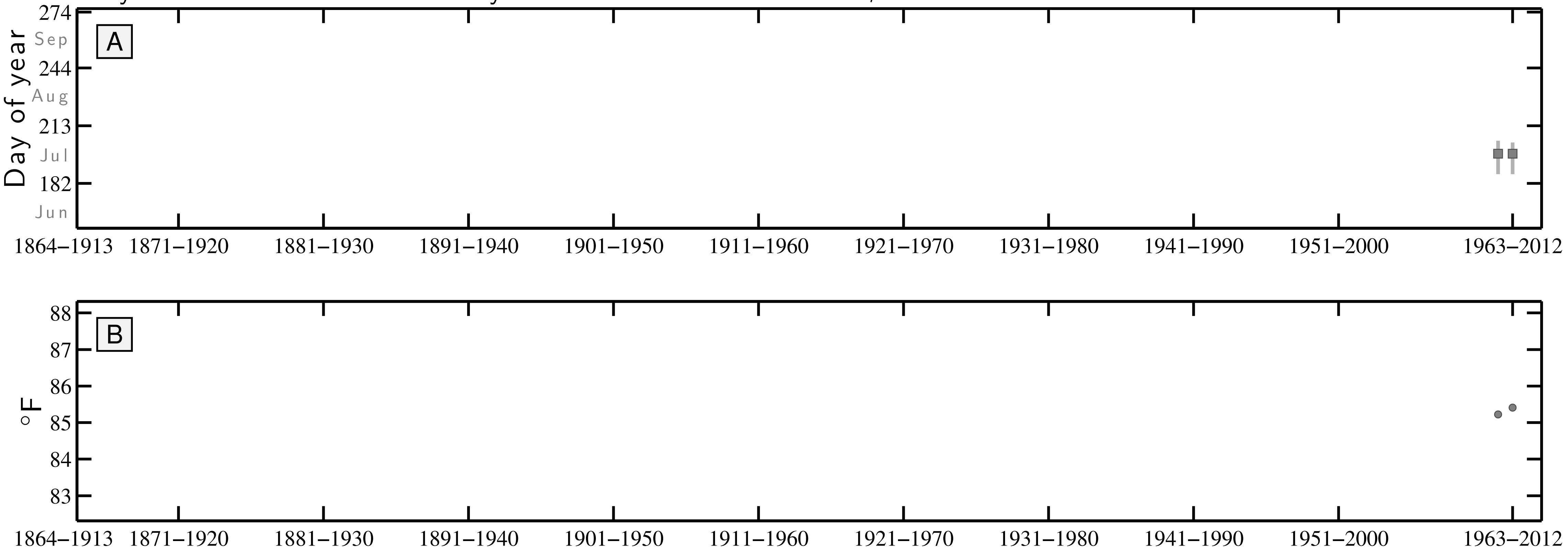

50 year Winter Teletherm dynamics for RENSSELAER, IN:

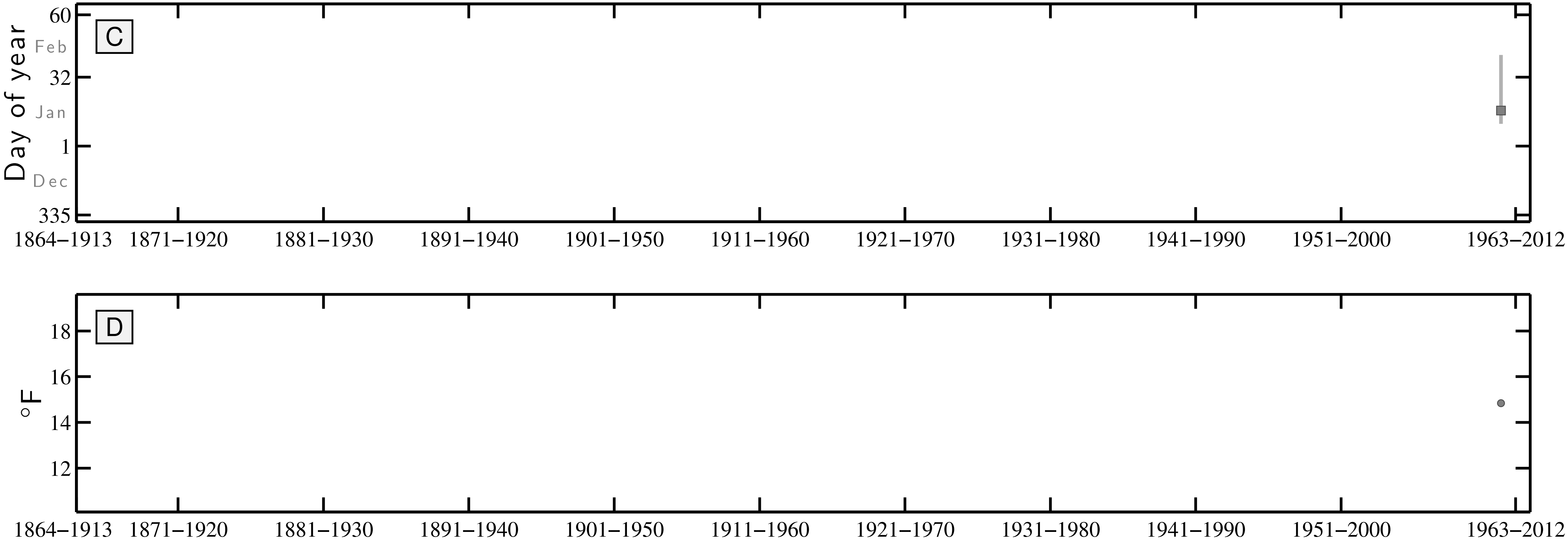

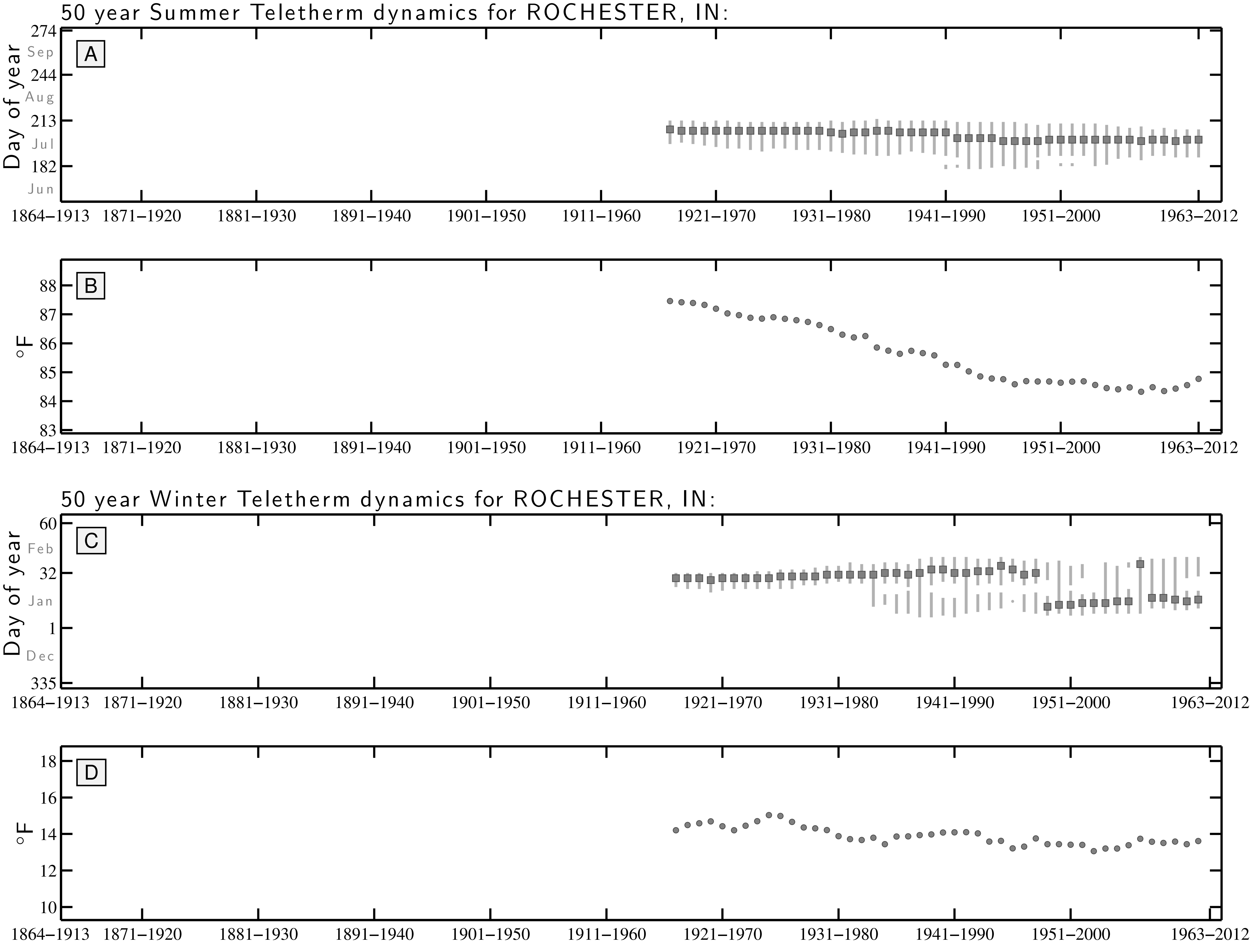
50 year Summer Teletherm dynamics for ROCHESTER, IN:
A
Day of year
274
Sep
244
Aug
213
Jul
182
Jun
1864–1913
1871–1920
1881–1930
1891–1940
1901–1950
1911–1960
1921–1970
1931–1980
1941–1990
1951–2000
1963–2012
B
°F
88
87
86
85
84
83
50 year Winter Teletherm dynamics for ROCHESTER, IN:
C
60
Feb
32
Jan
1
Dec
335
D
18
16
14
12
10

50 year Summer Teletherm dynamics for ROCKVILLE, IN:

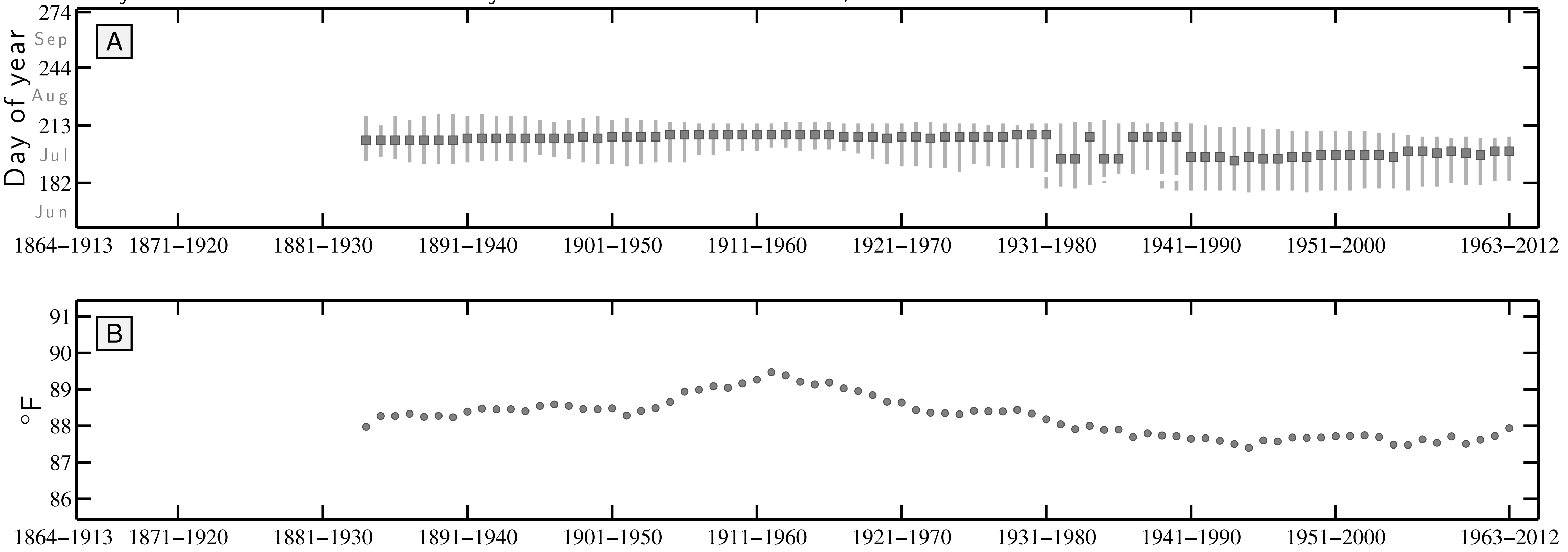

50 year Winter Teletherm dynamics for ROCKVILLE, IN:

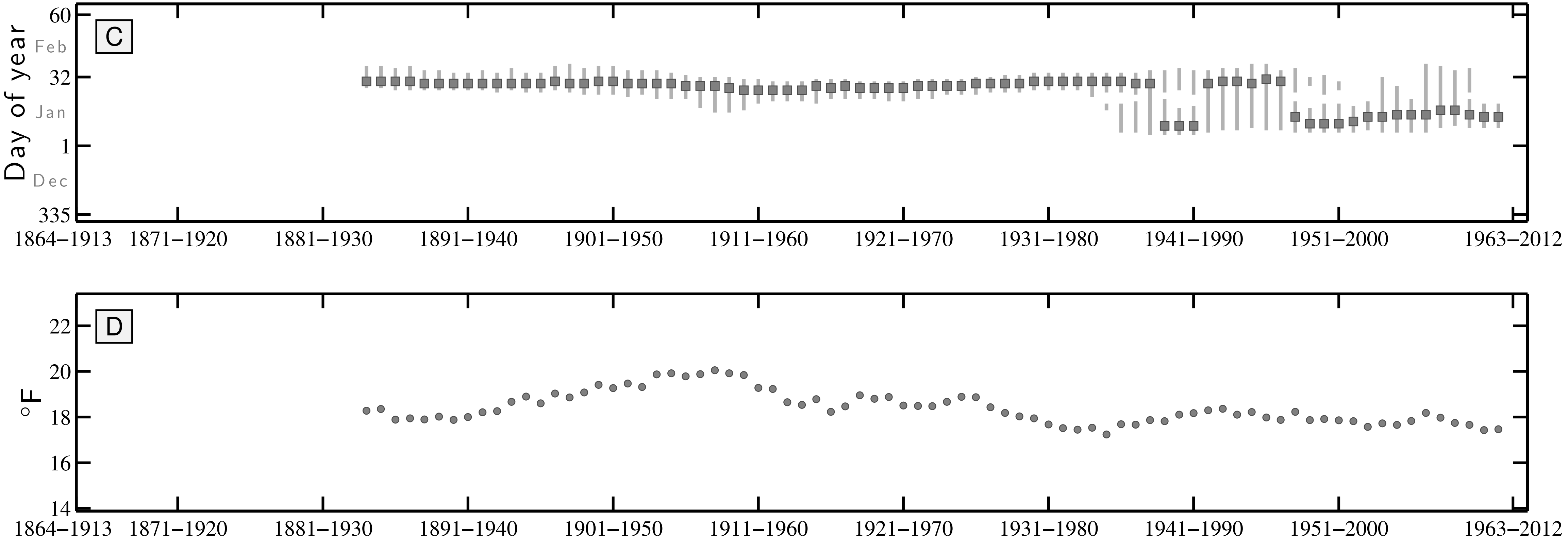

50 year Summer Teletherm dynamics for RUSHVILLE, IN:

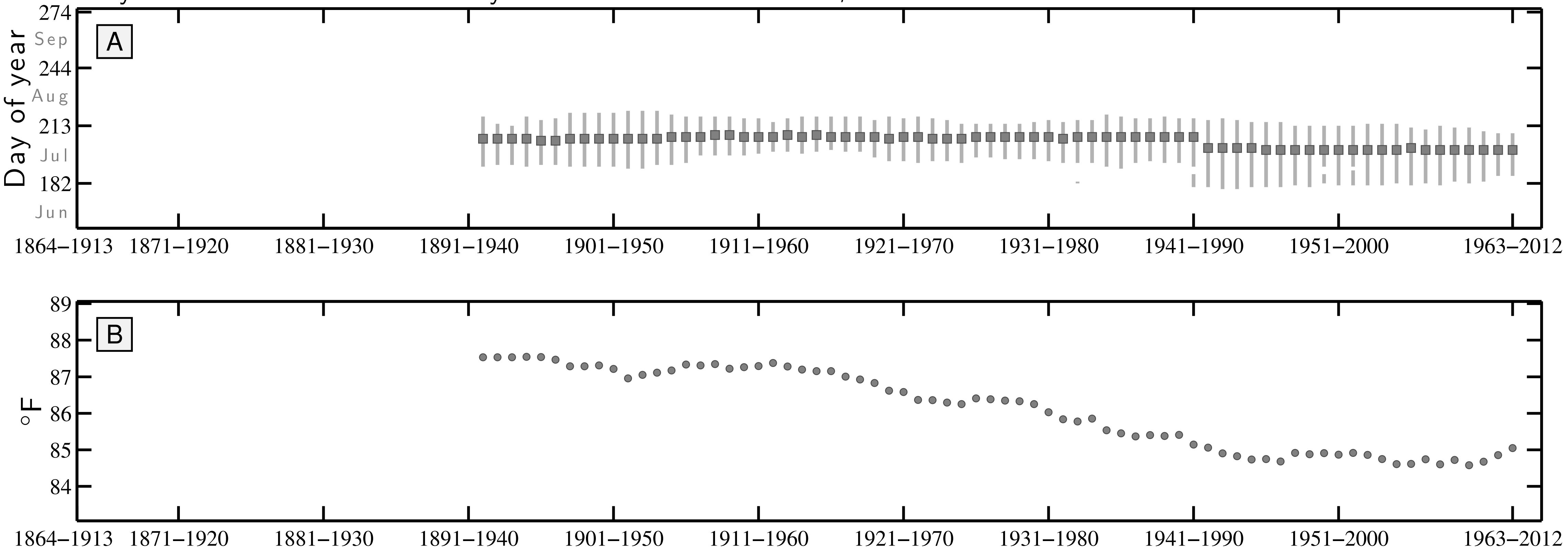

50 year Winter Teletherm dynamics for RUSHVILLE, IN:

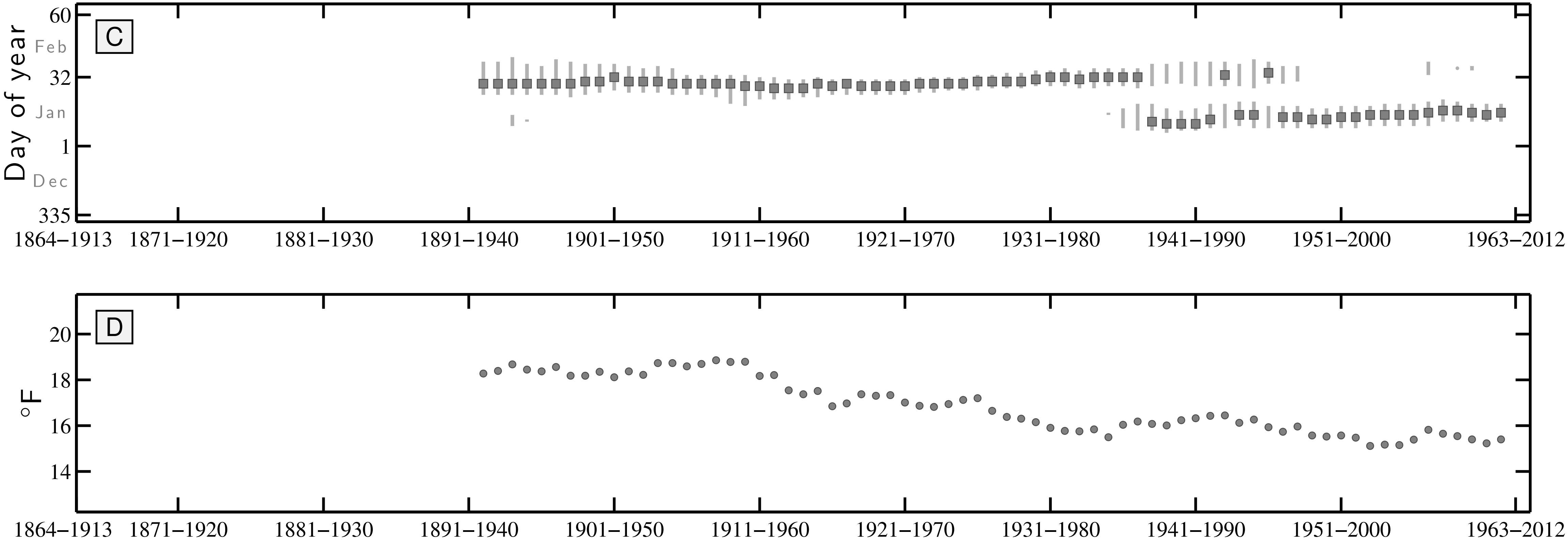

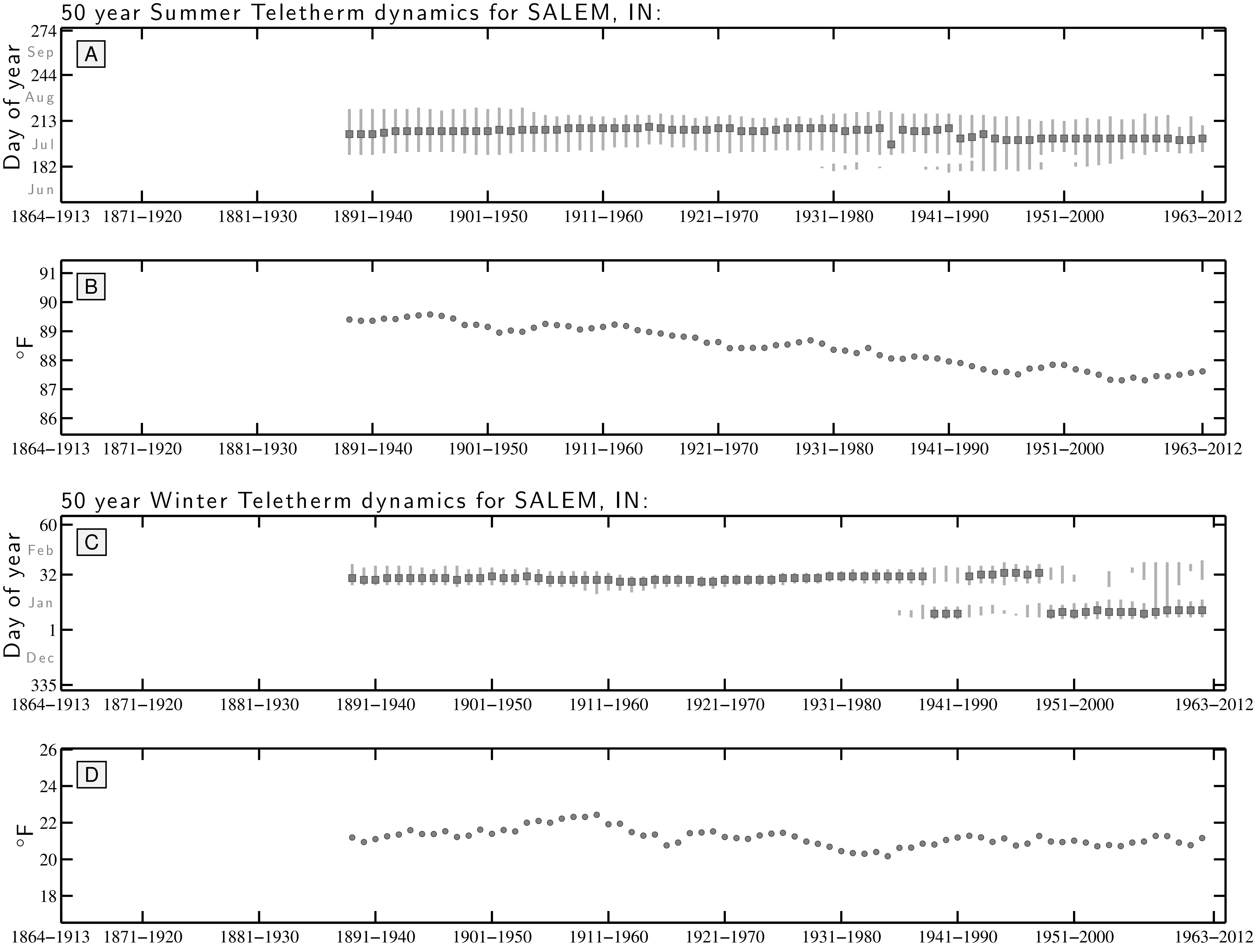

50 year Summer Teletherm dynamics for SALEM, IN:
A
B
Day of year
°F
274
244
213
182
Sep
Aug
Jul
Jun
91
90
89
88
87
86
1864–1913
1871–1920
1881–1930
1891–1940
1901–1950
1911–1960
1921–1970
1931–1980
1941–1990
1951–2000
1963–2012
50 year Winter Teletherm dynamics for SALEM, IN:
C
D
60
32
1
335
Feb
Jan
Dec
26
24
22
20
18

50 year Summer Teletherm dynamics for SCOTTSBURG, IN:

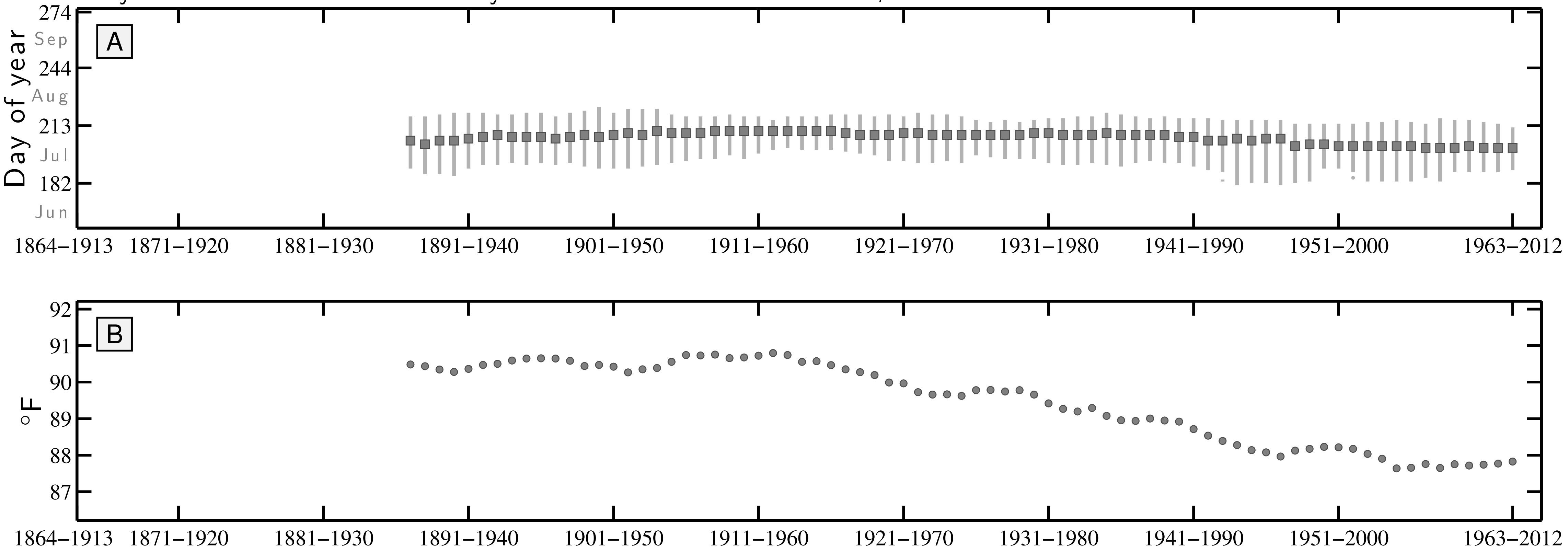

50 year Winter Teletherm dynamics for SCOTTSBURG, IN:

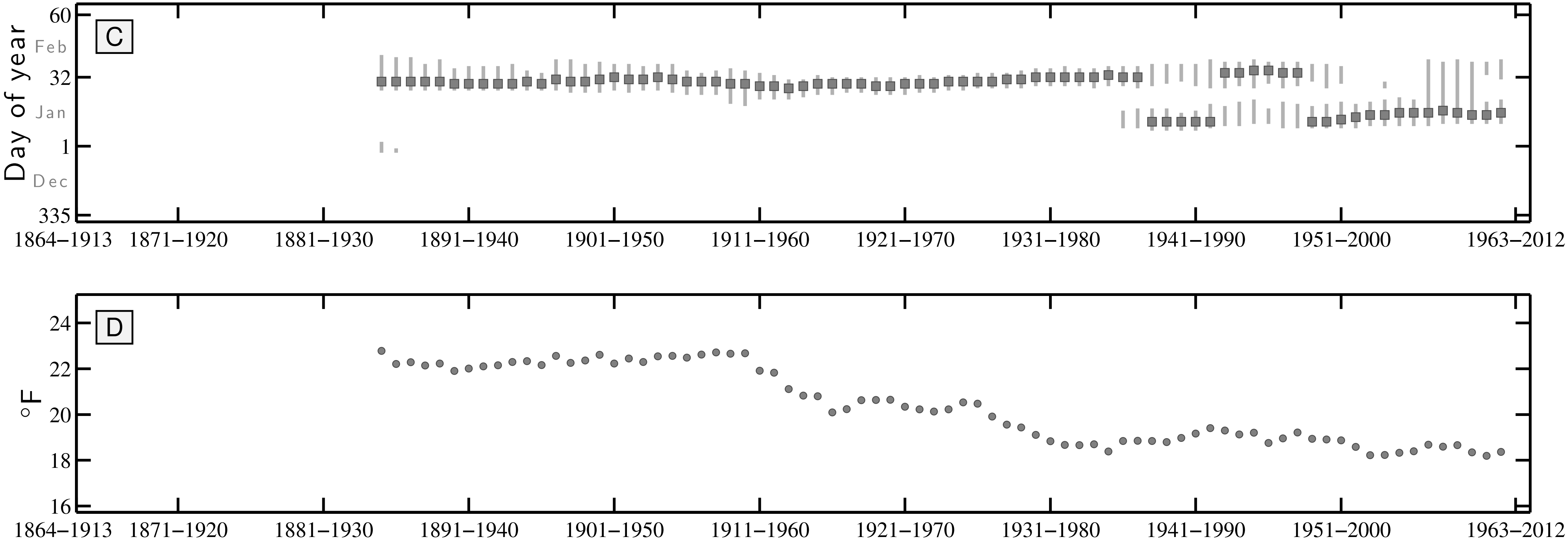

50 year Summer Teletherm dynamics for SEYMOUR 2 N, IN:

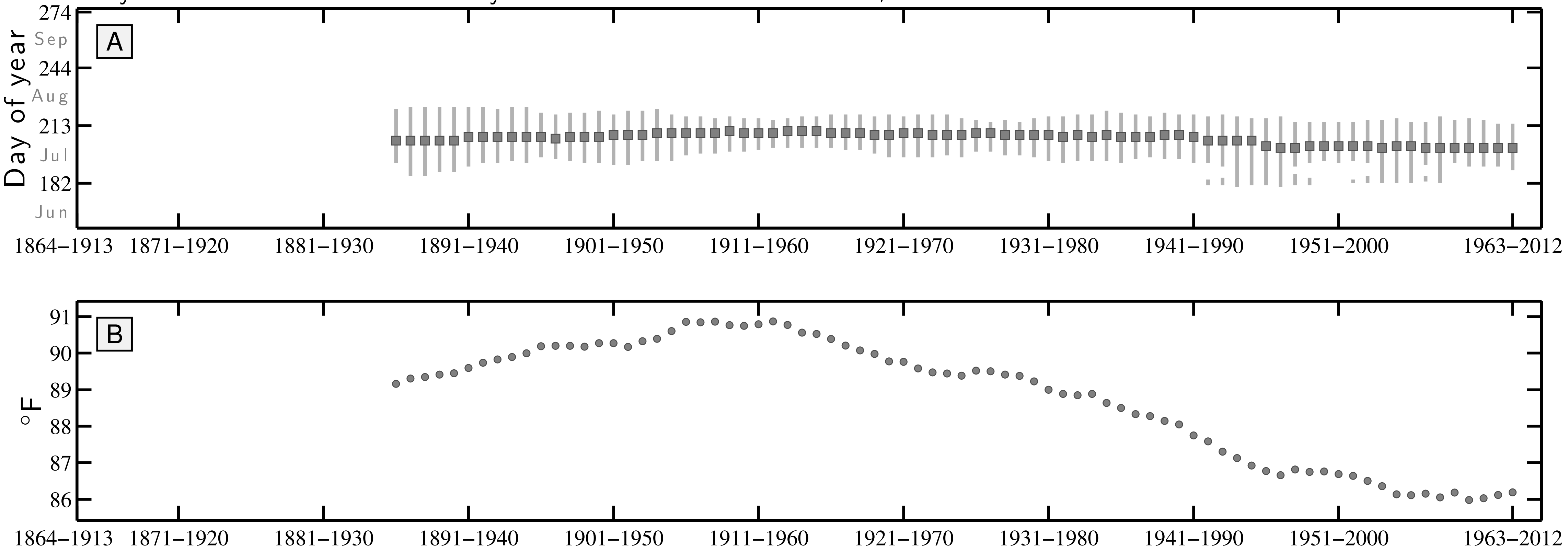

50 year Winter Teletherm dynamics for SEYMOUR 2 N, IN:

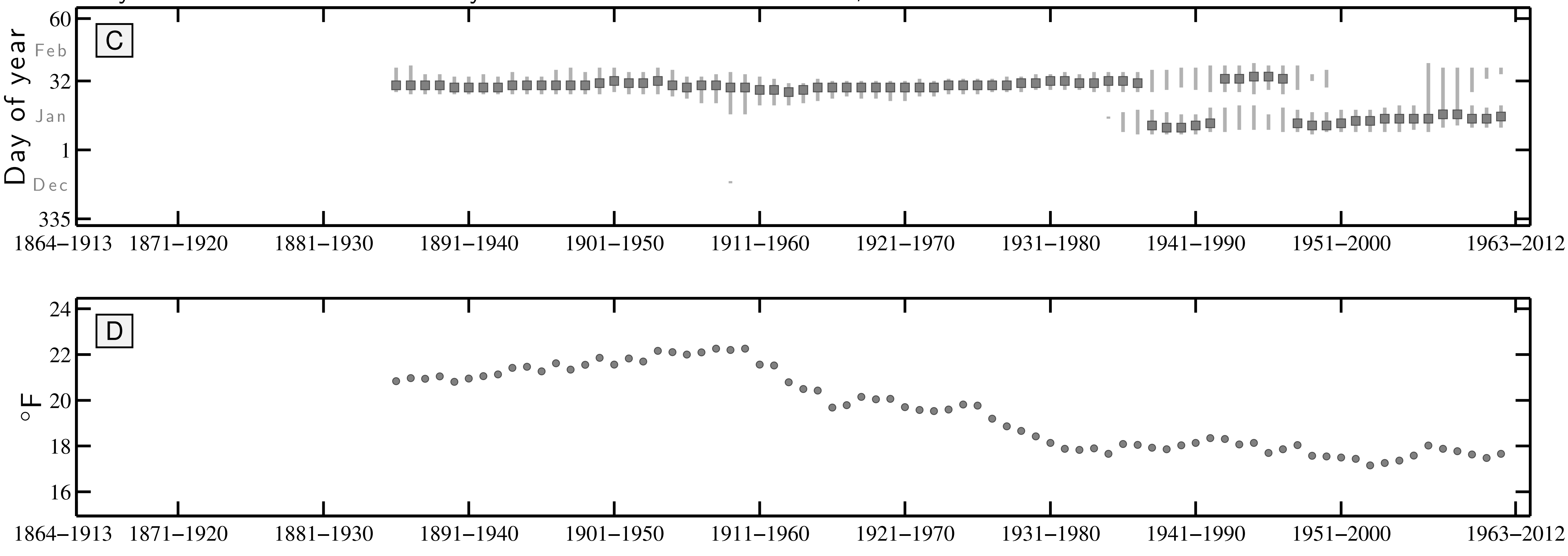

50 year Summer Teletherm dynamics for SHOALS 8 S, IN:

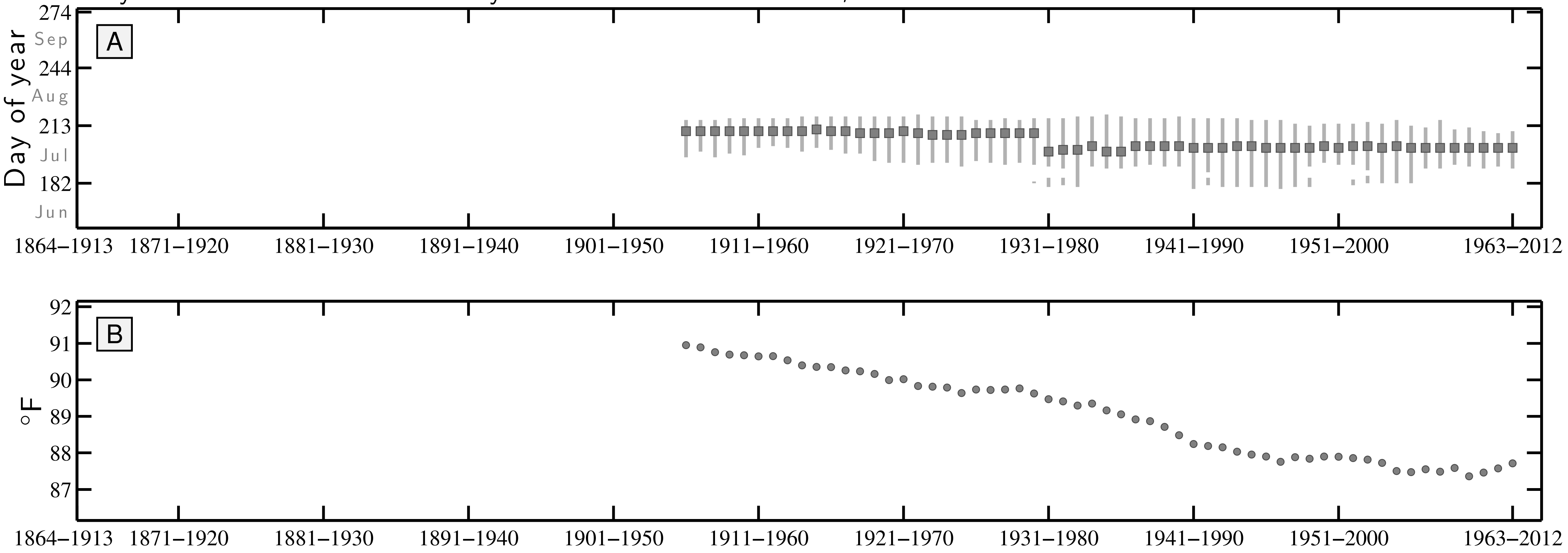

50 year Winter Teletherm dynamics for SHOALS 8 S, IN:

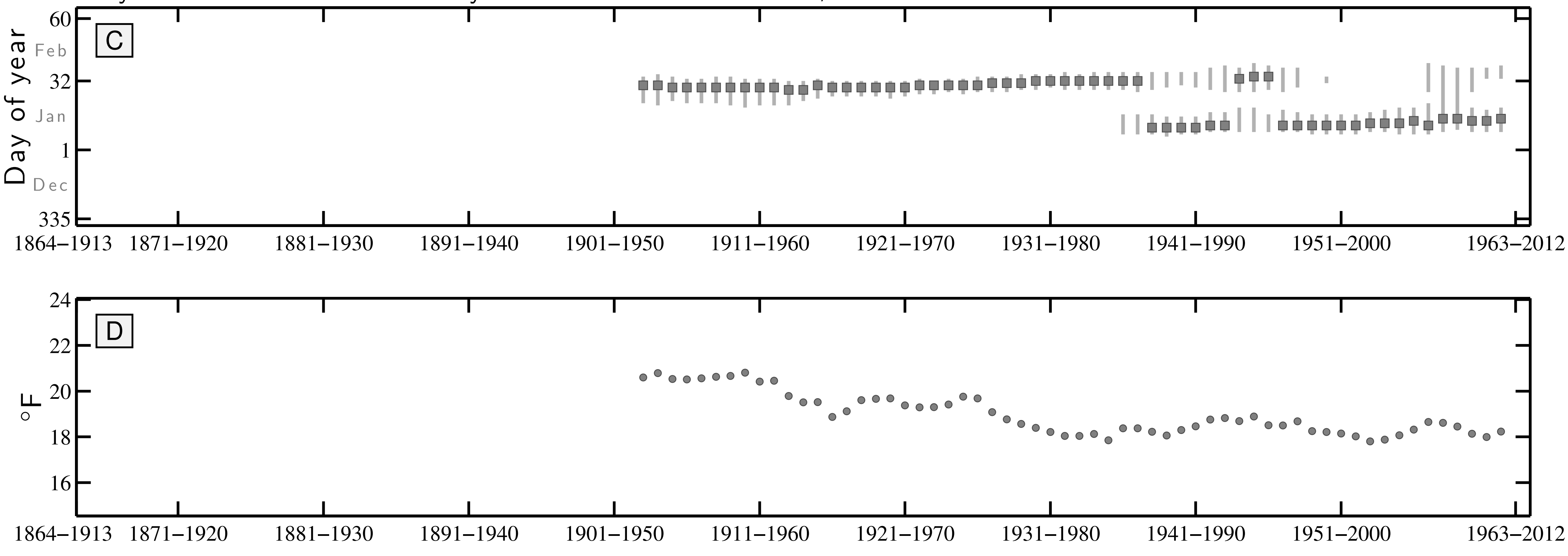

50 year Summer Teletherm dynamics for VEVAY, IN:

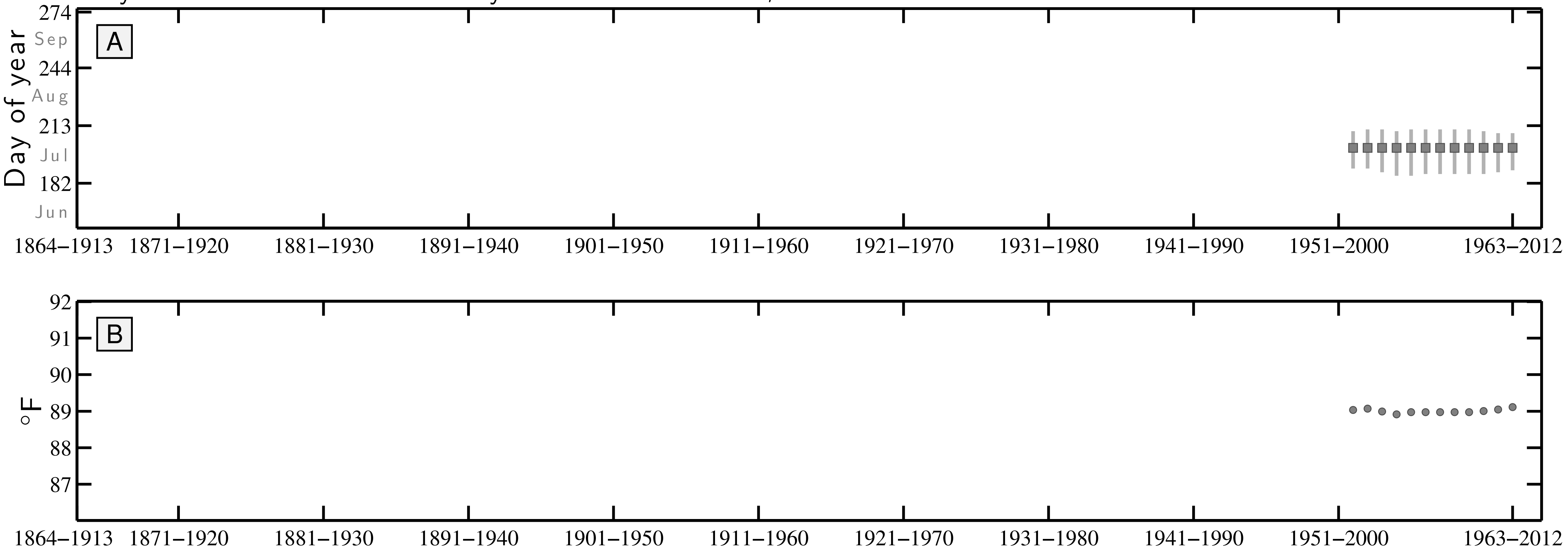

50 year Winter Teletherm dynamics for VEVAY, IN:

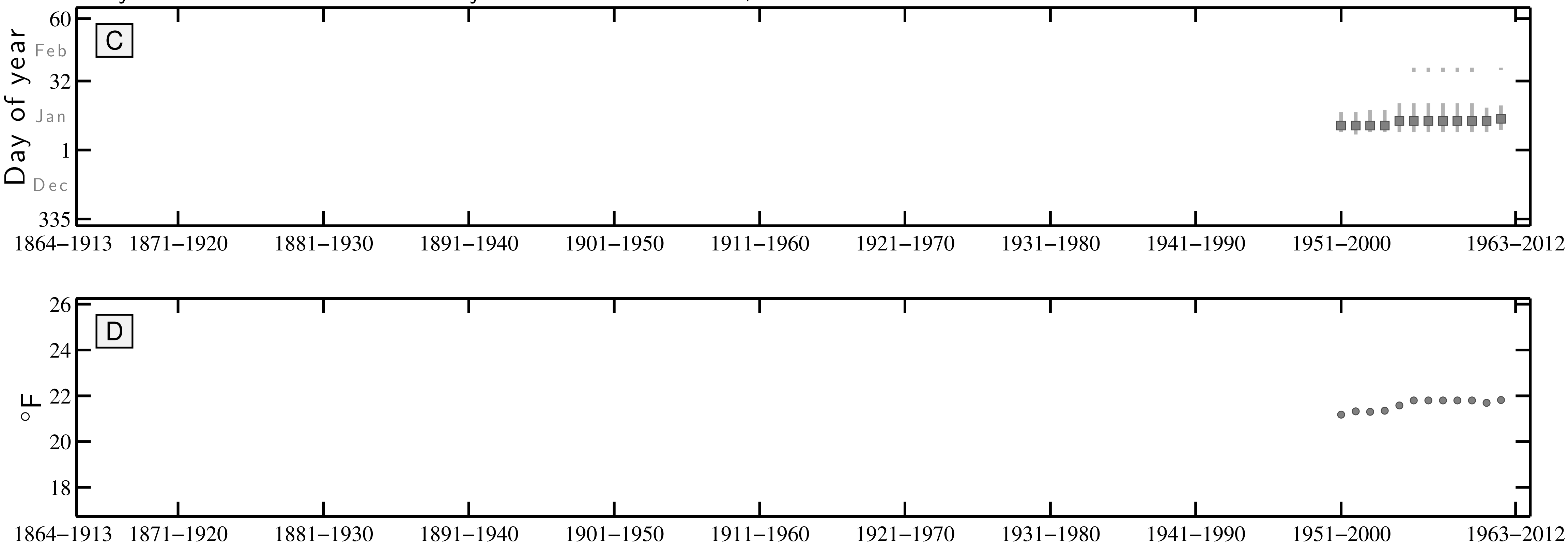

50 year Summer Teletherm dynamics for WASHINGTON 1 W, IN:

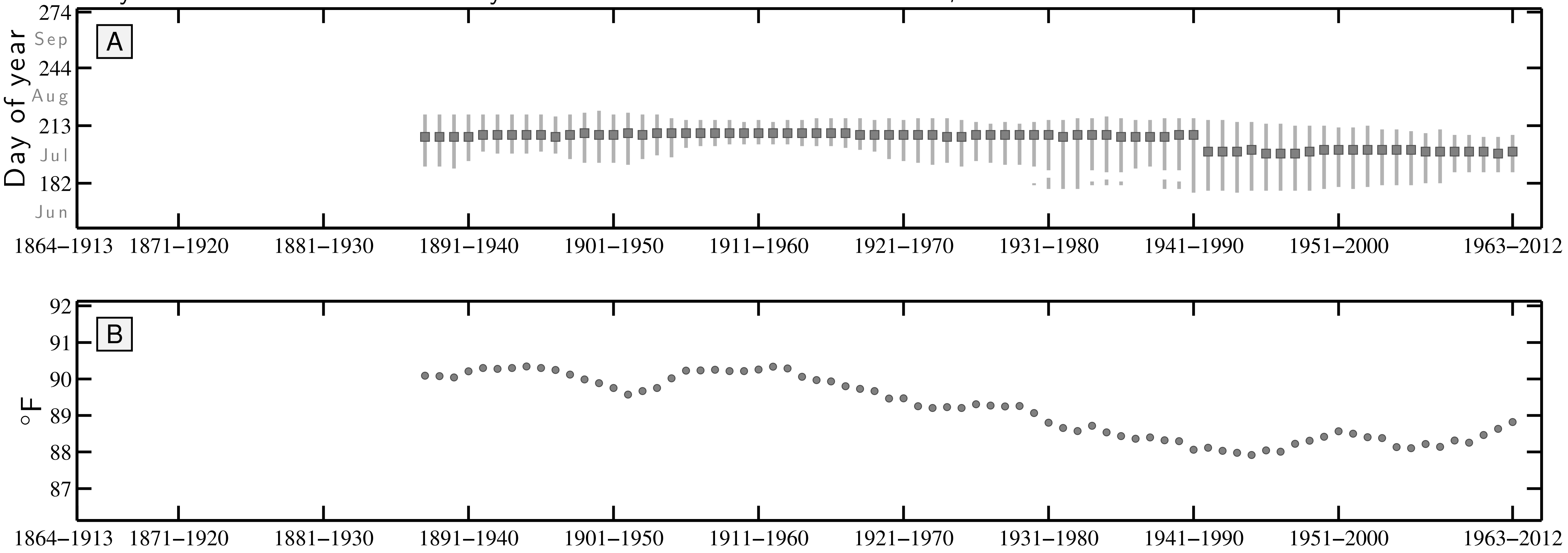

50 year Winter Teletherm dynamics for WASHINGTON 1 W, IN:

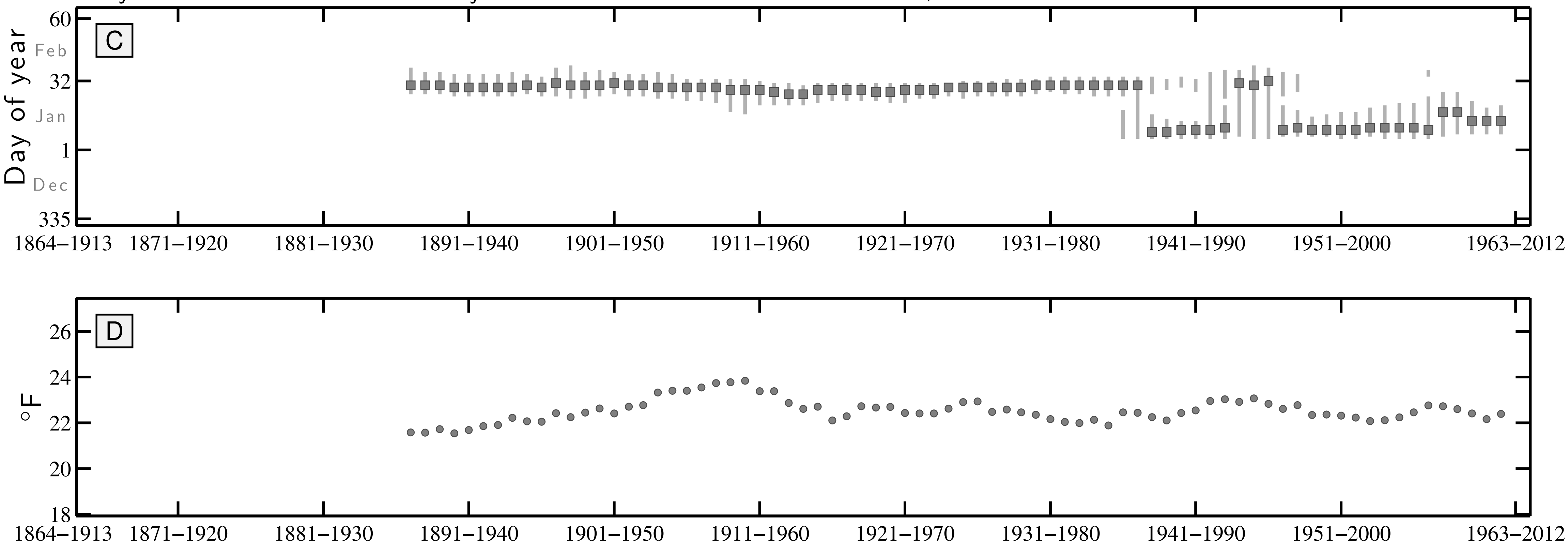

50 year Summer Teletherm dynamics for WHEATFIELD, IN:

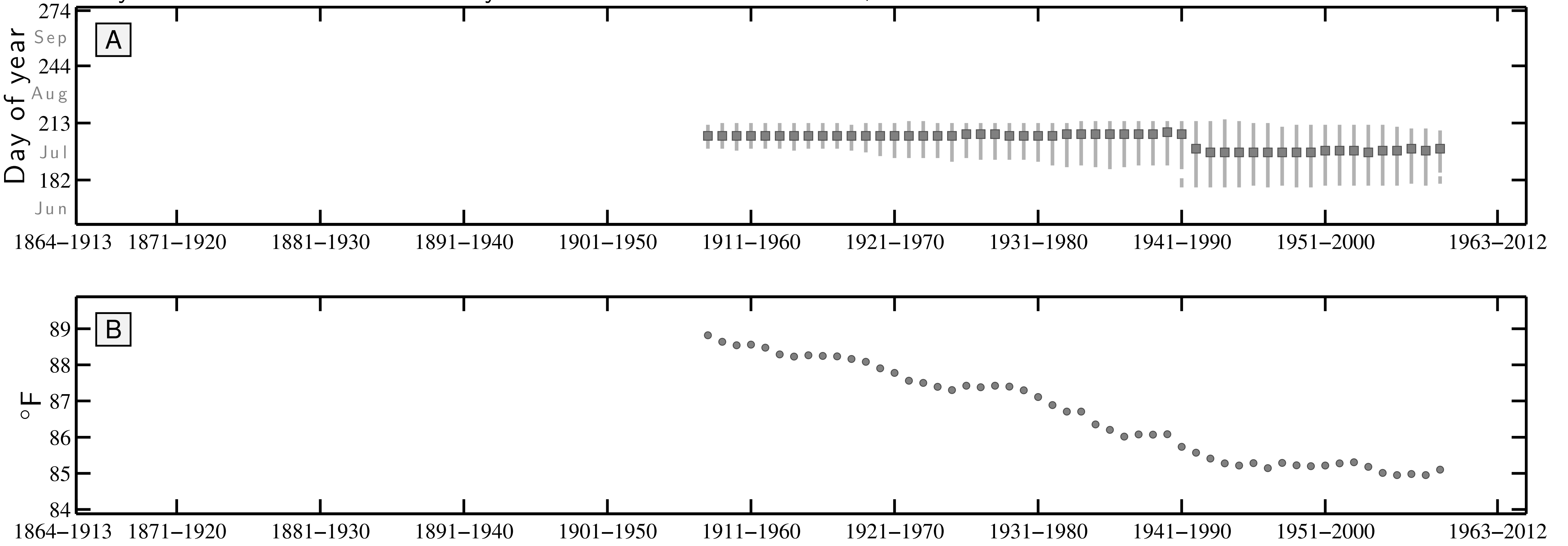

50 year Winter Teletherm dynamics for WHEATFIELD, IN:

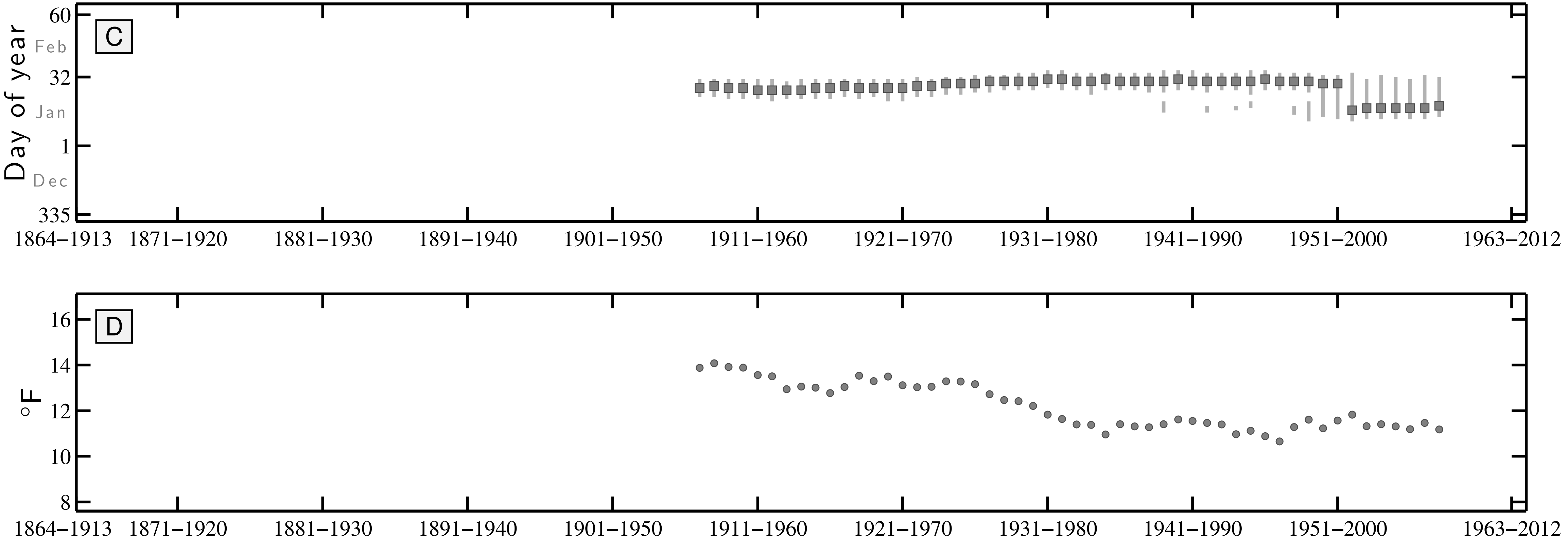

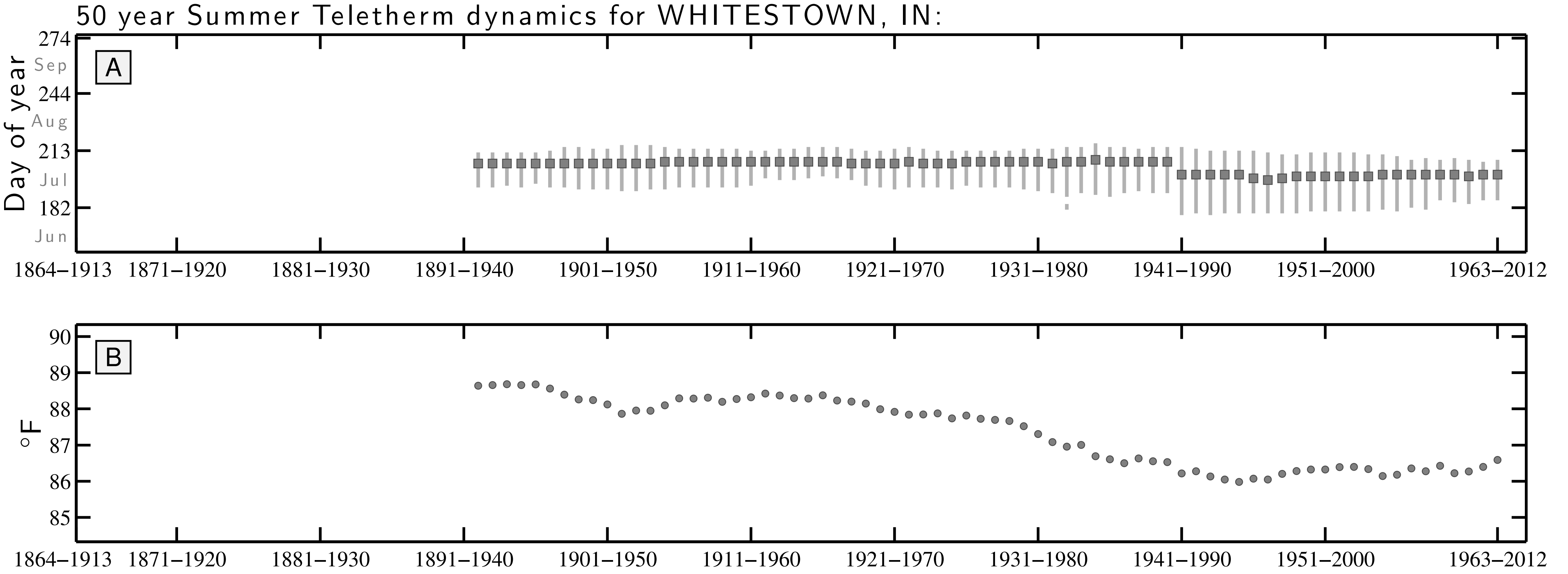

50 year Summer Teletherm dynamics for WHITESTOWN, IN:
A
B
Day of year
274
Sep
244
Aug
213
Jul
182
Jun
°F
90
89
88
87
86
85
1864–1913
1871–1920
1881–1930
1891–1940
1901–1950
1911–1960
1921–1970
1931–1980
1941–1990
1951–2000
1963–2012

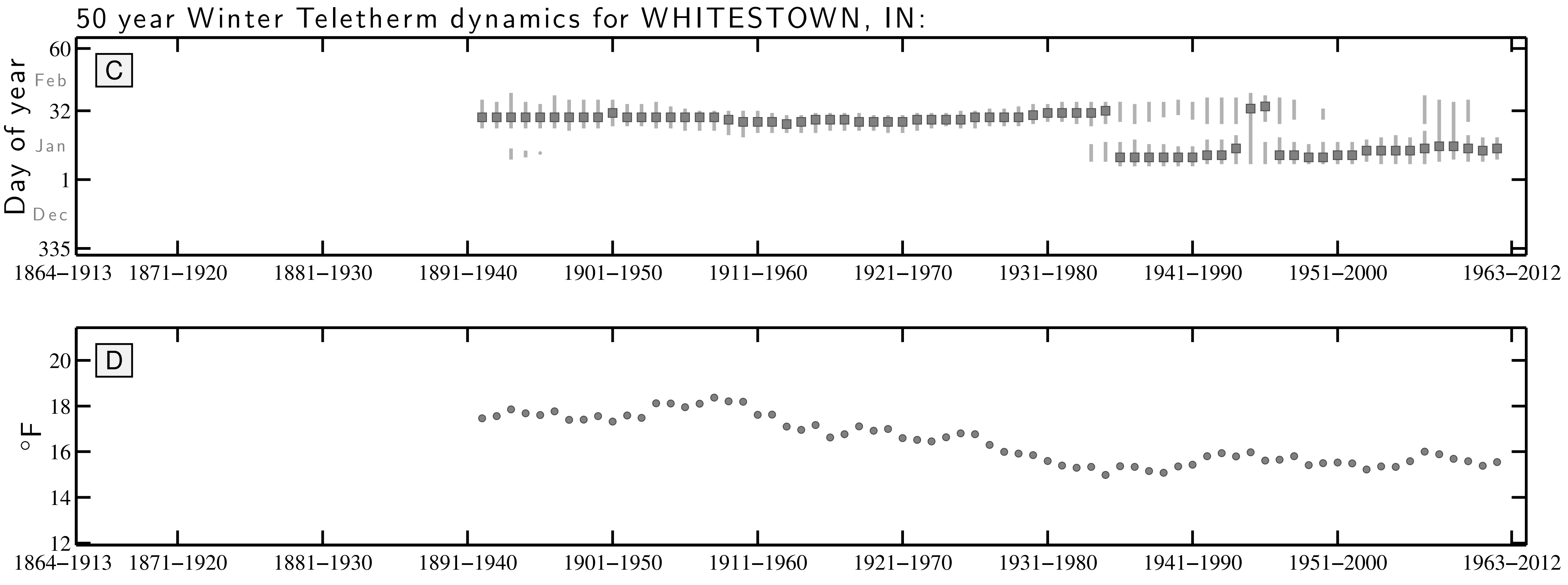

50 year Winter Teletherm dynamics for WHITESTOWN, IN:
C
D
Day of year
60
Feb
32
Jan
1
Dec
335
°F
20
18
16
14
12
1864–1913
1871–1920
1881–1930
1891–1940
1901–1950
1911–1960
1921–1970
1931–1980
1941–1990
1951–2000
1963–2012

50 year Summer Teletherm dynamics for WINAMAC 2SSE, IN:

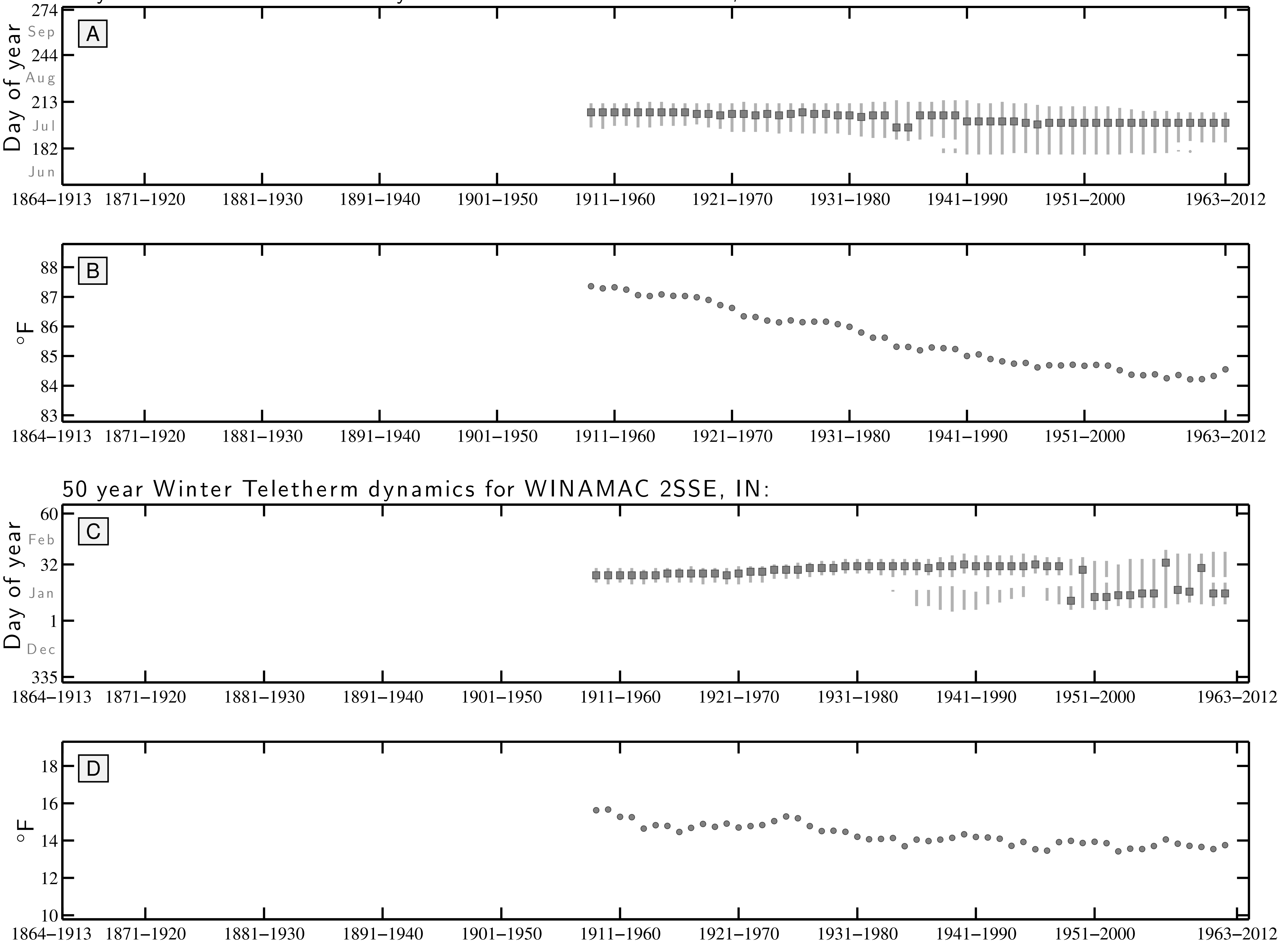

50 year Winter Teletherm dynamics for WINAMAC 2SSE, IN:

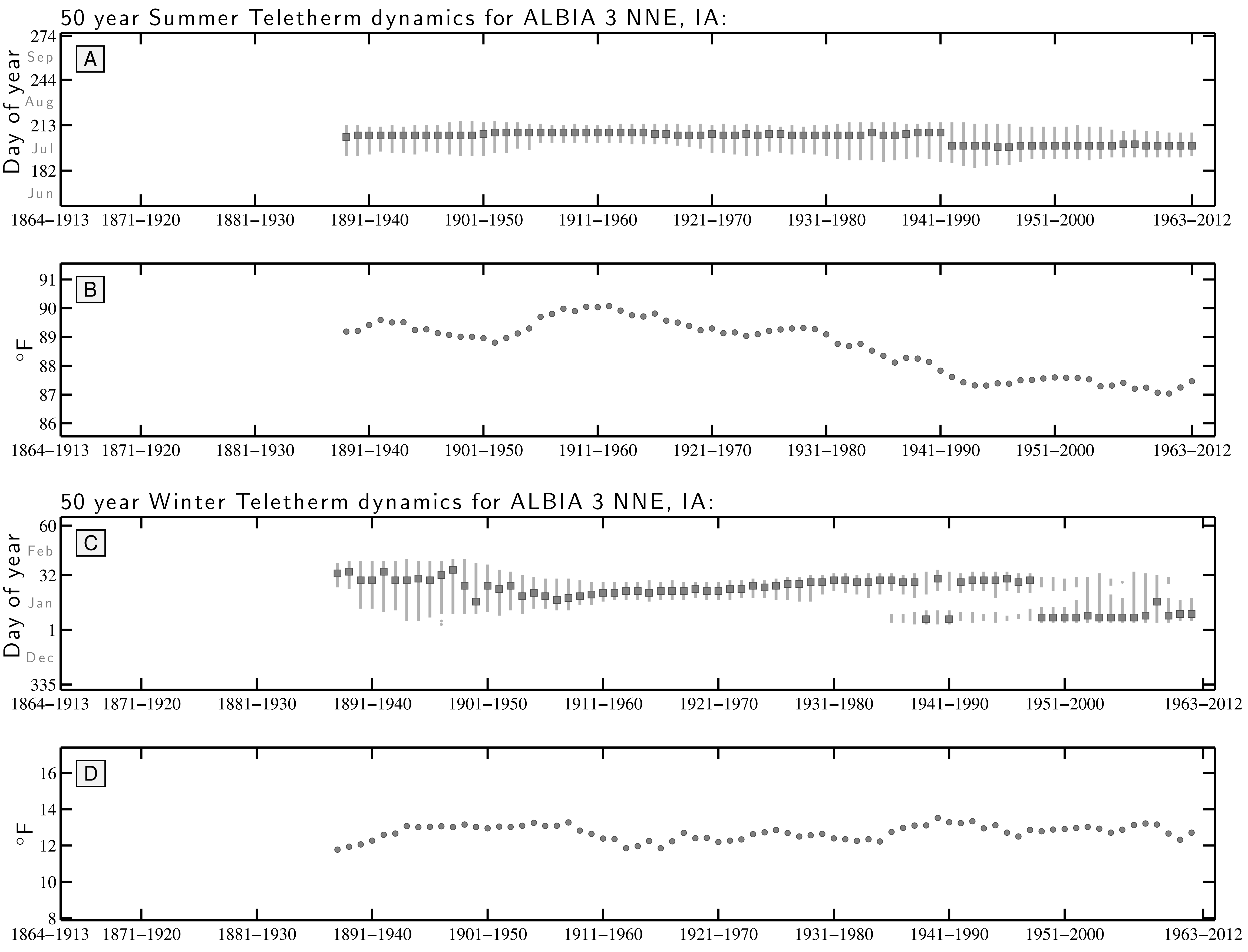
50 year Summer Teletherm dynamics for ALBIA 3 NNE, IA:
A
B
Day of year
°F
Sep
Aug
Jul
Jun
274
244
213
182
91
90
89
88
87
86
1864–1913
1871–1920
1881–1930
1891–1940
1901–1950
1911–1960
1921–1970
1931–1980
1941–1990
1951–2000
1963–2012
50 year Winter Teletherm dynamics for ALBIA 3 NNE, IA:
C
D
Feb
Jan
Dec
60
32
1
335
16
14
12
10
8

50 year Summer Teletherm dynamics for ALGONA 3 W, IA:

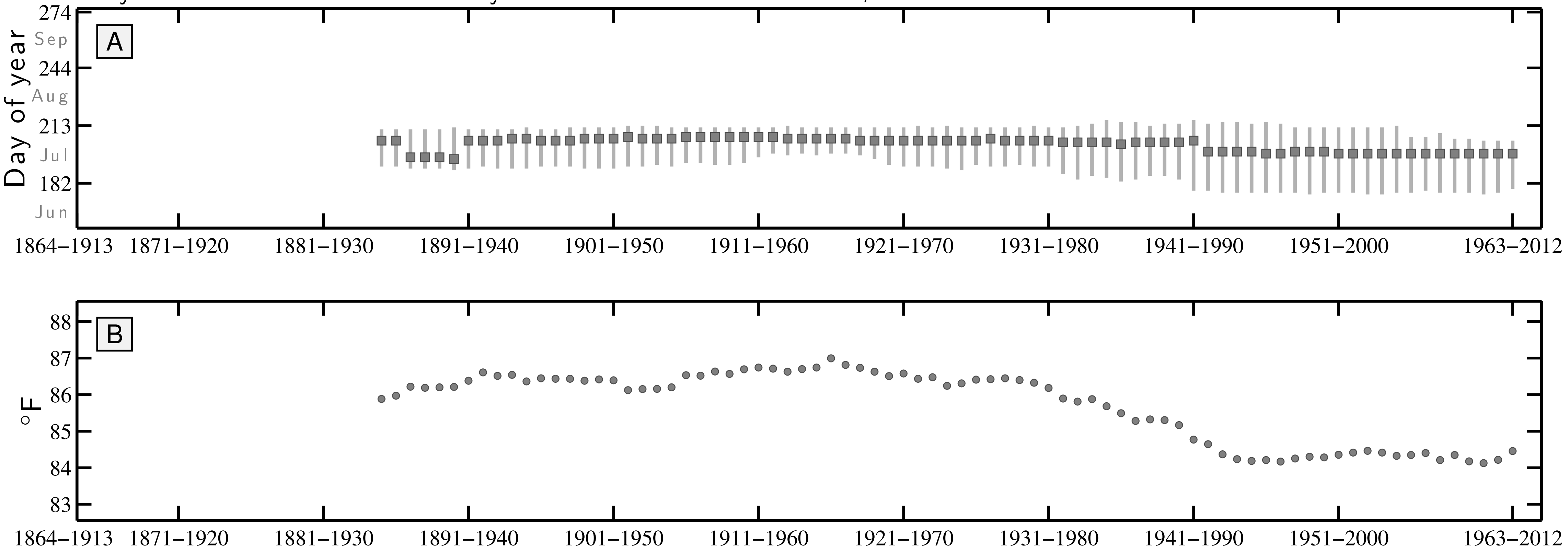

50 year Winter Teletherm dynamics for ALGONA 3 W, IA:

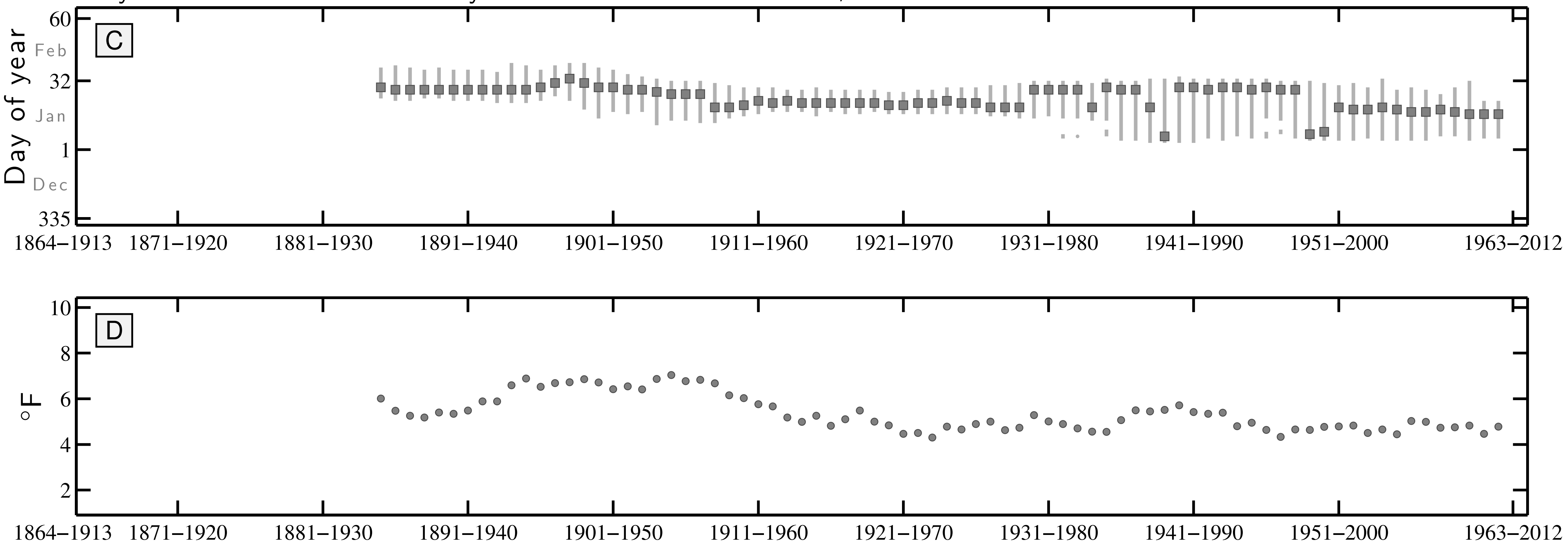

50 year Summer Teletherm dynamics for BELLE PLAINE, IA:

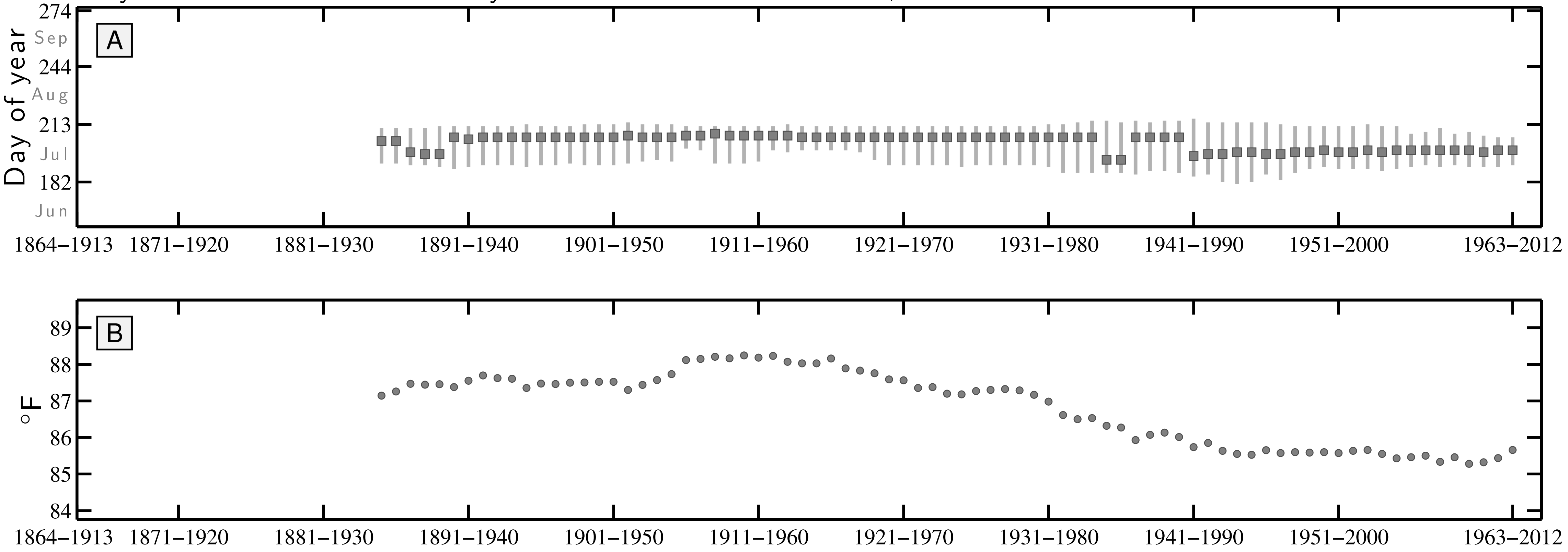

50 year Winter Teletherm dynamics for BELLE PLAINE, IA:

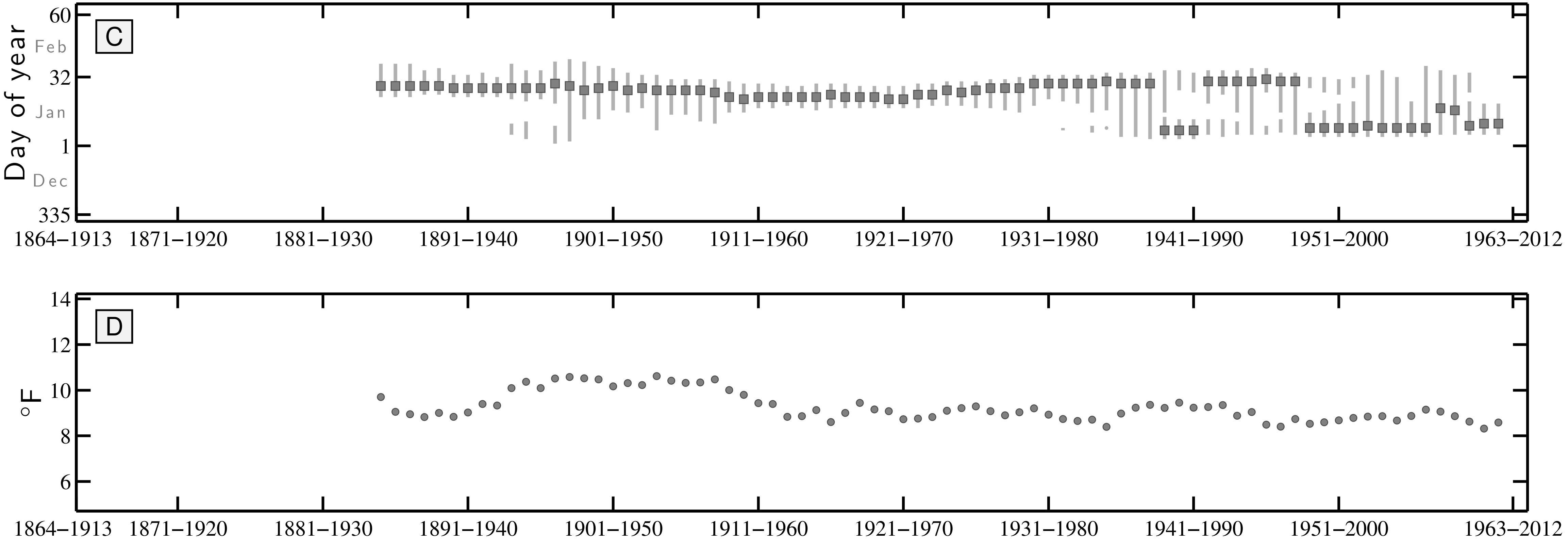

50 year Summer Teletherm dynamics for CHARLES CITY, IA:

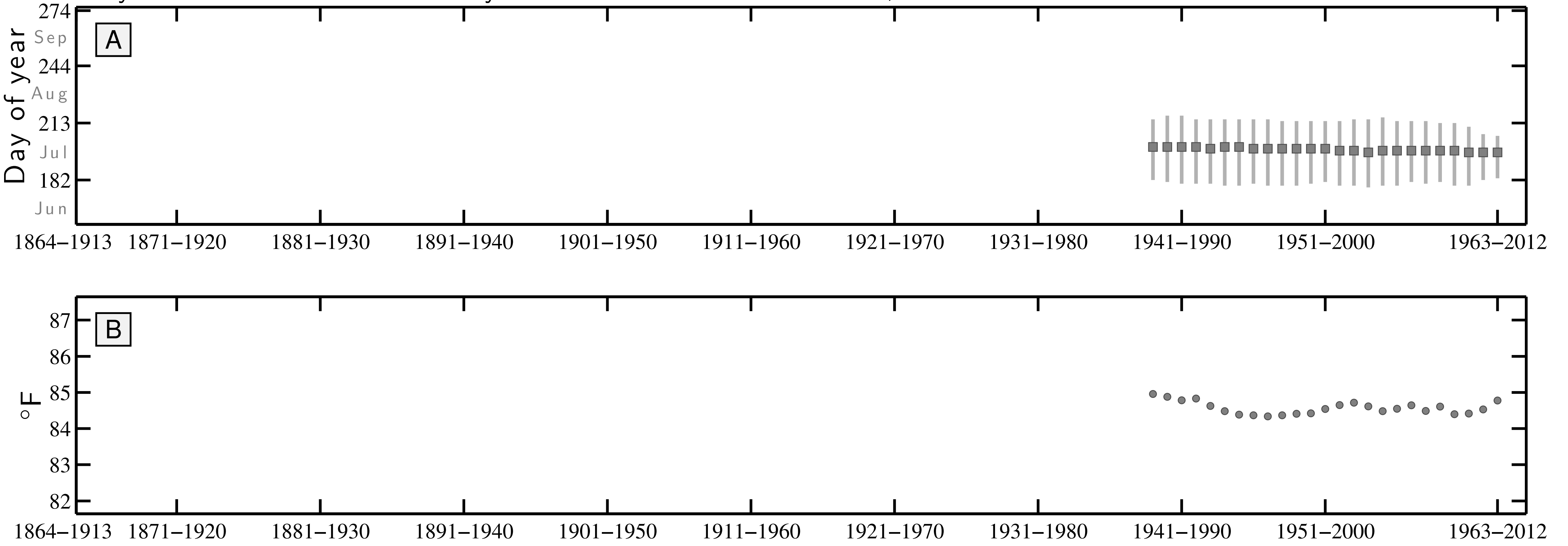

50 year Winter Teletherm dynamics for CHARLES CITY, IA:

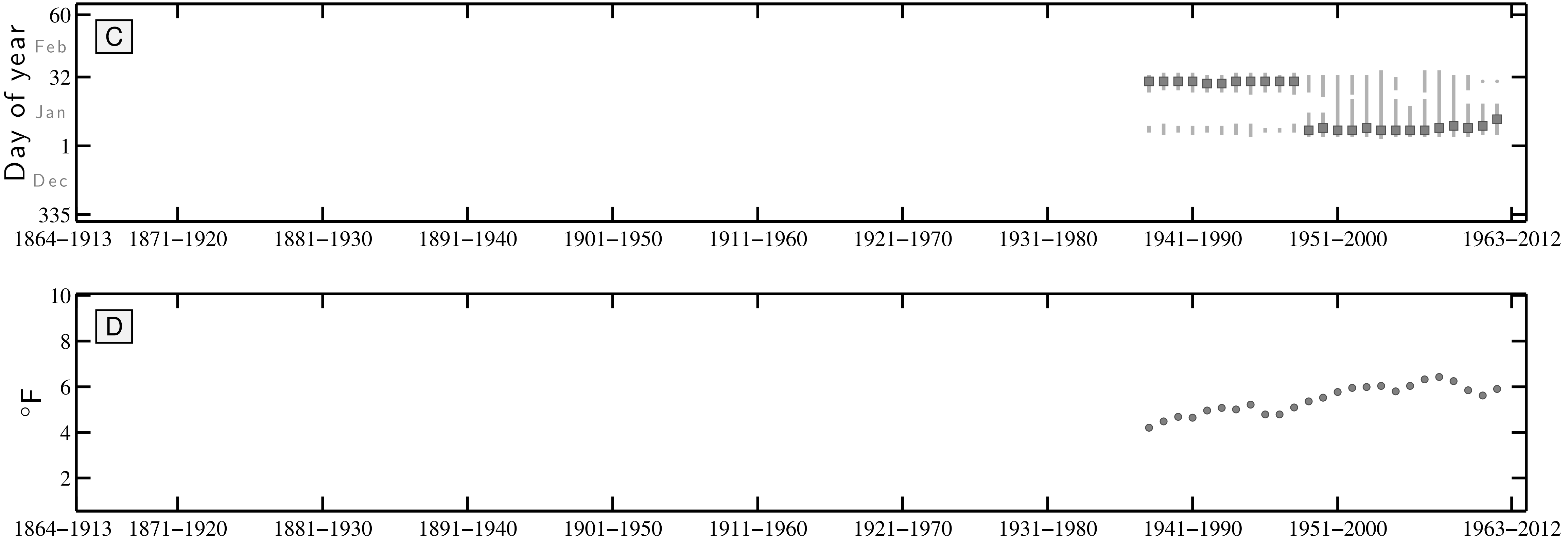

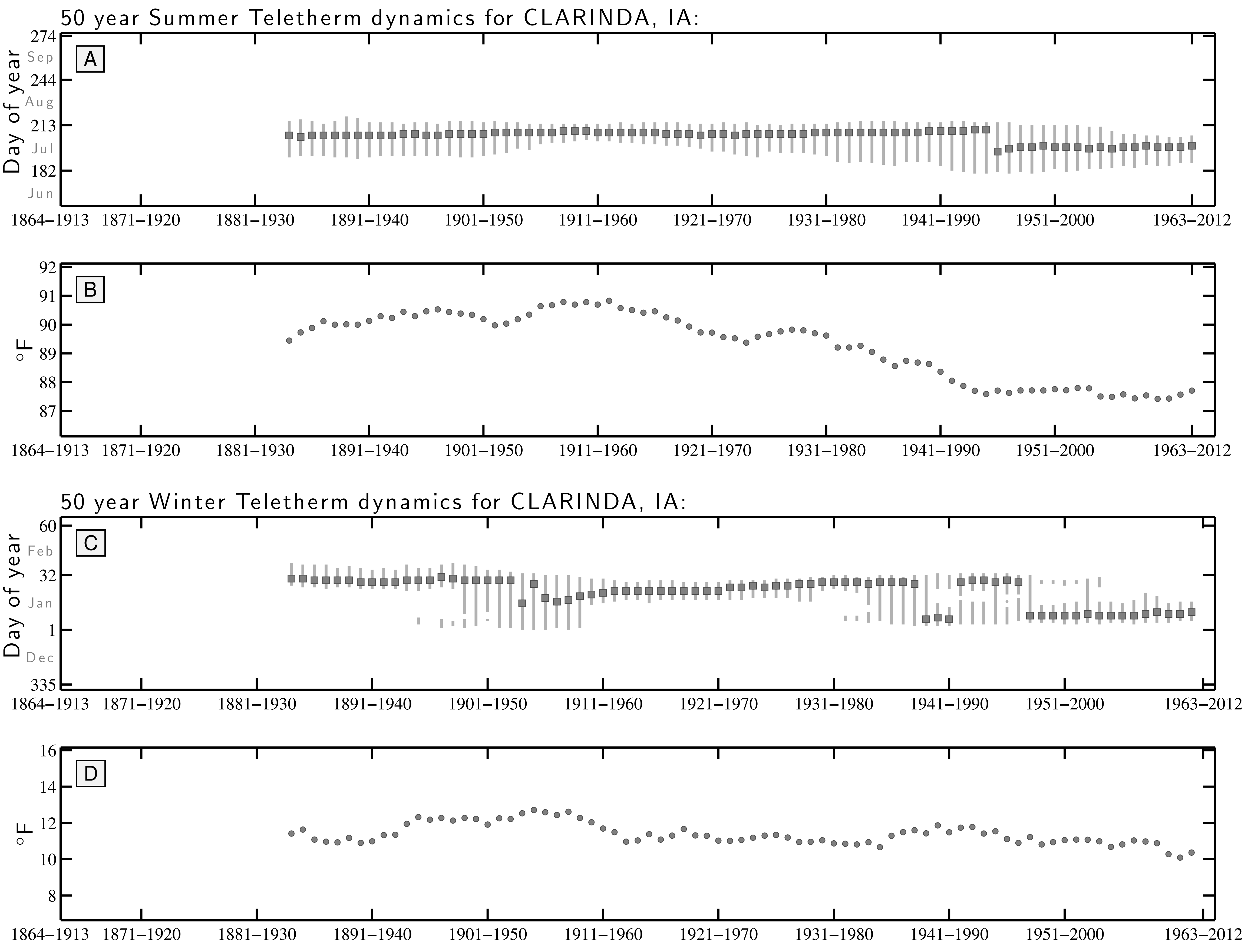

50 year Summer Teletherm dynamics for CLARINDA, IA:
A
Day of year
274
Sep
244
Aug
213
Jul
182
Jun
1864–1913
1871–1920
1881–1930
1891–1940
1901–1950
1911–1960
1921–1970
1931–1980
1941–1990
1951–2000
1963–2012
B
°F
92
91
90
89
88
87
50 year Winter Teletherm dynamics for CLARINDA, IA:
C
Day of year
60
Feb
32
Jan
1
Dec
335
D
°F
16
14
12
10
8

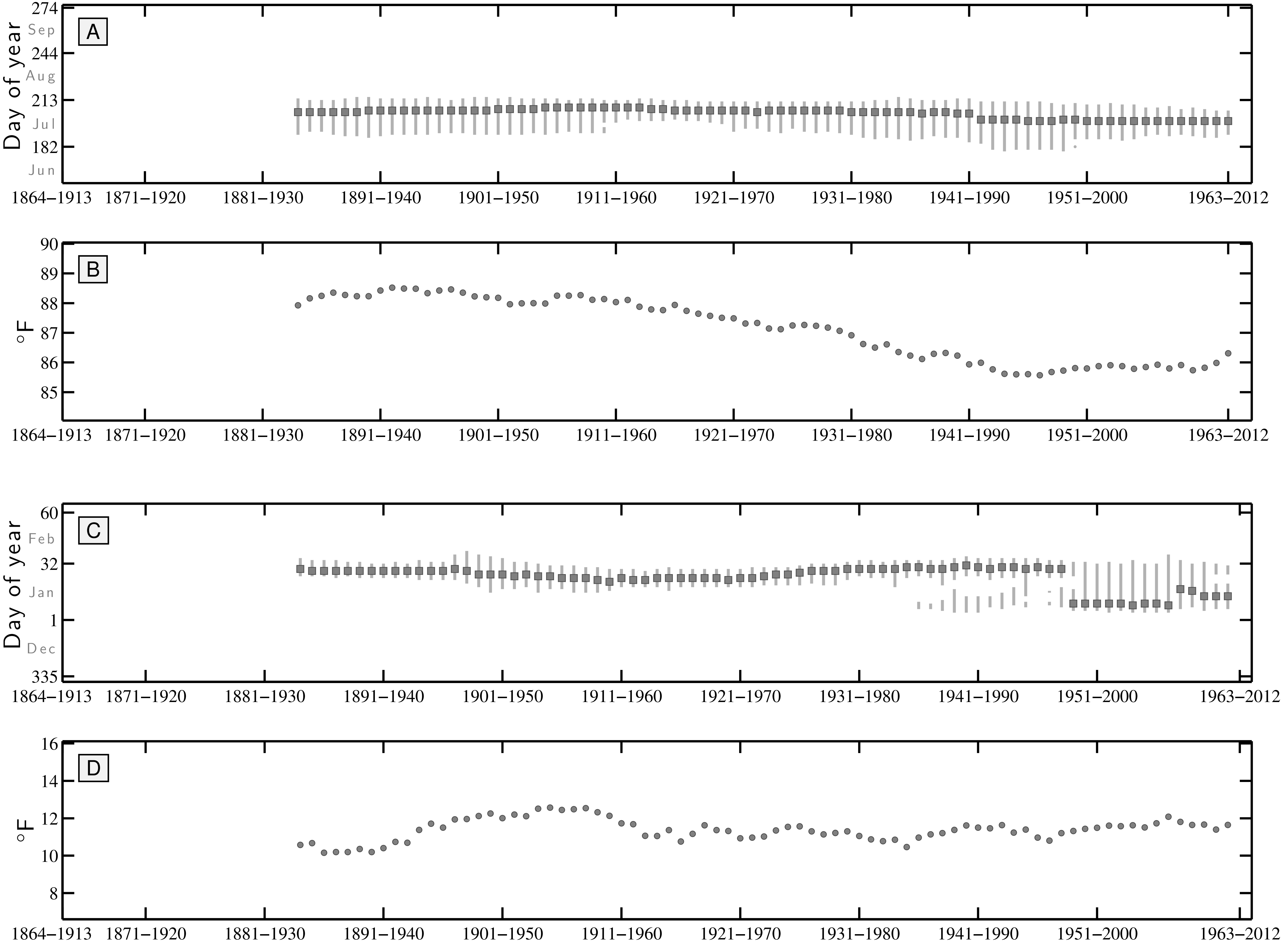
A
Day of year
274
Sep
244
Aug
213
Jul
182
Jun
B
°F
90
89
88
87
86
85
C
Day of year
60
Feb
32
Jan
1
Dec
335
D
°F
16
14
12
10
8
1864–1913
1871–1920
1881–1930
1891–1940
1901–1950
1911–1960
1921–1970
1931–1980
1941–1990
1951–2000
1963–2012

50 year Summer Teletherm dynamics for ESTHERVILLE 2 N, IA:

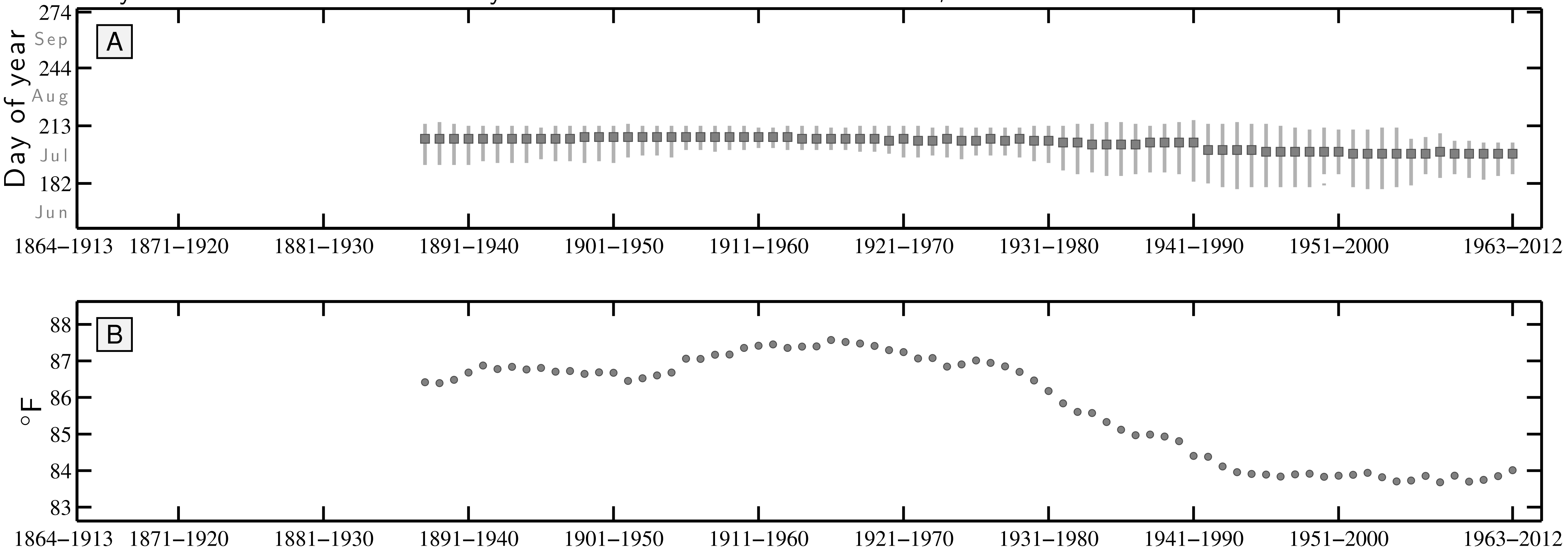

50 year Winter Teletherm dynamics for ESTHERVILLE 2 N, IA:

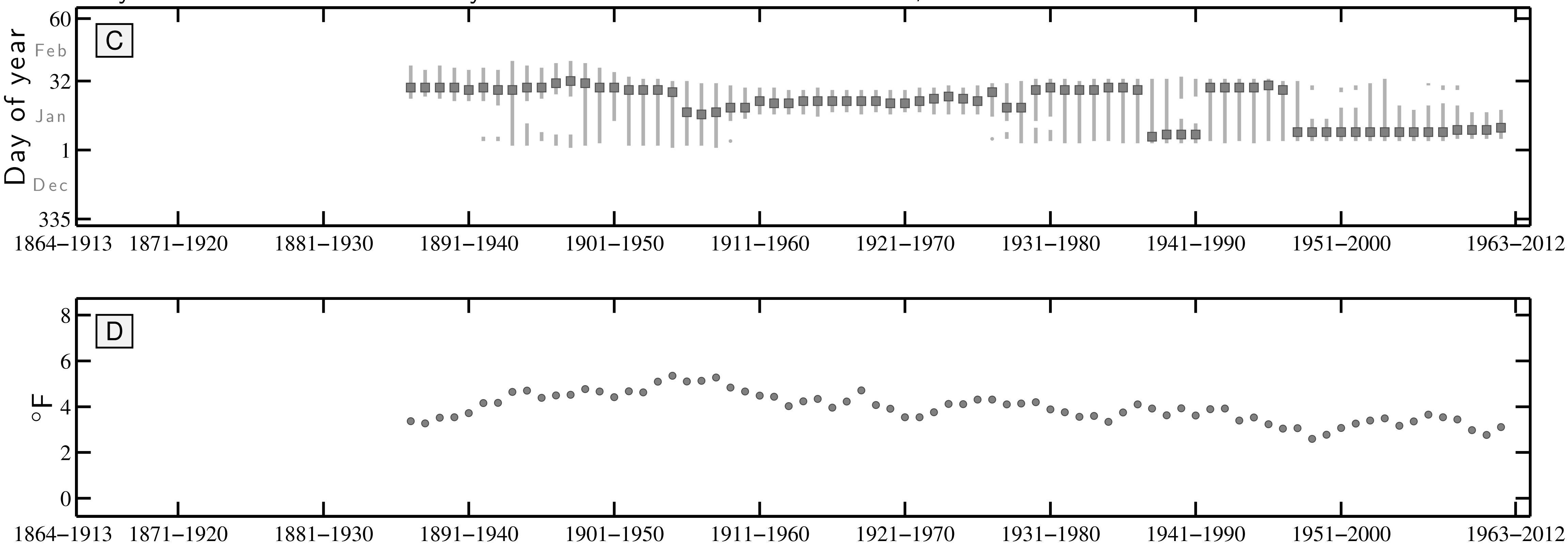

50 year Summer Teletherm dynamics for FAIRFIELD, IA:

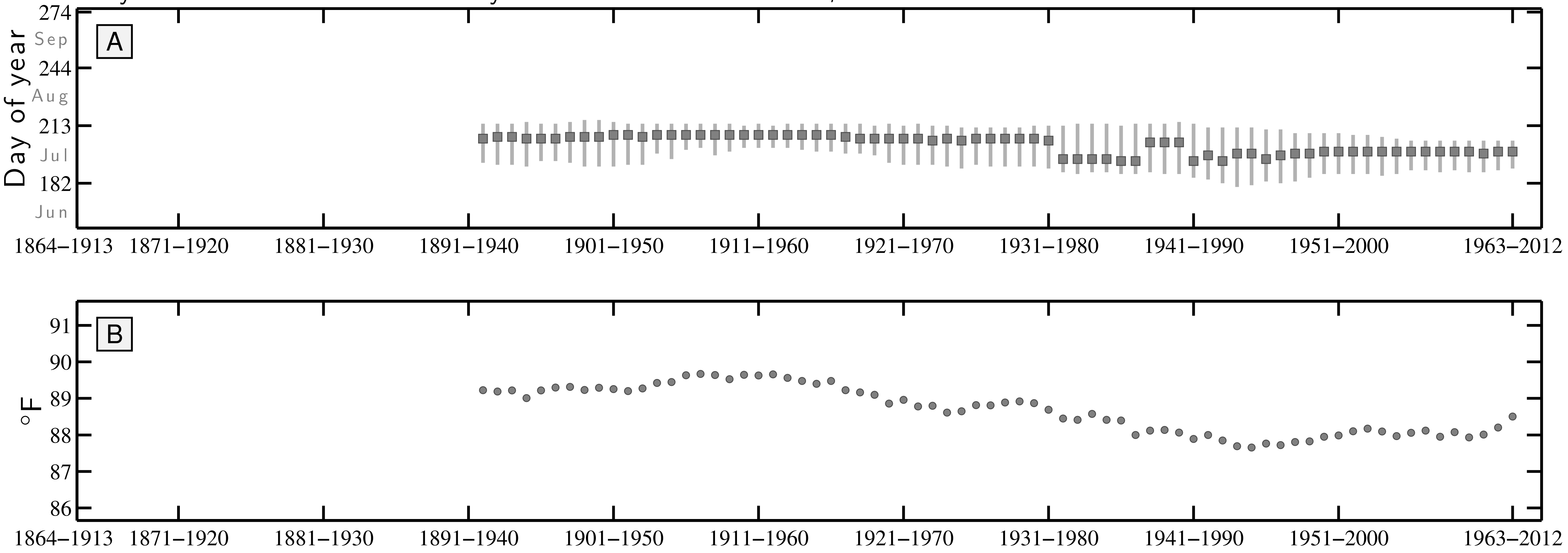

50 year Winter Teletherm dynamics for FAIRFIELD, IA:

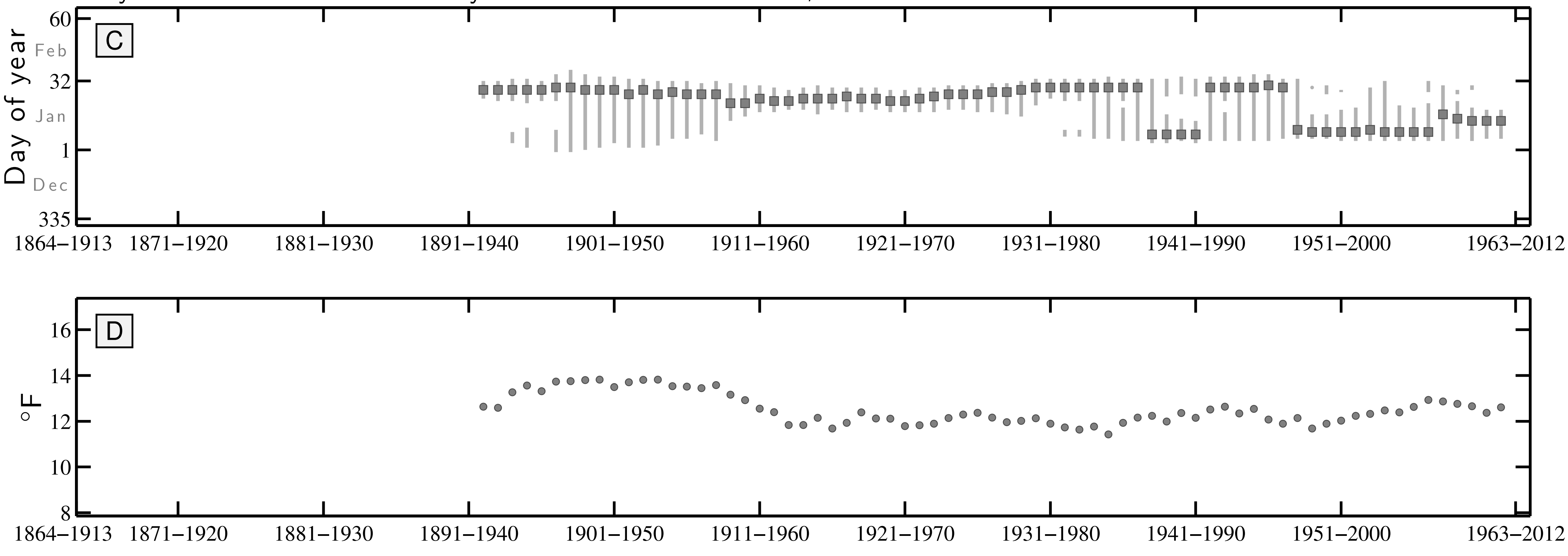

50 year Summer Teletherm dynamics for FAYETTE, IA:

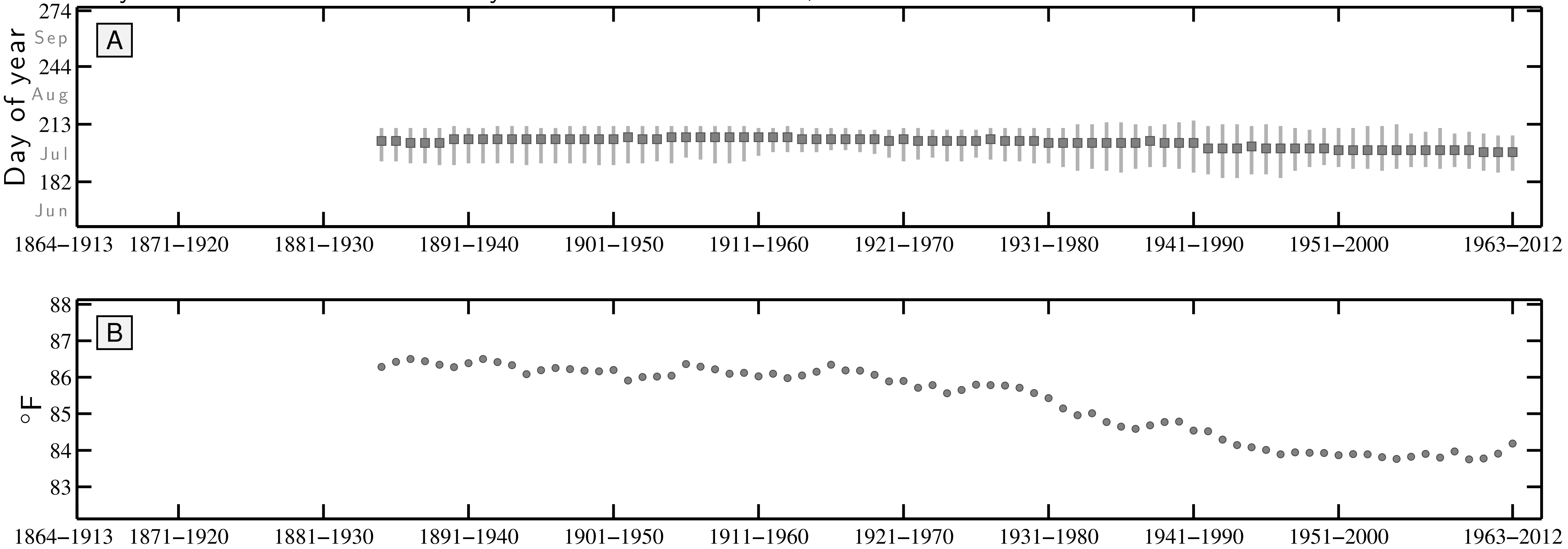

50 year Winter Teletherm dynamics for FAYETTE, IA:

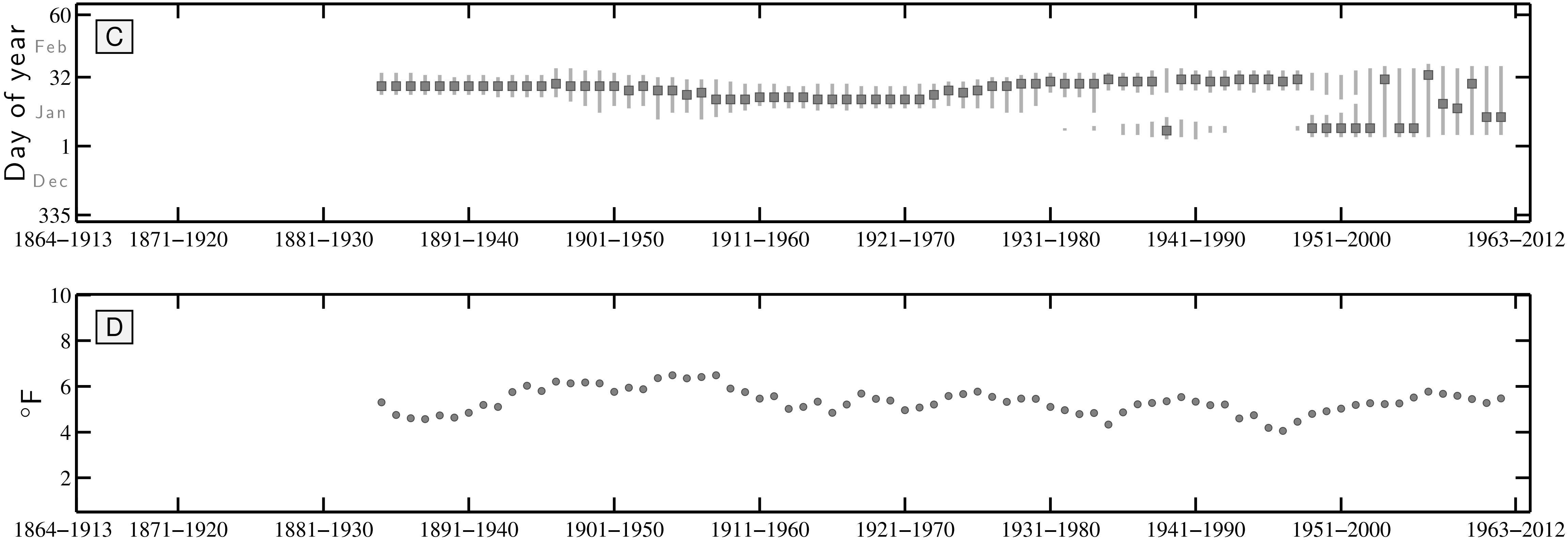

50 year Summer Teletherm dynamics for FOREST CITY 2 NNE, IA:

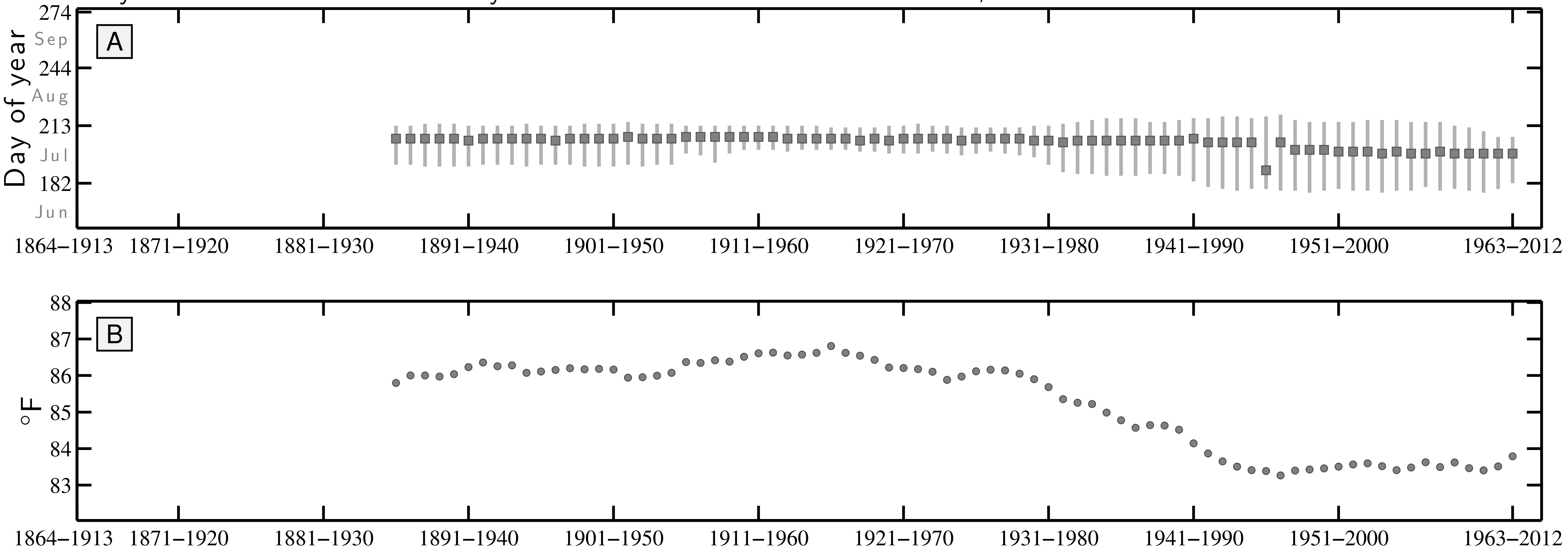

50 year Winter Teletherm dynamics for FOREST CITY 2 NNE, IA:

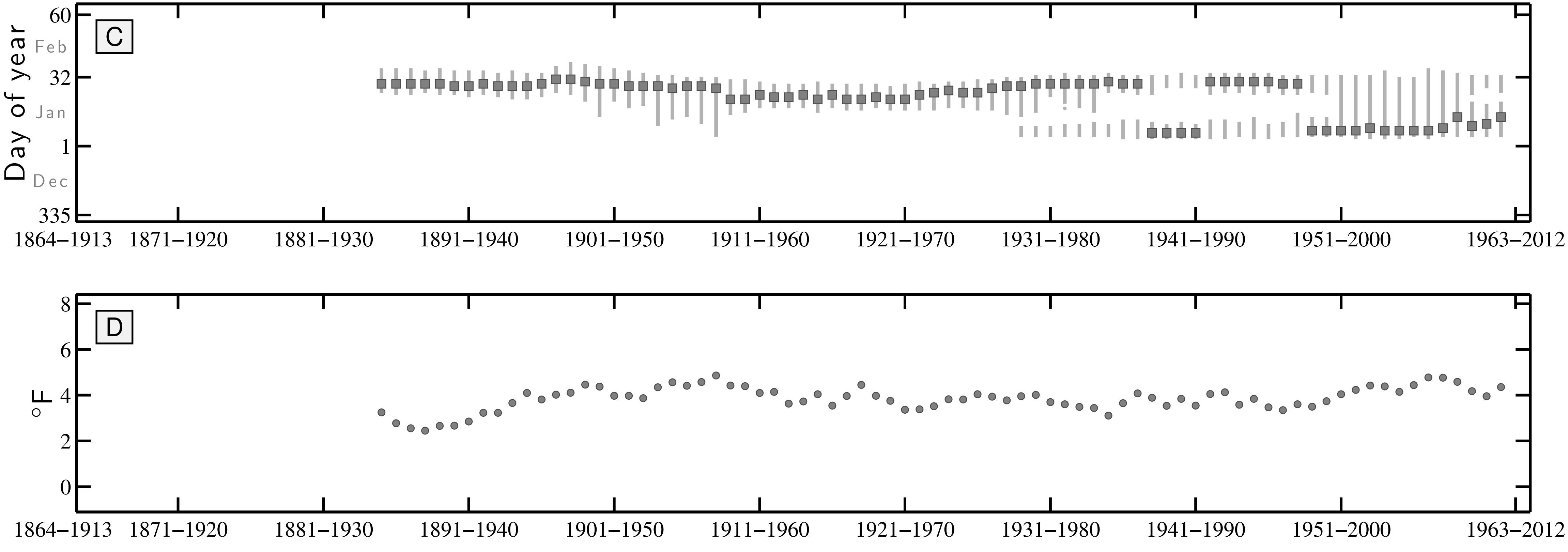

50 year Summer Teletherm dynamics for FORT DODGE 5NNW, IA:

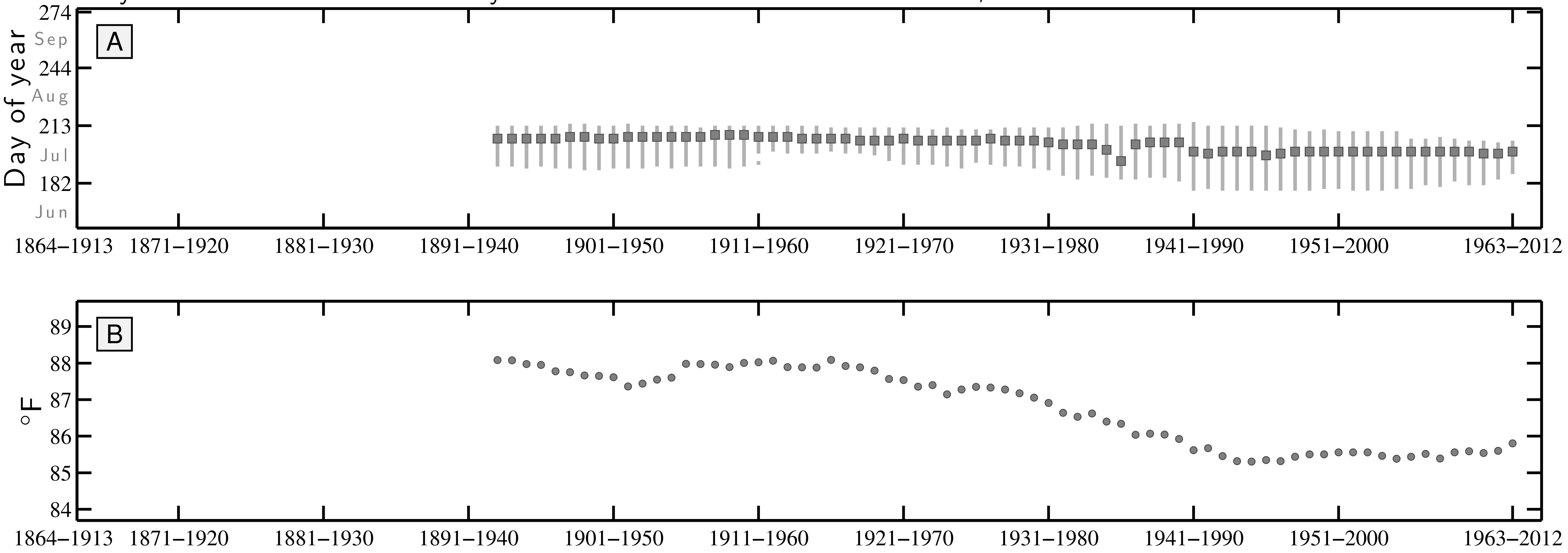

50 year Winter Teletherm dynamics for FORT DODGE 5NNW, IA:

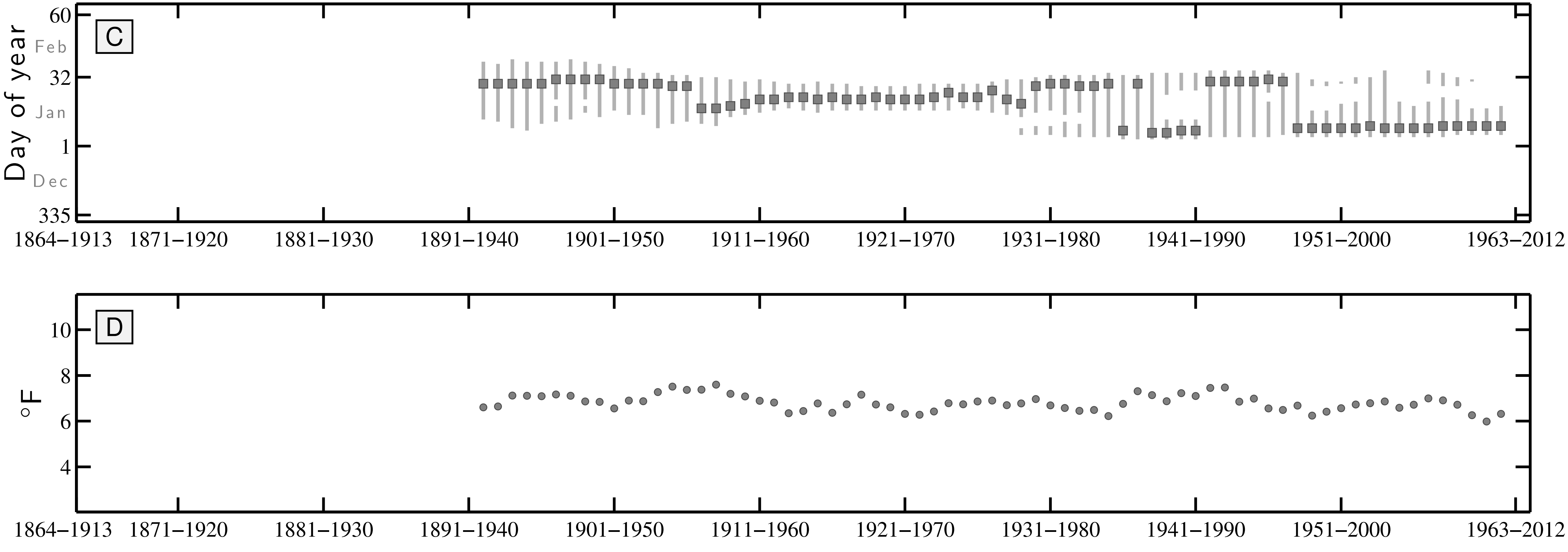

50 year Summer Teletherm dynamics for INDIANOLA 2W, IA:

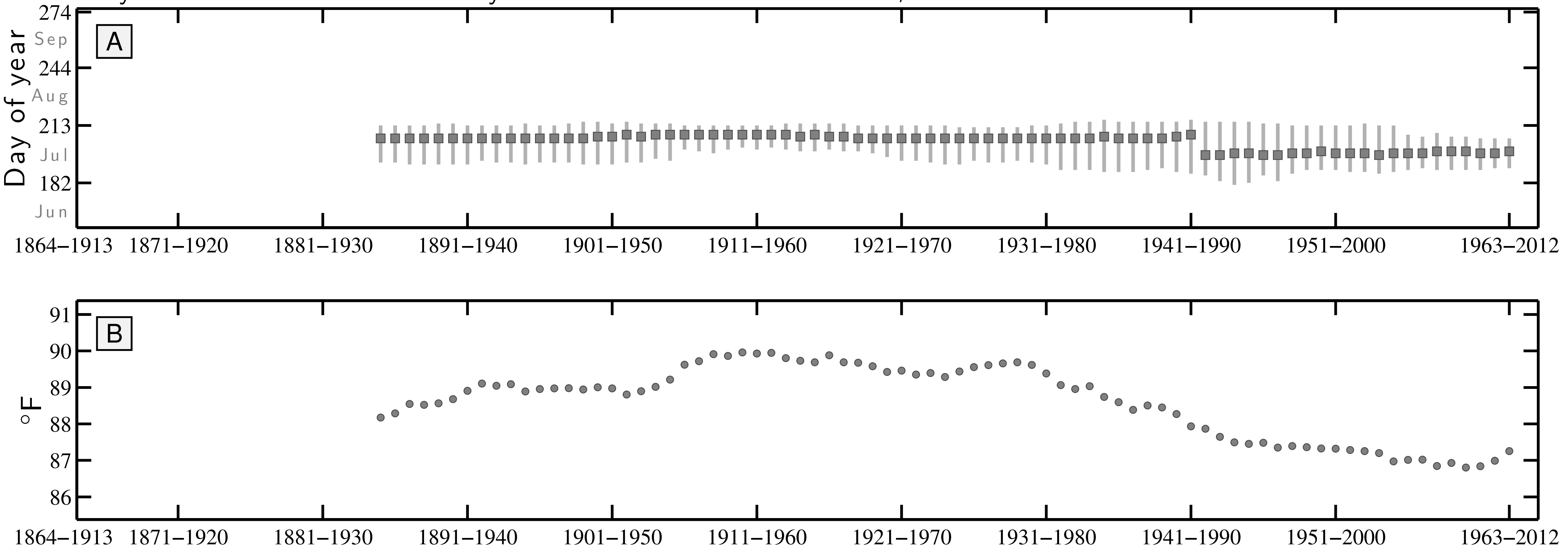

50 year Winter Teletherm dynamics for INDIANOLA 2W, IA:

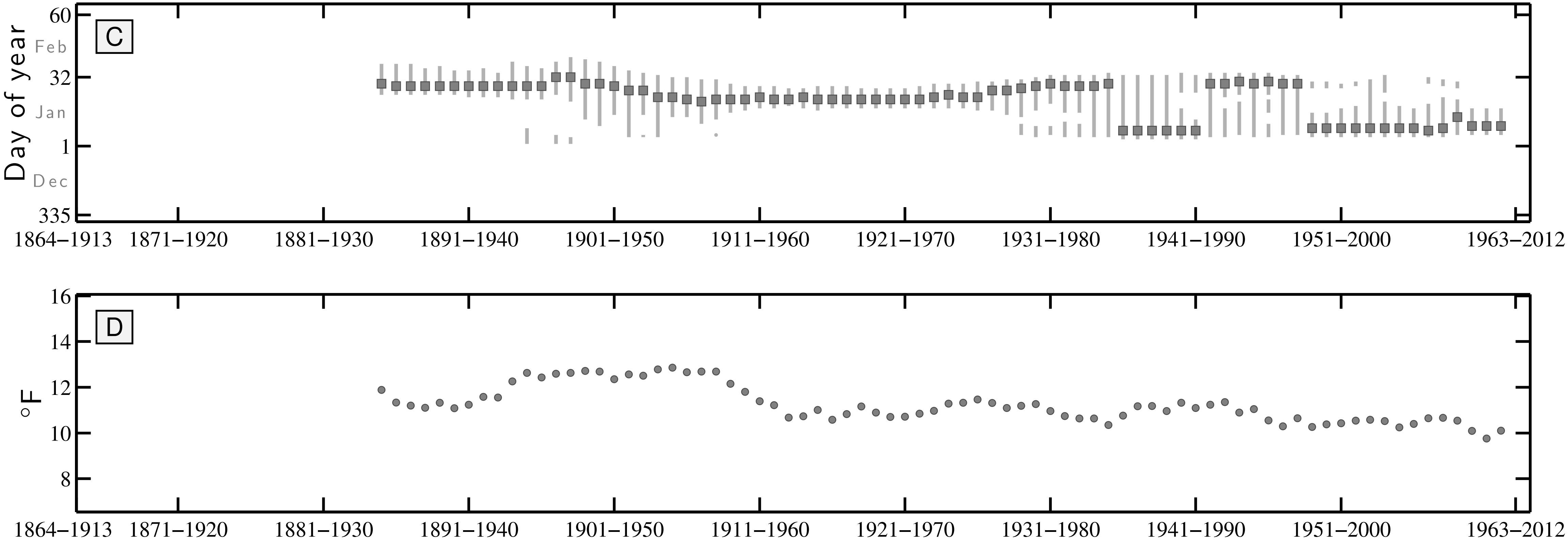

50 year Summer Teletherm dynamics for IOWA FALLS, IA:

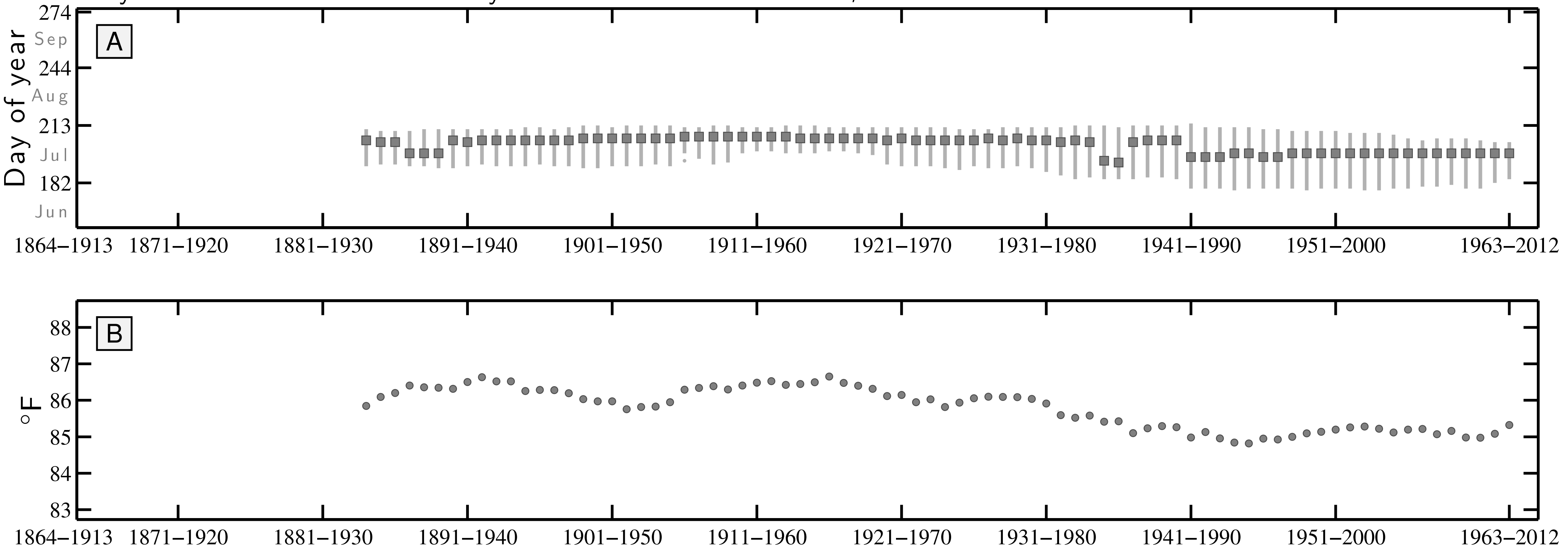

50 year Winter Teletherm dynamics for IOWA FALLS, IA:

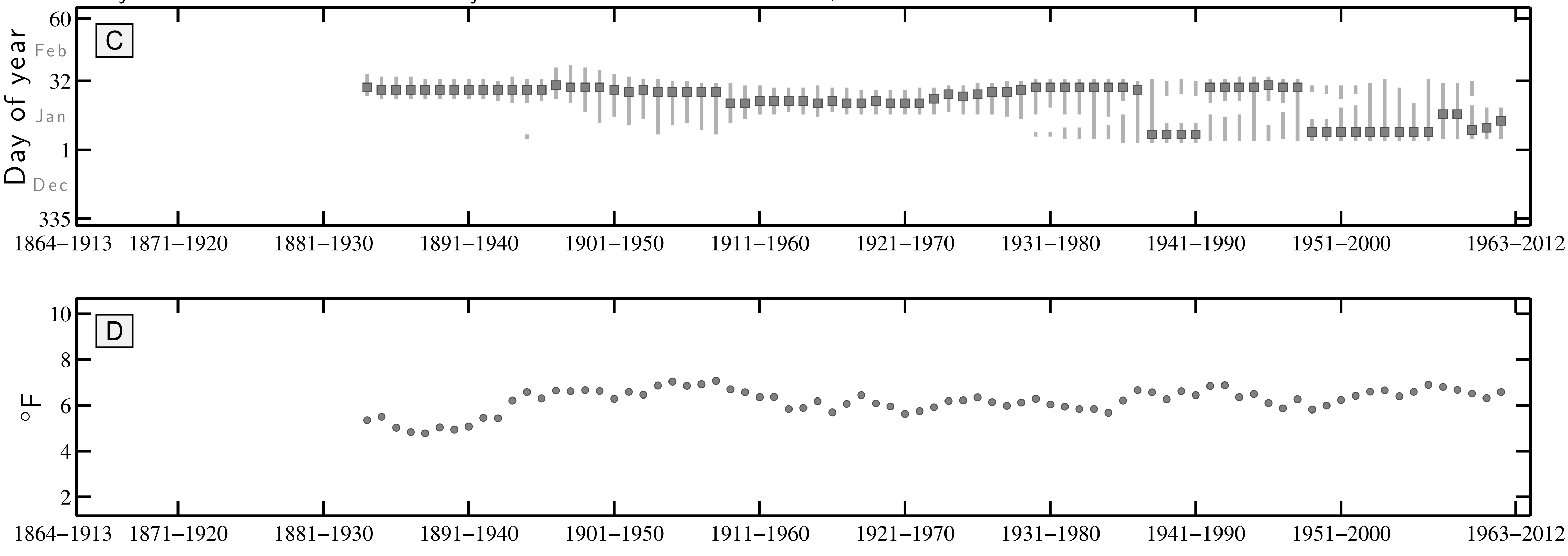

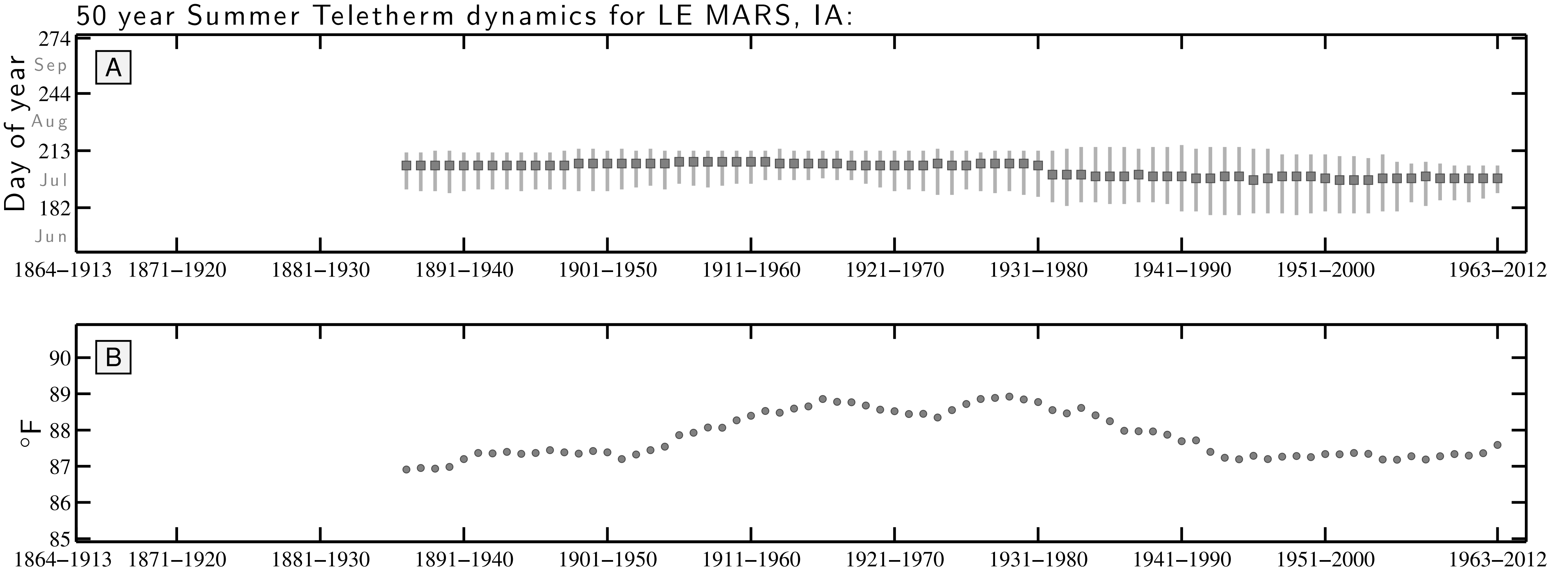
50 year Summer Teletherm dynamics for LE MARS, IA:
A
B
Day of year
°F
274
Sep
244
Aug
213
Jul
182
Jun
90
89
88
87
86
85
1864–1913
1871–1920
1881–1930
1891–1940
1901–1950
1911–1960
1921–1970
1931–1980
1941–1990
1951–2000
1963–2012

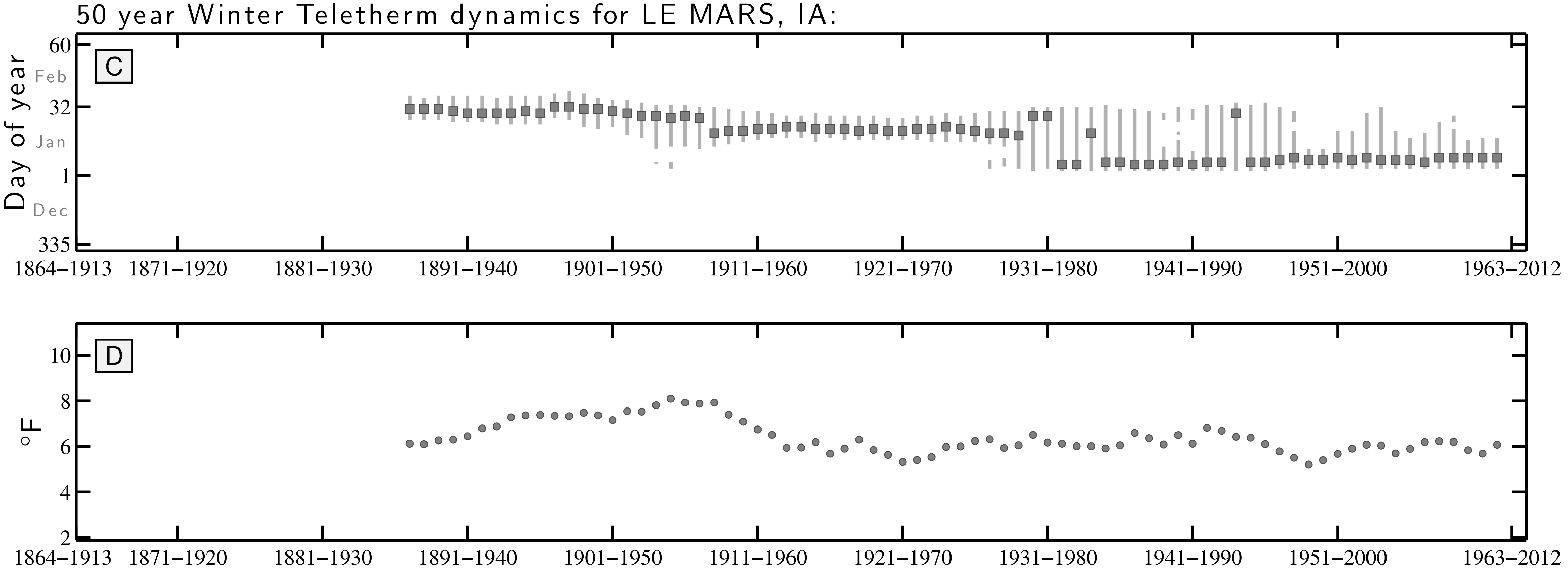
50 year Winter Teletherm dynamics for LE MARS, IA:
C
D
Day of year
°F
60
Feb
32
Jan
1
Dec
335
10
8
6
4
2
1864–1913
1871–1920
1881–1930
1891–1940
1901–1950
1911–1960
1921–1970
1931–1980
1941–1990
1951–2000
1963–2012

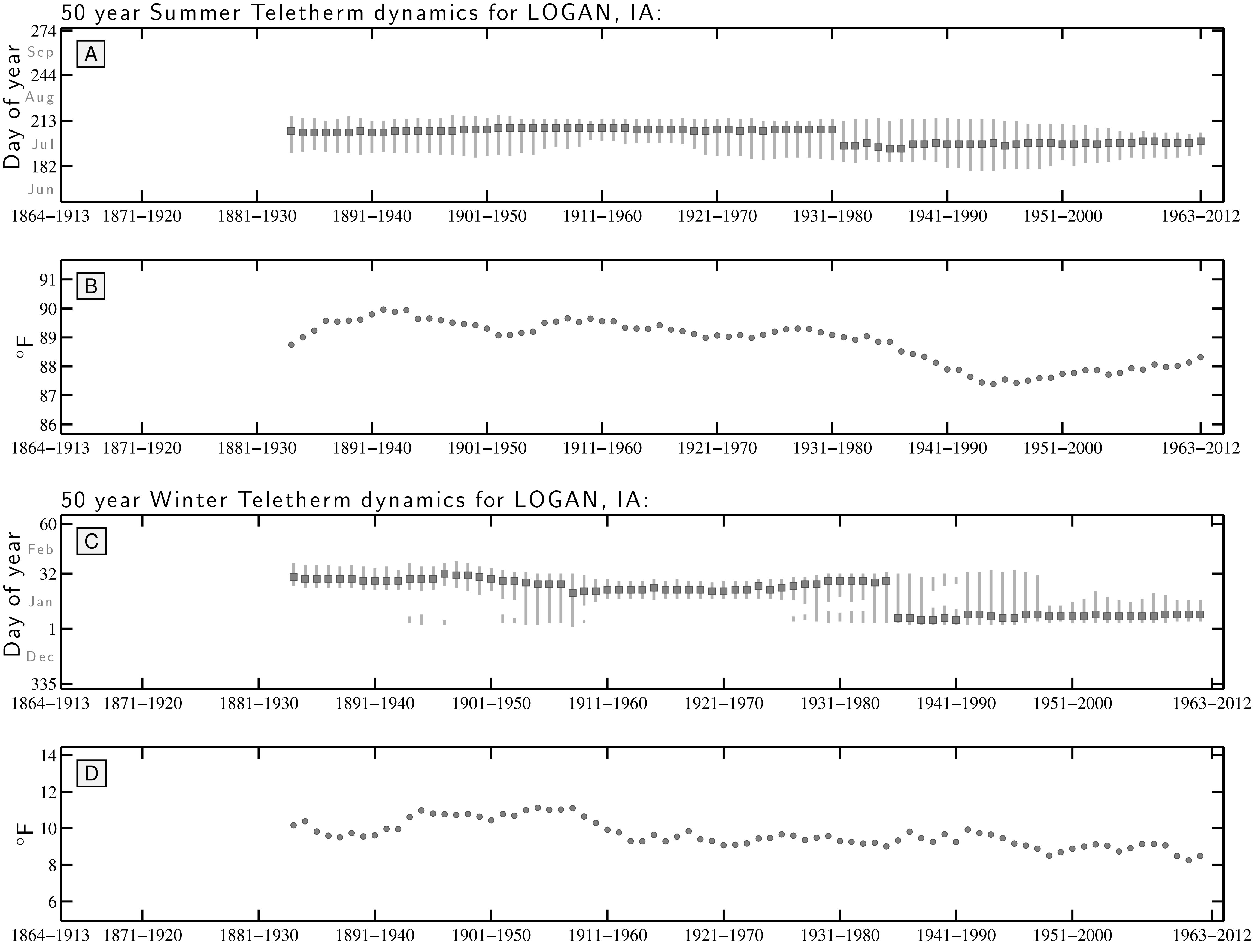

50 year Summer Teletherm dynamics for LOGAN, IA:
A
Day of year
274
Sep
244
Aug
213
Jul
182
Jun
B
°F
91
90
89
88
87
86
50 year Winter Teletherm dynamics for LOGAN, IA:
C
Day of year
60
Feb
32
Jan
1
Dec
335
D
°F
14
12
10
8
6
1864–1913
1871–1920
1881–1930
1891–1940
1901–1950
1911–1960
1921–1970
1931–1980
1941–1990
1951–2000
1963–2012

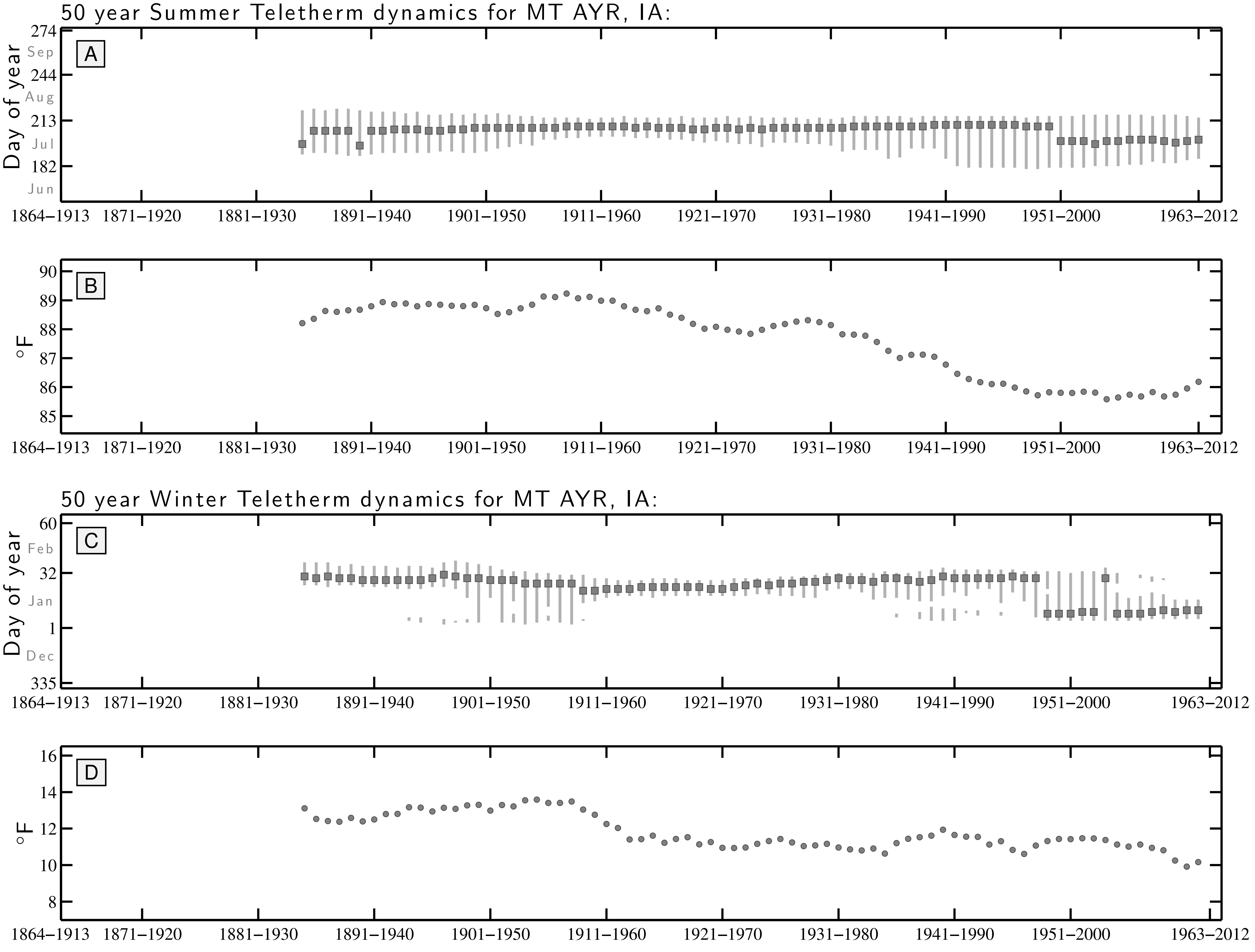
50 year Summer Teletherm dynamics for MT AYR, IA:
A
B
Day of year
°F
274
244
213
182
Sep
Aug
Jul
Jun
90
89
88
87
86
85
1864–1913
1871–1920
1881–1930
1891–1940
1901–1950
1911–1960
1921–1970
1931–1980
1941–1990
1951–2000
1963–2012
50 year Winter Teletherm dynamics for MT AYR, IA:
C
D
60
32
1
335
Feb
Jan
Dec
16
14
12
10
8

50 year Summer Teletherm dynamics for MT PLEASANT 1 SSW, IA:

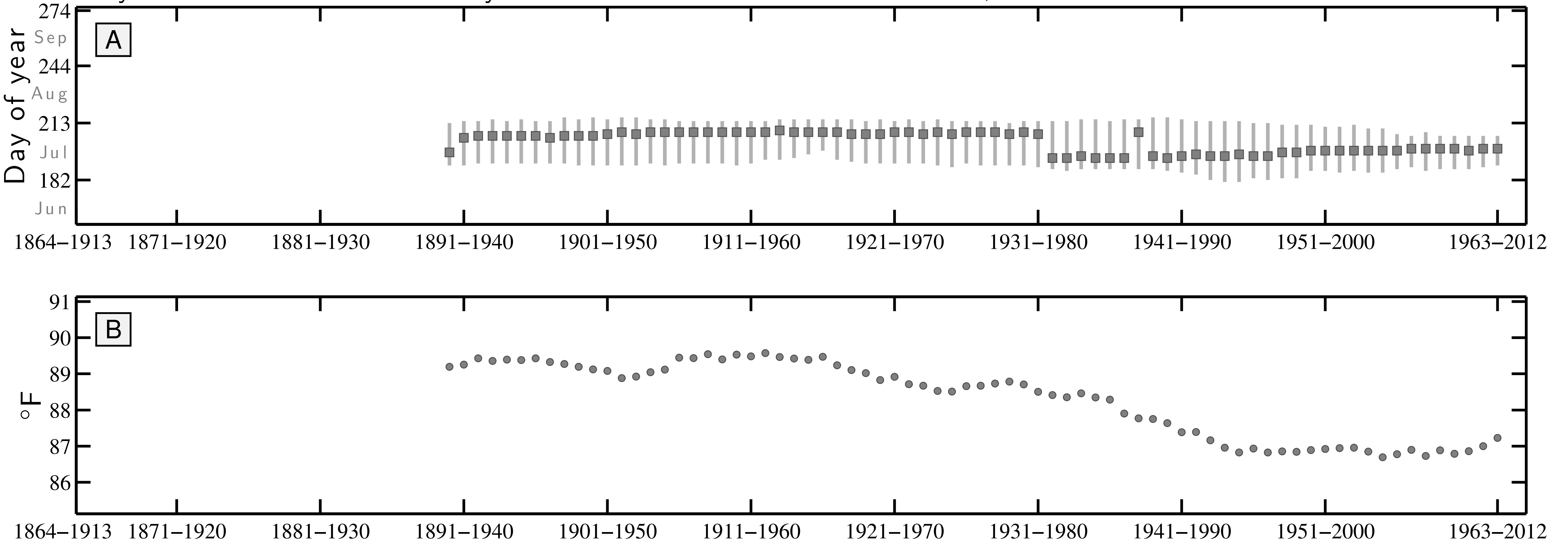

50 year Winter Teletherm dynamics for MT PLEASANT 1 SSW, IA:

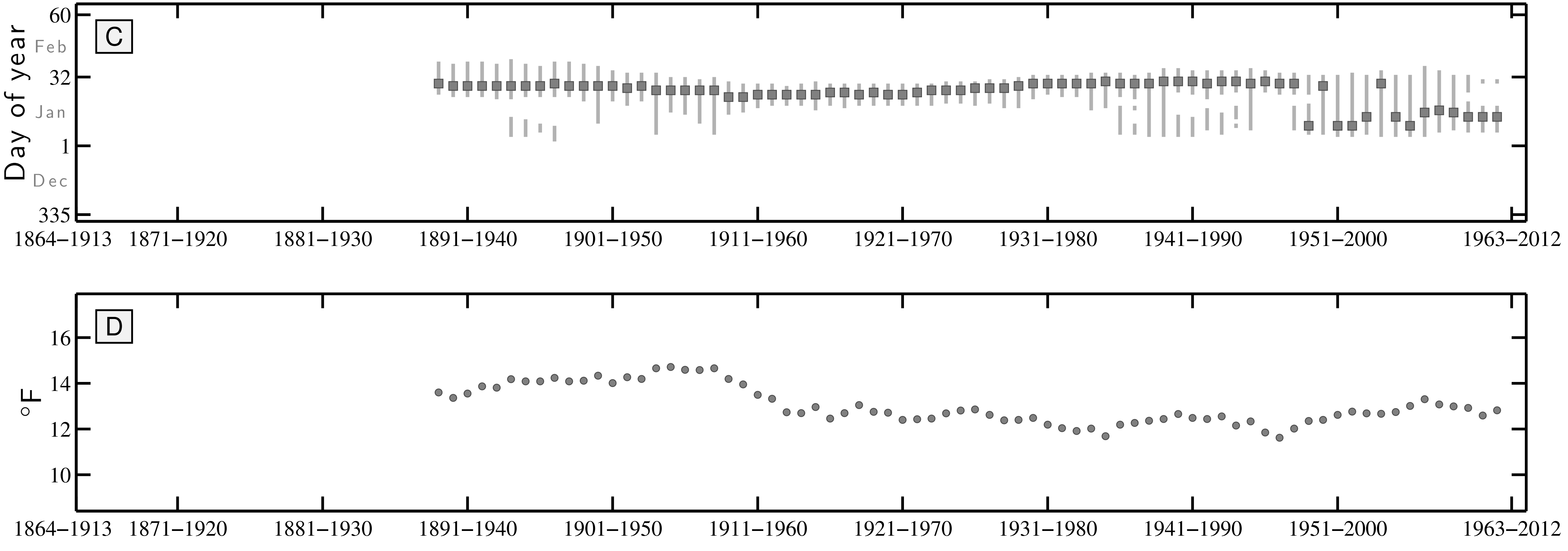

50 year Summer Teletherm dynamics for NEW HAMPTON, IA:

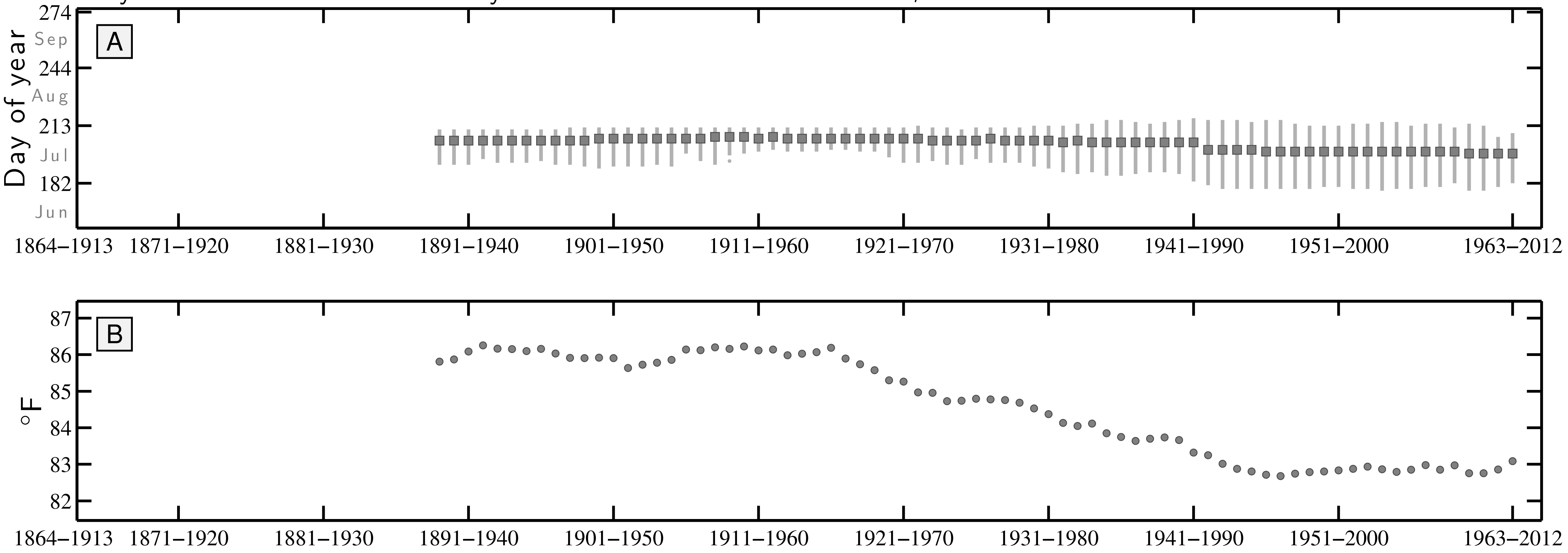

50 year Winter Teletherm dynamics for NEW HAMPTON, IA:

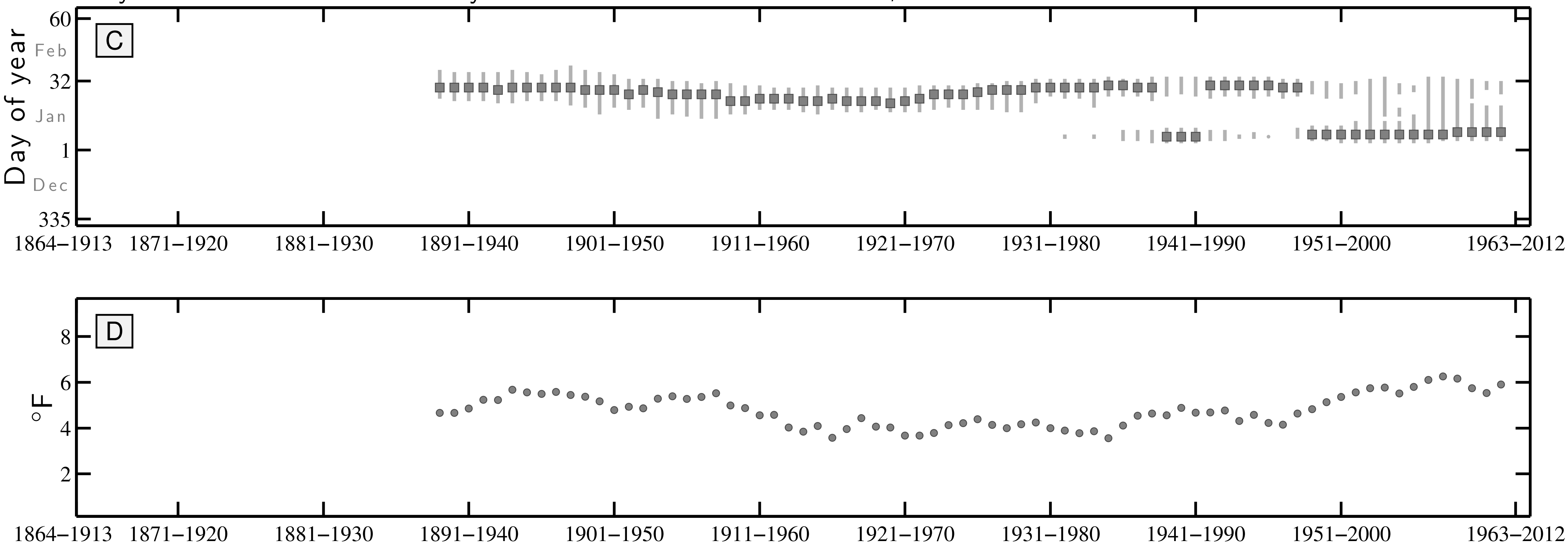

50 year Summer Teletherm dynamics for ROCK RAPIDS, IA:

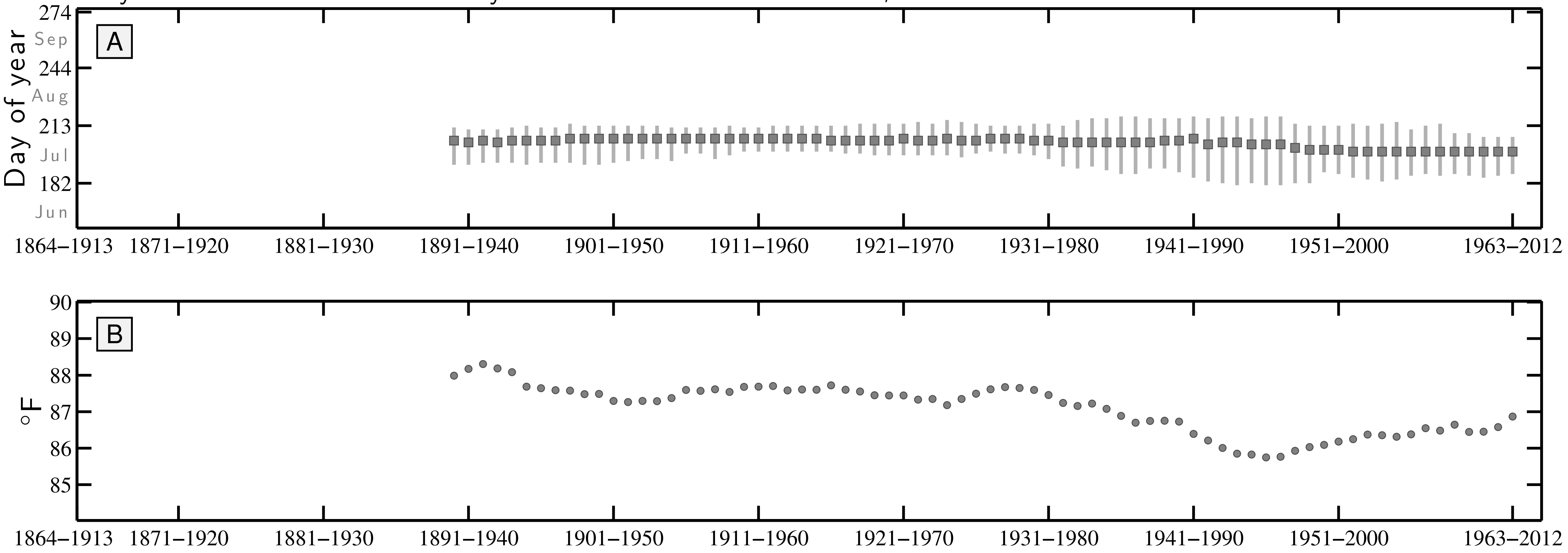

50 year Winter Teletherm dynamics for ROCK RAPIDS, IA:

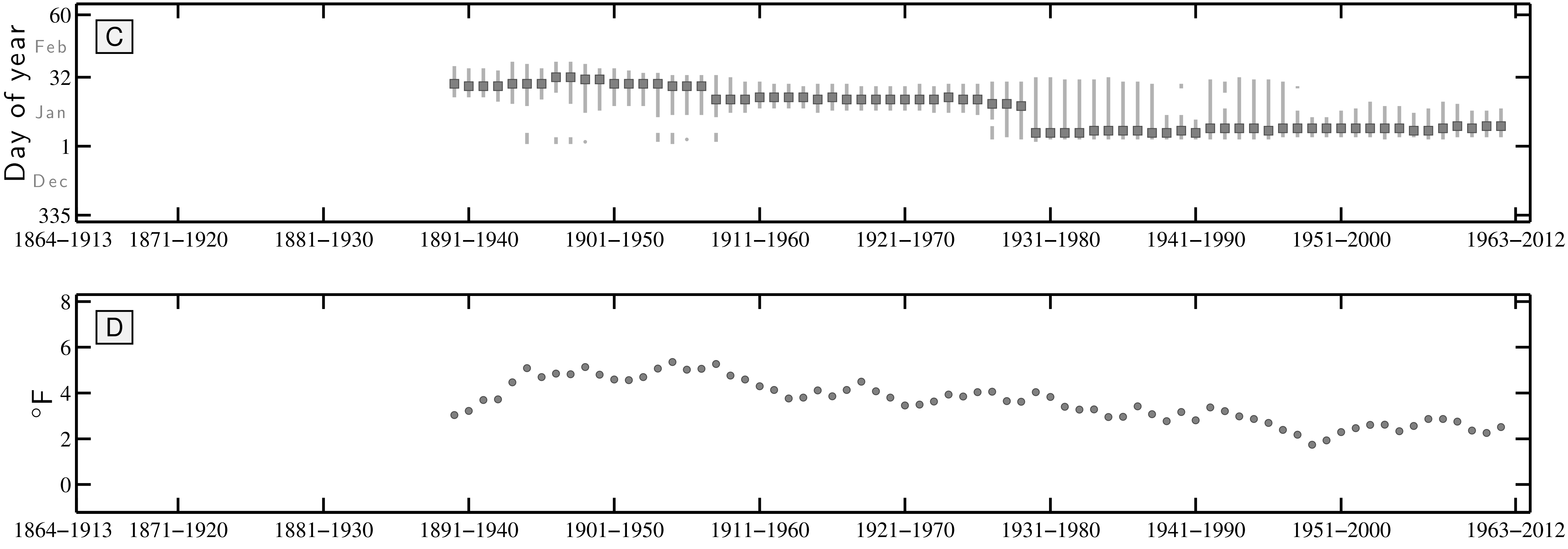

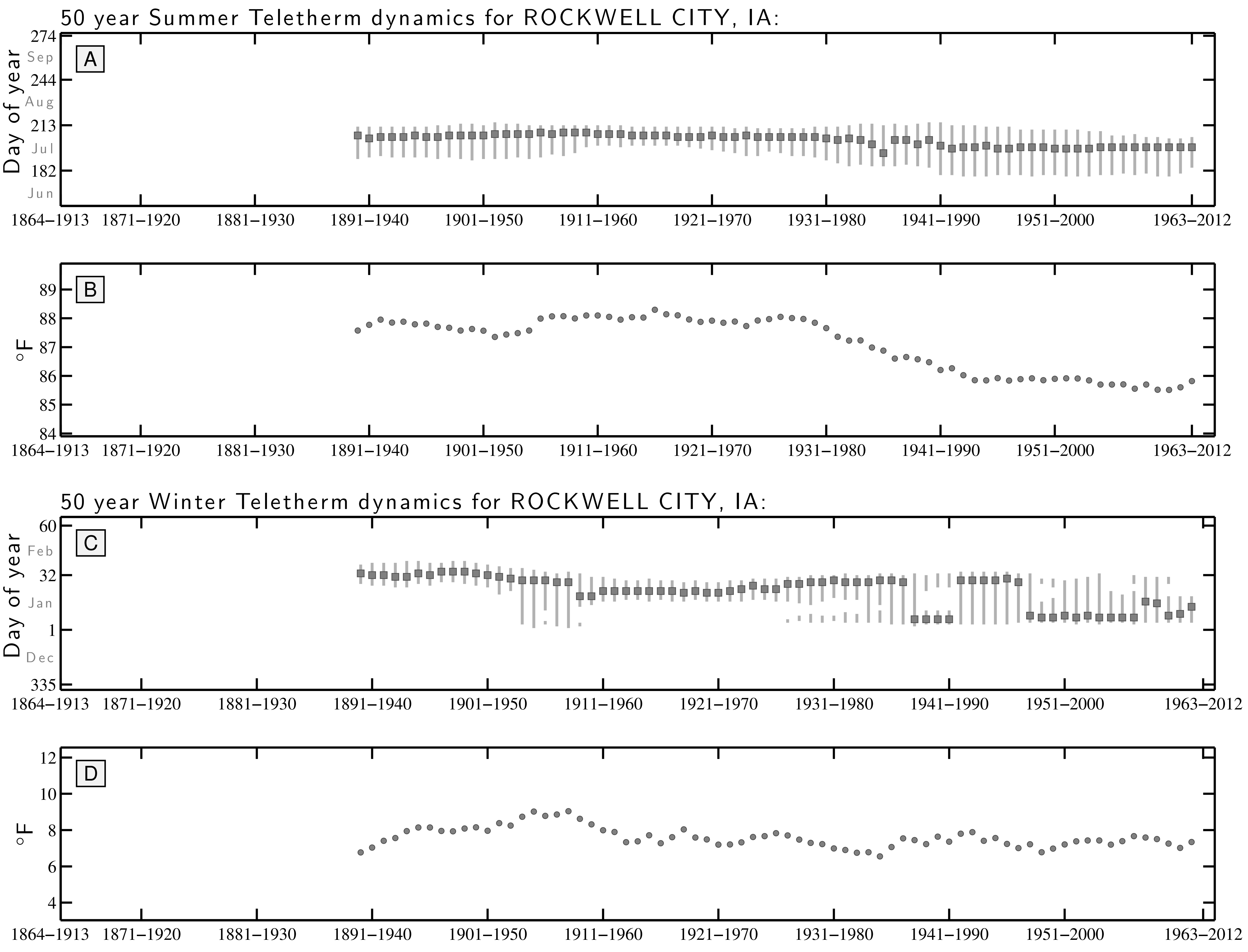
50 year Summer Teletherm dynamics for ROCKWELL CITY, IA:
A
B
Day of year
°F
274
Sep
244
Aug
213
Jul
182
Jun
89
88
87
86
85
84
1864–1913
1871–1920
1881–1930
1891–1940
1901–1950
1911–1960
1921–1970
1931–1980
1941–1990
1951–2000
1963–2012
50 year Winter Teletherm dynamics for ROCKWELL CITY, IA:
C
D
60
Feb
32
Jan
1
Dec
335
12
10
8
6
4

50 year Summer Teletherm dynamics for STORM LAKE 2 E, IA:

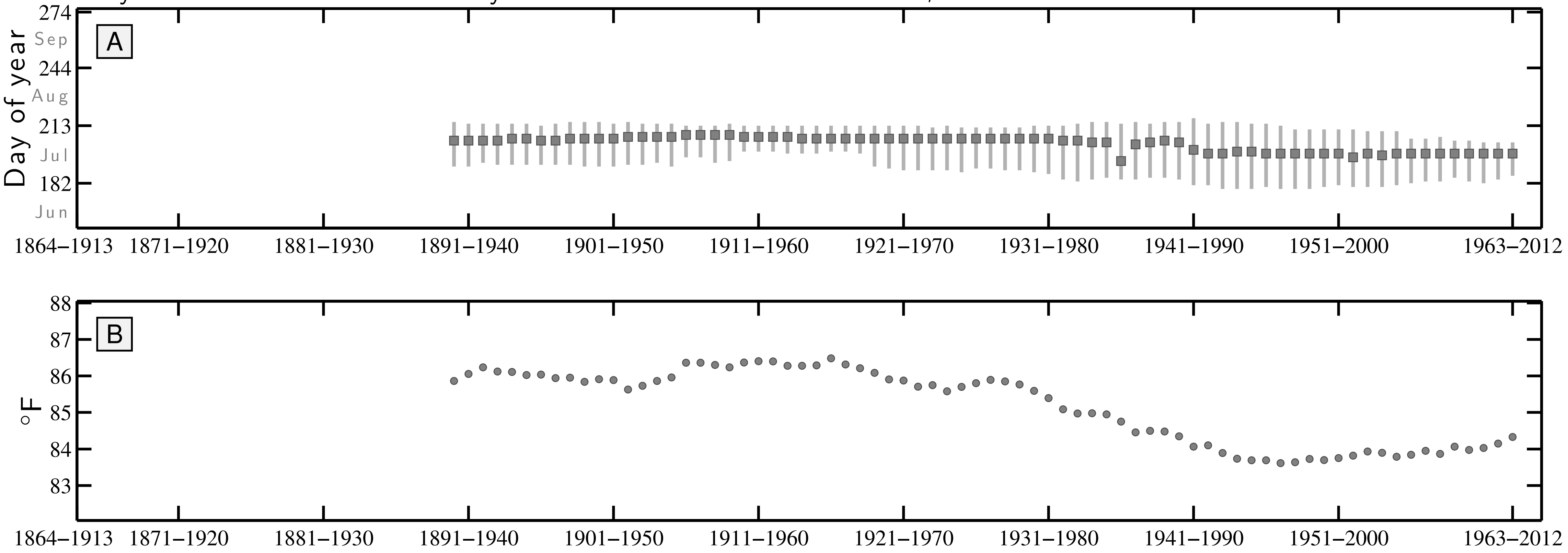

50 year Winter Teletherm dynamics for STORM LAKE 2 E, IA:

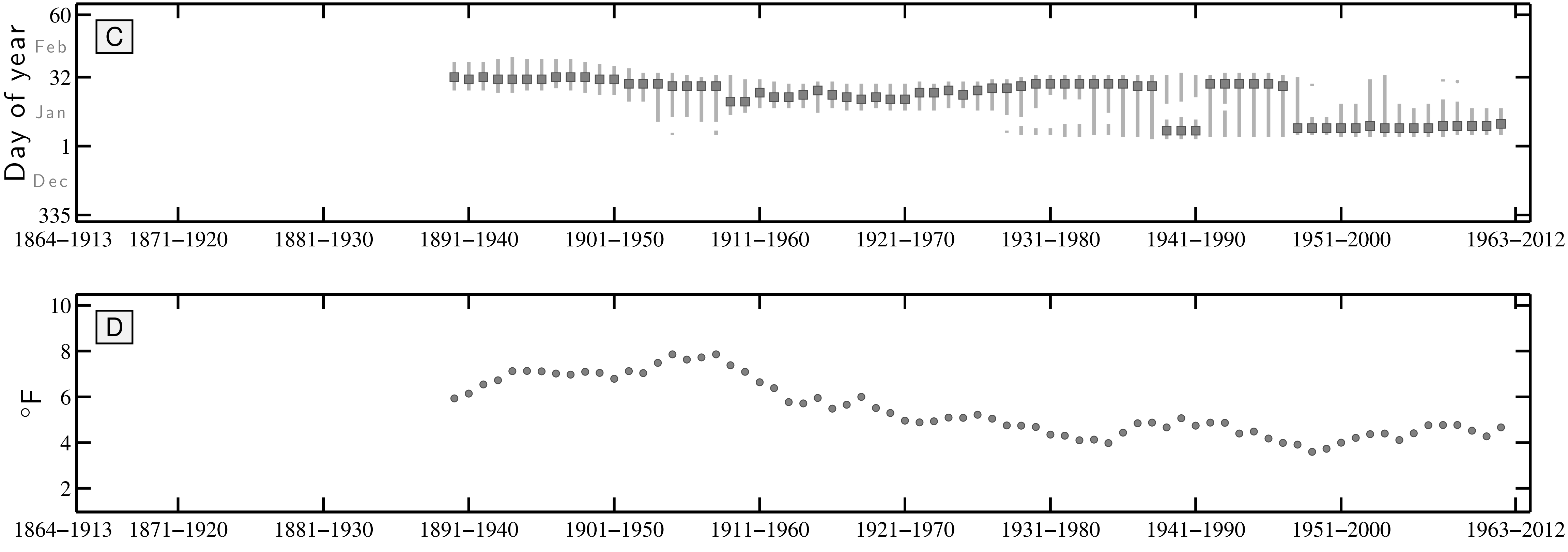

50 year Summer Teletherm dynamics for TOLEDO 3N, IA:

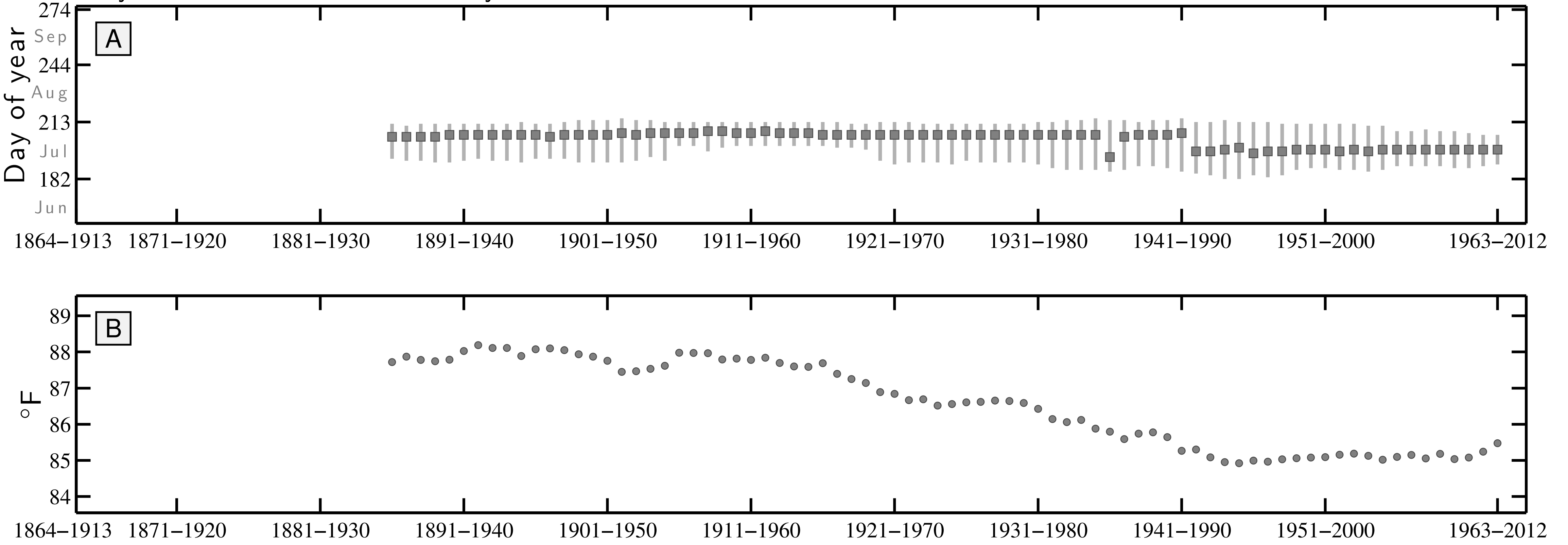

50 year Winter Teletherm dynamics for TOLEDO 3N, IA:

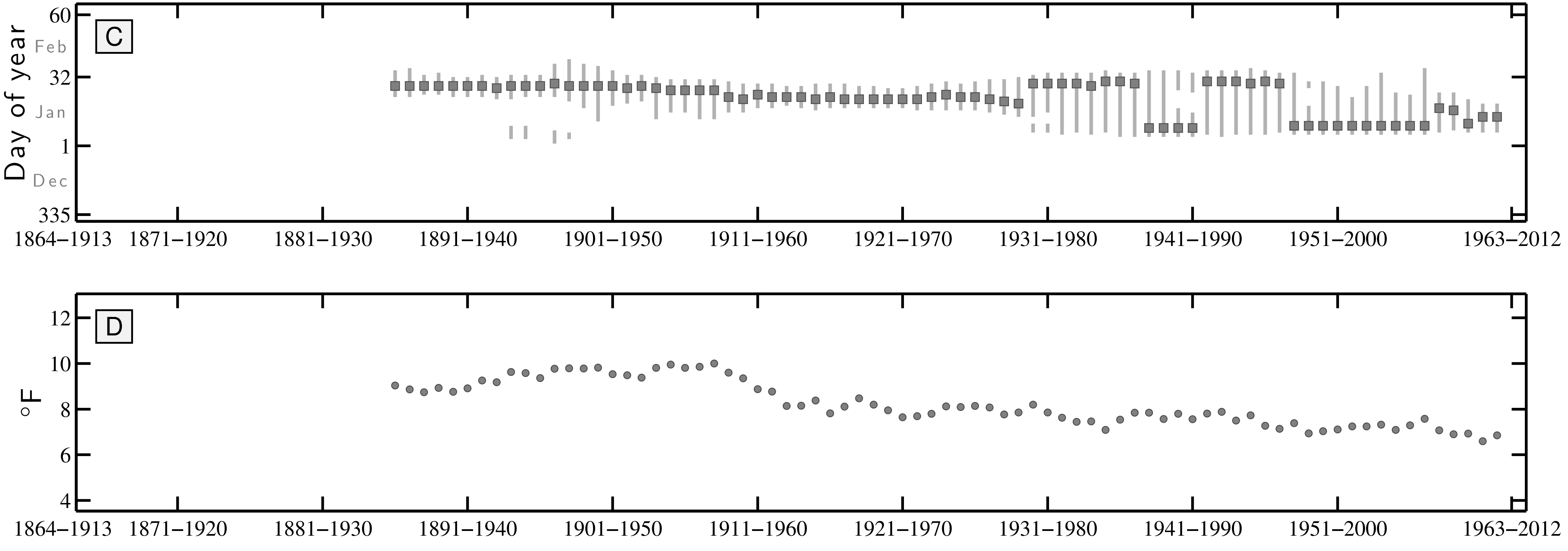

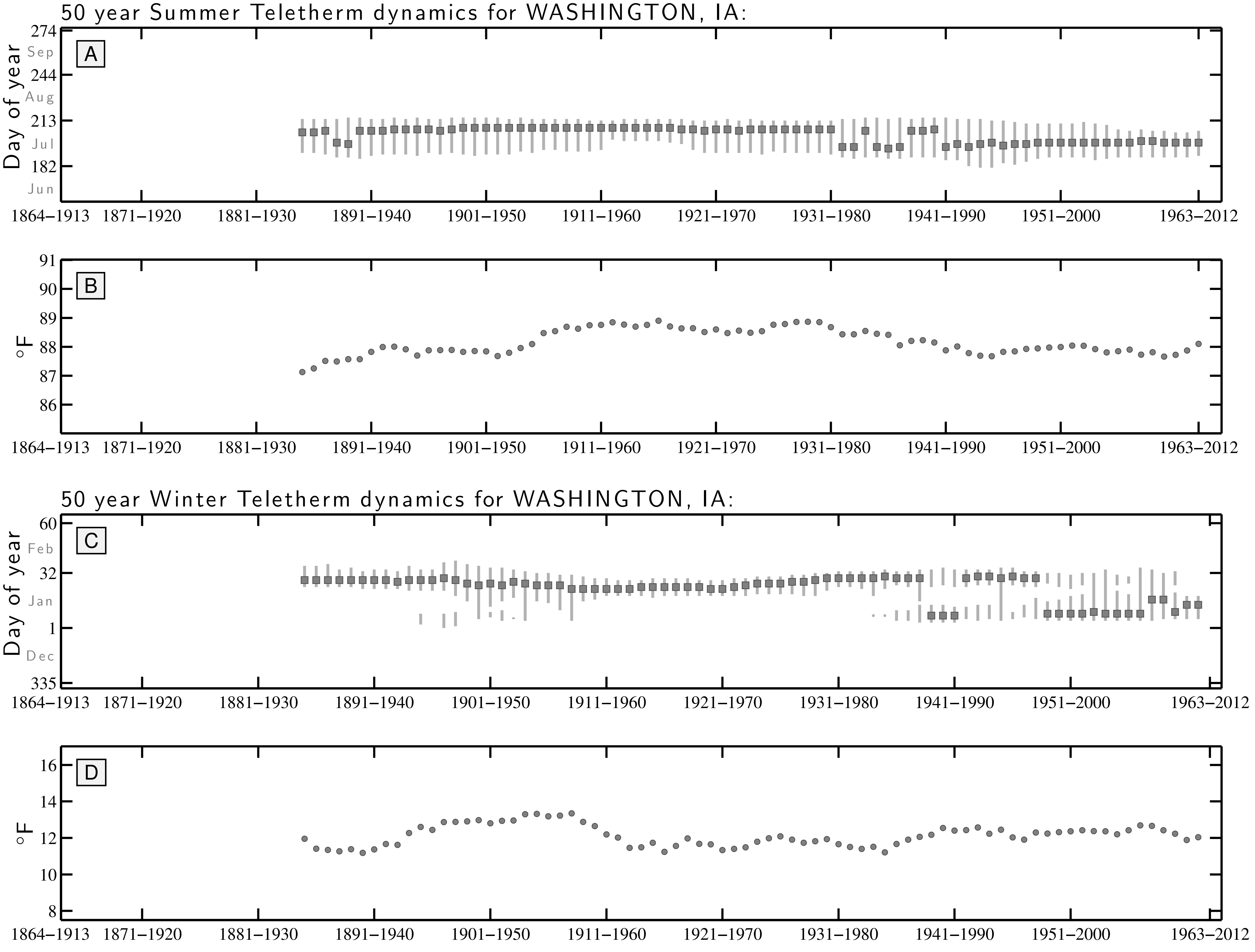

50 year Summer Teletherm dynamics for WASHINGTON, IA:
A
Day of year
274
Sep
244
Aug
213
Jul
182
Jun
1864–1913
1871–1920
1881–1930
1891–1940
1901–1950
1911–1960
1921–1970
1931–1980
1941–1990
1951–2000
1963–2012
B
°F
91
90
89
88
87
86
50 year Winter Teletherm dynamics for WASHINGTON, IA:
C
Day of year
60
Feb
32
Jan
1
Dec
335
D
°F
16
14
12
10
8

50 year Summer Teletherm dynamics for ANTHONY, KS:

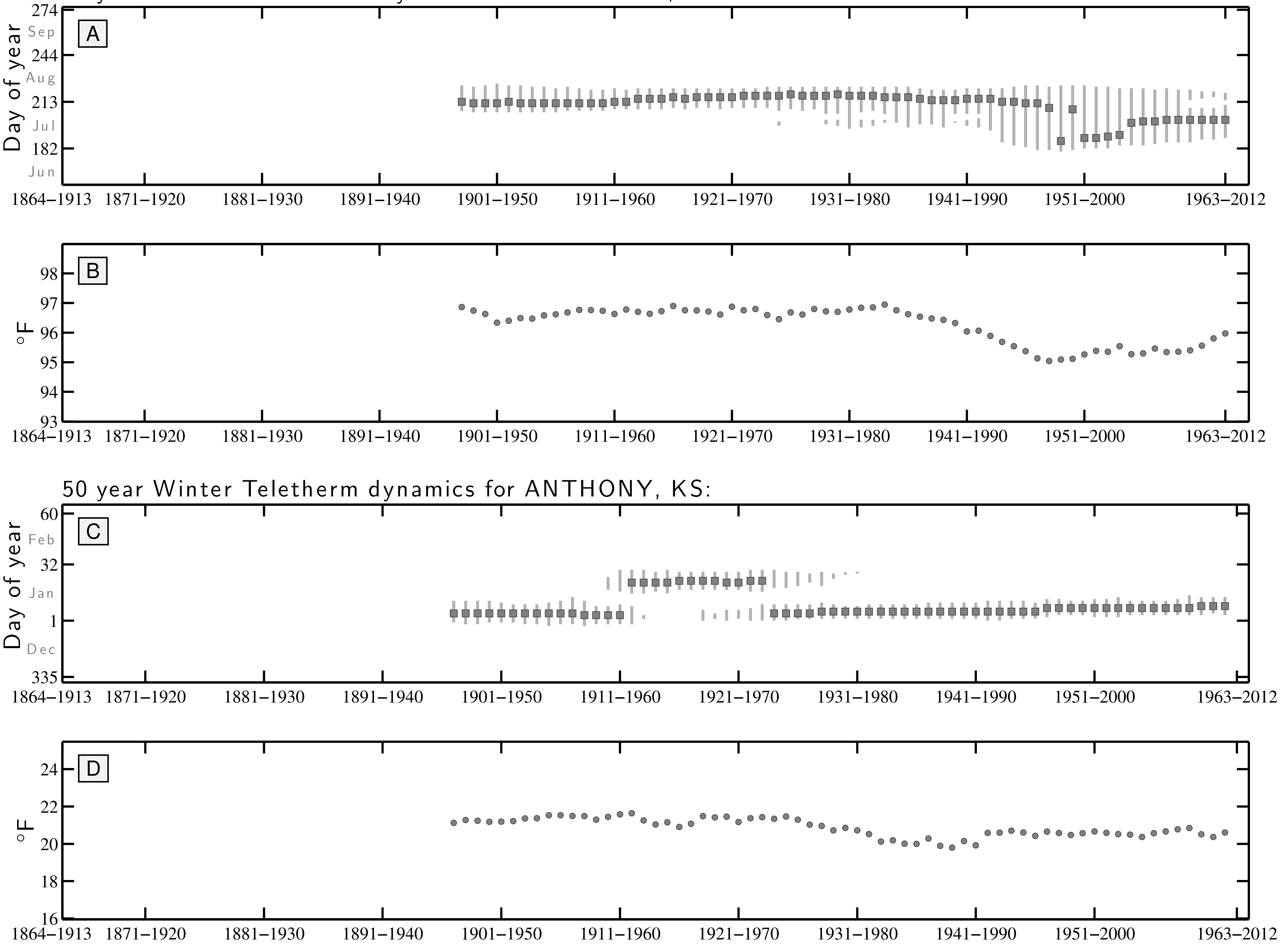

50 year Winter Teletherm dynamics for ANTHONY, KS:

50 year Summer Teletherm dynamics for ASHLAND, KS:

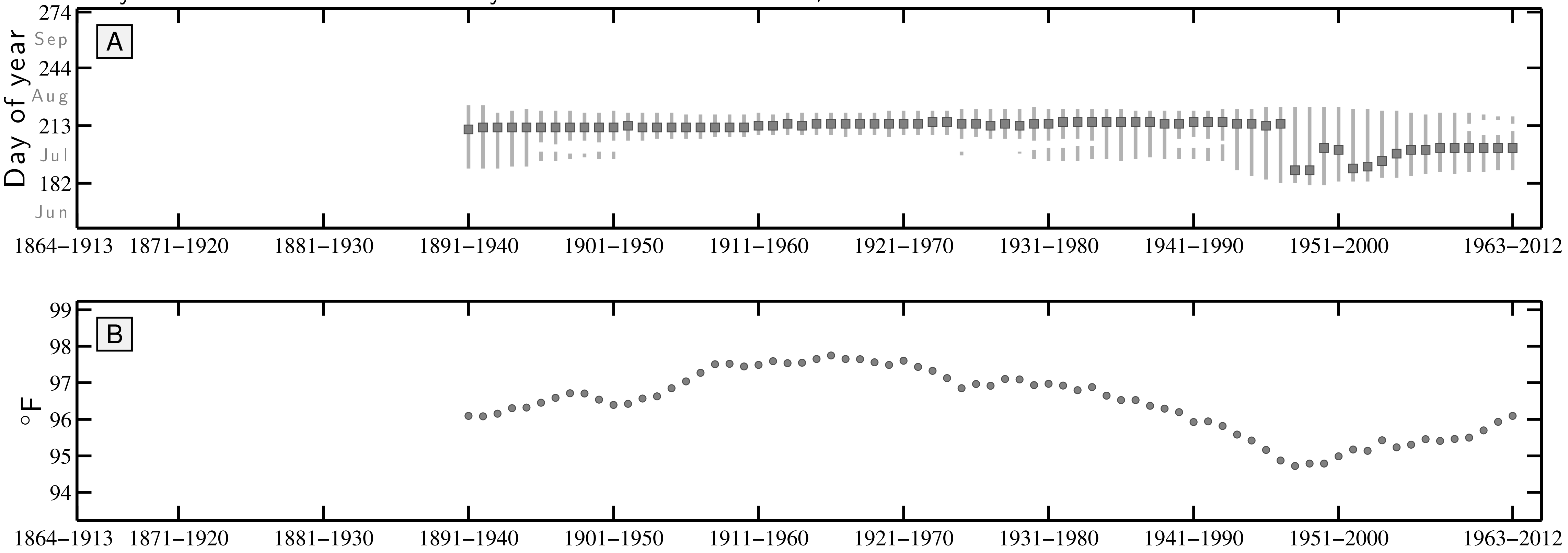

50 year Winter Teletherm dynamics for ASHLAND, KS:

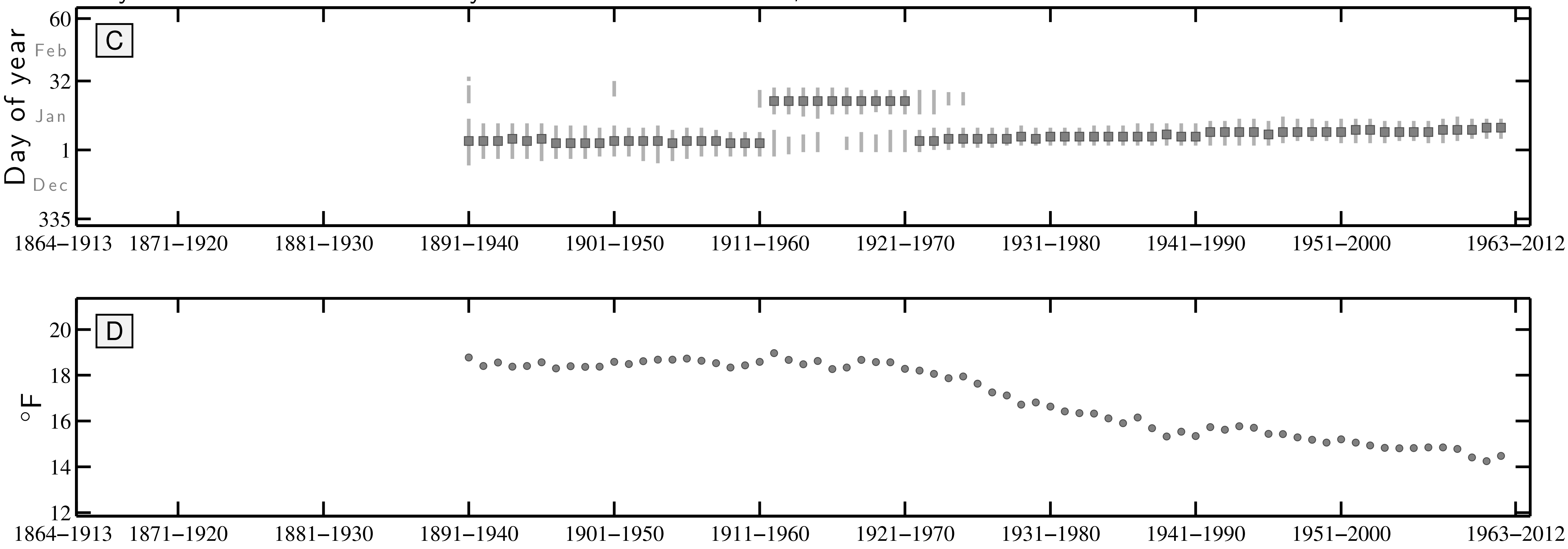

50 year Summer Teletherm dynamics for ATCHISON, KS:

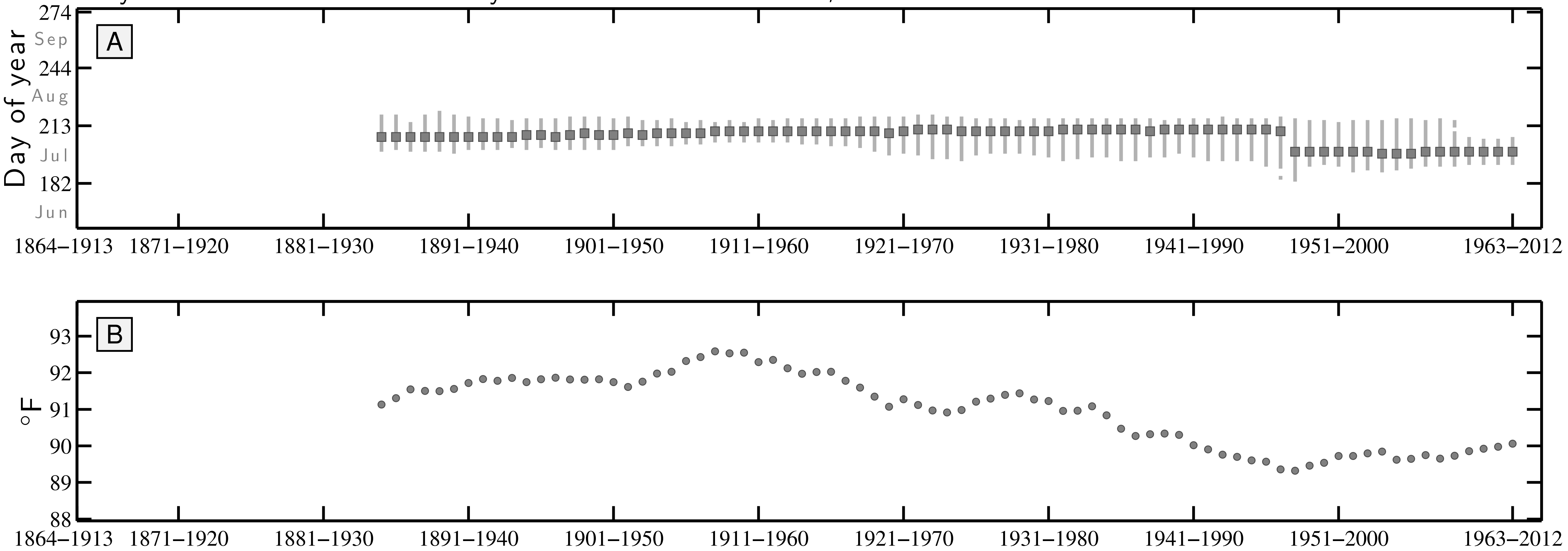

50 year Winter Teletherm dynamics for ATCHISON, KS:

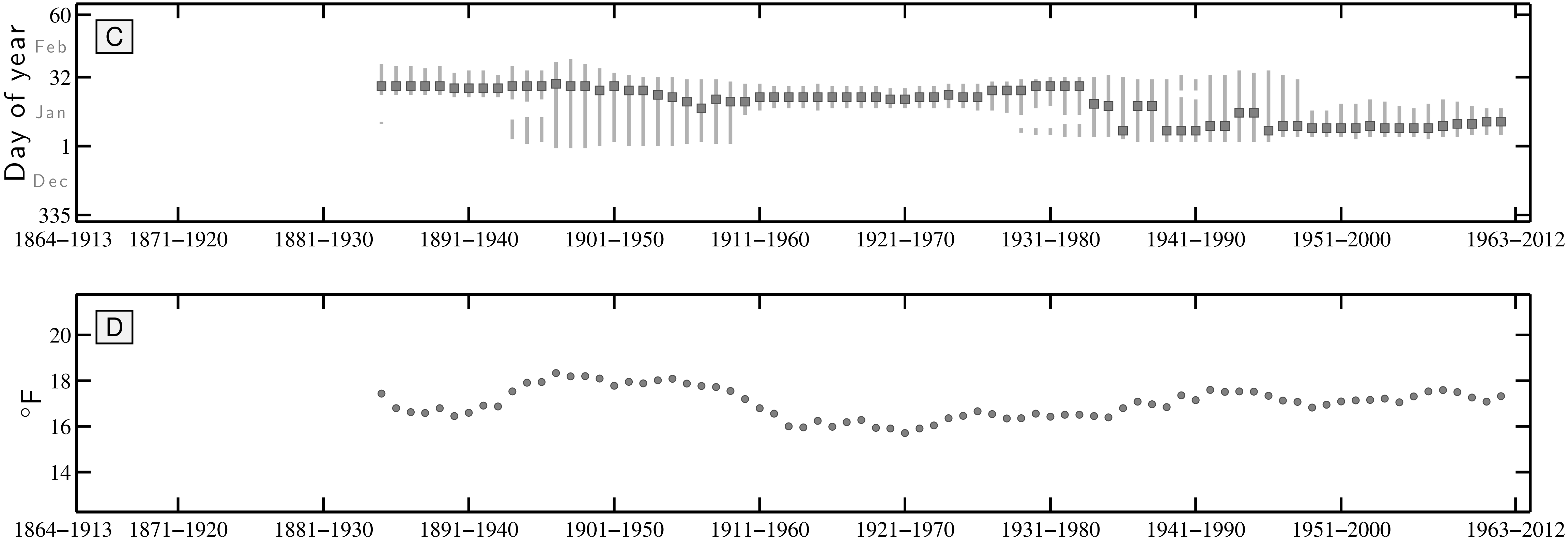

50 year Summer Teletherm dynamics for COLDWATER, KS:

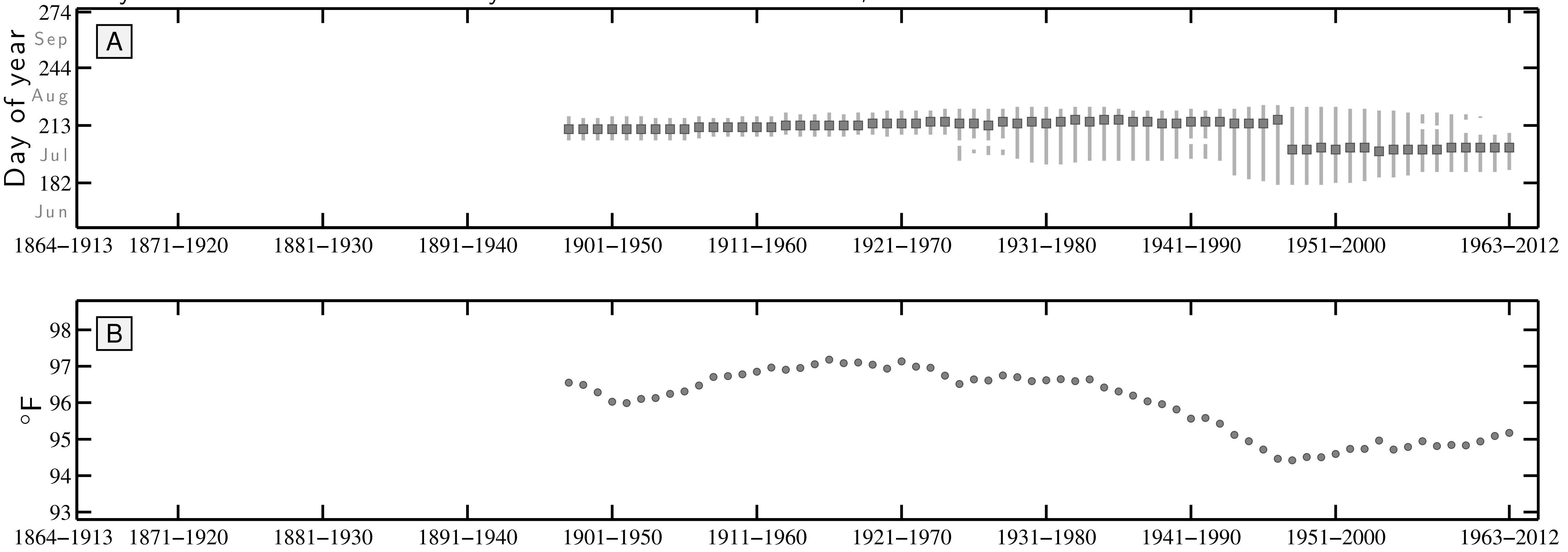

50 year Winter Teletherm dynamics for COLDWATER, KS:

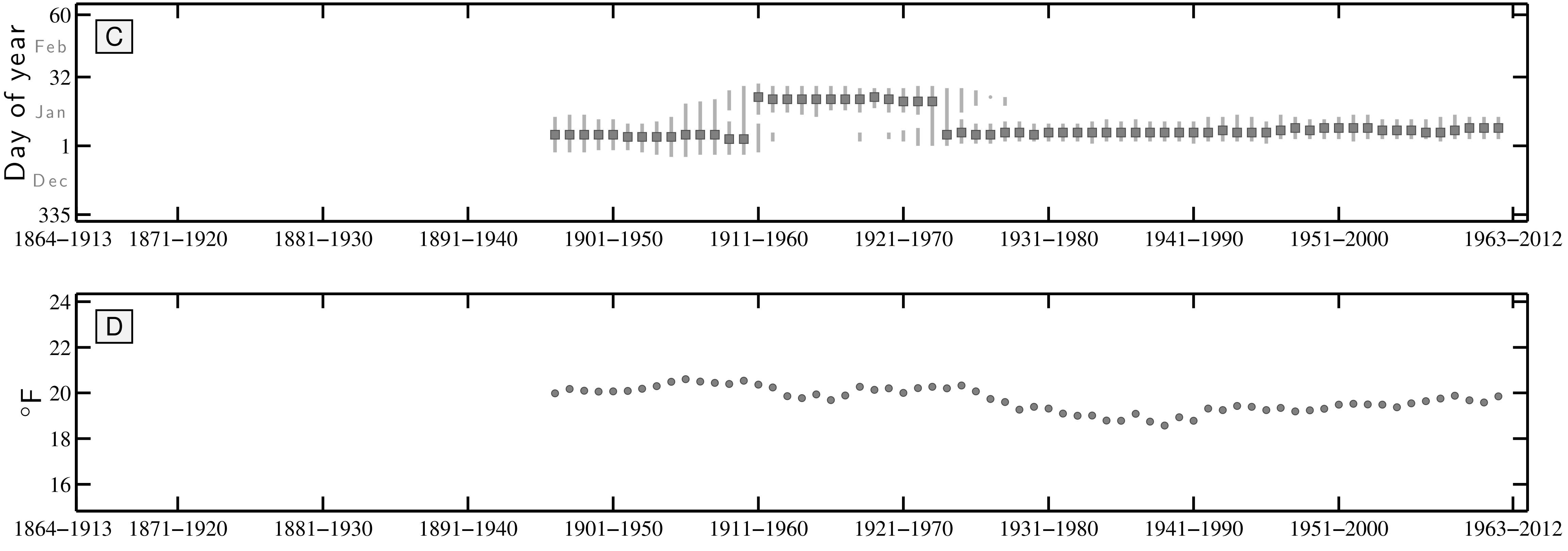

50 year Summer Teletherm dynamics for COLUMBUS, KS:

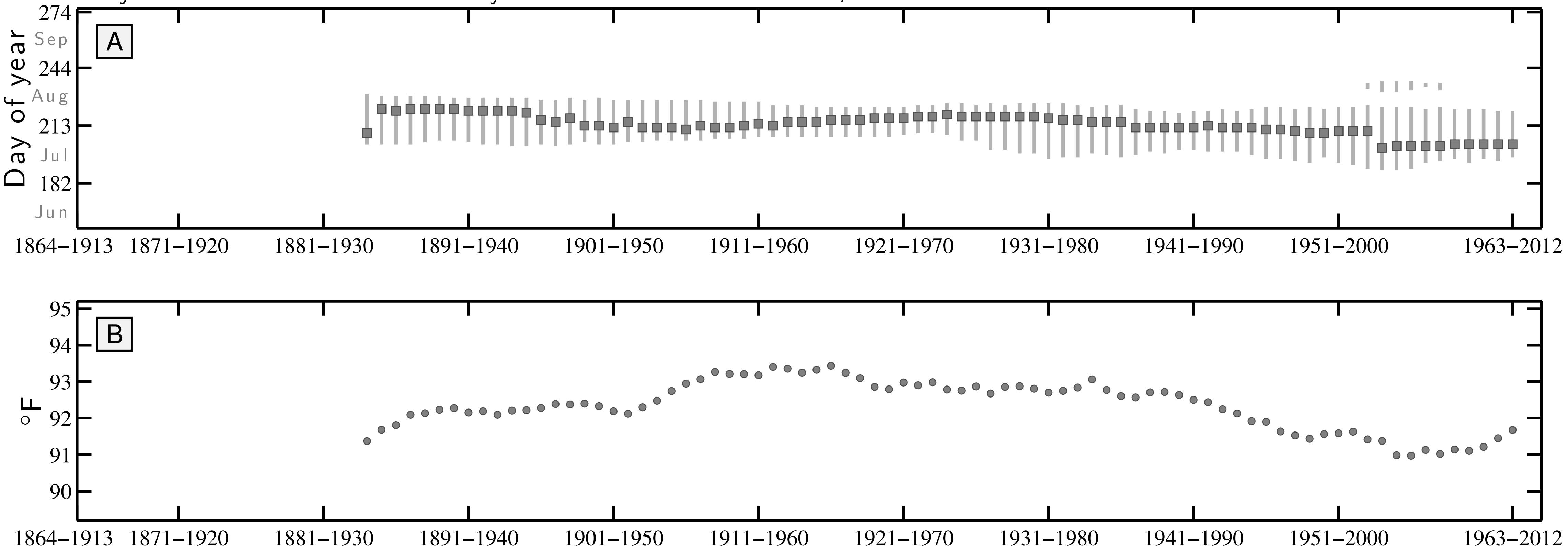

50 year Winter Teletherm dynamics for COLUMBUS, KS:

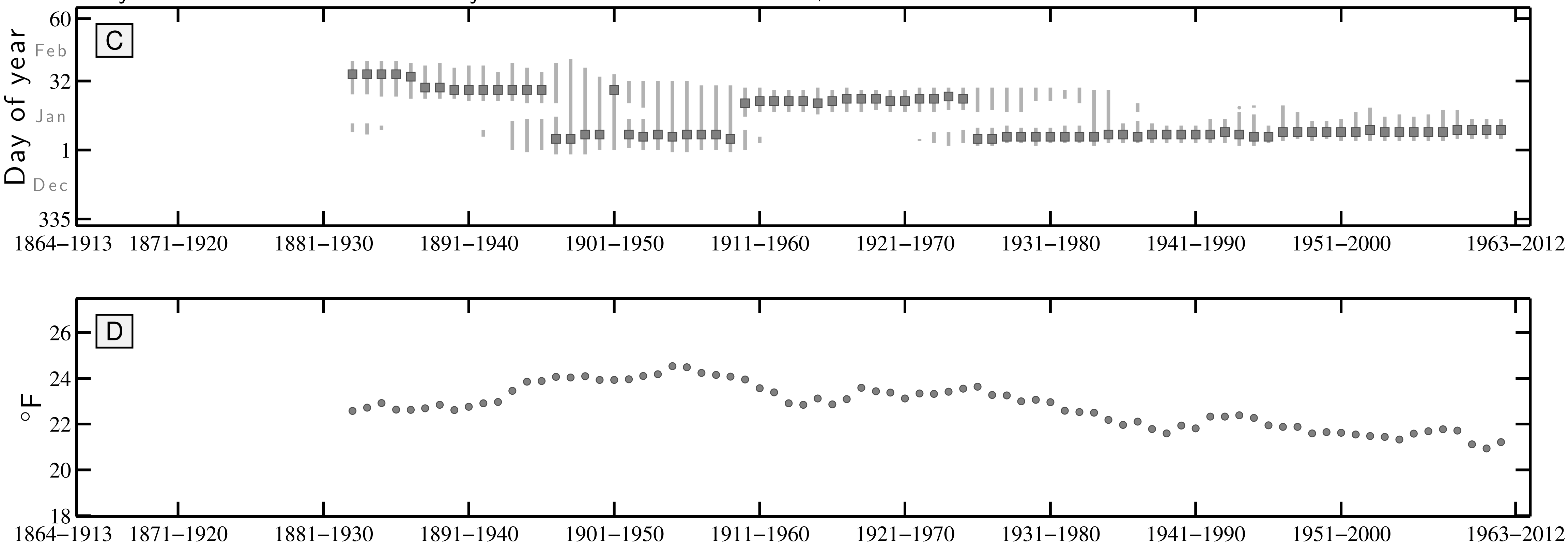

50 year Summer Teletherm dynamics for COUNCIL GROVE LAKE, KS:

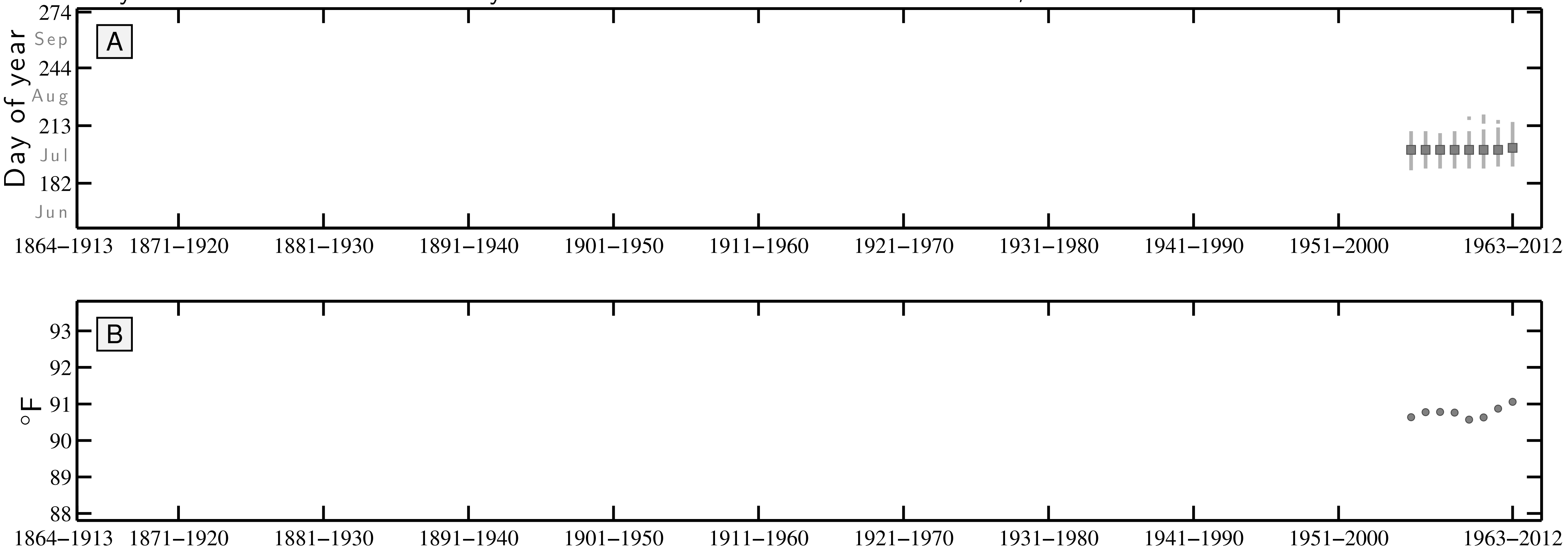

50 year Winter Teletherm dynamics for COUNCIL GROVE LAKE, KS:

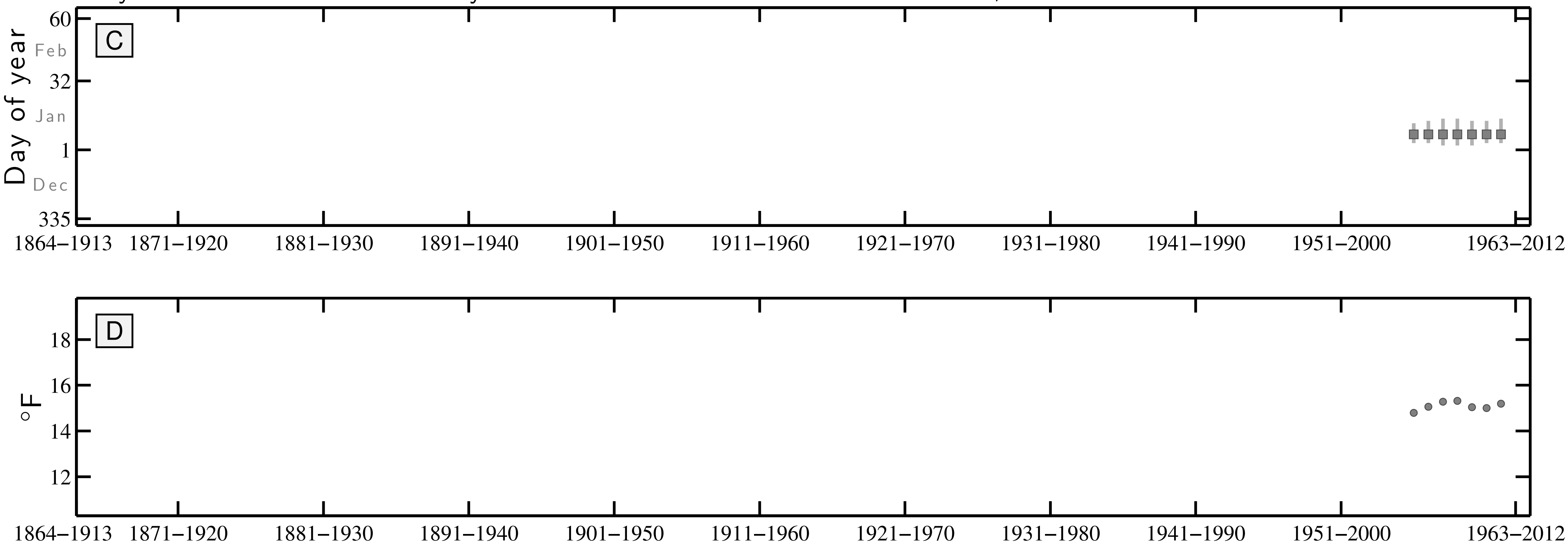

50 year Summer Teletherm dynamics for EL DORADO, KS:

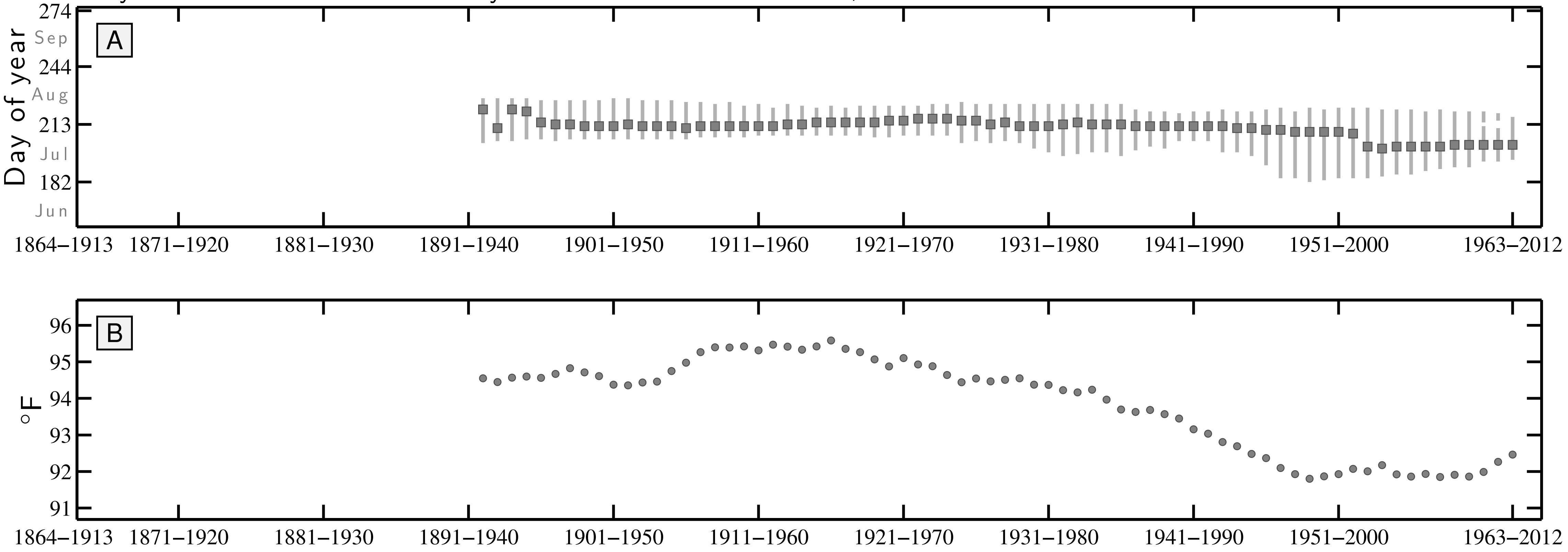

50 year Winter Teletherm dynamics for EL DORADO, KS:

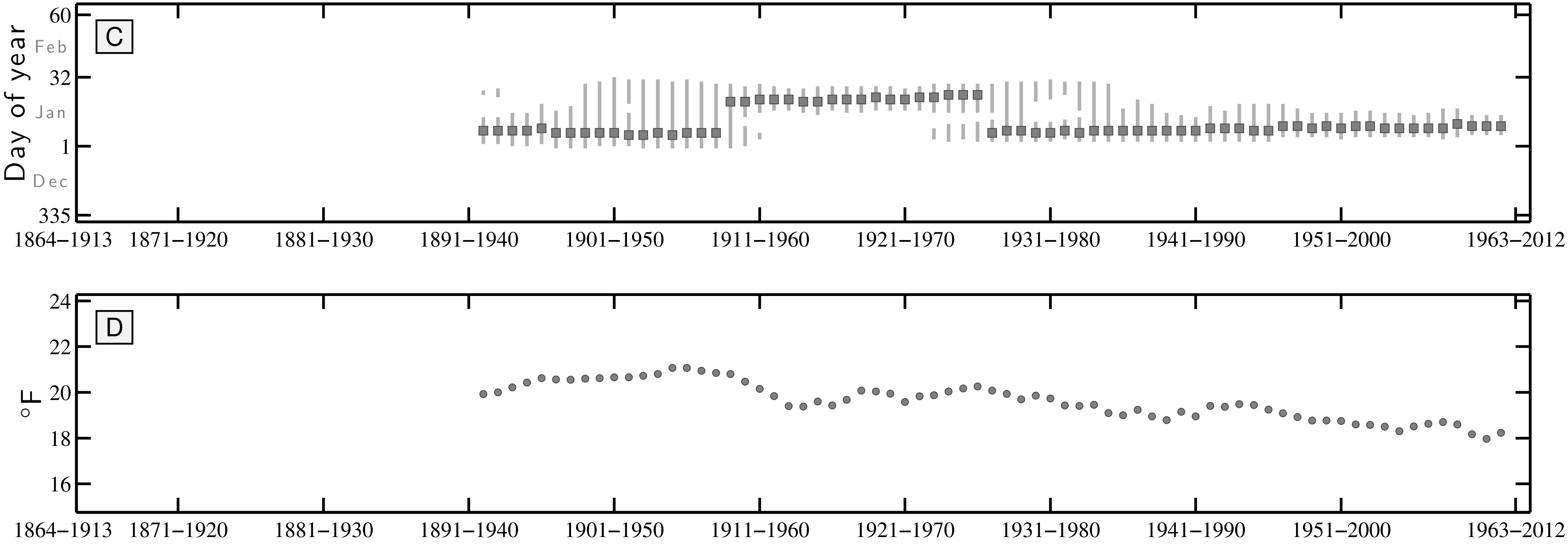

50 year Summer Teletherm dynamics for ELLSWORTH, KS:

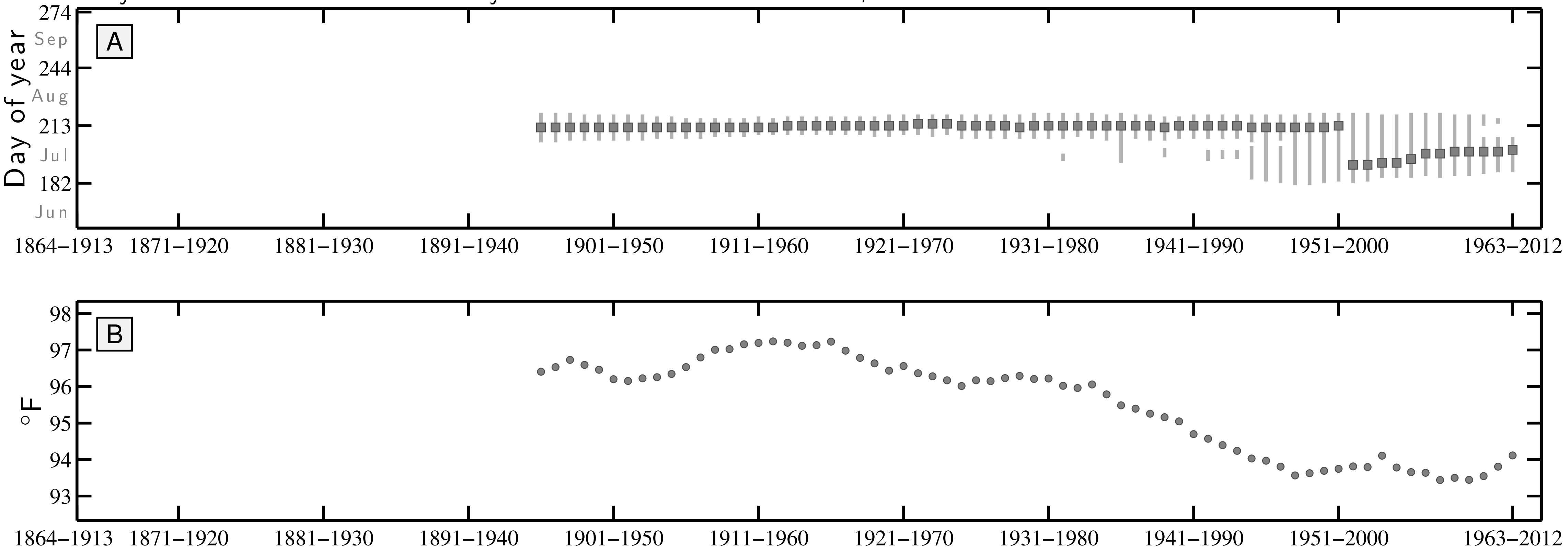

50 year Winter Teletherm dynamics for ELLSWORTH, KS:

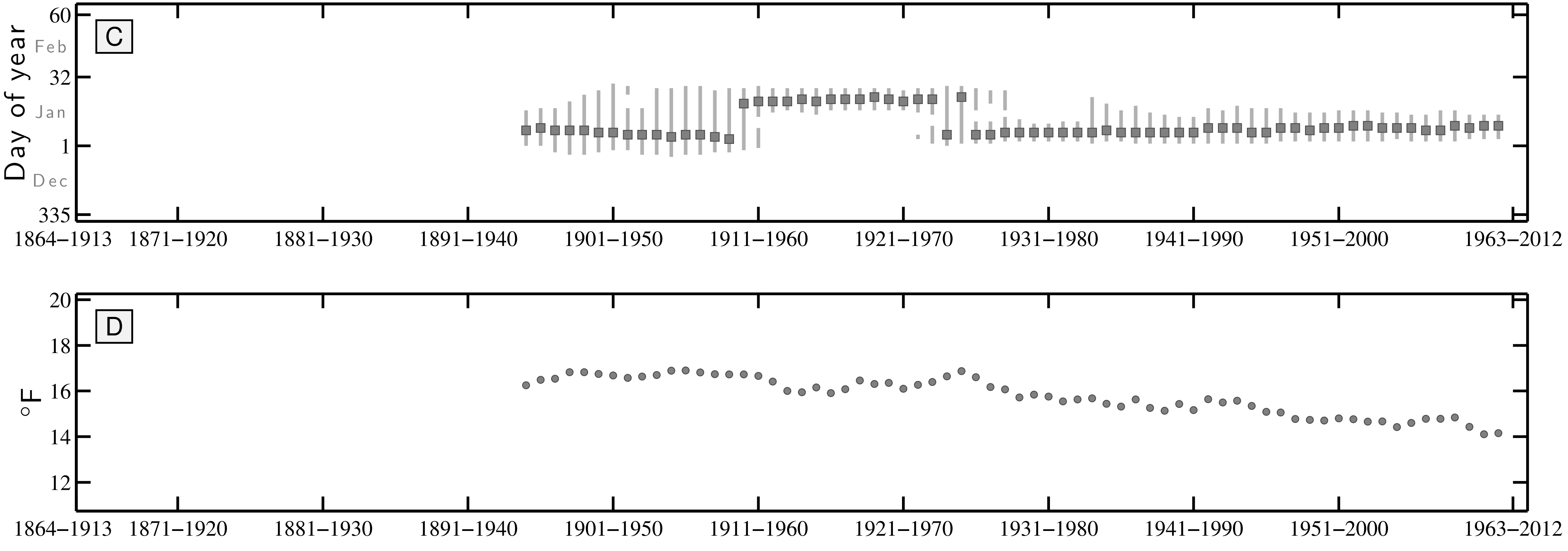

50 year Summer Teletherm dynamics for FT SCOTT, KS:

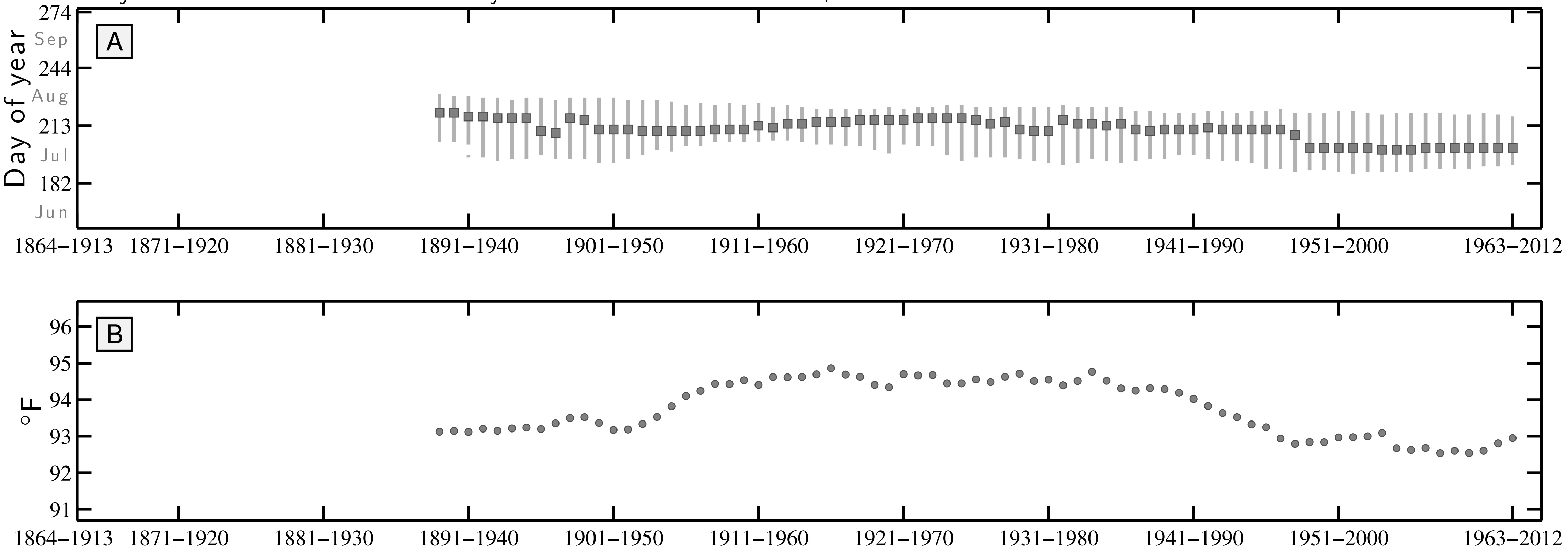

50 year Winter Teletherm dynamics for FT SCOTT, KS:

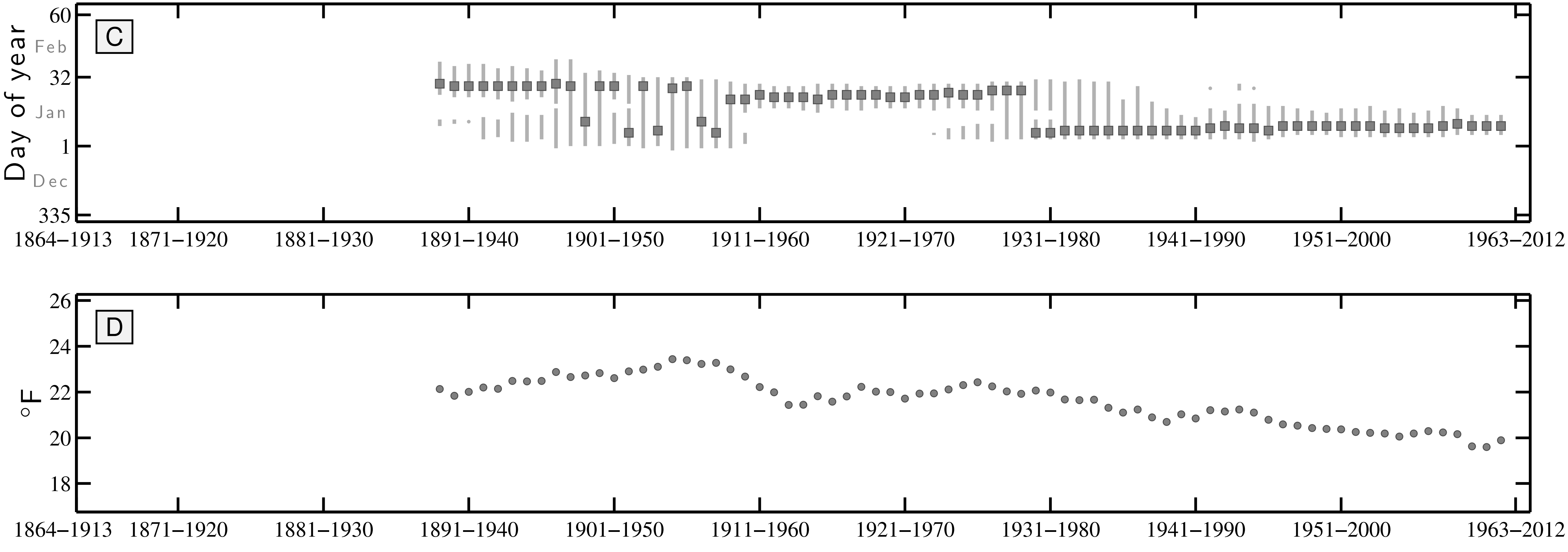

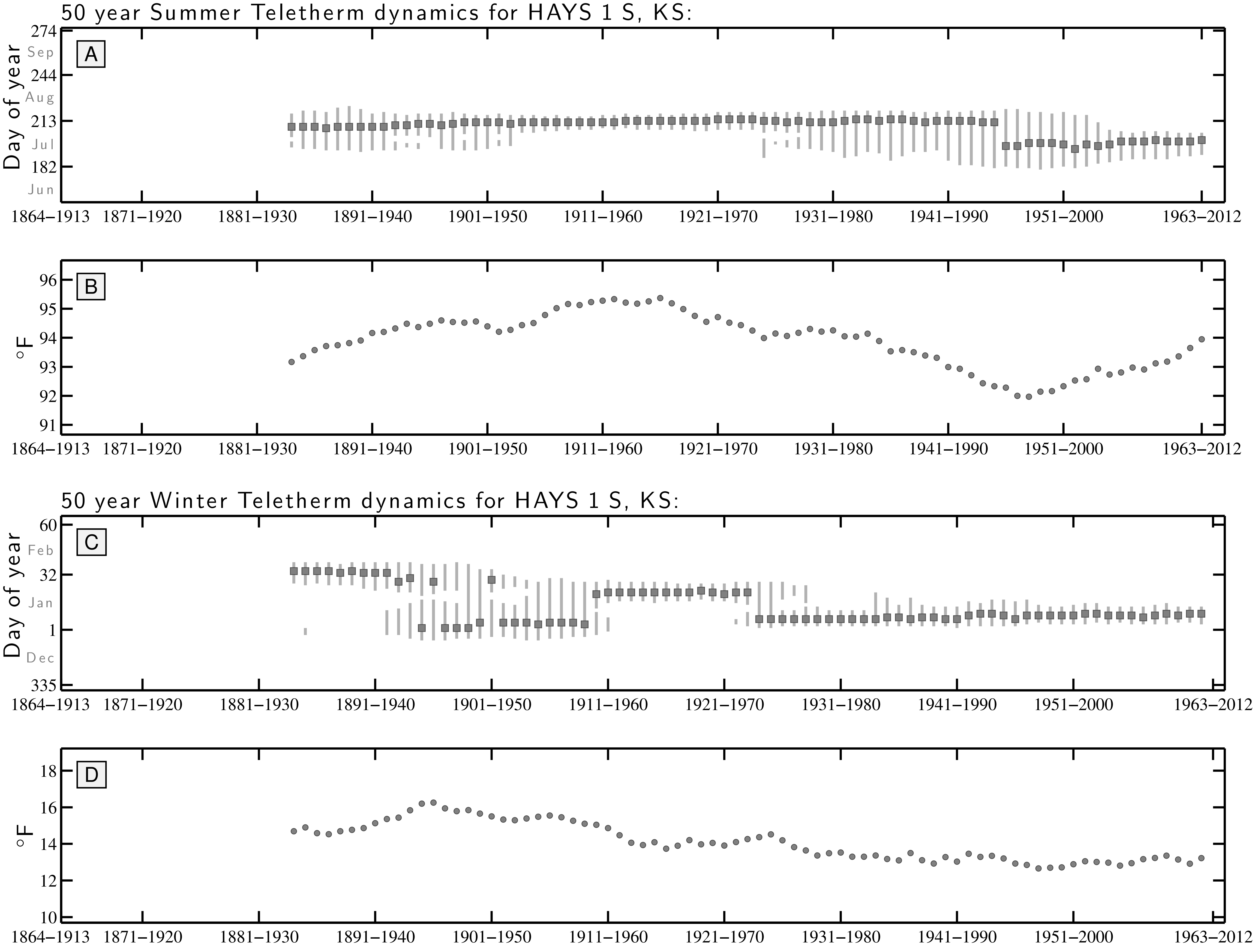

50 year Summer Teletherm dynamics for HAYS 1 S, KS:
A
B
50 year Winter Teletherm dynamics for HAYS 1 S, KS:
C
D
Day of year
°F
Jun
Jul
Aug
Sep
Dec
Jan
Feb
274
244
213
182
96
95
94
93
92
91
60
32
1
335
18
16
14
12
10
1864–1913
1871–1920
1881–1930
1891–1940
1901–1950
1911–1960
1921–1970
1931–1980
1941–1990
1951–2000
1963–2012

50 year Summer Teletherm dynamics for HORTON, KS:

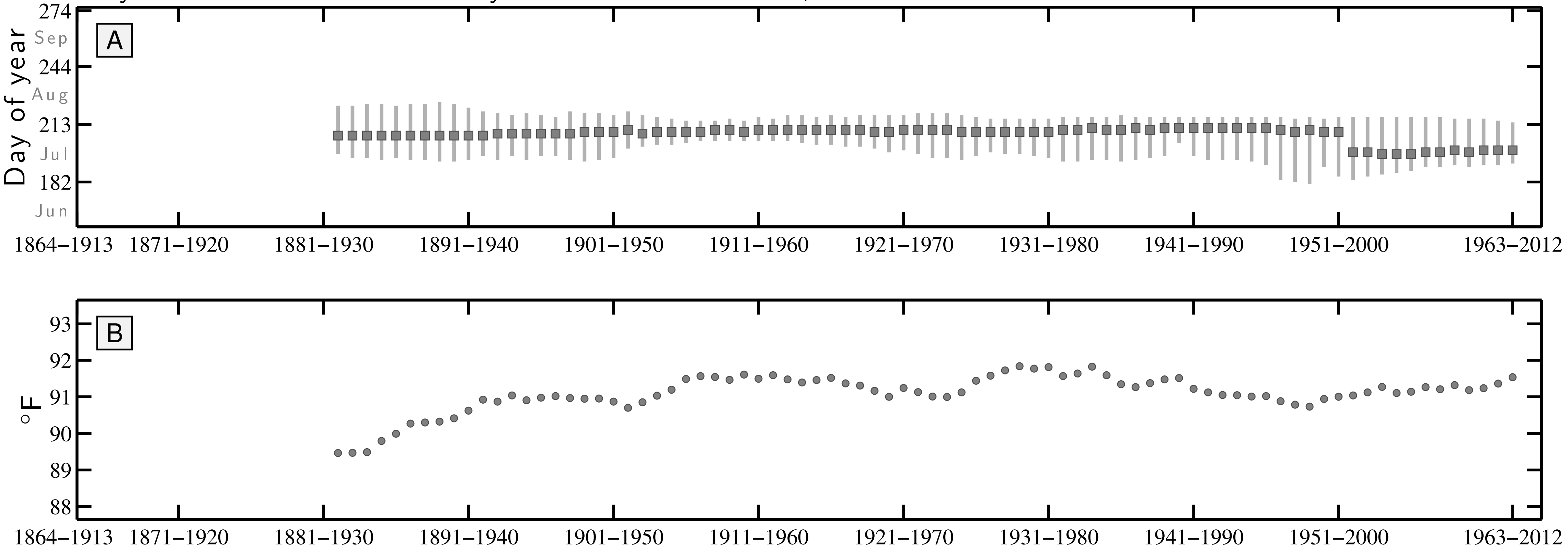

50 year Winter Teletherm dynamics for HORTON, KS:

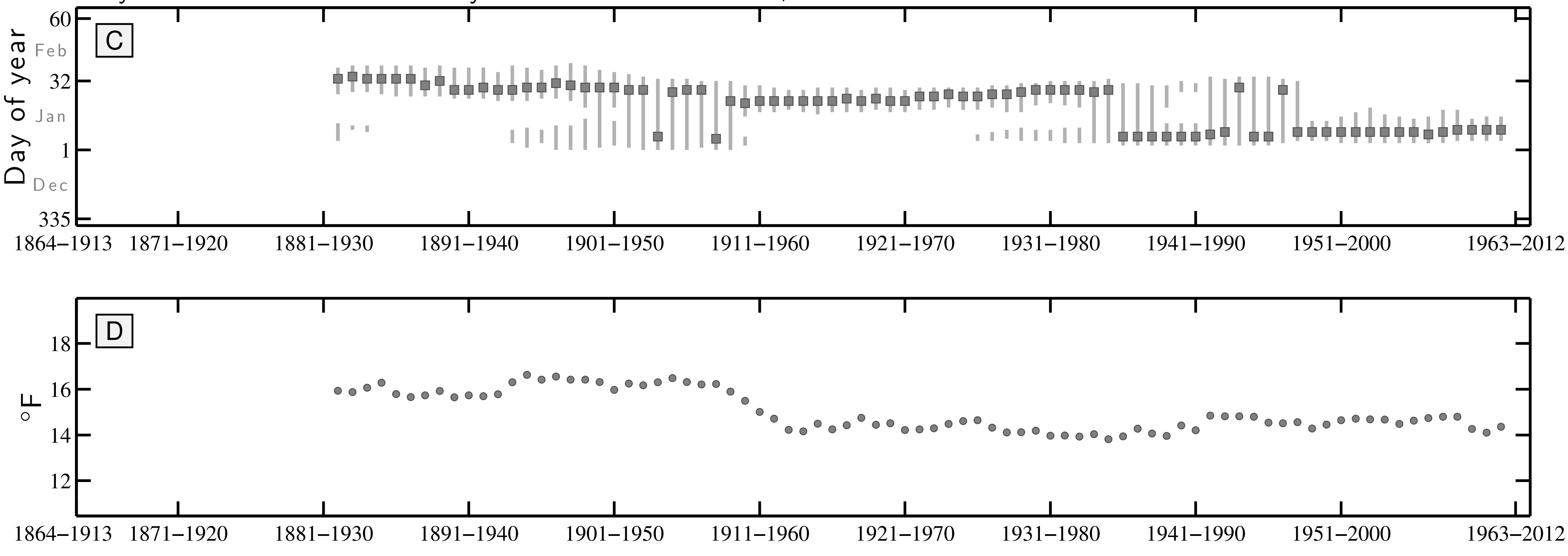

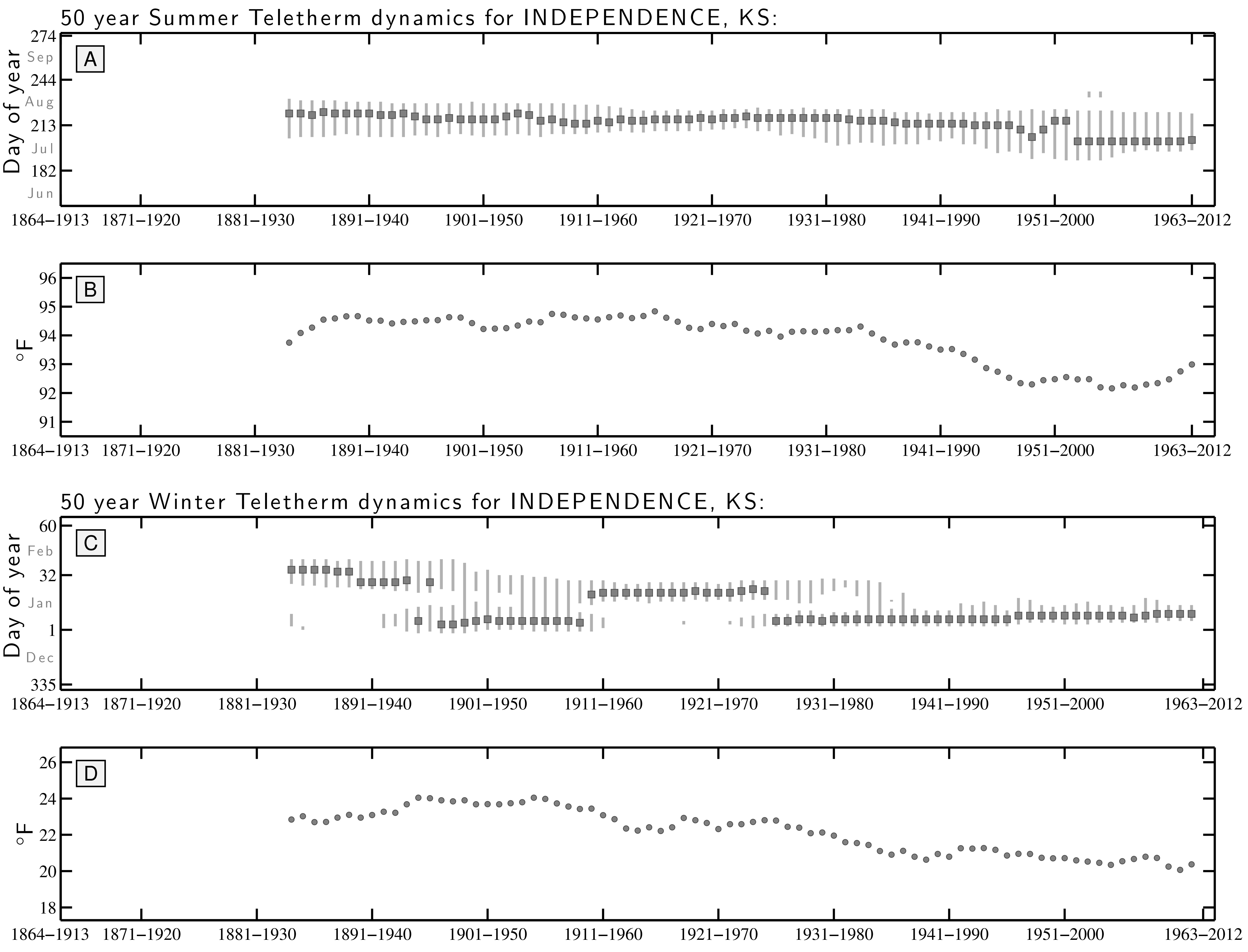

50 year Summer Teletherm dynamics for INDEPENDENCE, KS:
A
Day of year
274
Sep
244
Aug
213
Jul
182
Jun
1864–1913
1871–1920
1881–1930
1891–1940
1901–1950
1911–1960
1921–1970
1931–1980
1941–1990
1951–2000
1963–2012
B
°F
96
95
94
93
92
91
50 year Winter Teletherm dynamics for INDEPENDENCE, KS:
C
Day of year
60
Feb
32
Jan
1
Dec
335
D
°F
26
24
22
20
18

50 year Summer Teletherm dynamics for LAKIN, KS:

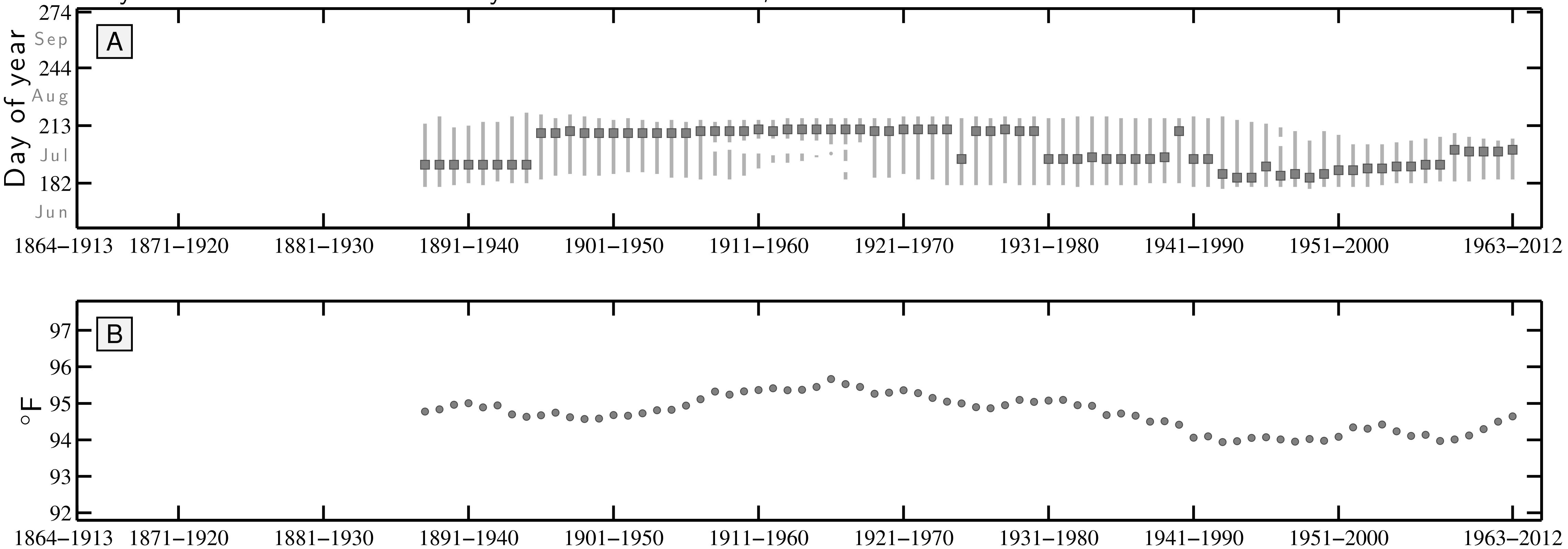

50 year Winter Teletherm dynamics for LAKIN, KS:

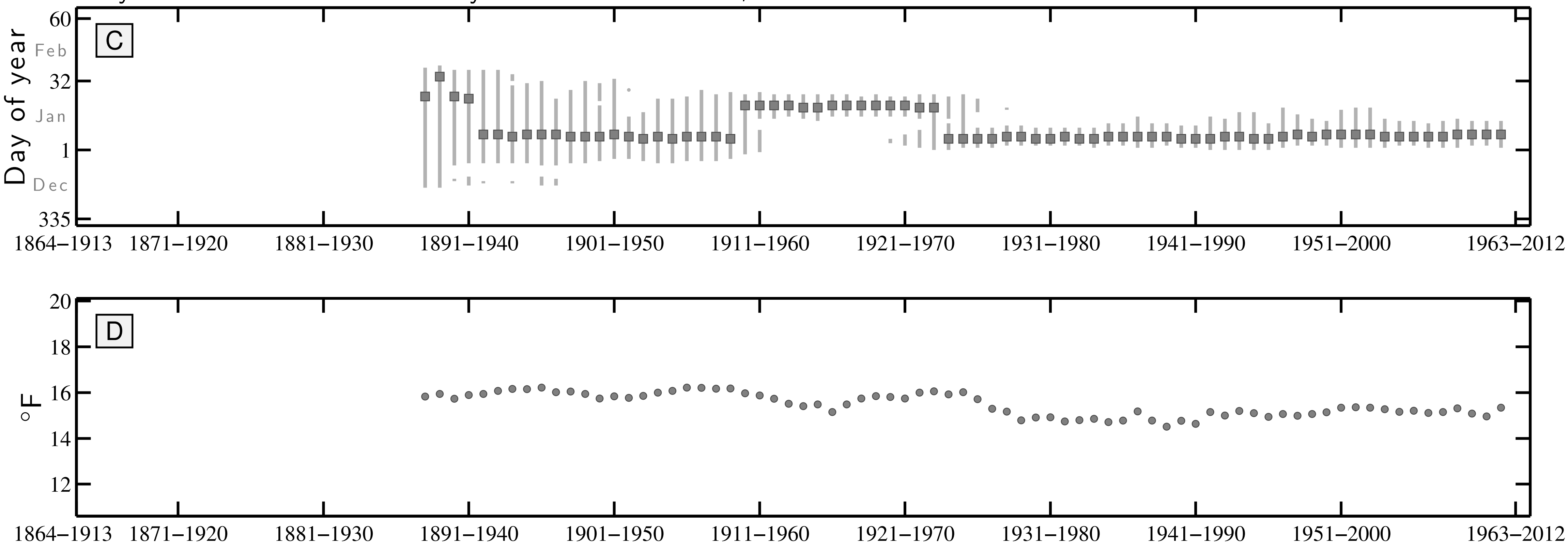

50 year Summer Teletherm dynamics for LARNED, KS:

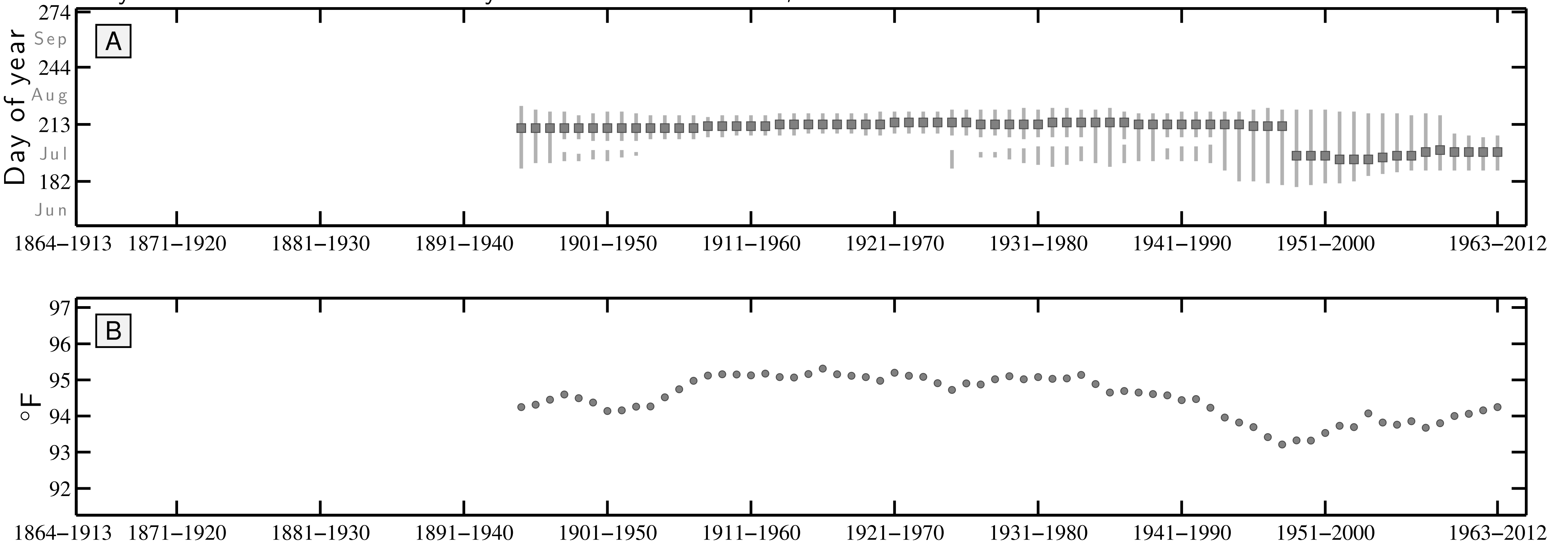

50 year Winter Teletherm dynamics for LARNED, KS:

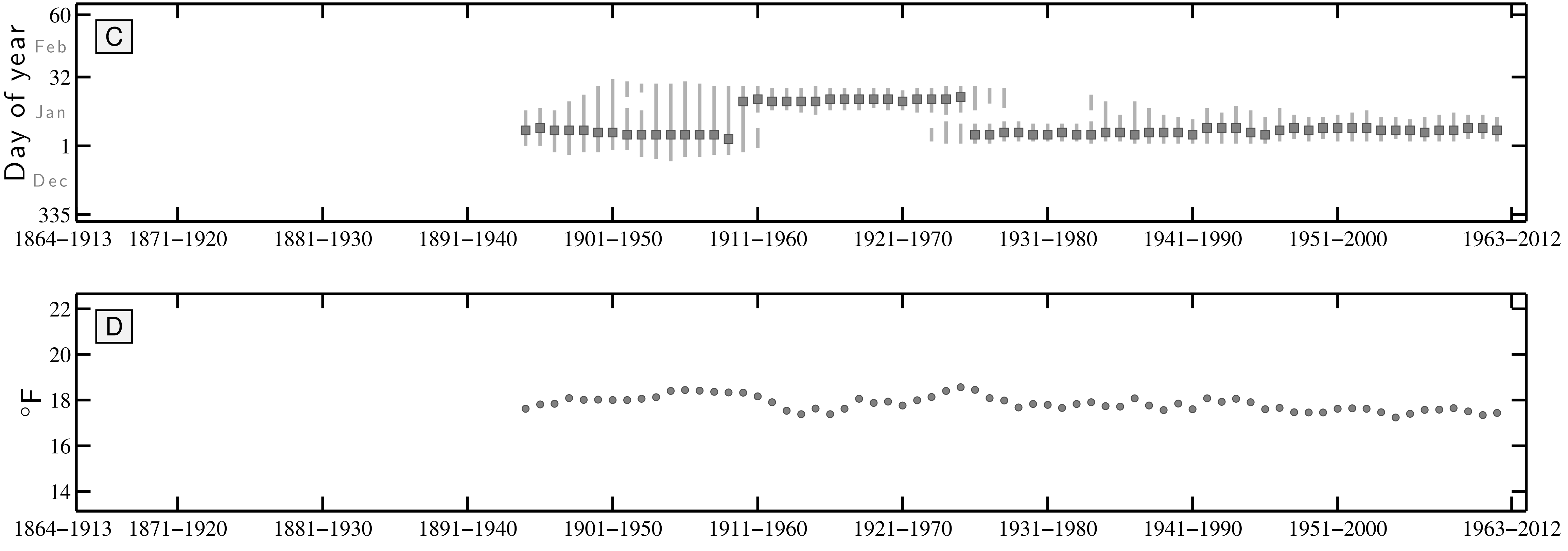

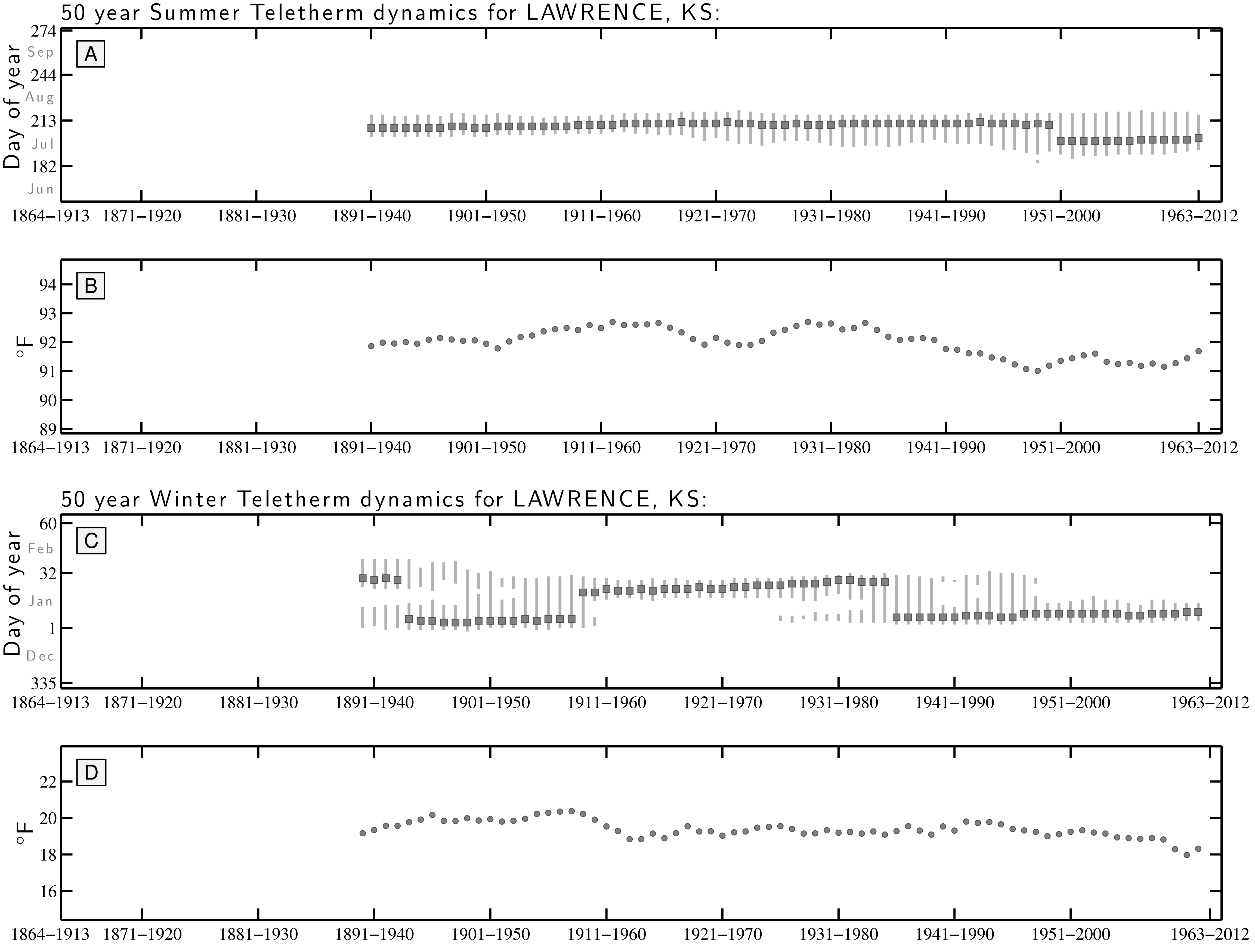
50 year Summer Teletherm dynamics for LAWRENCE, KS:
A
Day of year
274
Sep
244
Aug
213
Jul
182
Jun
B
°F
94
93
92
91
90
89
1864–1913
1871–1920
1881–1930
1891–1940
1901–1950
1911–1960
1921–1970
1931–1980
1941–1990
1951–2000
1963–2012
50 year Winter Teletherm dynamics for LAWRENCE, KS:
C
Day of year
60
Feb
32
Jan
1
Dec
335
D
°F
22
20
18
16

50 year Summer Teletherm dynamics for LEAVENWORTH, KS:

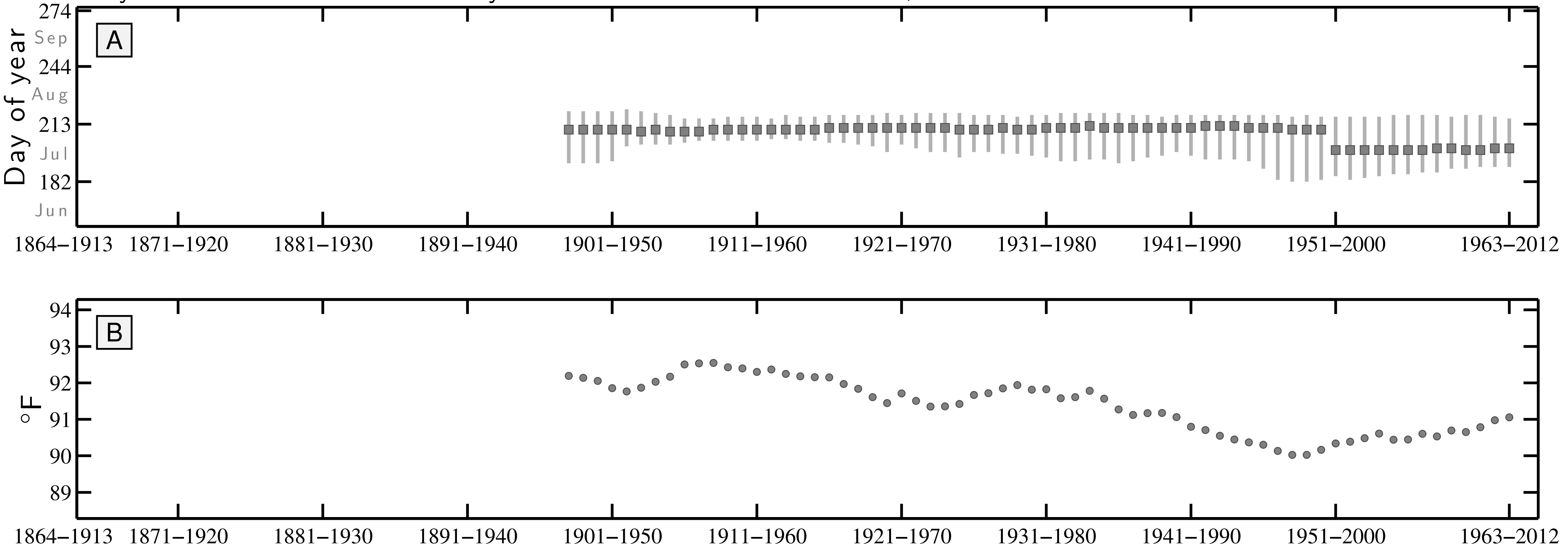

50 year Winter Teletherm dynamics for LEAVENWORTH, KS:

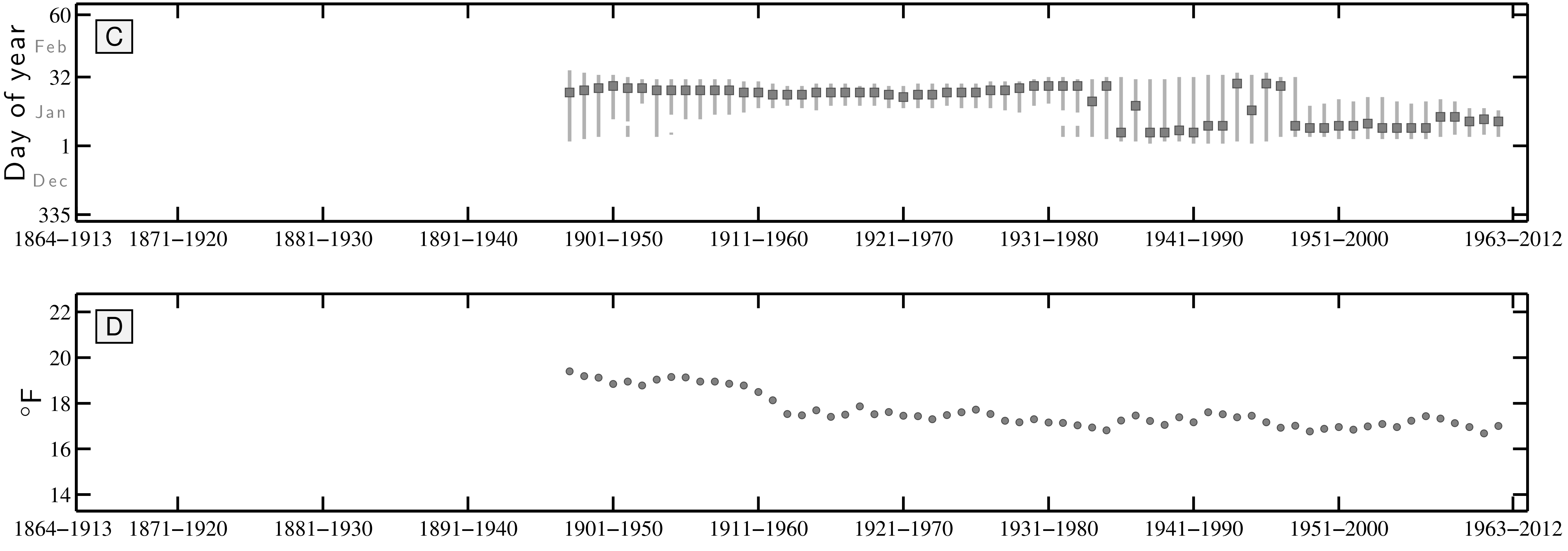

50 year Summer Teletherm dynamics for LIBERAL, KS:

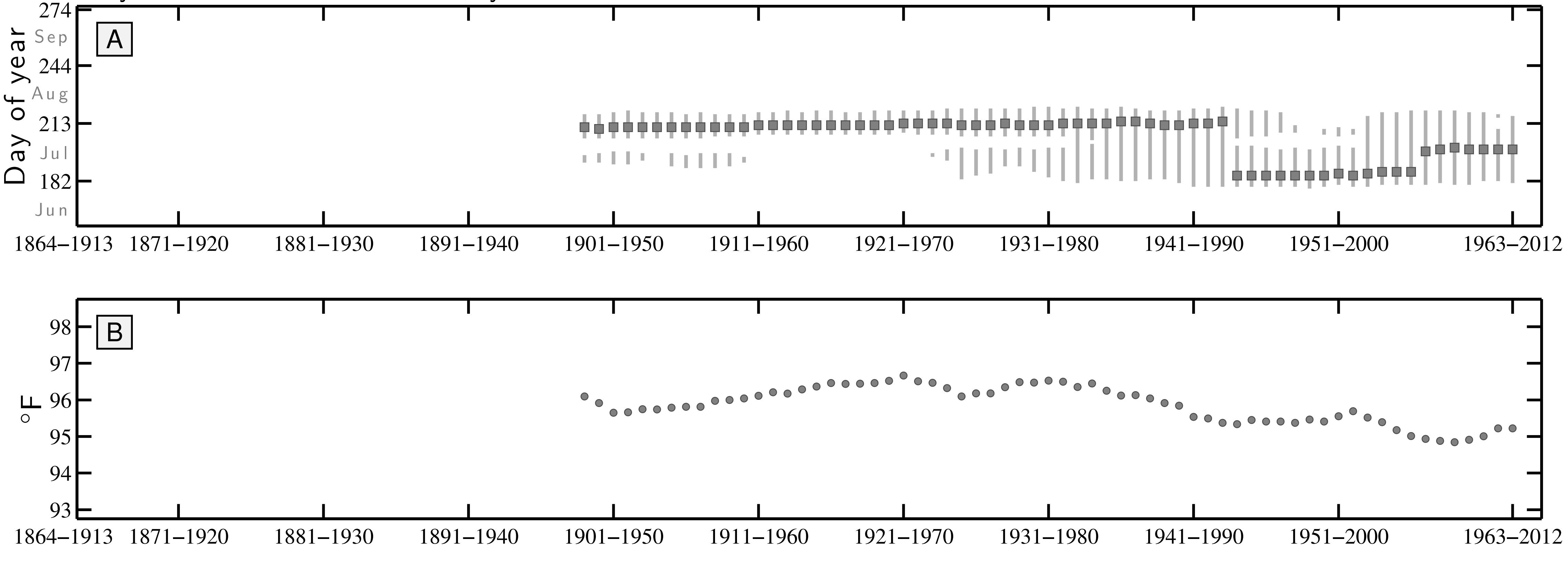

50 year Winter Teletherm dynamics for LIBERAL, KS:

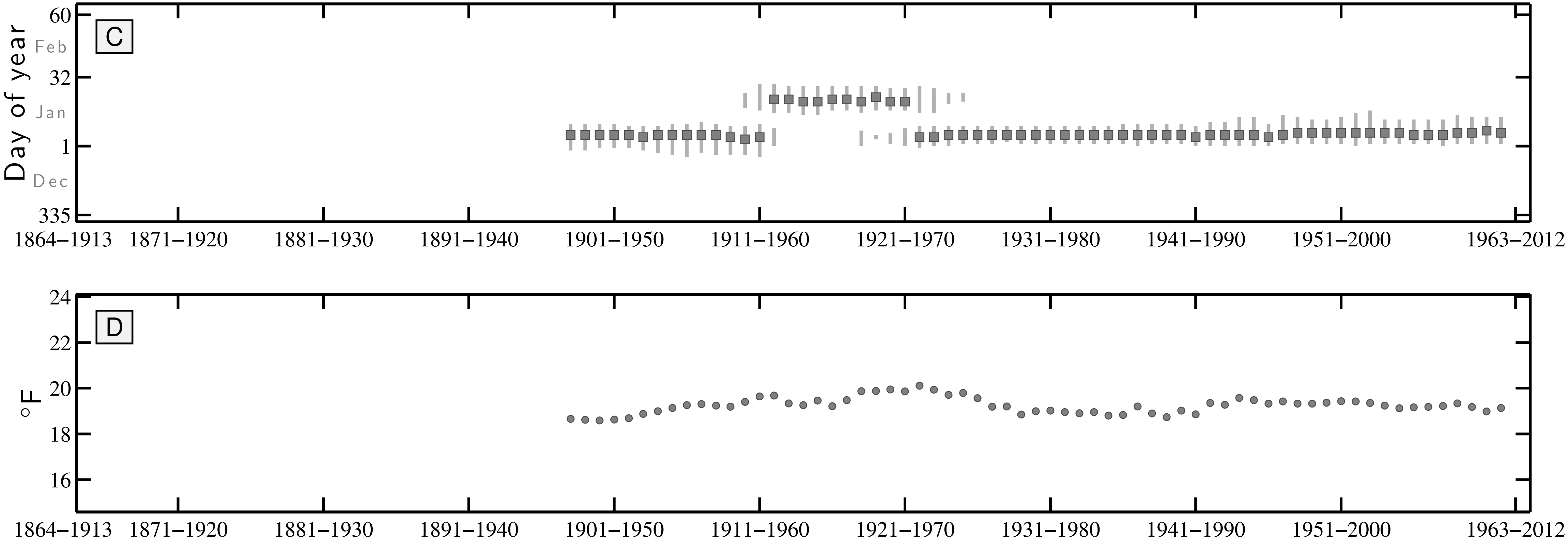

50 year Summer Teletherm dynamics for MANHATTAN, KS:

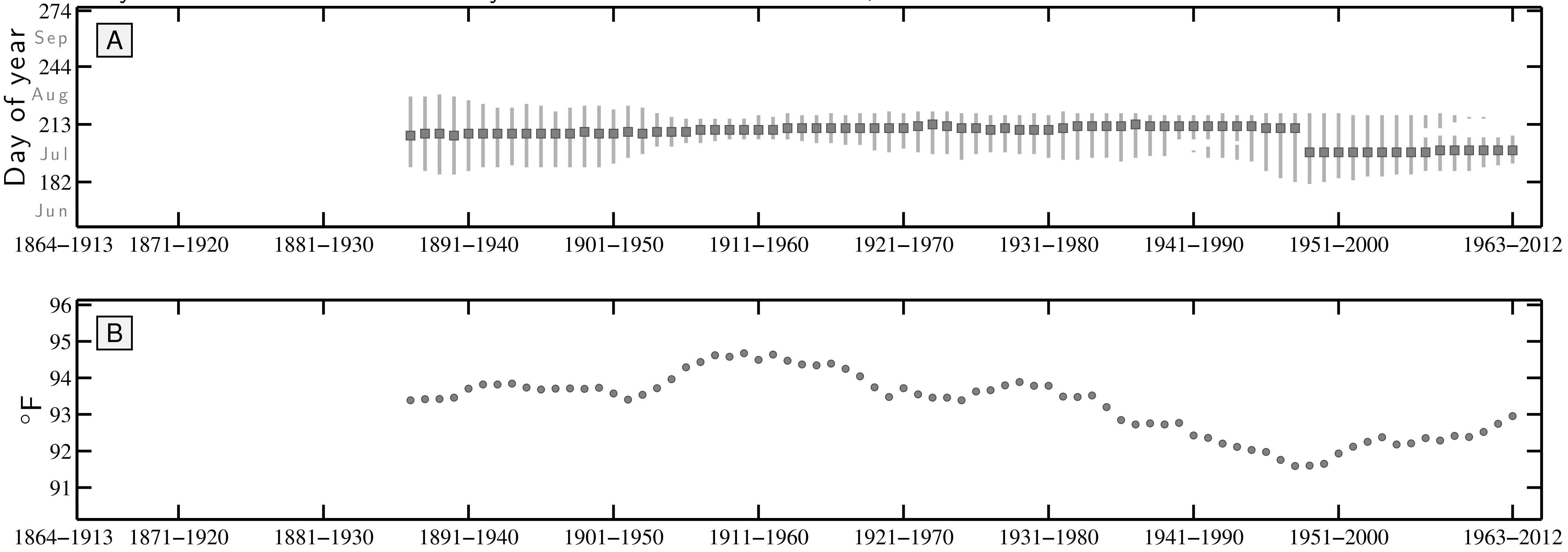

50 year Winter Teletherm dynamics for MANHATTAN, KS:

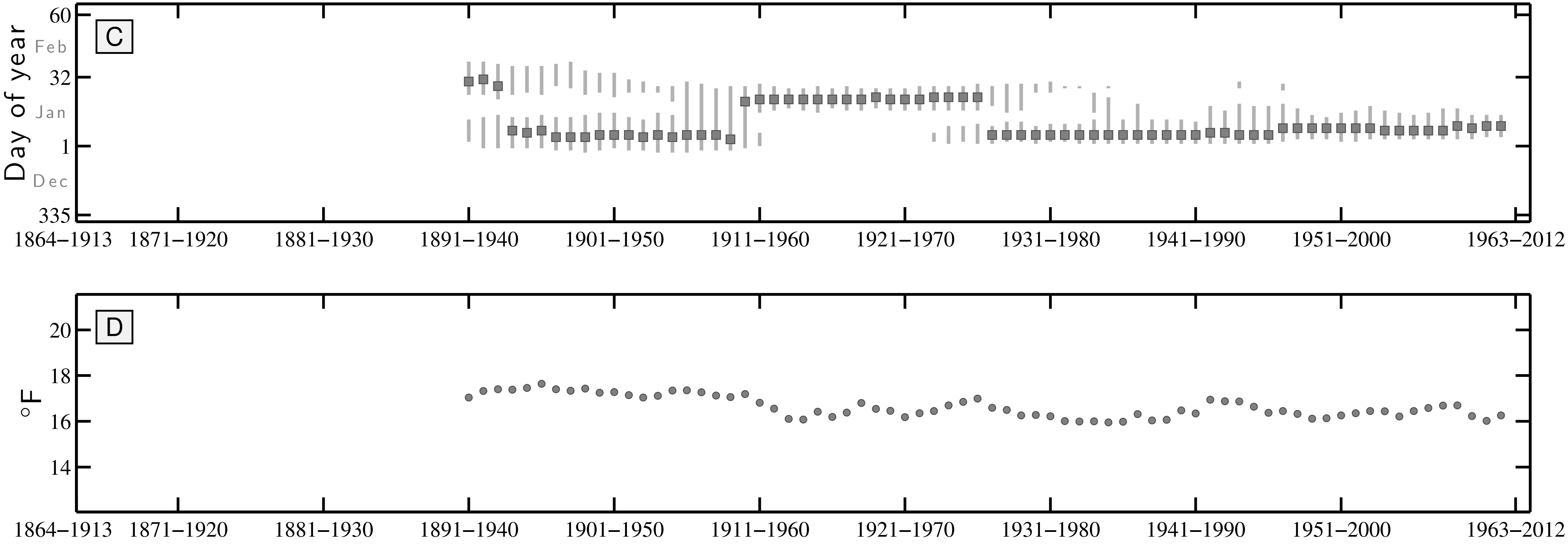

50 year Summer Teletherm dynamics for MCPHERSON, KS:

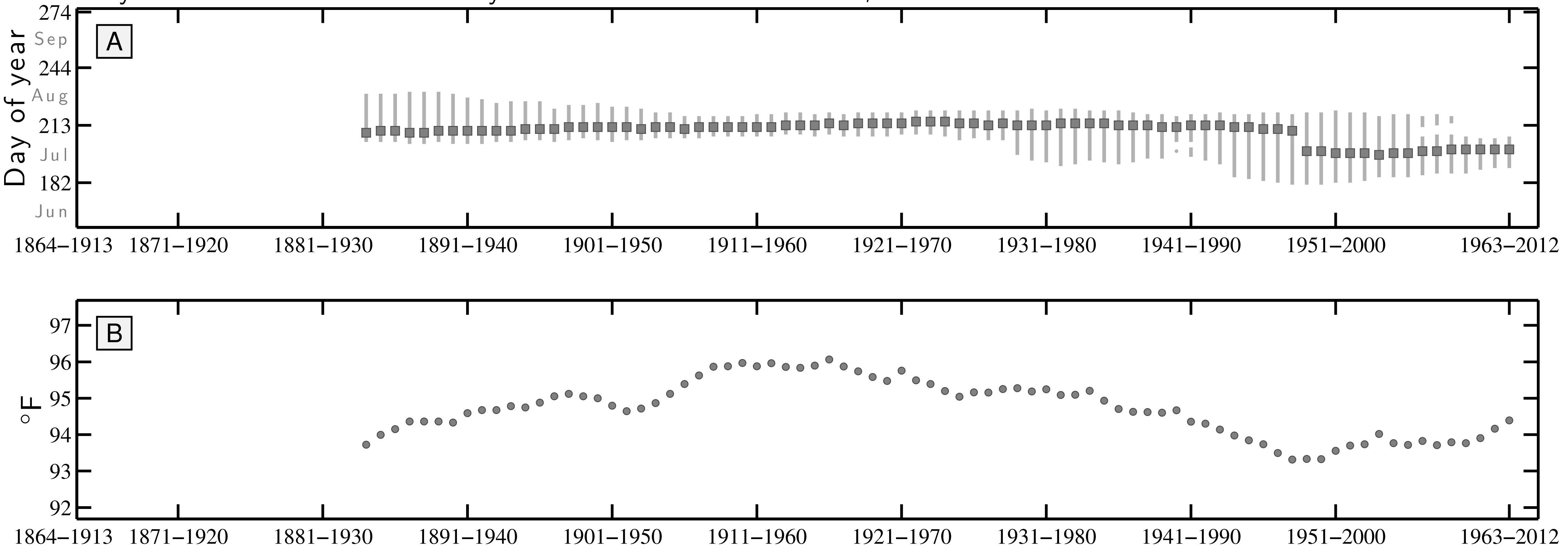

50 year Winter Teletherm dynamics for MCPHERSON, KS:

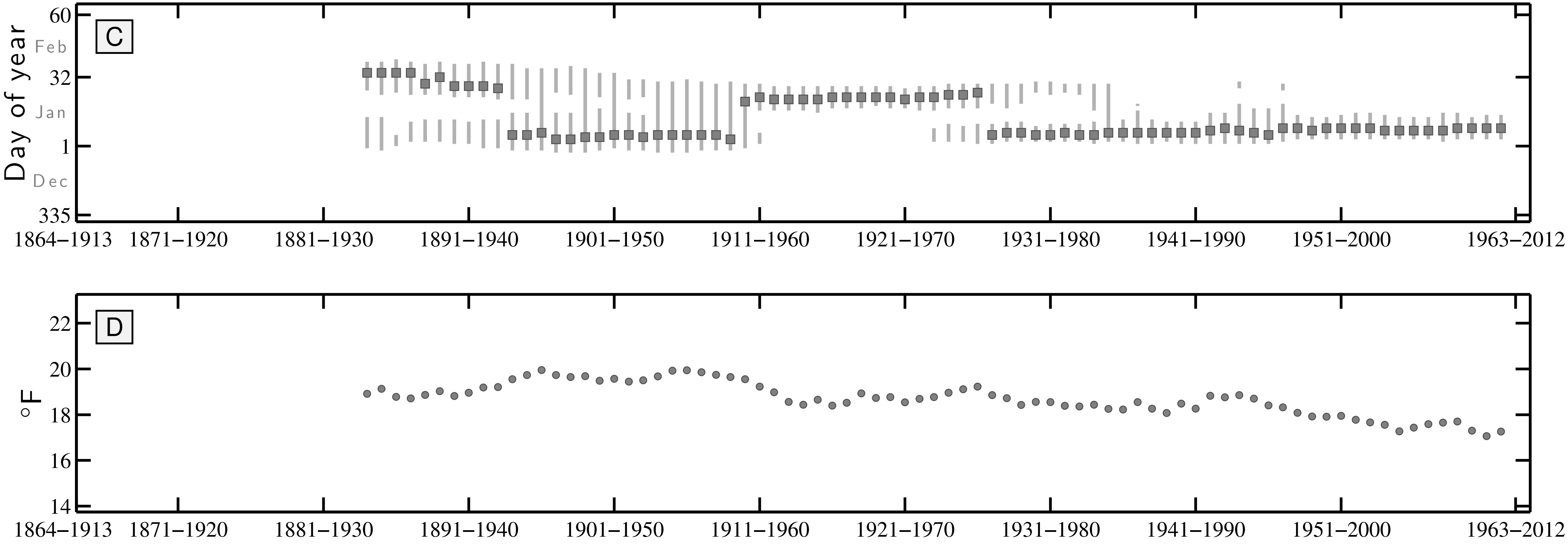

50 year Summer Teletherm dynamics for MEDICINE LODGE, KS:

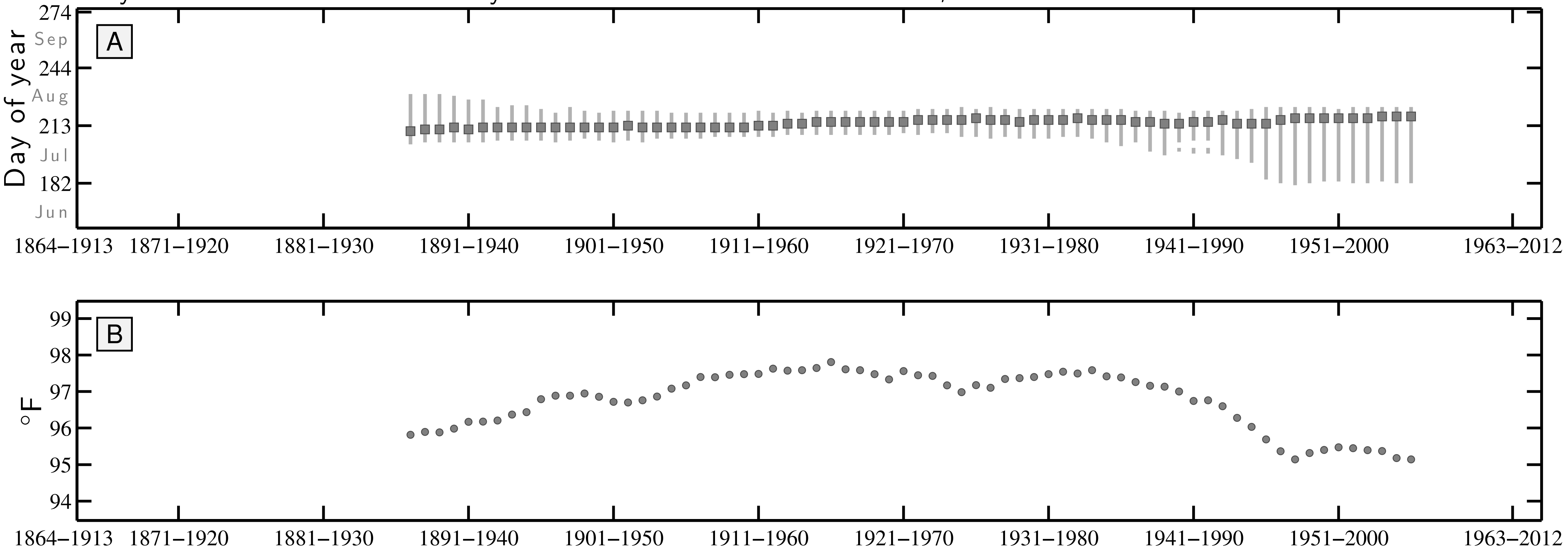

50 year Winter Teletherm dynamics for MEDICINE LODGE, KS:

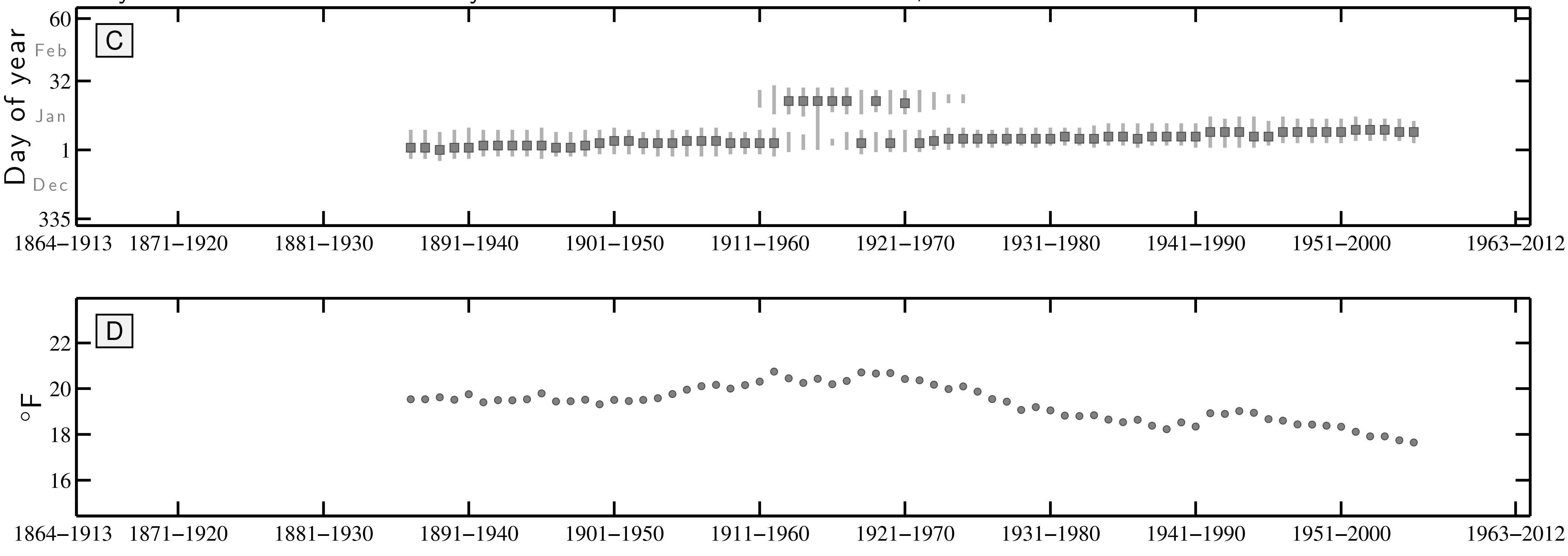

50 year Summer Teletherm dynamics for MINNEAPOLIS, KS:

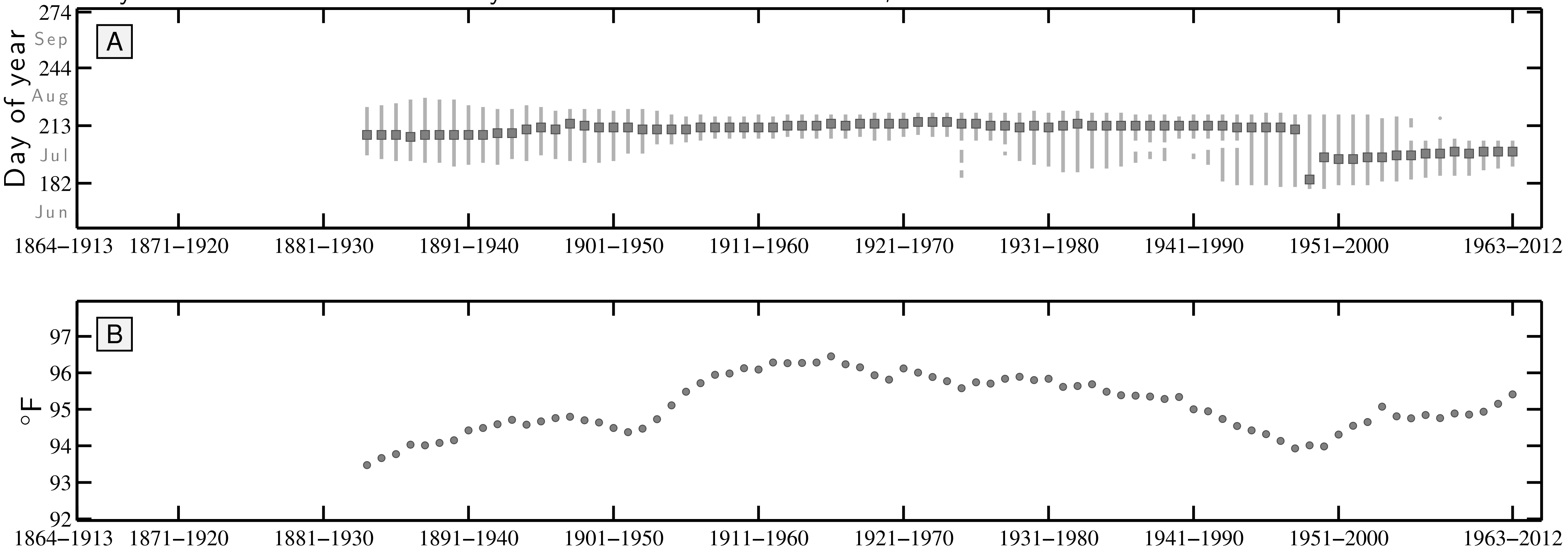

50 year Winter Teletherm dynamics for MINNEAPOLIS, KS:

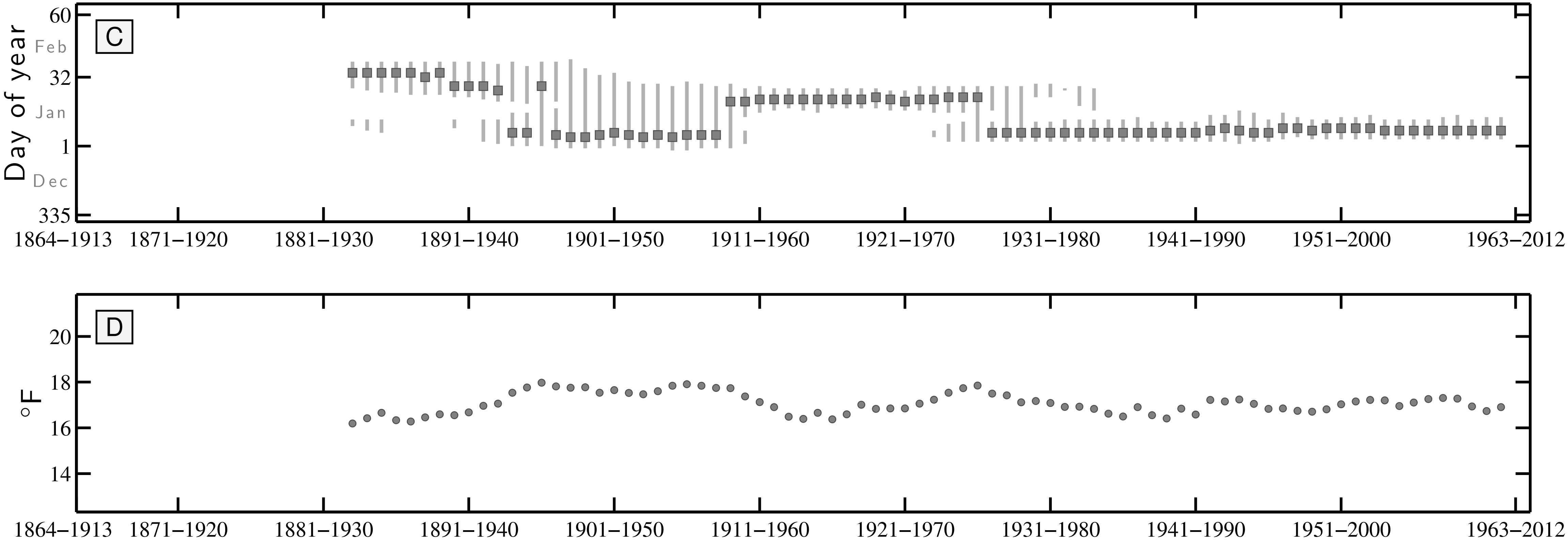

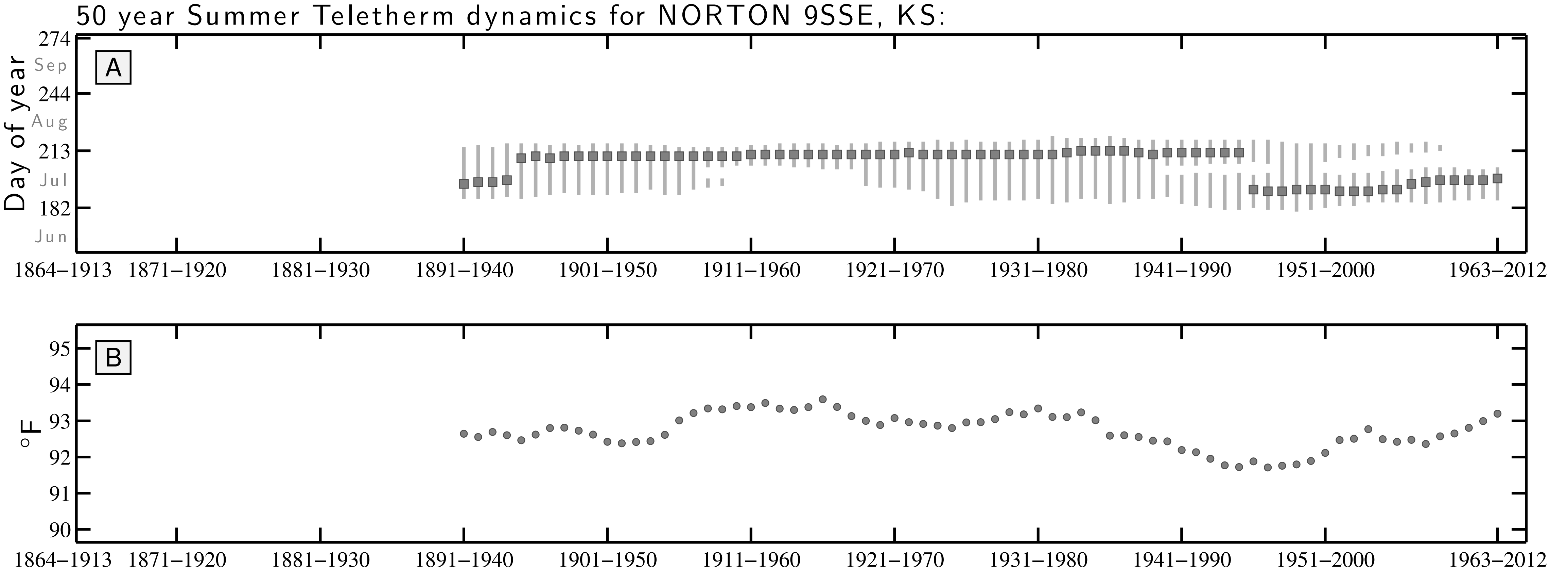
50 year Summer Teletherm dynamics for NORTON 9SSE, KS:
A
B
Day of year
°F
Sep
Aug
Jul
Jun
274
244
213
182
95
94
93
92
91
90
1864–1913
1871–1920
1881–1930
1891–1940
1901–1950
1911–1960
1921–1970
1931–1980
1941–1990
1951–2000
1963–2012

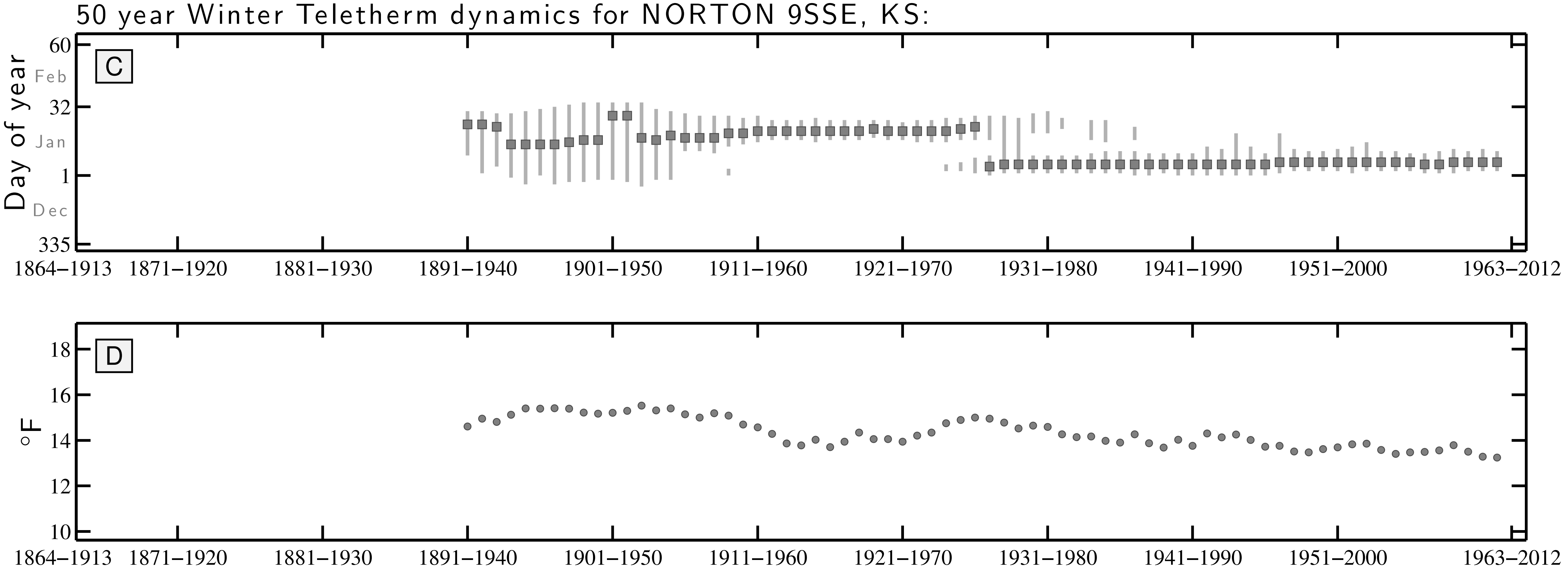
50 year Winter Teletherm dynamics for NORTON 9SSE, KS:
C
D
Day of year
°F
Feb
Jan
Dec
60
32
1
335
18
16
14
12
10
1864–1913
1871–1920
1881–1930
1891–1940
1901–1950
1911–1960
1921–1970
1931–1980
1941–1990
1951–2000
1963–2012

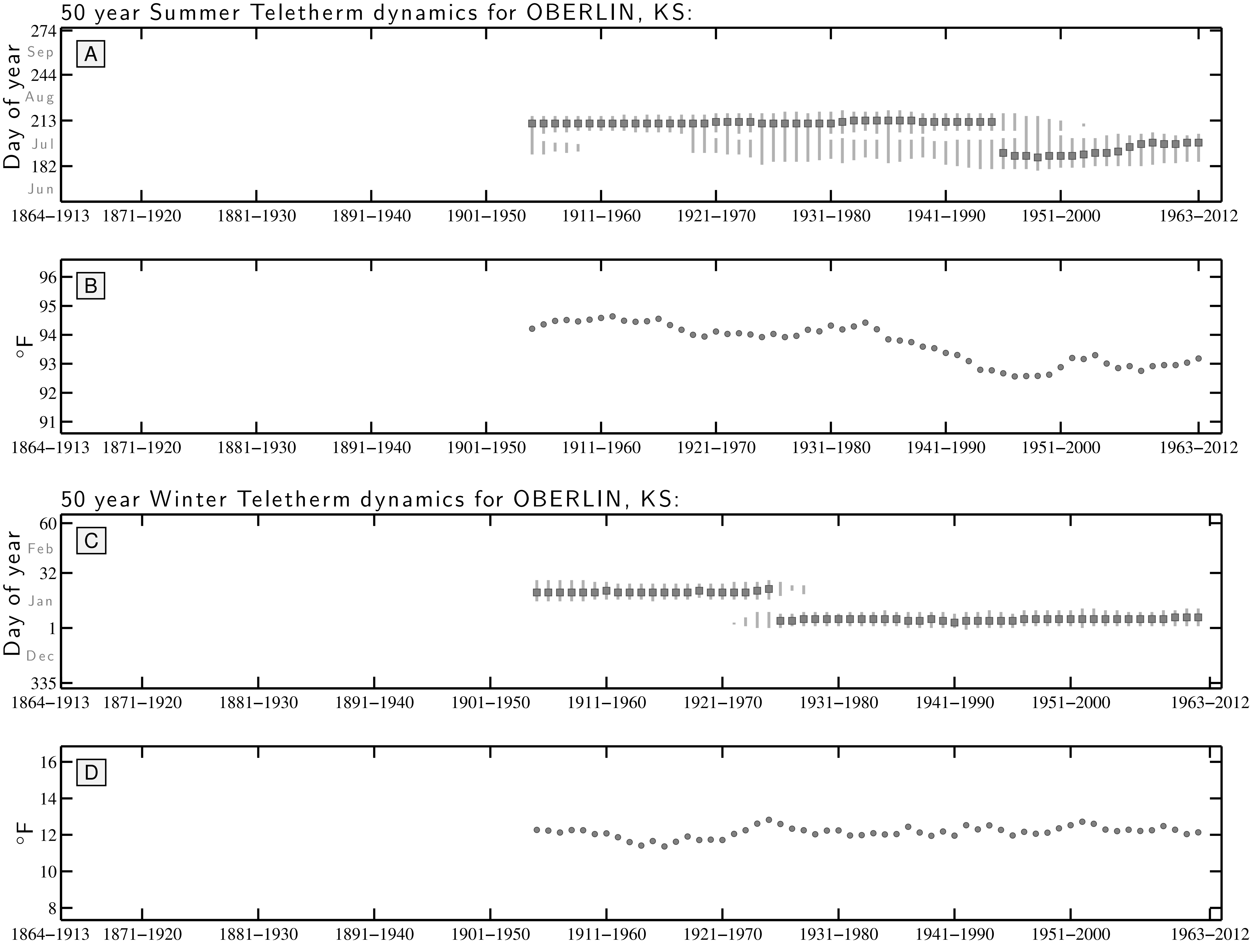

50 year Summer Teletherm dynamics for OBERLIN, KS:
A
Day of year
274
Sep
244
Aug
213
Jul
182
Jun
B
°F
96
95
94
93
92
91
1864–1913
1871–1920
1881–1930
1891–1940
1901–1950
1911–1960
1921–1970
1931–1980
1941–1990
1951–2000
1963–2012
50 year Winter Teletherm dynamics for OBERLIN, KS:
C
Day of year
60
Feb
32
Jan
1
Dec
335
D
°F
16
14
12
10
8

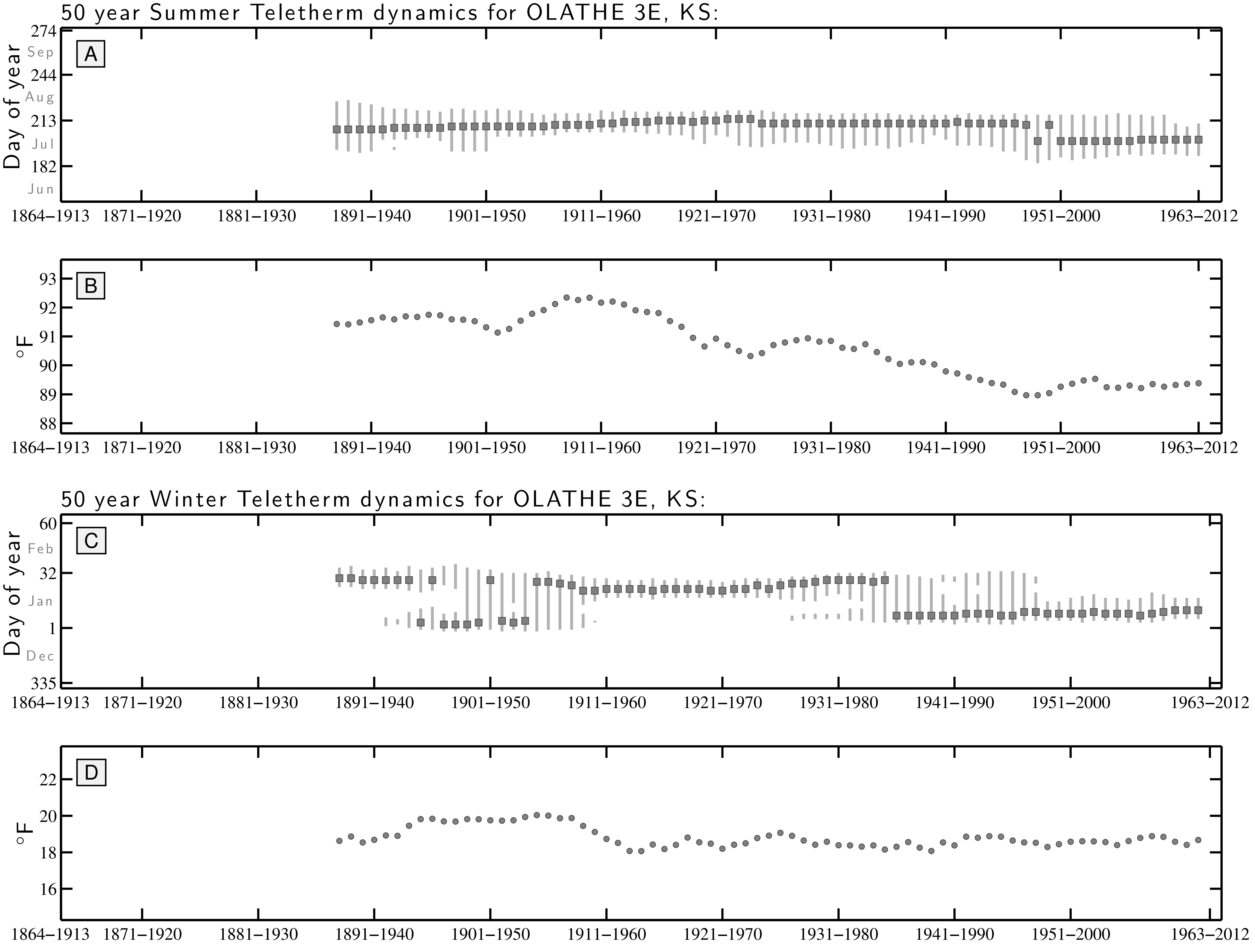
50 year Summer Teletherm dynamics for OLATHE 3E, KS:
A
B
Day of year
°F
Jun
Jul
Aug
Sep
182
213
244
274
88
89
90
91
92
93
50 year Winter Teletherm dynamics for OLATHE 3E, KS:
C
D
Dec
Jan
Feb
335
1
32
60
16
18
20
22
1864–1913
1871–1920
1881–1930
1891–1940
1901–1950
1911–1960
1921–1970
1931–1980
1941–1990
1951–2000
1963–2012

50 year Summer Teletherm dynamics for OTTAWA, KS:

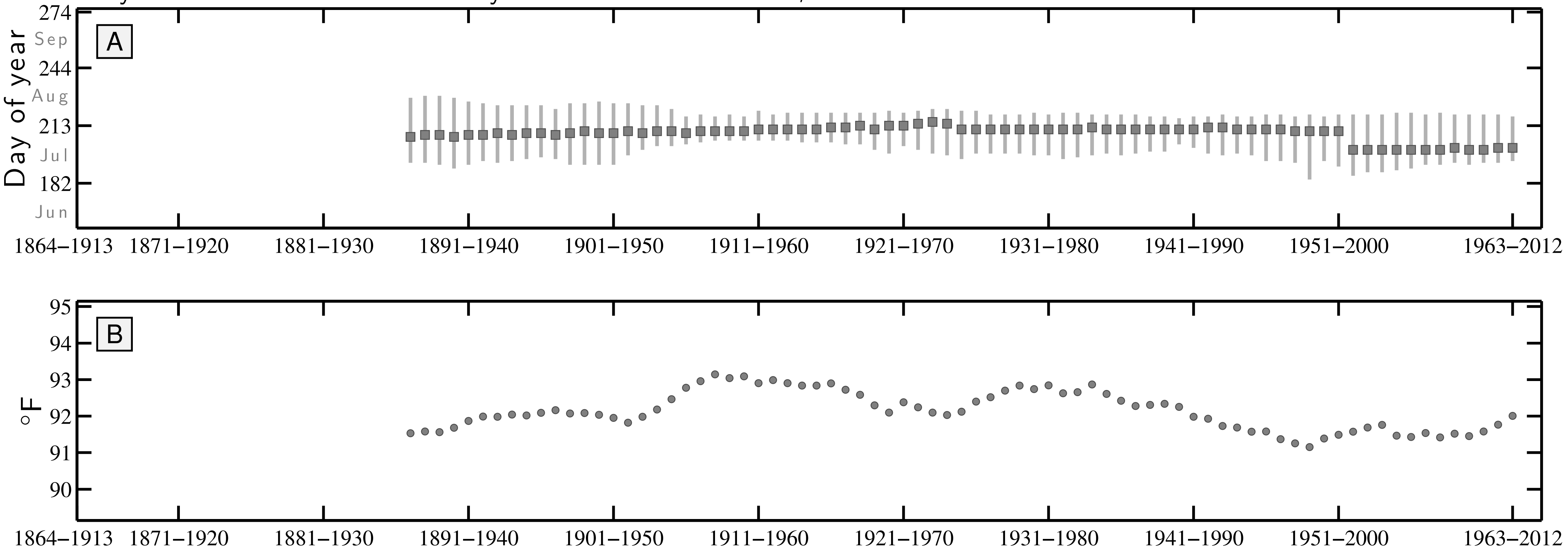

50 year Winter Teletherm dynamics for OTTAWA, KS:

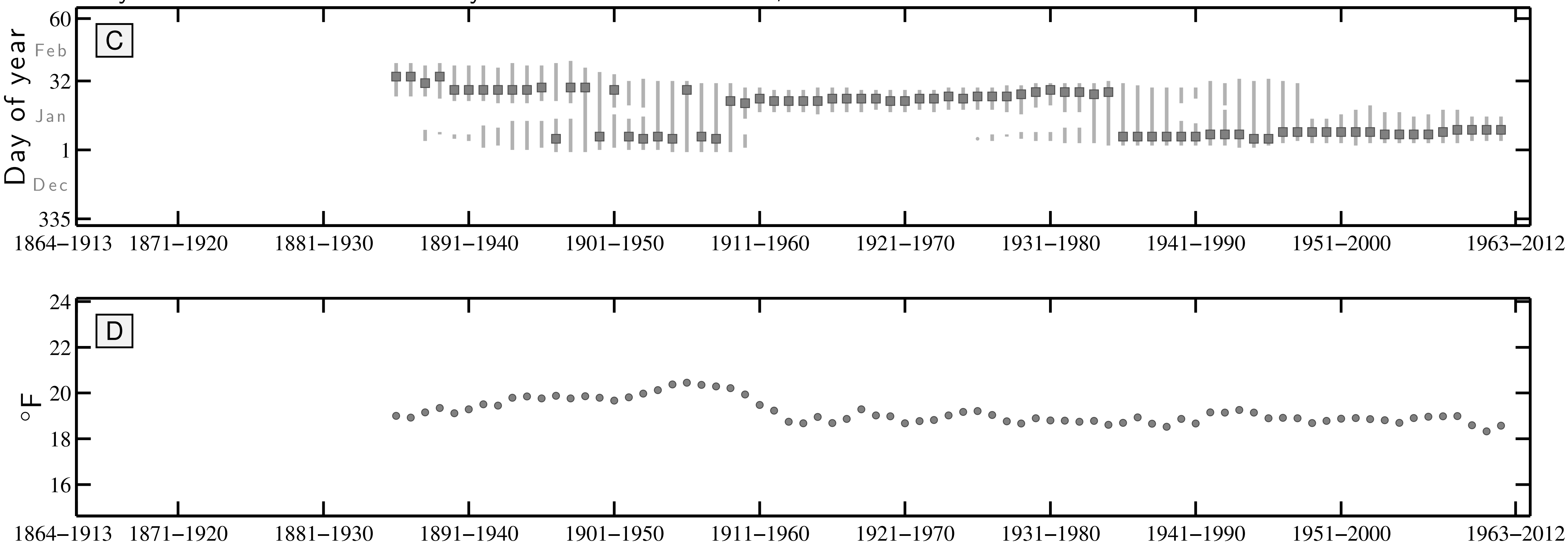

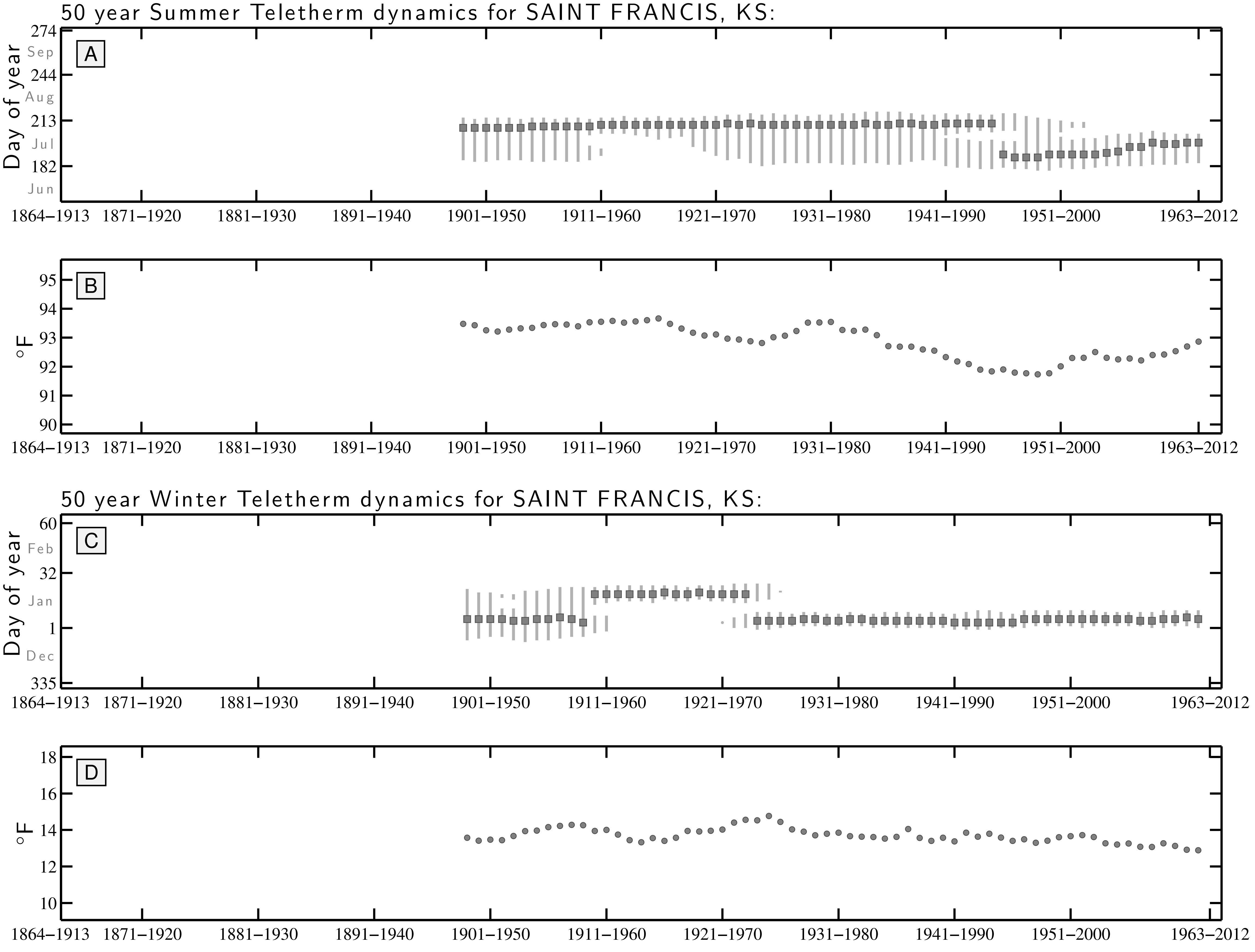

50 year Summer Teletherm dynamics for SAINT FRANCIS, KS:
A
Day of year
274
Sep
244
Aug
213
Jul
182
Jun
1864–1913
1871–1920
1881–1930
1891–1940
1901–1950
1911–1960
1921–1970
1931–1980
1941–1990
1951–2000
1963–2012
B
°F
95
94
93
92
91
90
50 year Winter Teletherm dynamics for SAINT FRANCIS, KS:
C
60
Feb
32
Jan
1
Dec
335
D
18
16
14
12
10

50 year Summer Teletherm dynamics for SCOTT CITY, KS:

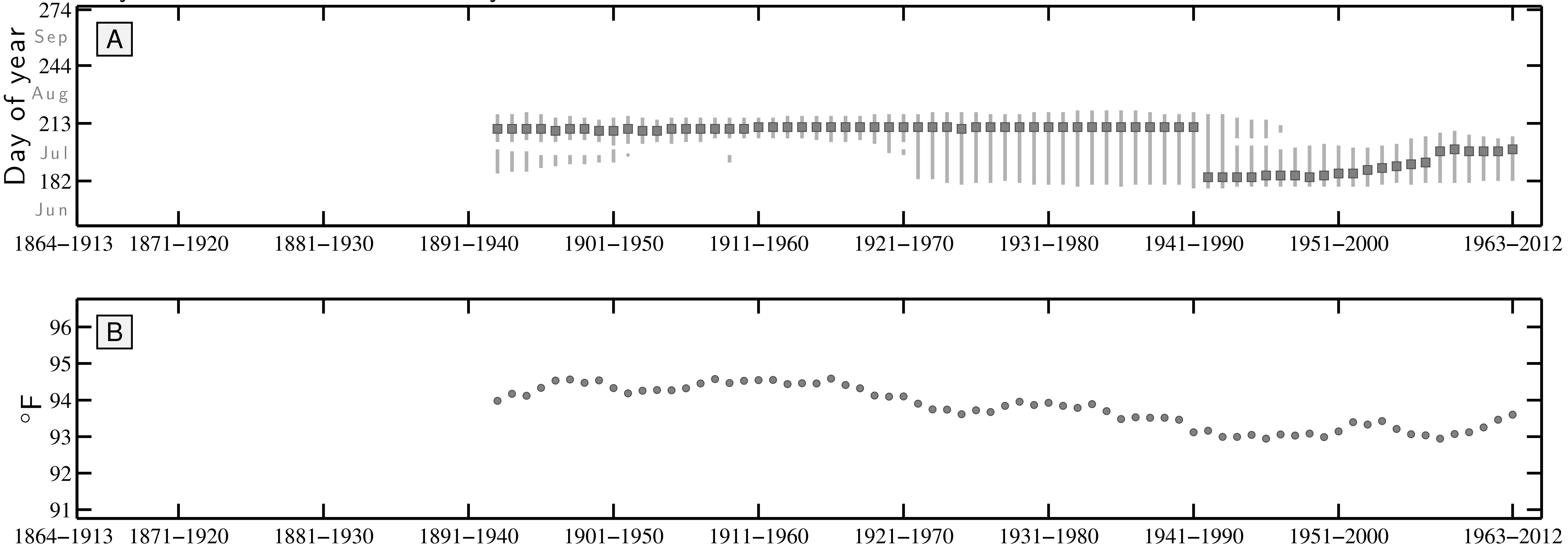

50 year Winter Teletherm dynamics for SCOTT CITY, KS:

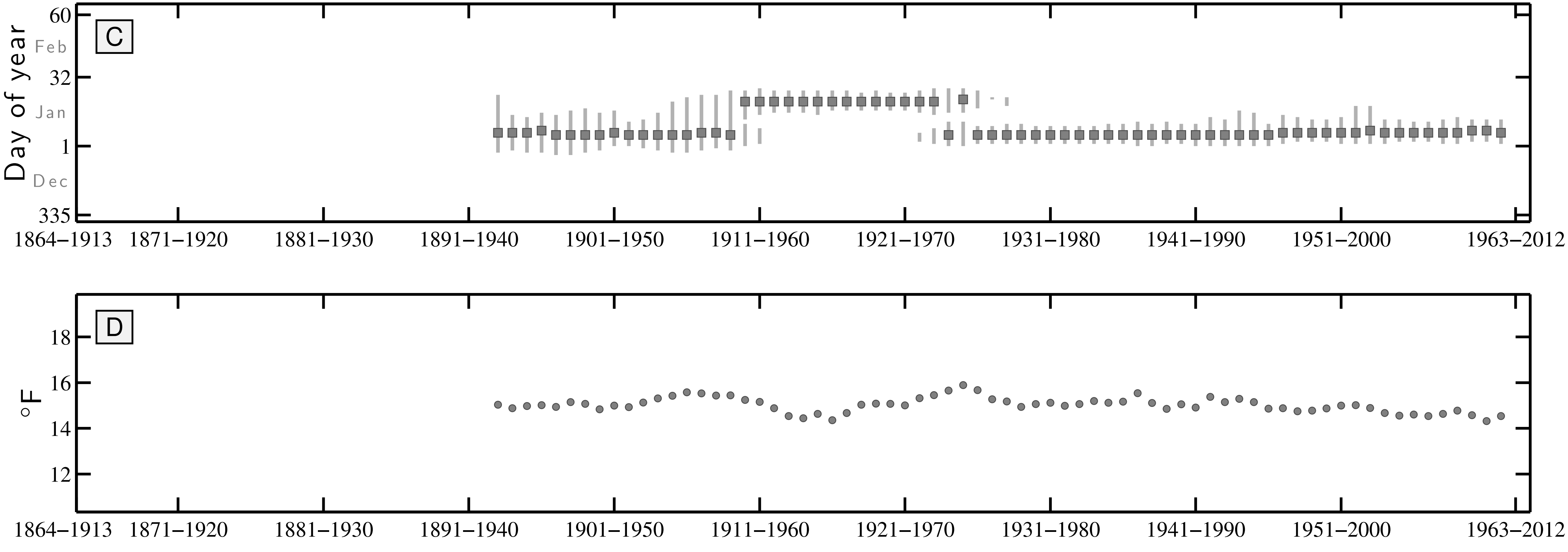

50 year Summer Teletherm dynamics for SEDAN, KS:

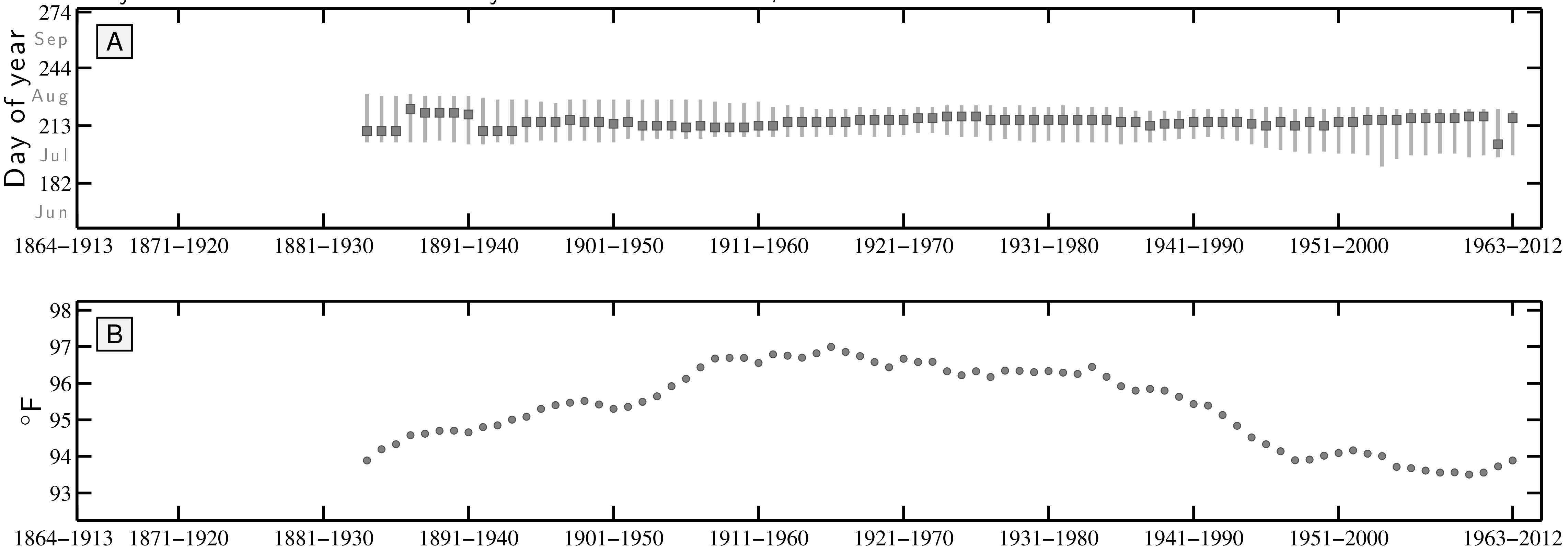

50 year Winter Teletherm dynamics for SEDAN, KS:

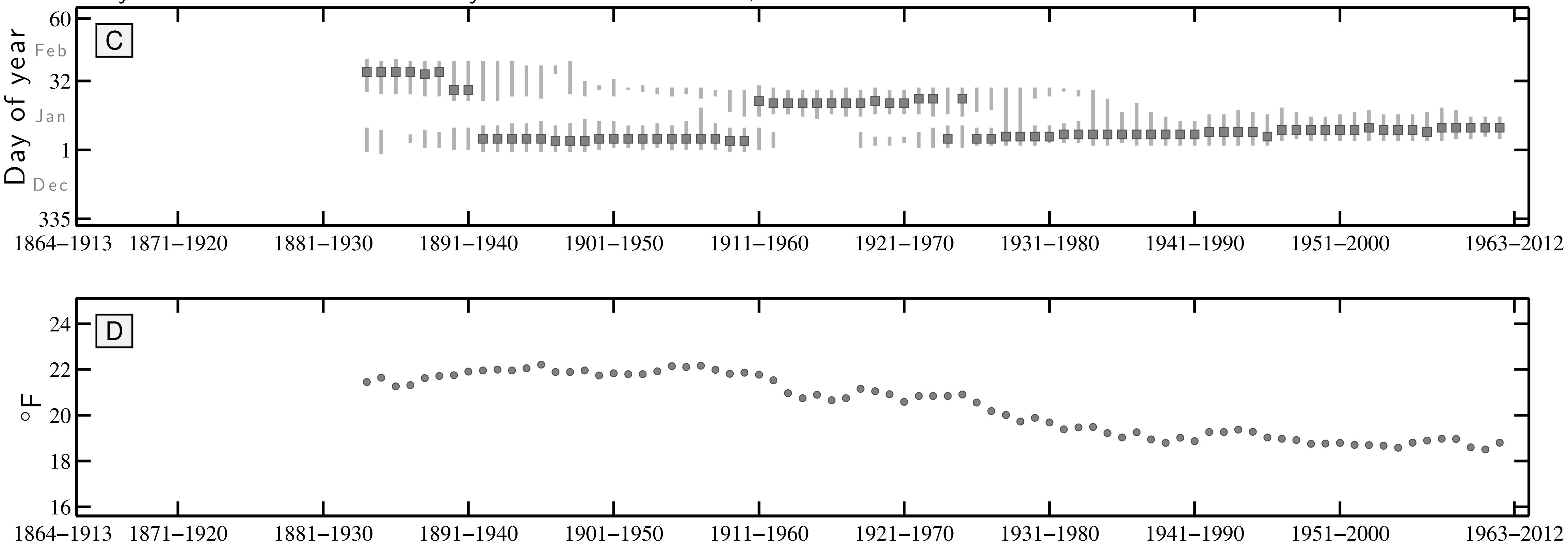

50 year Summer Teletherm dynamics for SMITH CTR, KS:

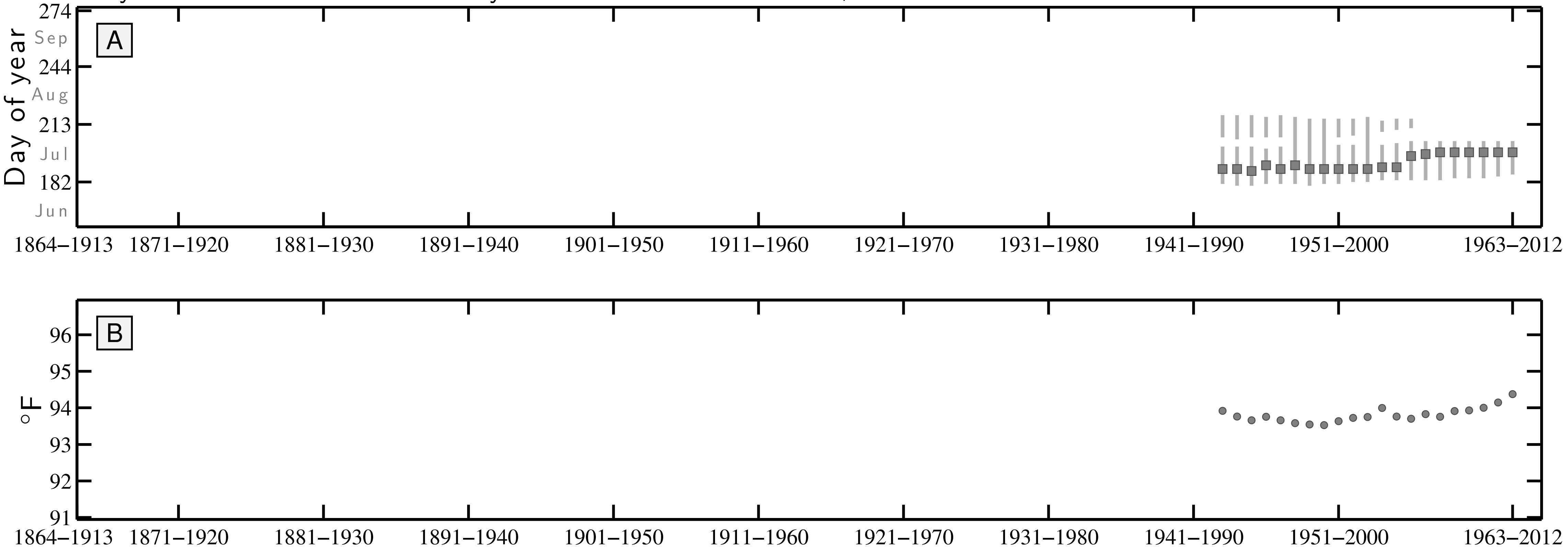

50 year Winter Teletherm dynamics for SMITH CTR, KS:

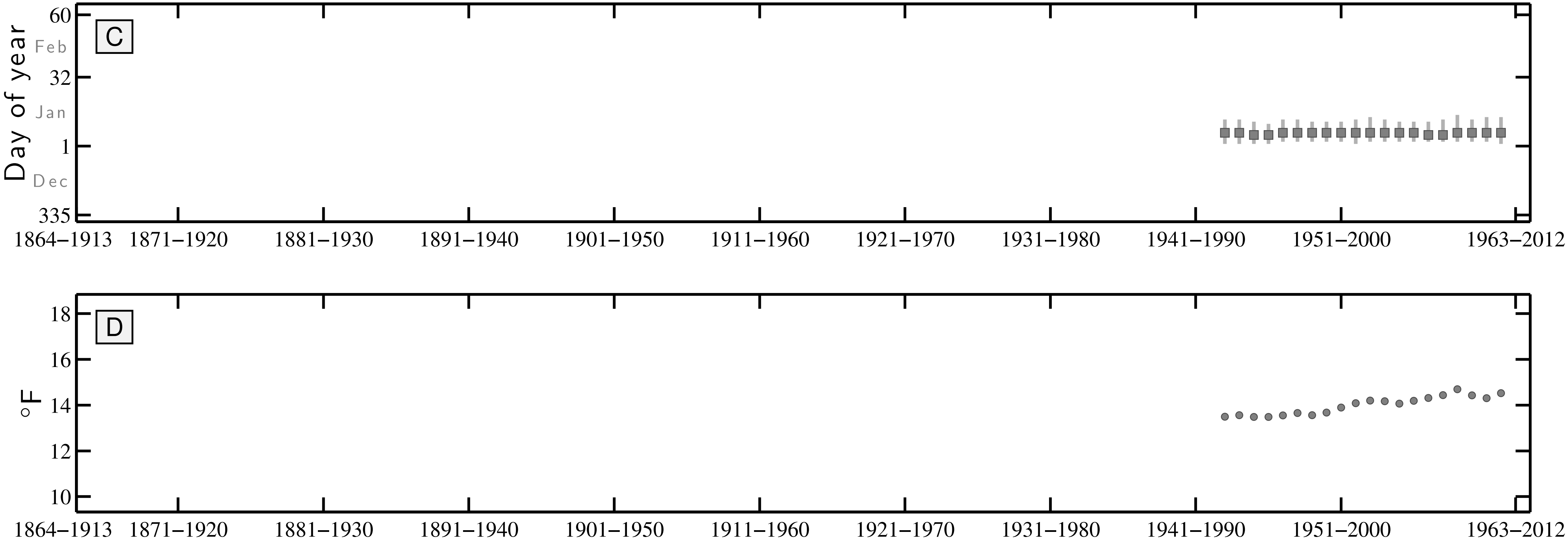

50 year Summer Teletherm dynamics for WAKEENEY, KS:

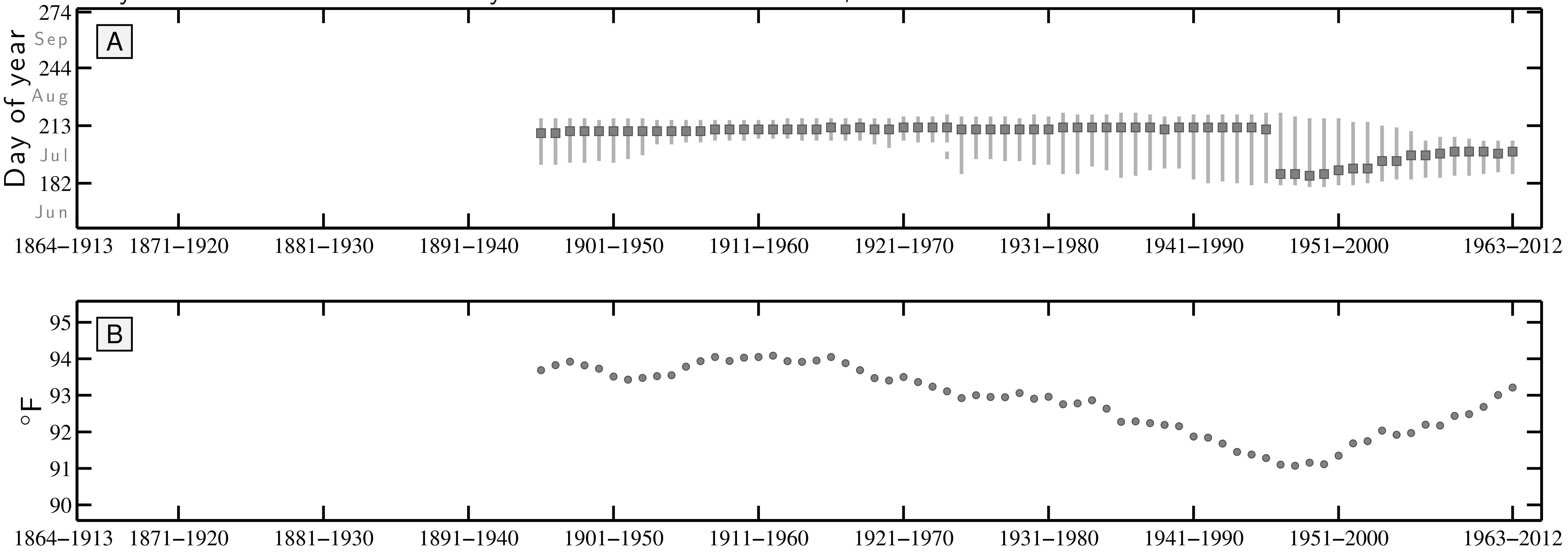

50 year Winter Teletherm dynamics for WAKEENEY, KS:

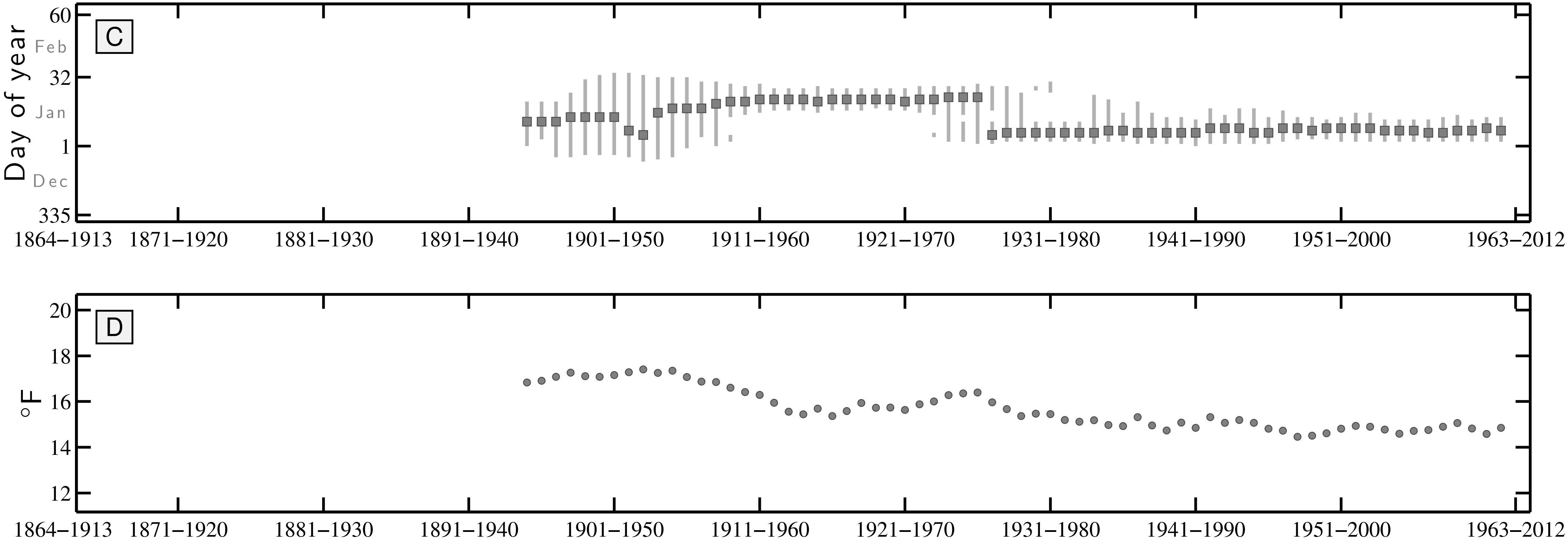

50 year Summer Teletherm dynamics for ASHLAND, KY:

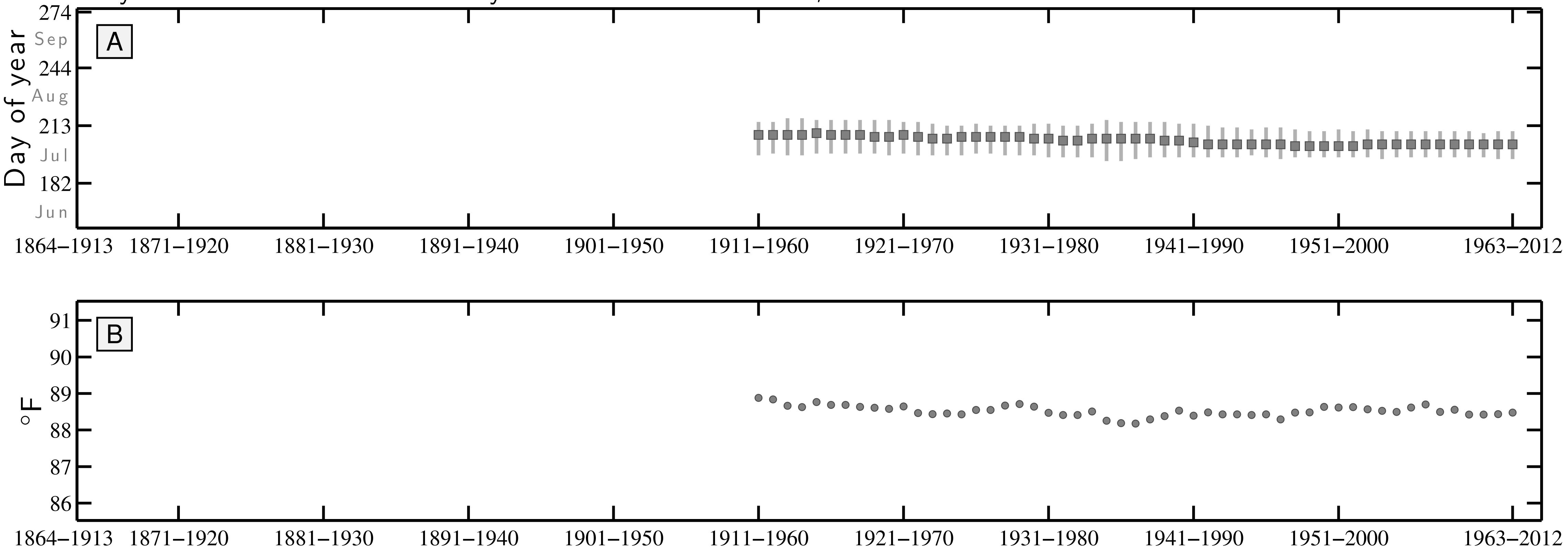

50 year Winter Teletherm dynamics for ASHLAND, KY:

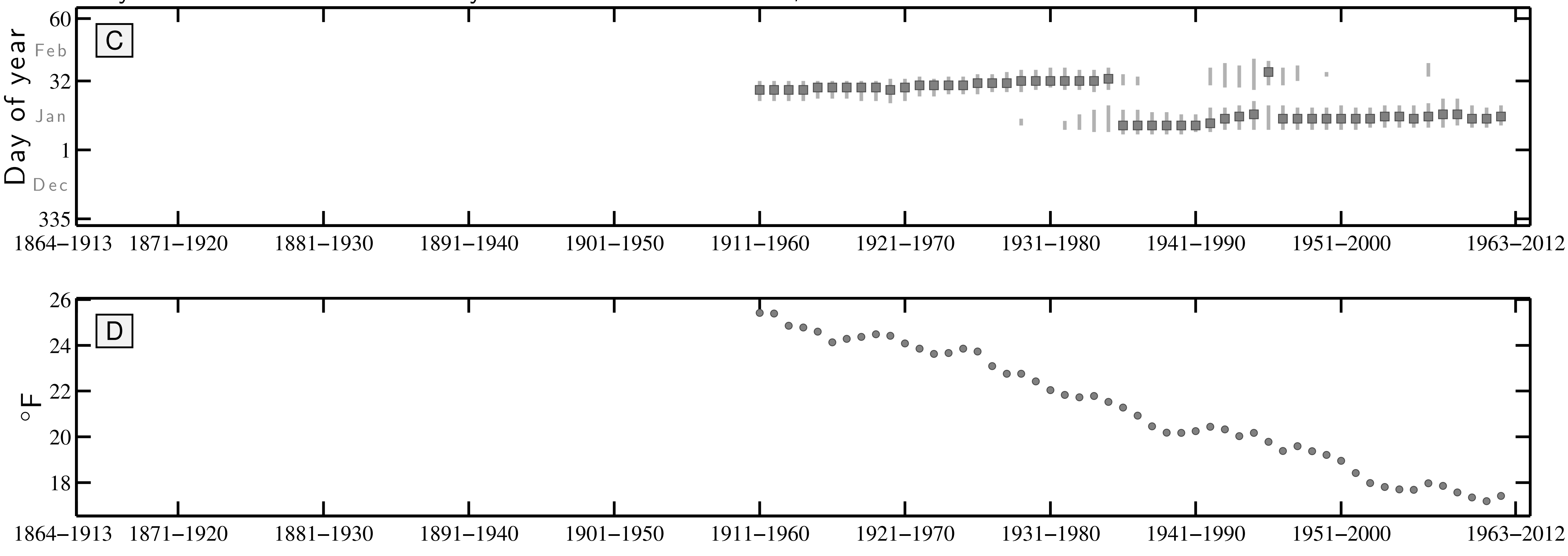

50 year Summer Teletherm dynamics for BARBOURVILLE, KY:

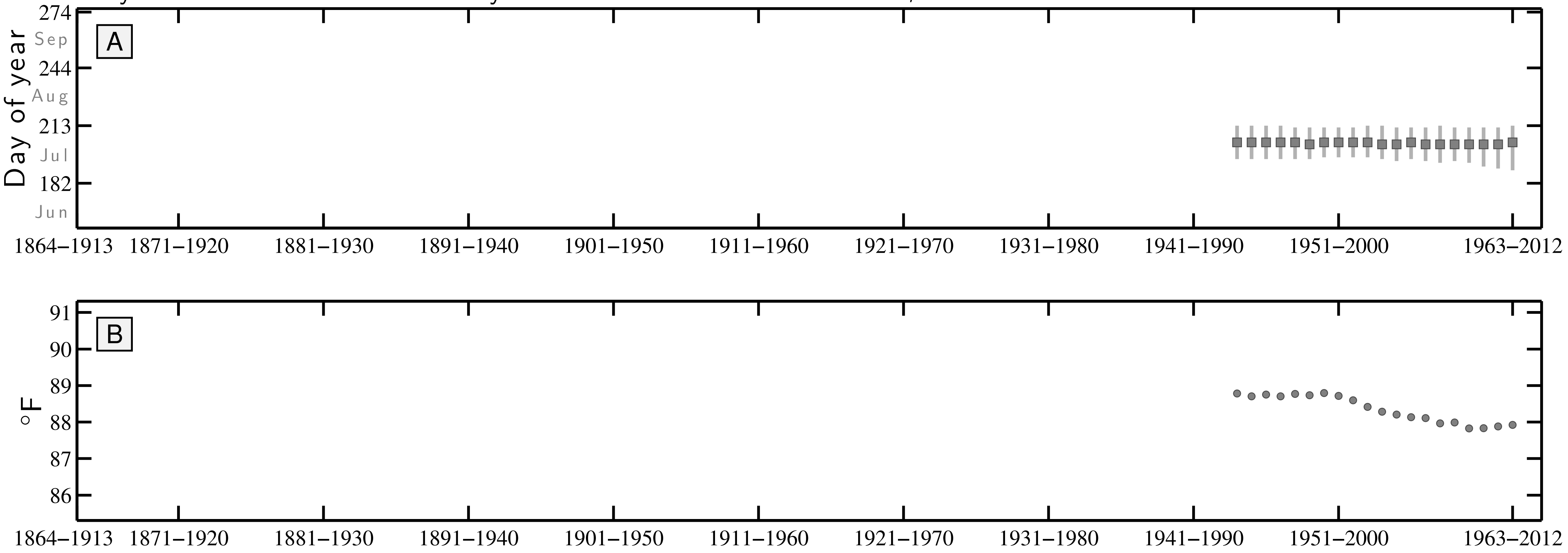

50 year Winter Teletherm dynamics for BARBOURVILLE, KY:

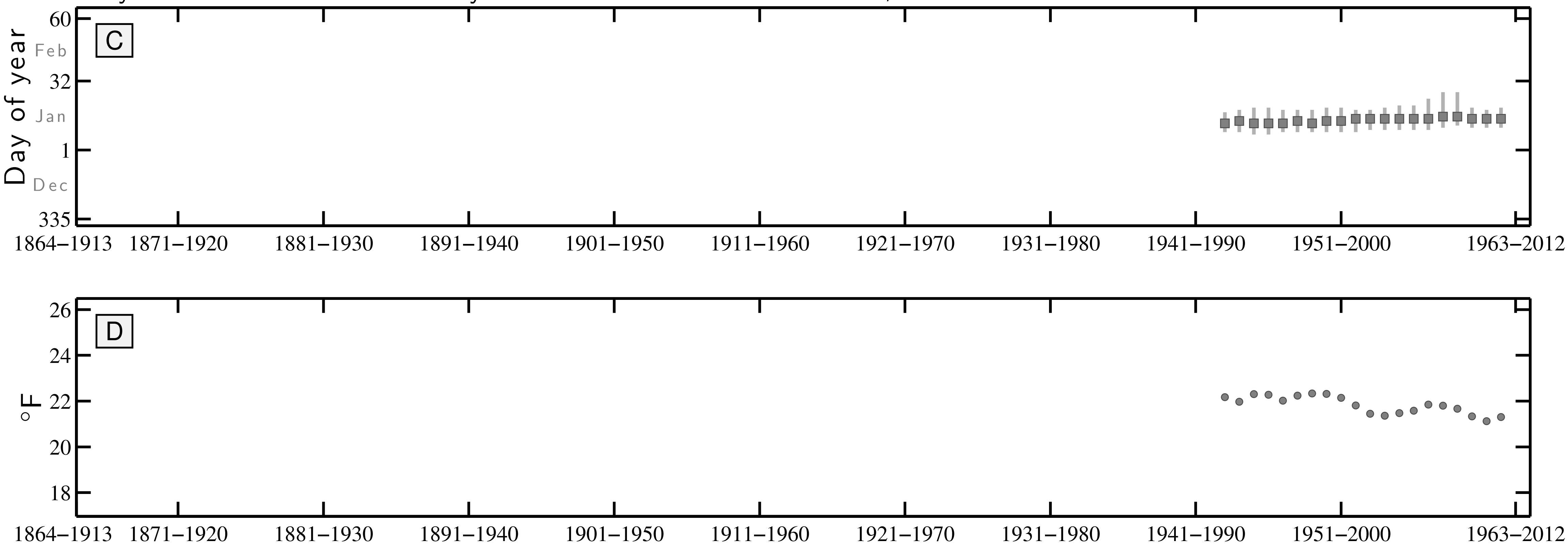

50 year Summer Teletherm dynamics for BEREA COLLEGE, KY:

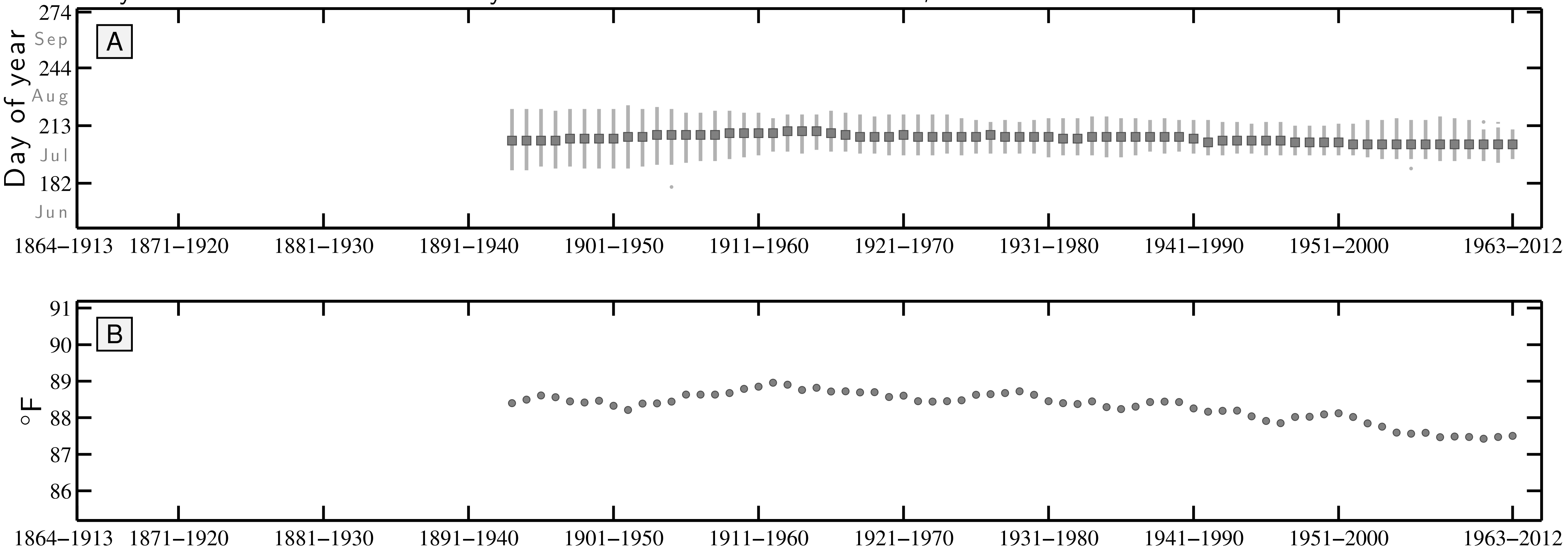

50 year Winter Teletherm dynamics for BEREA COLLEGE, KY:

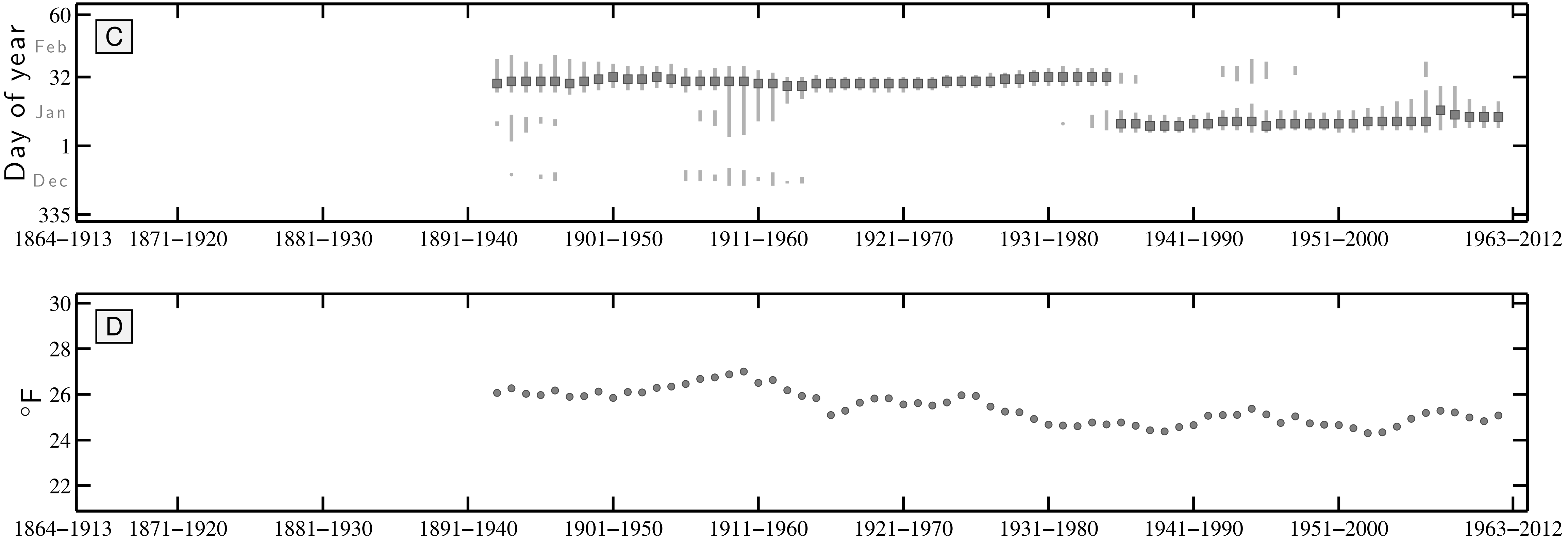

50 year Summer Teletherm dynamics for BOWLING GREEN RGNL AP, KY:

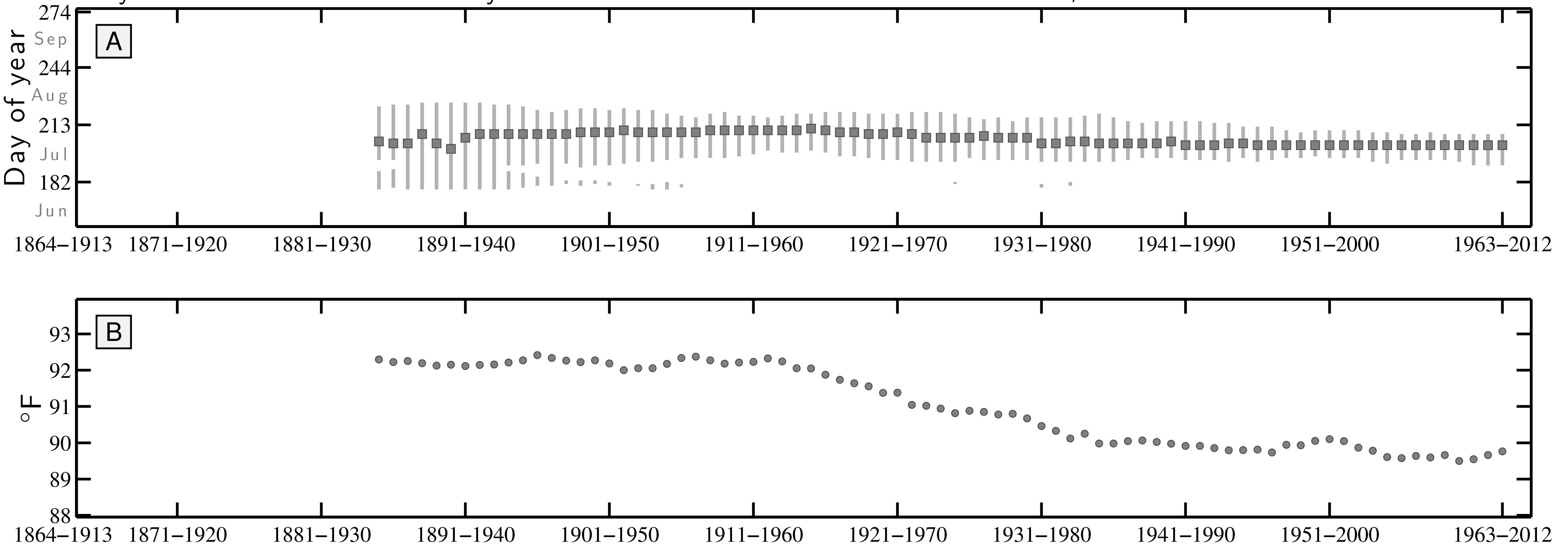

50 year Winter Teletherm dynamics for BOWLING GREEN RGNL AP, KY:

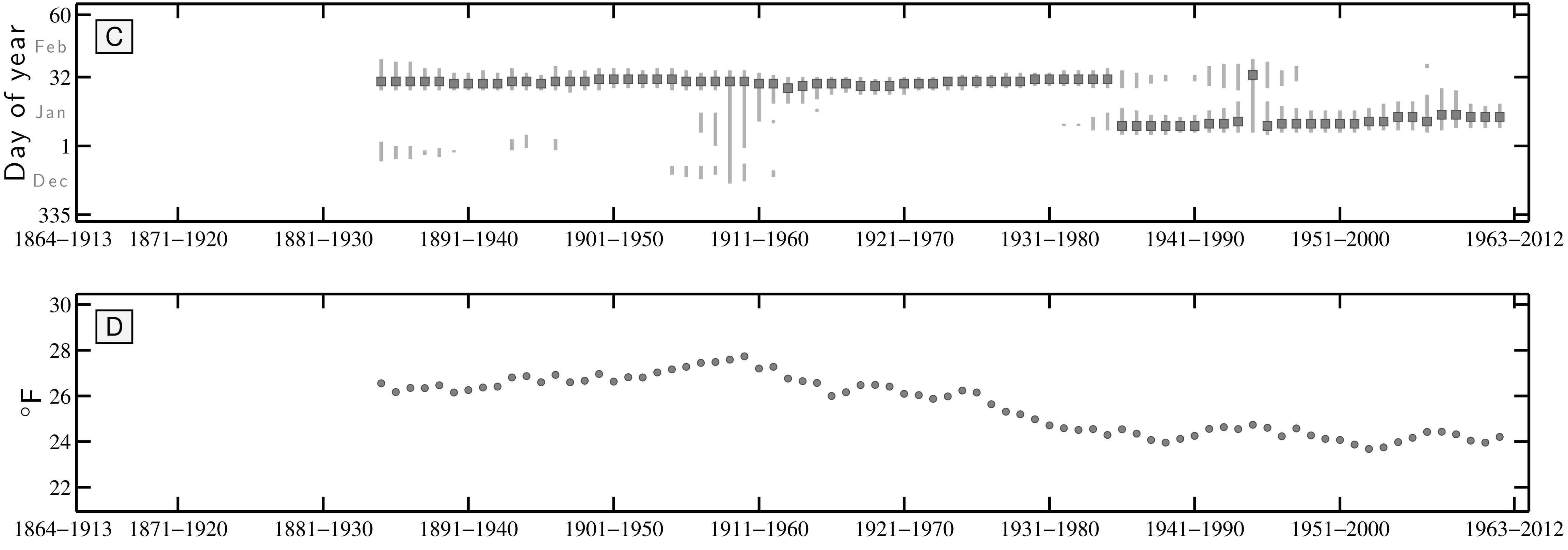

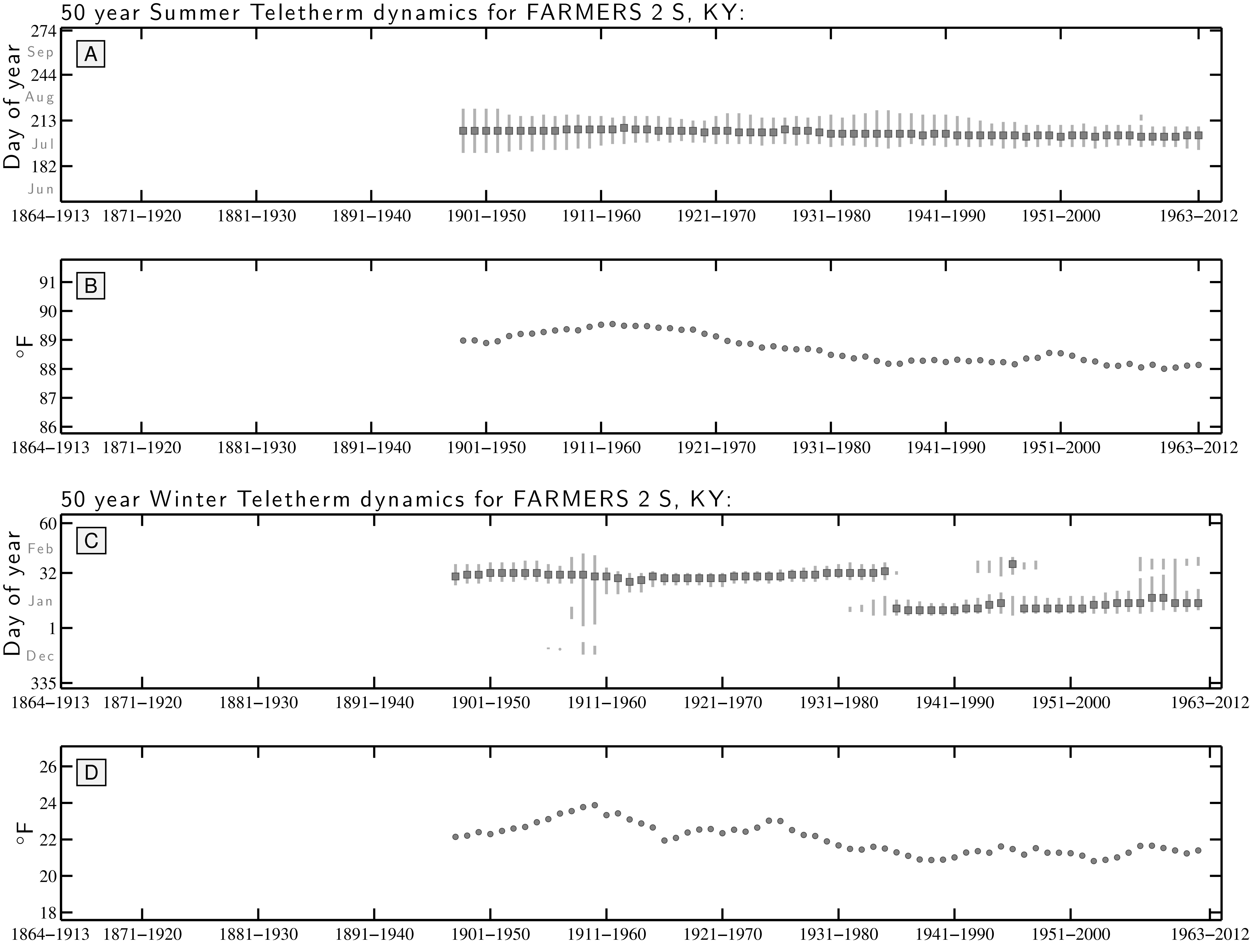
50 year Summer Teletherm dynamics for FARMERS 2 S, KY:
A
Day of year
274
Sep
244
Aug
213
Jul
182
Jun
1864–1913
1871–1920
1881–1930
1891–1940
1901–1950
1911–1960
1921–1970
1931–1980
1941–1990
1951–2000
1963–2012
B
°F
91
90
89
88
87
86
50 year Winter Teletherm dynamics for FARMERS 2 S, KY:
C
Day of year
60
Feb
32
Jan
1
Dec
335
D
°F
26
24
22
20
18

50 year Summer Teletherm dynamics for FRANKFORT DOWNTOWN, KY:

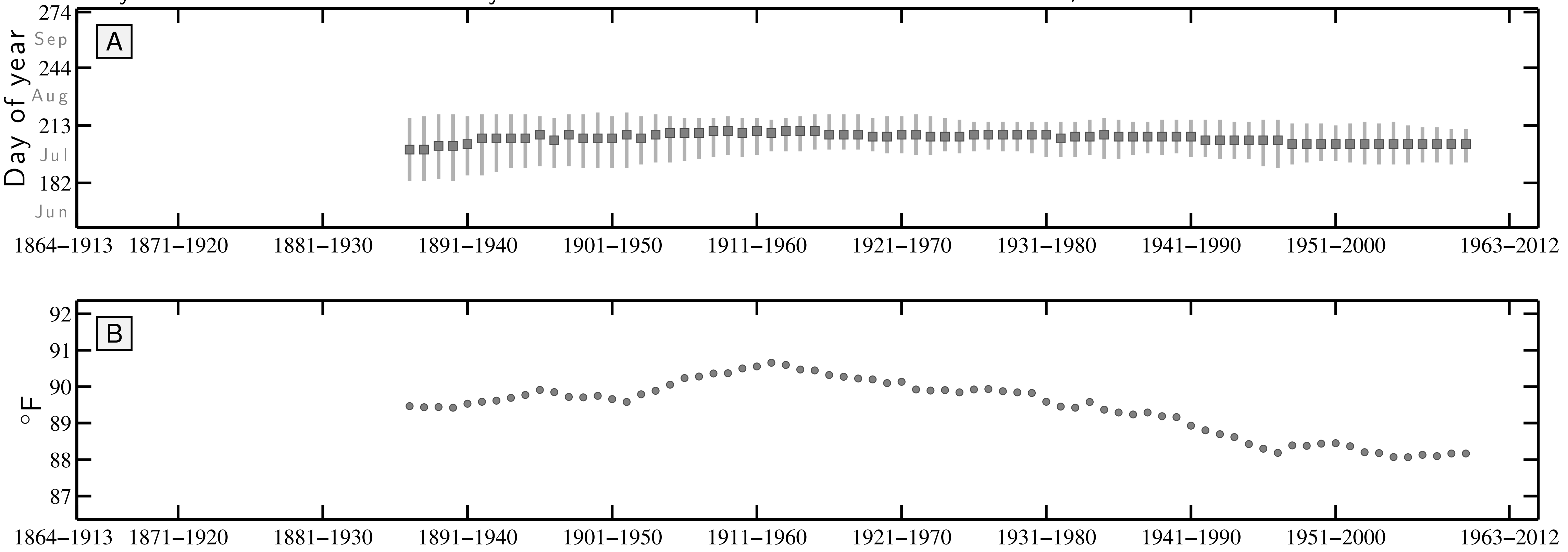

50 year Winter Teletherm dynamics for FRANKFORT DOWNTOWN, KY:

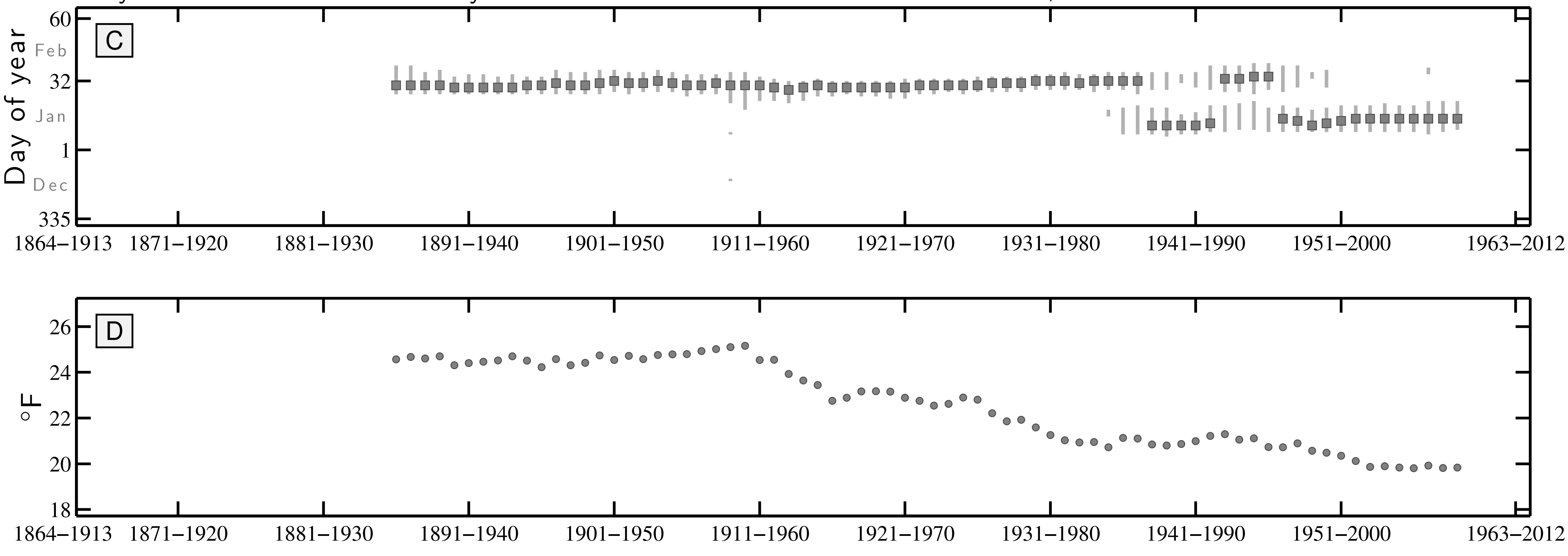

50 year Summer Teletherm dynamics for GREENSBURG, KY:

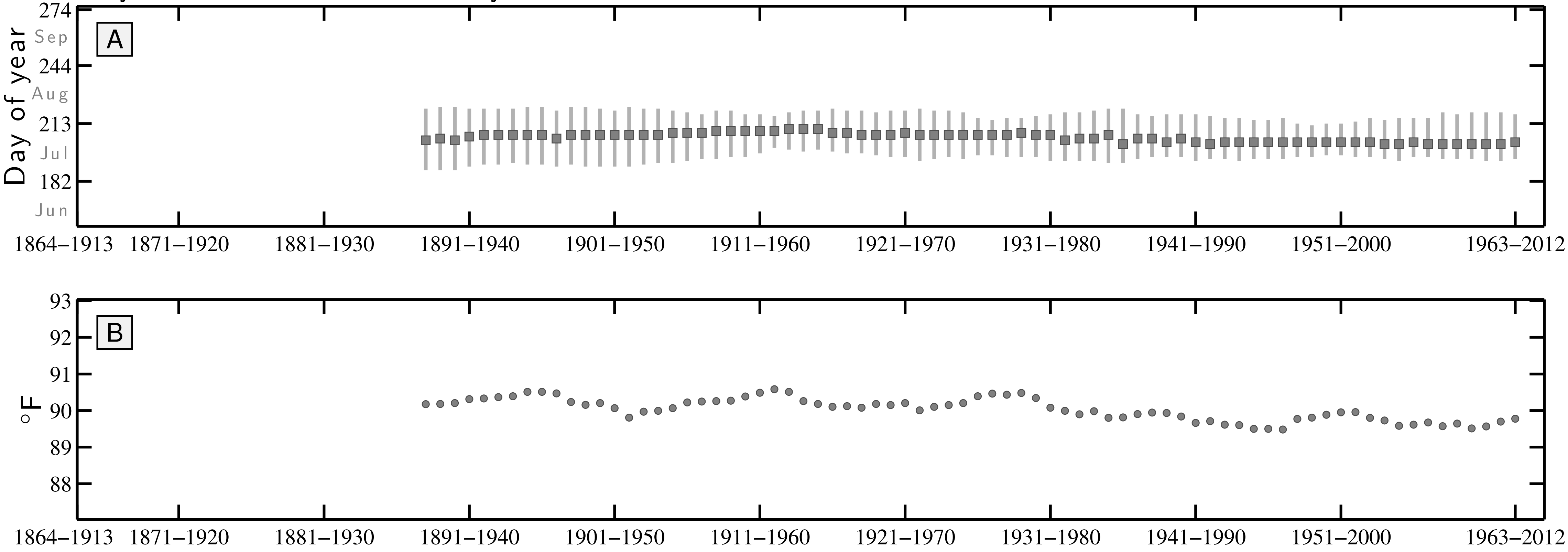

50 year Winter Teletherm dynamics for GREENSBURG, KY:

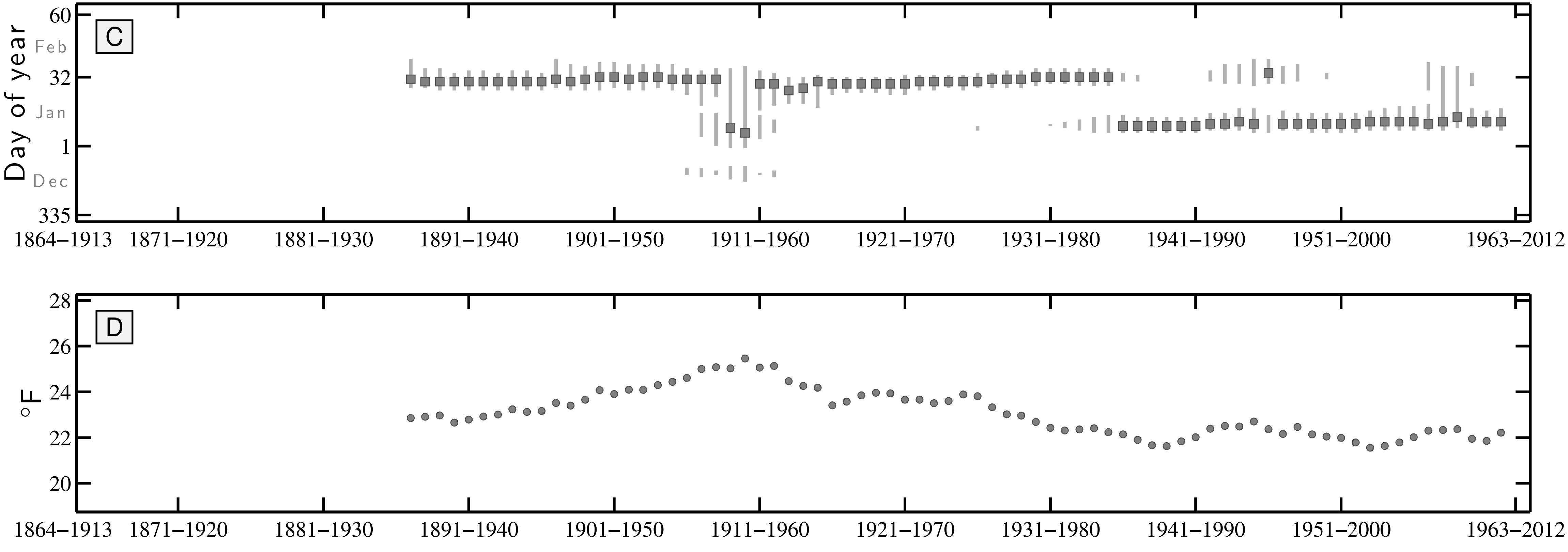

50 year Summer Teletherm dynamics for HENDERSON 8 SSW, KY:

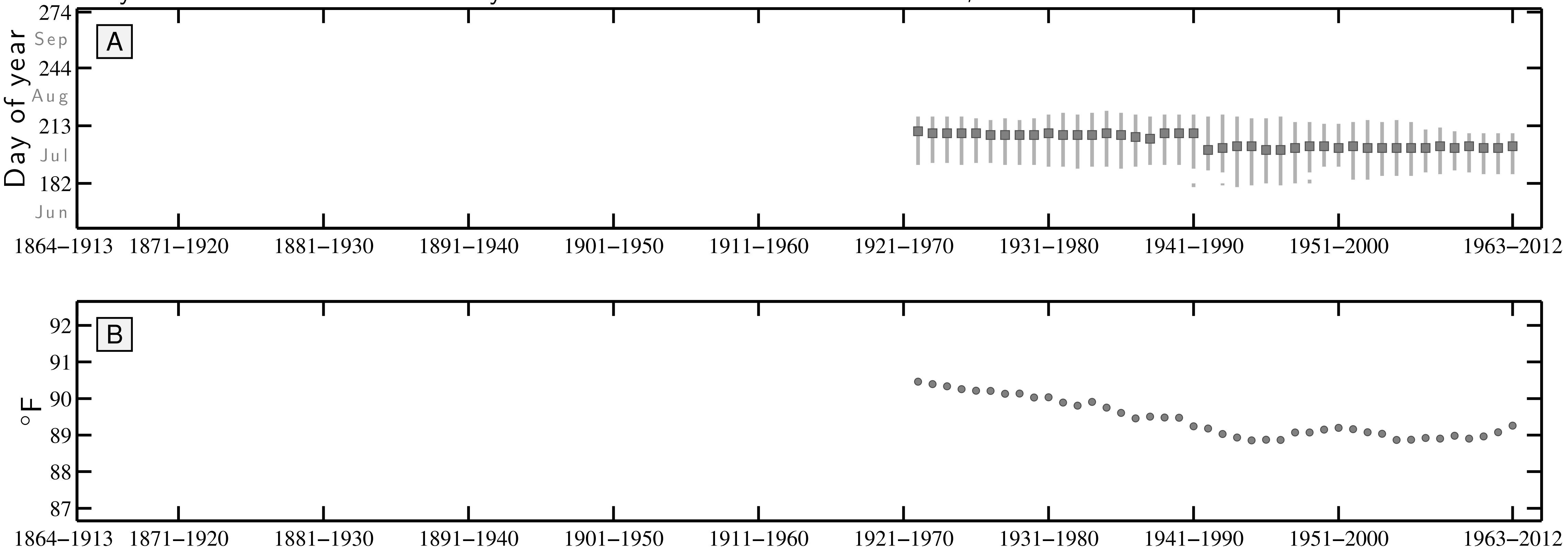

50 year Winter Teletherm dynamics for HENDERSON 8 SSW, KY:

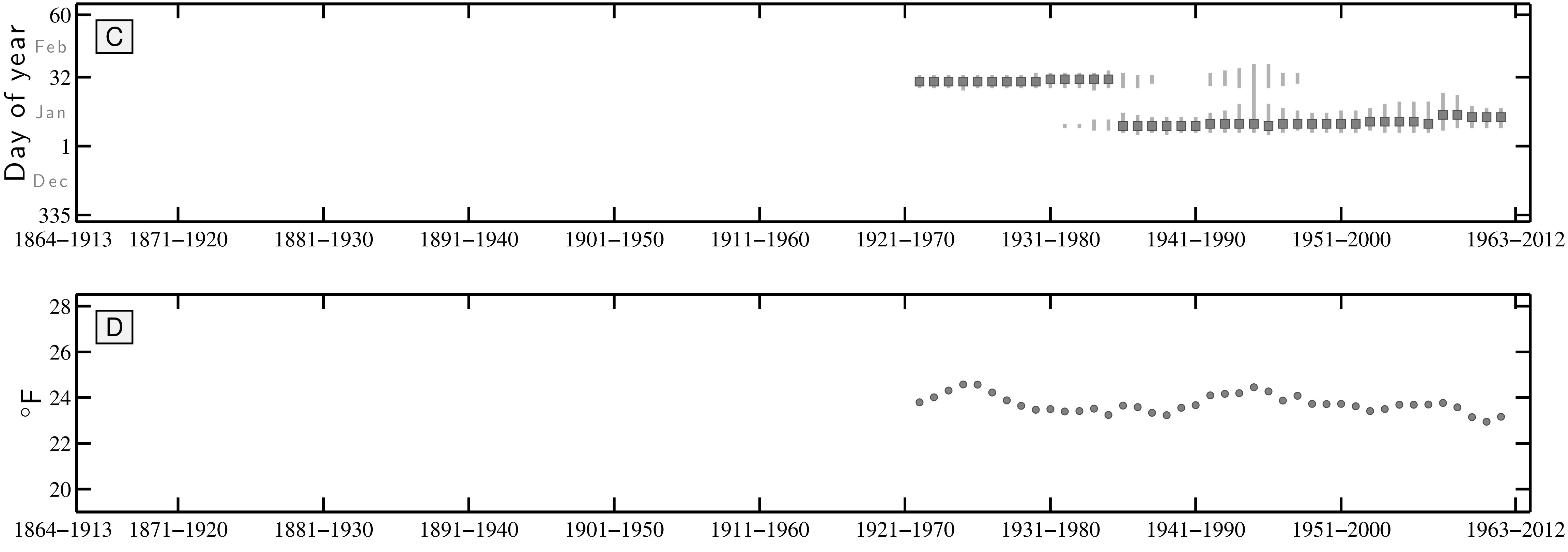

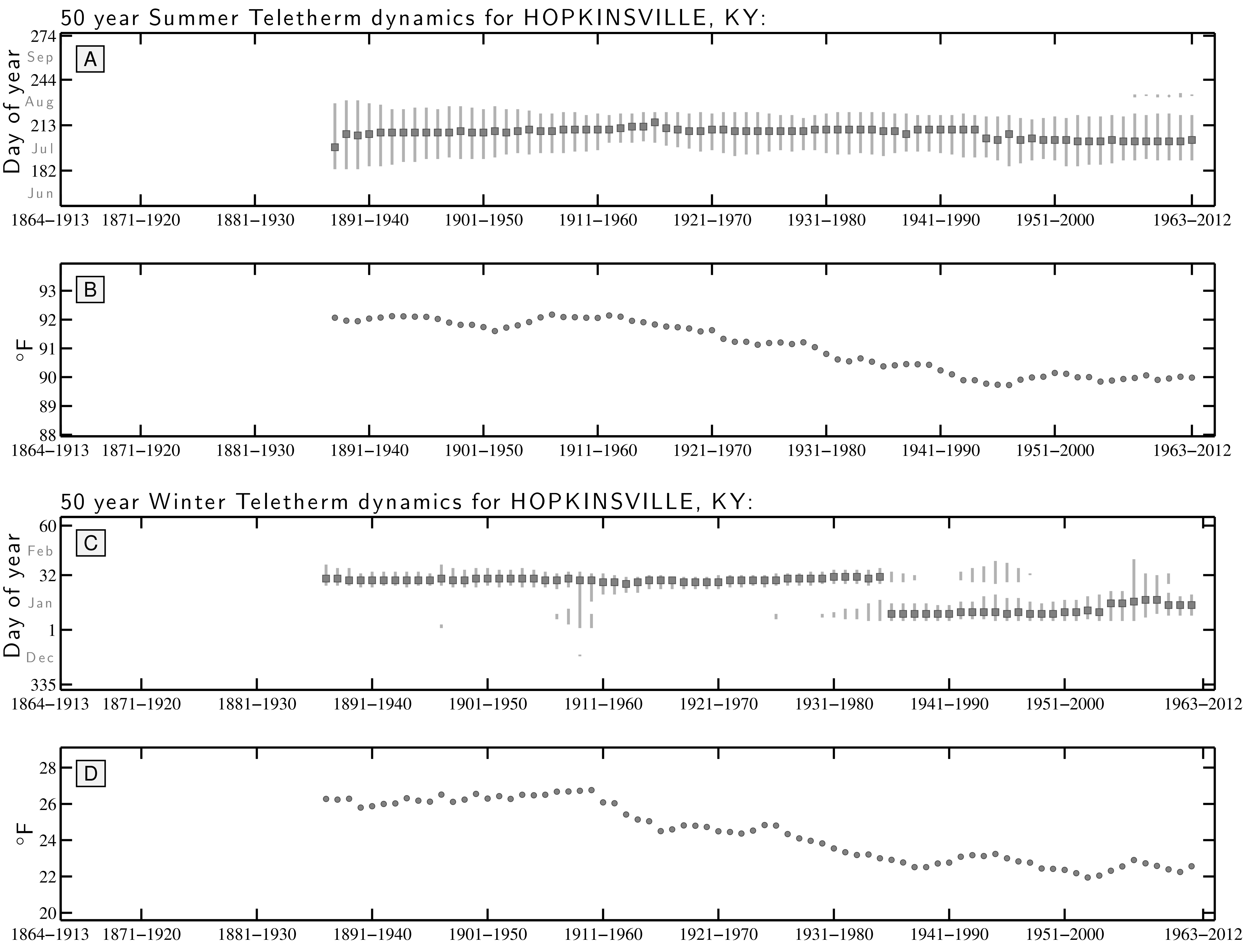
50 year Summer Teletherm dynamics for HOPKINSVILLE, KY:
A
Day of year
274
Sep
244
Aug
213
Jul
182
Jun
1864–1913
1871–1920
1881–1930
1891–1940
1901–1950
1911–1960
1921–1970
1931–1980
1941–1990
1951–2000
1963–2012
B
°F
93
92
91
90
89
88
50 year Winter Teletherm dynamics for HOPKINSVILLE, KY:
C
60
Feb
32
Jan
1
Dec
335
D
28
26
24
22
20

50 year Summer Teletherm dynamics for LEITCHFIELD 2 N, KY:

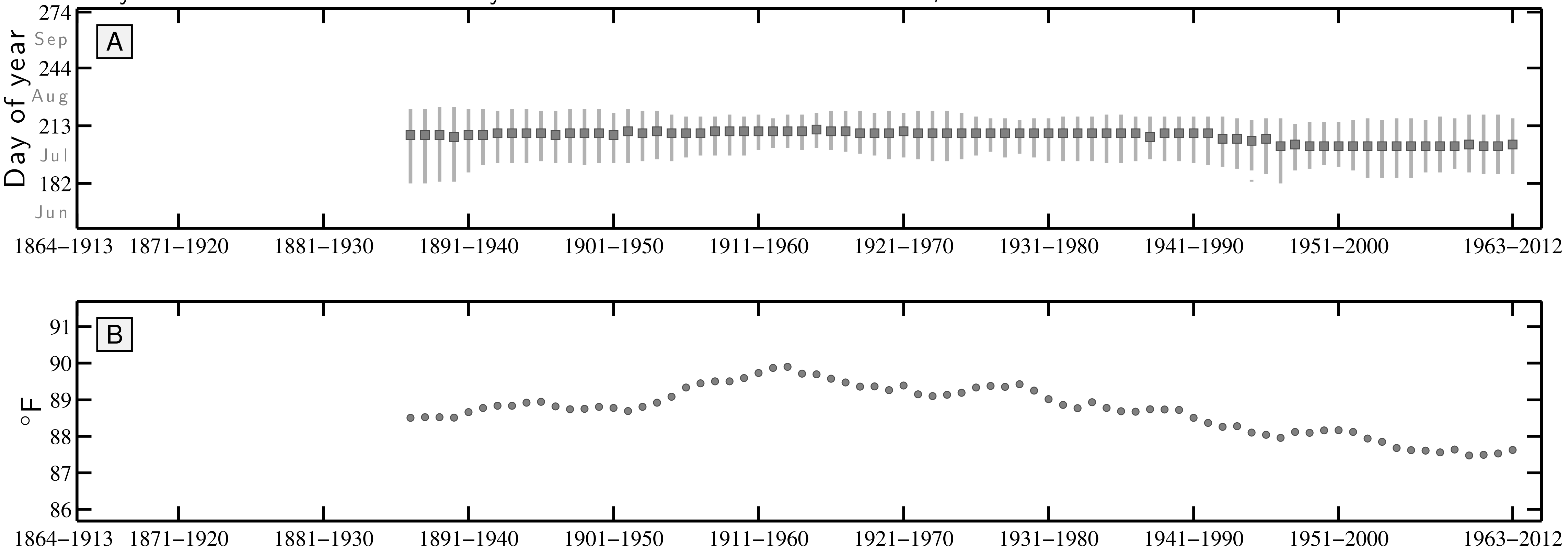

50 year Winter Teletherm dynamics for LEITCHFIELD 2 N, KY:

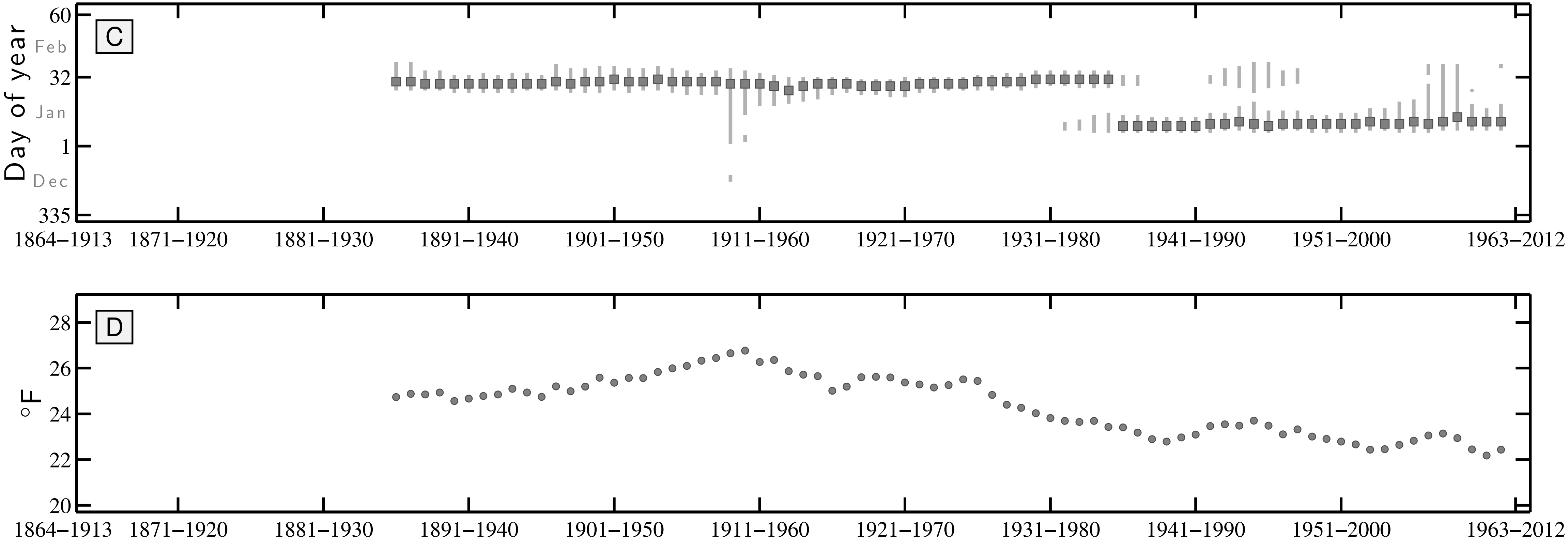

50 year Summer Teletherm dynamics for SHELBYVILLE 1 E, KY:

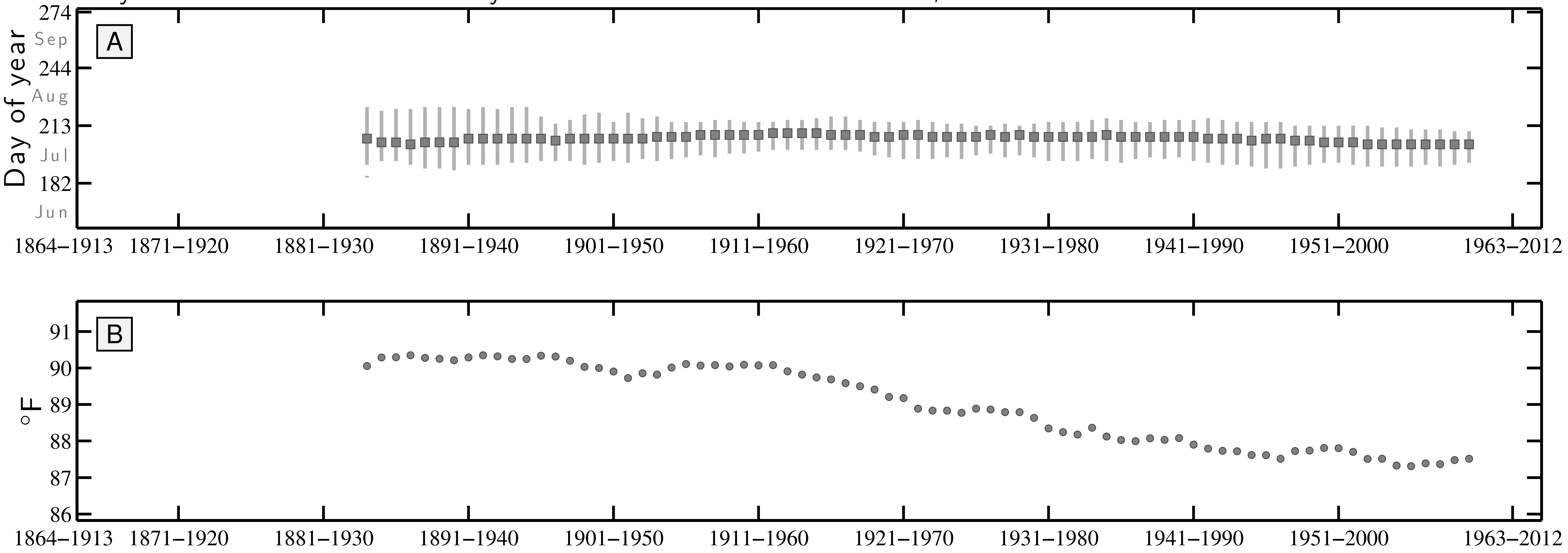

50 year Winter Teletherm dynamics for SHELBYVILLE 1 E, KY:

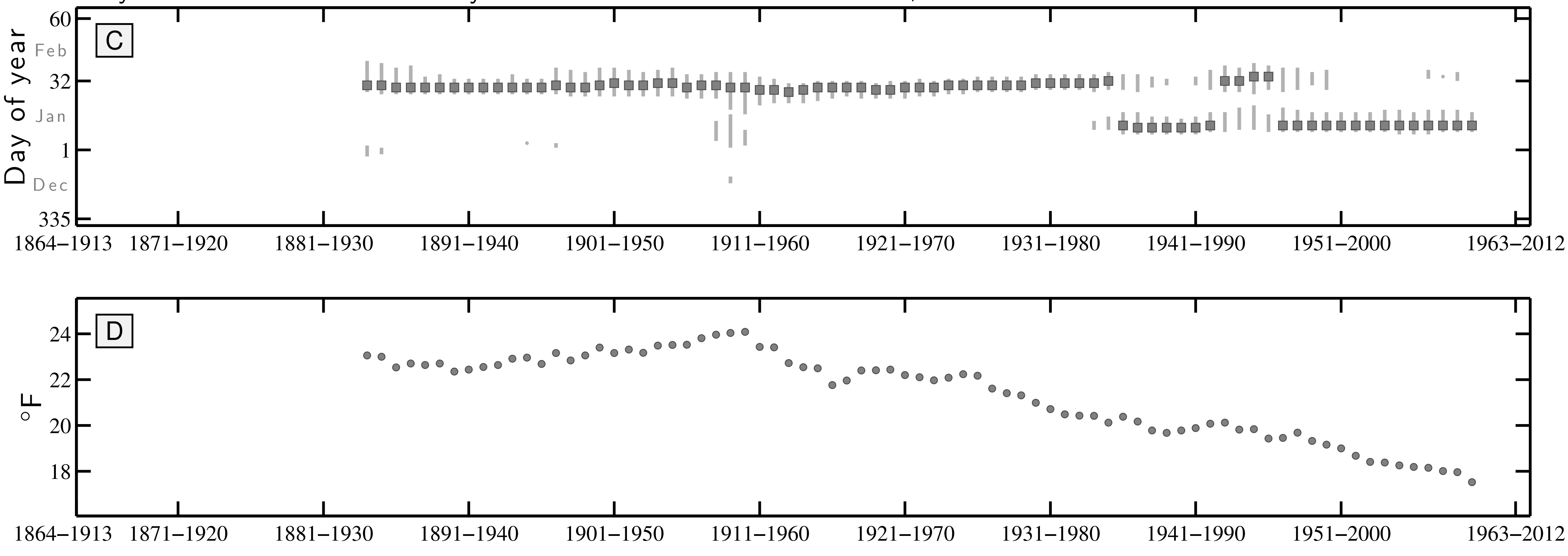

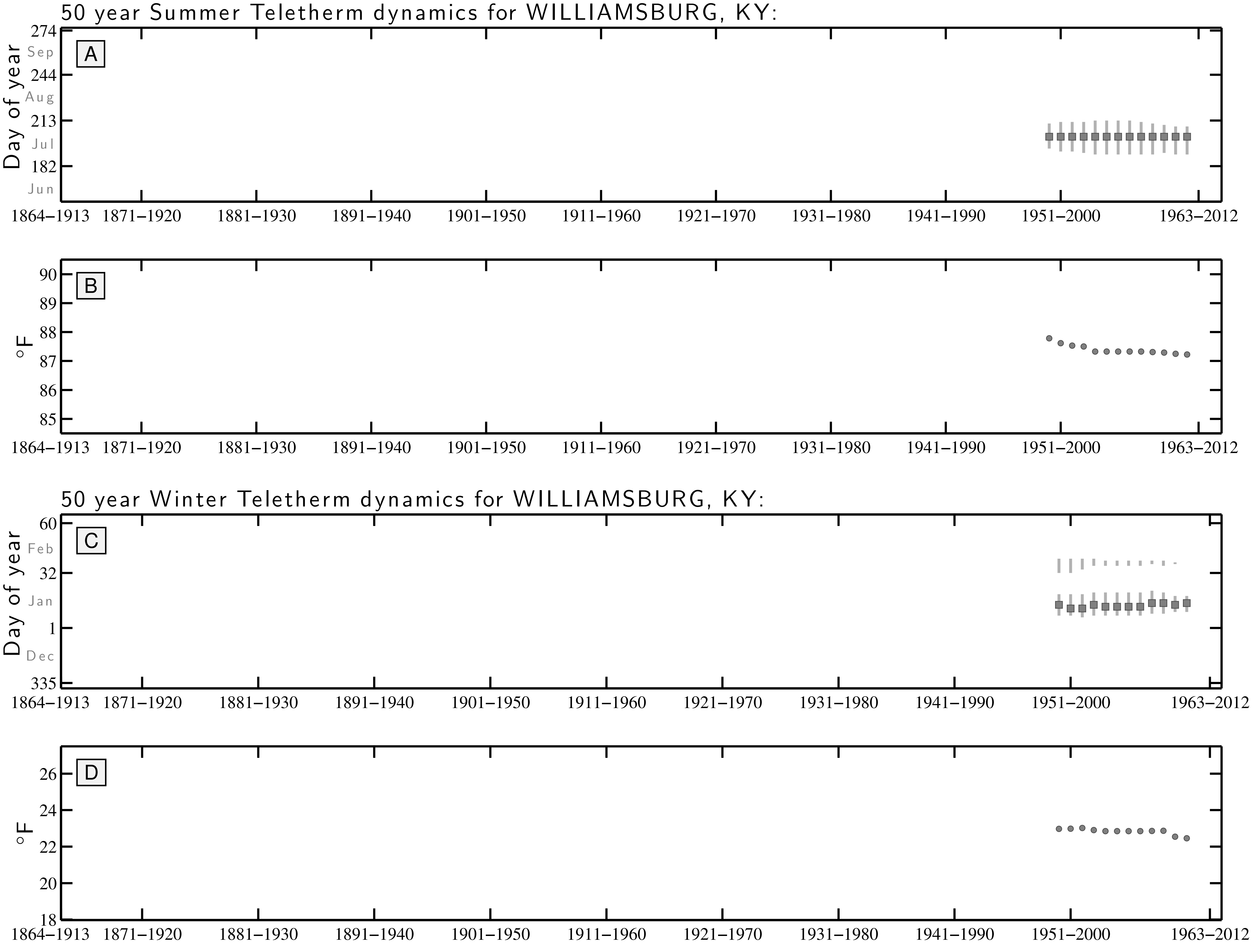

50 year Summer Teletherm dynamics for WILLIAMSBURG, KY:
A
B
Day of year
°F
274
Sep
244
Aug
213
Jul
182
Jun
90
89
88
87
86
85
1864–1913
1871–1920
1881–1930
1891–1940
1901–1950
1911–1960
1921–1970
1931–1980
1941–1990
1951–2000
1963–2012
50 year Winter Teletherm dynamics for WILLIAMSBURG, KY:
C
D
60
Feb
32
Jan
1
Dec
335
26
24
22
20
18

50 year Summer Teletherm dynamics for WILLIAMSTOWN 3 W, KY:

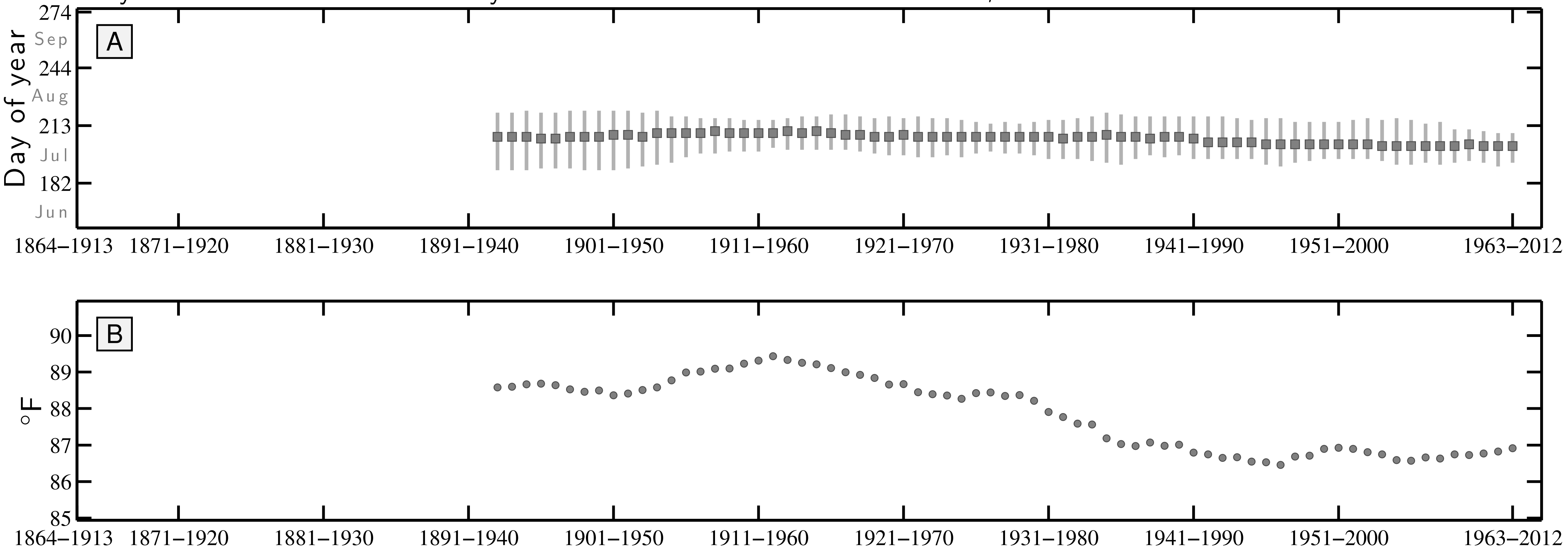

50 year Winter Teletherm dynamics for WILLIAMSTOWN 3 W, KY:

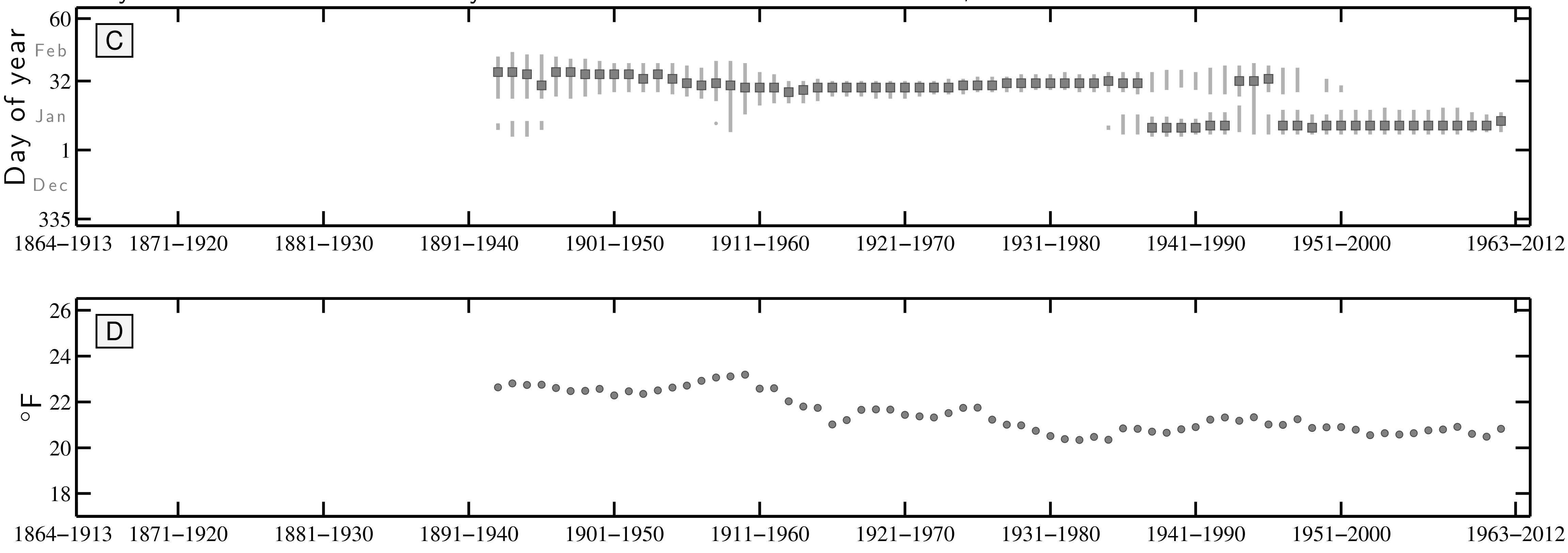

50 year Summer Teletherm dynamics for ALEXANDRIA, LA:

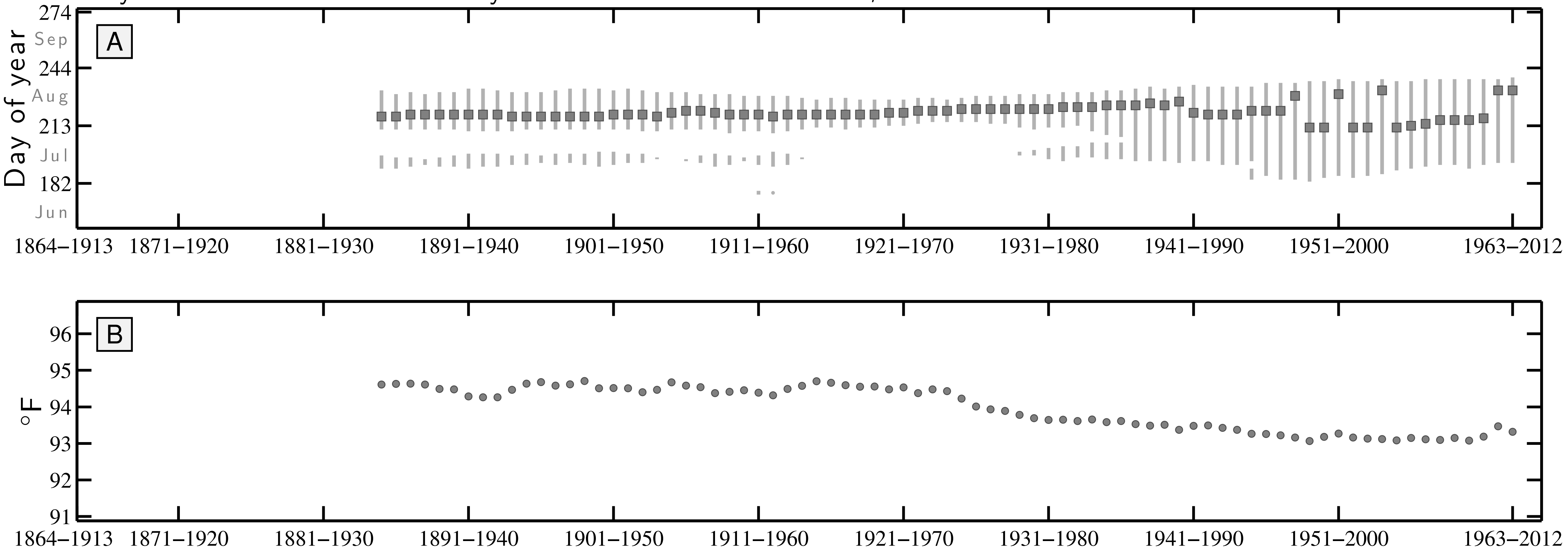

50 year Winter Teletherm dynamics for ALEXANDRIA, LA:

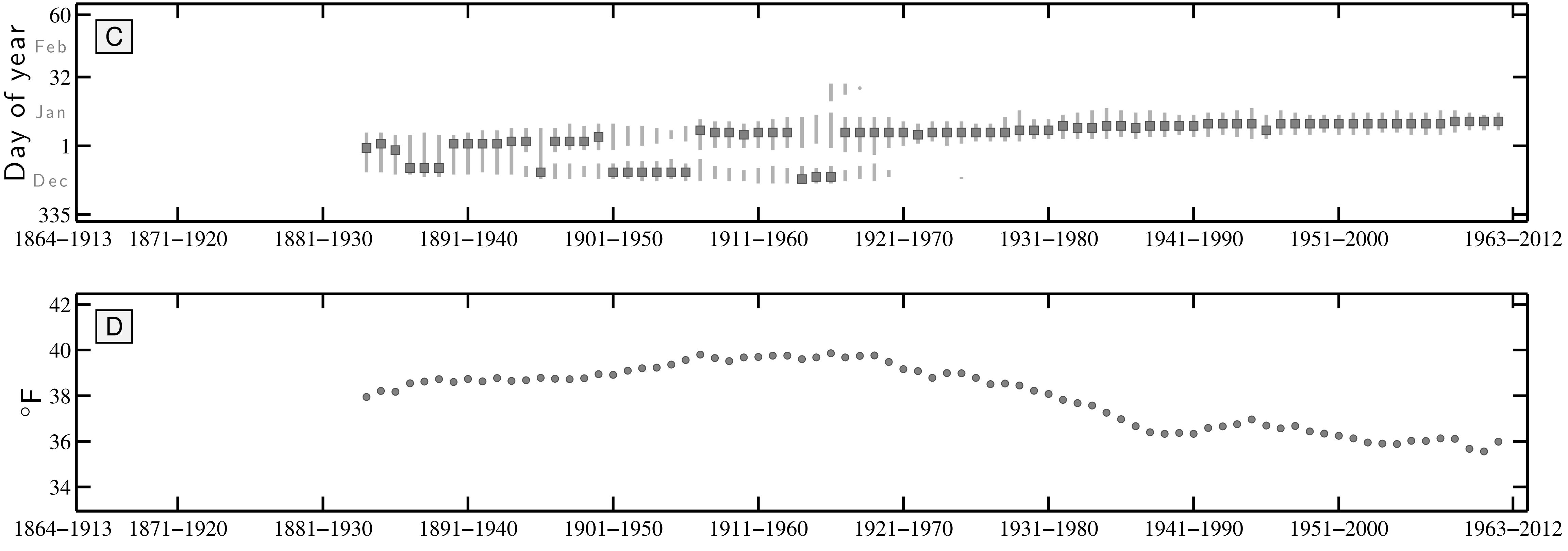

50 year Summer Teletherm dynamics for AMITE, LA:

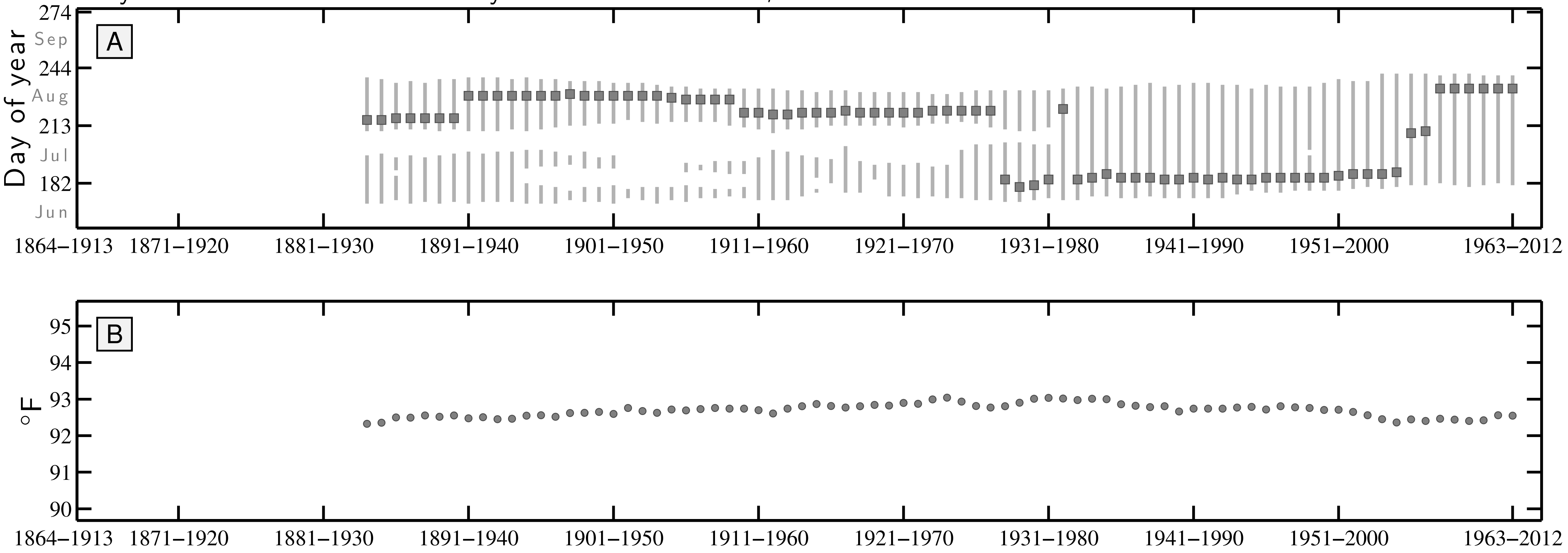

50 year Winter Teletherm dynamics for AMITE, LA:

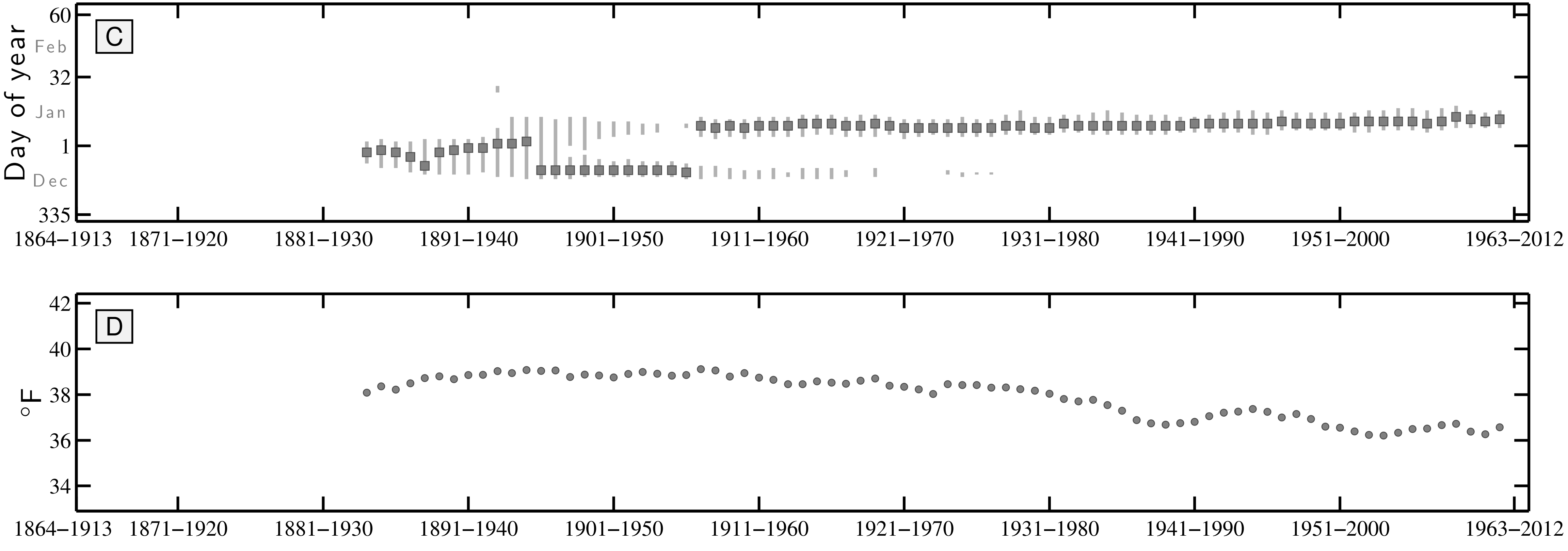

50 year Summer Teletherm dynamics for BASTROP, LA:

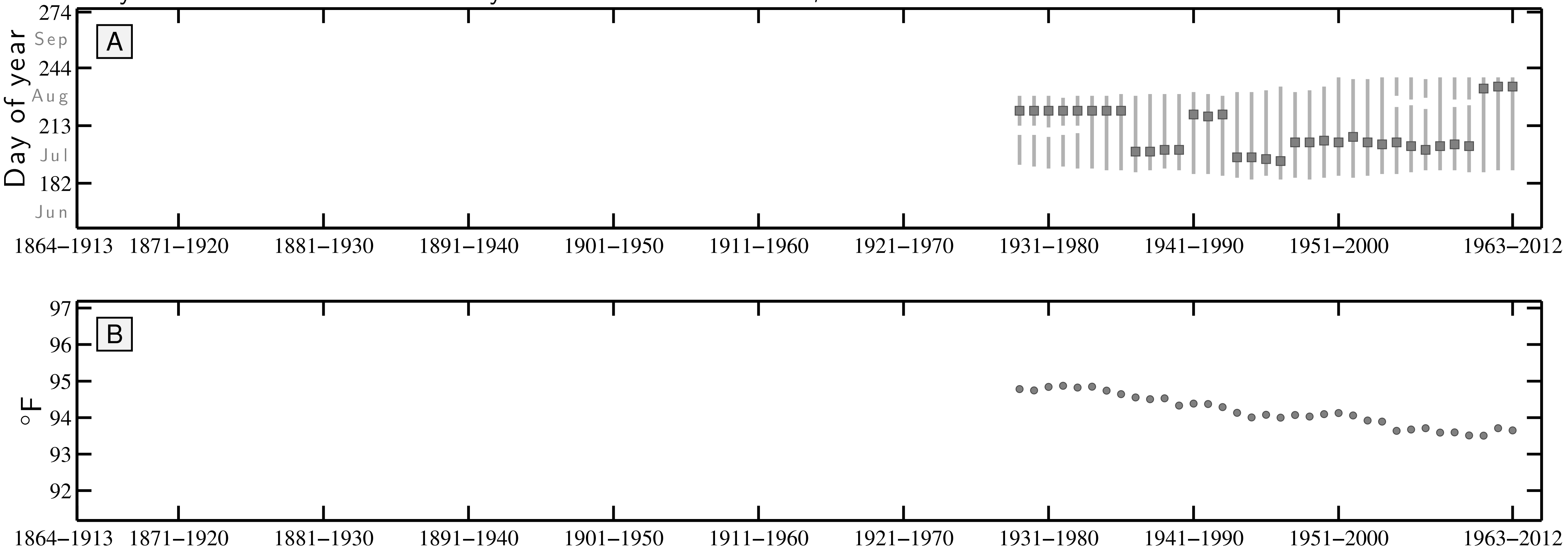

50 year Winter Teletherm dynamics for BASTROP, LA:

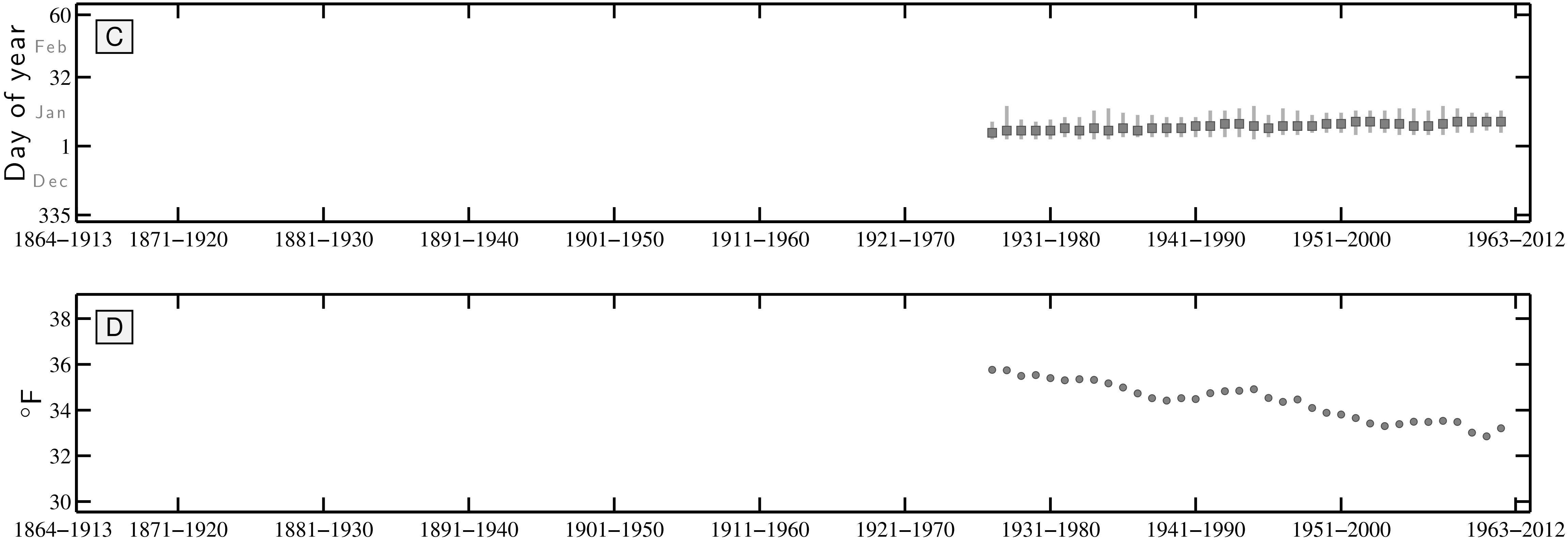

50 year Summer Teletherm dynamics for BATON ROUGE METRO AP, LA:

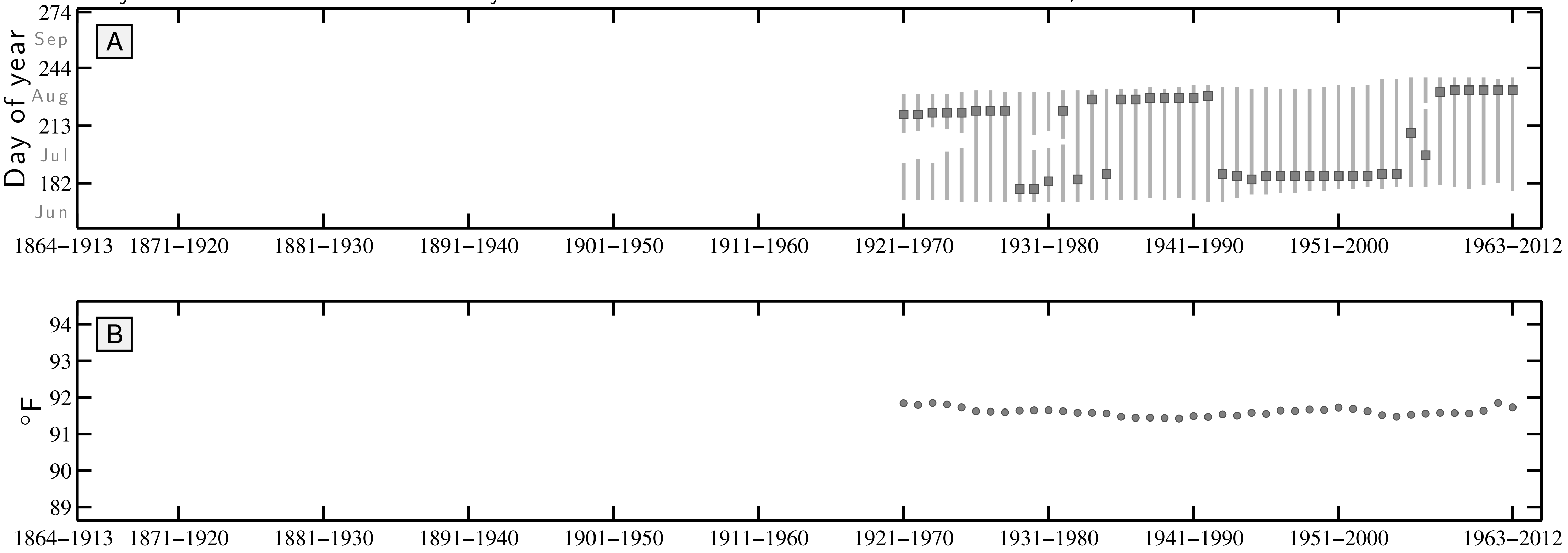

50 year Winter Teletherm dynamics for BATON ROUGE METRO AP, LA:

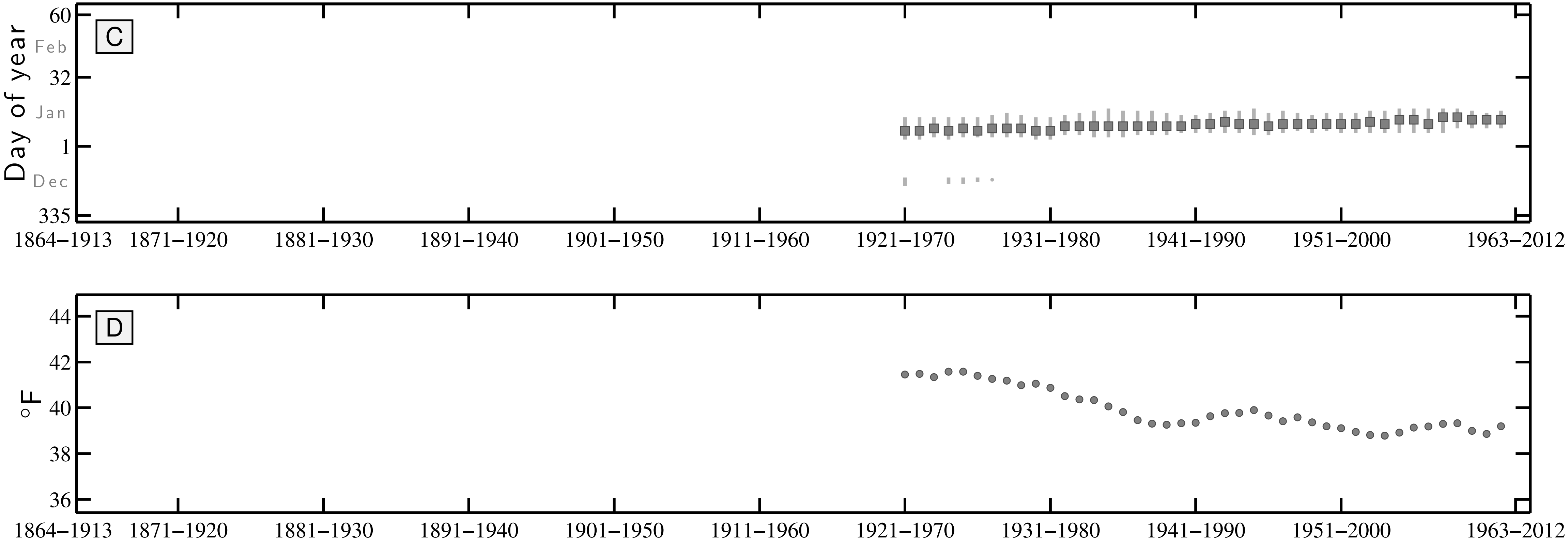

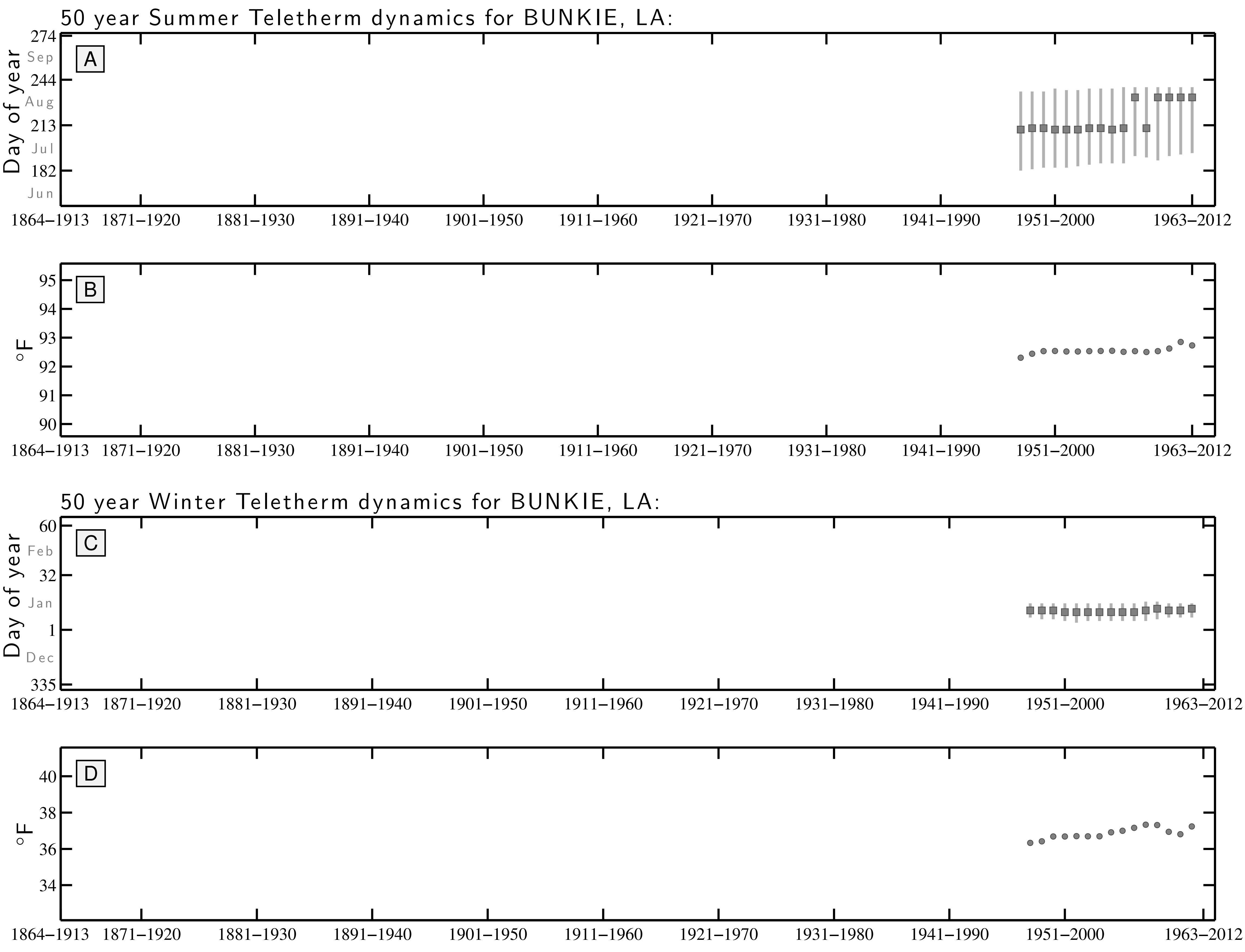

50 year Summer Teletherm dynamics for BUNKIE, LA:
A
Day of year
274
Sep
244
Aug
213
Jul
182
Jun
1864–1913
1871–1920
1881–1930
1891–1940
1901–1950
1911–1960
1921–1970
1931–1980
1941–1990
1951–2000
1963–2012
B
°F
95
94
93
92
91
90
50 year Winter Teletherm dynamics for BUNKIE, LA:
C
60
Feb
32
Jan
1
Dec
335
D
40
38
36
34

50 year Summer Teletherm dynamics for CALHOUN RSCH STN, LA:

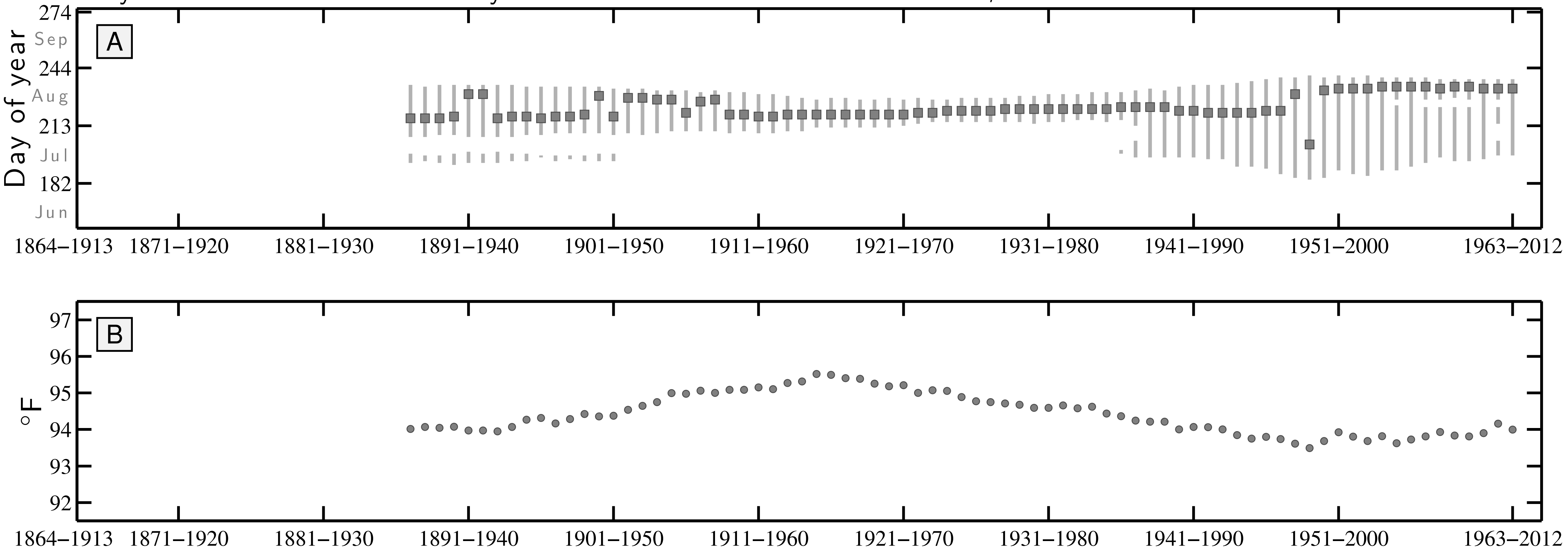

50 year Winter Teletherm dynamics for CALHOUN RSCH STN, LA:

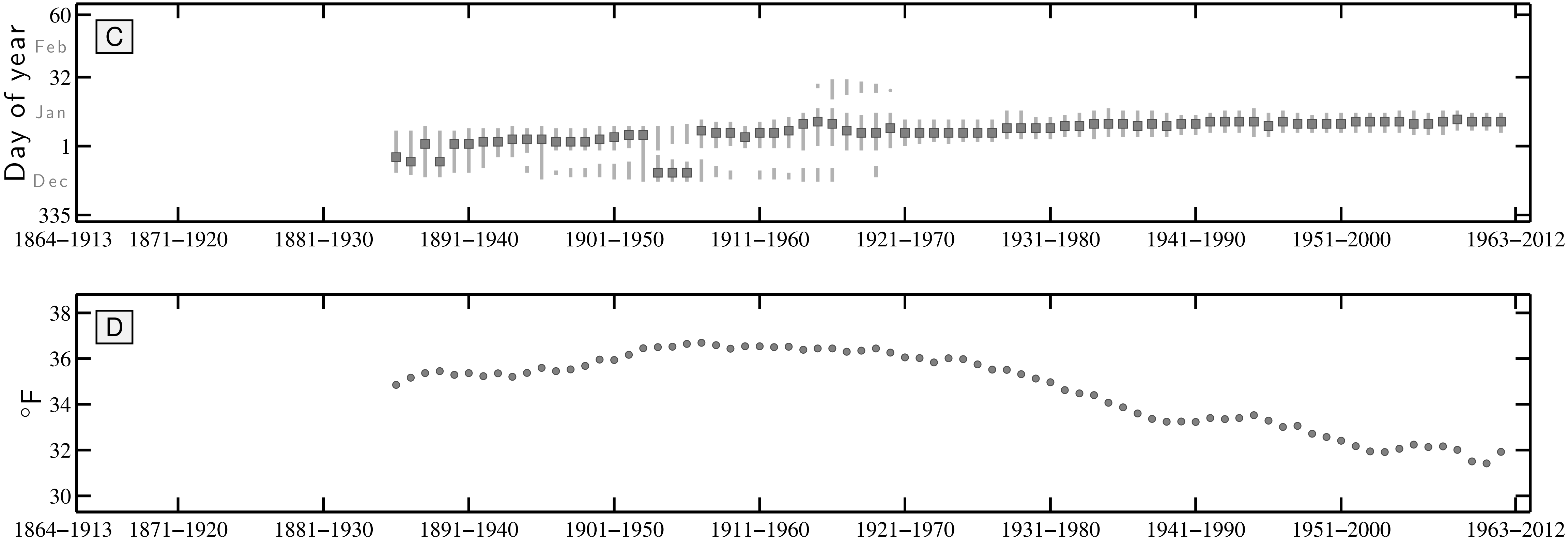

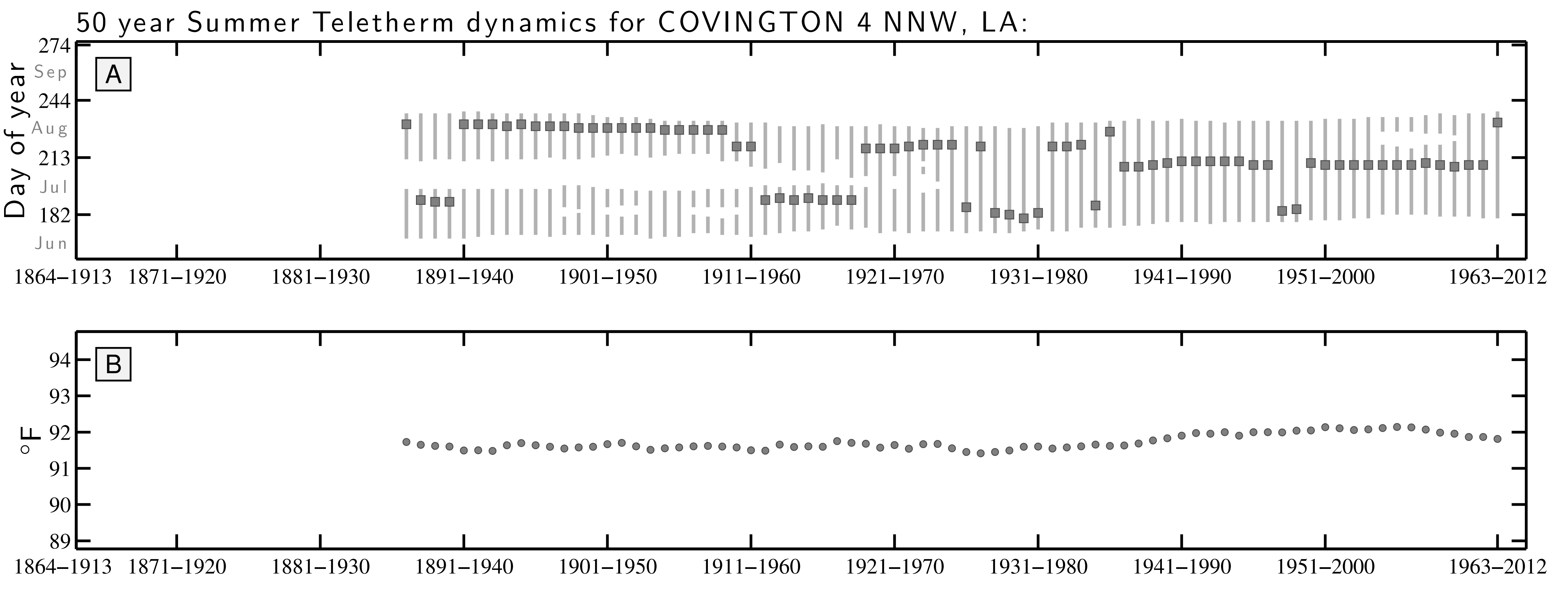
50 year Summer Teletherm dynamics for COVINGTON 4 NNW, LA:
A
B
Day of year
°F
274
Sep
244
Aug
213
Jul
182
Jun
94
93
92
91
90
89
1864–1913
1871–1920
1881–1930
1891–1940
1901–1950
1911–1960
1921–1970
1931–1980
1941–1990
1951–2000
1963–2012

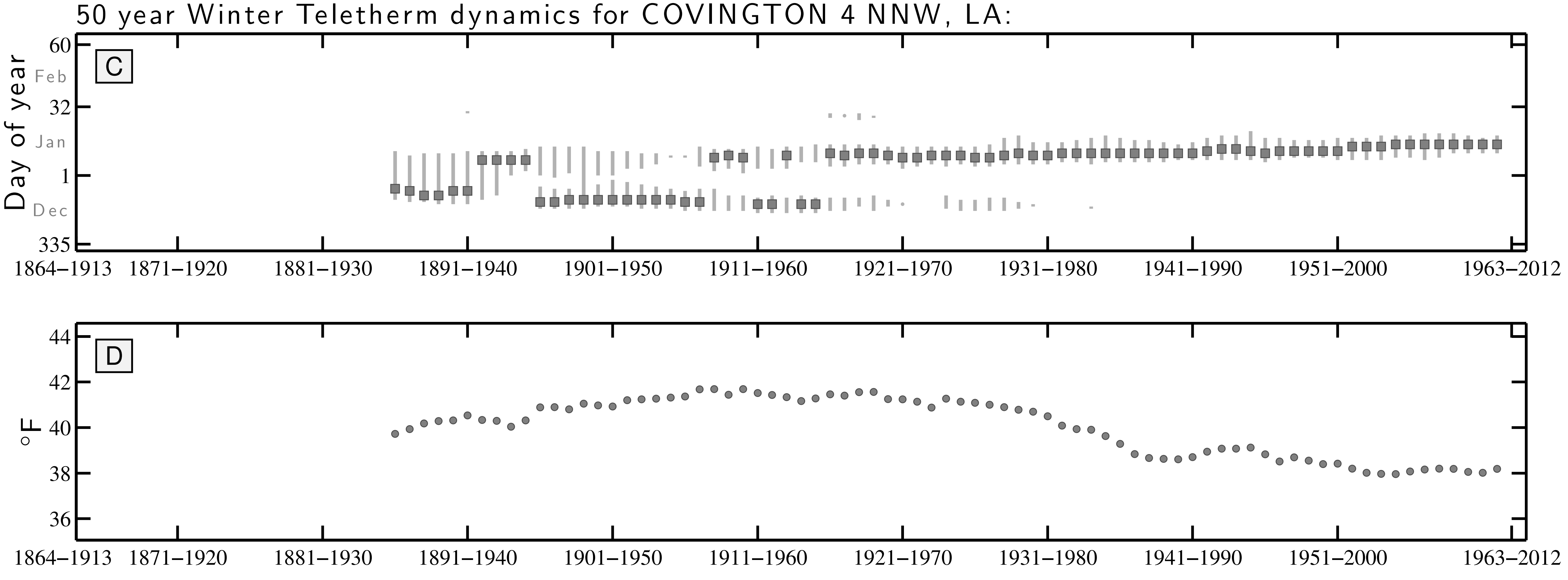
50 year Winter Teletherm dynamics for COVINGTON 4 NNW, LA:
C
D
Day of year
°F
60
Feb
32
Jan
1
Dec
335
44
42
40
38
36
1864–1913
1871–1920
1881–1930
1891–1940
1901–1950
1911–1960
1921–1970
1931–1980
1941–1990
1951–2000
1963–2012

50 year Summer Teletherm dynamics for DONALDSONVILLE 4 SW, LA:

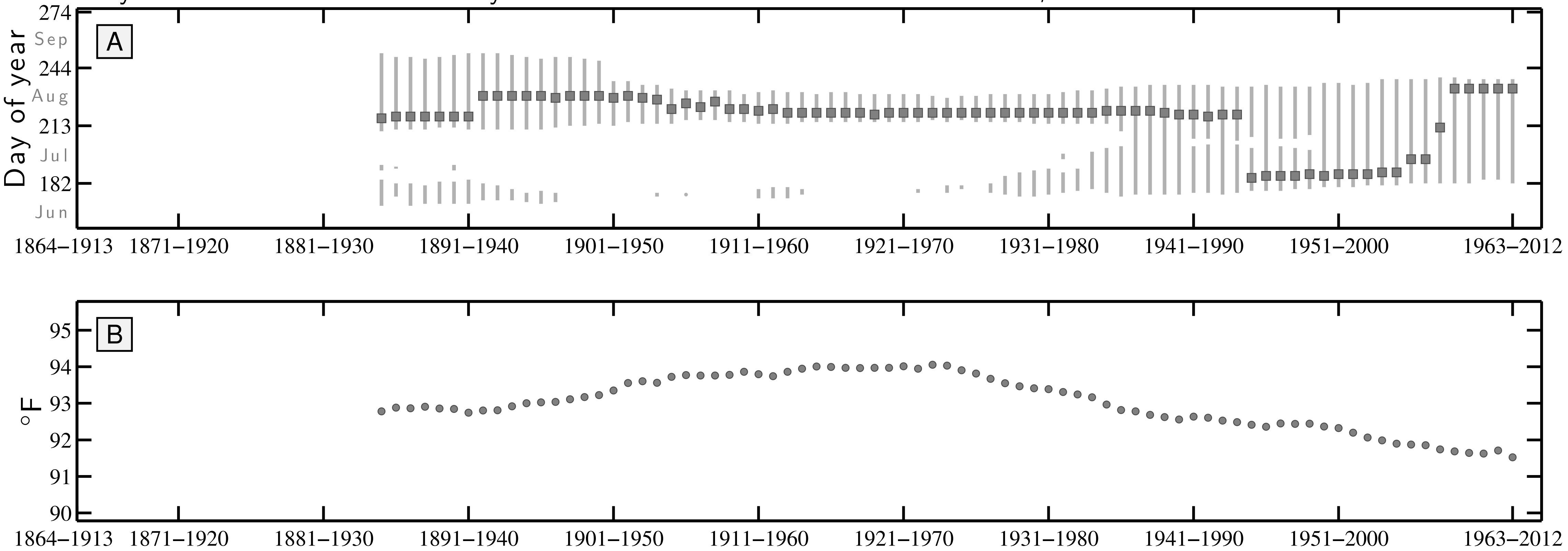

50 year Winter Teletherm dynamics for DONALDSONVILLE 4 SW, LA:

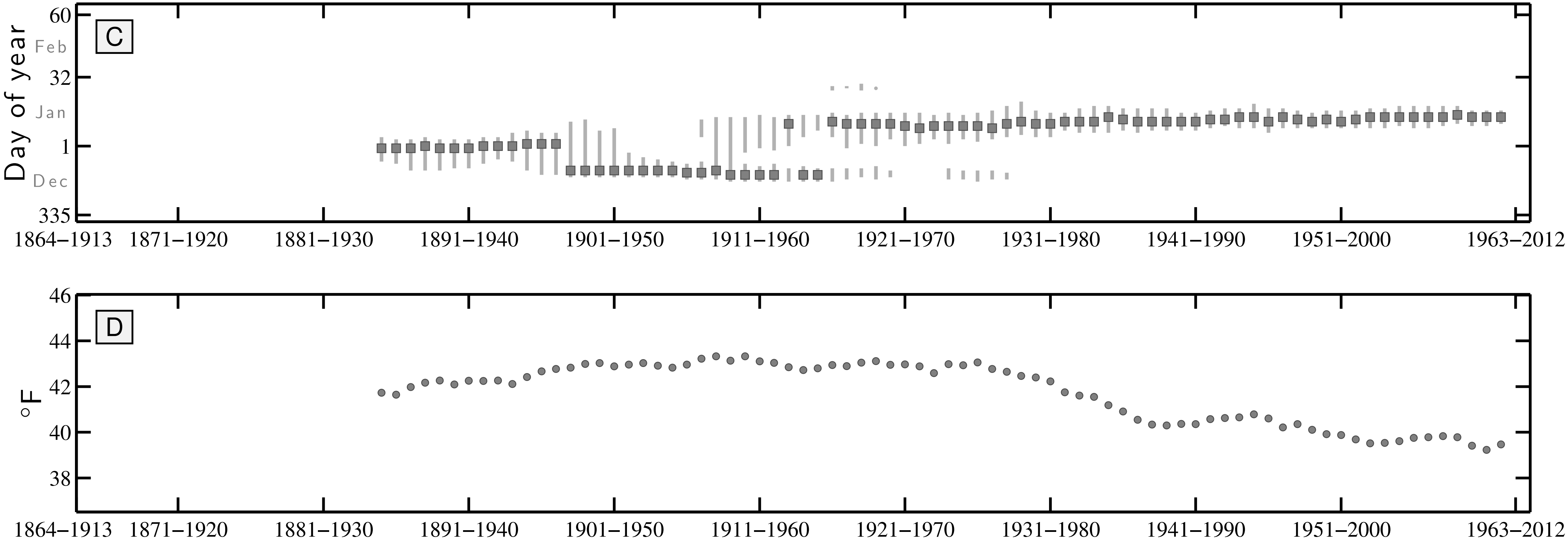

50 year Summer Teletherm dynamics for FRANKLIN 3 NW, LA:

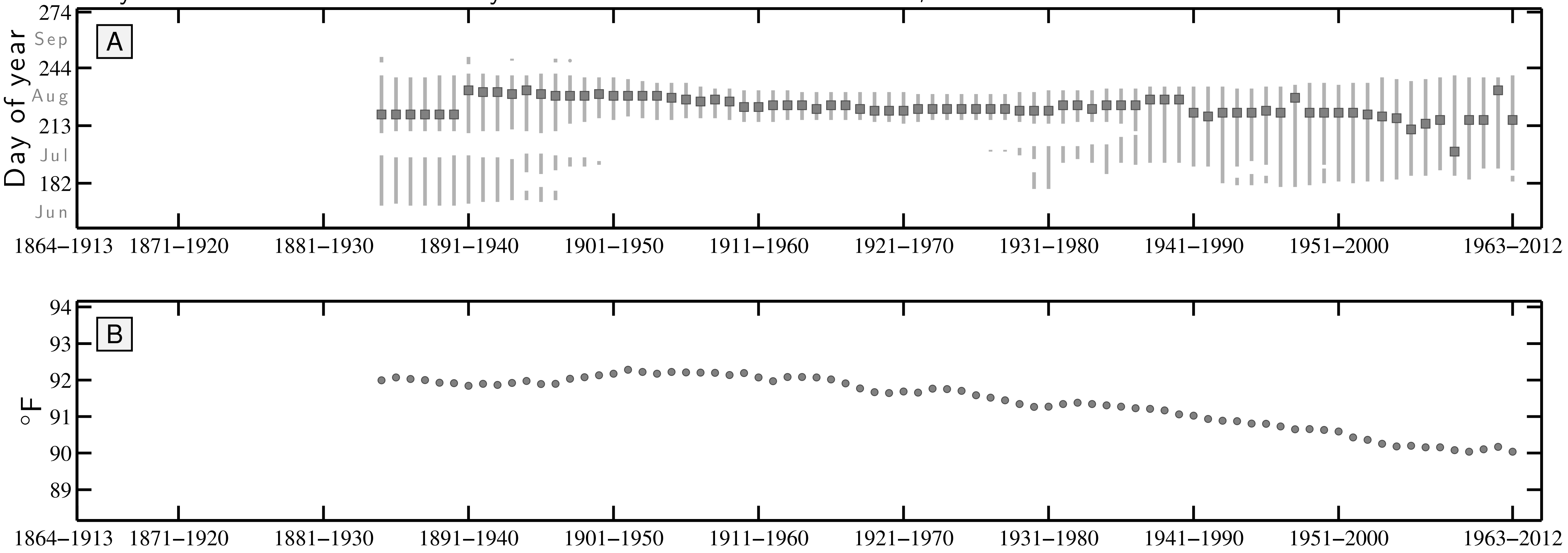

50 year Winter Teletherm dynamics for FRANKLIN 3 NW, LA:

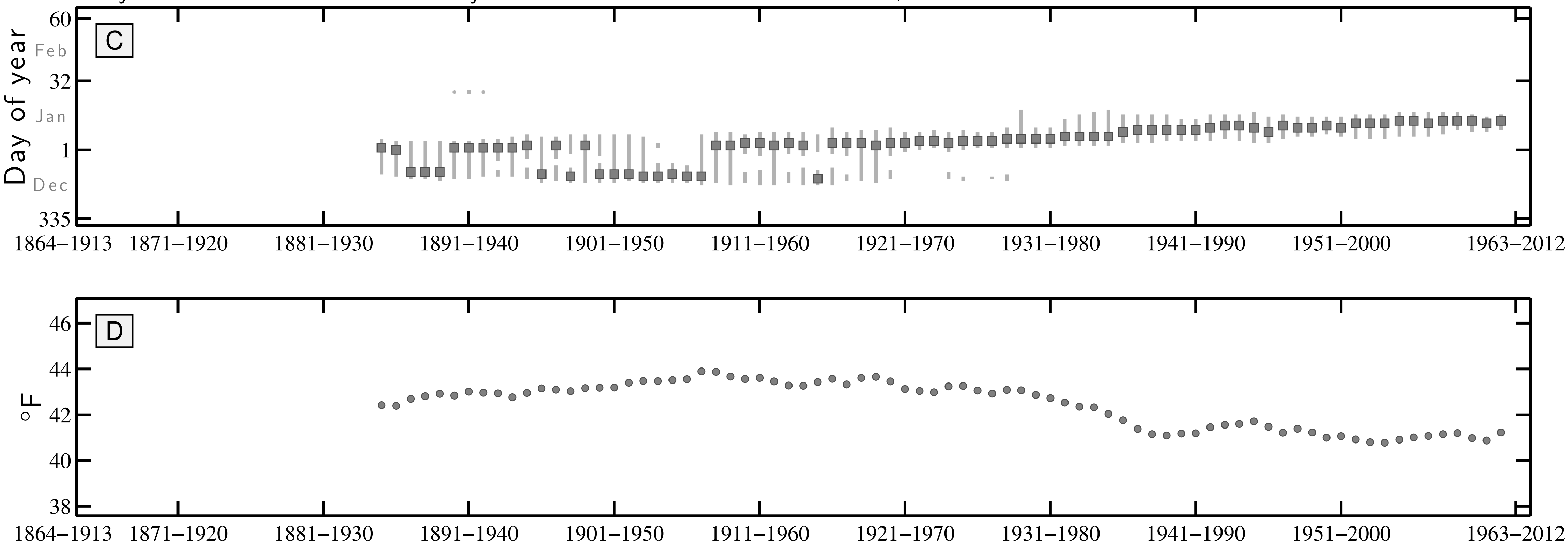

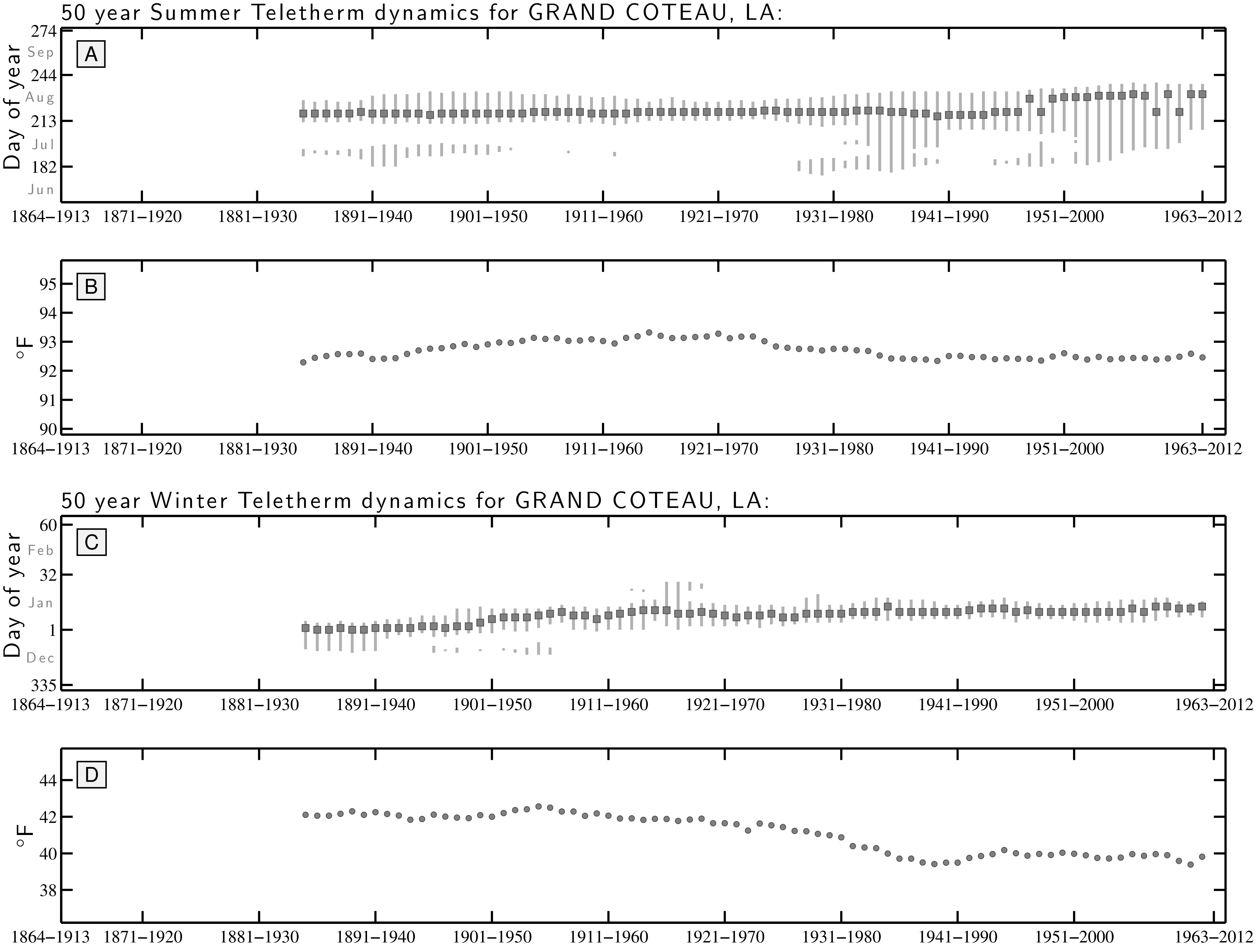
50 year Summer Teletherm dynamics for GRAND COTEAU, LA:
A
B
Day of year
274
Sep
244
Aug
213
Jul
182
Jun
°F
95
94
93
92
91
90
1864–1913
1871–1920
1881–1930
1891–1940
1901–1950
1911–1960
1921–1970
1931–1980
1941–1990
1951–2000
1963–2012
50 year Winter Teletherm dynamics for GRAND COTEAU, LA:
C
D
60
Feb
32
Jan
1
Dec
335
44
42
40
38

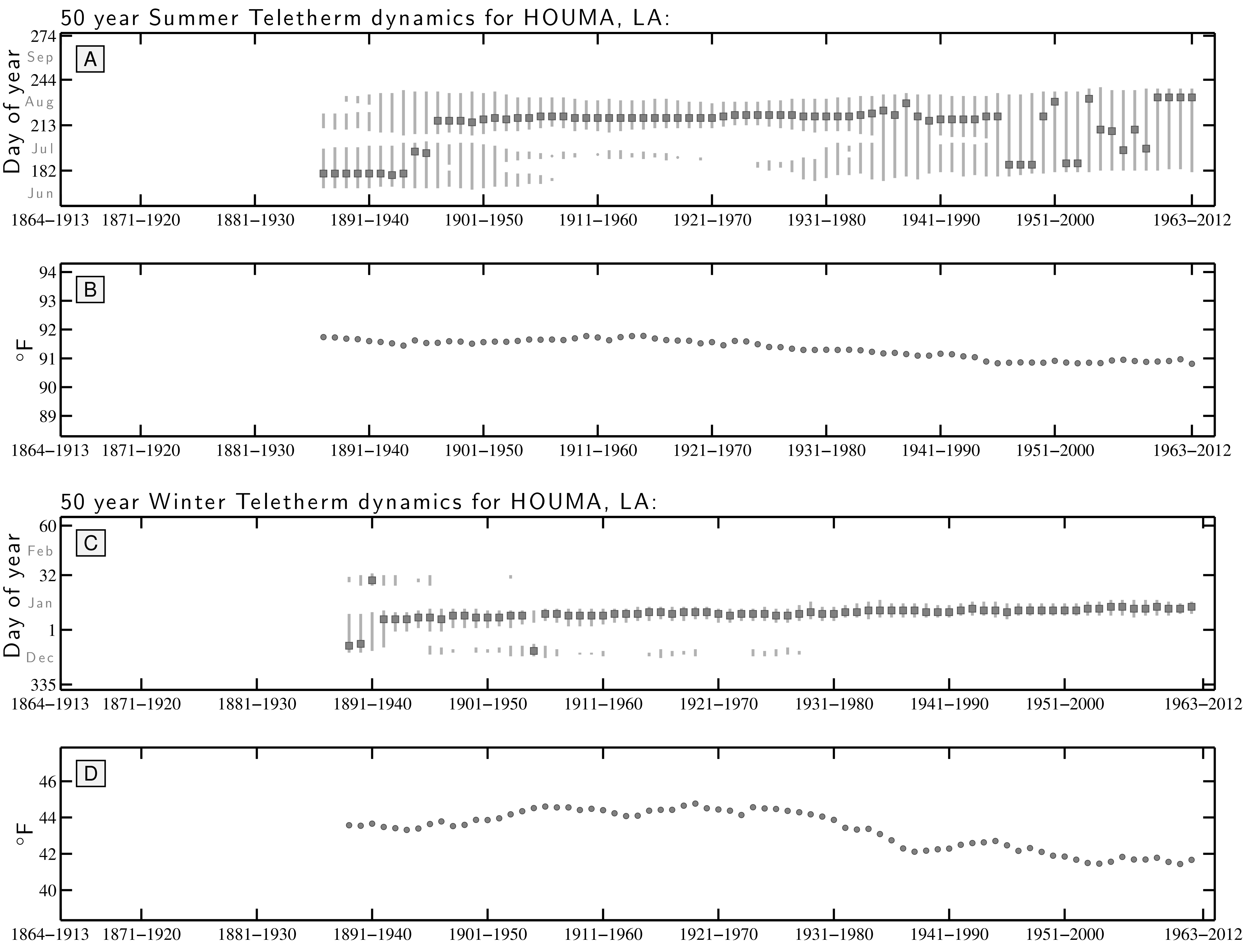
50 year Summer Teletherm dynamics for HOUMA, LA:
A
Day of year
274
Sep
244
Aug
213
Jul
182
Jun
1864–1913
1871–1920
1881–1930
1891–1940
1901–1950
1911–1960
1921–1970
1931–1980
1941–1990
1951–2000
1963–2012
B
°F
94
93
92
91
90
89
50 year Winter Teletherm dynamics for HOUMA, LA:
C
Day of year
60
Feb
32
Jan
1
Dec
335
D
°F
46
44
42
40

50 year Summer Teletherm dynamics for JENNINGS, LA:

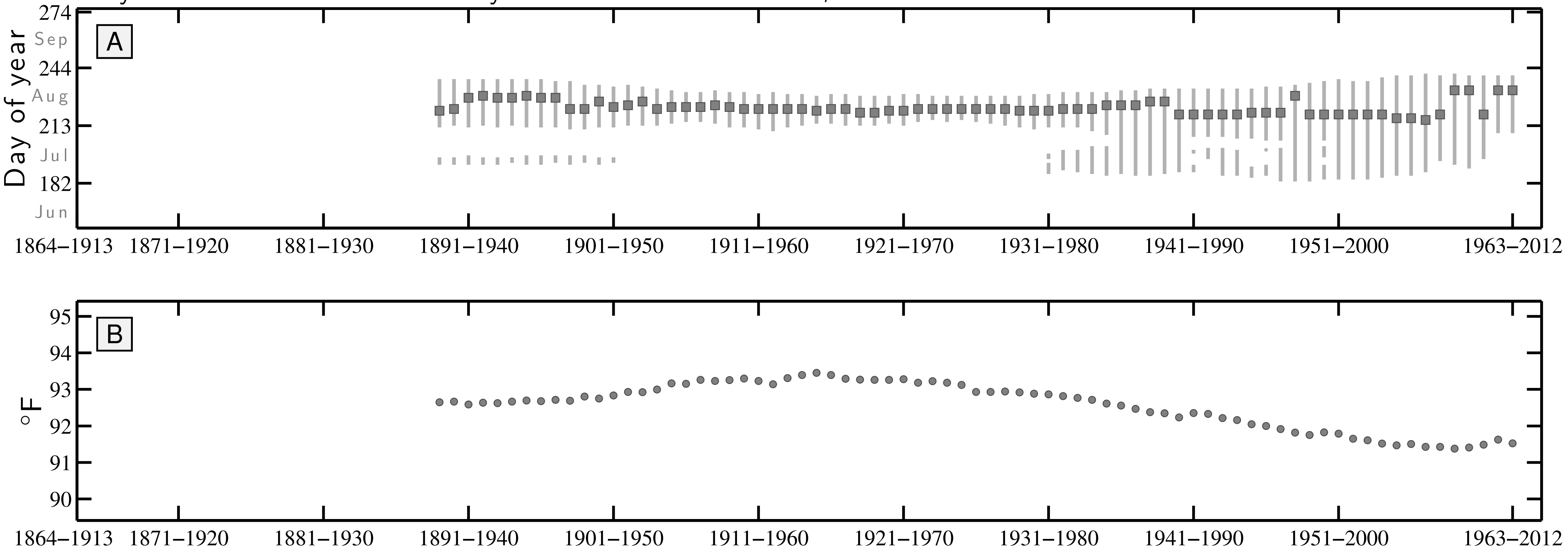

50 year Winter Teletherm dynamics for JENNINGS, LA:

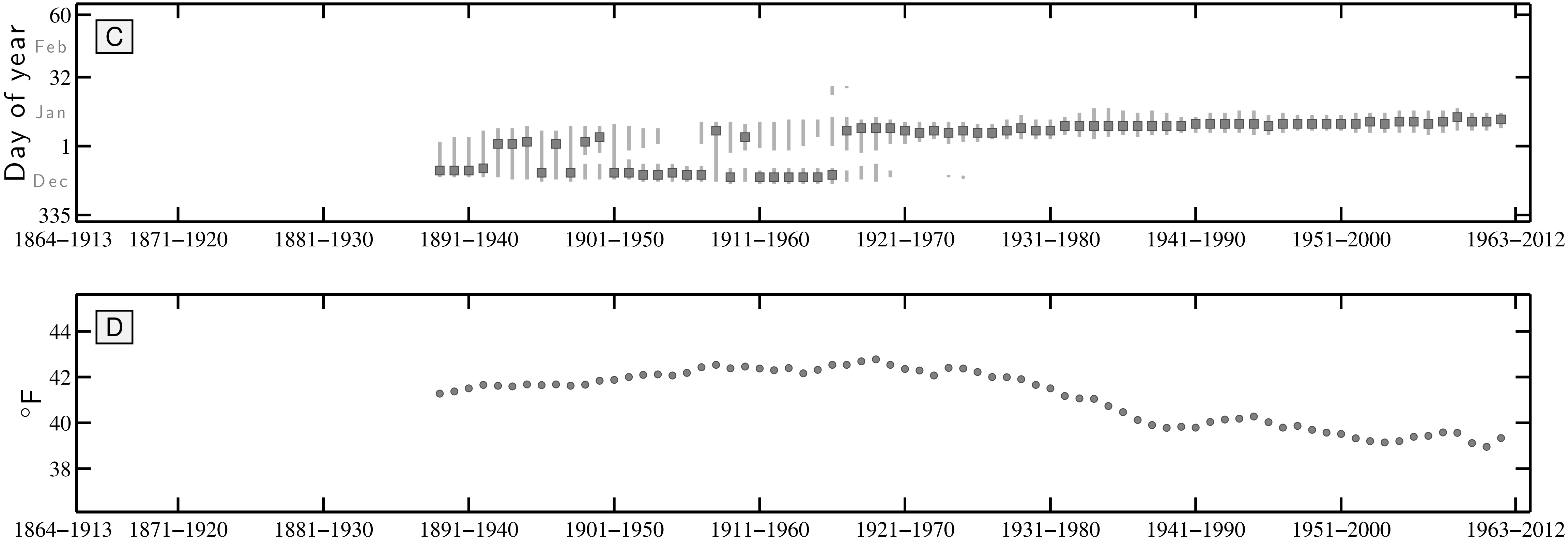

50 year Summer Teletherm dynamics for LAFAYETTE FCWOS, LA:

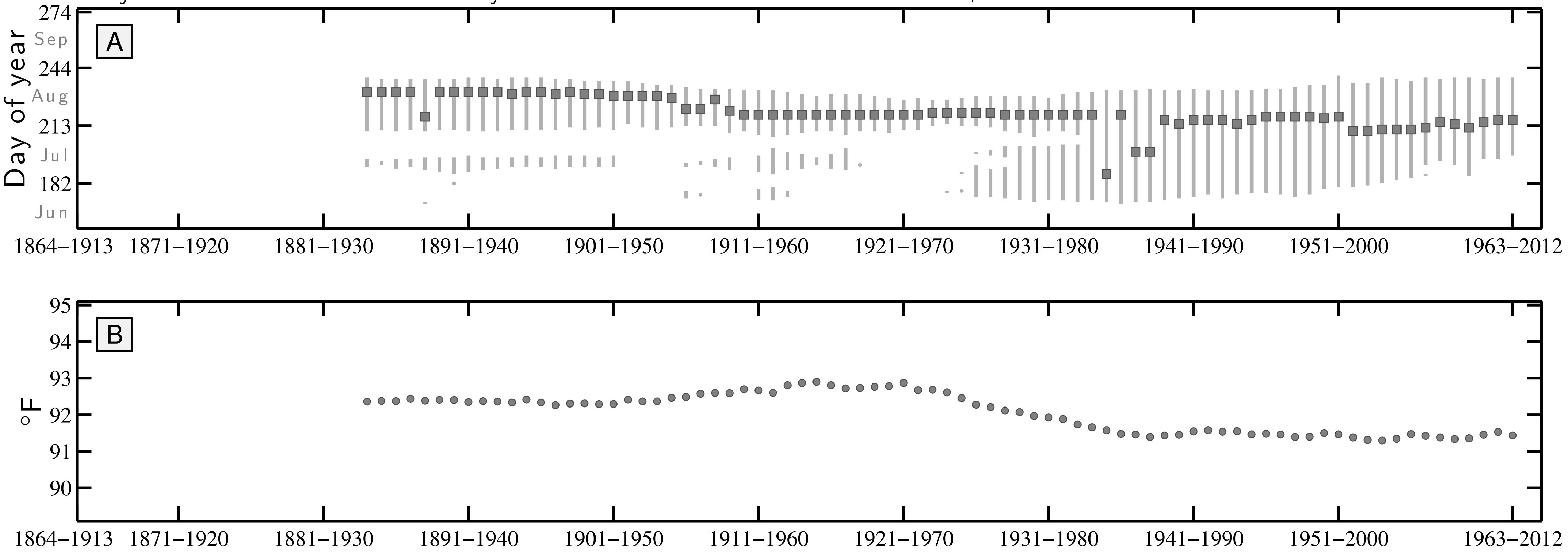

50 year Winter Teletherm dynamics for LAFAYETTE FCWOS, LA:

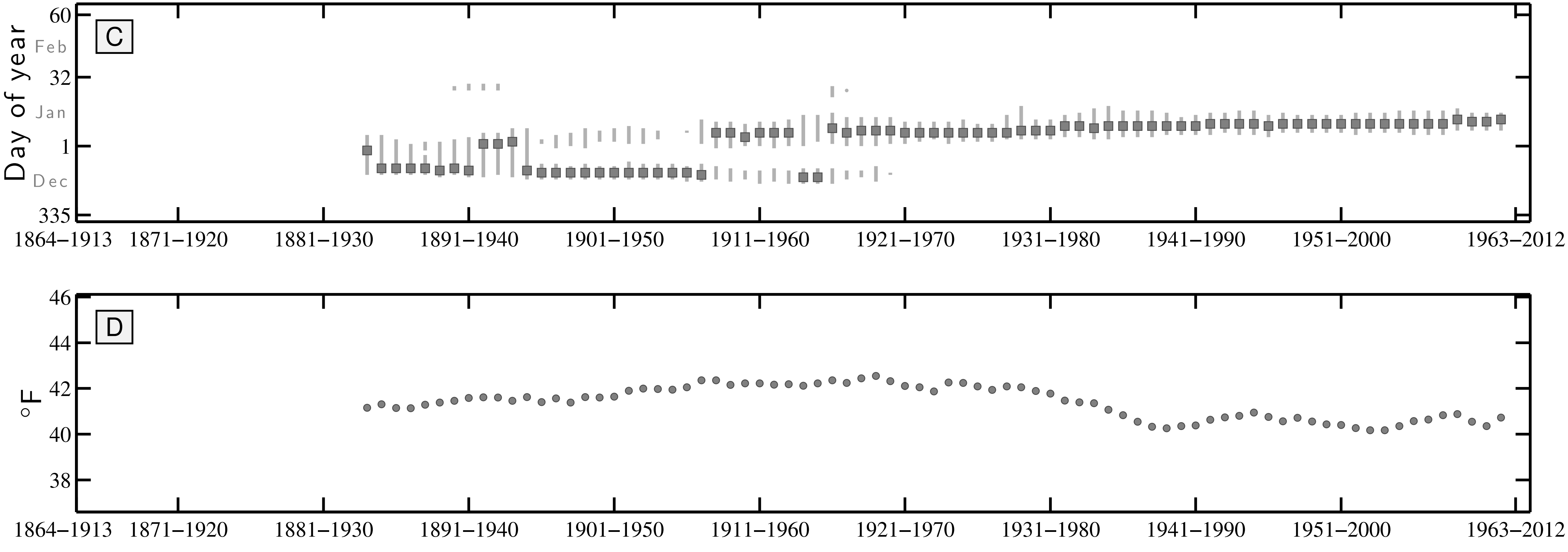

50 year Summer Teletherm dynamics for NEW ORLEANS AUDUBON, LA:

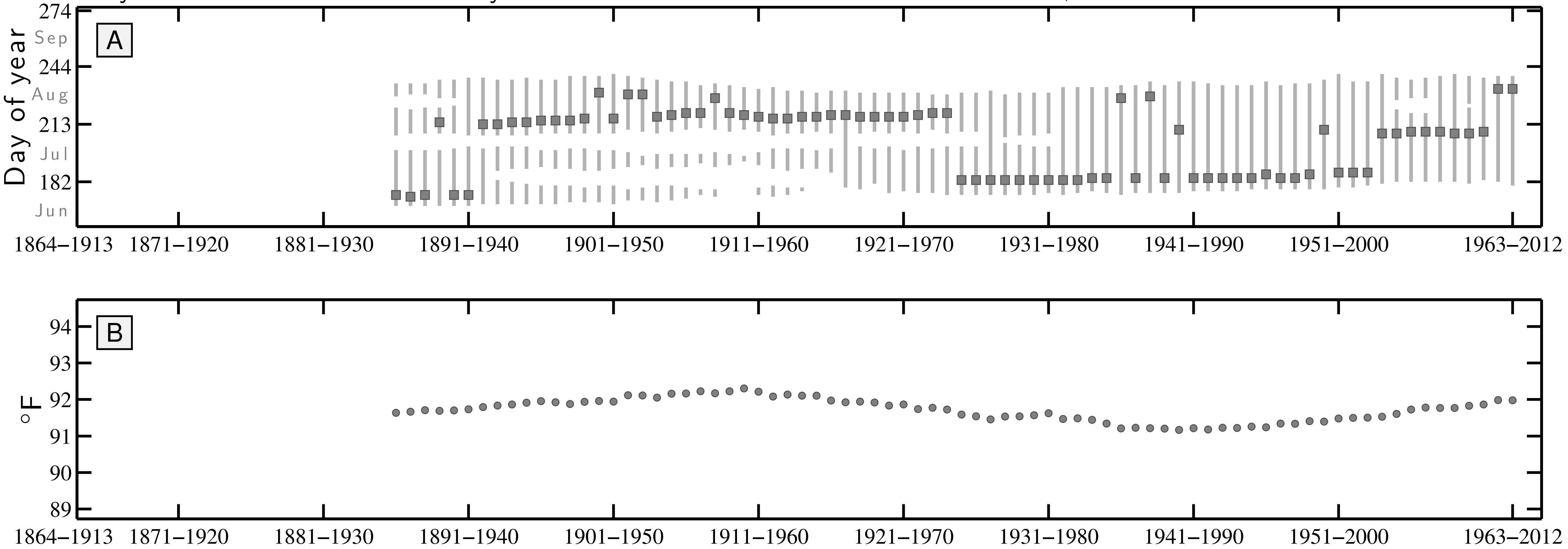

50 year Winter Teletherm dynamics for NEW ORLEANS AUDUBON, LA:

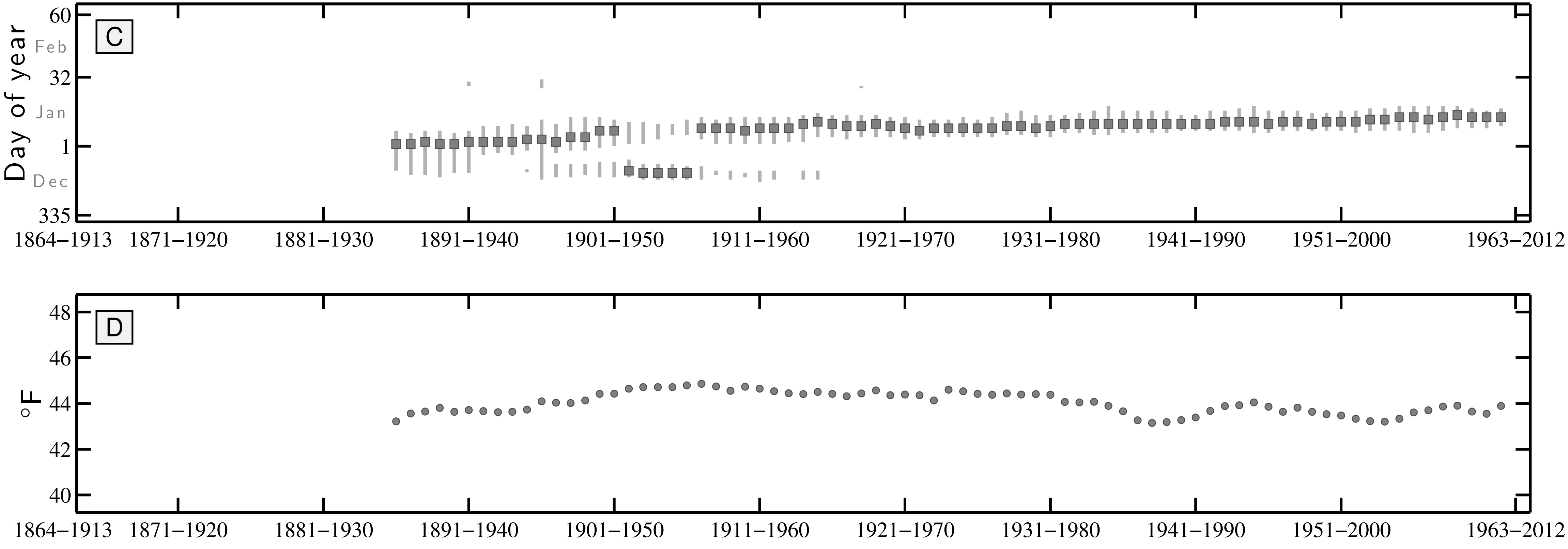

50 year Summer Teletherm dynamics for PLAIN DEALING 4 W, LA:

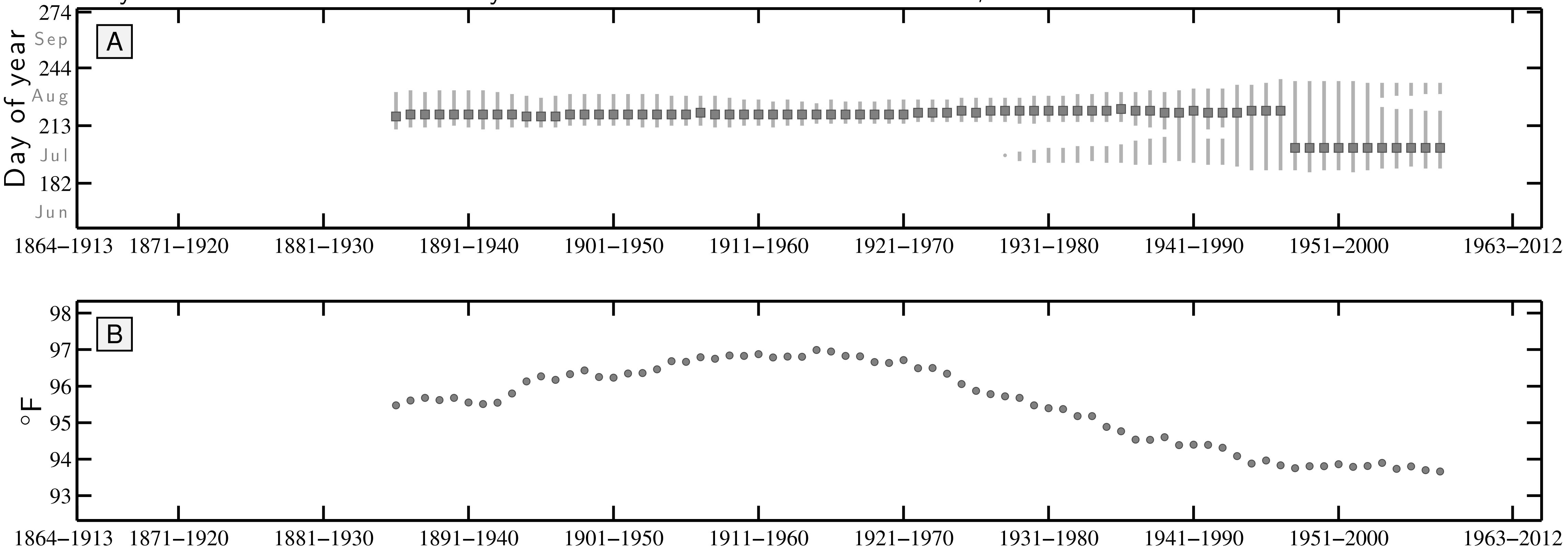

50 year Winter Teletherm dynamics for PLAIN DEALING 4 W, LA:

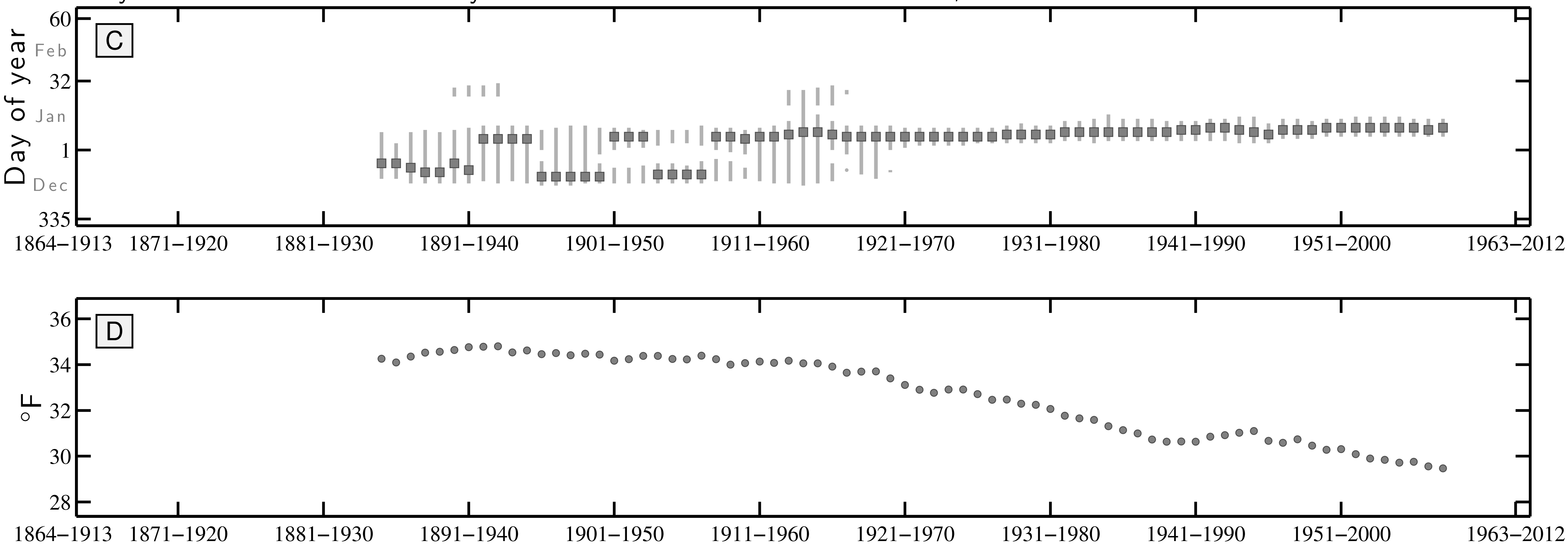

50 year Summer Teletherm dynamics for ST JOSEPH 3 N, LA:

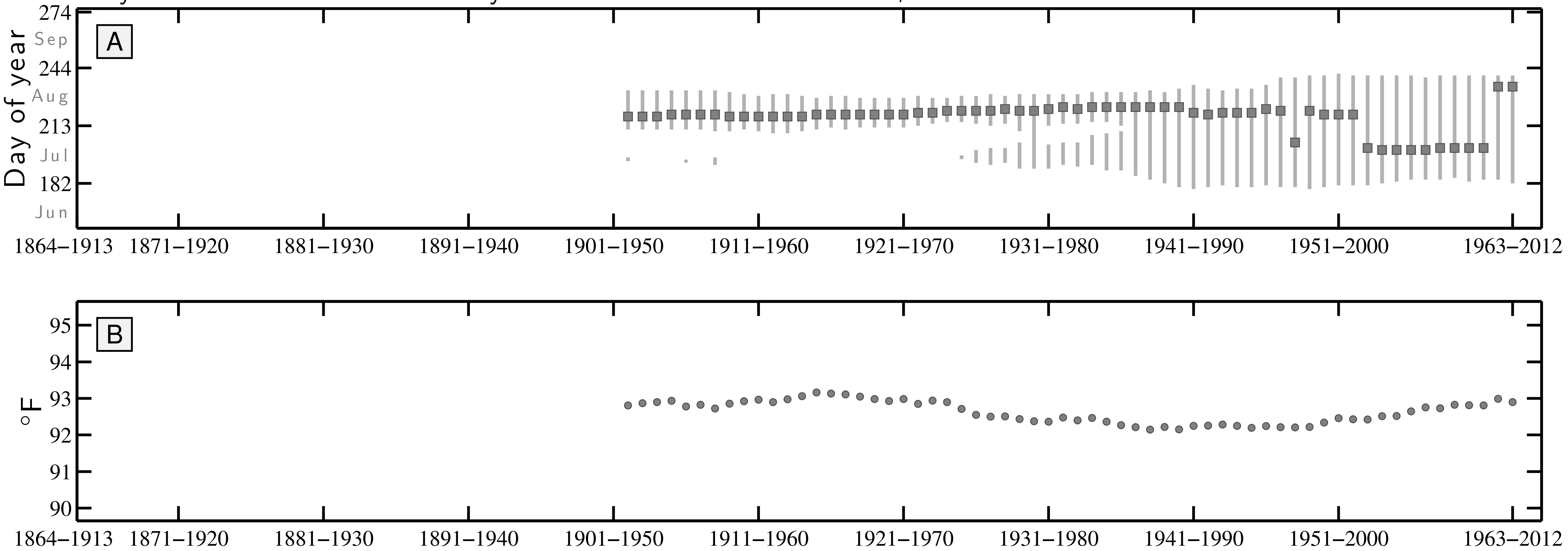

50 year Winter Teletherm dynamics for ST JOSEPH 3 N, LA:

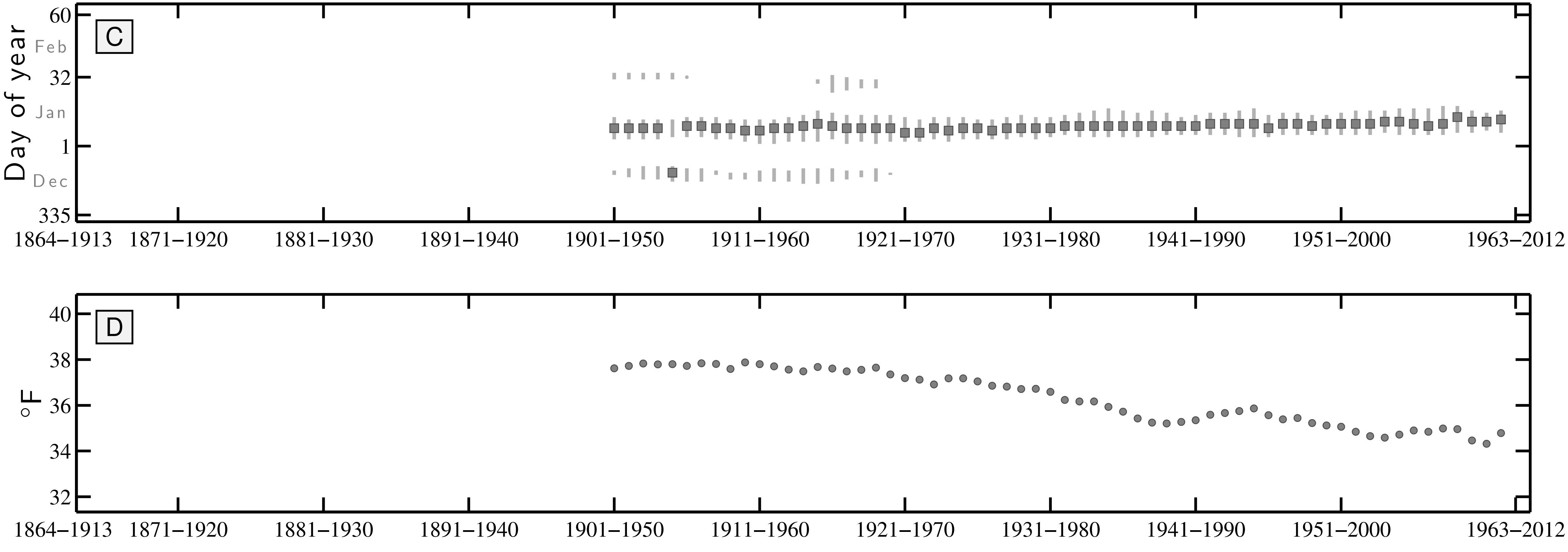

50 year Summer Teletherm dynamics for WINNSBORO 5 SSE, LA:

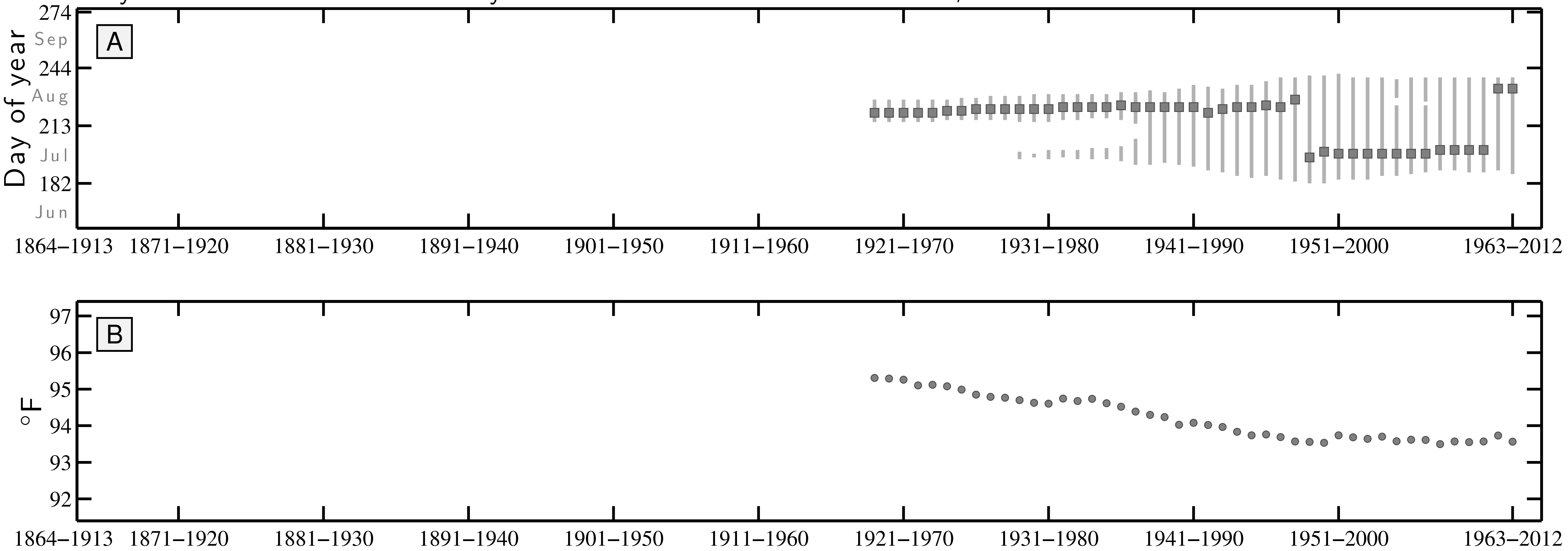

50 year Winter Teletherm dynamics for WINNSBORO 5 SSE, LA:

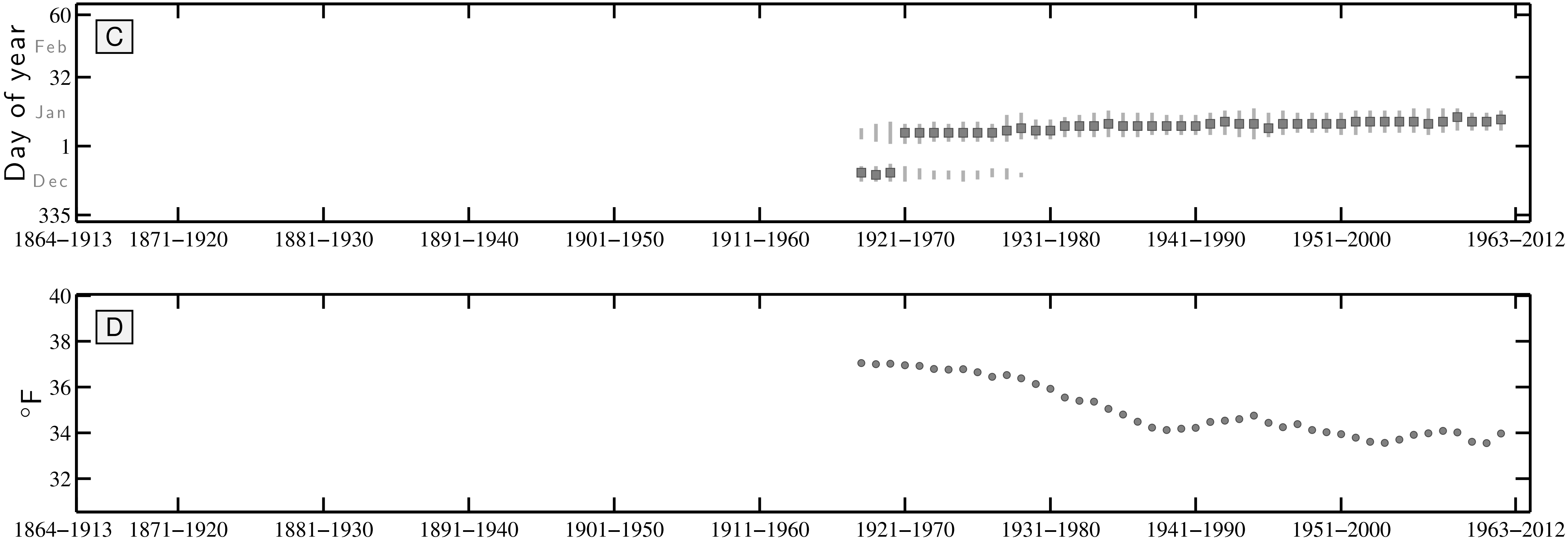

50 year Summer Teletherm dynamics for BRASSUA DAM, ME:

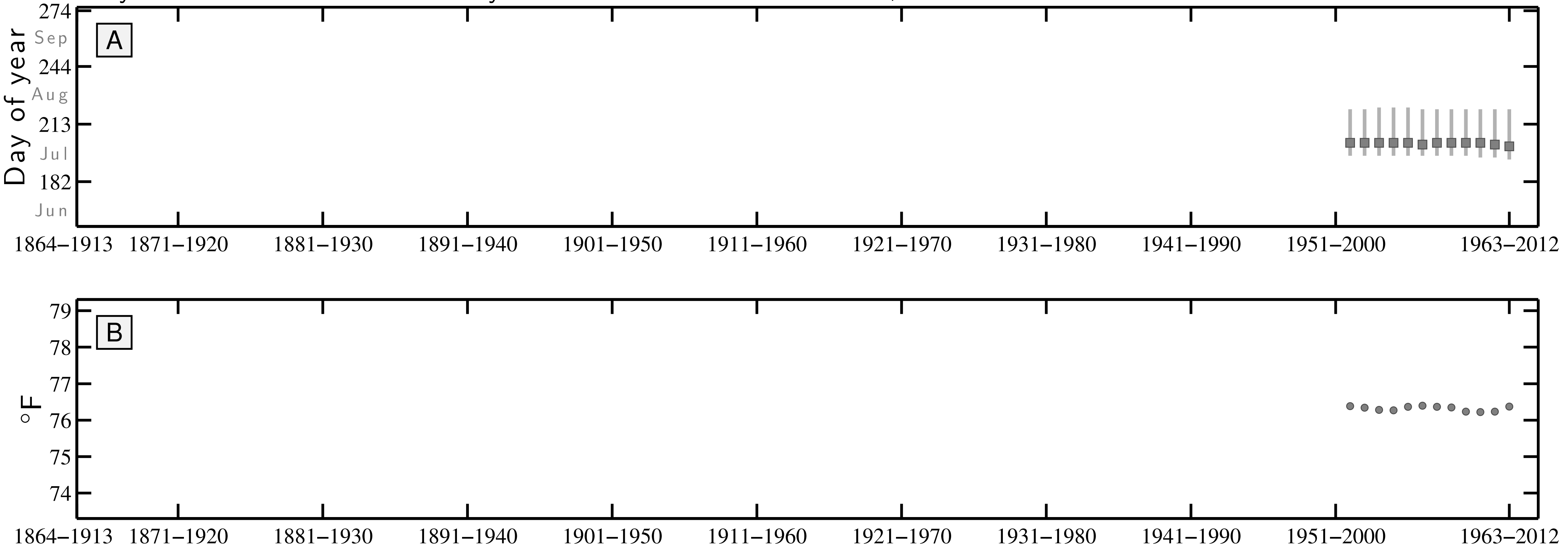

50 year Winter Teletherm dynamics for BRASSUA DAM, ME:

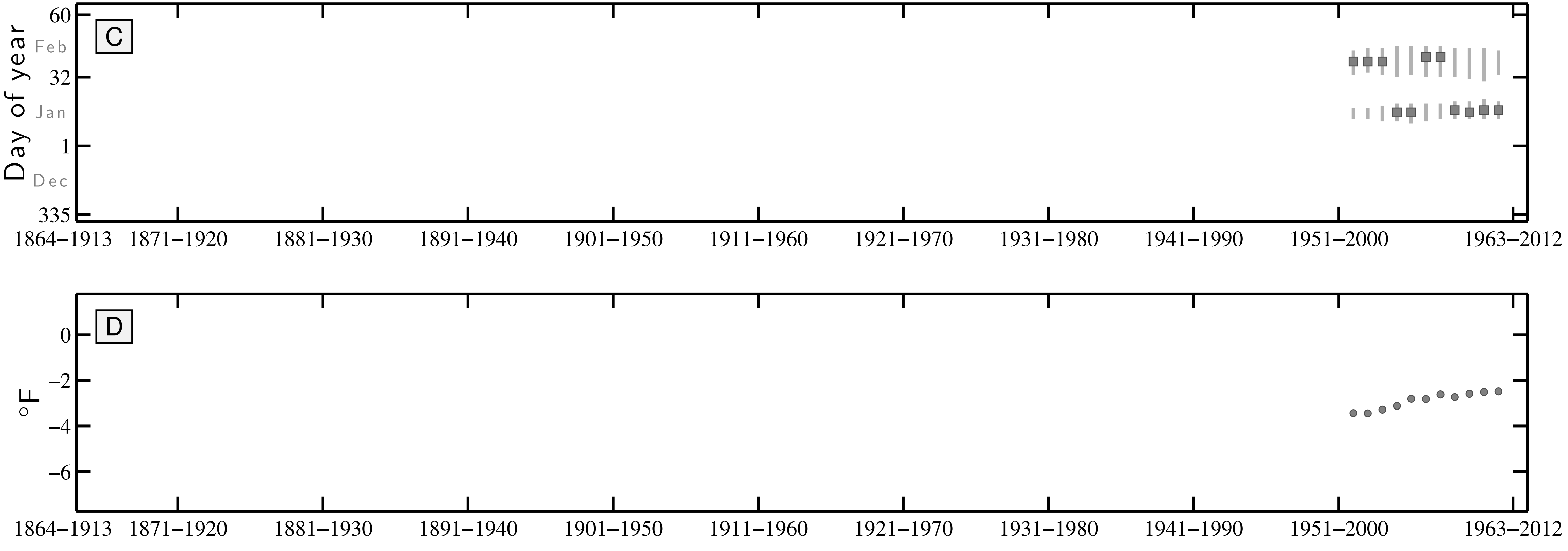

50 year Summer Teletherm dynamics for CORINNA, ME:

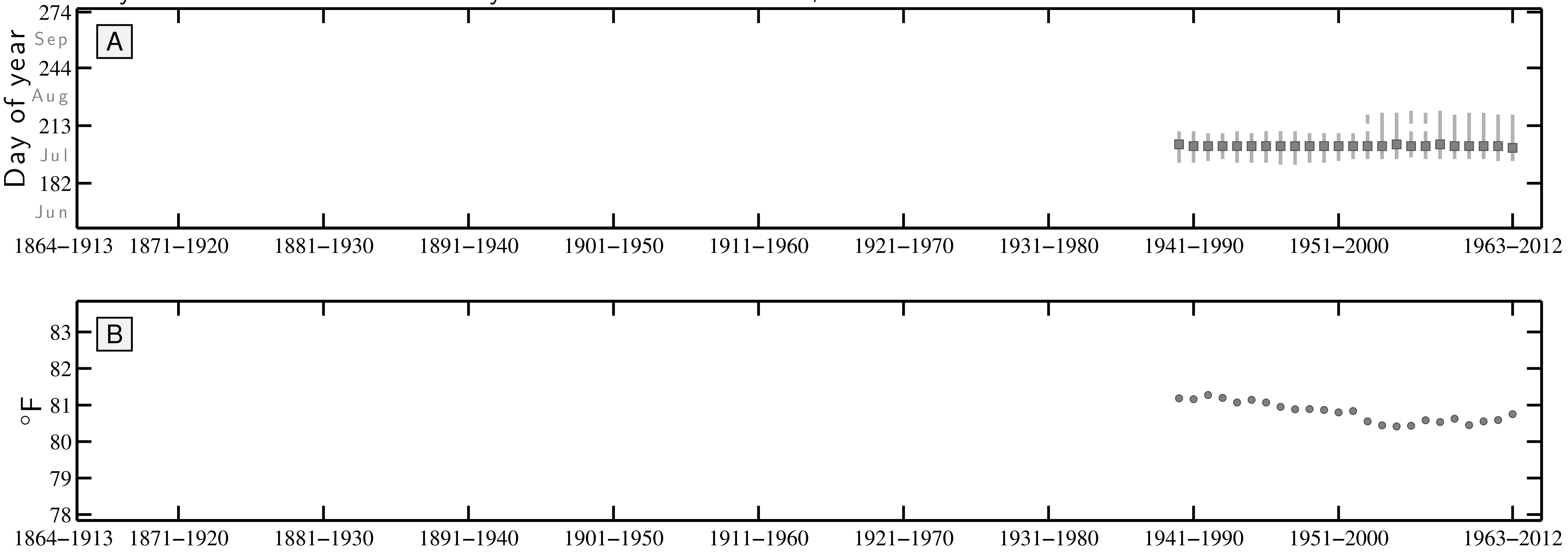

50 year Winter Teletherm dynamics for CORINNA, ME:

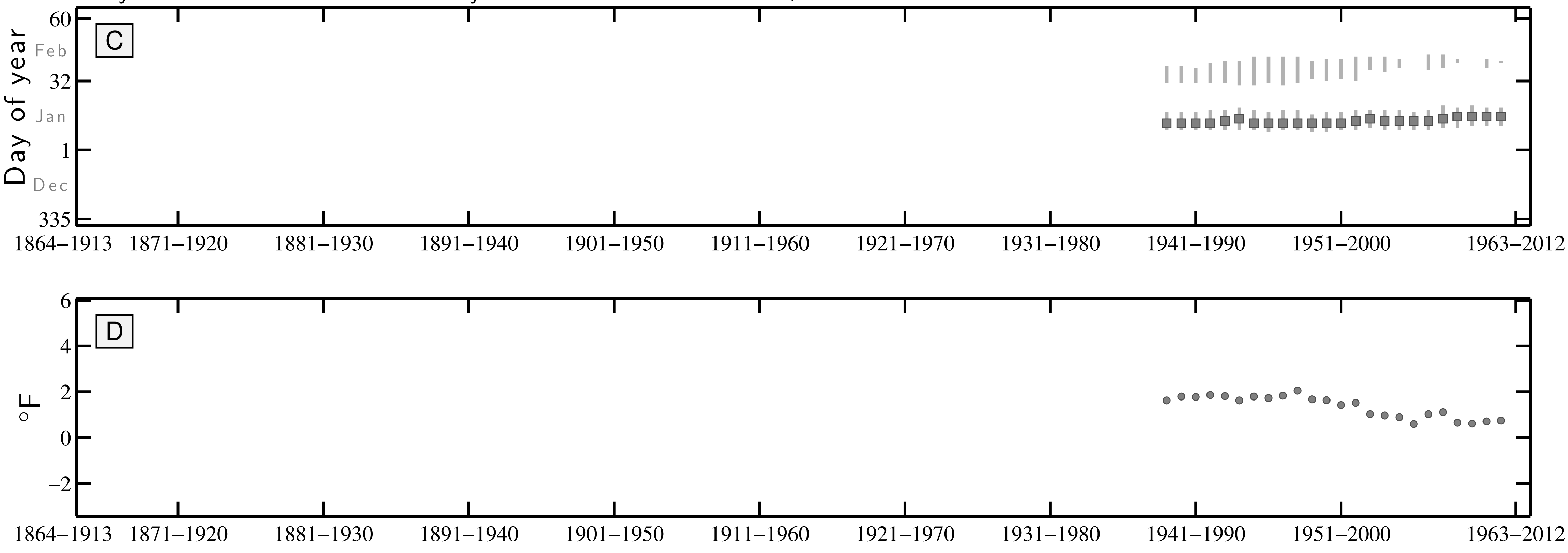

50 year Summer Teletherm dynamics for EASTPORT, ME:

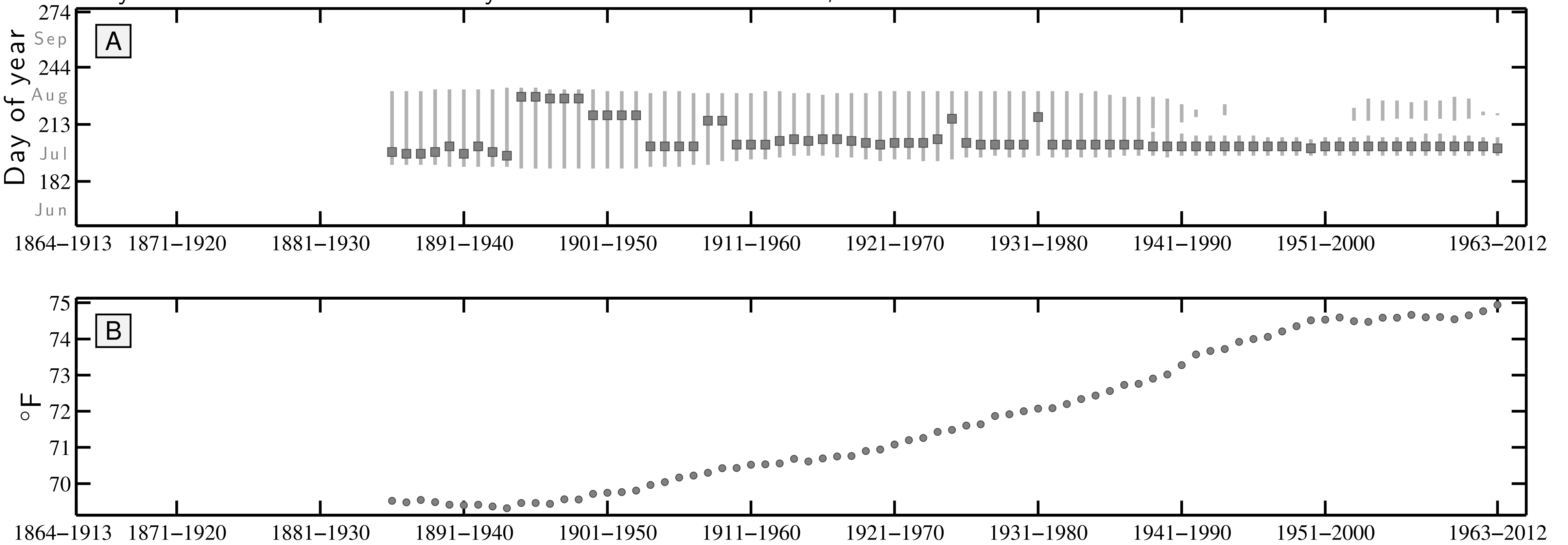

50 year Winter Teletherm dynamics for EASTPORT, ME:

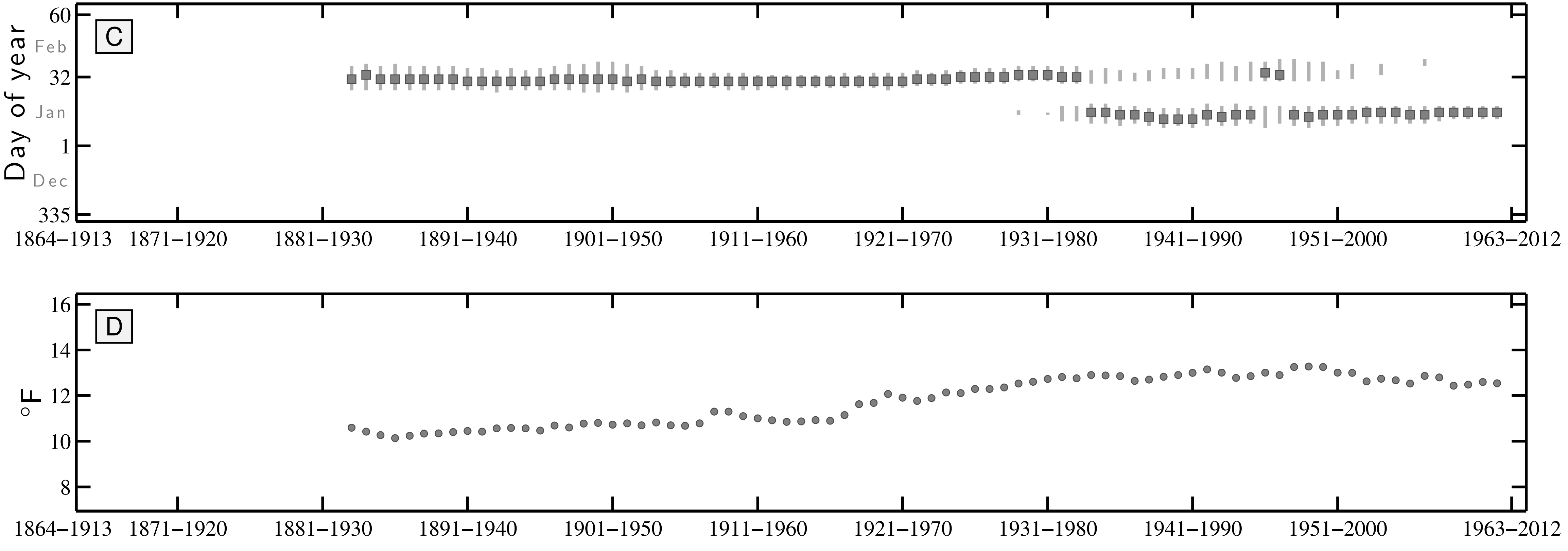

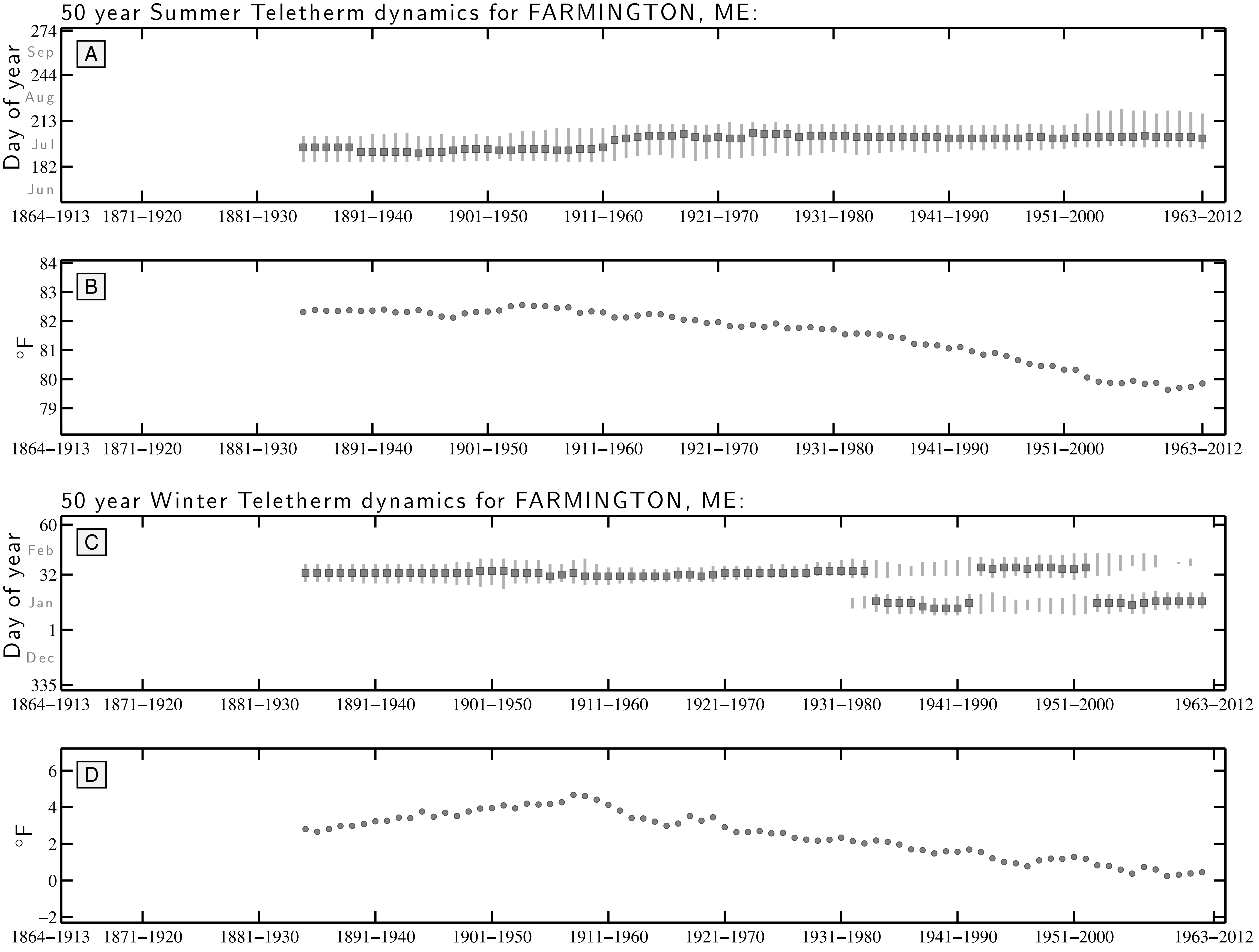

50 year Summer Teletherm dynamics for FARMINGTON, ME:
A
Day of year
274
Sep
244
Aug
213
Jul
182
Jun
1864–1913
1871–1920
1881–1930
1891–1940
1901–1950
1911–1960
1921–1970
1931–1980
1941–1990
1951–2000
1963–2012
B
°F
84
83
82
81
80
79
50 year Winter Teletherm dynamics for FARMINGTON, ME:
C
60
Feb
32
Jan
1
Dec
335
D
6
4
2
0
−2

50 year Summer Teletherm dynamics for GARDINER, ME:

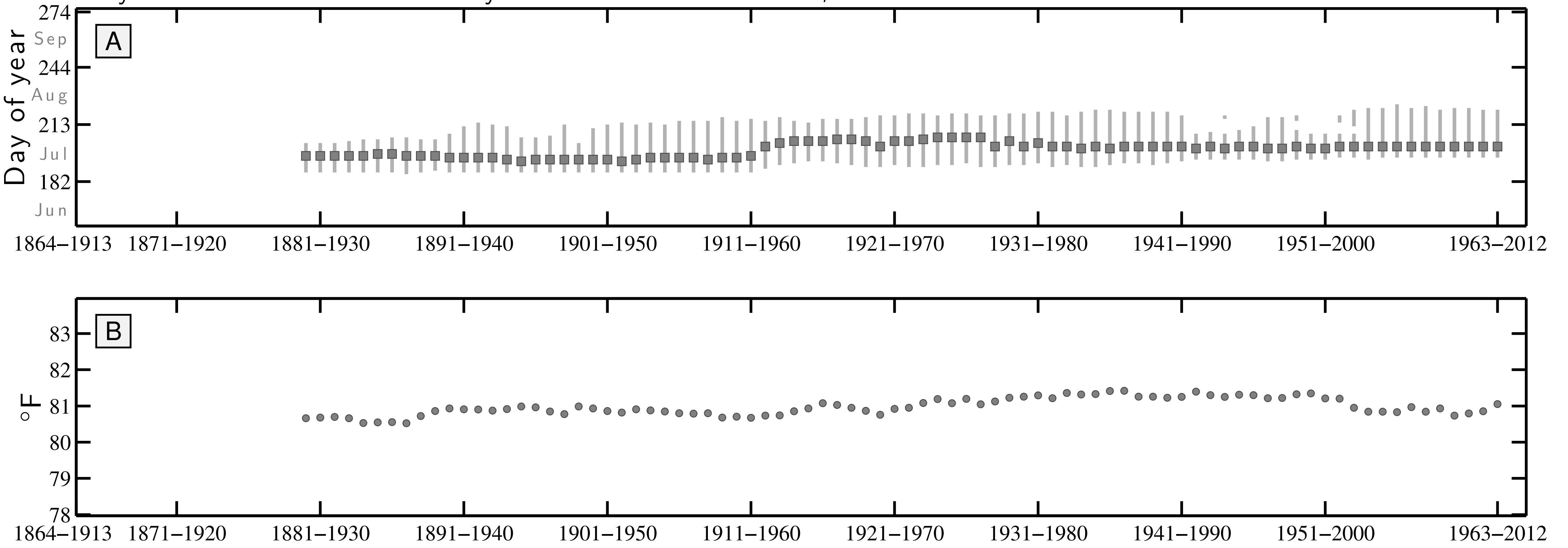

50 year Winter Teletherm dynamics for GARDINER, ME:

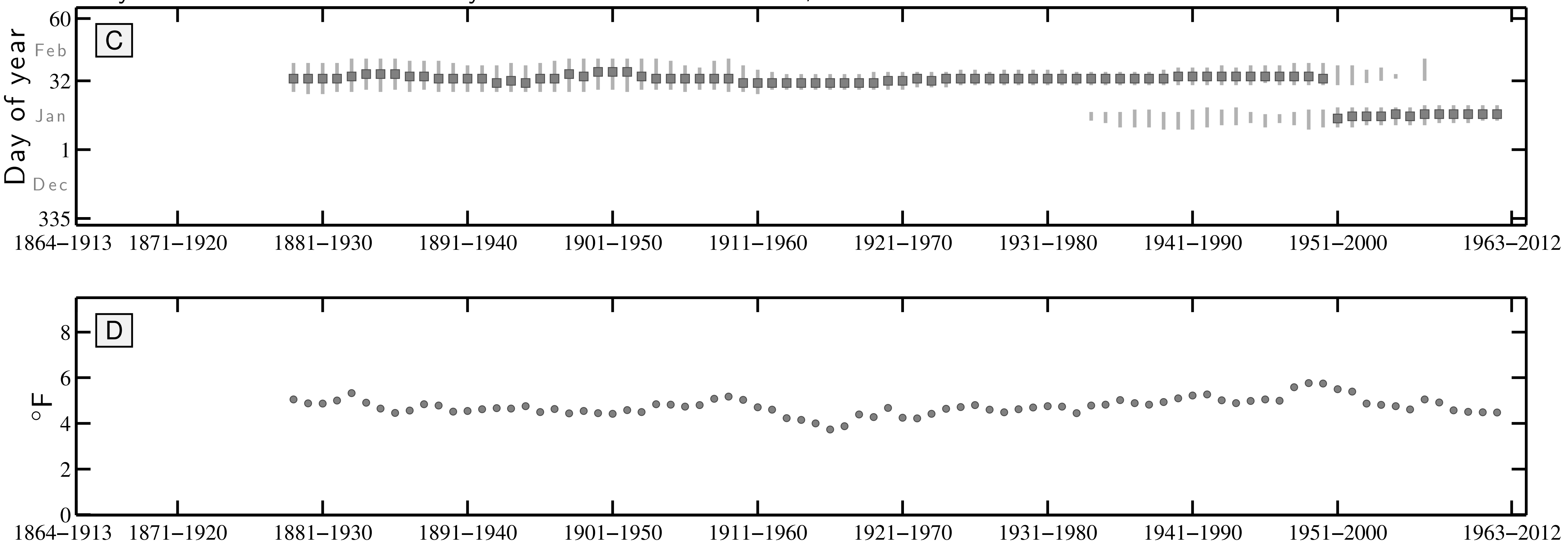

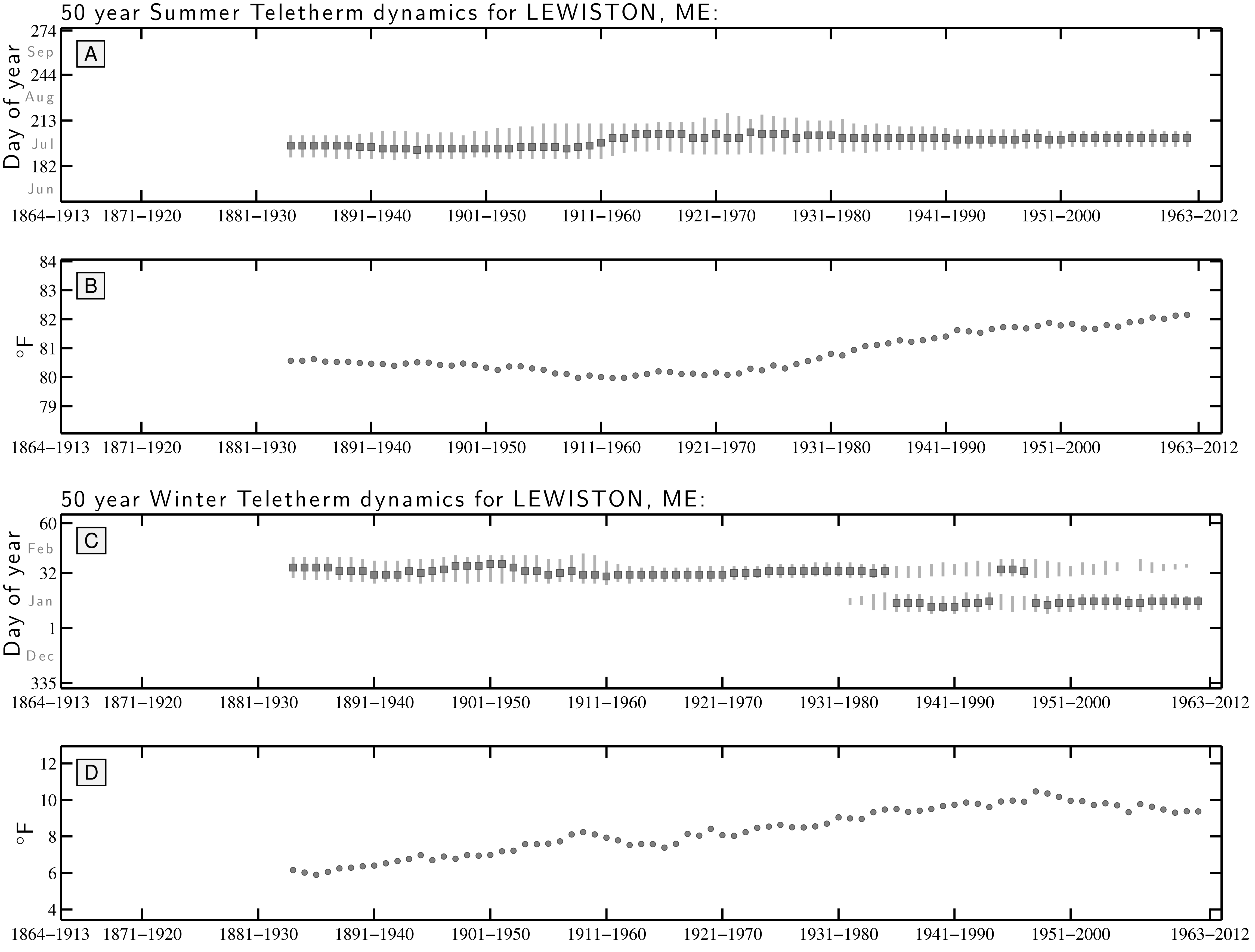
50 year Summer Teletherm dynamics for LEWISTON, ME:
A
B
Day of year
°F
274
Sep
244
Aug
213
Jul
182
Jun
84
83
82
81
80
79
1864–1913
1871–1920
1881–1930
1891–1940
1901–1950
1911–1960
1921–1970
1931–1980
1941–1990
1951–2000
1963–2012
50 year Winter Teletherm dynamics for LEWISTON, ME:
C
D
60
Feb
32
Jan
1
Dec
335
12
10
8
6
4

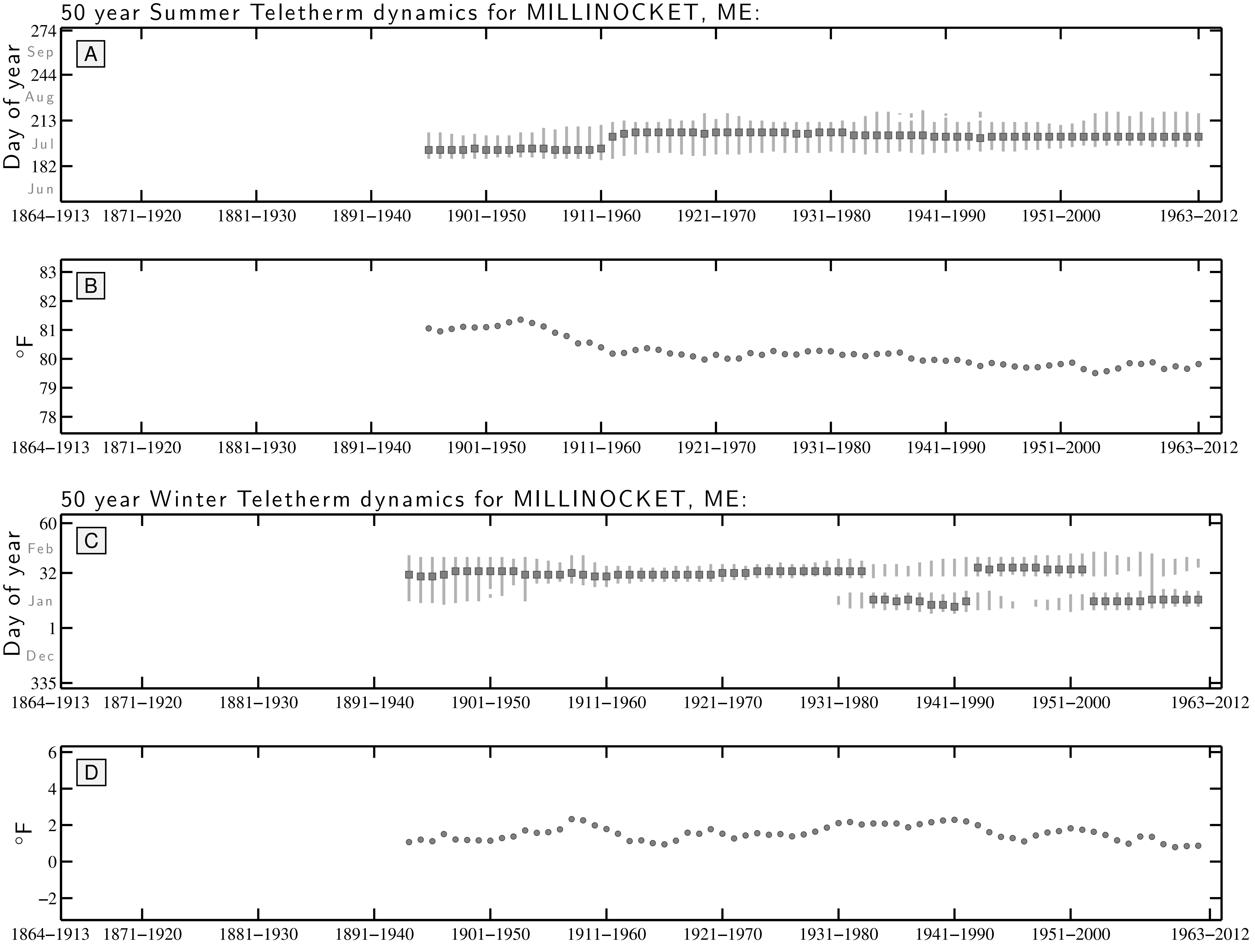

50 year Summer Teletherm dynamics for MILLINOCKET, ME:
A
Day of year
274
Sep
244
Aug
213
Jul
182
Jun
B
°F
83
82
81
80
79
78
1864–1913
1871–1920
1881–1930
1891–1940
1901–1950
1911–1960
1921–1970
1931–1980
1941–1990
1951–2000
1963–2012
50 year Winter Teletherm dynamics for MILLINOCKET, ME:
C
Day of year
60
Feb
32
Jan
1
Dec
335
D
°F
6
4
2
0
−2

50 year Summer Teletherm dynamics for PORTLAND JETPORT, ME:

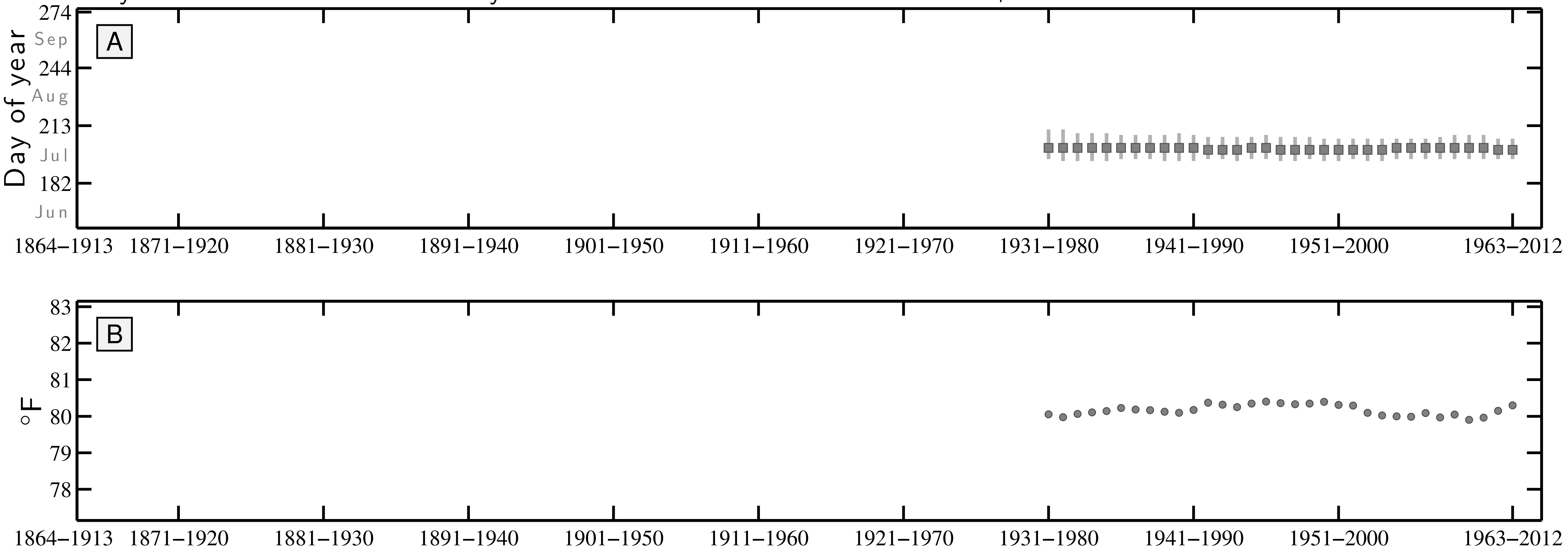

50 year Winter Teletherm dynamics for PORTLAND JETPORT, ME:

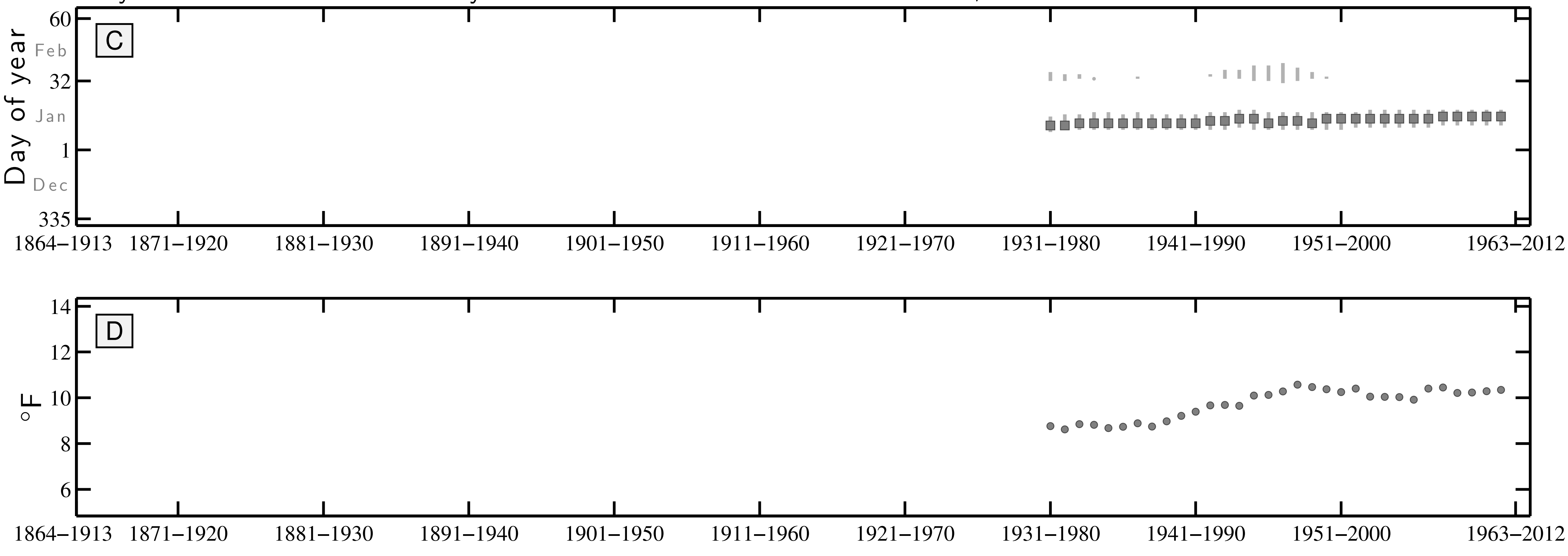

50 year Summer Teletherm dynamics for PRESQUE ISLE, ME:

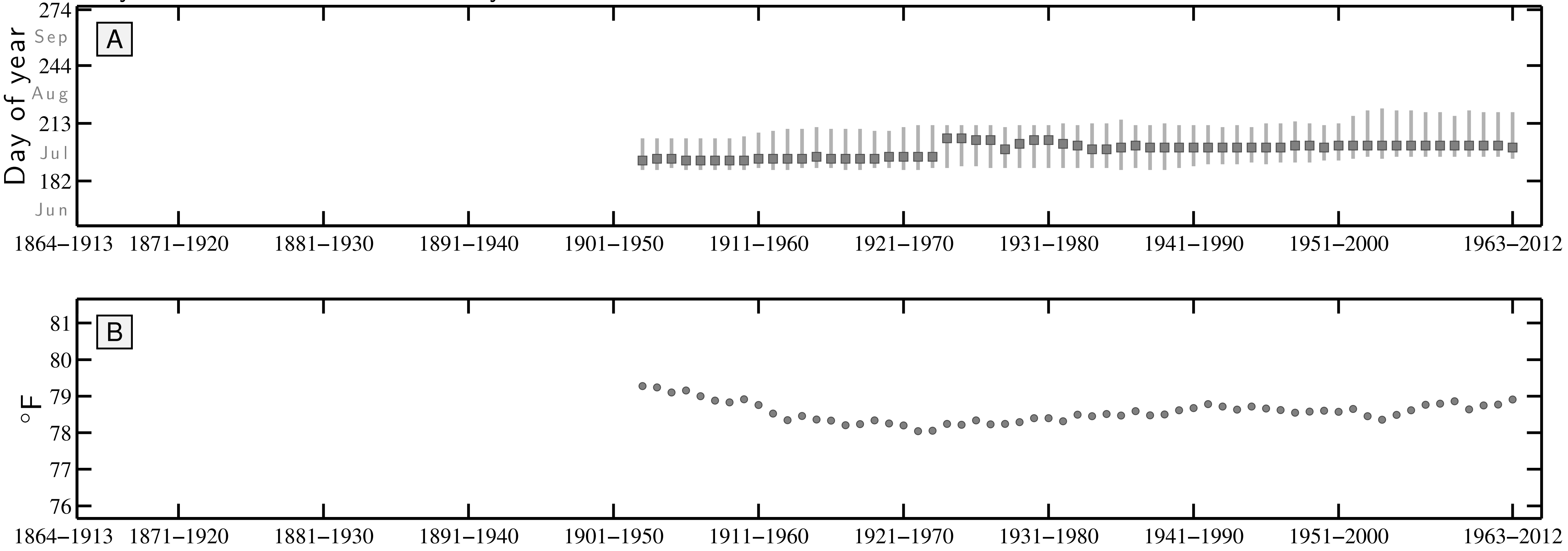

50 year Winter Teletherm dynamics for PRESQUE ISLE, ME:

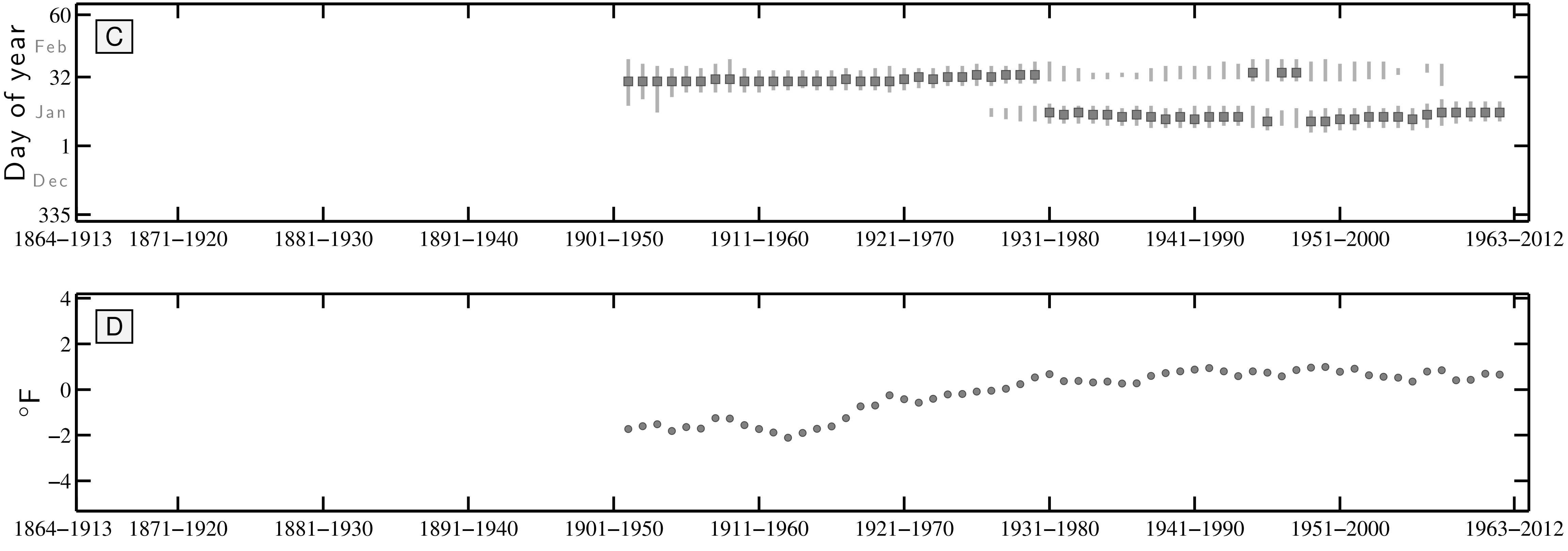

50 year Summer Teletherm dynamics for WOODLAND, ME:

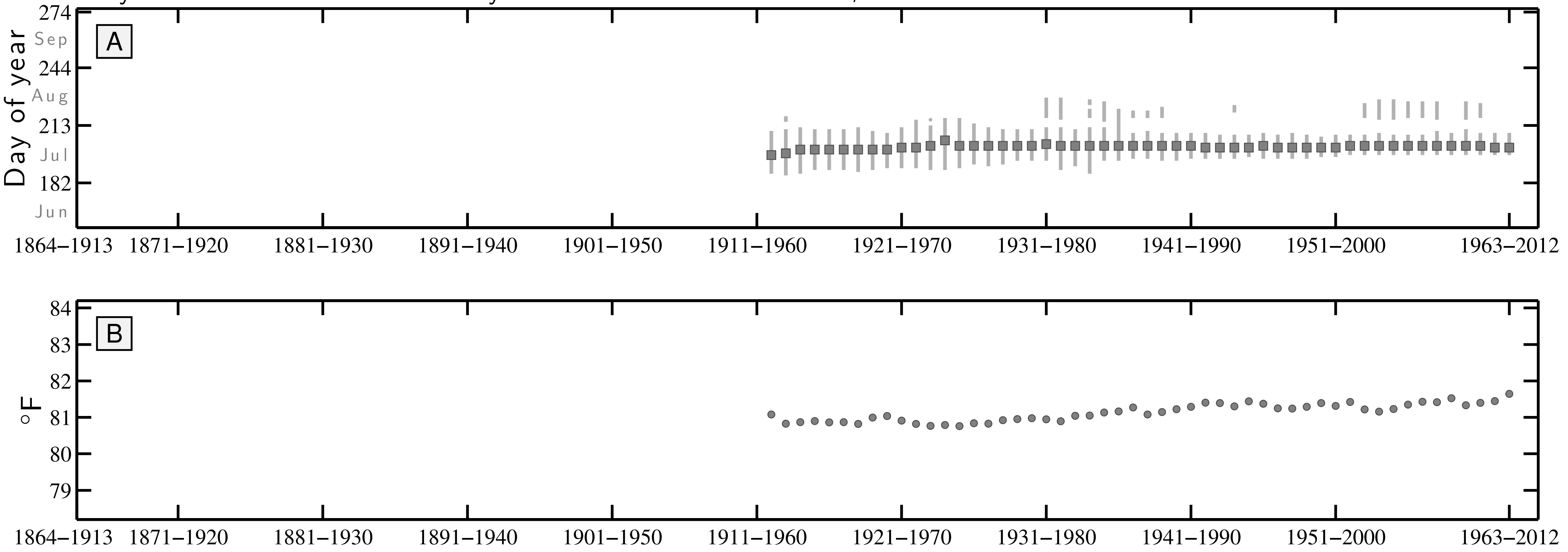

50 year Winter Teletherm dynamics for WOODLAND, ME:

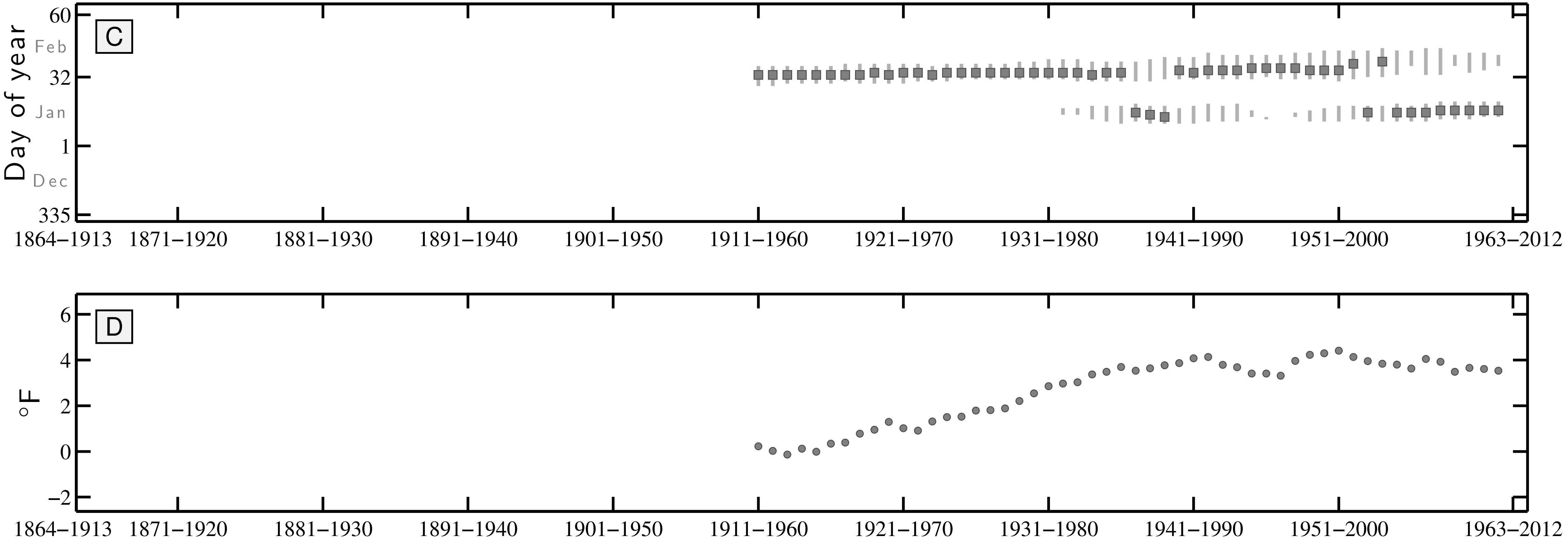

50 year Summer Teletherm dynamics for BELTSVILLE, MD:

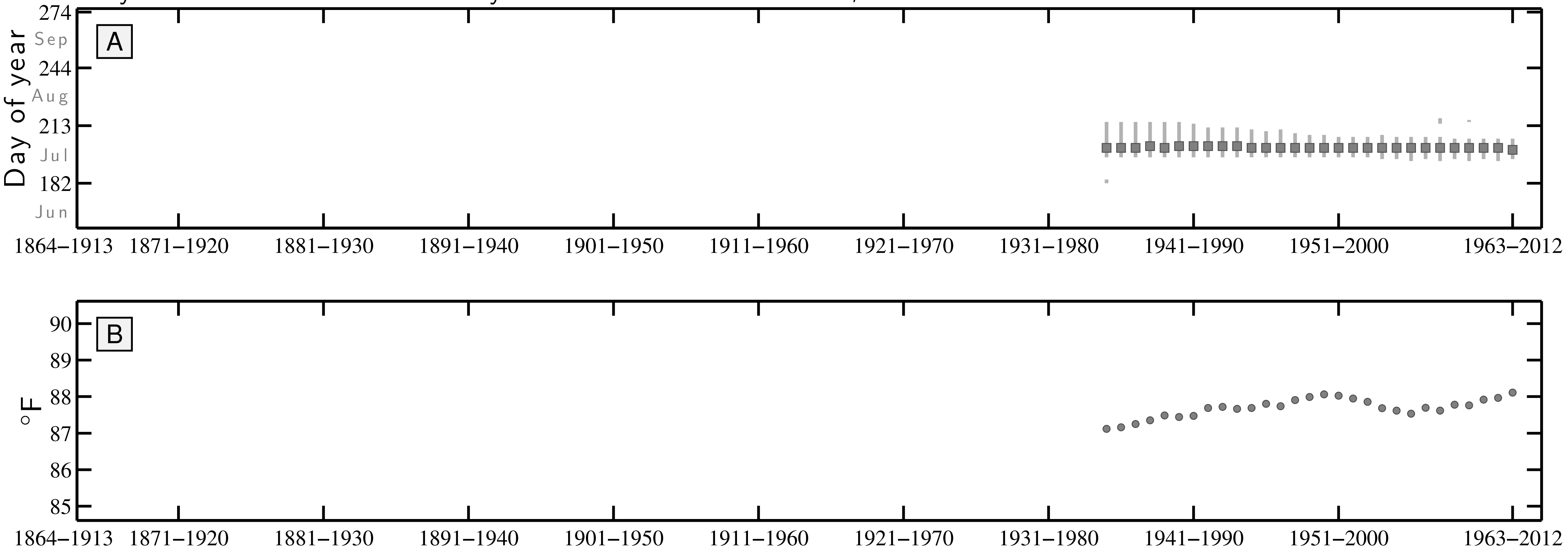

50 year Winter Teletherm dynamics for BELTSVILLE, MD:

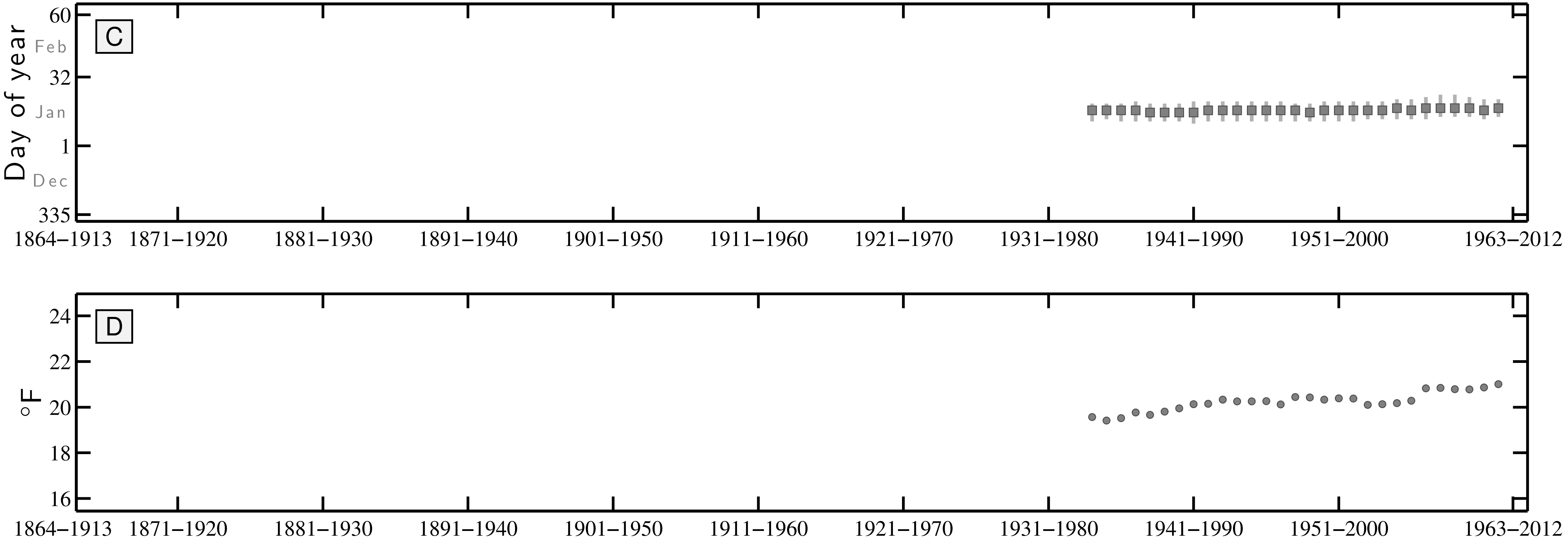

50 year Summer Teletherm dynamics for CAMBRIDGE WATER TRMT P, MD:

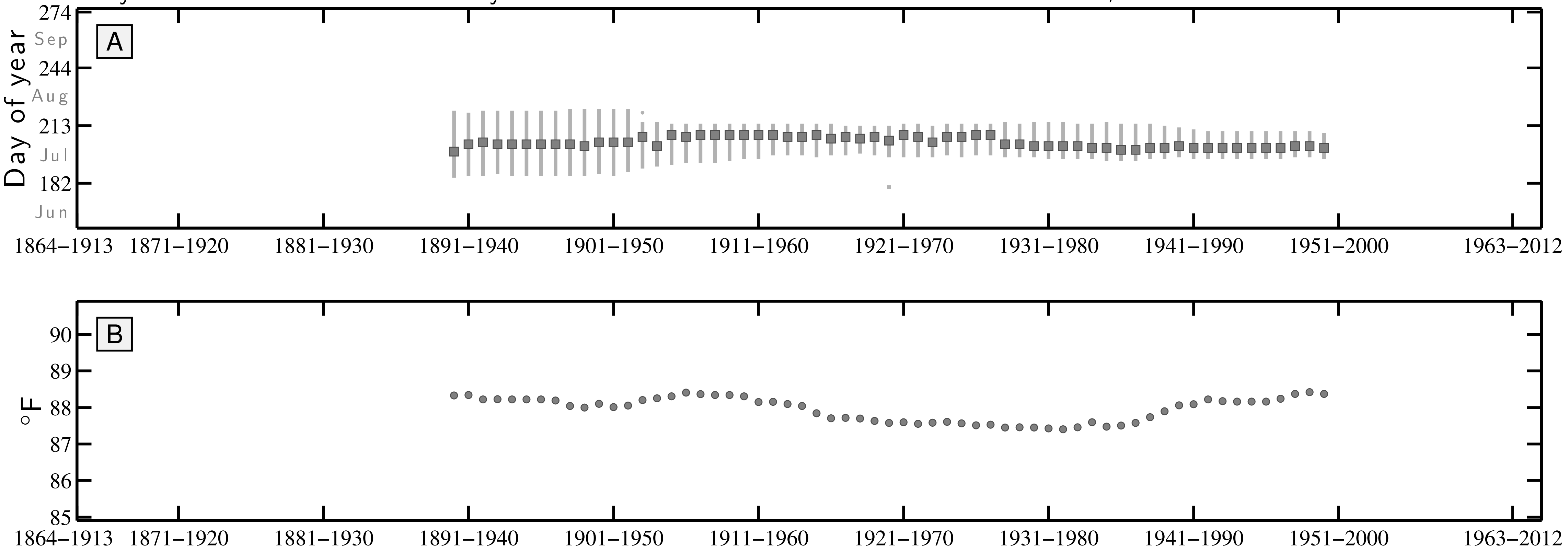

50 year Winter Teletherm dynamics for CAMBRIDGE WATER TRMT P, MD:

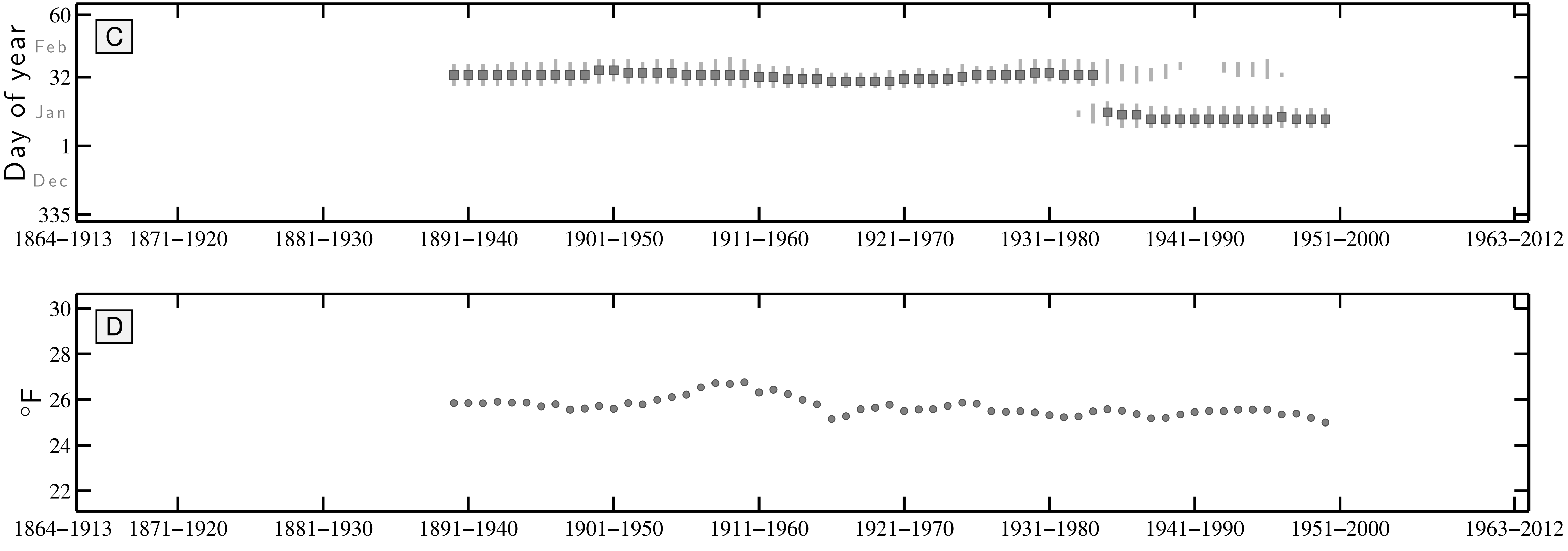

50 year Summer Teletherm dynamics for CHESTERTOWN, MD:

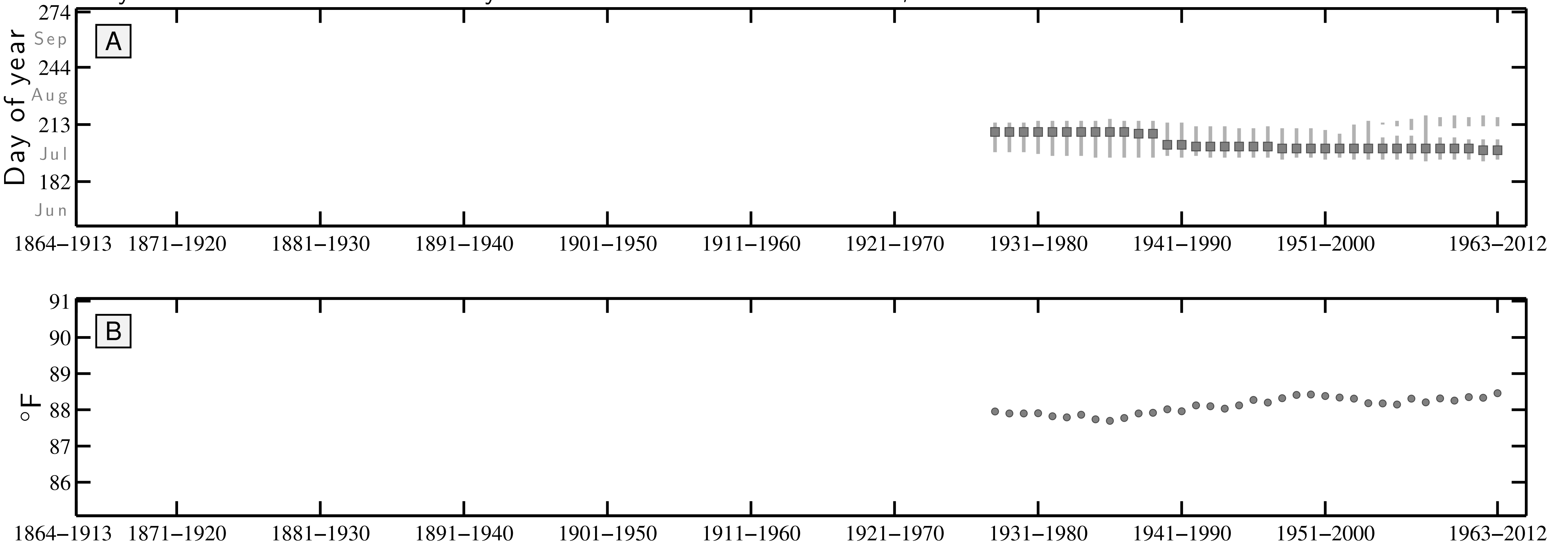

50 year Winter Teletherm dynamics for CHESTERTOWN, MD:

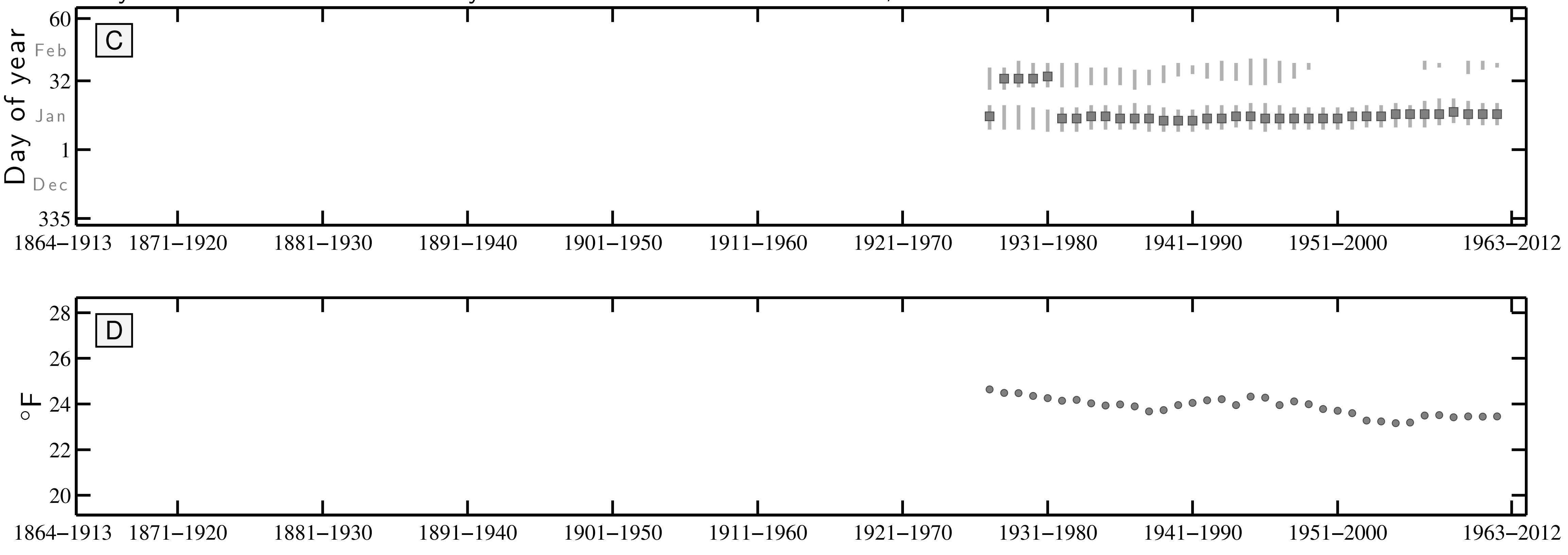

50 year Summer Teletherm dynamics for GLENN DALE BELL STN, MD:

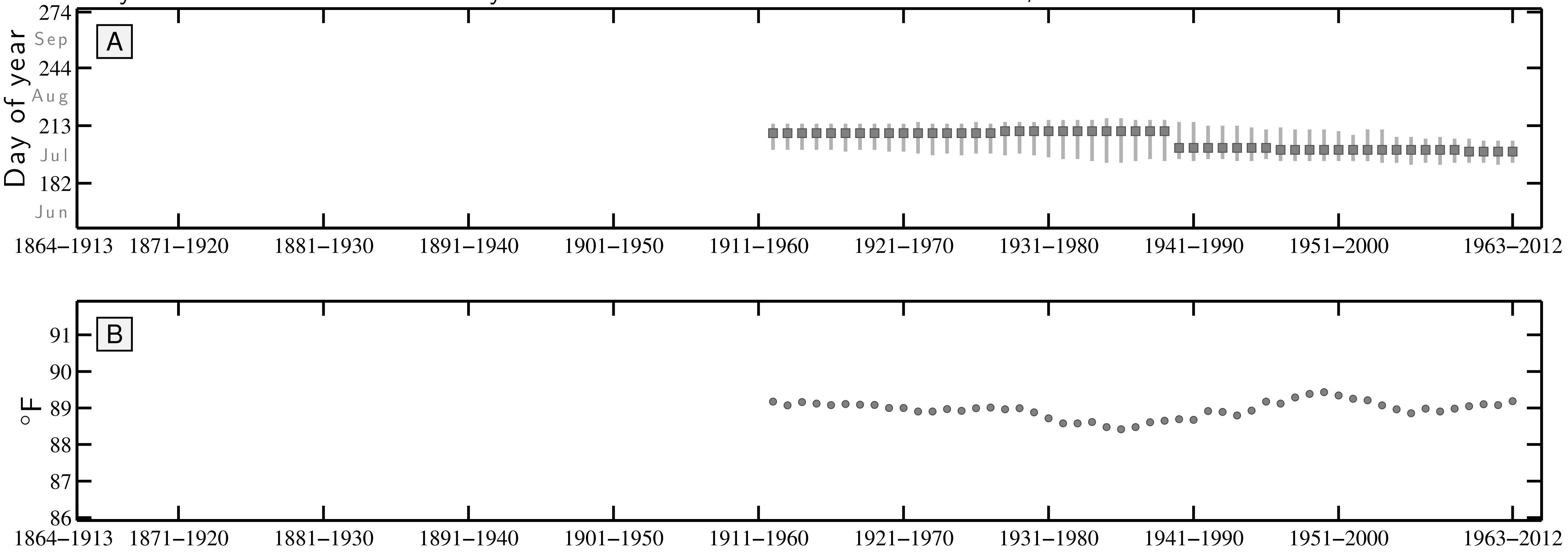

50 year Winter Teletherm dynamics for GLENN DALE BELL STN, MD:

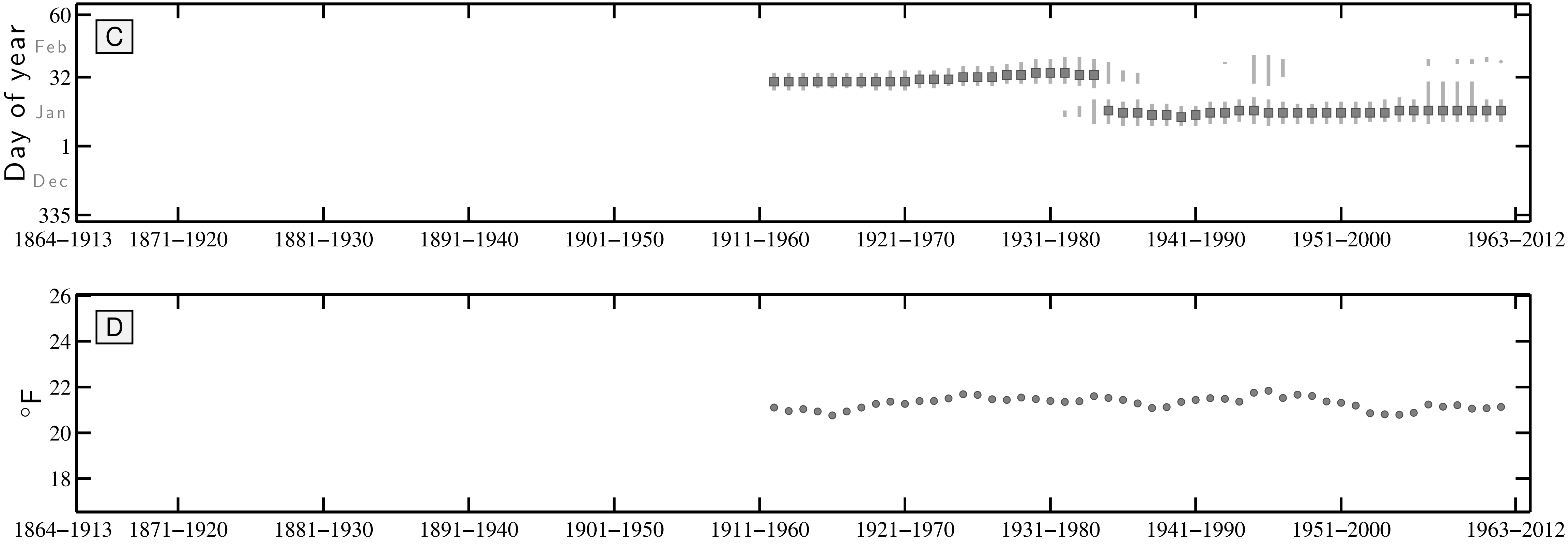

50 year Summer Teletherm dynamics for LAUREL 3 W, MD:

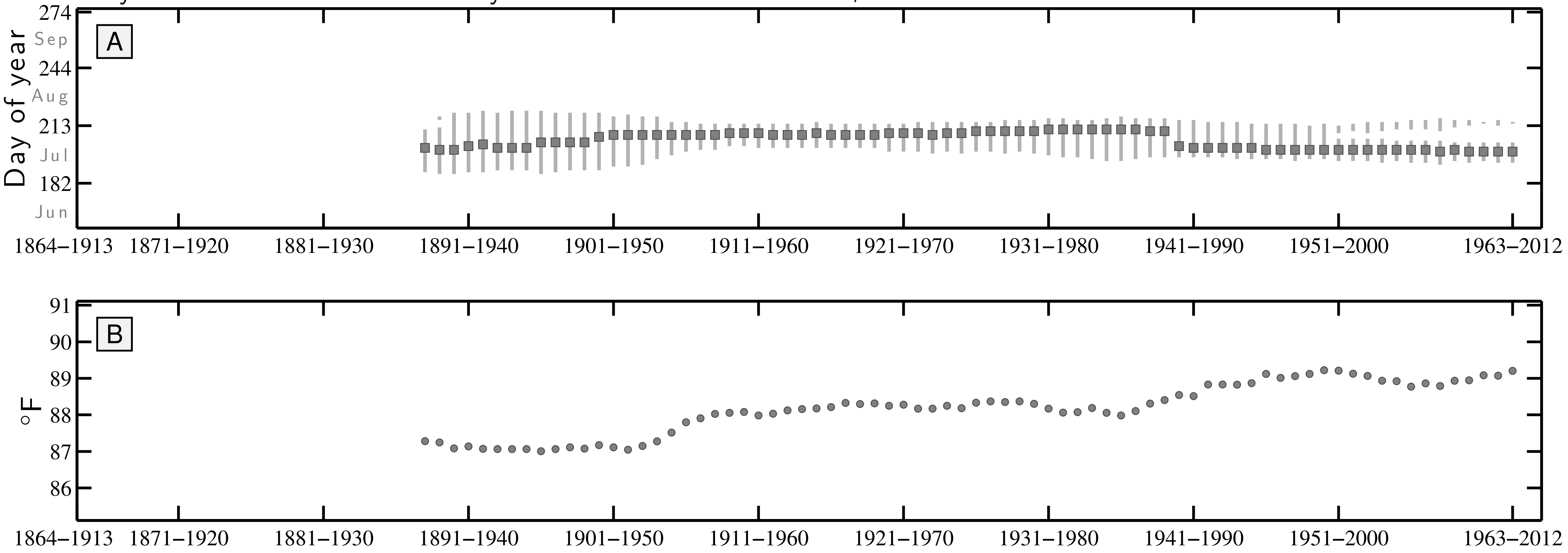

50 year Winter Teletherm dynamics for LAUREL 3 W, MD:

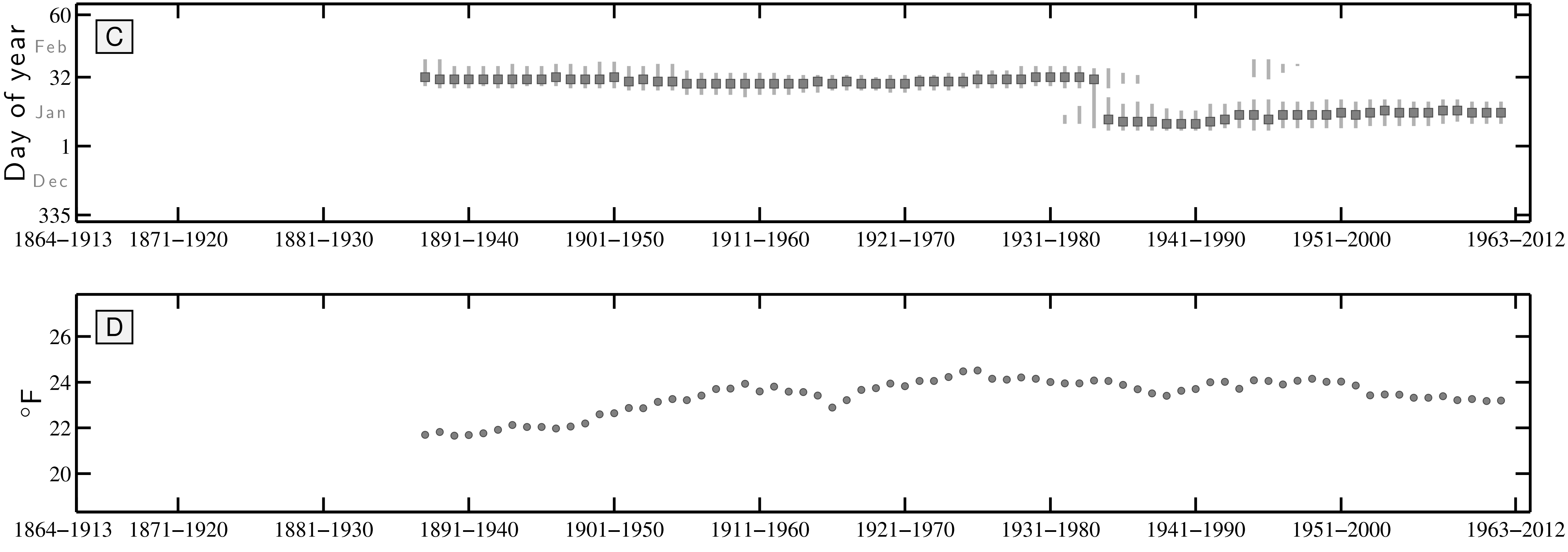

50 year Summer Teletherm dynamics for MILLINGTON 1 SE, MD:

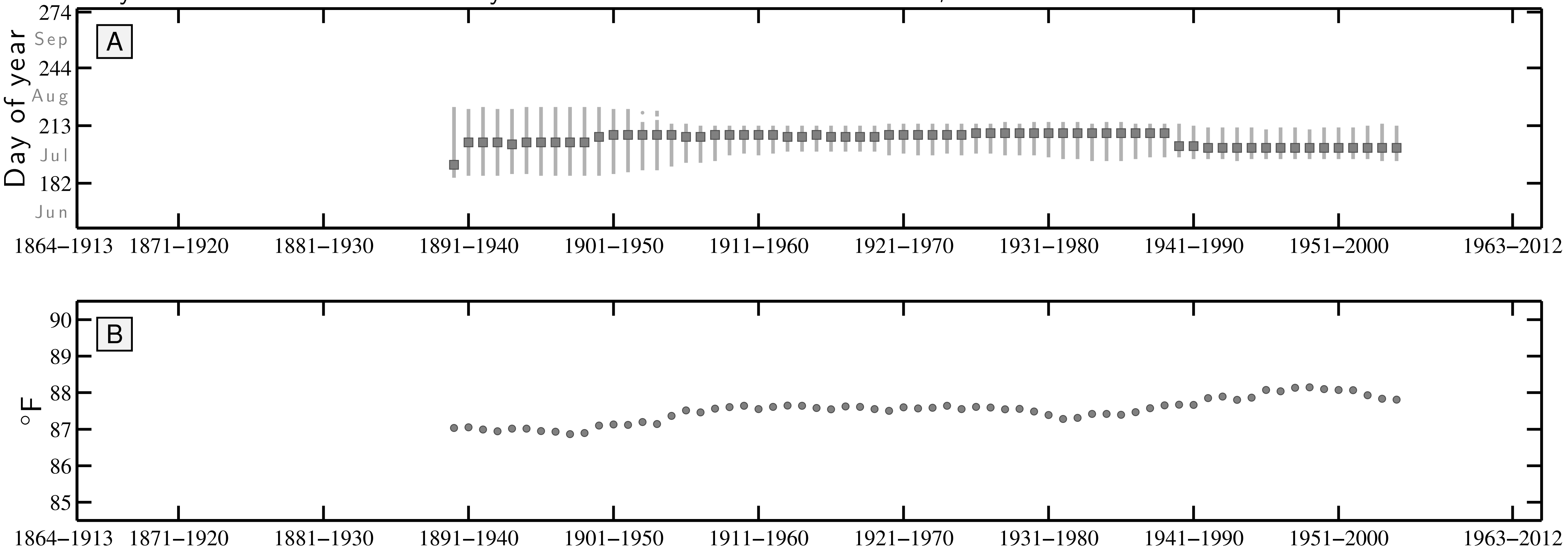

50 year Winter Teletherm dynamics for MILLINGTON 1 SE, MD:

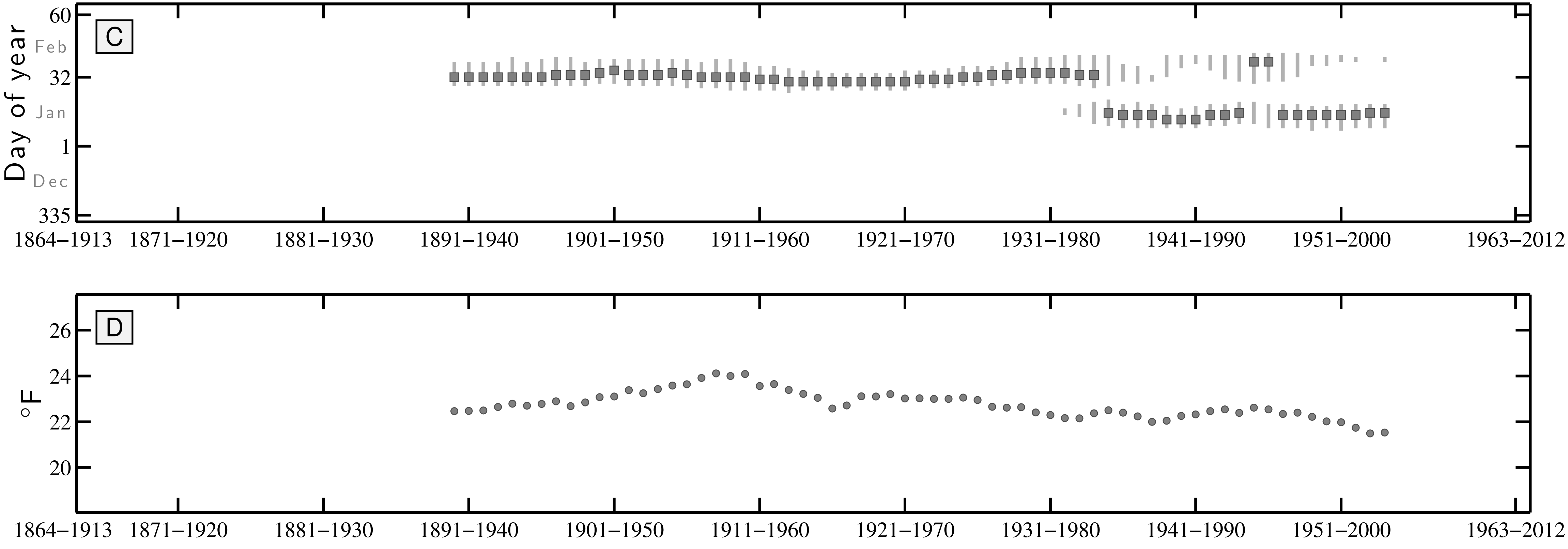

50 year Summer Teletherm dynamics for OAKLAND 1 SE, MD:

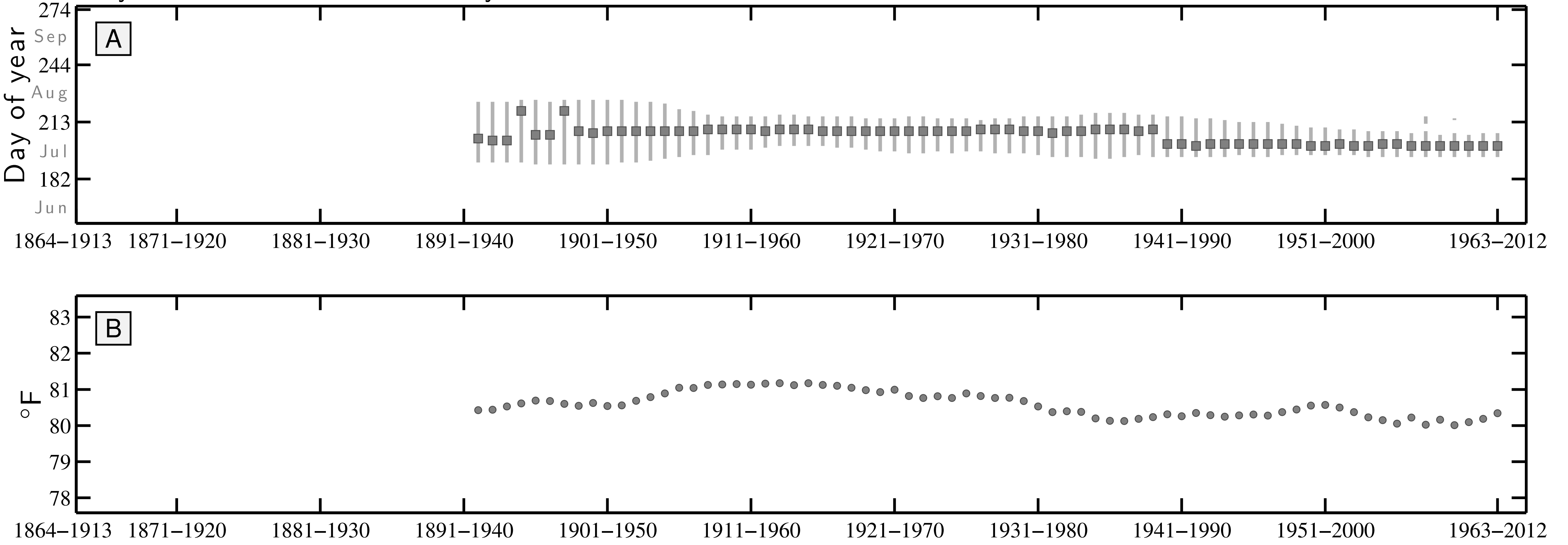

50 year Winter Teletherm dynamics for OAKLAND 1 SE, MD:

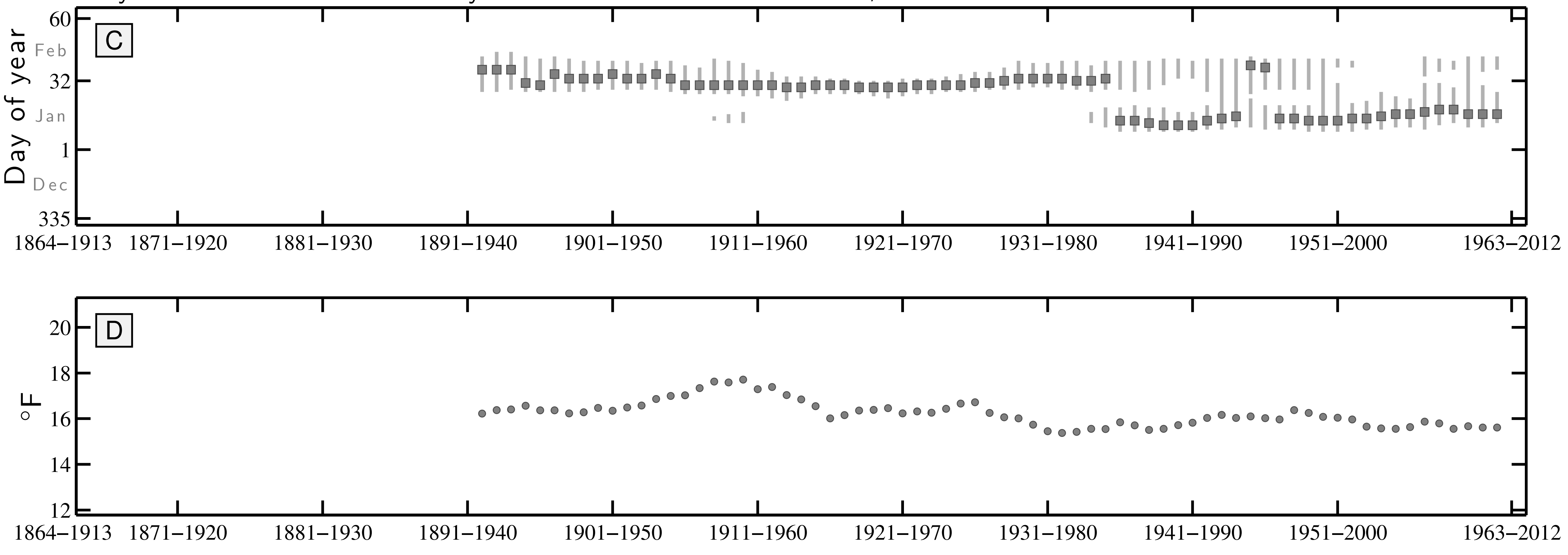

50 year Summer Teletherm dynamics for OWINGS FERRY LANDING, MD:

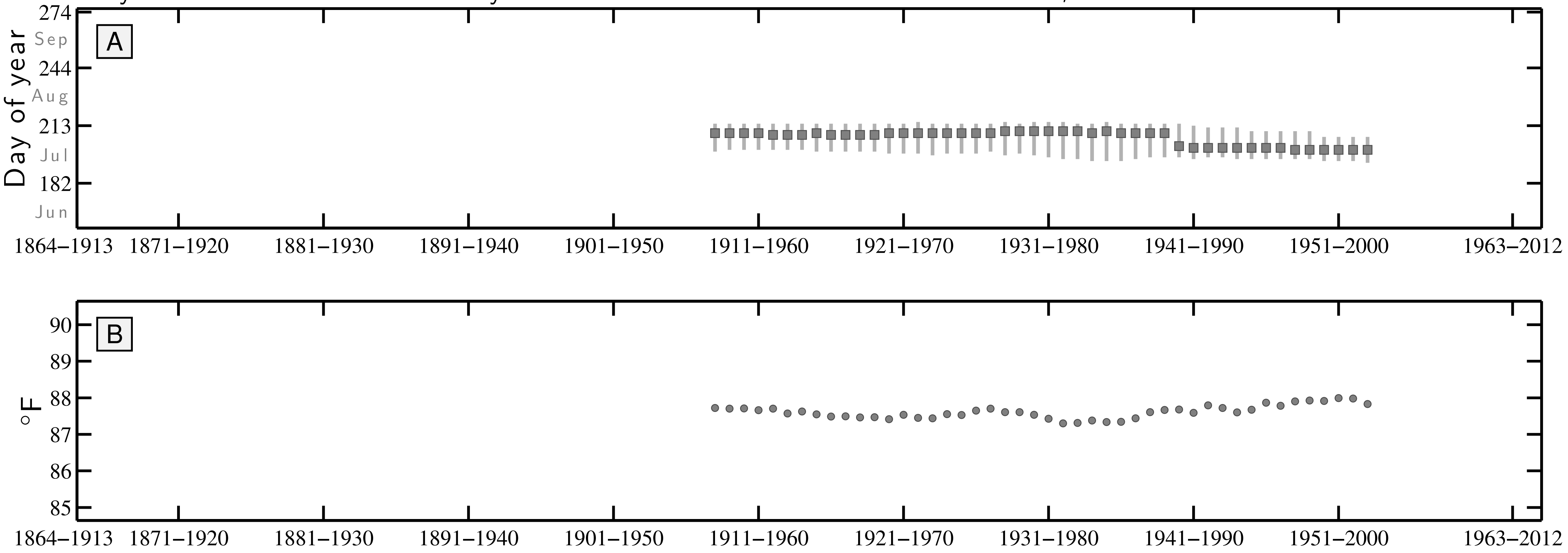

50 year Winter Teletherm dynamics for OWINGS FERRY LANDING, MD:

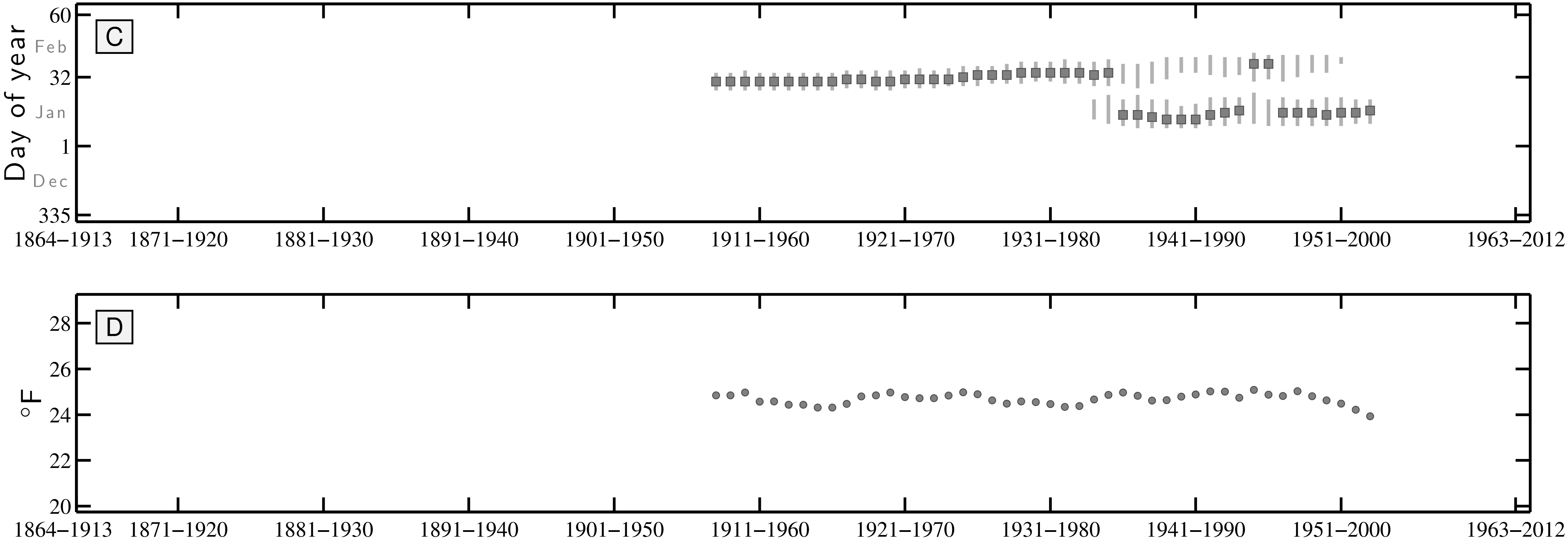

50 year Summer Teletherm dynamics for PRINCESS ANNE, MD:

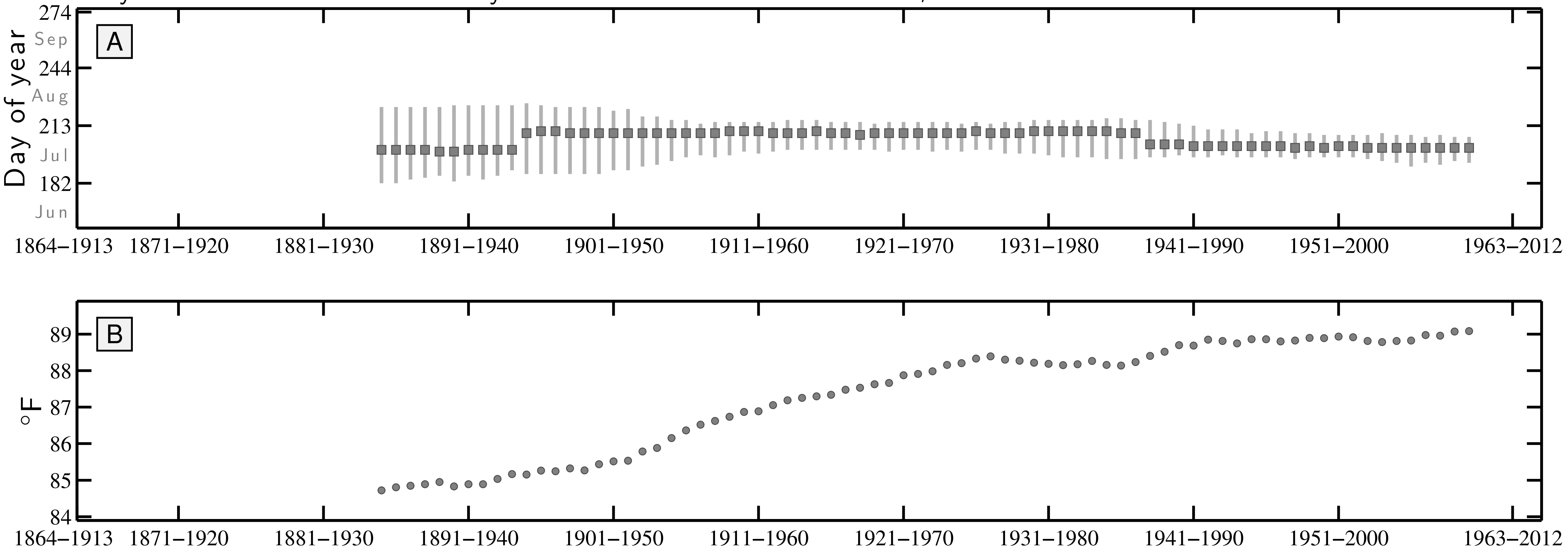

50 year Winter Teletherm dynamics for PRINCESS ANNE, MD:

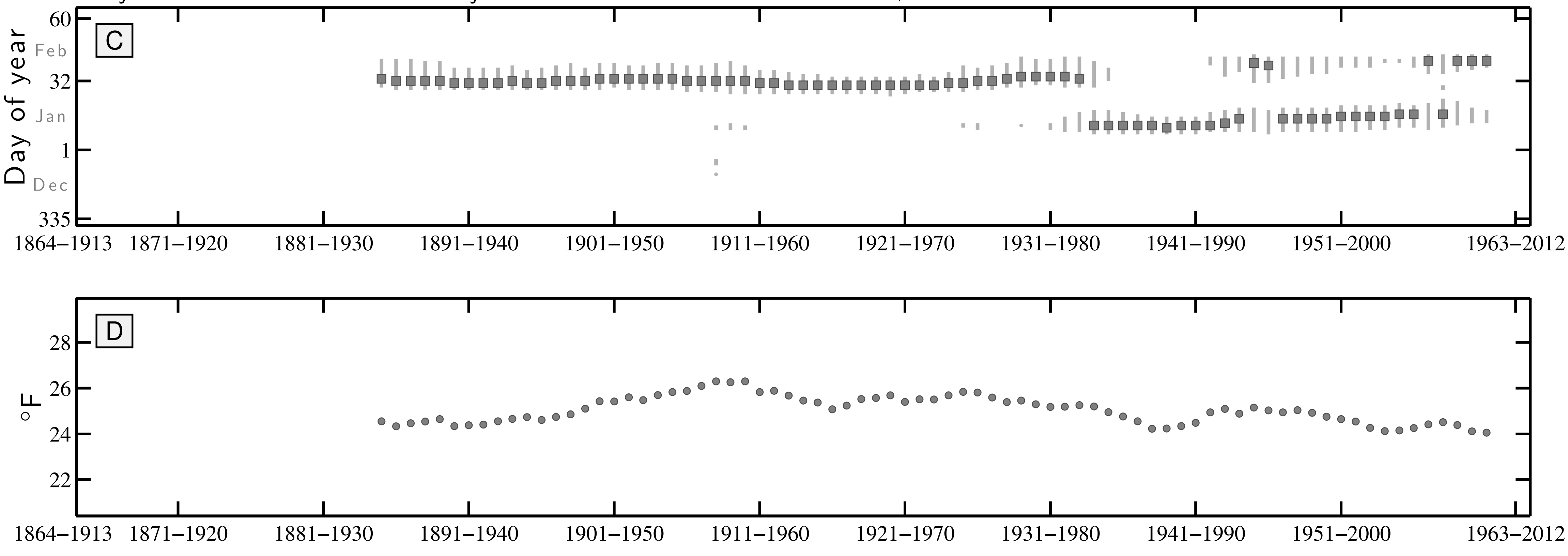

50 year Summer Teletherm dynamics for ROYAL OAK 2 SSW, MD:

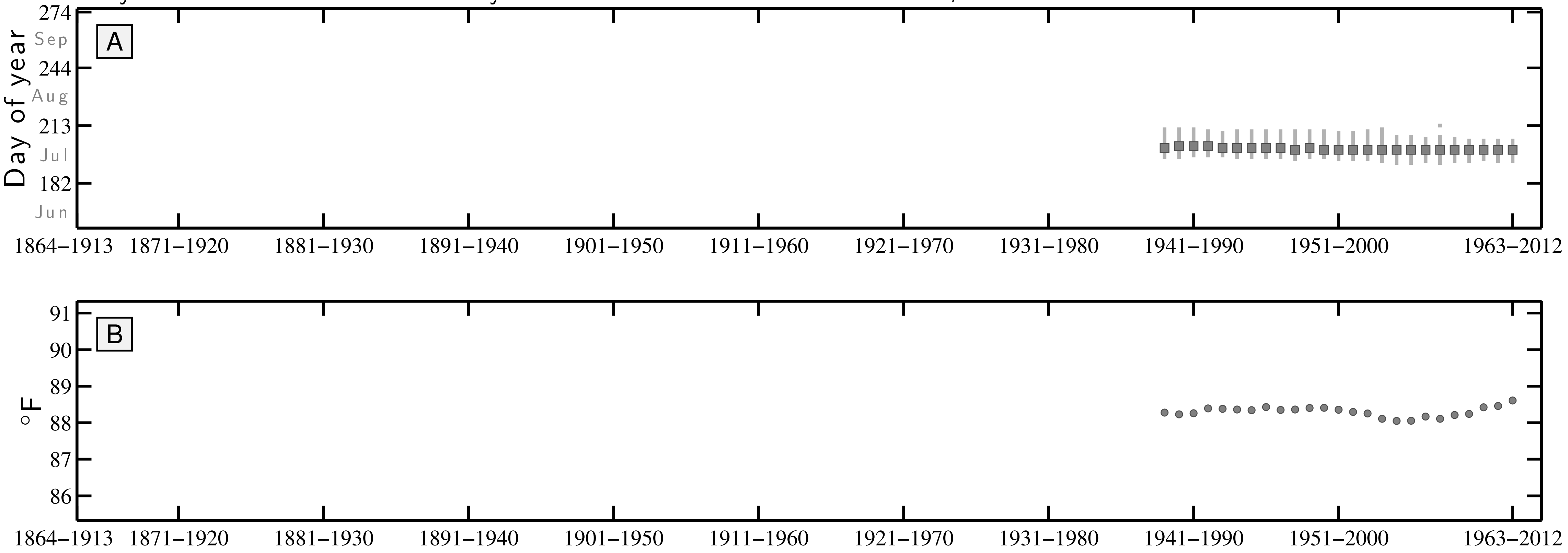

50 year Winter Teletherm dynamics for ROYAL OAK 2 SSW, MD:

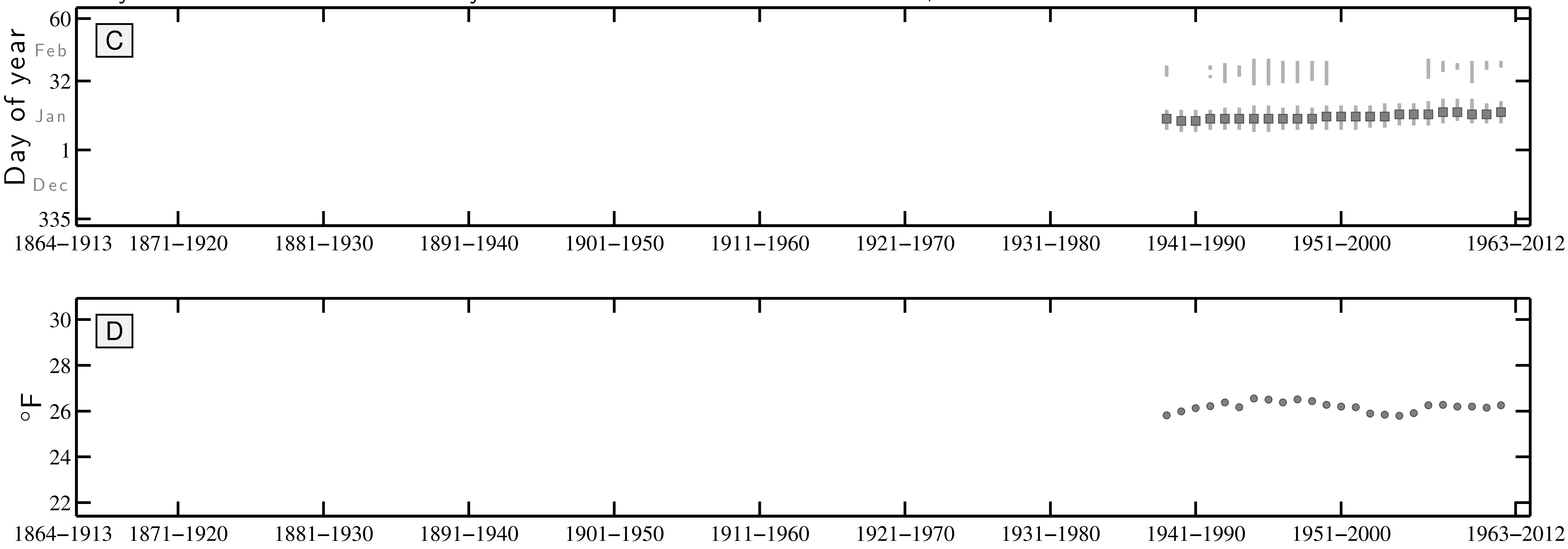

50 year Summer Teletherm dynamics for SALISBURY, MD:

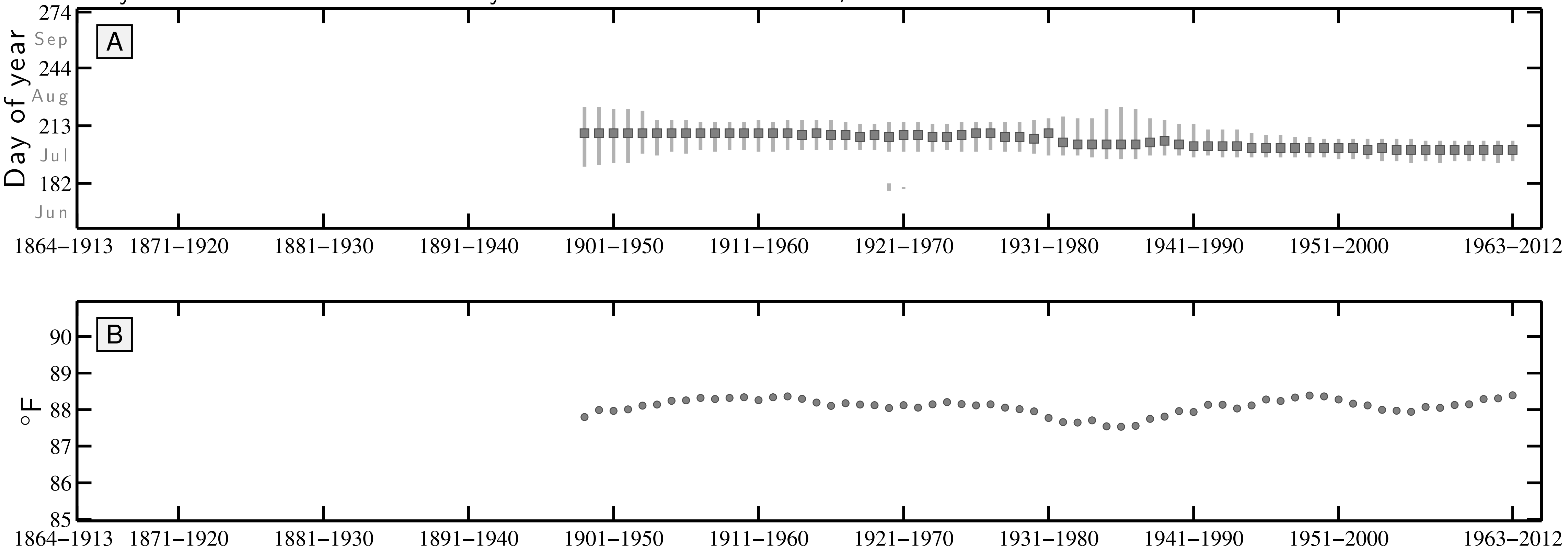

50 year Winter Teletherm dynamics for SALISBURY, MD:

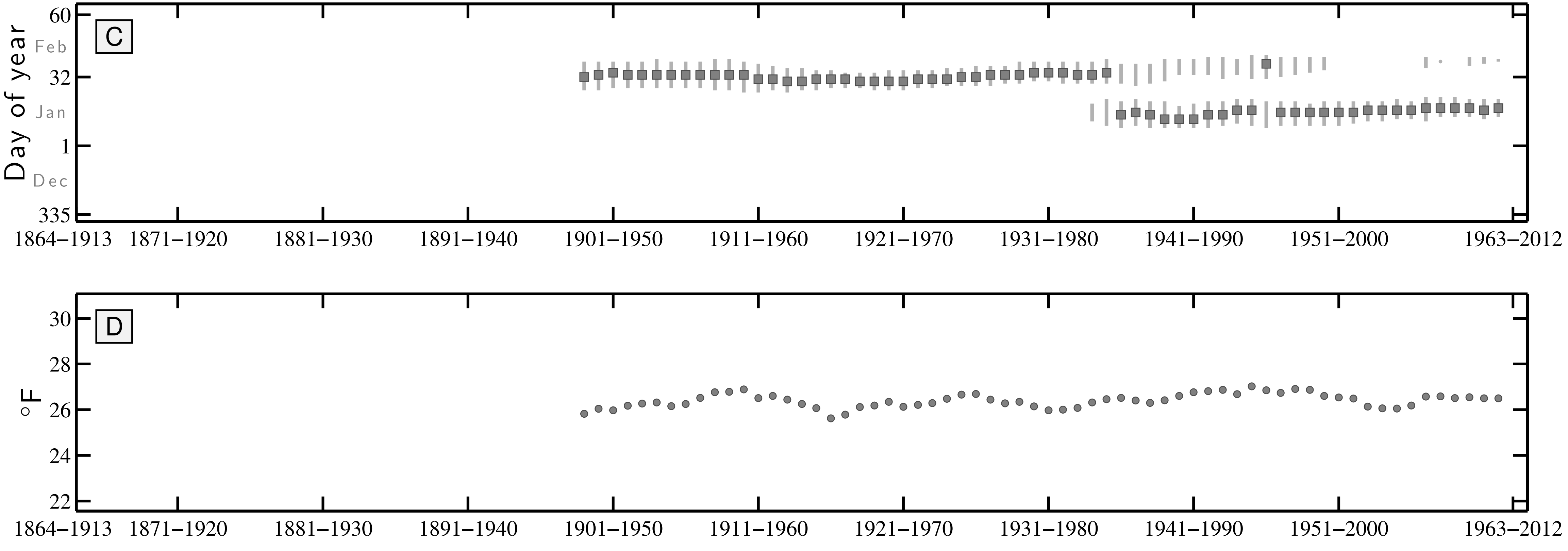

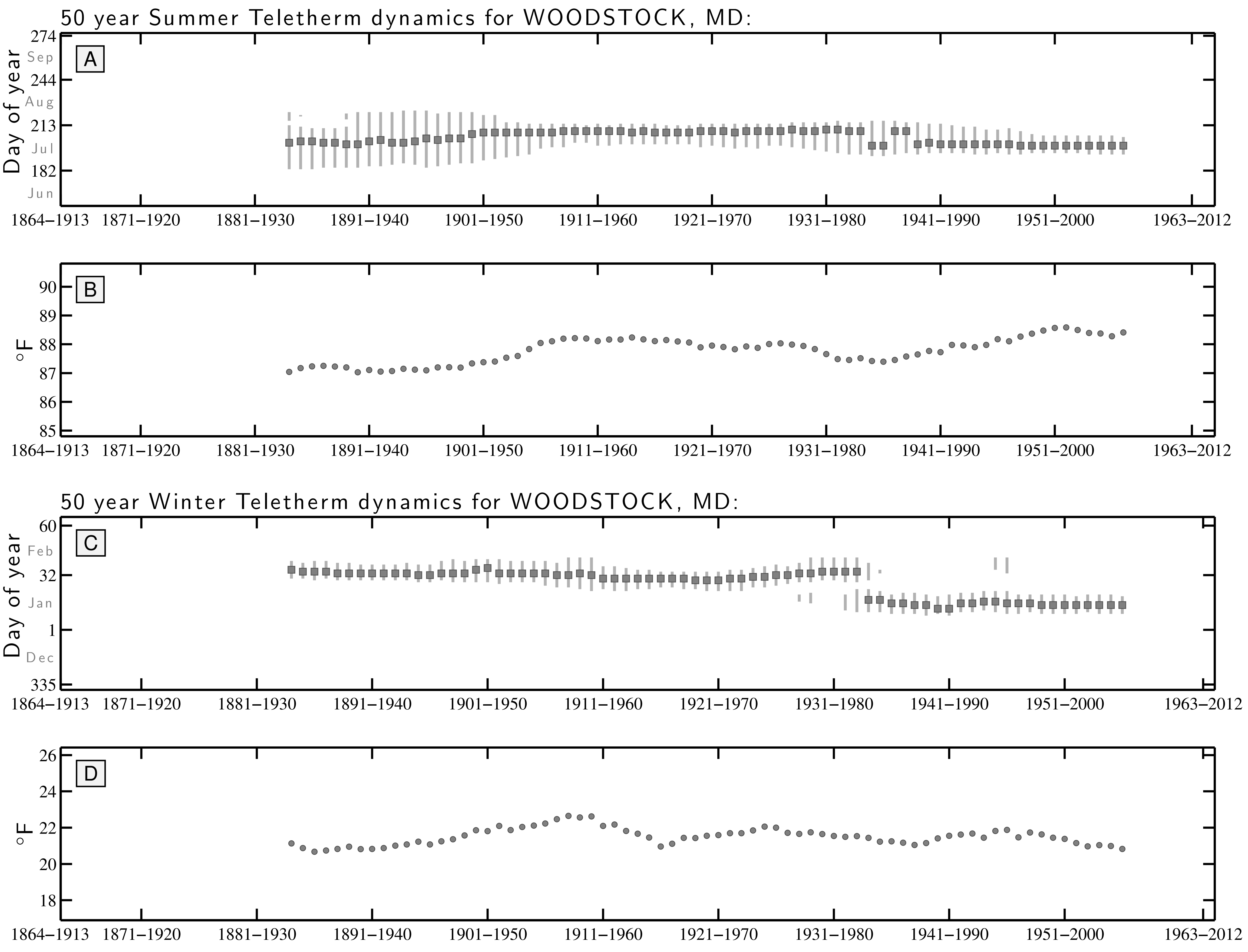

50 year Summer Teletherm dynamics for WOODSTOCK, MD:
A
B
Day of year
°F
274
244
213
182
Sep
Aug
Jul
Jun
90
89
88
87
86
85
50 year Winter Teletherm dynamics for WOODSTOCK, MD:
C
D
60
32
1
335
Feb
Jan
Dec
26
24
22
20
18
1864–1913
1871–1920
1881–1930
1891–1940
1901–1950
1911–1960
1921–1970
1931–1980
1941–1990
1951–2000
1963–2012

50 year Summer Teletherm dynamics for AMHERST, MA:

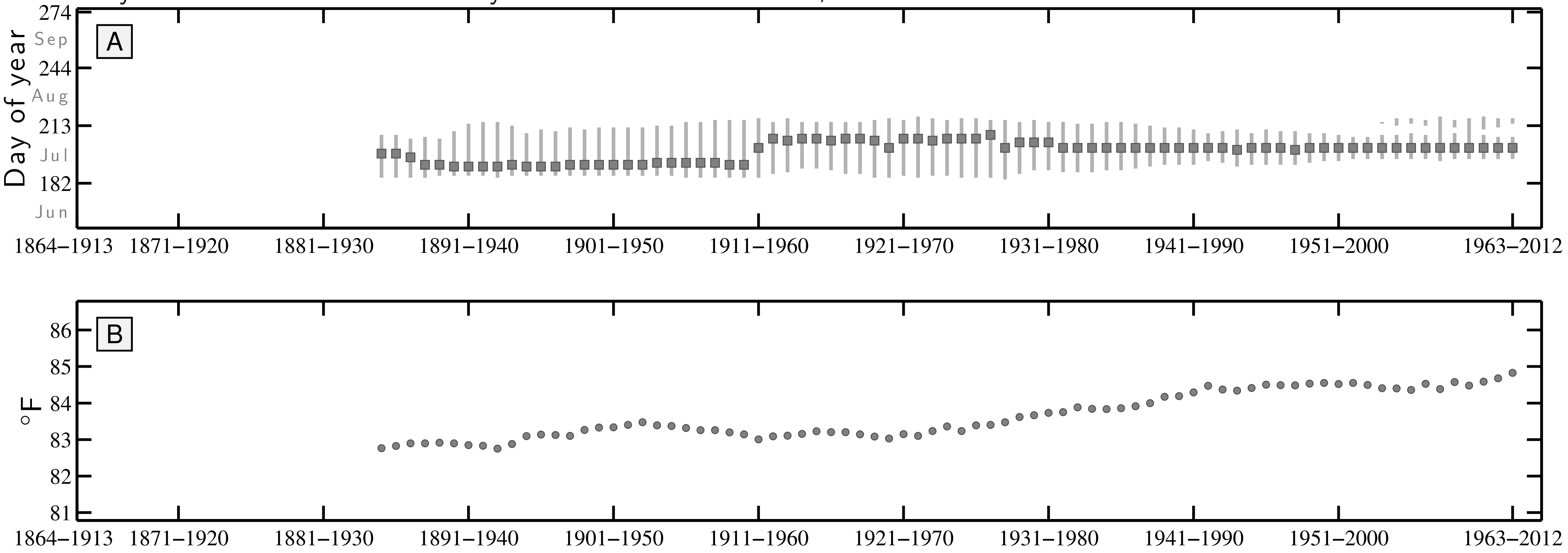

50 year Winter Teletherm dynamics for AMHERST, MA:

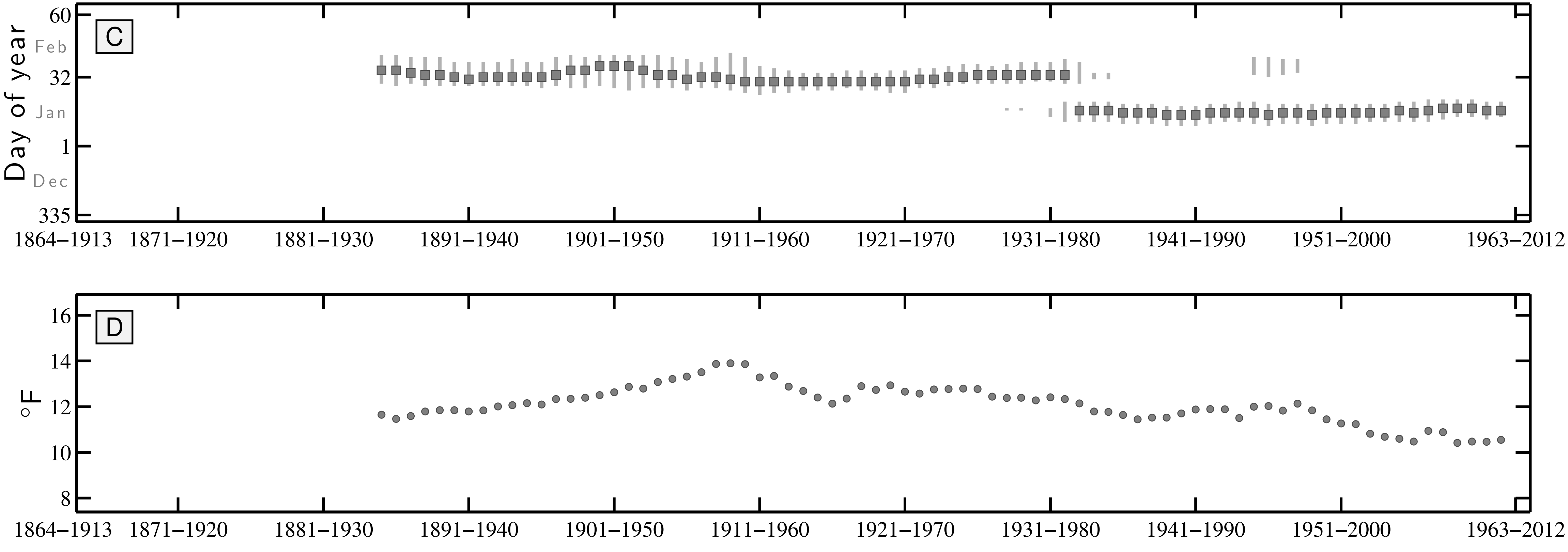

50 year Summer Teletherm dynamics for BEDFORD, MA:

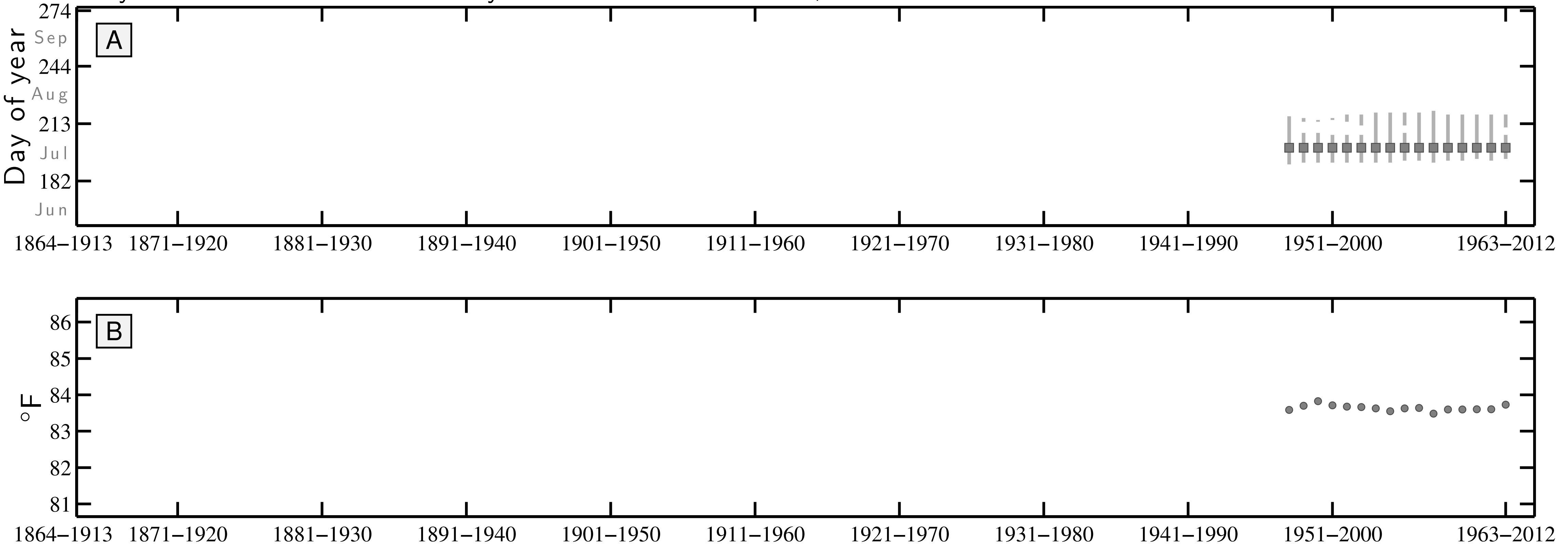

50 year Winter Teletherm dynamics for BEDFORD, MA:

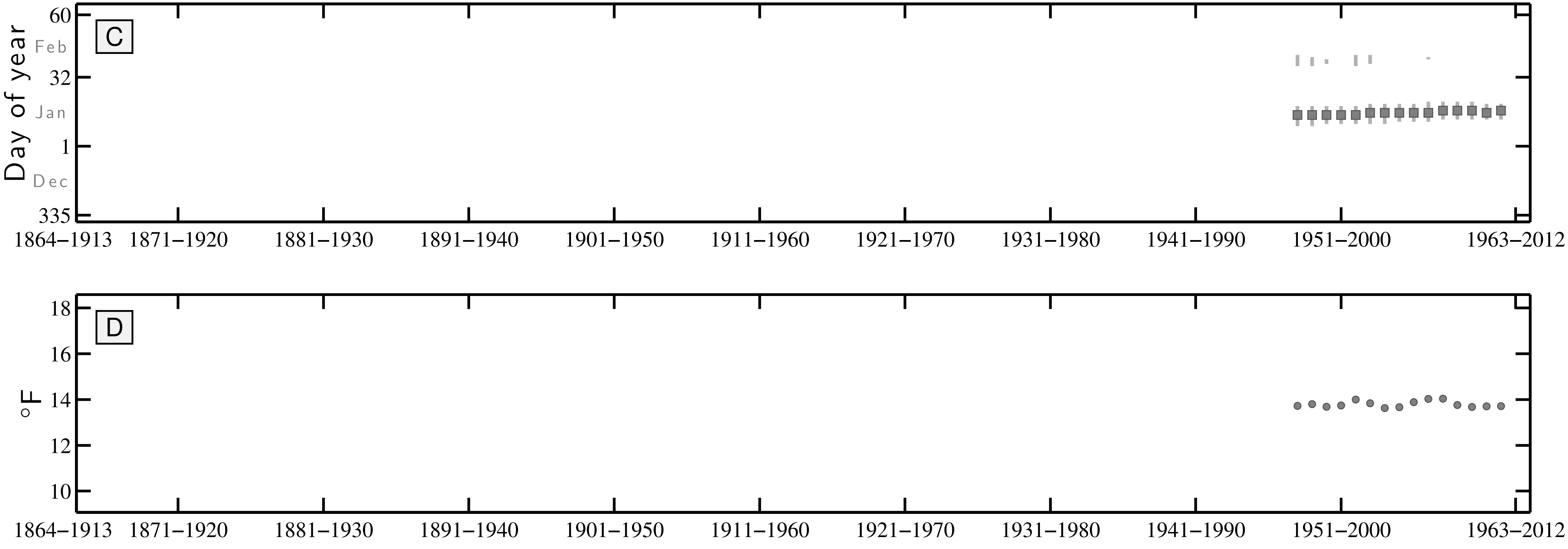

50 year Summer Teletherm dynamics for BLUE HILL, MA:

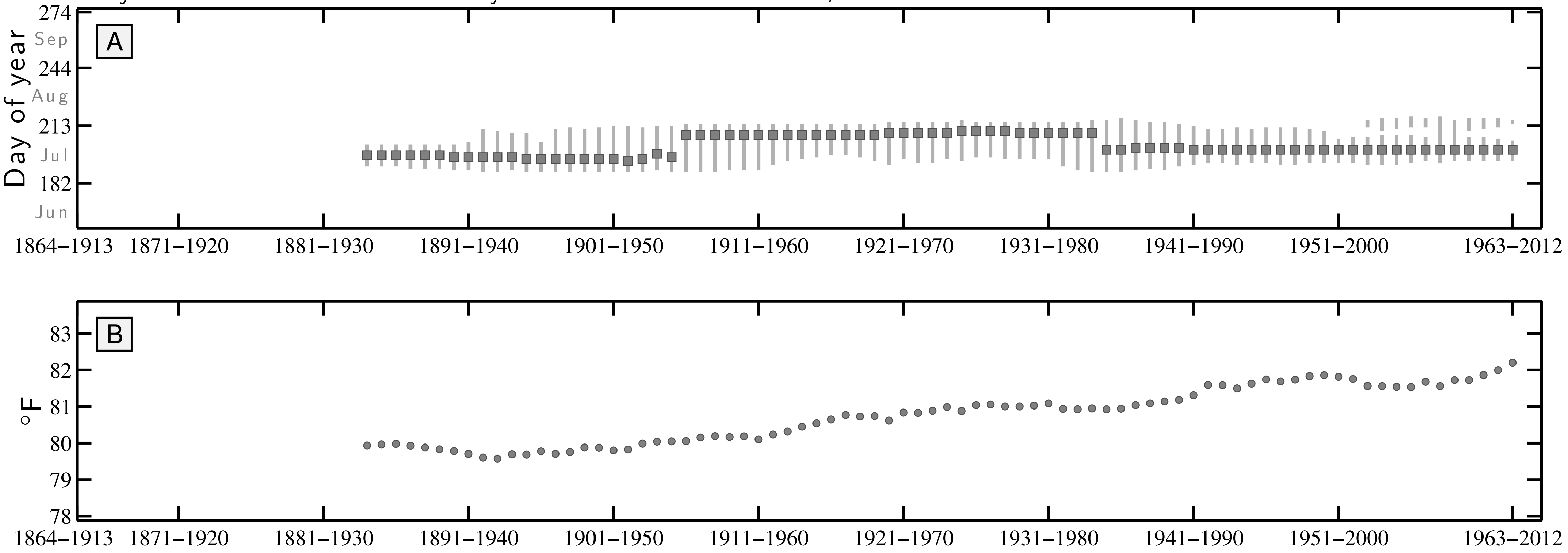

50 year Winter Teletherm dynamics for BLUE HILL, MA:

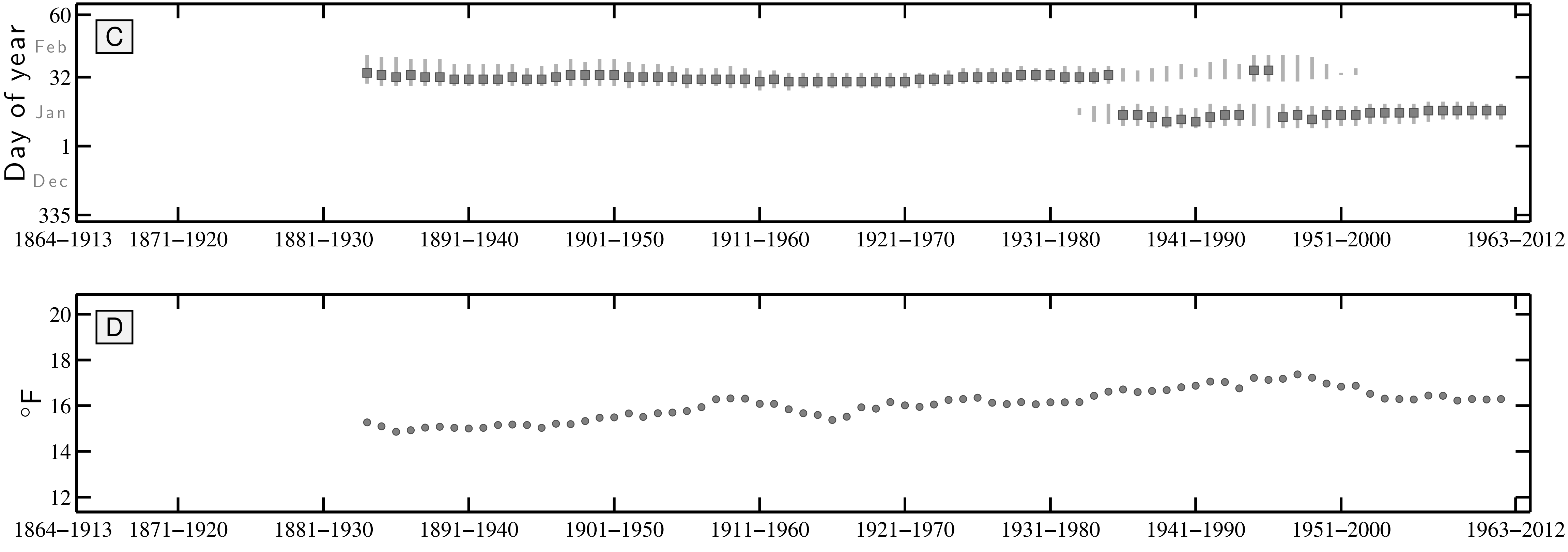

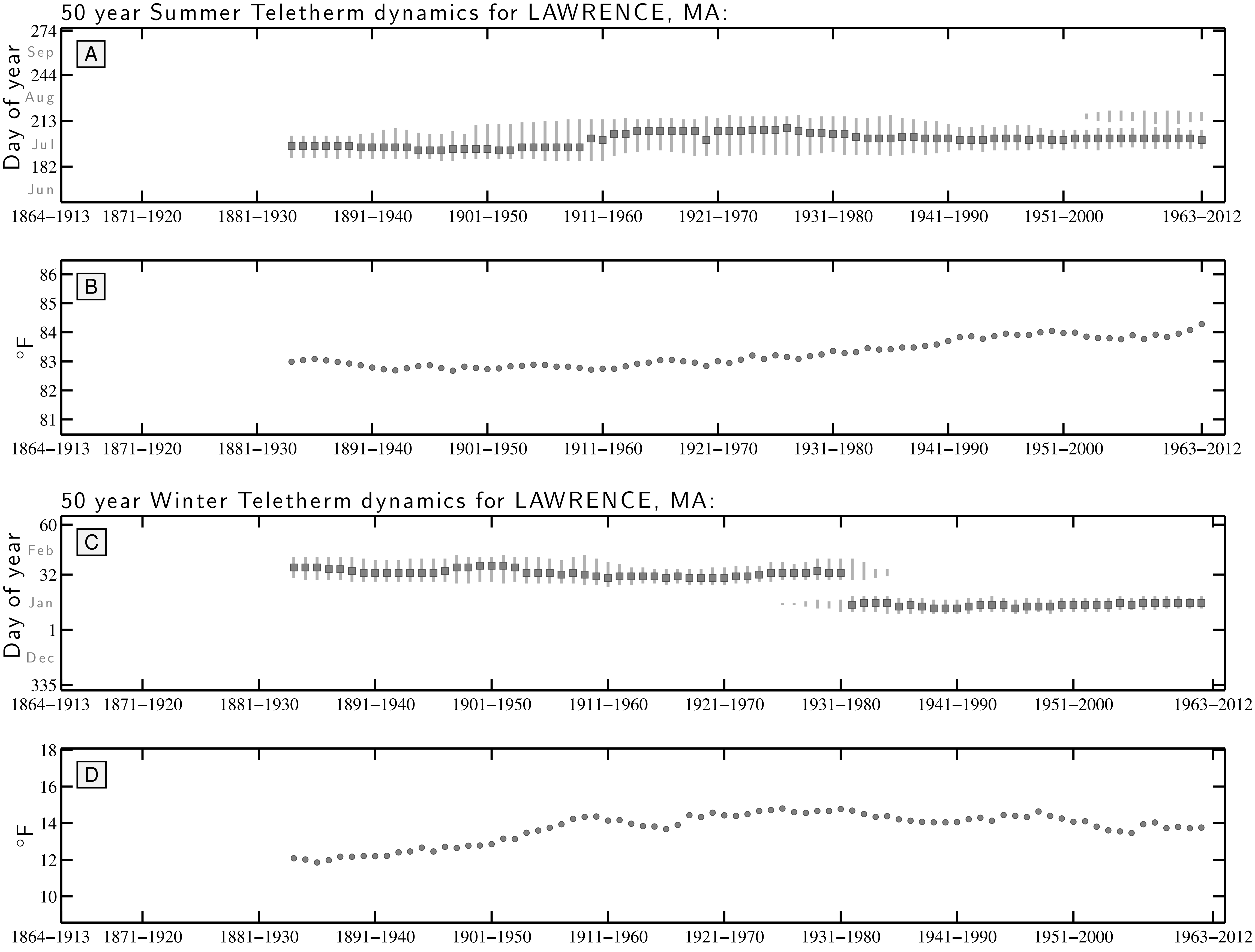

50 year Summer Teletherm dynamics for LAWRENCE, MA:
A
Day of year
274
Sep
244
Aug
213
Jul
182
Jun
1864–1913
1871–1920
1881–1930
1891–1940
1901–1950
1911–1960
1921–1970
1931–1980
1941–1990
1951–2000
1963–2012
B
°F
86
85
84
83
82
81
50 year Winter Teletherm dynamics for LAWRENCE, MA:
C
60
Feb
32
Jan
1
Dec
335
D
18
16
14
12
10

50 year Summer Teletherm dynamics for NEW BEDFORD, MA:

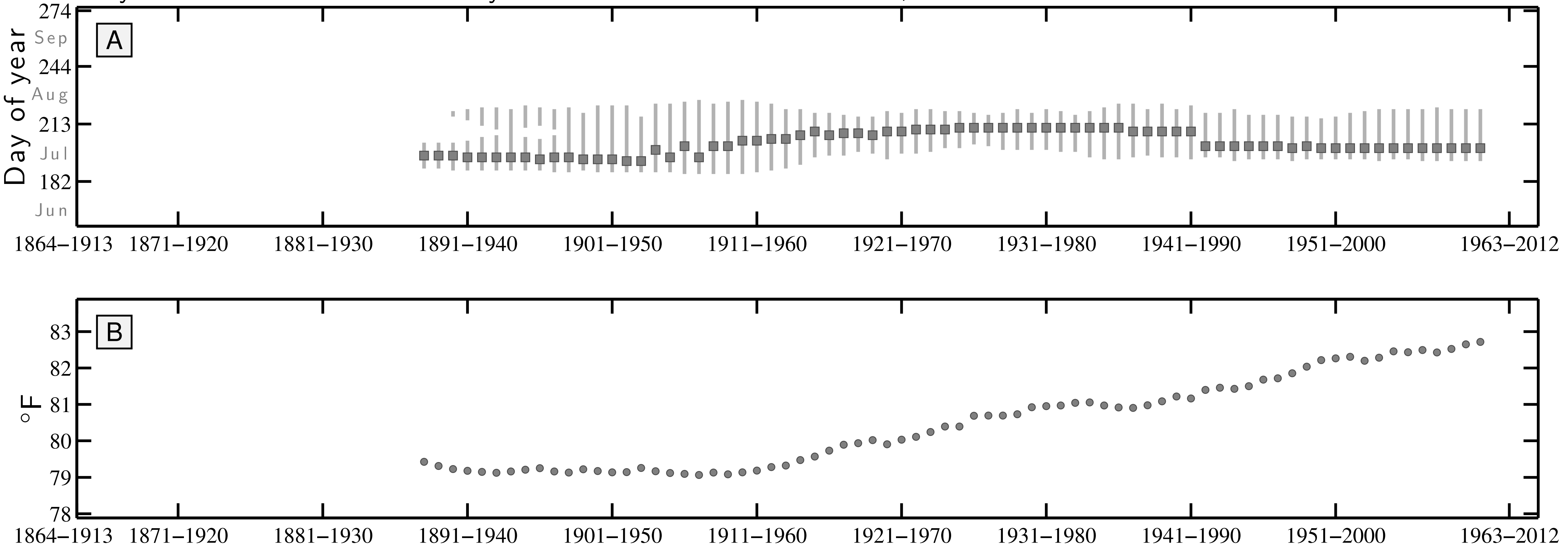

50 year Winter Teletherm dynamics for NEW BEDFORD, MA:

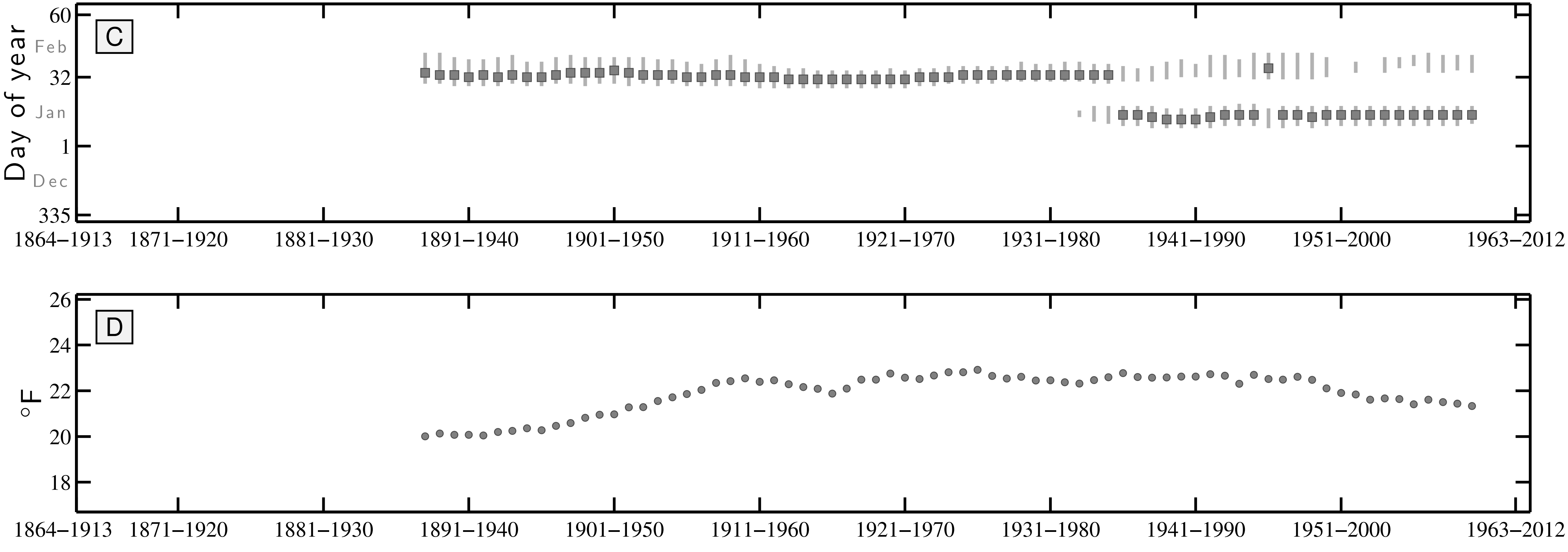

50 year Summer Teletherm dynamics for PLYMOUTH-KINGSTON, MA:

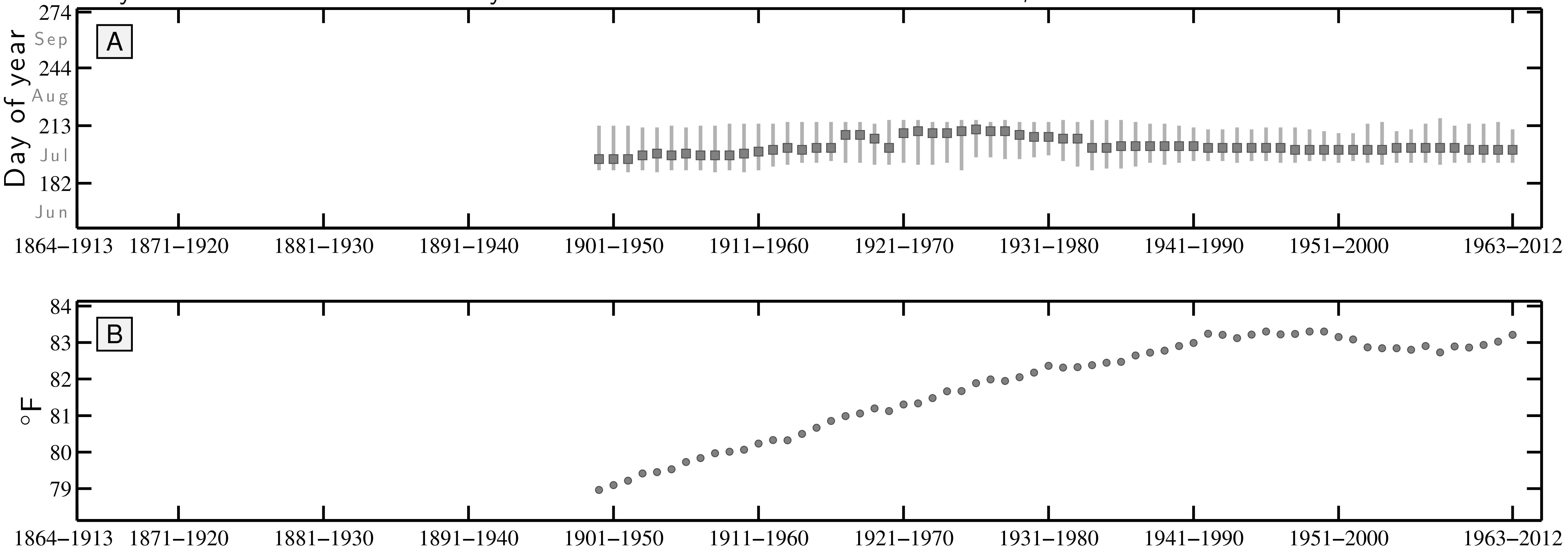

50 year Winter Teletherm dynamics for PLYMOUTH-KINGSTON, MA:

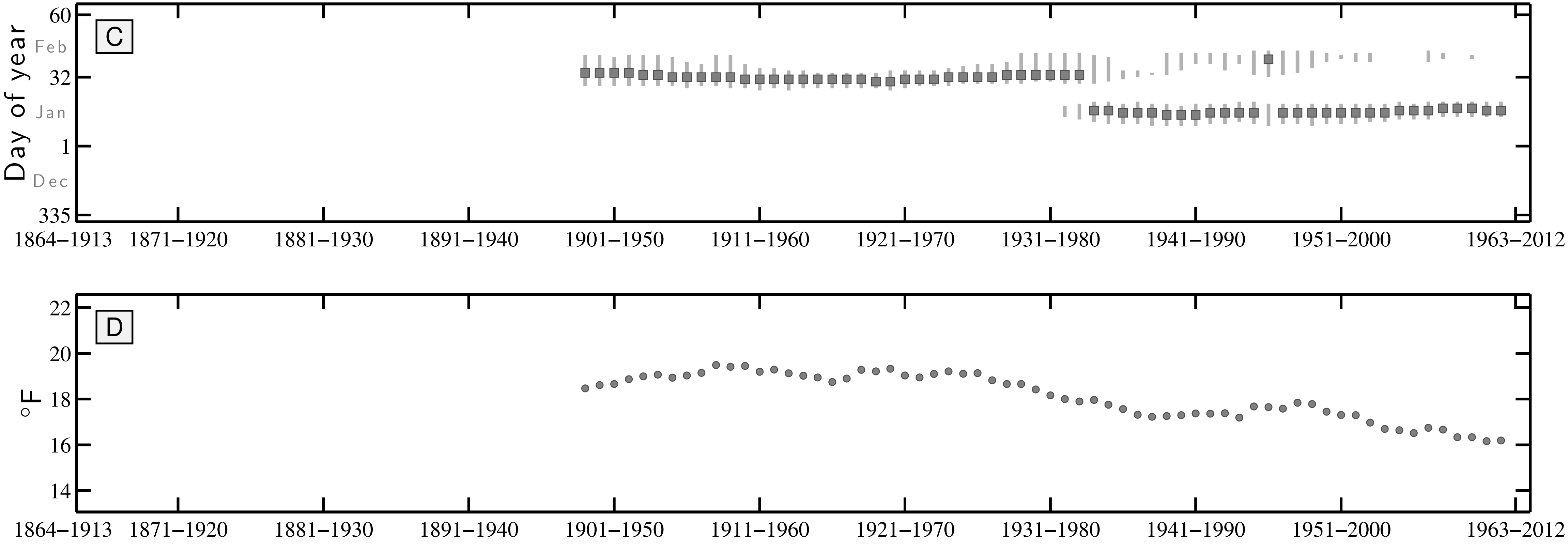

50 year Summer Teletherm dynamics for READING, MA:

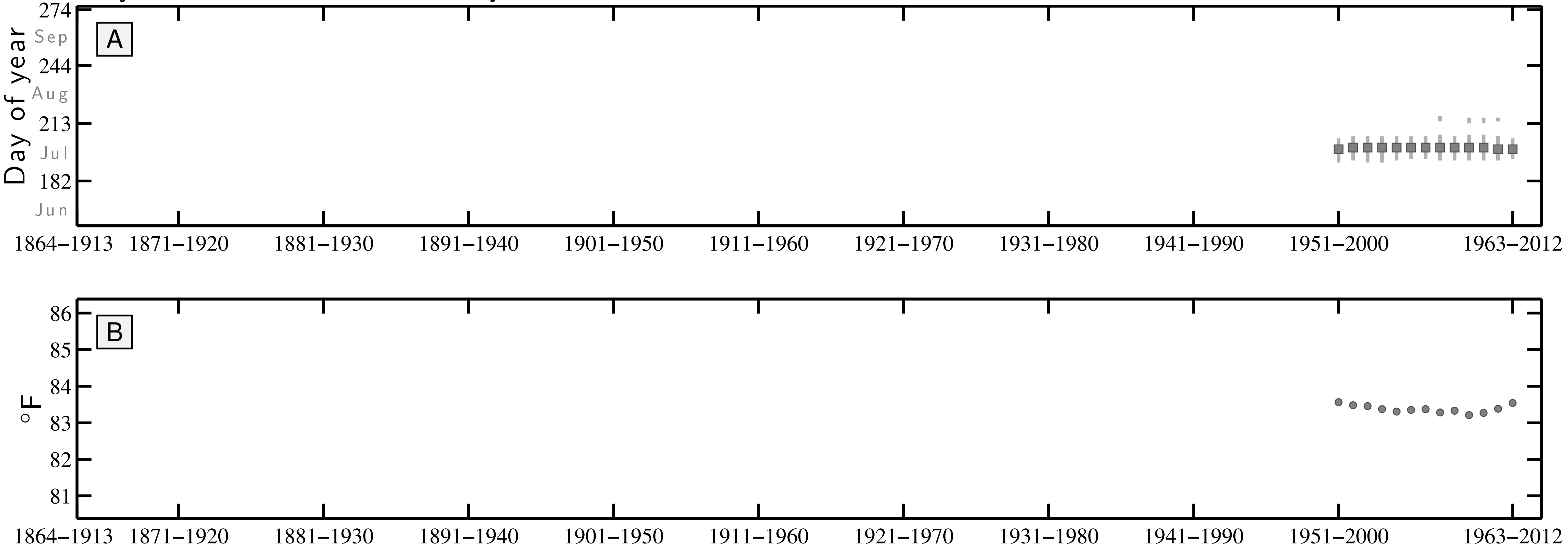

50 year Winter Teletherm dynamics for READING, MA:

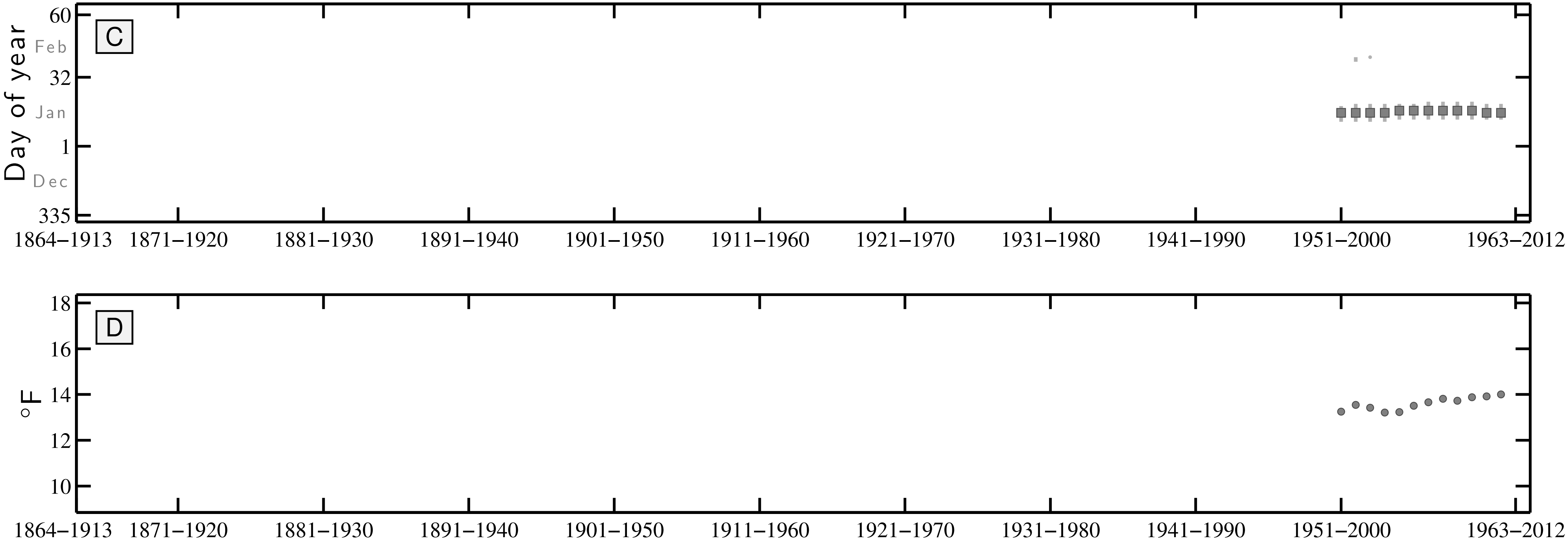

50 year Summer Teletherm dynamics for TAUNTON, MA:

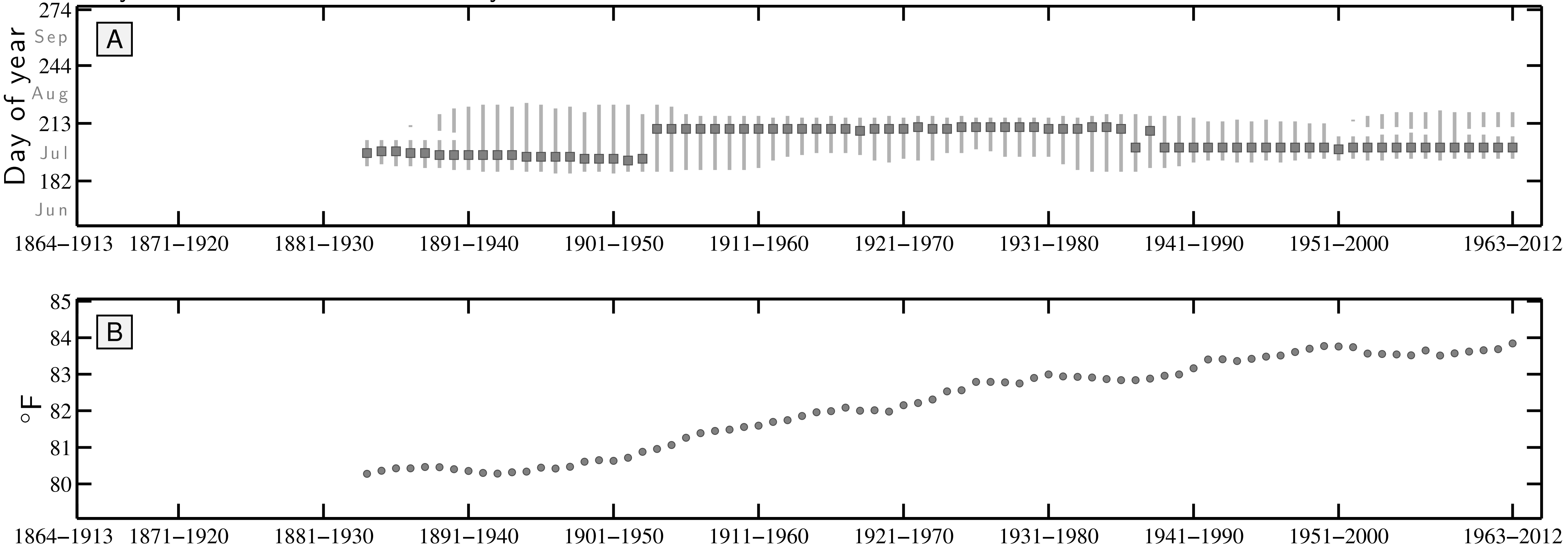

50 year Winter Teletherm dynamics for TAUNTON, MA:

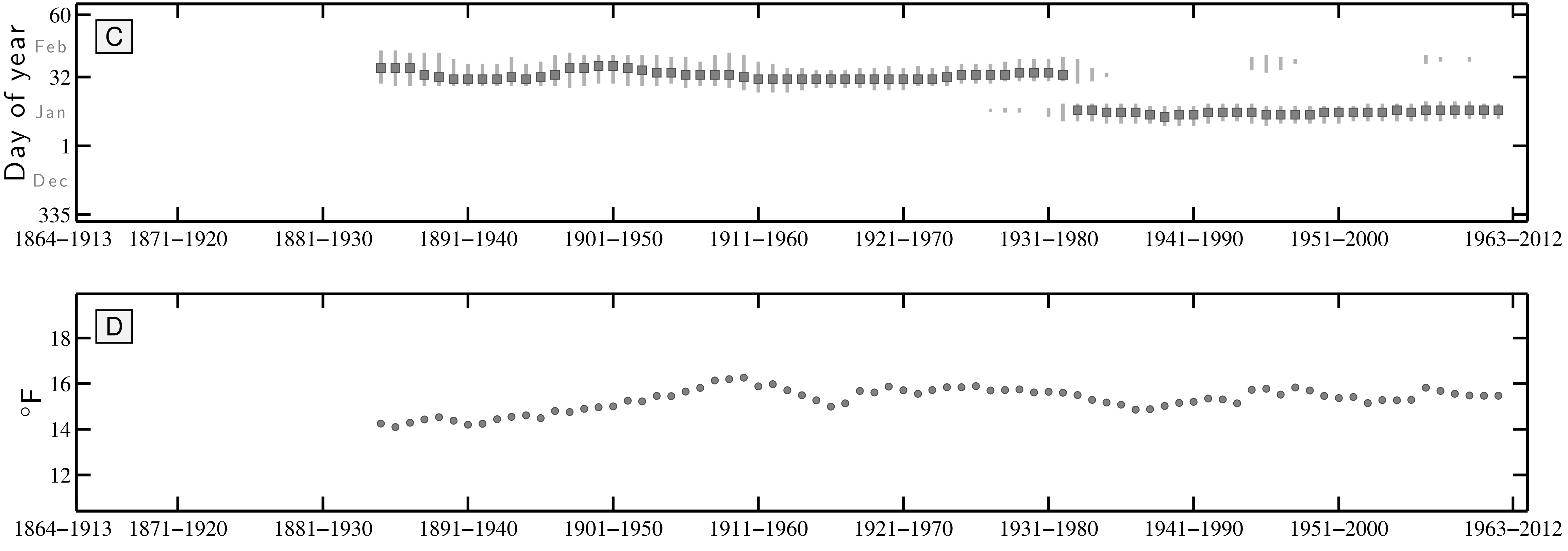

50 year Summer Teletherm dynamics for WEST MEDWAY, MA:

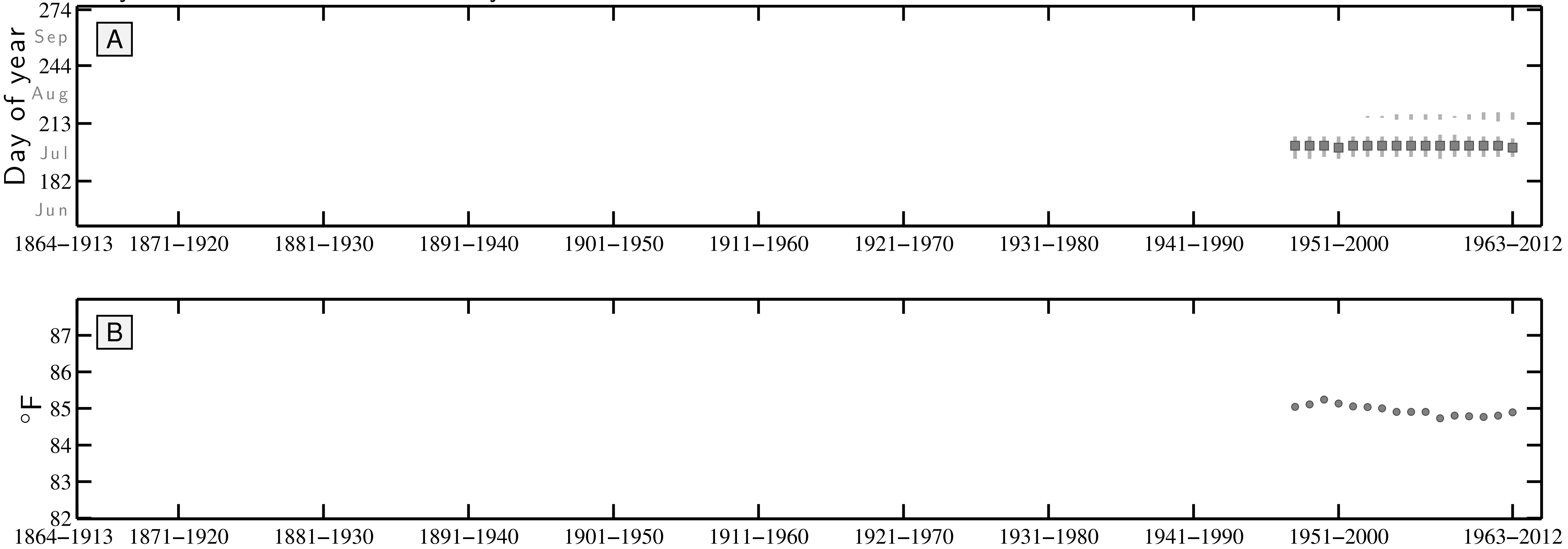

50 year Winter Teletherm dynamics for WEST MEDWAY, MA:

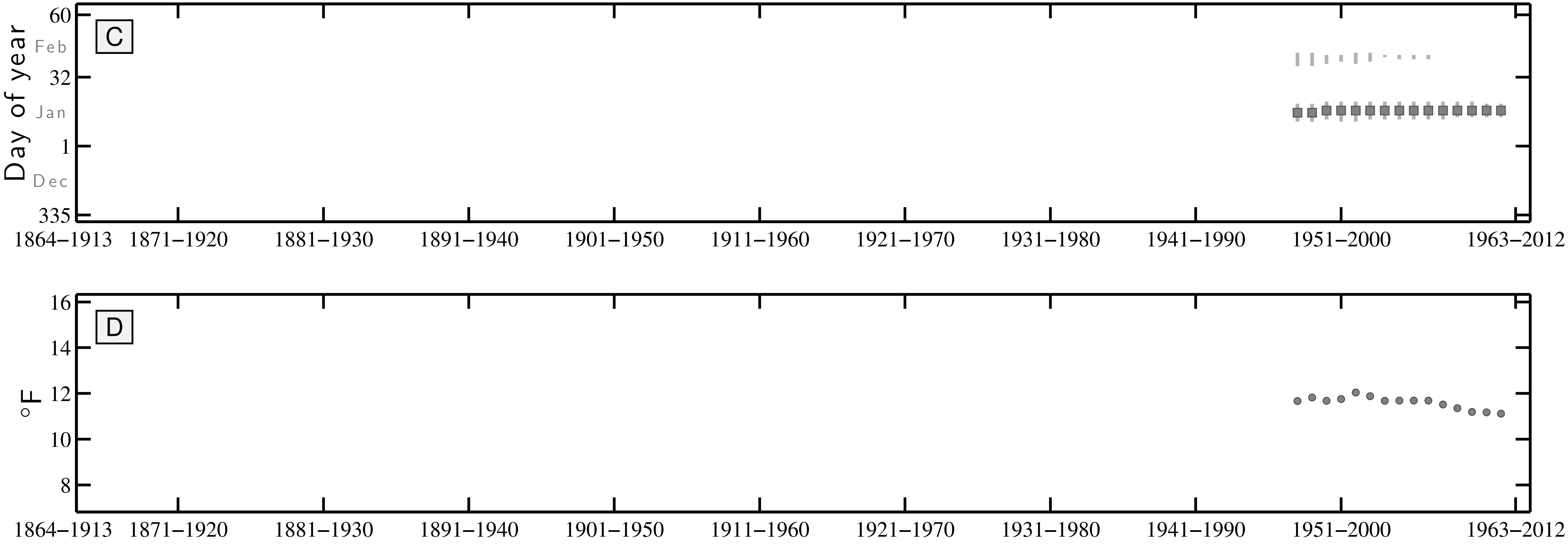

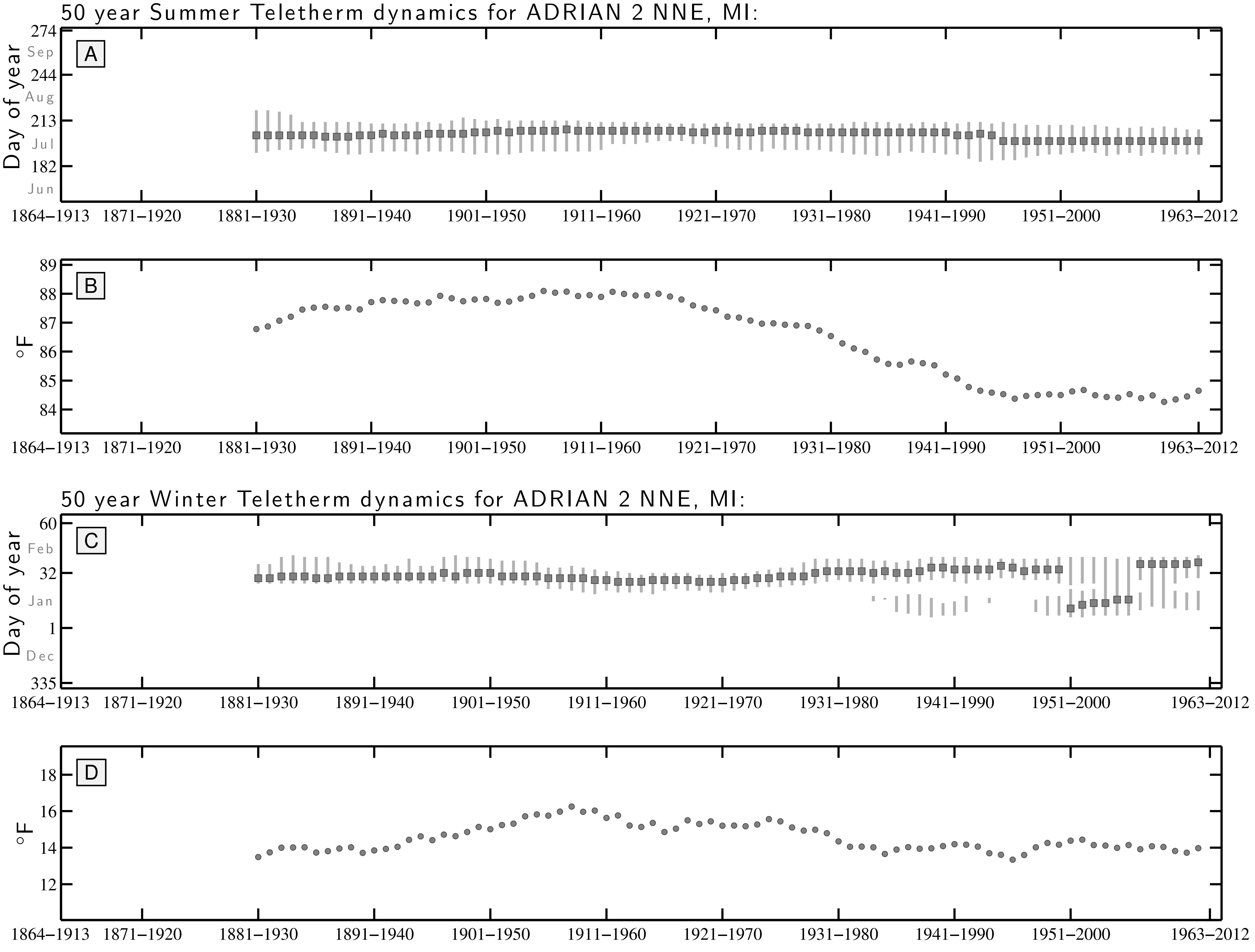
50 year Summer Teletherm dynamics for ADRIAN 2 NNE, MI:
A
Day of year
274
Sep
244
Aug
213
Jul
182
Jun
B
°F
89
88
87
86
85
84
1864–1913
1871–1920
1881–1930
1891–1940
1901–1950
1911–1960
1921–1970
1931–1980
1941–1990
1951–2000
1963–2012
50 year Winter Teletherm dynamics for ADRIAN 2 NNE, MI:
C
Day of year
60
Feb
32
Jan
1
Dec
335
D
°F
18
16
14
12

50 year Summer Teletherm dynamics for ALLEGAN 5NE, MI:

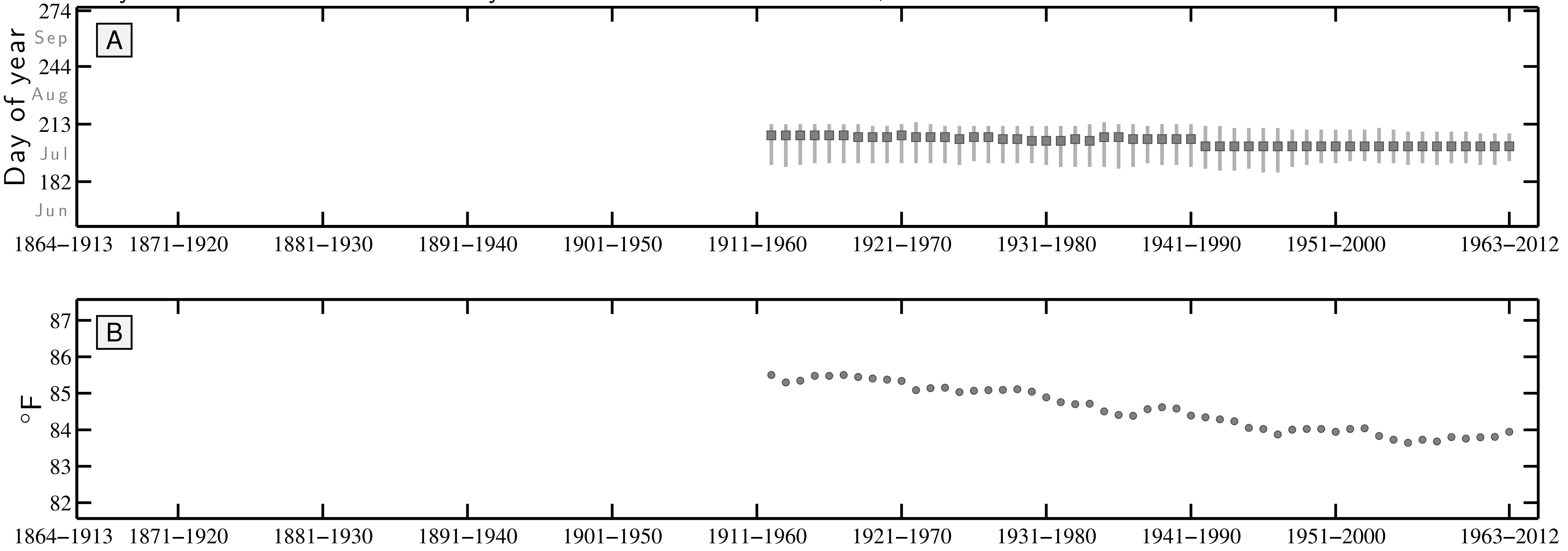

50 year Winter Teletherm dynamics for ALLEGAN 5NE, MI:

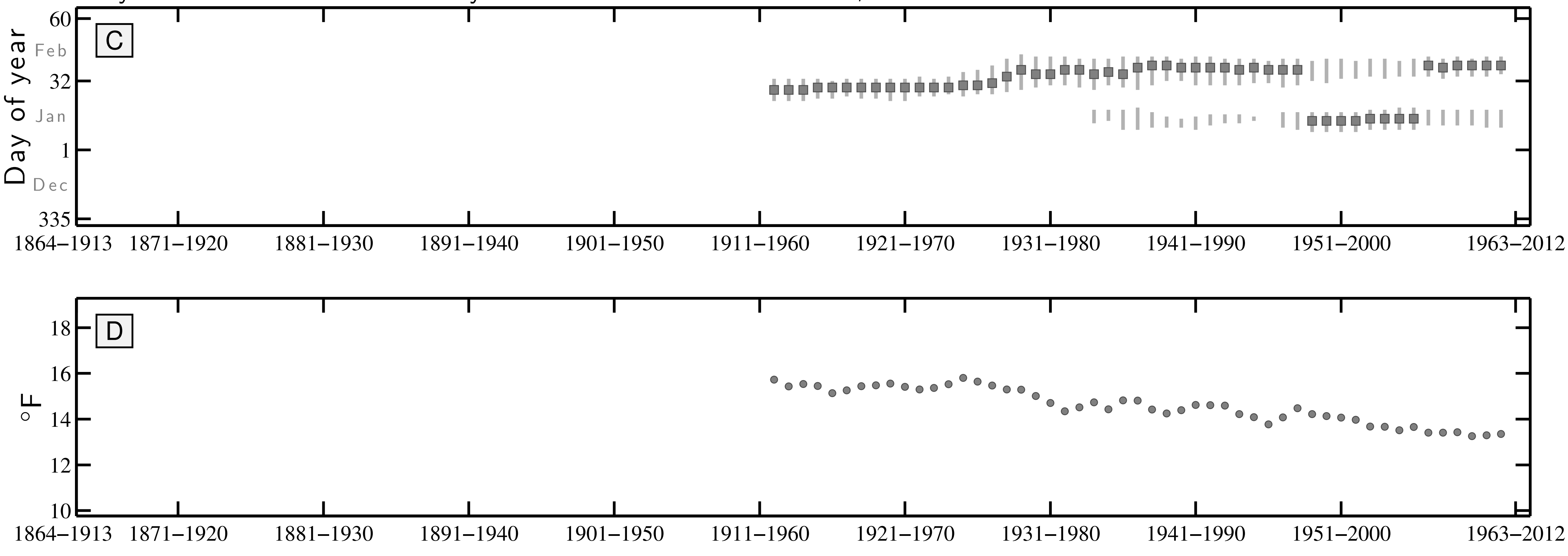

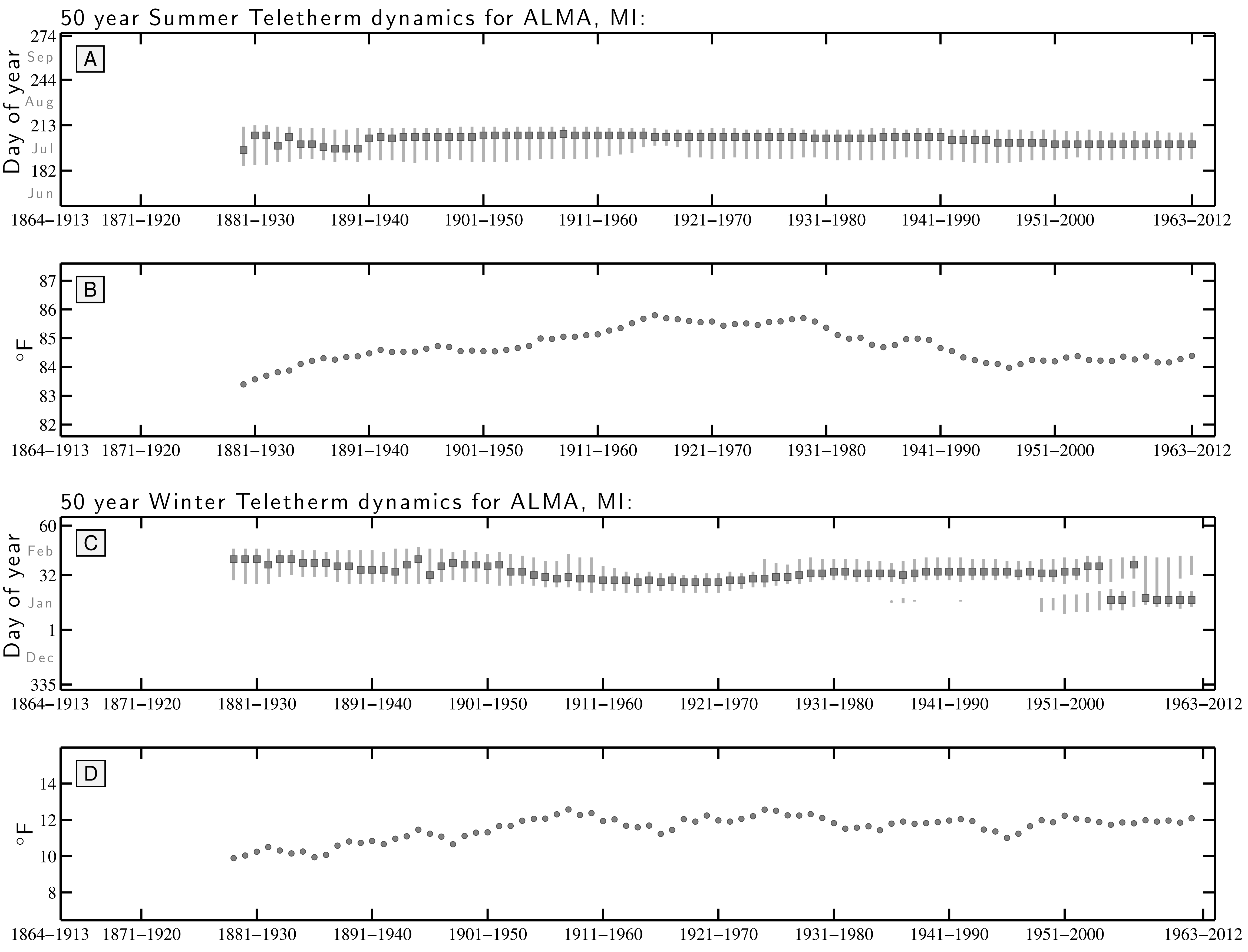
50 year Summer Teletherm dynamics for ALMA, MI:
A
Day of year
274
Sep
244
Aug
213
Jul
182
Jun
1864–1913
1871–1920
1881–1930
1891–1940
1901–1950
1911–1960
1921–1970
1931–1980
1941–1990
1951–2000
1963–2012
B
°F
87
86
85
84
83
82
50 year Winter Teletherm dynamics for ALMA, MI:
C
Day of year
60
Feb
32
Jan
1
Dec
335
D
°F
14
12
10
8

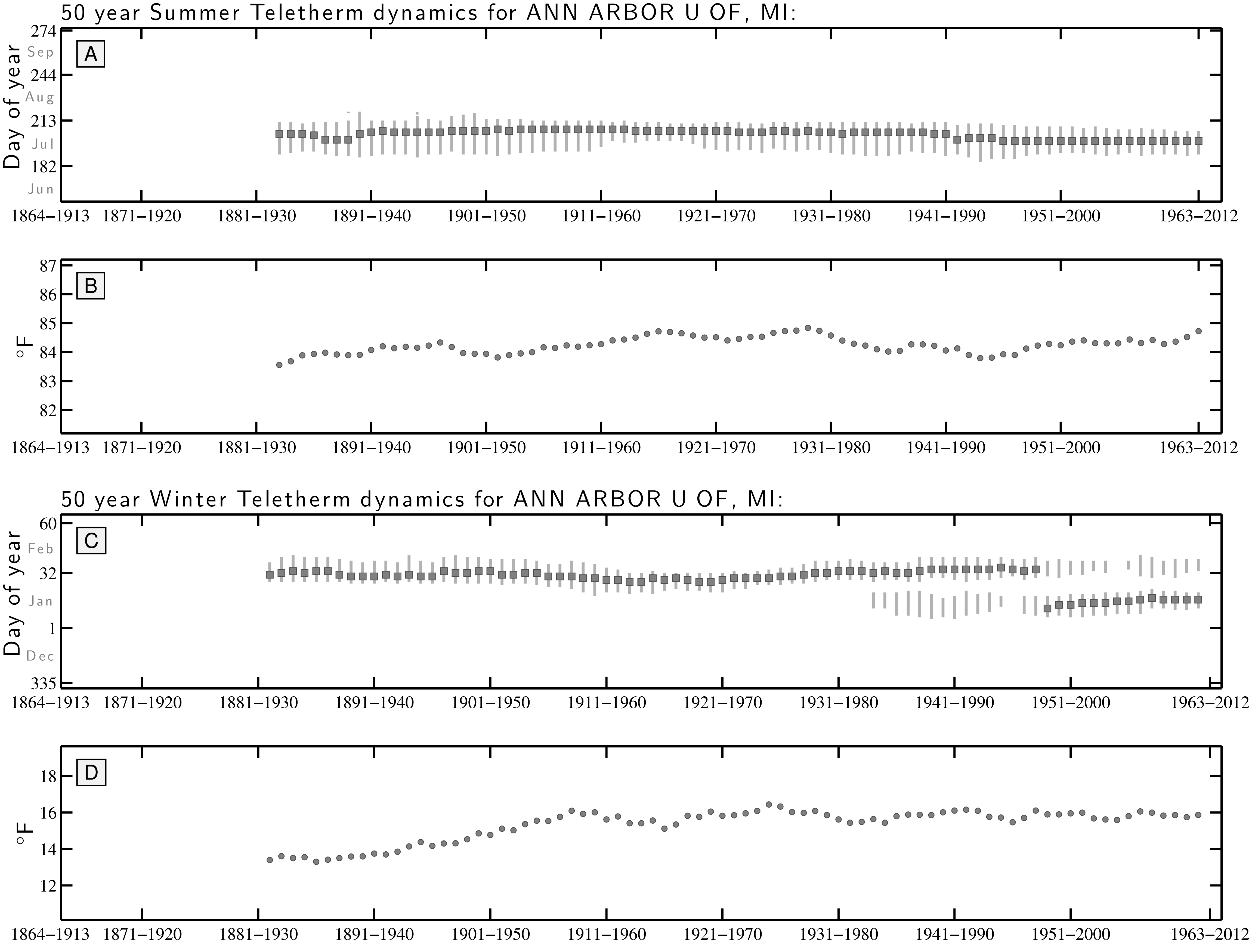
50 year Summer Teletherm dynamics for ANN ARBOR U OF, MI:
A
Day of year
274
Sep
244
Aug
213
Jul
182
Jun
1864–1913
1871–1920
1881–1930
1891–1940
1901–1950
1911–1960
1921–1970
1931–1980
1941–1990
1951–2000
1963–2012
B
°F
87
86
85
84
83
82
50 year Winter Teletherm dynamics for ANN ARBOR U OF, MI:
C
Day of year
60
Feb
32
Jan
1
Dec
335
D
°F
18
16
14
12

50 year Summer Teletherm dynamics for BIG RAPIDS WTR WKS, MI:

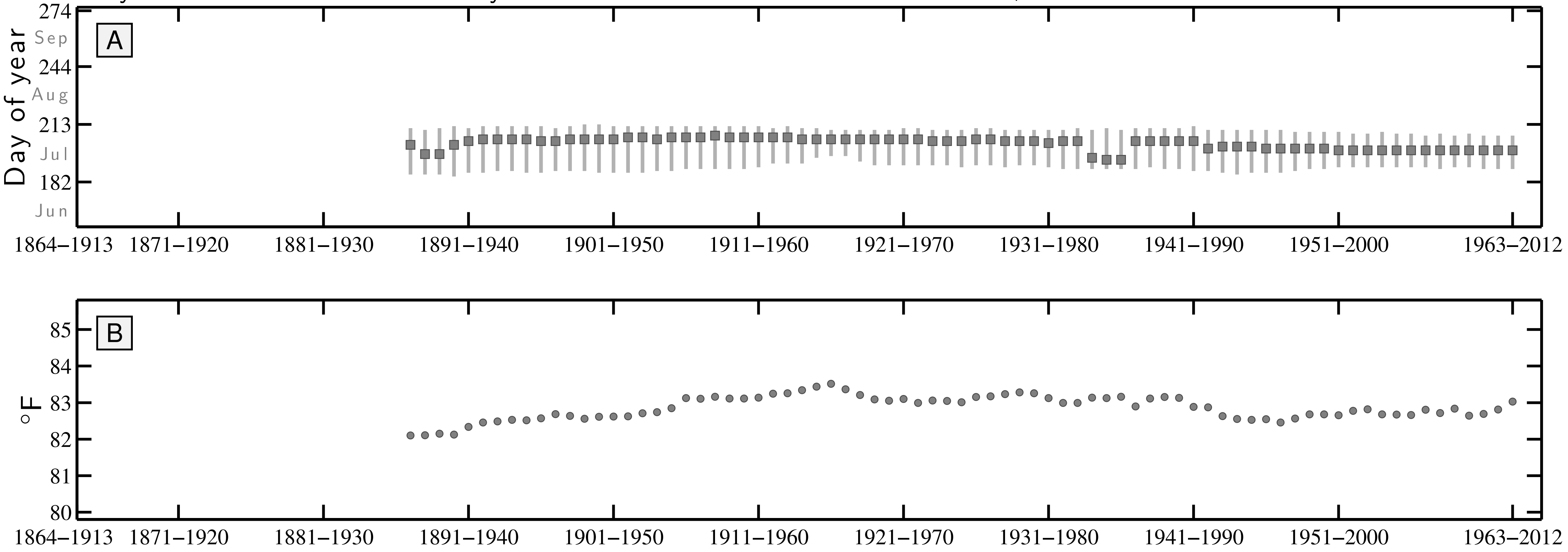

50 year Winter Teletherm dynamics for BIG RAPIDS WTR WKS, MI:

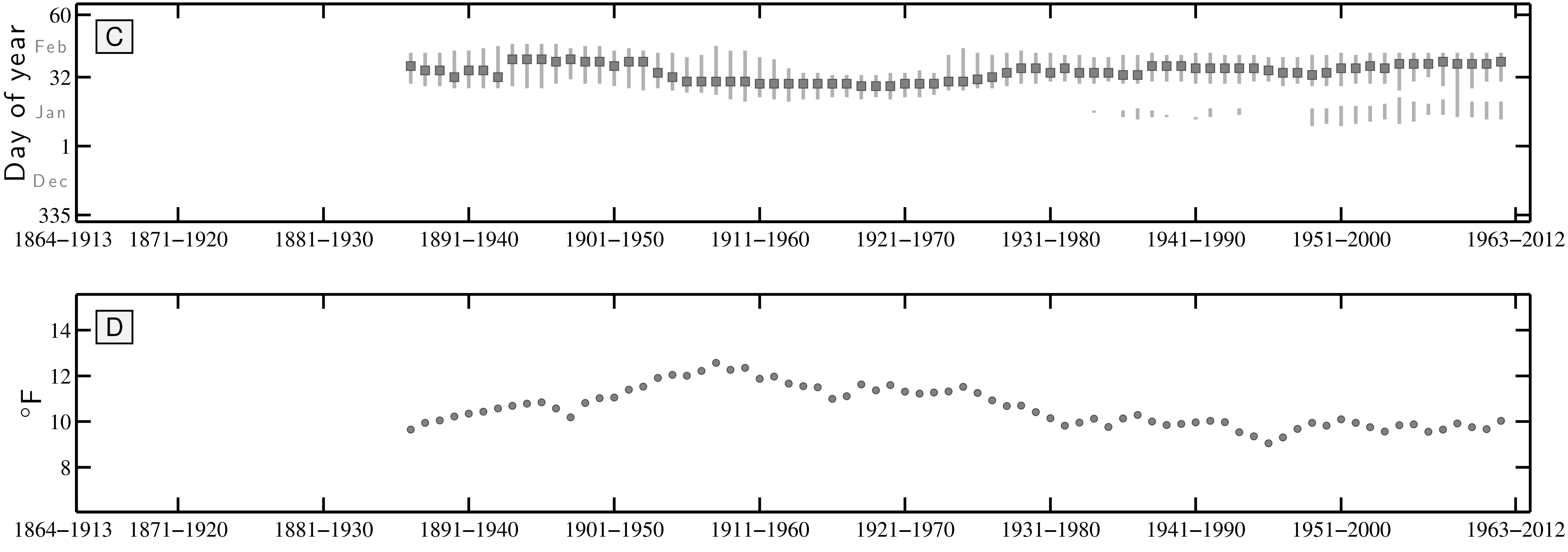

50 year Summer Teletherm dynamics for CHAMPION VAN RIPER PK, MI:

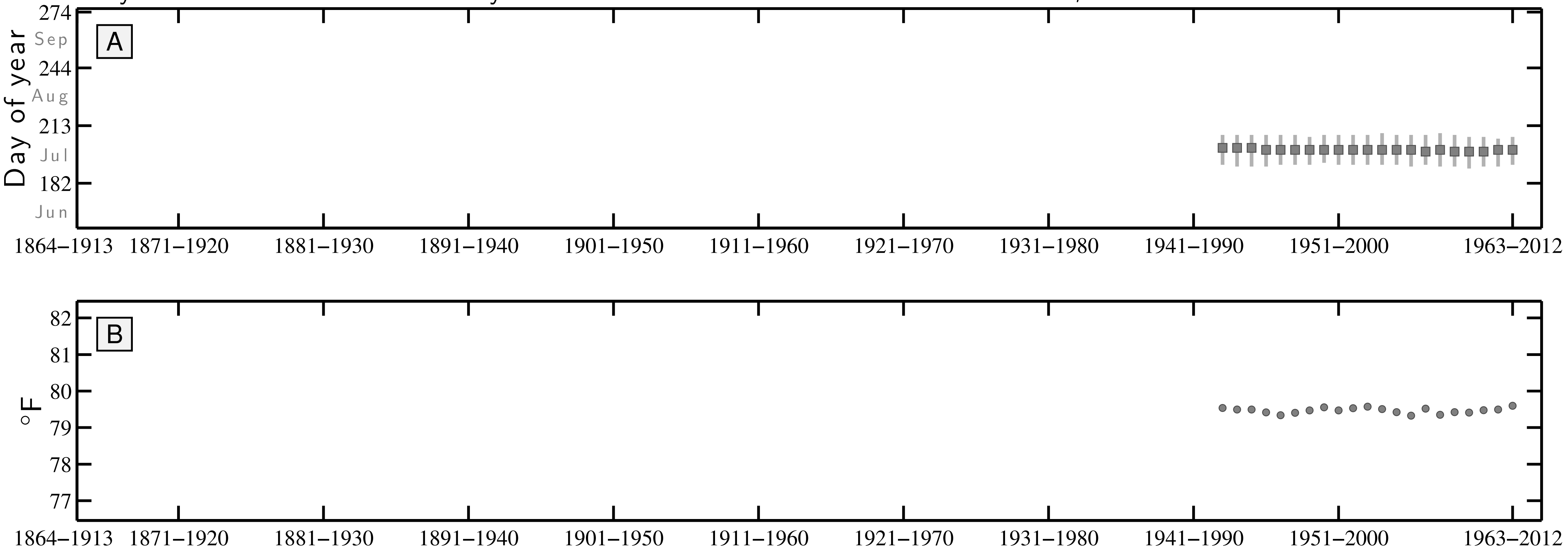

50 year Winter Teletherm dynamics for CHAMPION VAN RIPER PK, MI:

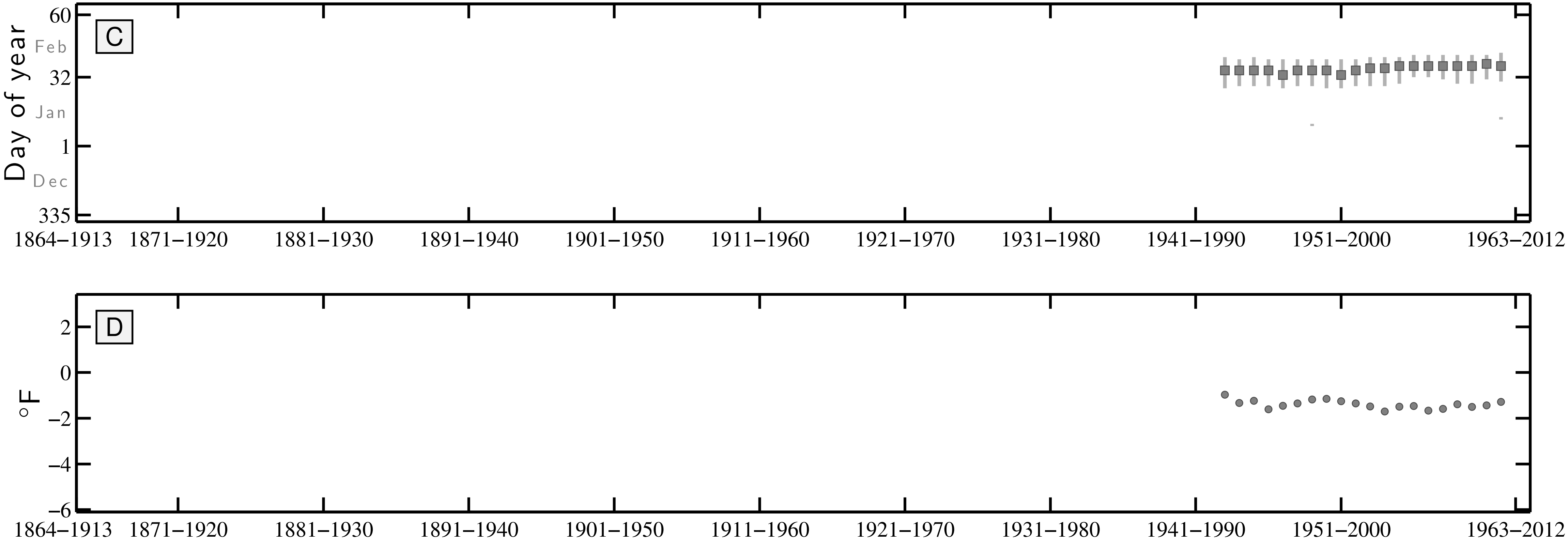

50 year Summer Teletherm dynamics for CHEBOYGAN, MI:

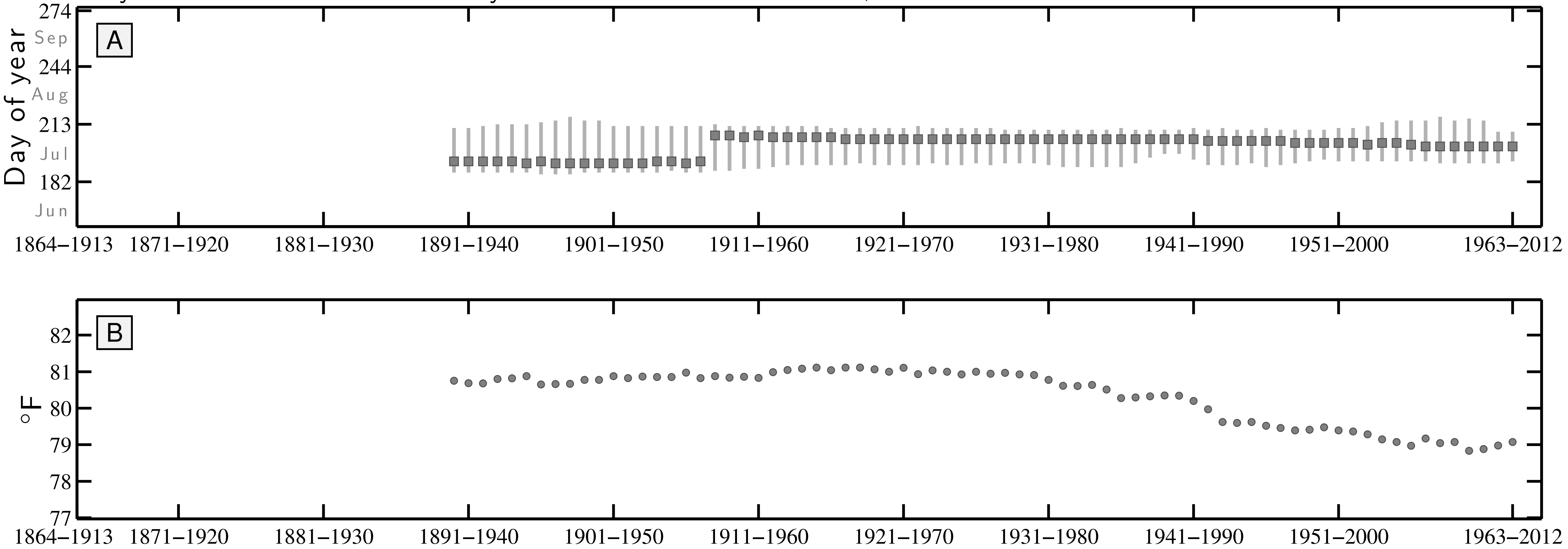

50 year Winter Teletherm dynamics for CHEBOYGAN, MI:

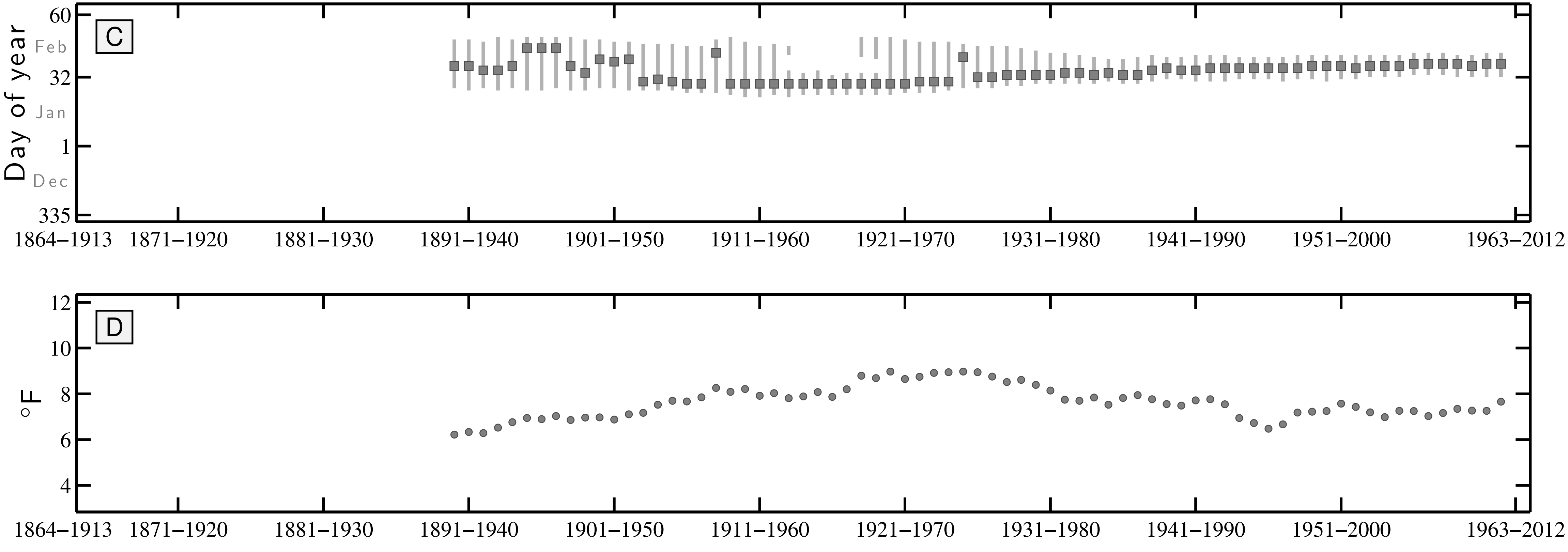

50 year Summer Teletherm dynamics for COLDWATER ST SCHOOL, MI:

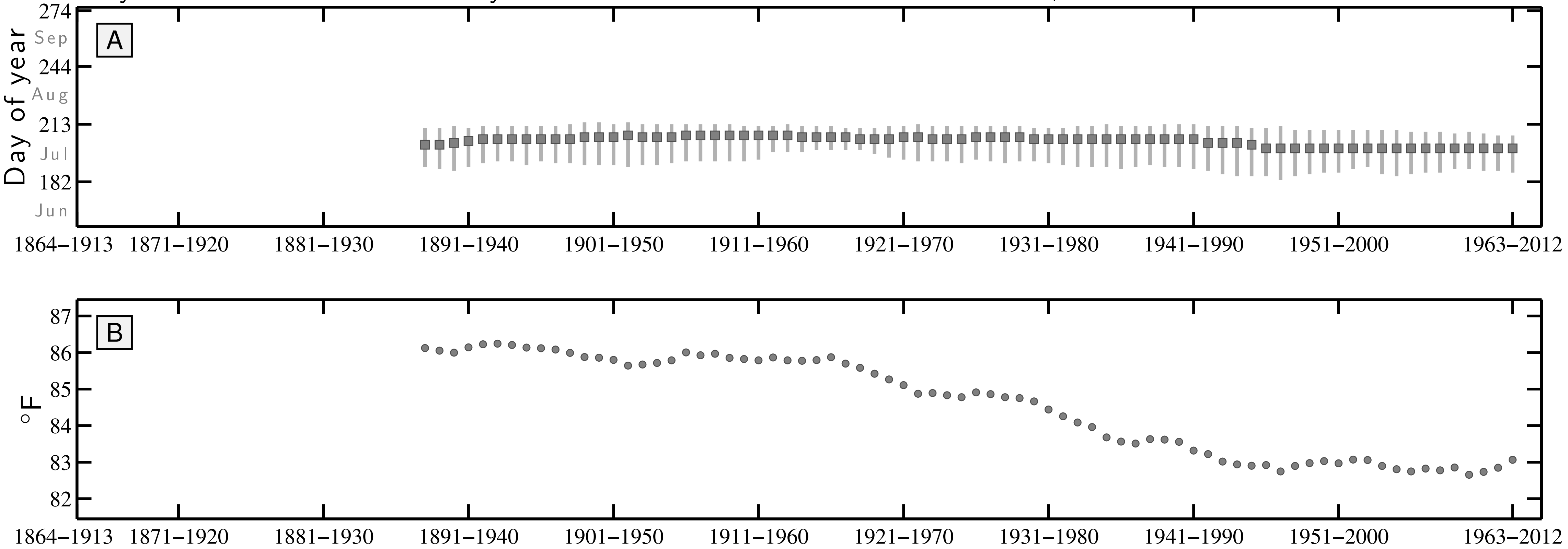

50 year Winter Teletherm dynamics for COLDWATER ST SCHOOL, MI:

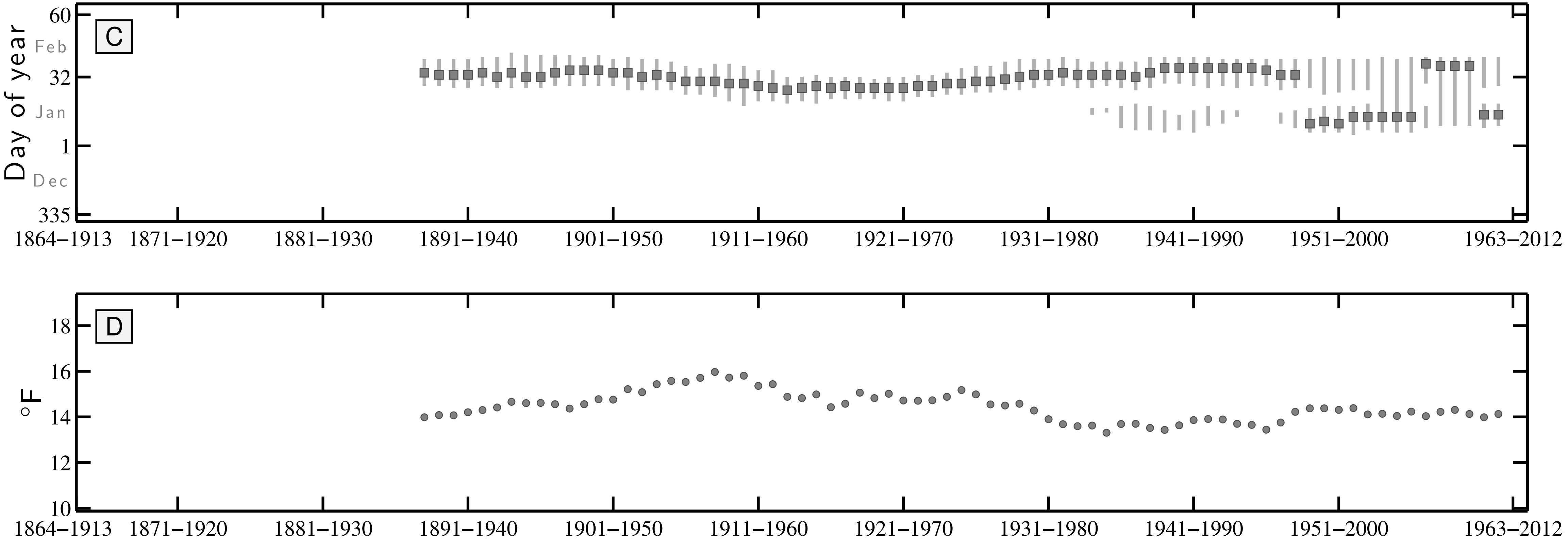

50 year Summer Teletherm dynamics for EAST TAWAS, MI:

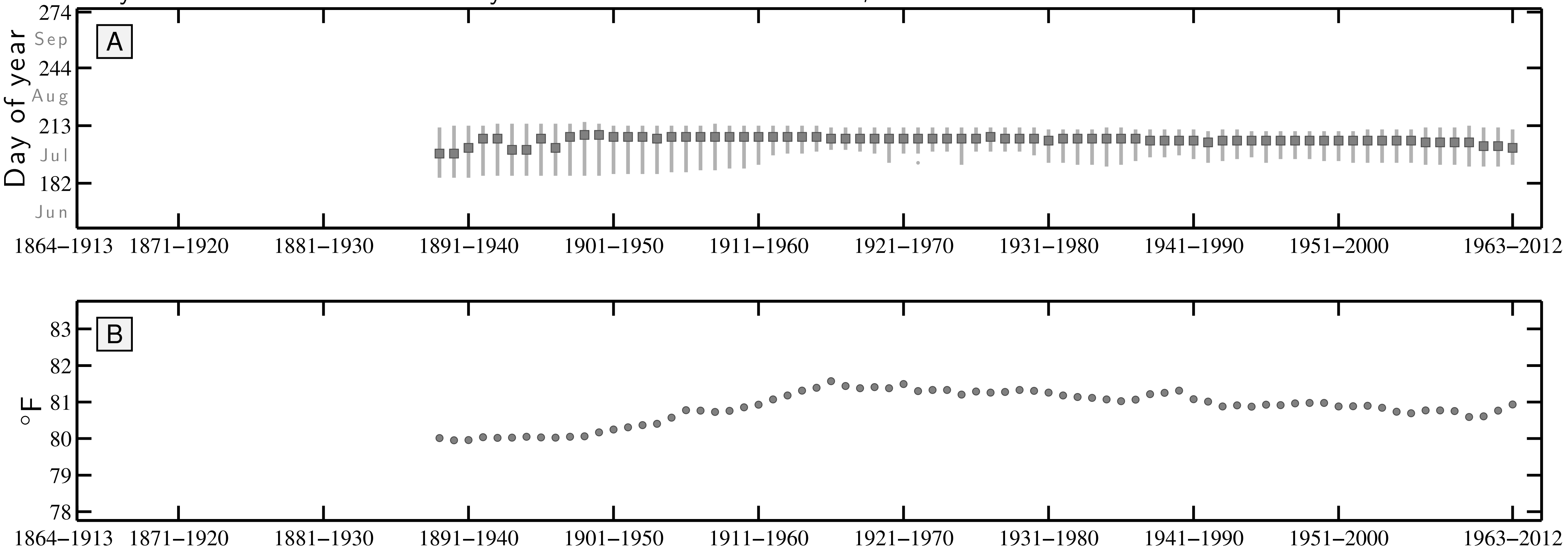

50 year Winter Teletherm dynamics for EAST TAWAS, MI:

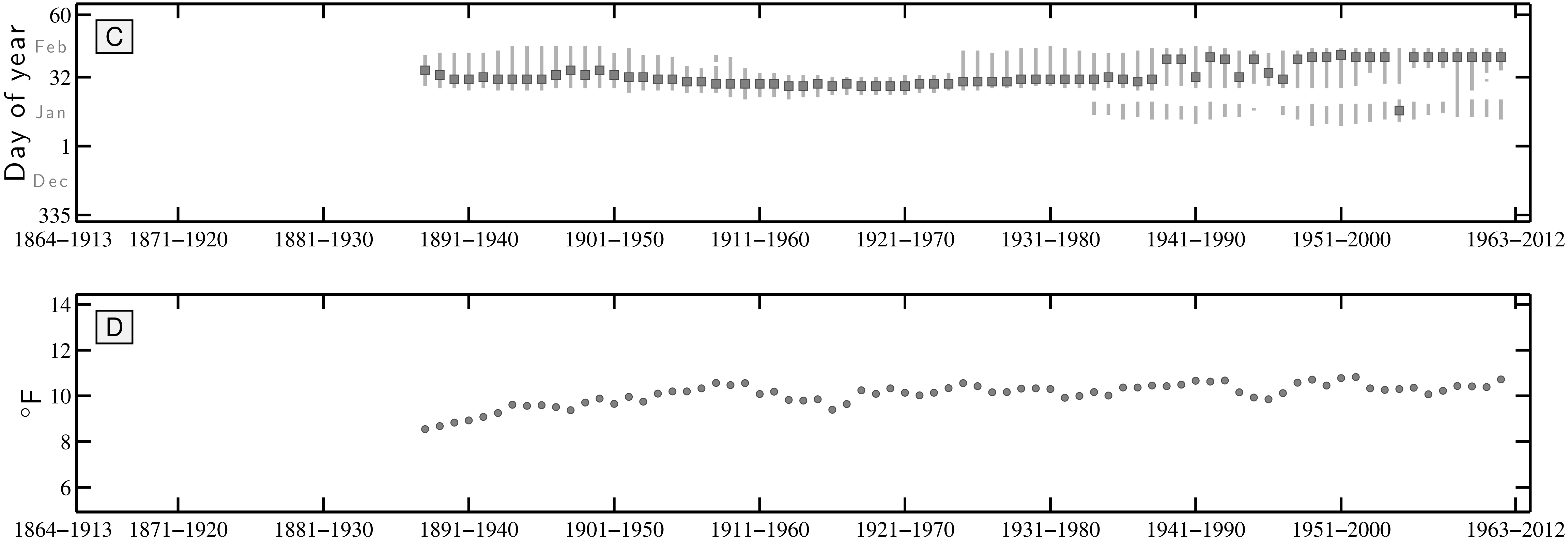

50 year Summer Teletherm dynamics for FAYETTE 4 SW, MI:

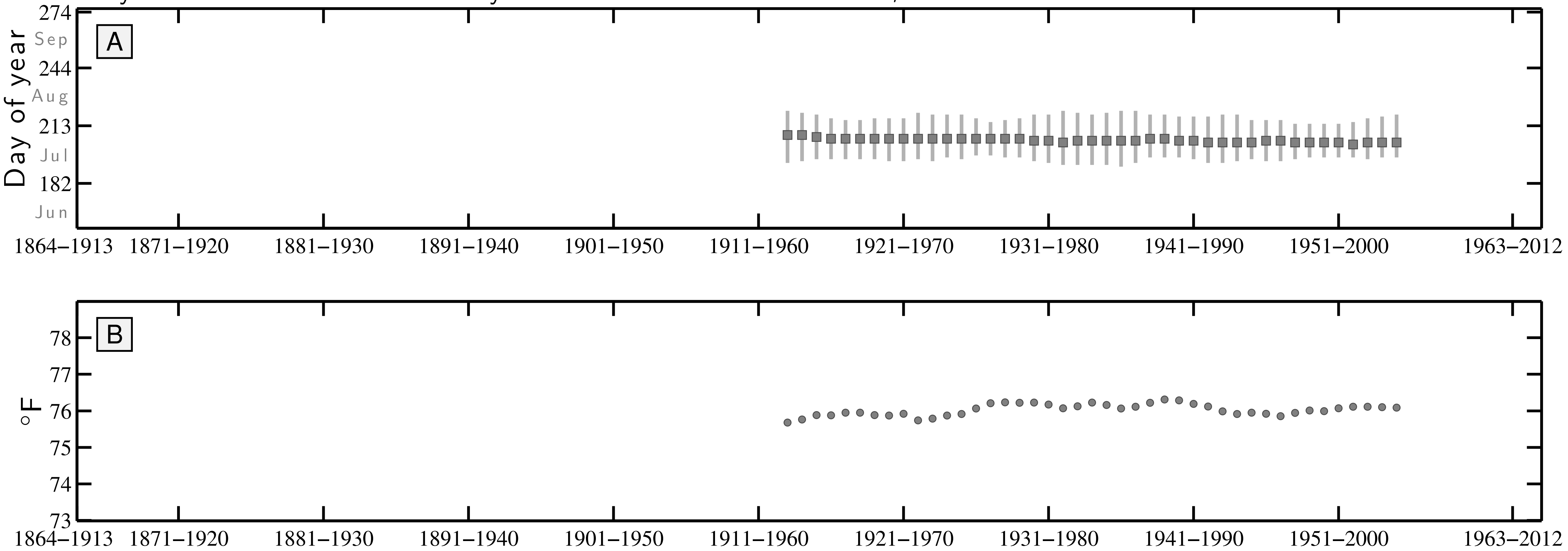

50 year Winter Teletherm dynamics for FAYETTE 4 SW, MI:

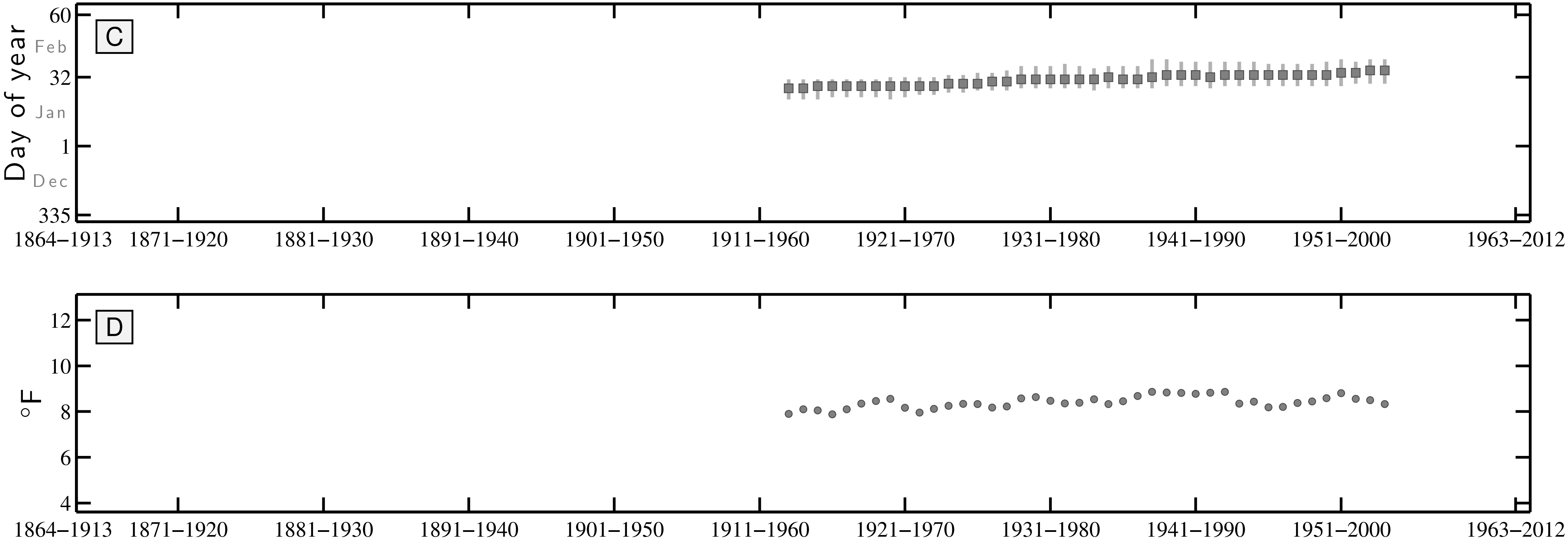

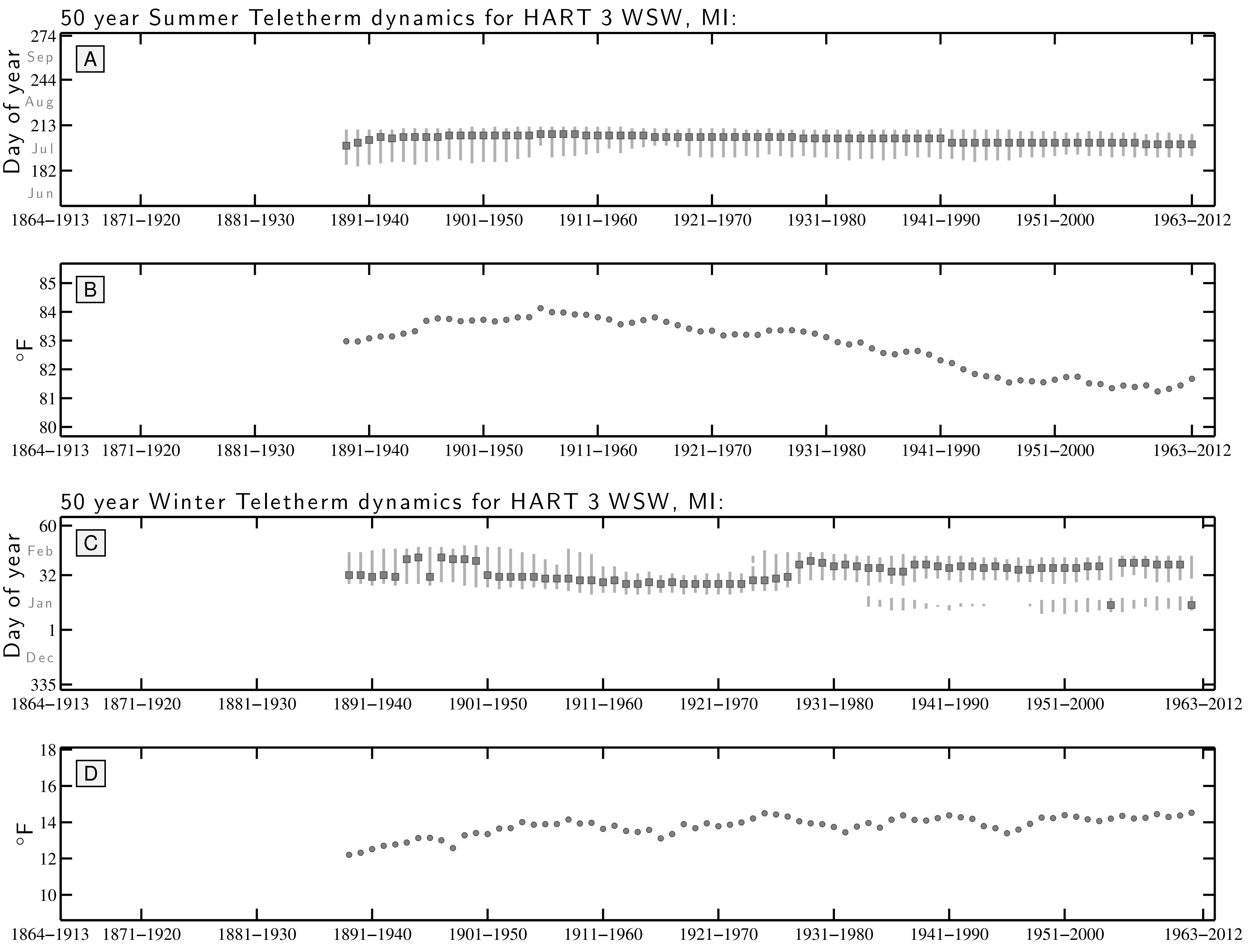
50 year Summer Teletherm dynamics for HART 3 WSW, MI:
A
Day of year
274
Sep
244
Aug
213
Jul
182
Jun
1864–1913
1871–1920
1881–1930
1891–1940
1901–1950
1911–1960
1921–1970
1931–1980
1941–1990
1951–2000
1963–2012
B
°F
85
84
83
82
81
80
50 year Winter Teletherm dynamics for HART 3 WSW, MI:
C
60
Feb
32
Jan
1
Dec
335
D
18
16
14
12
10

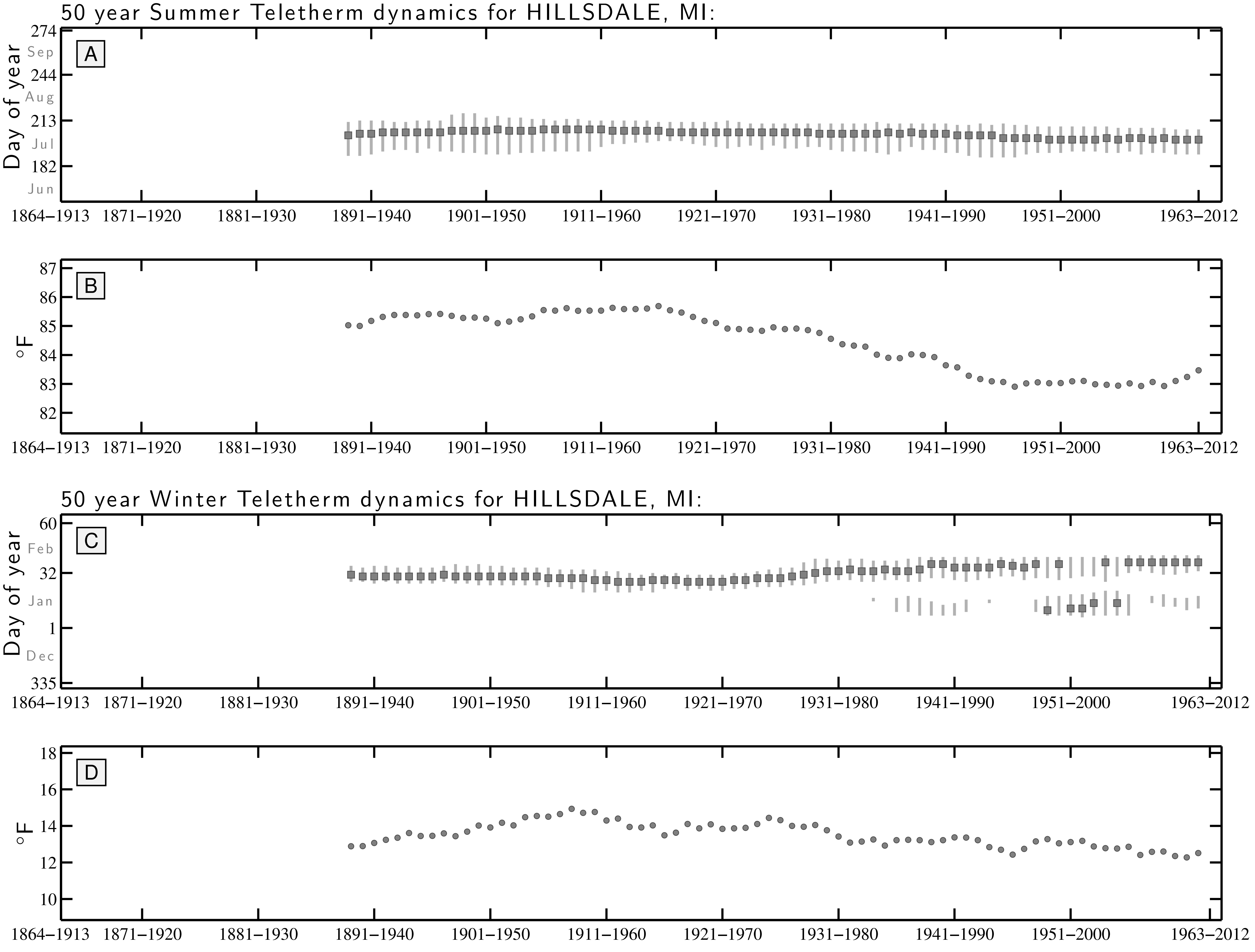

50 year Summer Teletherm dynamics for HILLSDALE, MI:
A
Day of year
274
Sep
244
Aug
213
Jul
182
Jun
1864–1913
1871–1920
1881–1930
1891–1940
1901–1950
1911–1960
1921–1970
1931–1980
1941–1990
1951–2000
1963–2012
B
°F
87
86
85
84
83
82
50 year Winter Teletherm dynamics for HILLSDALE, MI:
C
Day of year
60
Feb
32
Jan
1
Dec
335
D
°F
18
16
14
12
10

50 year Summer Teletherm dynamics for IRON MT KINGSFORD WWTP, MI:

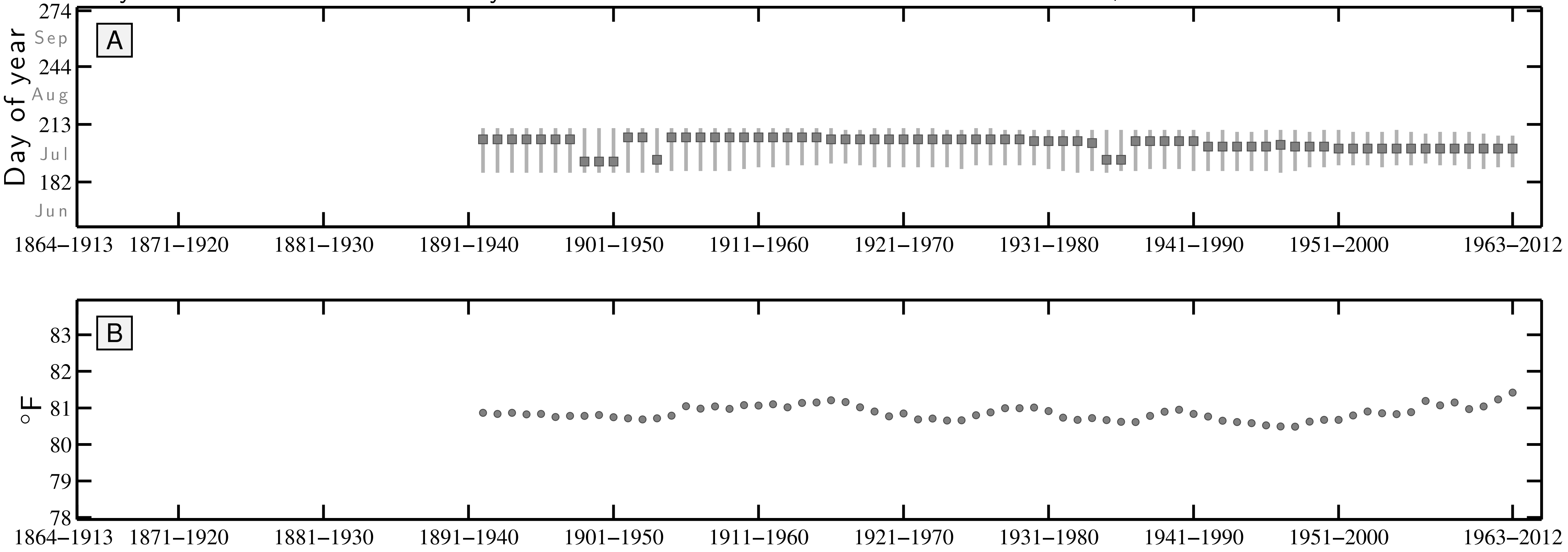

50 year Winter Teletherm dynamics for IRON MT KINGSFORD WWTP, MI:

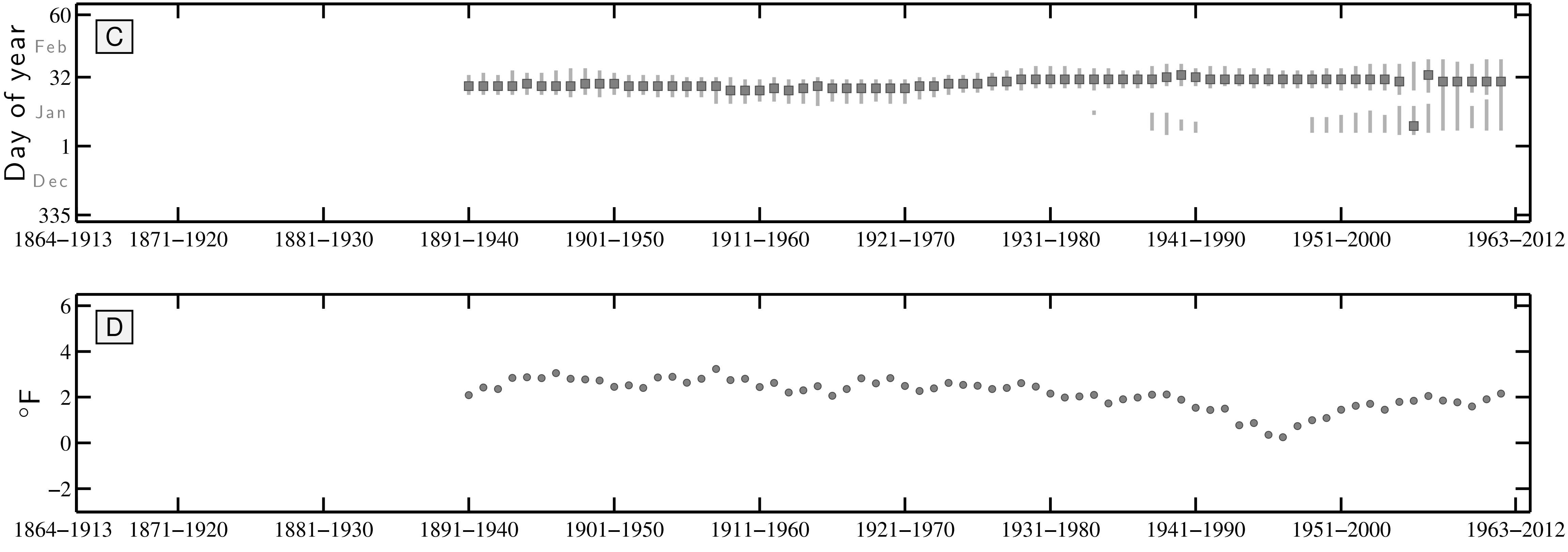

50 year Summer Teletherm dynamics for IRONWOOD, MI:

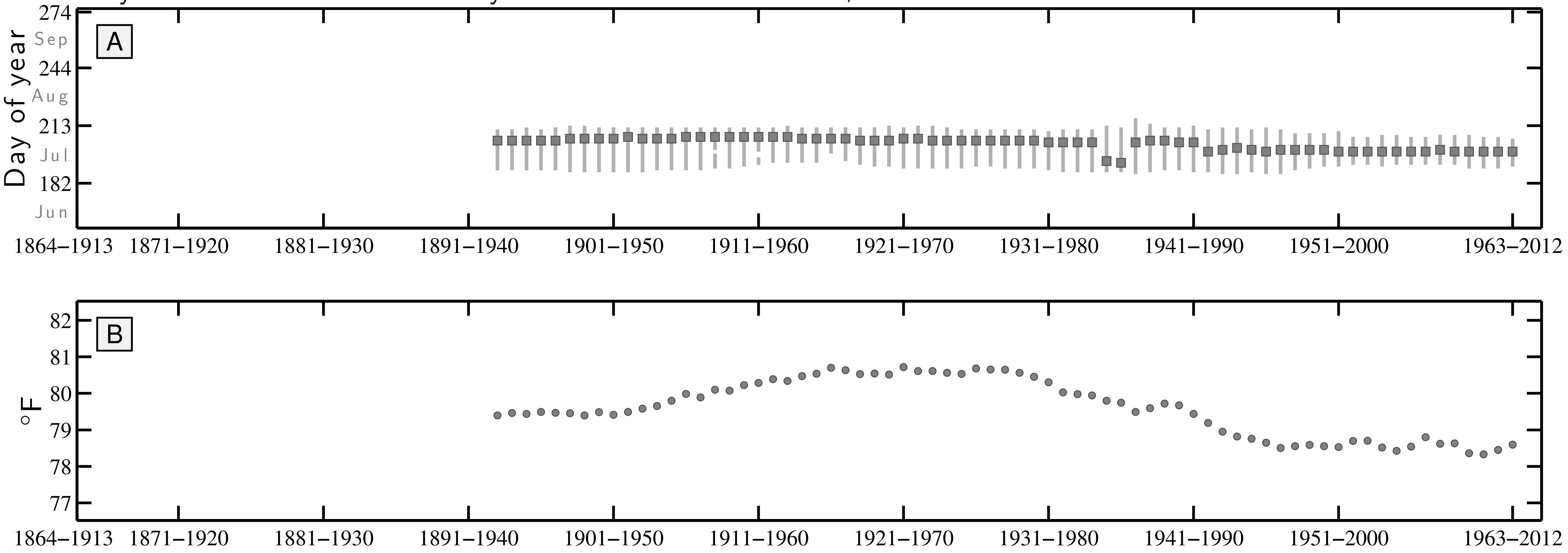

50 year Winter Teletherm dynamics for IRONWOOD, MI:

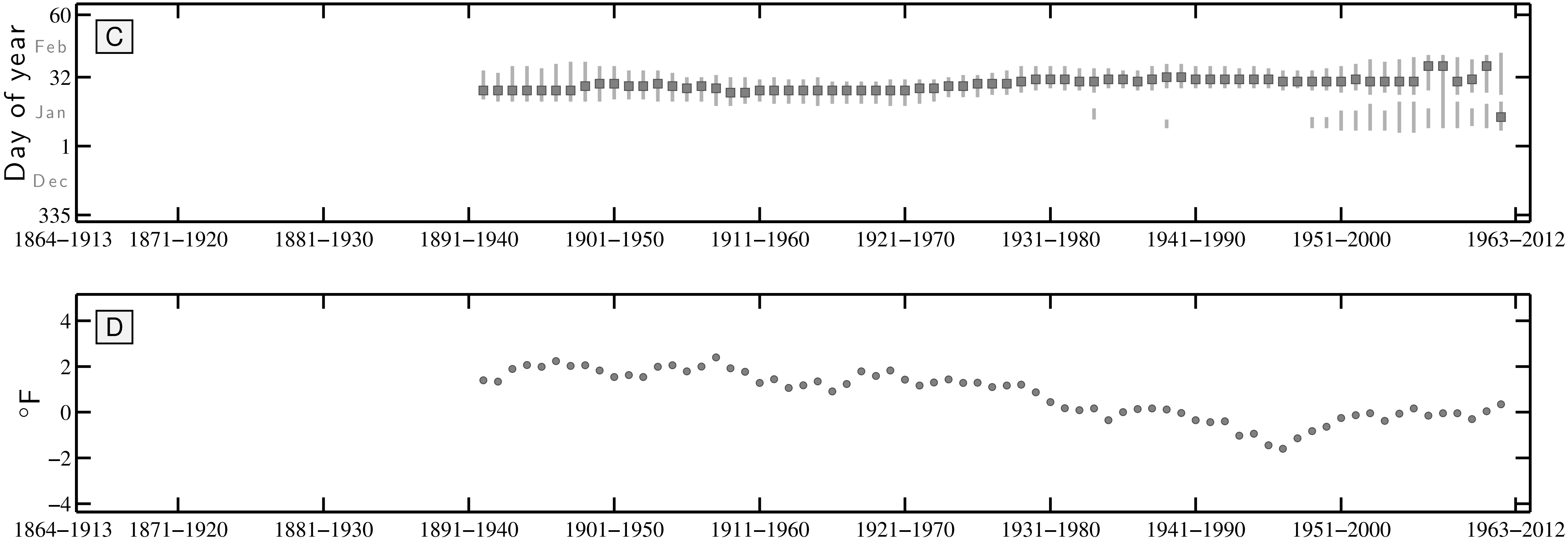

50 year Summer Teletherm dynamics for KALAMAZOO STATE HOSP, MI:

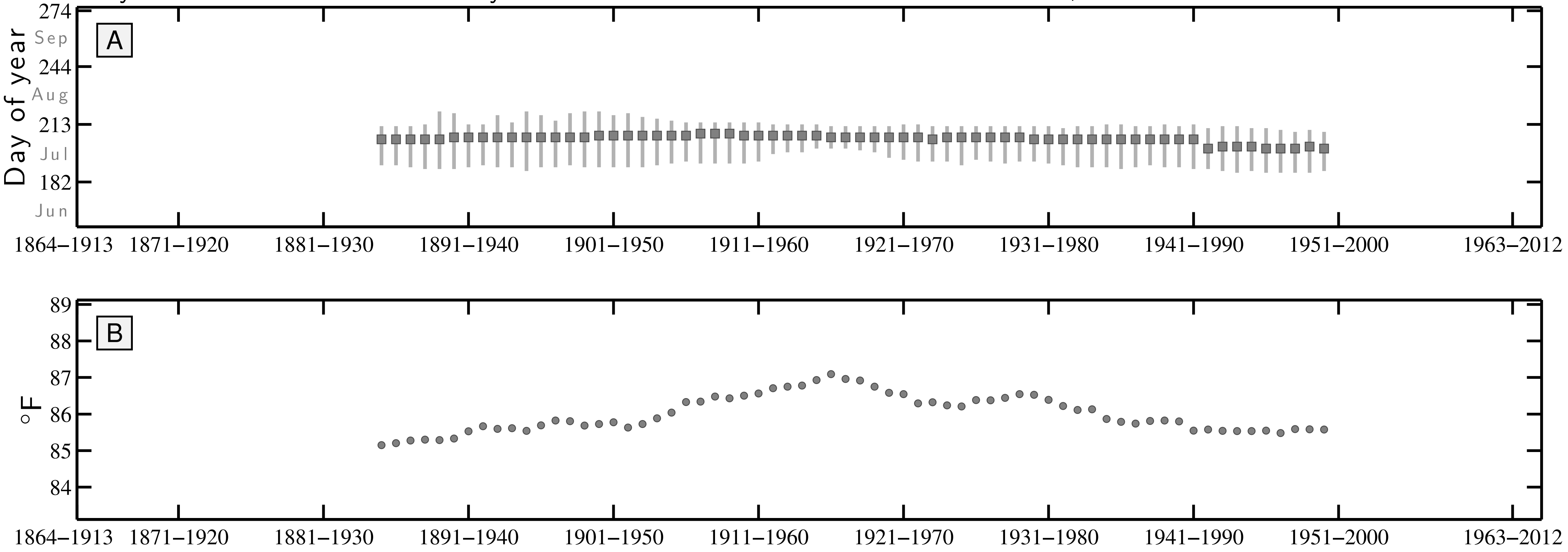

50 year Winter Teletherm dynamics for KALAMAZOO STATE HOSP, MI:

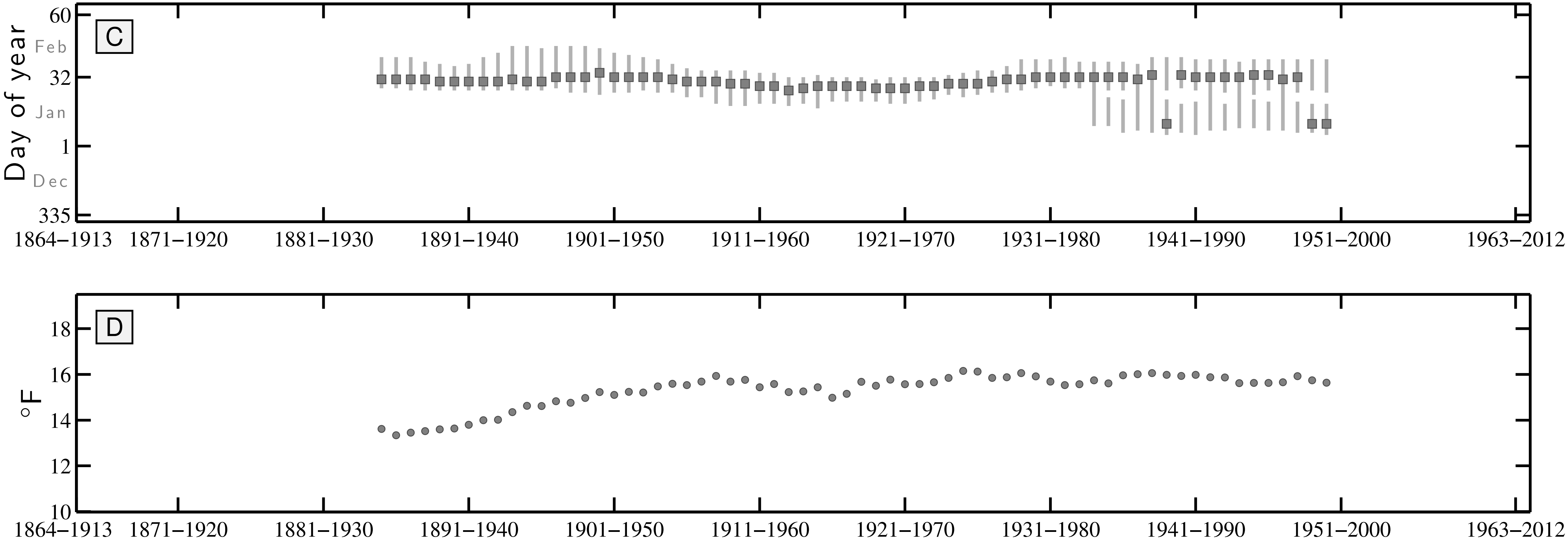

50 year Summer Teletherm dynamics for MIDLAND, MI:

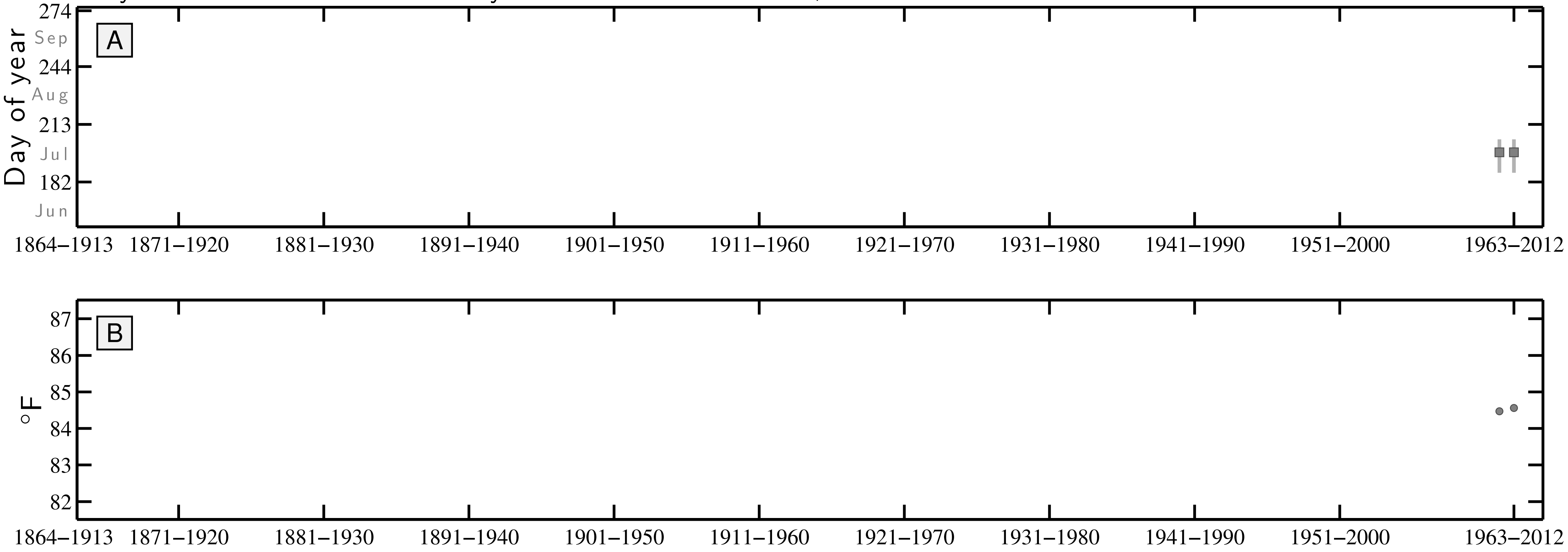

50 year Winter Teletherm dynamics for MIDLAND, MI:

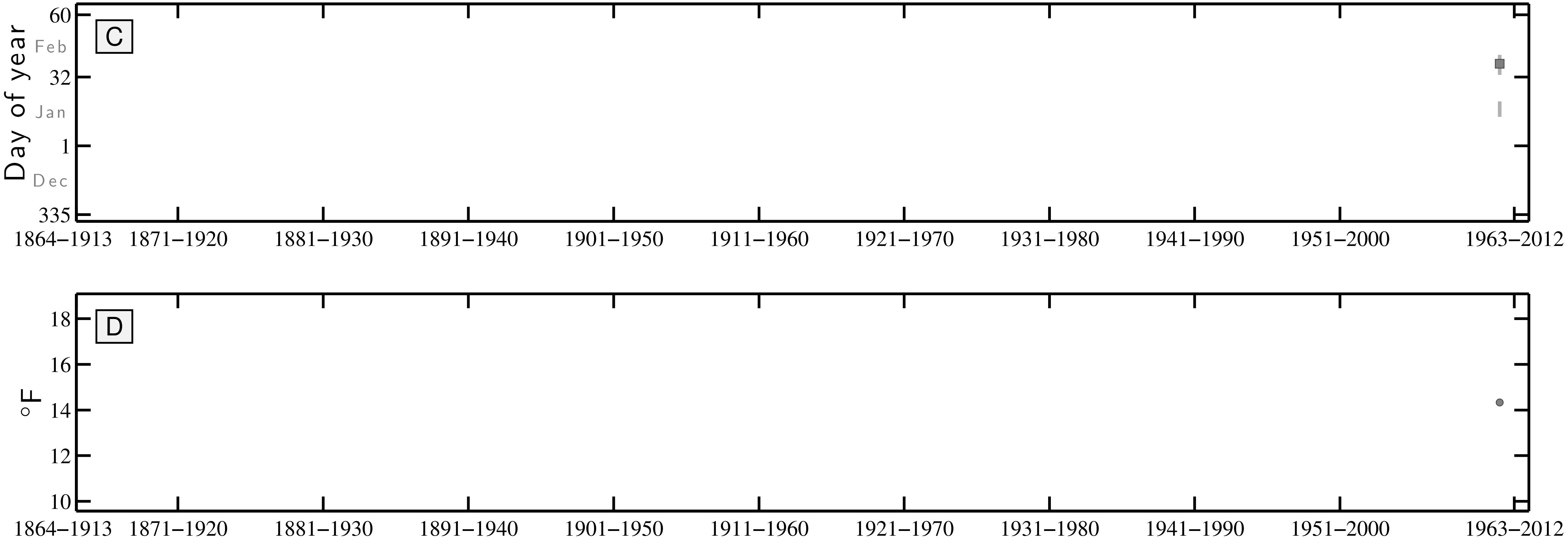

50 year Summer Teletherm dynamics for MT CLEMENS ANG BASE, MI:

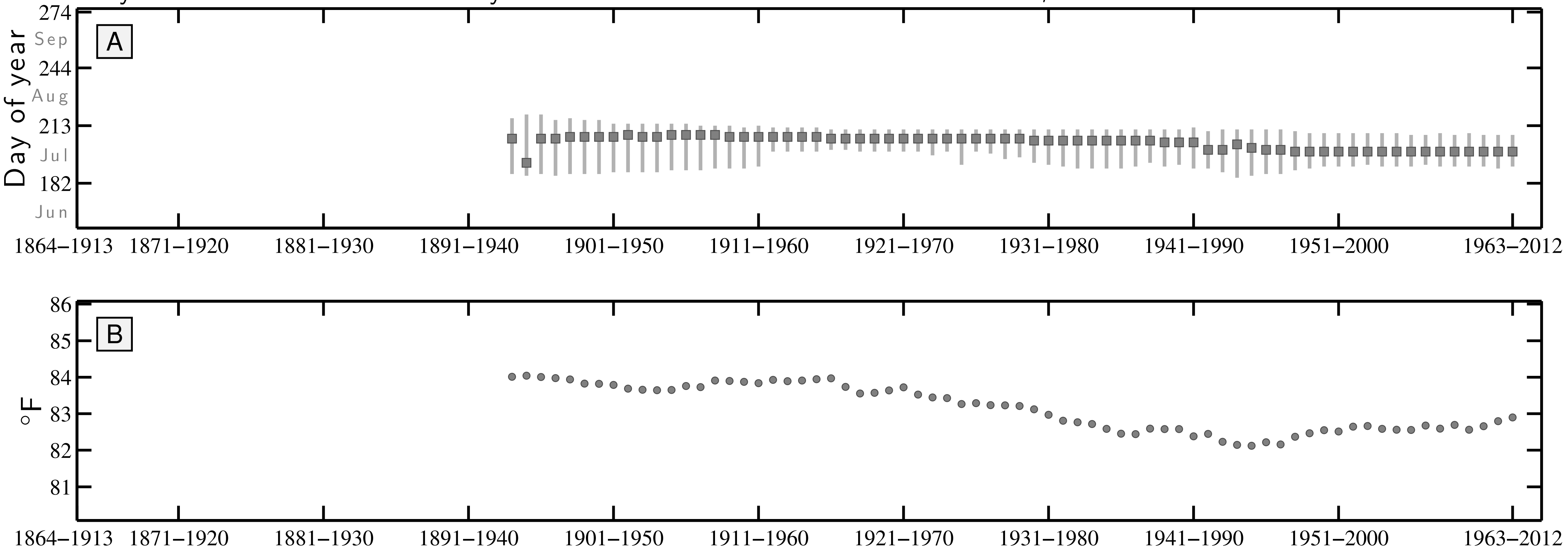

50 year Winter Teletherm dynamics for MT CLEMENS ANG BASE, MI:

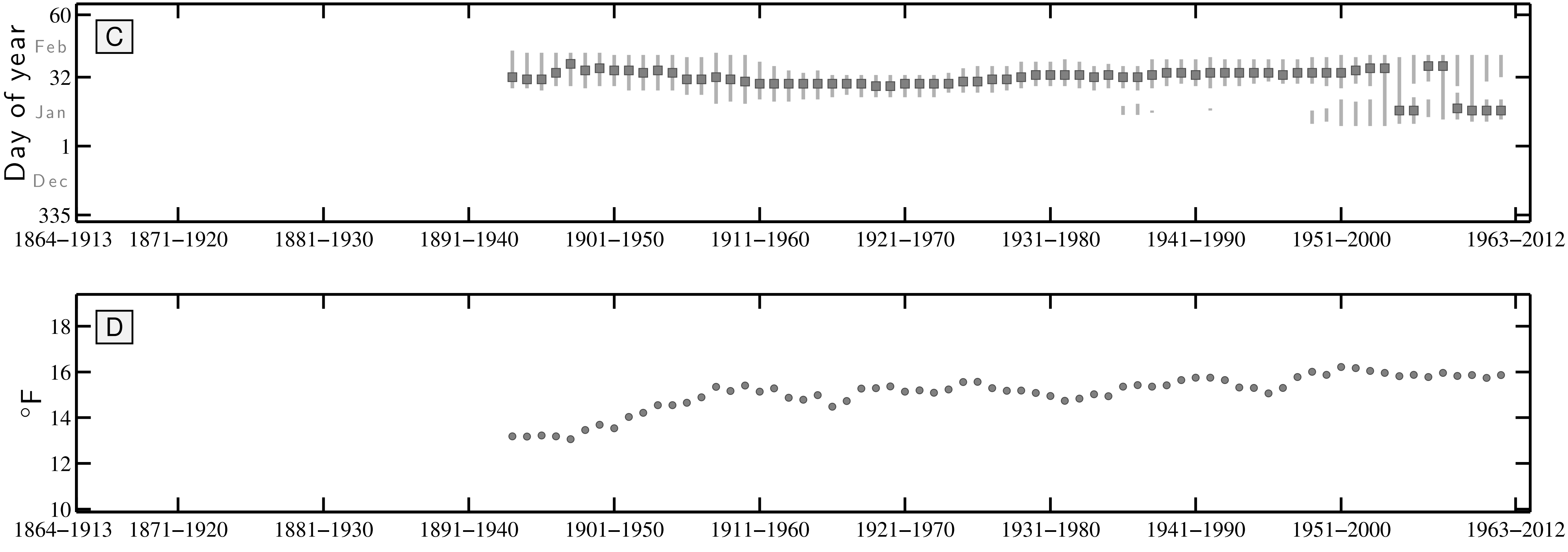

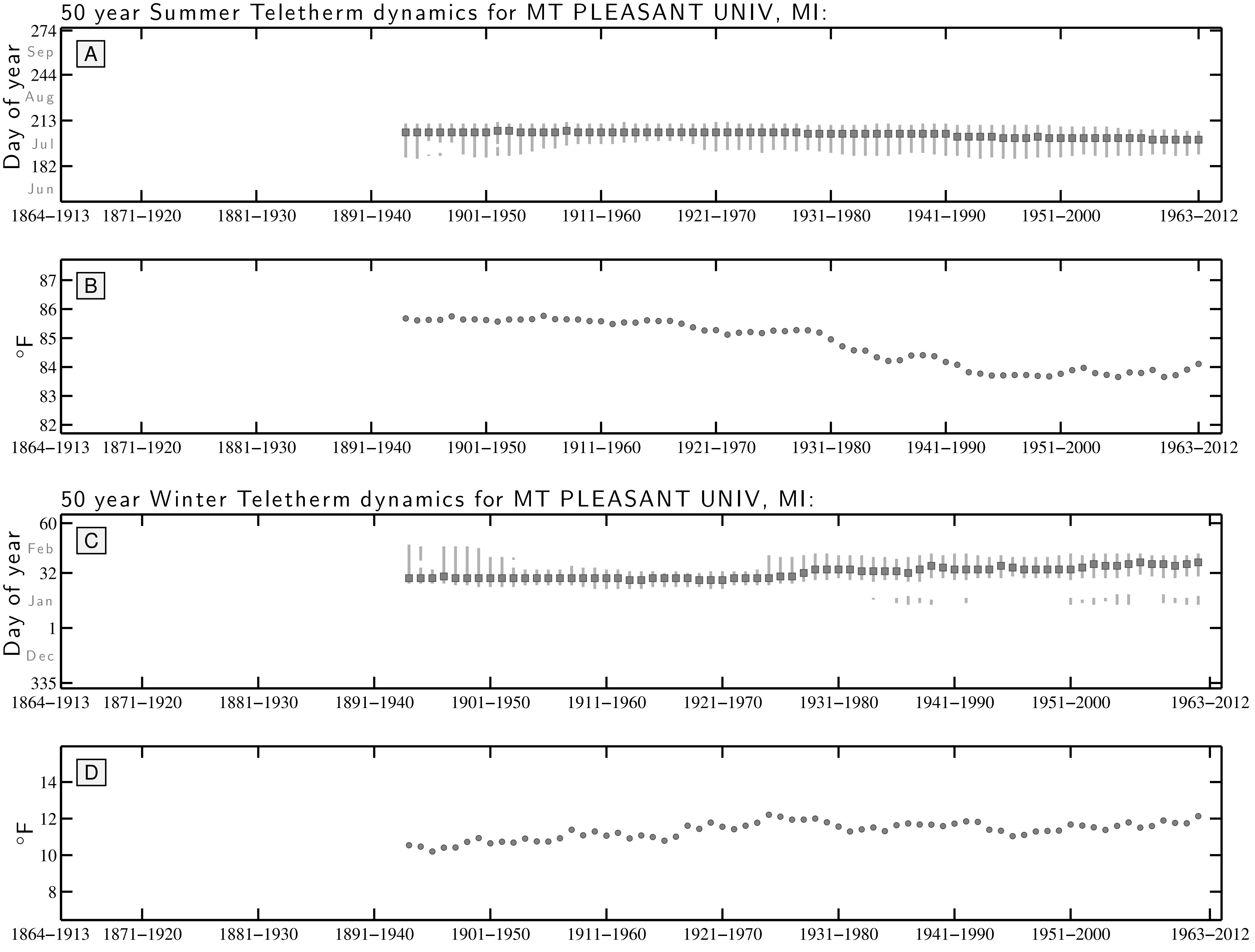
50 year Summer Teletherm dynamics for MT PLEASANT UNIV, MI:
A
B
Day of year
°F
274
Sep
244
Aug
213
Jul
182
Jun
87
86
85
84
83
82
1864–1913
1871–1920
1881–1930
1891–1940
1901–1950
1911–1960
1921–1970
1931–1980
1941–1990
1951–2000
1963–2012
50 year Winter Teletherm dynamics for MT PLEASANT UNIV, MI:
C
D
60
Feb
32
Jan
1
Dec
335
14
12
10
8

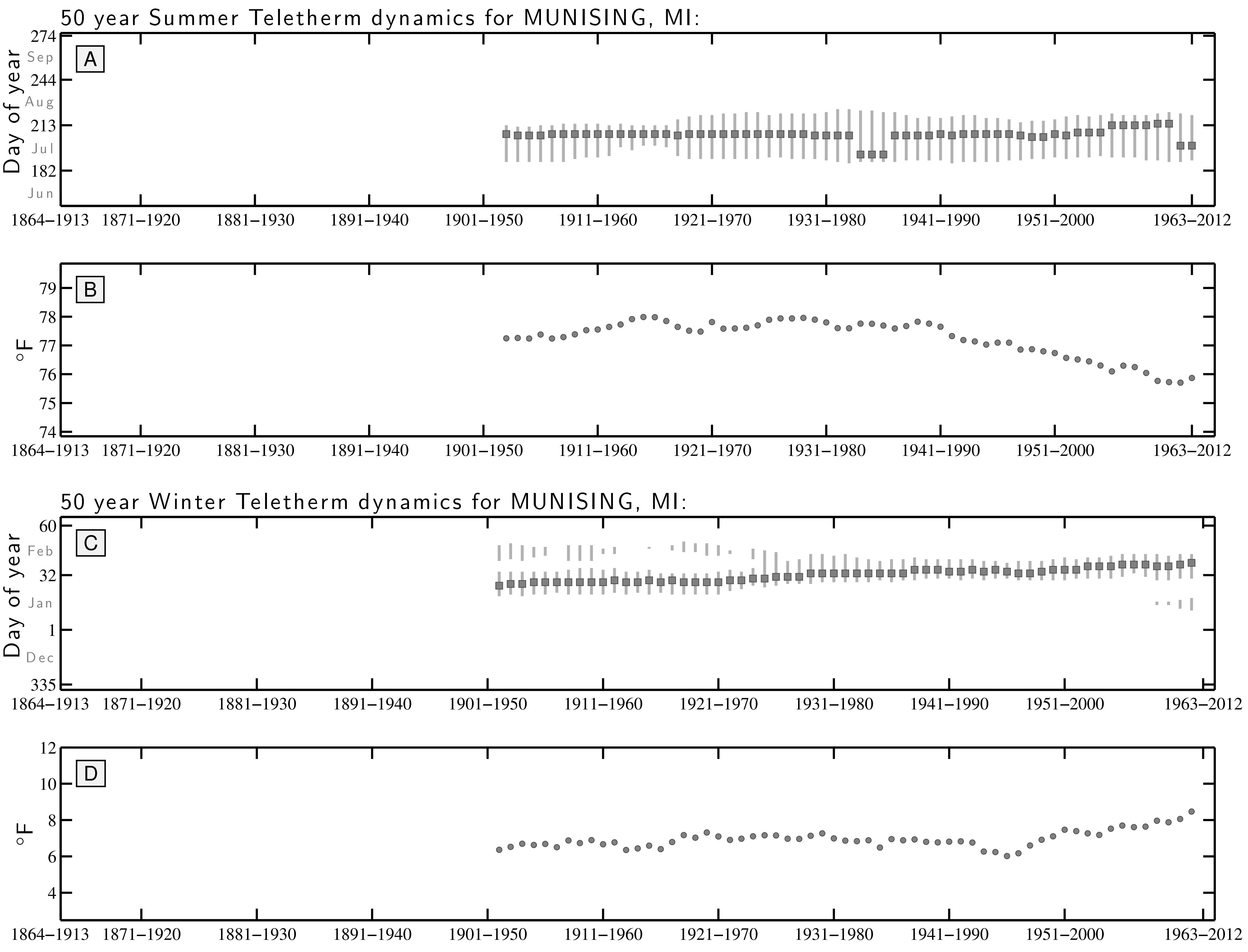
50 year Summer Teletherm dynamics for MUNISING, MI:
A
Day of year
274
Sep
244
Aug
213
Jul
182
Jun
1864–1913
1871–1920
1881–1930
1891–1940
1901–1950
1911–1960
1921–1970
1931–1980
1941–1990
1951–2000
1963–2012
B
°F
79
78
77
76
75
74
50 year Winter Teletherm dynamics for MUNISING, MI:
C
Day of year
60
Feb
32
Jan
1
Dec
335
D
°F
12
10
8
6
4

50 year Summer Teletherm dynamics for NEWBERRY 3S, MI:

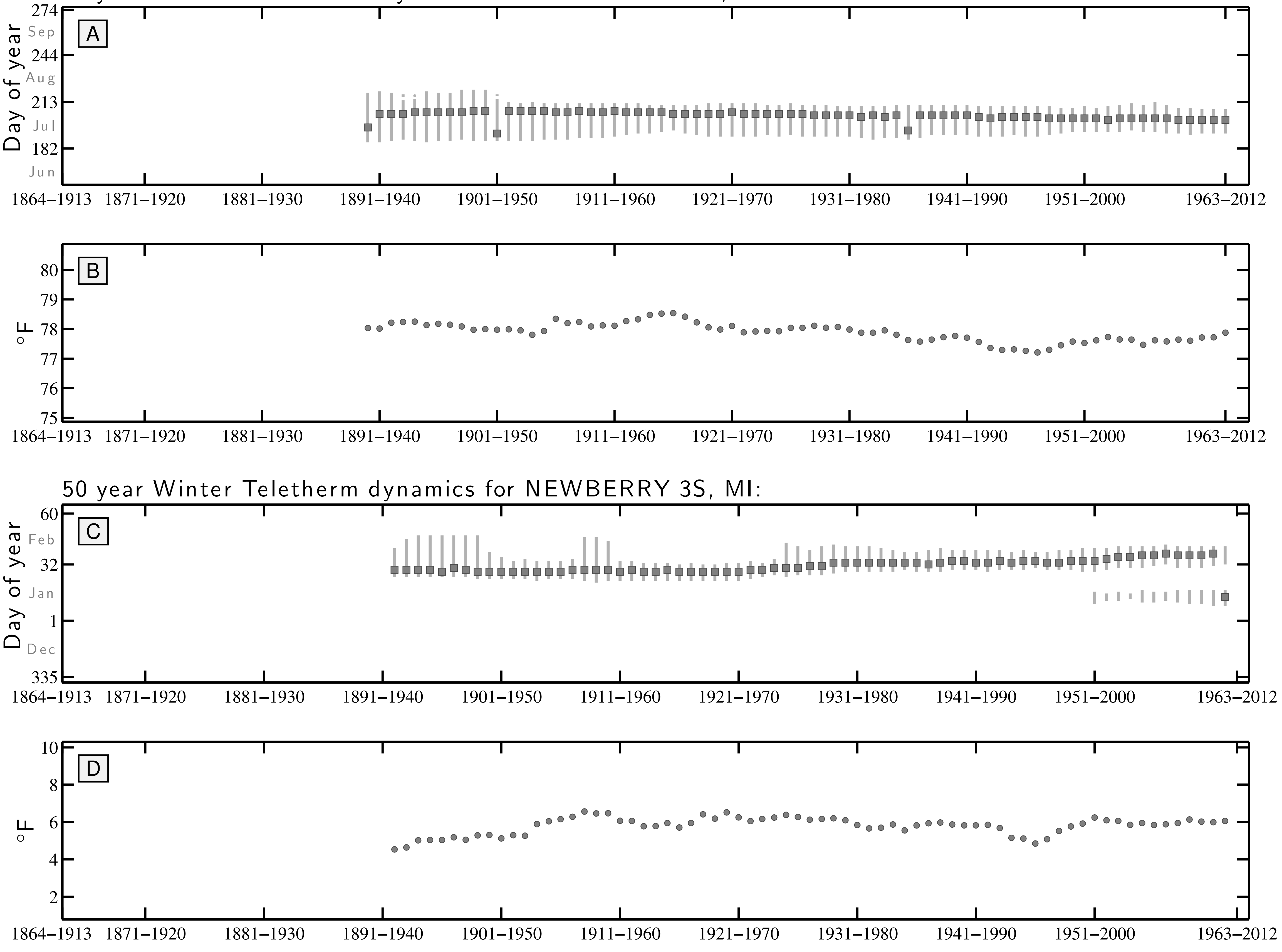

50 year Winter Teletherm dynamics for NEWBERRY 3S, MI:

50 year Summer Teletherm dynamics for OWOSSO WWTP, MI:

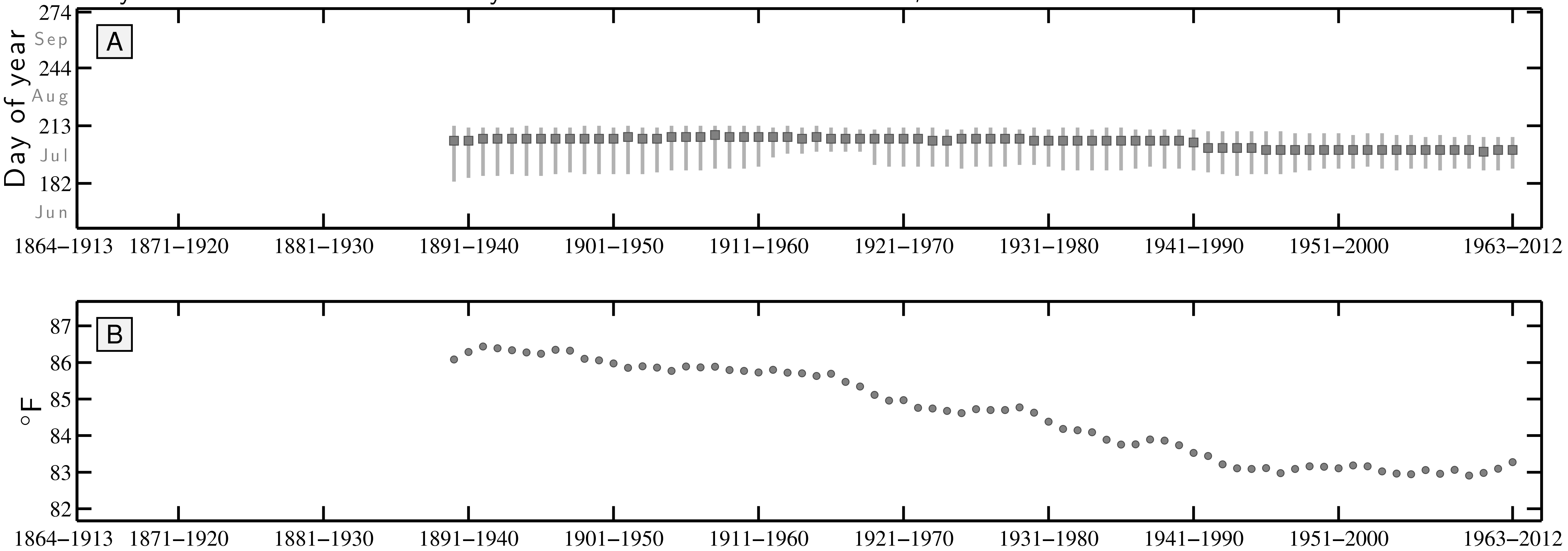

50 year Winter Teletherm dynamics for OWOSSO WWTP, MI:

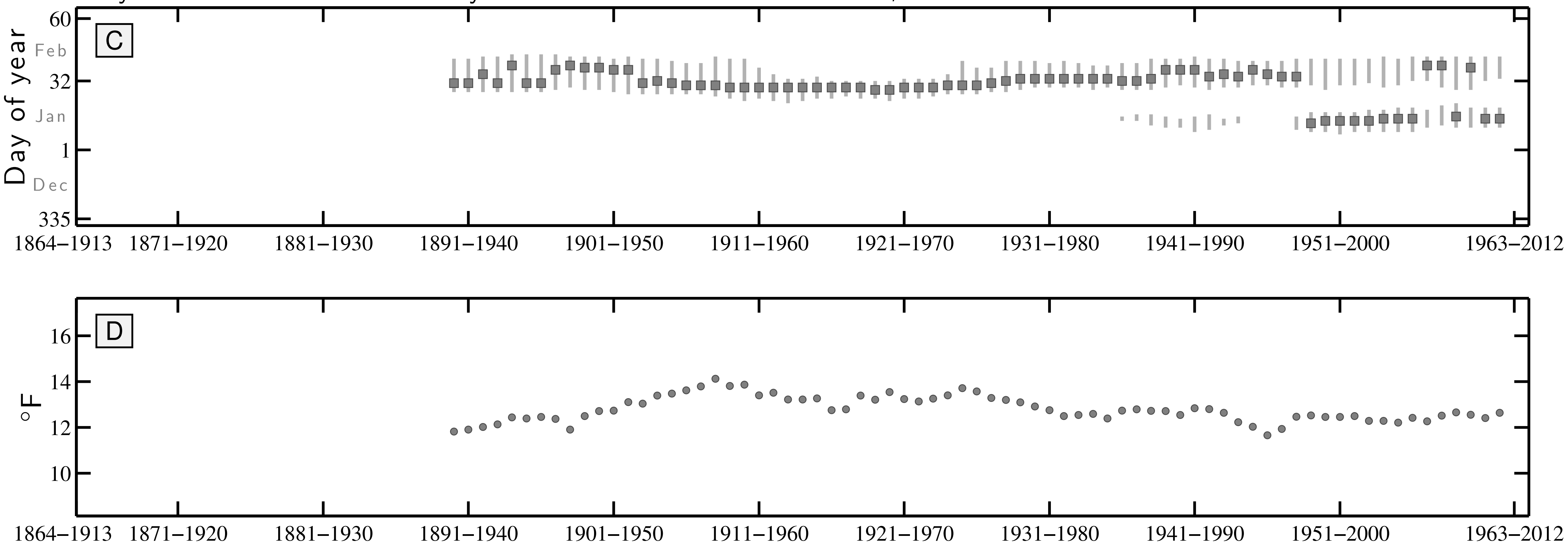

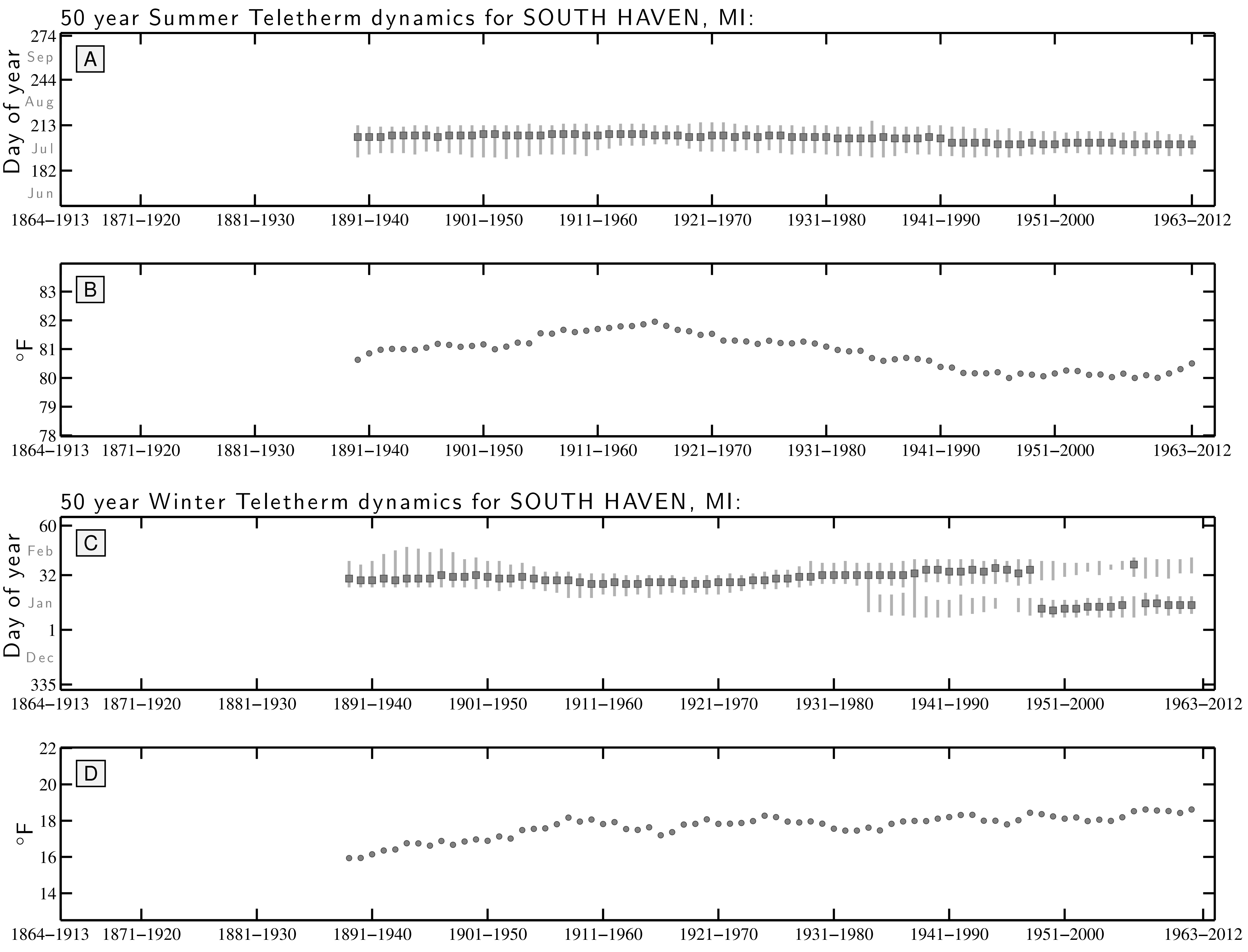

50 year Summer Teletherm dynamics for SOUTH HAVEN, MI:
A
B
Day of year
°F
Jun
Jul
Aug
Sep
182
213
244
274
78
79
80
81
82
83
1864–1913
1871–1920
1881–1930
1891–1940
1901–1950
1911–1960
1921–1970
1931–1980
1941–1990
1951–2000
1963–2012
50 year Winter Teletherm dynamics for SOUTH HAVEN, MI:
C
D
Day of year
°F
Dec
Jan
Feb
335
1
32
60
14
16
18
20
22

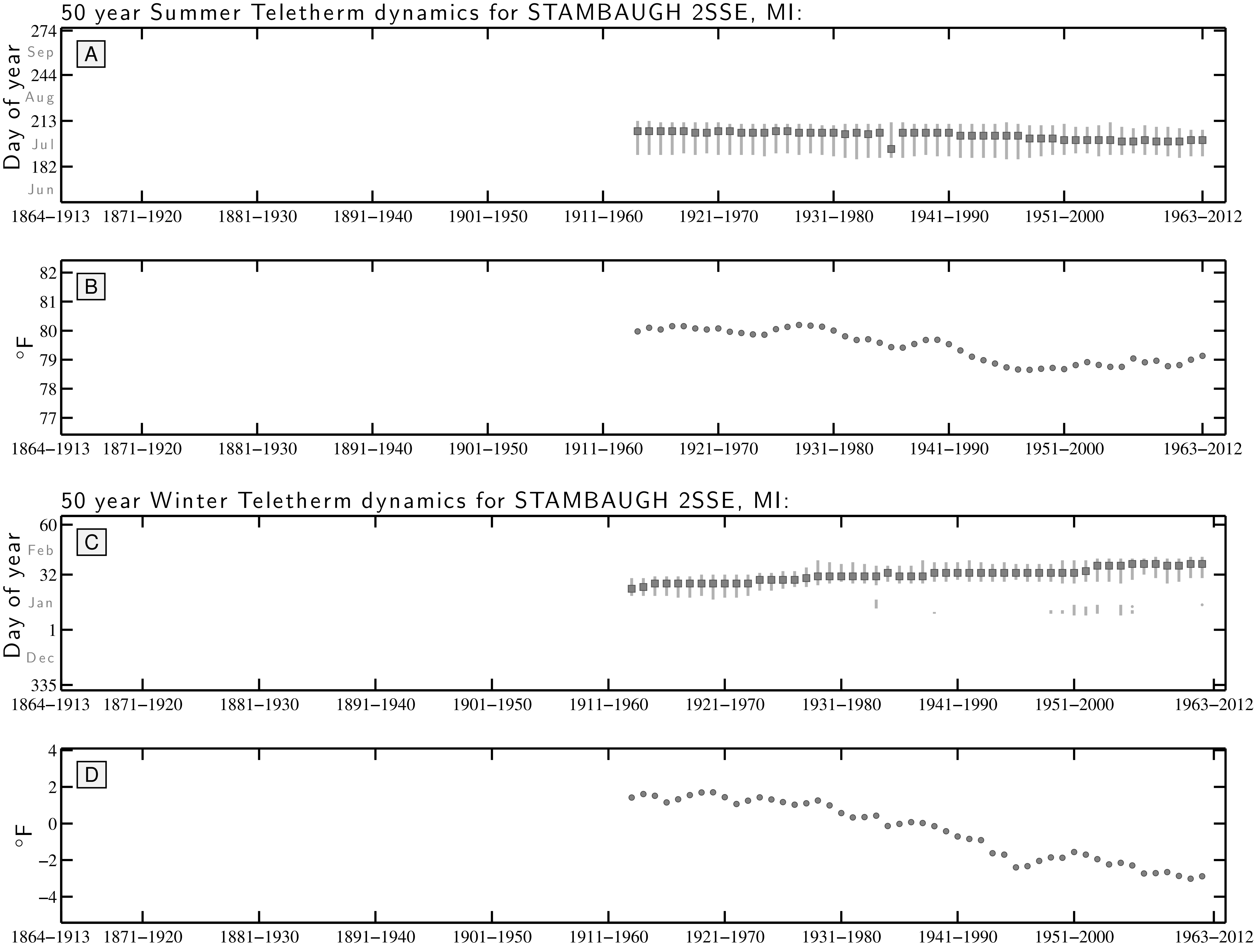

50 year Summer Teletherm dynamics for STAMBAUGH 2SSE, MI:
A
Day of year
274
Sep
244
Aug
213
Jul
182
Jun
B
°F
82
81
80
79
78
77
50 year Winter Teletherm dynamics for STAMBAUGH 2SSE, MI:
C
Day of year
60
Feb
32
Jan
1
Dec
335
D
°F
4
2
0
−2
−4
1864−1913
1871−1920
1881−1930
1891−1940
1901−1950
1911−1960
1921−1970
1931−1980
1941−1990
1951−2000
1963−2012

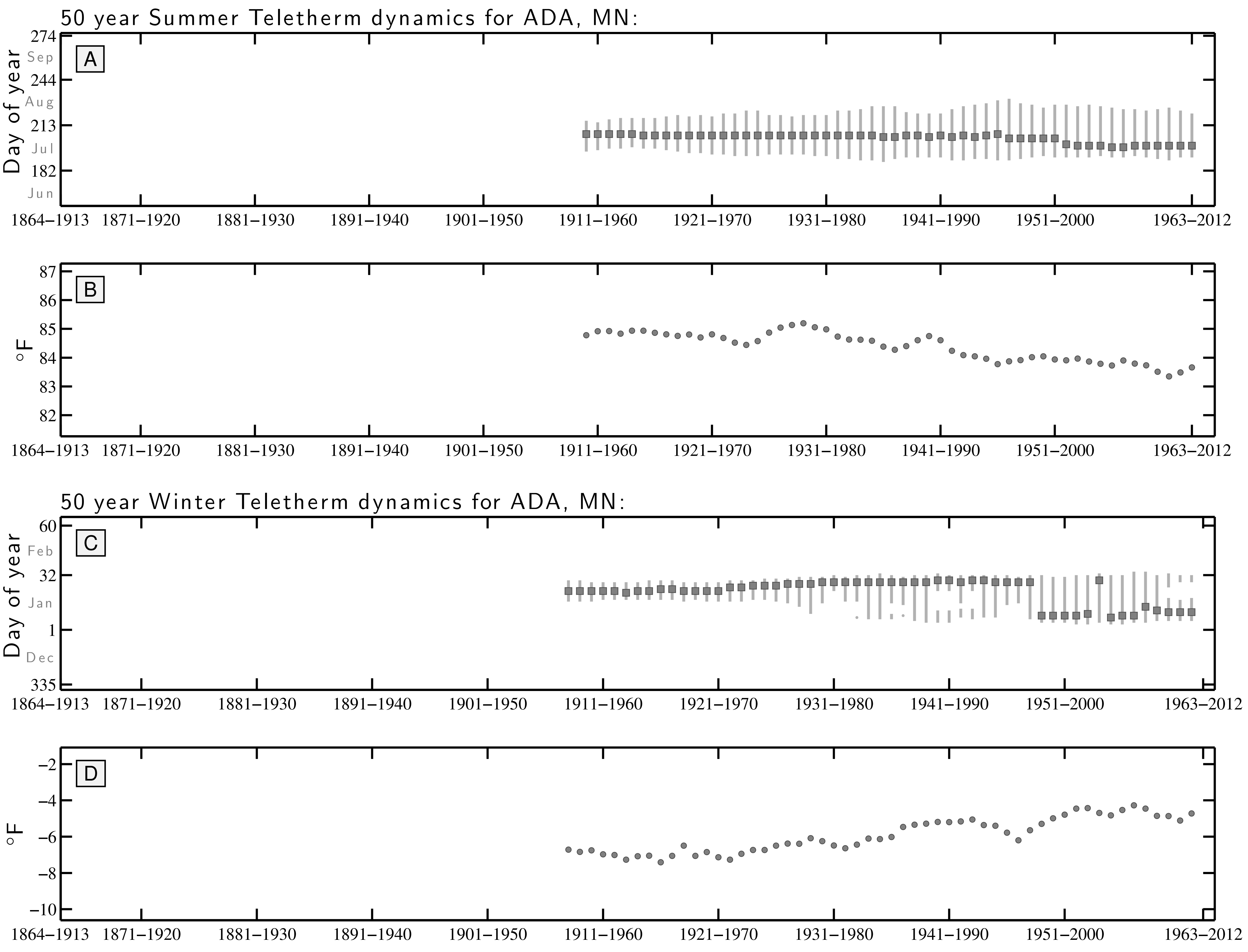
50 year Summer Teletherm dynamics for ADA, MN:
A
B
Day of year
°F
274
244
213
182
Sep
Aug
Jul
Jun
87
86
85
84
83
82
50 year Winter Teletherm dynamics for ADA, MN:
C
D
60
32
1
335
Feb
Jan
Dec
−2
−4
−6
−8
−10
1864–1913
1871–1920
1881–1930
1891–1940
1901–1950
1911–1960
1921–1970
1931–1980
1941–1990
1951–2000
1963–2012

50 year Summer Teletherm dynamics for ALBERT LEA 3 SE, MN:

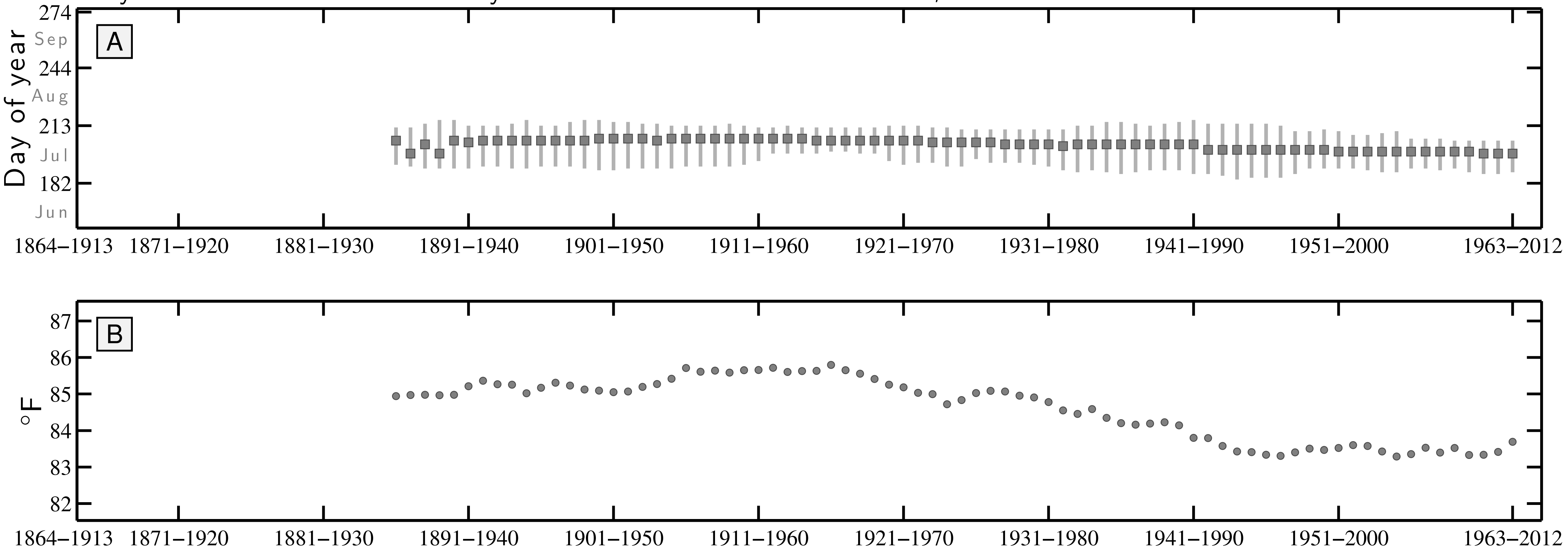

50 year Winter Teletherm dynamics for ALBERT LEA 3 SE, MN:

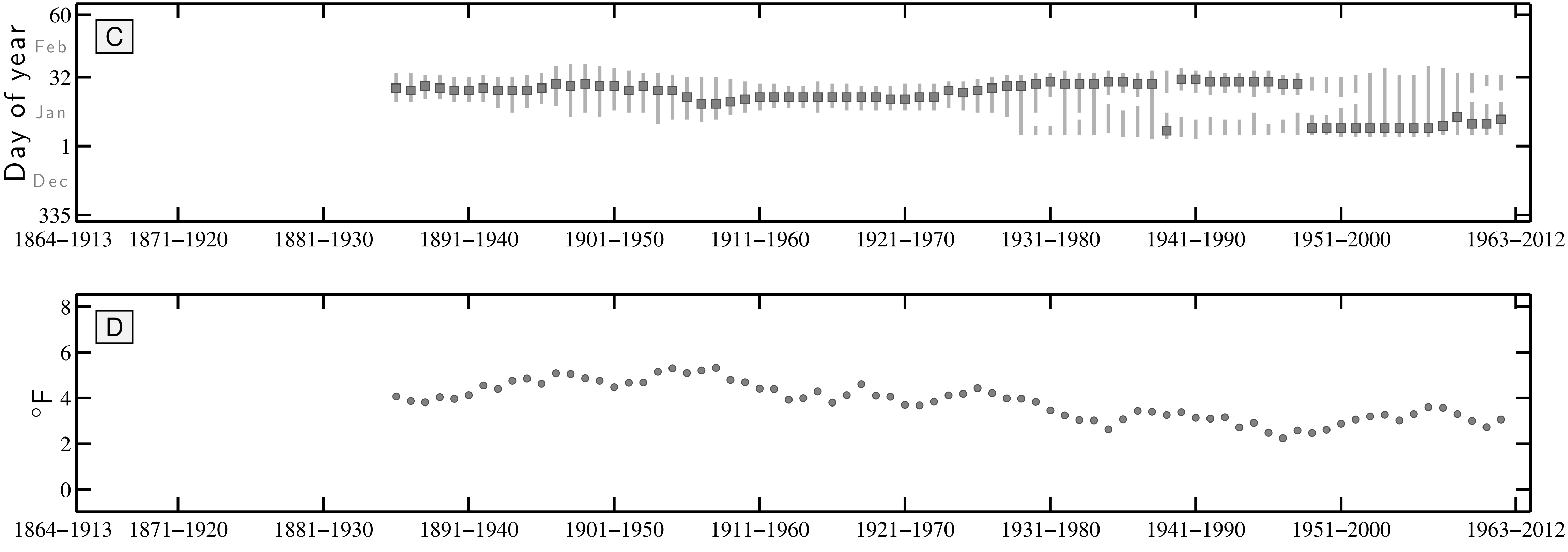

50 year Summer Teletherm dynamics for ARGYLE, MN:

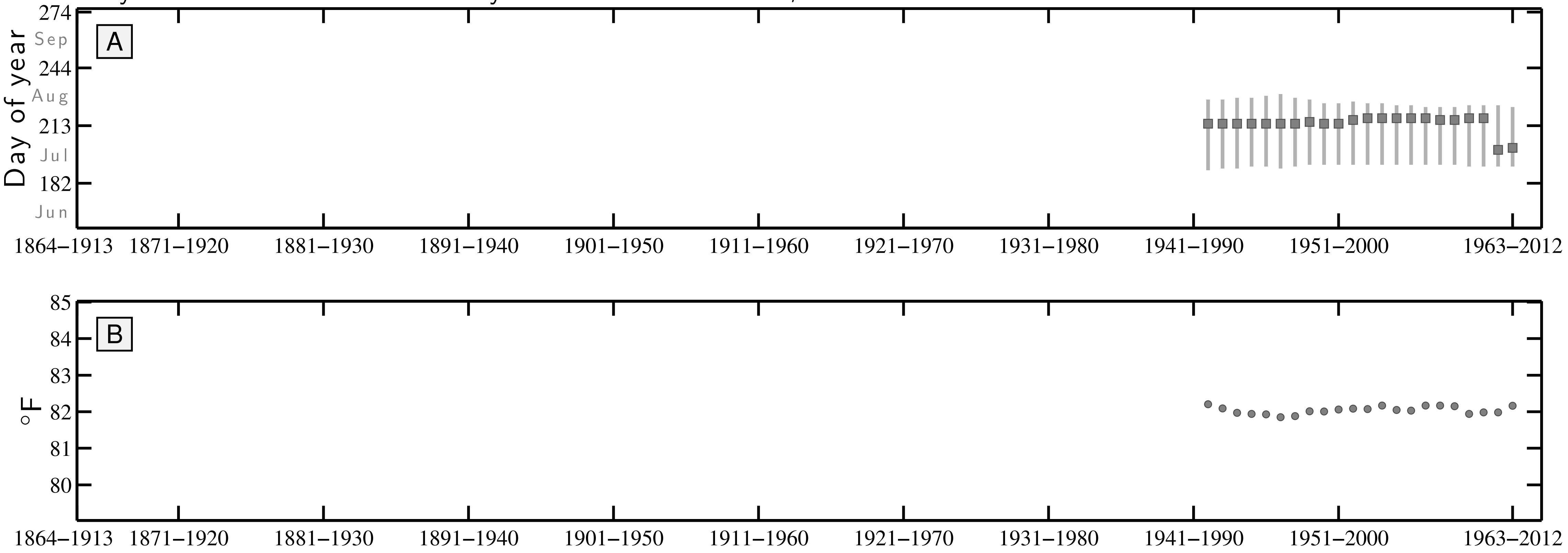

50 year Winter Teletherm dynamics for ARGYLE, MN:

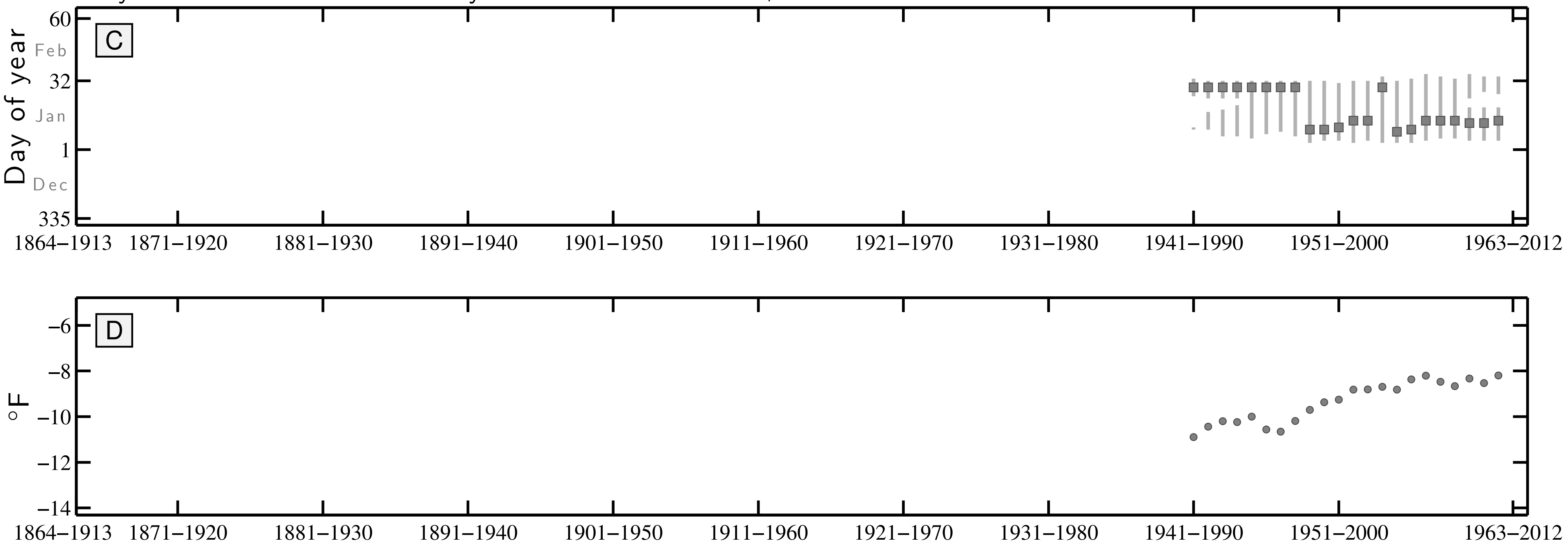

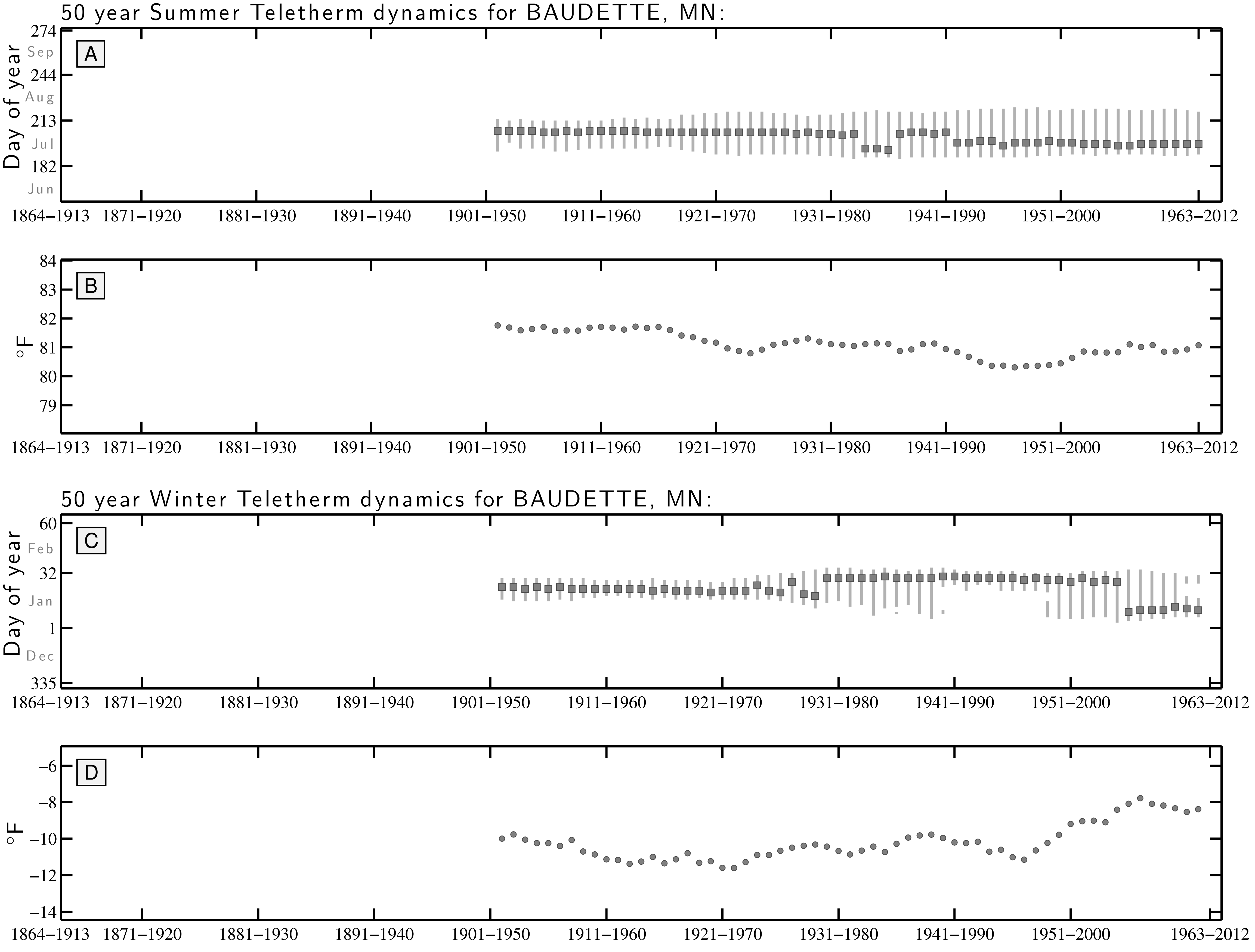
50 year Summer Teletherm dynamics for BAUDETTE, MN:
A
B
Day of year
°F
274
244
213
182
Sep
Aug
Jul
Jun
84
83
82
81
80
79
50 year Winter Teletherm dynamics for BAUDETTE, MN:
C
D
60
32
1
335
Feb
Jan
Dec
−6
−8
−10
−12
−14
1864–1913
1871–1920
1881–1930
1891–1940
1901–1950
1911–1960
1921–1970
1931–1980
1941–1990
1951–2000
1963–2012

50 year Summer Teletherm dynamics for CHASKA, MN:

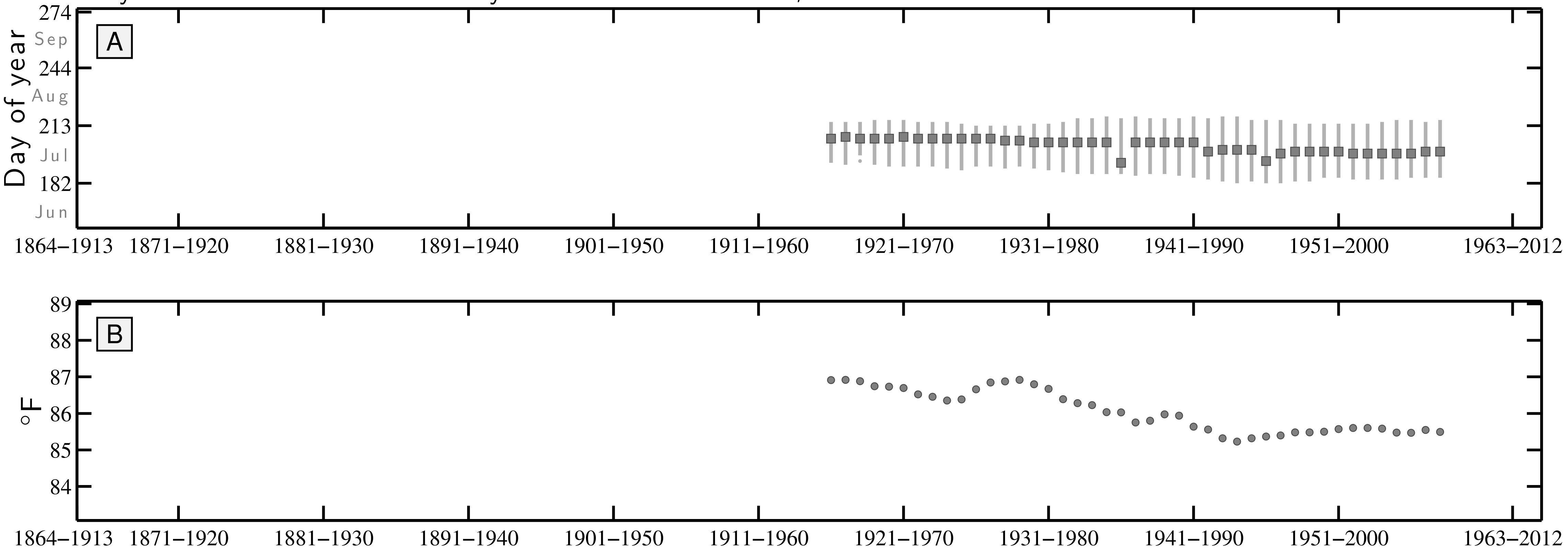

50 year Winter Teletherm dynamics for CHASKA, MN:

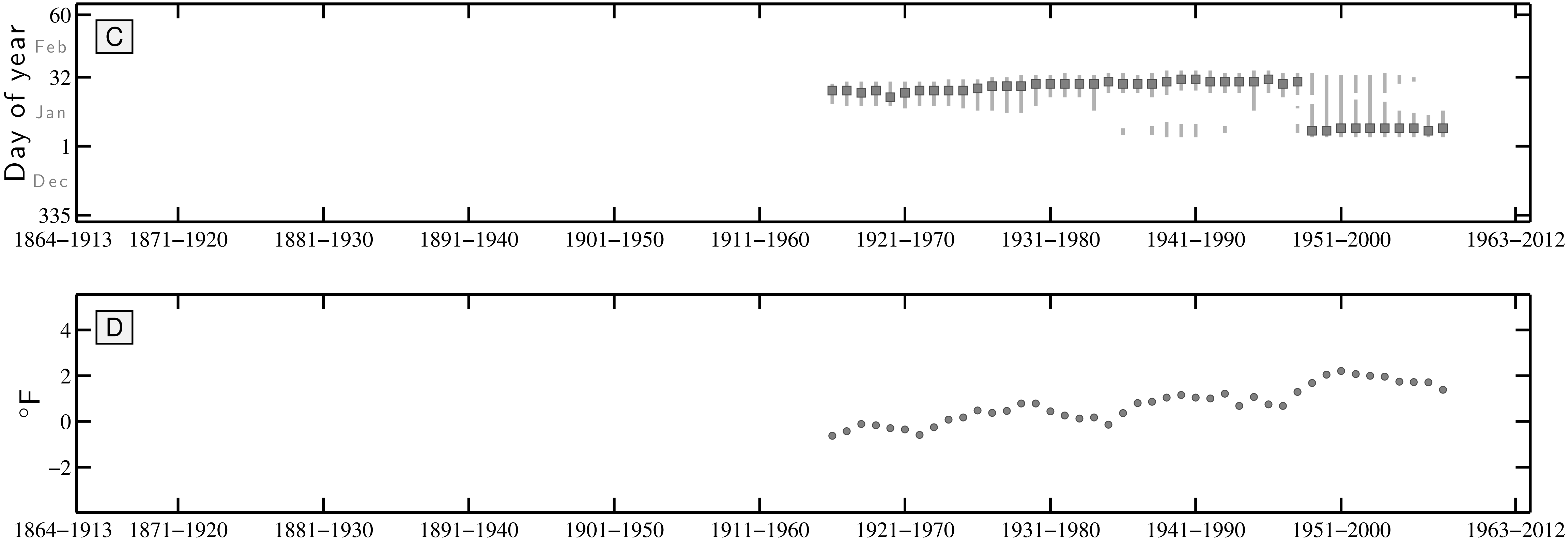

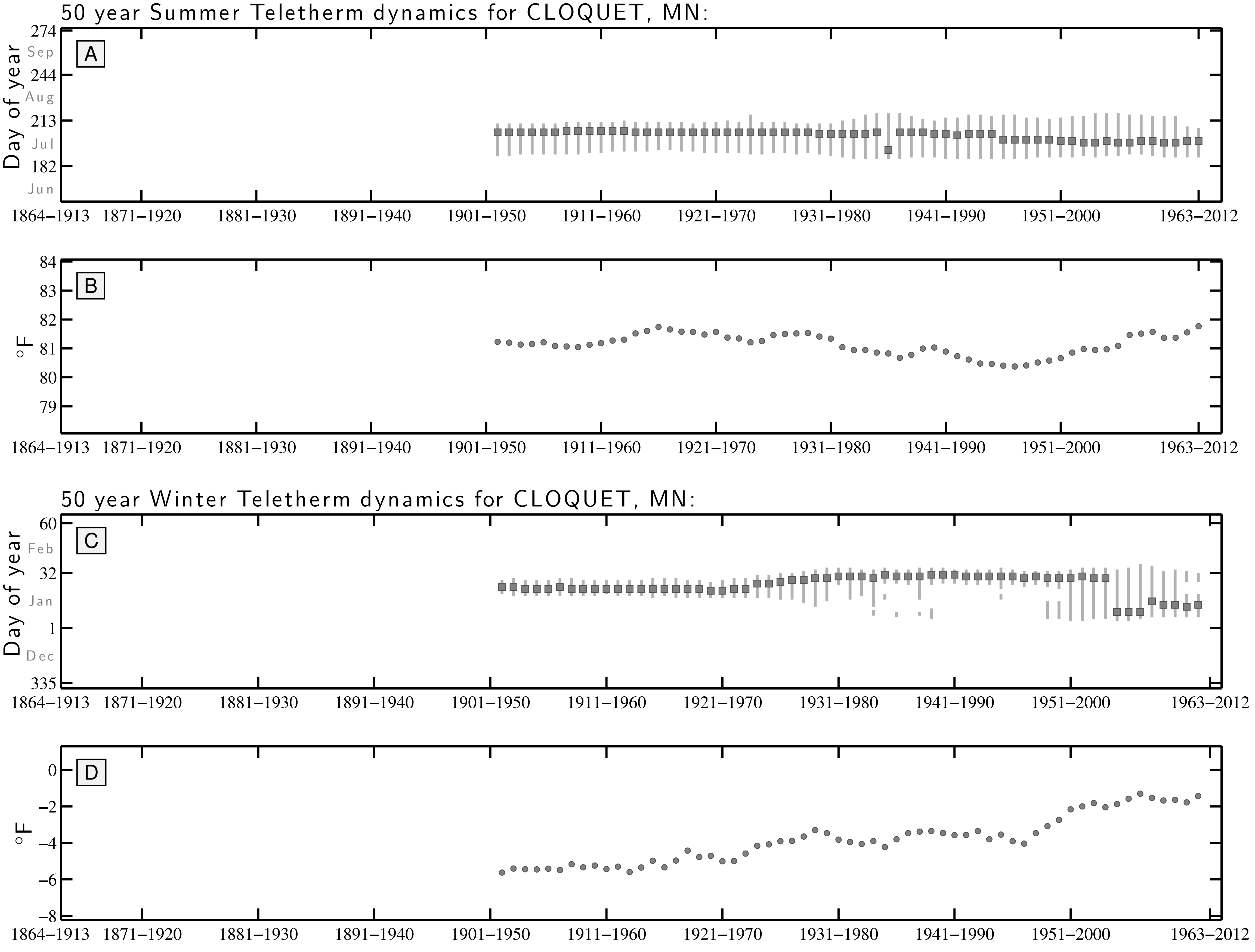

50 year Summer Teletherm dynamics for CLOQUET, MN:
A
Day of year
274
Sep
244
Aug
213
Jul
182
Jun
B
°F
84
83
82
81
80
79
50 year Winter Teletherm dynamics for CLOQUET, MN:
C
Day of year
60
Feb
32
Jan
1
Dec
335
D
°F
0
−2
−4
−6
−8
1864–1913
1871–1920
1881–1930
1891–1940
1901–1950
1911–1960
1921–1970
1931–1980
1941–1990
1951–2000
1963–2012

50 year Summer Teletherm dynamics for DETROIT LAKES 1 NNE, MN:

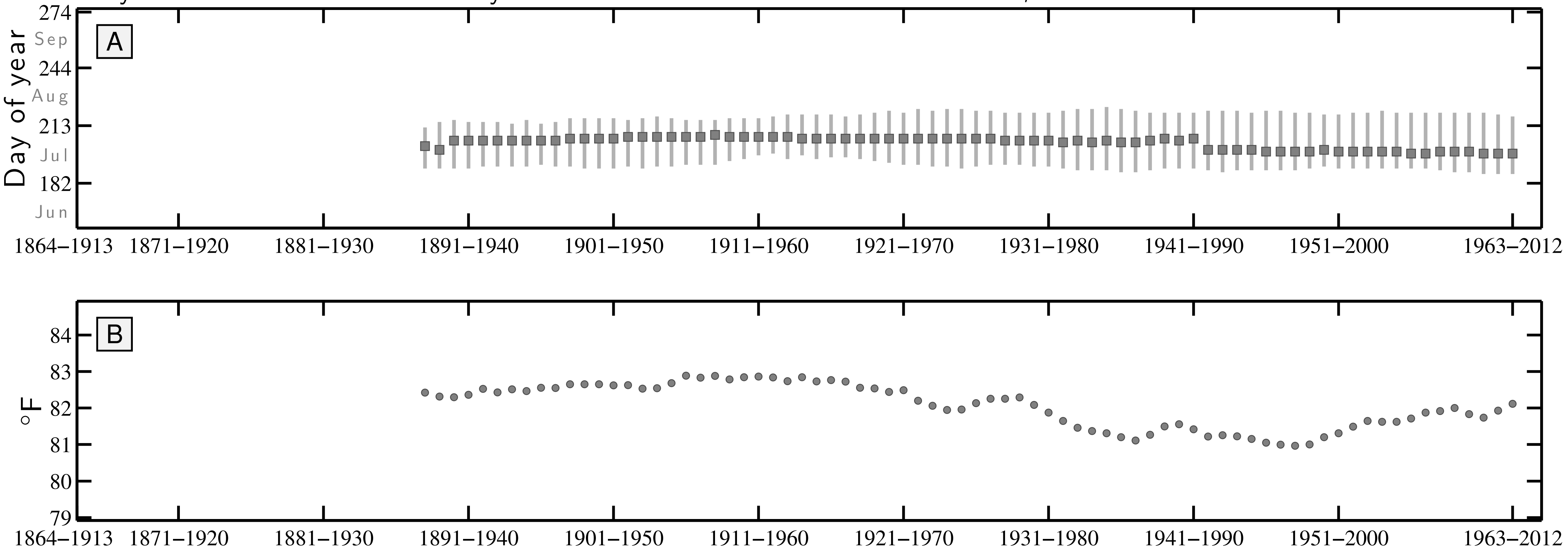

50 year Winter Teletherm dynamics for DETROIT LAKES 1 NNE, MN:

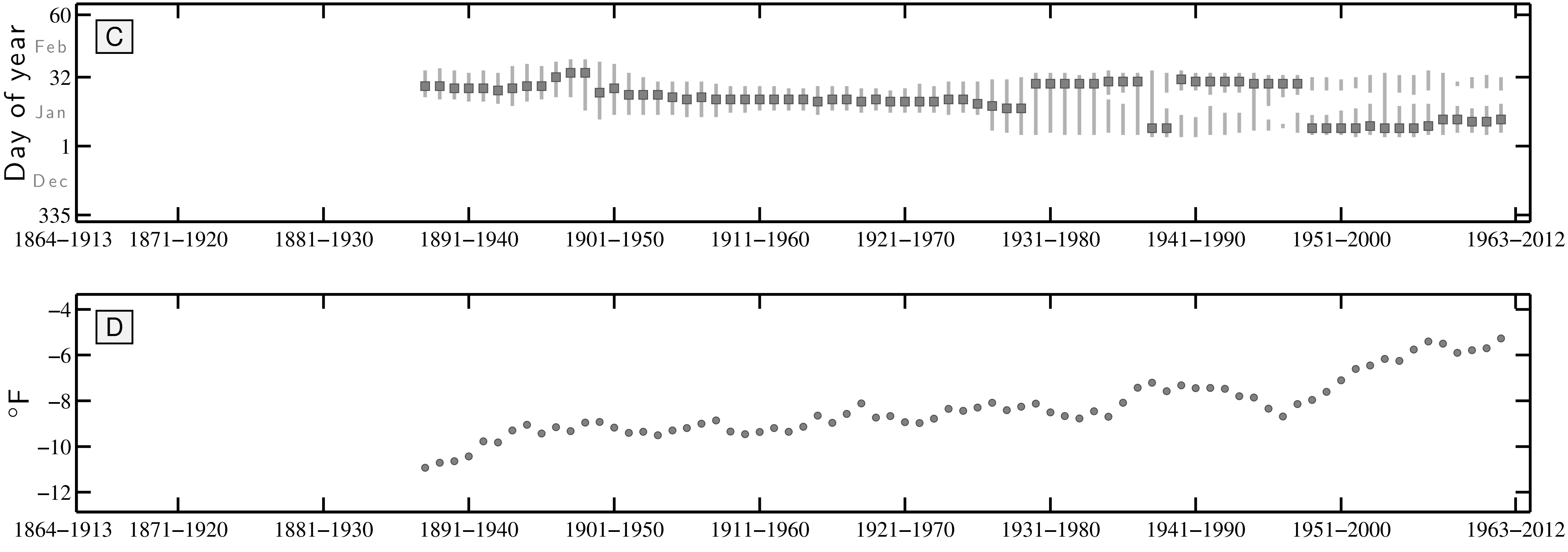

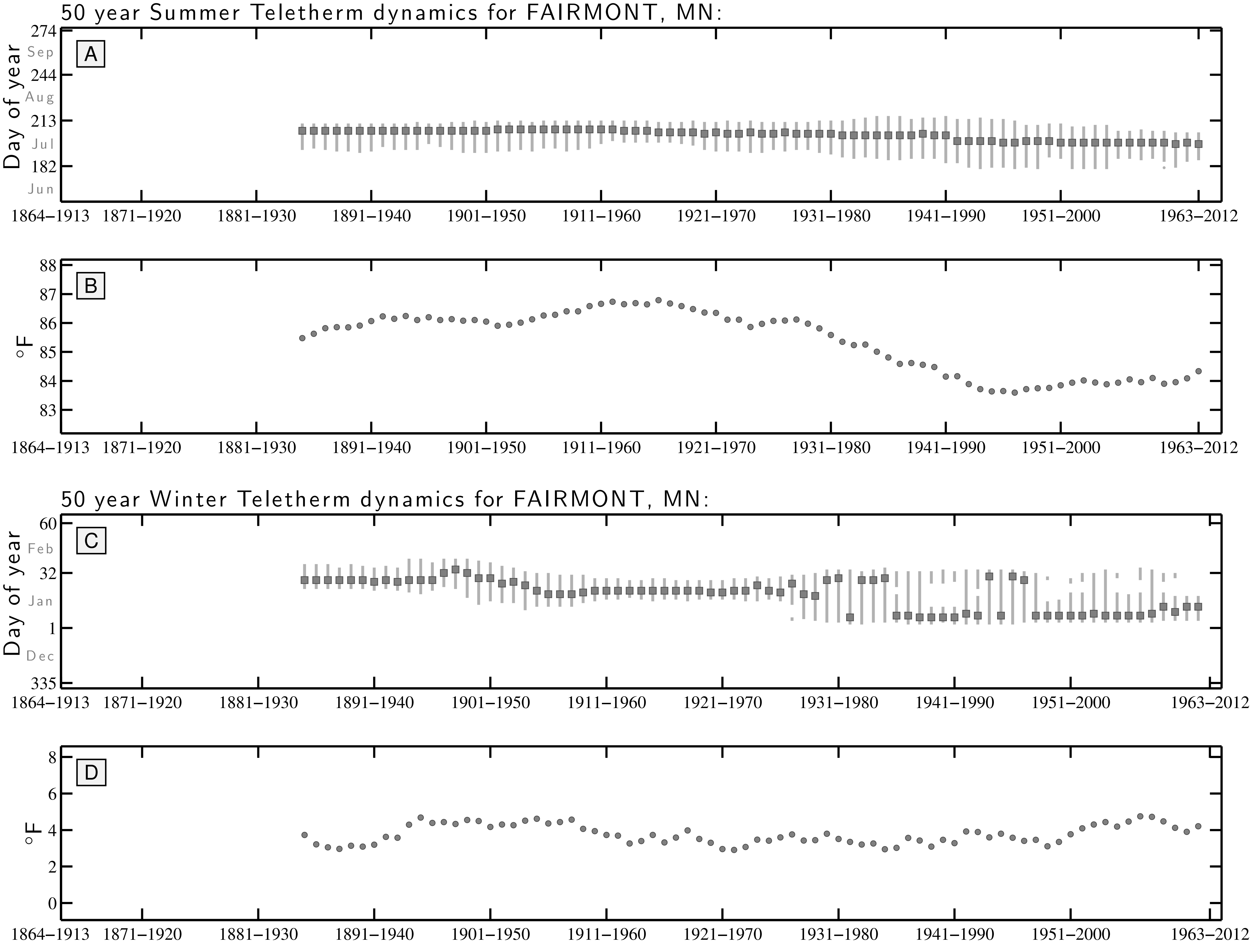

50 year Summer Teletherm dynamics for FAIRMONT, MN:
A
B
Day of year
°F
Sep
Aug
Jul
Jun
274
244
213
182
88
87
86
85
84
83
50 year Winter Teletherm dynamics for FAIRMONT, MN:
C
D
Feb
Jan
Dec
60
32
1
335
8
6
4
2
0
1864–1913
1871–1920
1881–1930
1891–1940
1901–1950
1911–1960
1921–1970
1931–1980
1941–1990
1951–2000
1963–2012

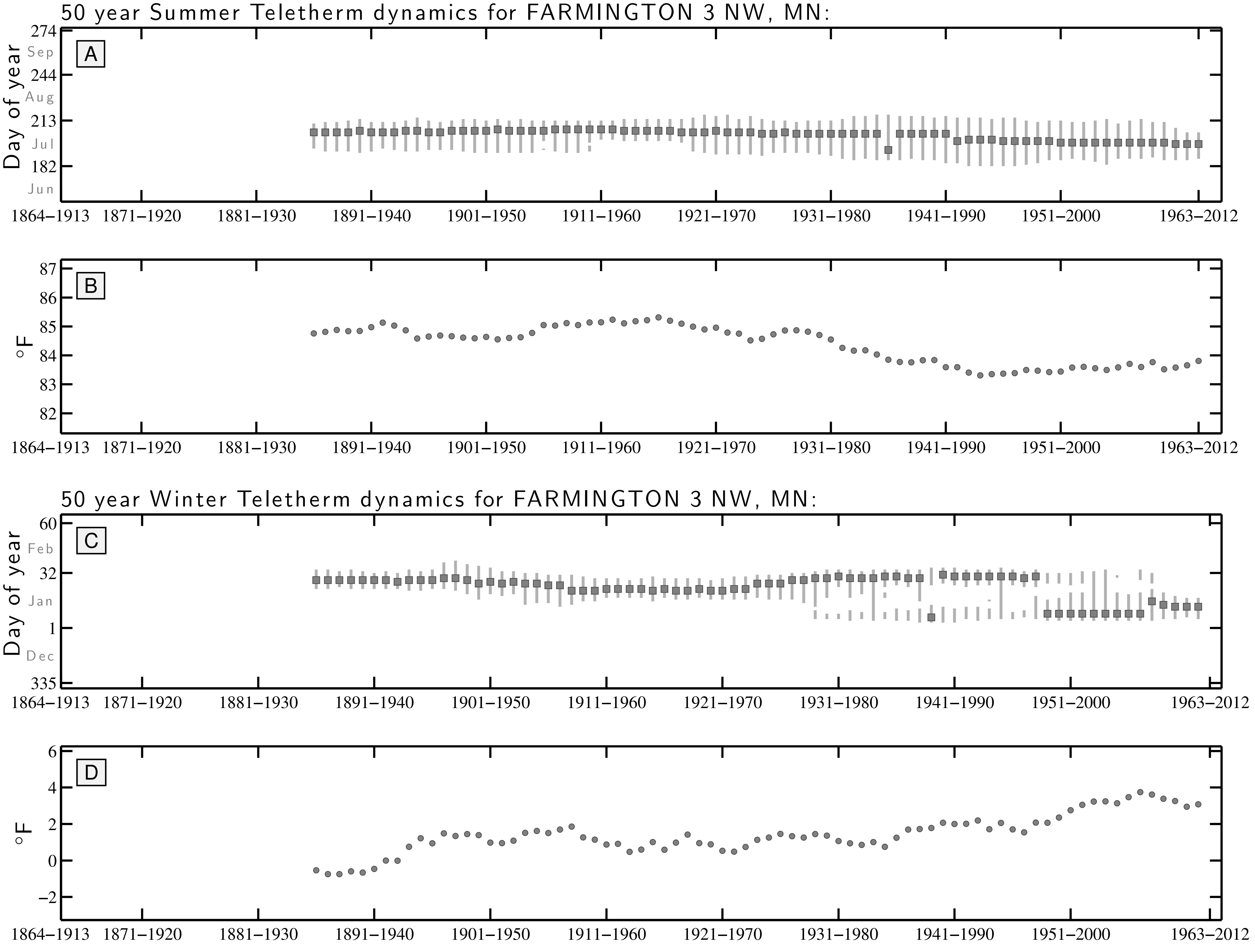

50 year Summer Teletherm dynamics for FARMINGTON 3 NW, MN:
A
B
Day of year
°F
Jun
Jul
Aug
Sep
182
213
244
274
82
83
84
85
86
87
50 year Winter Teletherm dynamics for FARMINGTON 3 NW, MN:
C
D
Dec
Jan
Feb
335
1
32
60
−2
0
2
4
6
1864–1913
1871–1920
1881–1930
1891–1940
1901–1950
1911–1960
1921–1970
1931–1980
1941–1990
1951–2000
1963–2012

50 year Summer Teletherm dynamics for FOSSTON 1 E, MN:

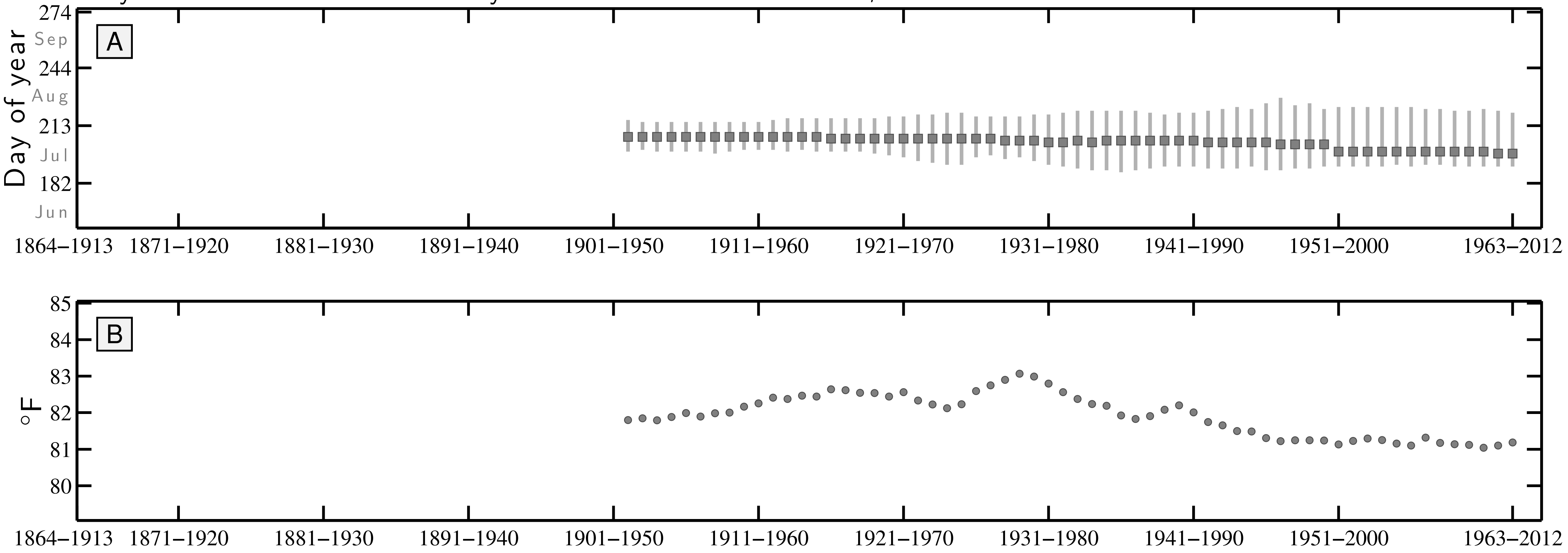

50 year Winter Teletherm dynamics for FOSSTON 1 E, MN:

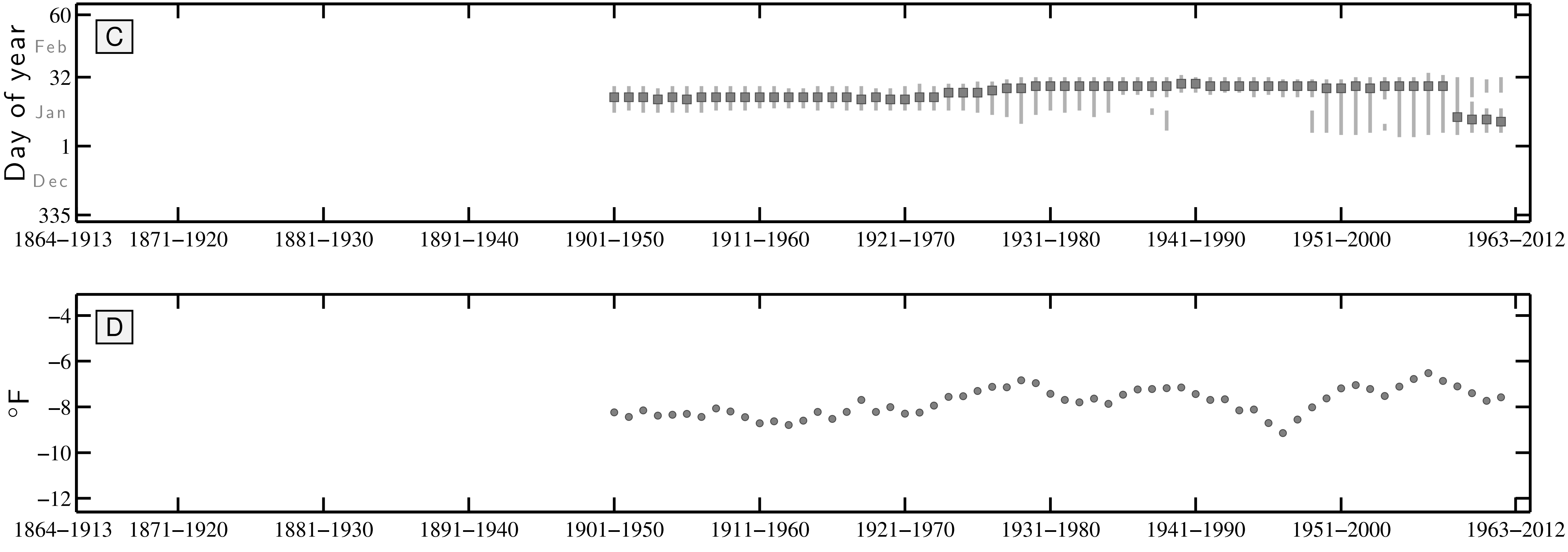

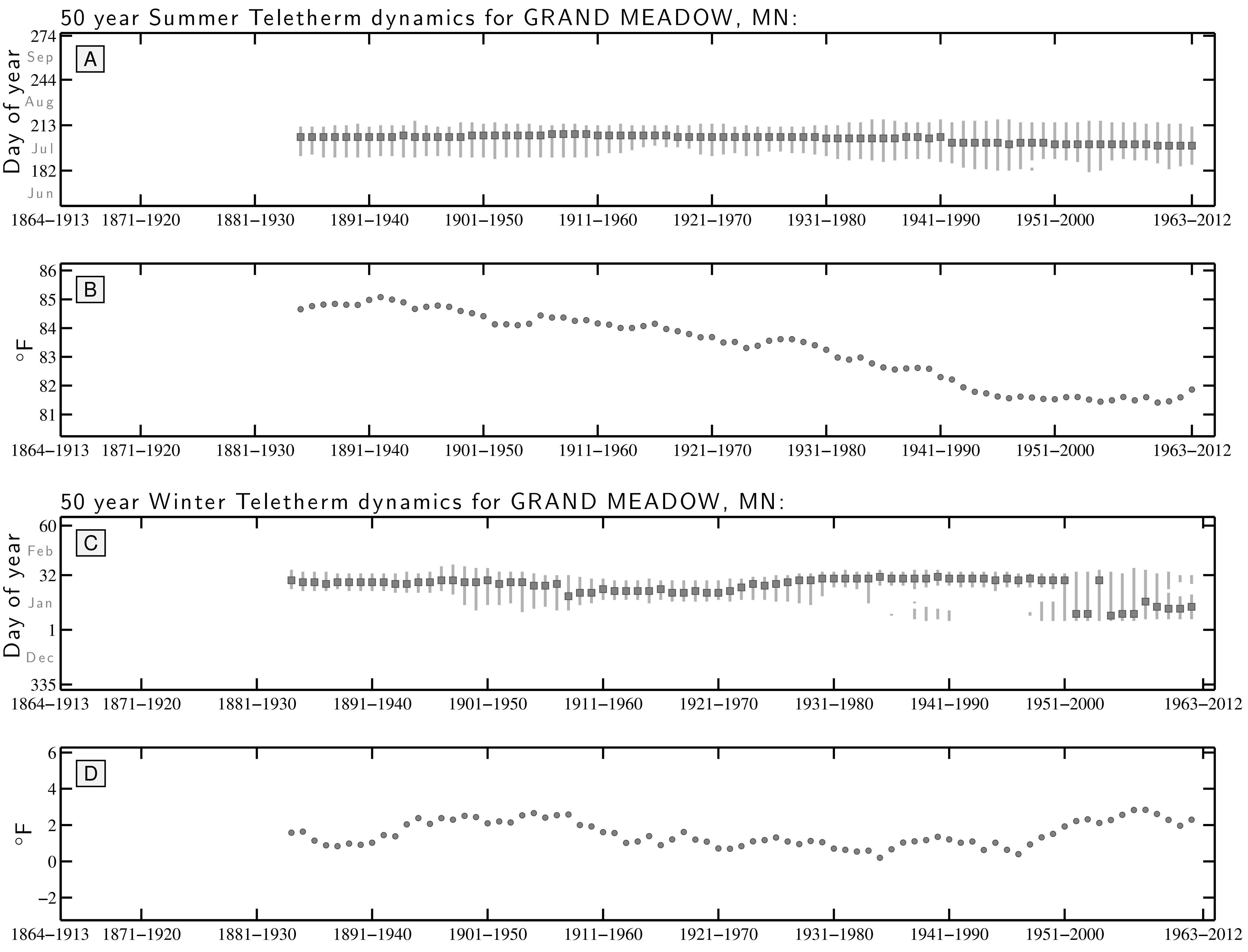

50 year Summer Teletherm dynamics for GRAND MEADOW, MN:
A
B
Day of year
°F
Sep
Aug
Jul
Jun
274
244
213
182
86
85
84
83
82
81
1864–1913
1871–1920
1881–1930
1891–1940
1901–1950
1911–1960
1921–1970
1931–1980
1941–1990
1951–2000
1963–2012
50 year Winter Teletherm dynamics for GRAND MEADOW, MN:
C
D
Feb
Jan
Dec
60
32
1
335
6
4
2
0
−2

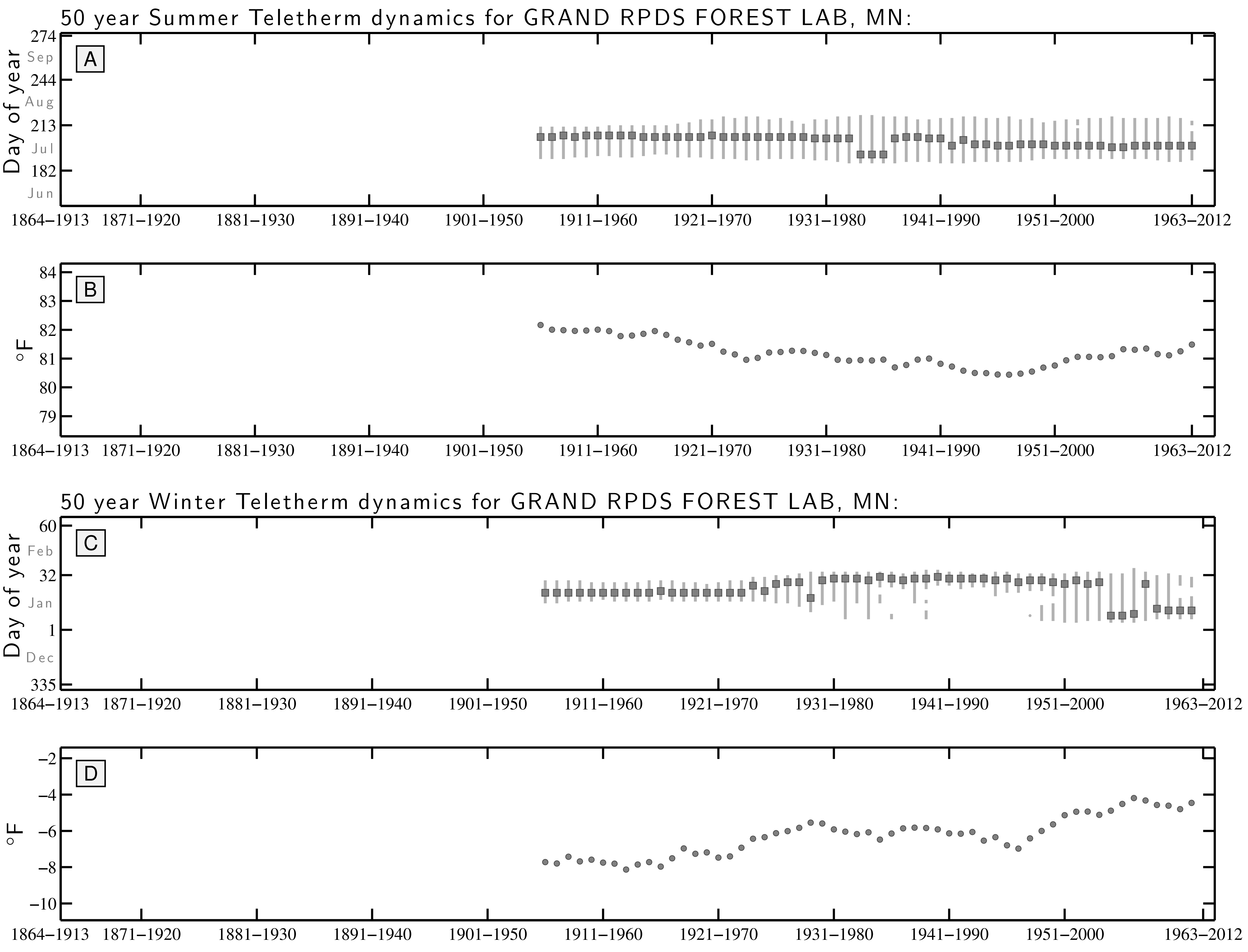
50 year Summer Teletherm dynamics for GRAND RPDS FOREST LAB, MN:
A
B
Day of year
°F
274
Sep
244
Aug
213
Jul
182
Jun
84
83
82
81
80
79
1864–1913
1871–1920
1881–1930
1891–1940
1901–1950
1911–1960
1921–1970
1931–1980
1941–1990
1951–2000
1963–2012
50 year Winter Teletherm dynamics for GRAND RPDS FOREST LAB, MN:
C
D
60
Feb
32
Jan
1
Dec
335
−2
−4
−6
−8
−10

50 year Summer Teletherm dynamics for ITASCA UNIV OF MINN, MN:

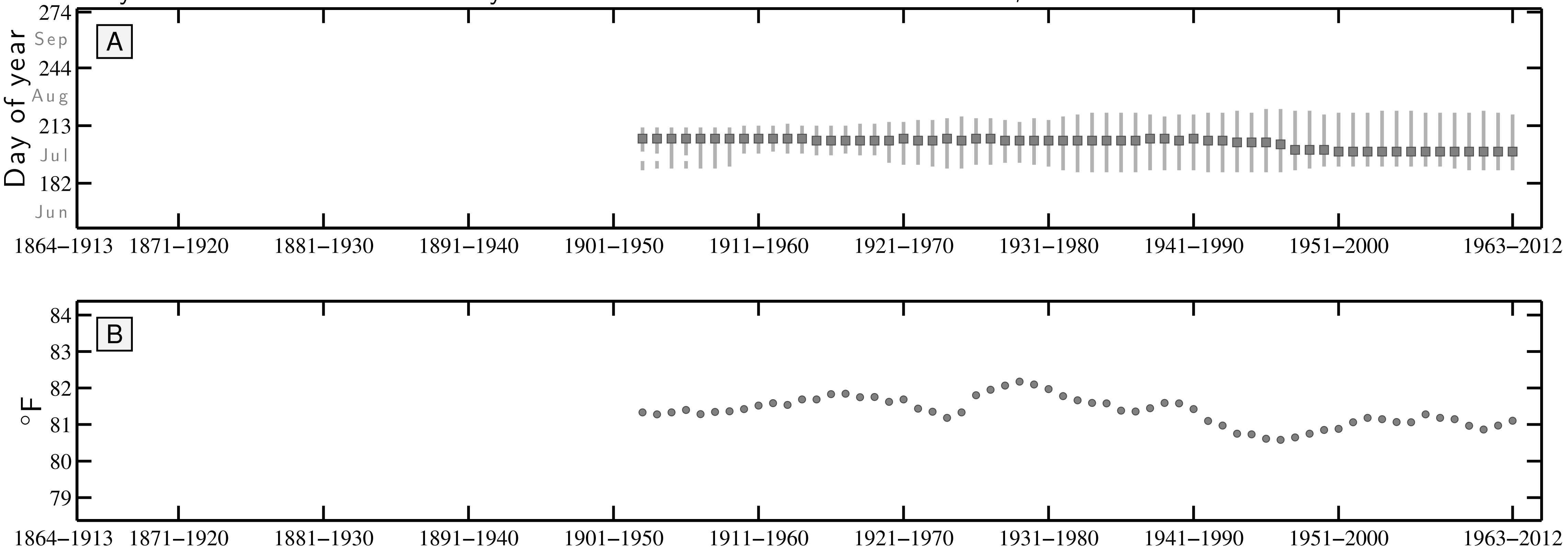

50 year Winter Teletherm dynamics for ITASCA UNIV OF MINN, MN:

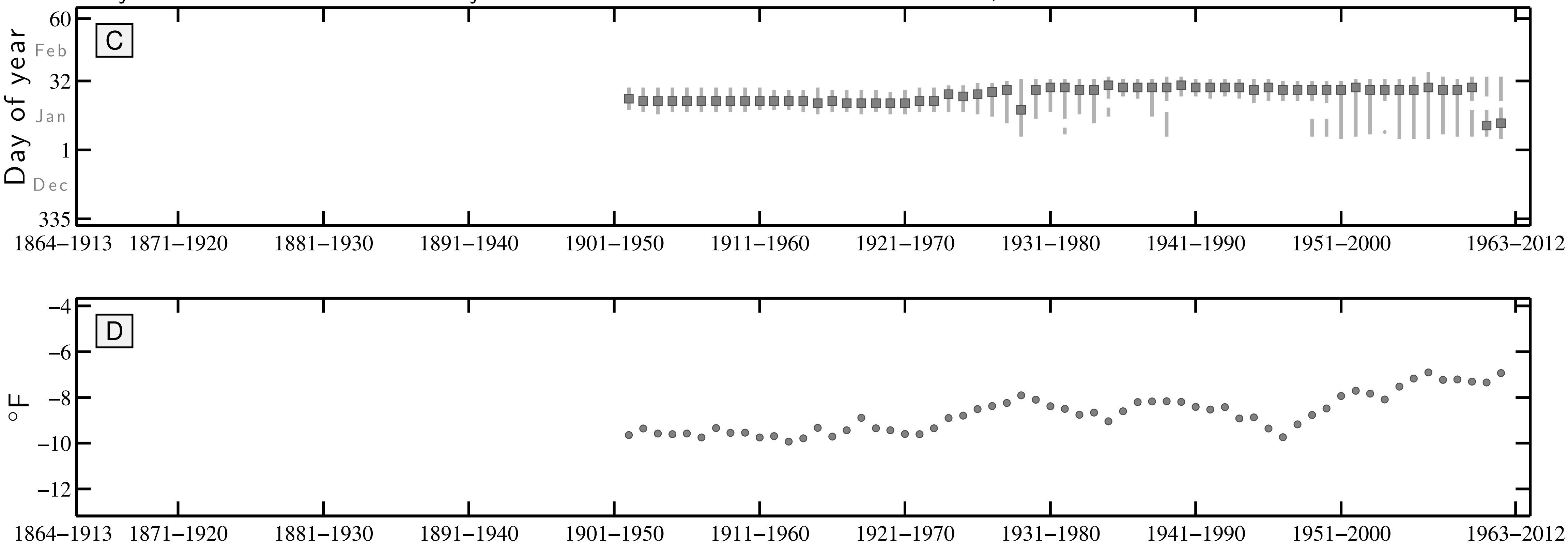

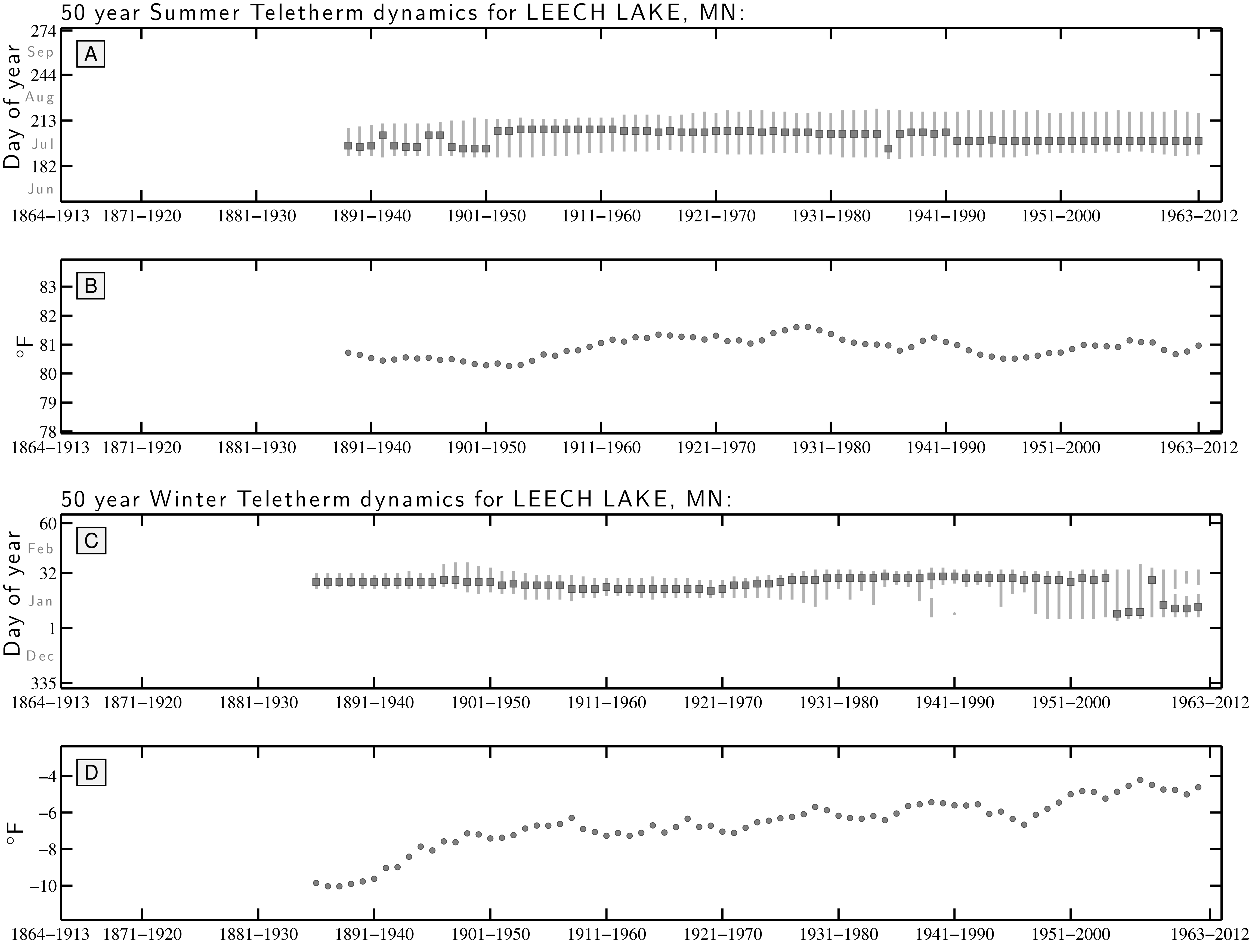

50 year Summer Teletherm dynamics for LEECH LAKE, MN:
A
Day of year
274
Sep
244
Aug
213
Jul
182
Jun
1864–1913
1871–1920
1881–1930
1891–1940
1901–1950
1911–1960
1921–1970
1931–1980
1941–1990
1951–2000
1963–2012
B
°F
83
82
81
80
79
78
50 year Winter Teletherm dynamics for LEECH LAKE, MN:
C
Day of year
60
Feb
32
Jan
1
Dec
335
D
°F
-4
-6
-8
-10

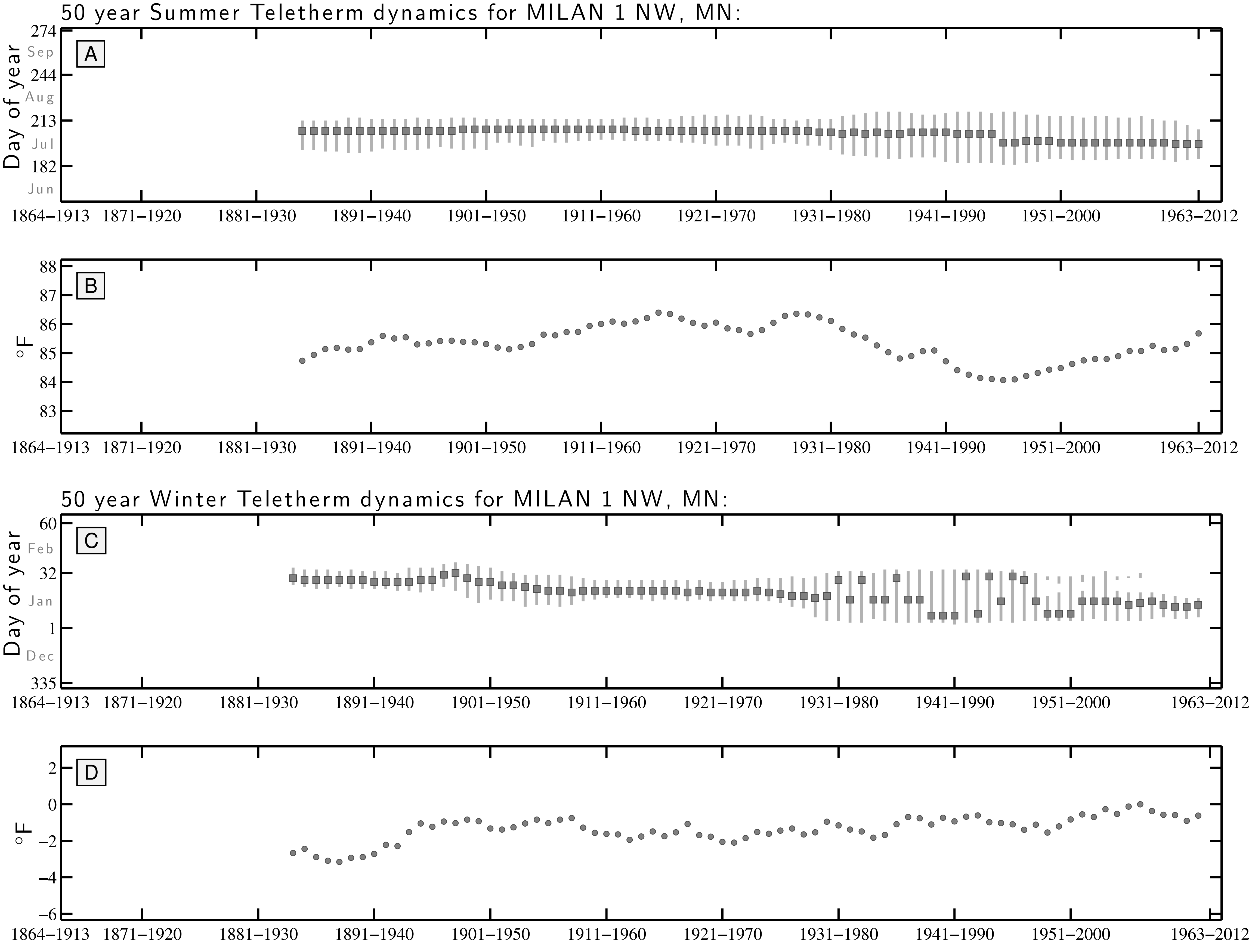
50 year Summer Teletherm dynamics for MILAN 1 NW, MN:
A
Day of year
274
Sep
244
Aug
213
Jul
182
Jun
B
°F
88
87
86
85
84
83
1864–1913
1871–1920
1881–1930
1891–1940
1901–1950
1911–1960
1921–1970
1931–1980
1941–1990
1951–2000
1963–2012
50 year Winter Teletherm dynamics for MILAN 1 NW, MN:
C
Day of year
60
Feb
32
Jan
1
Dec
335
D
°F
2
0
−2
−4
−6

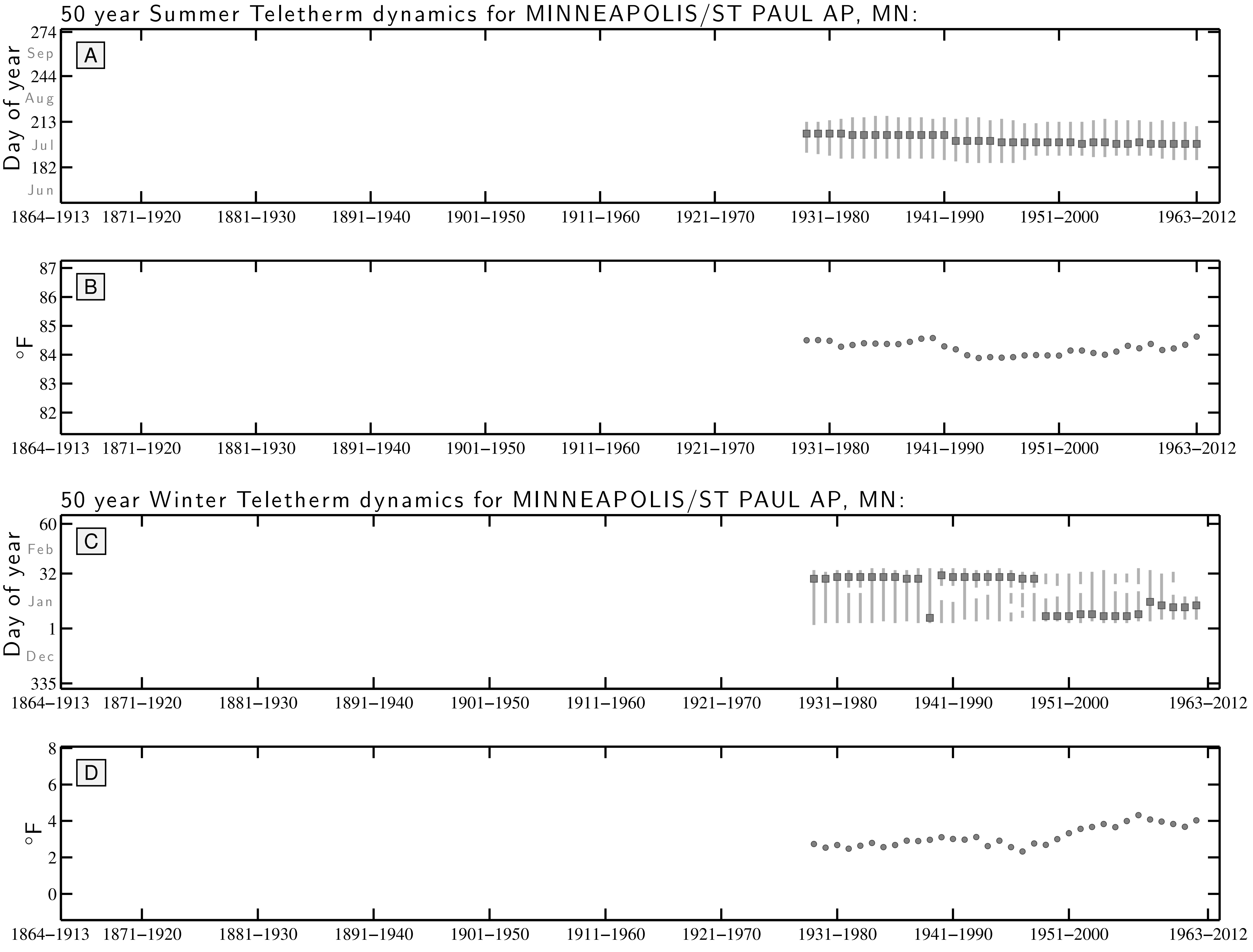

50 year Summer Teletherm dynamics for MINNEAPOLIS/ST PAUL AP, MN:
A
Day of year
274
Sep
244
Aug
213
Jul
182
Jun
1864–1913
1871–1920
1881–1930
1891–1940
1901–1950
1911–1960
1921–1970
1931–1980
1941–1990
1951–2000
1963–2012
B
°F
87
86
85
84
83
82
50 year Winter Teletherm dynamics for MINNEAPOLIS/ST PAUL AP, MN:
C
Day of year
60
Feb
32
Jan
1
Dec
335
D
°F
8
6
4
2
0

50 year Summer Teletherm dynamics for MONTEVIDEO 1 SW, MN:

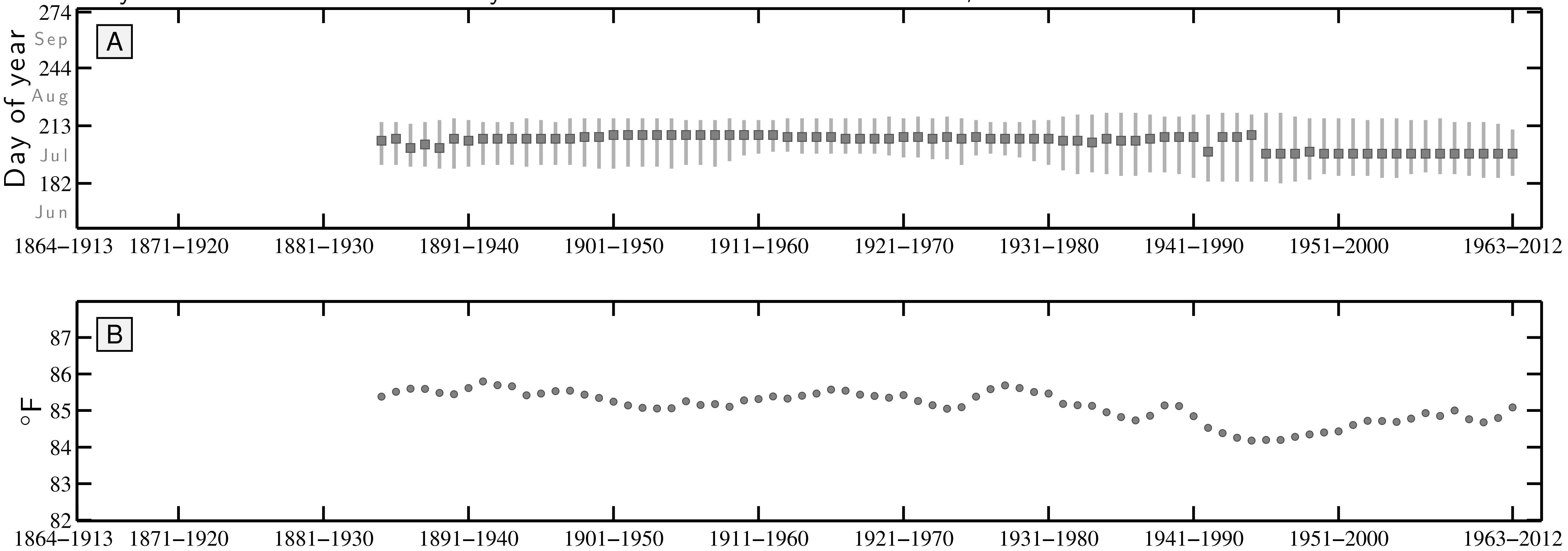

50 year Winter Teletherm dynamics for MONTEVIDEO 1 SW, MN:

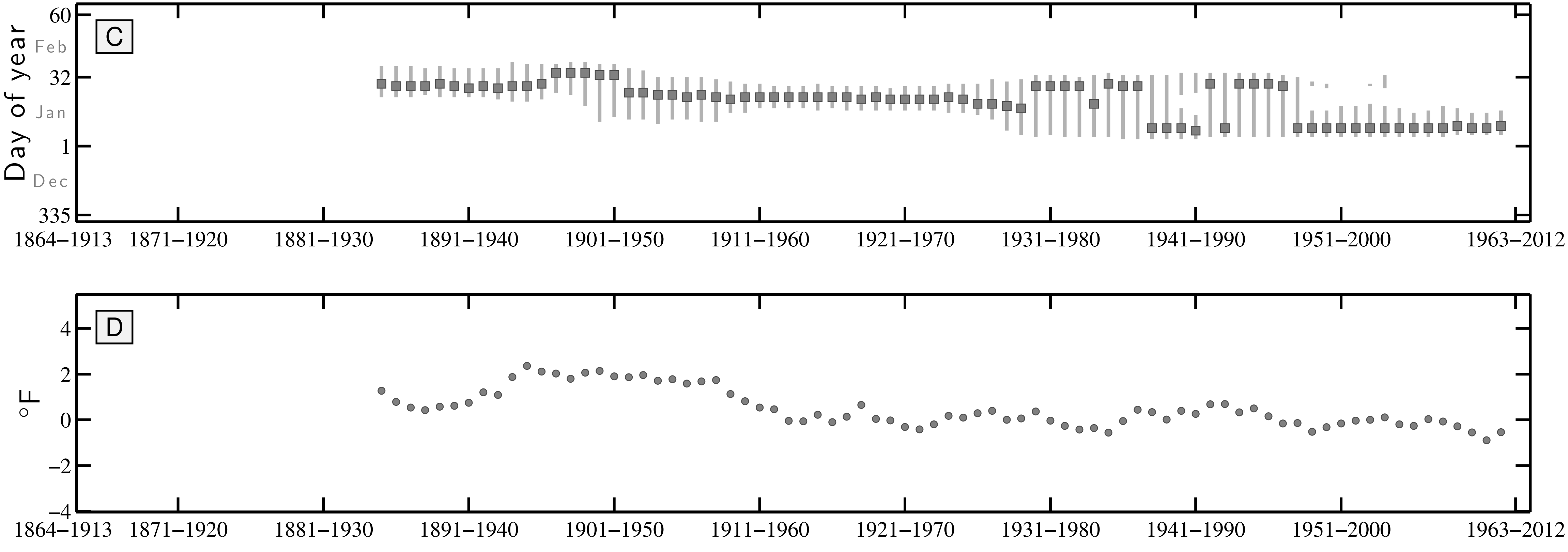

50 year Summer Teletherm dynamics for MORA, MN:

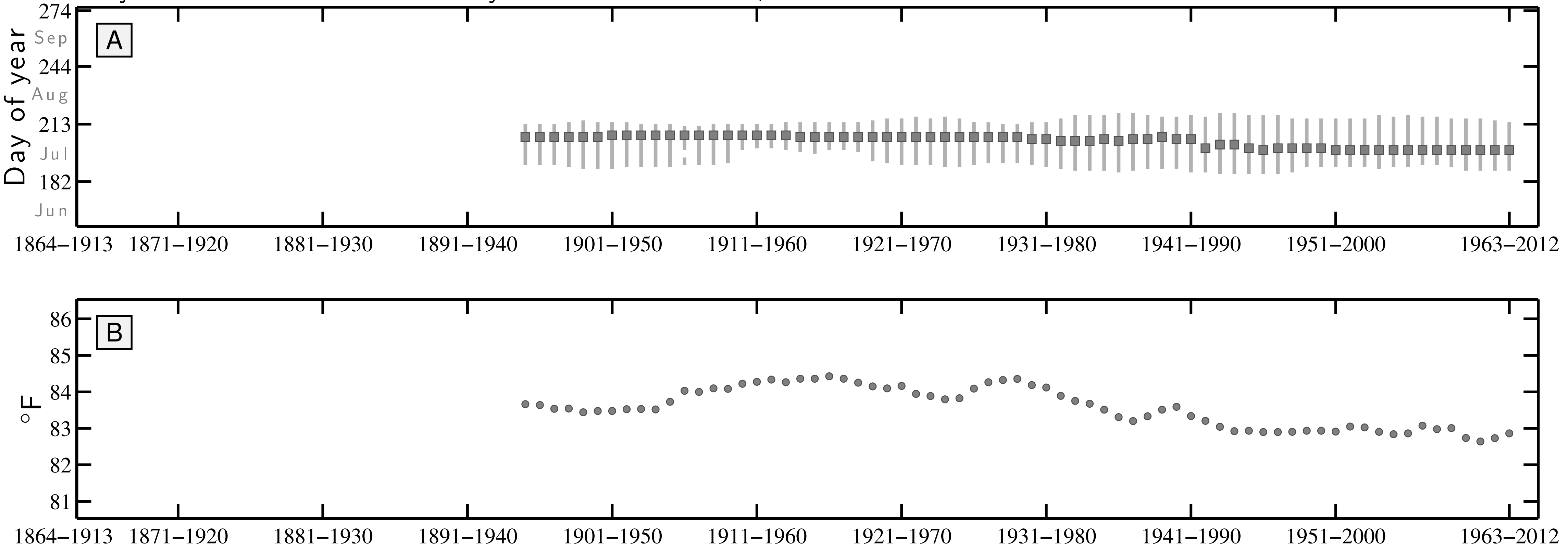

50 year Winter Teletherm dynamics for MORA, MN:

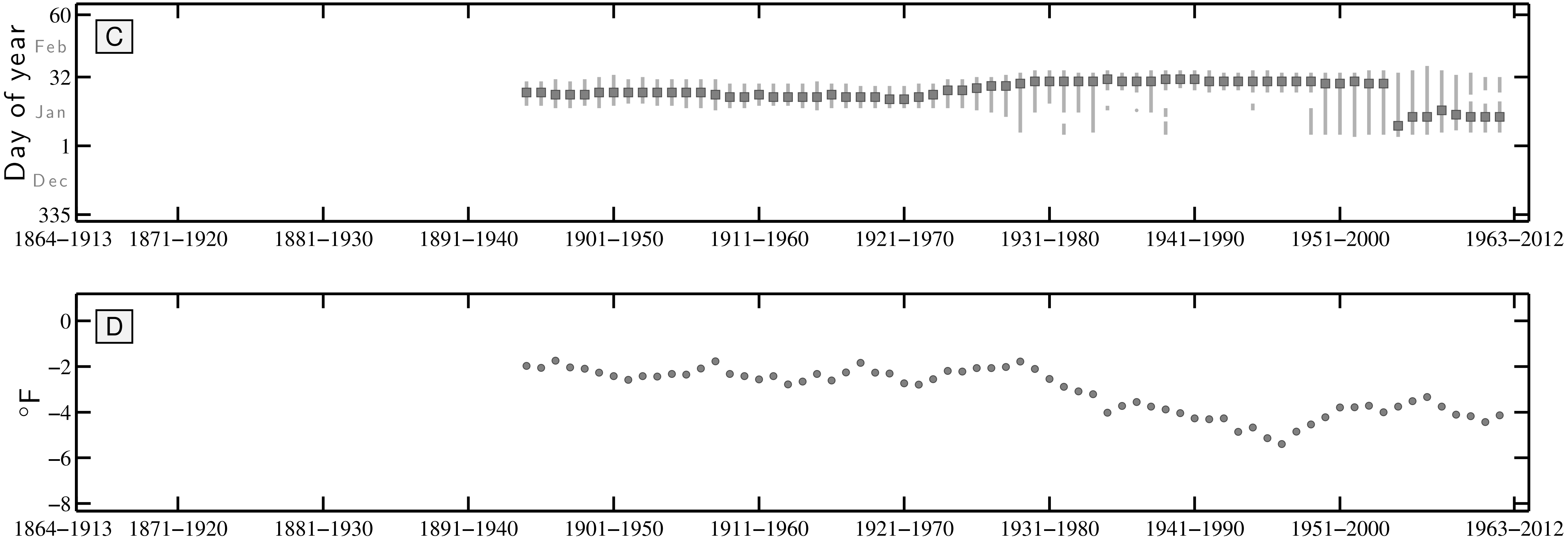

50 year Summer Teletherm dynamics for MORRIS WC EXP STN, MN:

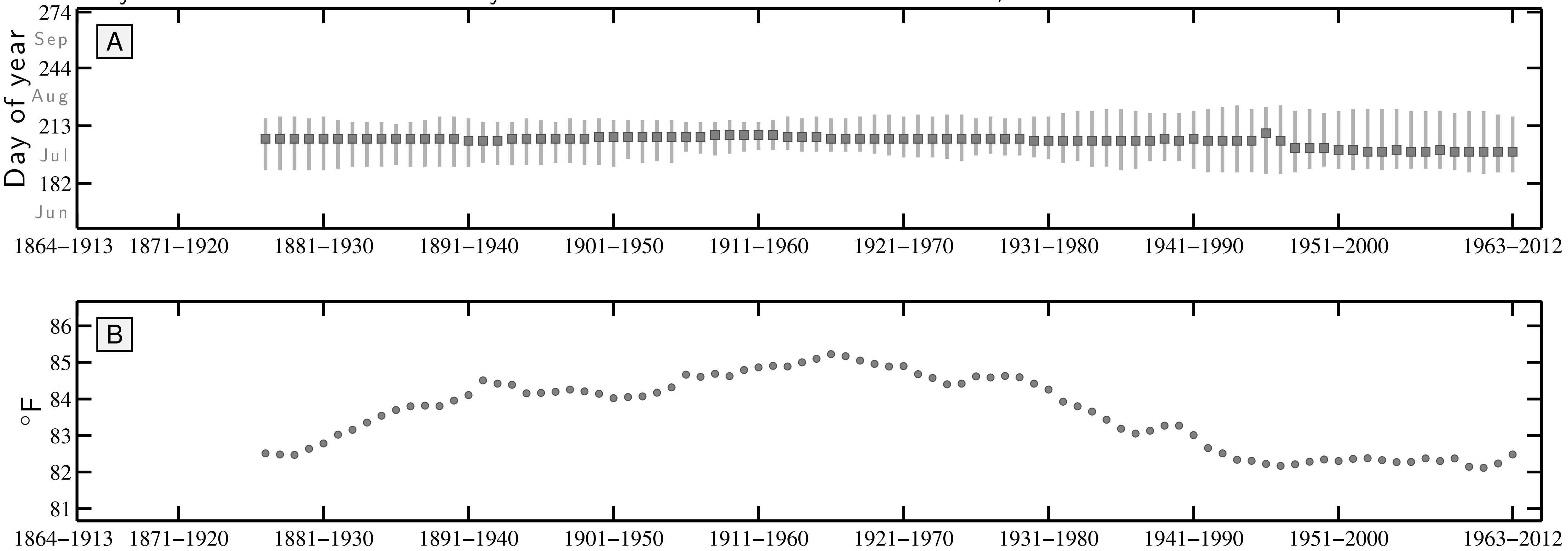

50 year Winter Teletherm dynamics for MORRIS WC EXP STN, MN:

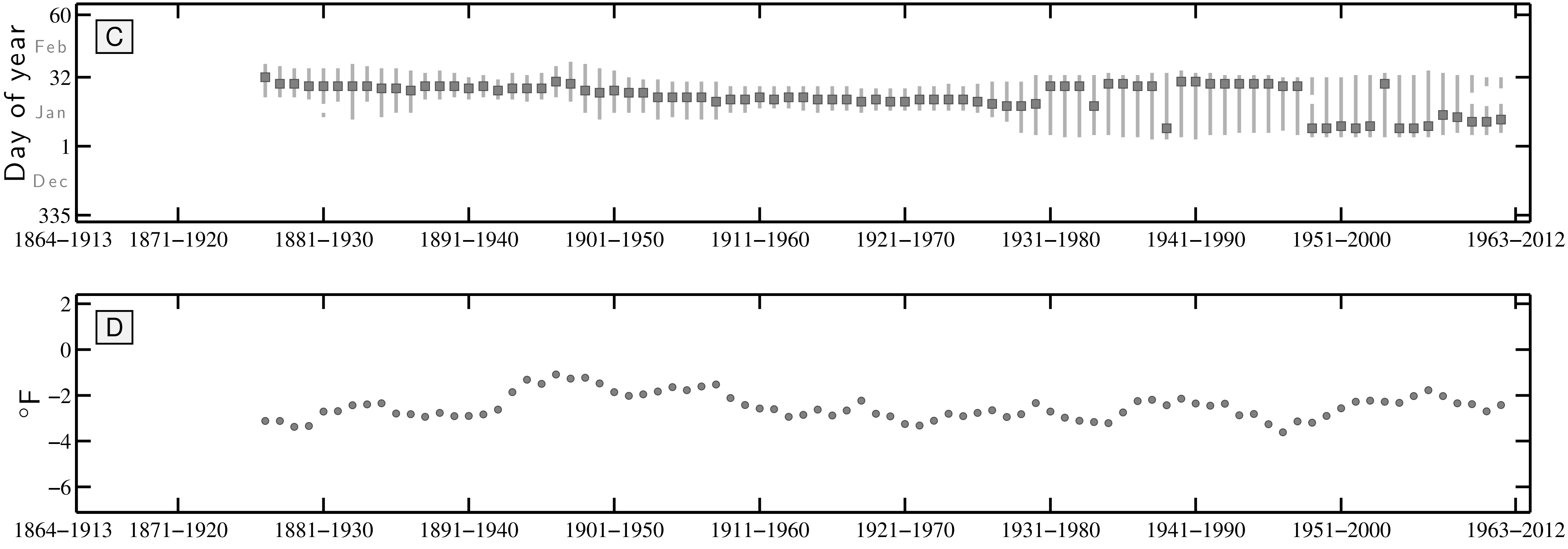

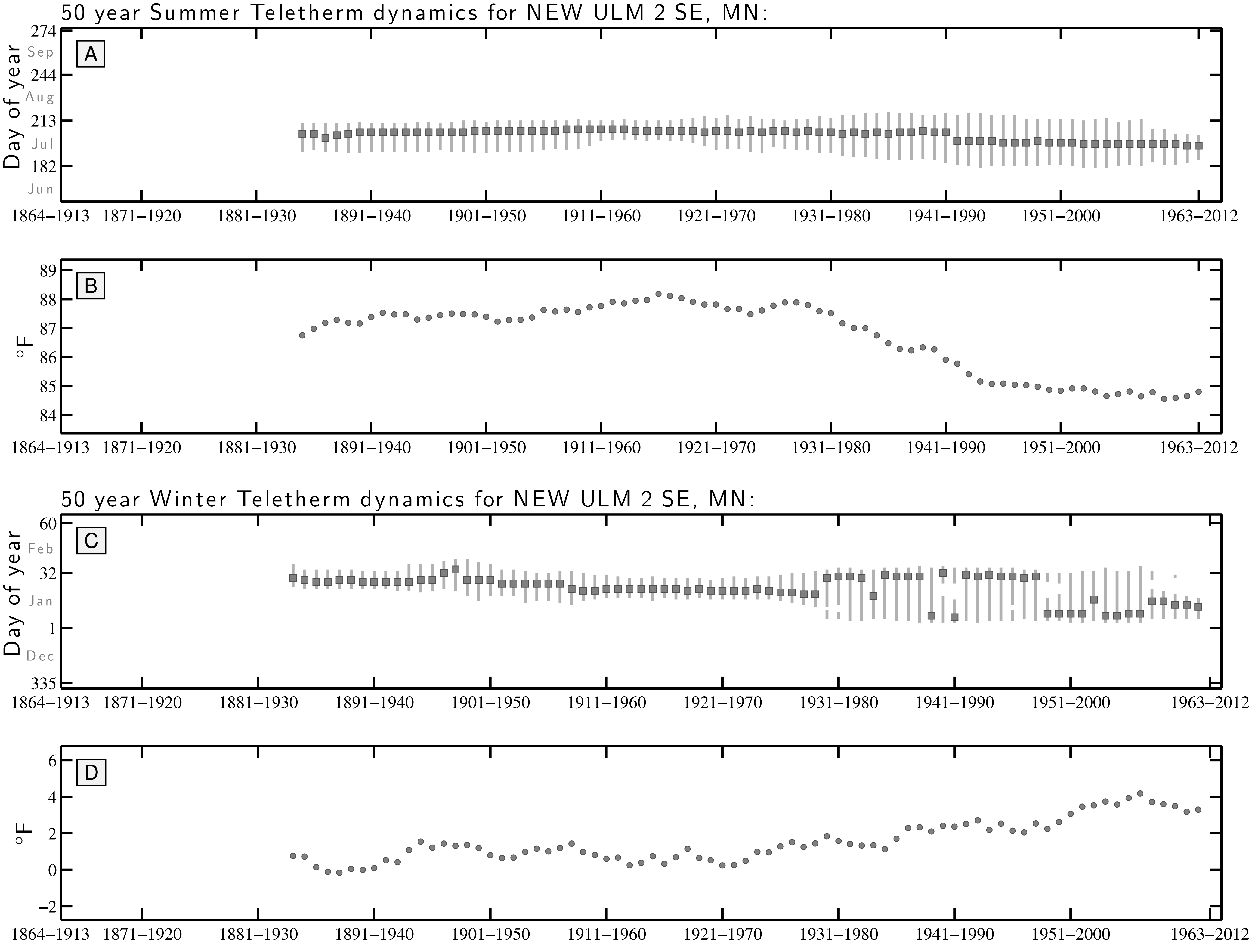
50 year Summer Teletherm dynamics for NEW ULM 2 SE, MN:
A
Day of year
274
Sep
244
Aug
213
Jul
182
Jun
1864–1913
1871–1920
1881–1930
1891–1940
1901–1950
1911–1960
1921–1970
1931–1980
1941–1990
1951–2000
1963–2012
B
°F
89
88
87
86
85
84
50 year Winter Teletherm dynamics for NEW ULM 2 SE, MN:
C
Day of year
60
Feb
32
Jan
1
Dec
335
D
°F
6
4
2
0
−2

50 year Summer Teletherm dynamics for PARK RAPIDS 2 S, MN:

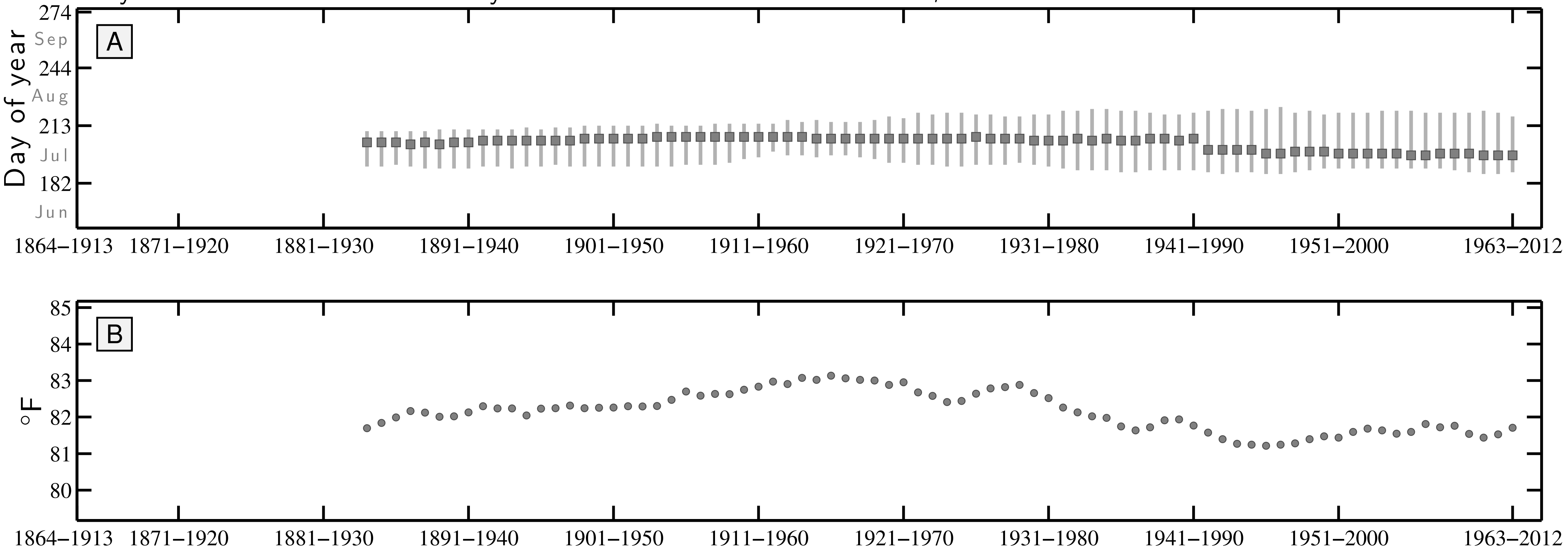

50 year Winter Teletherm dynamics for PARK RAPIDS 2 S, MN:

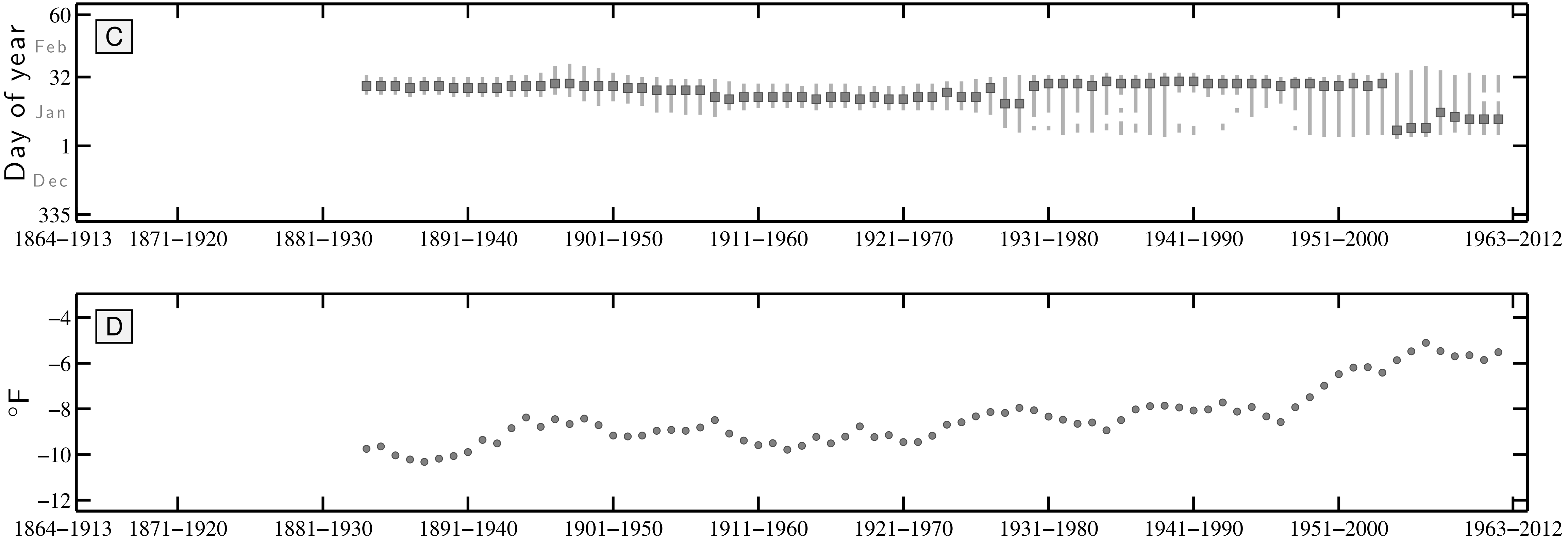

50 year Summer Teletherm dynamics for PINE RIVER DAM, MN:

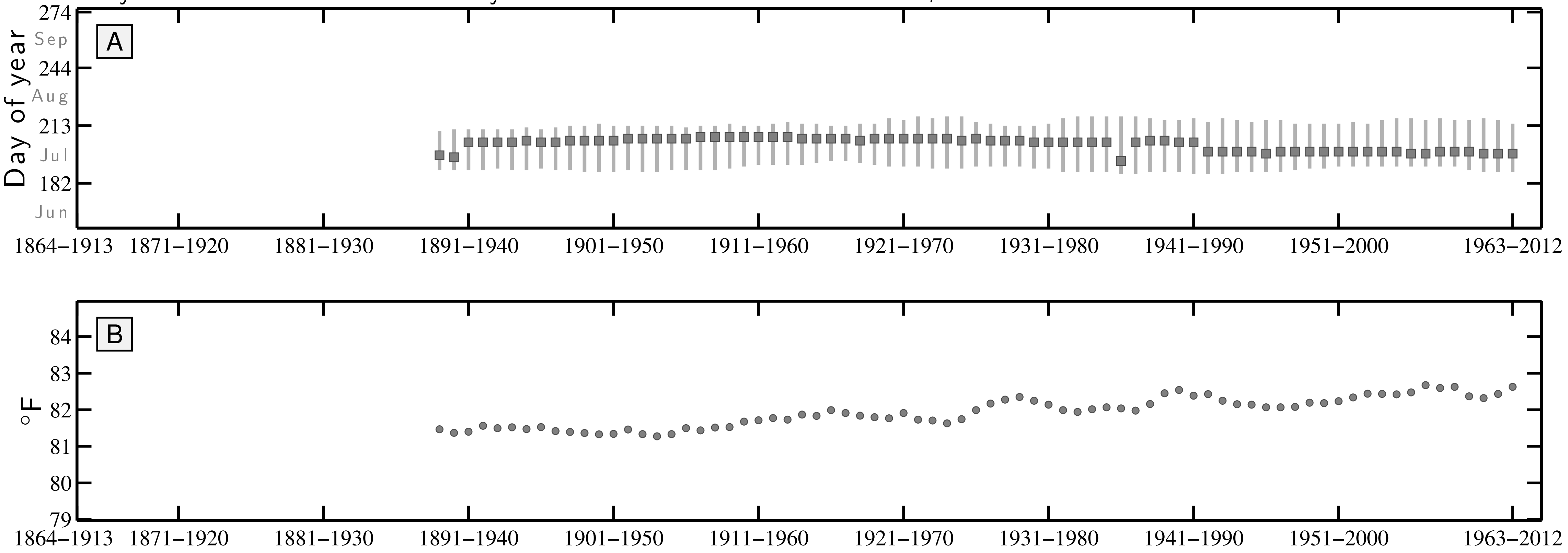

50 year Winter Teletherm dynamics for PINE RIVER DAM, MN:

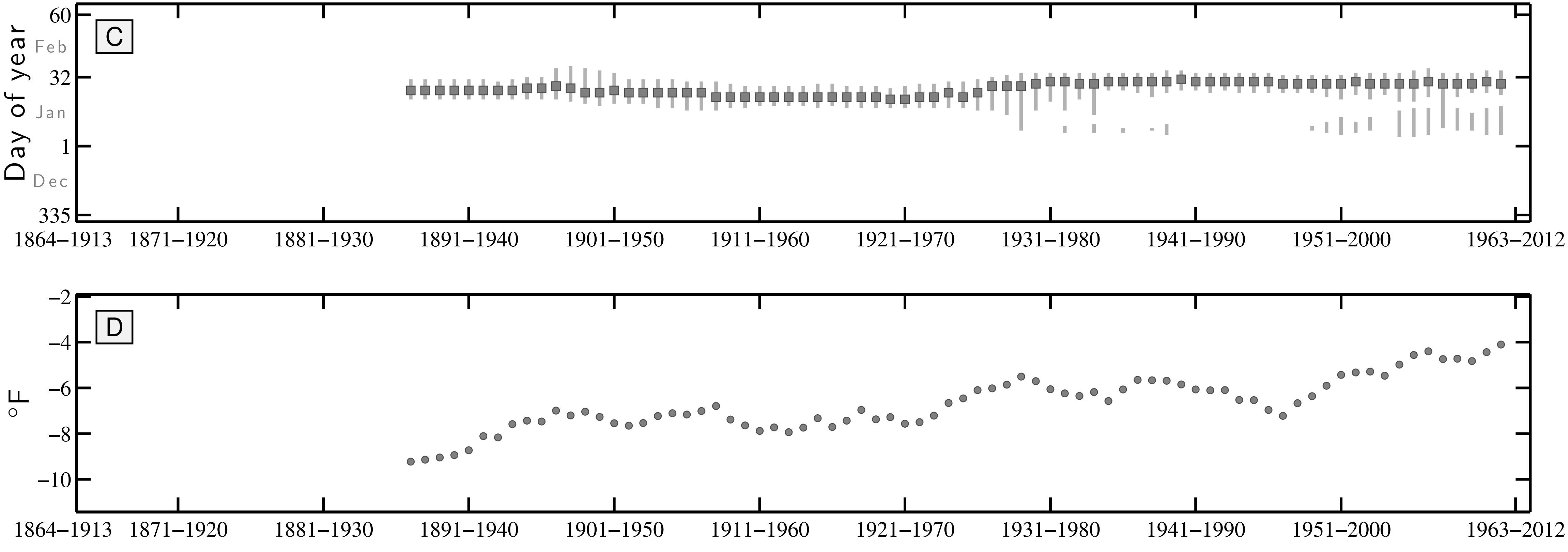

50 year Summer Teletherm dynamics for PIPESTONE, MN:

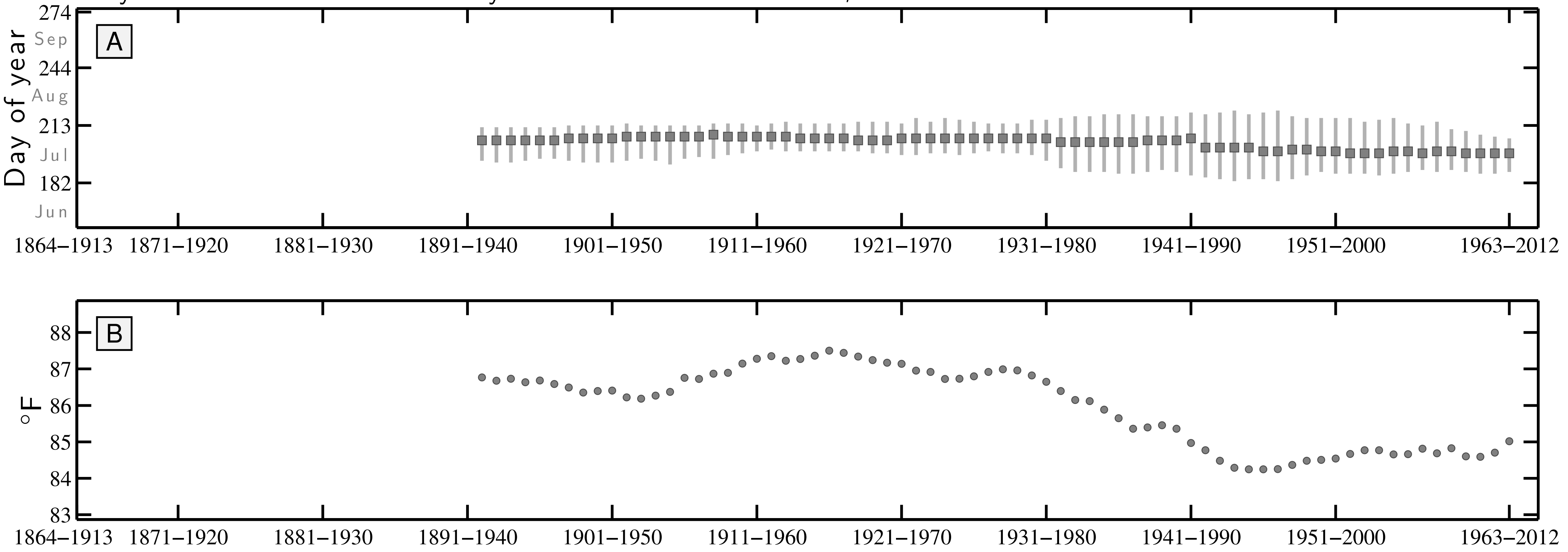

50 year Winter Teletherm dynamics for PIPESTONE, MN:

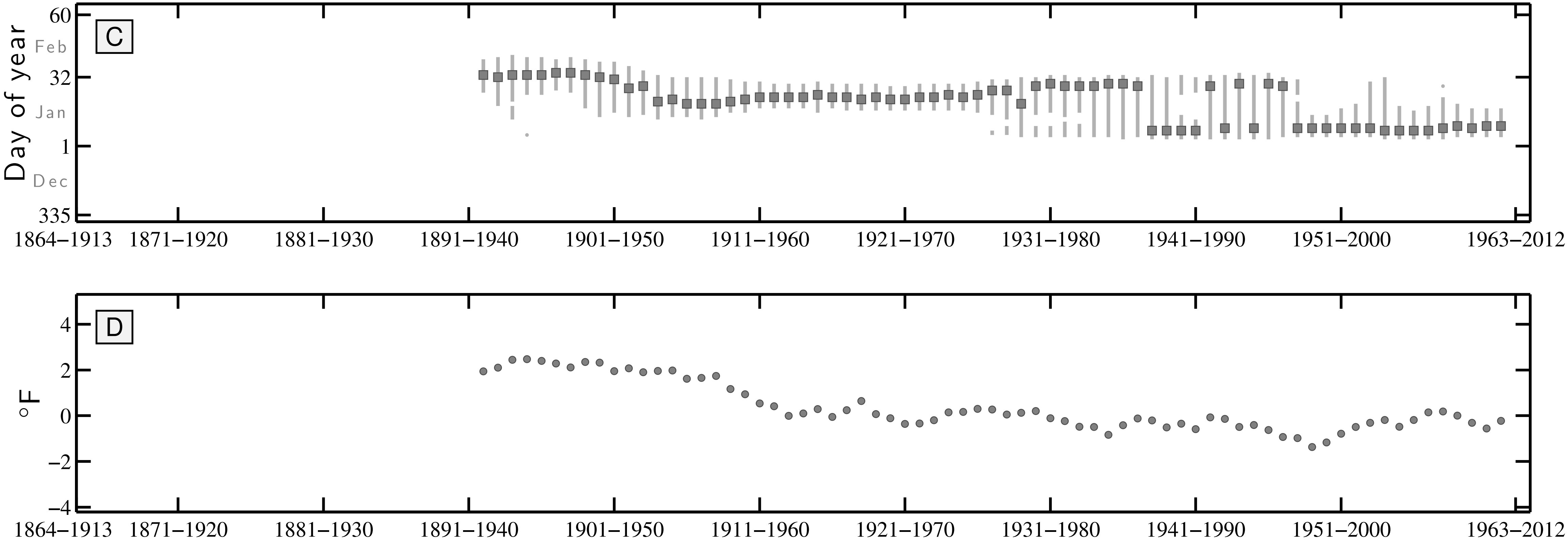

50 year Summer Teletherm dynamics for ROSEAU, MN:

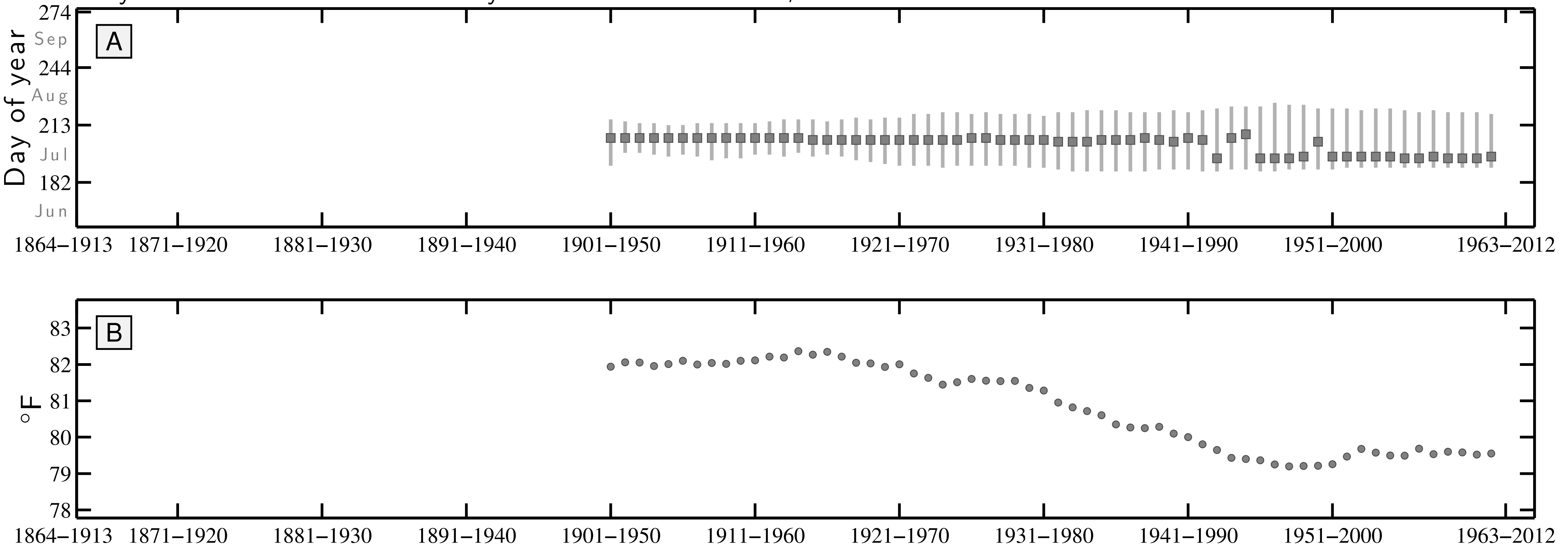

50 year Winter Teletherm dynamics for ROSEAU, MN:

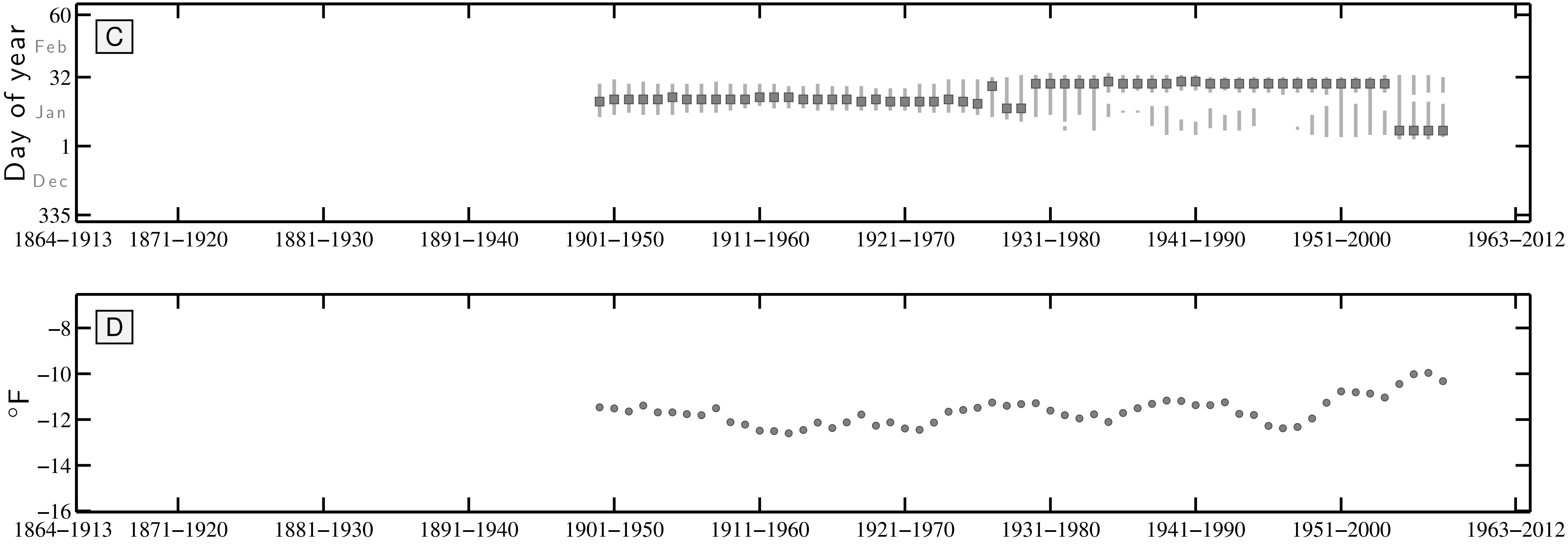

50 year Summer Teletherm dynamics for ST PETER, MN:

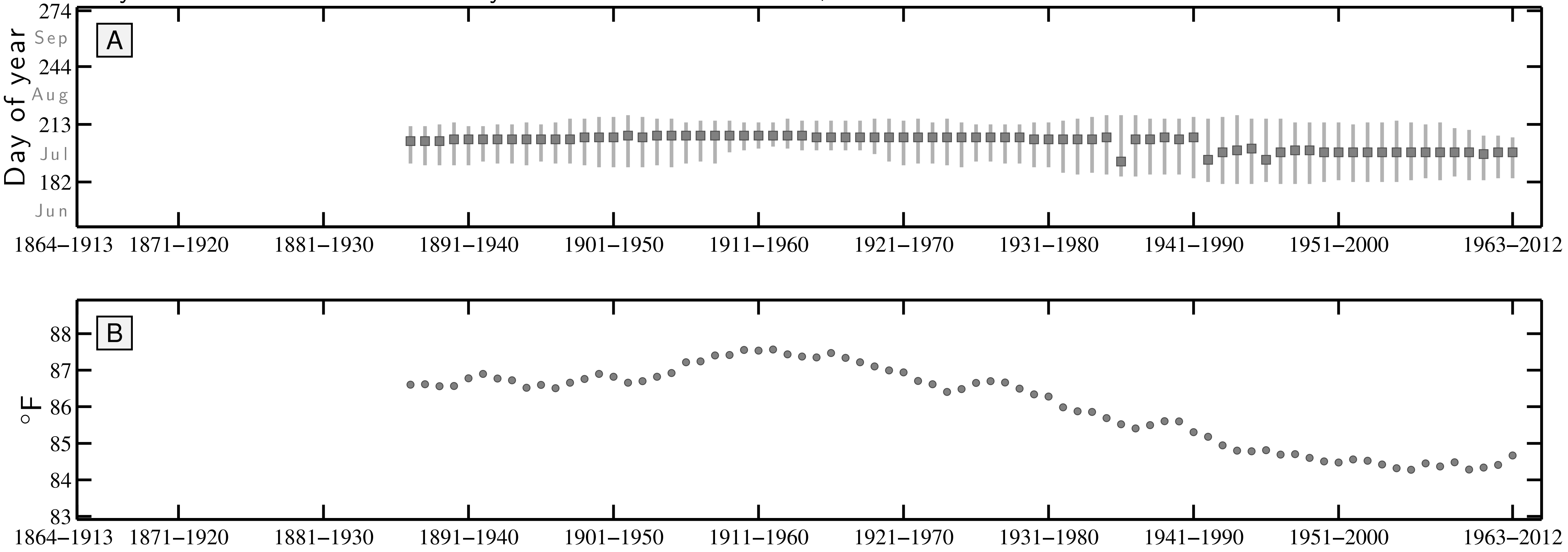

50 year Winter Teletherm dynamics for ST PETER, MN:

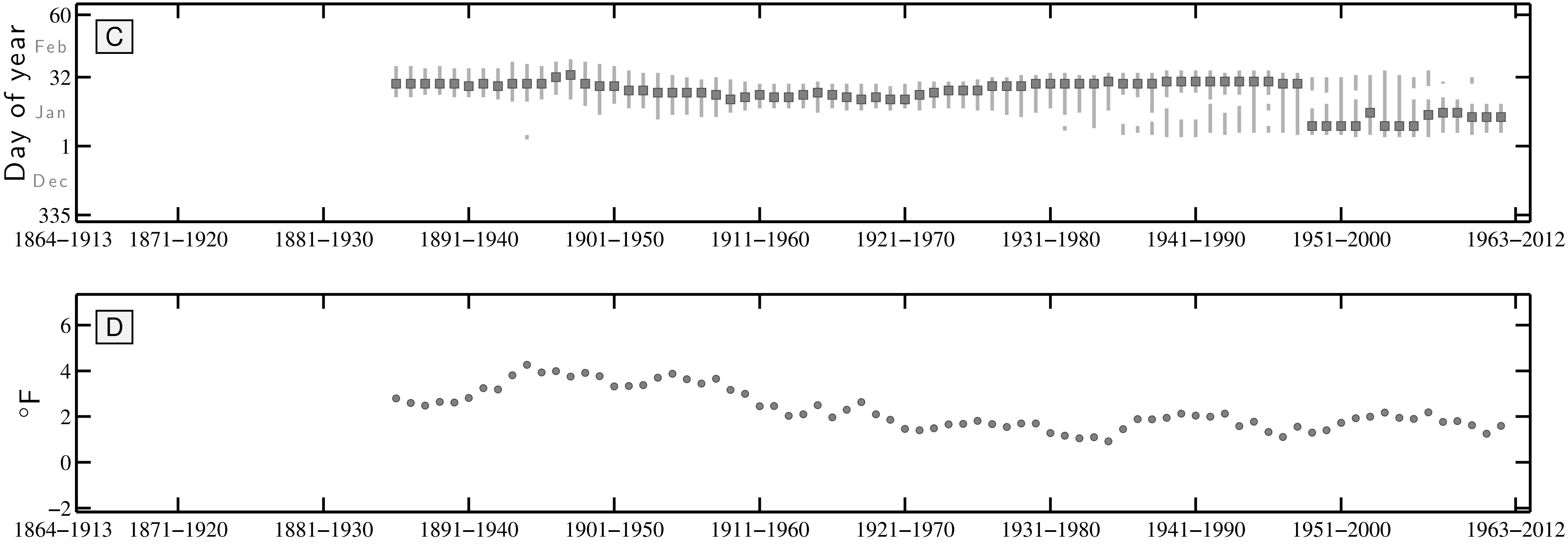

50 year Summer Teletherm dynamics for SANDY LAKE DAM LIBBY, MN:

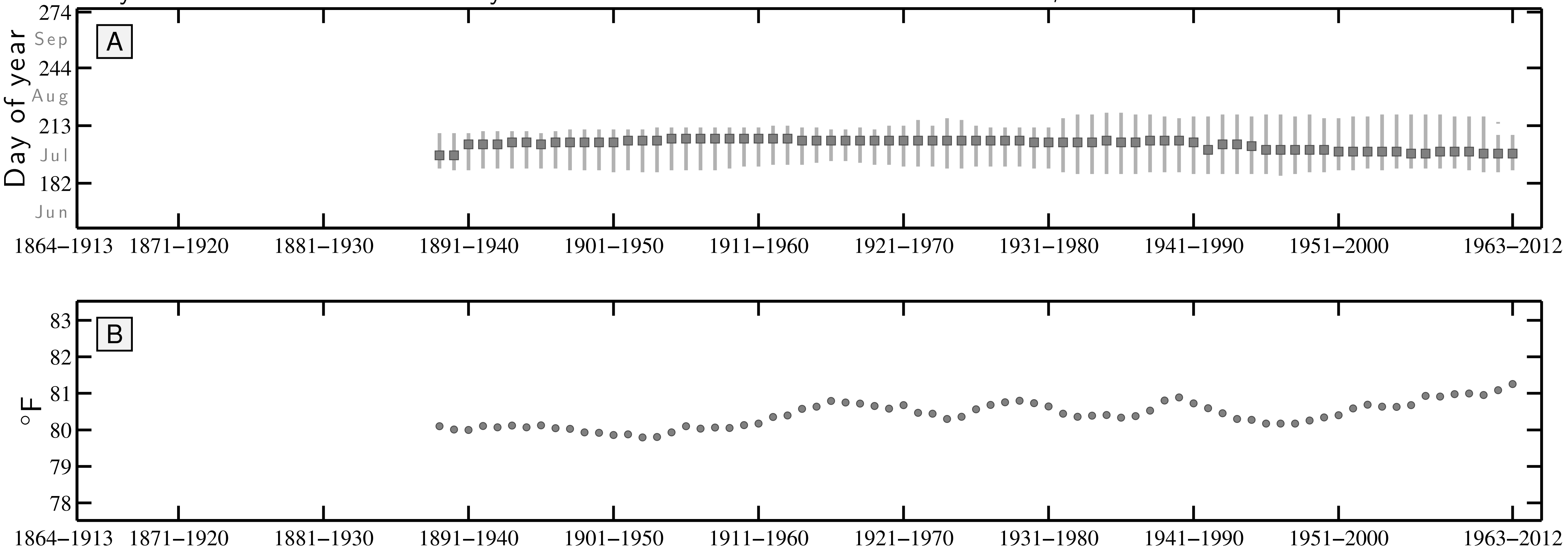

50 year Winter Teletherm dynamics for SANDY LAKE DAM LIBBY, MN:

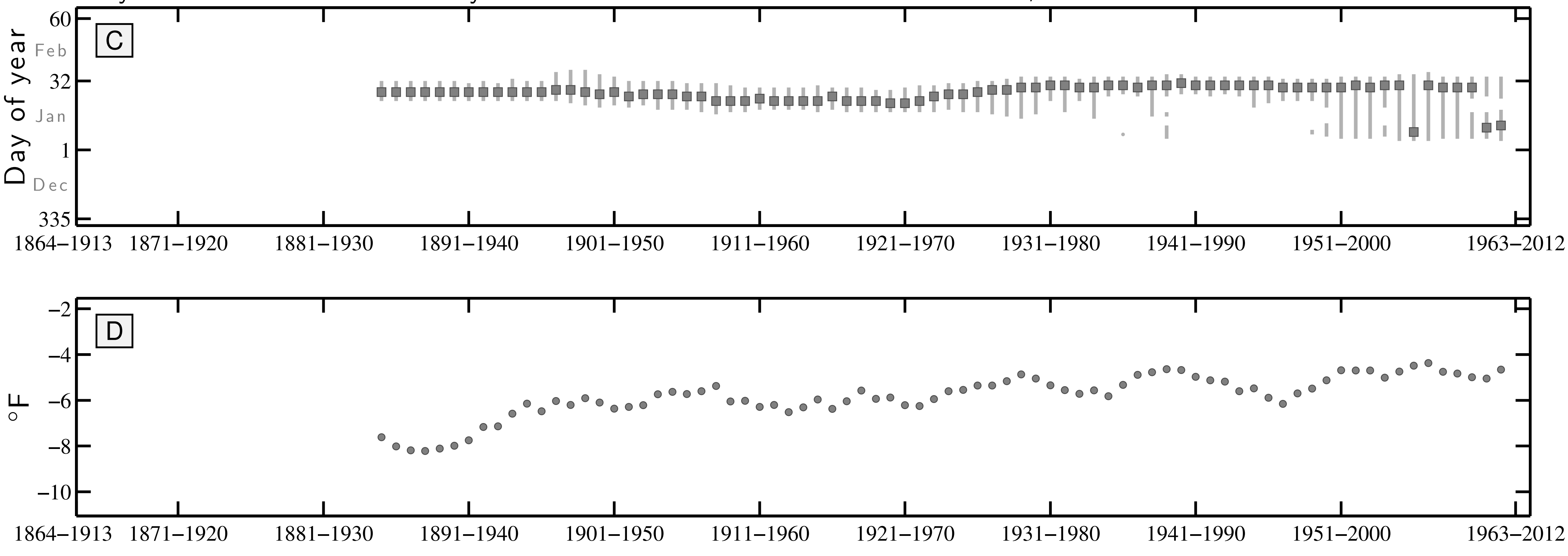

50 year Summer Teletherm dynamics for TWO HARBORS, MN:

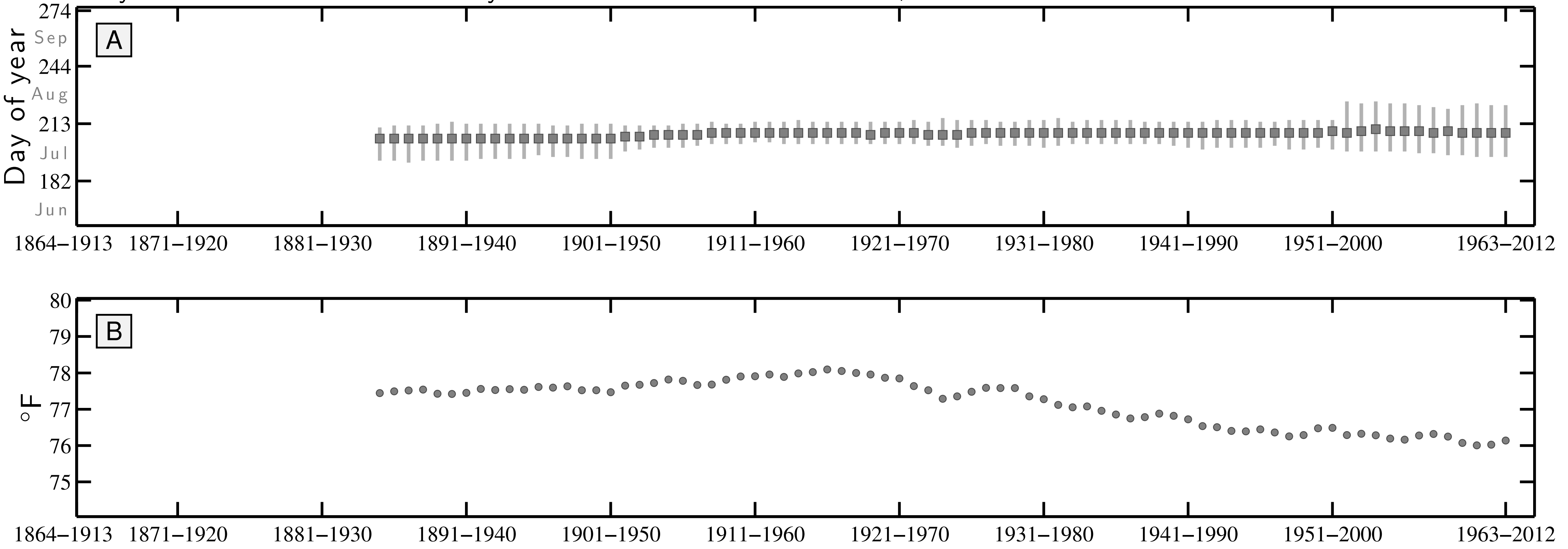

50 year Winter Teletherm dynamics for TWO HARBORS, MN:

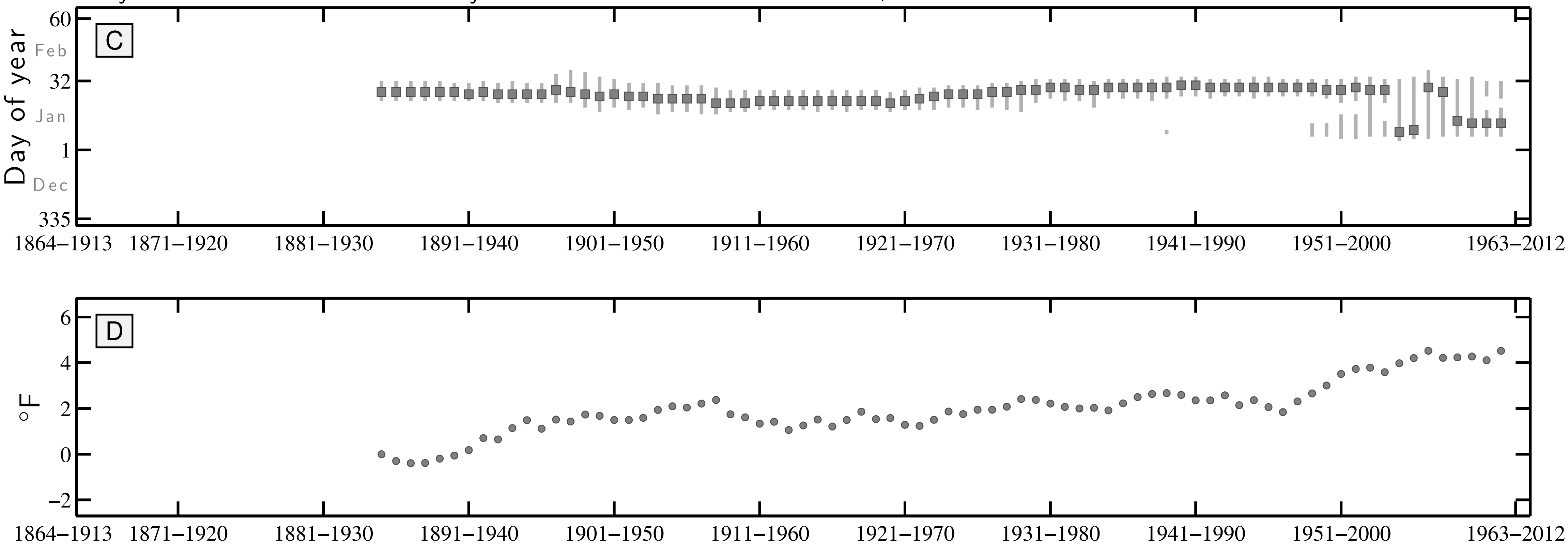

50 year Summer Teletherm dynamics for WALKER AH GWAH CHING, MN:

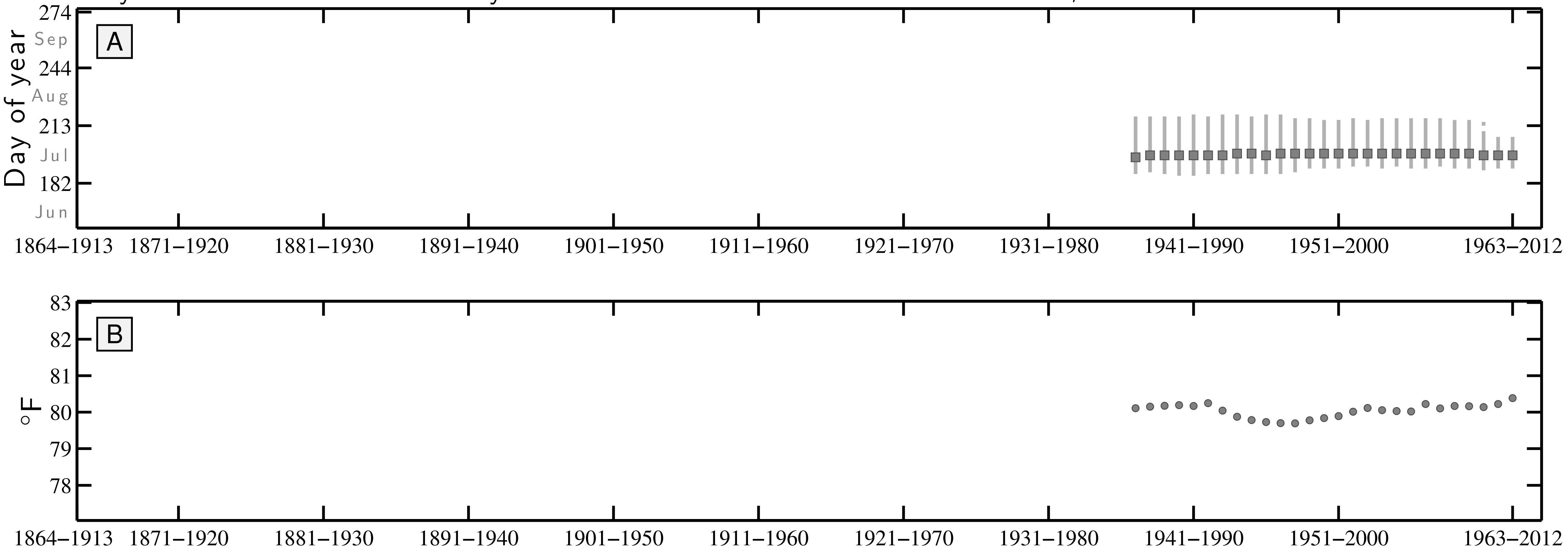

50 year Winter Teletherm dynamics for WALKER AH GWAH CHING, MN:

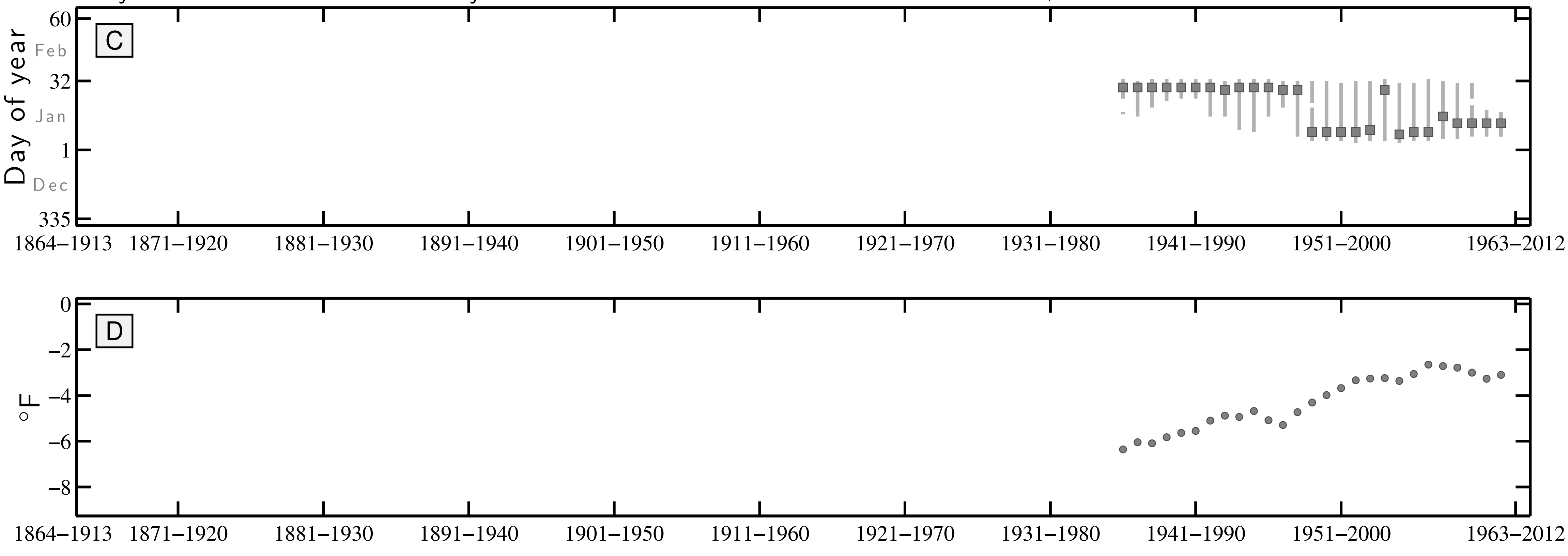

50 year Summer Teletherm dynamics for WINNEBAGO, MN:

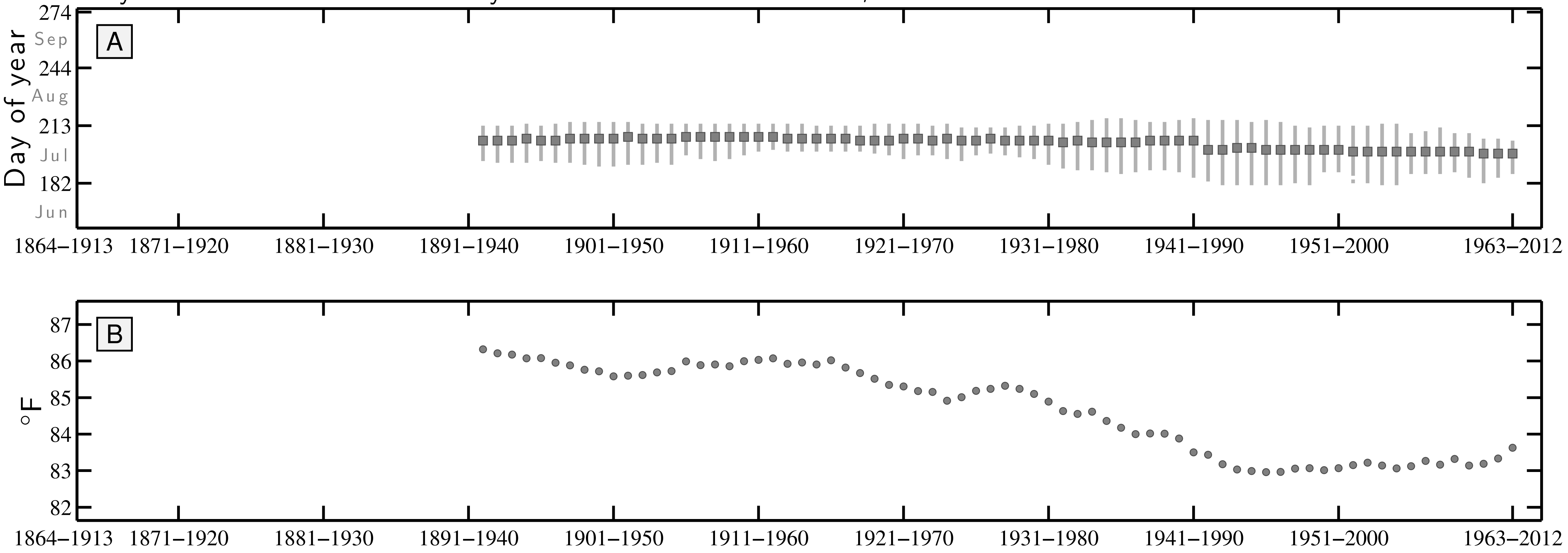

50 year Winter Teletherm dynamics for WINNEBAGO, MN:

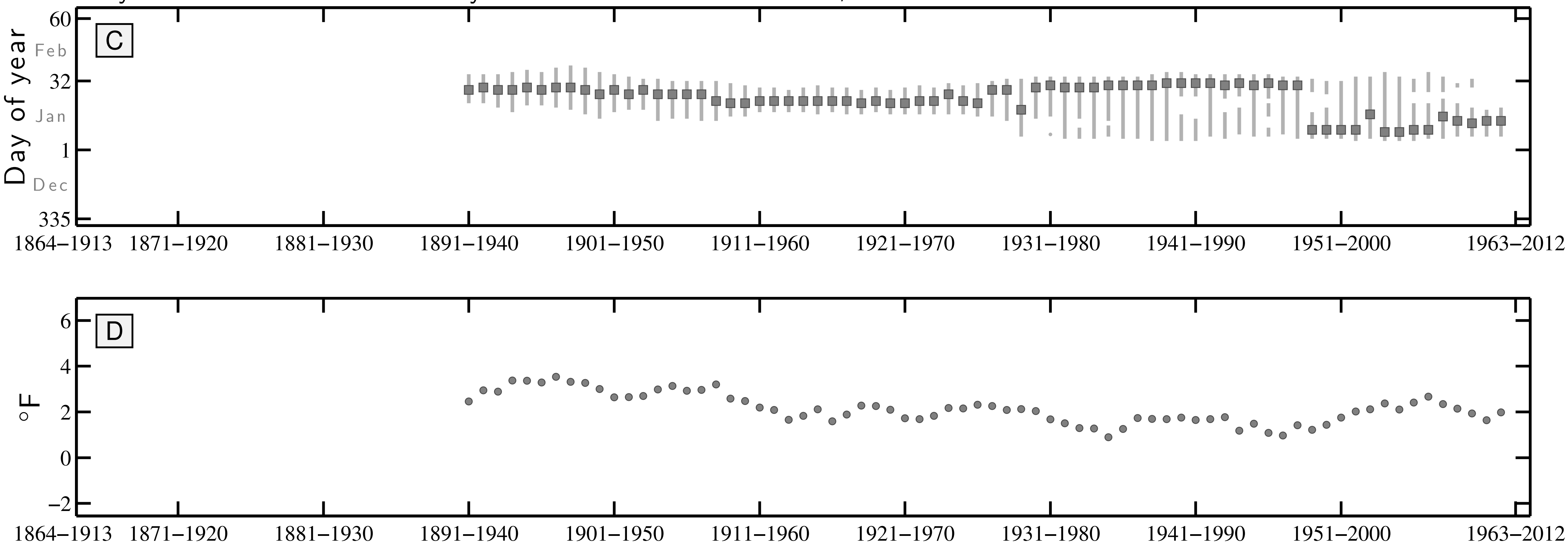

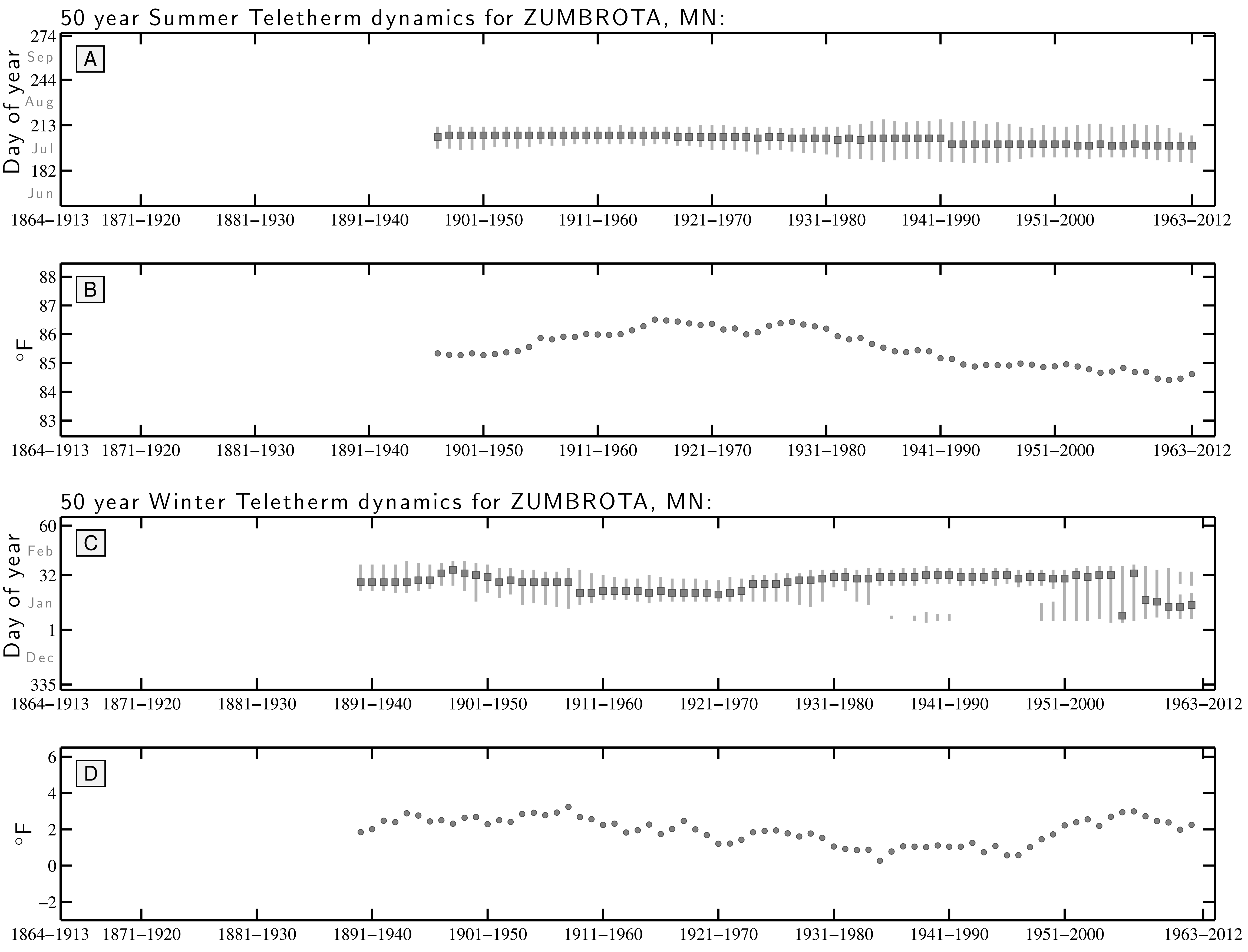
50 year Summer Teletherm dynamics for ZUMBROTA, MN:
A
Day of year
274
Sep
244
Aug
213
Jul
182
Jun
1864–1913
1871–1920
1881–1930
1891–1940
1901–1950
1911–1960
1921–1970
1931–1980
1941–1990
1951–2000
1963–2012
B
°F
88
87
86
85
84
83
50 year Winter Teletherm dynamics for ZUMBROTA, MN:
C
Day of year
60
Feb
32
Jan
1
Dec
335
D
°F
6
4
2
0
−2

50 year Summer Teletherm dynamics for ABERDEEN, MS:

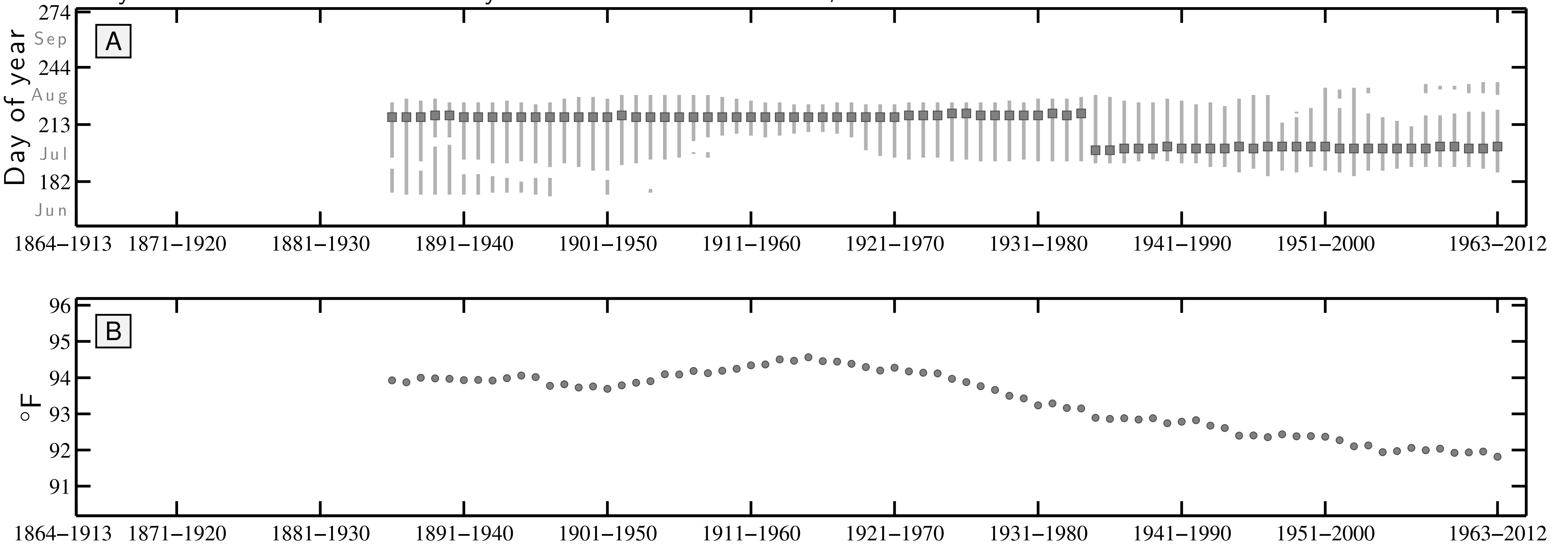

50 year Winter Teletherm dynamics for ABERDEEN, MS:

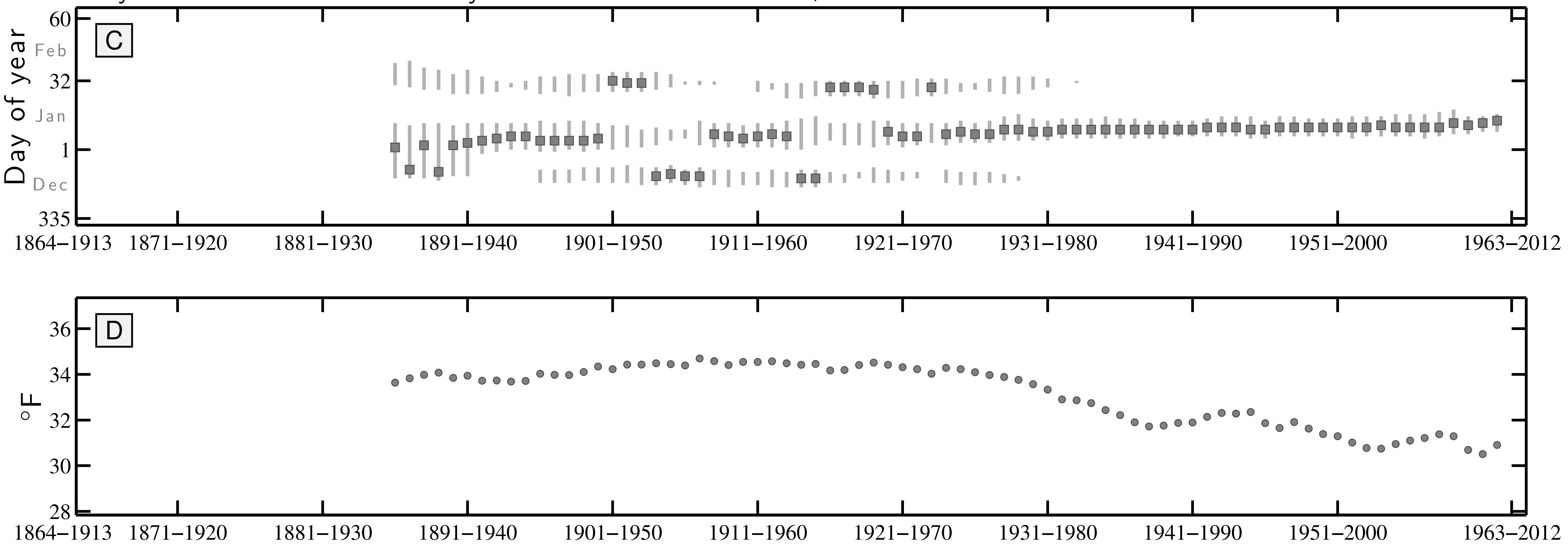

50 year Summer Teletherm dynamics for BATESVILLE 2 SW, MS:

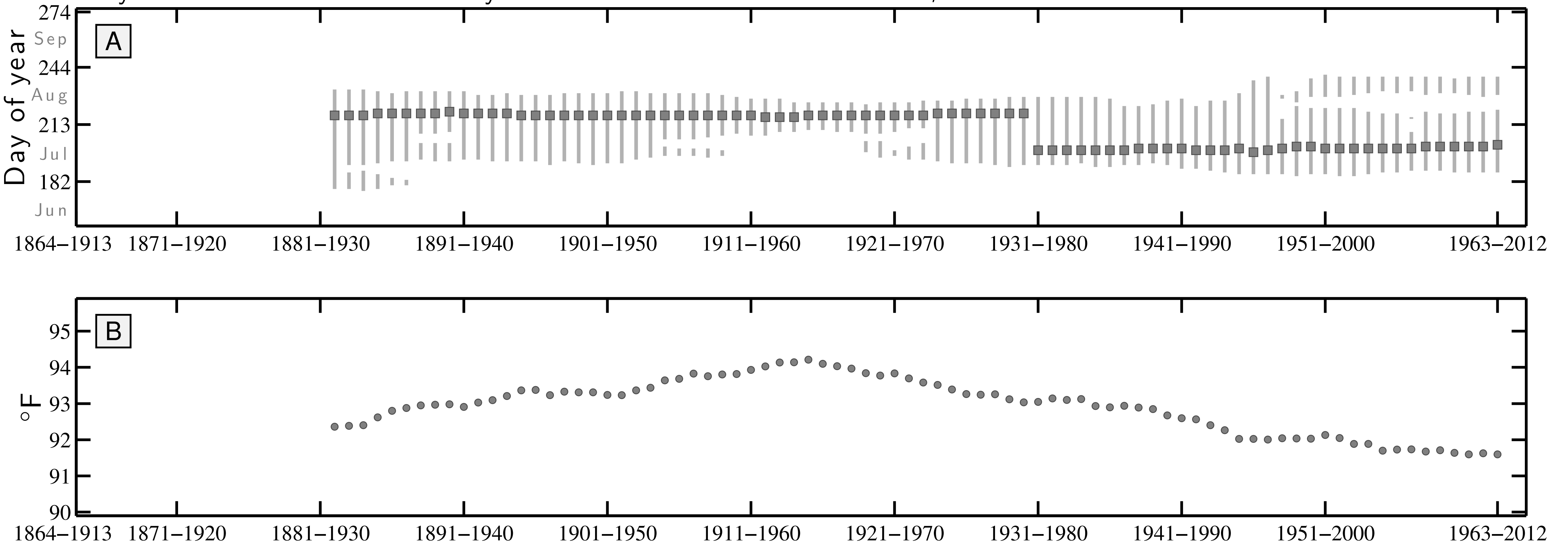

50 year Winter Teletherm dynamics for BATESVILLE 2 SW, MS:

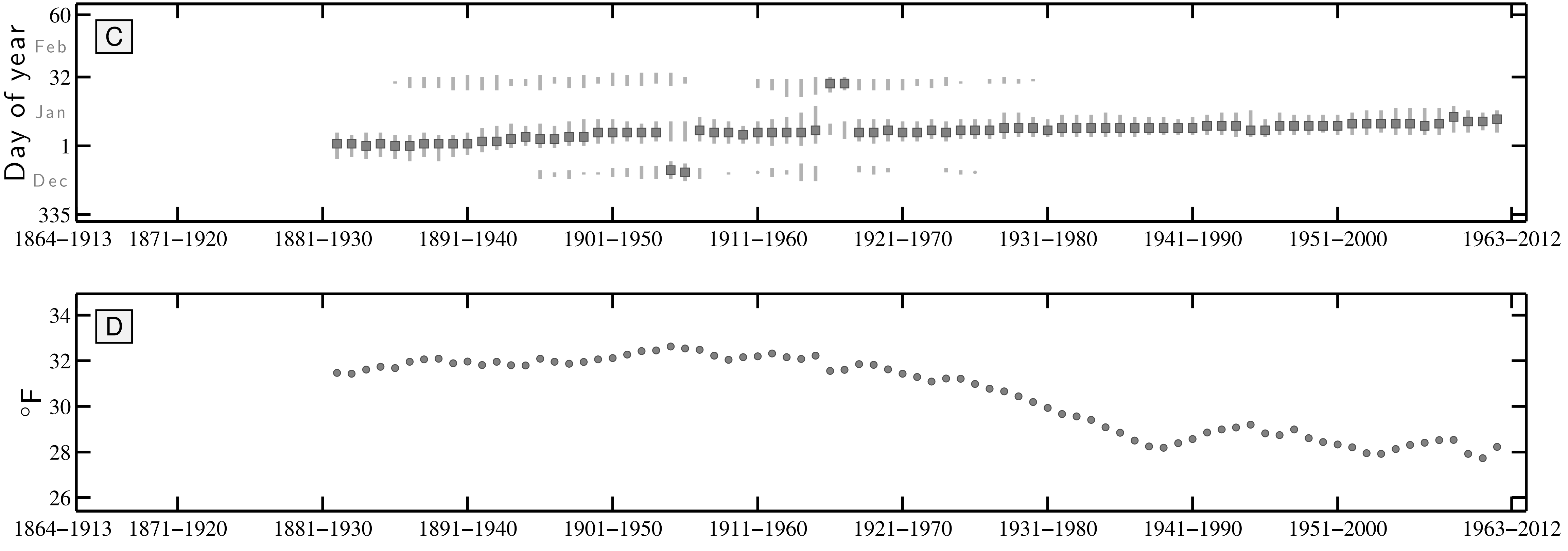

50 year Summer Teletherm dynamics for BOONEVILLE, MS:

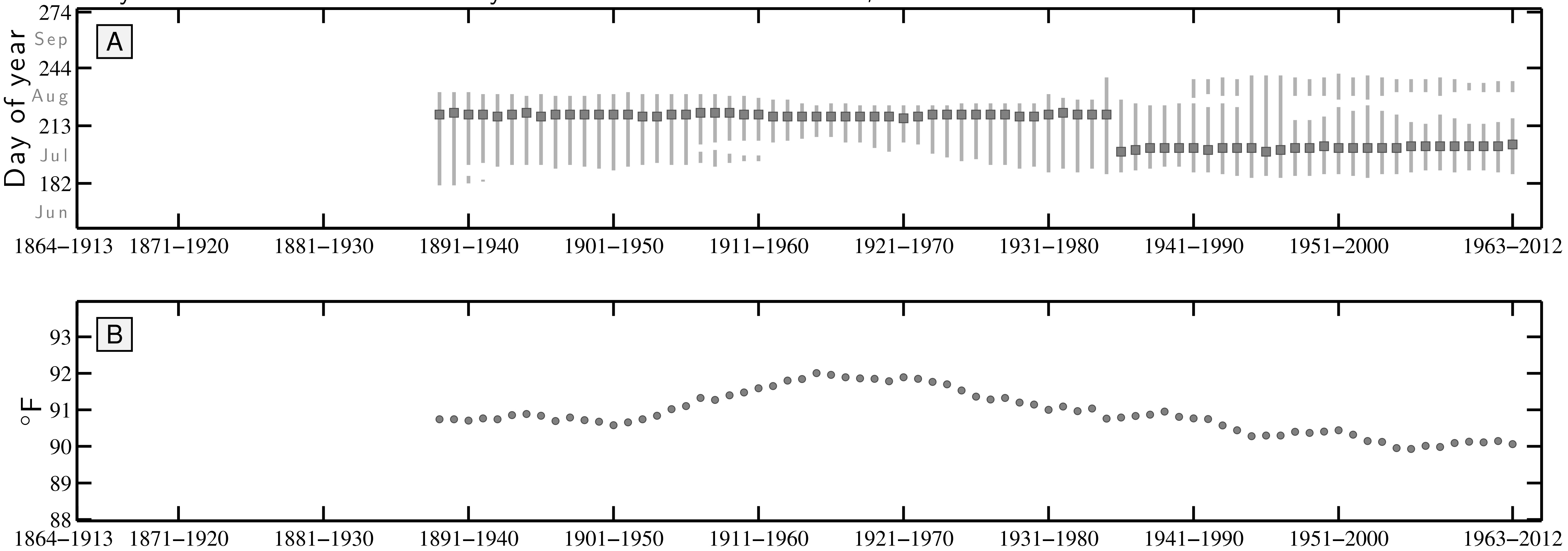

50 year Winter Teletherm dynamics for BOONEVILLE, MS:

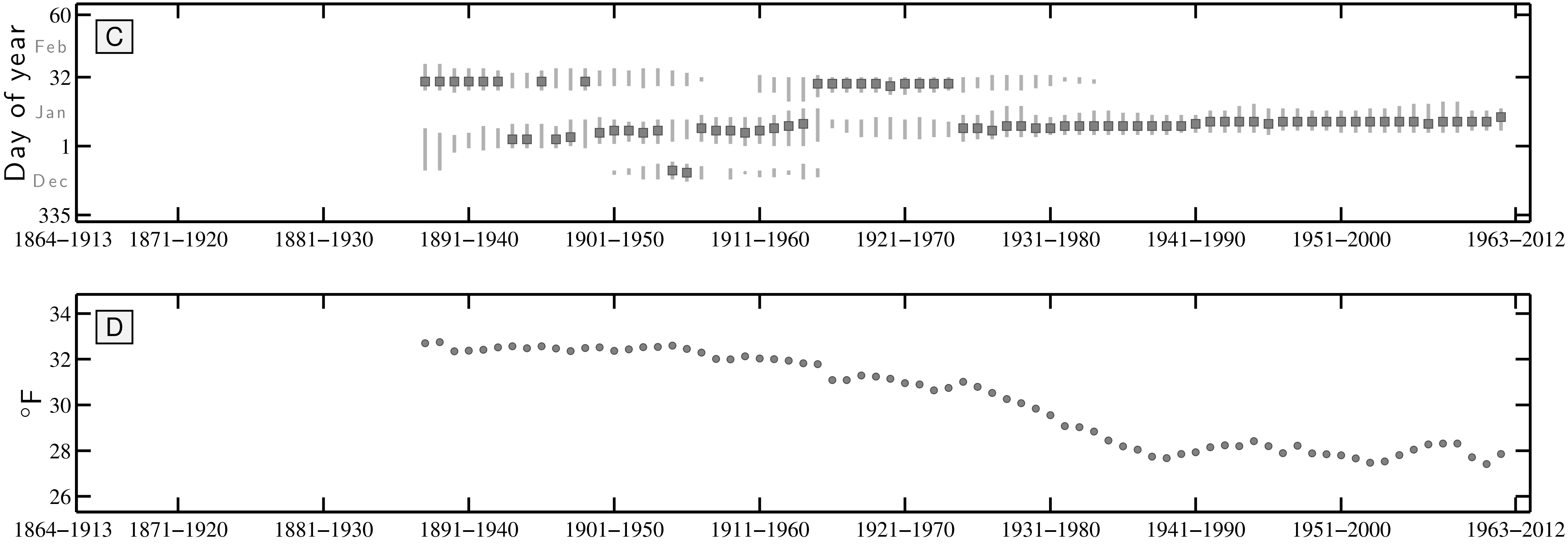

50 year Summer Teletherm dynamics for BROOKHAVEN CITY, MS:

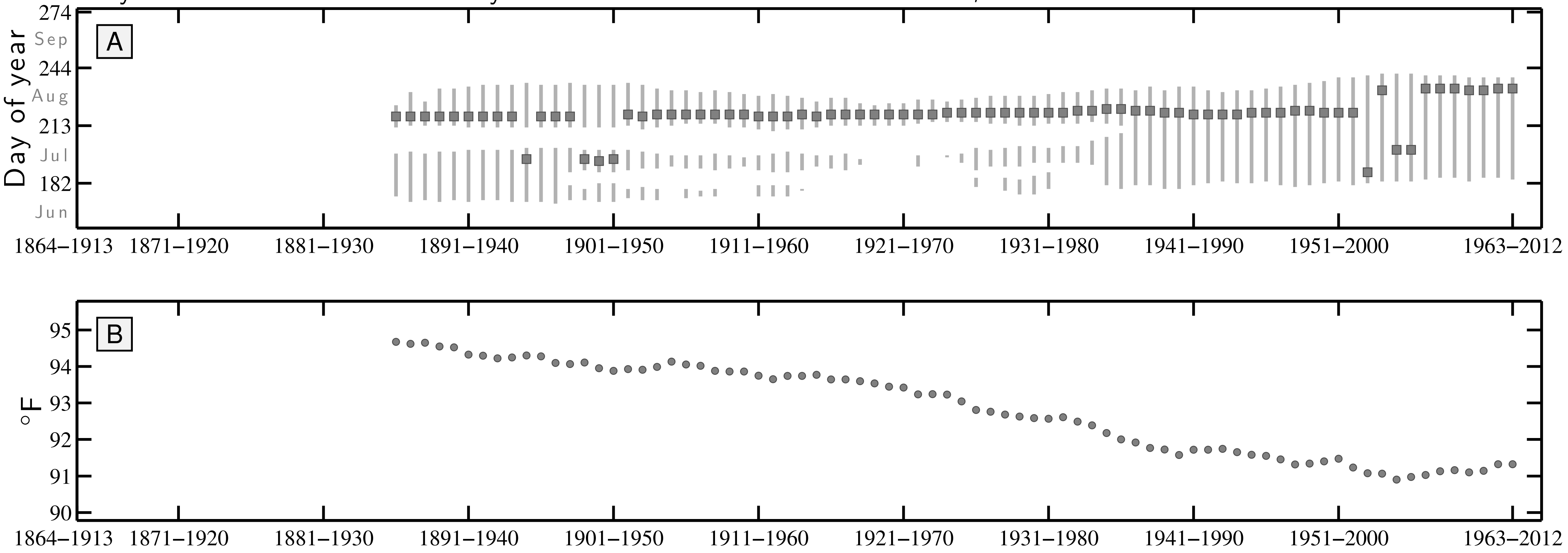

50 year Winter Teletherm dynamics for BROOKHAVEN CITY, MS:

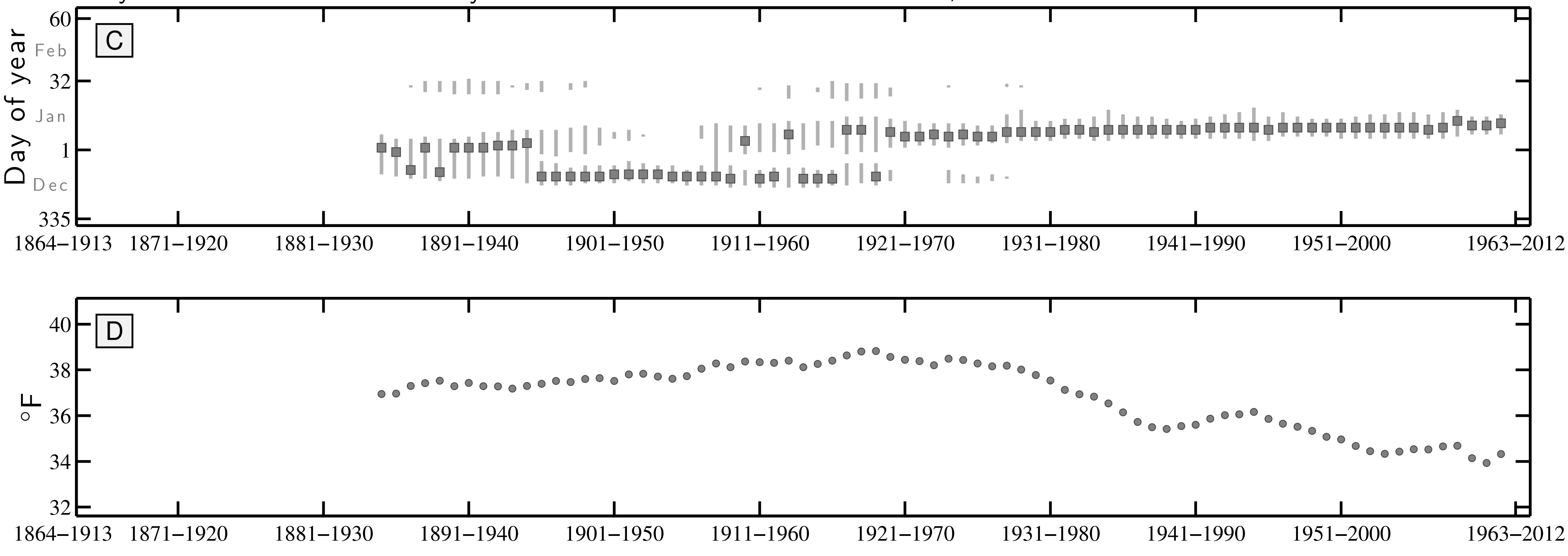

50 year Summer Teletherm dynamics for CANTON 4N, MS:

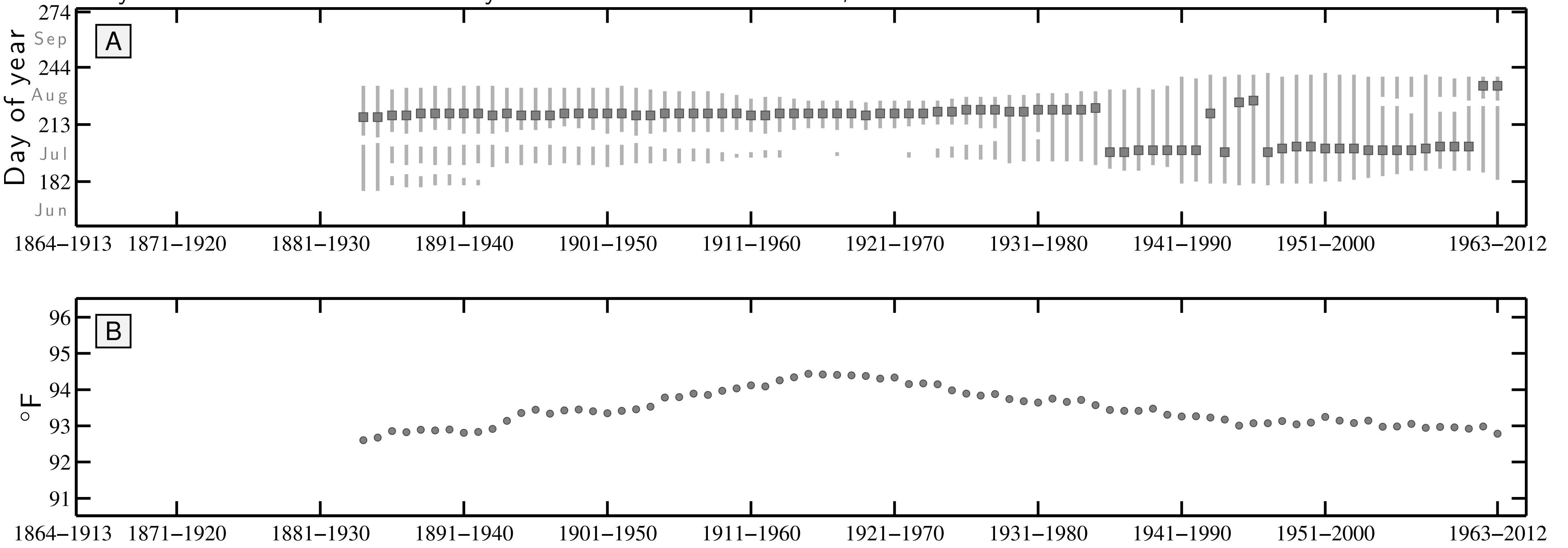

50 year Winter Teletherm dynamics for CANTON 4N, MS:

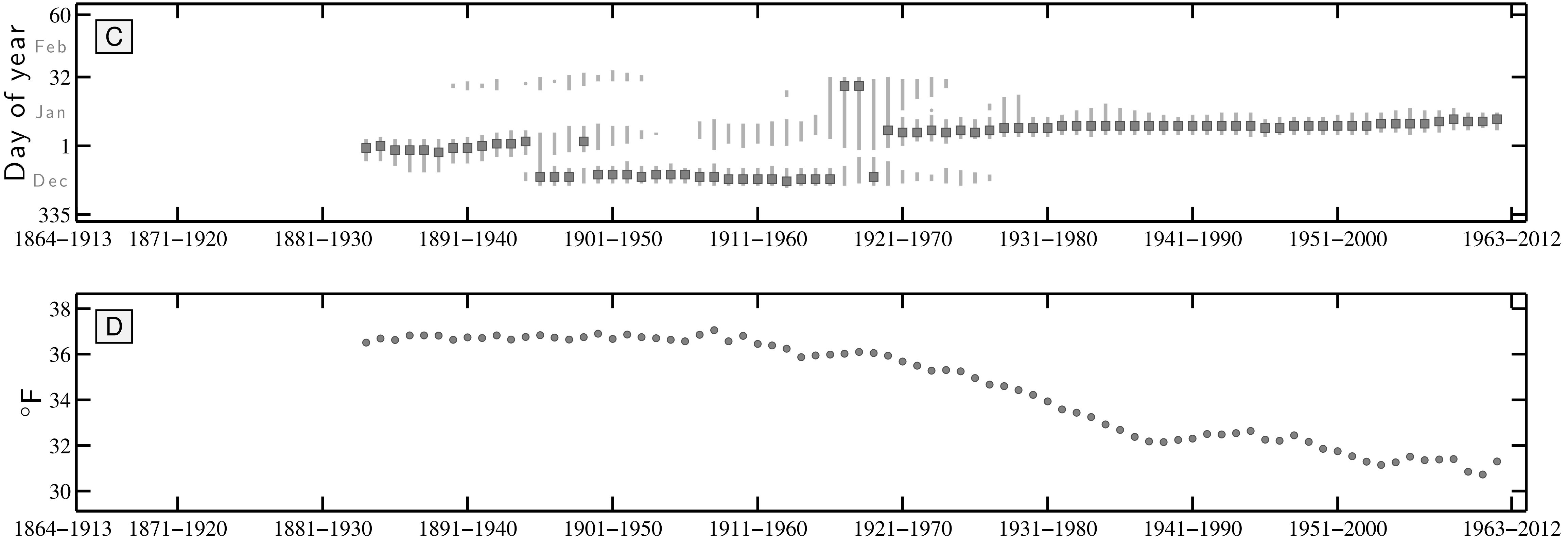

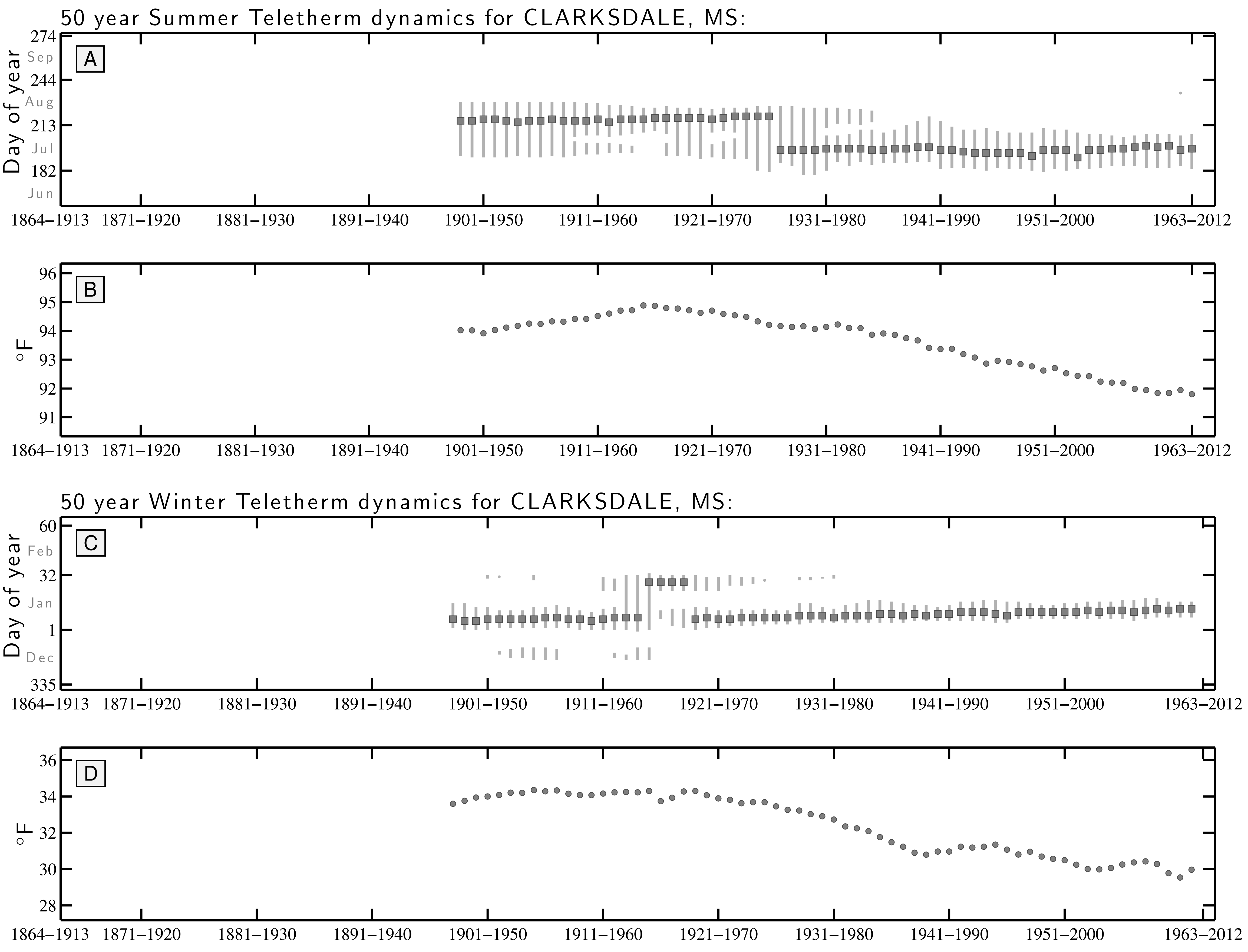

50 year Summer Teletherm dynamics for CLARKSDALE, MS:
A
Day of year
274
Sep
244
Aug
213
Jul
182
Jun
1864–1913
1871–1920
1881–1930
1891–1940
1901–1950
1911–1960
1921–1970
1931–1980
1941–1990
1951–2000
1963–2012
B
°F
96
95
94
93
92
91
50 year Winter Teletherm dynamics for CLARKSDALE, MS:
C
60
Feb
32
Jan
1
Dec
335
D
36
34
32
30
28

50 year Summer Teletherm dynamics for COLUMBIA, MS:

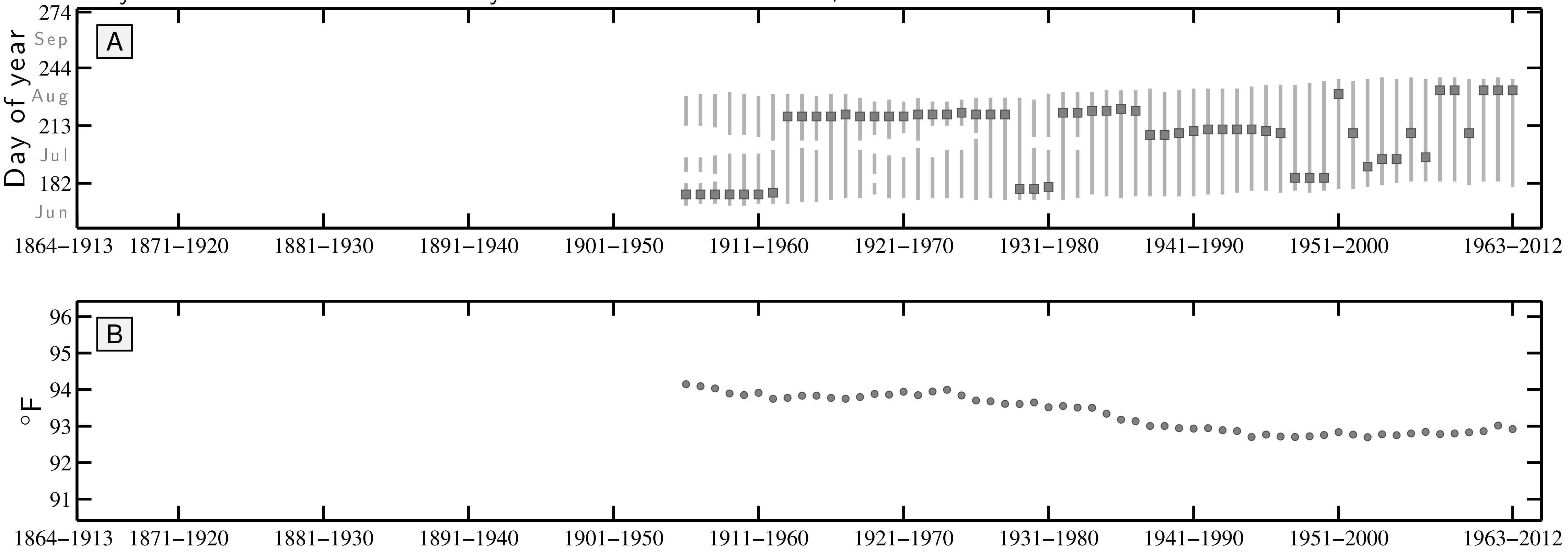

50 year Winter Teletherm dynamics for COLUMBIA, MS:

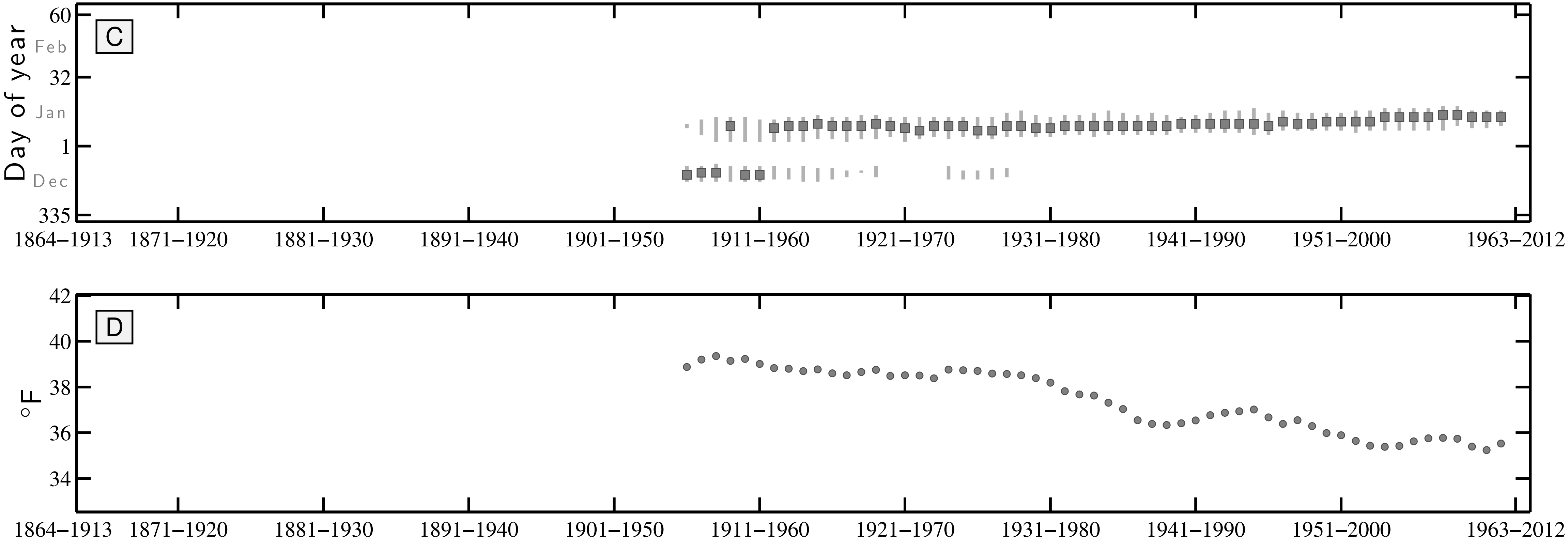

50 year Summer Teletherm dynamics for CORINTH 7 SW, MS:

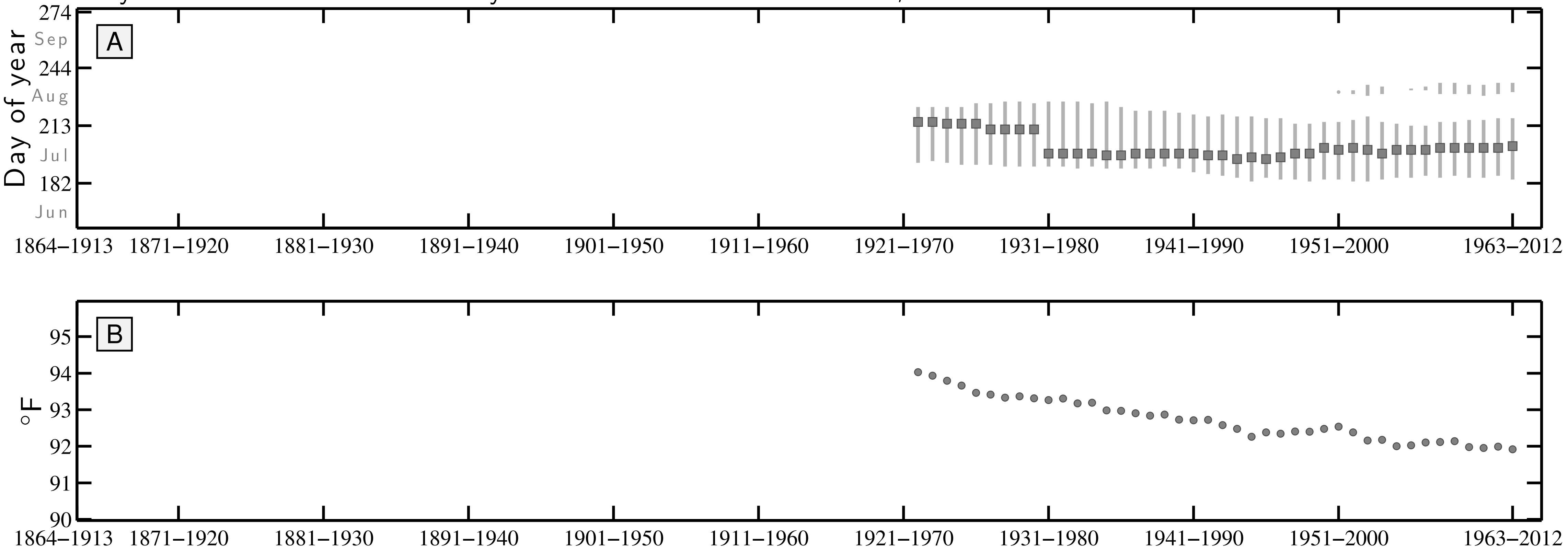

50 year Winter Teletherm dynamics for CORINTH 7 SW, MS:

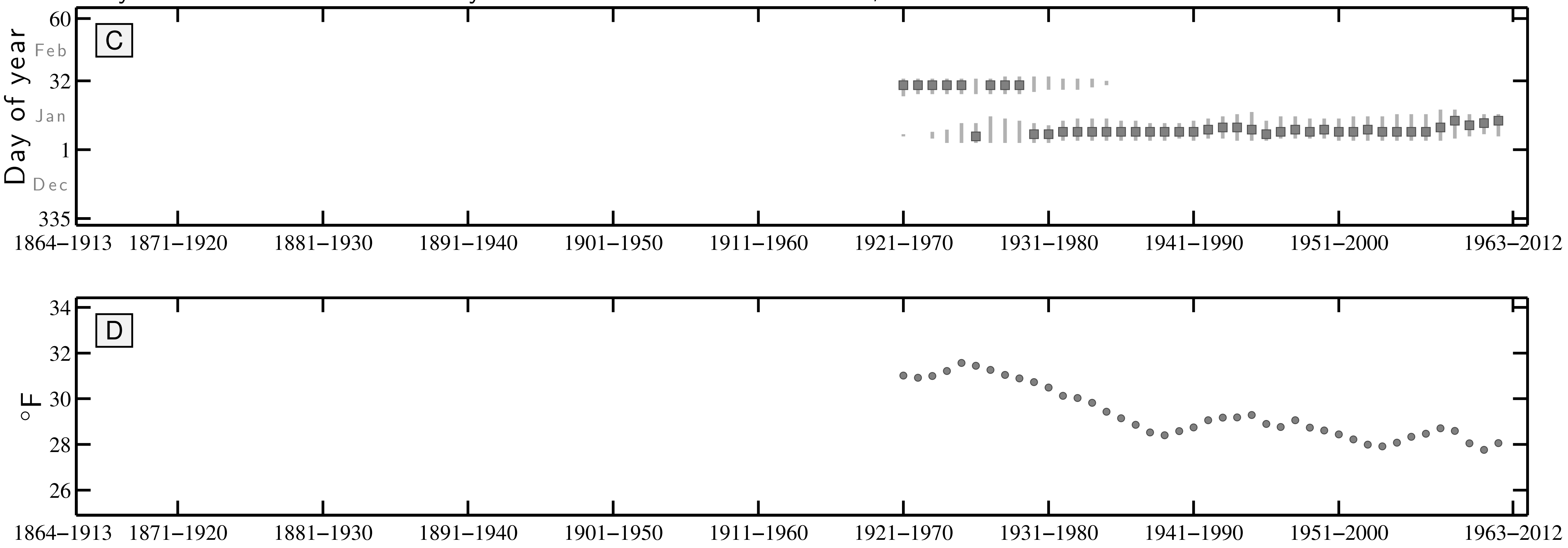

50 year Summer Teletherm dynamics for CRYSTAL SPGS EXP STN, MS:

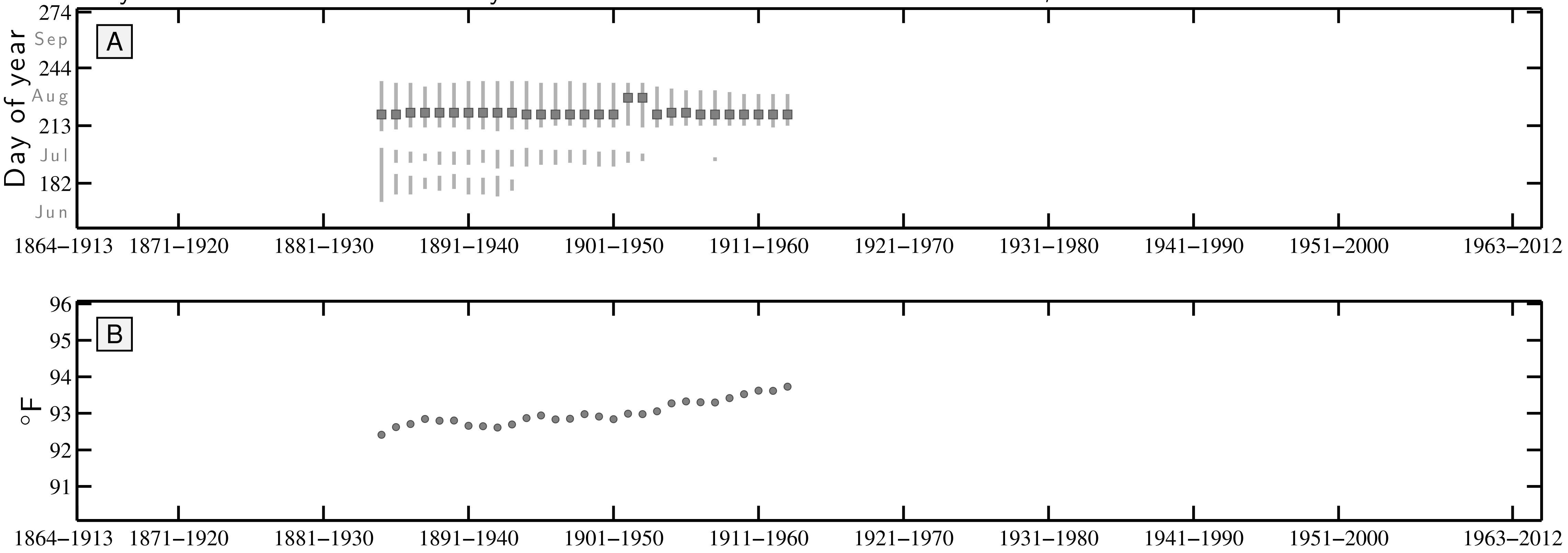

50 year Winter Teletherm dynamics for CRYSTAL SPGS EXP STN, MS:

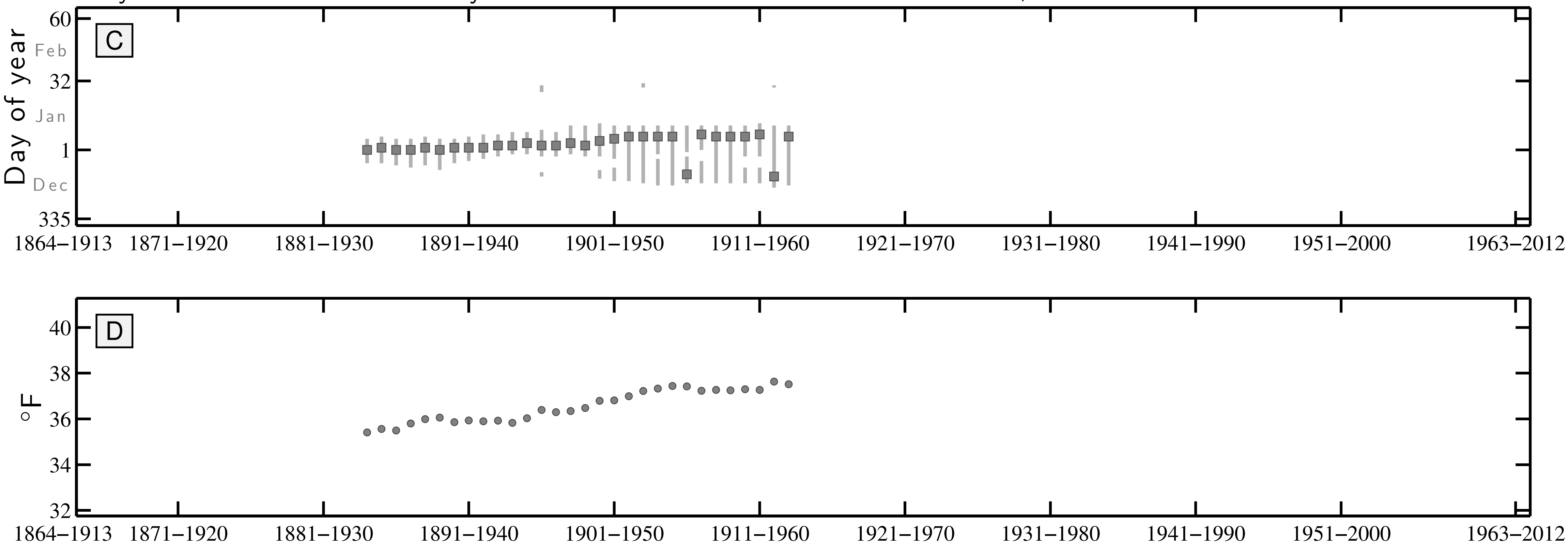

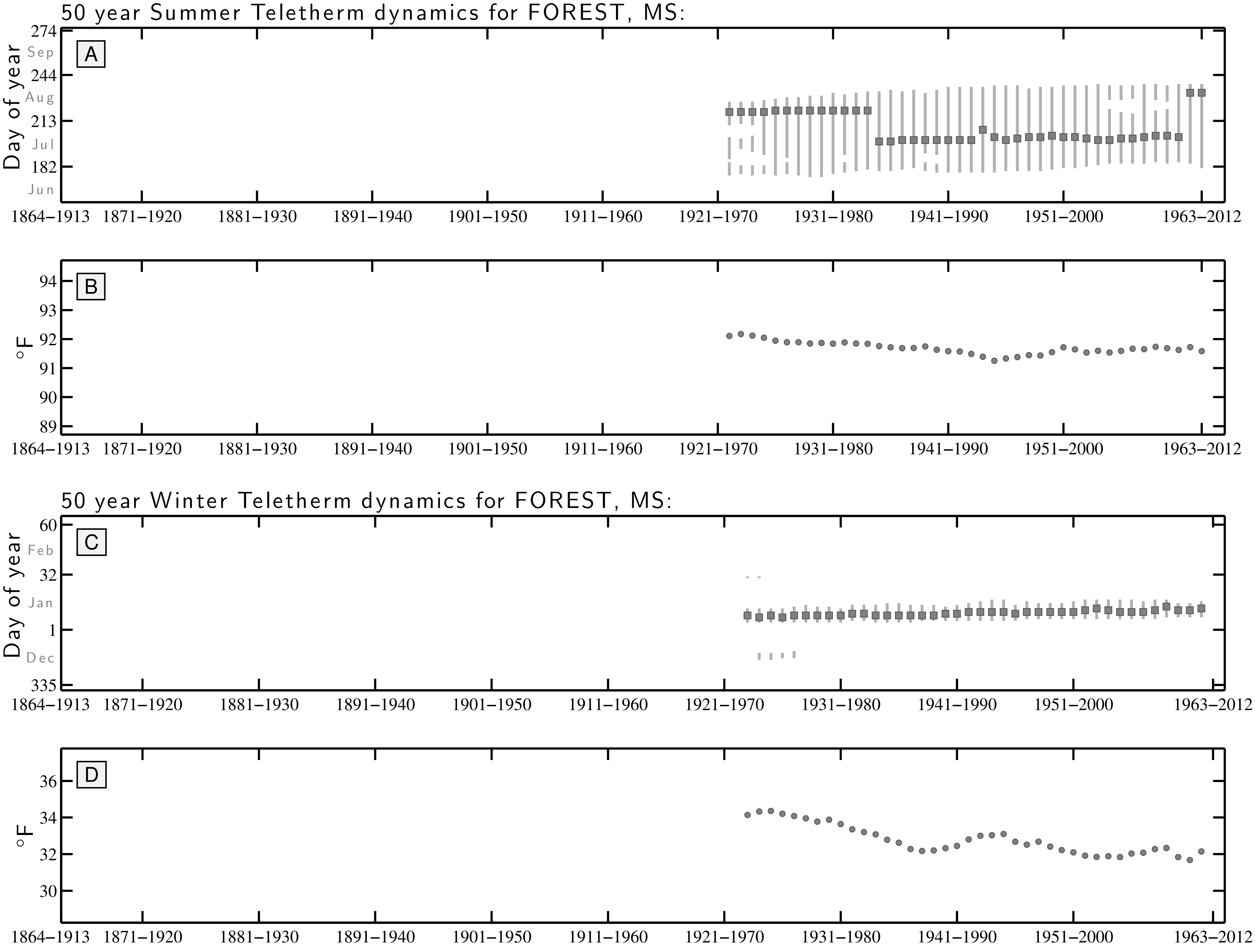

50 year Summer Teletherm dynamics for FOREST, MS:
A
Day of year
274
Sep
244
Aug
213
Jul
182
Jun
1864–1913
1871–1920
1881–1930
1891–1940
1901–1950
1911–1960
1921–1970
1931–1980
1941–1990
1951–2000
1963–2012
B
°F
94
93
92
91
90
89
50 year Winter Teletherm dynamics for FOREST, MS:
C
Day of year
60
Feb
32
Jan
1
Dec
335
D
°F
36
34
32
30

50 year Summer Teletherm dynamics for GREENVILLE, MS:

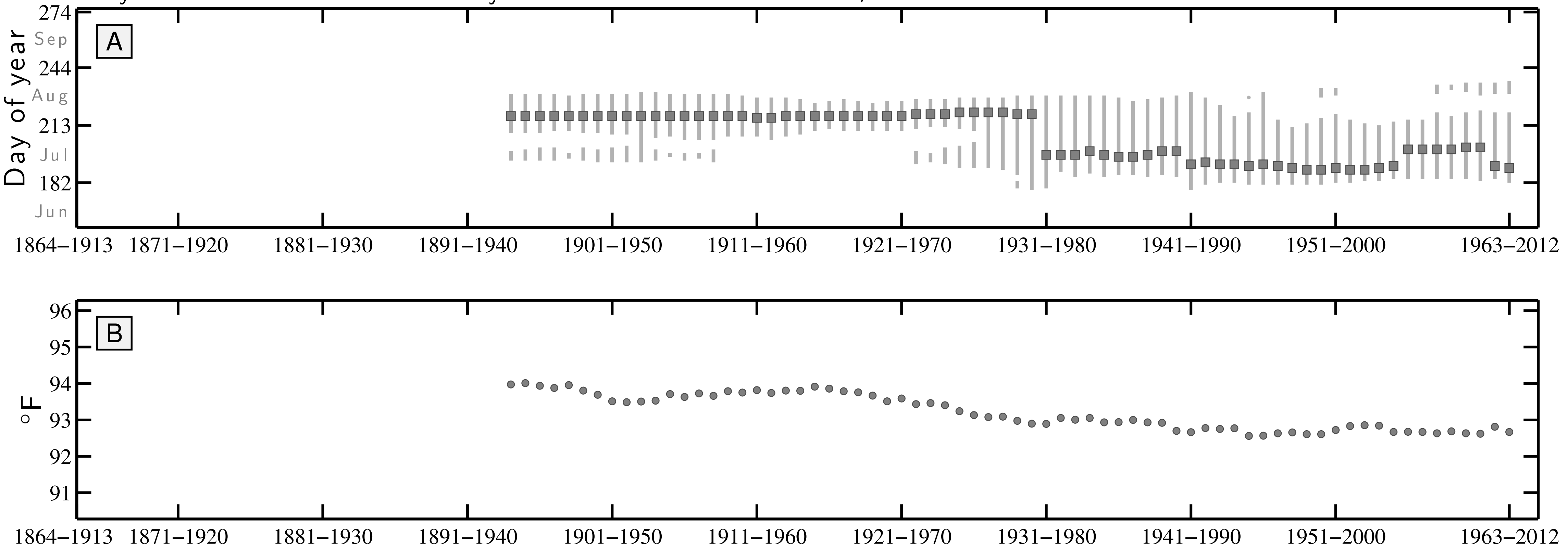

50 year Winter Teletherm dynamics for GREENVILLE, MS:

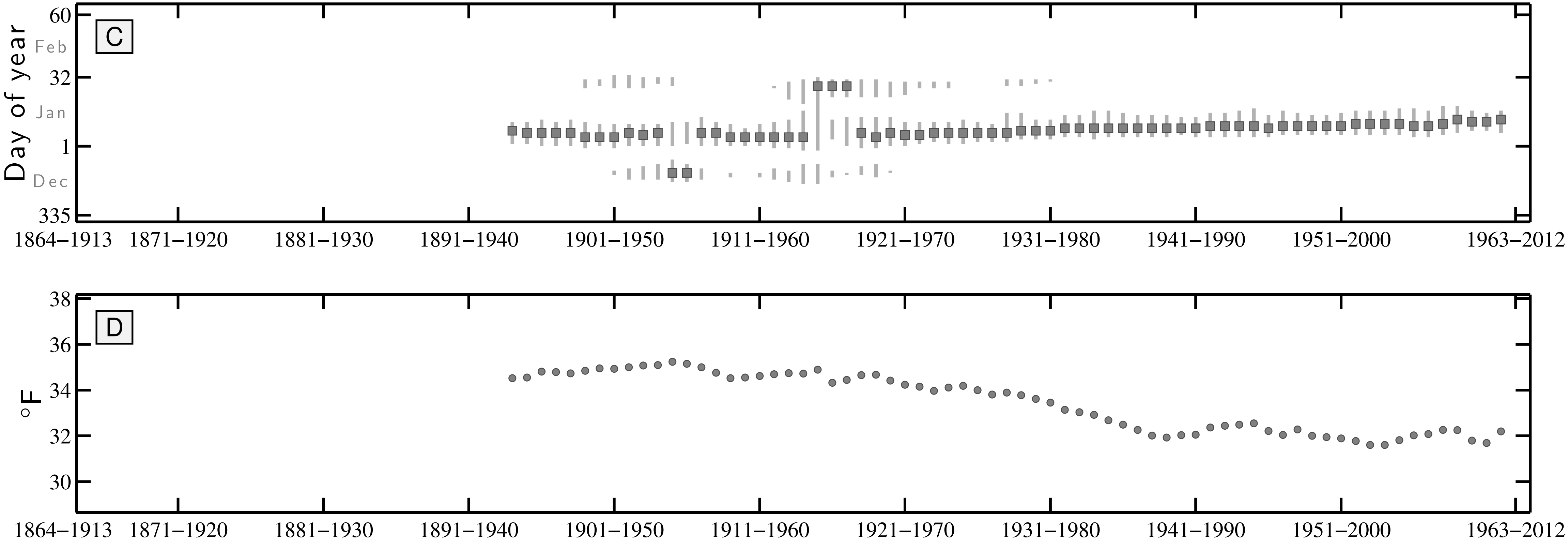

50 year Summer Teletherm dynamics for HATTIESBURG 5SW, MS:

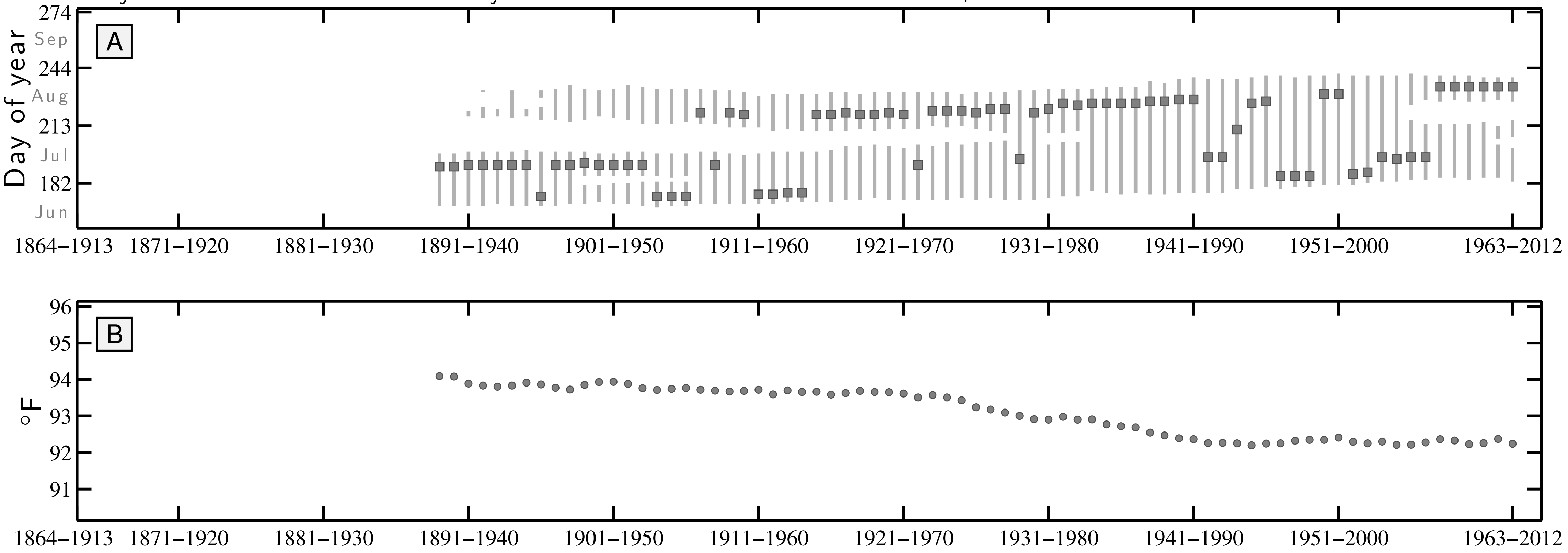

50 year Winter Teletherm dynamics for HATTIESBURG 5SW, MS:

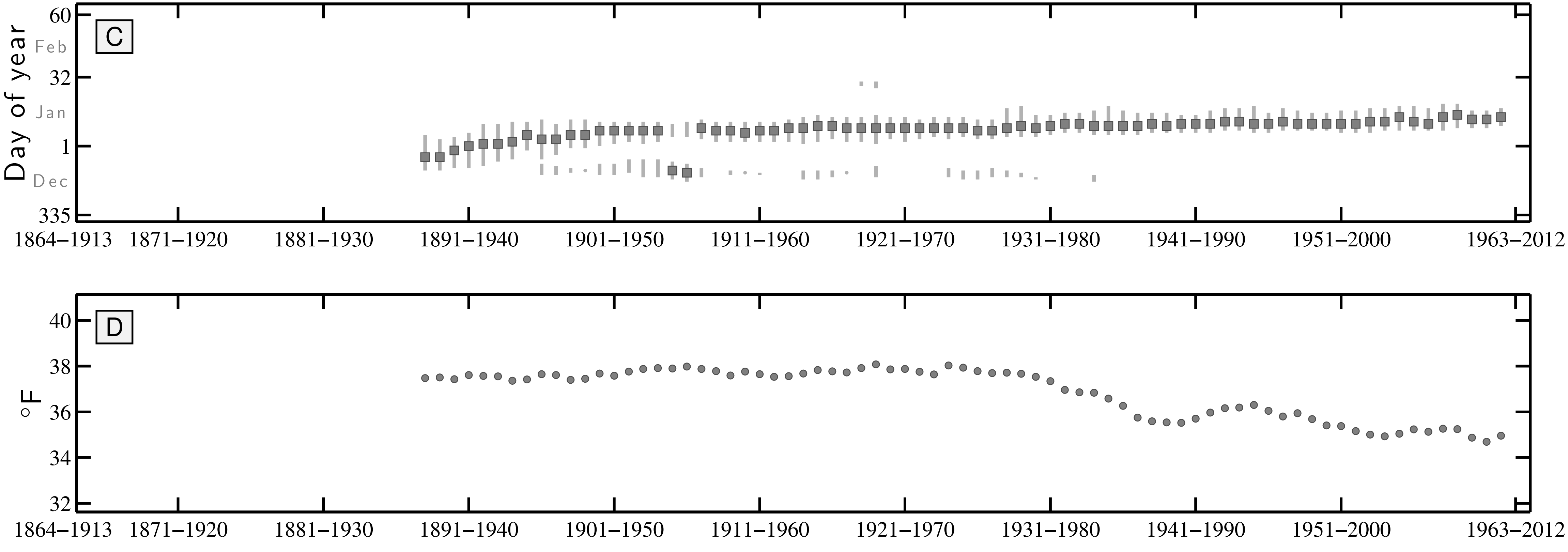

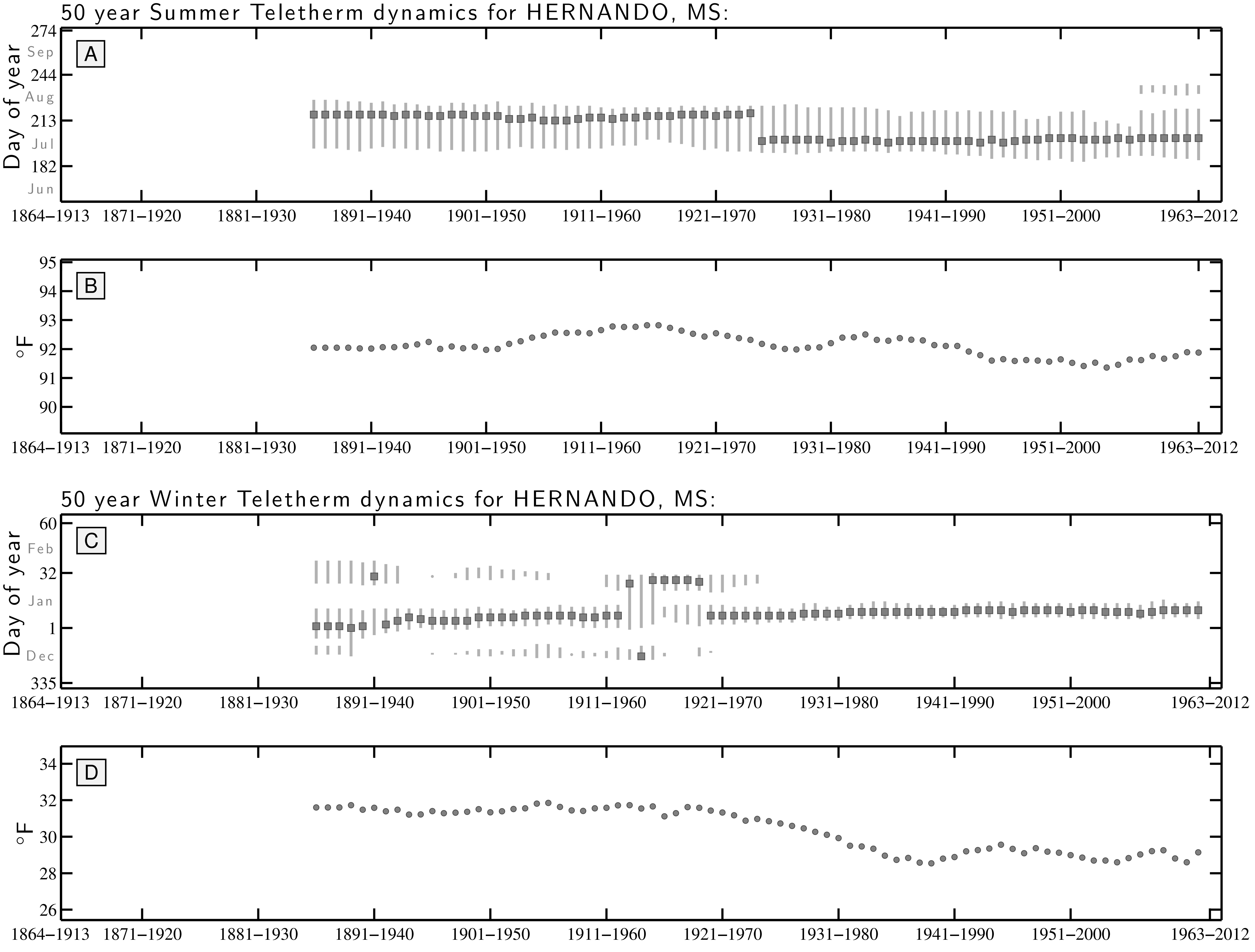

50 year Summer Teletherm dynamics for HERNANDO, MS:
A
Day of year
274
Sep
244
Aug
213
Jul
182
Jun
1864–1913
1871–1920
1881–1930
1891–1940
1901–1950
1911–1960
1921–1970
1931–1980
1941–1990
1951–2000
1963–2012
B
°F
95
94
93
92
91
90
50 year Winter Teletherm dynamics for HERNANDO, MS:
C
Day of year
60
Feb
32
Jan
1
Dec
335
D
°F
34
32
30
28
26

50 year Summer Teletherm dynamics for HOLLY SPRINGS 4 N, MS:

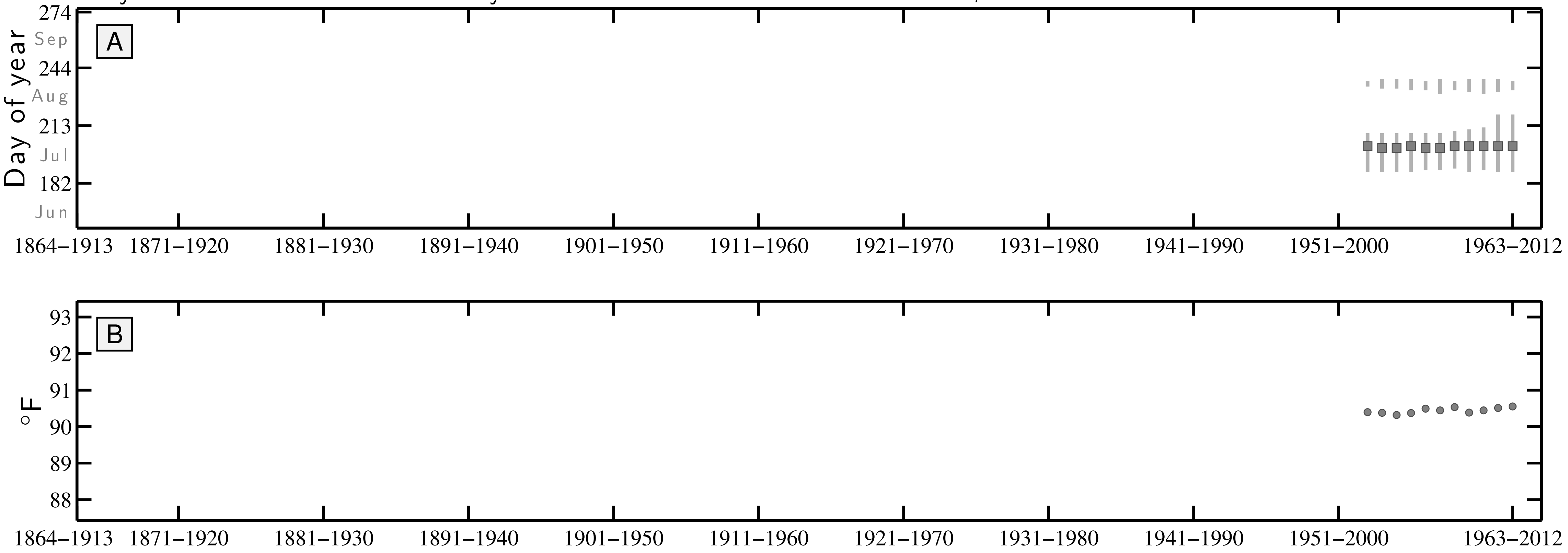

50 year Winter Teletherm dynamics for HOLLY SPRINGS 4 N, MS:

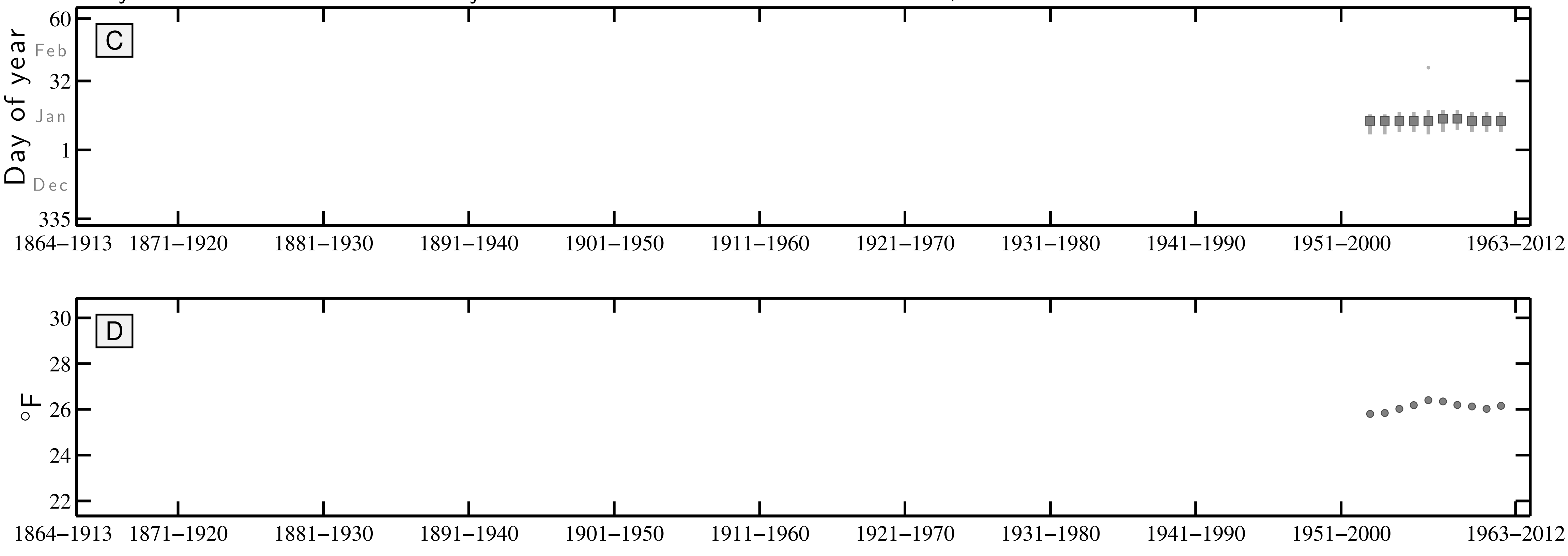

50 year Summer Teletherm dynamics for KOSCIUSKO, MS:

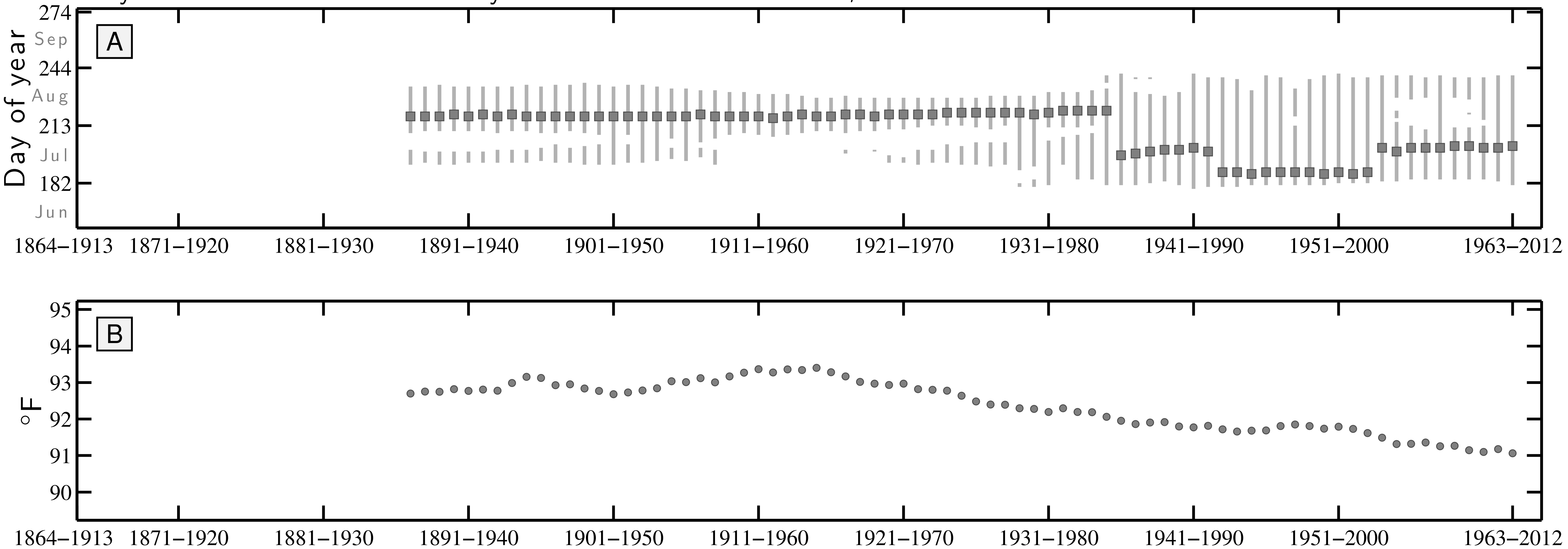

50 year Winter Teletherm dynamics for KOSCIUSKO, MS:

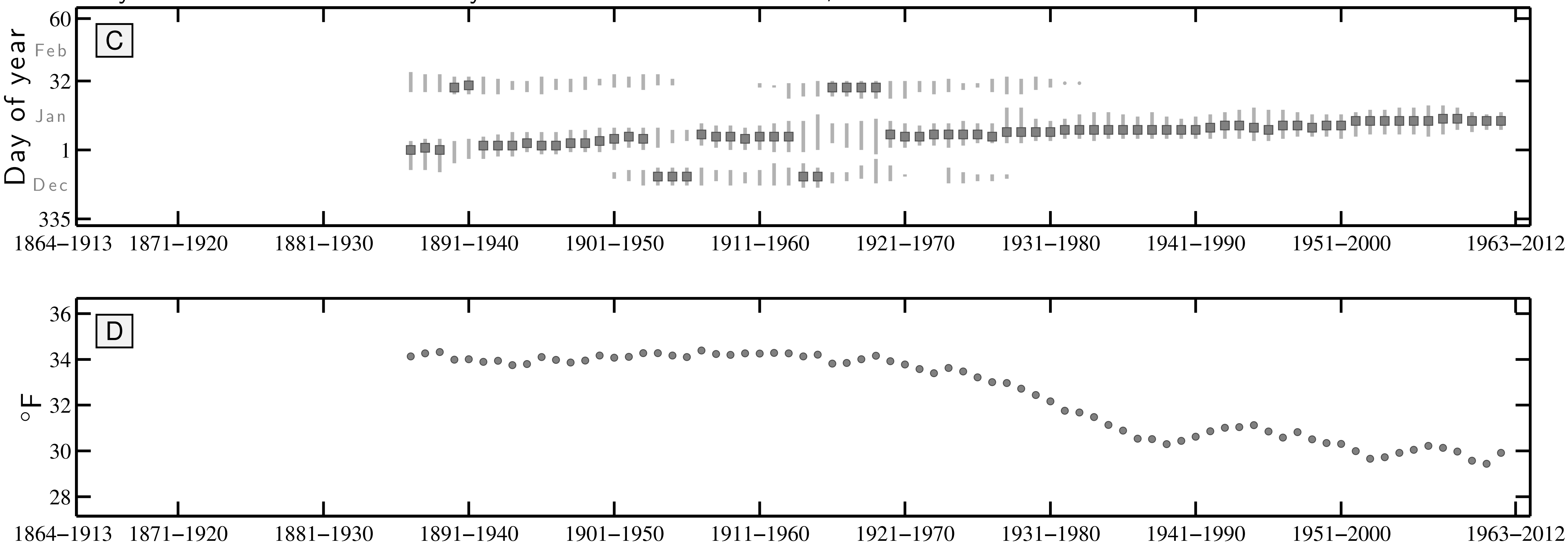

50 year Summer Teletherm dynamics for LAUREL, MS:

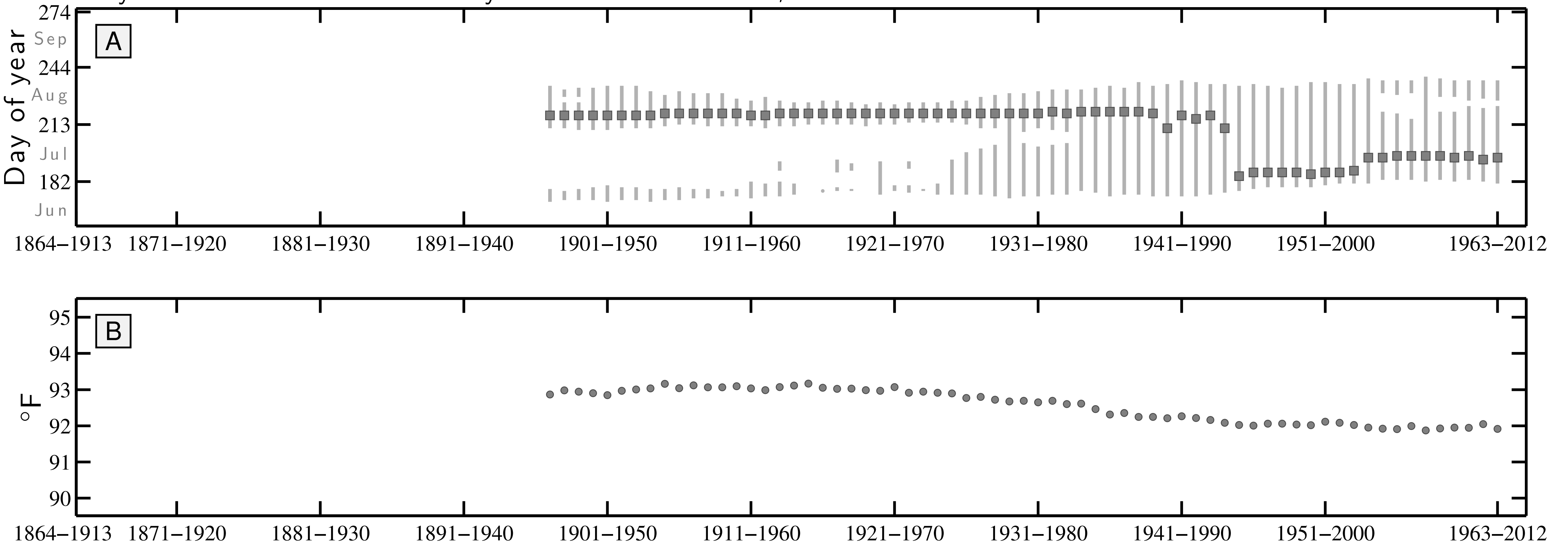

50 year Winter Teletherm dynamics for LAUREL, MS:

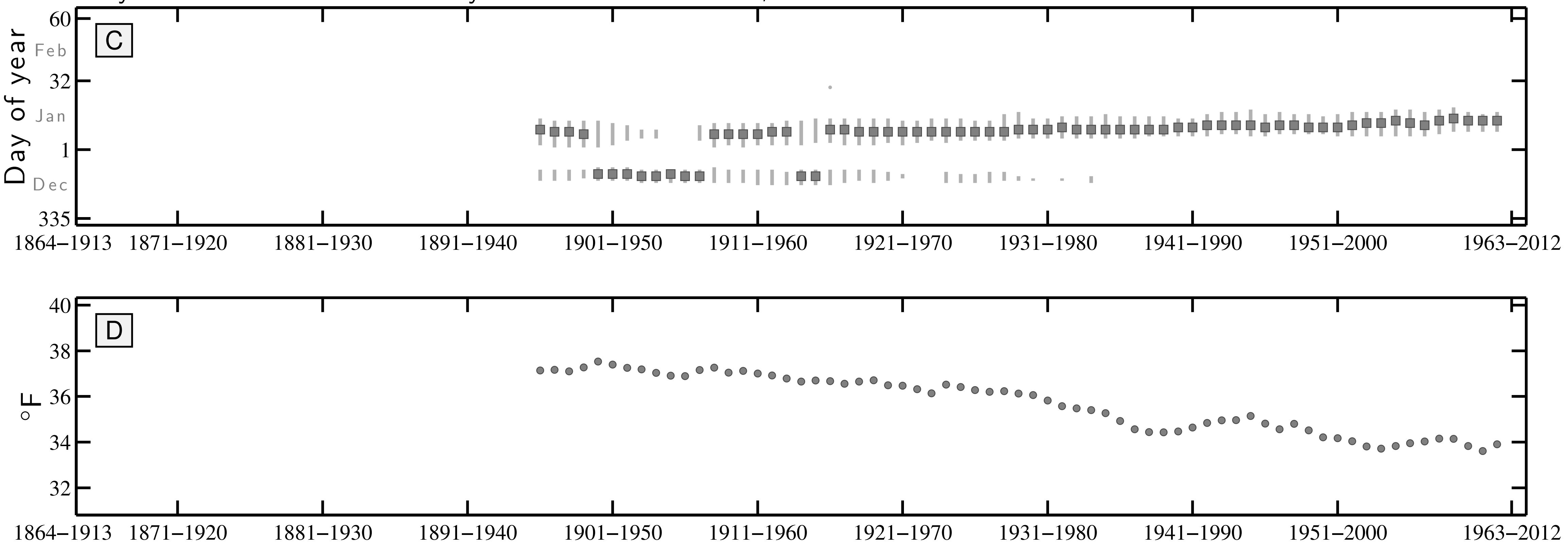

50 year Summer Teletherm dynamics for LOUISVILLE, MS:

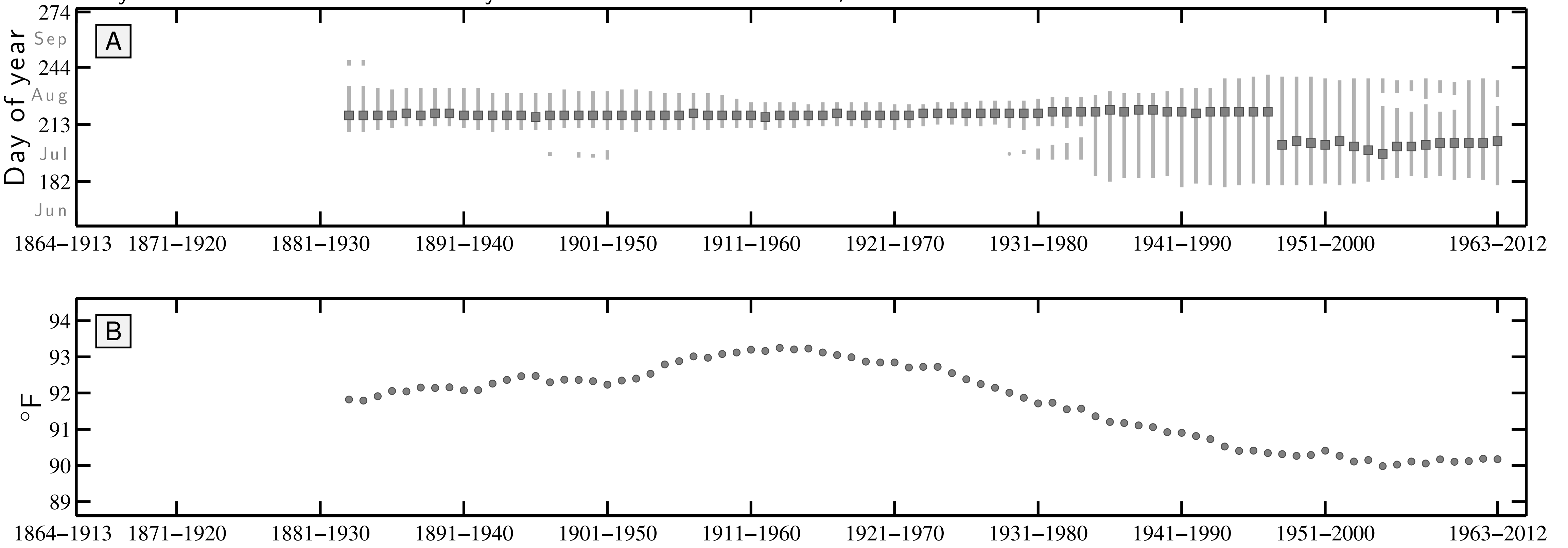

50 year Winter Teletherm dynamics for LOUISVILLE, MS:

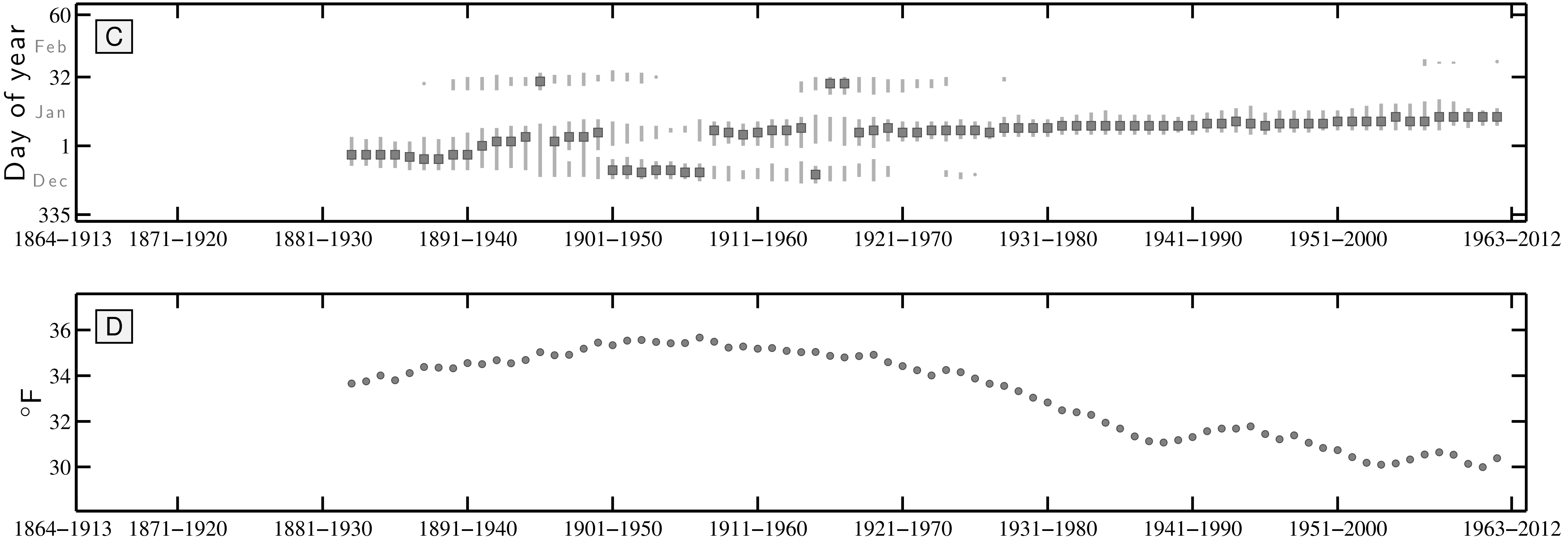

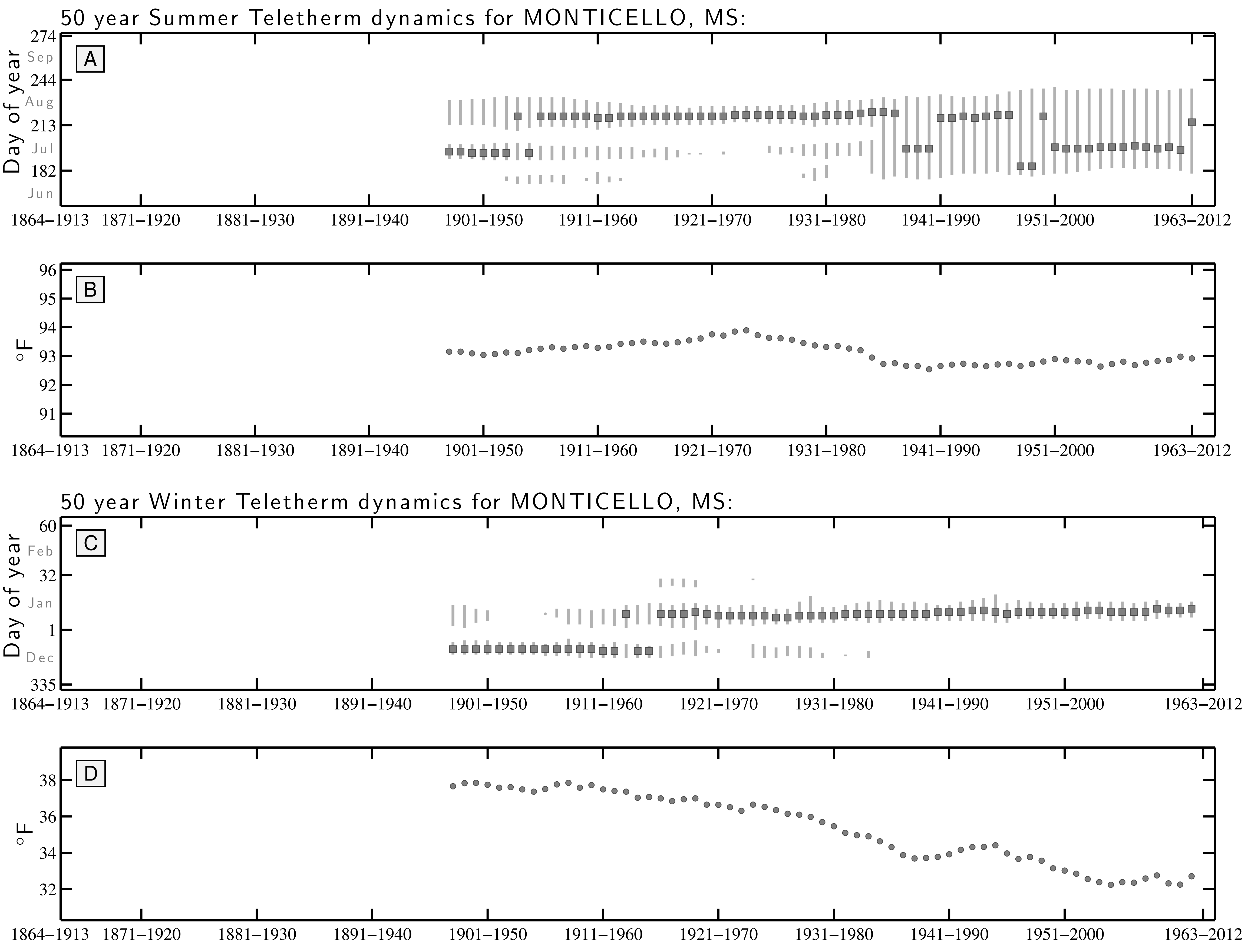
50 year Summer Teletherm dynamics for MONTICELLO, MS:
A
B
Day of year
°F
274
Sep
244
Aug
213
Jul
182
Jun
96
95
94
93
92
91
1864–1913
1871–1920
1881–1930
1891–1940
1901–1950
1911–1960
1921–1970
1931–1980
1941–1990
1951–2000
1963–2012
50 year Winter Teletherm dynamics for MONTICELLO, MS:
C
D
60
Feb
32
Jan
1
Dec
335
38
36
34
32

50 year Summer Teletherm dynamics for MOORHEAD, MS:

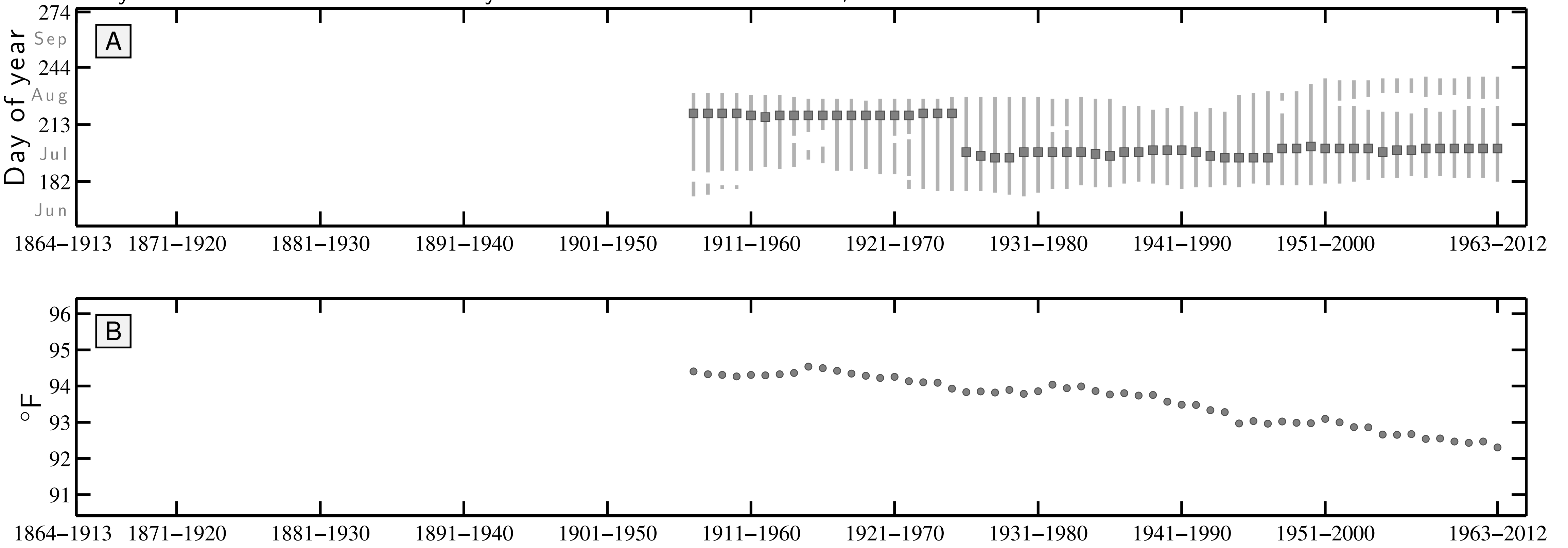

50 year Winter Teletherm dynamics for MOORHEAD, MS:

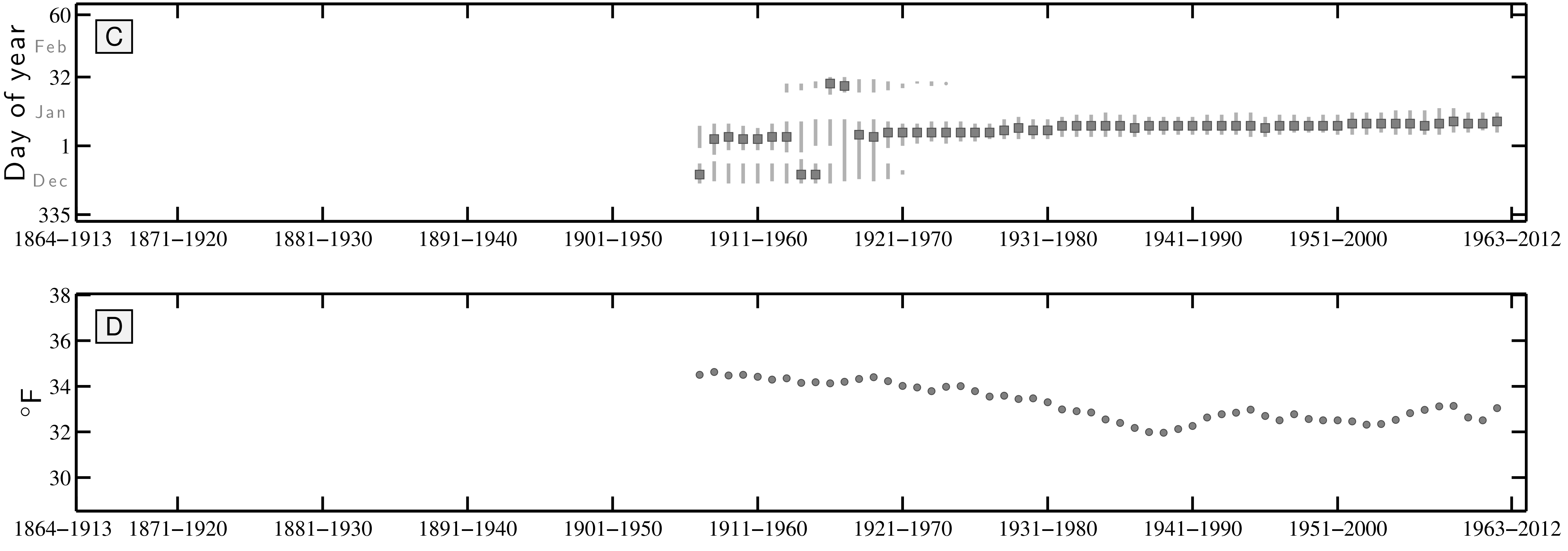

50 year Summer Teletherm dynamics for NATCHEZ, MS:

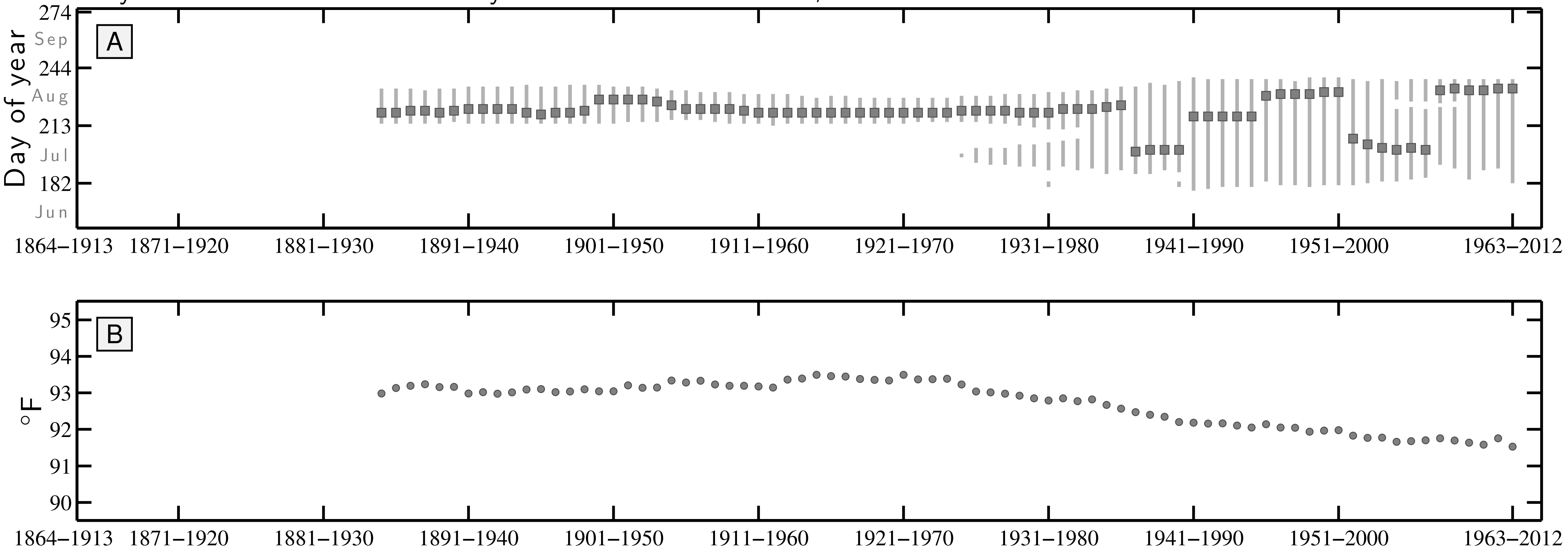

50 year Winter Teletherm dynamics for NATCHEZ, MS:

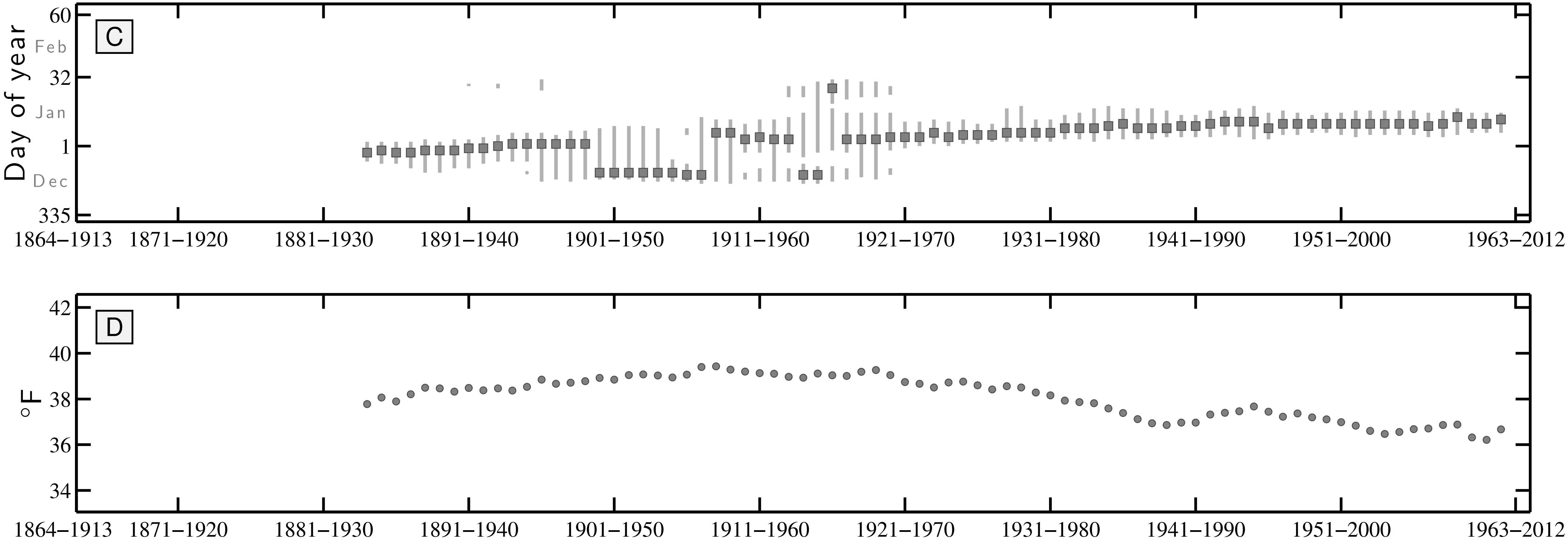

50 year Summer Teletherm dynamics for PASCAGOULA 3 NE, MS:

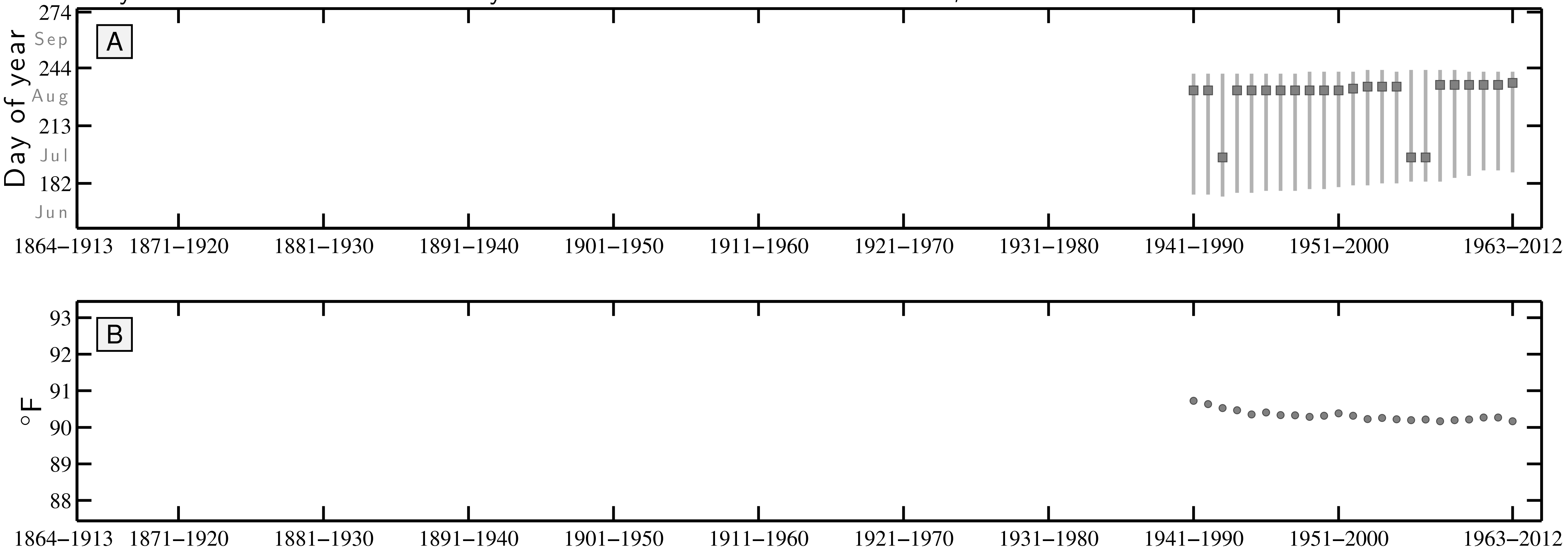

50 year Winter Teletherm dynamics for PASCAGOULA 3 NE, MS:

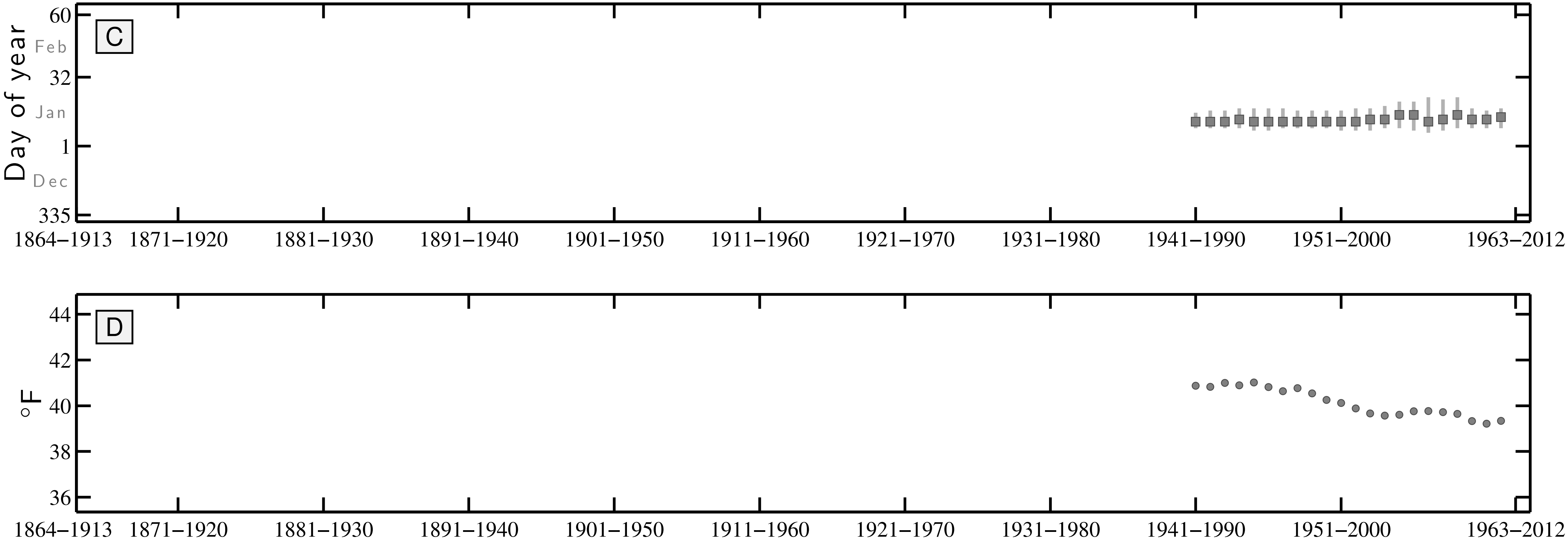

50 year Summer Teletherm dynamics for PONTOTOC EXP STN, MS:

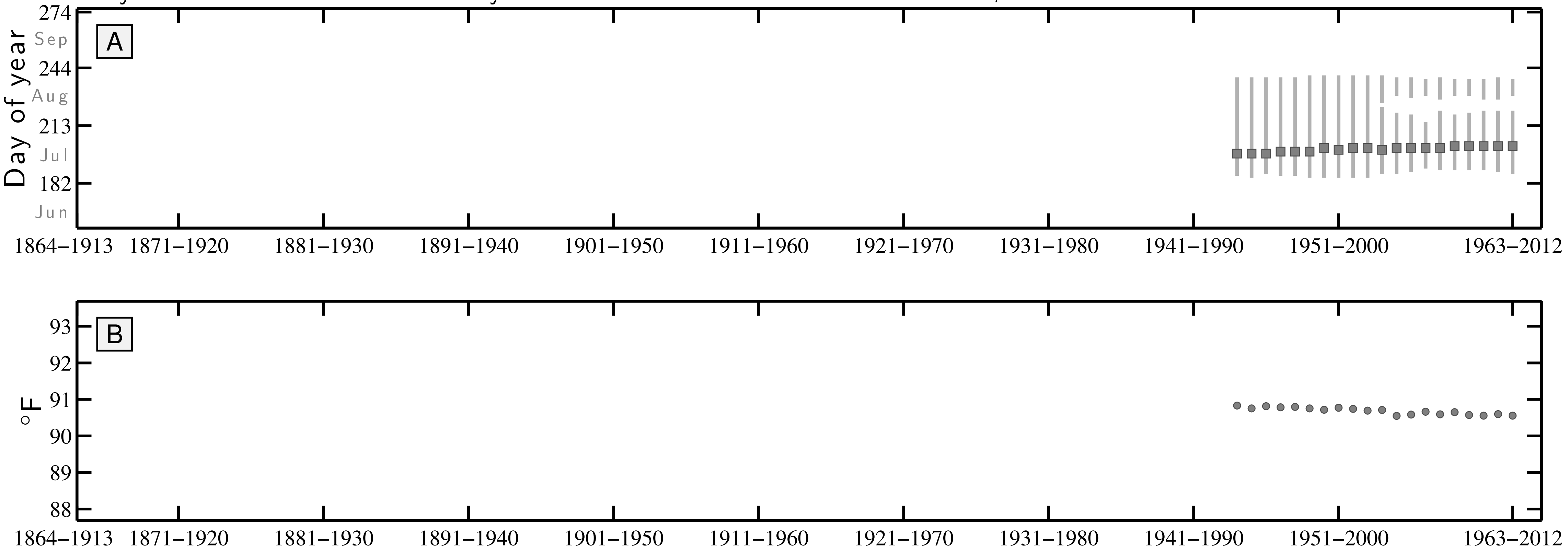

50 year Winter Teletherm dynamics for PONTOTOC EXP STN, MS:

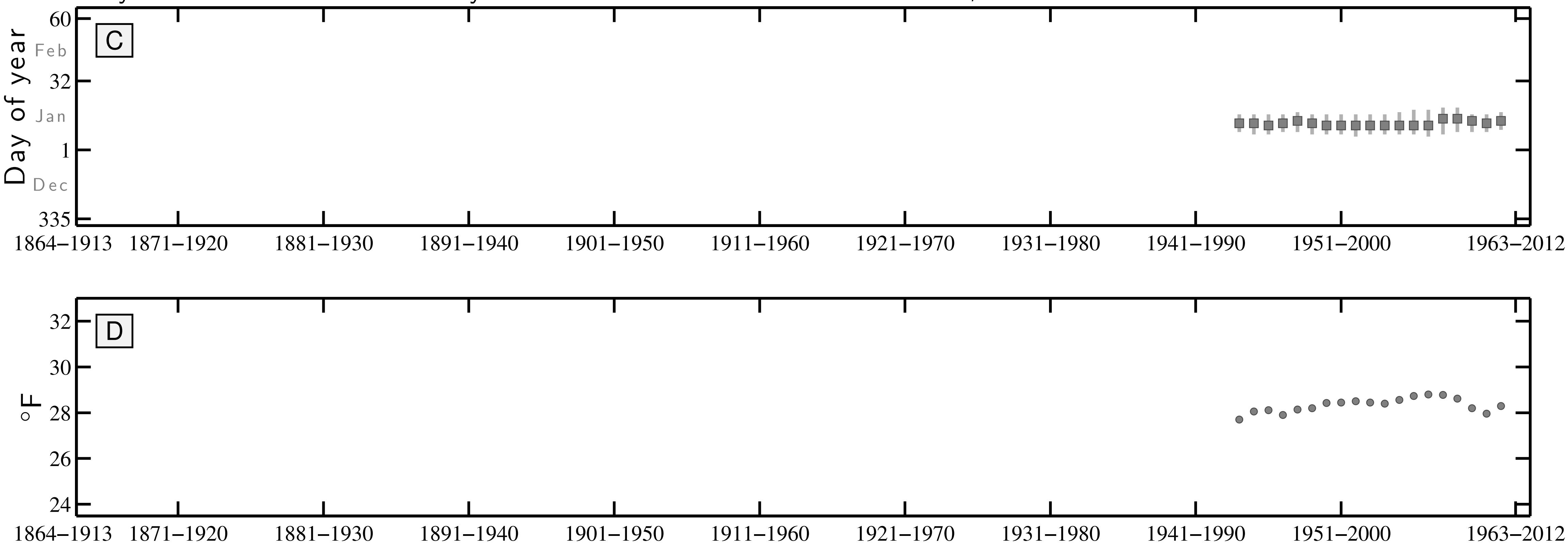

50 year Summer Teletherm dynamics for POPLARVILLE EXP STN, MS:

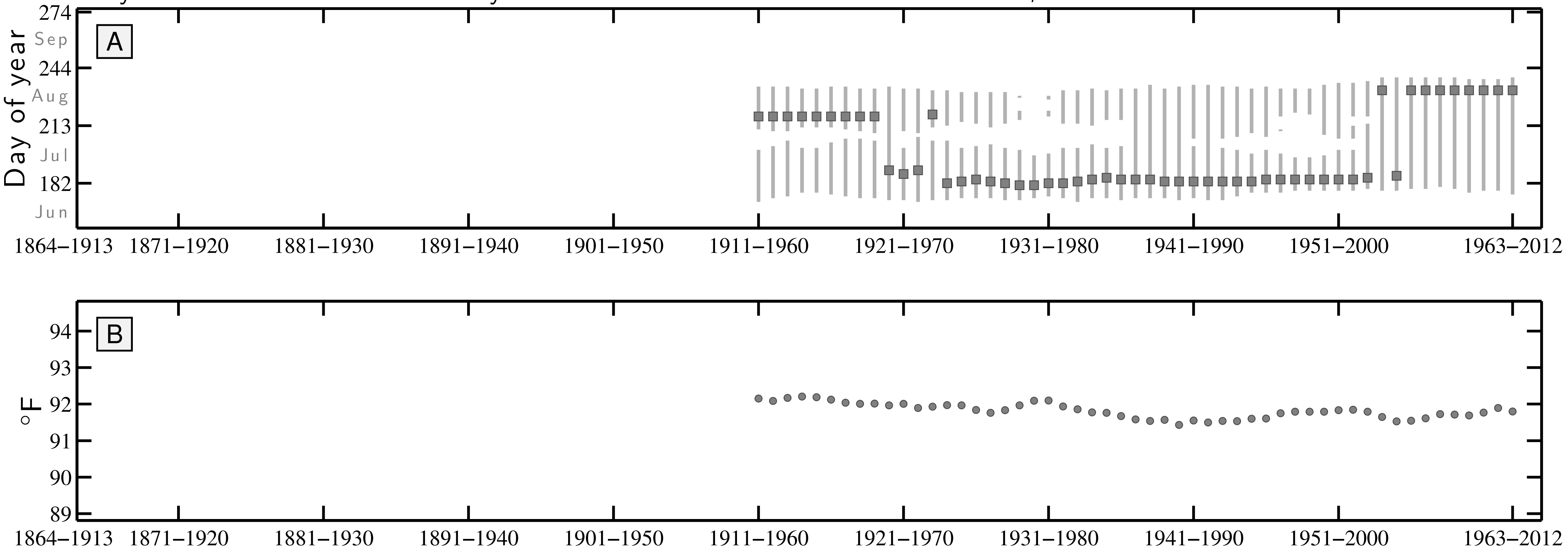

50 year Winter Teletherm dynamics for POPLARVILLE EXP STN, MS:

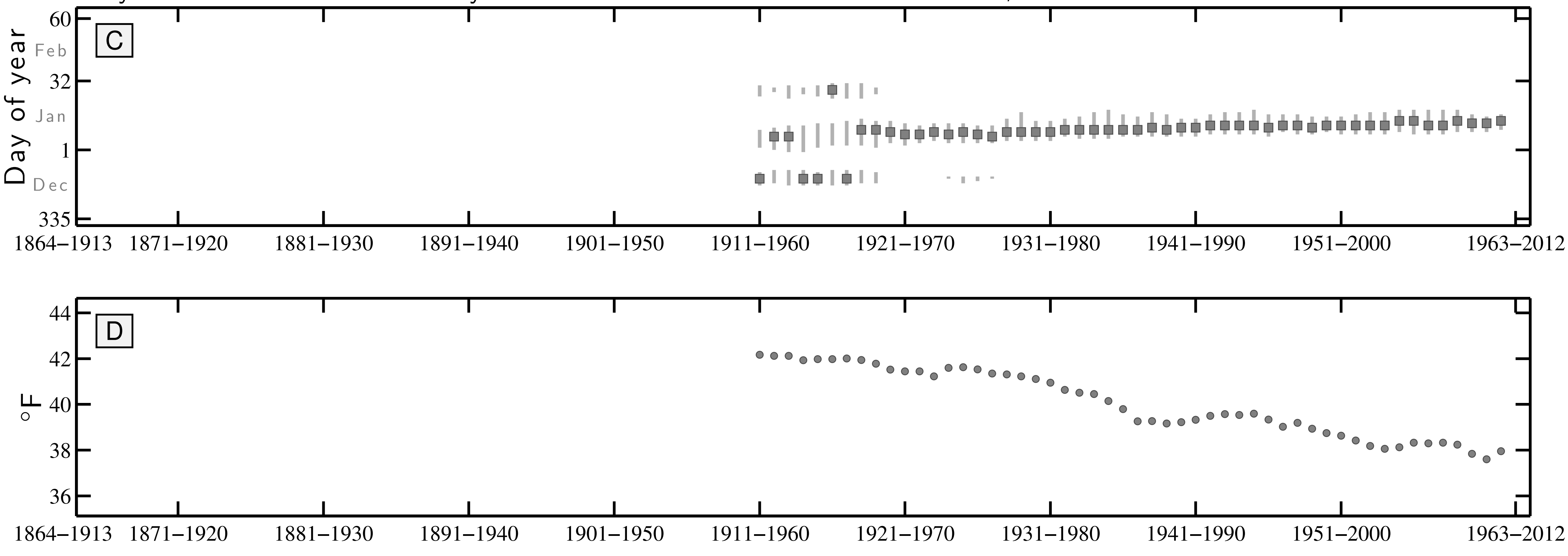

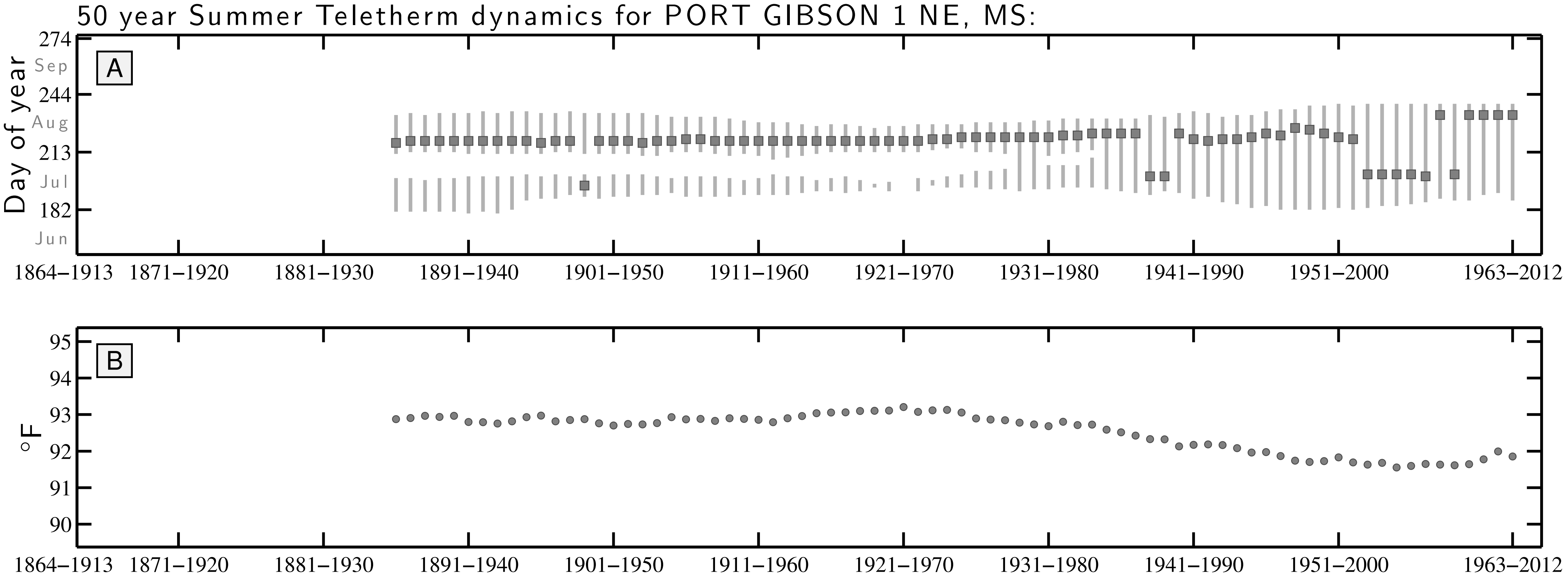
50 year Summer Teletherm dynamics for PORT GIBSON 1 NE, MS:
A
B
Day of year
274
Sep
244
Aug
213
Jul
182
Jun
°F
95
94
93
92
91
90
1864–1913
1871–1920
1881–1930
1891–1940
1901–1950
1911–1960
1921–1970
1931–1980
1941–1990
1951–2000
1963–2012

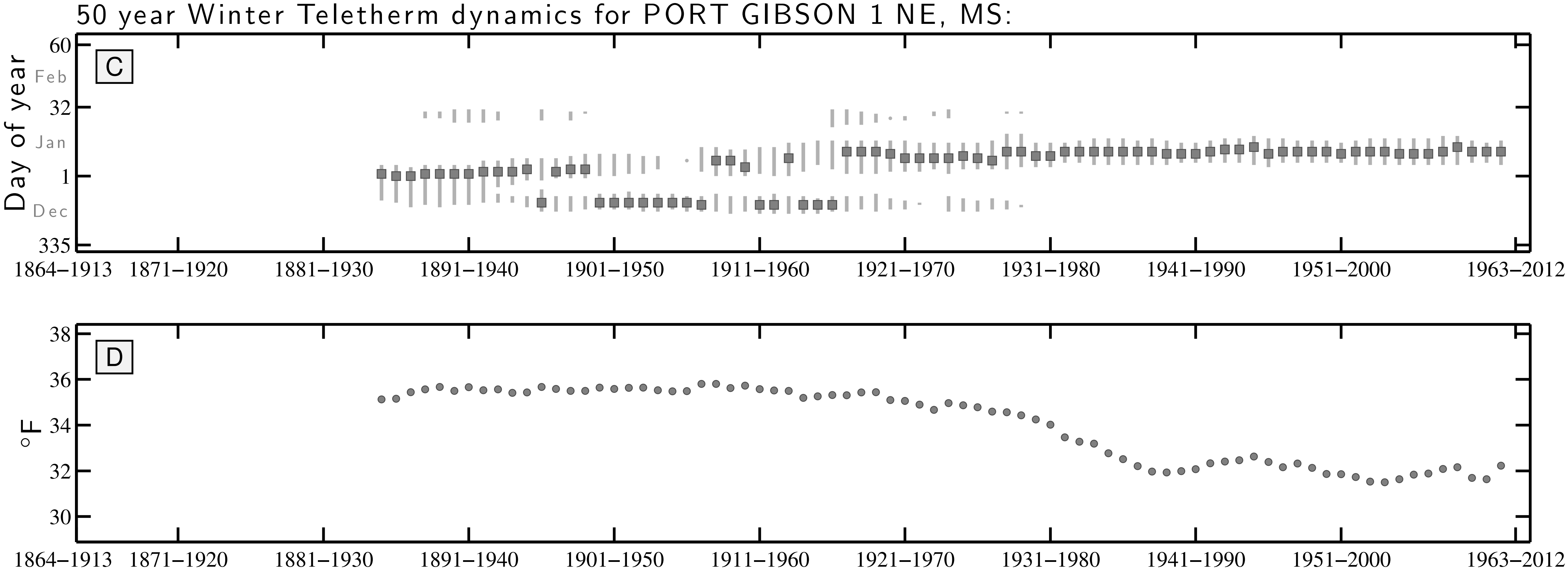
50 year Winter Teletherm dynamics for PORT GIBSON 1 NE, MS:
C
D
Day of year
60
Feb
32
Jan
1
Dec
335
°F
38
36
34
32
30
1864–1913
1871–1920
1881–1930
1891–1940
1901–1950
1911–1960
1921–1970
1931–1980
1941–1990
1951–2000
1963–2012

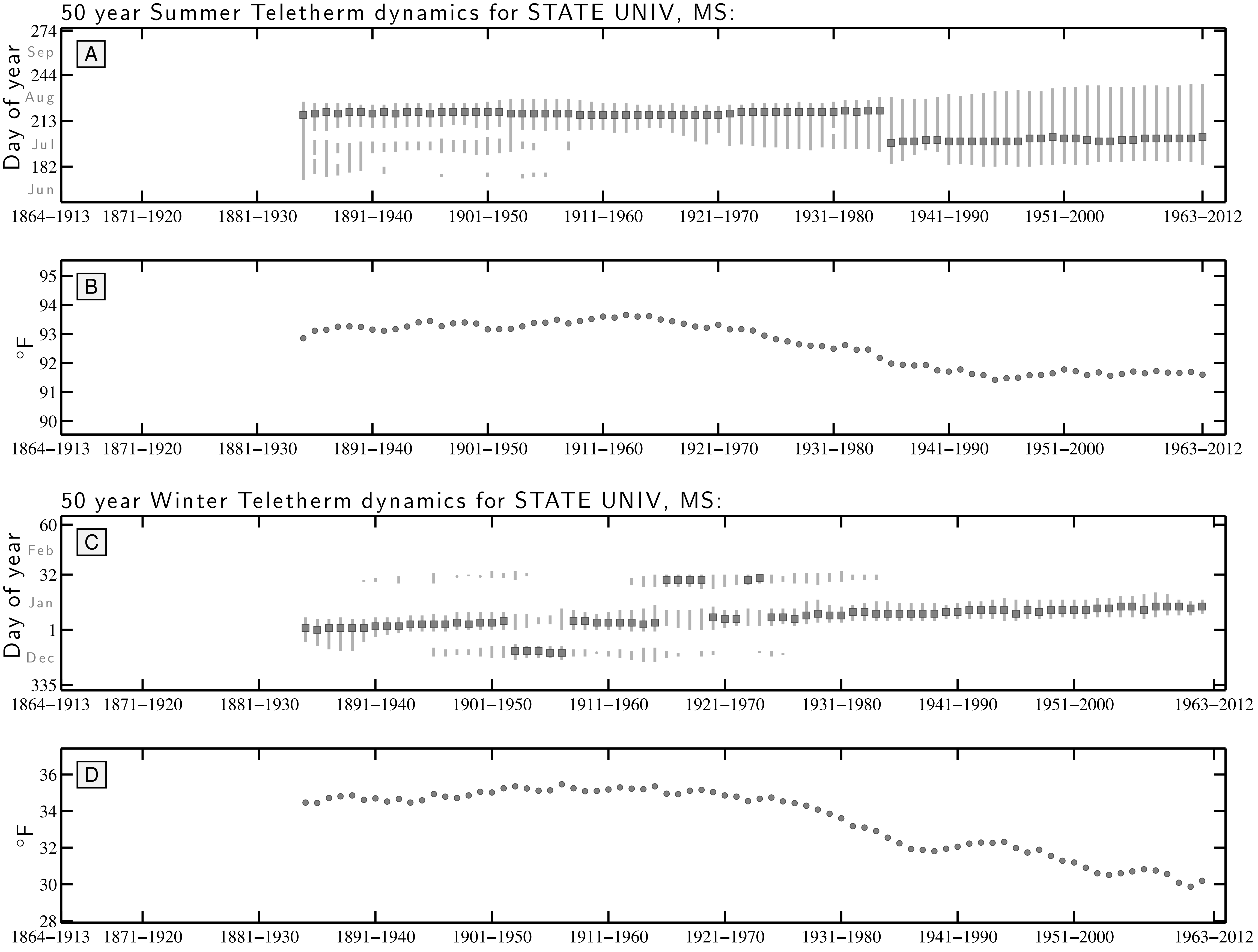

50 year Summer Teletherm dynamics for STATE UNIV, MS:
A
B
50 year Winter Teletherm dynamics for STATE UNIV, MS:
C
D
Day of year
°F
274
244
213
182
Sep
Aug
Jul
Jun
95
94
93
92
91
90
60
32
1
335
Feb
Jan
Dec
36
34
32
30
28
1864–1913
1871–1920
1881–1930
1891–1940
1901–1950
1911–1960
1921–1970
1931–1980
1941–1990
1951–2000
1963–2012

50 year Summer Teletherm dynamics for UNIVERSITY, MS:

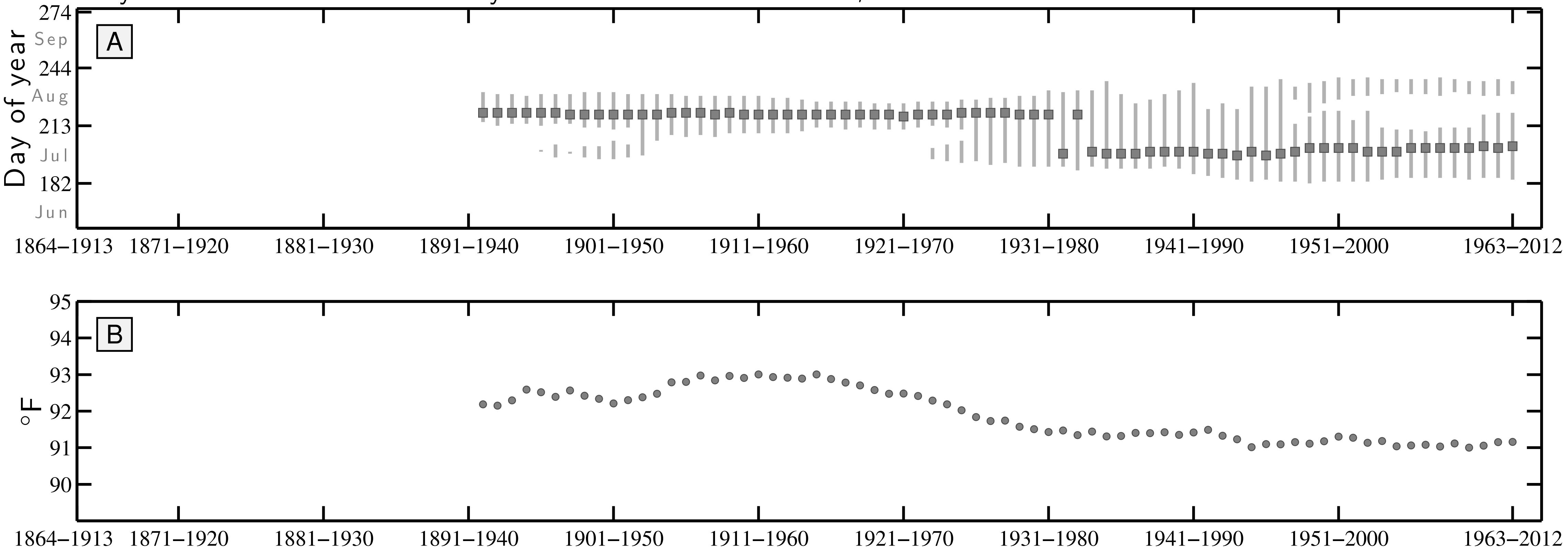

50 year Winter Teletherm dynamics for UNIVERSITY, MS:

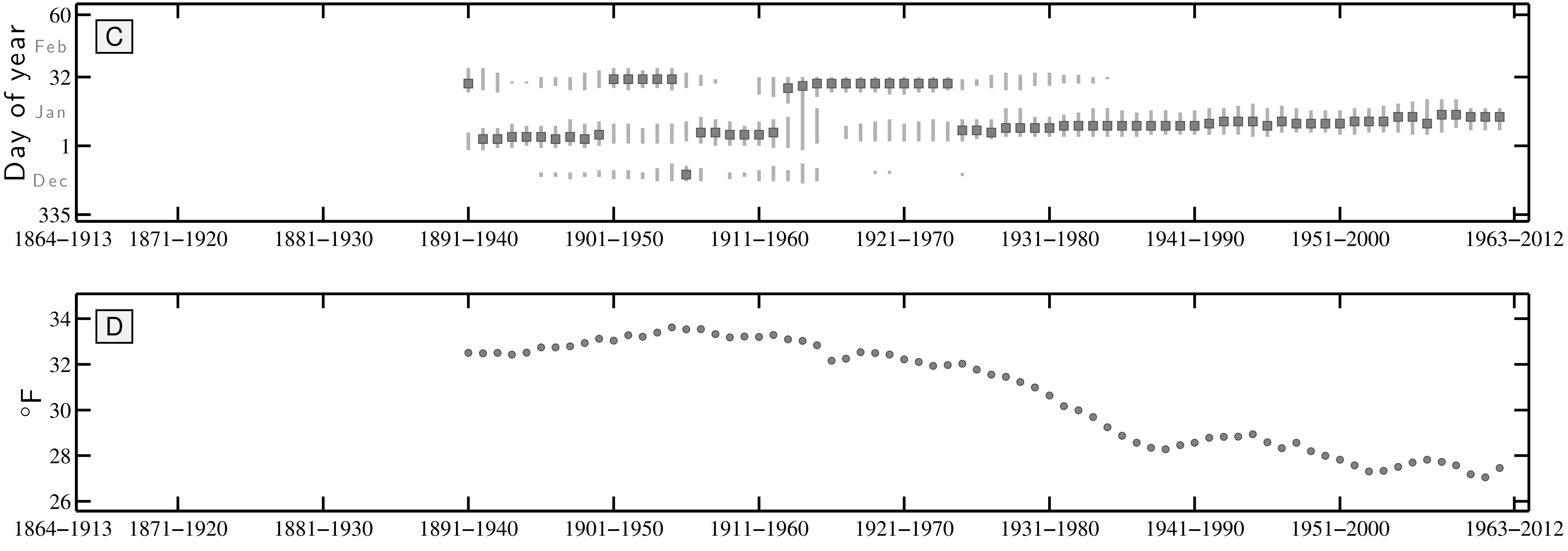

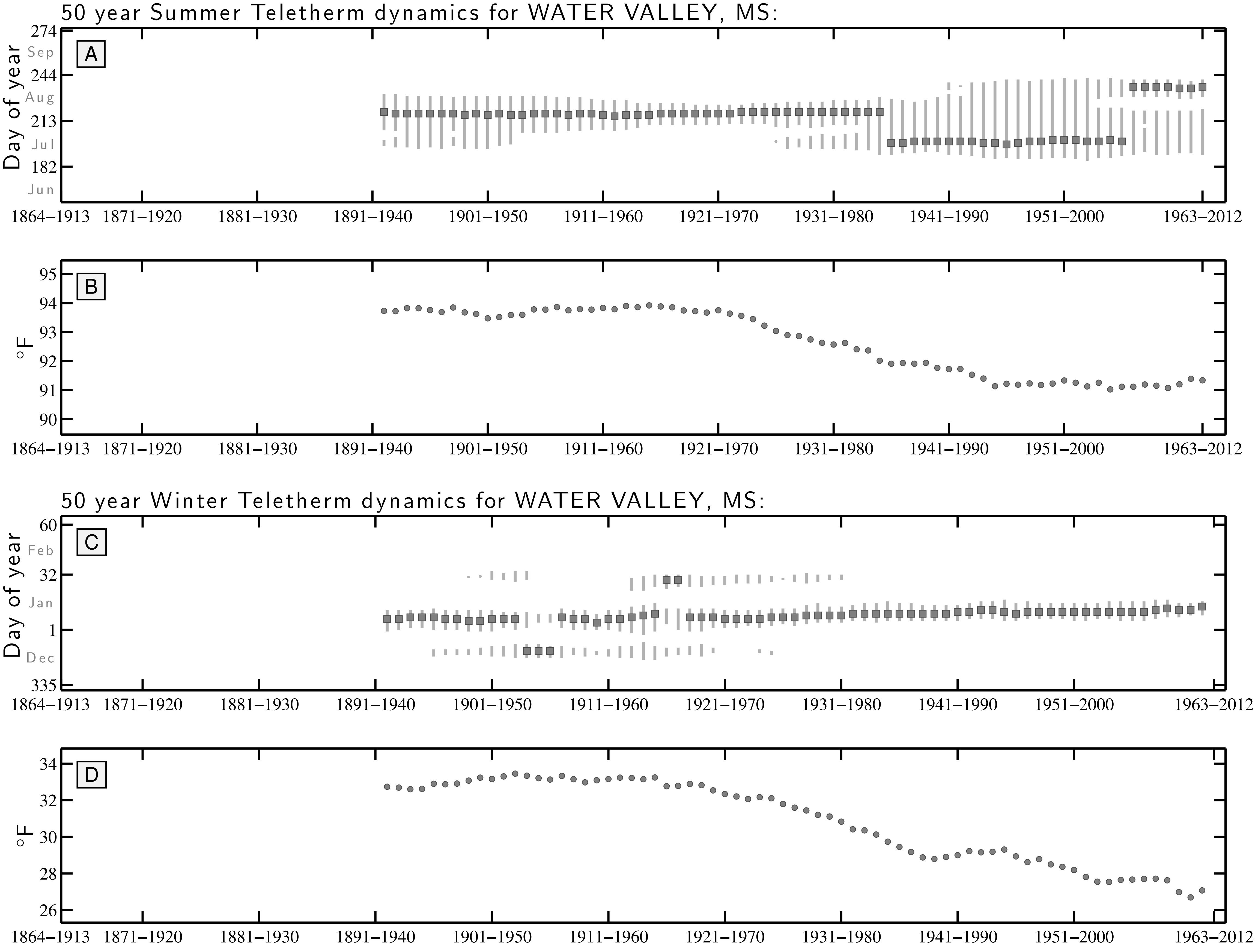

50 year Summer Teletherm dynamics for WATER VALLEY, MS:
A
B
Day of year
°F
Sep
Aug
Jul
Jun
274
244
213
182
95
94
93
92
91
90
1864–1913
1871–1920
1881–1930
1891–1940
1901–1950
1911–1960
1921–1970
1931–1980
1941–1990
1951–2000
1963–2012
50 year Winter Teletherm dynamics for WATER VALLEY, MS:
C
D
Feb
Jan
Dec
60
32
1
335
34
32
30
28
26

50 year Summer Teletherm dynamics for WAYNESBORO 2 W, MS:

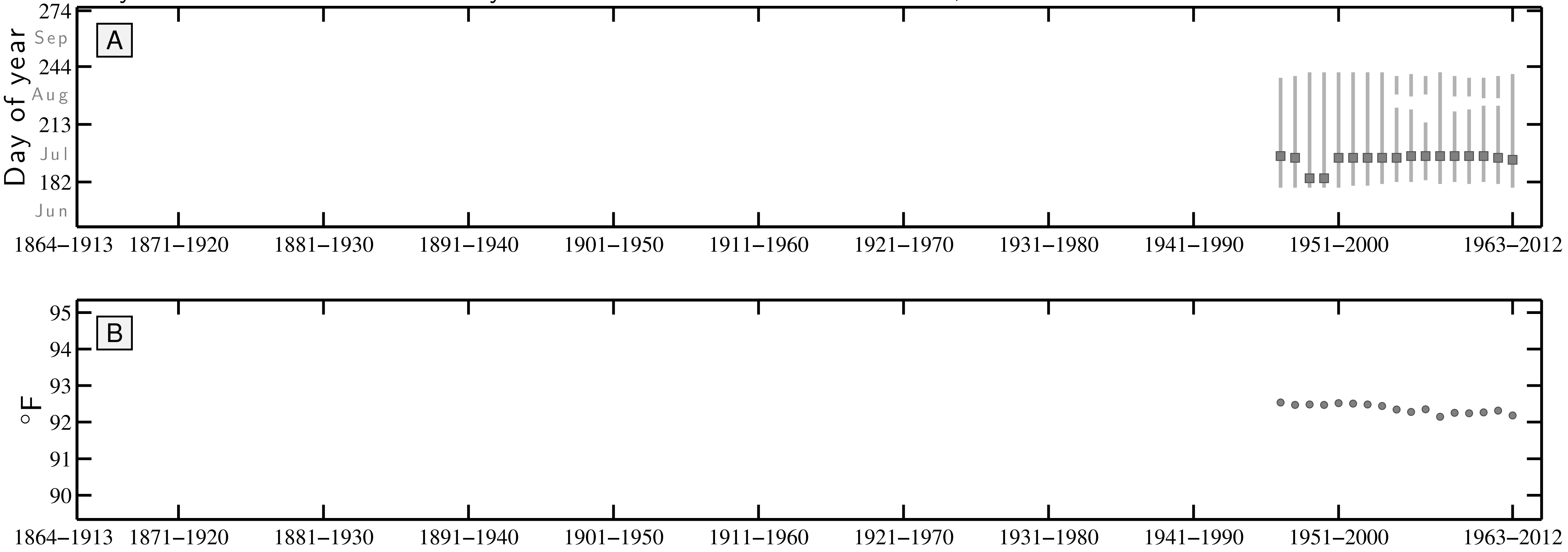

50 year Winter Teletherm dynamics for WAYNESBORO 2 W, MS:

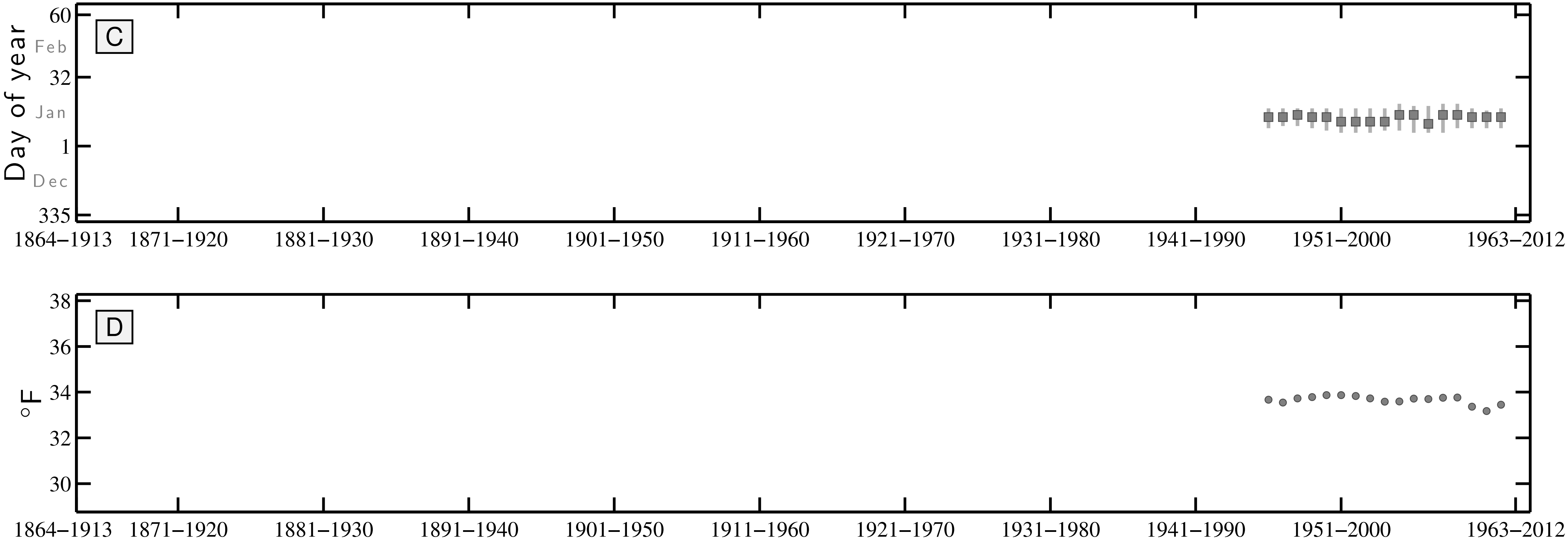

50 year Summer Teletherm dynamics for WOODVILLE 4 ESE, MS:

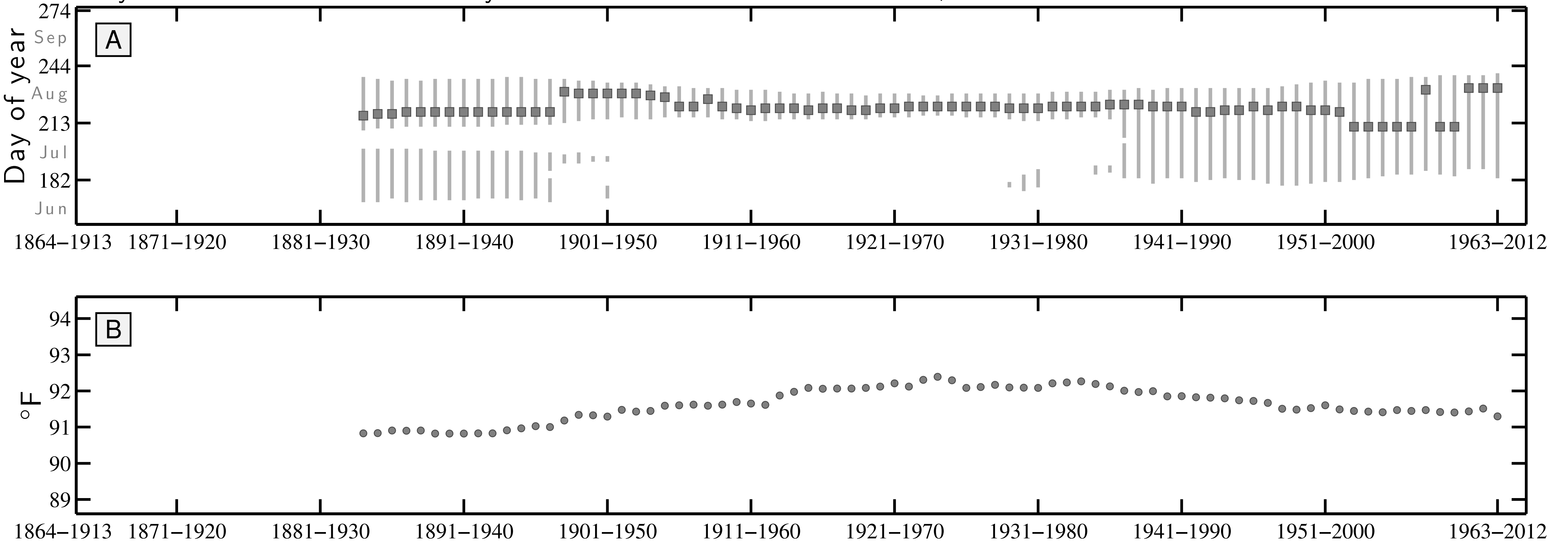

50 year Winter Teletherm dynamics for WOODVILLE 4 ESE, MS:

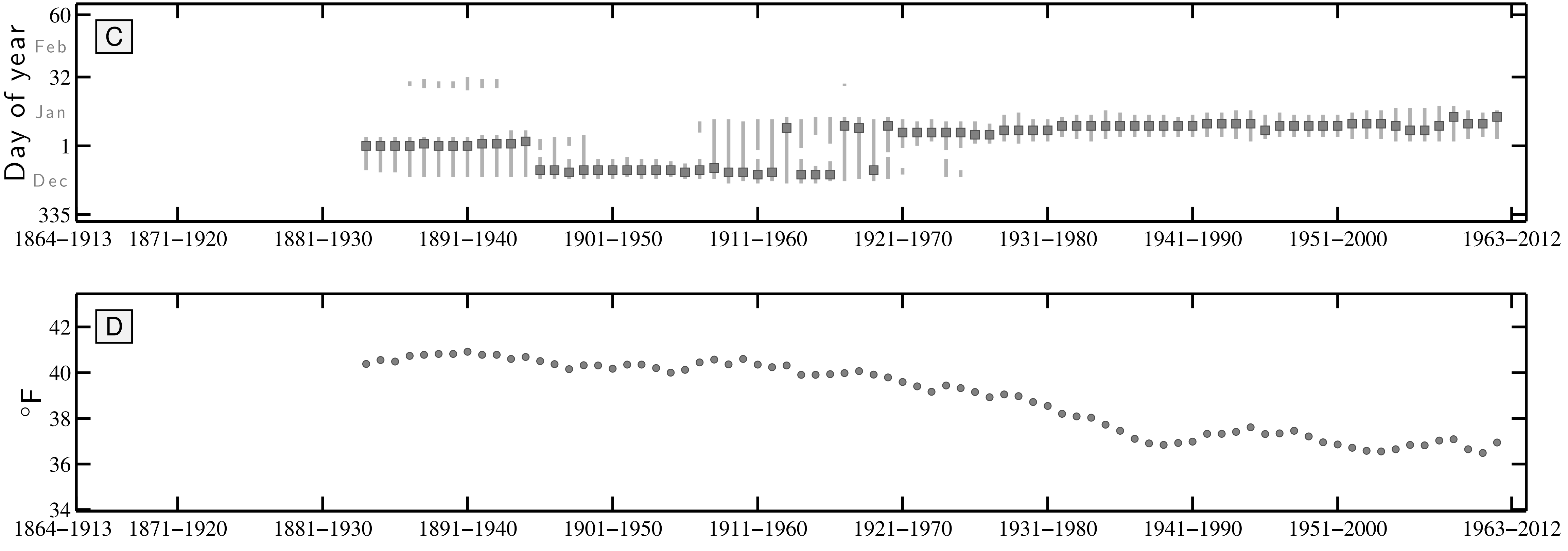

50 year Summer Teletherm dynamics for YAZOO CITY 5 NNE, MS:

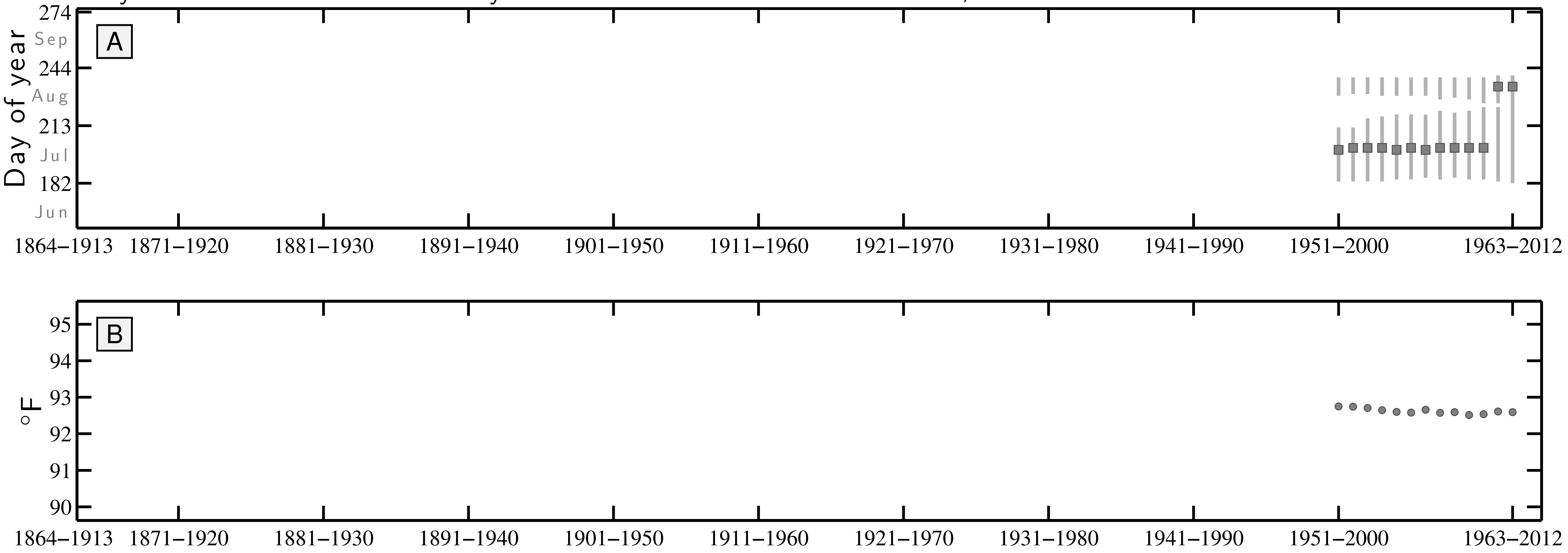

50 year Winter Teletherm dynamics for YAZOO CITY 5 NNE, MS:

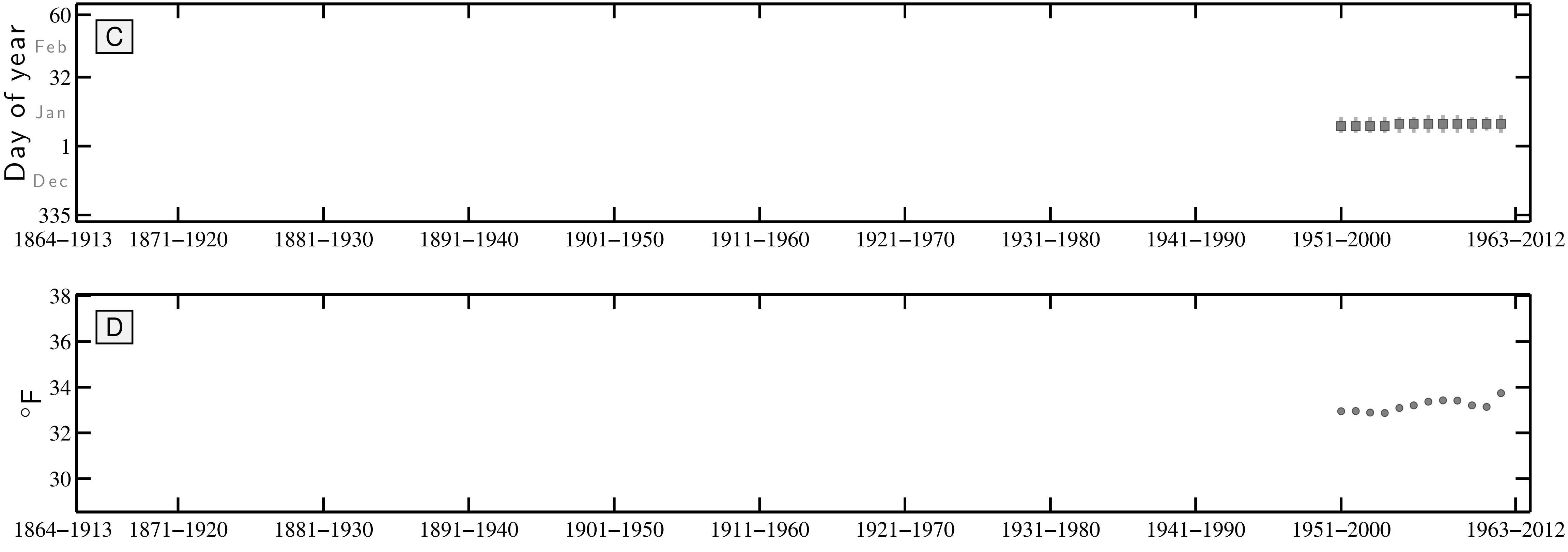

50 year Summer Teletherm dynamics for APPLETON CITY, MO:

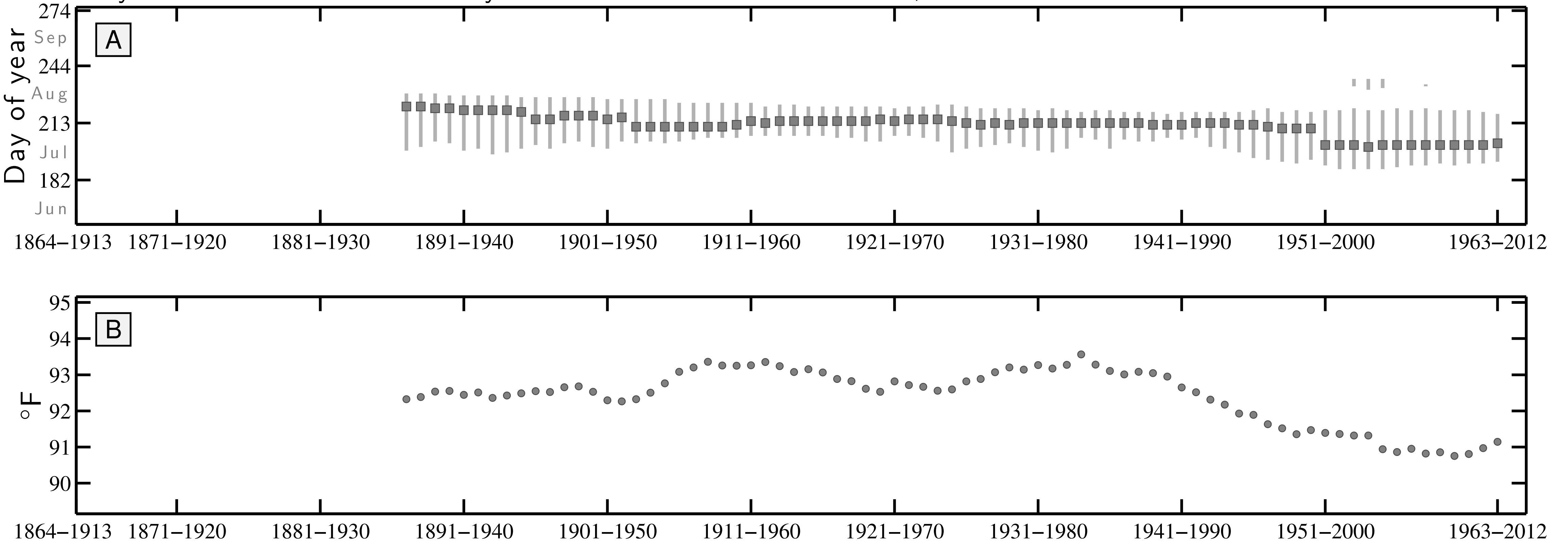

50 year Winter Teletherm dynamics for APPLETON CITY, MO:

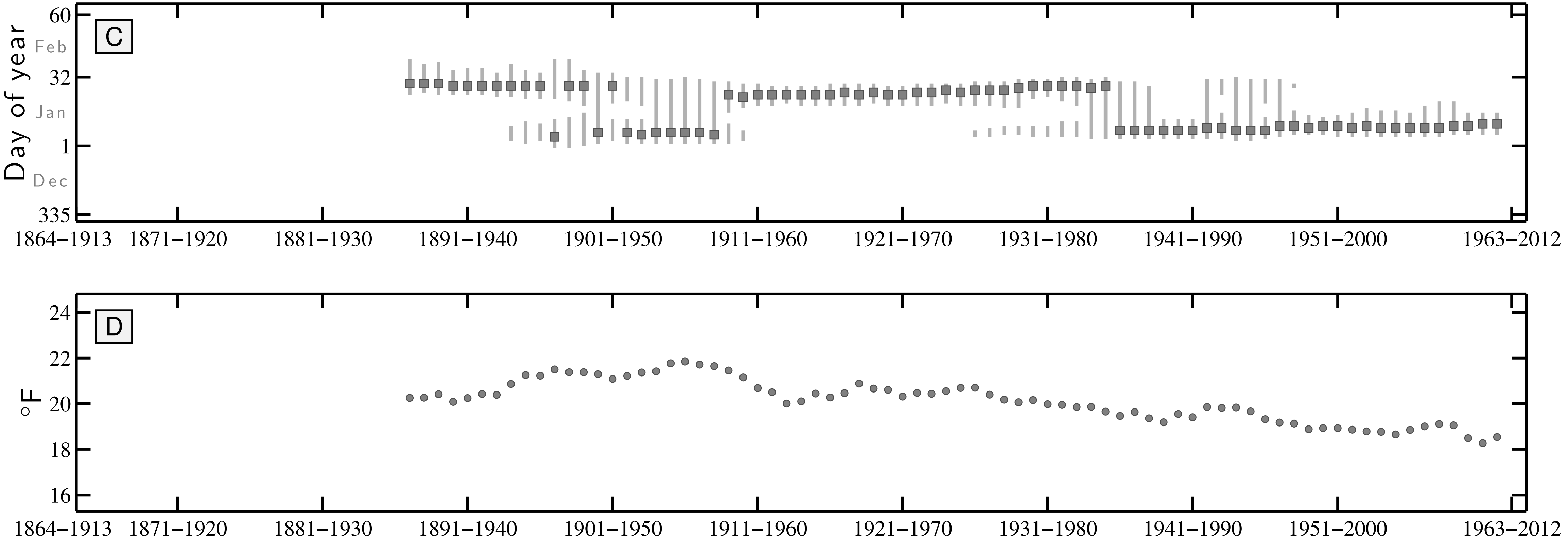

50 year Summer Teletherm dynamics for BRUNSWICK, MO:

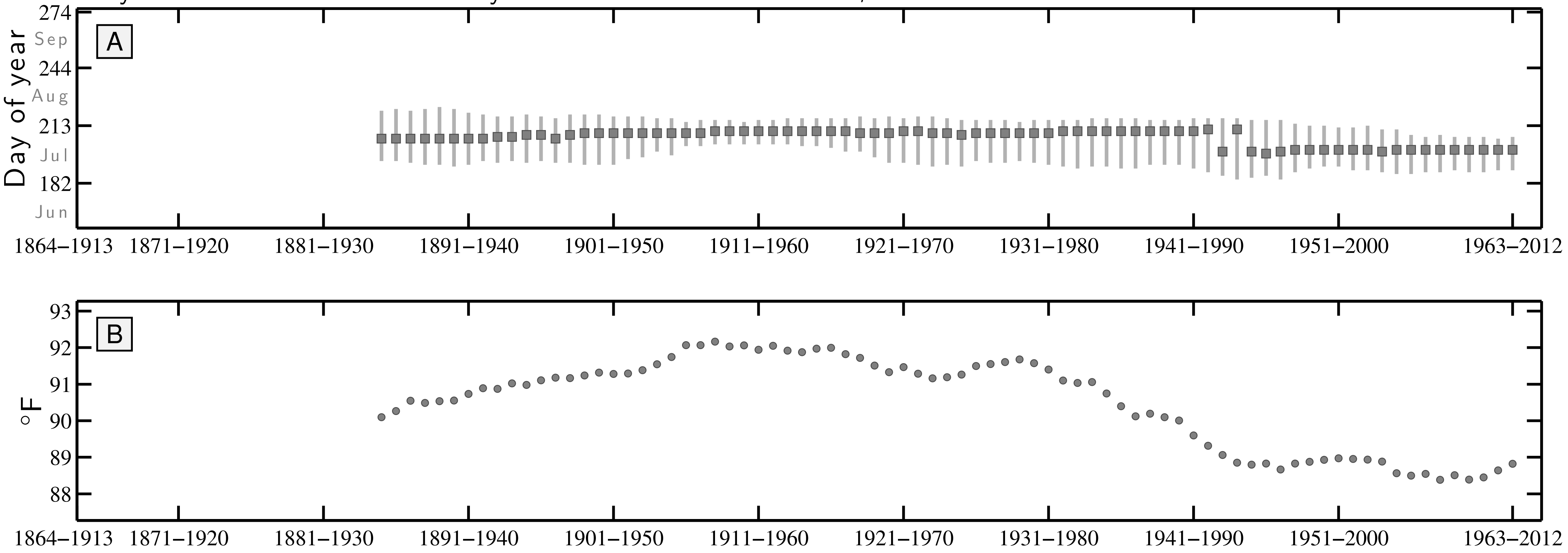

50 year Winter Teletherm dynamics for BRUNSWICK, MO:

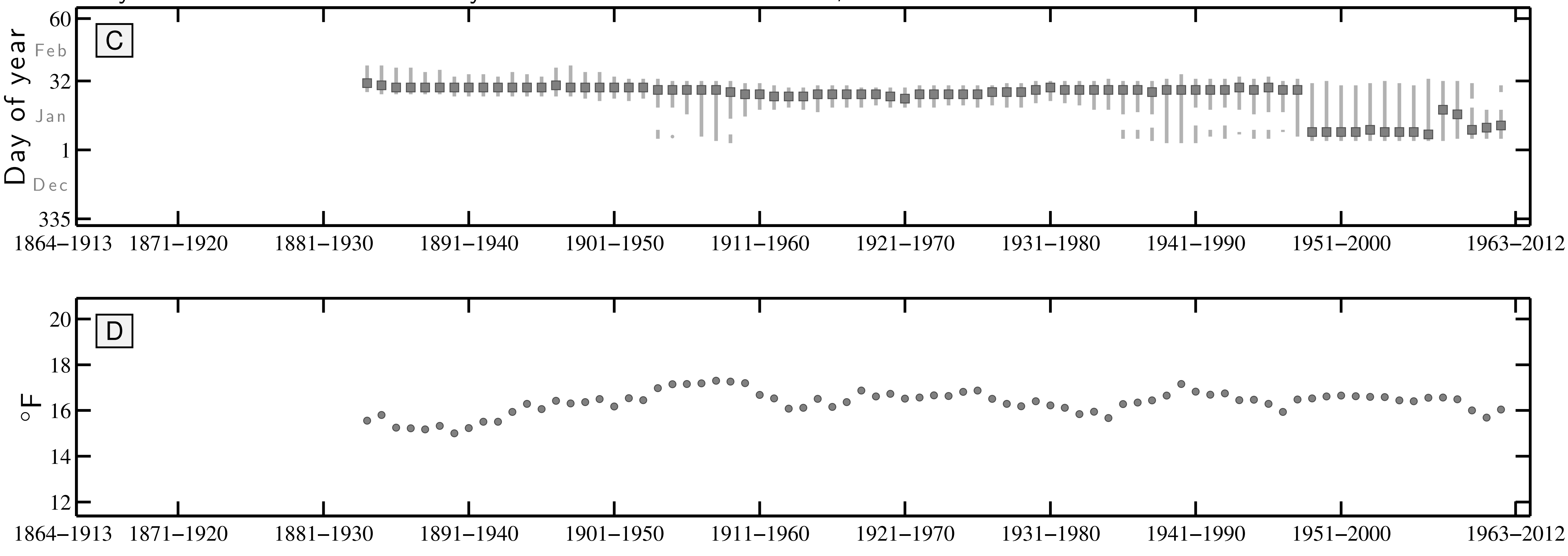

50 year Summer Teletherm dynamics for CARUTHERSVILLE, MO:

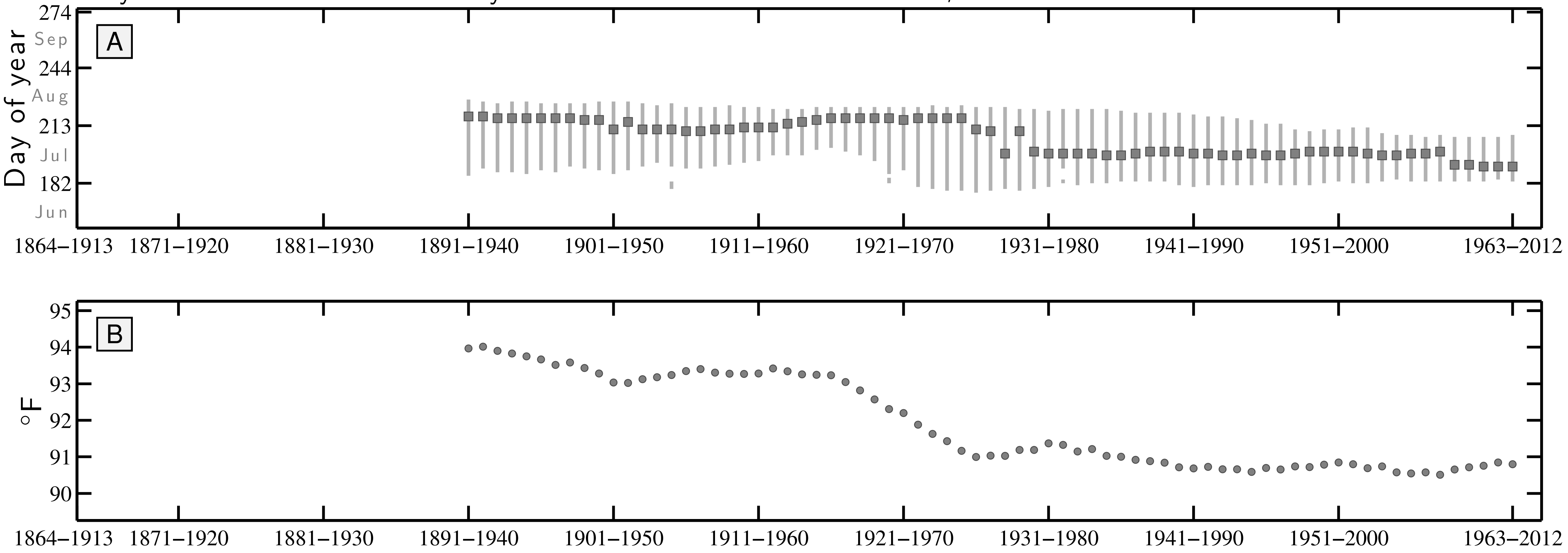

50 year Winter Teletherm dynamics for CARUTHERSVILLE, MO:

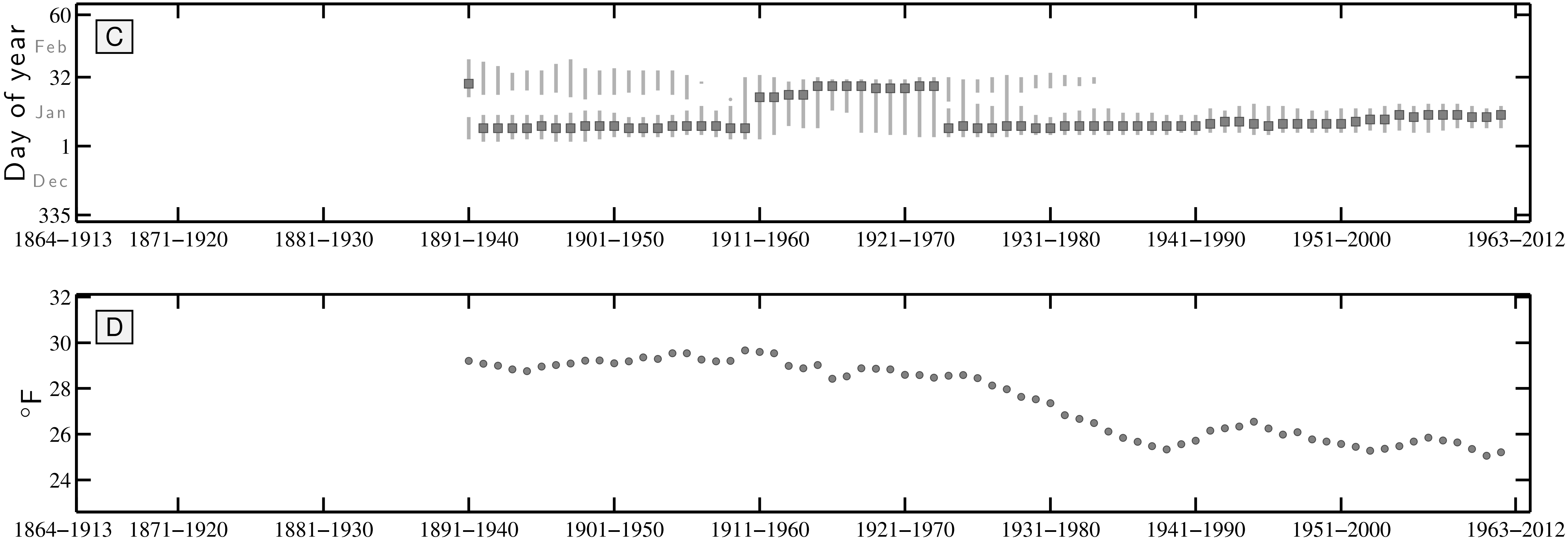

50 year Summer Teletherm dynamics for CLINTON, MO:

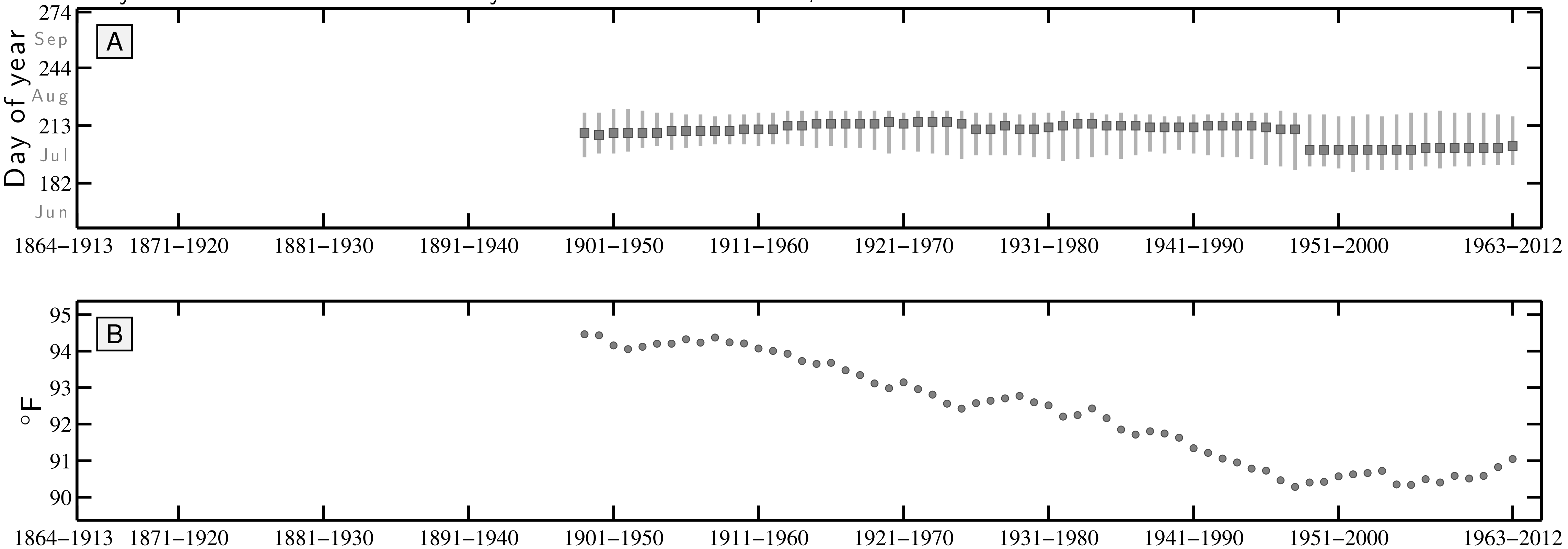

50 year Winter Teletherm dynamics for CLINTON, MO:

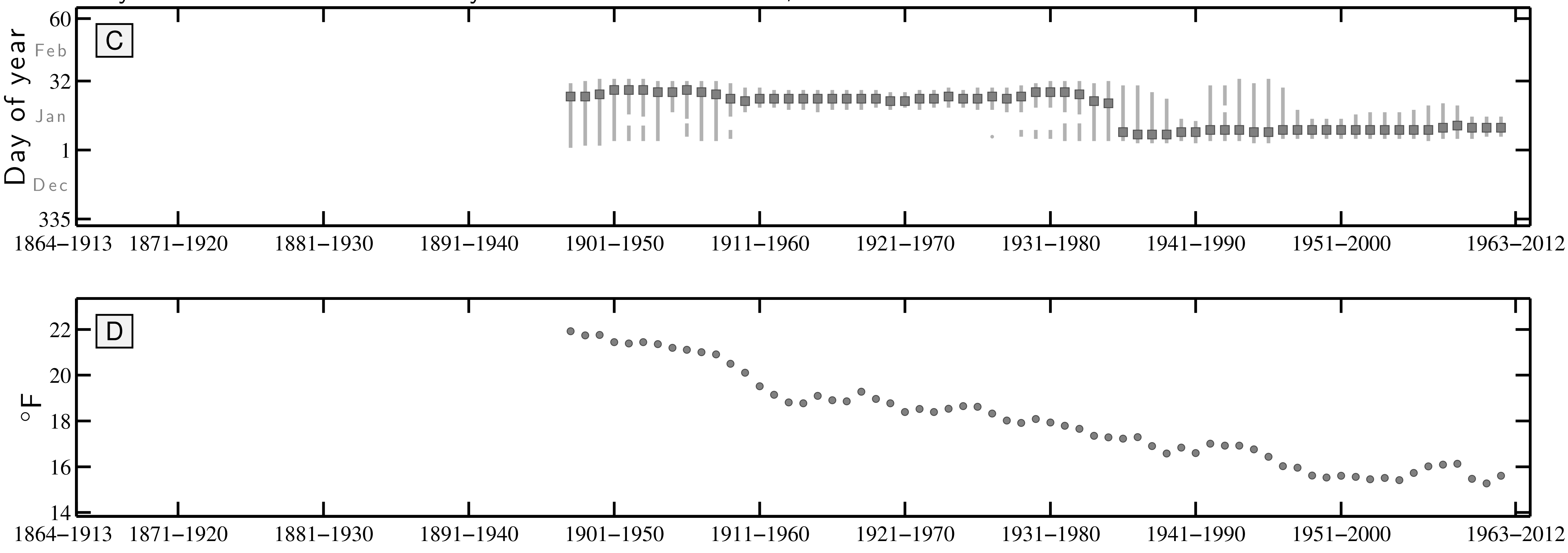

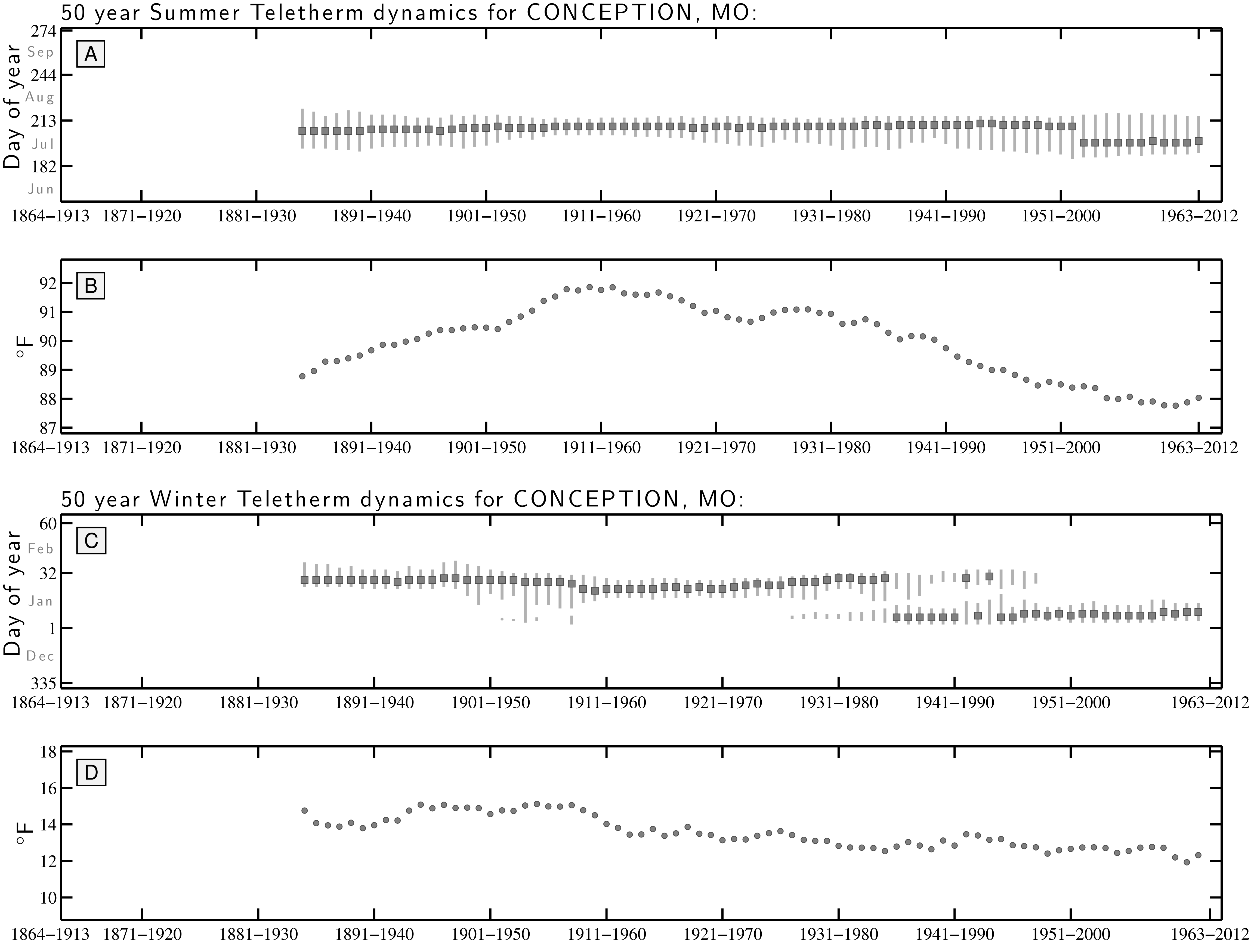

50 year Summer Teletherm dynamics for CONCEPTION, MO:
A
Day of year
274
Sep
244
Aug
213
Jul
182
Jun
1864–1913
1871–1920
1881–1930
1891–1940
1901–1950
1911–1960
1921–1970
1931–1980
1941–1990
1951–2000
1963–2012
B
°F
92
91
90
89
88
87
50 year Winter Teletherm dynamics for CONCEPTION, MO:
C
60
Feb
32
Jan
1
Dec
335
D
18
16
14
12
10

50 year Summer Teletherm dynamics for DONIPHAN, MO:

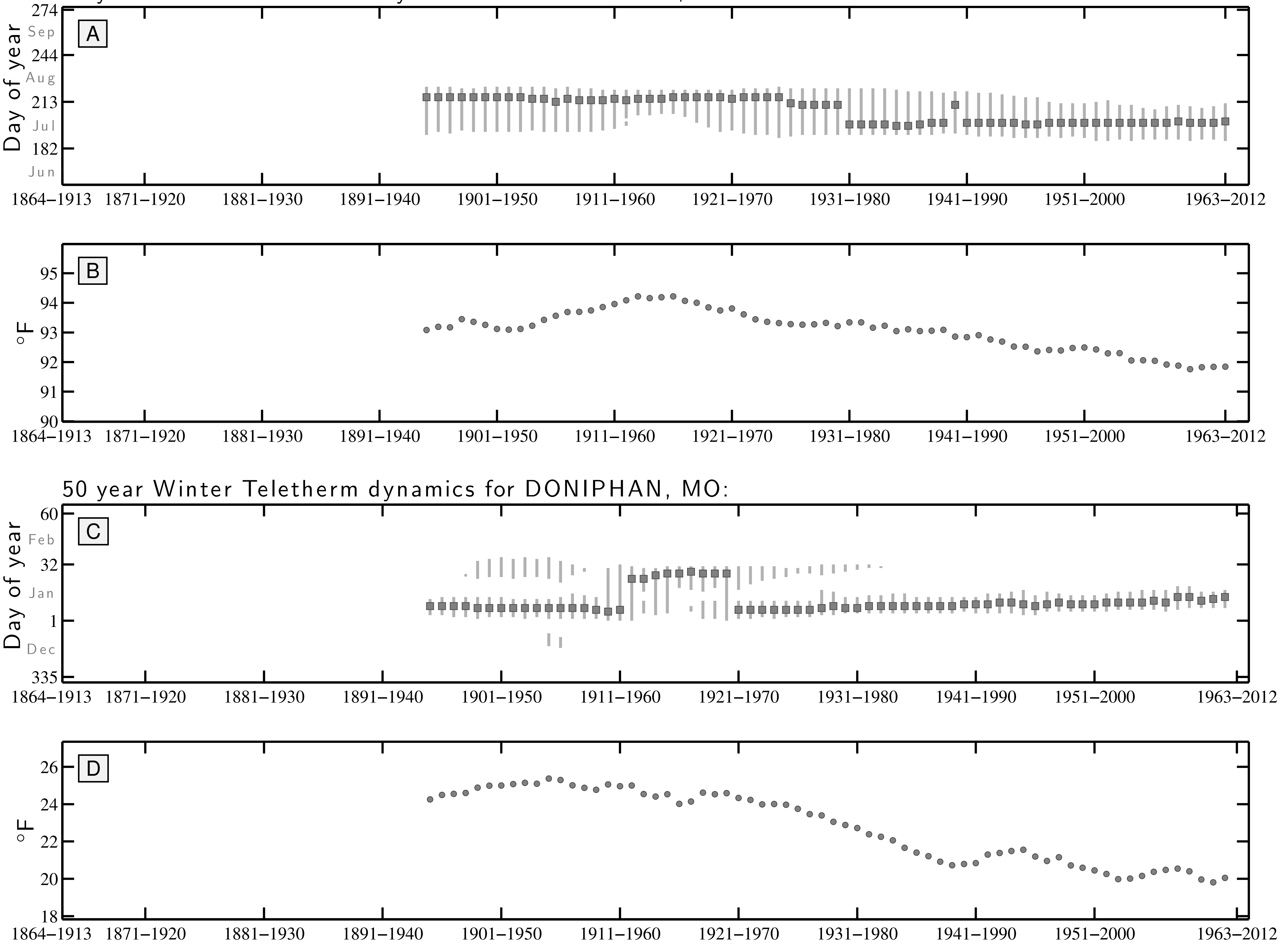

50 year Winter Teletherm dynamics for DONIPHAN, MO:

50 year Summer Teletherm dynamics for FARMINGTON, MO:

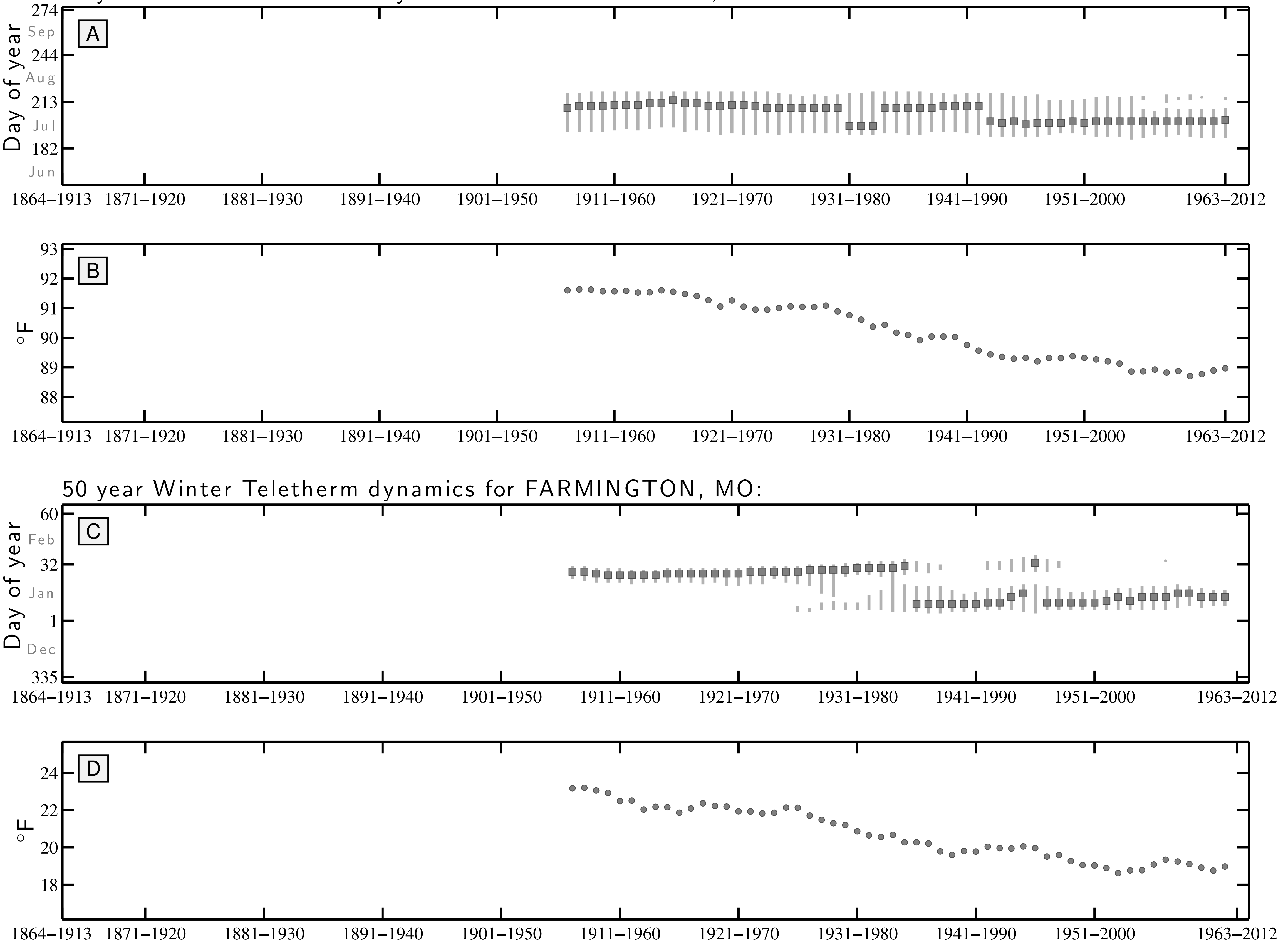

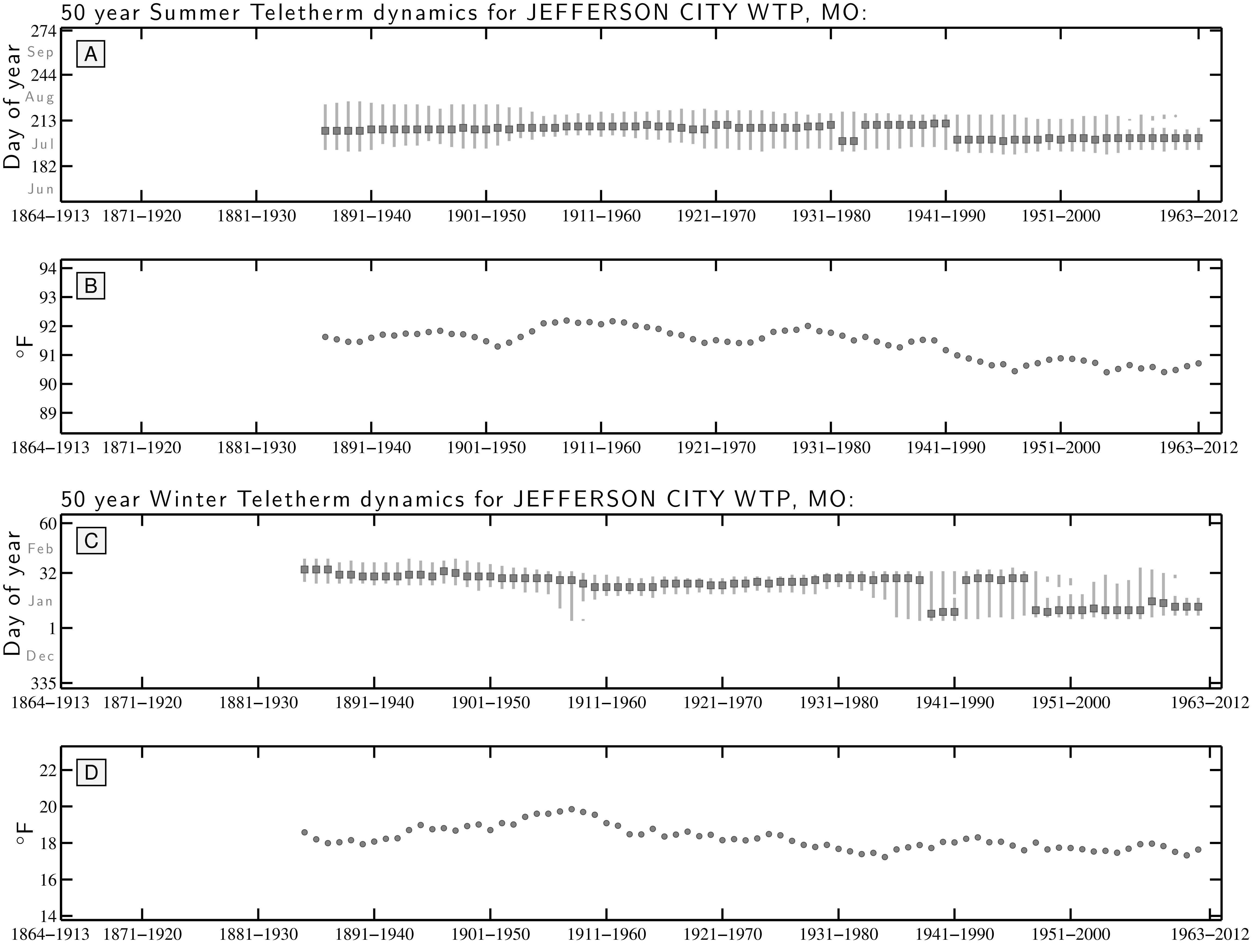
50 year Summer Teletherm dynamics for JEFFERSON CITY WTP, MO:
A
B
Day of year
°F
274
244
213
182
Sep
Aug
Jul
Jun
94
93
92
91
90
89
1864–1913
1871–1920
1881–1930
1891–1940
1901–1950
1911–1960
1921–1970
1931–1980
1941–1990
1951–2000
1963–2012
50 year Winter Teletherm dynamics for JEFFERSON CITY WTP, MO:
C
D
60
32
1
335
Feb
Jan
Dec
22
20
18
16
14

50 year Summer Teletherm dynamics for LAMAR, MO:

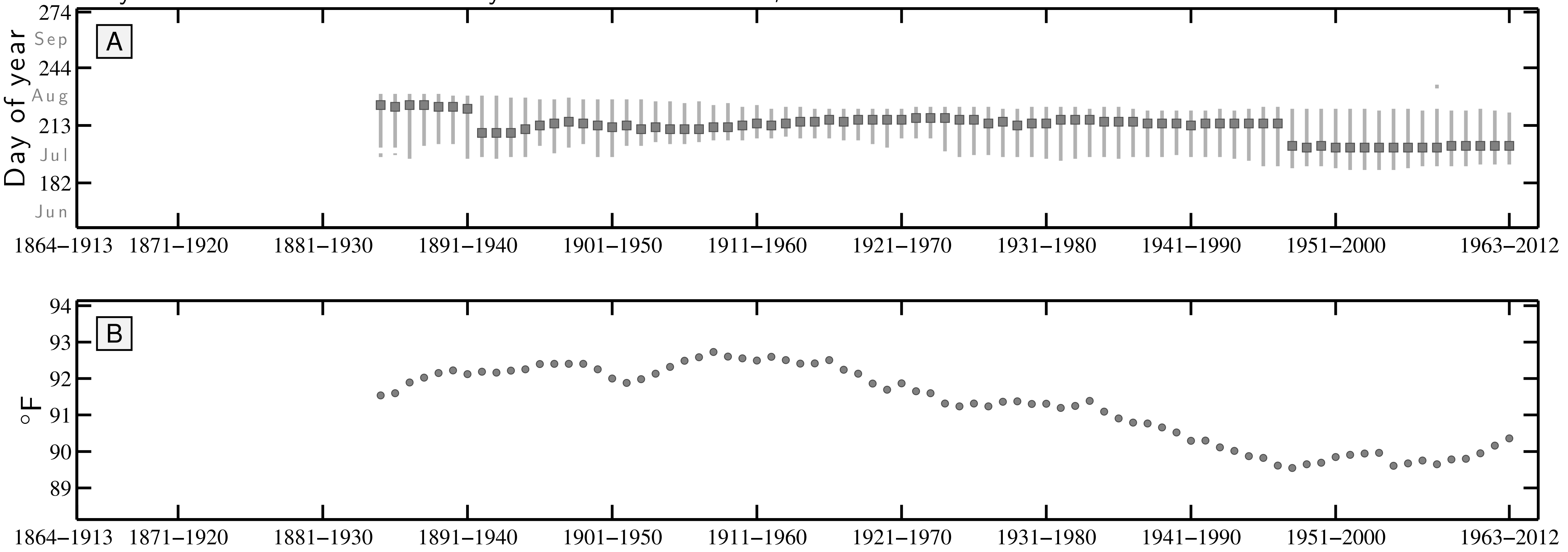

50 year Winter Teletherm dynamics for LAMAR, MO:

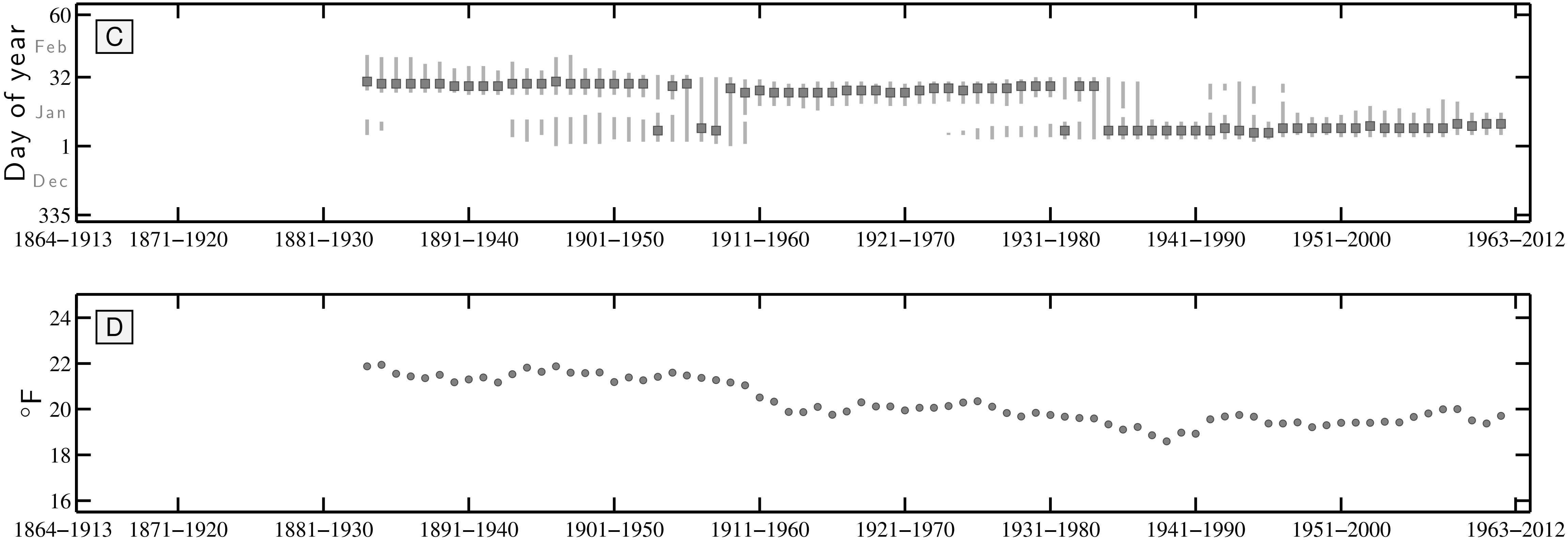

50 year Summer Teletherm dynamics for LEBANON 2W, MO:

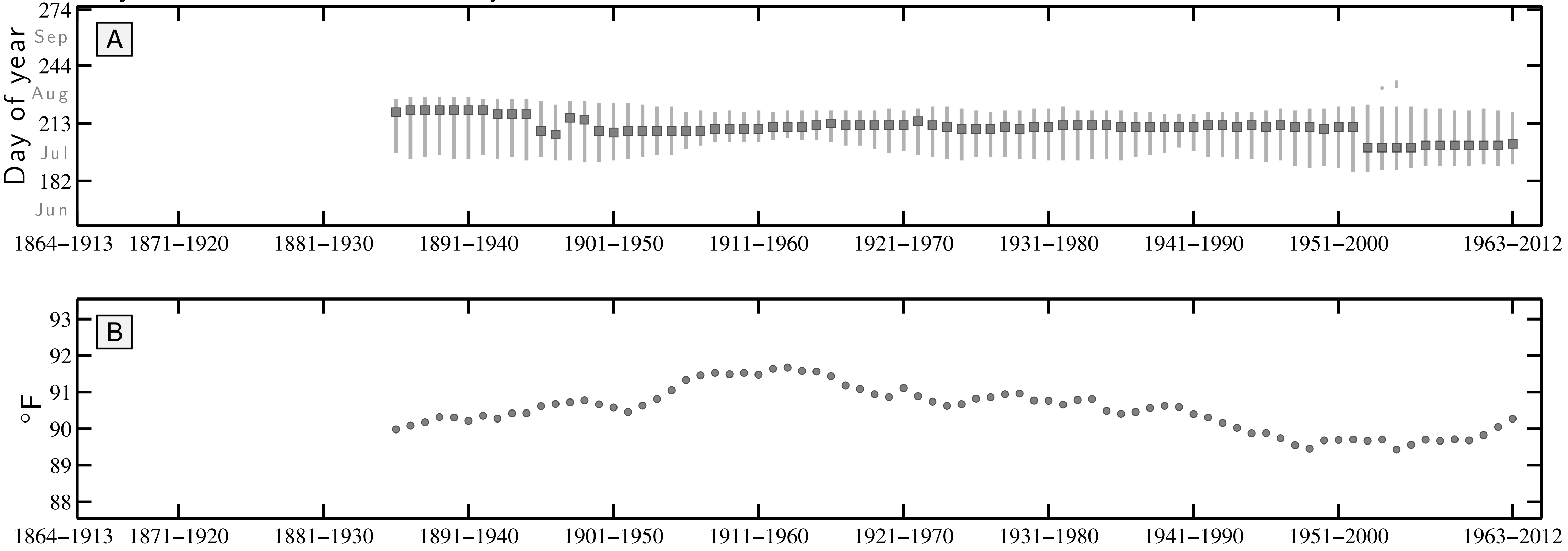

50 year Winter Teletherm dynamics for LEBANON 2W, MO:

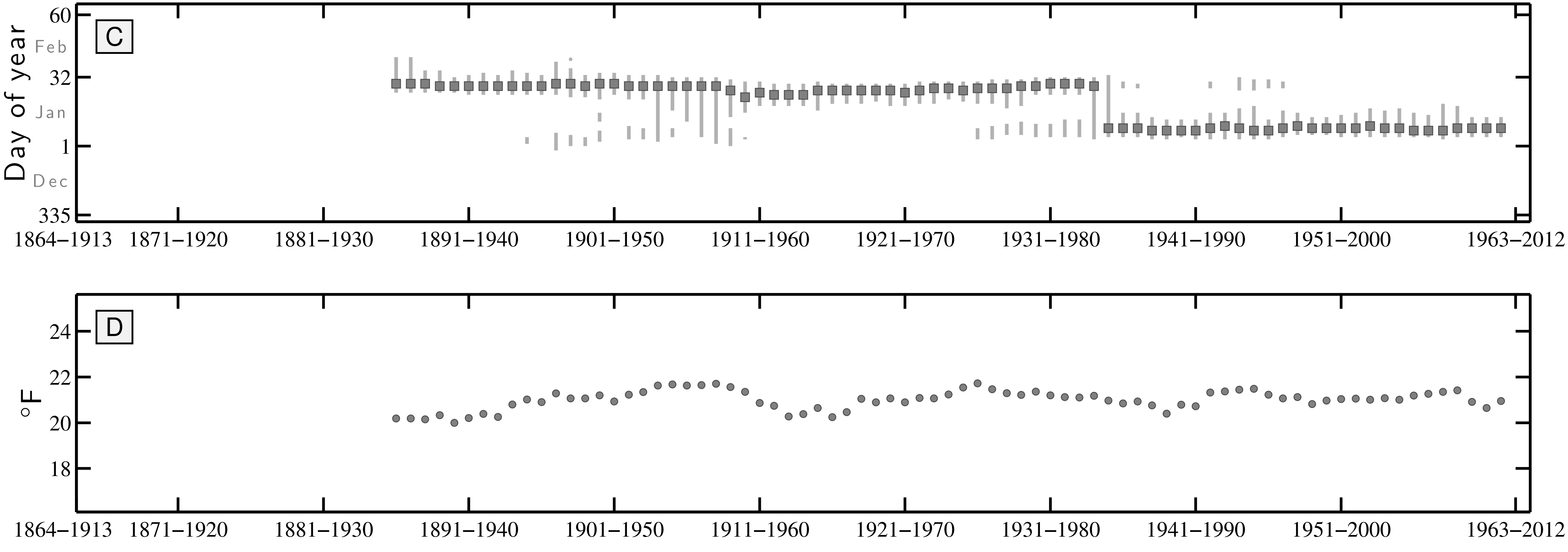

50 year Summer Teletherm dynamics for LEES SUMMIT REED WR, MO:

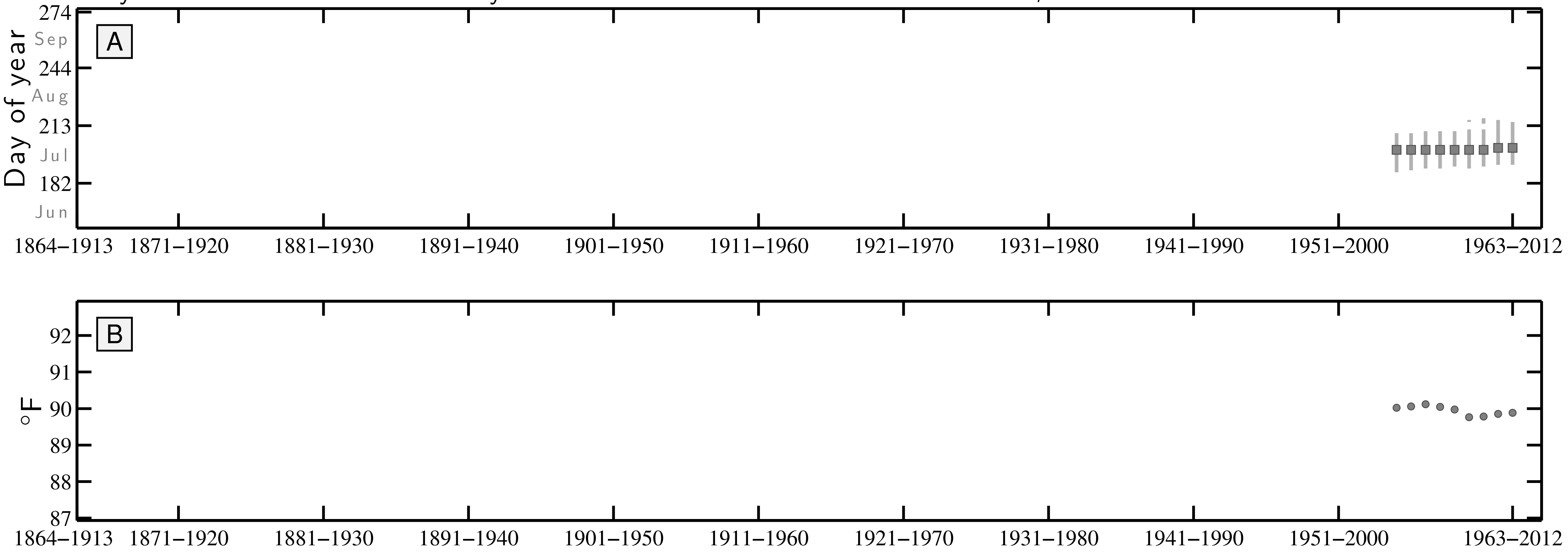

50 year Winter Teletherm dynamics for LEES SUMMIT REED WR, MO:

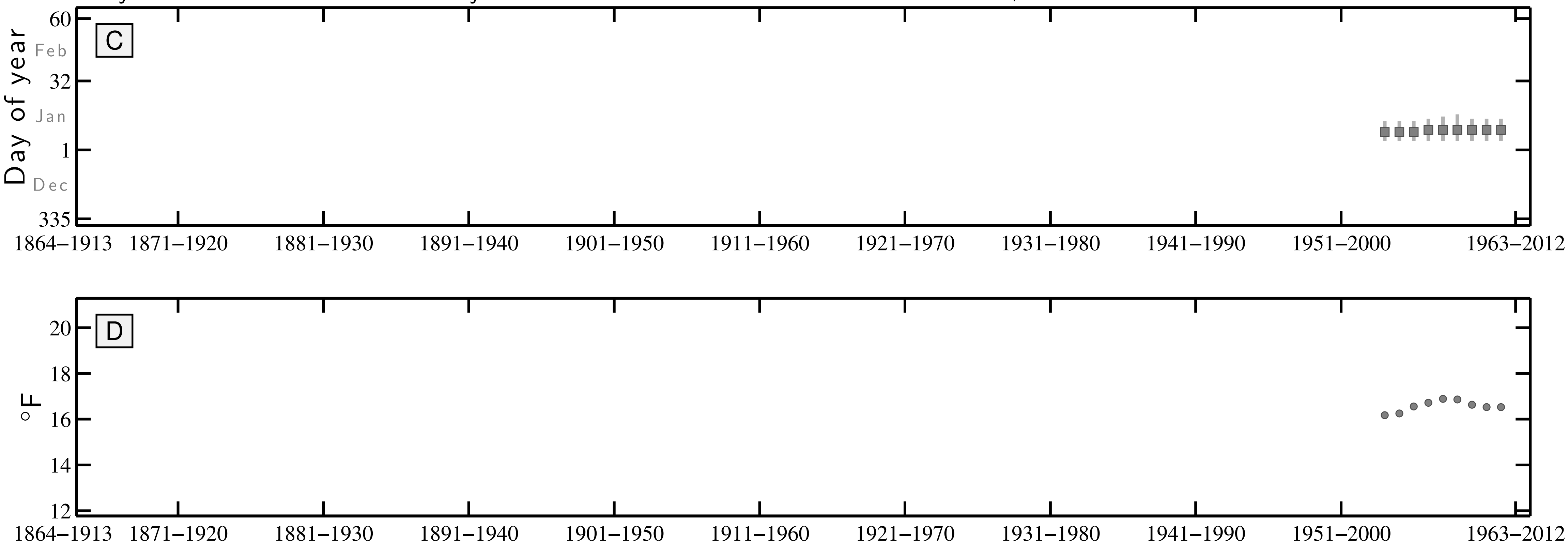

50 year Summer Teletherm dynamics for LEXINGTON 3E, MO:

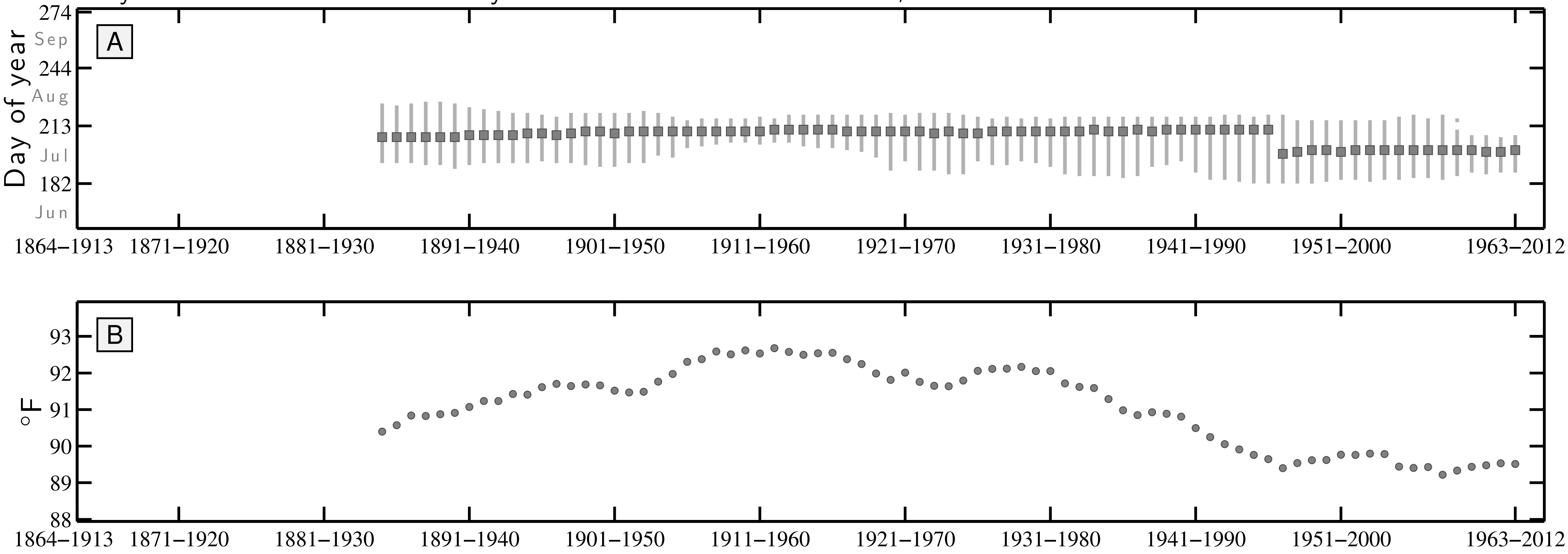

50 year Winter Teletherm dynamics for LEXINGTON 3E, MO:

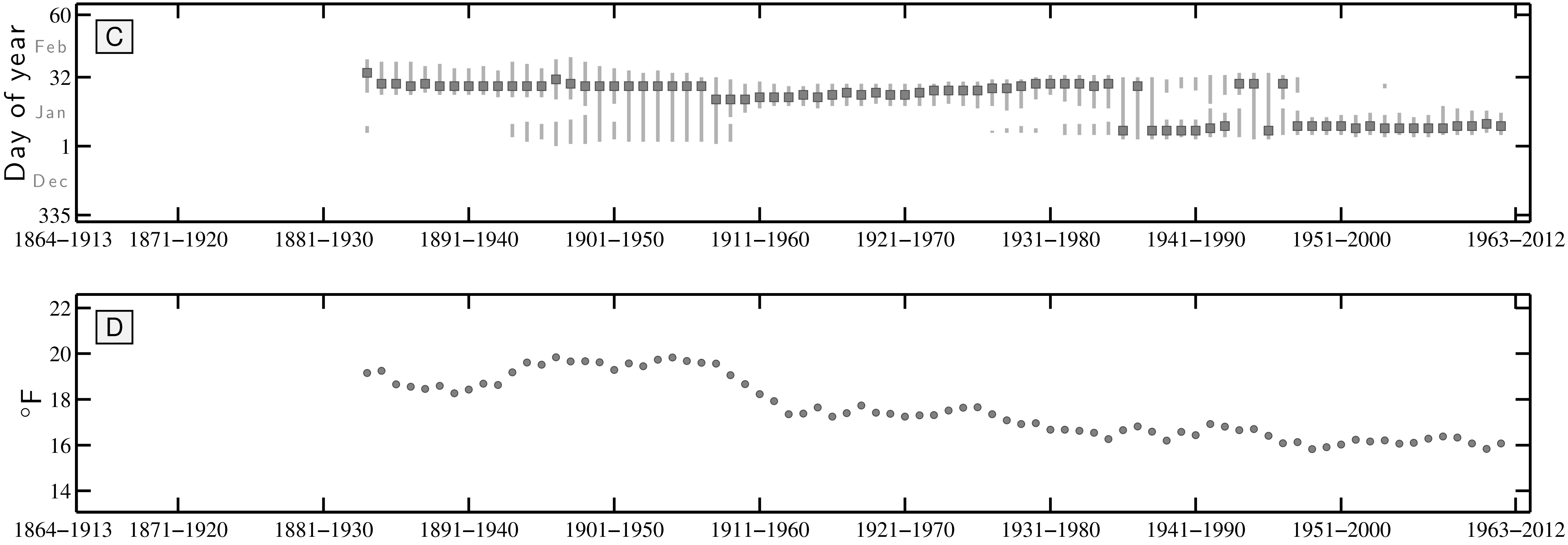

50 year Summer Teletherm dynamics for LOCKWOOD, MO:

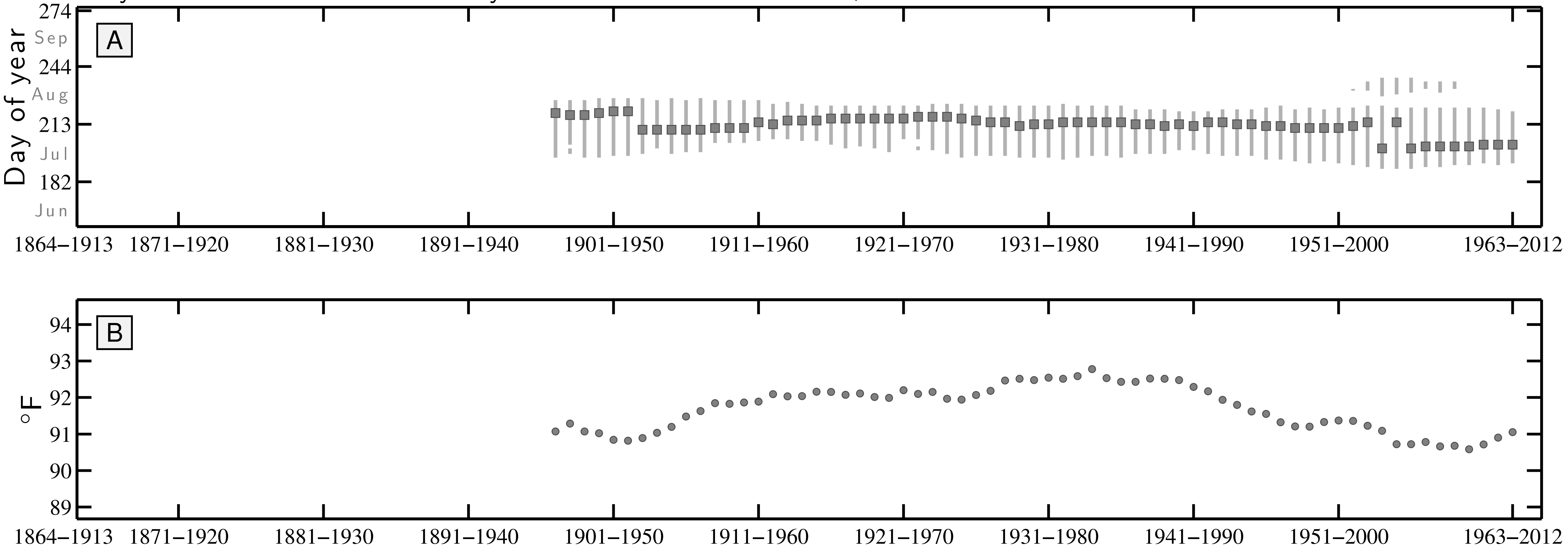

50 year Winter Teletherm dynamics for LOCKWOOD, MO:

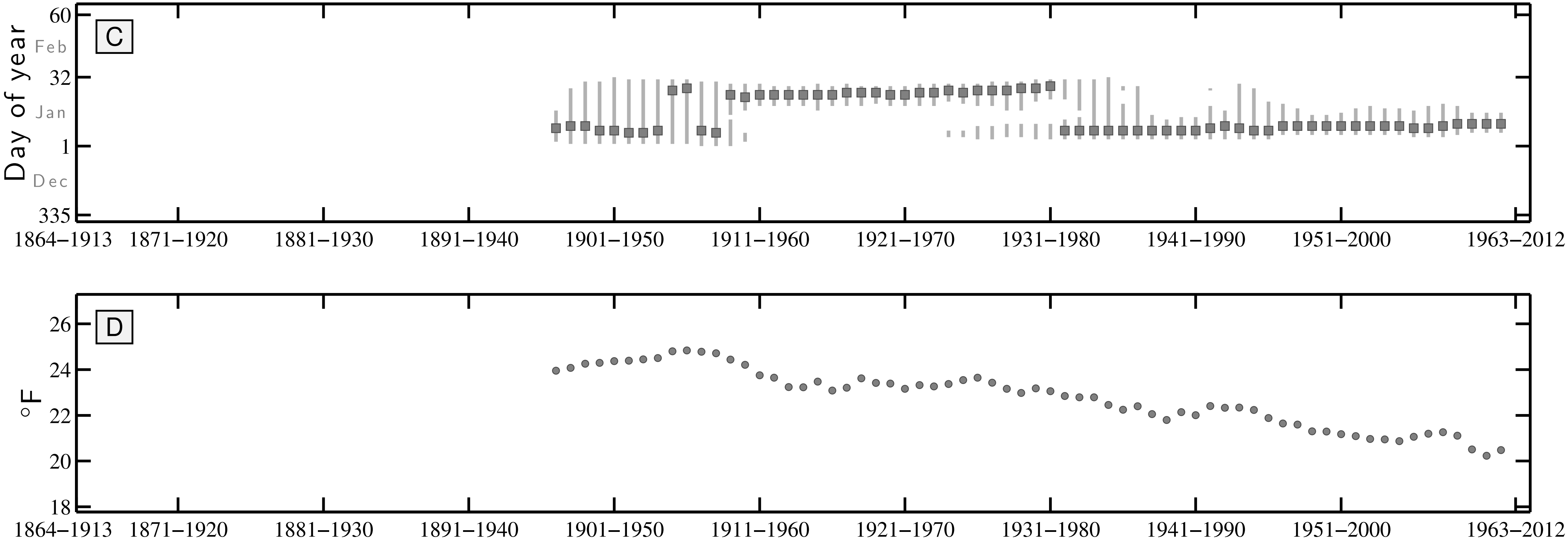

50 year Summer Teletherm dynamics for MARBLE HILL, MO:

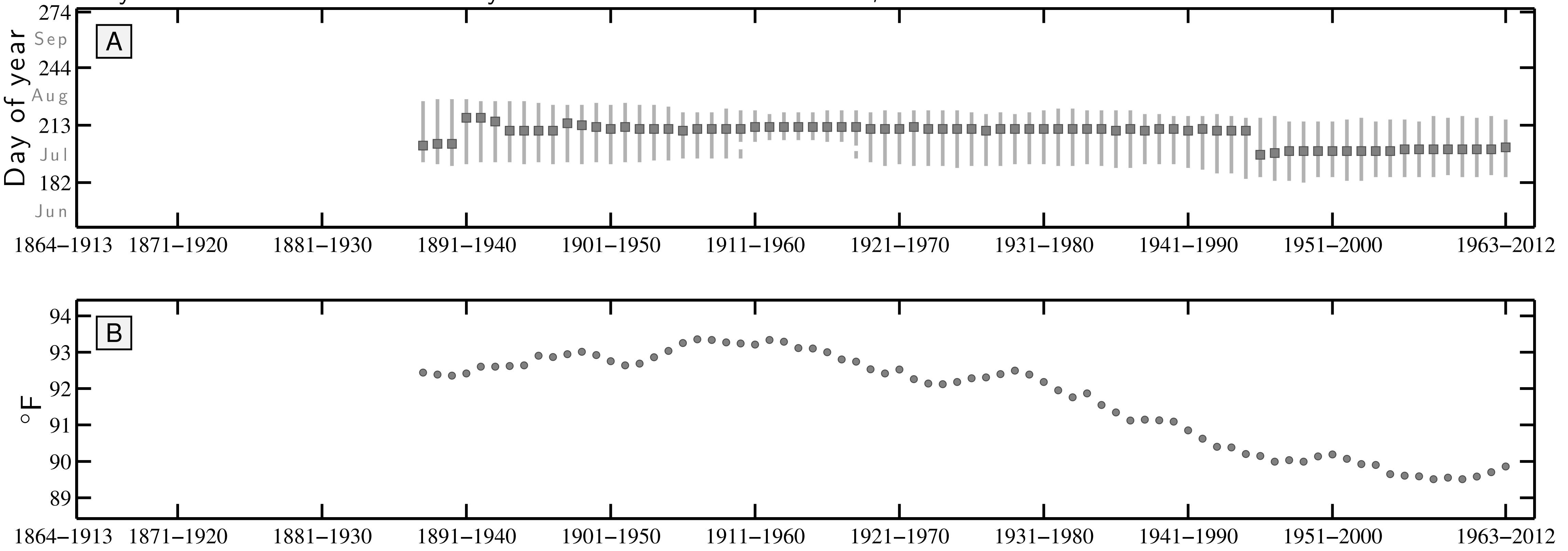

50 year Winter Teletherm dynamics for MARBLE HILL, MO:

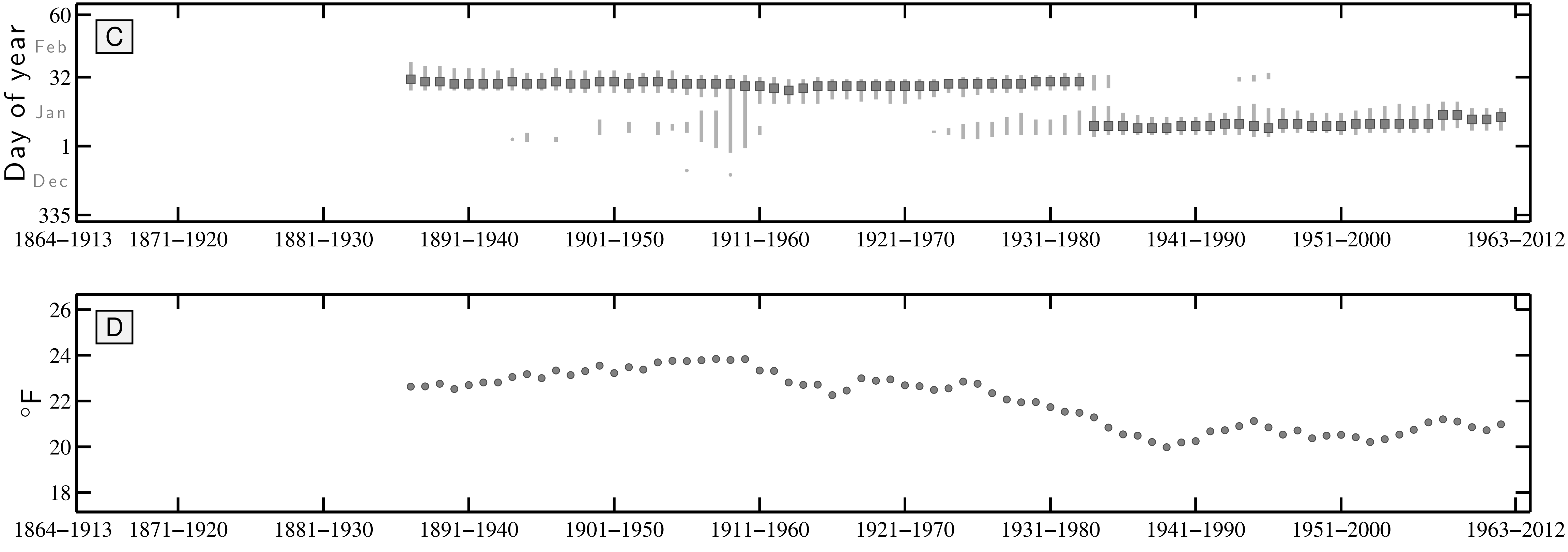

50 year Summer Teletherm dynamics for MEXICO, MO:

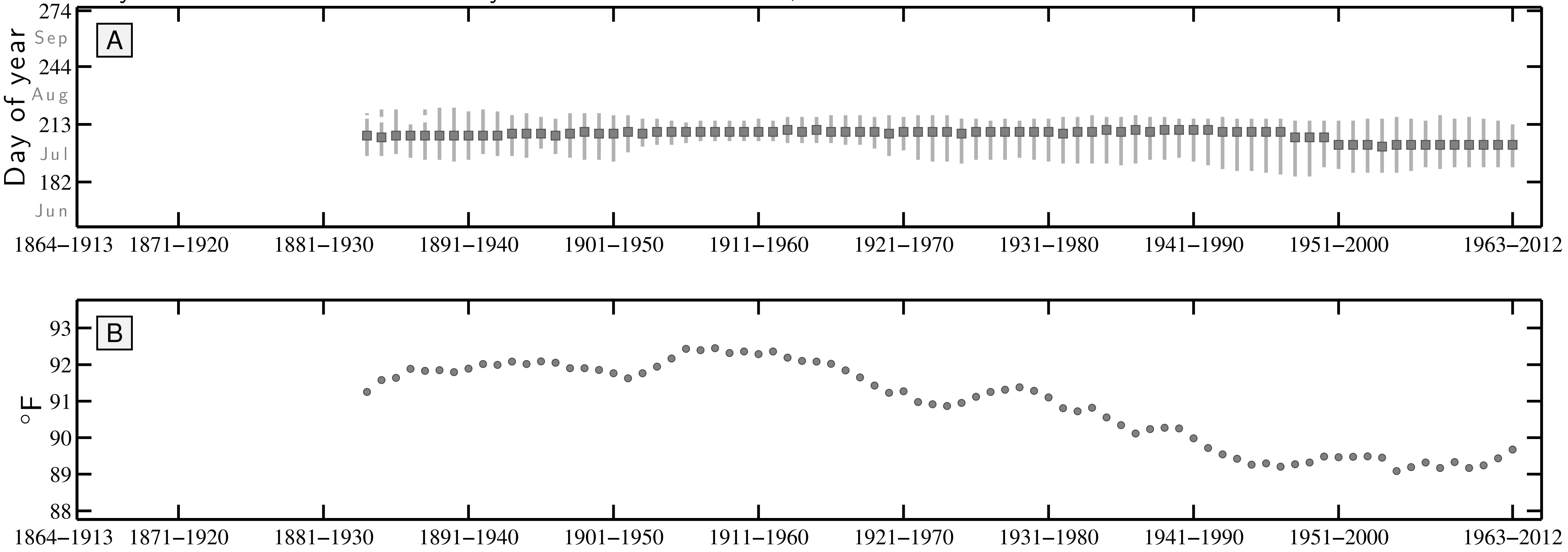

50 year Winter Teletherm dynamics for MEXICO, MO:

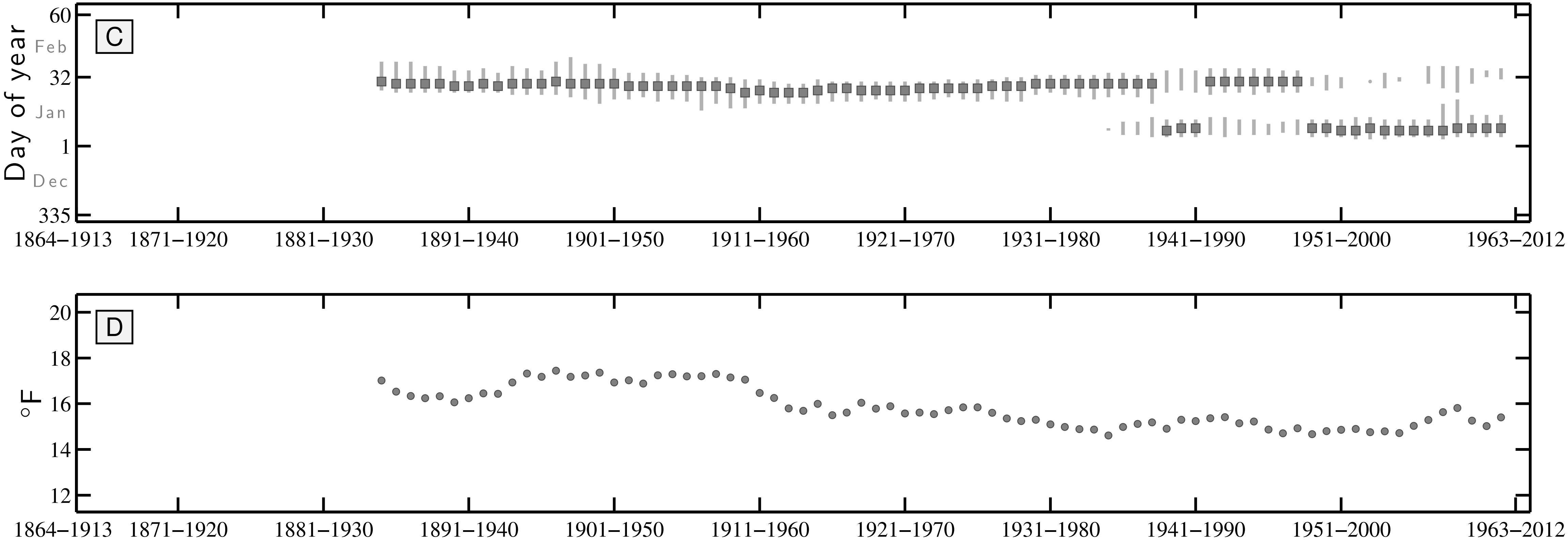

50 year Summer Teletherm dynamics for MOBERLY, MO:

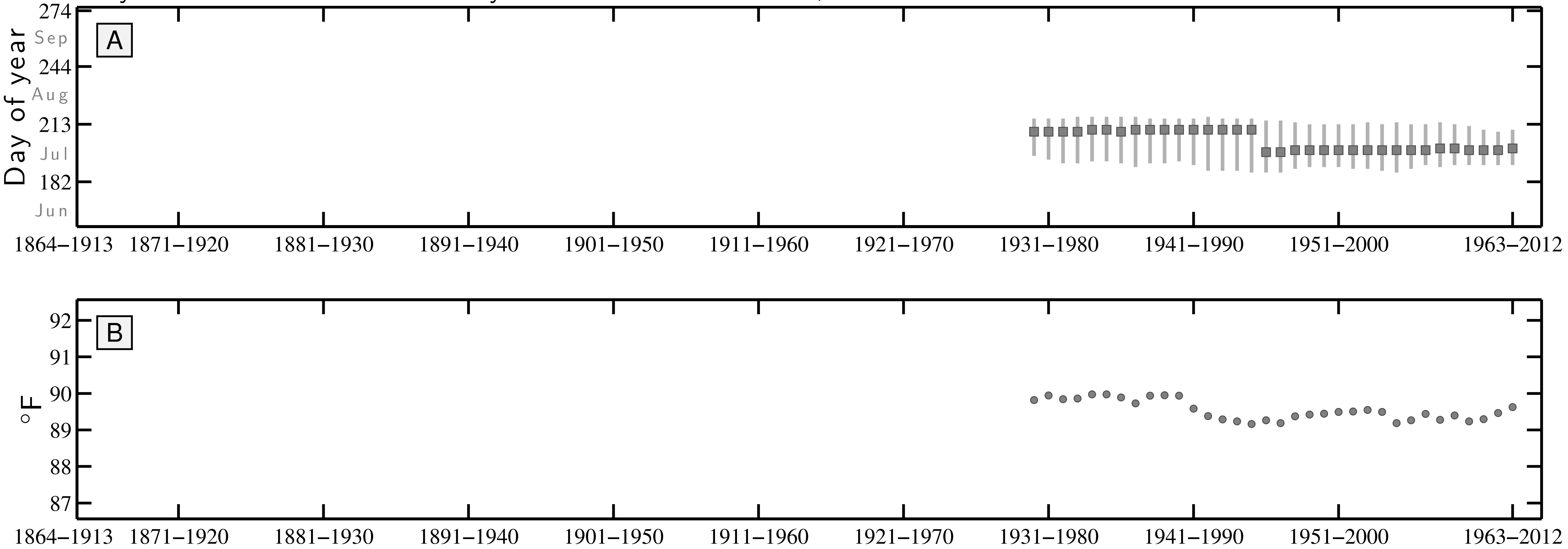

50 year Winter Teletherm dynamics for MOBERLY, MO:

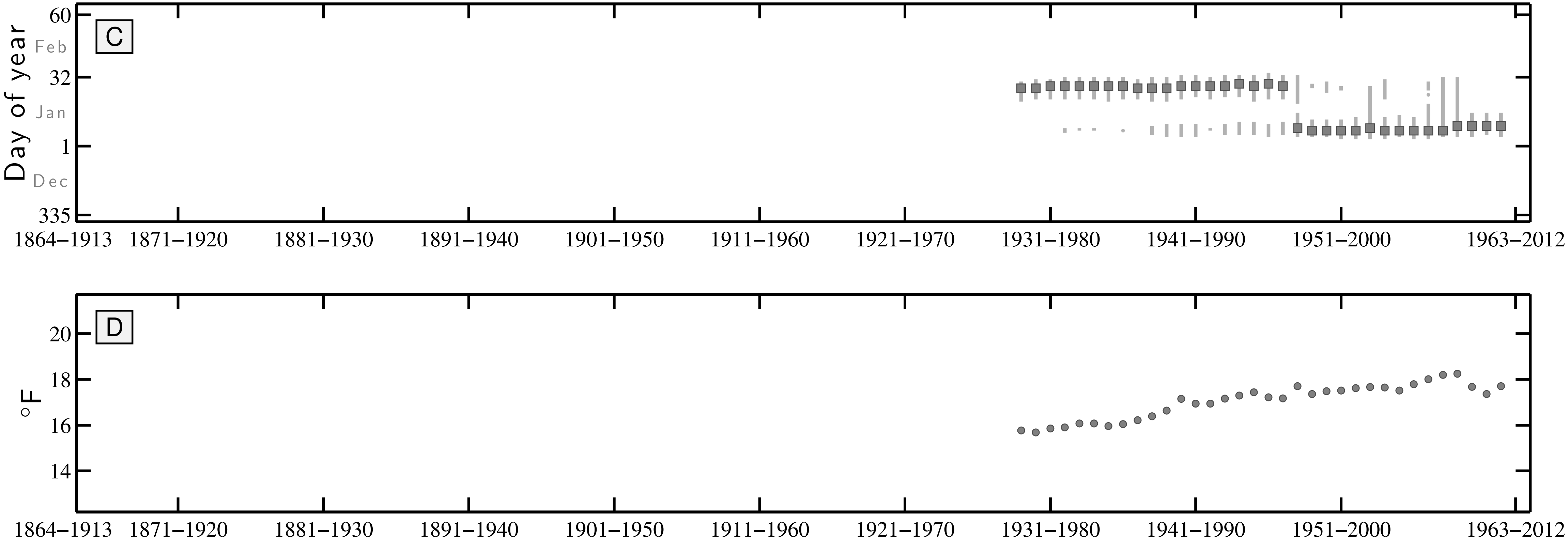

50 year Summer Teletherm dynamics for MTN GROVE 2 N, MO:

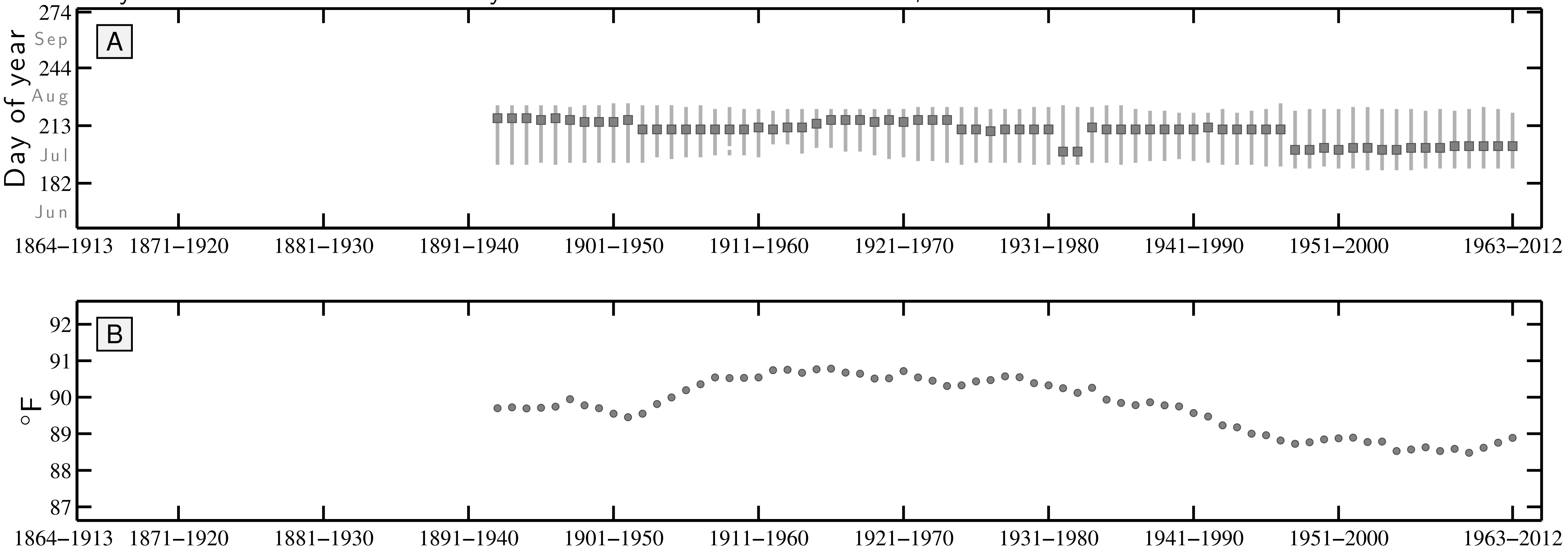

50 year Winter Teletherm dynamics for MTN GROVE 2 N, MO:

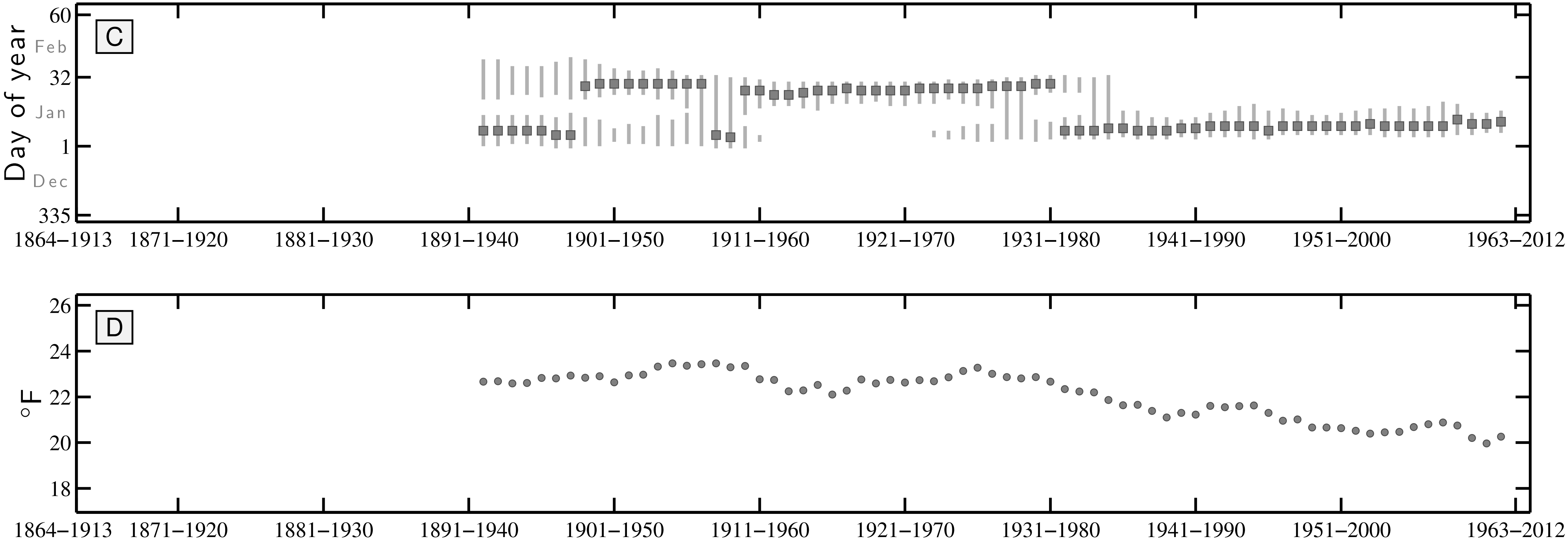

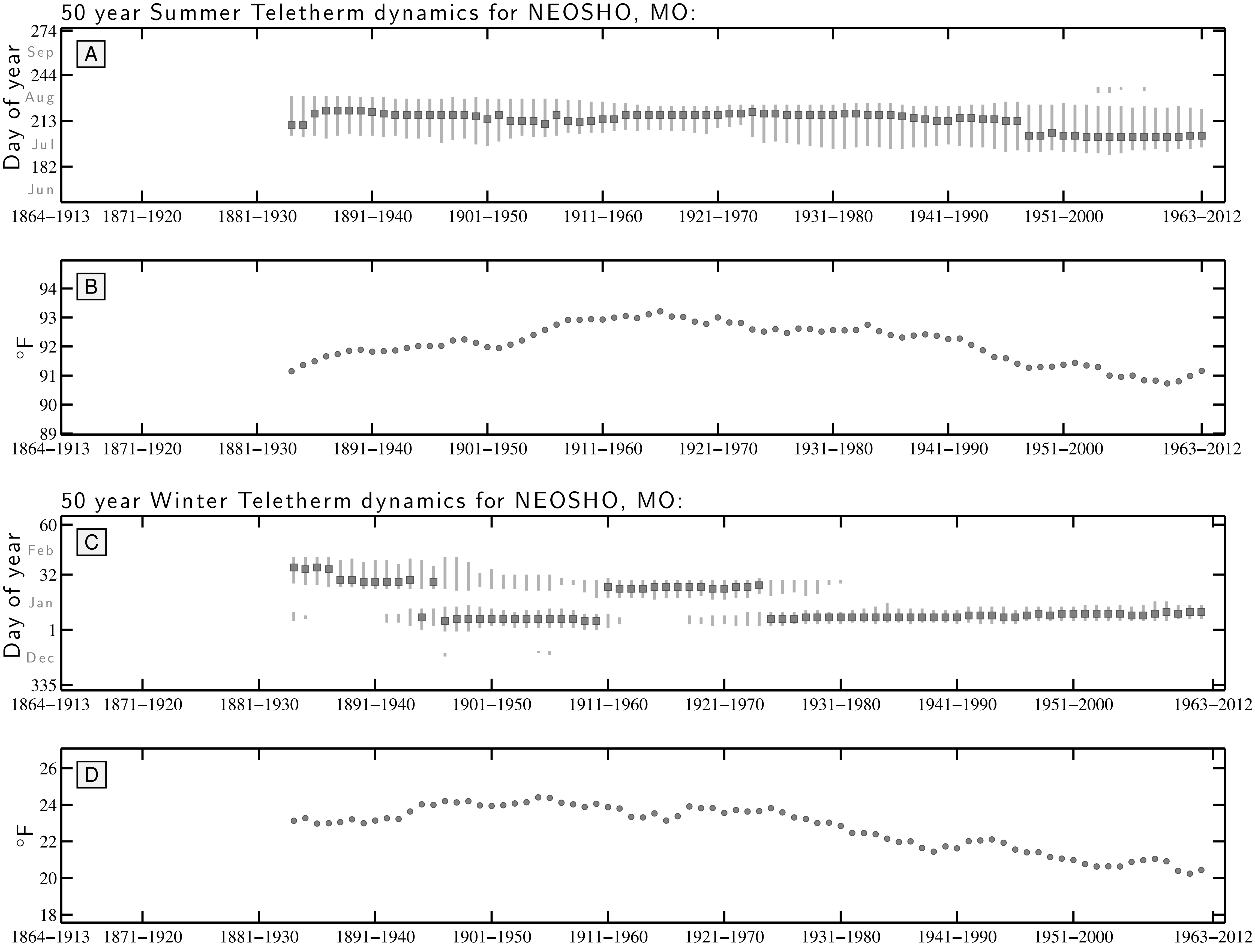

50 year Summer Teletherm dynamics for NEOSHO, MO:
A
Day of year
274
Sep
244
Aug
213
Jul
182
Jun
1864–1913
1871–1920
1881–1930
1891–1940
1901–1950
1911–1960
1921–1970
1931–1980
1941–1990
1951–2000
1963–2012
B
°F
94
93
92
91
90
89
50 year Winter Teletherm dynamics for NEOSHO, MO:
C
Day of year
60
Feb
32
Jan
1
Dec
335
D
°F
26
24
22
20
18

50 year Summer Teletherm dynamics for ROLLA UNI OF MISSOURI, MO:

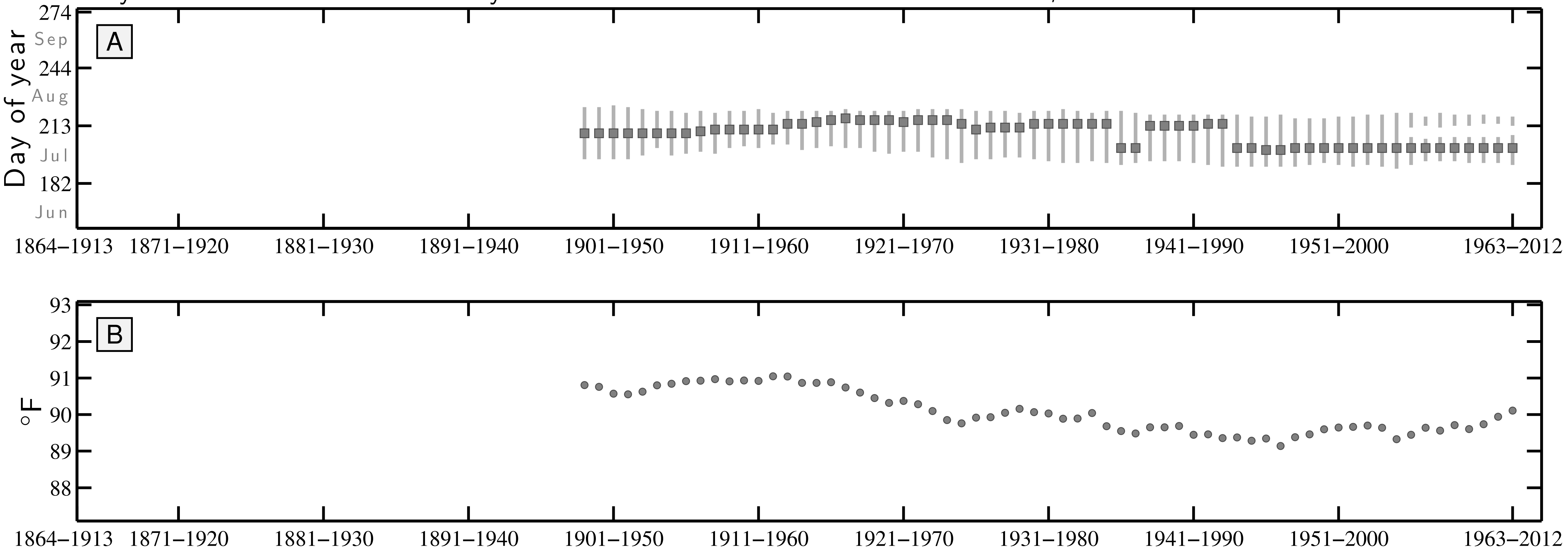

50 year Winter Teletherm dynamics for ROLLA UNI OF MISSOURI, MO:

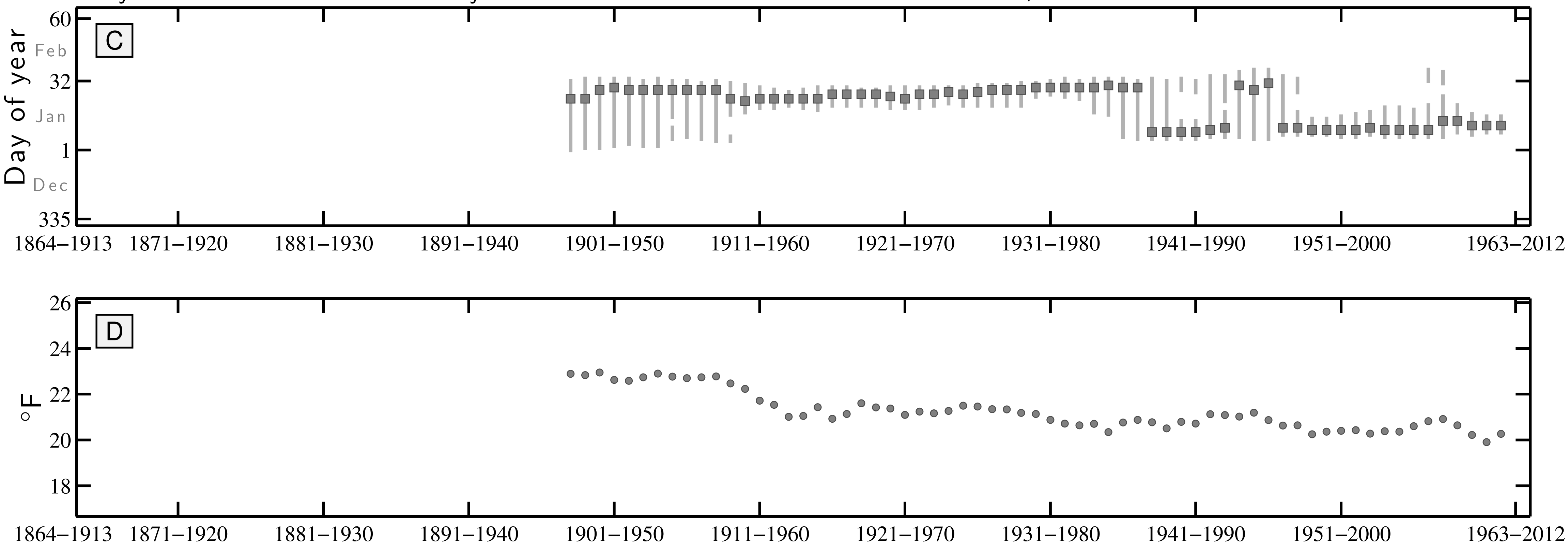

50 year Summer Teletherm dynamics for SPICKARD 7 W, MO:

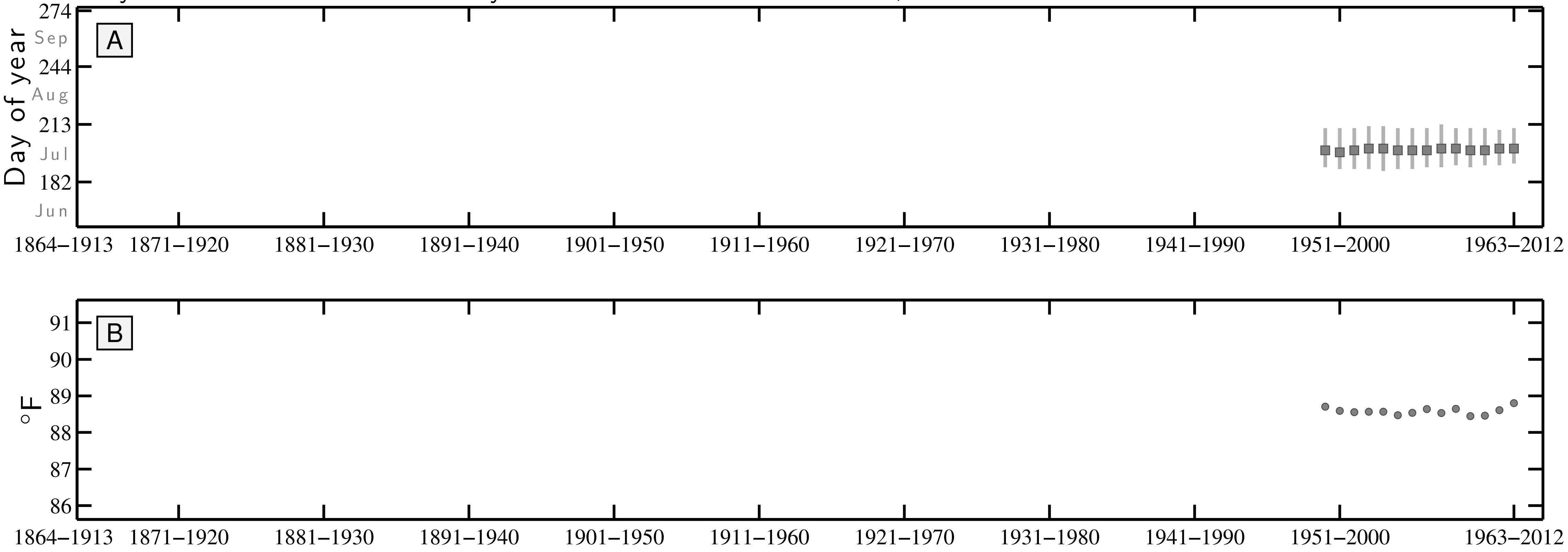

50 year Winter Teletherm dynamics for SPICKARD 7 W, MO:

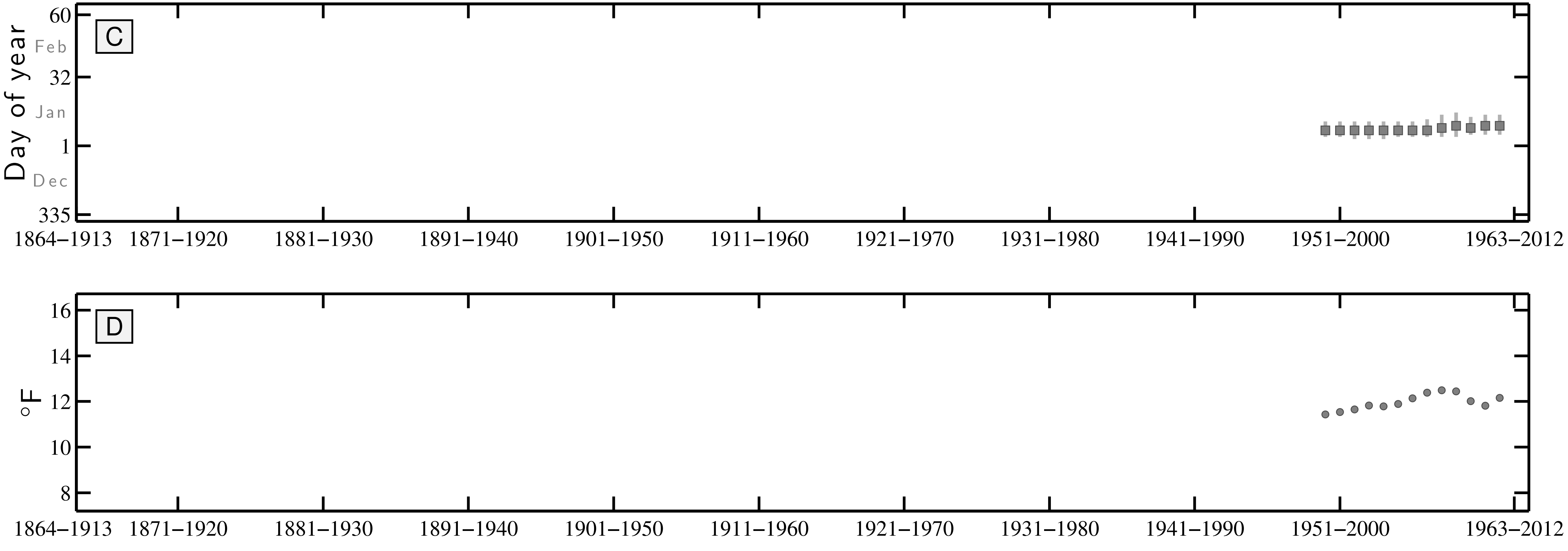

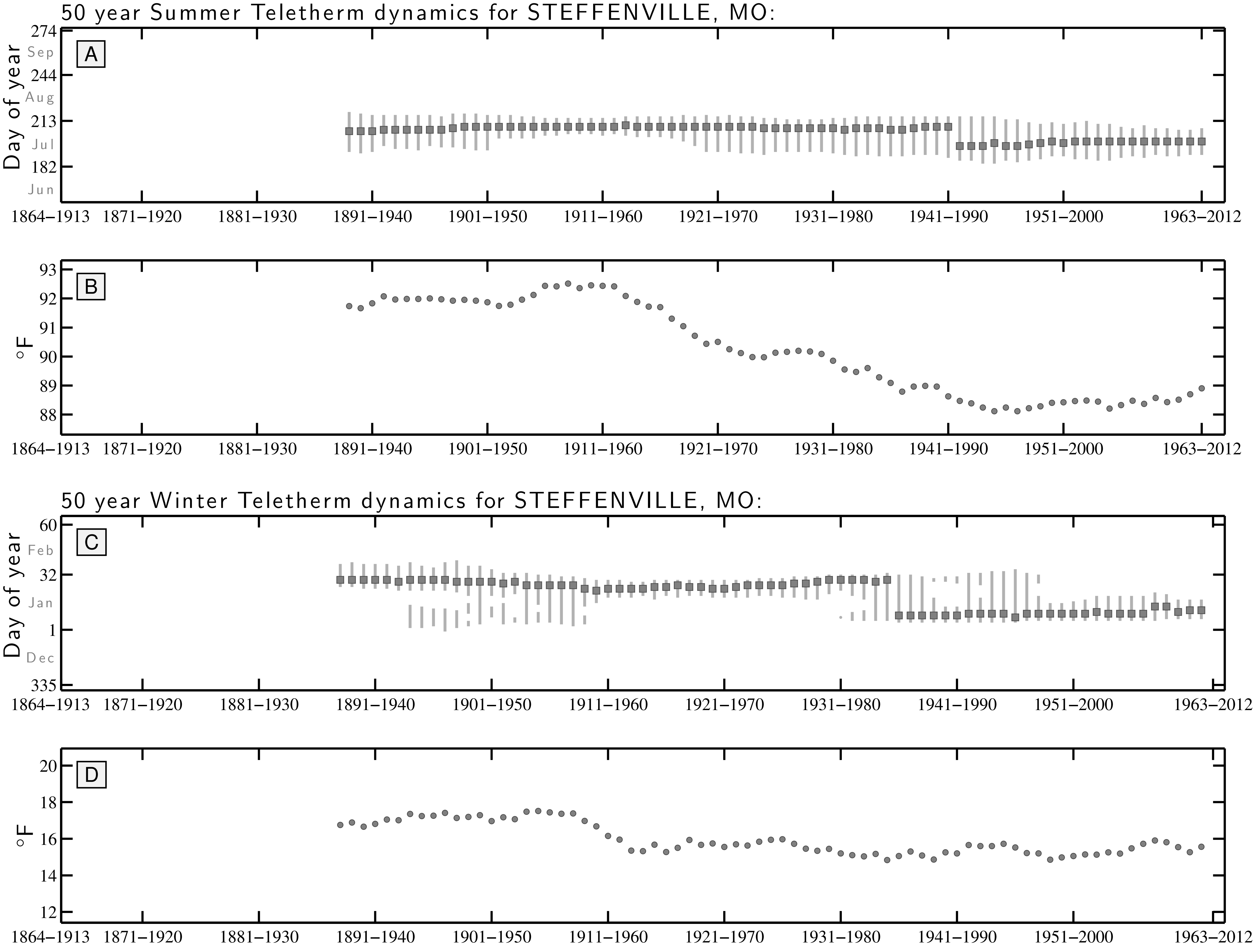

50 year Summer Teletherm dynamics for STEFFENVILLE, MO:
A
Day of year
274
Sep
244
Aug
213
Jul
182
Jun
1864–1913
1871–1920
1881–1930
1891–1940
1901–1950
1911–1960
1921–1970
1931–1980
1941–1990
1951–2000
1963–2012
B
°F
93
92
91
90
89
88
50 year Winter Teletherm dynamics for STEFFENVILLE, MO:
C
Day of year
60
Feb
32
Jan
1
Dec
335
D
°F
20
18
16
14
12

50 year Summer Teletherm dynamics for SWEET SPRINGS, MO:

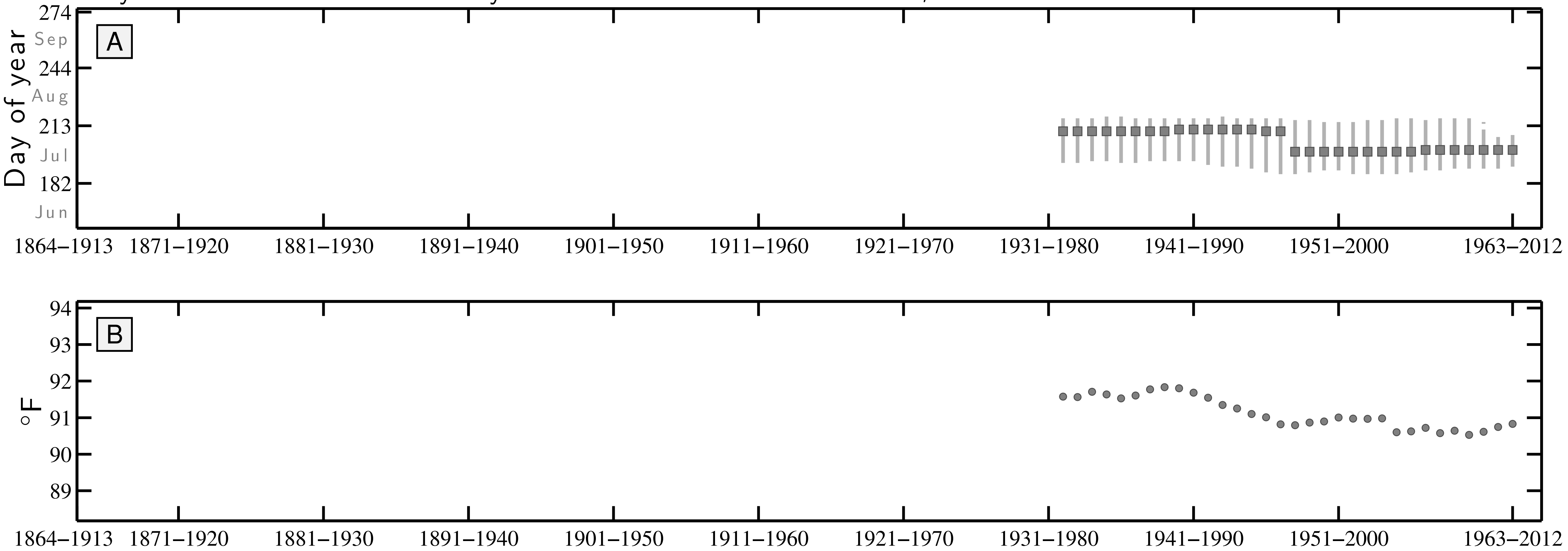

50 year Winter Teletherm dynamics for SWEET SPRINGS, MO:

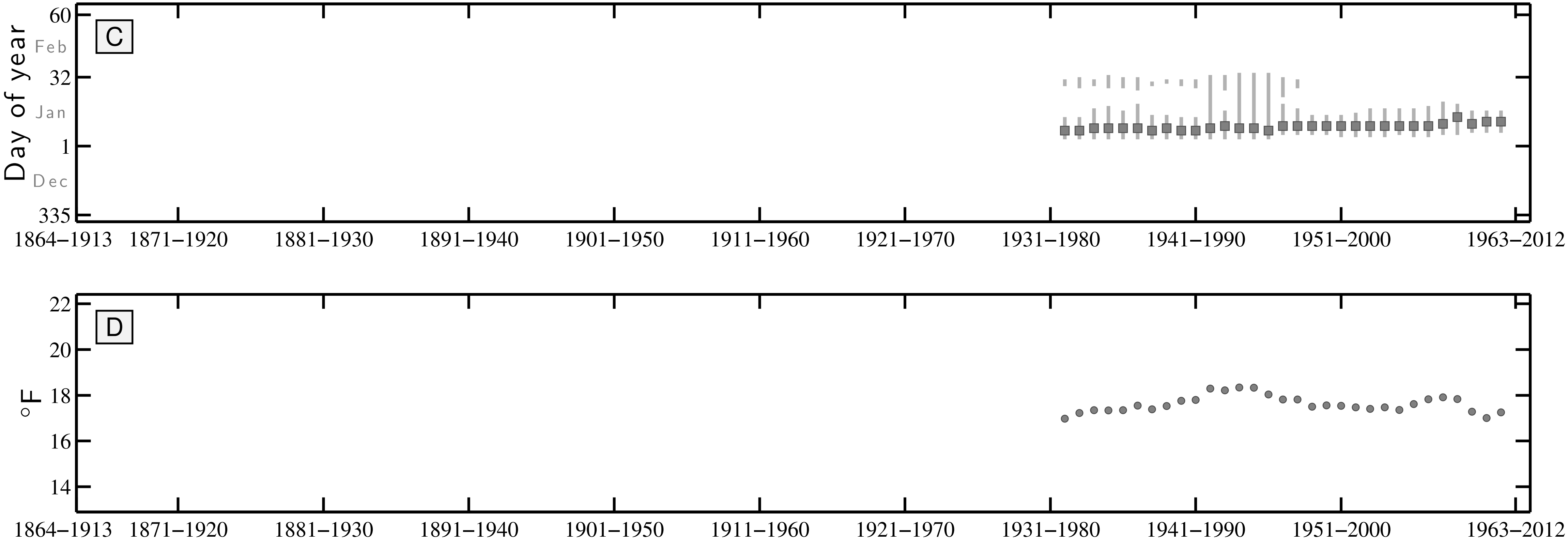

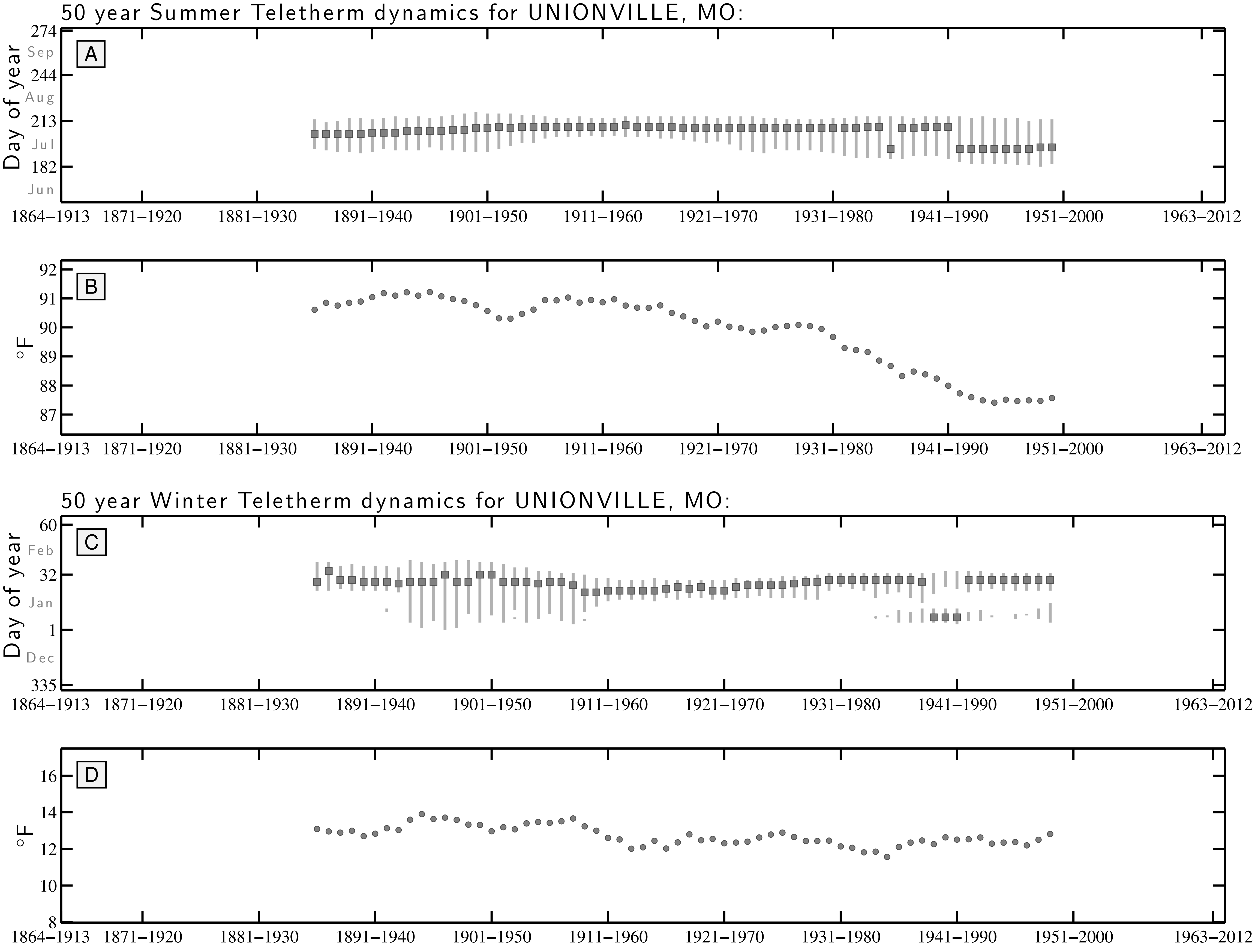

50 year Summer Teletherm dynamics for UNIONVILLE, MO:
A
Day of year
274
Sep
244
Aug
213
Jul
182
Jun
B
°F
92
91
90
89
88
87
50 year Winter Teletherm dynamics for UNIONVILLE, MO:
C
Day of year
60
Feb
32
Jan
1
Dec
335
D
°F
16
14
12
10
8
1864–1913
1871–1920
1881–1930
1891–1940
1901–1950
1911–1960
1921–1970
1931–1980
1941–1990
1951–2000
1963–2012

50 year Summer Teletherm dynamics for WARRENTON 1 N, MO:

50 year Winter Teletherm dynamics for WARRENTON 1 N, MO:

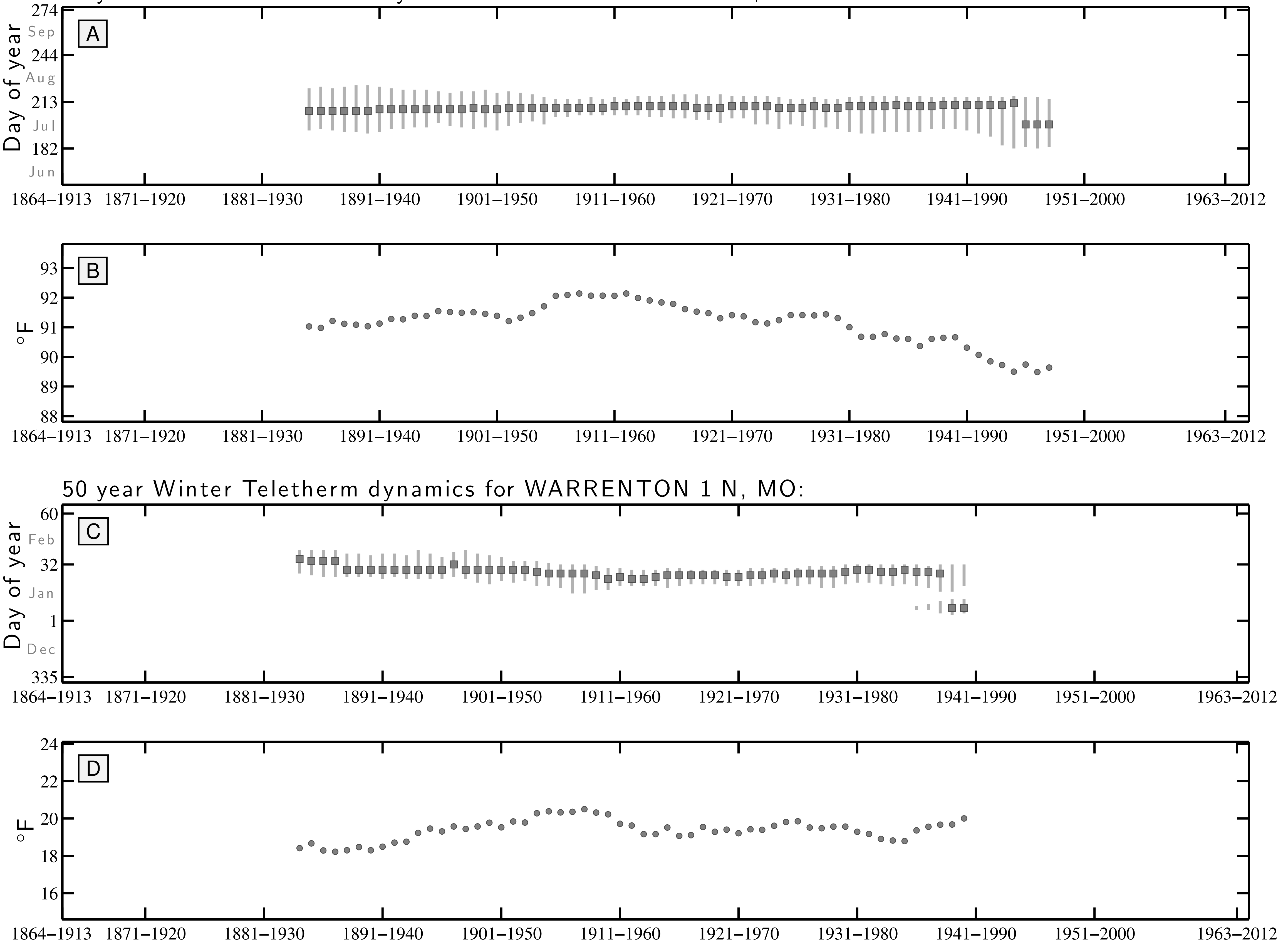

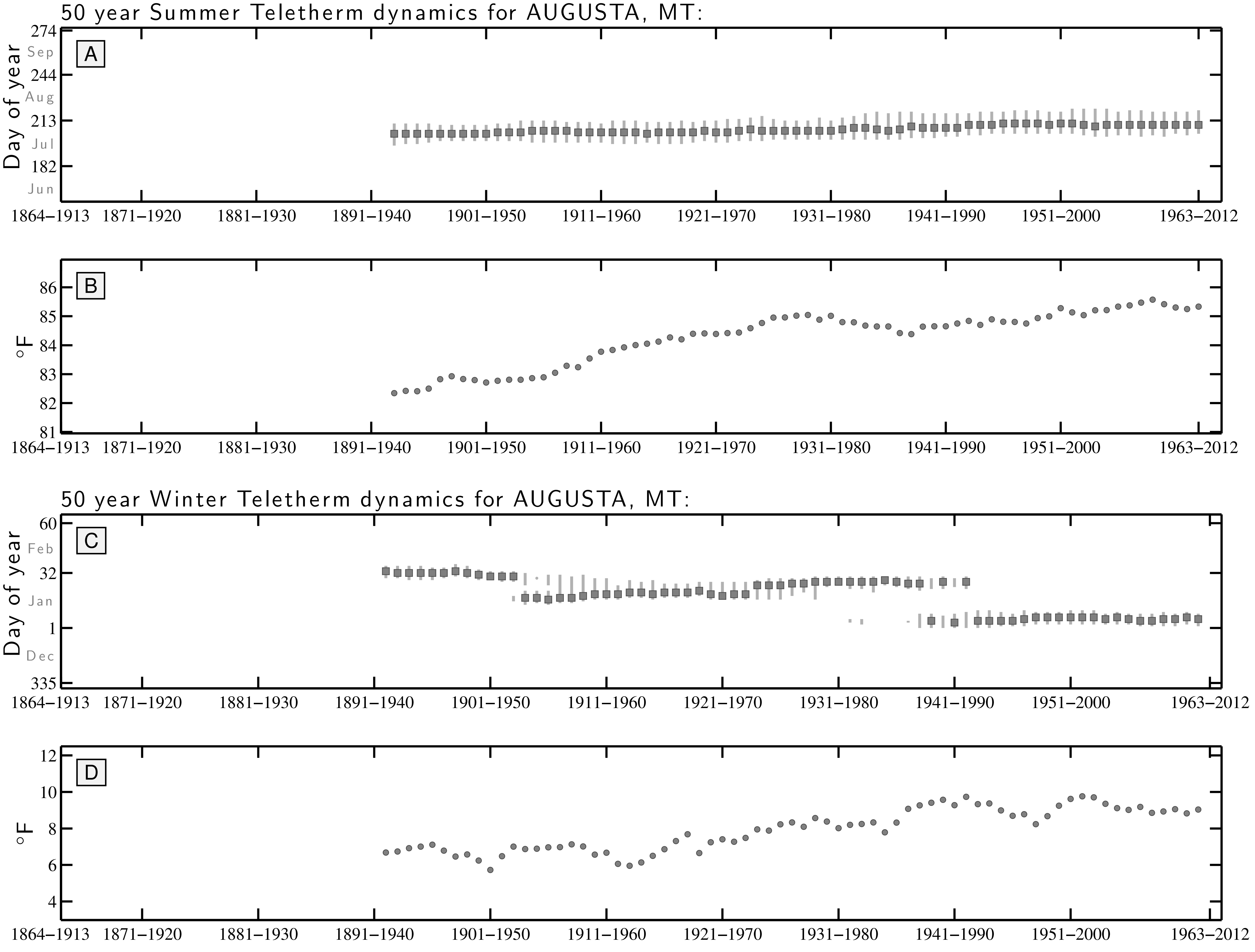
50 year Summer Teletherm dynamics for AUGUSTA, MT:
A
B
Day of year
°F
274
Sep
244
Aug
213
Jul
182
Jun
86
85
84
83
82
81
1864–1913
1871–1920
1881–1930
1891–1940
1901–1950
1911–1960
1921–1970
1931–1980
1941–1990
1951–2000
1963–2012
50 year Winter Teletherm dynamics for AUGUSTA, MT:
C
D
60
Feb
32
Jan
1
Dec
335
12
10
8
6
4

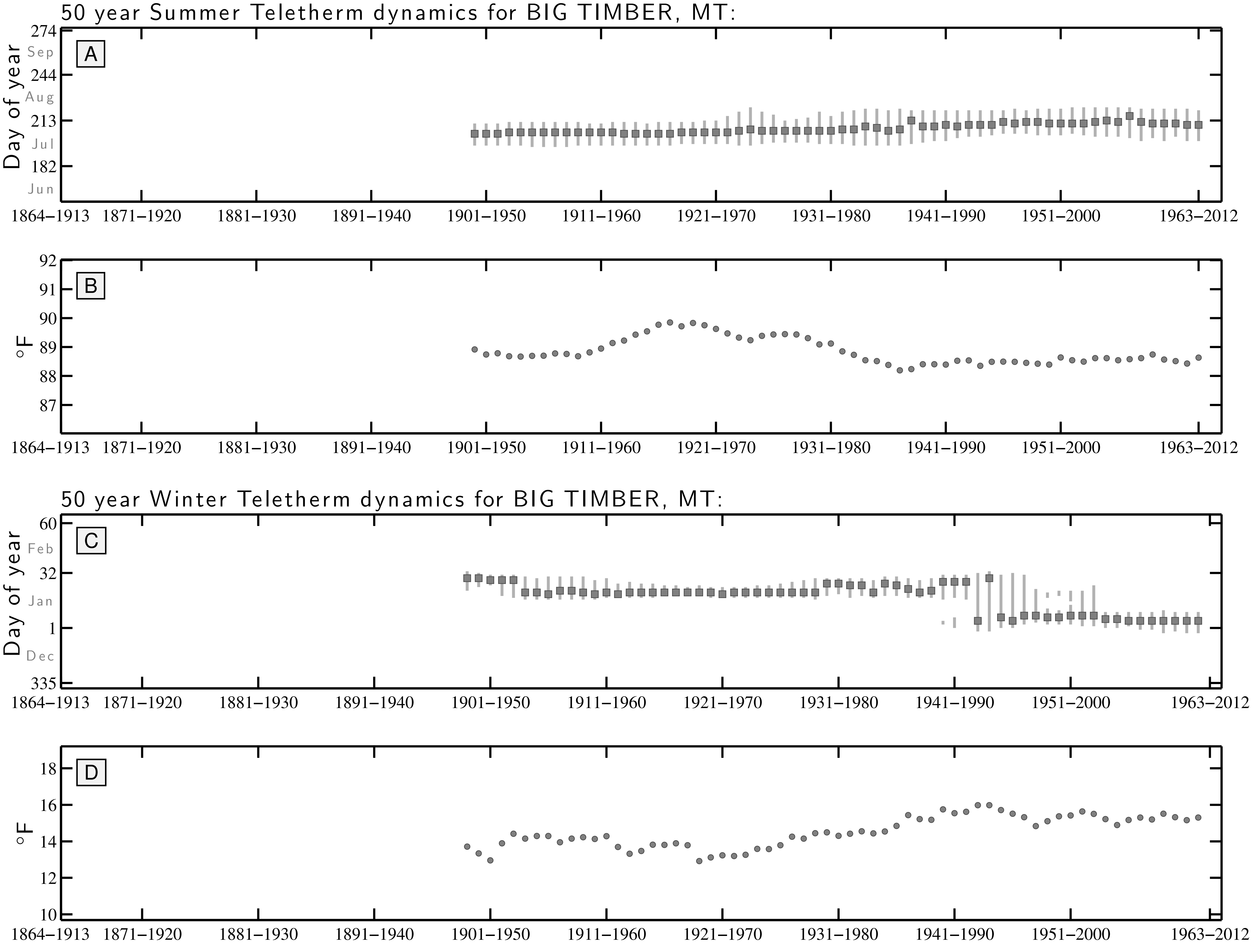
50 year Summer Teletherm dynamics for BIG TIMBER, MT:
A
B
Day of year
°F
Sep
Aug
Jul
Jun
274
244
213
182
92
91
90
89
88
87
1864–1913
1871–1920
1881–1930
1891–1940
1901–1950
1911–1960
1921–1970
1931–1980
1941–1990
1951–2000
1963–2012
50 year Winter Teletherm dynamics for BIG TIMBER, MT:
C
D
Feb
Jan
Dec
60
32
1
335
18
16
14
12
10

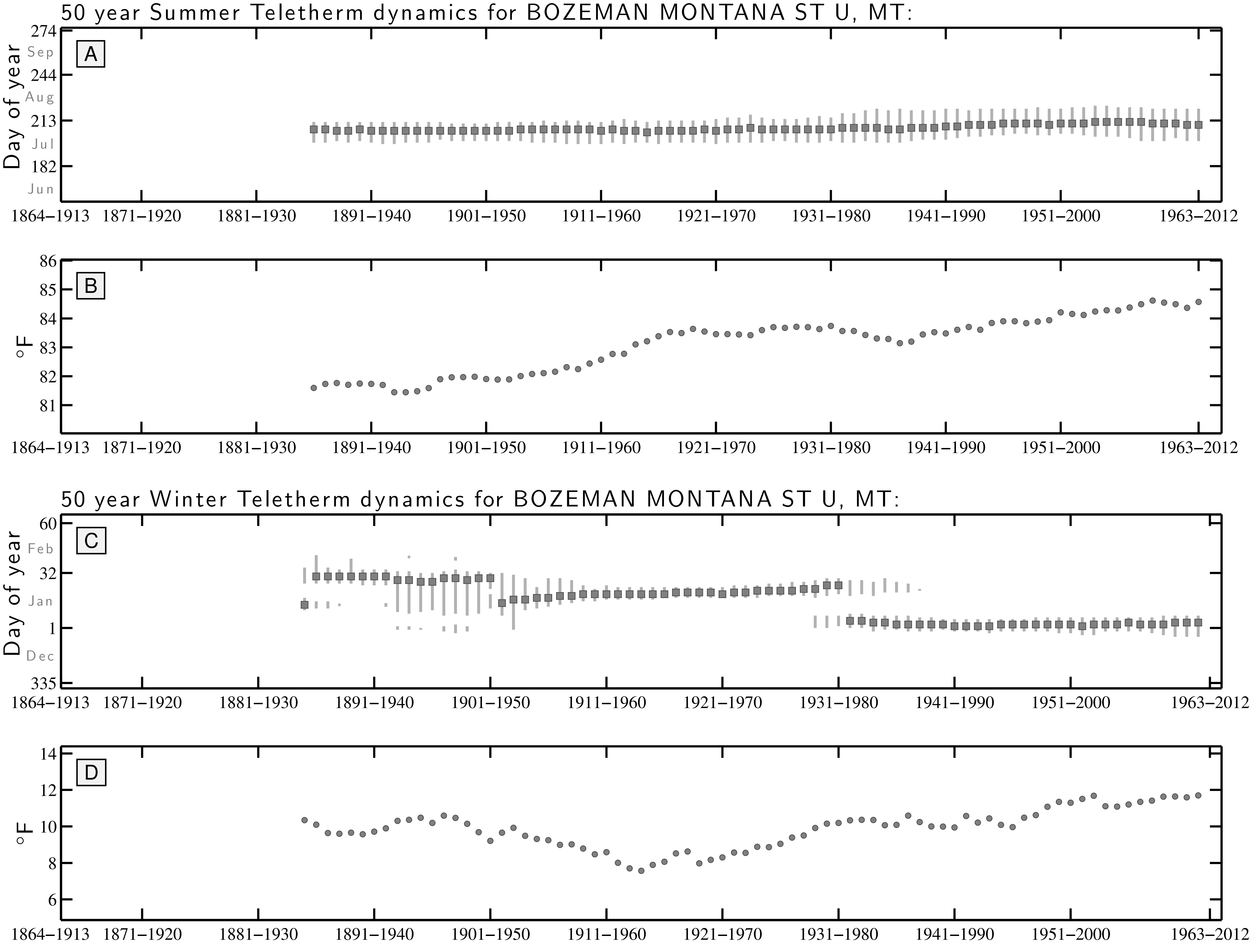
50 year Summer Teletherm dynamics for BOZEMAN MONTANA ST U, MT:
A
B
Day of year
274
Sep
244
Aug
213
Jul
182
Jun
86
85
84
83
82
81
°F
1864–1913
1871–1920
1881–1930
1891–1940
1901–1950
1911–1960
1921–1970
1931–1980
1941–1990
1951–2000
1963–2012
50 year Winter Teletherm dynamics for BOZEMAN MONTANA ST U, MT:
C
D
Day of year
60
Feb
32
Jan
1
Dec
335
14
12
10
8
6
°F

50 year Summer Teletherm dynamics for CASCADE 5 S, MT:

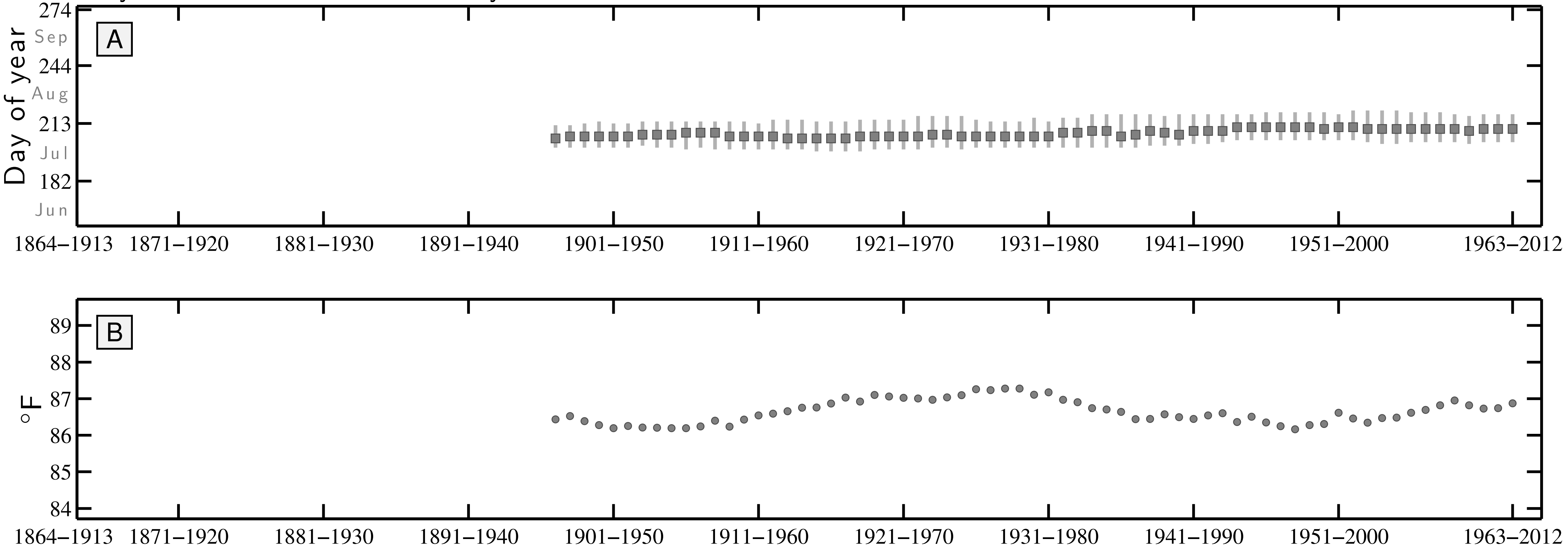

50 year Winter Teletherm dynamics for CASCADE 5 S, MT:

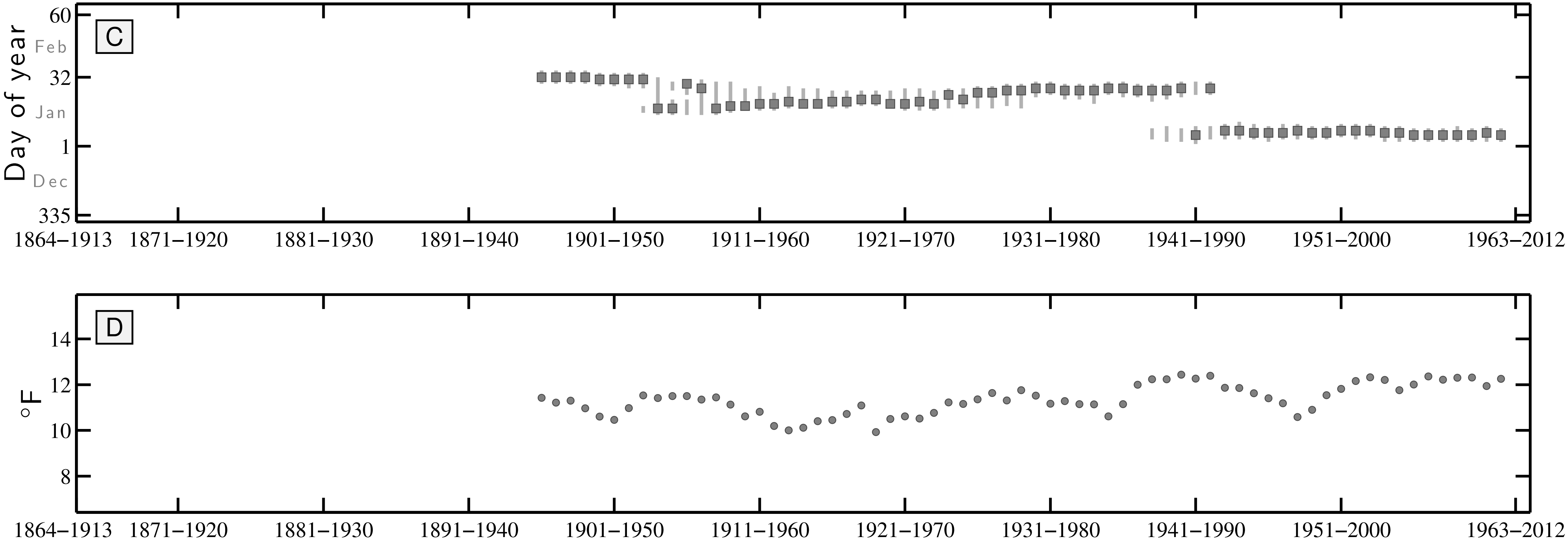

50 year Summer Teletherm dynamics for CHINOOK, MT:

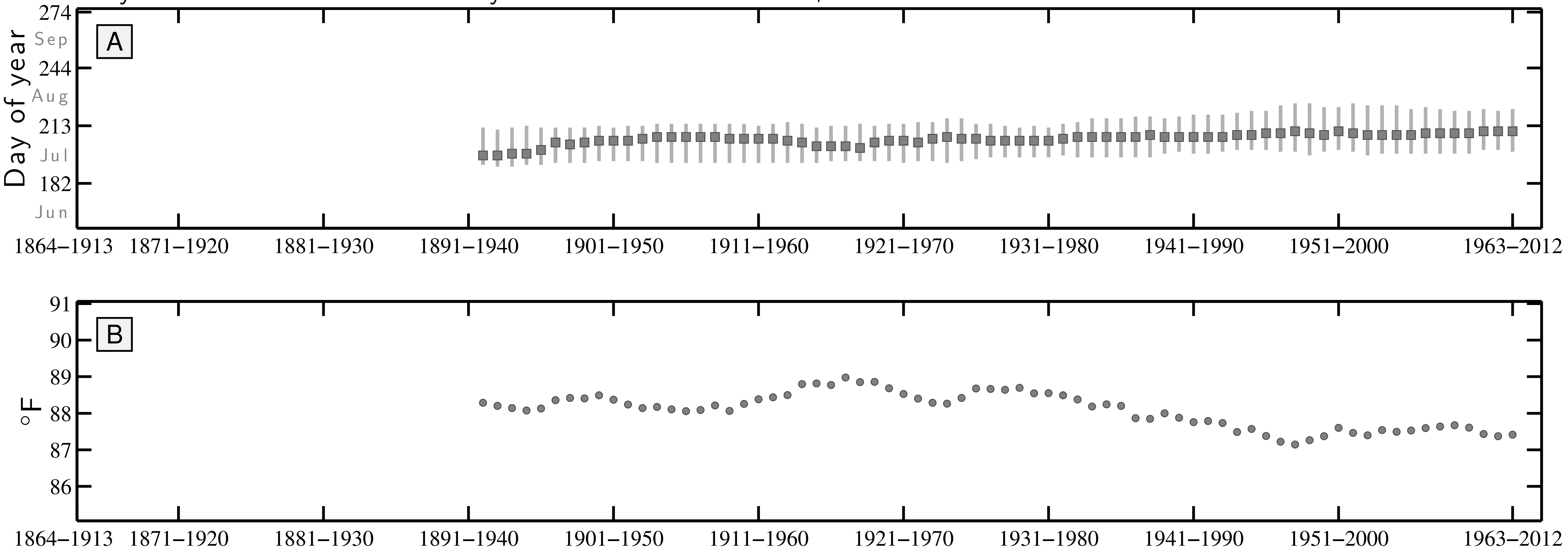

50 year Winter Teletherm dynamics for CHINOOK, MT:

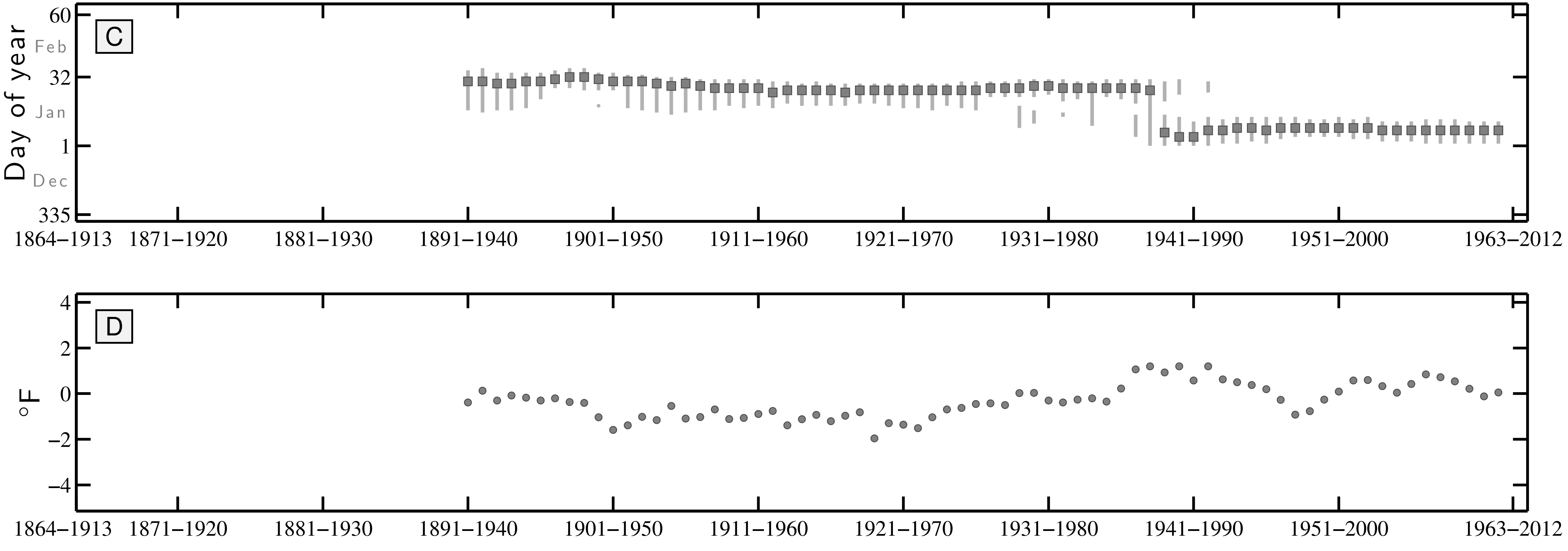

50 year Summer Teletherm dynamics for CHOTEAU, MT:

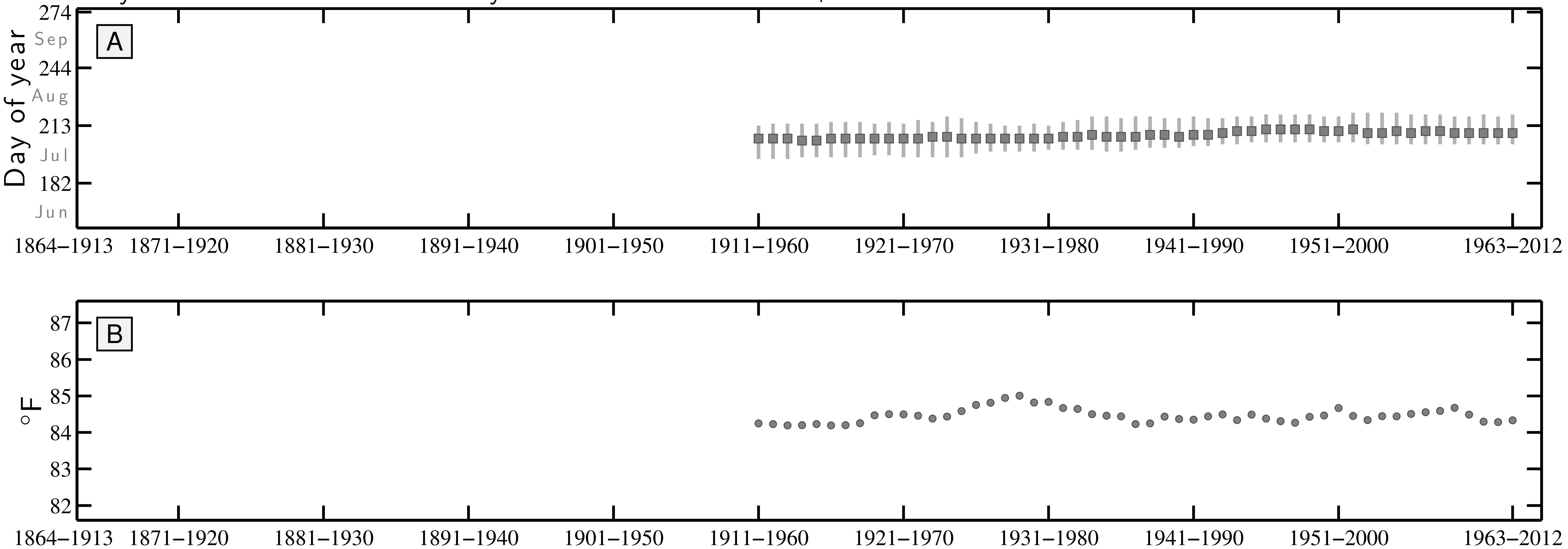

50 year Winter Teletherm dynamics for CHOTEAU, MT:

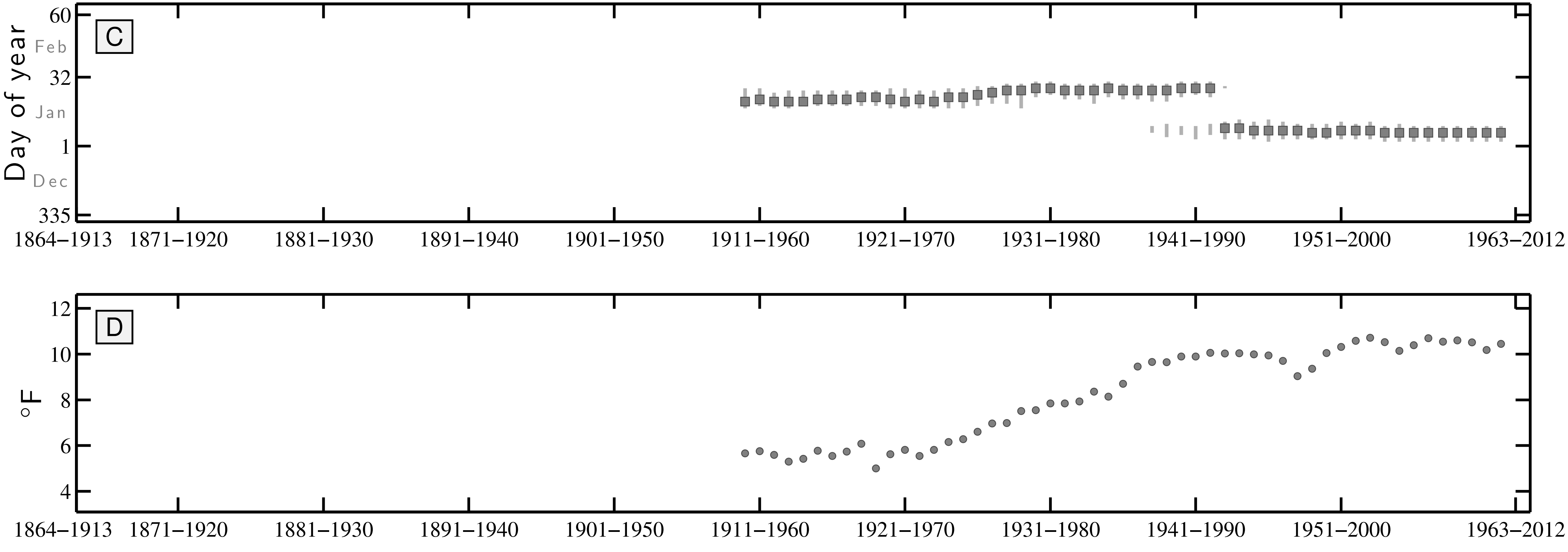

50 year Summer Teletherm dynamics for CUT BANK AP, MT:

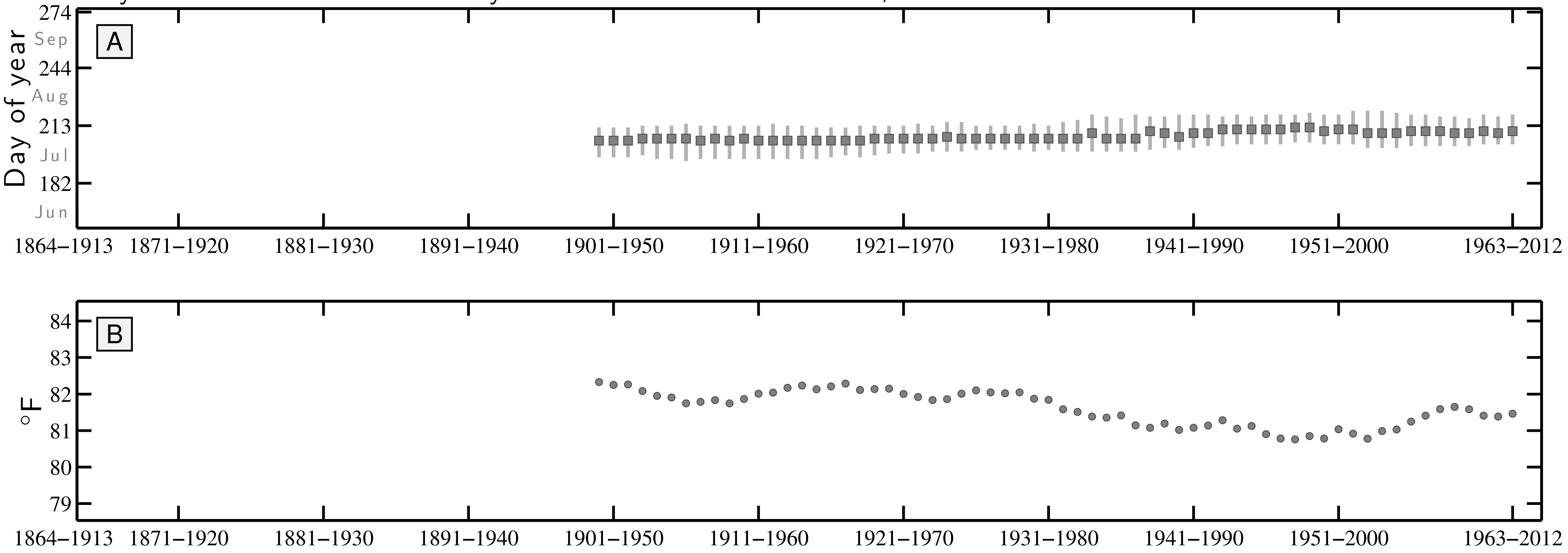

50 year Winter Teletherm dynamics for CUT BANK AP, MT:

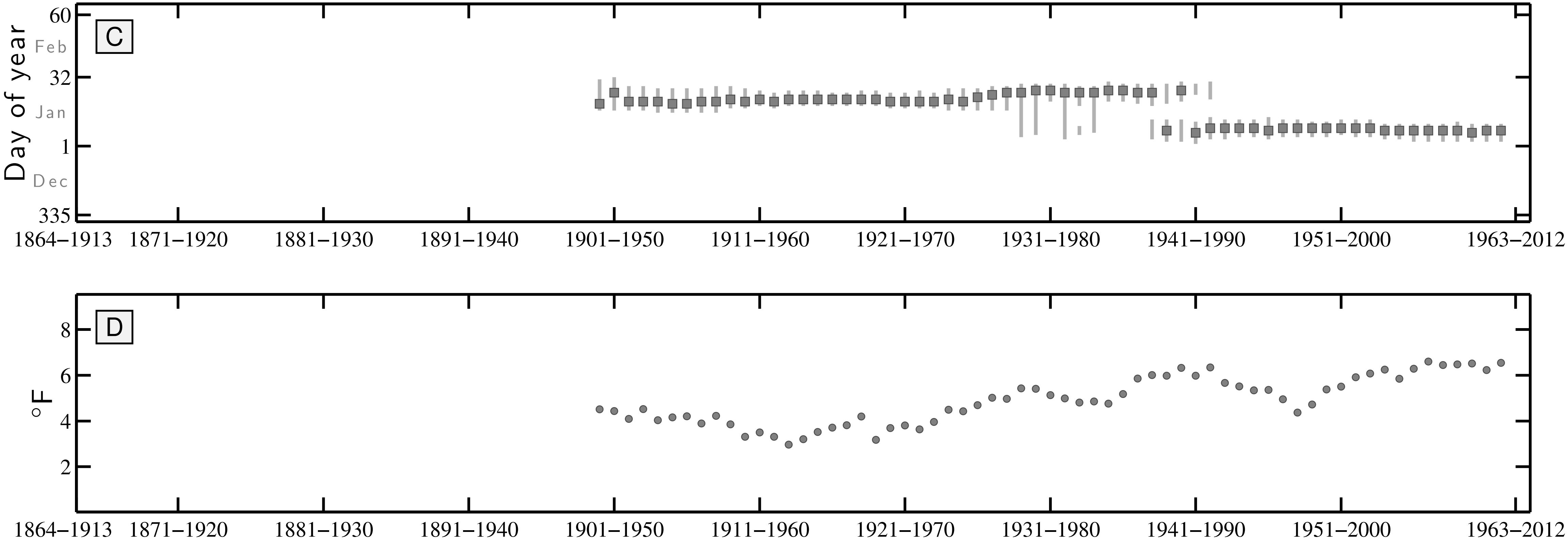

50 year Summer Teletherm dynamics for DILLON WMCE, MT:

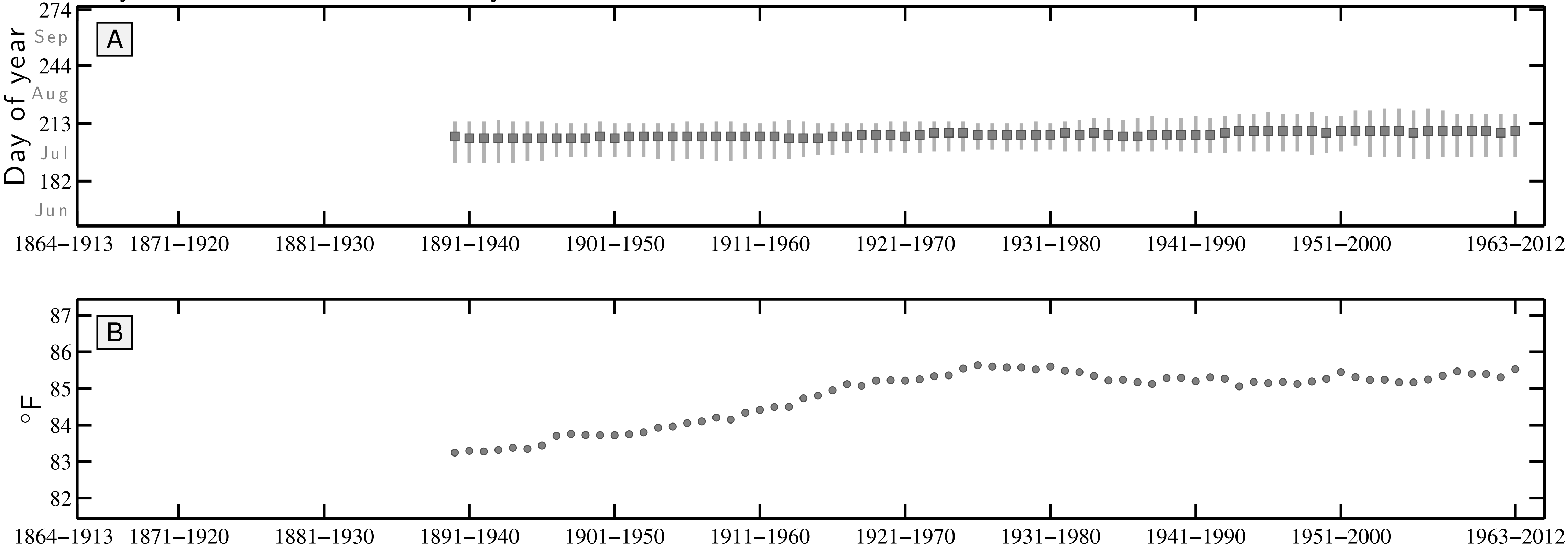

50 year Winter Teletherm dynamics for DILLON WMCE, MT:

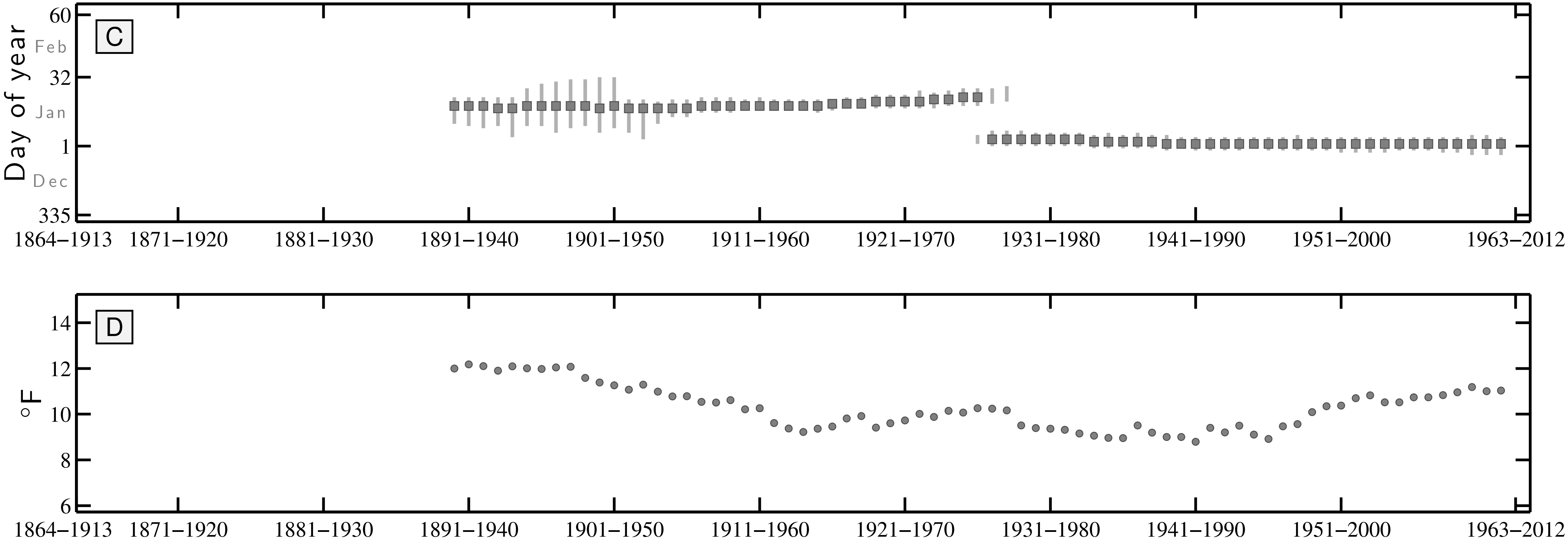

50 year Summer Teletherm dynamics for EKALAKA, MT:

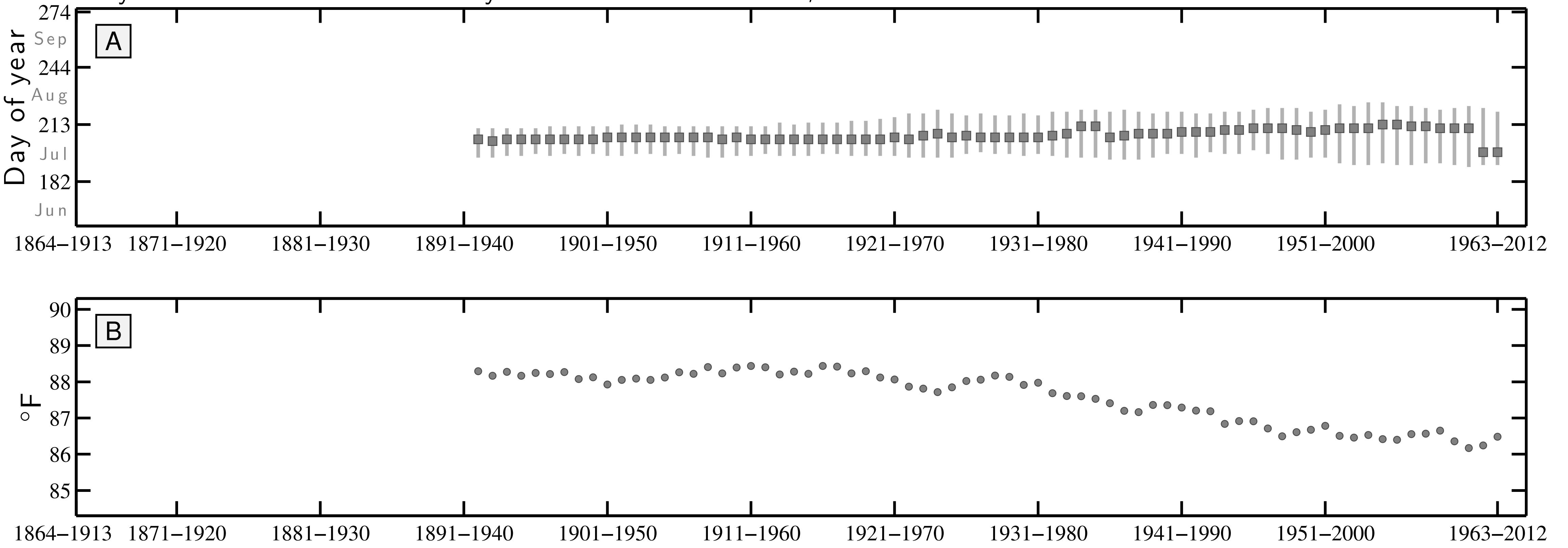

50 year Winter Teletherm dynamics for EKALAKA, MT:

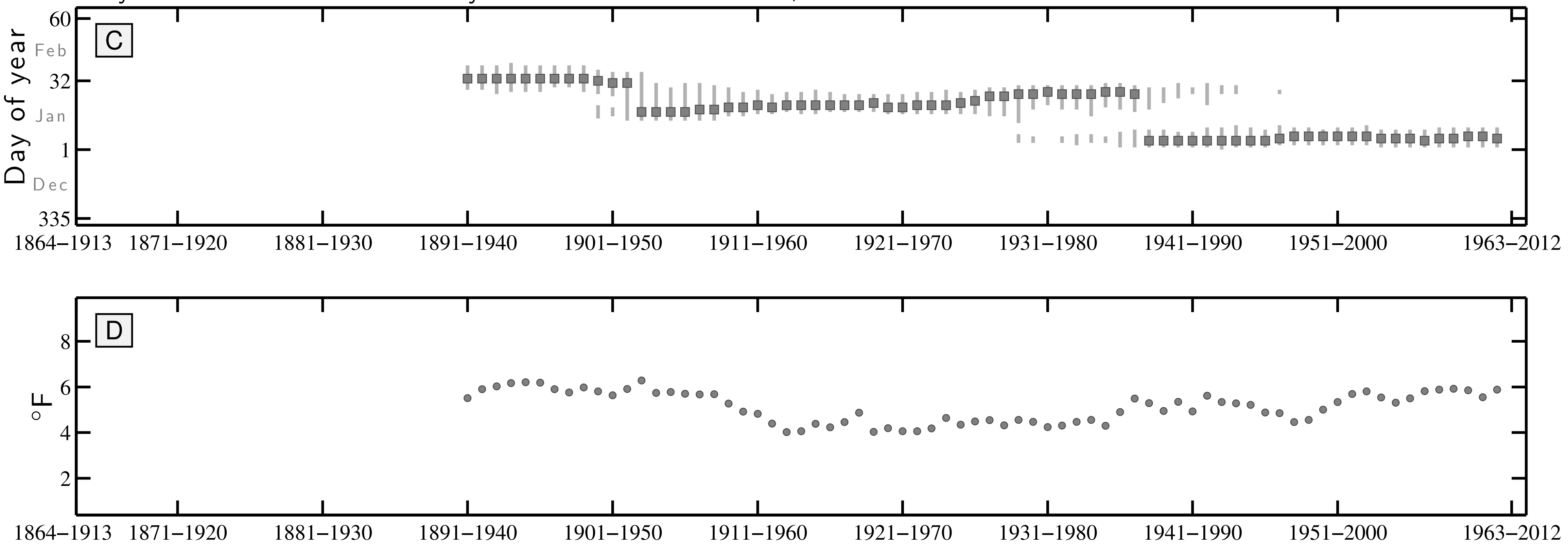

50 year Summer Teletherm dynamics for ENNIS, MT:

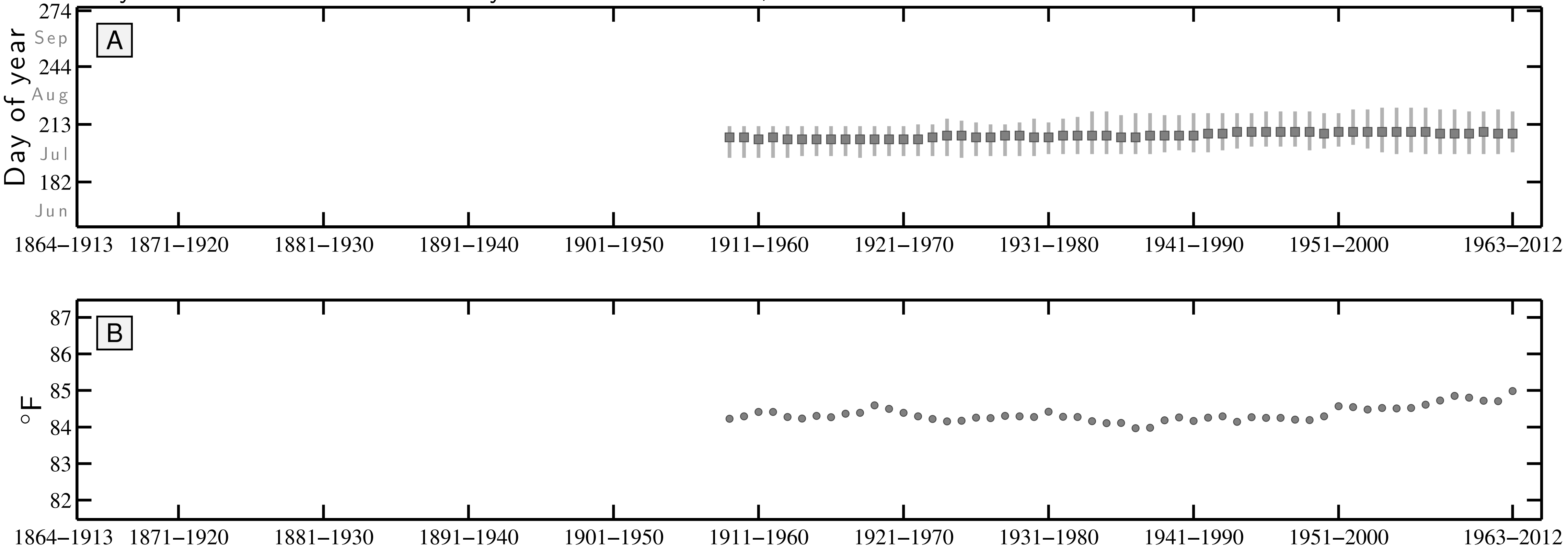

50 year Winter Teletherm dynamics for ENNIS, MT:

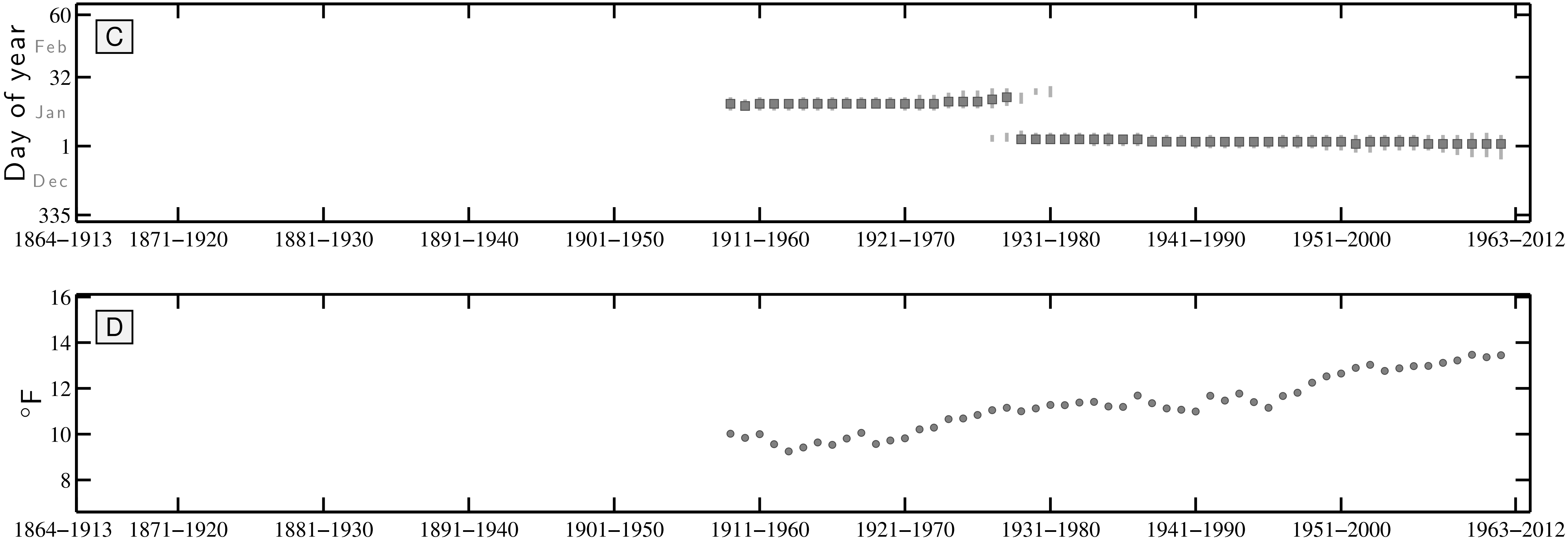

50 year Summer Teletherm dynamics for FLATWILLOW 4 ENE, MT:

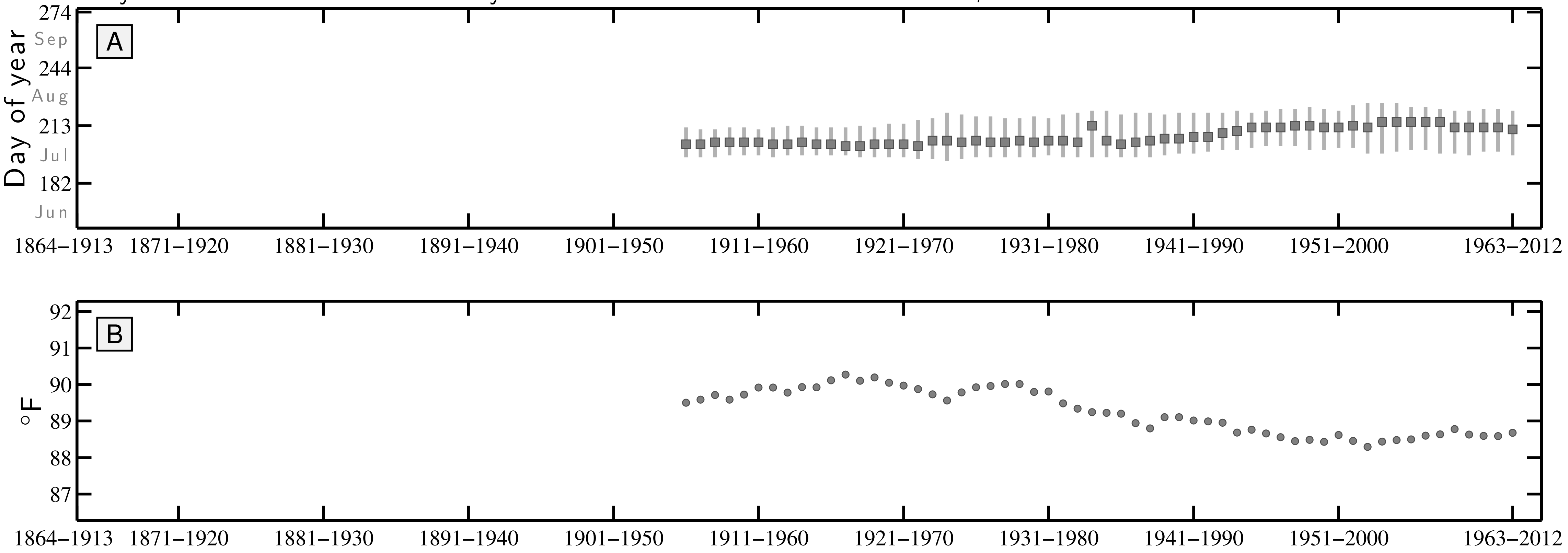

50 year Winter Teletherm dynamics for FLATWILLOW 4 ENE, MT:

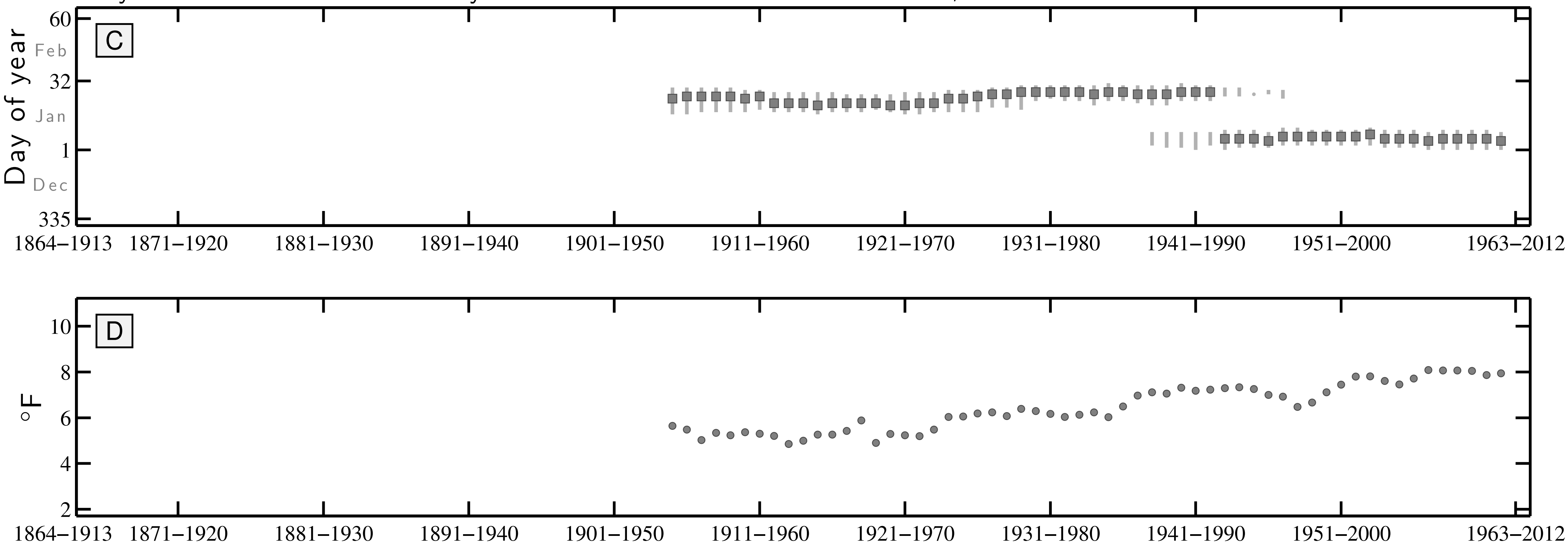

50 year Summer Teletherm dynamics for FORKS 4 NNE, MT:

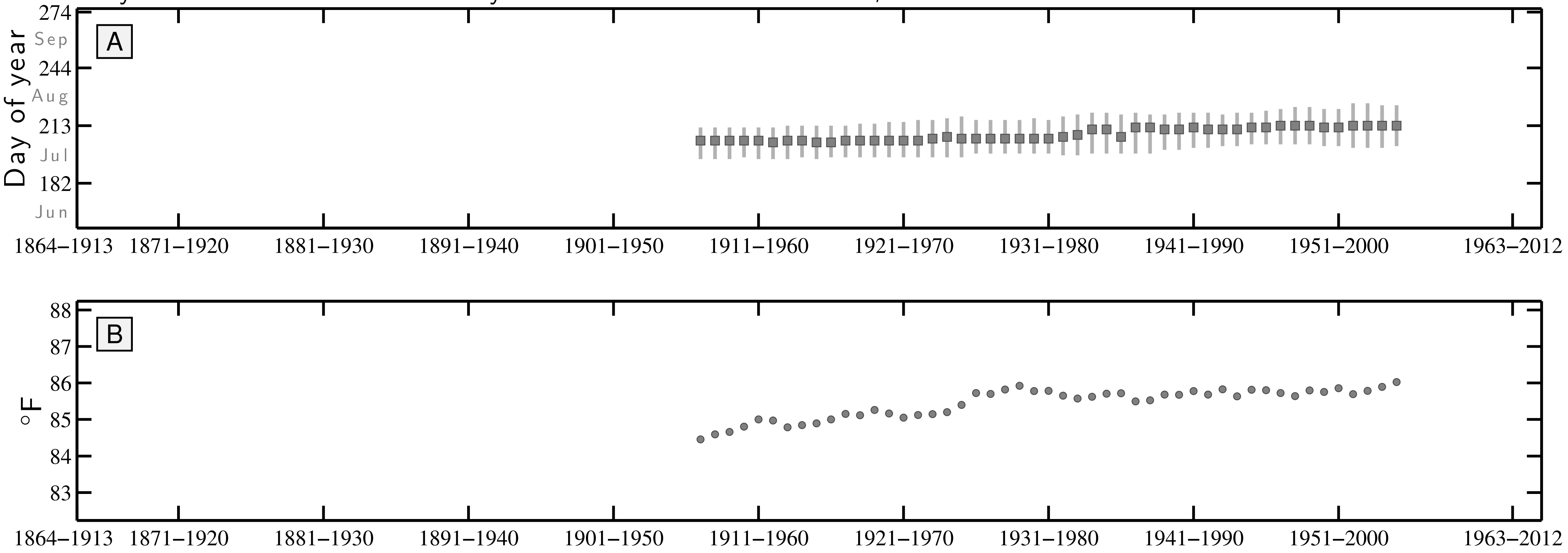

50 year Winter Teletherm dynamics for FORKS 4 NNE, MT:

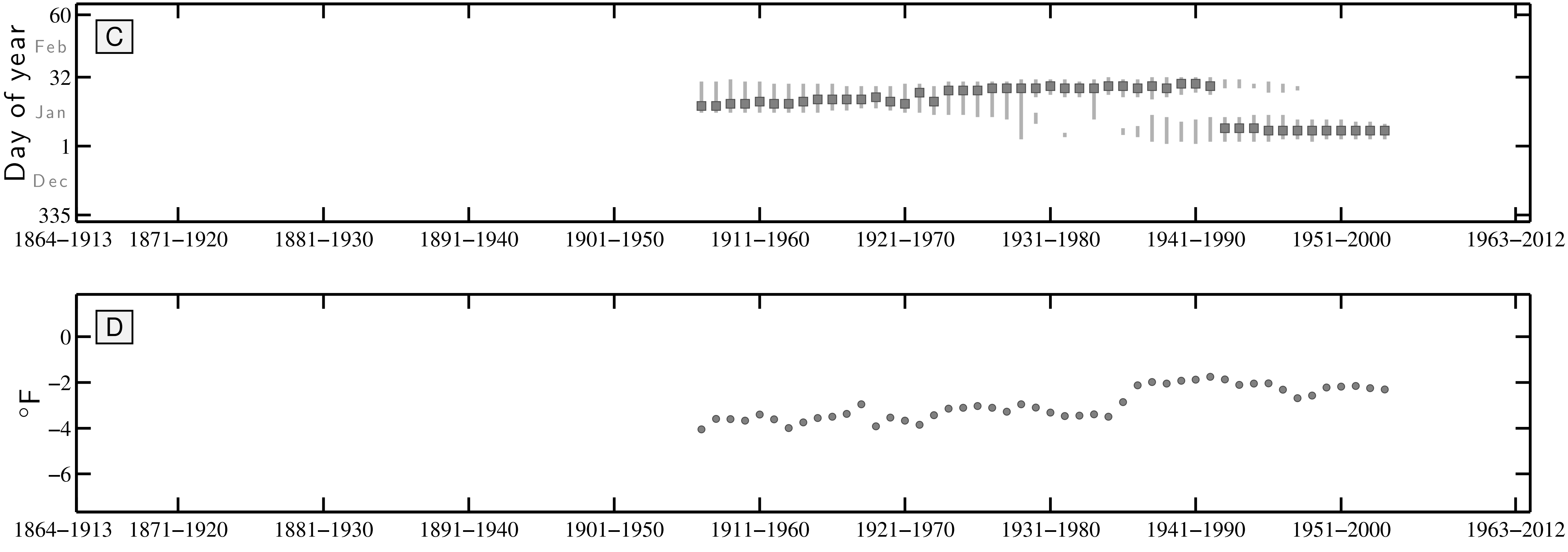

50 year Summer Teletherm dynamics for FT ASSINNIBOINE, MT:

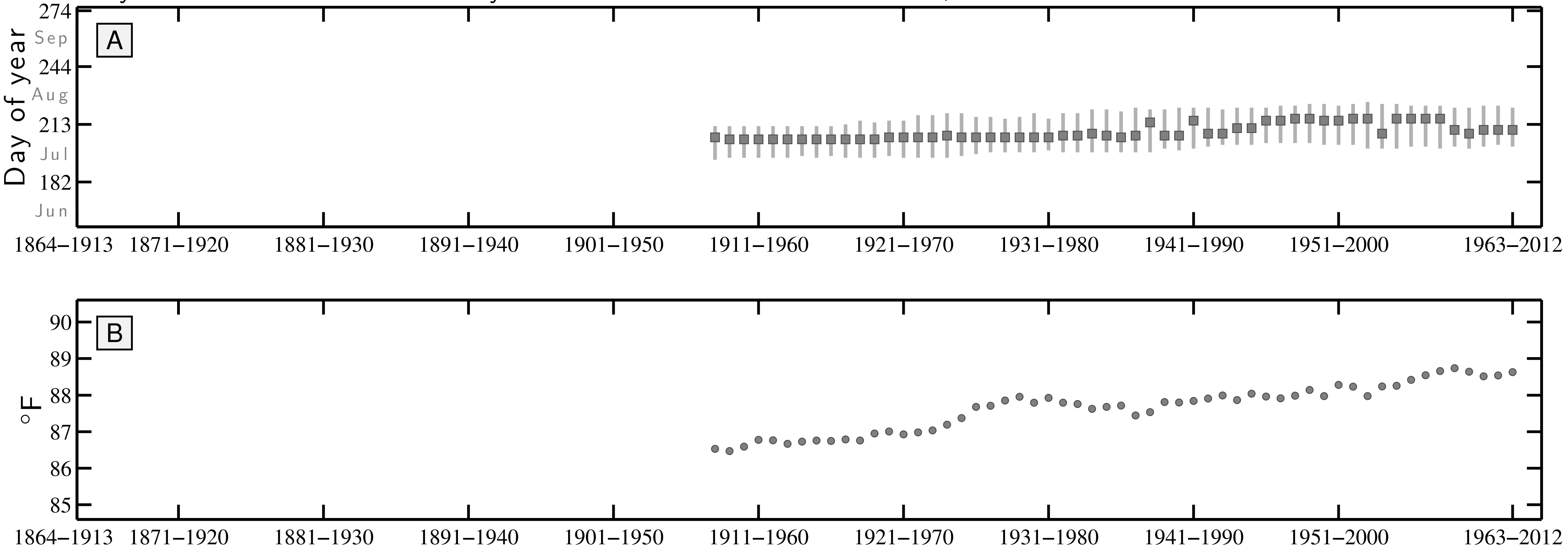

50 year Winter Teletherm dynamics for FT ASSINNIBOINE, MT:

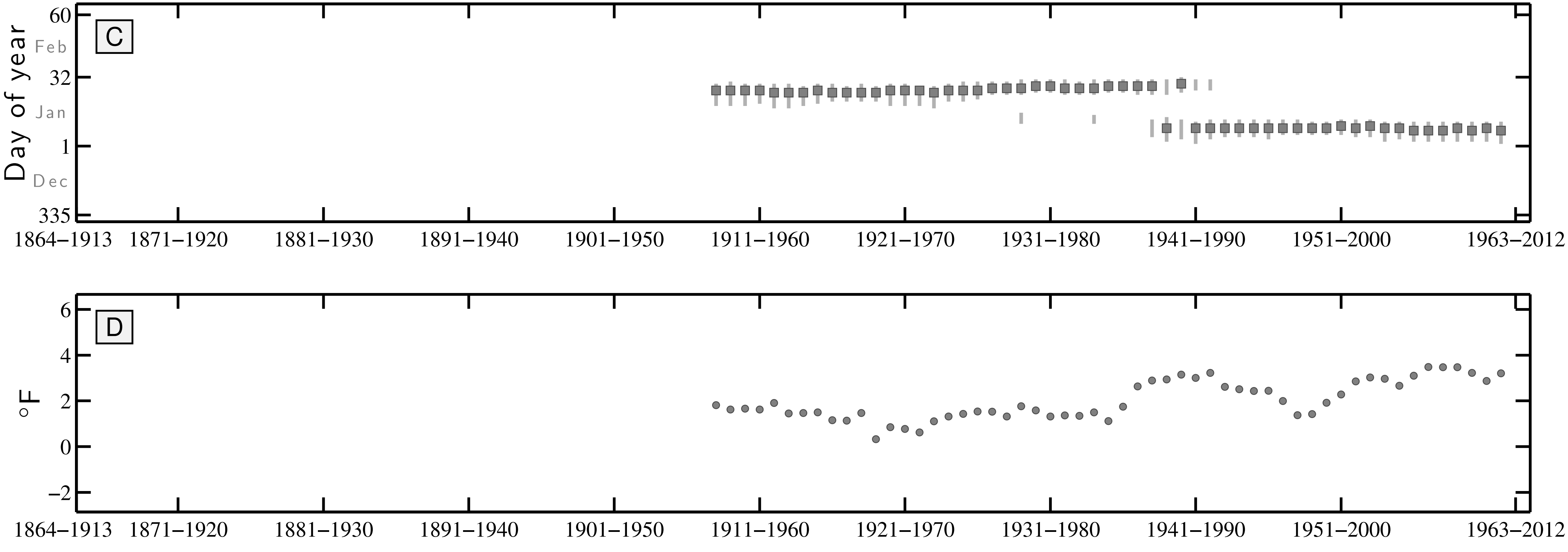

50 year Summer Teletherm dynamics for FORTINE 1 N, MT:

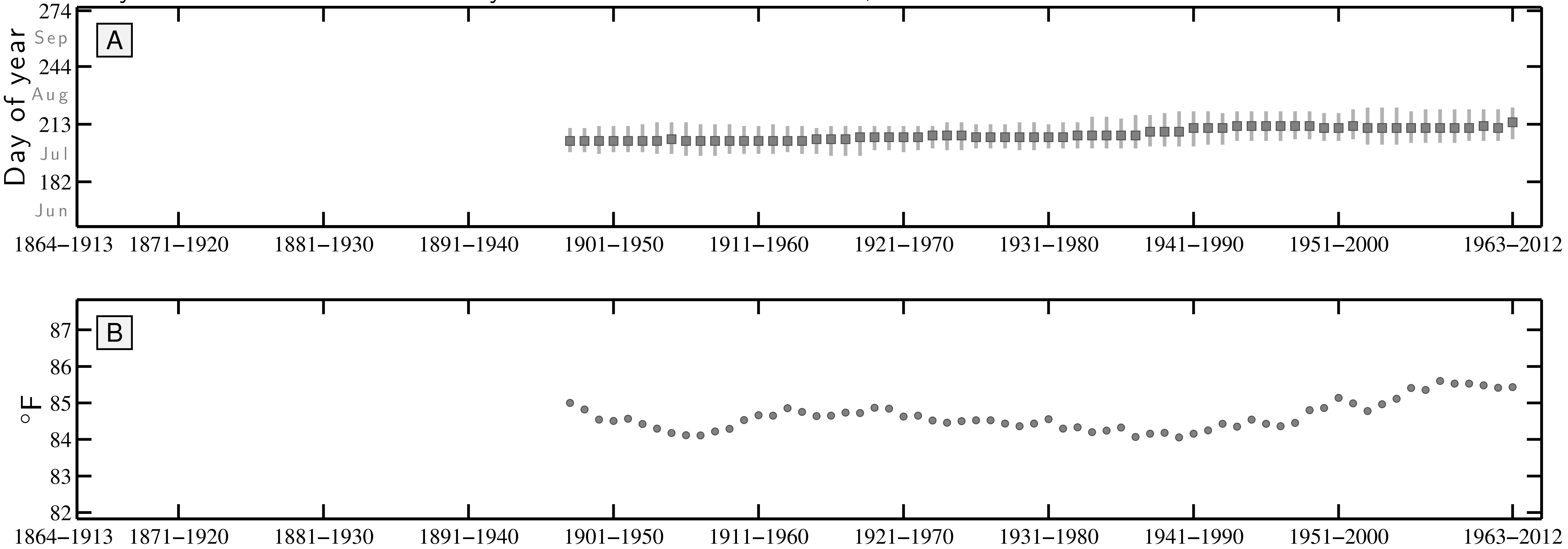

50 year Winter Teletherm dynamics for FORTINE 1 N, MT:

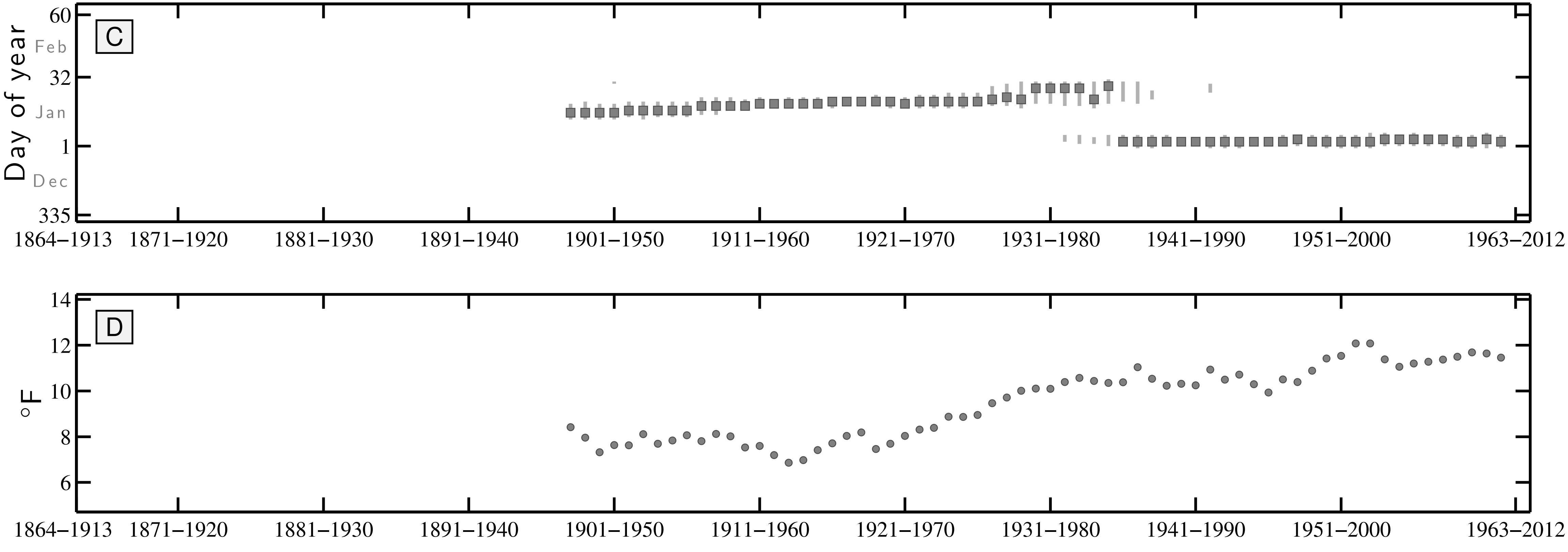

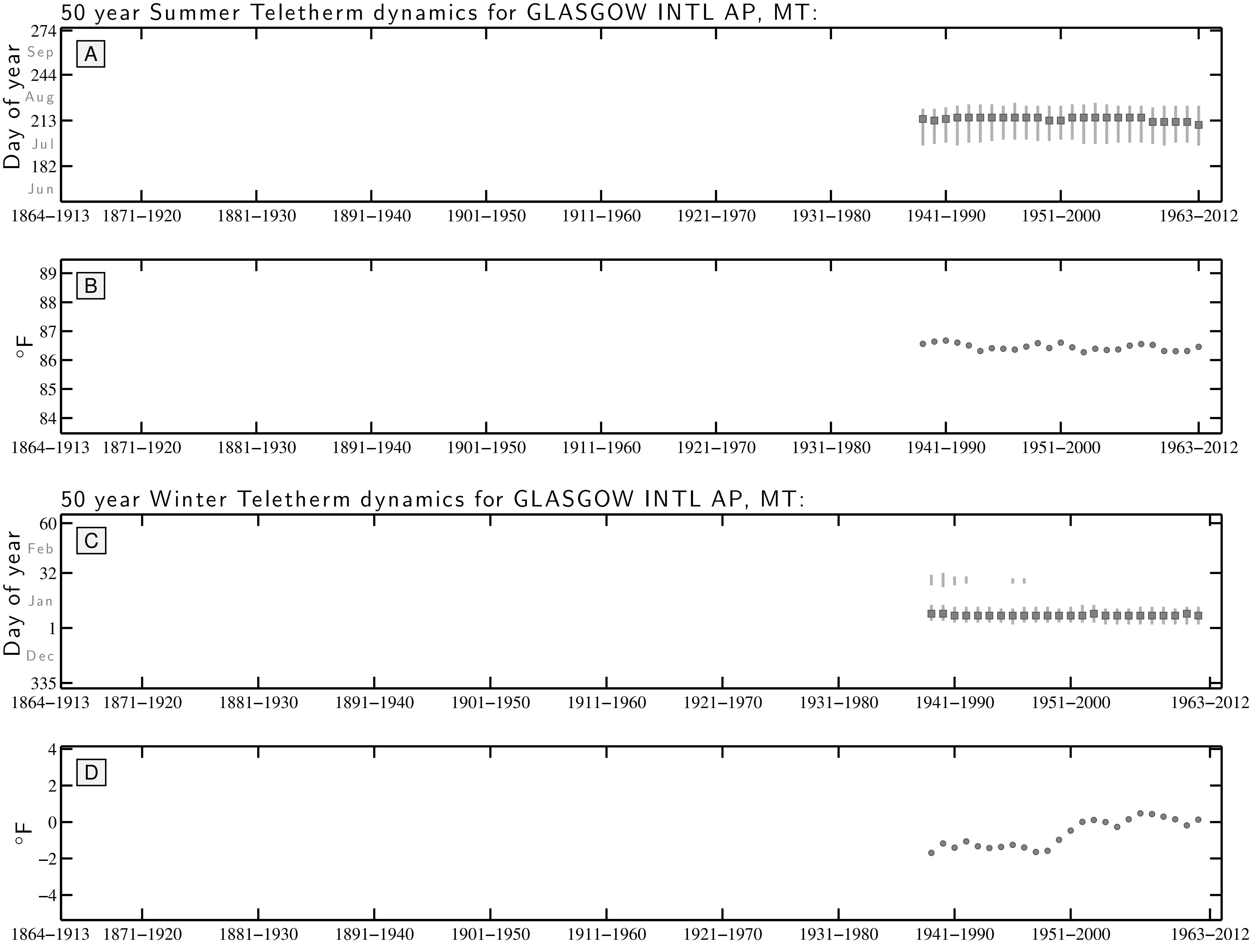

50 year Summer Teletherm dynamics for GLASGOW INTL AP, MT:
A
Day of year
274
Sep
244
Aug
213
Jul
182
Jun
1864–1913
1871–1920
1881–1930
1891–1940
1901–1950
1911–1960
1921–1970
1931–1980
1941–1990
1951–2000
1963–2012
B
°F
89
88
87
86
85
84
50 year Winter Teletherm dynamics for GLASGOW INTL AP, MT:
C
60
Feb
32
Jan
1
Dec
335
D
4
2
0
−2
−4

50 year Summer Teletherm dynamics for GLENDIVE, MT:

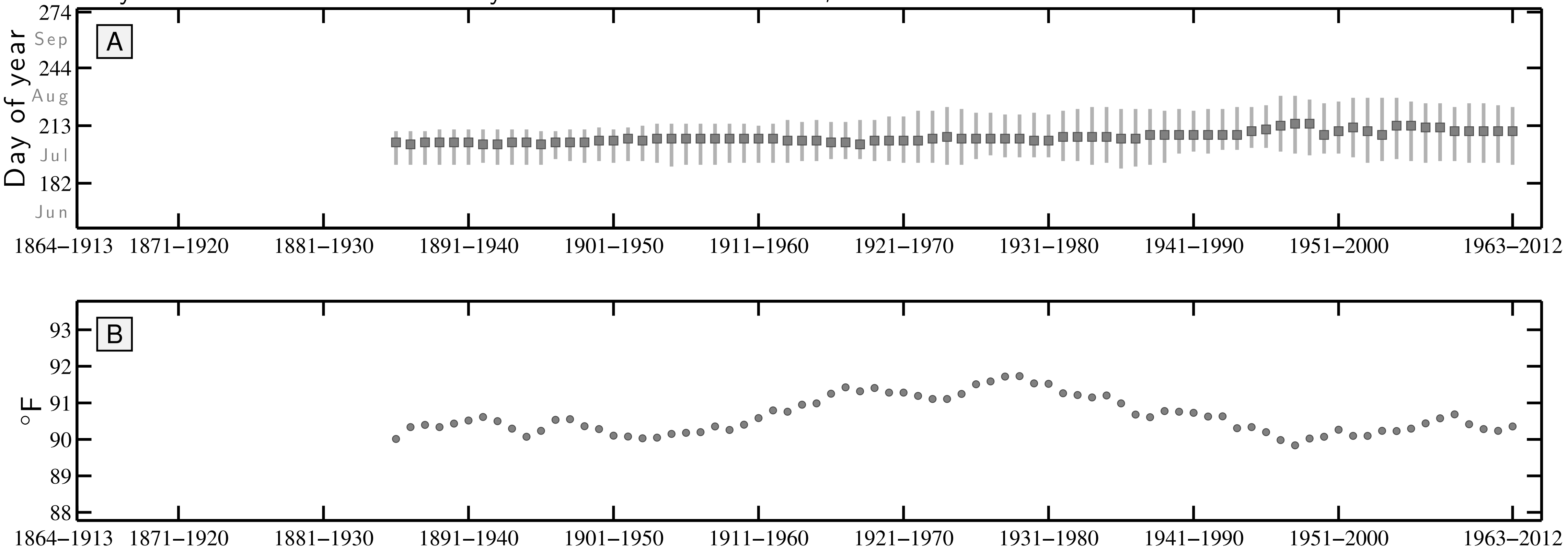

50 year Winter Teletherm dynamics for GLENDIVE, MT:

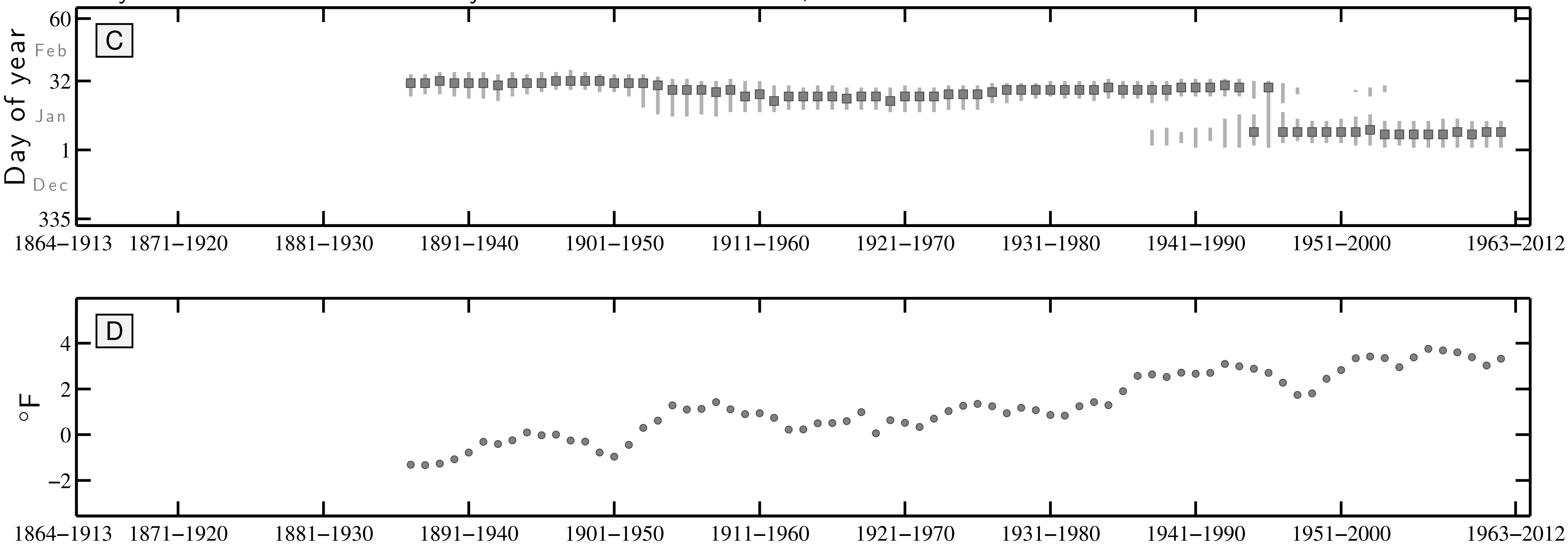

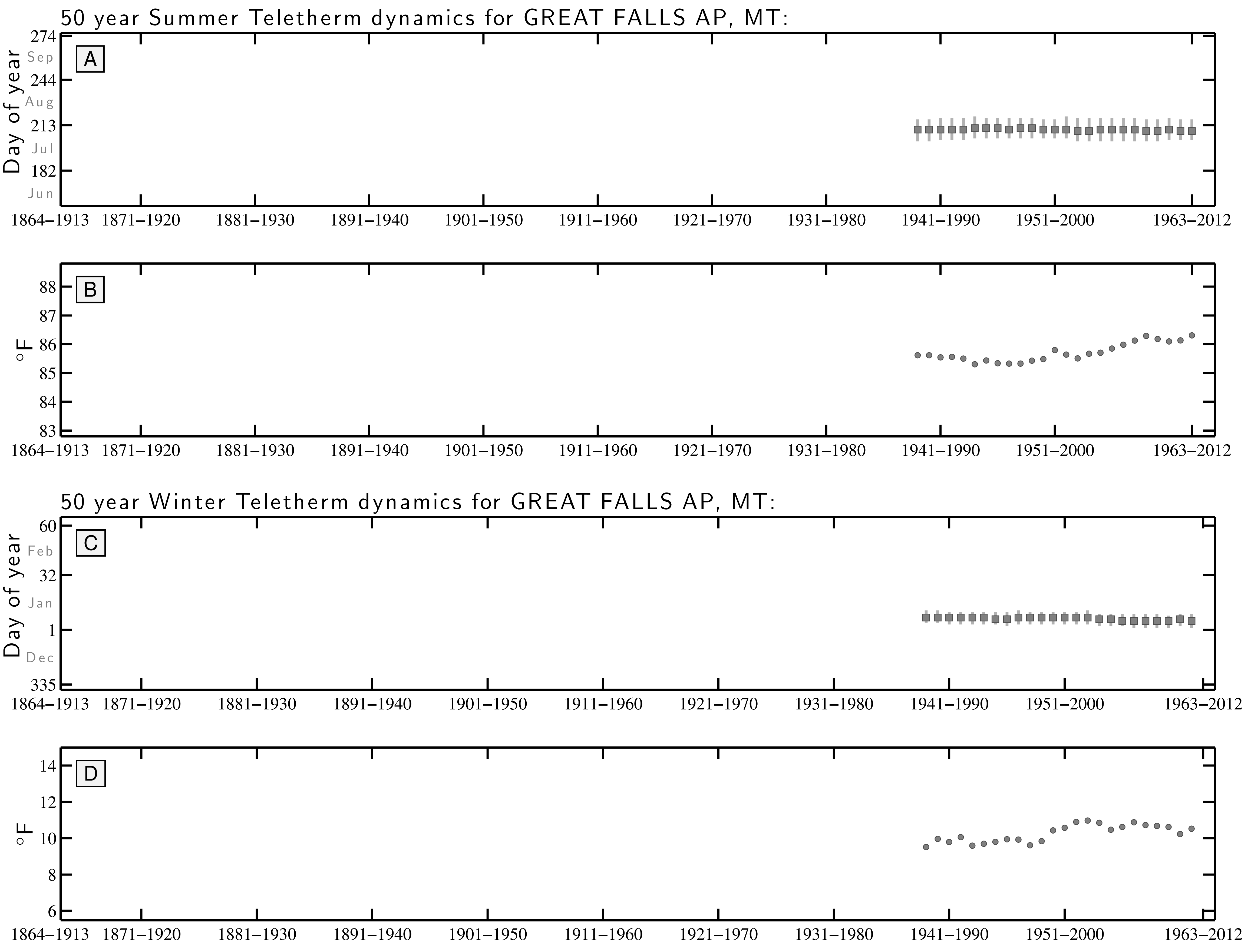

50 year Summer Teletherm dynamics for GREAT FALLS AP, MT:
A
Day of year
274
Sep
244
Aug
213
Jul
182
Jun
B
°F
88
87
86
85
84
83
50 year Winter Teletherm dynamics for GREAT FALLS AP, MT:
C
Day of year
60
Feb
32
Jan
1
Dec
335
D
°F
14
12
10
8
6
1864–1913
1871–1920
1881–1930
1891–1940
1901–1950
1911–1960
1921–1970
1931–1980
1941–1990
1951–2000
1963–2012

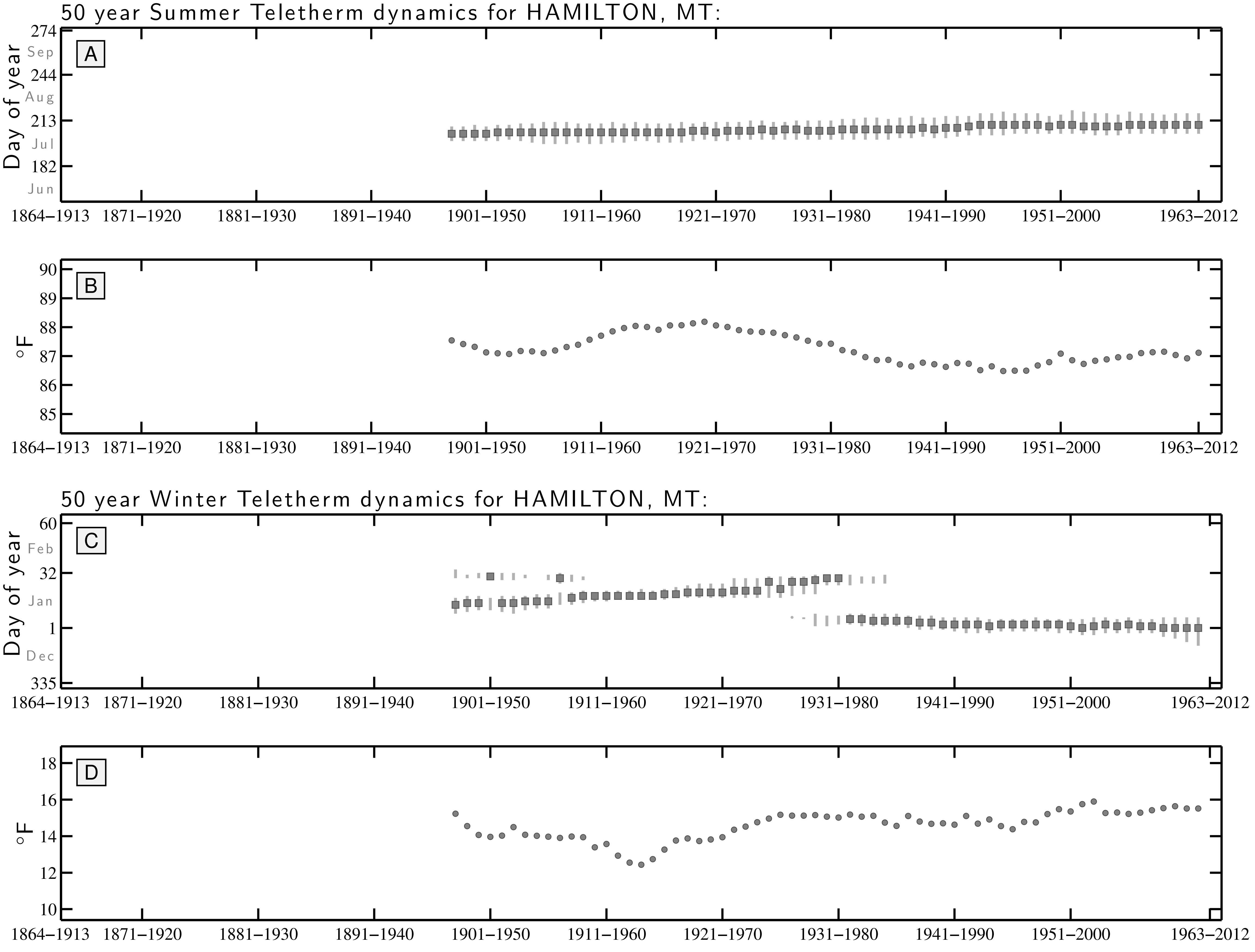

50 year Summer Teletherm dynamics for HAMILTON, MT:
A
Day of year
274
Sep
244
Aug
213
Jul
182
Jun
1864–1913
1871–1920
1881–1930
1891–1940
1901–1950
1911–1960
1921–1970
1931–1980
1941–1990
1951–2000
1963–2012
B
°F
90
89
88
87
86
85
50 year Winter Teletherm dynamics for HAMILTON, MT:
C
Day of year
60
Feb
32
Jan
1
Dec
335
D
°F
18
16
14
12
10

50 year Summer Teletherm dynamics for HEBGEN DAM, MT:

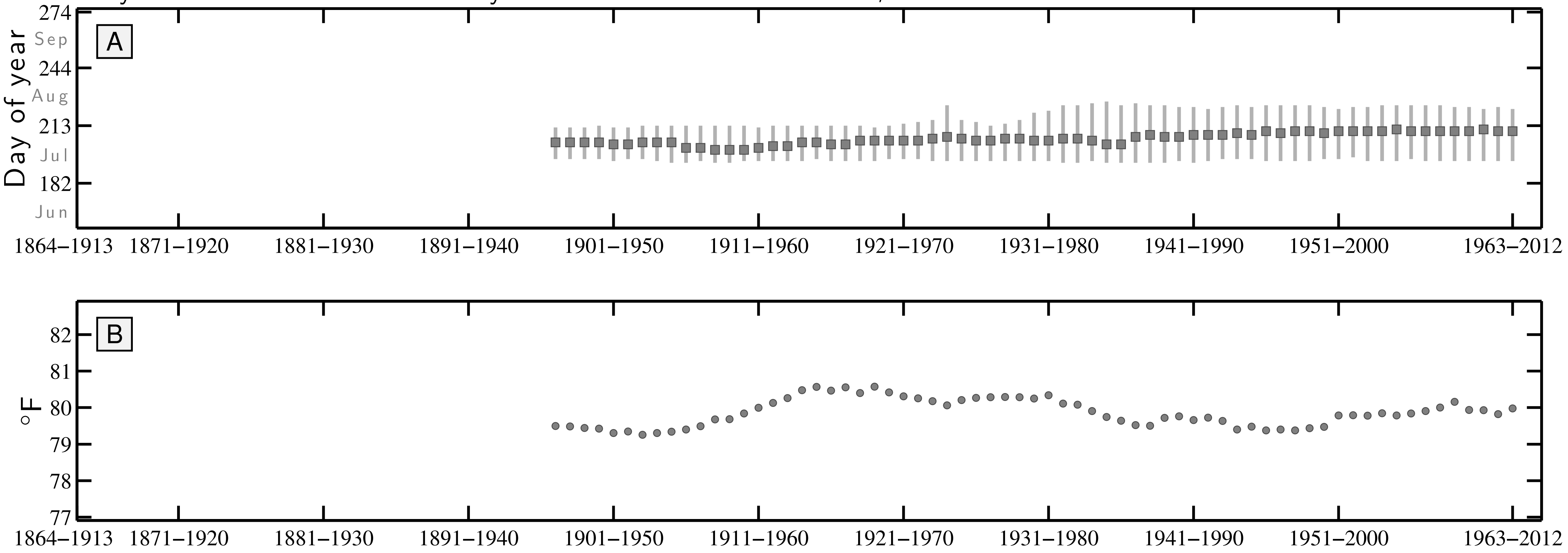

50 year Winter Teletherm dynamics for HEBGEN DAM, MT:

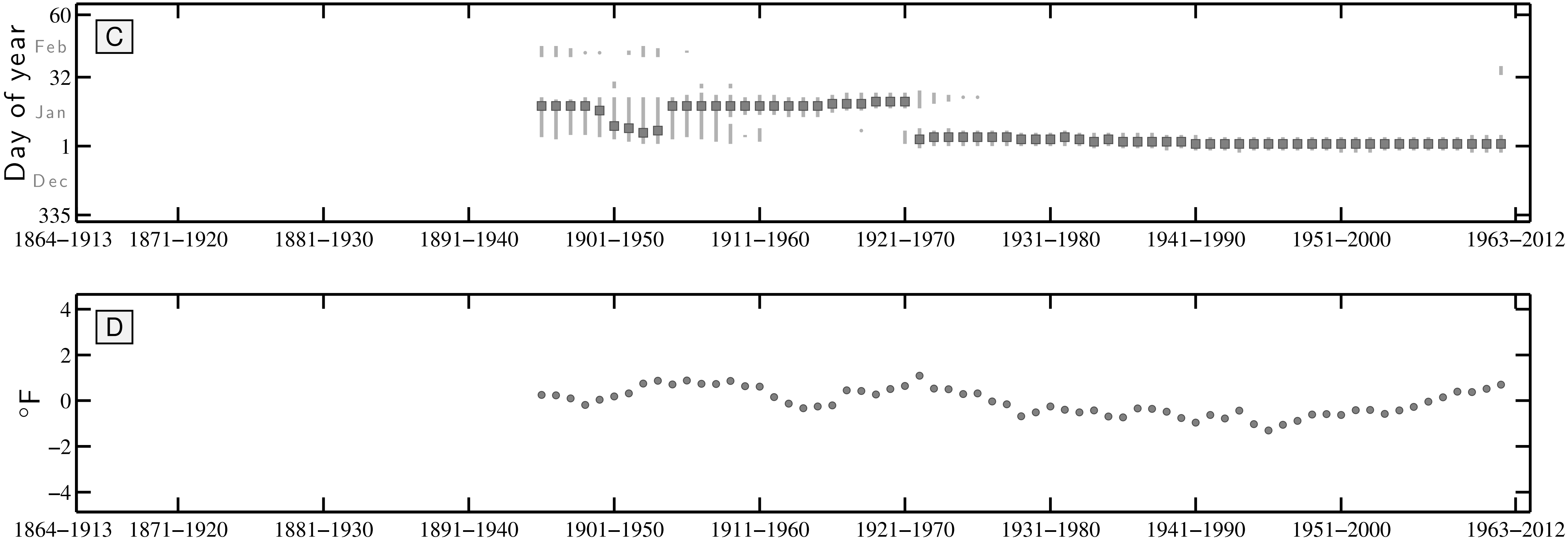

50 year Summer Teletherm dynamics for HELENA AP ASOS, MT:

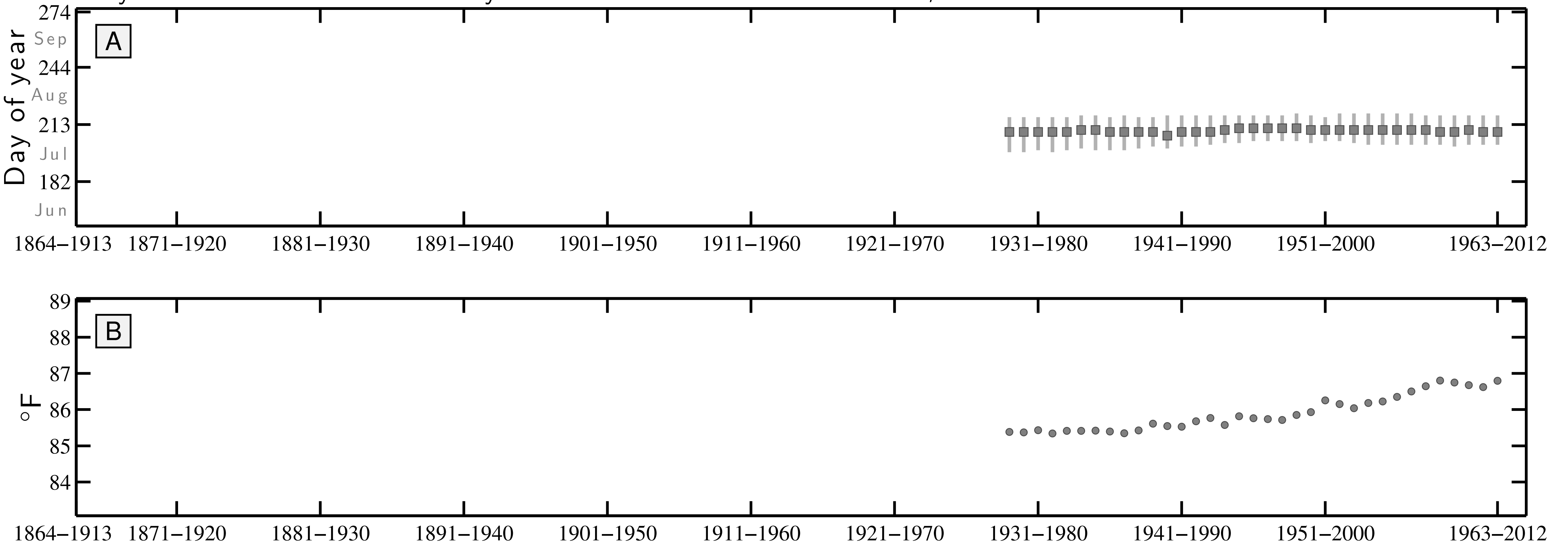

50 year Winter Teletherm dynamics for HELENA AP ASOS, MT:

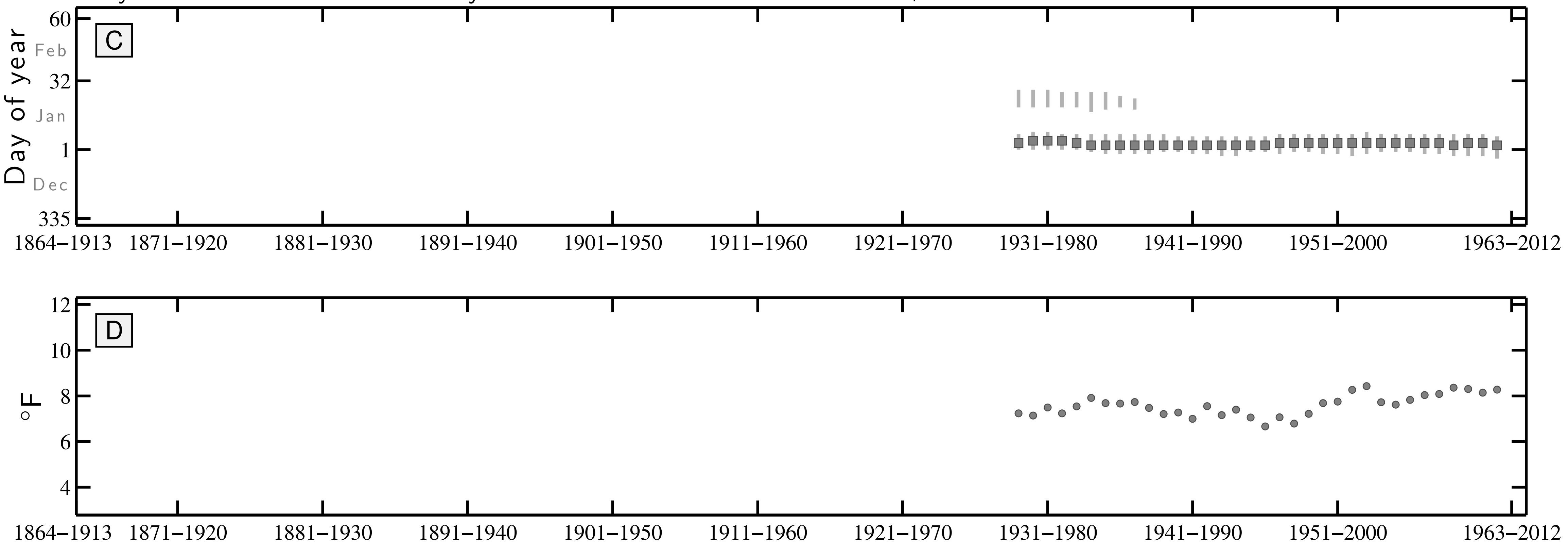

50 year Summer Teletherm dynamics for HUNTLEY EXP STN, MT:

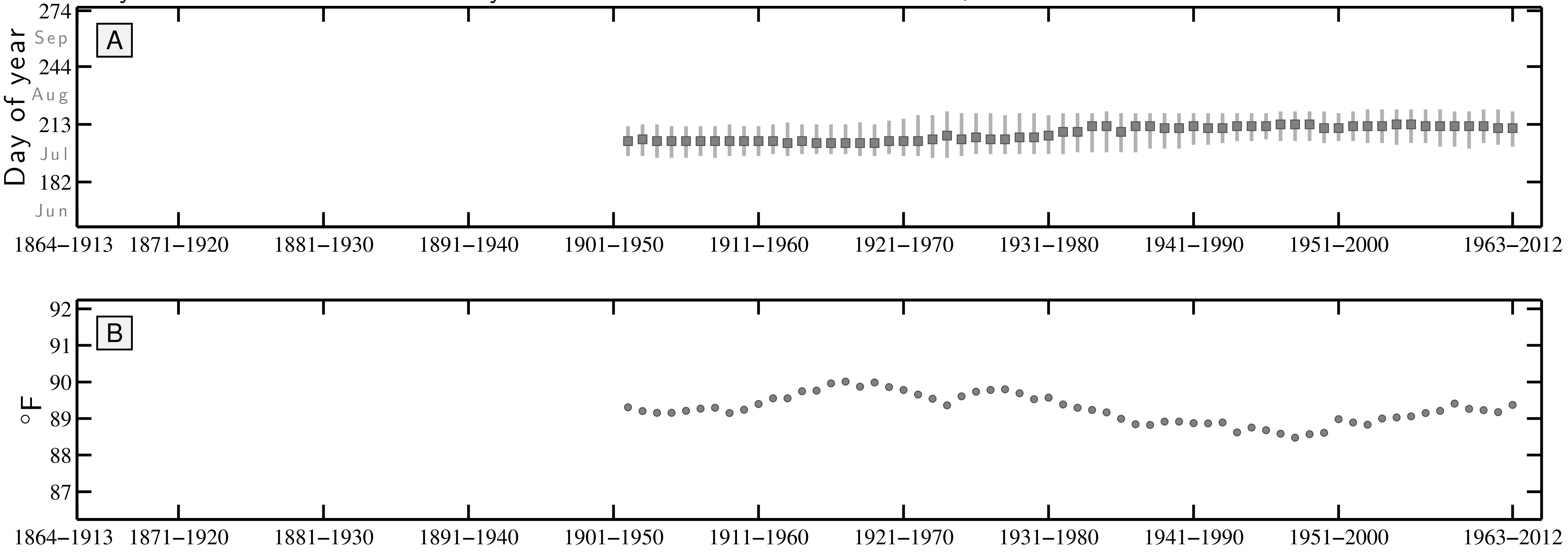

50 year Winter Teletherm dynamics for HUNTLEY EXP STN, MT:

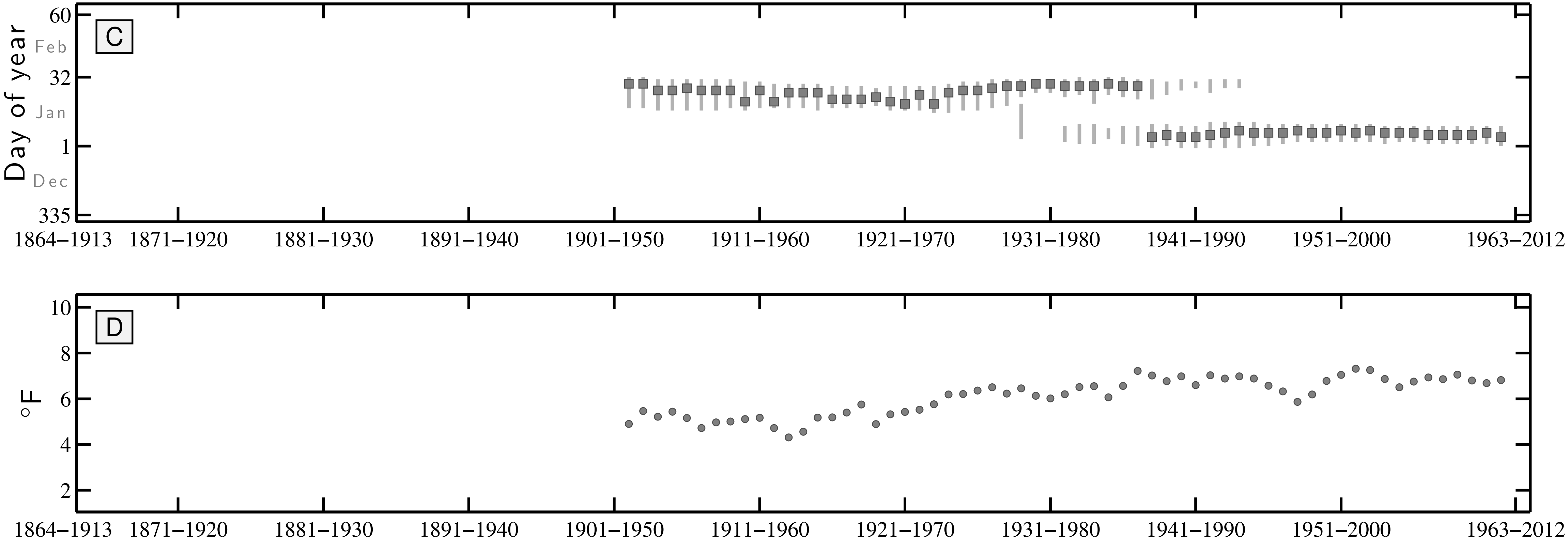

50 year Summer Teletherm dynamics for HYSHAM 25 SSE, MT:

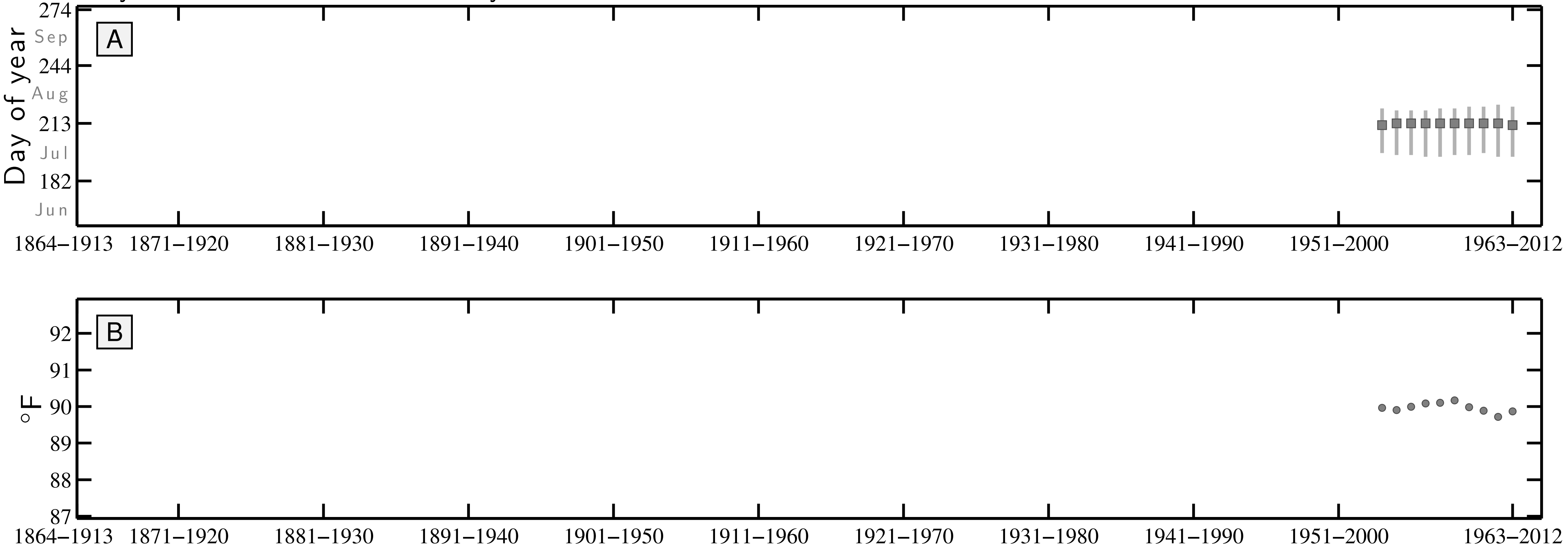

50 year Winter Teletherm dynamics for HYSHAM 25 SSE, MT:

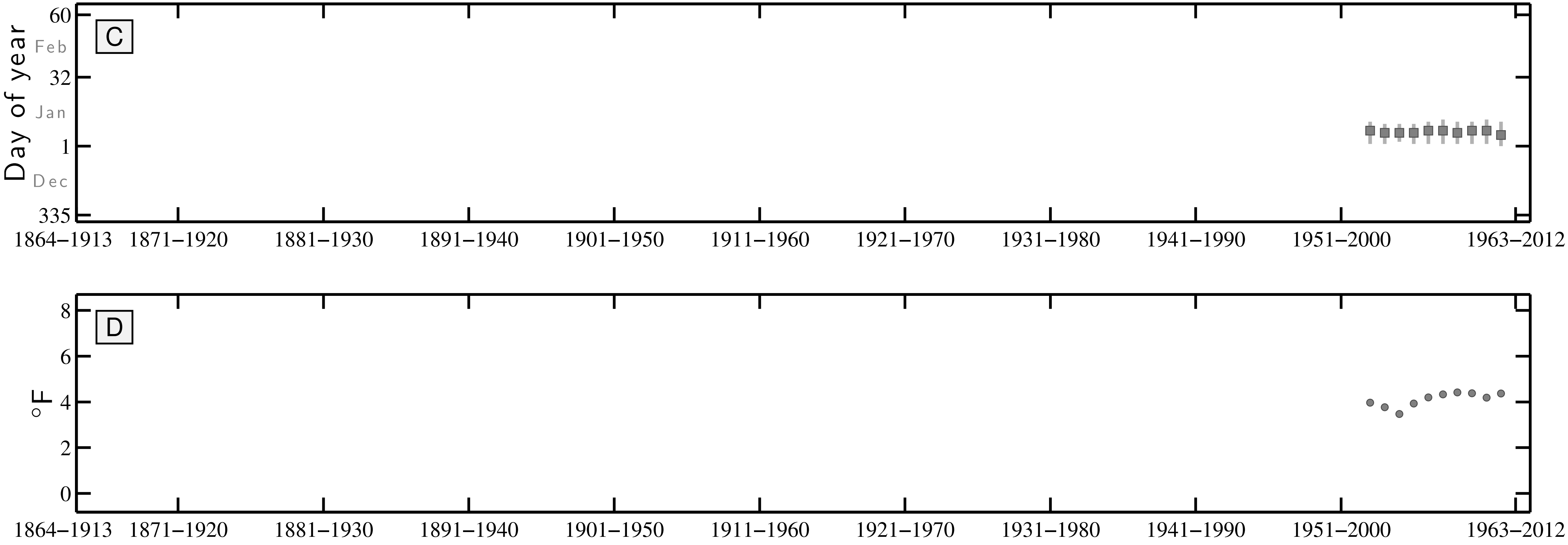

50 year Summer Teletherm dynamics for JORDAN, MT:

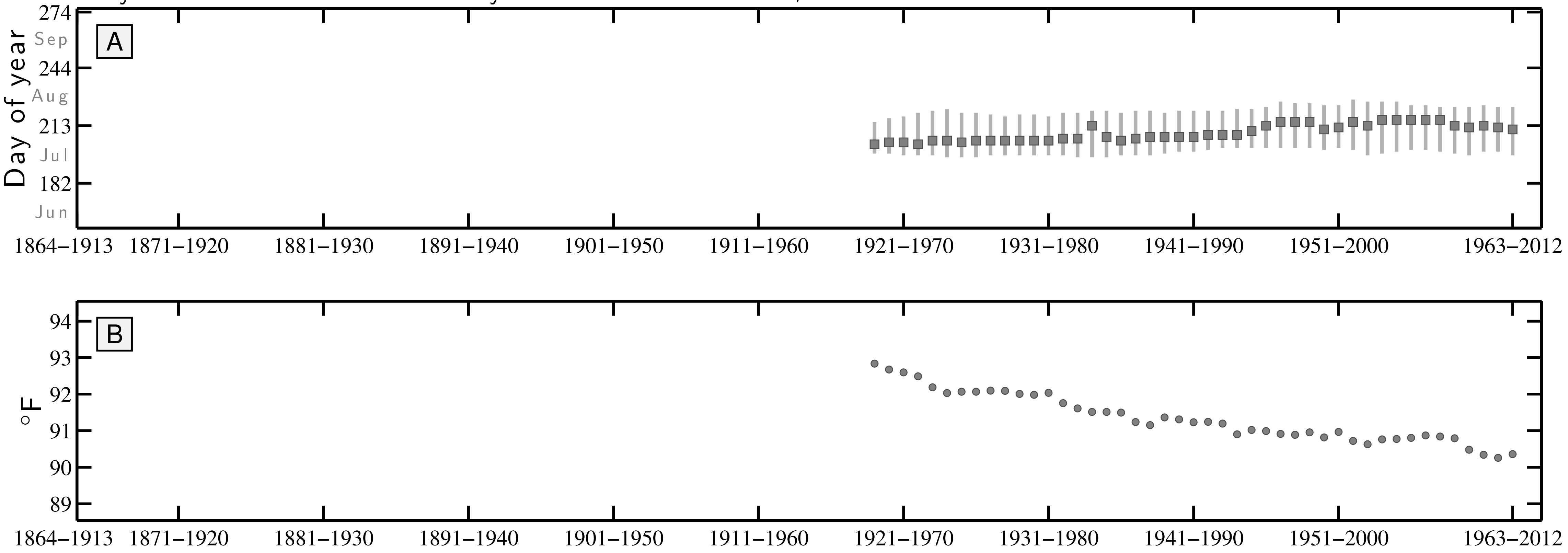

50 year Winter Teletherm dynamics for JORDAN, MT:

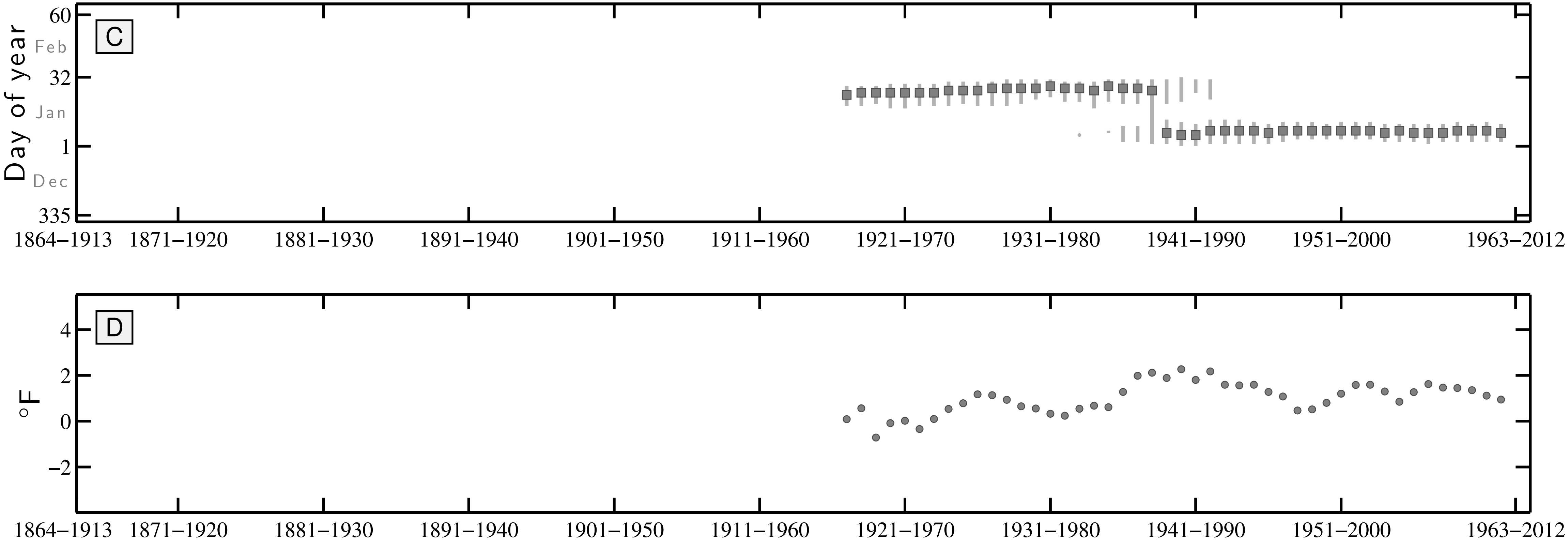

50 year Summer Teletherm dynamics for KALISPELL GLACIER AP, MT:

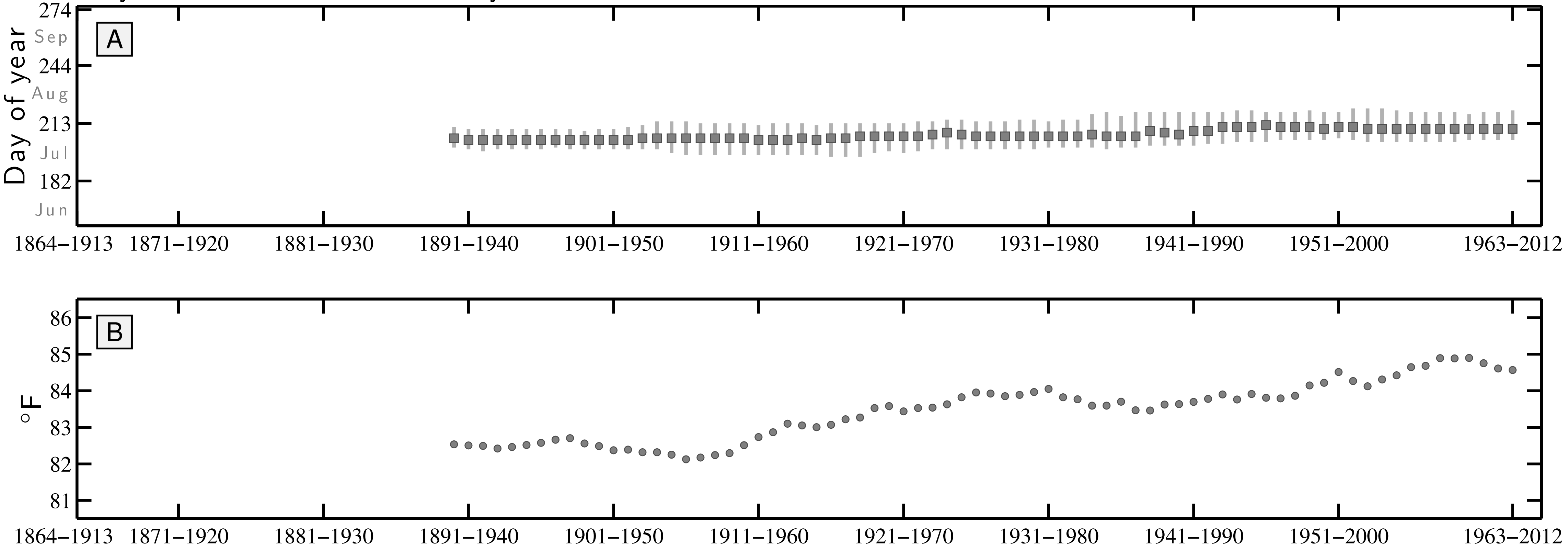

50 year Winter Teletherm dynamics for KALISPELL GLACIER AP, MT:

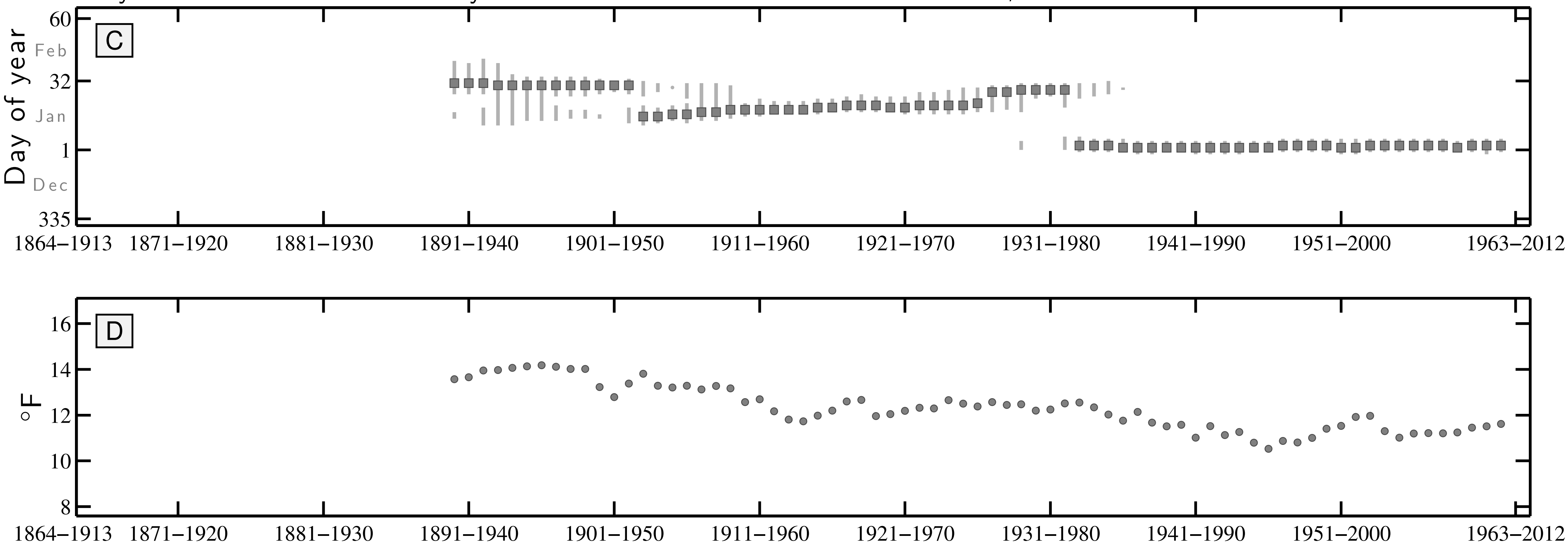

50 year Summer Teletherm dynamics for LIBBY 1 NE RS, MT:

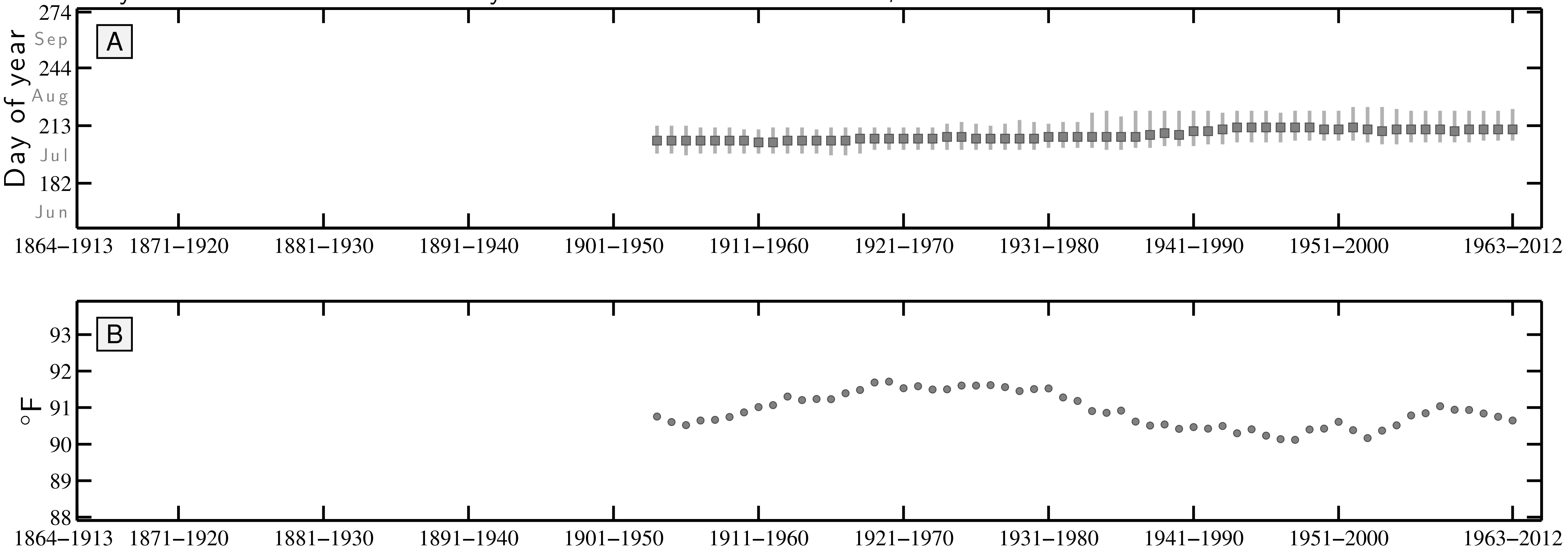

50 year Winter Teletherm dynamics for LIBBY 1 NE RS, MT:

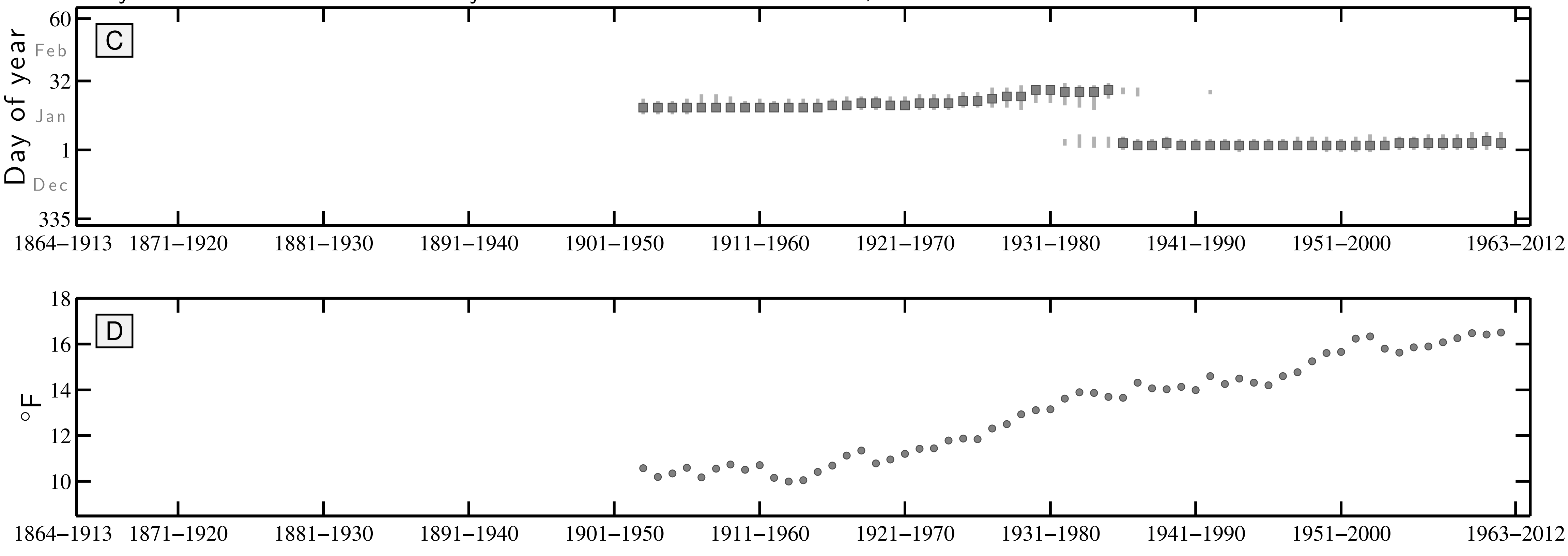

50 year Summer Teletherm dynamics for MEDICINE LAKE 3 SE, MT:

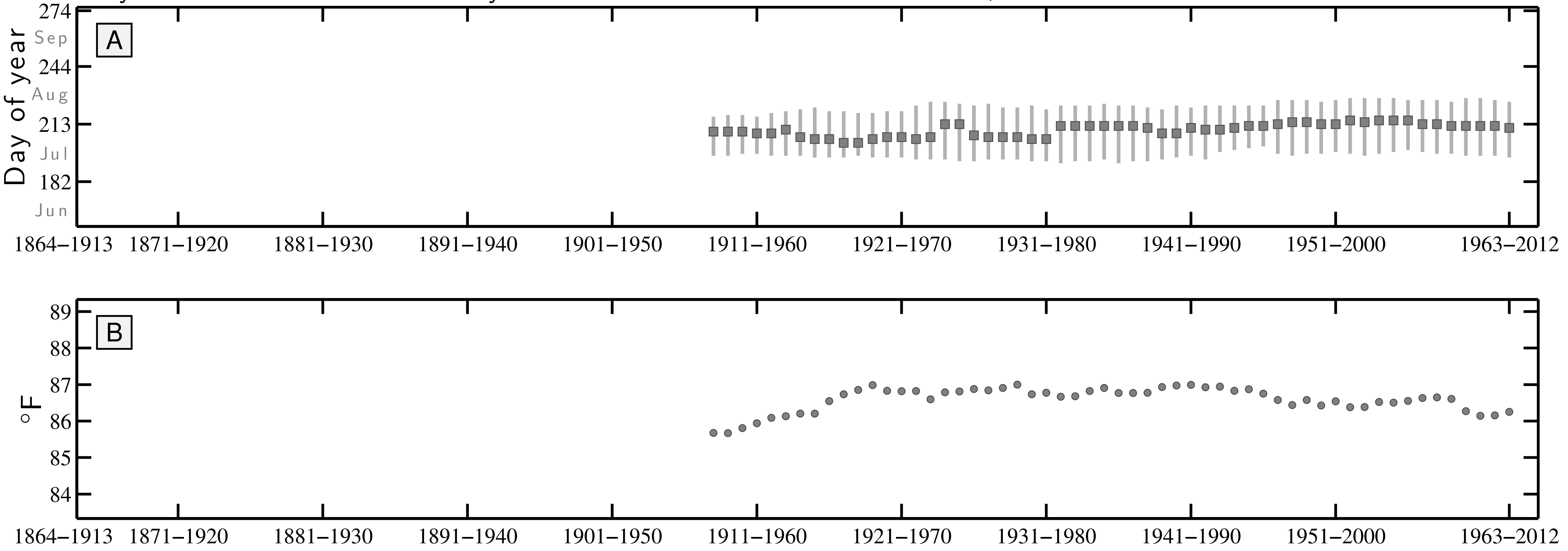

50 year Winter Teletherm dynamics for MEDICINE LAKE 3 SE, MT:

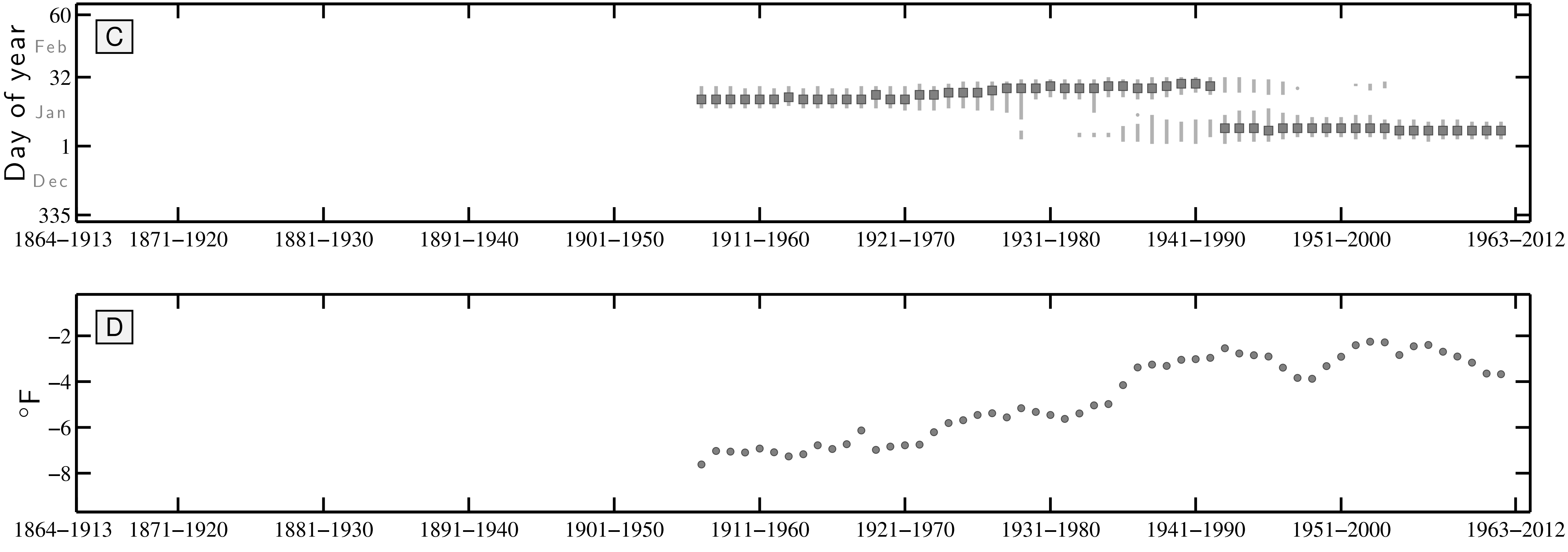

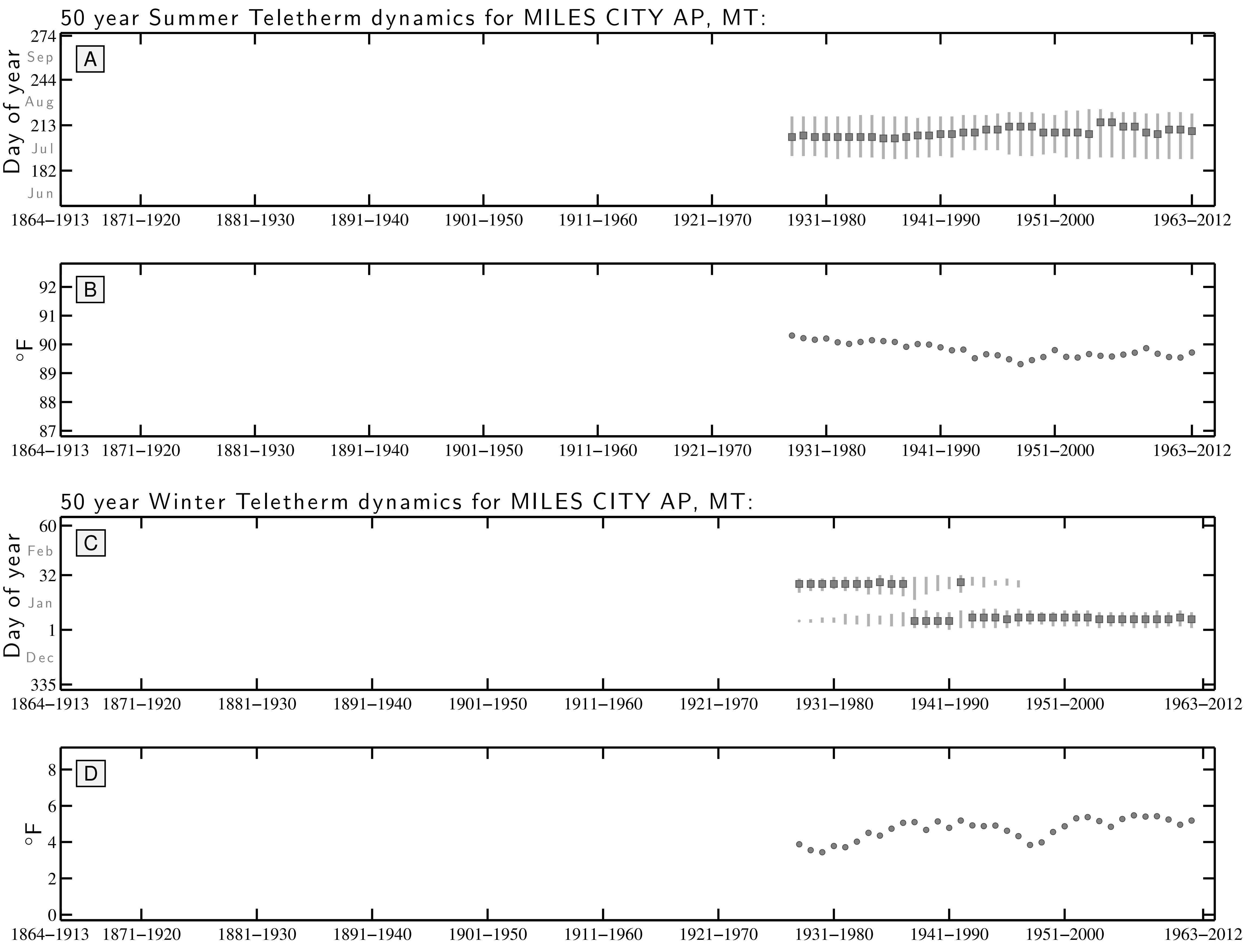
50 year Summer Teletherm dynamics for MILES CITY AP, MT:
A
Day of year
274
Sep
244
Aug
213
Jul
182
Jun
1864–1913
1871–1920
1881–1930
1891–1940
1901–1950
1911–1960
1921–1970
1931–1980
1941–1990
1951–2000
1963–2012
B
°F
92
91
90
89
88
87
50 year Winter Teletherm dynamics for MILES CITY AP, MT:
C
Day of year
60
Feb
32
Jan
1
Dec
335
D
°F
8
6
4
2
0

50 year Summer Teletherm dynamics for MOCCASIN EXP STN, MT:

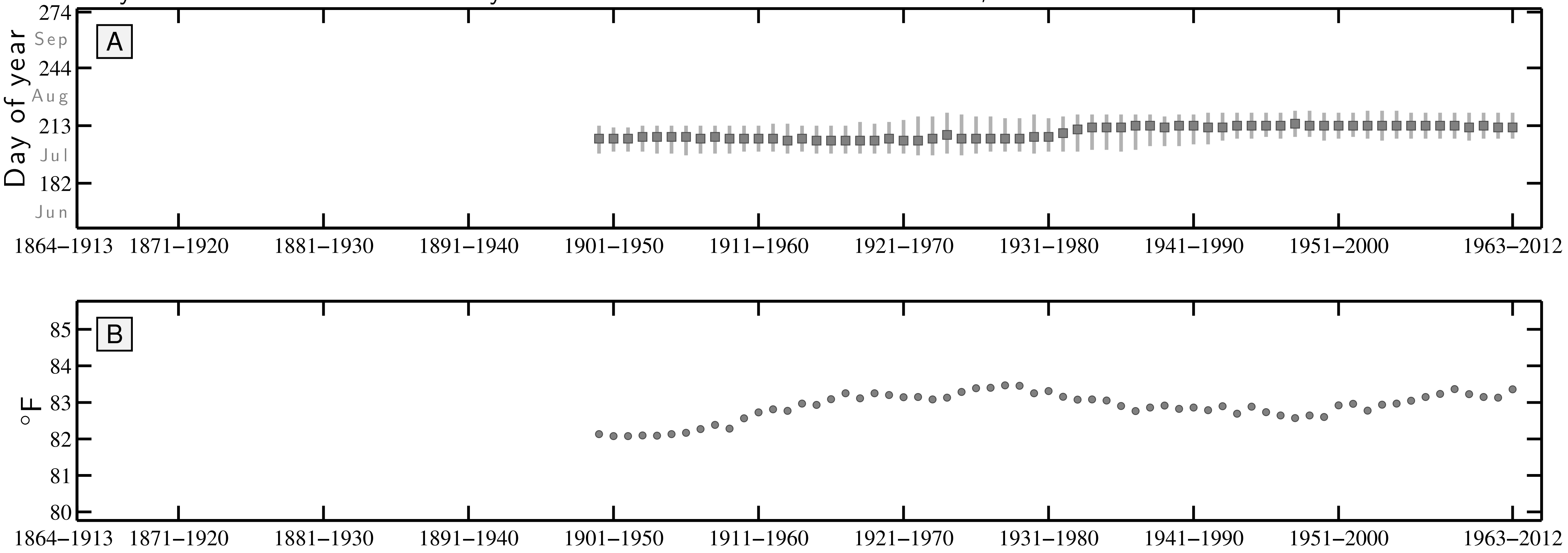

50 year Winter Teletherm dynamics for MOCCASIN EXP STN, MT:

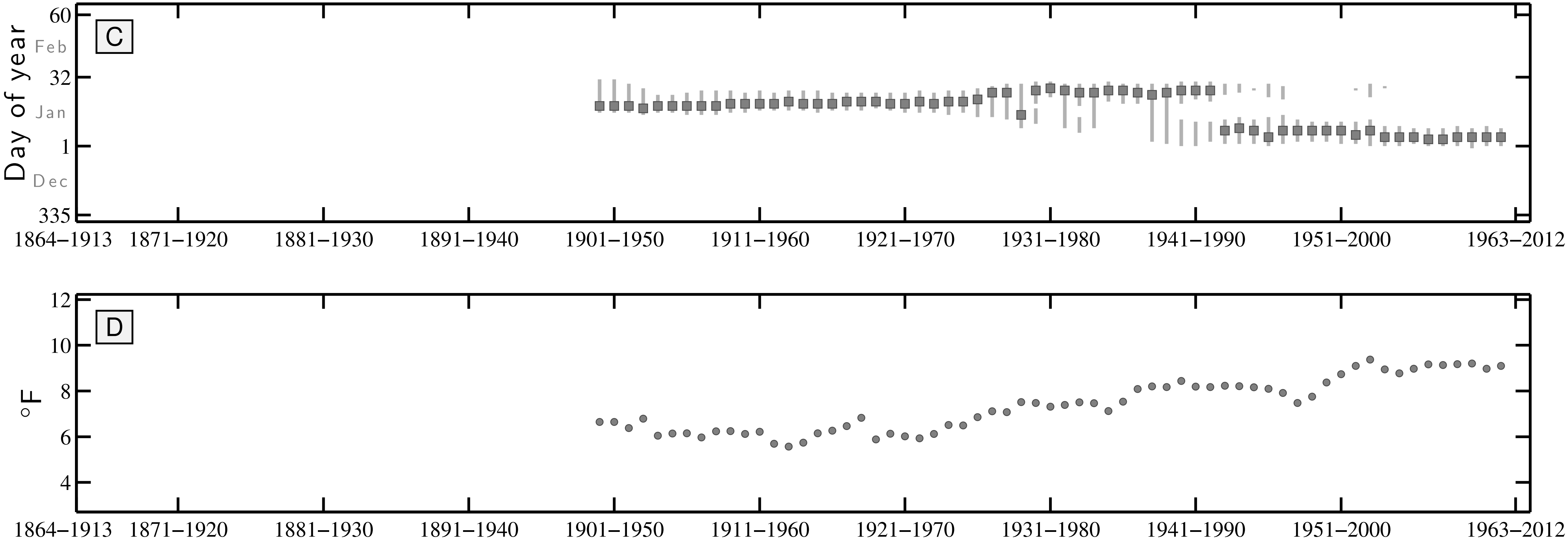

50 year Summer Teletherm dynamics for NORRIS MADISON PH, MT:

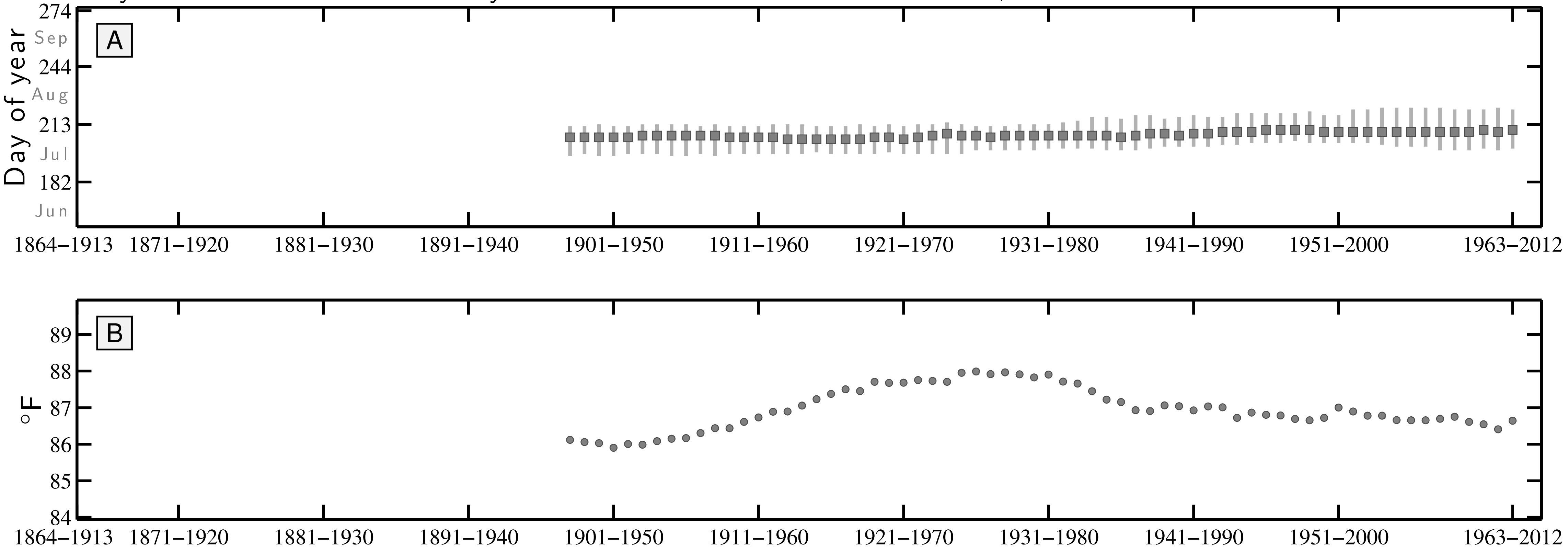

50 year Winter Teletherm dynamics for NORRIS MADISON PH, MT:

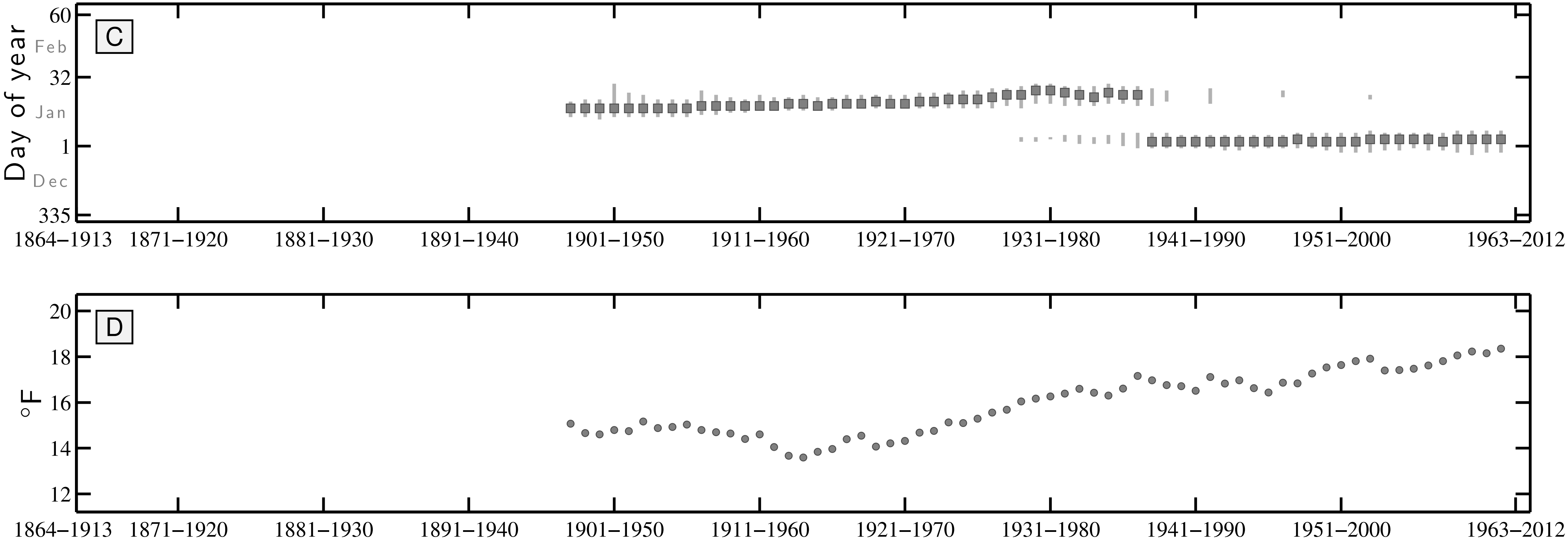

50 year Summer Teletherm dynamics for PHILIPSBURG RS, MT:

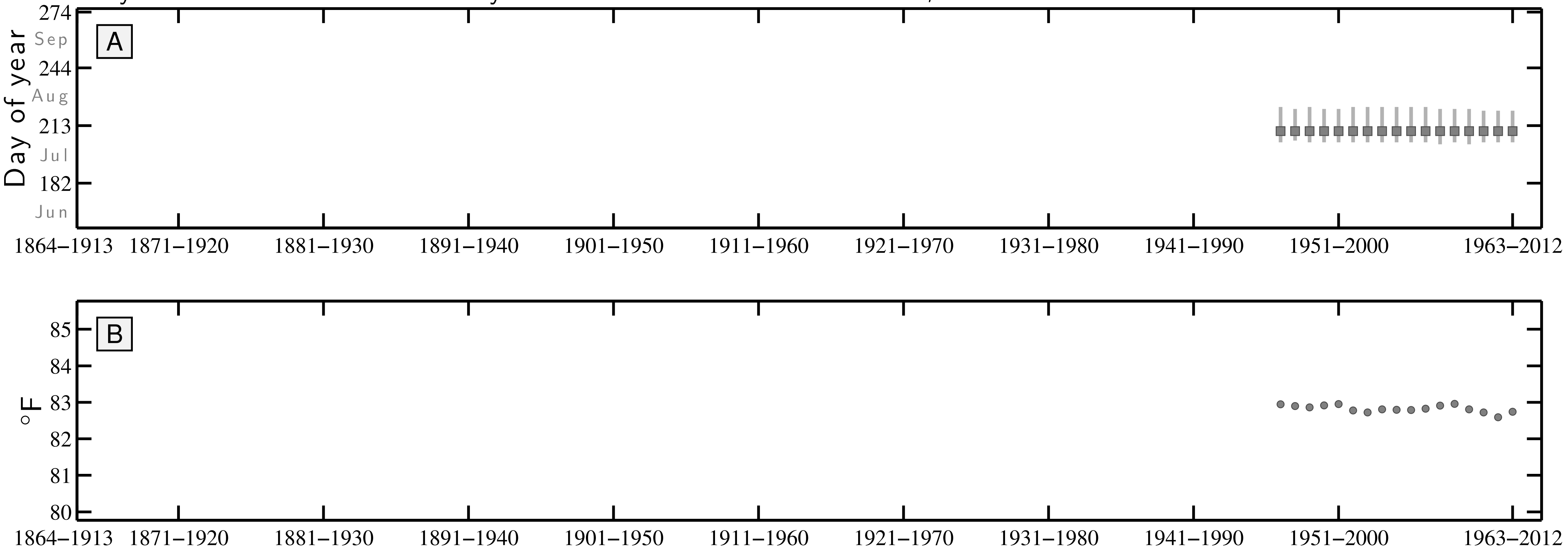

50 year Winter Teletherm dynamics for PHILIPSBURG RS, MT:

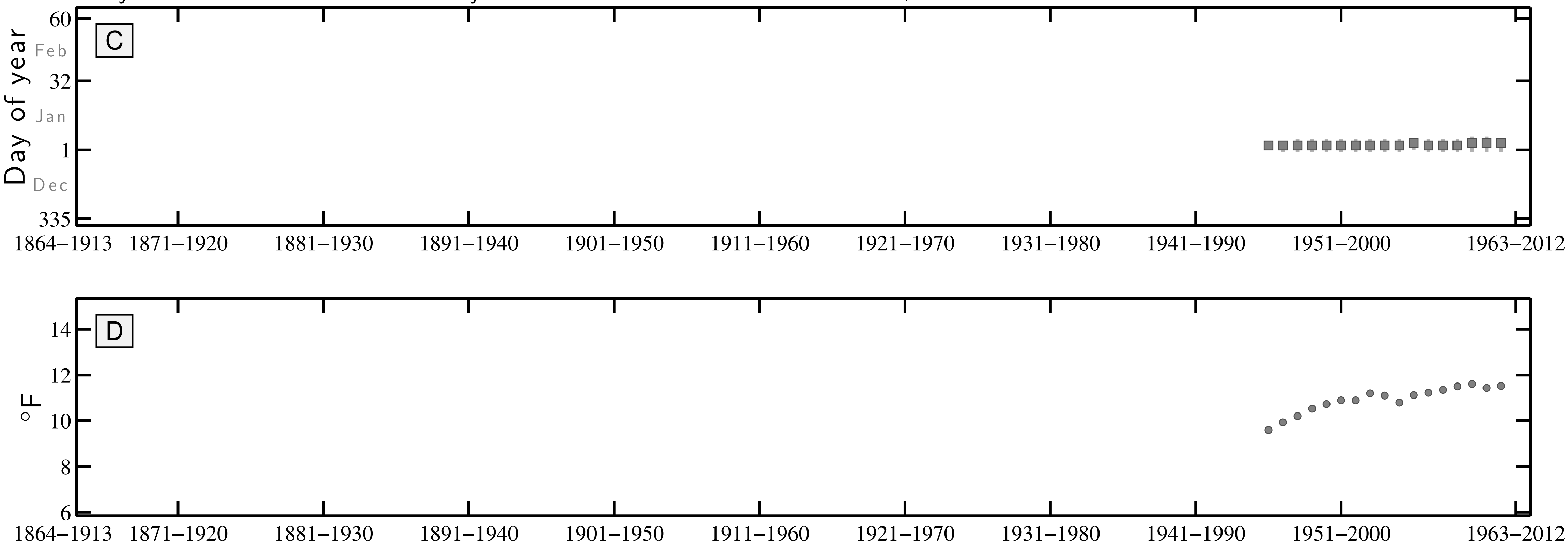

50 year Summer Teletherm dynamics for PLEVNA, MT:

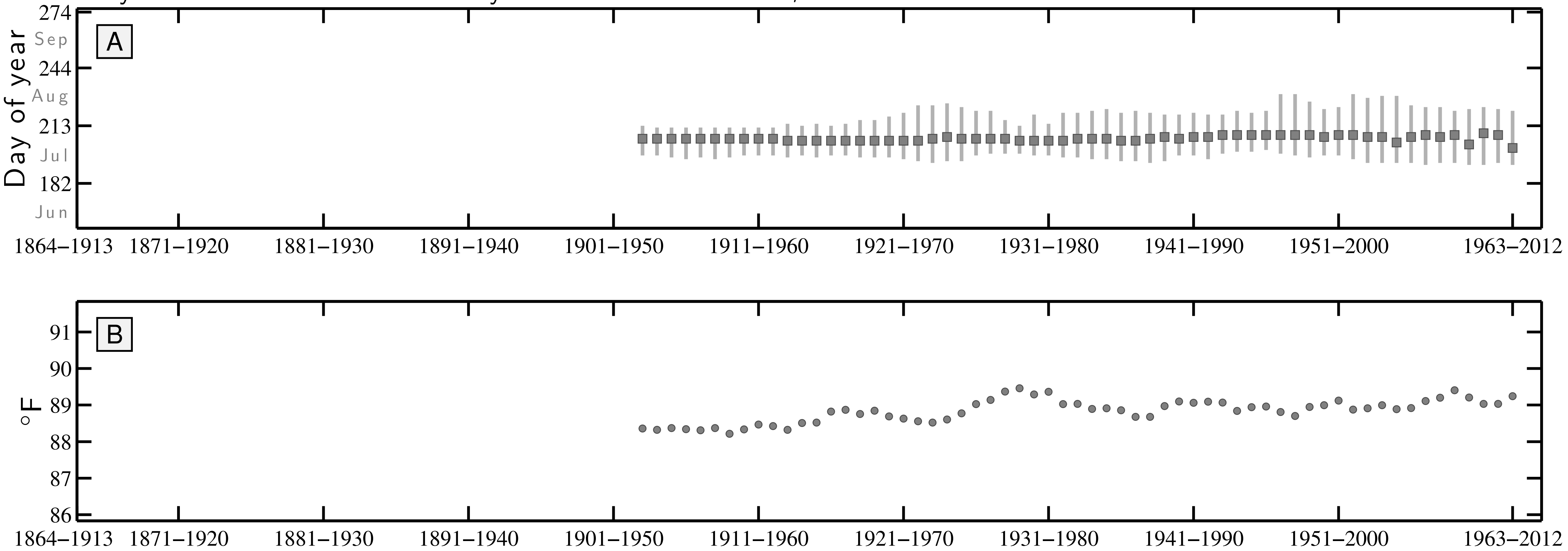

50 year Winter Teletherm dynamics for PLEVNA, MT:

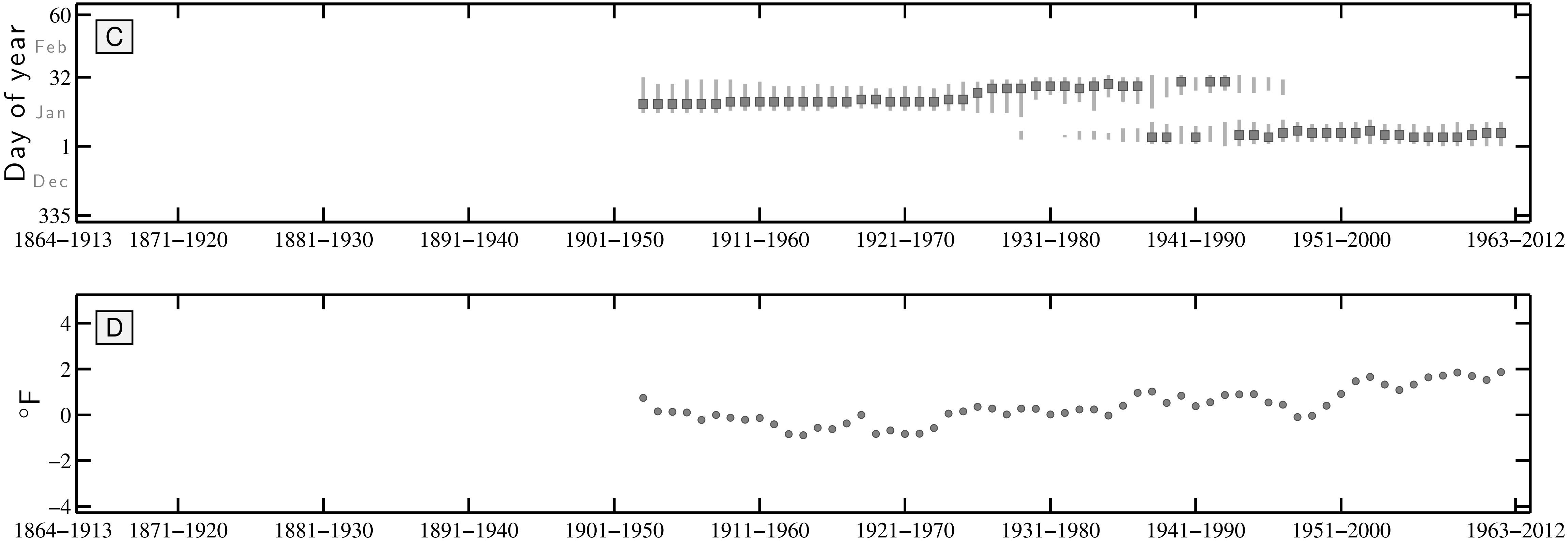

50 year Summer Teletherm dynamics for RED LODGE, MT:

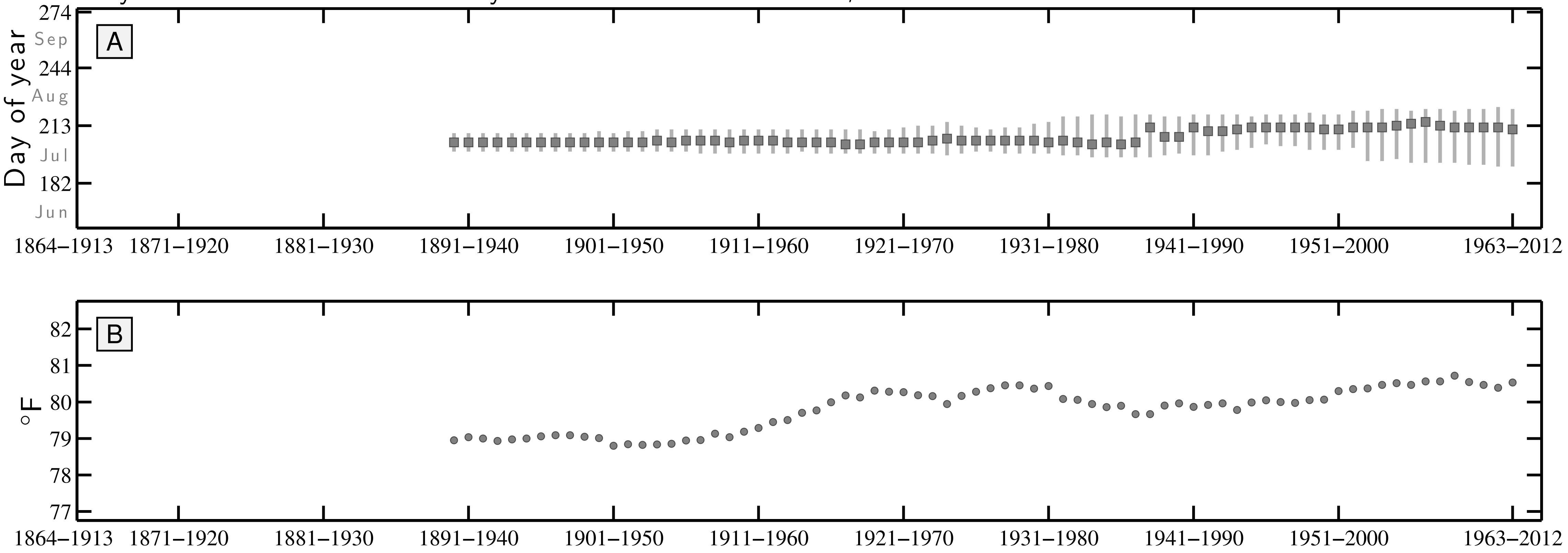

50 year Winter Teletherm dynamics for RED LODGE, MT:

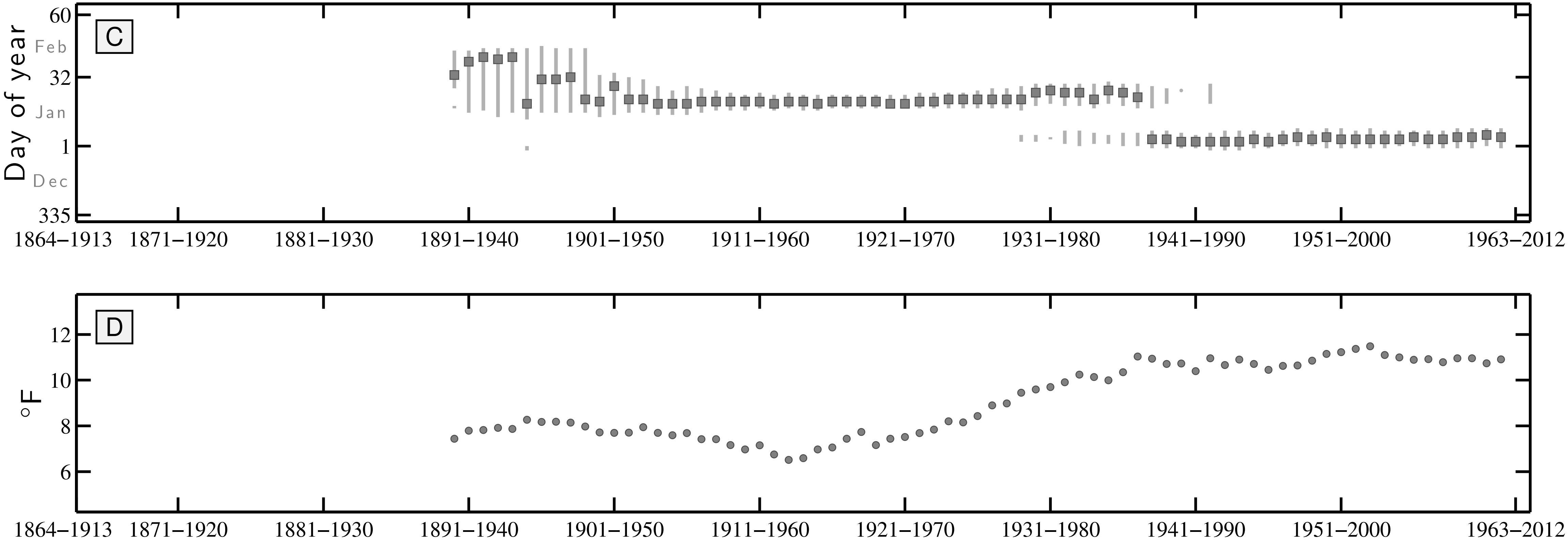

50 year Summer Teletherm dynamics for SAINT IGNATIUS, MT:

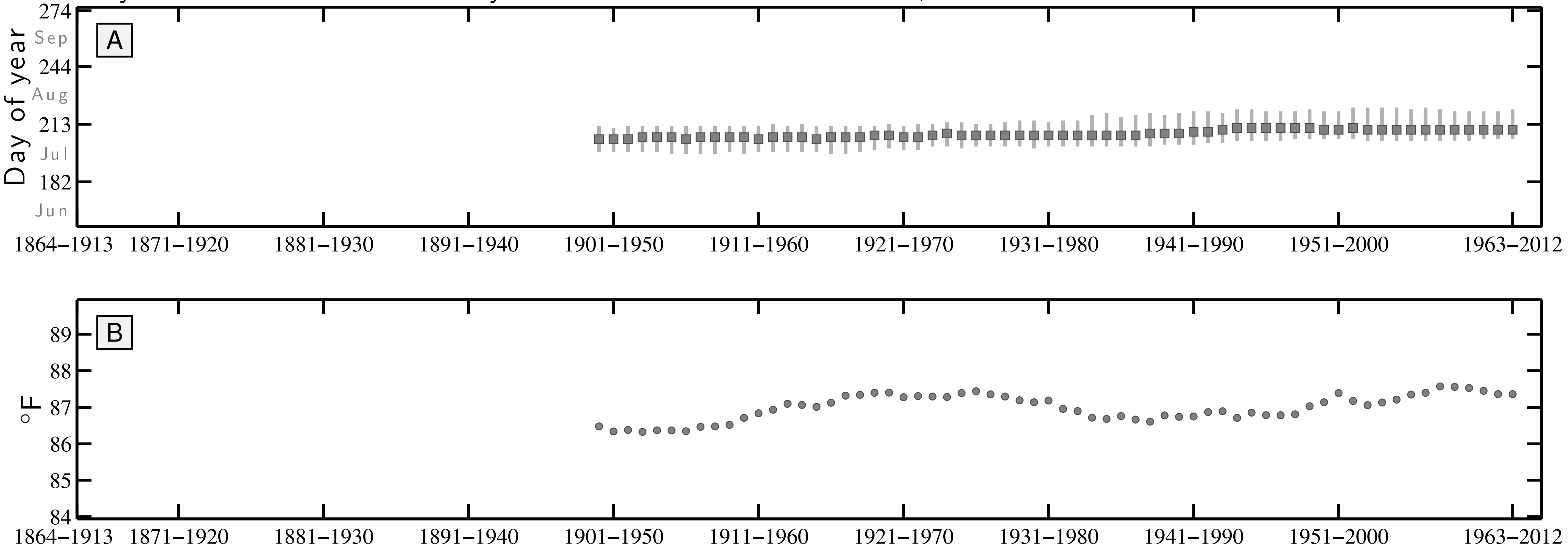

50 year Winter Teletherm dynamics for SAINT IGNATIUS, MT:

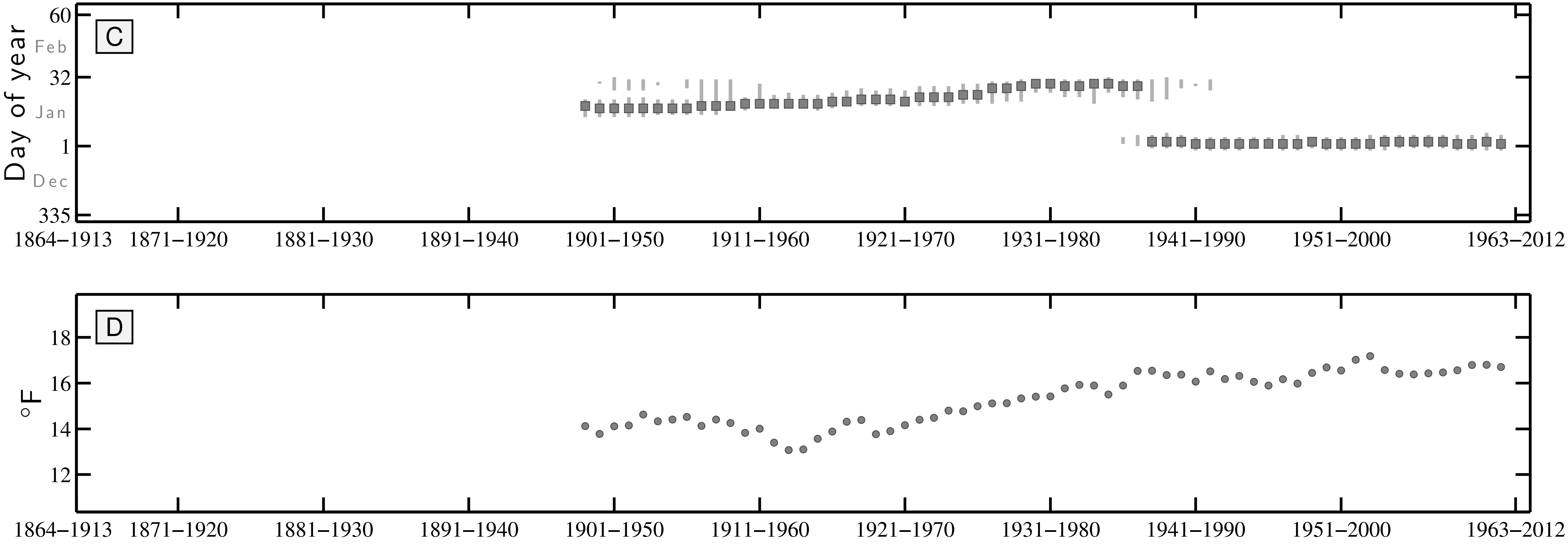

50 year Summer Teletherm dynamics for SAINT REGIS 1 NE, MT:

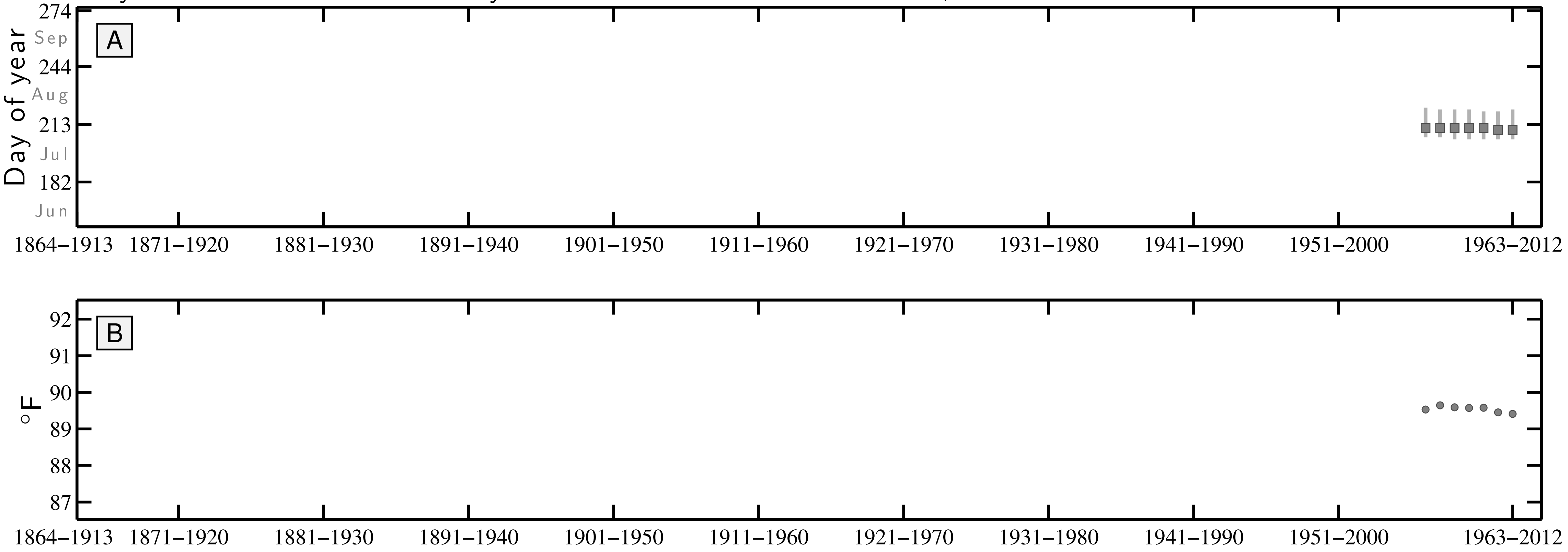

50 year Winter Teletherm dynamics for SAINT REGIS 1 NE, MT:

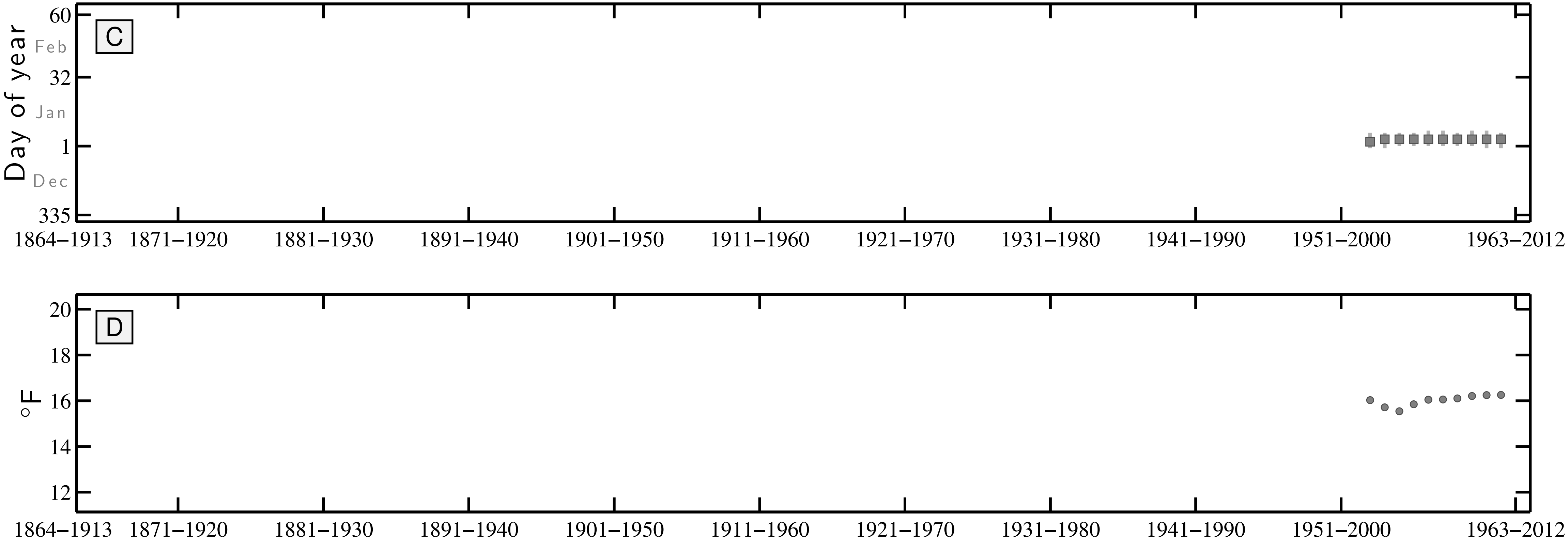

50 year Summer Teletherm dynamics for SAVAGE, MT:

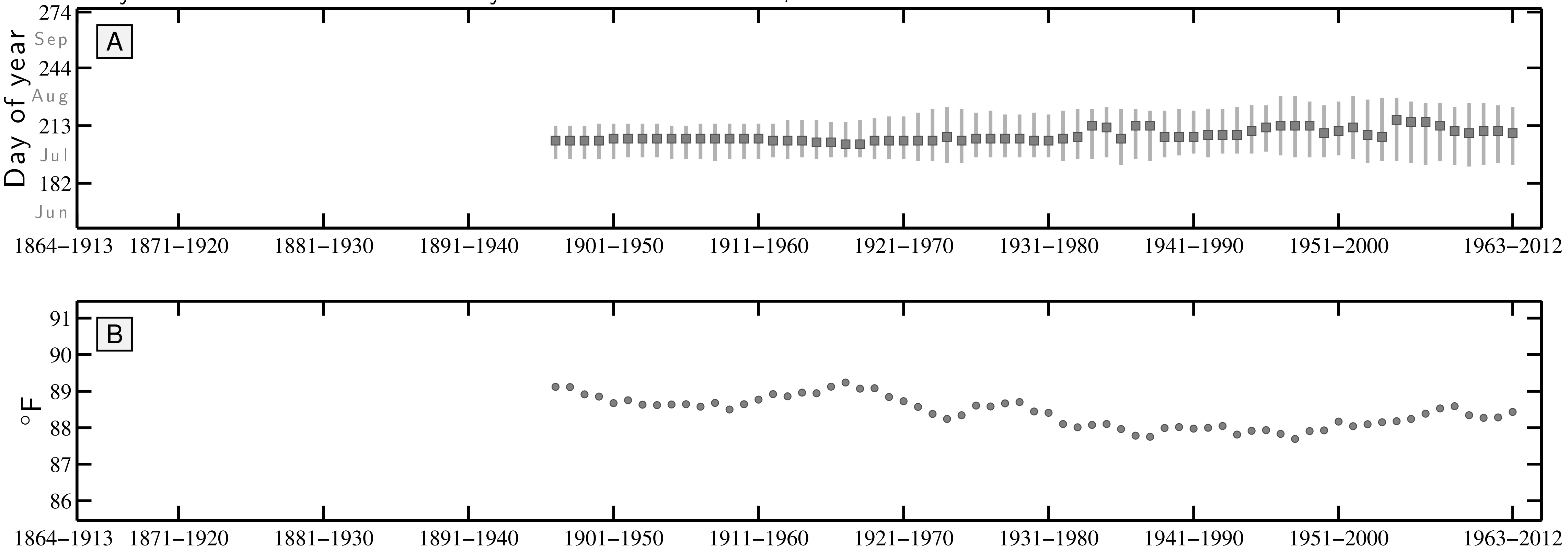

50 year Winter Teletherm dynamics for SAVAGE, MT:

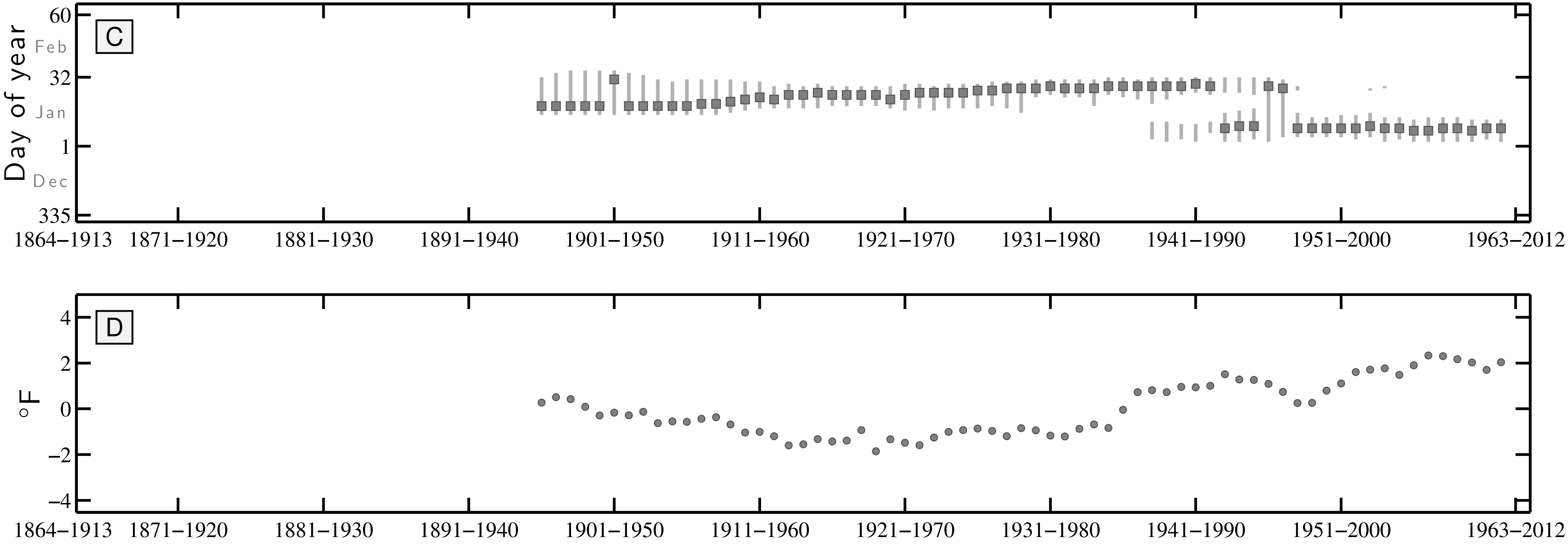

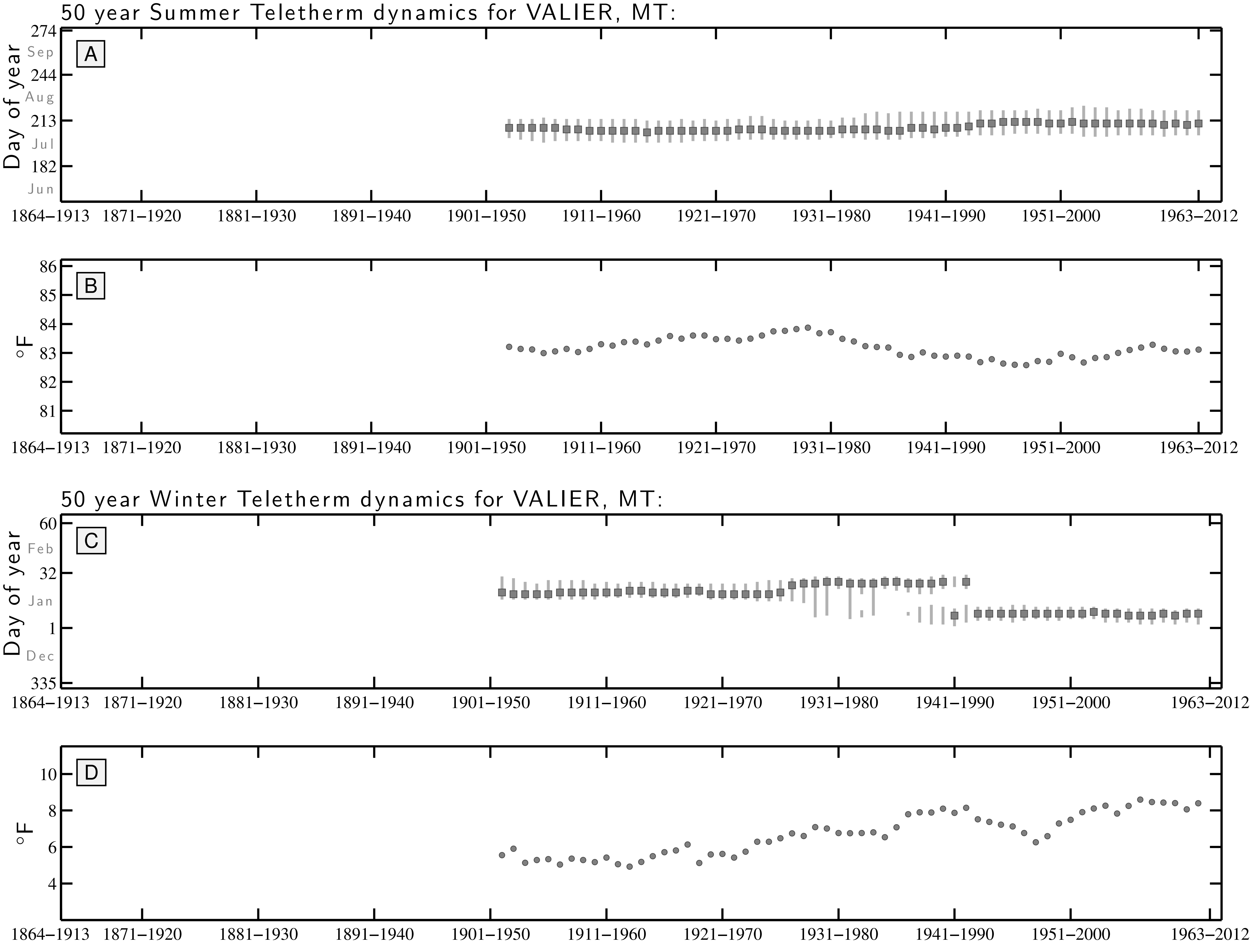
50 year Summer Teletherm dynamics for VALIER, MT:
A
Day of year
274
Sep
244
Aug
213
Jul
182
Jun
1864–1913
1871–1920
1881–1930
1891–1940
1901–1950
1911–1960
1921–1970
1931–1980
1941–1990
1951–2000
1963–2012
B
°F
86
85
84
83
82
81
50 year Winter Teletherm dynamics for VALIER, MT:
C
Day of year
60
Feb
32
Jan
1
Dec
335
D
°F
10
8
6
4

50 year Summer Teletherm dynamics for VIDA 6 NE, MT:

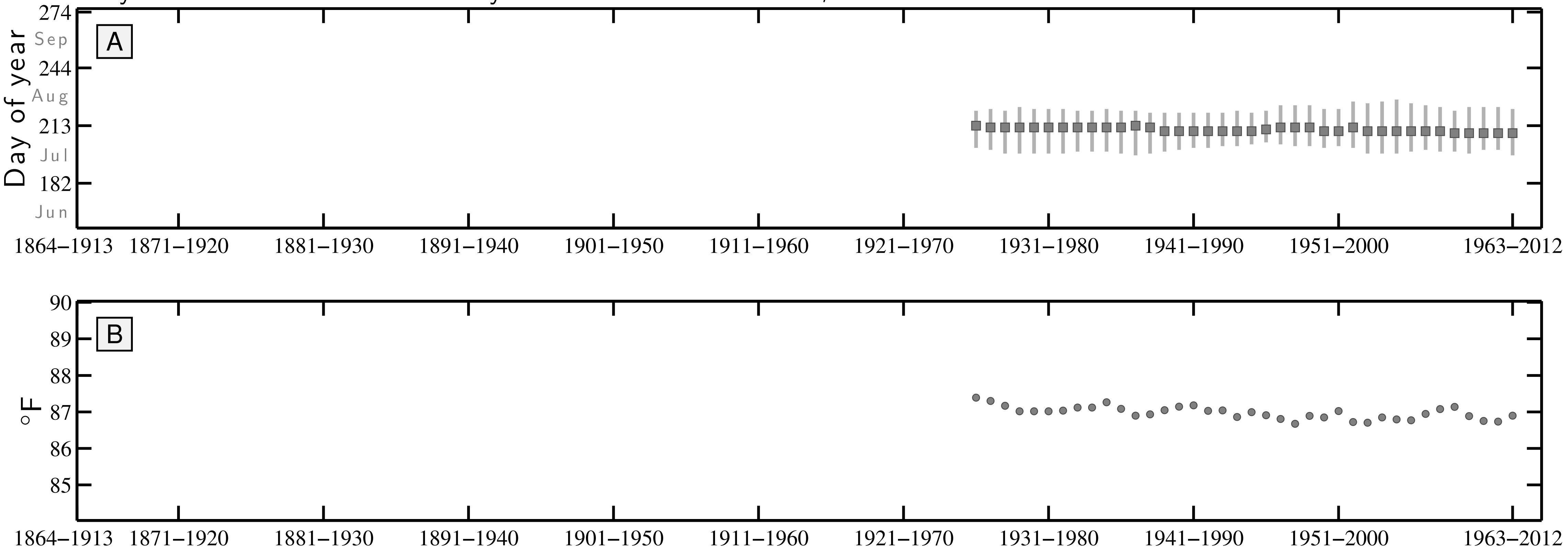

50 year Winter Teletherm dynamics for VIDA 6 NE, MT:

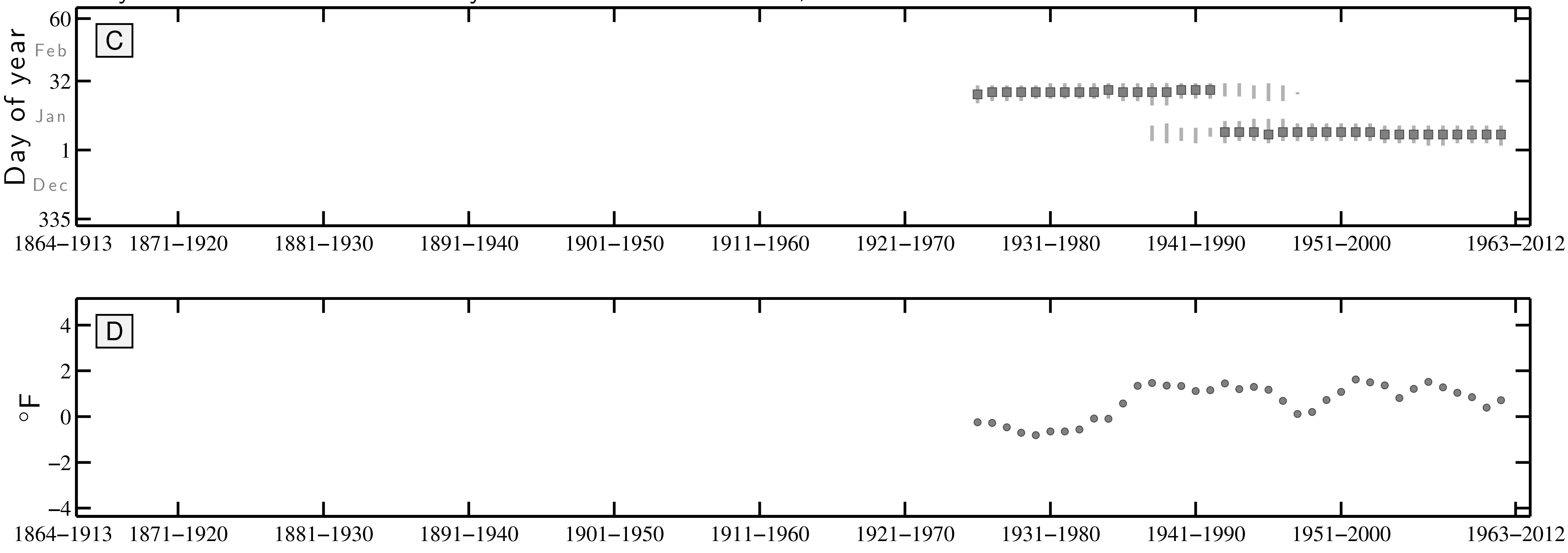

50 year Summer Teletherm dynamics for VIRGINIA CITY, MT:

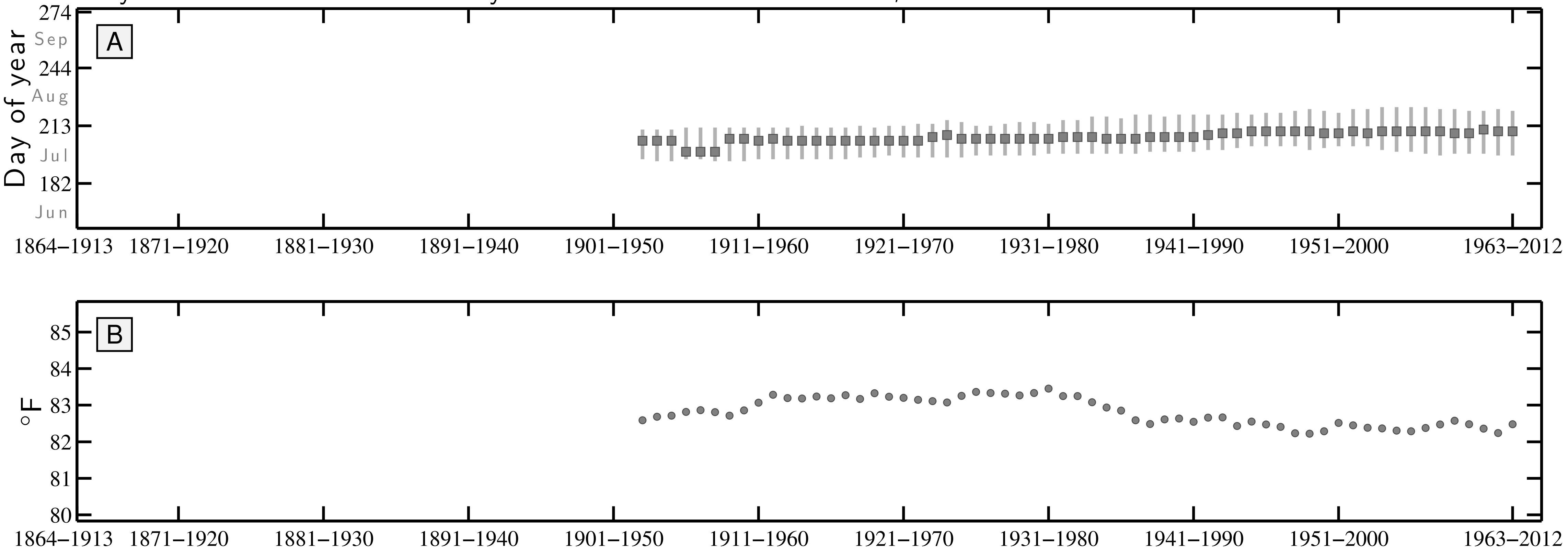

50 year Winter Teletherm dynamics for VIRGINIA CITY, MT:

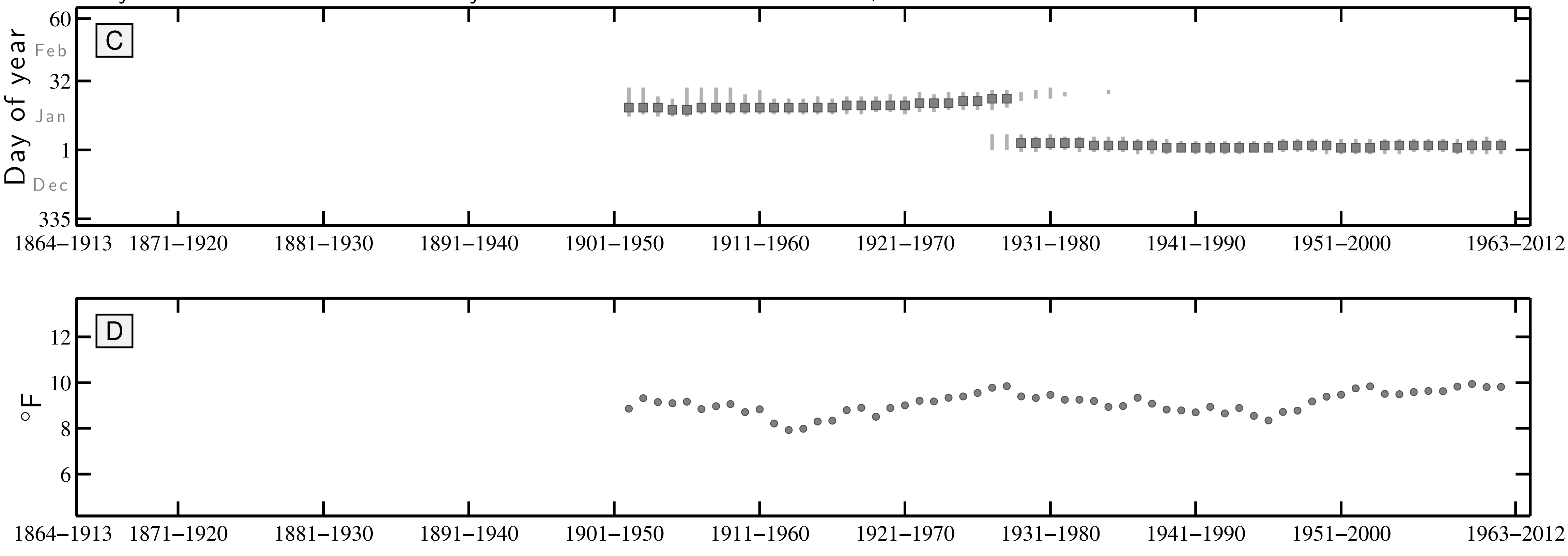

50 year Summer Teletherm dynamics for WEST YELLOWSTONE, MT:

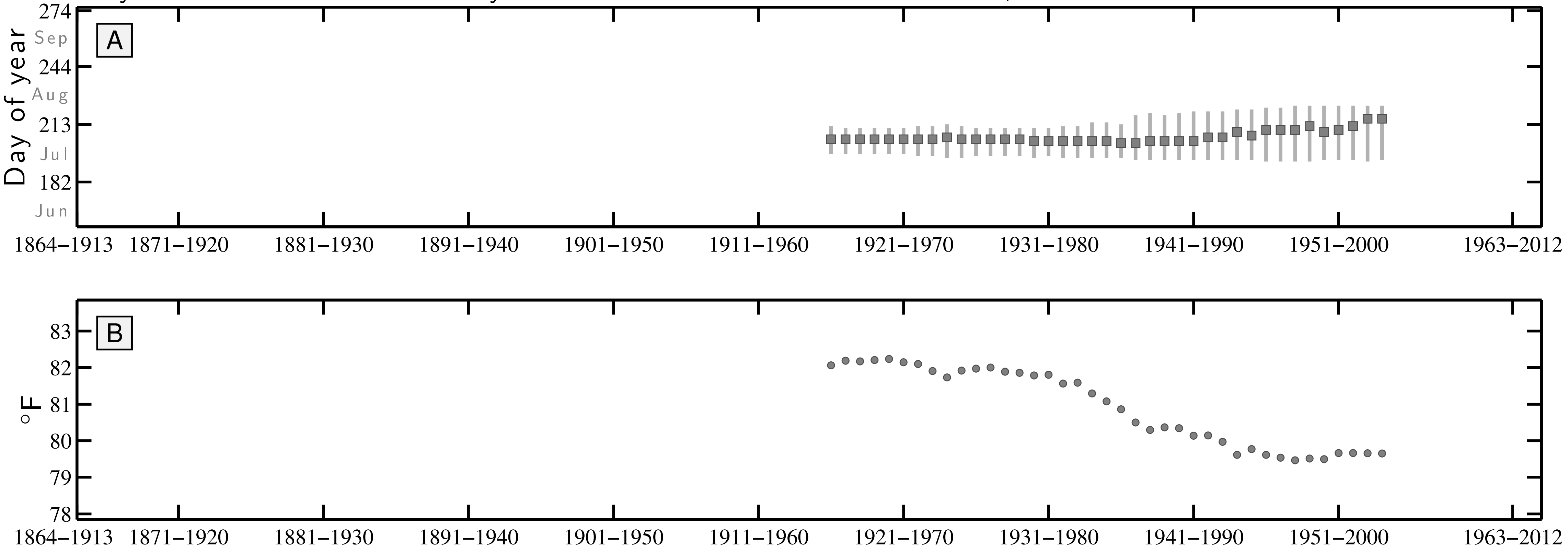

50 year Winter Teletherm dynamics for WEST YELLOWSTONE, MT:

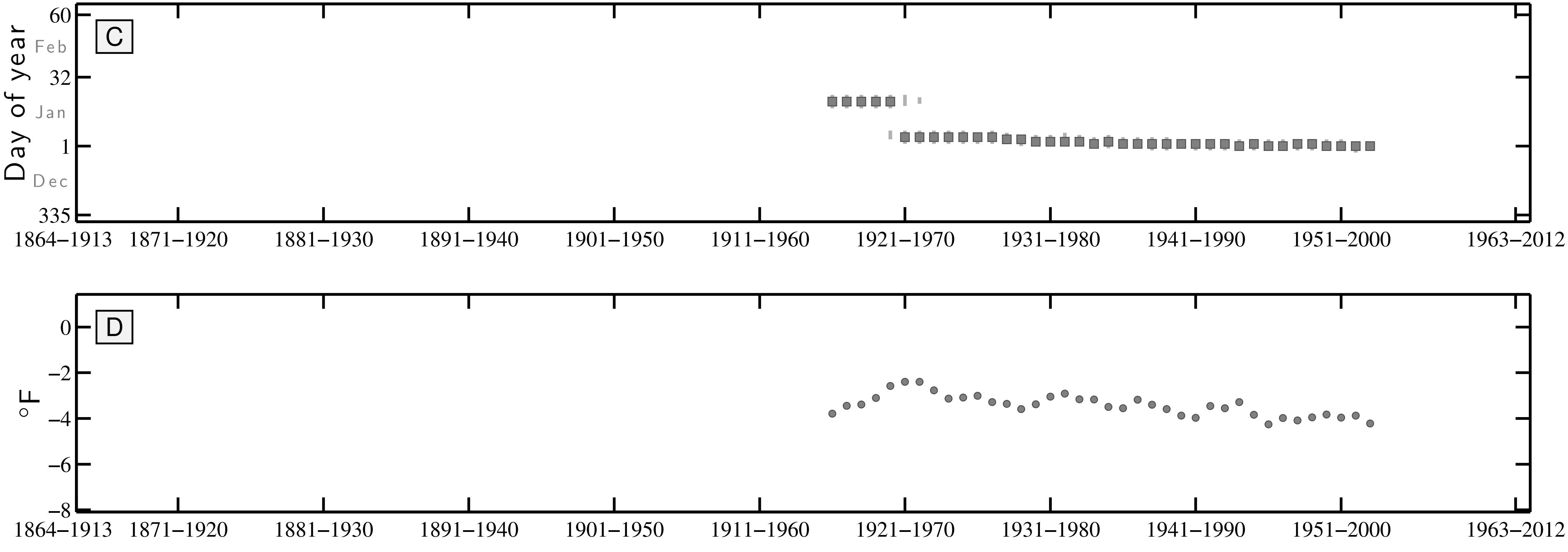

50 year Summer Teletherm dynamics for ALBION, NE:

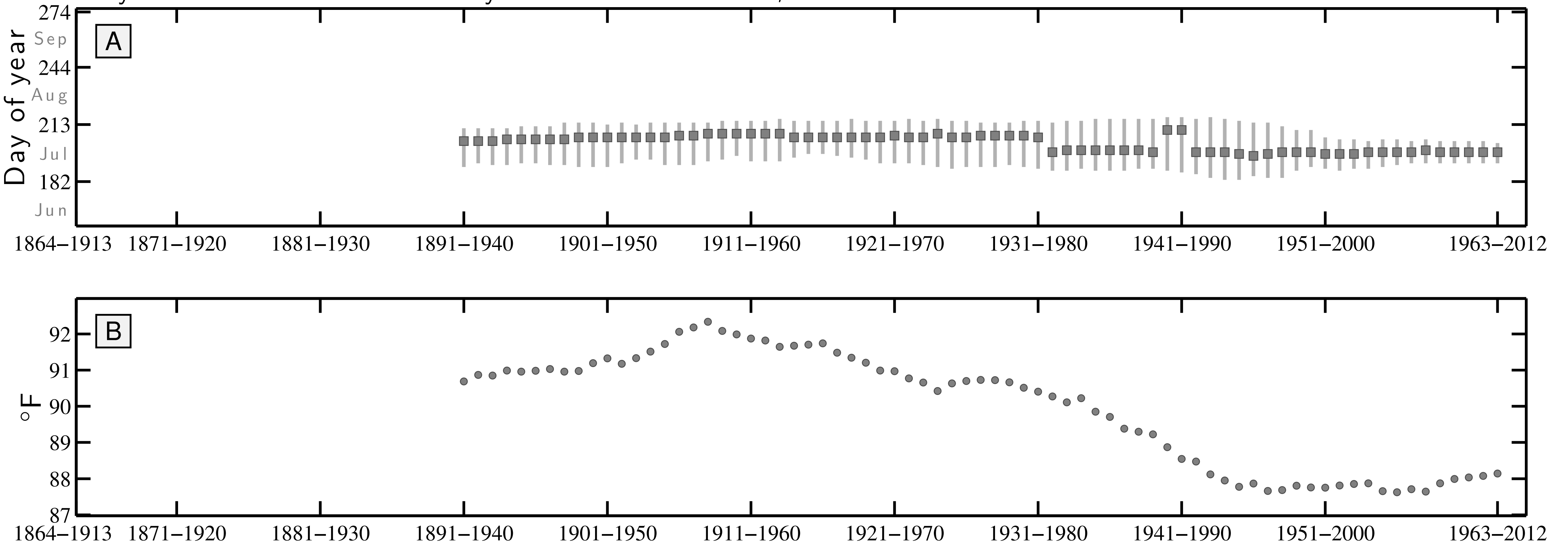

50 year Winter Teletherm dynamics for ALBION, NE:

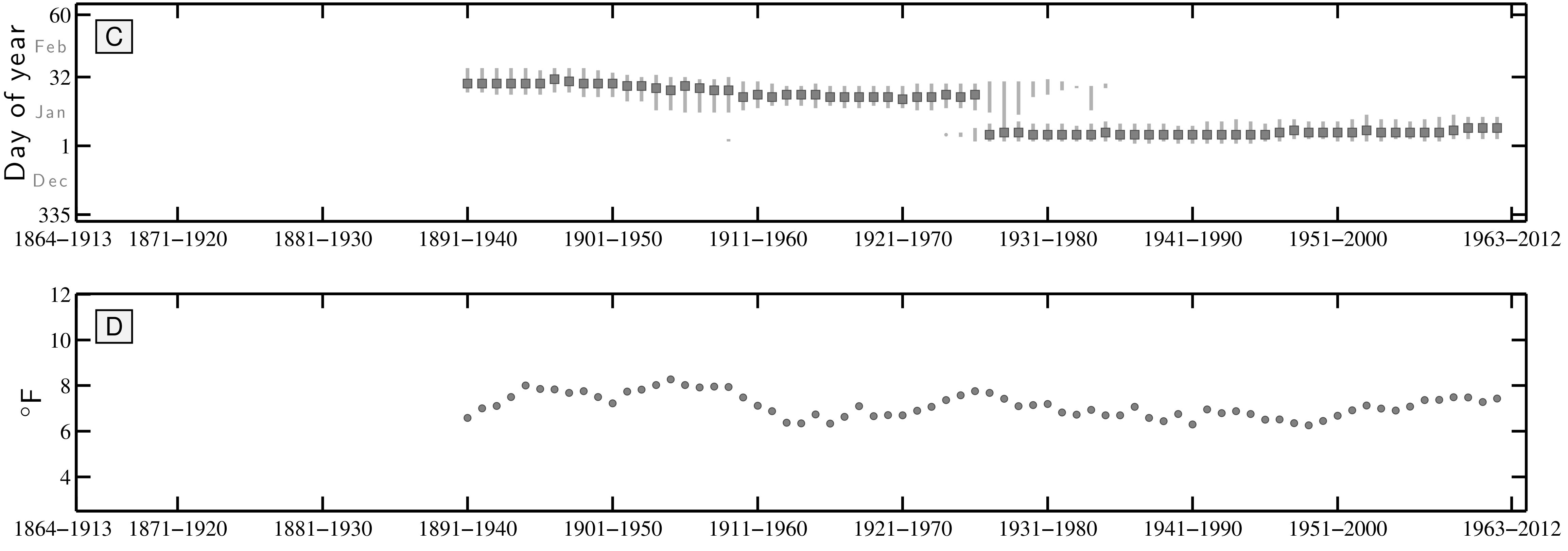

50 year Summer Teletherm dynamics for ALLIANCE 1WNW, NE:

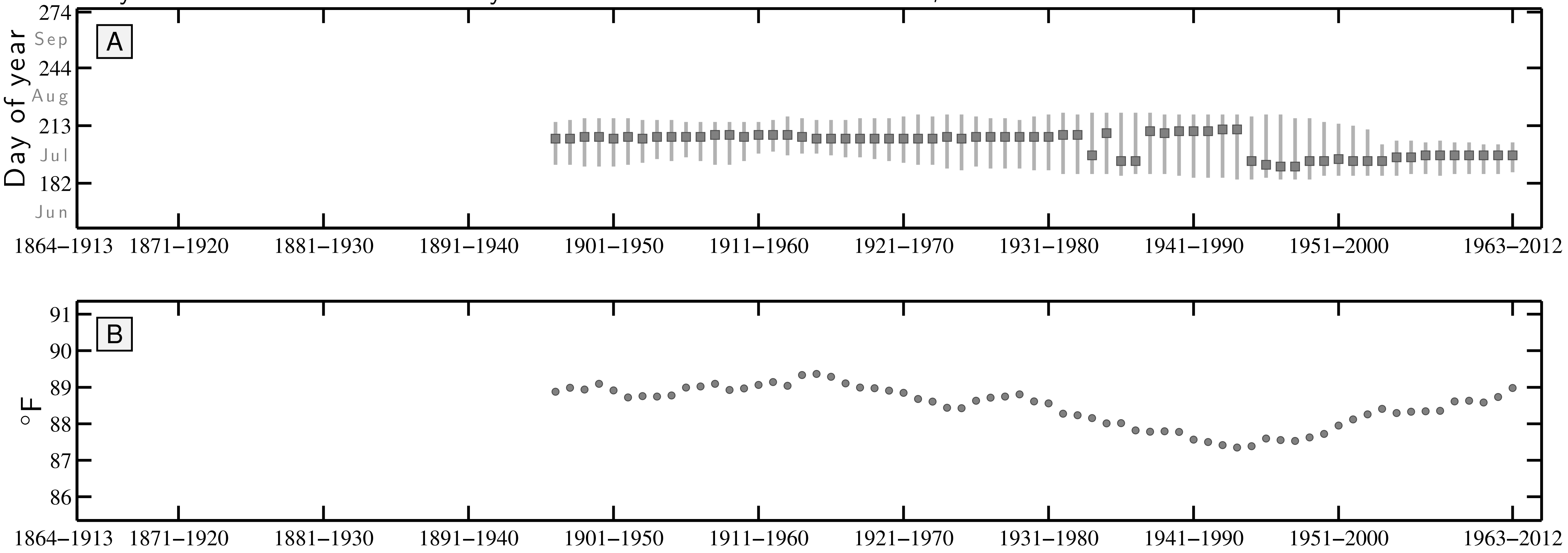

50 year Winter Teletherm dynamics for ALLIANCE 1WNW, NE:

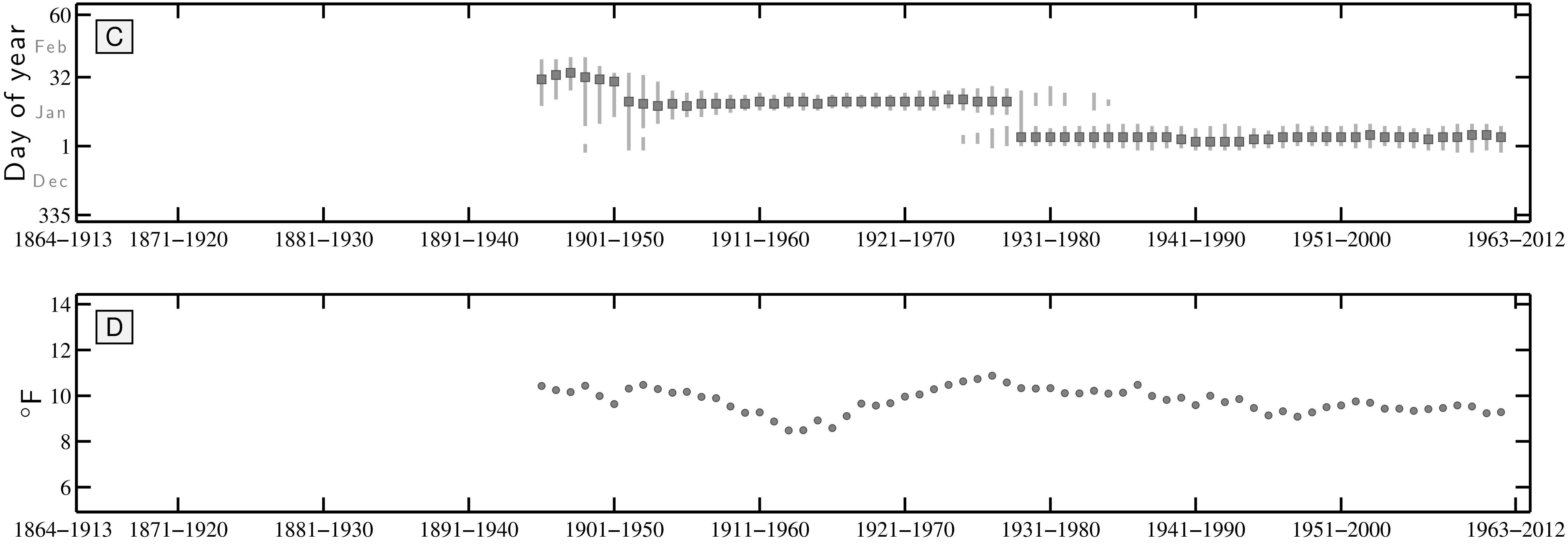

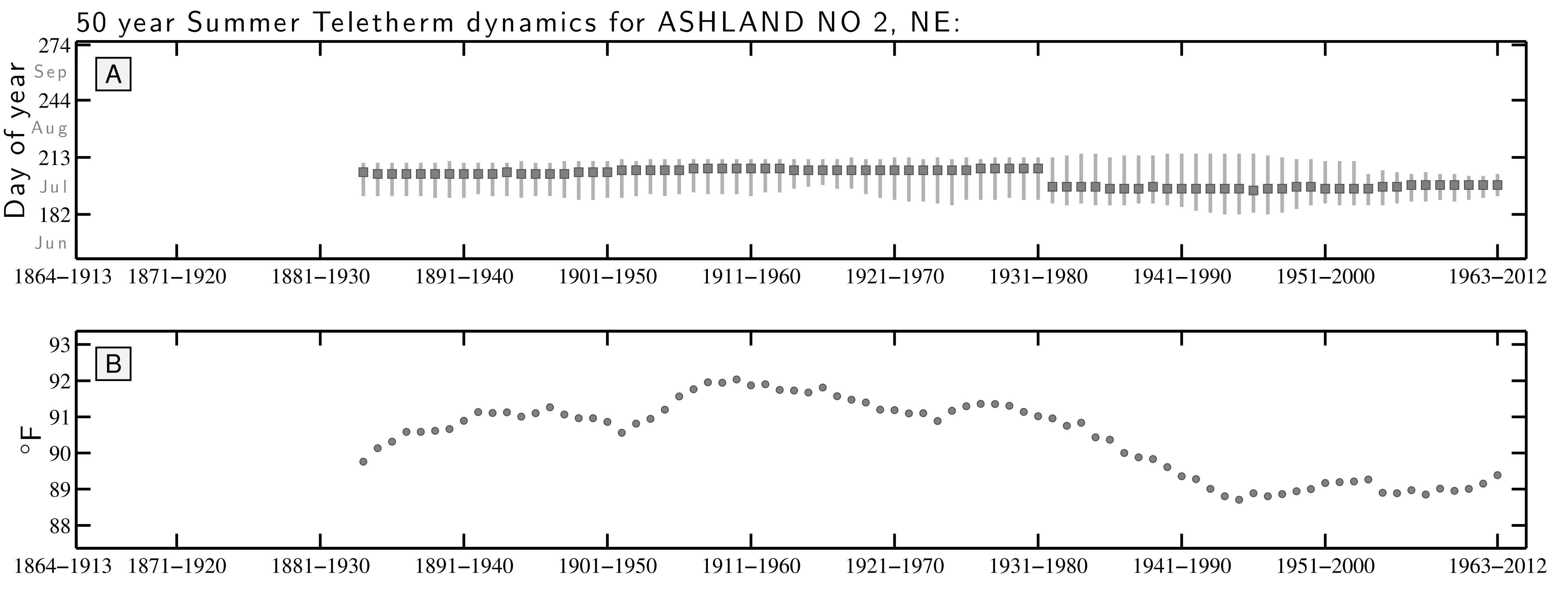
50 year Summer Teletherm dynamics for ASHLAND NO 2, NE:
A
B
Day of year
°F
274
244
213
182
Sep
Aug
Jul
Jun
93
92
91
90
89
88
1864–1913
1871–1920
1881–1930
1891–1940
1901–1950
1911–1960
1921–1970
1931–1980
1941–1990
1951–2000
1963–2012

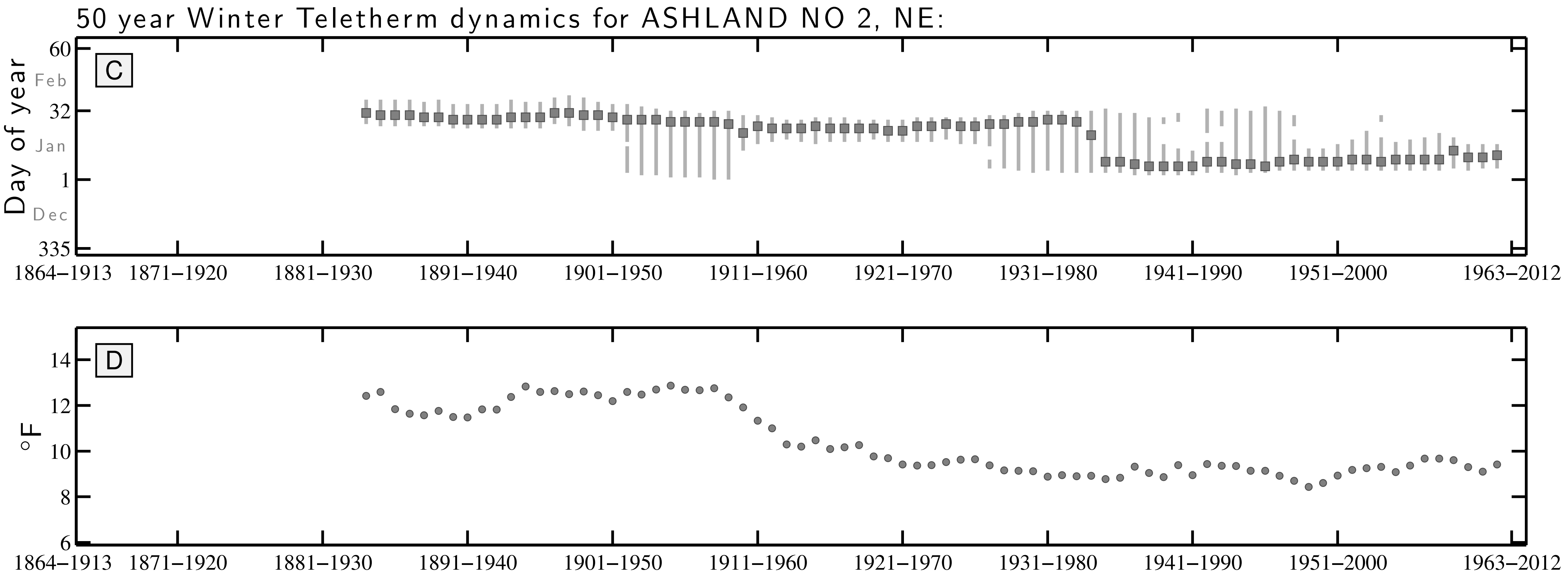
50 year Winter Teletherm dynamics for ASHLAND NO 2, NE:
C
D
Day of year
°F
60
32
1
335
Feb
Jan
Dec
14
12
10
8
6
1864–1913
1871–1920
1881–1930
1891–1940
1901–1950
1911–1960
1921–1970
1931–1980
1941–1990
1951–2000
1963–2012

50 year Summer Teletherm dynamics for ATKINSON 3SW, NE:

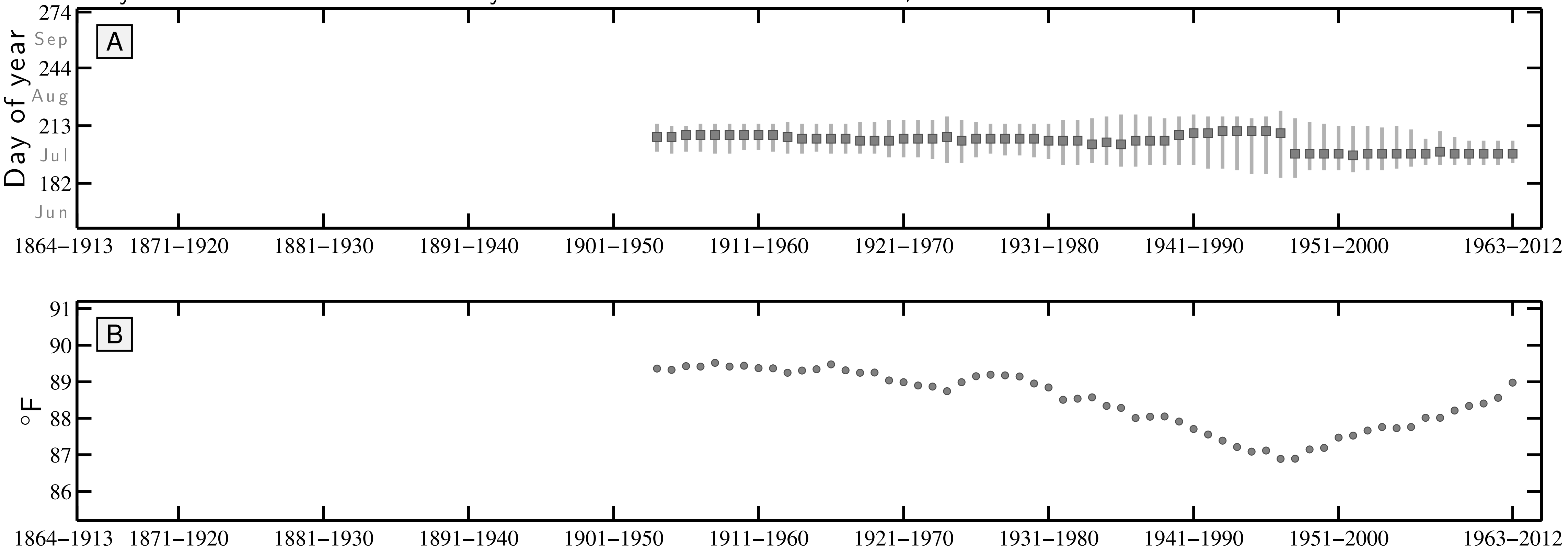

50 year Winter Teletherm dynamics for ATKINSON 3SW, NE:

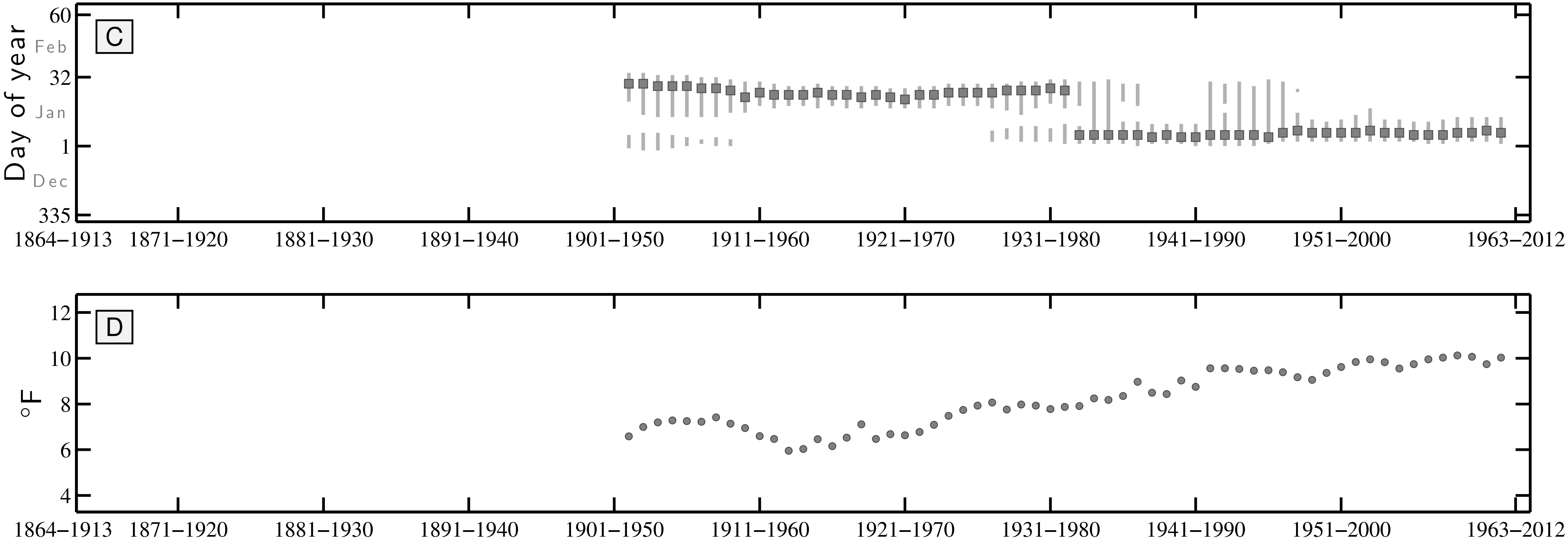

50 year Summer Teletherm dynamics for AUBURN 5 ESE, NE:

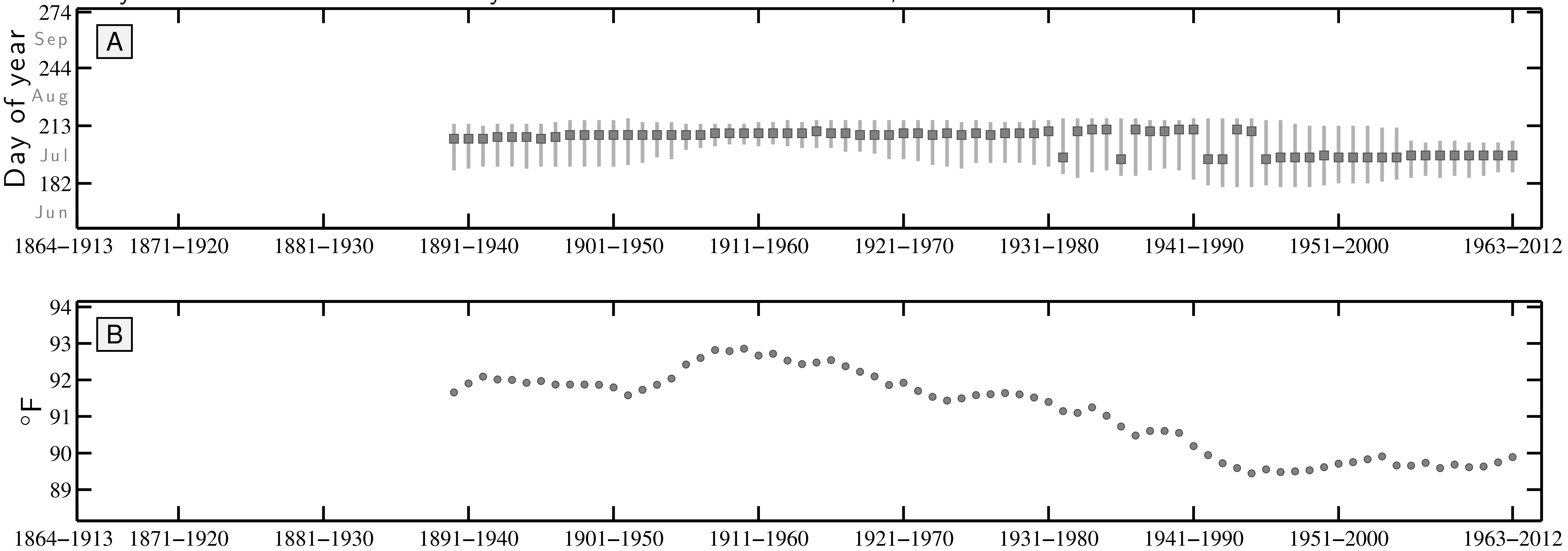

50 year Winter Teletherm dynamics for AUBURN 5 ESE, NE:

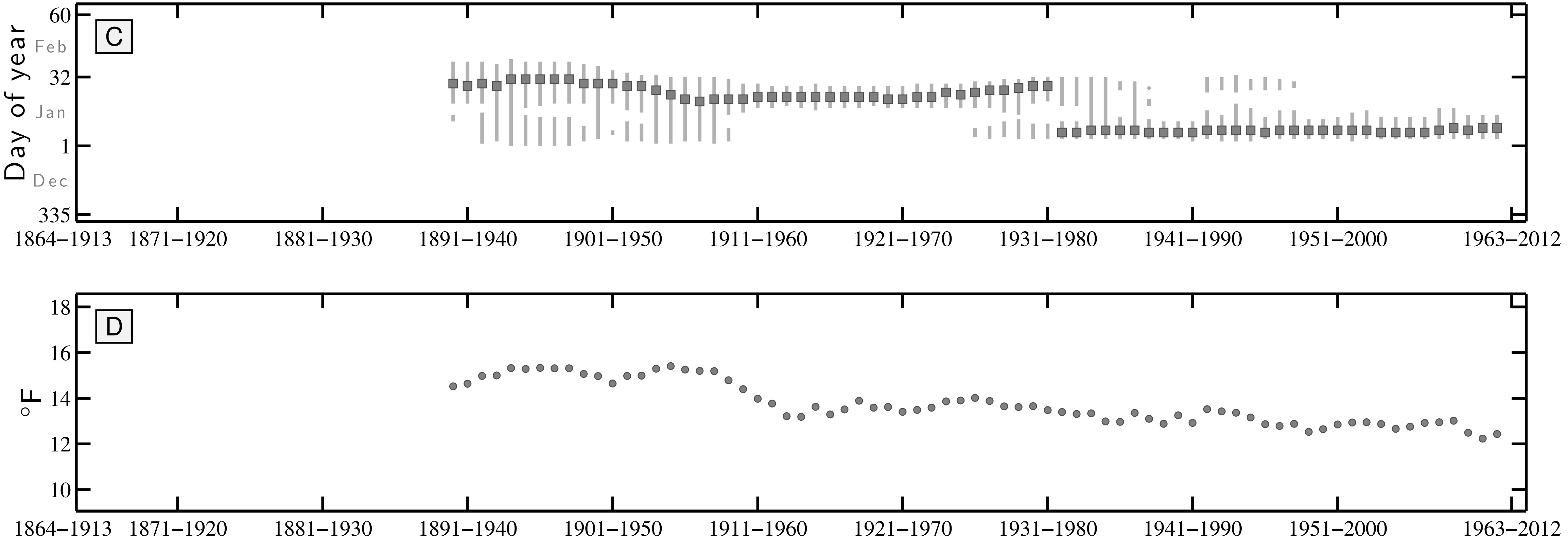

50 year Summer Teletherm dynamics for BEAVER CITY, NE:

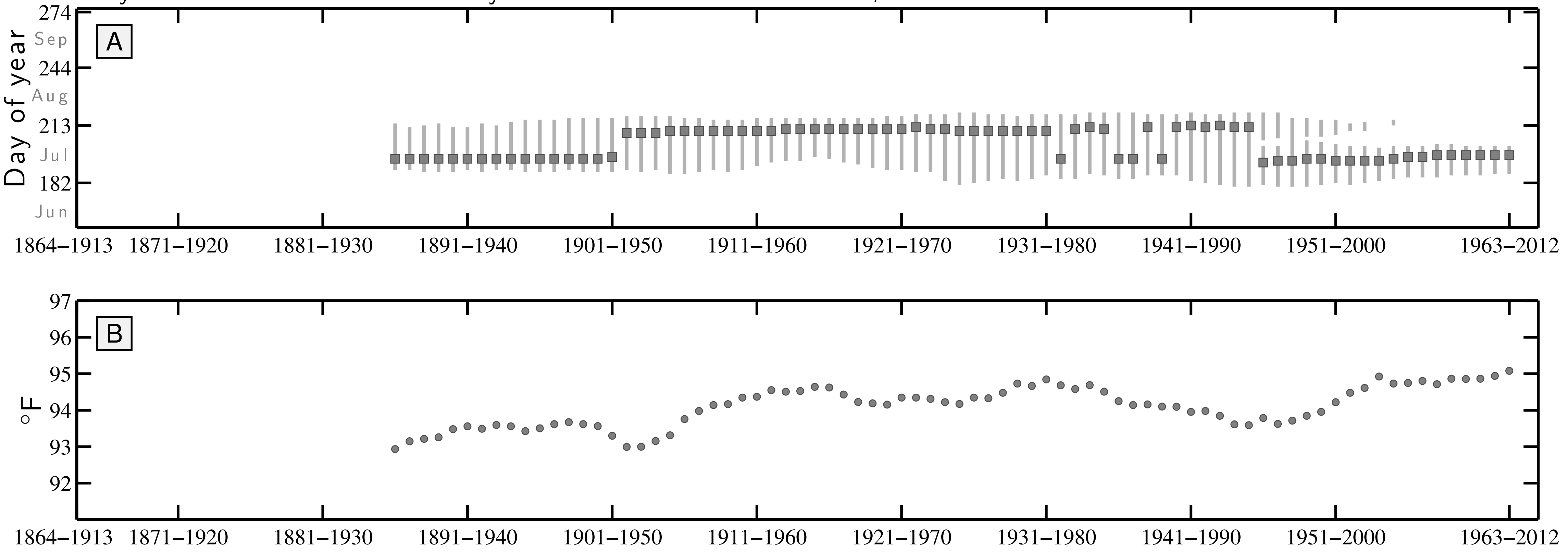

50 year Winter Teletherm dynamics for BEAVER CITY, NE:

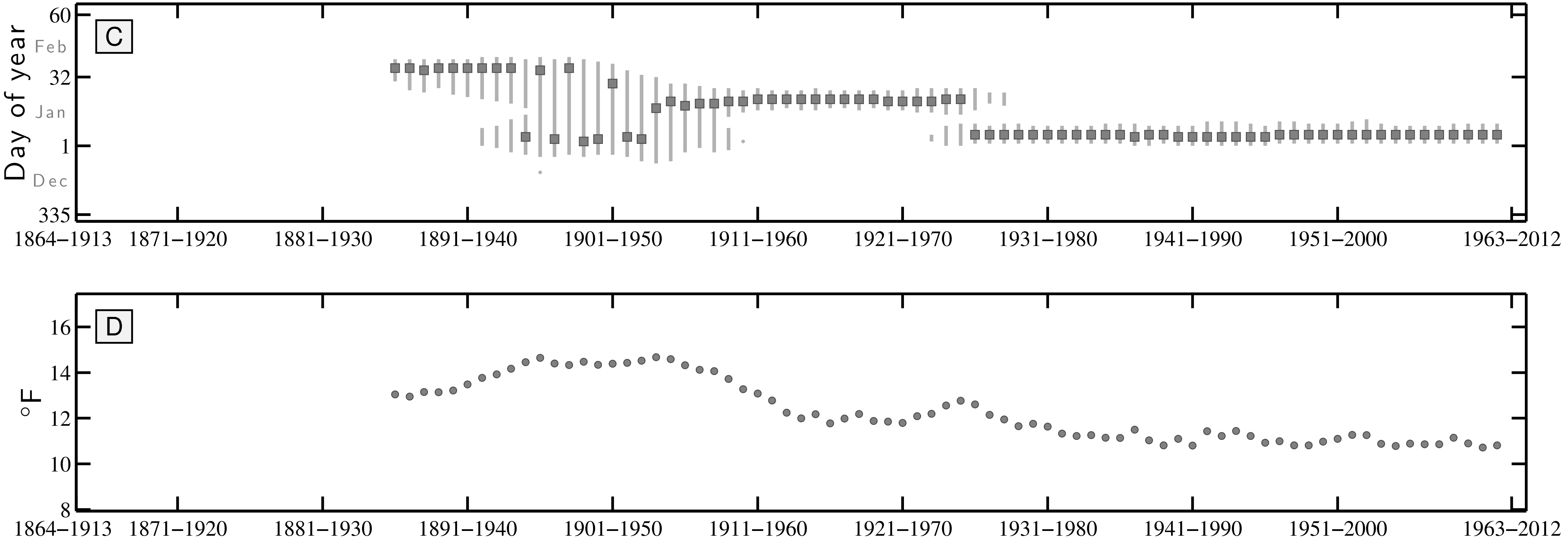

50 year Summer Teletherm dynamics for BRIDGEPORT, NE:

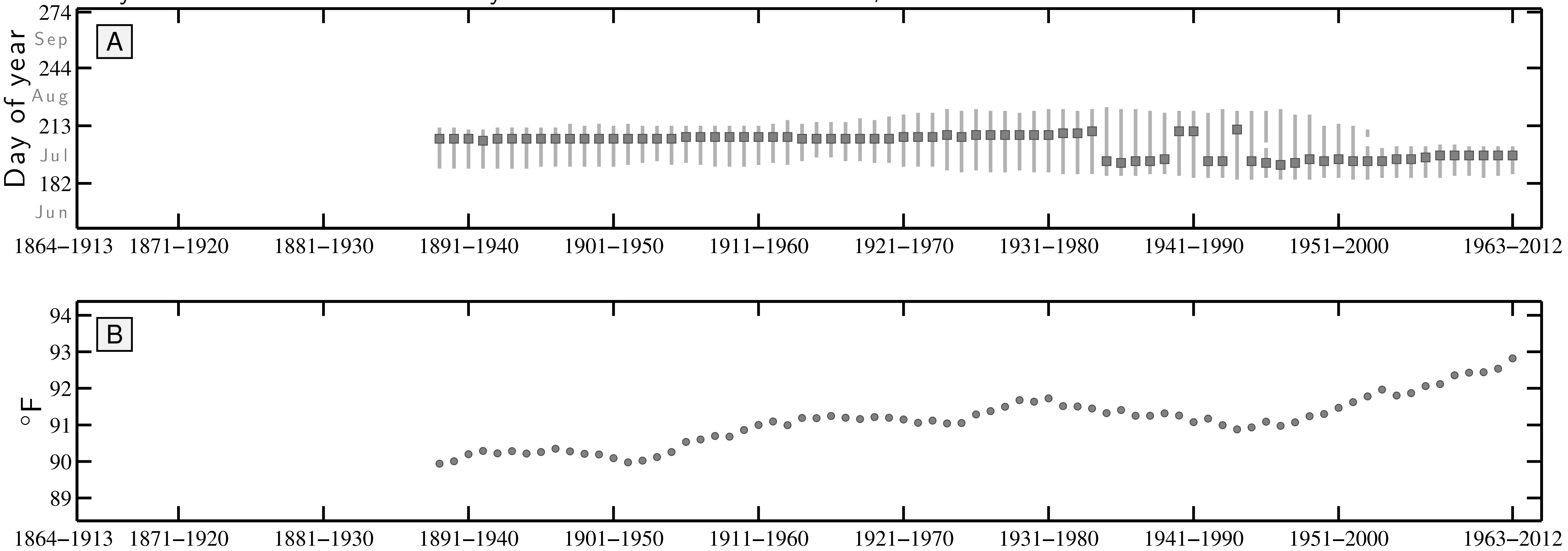

50 year Winter Teletherm dynamics for BRIDGEPORT, NE:

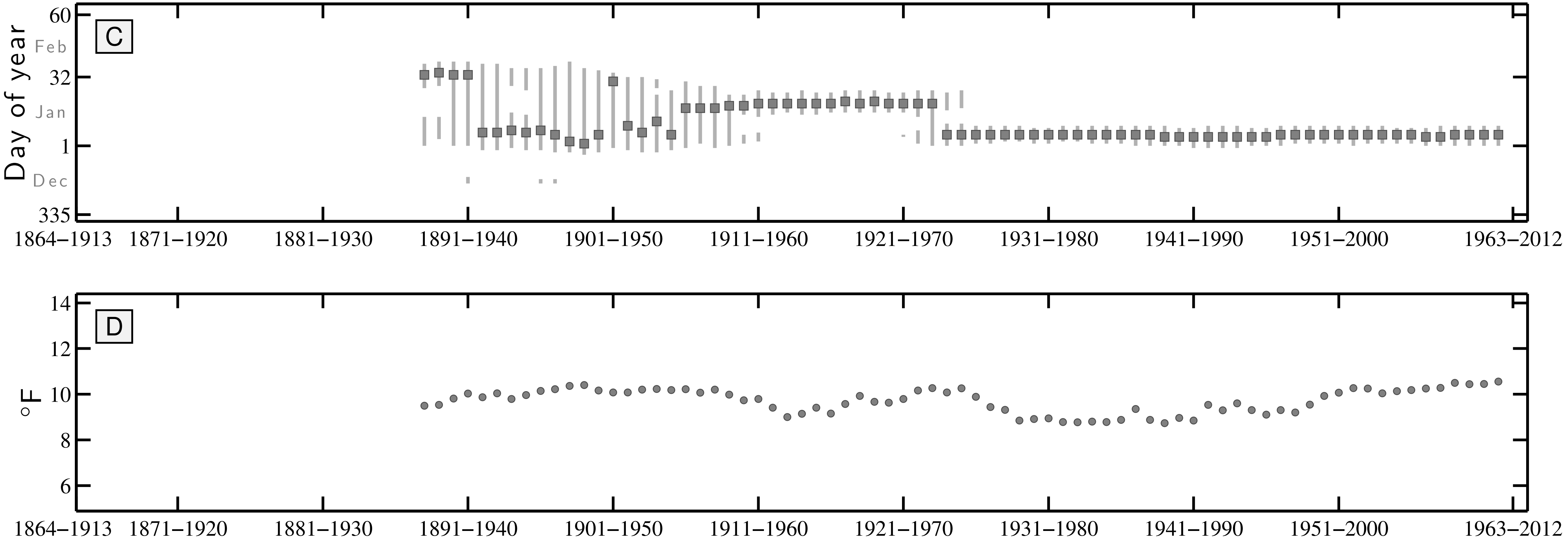

50 year Summer Teletherm dynamics for BROKEN BOW 2 W, NE:

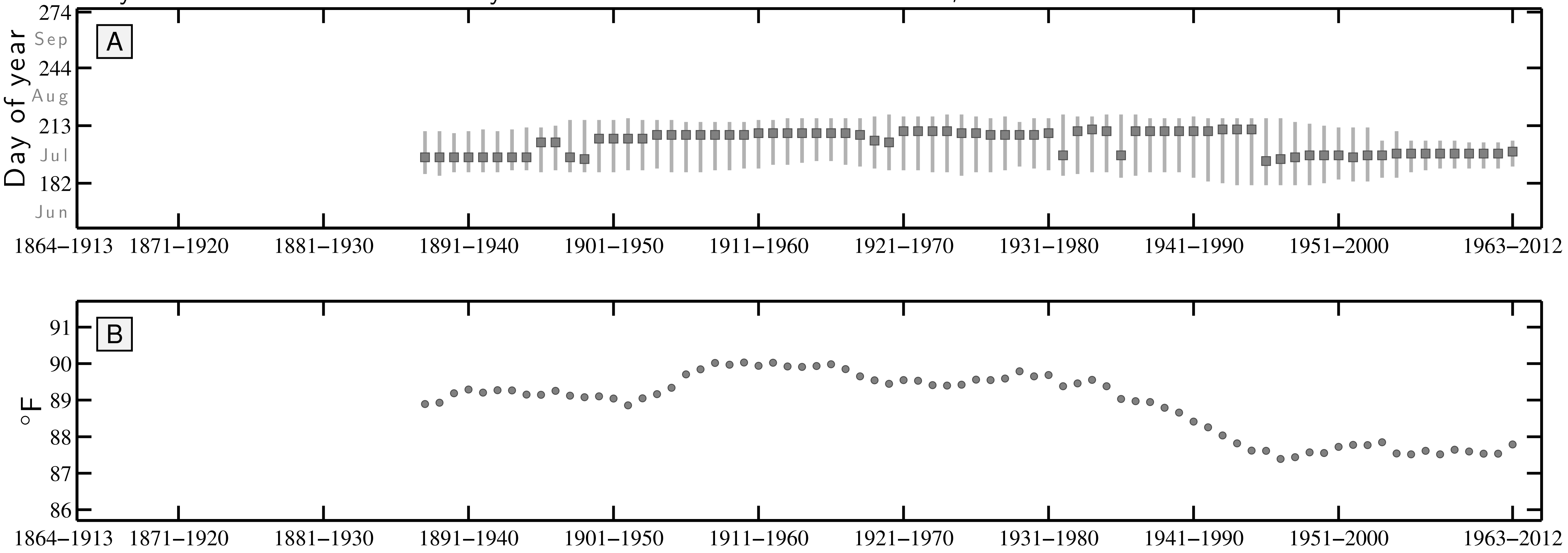

50 year Winter Teletherm dynamics for BROKEN BOW 2 W, NE:

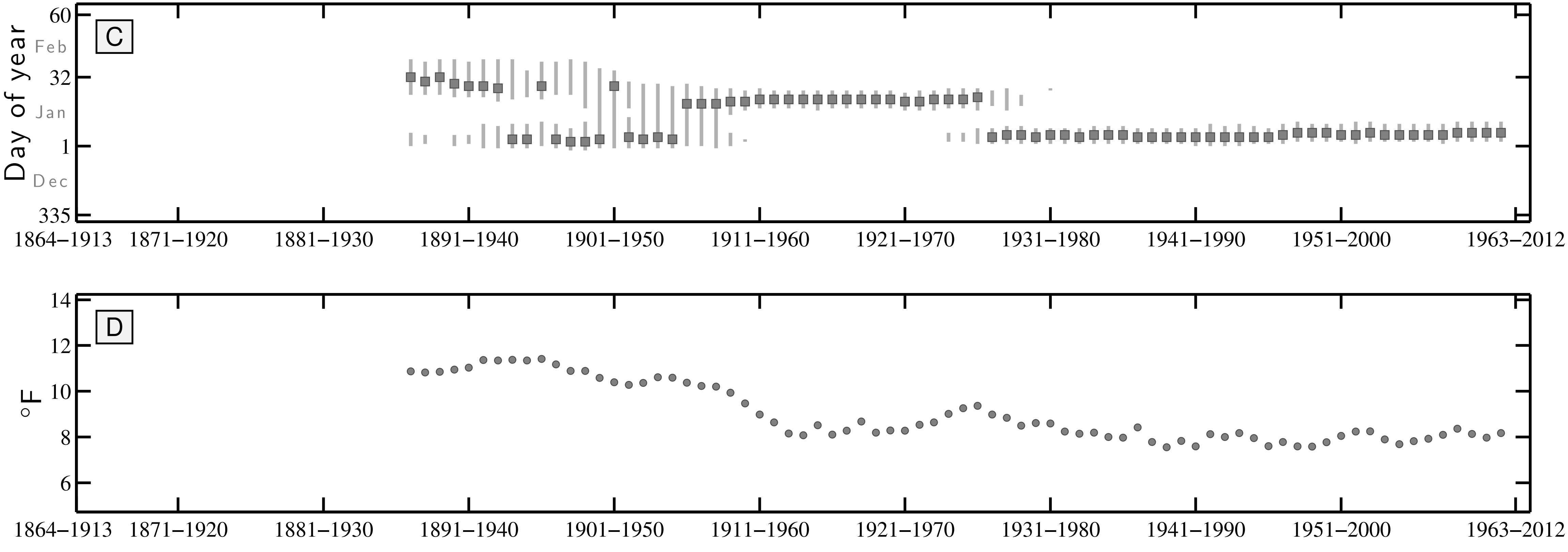

50 year Summer Teletherm dynamics for CRETE, NE:

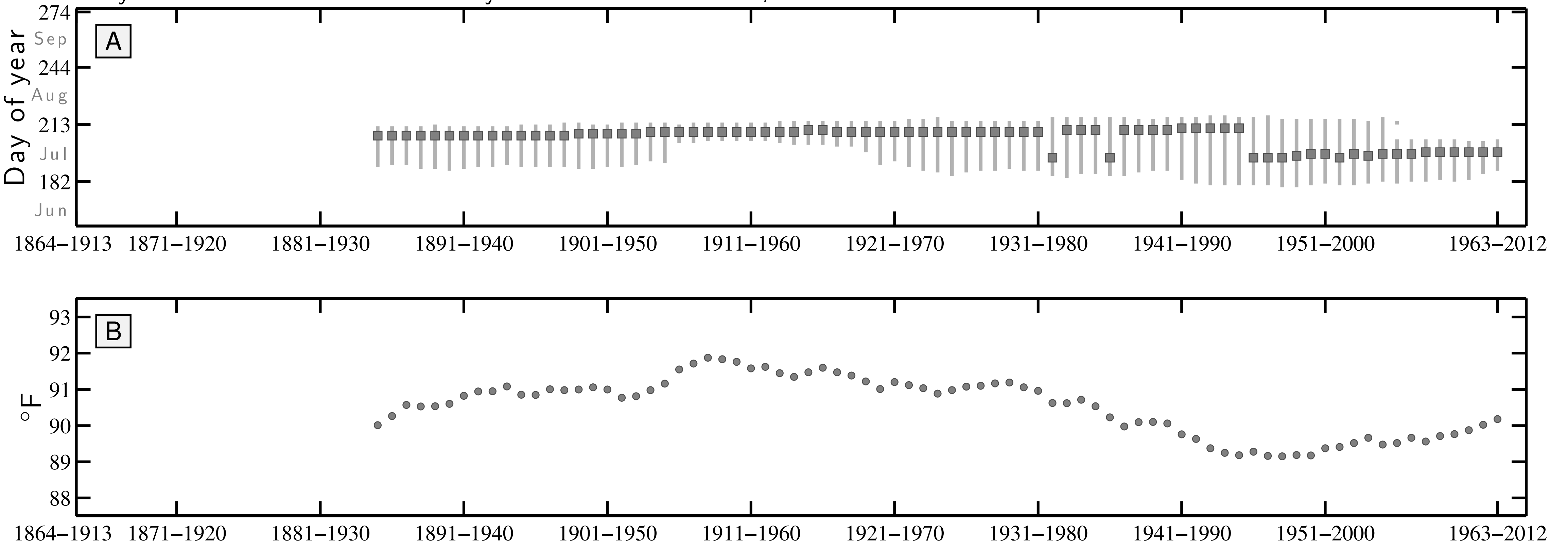

50 year Winter Teletherm dynamics for CRETE, NE:

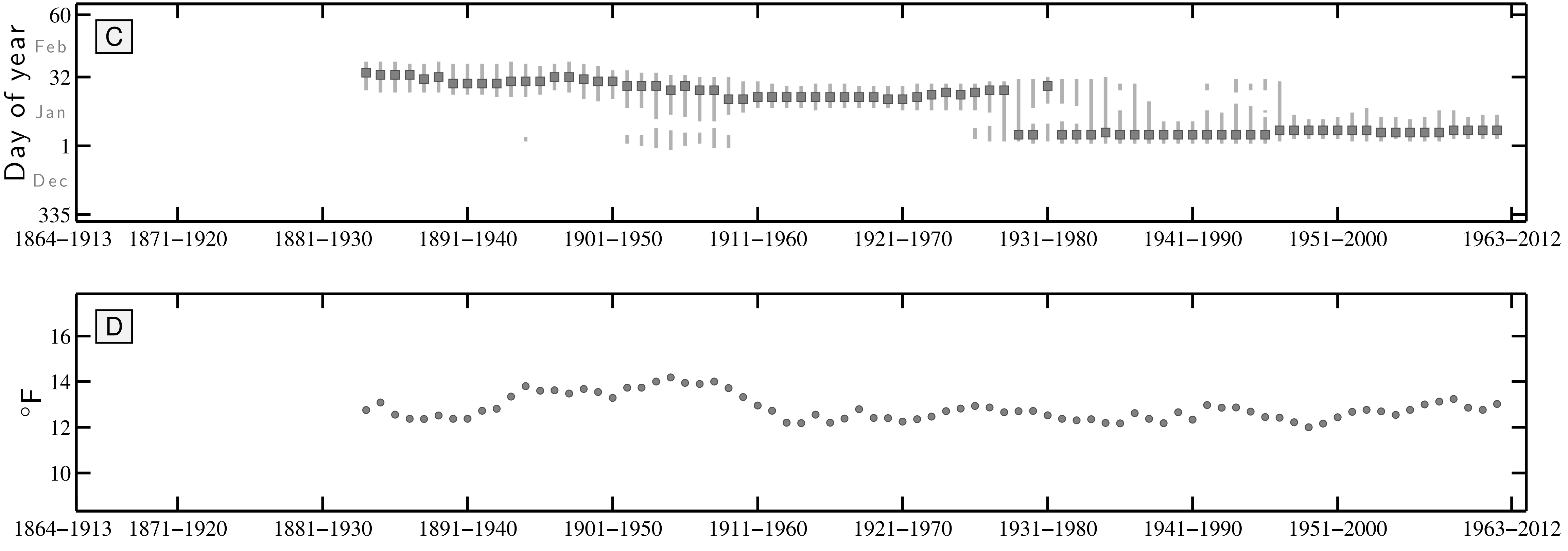

50 year Summer Teletherm dynamics for CURTIS 3NNE, NE:

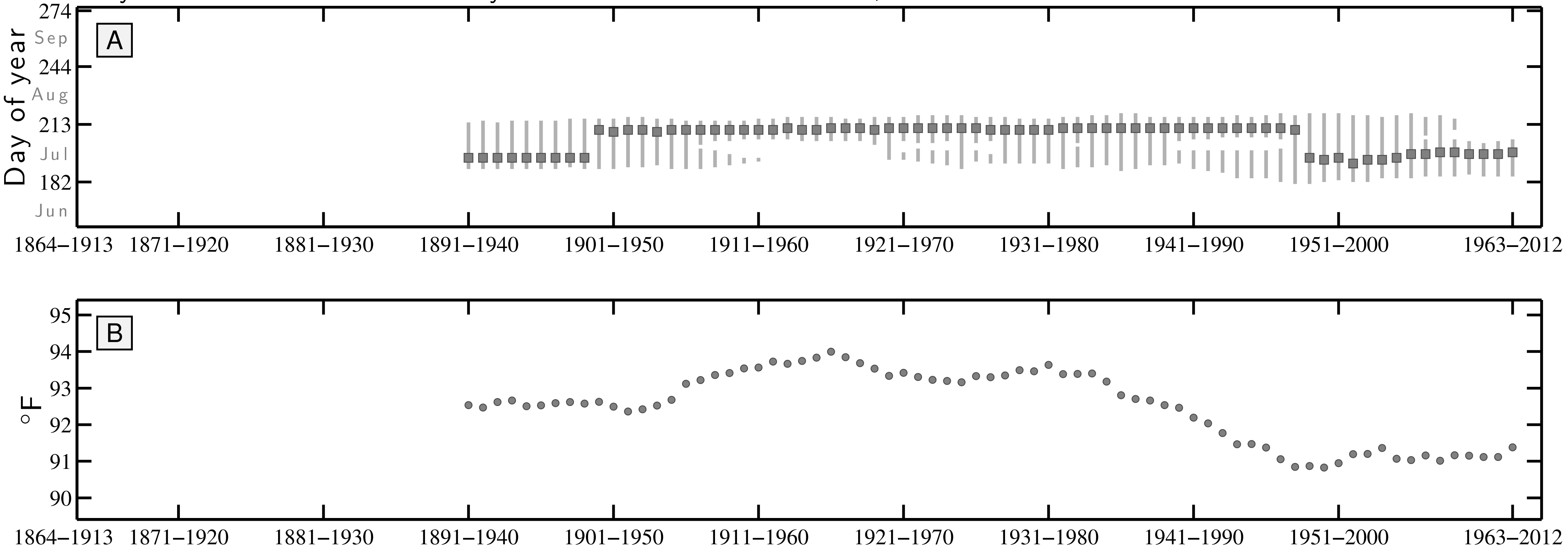

50 year Winter Teletherm dynamics for CURTIS 3NNE, NE:

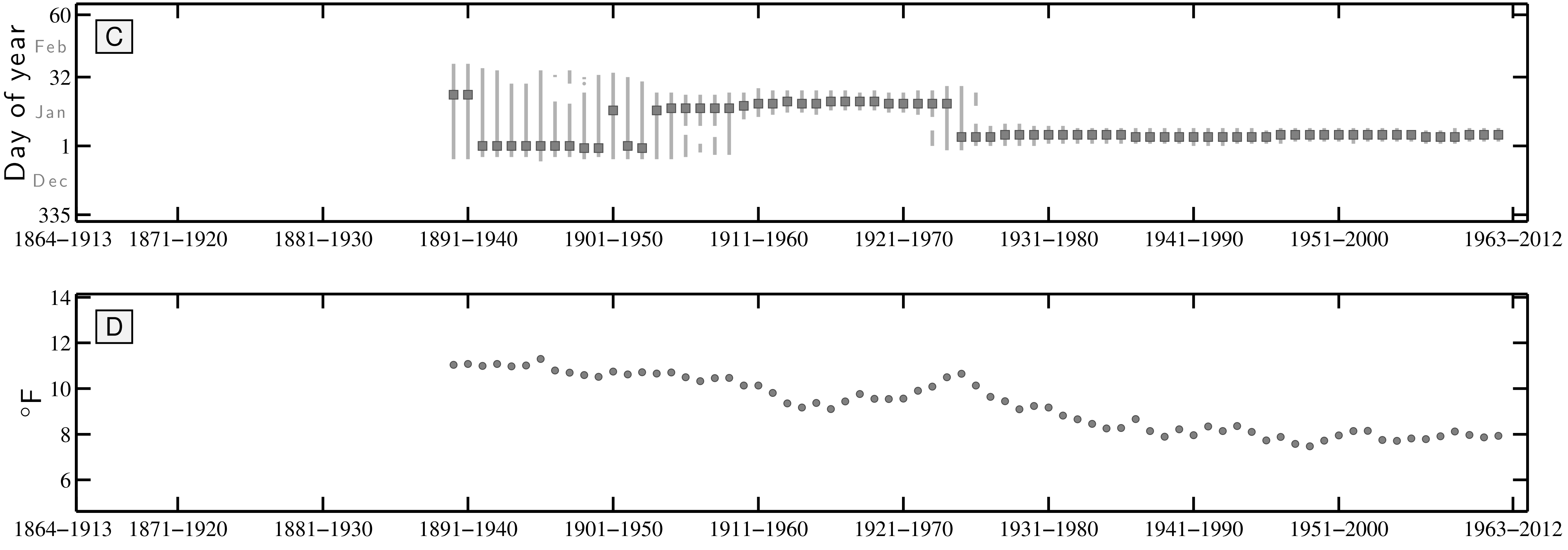

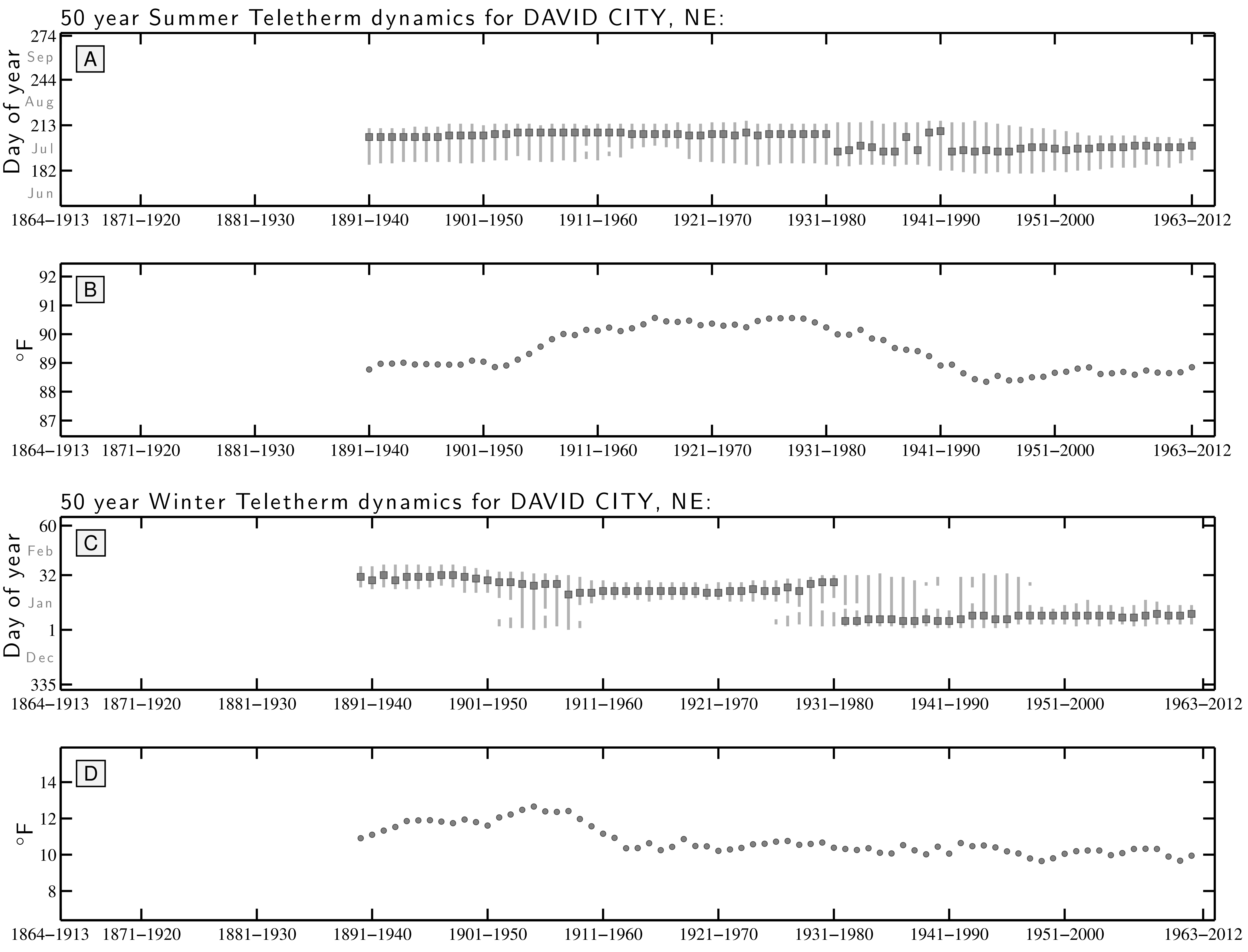
50 year Summer Teletherm dynamics for DAVID CITY, NE:
A
Day of year
274
Sep
244
Aug
213
Jul
182
Jun
1864–1913
1871–1920
1881–1930
1891–1940
1901–1950
1911–1960
1921–1970
1931–1980
1941–1990
1951–2000
1963–2012
B
°F
92
91
90
89
88
87
50 year Winter Teletherm dynamics for DAVID CITY, NE:
C
Day of year
60
Feb
32
Jan
1
Dec
335
D
°F
14
12
10
8

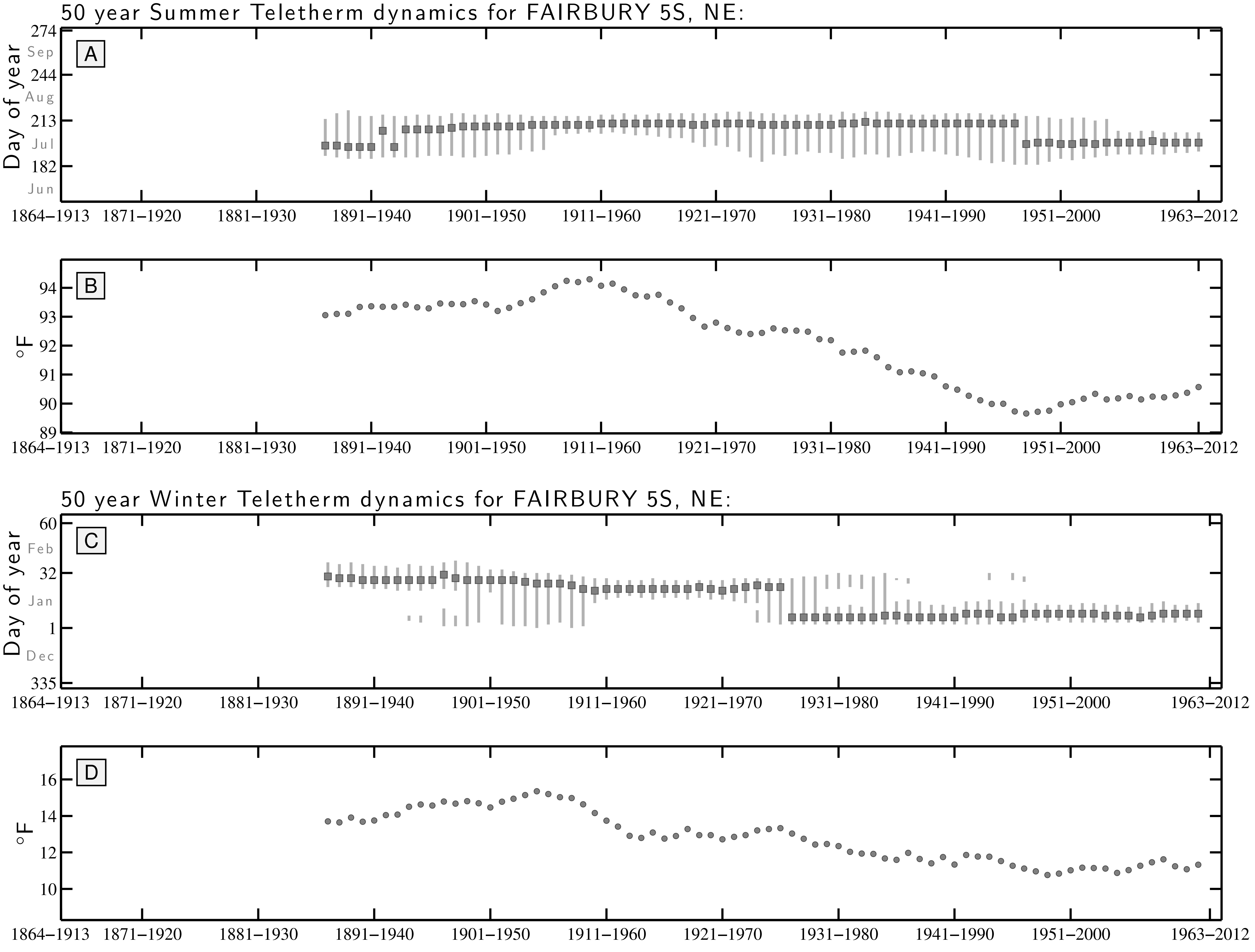

50 year Summer Teletherm dynamics for FAIRBURY 5S, NE:
A
Day of year
274
Sep
244
Aug
213
Jul
182
Jun
B
°F
94
93
92
91
90
89
1864–1913
1871–1920
1881–1930
1891–1940
1901–1950
1911–1960
1921–1970
1931–1980
1941–1990
1951–2000
1963–2012
50 year Winter Teletherm dynamics for FAIRBURY 5S, NE:
C
Day of year
60
Feb
32
Jan
1
Dec
335
D
°F
16
14
12
10

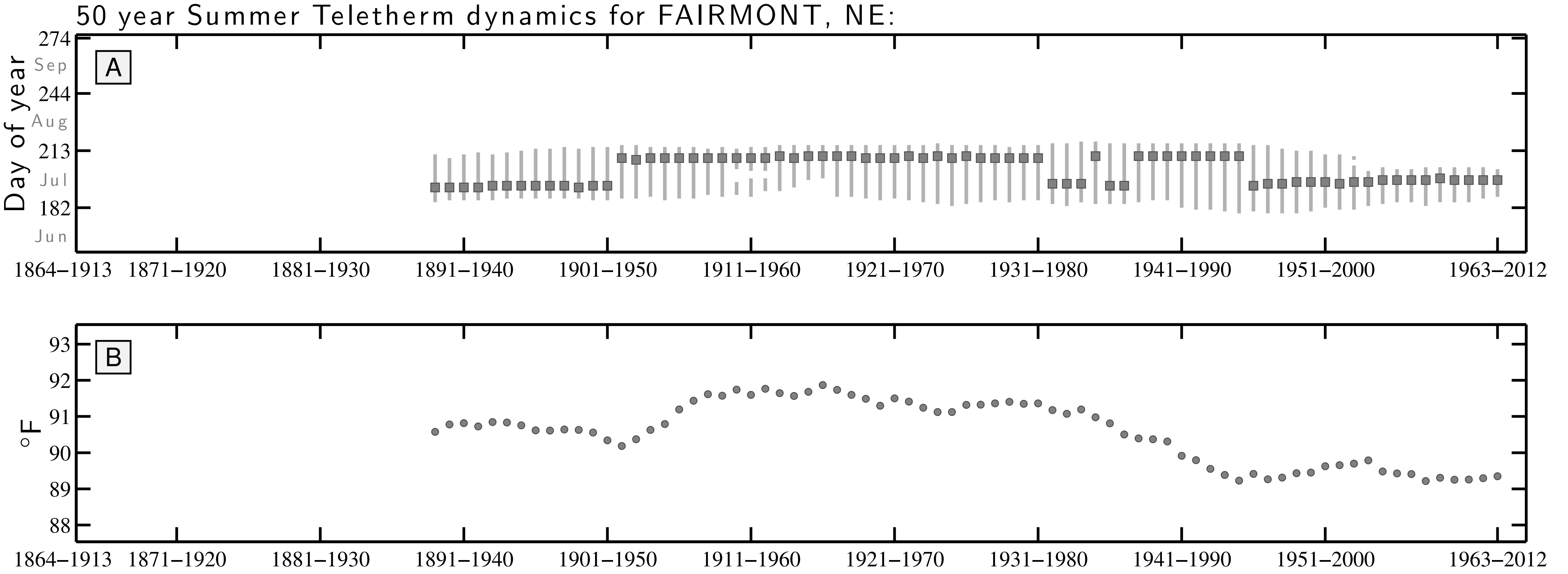
50 year Summer Teletherm dynamics for FAIRMONT, NE:
A
Day of year
274
Sep
244
Aug
213
Jul
182
Jun
B
°F
93
92
91
90
89
88
1864–1913
1871–1920
1881–1930
1891–1940
1901–1950
1911–1960
1921–1970
1931–1980
1941–1990
1951–2000
1963–2012

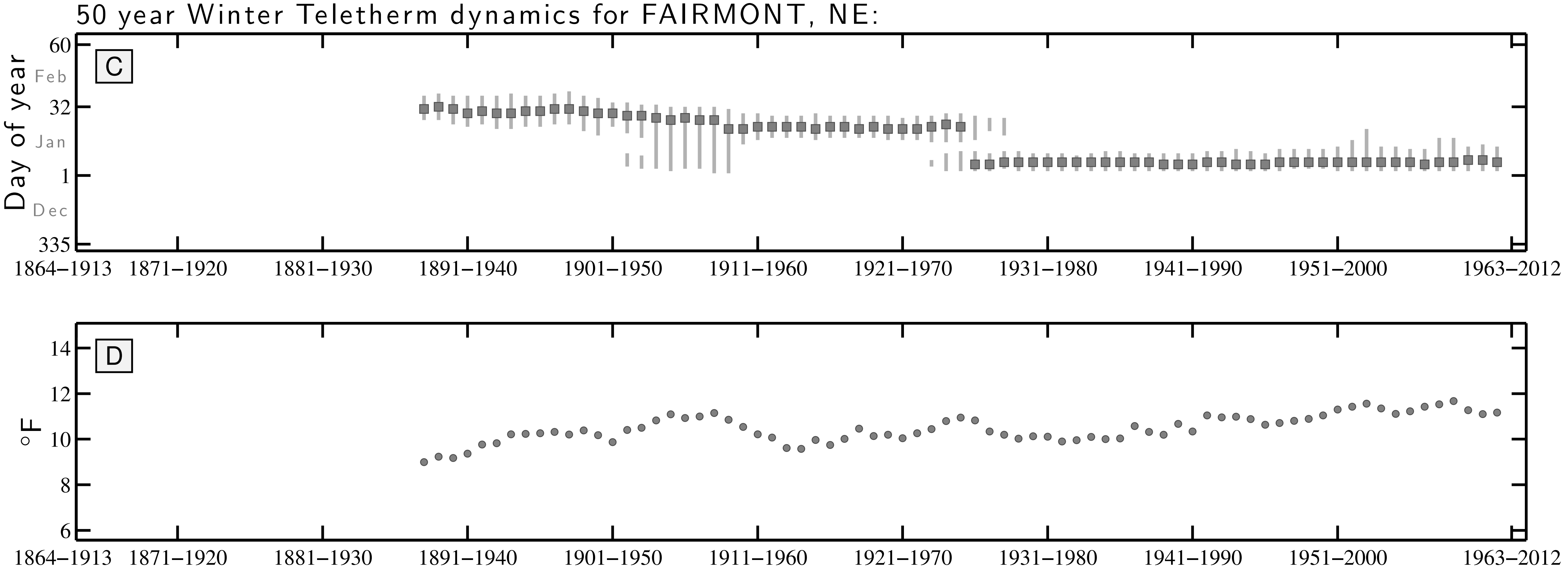
50 year Winter Teletherm dynamics for FAIRMONT, NE:
C
Day of year
60
Feb
32
Jan
1
Dec
335
D
°F
14
12
10
8
6
1864–1913
1871–1920
1881–1930
1891–1940
1901–1950
1911–1960
1921–1970
1931–1980
1941–1990
1951–2000
1963–2012

50 year Summer Teletherm dynamics for FRANKLIN, NE:

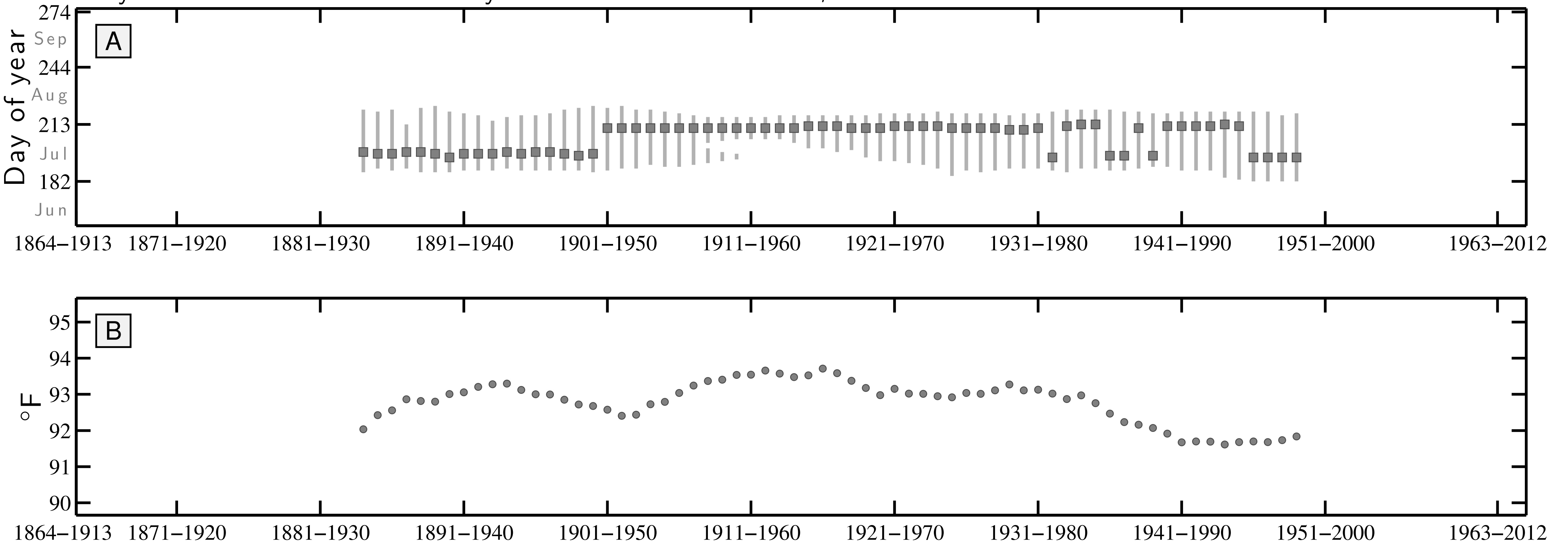

50 year Winter Teletherm dynamics for FRANKLIN, NE:

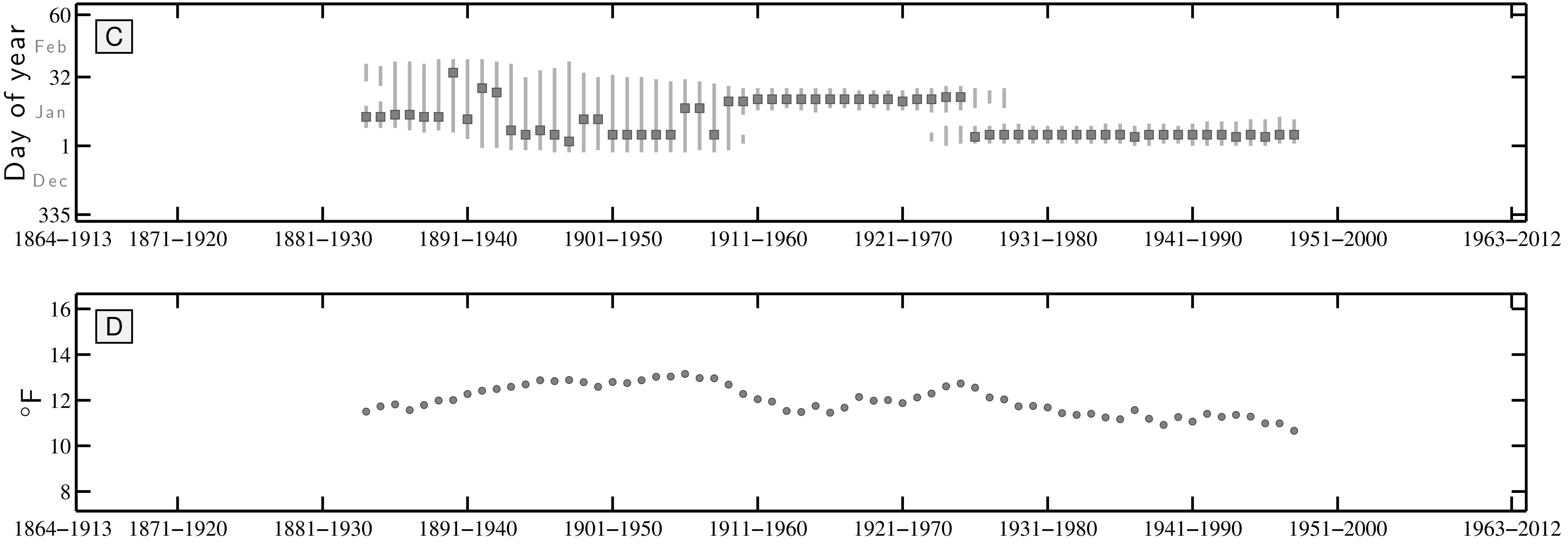

50 year Summer Teletherm dynamics for GENEVA, NE:

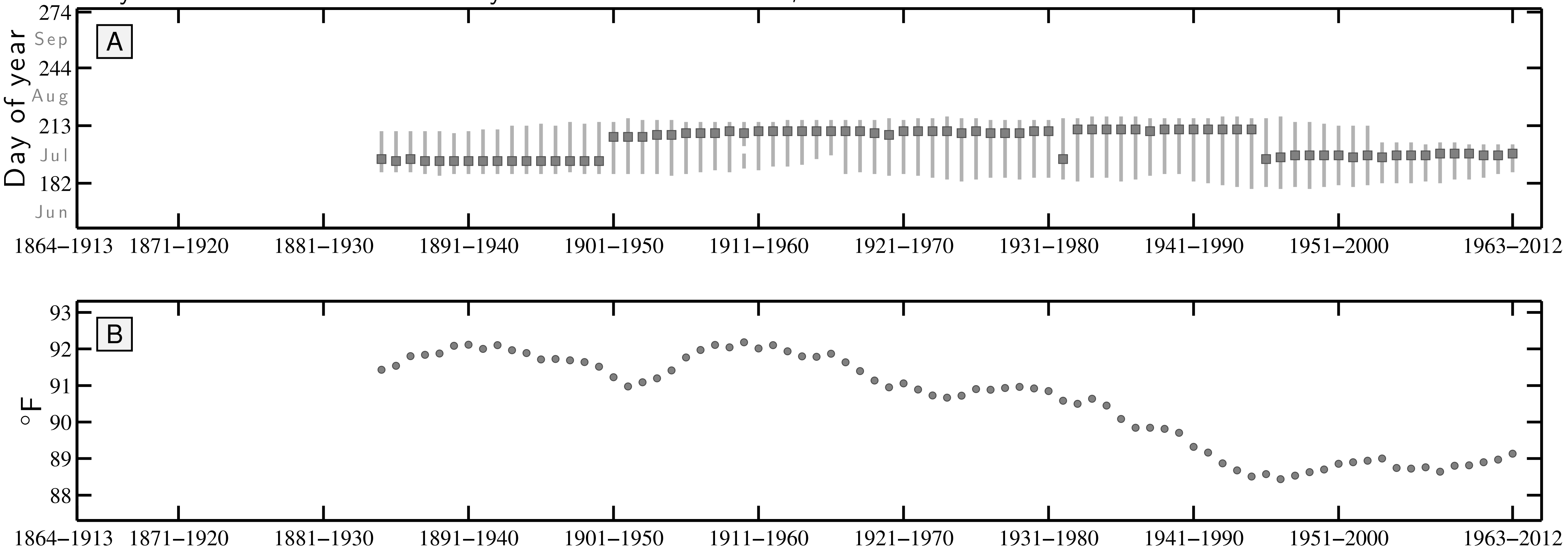

50 year Winter Teletherm dynamics for GENEVA, NE:

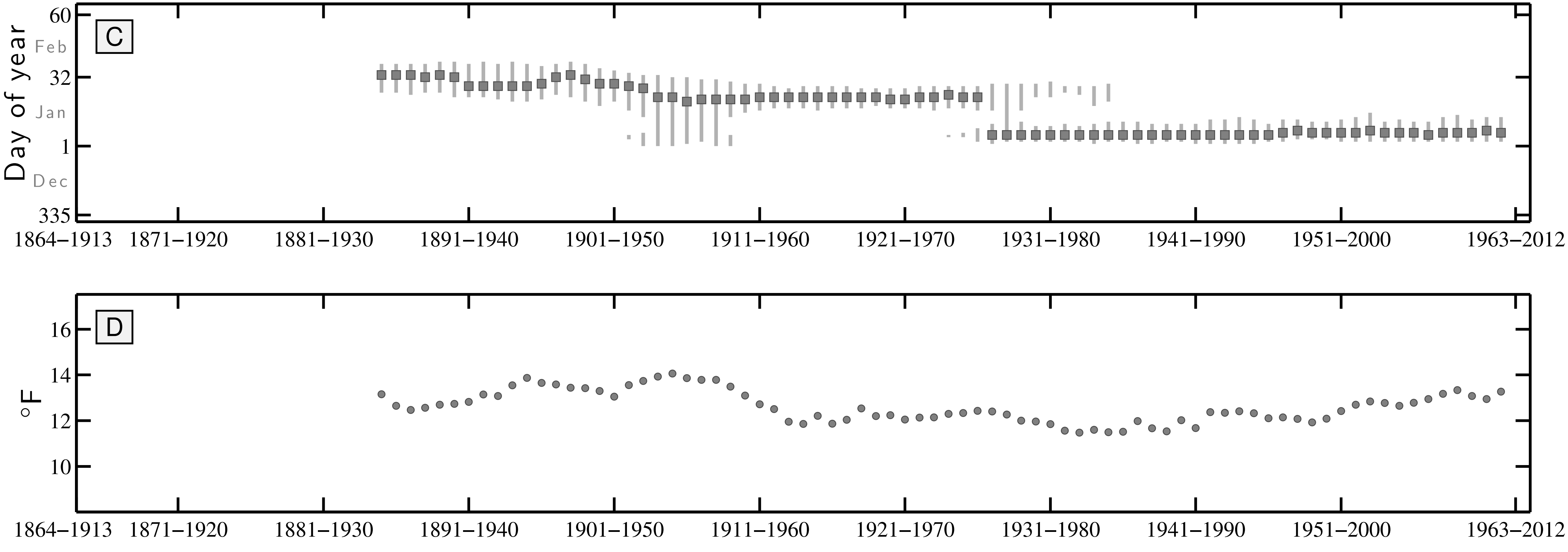

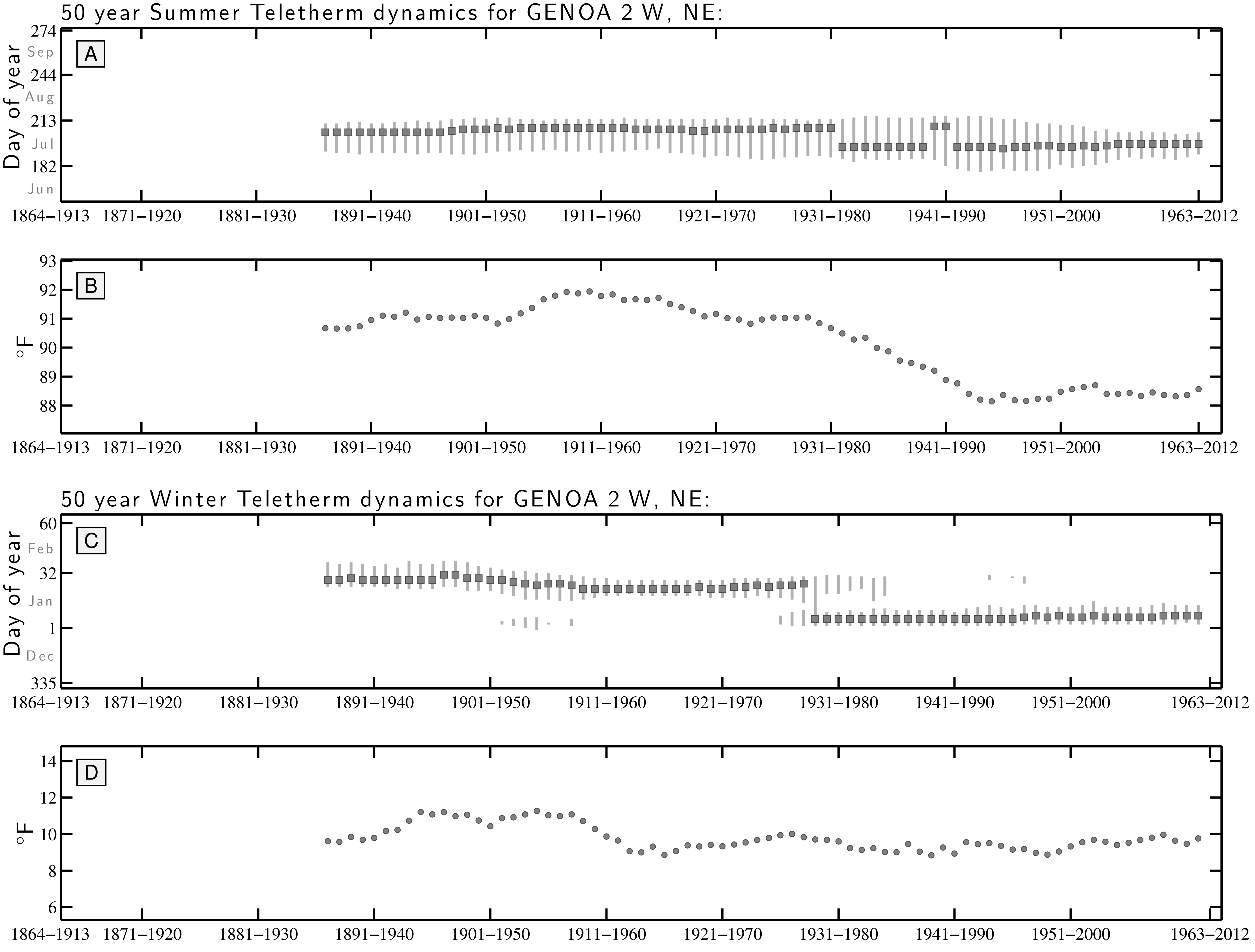
50 year Summer Teletherm dynamics for GENOA 2 W, NE:
A
Day of year
274
Sep
244
Aug
213
Jul
182
Jun
B
°F
93
92
91
90
89
88
1864–1913
1871–1920
1881–1930
1891–1940
1901–1950
1911–1960
1921–1970
1931–1980
1941–1990
1951–2000
1963–2012
50 year Winter Teletherm dynamics for GENOA 2 W, NE:
C
Day of year
60
Feb
32
Jan
1
Dec
335
D
°F
14
12
10
8
6

50 year Summer Teletherm dynamics for GOTHENBURG, NE:

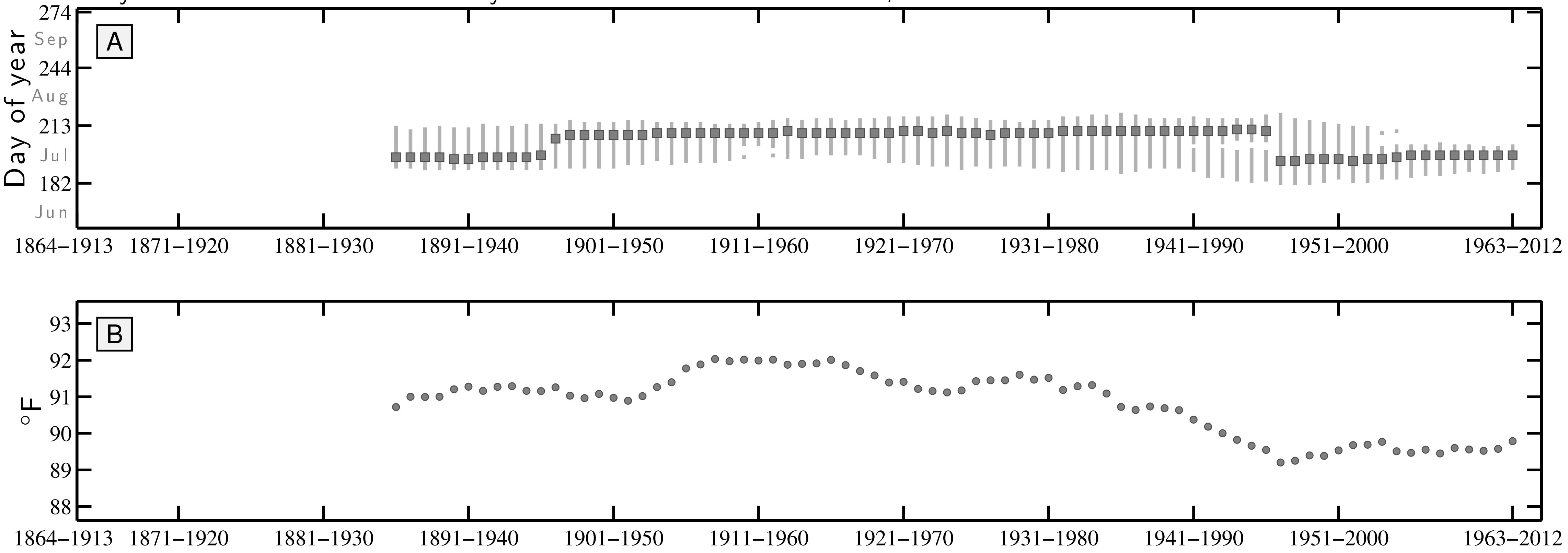

50 year Winter Teletherm dynamics for GOTHENBURG, NE:

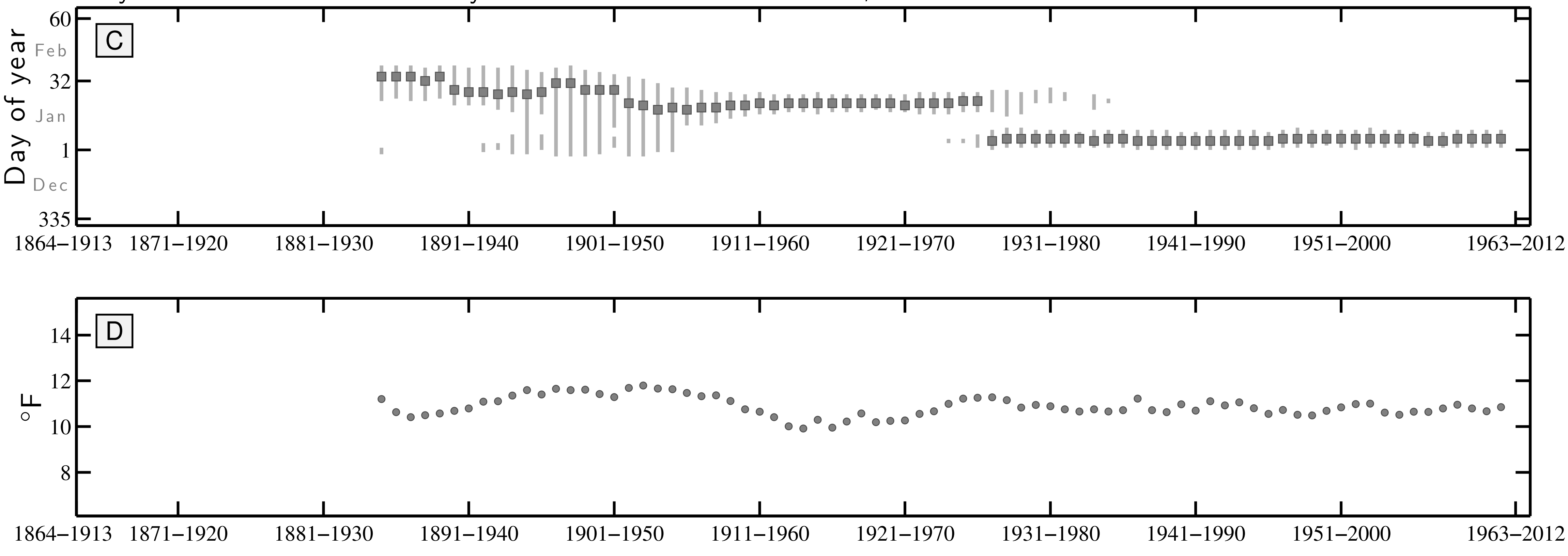

50 year Summer Teletherm dynamics for HARRISON, NE:

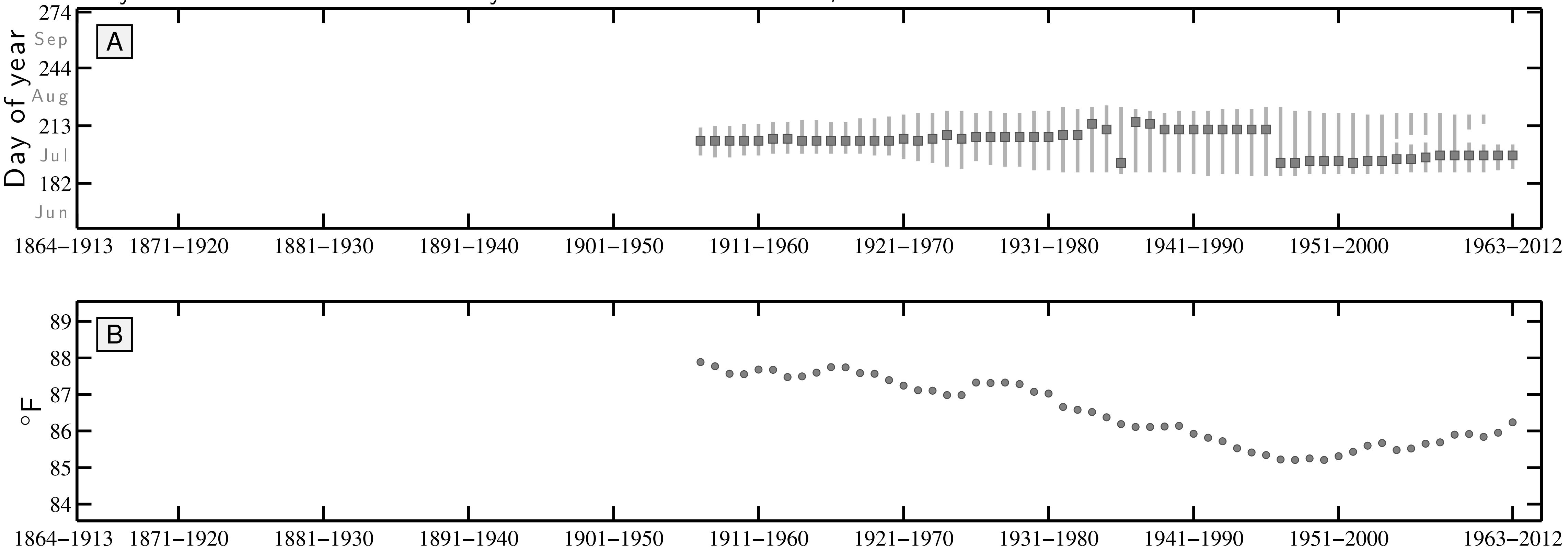

50 year Winter Teletherm dynamics for HARRISON, NE:

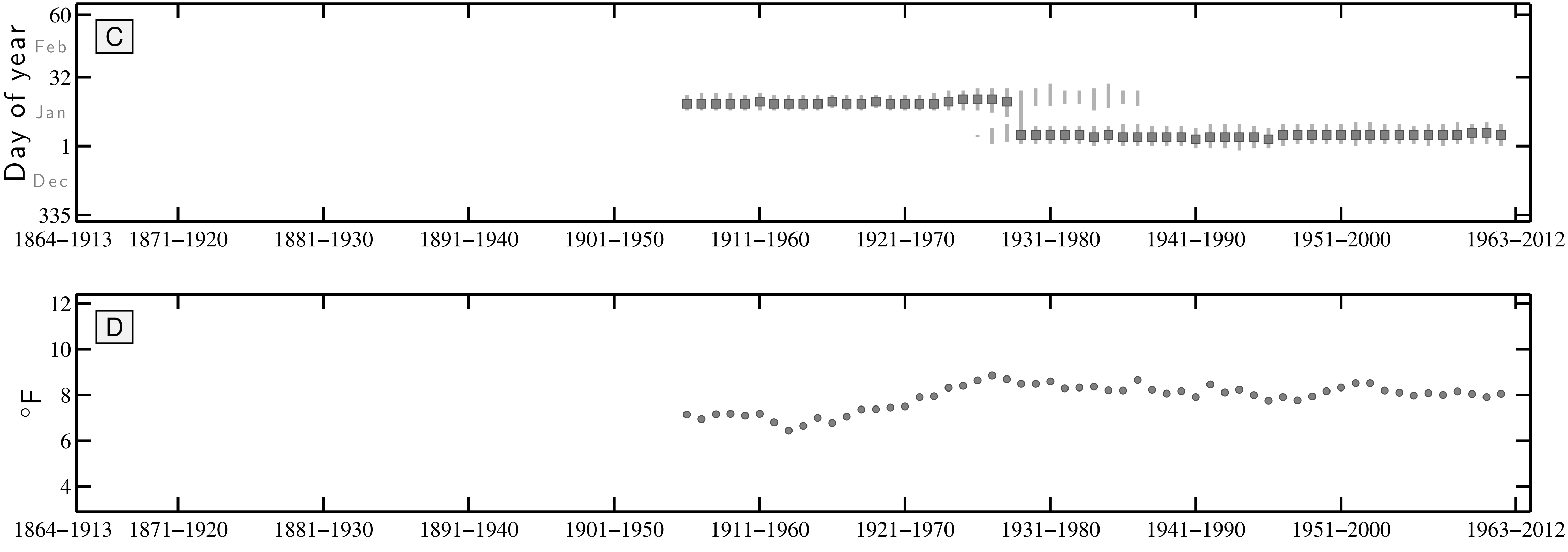

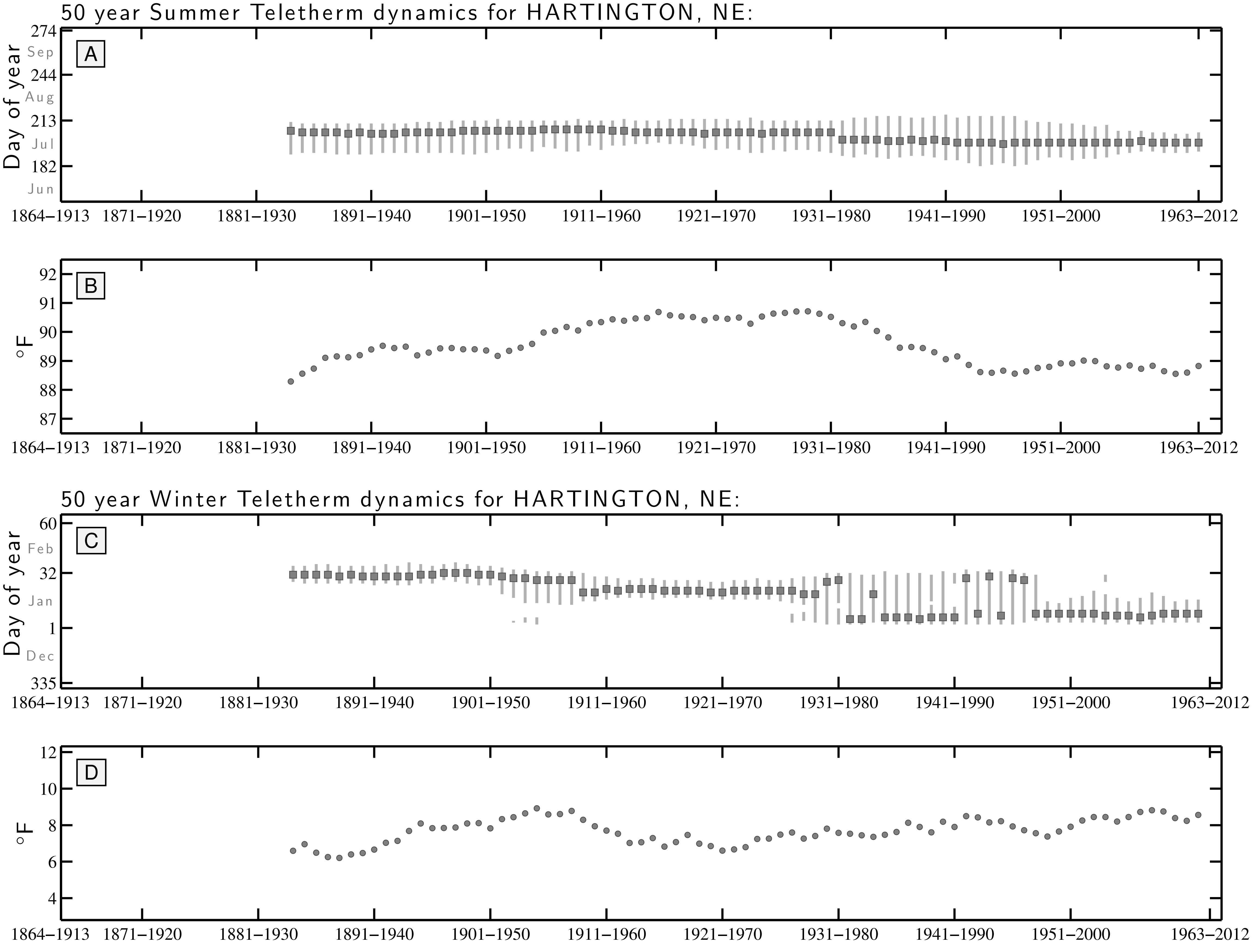

50 year Summer Teletherm dynamics for HARTINGTON, NE:
A
Day of year
274
Sep
244
Aug
213
Jul
182
Jun
1864–1913
1871–1920
1881–1930
1891–1940
1901–1950
1911–1960
1921–1970
1931–1980
1941–1990
1951–2000
1963–2012
B
°F
92
91
90
89
88
87
50 year Winter Teletherm dynamics for HARTINGTON, NE:
C
60
Feb
32
Jan
1
Dec
335
D
12
10
8
6
4

50 year Summer Teletherm dynamics for HASTINGS 4N, NE:

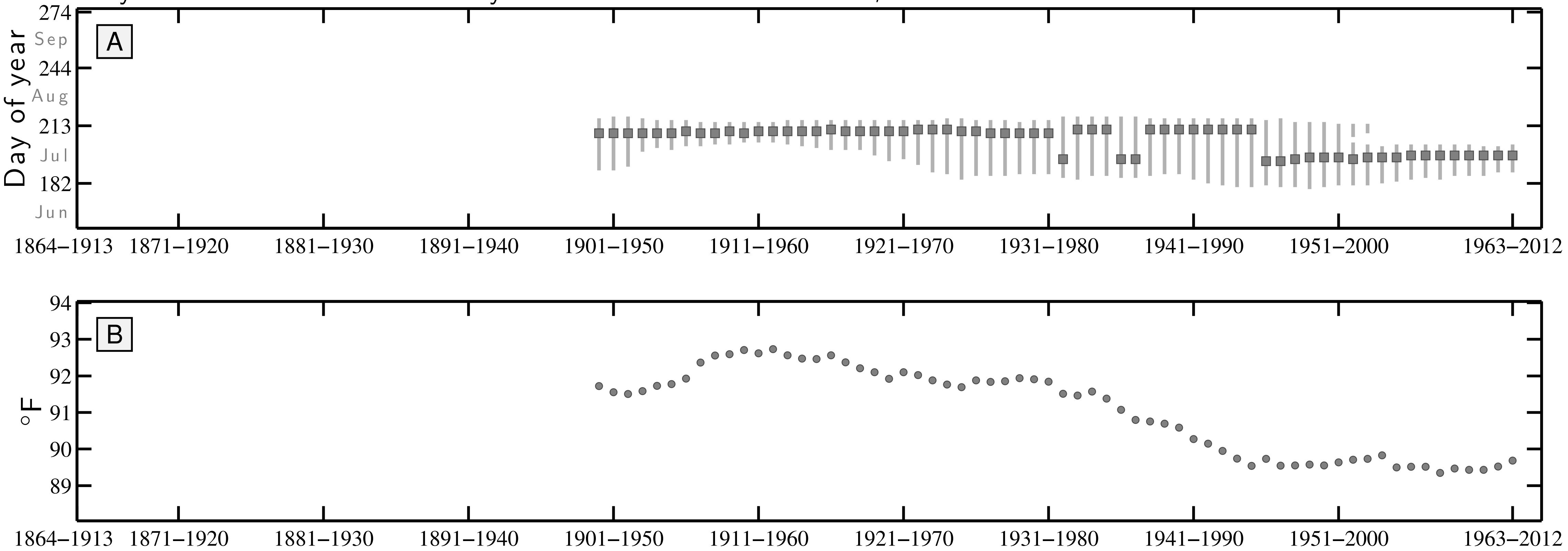

50 year Winter Teletherm dynamics for HASTINGS 4N, NE:

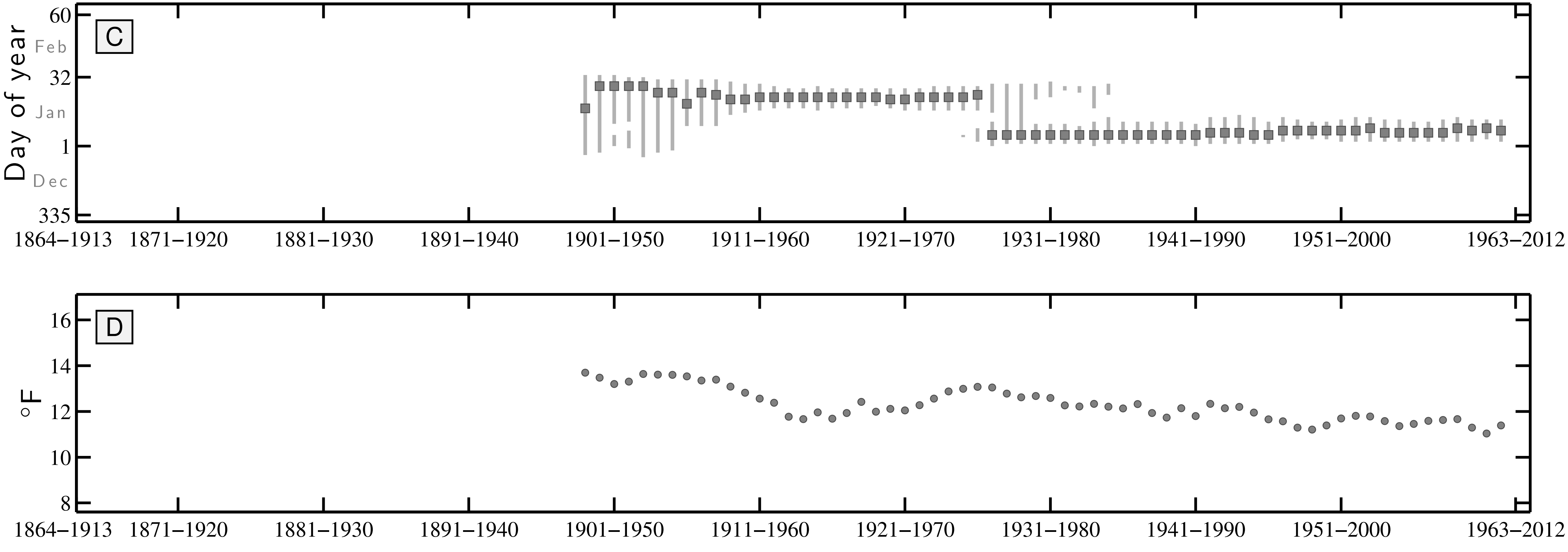

50 year Summer Teletherm dynamics for HAY SPRINGS 12 S, NE:

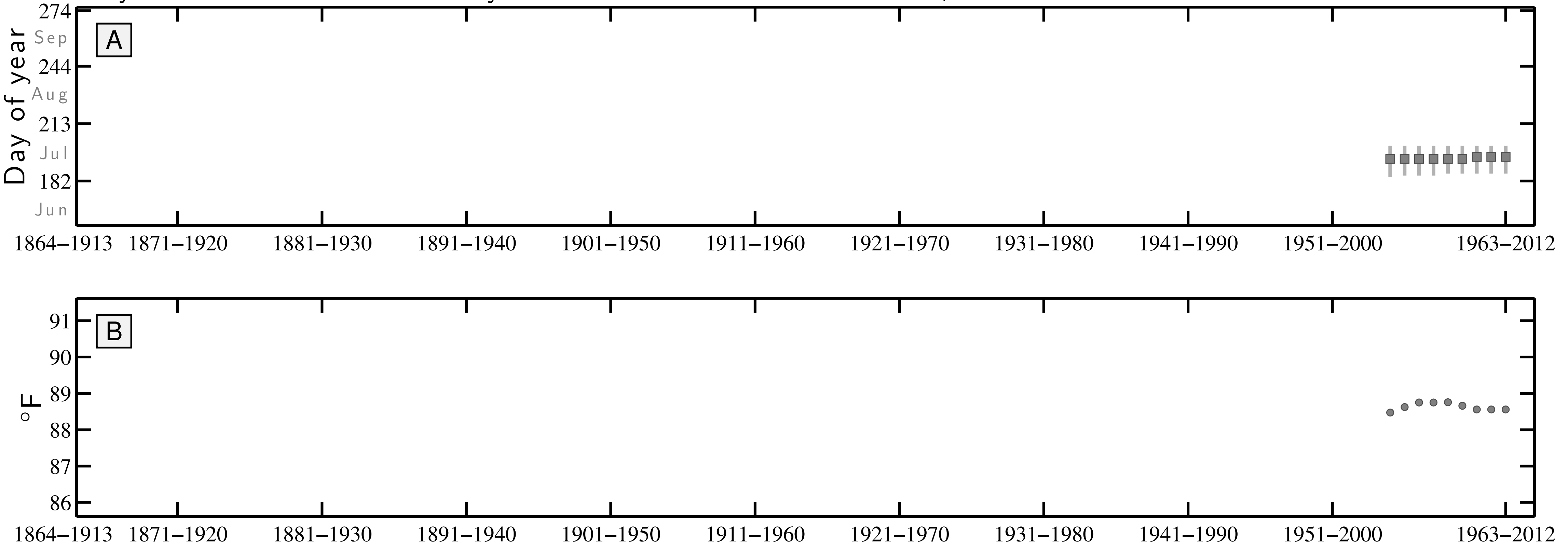

50 year Winter Teletherm dynamics for HAY SPRINGS 12 S, NE:

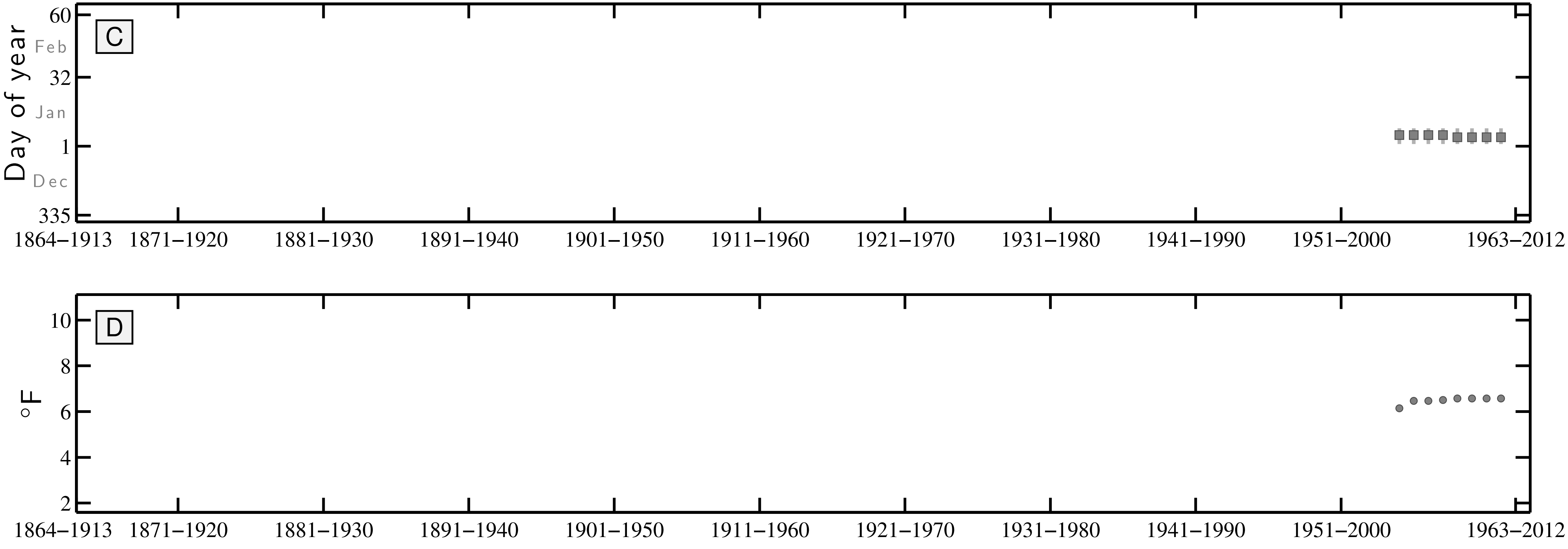

50 year Summer Teletherm dynamics for HEBRON, NE:

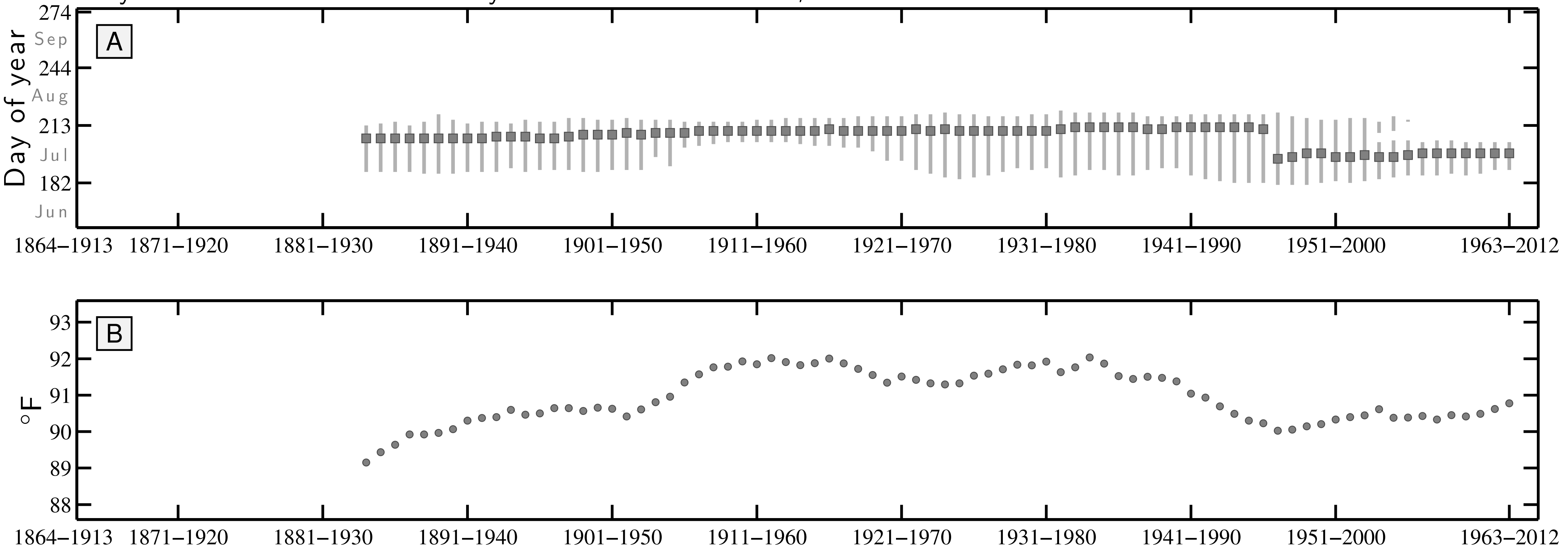

50 year Winter Teletherm dynamics for HEBRON, NE:

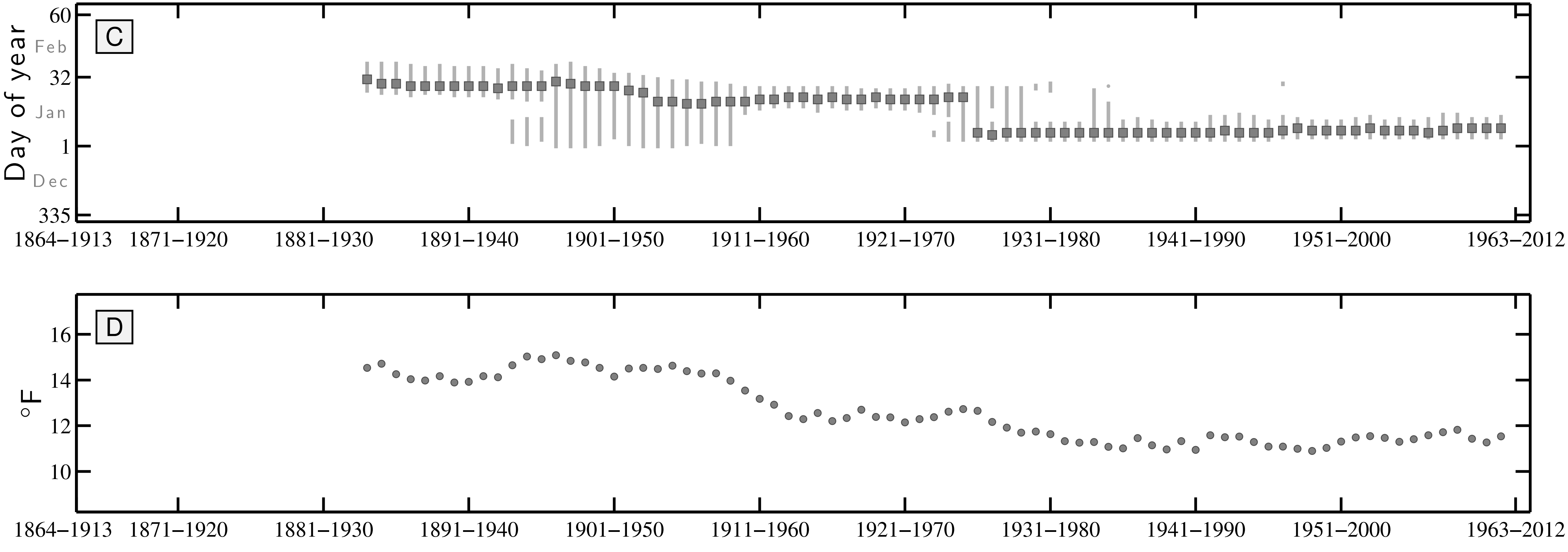

50 year Summer Teletherm dynamics for HOLDREGE, NE:

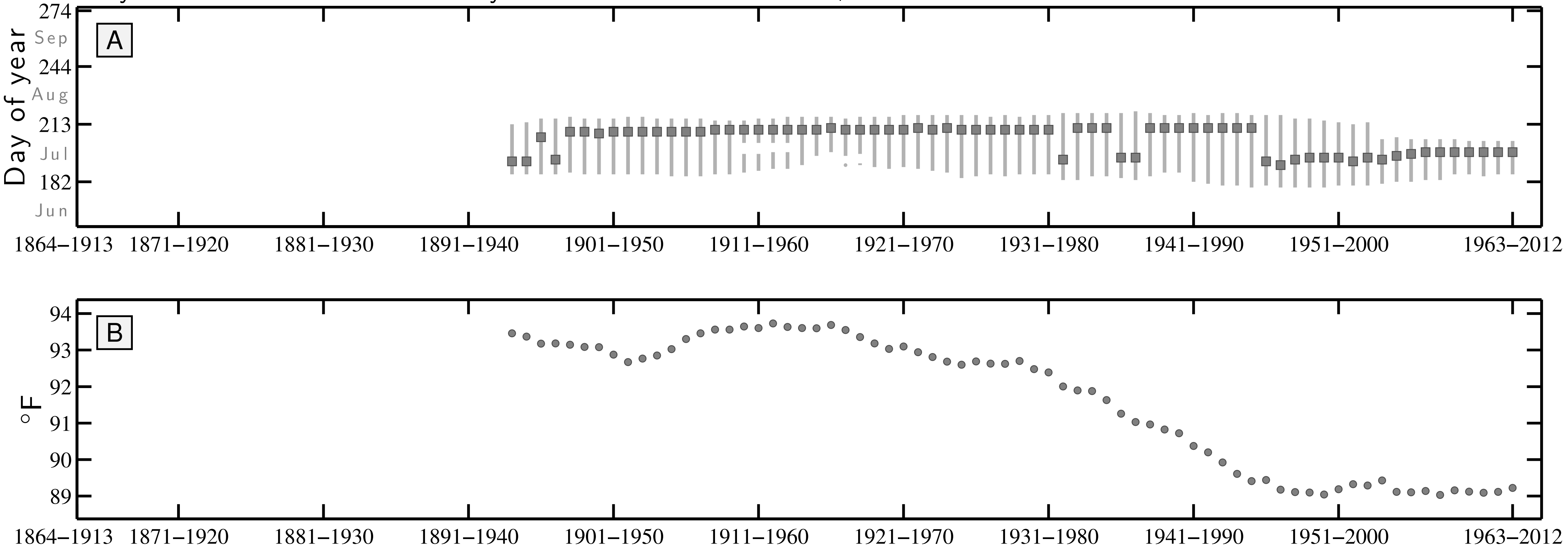

50 year Winter Teletherm dynamics for HOLDREGE, NE:

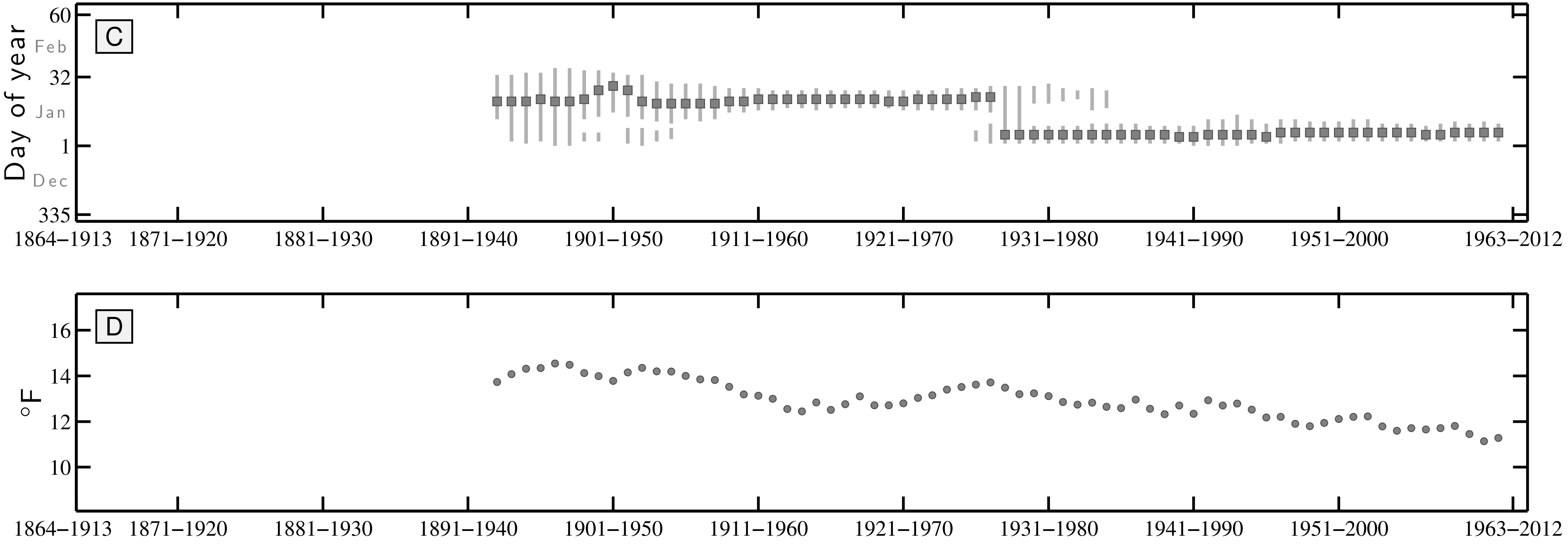

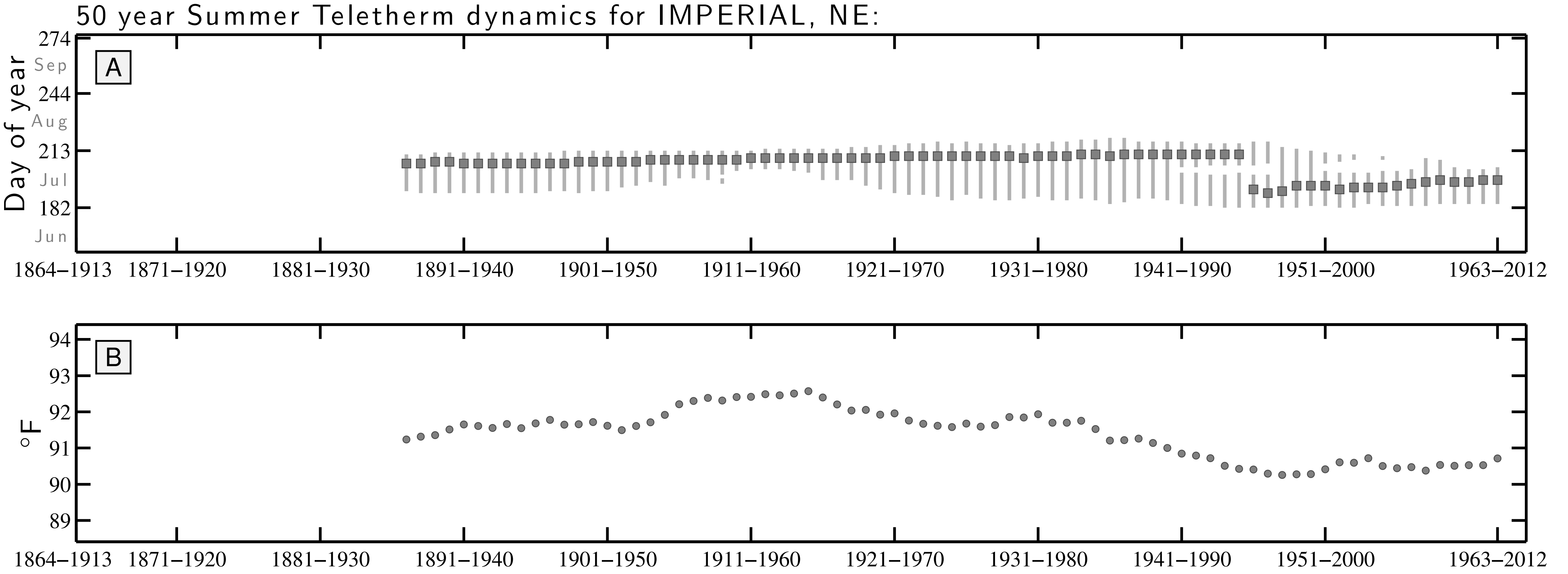
50 year Summer Teletherm dynamics for IMPERIAL, NE:
A
Day of year
274
Sep
244
Aug
213
Jul
182
Jun
B
°F
94
93
92
91
90
89
1864–1913
1871–1920
1881–1930
1891–1940
1901–1950
1911–1960
1921–1970
1931–1980
1941–1990
1951–2000
1963–2012

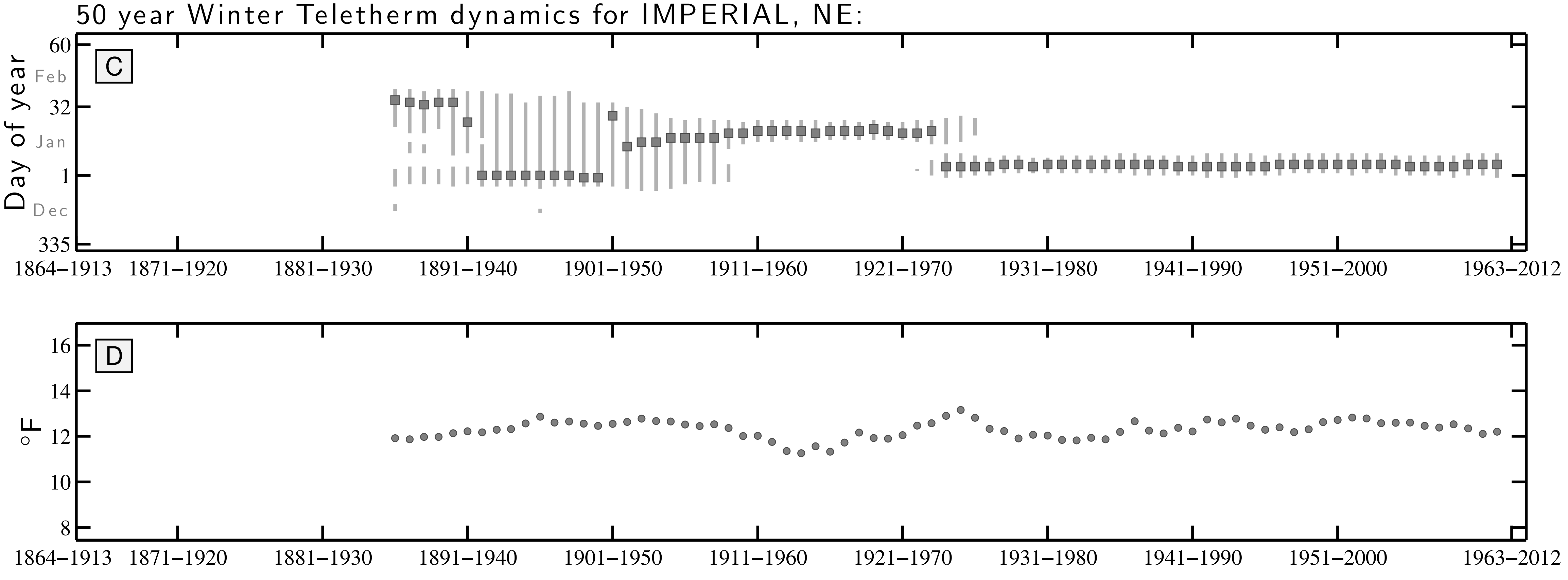
50 year Winter Teletherm dynamics for IMPERIAL, NE:
C
Day of year
60
Feb
32
Jan
1
Dec
335
D
°F
16
14
12
10
8
1864–1913
1871–1920
1881–1930
1891–1940
1901–1950
1911–1960
1921–1970
1931–1980
1941–1990
1951–2000
1963–2012

50 year Summer Teletherm dynamics for KIMBALL 2NE, NE:

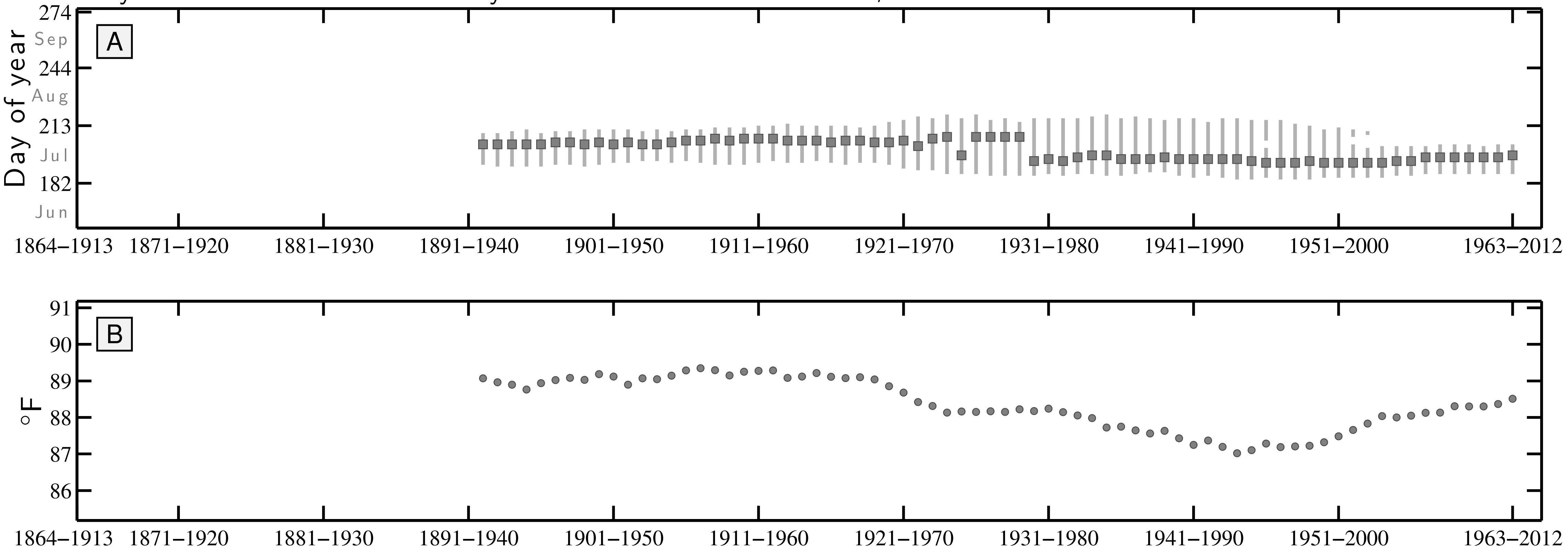

50 year Winter Teletherm dynamics for KIMBALL 2NE, NE:

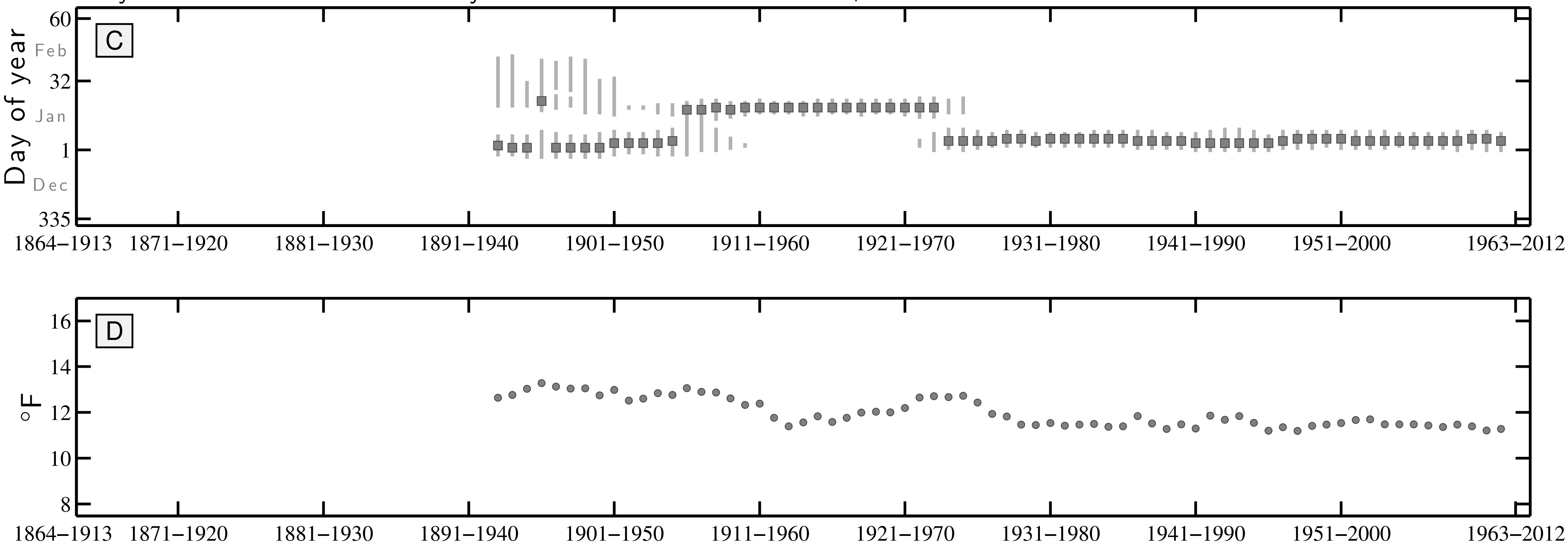

50 year Summer Teletherm dynamics for LODGEPOLE, NE:

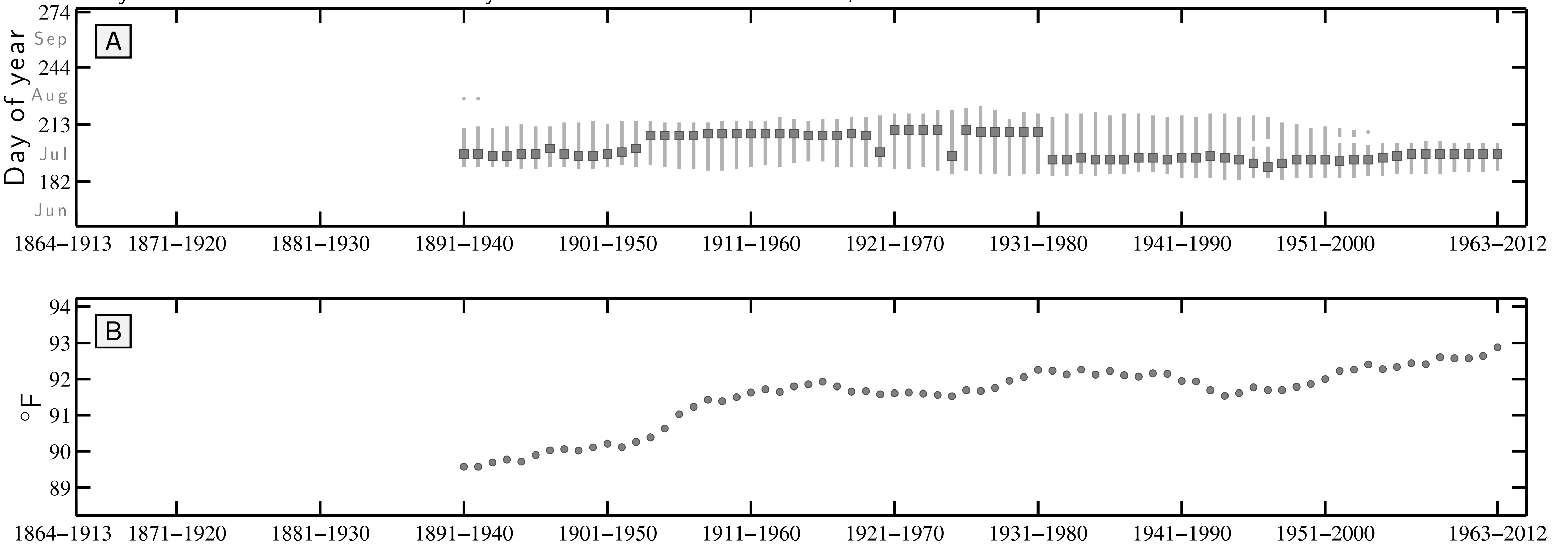

50 year Winter Teletherm dynamics for LODGEPOLE, NE:

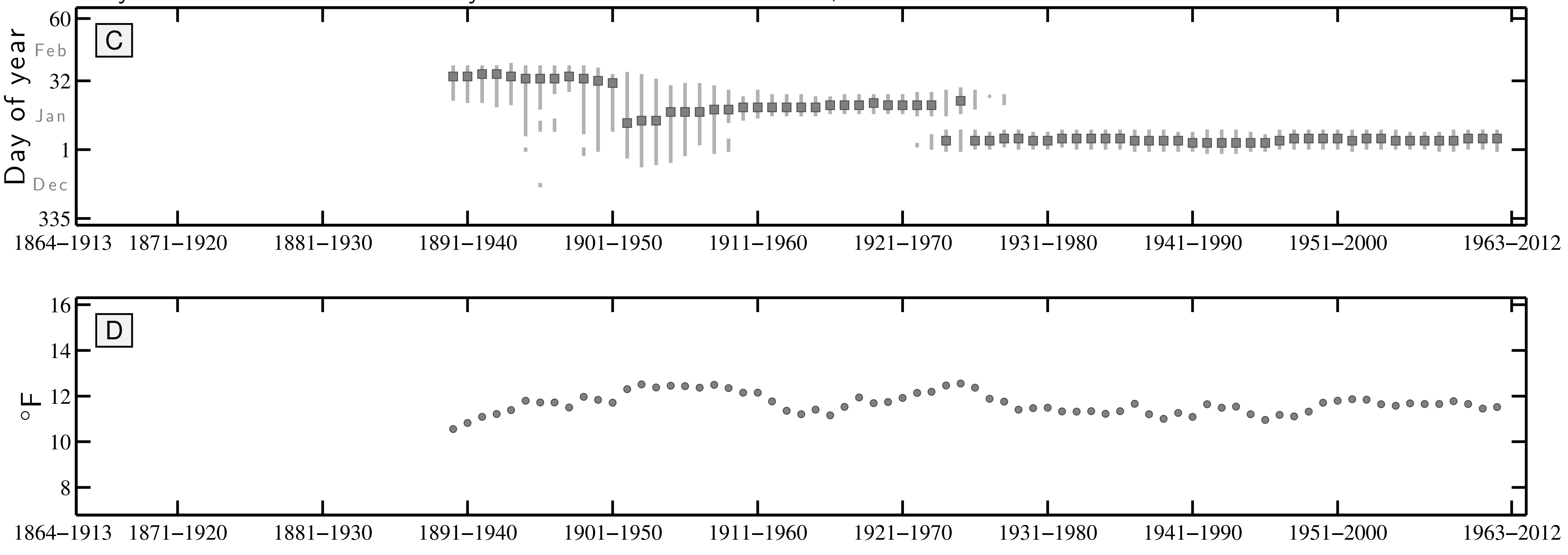

50 year Summer Teletherm dynamics for LOUP CITY, NE:

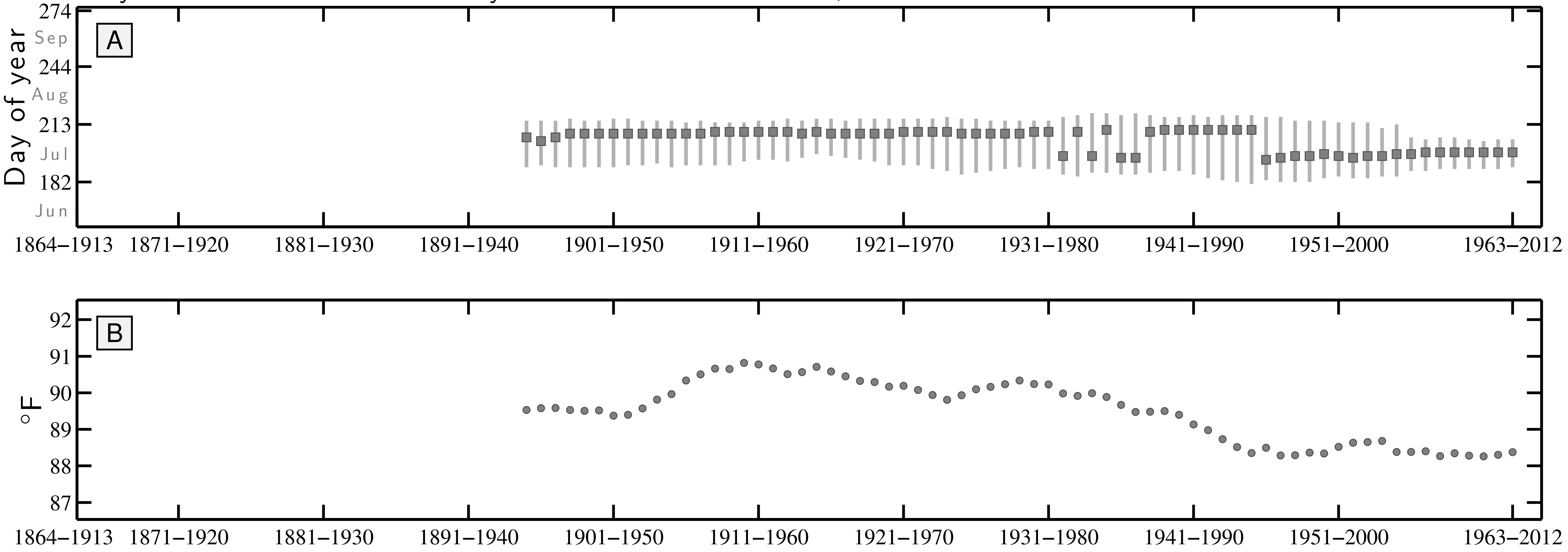

50 year Winter Teletherm dynamics for LOUP CITY, NE:

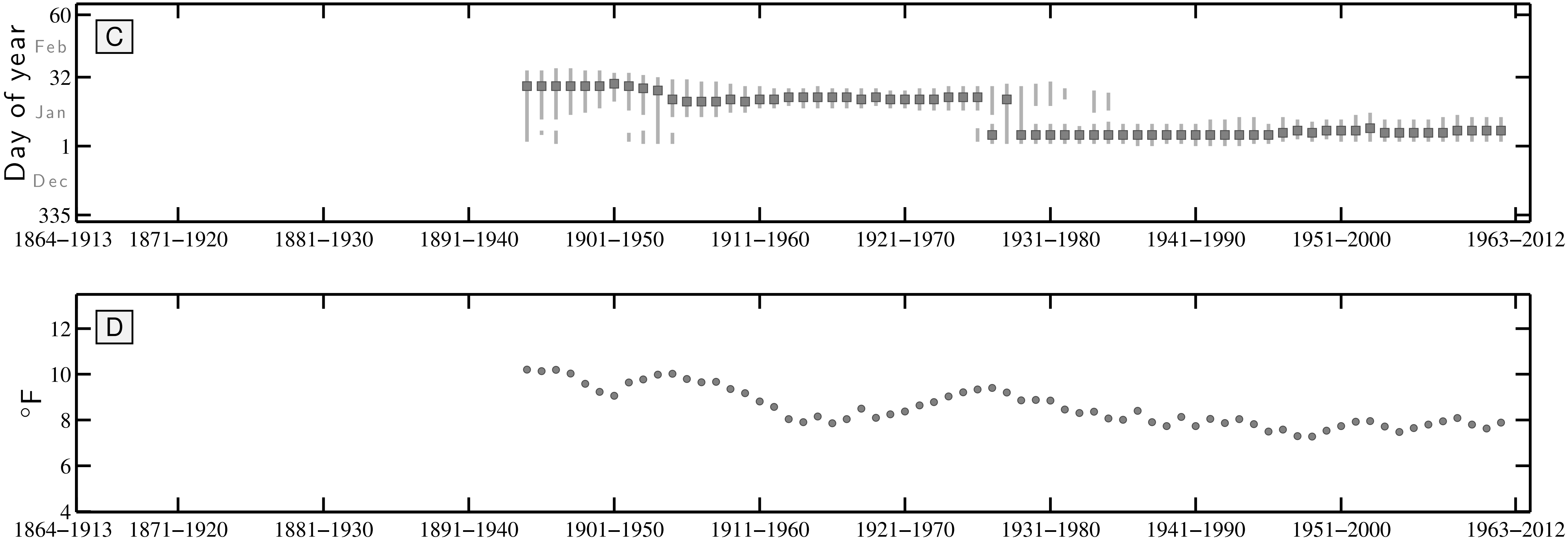

50 year Summer Teletherm dynamics for MADISON, NE:

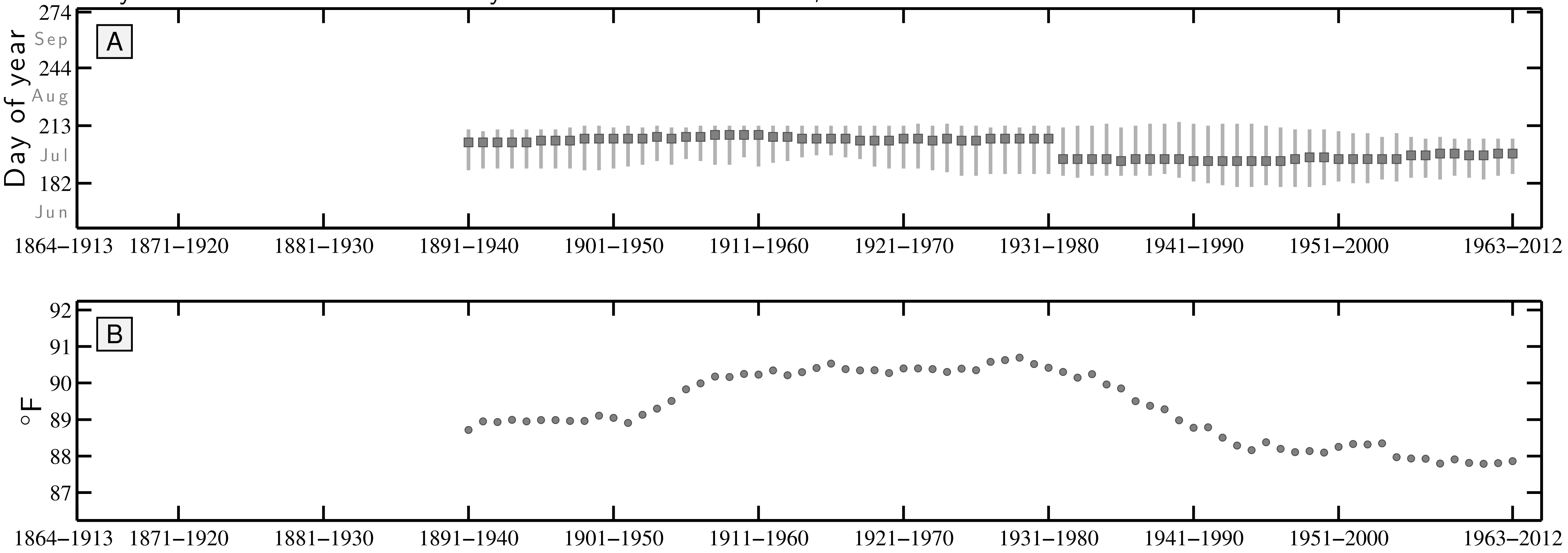

50 year Winter Teletherm dynamics for MADISON, NE:

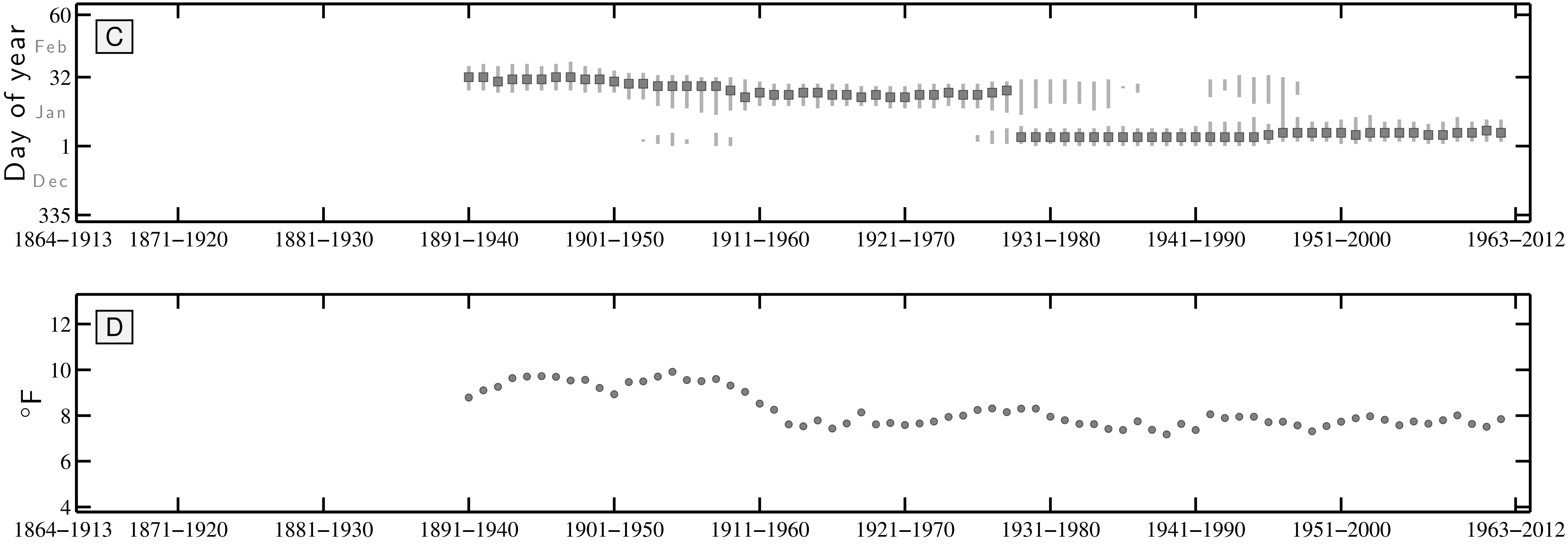

50 year Summer Teletherm dynamics for MC COOK, NE:

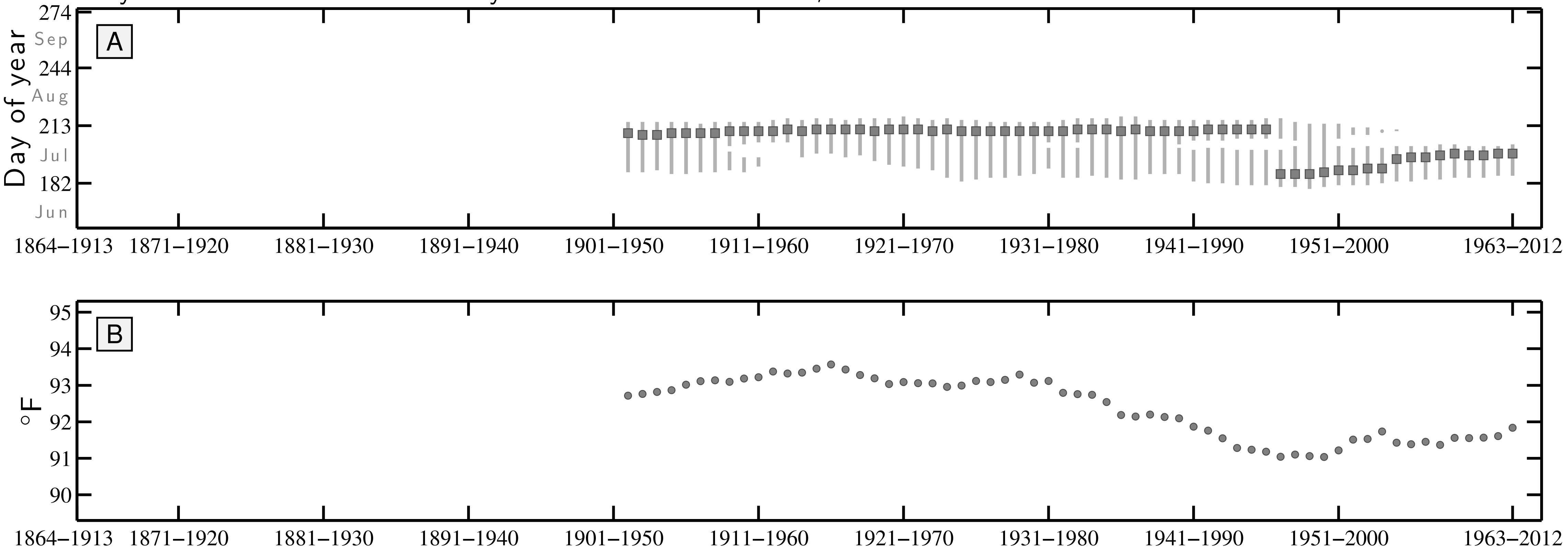

50 year Winter Teletherm dynamics for MC COOK, NE:

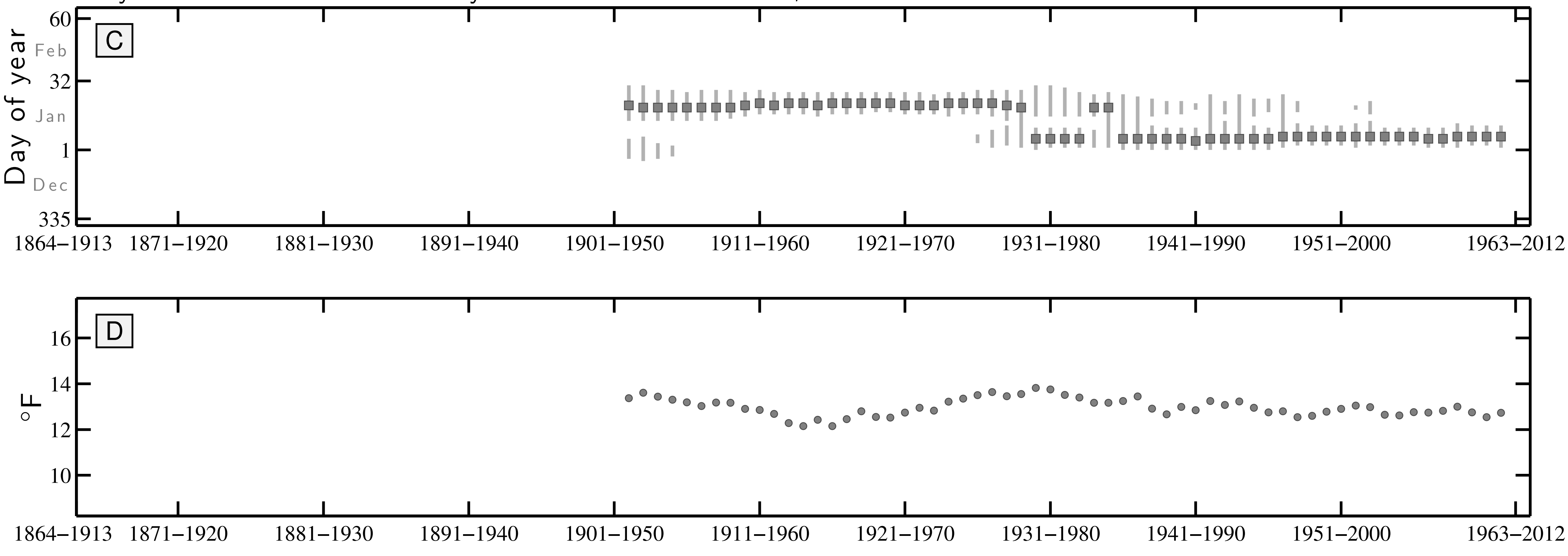

50 year Summer Teletherm dynamics for MERRIMAN, NE:

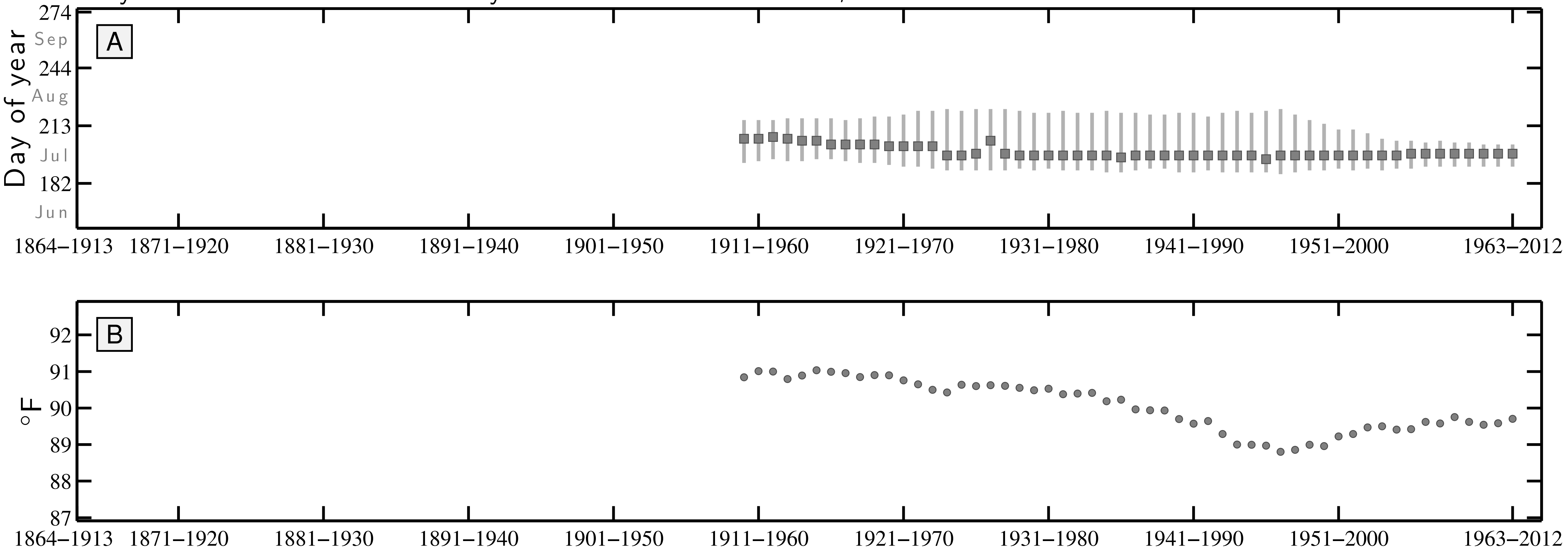

50 year Winter Teletherm dynamics for MERRIMAN, NE:

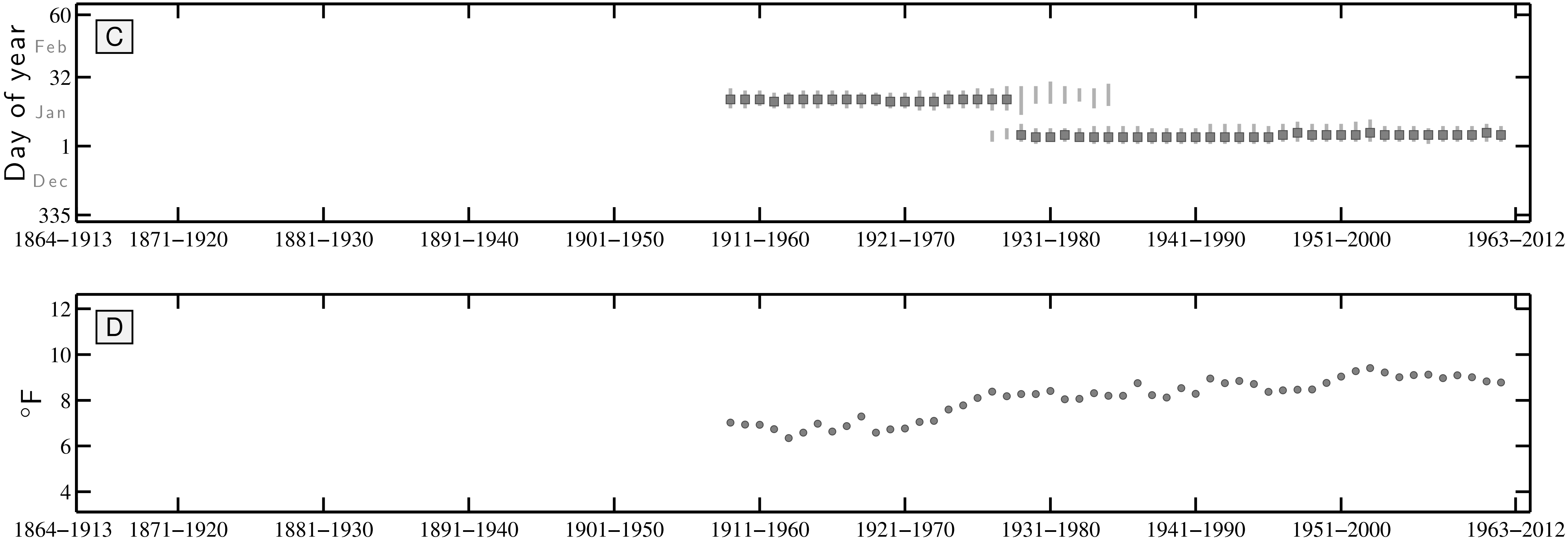

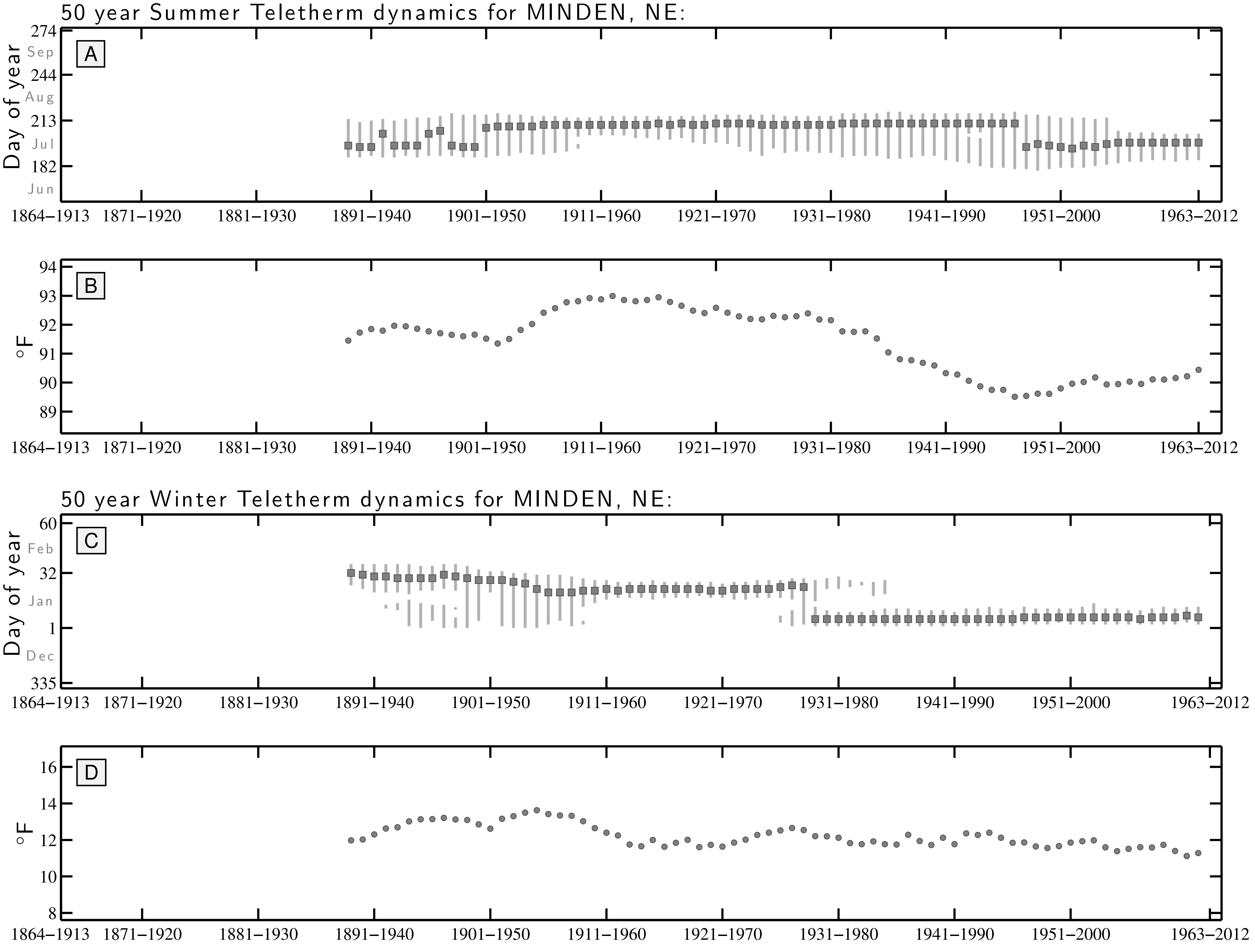
50 year Summer Teletherm dynamics for MINDEN, NE:
A
B
50 year Winter Teletherm dynamics for MINDEN, NE:
C
D
Day of year
°F
274
Sep
244
Aug
213
Jul
182
Jun
94
93
92
91
90
89
60
Feb
32
Jan
1
Dec
335
16
14
12
10
8
1864–1913
1871–1920
1881–1930
1891–1940
1901–1950
1911–1960
1921–1970
1931–1980
1941–1990
1951–2000
1963–2012

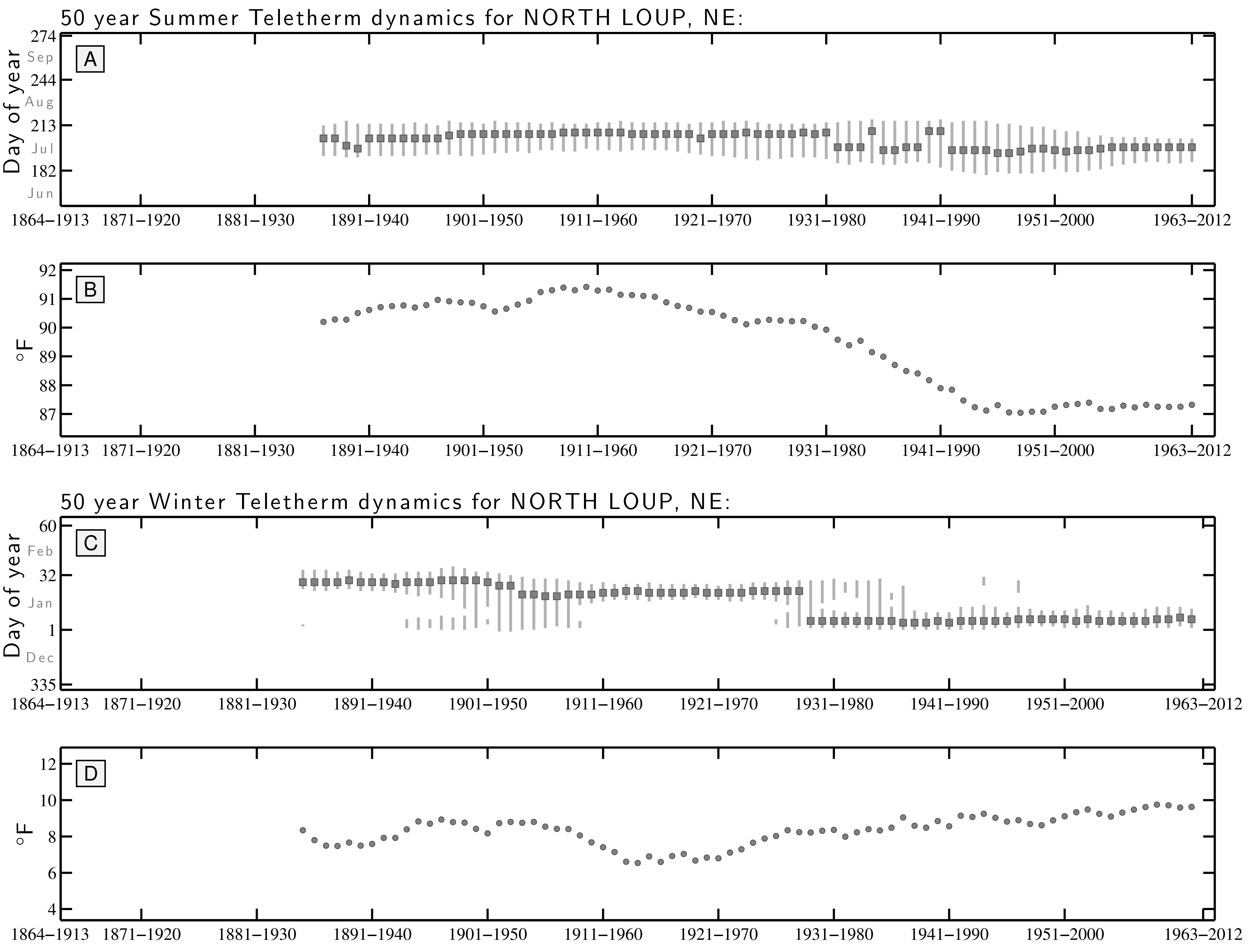

50 year Summer Teletherm dynamics for NORTH LOUP, NE:
A
Day of year
274
Sep
244
Aug
213
Jul
182
Jun
1864–1913
1871–1920
1881–1930
1891–1940
1901–1950
1911–1960
1921–1970
1931–1980
1941–1990
1951–2000
1963–2012
B
°F
92
91
90
89
88
87
50 year Winter Teletherm dynamics for NORTH LOUP, NE:
C
Day of year
60
Feb
32
Jan
1
Dec
335
D
°F
12
10
8
6
4

50 year Summer Teletherm dynamics for OAKDALE, NE:

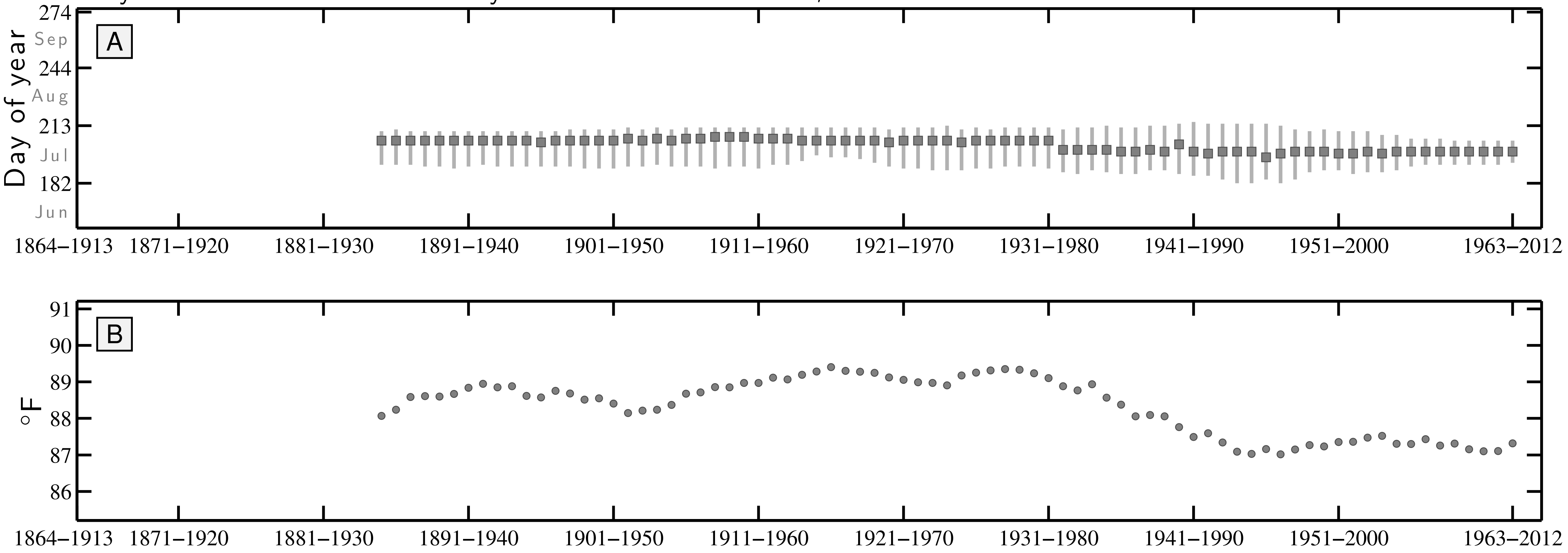

50 year Winter Teletherm dynamics for OAKDALE, NE:

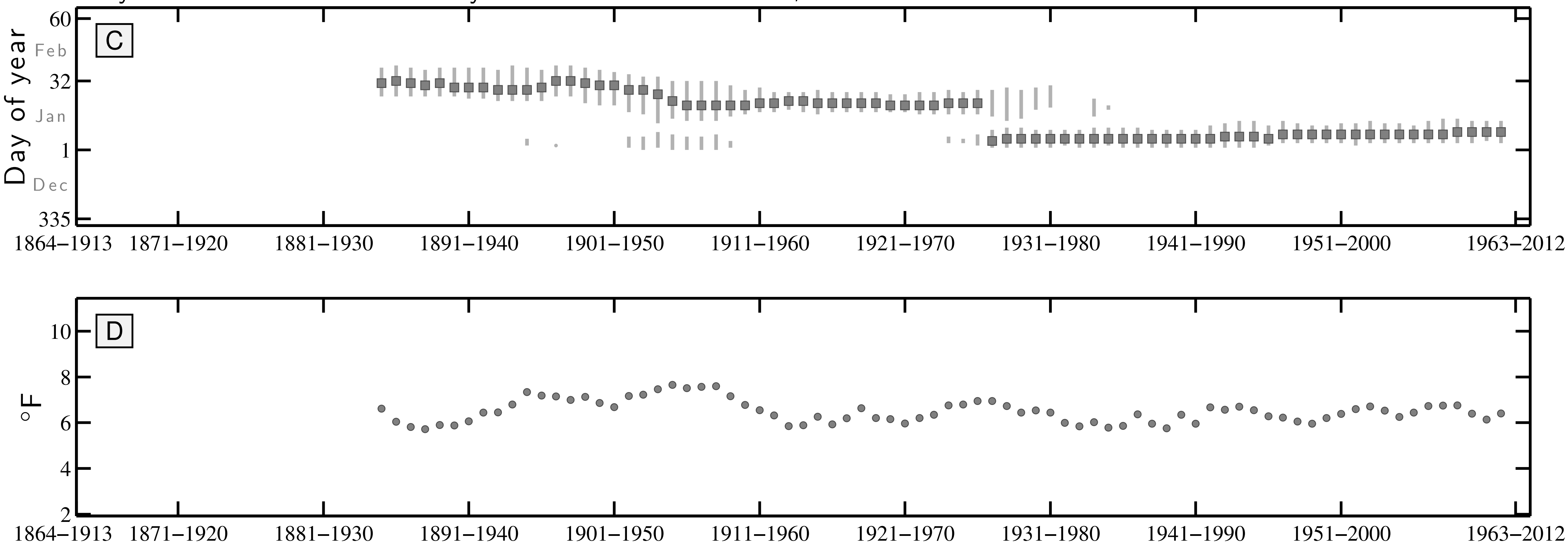

50 year Summer Teletherm dynamics for PAWNEE CITY, NE:

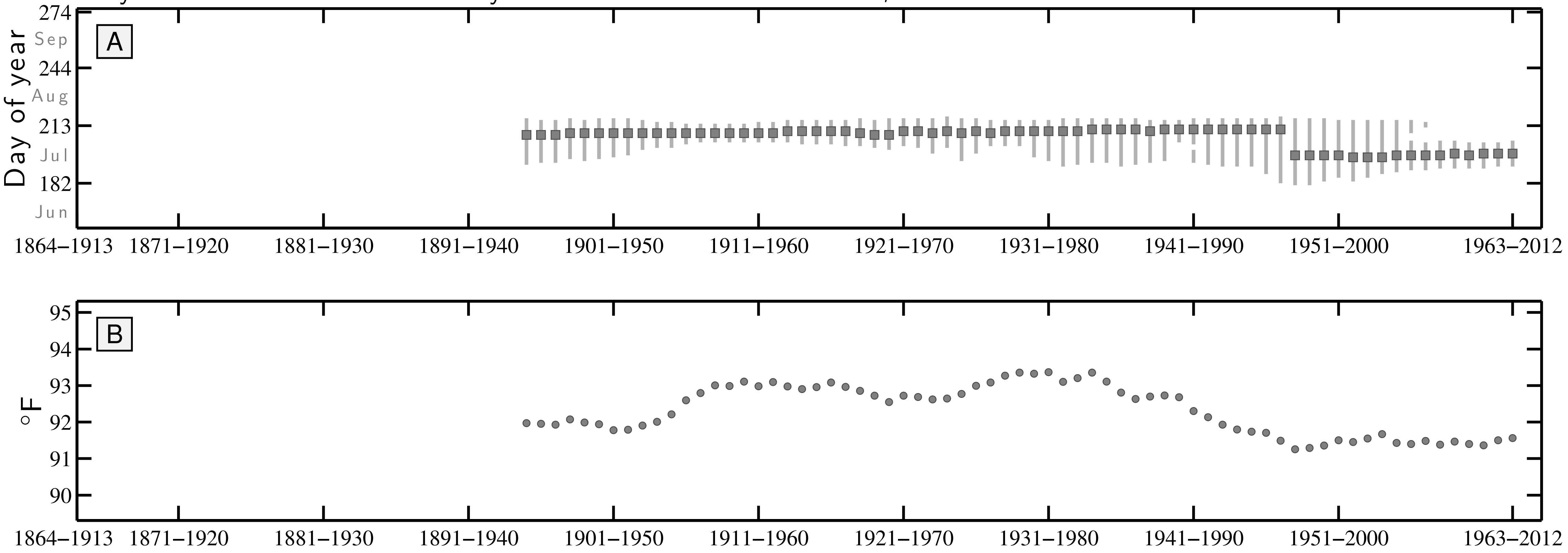

50 year Winter Teletherm dynamics for PAWNEE CITY, NE:

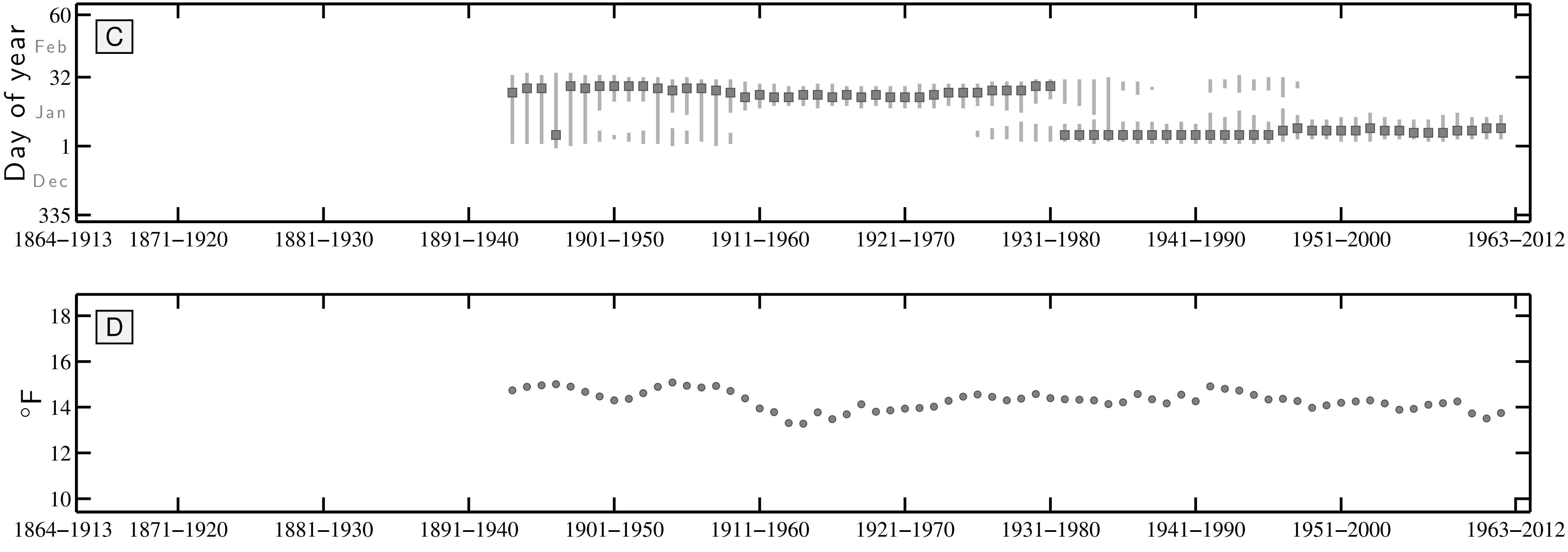

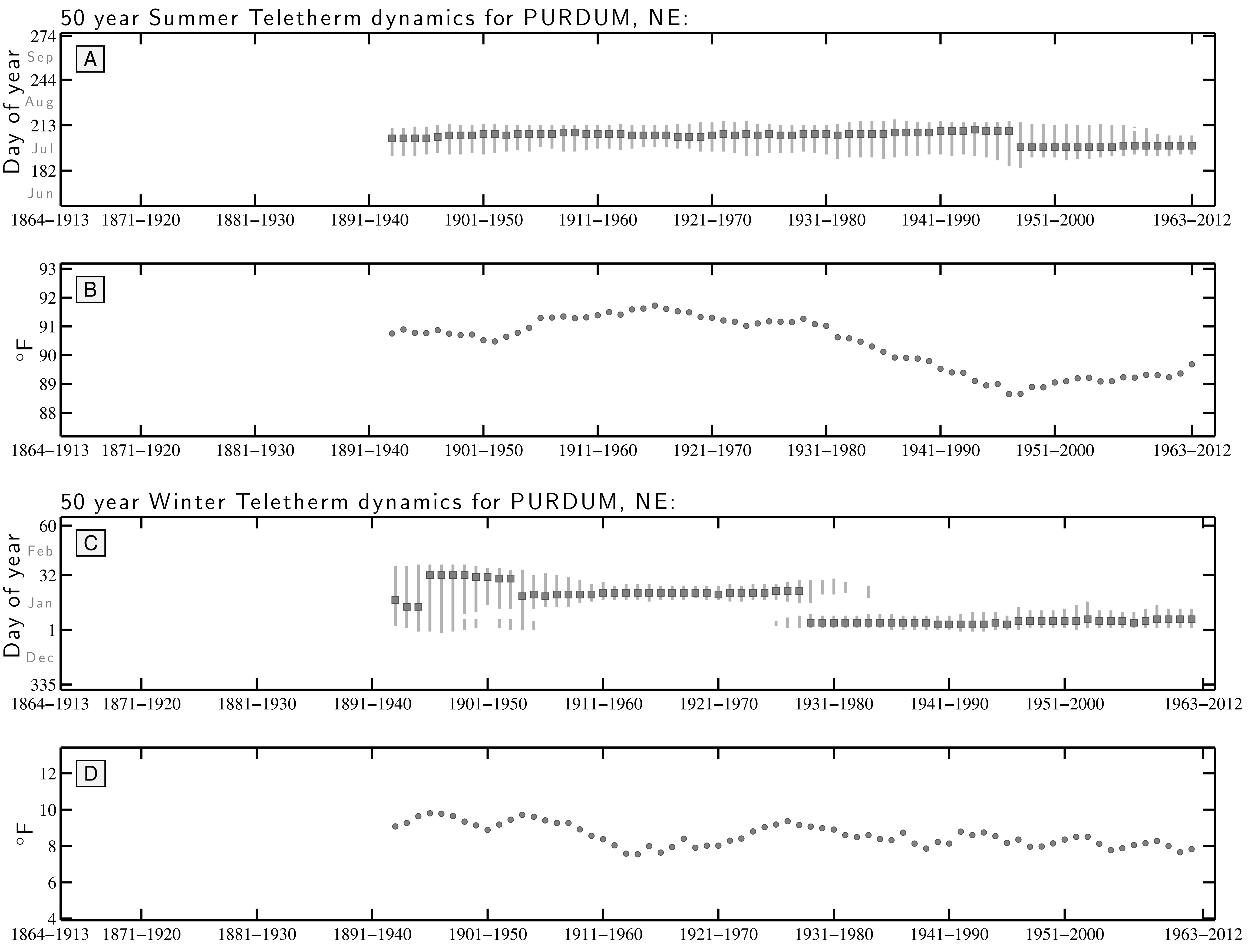
50 year Summer Teletherm dynamics for PURDUM, NE:
A
Day of year
274
Sep
244
Aug
213
Jul
182
Jun
1864–1913
1871–1920
1881–1930
1891–1940
1901–1950
1911–1960
1921–1970
1931–1980
1941–1990
1951–2000
1963–2012
B
°F
93
92
91
90
89
88
50 year Winter Teletherm dynamics for PURDUM, NE:
C
Day of year
60
Feb
32
Jan
1
Dec
335
D
°F
12
10
8
6
4

50 year Summer Teletherm dynamics for RED CLOUD, NE:

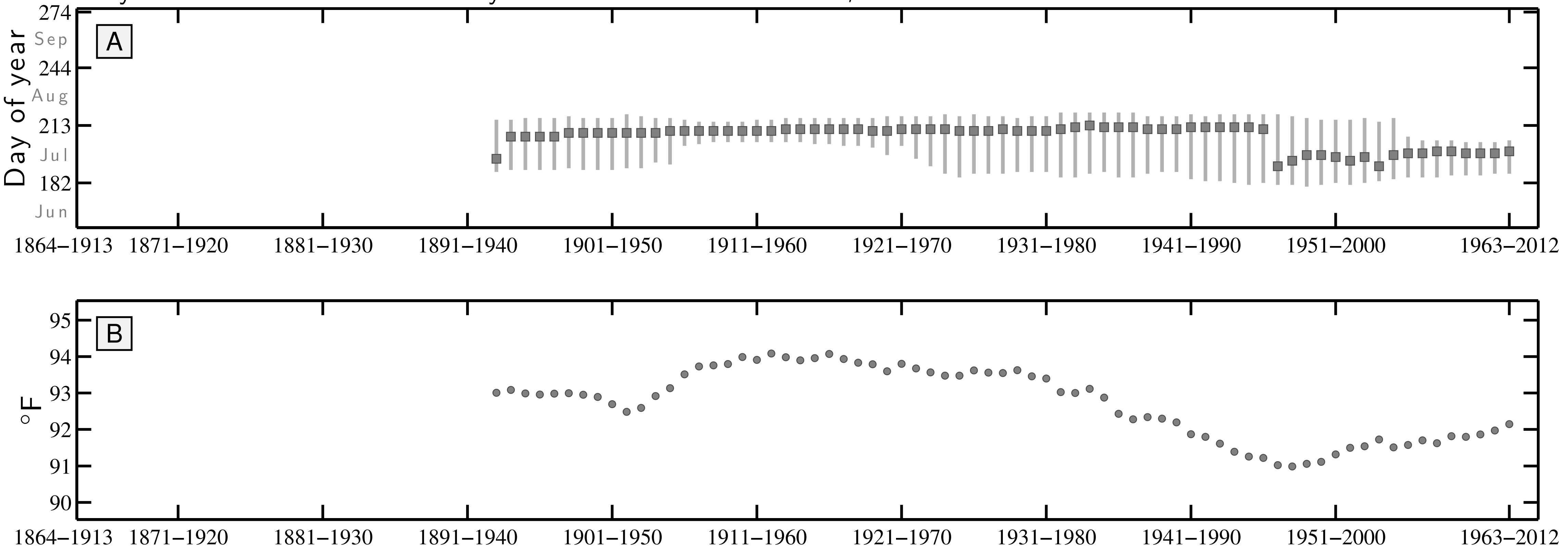

50 year Winter Teletherm dynamics for RED CLOUD, NE:

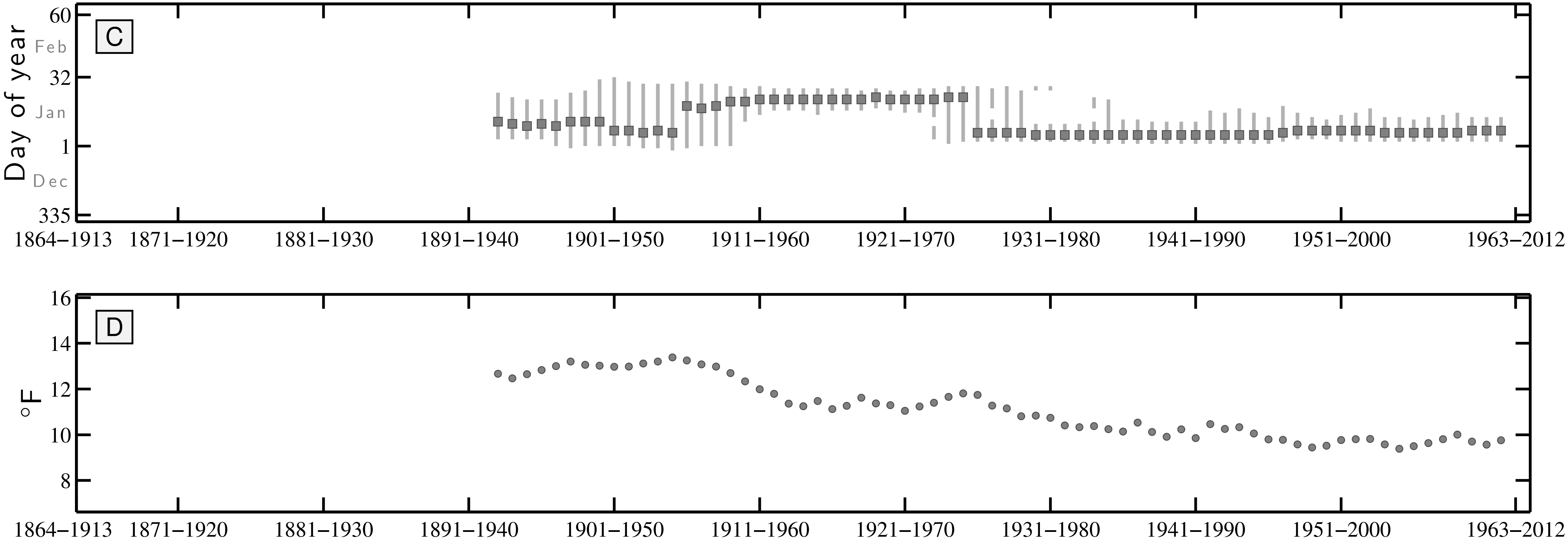

50 year Summer Teletherm dynamics for SAINT PAUL 4N, NE:

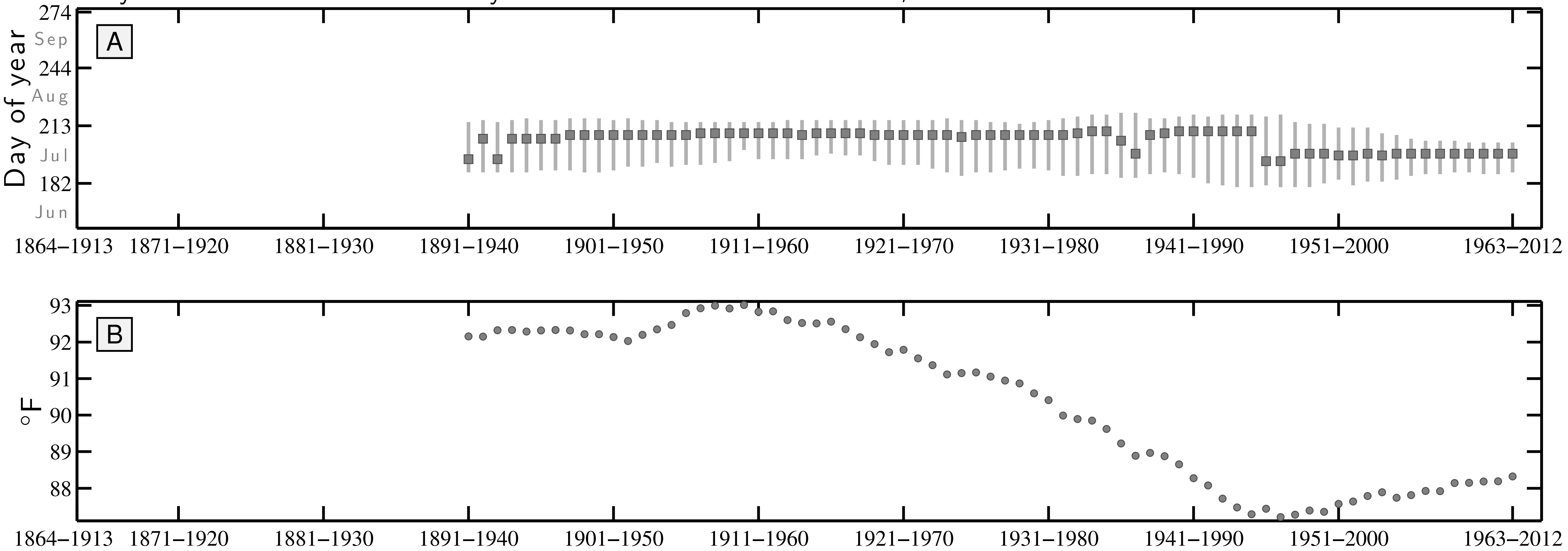

50 year Winter Teletherm dynamics for SAINT PAUL 4N, NE:

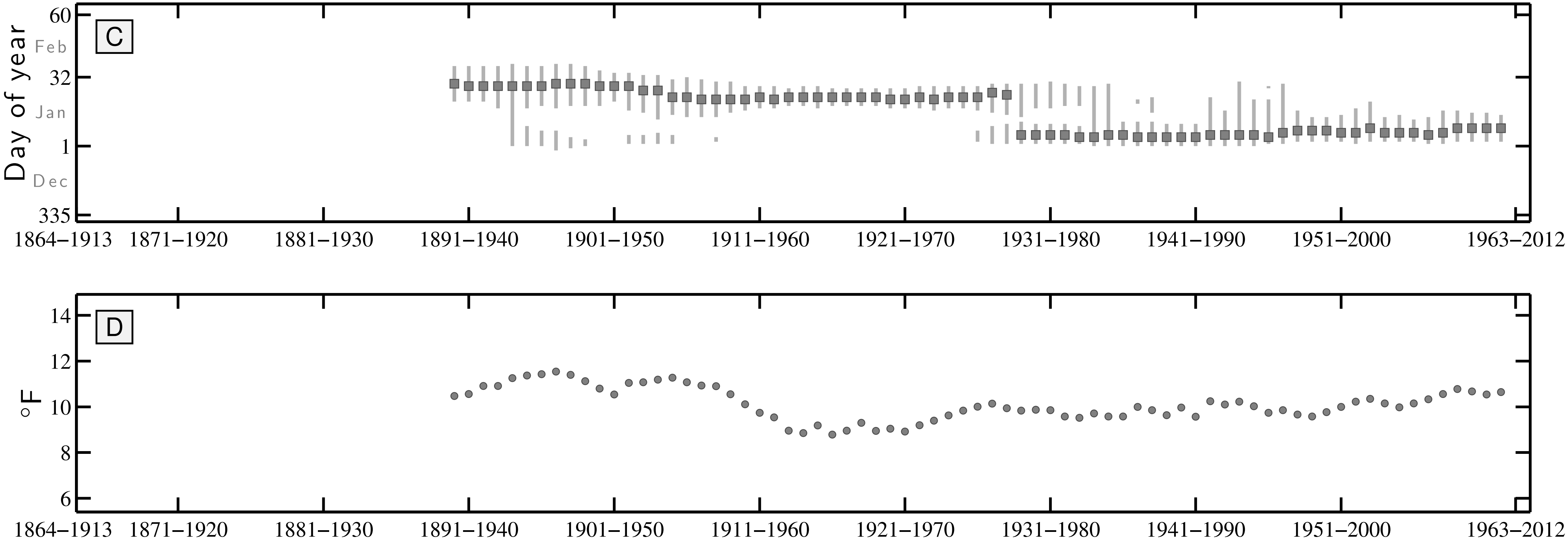

50 year Summer Teletherm dynamics for SEWARD, NE:

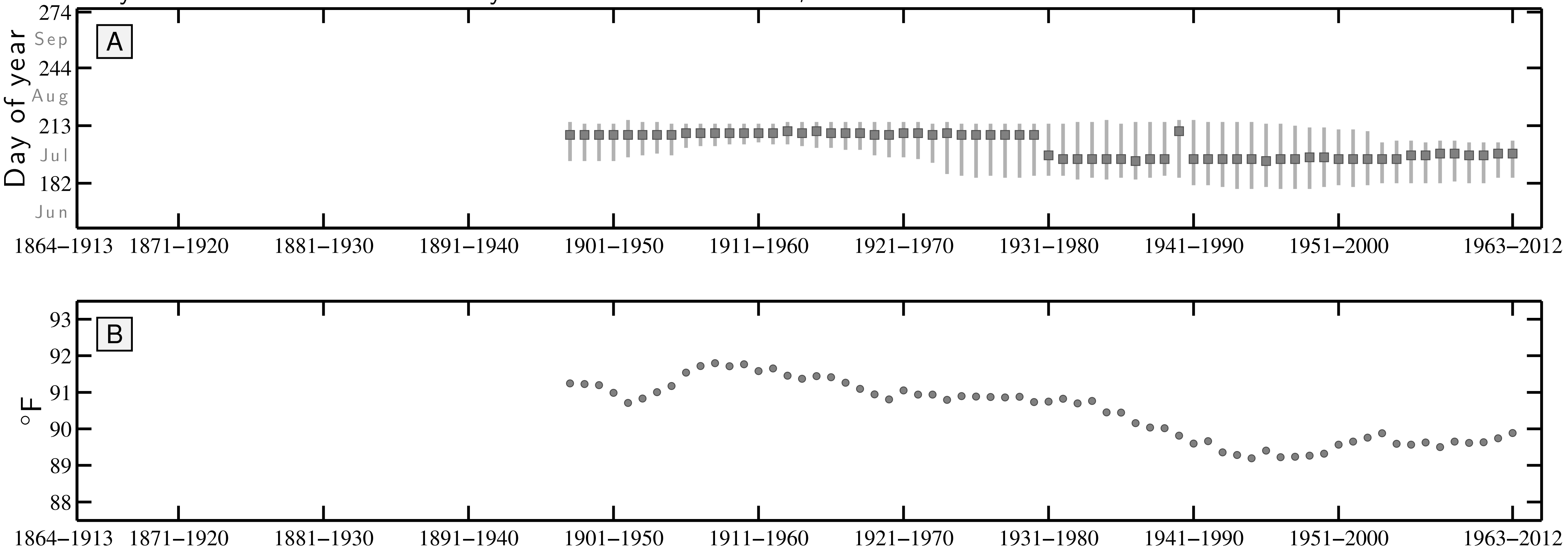

50 year Winter Teletherm dynamics for SEWARD, NE:

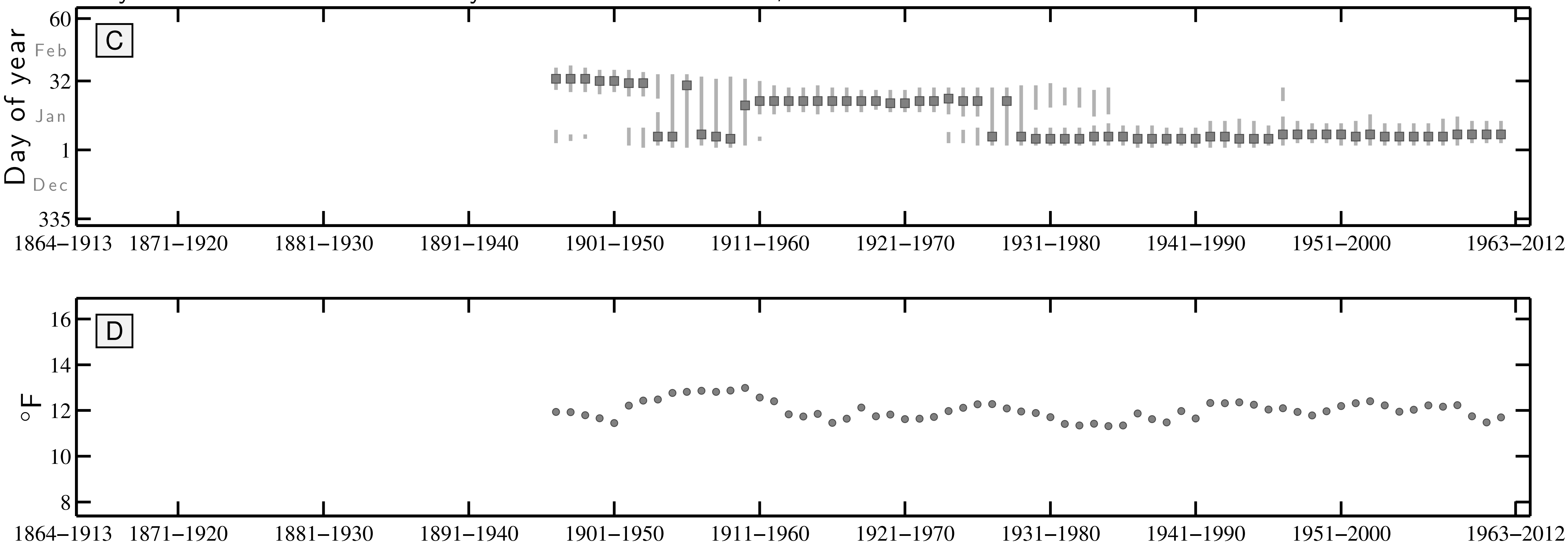

50 year Summer Teletherm dynamics for SYRACUSE, NE:

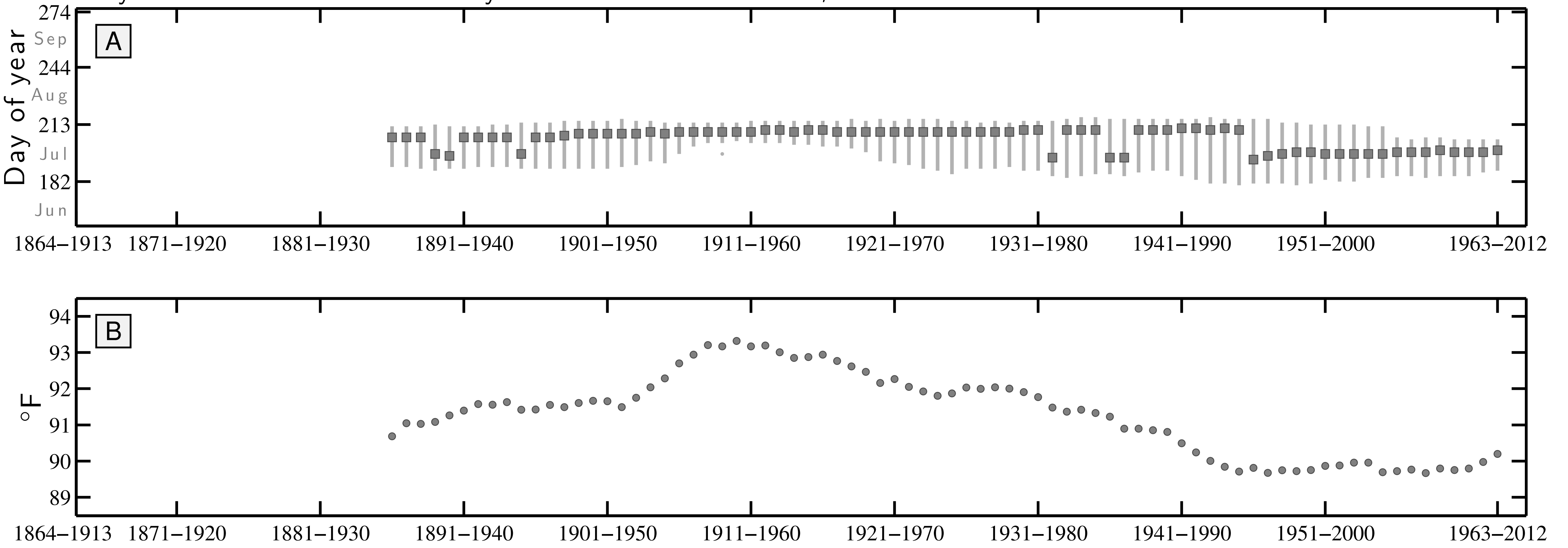

50 year Winter Teletherm dynamics for SYRACUSE, NE:

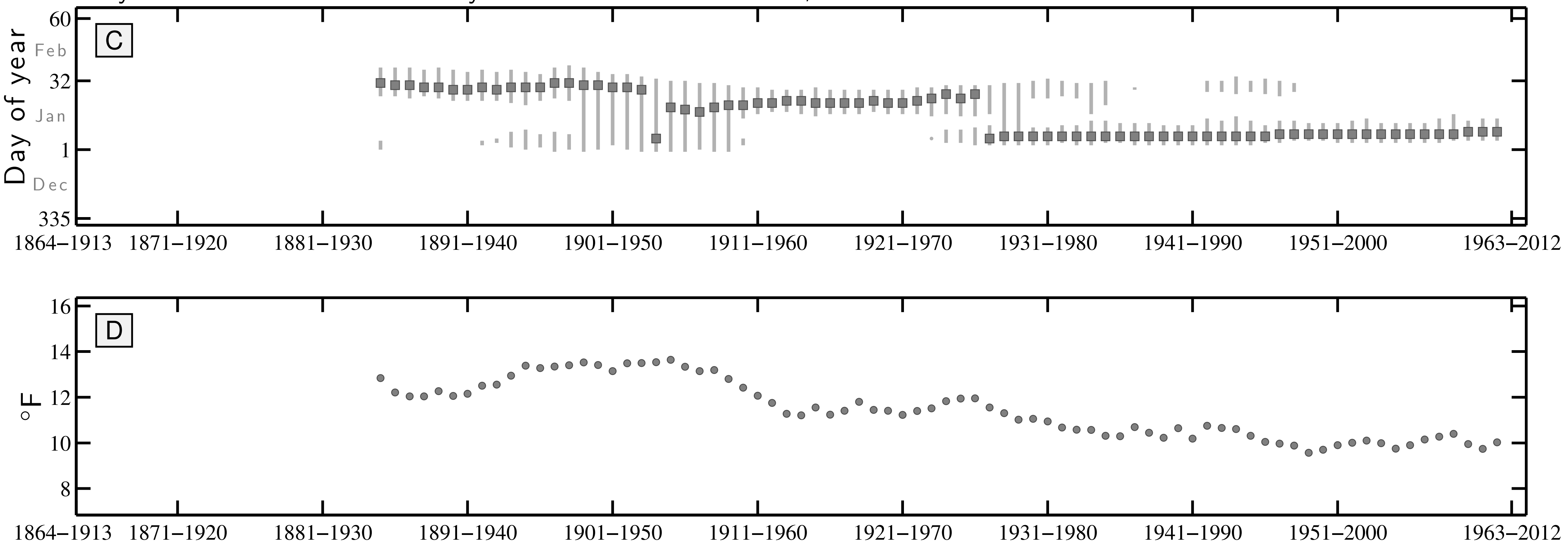

50 year Summer Teletherm dynamics for TECUMSEH 1S, NE:

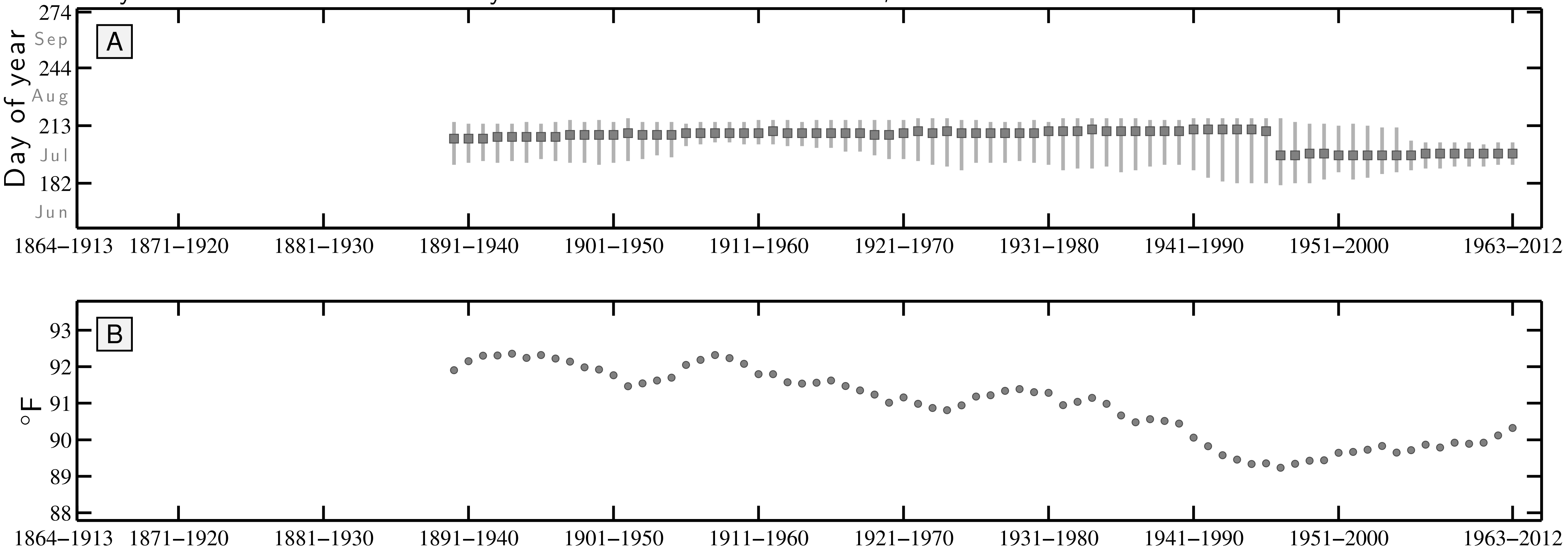

50 year Winter Teletherm dynamics for TECUMSEH 1S, NE:

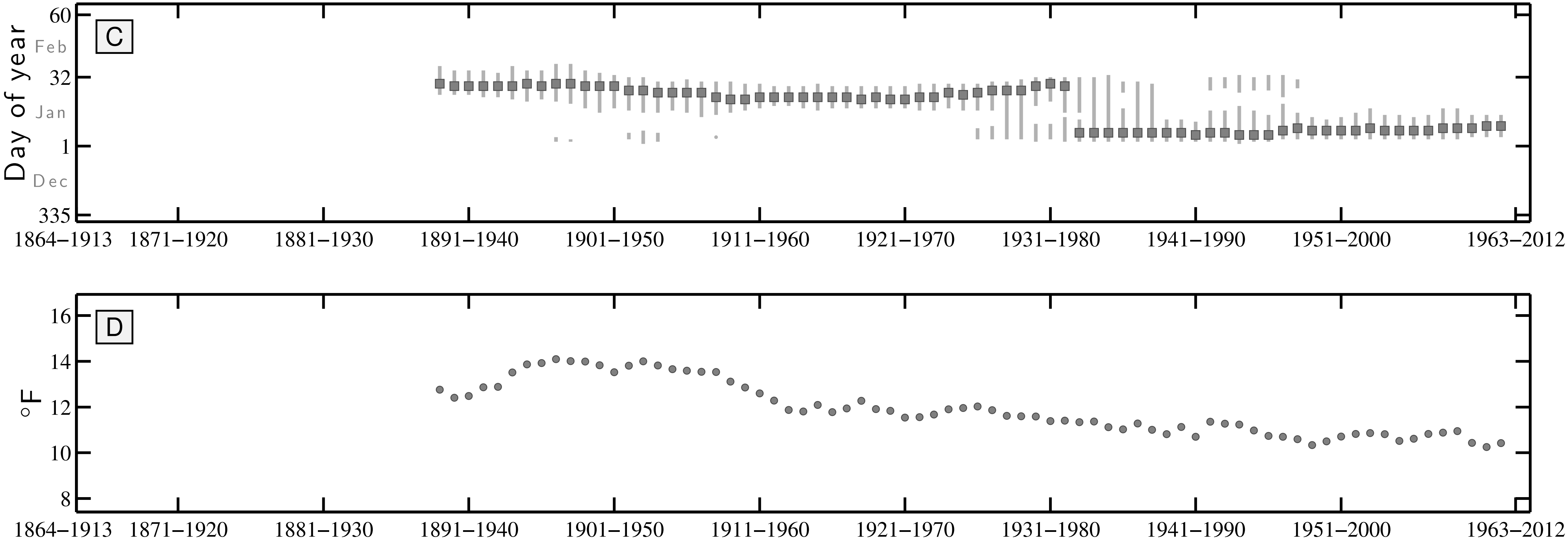

50 year Summer Teletherm dynamics for TEKAMAH, NE:

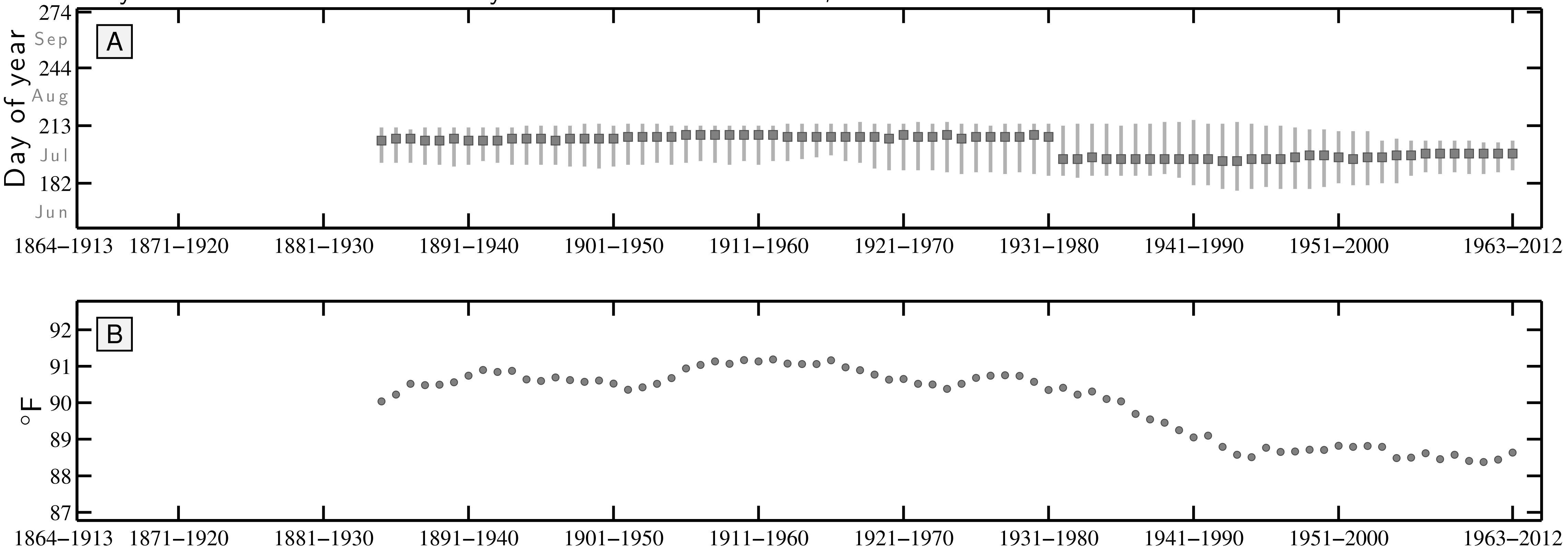

50 year Winter Teletherm dynamics for TEKAMAH, NE:

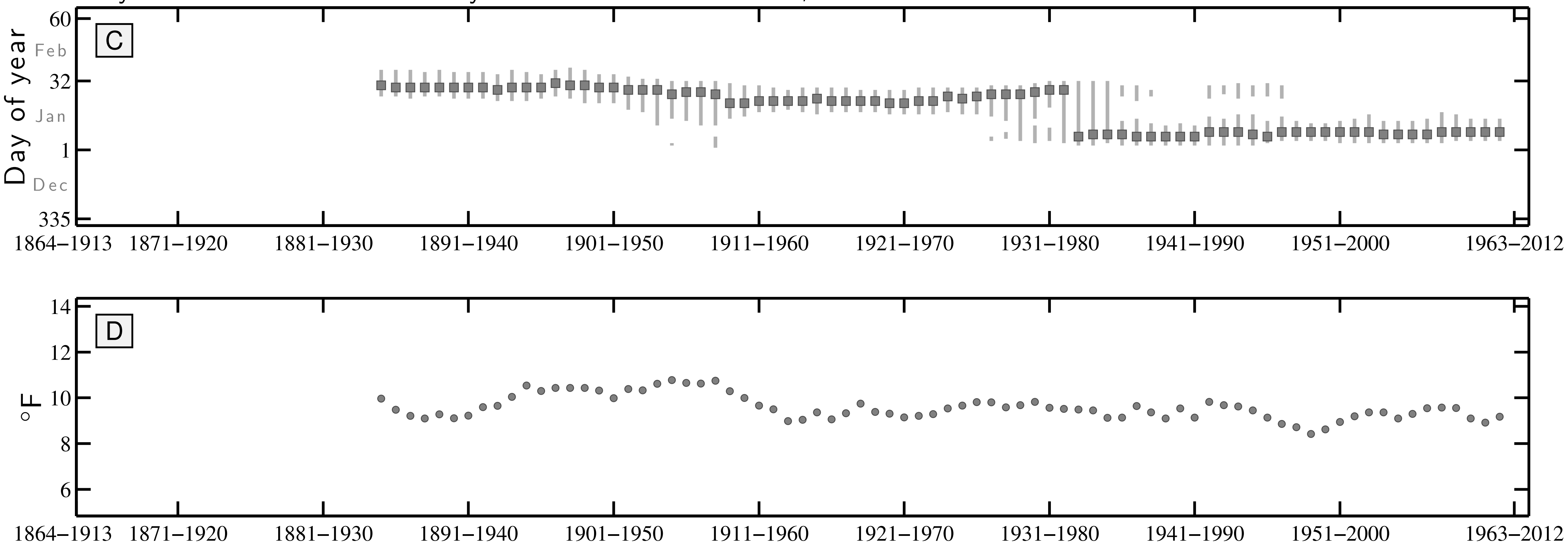

50 year Summer Teletherm dynamics for WAKEFIELD, NE:

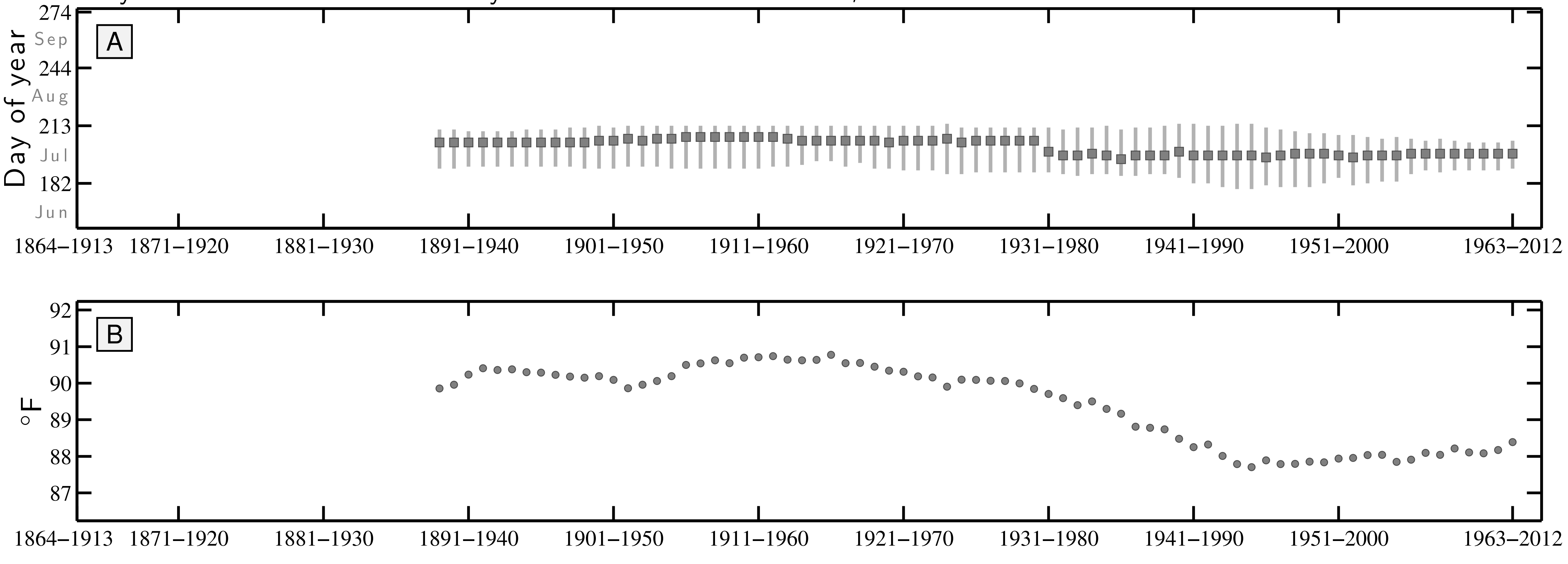

50 year Winter Teletherm dynamics for WAKEFIELD, NE:

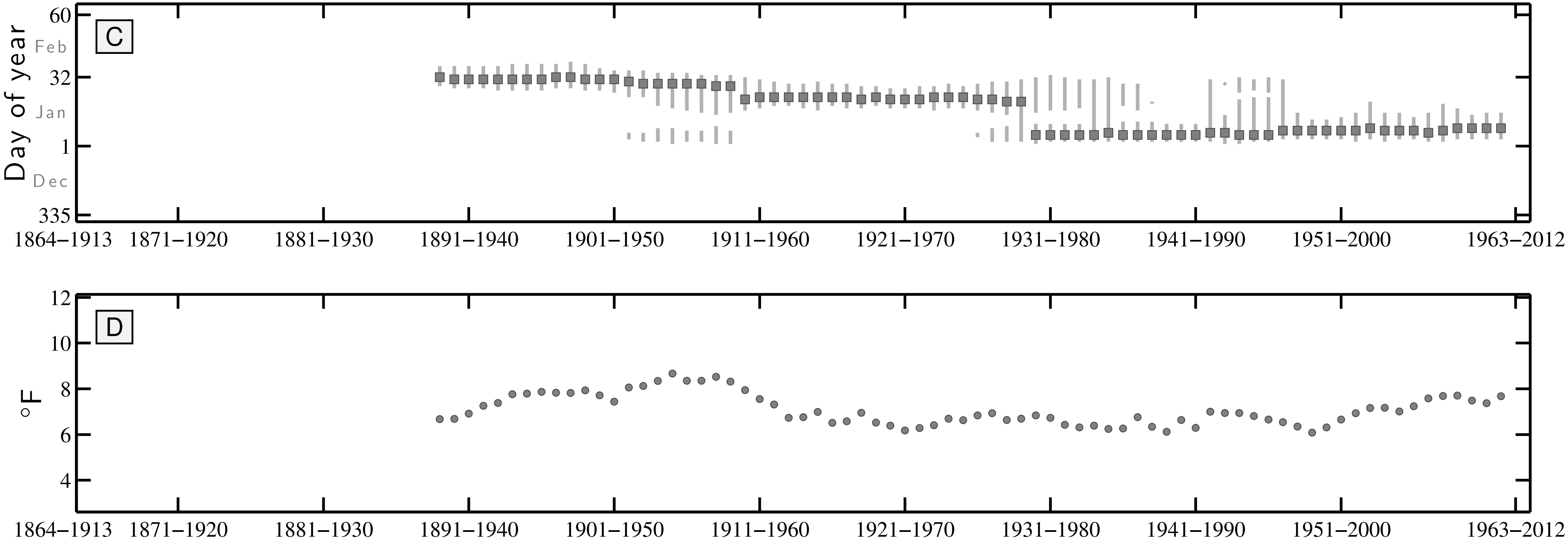

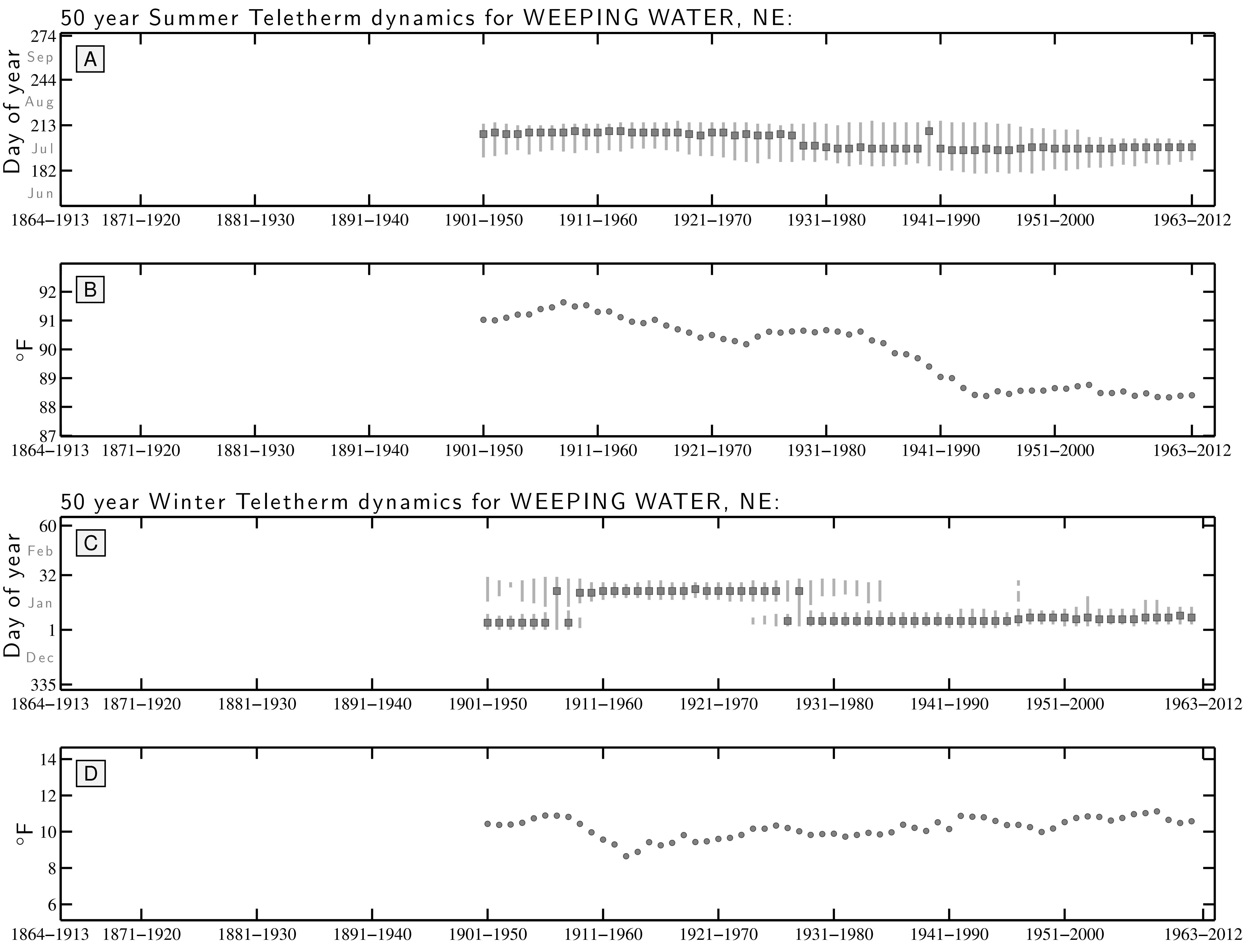
50 year Summer Teletherm dynamics for WEEPING WATER, NE:
A
Day of year
274
Sep
244
Aug
213
Jul
182
Jun
B
°F
92
91
90
89
88
87
1864–1913
1871–1920
1881–1930
1891–1940
1901–1950
1911–1960
1921–1970
1931–1980
1941–1990
1951–2000
1963–2012
50 year Winter Teletherm dynamics for WEEPING WATER, NE:
C
Day of year
60
Feb
32
Jan
1
Dec
335
D
°F
14
12
10
8
6

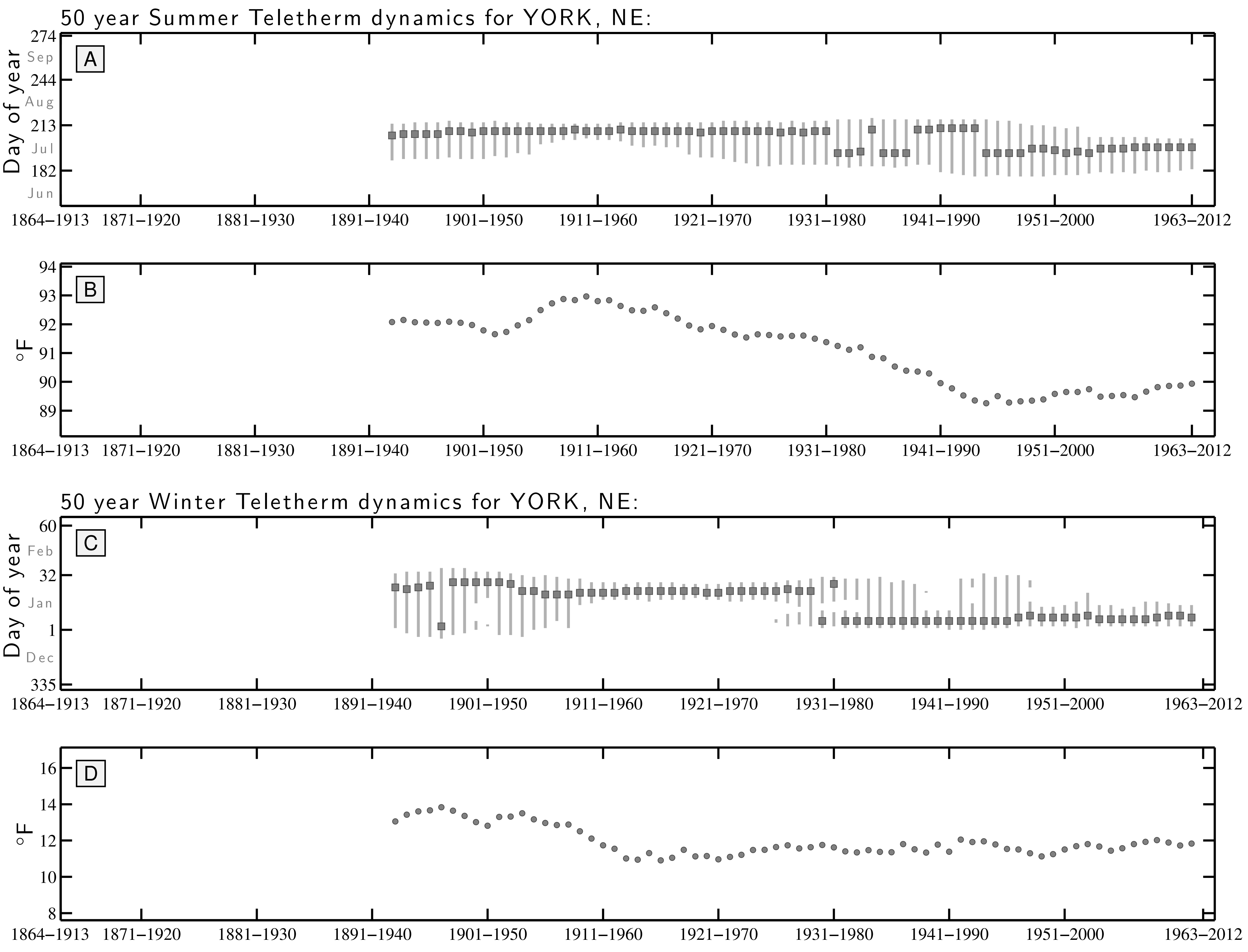

50 year Summer Teletherm dynamics for YORK, NE:
A
Day of year
274
Sep
244
Aug
213
Jul
182
Jun
1864–1913
1871–1920
1881–1930
1891–1940
1901–1950
1911–1960
1921–1970
1931–1980
1941–1990
1951–2000
1963–2012
B
°F
94
93
92
91
90
89
50 year Winter Teletherm dynamics for YORK, NE:
C
Day of year
60
Feb
32
Jan
1
Dec
335
D
°F
16
14
12
10
8

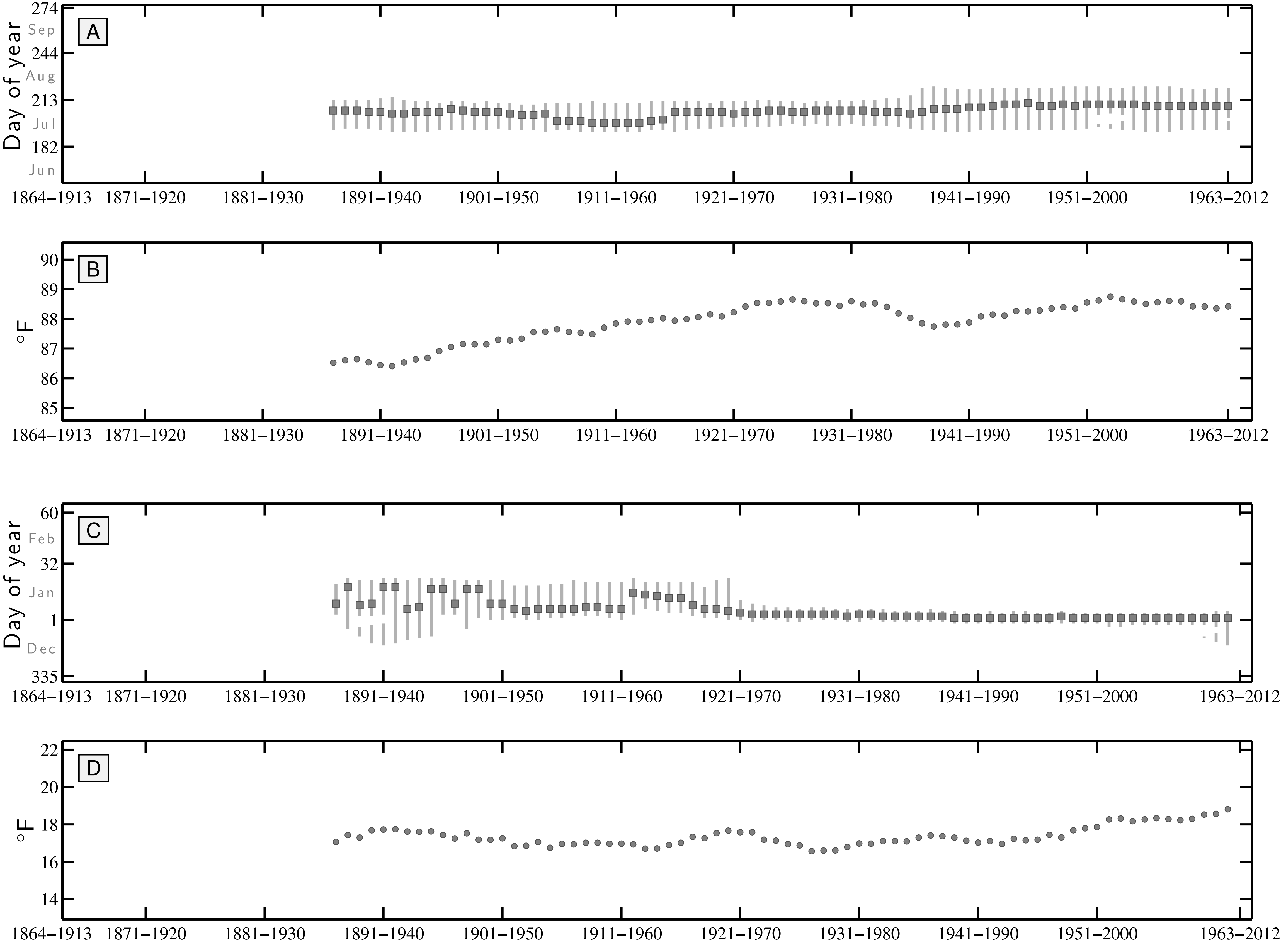

A
B
C
D
Day of year
°F
Sep
Aug
Jul
Jun
Feb
Jan
Dec
274
244
213
182
90
89
88
87
86
85
60
32
1
335
22
20
18
16
14
1864–1913
1871–1920
1881–1930
1891–1940
1901–1950
1911–1960
1921–1970
1931–1980
1941–1990
1951–2000
1963–2012

50 year Summer Teletherm dynamics for BATTLE MOUNTAIN 4SE, NV:

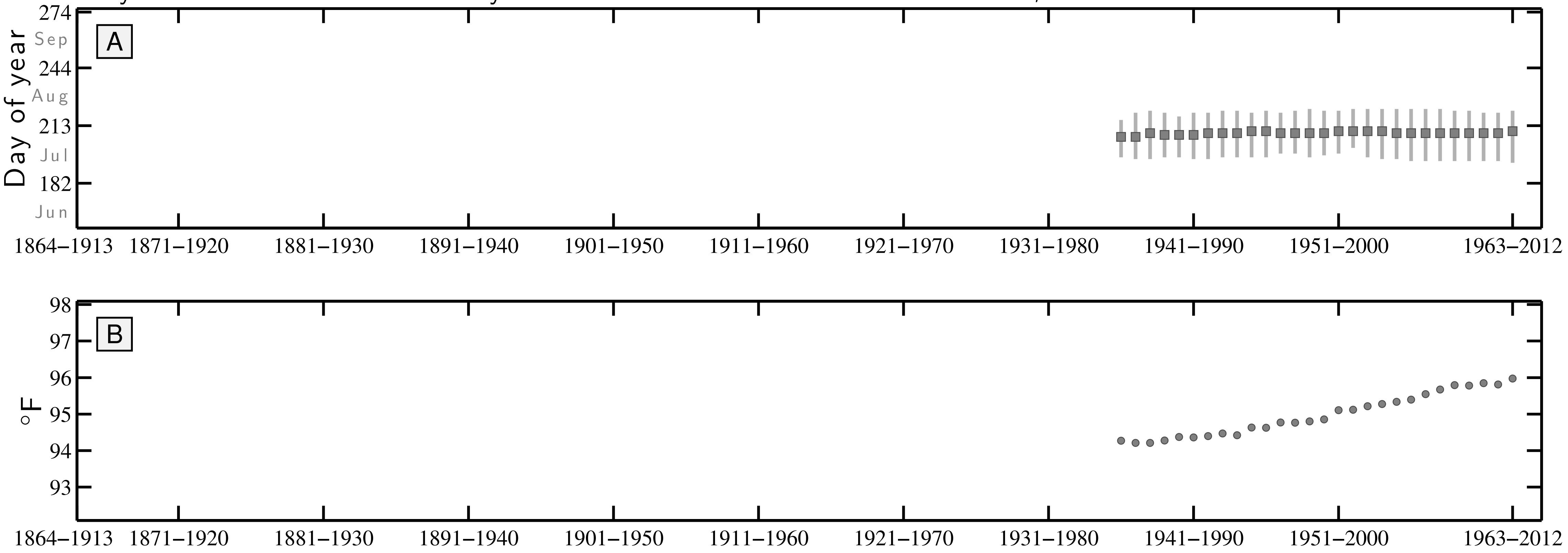

50 year Winter Teletherm dynamics for BATTLE MOUNTAIN 4SE, NV:

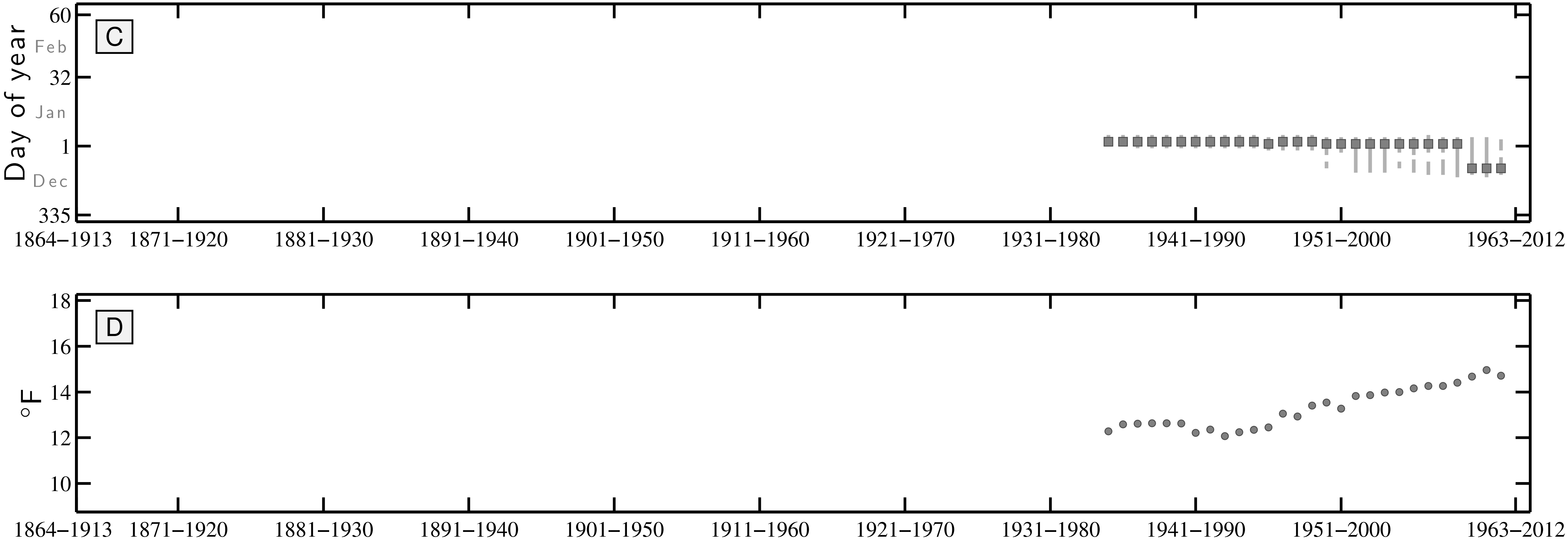

50 year Summer Teletherm dynamics for BOULDER CITY, NV:

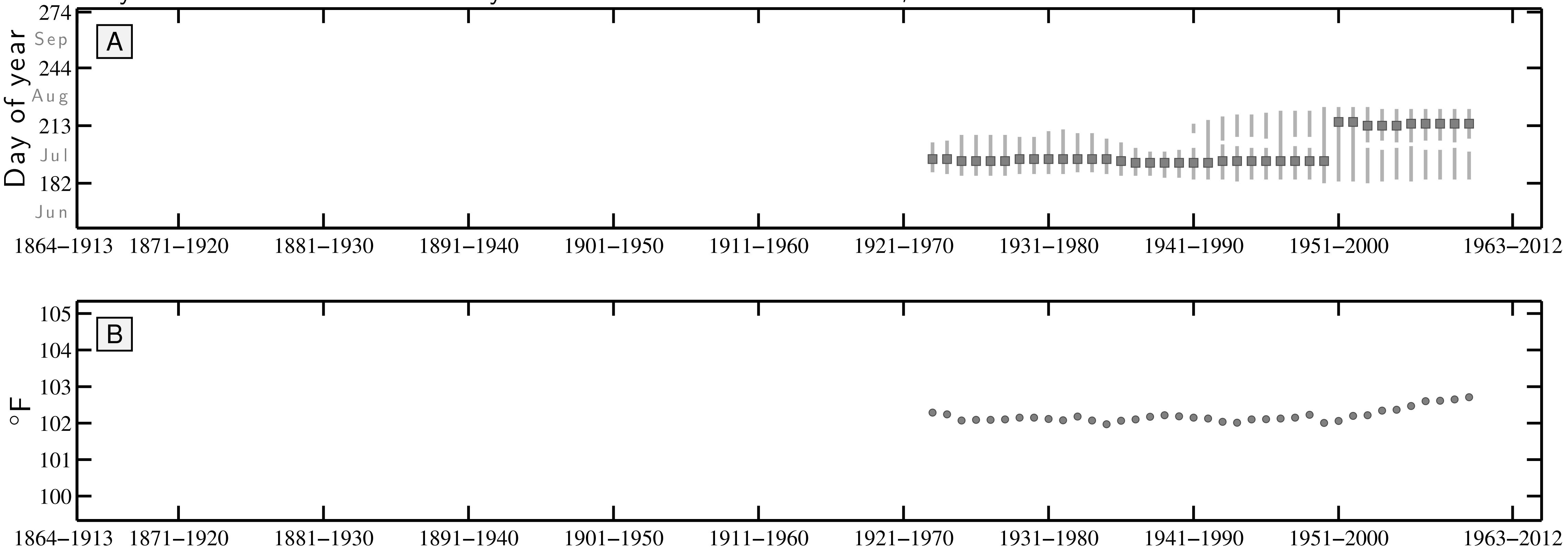

50 year Winter Teletherm dynamics for BOULDER CITY, NV:

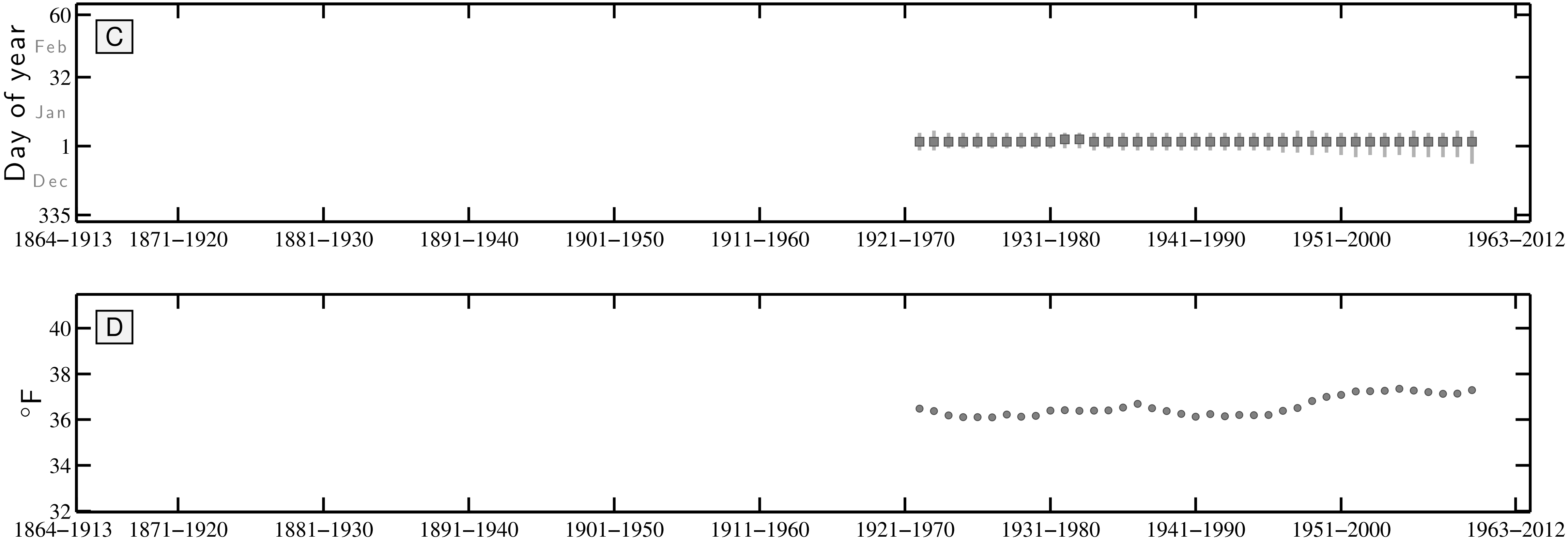

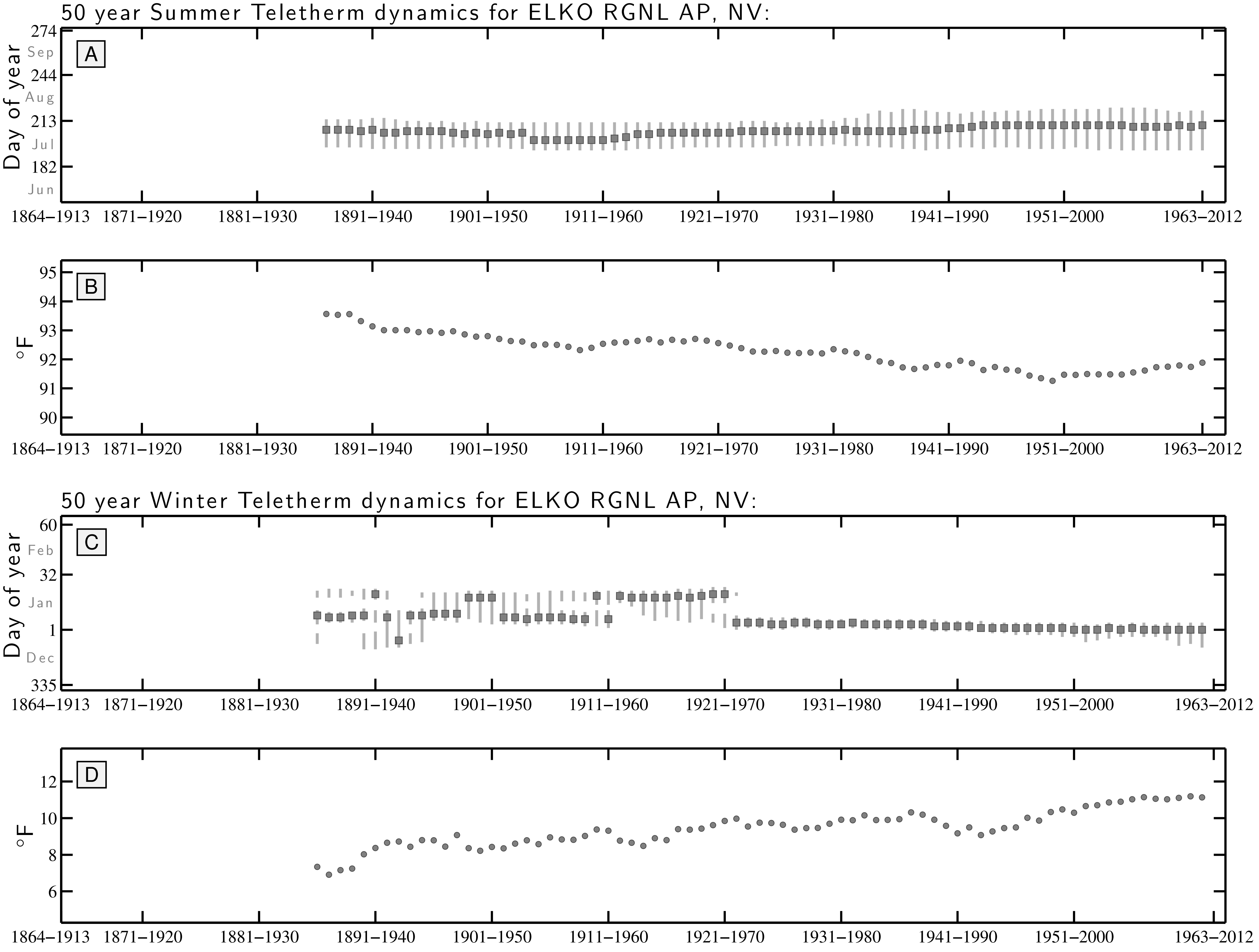
50 year Summer Teletherm dynamics for ELKO RGNL AP, NV:
A
B
Day of year
°F
274
244
213
182
Sep
Aug
Jul
Jun
95
94
93
92
91
90
1864–1913
1871–1920
1881–1930
1891–1940
1901–1950
1911–1960
1921–1970
1931–1980
1941–1990
1951–2000
1963–2012
50 year Winter Teletherm dynamics for ELKO RGNL AP, NV:
C
D
60
32
1
335
Feb
Jan
Dec
12
10
8
6

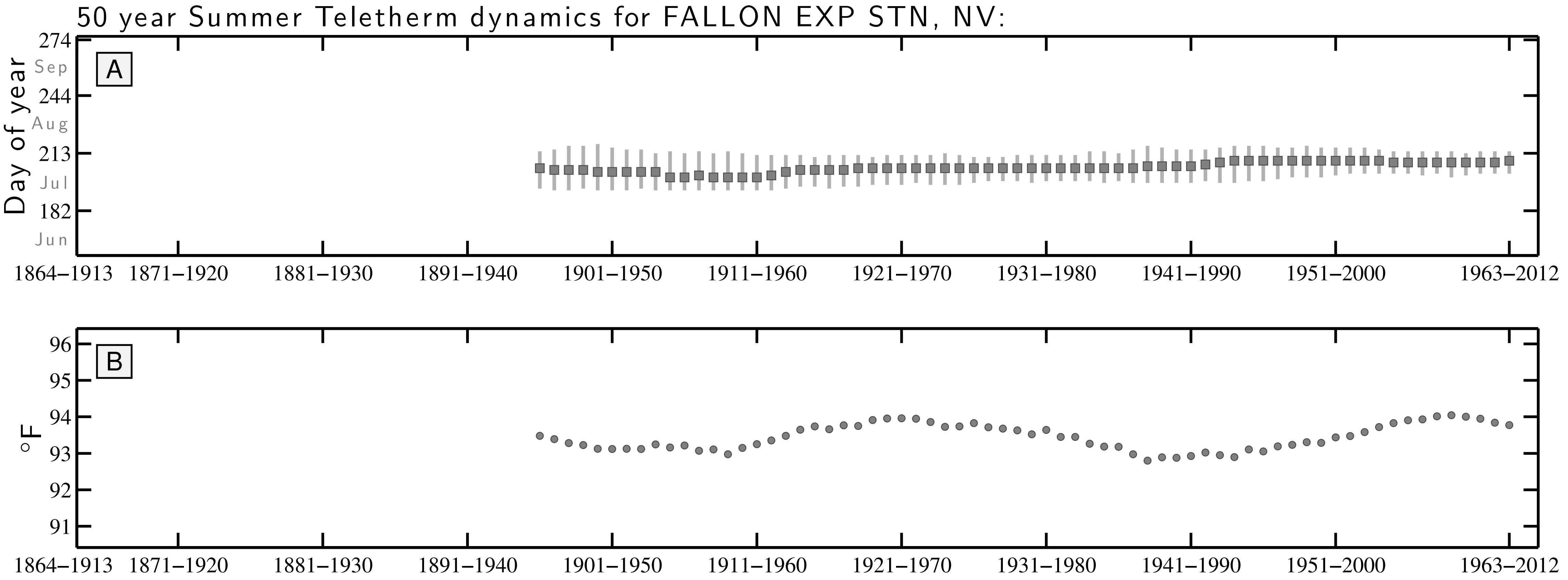

50 year Summer Teletherm dynamics for FALLON EXP STN, NV:
A
B
Day of year
274
Sep
244
Aug
213
Jul
182
Jun
°F
96
95
94
93
92
91
1864–1913
1871–1920
1881–1930
1891–1940
1901–1950
1911–1960
1921–1970
1931–1980
1941–1990
1951–2000
1963–2012

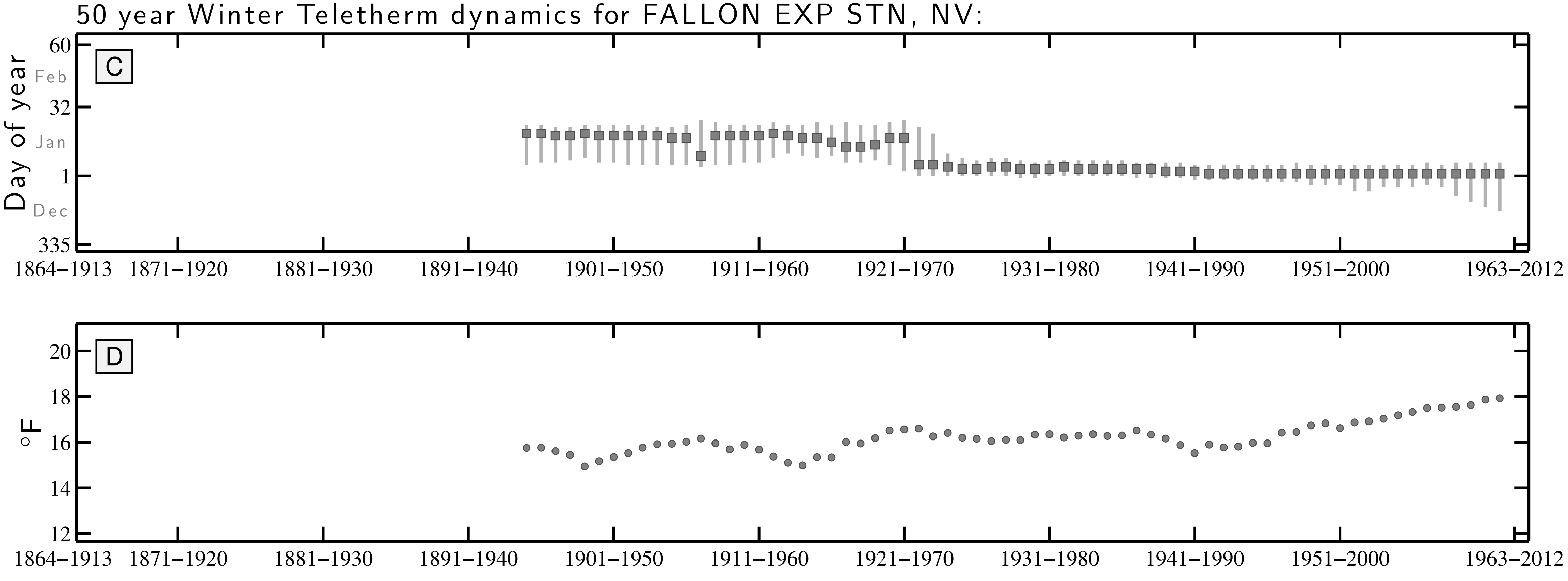

50 year Winter Teletherm dynamics for FALLON EXP STN, NV:
C
D
Day of year
60
Feb
32
Jan
1
Dec
335
°F
20
18
16
14
12
1864–1913
1871–1920
1881–1930
1891–1940
1901–1950
1911–1960
1921–1970
1931–1980
1941–1990
1951–2000
1963–2012

50 year Summer Teletherm dynamics for GOLCONDA, NV:

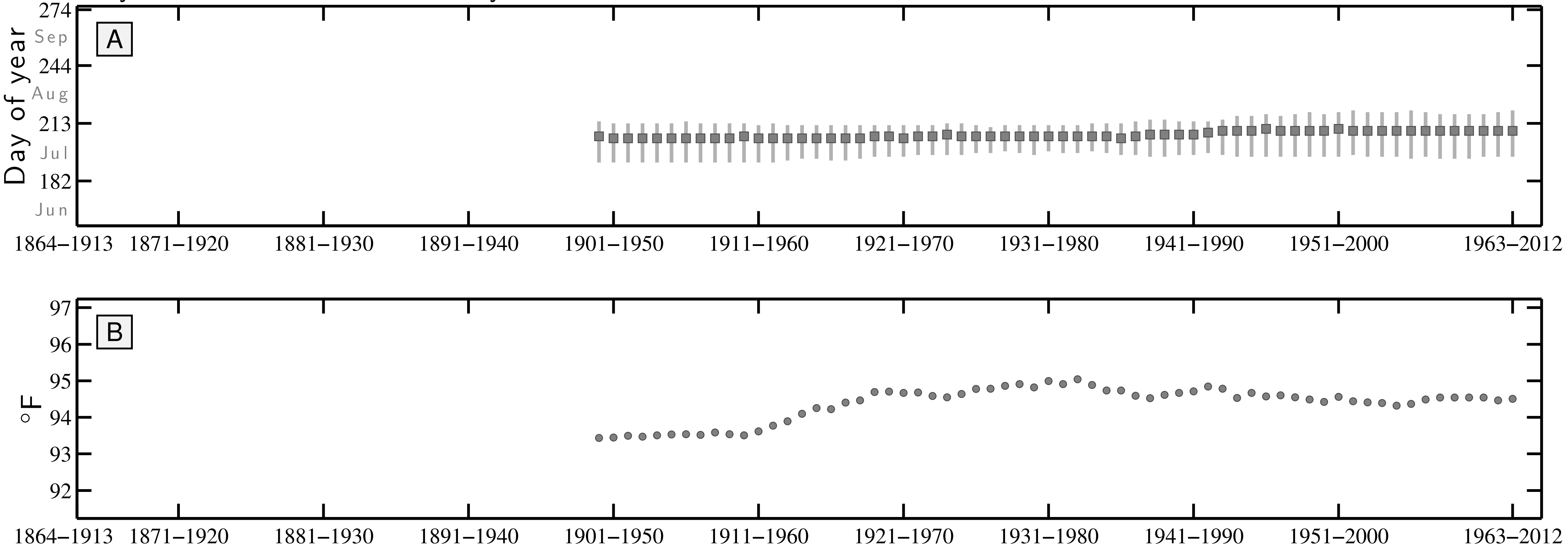

50 year Winter Teletherm dynamics for GOLCONDA, NV:

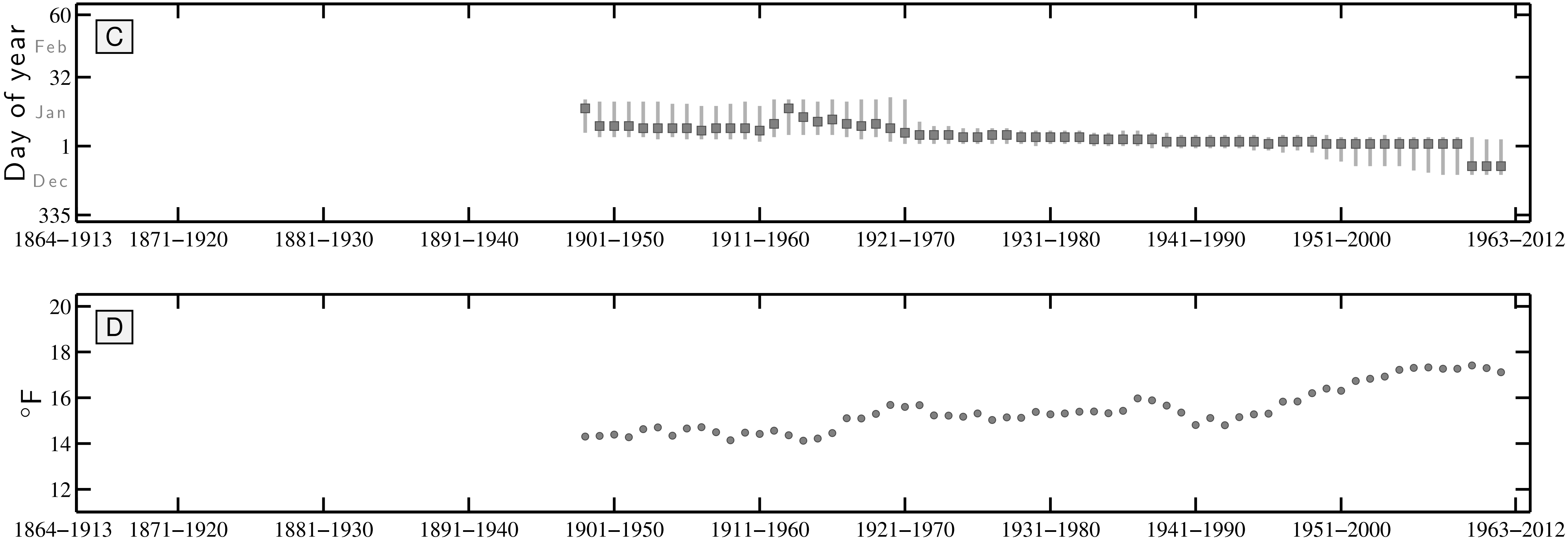

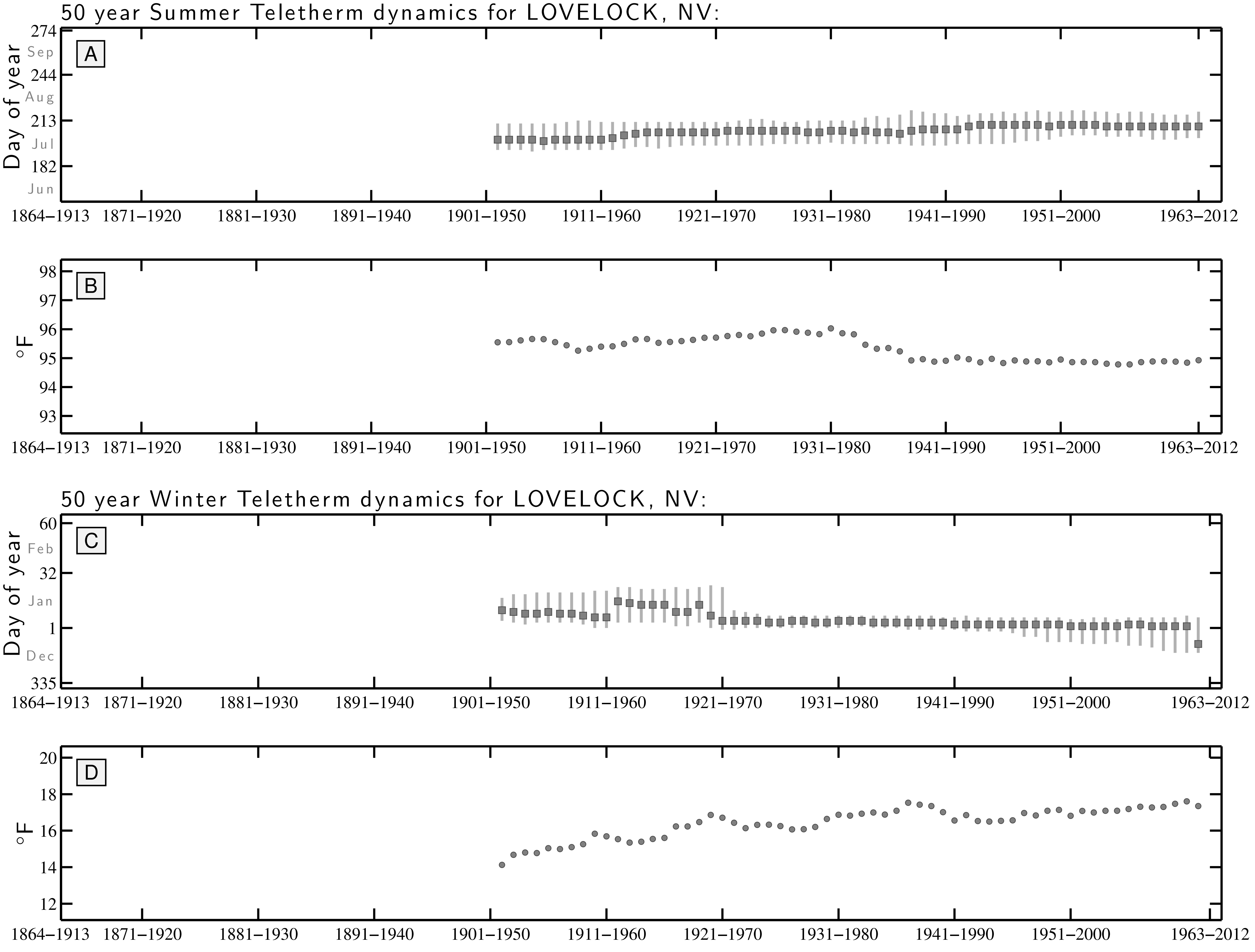
50 year Summer Teletherm dynamics for LOVELOCK, NV:
A
B
Day of year
°F
Sep
Aug
Jul
Jun
274
244
213
182
98
97
96
95
94
93
1864–1913
1871–1920
1881–1930
1891–1940
1901–1950
1911–1960
1921–1970
1931–1980
1941–1990
1951–2000
1963–2012
50 year Winter Teletherm dynamics for LOVELOCK, NV:
C
D
Feb
Jan
Dec
60
32
1
335
20
18
16
14
12

50 year Summer Teletherm dynamics for MCGILL, NV:

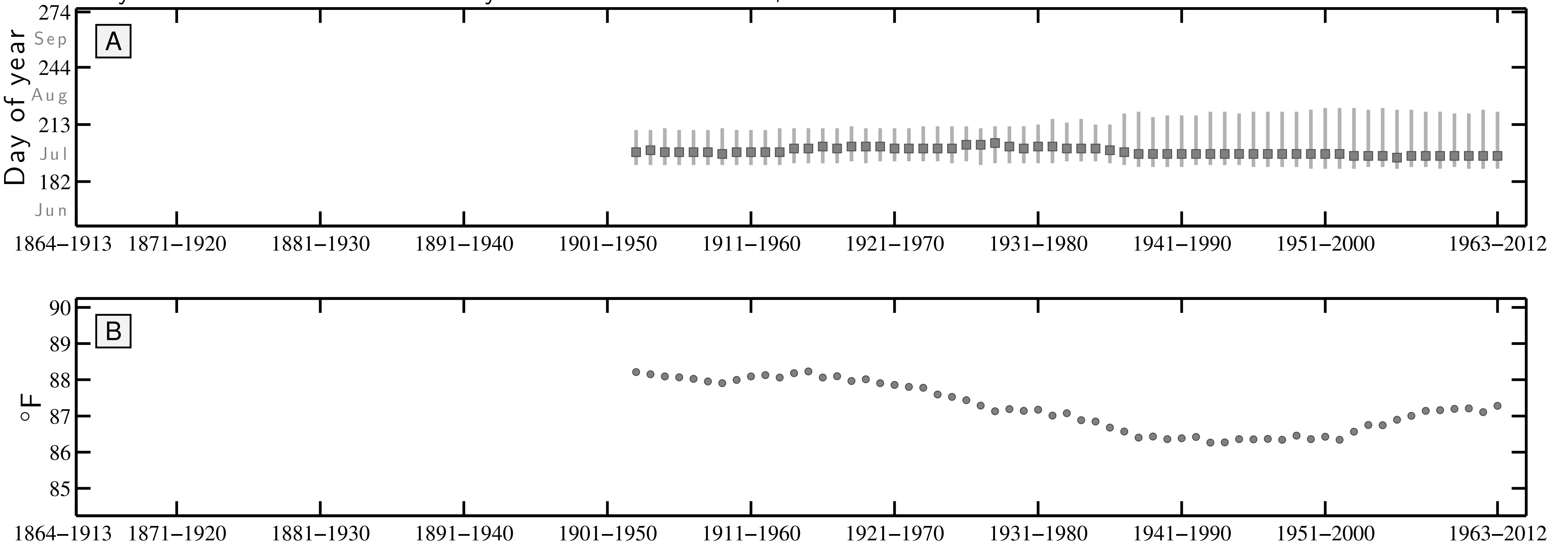

50 year Winter Teletherm dynamics for MCGILL, NV:

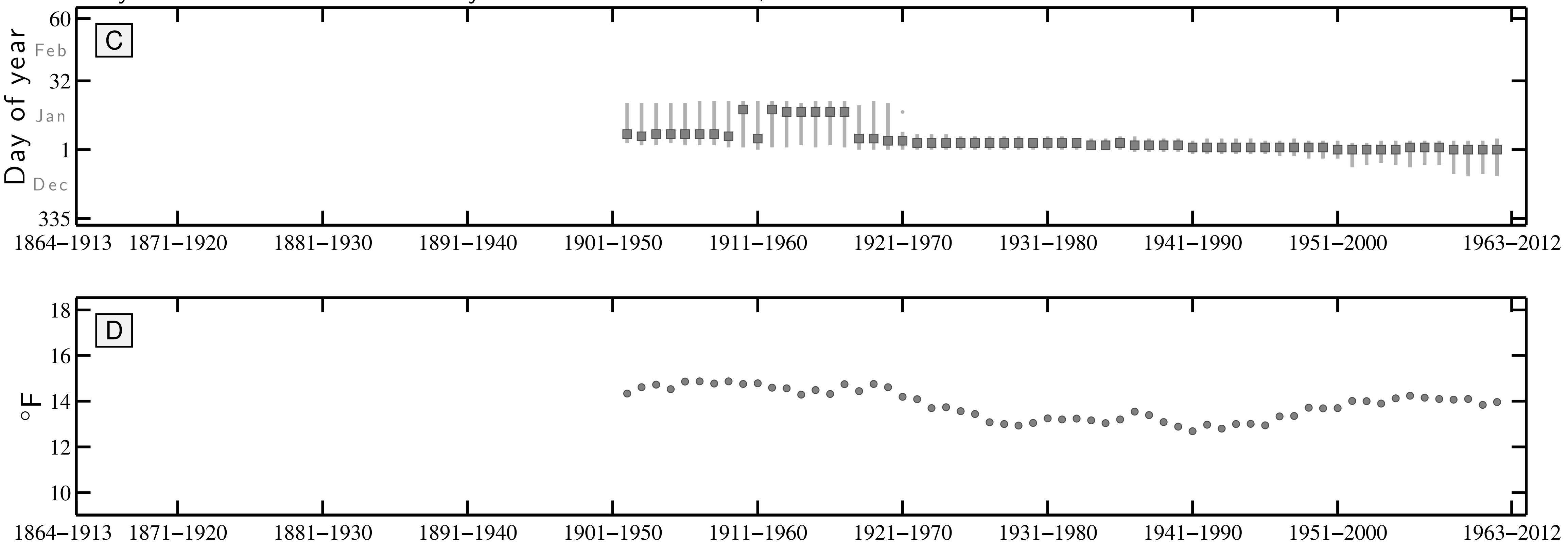

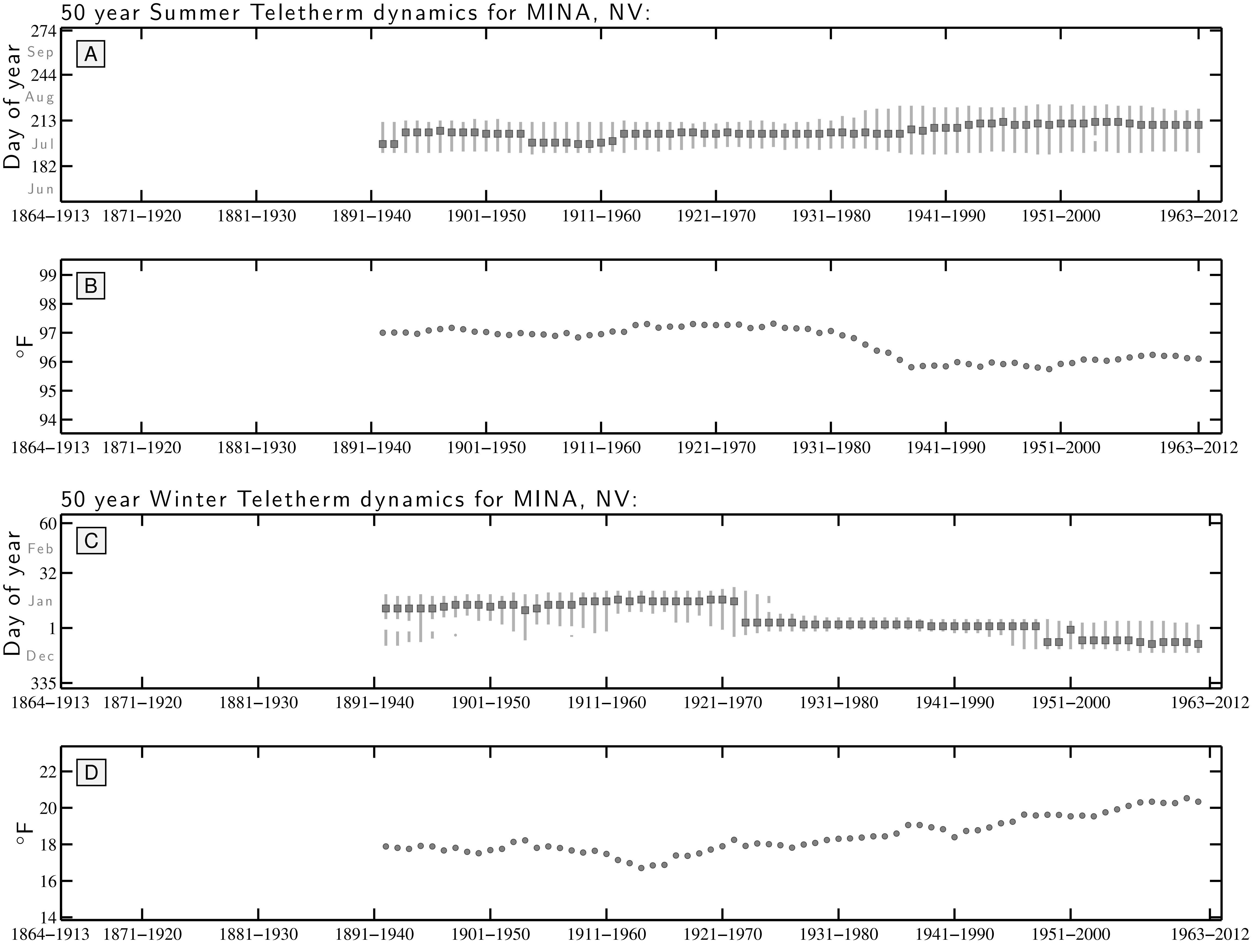

50 year Summer Teletherm dynamics for MINA, NV:
A
B
Day of year
°F
274
Sep
244
Aug
213
Jul
182
Jun
99
98
97
96
95
94
1864–1913
1871–1920
1881–1930
1891–1940
1901–1950
1911–1960
1921–1970
1931–1980
1941–1990
1951–2000
1963–2012
50 year Winter Teletherm dynamics for MINA, NV:
C
D
60
Feb
32
Jan
1
Dec
335
22
20
18
16
14

50 year Summer Teletherm dynamics for RENO AP, NV:

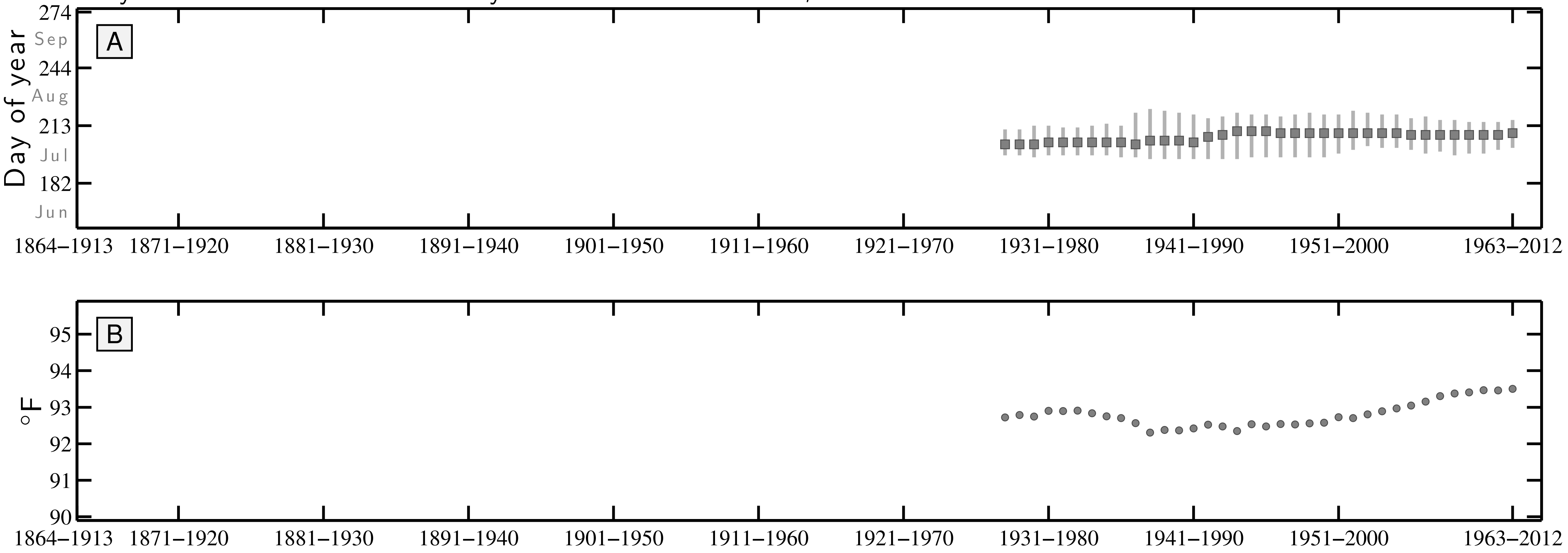

50 year Winter Teletherm dynamics for RENO AP, NV:

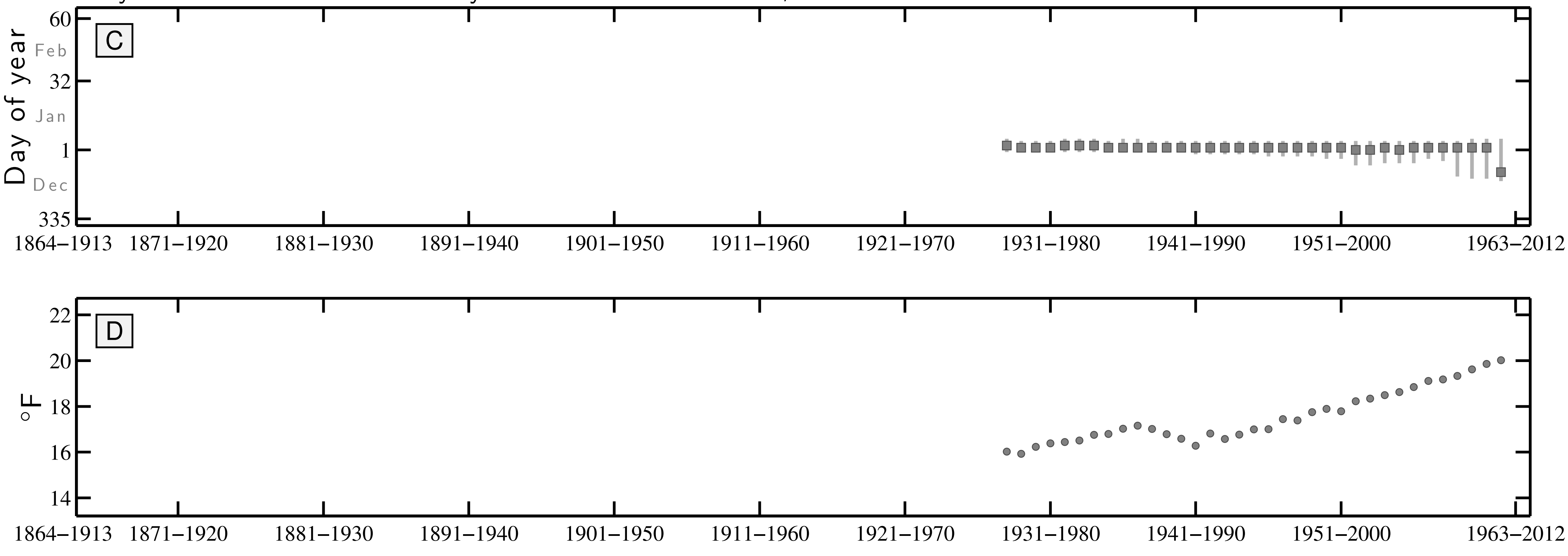

50 year Summer Teletherm dynamics for SEARCHLIGHT, NV:

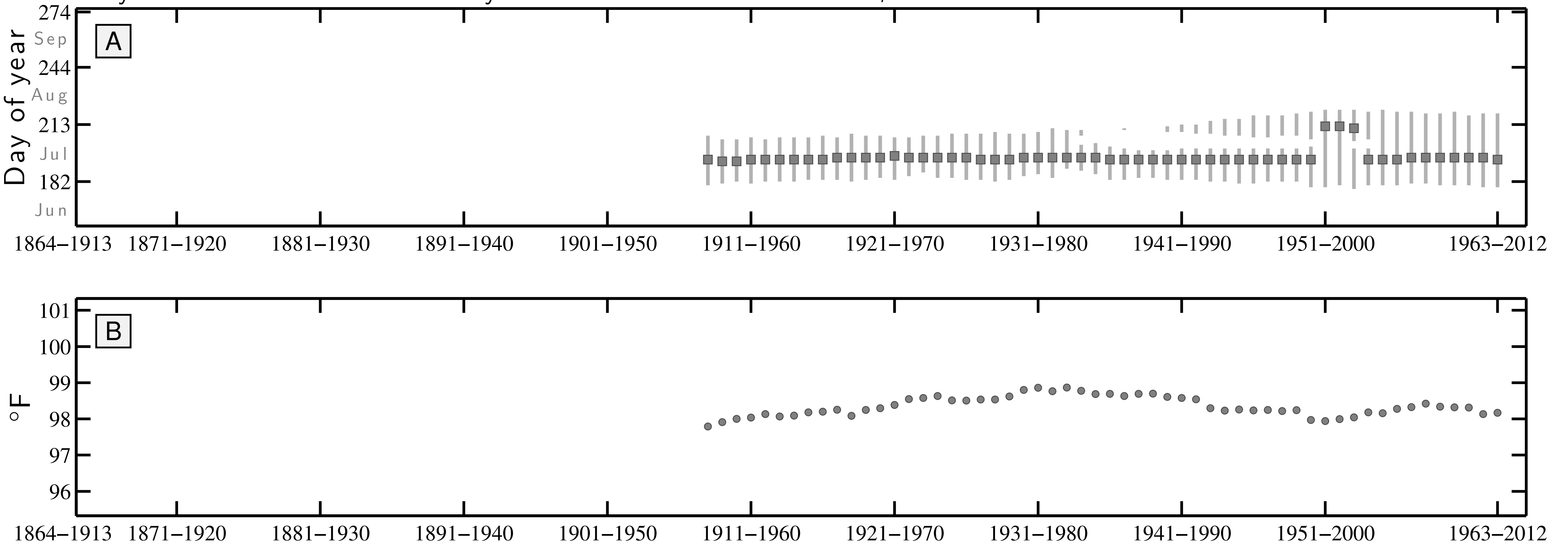

50 year Winter Teletherm dynamics for SEARCHLIGHT, NV:

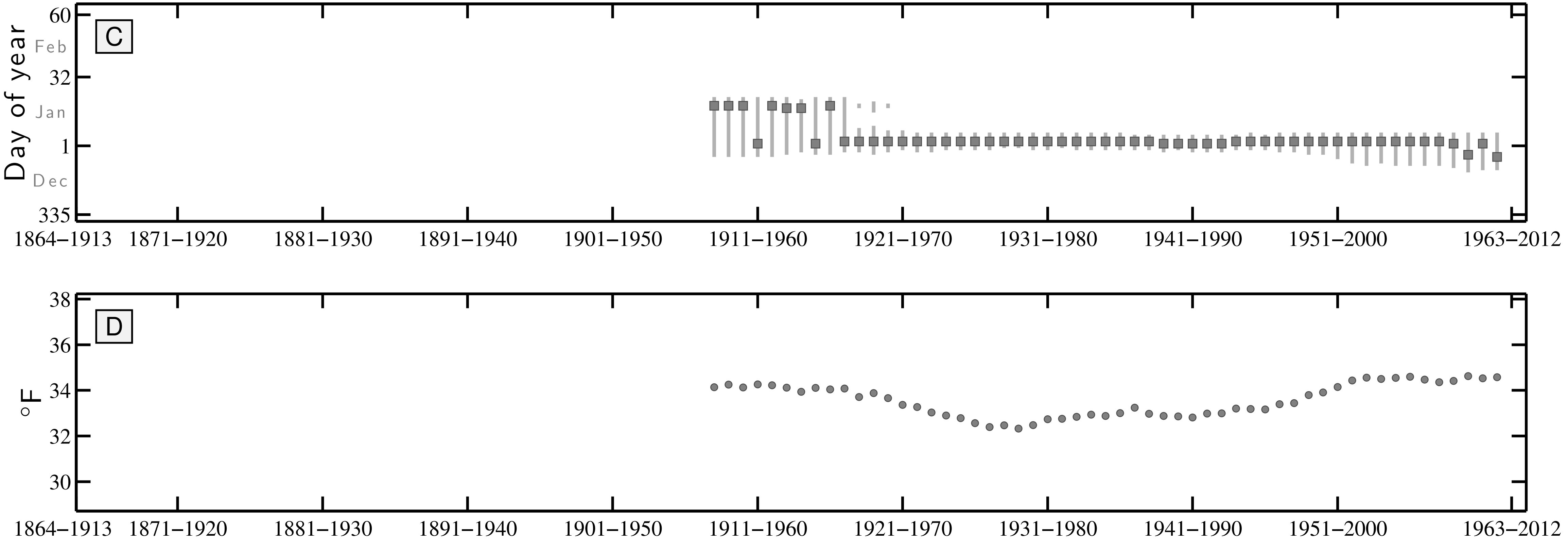

50 year Summer Teletherm dynamics for WELLS, NV:

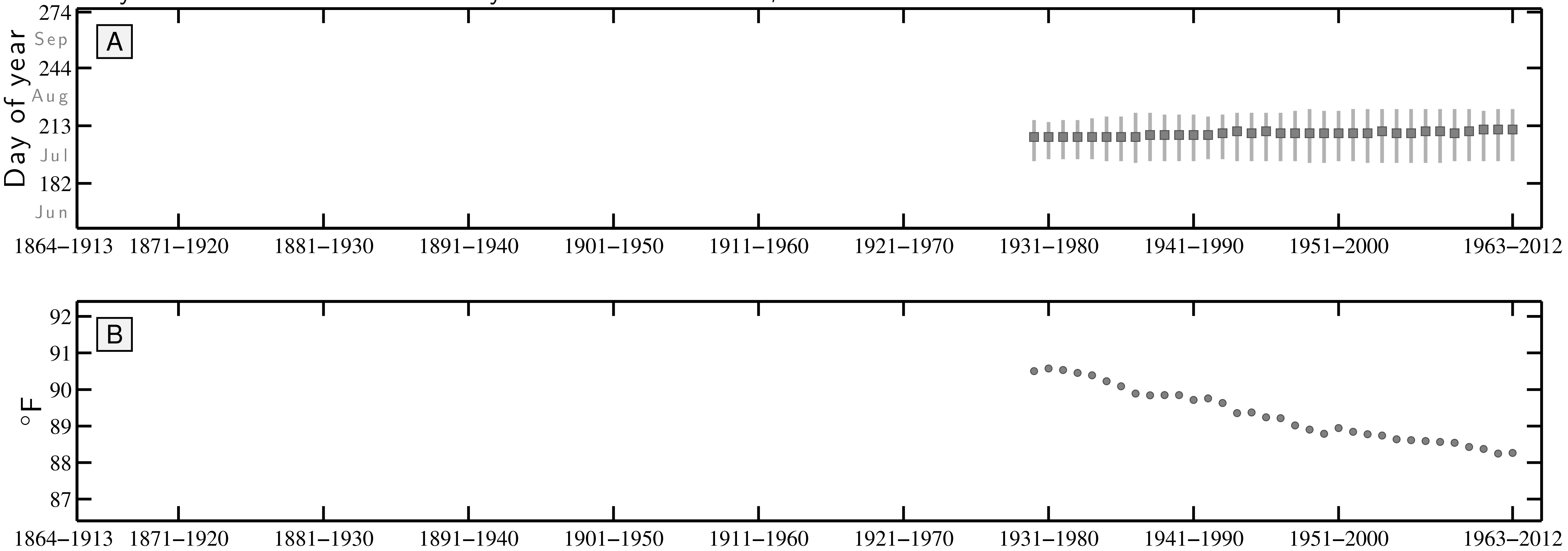

50 year Winter Teletherm dynamics for WELLS, NV:

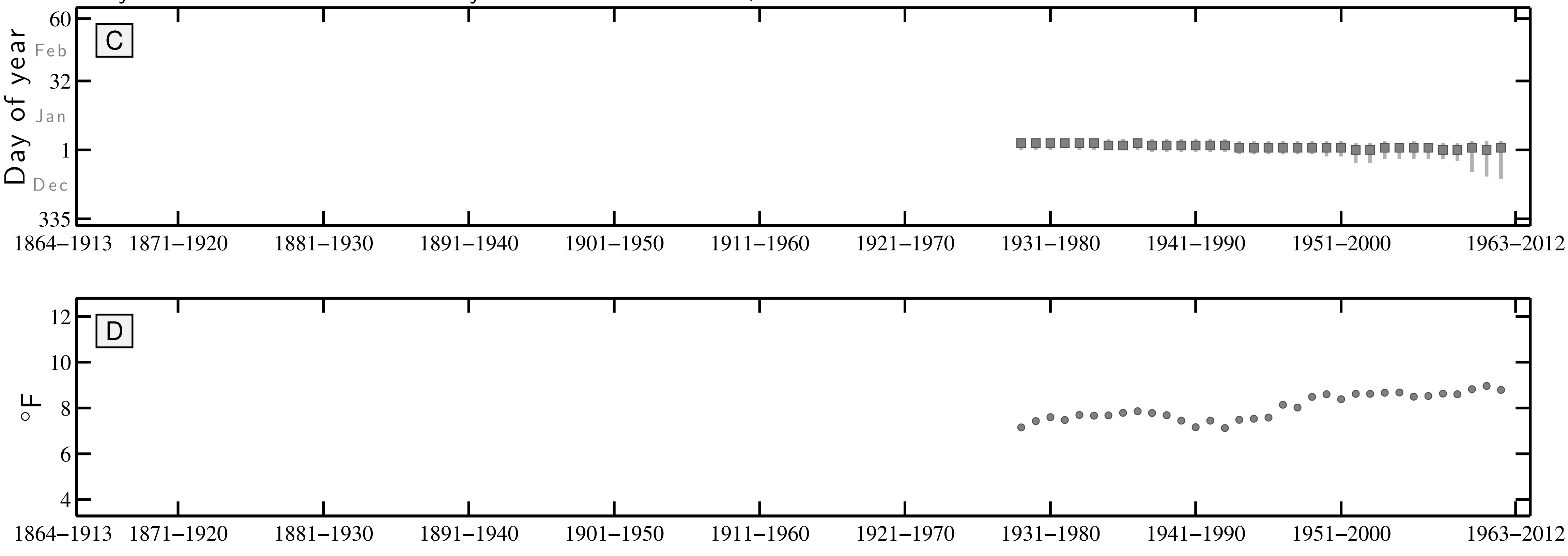

50 year Summer Teletherm dynamics for WINNEMUCCA AP, NV:

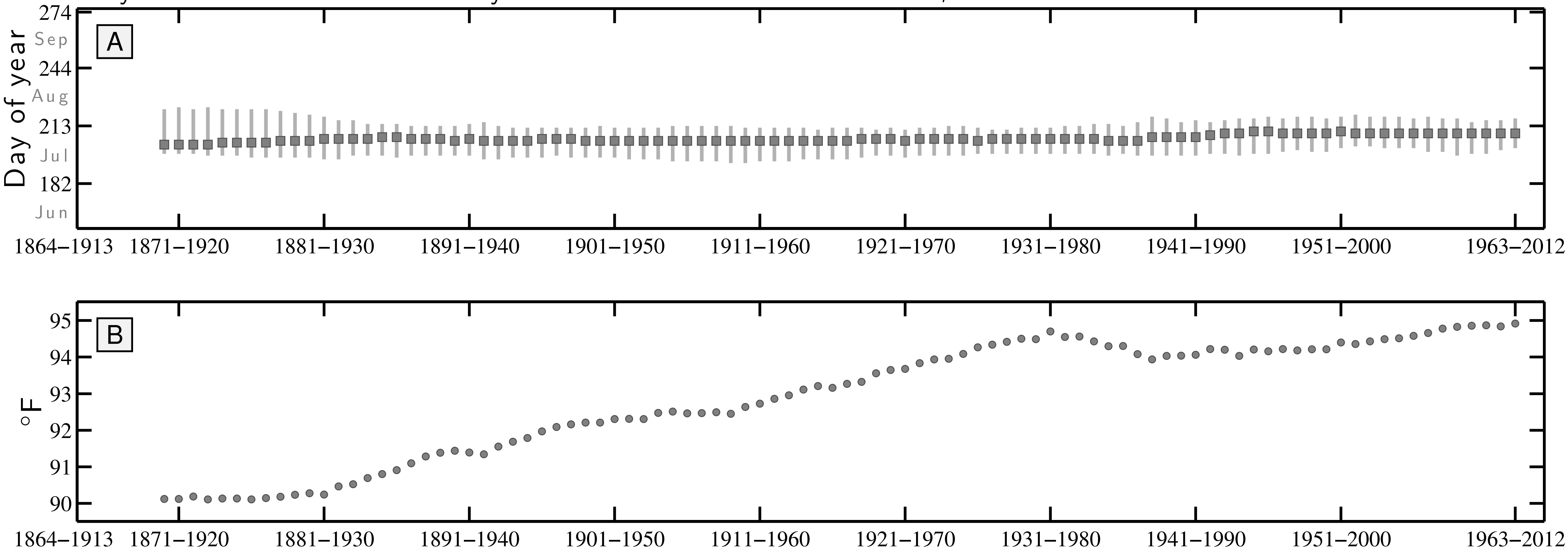

50 year Winter Teletherm dynamics for WINNEMUCCA AP, NV:

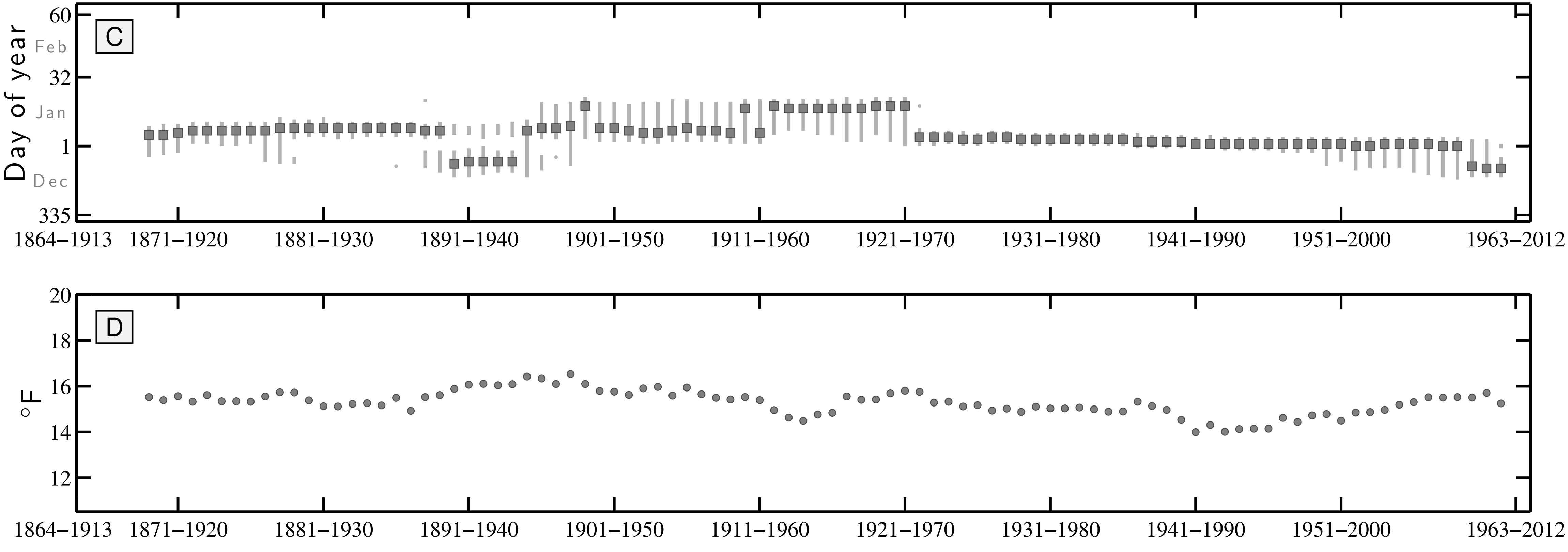

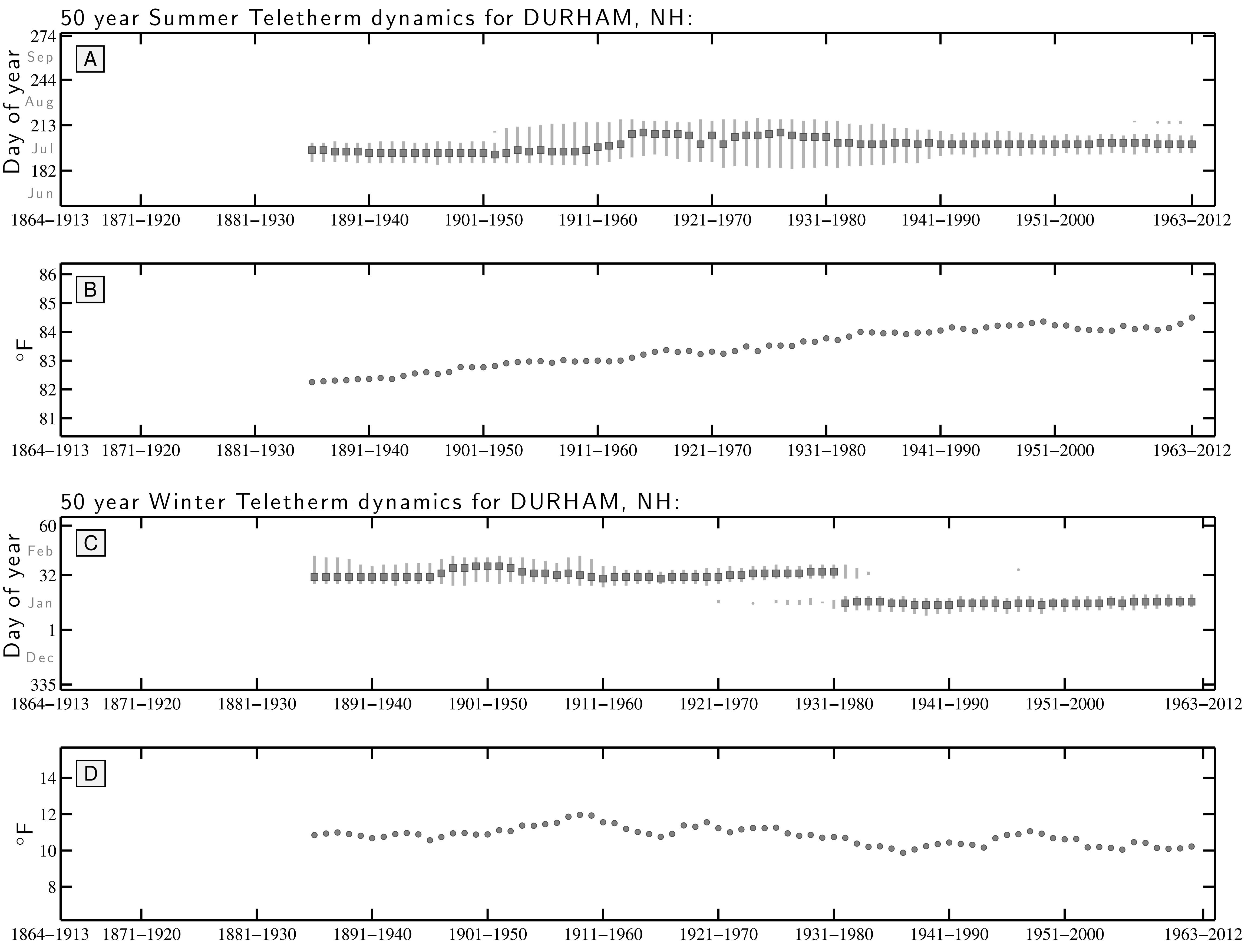
50 year Summer Teletherm dynamics for DURHAM, NH:
A
B
Day of year
°F
274
Sep
244
Aug
213
Jul
182
Jun
86
85
84
83
82
81
1864–1913
1871–1920
1881–1930
1891–1940
1901–1950
1911–1960
1921–1970
1931–1980
1941–1990
1951–2000
1963–2012
50 year Winter Teletherm dynamics for DURHAM, NH:
C
D
60
Feb
32
Jan
1
Dec
335
14
12
10
8

50 year Summer Teletherm dynamics for FIRST CONNECTICUT LAKE, NH:

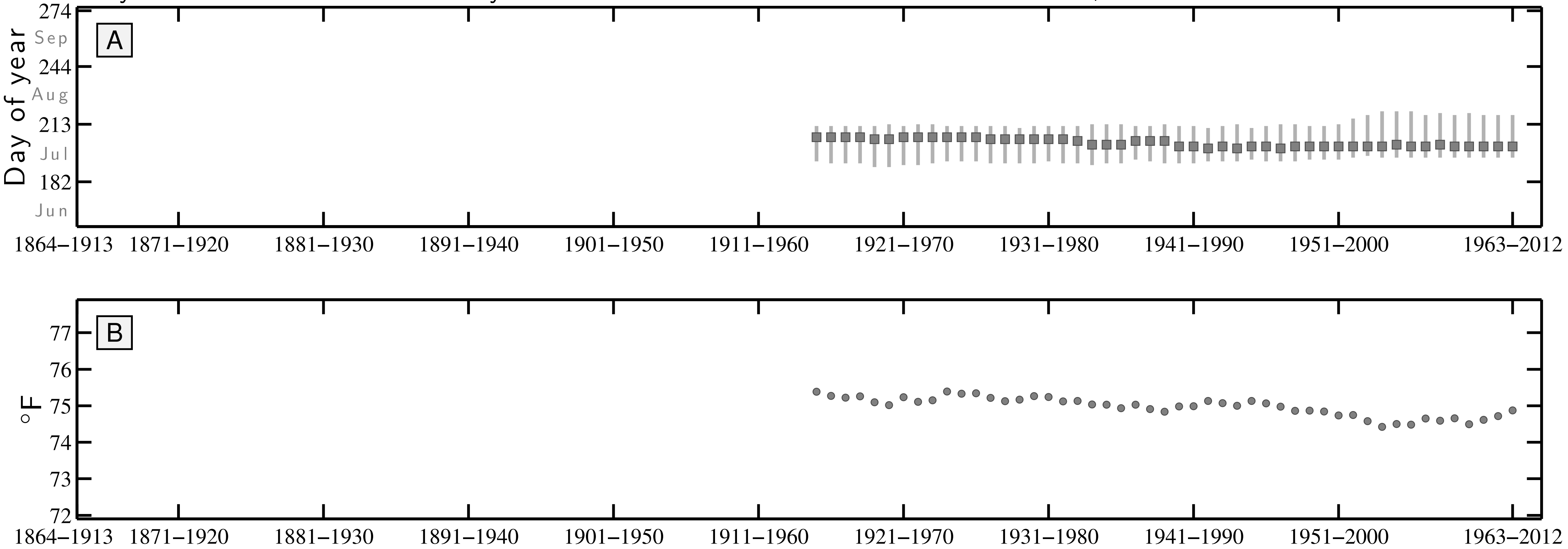

50 year Winter Teletherm dynamics for FIRST CONNECTICUT LAKE, NH:

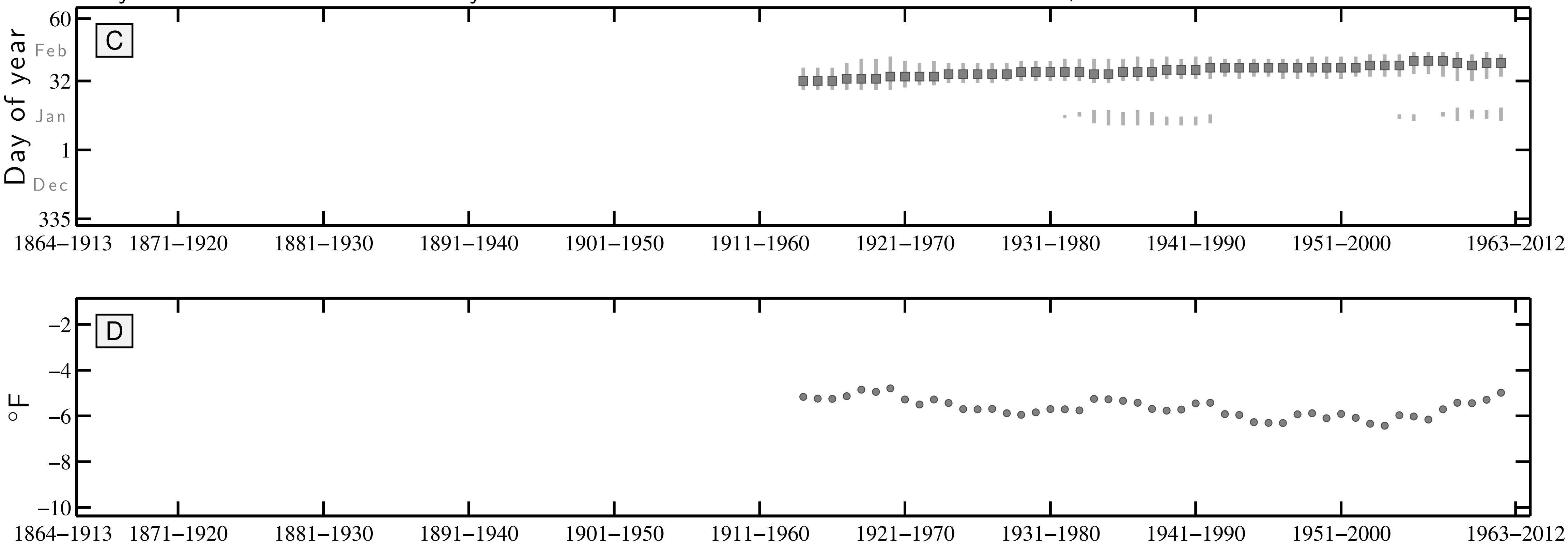

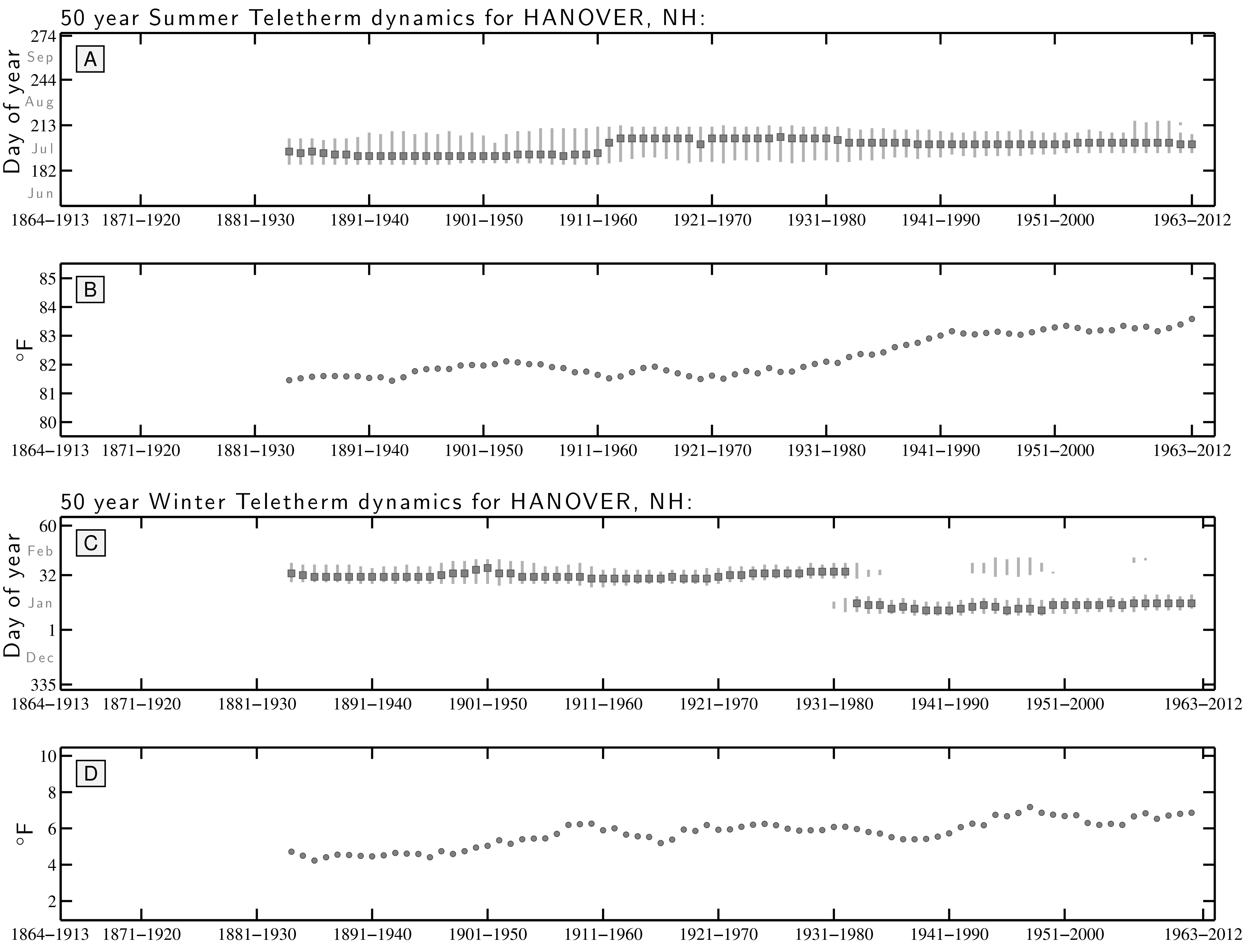

50 year Summer Teletherm dynamics for HANOVER, NH:
A
Day of year
274
Sep
244
Aug
213
Jul
182
Jun
1864–1913
1871–1920
1881–1930
1891–1940
1901–1950
1911–1960
1921–1970
1931–1980
1941–1990
1951–2000
1963–2012
B
°F
85
84
83
82
81
80
50 year Winter Teletherm dynamics for HANOVER, NH:
C
Day of year
60
Feb
32
Jan
1
Dec
335
D
°F
10
8
6
4
2

50 year Summer Teletherm dynamics for KEENE, NH:

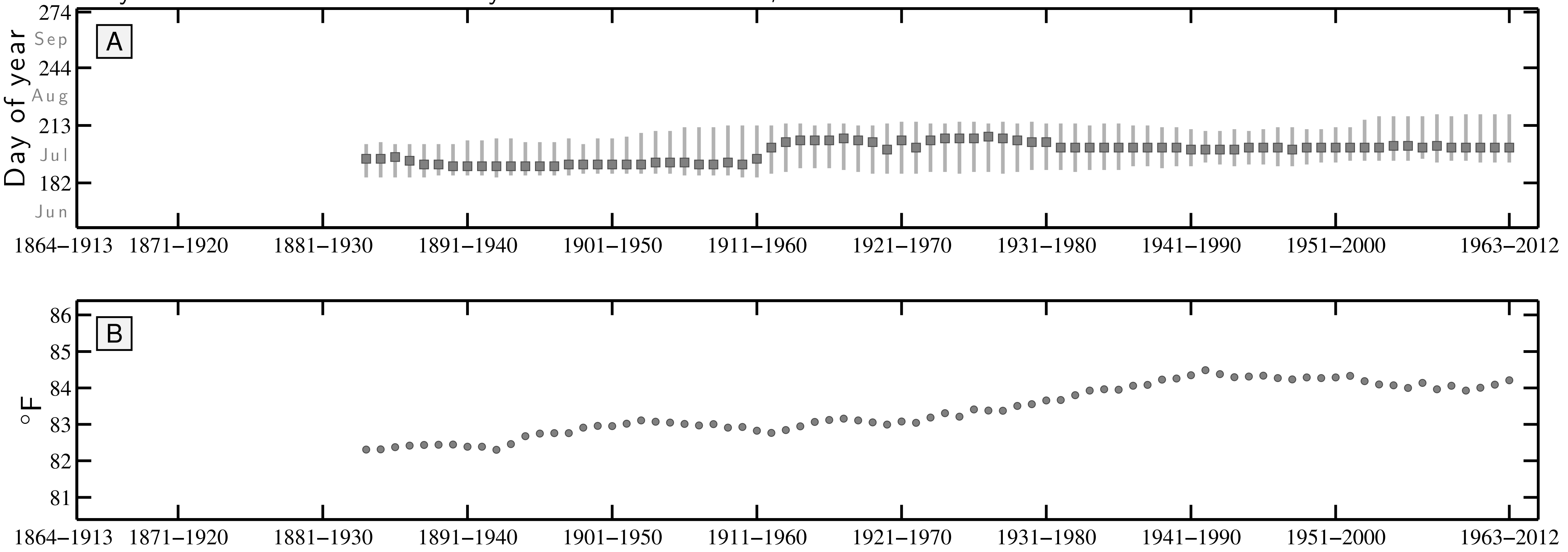

50 year Winter Teletherm dynamics for KEENE, NH:

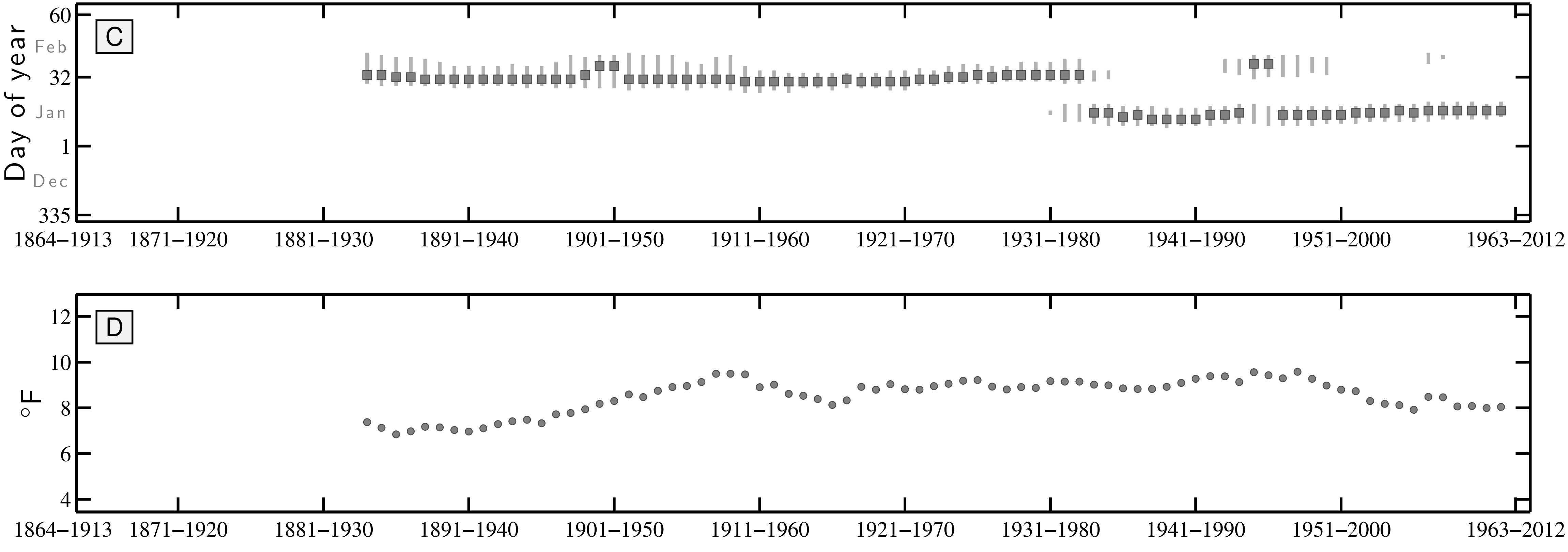

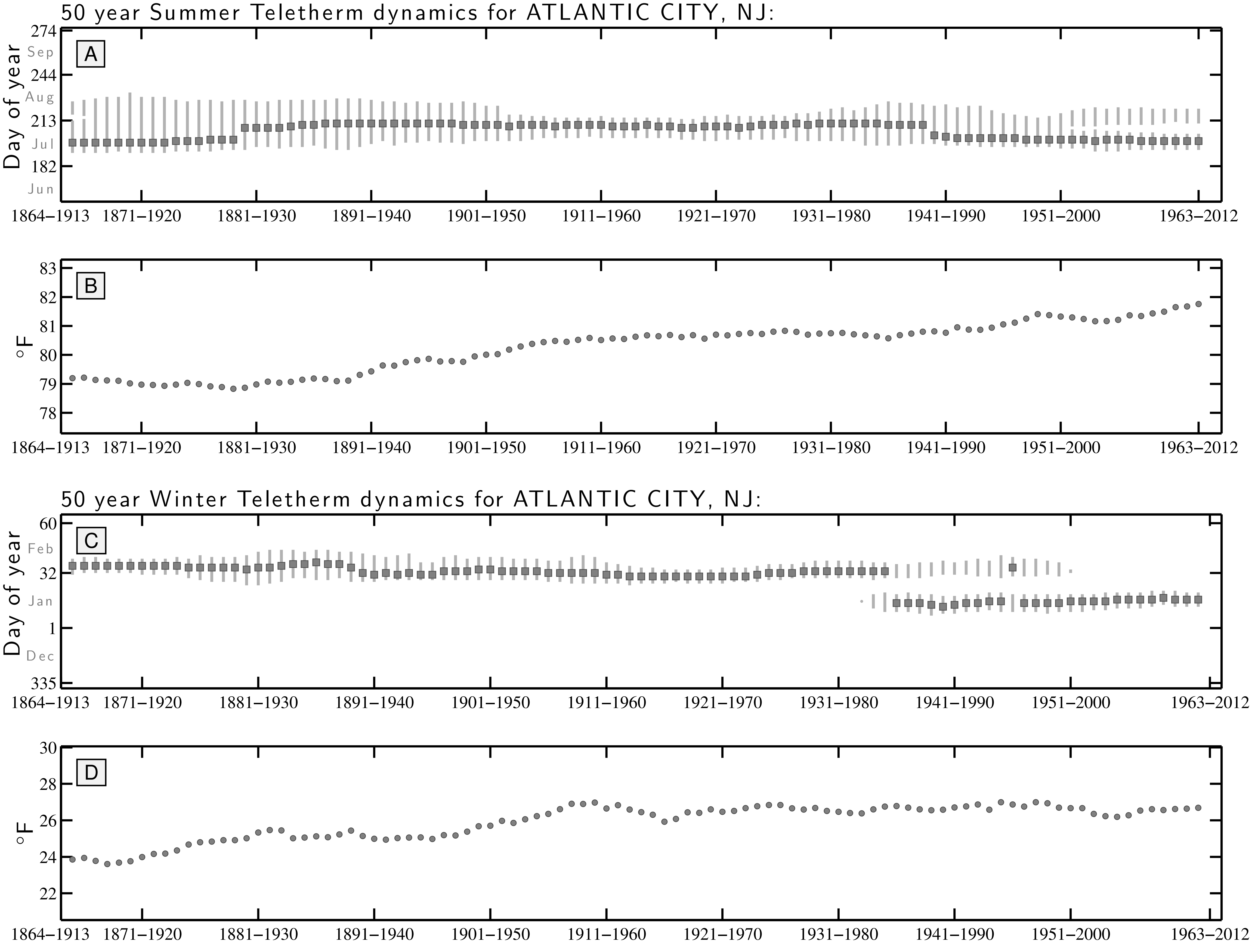
50 year Summer Teletherm dynamics for ATLANTIC CITY, NJ:
A
Day of year
274
Sep
244
Aug
213
Jul
182
Jun
B
°F
83
82
81
80
79
78
50 year Winter Teletherm dynamics for ATLANTIC CITY, NJ:
C
Day of year
60
Feb
32
Jan
1
Dec
335
D
°F
30
28
26
24
22
1864–1913
1871–1920
1881–1930
1891–1940
1901–1950
1911–1960
1921–1970
1931–1980
1941–1990
1951–2000
1963–2012

50 year Summer Teletherm dynamics for BOONTON 1 SE, NJ:

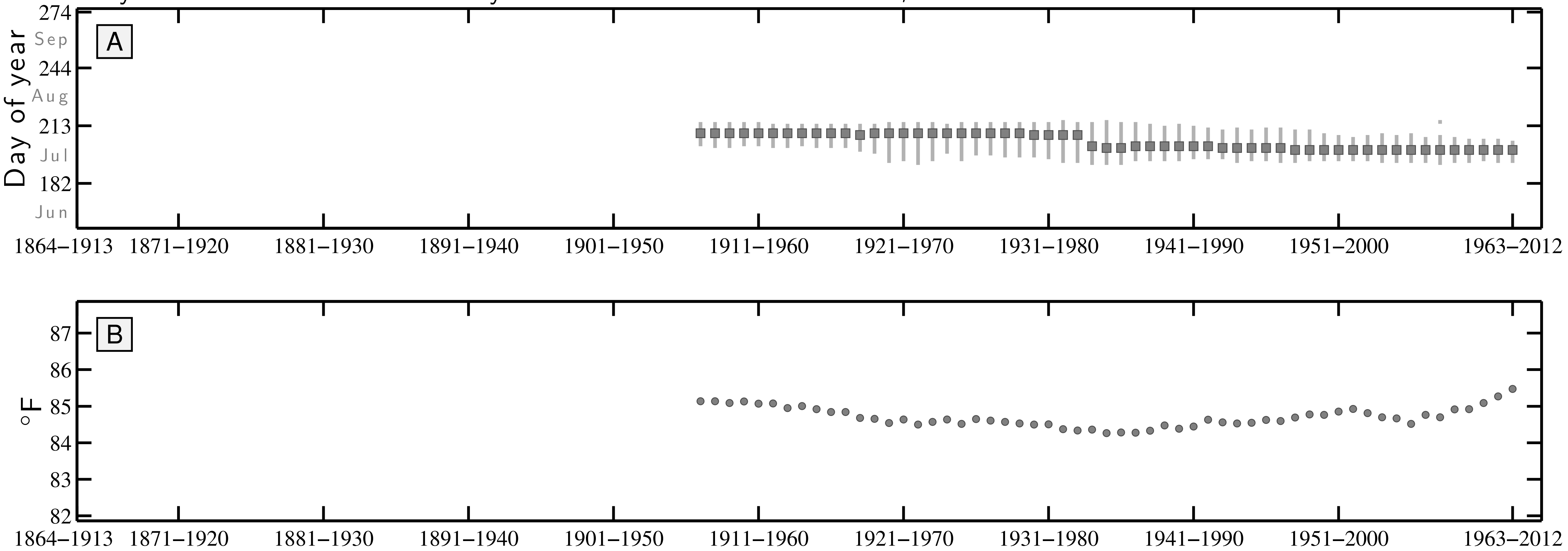

50 year Winter Teletherm dynamics for BOONTON 1 SE, NJ:

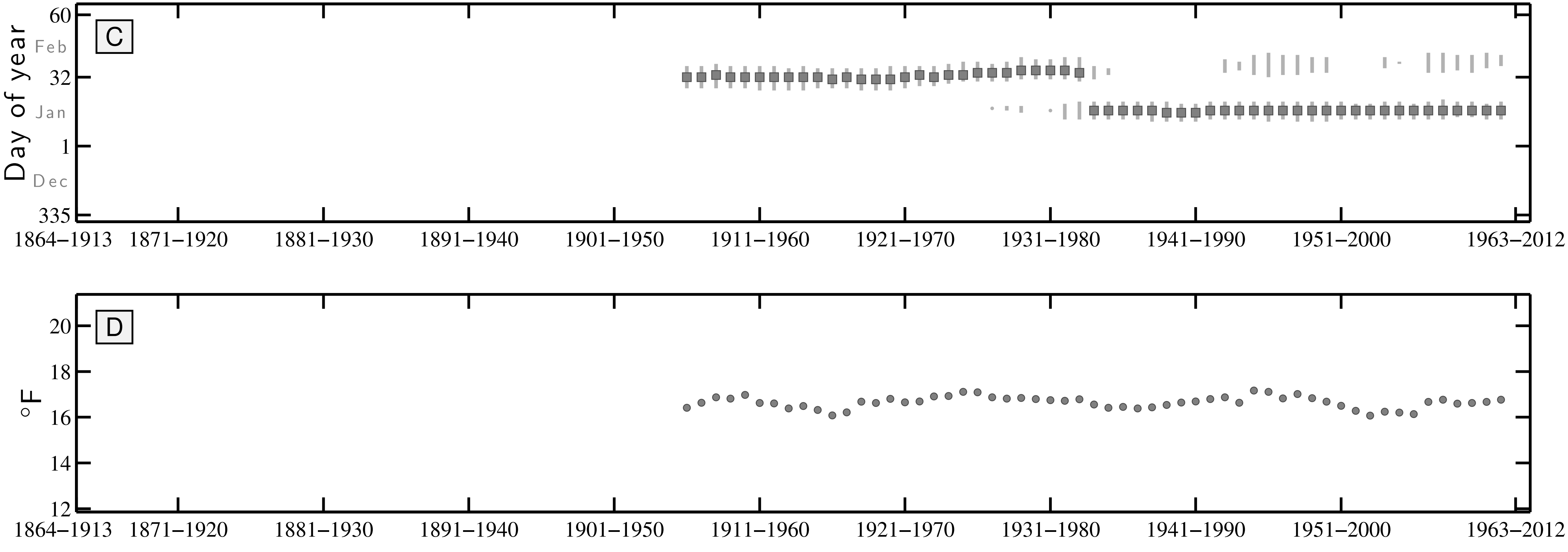

50 year Summer Teletherm dynamics for CHARLOTTEBURG RSVR, NJ:

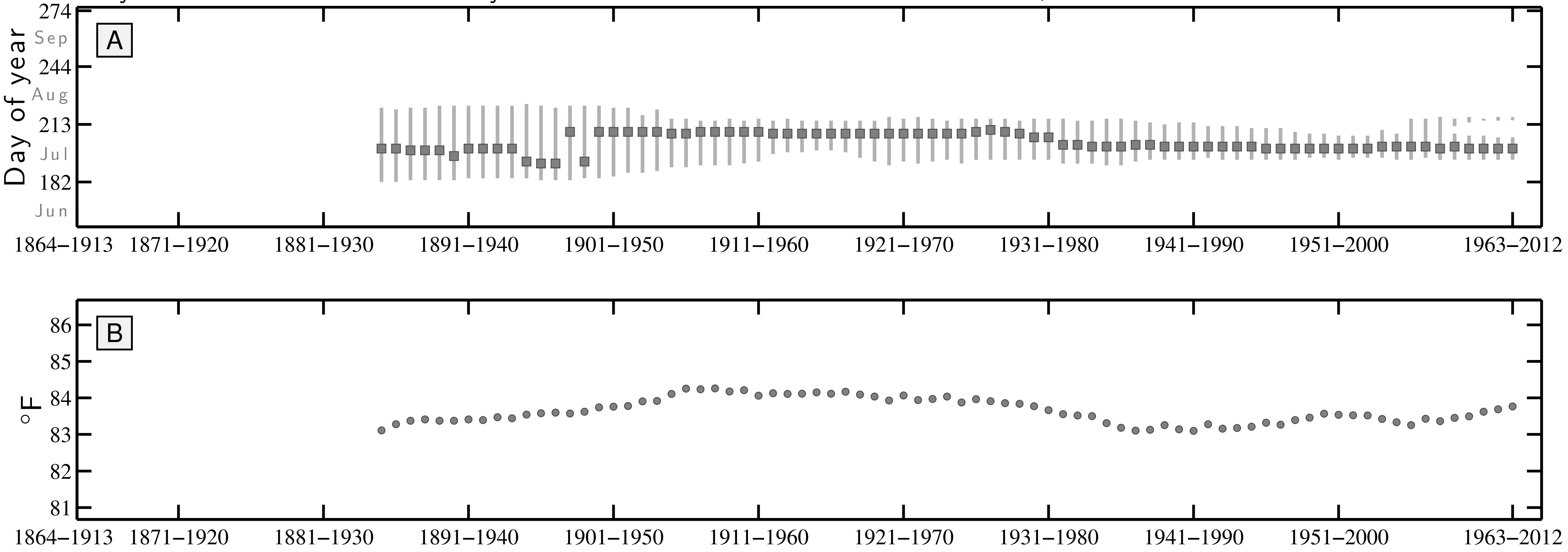

50 year Winter Teletherm dynamics for CHARLOTTEBURG RSVR, NJ:

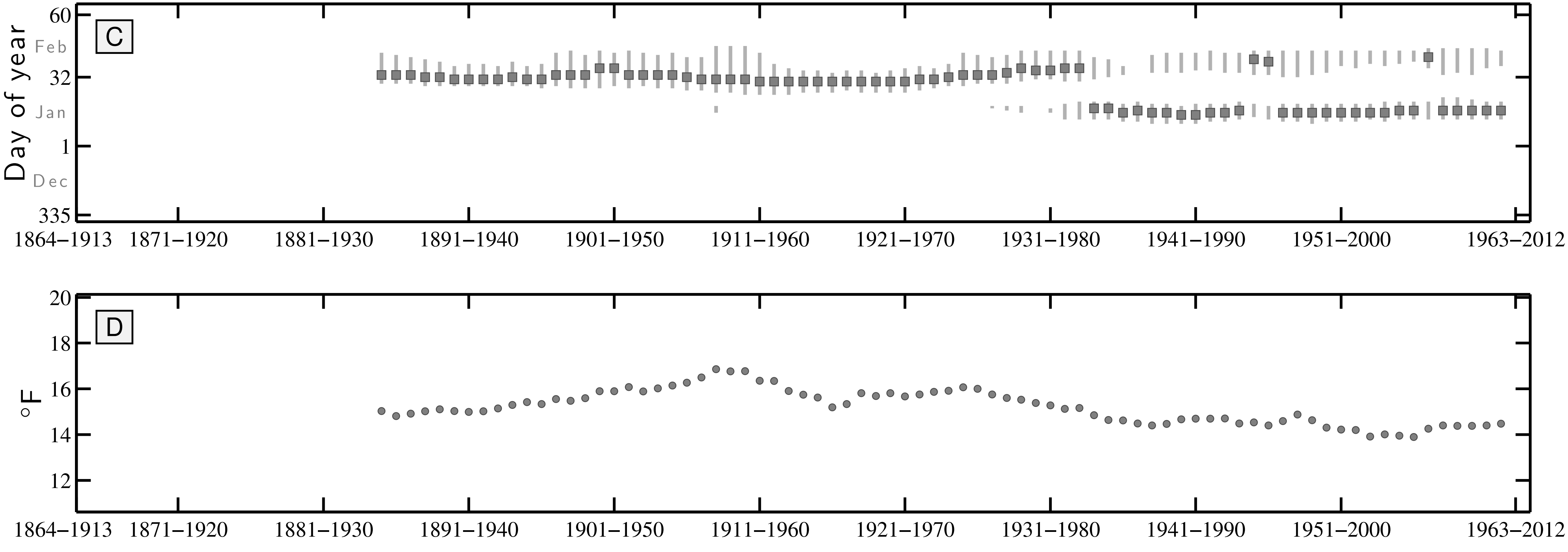

50 year Summer Teletherm dynamics for FLEMINGTON 5 NNW, NJ:

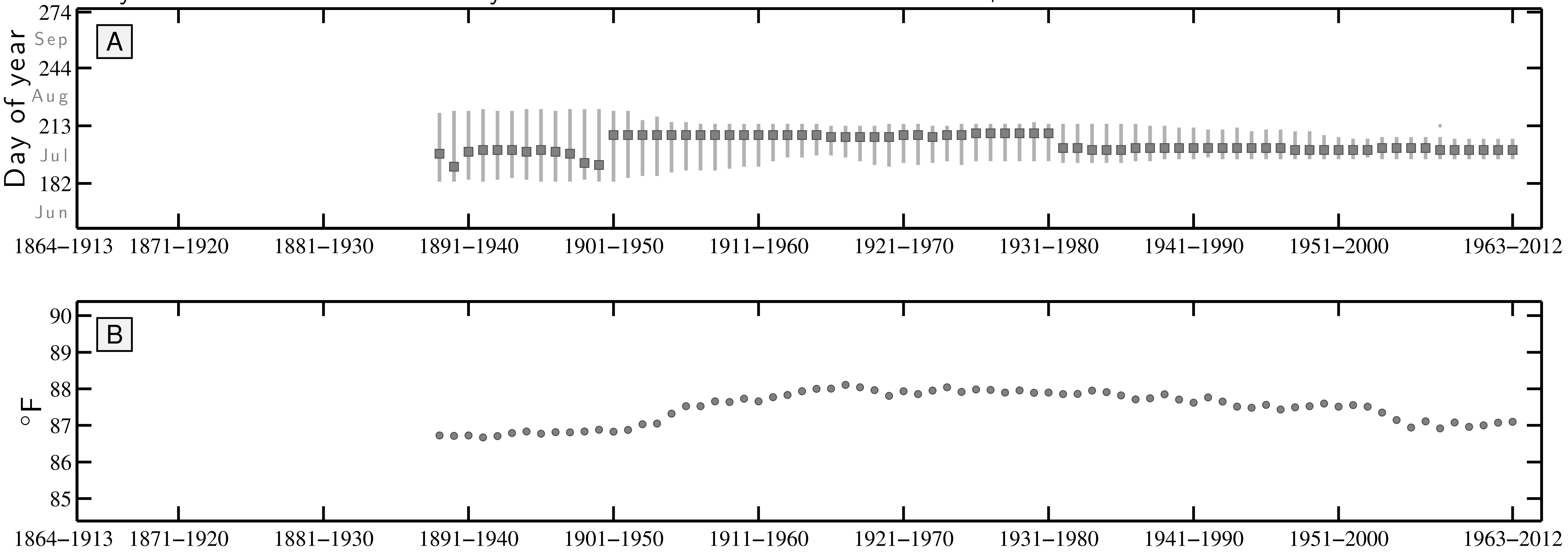

50 year Winter Teletherm dynamics for FLEMINGTON 5 NNW, NJ:

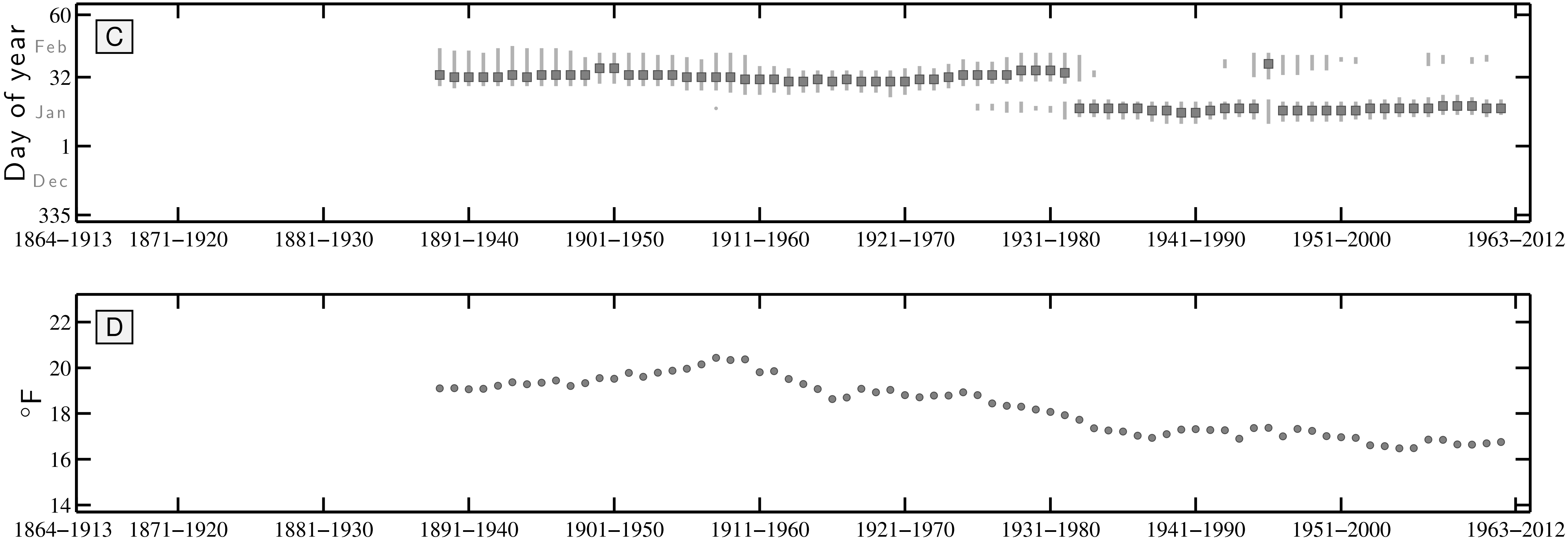

50 year Summer Teletherm dynamics for HIGHTSTOWN 2 W, NJ:

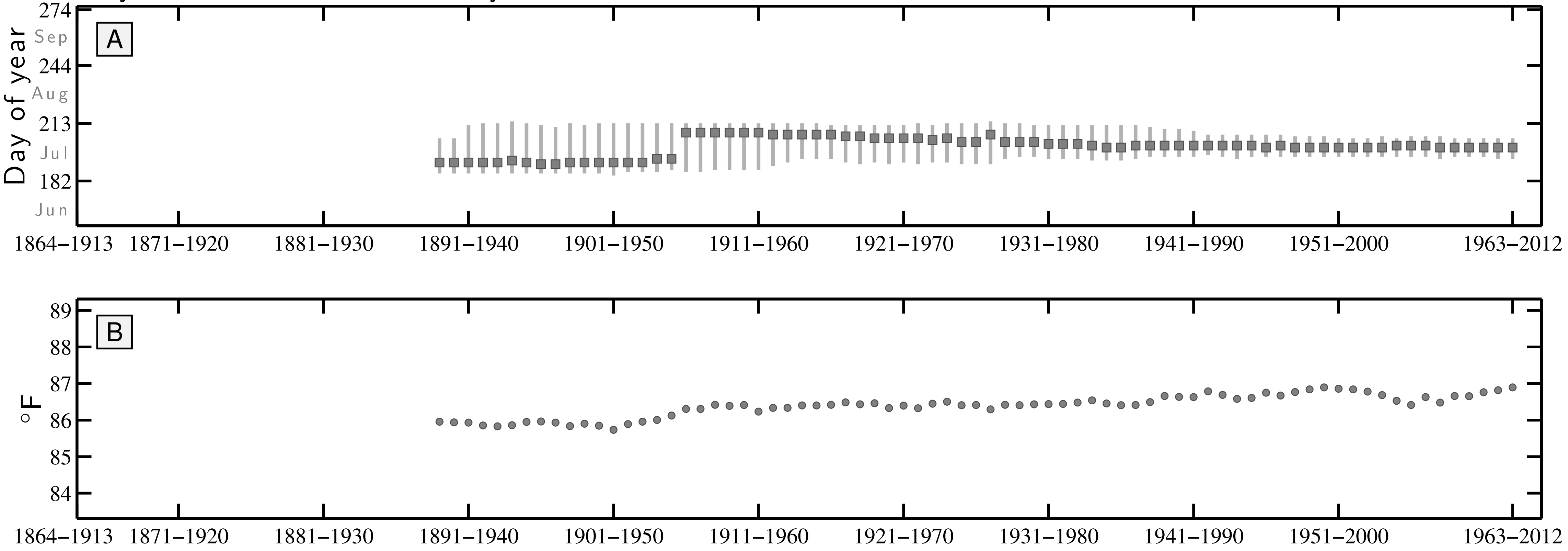

50 year Winter Teletherm dynamics for HIGHTSTOWN 2 W, NJ:

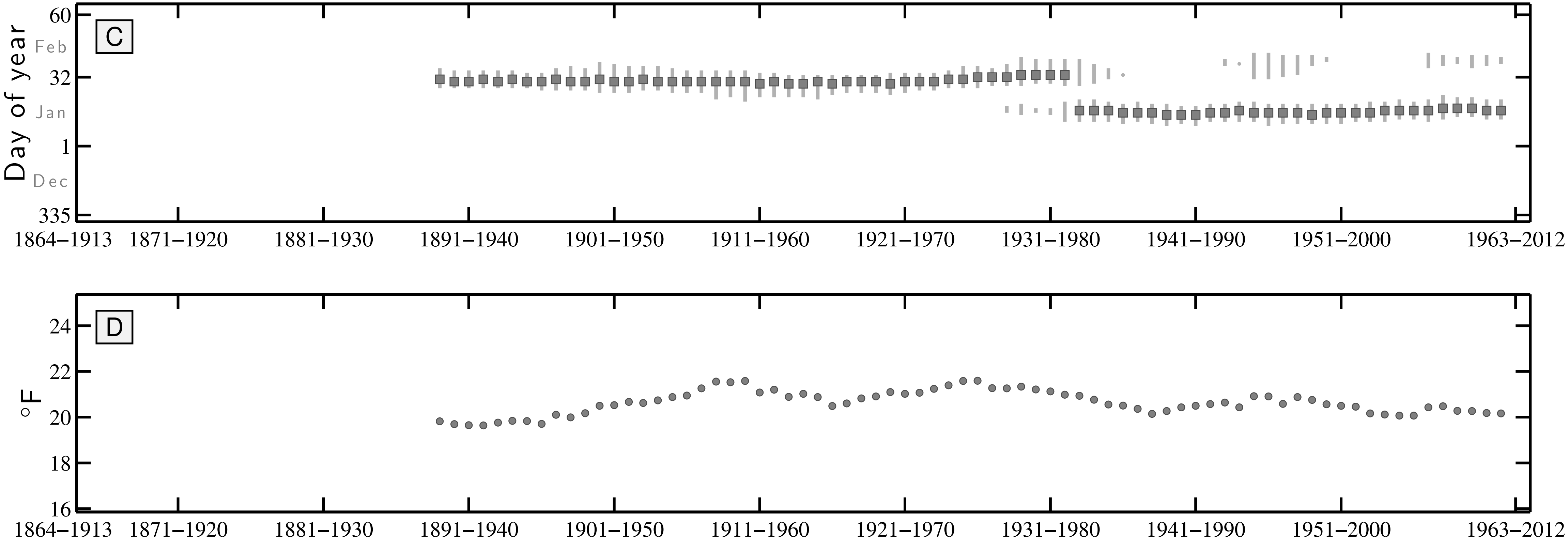

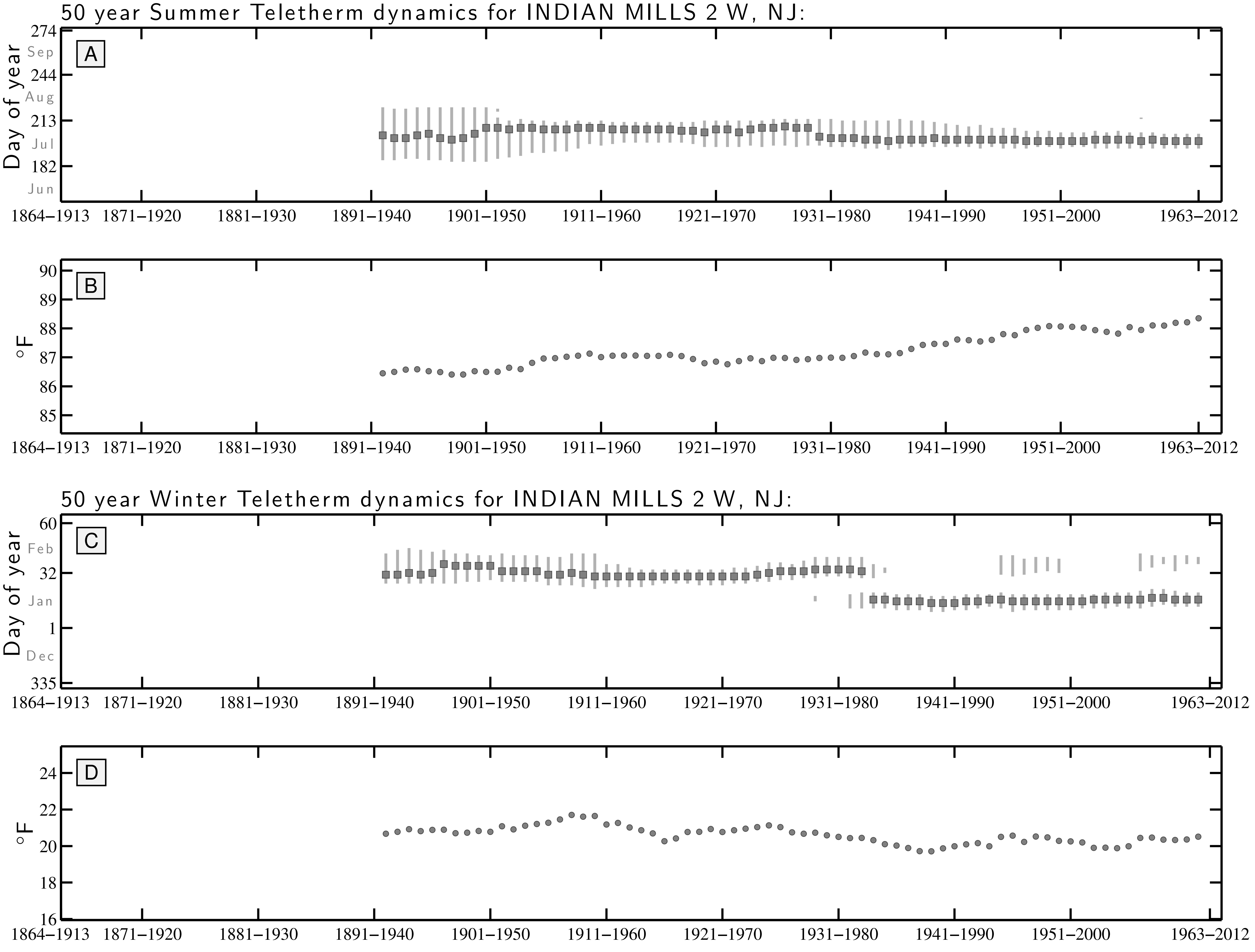

50 year Summer Teletherm dynamics for INDIAN MILLS 2 W, NJ:
A
Day of year
274
Sep
244
Aug
213
Jul
182
Jun
1864–1913
1871–1920
1881–1930
1891–1940
1901–1950
1911–1960
1921–1970
1931–1980
1941–1990
1951–2000
1963–2012
B
°F
90
89
88
87
86
85
50 year Winter Teletherm dynamics for INDIAN MILLS 2 W, NJ:
C
60
Feb
32
Jan
1
Dec
335
D
24
22
20
18
16

50 year Summer Teletherm dynamics for LONG BRANCH OAKHURST, NJ:

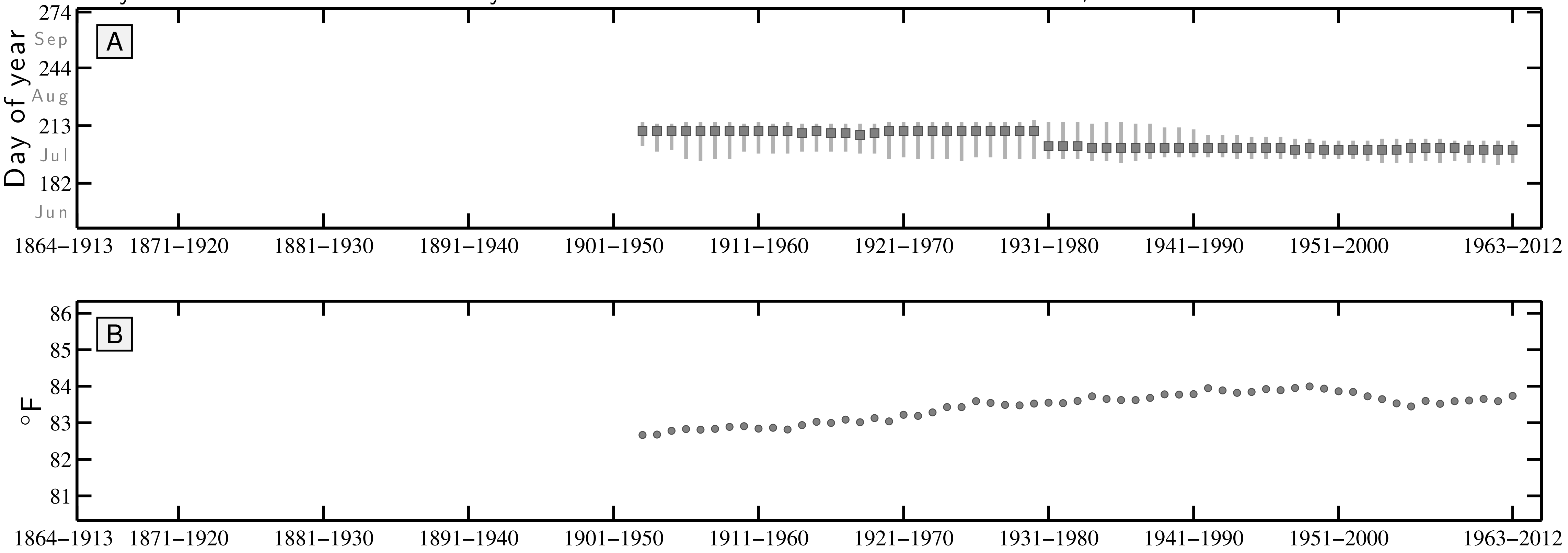

50 year Winter Teletherm dynamics for LONG BRANCH OAKHURST, NJ:

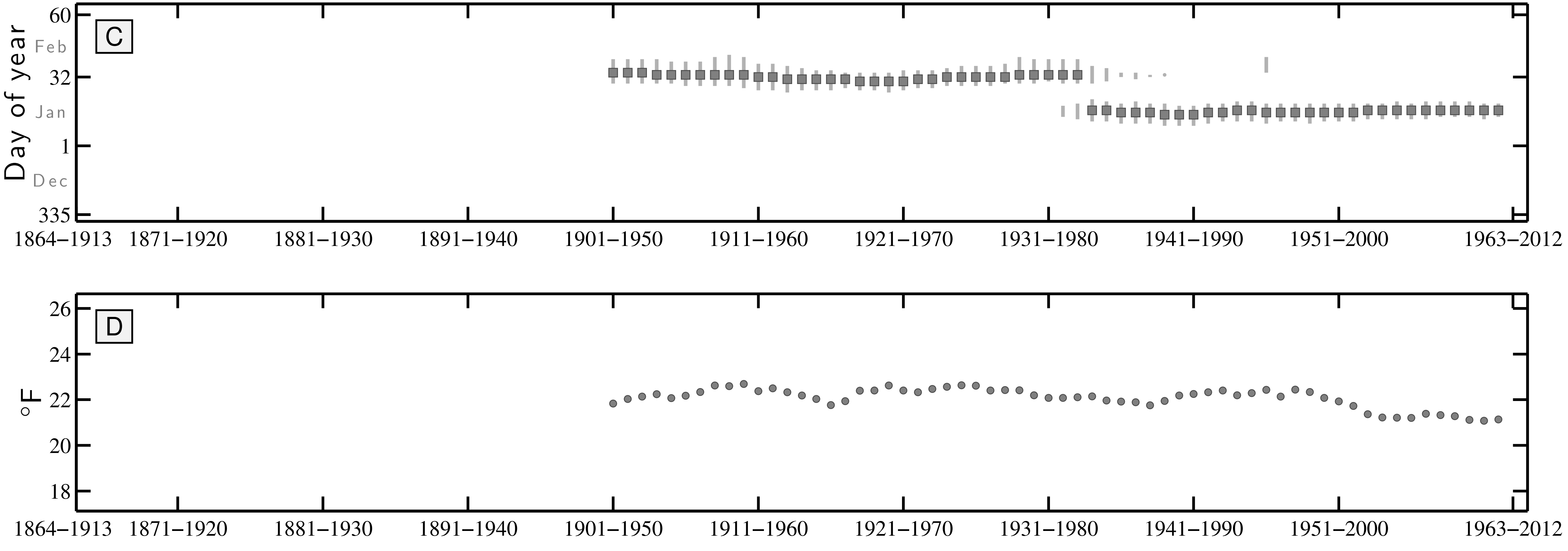

50 year Summer Teletherm dynamics for MOORESTOWN, NJ:

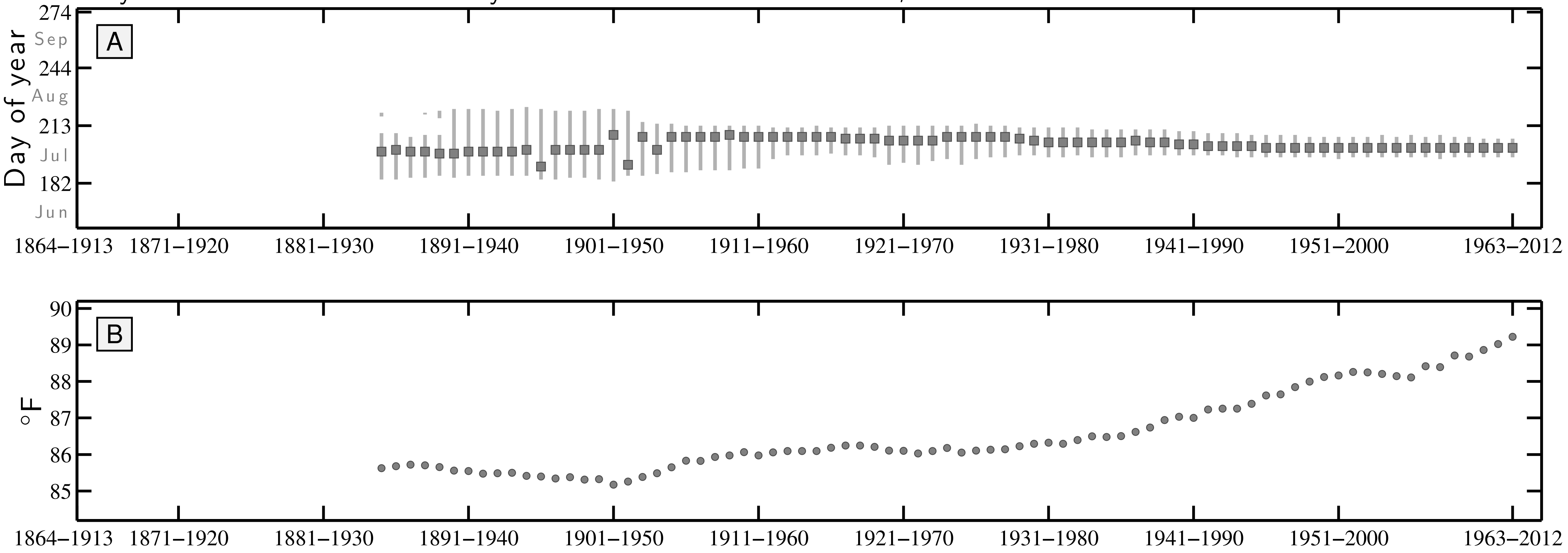

50 year Winter Teletherm dynamics for MOORESTOWN, NJ:

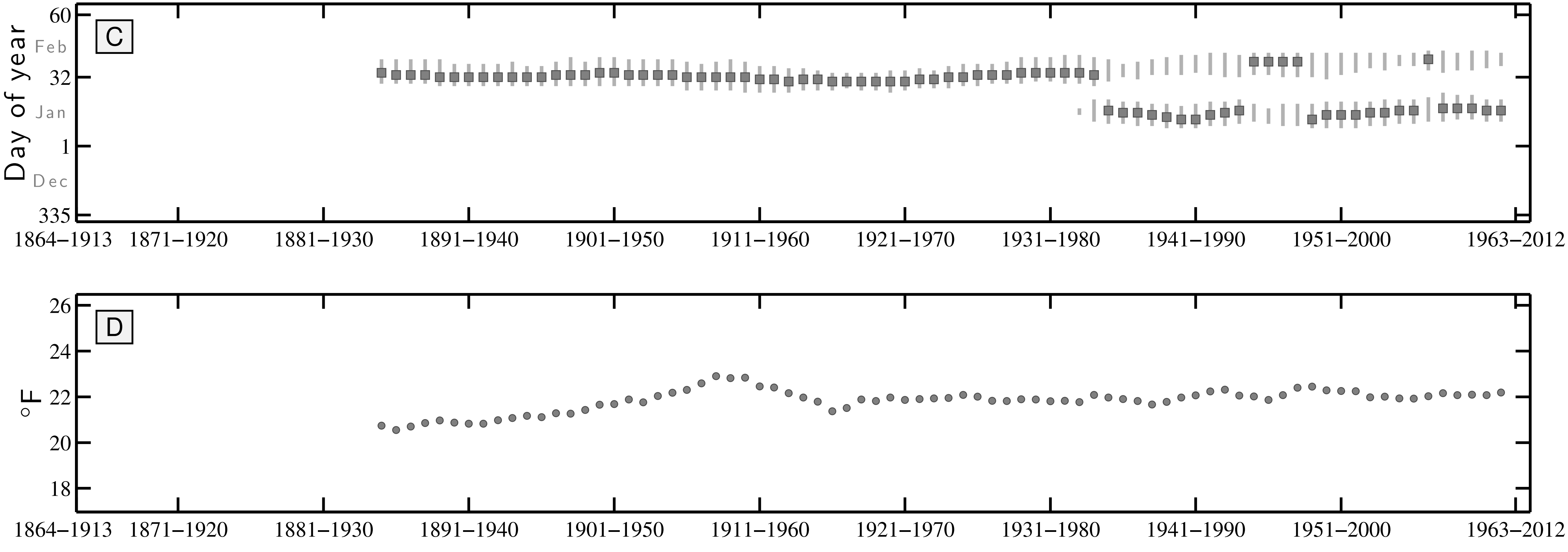

50 year Summer Teletherm dynamics for NEW BRUNSWICK 3 SE, NJ:

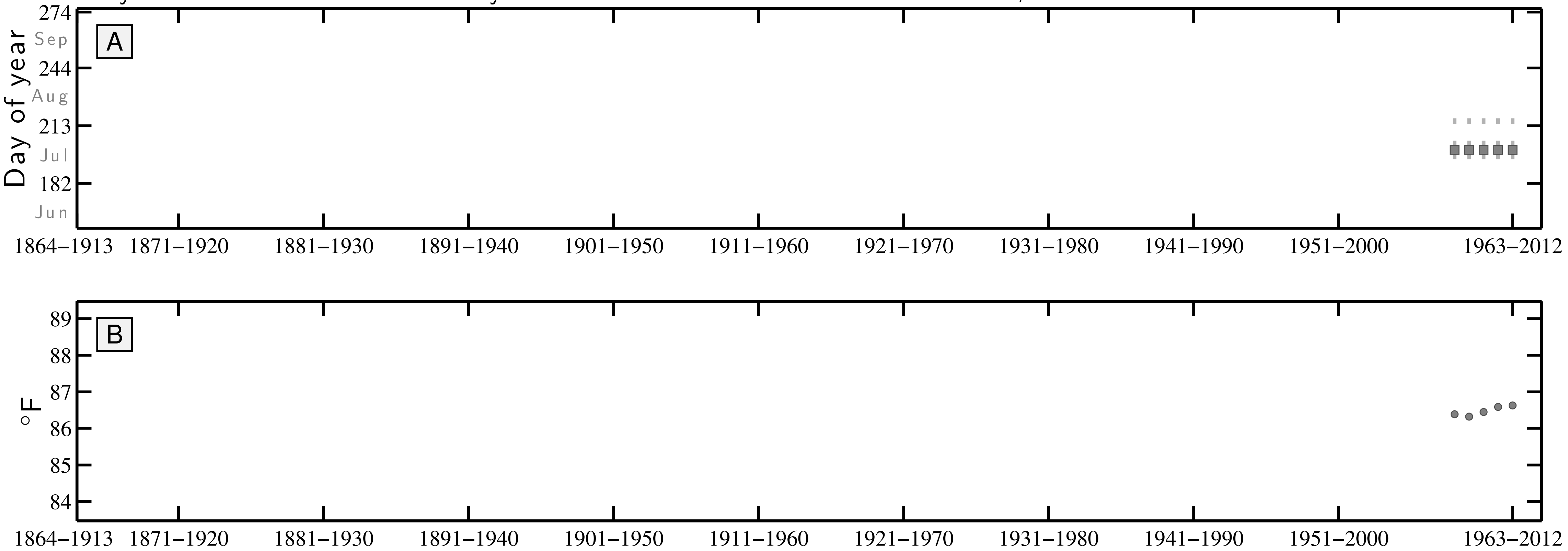

50 year Winter Teletherm dynamics for NEW BRUNSWICK 3 SE, NJ:

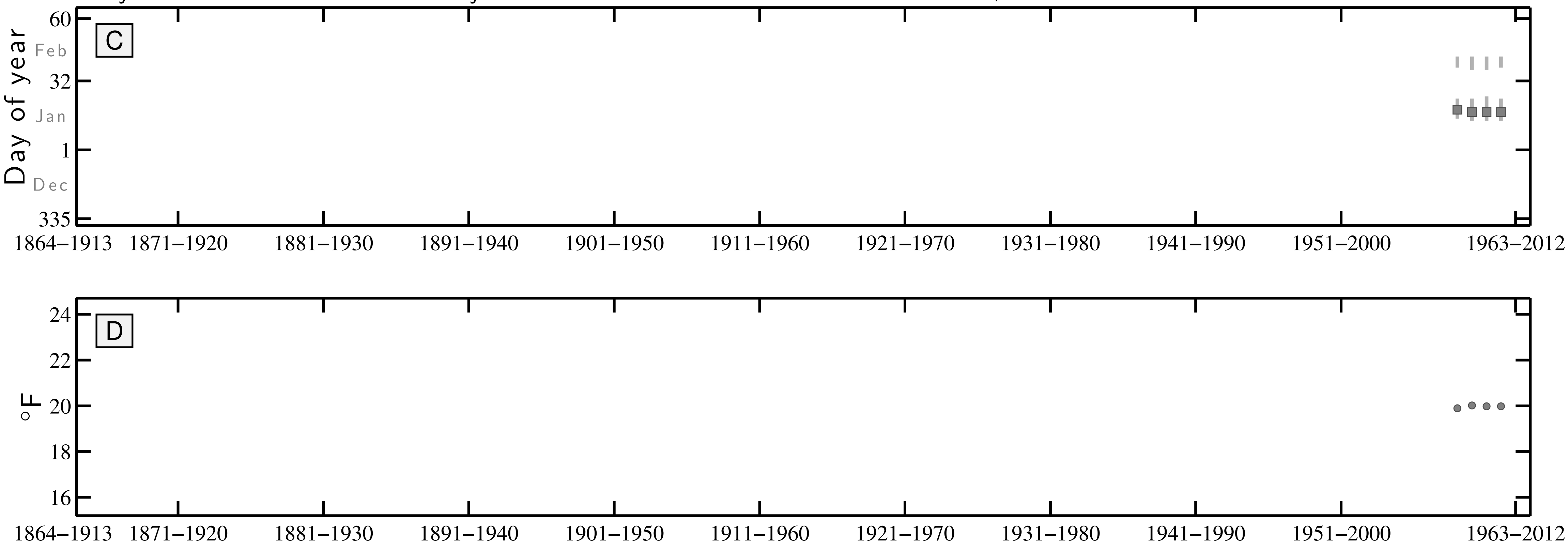

50 year Summer Teletherm dynamics for PLAINFIELD, NJ:

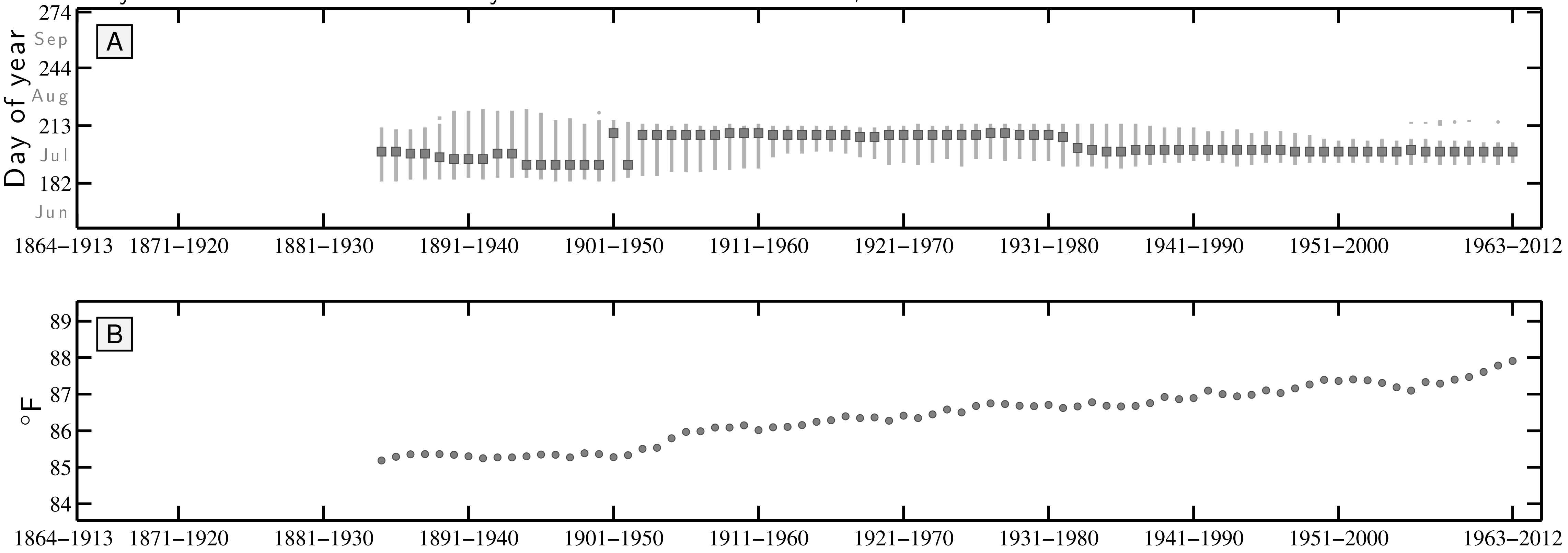

50 year Winter Teletherm dynamics for PLAINFIELD, NJ:

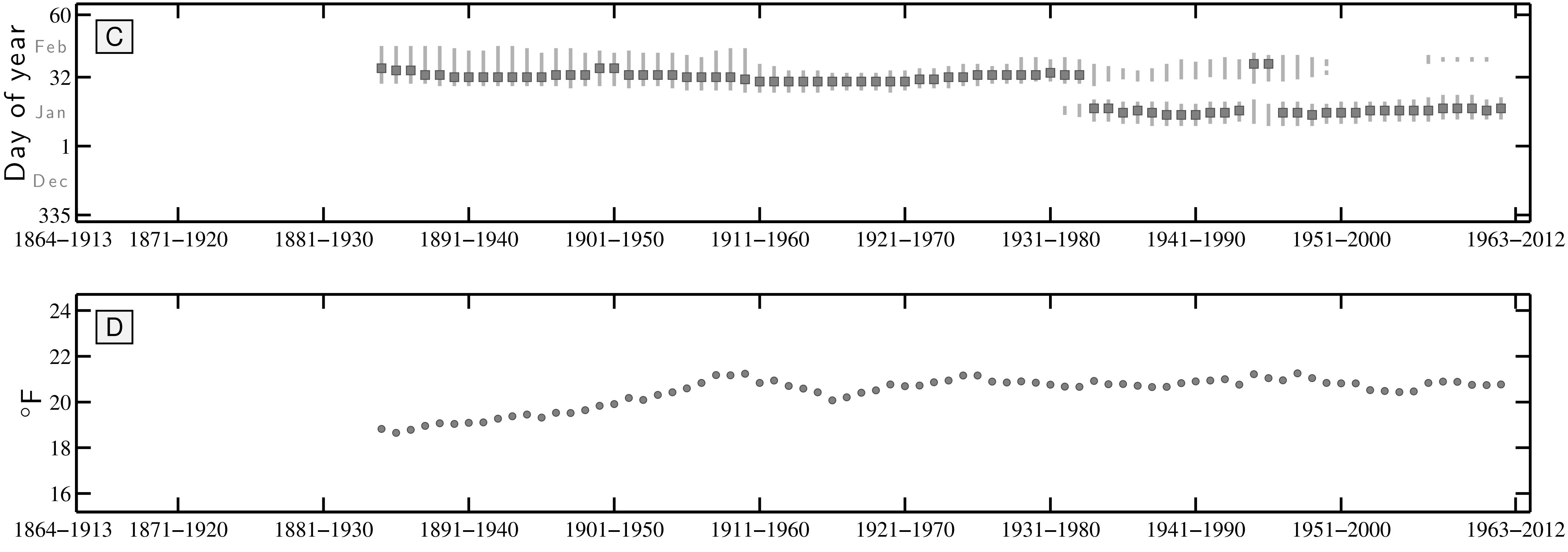

50 year Summer Teletherm dynamics for TOMS RIVER, NJ:

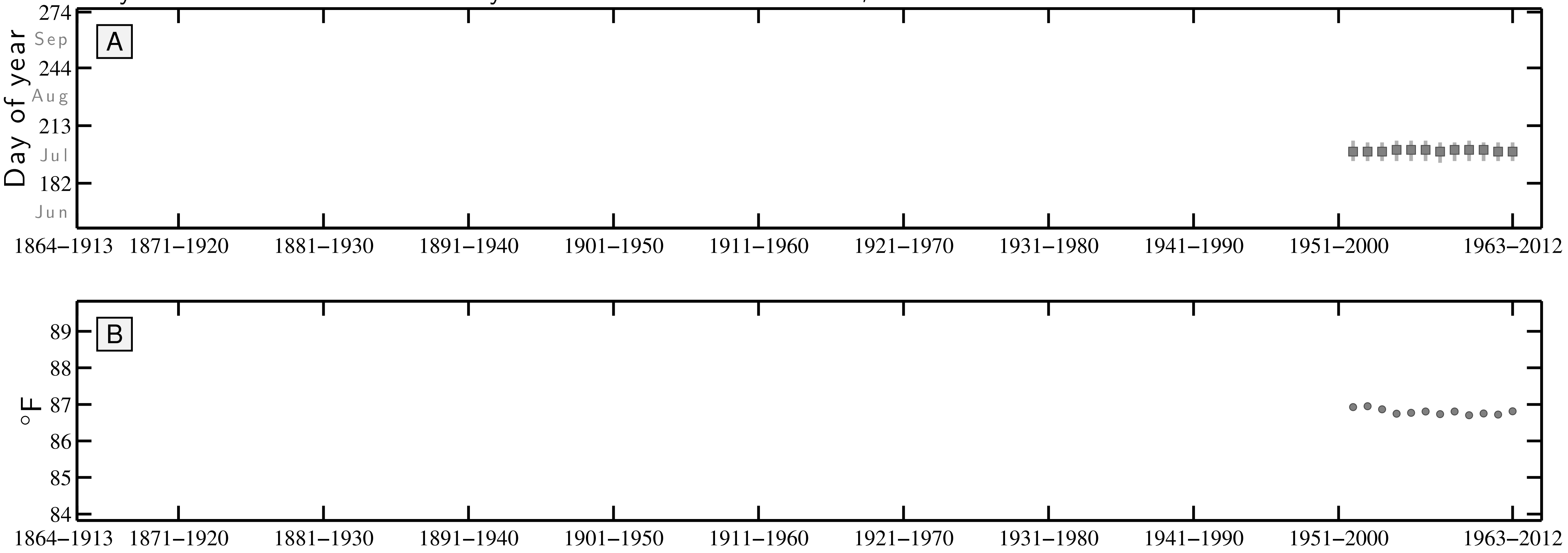

50 year Winter Teletherm dynamics for TOMS RIVER, NJ:

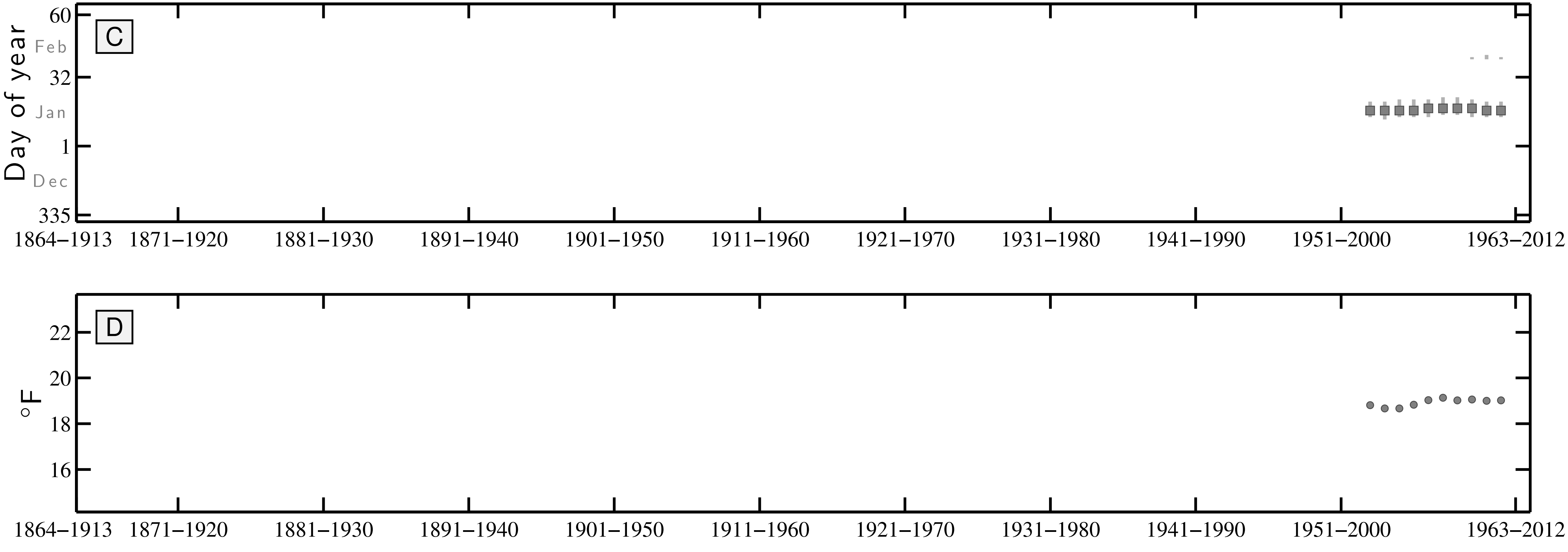

50 year Summer Teletherm dynamics for AZTEC RUINS NM, NM:

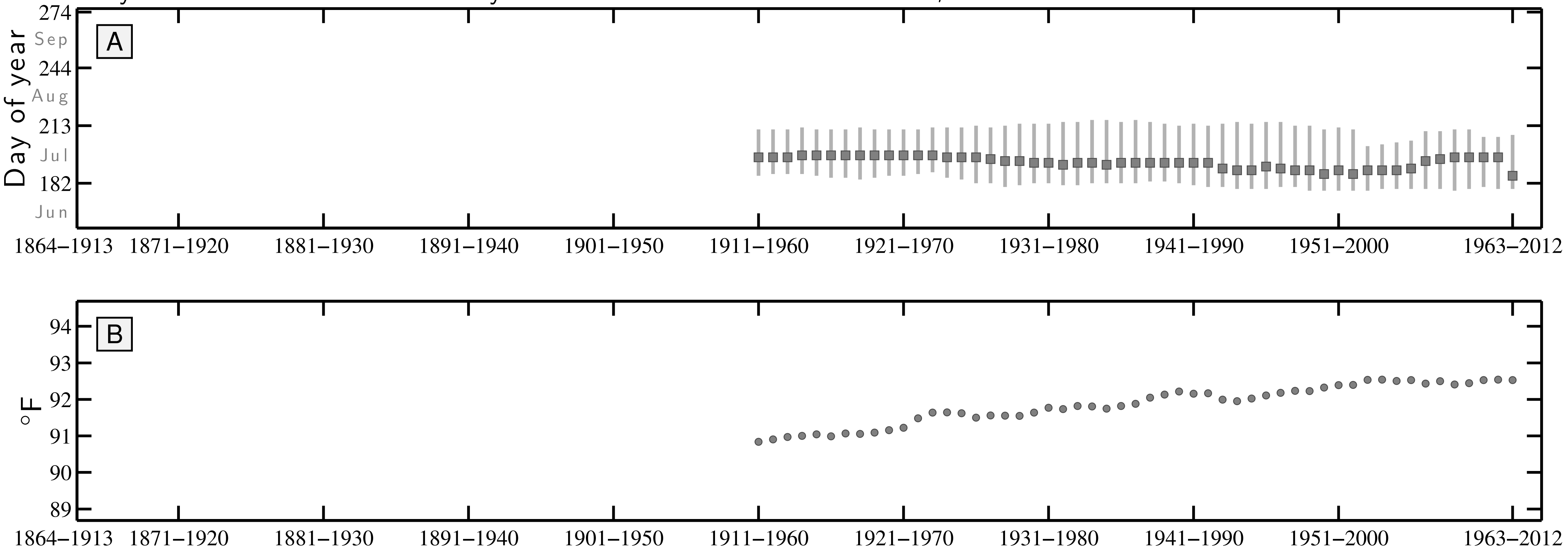

50 year Winter Teletherm dynamics for AZTEC RUINS NM, NM:

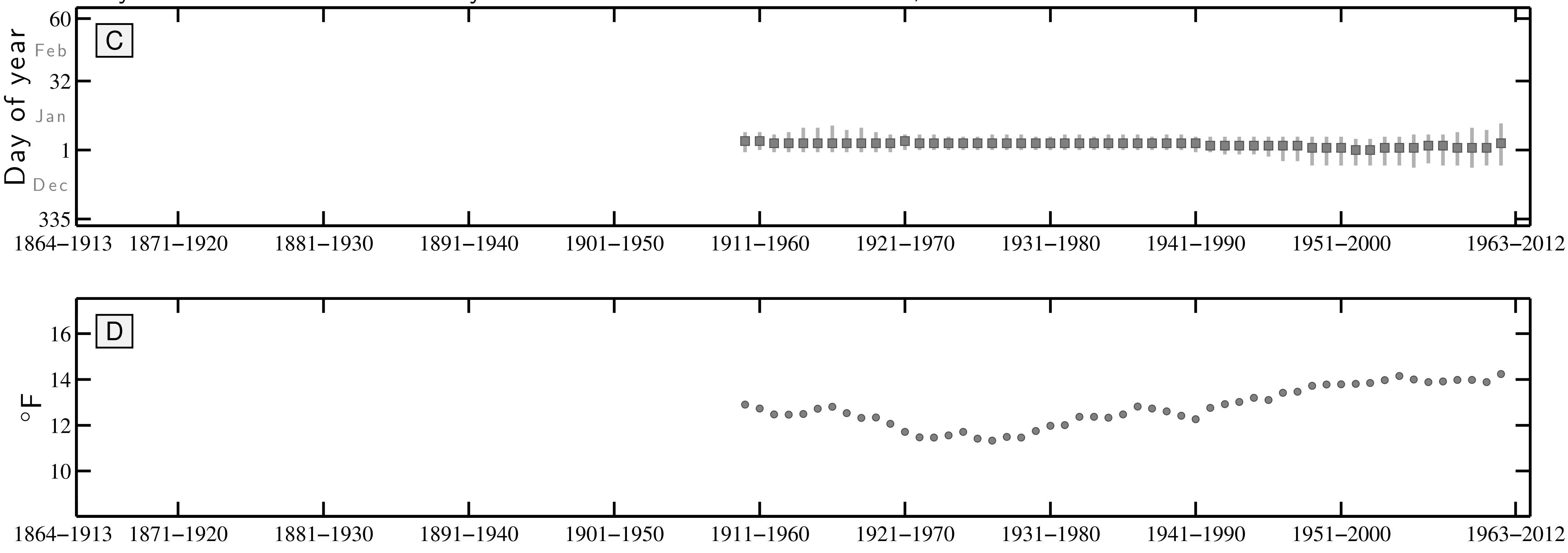

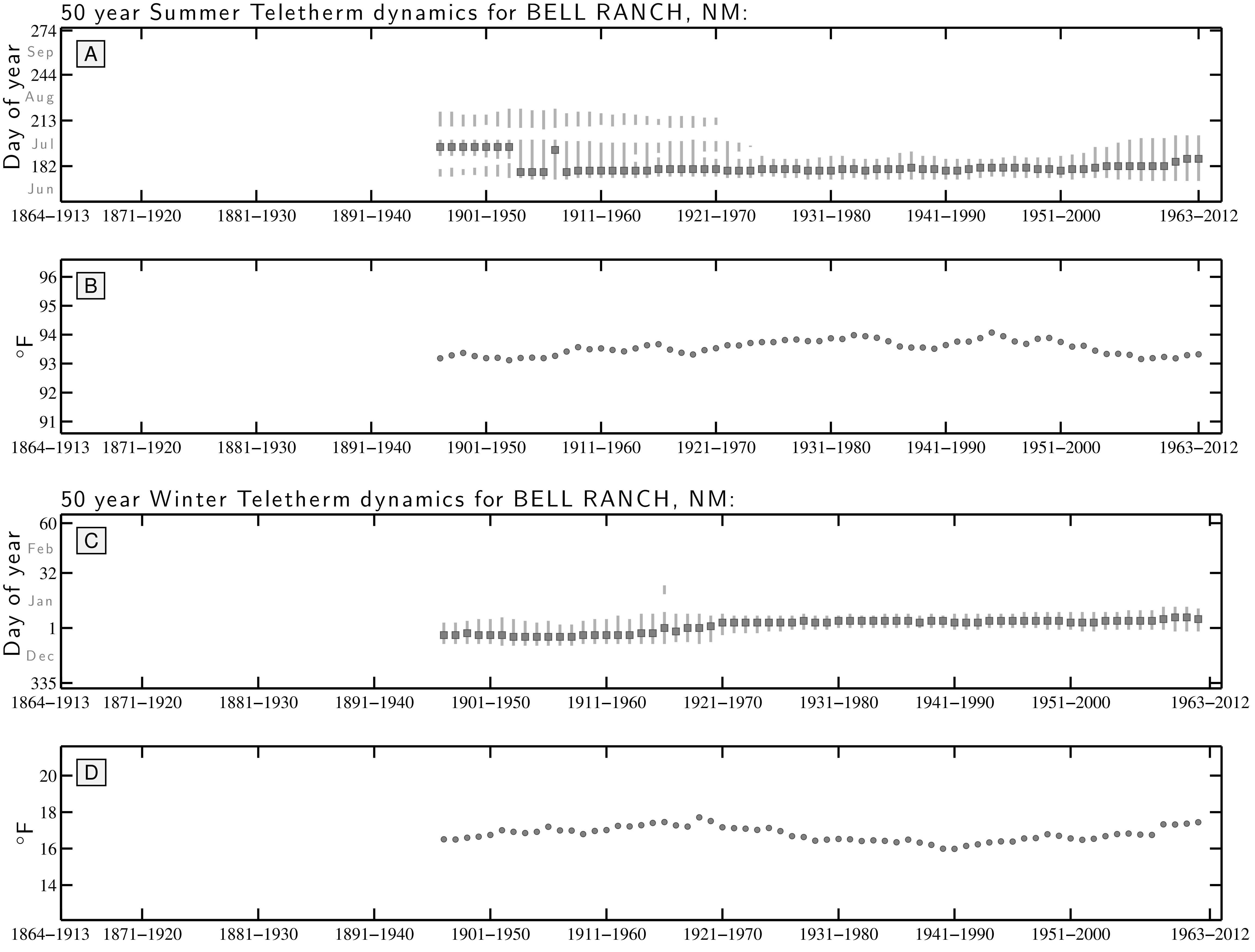
50 year Summer Teletherm dynamics for BELL RANCH, NM:
A
Day of year
274
Sep
244
Aug
213
Jul
182
Jun
1864–1913
1871–1920
1881–1930
1891–1940
1901–1950
1911–1960
1921–1970
1931–1980
1941–1990
1951–2000
1963–2012
B
°F
96
95
94
93
92
91
50 year Winter Teletherm dynamics for BELL RANCH, NM:
C
Day of year
60
Feb
32
Jan
1
Dec
335
D
°F
20
18
16
14

50 year Summer Teletherm dynamics for CARLSBAD, NM:

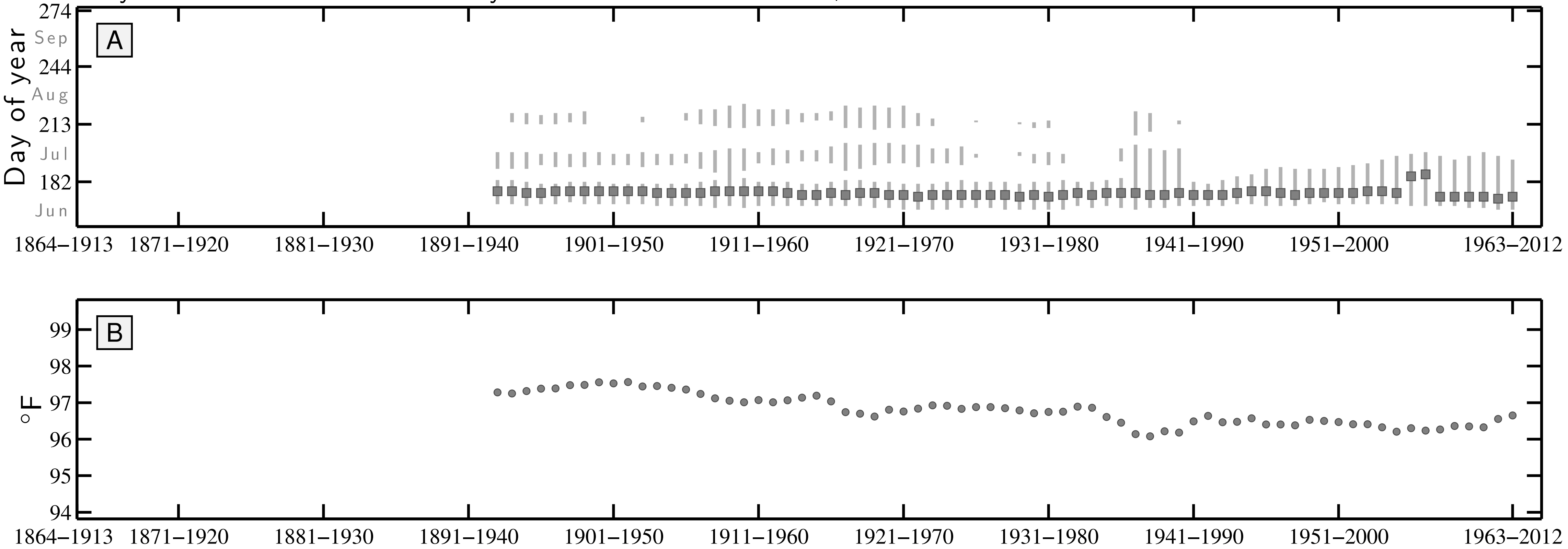

50 year Winter Teletherm dynamics for CARLSBAD, NM:

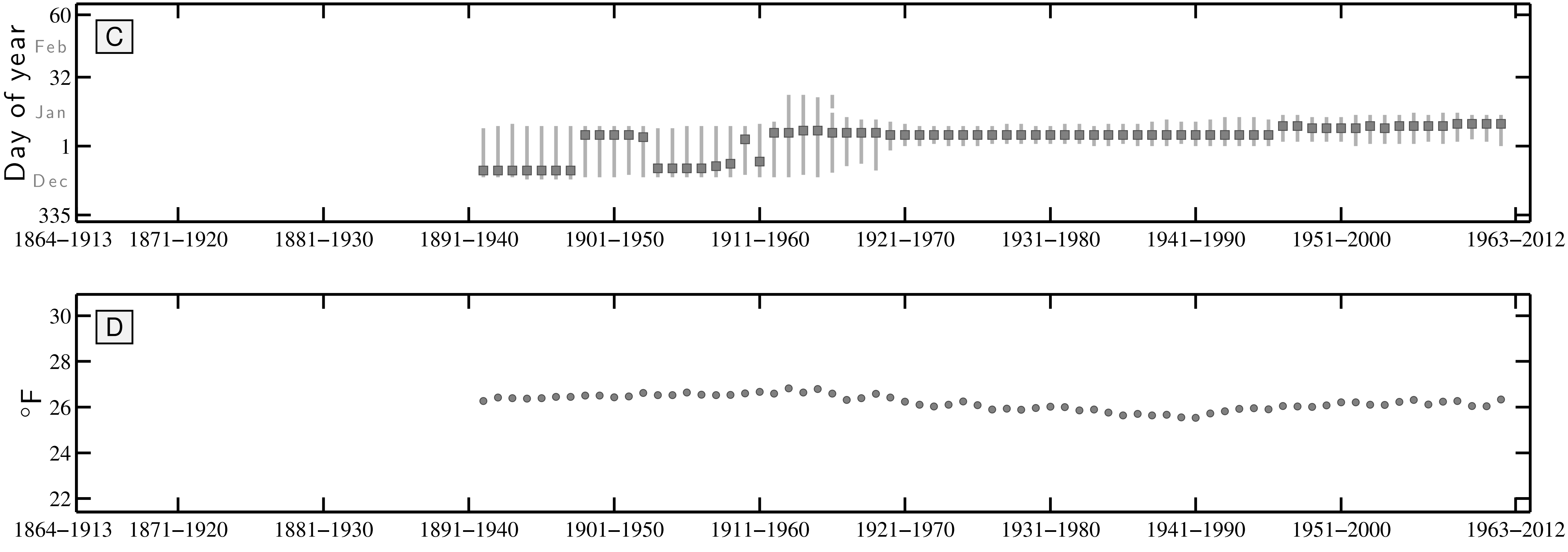

50 year Summer Teletherm dynamics for CARRIZOZO 1SW, NM:

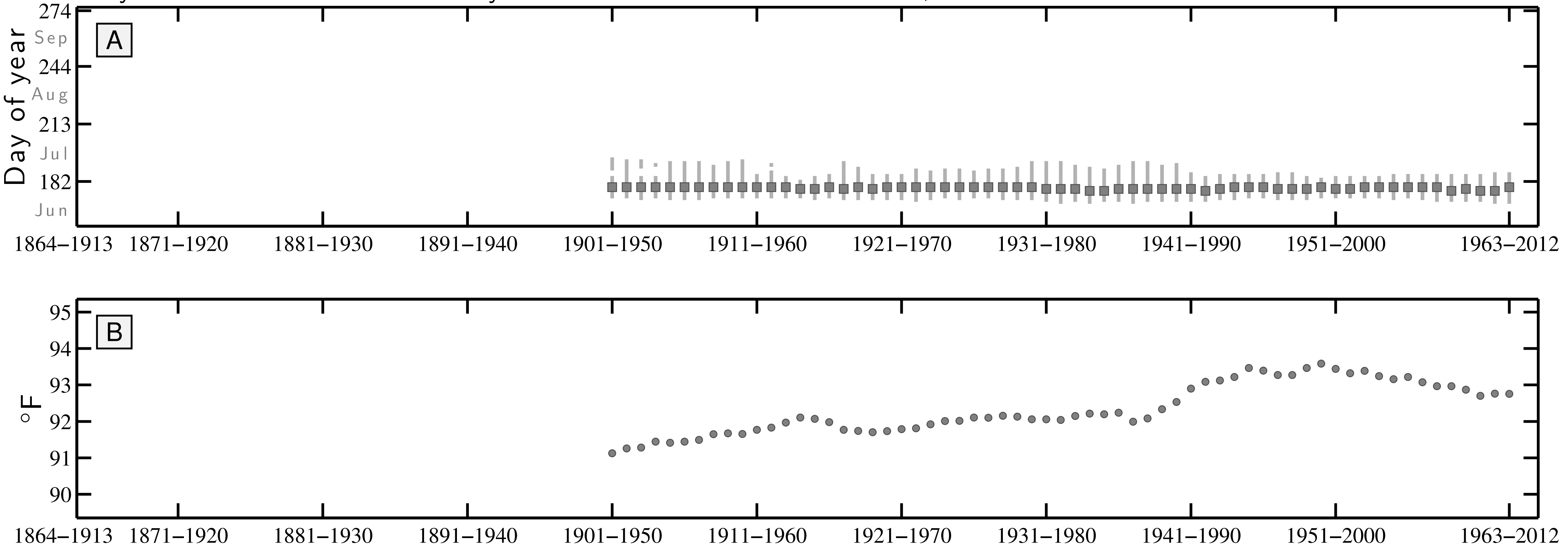

50 year Winter Teletherm dynamics for CARRIZOZO 1SW, NM:

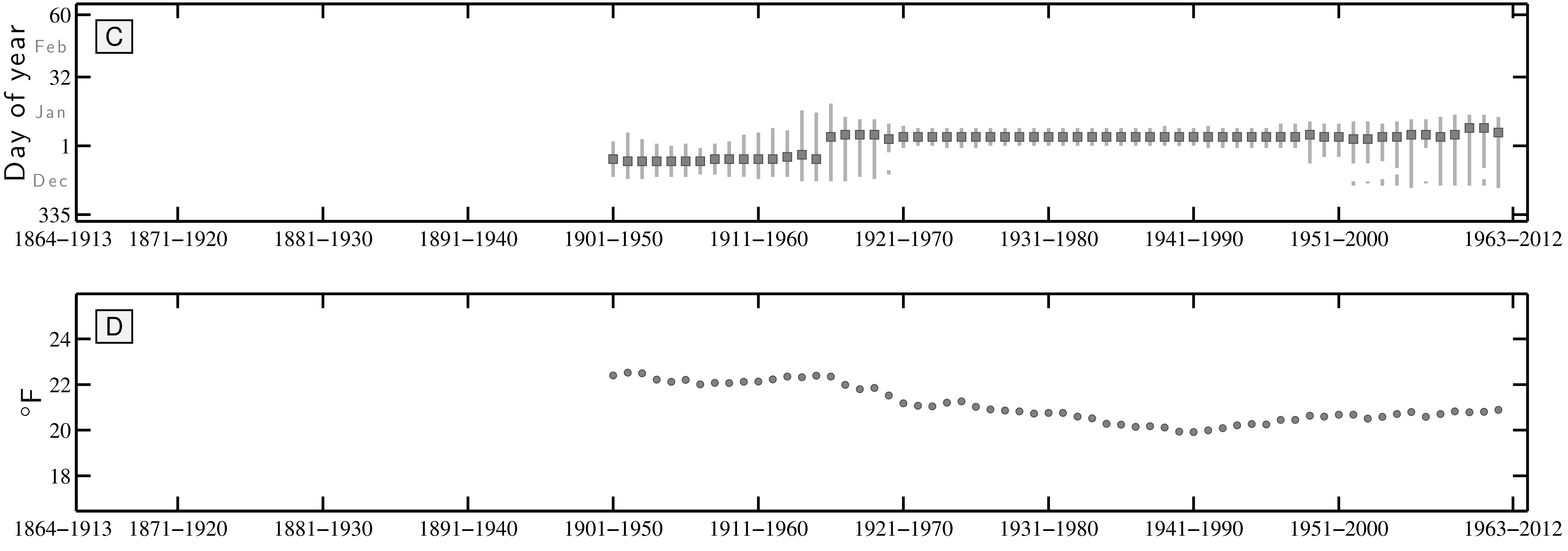

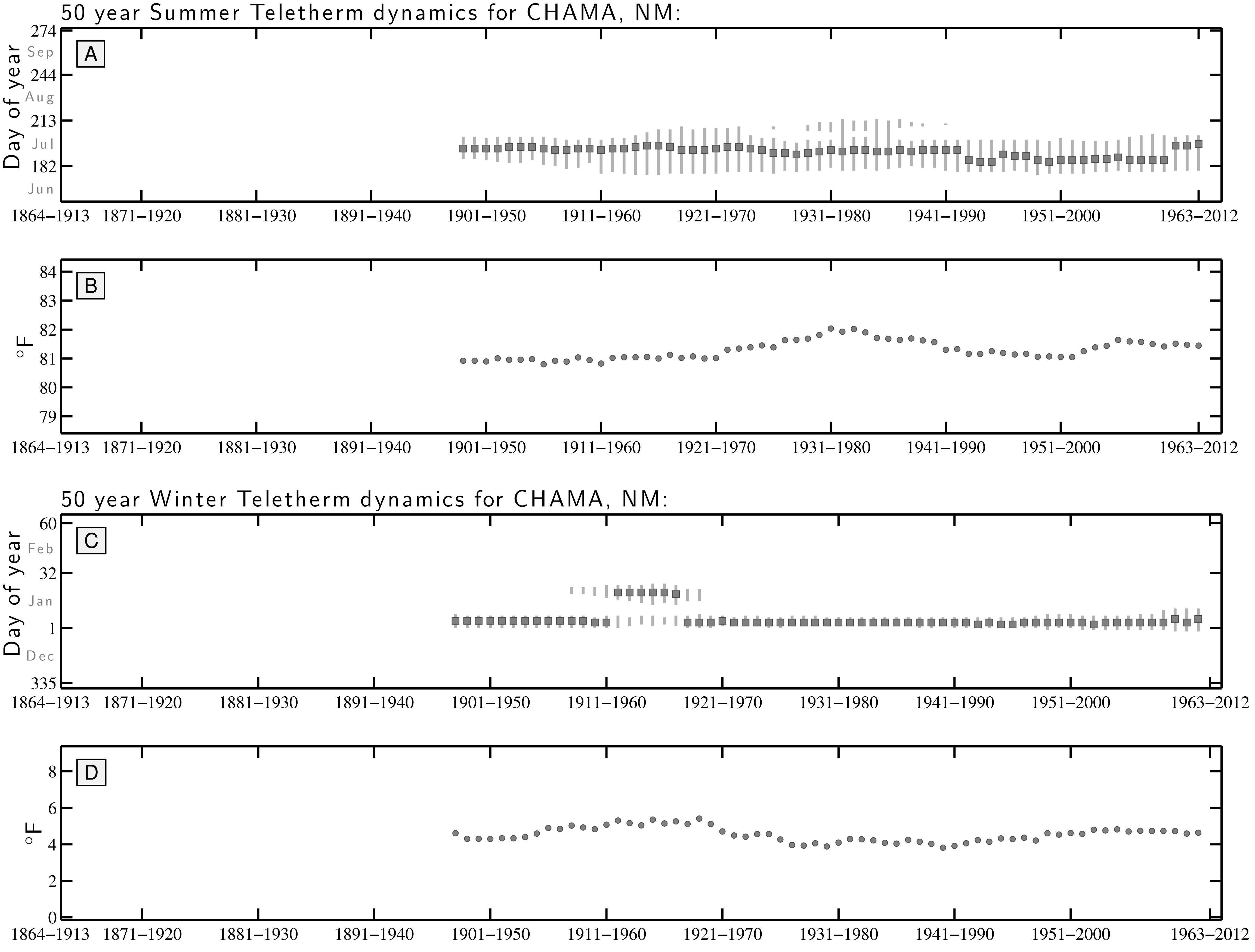
50 year Summer Teletherm dynamics for CHAMA, NM:
A
B
Day of year
°F
Jun
Jul
Aug
Sep
182
213
244
274
79
80
81
82
83
84
1864–1913
1871–1920
1881–1930
1891–1940
1901–1950
1911–1960
1921–1970
1931–1980
1941–1990
1951–2000
1963–2012
50 year Winter Teletherm dynamics for CHAMA, NM:
C
D
Dec
Jan
Feb
335
1
32
60
0
2
4
6
8

50 year Summer Teletherm dynamics for CIMARRON 4 SW, NM:

50 year Winter Teletherm dynamics for CIMARRON 4 SW, NM:

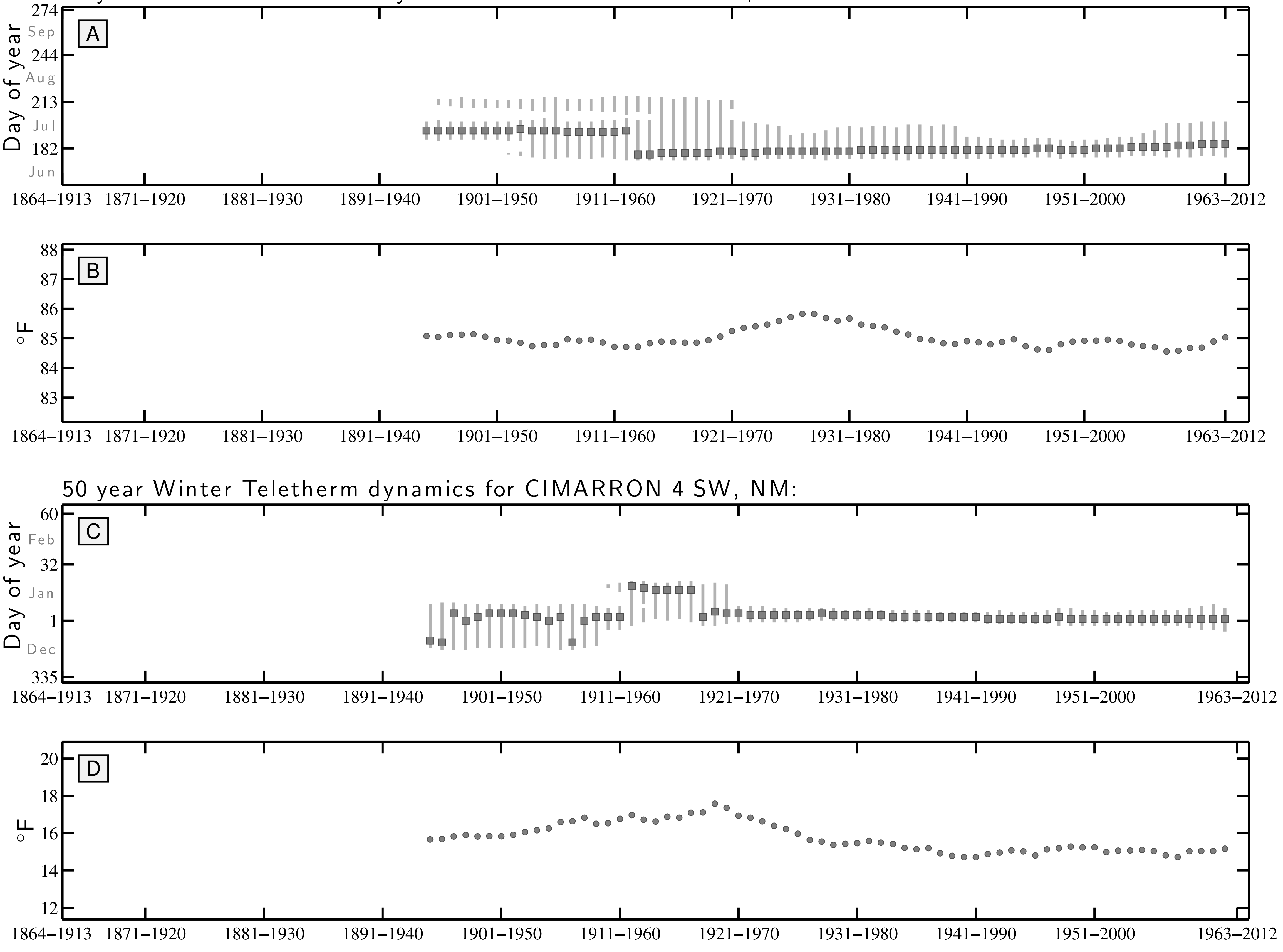

50 year Summer Teletherm dynamics for CLAYTON MUNI ARPK AP, NM:

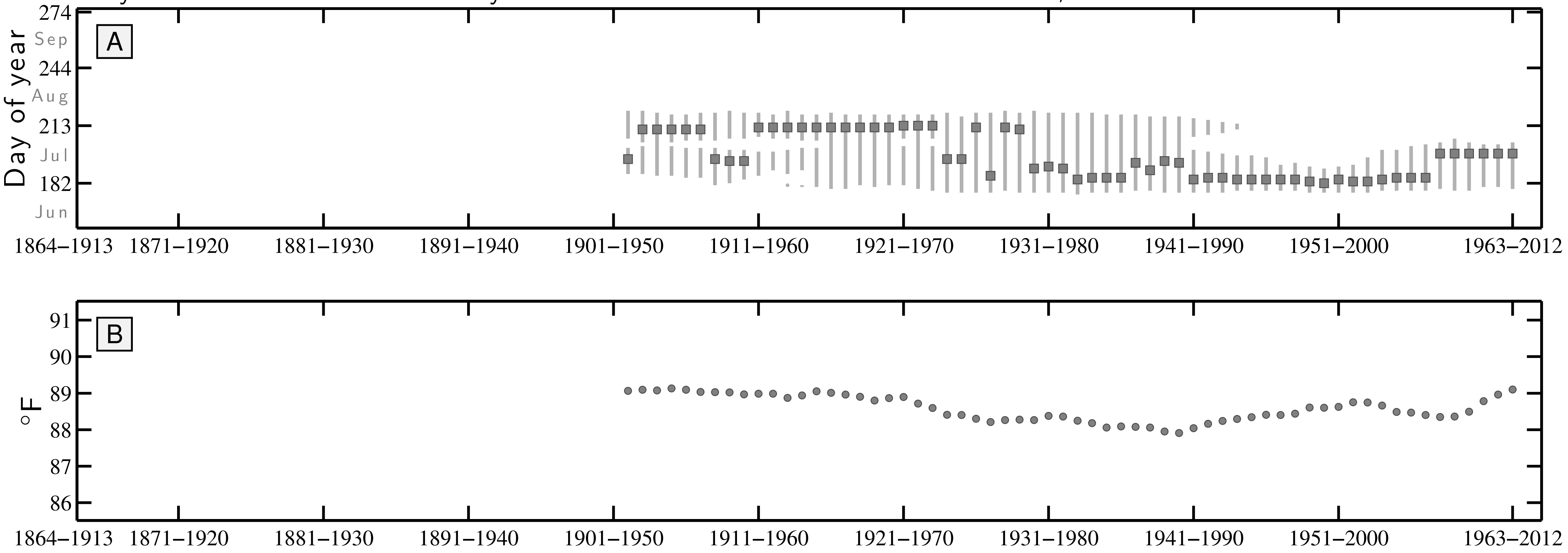

50 year Winter Teletherm dynamics for CLAYTON MUNI ARPK AP, NM:

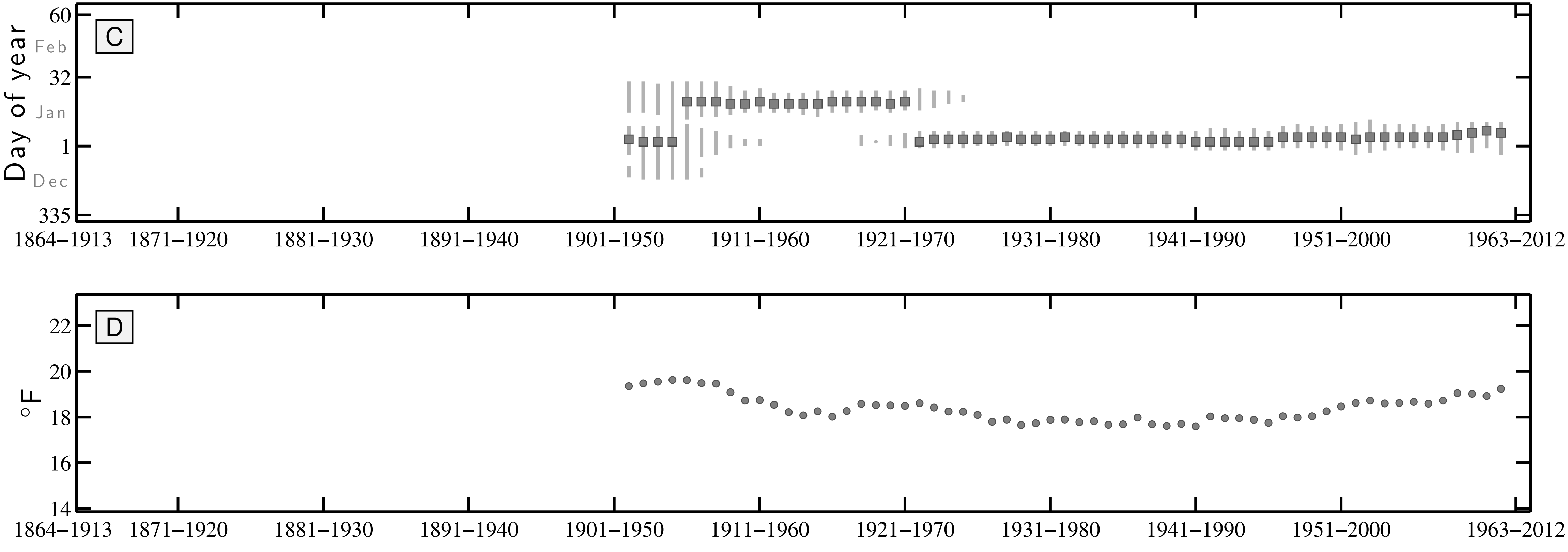

50 year Summer Teletherm dynamics for DULCE, NM:

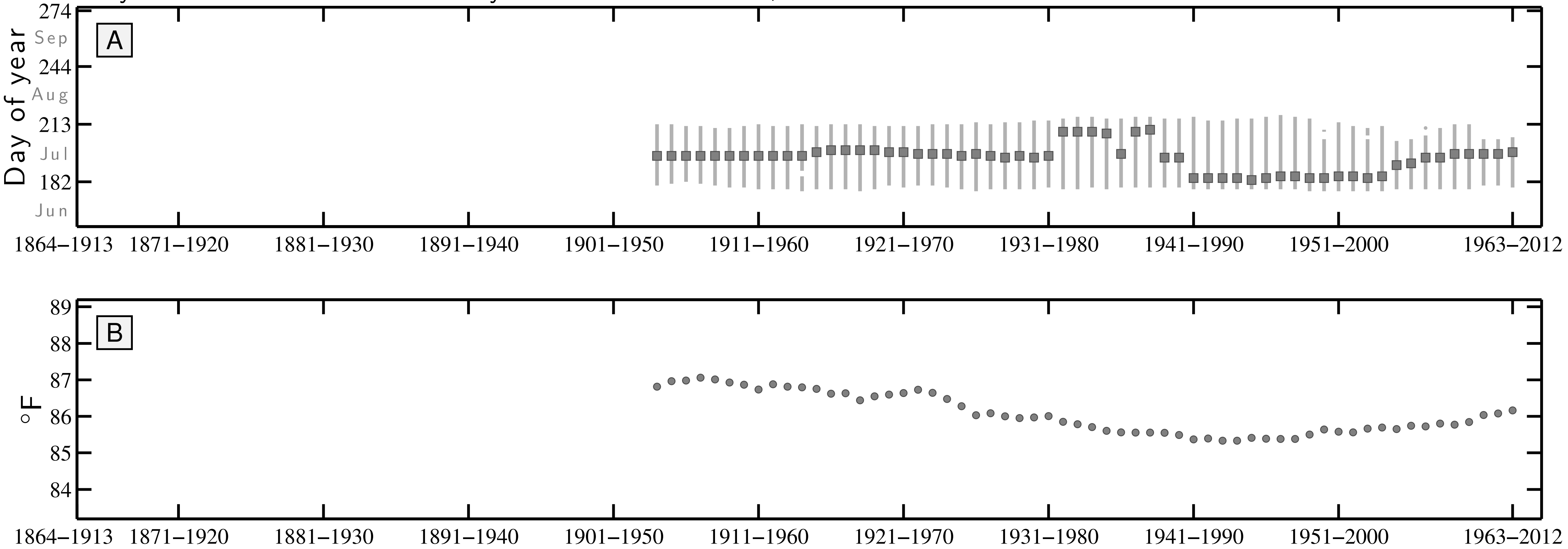

50 year Winter Teletherm dynamics for DULCE, NM:

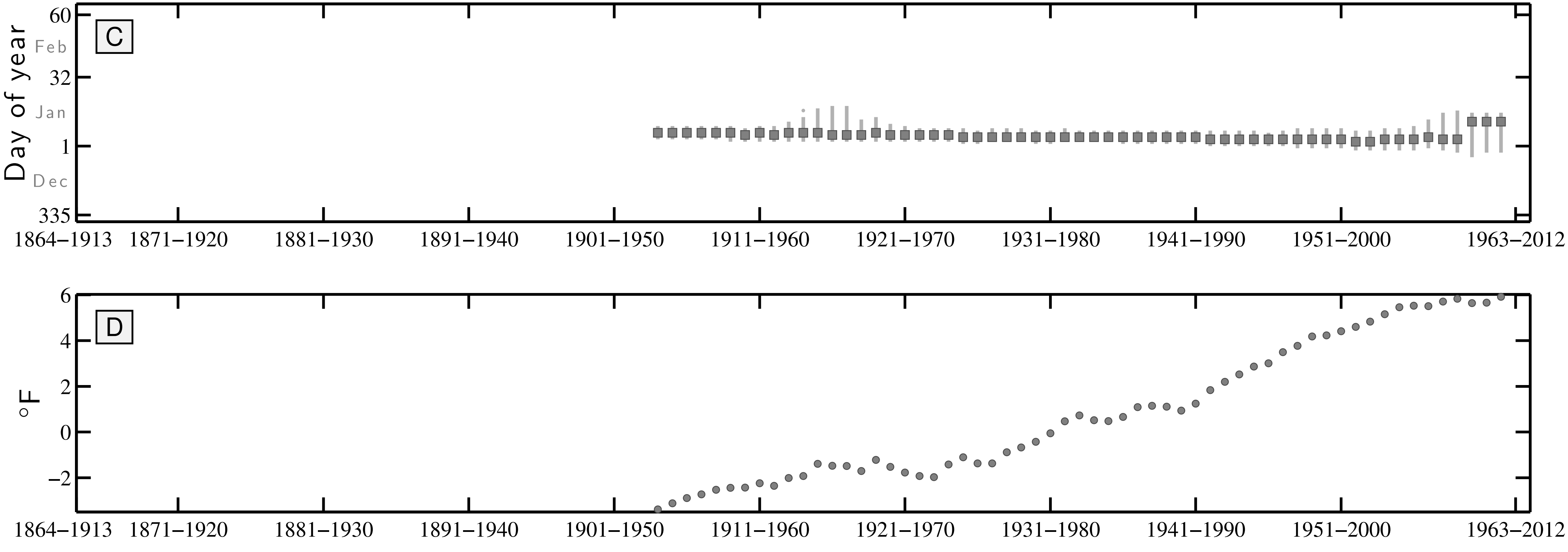

50 year Summer Teletherm dynamics for ELEPHANT BUTTE DAM, NM:

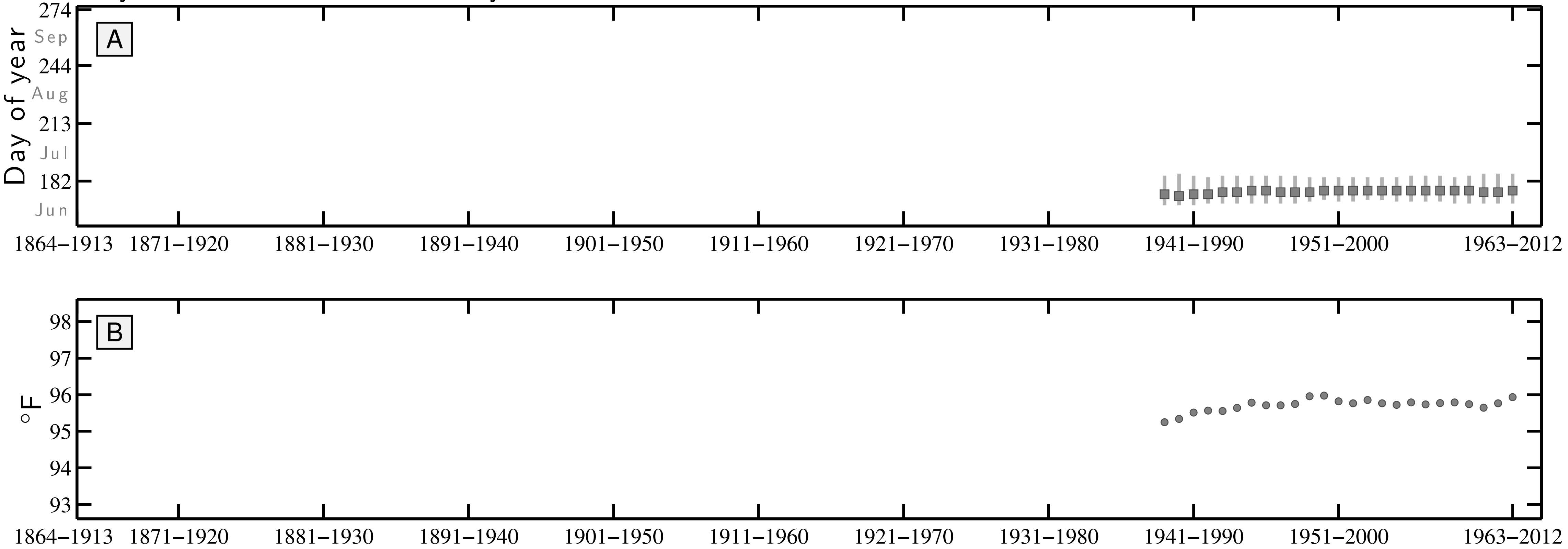

50 year Winter Teletherm dynamics for ELEPHANT BUTTE DAM, NM:

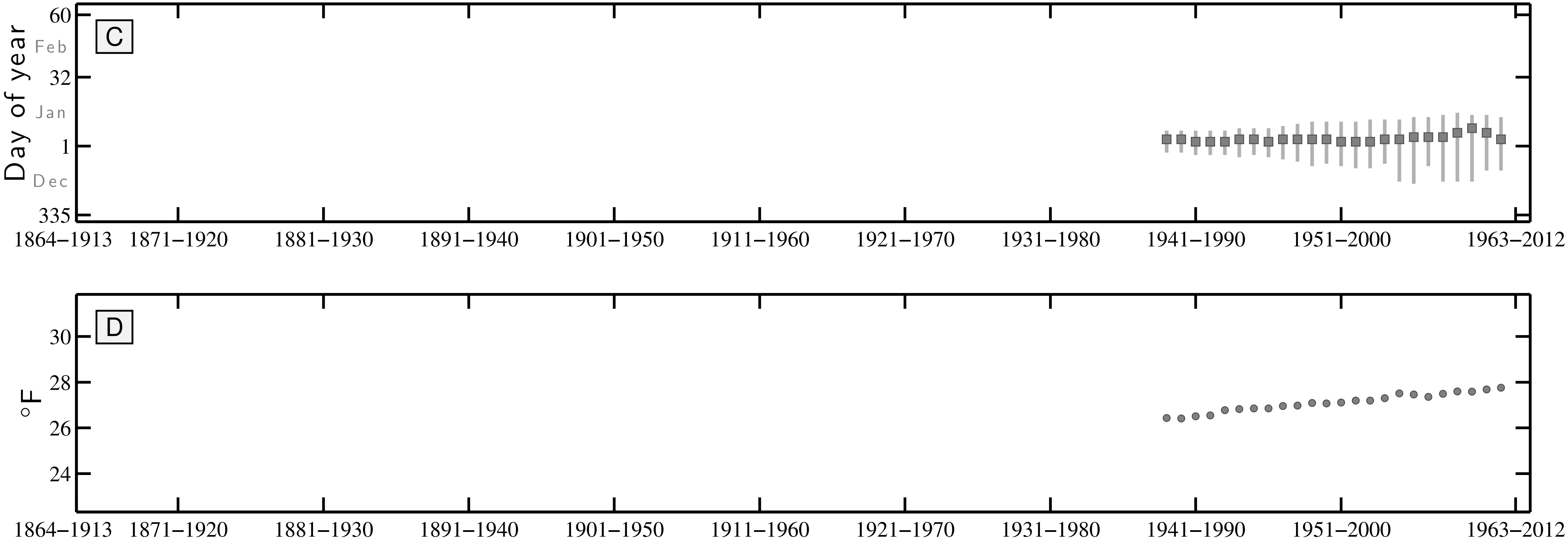

50 year Summer Teletherm dynamics for FT BAYARD, NM:

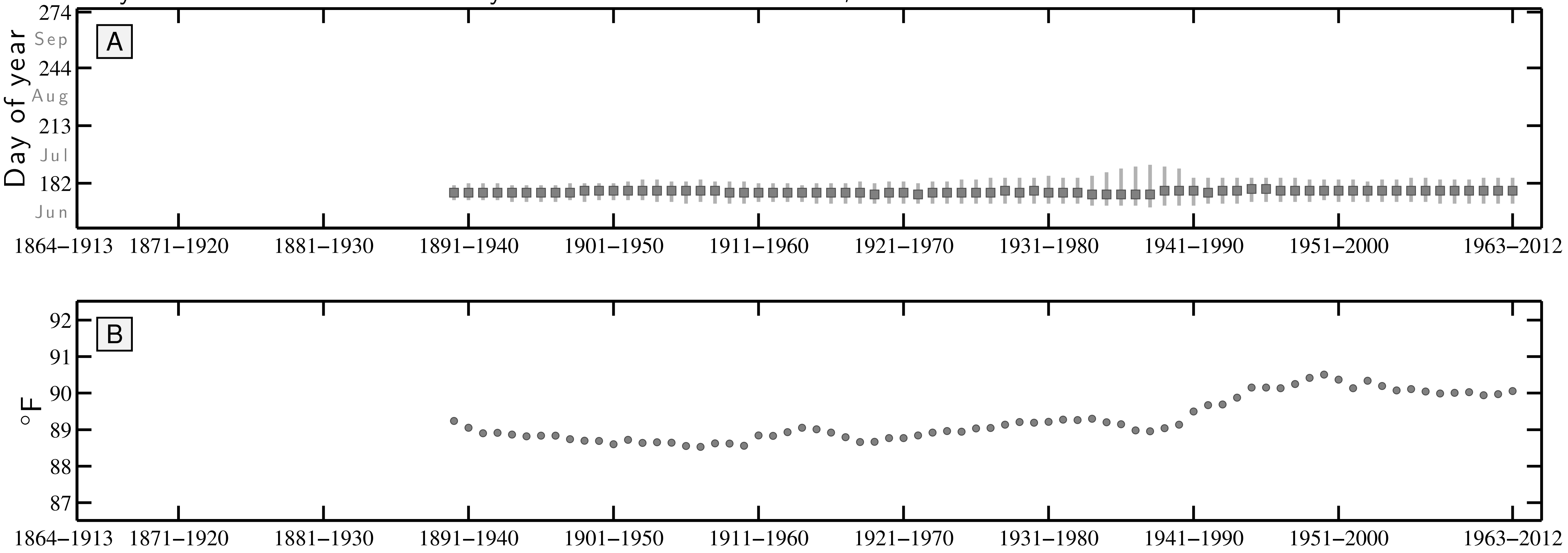

50 year Winter Teletherm dynamics for FT BAYARD, NM:

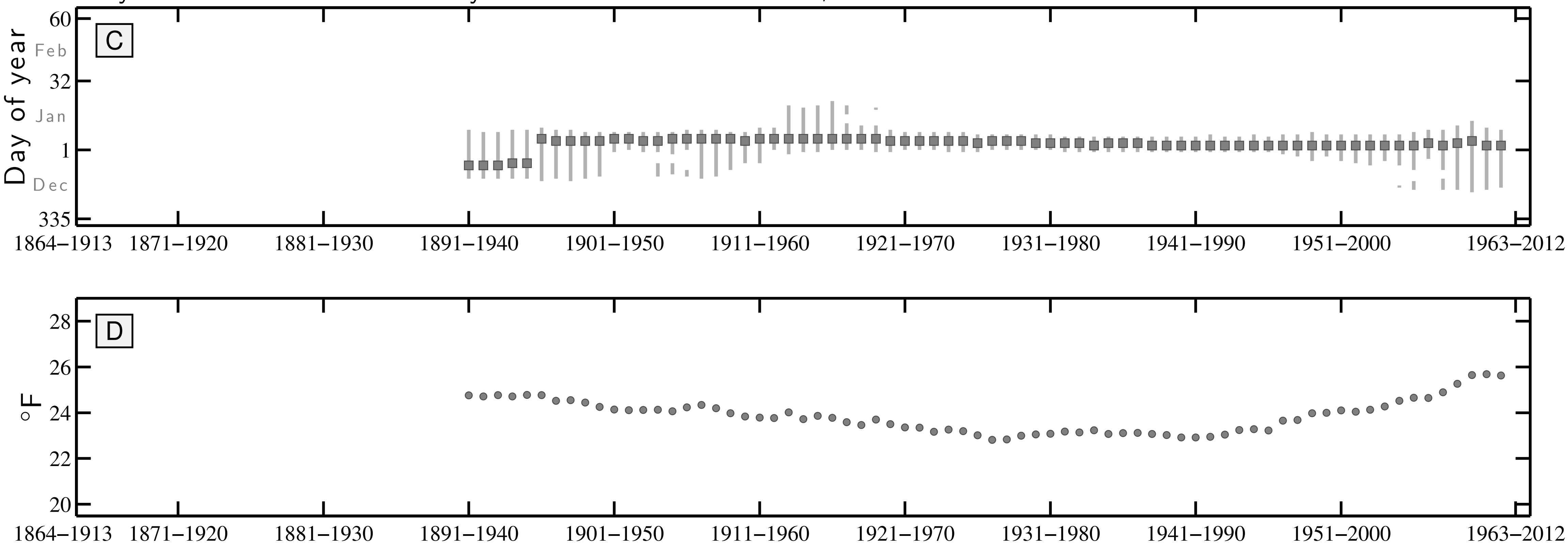

50 year Summer Teletherm dynamics for FT SUMNER, NM:

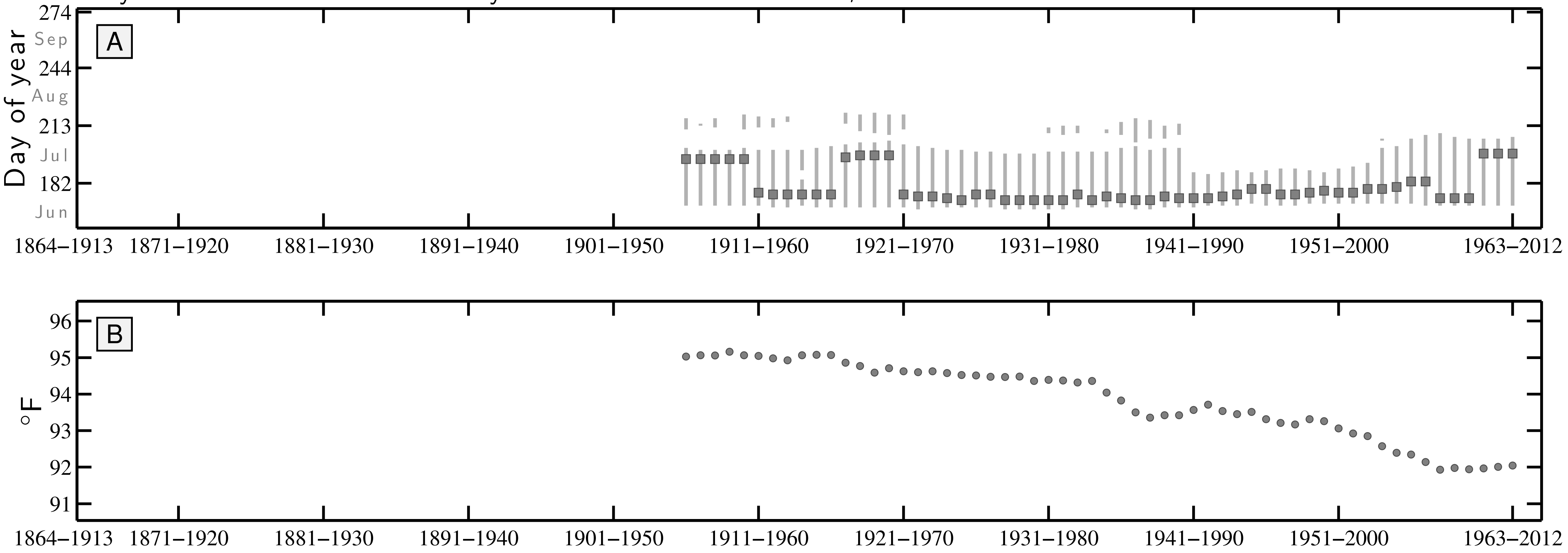

50 year Winter Teletherm dynamics for FT SUMNER, NM:

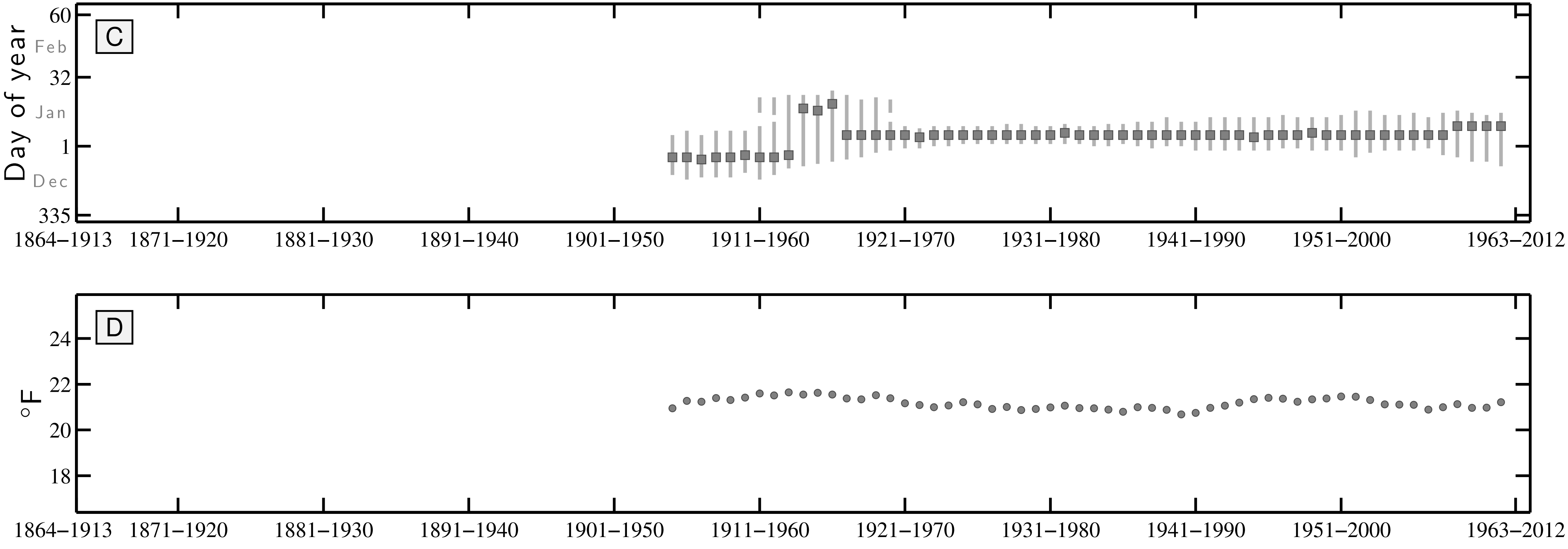

50 year Summer Teletherm dynamics for GAGE, NM:

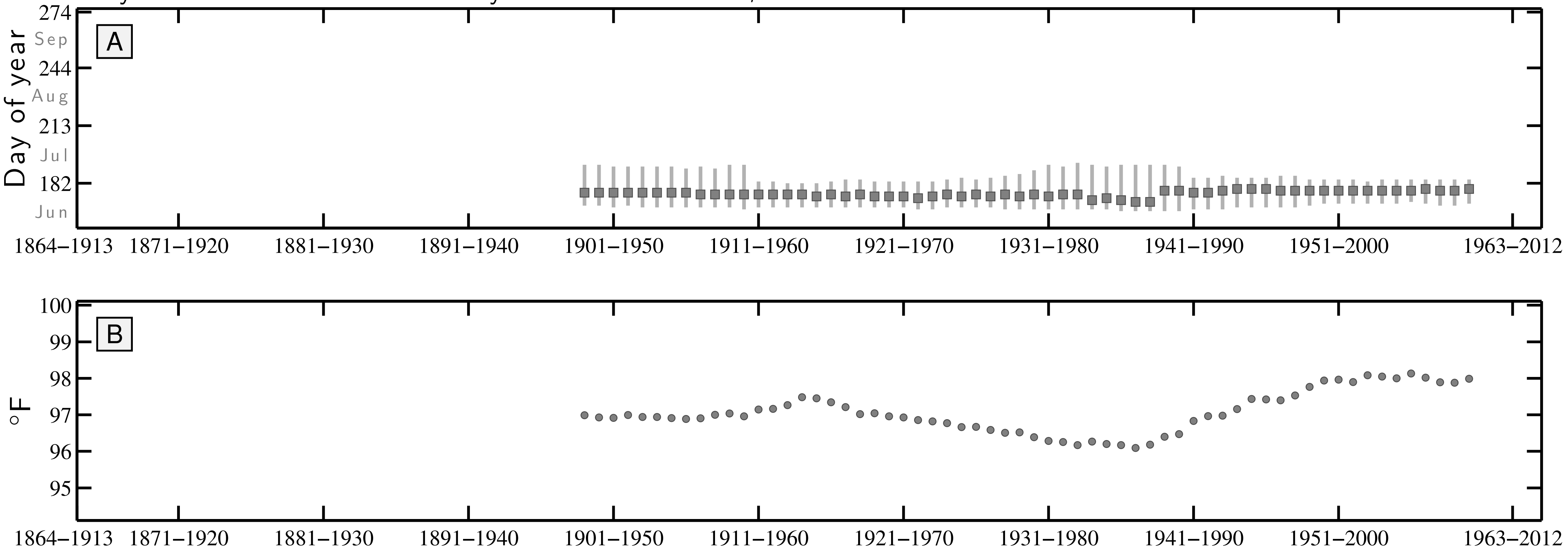

50 year Winter Teletherm dynamics for GAGE, NM:

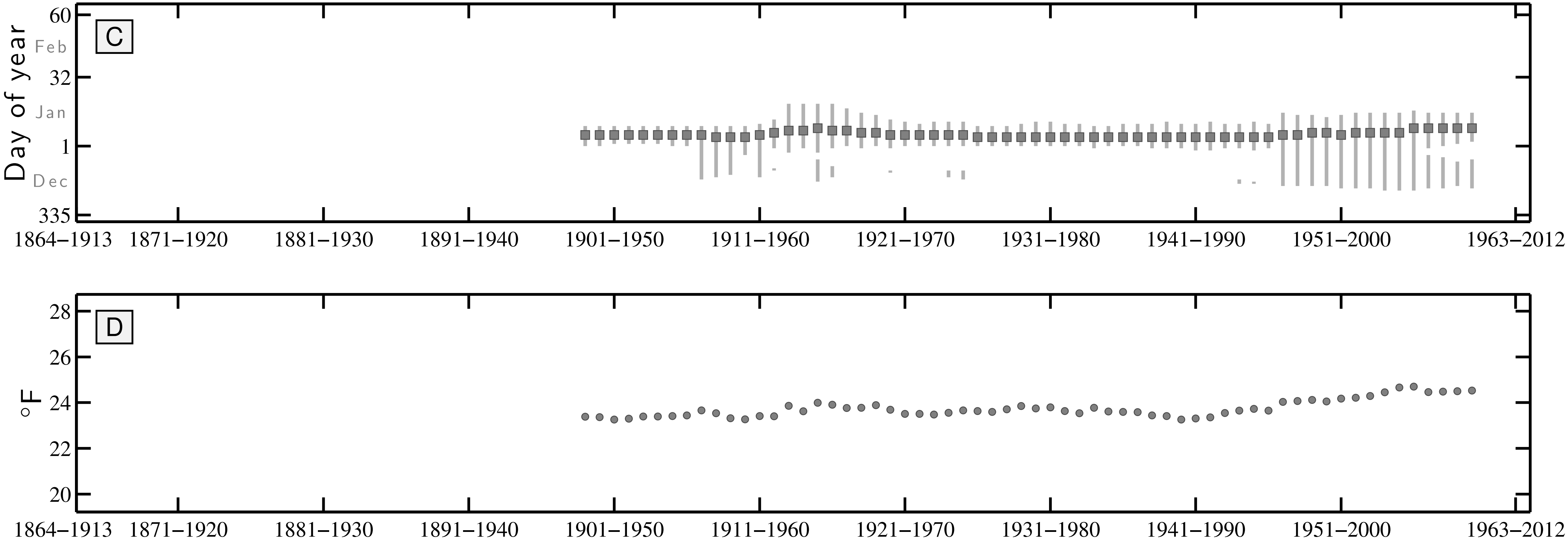

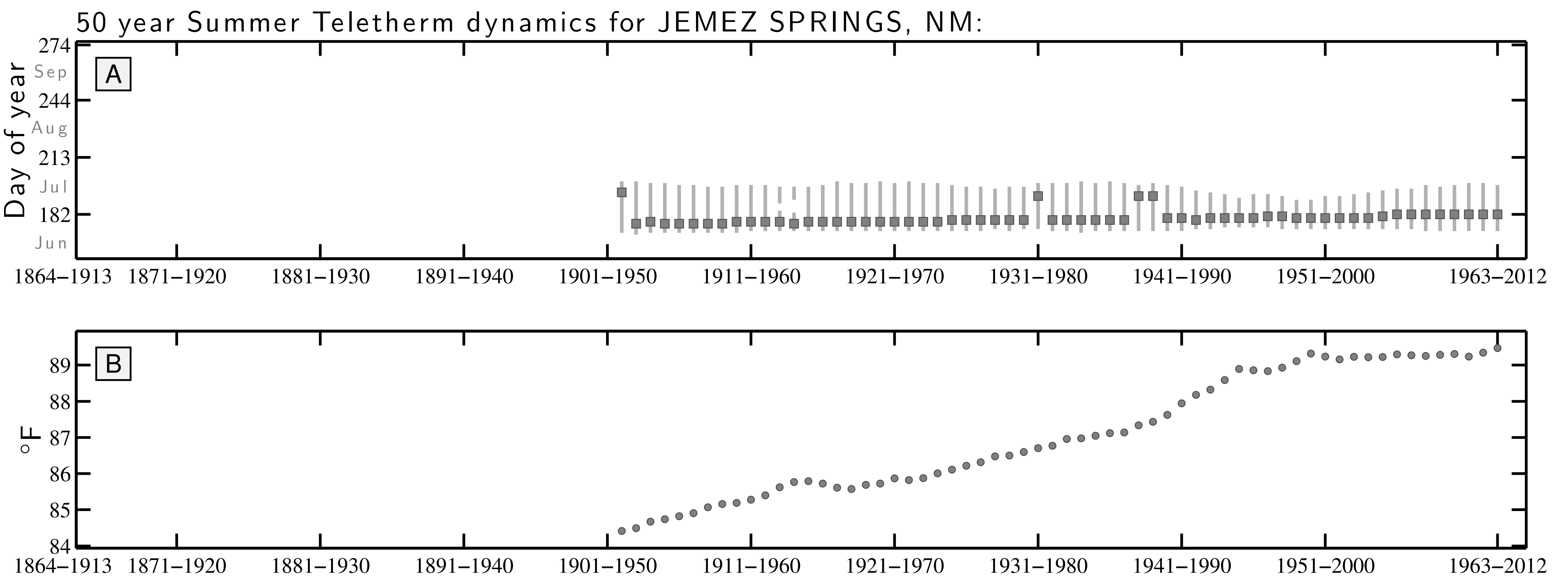
50 year Summer Teletherm dynamics for JEMEZ SPRINGS, NM:
A
B
Day of year
°F
274
Sep
244
Aug
213
Jul
182
Jun
89
88
87
86
85
84
1864–1913
1871–1920
1881–1930
1891–1940
1901–1950
1911–1960
1921–1970
1931–1980
1941–1990
1951–2000
1963–2012

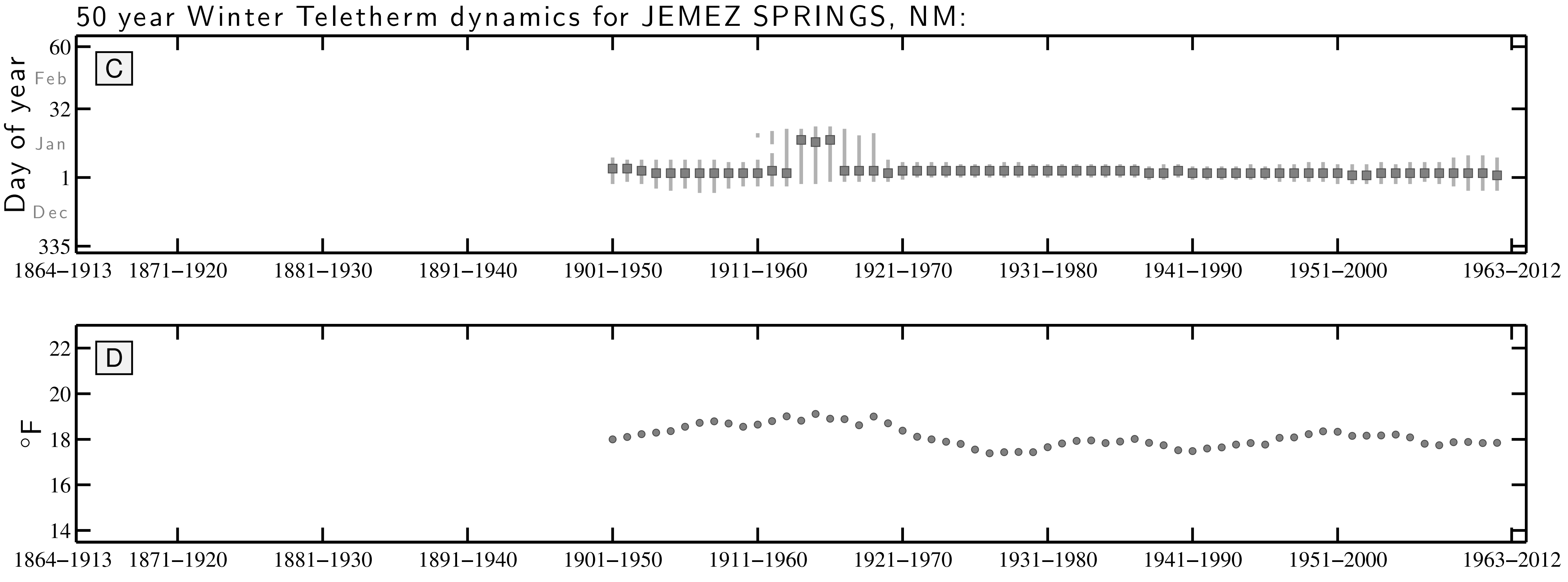
50 year Winter Teletherm dynamics for JEMEZ SPRINGS, NM:
C
D
Day of year
°F
60
Feb
32
Jan
1
Dec
335
22
20
18
16
14
1864–1913
1871–1920
1881–1930
1891–1940
1901–1950
1911–1960
1921–1970
1931–1980
1941–1990
1951–2000
1963–2012

50 year Summer Teletherm dynamics for JORNADA EXP RANGE, NM:

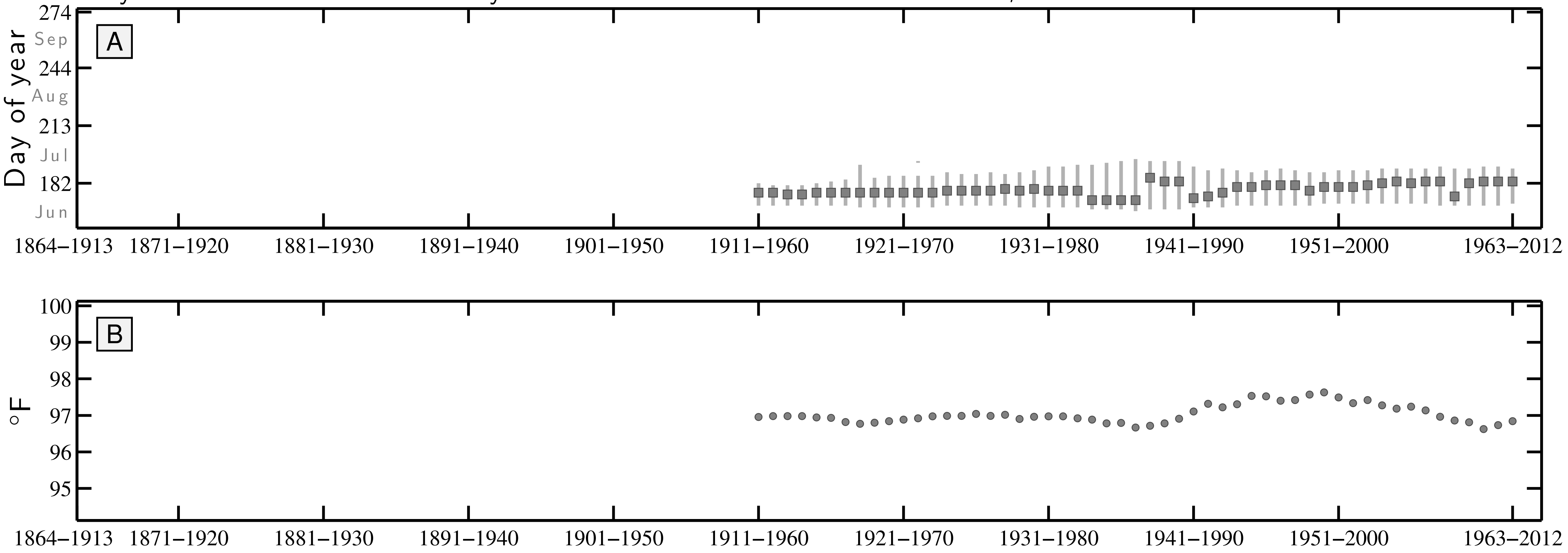

50 year Winter Teletherm dynamics for JORNADA EXP RANGE, NM:

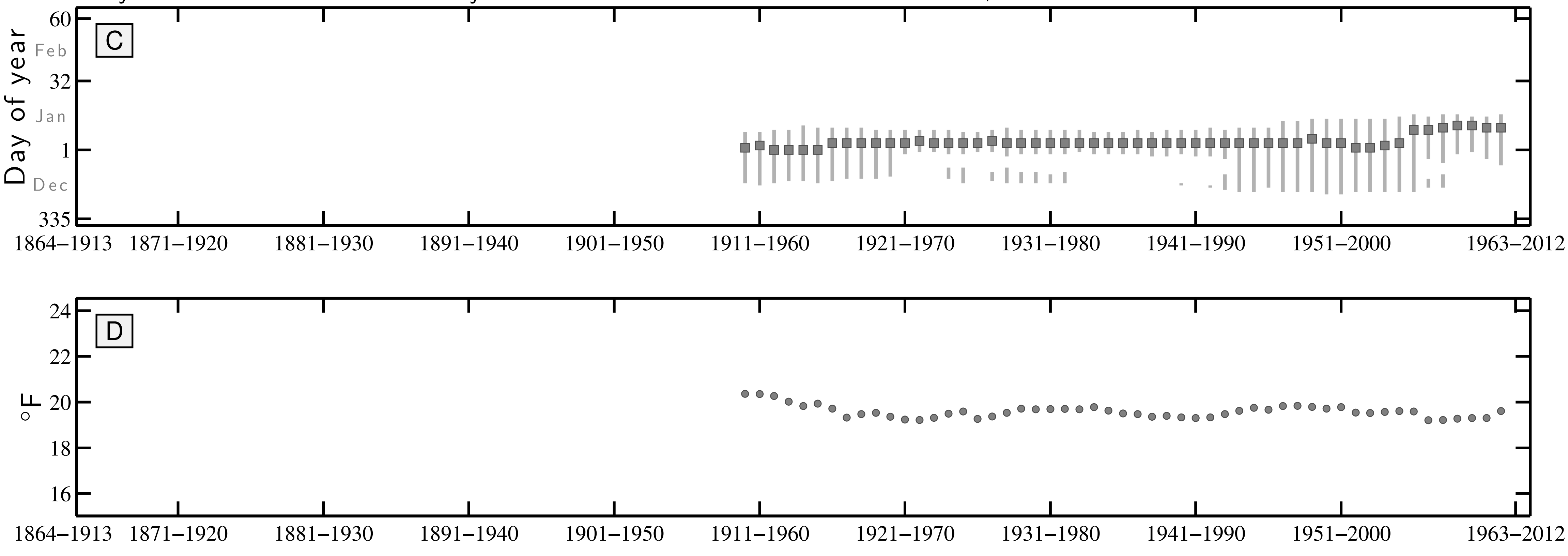

50 year Summer Teletherm dynamics for LOS LUNAS 3 SSW, NM:

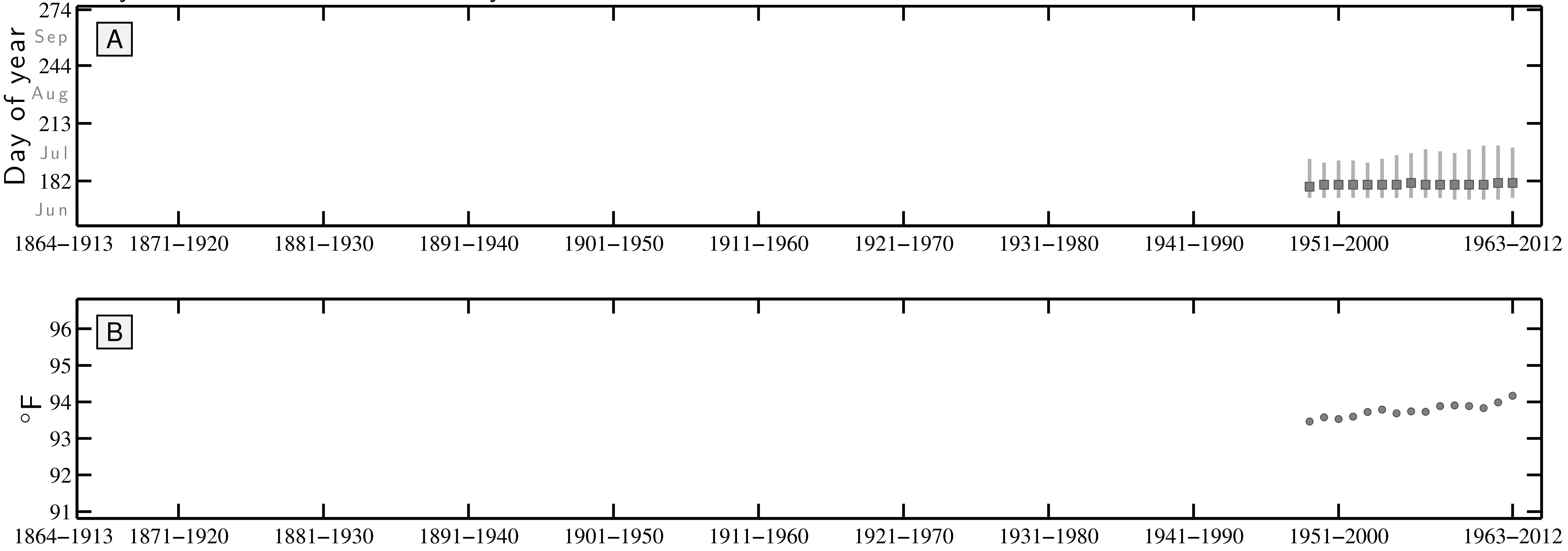

50 year Winter Teletherm dynamics for LOS LUNAS 3 SSW, NM:

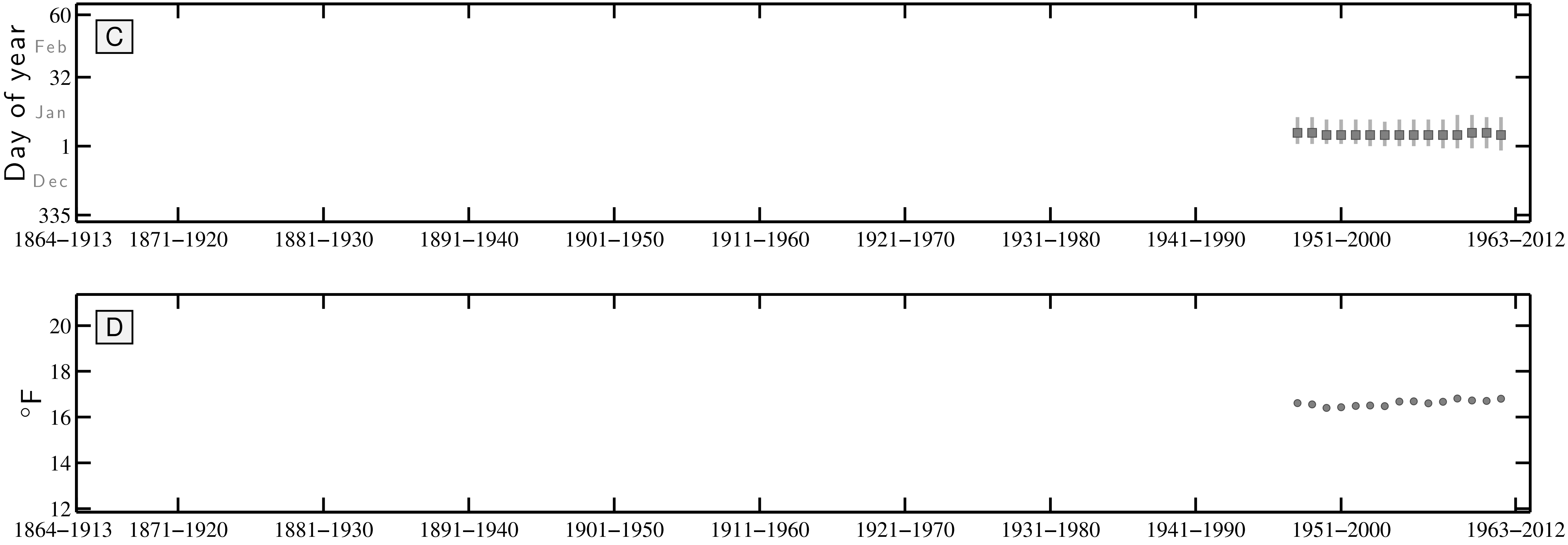

50 year Summer Teletherm dynamics for LUNA RS, NM:

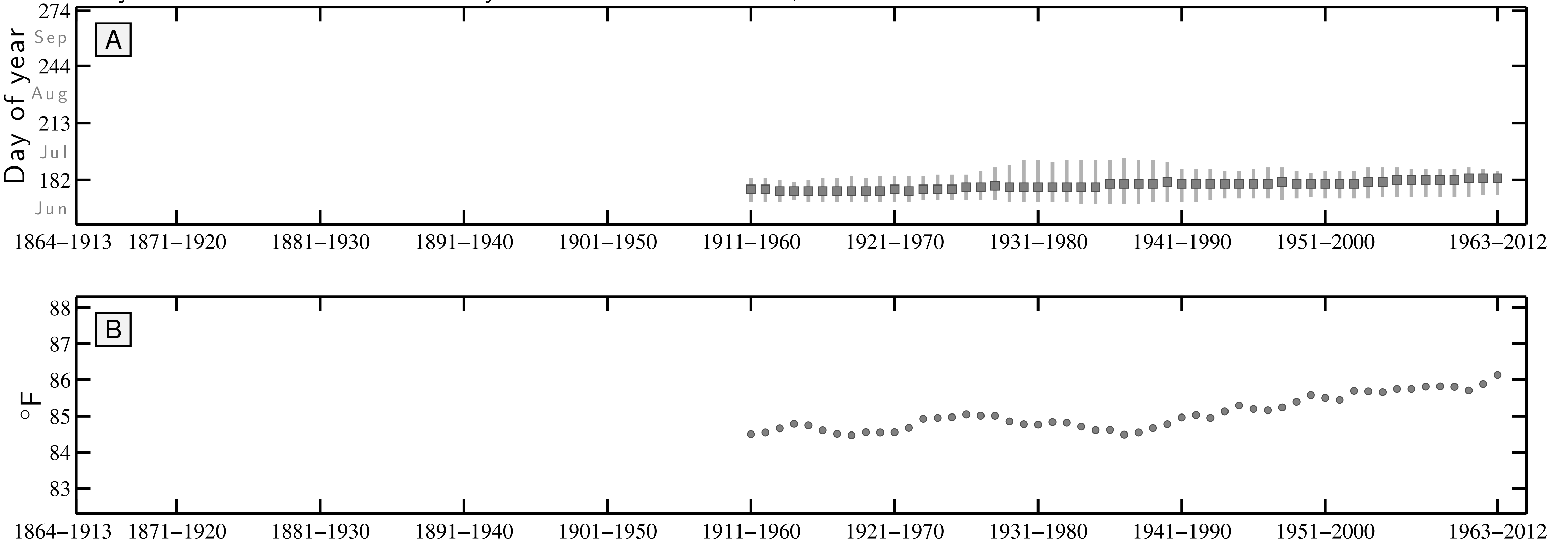

50 year Winter Teletherm dynamics for LUNA RS, NM:

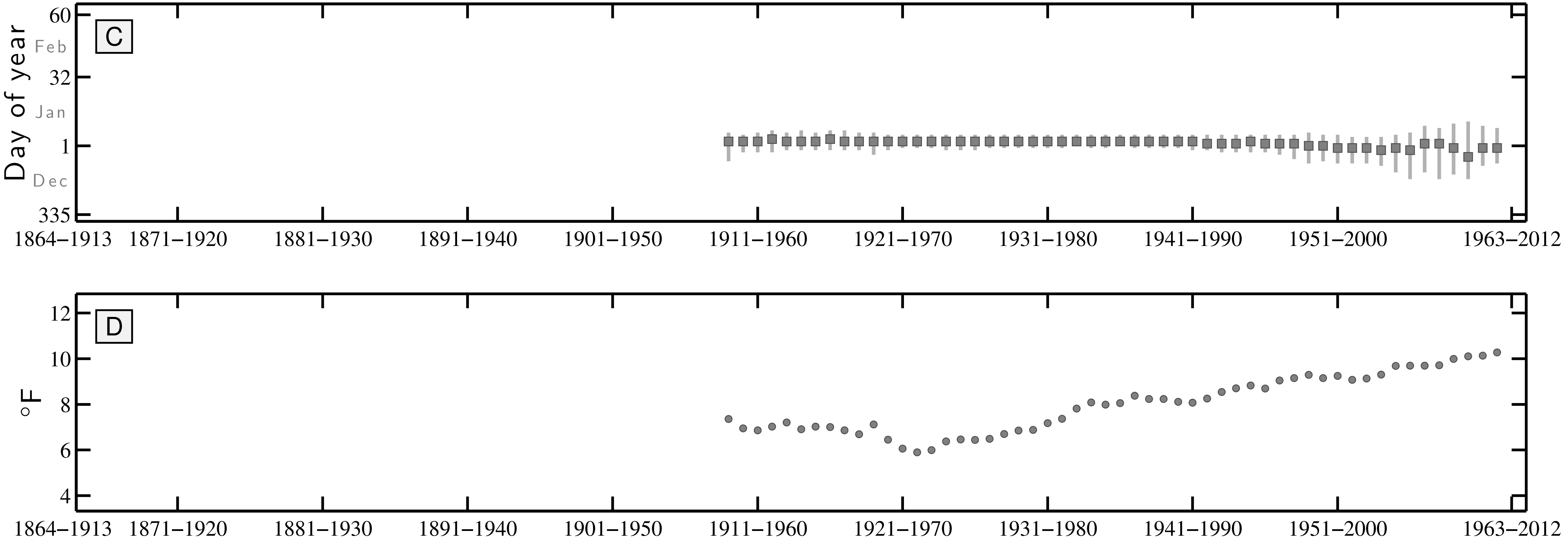

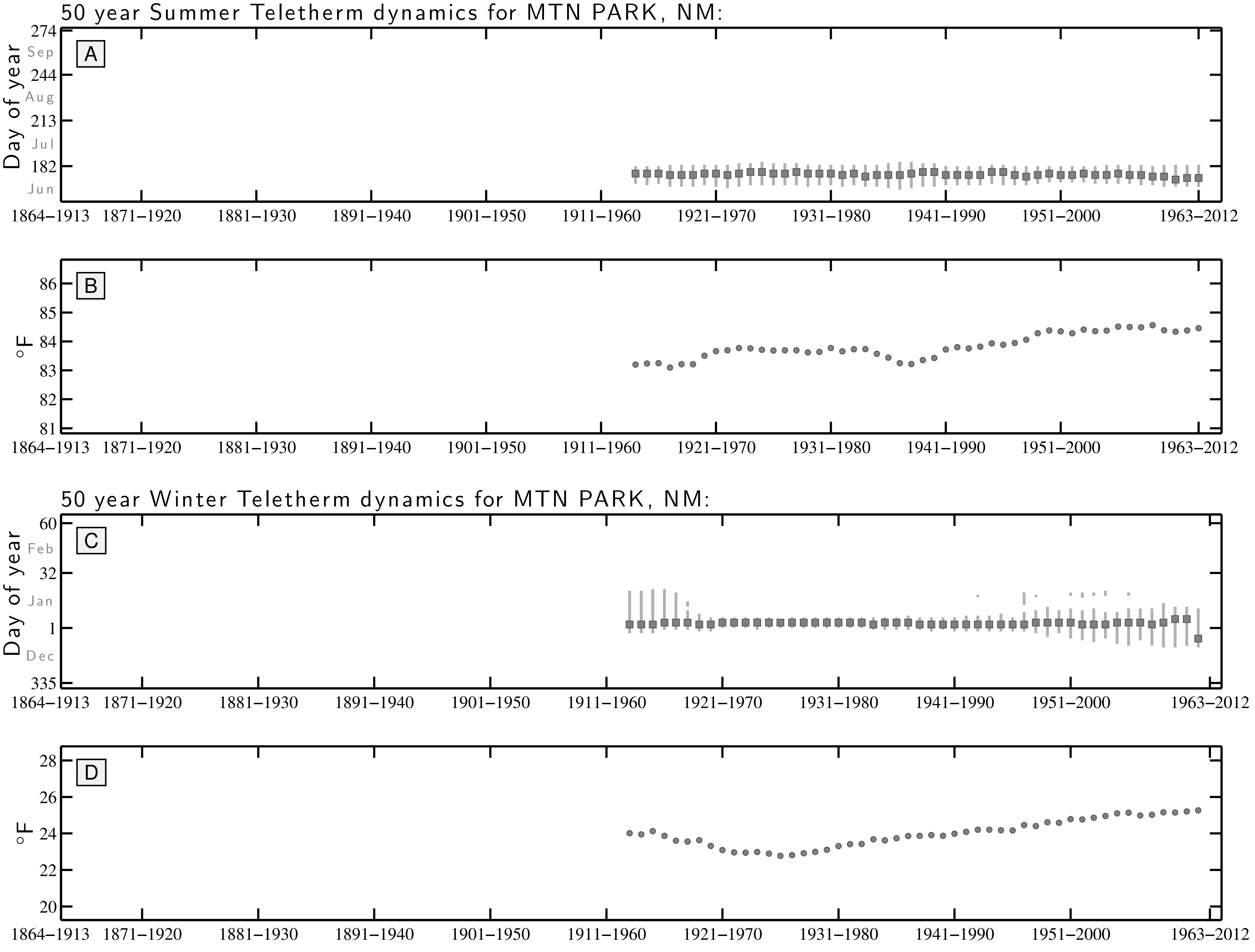

50 year Summer Teletherm dynamics for MTN PARK, NM:
A
Day of year
274
Sep
244
Aug
213
Jul
182
Jun
1864–1913
1871–1920
1881–1930
1891–1940
1901–1950
1911–1960
1921–1970
1931–1980
1941–1990
1951–2000
1963–2012
B
°F
86
85
84
83
82
81
50 year Winter Teletherm dynamics for MTN PARK, NM:
C
Day of year
60
Feb
32
Jan
1
Dec
335
D
°F
28
26
24
22
20

50 year Summer Teletherm dynamics for MOUNTAINAIR, NM:

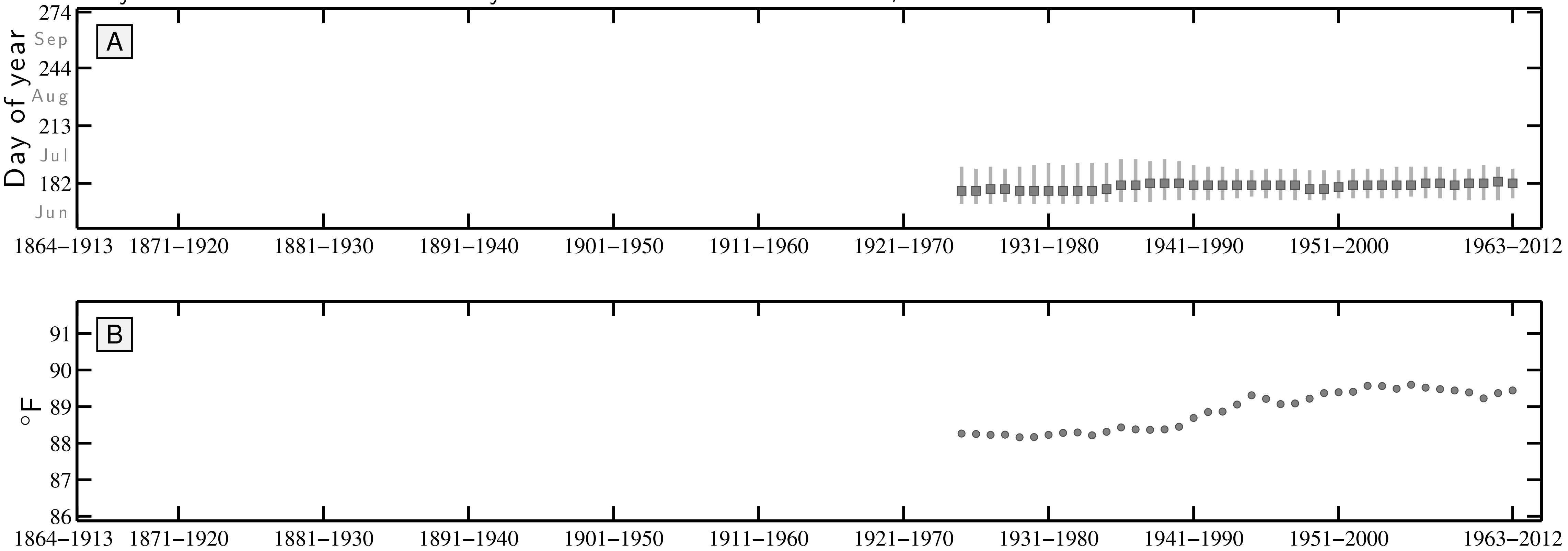

50 year Winter Teletherm dynamics for MOUNTAINAIR, NM:

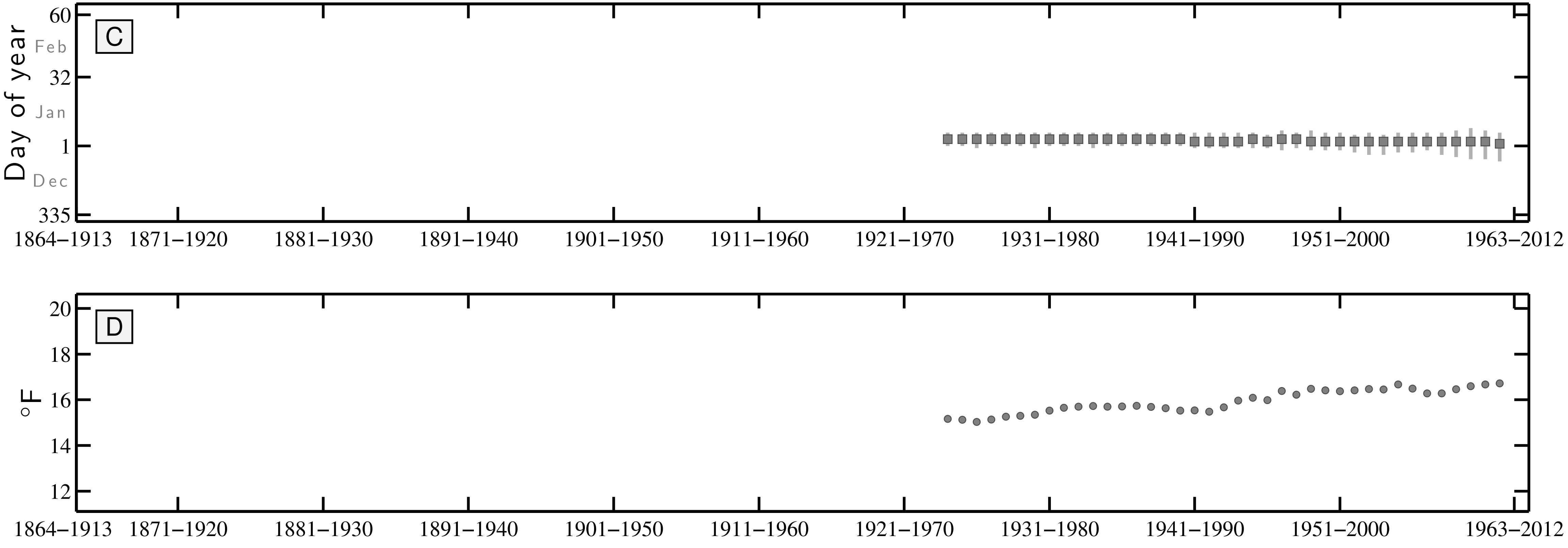

50 year Summer Teletherm dynamics for OROGRANDE, NM:

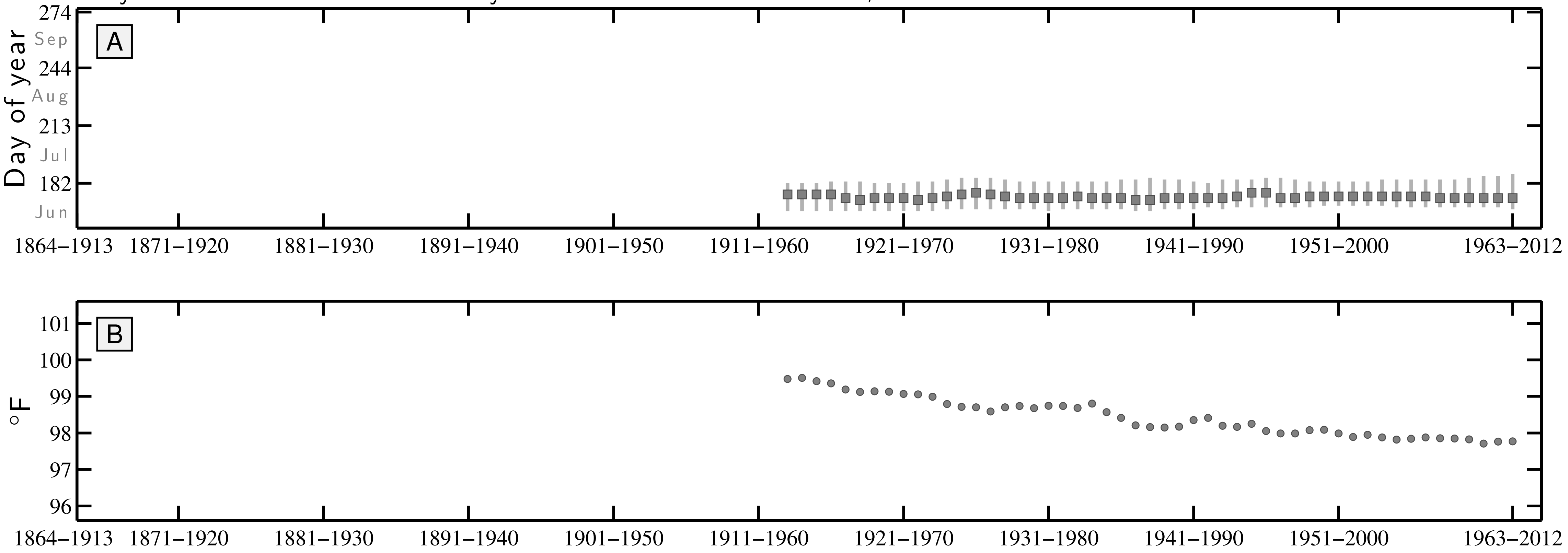

50 year Winter Teletherm dynamics for OROGRANDE, NM:

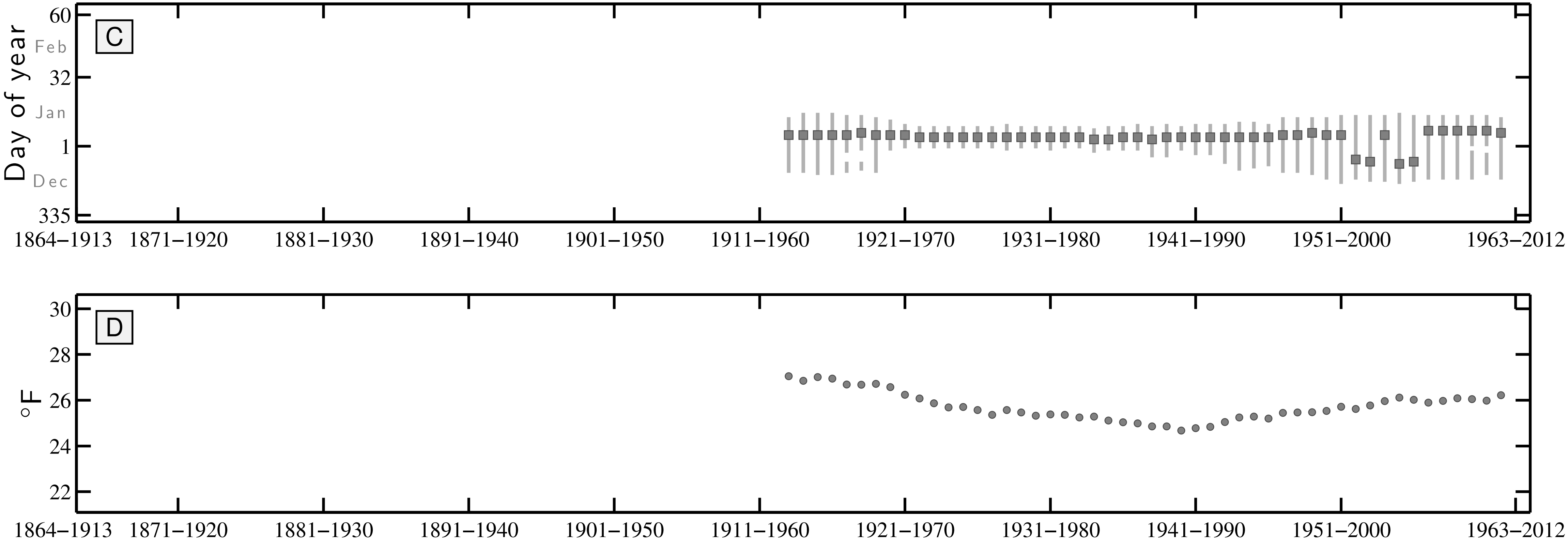

50 year Summer Teletherm dynamics for RED RIVER, NM:

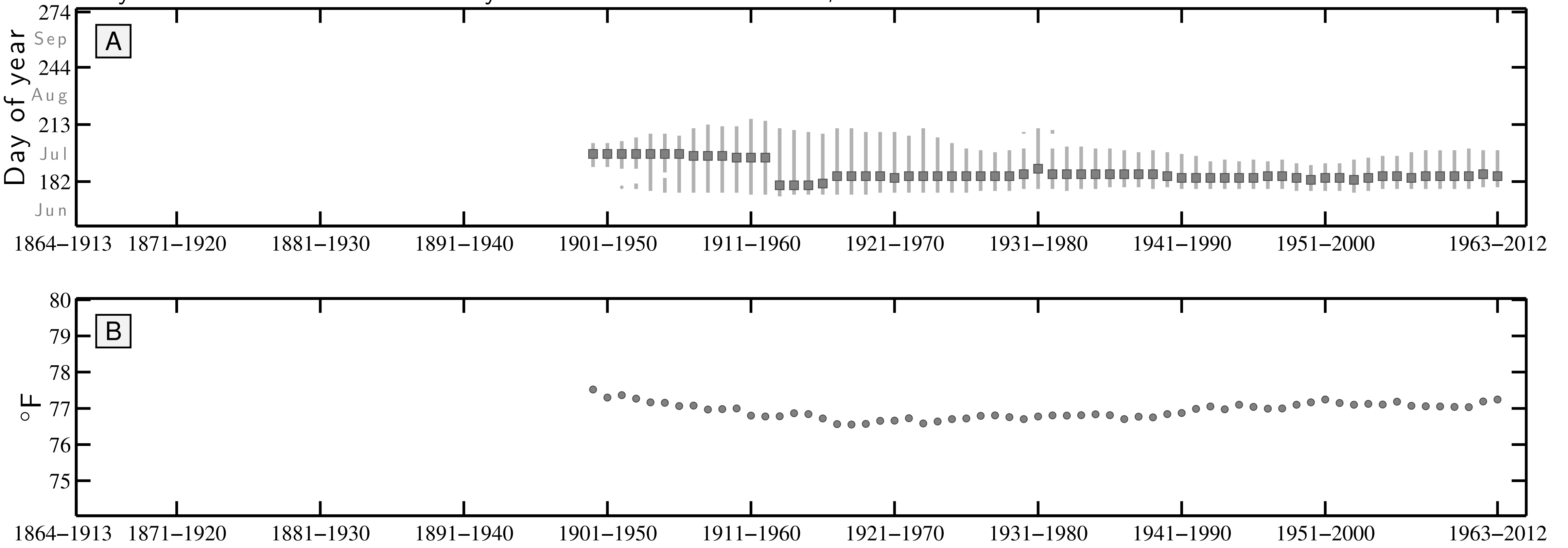

50 year Winter Teletherm dynamics for RED RIVER, NM:

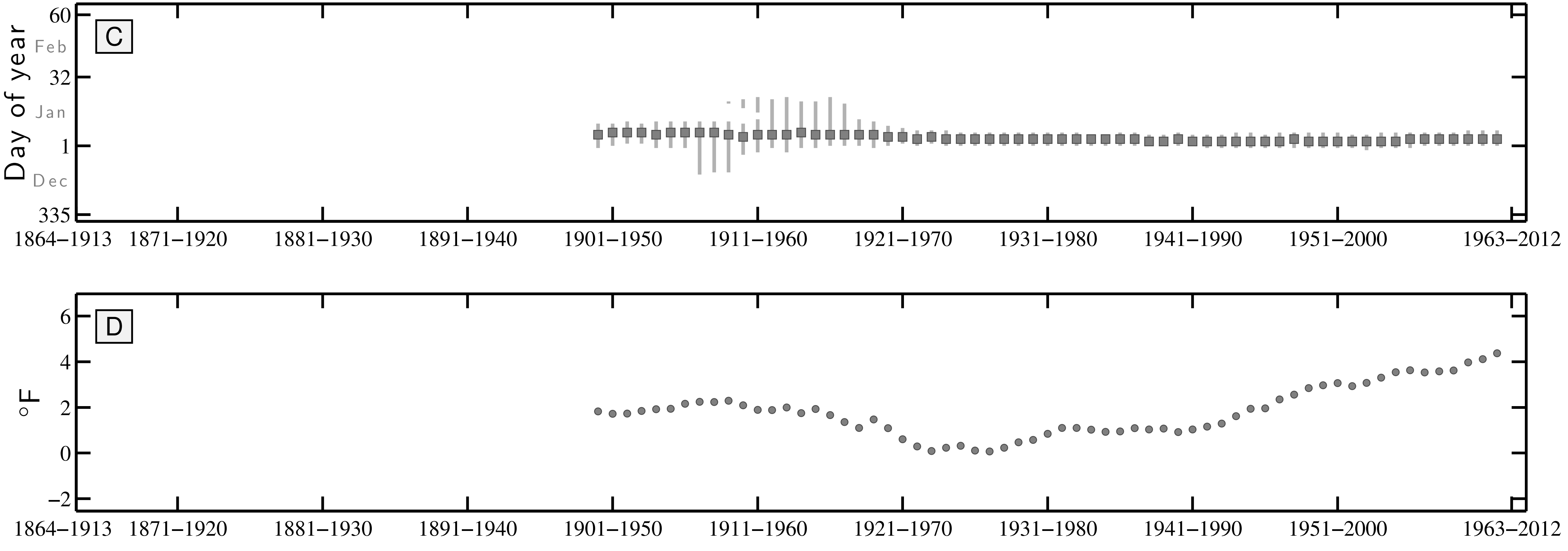

50 year Summer Teletherm dynamics for ROSWELL IND AP, NM:

50 year Winter Teletherm dynamics for ROSWELL IND AP, NM:

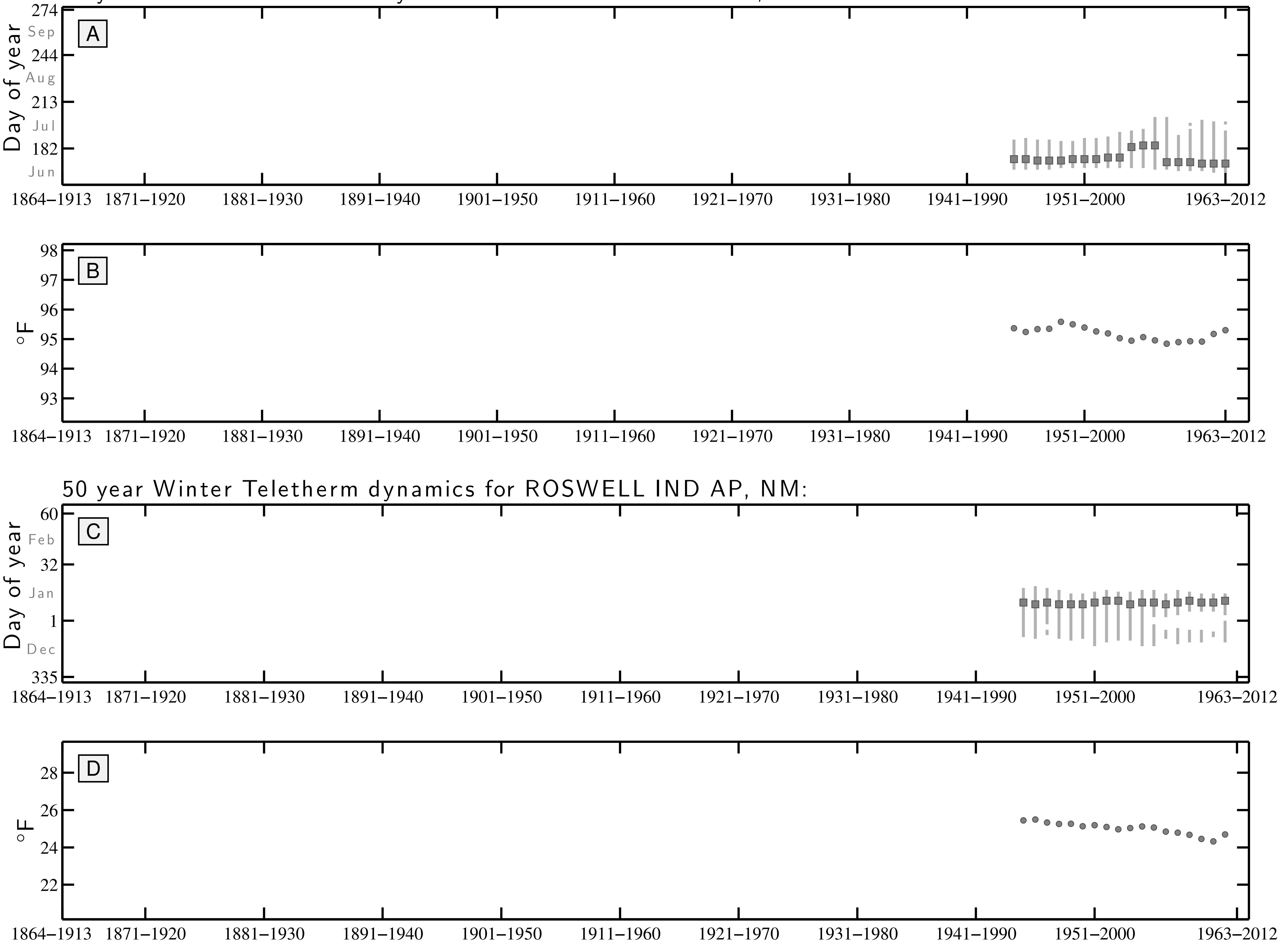

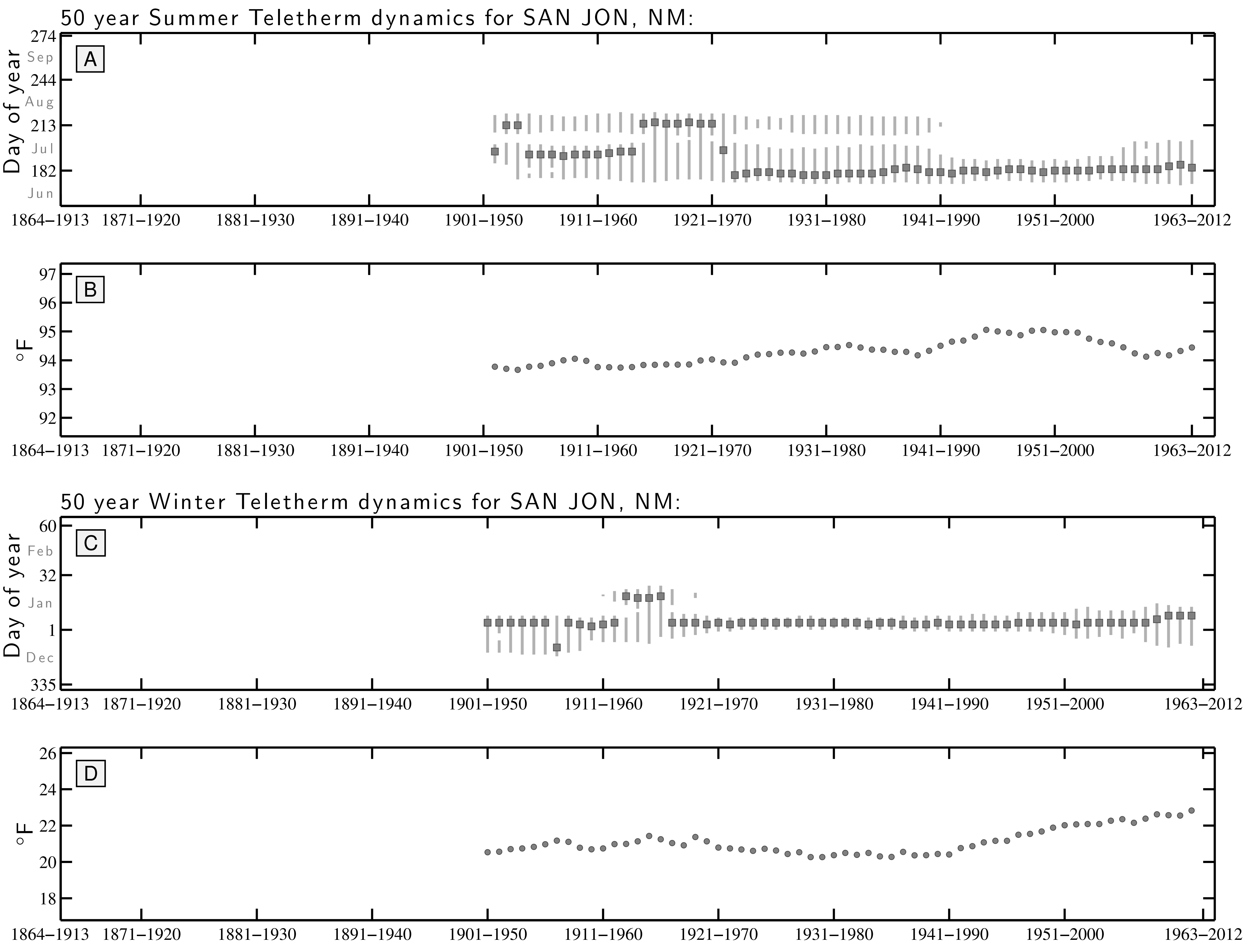

50 year Summer Teletherm dynamics for SAN JON, NM:
A
B
Day of year
°F
274
Sep
244
Aug
213
Jul
182
Jun
97
96
95
94
93
92
1864–1913
1871–1920
1881–1930
1891–1940
1901–1950
1911–1960
1921–1970
1931–1980
1941–1990
1951–2000
1963–2012
50 year Winter Teletherm dynamics for SAN JON, NM:
C
D
60
Feb
32
Jan
1
Dec
335
26
24
22
20
18

50 year Summer Teletherm dynamics for SANTA ROSA, NM:

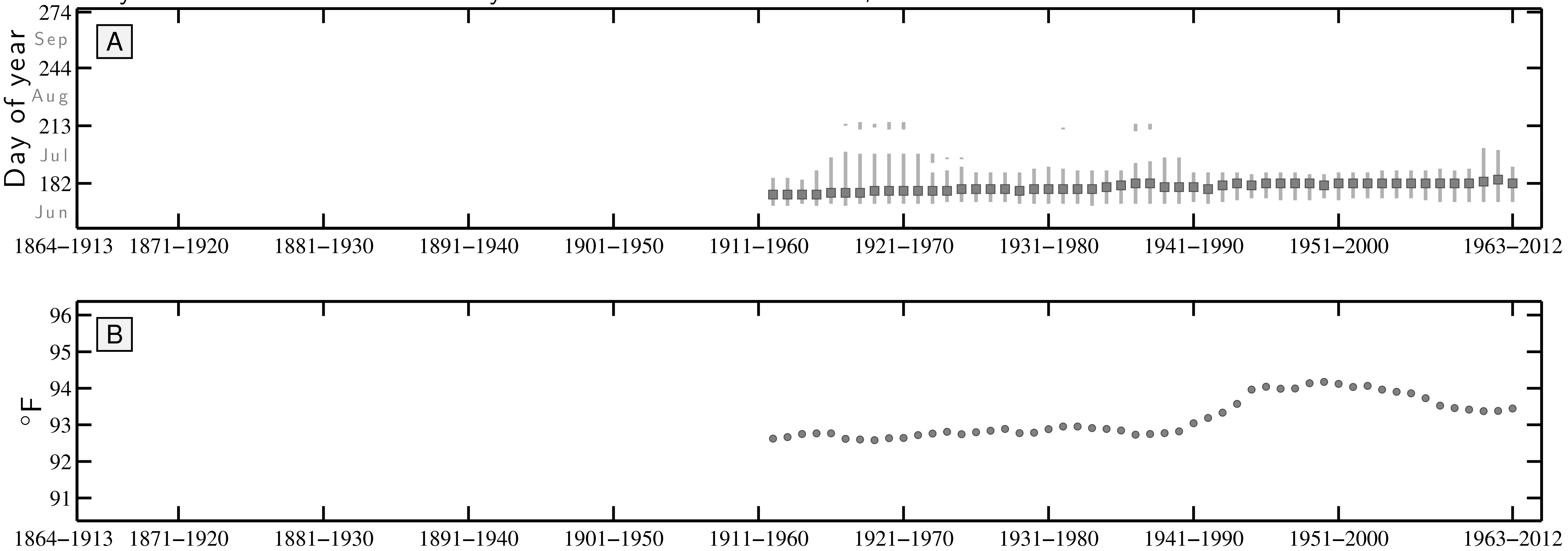

50 year Winter Teletherm dynamics for SANTA ROSA, NM:

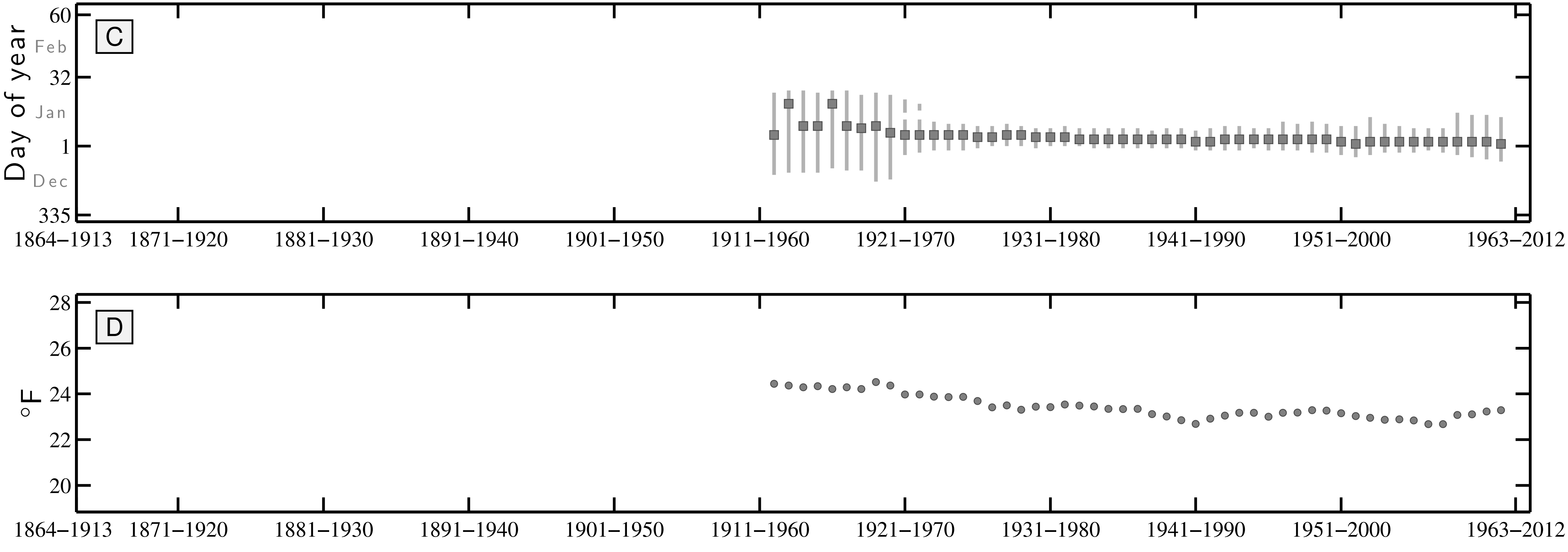

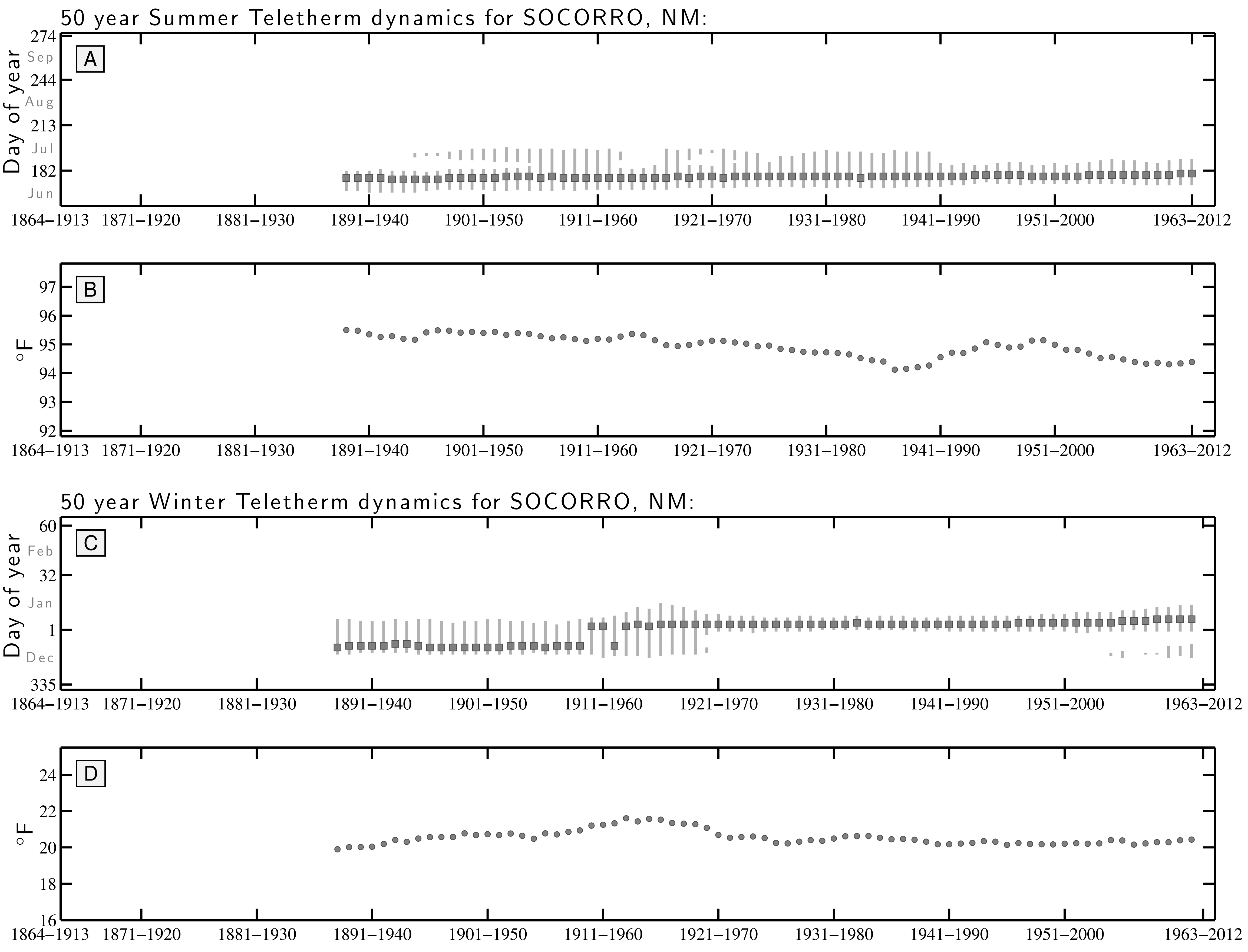

50 year Summer Teletherm dynamics for SOCORRO, NM:
A
B
50 year Winter Teletherm dynamics for SOCORRO, NM:
C
D
Day of year
°F
Sep
Aug
Jul
Jun
Feb
Jan
Dec
274
244
213
182
97
96
95
94
93
92
60
32
1
335
24
22
20
18
16
1864–1913
1871–1920
1881–1930
1891–1940
1901–1950
1911–1960
1921–1970
1931–1980
1941–1990
1951–2000
1963–2012

50 year Summer Teletherm dynamics for SPRINGER, NM:

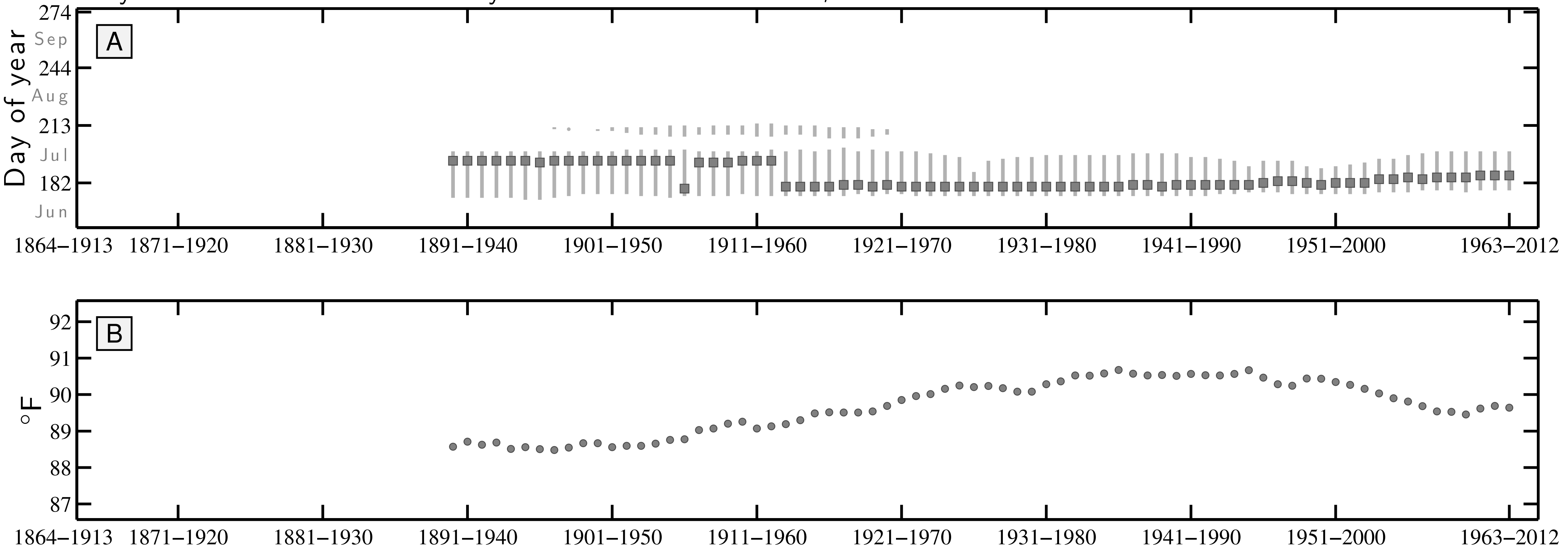

50 year Winter Teletherm dynamics for SPRINGER, NM:

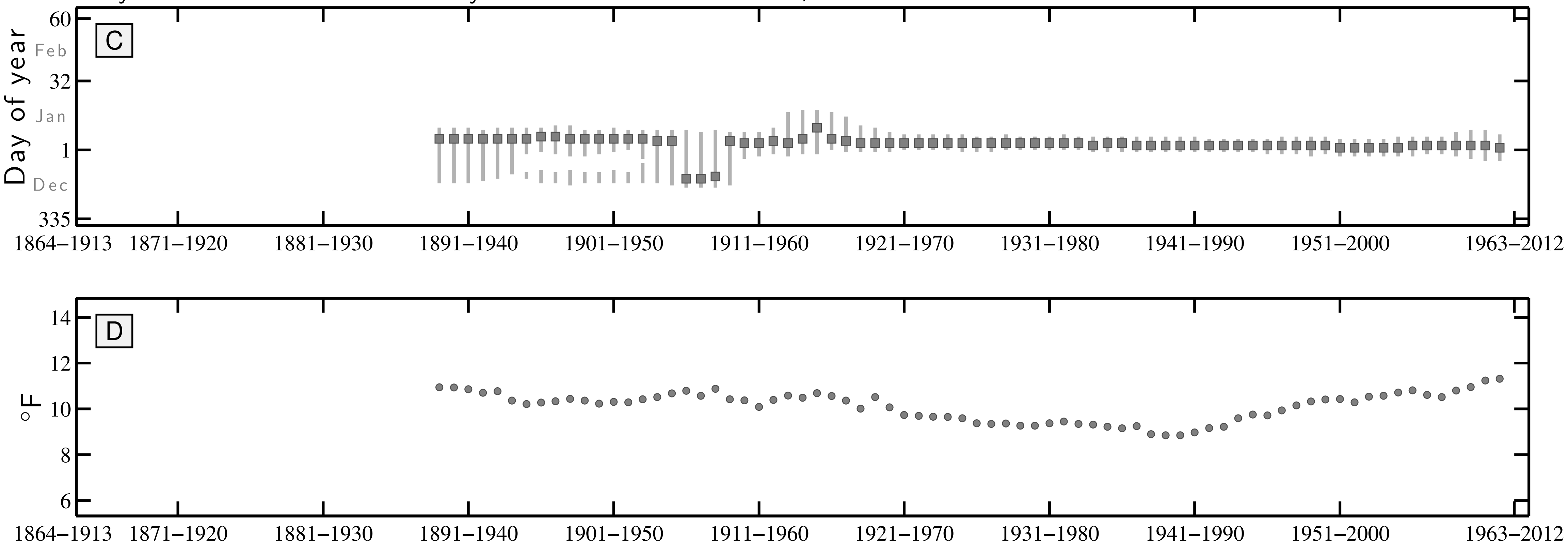

50 year Summer Teletherm dynamics for STATE UNIV, NM:

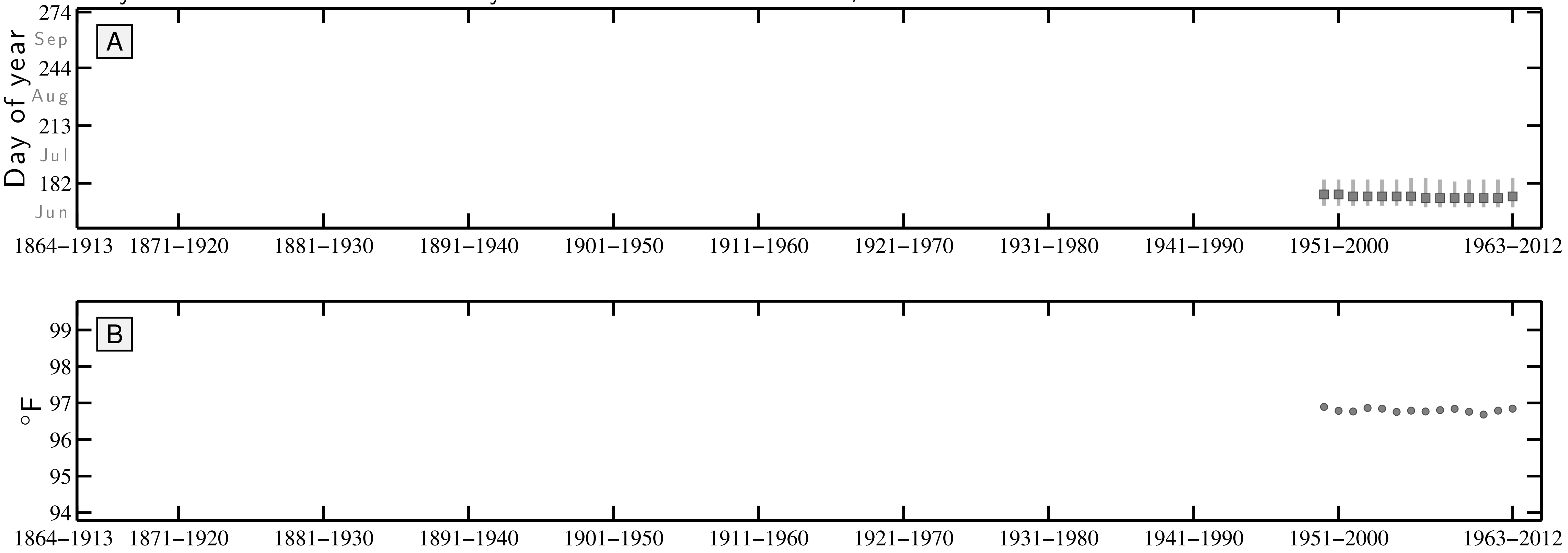

50 year Winter Teletherm dynamics for STATE UNIV, NM:

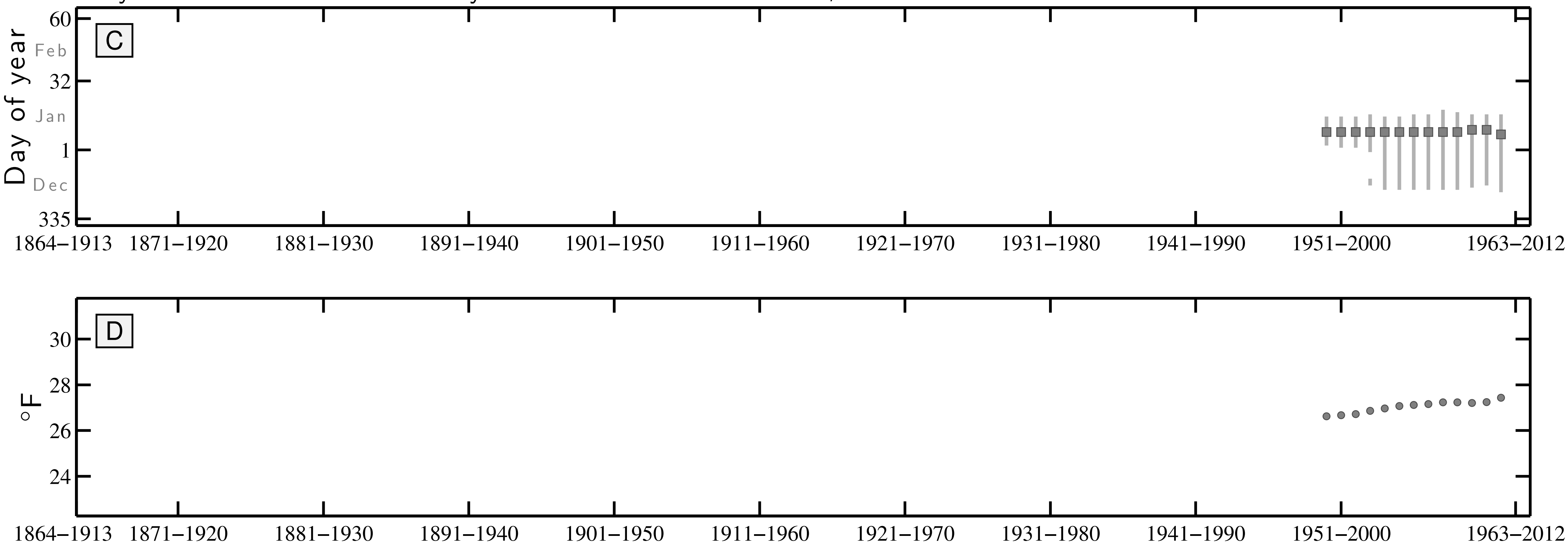

50 year Summer Teletherm dynamics for TUCUMCARI 4 NE, NM:

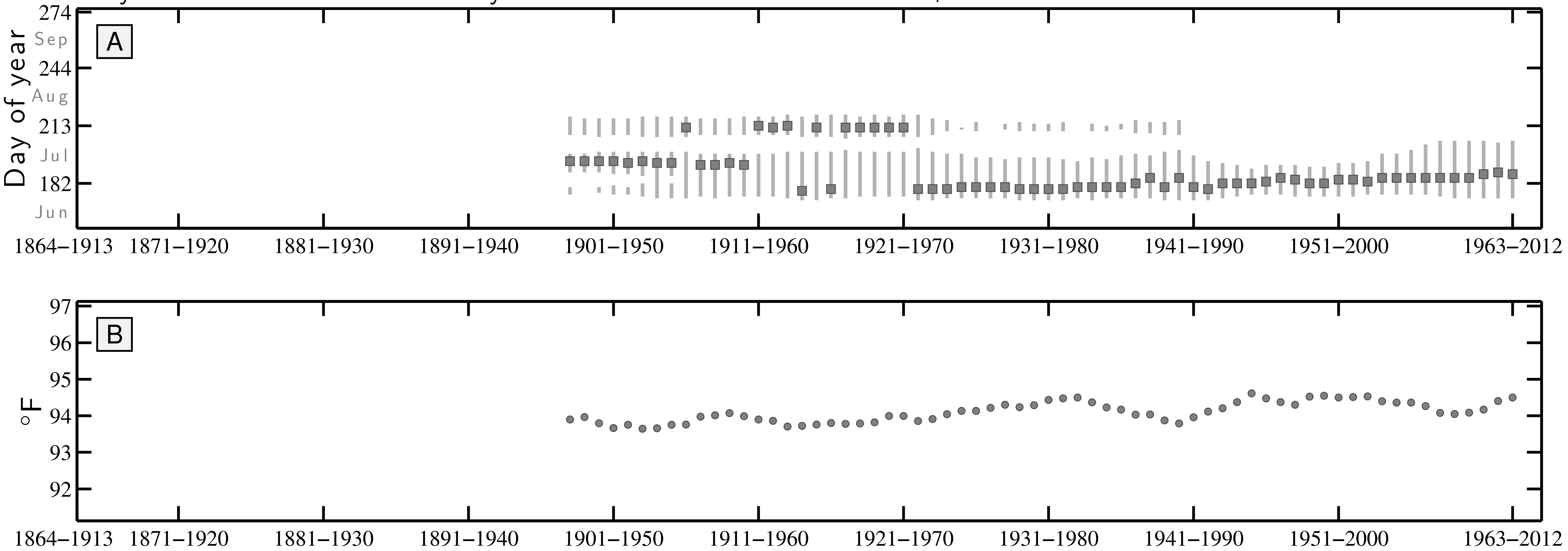

50 year Winter Teletherm dynamics for TUCUMCARI 4 NE, NM:

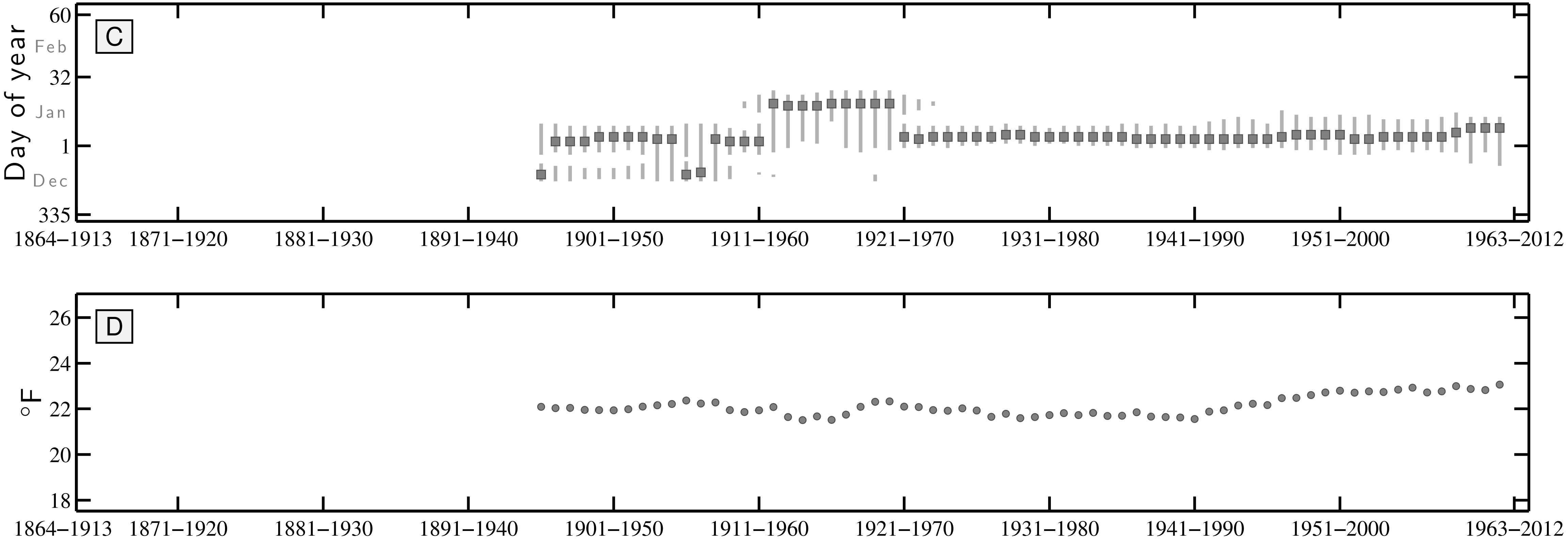

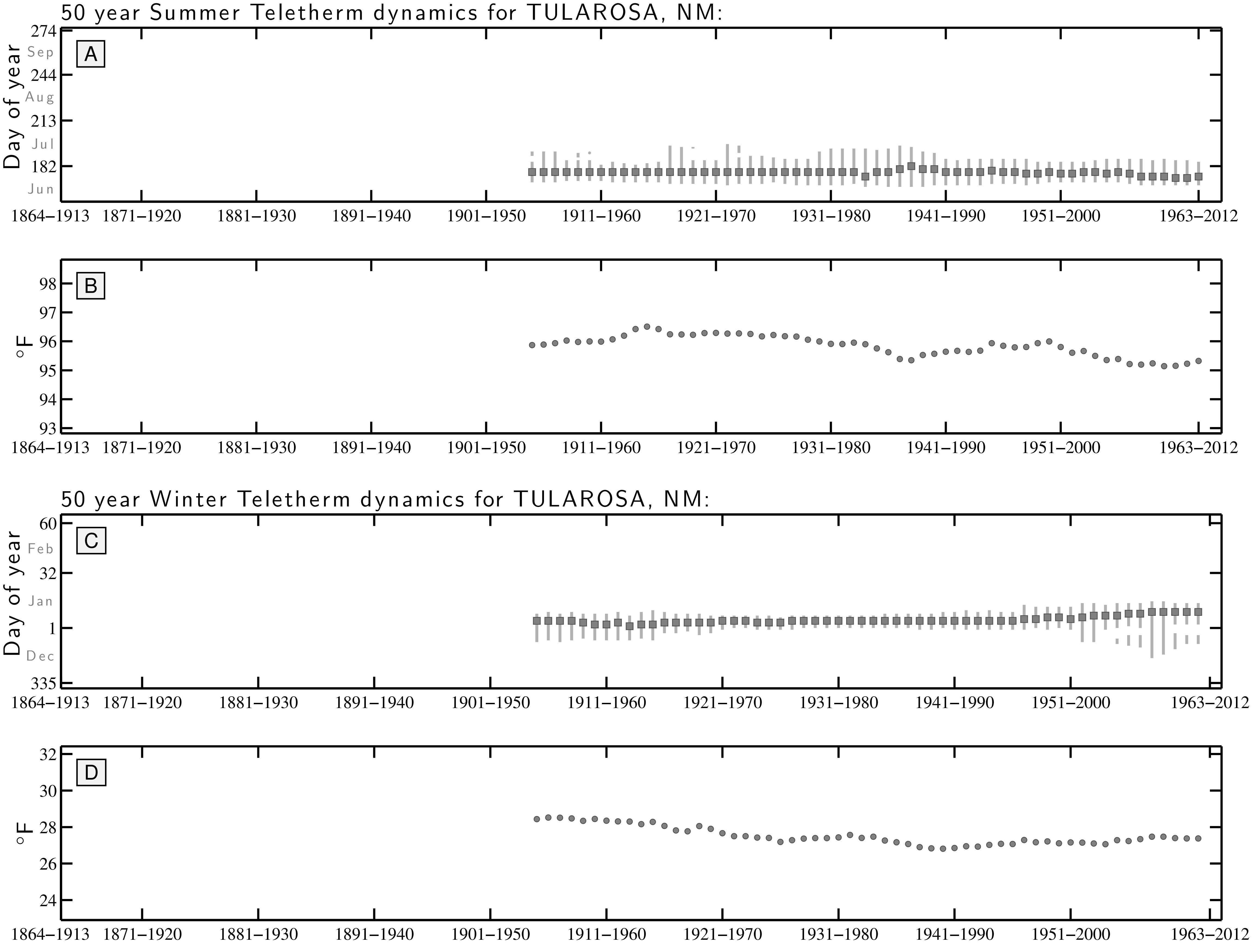
50 year Summer Teletherm dynamics for TULAROSA, NM:
A
Day of year
274
Sep
244
Aug
213
Jul
182
Jun
B
°F
98
97
96
95
94
93
50 year Winter Teletherm dynamics for TULAROSA, NM:
C
Day of year
60
Feb
32
Jan
1
Dec
335
D
°F
32
30
28
26
24
1864–1913
1871–1920
1881–1930
1891–1940
1901–1950
1911–1960
1921–1970
1931–1980
1941–1990
1951–2000
1963–2012

50 year Summer Teletherm dynamics for ADDISON, NY:

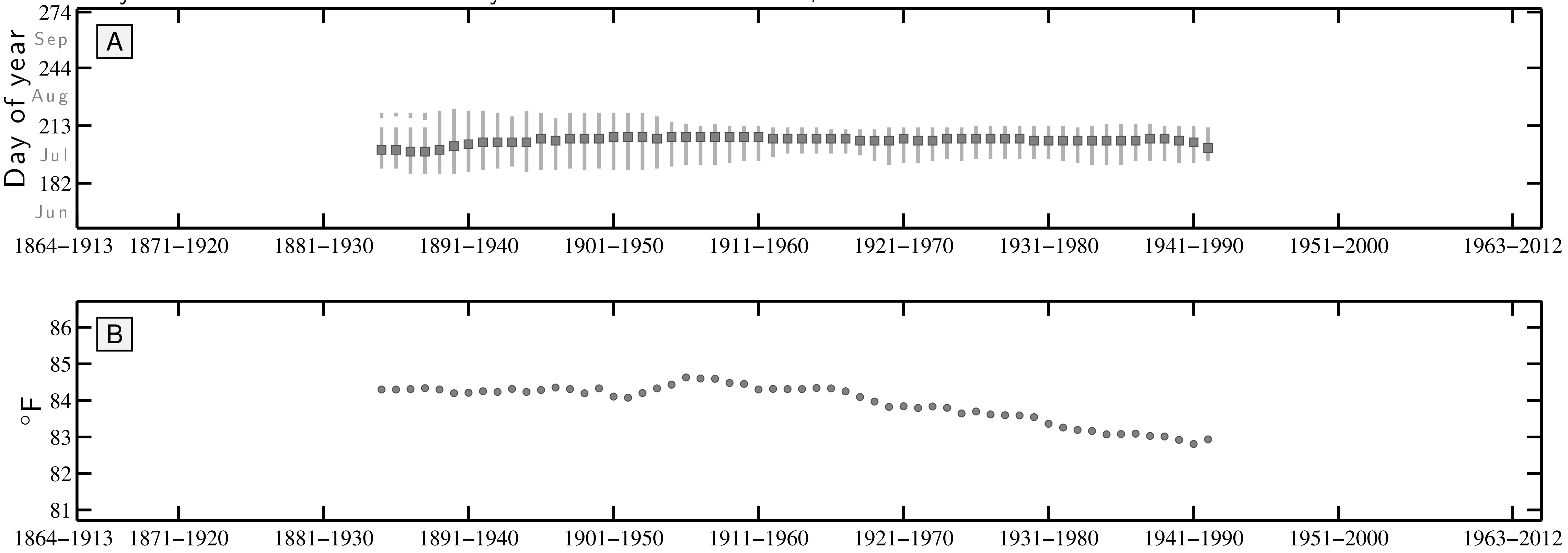

50 year Winter Teletherm dynamics for ADDISON, NY:

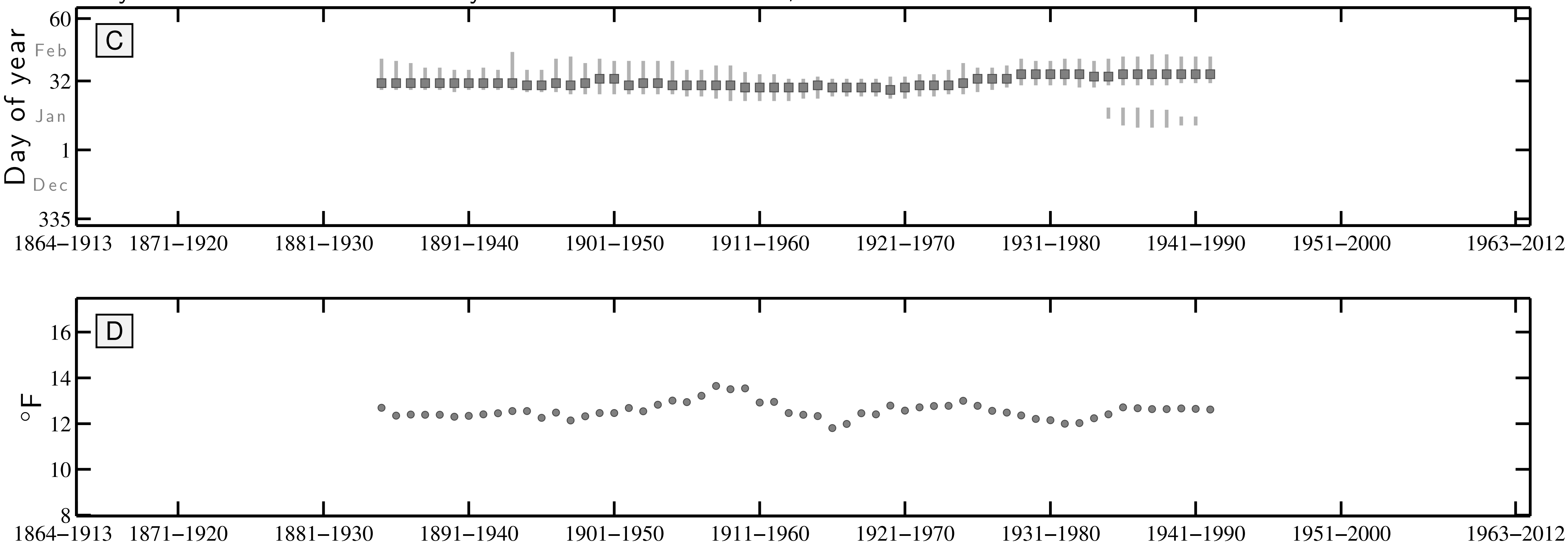

50 year Summer Teletherm dynamics for ALBANY INTL AP, NY:

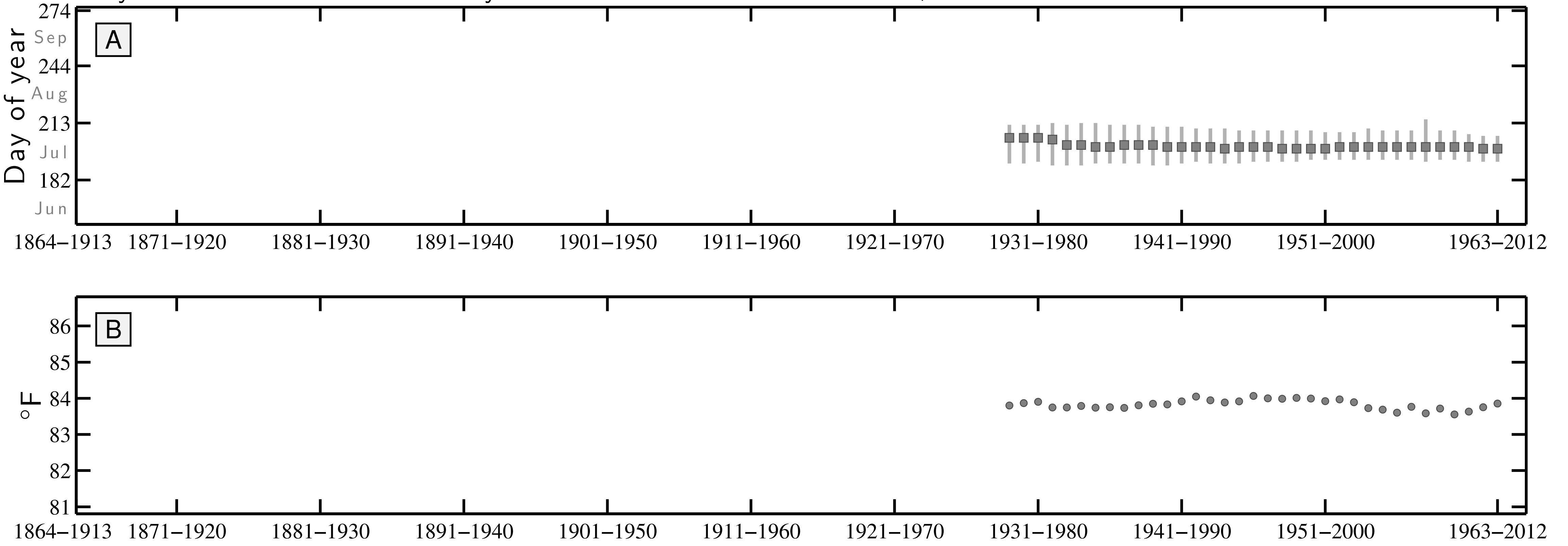

50 year Winter Teletherm dynamics for ALBANY INTL AP, NY:

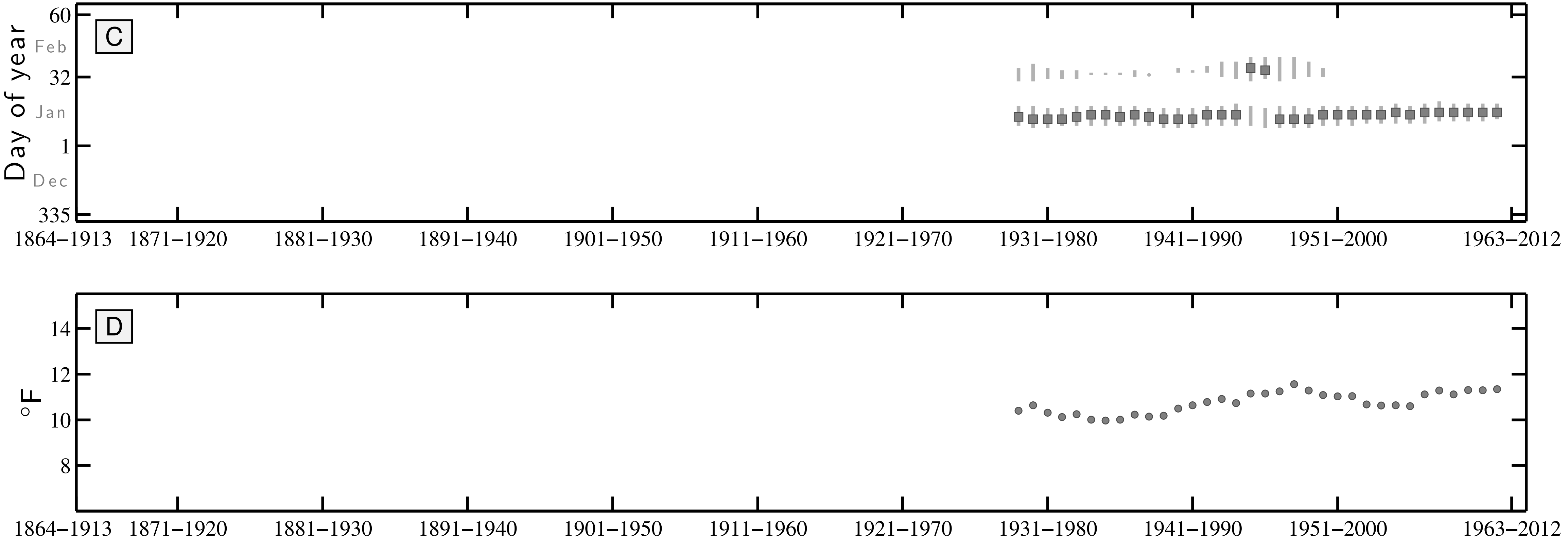

50 year Summer Teletherm dynamics for ALFRED, NY:

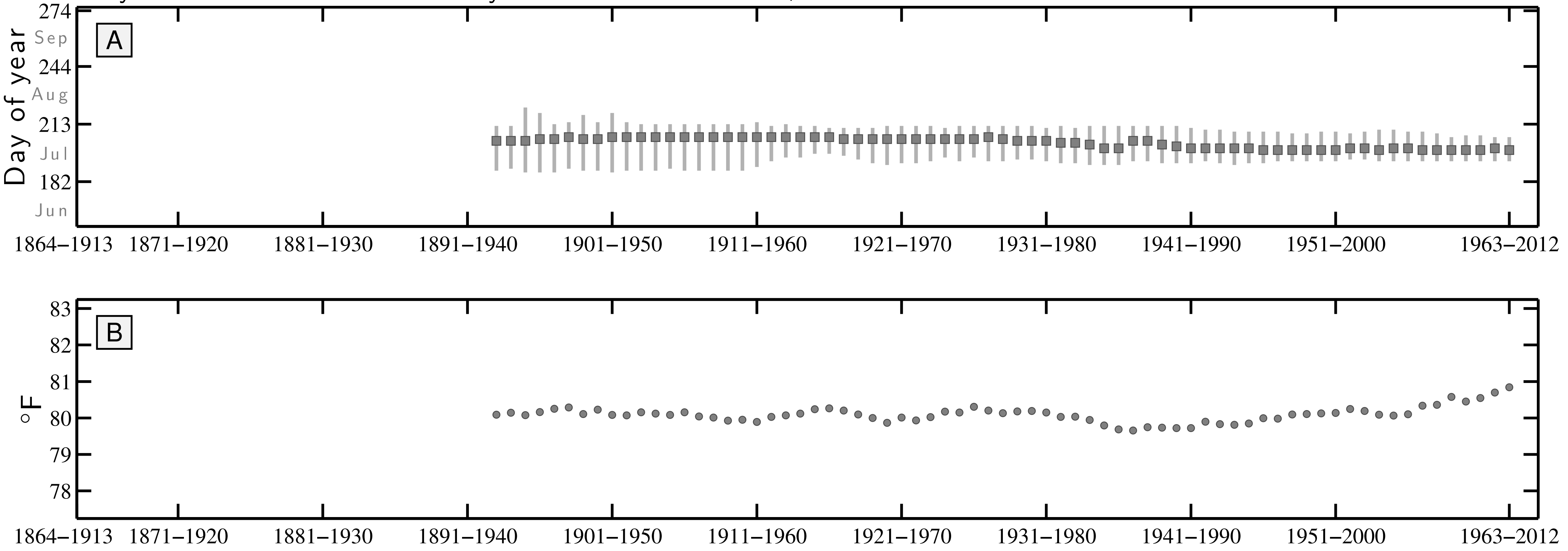

50 year Winter Teletherm dynamics for ALFRED, NY:

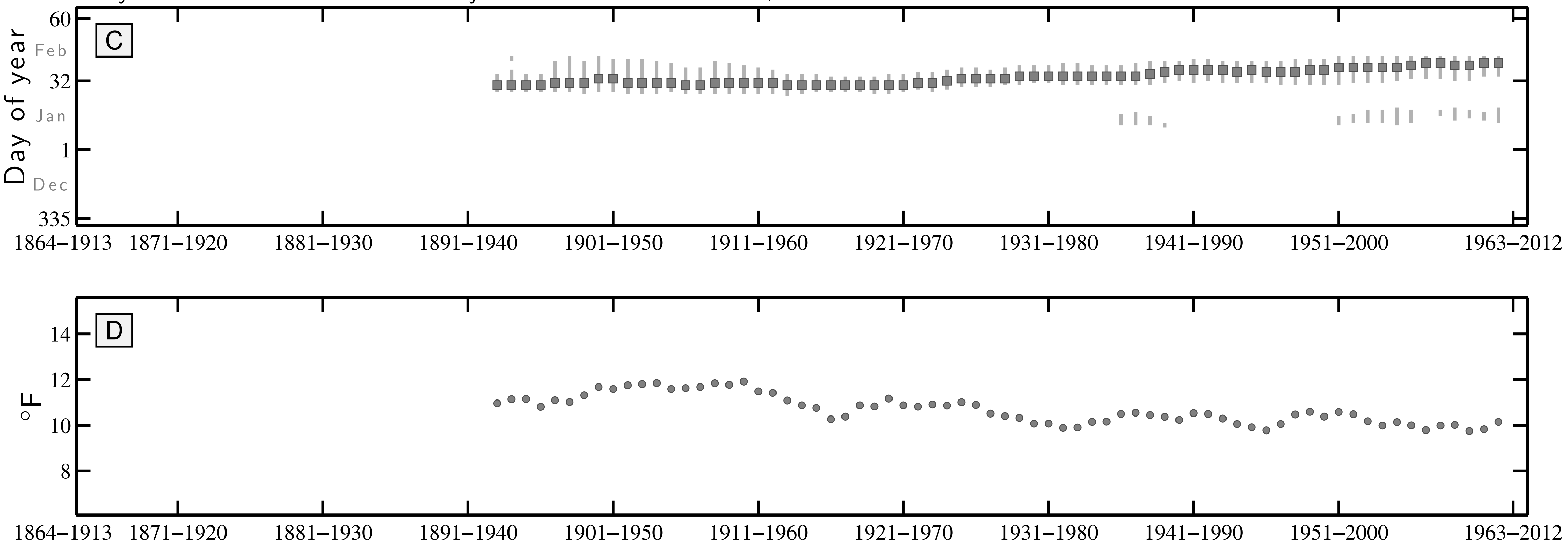

50 year Summer Teletherm dynamics for ALLEGANY SP, NY:

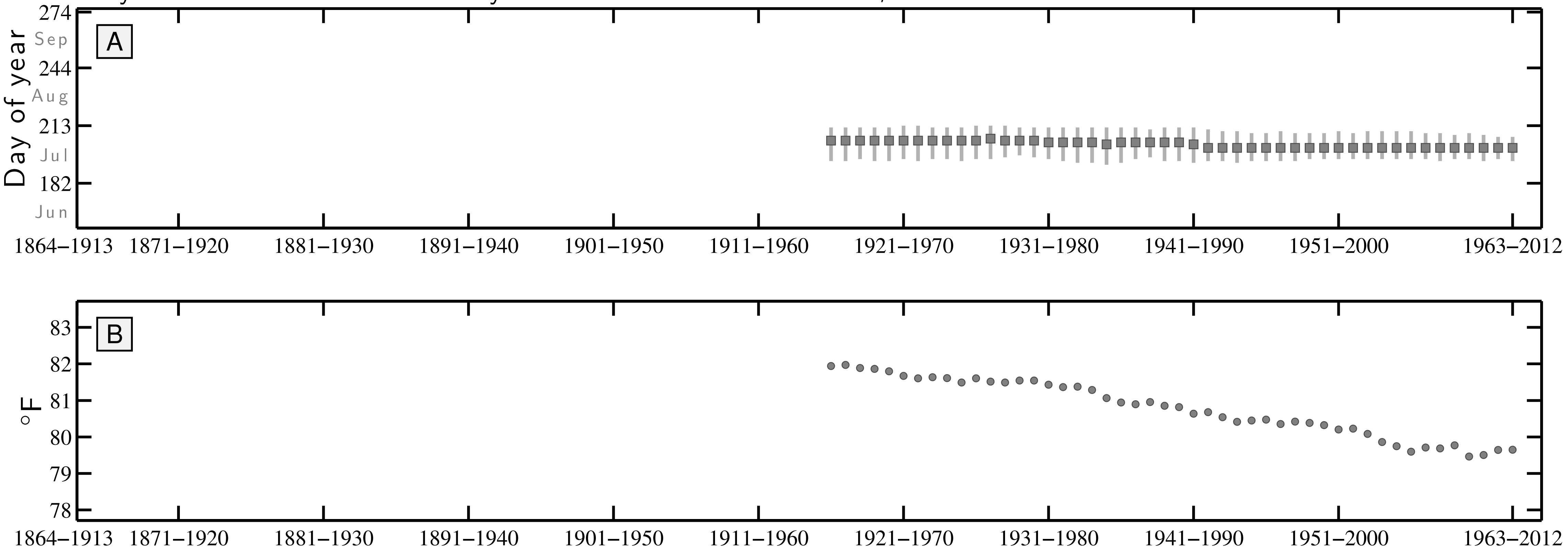

50 year Winter Teletherm dynamics for ALLEGANY SP, NY:

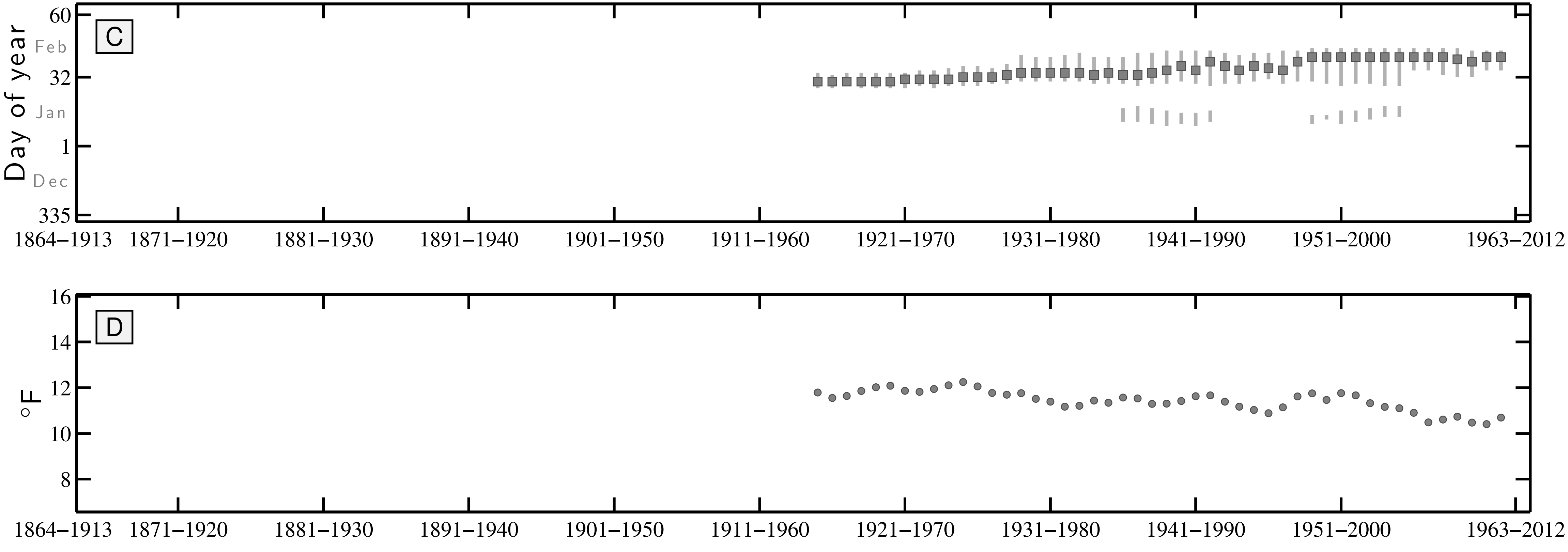

50 year Summer Teletherm dynamics for ANGELICA, NY:

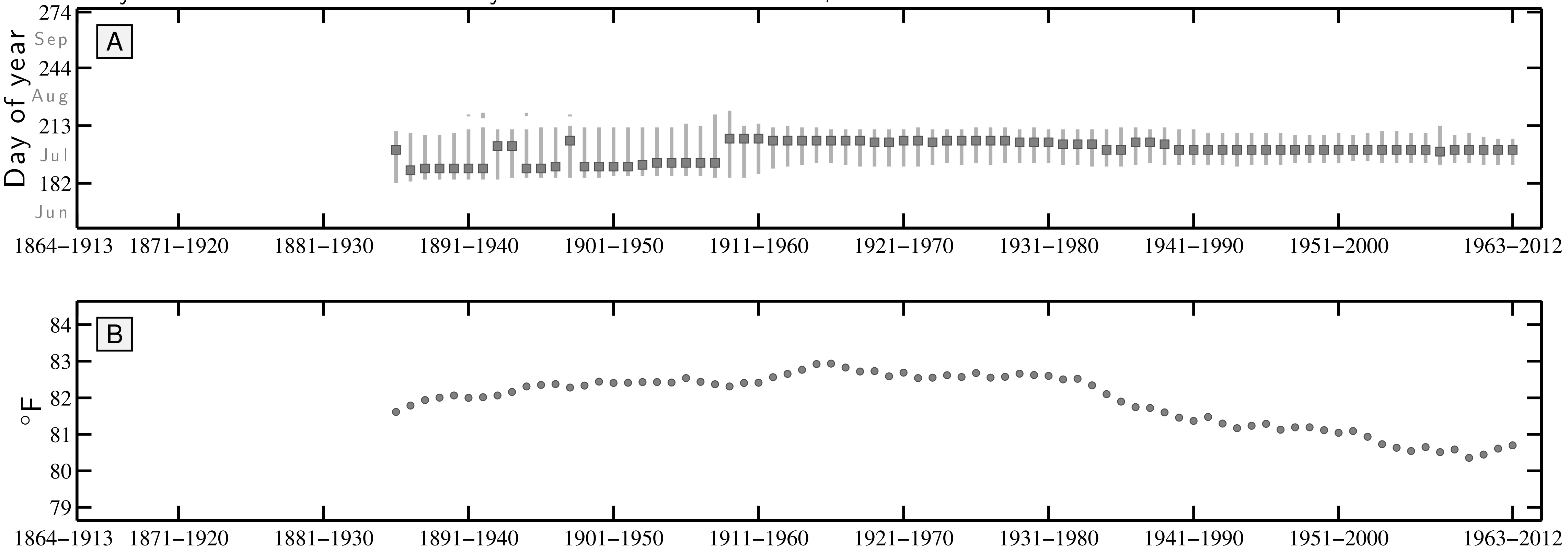

50 year Winter Teletherm dynamics for ANGELICA, NY:

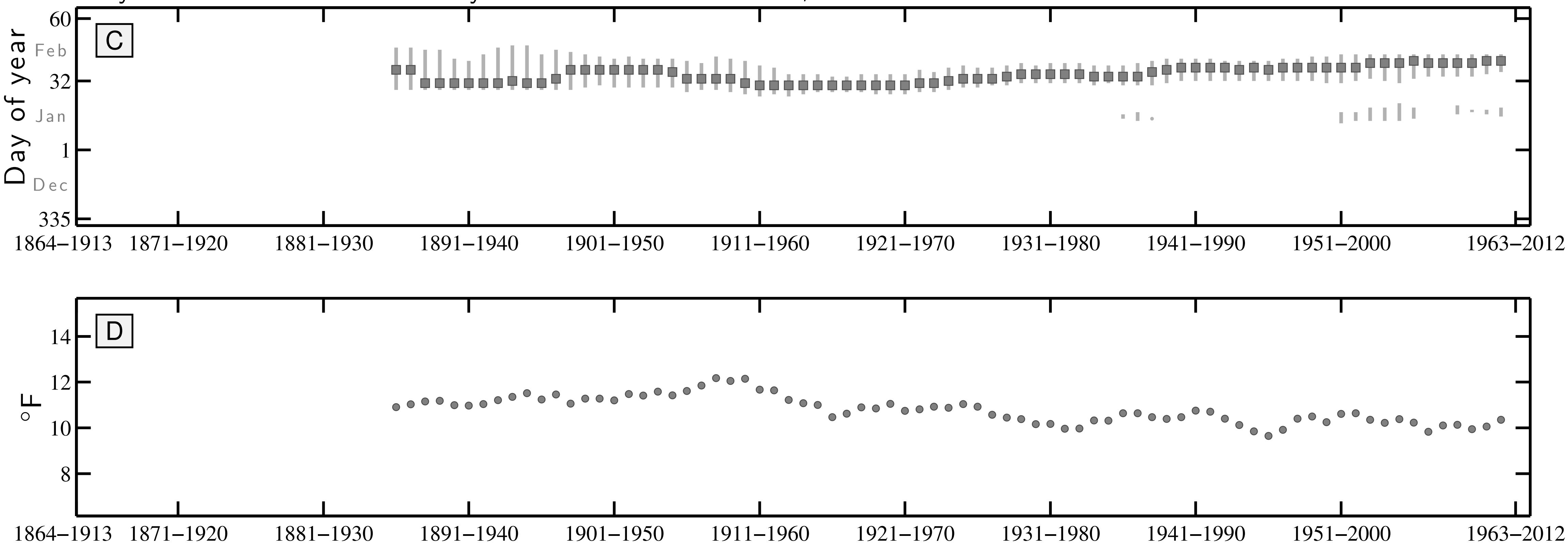

50 year Summer Teletherm dynamics for AUBURN, NY:

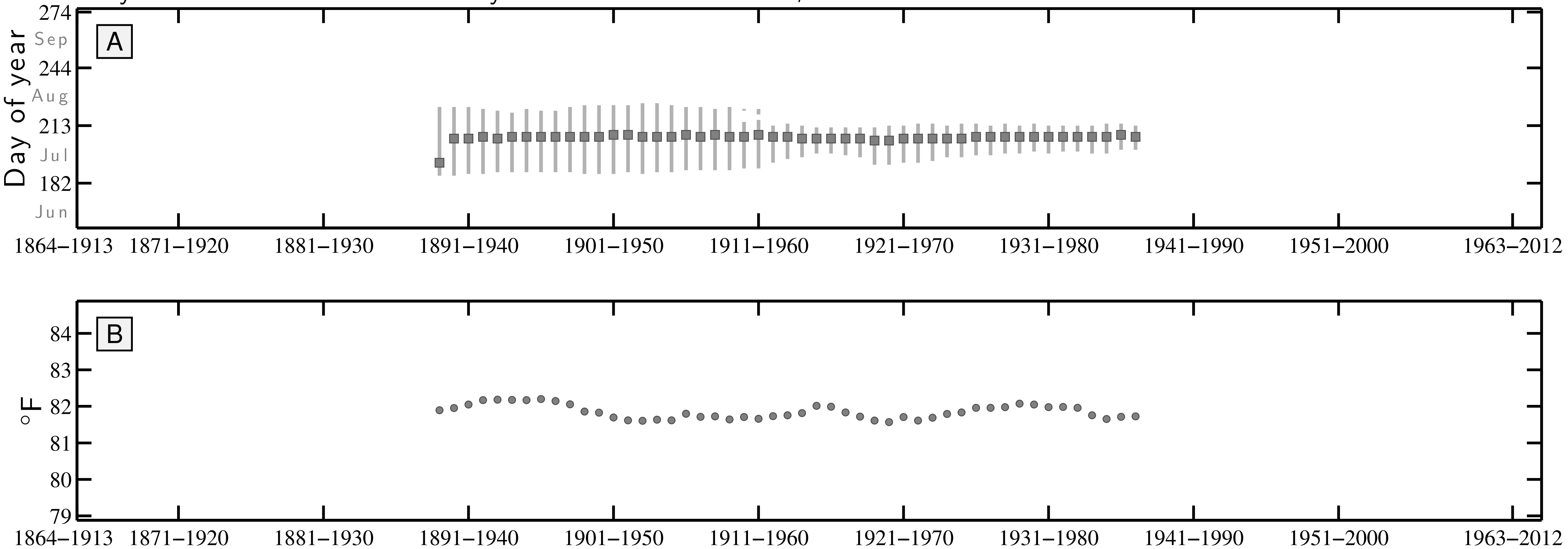

50 year Winter Teletherm dynamics for AUBURN, NY:

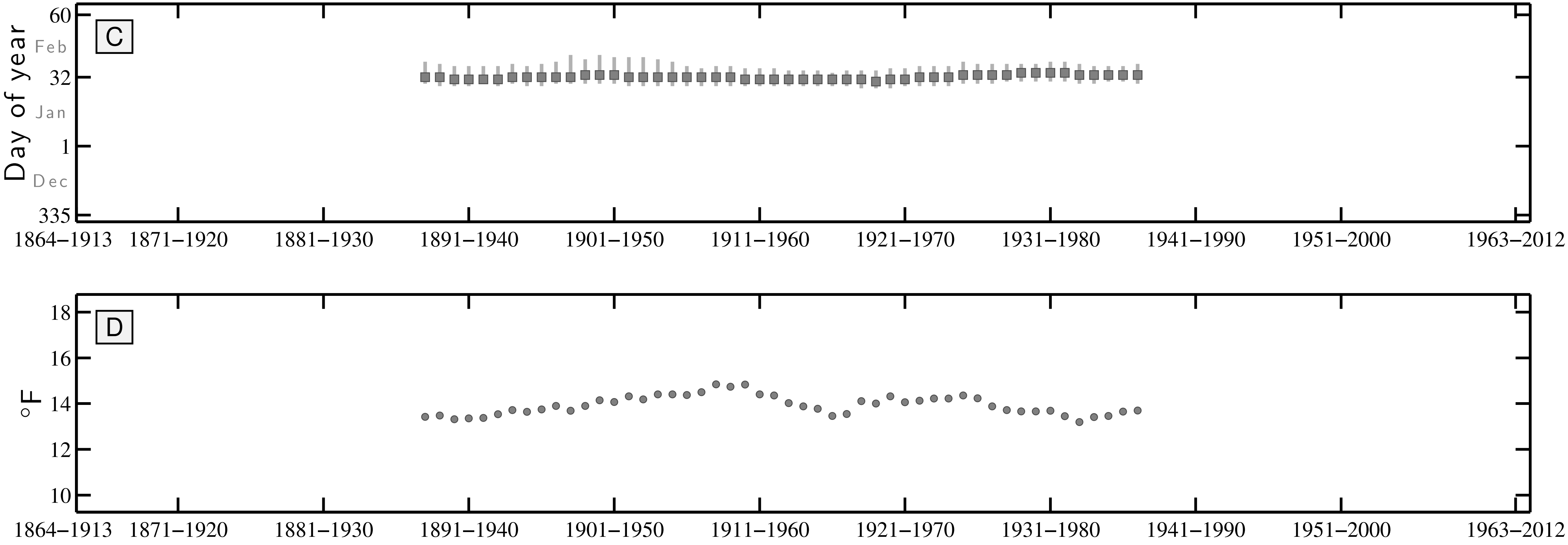

50 year Summer Teletherm dynamics for BATAVIA, NY:

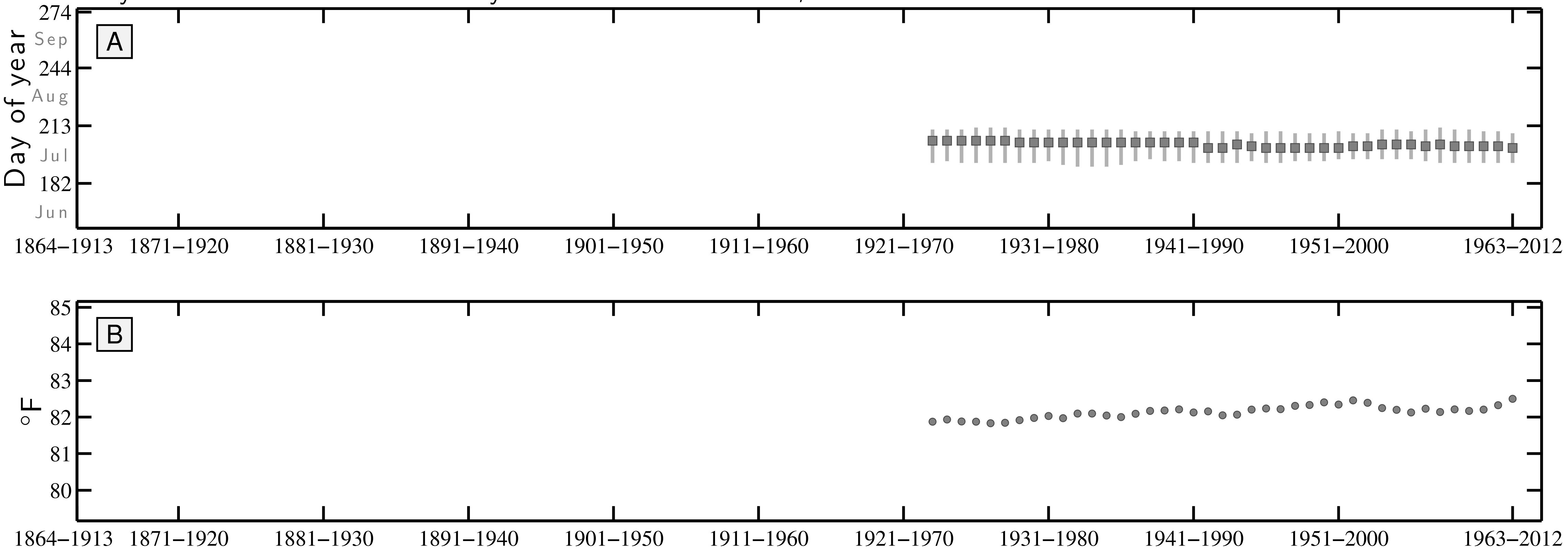

50 year Winter Teletherm dynamics for BATAVIA, NY:

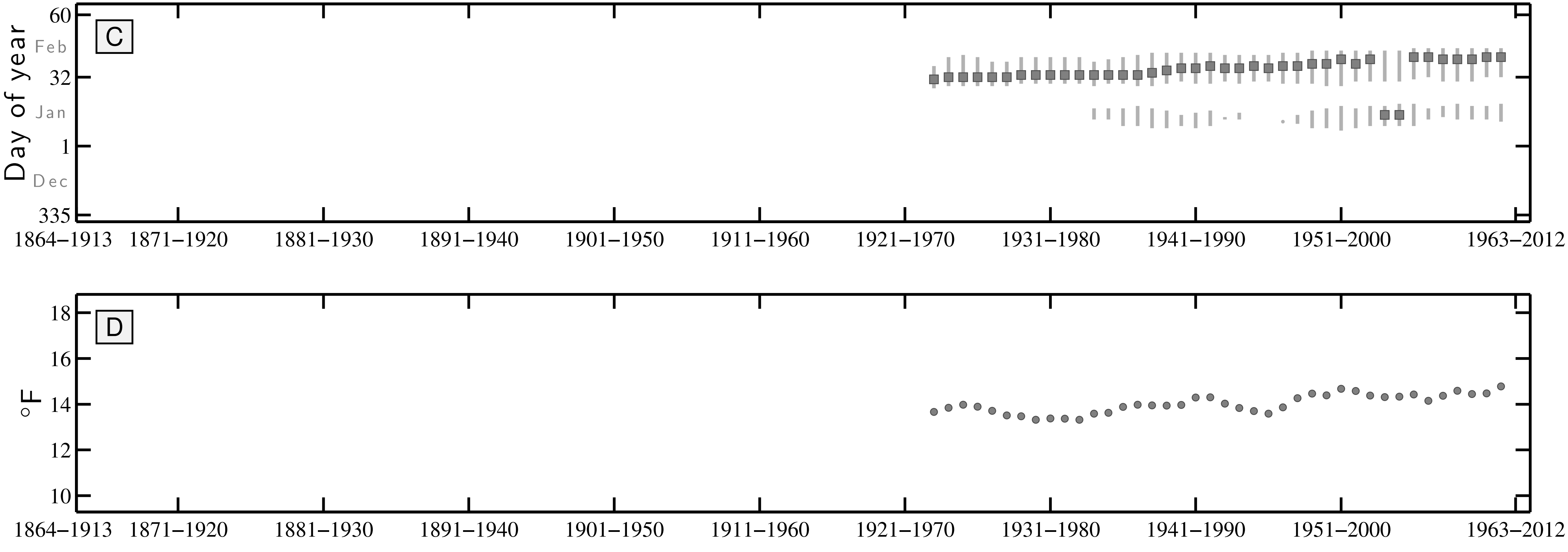

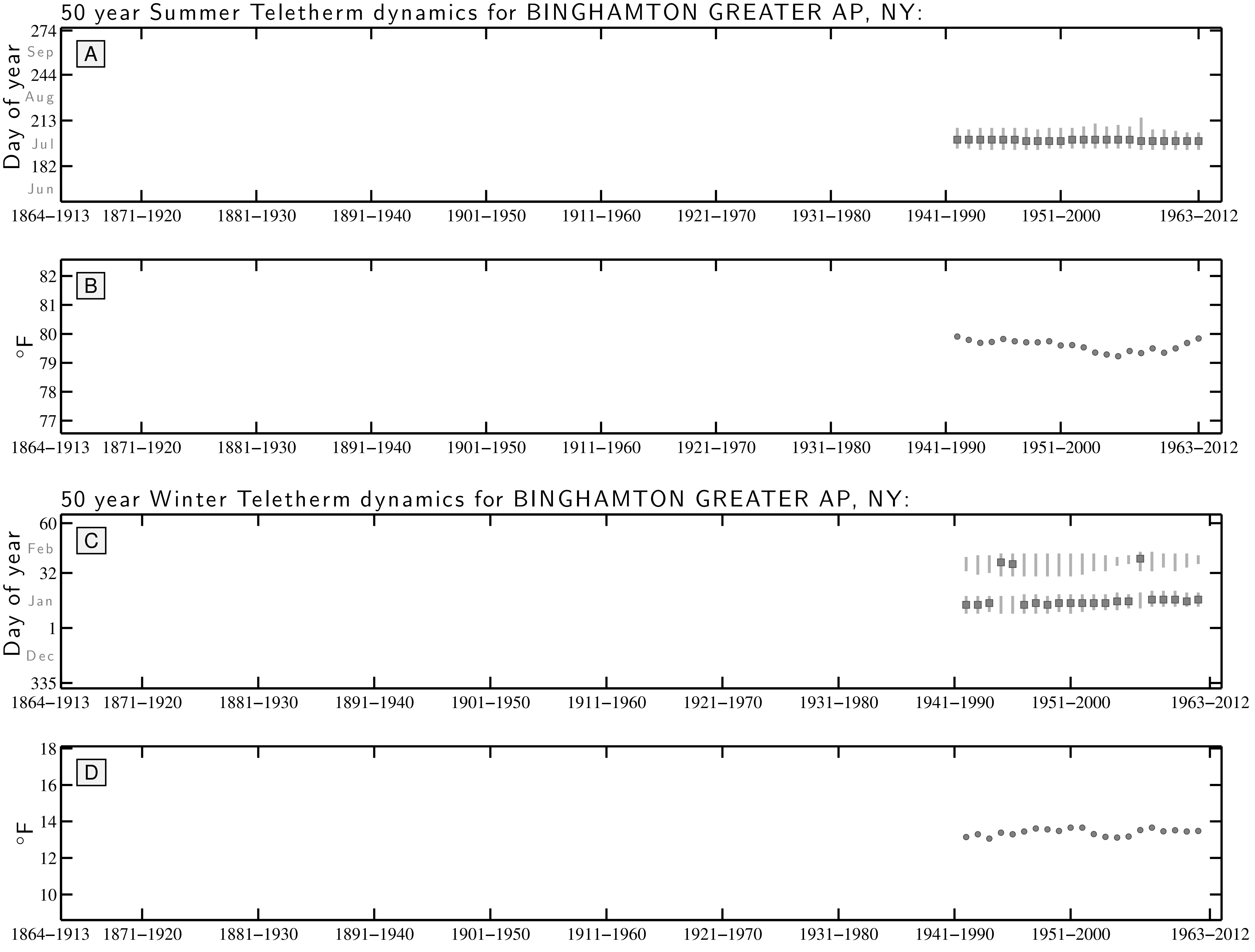

50 year Summer Teletherm dynamics for BINGHAMTON GREATER AP, NY:
A
Day of year
274
Sep
244
Aug
213
Jul
182
Jun
1864–1913
1871–1920
1881–1930
1891–1940
1901–1950
1911–1960
1921–1970
1931–1980
1941–1990
1951–2000
1963–2012
B
°F
82
81
80
79
78
77
50 year Winter Teletherm dynamics for BINGHAMTON GREATER AP, NY:
C
Day of year
60
Feb
32
Jan
1
Dec
335
D
°F
18
16
14
12
10

50 year Summer Teletherm dynamics for BRIDGEHAMPTON, NY:

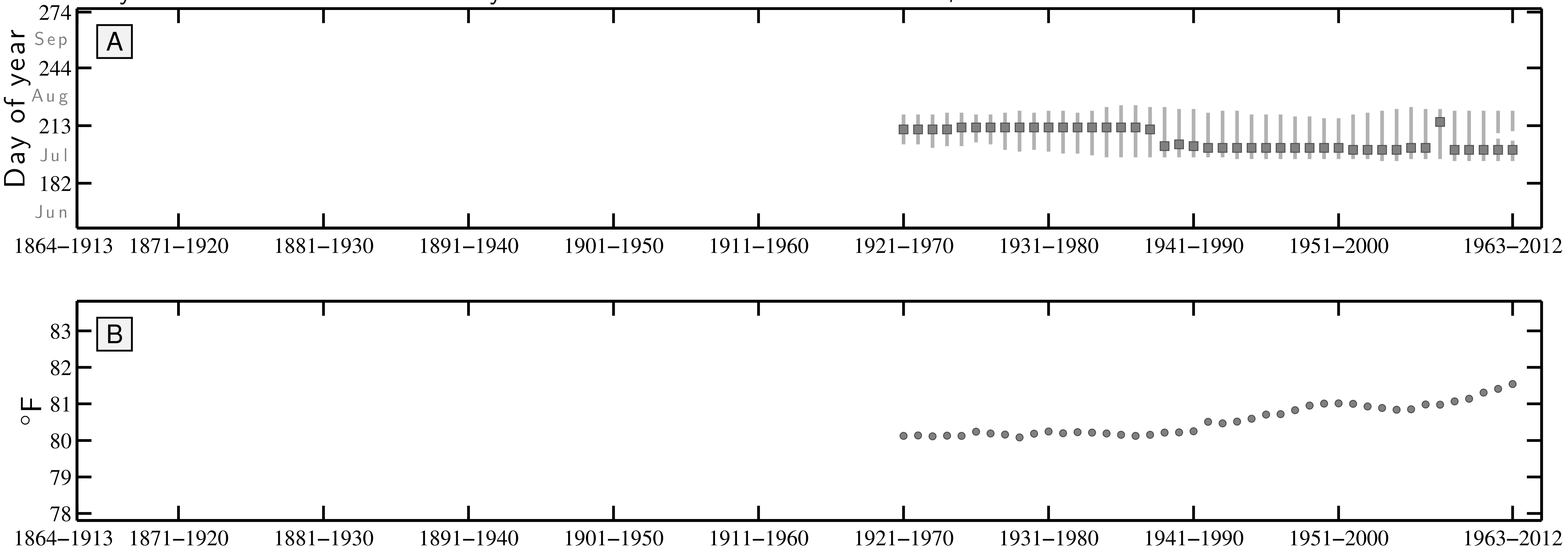

50 year Winter Teletherm dynamics for BRIDGEHAMPTON, NY:

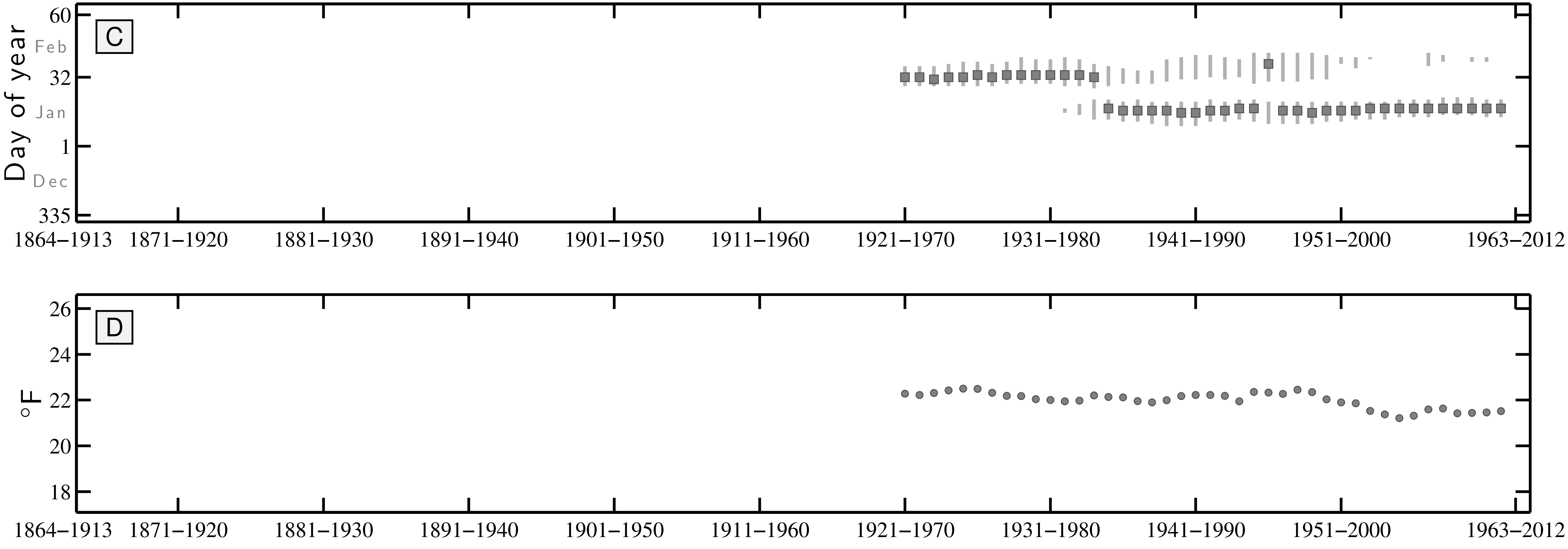

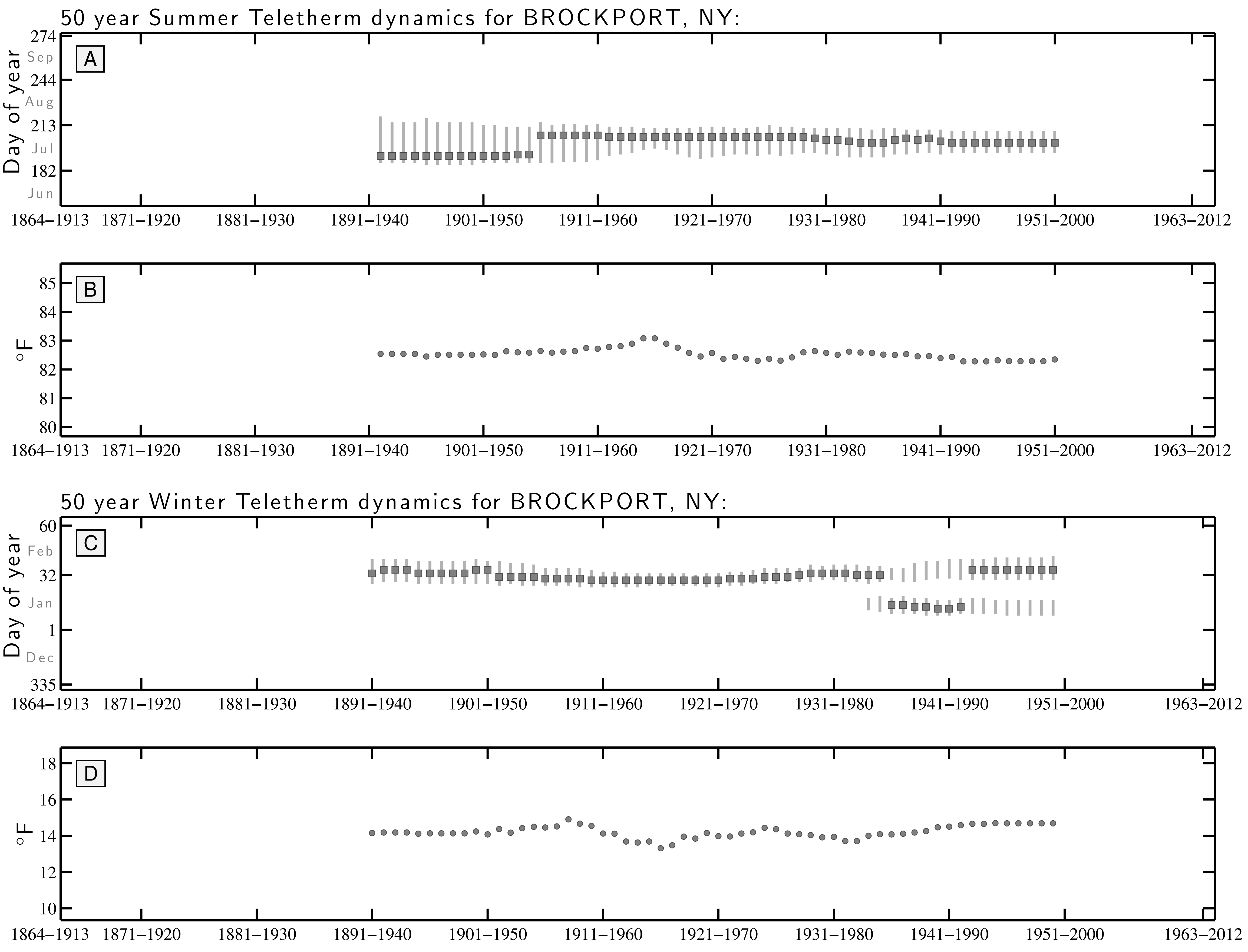
50 year Summer Teletherm dynamics for BROCKPORT, NY:
A
Day of year
274
Sep
244
Aug
213
Jul
182
Jun
1864–1913
1871–1920
1881–1930
1891–1940
1901–1950
1911–1960
1921–1970
1931–1980
1941–1990
1951–2000
1963–2012
B
°F
85
84
83
82
81
80
1864–1913
1871–1920
1881–1930
1891–1940
1901–1950
1911–1960
1921–1970
1931–1980
1941–1990
1951–2000
1963–2012
50 year Winter Teletherm dynamics for BROCKPORT, NY:
C
Day of year
60
Feb
32
Jan
1
Dec
335
1864–1913
1871–1920
1881–1930
1891–1940
1901–1950
1911–1960
1921–1970
1931–1980
1941–1990
1951–2000
1963–2012
D
°F
18
16
14
12
10
1864–1913
1871–1920
1881–1930
1891–1940
1901–1950
1911–1960
1921–1970
1931–1980
1941–1990
1951–2000
1963–2012

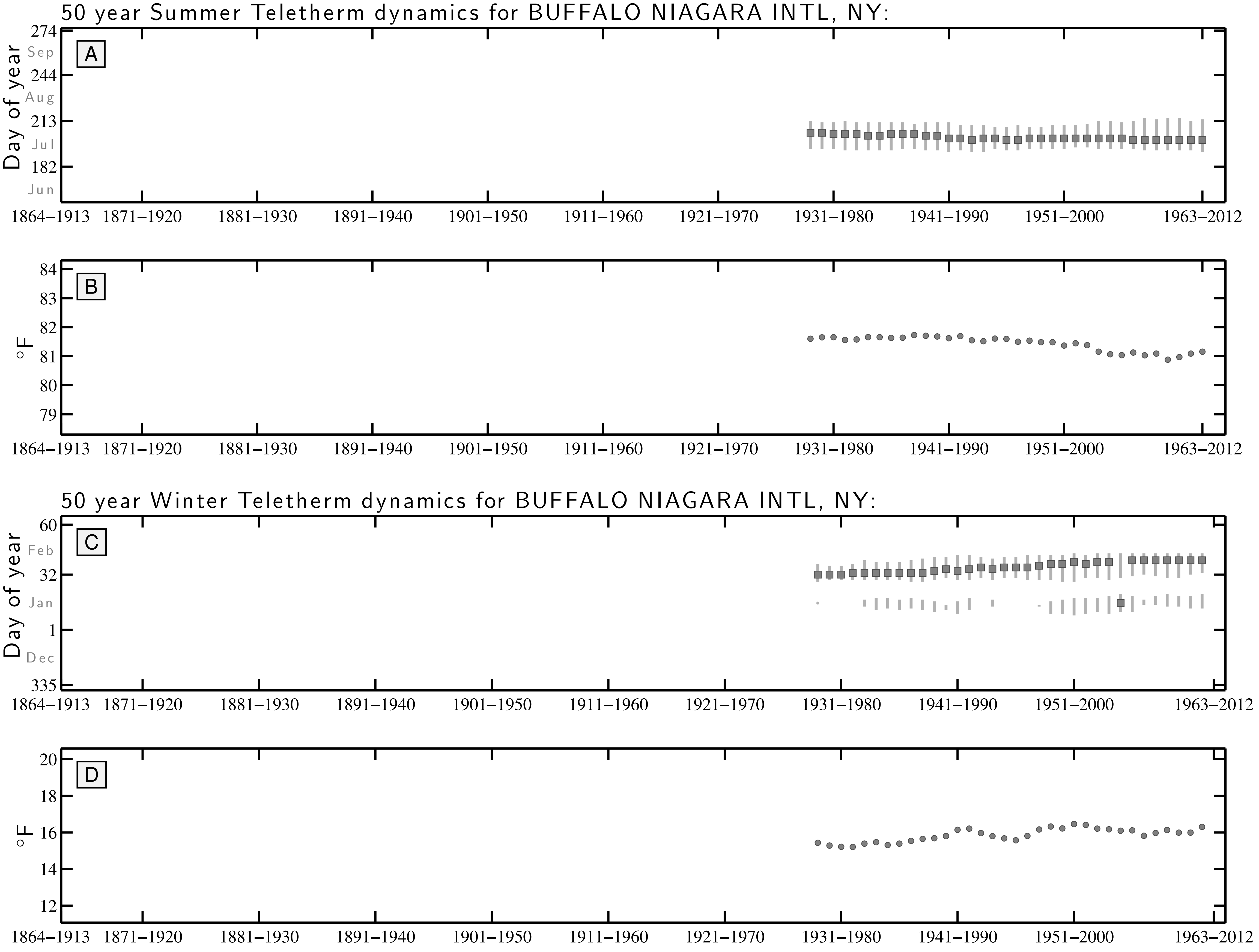
50 year Summer Teletherm dynamics for BUFFALO NIAGARA INTL, NY:
A
B
Day of year
°F
274
Sep
244
Aug
213
Jul
182
Jun
84
83
82
81
80
79
50 year Winter Teletherm dynamics for BUFFALO NIAGARA INTL, NY:
C
D
60
Feb
32
Jan
1
Dec
335
20
18
16
14
12
1864–1913
1871–1920
1881–1930
1891–1940
1901–1950
1911–1960
1921–1970
1931–1980
1941–1990
1951–2000
1963–2012

50 year Summer Teletherm dynamics for CANTON 4 SE, NY:

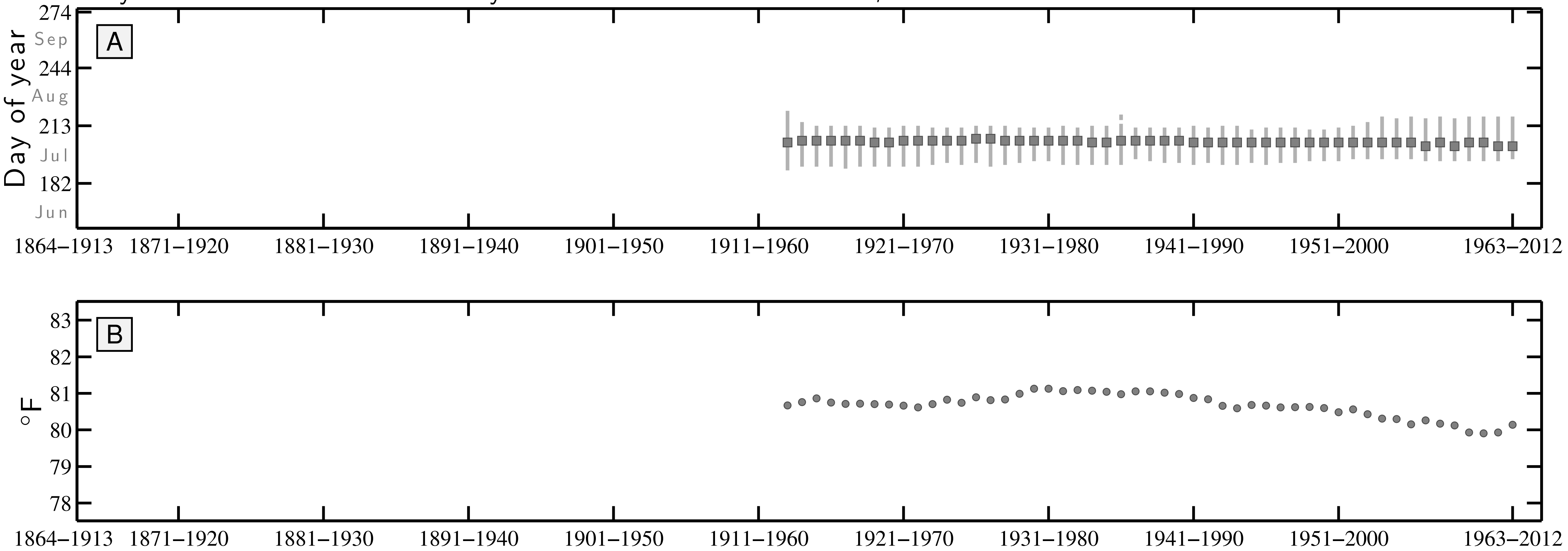

50 year Winter Teletherm dynamics for CANTON 4 SE, NY:

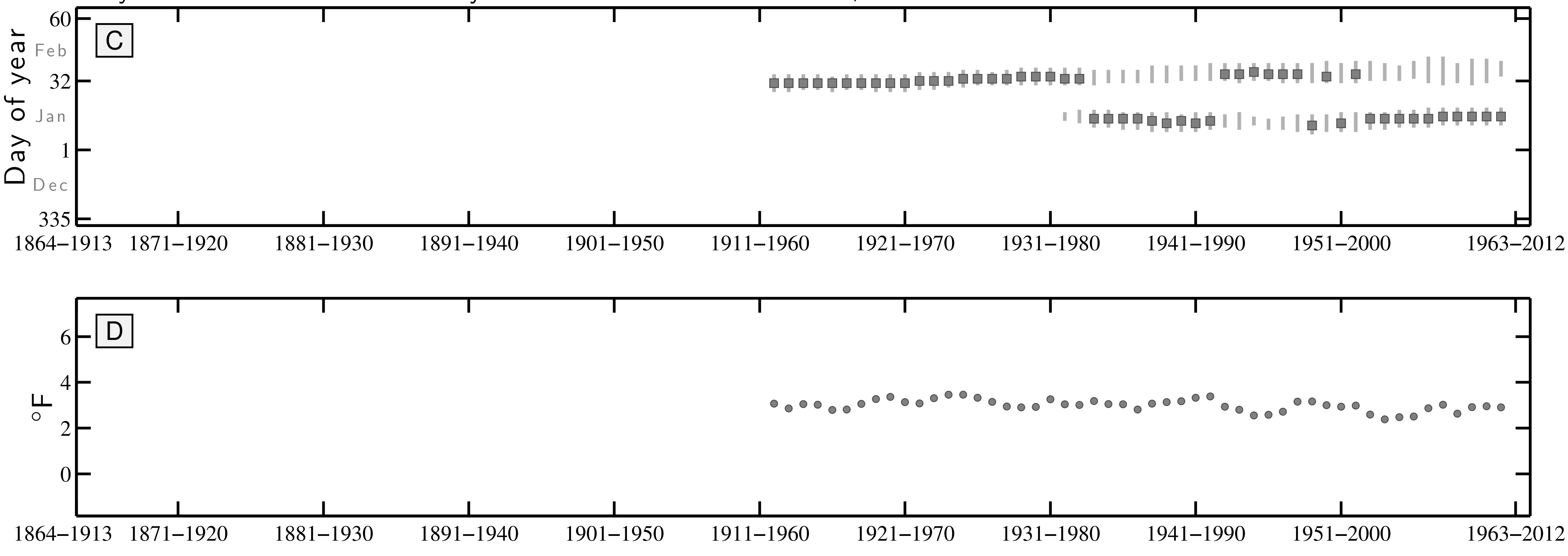

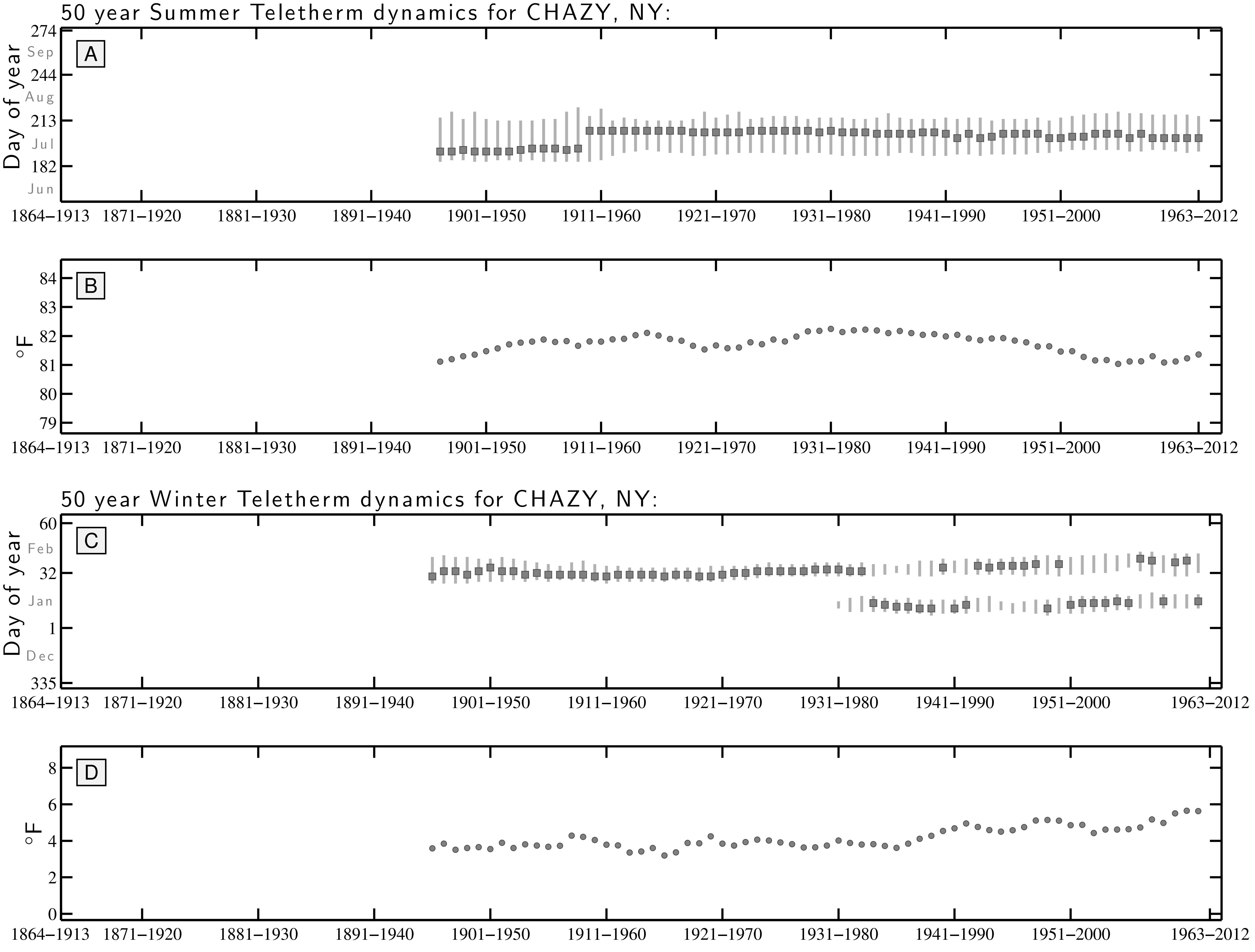
50 year Summer Teletherm dynamics for CHAZY, NY:
A
Day of year
274
Sep
244
Aug
213
Jul
182
Jun
1864–1913
1871–1920
1881–1930
1891–1940
1901–1950
1911–1960
1921–1970
1931–1980
1941–1990
1951–2000
1963–2012
B
°F
84
83
82
81
80
79
50 year Winter Teletherm dynamics for CHAZY, NY:
C
60
Feb
32
Jan
1
Dec
335
D
8
6
4
2
0

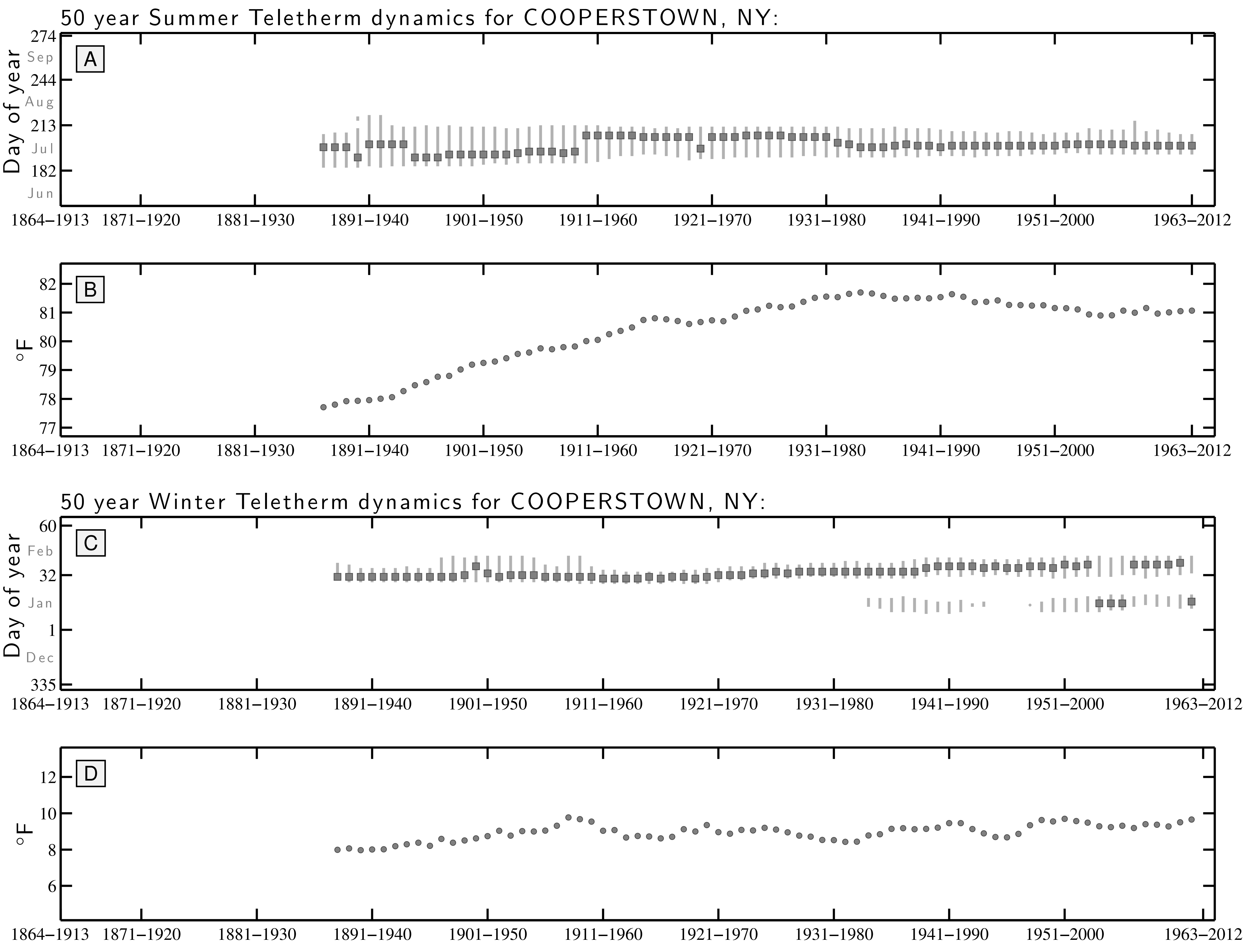
50 year Summer Teletherm dynamics for COOPERSTOWN, NY:
A
Day of year
274
Sep
244
Aug
213
Jul
182
Jun
1864–1913
1871–1920
1881–1930
1891–1940
1901–1950
1911–1960
1921–1970
1931–1980
1941–1990
1951–2000
1963–2012
B
°F
82
81
80
79
78
77
50 year Winter Teletherm dynamics for COOPERSTOWN, NY:
C
60
Feb
32
Jan
1
Dec
335
D
12
10
8
6

50 year Summer Teletherm dynamics for CORTLAND, NY:

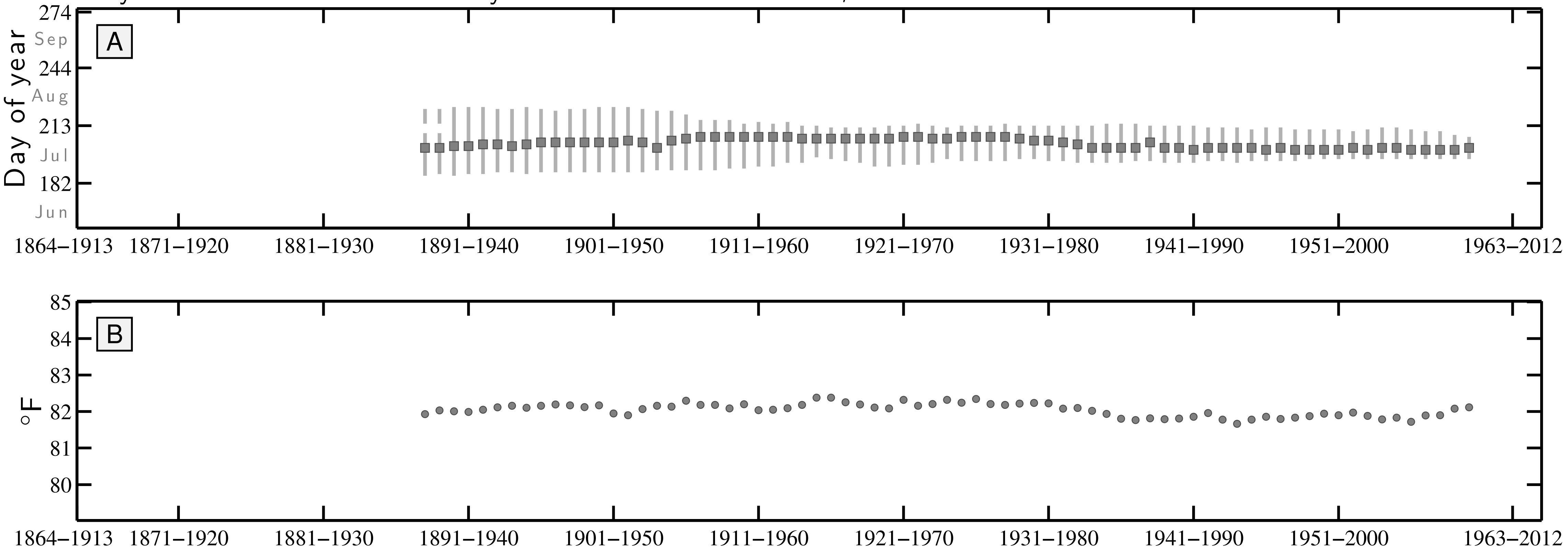

50 year Winter Teletherm dynamics for CORTLAND, NY:

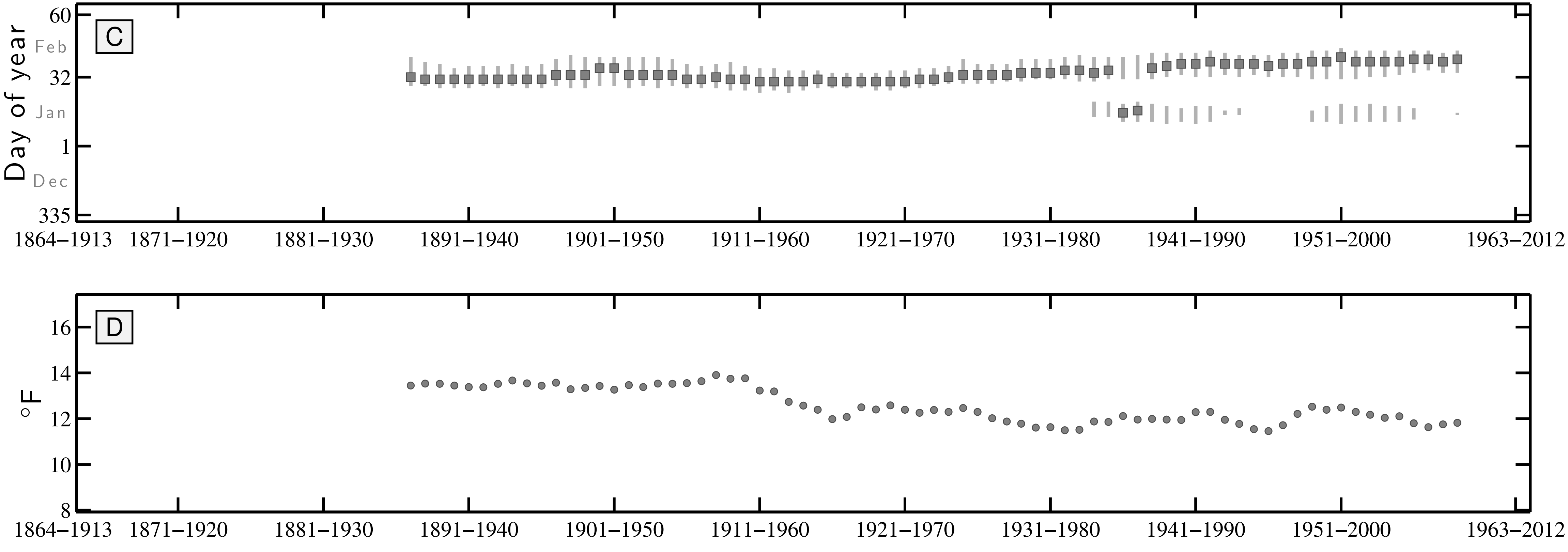

50 year Summer Teletherm dynamics for DANNEMORA, NY:

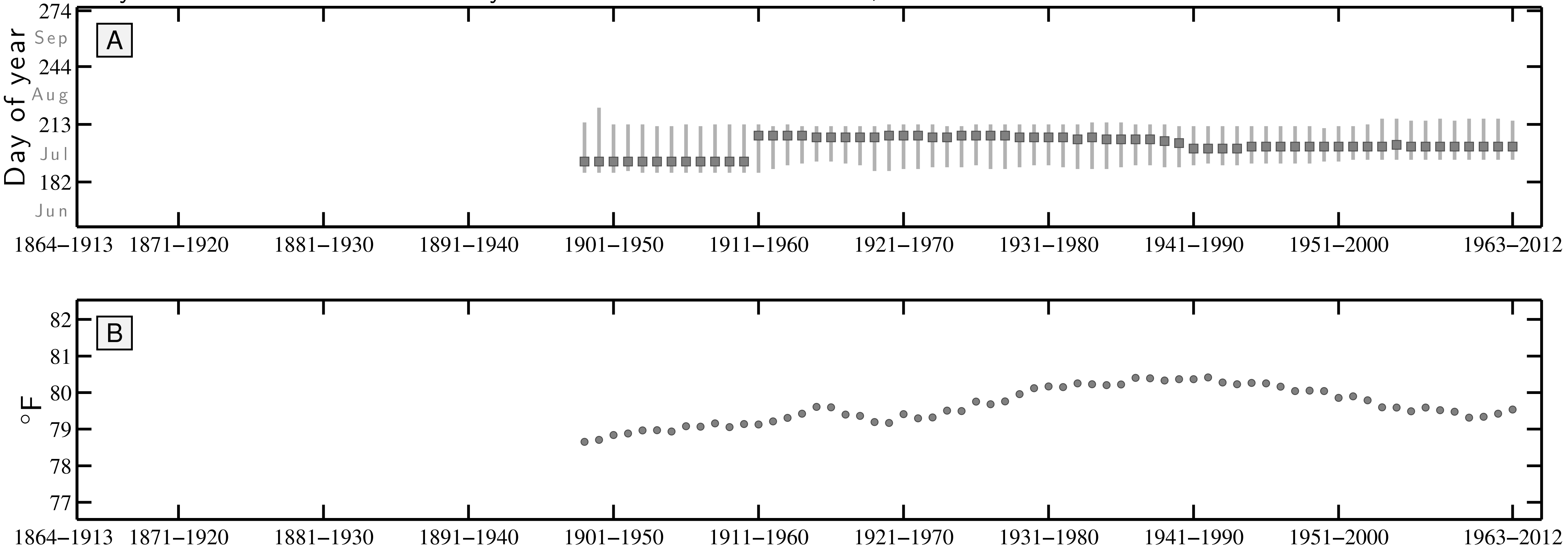

50 year Winter Teletherm dynamics for DANNEMORA, NY:

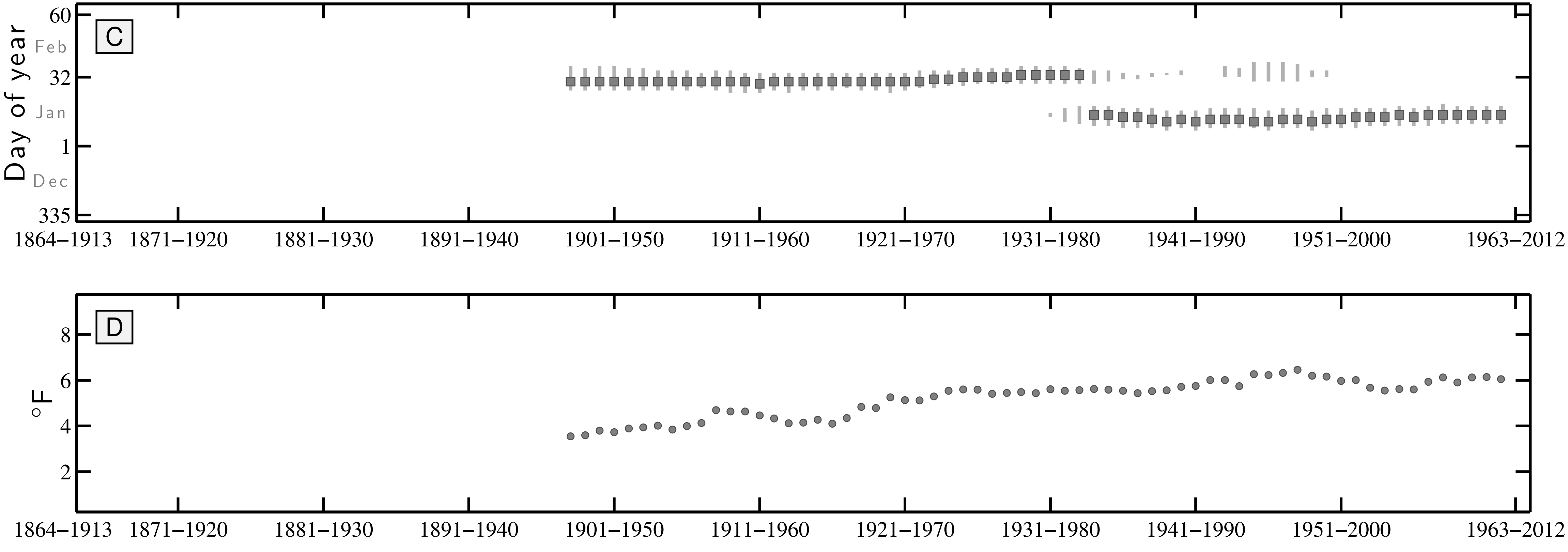

50 year Summer Teletherm dynamics for DANSVILLE, NY:

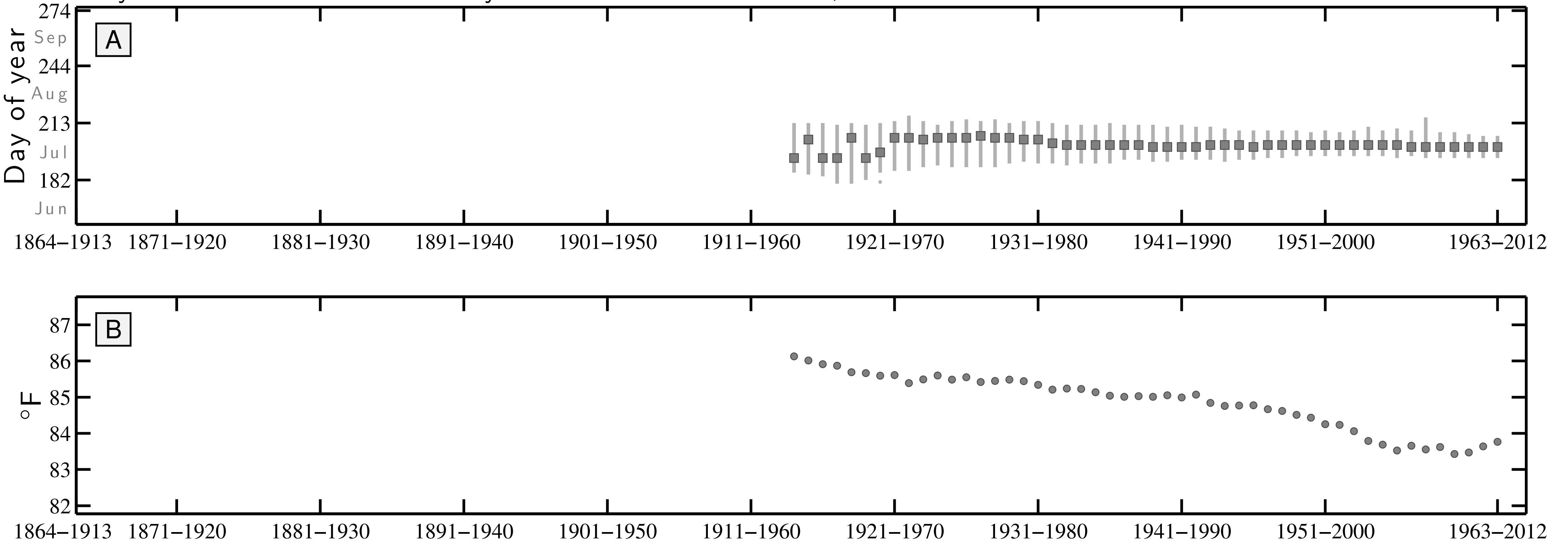

50 year Winter Teletherm dynamics for DANSVILLE, NY:

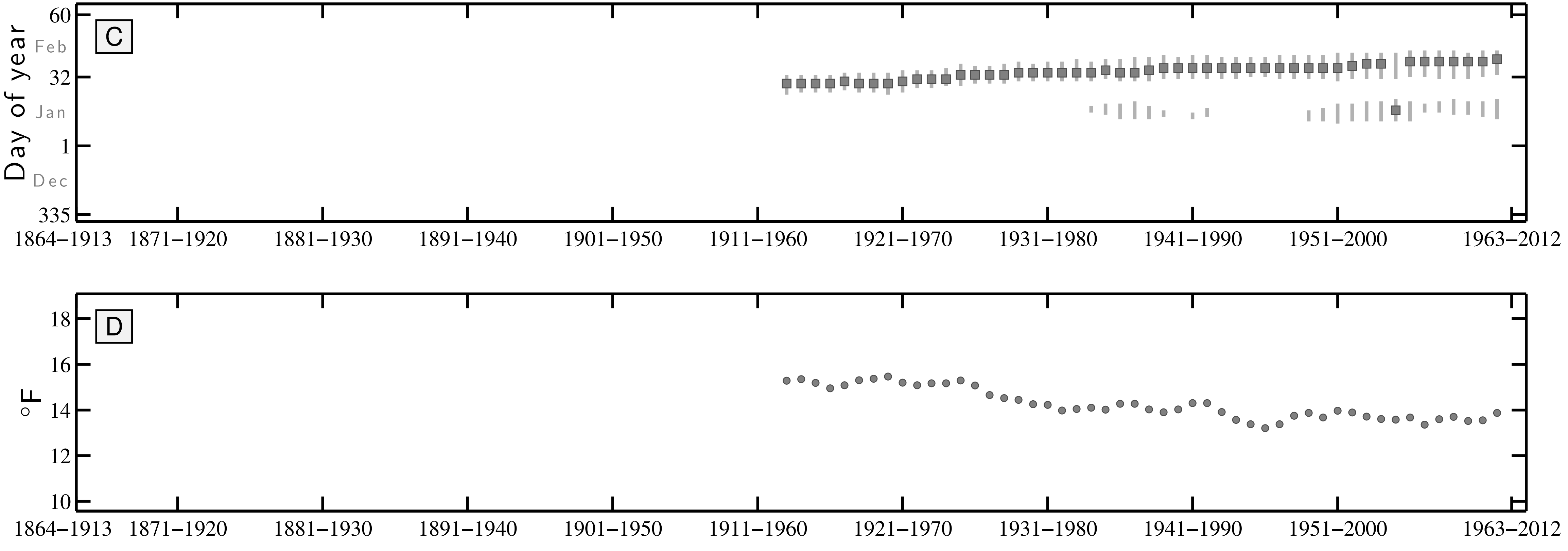

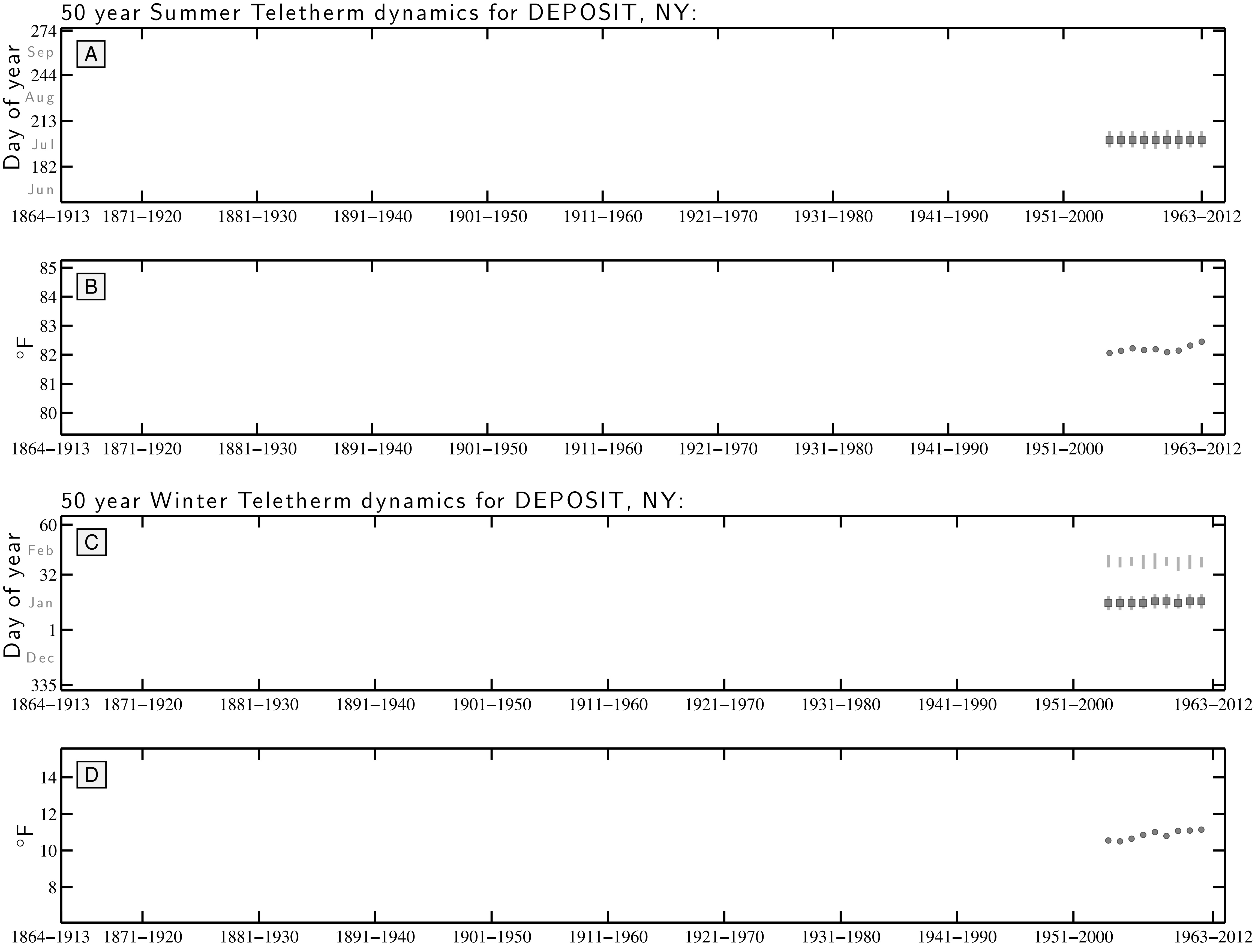

50 year Summer Teletherm dynamics for DEPOSIT, NY:
A
Day of year
274
Sep
244
Aug
213
Jul
182
Jun
1864–1913
1871–1920
1881–1930
1891–1940
1901–1950
1911–1960
1921–1970
1931–1980
1941–1990
1951–2000
1963–2012
B
°F
85
84
83
82
81
80
50 year Winter Teletherm dynamics for DEPOSIT, NY:
C
Day of year
60
Feb
32
Jan
1
Dec
335
D
°F
14
12
10
8

50 year Summer Teletherm dynamics for DOBBS FERRY ARDSLEY, NY:

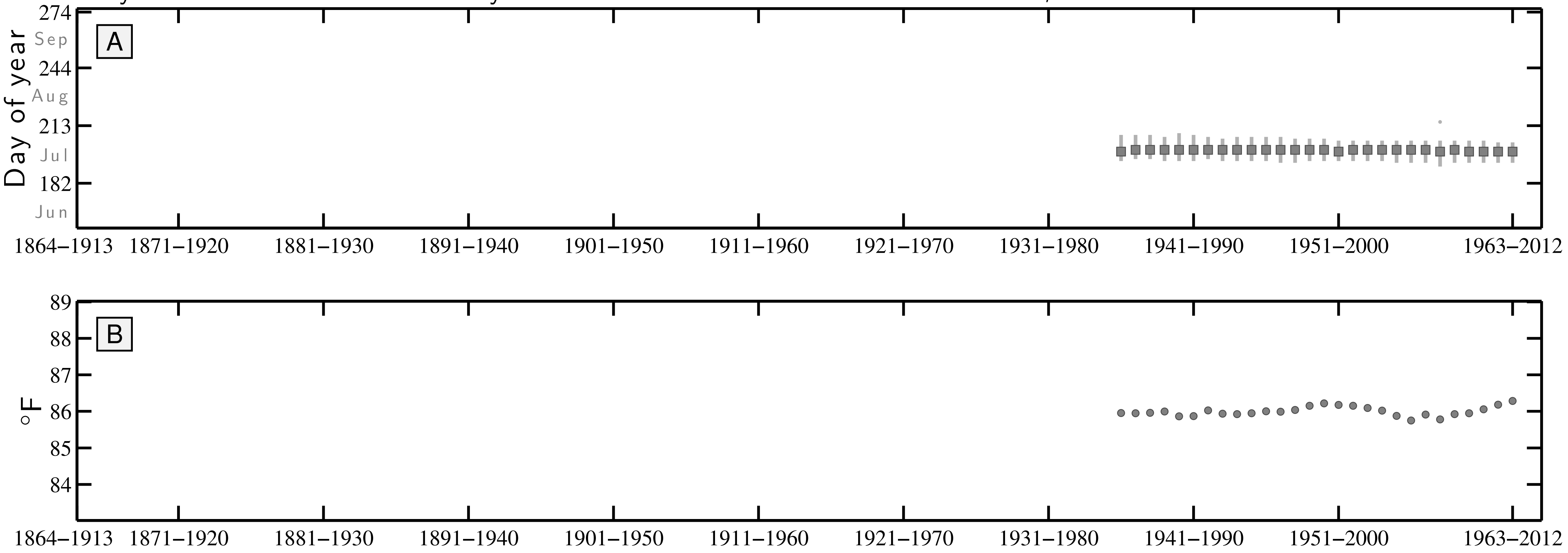

50 year Winter Teletherm dynamics for DOBBS FERRY ARDSLEY, NY:

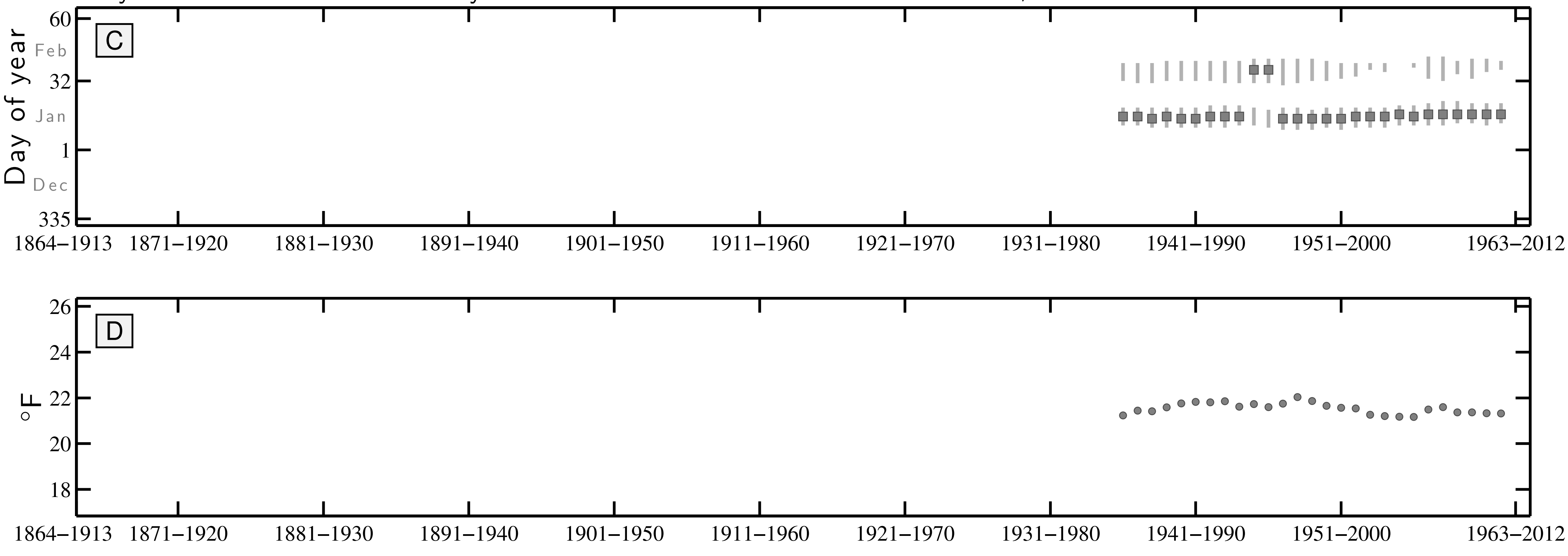

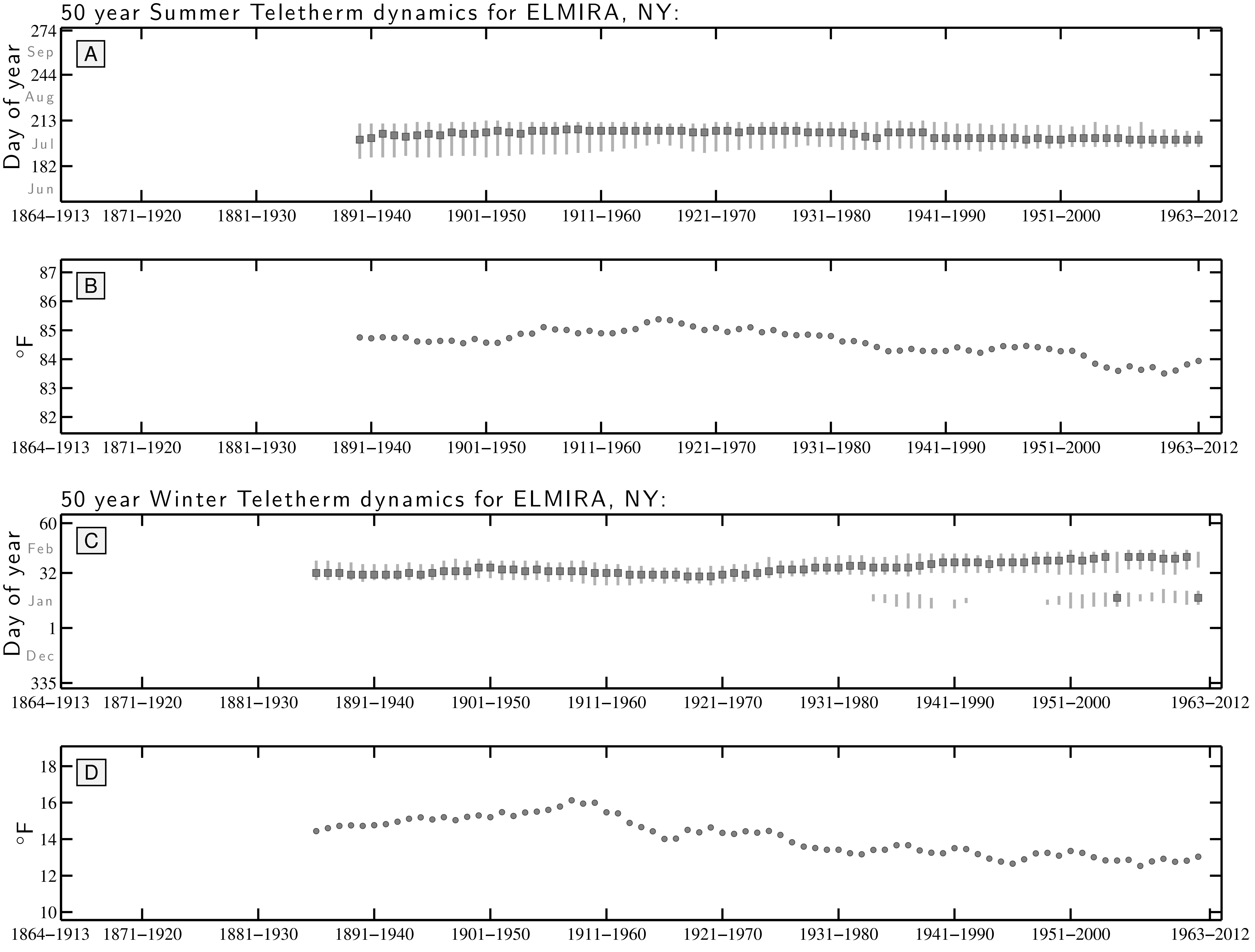
50 year Summer Teletherm dynamics for ELMIRA, NY:
A
B
Day of year
°F
274
Sep
244
Aug
213
Jul
182
Jun
87
86
85
84
83
82
1864–1913
1871–1920
1881–1930
1891–1940
1901–1950
1911–1960
1921–1970
1931–1980
1941–1990
1951–2000
1963–2012
50 year Winter Teletherm dynamics for ELMIRA, NY:
C
D
60
Feb
32
Jan
1
Dec
335
18
16
14
12
10

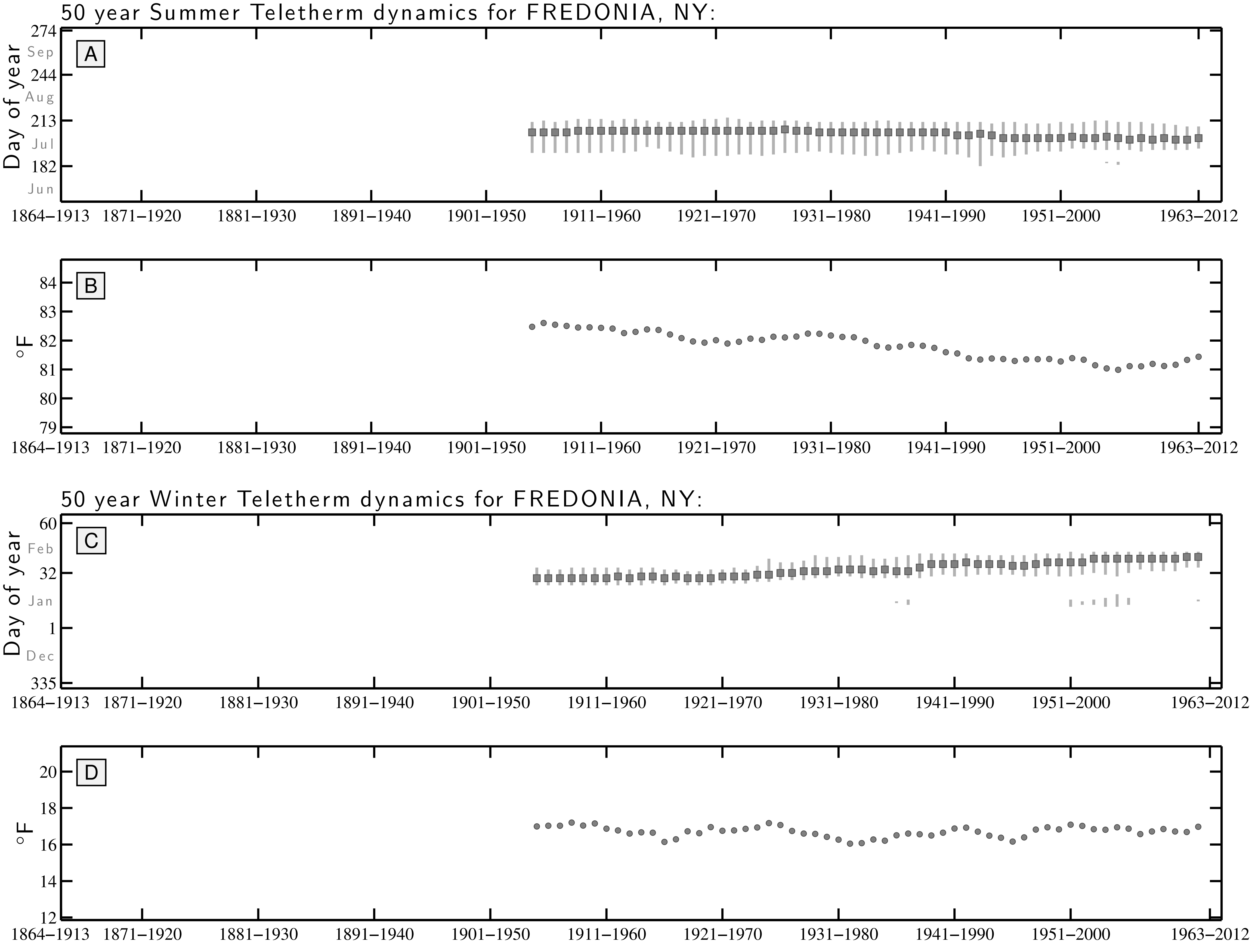
50 year Summer Teletherm dynamics for FREDONIA, NY:
A
Day of year
274
Sep
244
Aug
213
Jul
182
Jun
B
°F
84
83
82
81
80
79
50 year Winter Teletherm dynamics for FREDONIA, NY:
C
Day of year
60
Feb
32
Jan
1
Dec
335
D
°F
20
18
16
14
12
1864–1913
1871–1920
1881–1930
1891–1940
1901–1950
1911–1960
1921–1970
1931–1980
1941–1990
1951–2000
1963–2012

50 year Summer Teletherm dynamics for GENEVA RSCH FARM, NY:

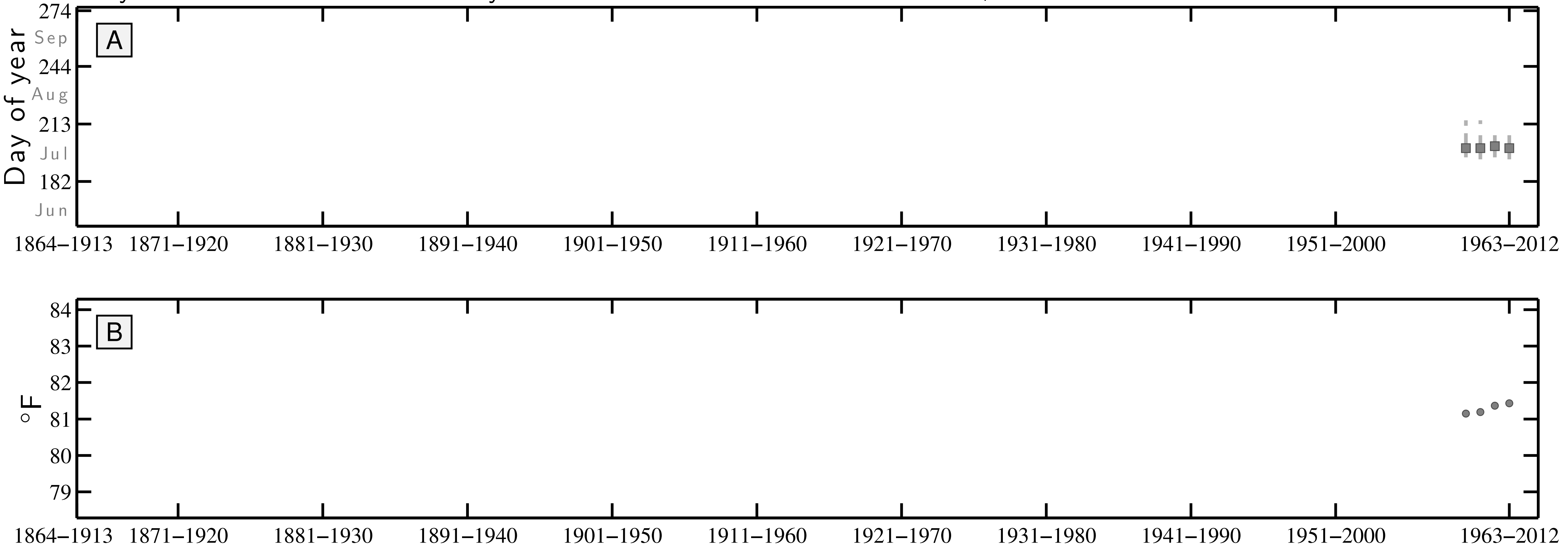

50 year Winter Teletherm dynamics for GENEVA RSCH FARM, NY:

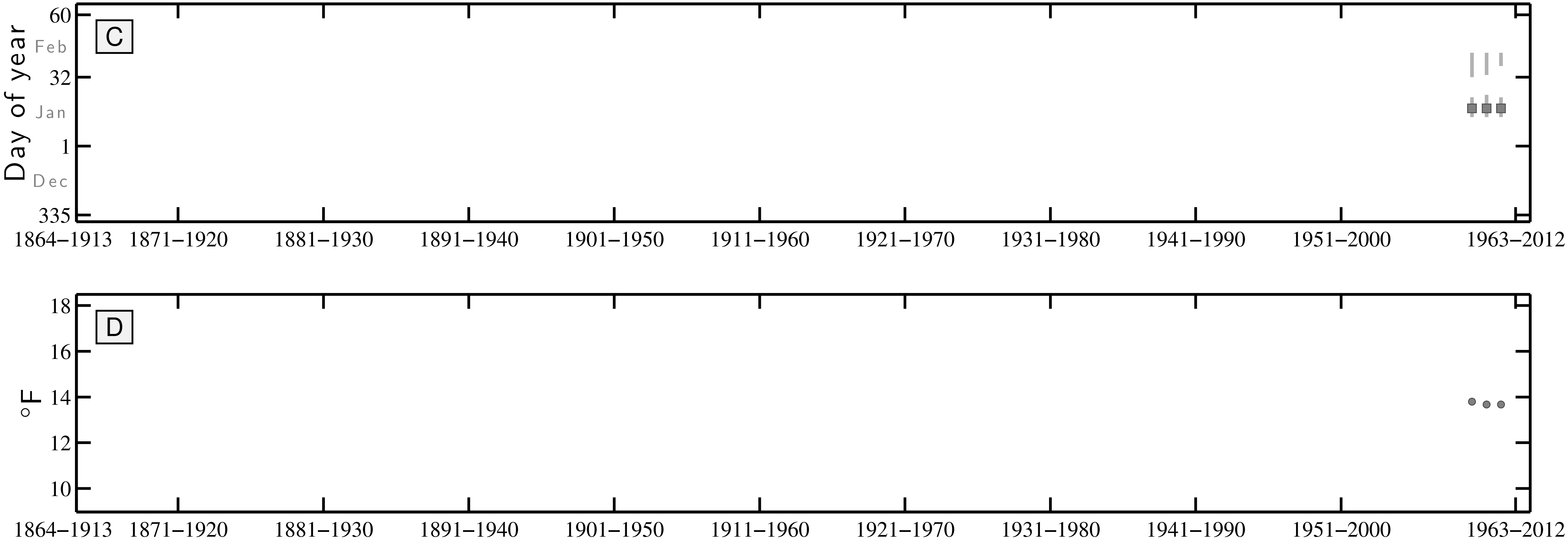

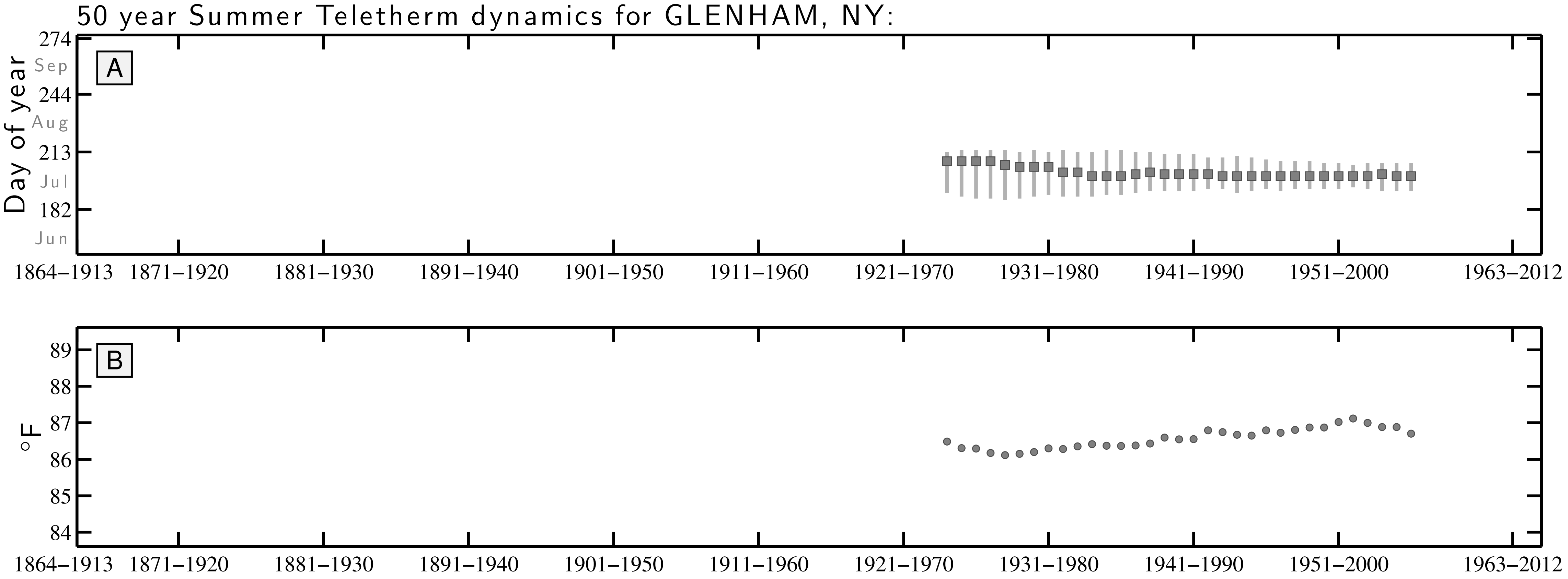
50 year Summer Teletherm dynamics for GLENHAM, NY:
A
B
Day of year
°F
Sep
Aug
Jul
Jun
274
244
213
182
89
88
87
86
85
84
1864–1913
1871–1920
1881–1930
1891–1940
1901–1950
1911–1960
1921–1970
1931–1980
1941–1990
1951–2000
1963–2012

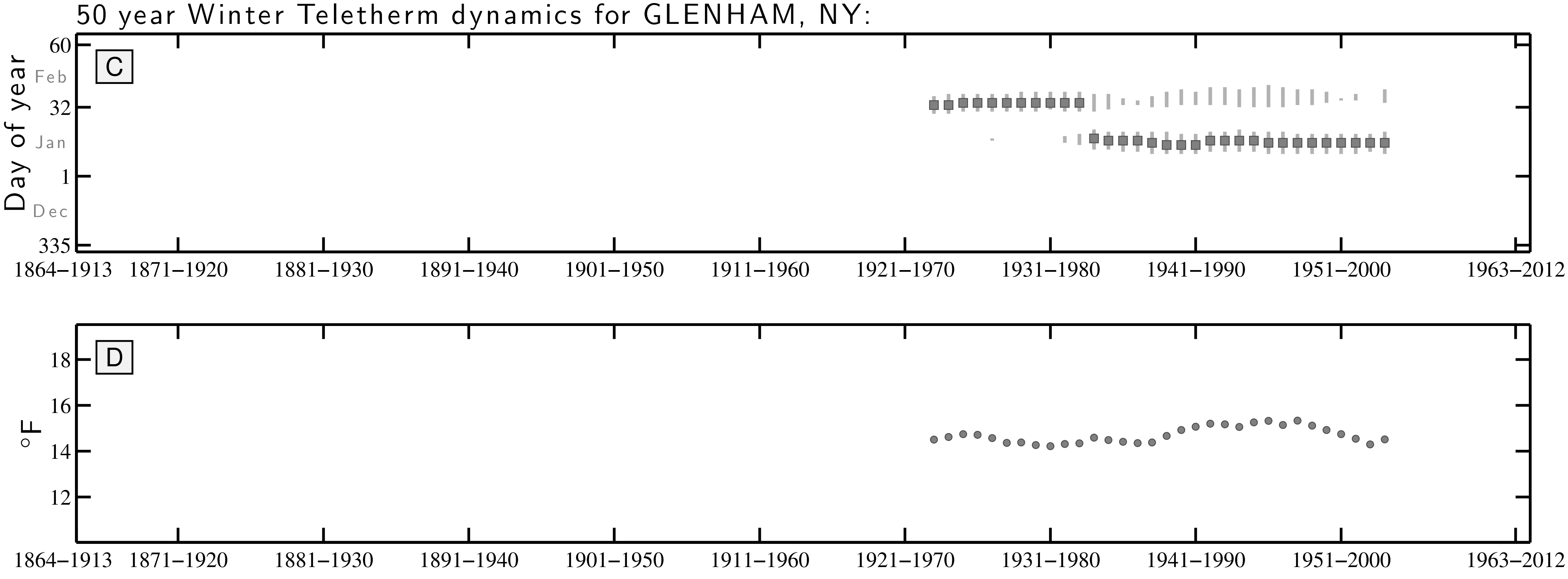
50 year Winter Teletherm dynamics for GLENHAM, NY:
C
D
Day of year
°F
Feb
Jan
Dec
60
32
1
335
18
16
14
12
1864–1913
1871–1920
1881–1930
1891–1940
1901–1950
1911–1960
1921–1970
1931–1980
1941–1990
1951–2000
1963–2012

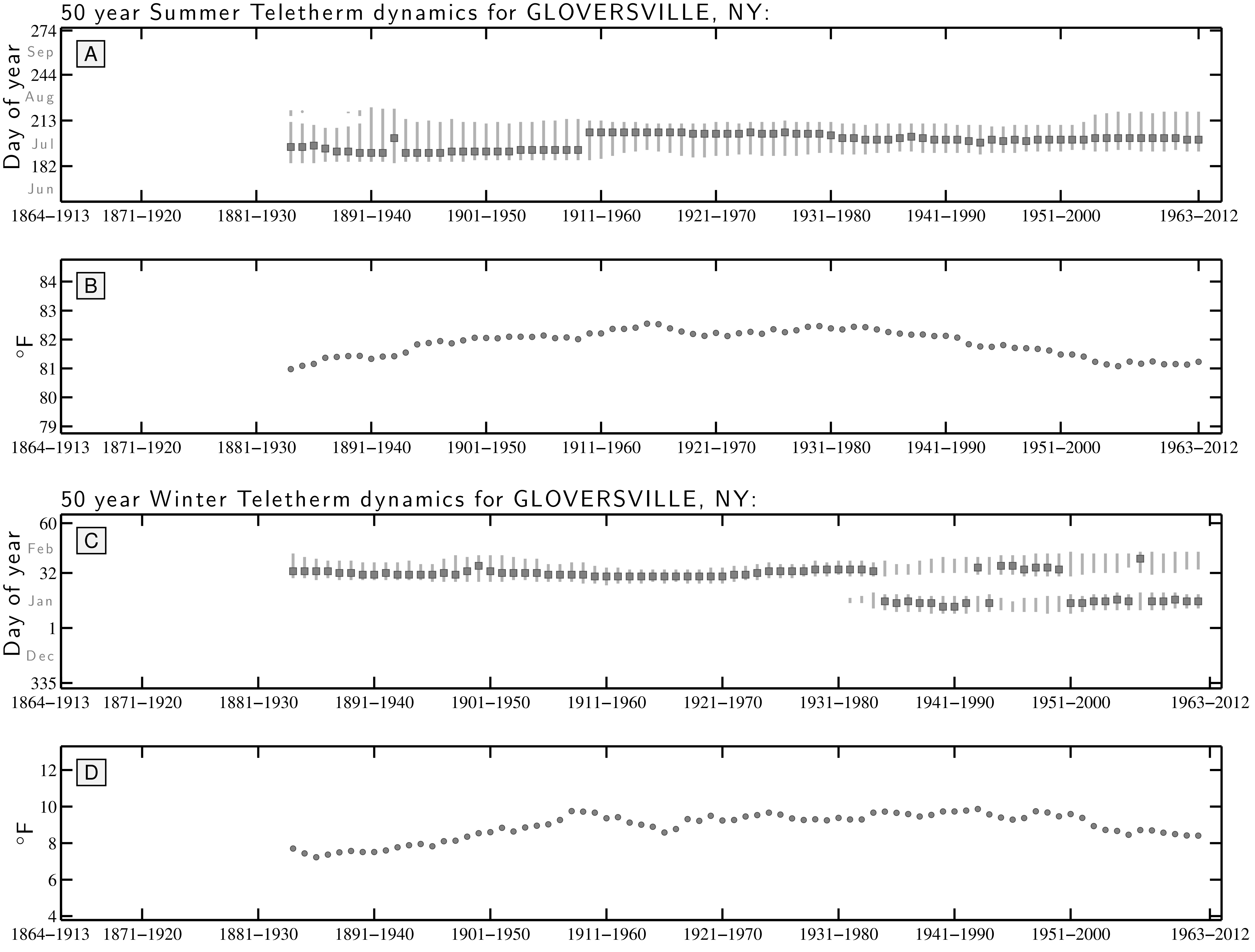
50 year Summer Teletherm dynamics for GLOVERSVILLE, NY:
A
Day of year
274
Sep
244
Aug
213
Jul
182
Jun
1864–1913
1871–1920
1881–1930
1891–1940
1901–1950
1911–1960
1921–1970
1931–1980
1941–1990
1951–2000
1963–2012
B
°F
84
83
82
81
80
79
50 year Winter Teletherm dynamics for GLOVERSVILLE, NY:
C
Day of year
60
Feb
32
Jan
1
Dec
335
D
°F
12
10
8
6
4

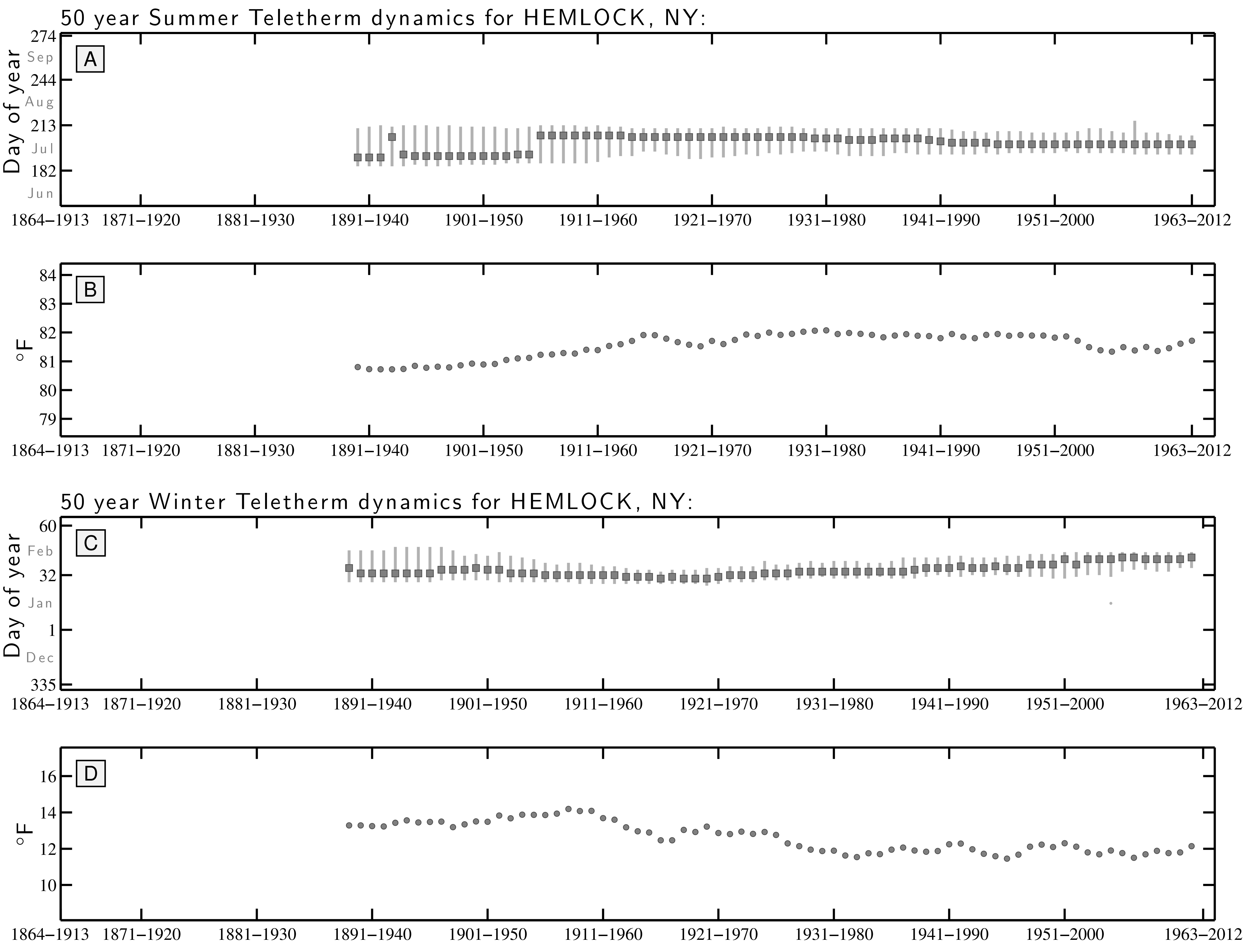

50 year Summer Teletherm dynamics for HEMLOCK, NY:
A
B
Day of year
°F
274
Sep
244
Aug
213
Jul
182
Jun
84
83
82
81
80
79
1864–1913
1871–1920
1881–1930
1891–1940
1901–1950
1911–1960
1921–1970
1931–1980
1941–1990
1951–2000
1963–2012
50 year Winter Teletherm dynamics for HEMLOCK, NY:
C
D
60
Feb
32
Jan
1
Dec
335
16
14
12
10

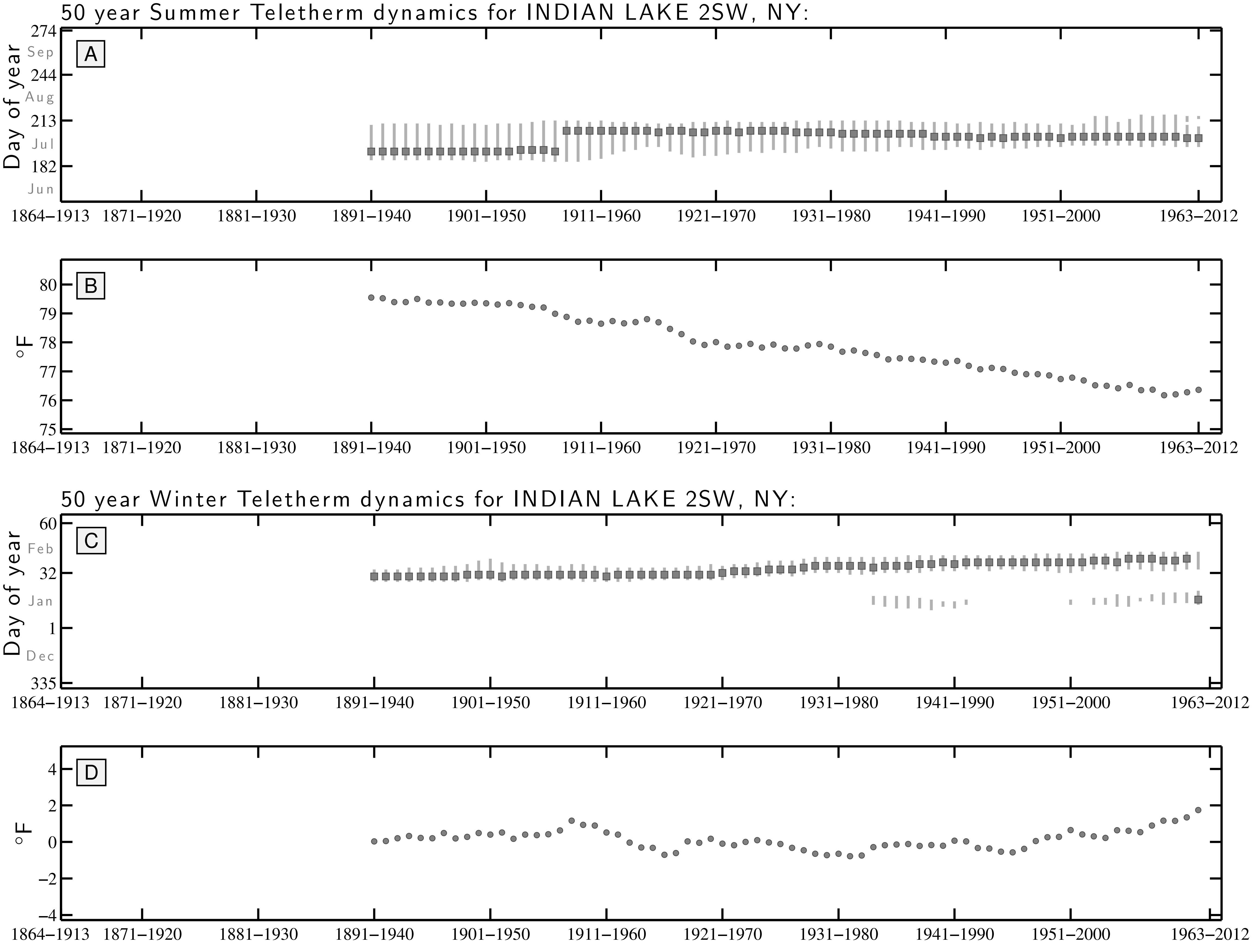

50 year Summer Teletherm dynamics for INDIAN LAKE 2SW, NY:
A
Day of year
274
Sep
244
Aug
213
Jul
182
Jun
1864–1913
1871–1920
1881–1930
1891–1940
1901–1950
1911–1960
1921–1970
1931–1980
1941–1990
1951–2000
1963–2012
B
°F
80
79
78
77
76
75
50 year Winter Teletherm dynamics for INDIAN LAKE 2SW, NY:
C
60
Feb
32
Jan
1
Dec
335
D
4
2
0
−2
−4

50 year Summer Teletherm dynamics for ITHACA CORNELL UNIV, NY:

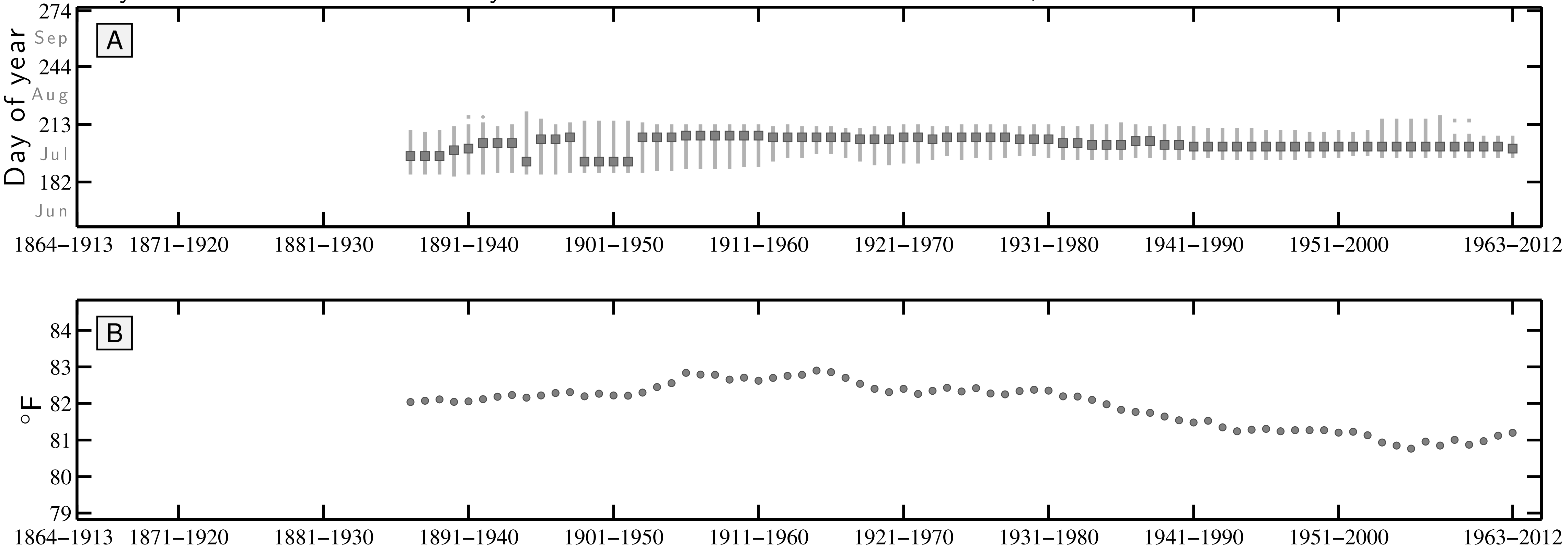

50 year Winter Teletherm dynamics for ITHACA CORNELL UNIV, NY:

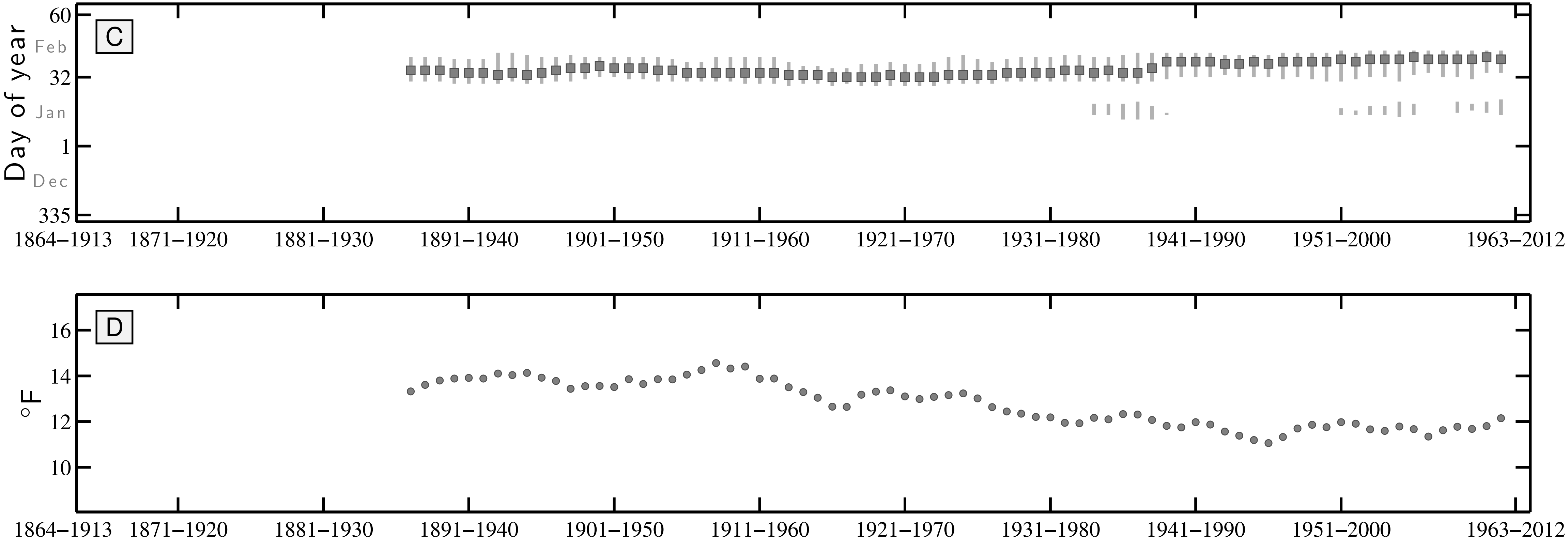

50 year Summer Teletherm dynamics for LAKE PLACID 2 S, NY:

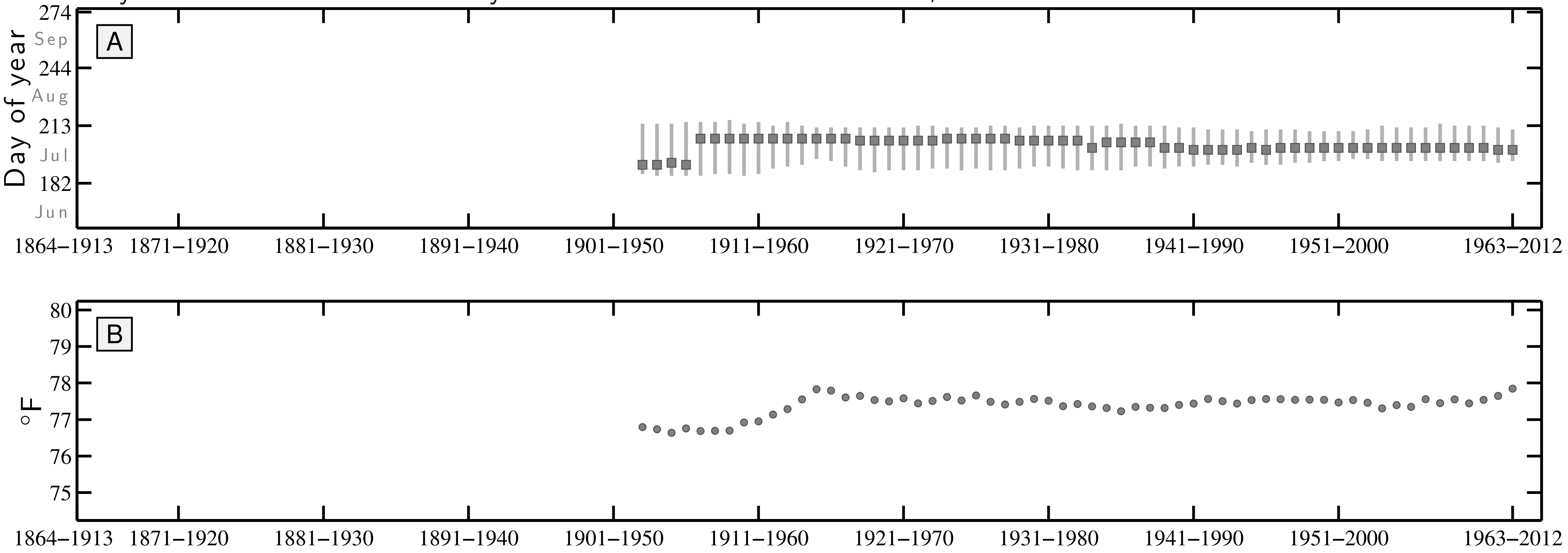

50 year Winter Teletherm dynamics for LAKE PLACID 2 S, NY:

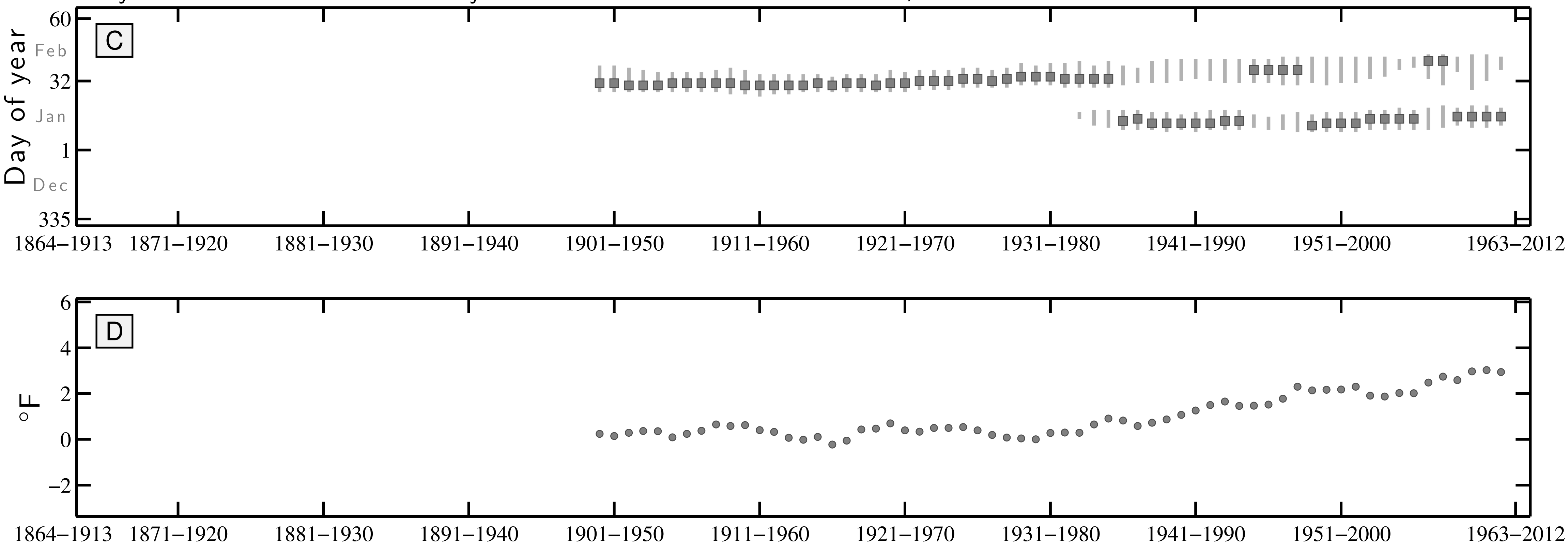

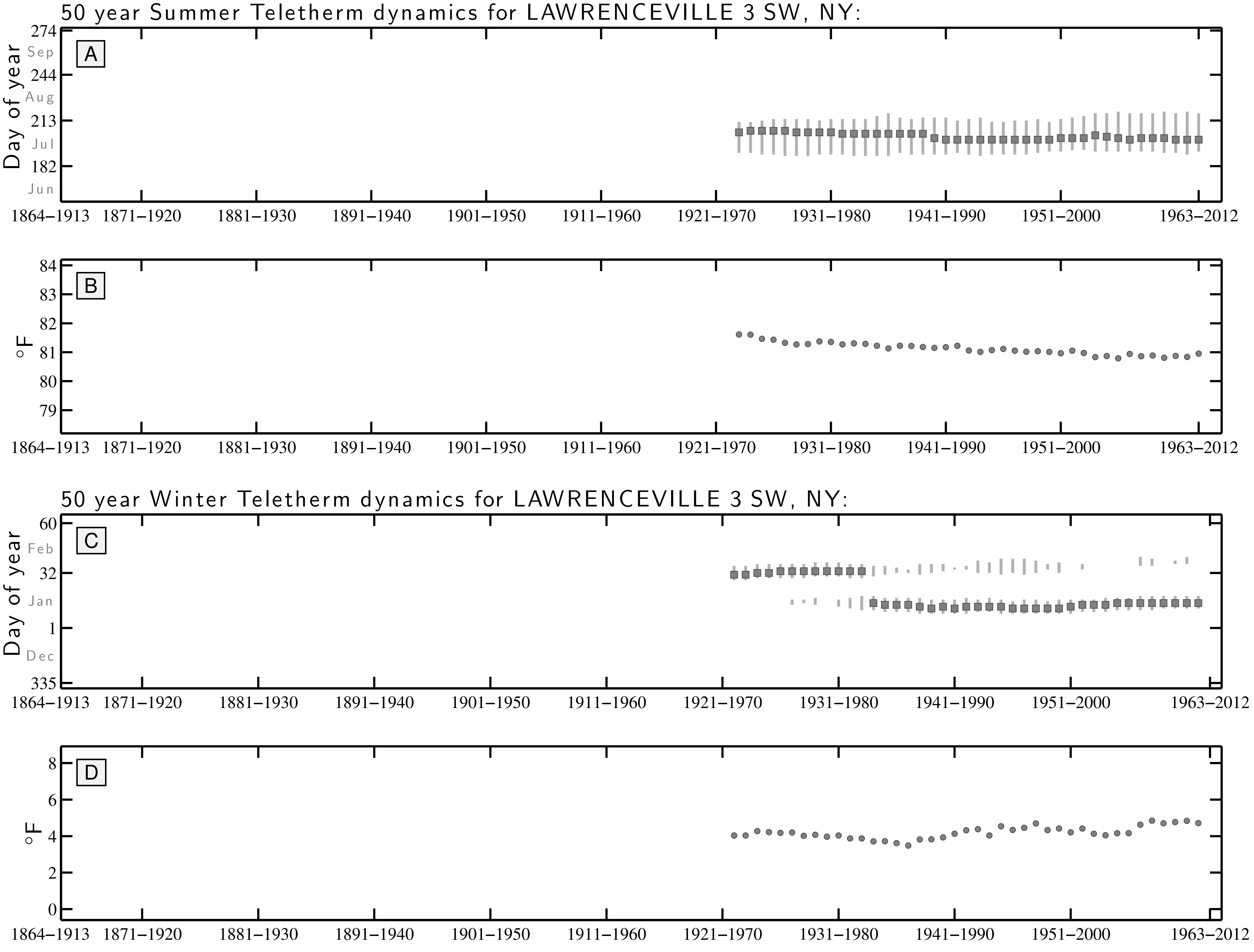
50 year Summer Teletherm dynamics for LAWRENCEVILLE 3 SW, NY:
A
B
Day of year
°F
274
Sep
244
Aug
213
Jul
182
Jun
84
83
82
81
80
79
50 year Winter Teletherm dynamics for LAWRENCEVILLE 3 SW, NY:
C
D
60
Feb
32
Jan
1
Dec
335
8
6
4
2
0
1864–1913
1871–1920
1881–1930
1891–1940
1901–1950
1911–1960
1921–1970
1931–1980
1941–1990
1951–2000
1963–2012

50 year Summer Teletherm dynamics for LITTLE FALLS CITY RSVR, NY:

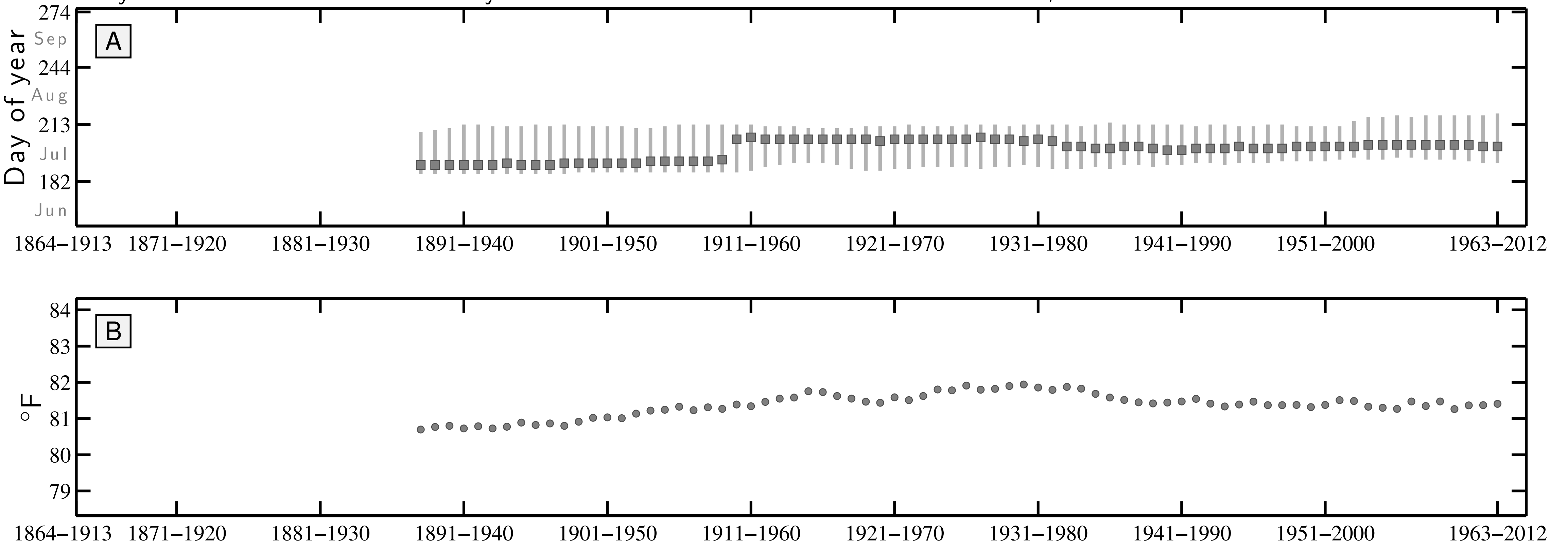

50 year Winter Teletherm dynamics for LITTLE FALLS CITY RSVR, NY:

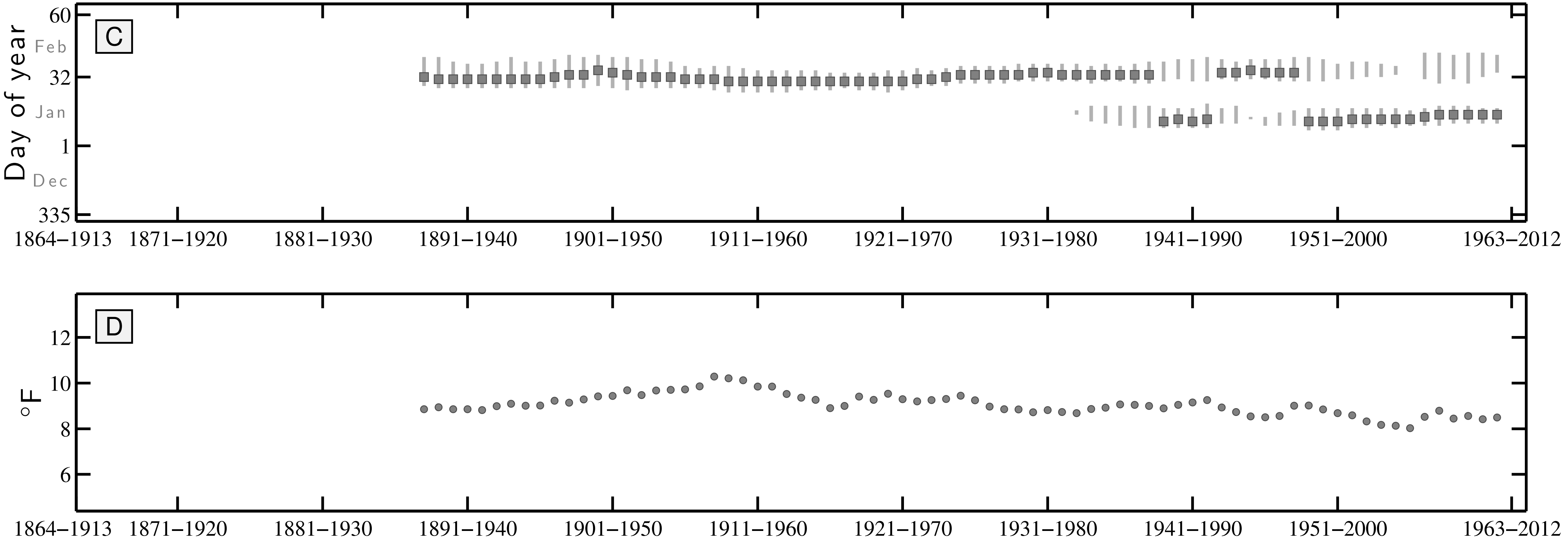

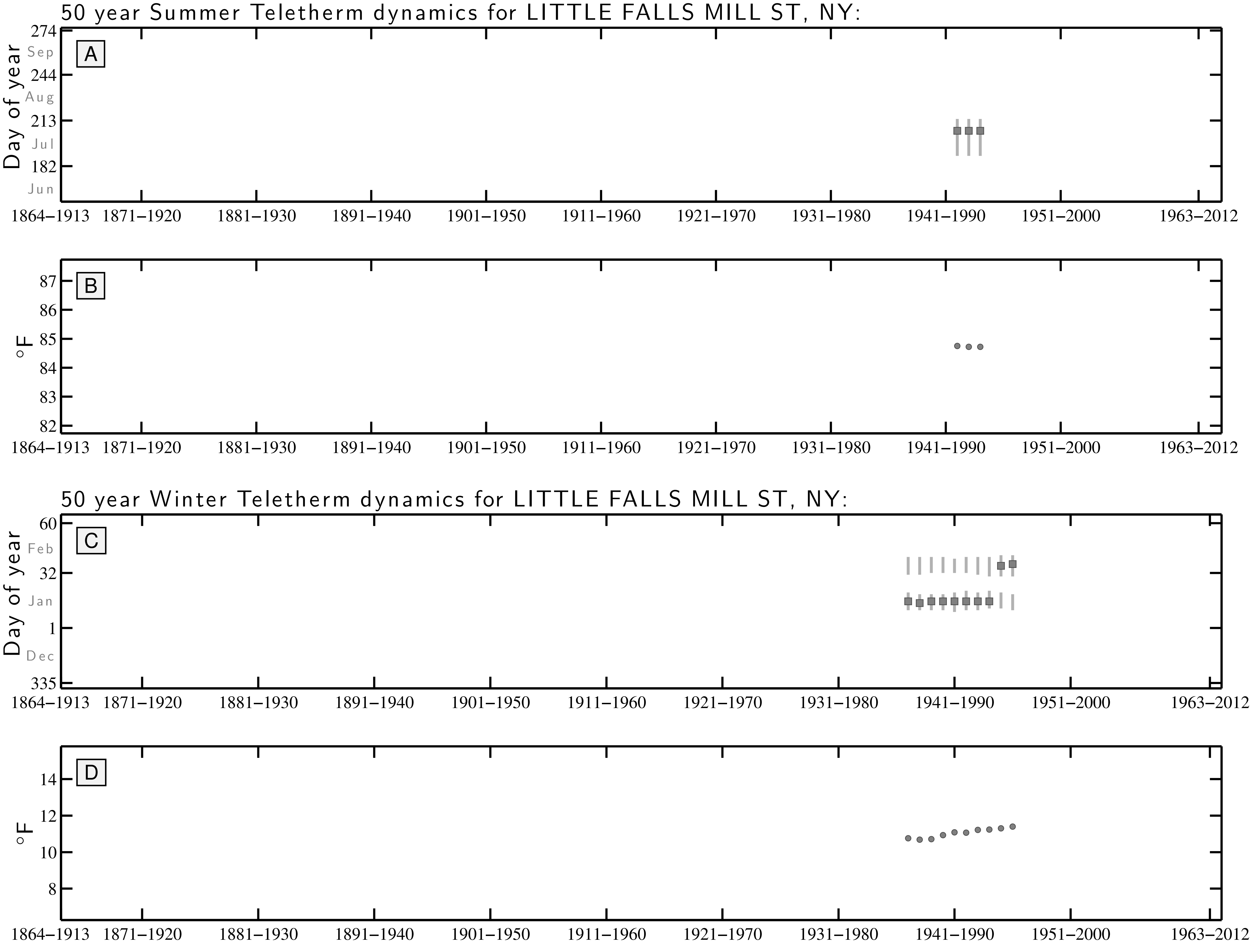
50 year Summer Teletherm dynamics for LITTLE FALLS MILL ST, NY:
A
Day of year
274
Sep
244
Aug
213
Jul
182
Jun
B
°F
87
86
85
84
83
82
50 year Winter Teletherm dynamics for LITTLE FALLS MILL ST, NY:
C
Day of year
60
Feb
32
Jan
1
Dec
335
D
°F
14
12
10
8
1864–1913
1871–1920
1881–1930
1891–1940
1901–1950
1911–1960
1921–1970
1931–1980
1941–1990
1951–2000
1963–2012

50 year Summer Teletherm dynamics for LOCKPORT 3 S, NY:

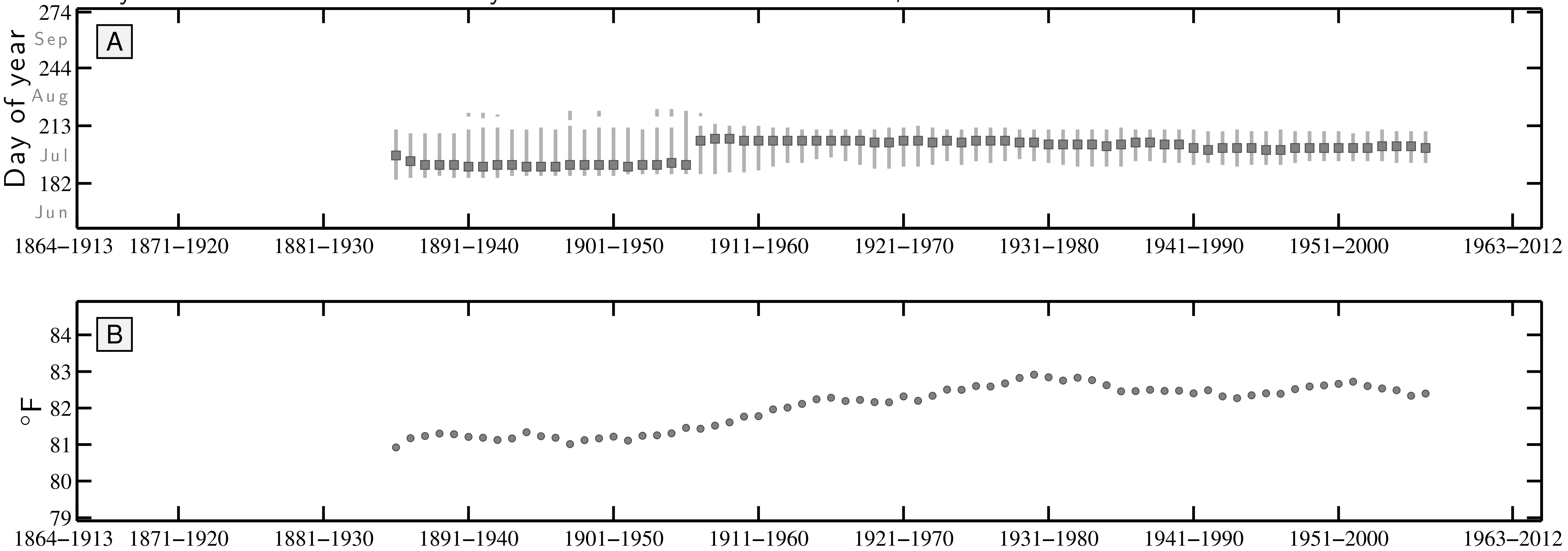

50 year Winter Teletherm dynamics for LOCKPORT 3 S, NY:

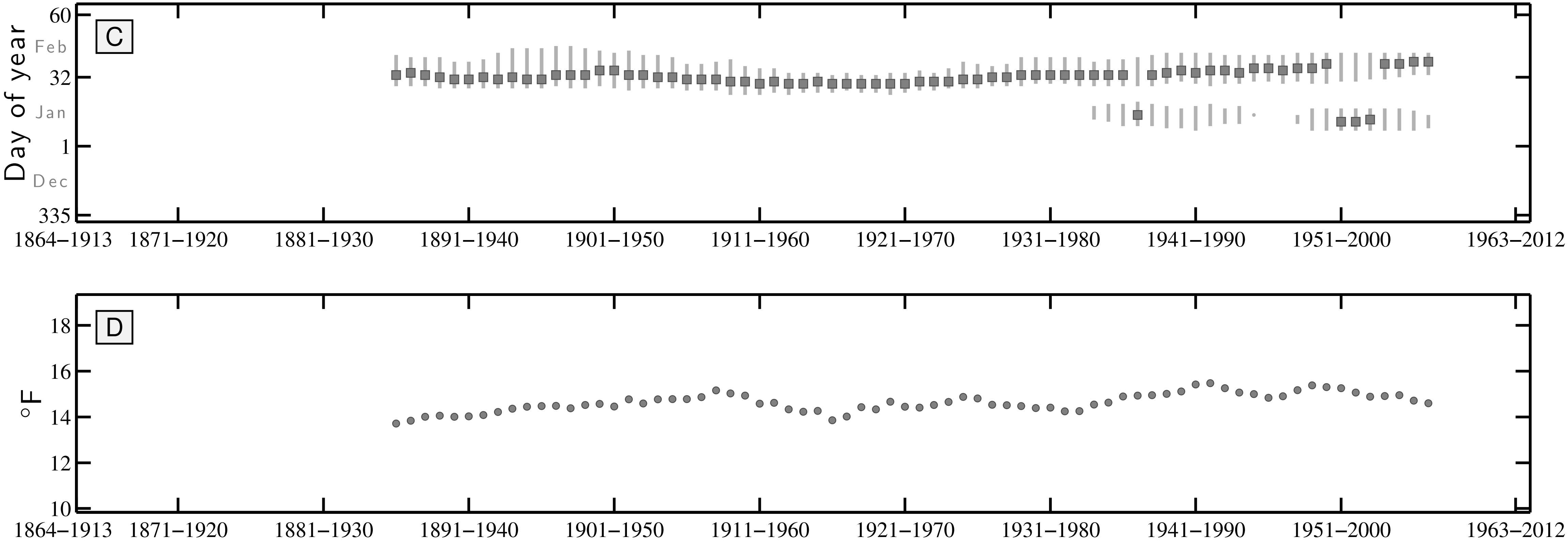

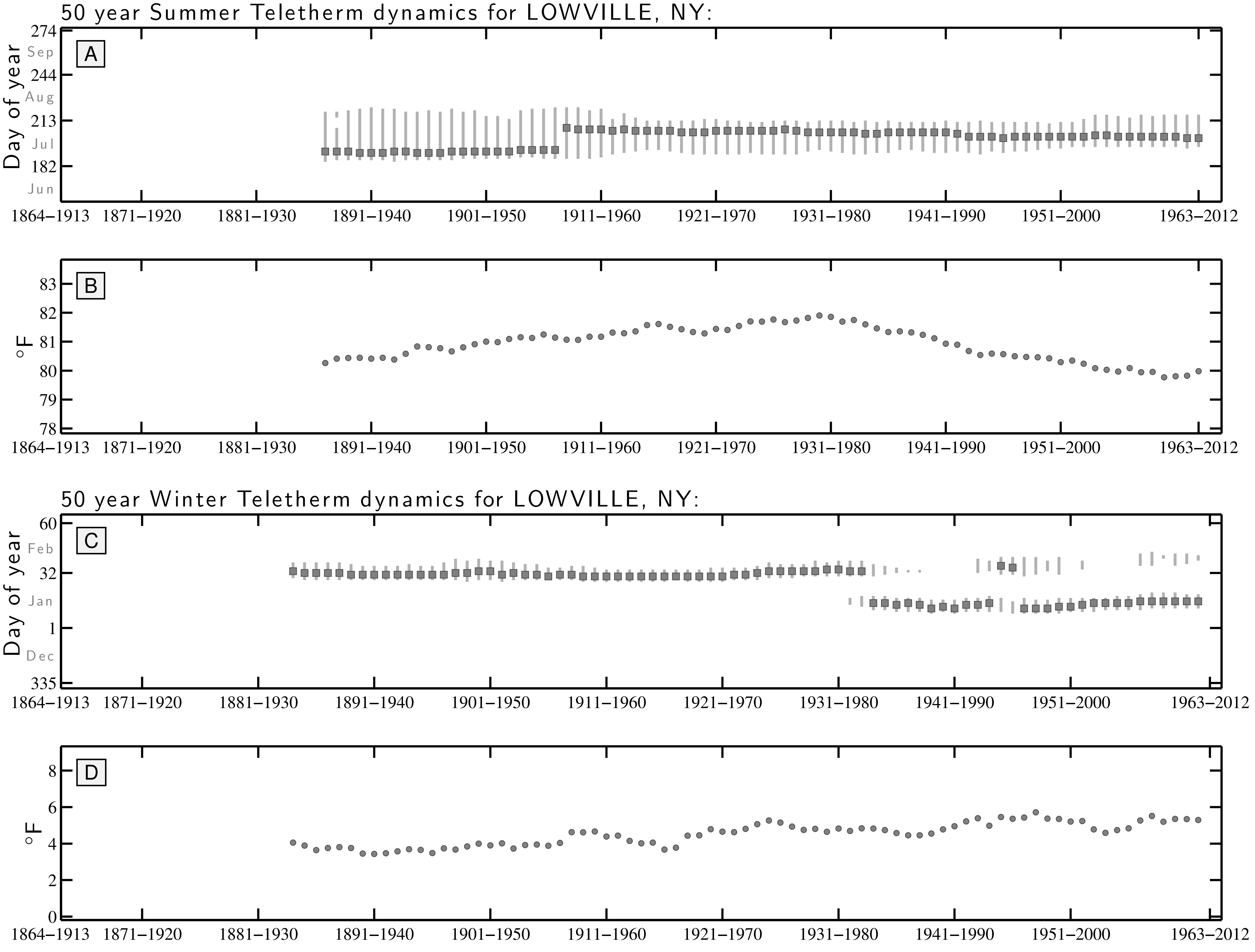
50 year Summer Teletherm dynamics for LOWVILLE, NY:
A
Day of year
274
Sep
244
Aug
213
Jul
182
Jun
B
°F
83
82
81
80
79
78
50 year Winter Teletherm dynamics for LOWVILLE, NY:
C
Day of year
60
Feb
32
Jan
1
Dec
335
D
°F
8
6
4
2
0
1864–1913
1871–1920
1881–1930
1891–1940
1901–1950
1911–1960
1921–1970
1931–1980
1941–1990
1951–2000
1963–2012

50 year Summer Teletherm dynamics for MOHONK LAKE, NY:

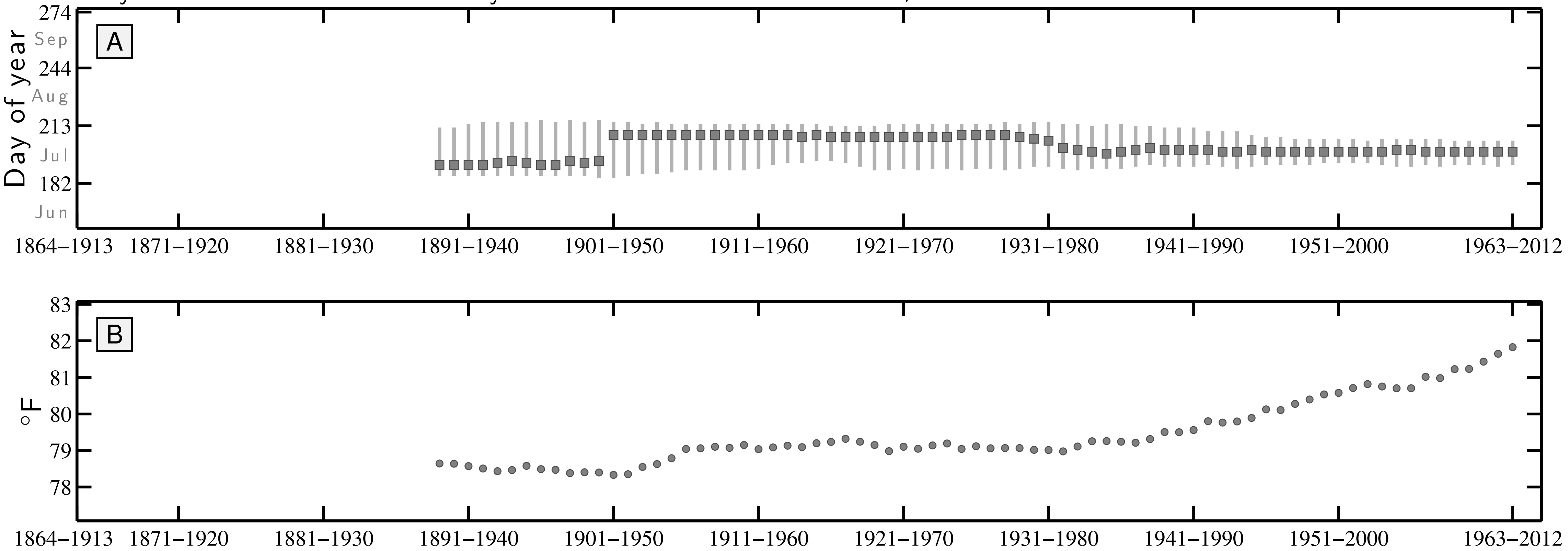

50 year Winter Teletherm dynamics for MOHONK LAKE, NY:

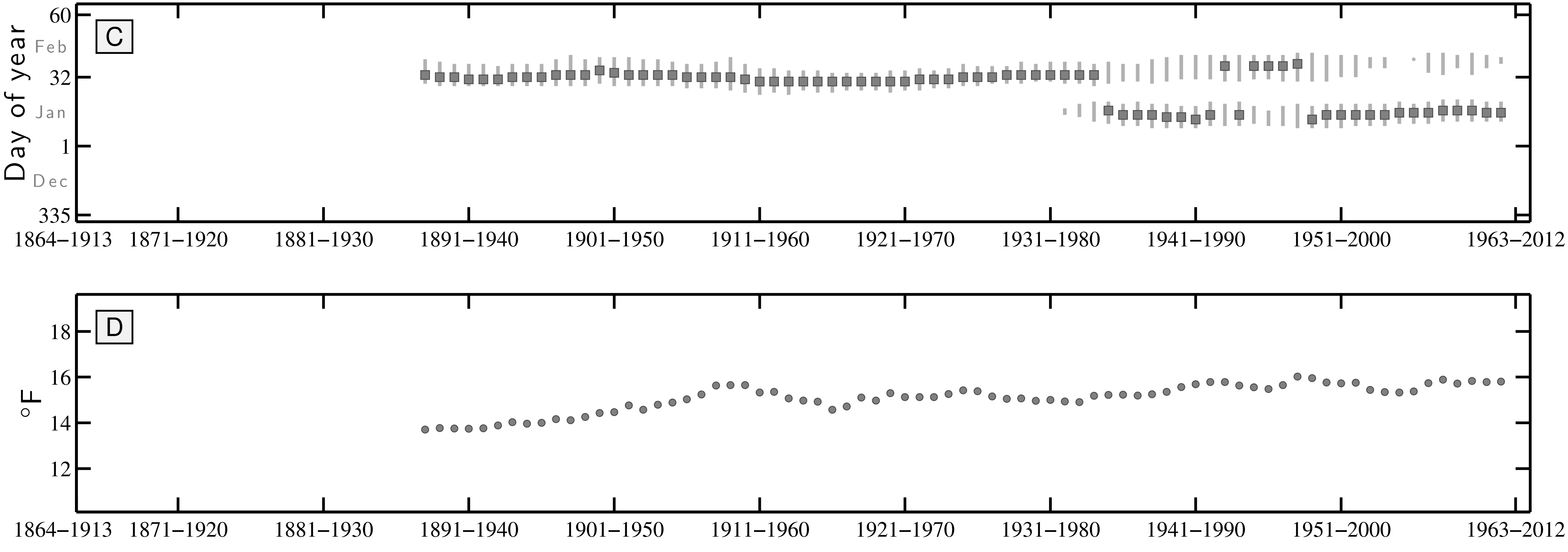

50 year Summer Teletherm dynamics for MORRISVILLE 6 SW, NY:

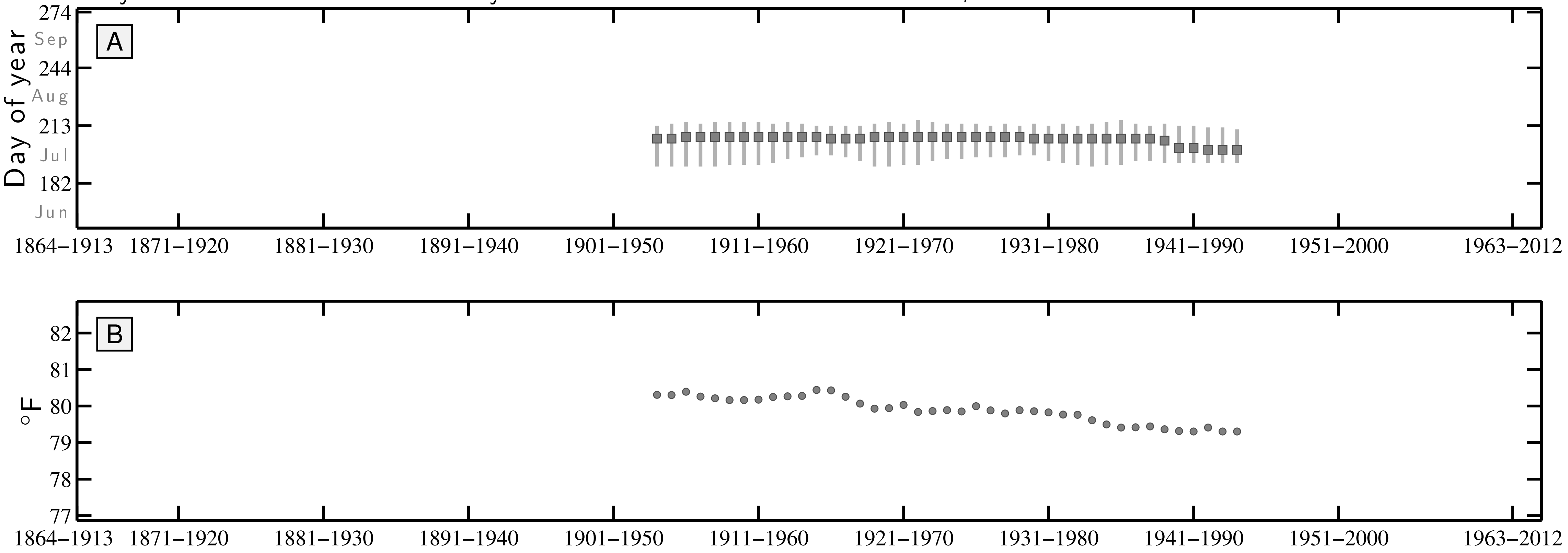

50 year Winter Teletherm dynamics for MORRISVILLE 6 SW, NY:

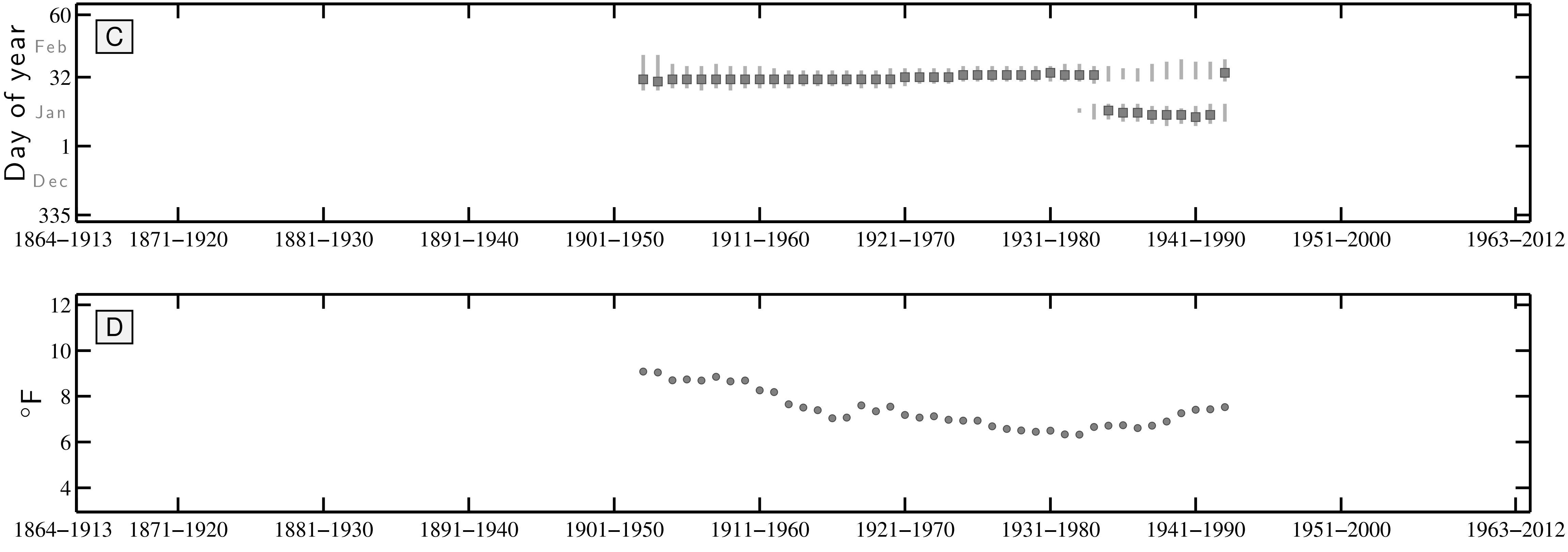

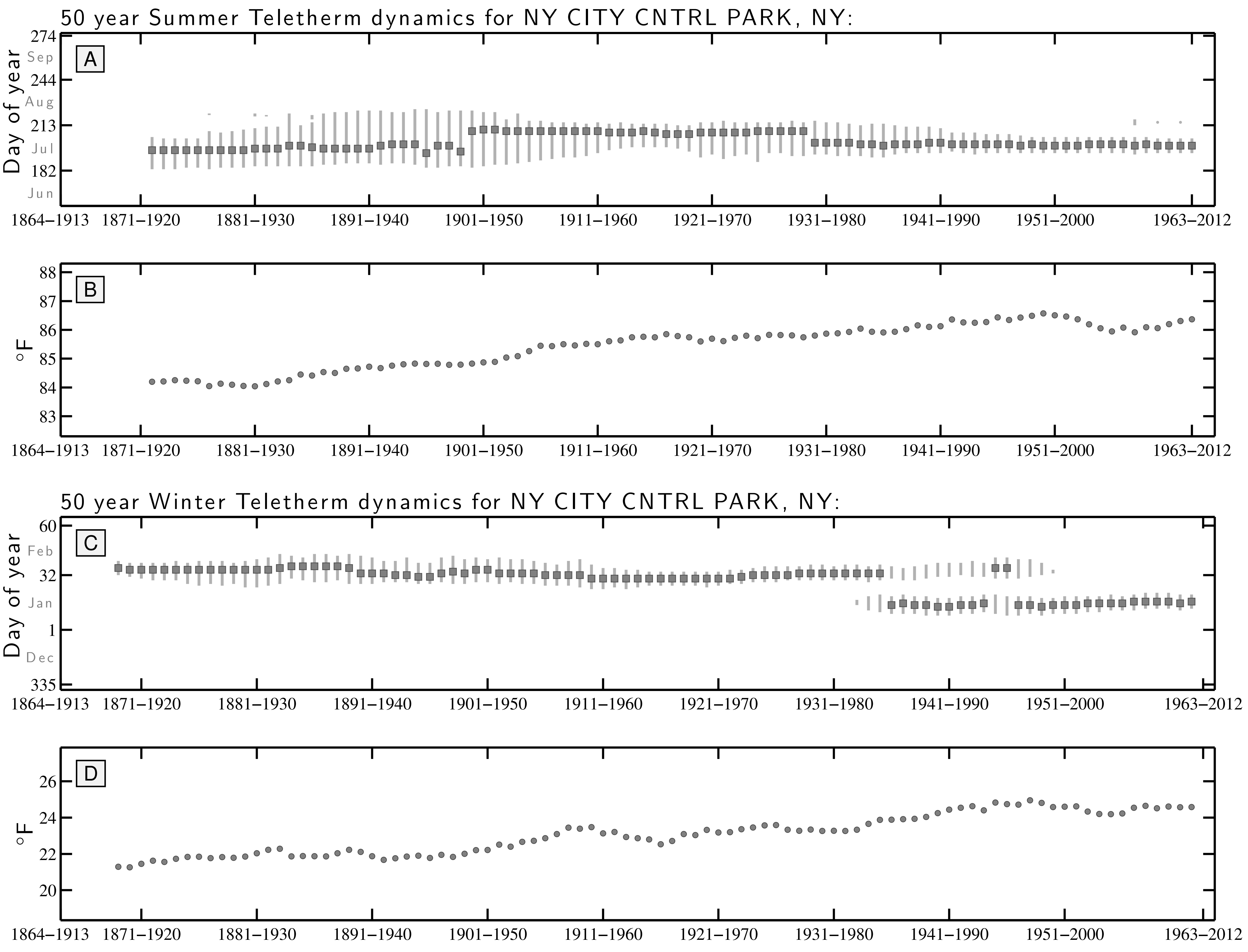

50 year Summer Teletherm dynamics for NY CITY CNTRL PARK, NY:
A
B
Day of year
°F
274
244
213
182
Sep
Aug
Jul
Jun
88
87
86
85
84
83
1864–1913
1871–1920
1881–1930
1891–1940
1901–1950
1911–1960
1921–1970
1931–1980
1941–1990
1951–2000
1963–2012
50 year Winter Teletherm dynamics for NY CITY CNTRL PARK, NY:
C
D
60
32
1
335
Feb
Jan
Dec
26
24
22
20

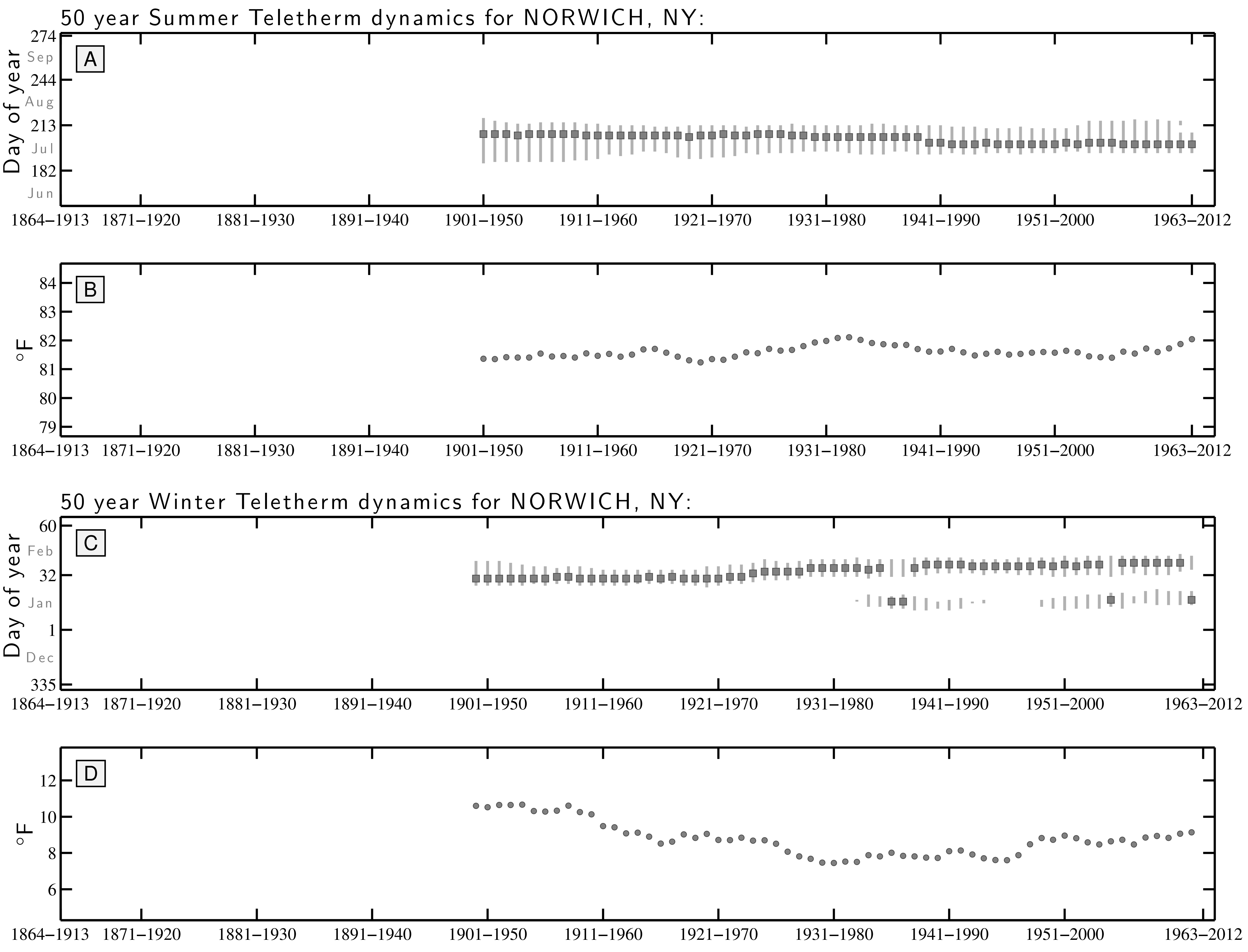
50 year Summer Teletherm dynamics for NORWICH, NY:
A
Day of year
274
Sep
244
Aug
213
Jul
182
Jun
1864–1913
1871–1920
1881–1930
1891–1940
1901–1950
1911–1960
1921–1970
1931–1980
1941–1990
1951–2000
1963–2012
B
°F
84
83
82
81
80
79
50 year Winter Teletherm dynamics for NORWICH, NY:
C
Day of year
60
Feb
32
Jan
1
Dec
335
D
°F
12
10
8
6

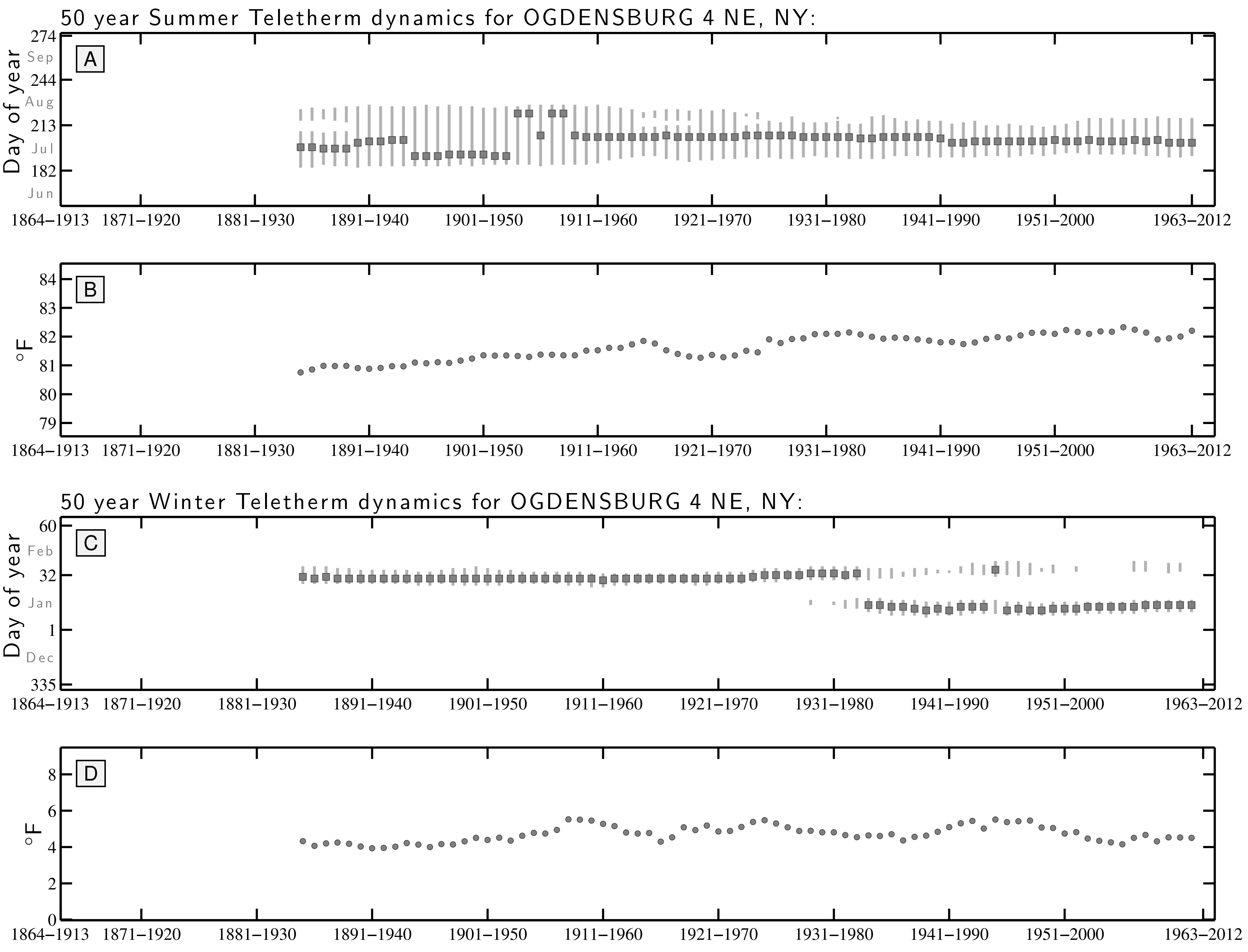
50 year Summer Teletherm dynamics for OGDENSBURG 4 NE, NY:
A
B
Day of year
°F
274
Sep
244
Aug
213
Jul
182
Jun
84
83
82
81
80
79
1864–1913
1871–1920
1881–1930
1891–1940
1901–1950
1911–1960
1921–1970
1931–1980
1941–1990
1951–2000
1963–2012
50 year Winter Teletherm dynamics for OGDENSBURG 4 NE, NY:
C
D
60
Feb
32
Jan
1
Dec
335
8
6
4
2
0

50 year Summer Teletherm dynamics for OSWEGO EAST, NY:

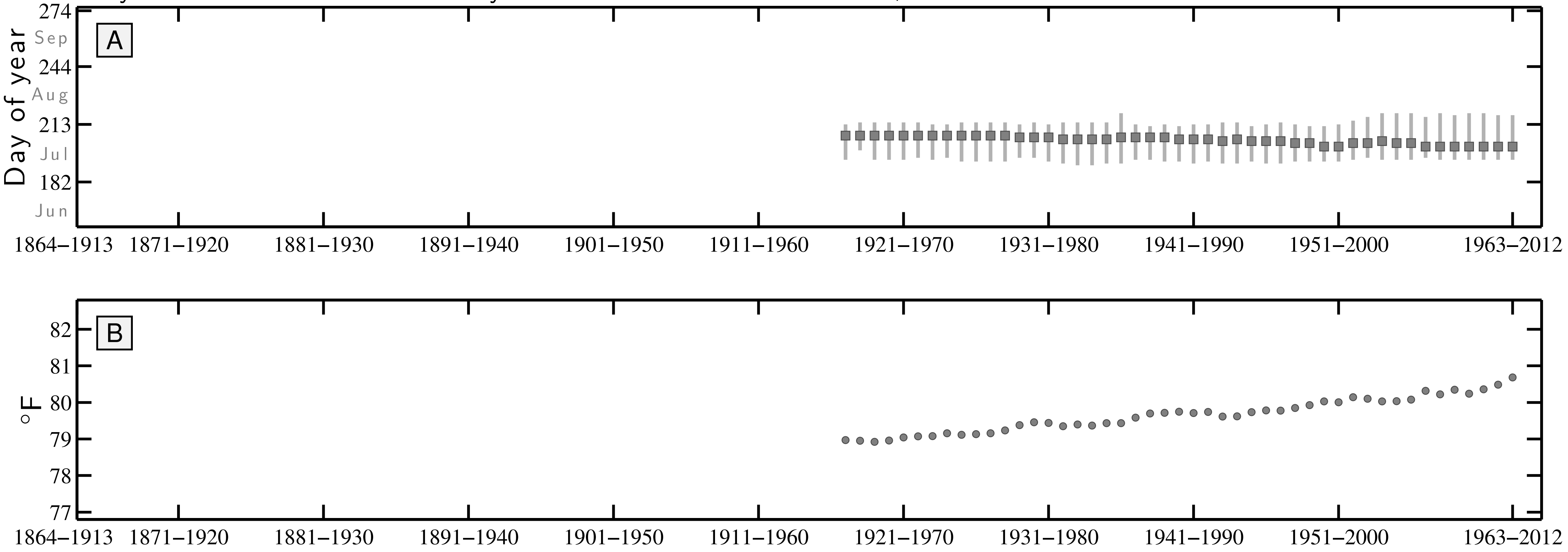

50 year Winter Teletherm dynamics for OSWEGO EAST, NY:

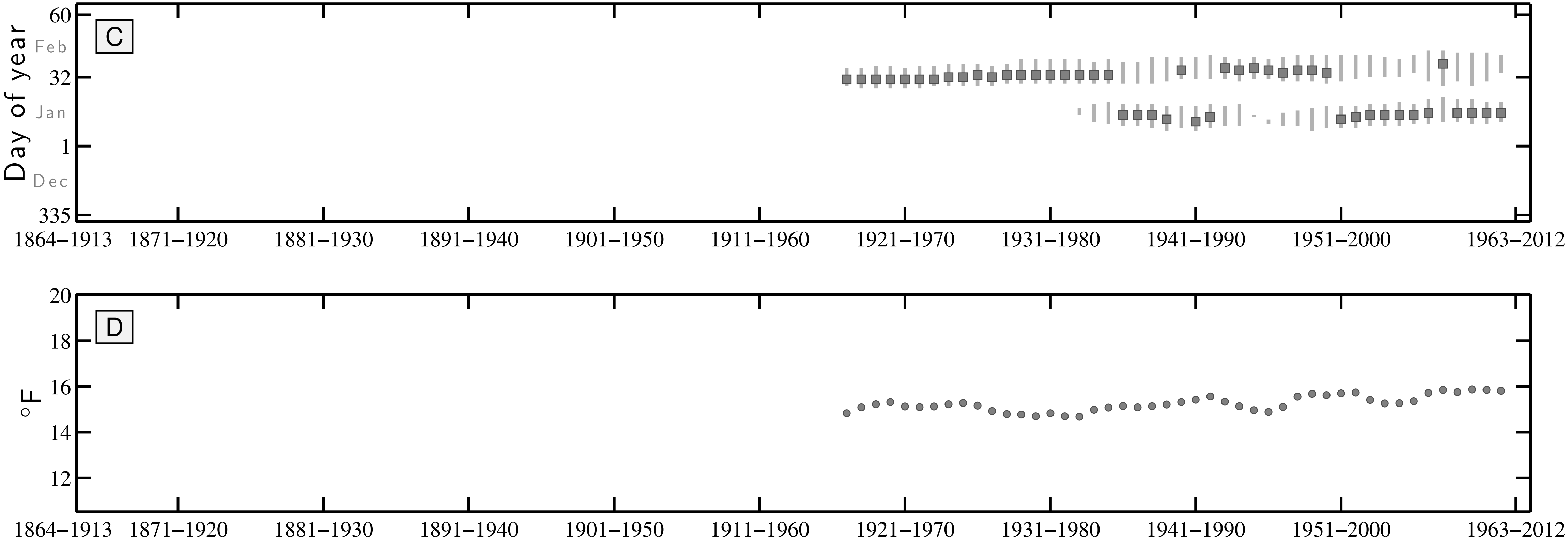

50 year Summer Teletherm dynamics for PORT JERVIS, NY:

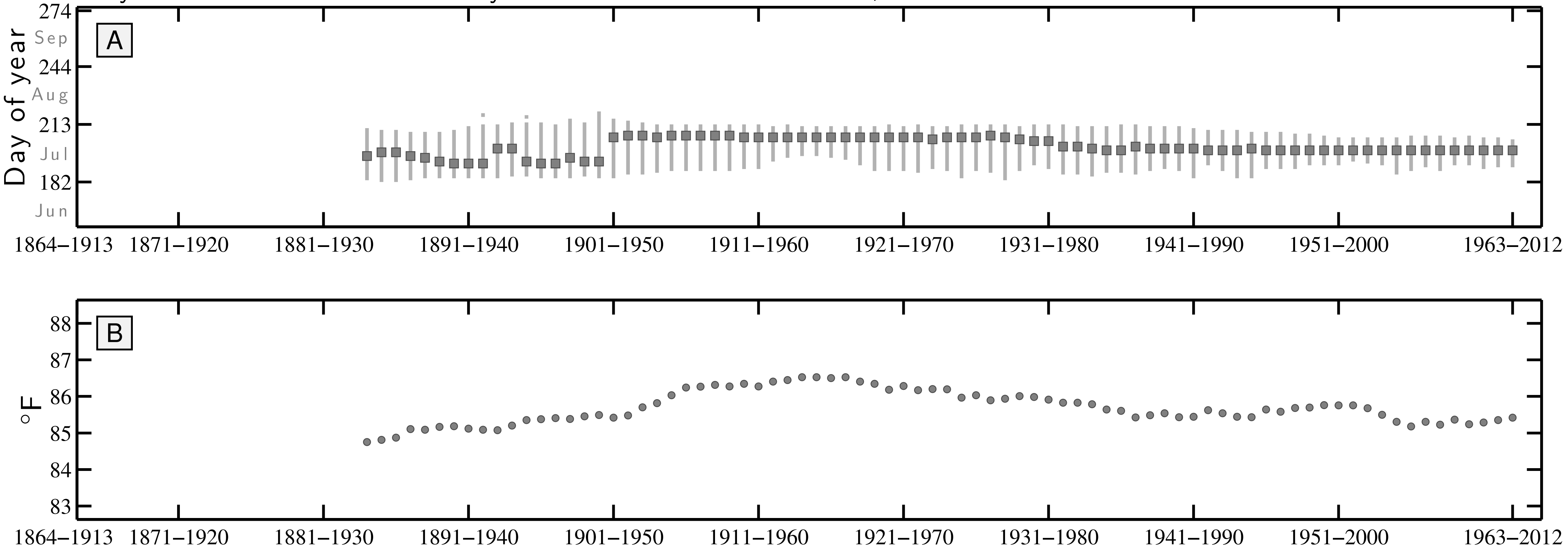

50 year Winter Teletherm dynamics for PORT JERVIS, NY:

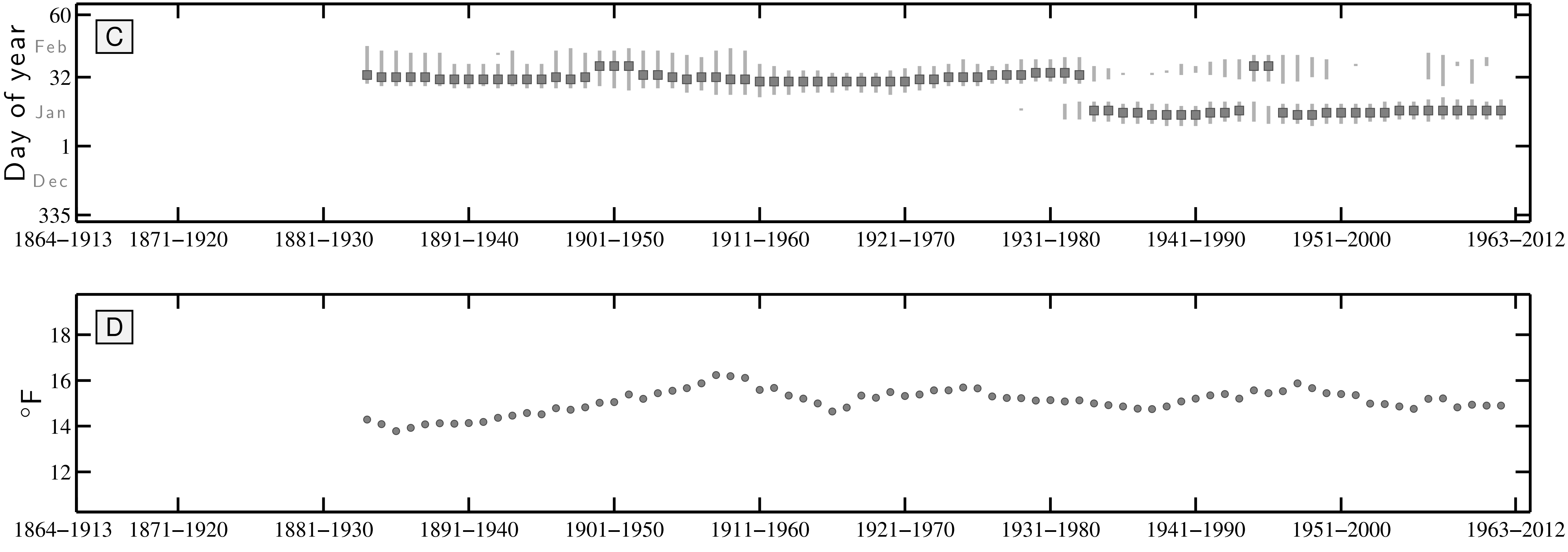

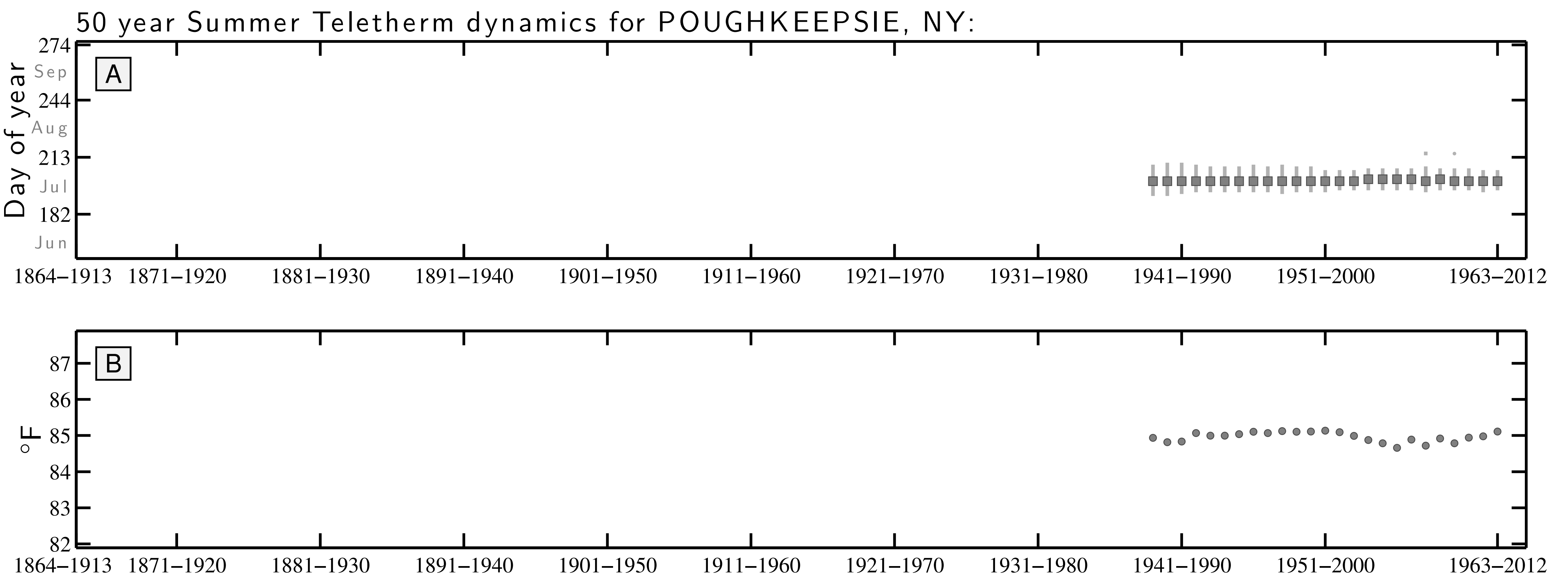

50 year Summer Teletherm dynamics for POUGHKEEPSIE, NY:
A
B
Day of year
°F
274
Sep
244
Aug
213
Jul
182
Jun
87
86
85
84
83
82
1864–1913
1871–1920
1881–1930
1891–1940
1901–1950
1911–1960
1921–1970
1931–1980
1941–1990
1951–2000
1963–2012

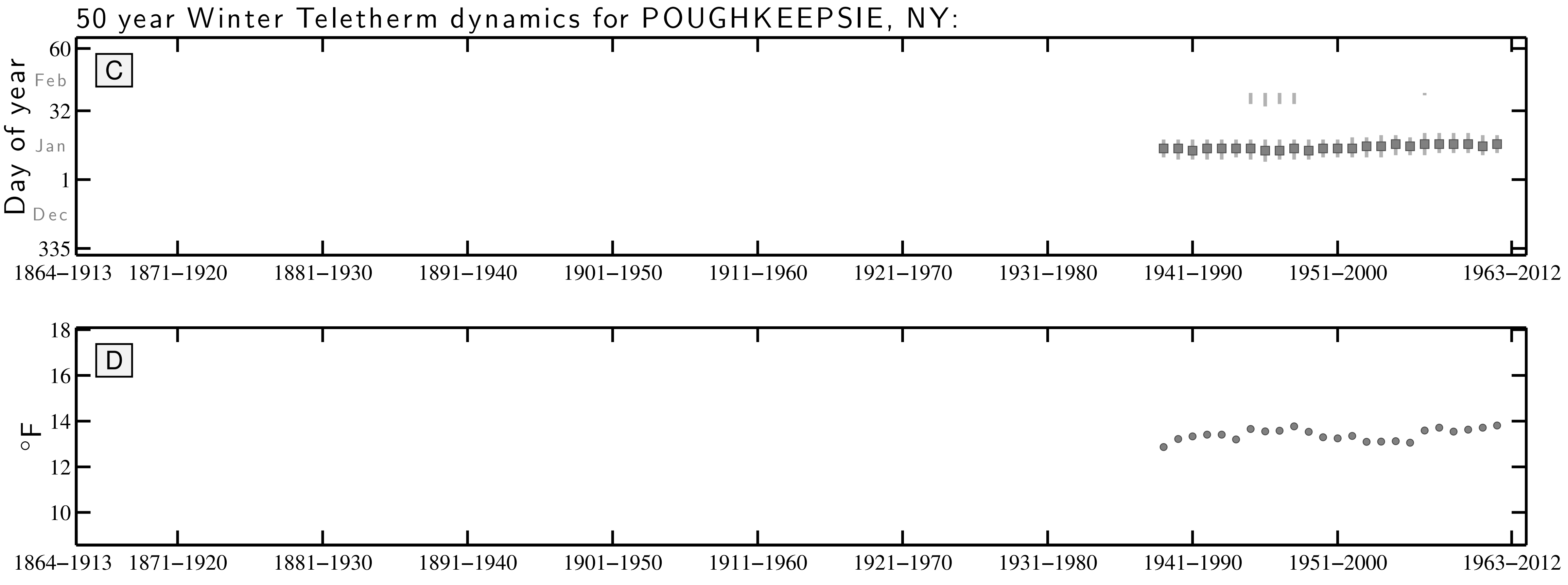

50 year Winter Teletherm dynamics for POUGHKEEPSIE, NY:
C
D
Day of year
°F
60
Feb
32
Jan
1
Dec
335
18
16
14
12
10
1864–1913
1871–1920
1881–1930
1891–1940
1901–1950
1911–1960
1921–1970
1931–1980
1941–1990
1951–2000
1963–2012

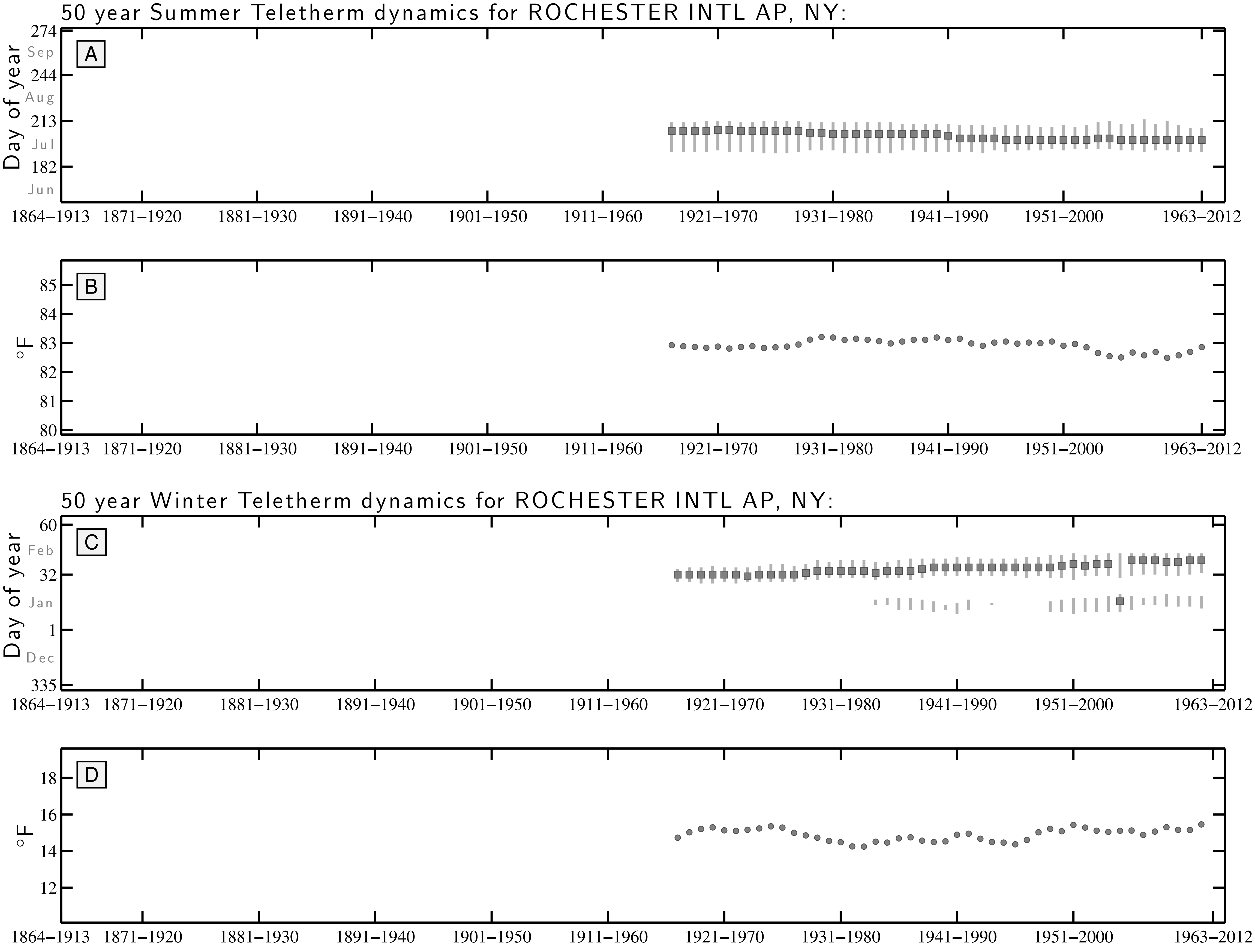
50 year Summer Teletherm dynamics for ROCHESTER INTL AP, NY:
A
Day of year
274
Sep
244
Aug
213
Jul
182
Jun
1864–1913
1871–1920
1881–1930
1891–1940
1901–1950
1911–1960
1921–1970
1931–1980
1941–1990
1951–2000
1963–2012
B
°F
85
84
83
82
81
80
50 year Winter Teletherm dynamics for ROCHESTER INTL AP, NY:
C
60
Feb
32
Jan
1
Dec
335
D
18
16
14
12

50 year Summer Teletherm dynamics for SARATOGA SPRINGS 4 SW, NY:

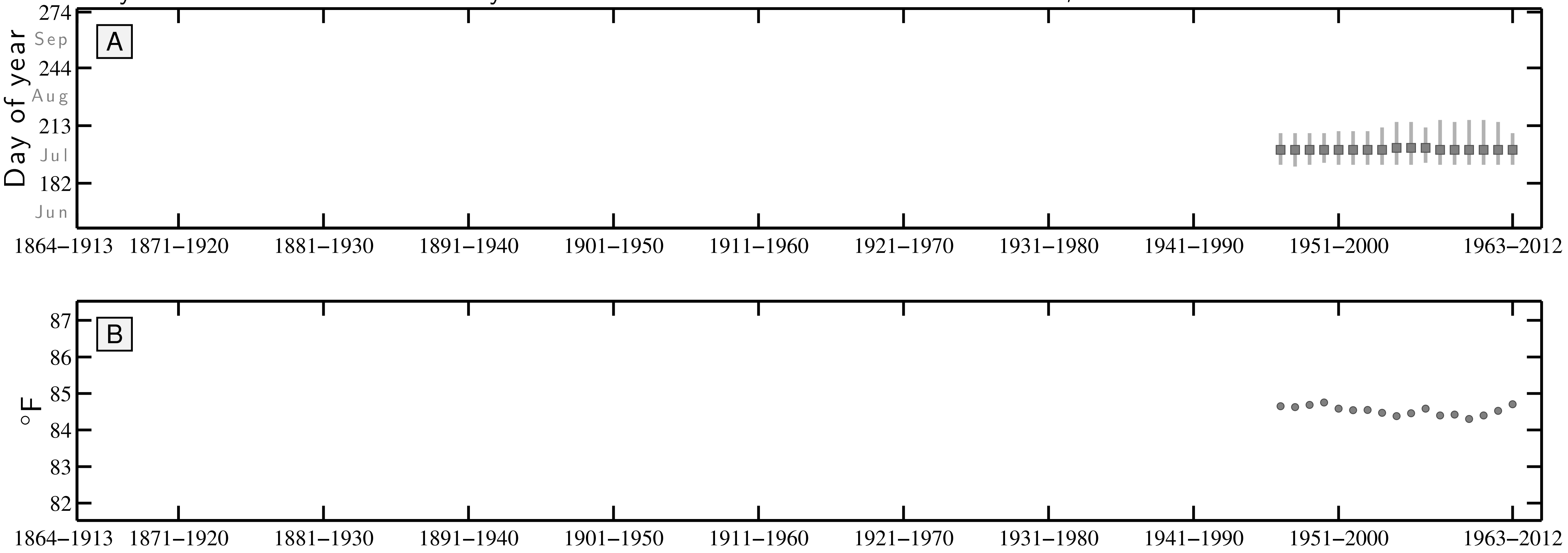

50 year Winter Teletherm dynamics for SARATOGA SPRINGS 4 SW, NY:

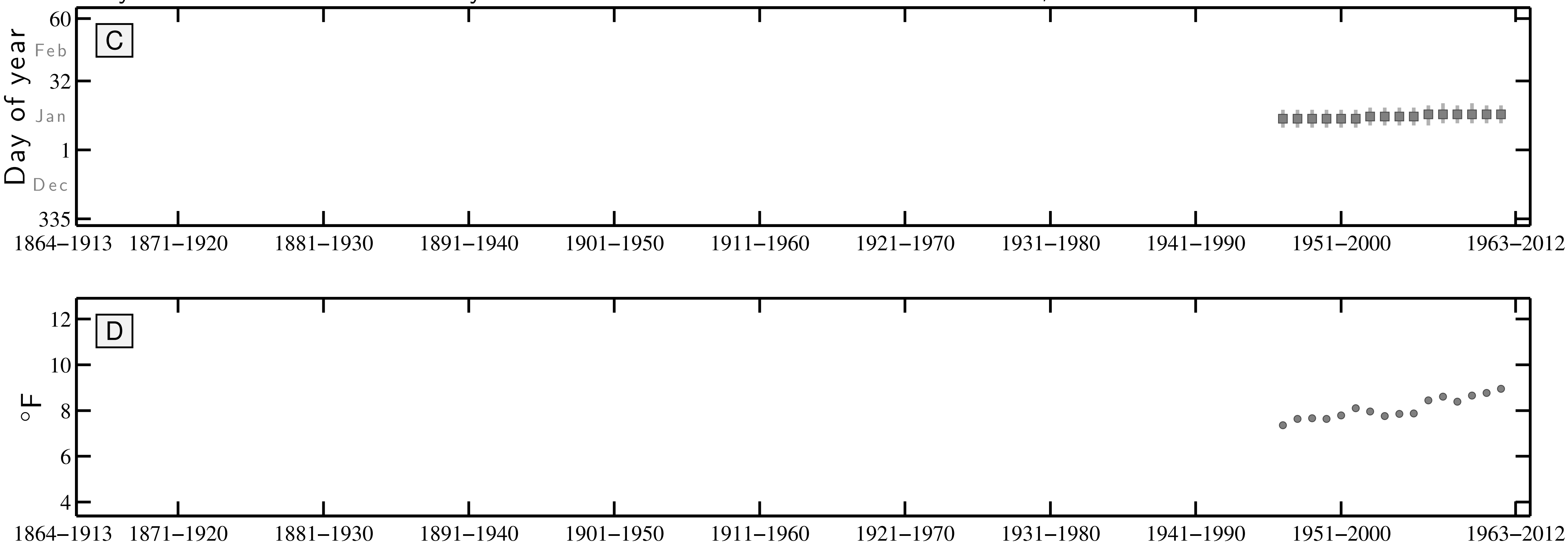

50 year Summer Teletherm dynamics for SETAUKET STRONG, NY:

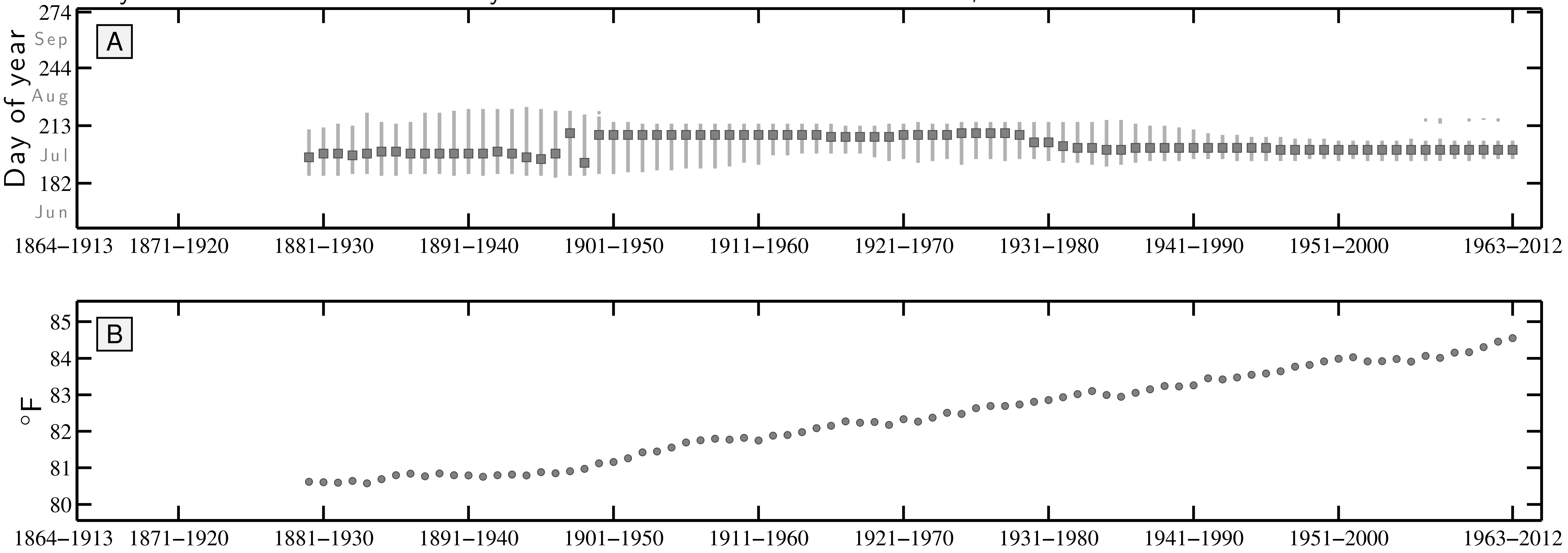

50 year Winter Teletherm dynamics for SETAUKET STRONG, NY:

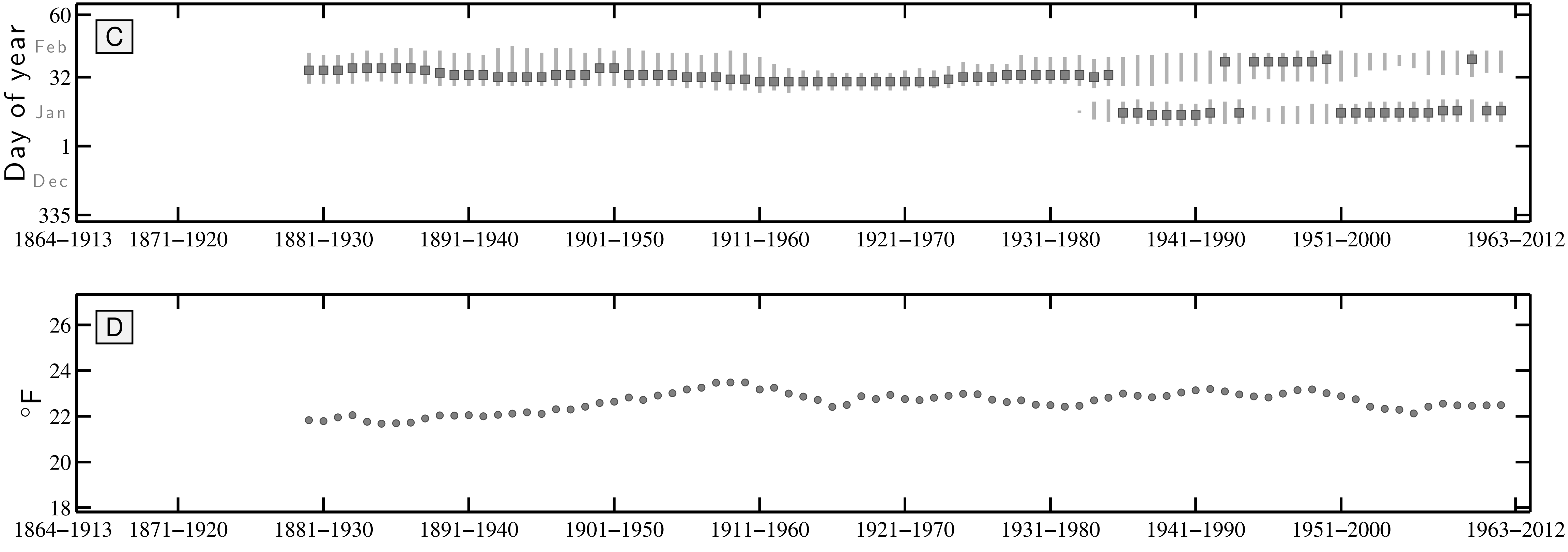

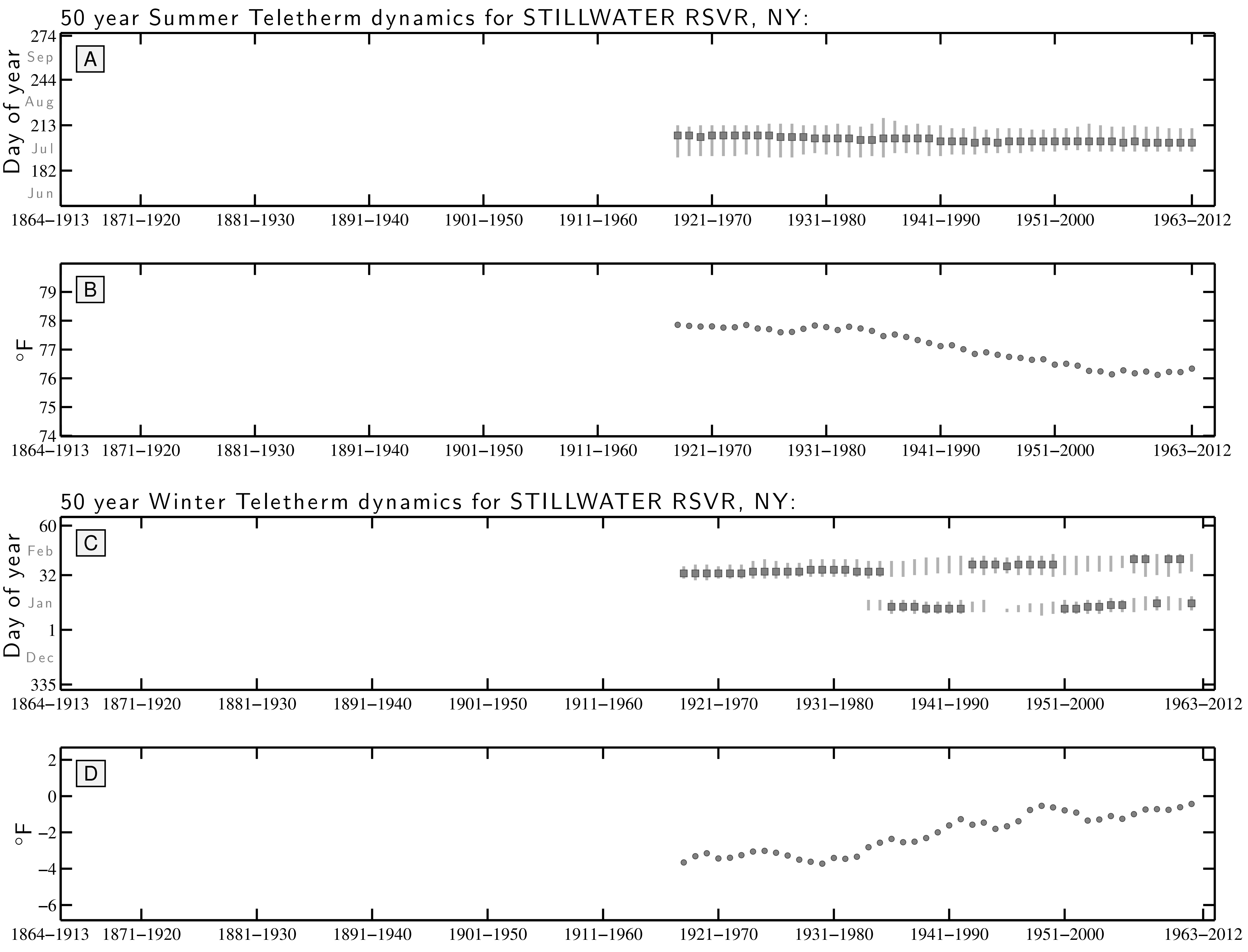
50 year Summer Teletherm dynamics for STILLWATER RSVR, NY:
A
Day of year
274
Sep
244
Aug
213
Jul
182
Jun
1864–1913
1871–1920
1881–1930
1891–1940
1901–1950
1911–1960
1921–1970
1931–1980
1941–1990
1951–2000
1963–2012
B
°F
79
78
77
76
75
74
50 year Winter Teletherm dynamics for STILLWATER RSVR, NY:
C
60
Feb
32
Jan
1
Dec
335
D
2
0
−2
−4
−6

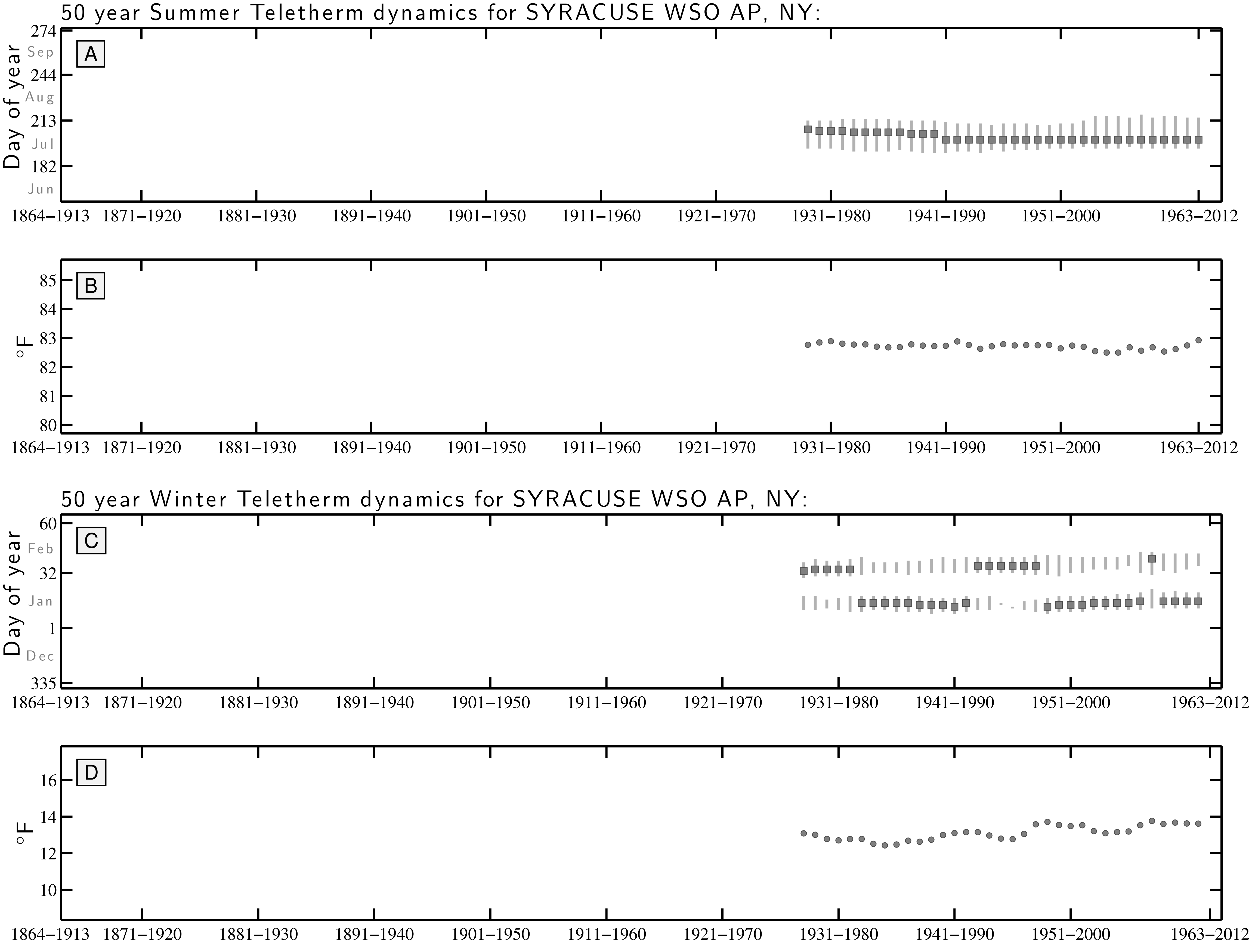
50 year Summer Teletherm dynamics for SYRACUSE WSO AP, NY:
A
Day of year
274
Sep
244
Aug
213
Jul
182
Jun
B
°F
85
84
83
82
81
80
50 year Winter Teletherm dynamics for SYRACUSE WSO AP, NY:
C
Day of year
60
Feb
32
Jan
1
Dec
335
D
°F
16
14
12
10
1864–1913
1871–1920
1881–1930
1891–1940
1901–1950
1911–1960
1921–1970
1931–1980
1941–1990
1951–2000
1963–2012

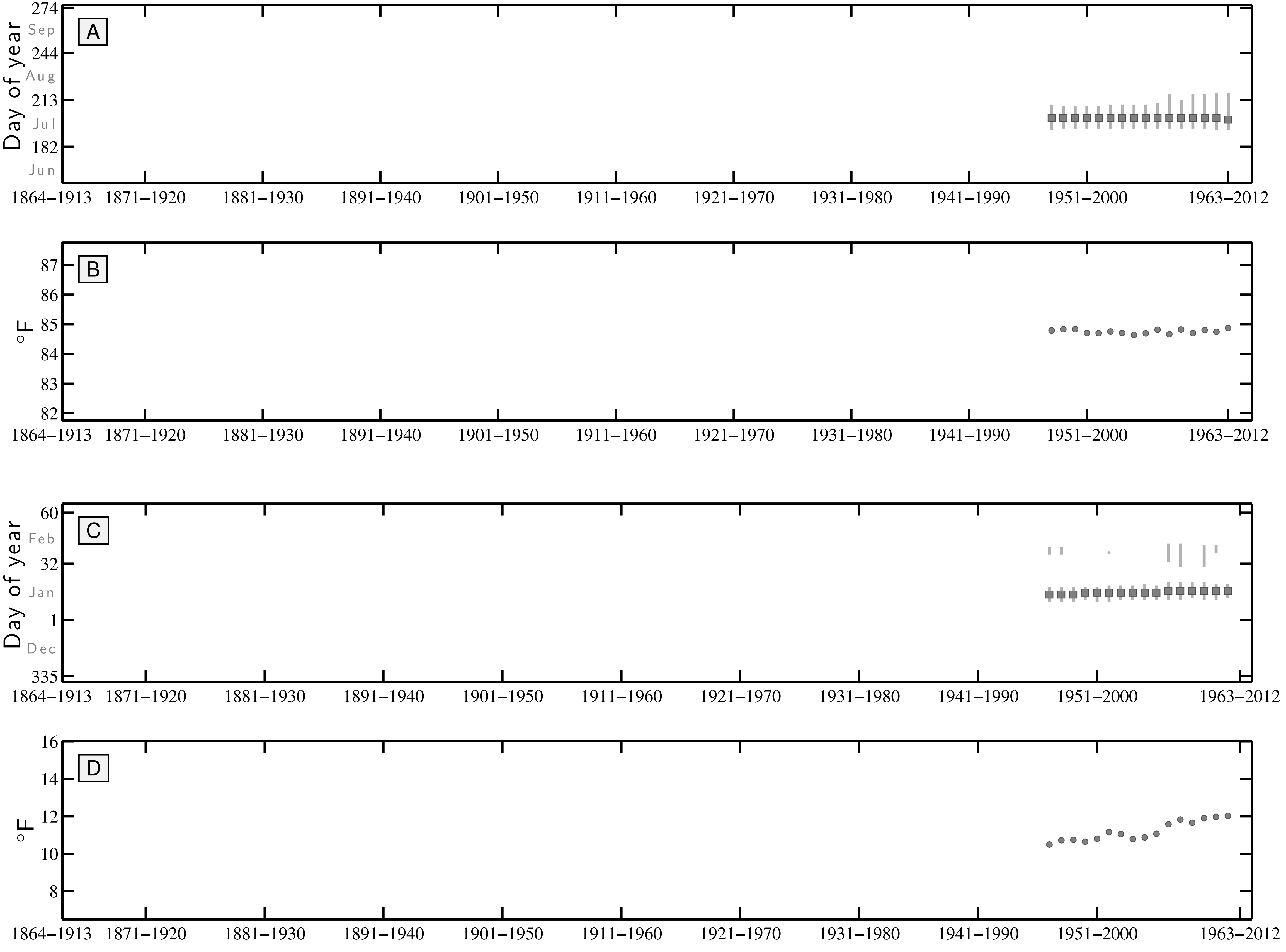
A
Day of year
274
Sep
244
Aug
213
Jul
182
Jun
B
°F
87
86
85
84
83
82
C
Day of year
60
Feb
32
Jan
1
Dec
335
D
°F
16
14
12
10
8
1864–1913
1871–1920
1881–1930
1891–1940
1901–1950
1911–1960
1921–1970
1931–1980
1941–1990
1951–2000
1963–2012

50 year Summer Teletherm dynamics for TUPPER LAKE SUNMOUNT, NY:

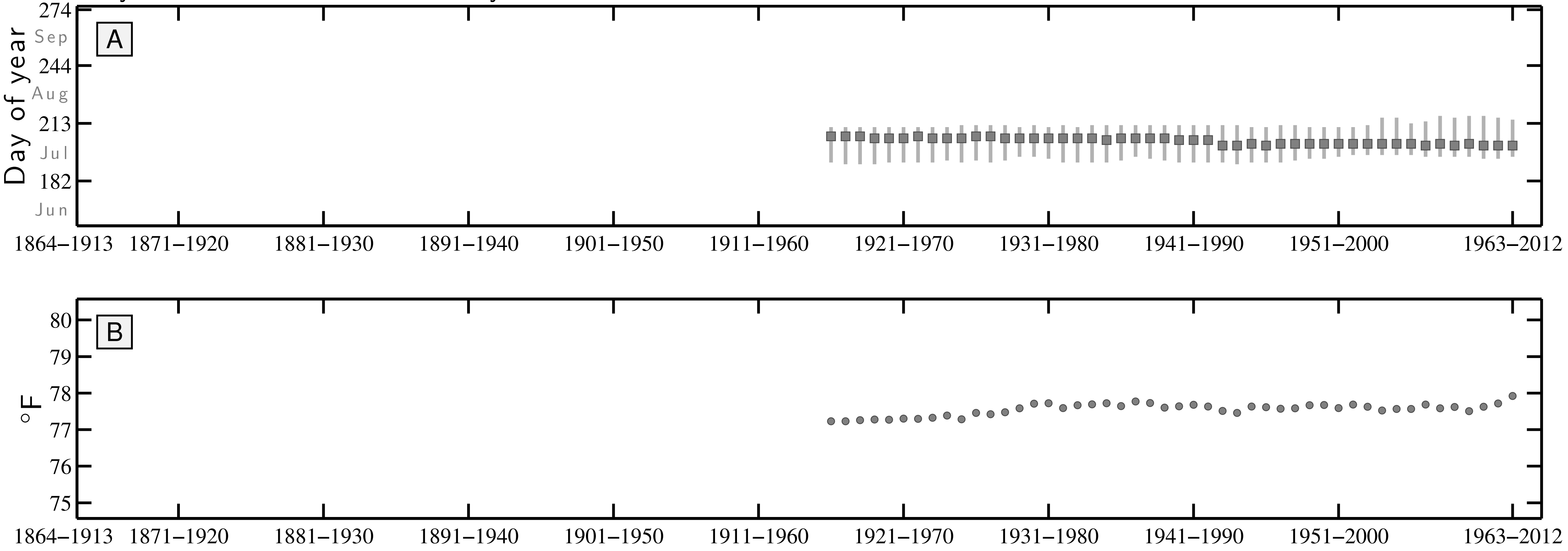

50 year Winter Teletherm dynamics for TUPPER LAKE SUNMOUNT, NY:

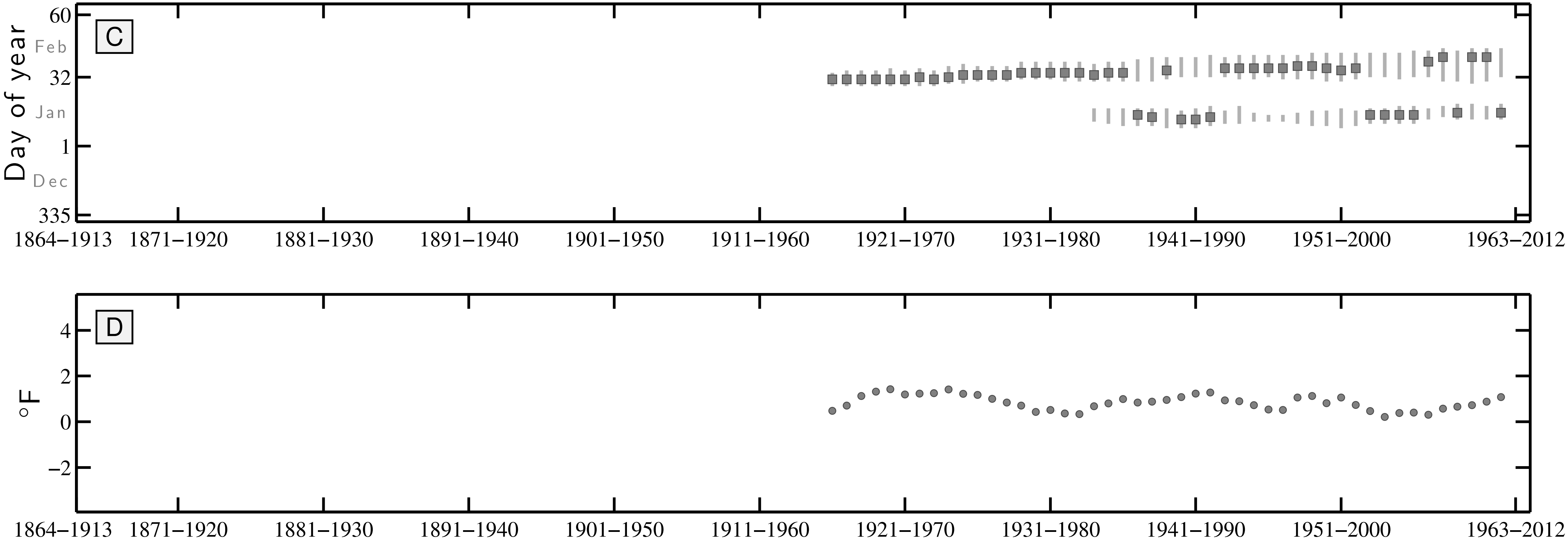

50 year Summer Teletherm dynamics for UTICA FAA AP, NY:

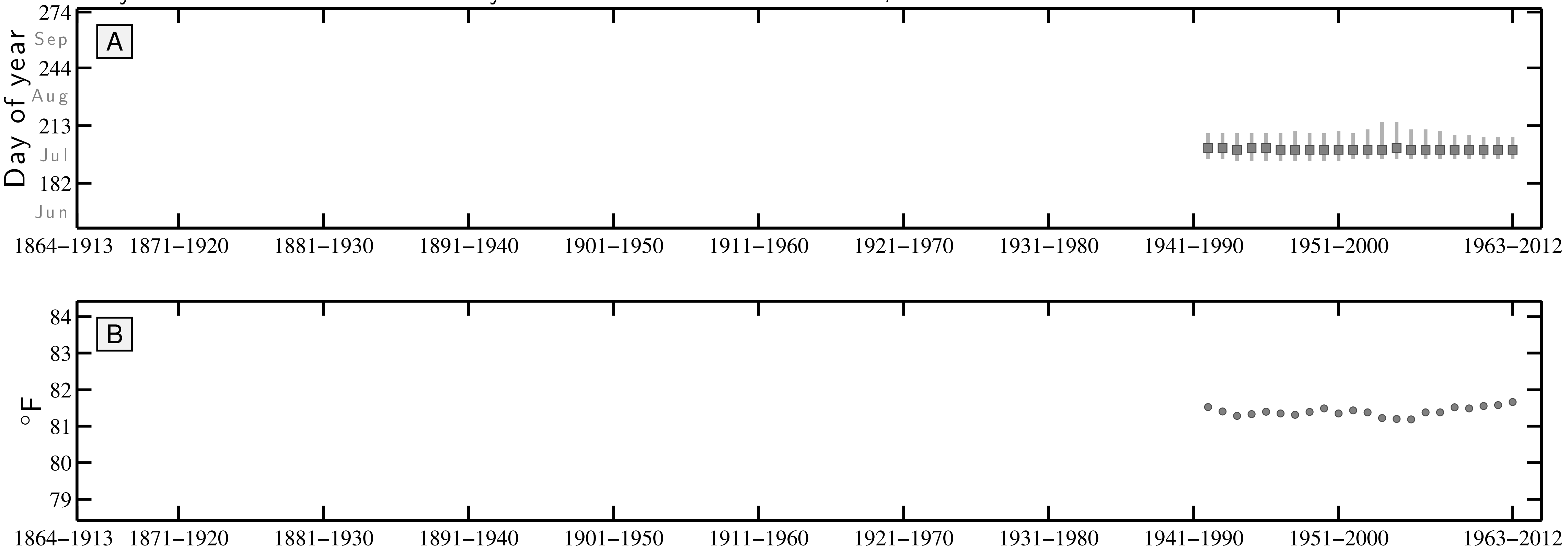

50 year Winter Teletherm dynamics for UTICA FAA AP, NY:

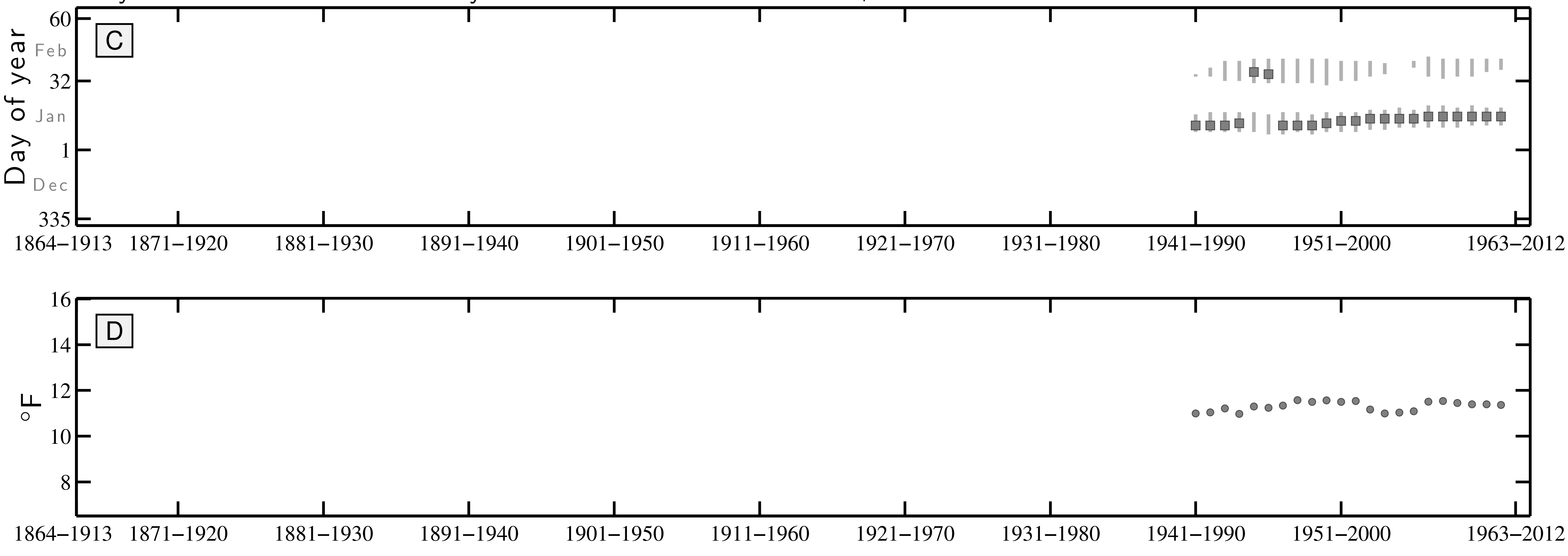

50 year Summer Teletherm dynamics for WANAKENA RNGR SCHOOL, NY:

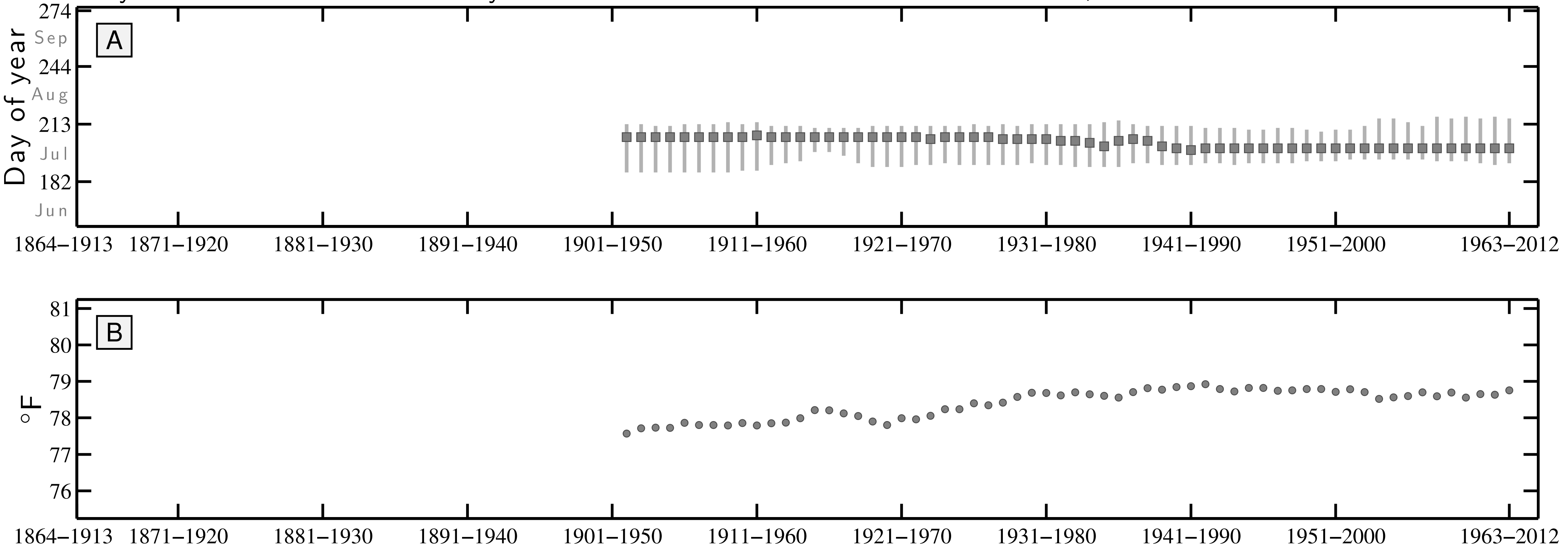

50 year Winter Teletherm dynamics for WANAKENA RNGR SCHOOL, NY:

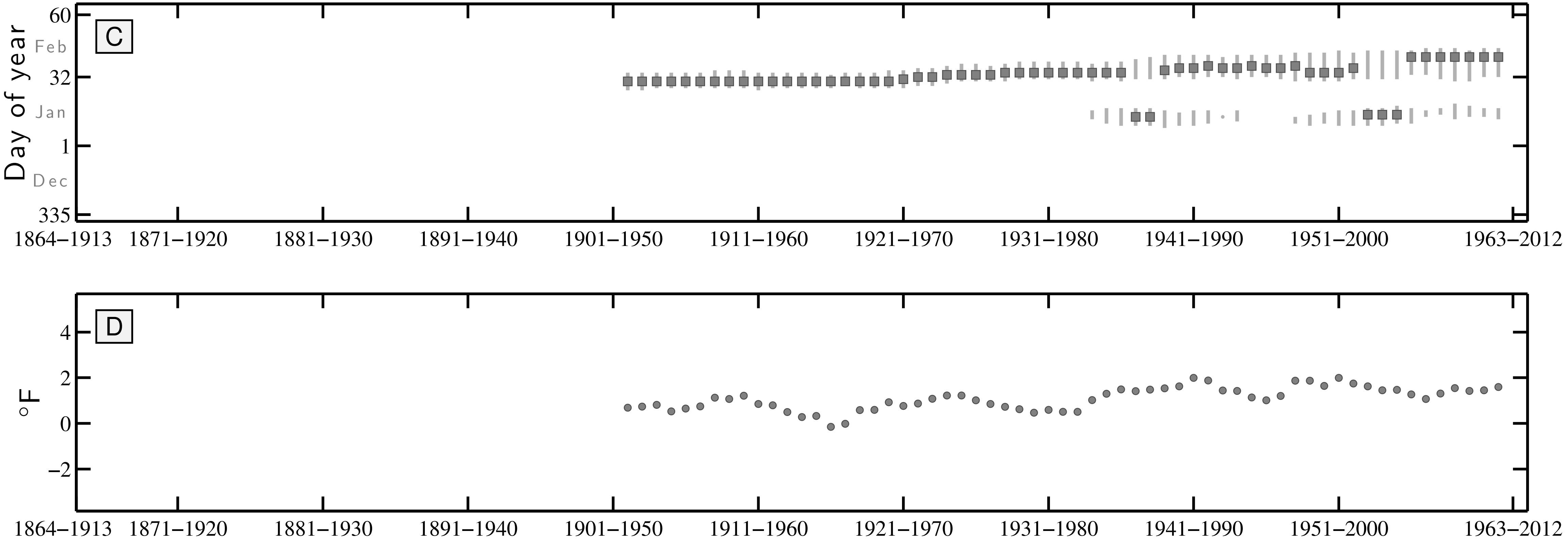

50 year Summer Teletherm dynamics for WATERTOWN, NY:

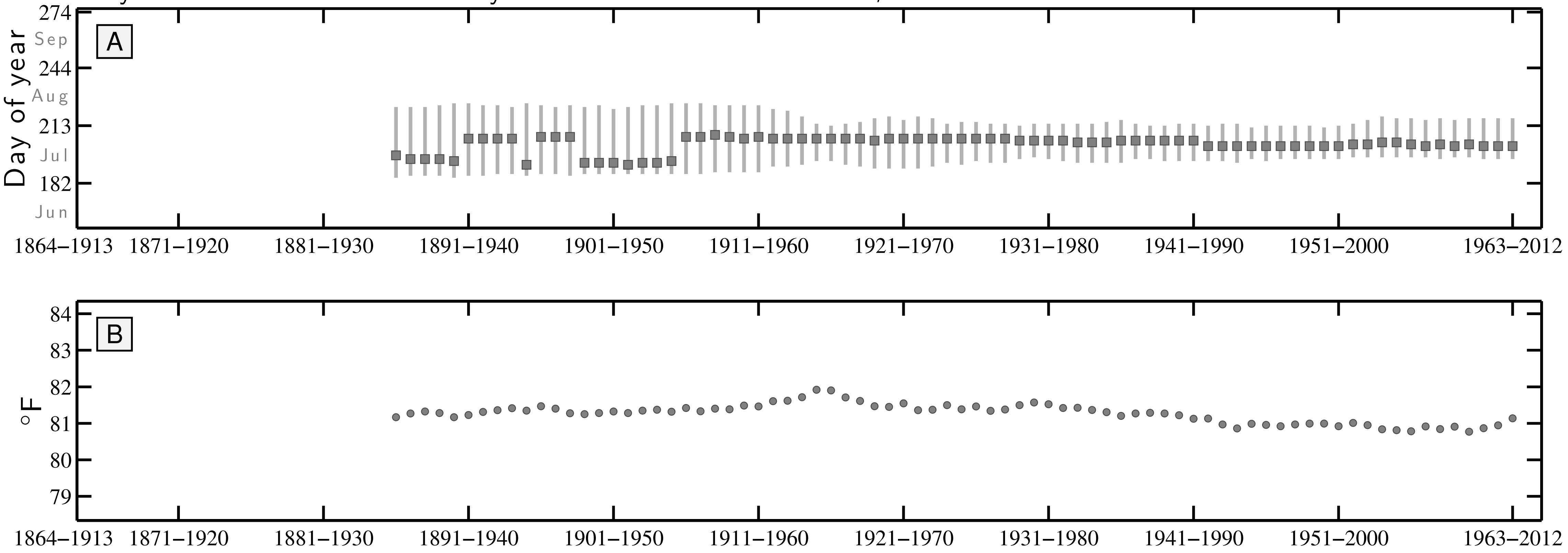

50 year Winter Teletherm dynamics for WATERTOWN, NY:

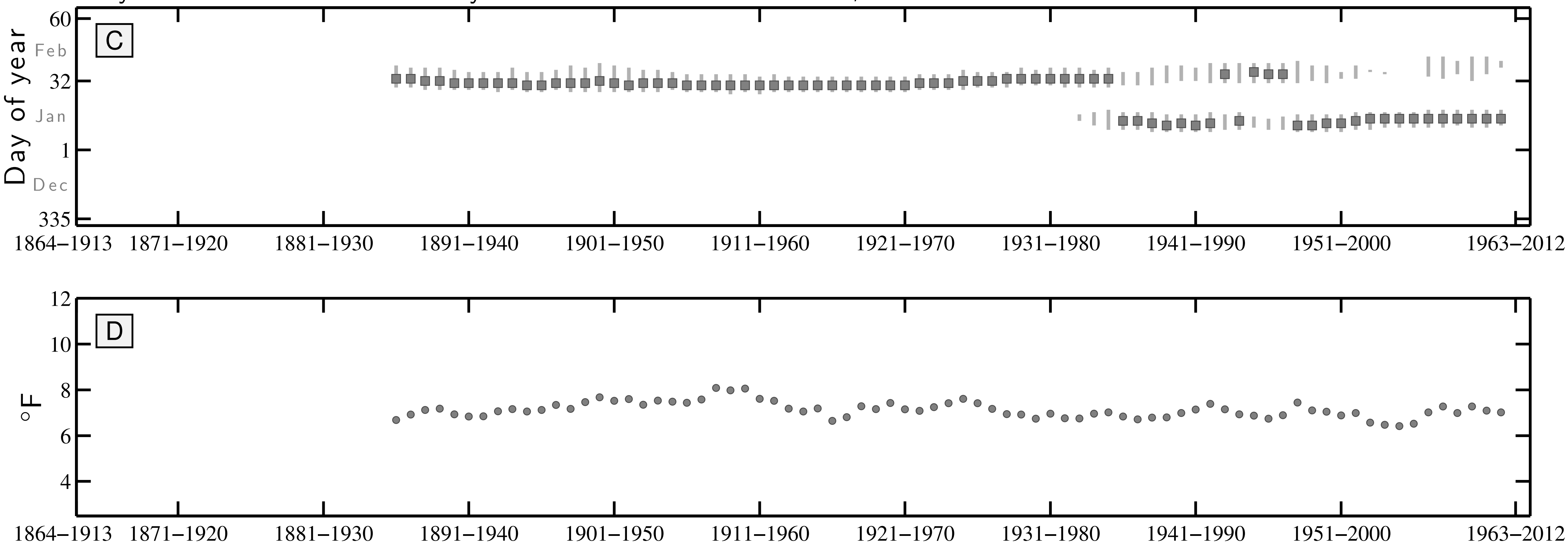

50 year Summer Teletherm dynamics for WEST POINT, NY:

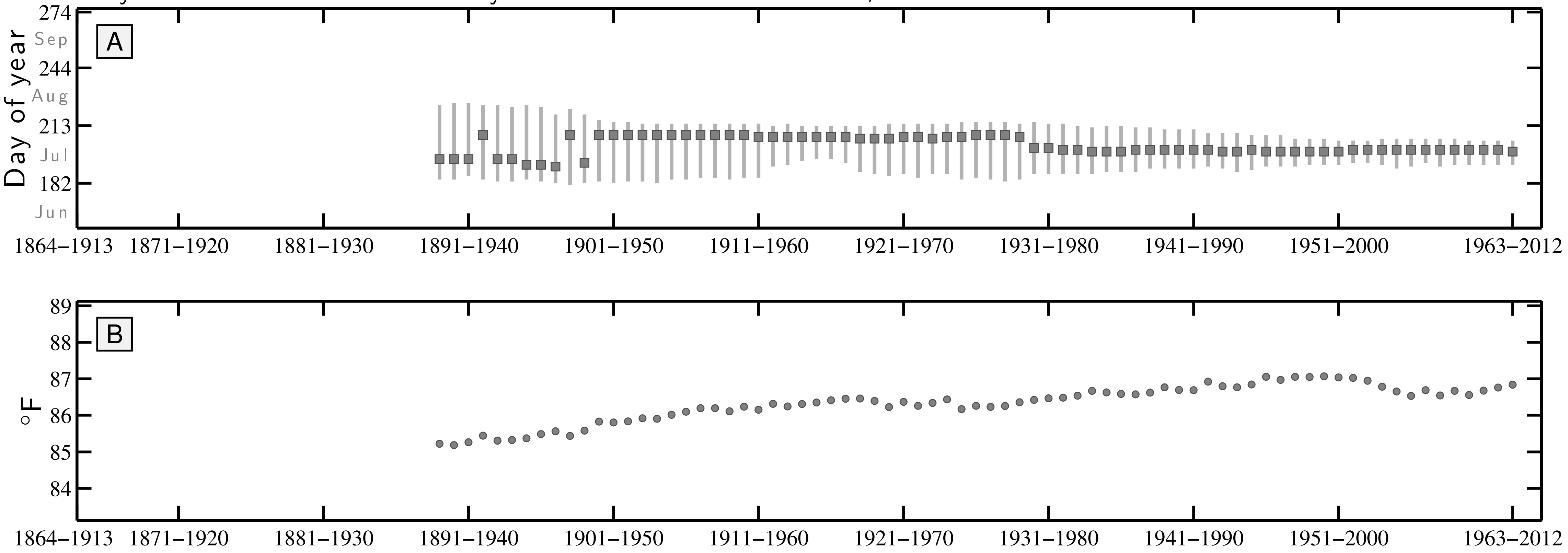

50 year Winter Teletherm dynamics for WEST POINT, NY:

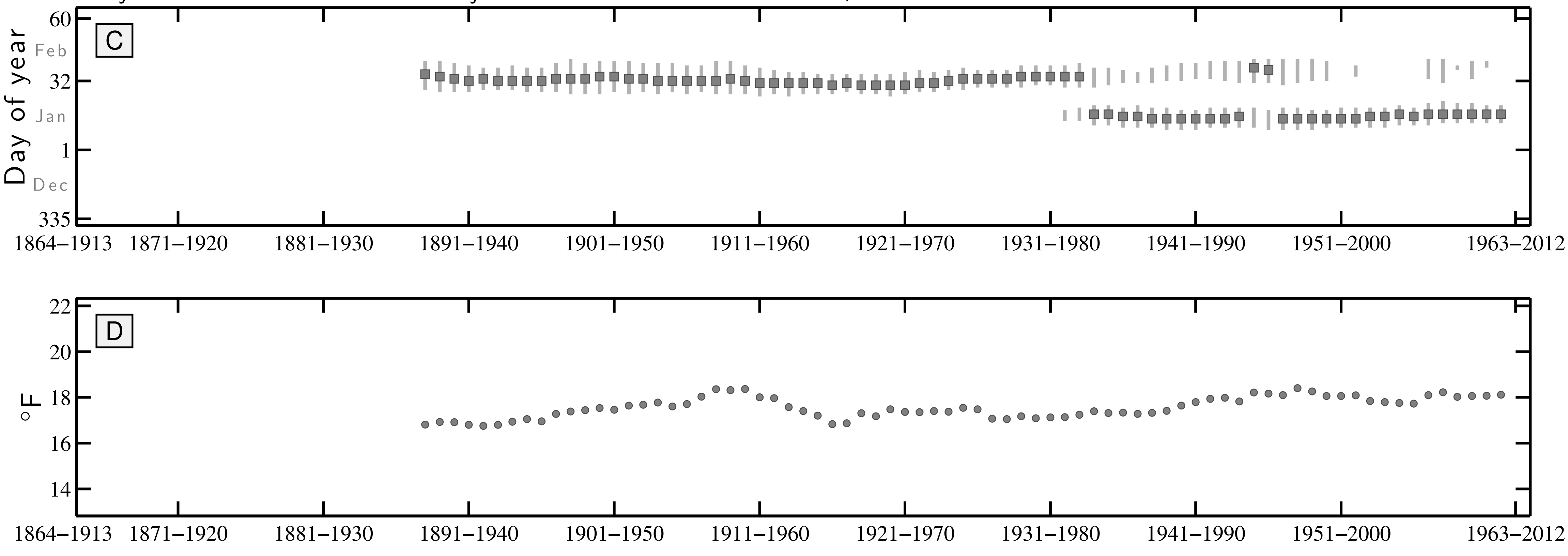

50 year Summer Teletherm dynamics for YORKTOWN HEIGHTS 1 W, NY:

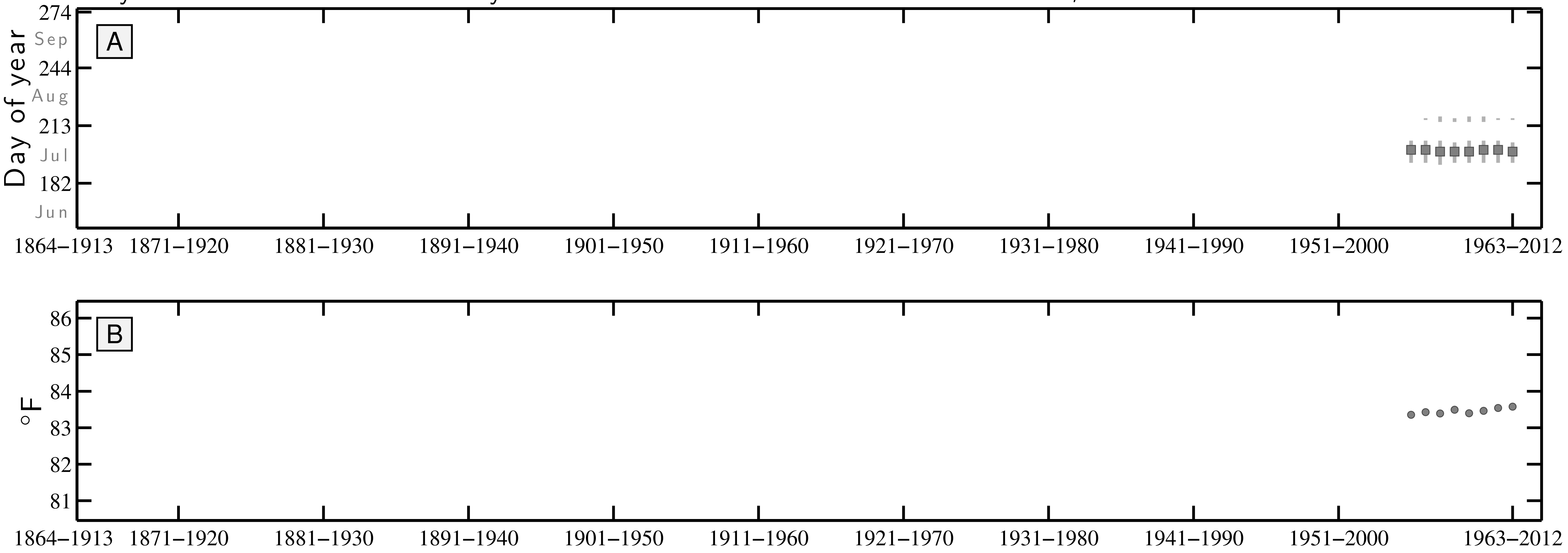

50 year Winter Teletherm dynamics for YORKTOWN HEIGHTS 1 W, NY:

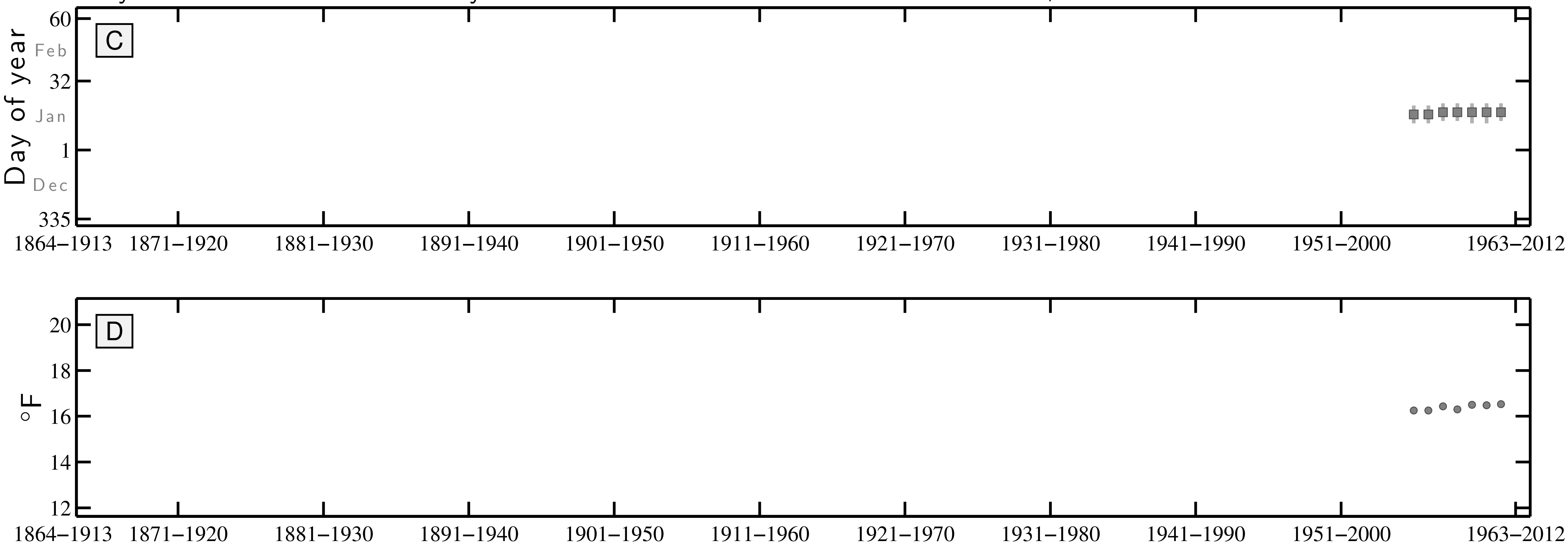

50 year Summer Teletherm dynamics for ALBEMARLE, NC:

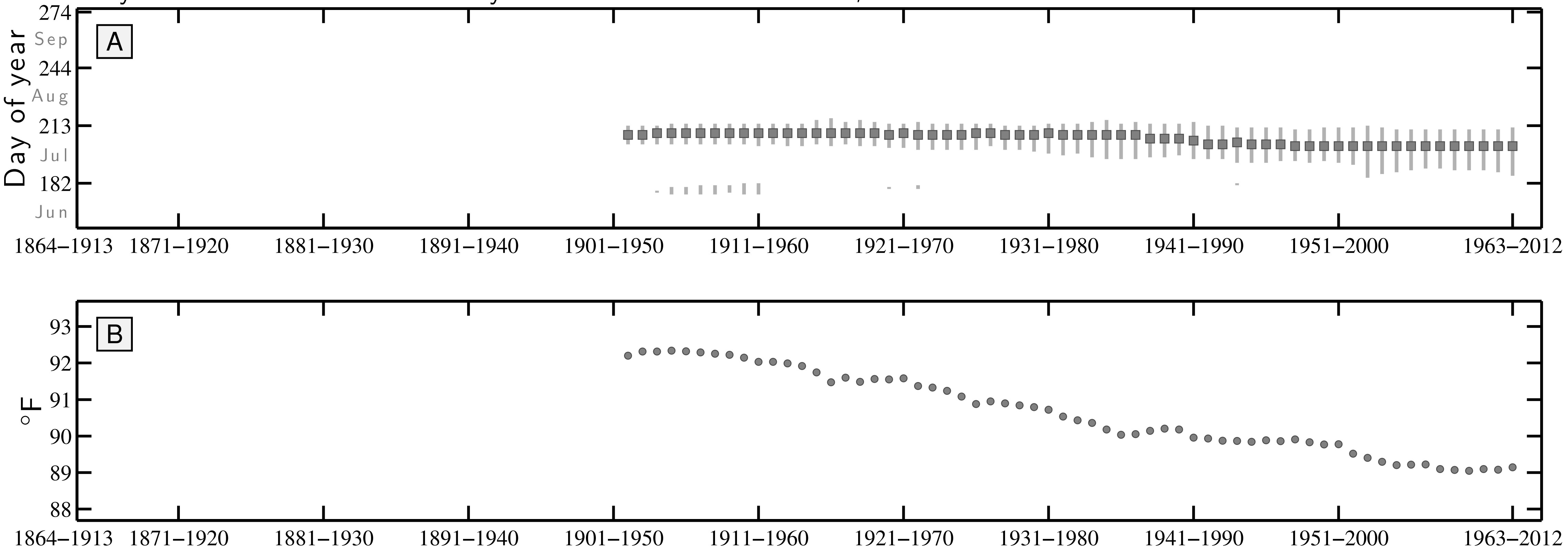

50 year Winter Teletherm dynamics for ALBEMARLE, NC:

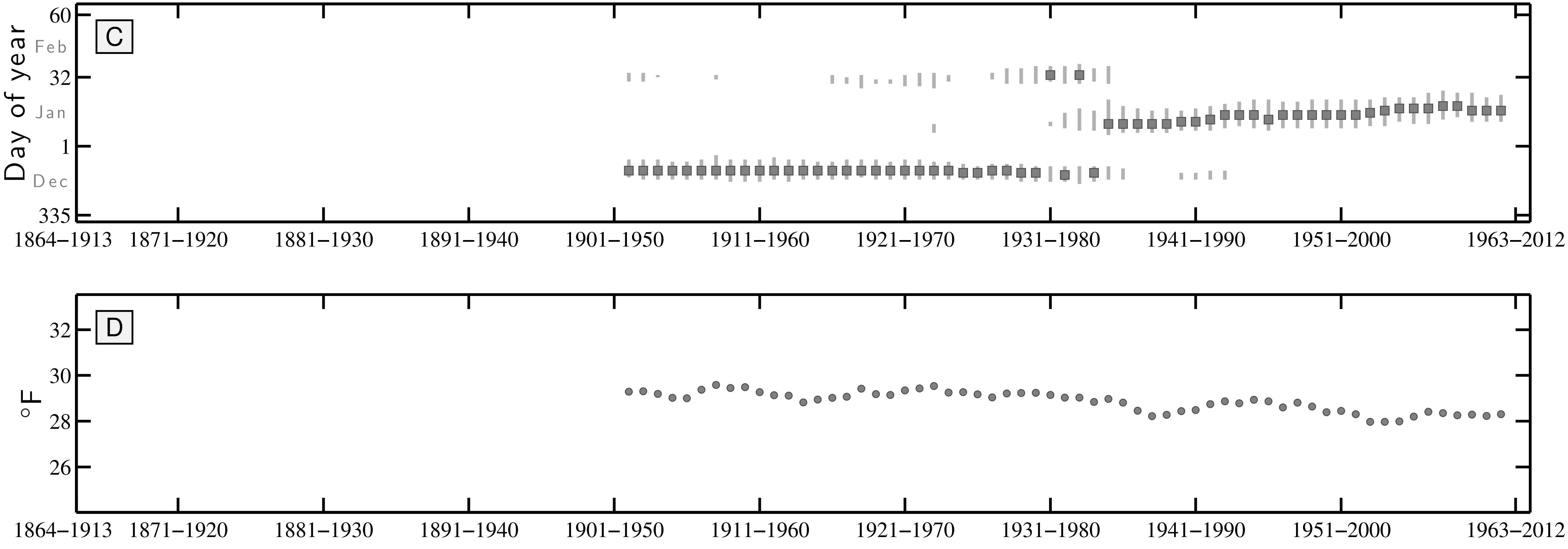

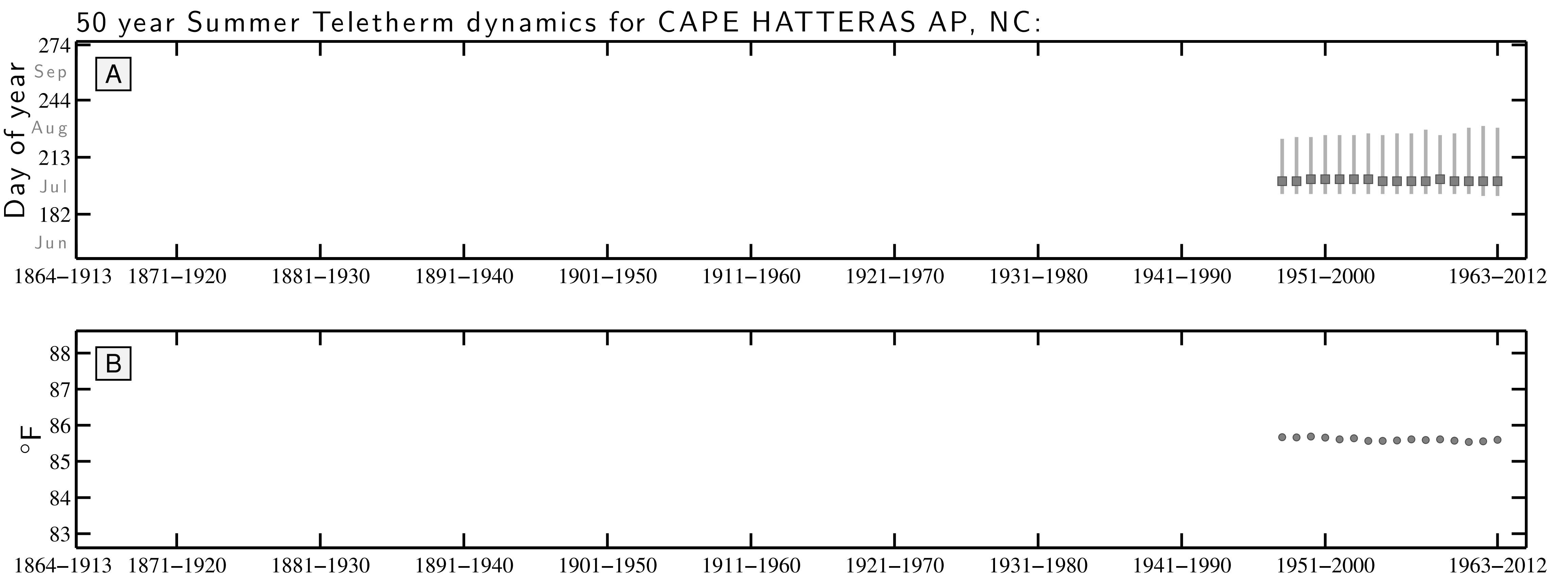

50 year Summer Teletherm dynamics for CAPE HATTERAS AP, NC:
A
B
Day of year
274
Sep
244
Aug
213
Jul
182
Jun
°F
88
87
86
85
84
83
1864–1913
1871–1920
1881–1930
1891–1940
1901–1950
1911–1960
1921–1970
1931–1980
1941–1990
1951–2000
1963–2012

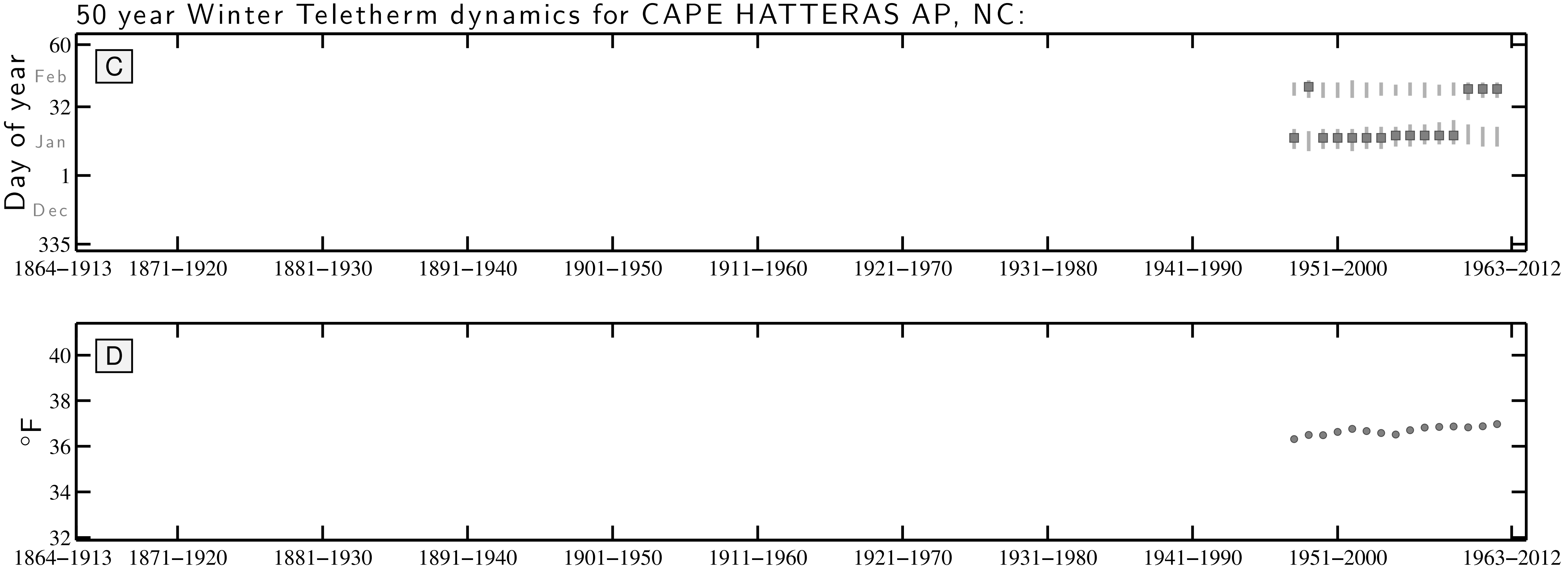

50 year Winter Teletherm dynamics for CAPE HATTERAS AP, NC:
C
D
Day of year
60
Feb
32
Jan
1
Dec
335
°F
40
38
36
34
32
1864–1913
1871–1920
1881–1930
1891–1940
1901–1950
1911–1960
1921–1970
1931–1980
1941–1990
1951–2000
1963–2012

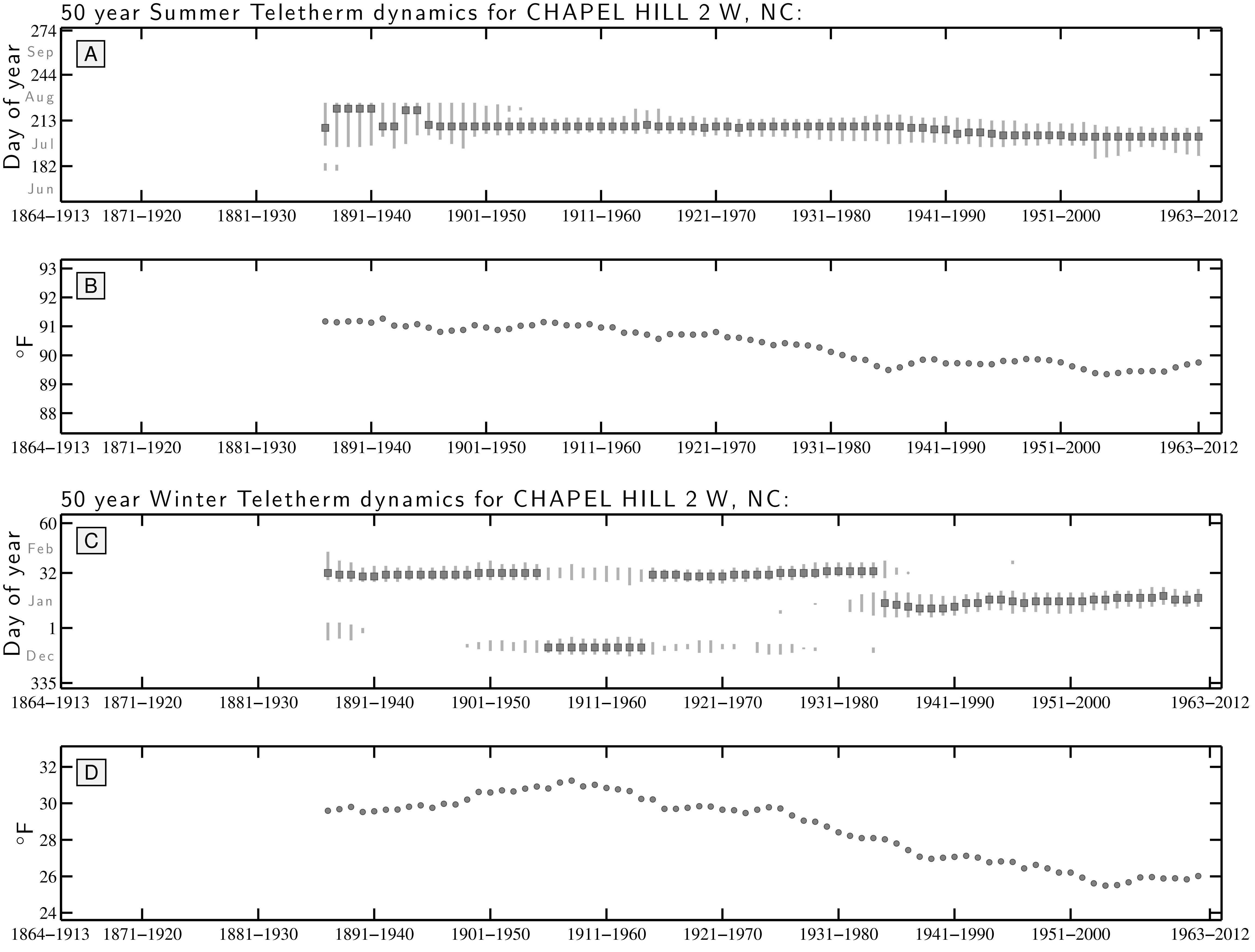
50 year Summer Teletherm dynamics for CHAPEL HILL 2 W, NC:
A
Day of year
274
Sep
244
Aug
213
Jul
182
Jun
1864–1913
1871–1920
1881–1930
1891–1940
1901–1950
1911–1960
1921–1970
1931–1980
1941–1990
1951–2000
1963–2012
B
°F
93
92
91
90
89
88
50 year Winter Teletherm dynamics for CHAPEL HILL 2 W, NC:
C
60
Feb
32
Jan
1
Dec
335
D
32
30
28
26
24

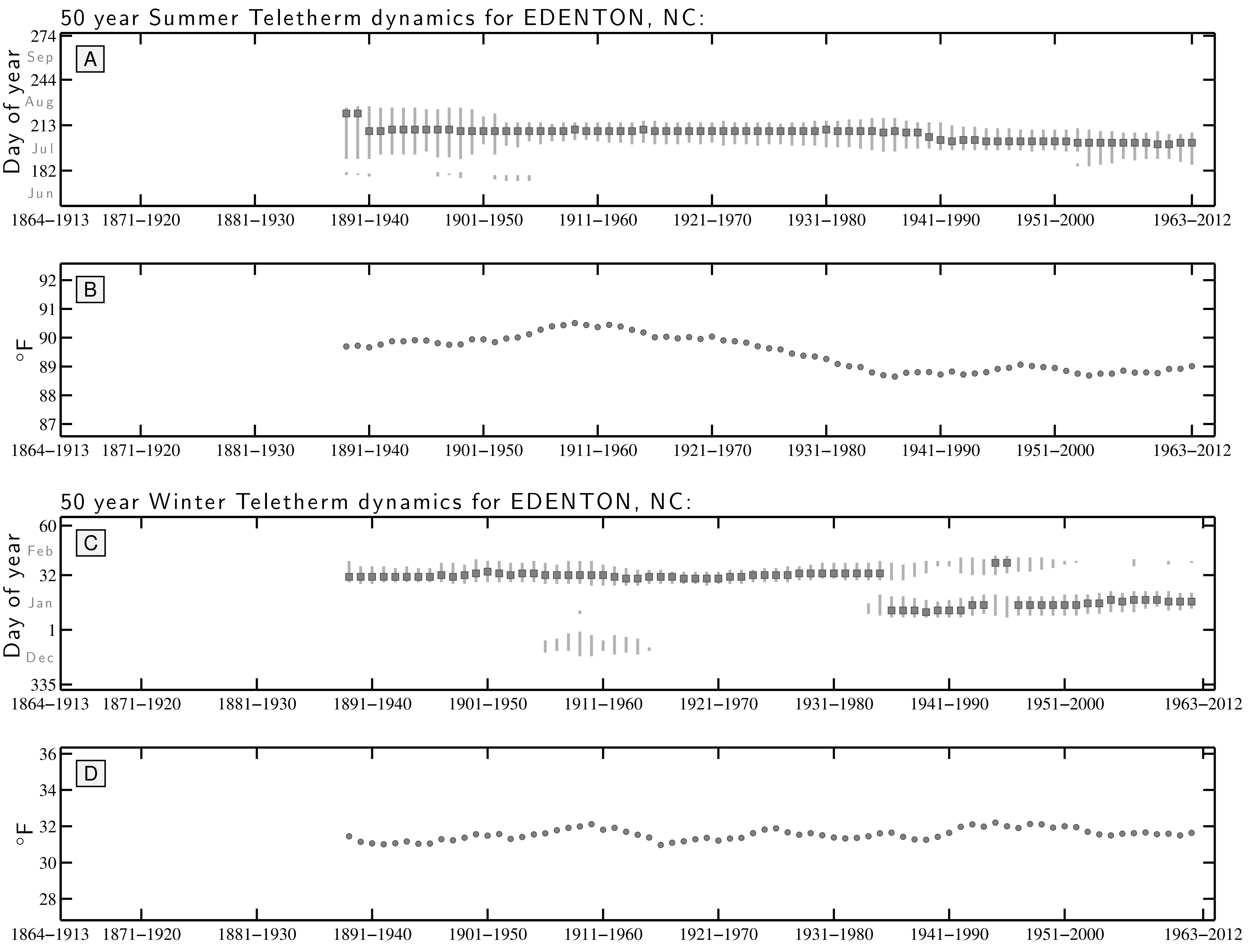
50 year Summer Teletherm dynamics for EDENTON, NC:
A
Day of year
274
Sep
244
Aug
213
Jul
182
Jun
1864–1913
1871–1920
1881–1930
1891–1940
1901–1950
1911–1960
1921–1970
1931–1980
1941–1990
1951–2000
1963–2012
B
°F
92
91
90
89
88
87
50 year Winter Teletherm dynamics for EDENTON, NC:
C
Day of year
60
Feb
32
Jan
1
Dec
335
D
°F
36
34
32
30
28

50 year Summer Teletherm dynamics for ELIZABETH CITY, NC:

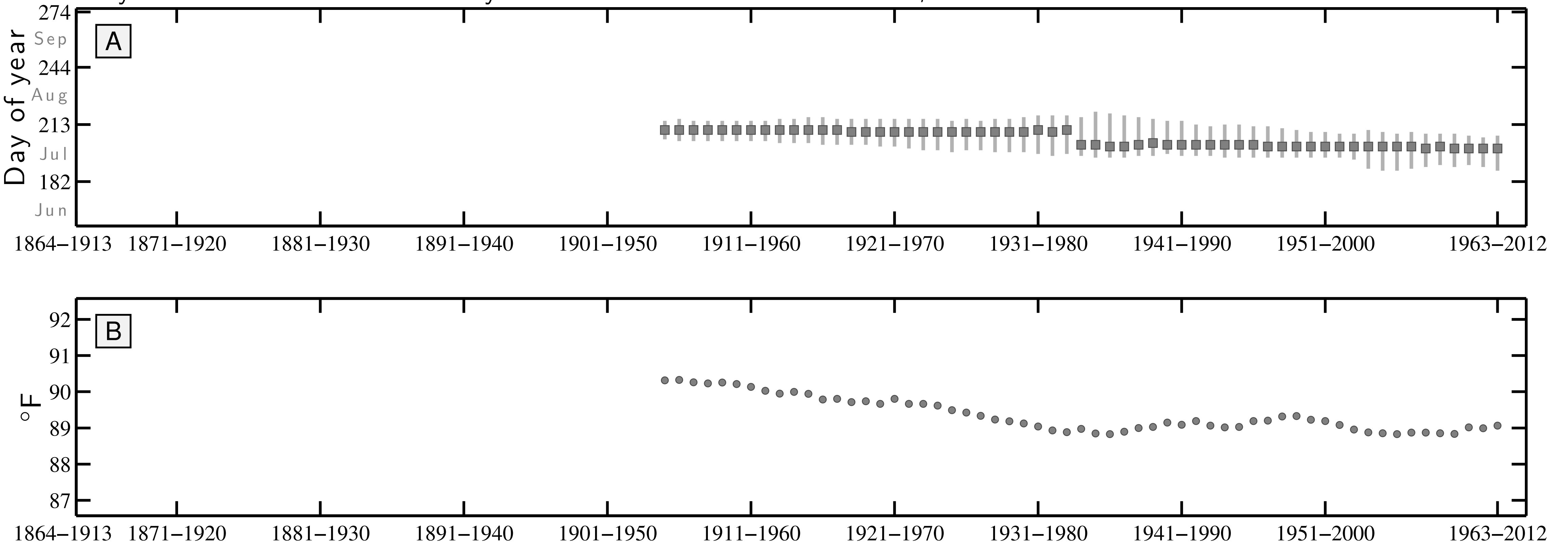

50 year Winter Teletherm dynamics for ELIZABETH CITY, NC:

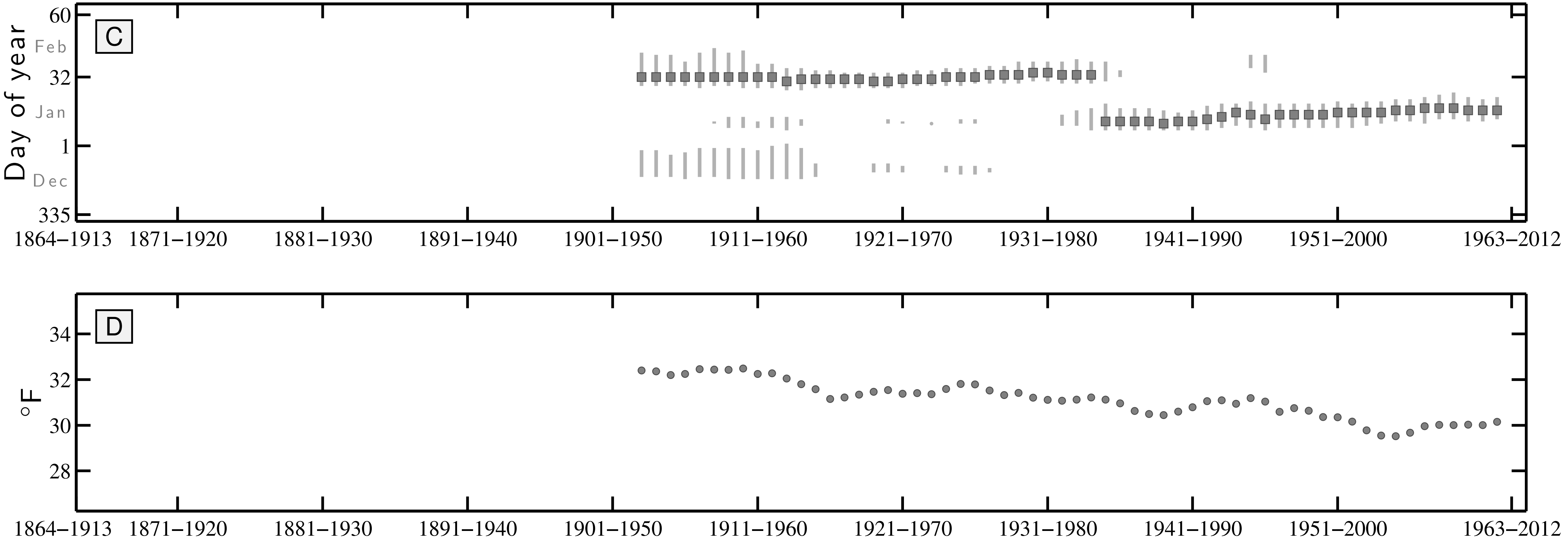

50 year Summer Teletherm dynamics for FAYETTEVILLE PWC, NC:

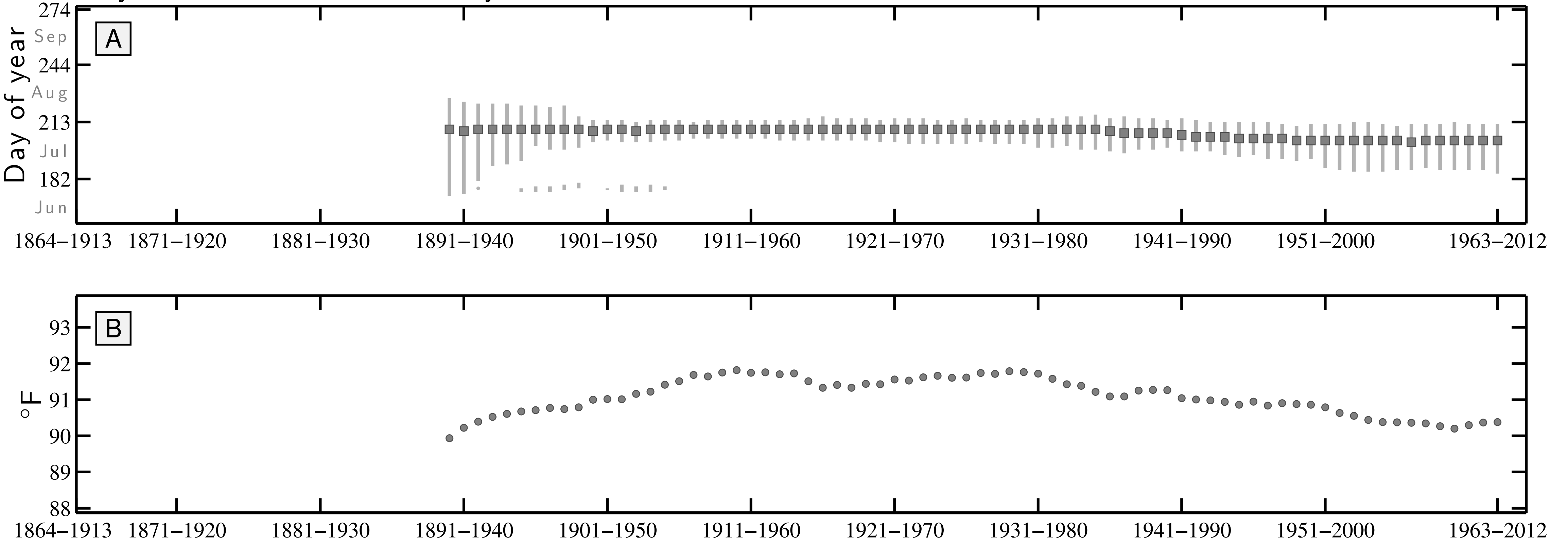

50 year Winter Teletherm dynamics for FAYETTEVILLE PWC, NC:

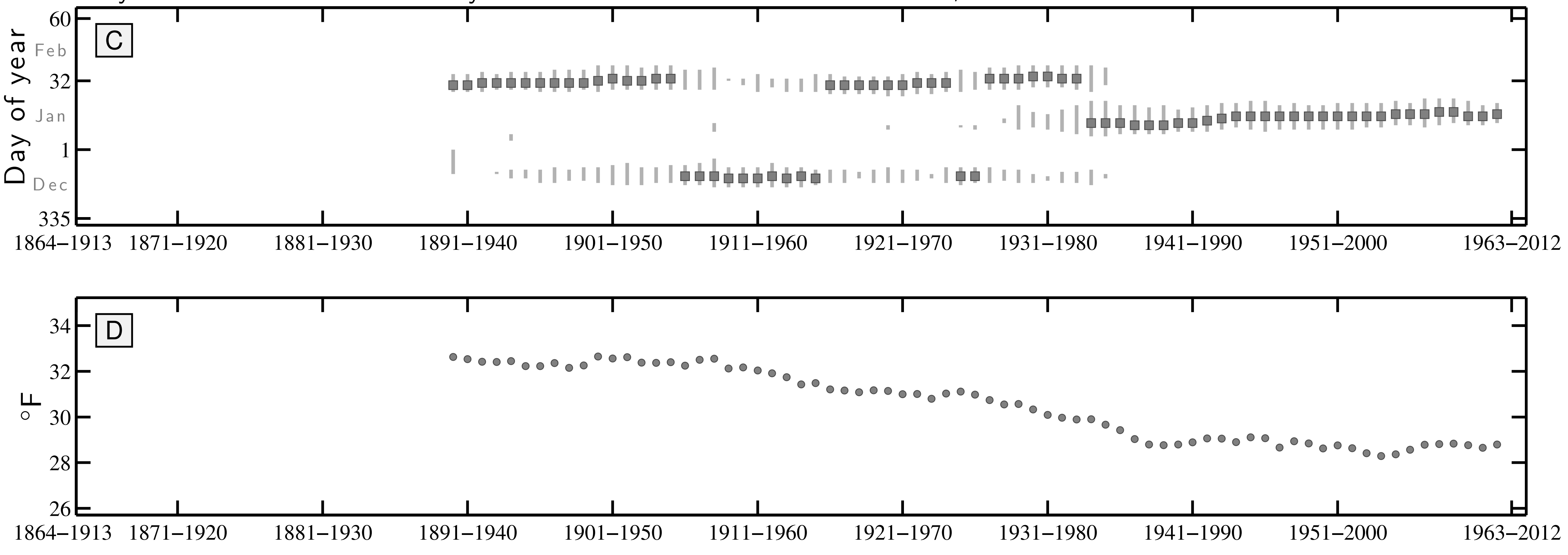

50 year Summer Teletherm dynamics for GOLDSBORO 4 SE, NC:

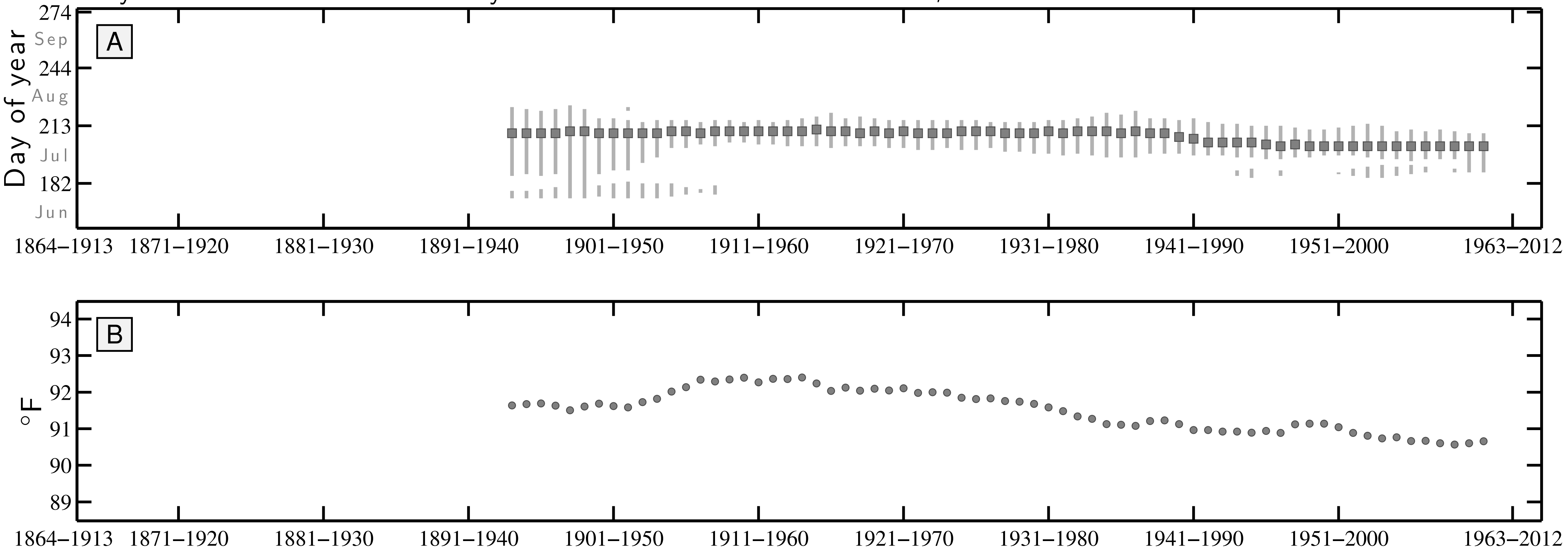

50 year Winter Teletherm dynamics for GOLDSBORO 4 SE, NC:

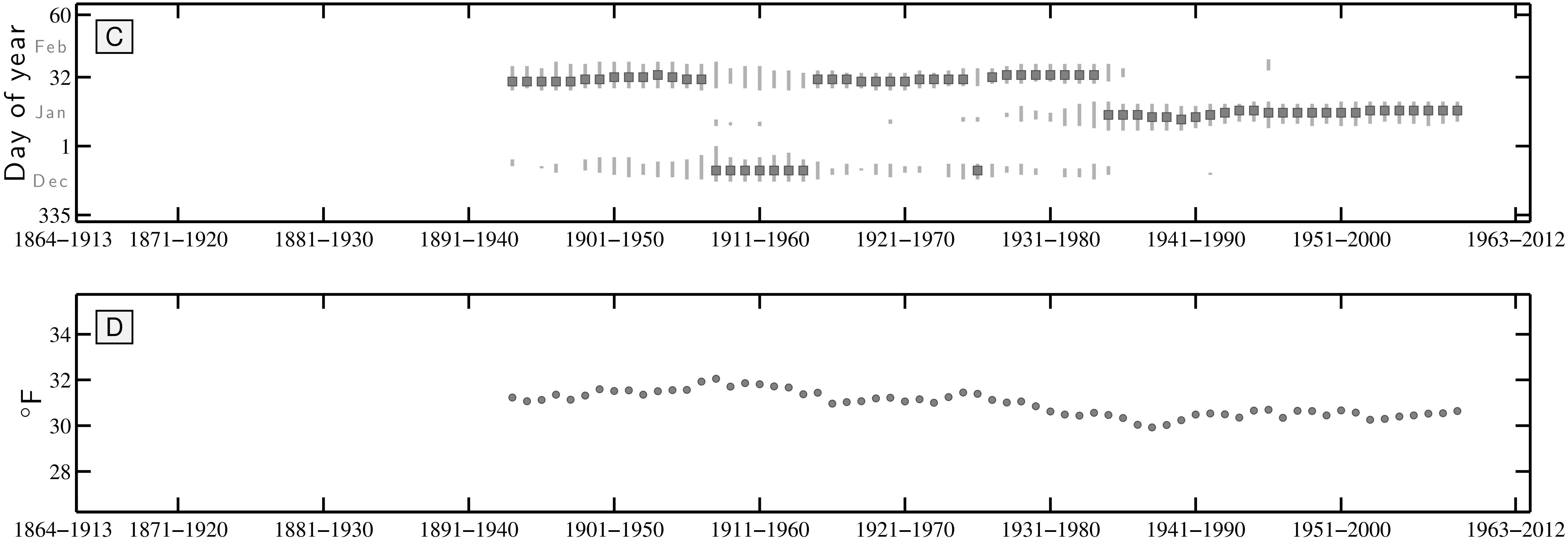

50 year Summer Teletherm dynamics for HENDERSON 2 NNW, NC:

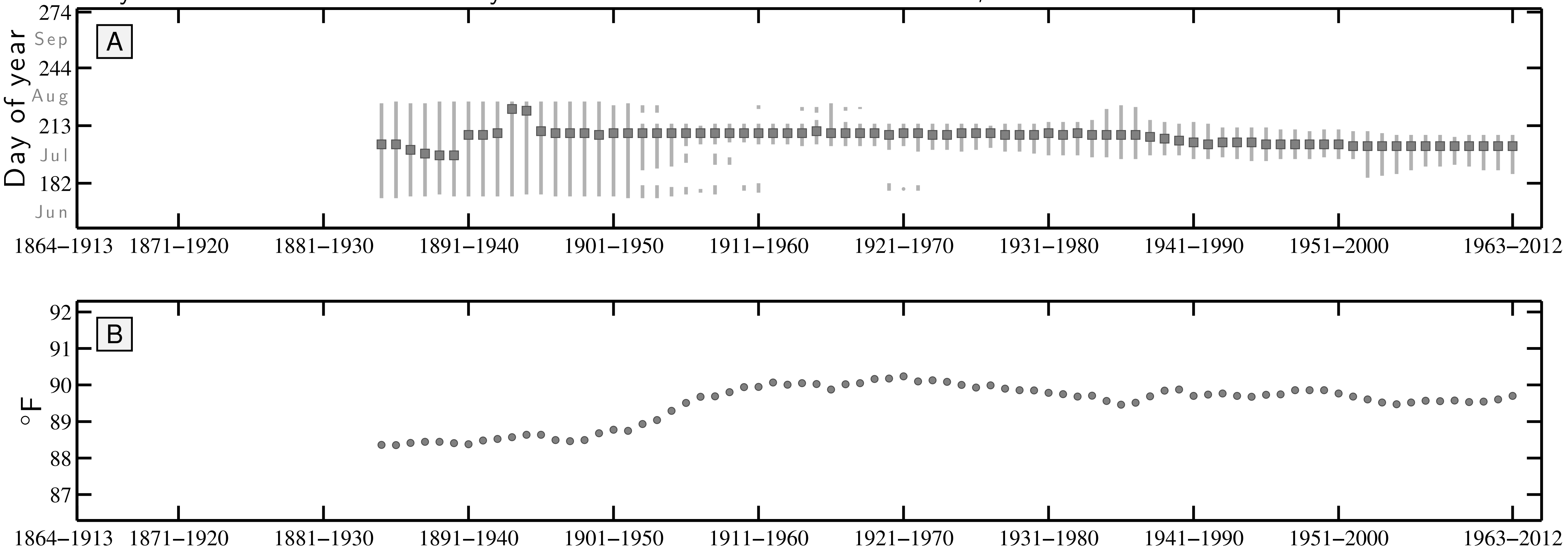

50 year Winter Teletherm dynamics for HENDERSON 2 NNW, NC:

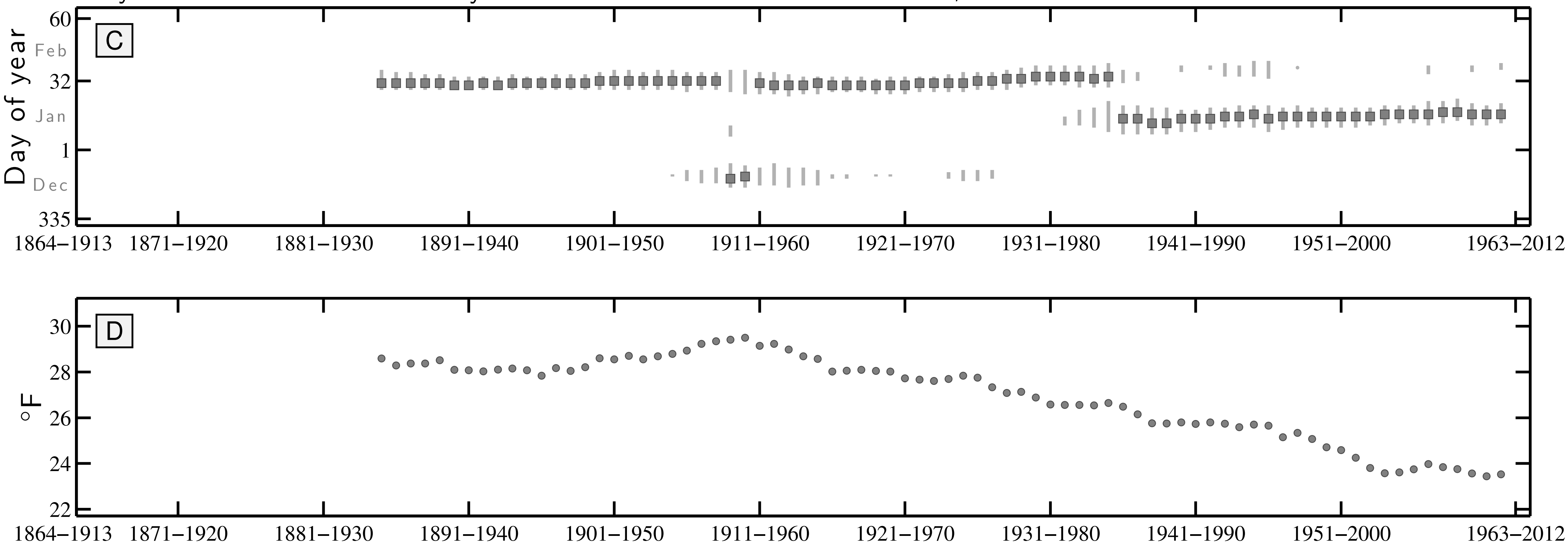

50 year Summer Teletherm dynamics for HENDERSONVILLE 1 NE, NC:

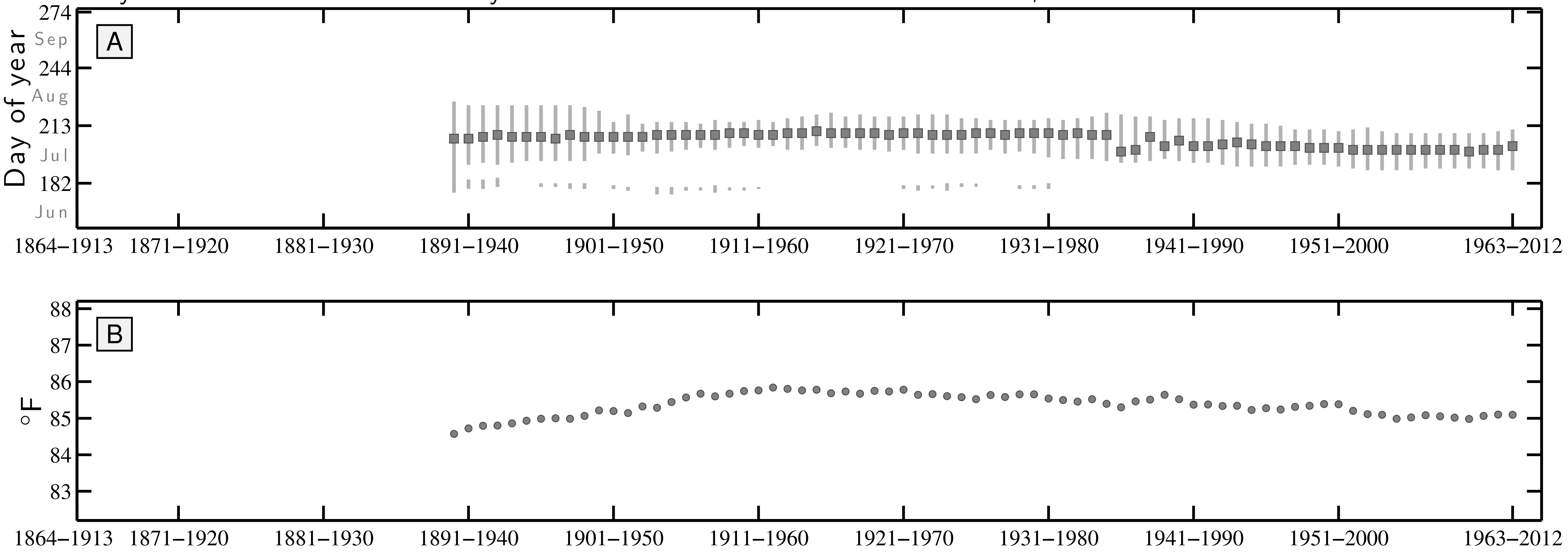

50 year Winter Teletherm dynamics for HENDERSONVILLE 1 NE, NC:

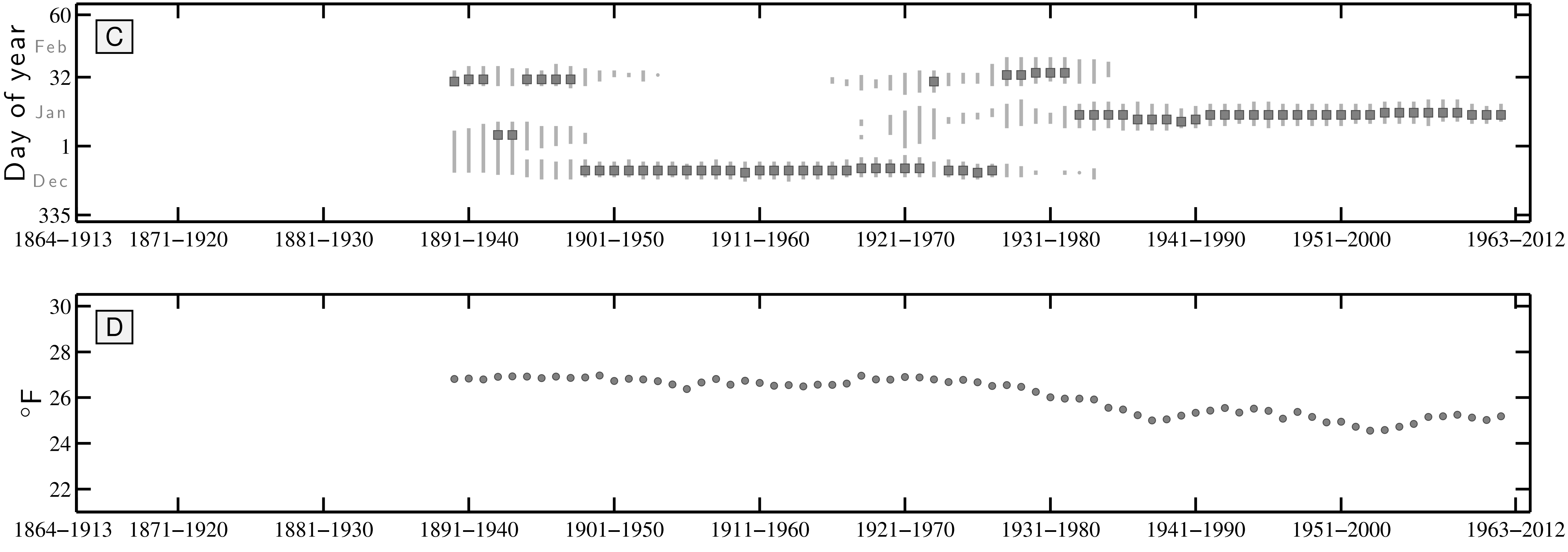

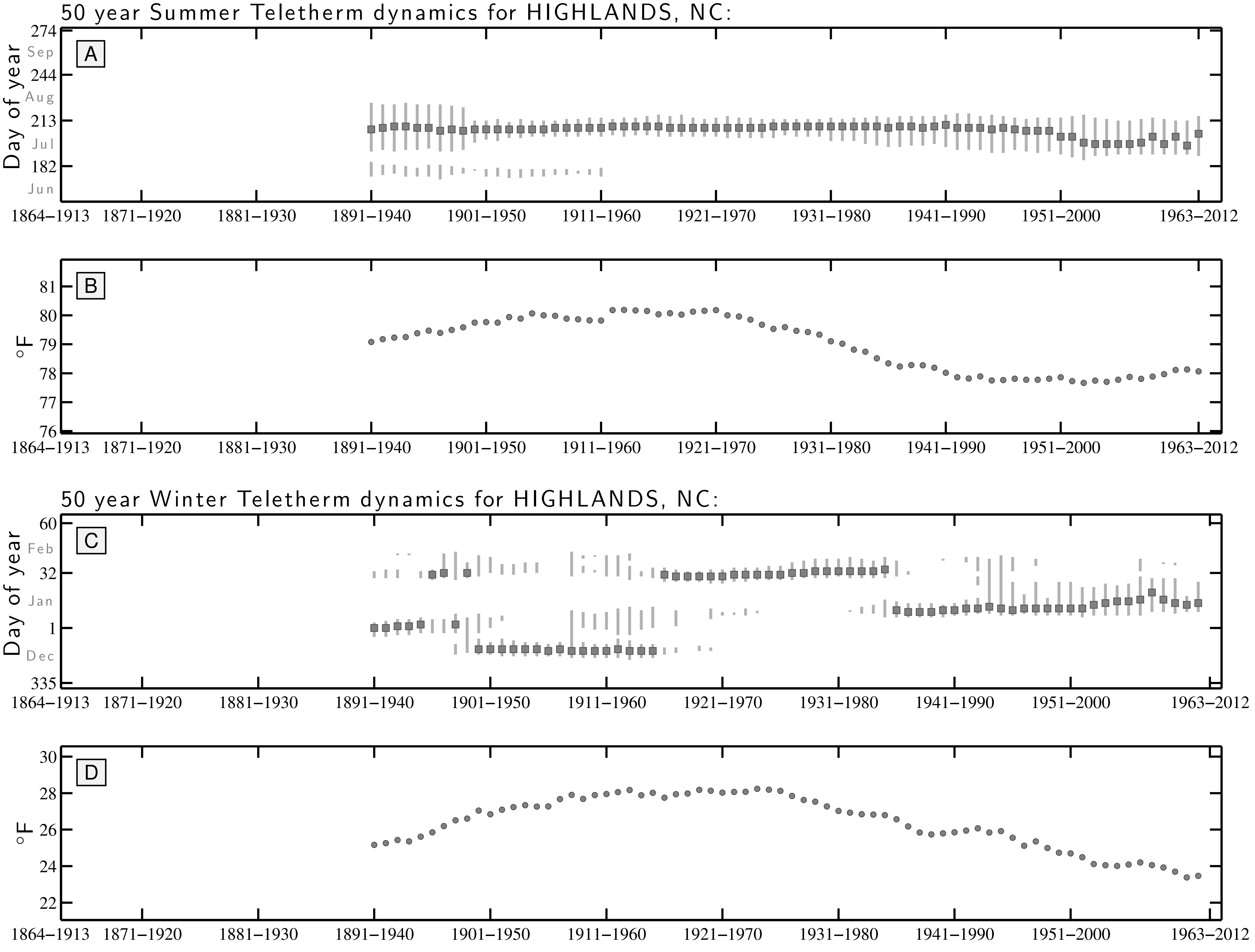
50 year Summer Teletherm dynamics for HIGHLANDS, NC:
A
B
Day of year
274
Sep
244
Aug
213
Jul
182
Jun
°F
81
80
79
78
77
76
1864–1913
1871–1920
1881–1930
1891–1940
1901–1950
1911–1960
1921–1970
1931–1980
1941–1990
1951–2000
1963–2012
50 year Winter Teletherm dynamics for HIGHLANDS, NC:
C
D
Day of year
60
Feb
32
Jan
1
Dec
335
30
28
26
24
22

50 year Summer Teletherm dynamics for KINSTON 7 SE, NC:

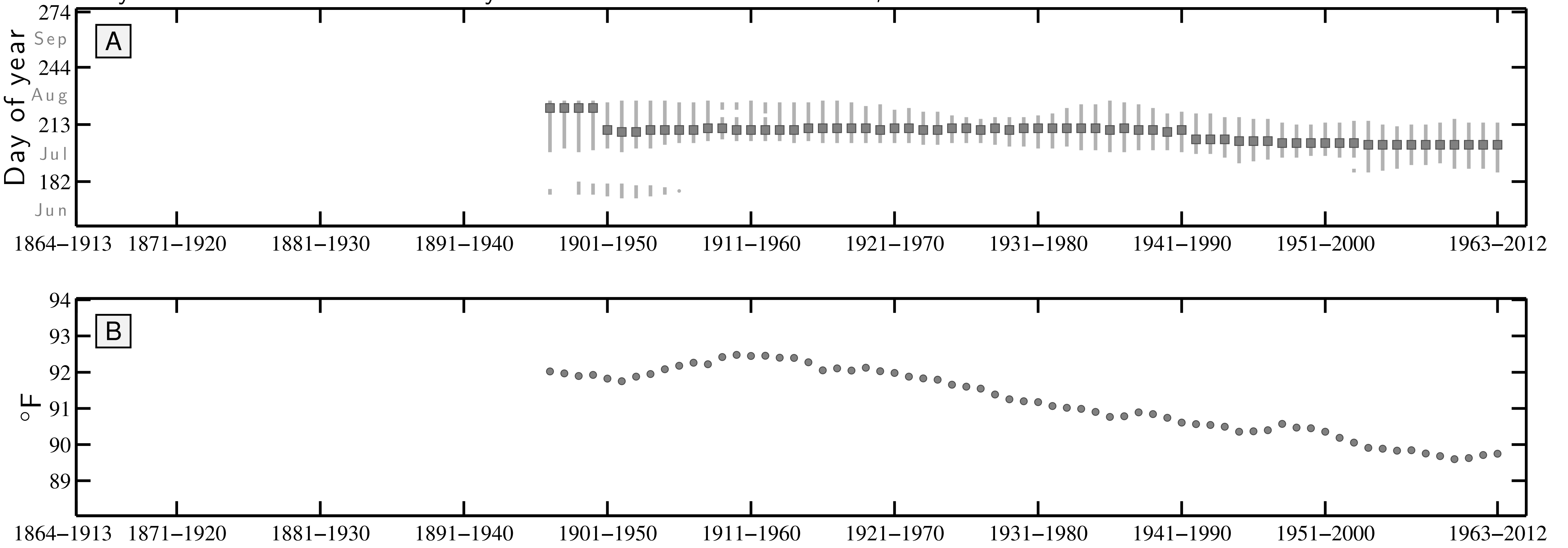

50 year Winter Teletherm dynamics for KINSTON 7 SE, NC:

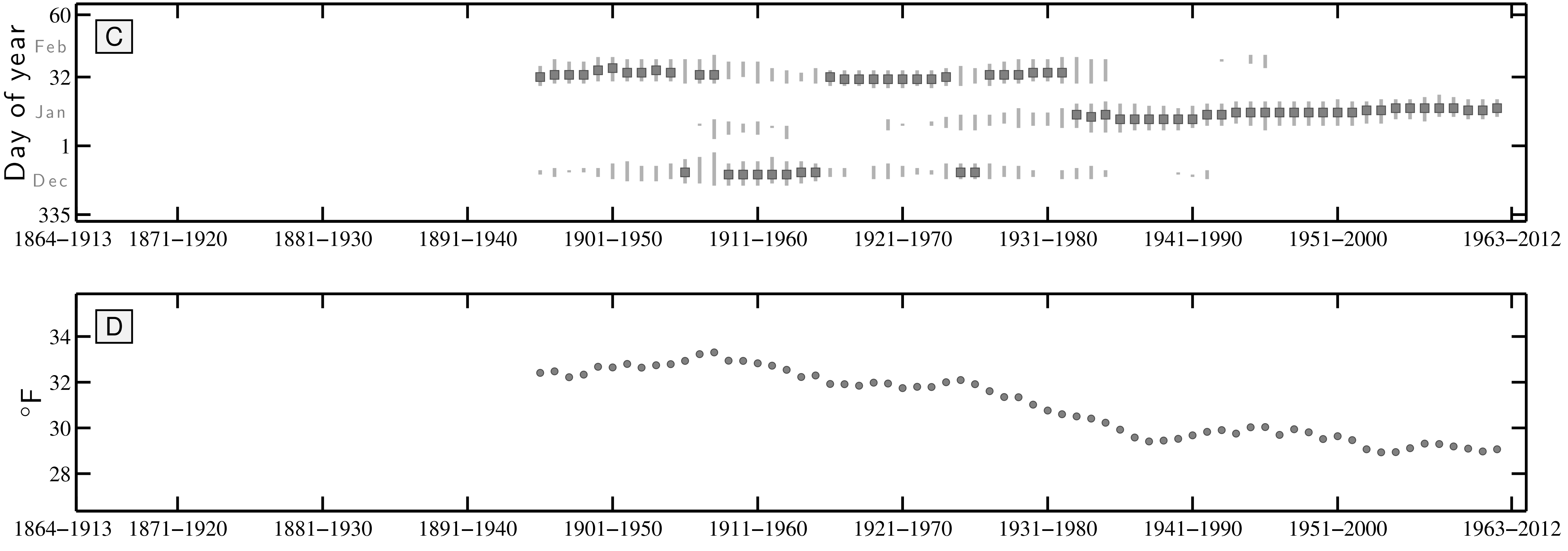

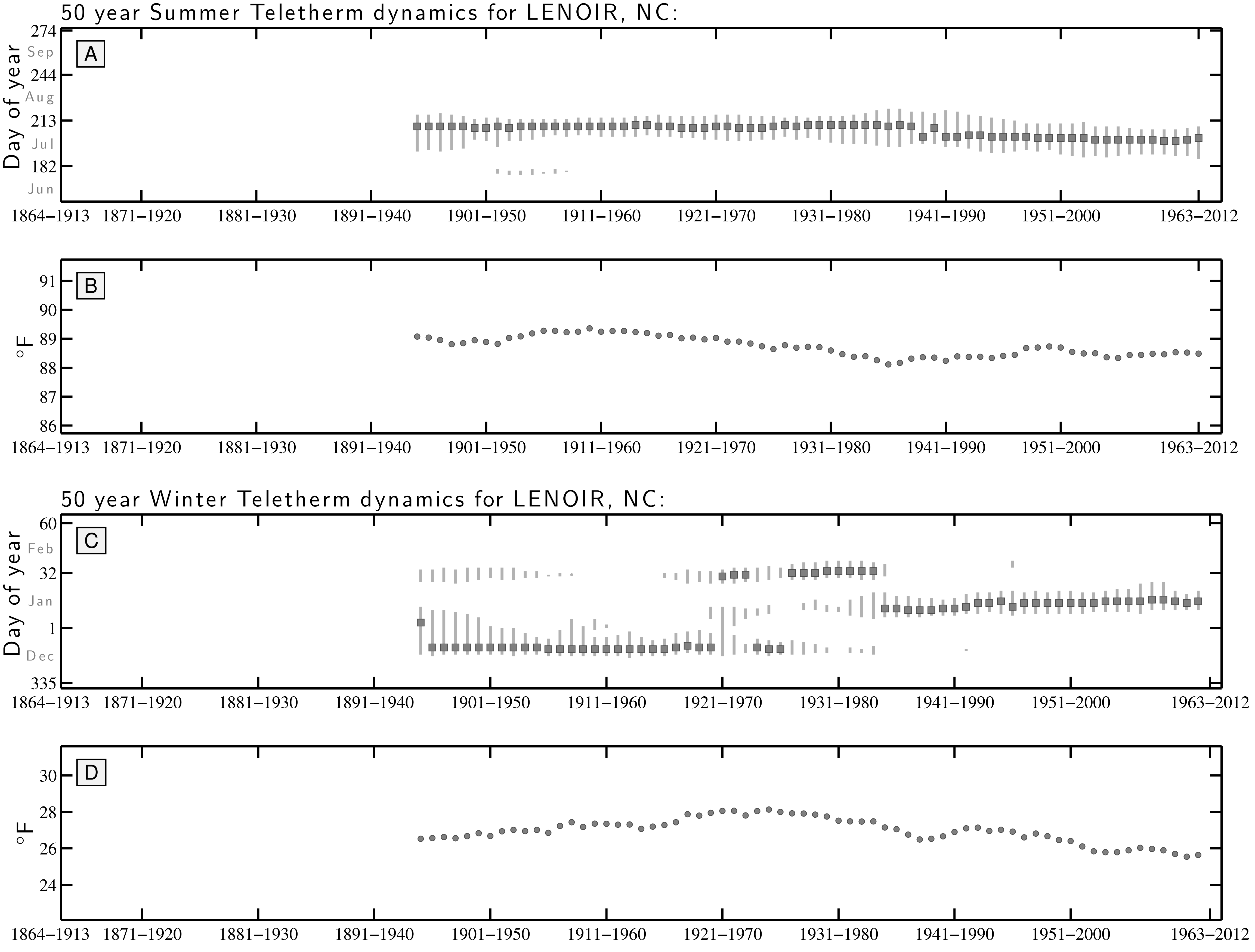
50 year Summer Teletherm dynamics for LENOIR, NC:
A
Day of year
274
Sep
244
Aug
213
Jul
182
Jun
1864–1913
1871–1920
1881–1930
1891–1940
1901–1950
1911–1960
1921–1970
1931–1980
1941–1990
1951–2000
1963–2012
B
°F
91
90
89
88
87
86
50 year Winter Teletherm dynamics for LENOIR, NC:
C
60
Feb
32
Jan
1
Dec
335
D
30
28
26
24

50 year Summer Teletherm dynamics for LOUISBURG, NC:

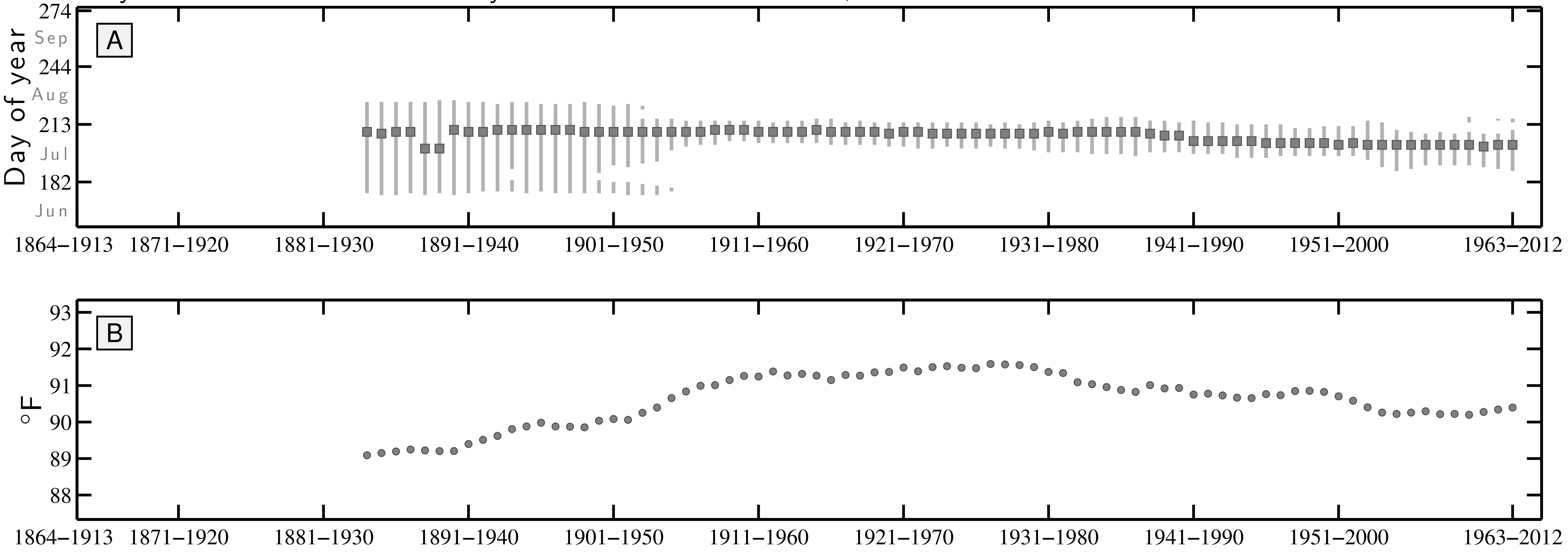

50 year Winter Teletherm dynamics for LOUISBURG, NC:

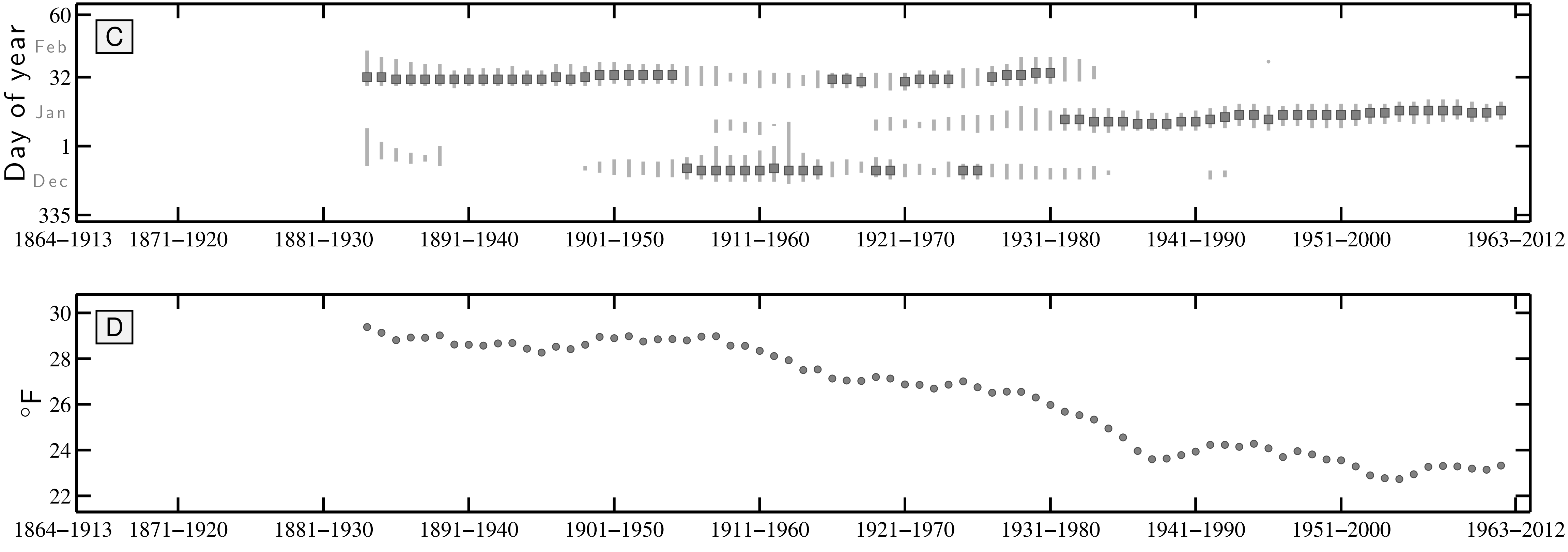

50 year Summer Teletherm dynamics for LUMBERTON, NC:

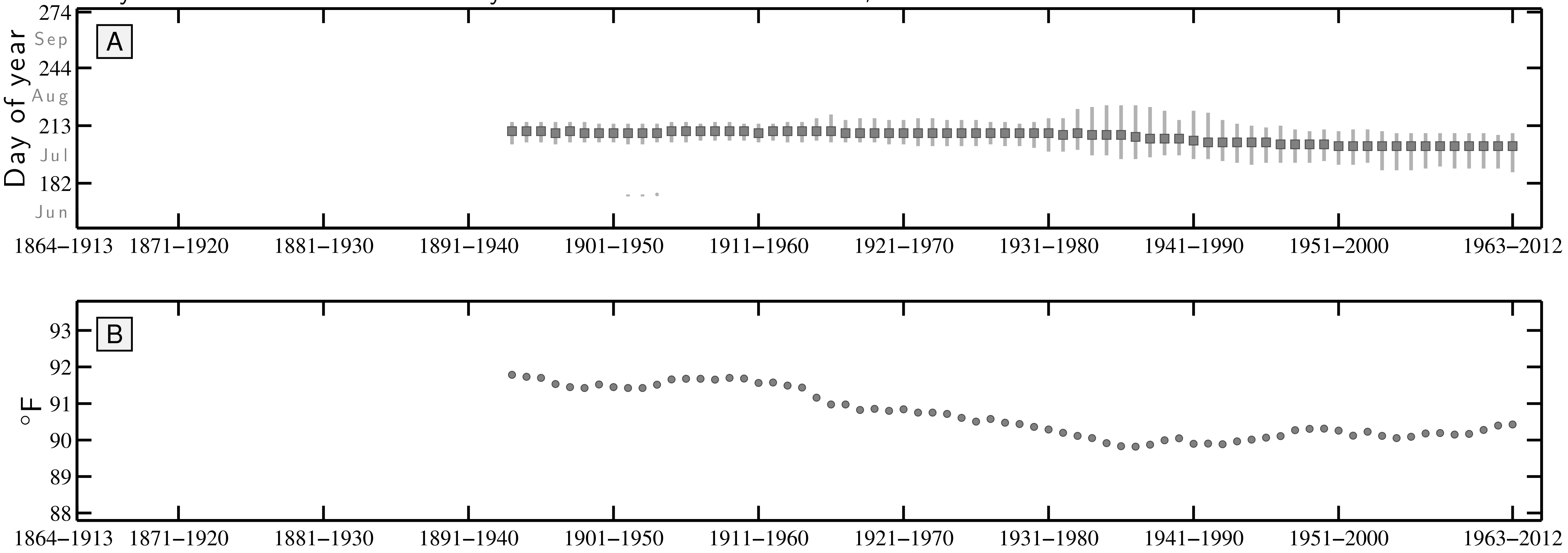

50 year Winter Teletherm dynamics for LUMBERTON, NC:

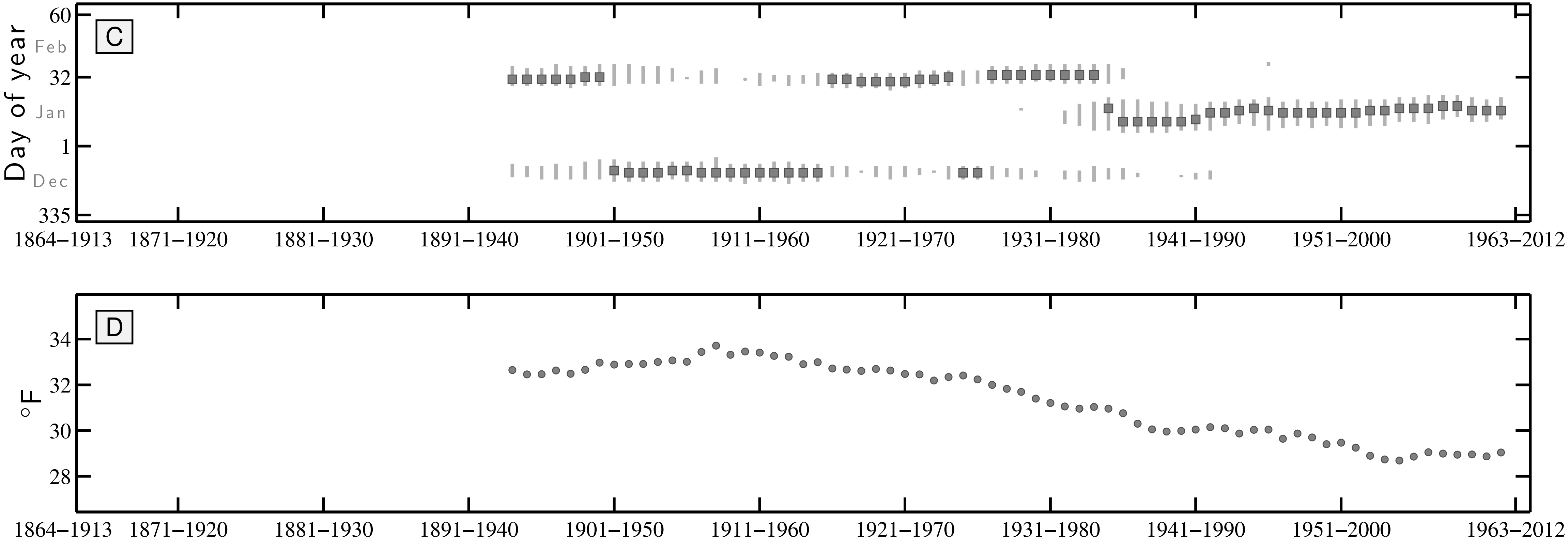

50 year Summer Teletherm dynamics for MARION 2 NW, NC:

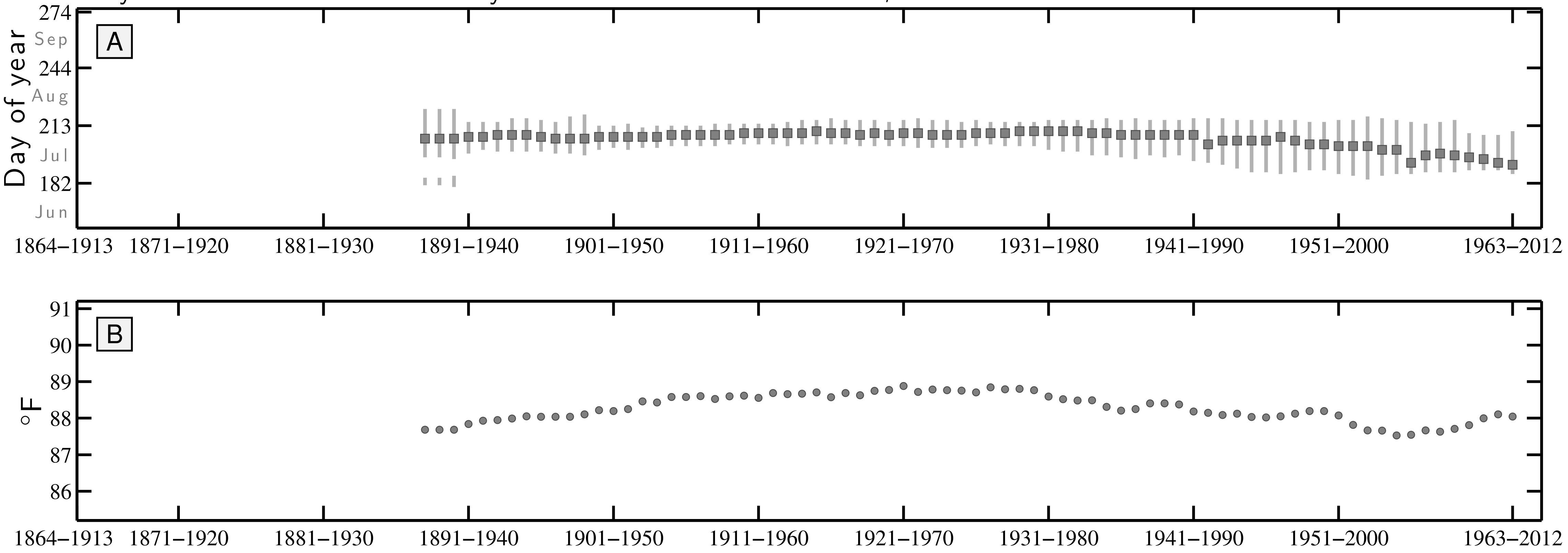

50 year Winter Teletherm dynamics for MARION 2 NW, NC:

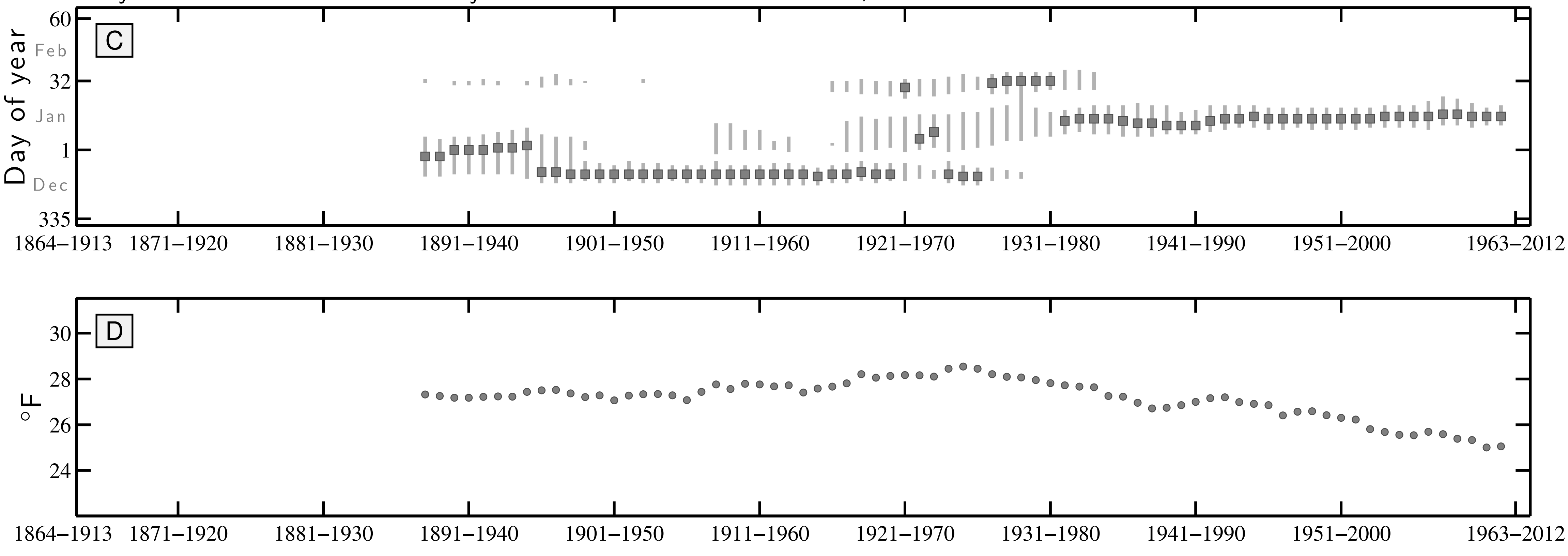

50 year Summer Teletherm dynamics for MARSHALL, NC:

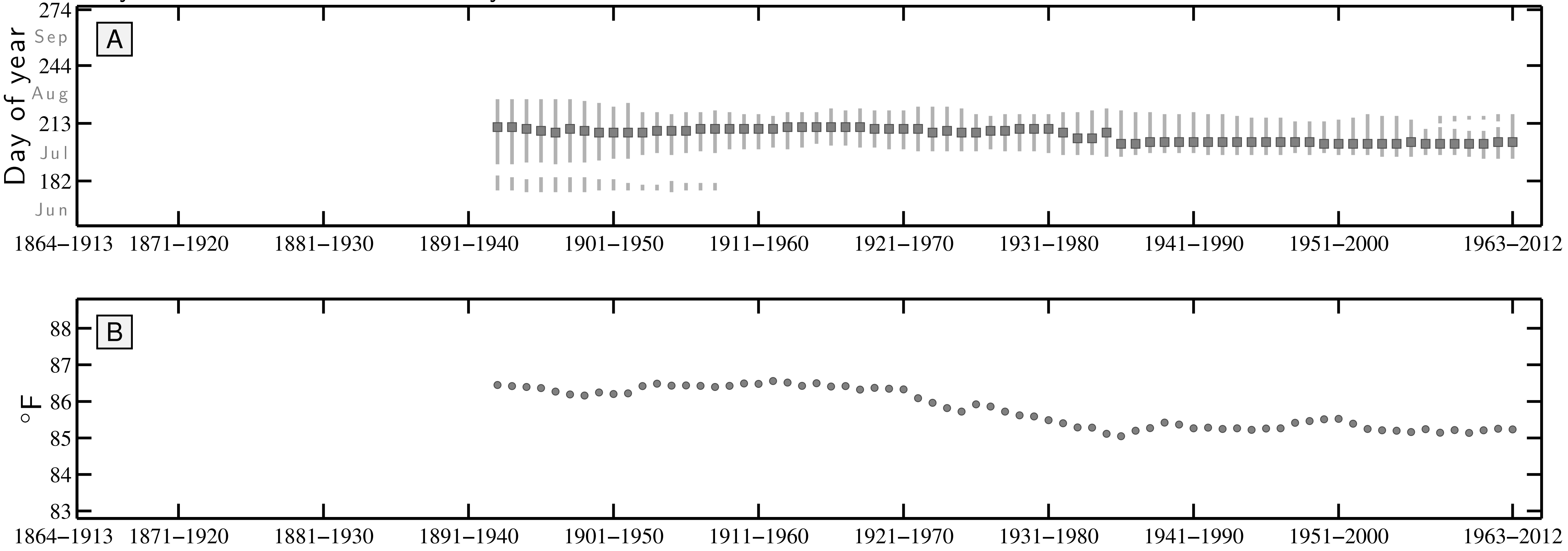

50 year Winter Teletherm dynamics for MARSHALL, NC:

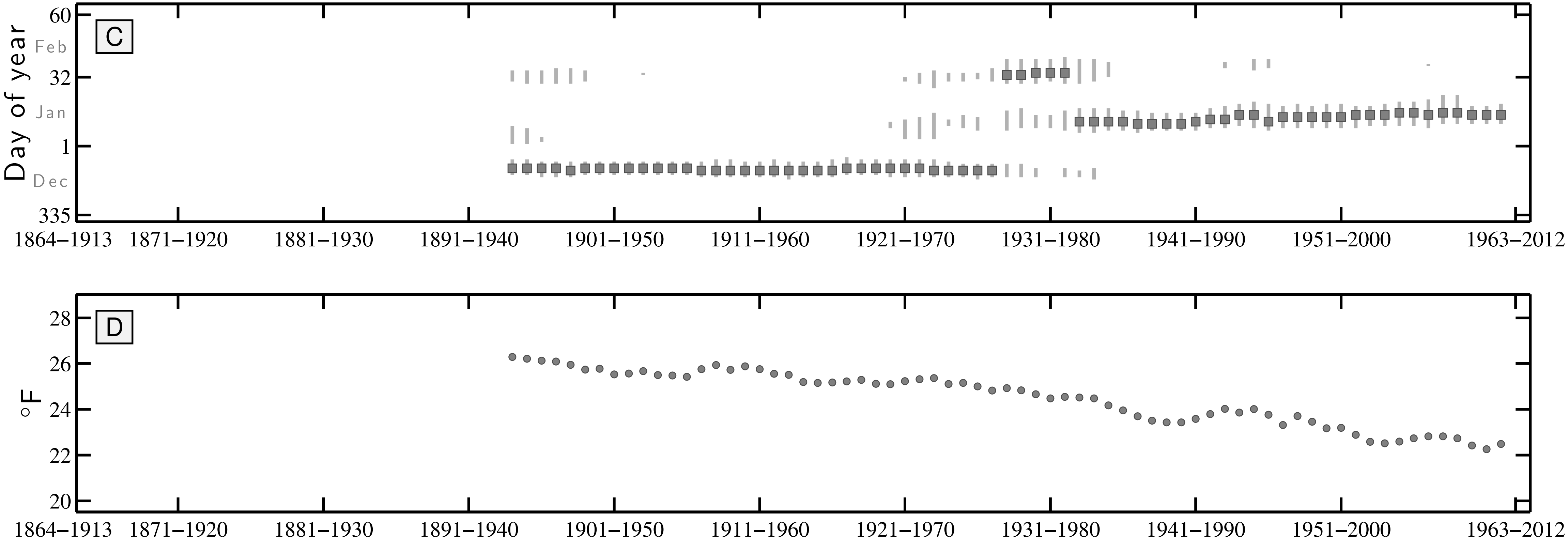

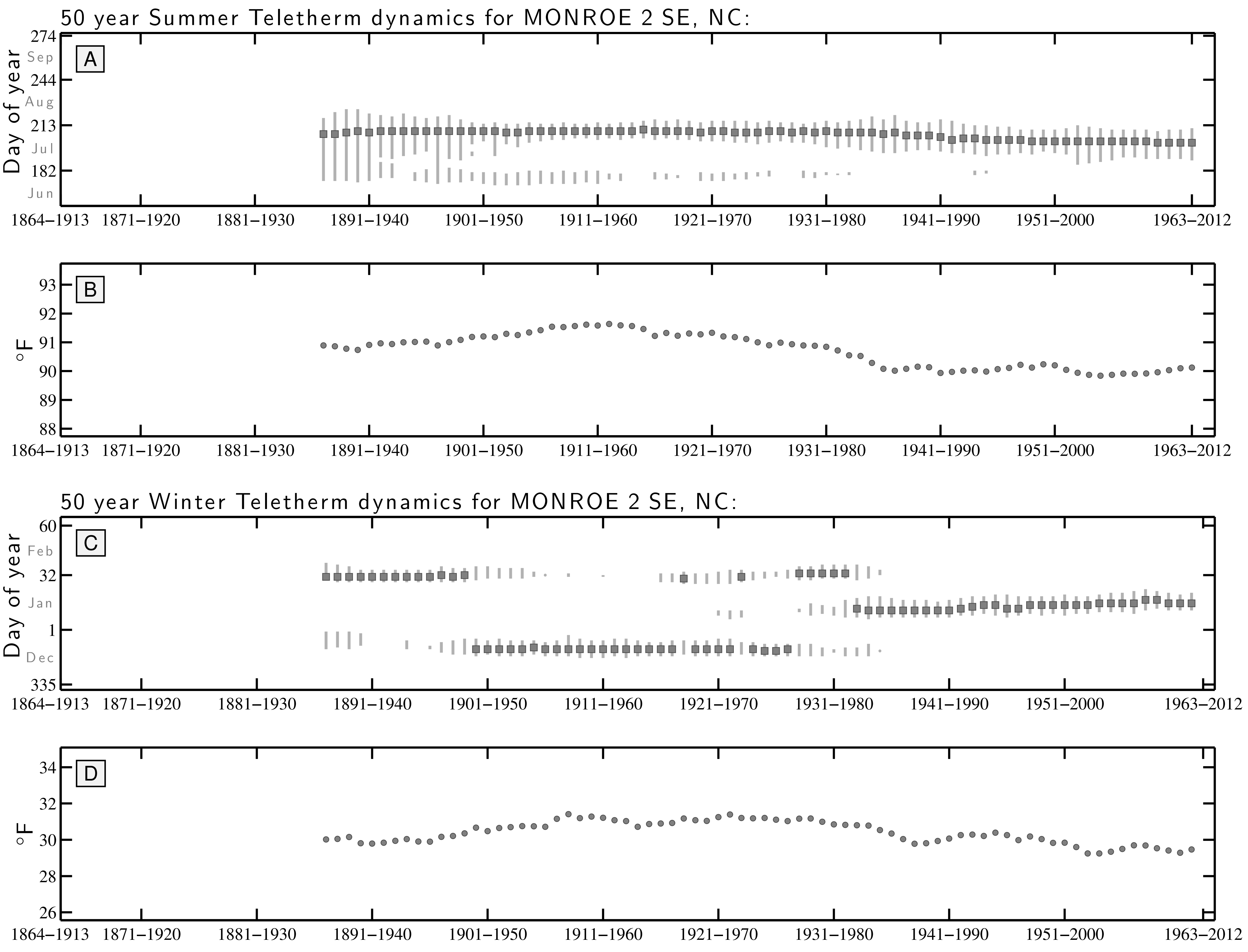

50 year Summer Teletherm dynamics for MONROE 2 SE, NC:
A
B
Day of year
274
Sep
244
Aug
213
Jul
182
Jun
°F
93
92
91
90
89
88
50 year Winter Teletherm dynamics for MONROE 2 SE, NC:
C
D
60
Feb
32
Jan
1
Dec
335
34
32
30
28
26
1864–1913
1871–1920
1881–1930
1891–1940
1901–1950
1911–1960
1921–1970
1931–1980
1941–1990
1951–2000
1963–2012

50 year Summer Teletherm dynamics for MOREHEAD CITY 2 WNW, NC:

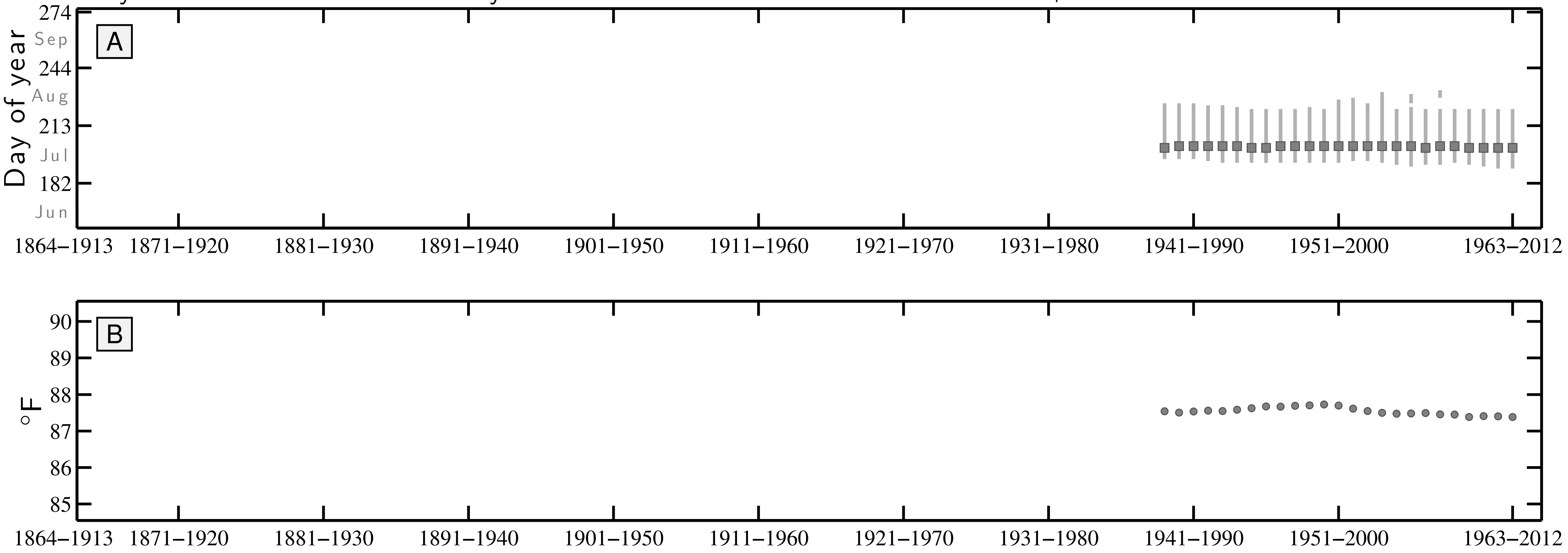

50 year Winter Teletherm dynamics for MOREHEAD CITY 2 WNW, NC:

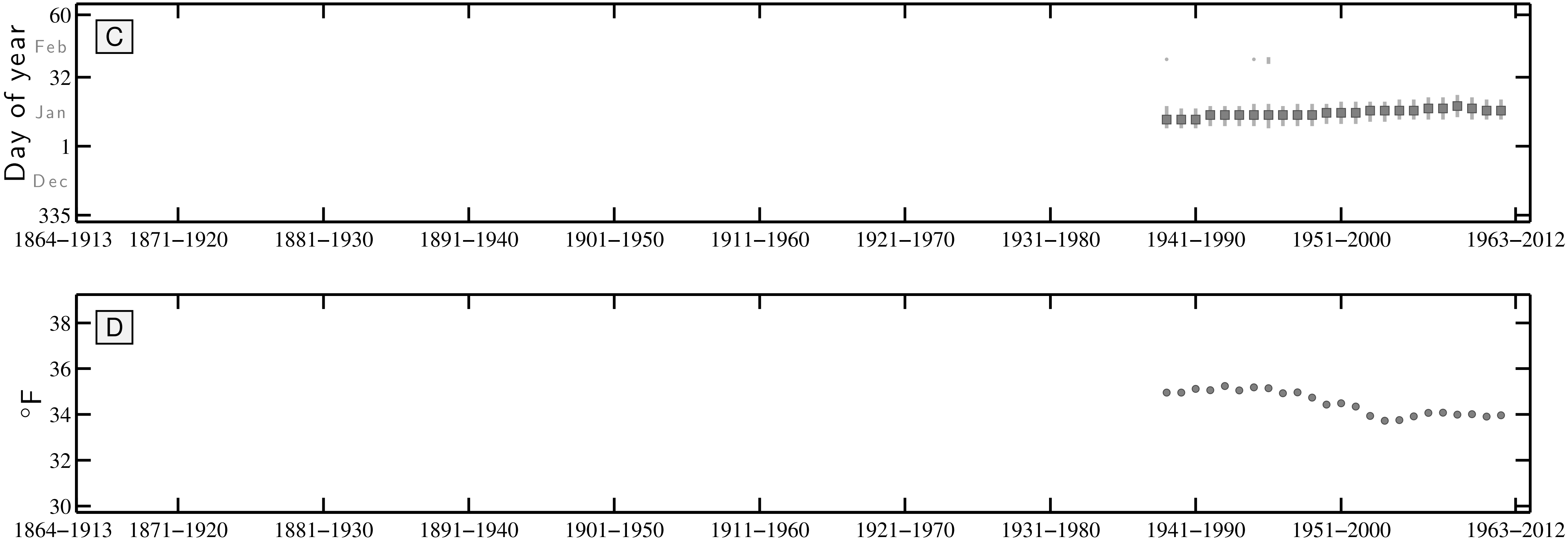

50 year Summer Teletherm dynamics for MORGANTON, NC:

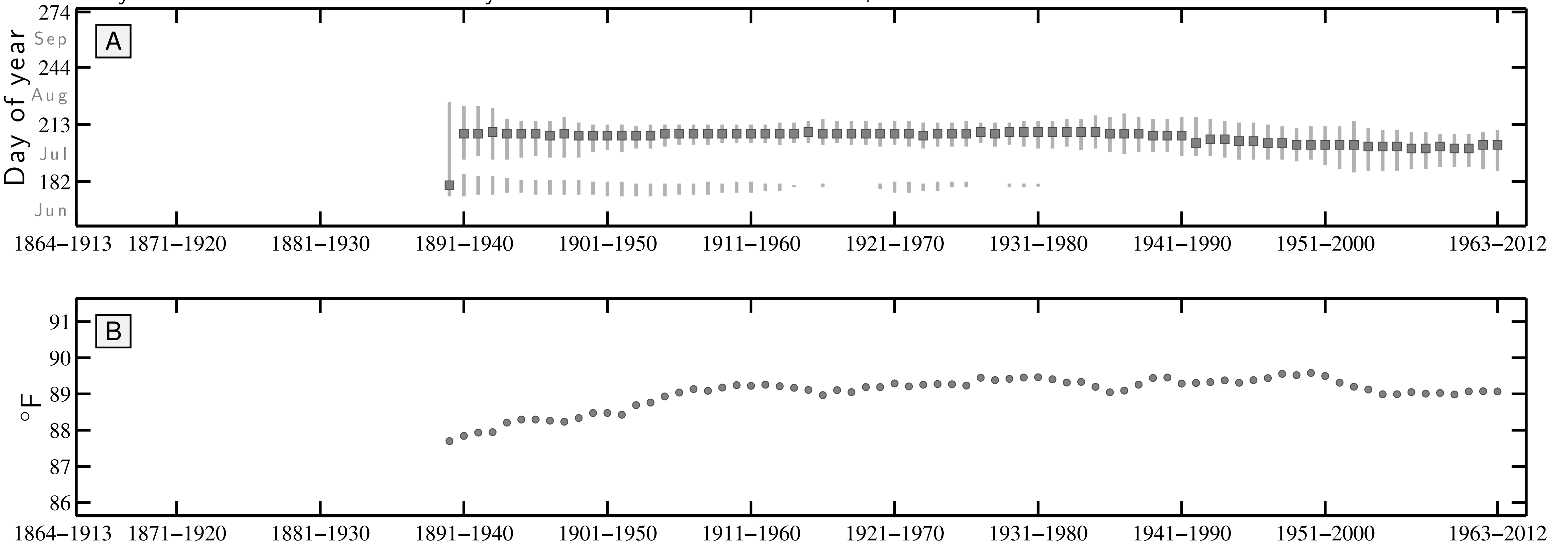

50 year Winter Teletherm dynamics for MORGANTON, NC:

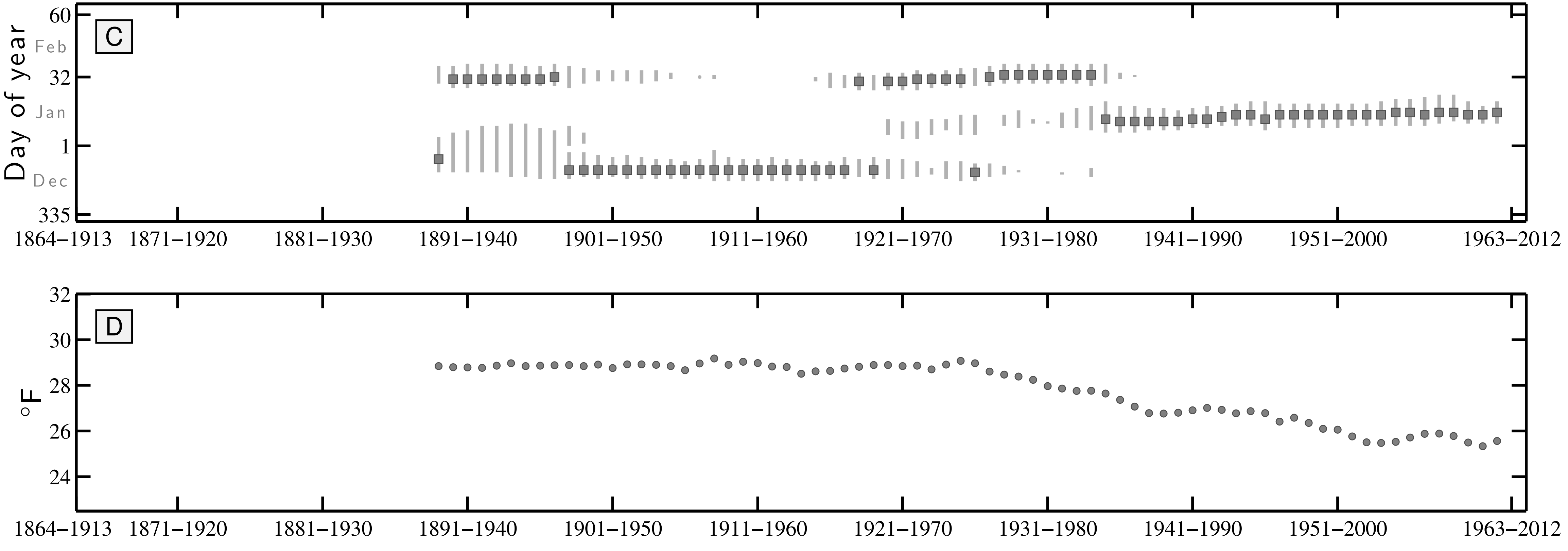

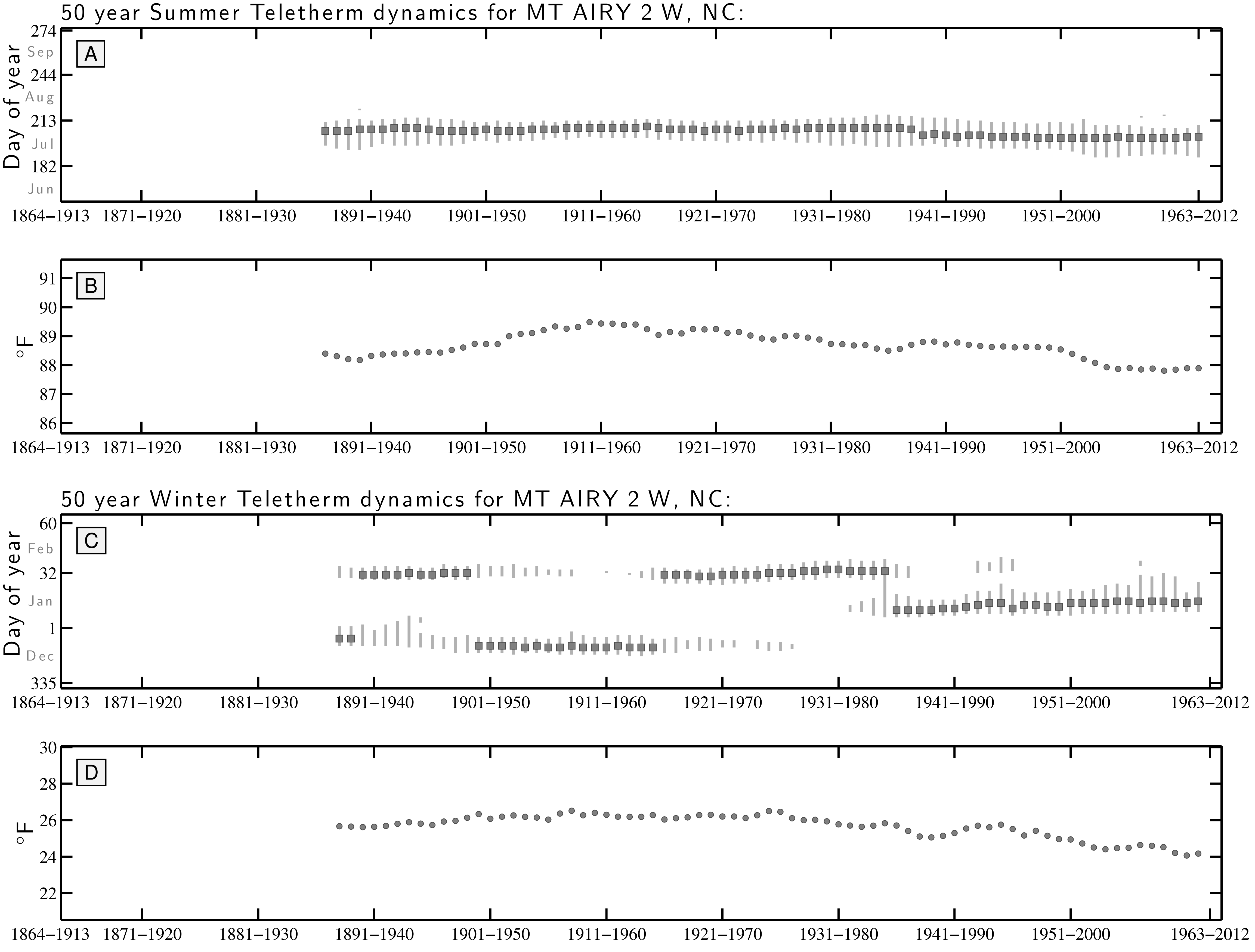

50 year Summer Teletherm dynamics for MT AIRY 2 W, NC:
A
Day of year
274
Sep
244
Aug
213
Jul
182
Jun
1864–1913
1871–1920
1881–1930
1891–1940
1901–1950
1911–1960
1921–1970
1931–1980
1941–1990
1951–2000
1963–2012
B
°F
91
90
89
88
87
86
50 year Winter Teletherm dynamics for MT AIRY 2 W, NC:
C
60
Feb
32
Jan
1
Dec
335
D
30
28
26
24
22

50 year Summer Teletherm dynamics for REIDSVILLE 2 NW, NC:

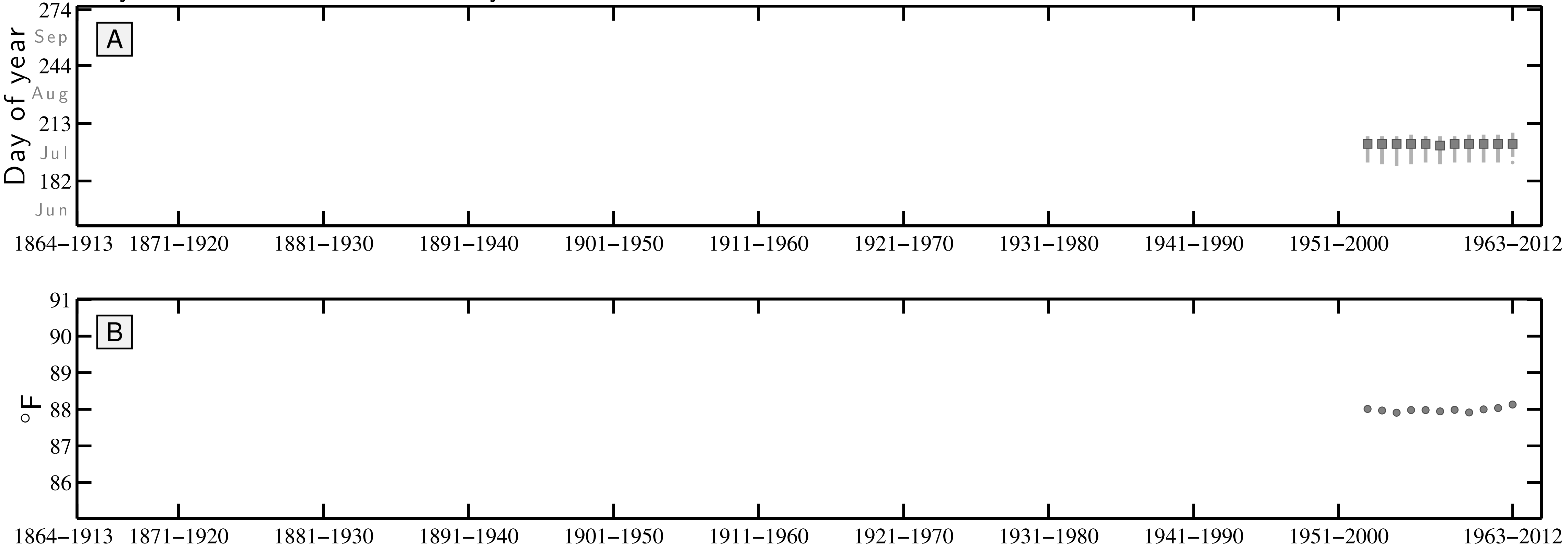

50 year Winter Teletherm dynamics for REIDSVILLE 2 NW, NC:

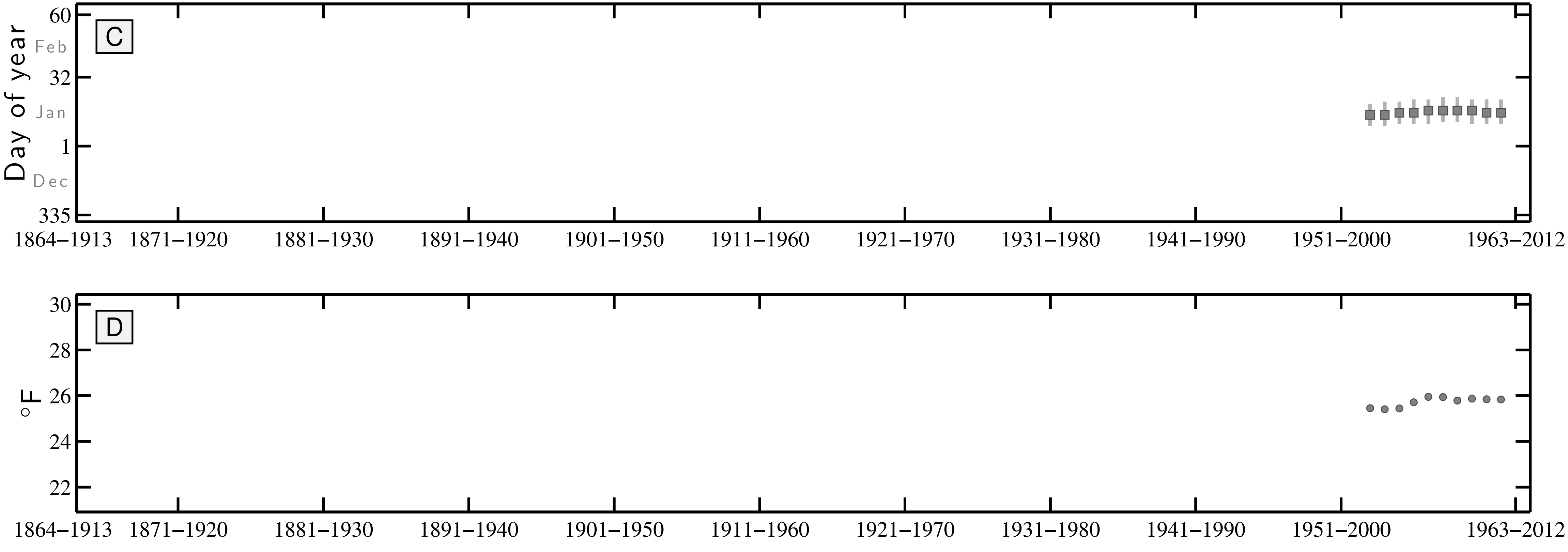

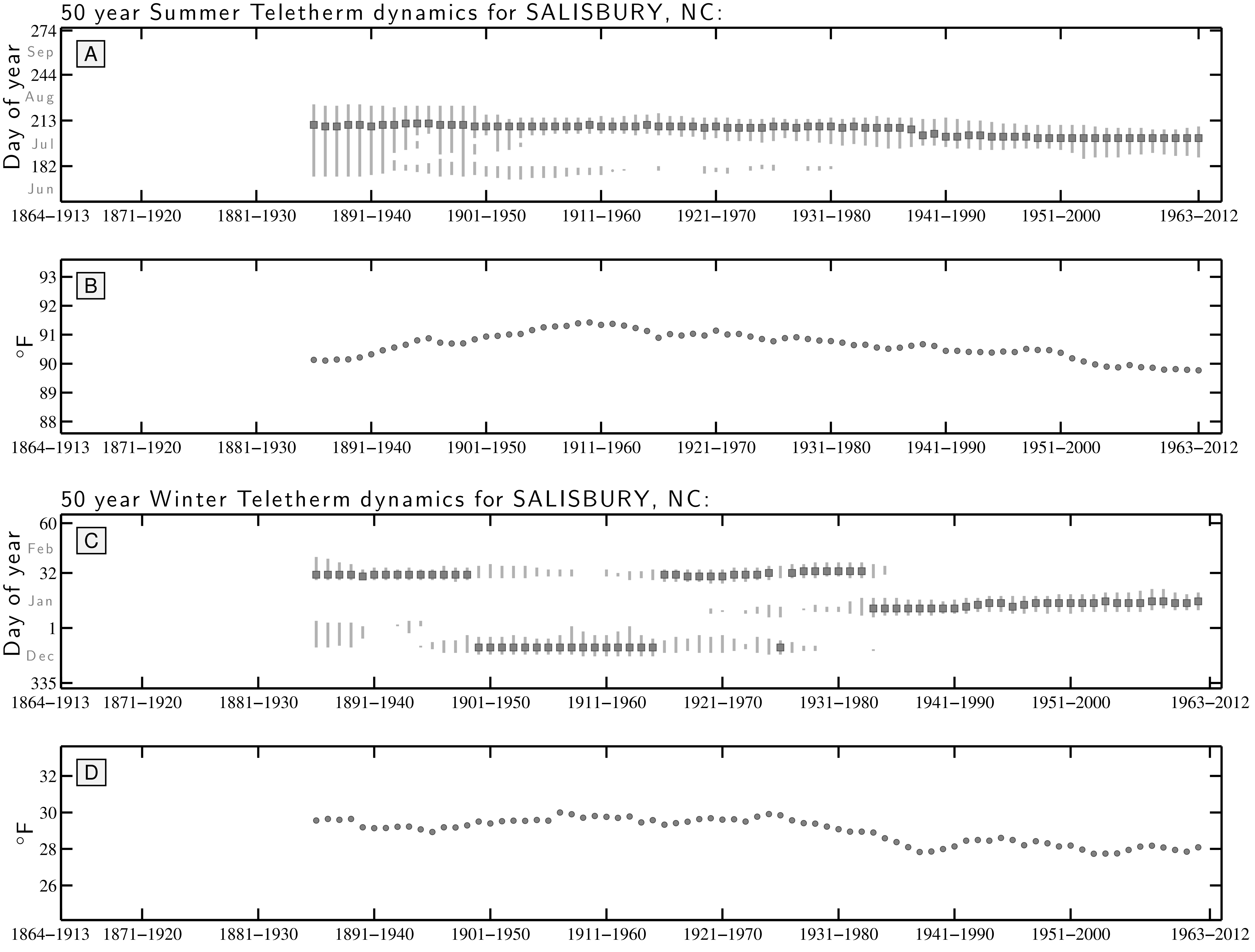
50 year Summer Teletherm dynamics for SALISBURY, NC:
A
Day of year
274
Sep
244
Aug
213
Jul
182
Jun
1864–1913
1871–1920
1881–1930
1891–1940
1901–1950
1911–1960
1921–1970
1931–1980
1941–1990
1951–2000
1963–2012
B
°F
93
92
91
90
89
88
50 year Winter Teletherm dynamics for SALISBURY, NC:
C
60
Feb
32
Jan
1
Dec
335
D
32
30
28
26

50 year Summer Teletherm dynamics for SMITHFIELD, NC:

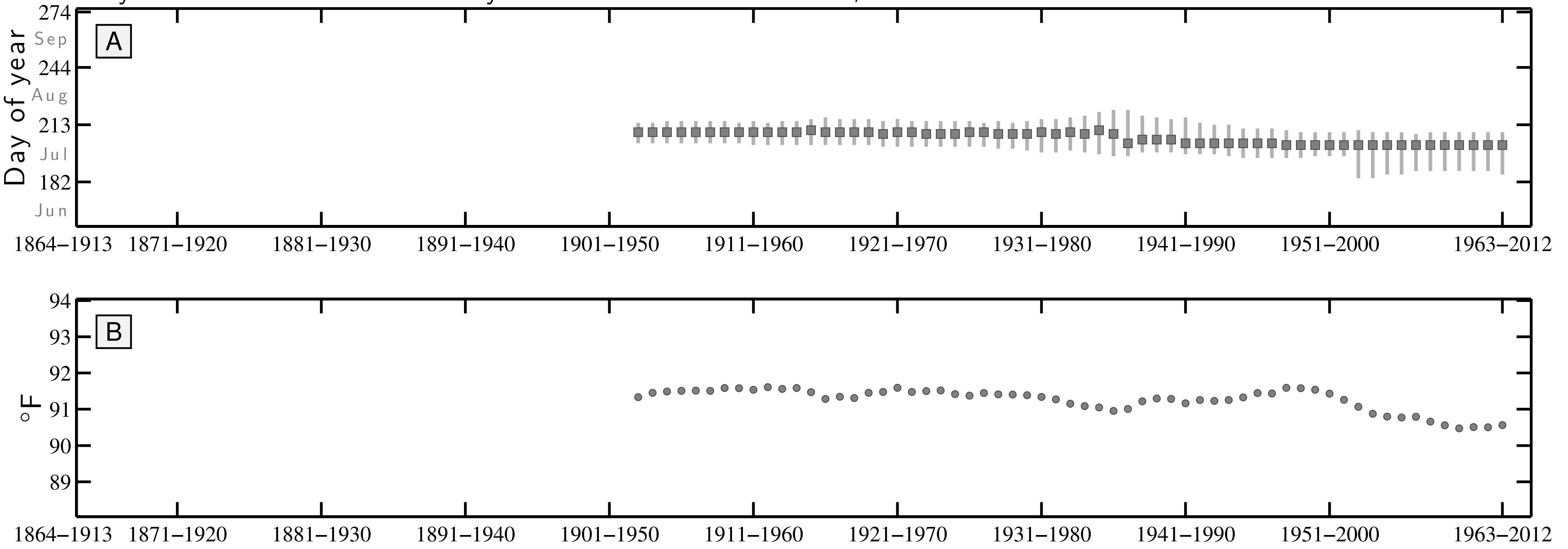

50 year Winter Teletherm dynamics for SMITHFIELD, NC:

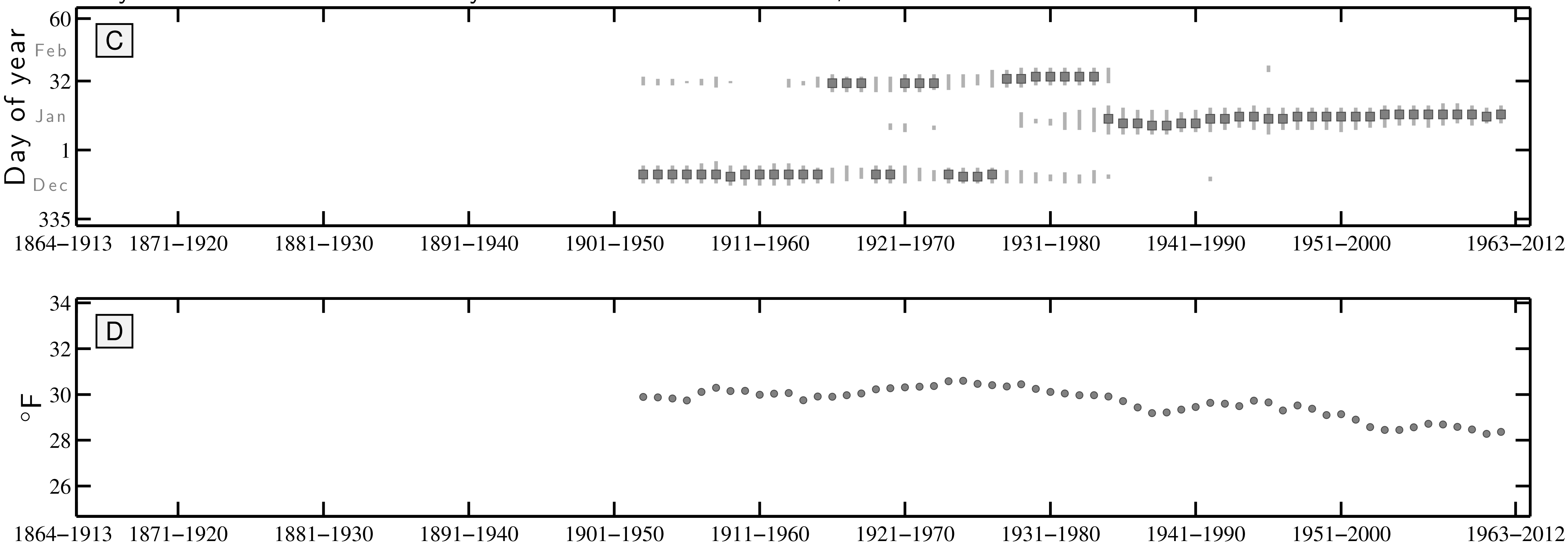

50 year Summer Teletherm dynamics for SOUTHPORT 5 N, NC:

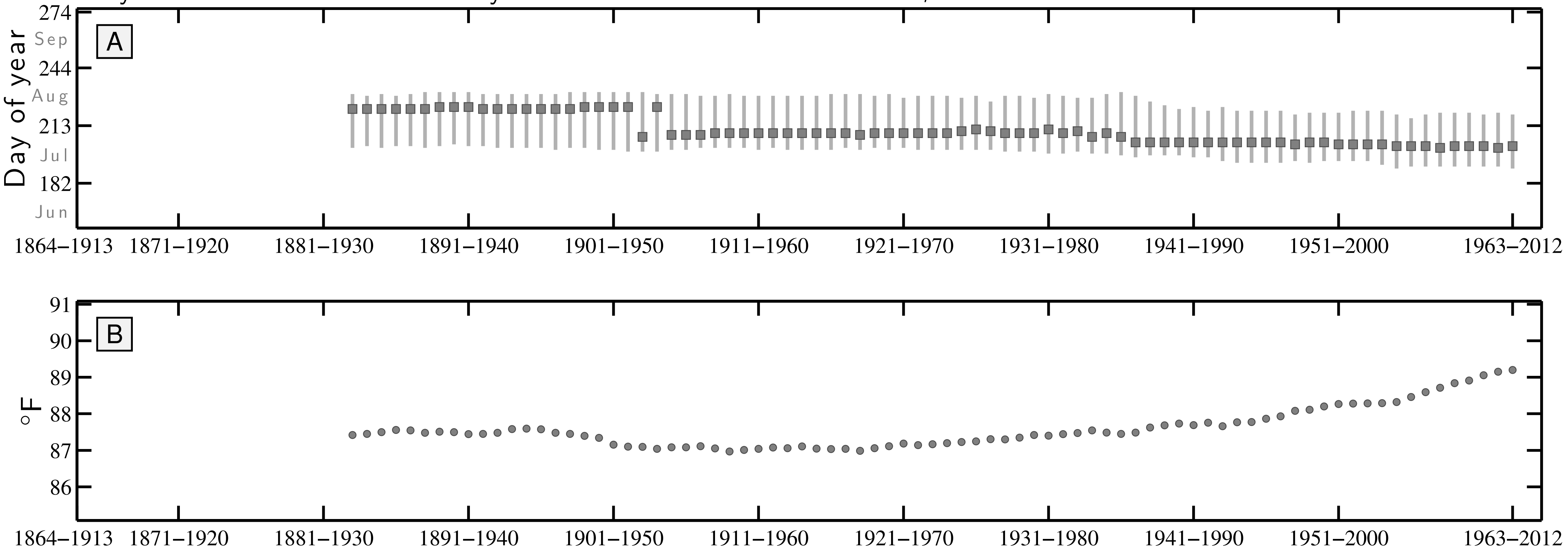

50 year Winter Teletherm dynamics for SOUTHPORT 5 N, NC:

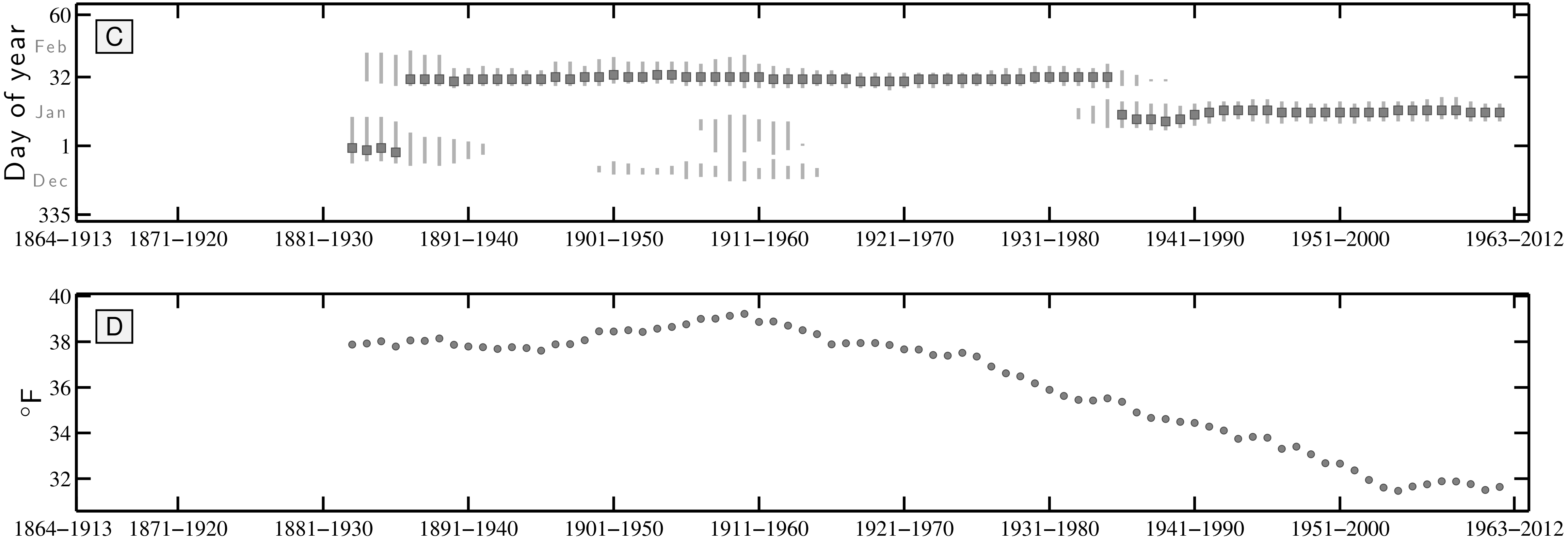

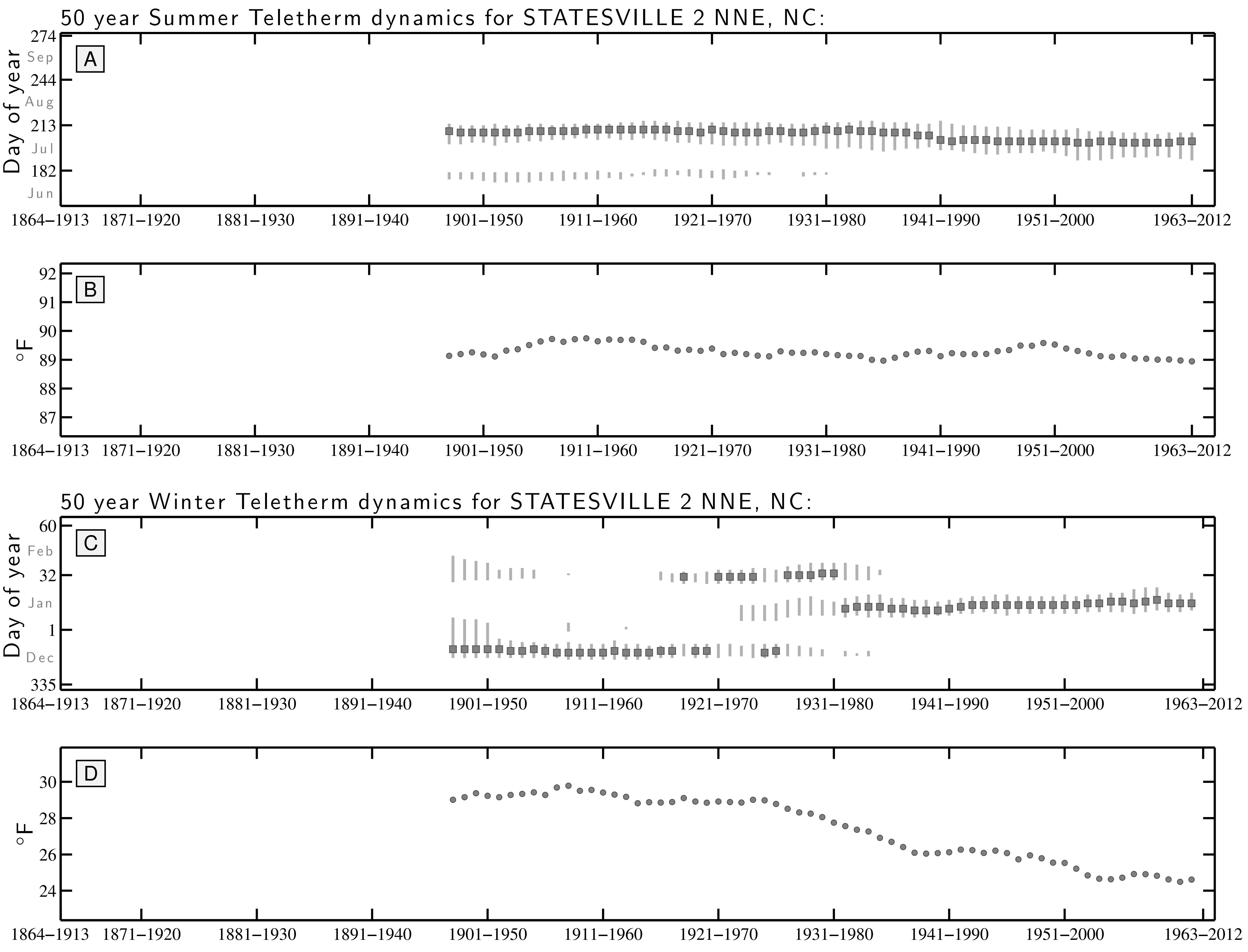
50 year Summer Teletherm dynamics for STATESVILLE 2 NNE, NC:
A
B
Day of year
°F
274
Sep
244
Aug
213
Jul
182
Jun
92
91
90
89
88
87
1864–1913
1871–1920
1881–1930
1891–1940
1901–1950
1911–1960
1921–1970
1931–1980
1941–1990
1951–2000
1963–2012
50 year Winter Teletherm dynamics for STATESVILLE 2 NNE, NC:
C
D
60
Feb
32
Jan
1
Dec
335
30
28
26
24

50 year Summer Teletherm dynamics for TARBORO 1 S, NC:

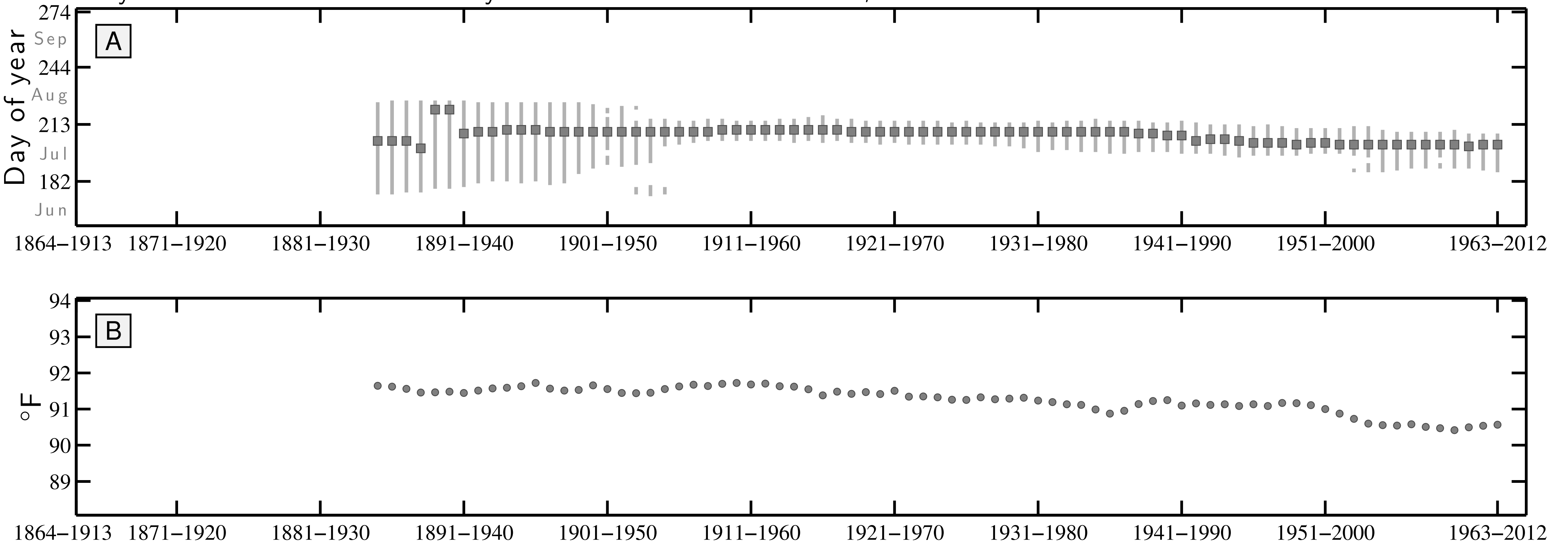

50 year Winter Teletherm dynamics for TARBORO 1 S, NC:

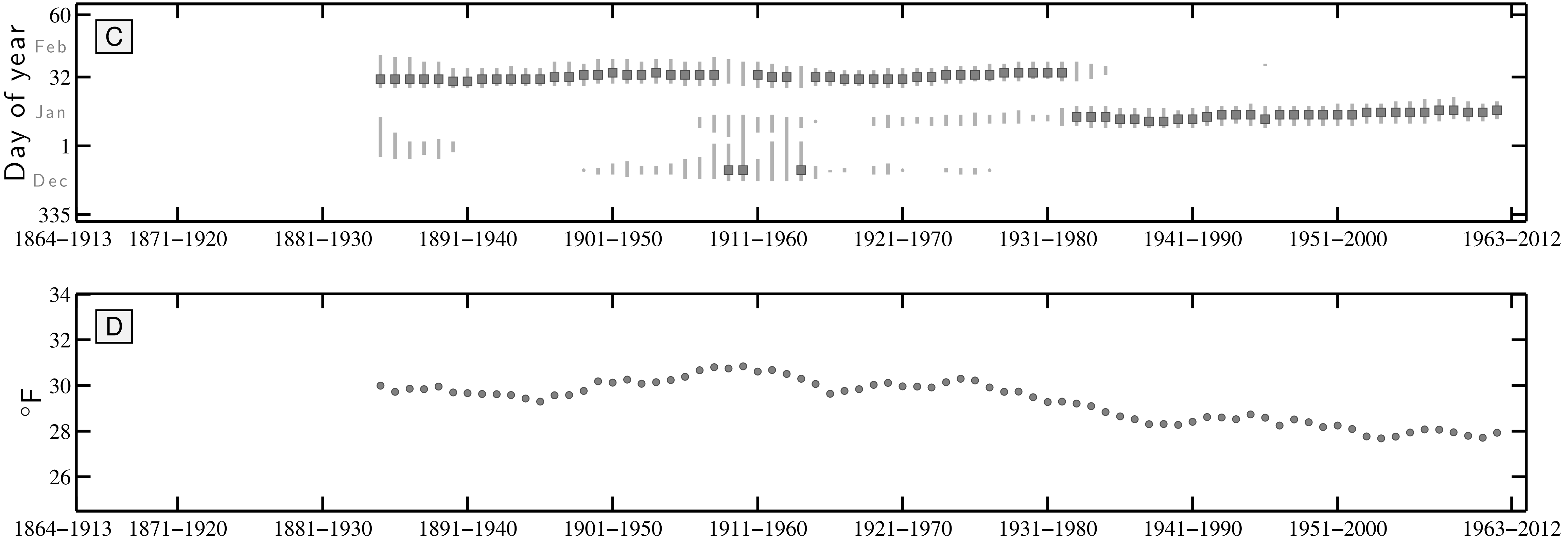

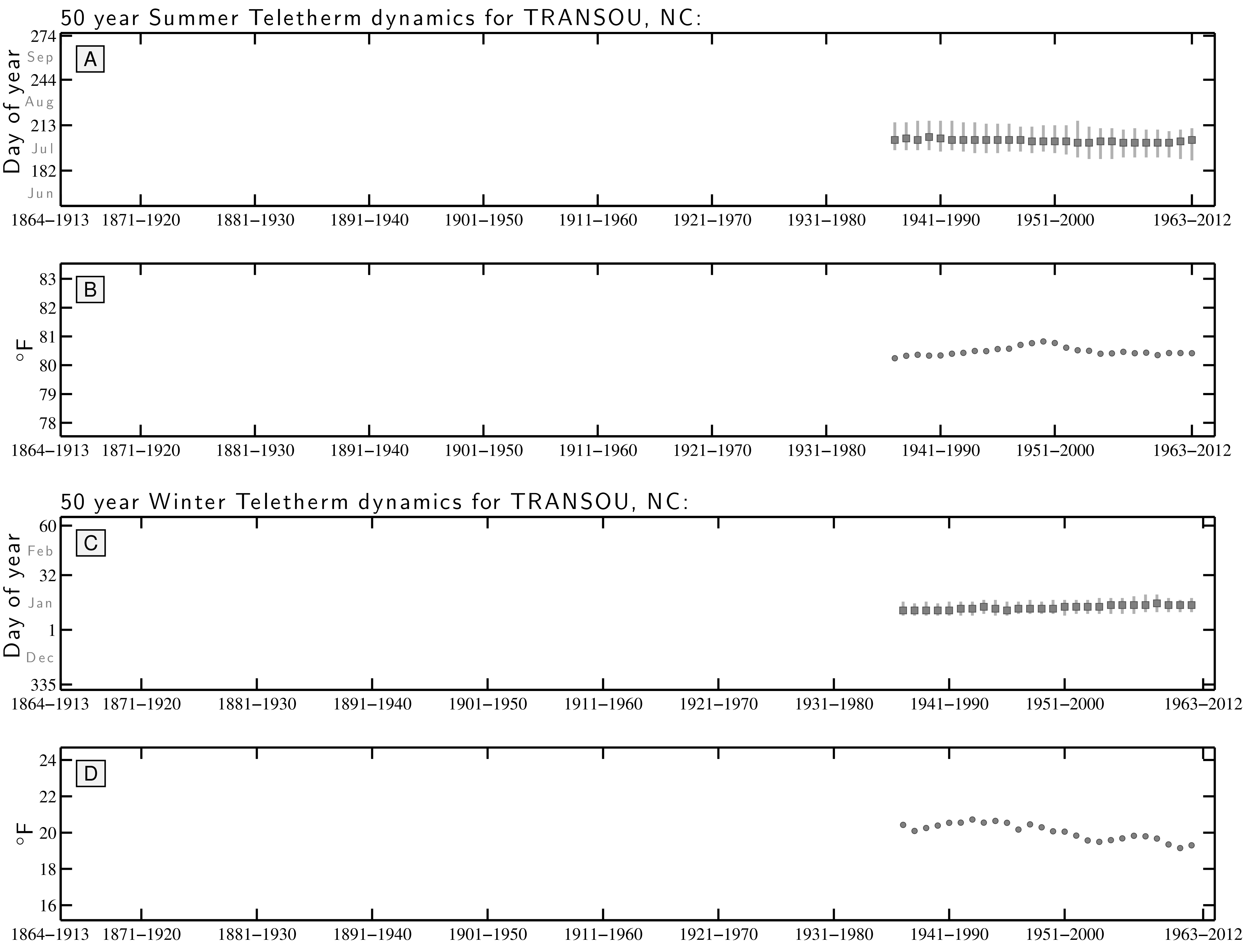

50 year Summer Teletherm dynamics for TRANSOU, NC:
A
B
Day of year
°F
274
Sep
244
Aug
213
Jul
182
Jun
83
82
81
80
79
78
1864–1913
1871–1920
1881–1930
1891–1940
1901–1950
1911–1960
1921–1970
1931–1980
1941–1990
1951–2000
1963–2012
50 year Winter Teletherm dynamics for TRANSOU, NC:
C
D
60
Feb
32
Jan
1
Dec
335
24
22
20
18
16

50 year Summer Teletherm dynamics for WAYNESVILLE 1 E, NC:

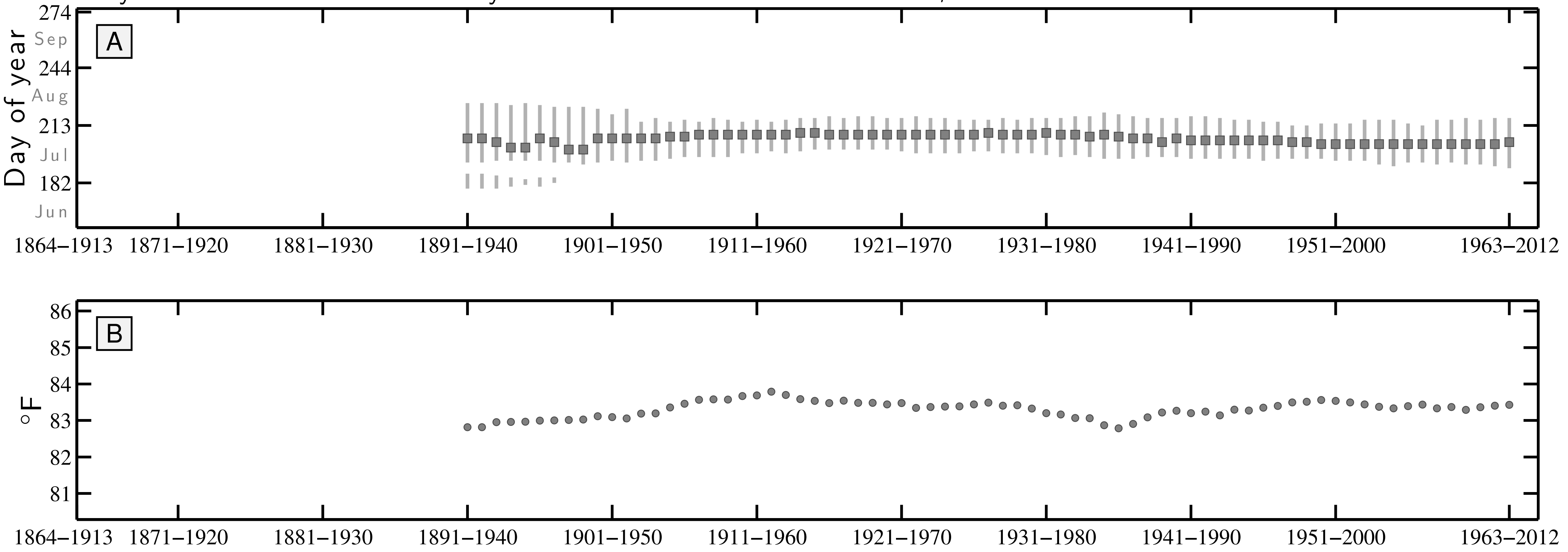

50 year Winter Teletherm dynamics for WAYNESVILLE 1 E, NC:

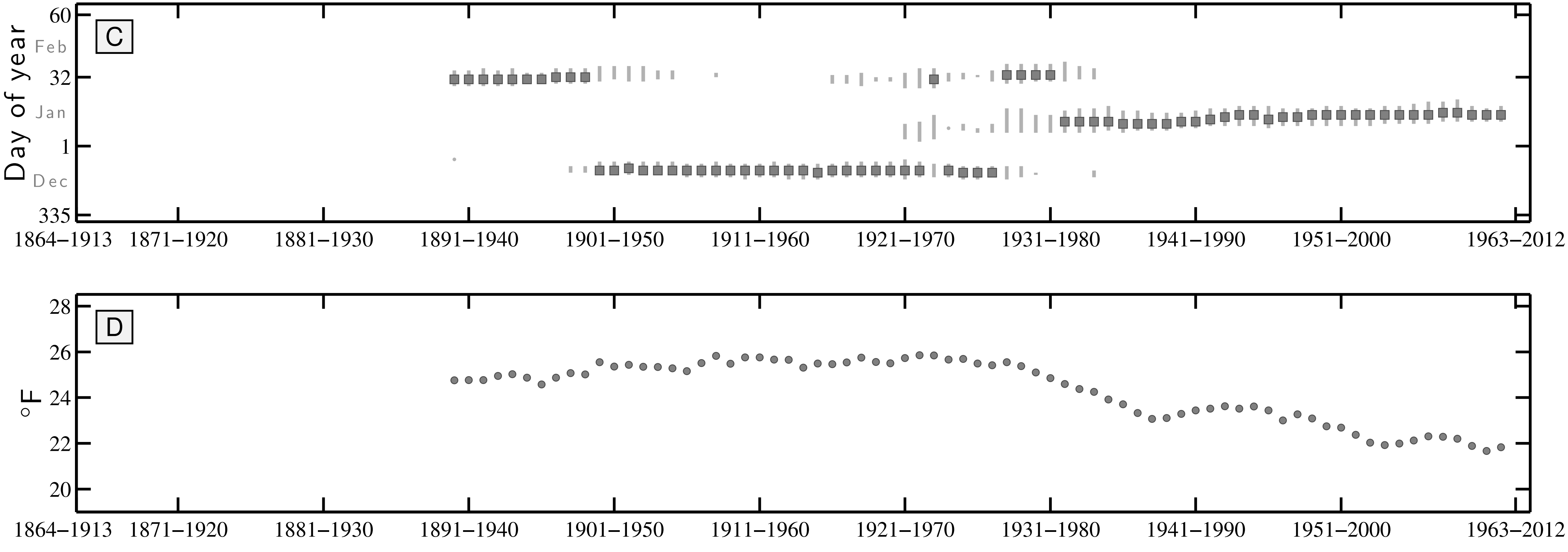

50 year Summer Teletherm dynamics for WILSON 3 SW, NC:

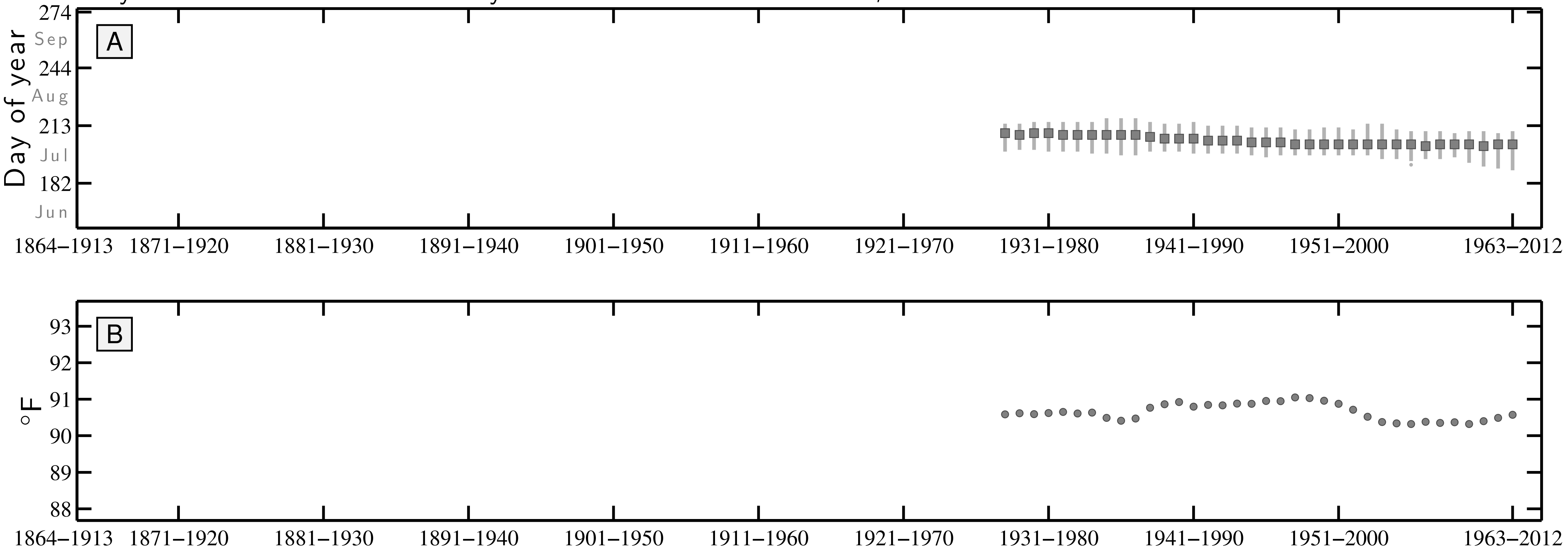

50 year Winter Teletherm dynamics for WILSON 3 SW, NC:

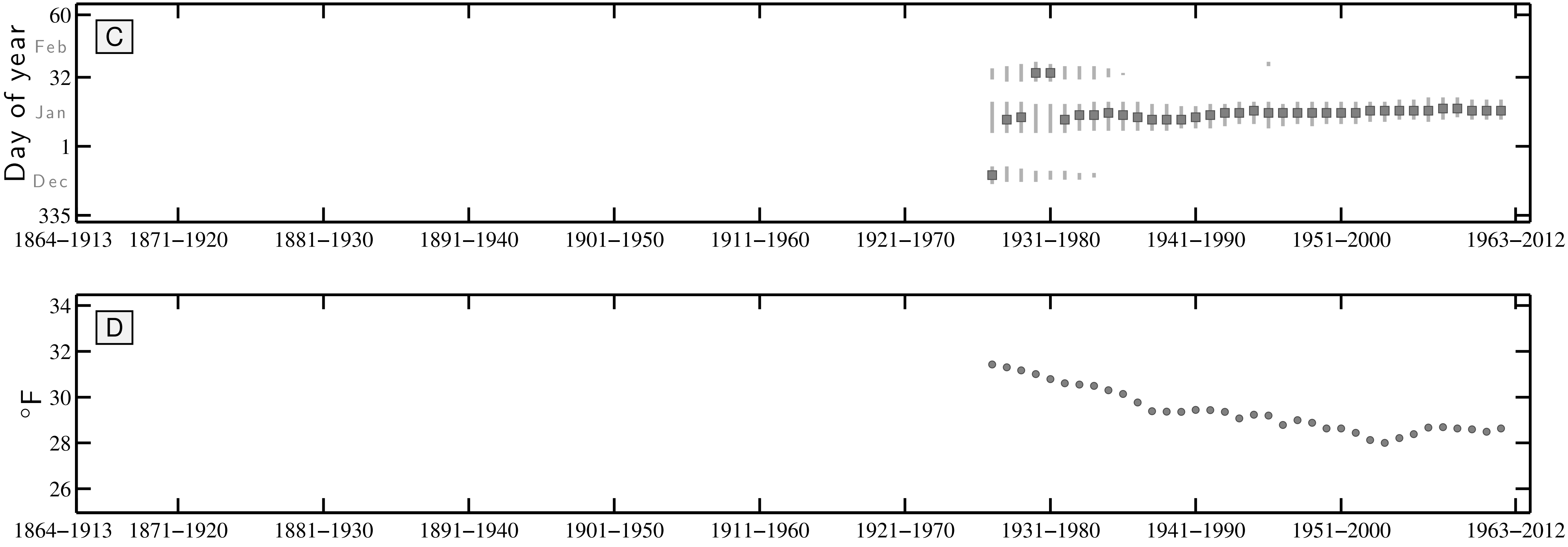

50 year Summer Teletherm dynamics for BOTTINEAU, ND:

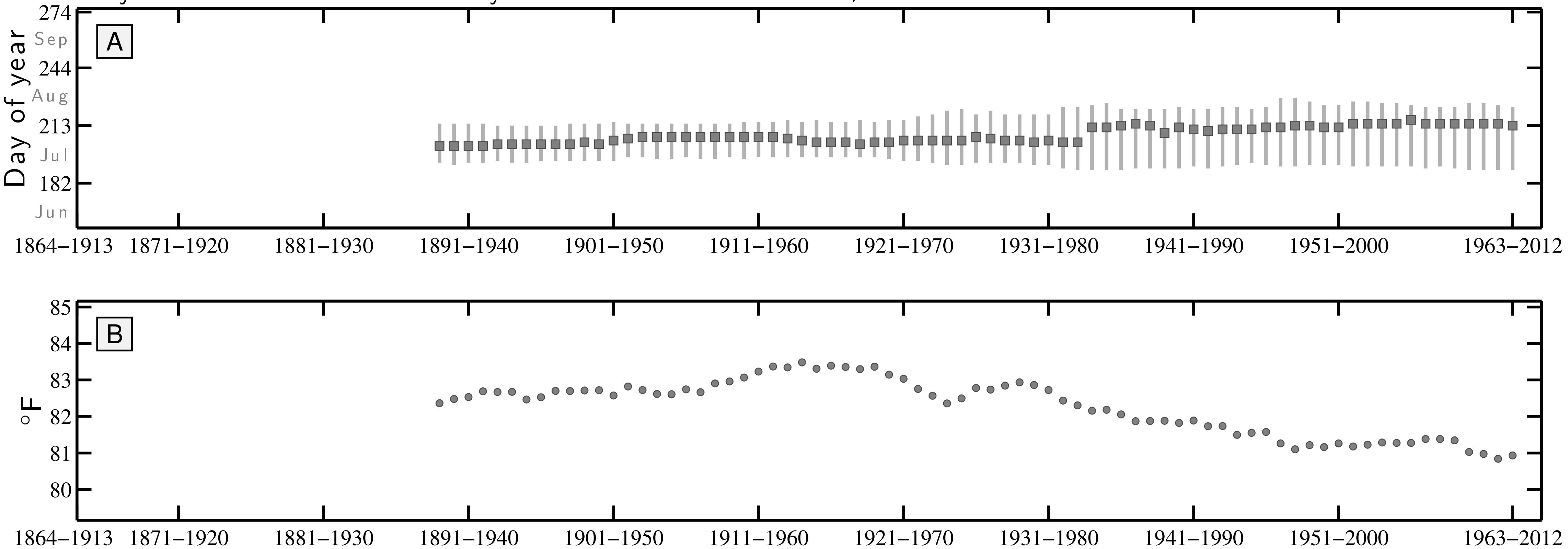

50 year Winter Teletherm dynamics for BOTTINEAU, ND:

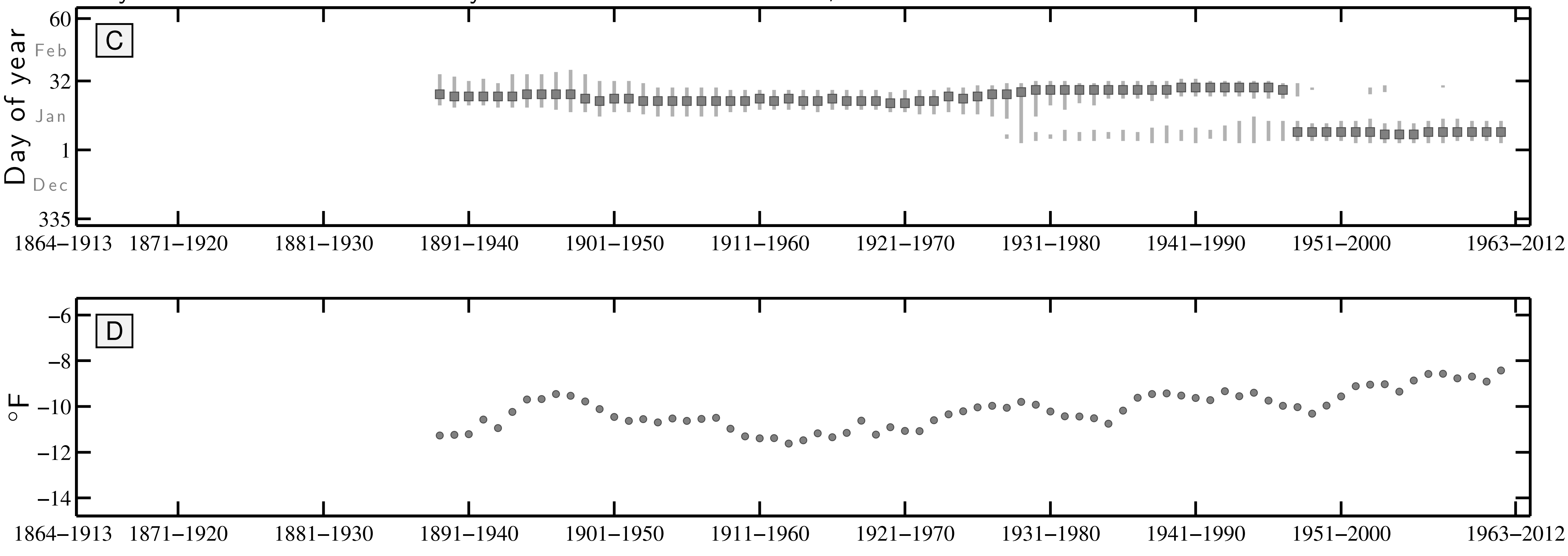

50 year Summer Teletherm dynamics for CROSBY, ND:

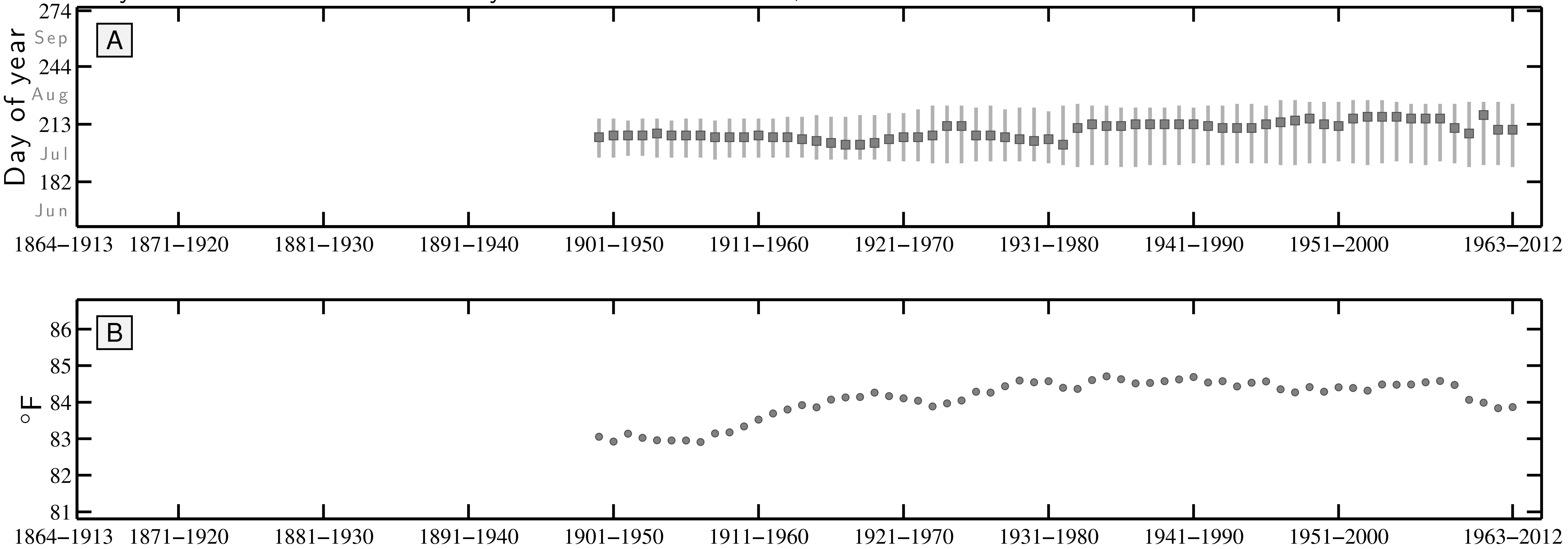

50 year Winter Teletherm dynamics for CROSBY, ND:

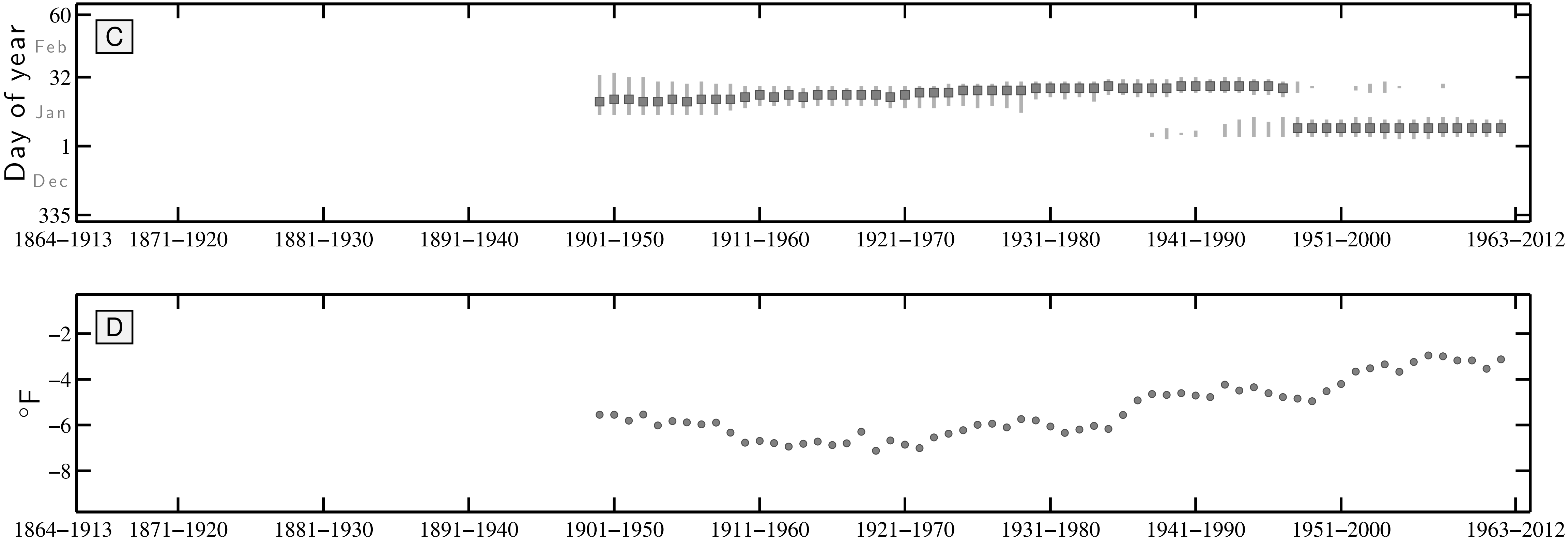

50 year Summer Teletherm dynamics for DICKINSON EXP STN, ND:

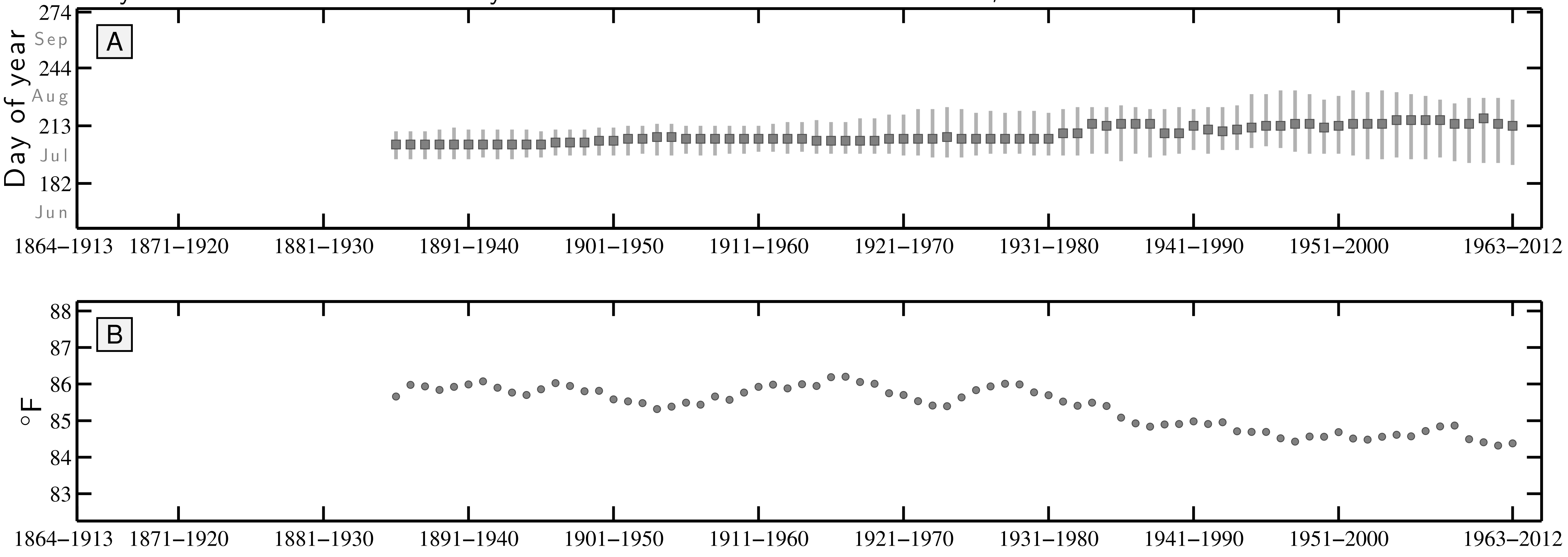

50 year Winter Teletherm dynamics for DICKINSON EXP STN, ND:

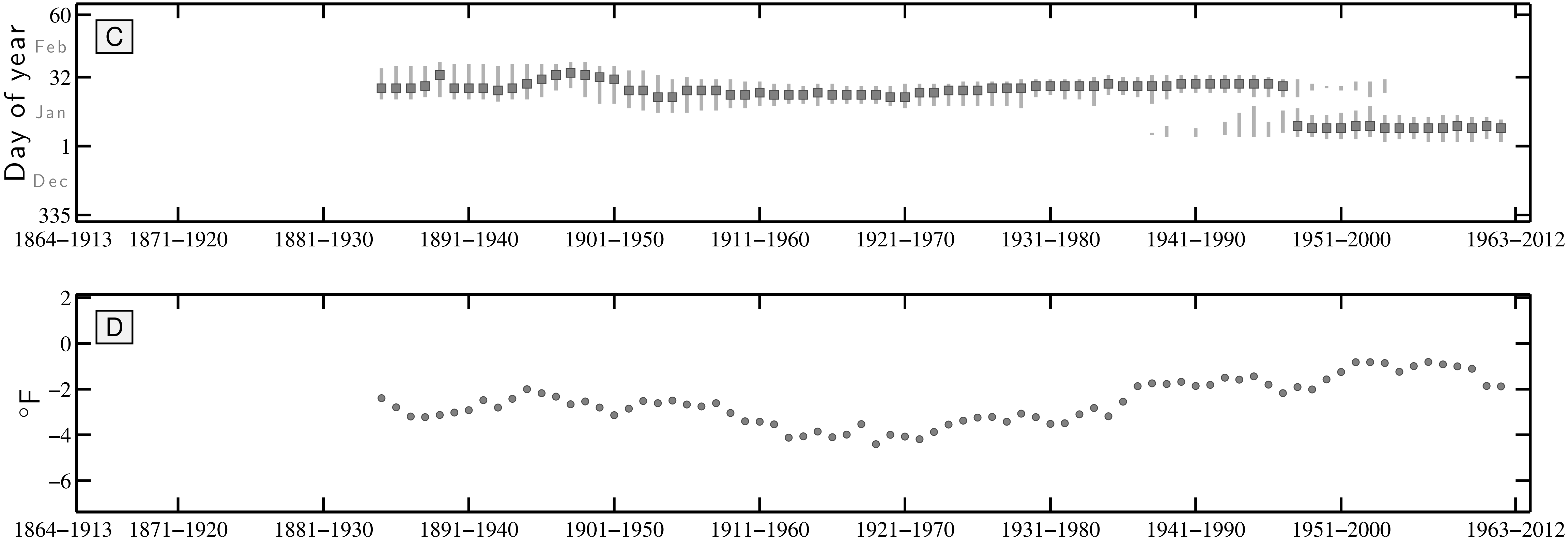

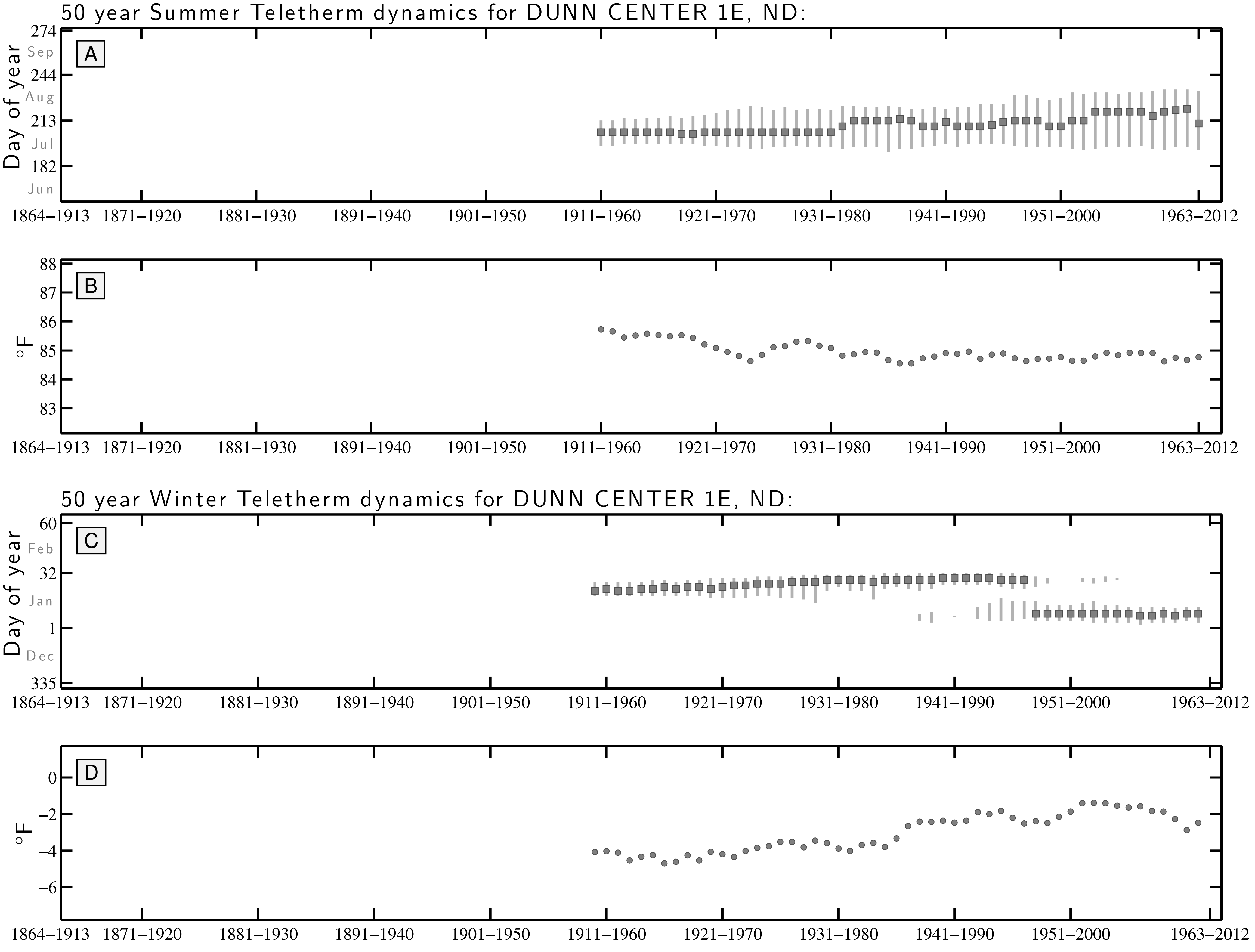
50 year Summer Teletherm dynamics for DUNN CENTER 1E, ND:
A
Day of year
274
Sep
244
Aug
213
Jul
182
Jun
1864–1913
1871–1920
1881–1930
1891–1940
1901–1950
1911–1960
1921–1970
1931–1980
1941–1990
1951–2000
1963–2012
B
°F
88
87
86
85
84
83
50 year Winter Teletherm dynamics for DUNN CENTER 1E, ND:
C
Day of year
60
Feb
32
Jan
1
Dec
335
D
°F
0
−2
−4
−6

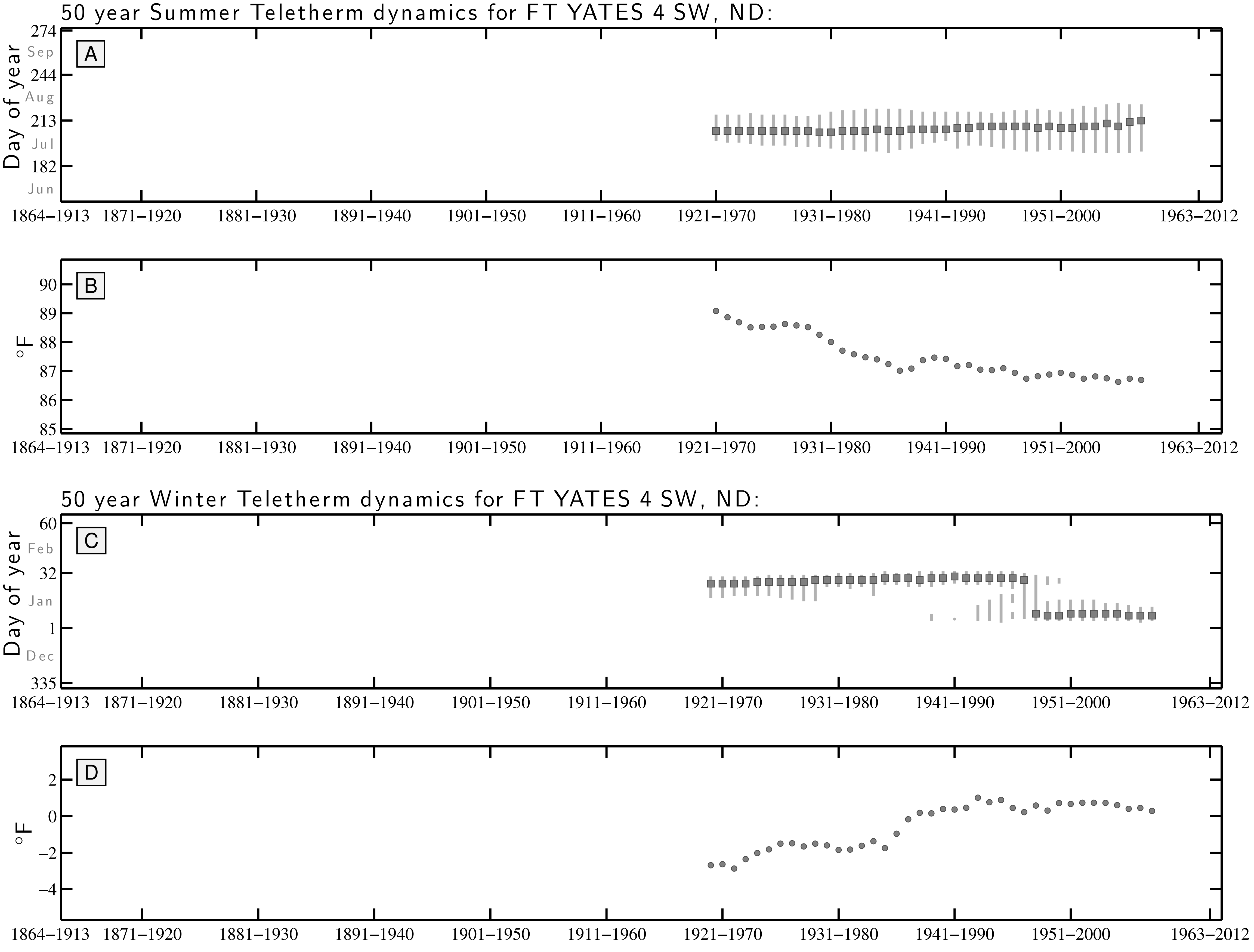

50 year Summer Teletherm dynamics for FT YATES 4 SW, ND:
A
Day of year
274
Sep
244
Aug
213
Jul
182
Jun
1864–1913
1871–1920
1881–1930
1891–1940
1901–1950
1911–1960
1921–1970
1931–1980
1941–1990
1951–2000
1963–2012
B
°F
90
89
88
87
86
85
50 year Winter Teletherm dynamics for FT YATES 4 SW, ND:
C
60
Feb
32
Jan
1
Dec
335
D
2
0
−2
−4

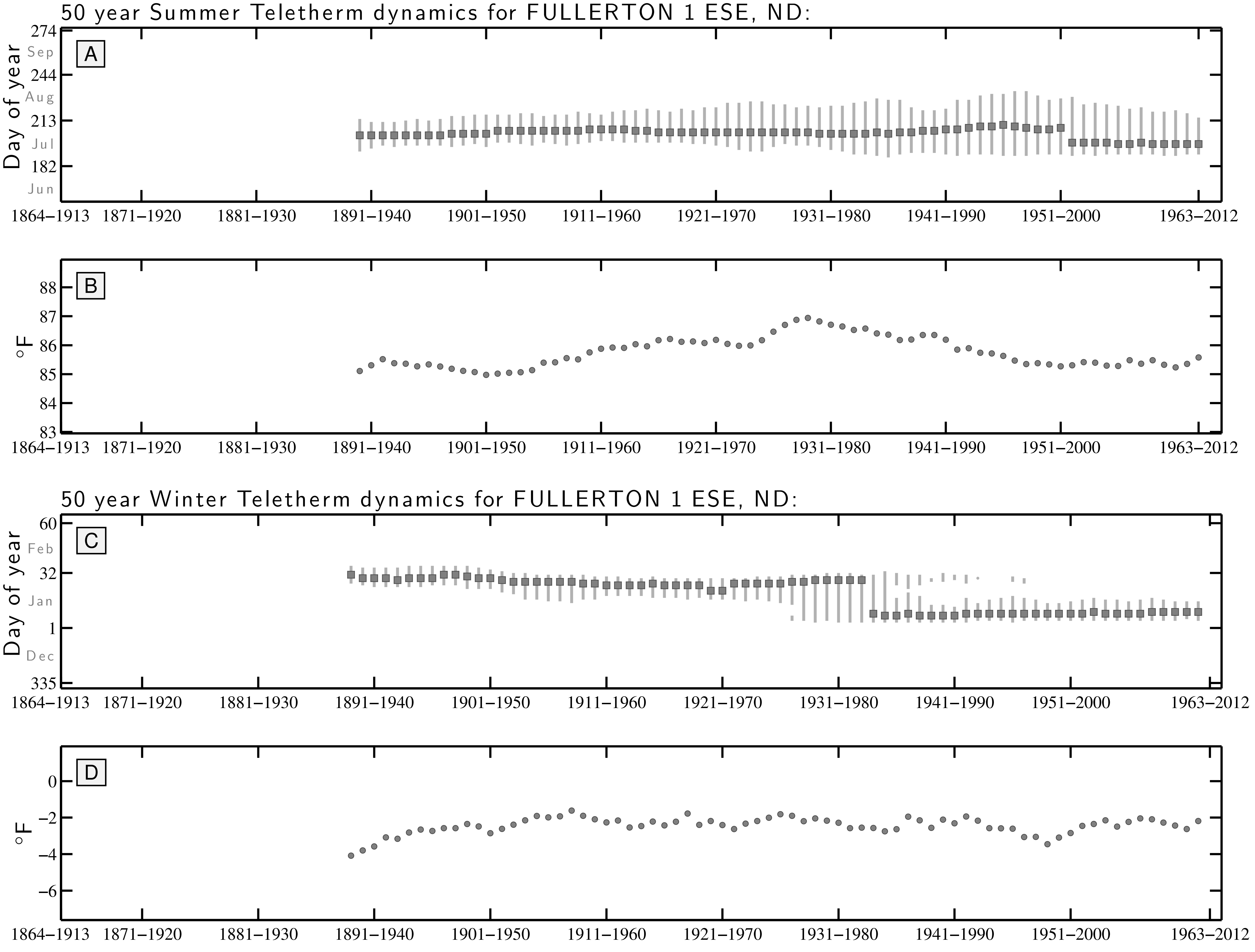
50 year Summer Teletherm dynamics for FULLERTON 1 ESE, ND:
A
Day of year
274
Sep
244
Aug
213
Jul
182
Jun
1864–1913
1871–1920
1881–1930
1891–1940
1901–1950
1911–1960
1921–1970
1931–1980
1941–1990
1951–2000
1963–2012
B
°F
88
87
86
85
84
83
50 year Winter Teletherm dynamics for FULLERTON 1 ESE, ND:
C
60
Feb
32
Jan
1
Dec
335
D
0
−2
−4
−6

50 year Summer Teletherm dynamics for GRAFTON, ND:

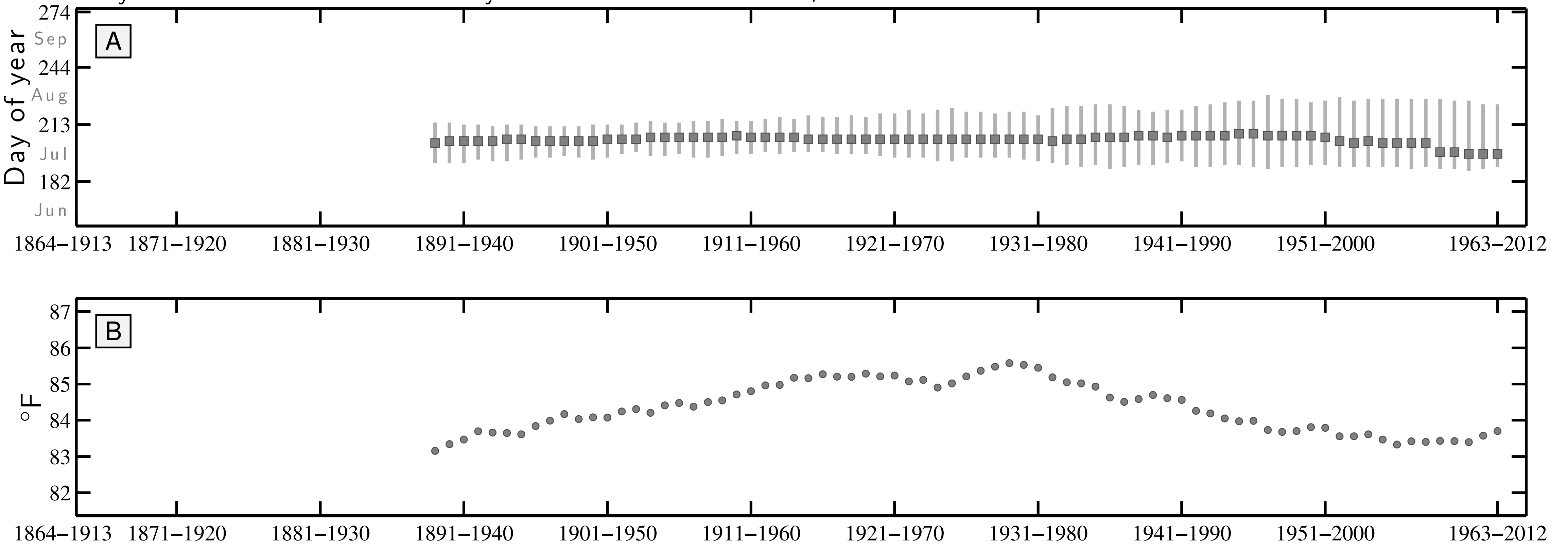

50 year Winter Teletherm dynamics for GRAFTON, ND:

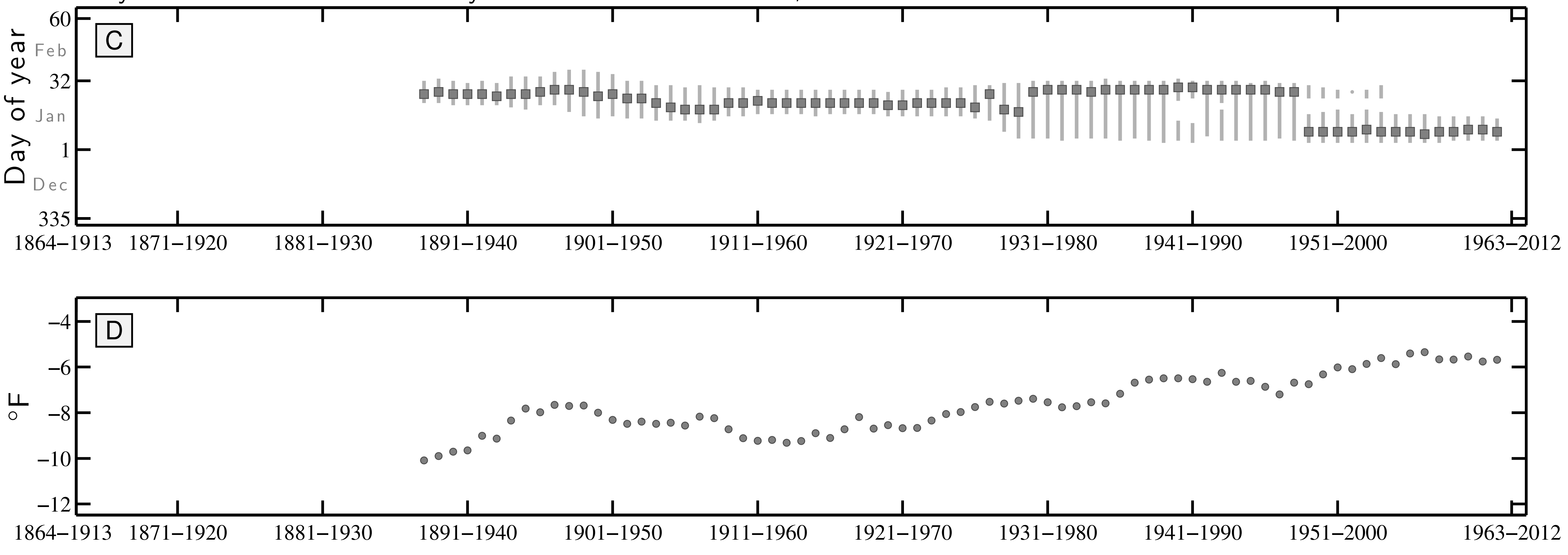

50 year Summer Teletherm dynamics for GRAND FORKS UNIV NWS, ND:

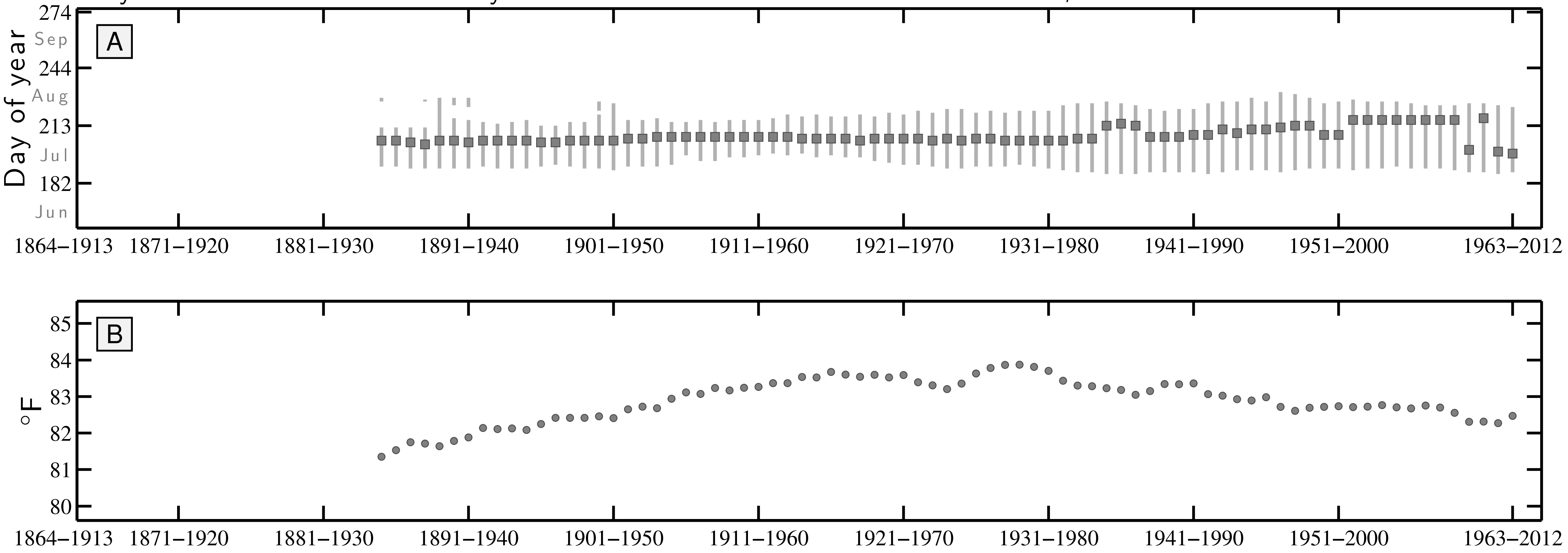

50 year Winter Teletherm dynamics for GRAND FORKS UNIV NWS, ND:

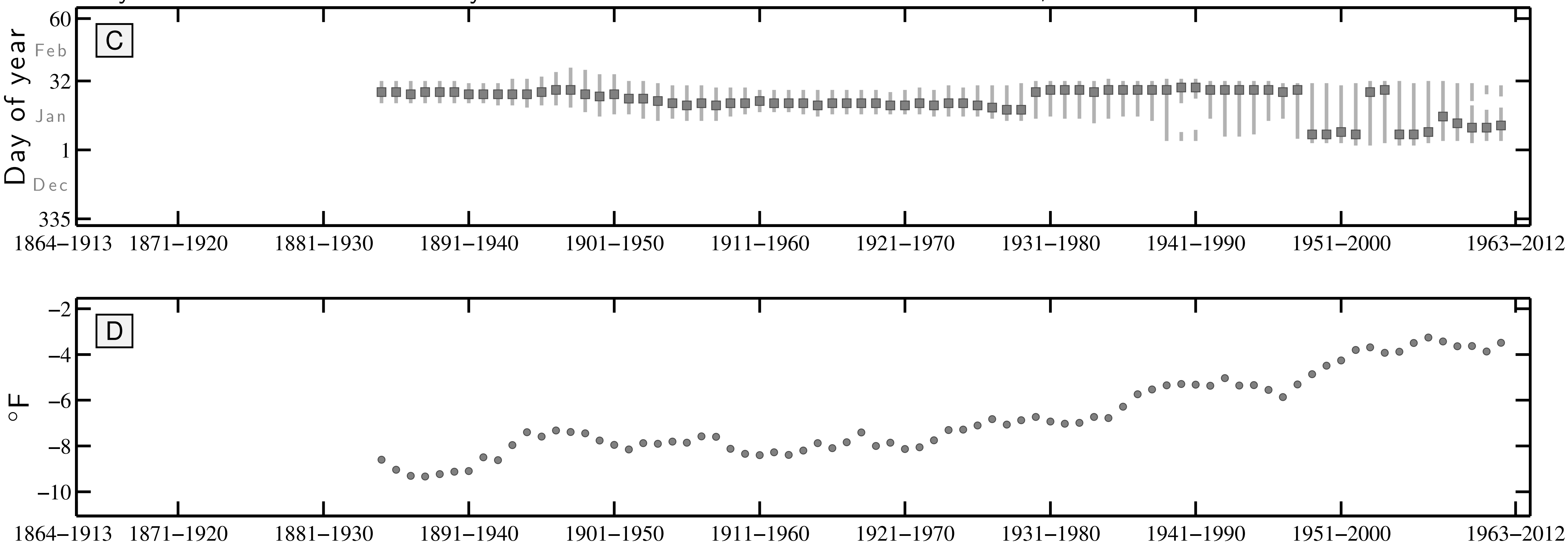

50 year Summer Teletherm dynamics for HETTINGER, ND:

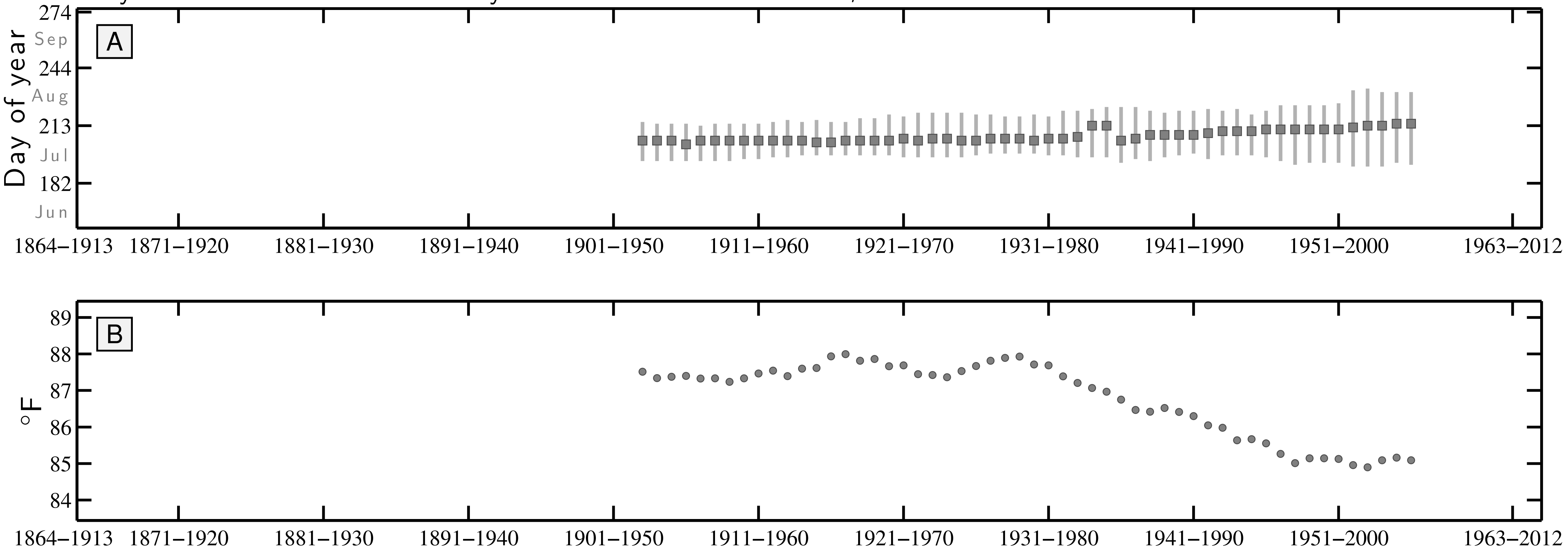

50 year Winter Teletherm dynamics for HETTINGER, ND:

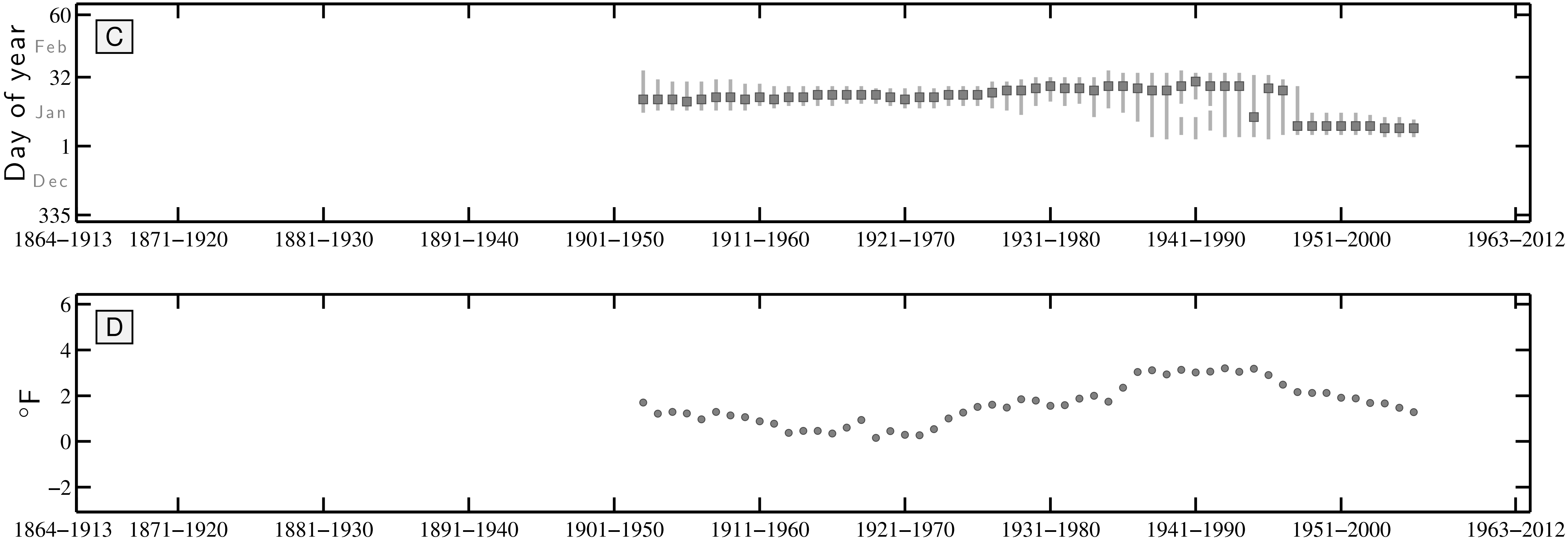

50 year Summer Teletherm dynamics for HILLSBORO 3 N, ND:

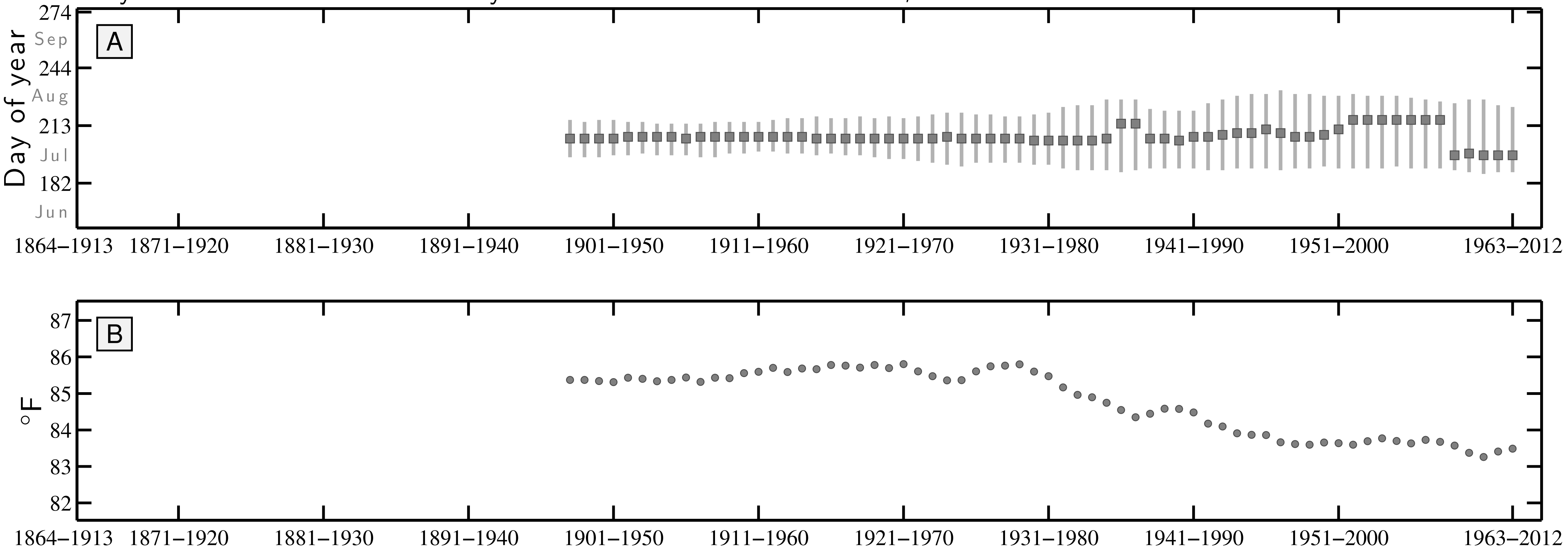

50 year Winter Teletherm dynamics for HILLSBORO 3 N, ND:

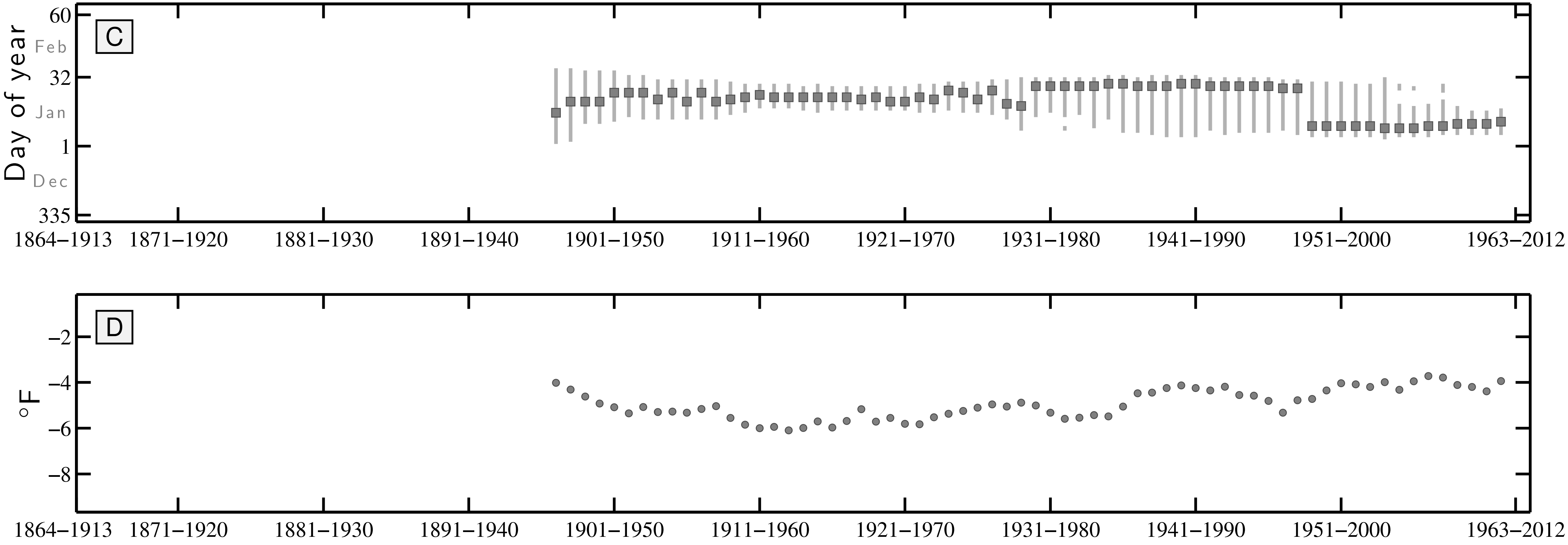

50 year Summer Teletherm dynamics for JAMESTOWN STATE HOSP, ND:

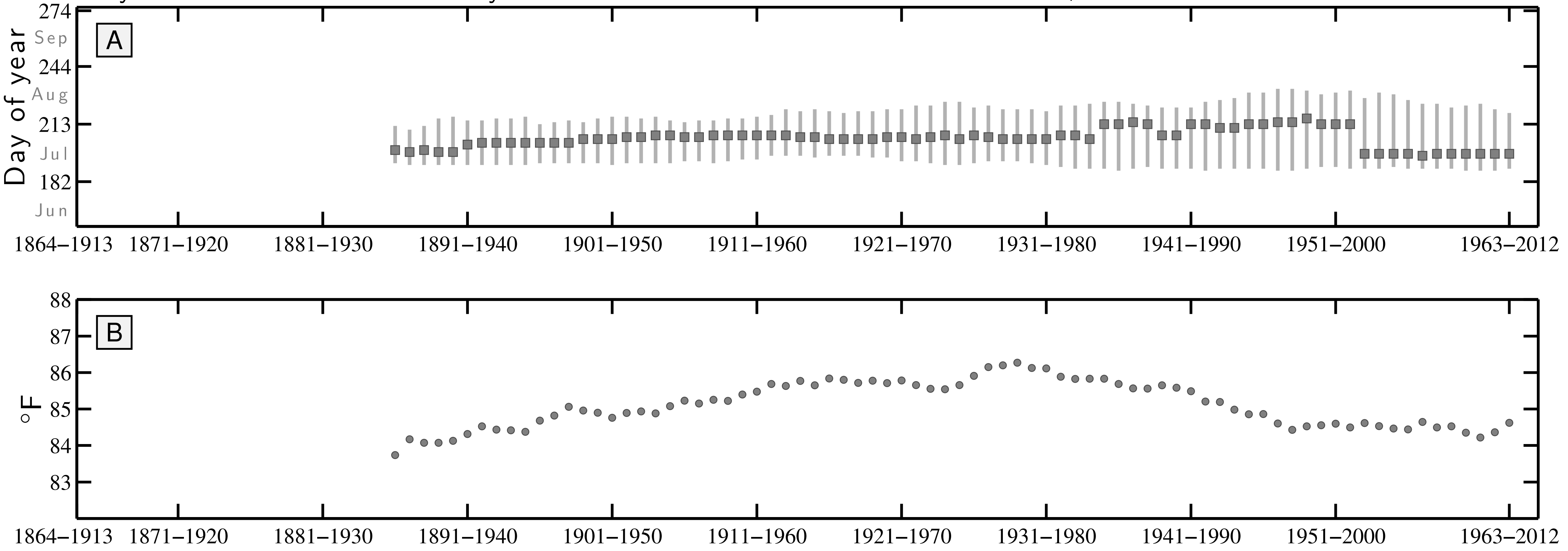

50 year Winter Teletherm dynamics for JAMESTOWN STATE HOSP, ND:

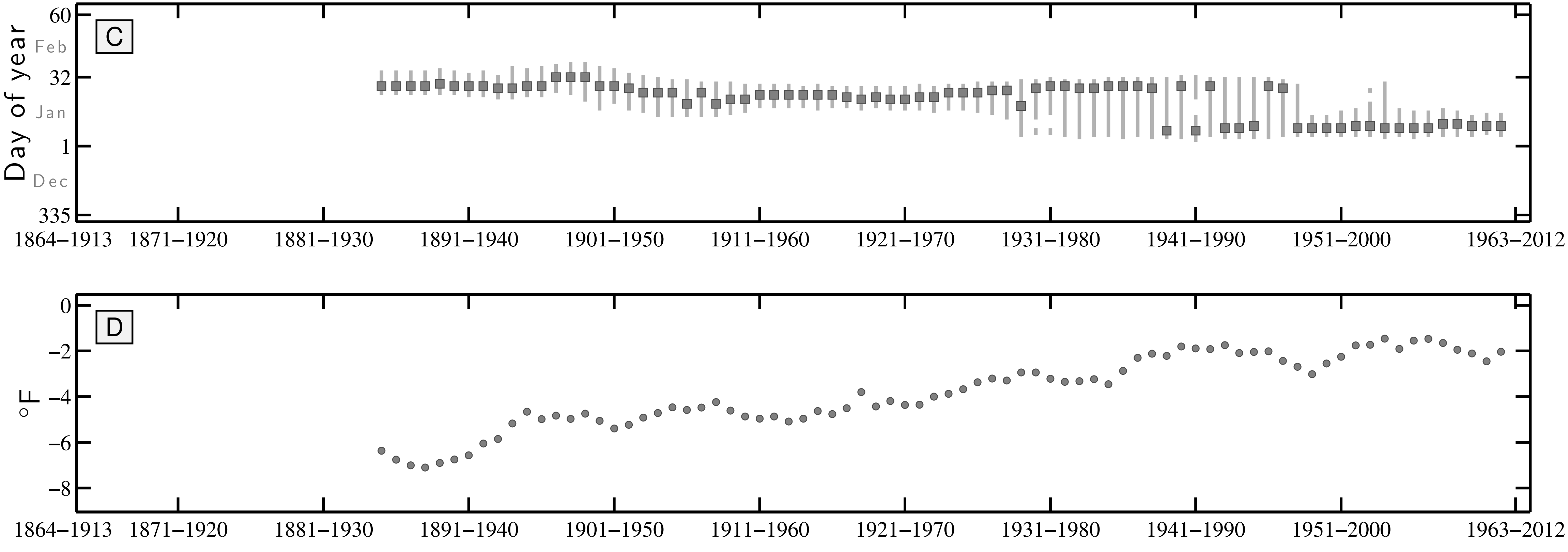

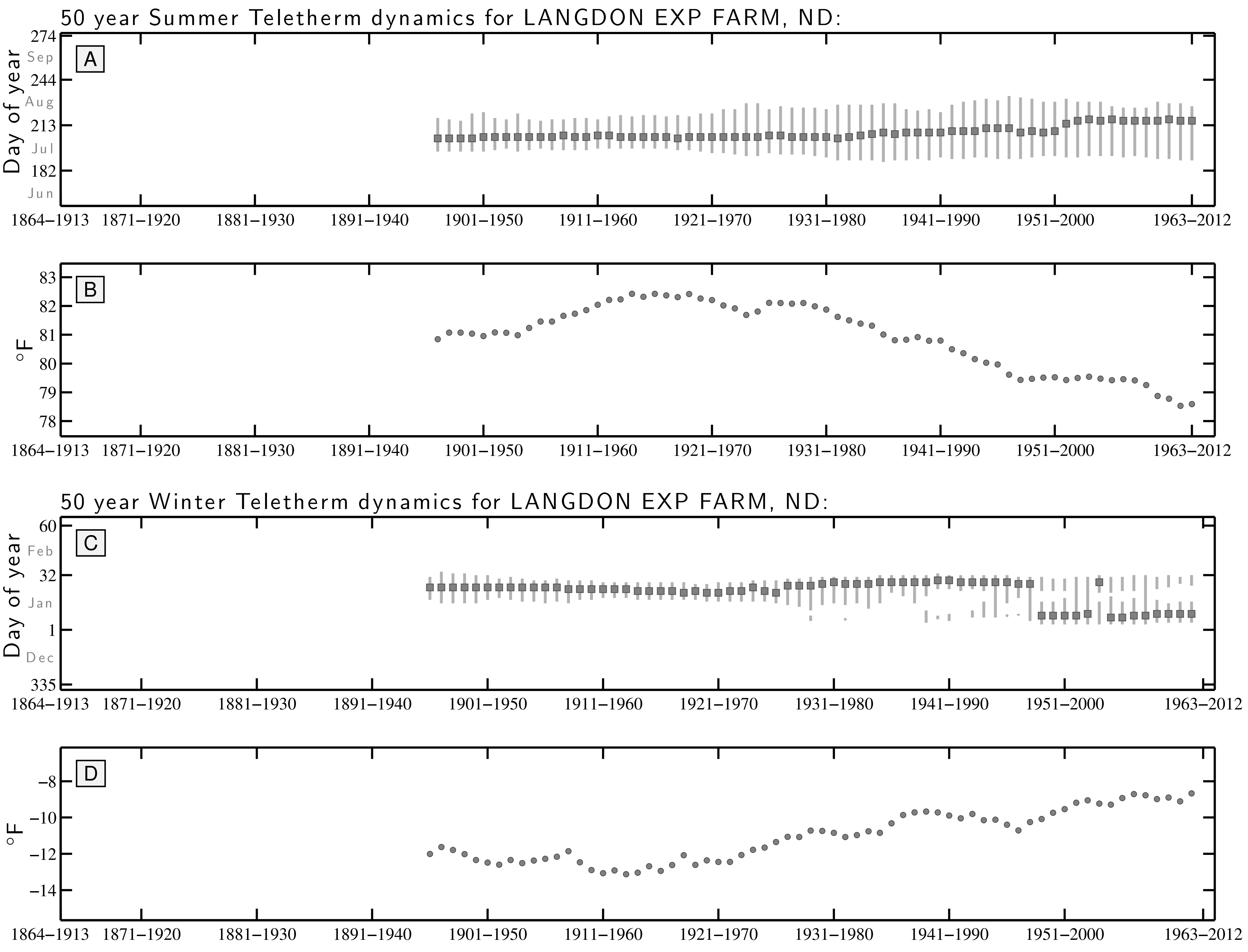
50 year Summer Teletherm dynamics for LANGDON EXP FARM, ND:
A
B
Day of year
274
Sep
244
Aug
213
Jul
182
Jun
°F
83
82
81
80
79
78
50 year Winter Teletherm dynamics for LANGDON EXP FARM, ND:
C
D
60
Feb
32
Jan
1
Dec
335
−8
−10
−12
−14
1864–1913
1871–1920
1881–1930
1891–1940
1901–1950
1911–1960
1921–1970
1931–1980
1941–1990
1951–2000
1963–2012

50 year Summer Teletherm dynamics for LISBON, ND:

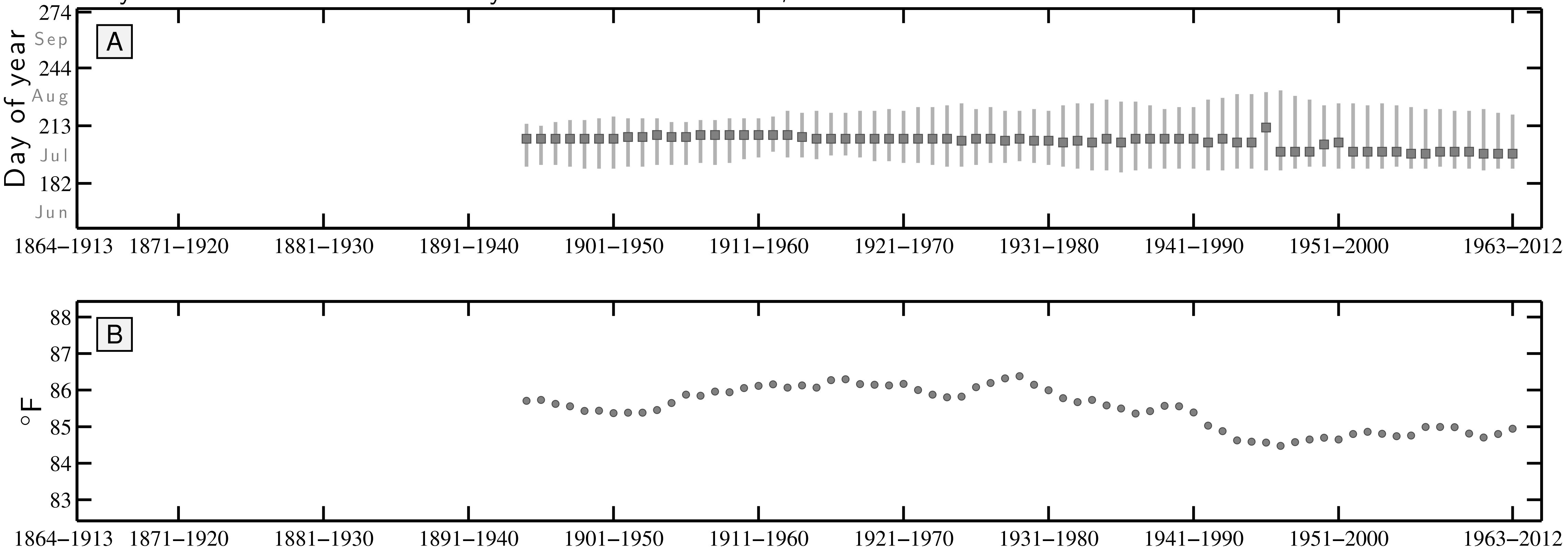

50 year Winter Teletherm dynamics for LISBON, ND:

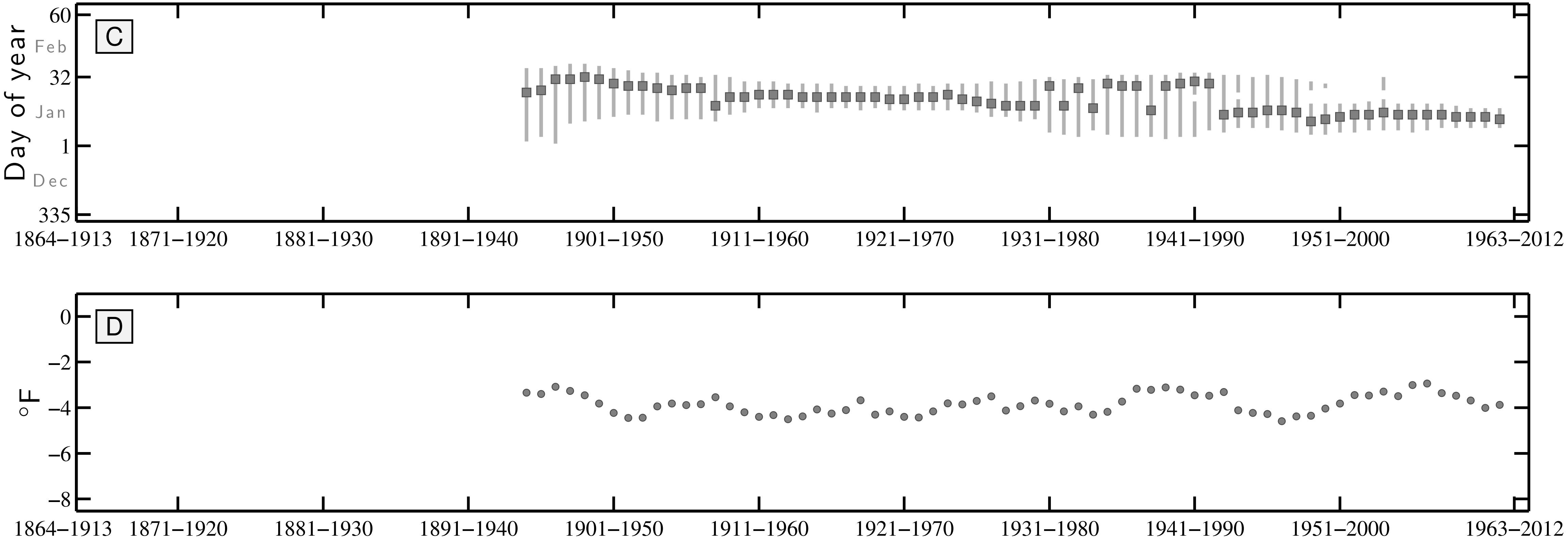

50 year Summer Teletherm dynamics for MANDAN EXP STN, ND:

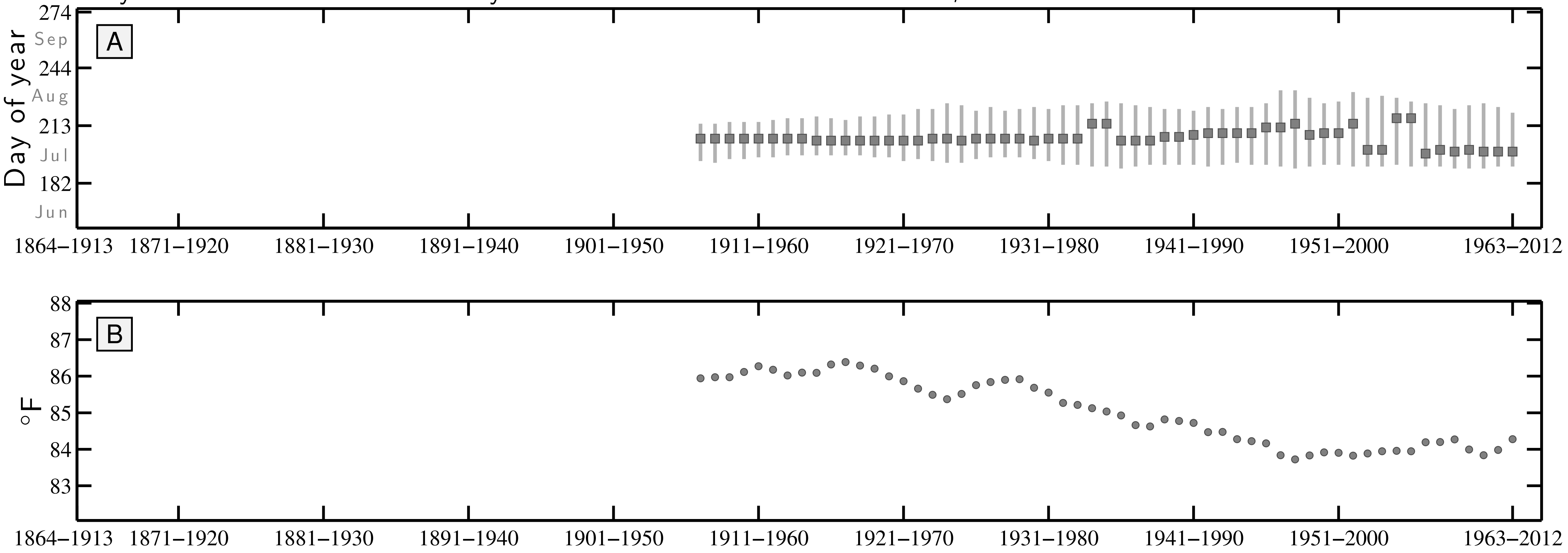

50 year Winter Teletherm dynamics for MANDAN EXP STN, ND:

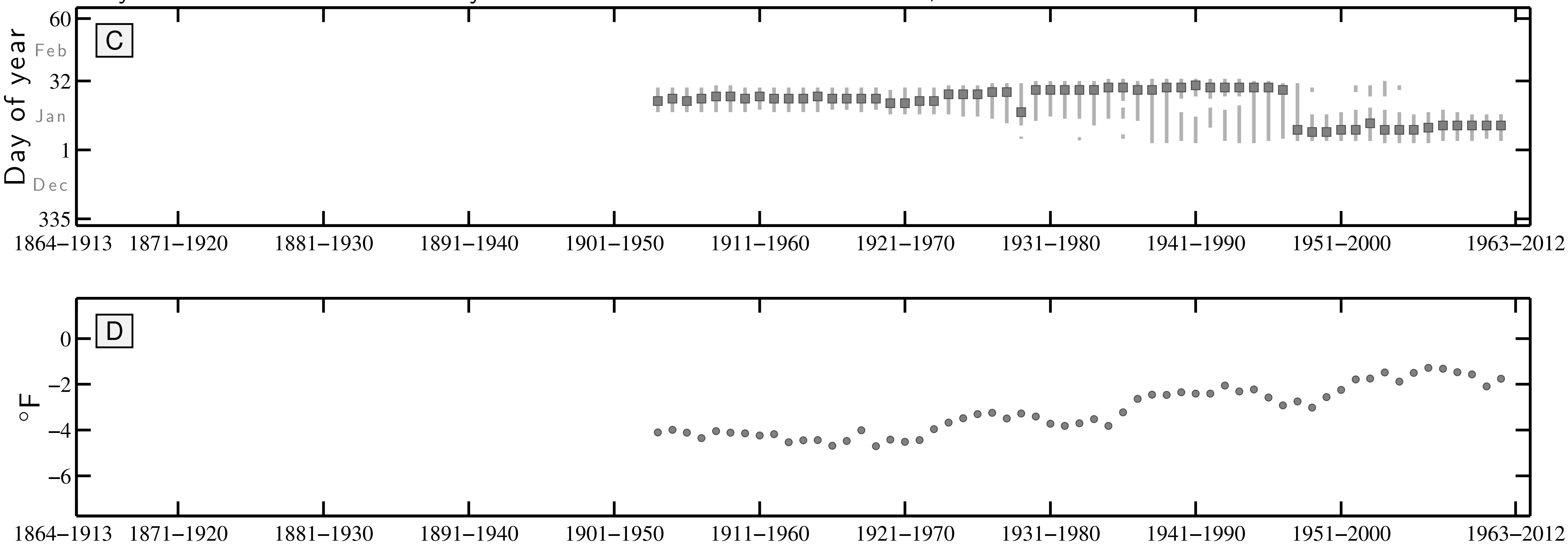

50 year Summer Teletherm dynamics for MOFFIT 3 SE, ND:

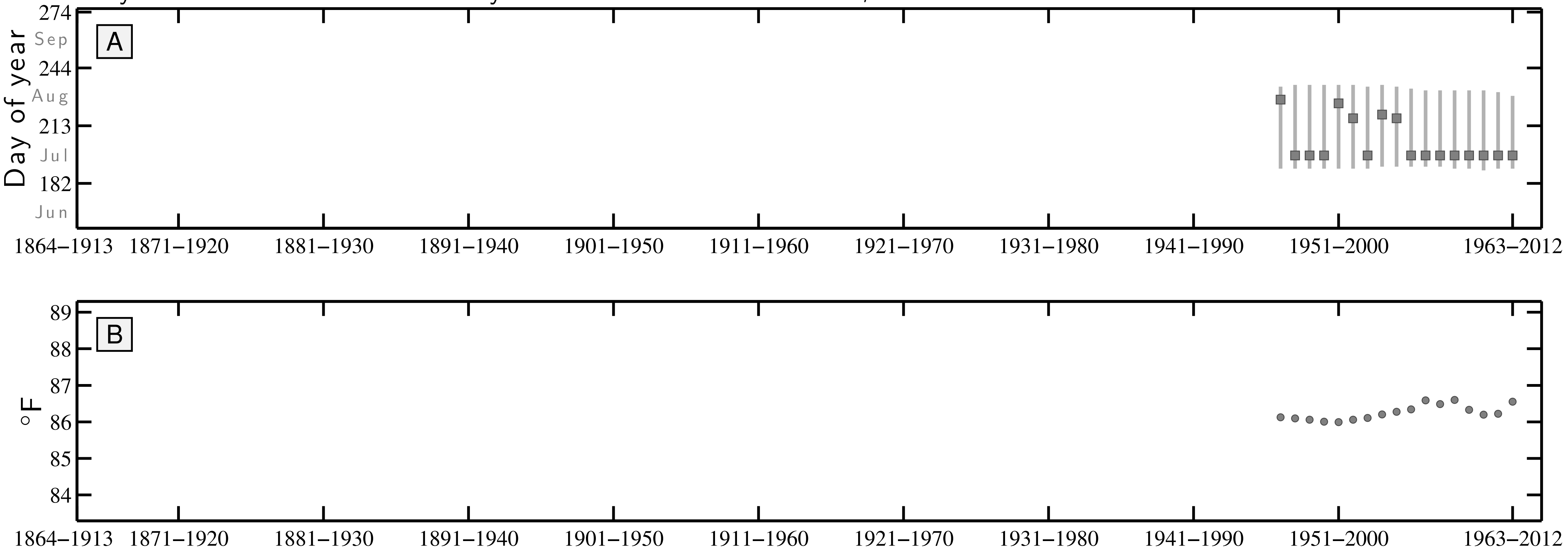

50 year Winter Teletherm dynamics for MOFFIT 3 SE, ND:

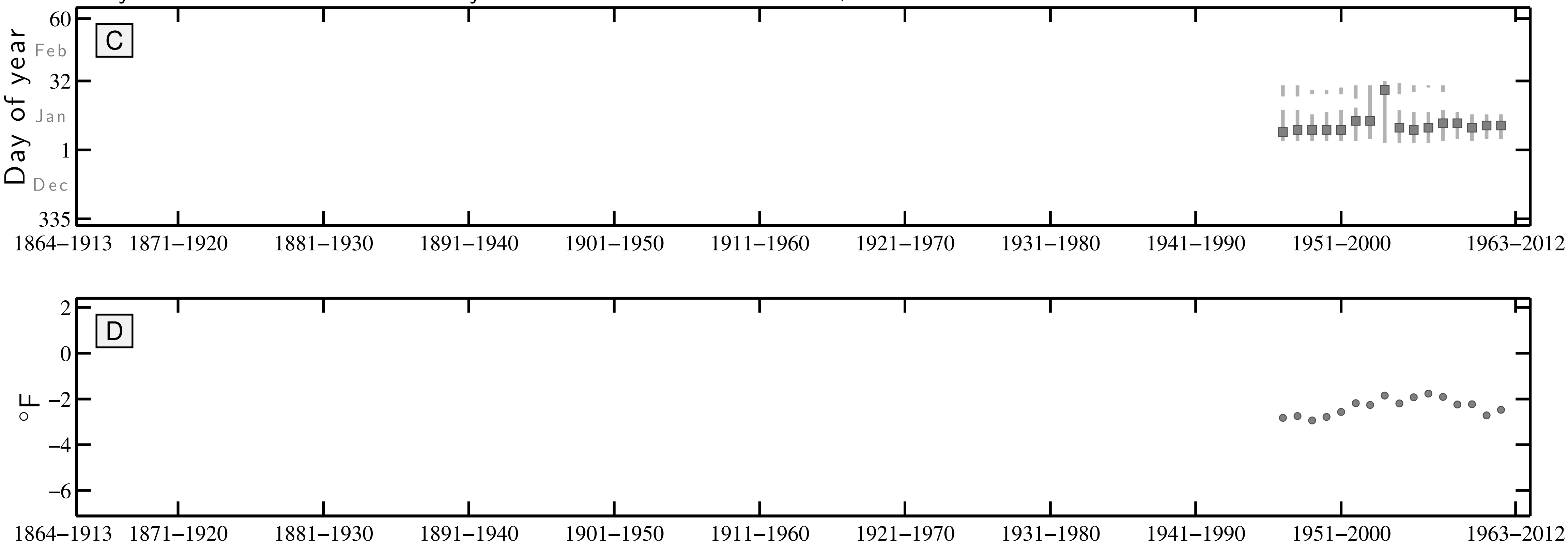

50 year Summer Teletherm dynamics for MOTT, ND:

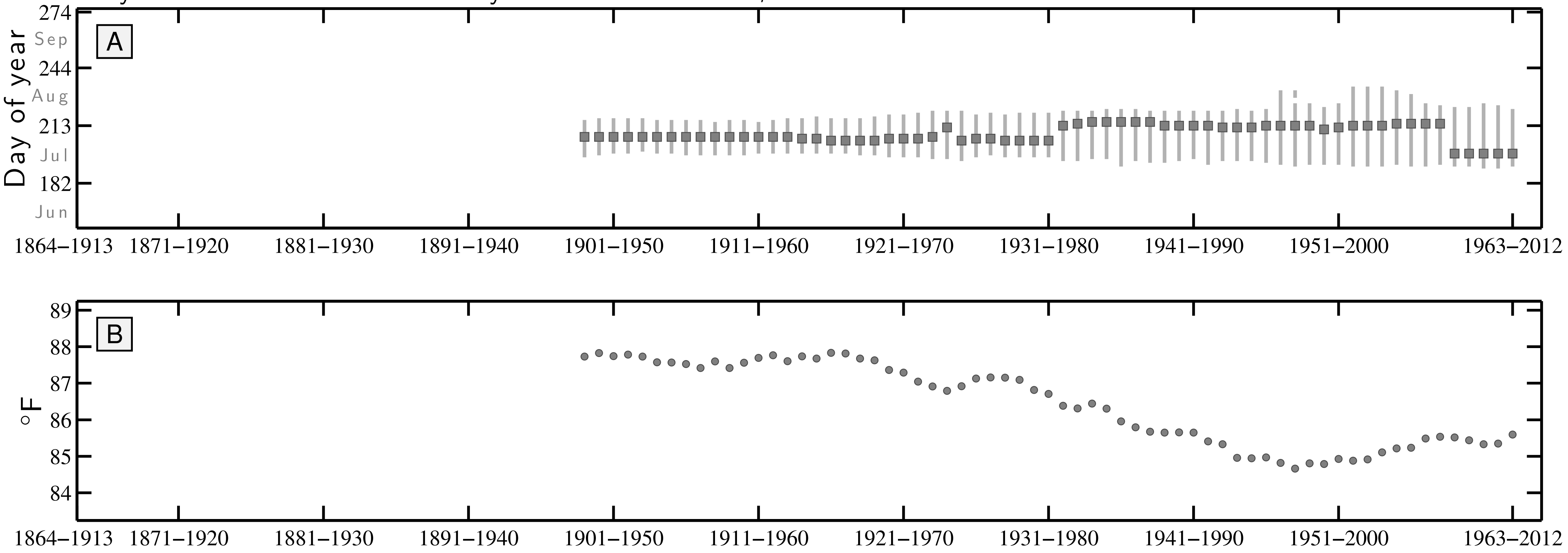

50 year Winter Teletherm dynamics for MOTT, ND:

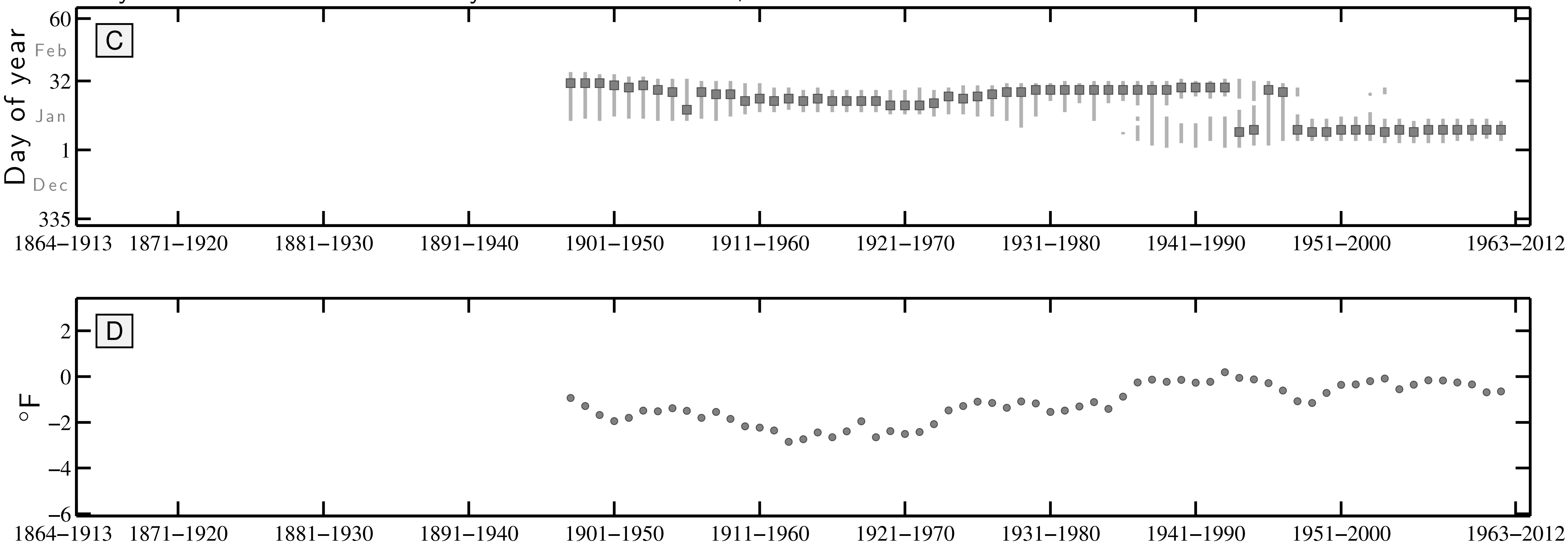

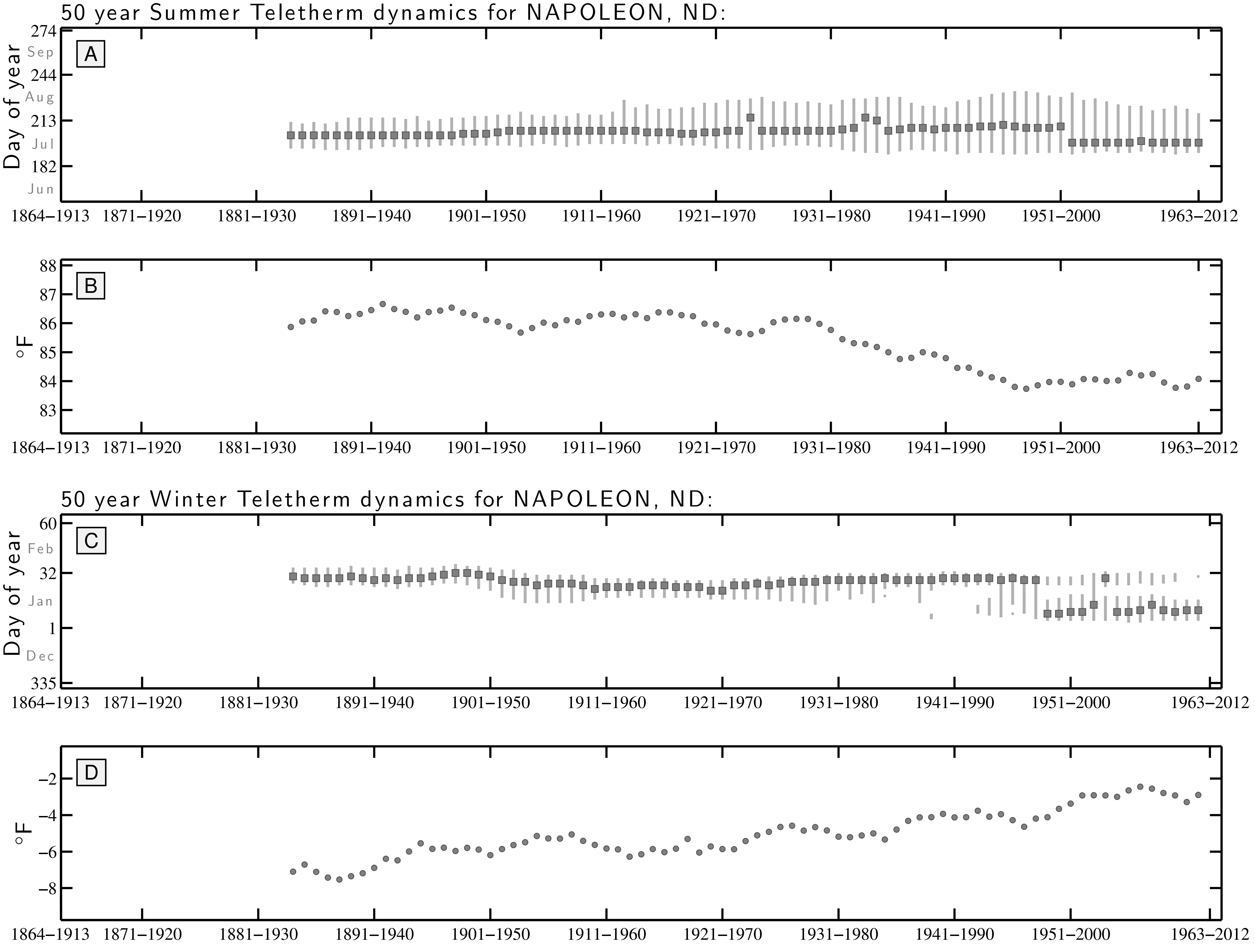
50 year Summer Teletherm dynamics for NAPOLEON, ND:
A
B
Day of year
°F
274
Sep
244
Aug
213
Jul
182
Jun
88
87
86
85
84
83
1864–1913
1871–1920
1881–1930
1891–1940
1901–1950
1911–1960
1921–1970
1931–1980
1941–1990
1951–2000
1963–2012
50 year Winter Teletherm dynamics for NAPOLEON, ND:
C
D
60
Feb
32
Jan
1
Dec
335
−2
−4
−6
−8

50 year Summer Teletherm dynamics for NEW ENGLAND, ND:

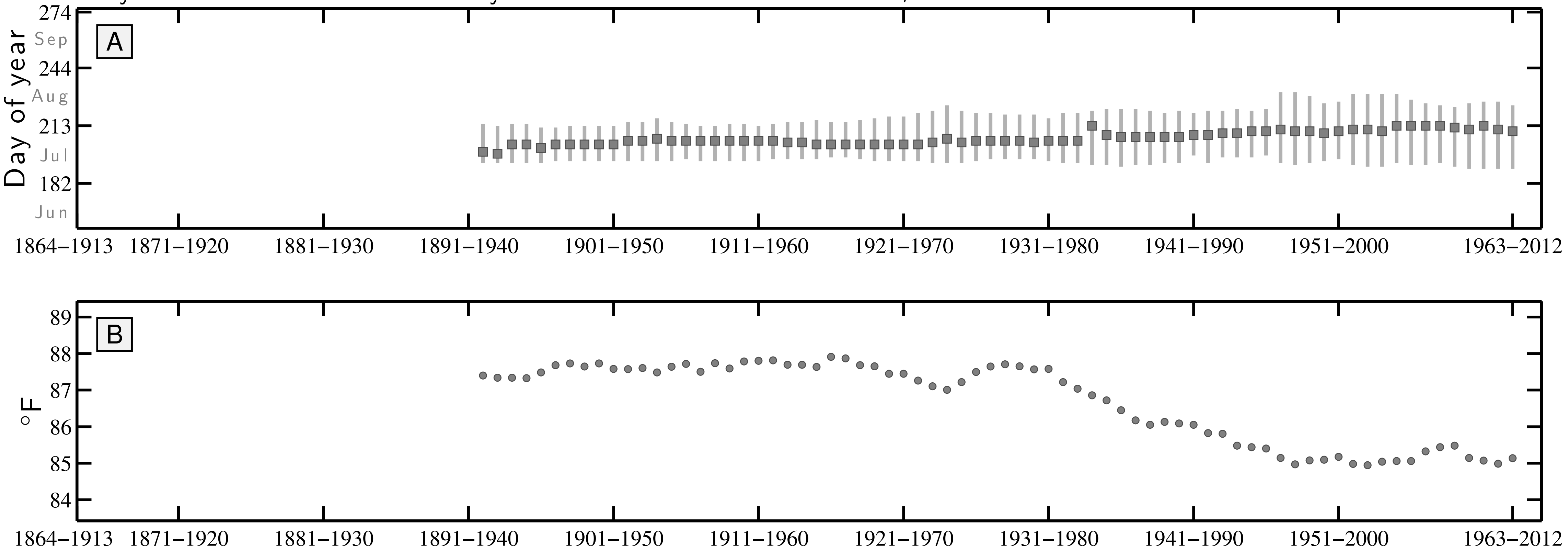

50 year Winter Teletherm dynamics for NEW ENGLAND, ND:

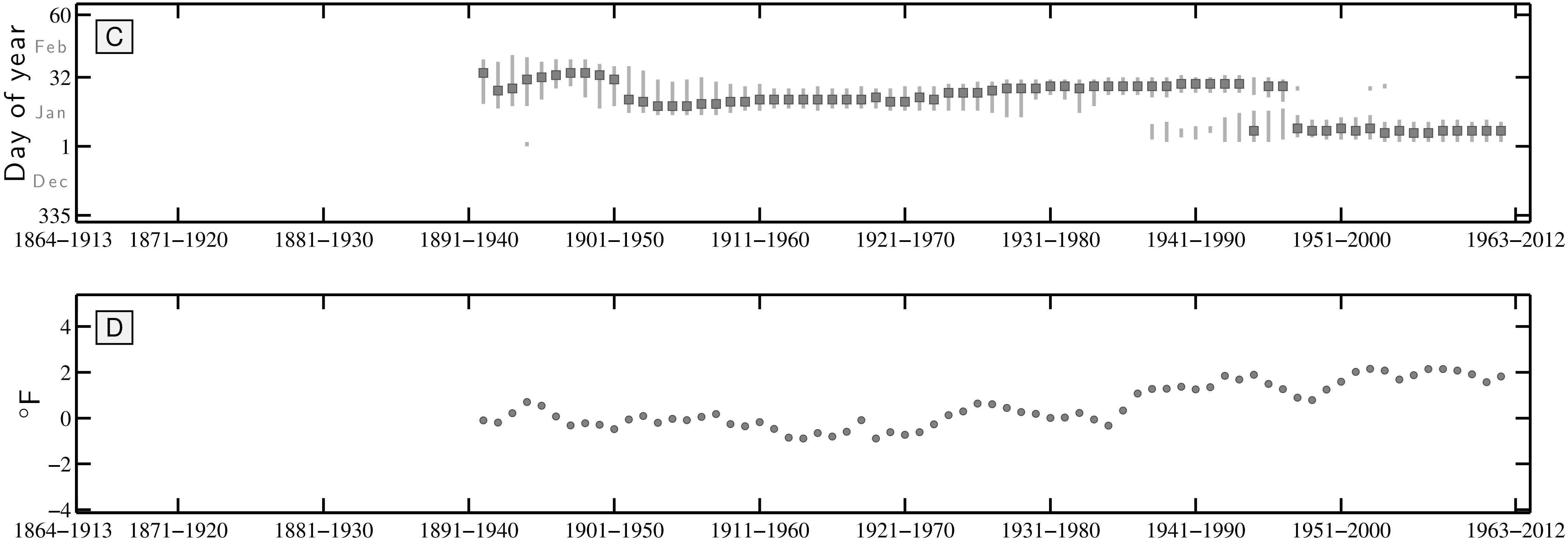

50 year Summer Teletherm dynamics for PEMBINA, ND:

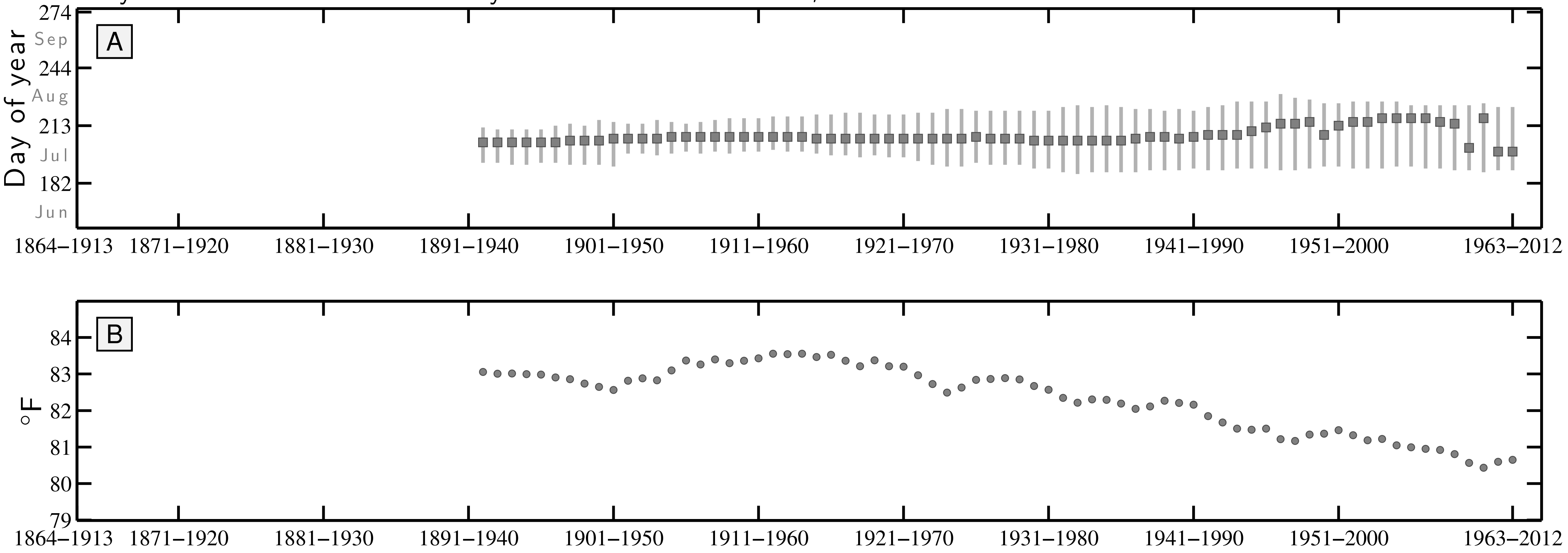

50 year Winter Teletherm dynamics for PEMBINA, ND:

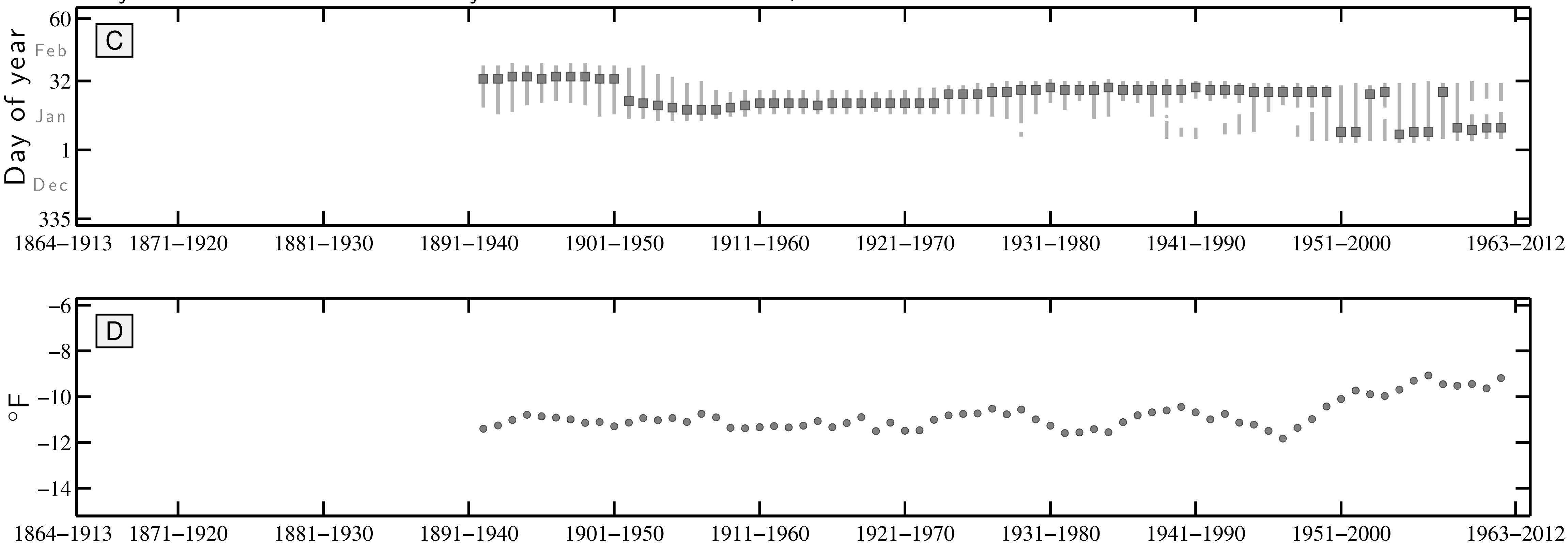

50 year Summer Teletherm dynamics for RICHARDTON ABBEY, ND:

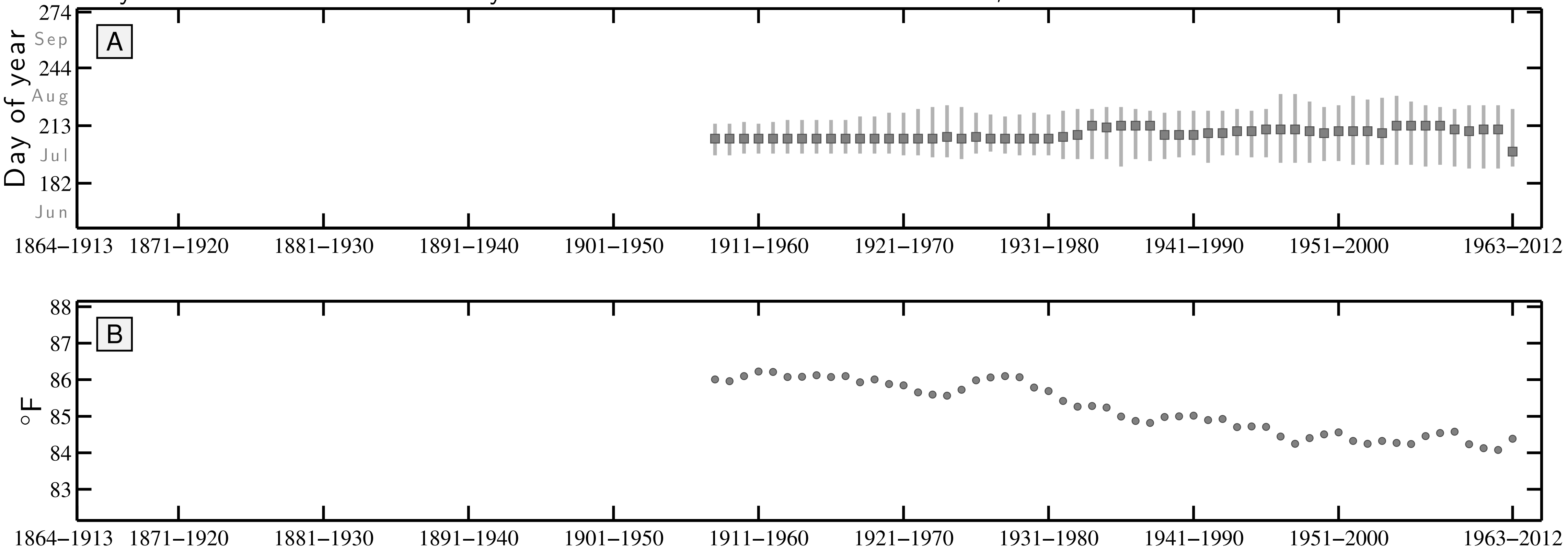

50 year Winter Teletherm dynamics for RICHARDTON ABBEY, ND:

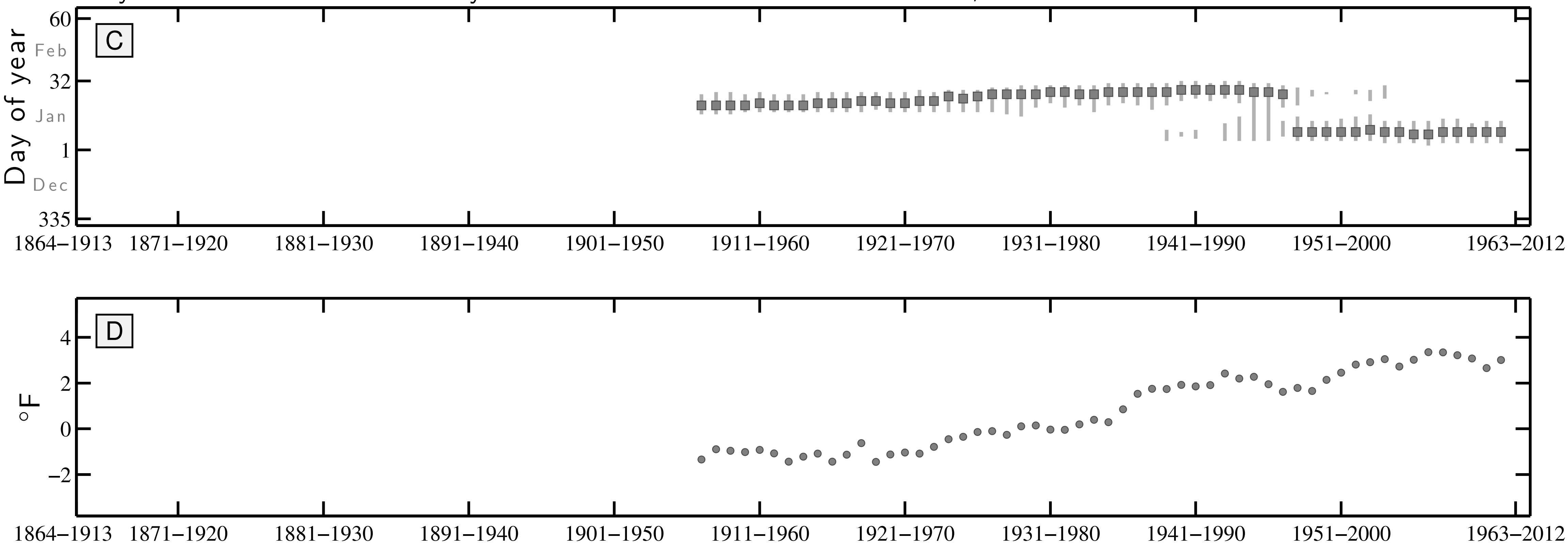

50 year Summer Teletherm dynamics for TOWNER 2 NE, ND:

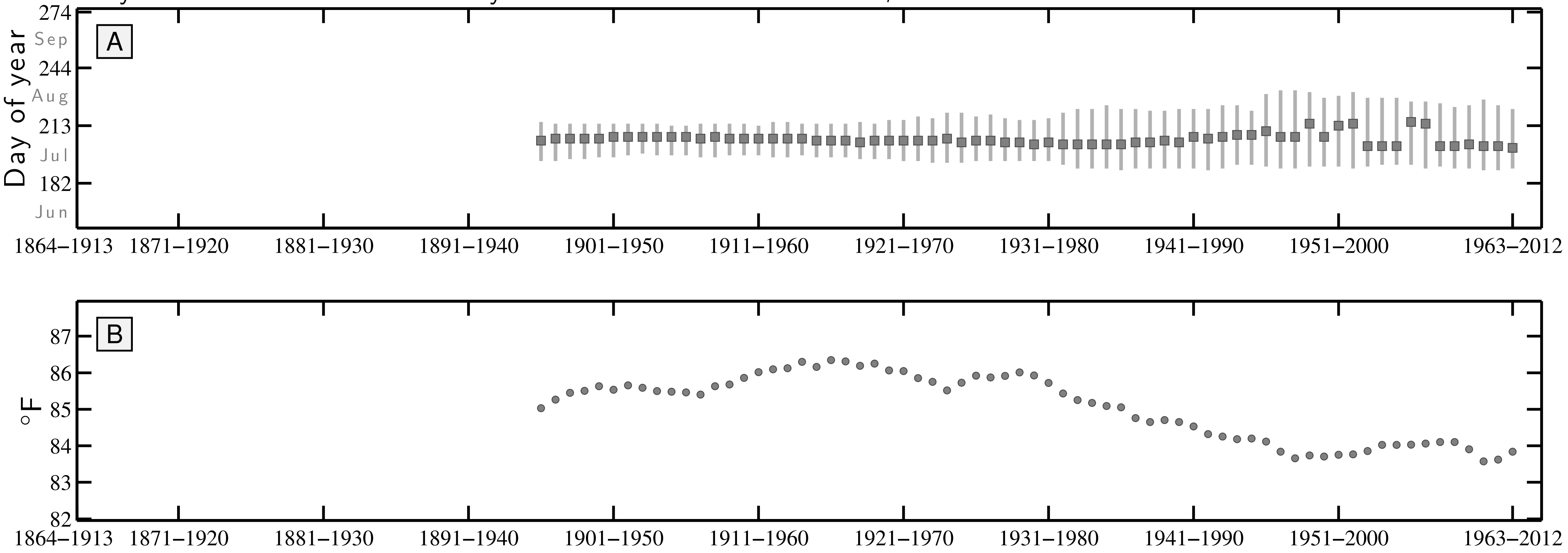

50 year Winter Teletherm dynamics for TOWNER 2 NE, ND:

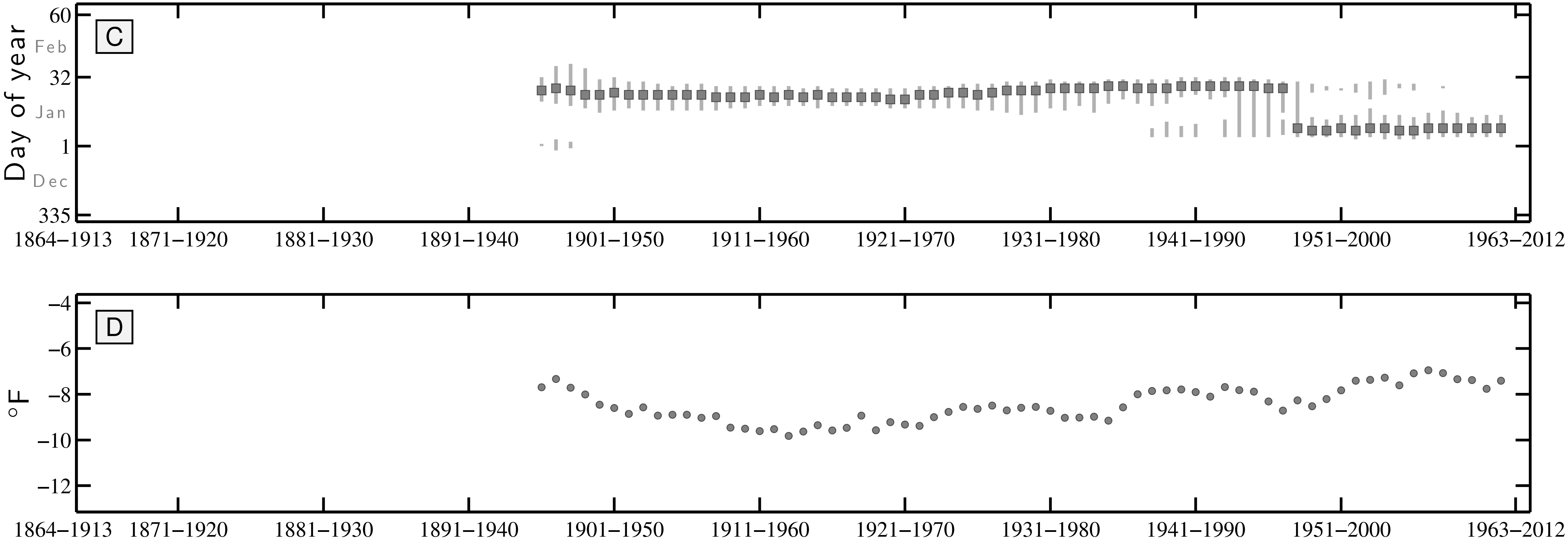

50 year Summer Teletherm dynamics for WAHPETON 3 N, ND:

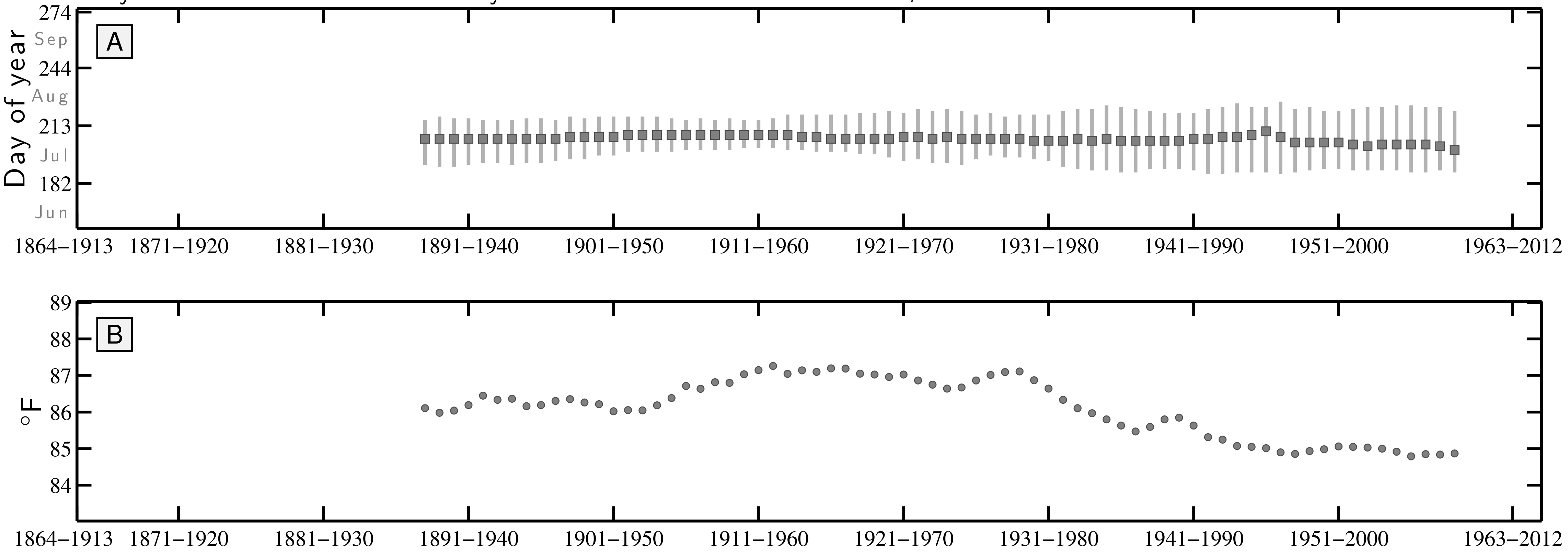

50 year Winter Teletherm dynamics for WAHPETON 3 N, ND:

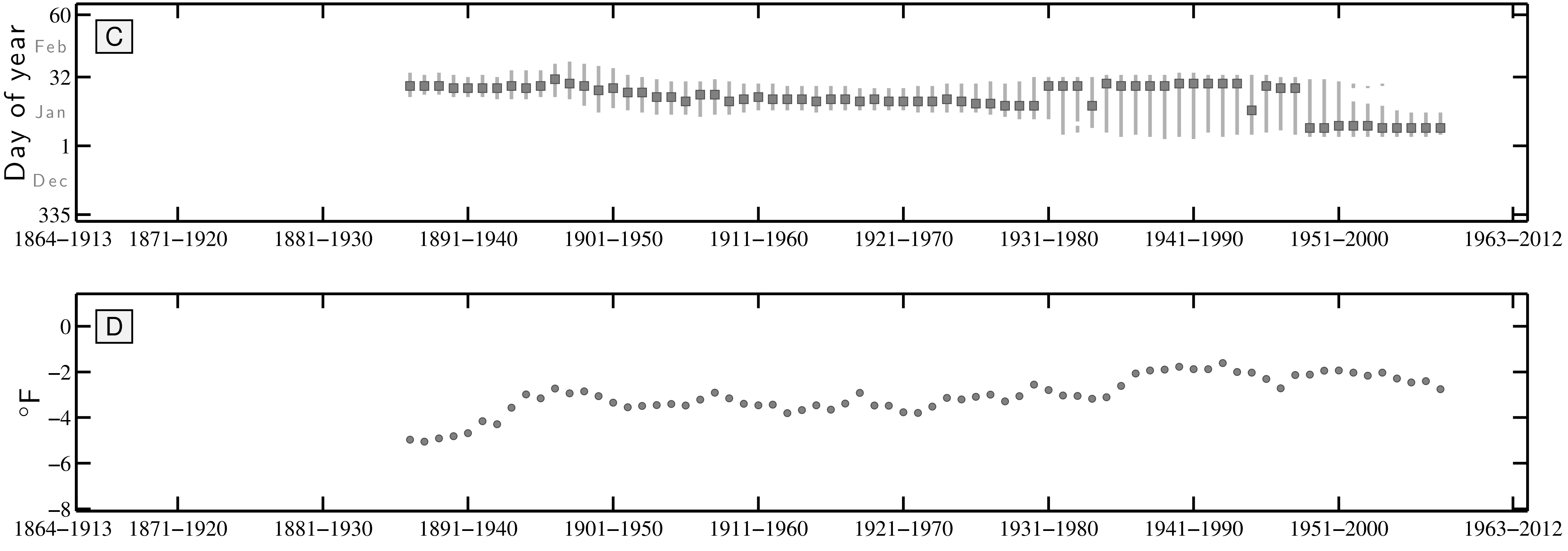

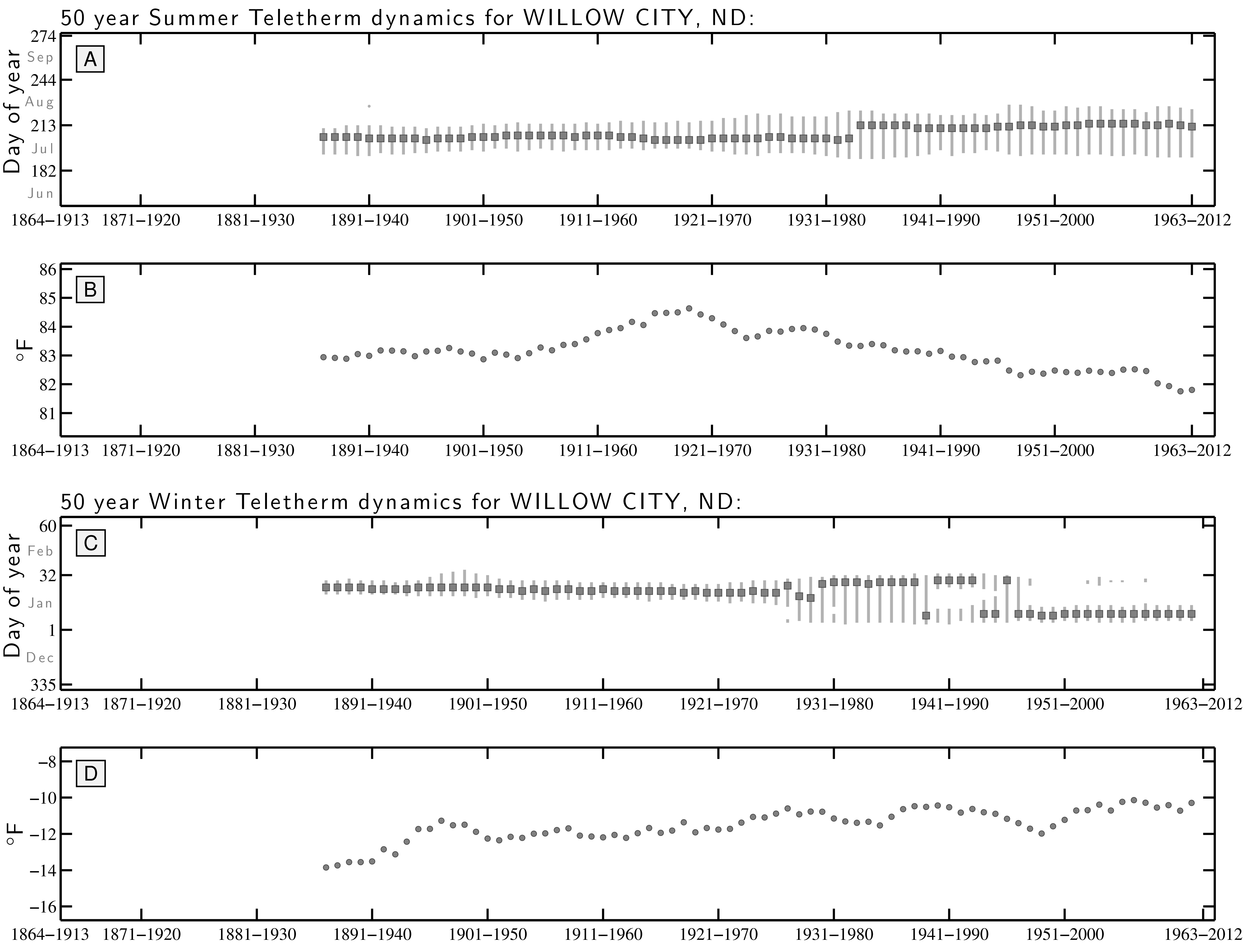

50 year Summer Teletherm dynamics for WILLOW CITY, ND:
A
Day of year
274
Sep
244
Aug
213
Jul
182
Jun
1864–1913
1871–1920
1881–1930
1891–1940
1901–1950
1911–1960
1921–1970
1931–1980
1941–1990
1951–2000
1963–2012
B
°F
86
85
84
83
82
81
50 year Winter Teletherm dynamics for WILLOW CITY, ND:
C
Day of year
60
Feb
32
Jan
1
Dec
335
D
°F
−8
−10
−12
−14
−16

50 year Summer Teletherm dynamics for BUCYRUS, OH:

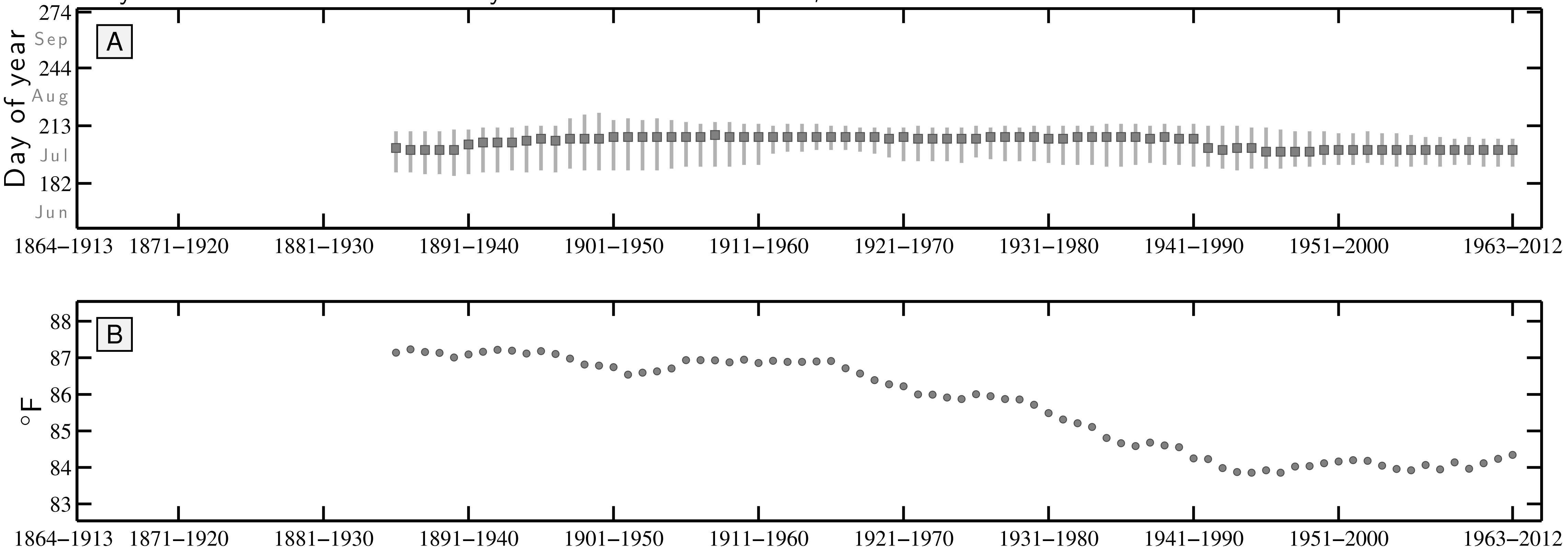

50 year Winter Teletherm dynamics for BUCYRUS, OH:

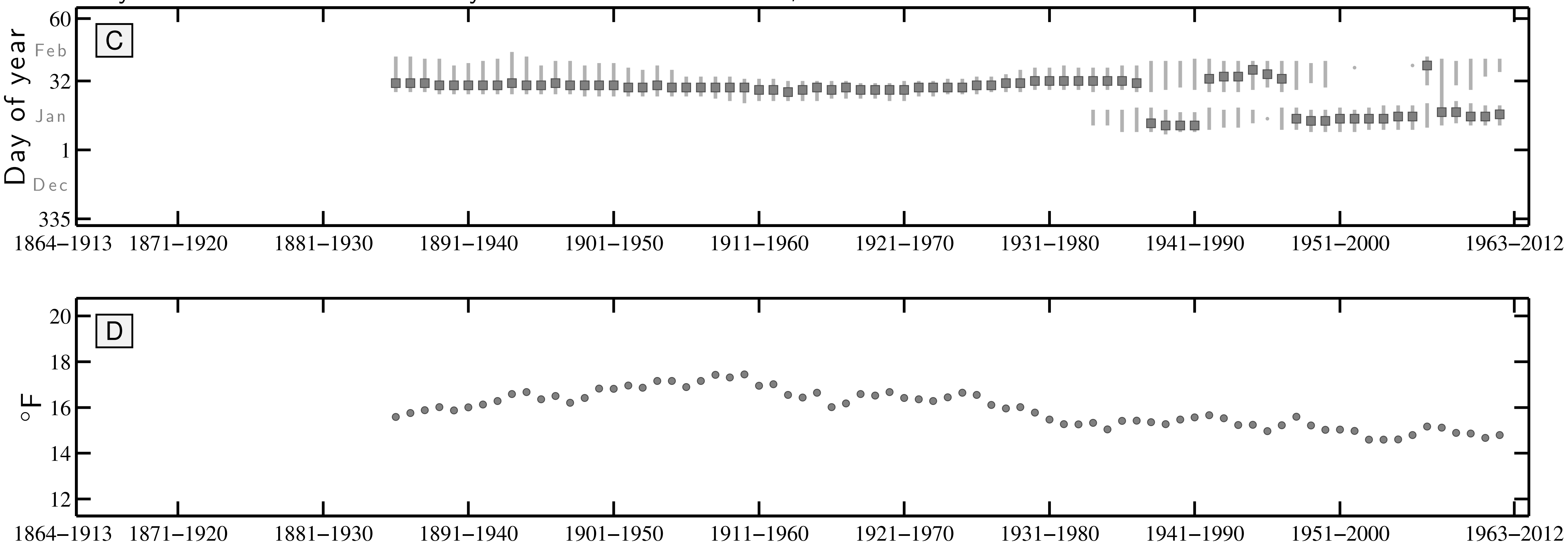

50 year Summer Teletherm dynamics for CADIZ, OH:

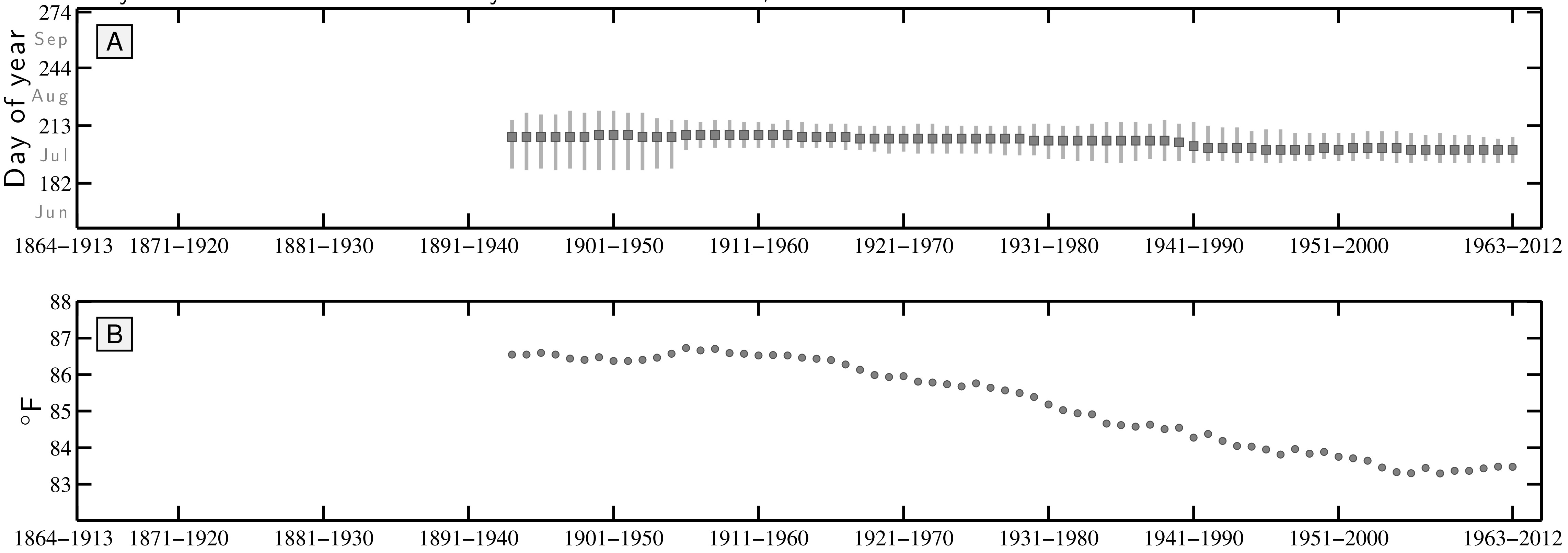

50 year Winter Teletherm dynamics for CADIZ, OH:

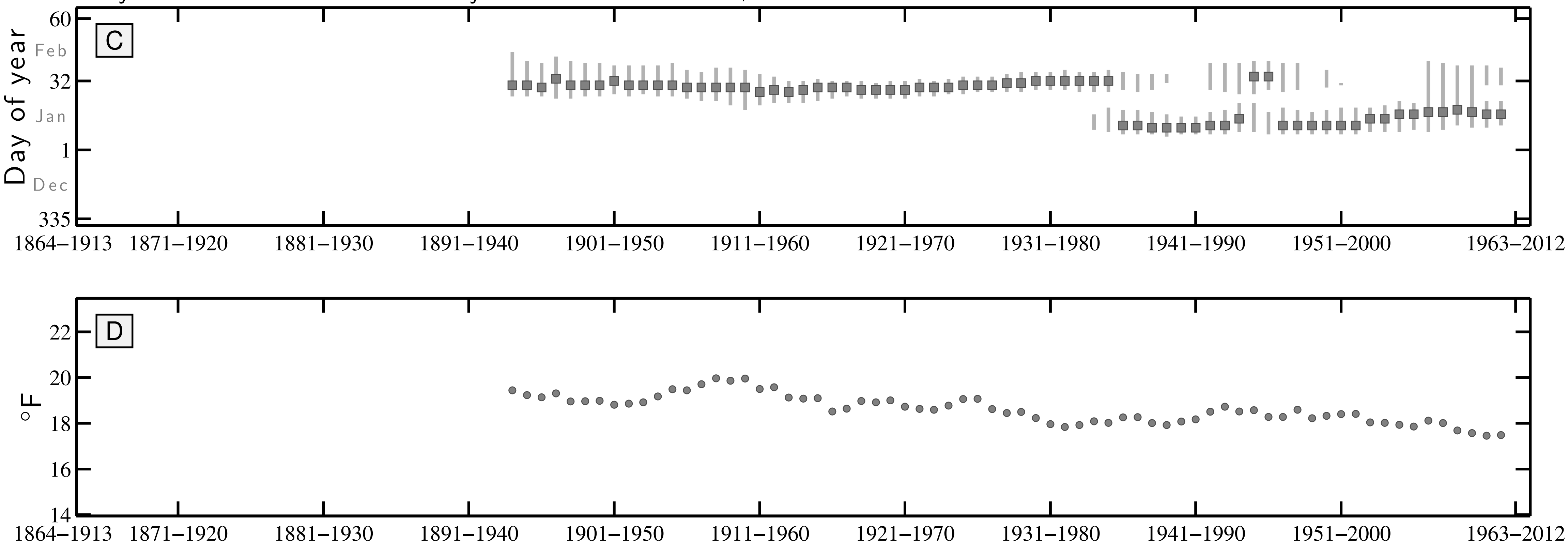

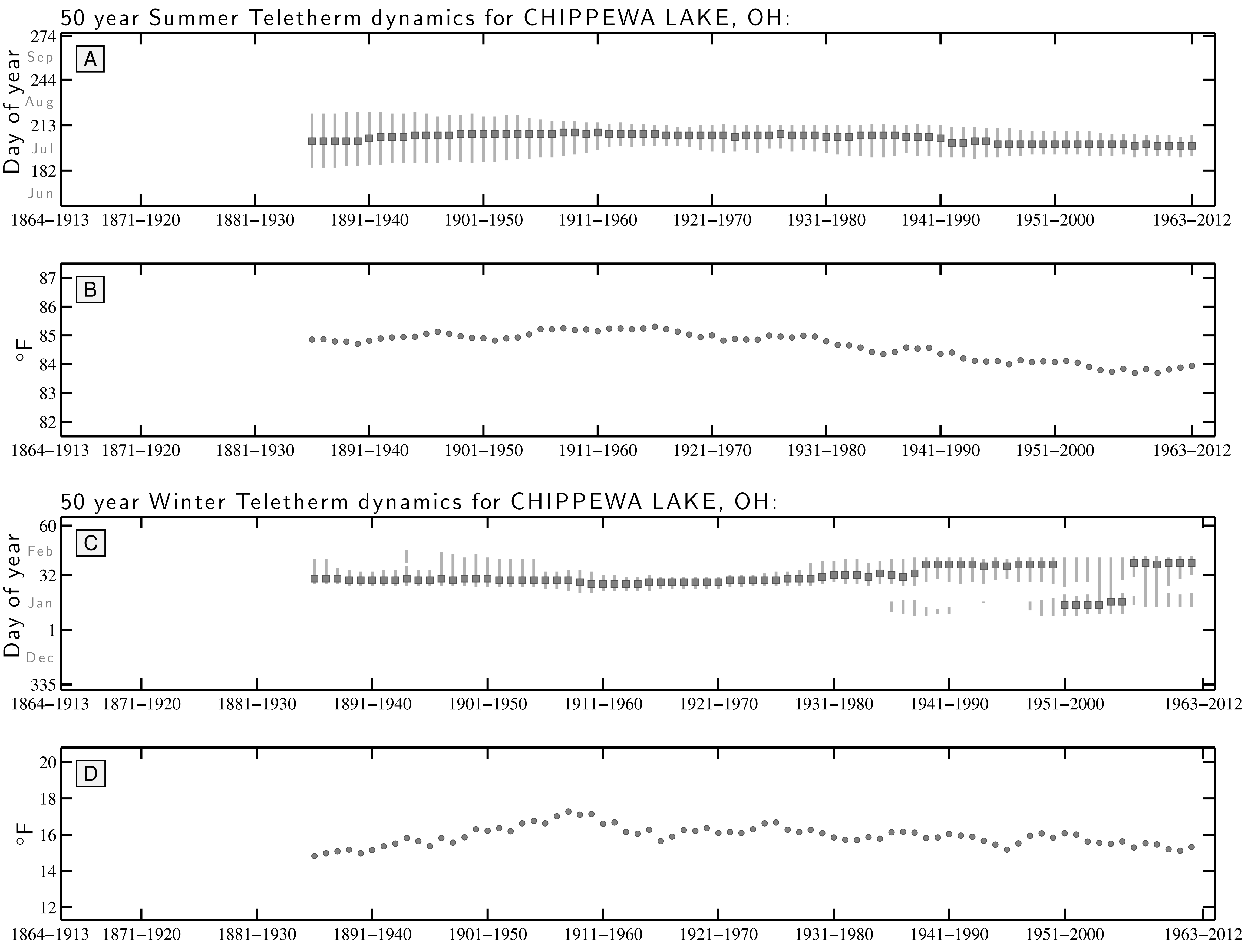
50 year Summer Teletherm dynamics for CHIPPEWA LAKE, OH:
A
Day of year
274
Sep
244
Aug
213
Jul
182
Jun
1864–1913
1871–1920
1881–1930
1891–1940
1901–1950
1911–1960
1921–1970
1931–1980
1941–1990
1951–2000
1963–2012
B
°F
87
86
85
84
83
82
50 year Winter Teletherm dynamics for CHIPPEWA LAKE, OH:
C
60
Feb
32
Jan
1
Dec
335
D
20
18
16
14
12

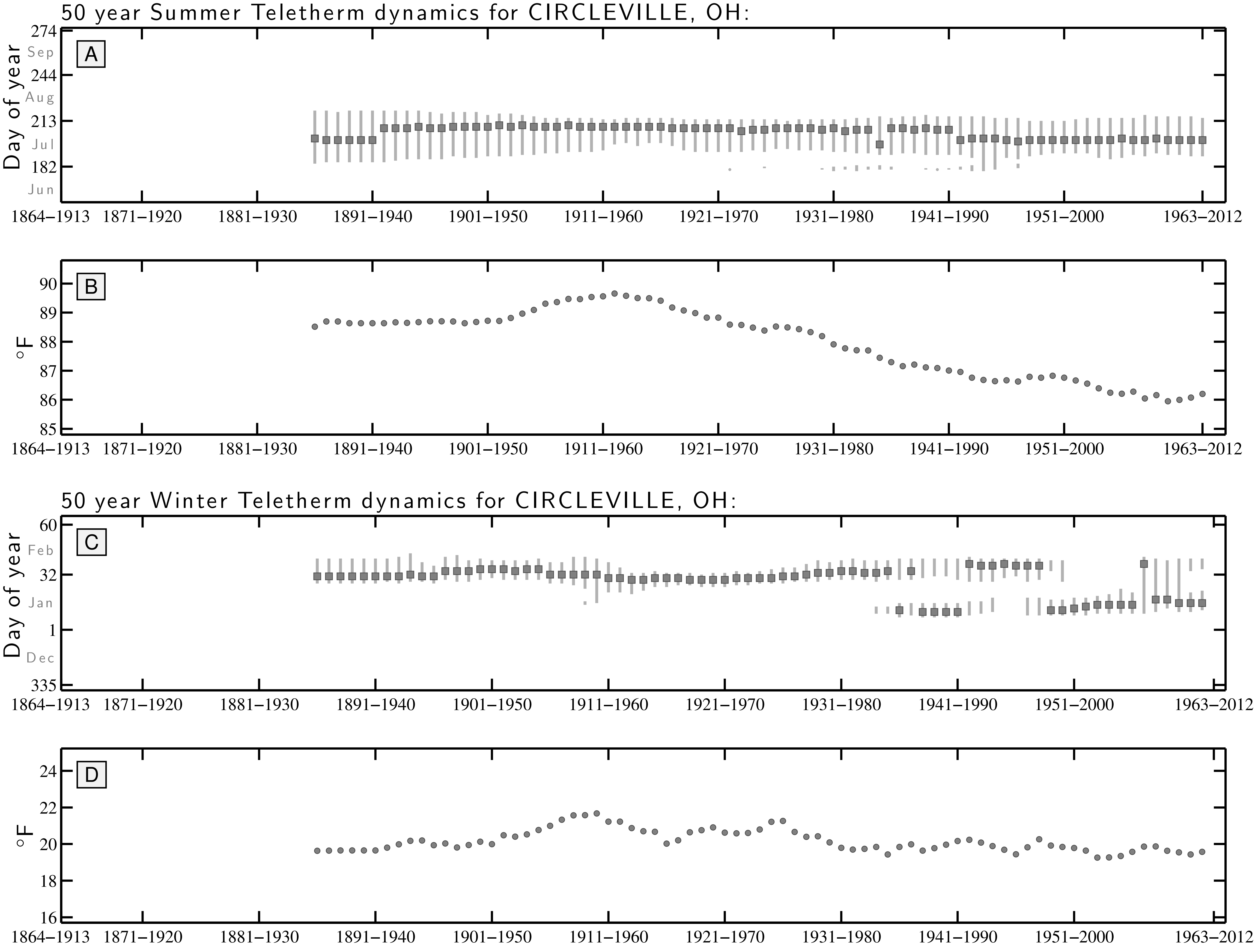

50 year Summer Teletherm dynamics for CIRCLEVILLE, OH:
A
B
Day of year
°F
274
Sep
244
Aug
213
Jul
182
Jun
90
89
88
87
86
85
1864–1913
1871–1920
1881–1930
1891–1940
1901–1950
1911–1960
1921–1970
1931–1980
1941–1990
1951–2000
1963–2012
50 year Winter Teletherm dynamics for CIRCLEVILLE, OH:
C
D
60
Feb
32
Jan
1
Dec
335
24
22
20
18
16

50 year Summer Teletherm dynamics for COSHOCTON WPC PLT, OH:

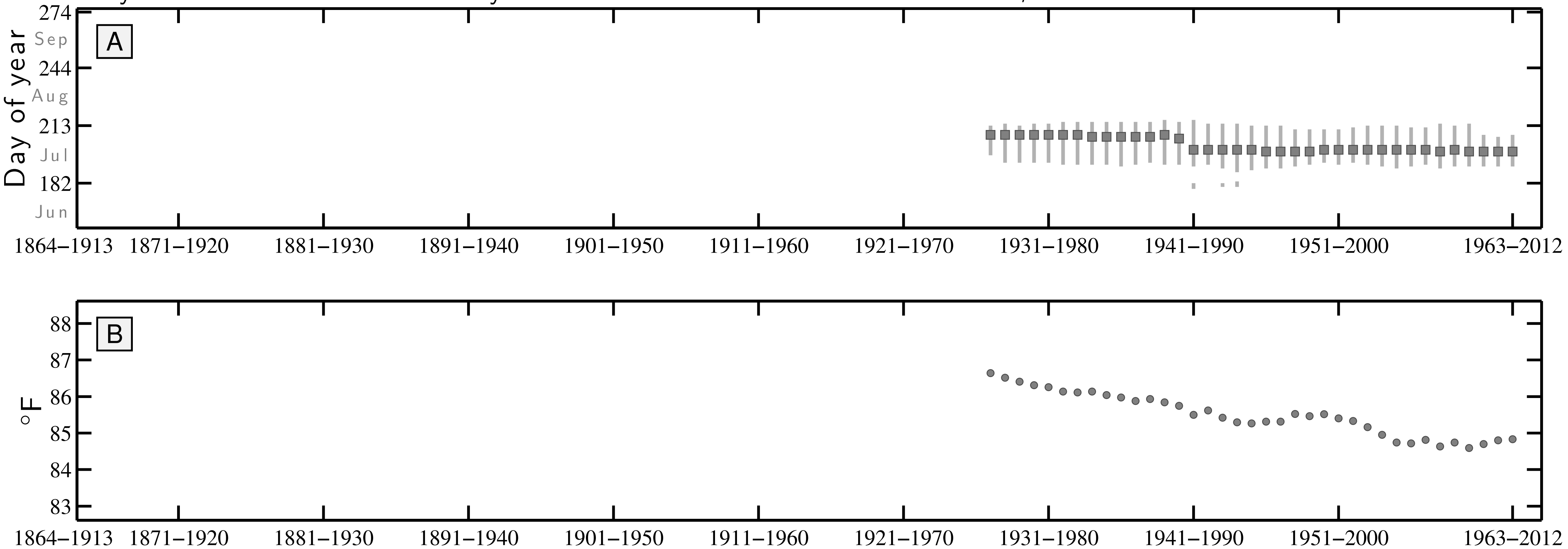

50 year Winter Teletherm dynamics for COSHOCTON WPC PLT, OH:

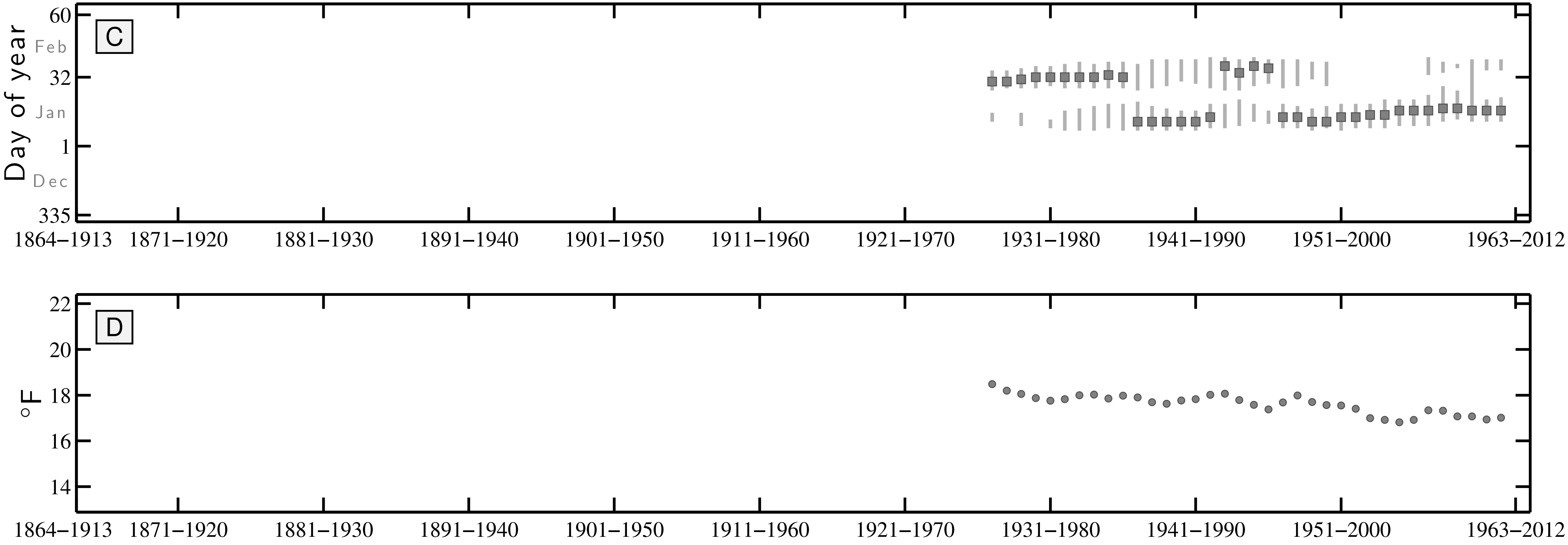

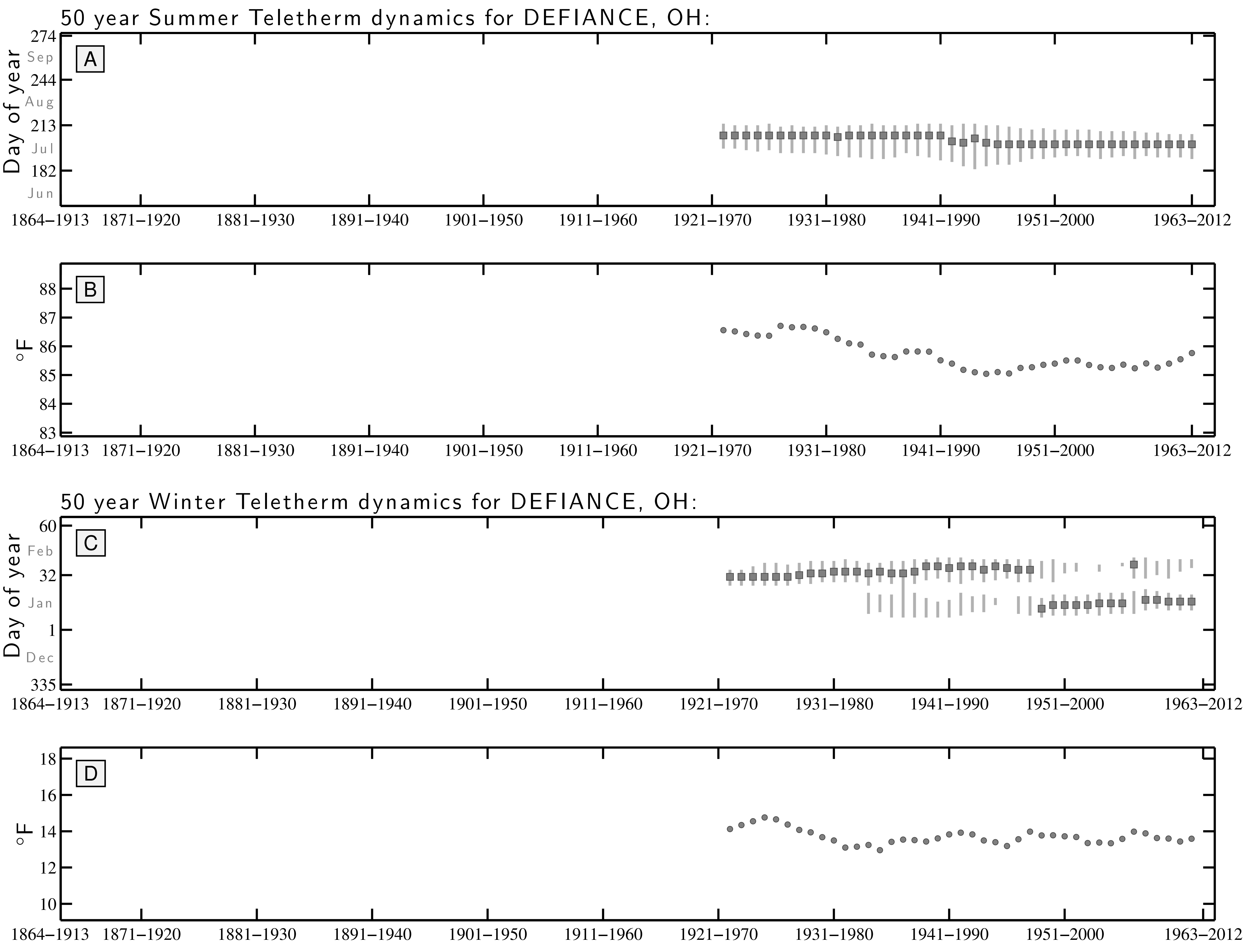

50 year Summer Teletherm dynamics for DEFIANCE, OH:
A
Day of year
274
Sep
244
Aug
213
Jul
182
Jun
1864–1913
1871–1920
1881–1930
1891–1940
1901–1950
1911–1960
1921–1970
1931–1980
1941–1990
1951–2000
1963–2012
B
°F
88
87
86
85
84
83
50 year Winter Teletherm dynamics for DEFIANCE, OH:
C
Day of year
60
Feb
32
Jan
1
Dec
335
D
°F
18
16
14
12
10

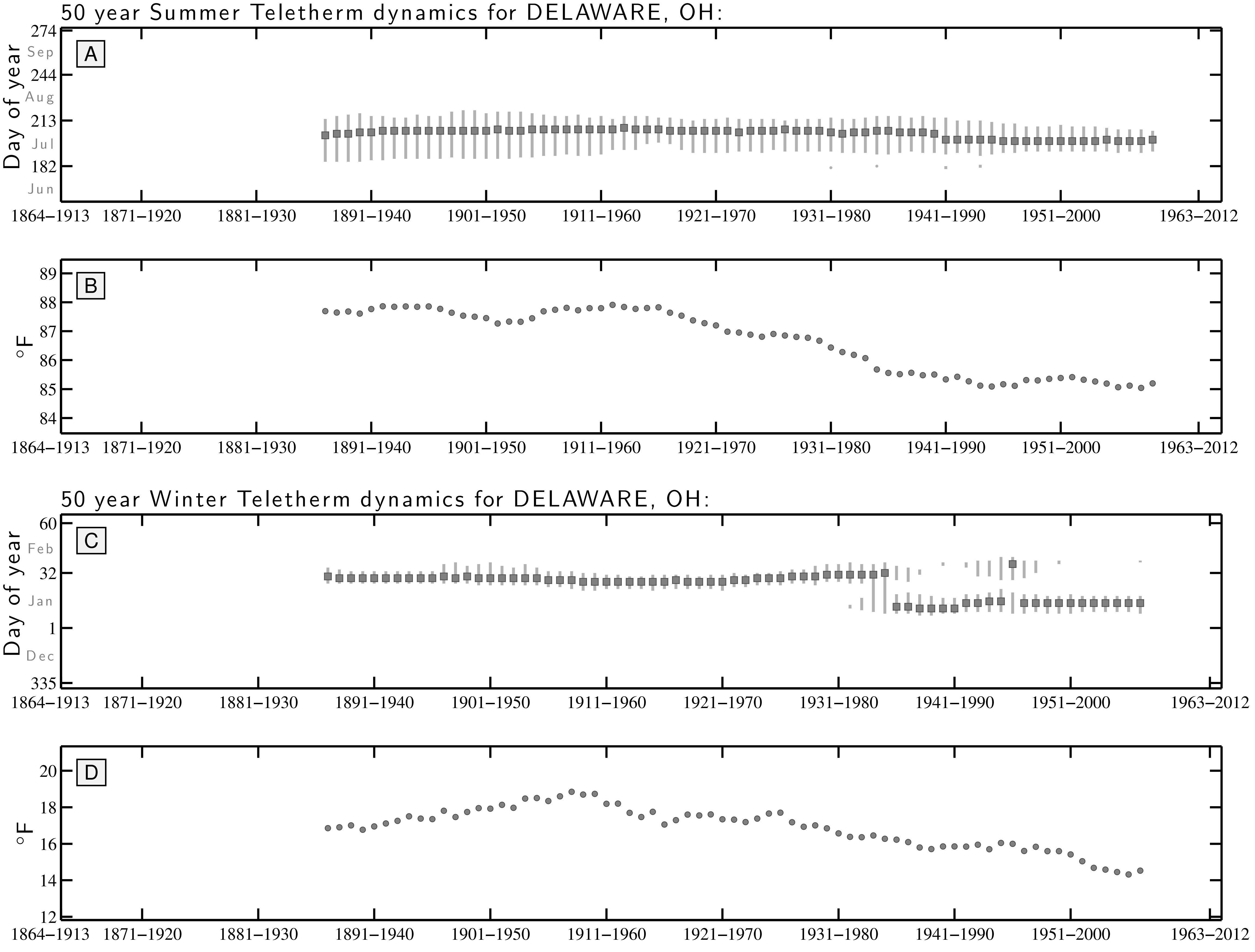

50 year Summer Teletherm dynamics for DELAWARE, OH:
A
Day of year
274
Sep
244
Aug
213
Jul
182
Jun
B
°F
89
88
87
86
85
84
50 year Winter Teletherm dynamics for DELAWARE, OH:
C
Day of year
60
Feb
32
Jan
1
Dec
335
D
°F
20
18
16
14
12
1864–1913
1871–1920
1881–1930
1891–1940
1901–1950
1911–1960
1921–1970
1931–1980
1941–1990
1951–2000
1963–2012

50 year Summer Teletherm dynamics for FINDLAY WPCC, OH:

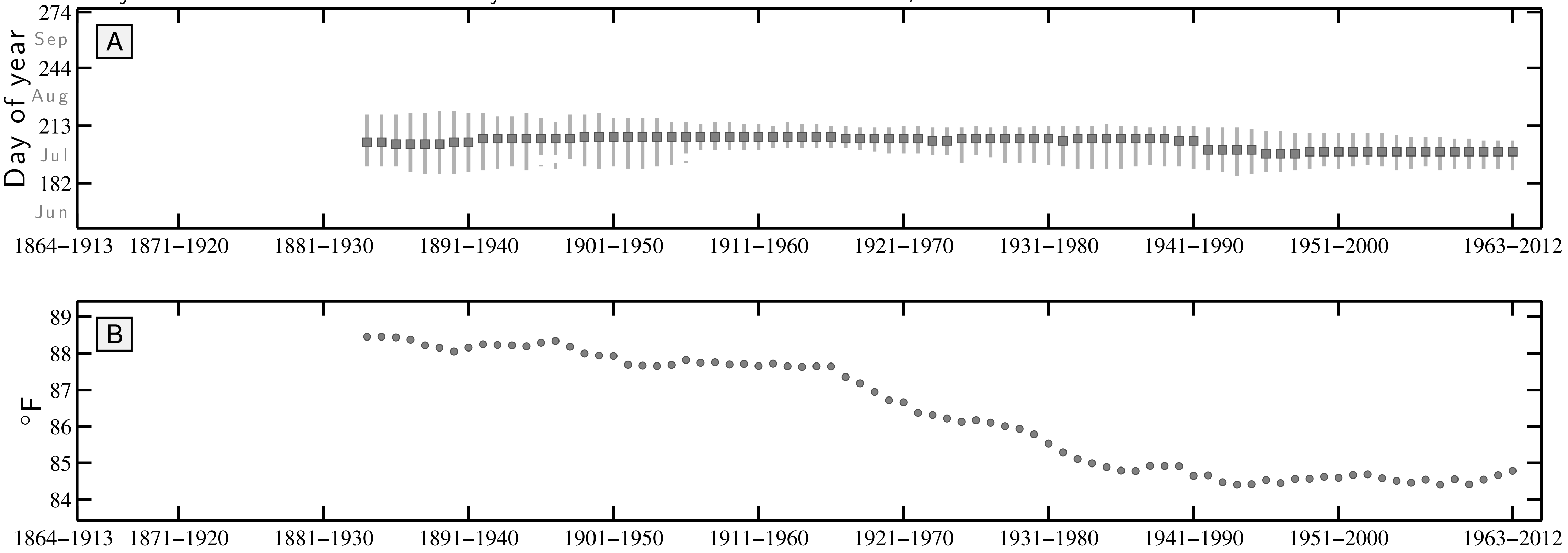

50 year Winter Teletherm dynamics for FINDLAY WPCC, OH:

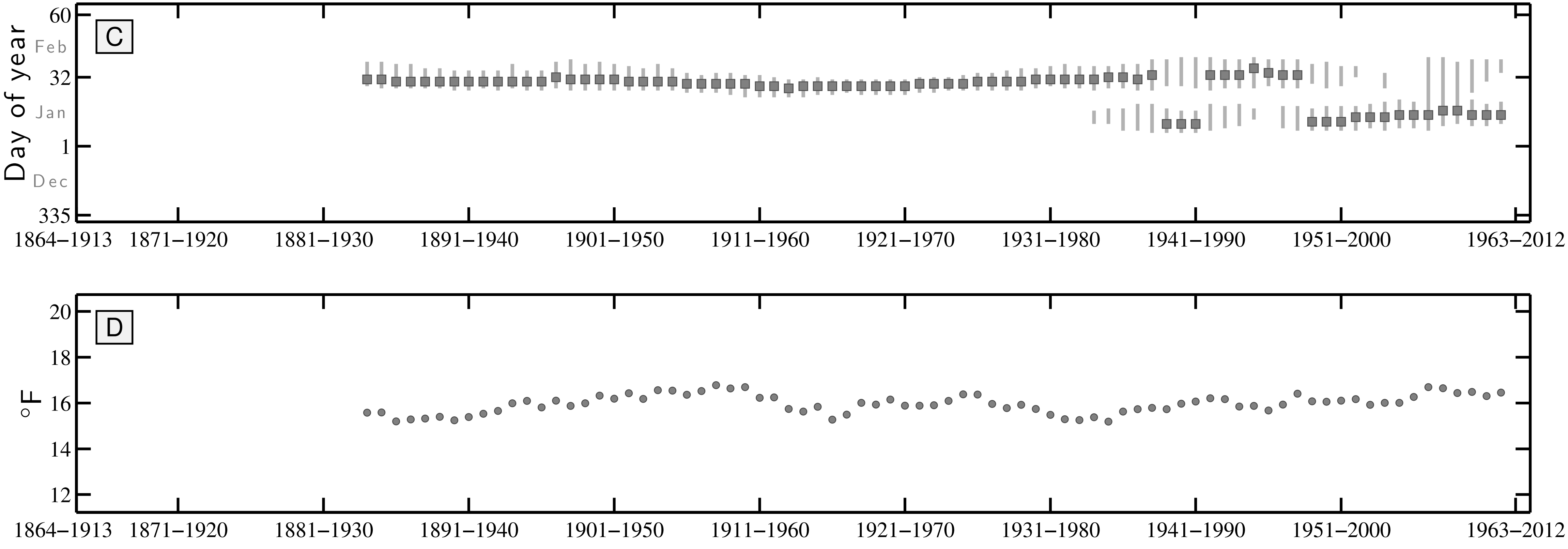

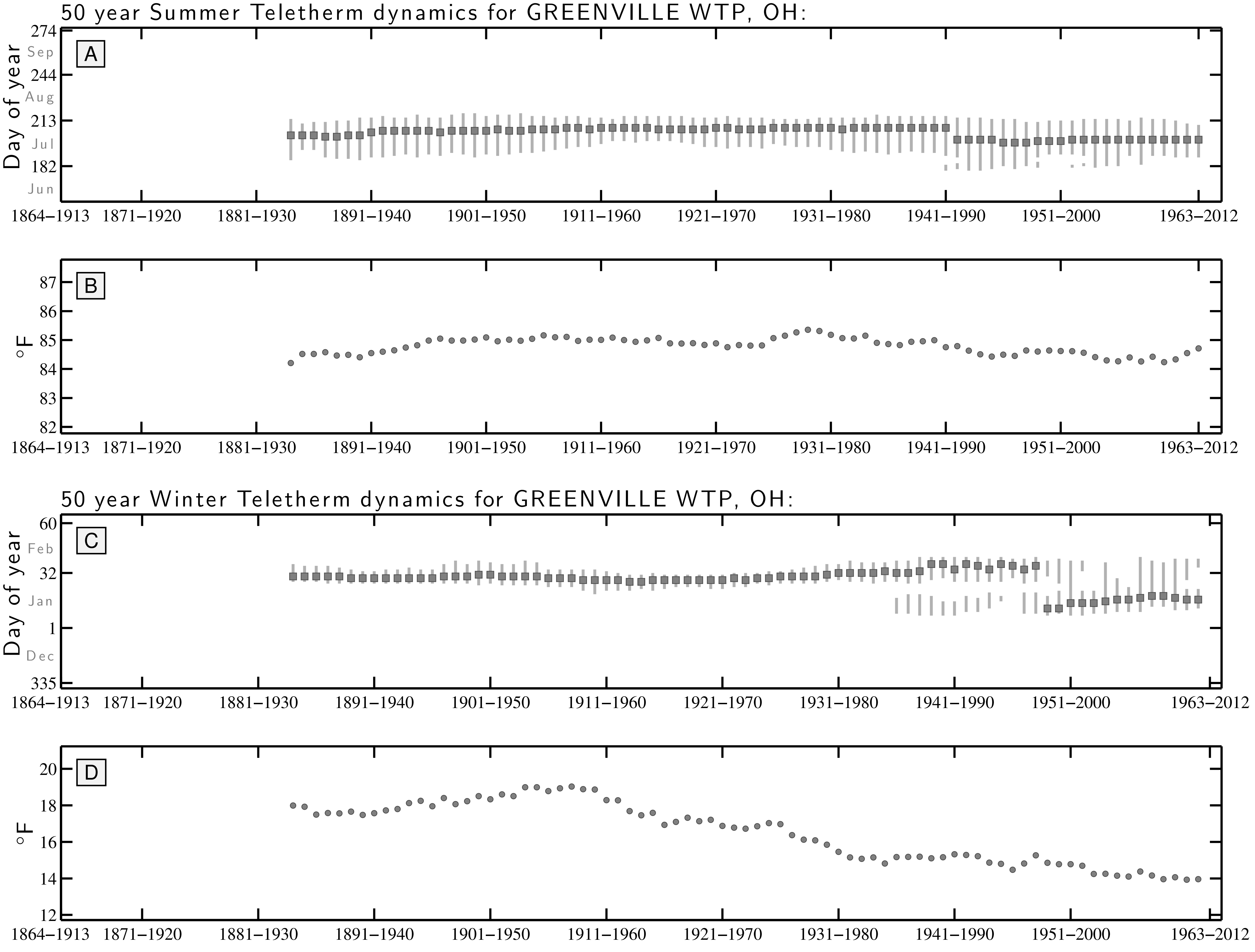
50 year Summer Teletherm dynamics for GREENVILLE WTP, OH:
A
B
Day of year
°F
274
244
213
182
Sep
Aug
Jul
Jun
87
86
85
84
83
82
1864–1913
1871–1920
1881–1930
1891–1940
1901–1950
1911–1960
1921–1970
1931–1980
1941–1990
1951–2000
1963–2012
50 year Winter Teletherm dynamics for GREENVILLE WTP, OH:
C
D
60
32
1
335
Feb
Jan
Dec
20
18
16
14
12

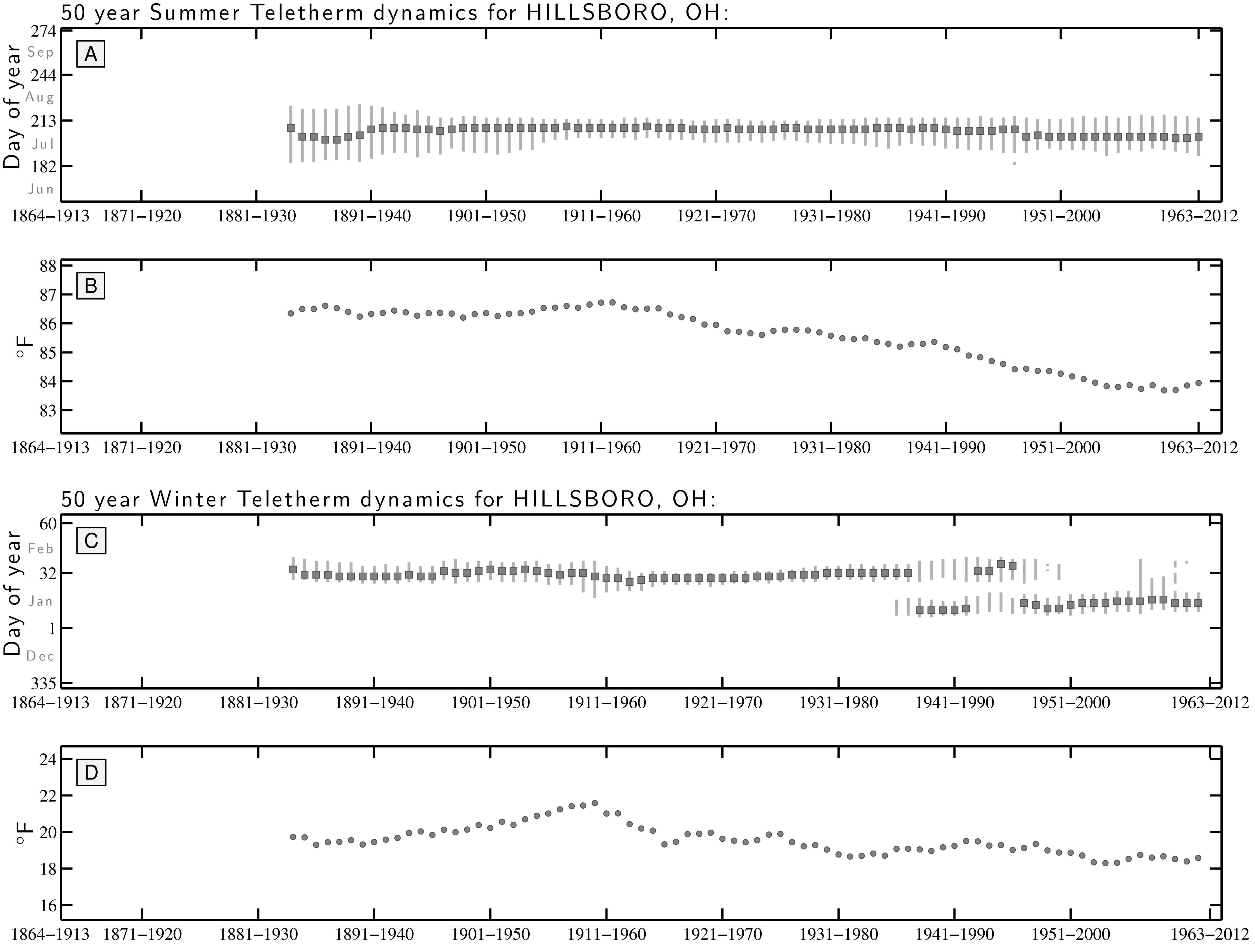

50 year Summer Teletherm dynamics for HILLSBORO, OH:
A
Day of year
274
Sep
244
Aug
213
Jul
182
Jun
B
°F
88
87
86
85
84
83
50 year Winter Teletherm dynamics for HILLSBORO, OH:
C
Day of year
60
Feb
32
Jan
1
Dec
335
D
°F
24
22
20
18
16
1864–1913
1871–1920
1881–1930
1891–1940
1901–1950
1911–1960
1921–1970
1931–1980
1941–1990
1951–2000
1963–2012

50 year Summer Teletherm dynamics for HIRAM, OH:

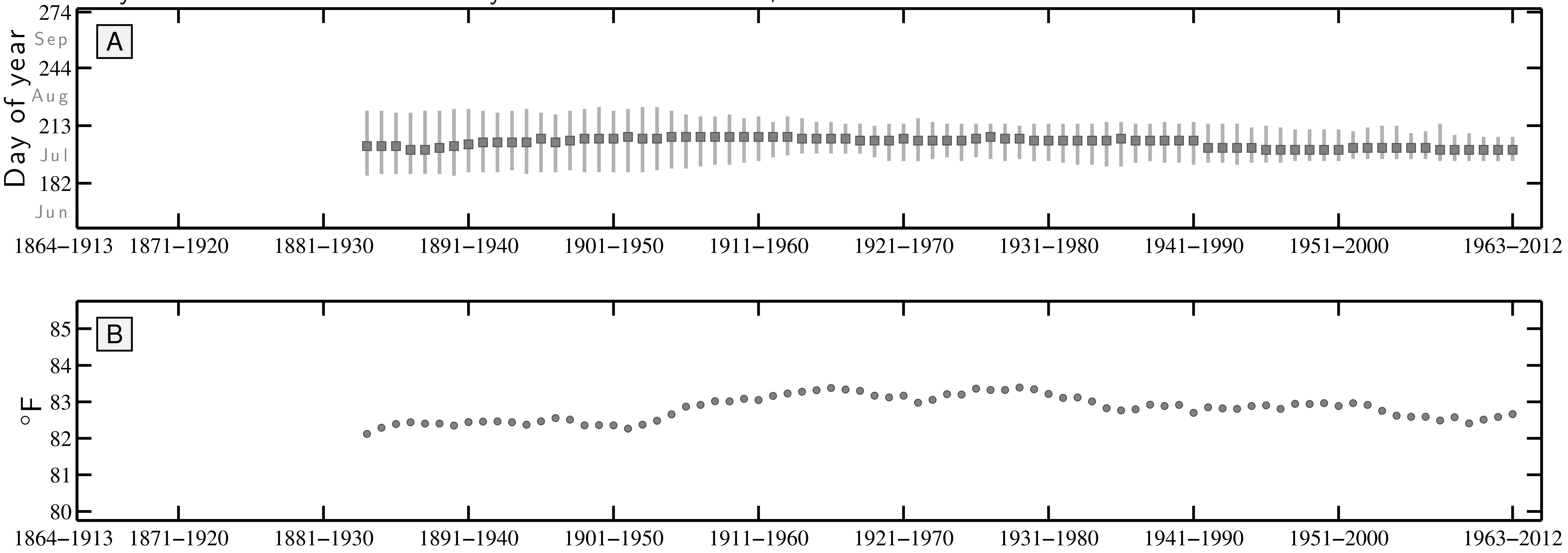

50 year Winter Teletherm dynamics for HIRAM, OH:

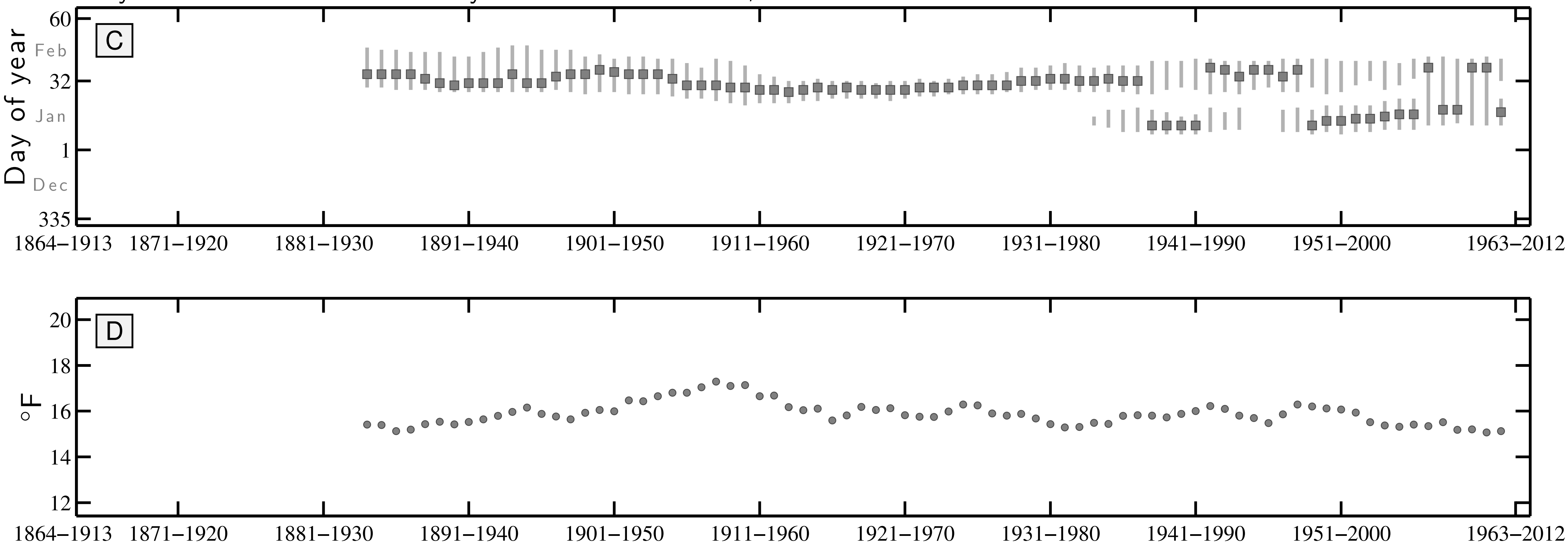

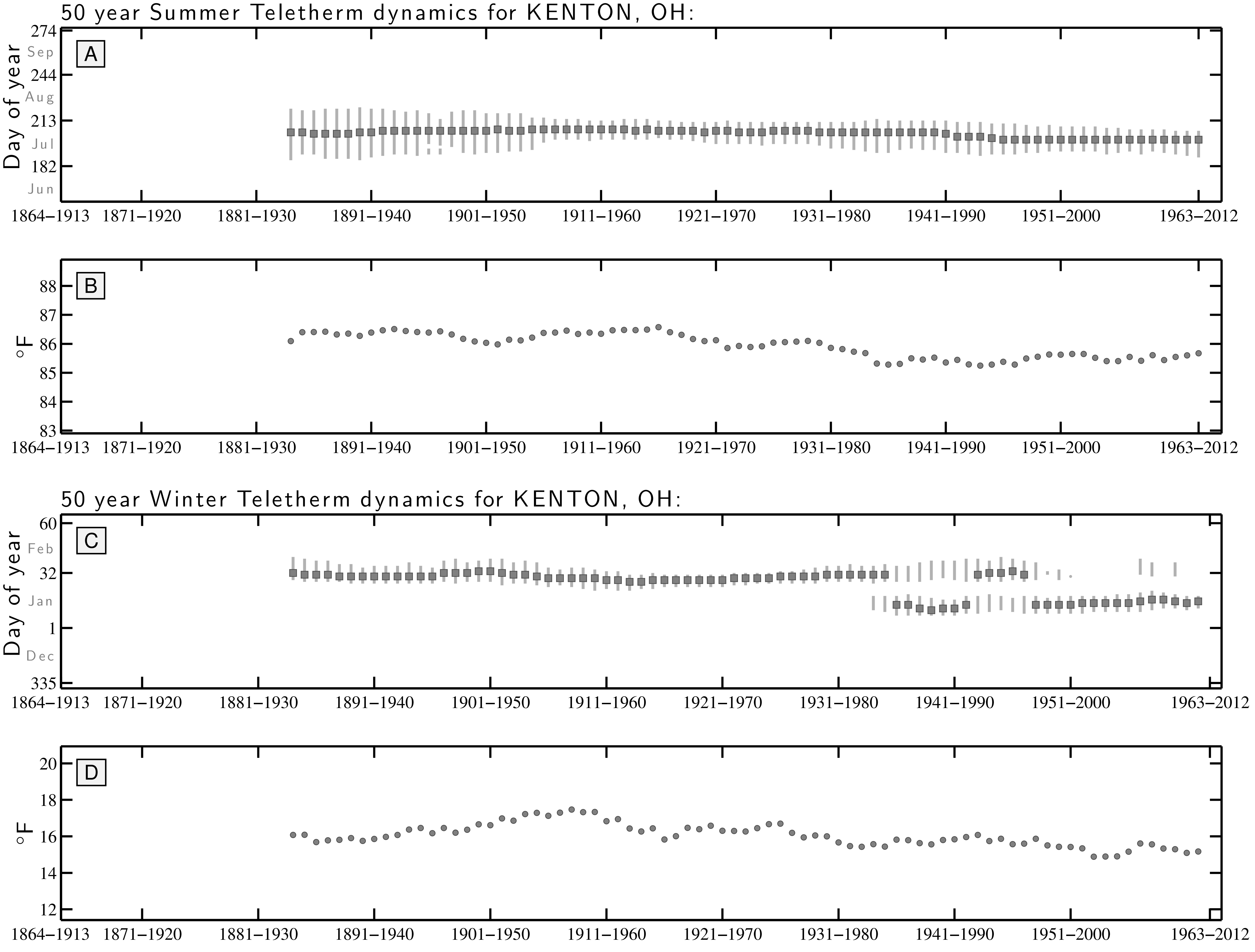
50 year Summer Teletherm dynamics for KENTON, OH:
A
B
Day of year
274
Sep
244
Aug
213
Jul
182
Jun
°F
88
87
86
85
84
83
1864–1913
1871–1920
1881–1930
1891–1940
1901–1950
1911–1960
1921–1970
1931–1980
1941–1990
1951–2000
1963–2012
50 year Winter Teletherm dynamics for KENTON, OH:
C
D
60
Feb
32
Jan
1
Dec
335
20
18
16
14
12

50 year Summer Teletherm dynamics for MC CONNELLSVILLE LK 7, OH:

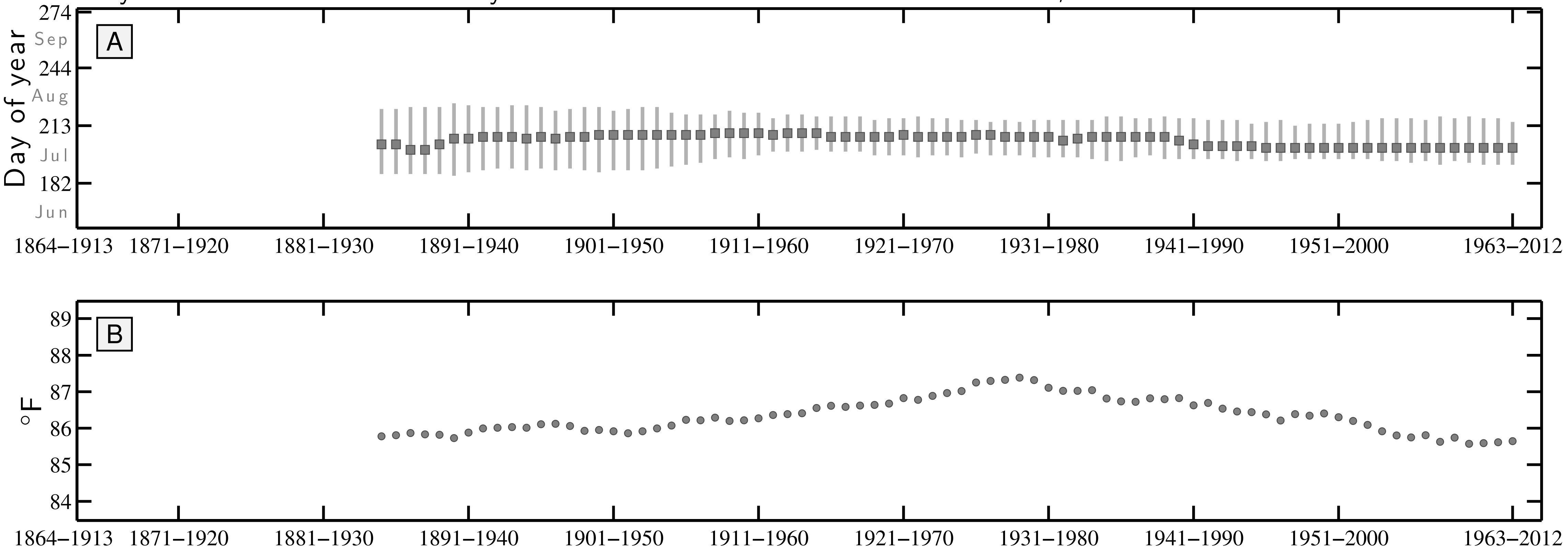

50 year Winter Teletherm dynamics for MC CONNELLSVILLE LK 7, OH:

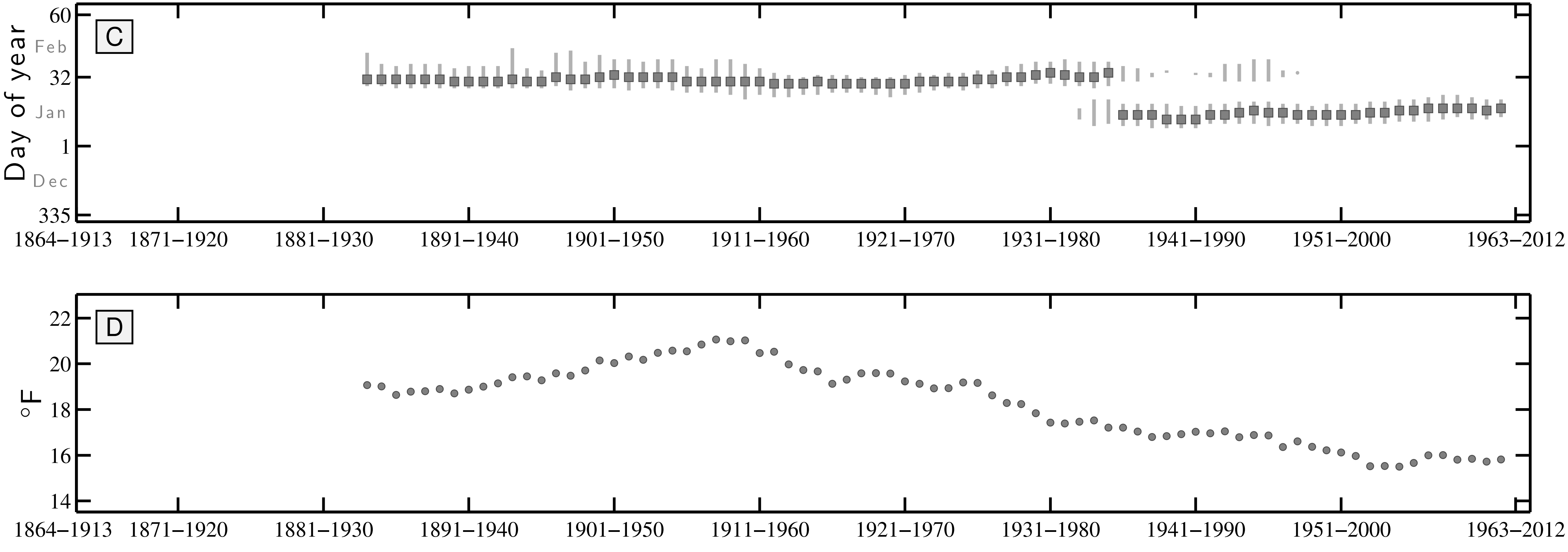

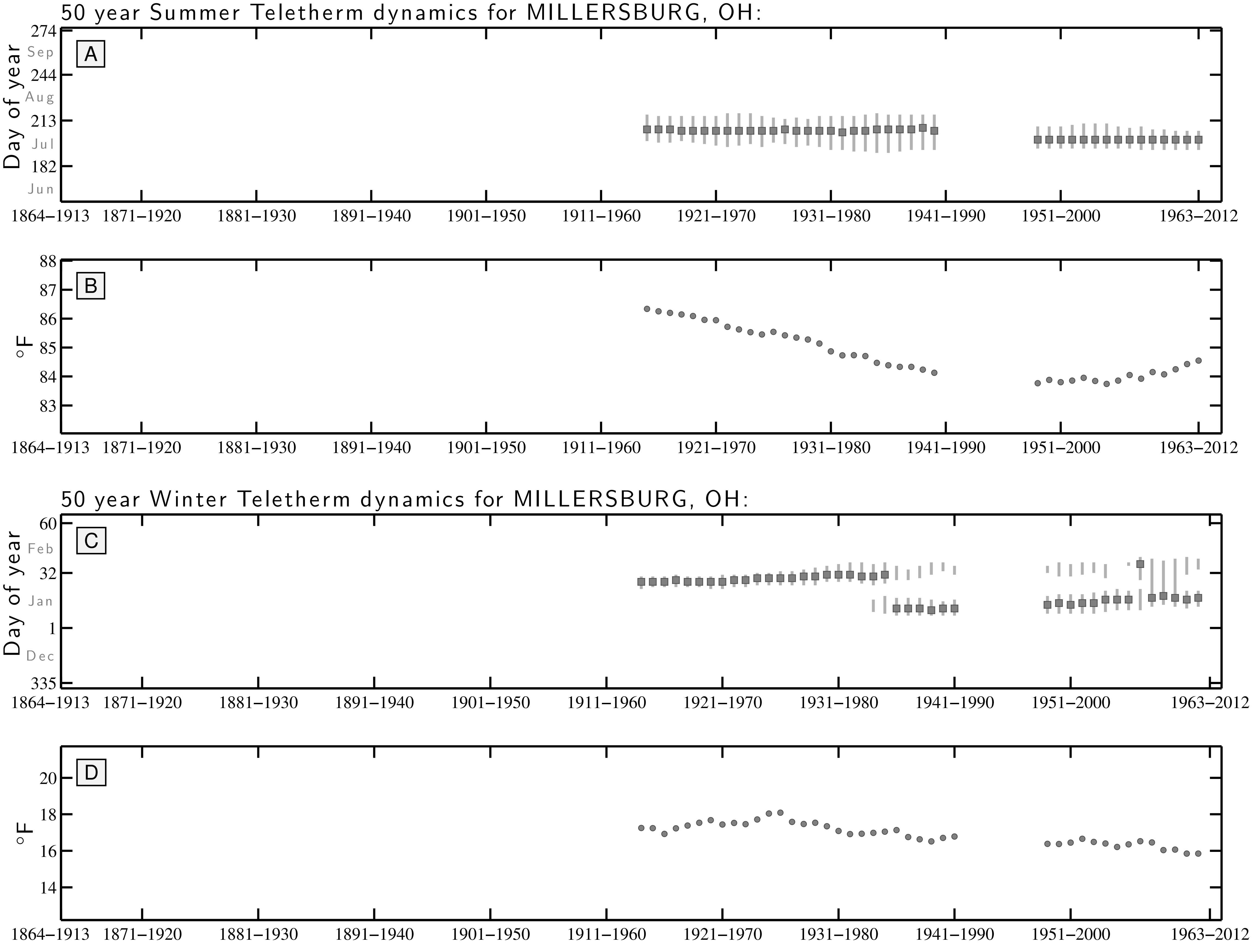

50 year Summer Teletherm dynamics for MILLERSBURG, OH:
A
B
Day of year
°F
274
Sep
244
Aug
213
Jul
182
Jun
88
87
86
85
84
83
50 year Winter Teletherm dynamics for MILLERSBURG, OH:
C
D
60
Feb
32
Jan
1
Dec
335
20
18
16
14
1864–1913
1871–1920
1881–1930
1891–1940
1901–1950
1911–1960
1921–1970
1931–1980
1941–1990
1951–2000
1963–2012

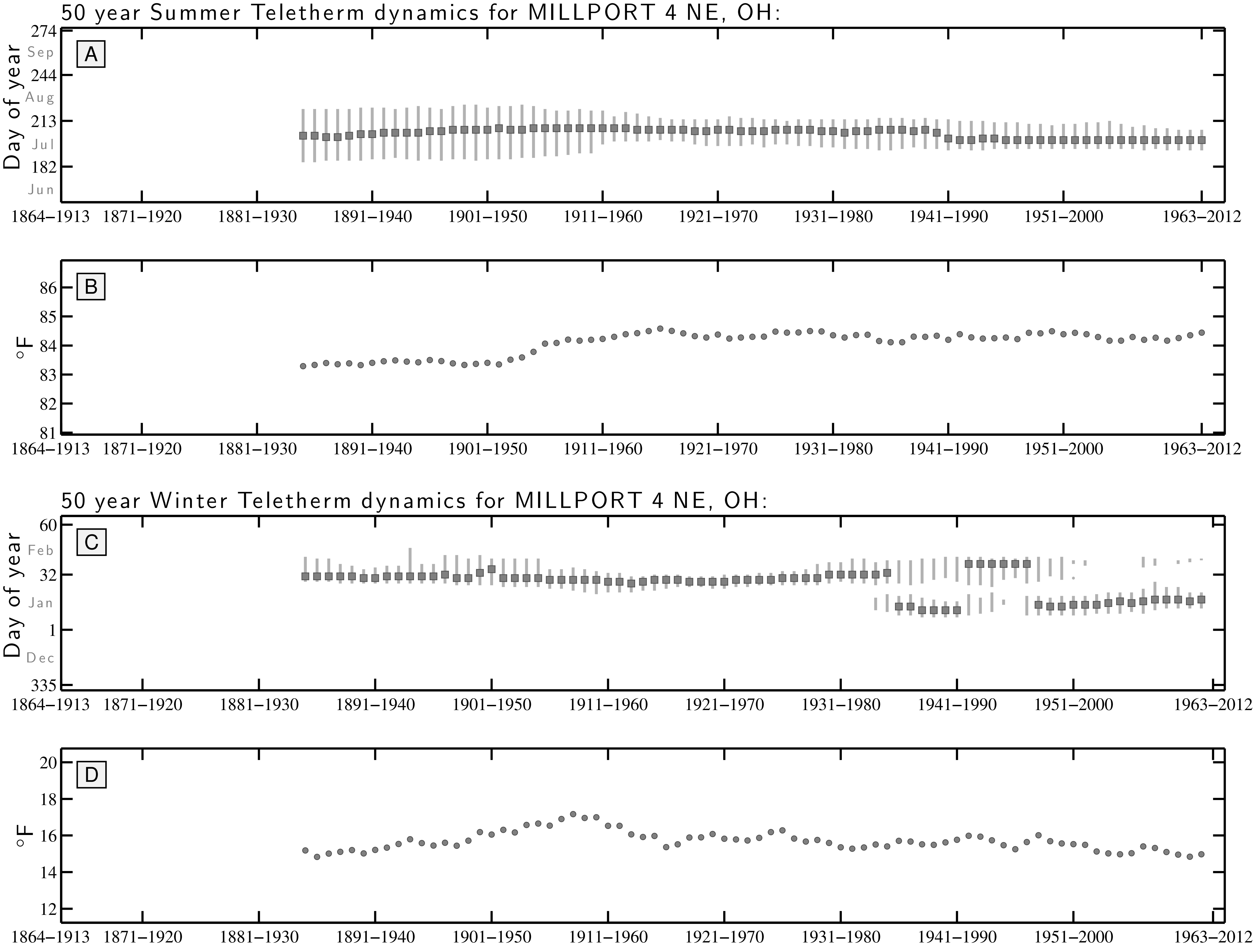

50 year Summer Teletherm dynamics for MILLPORT 4 NE, OH:
A
Day of year
274
Sep
244
Aug
213
Jul
182
Jun
B
°F
86
85
84
83
82
81
50 year Winter Teletherm dynamics for MILLPORT 4 NE, OH:
C
Day of year
60
Feb
32
Jan
1
Dec
335
D
°F
20
18
16
14
12
1864–1913
1871–1920
1881–1930
1891–1940
1901–1950
1911–1960
1921–1970
1931–1980
1941–1990
1951–2000
1963–2012

50 year Summer Teletherm dynamics for NORWALK WWTP, OH:

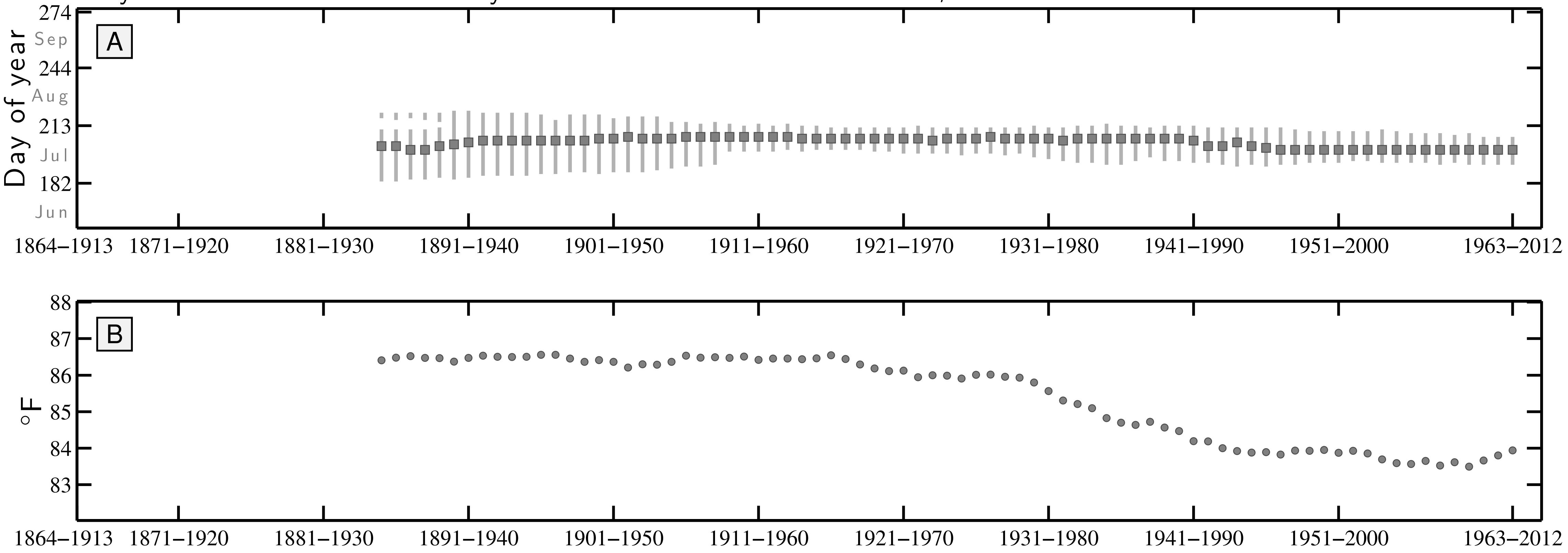

50 year Winter Teletherm dynamics for NORWALK WWTP, OH:

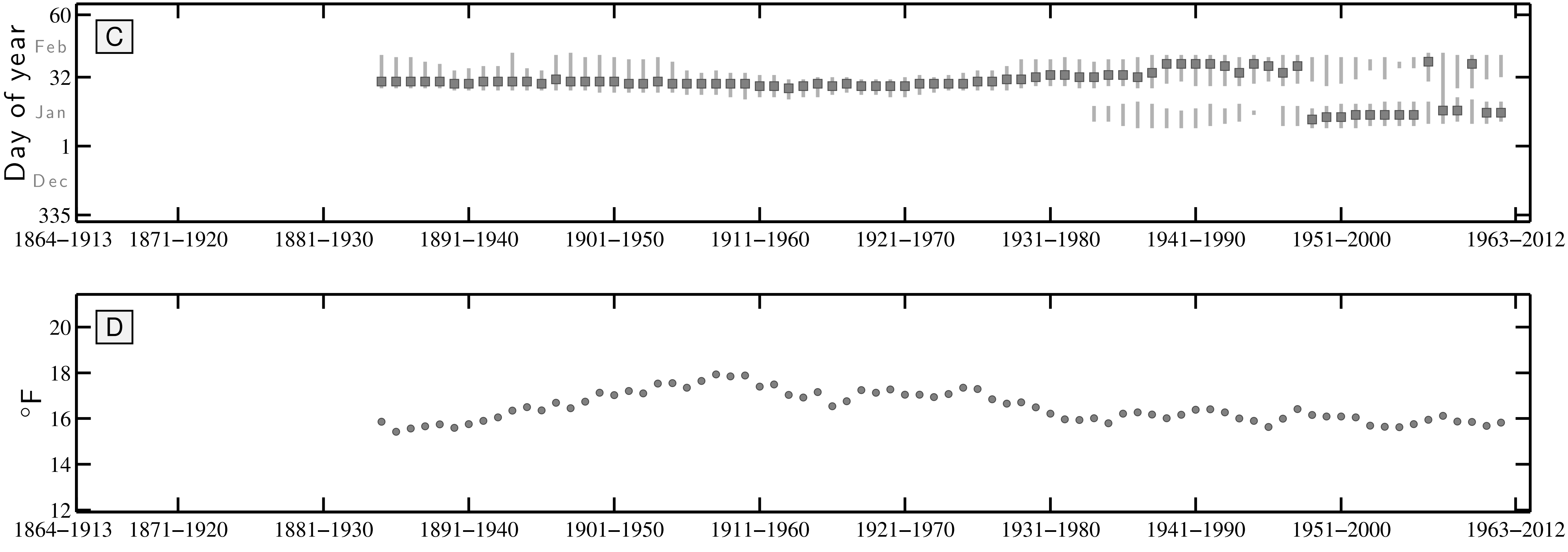

50 year Summer Teletherm dynamics for OBERLIN, OH:

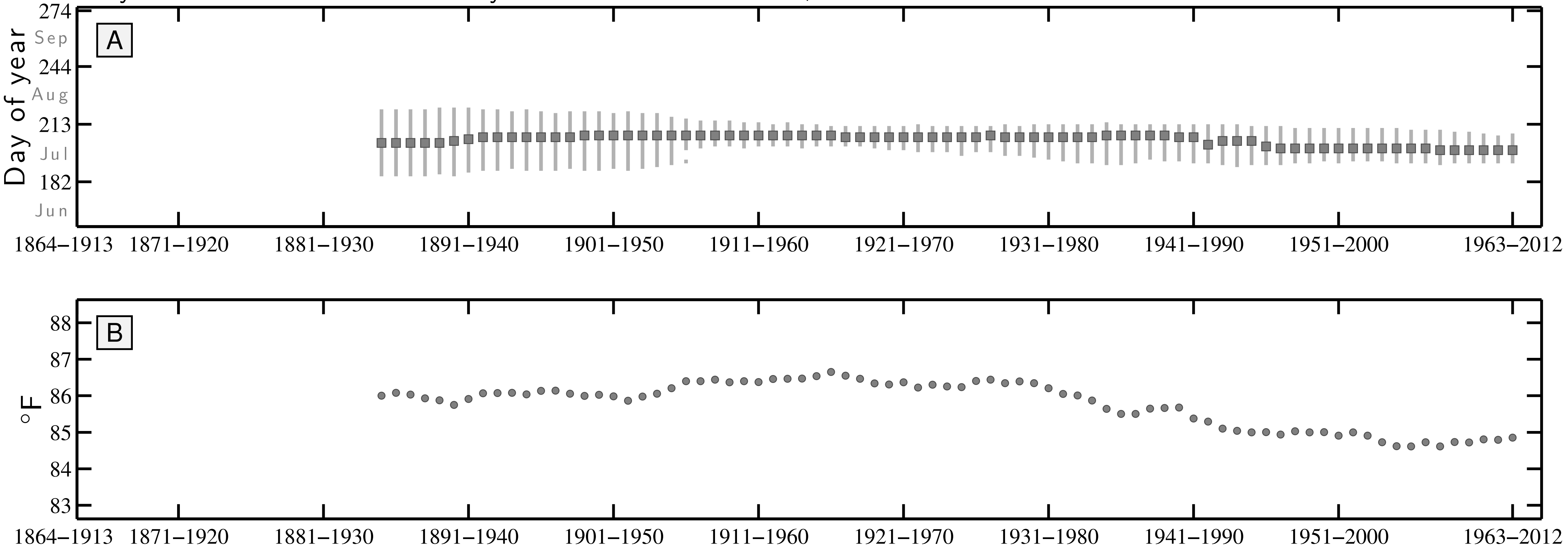

50 year Winter Teletherm dynamics for OBERLIN, OH:

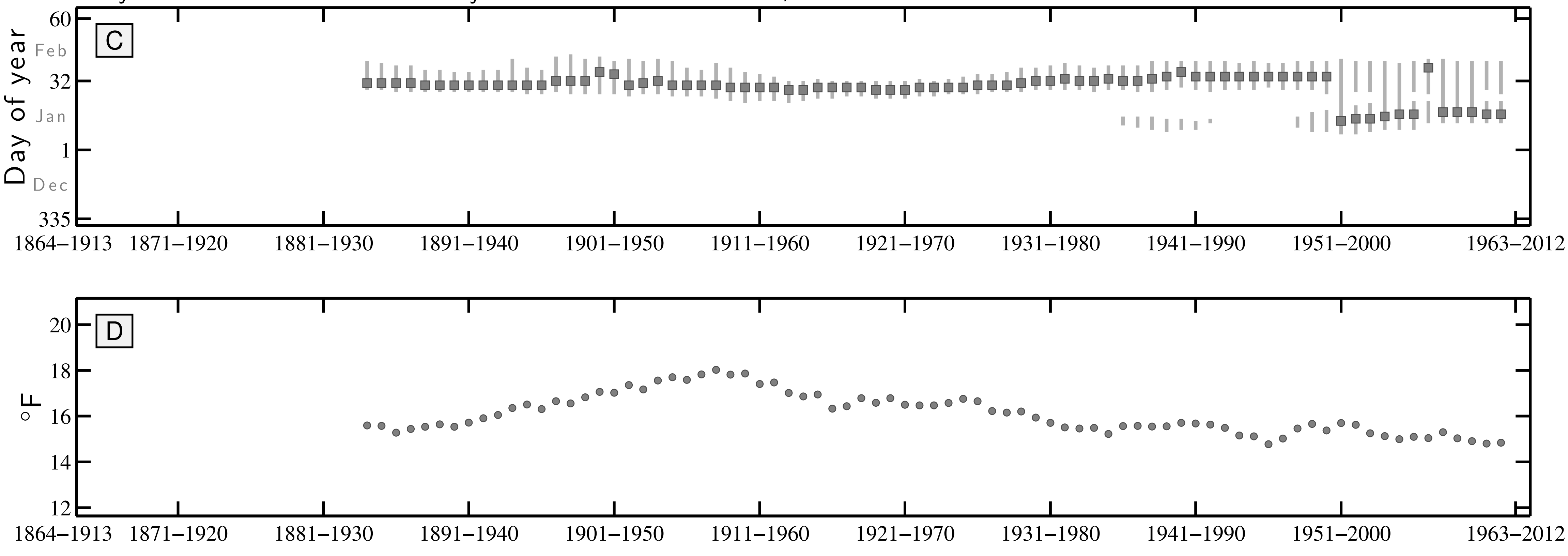

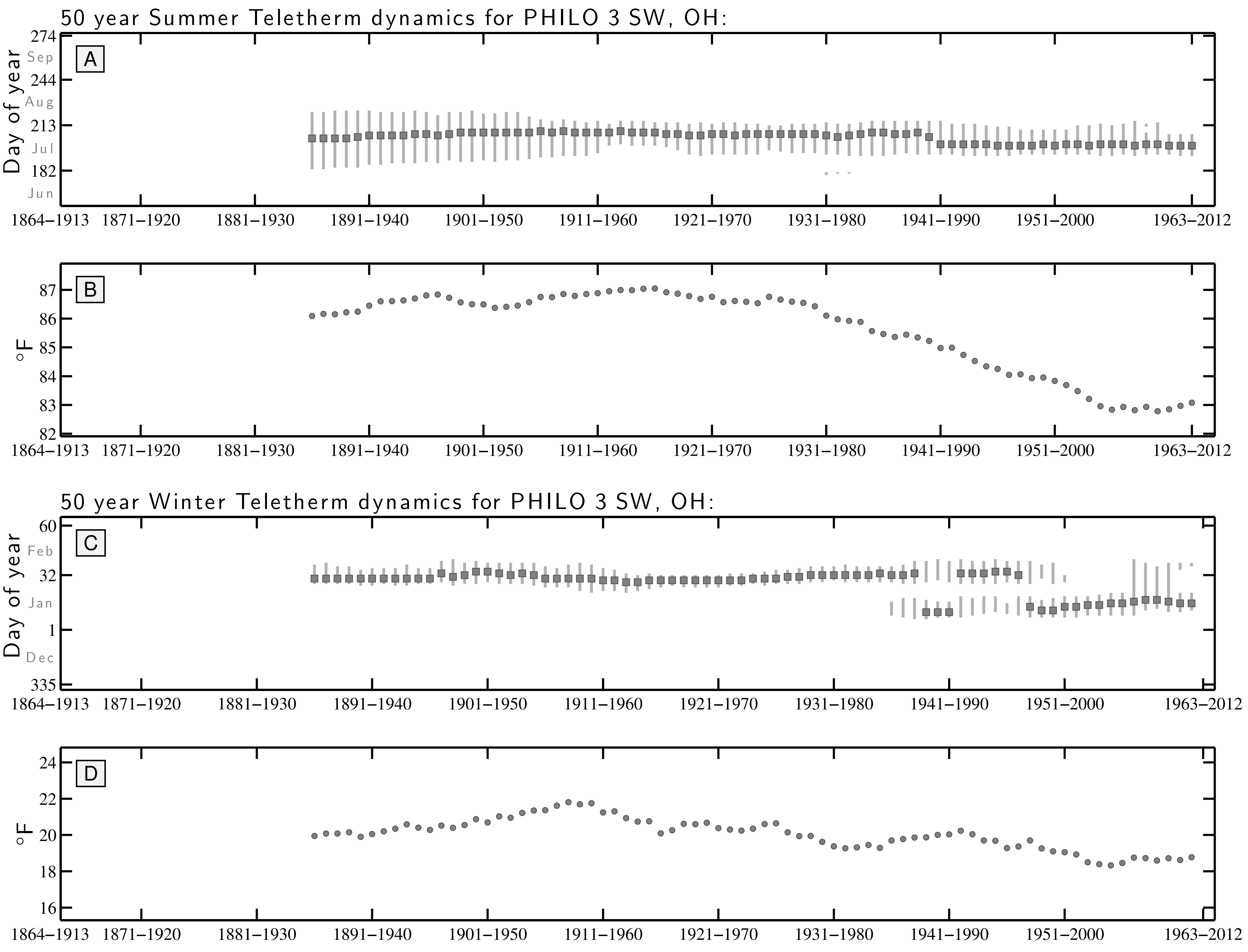

50 year Summer Teletherm dynamics for PHILO 3 SW, OH:
A
B
Day of year
°F
274
244
213
182
Sep
Aug
Jul
Jun
87
86
85
84
83
82
1864–1913
1871–1920
1881–1930
1891–1940
1901–1950
1911–1960
1921–1970
1931–1980
1941–1990
1951–2000
1963–2012
50 year Winter Teletherm dynamics for PHILO 3 SW, OH:
C
D
60
32
1
335
Feb
Jan
Dec
24
22
20
18
16

50 year Summer Teletherm dynamics for PORTSMOUTH-SCIOTOVILLE, OH:

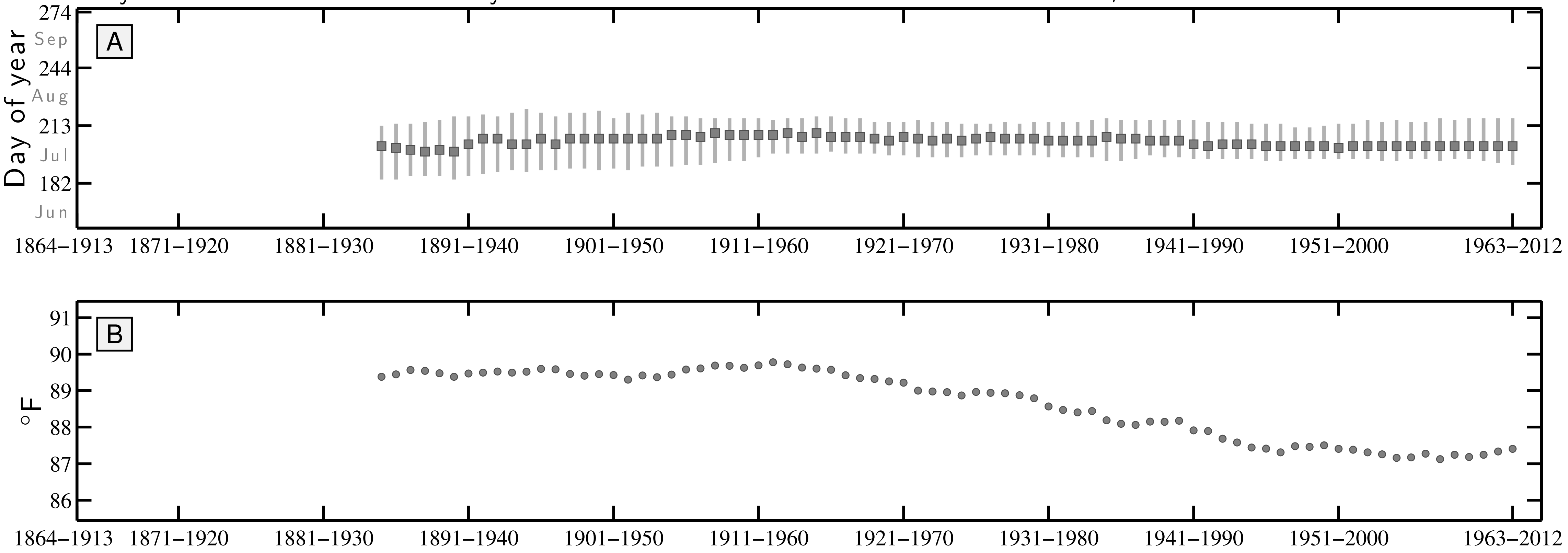

50 year Winter Teletherm dynamics for PORTSMOUTH-SCIOTOVILLE, OH:

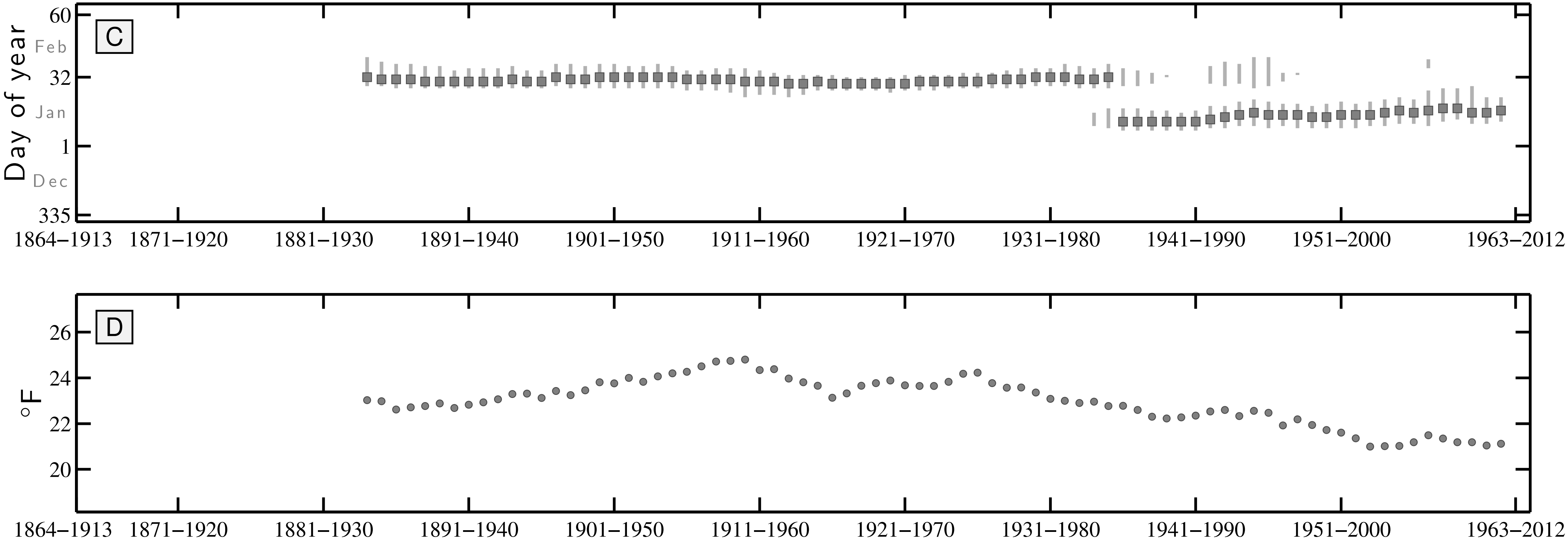

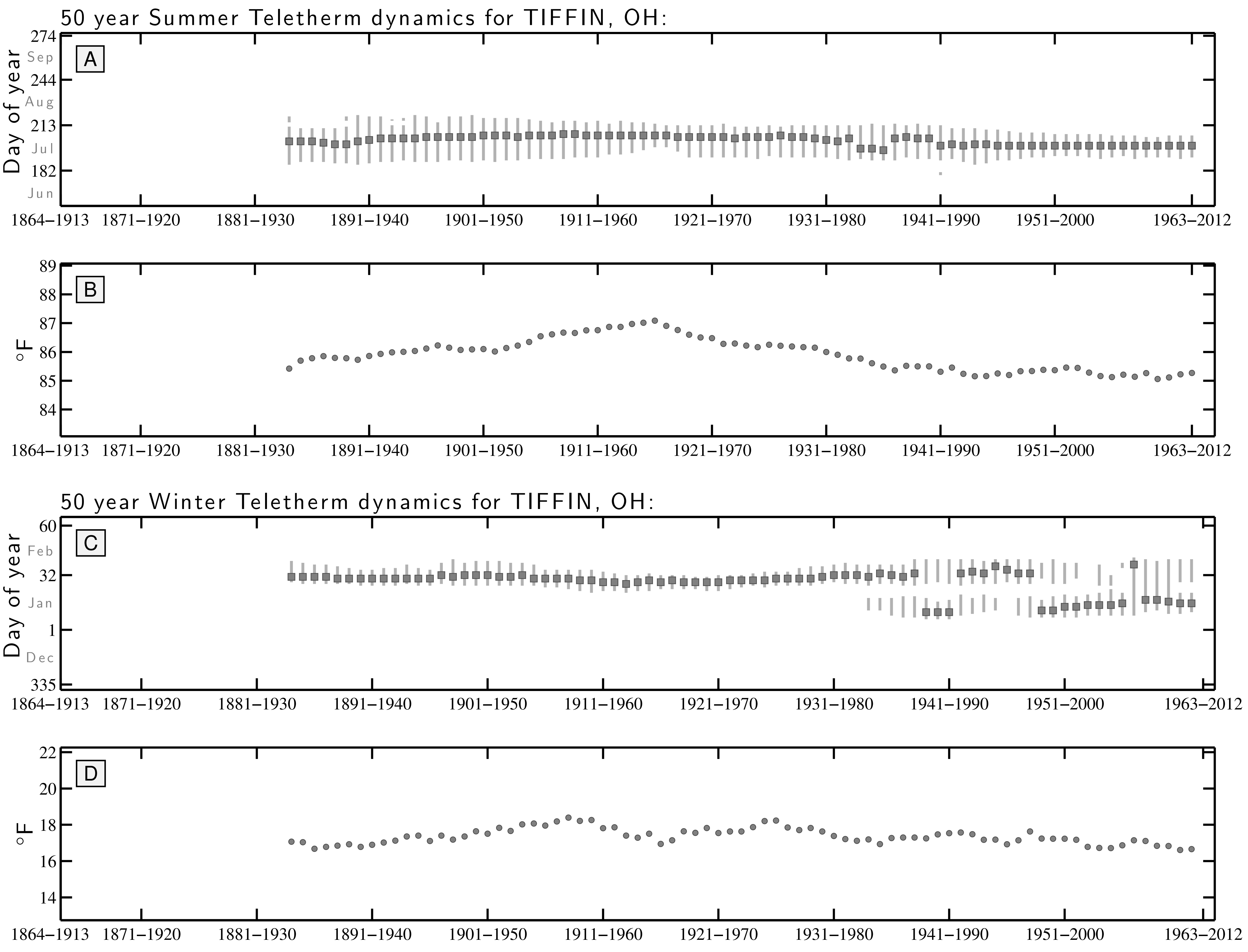

50 year Summer Teletherm dynamics for TIFFIN, OH:
A
Day of year
274
Sep
244
Aug
213
Jul
182
Jun
1864–1913
1871–1920
1881–1930
1891–1940
1901–1950
1911–1960
1921–1970
1931–1980
1941–1990
1951–2000
1963–2012
B
°F
89
88
87
86
85
84
50 year Winter Teletherm dynamics for TIFFIN, OH:
C
60
Feb
32
Jan
1
Dec
335
D
22
20
18
16
14

50 year Summer Teletherm dynamics for UPPER SANDUSKY, OH:

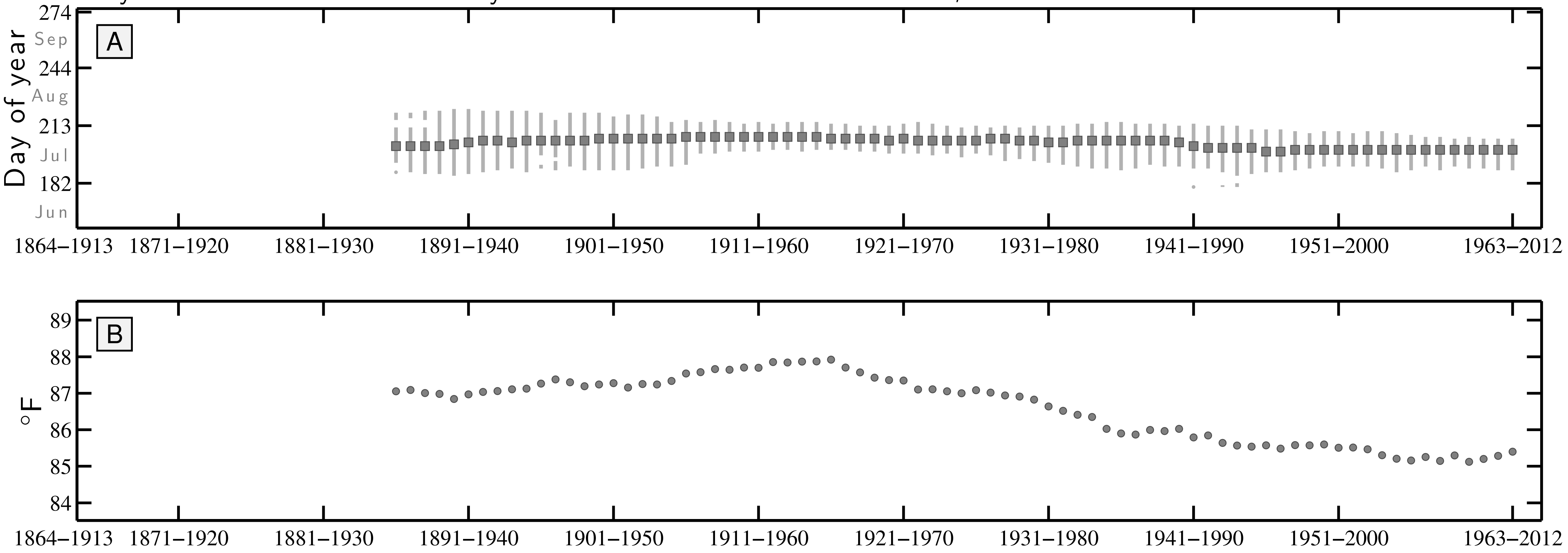

50 year Winter Teletherm dynamics for UPPER SANDUSKY, OH:

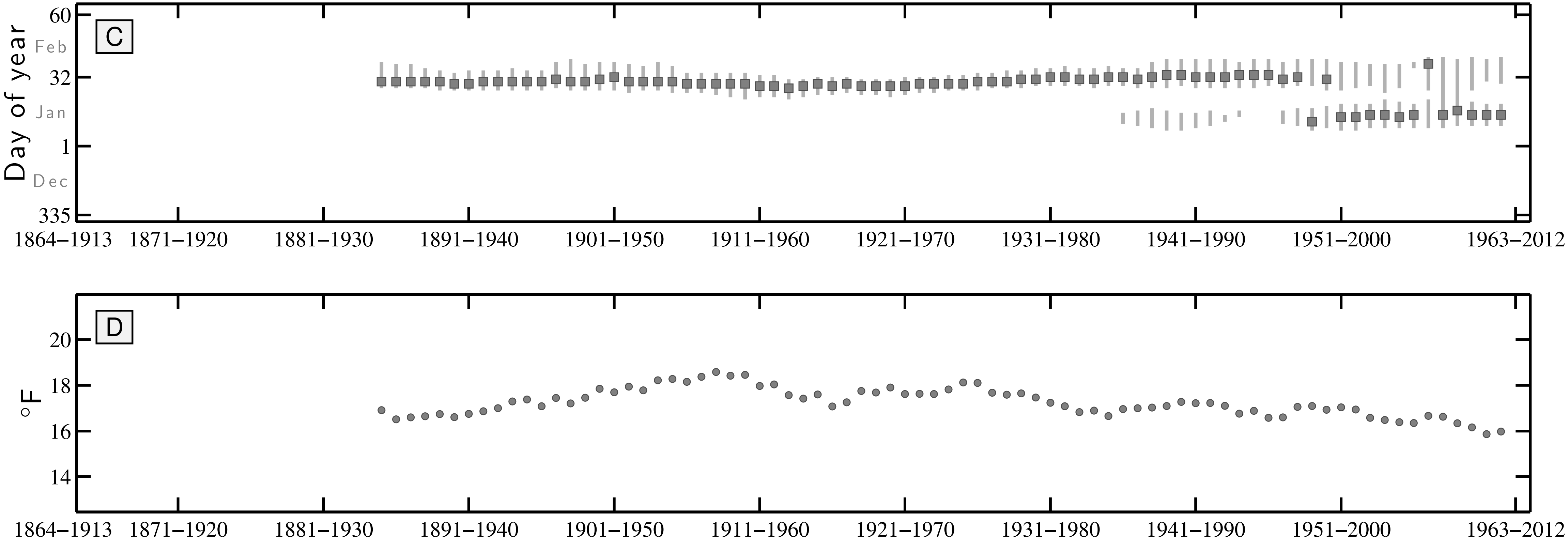

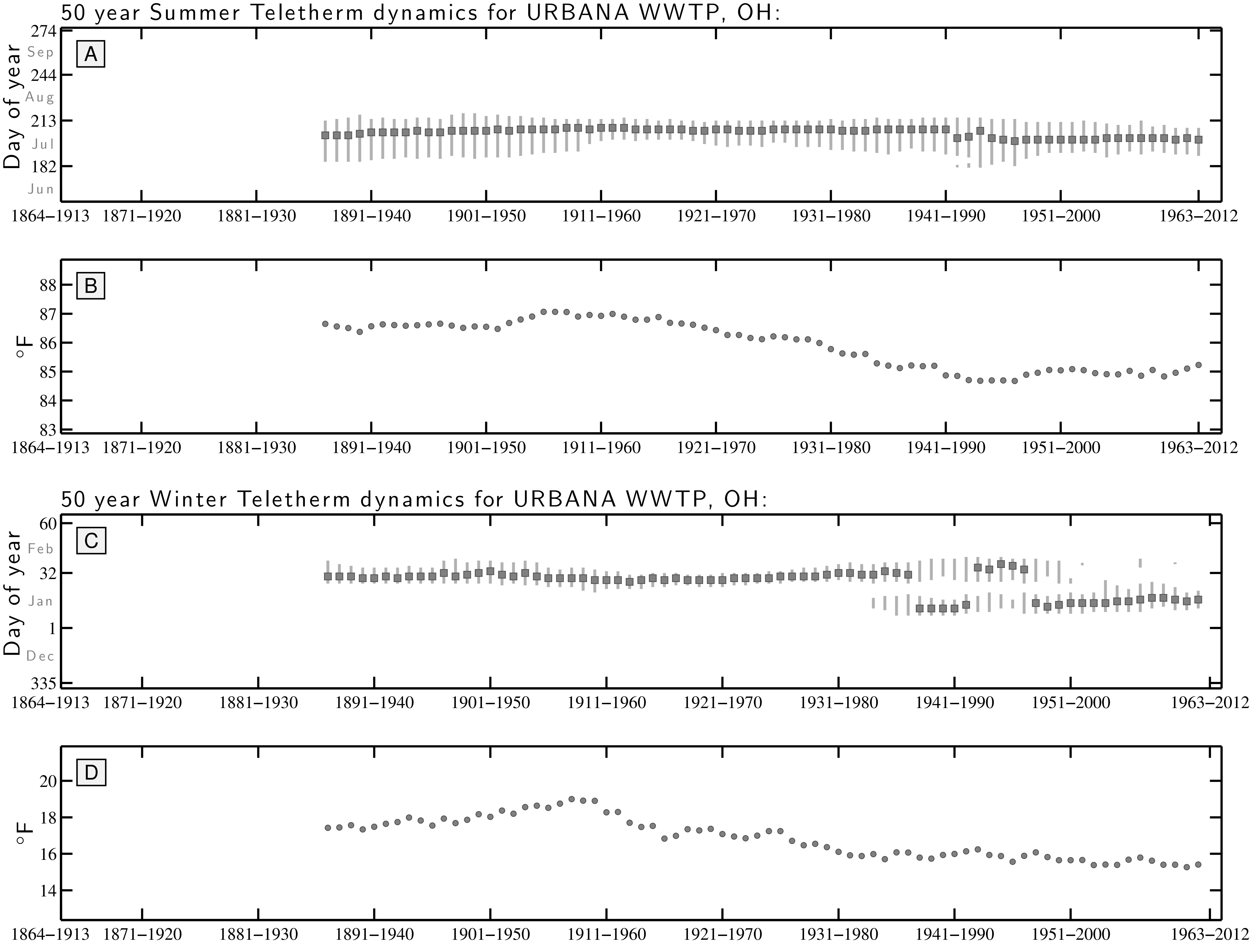
50 year Summer Teletherm dynamics for URBANA WWTP, OH:
A
Day of year
274
Sep
244
Aug
213
Jul
182
Jun
B
°F
88
87
86
85
84
83
50 year Winter Teletherm dynamics for URBANA WWTP, OH:
C
Day of year
60
Feb
32
Jan
1
Dec
335
D
°F
20
18
16
14
1864–1913
1871–1920
1881–1930
1891–1940
1901–1950
1911–1960
1921–1970
1931–1980
1941–1990
1951–2000
1963–2012

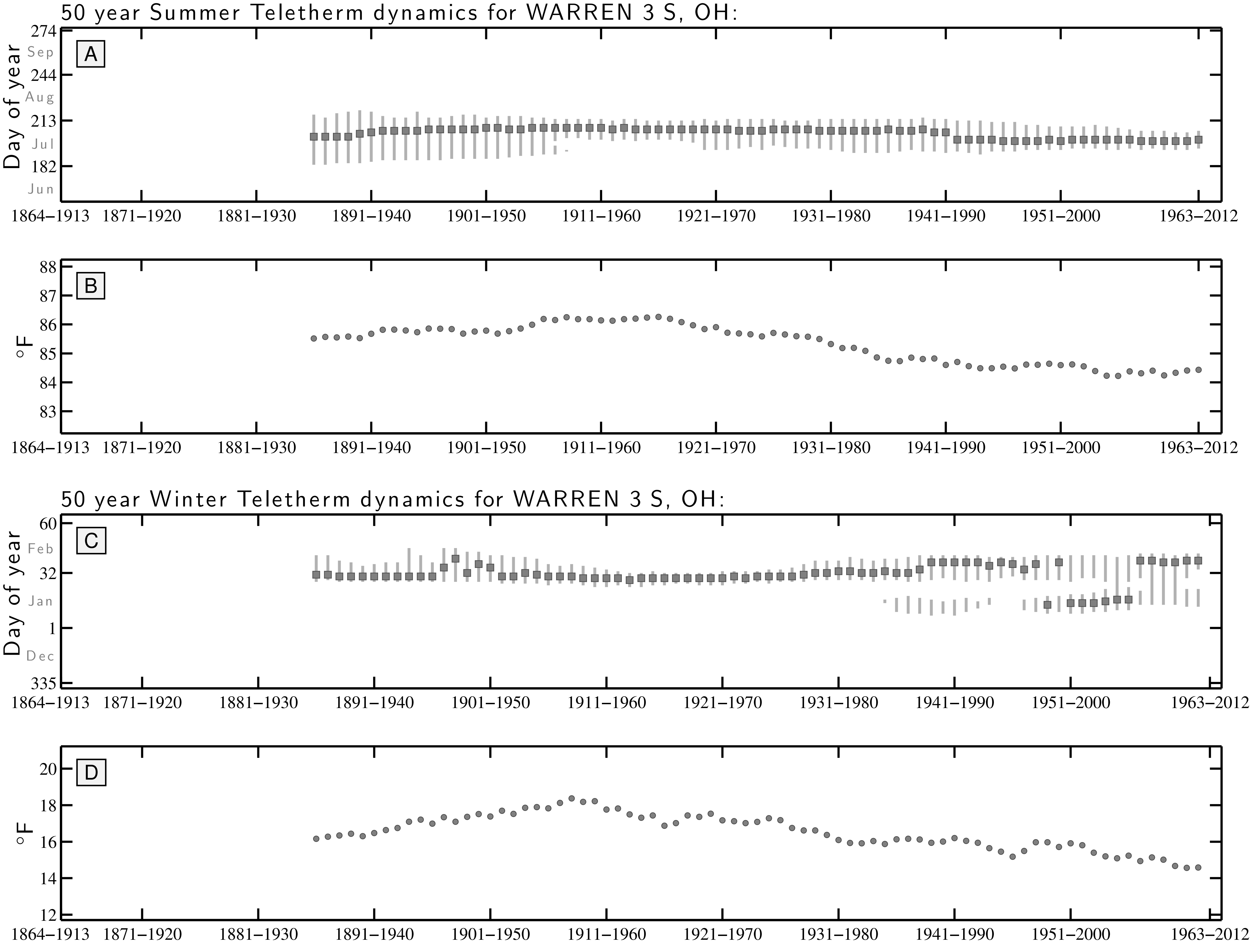
50 year Summer Teletherm dynamics for WARREN 3 S, OH:
A
Day of year
274
Sep
244
Aug
213
Jul
182
Jun
1864–1913
1871–1920
1881–1930
1891–1940
1901–1950
1911–1960
1921–1970
1931–1980
1941–1990
1951–2000
1963–2012
B
°F
88
87
86
85
84
83
50 year Winter Teletherm dynamics for WARREN 3 S, OH:
C
Day of year
60
Feb
32
Jan
1
Dec
335
D
°F
20
18
16
14
12

50 year Summer Teletherm dynamics for WAUSEON WTP, OH:

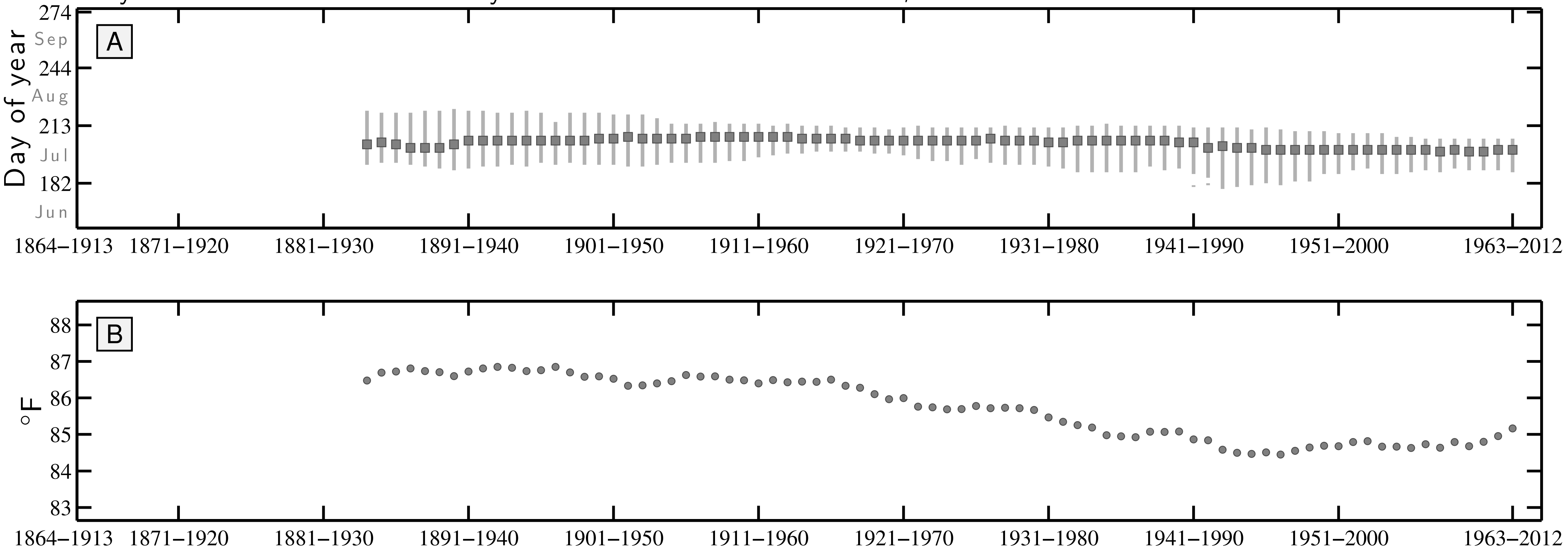

50 year Winter Teletherm dynamics for WAUSEON WTP, OH:

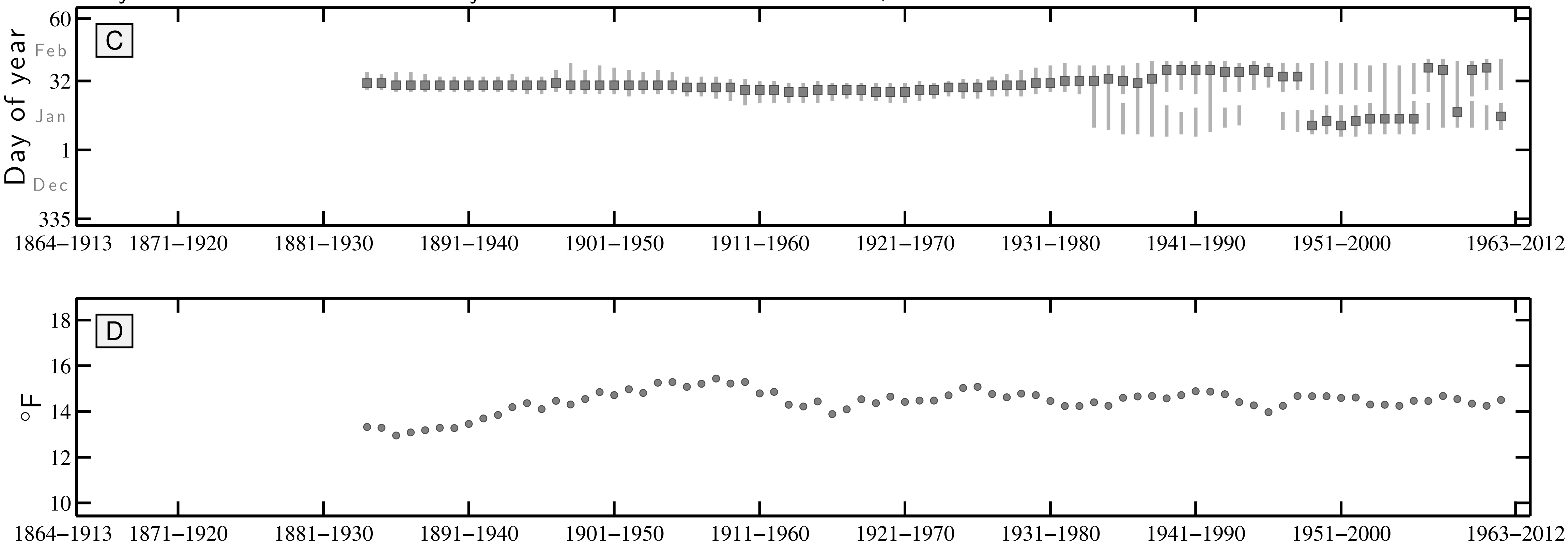

50 year Summer Teletherm dynamics for WAVERLY, OH:

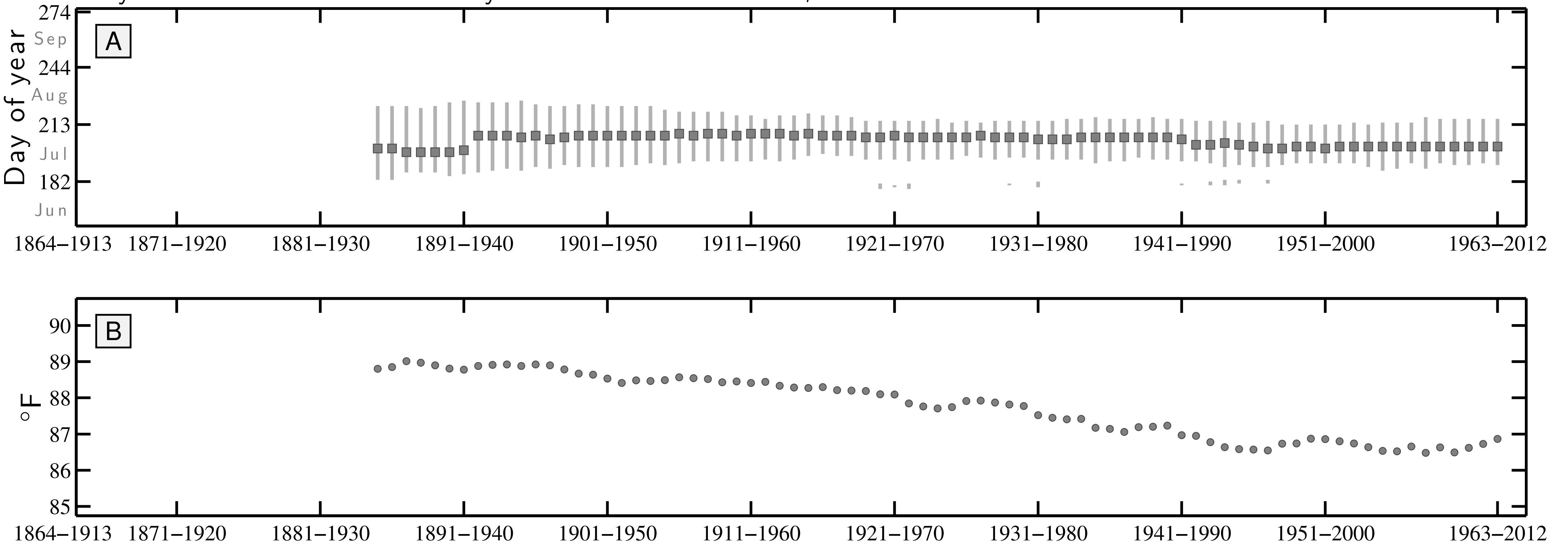

50 year Winter Teletherm dynamics for WAVERLY, OH:

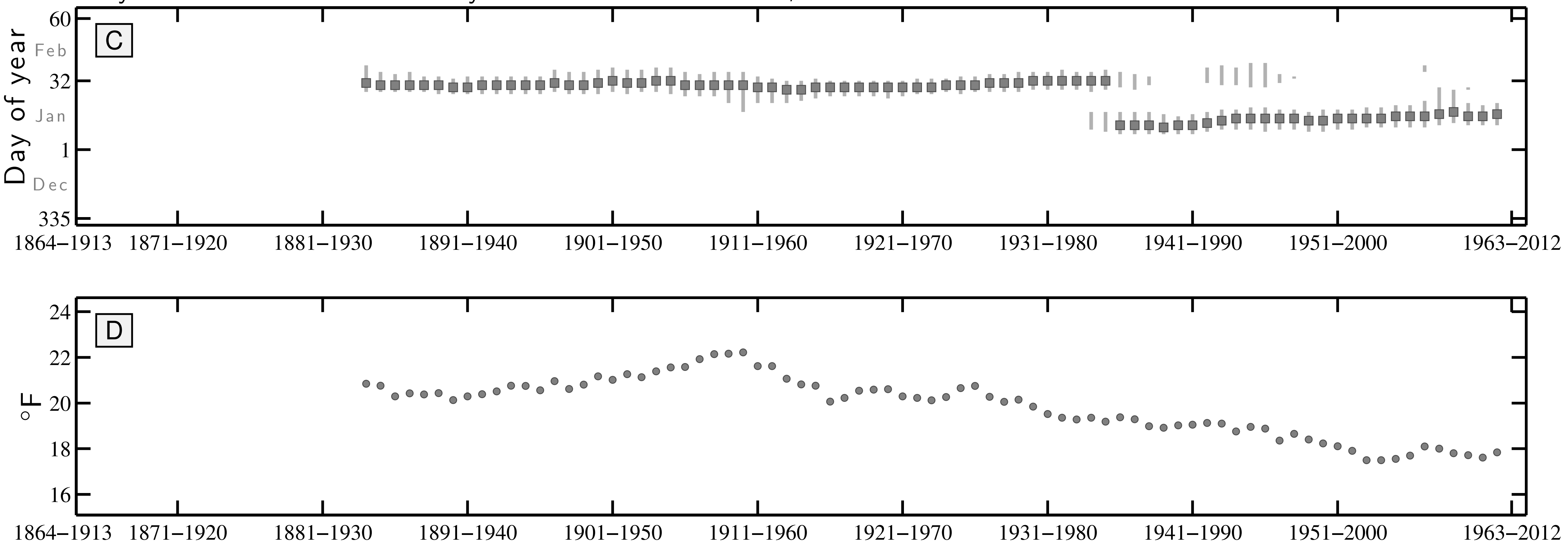

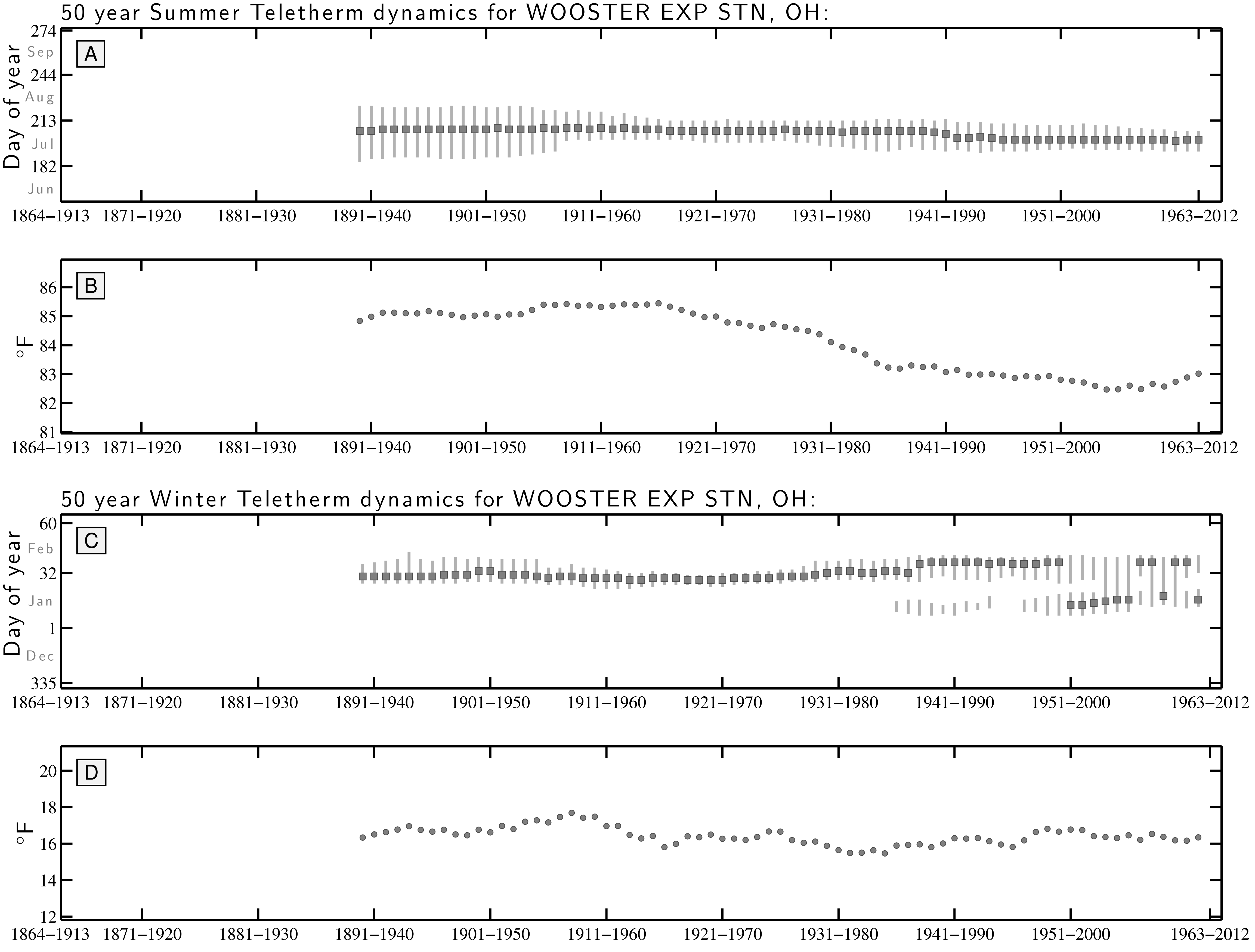
50 year Summer Teletherm dynamics for WOOSTER EXP STN, OH:
A
Day of year
274
Sep
244
Aug
213
Jul
182
Jun
1864–1913
1871–1920
1881–1930
1891–1940
1901–1950
1911–1960
1921–1970
1931–1980
1941–1990
1951–2000
1963–2012
B
°F
86
85
84
83
82
81
50 year Winter Teletherm dynamics for WOOSTER EXP STN, OH:
C
Day of year
60
Feb
32
Jan
1
Dec
335
D
°F
20
18
16
14
12

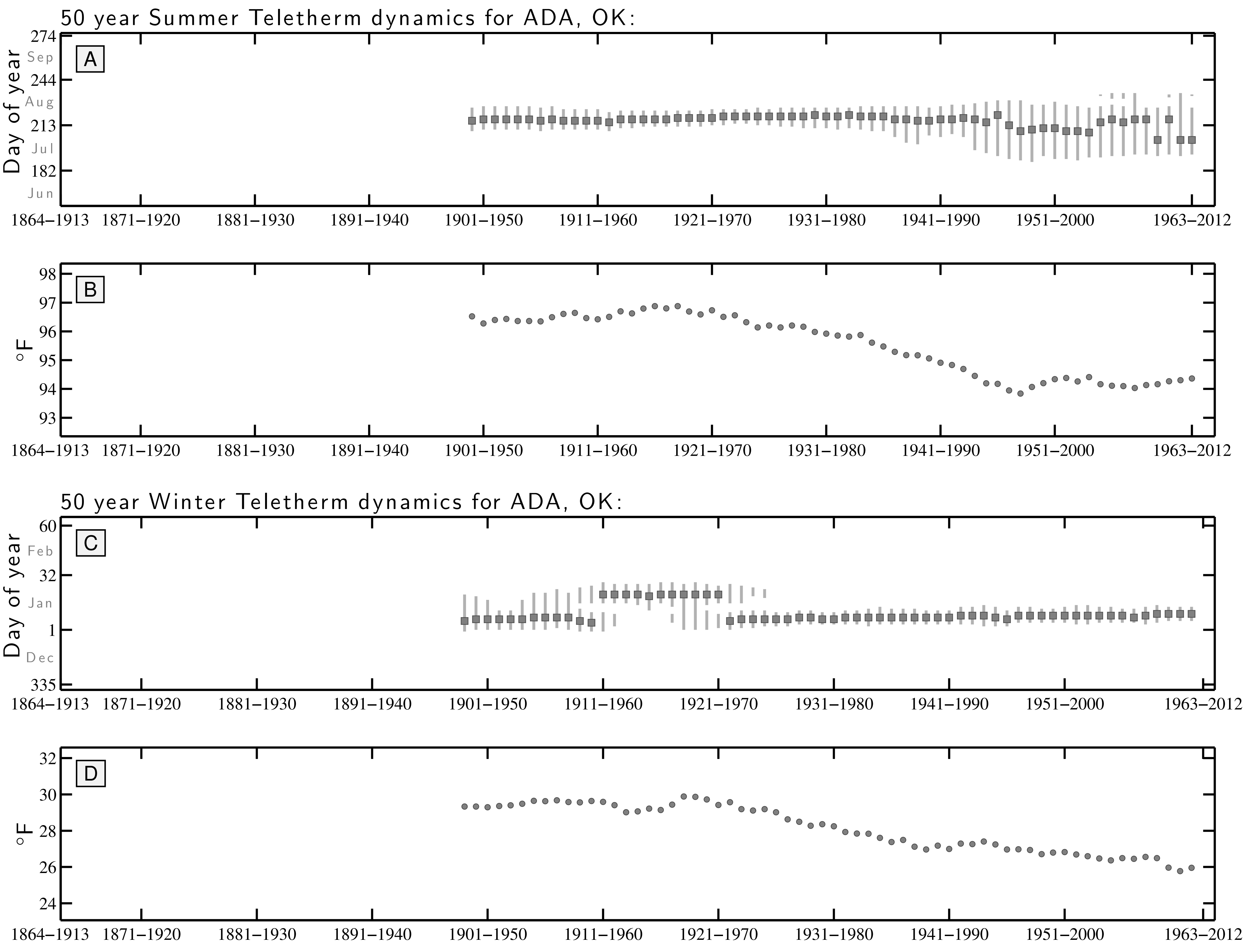

50 year Summer Teletherm dynamics for ADA, OK:
A
Day of year
274
Sep
244
Aug
213
Jul
182
Jun
1864–1913
1871–1920
1881–1930
1891–1940
1901–1950
1911–1960
1921–1970
1931–1980
1941–1990
1951–2000
1963–2012
B
°F
98
97
96
95
94
93
50 year Winter Teletherm dynamics for ADA, OK:
C
Day of year
60
Feb
32
Jan
1
Dec
335
D
°F
32
30
28
26
24

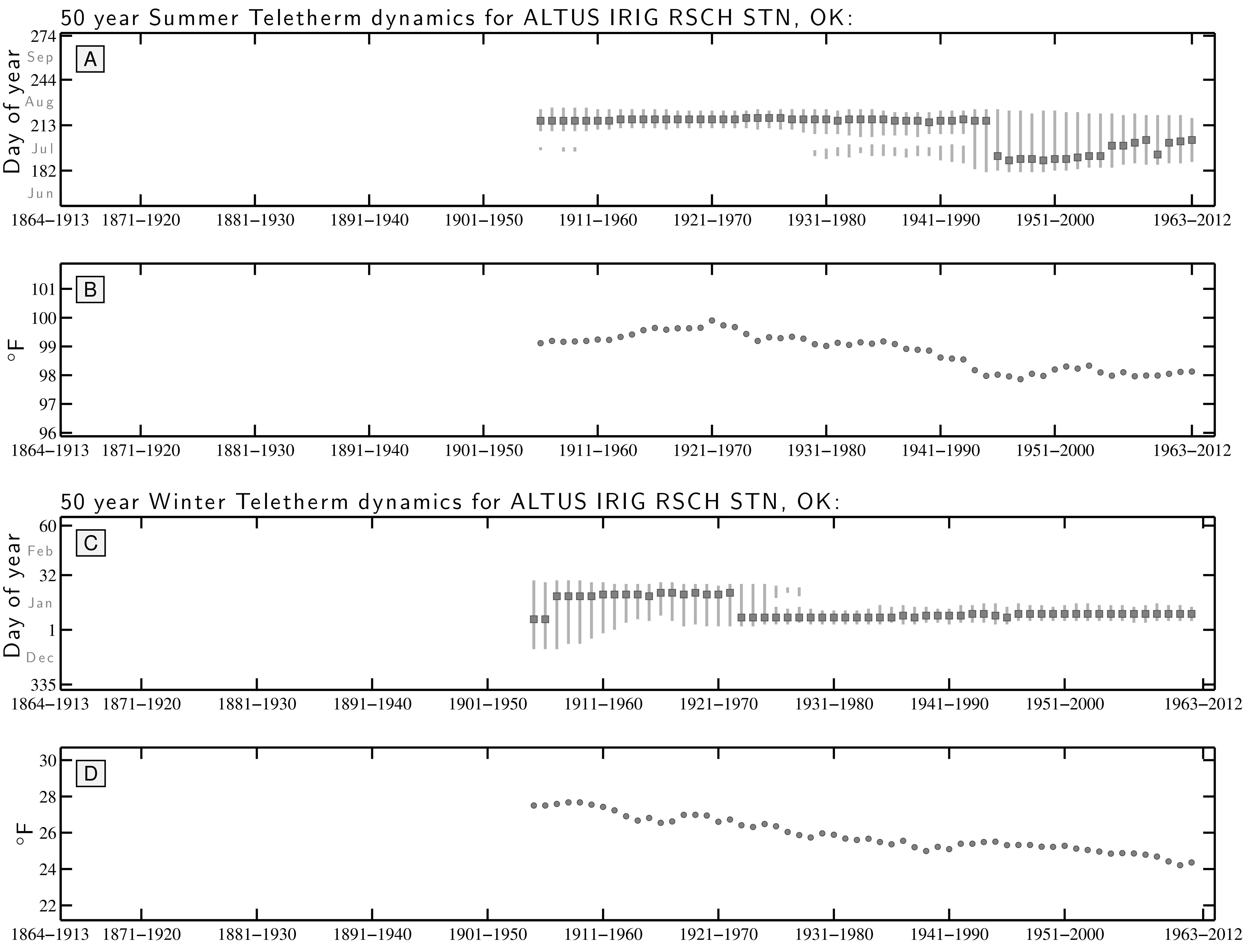
50 year Summer Teletherm dynamics for ALTUS IRIG RSCH STN, OK:
A
Day of year
274
Sep
244
Aug
213
Jul
182
Jun
1864–1913
1871–1920
1881–1930
1891–1940
1901–1950
1911–1960
1921–1970
1931–1980
1941–1990
1951–2000
1963–2012
B
°F
101
100
99
98
97
96
50 year Winter Teletherm dynamics for ALTUS IRIG RSCH STN, OK:
C
Day of year
60
Feb
32
Jan
1
Dec
335
D
°F
30
28
26
24
22

50 year Summer Teletherm dynamics for ANTLERS, OK:

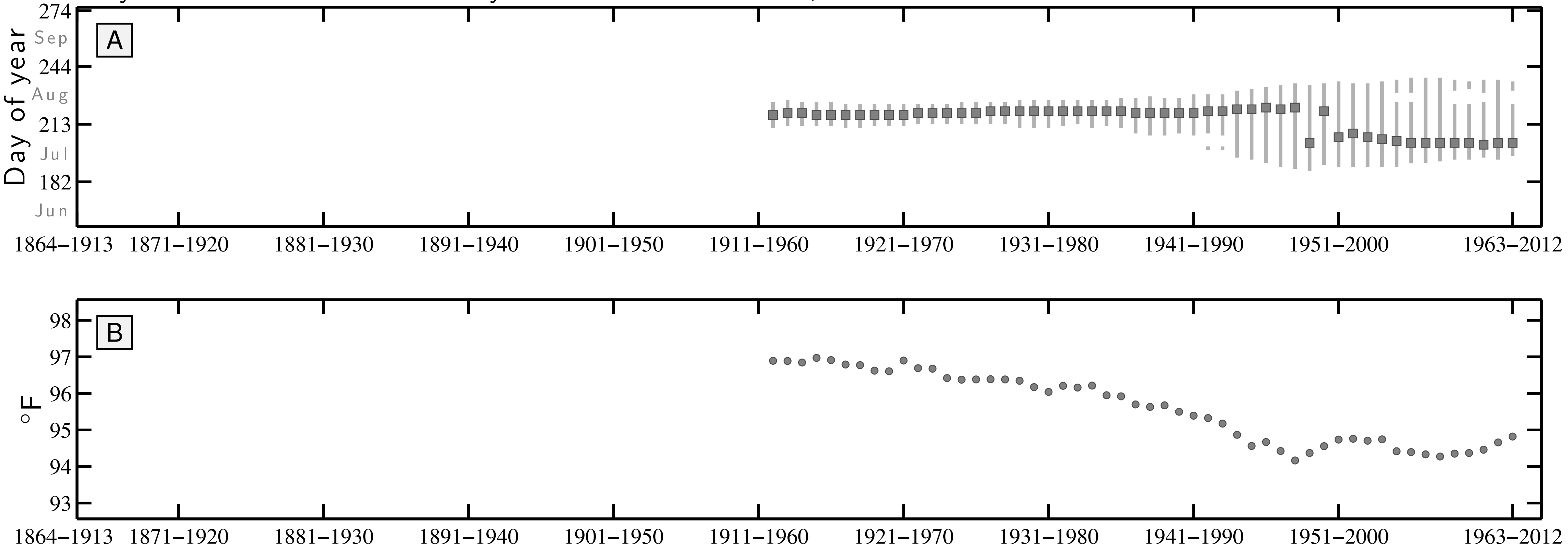

50 year Winter Teletherm dynamics for ANTLERS, OK:

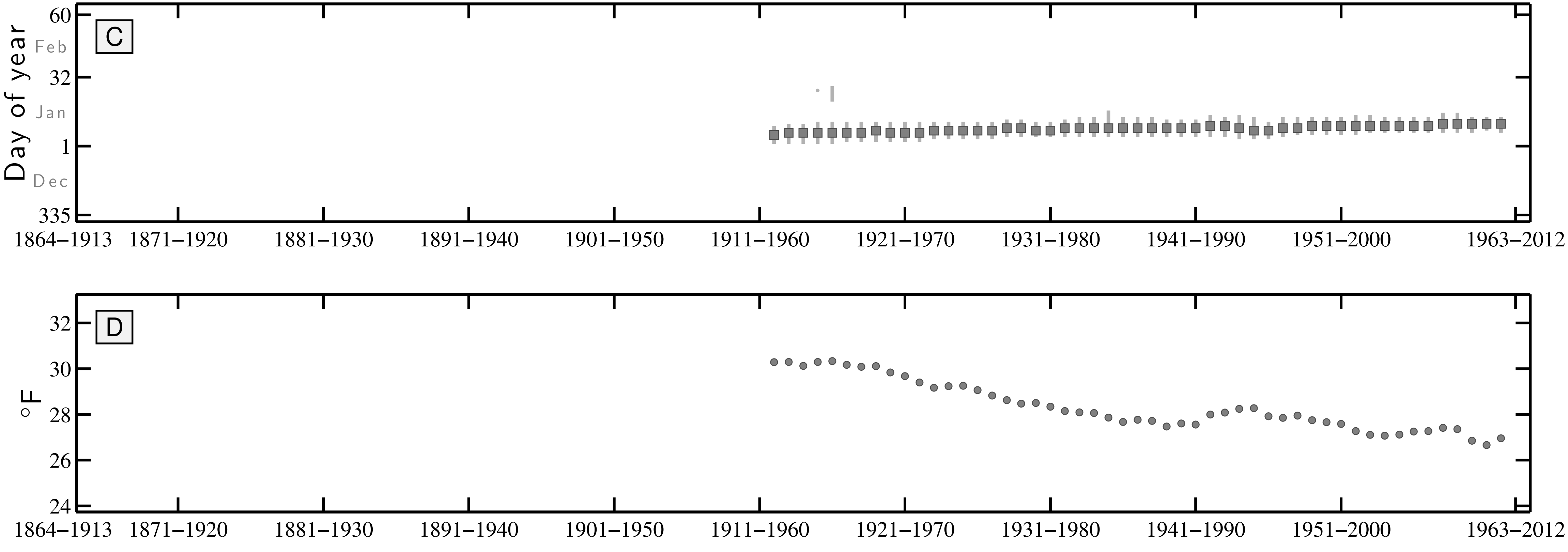

50 year Summer Teletherm dynamics for ARDMORE, OK:

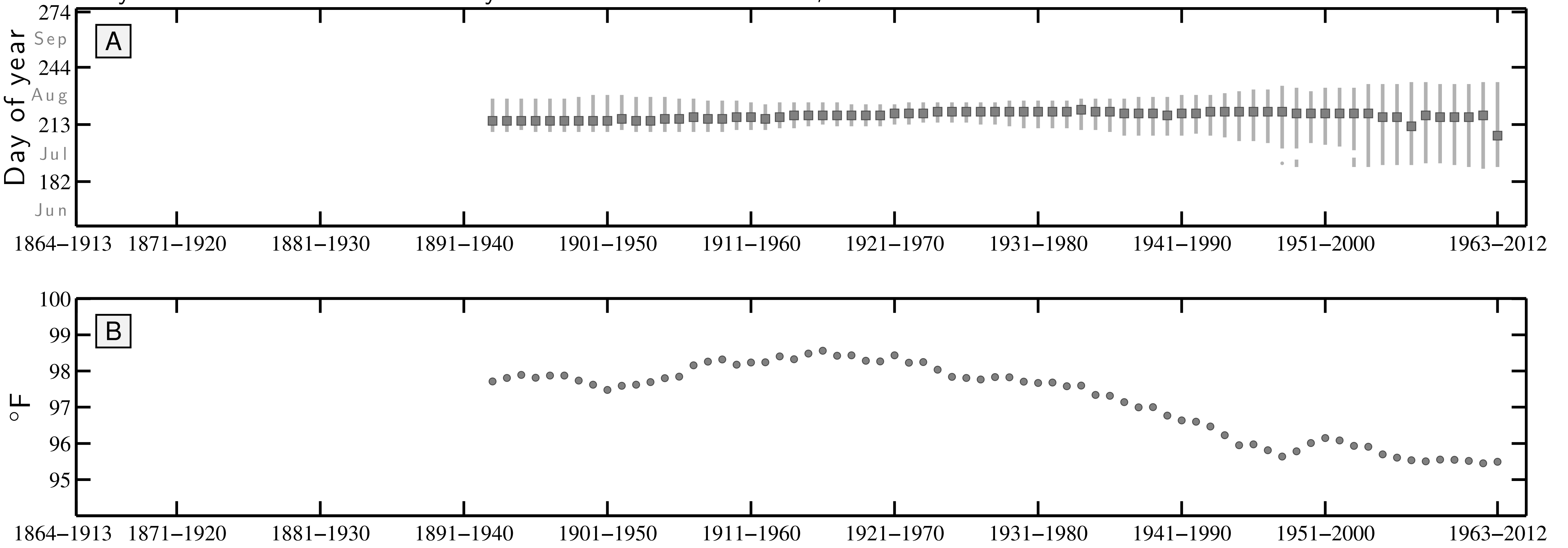

50 year Winter Teletherm dynamics for ARDMORE, OK:

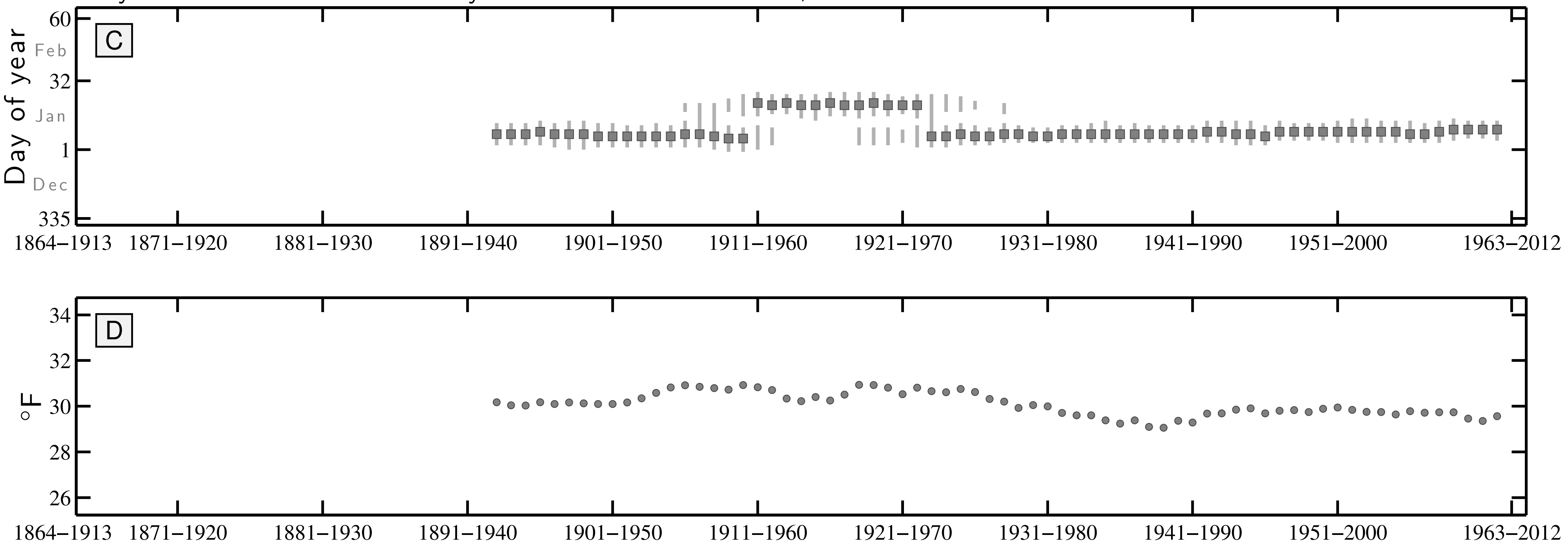

50 year Summer Teletherm dynamics for BARTLESVILLE MUNI AP, OK:

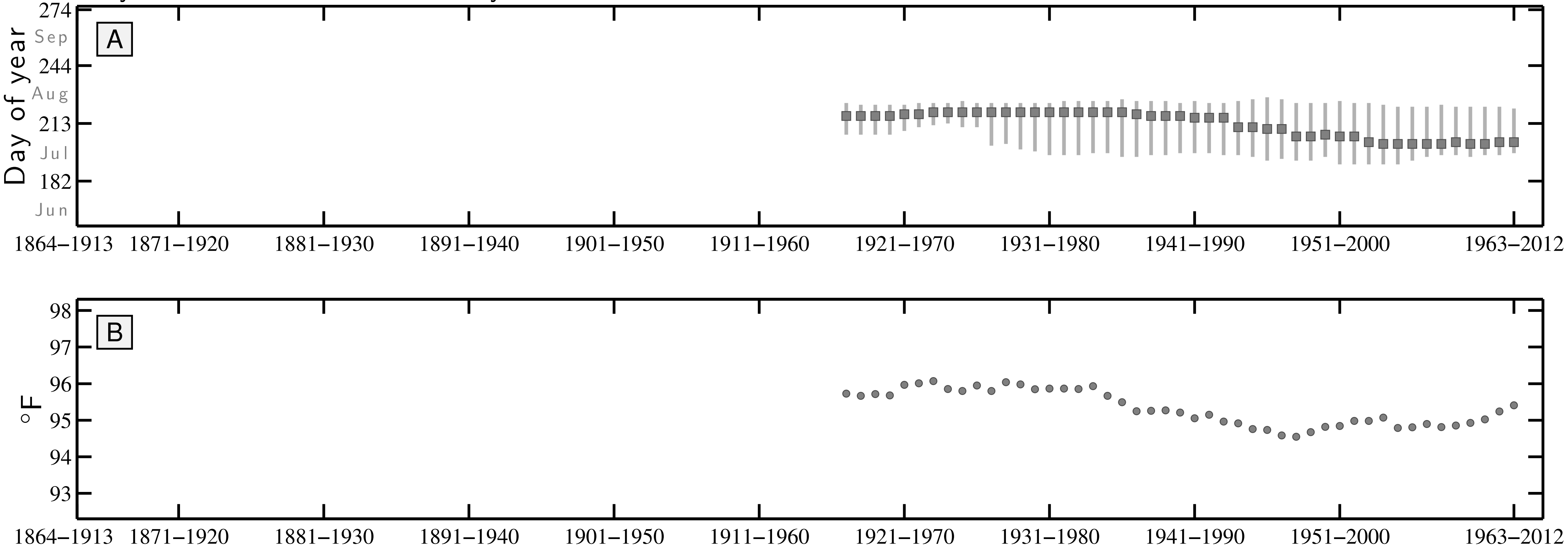

50 year Winter Teletherm dynamics for BARTLESVILLE MUNI AP, OK:

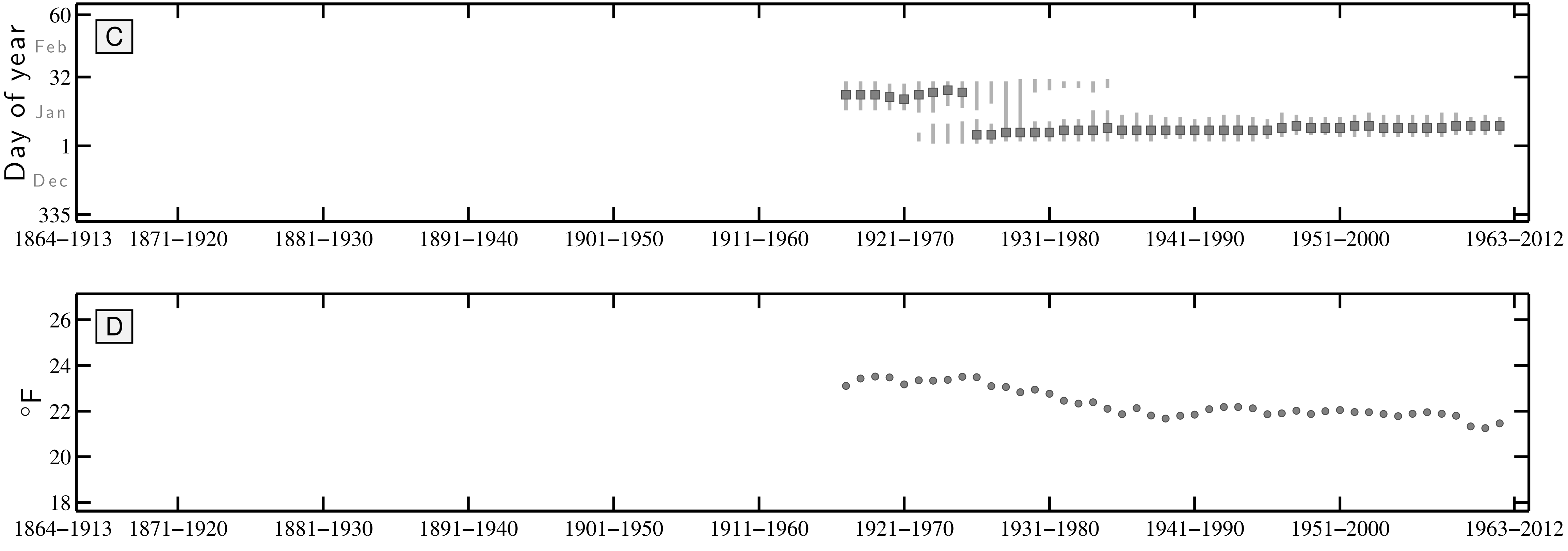

50 year Summer Teletherm dynamics for BEAVER, OK:

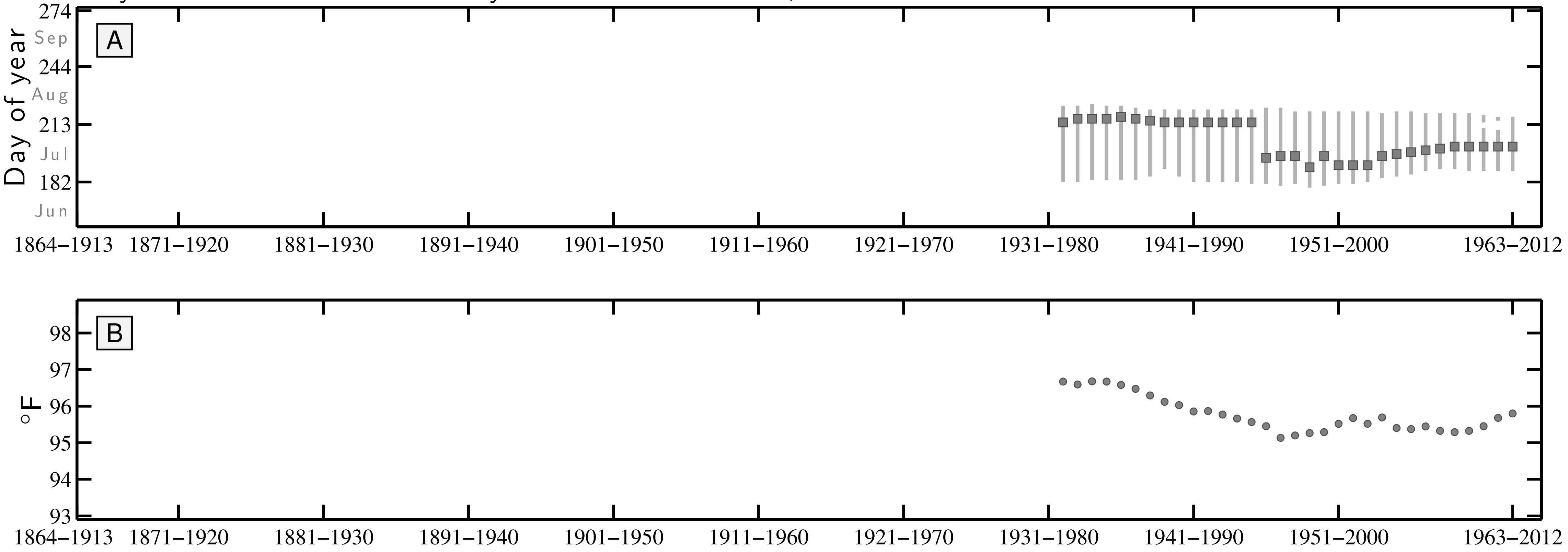

50 year Winter Teletherm dynamics for BEAVER, OK:

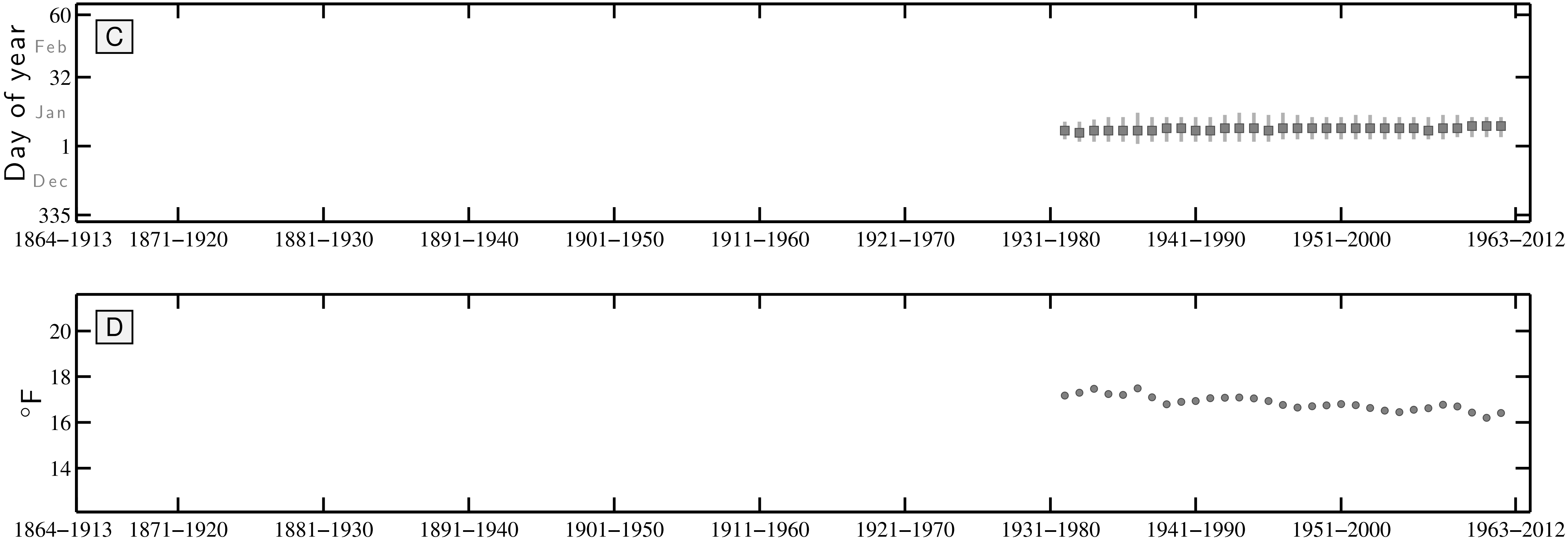

50 year Summer Teletherm dynamics for BOISE CITY 2 E, OK:

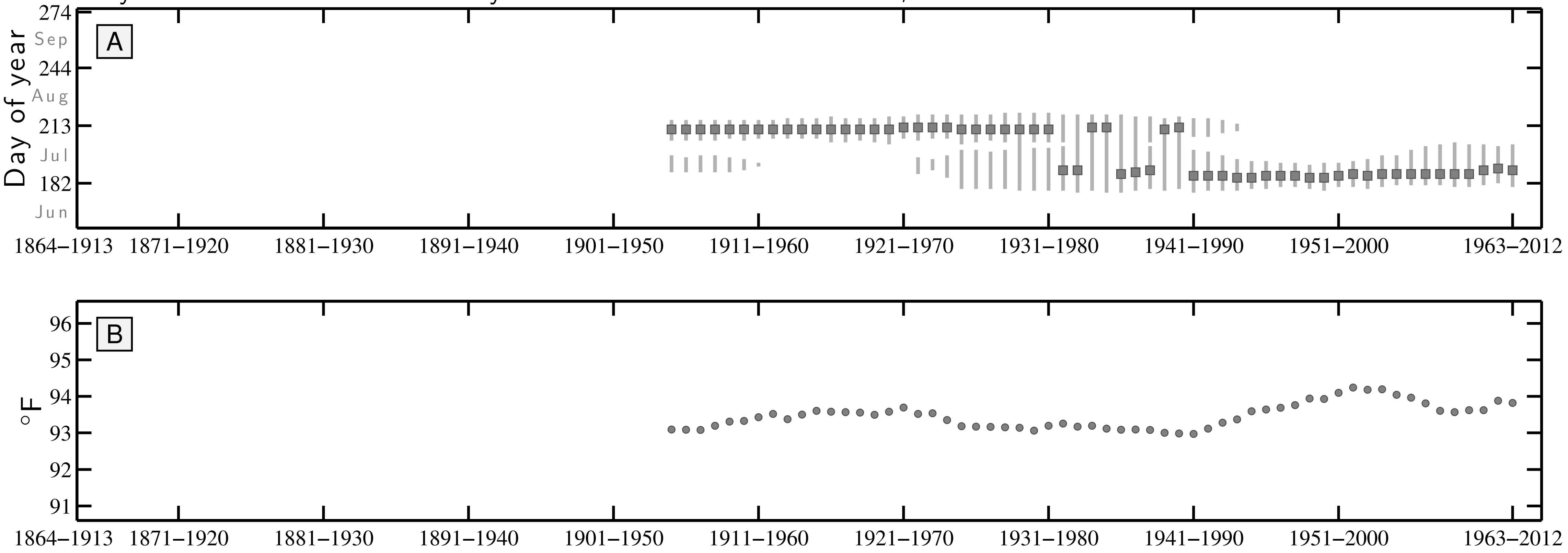

50 year Winter Teletherm dynamics for BOISE CITY 2 E, OK:

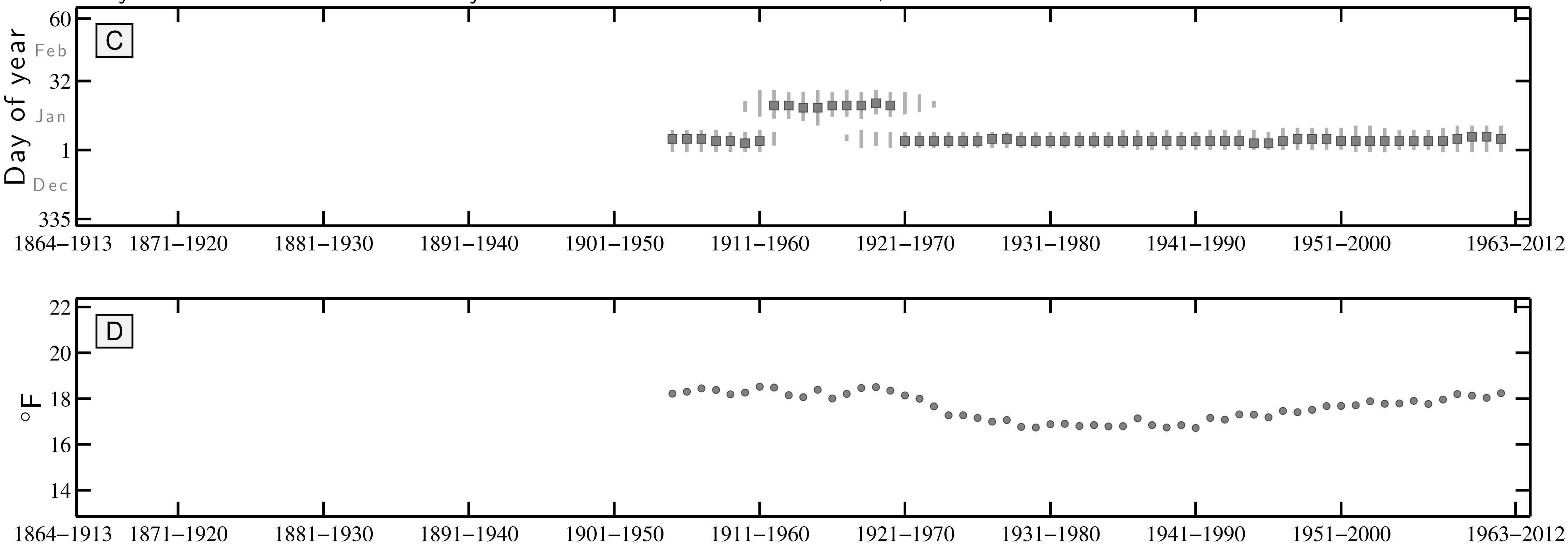

50 year Summer Teletherm dynamics for BUFFALO 2 SSW, OK:

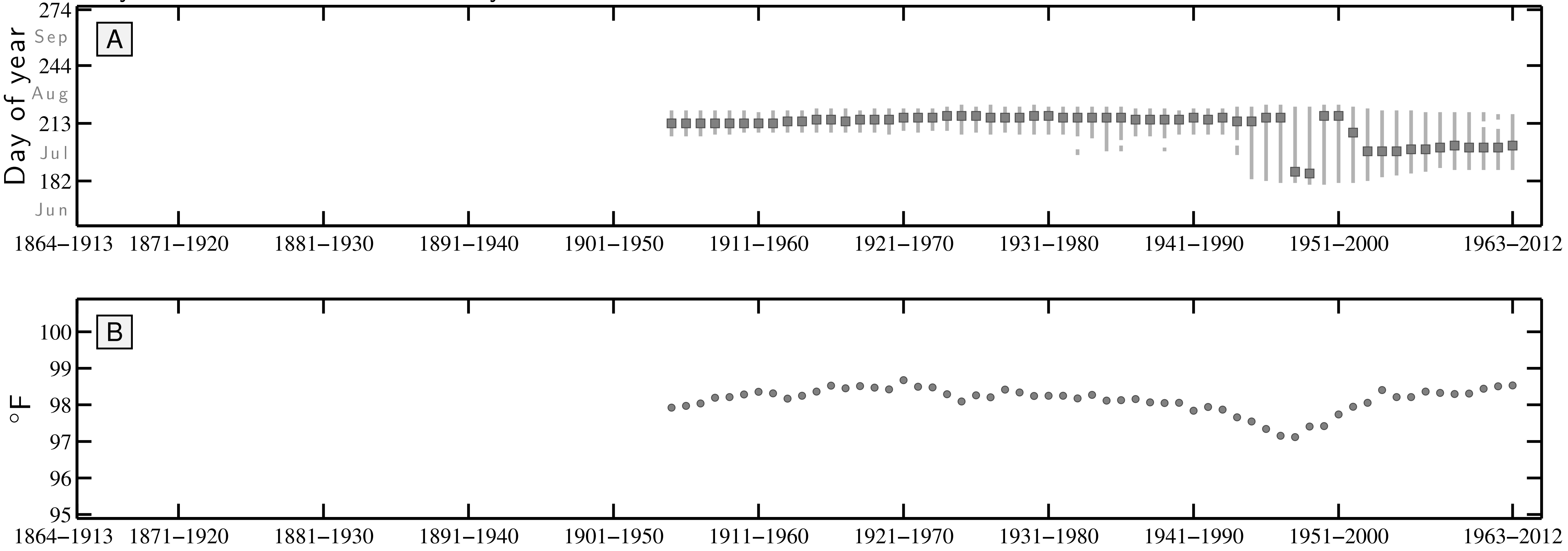

50 year Winter Teletherm dynamics for BUFFALO 2 SSW, OK:

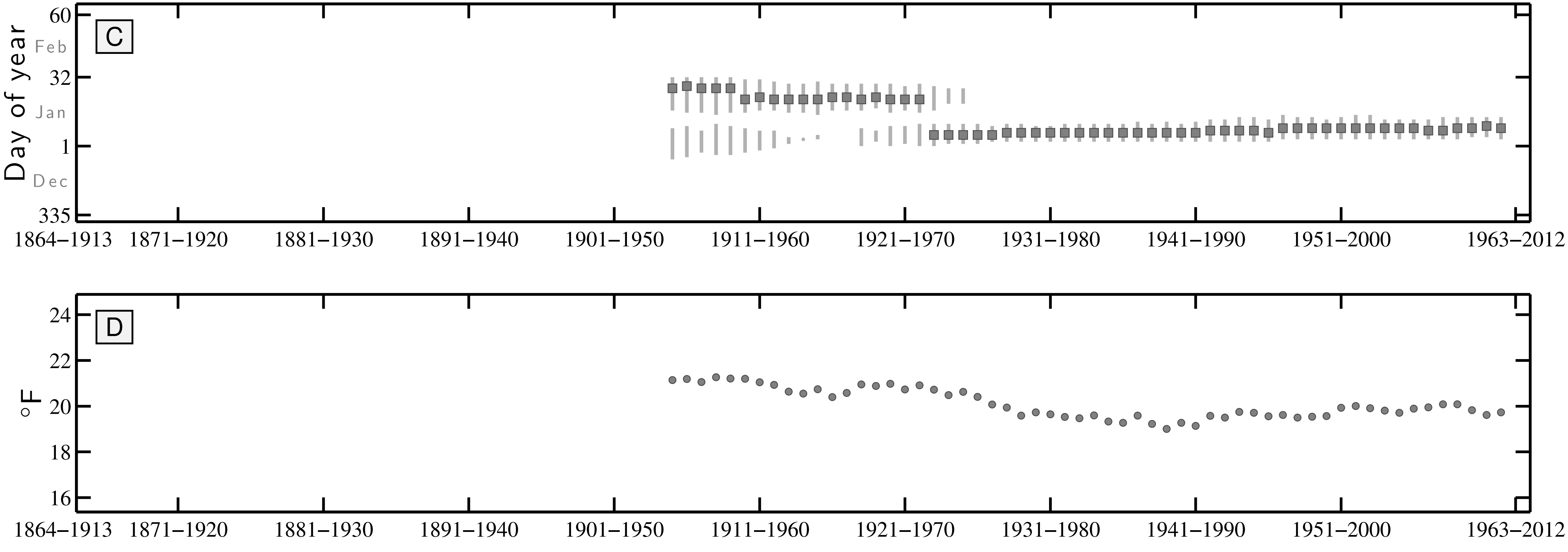

50 year Summer Teletherm dynamics for CARNEGIE 5 NE, OK:

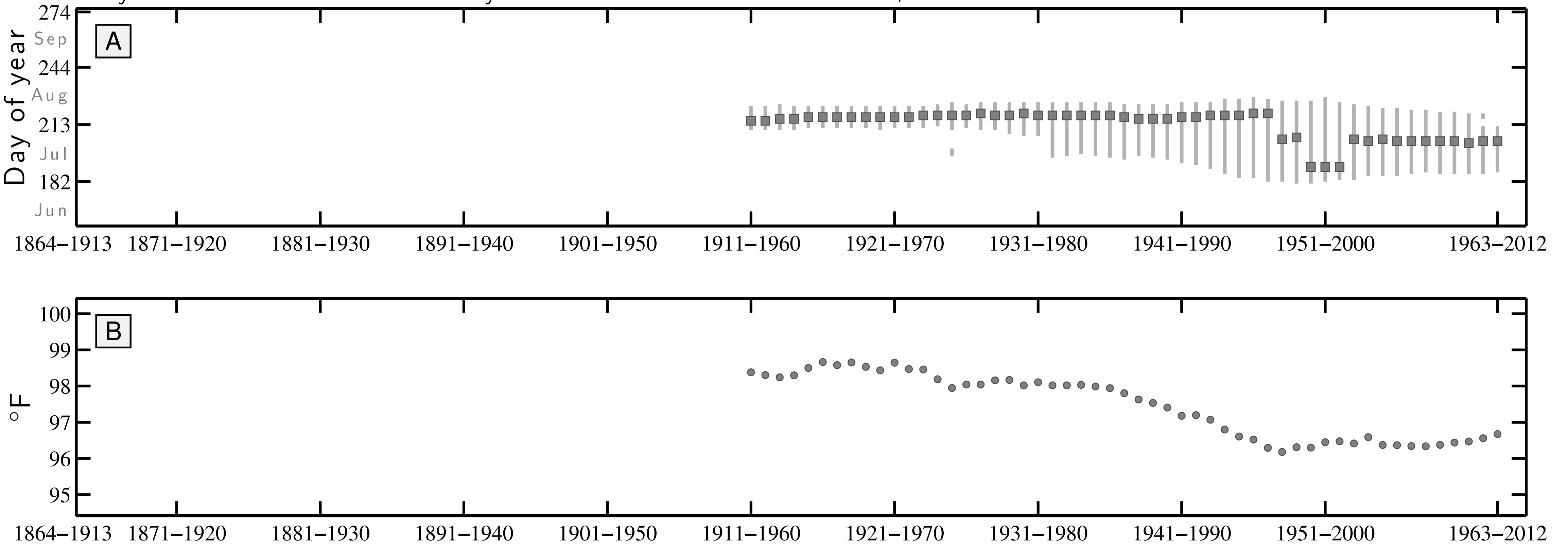

50 year Winter Teletherm dynamics for CARNEGIE 5 NE, OK:

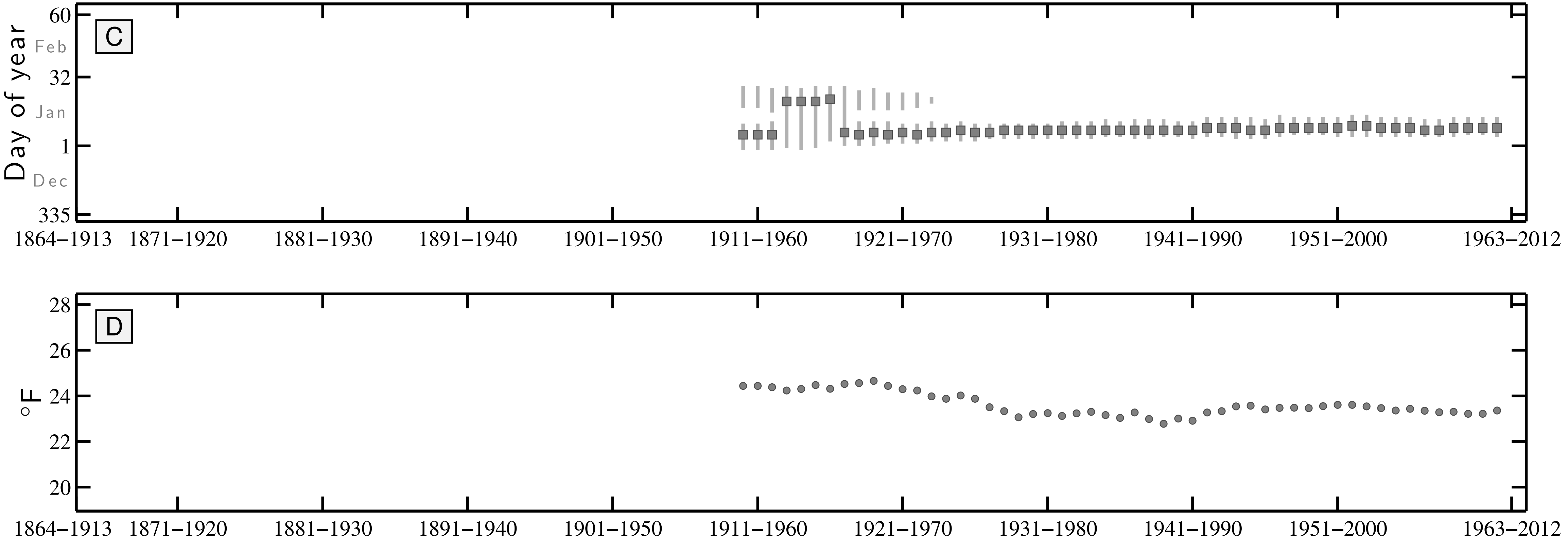

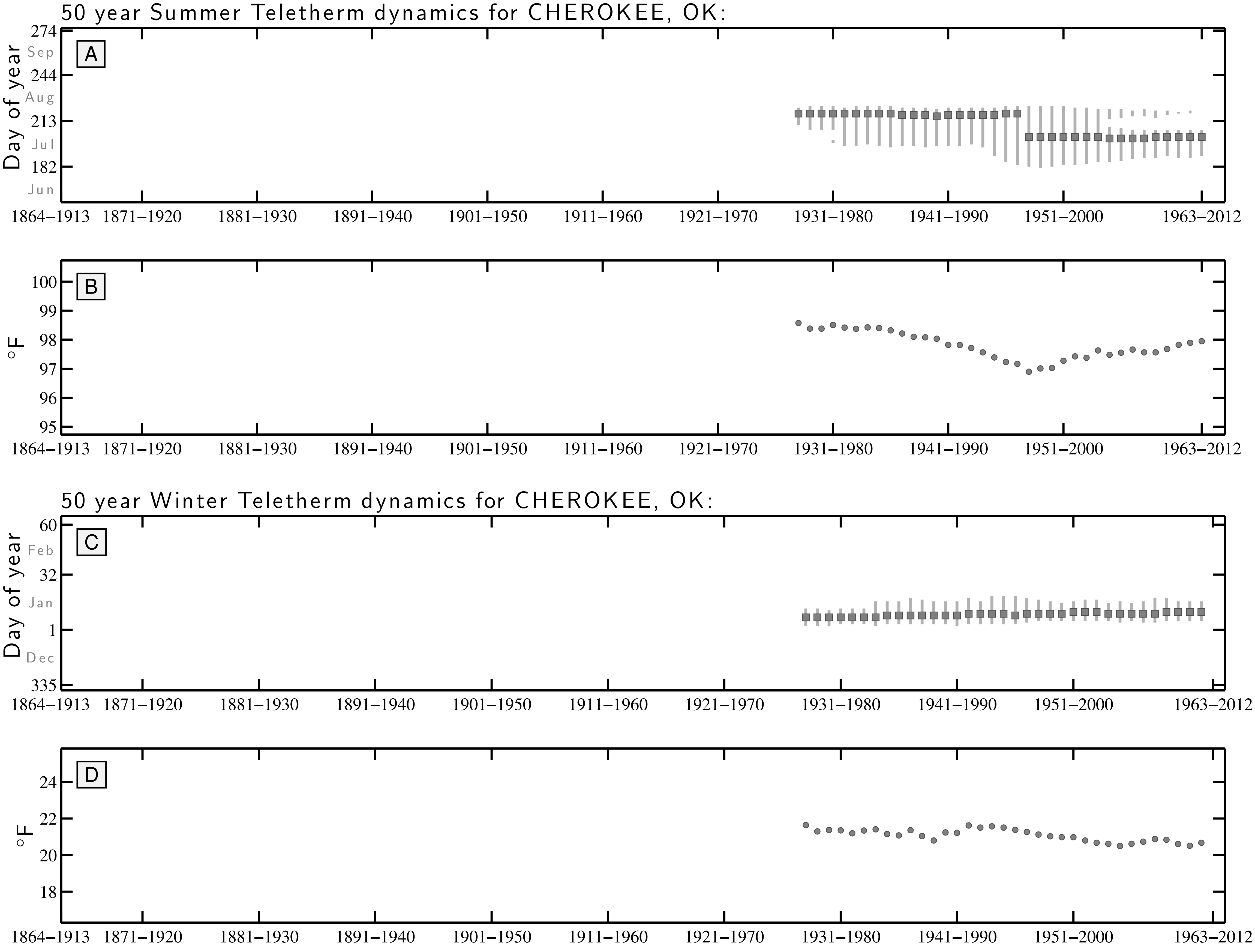
50 year Summer Teletherm dynamics for CHEROKEE, OK:
A
Day of year
274
Sep
244
Aug
213
Jul
182
Jun
1864–1913
1871–1920
1881–1930
1891–1940
1901–1950
1911–1960
1921–1970
1931–1980
1941–1990
1951–2000
1963–2012
B
°F
100
99
98
97
96
95
50 year Winter Teletherm dynamics for CHEROKEE, OK:
C
Day of year
60
Feb
32
Jan
1
Dec
335
D
°F
24
22
20
18

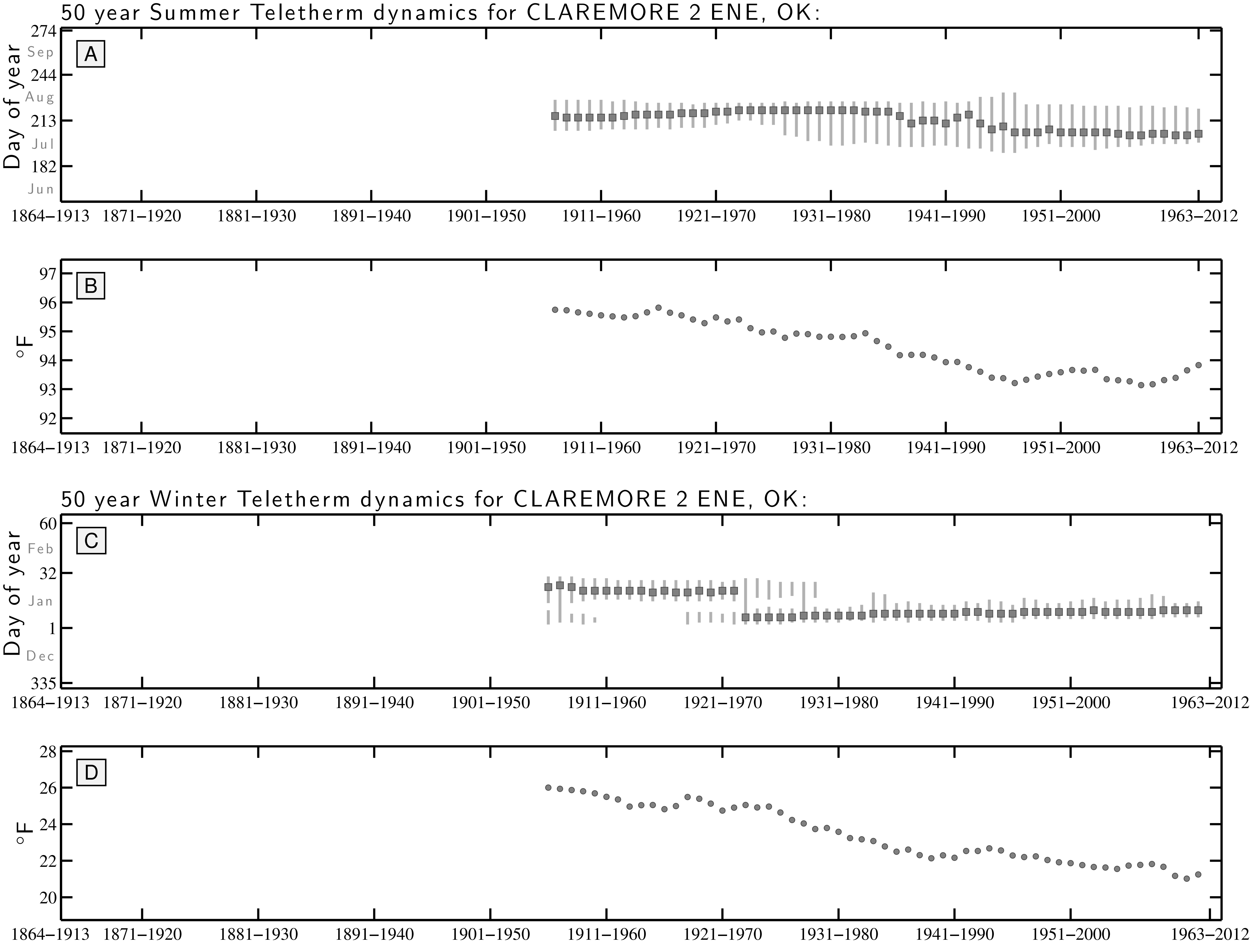
50 year Summer Teletherm dynamics for CLAREMORE 2 ENE, OK:
A
B
Day of year
°F
Jun
Jul
Aug
Sep
182
213
244
274
92
93
94
95
96
97
1864–1913
1871–1920
1881–1930
1891–1940
1901–1950
1911–1960
1921–1970
1931–1980
1941–1990
1951–2000
1963–2012
50 year Winter Teletherm dynamics for CLAREMORE 2 ENE, OK:
C
D
Dec
Jan
Feb
335
1
32
60
20
22
24
26
28

50 year Summer Teletherm dynamics for DURANT, OK:

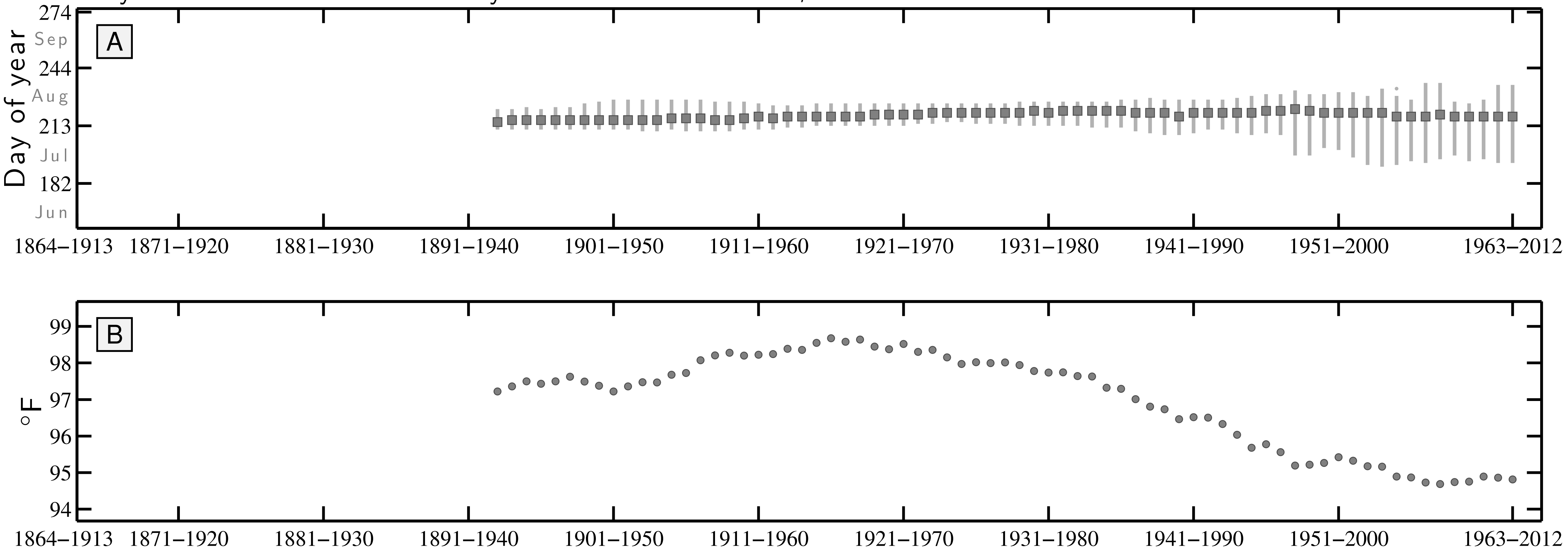

50 year Winter Teletherm dynamics for DURANT, OK:

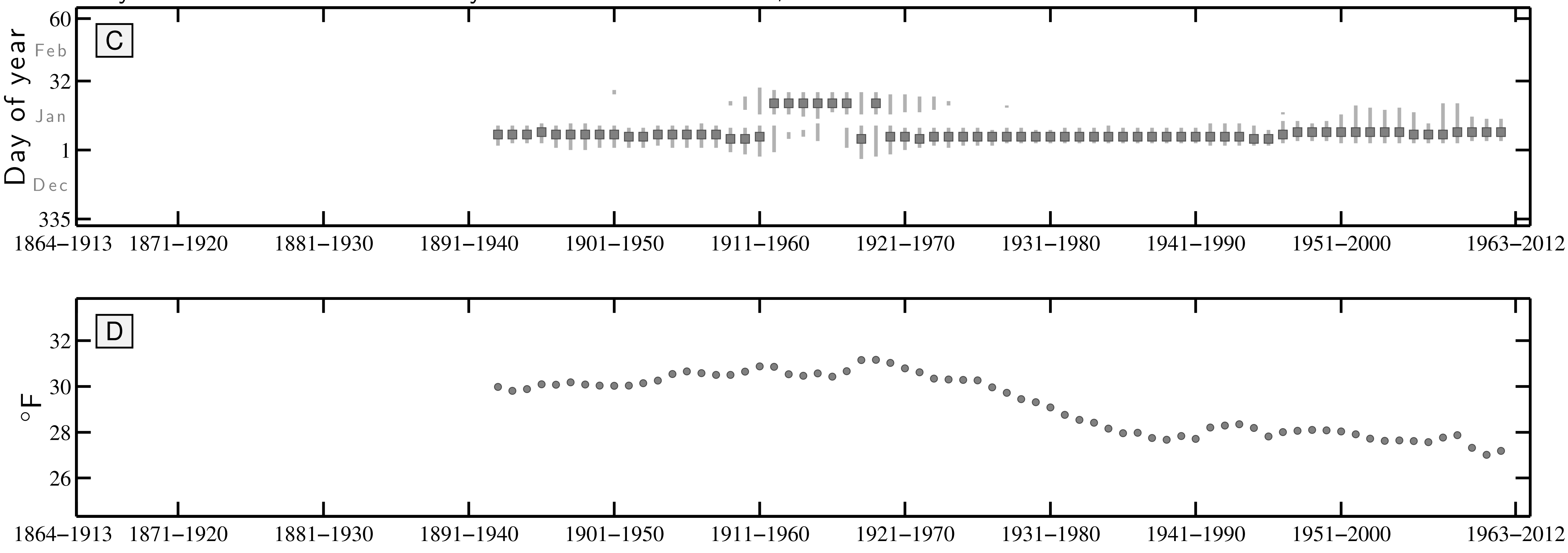

50 year Summer Teletherm dynamics for ENID, OK:

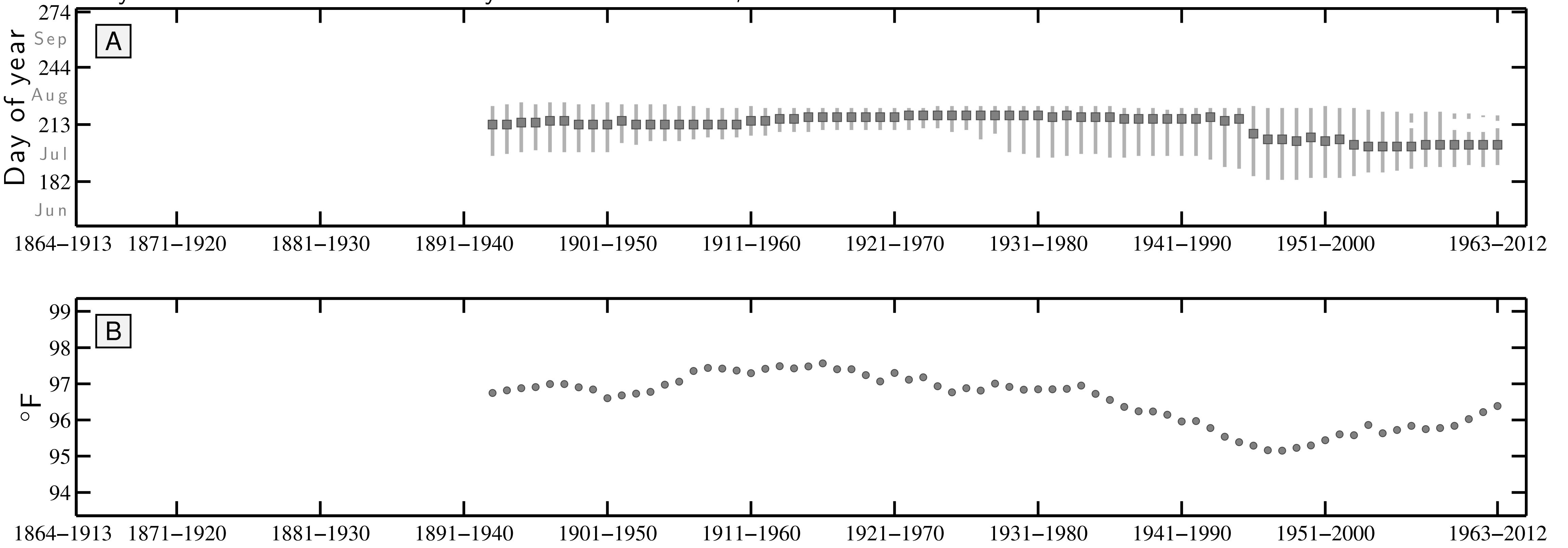

50 year Winter Teletherm dynamics for ENID, OK:

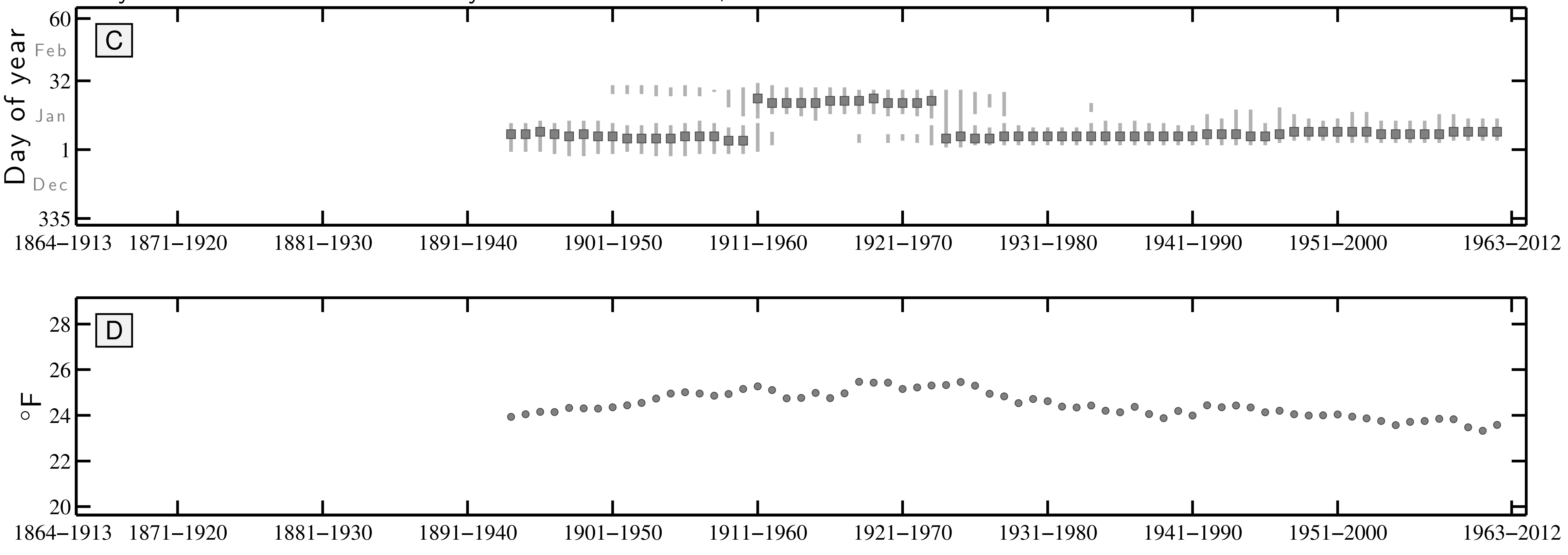

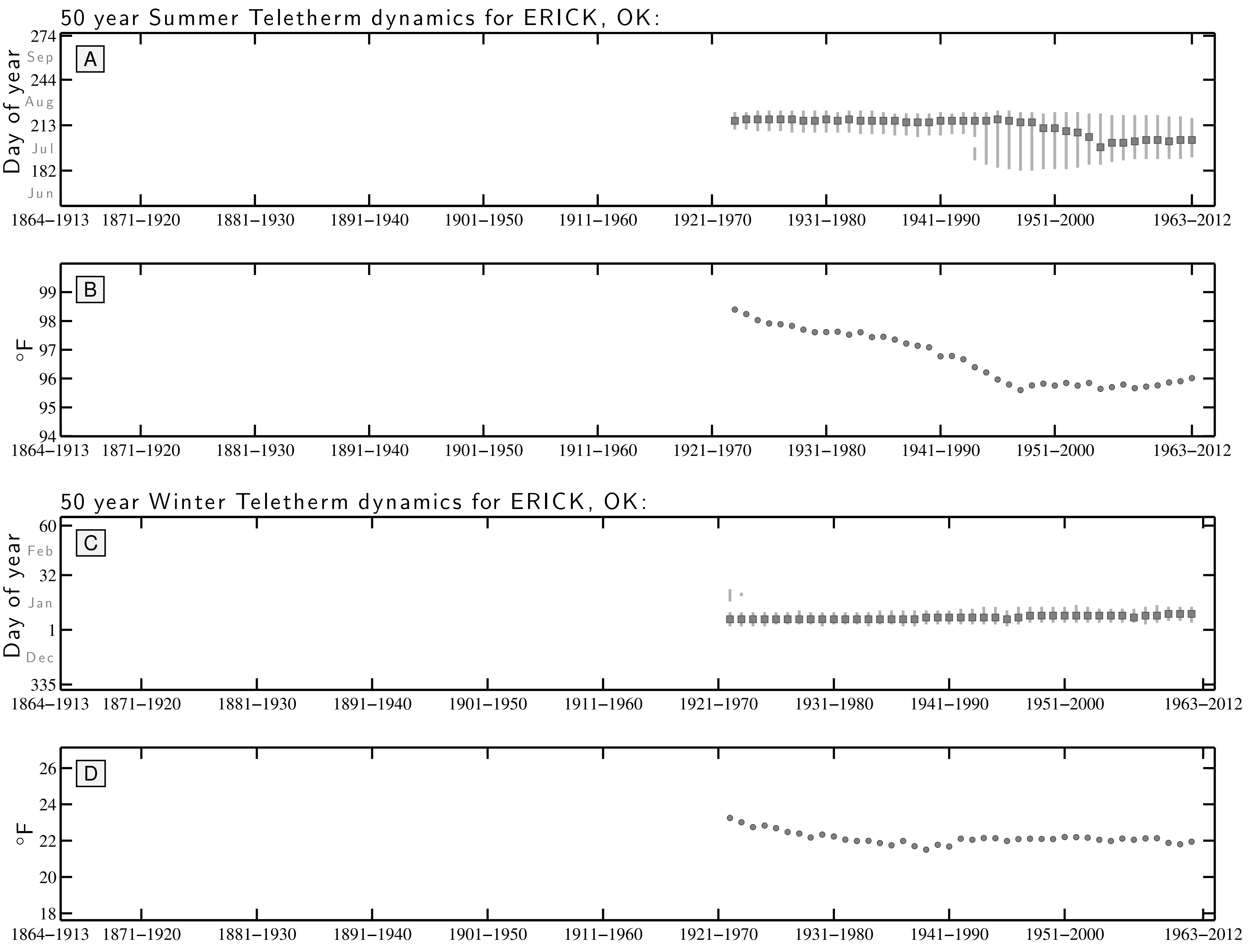

50 year Summer Teletherm dynamics for ERICK, OK:
A
Day of year
274
Sep
244
Aug
213
Jul
182
Jun
B
°F
99
98
97
96
95
94
50 year Winter Teletherm dynamics for ERICK, OK:
C
Day of year
60
Feb
32
Jan
1
Dec
335
D
°F
26
24
22
20
18
1864–1913
1871–1920
1881–1930
1891–1940
1901–1950
1911–1960
1921–1970
1931–1980
1941–1990
1951–2000
1963–2012

50 year Summer Teletherm dynamics for GEARY, OK:

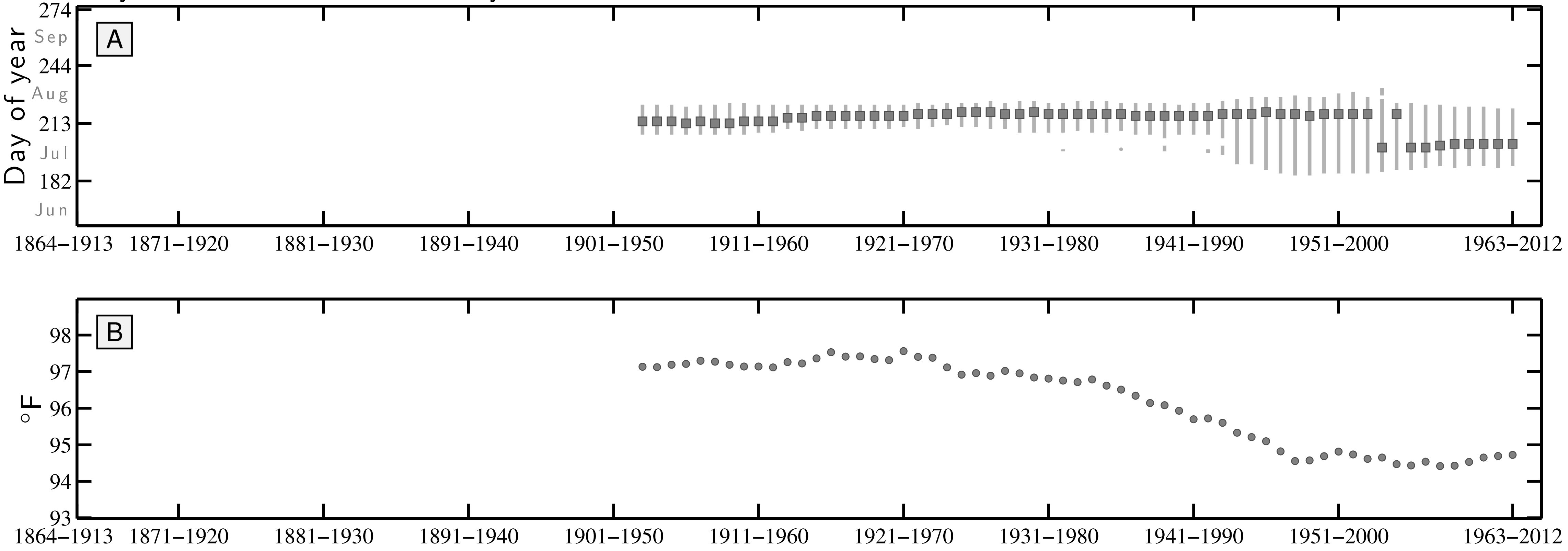

50 year Winter Teletherm dynamics for GEARY, OK:

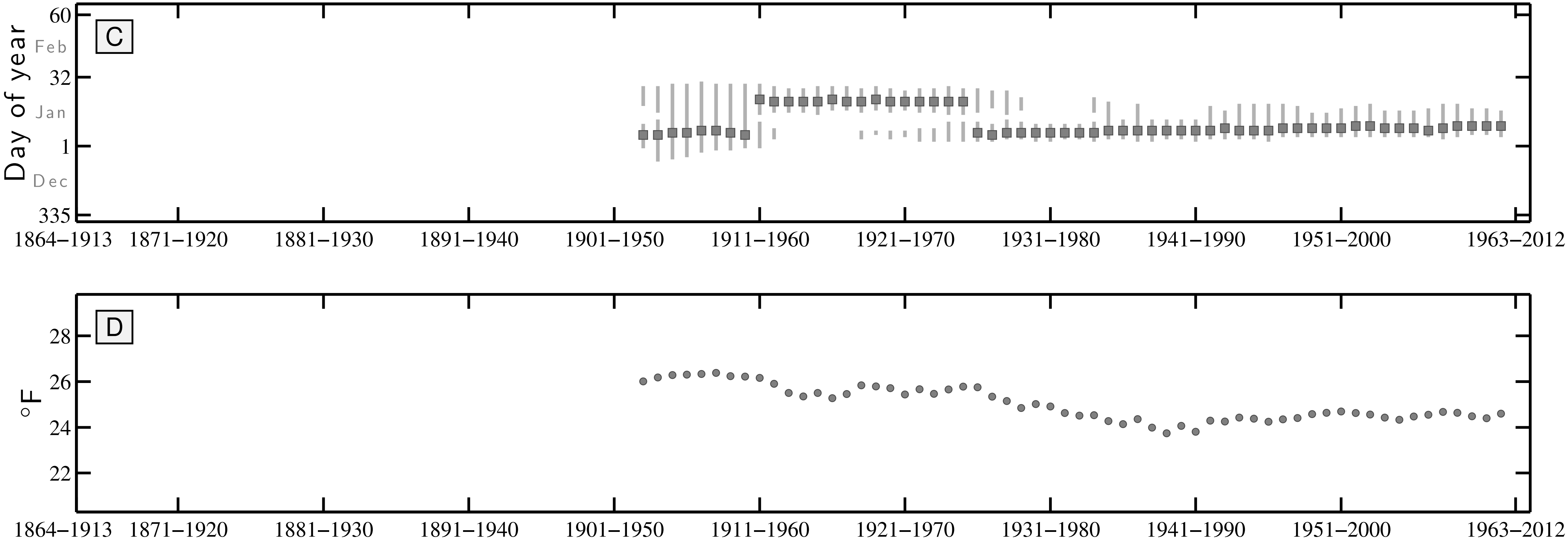

50 year Summer Teletherm dynamics for GOODWELL RSCH STN, OK:

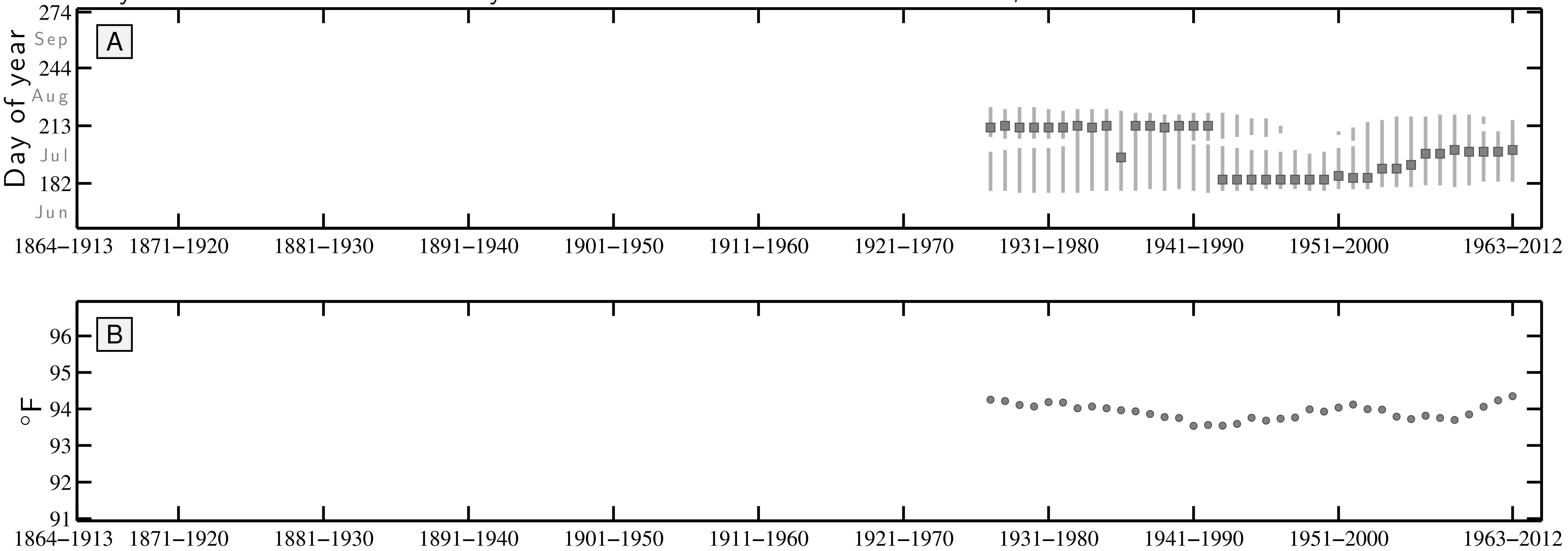

50 year Winter Teletherm dynamics for GOODWELL RSCH STN, OK:

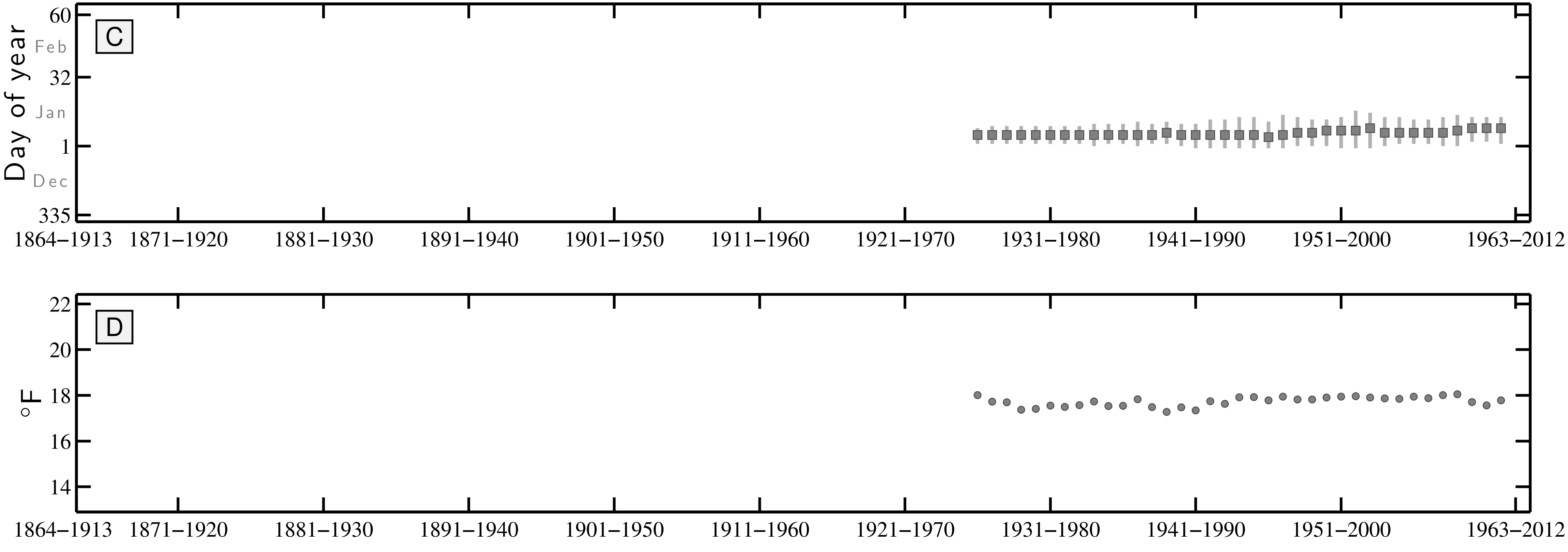

50 year Summer Teletherm dynamics for GUTHRIE 5S, OK:

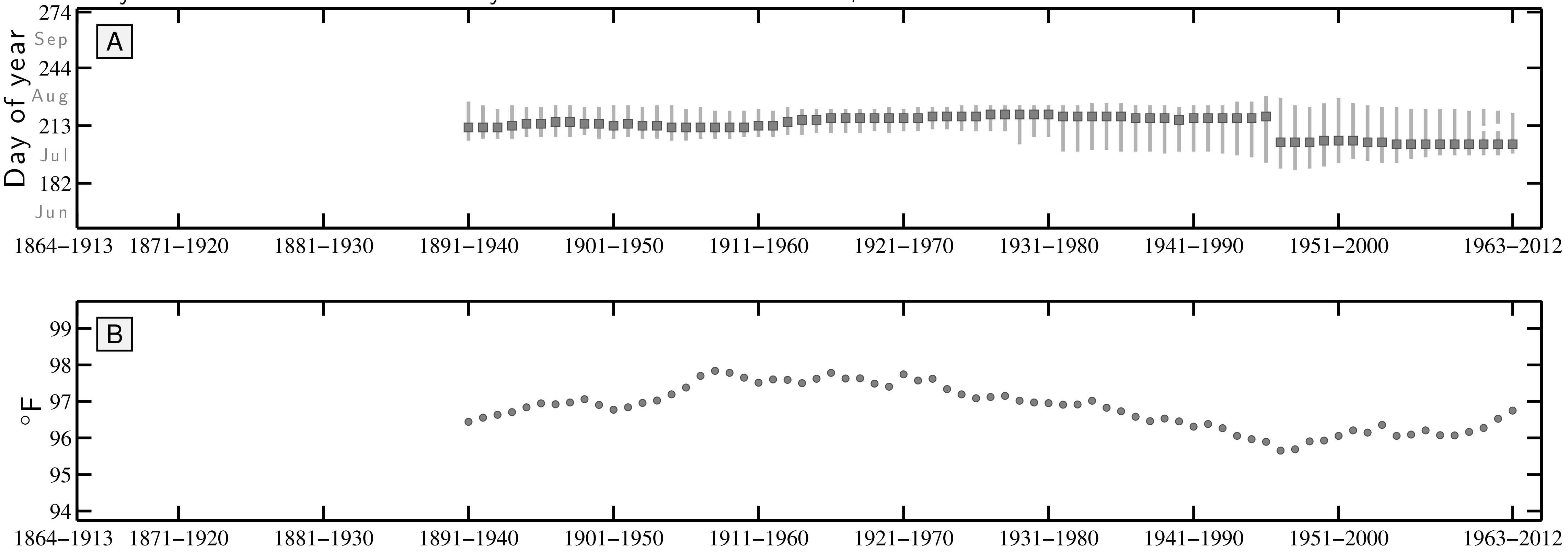

50 year Winter Teletherm dynamics for GUTHRIE 5S, OK:

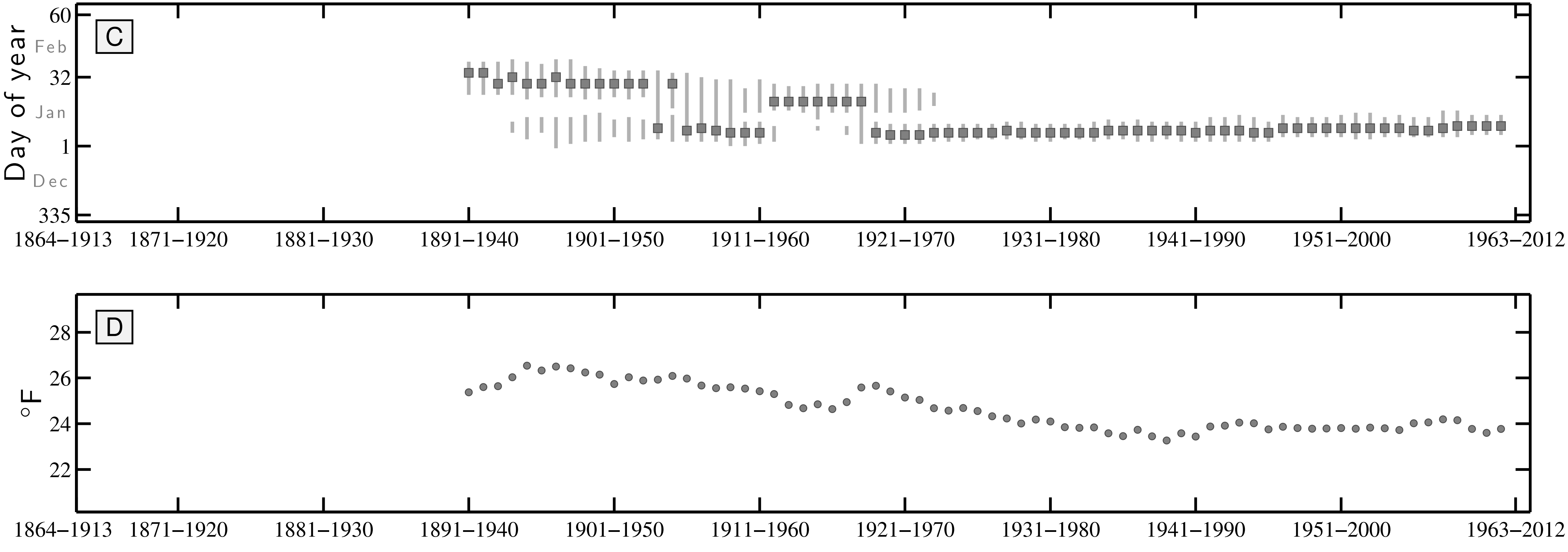

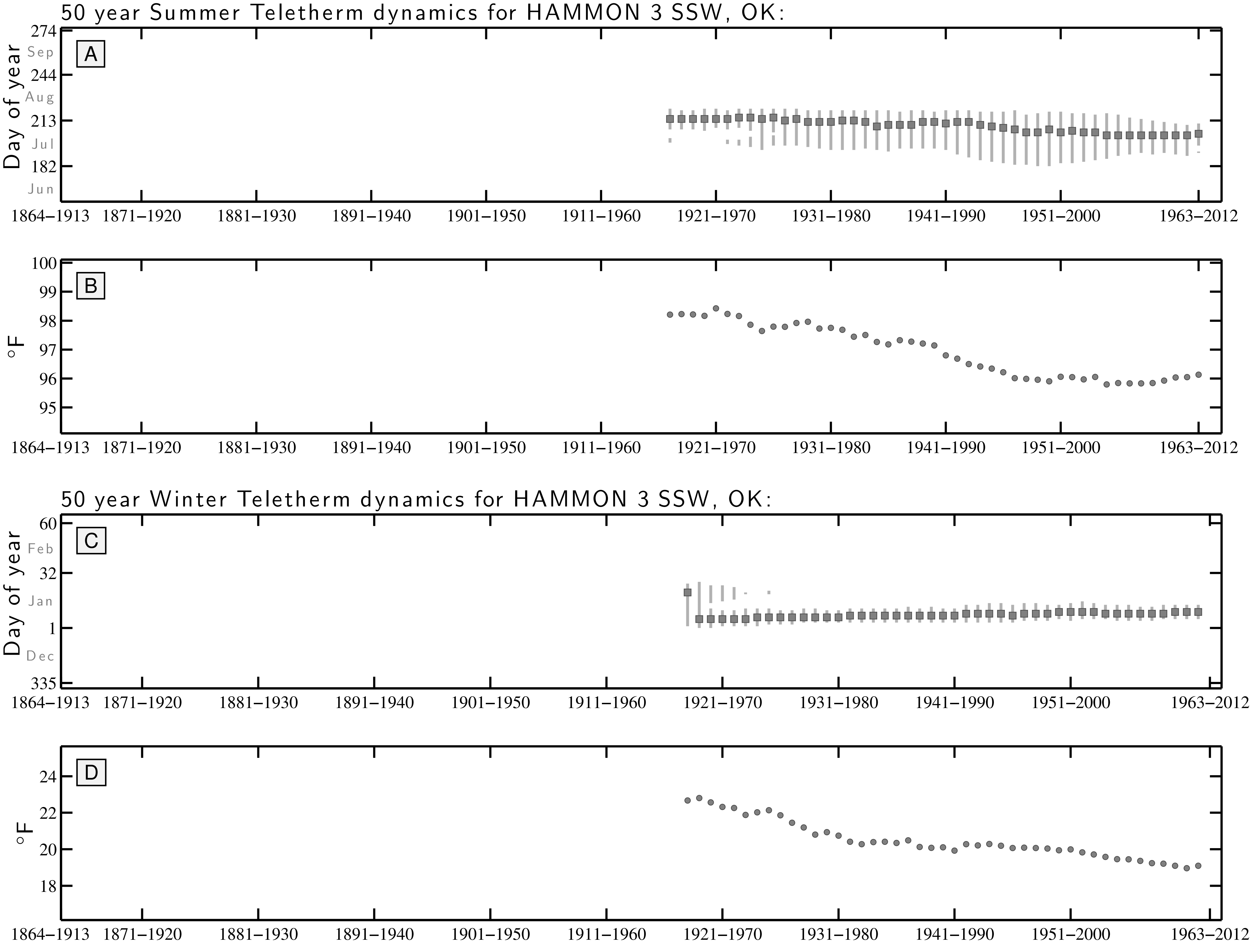
50 year Summer Teletherm dynamics for HAMMON 3 SSW, OK:
A
Day of year
274
Sep
244
Aug
213
Jul
182
Jun
1864–1913
1871–1920
1881–1930
1891–1940
1901–1950
1911–1960
1921–1970
1931–1980
1941–1990
1951–2000
1963–2012
B
°F
100
99
98
97
96
95
50 year Winter Teletherm dynamics for HAMMON 3 SSW, OK:
C
60
Feb
32
Jan
1
Dec
335
D
24
22
20
18

50 year Summer Teletherm dynamics for HENNESSEY 4 ESE, OK:

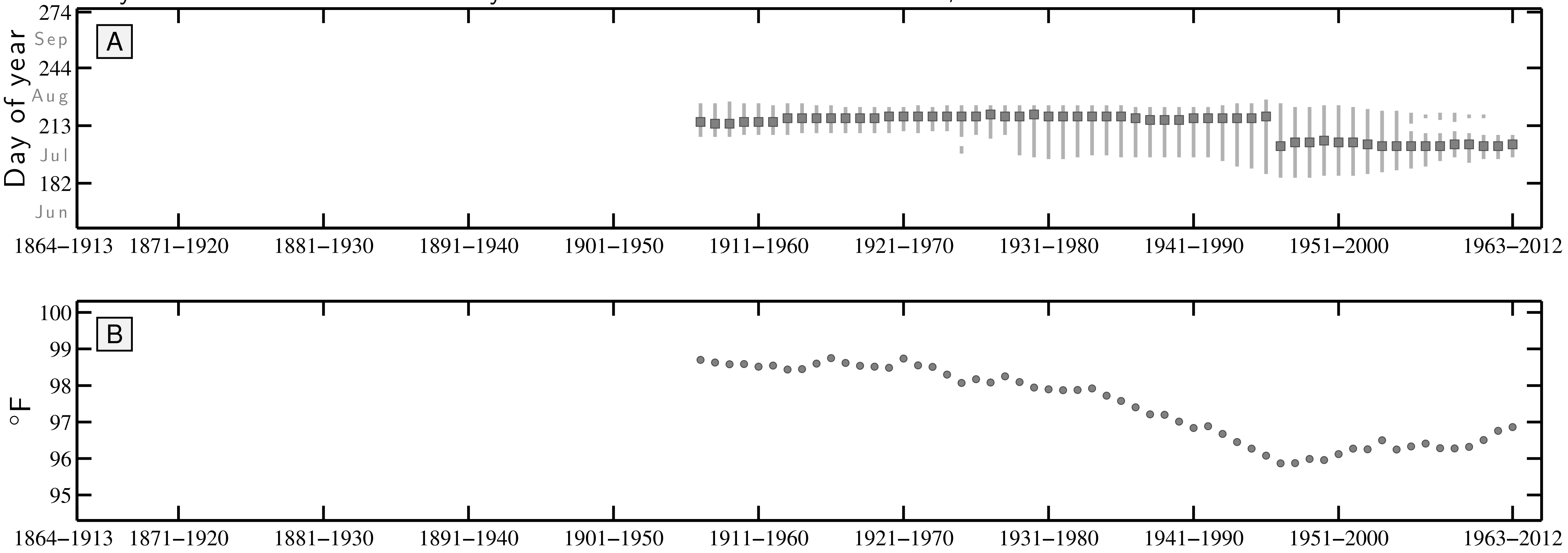

50 year Winter Teletherm dynamics for HENNESSEY 4 ESE, OK:

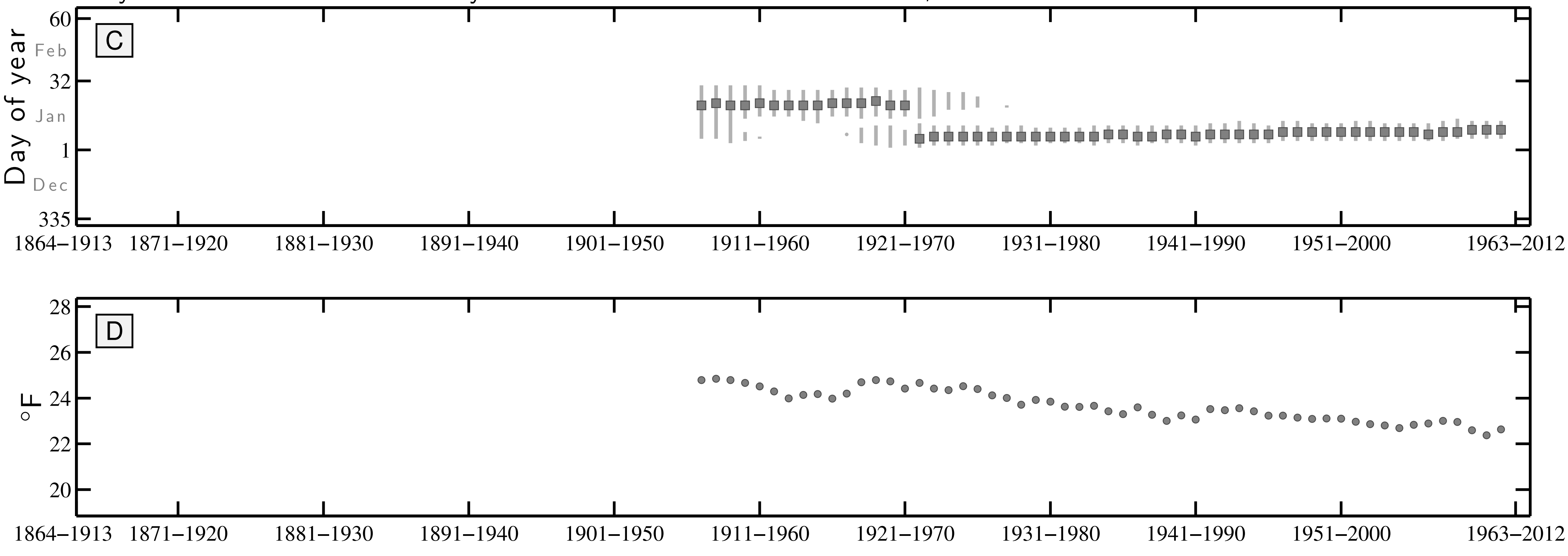

50 year Summer Teletherm dynamics for HOBART MUNI AP, OK:

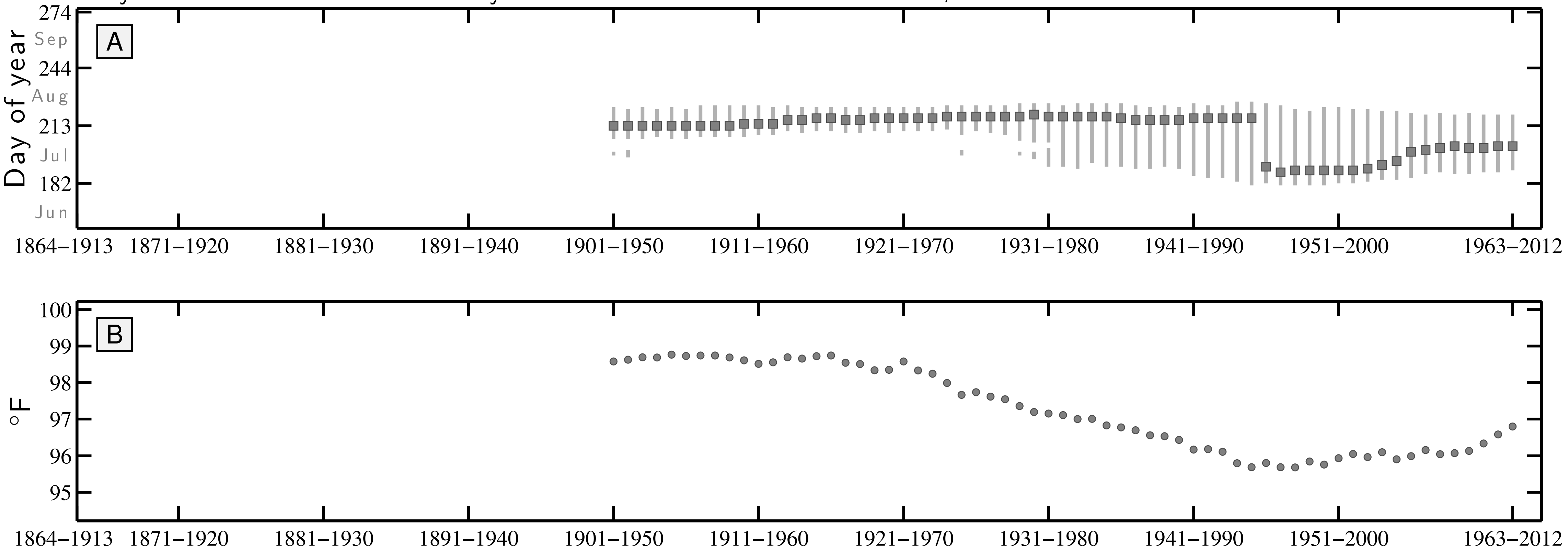

50 year Winter Teletherm dynamics for HOBART MUNI AP, OK:

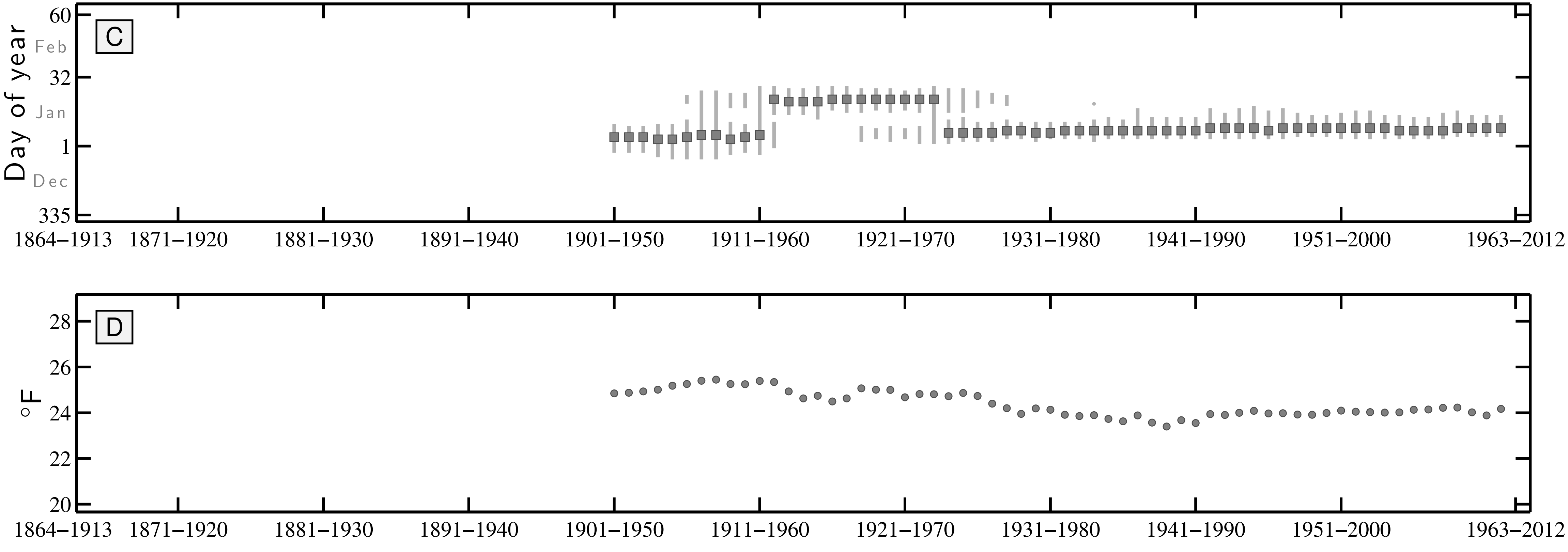

50 year Summer Teletherm dynamics for HOLDENVILLE 2SSE, OK:

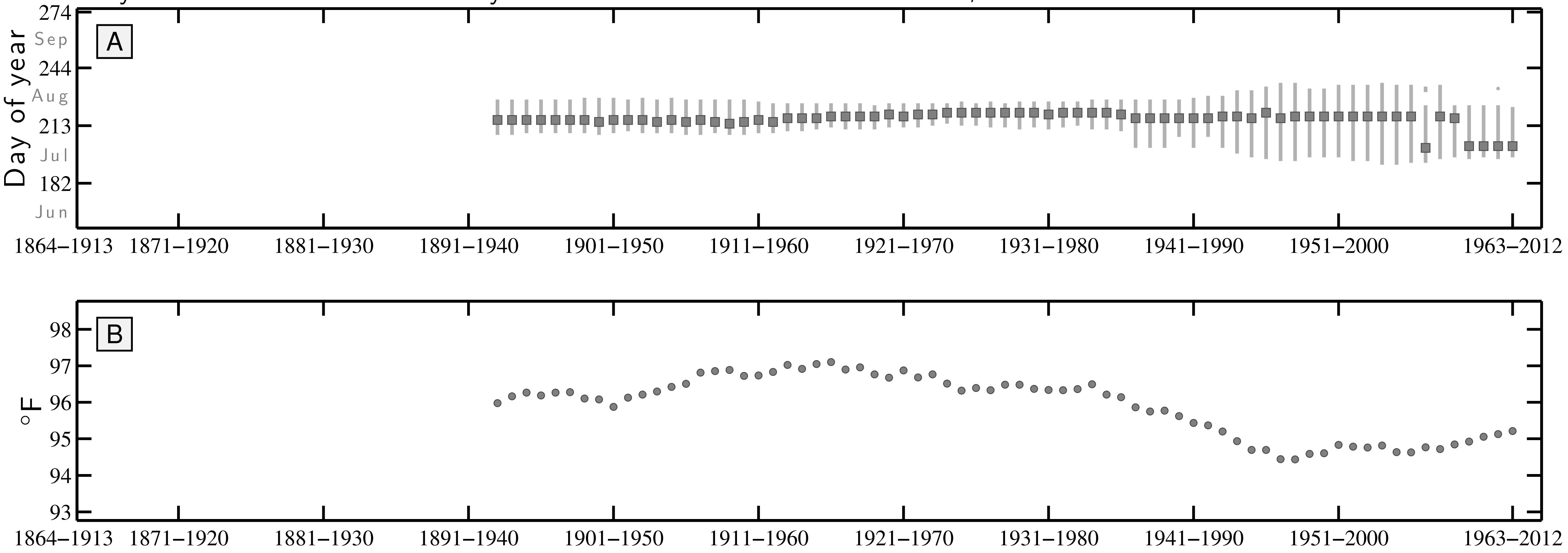

50 year Winter Teletherm dynamics for HOLDENVILLE 2SSE, OK:

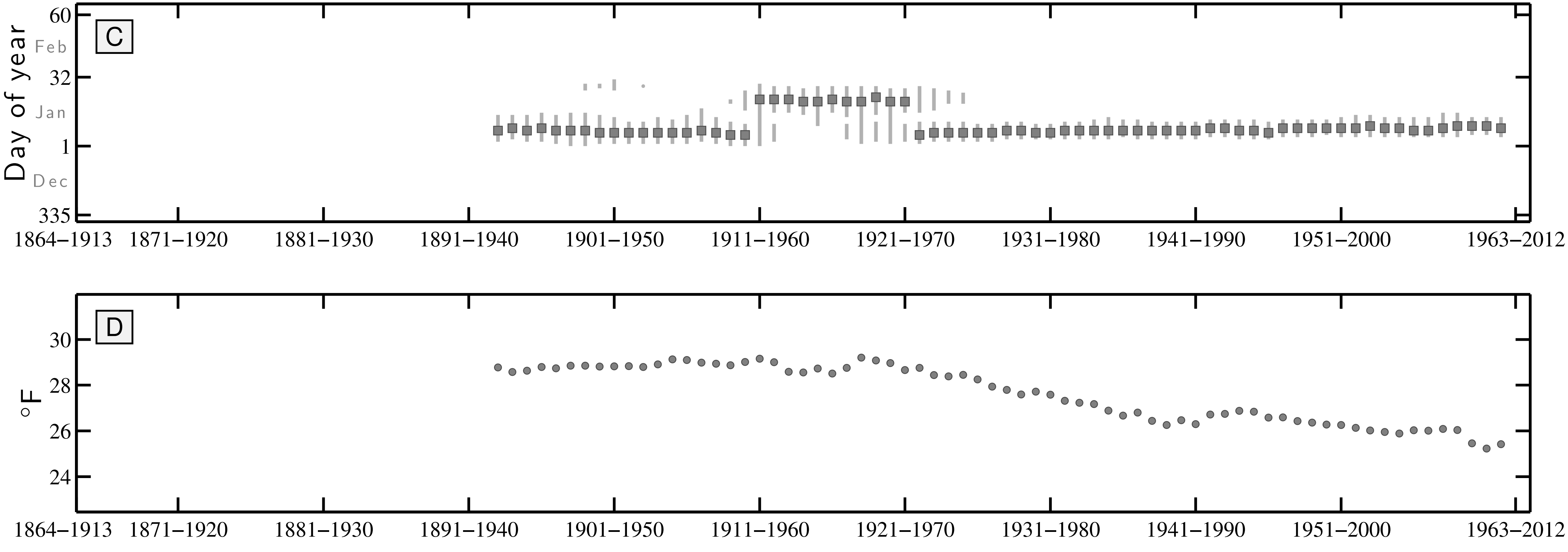

50 year Summer Teletherm dynamics for HOOKER, OK:

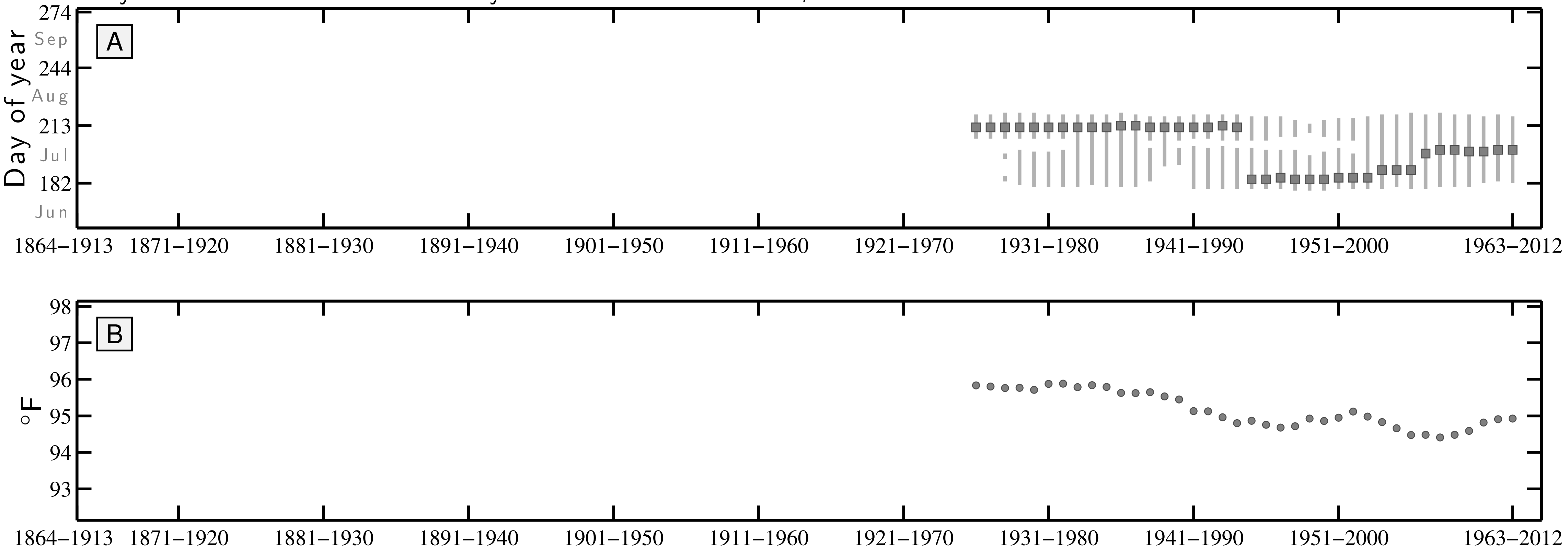

50 year Winter Teletherm dynamics for HOOKER, OK:

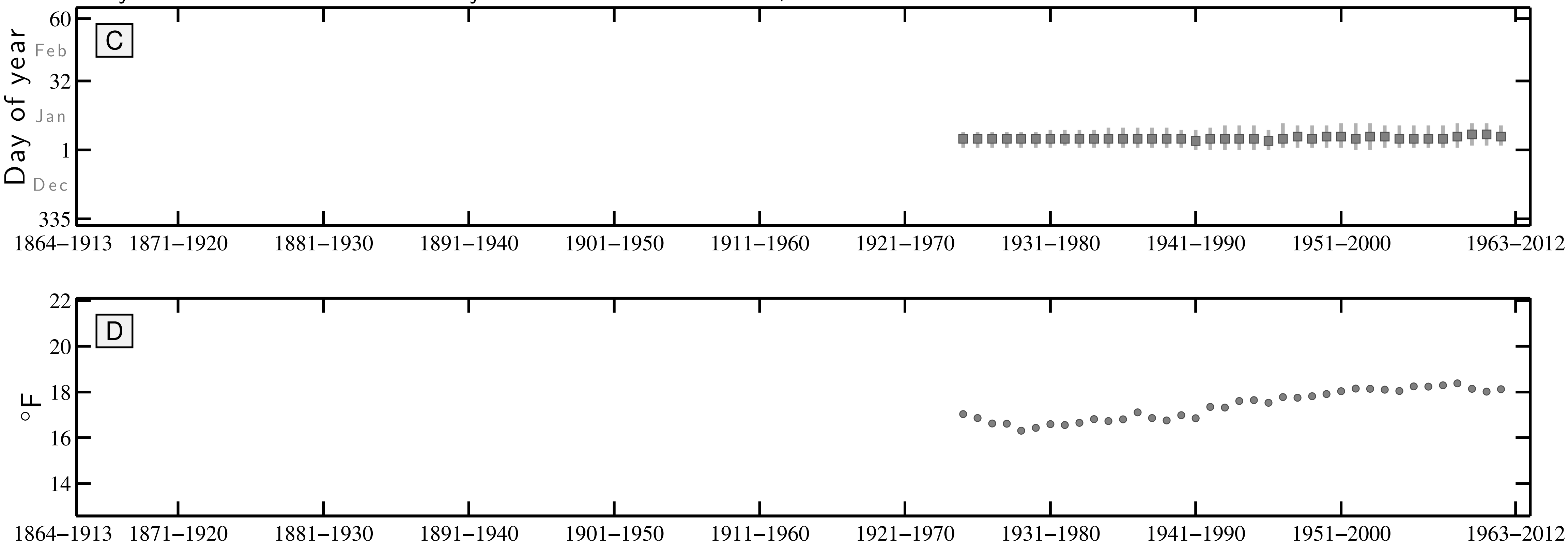

50 year Summer Teletherm dynamics for JEFFERSON, OK:

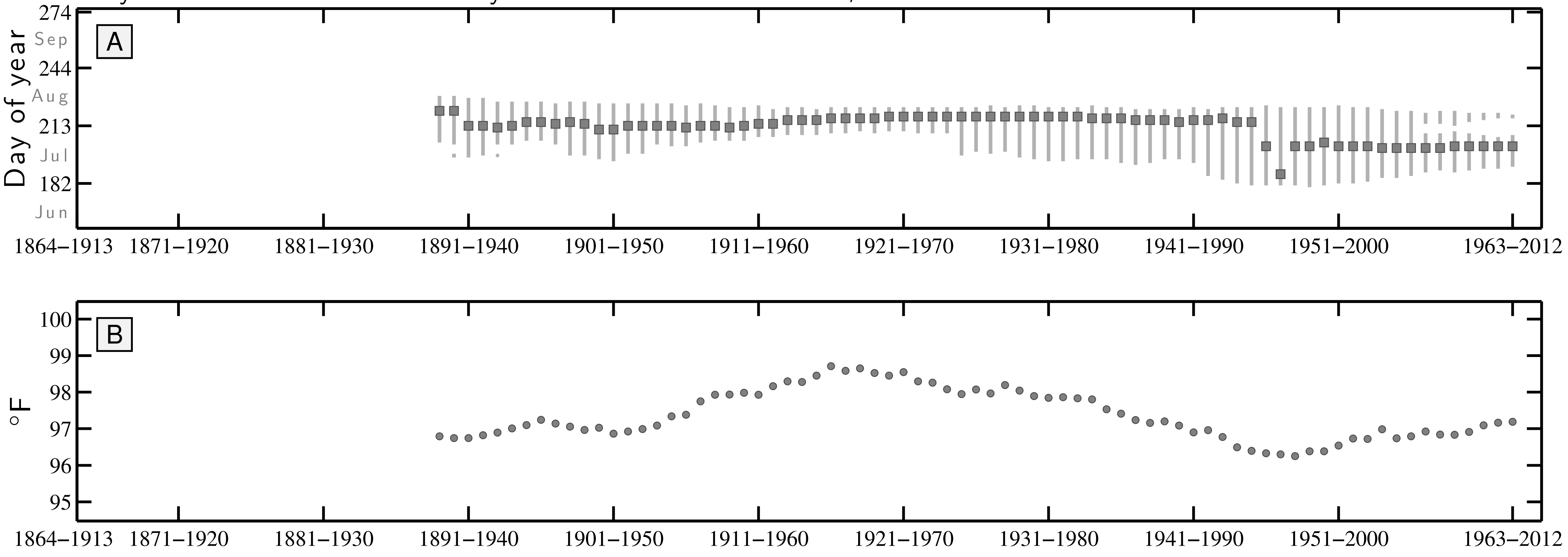

50 year Winter Teletherm dynamics for JEFFERSON, OK:

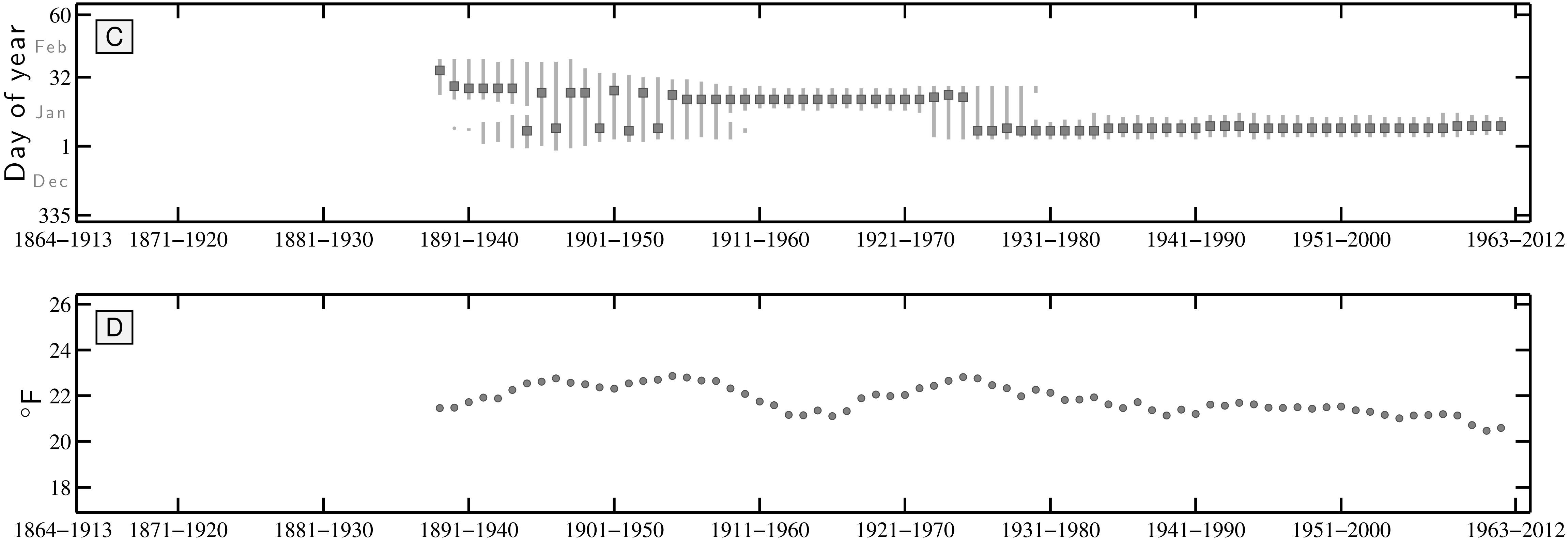

50 year Summer Teletherm dynamics for KENTON, OK:

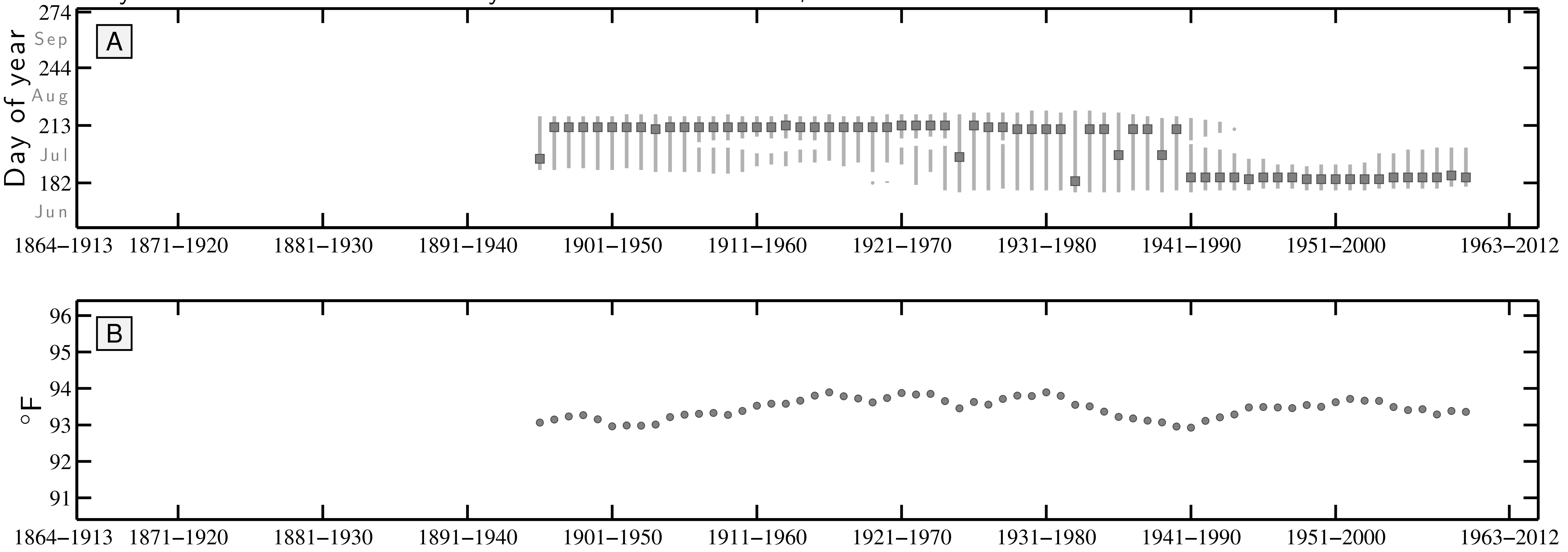

50 year Winter Teletherm dynamics for KENTON, OK:

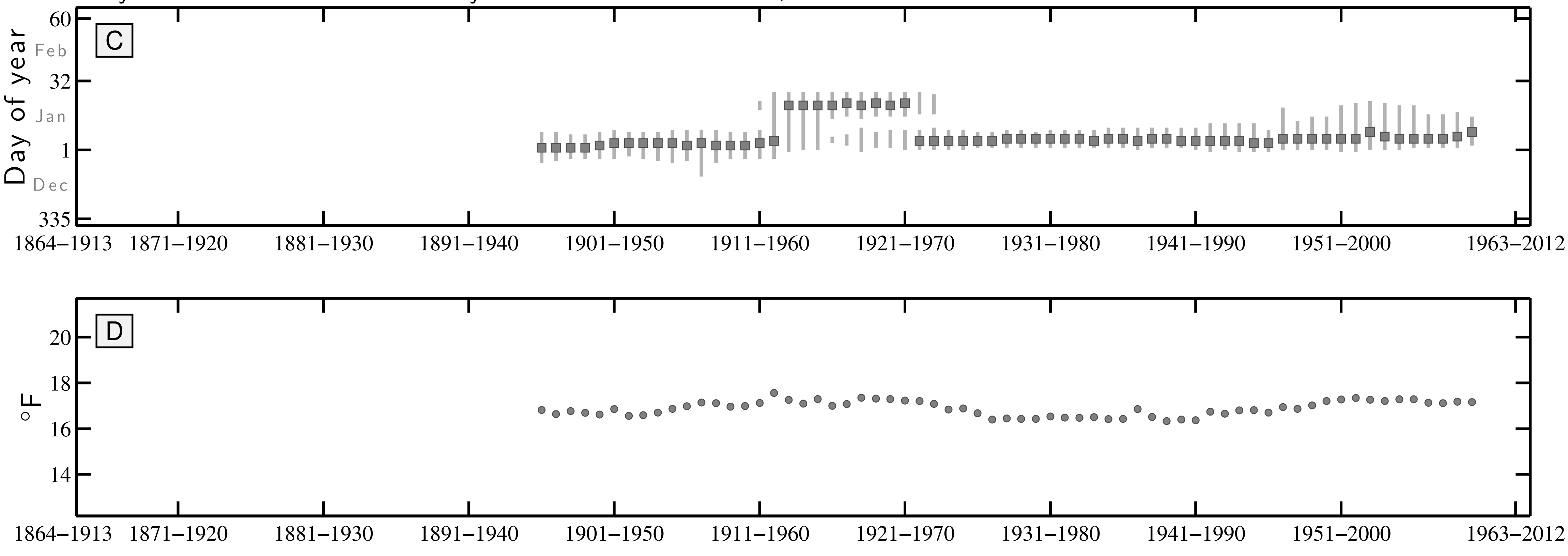

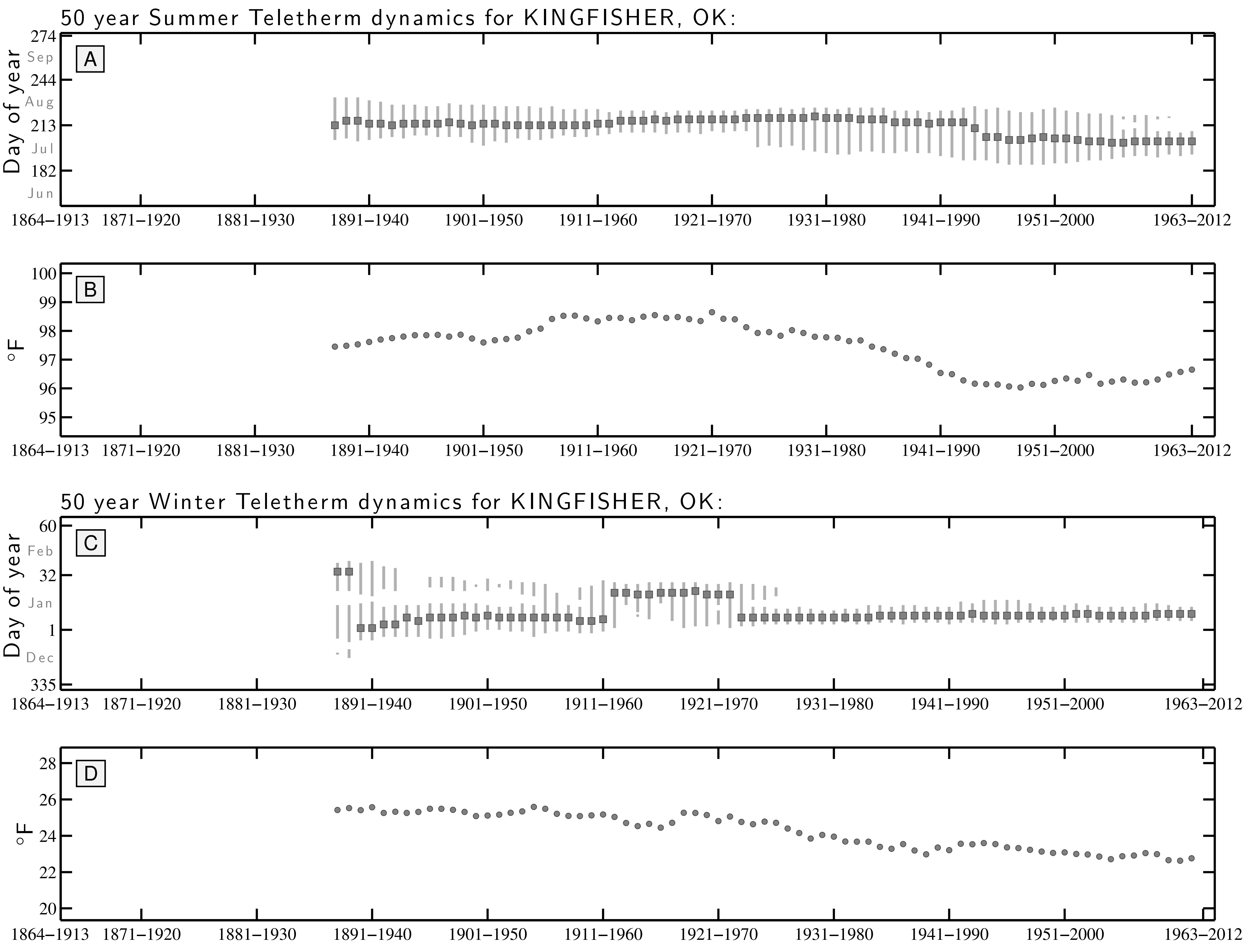

50 year Summer Teletherm dynamics for KINGFISHER, OK:
A
B
50 year Winter Teletherm dynamics for KINGFISHER, OK:
C
D
Day of year
°F
274
Sep
244
Aug
213
Jul
182
Jun
100
99
98
97
96
95
60
Feb
32
Jan
1
Dec
335
28
26
24
22
20
1864–1913
1871–1920
1881–1930
1891–1940
1901–1950
1911–1960
1921–1970
1931–1980
1941–1990
1951–2000
1963–2012

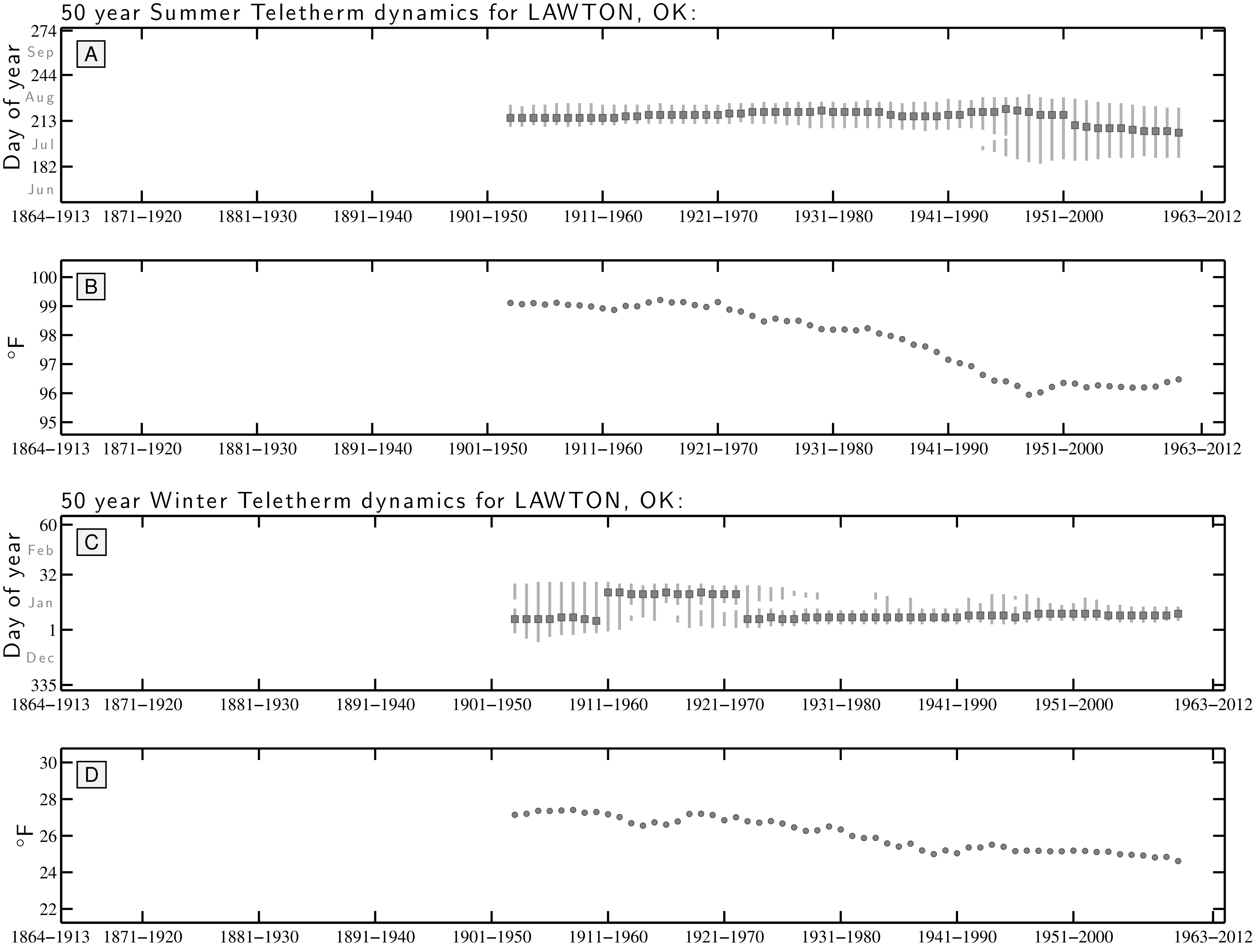
50 year Summer Teletherm dynamics for LAWTON, OK:
A
Day of year
274
Sep
244
Aug
213
Jul
182
Jun
1864–1913
1871–1920
1881–1930
1891–1940
1901–1950
1911–1960
1921–1970
1931–1980
1941–1990
1951–2000
1963–2012
B
°F
100
99
98
97
96
95
50 year Winter Teletherm dynamics for LAWTON, OK:
C
Day of year
60
Feb
32
Jan
1
Dec
335
D
°F
30
28
26
24
22

50 year Summer Teletherm dynamics for MANGUM, OK:

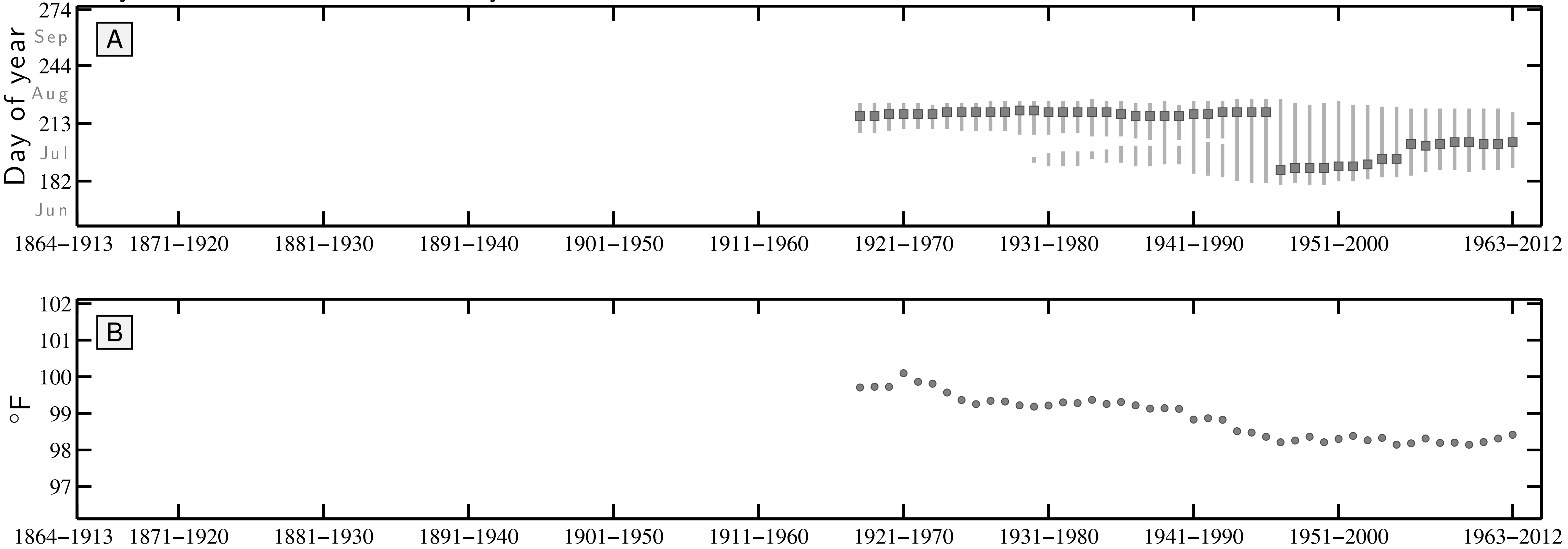

50 year Winter Teletherm dynamics for MANGUM, OK:

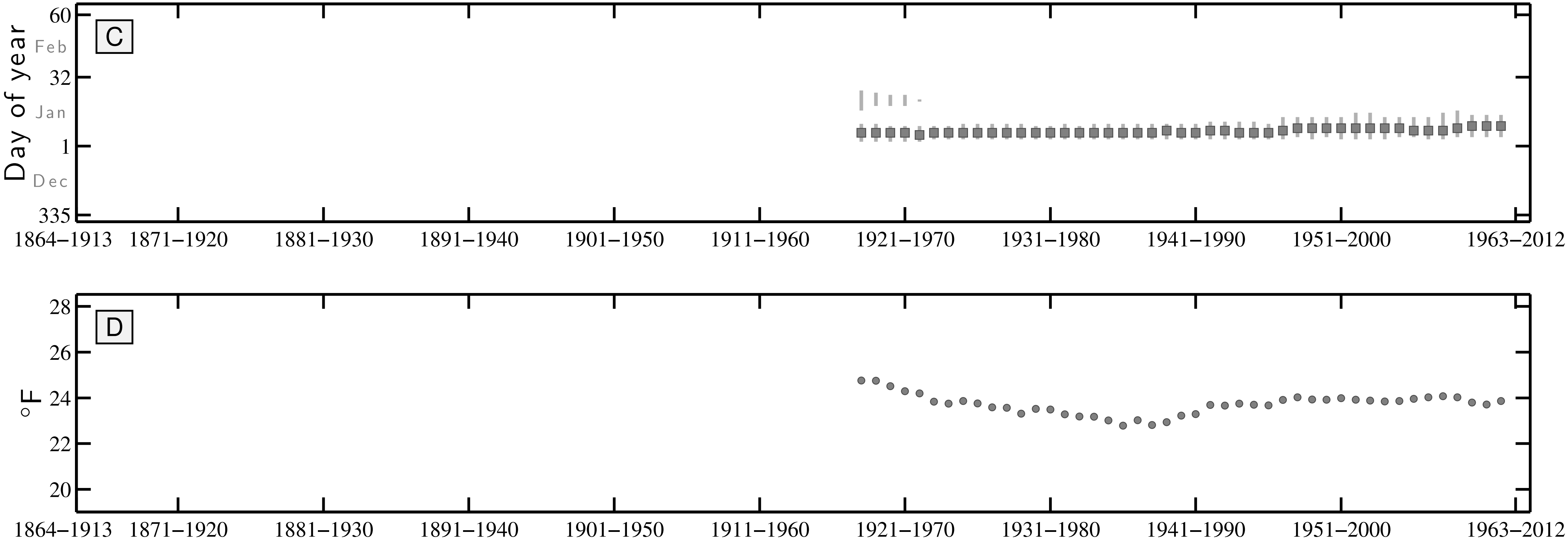

50 year Summer Teletherm dynamics for MEEKER 5 W, OK:

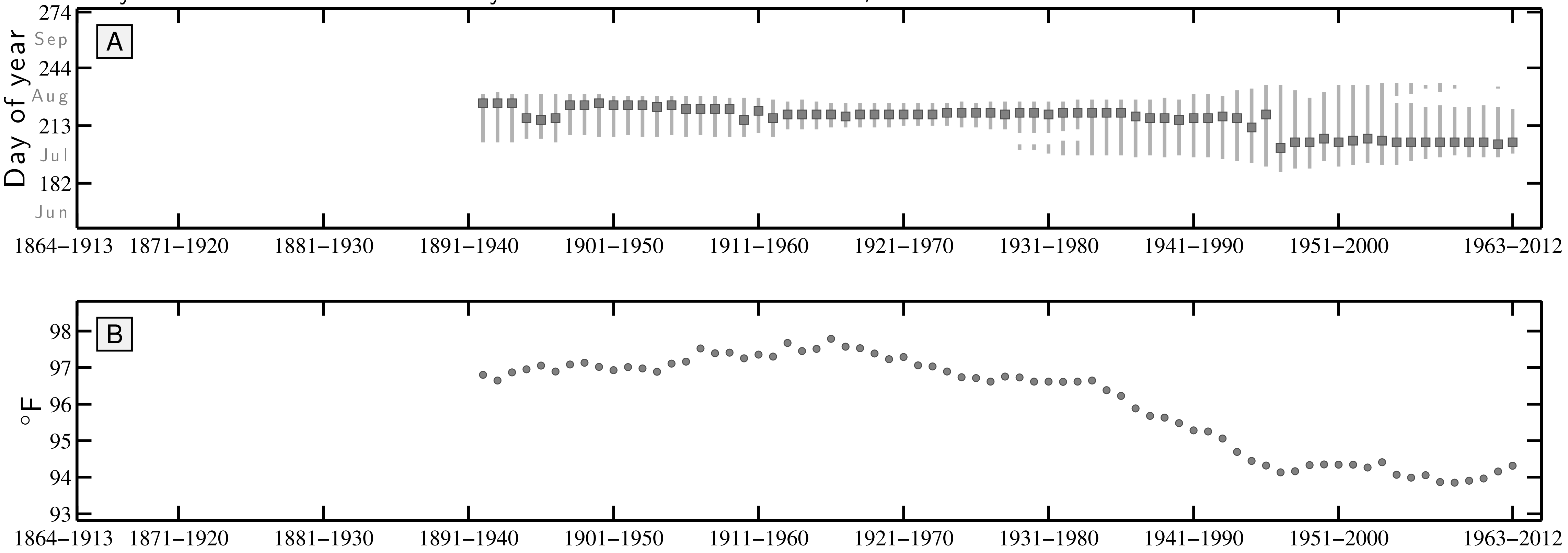

50 year Winter Teletherm dynamics for MEEKER 5 W, OK:

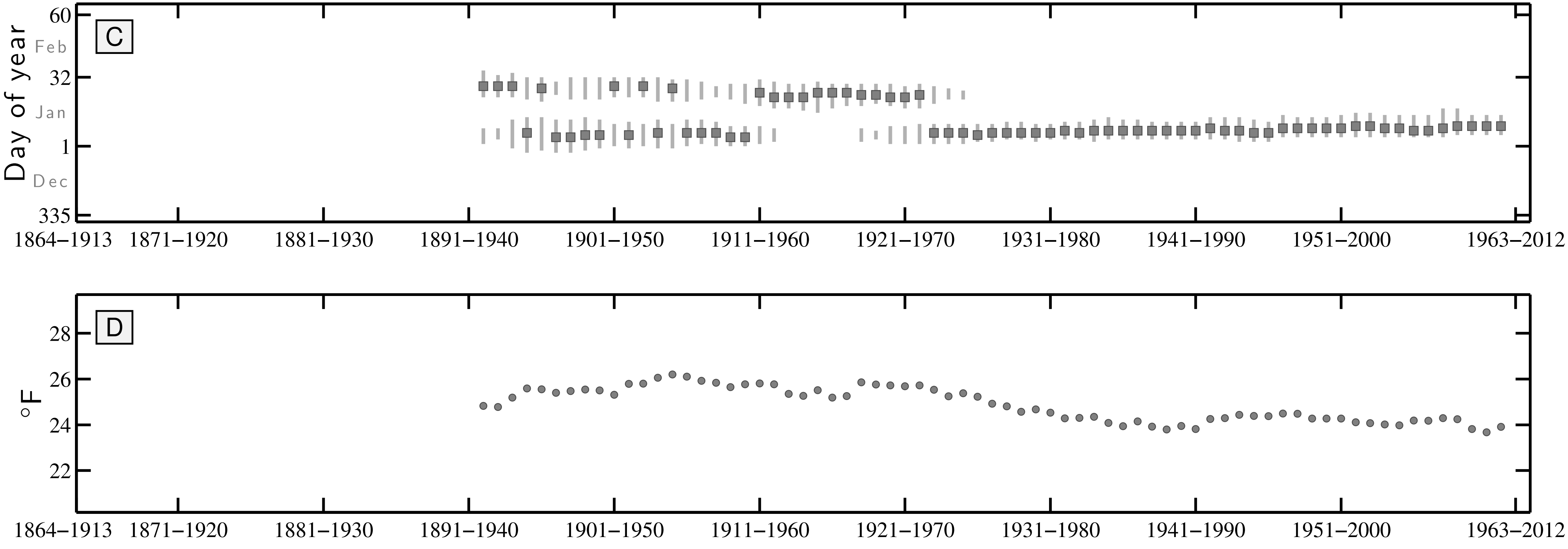

50 year Summer Teletherm dynamics for MIAMI, OK:

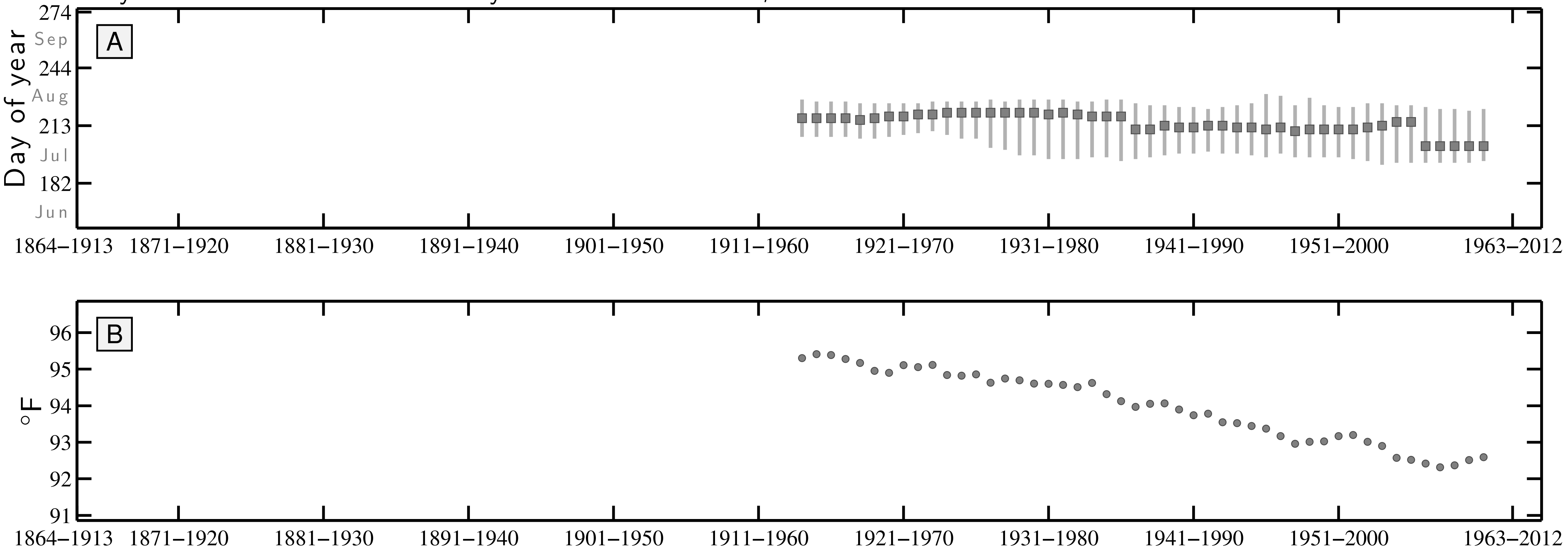

50 year Winter Teletherm dynamics for MIAMI, OK:

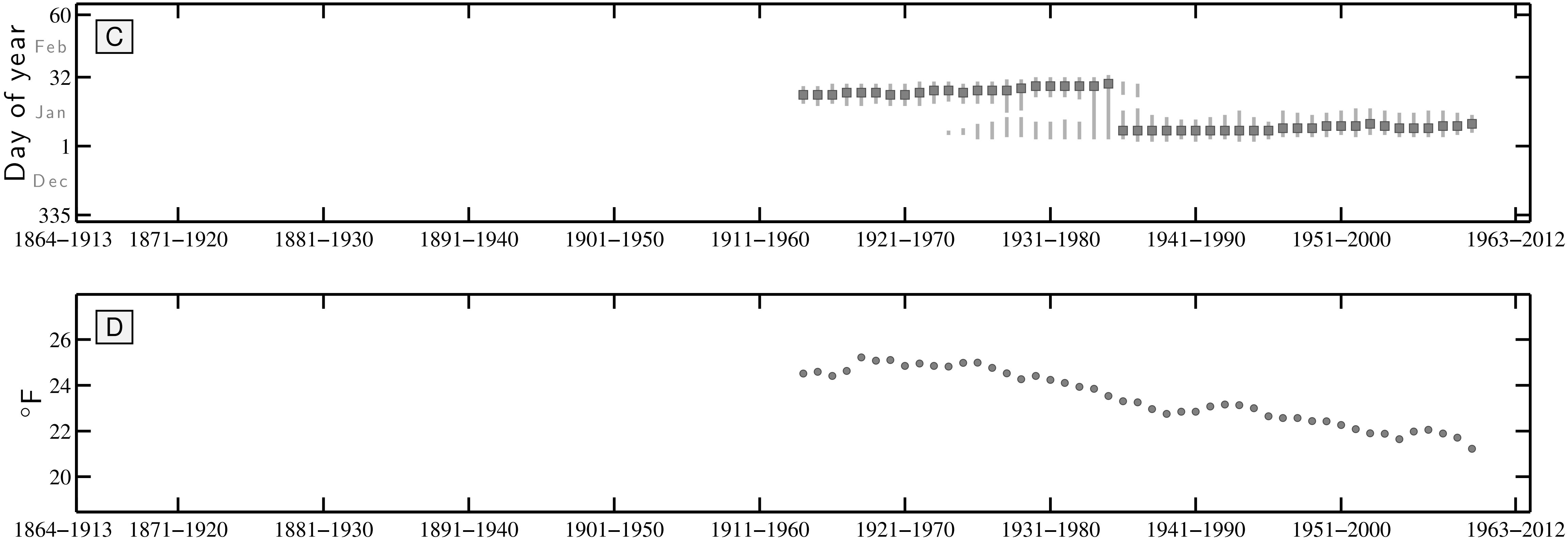

50 year Summer Teletherm dynamics for MUSKOGEE, OK:

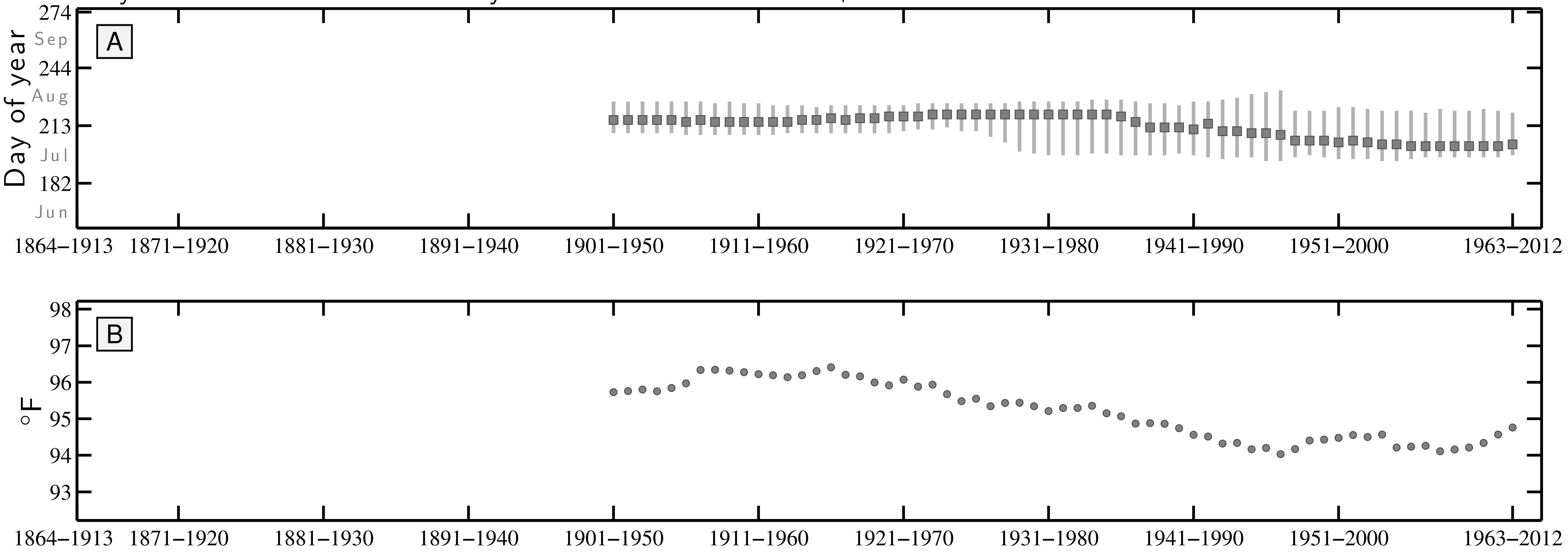

50 year Winter Teletherm dynamics for MUSKOGEE, OK:

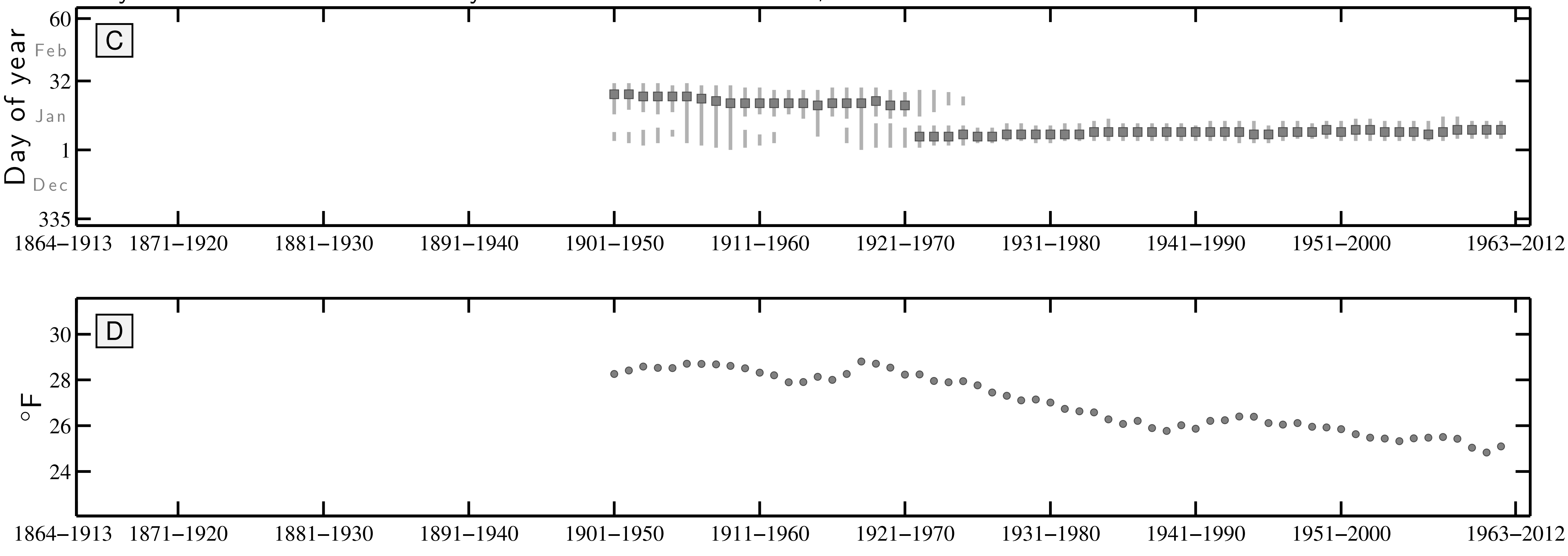

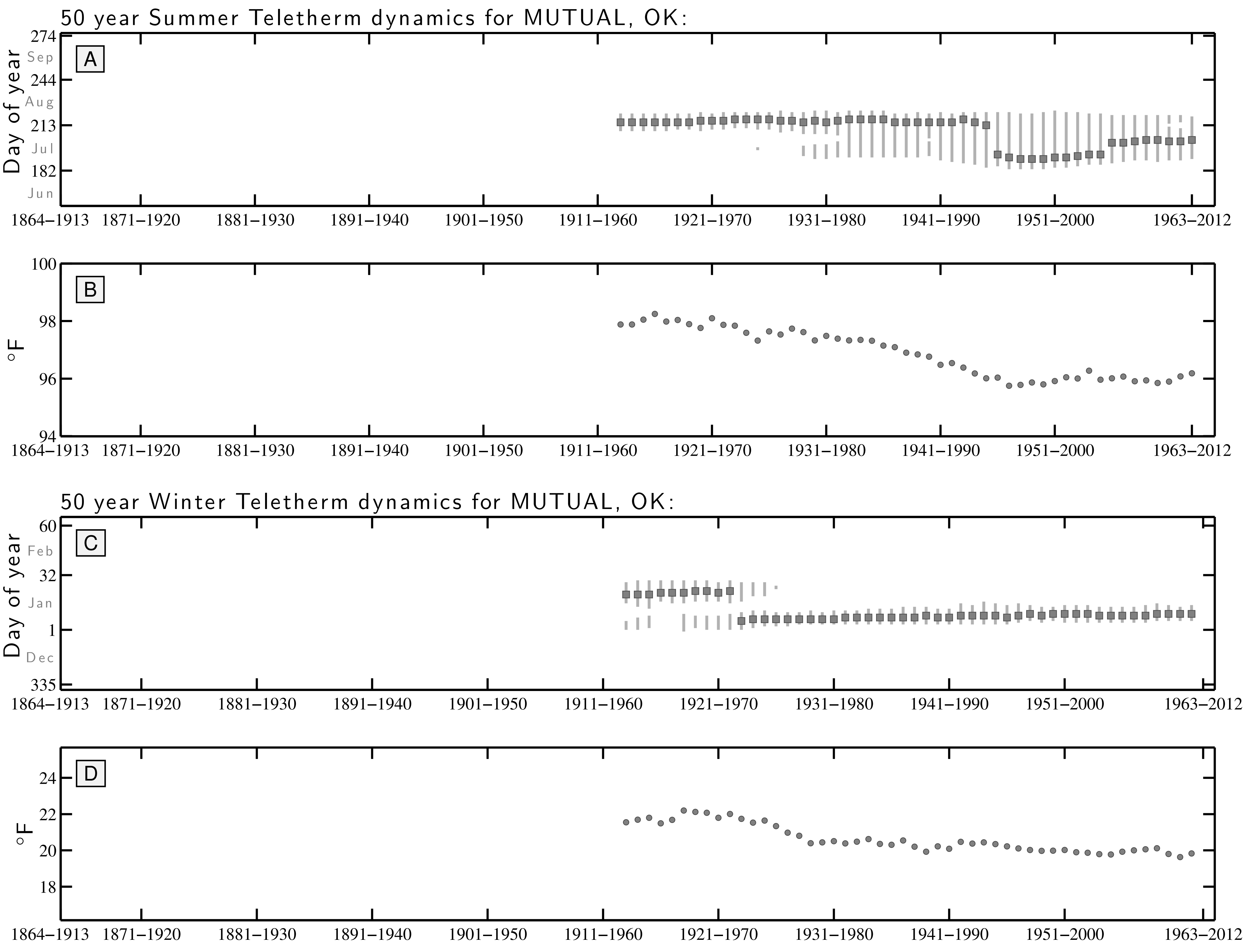
50 year Summer Teletherm dynamics for MUTUAL, OK:
A
Day of year
274
Sep
244
Aug
213
Jul
182
Jun
1864–1913
1871–1920
1881–1930
1891–1940
1901–1950
1911–1960
1921–1970
1931–1980
1941–1990
1951–2000
1963–2012
B
°F
100
98
96
94
50 year Winter Teletherm dynamics for MUTUAL, OK:
C
60
Feb
32
Jan
1
Dec
335
D
24
22
20
18

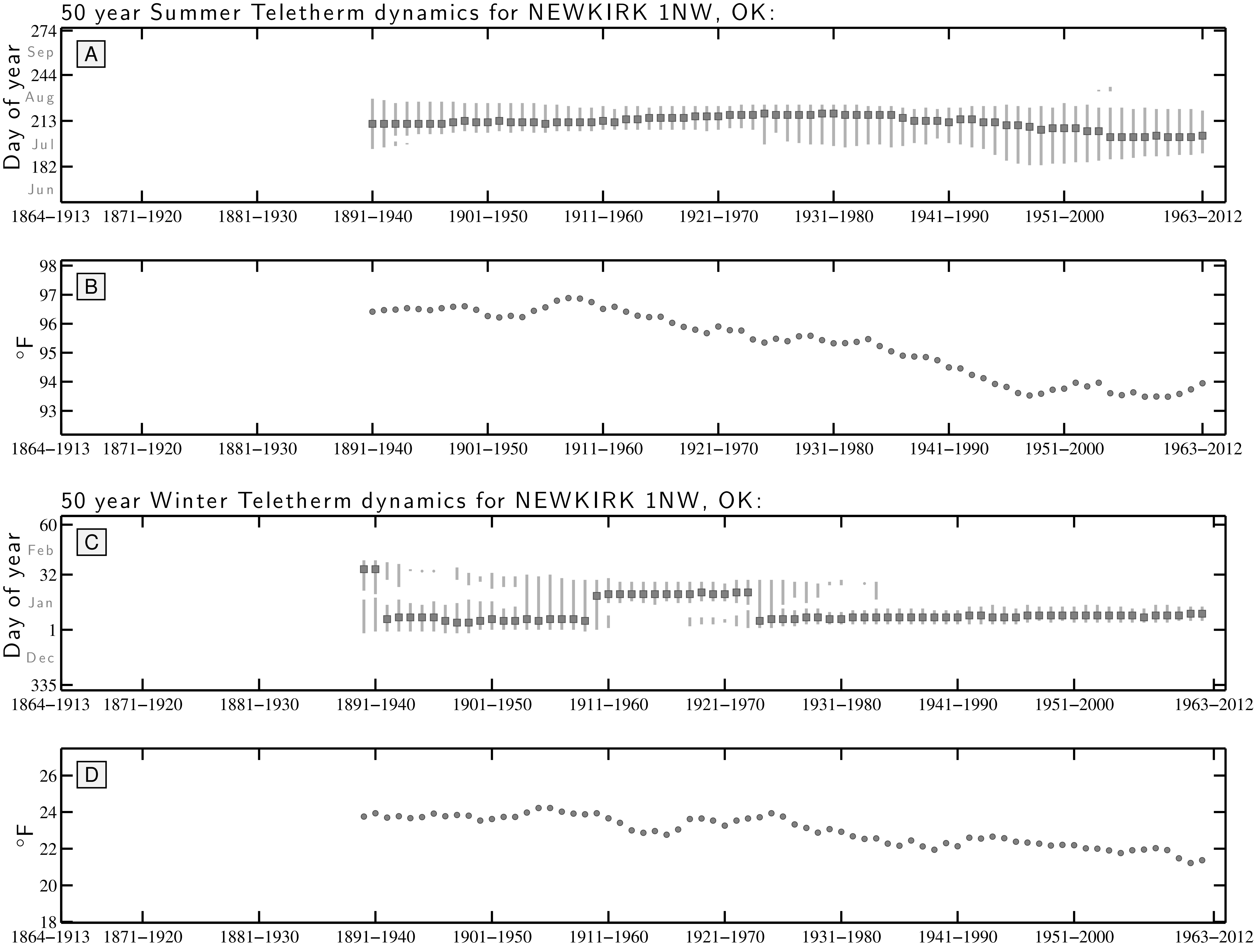
50 year Summer Teletherm dynamics for NEWKIRK 1NW, OK:
A
B
Day of year
°F
274
244
213
182
Sep
Aug
Jul
Jun
98
97
96
95
94
93
1864–1913
1871–1920
1881–1930
1891–1940
1901–1950
1911–1960
1921–1970
1931–1980
1941–1990
1951–2000
1963–2012
50 year Winter Teletherm dynamics for NEWKIRK 1NW, OK:
C
D
60
32
1
335
Feb
Jan
Dec
26
24
22
20
18

50 year Summer Teletherm dynamics for OKEENE, OK:

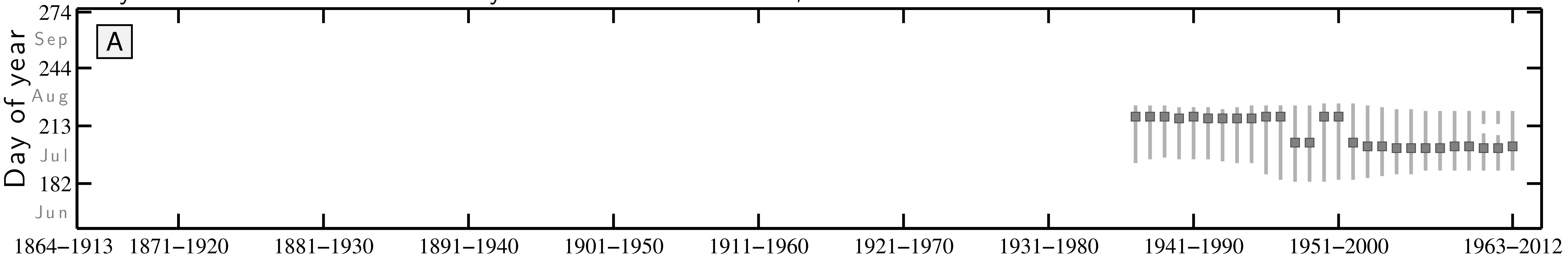

50 year Winter Teletherm dynamics for OKEENE, OK:

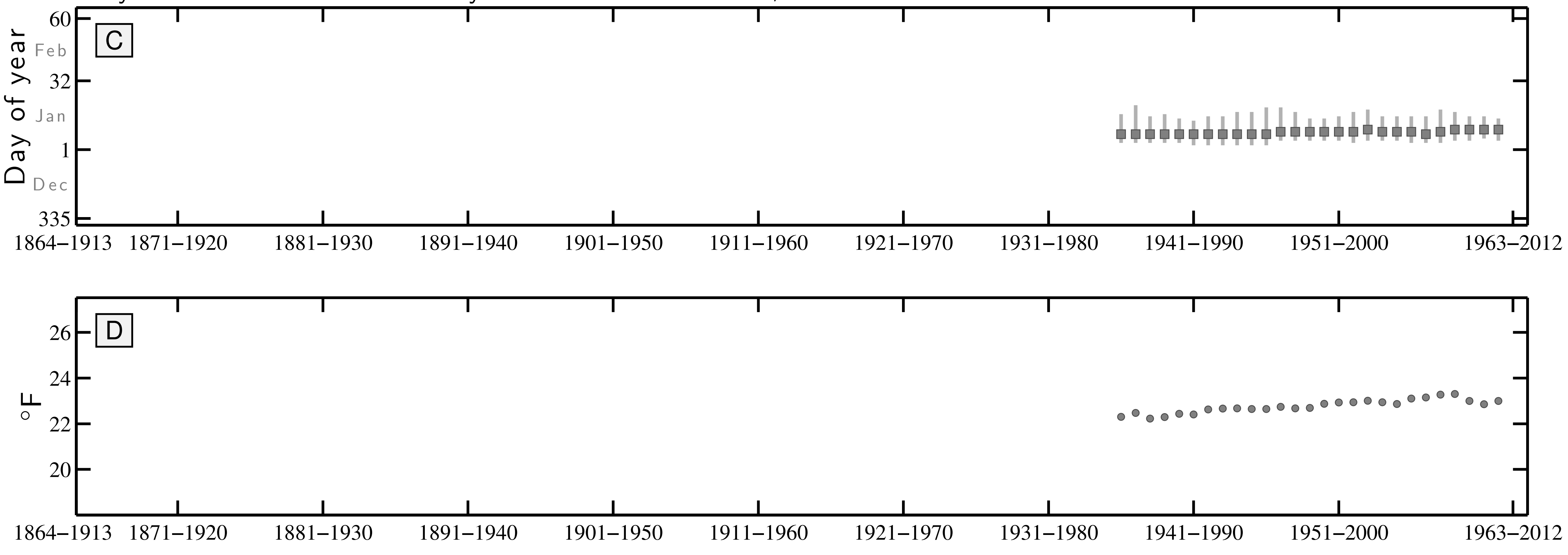

50 year Summer Teletherm dynamics for OKEMAH, OK:

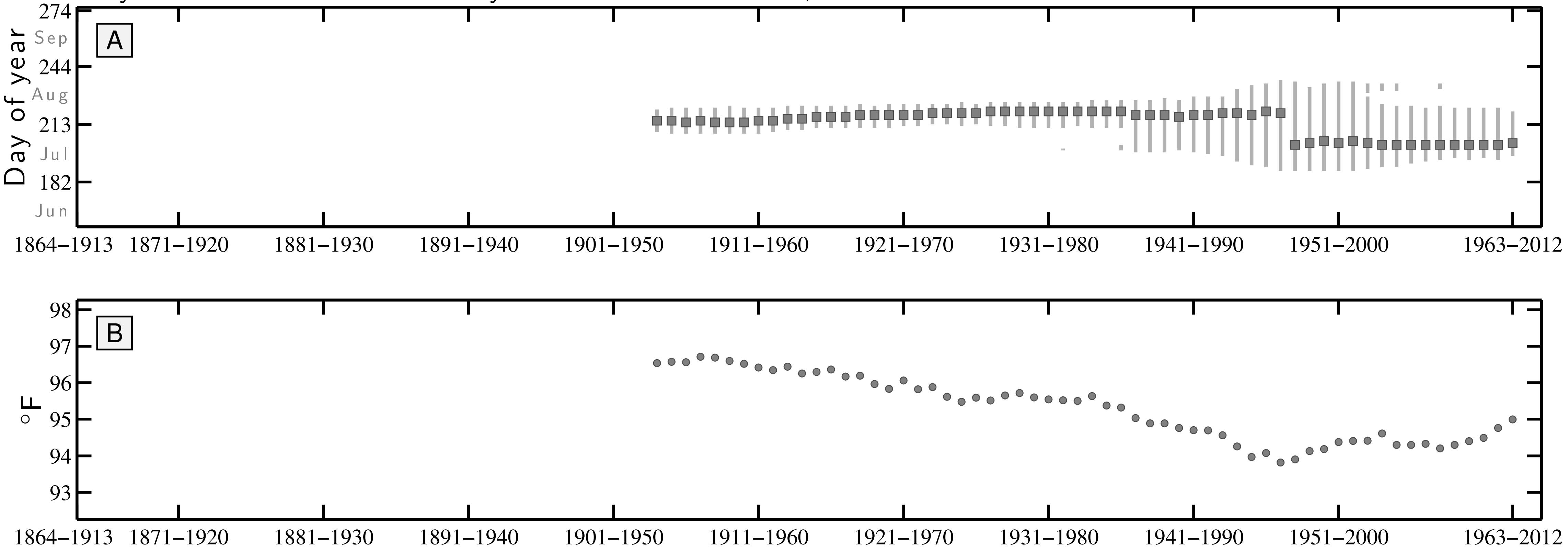

50 year Winter Teletherm dynamics for OKEMAH, OK:

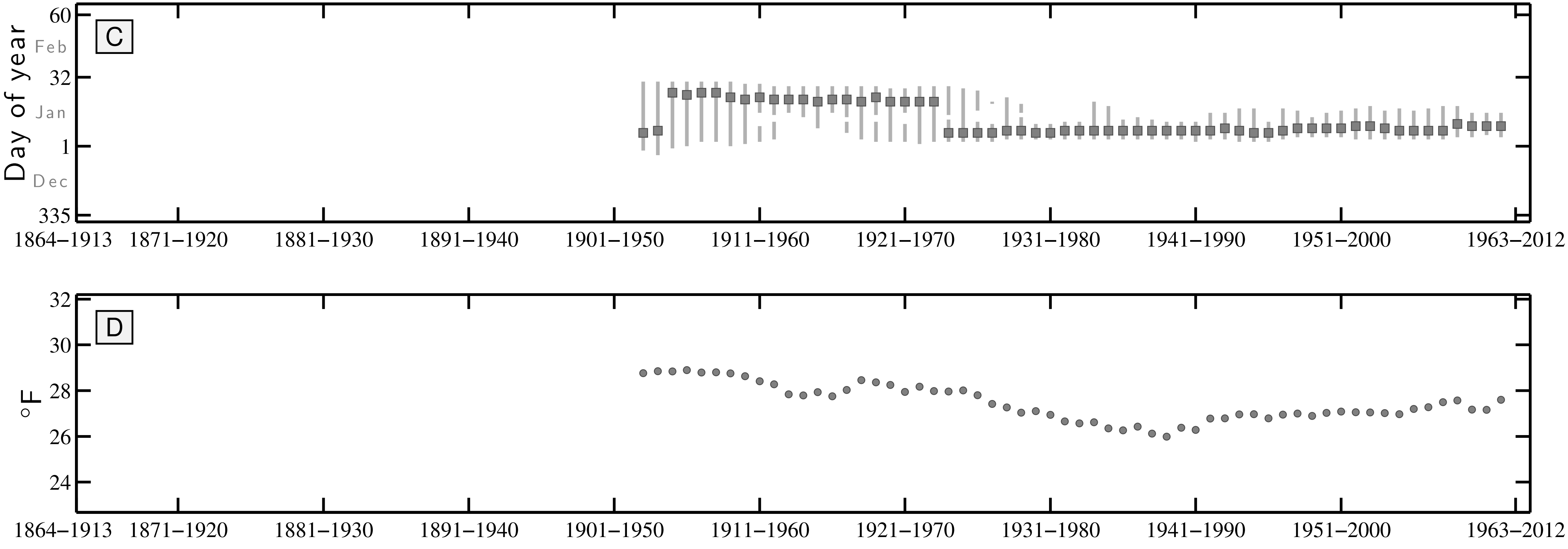

50 year Summer Teletherm dynamics for OKMULGEE WTR WKS, OK:

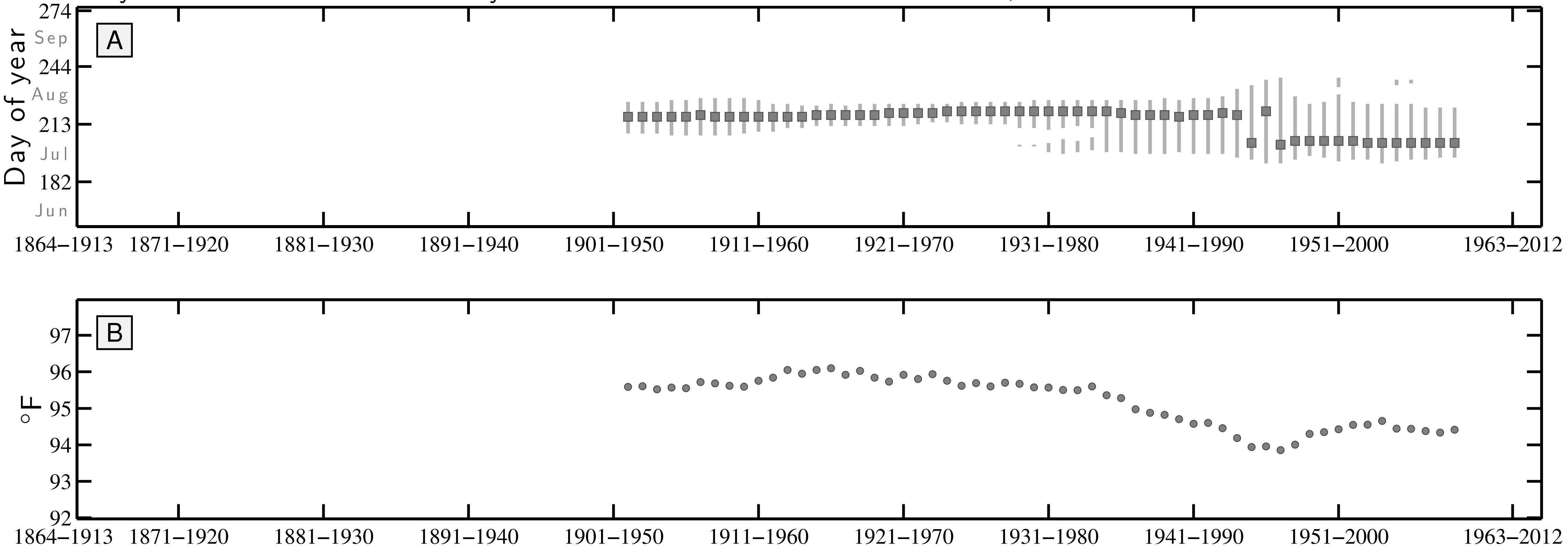

50 year Winter Teletherm dynamics for OKMULGEE WTR WKS, OK:

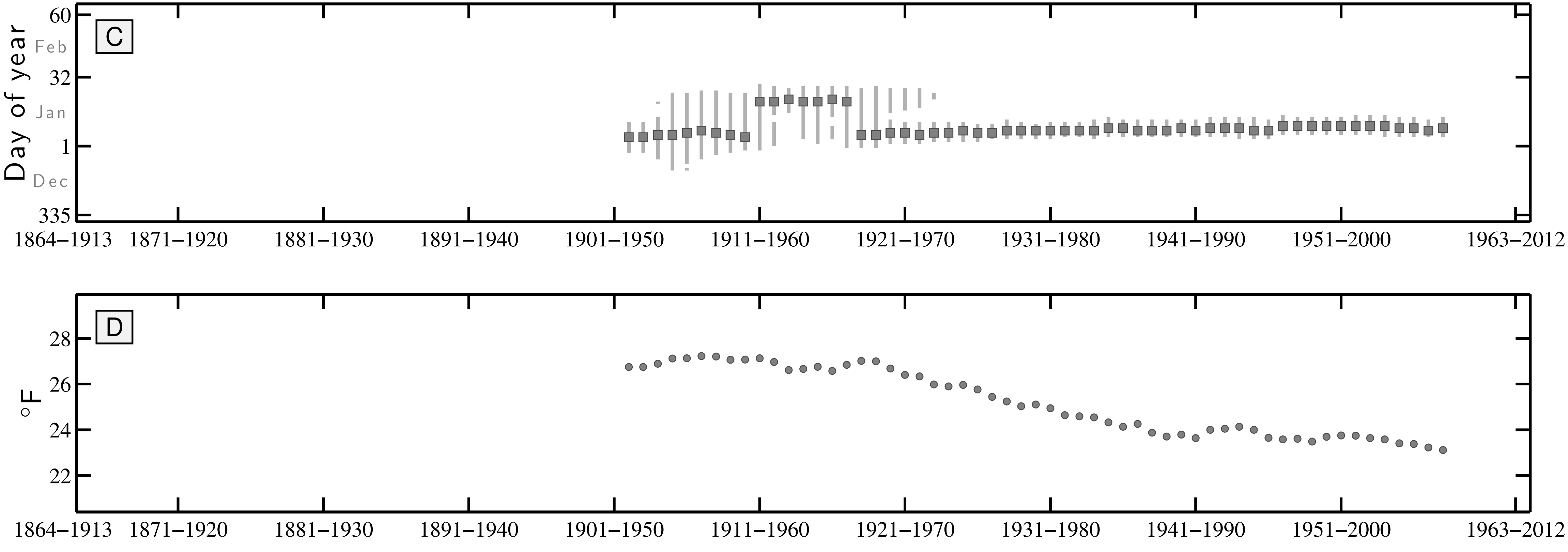

50 year Summer Teletherm dynamics for PAULS VALLEY 4 WSW, OK:

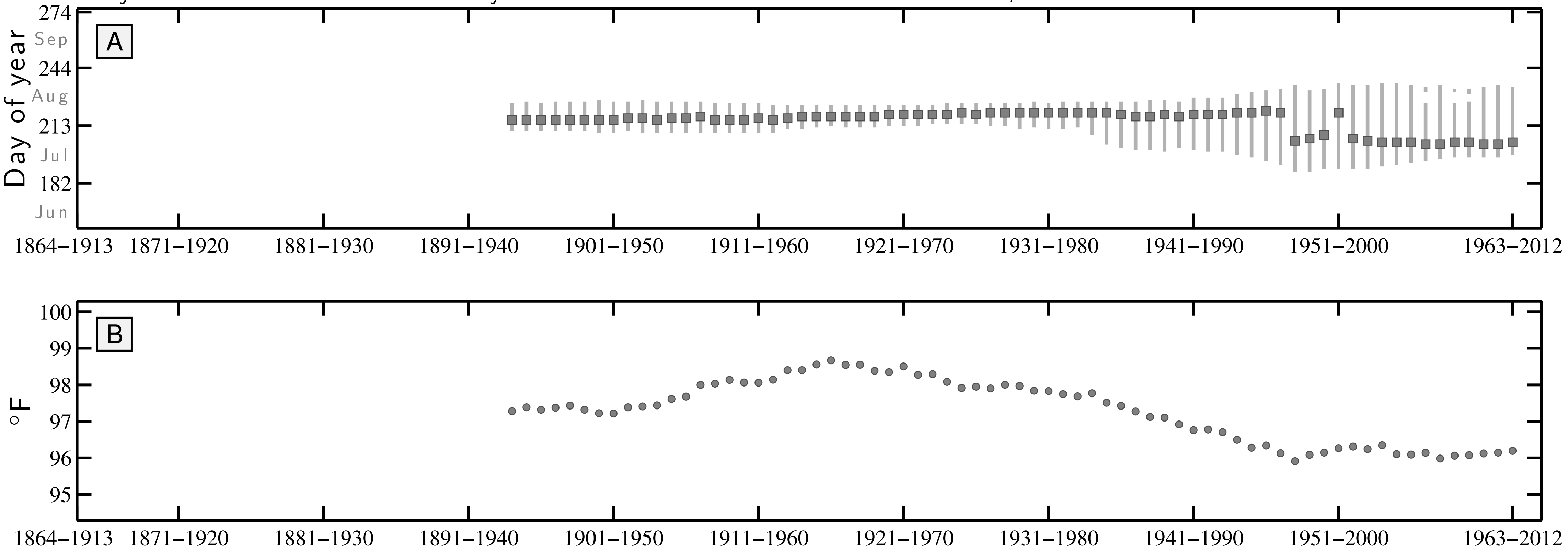

50 year Winter Teletherm dynamics for PAULS VALLEY 4 WSW, OK:

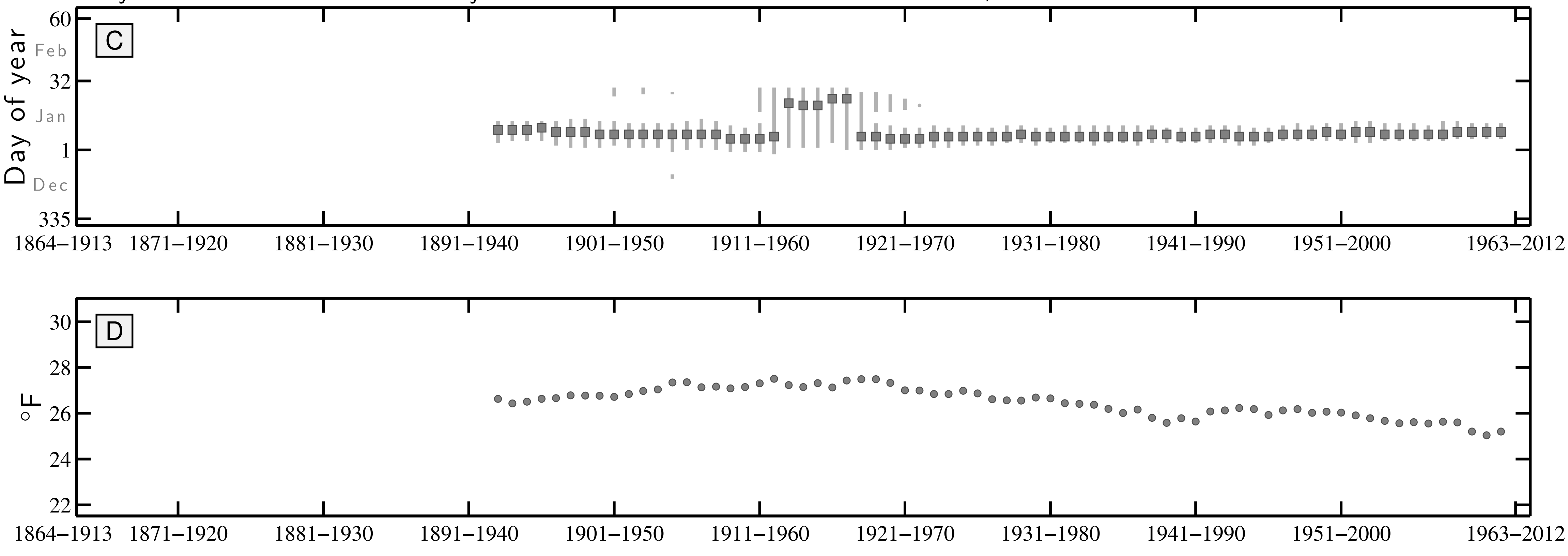

50 year Summer Teletherm dynamics for PAWHUSKA, OK:

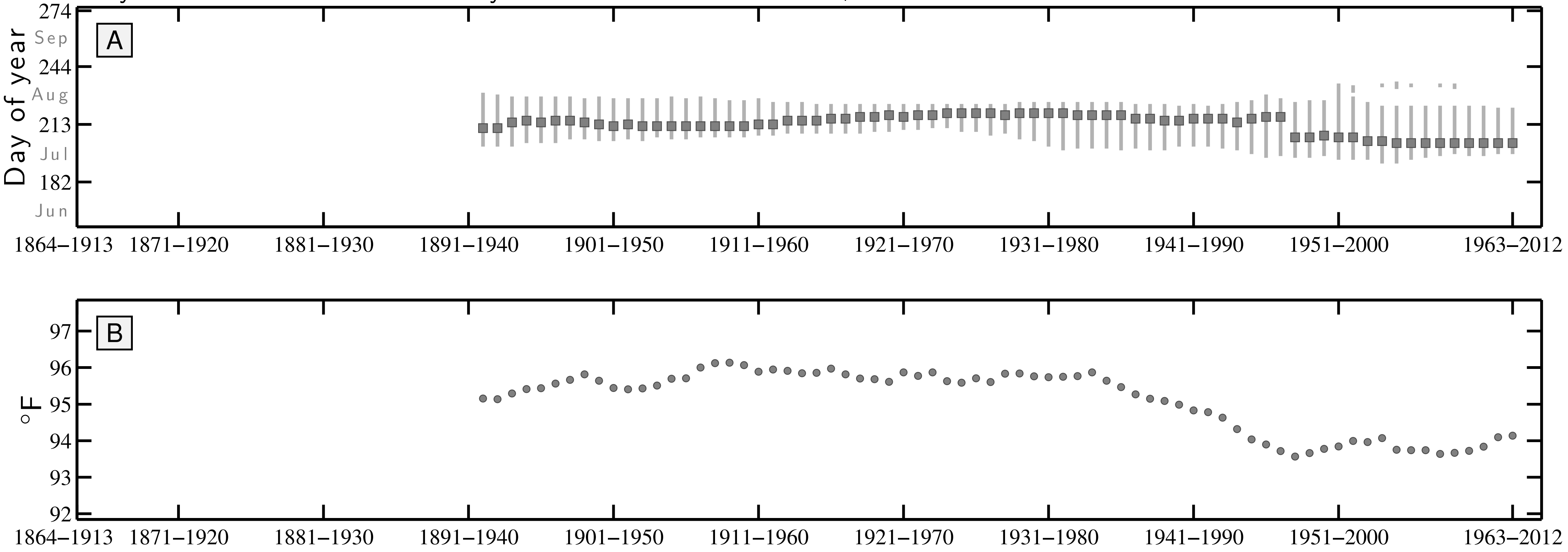

50 year Winter Teletherm dynamics for PAWHUSKA, OK:

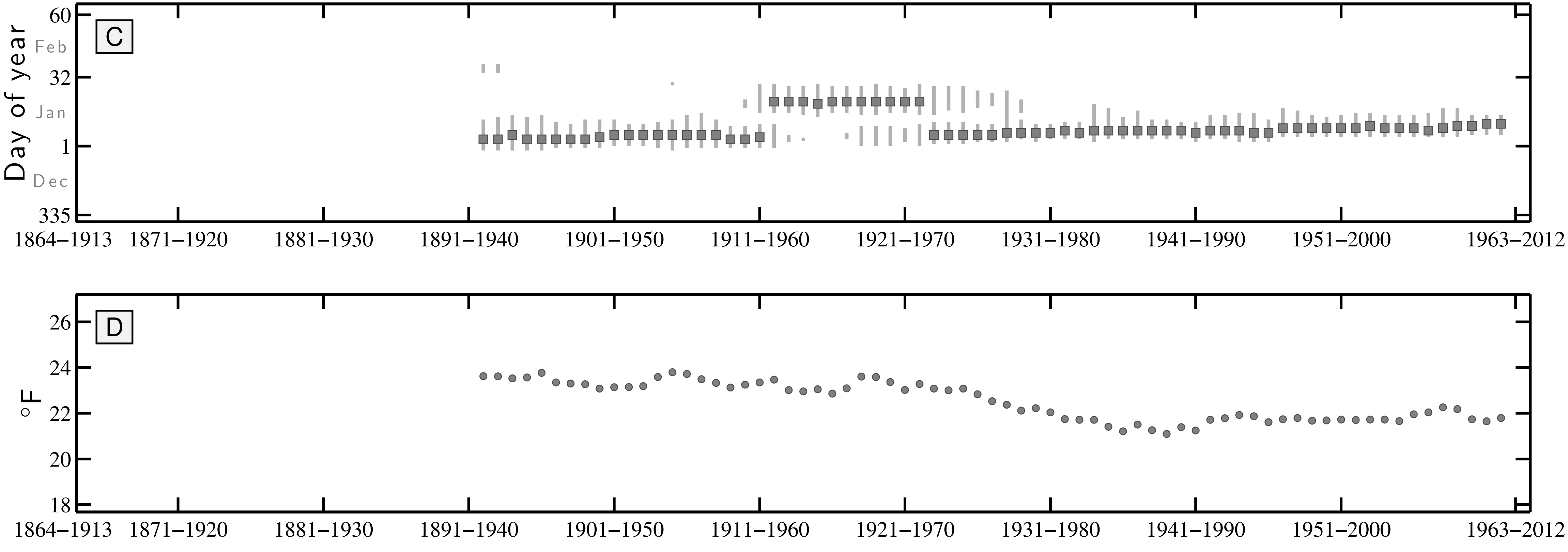

50 year Summer Teletherm dynamics for PERRY, OK:

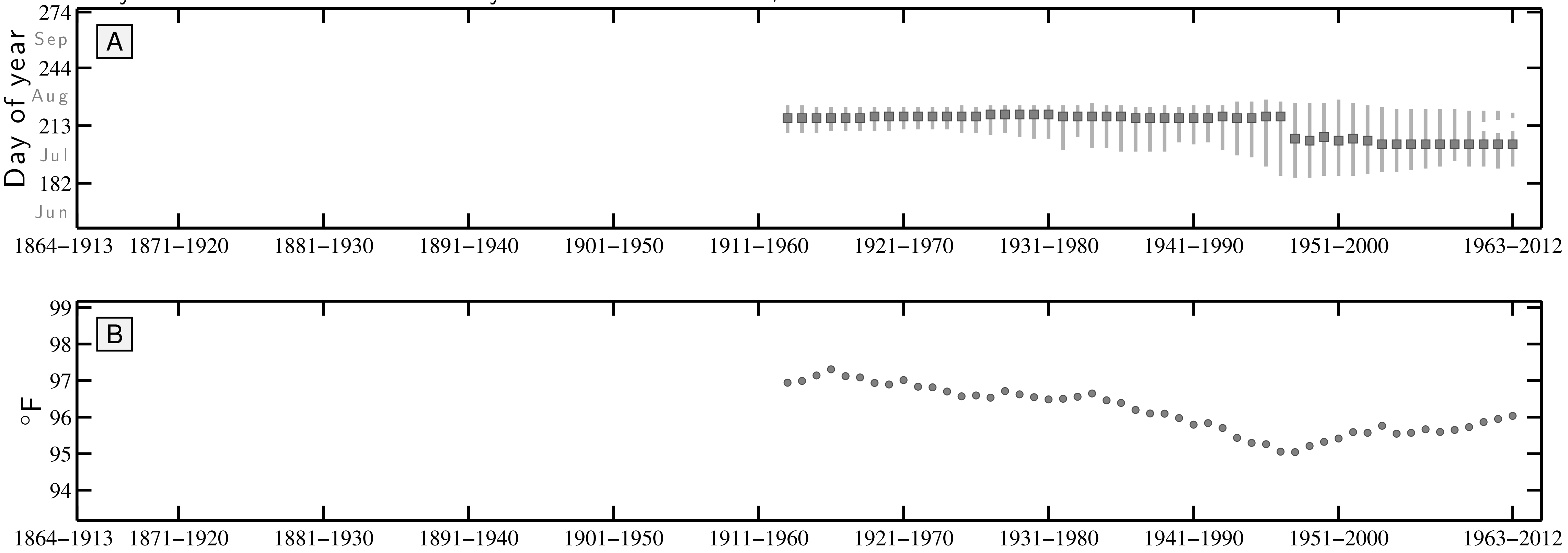

50 year Winter Teletherm dynamics for PERRY, OK:

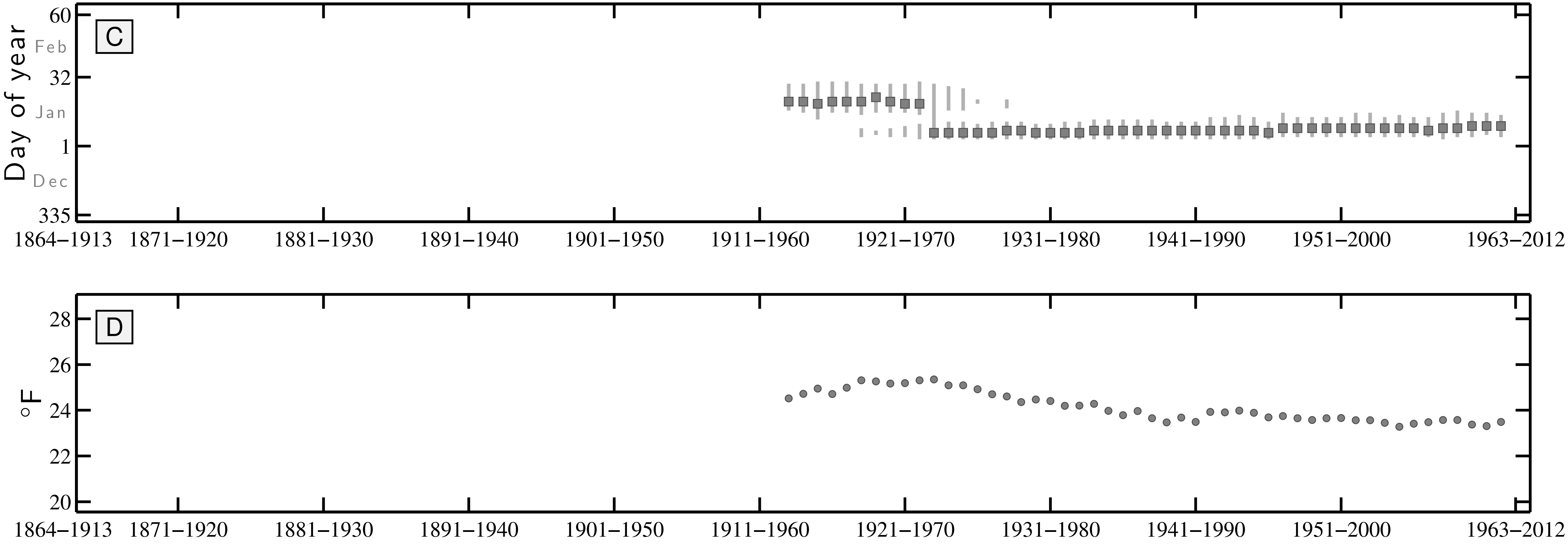

50 year Summer Teletherm dynamics for STILLWATER 2 W, OK:

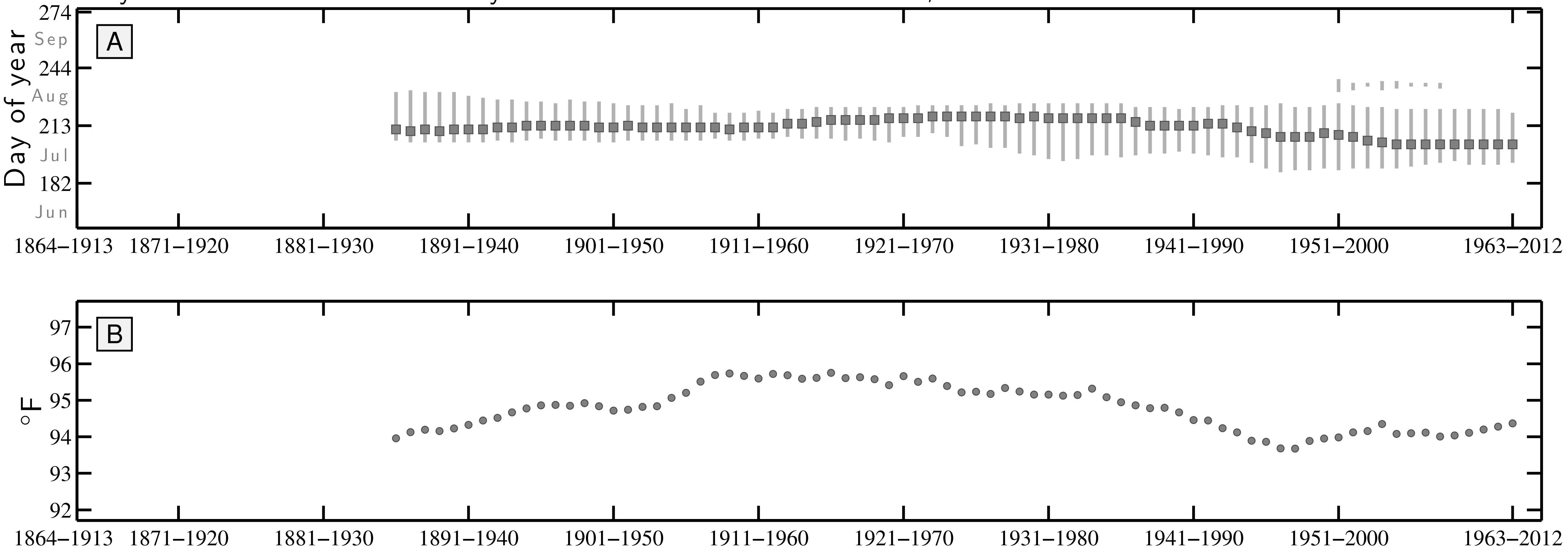

50 year Winter Teletherm dynamics for STILLWATER 2 W, OK:

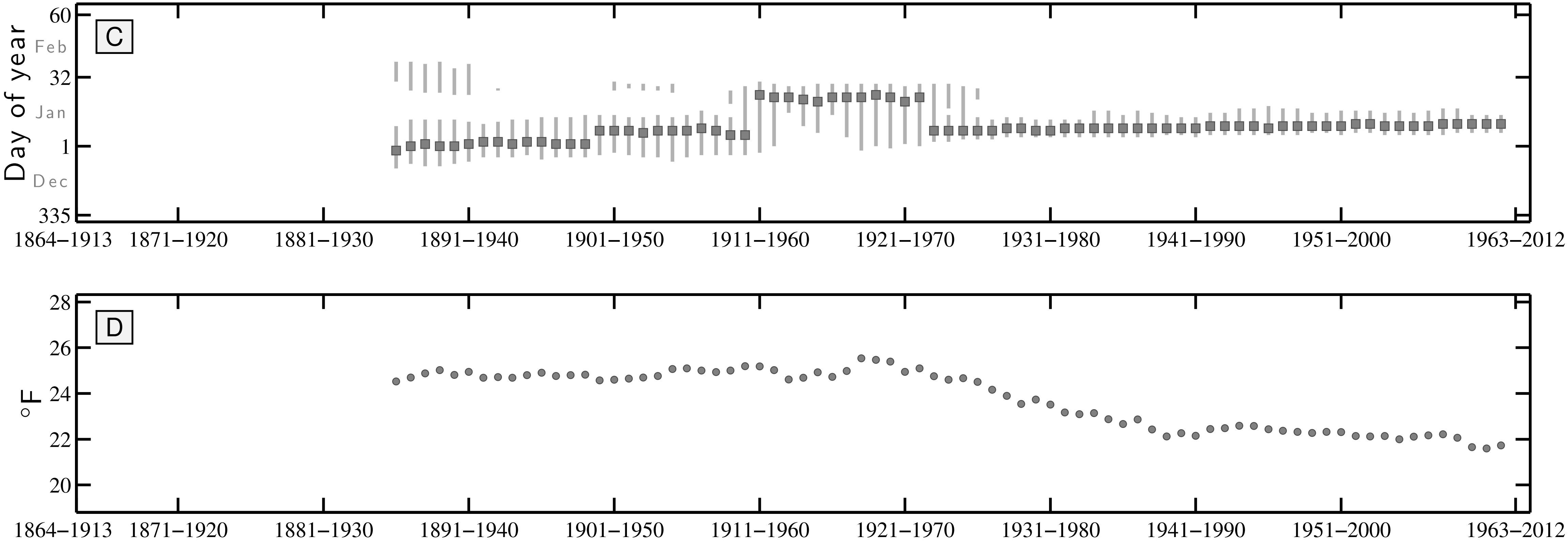

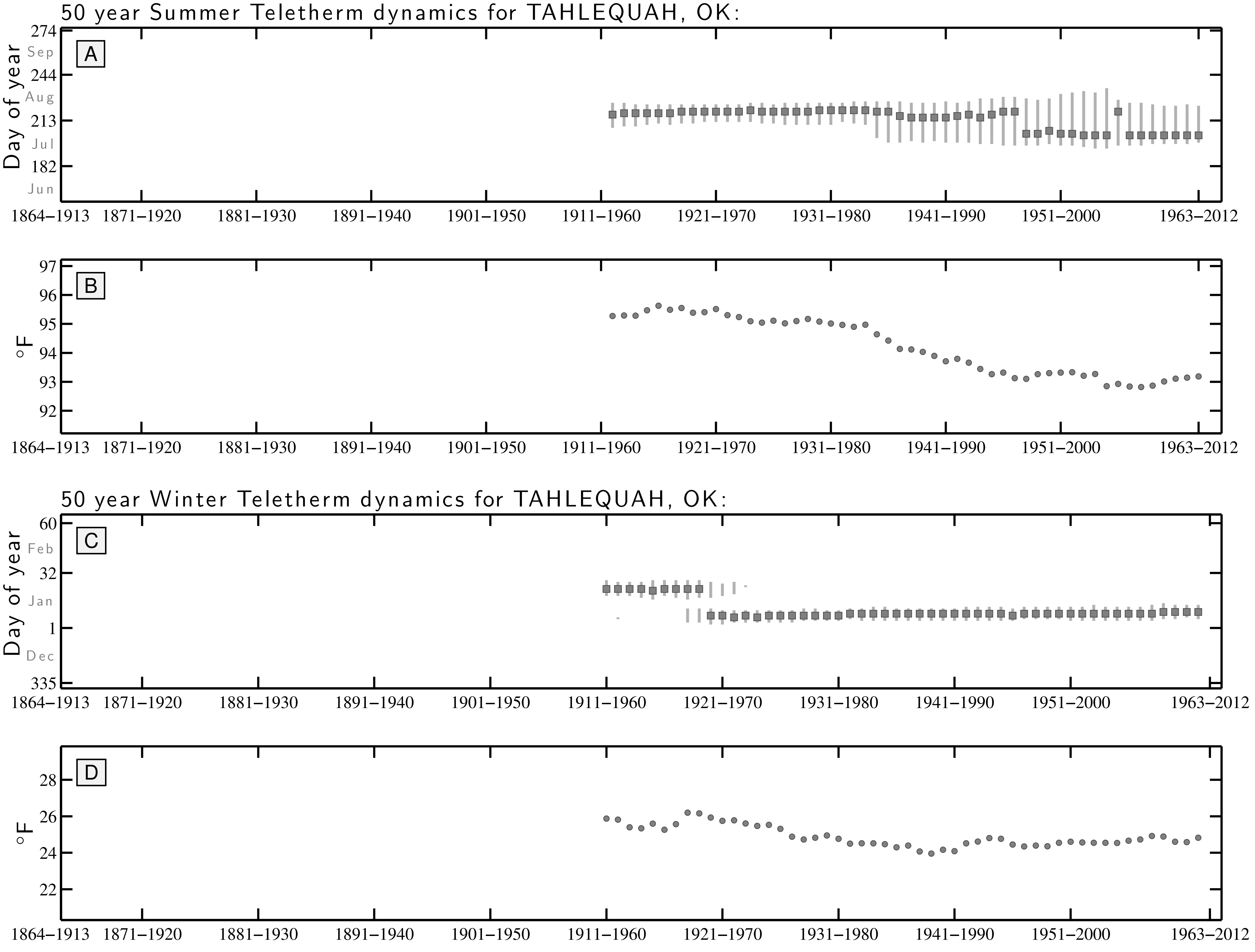

50 year Summer Teletherm dynamics for TAHLEQUAH, OK:
A
Day of year
274
Sep
244
Aug
213
Jul
182
Jun
1864–1913
1871–1920
1881–1930
1891–1940
1901–1950
1911–1960
1921–1970
1931–1980
1941–1990
1951–2000
1963–2012
B
°F
97
96
95
94
93
92
50 year Winter Teletherm dynamics for TAHLEQUAH, OK:
C
60
Feb
32
Jan
1
Dec
335
D
28
26
24
22

50 year Summer Teletherm dynamics for WAURIKA, OK:

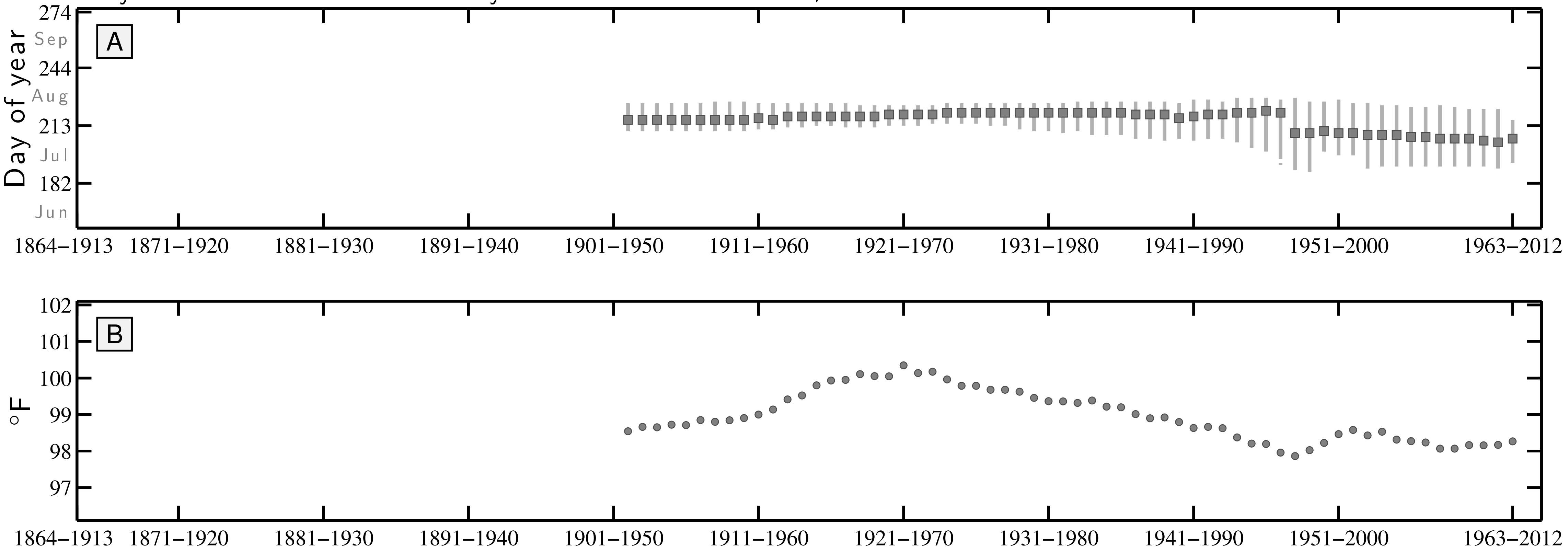

50 year Winter Teletherm dynamics for WAURIKA, OK:

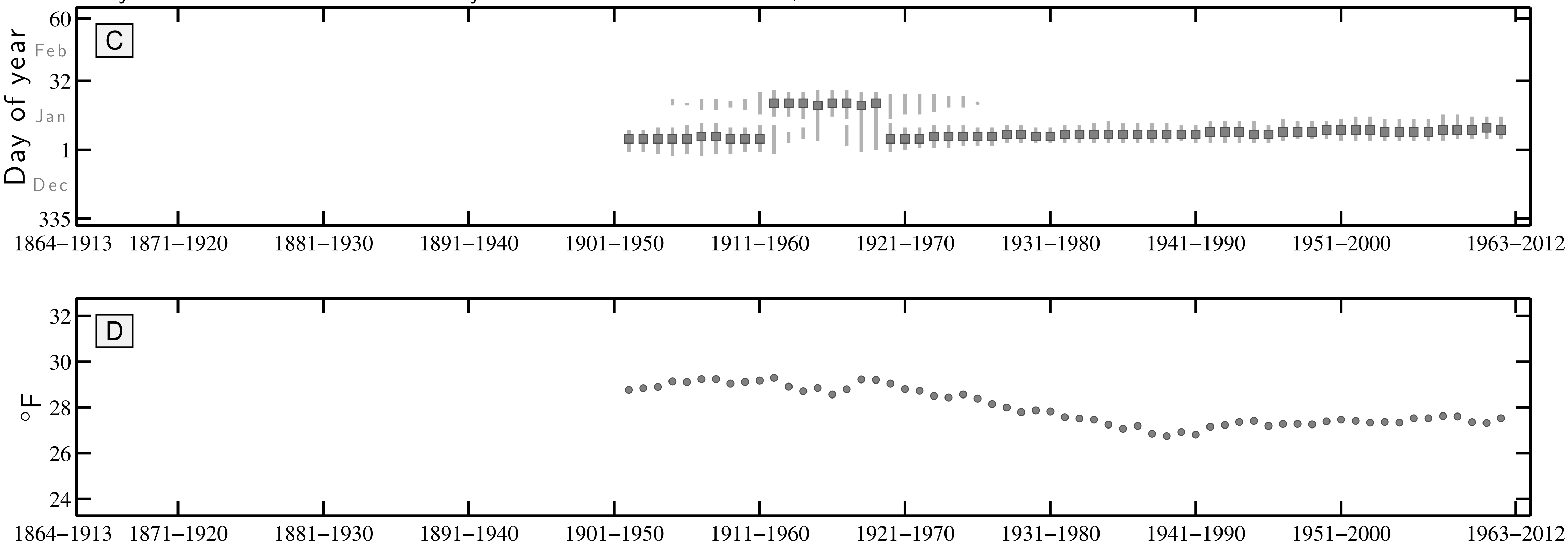

50 year Summer Teletherm dynamics for WEATHERFORD, OK:

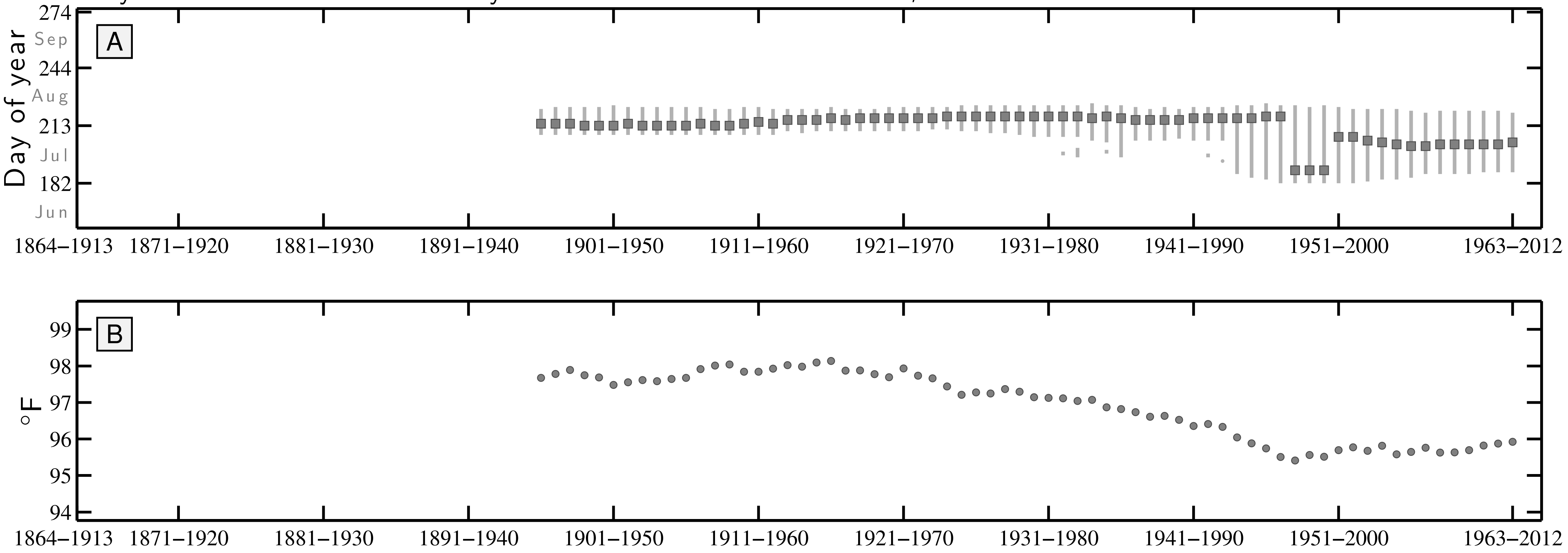

50 year Winter Teletherm dynamics for WEATHERFORD, OK:

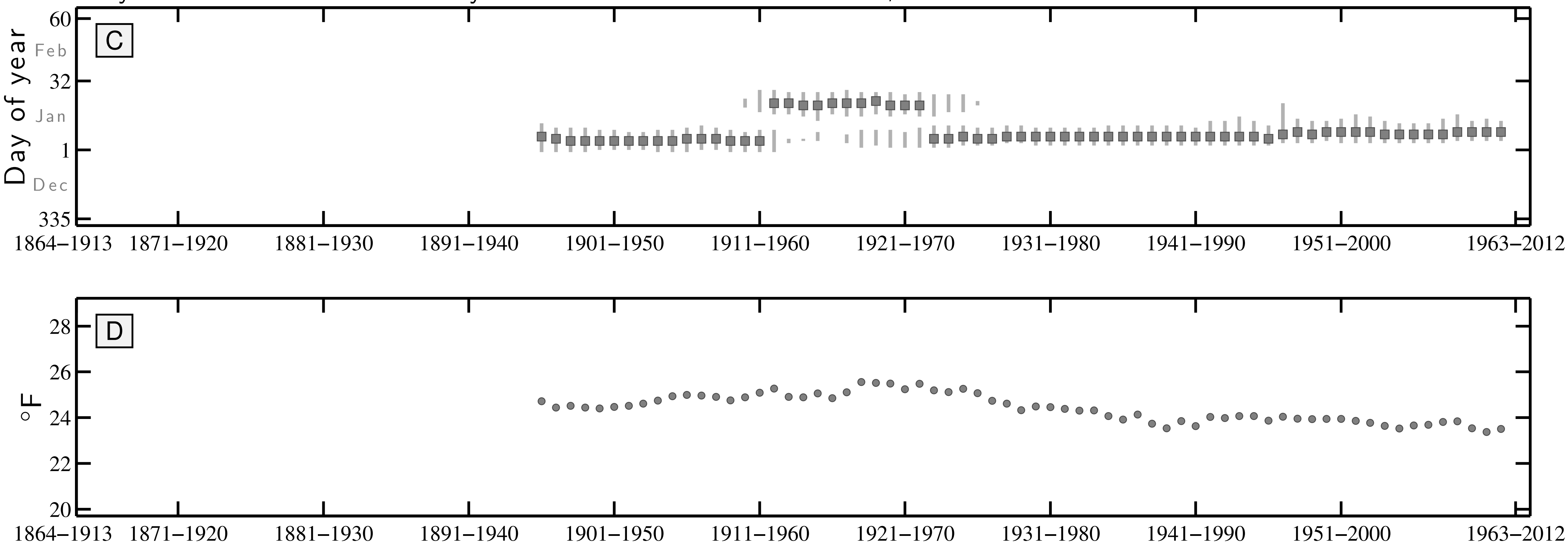

50 year Summer Teletherm dynamics for WEBBERS FALLS 5 WSW, OK:

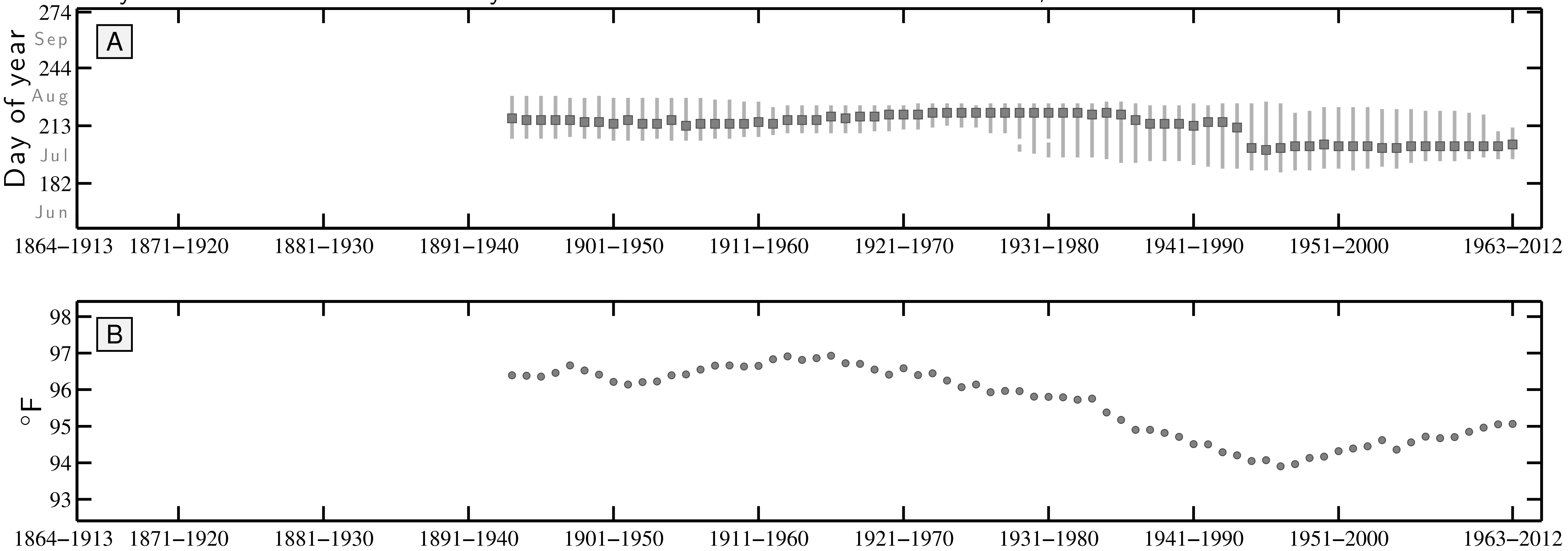

50 year Winter Teletherm dynamics for WEBBERS FALLS 5 WSW, OK:

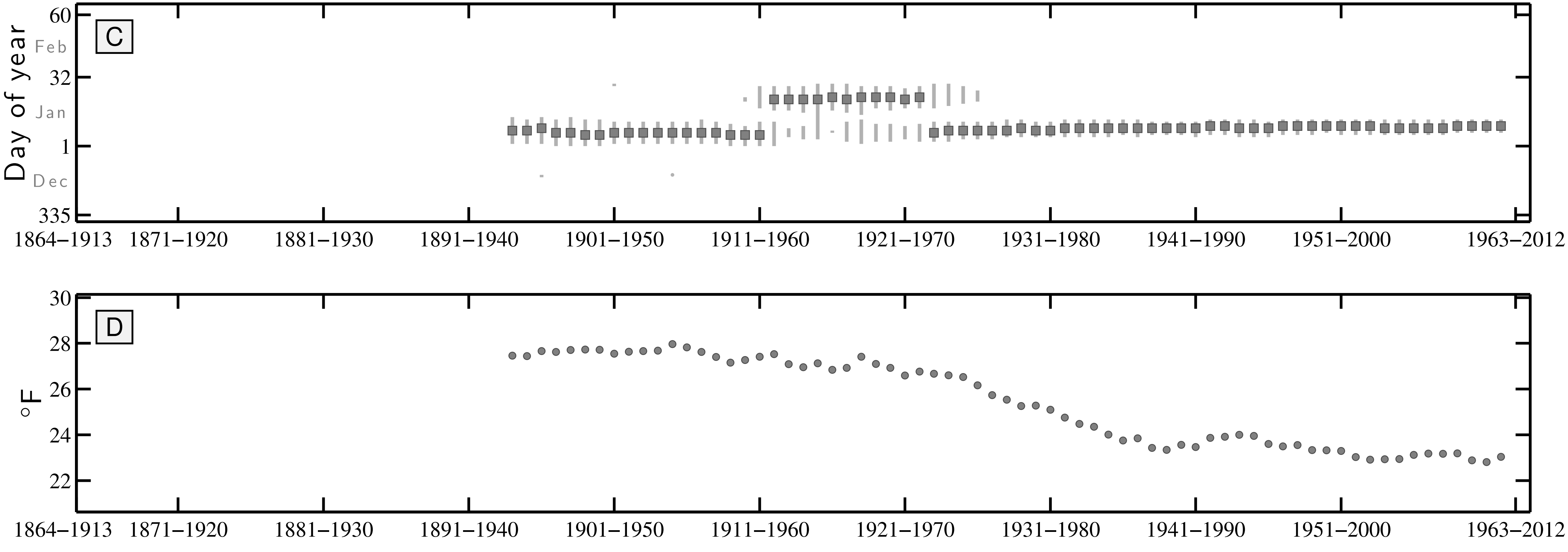

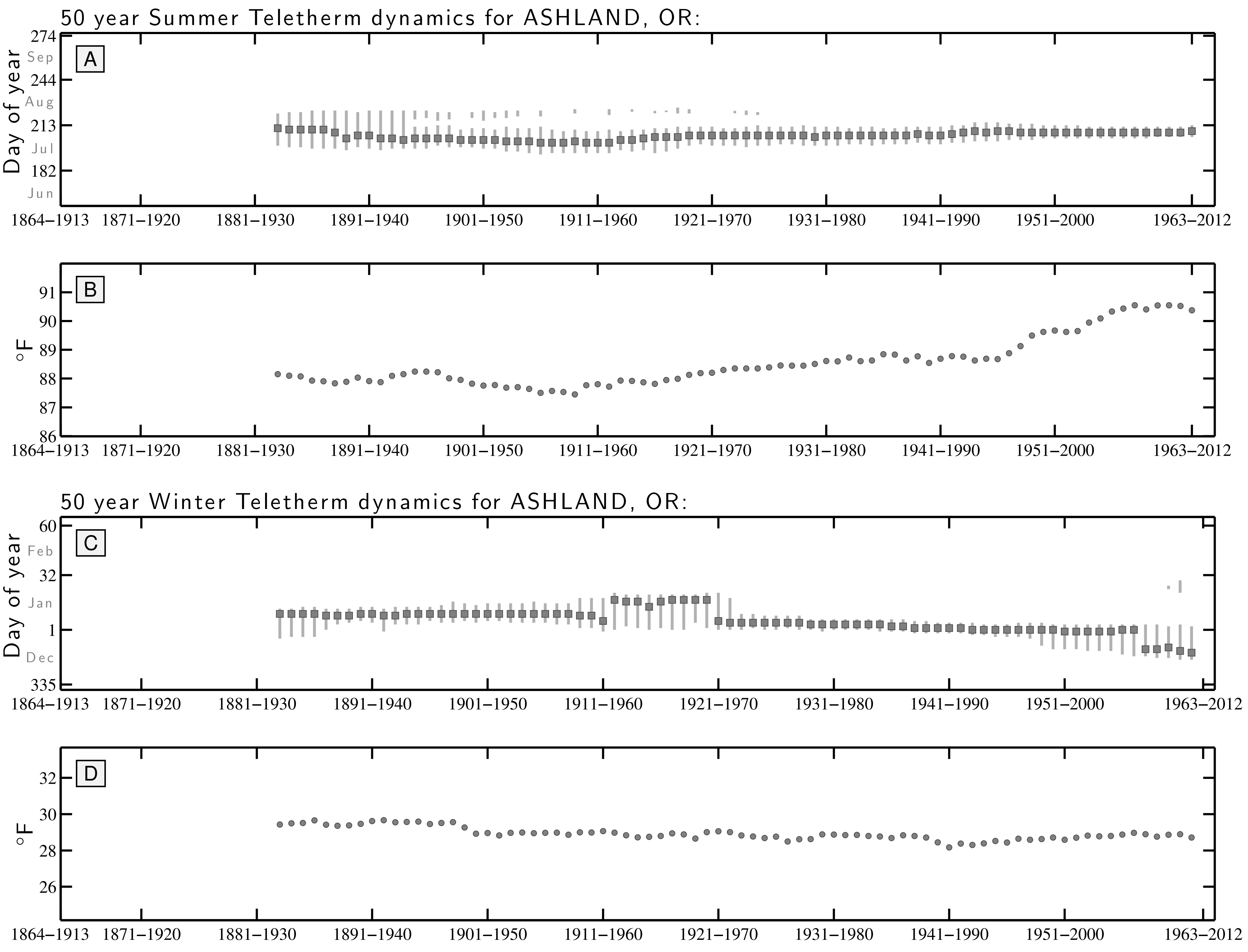

50 year Summer Teletherm dynamics for ASHLAND, OR:
A
Day of year
274
Sep
244
Aug
213
Jul
182
Jun
1864–1913
1871–1920
1881–1930
1891–1940
1901–1950
1911–1960
1921–1970
1931–1980
1941–1990
1951–2000
1963–2012
B
°F
91
90
89
88
87
86
50 year Winter Teletherm dynamics for ASHLAND, OR:
C
Day of year
60
Feb
32
Jan
1
Dec
335
D
°F
32
30
28
26

50 year Summer Teletherm dynamics for ASTORIA AP PORT OF, OR:

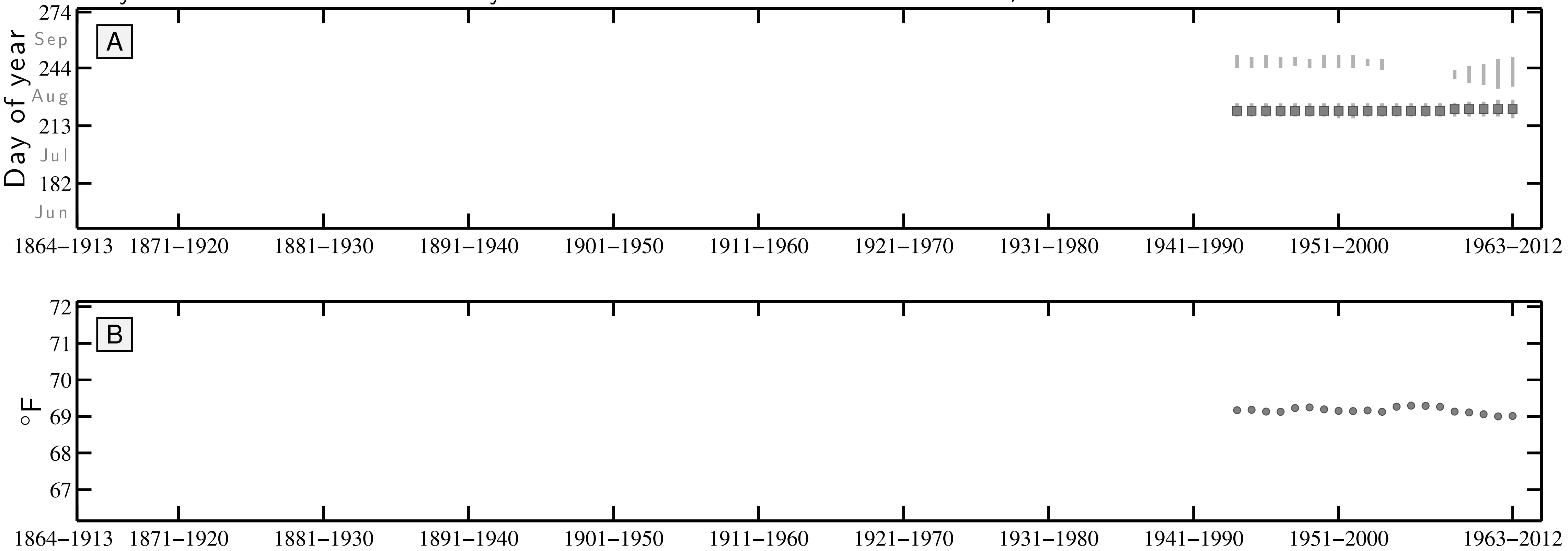

50 year Winter Teletherm dynamics for ASTORIA AP PORT OF, OR:

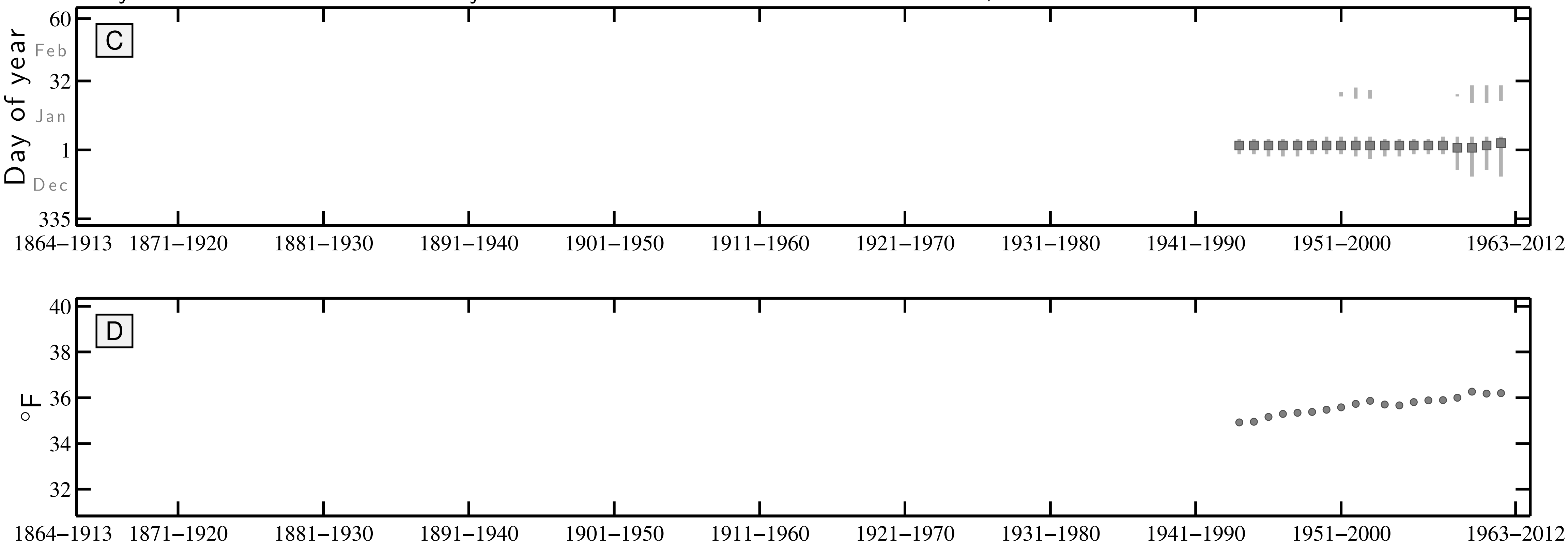

50 year Summer Teletherm dynamics for BAKER CITY AP, OR:

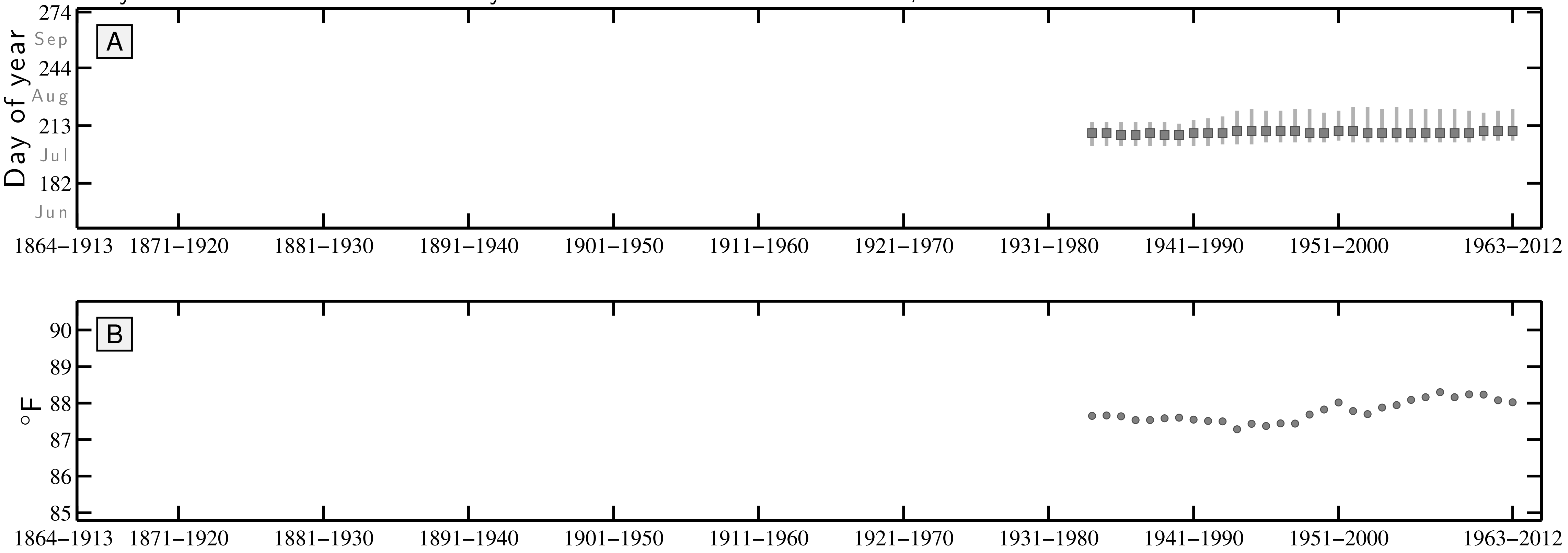

50 year Winter Teletherm dynamics for BAKER CITY AP, OR:

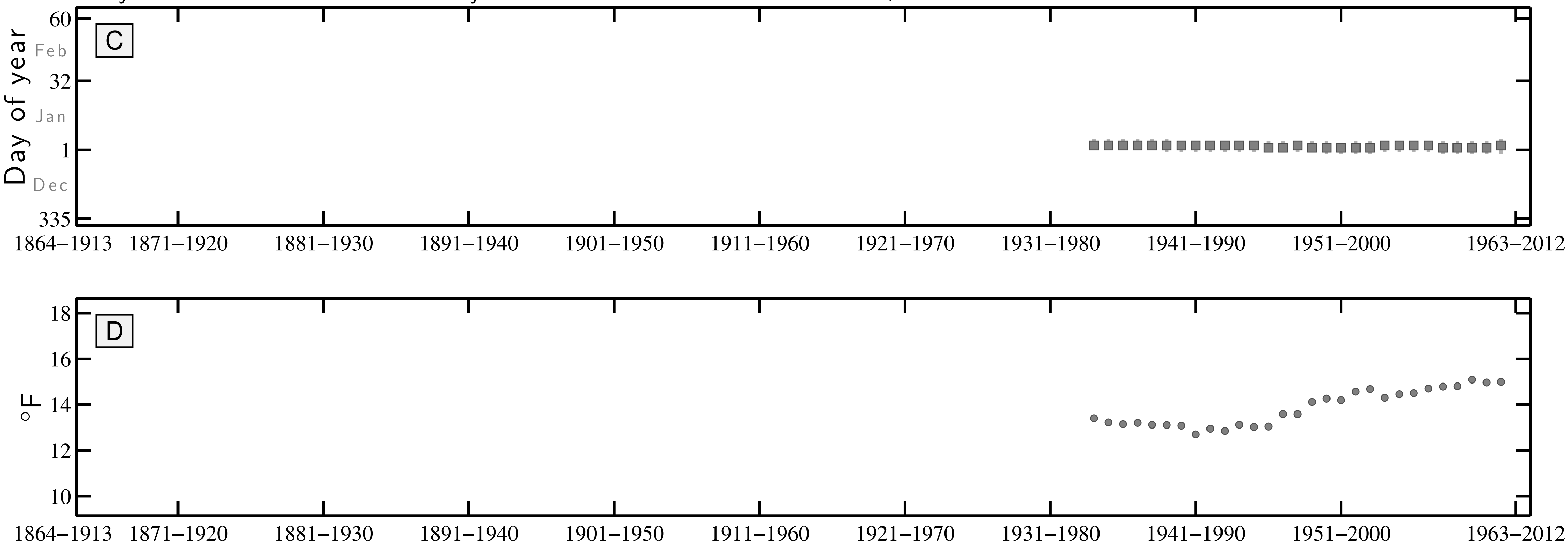

50 year Summer Teletherm dynamics for BEND, OR:

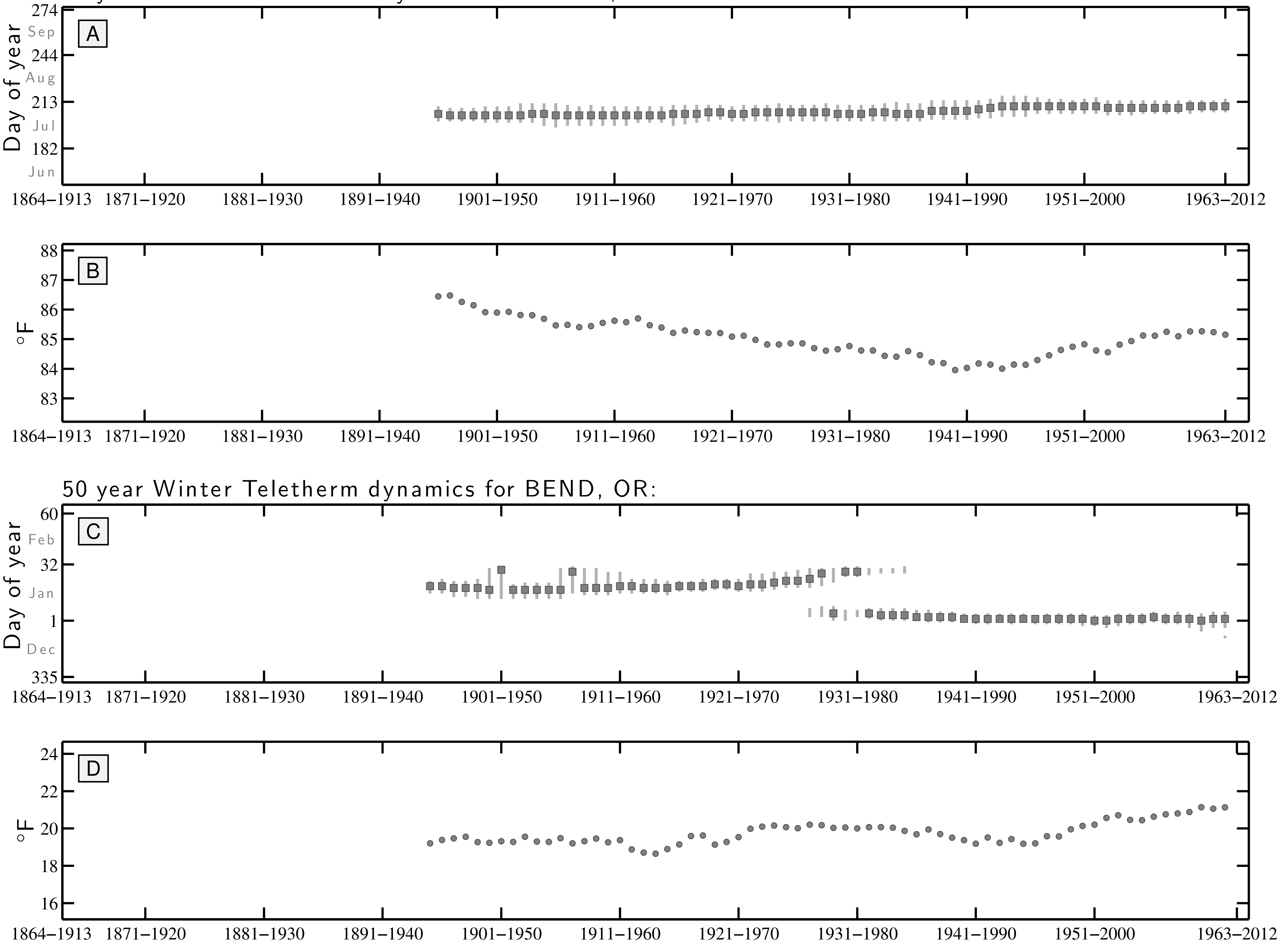

50 year Summer Teletherm dynamics for BROOKINGS 2 SE, OR:

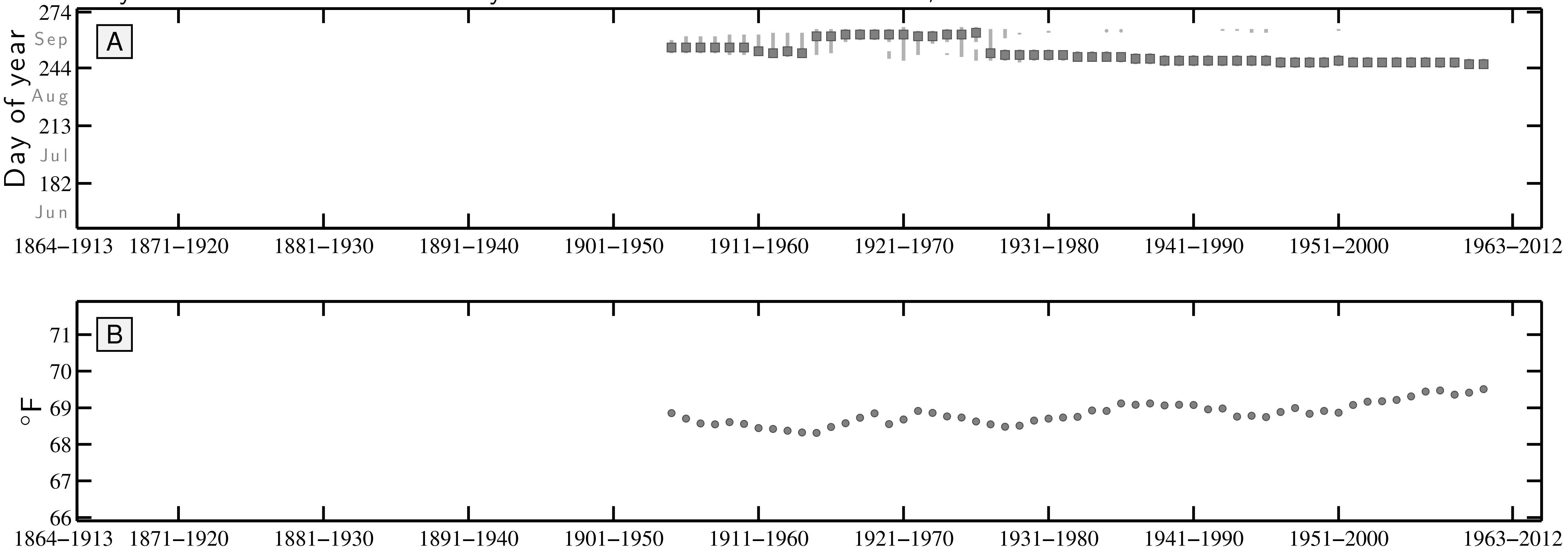

50 year Winter Teletherm dynamics for BROOKINGS 2 SE, OR:

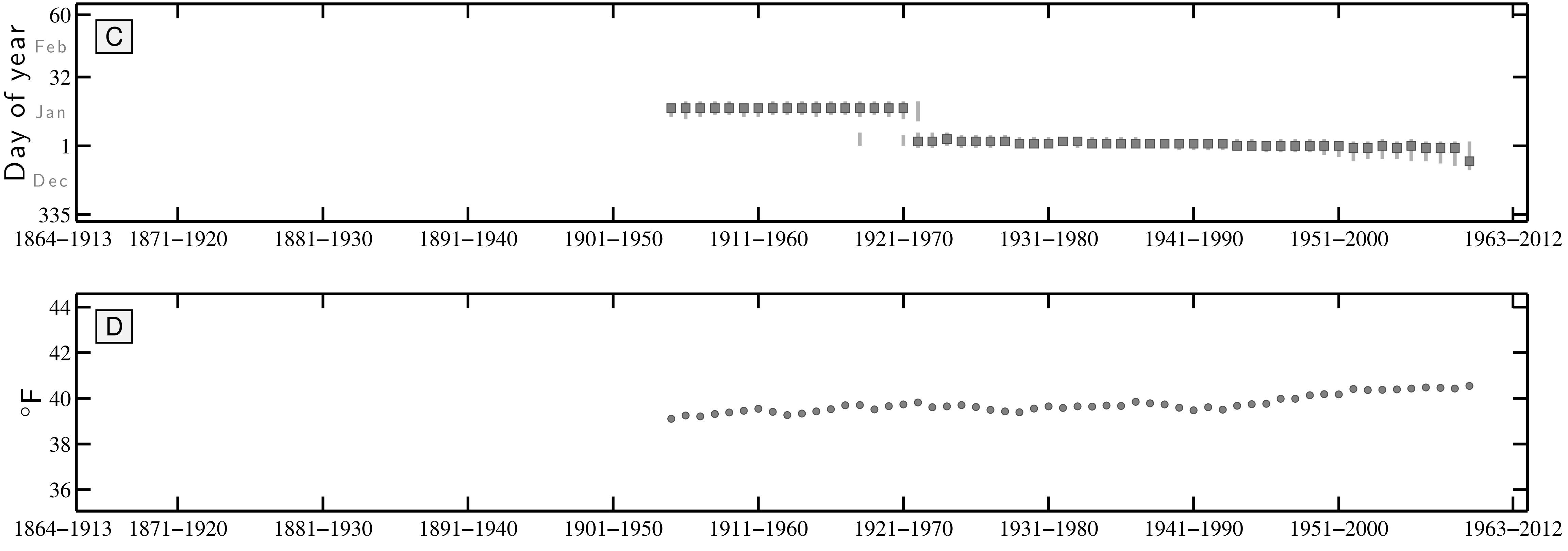

50 year Summer Teletherm dynamics for CASCADIA, OR:

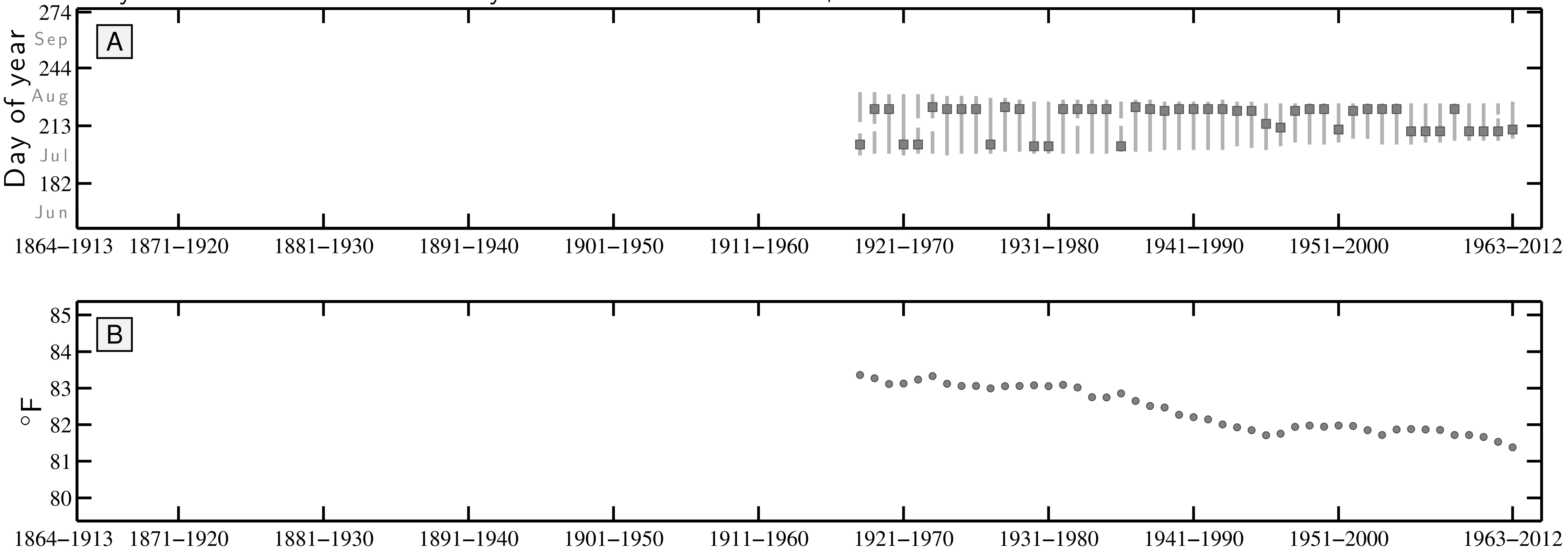

50 year Winter Teletherm dynamics for CASCADIA, OR:

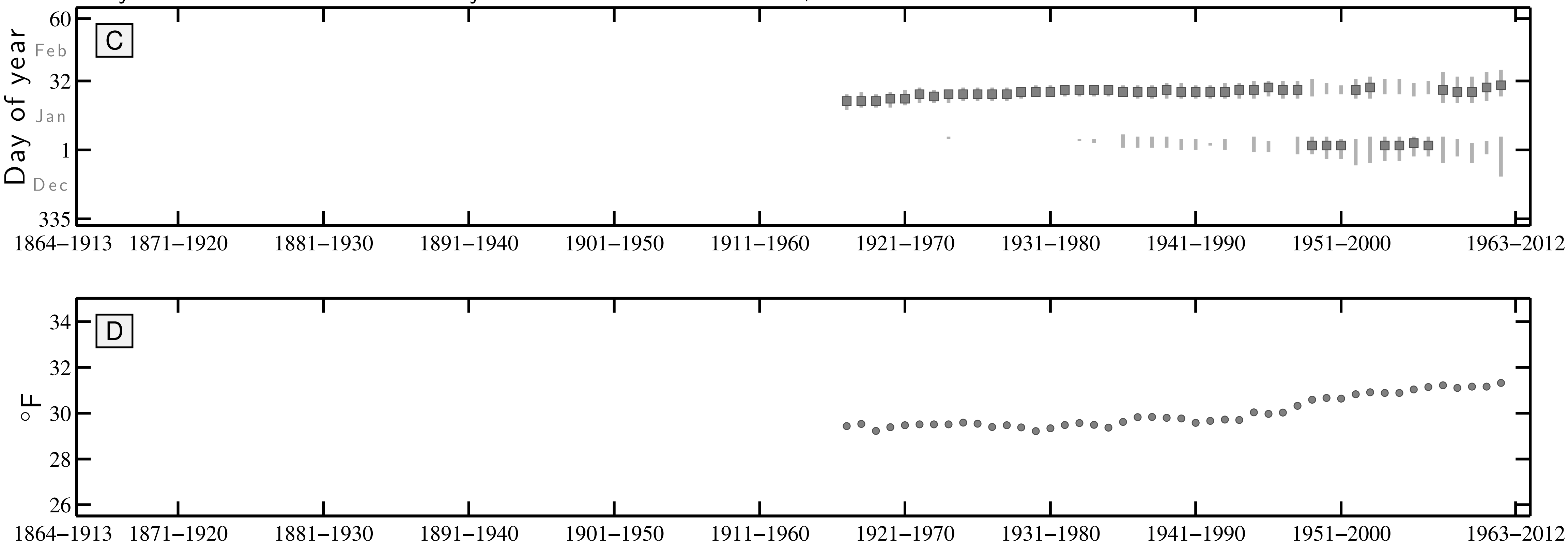

50 year Summer Teletherm dynamics for CONDON, OR:

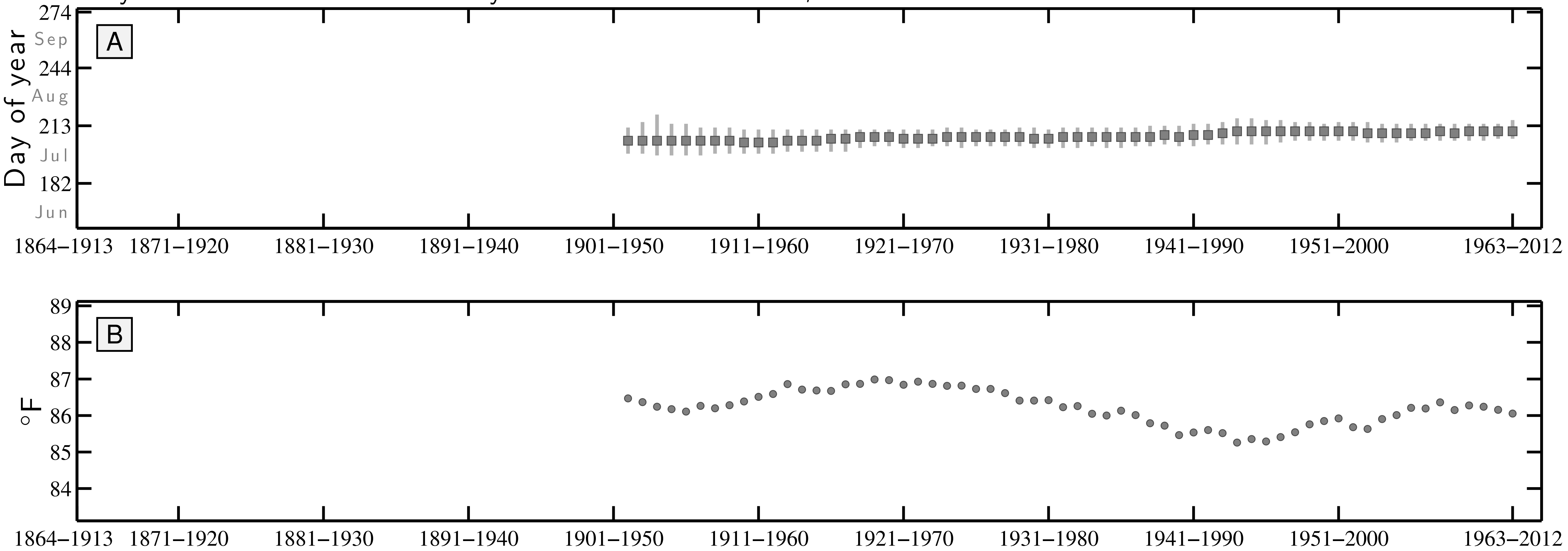

50 year Winter Teletherm dynamics for CONDON, OR:

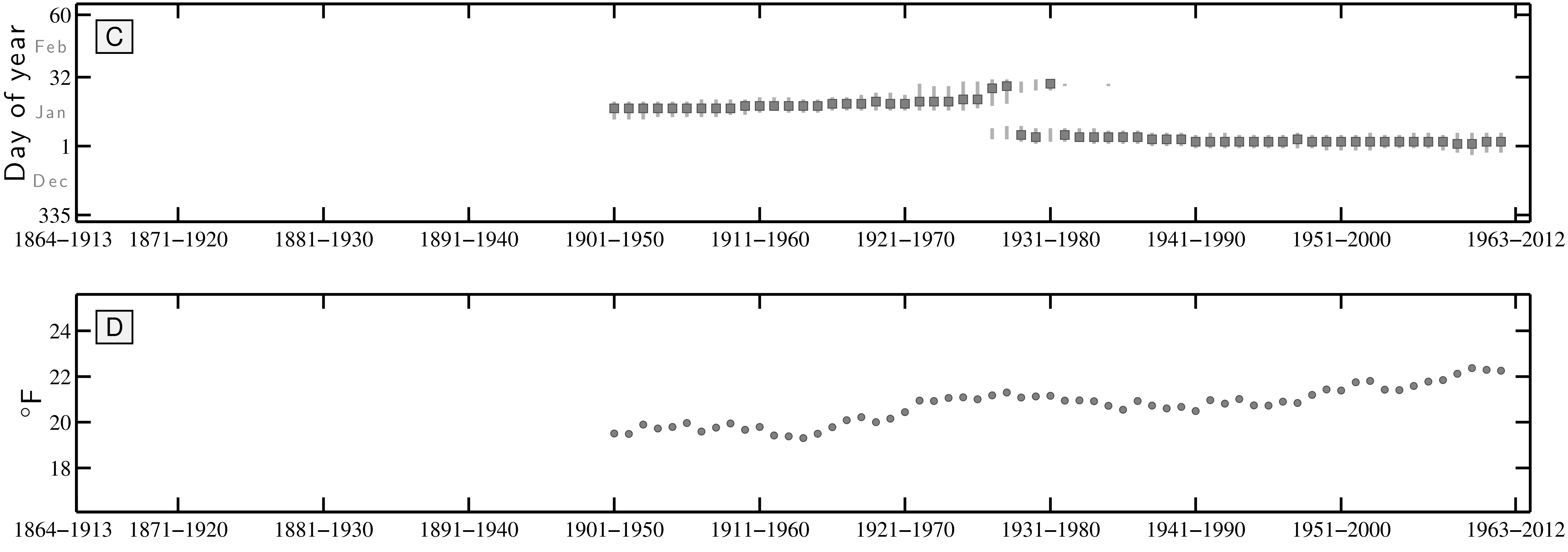

50 year Summer Teletherm dynamics for CORVALLIS STATE UNIV, OR:

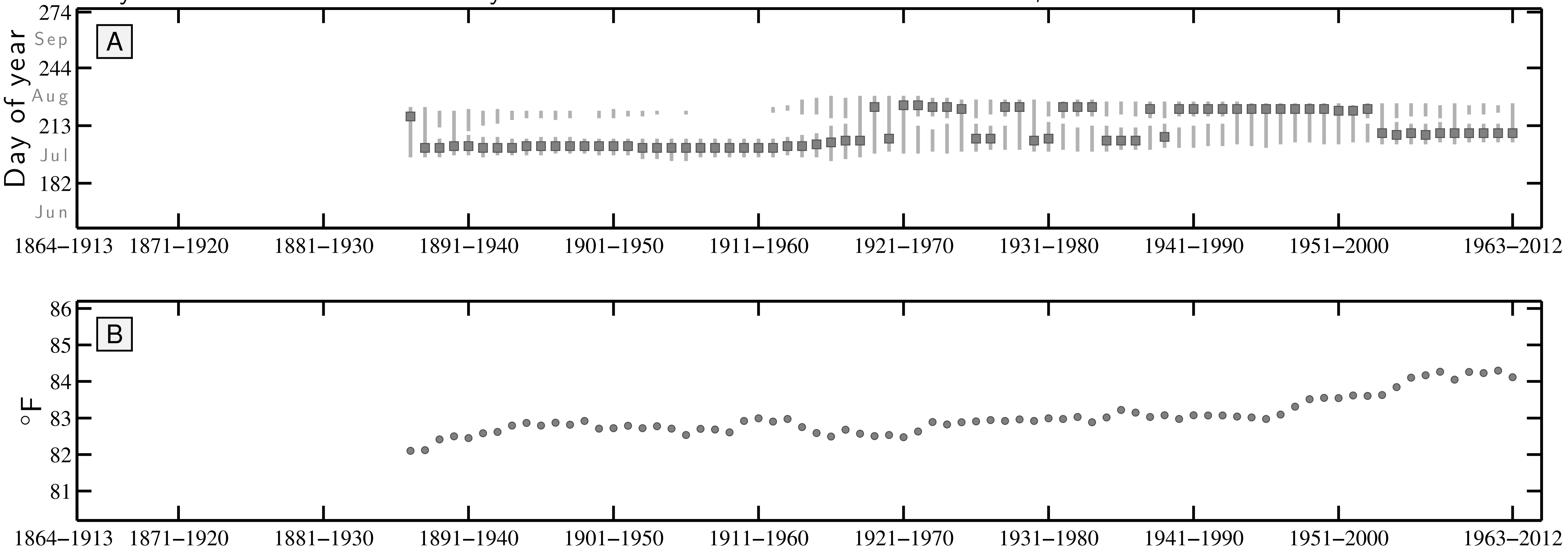

50 year Winter Teletherm dynamics for CORVALLIS STATE UNIV, OR:

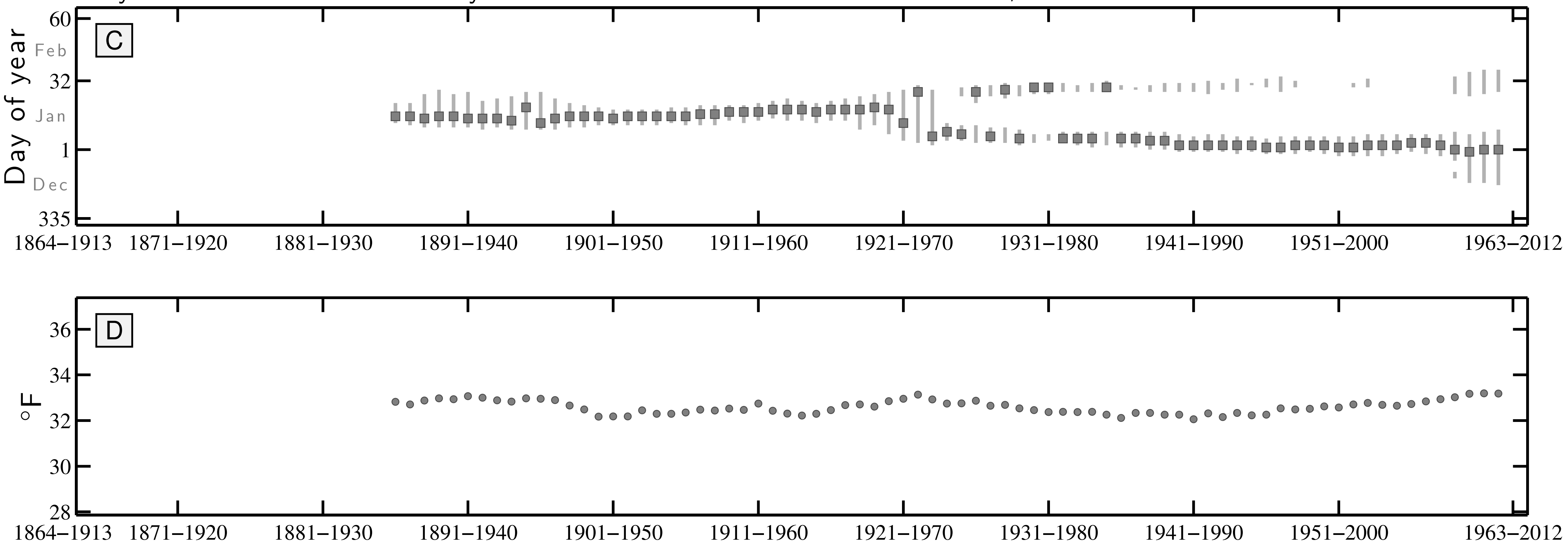

50 year Summer Teletherm dynamics for COTTAGE GROVE 1 NNE, OR:

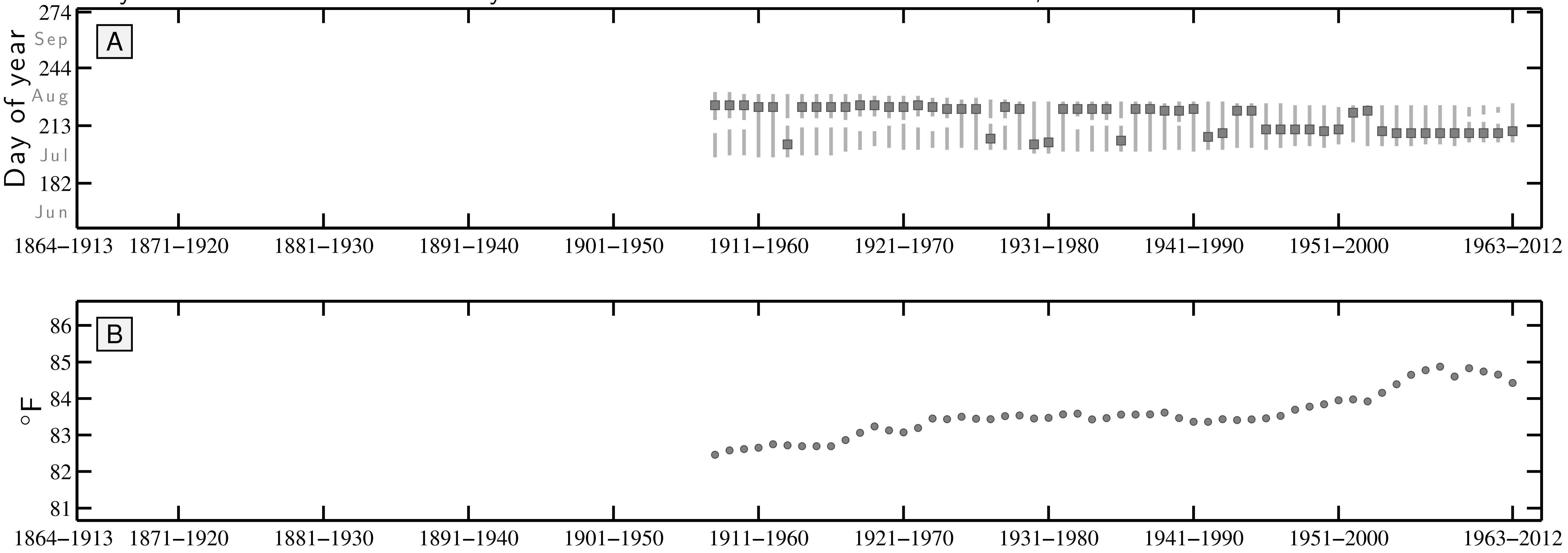

50 year Winter Teletherm dynamics for COTTAGE GROVE 1 NNE, OR:

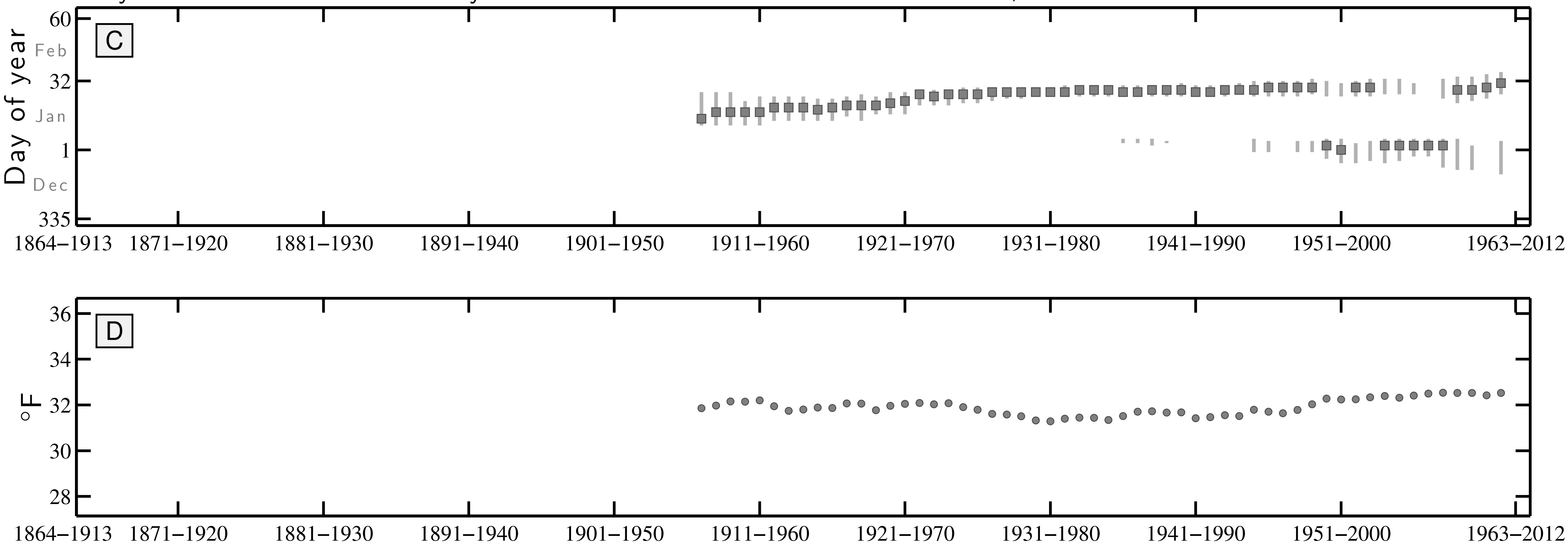

50 year Summer Teletherm dynamics for CRATER LAKE NPS HQ, OR:

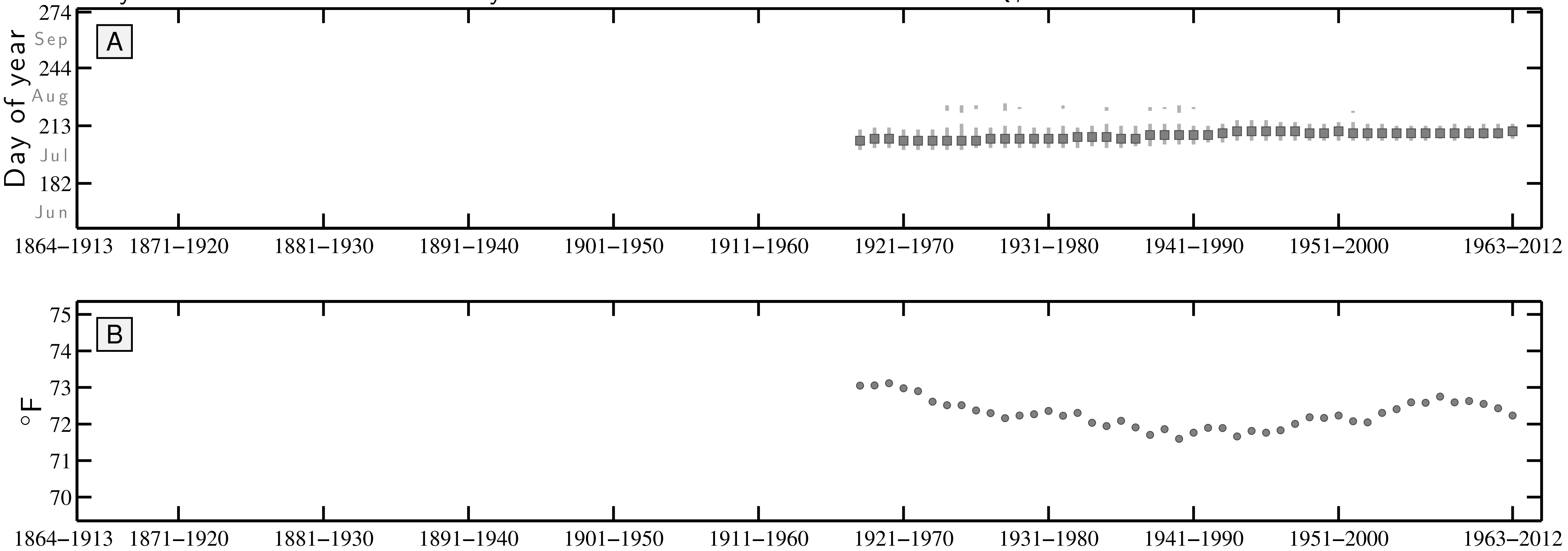

50 year Winter Teletherm dynamics for CRATER LAKE NPS HQ, OR:

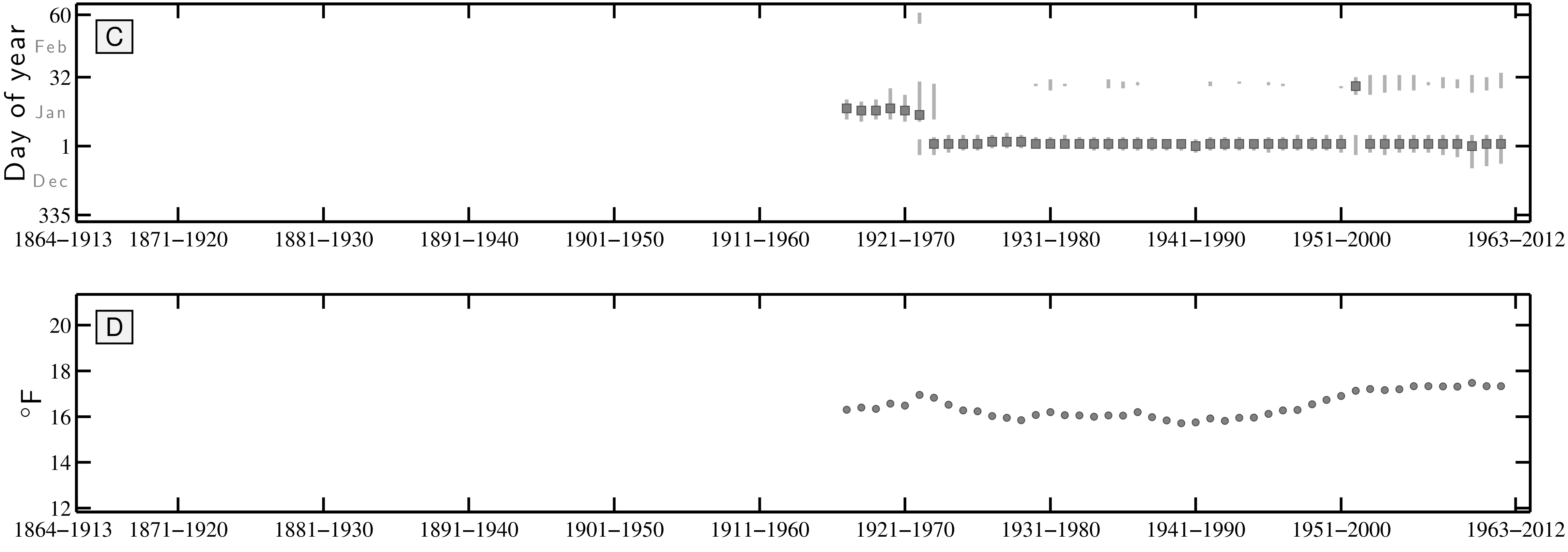

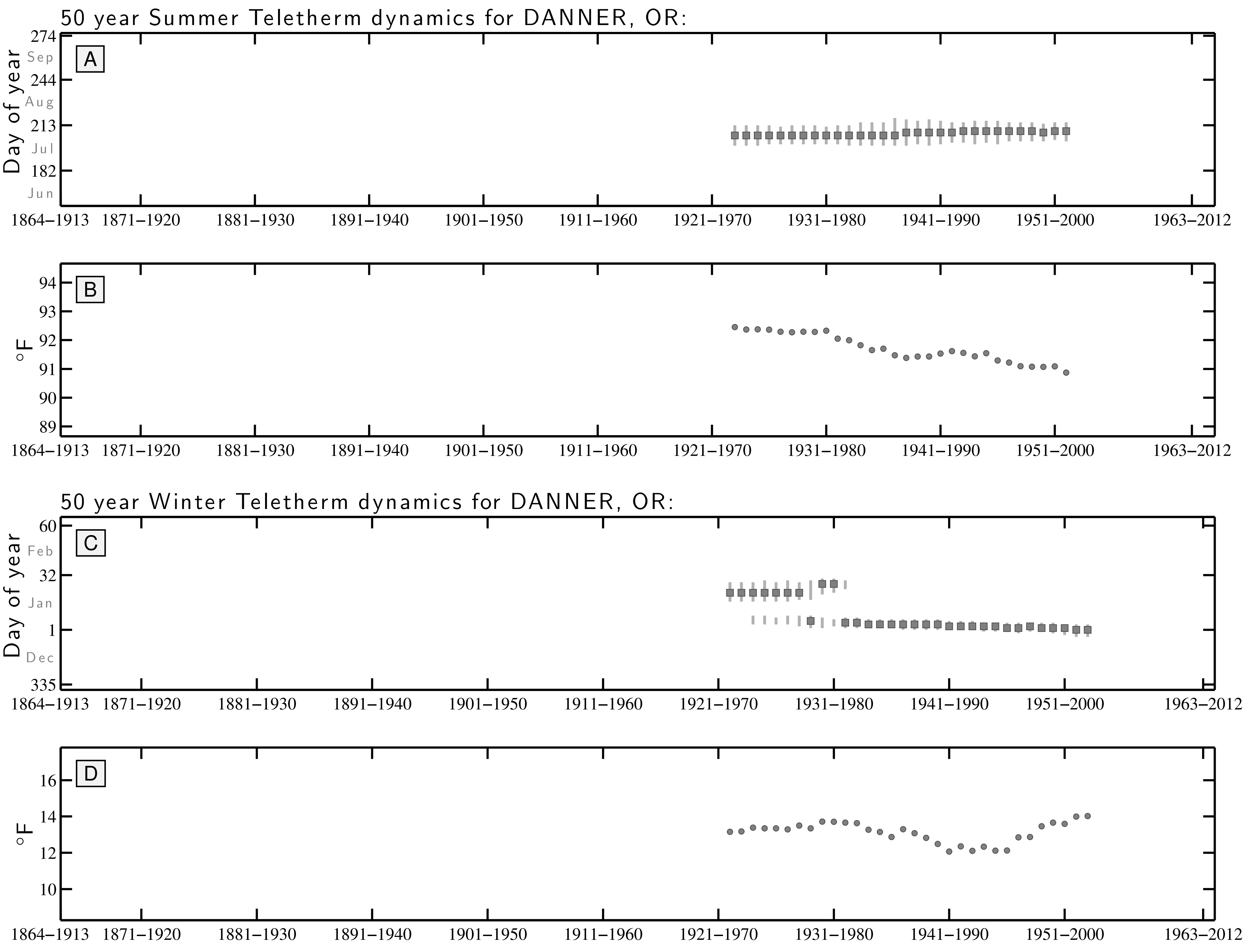
50 year Summer Teletherm dynamics for DANNER, OR:
A
B
Day of year
°F
274
Sep
244
Aug
213
Jul
182
Jun
94
93
92
91
90
89
1864–1913
1871–1920
1881–1930
1891–1940
1901–1950
1911–1960
1921–1970
1931–1980
1941–1990
1951–2000
1963–2012
50 year Winter Teletherm dynamics for DANNER, OR:
C
D
60
Feb
32
Jan
1
Dec
335
16
14
12
10

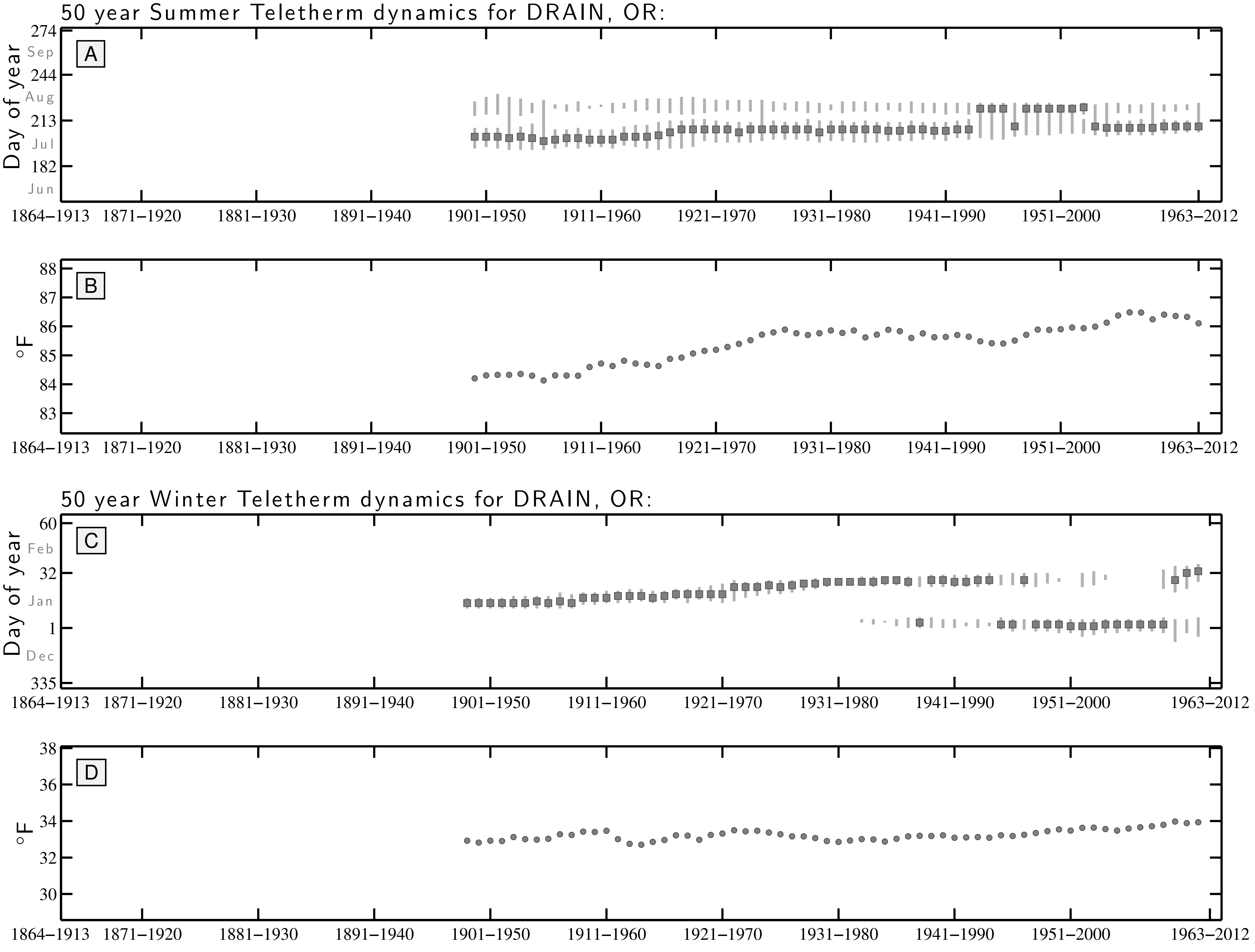

50 year Summer Teletherm dynamics for DRAIN, OR:
A
Day of year
274
Sep
244
Aug
213
Jul
182
Jun
1864–1913
1871–1920
1881–1930
1891–1940
1901–1950
1911–1960
1921–1970
1931–1980
1941–1990
1951–2000
1963–2012
B
°F
88
87
86
85
84
83
50 year Winter Teletherm dynamics for DRAIN, OR:
C
60
Feb
32
Jan
1
Dec
335
D
38
36
34
32
30

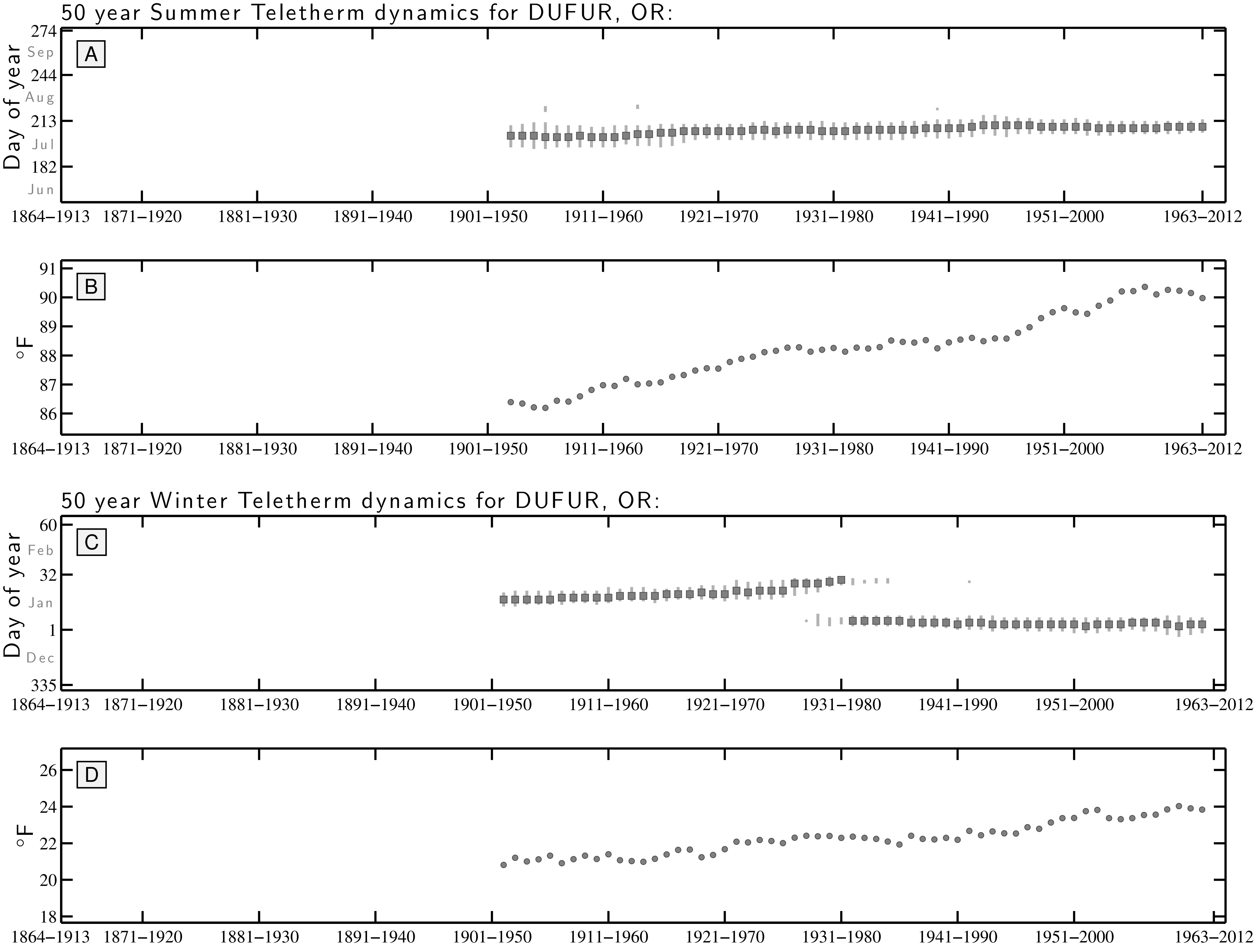
50 year Summer Teletherm dynamics for DUFUR, OR:
A
Day of year
274
Sep
244
Aug
213
Jul
182
Jun
1864–1913
1871–1920
1881–1930
1891–1940
1901–1950
1911–1960
1921–1970
1931–1980
1941–1990
1951–2000
1963–2012
B
°F
91
90
89
88
87
86
50 year Winter Teletherm dynamics for DUFUR, OR:
C
60
Feb
32
Jan
1
Dec
335
D
26
24
22
20
18

50 year Summer Teletherm dynamics for FOREST GROVE, OR:

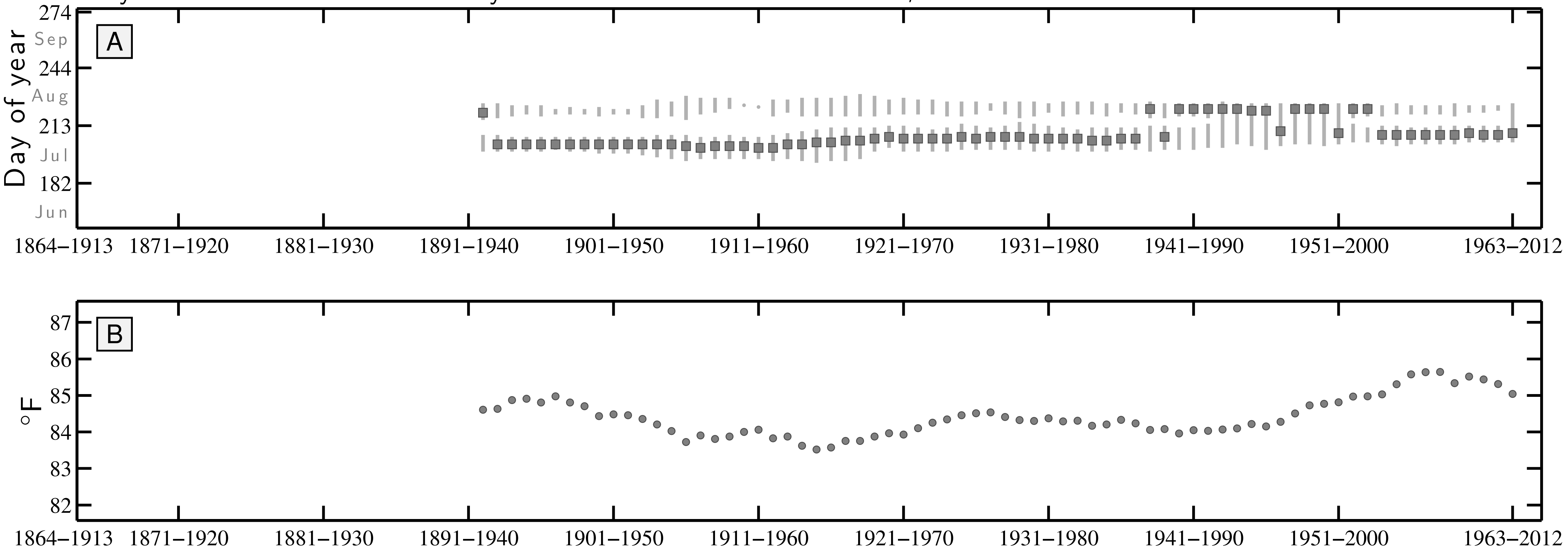

50 year Winter Teletherm dynamics for FOREST GROVE, OR:

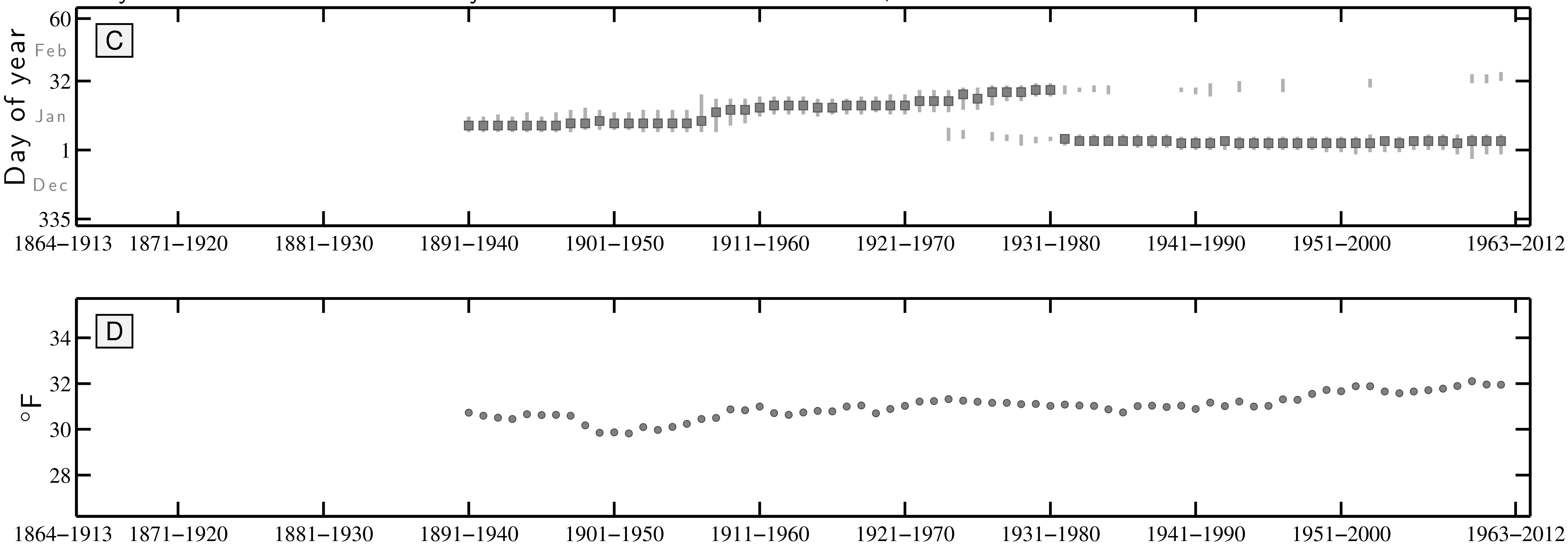

50 year Summer Teletherm dynamics for FREMONT 5 NW, OR:

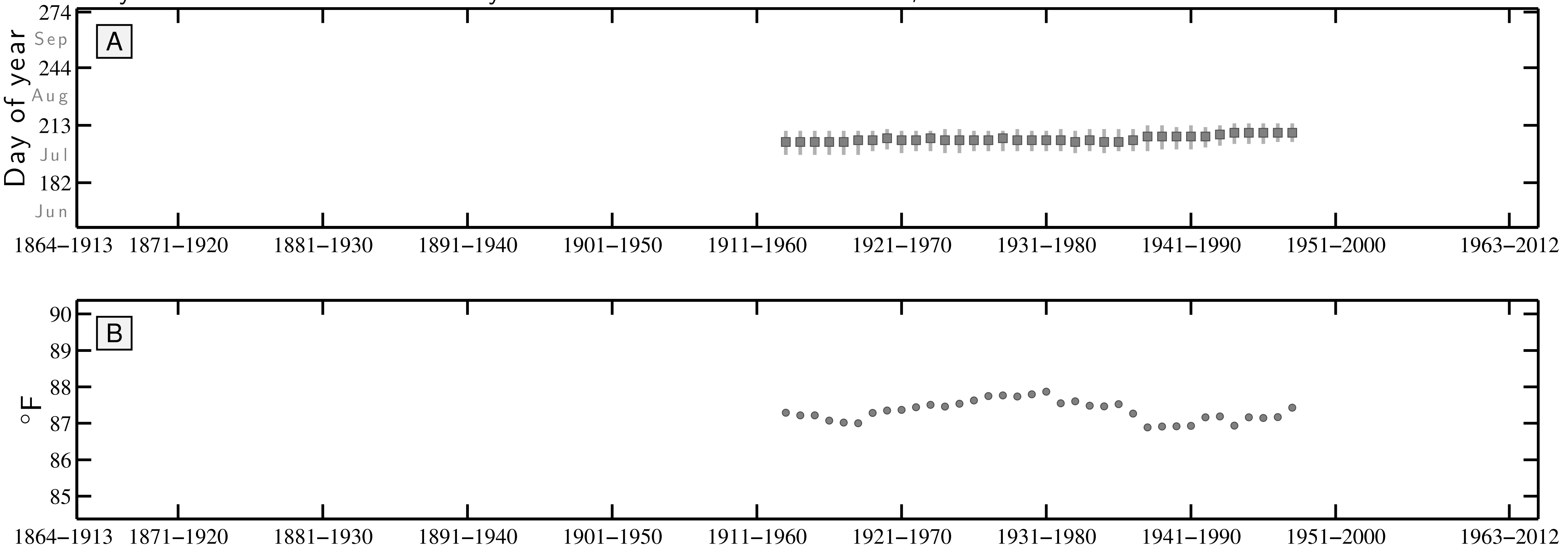

50 year Winter Teletherm dynamics for FREMONT 5 NW, OR:

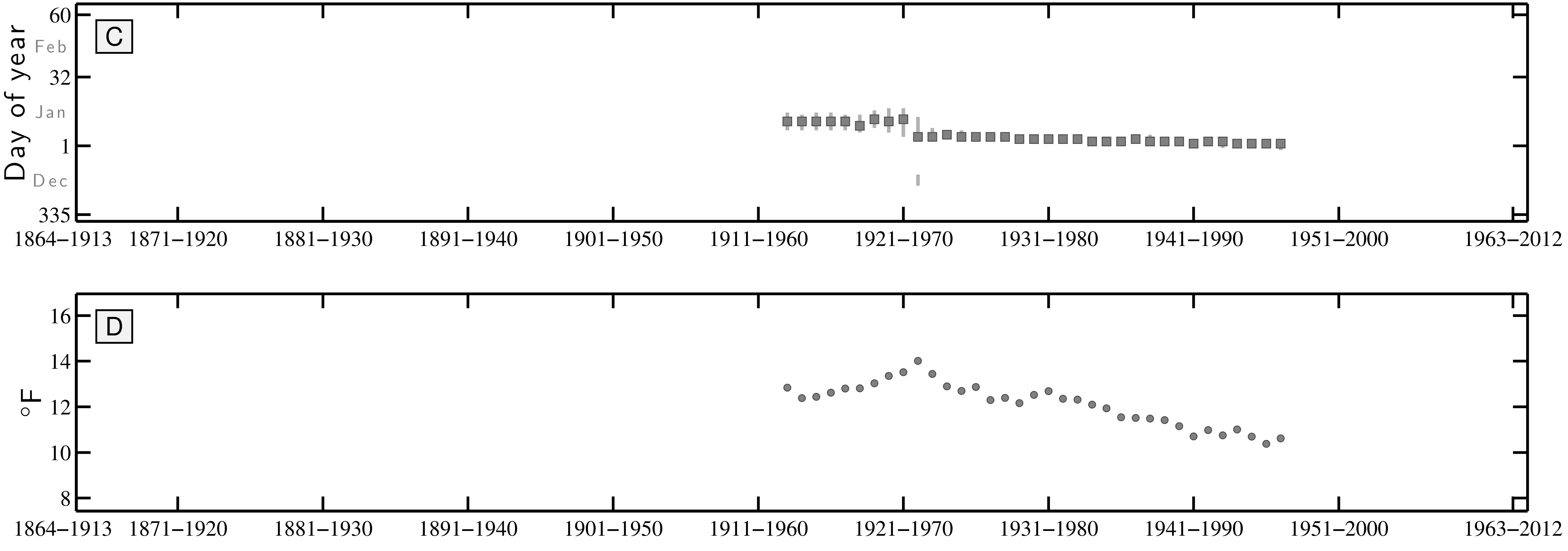

50 year Summer Teletherm dynamics for GRANTS PASS, OR:

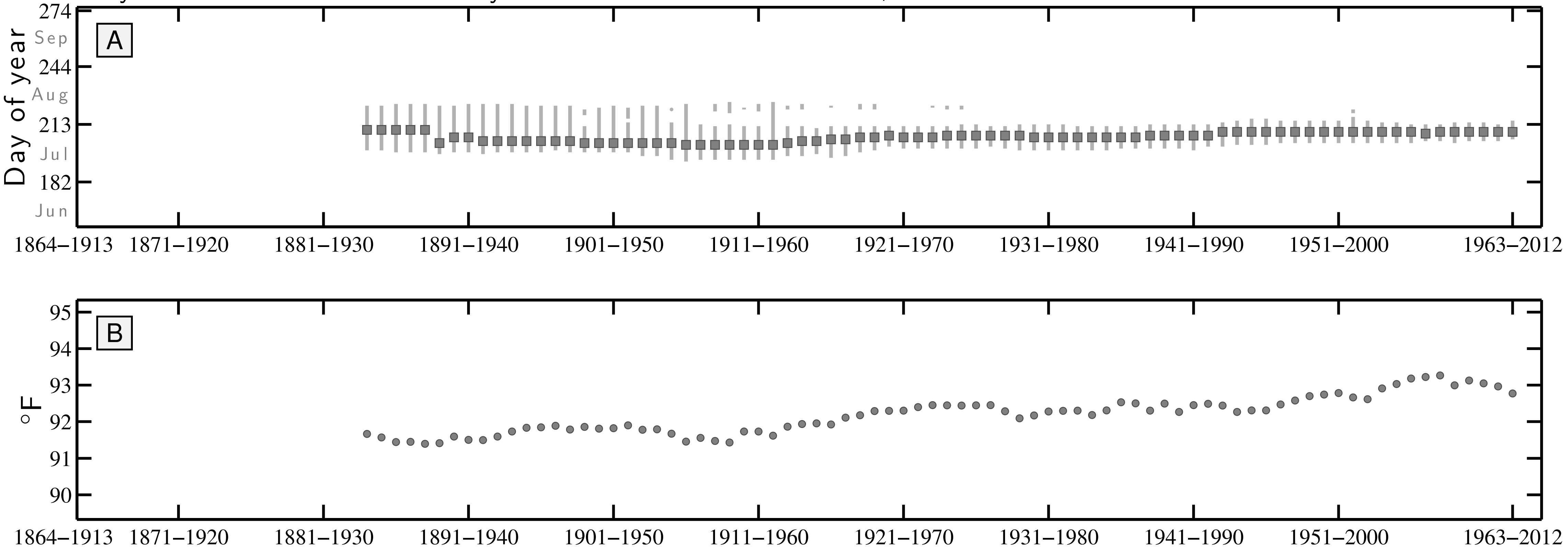

50 year Winter Teletherm dynamics for GRANTS PASS, OR:

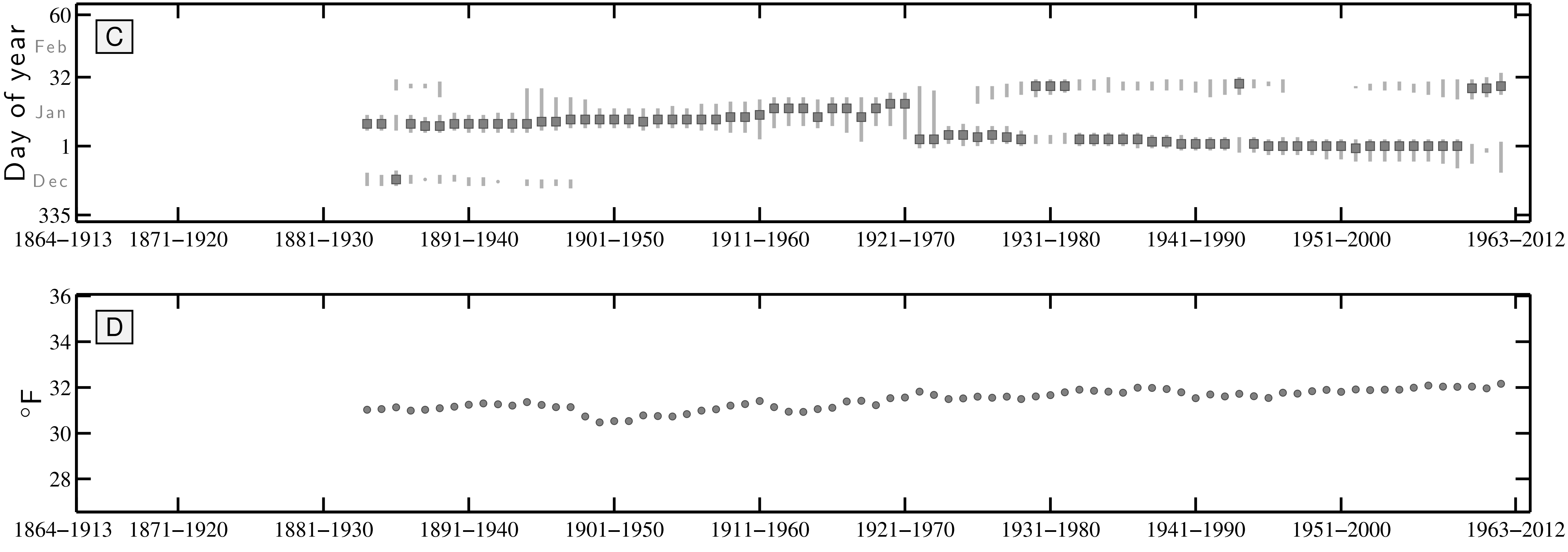

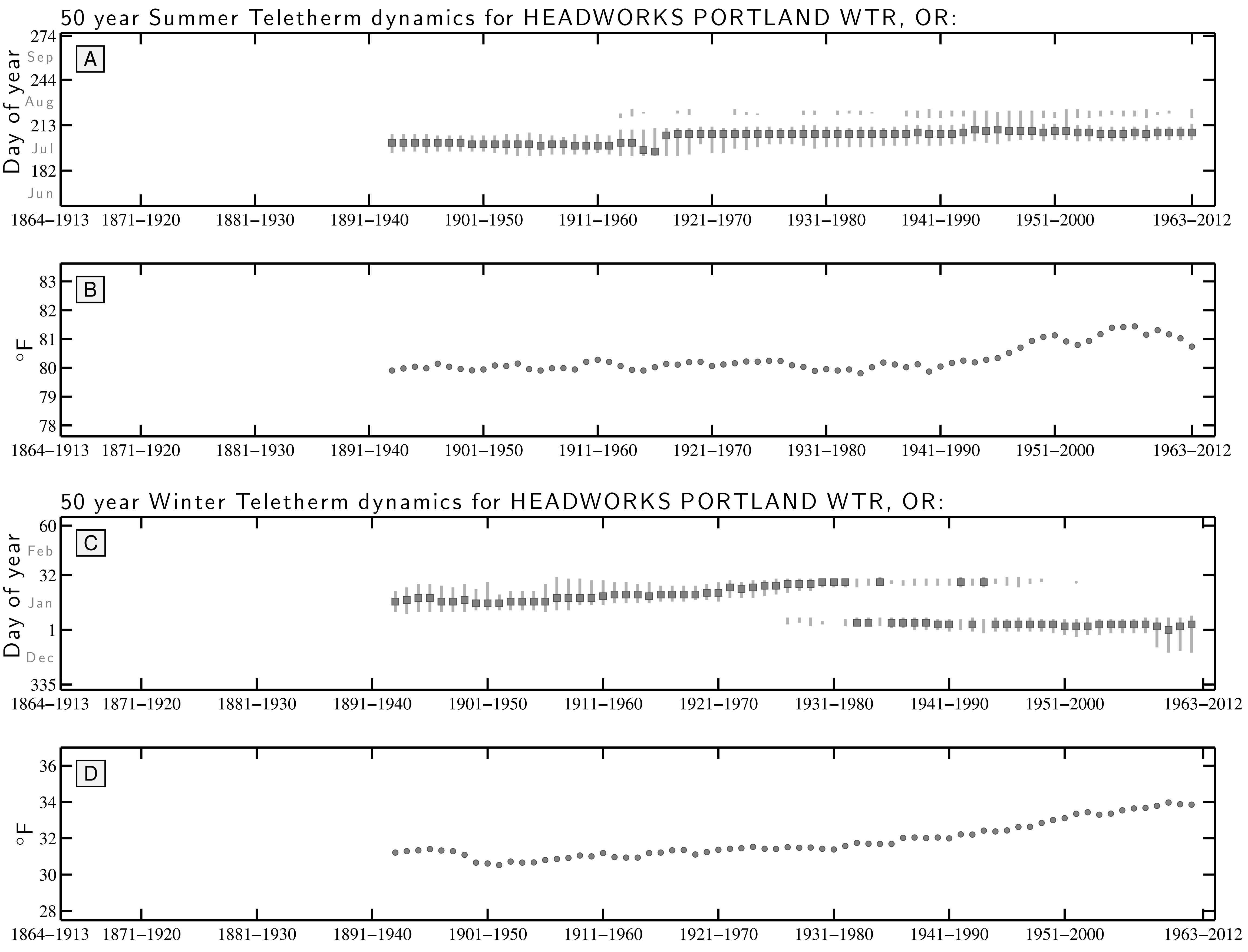
50 year Summer Teletherm dynamics for HEADWORKS PORTLAND WTR, OR:
A
B
Day of year
°F
274
Sep
244
Aug
213
Jul
182
Jun
83
82
81
80
79
78
1864–1913
1871–1920
1881–1930
1891–1940
1901–1950
1911–1960
1921–1970
1931–1980
1941–1990
1951–2000
1963–2012
50 year Winter Teletherm dynamics for HEADWORKS PORTLAND WTR, OR:
C
D
Day of year
°F
60
Feb
32
Jan
1
Dec
335
36
34
32
30
28

50 year Summer Teletherm dynamics for HEPPNER, OR:

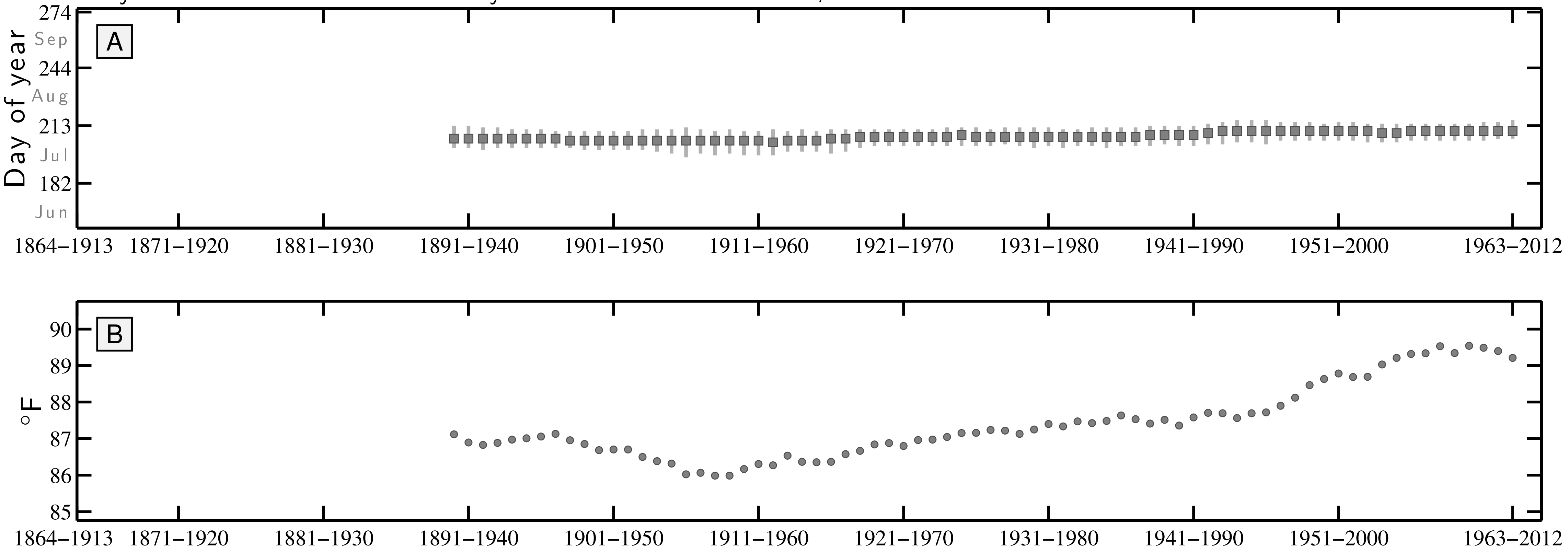

50 year Winter Teletherm dynamics for HEPPNER, OR:

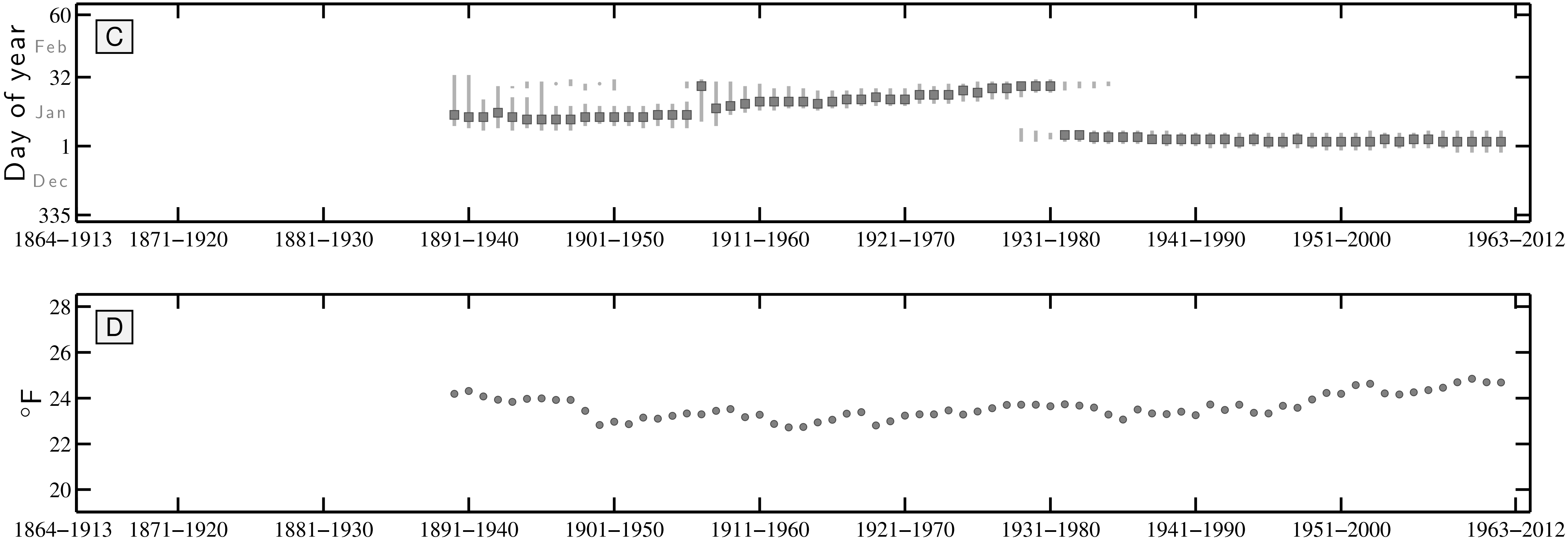

50 year Summer Teletherm dynamics for HERMISTON 1 SE, OR:

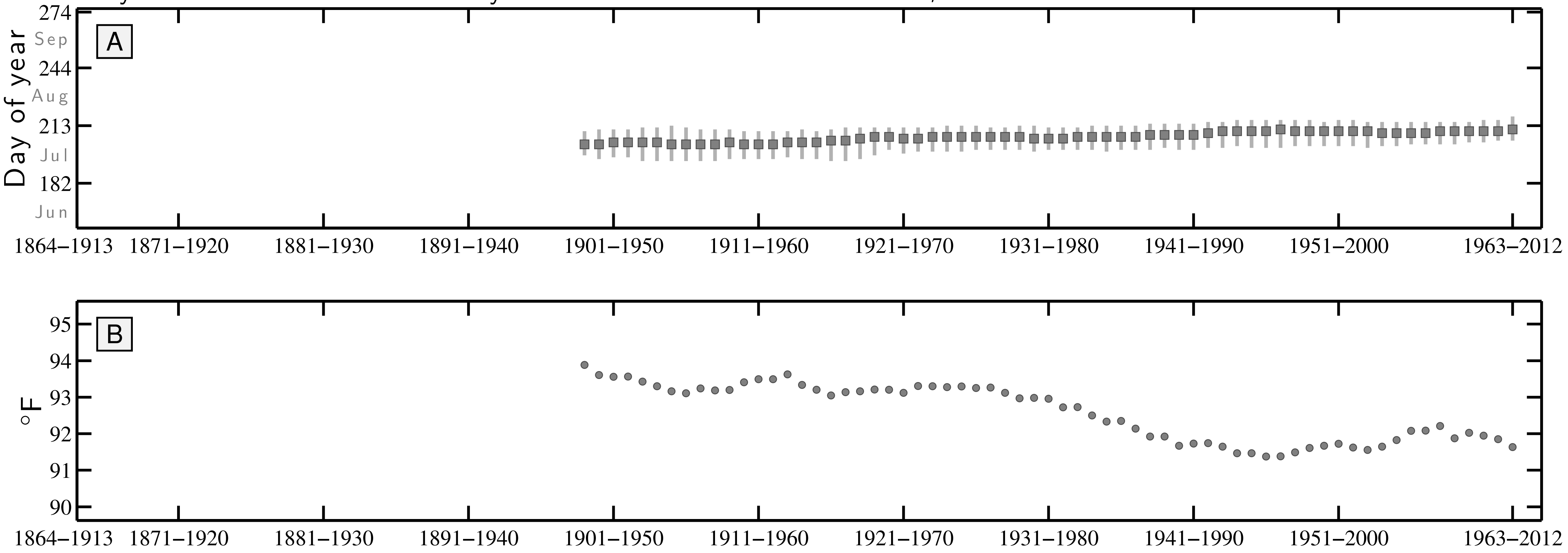

50 year Winter Teletherm dynamics for HERMISTON 1 SE, OR:

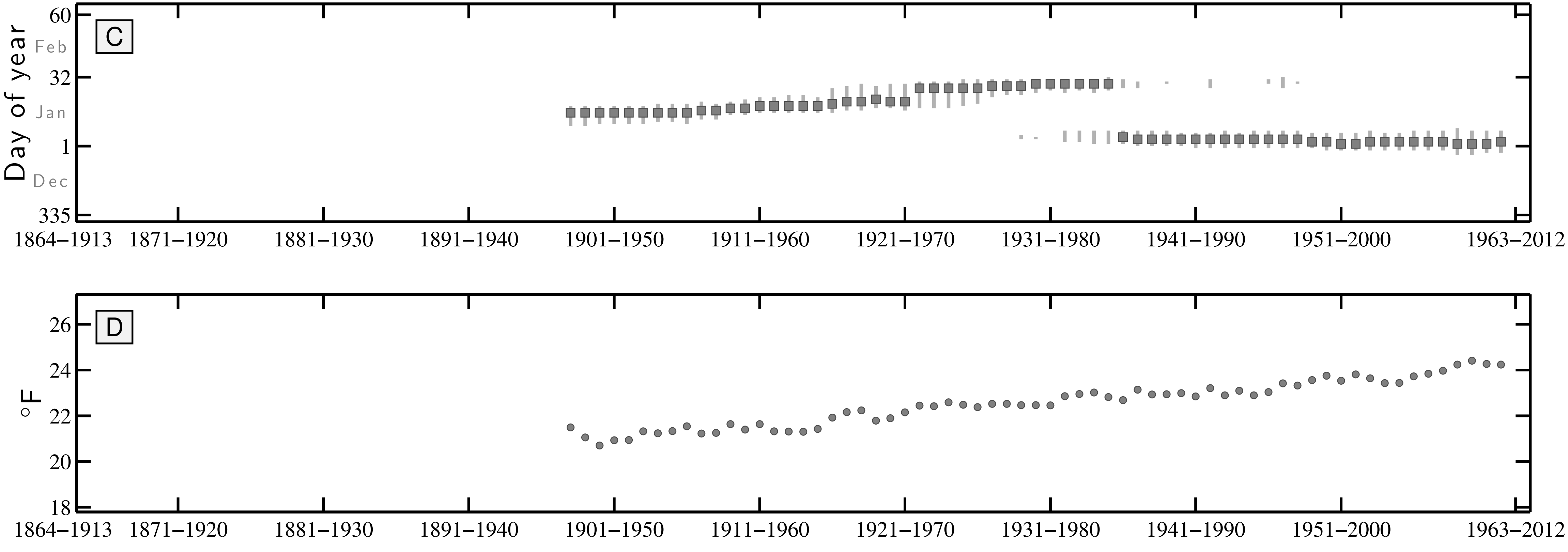

50 year Summer Teletherm dynamics for HOOD RIVER EXP STN, OR:

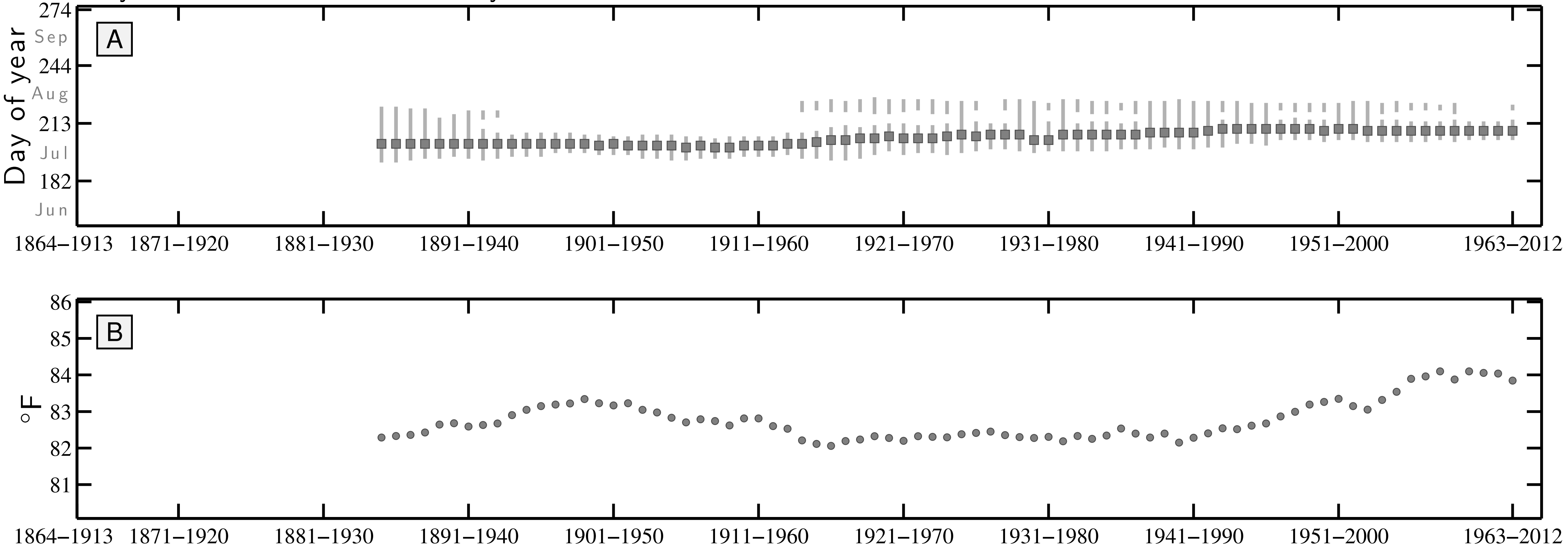

50 year Winter Teletherm dynamics for HOOD RIVER EXP STN, OR:

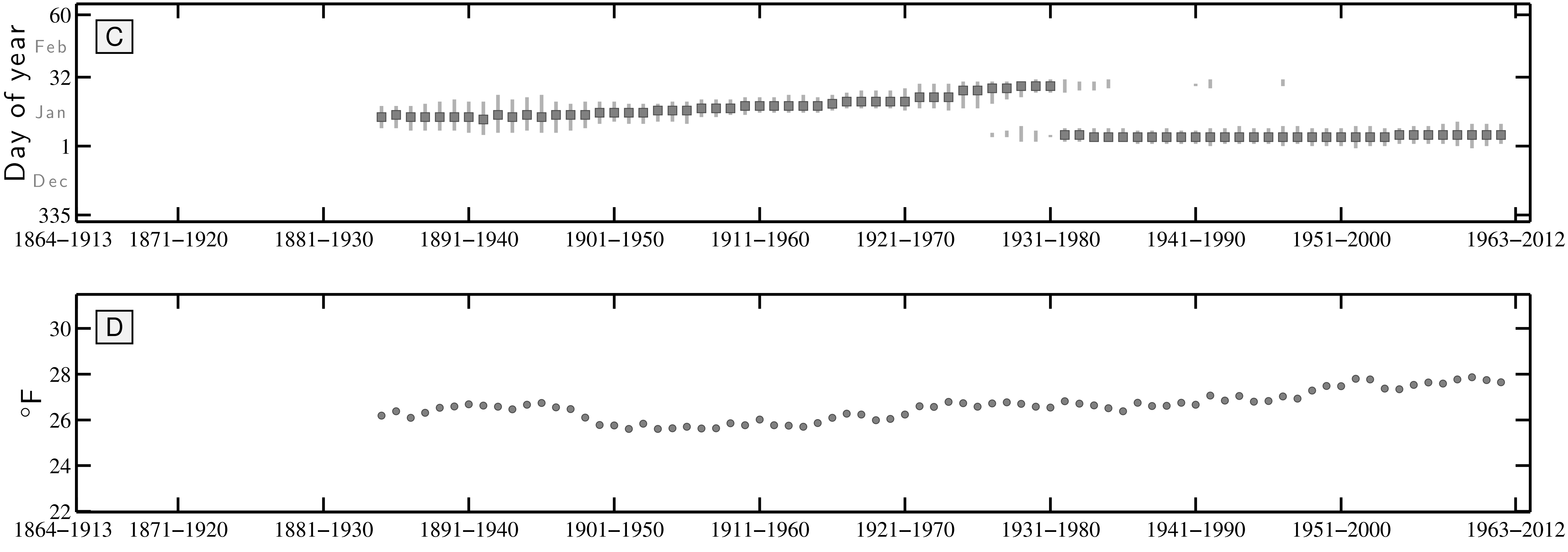

50 year Summer Teletherm dynamics for KLAMATH FALLS 2 SSW, OR:

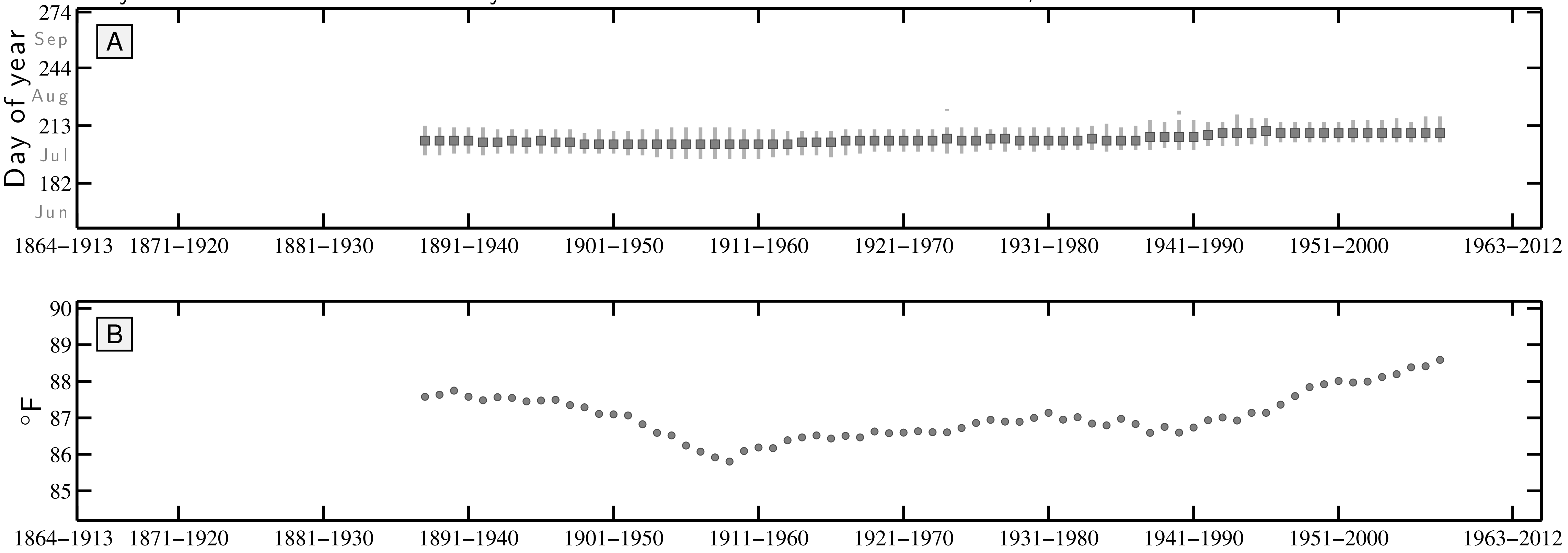

50 year Winter Teletherm dynamics for KLAMATH FALLS 2 SSW, OR:

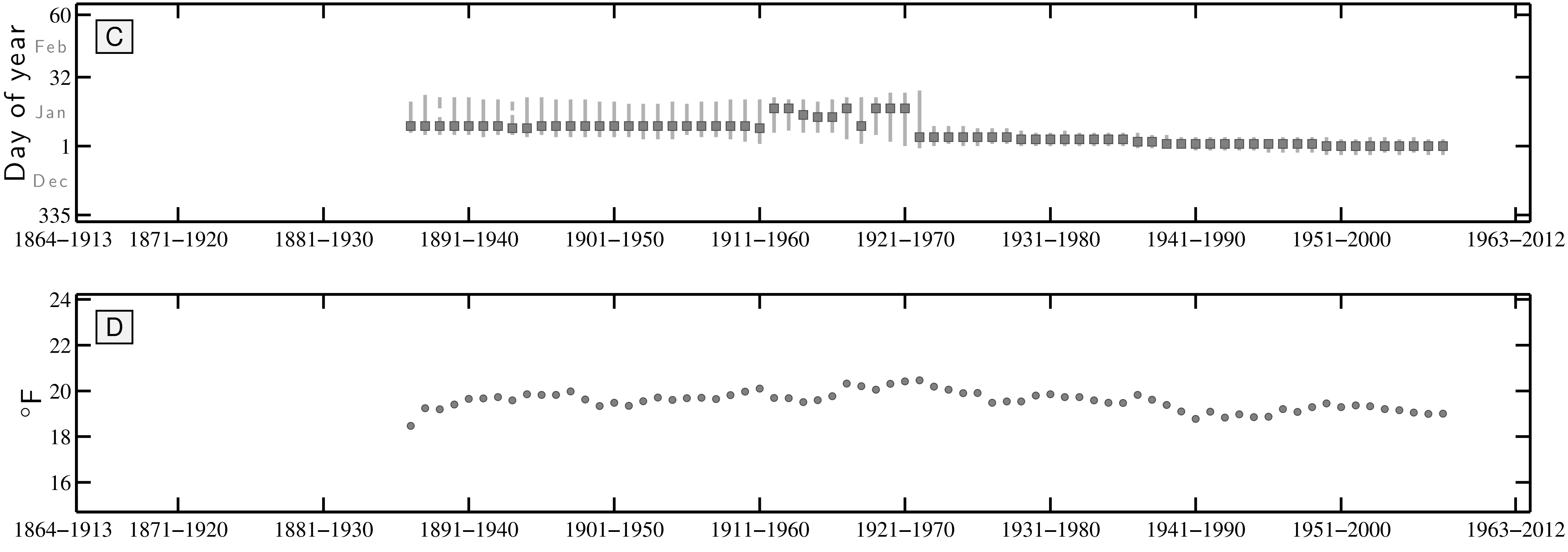

50 year Summer Teletherm dynamics for LAKEVIEW 2 NNW, OR:

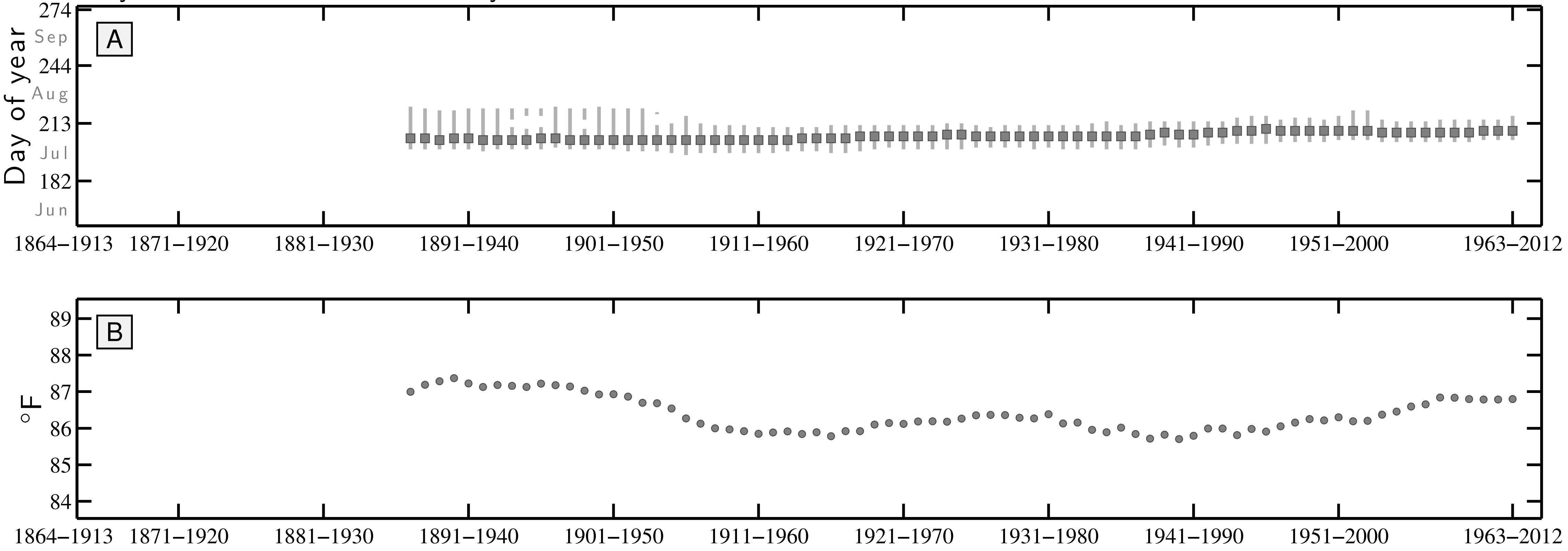

50 year Winter Teletherm dynamics for LAKEVIEW 2 NNW, OR:

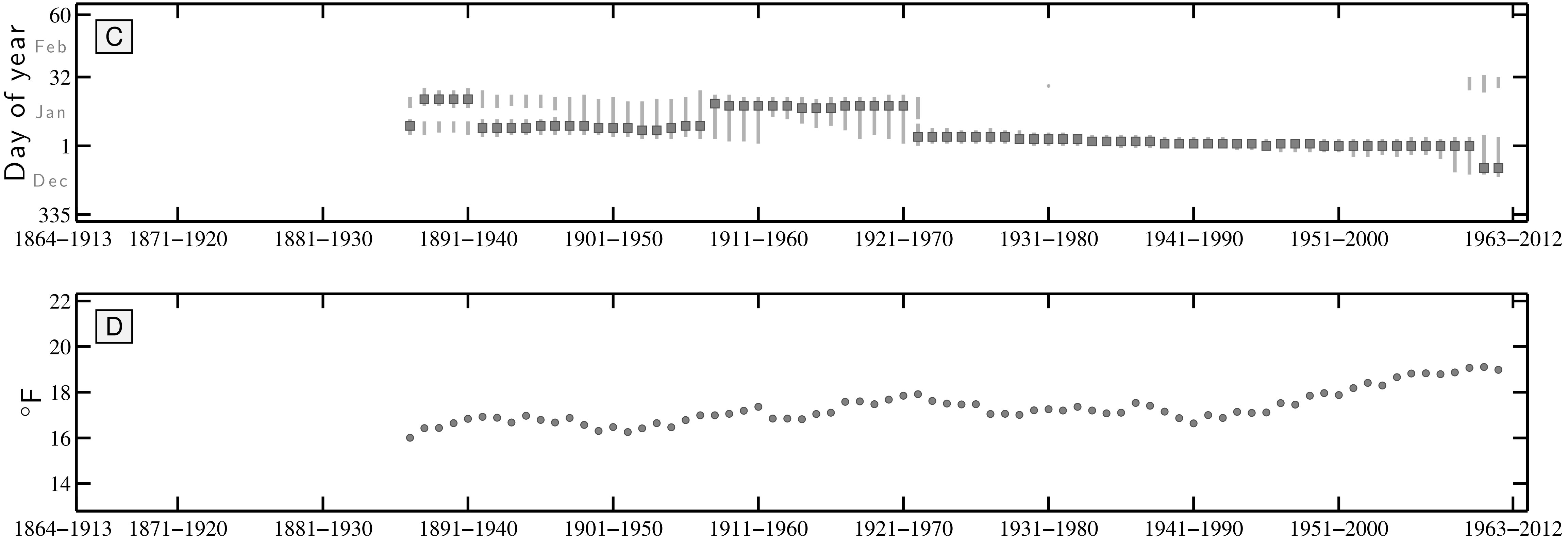

50 year Summer Teletherm dynamics for MALHEUR REFUGE HQ, OR:

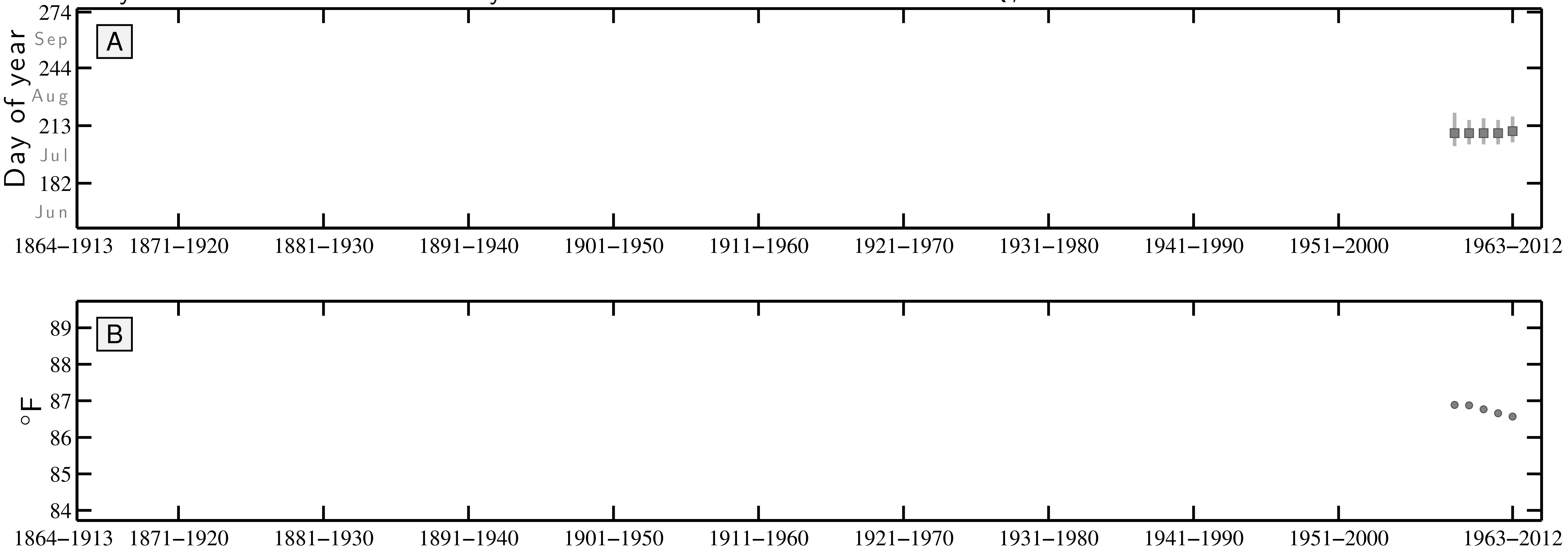

50 year Winter Teletherm dynamics for MALHEUR REFUGE HQ, OR:

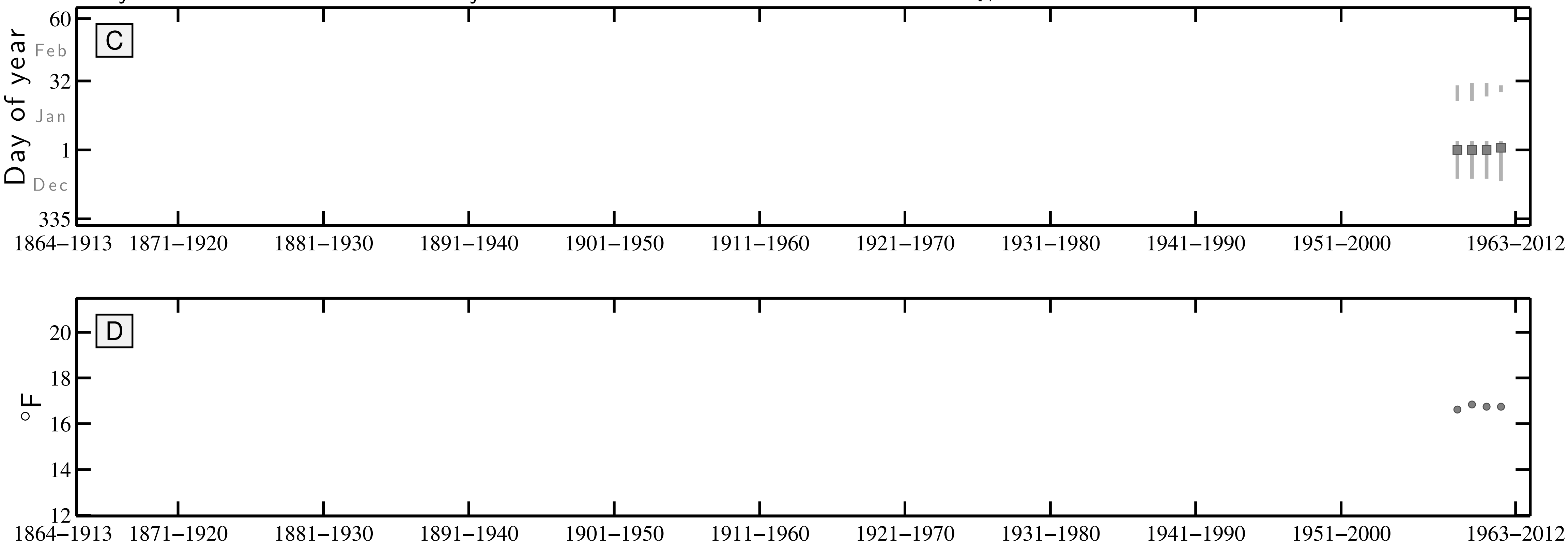

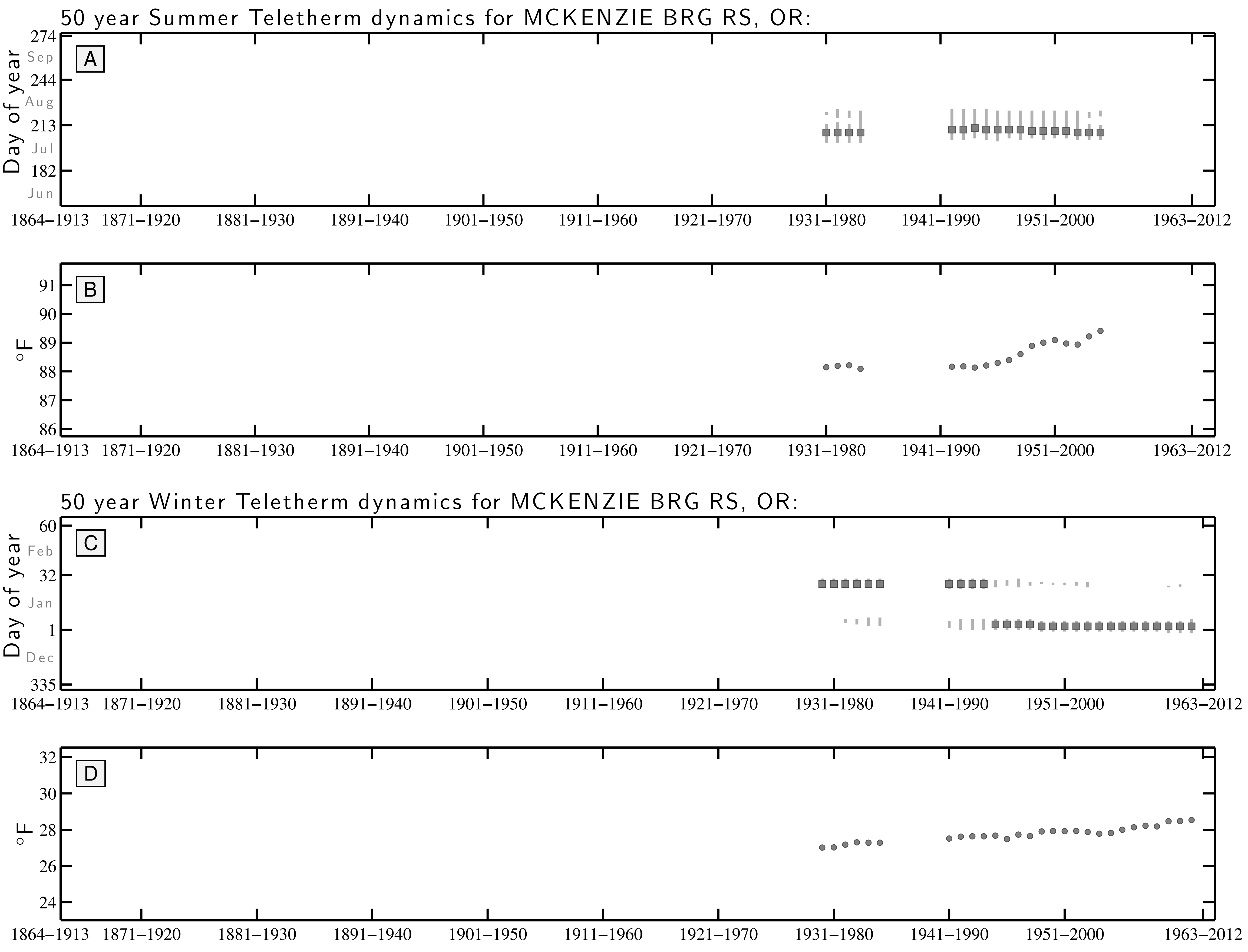

50 year Summer Teletherm dynamics for MCKENZIE BRG RS, OR:
A
Day of year
274
Sep
244
Aug
213
Jul
182
Jun
1864–1913
1871–1920
1881–1930
1891–1940
1901–1950
1911–1960
1921–1970
1931–1980
1941–1990
1951–2000
1963–2012
B
°F
91
90
89
88
87
86
50 year Winter Teletherm dynamics for MCKENZIE BRG RS, OR:
C
Day of year
60
Feb
32
Jan
1
Dec
335
D
°F
32
30
28
26
24

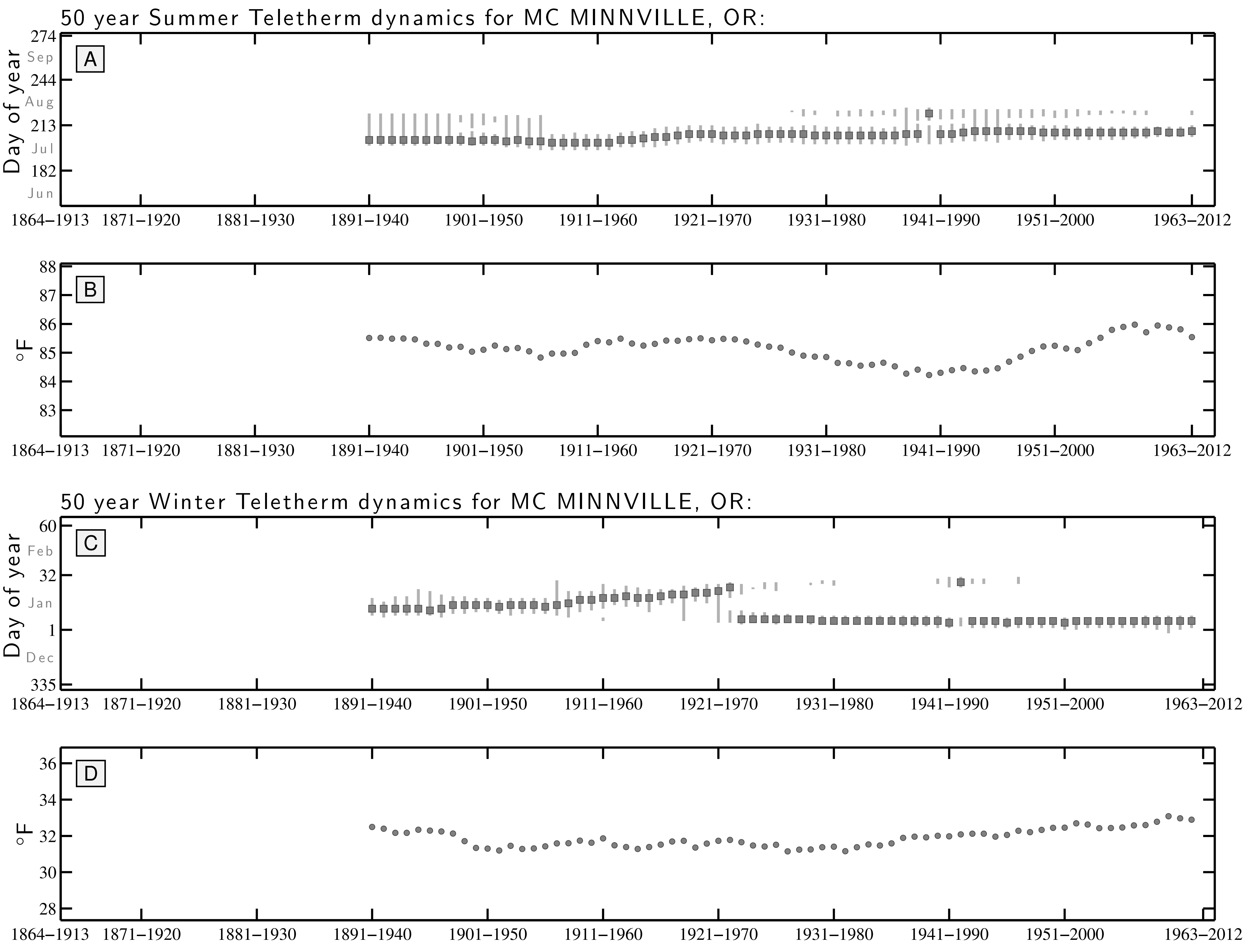

50 year Summer Teletherm dynamics for MC MINNVILLE, OR:
A
Day of year
274
Sep
244
Aug
213
Jul
182
Jun
1864–1913
1871–1920
1881–1930
1891–1940
1901–1950
1911–1960
1921–1970
1931–1980
1941–1990
1951–2000
1963–2012
B
°F
88
87
86
85
84
83
50 year Winter Teletherm dynamics for MC MINNVILLE, OR:
C
60
Feb
32
Jan
1
Dec
335
D
36
34
32
30
28

50 year Summer Teletherm dynamics for MILTON FREEWATER, OR:

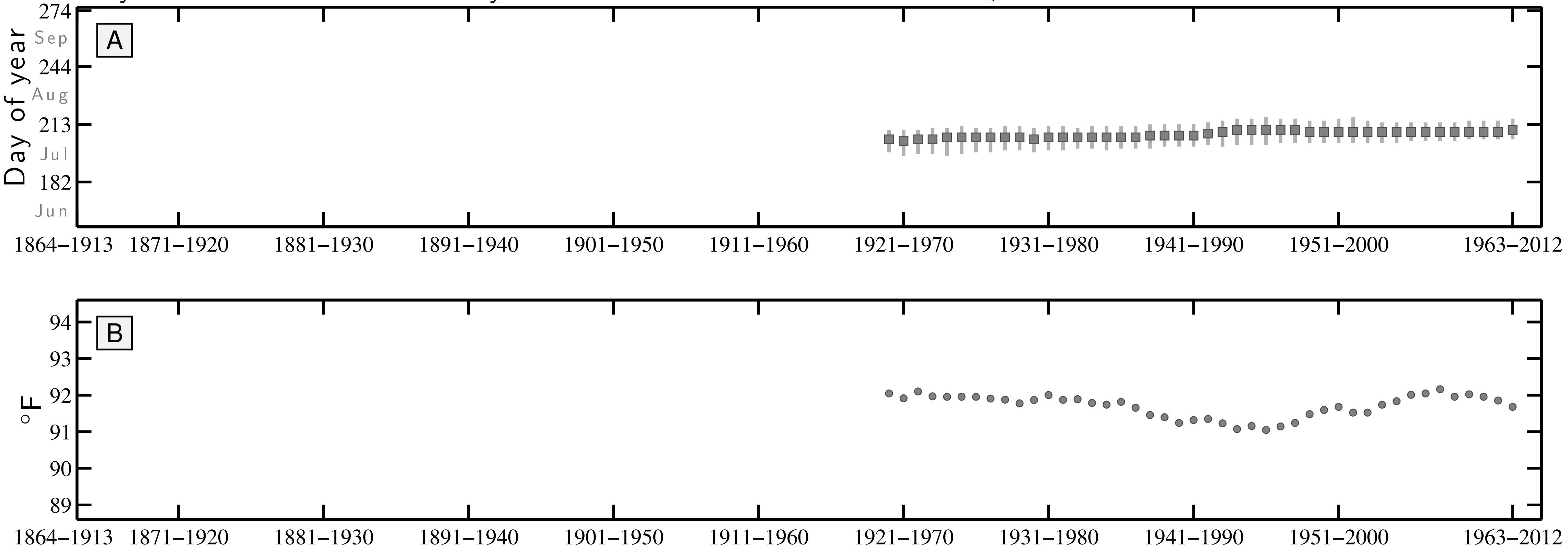

50 year Winter Teletherm dynamics for MILTON FREEWATER, OR:

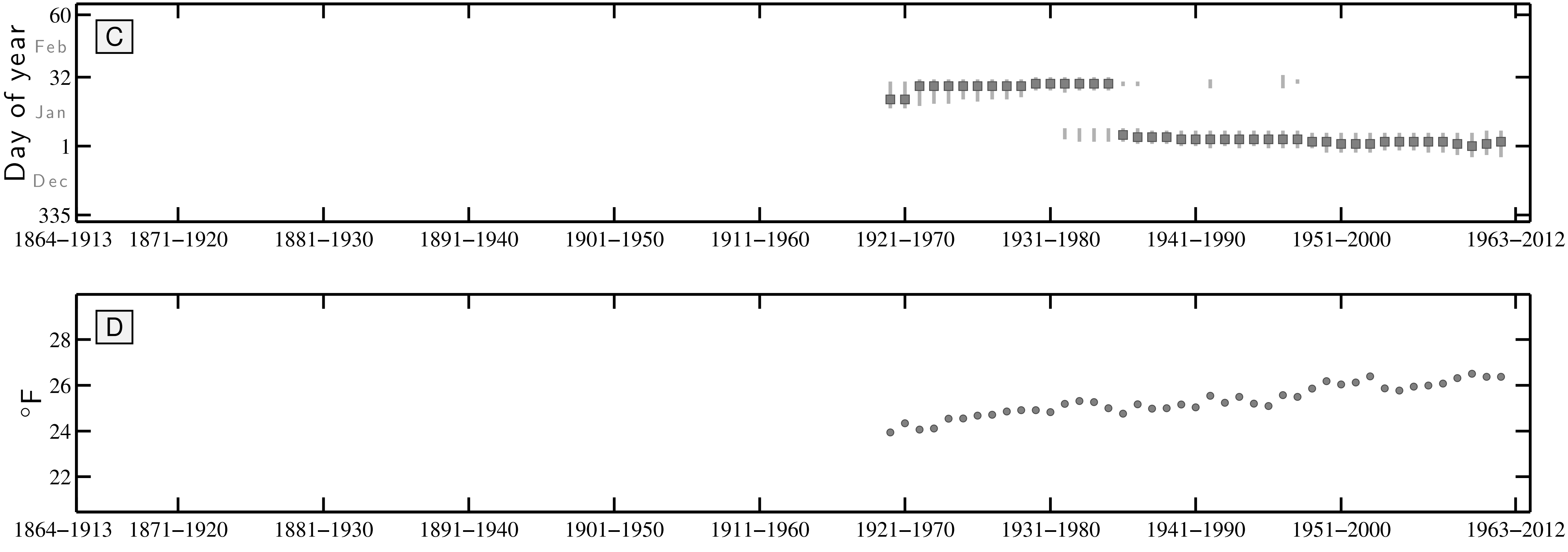

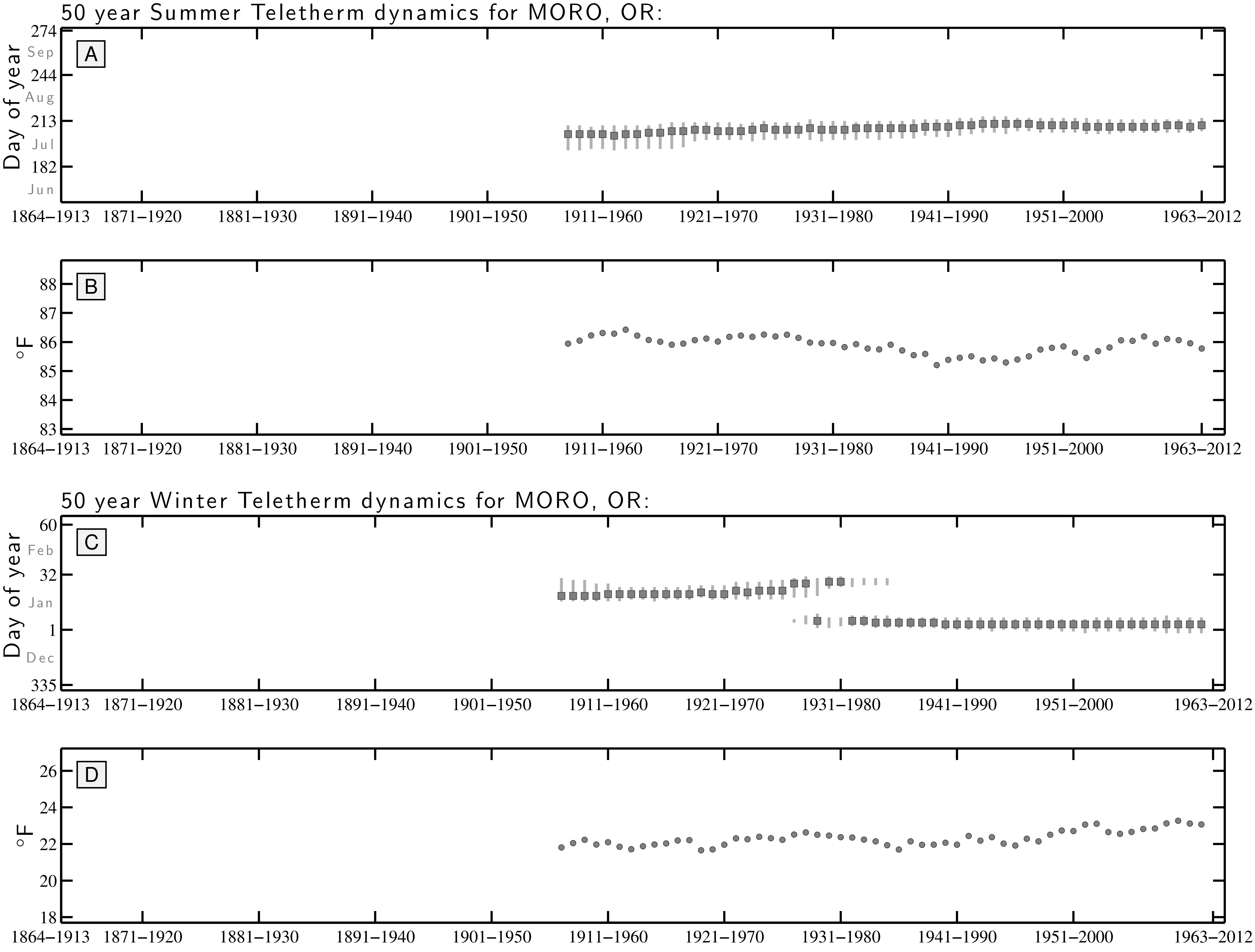
50 year Summer Teletherm dynamics for MORO, OR:
A
Day of year
274
Sep
244
Aug
213
Jul
182
Jun
1864–1913
1871–1920
1881–1930
1891–1940
1901–1950
1911–1960
1921–1970
1931–1980
1941–1990
1951–2000
1963–2012
B
°F
88
87
86
85
84
83
50 year Winter Teletherm dynamics for MORO, OR:
C
Day of year
60
Feb
32
Jan
1
Dec
335
D
°F
26
24
22
20
18

50 year Summer Teletherm dynamics for NEWPORT, OR:

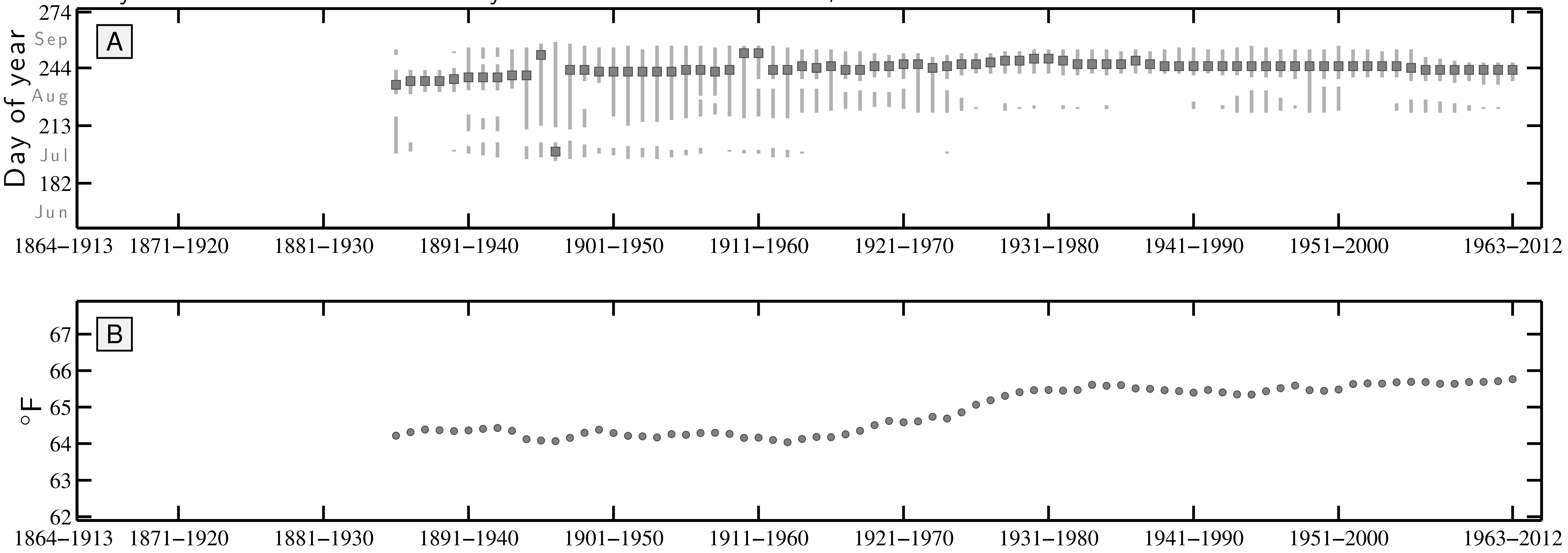

50 year Winter Teletherm dynamics for NEWPORT, OR:

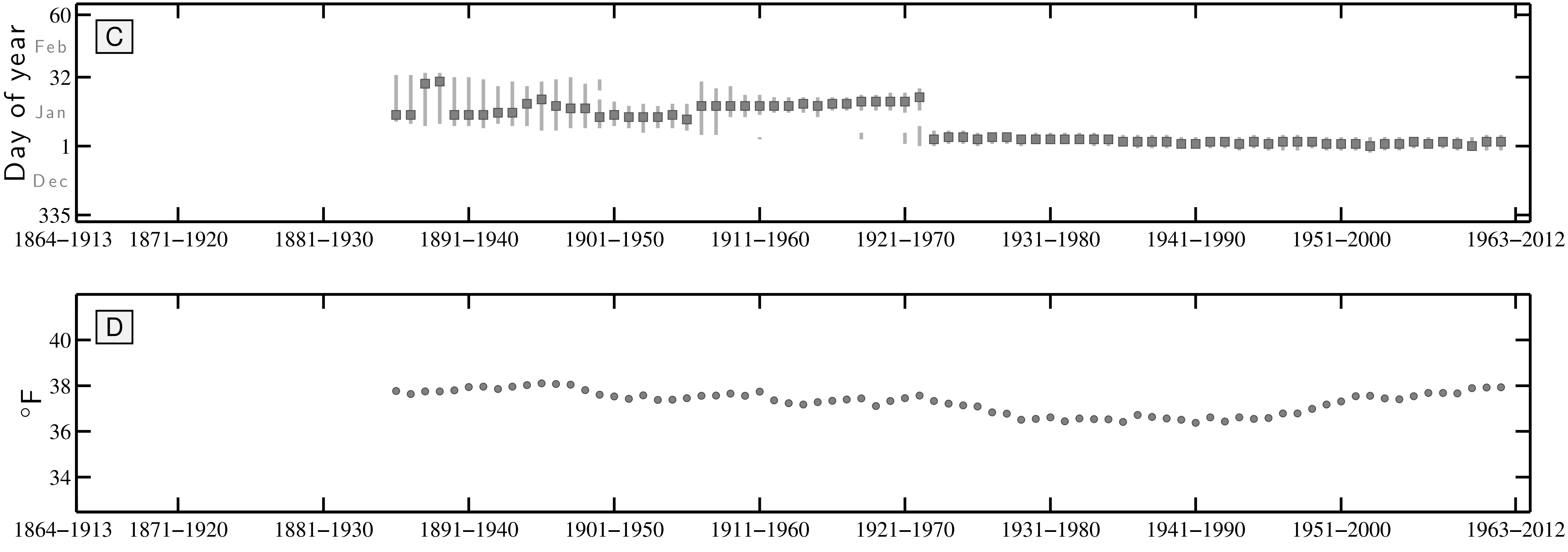

50 year Summer Teletherm dynamics for NORTH BEND FCWOS, OR:

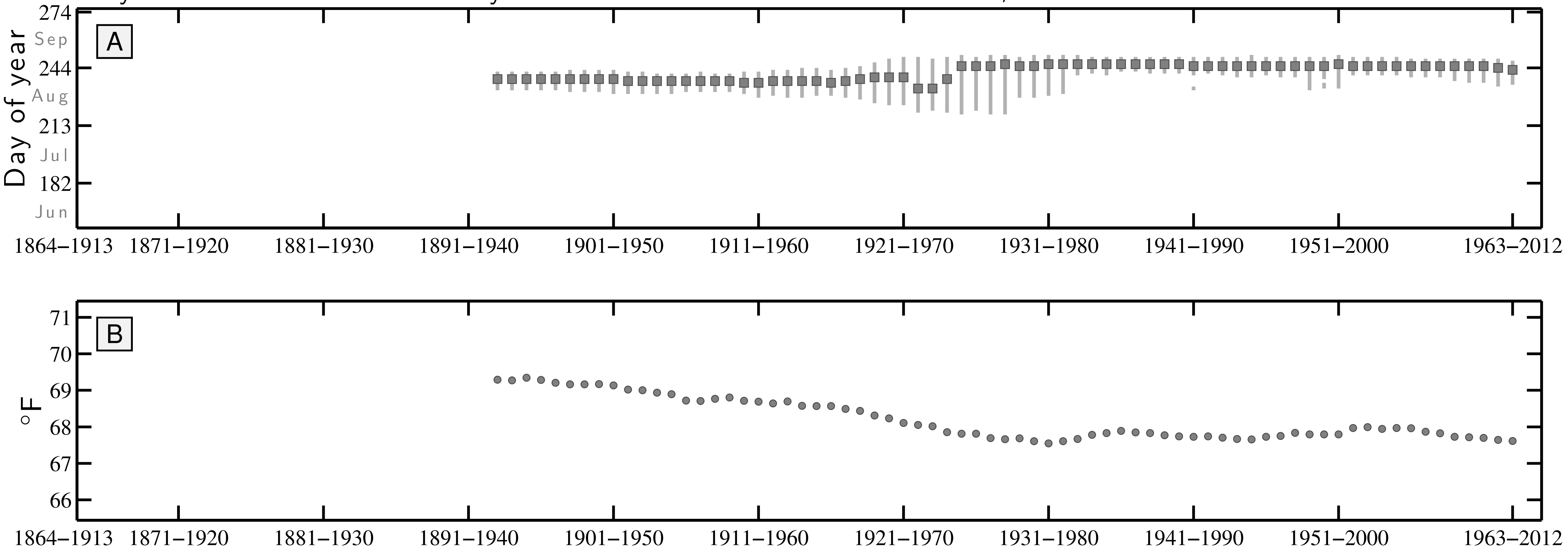

50 year Winter Teletherm dynamics for NORTH BEND FCWOS, OR:

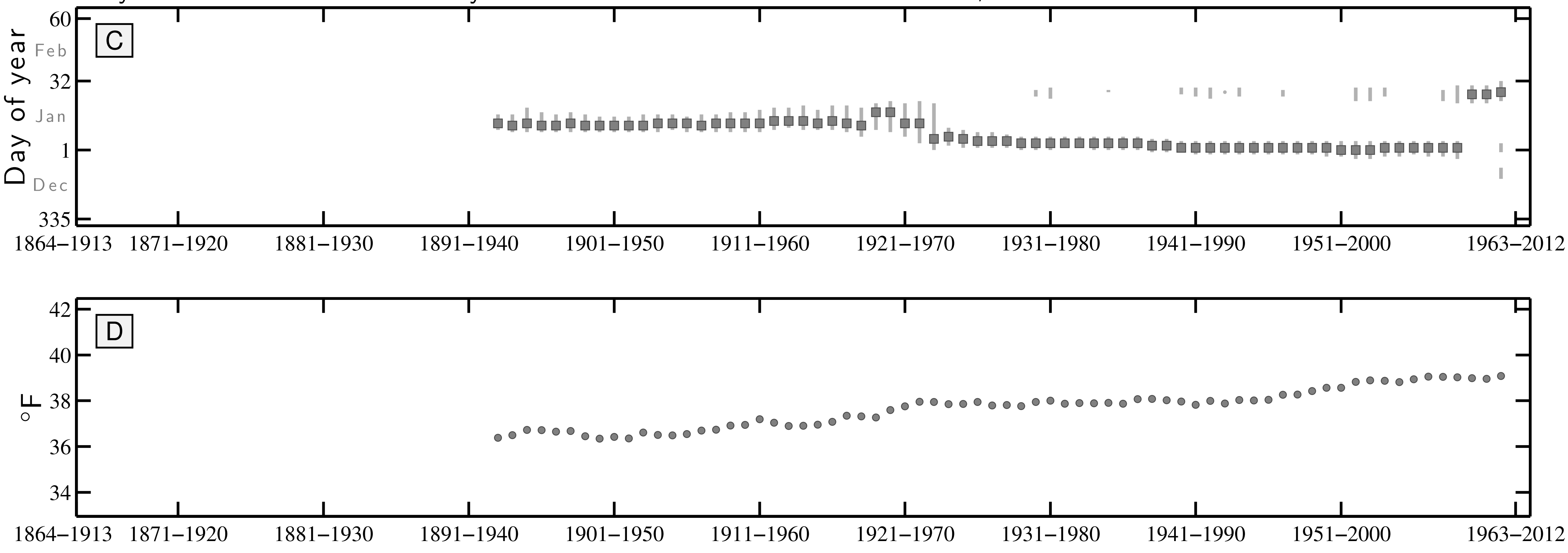

50 year Summer Teletherm dynamics for PAISLEY, OR:

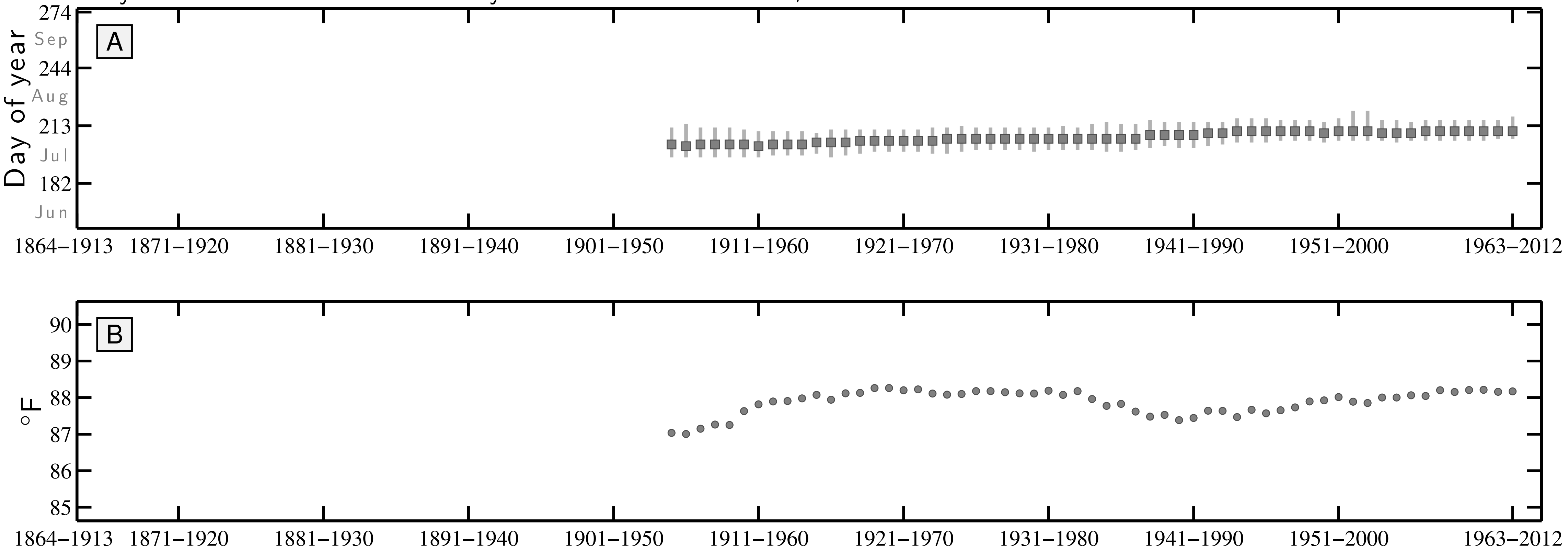

50 year Winter Teletherm dynamics for PAISLEY, OR:

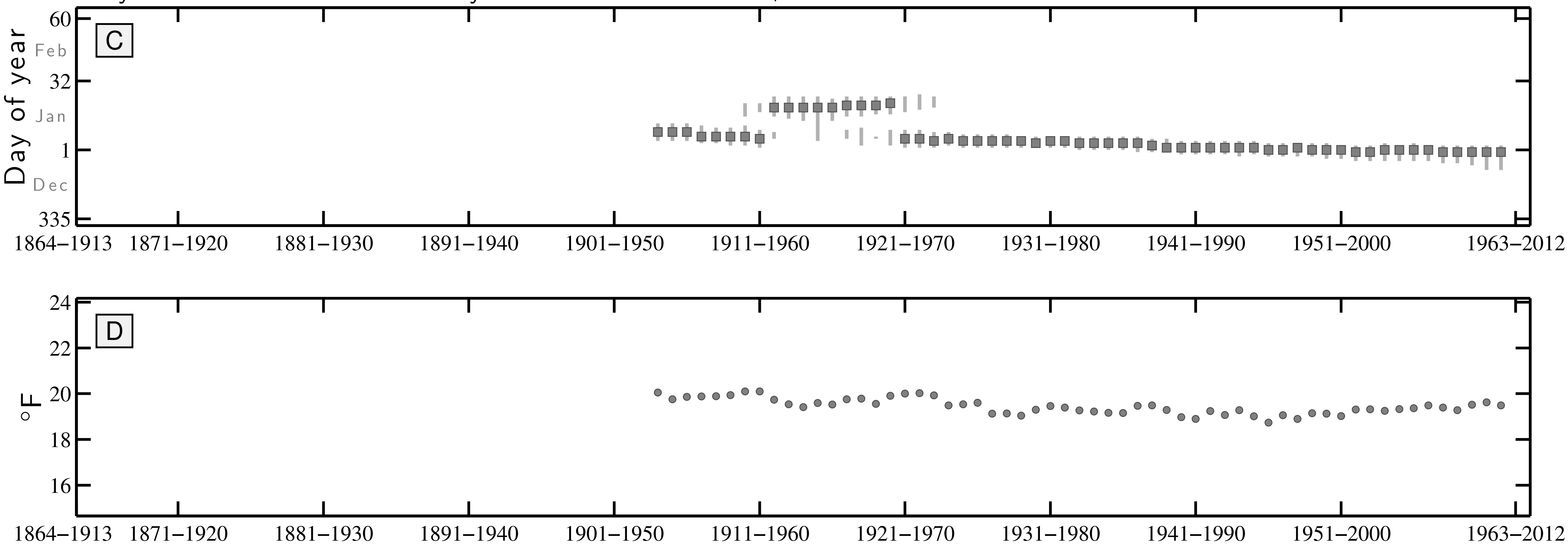

50 year Summer Teletherm dynamics for PILOT ROCK 1 SE, OR:

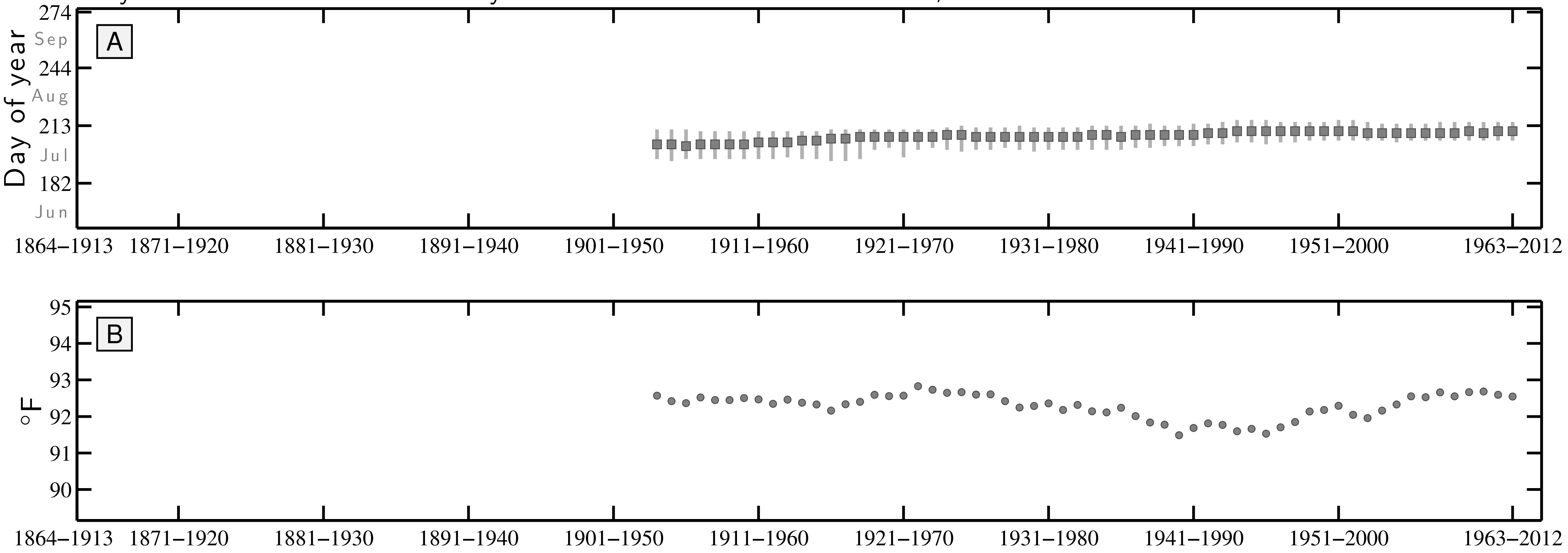

50 year Winter Teletherm dynamics for PILOT ROCK 1 SE, OR:

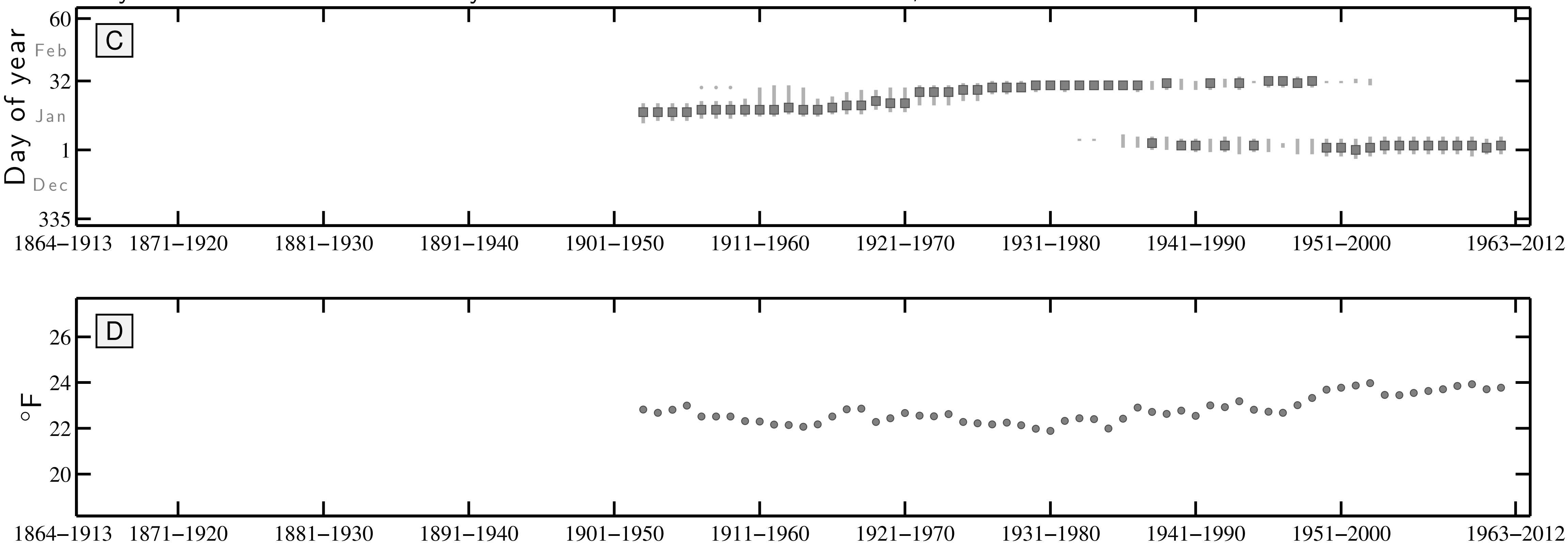

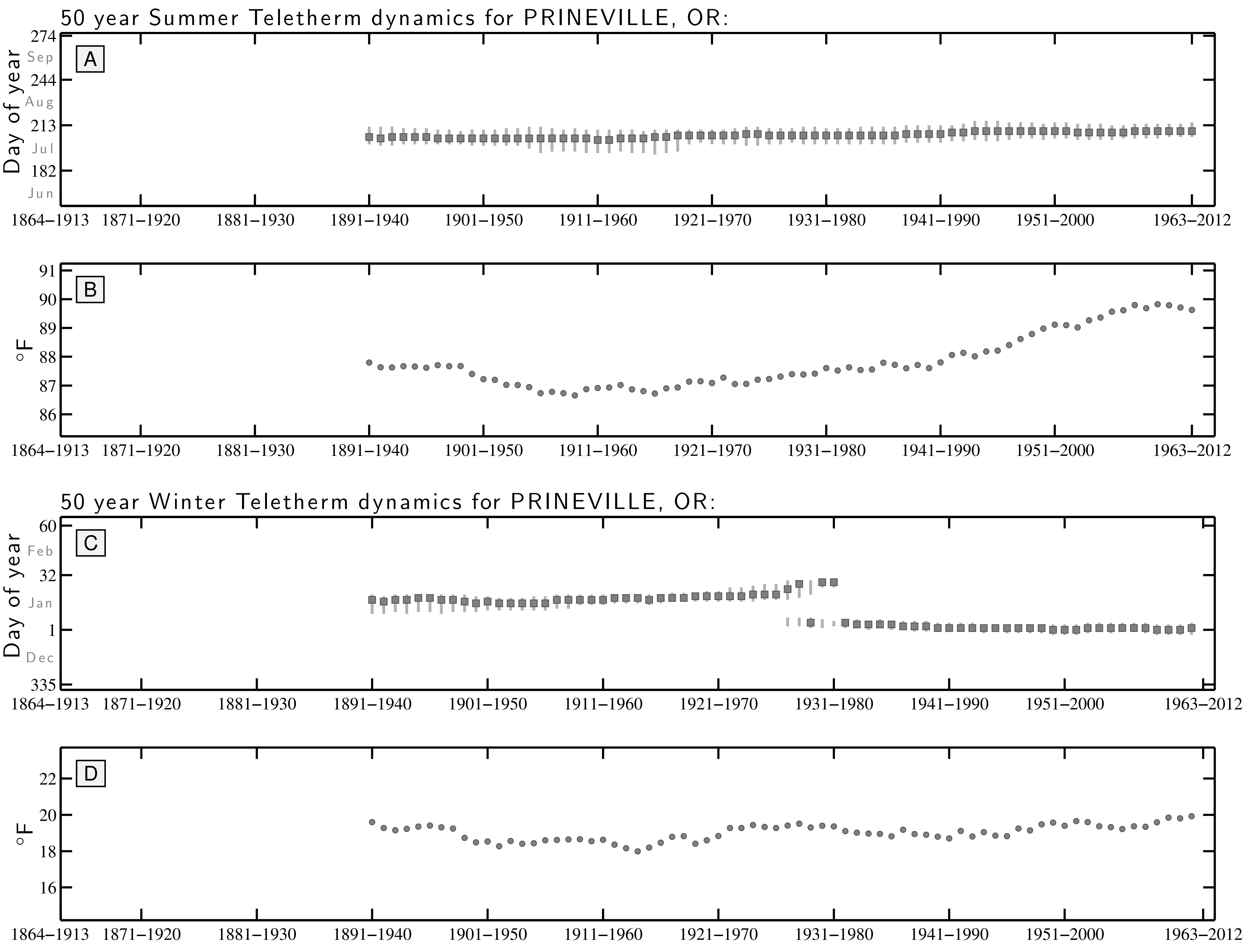
50 year Summer Teletherm dynamics for PRINEVILLE, OR:
A
Day of year
274
Sep
244
Aug
213
Jul
182
Jun
1864–1913
1871–1920
1881–1930
1891–1940
1901–1950
1911–1960
1921–1970
1931–1980
1941–1990
1951–2000
1963–2012
B
°F
91
90
89
88
87
86
50 year Winter Teletherm dynamics for PRINEVILLE, OR:
C
Day of year
60
Feb
32
Jan
1
Dec
335
D
°F
22
20
18
16

50 year Summer Teletherm dynamics for PROSPECT 2 SW, OR:

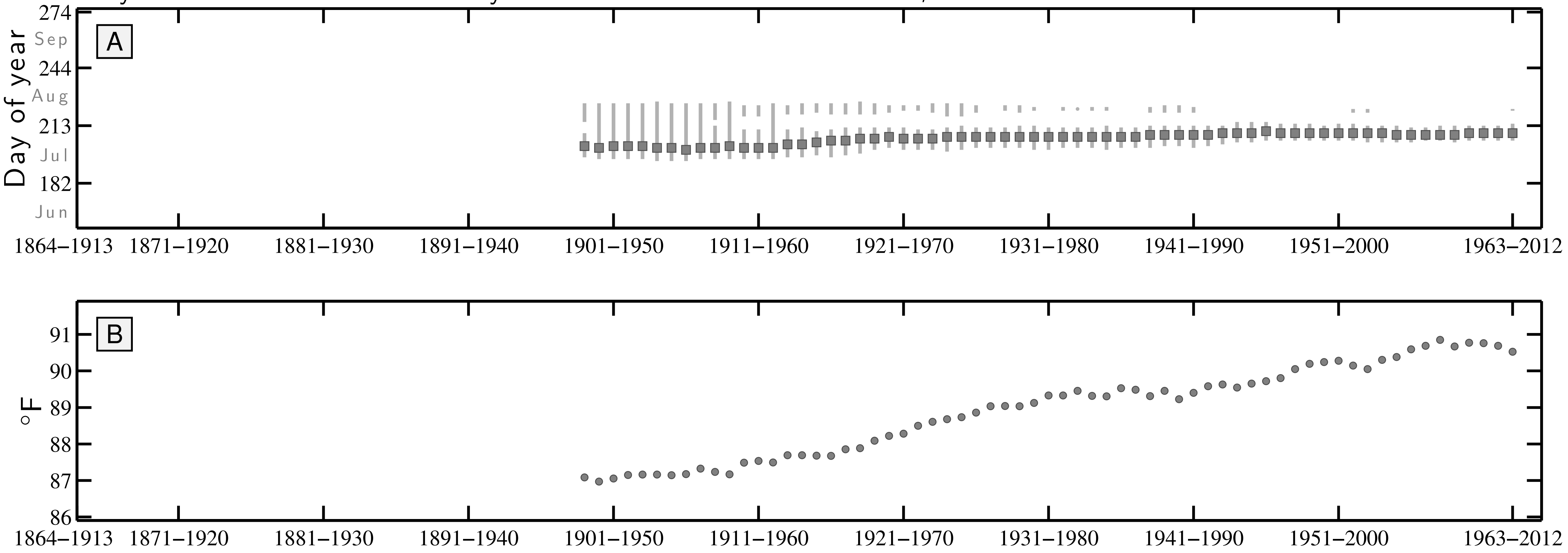

50 year Winter Teletherm dynamics for PROSPECT 2 SW, OR:

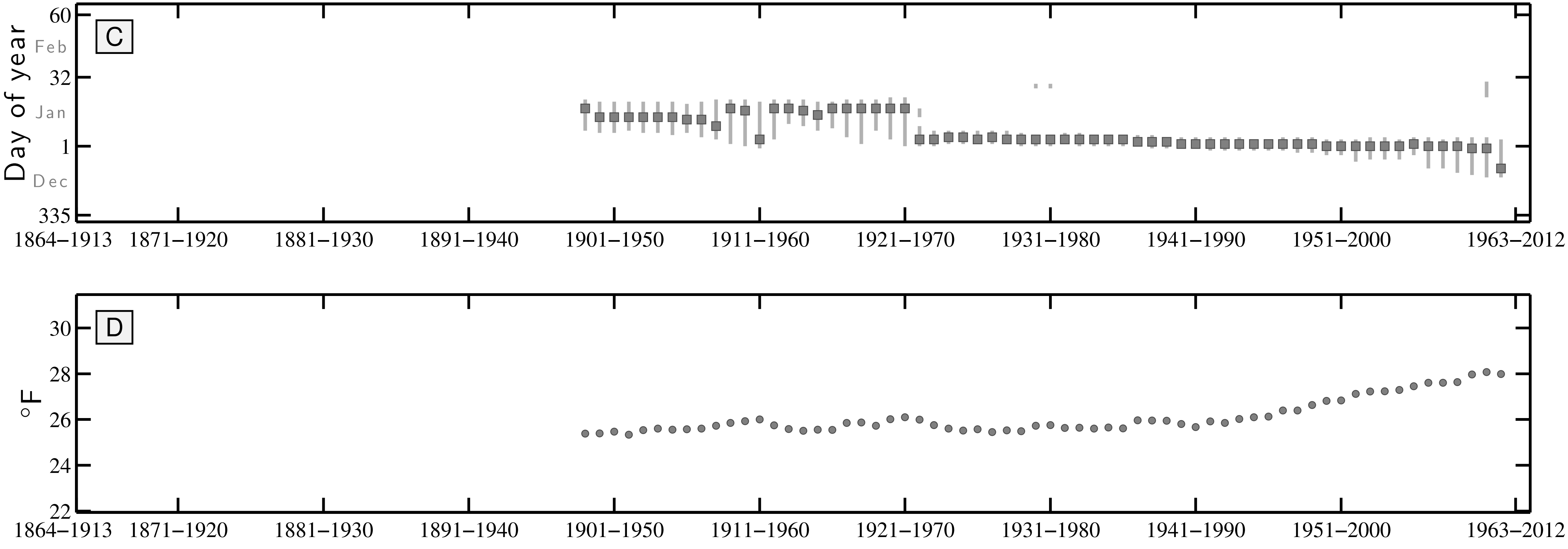

50 year Summer Teletherm dynamics for RIDDLE, OR:

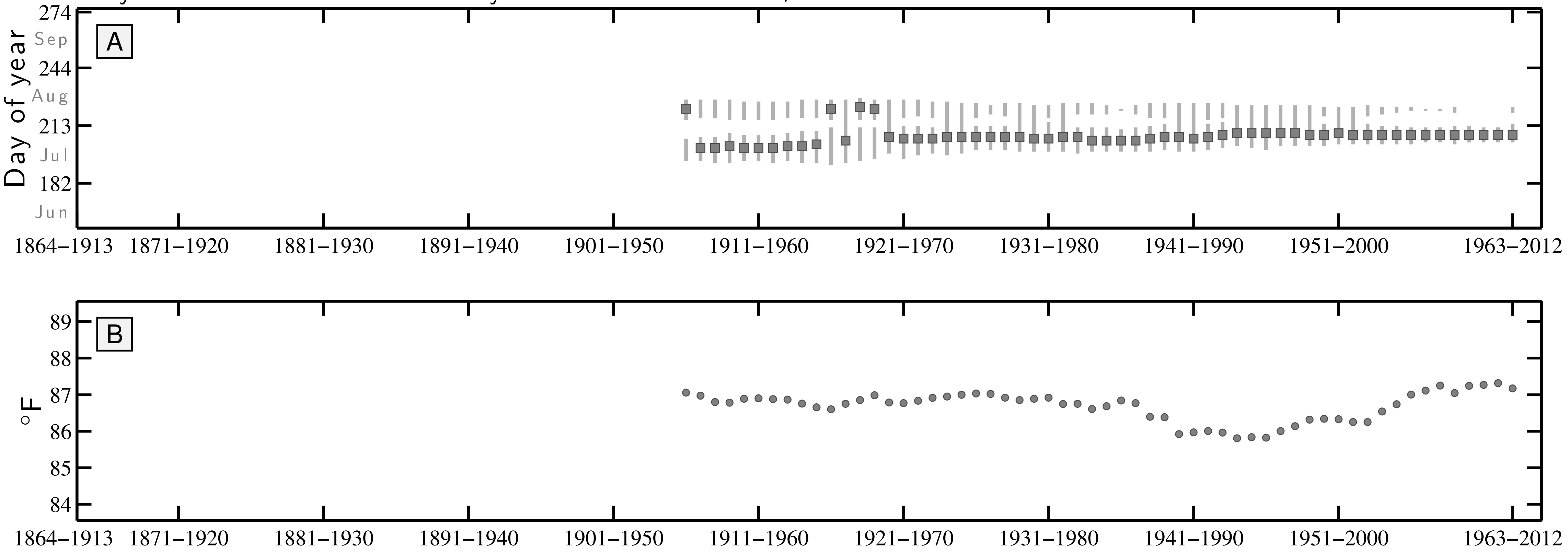

50 year Winter Teletherm dynamics for RIDDLE, OR:

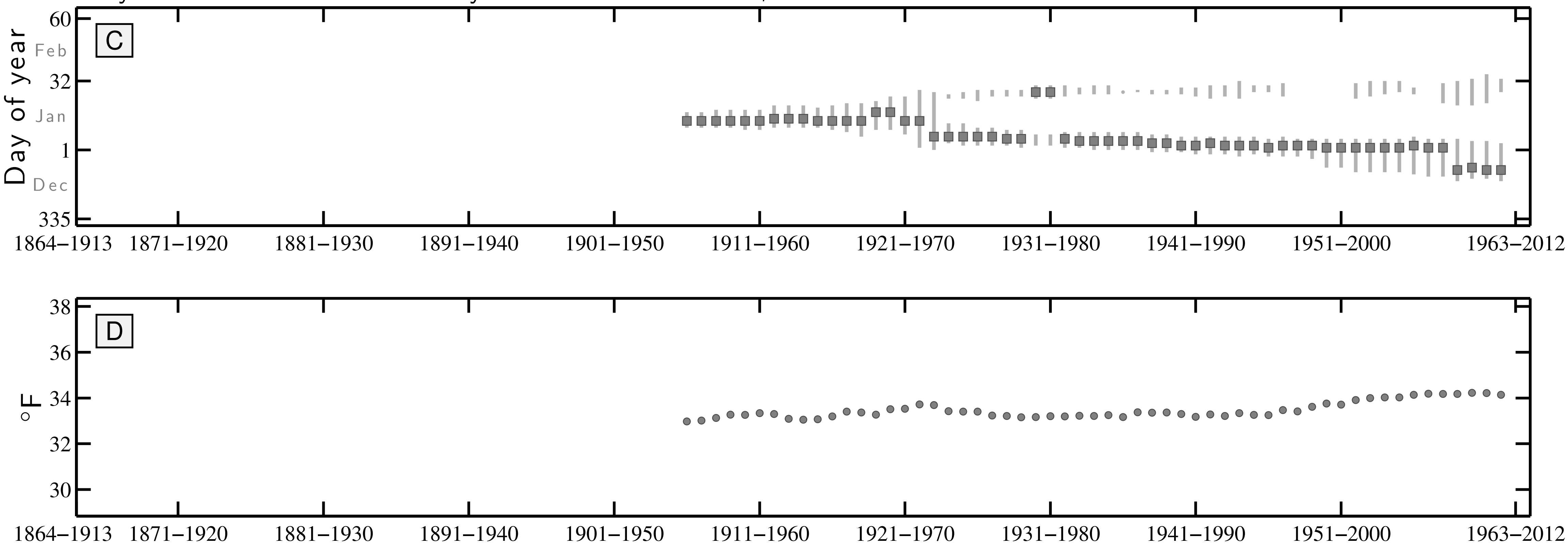

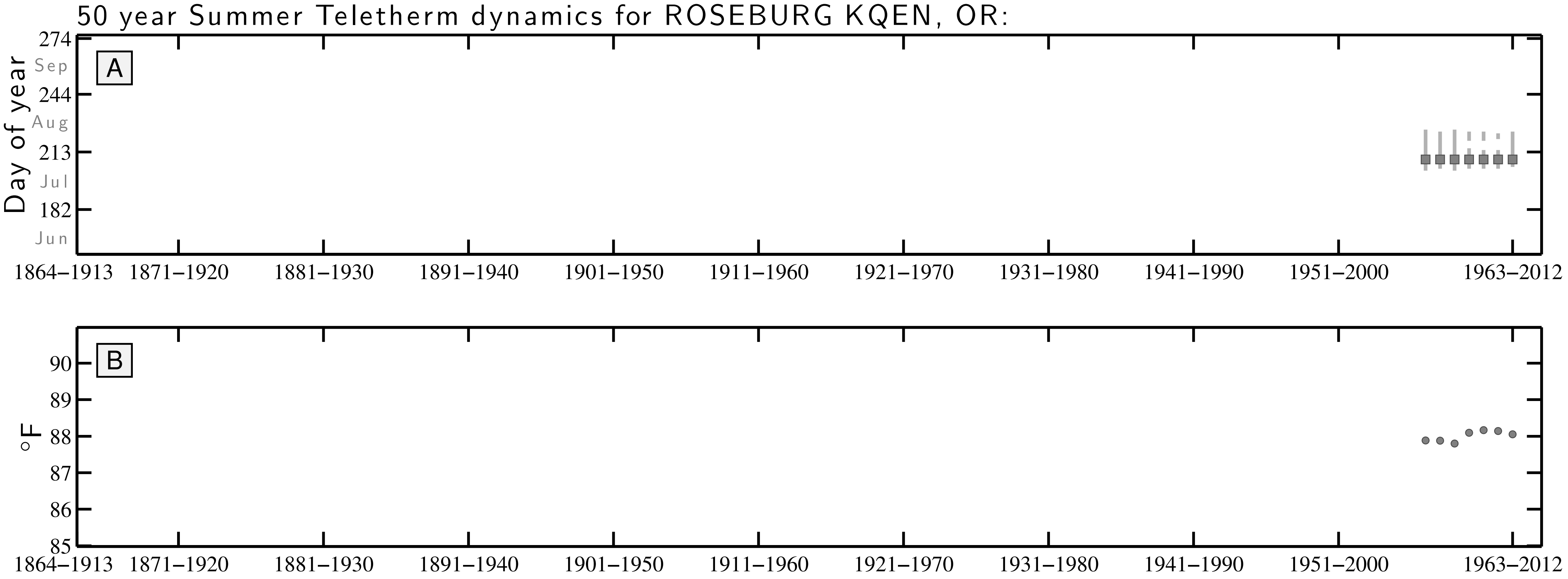
50 year Summer Teletherm dynamics for ROSEBURG KQEN, OR:
A
B
Day of year
°F
274
Sep
244
Aug
213
Jul
182
Jun
90
89
88
87
86
85
1864–1913
1871–1920
1881–1930
1891–1940
1901–1950
1911–1960
1921–1970
1931–1980
1941–1990
1951–2000
1963–2012

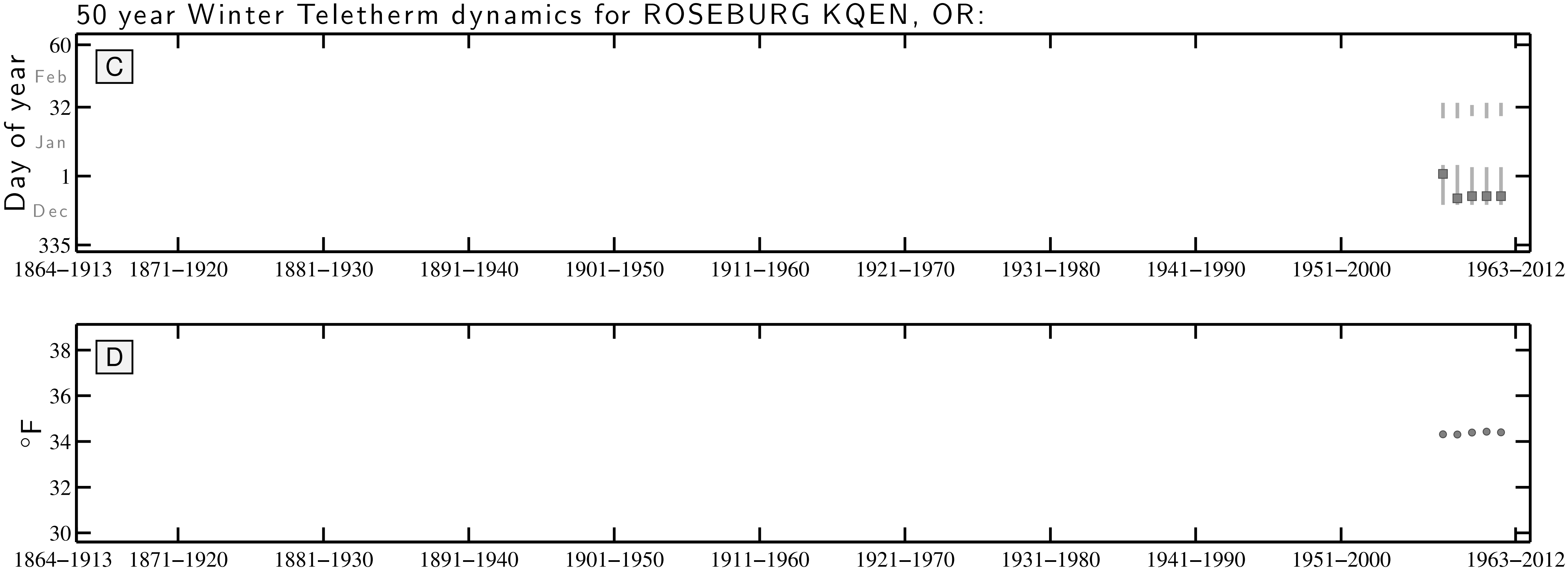
50 year Winter Teletherm dynamics for ROSEBURG KQEN, OR:
C
D
Day of year
°F
60
Feb
32
Jan
1
Dec
335
38
36
34
32
30
1864–1913
1871–1920
1881–1930
1891–1940
1901–1950
1911–1960
1921–1970
1931–1980
1941–1990
1951–2000
1963–2012

50 year Summer Teletherm dynamics for THREE LYNX, OR:

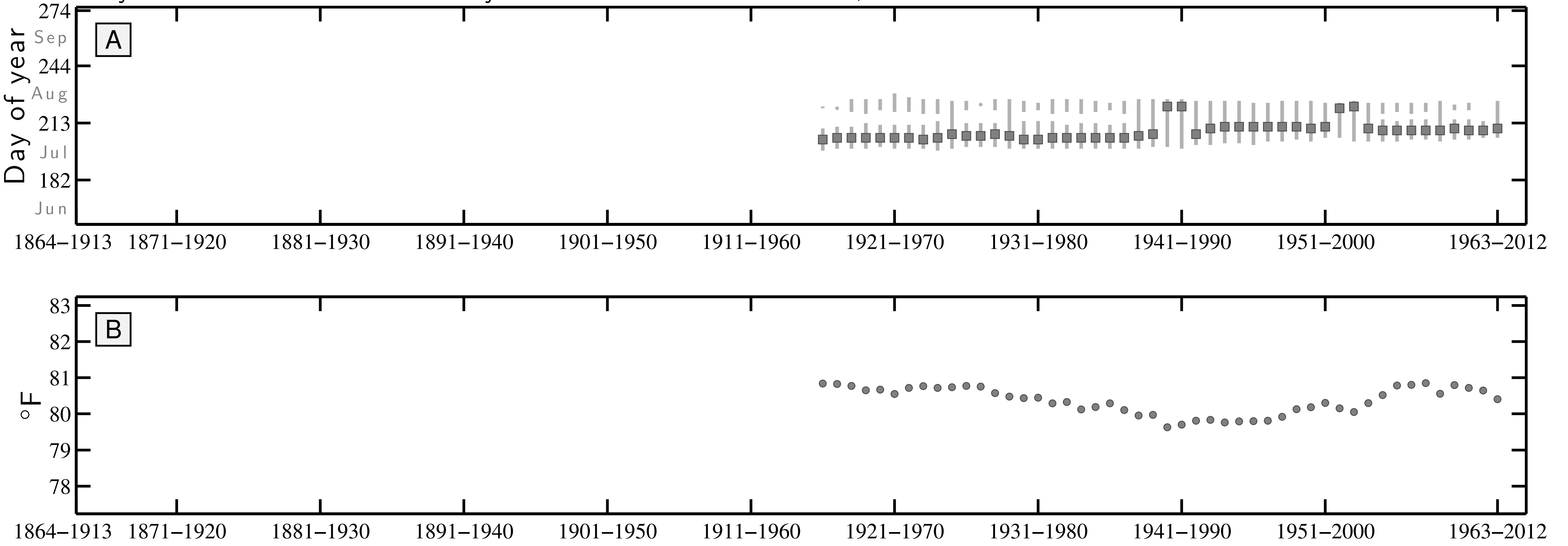

50 year Winter Teletherm dynamics for THREE LYNX, OR:

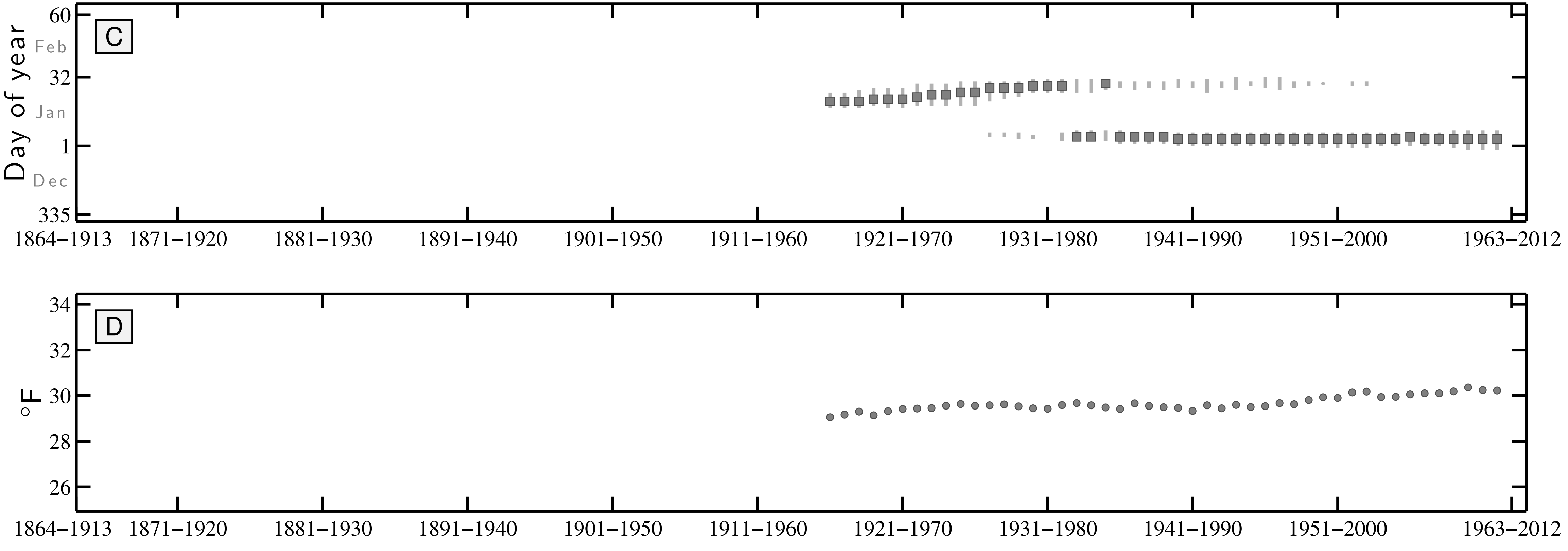

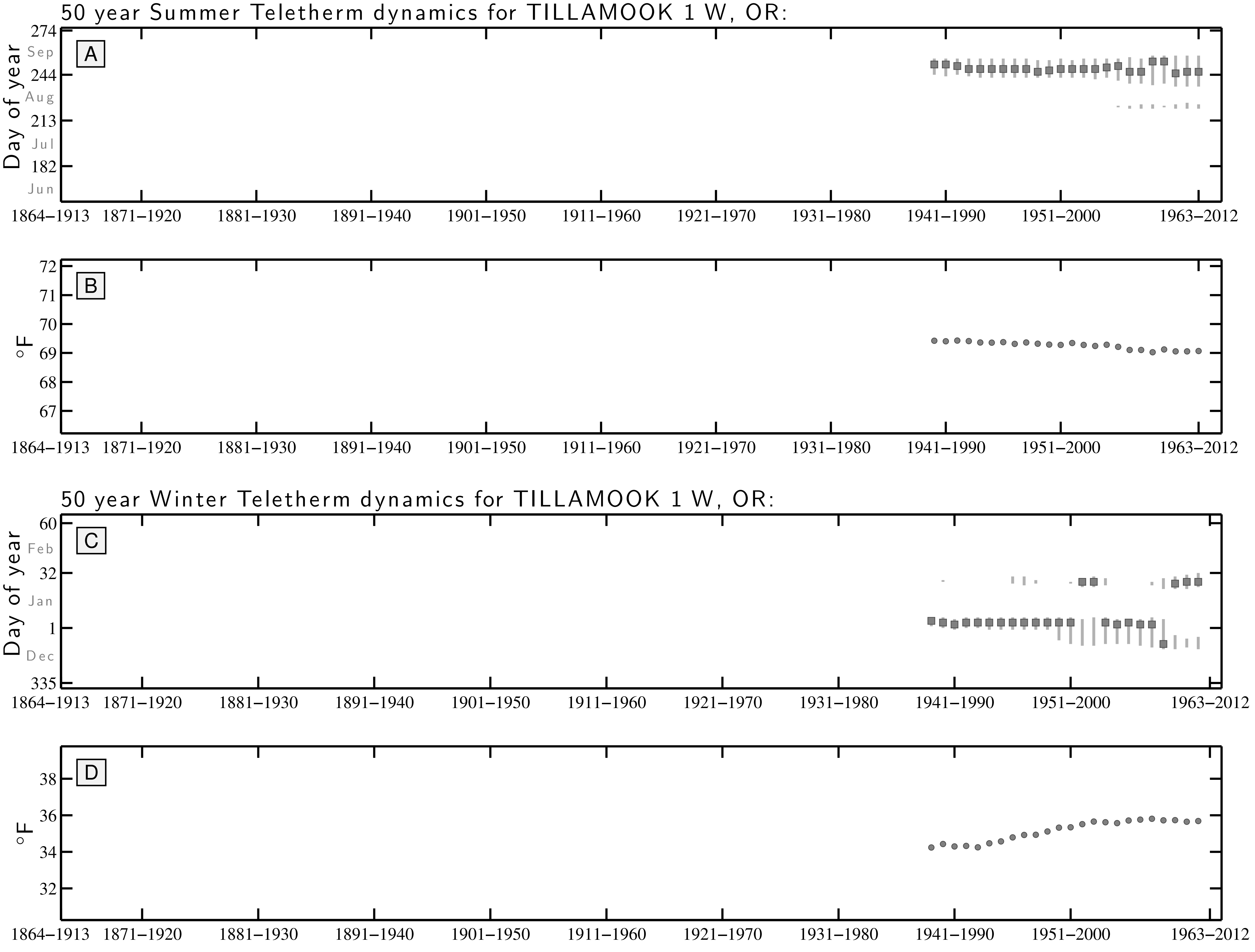
50 year Summer Teletherm dynamics for TILLAMOOK 1 W, OR:
A
B
Day of year
°F
274
Sep
244
Aug
213
Jul
182
Jun
72
71
70
69
68
67
1864–1913
1871–1920
1881–1930
1891–1940
1901–1950
1911–1960
1921–1970
1931–1980
1941–1990
1951–2000
1963–2012
50 year Winter Teletherm dynamics for TILLAMOOK 1 W, OR:
C
D
60
Feb
32
Jan
1
Dec
335
38
36
34
32

50 year Summer Teletherm dynamics for UNION EXP STN, OR:

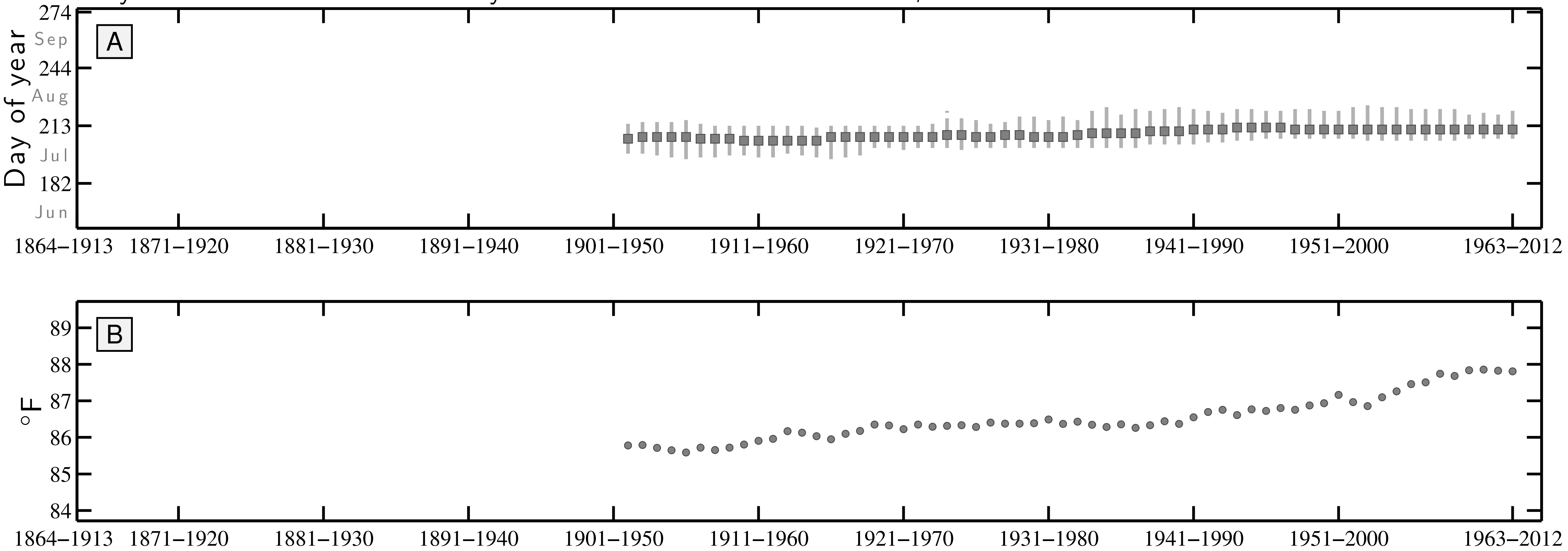

50 year Winter Teletherm dynamics for UNION EXP STN, OR:

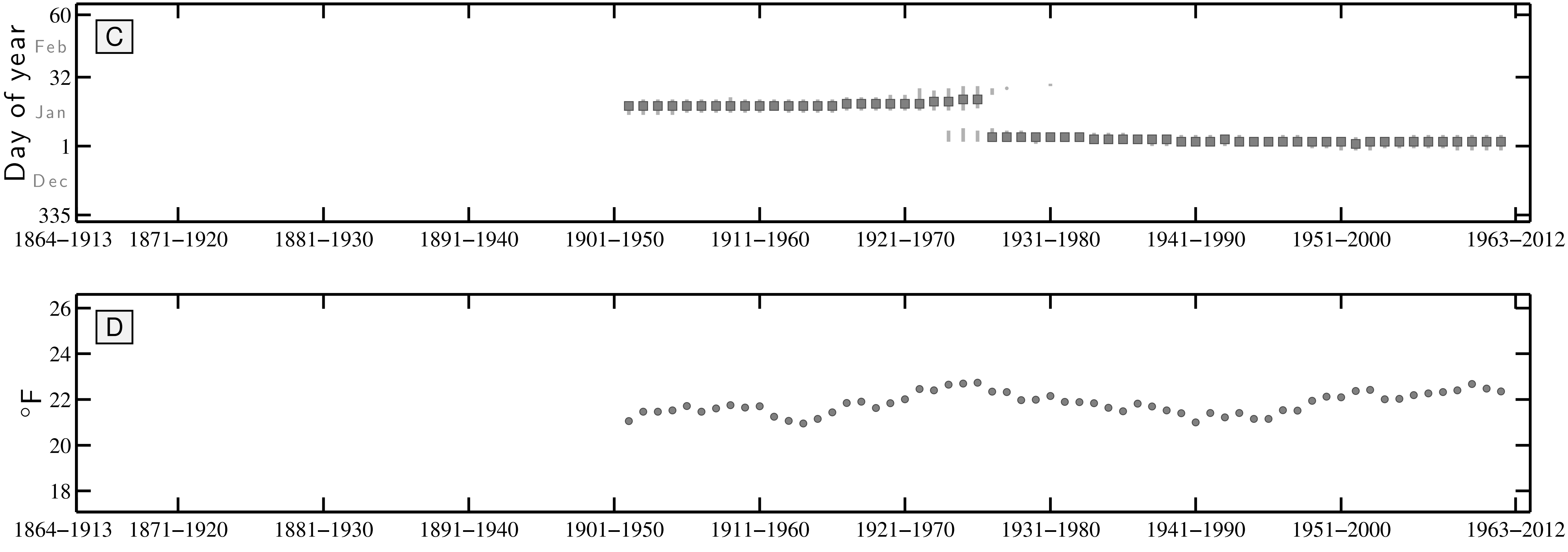

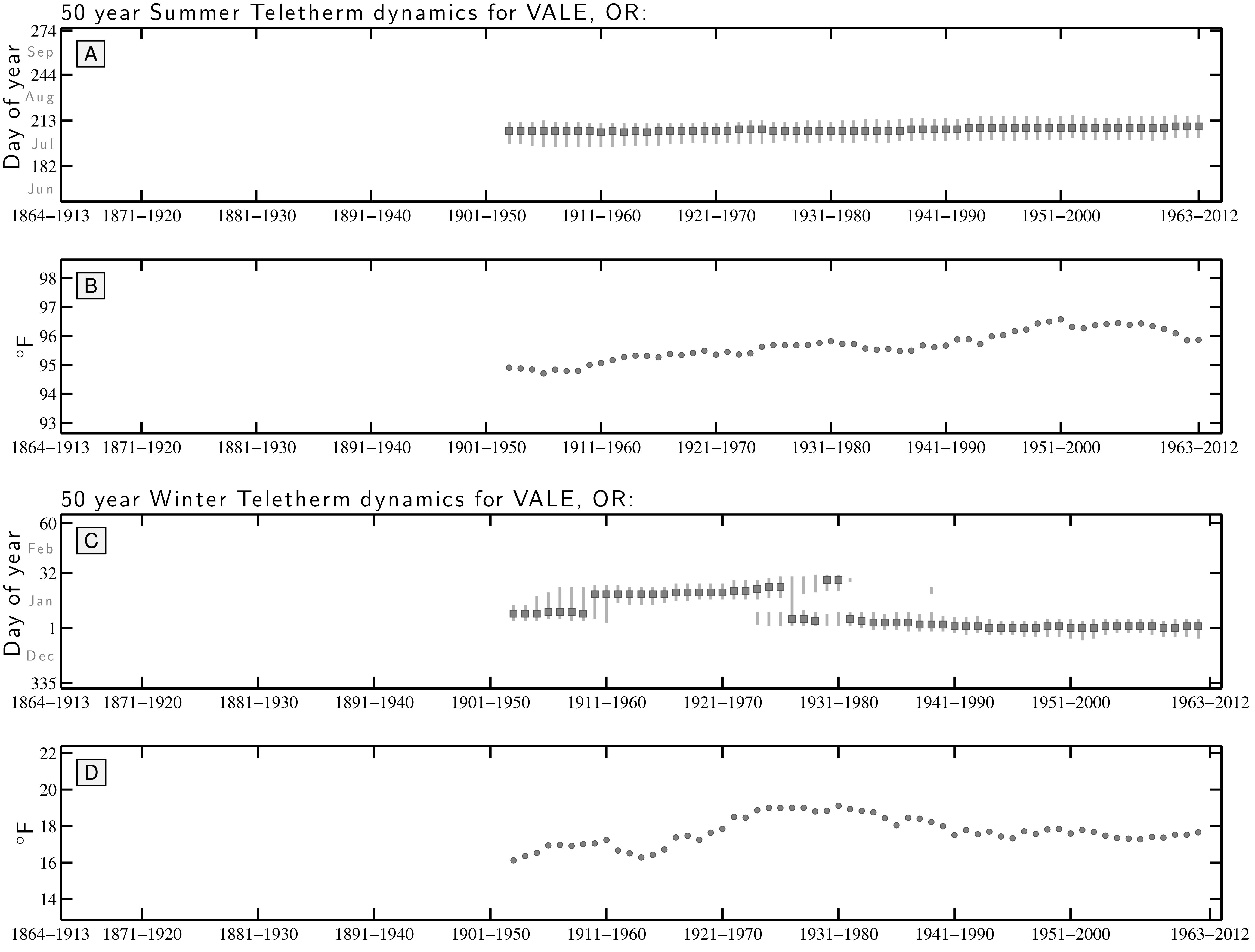

50 year Summer Teletherm dynamics for VALE, OR:
A
Day of year
274
Sep
244
Aug
213
Jul
182
Jun
1864–1913
1871–1920
1881–1930
1891–1940
1901–1950
1911–1960
1921–1970
1931–1980
1941–1990
1951–2000
1963–2012
B
°F
98
97
96
95
94
93
50 year Winter Teletherm dynamics for VALE, OR:
C
Day of year
60
Feb
32
Jan
1
Dec
335
D
°F
22
20
18
16
14

50 year Summer Teletherm dynamics for WALLOWA, OR:

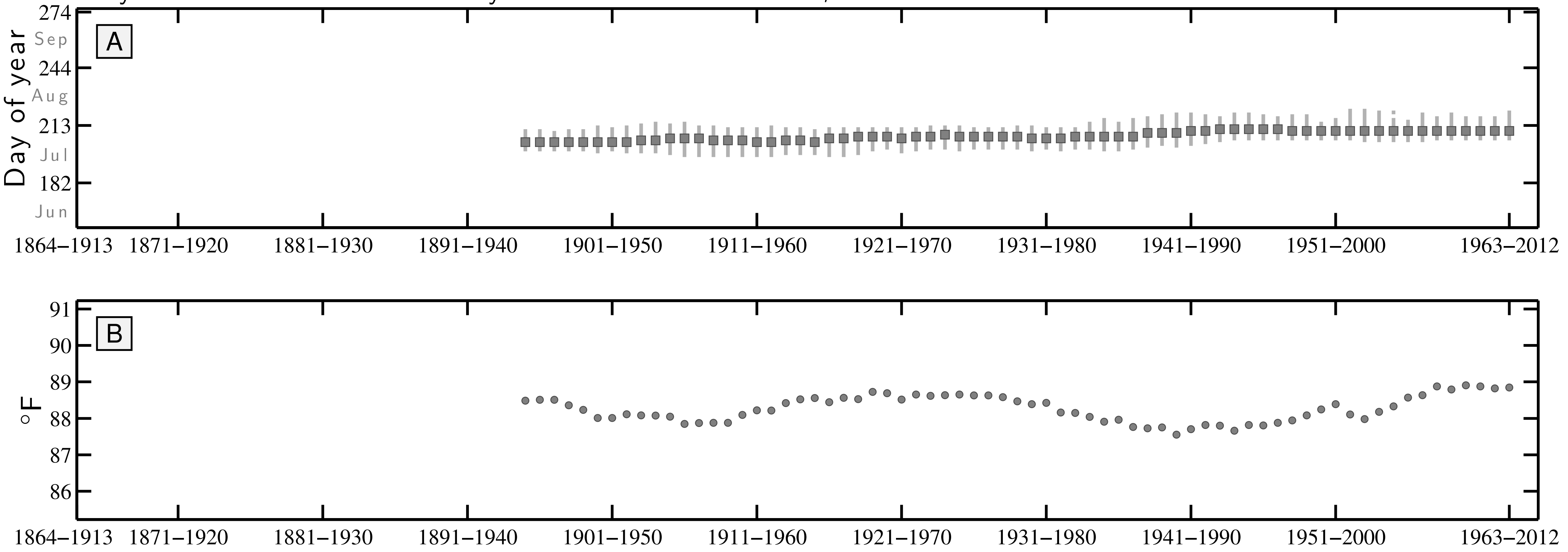

50 year Winter Teletherm dynamics for WALLOWA, OR:

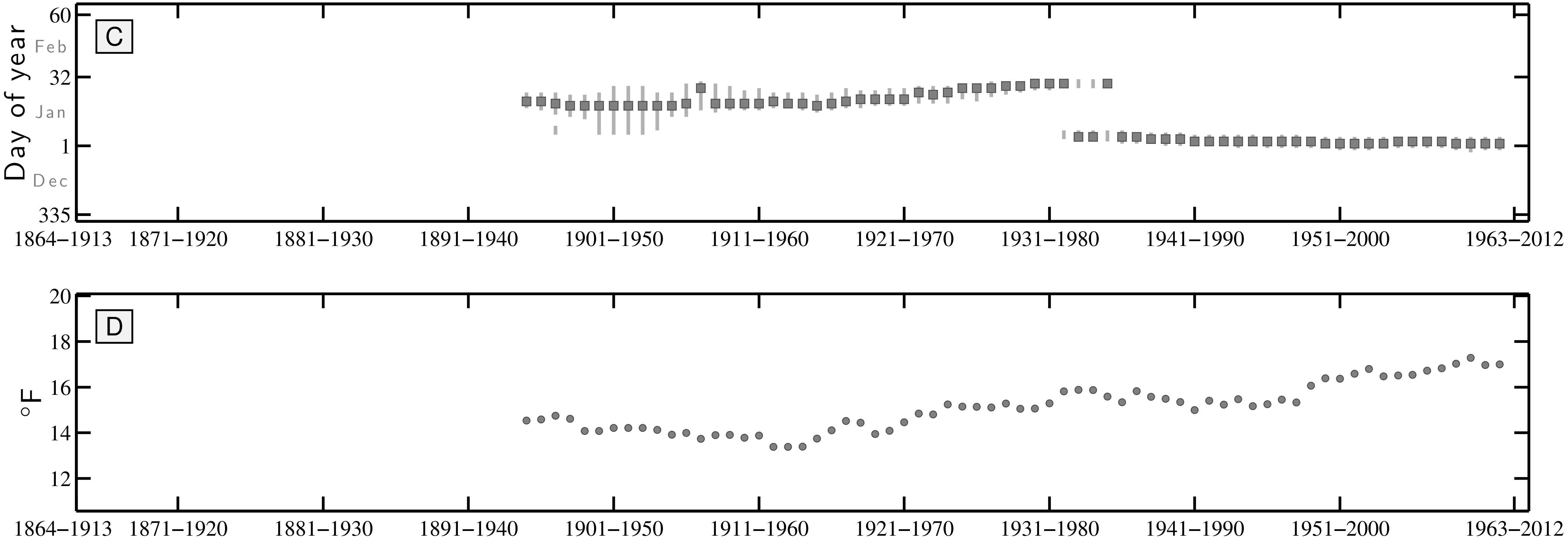

50 year Summer Teletherm dynamics for ALLENTOWN AP, PA:

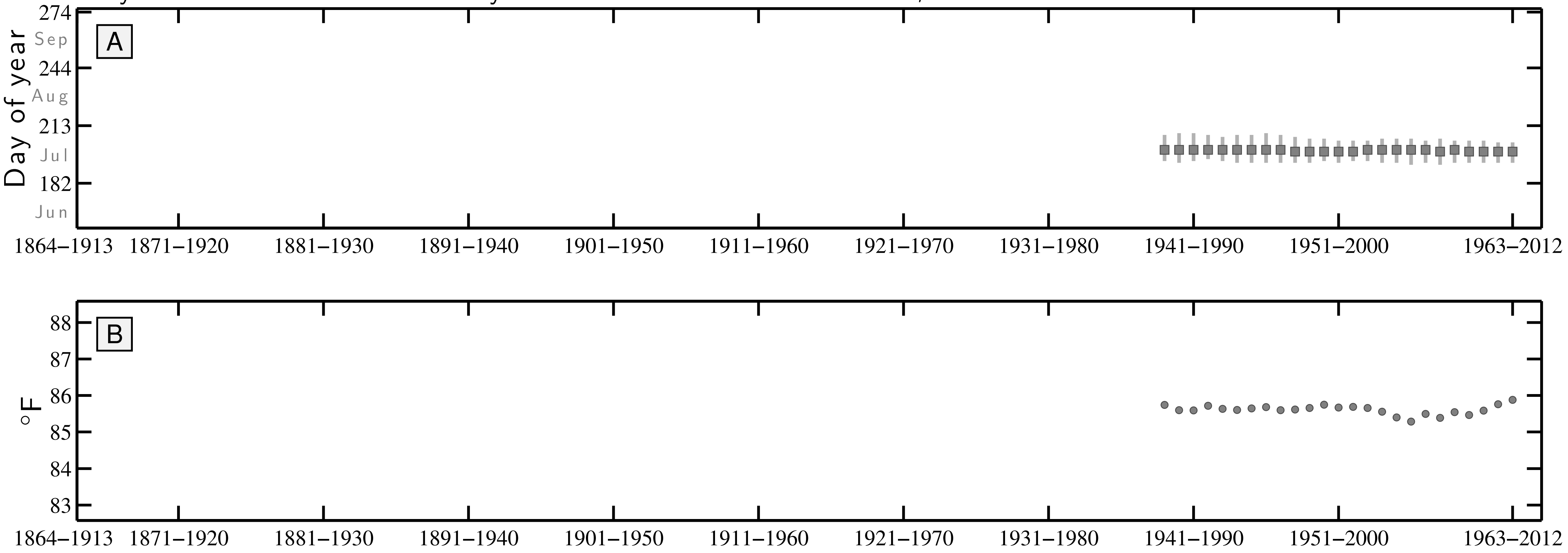

50 year Winter Teletherm dynamics for ALLENTOWN AP, PA:

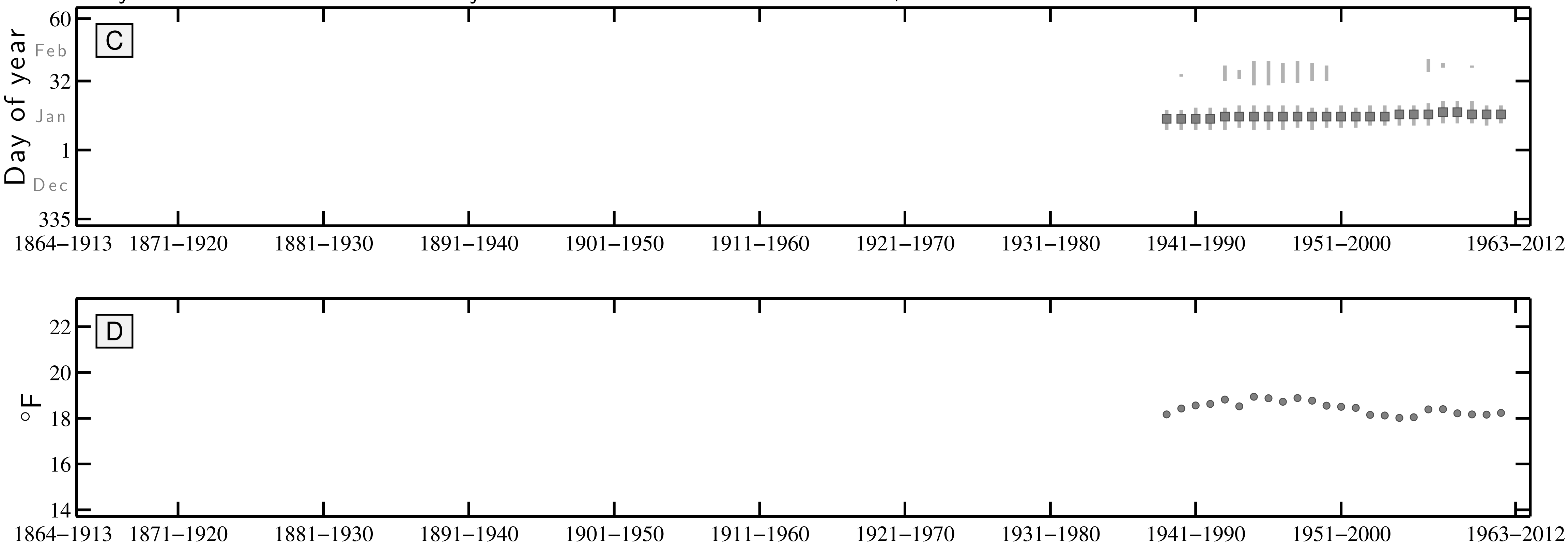

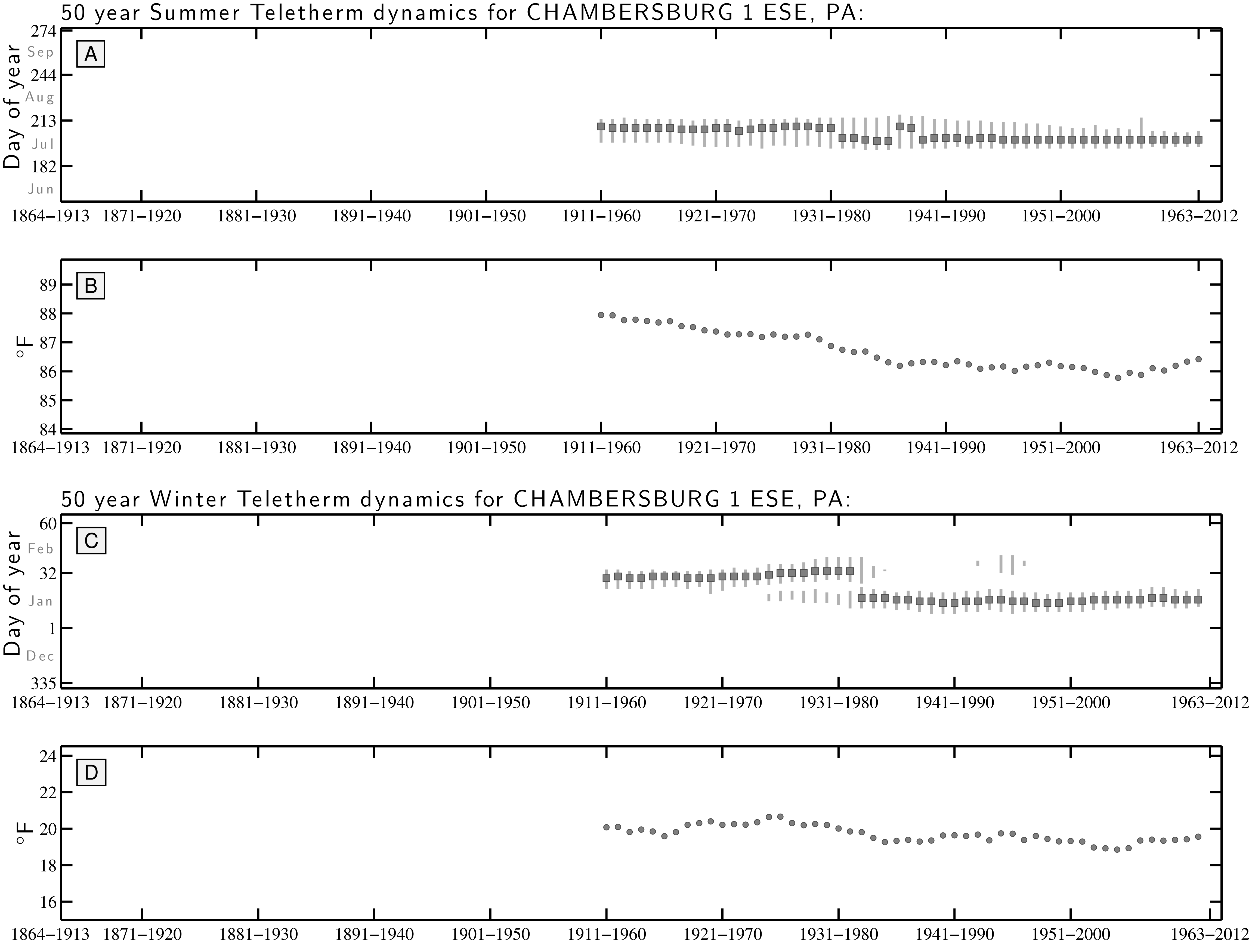
50 year Summer Teletherm dynamics for CHAMBERSBURG 1 ESE, PA:
A
B
Day of year
°F
274
Sep
244
Aug
213
Jul
182
Jun
89
88
87
86
85
84
1864–1913
1871–1920
1881–1930
1891–1940
1901–1950
1911–1960
1921–1970
1931–1980
1941–1990
1951–2000
1963–2012
50 year Winter Teletherm dynamics for CHAMBERSBURG 1 ESE, PA:
C
D
60
Feb
32
Jan
1
Dec
335
24
22
20
18
16

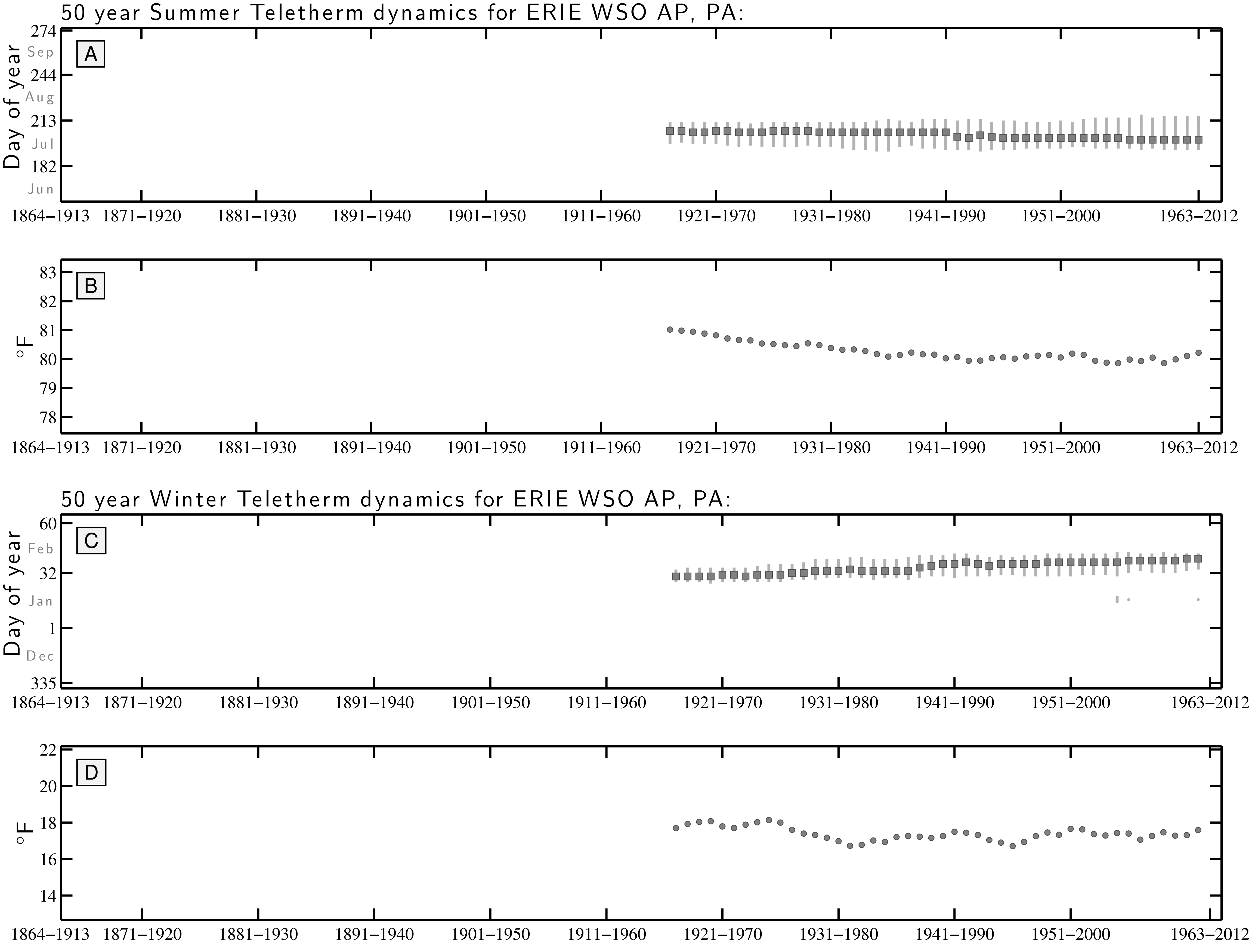

50 year Summer Teletherm dynamics for ERIE WSO AP, PA:
A
B
Day of year
°F
274
Sep
244
Aug
213
Jul
182
Jun
83
82
81
80
79
78
1864–1913
1871–1920
1881–1930
1891–1940
1901–1950
1911–1960
1921–1970
1931–1980
1941–1990
1951–2000
1963–2012
50 year Winter Teletherm dynamics for ERIE WSO AP, PA:
C
D
60
Feb
32
Jan
1
Dec
335
22
20
18
16
14

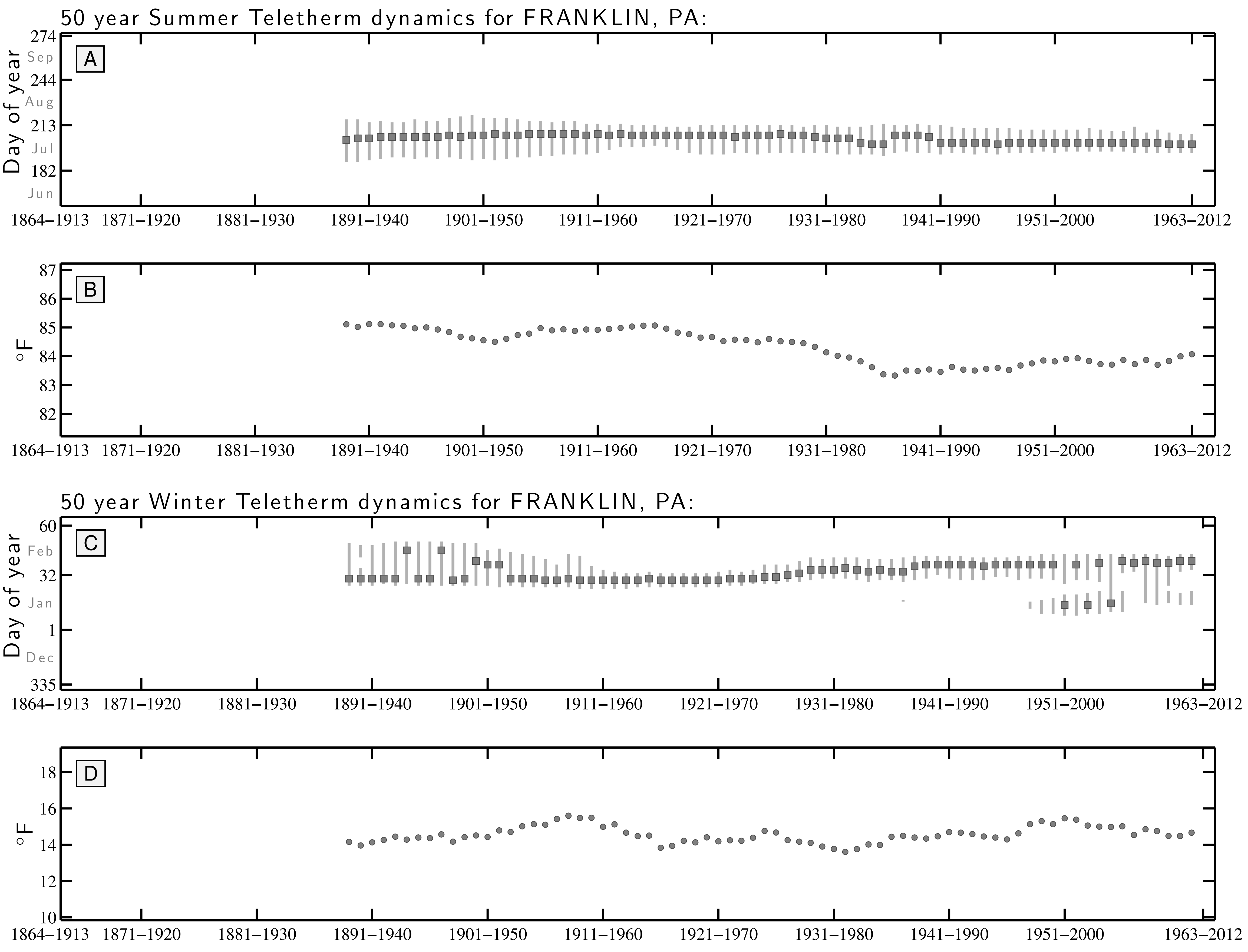
50 year Summer Teletherm dynamics for FRANKLIN, PA:
A
Day of year
274
Sep
244
Aug
213
Jul
182
Jun
B
°F
87
86
85
84
83
82
1864–1913
1871–1920
1881–1930
1891–1940
1901–1950
1911–1960
1921–1970
1931–1980
1941–1990
1951–2000
1963–2012
50 year Winter Teletherm dynamics for FRANKLIN, PA:
C
Day of year
60
Feb
32
Jan
1
Dec
335
D
°F
18
16
14
12
10

50 year Summer Teletherm dynamics for GREENVILLE 2 NE, PA:

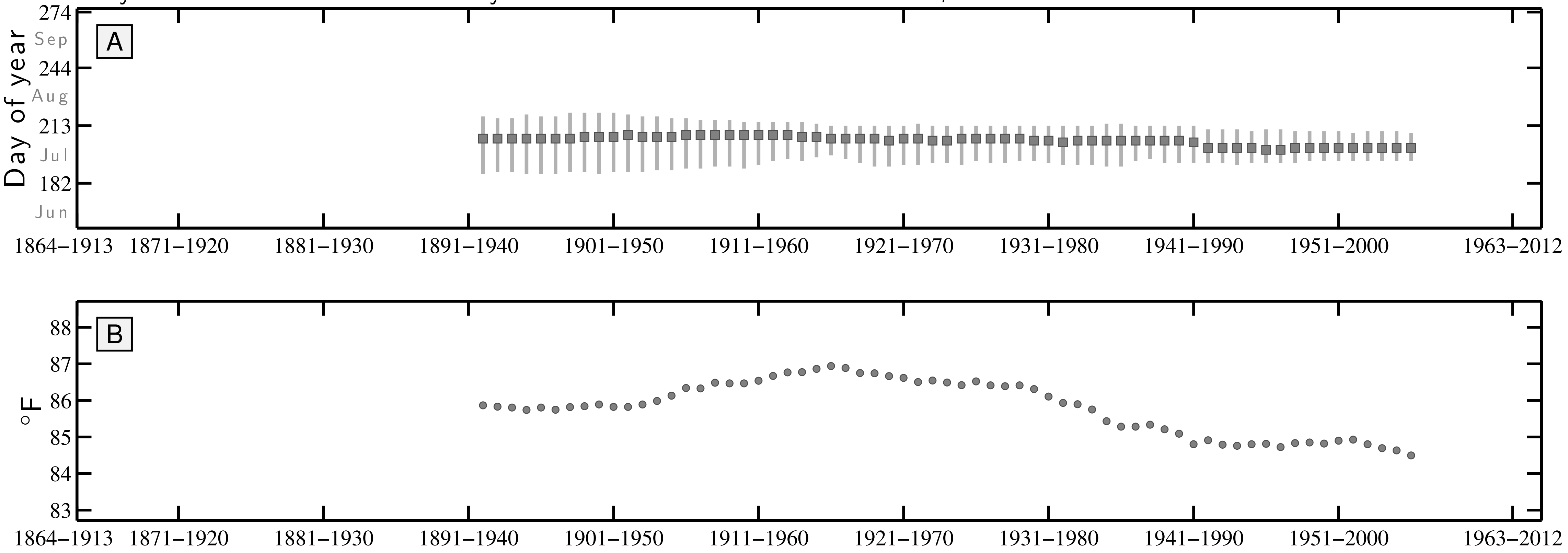

50 year Winter Teletherm dynamics for GREENVILLE 2 NE, PA:

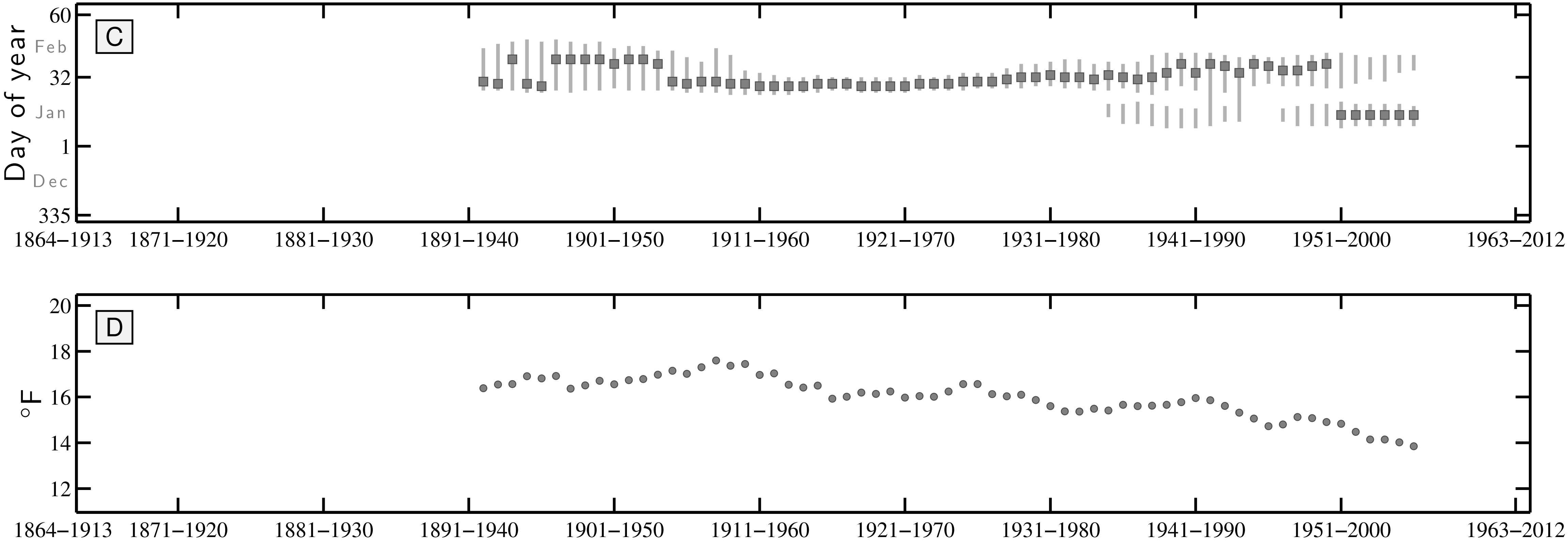

50 year Summer Teletherm dynamics for JOHNSTOWN, PA:

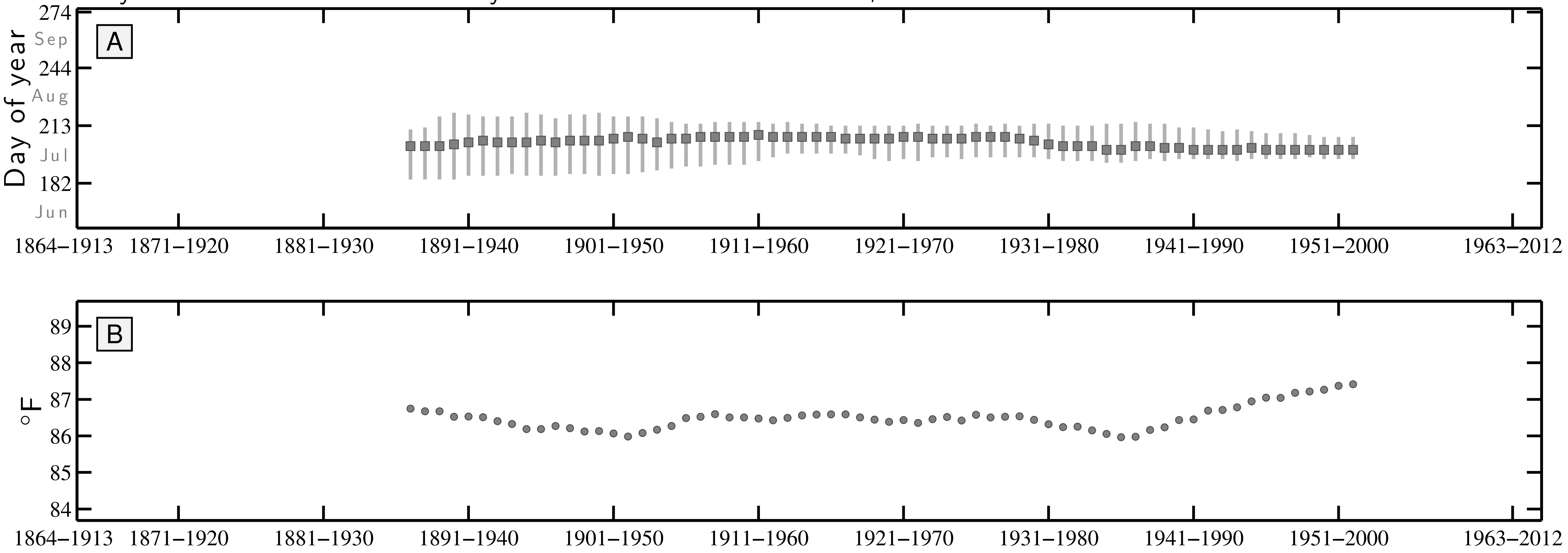

50 year Winter Teletherm dynamics for JOHNSTOWN, PA:

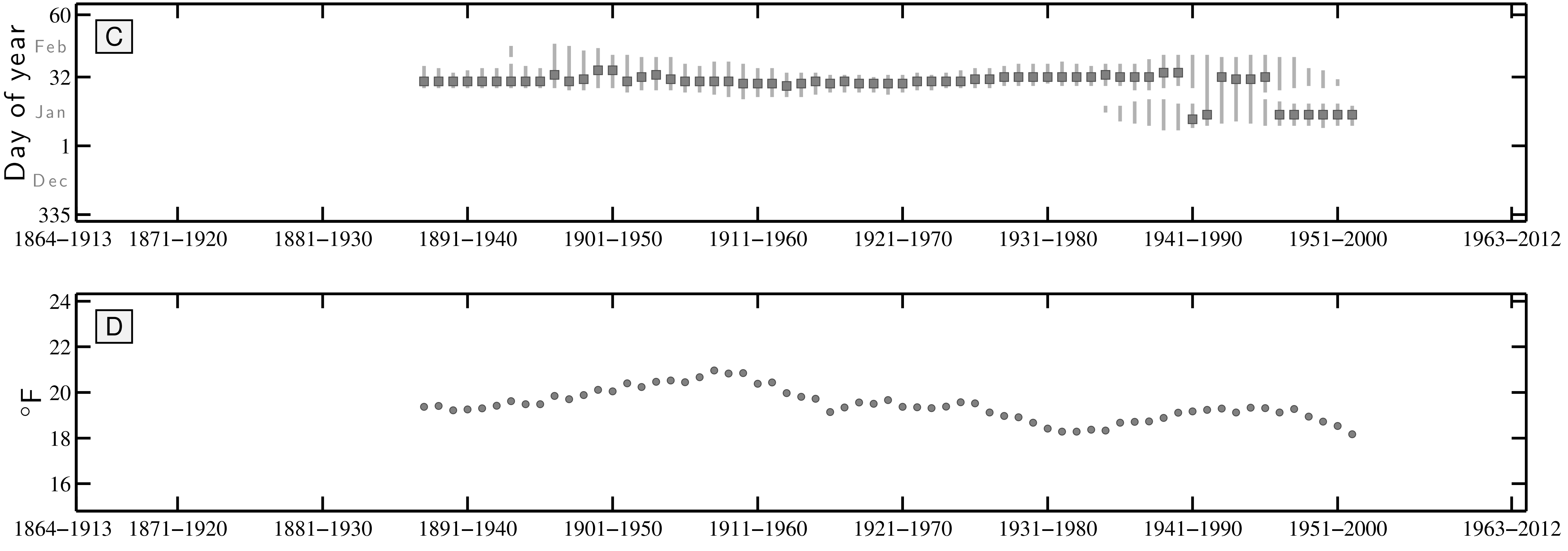

50 year Summer Teletherm dynamics for LEBANON 2 W, PA:

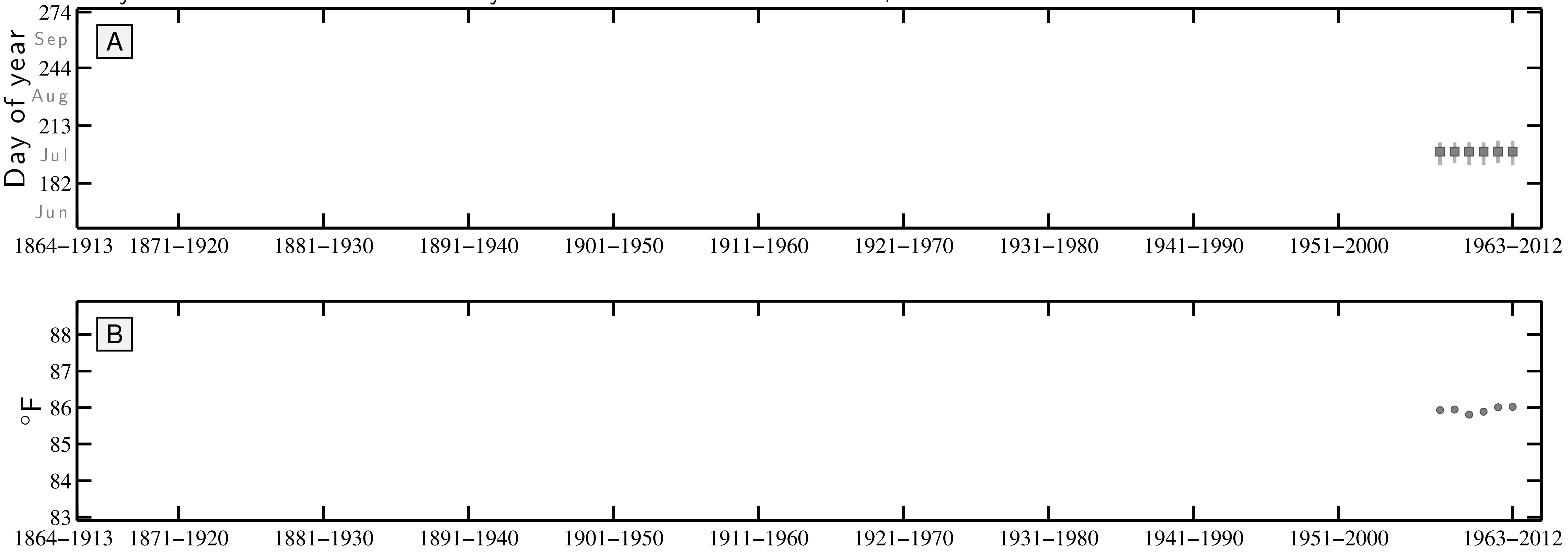

50 year Winter Teletherm dynamics for LEBANON 2 W, PA:

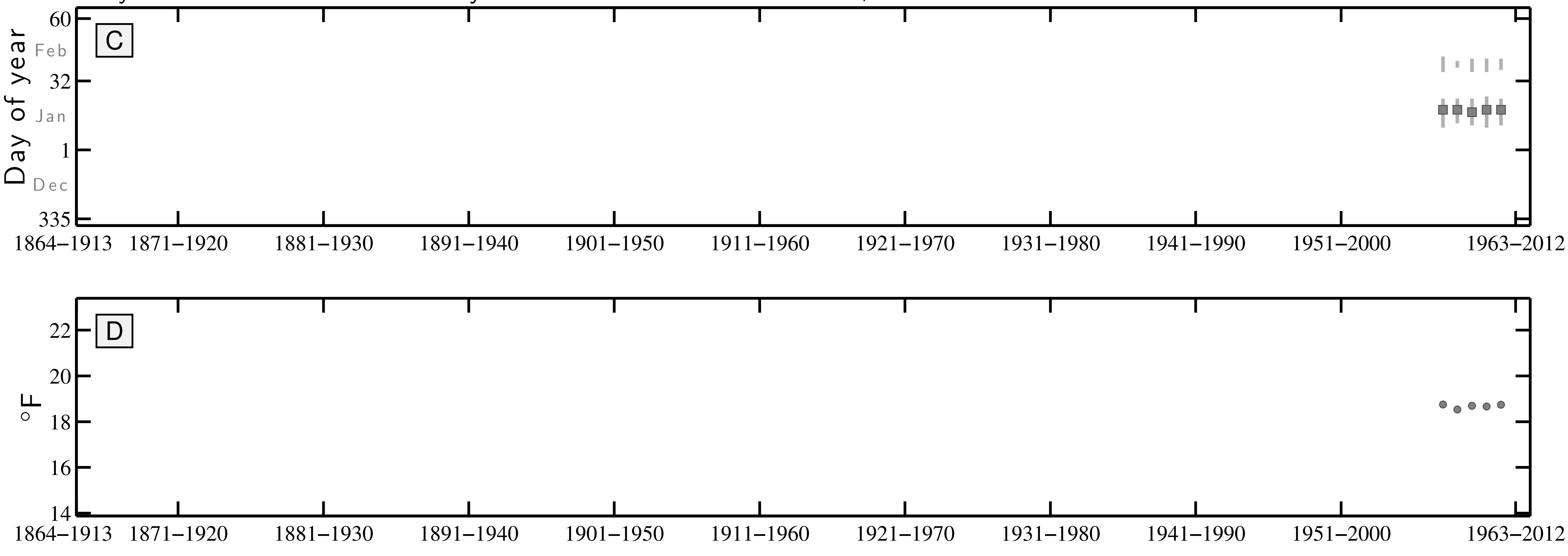

50 year Summer Teletherm dynamics for MONTROSE, PA:

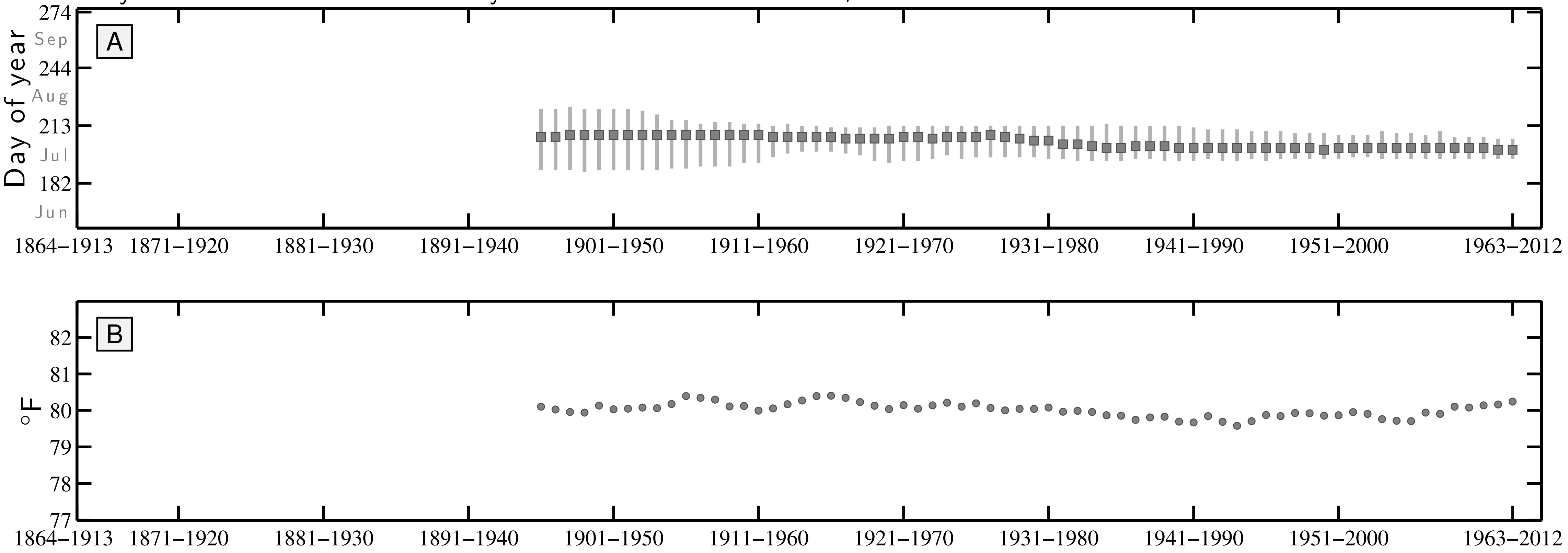

50 year Winter Teletherm dynamics for MONTROSE, PA:

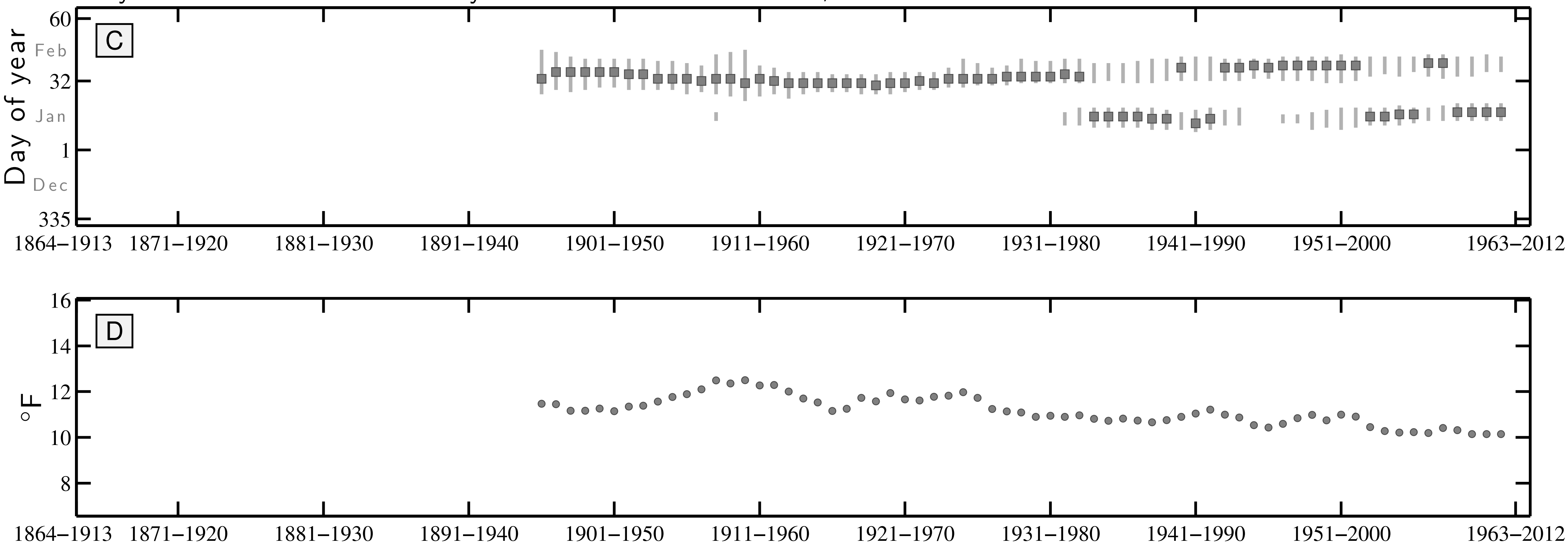

50 year Summer Teletherm dynamics for NEW CASTLE 1 N, PA:

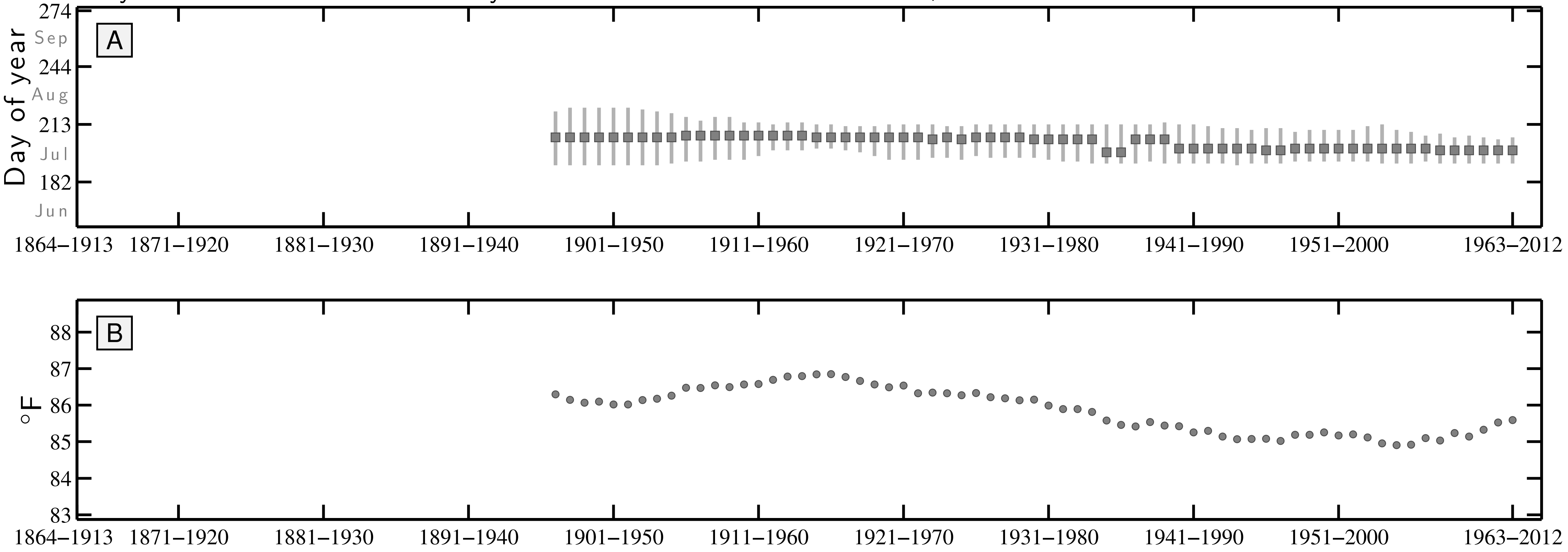

50 year Winter Teletherm dynamics for NEW CASTLE 1 N, PA:

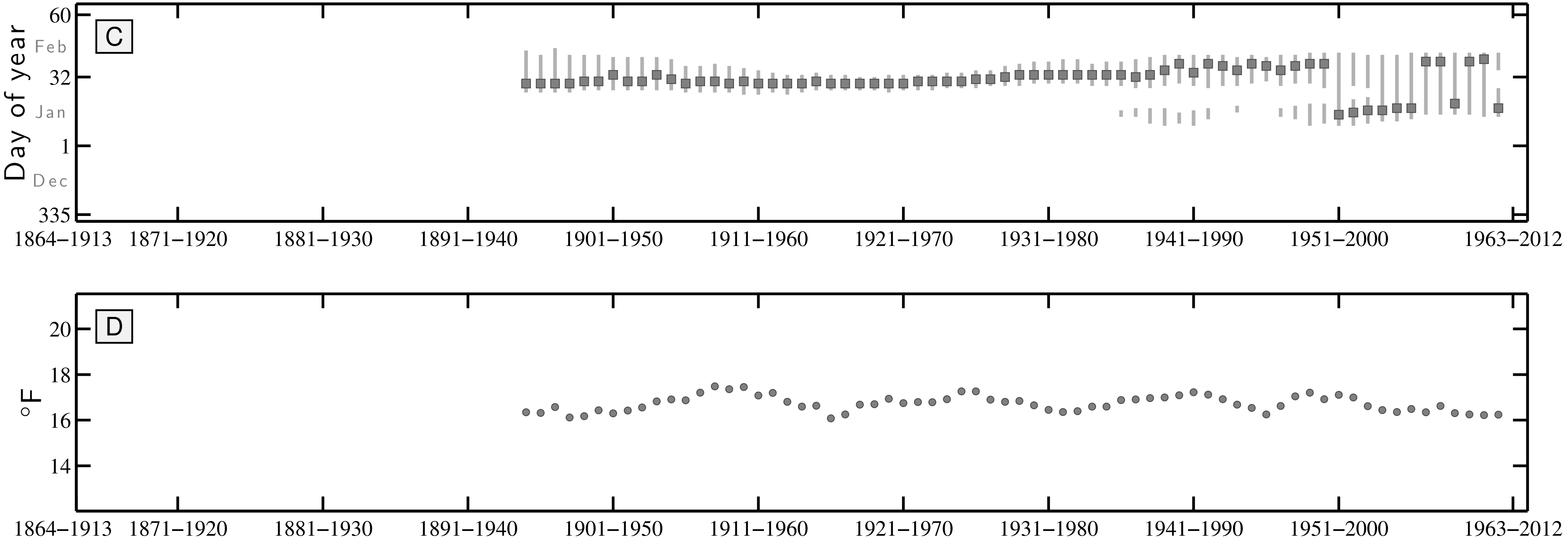

50 year Summer Teletherm dynamics for PALMERTON, PA:

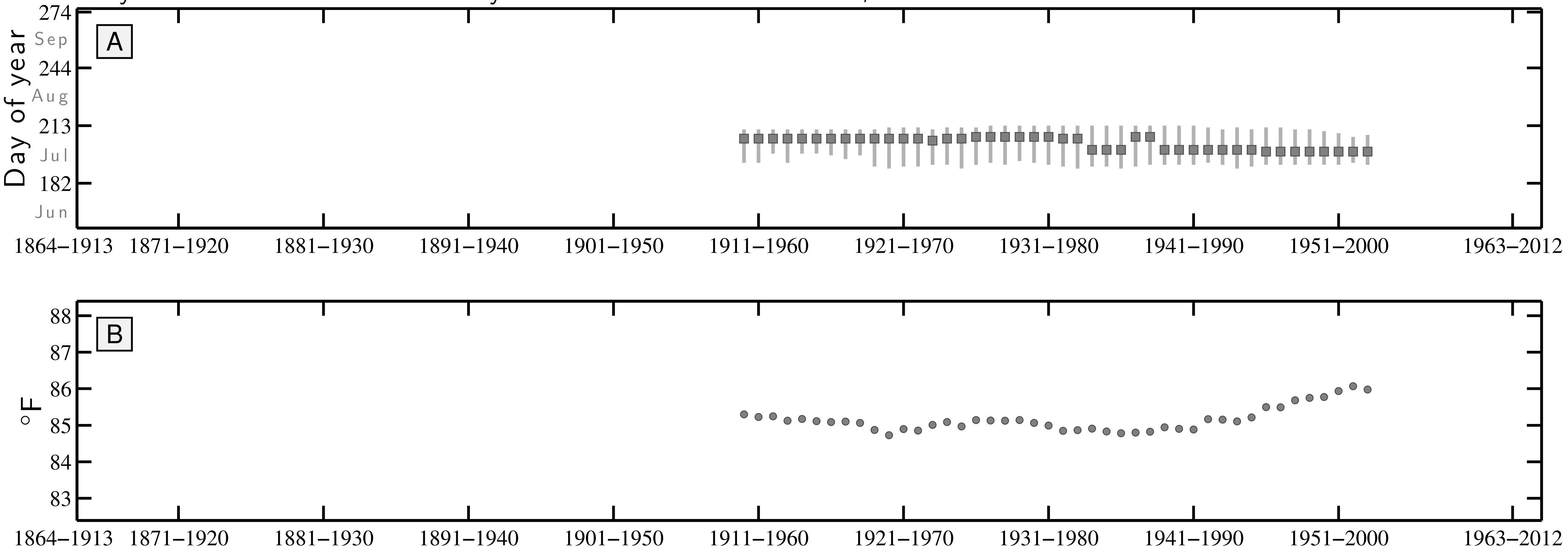

50 year Winter Teletherm dynamics for PALMERTON, PA:

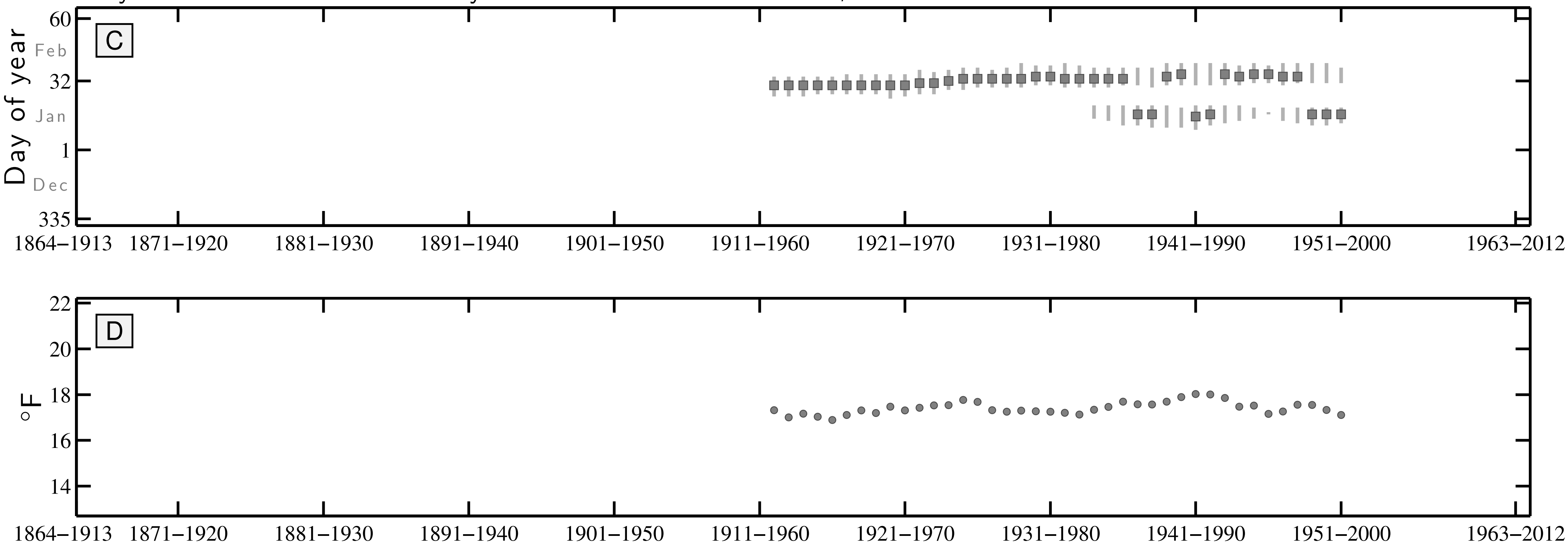

50 year Summer Teletherm dynamics for PLEASANT MT 1 W, PA:

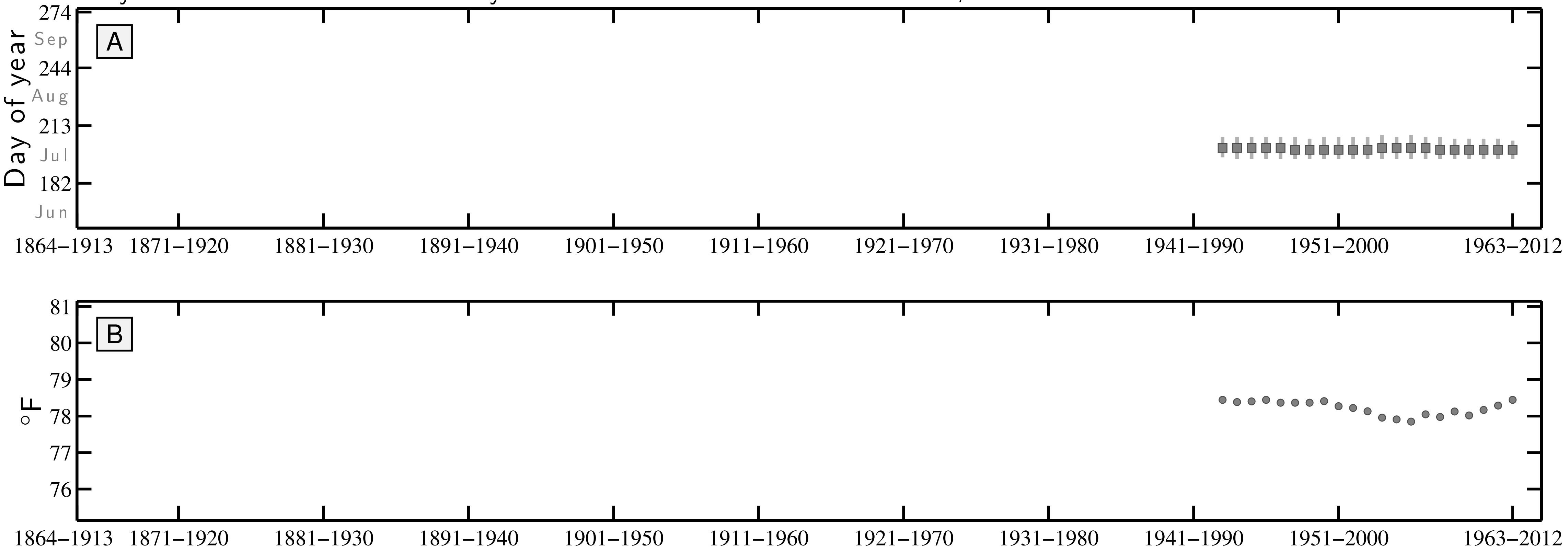

50 year Winter Teletherm dynamics for PLEASANT MT 1 W, PA:

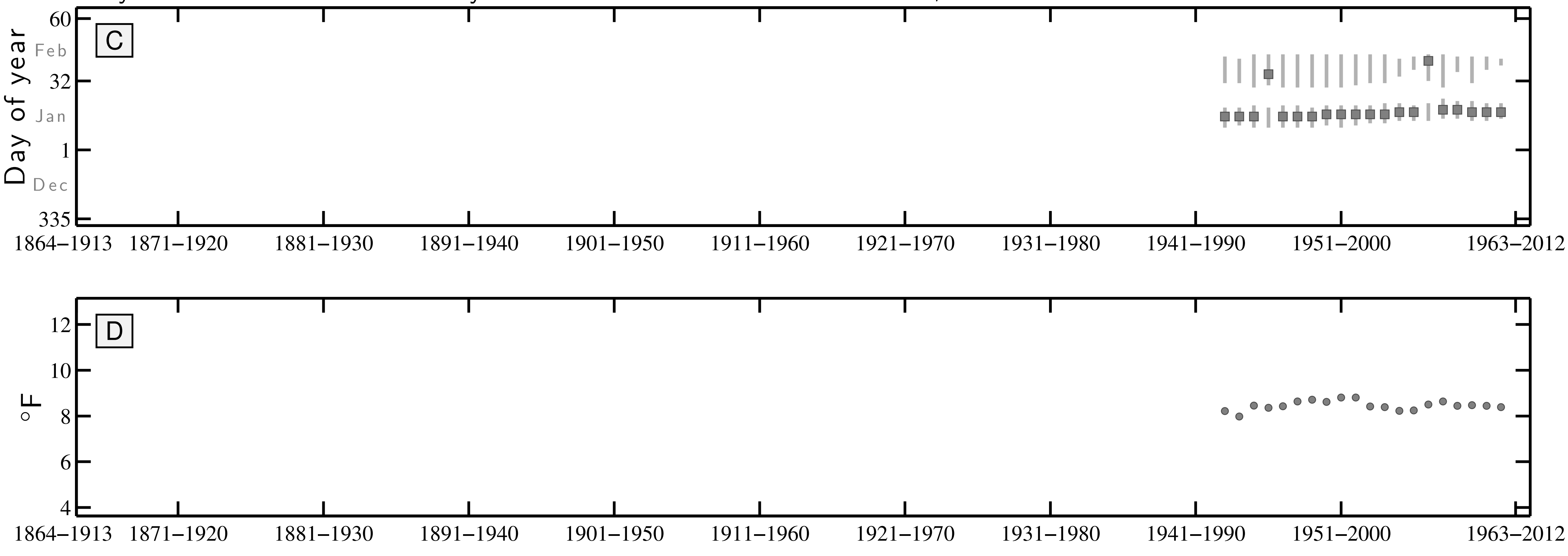

50 year Summer Teletherm dynamics for RIDGWAY, PA:

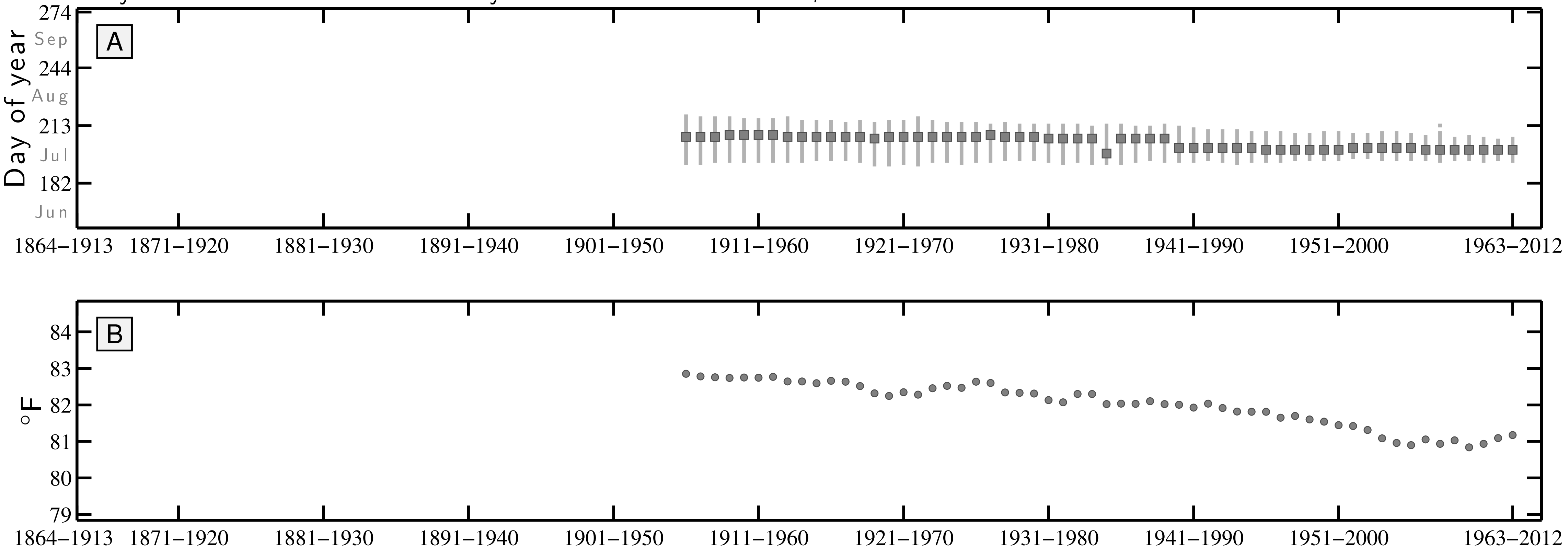

50 year Winter Teletherm dynamics for RIDGWAY, PA:

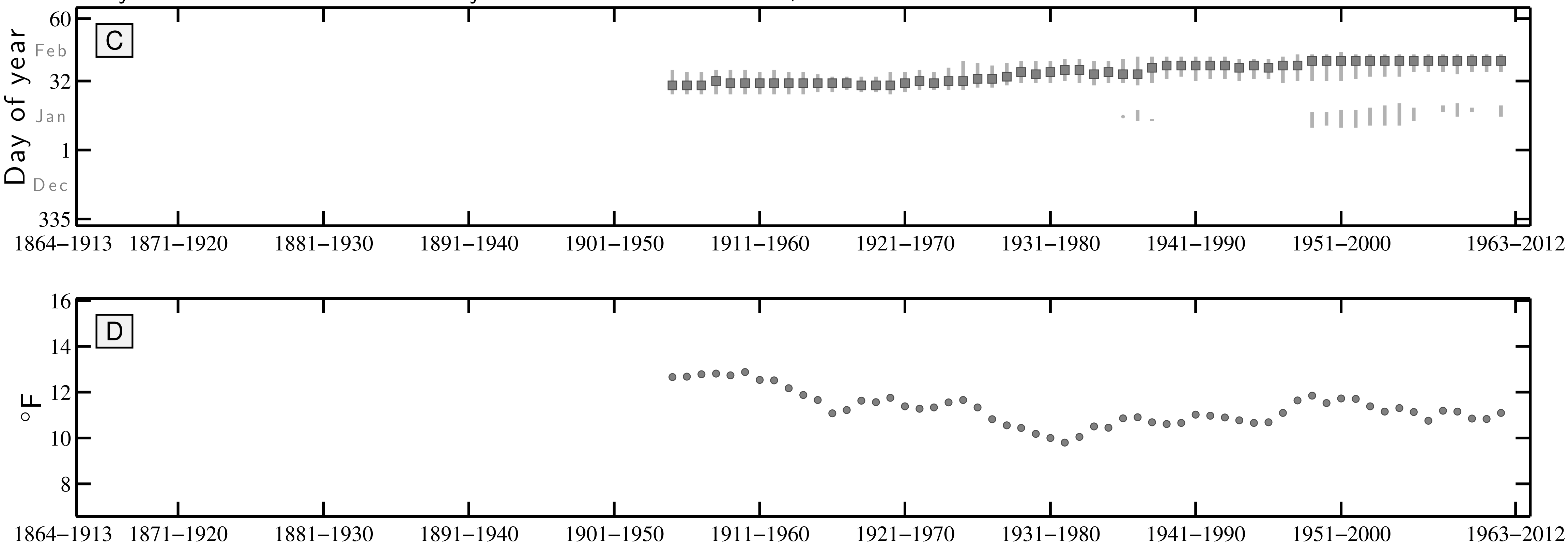

50 year Summer Teletherm dynamics for SELINSGROVE 2 S, PA:

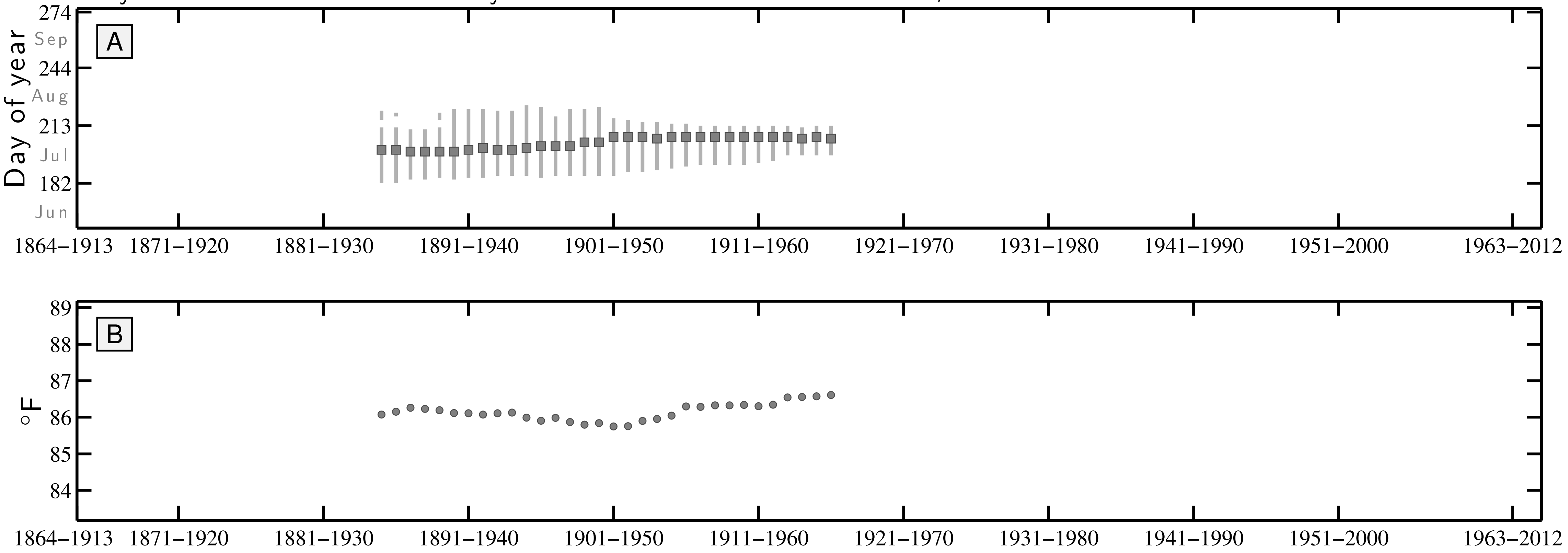

50 year Winter Teletherm dynamics for SELINSGROVE 2 S, PA:

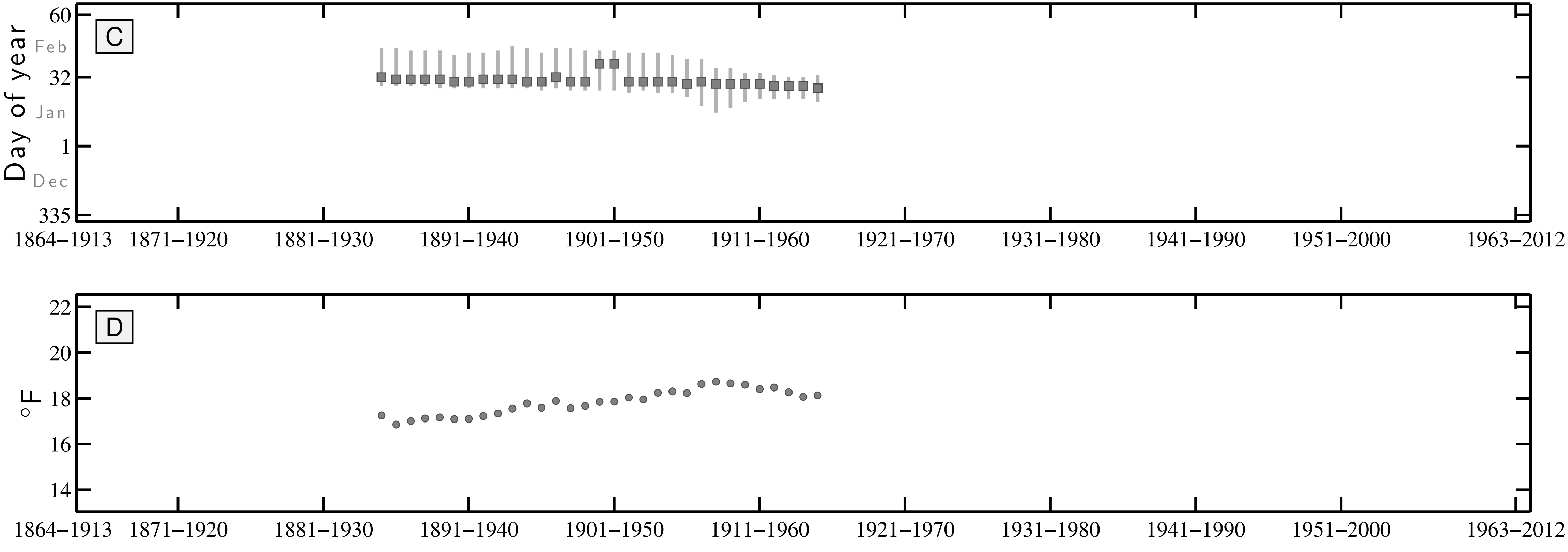

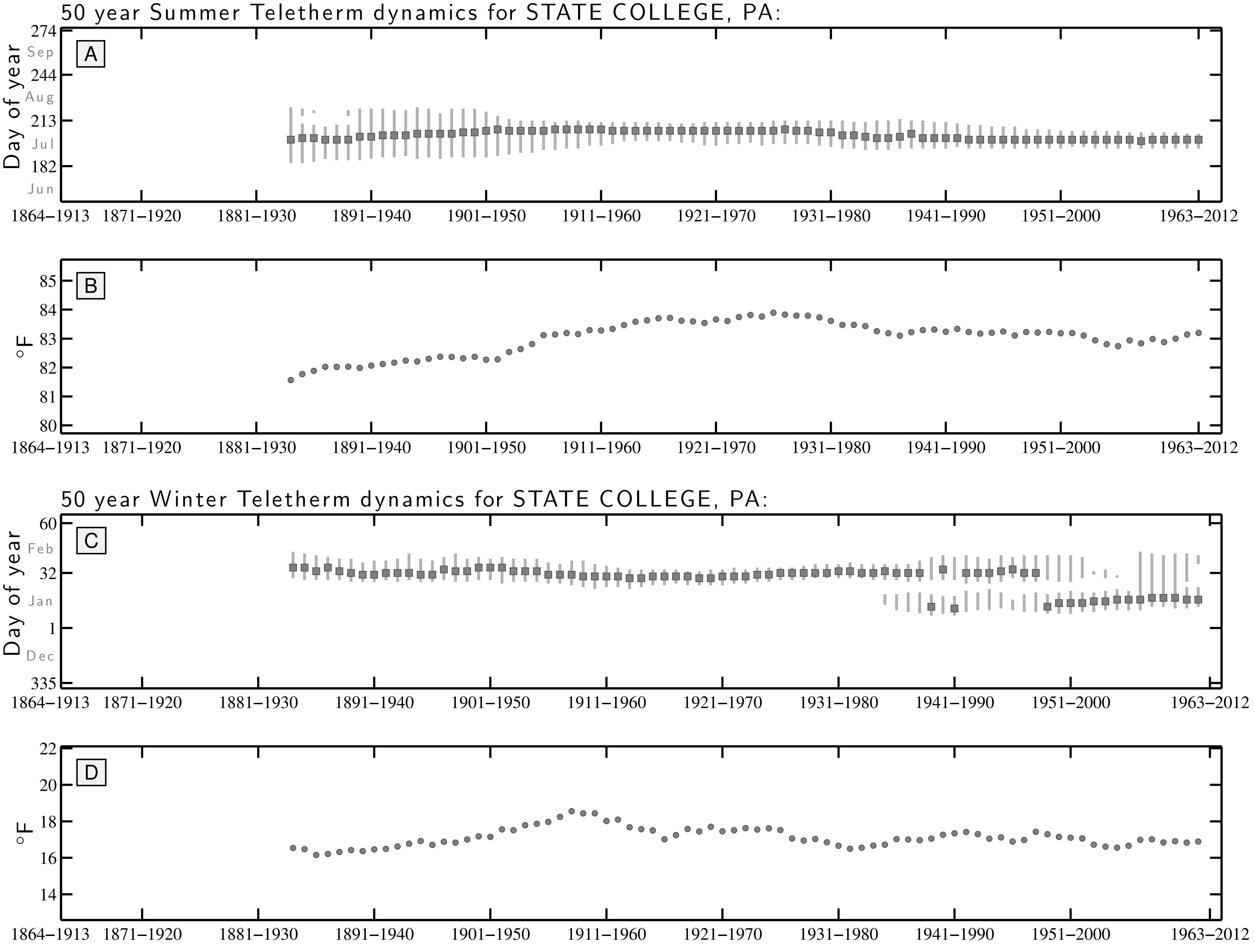

50 year Summer Teletherm dynamics for STATE COLLEGE, PA:
A
Day of year
274
Sep
244
Aug
213
Jul
182
Jun
B
°F
85
84
83
82
81
80
50 year Winter Teletherm dynamics for STATE COLLEGE, PA:
C
Day of year
60
Feb
32
Jan
1
Dec
335
D
°F
22
20
18
16
14
1864–1913
1871–1920
1881–1930
1891–1940
1901–1950
1911–1960
1921–1970
1931–1980
1941–1990
1951–2000
1963–2012

50 year Summer Teletherm dynamics for STROUDSBURG, PA:

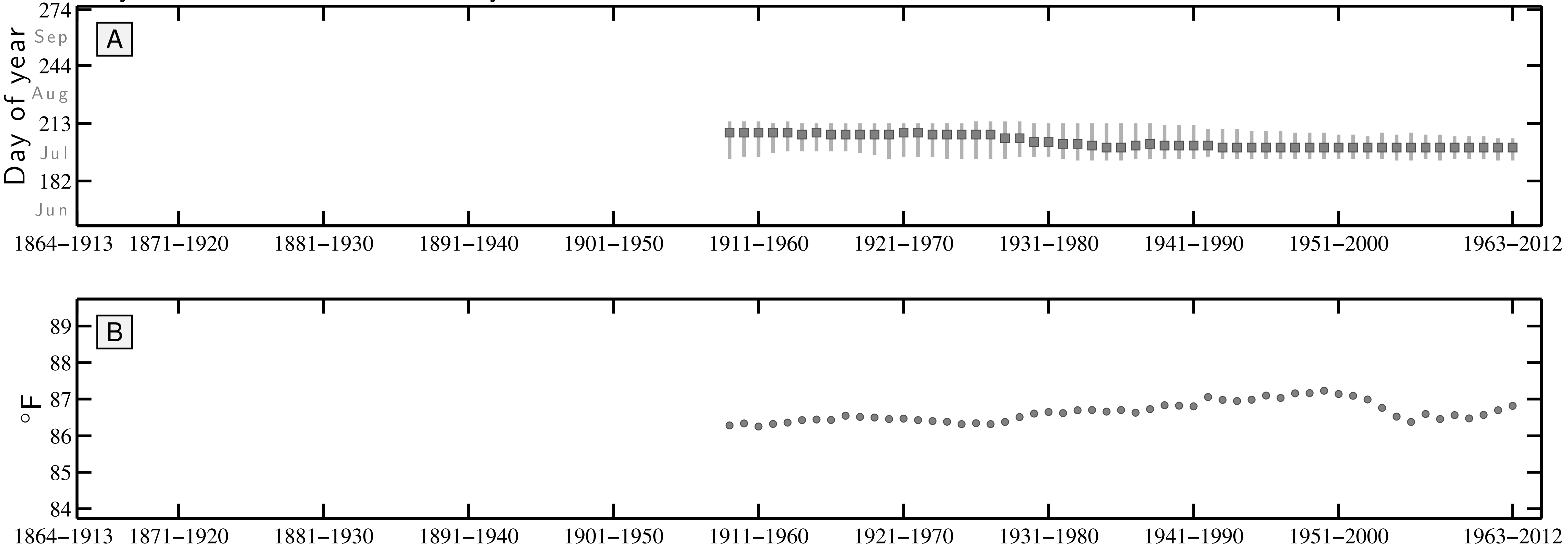

50 year Winter Teletherm dynamics for STROUDSBURG, PA:

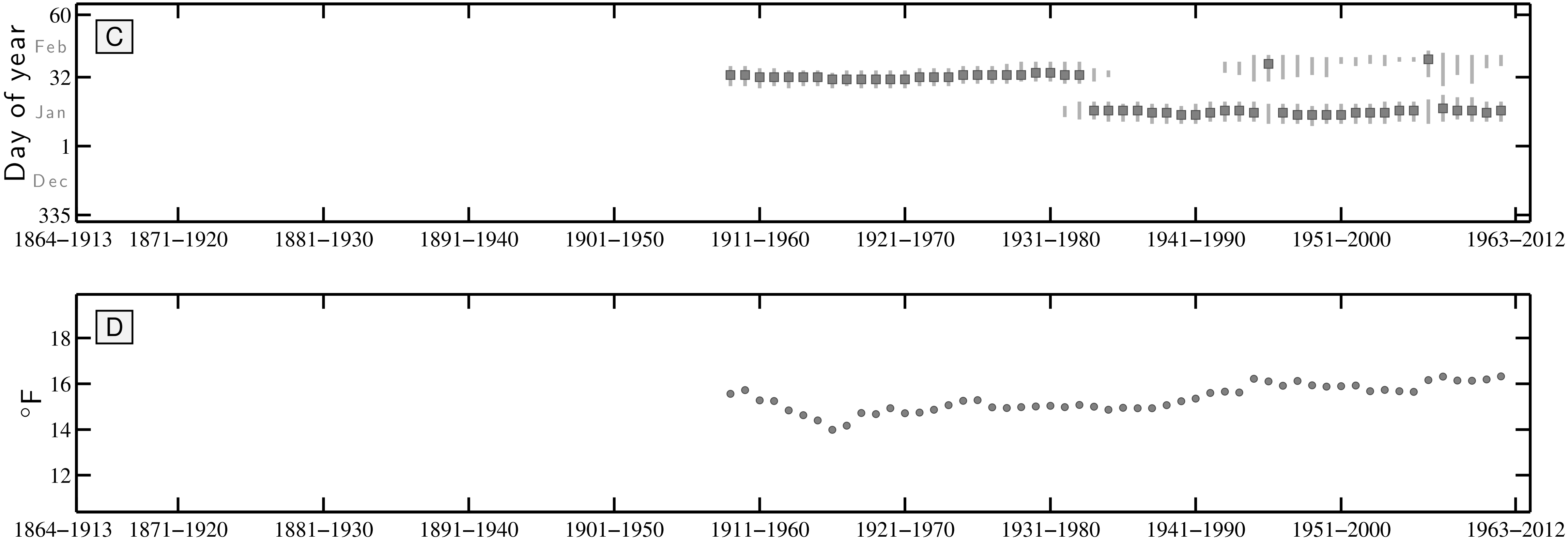

50 year Summer Teletherm dynamics for TOWANDA 1 S, PA:

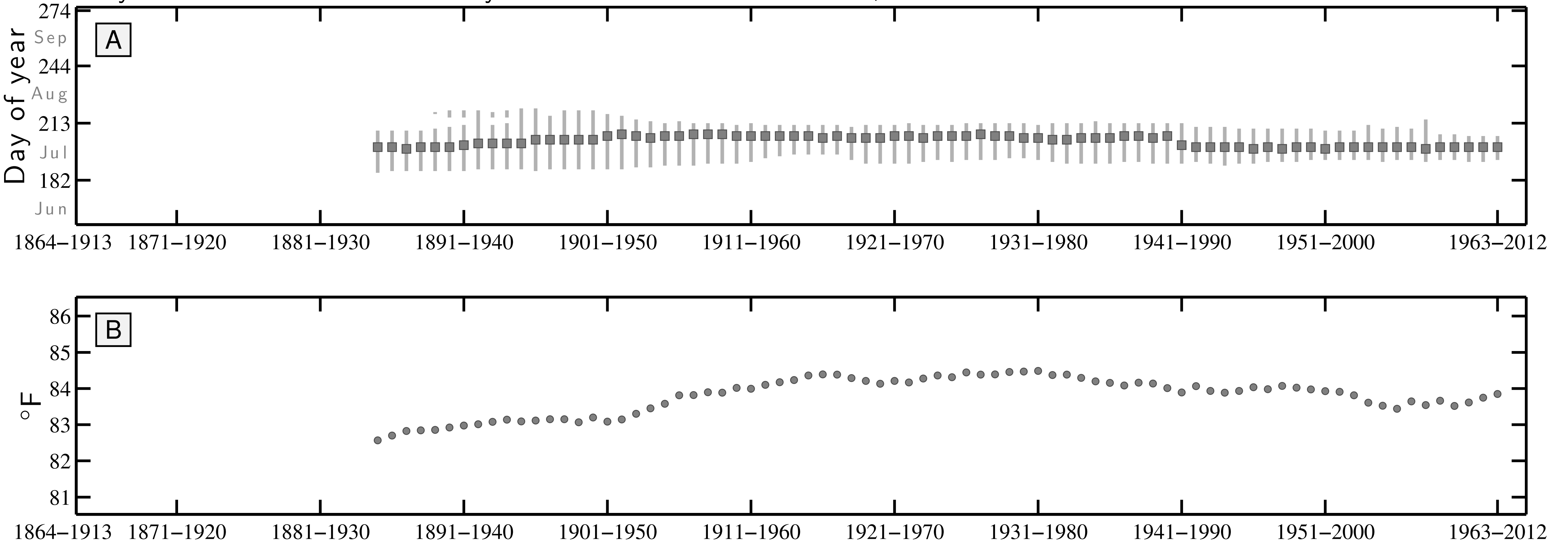

50 year Winter Teletherm dynamics for TOWANDA 1 S, PA:

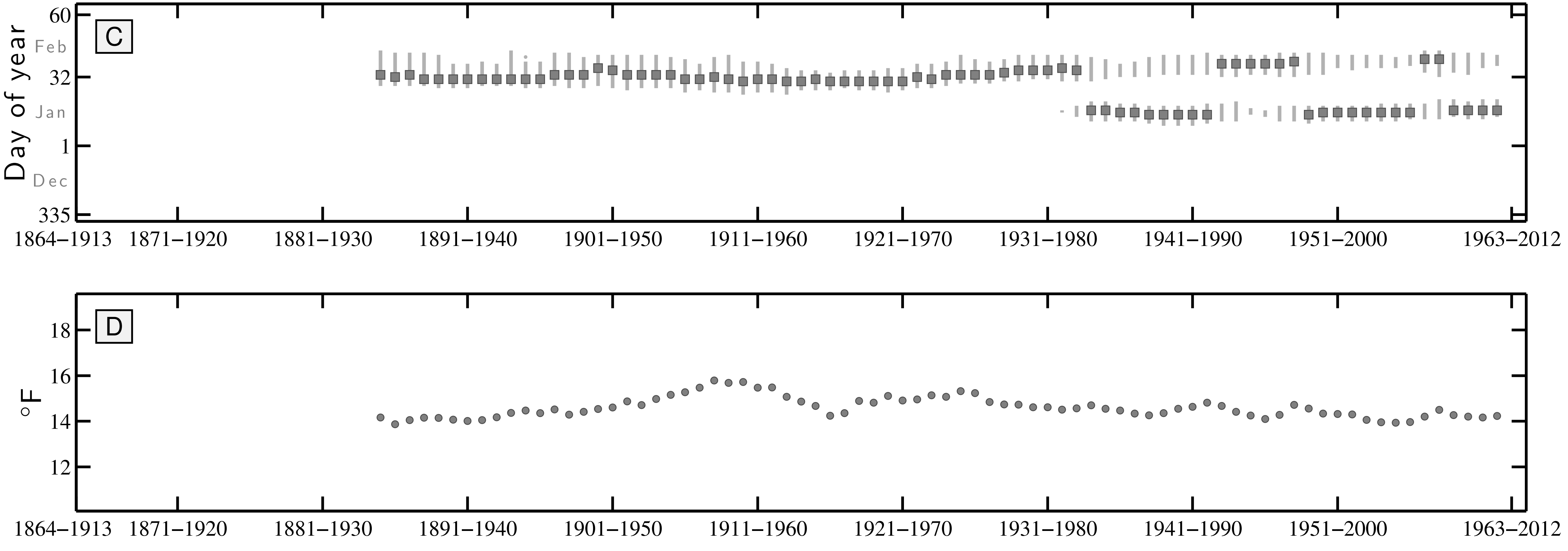

50 year Summer Teletherm dynamics for UNIONTOWN 1 NE, PA:

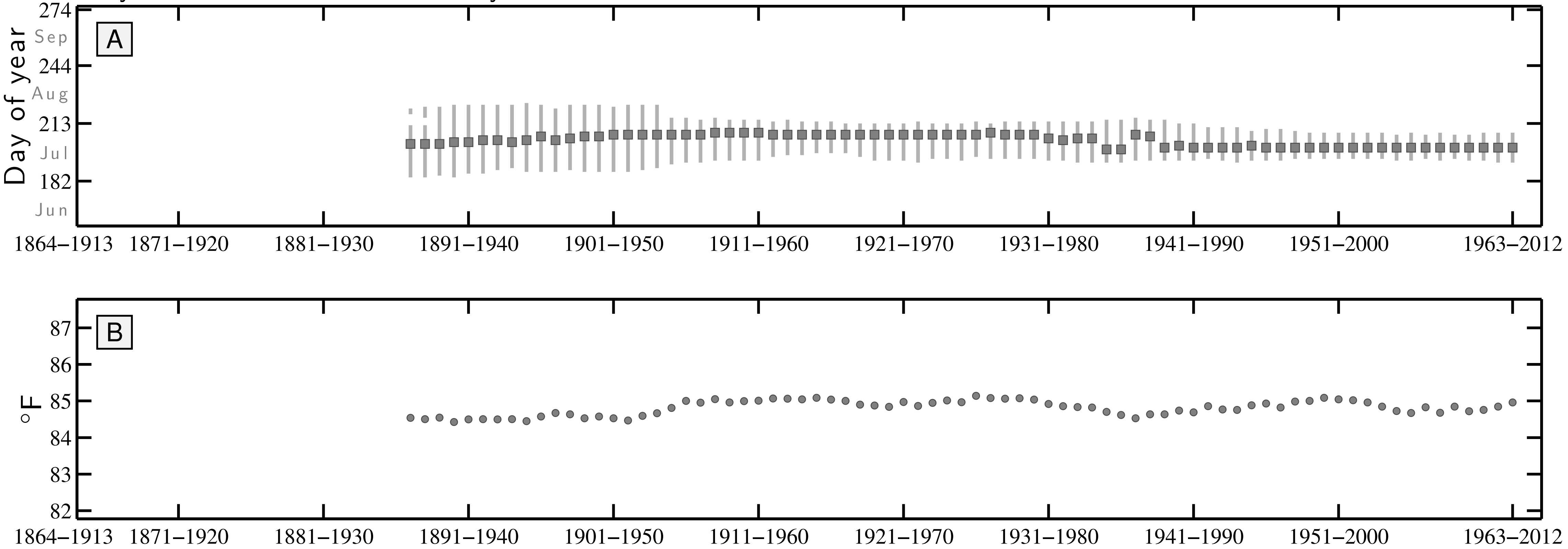

50 year Winter Teletherm dynamics for UNIONTOWN 1 NE, PA:

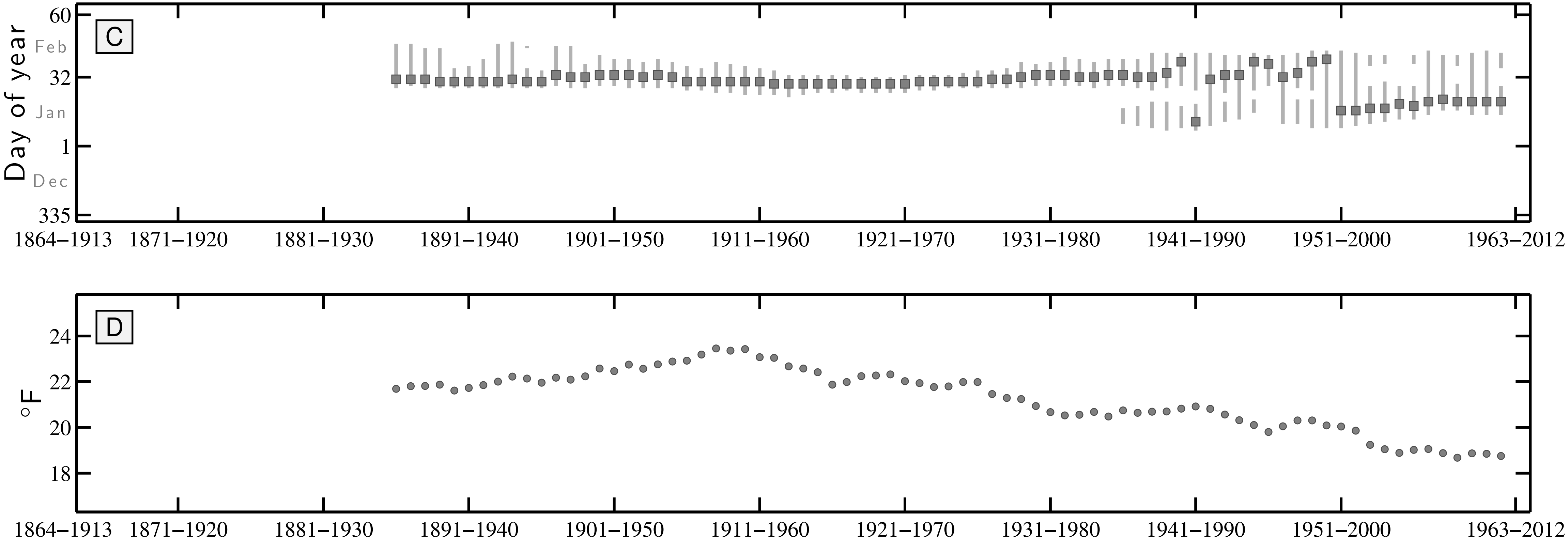

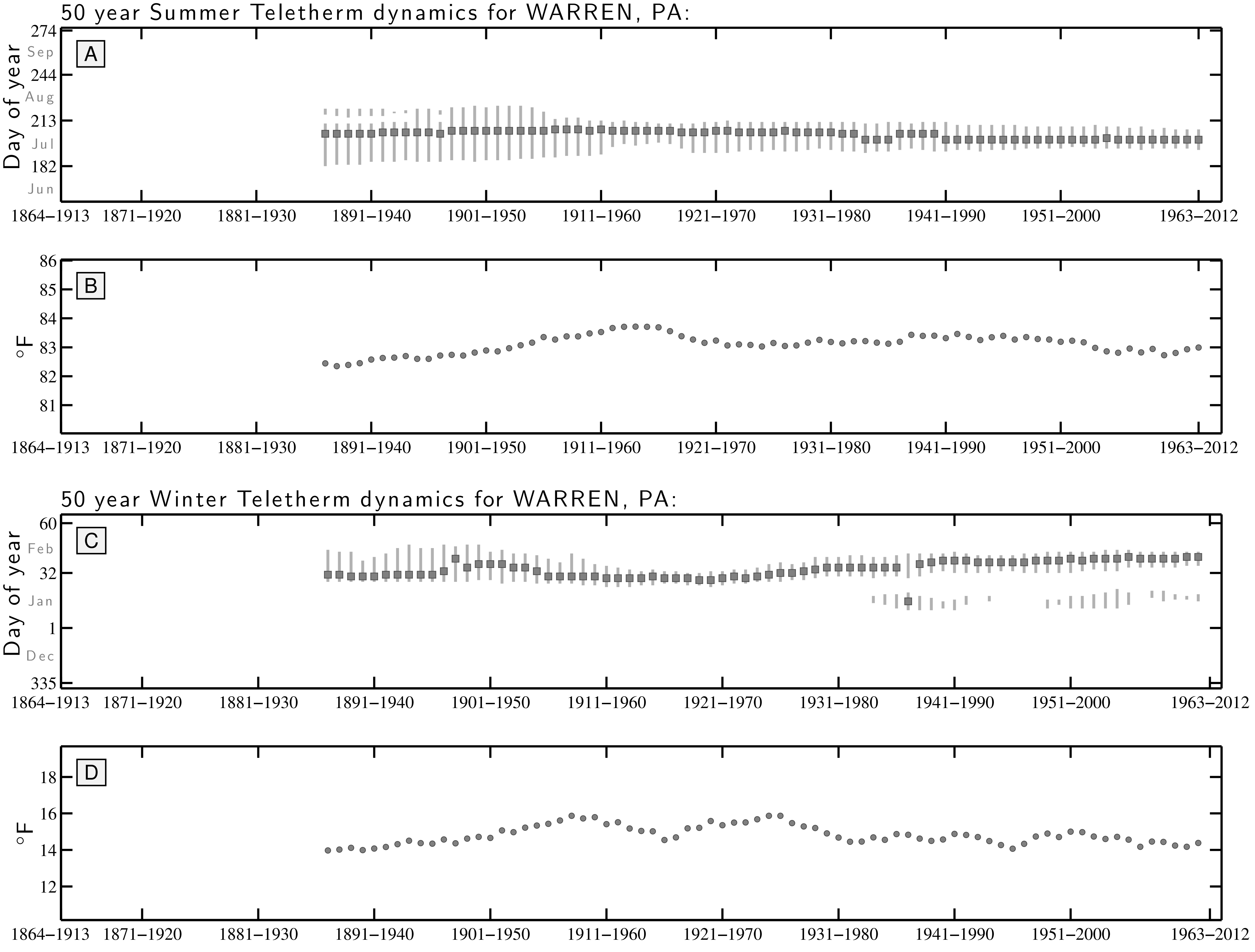
50 year Summer Teletherm dynamics for WARREN, PA:
A
B
Day of year
274
Sep
244
Aug
213
Jul
182
Jun
°F
86
85
84
83
82
81
50 year Winter Teletherm dynamics for WARREN, PA:
C
D
60
Feb
32
Jan
1
Dec
335
18
16
14
12
1864–1913
1871–1920
1881–1930
1891–1940
1901–1950
1911–1960
1921–1970
1931–1980
1941–1990
1951–2000
1963–2012

50 year Summer Teletherm dynamics for WELLSBORO 4 SW, PA:

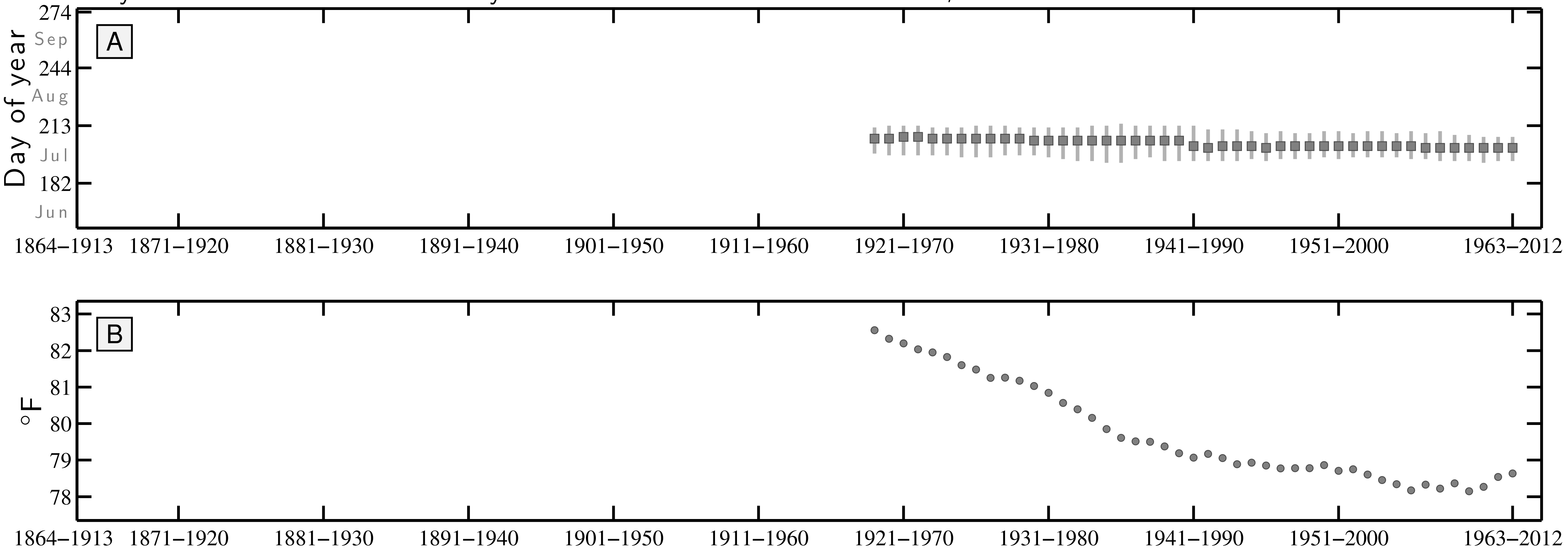

50 year Winter Teletherm dynamics for WELLSBORO 4 SW, PA:

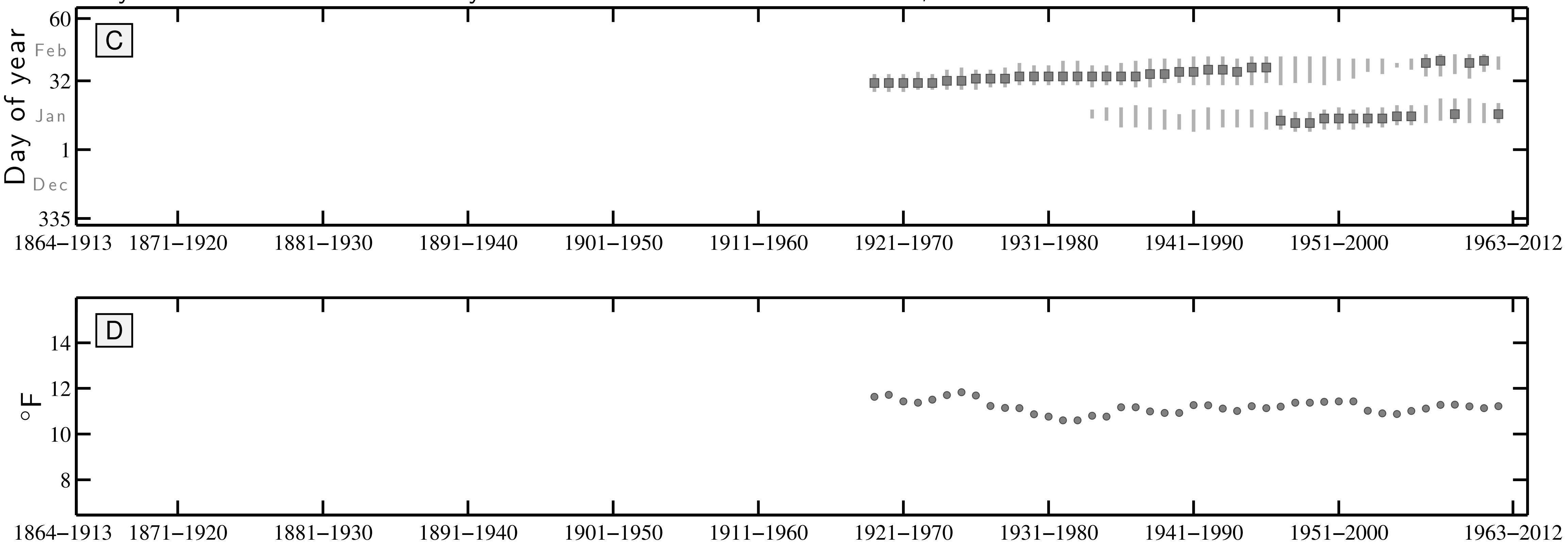

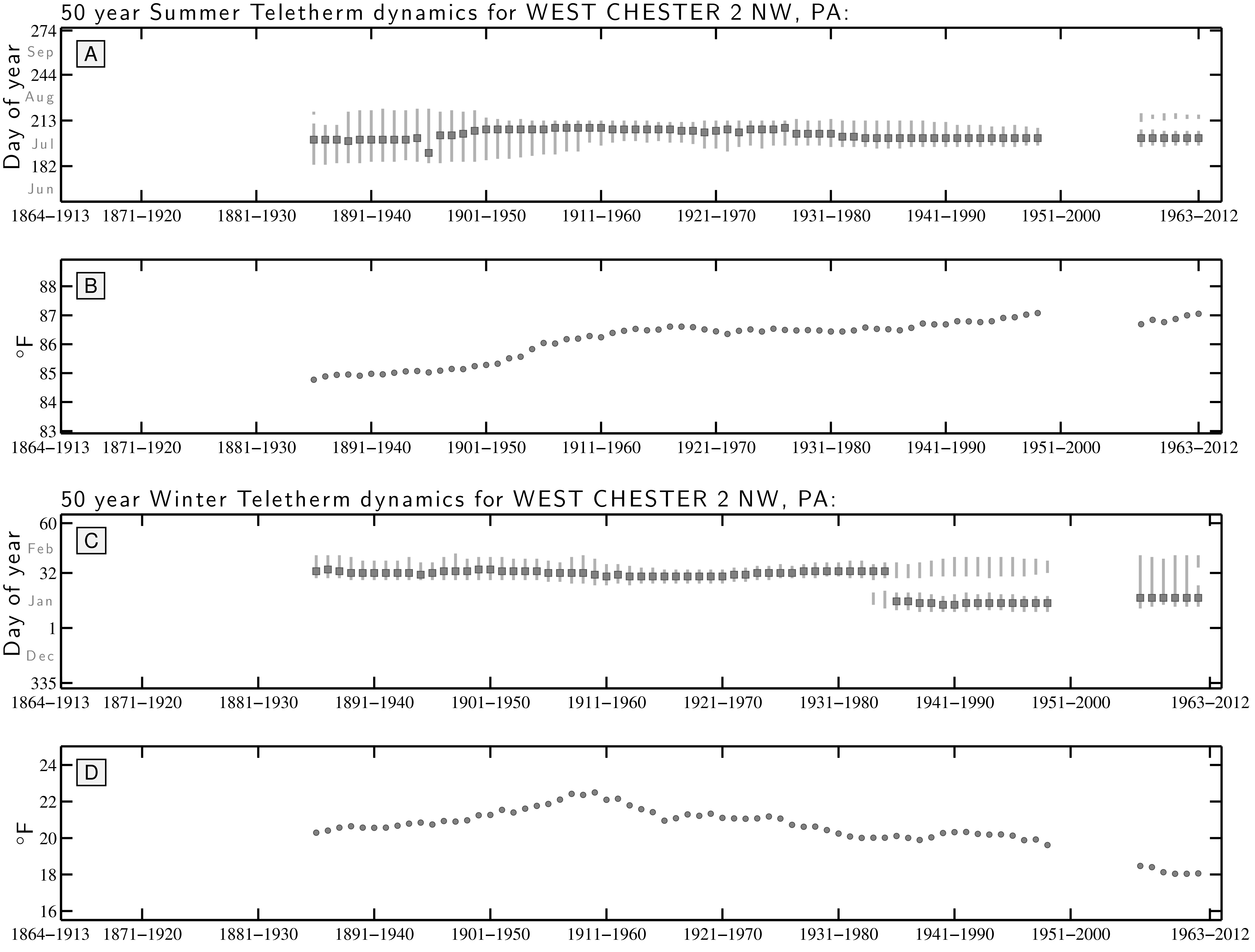
50 year Summer Teletherm dynamics for WEST CHESTER 2 NW, PA:
A
Day of year
274
Sep
244
Aug
213
Jul
182
Jun
B
°F
88
87
86
85
84
83
1864–1913
1871–1920
1881–1930
1891–1940
1901–1950
1911–1960
1921–1970
1931–1980
1941–1990
1951–2000
1963–2012
50 year Winter Teletherm dynamics for WEST CHESTER 2 NW, PA:
C
Day of year
60
Feb
32
Jan
1
Dec
335
D
°F
24
22
20
18
16

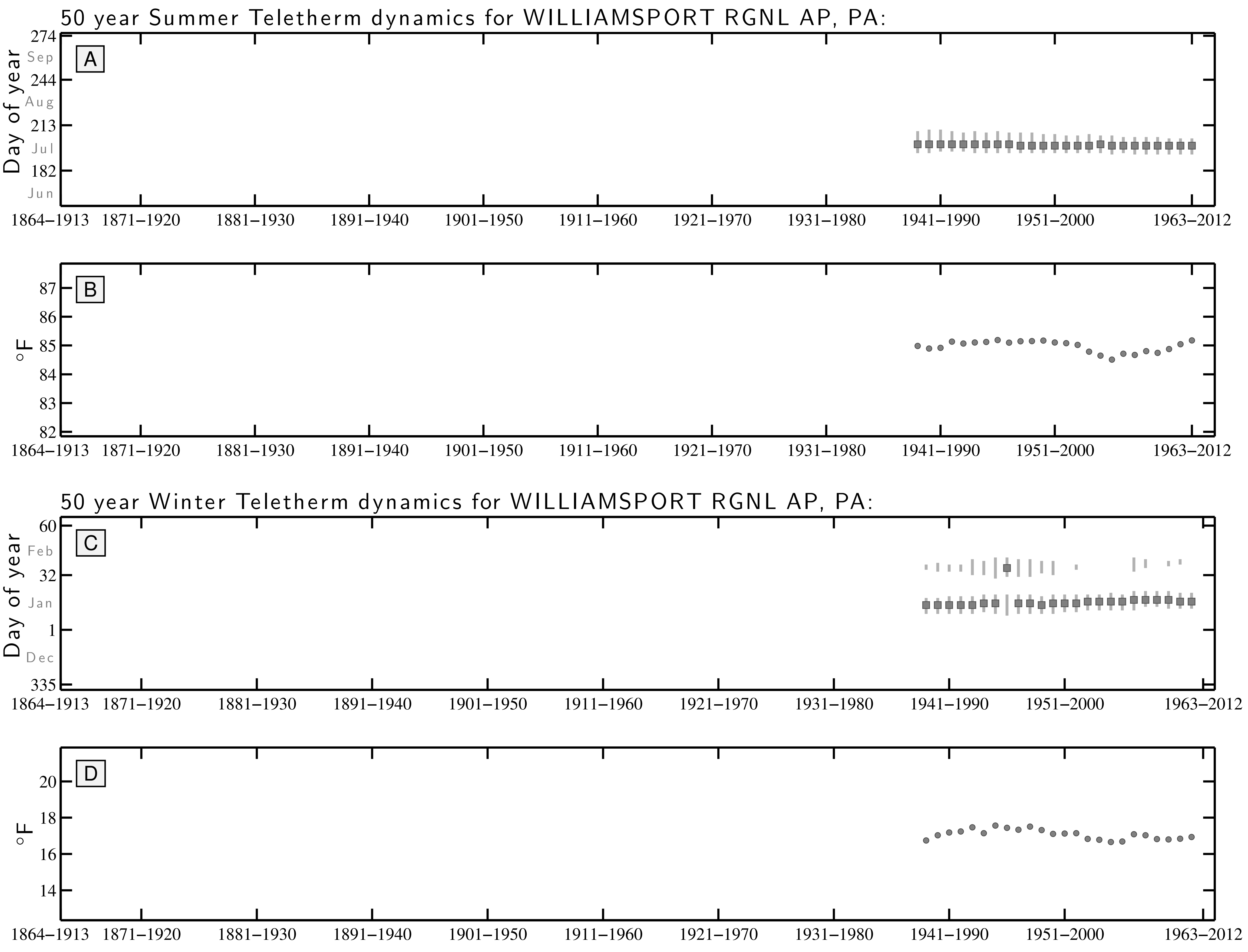

50 year Summer Teletherm dynamics for WILLIAMSPORT RGNL AP, PA:
A
B
Day of year
°F
274
Sep
244
Aug
213
Jul
182
Jun
87
86
85
84
83
82
1864–1913
1871–1920
1881–1930
1891–1940
1901–1950
1911–1960
1921–1970
1931–1980
1941–1990
1951–2000
1963–2012
50 year Winter Teletherm dynamics for WILLIAMSPORT RGNL AP, PA:
C
D
60
Feb
32
Jan
1
Dec
335
20
18
16
14

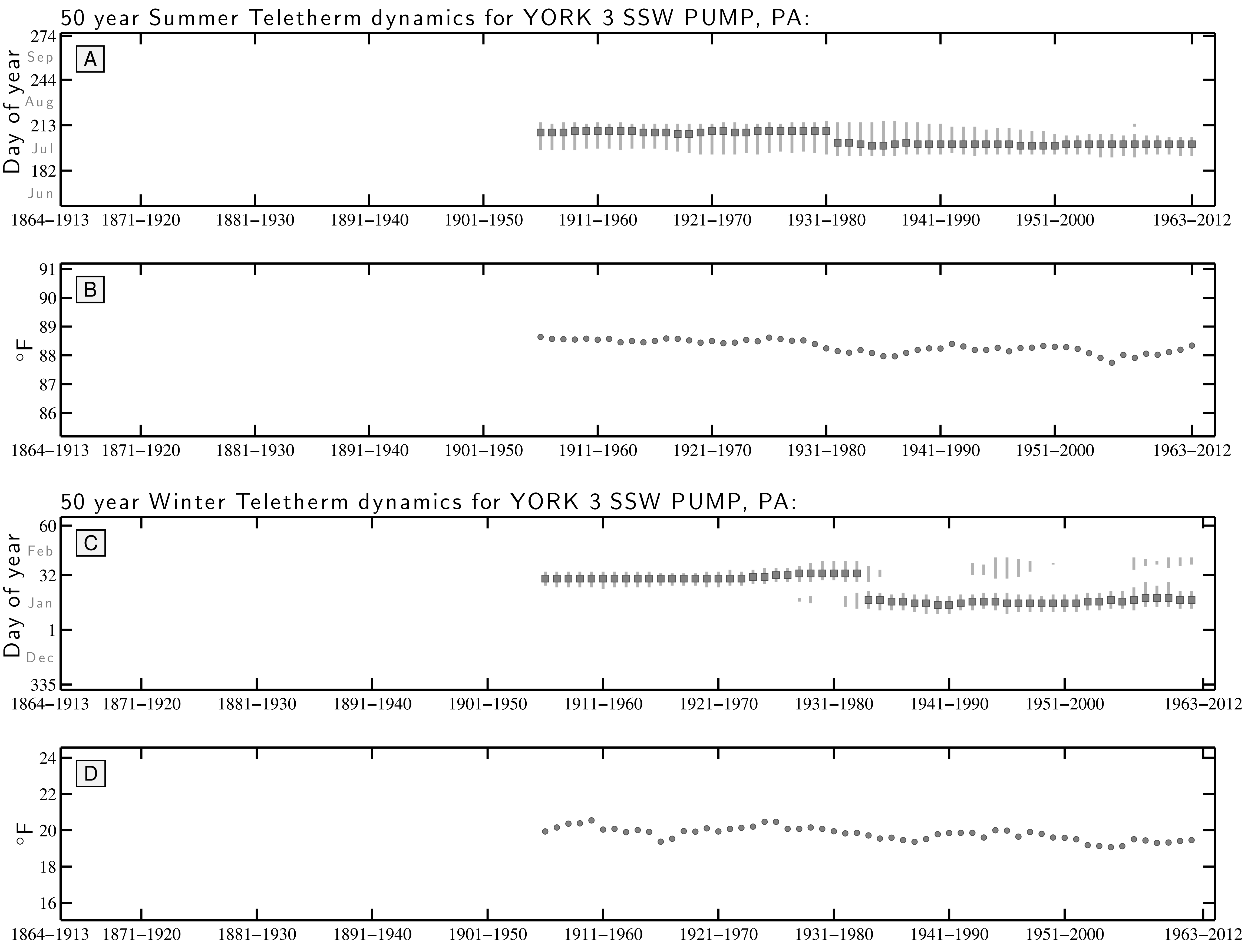
50 year Summer Teletherm dynamics for YORK 3 SSW PUMP, PA:
A
B
Day of year
°F
Jun
Jul
Aug
Sep
182
213
244
274
86
87
88
89
90
91
1864–1913
1871–1920
1881–1930
1891–1940
1901–1950
1911–1960
1921–1970
1931–1980
1941–1990
1951–2000
1963–2012
50 year Winter Teletherm dynamics for YORK 3 SSW PUMP, PA:
C
D
Dec
Jan
Feb
335
1
32
60
16
18
20
22
24

50 year Summer Teletherm dynamics for BLOCK ISLAND STATE AP, RI:

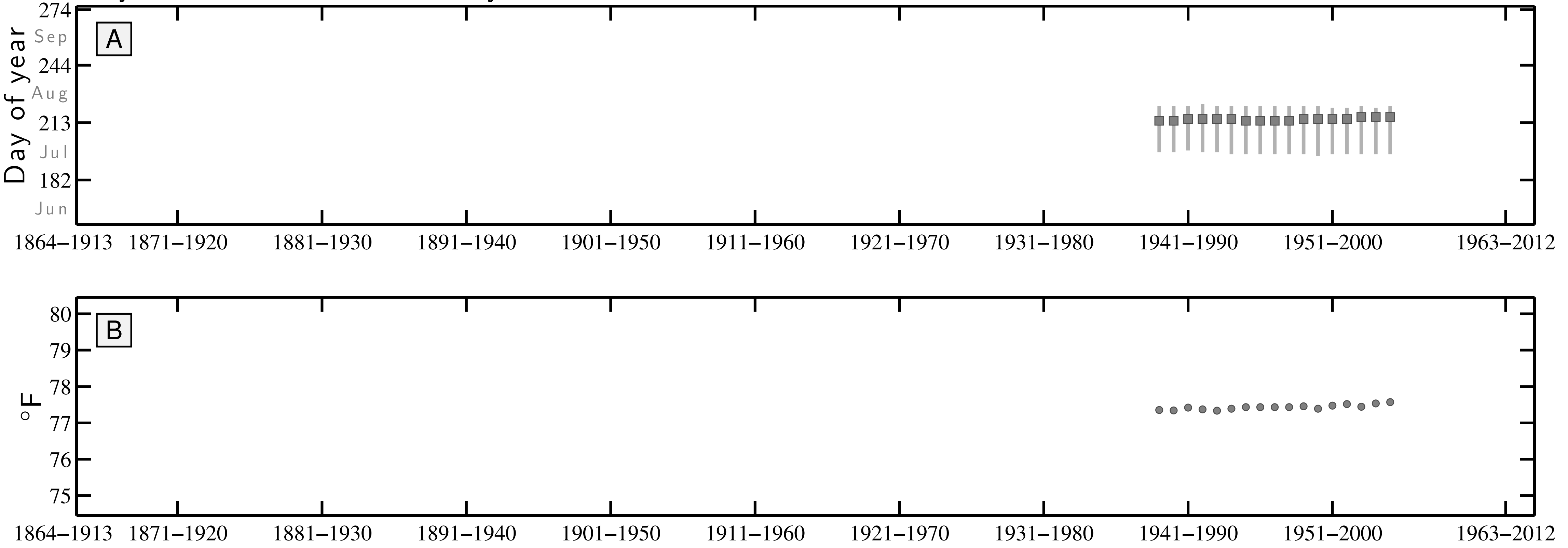

50 year Winter Teletherm dynamics for BLOCK ISLAND STATE AP, RI:

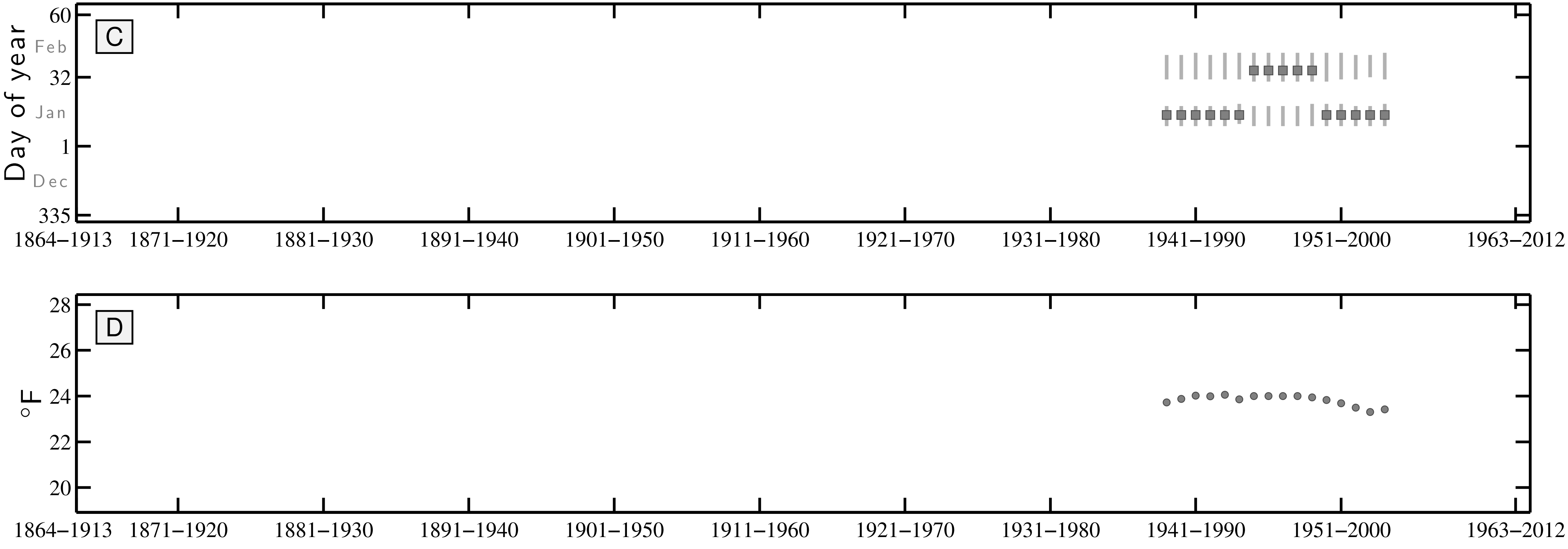

50 year Summer Teletherm dynamics for KINGSTON, RI:

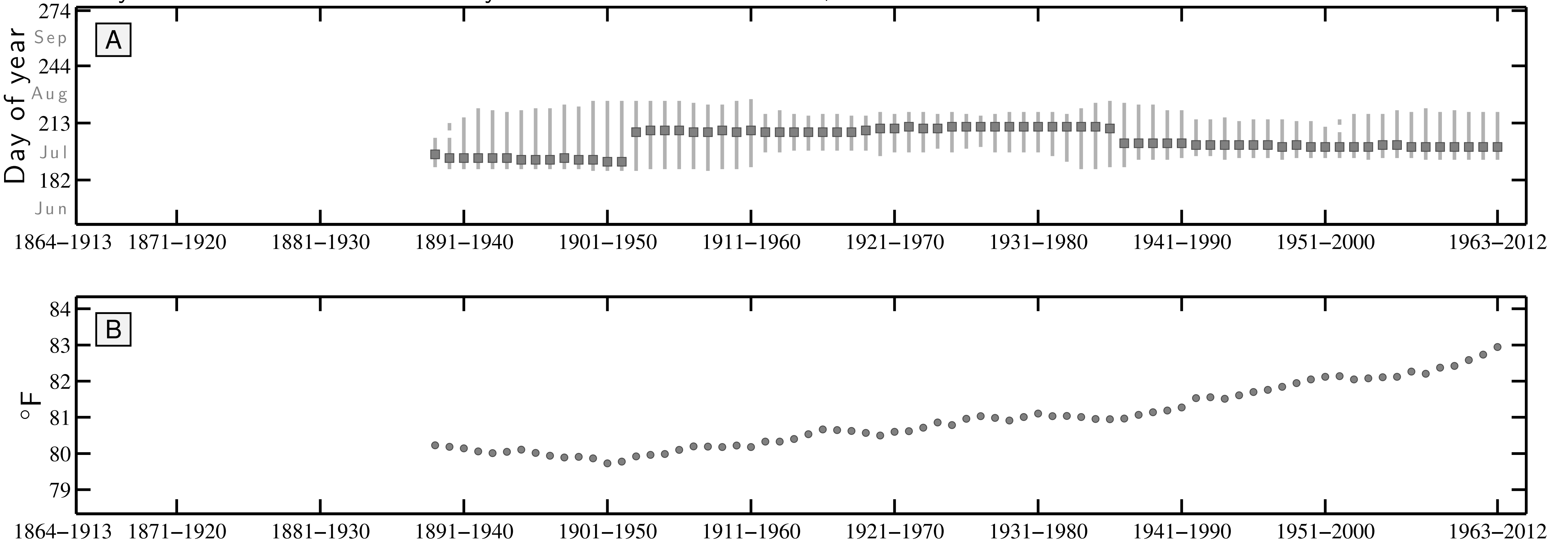

50 year Winter Teletherm dynamics for KINGSTON, RI:

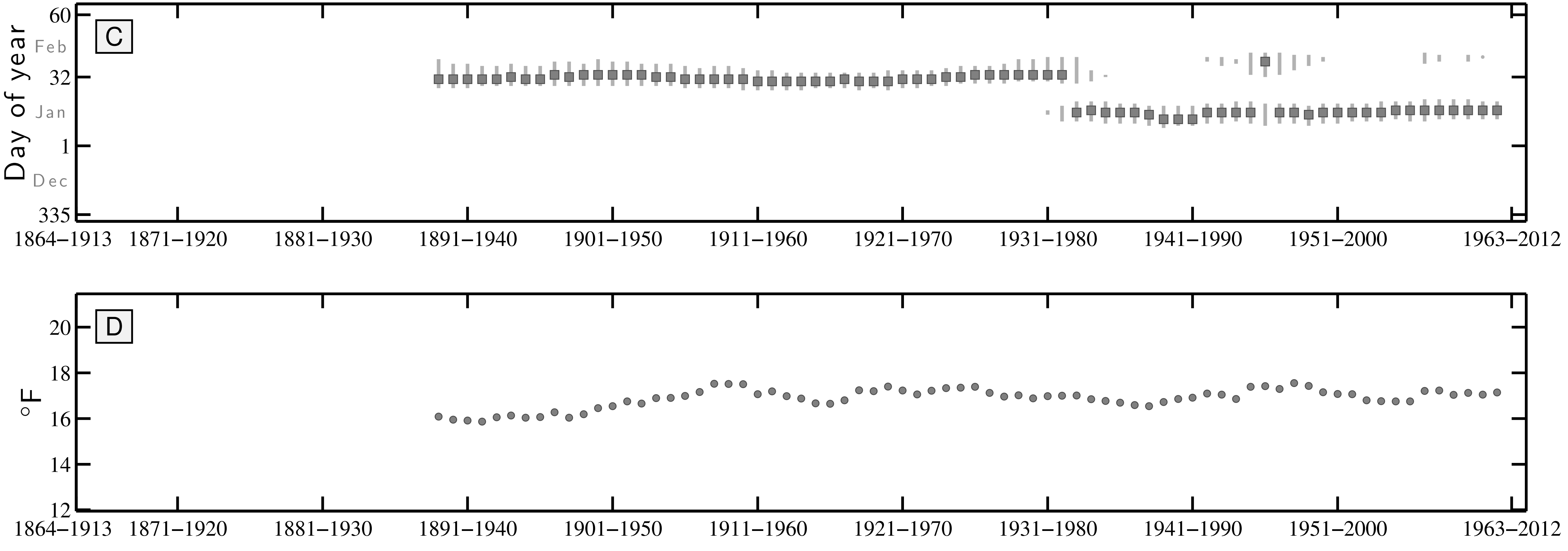

50 year Summer Teletherm dynamics for PROVIDENCE WSO AP, RI:

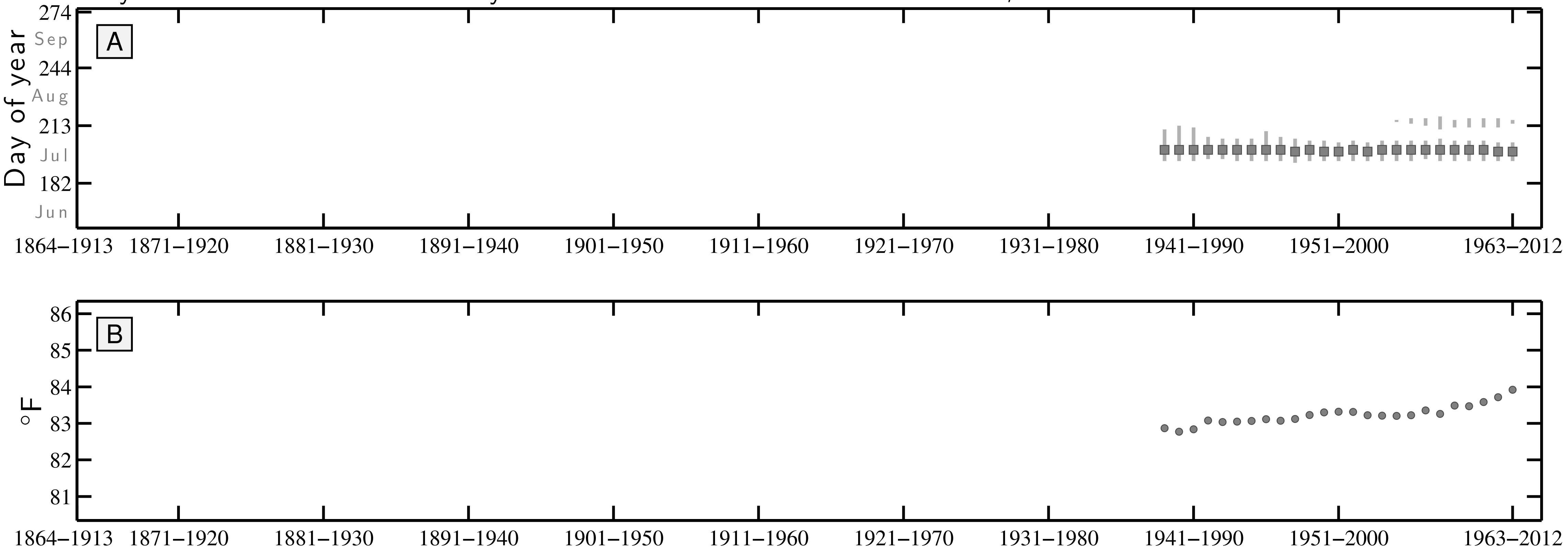

50 year Winter Teletherm dynamics for PROVIDENCE WSO AP, RI:

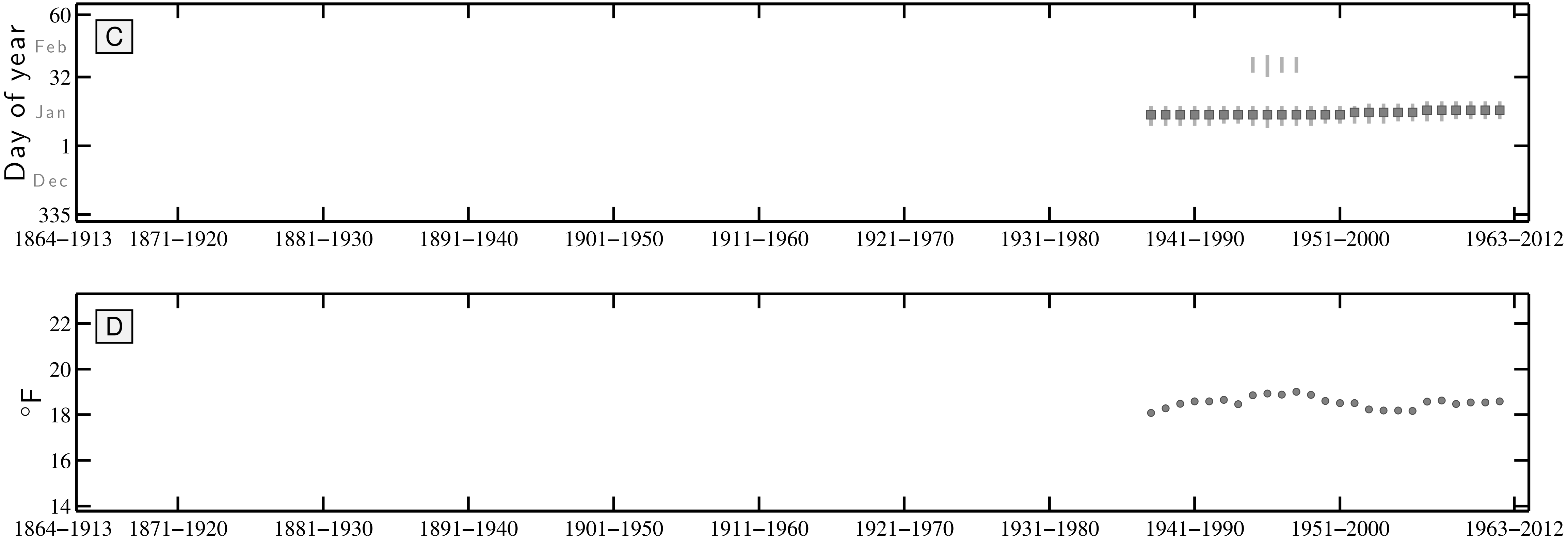

50 year Summer Teletherm dynamics for AIKEN 5SE, SC:

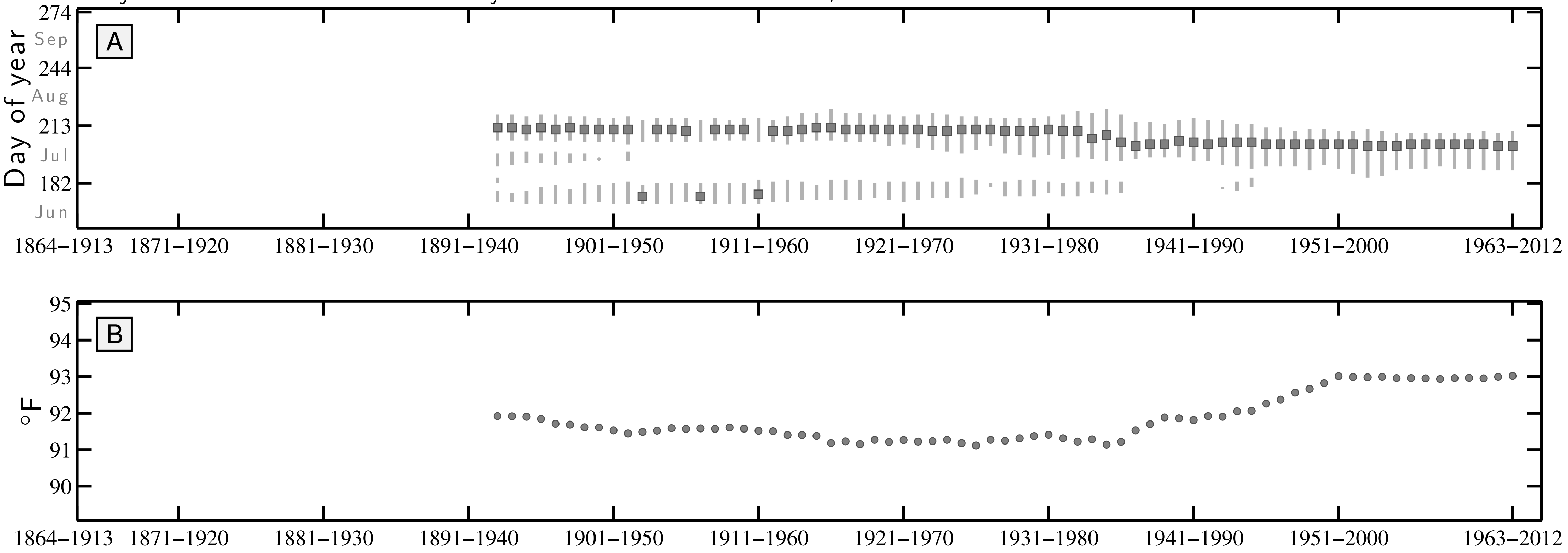

50 year Winter Teletherm dynamics for AIKEN 5SE, SC:

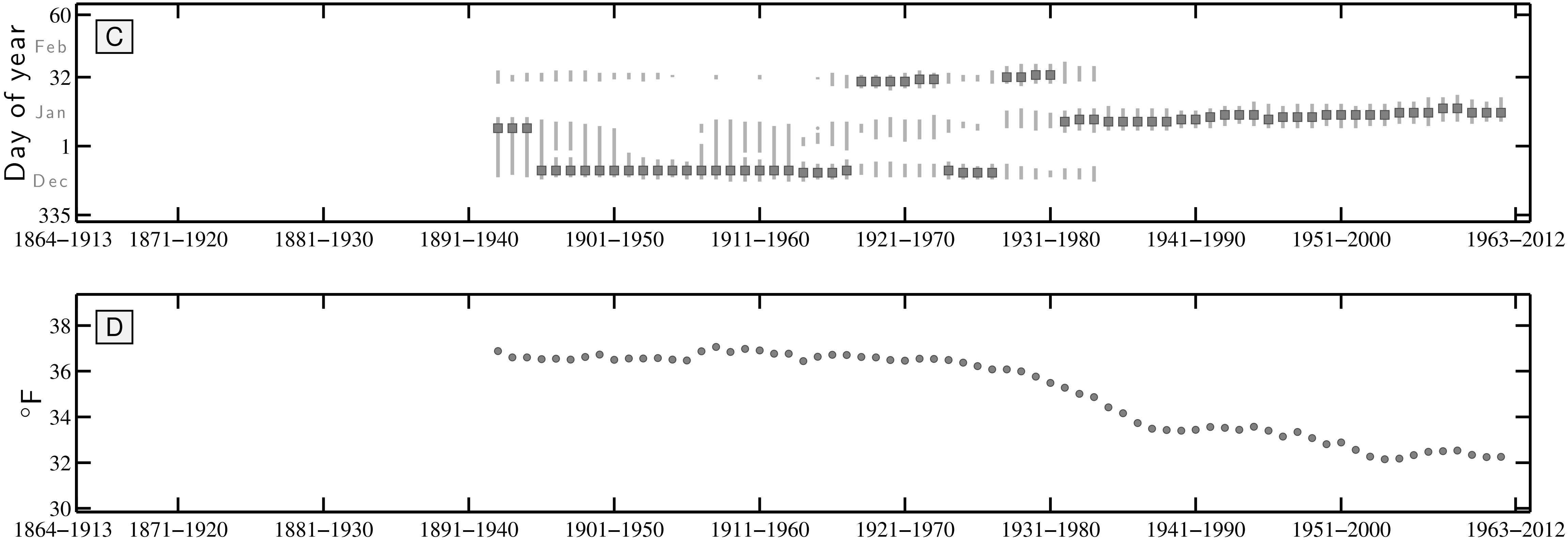

50 year Summer Teletherm dynamics for ANDERSON, SC:

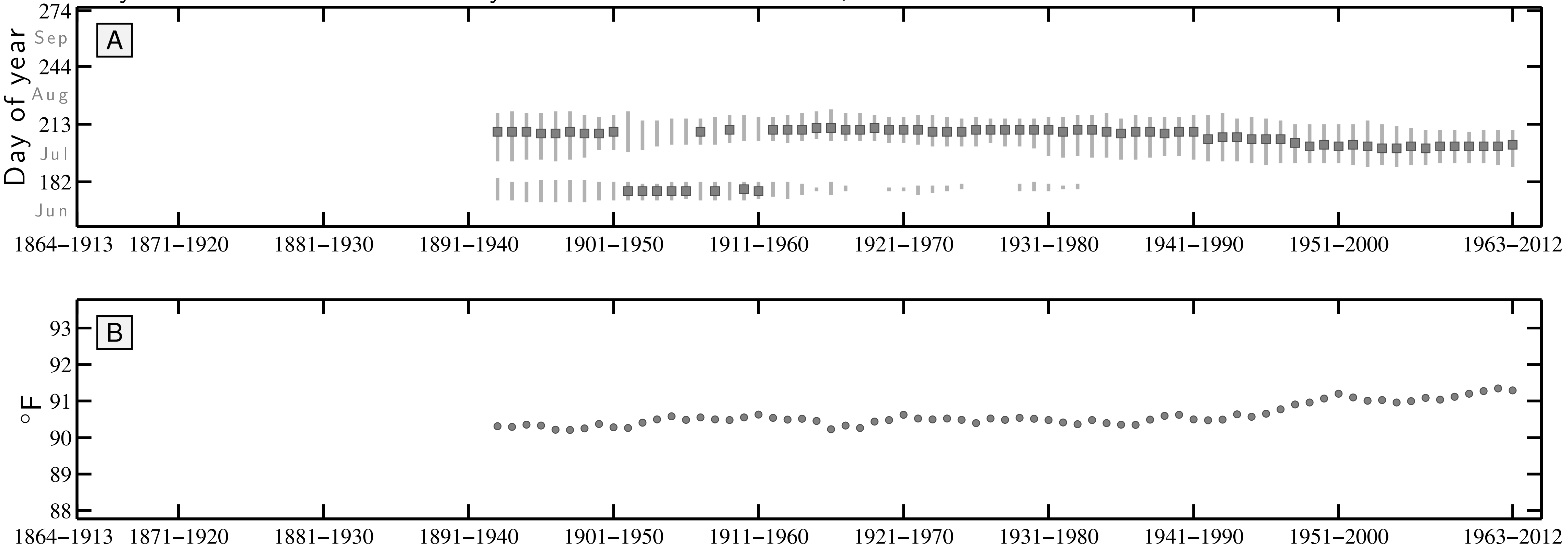

50 year Winter Teletherm dynamics for ANDERSON, SC:

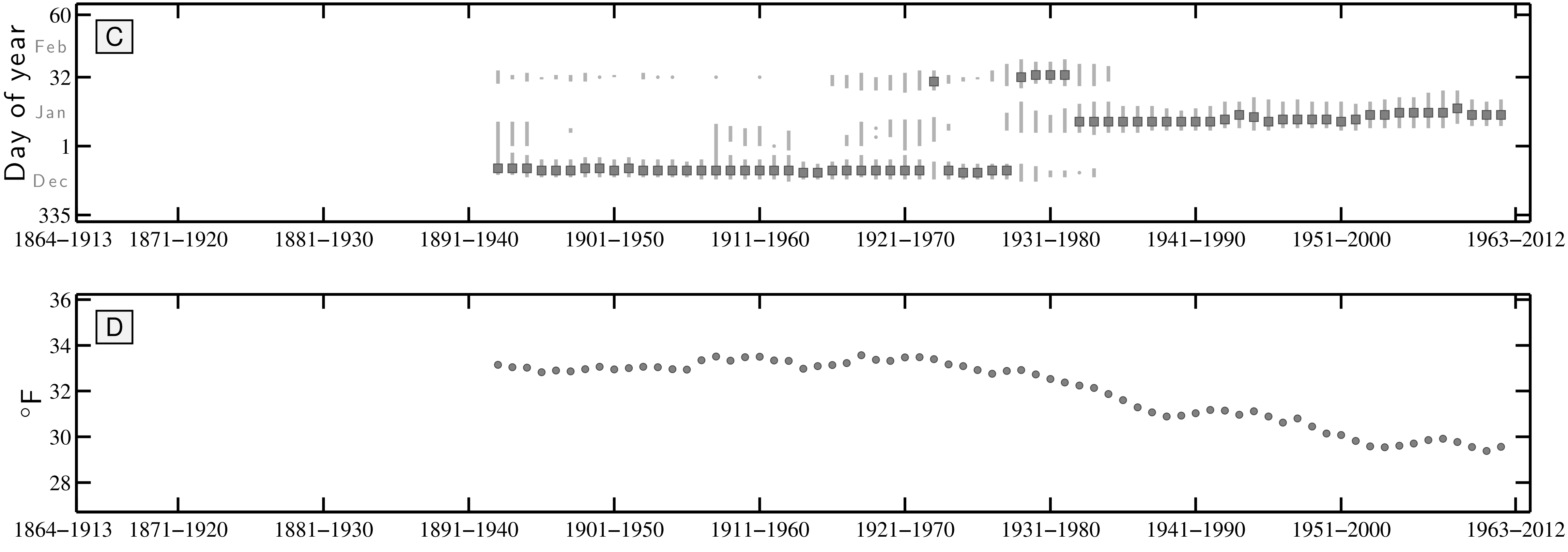

50 year Summer Teletherm dynamics for BEAUFORT WWTP, SC:

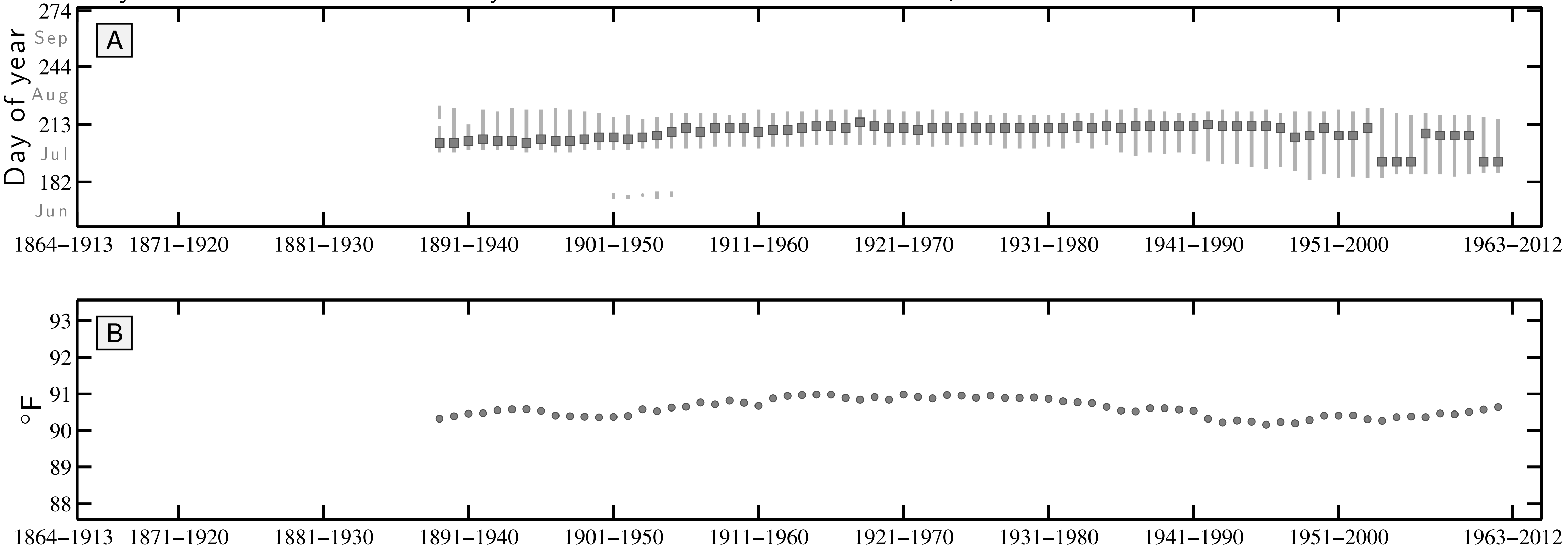

50 year Winter Teletherm dynamics for BEAUFORT WWTP, SC:

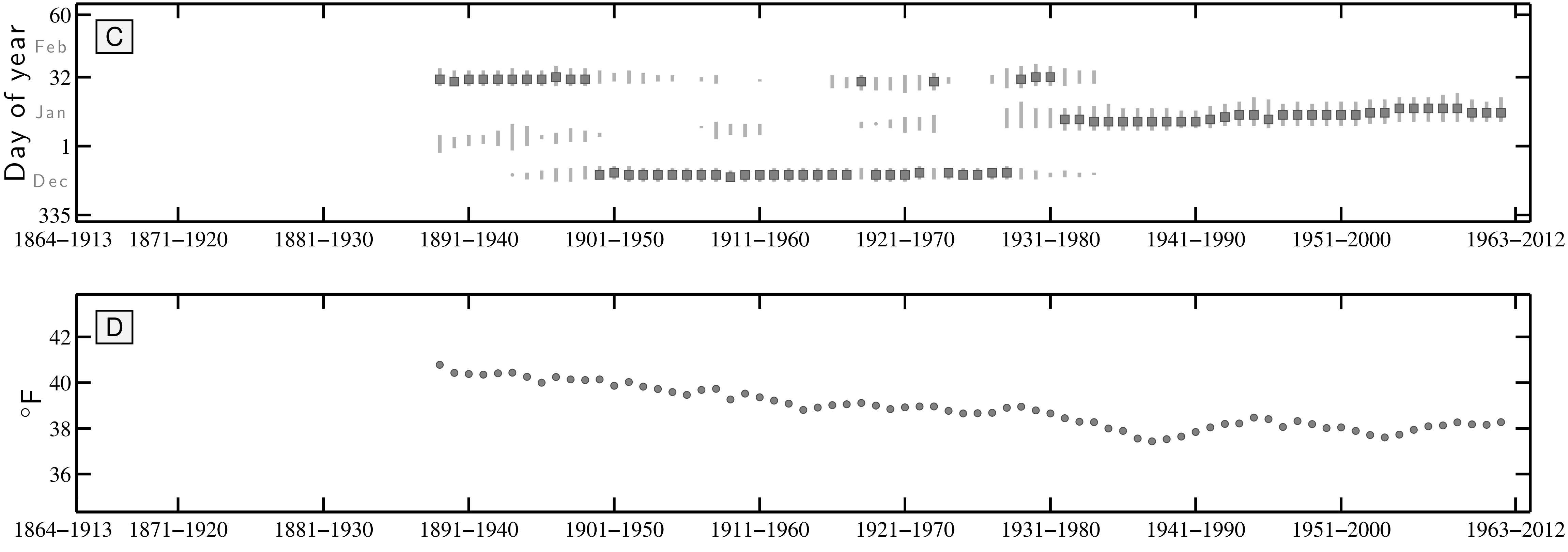

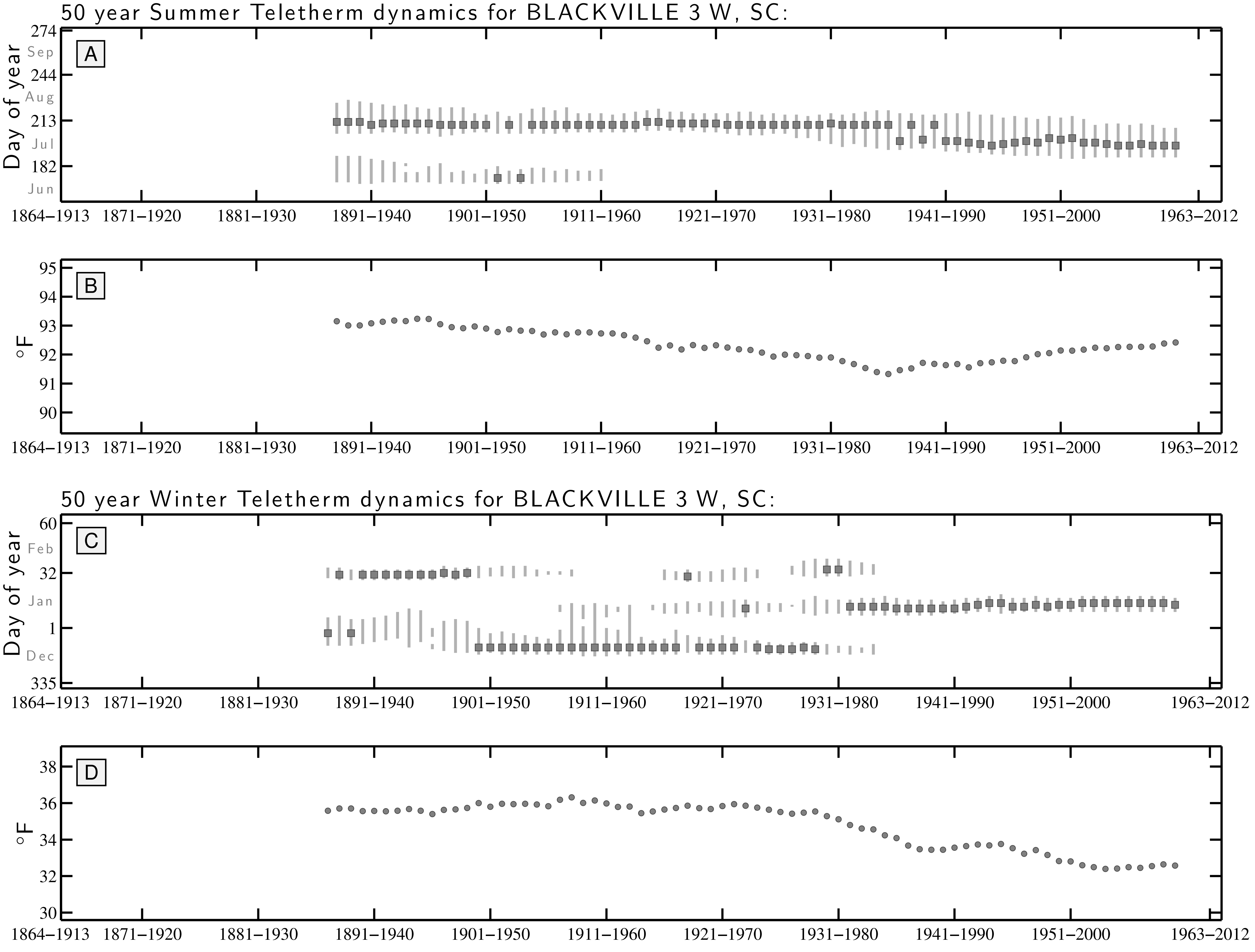

50 year Summer Teletherm dynamics for BLACKVILLE 3 W, SC:
A
Day of year
274
Sep
244
Aug
213
Jul
182
Jun
1864–1913
1871–1920
1881–1930
1891–1940
1901–1950
1911–1960
1921–1970
1931–1980
1941–1990
1951–2000
1963–2012
B
°F
95
94
93
92
91
90
50 year Winter Teletherm dynamics for BLACKVILLE 3 W, SC:
C
Day of year
60
Feb
32
Jan
1
Dec
335
D
°F
38
36
34
32
30

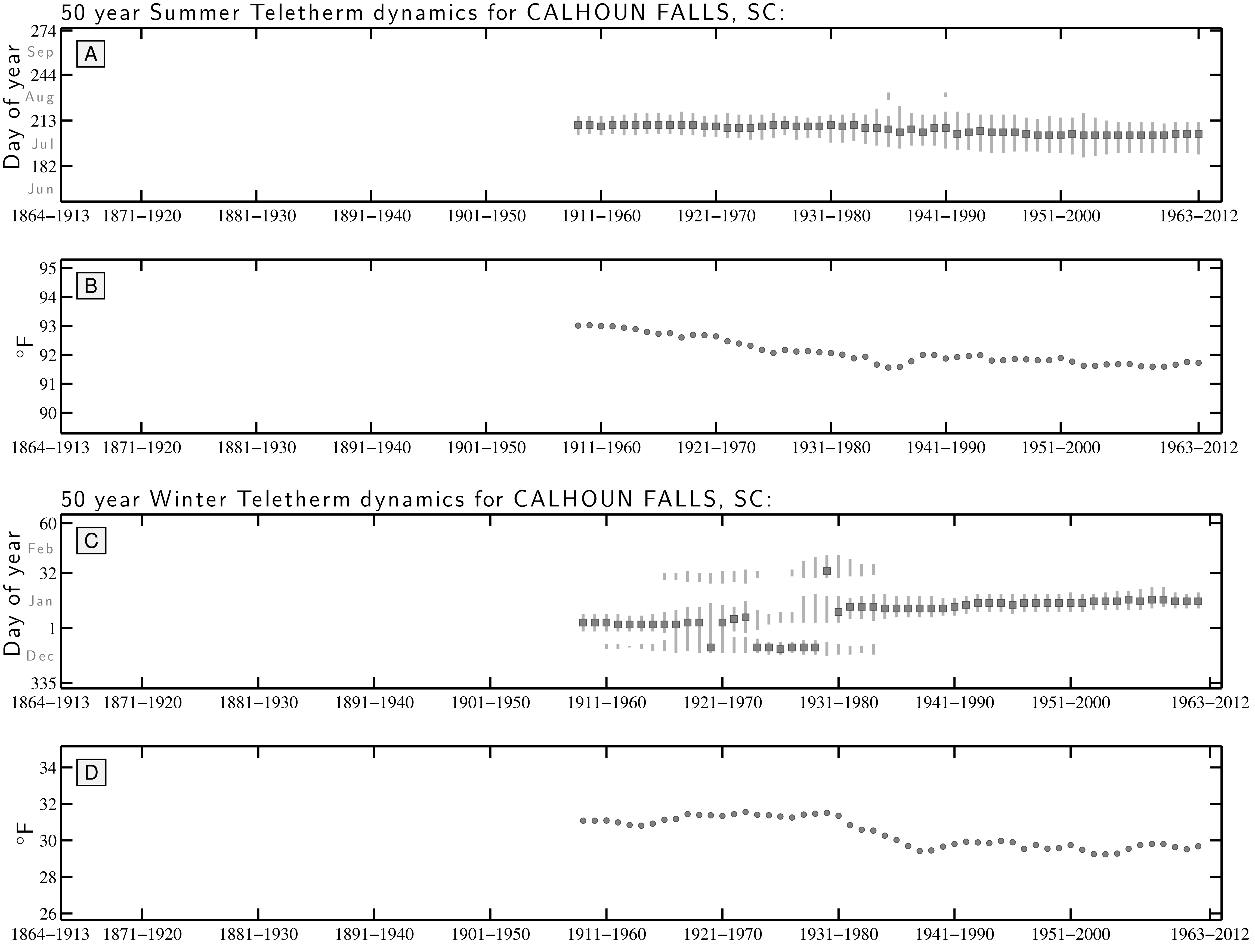

50 year Summer Teletherm dynamics for CALHOUN FALLS, SC:
A
Day of year
274
Sep
244
Aug
213
Jul
182
Jun
B
°F
95
94
93
92
91
90
1864–1913
1871–1920
1881–1930
1891–1940
1901–1950
1911–1960
1921–1970
1931–1980
1941–1990
1951–2000
1963–2012
50 year Winter Teletherm dynamics for CALHOUN FALLS, SC:
C
Day of year
60
Feb
32
Jan
1
Dec
335
D
°F
34
32
30
28
26

50 year Summer Teletherm dynamics for CAMDEN 3 W, SC:

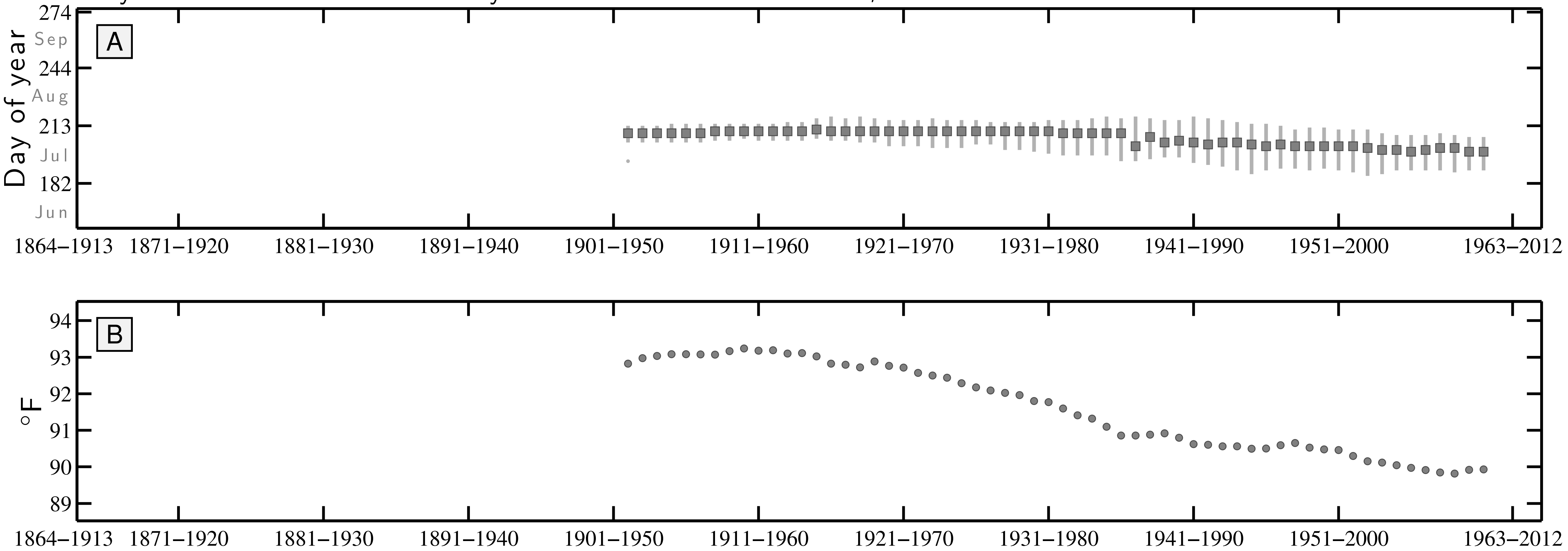

50 year Winter Teletherm dynamics for CAMDEN 3 W, SC:

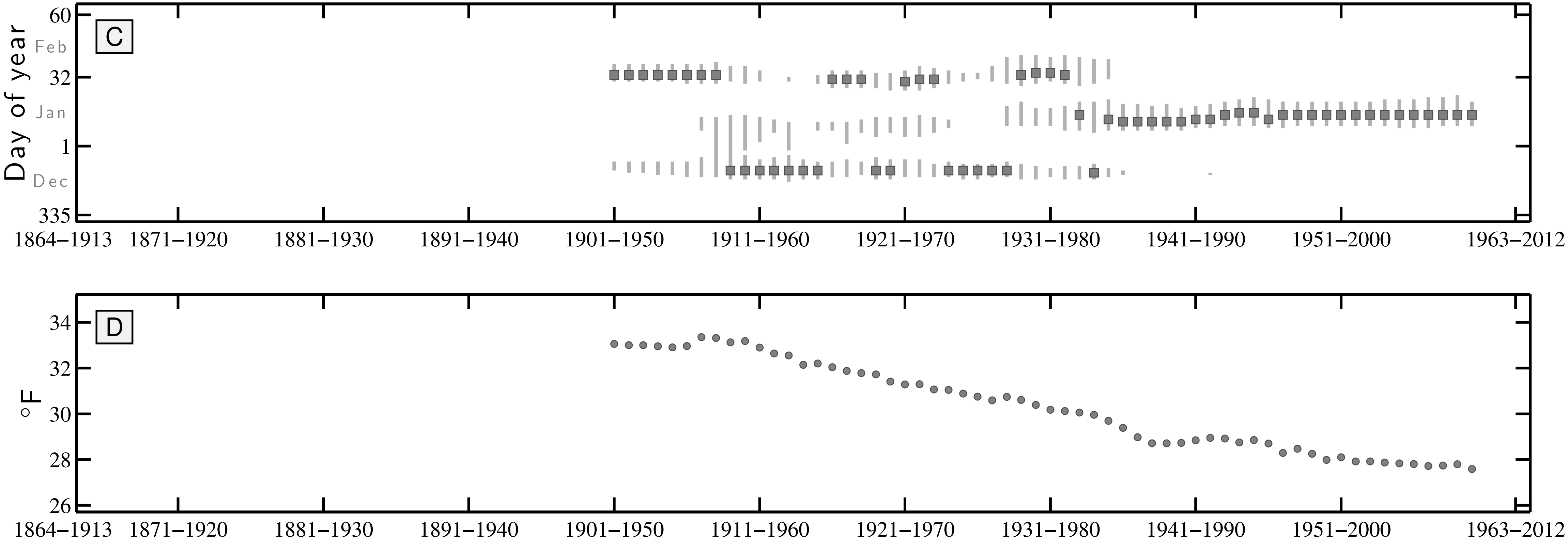

50 year Summer Teletherm dynamics for CHARLESTON CITY, SC:

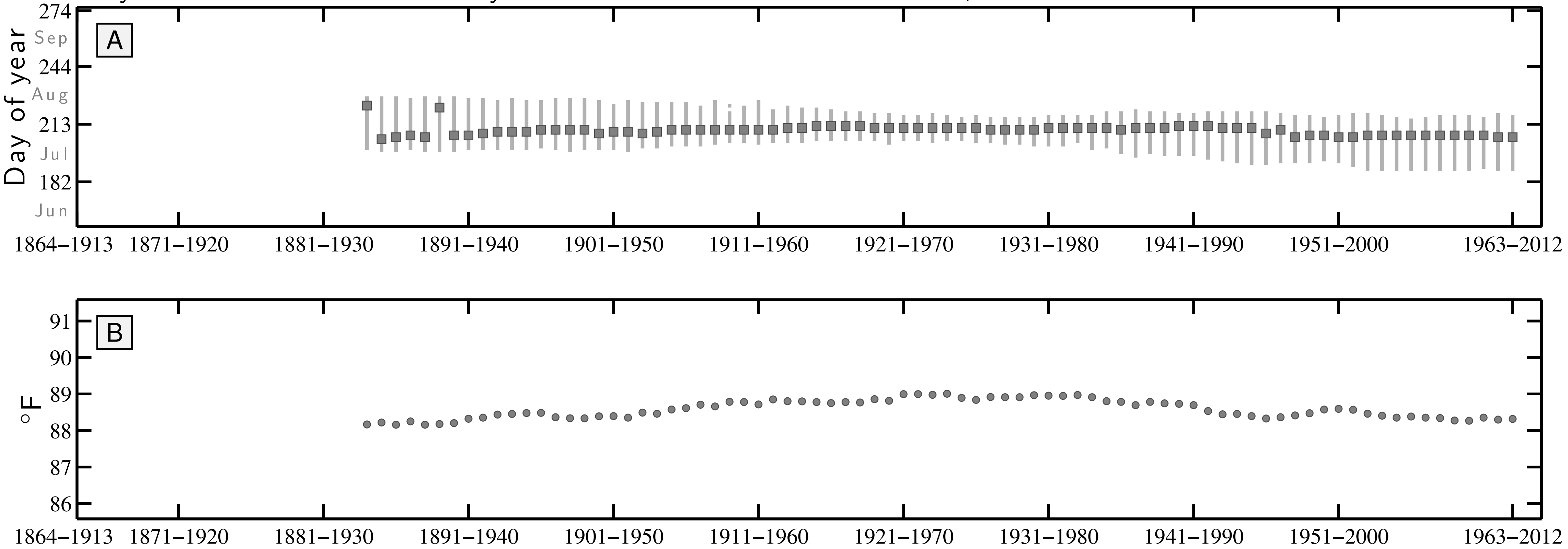

50 year Winter Teletherm dynamics for CHARLESTON CITY, SC:

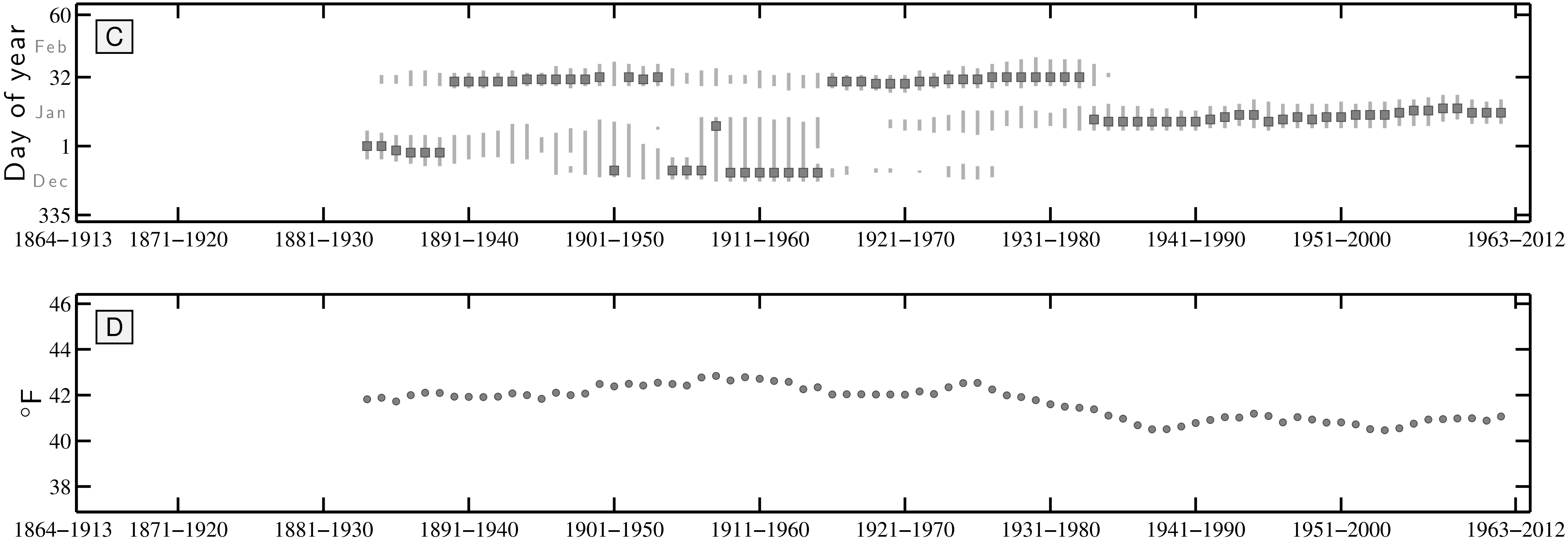

50 year Summer Teletherm dynamics for CHERAW, SC:

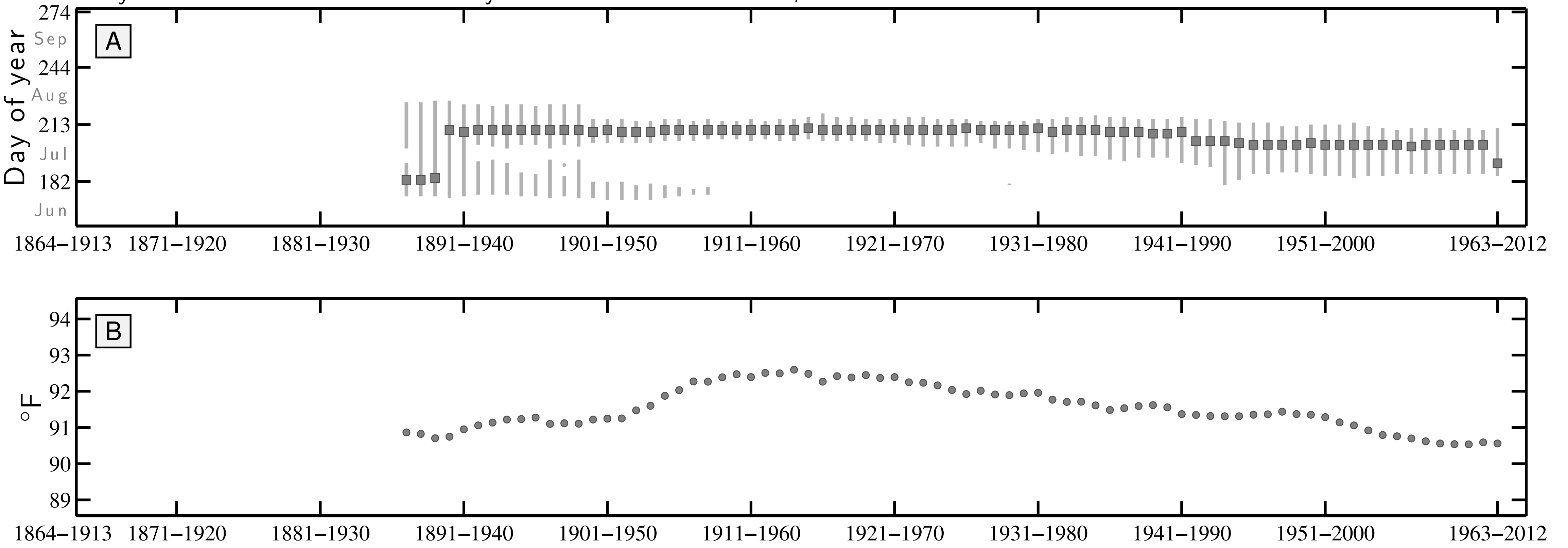

50 year Winter Teletherm dynamics for CHERAW, SC:

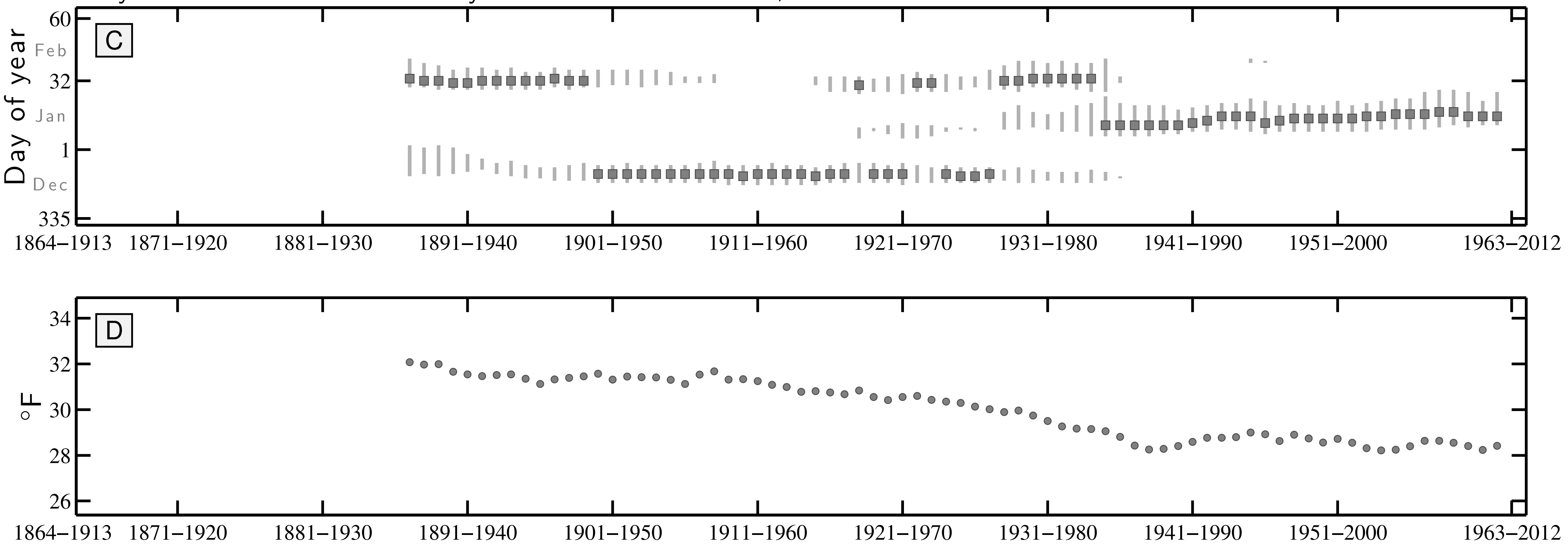

50 year Summer Teletherm dynamics for CLEMSON UNIV, SC:

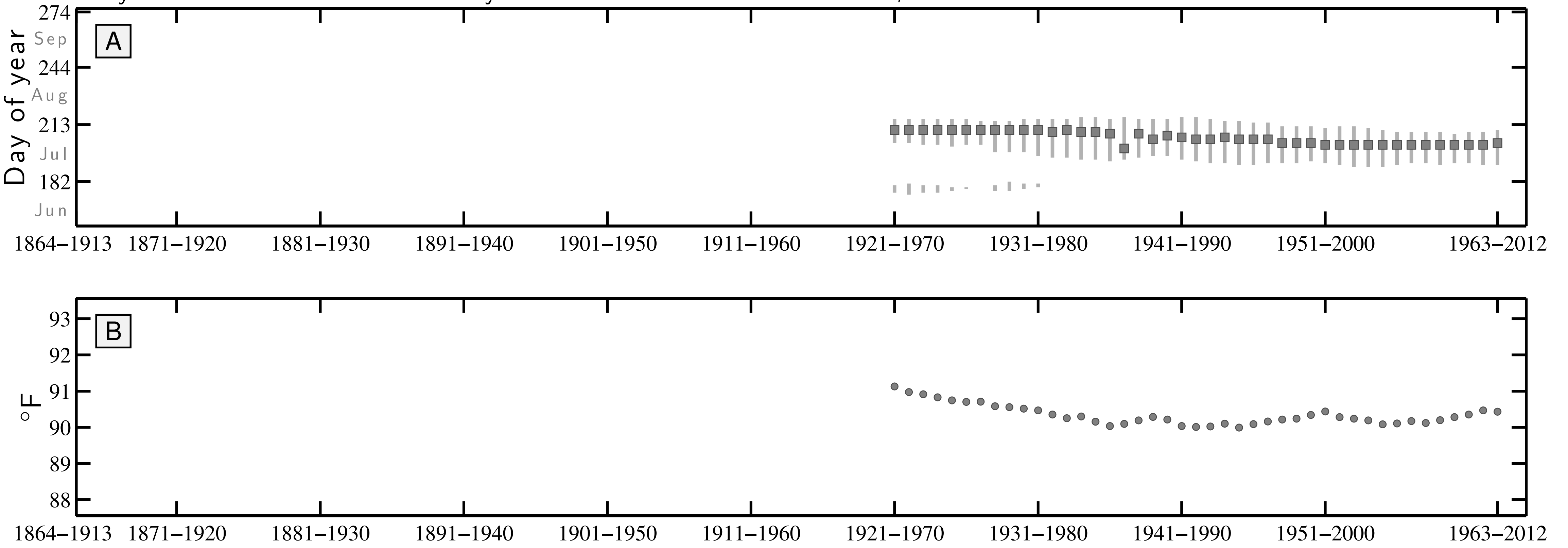

50 year Winter Teletherm dynamics for CLEMSON UNIV, SC:

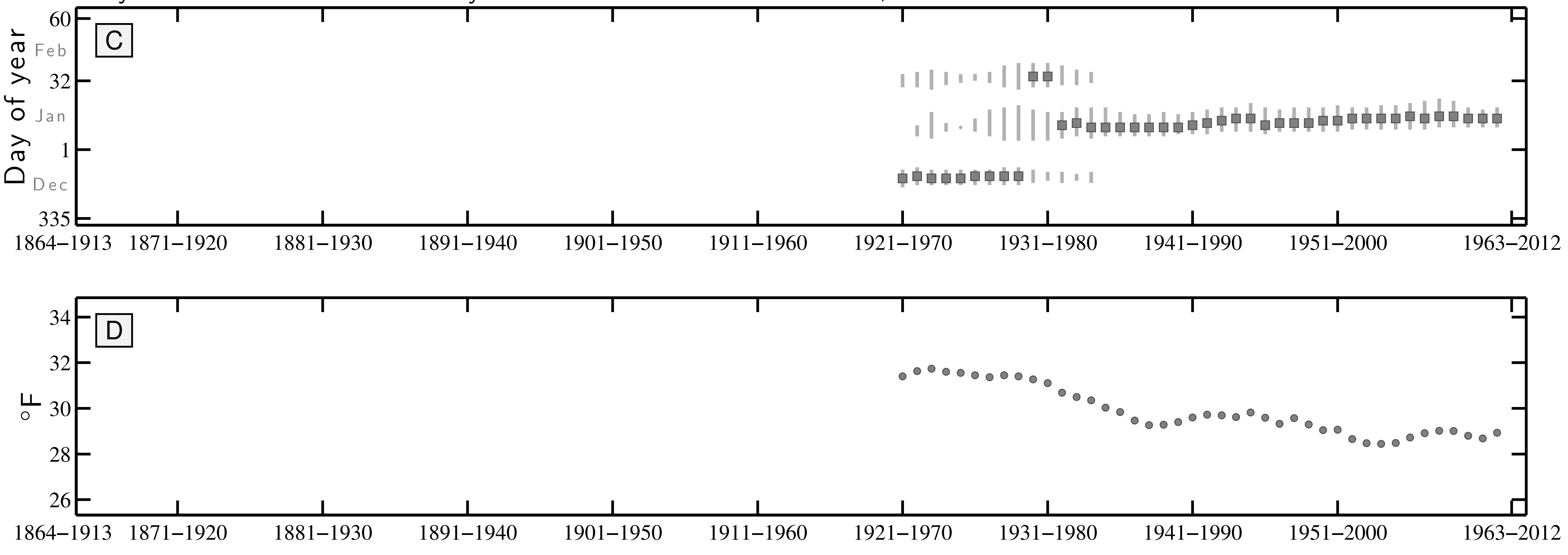

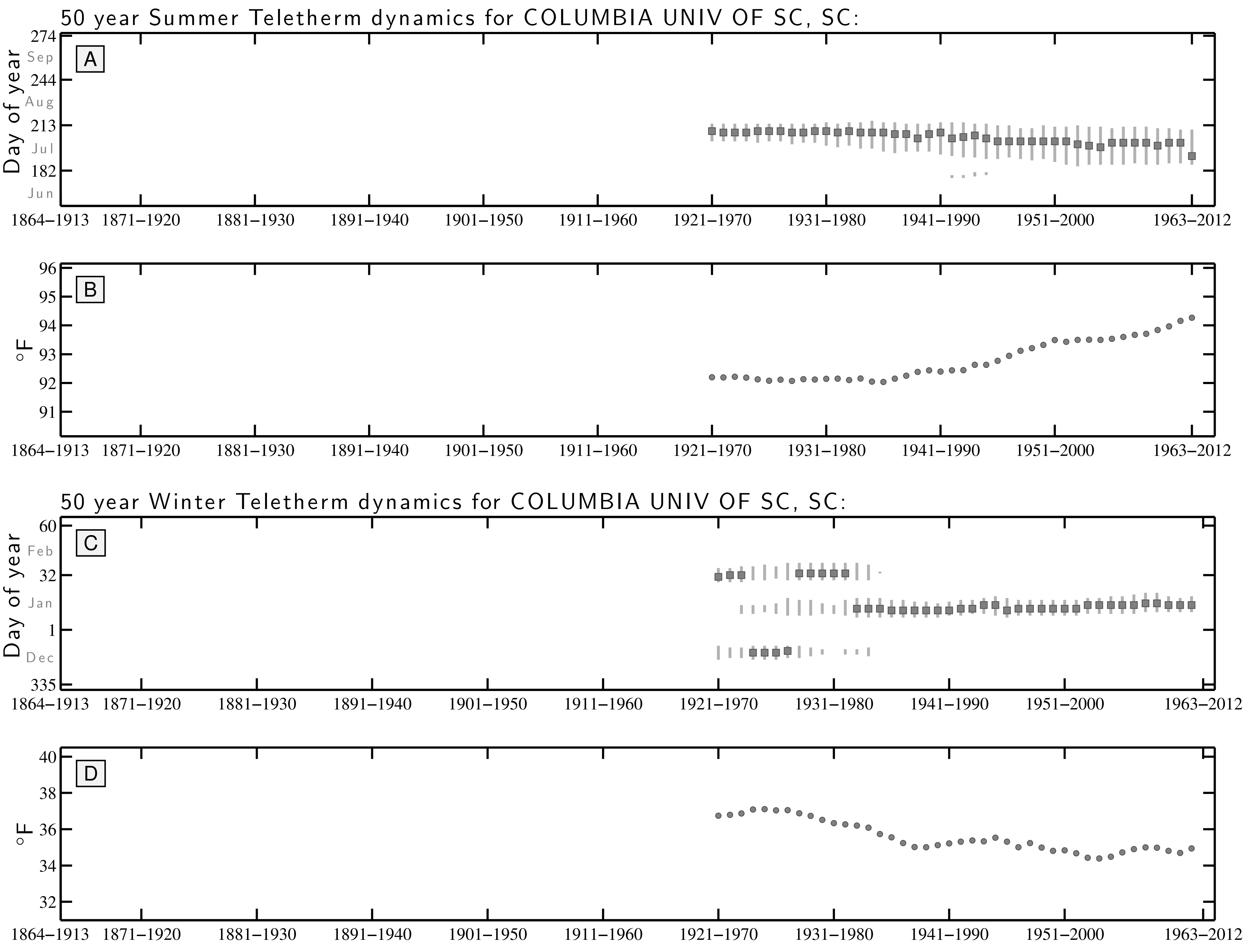

50 year Summer Teletherm dynamics for COLUMBIA UNIV OF SC, SC:
A
Day of year
274
Sep
244
Aug
213
Jul
182
Jun
1864–1913
1871–1920
1881–1930
1891–1940
1901–1950
1911–1960
1921–1970
1931–1980
1941–1990
1951–2000
1963–2012
B
°F
96
95
94
93
92
91
50 year Winter Teletherm dynamics for COLUMBIA UNIV OF SC, SC:
C
Day of year
60
Feb
32
Jan
1
Dec
335
D
°F
40
38
36
34
32

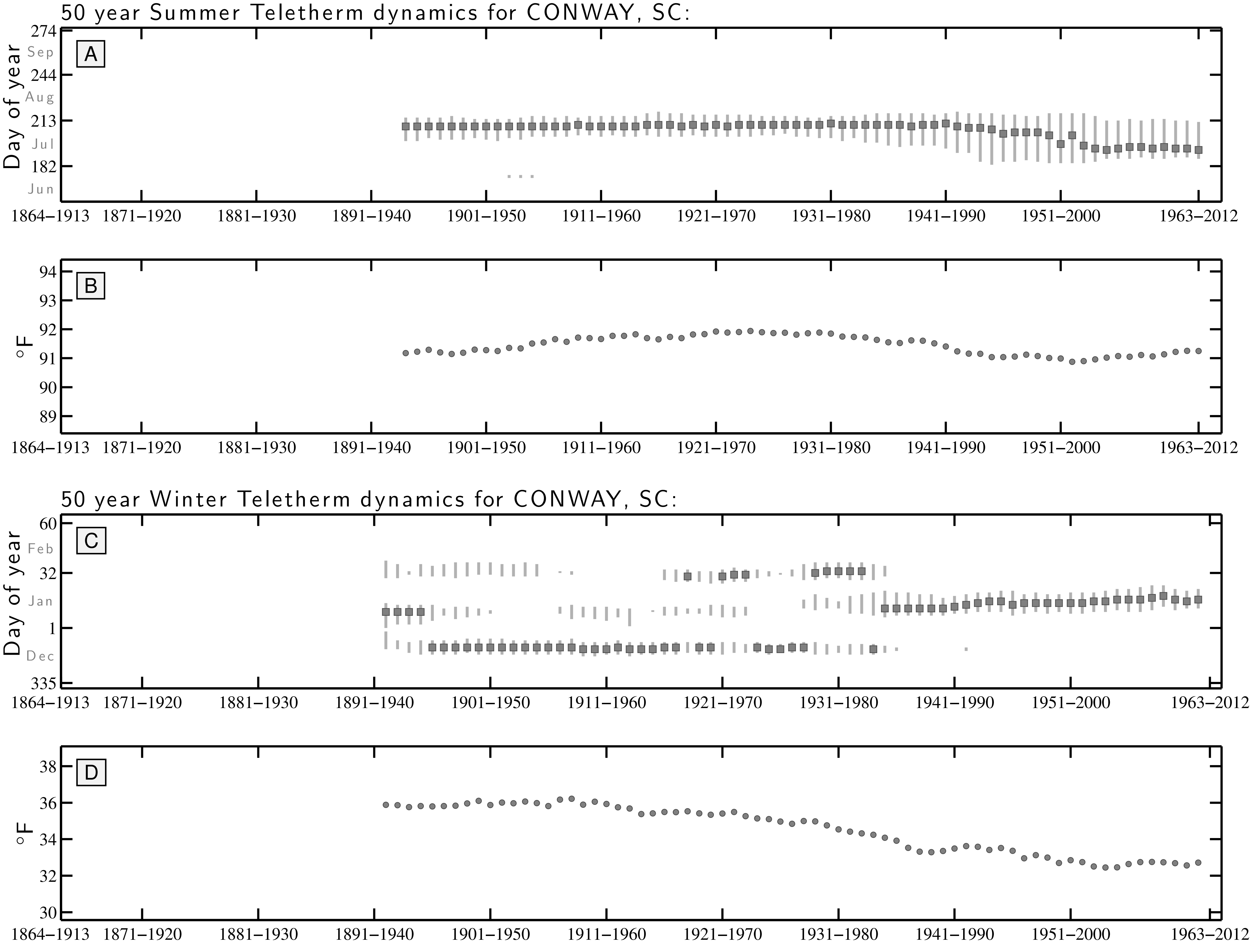
50 year Summer Teletherm dynamics for CONWAY, SC:
A
B
Day of year
°F
274
244
213
182
Sep
Aug
Jul
Jun
94
93
92
91
90
89
1864–1913
1871–1920
1881–1930
1891–1940
1901–1950
1911–1960
1921–1970
1931–1980
1941–1990
1951–2000
1963–2012
50 year Winter Teletherm dynamics for CONWAY, SC:
C
D
60
32
1
335
Feb
Jan
Dec
38
36
34
32
30

50 year Summer Teletherm dynamics for DARLINGTON, SC:

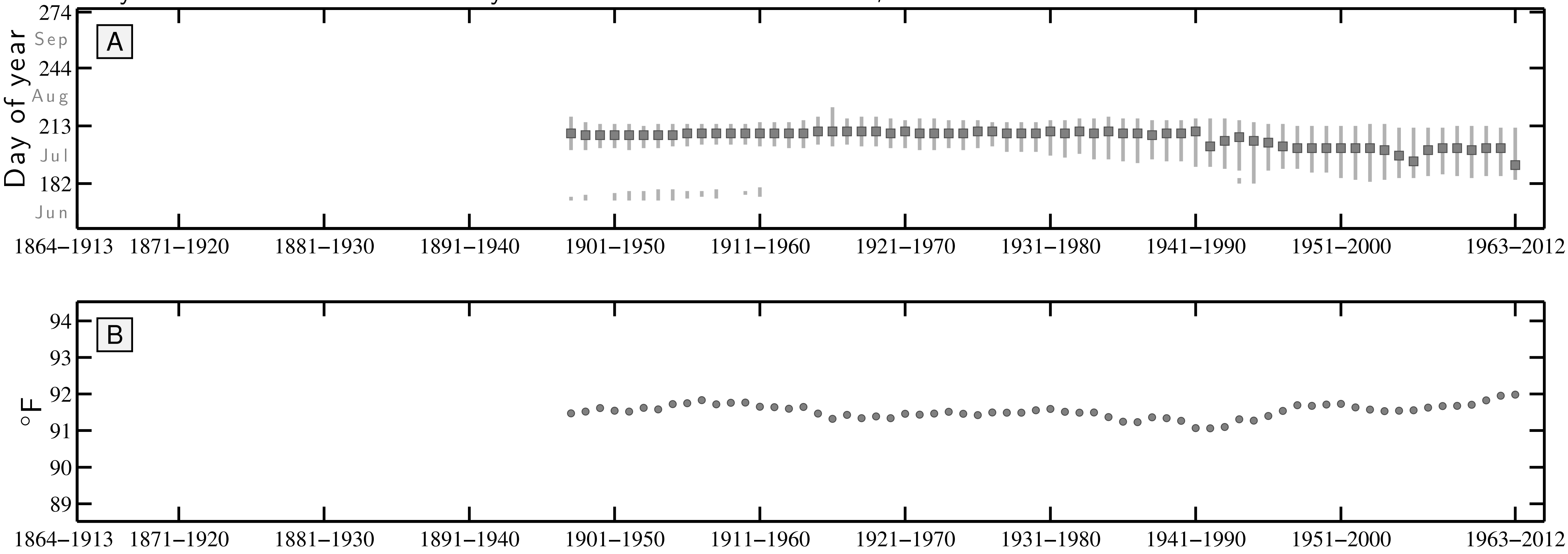

50 year Winter Teletherm dynamics for DARLINGTON, SC:

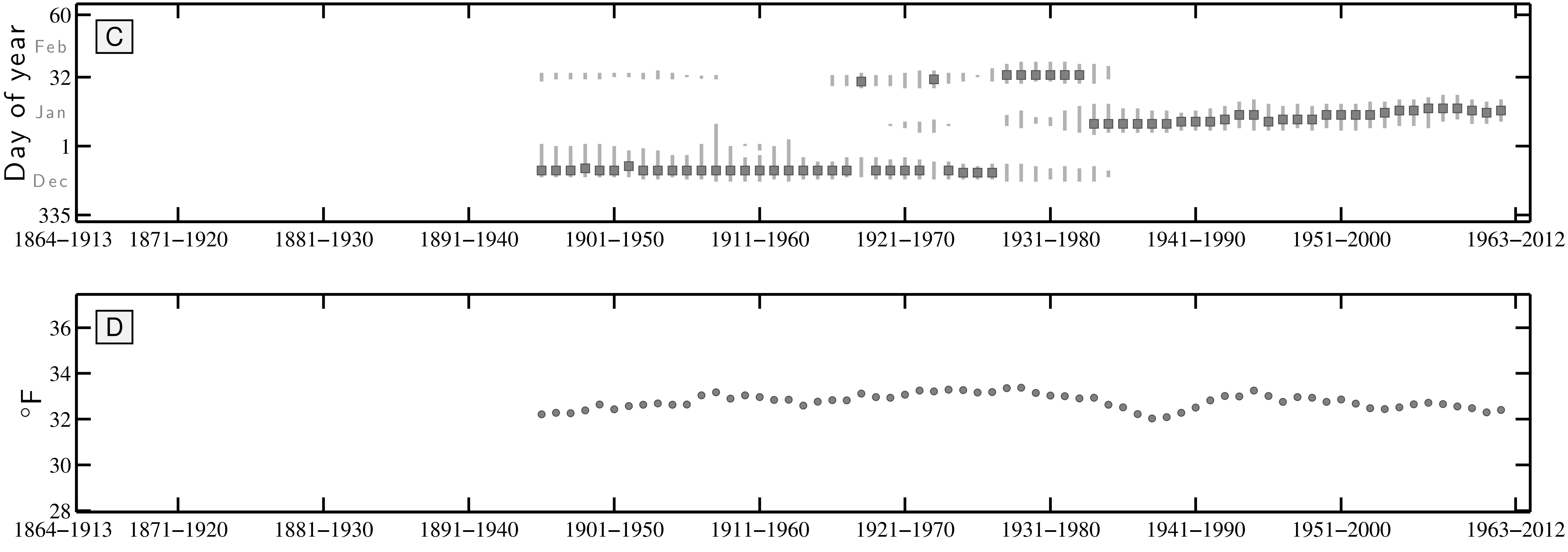

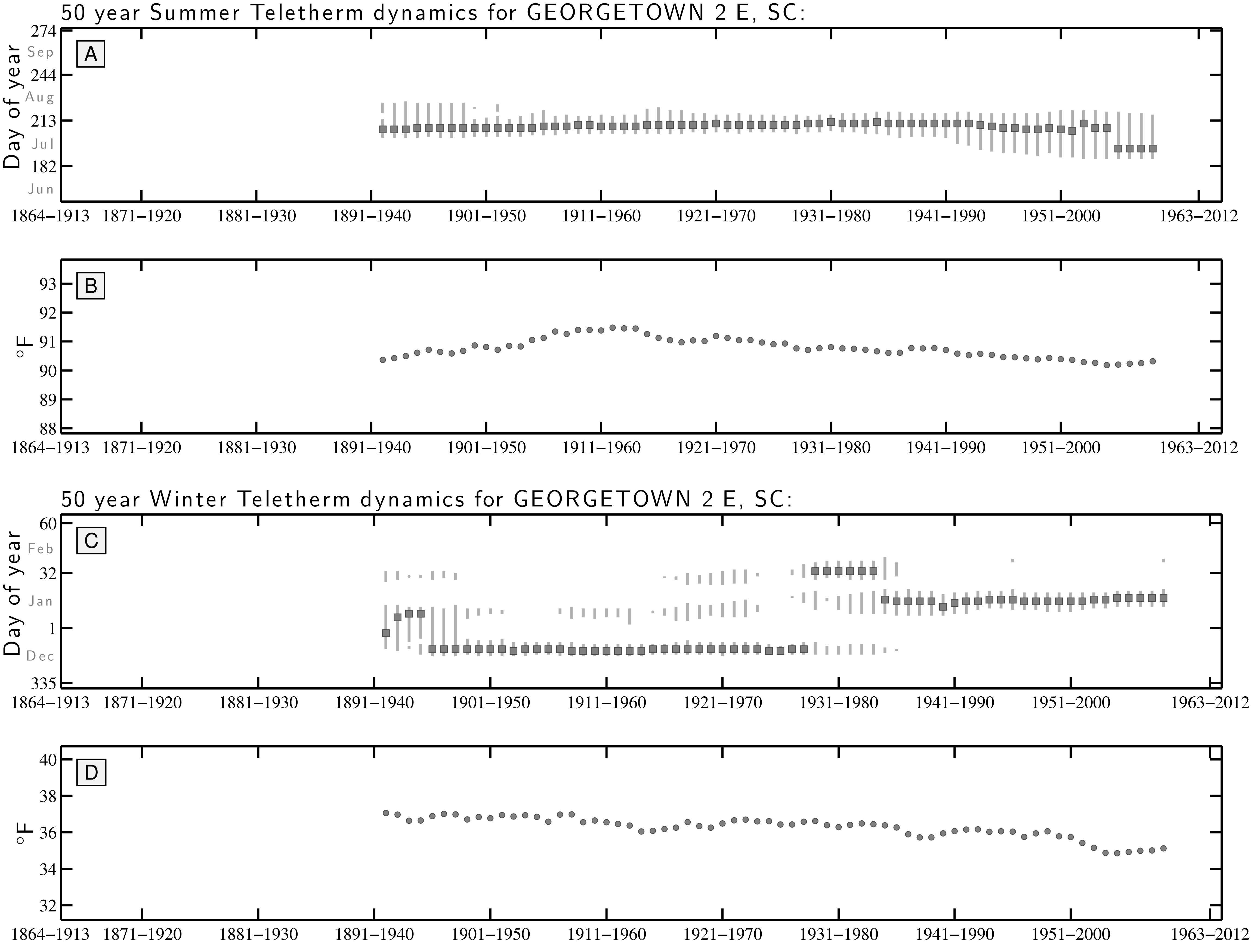
50 year Summer Teletherm dynamics for GEORGETOWN 2 E, SC:
A
Day of year
274
Sep
244
Aug
213
Jul
182
Jun
1864–1913
1871–1920
1881–1930
1891–1940
1901–1950
1911–1960
1921–1970
1931–1980
1941–1990
1951–2000
1963–2012
B
°F
93
92
91
90
89
88
50 year Winter Teletherm dynamics for GEORGETOWN 2 E, SC:
C
Day of year
60
Feb
32
Jan
1
Dec
335
D
°F
40
38
36
34
32

50 year Summer Teletherm dynamics for GRNVL SPART INTL AP, SC:

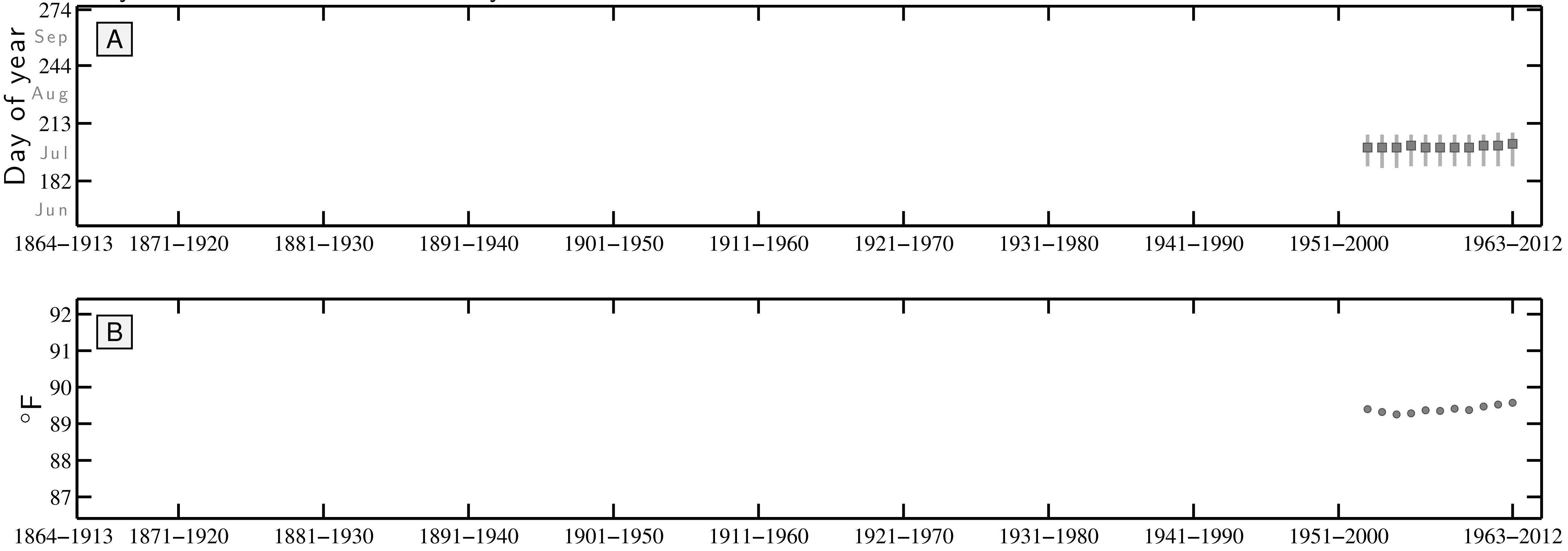

50 year Winter Teletherm dynamics for GRNVL SPART INTL AP, SC:

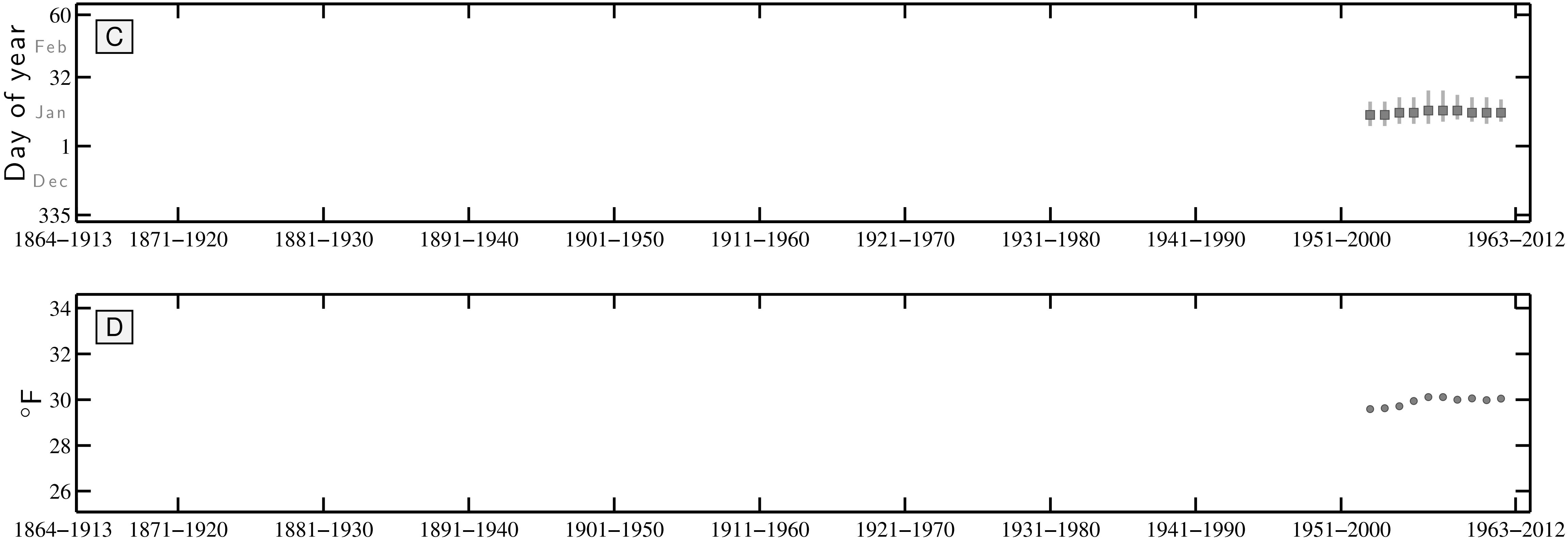

50 year Summer Teletherm dynamics for GREENWOOD, SC:

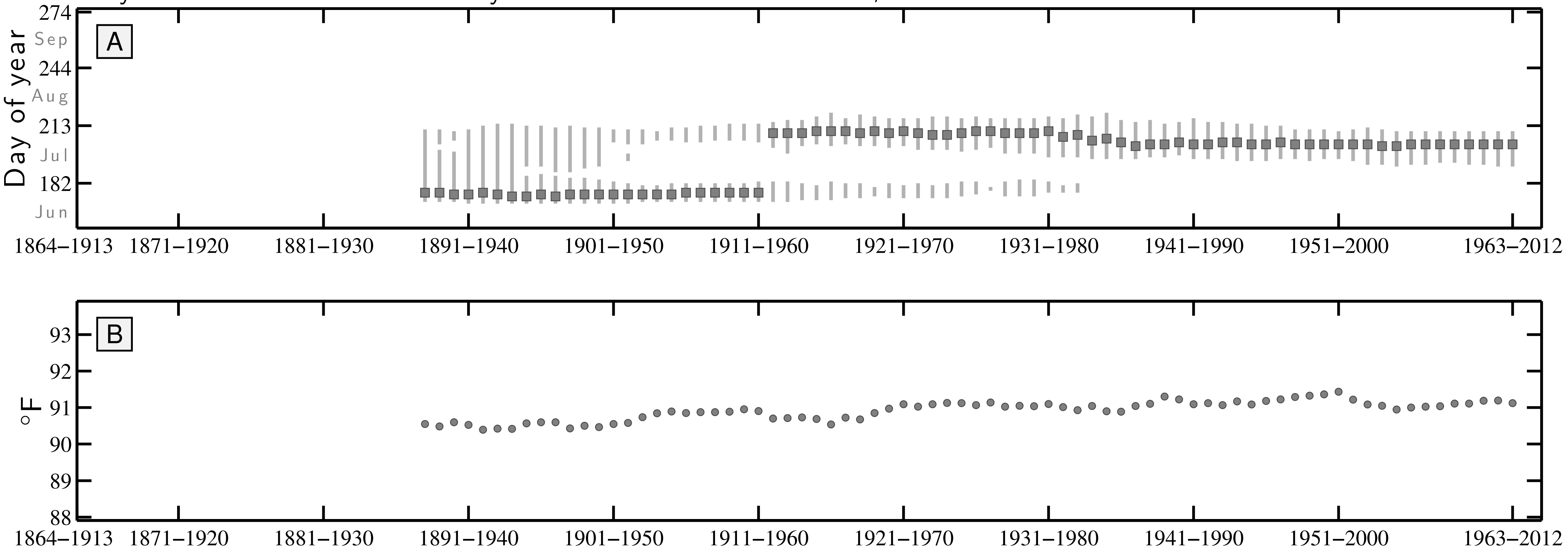

50 year Winter Teletherm dynamics for GREENWOOD, SC:

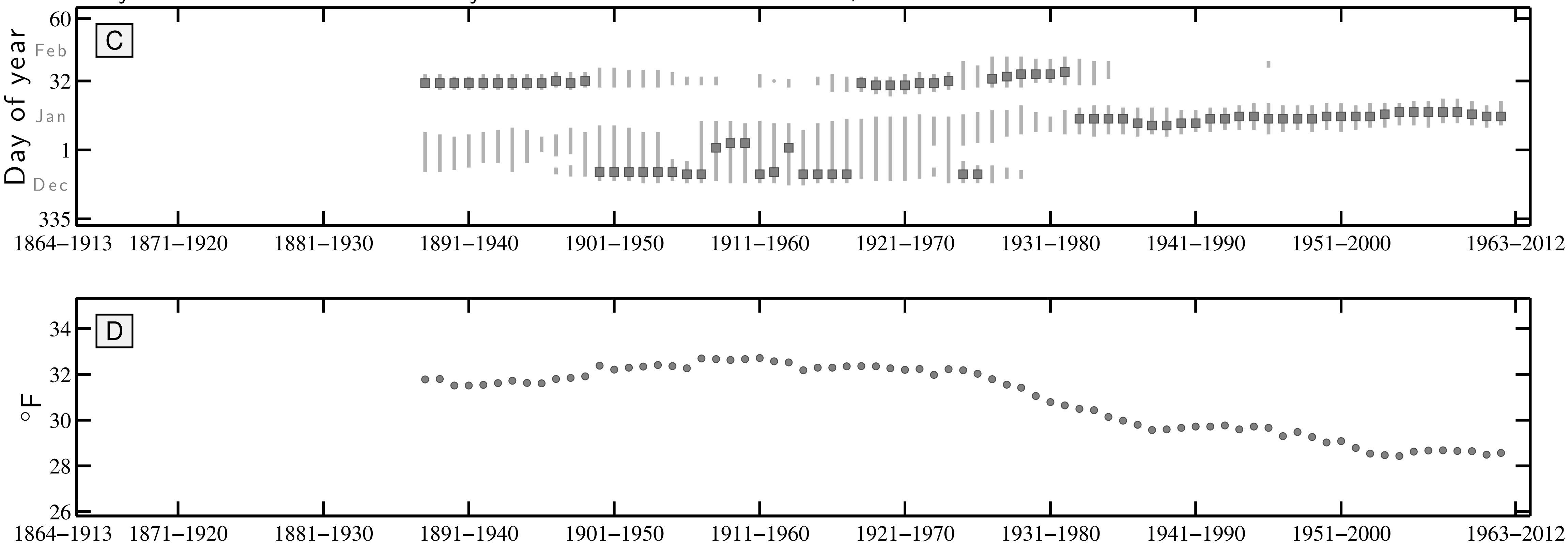

50 year Summer Teletherm dynamics for KERSHAW 1SW, SC:

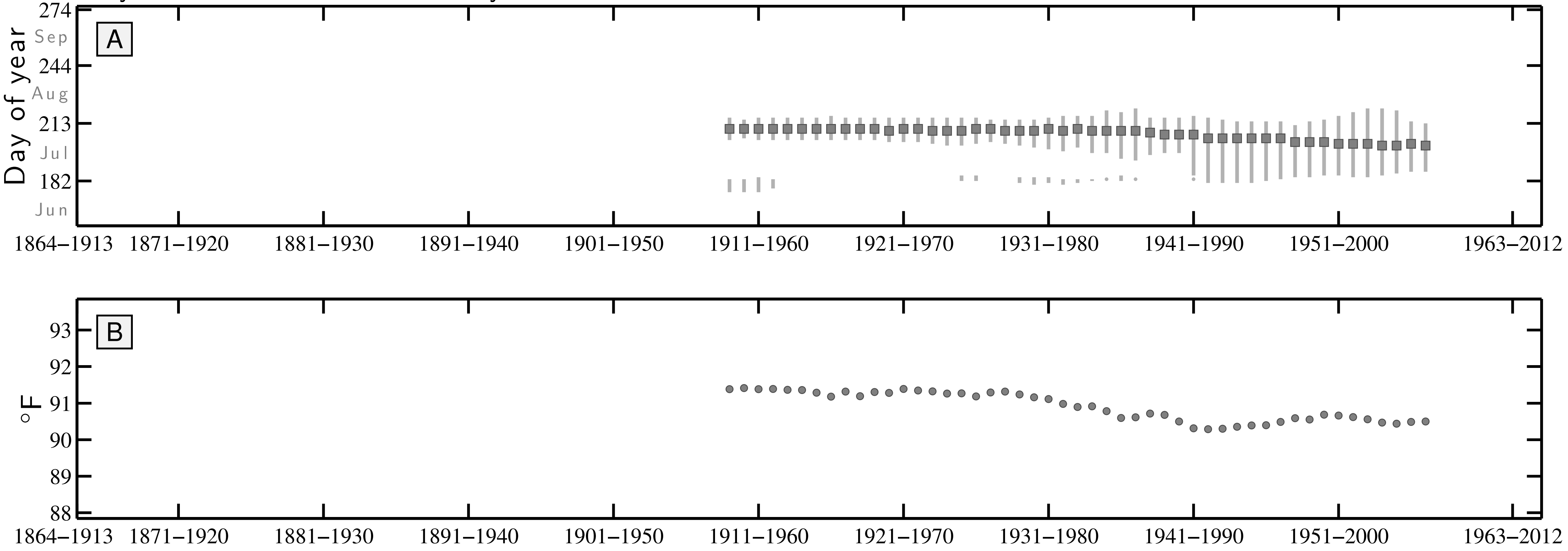

50 year Winter Teletherm dynamics for KERSHAW 1SW, SC:

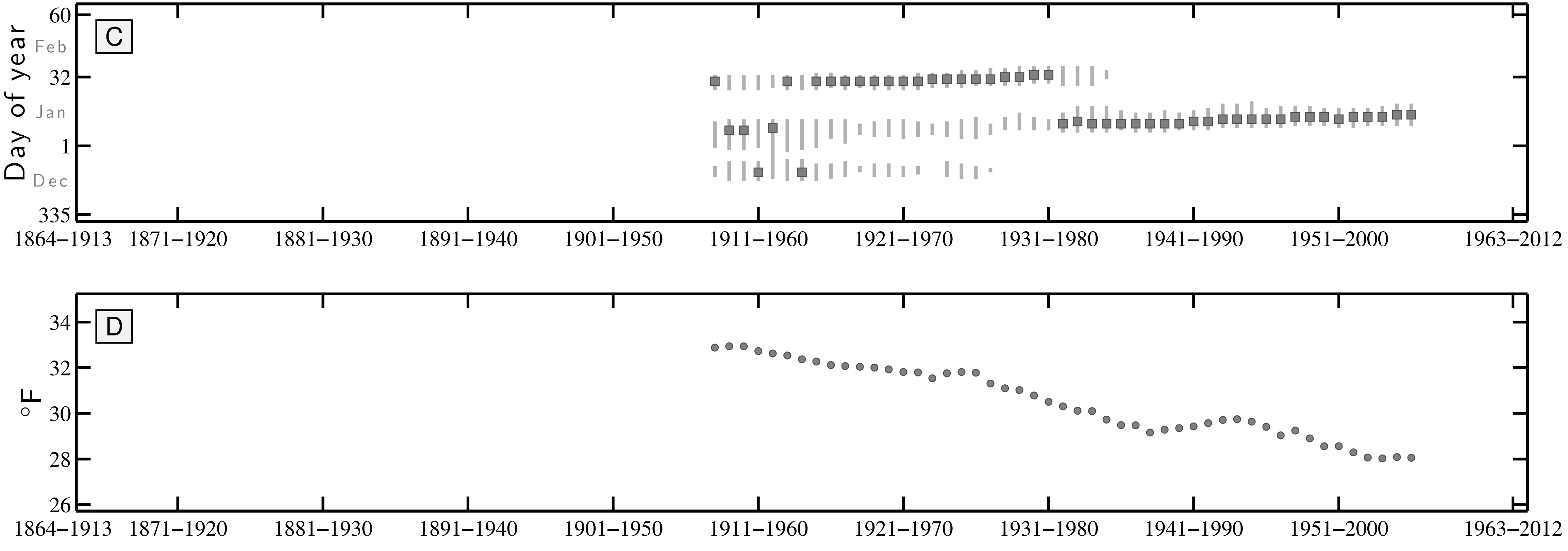

50 year Summer Teletherm dynamics for KINGSTREE, SC:

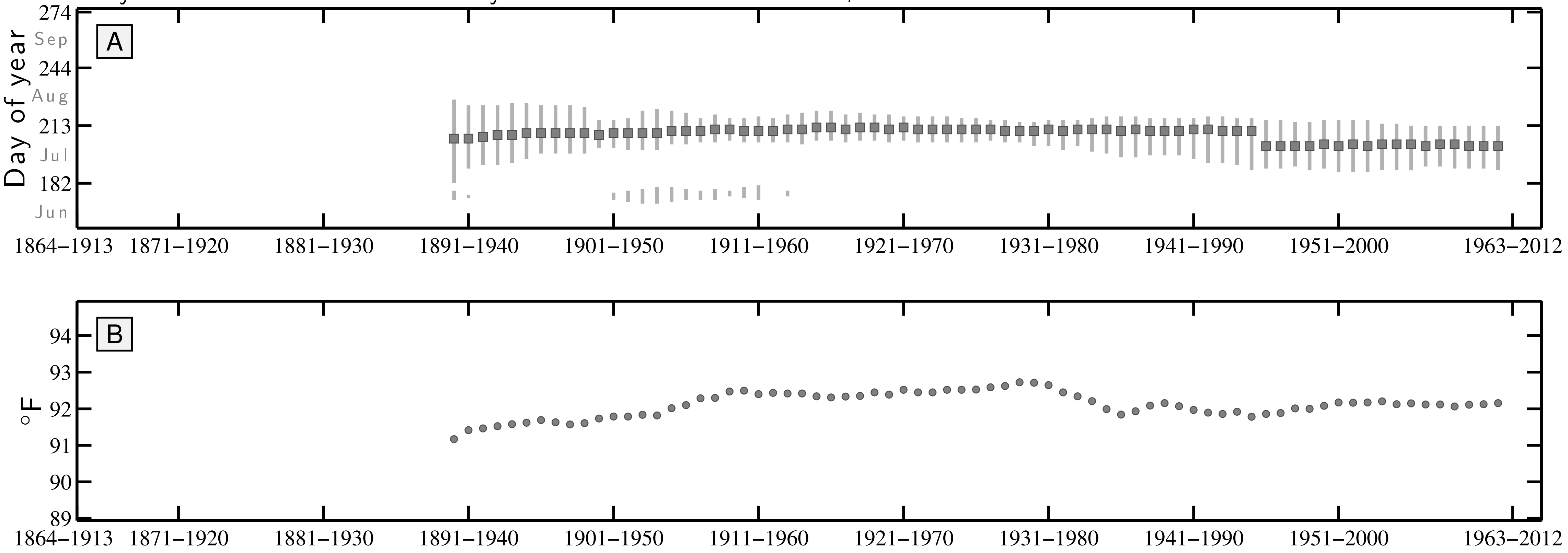

50 year Winter Teletherm dynamics for KINGSTREE, SC:

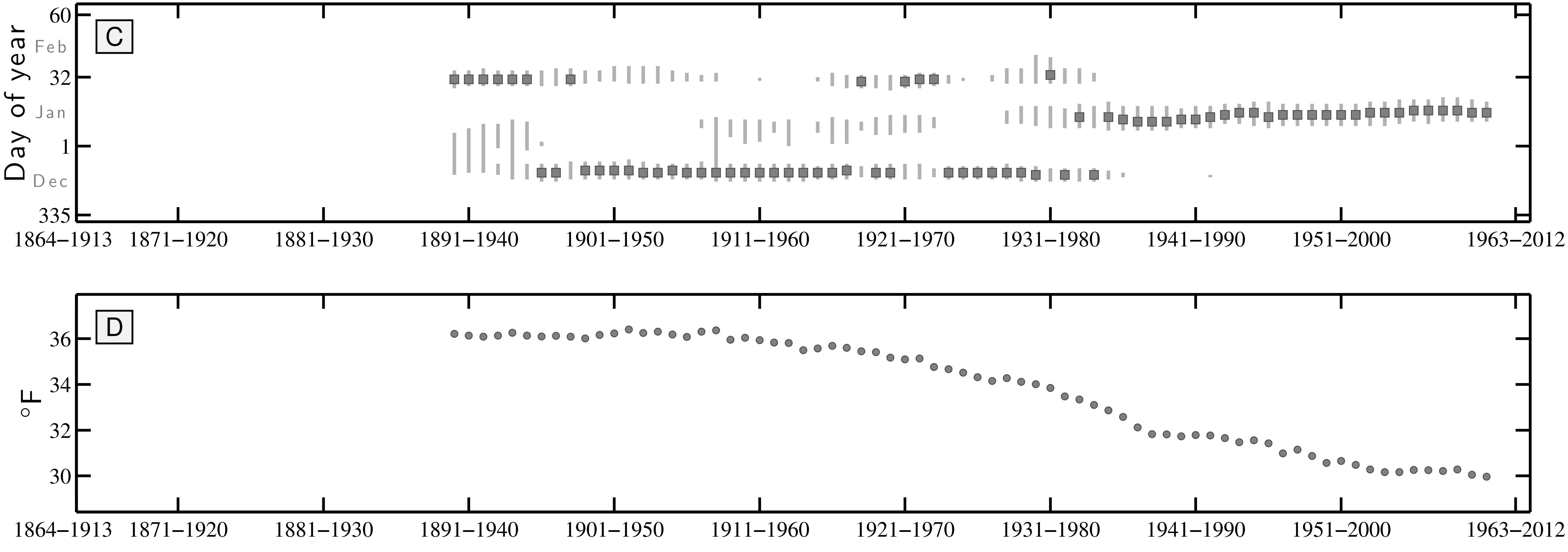

50 year Summer Teletherm dynamics for LAURENS, SC:

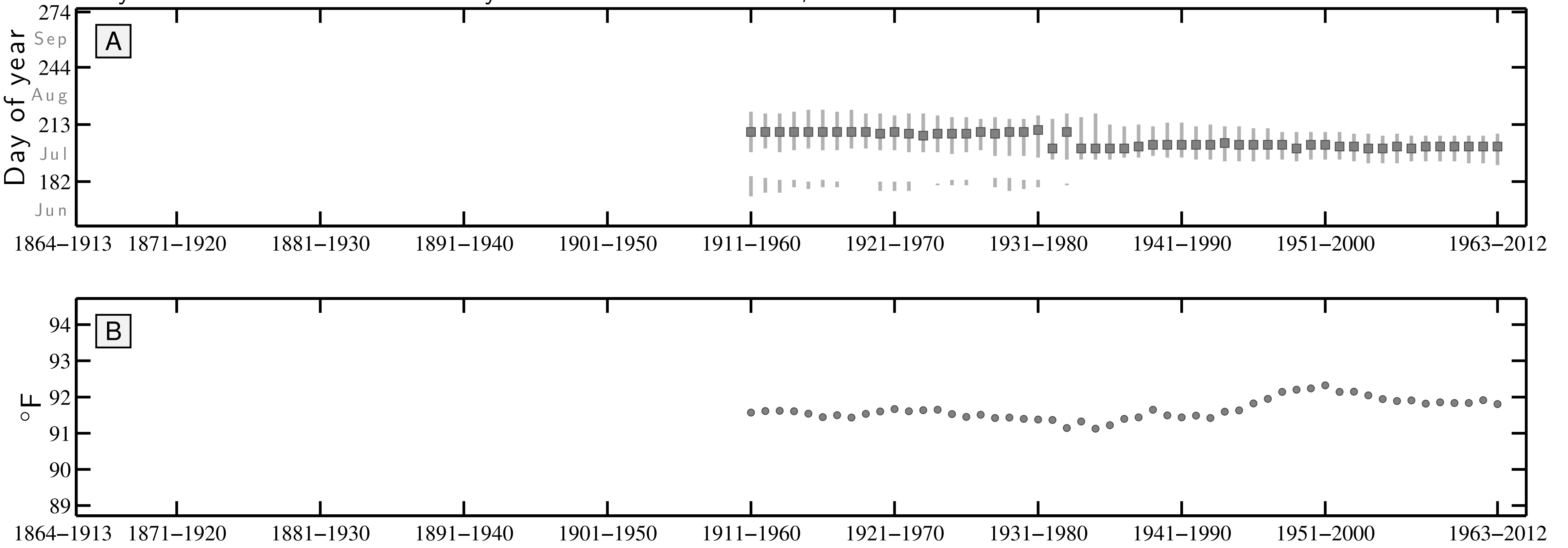

50 year Winter Teletherm dynamics for LAURENS, SC:

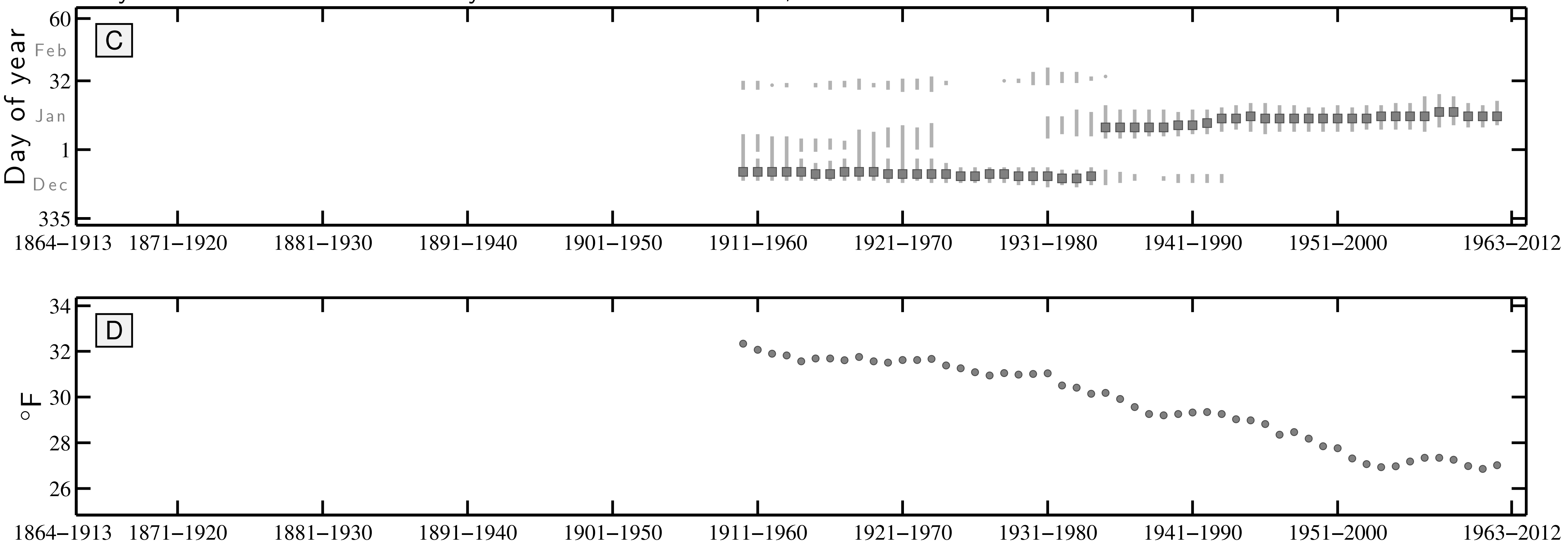

50 year Summer Teletherm dynamics for LITTLE MTN, SC:

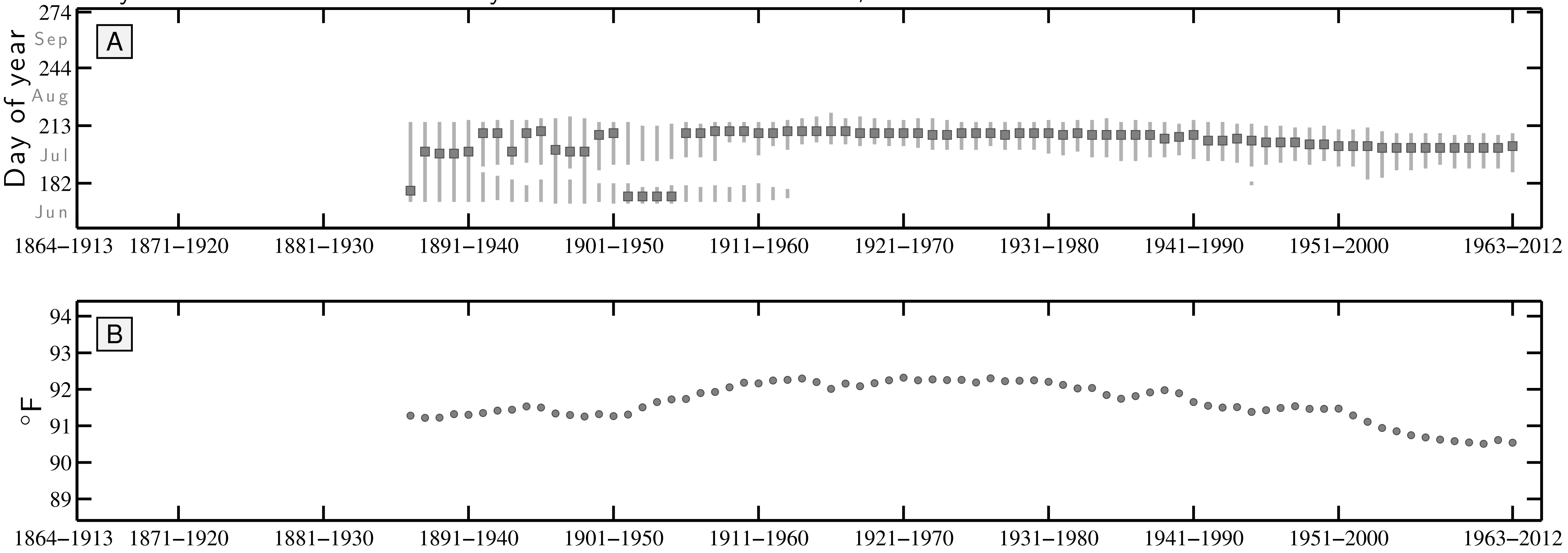

50 year Winter Teletherm dynamics for LITTLE MTN, SC:

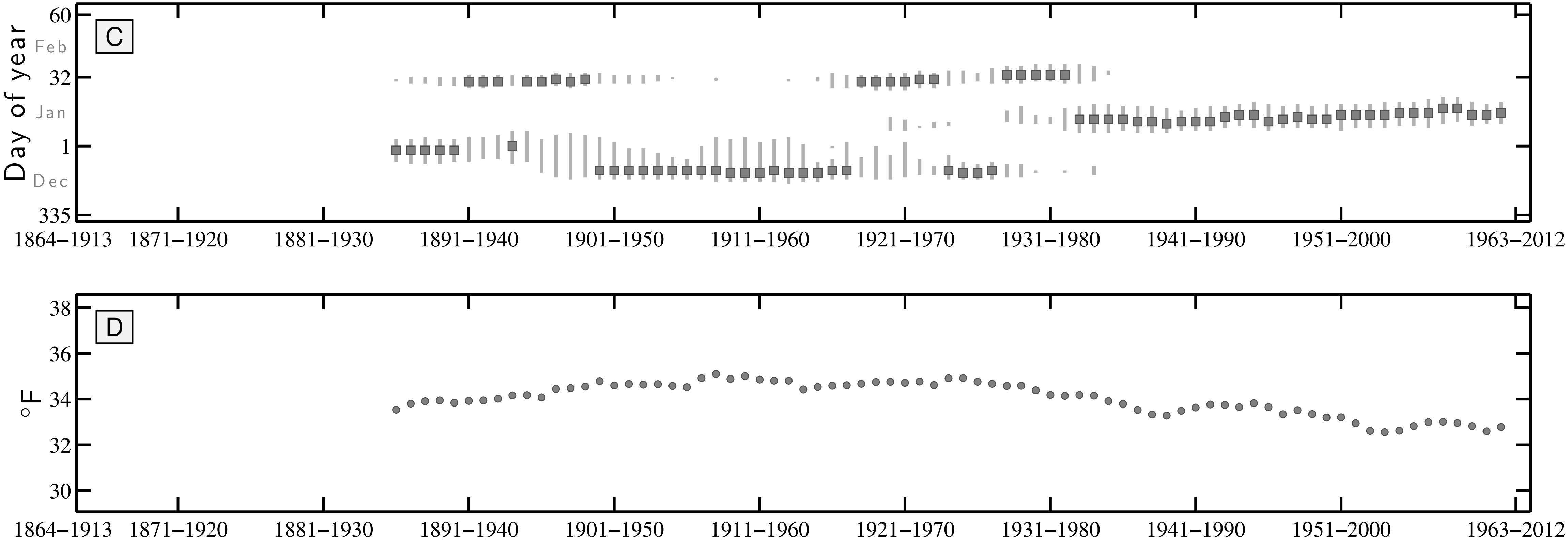

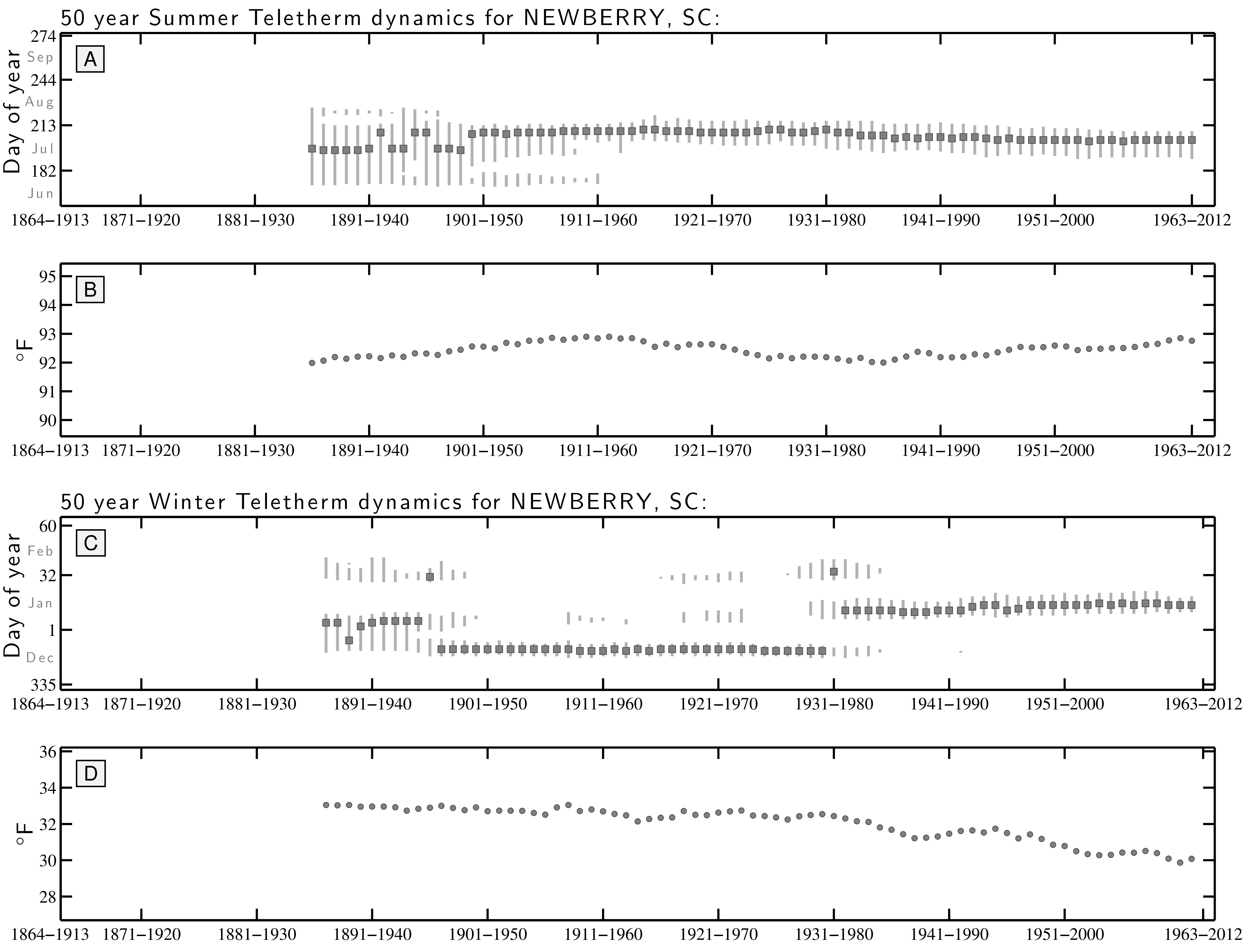
50 year Summer Teletherm dynamics for NEWBERRY, SC:
A
Day of year
274
Sep
244
Aug
213
Jul
182
Jun
1864–1913
1871–1920
1881–1930
1891–1940
1901–1950
1911–1960
1921–1970
1931–1980
1941–1990
1951–2000
1963–2012
B
°F
95
94
93
92
91
90
50 year Winter Teletherm dynamics for NEWBERRY, SC:
C
Day of year
60
Feb
32
Jan
1
Dec
335
D
°F
36
34
32
30
28

50 year Summer Teletherm dynamics for ORANGEBURG 2, SC:

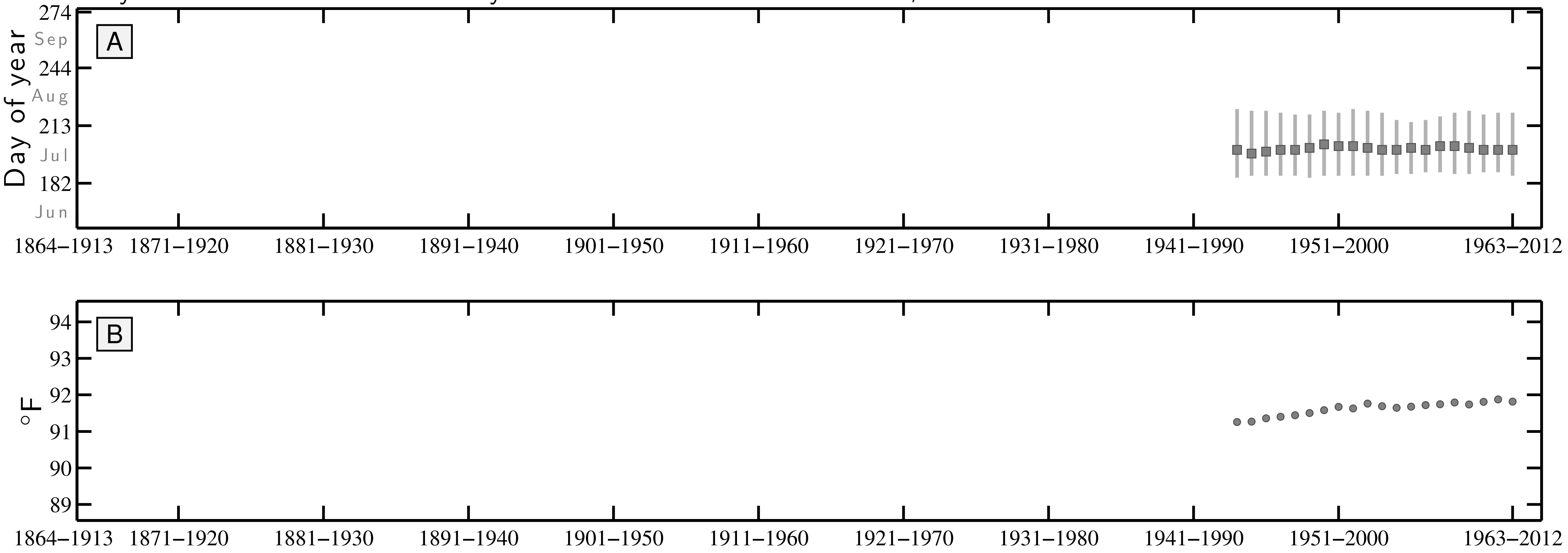

50 year Winter Teletherm dynamics for ORANGEBURG 2, SC:

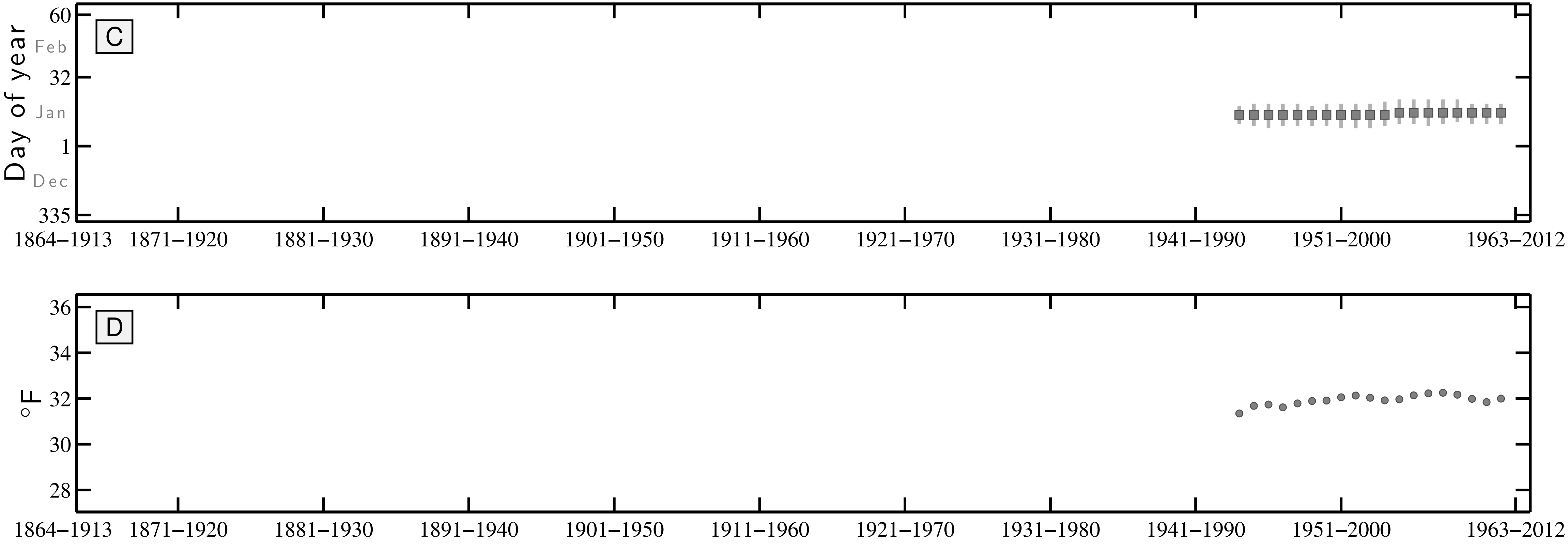

50 year Summer Teletherm dynamics for SALUDA, SC:

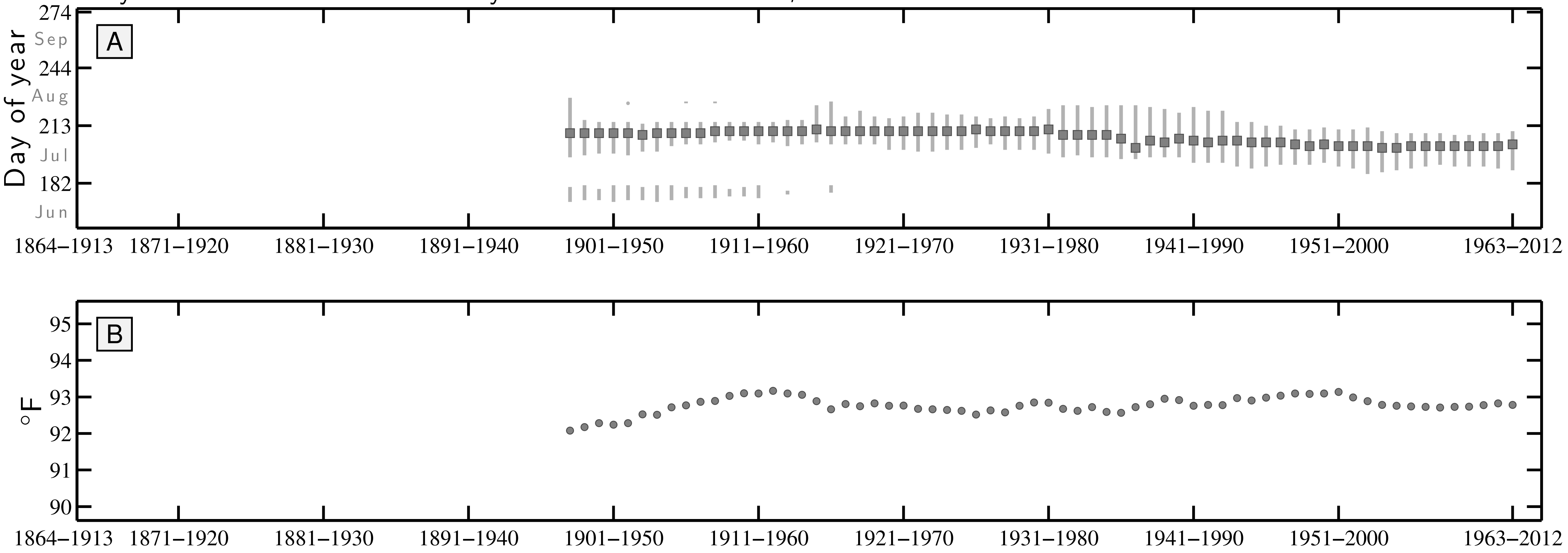

50 year Winter Teletherm dynamics for SALUDA, SC:

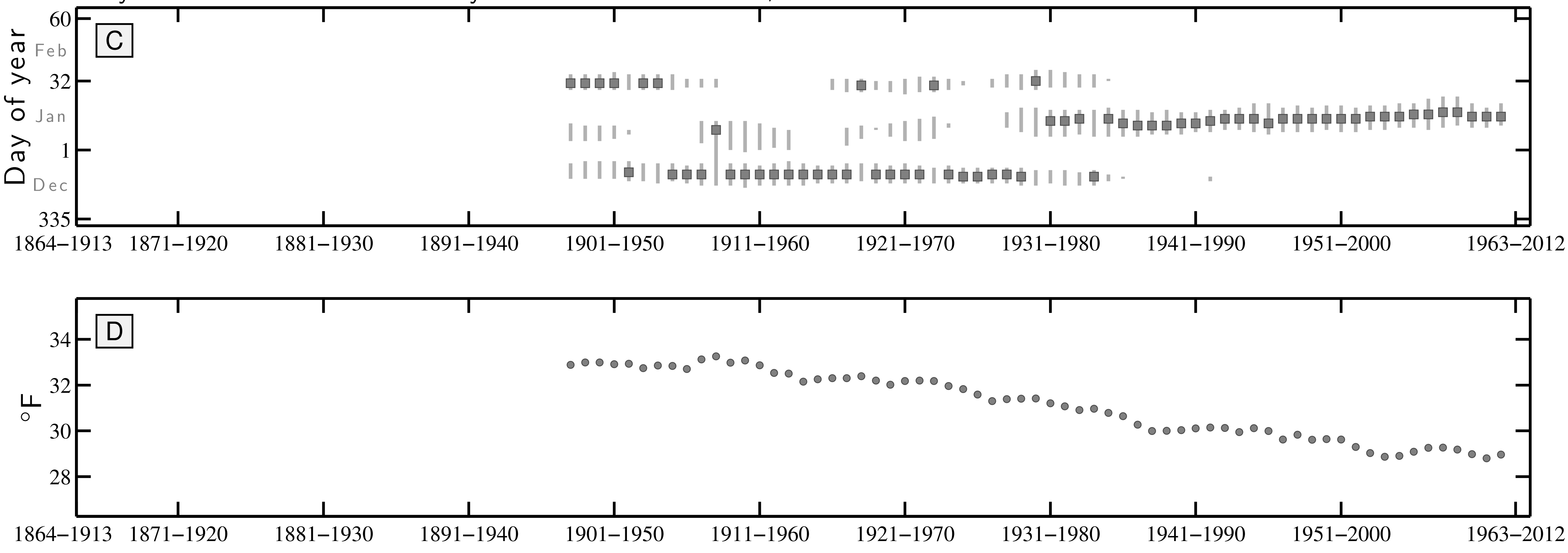

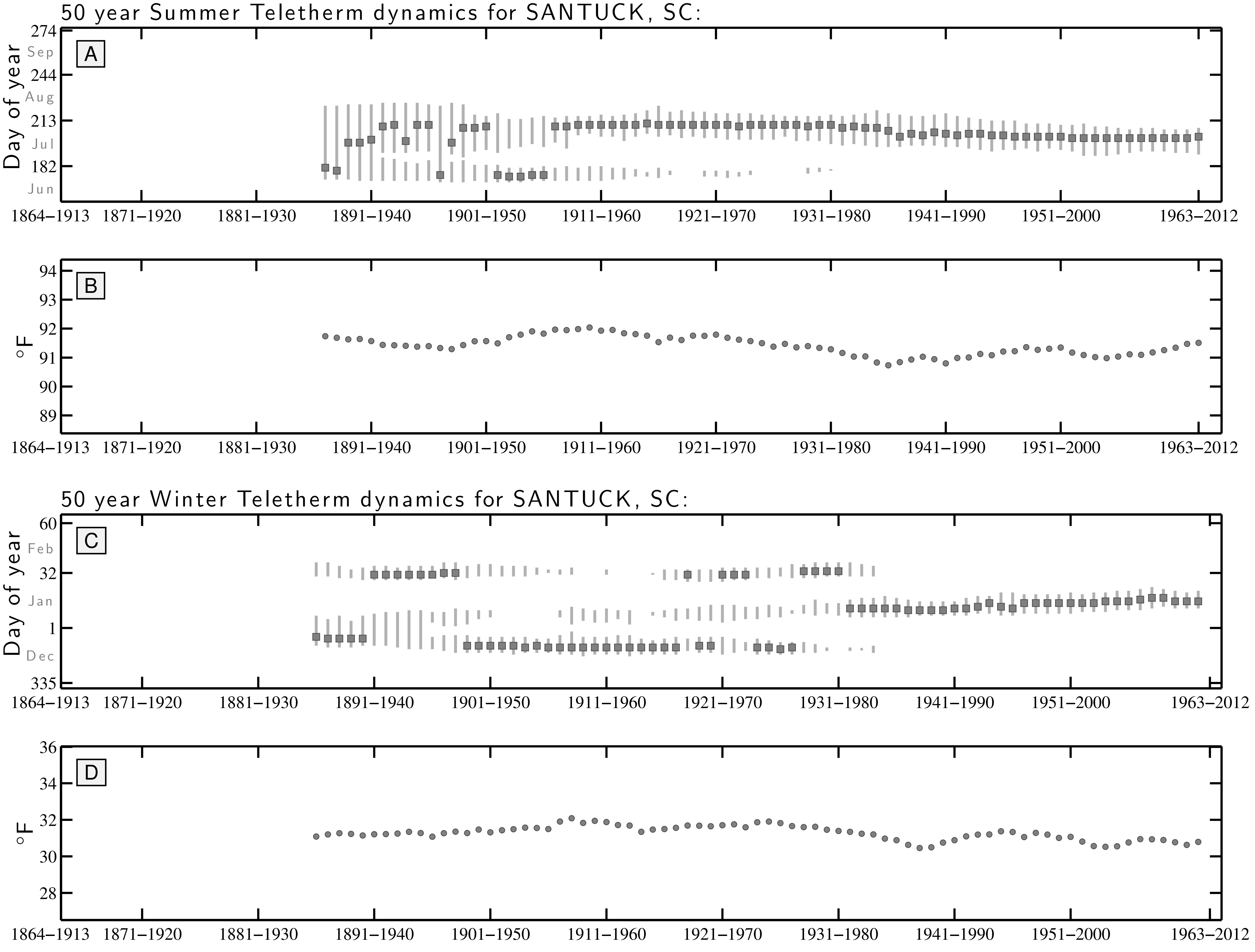

50 year Summer Teletherm dynamics for SANTUCK, SC:
A
B
Day of year
°F
Sep
Aug
Jul
Jun
274
244
213
182
94
93
92
91
90
89
1864–1913
1871–1920
1881–1930
1891–1940
1901–1950
1911–1960
1921–1970
1931–1980
1941–1990
1951–2000
1963–2012
50 year Winter Teletherm dynamics for SANTUCK, SC:
C
D
Feb
Jan
Dec
60
32
1
335
36
34
32
30
28

50 year Summer Teletherm dynamics for SUMMERVILLE 4W, SC:

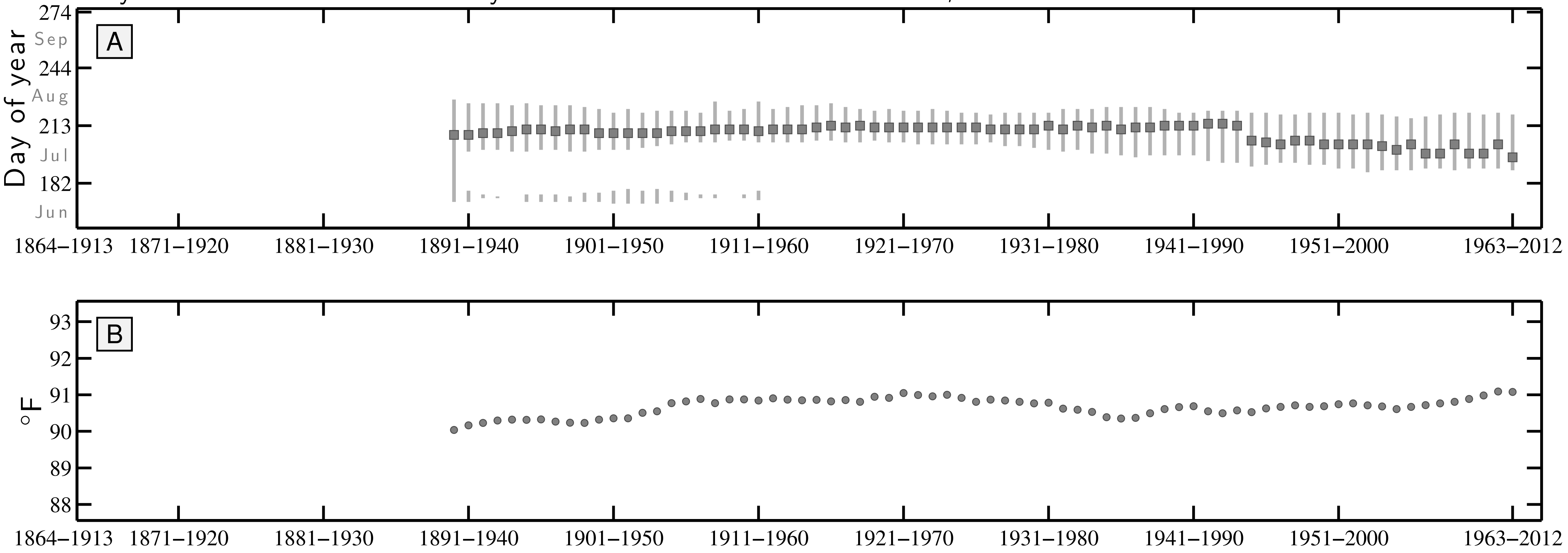

50 year Winter Teletherm dynamics for SUMMERVILLE 4W, SC:

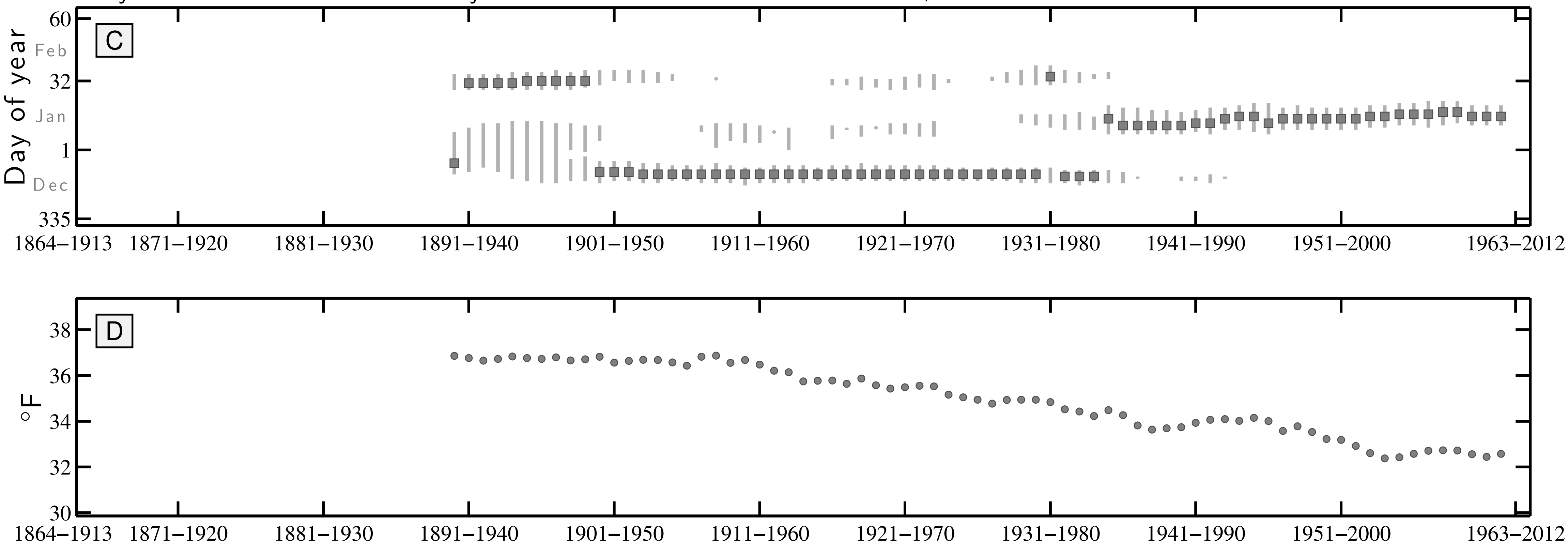

50 year Summer Teletherm dynamics for SUMTER, SC:

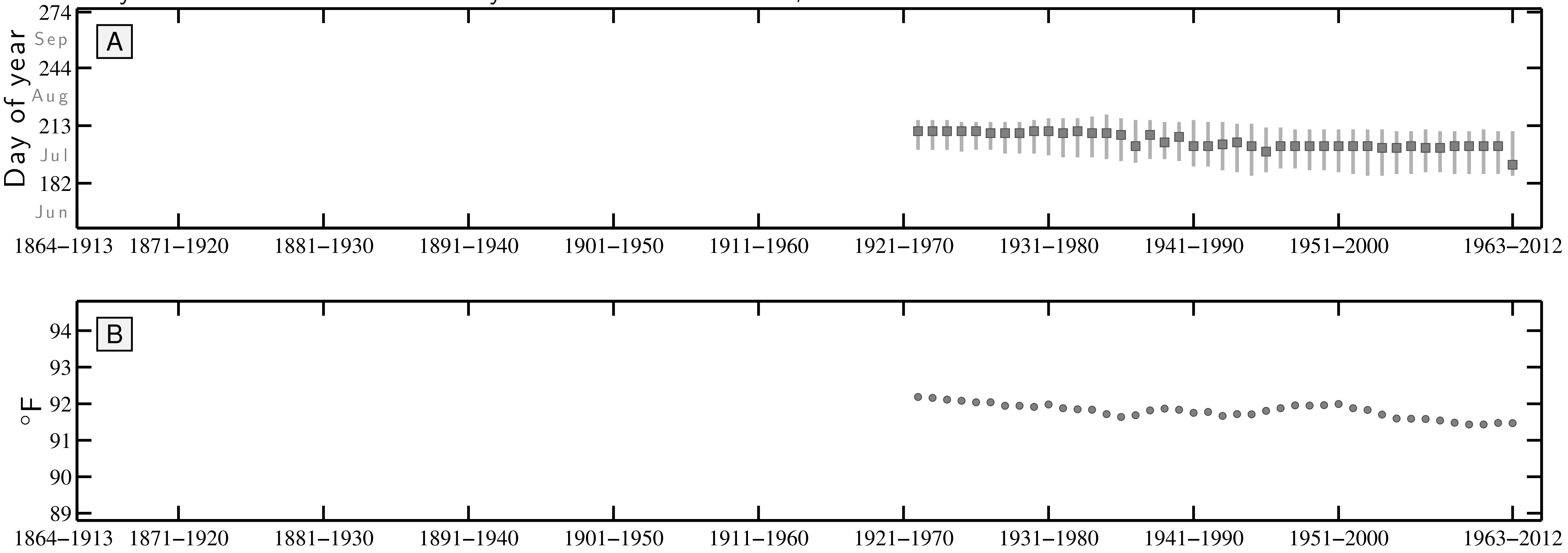

50 year Winter Teletherm dynamics for SUMTER, SC:

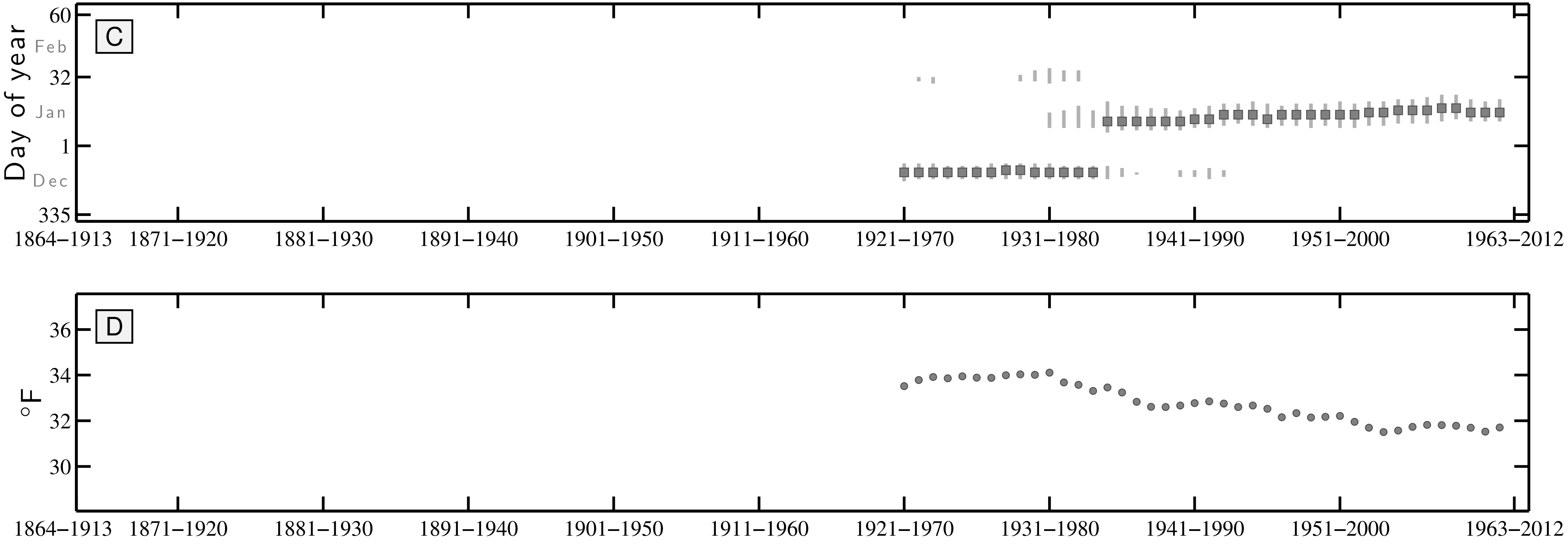

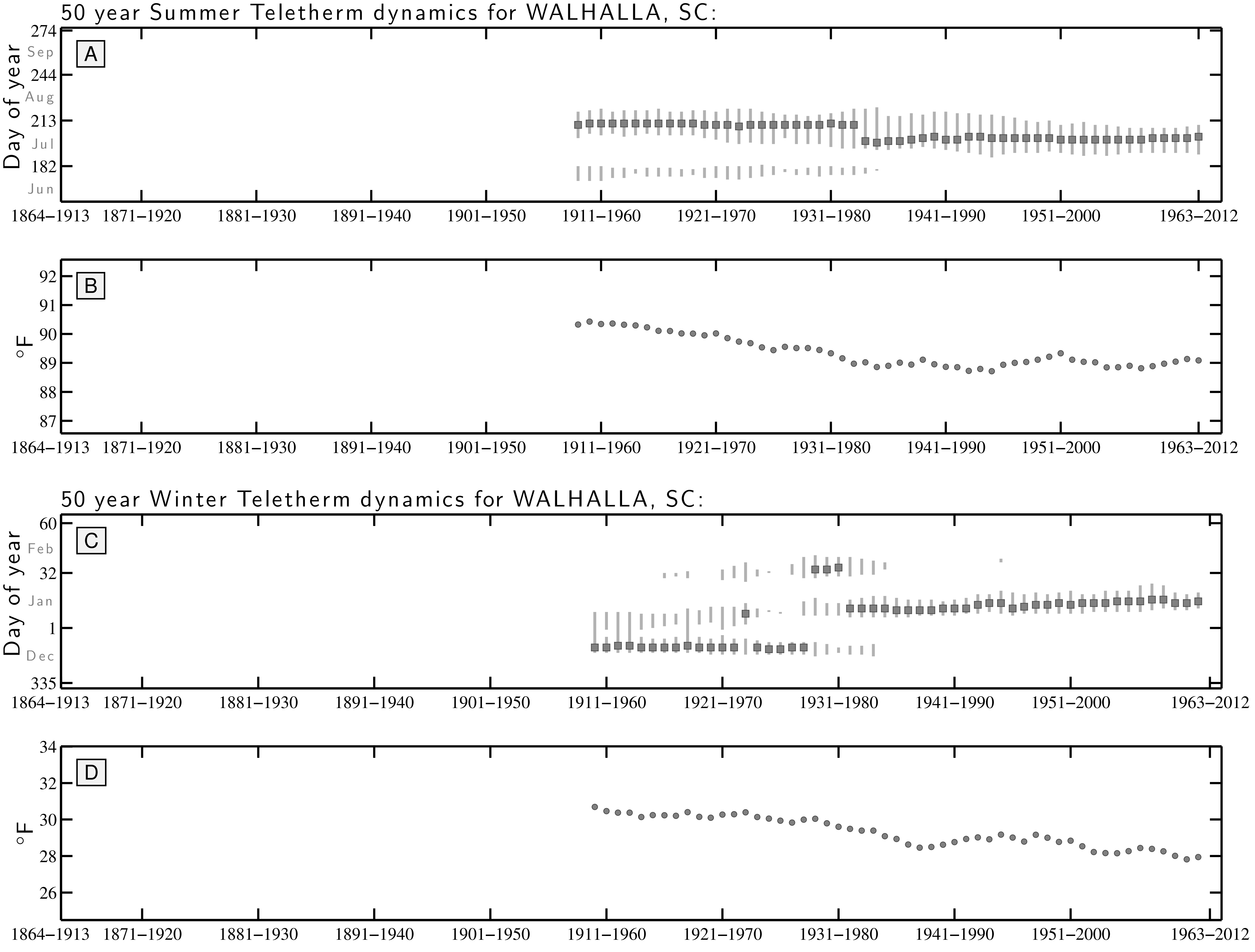
50 year Summer Teletherm dynamics for WALHALLA, SC:
A
Day of year
274
Sep
244
Aug
213
Jul
182
Jun
1864–1913
1871–1920
1881–1930
1891–1940
1901–1950
1911–1960
1921–1970
1931–1980
1941–1990
1951–2000
1963–2012
B
°F
92
91
90
89
88
87
50 year Winter Teletherm dynamics for WALHALLA, SC:
C
Day of year
60
Feb
32
Jan
1
Dec
335
D
°F
34
32
30
28
26

50 year Summer Teletherm dynamics for WINNSBORO, SC:

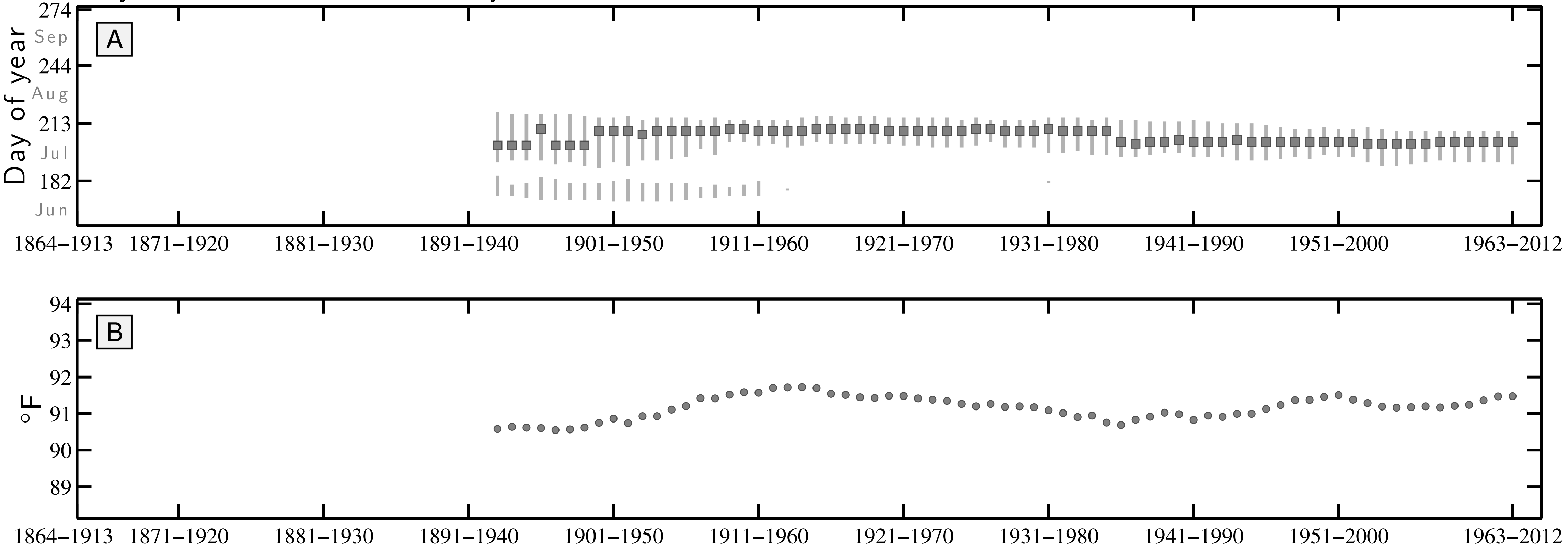

50 year Winter Teletherm dynamics for WINNSBORO, SC:

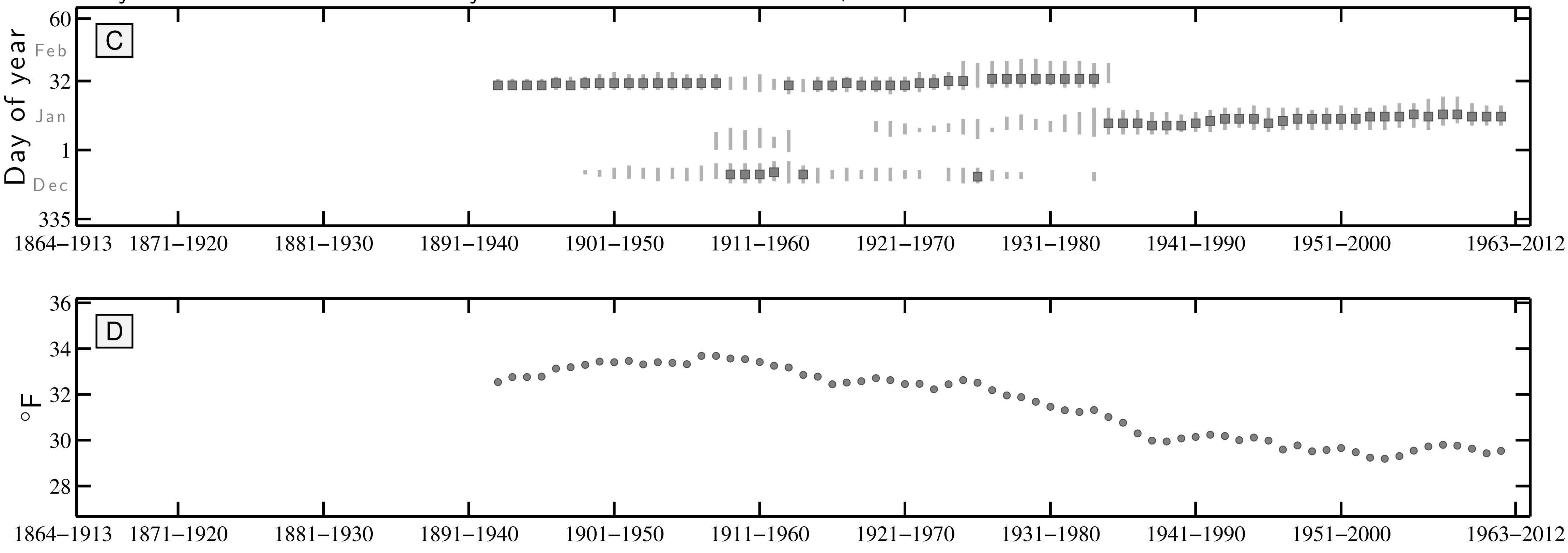

50 year Summer Teletherm dynamics for WINTHROP UNIV, SC:

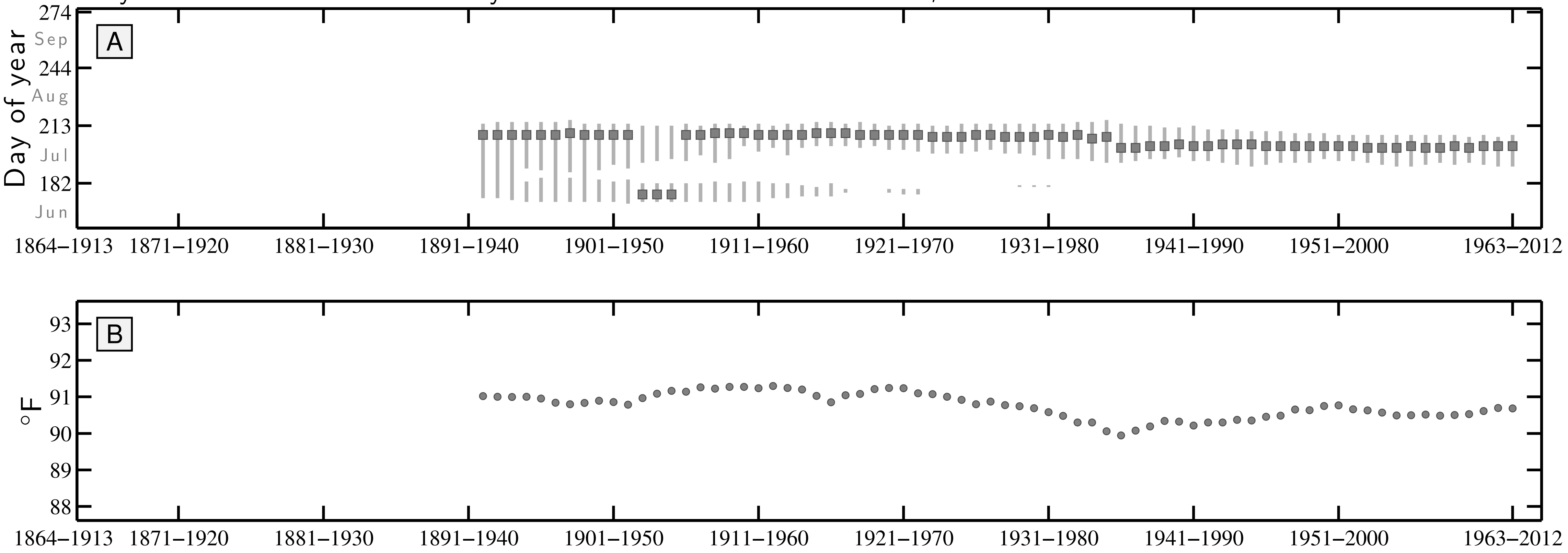

50 year Winter Teletherm dynamics for WINTHROP UNIV, SC:

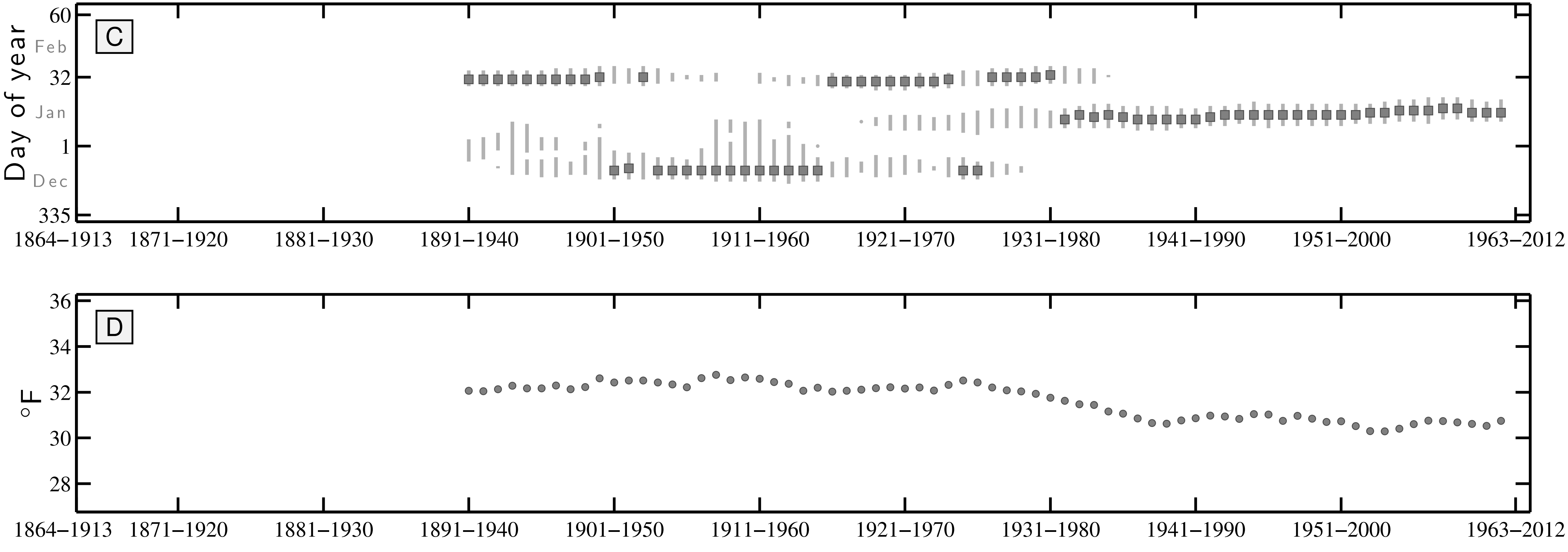

50 year Summer Teletherm dynamics for YEMASSEE, SC:

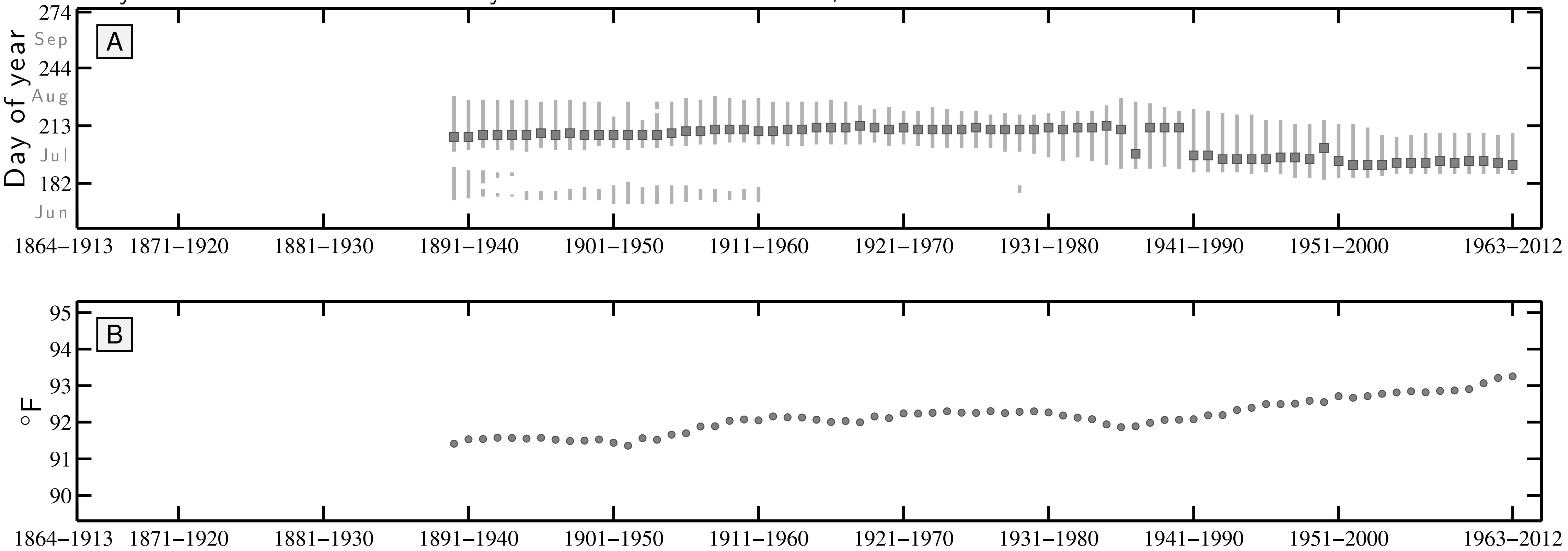

50 year Winter Teletherm dynamics for YEMASSEE, SC:

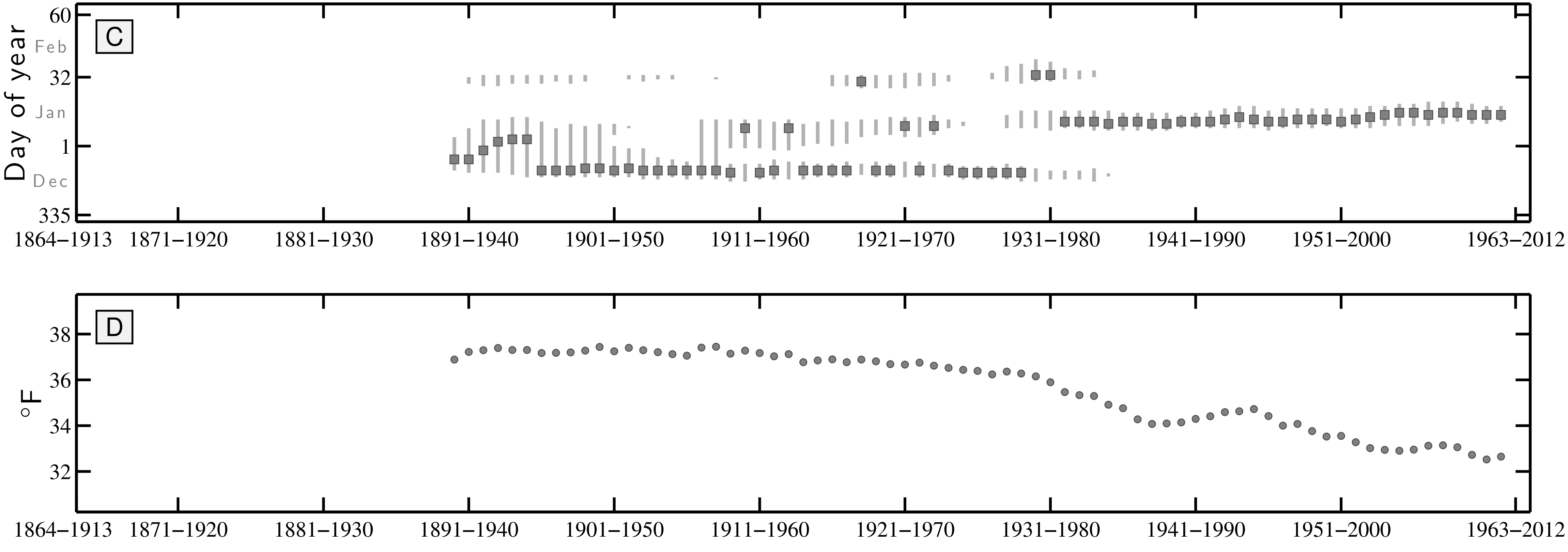

50 year Summer Teletherm dynamics for ABERDEEN RGNL AP, SD:

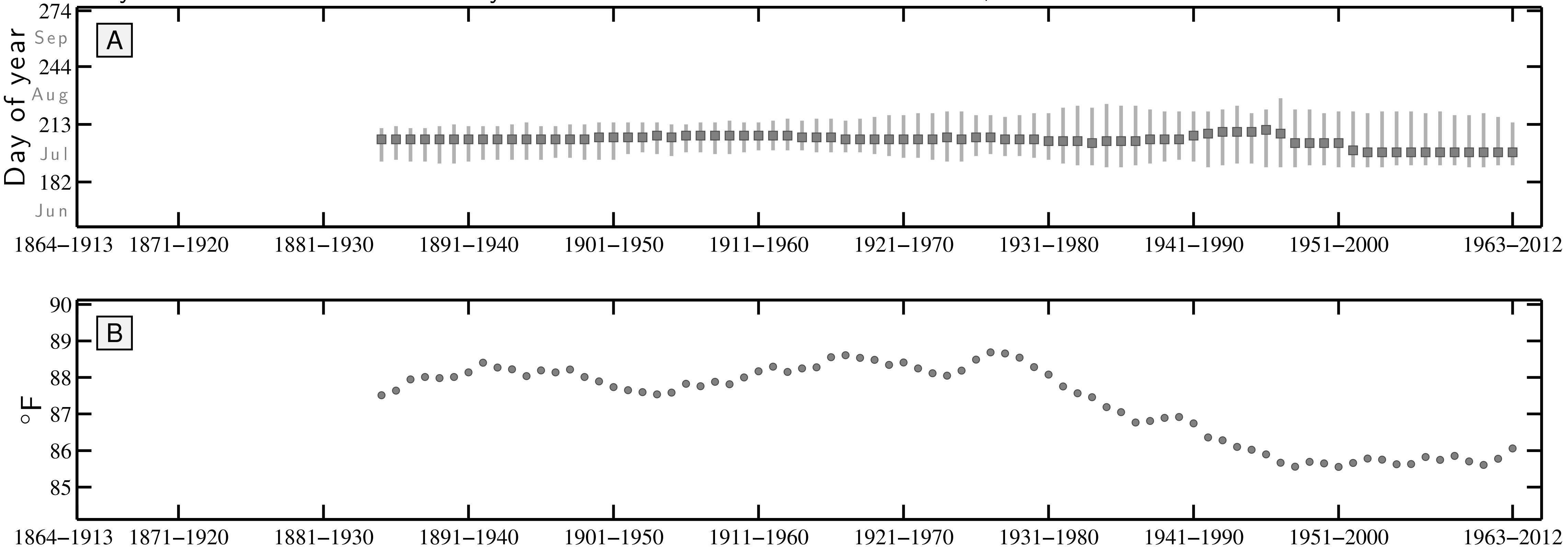

50 year Winter Teletherm dynamics for ABERDEEN RGNL AP, SD:

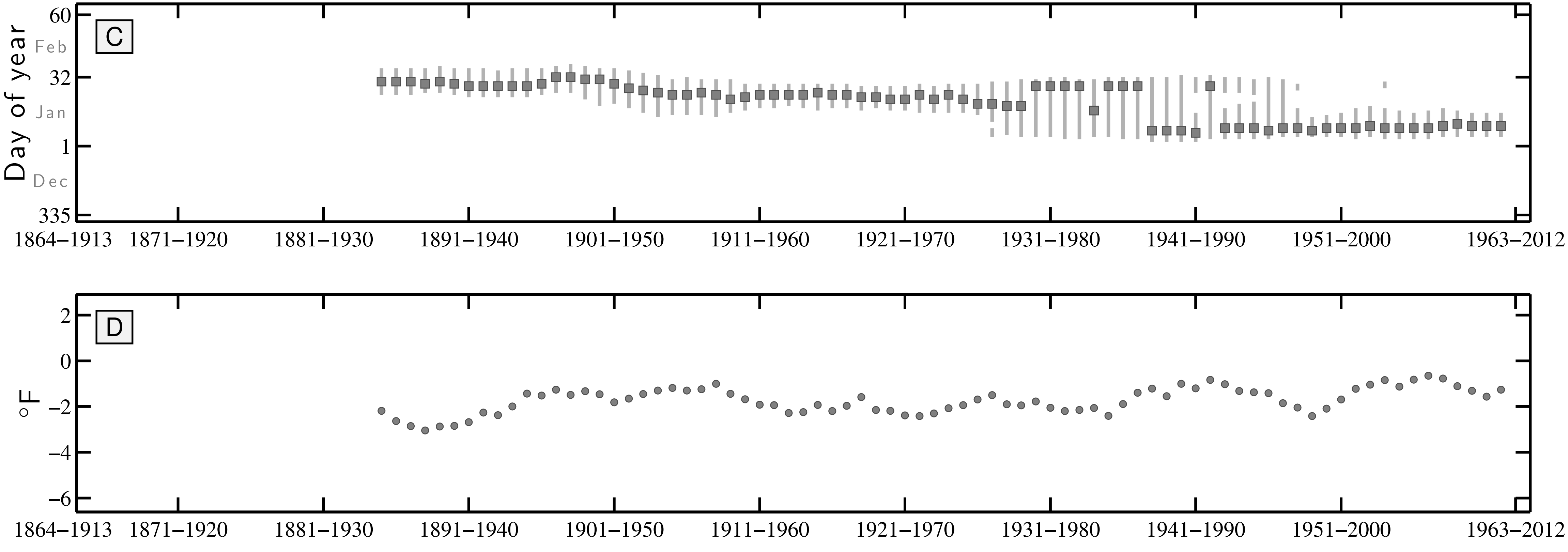

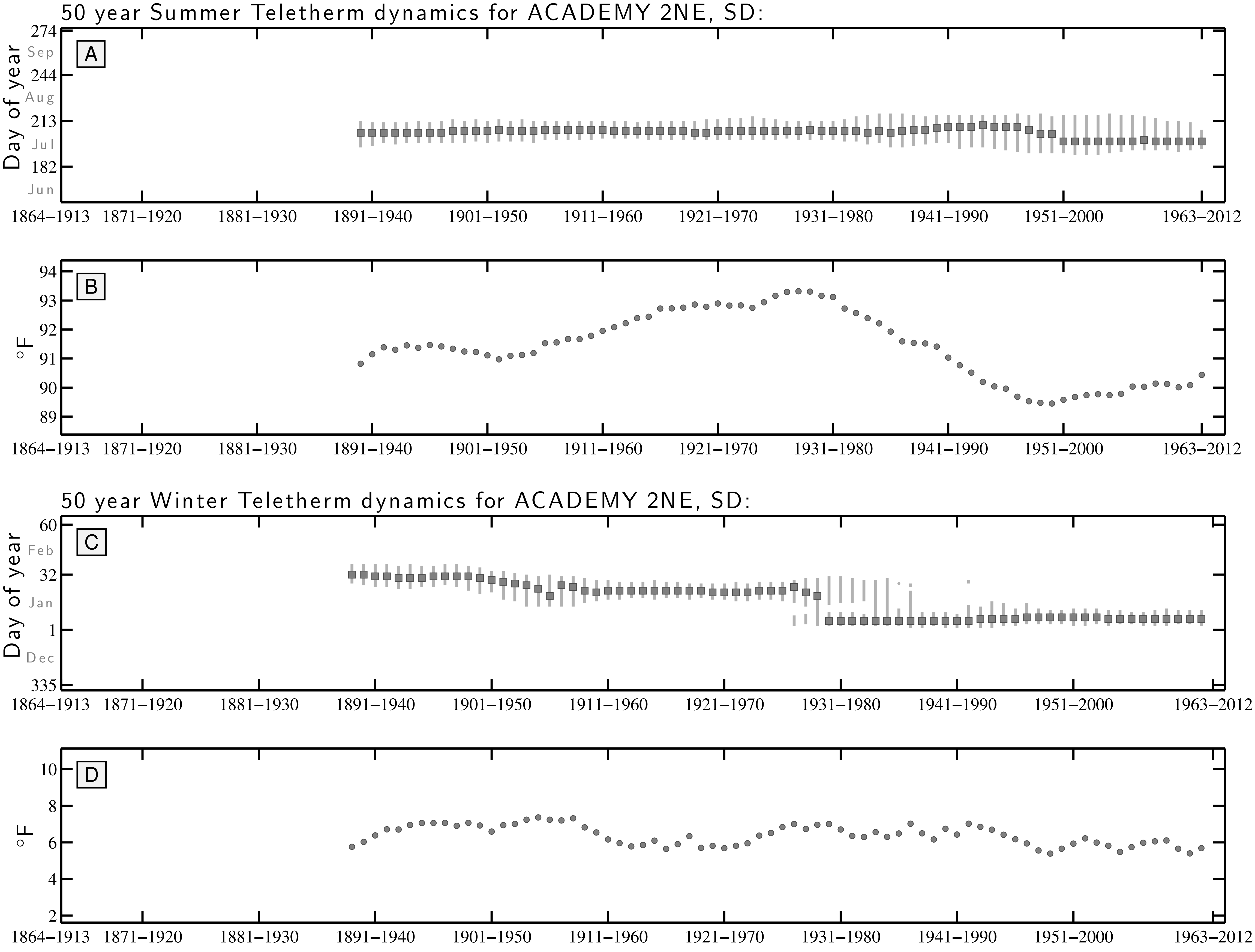

50 year Summer Teletherm dynamics for ACADEMY 2NE, SD:
A
Day of year
274
Sep
244
Aug
213
Jul
182
Jun
B
°F
94
93
92
91
90
89
50 year Winter Teletherm dynamics for ACADEMY 2NE, SD:
C
Day of year
60
Feb
32
Jan
1
Dec
335
D
°F
10
8
6
4
2
1864–1913
1871–1920
1881–1930
1891–1940
1901–1950
1911–1960
1921–1970
1931–1980
1941–1990
1951–2000
1963–2012

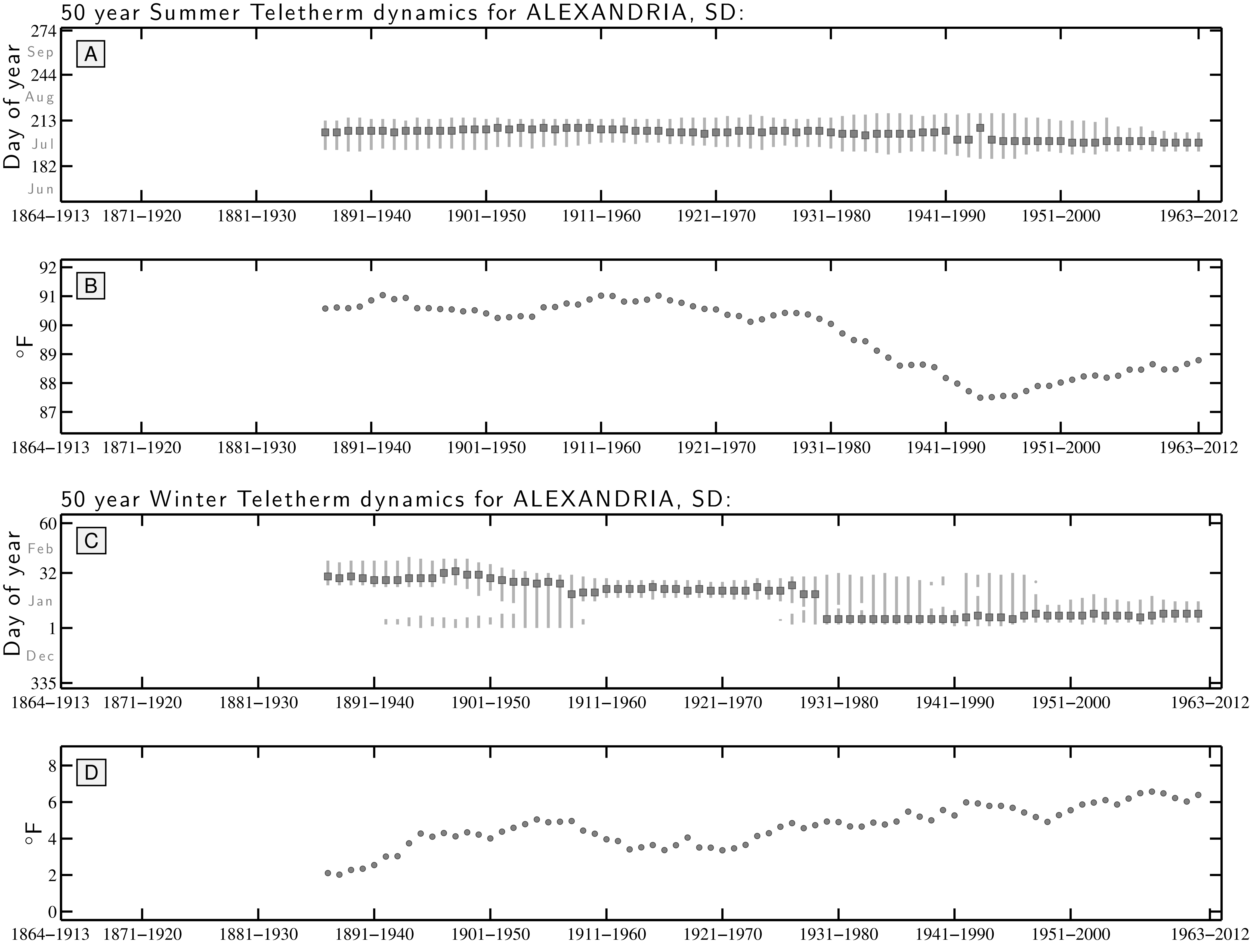

50 year Summer Teletherm dynamics for ALEXANDRIA, SD:
A
Day of year
274
Sep
244
Aug
213
Jul
182
Jun
1864–1913
1871–1920
1881–1930
1891–1940
1901–1950
1911–1960
1921–1970
1931–1980
1941–1990
1951–2000
1963–2012
B
°F
92
91
90
89
88
87
50 year Winter Teletherm dynamics for ALEXANDRIA, SD:
C
Day of year
60
Feb
32
Jan
1
Dec
335
D
°F
8
6
4
2
0

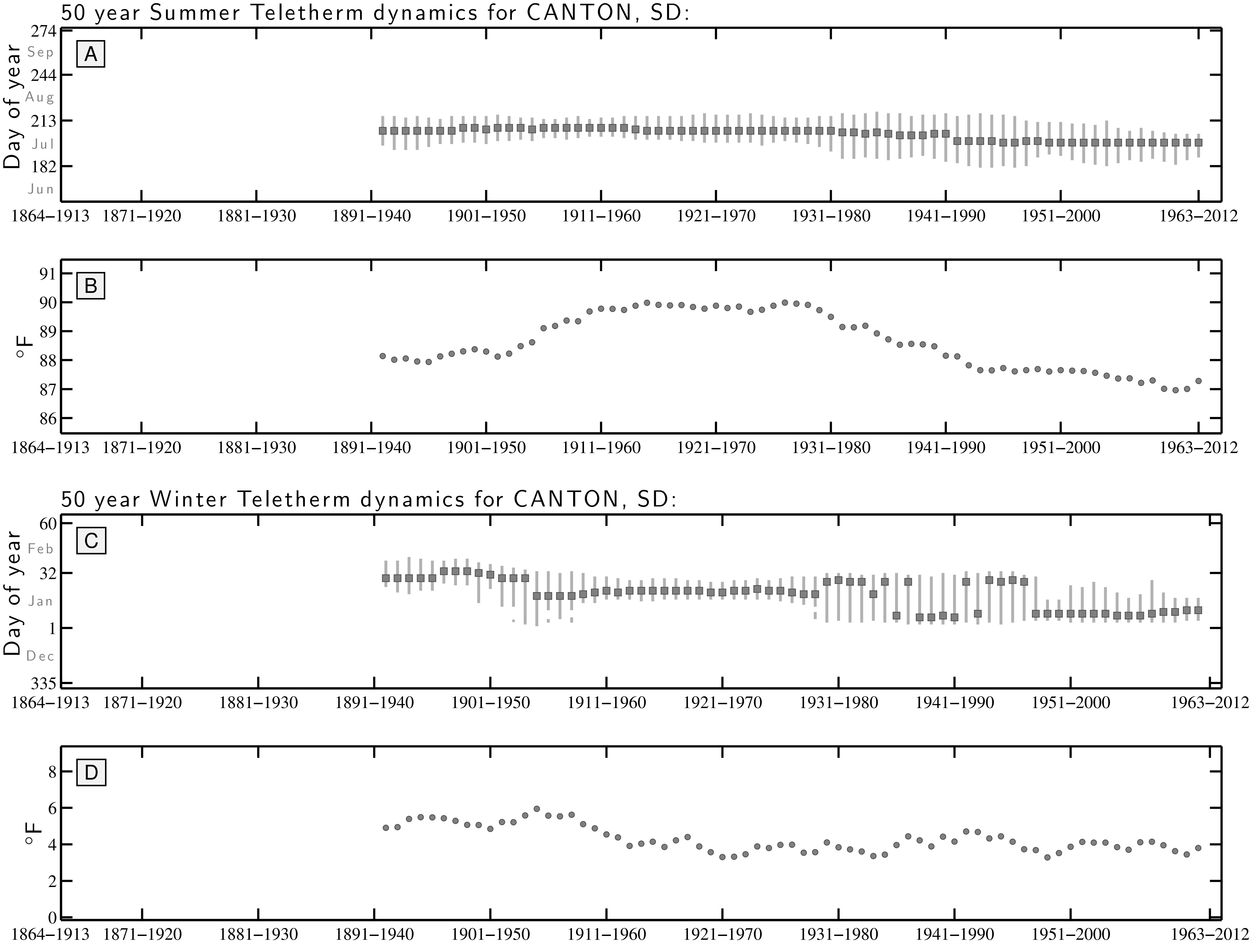

50 year Summer Teletherm dynamics for CANTON, SD:
A
Day of year
274
Sep
244
Aug
213
Jul
182
Jun
1864–1913
1871–1920
1881–1930
1891–1940
1901–1950
1911–1960
1921–1970
1931–1980
1941–1990
1951–2000
1963–2012
B
°F
91
90
89
88
87
86
50 year Winter Teletherm dynamics for CANTON, SD:
C
60
Feb
32
Jan
1
Dec
335
D
8
6
4
2
0

50 year Summer Teletherm dynamics for CLARK, SD:

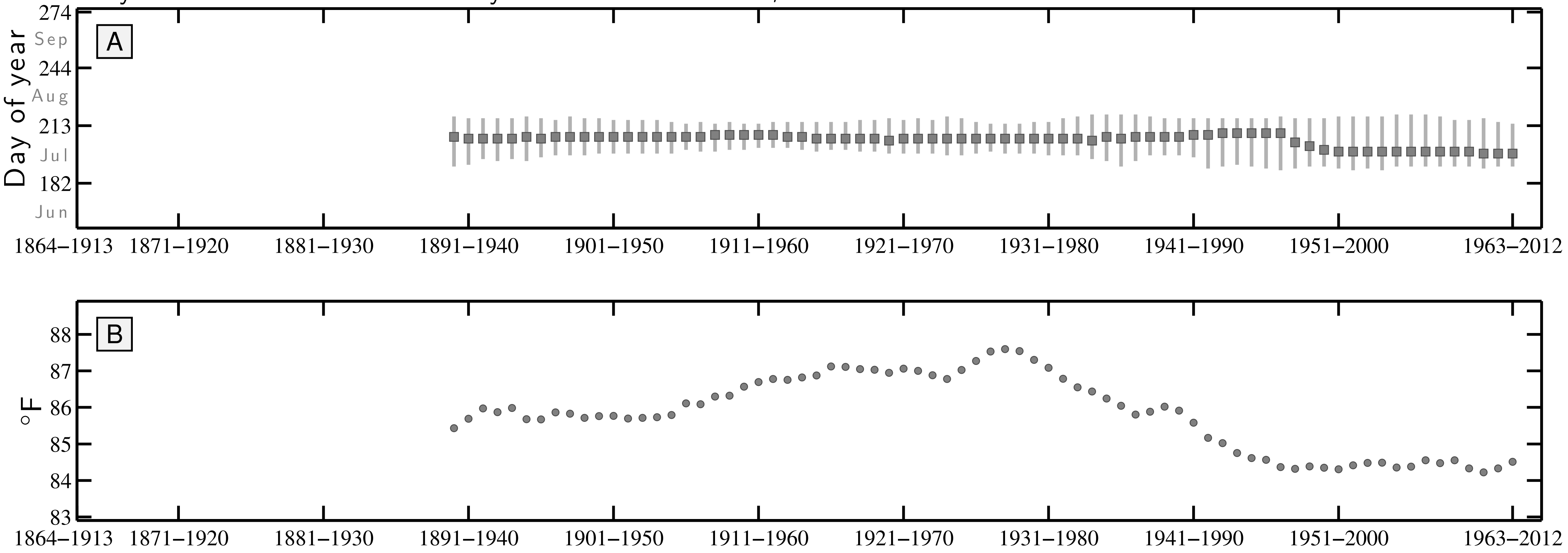

50 year Winter Teletherm dynamics for CLARK, SD:

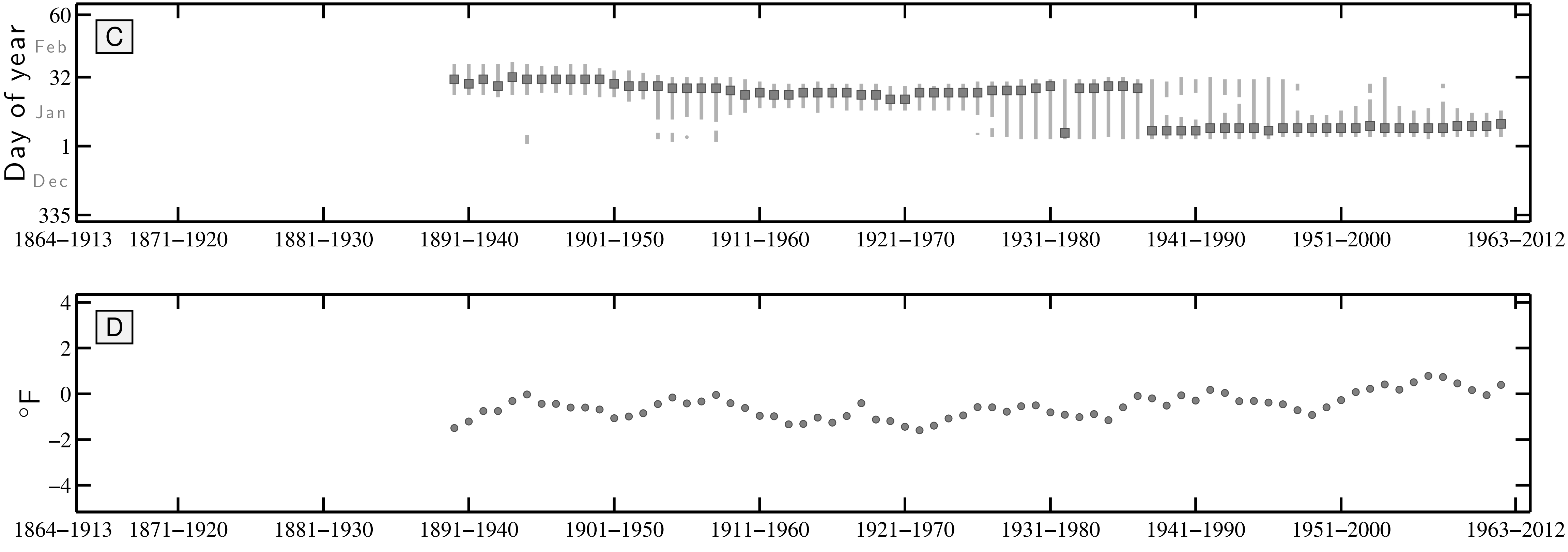

50 year Summer Teletherm dynamics for COTTONWOOD 2 E, SD:

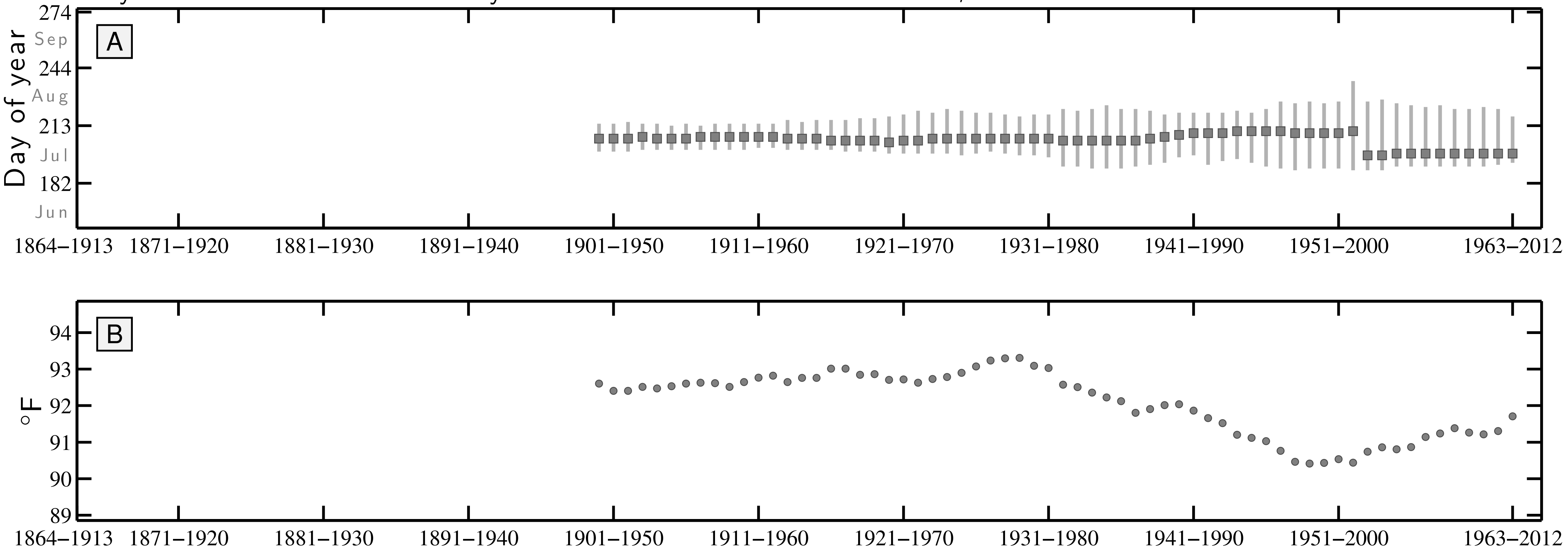

50 year Winter Teletherm dynamics for COTTONWOOD 2 E, SD:

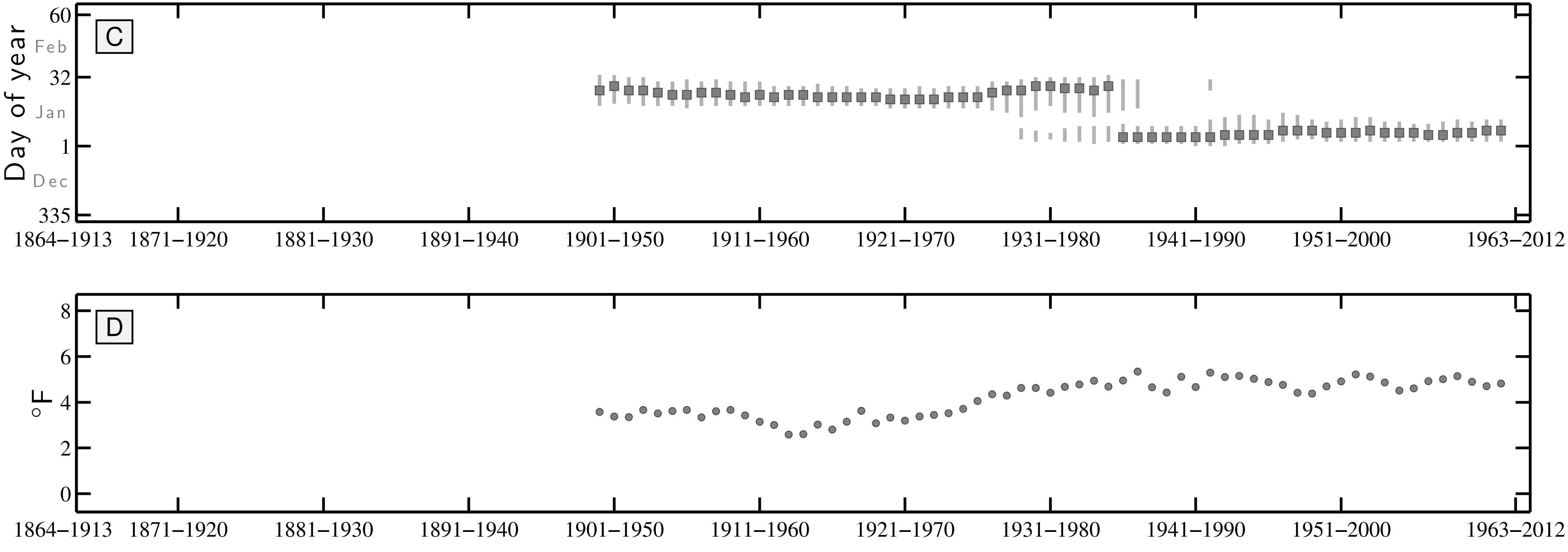

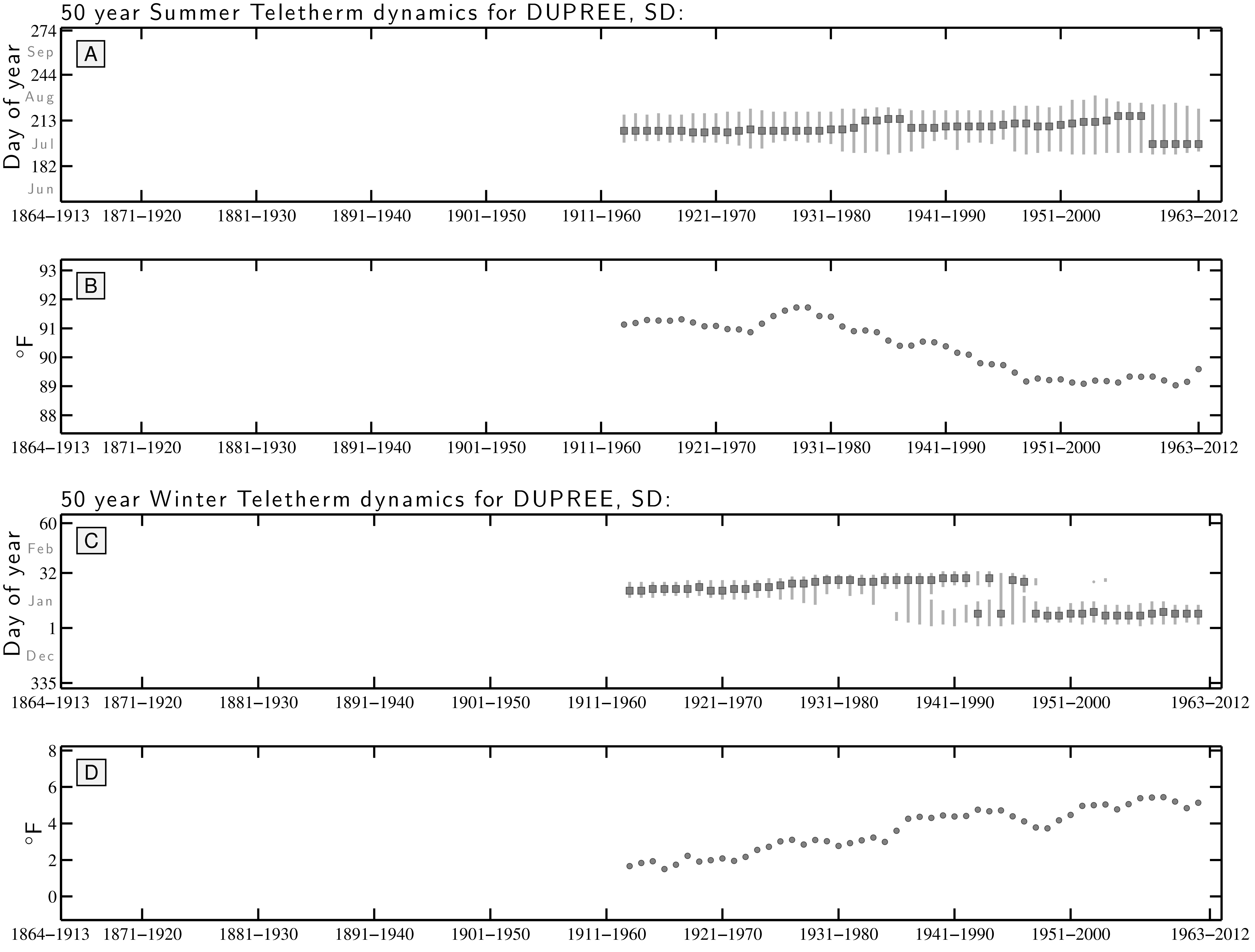

50 year Summer Teletherm dynamics for DUPREE, SD:
A
Day of year
274
Sep
244
Aug
213
Jul
182
Jun
B
°F
93
92
91
90
89
88
50 year Winter Teletherm dynamics for DUPREE, SD:
C
Day of year
60
Feb
32
Jan
1
Dec
335
D
°F
8
6
4
2
0
1864–1913
1871–1920
1881–1930
1891–1940
1901–1950
1911–1960
1921–1970
1931–1980
1941–1990
1951–2000
1963–2012

50 year Summer Teletherm dynamics for EUREKA, SD:

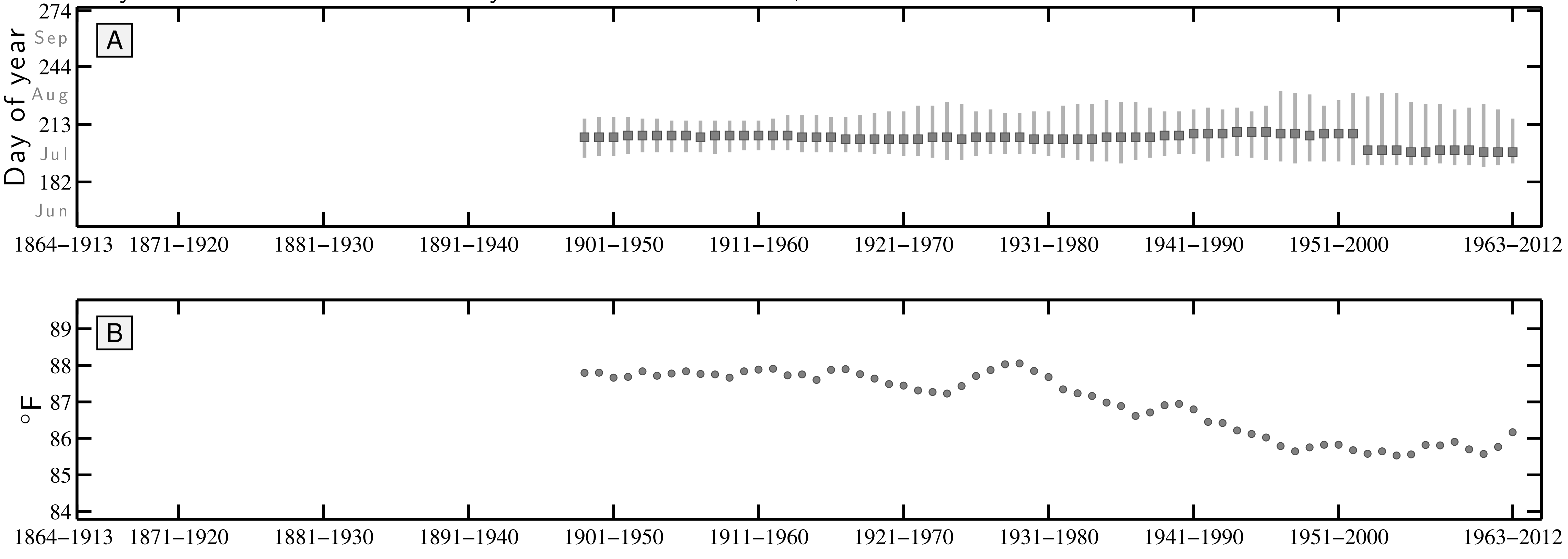

50 year Winter Teletherm dynamics for EUREKA, SD:

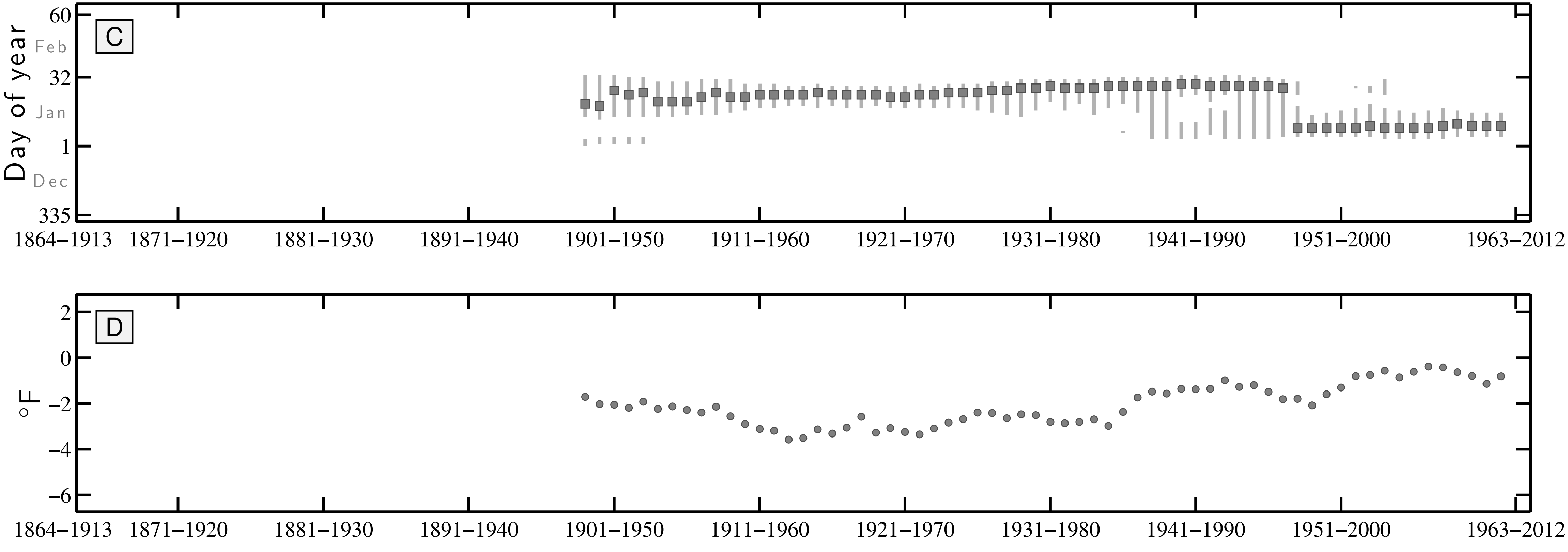

50 year Summer Teletherm dynamics for FAULKTON 1 NW, SD:

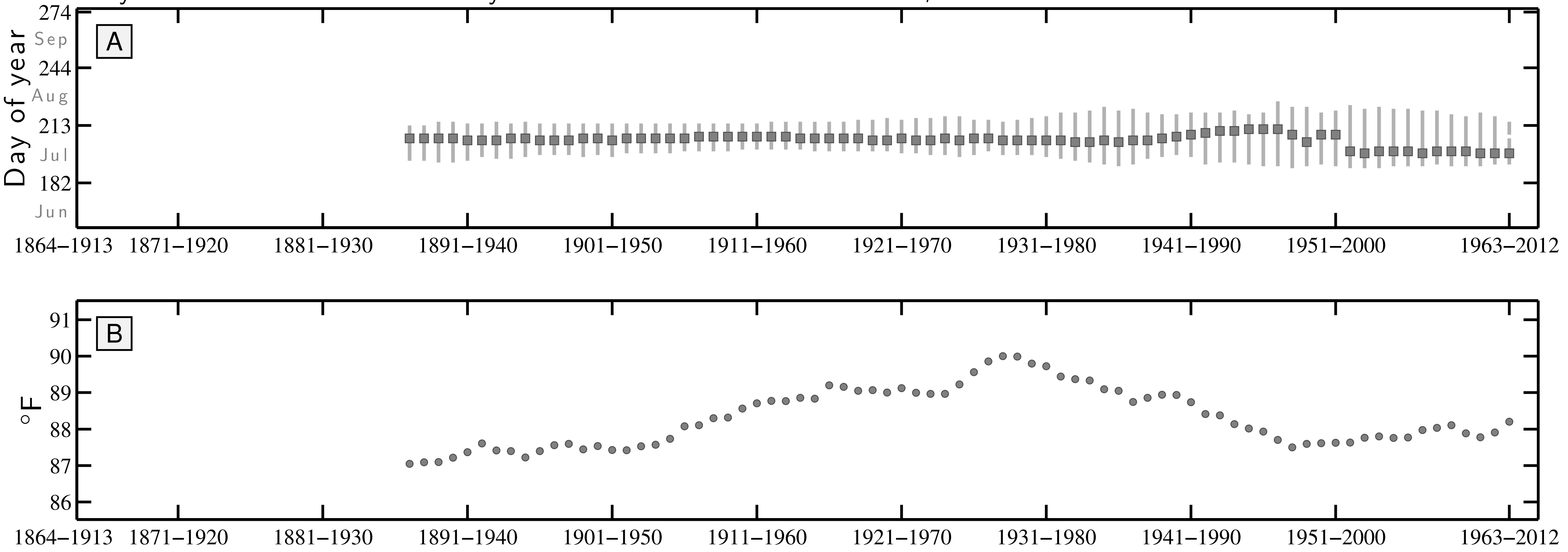

50 year Winter Teletherm dynamics for FAULKTON 1 NW, SD:

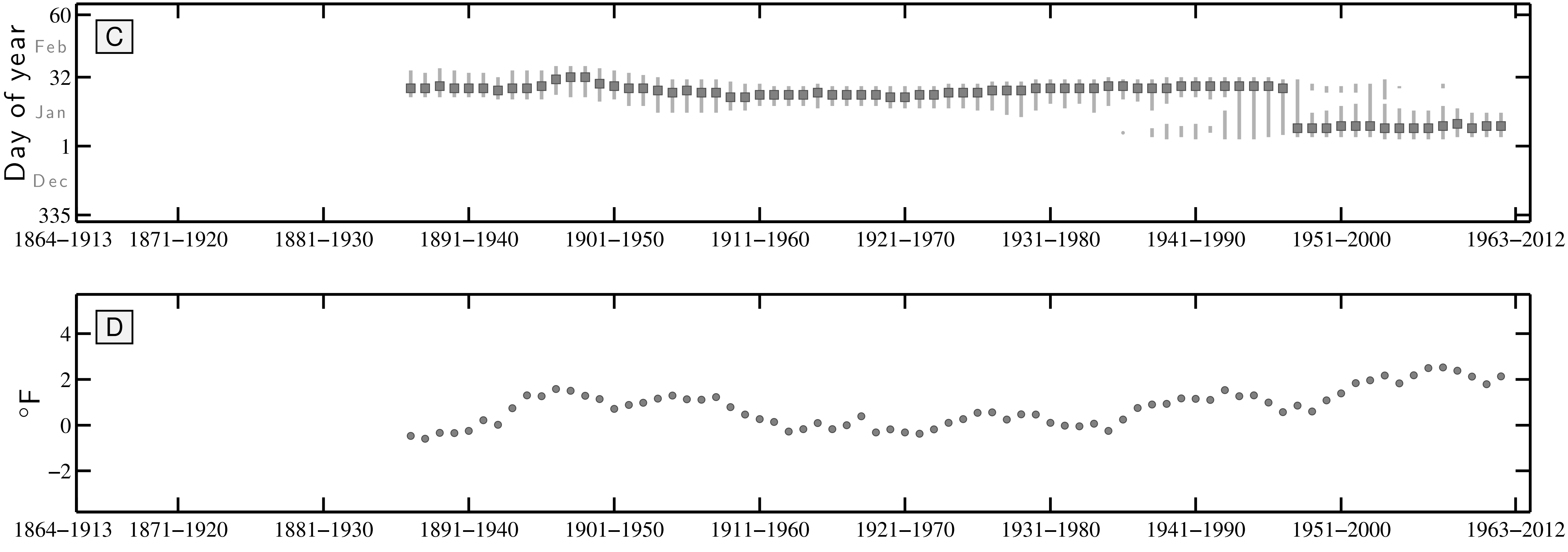

50 year Summer Teletherm dynamics for FORESTBURG 3 NE, SD:

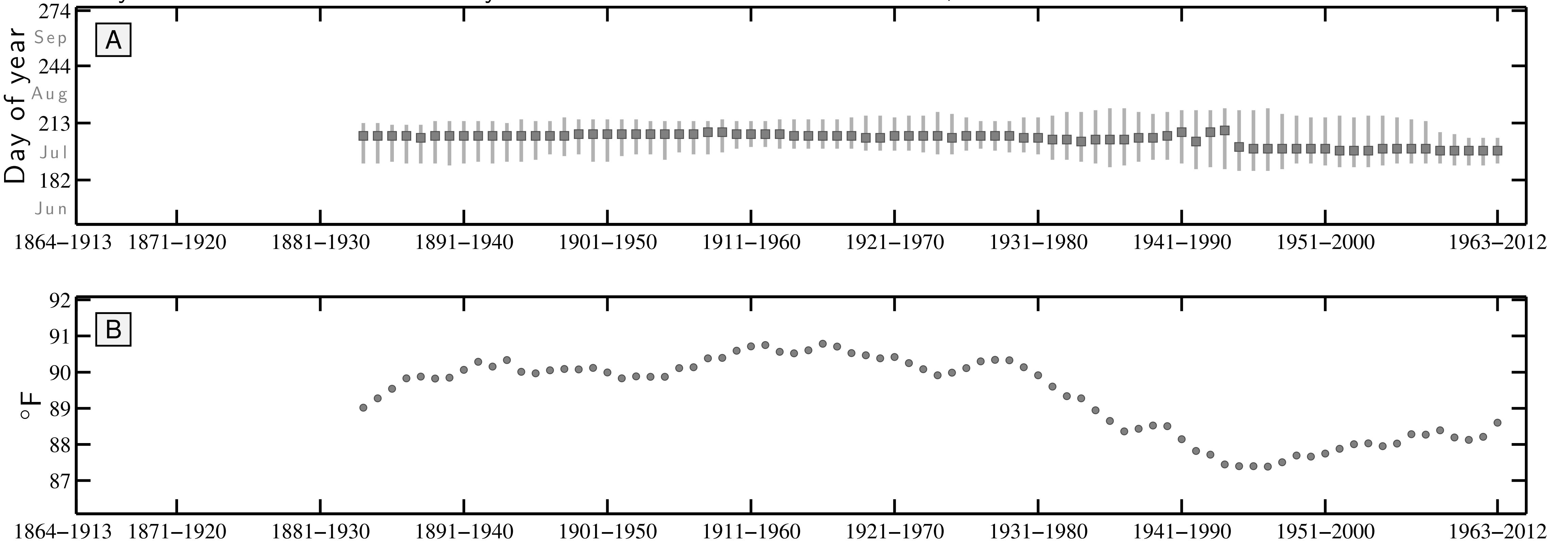

50 year Winter Teletherm dynamics for FORESTBURG 3 NE, SD:

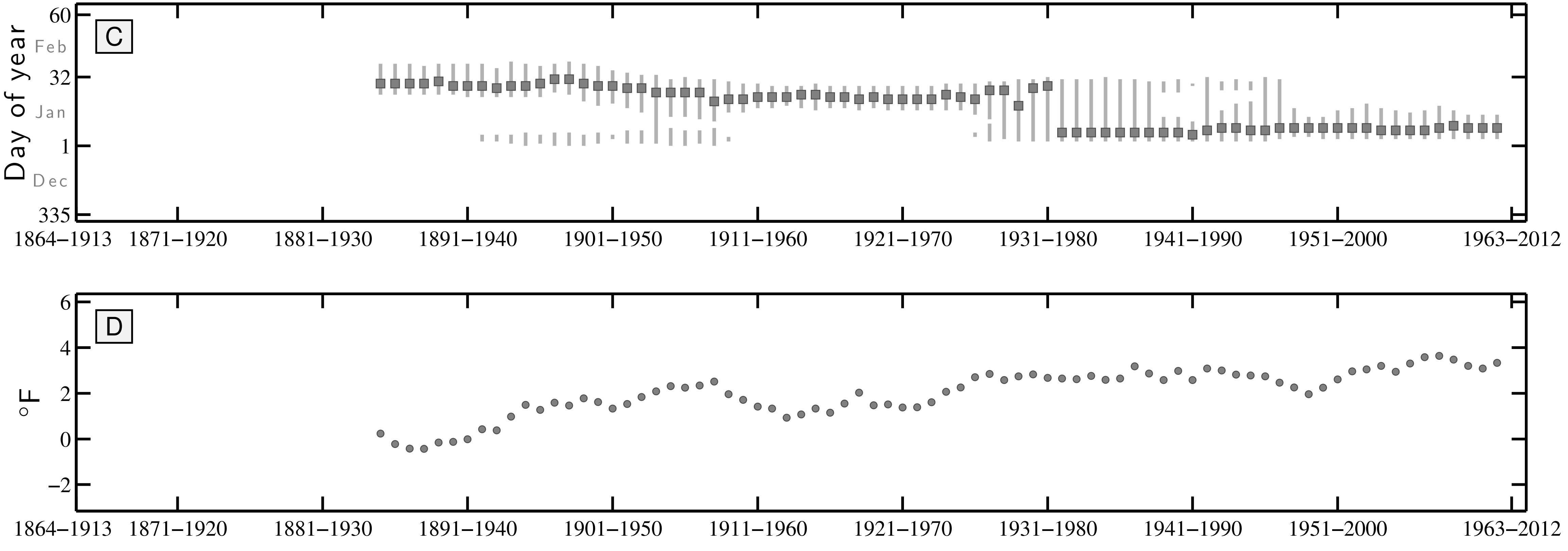

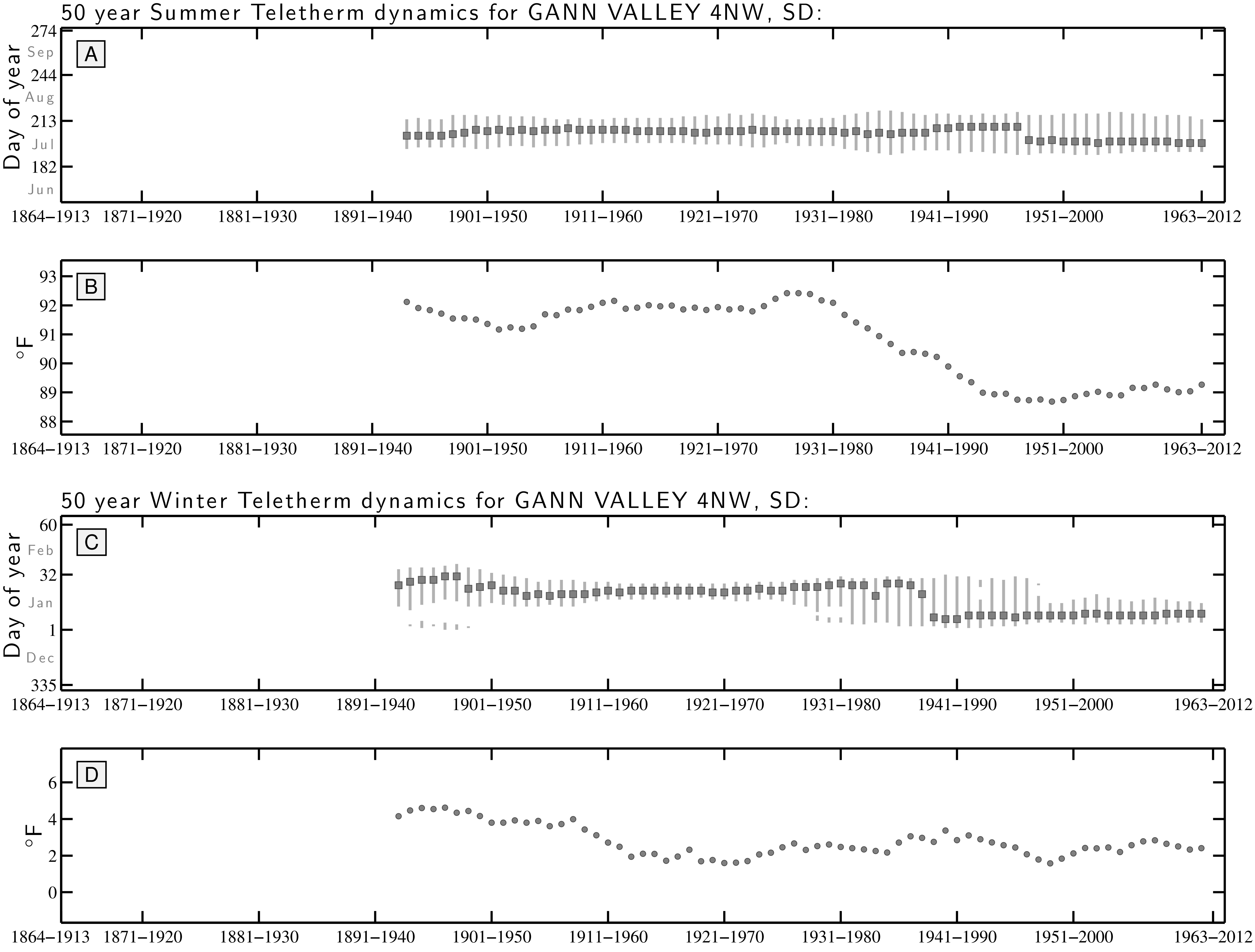

50 year Summer Teletherm dynamics for GANN VALLEY 4NW, SD:
A
B
Day of year
°F
274
244
213
182
Sep
Aug
Jul
Jun
93
92
91
90
89
88
1864–1913
1871–1920
1881–1930
1891–1940
1901–1950
1911–1960
1921–1970
1931–1980
1941–1990
1951–2000
1963–2012
50 year Winter Teletherm dynamics for GANN VALLEY 4NW, SD:
C
D
60
32
1
335
Feb
Jan
Dec
6
4
2
0

50 year Summer Teletherm dynamics for HIGHMORE 1 W, SD:

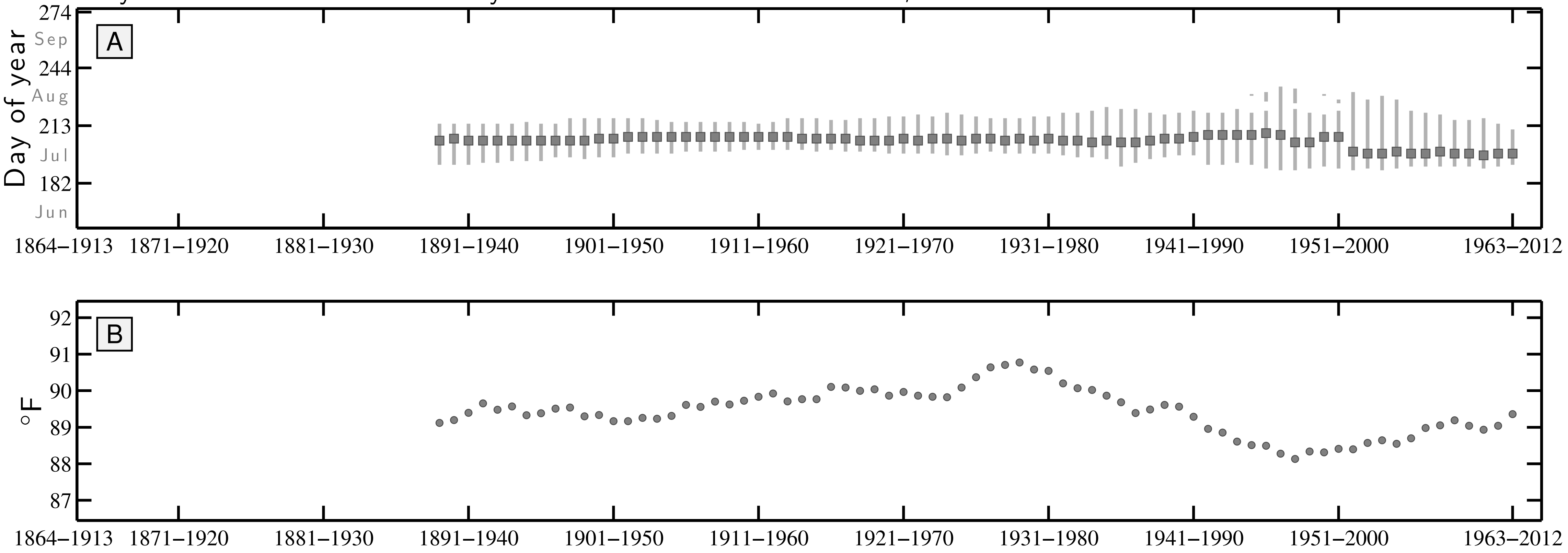

50 year Winter Teletherm dynamics for HIGHMORE 1 W, SD:

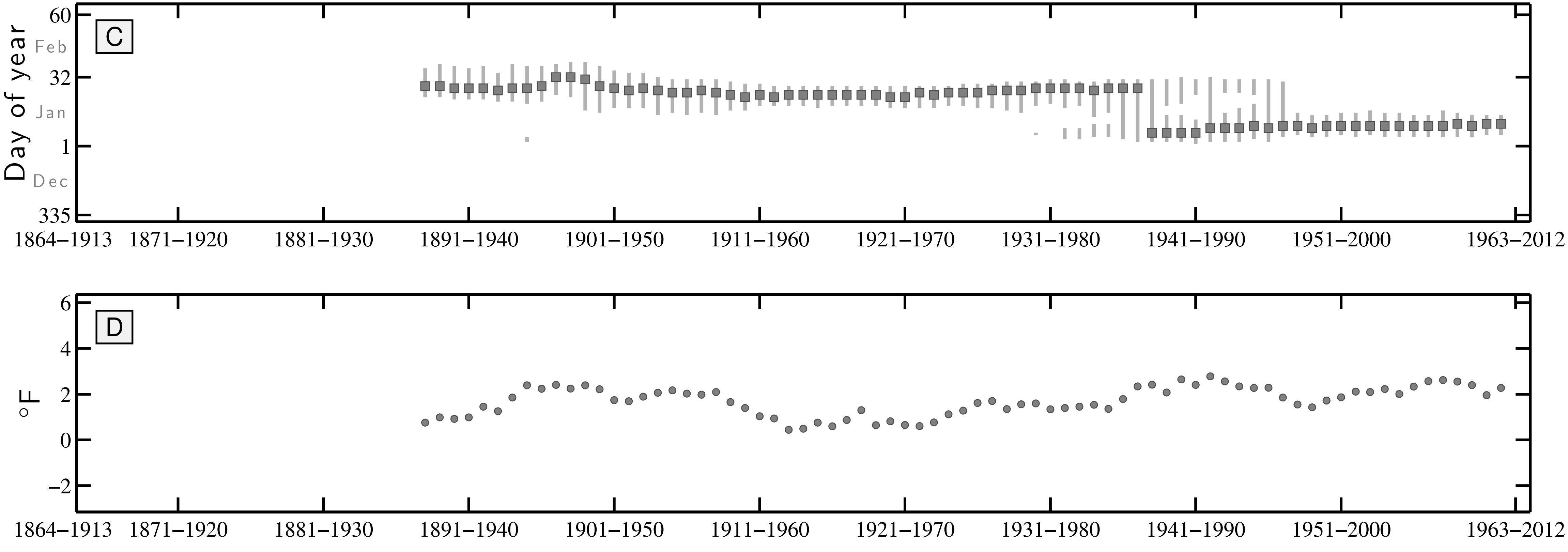

50 year Summer Teletherm dynamics for HOT SPRINGS, SD:

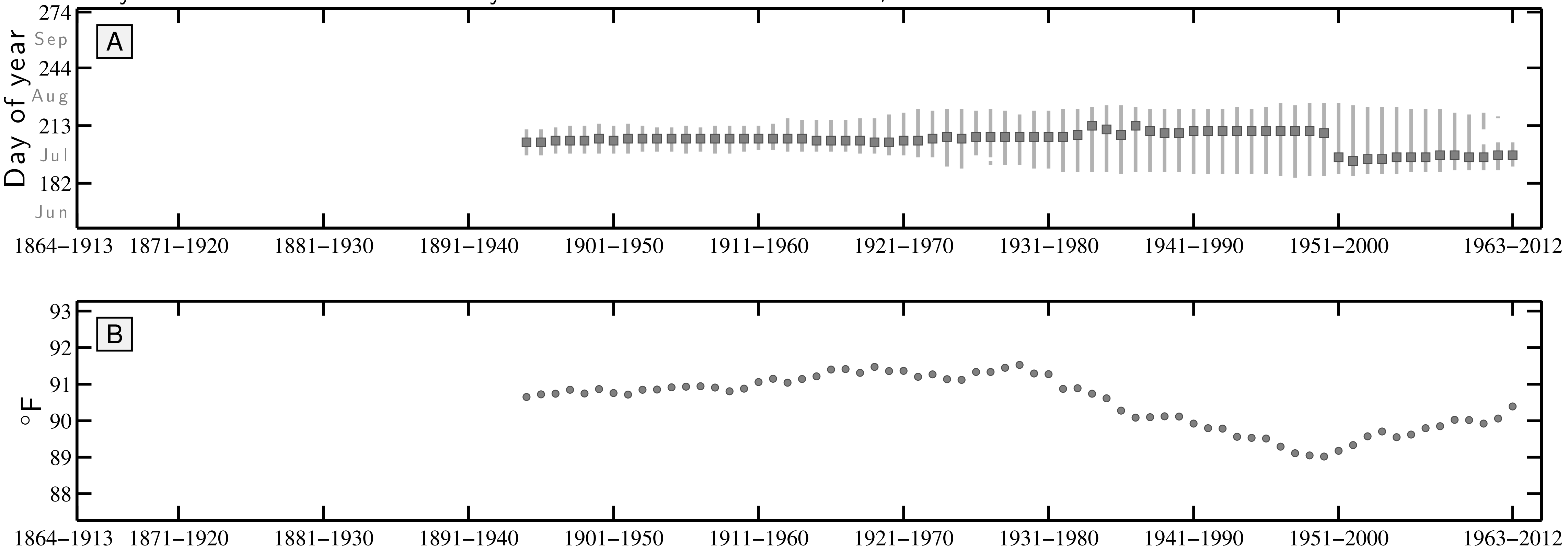

50 year Winter Teletherm dynamics for HOT SPRINGS, SD:

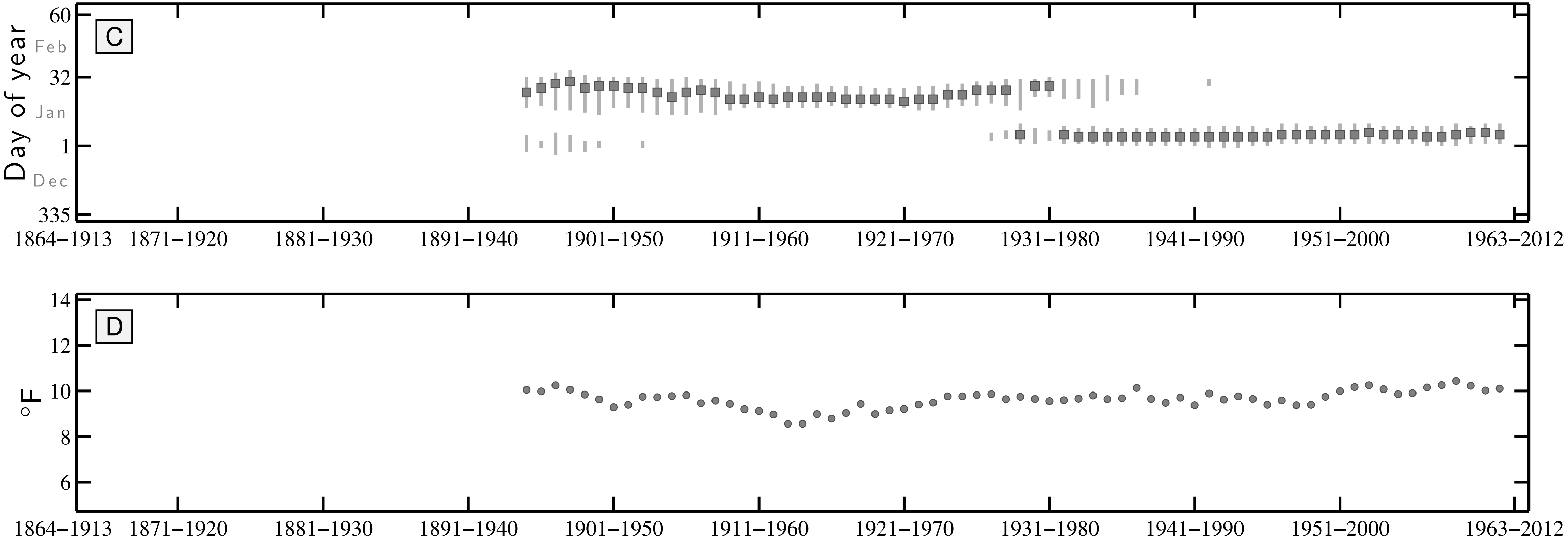

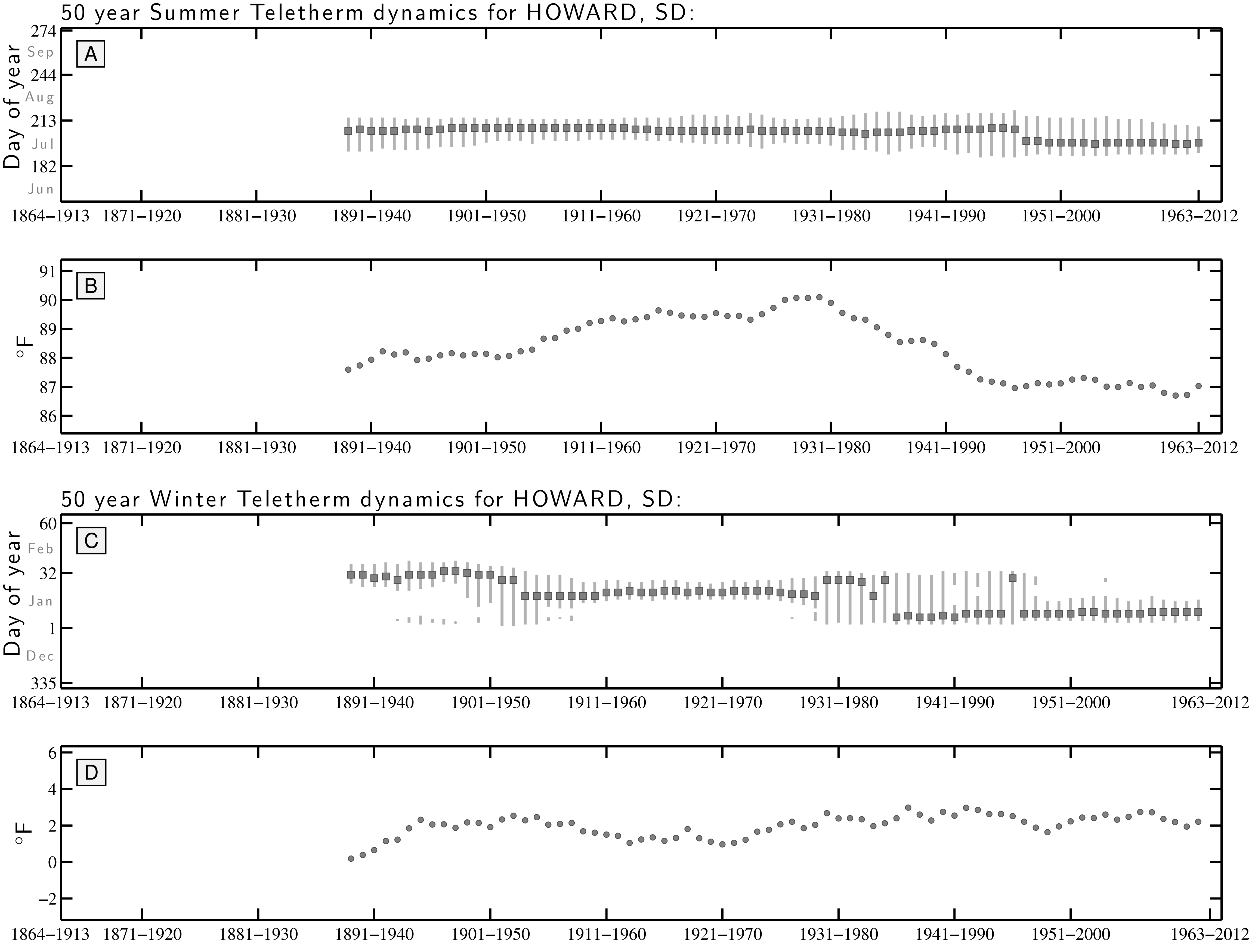
50 year Summer Teletherm dynamics for HOWARD, SD:
A
B
Day of year
°F
274
Sep
244
Aug
213
Jul
182
Jun
91
90
89
88
87
86
1864–1913
1871–1920
1881–1930
1891–1940
1901–1950
1911–1960
1921–1970
1931–1980
1941–1990
1951–2000
1963–2012
50 year Winter Teletherm dynamics for HOWARD, SD:
C
D
60
Feb
32
Jan
1
Dec
335
6
4
2
0
−2

50 year Summer Teletherm dynamics for KENNEBEC, SD:

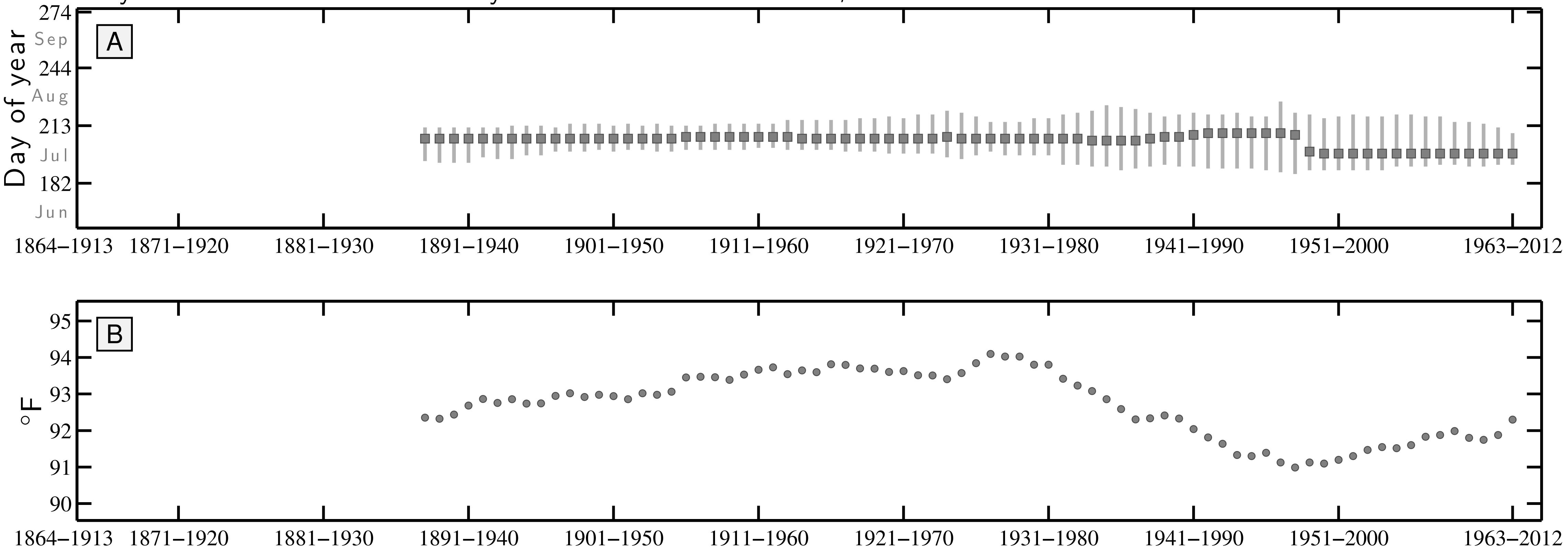

50 year Winter Teletherm dynamics for KENNEBEC, SD:

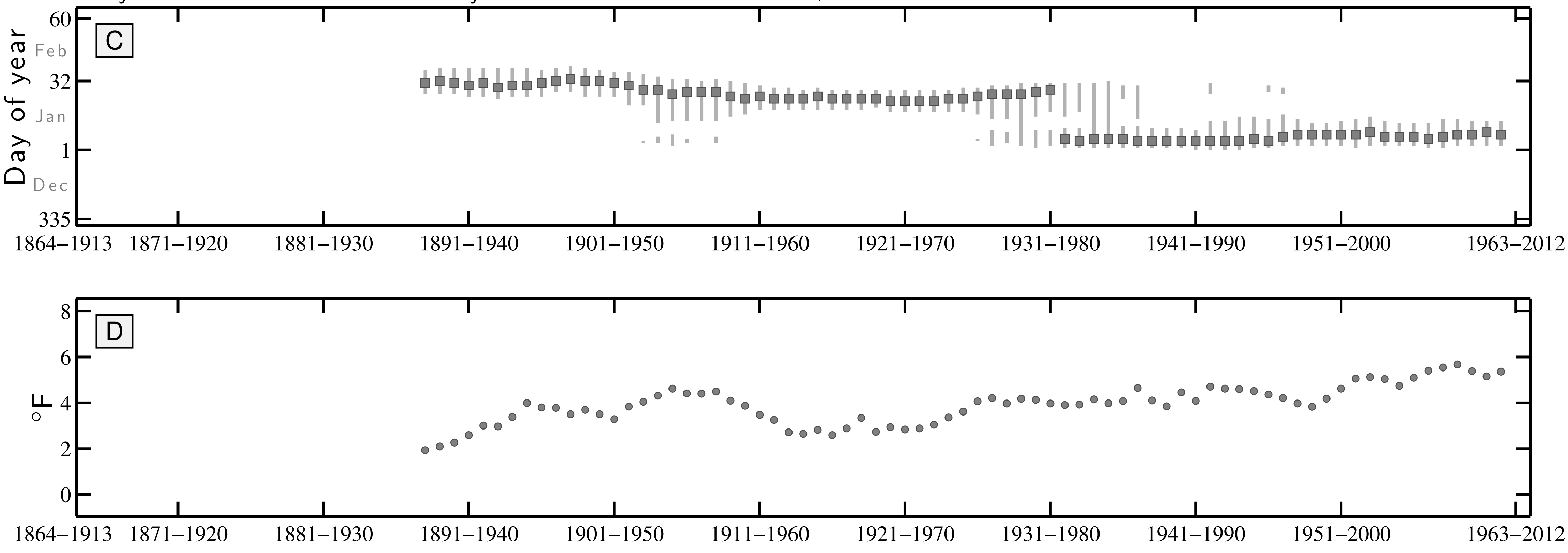

50 year Summer Teletherm dynamics for MELLETTE 4 W, SD:

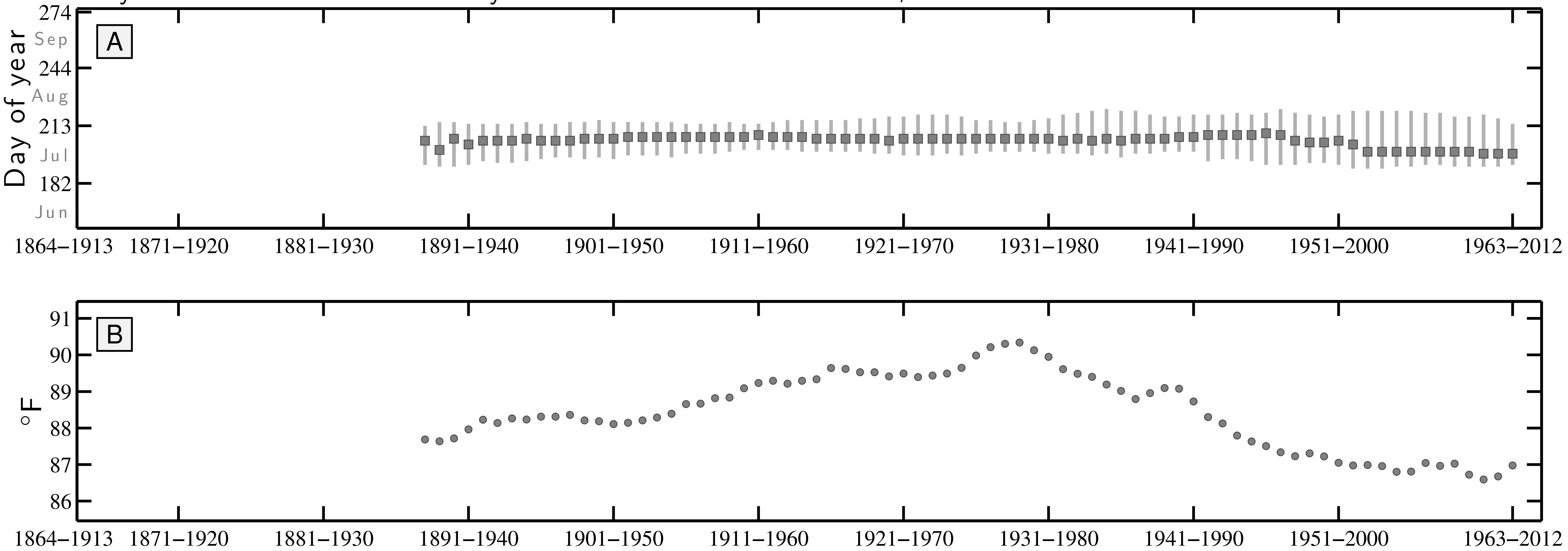

50 year Winter Teletherm dynamics for MELLETTE 4 W, SD:

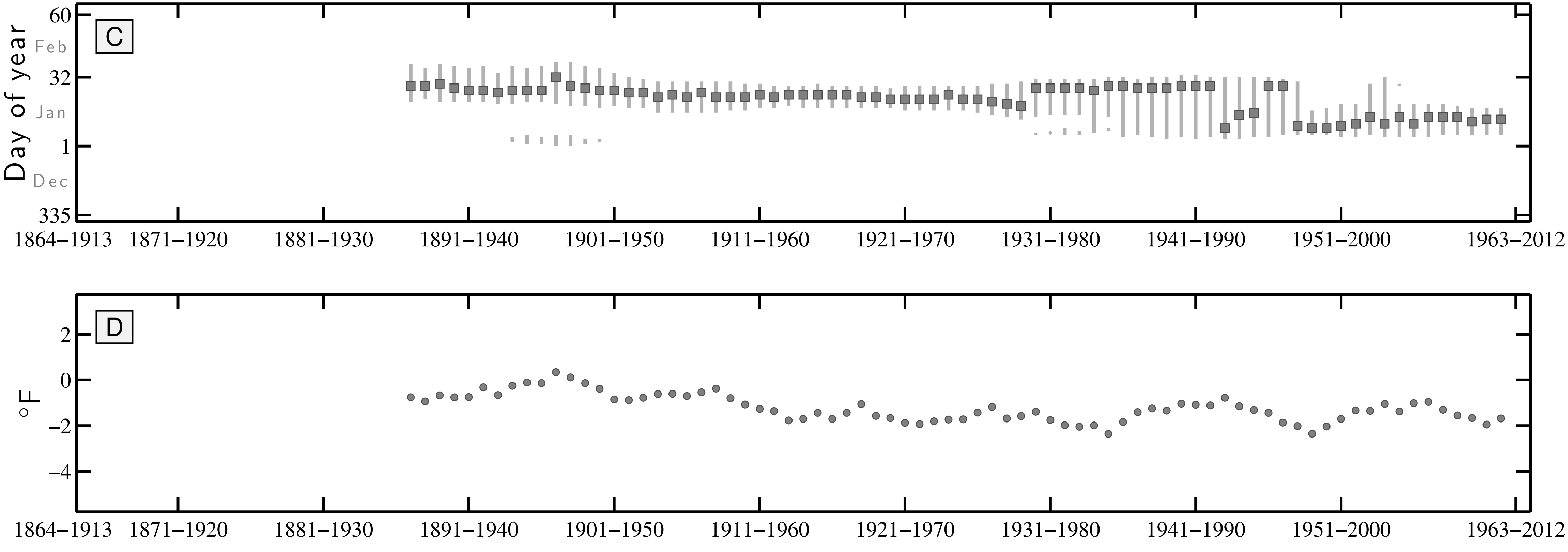

50 year Summer Teletherm dynamics for MENNO, SD:

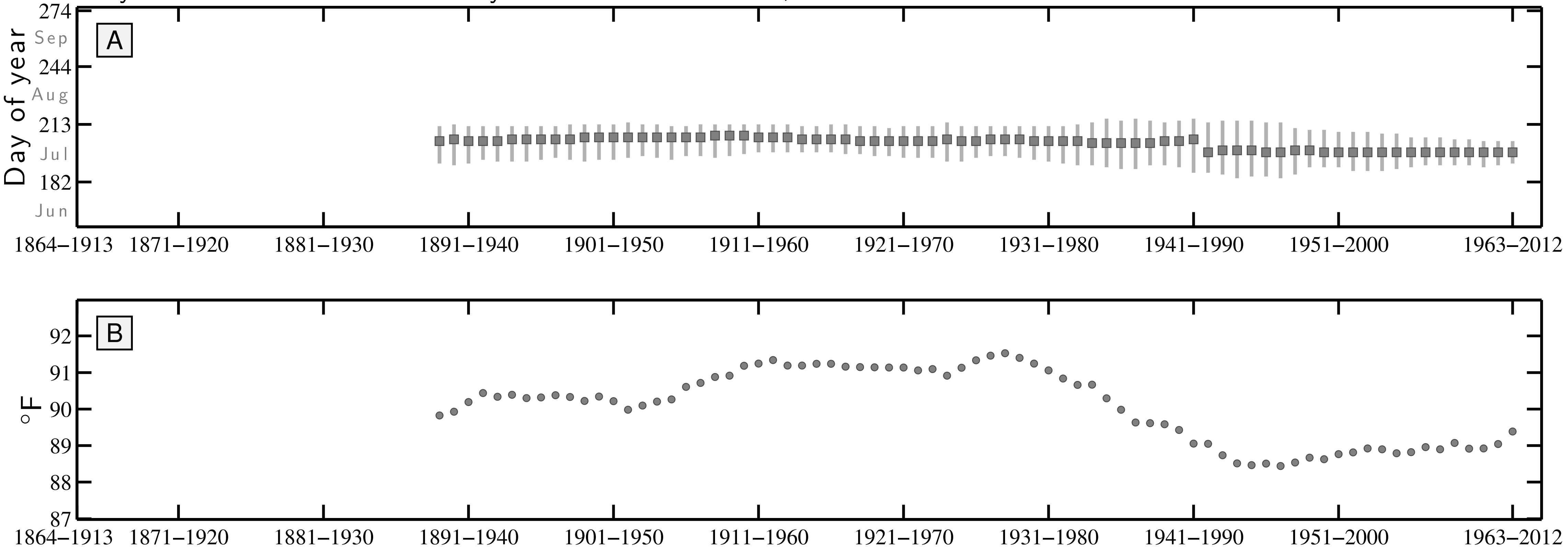

50 year Winter Teletherm dynamics for MENNO, SD:

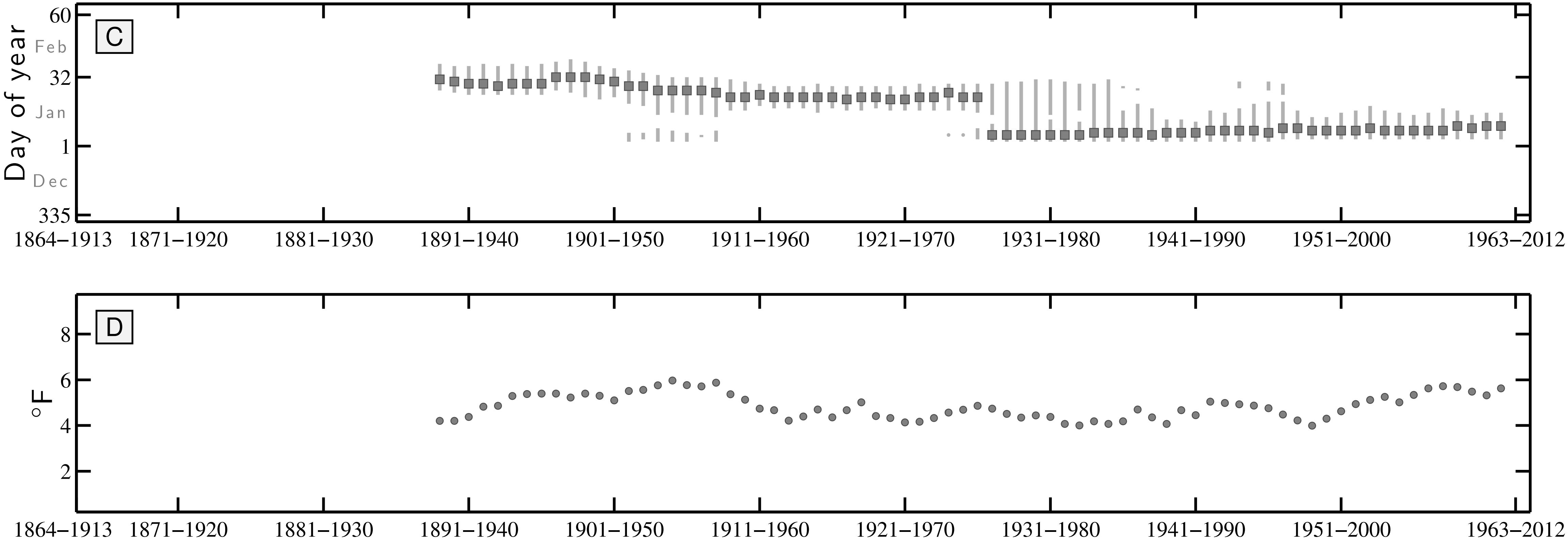

50 year Summer Teletherm dynamics for MILBANK 4 NW, SD:

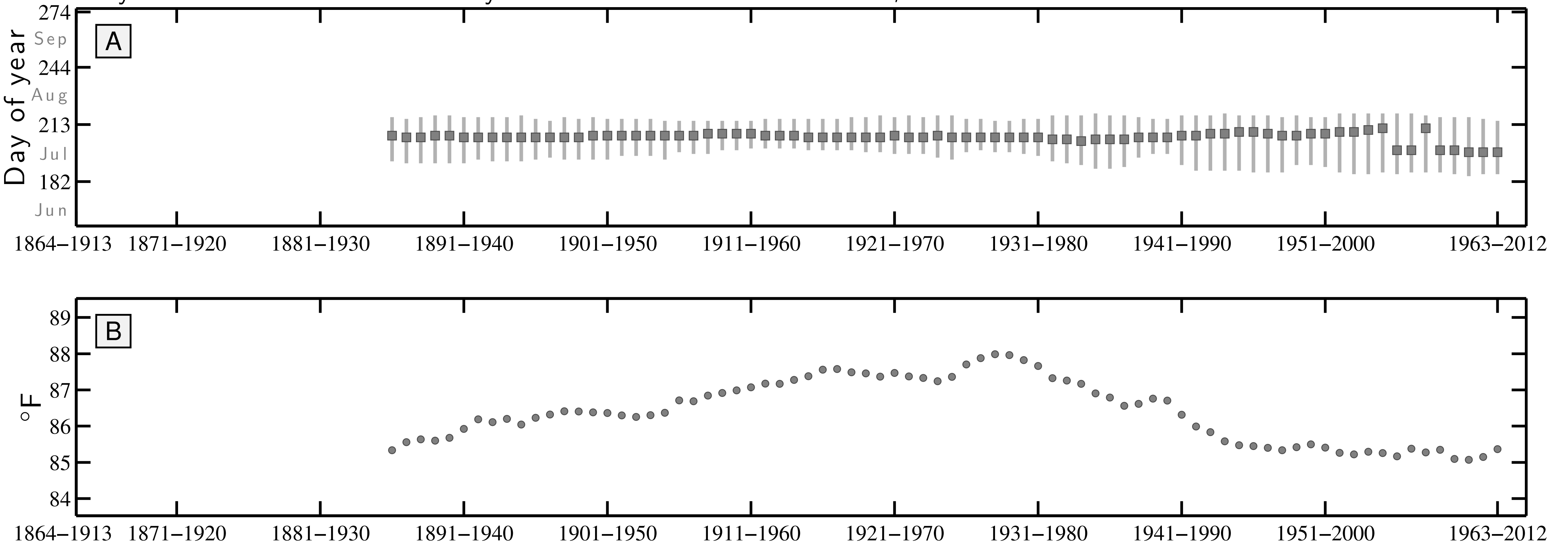

50 year Winter Teletherm dynamics for MILBANK 4 NW, SD:

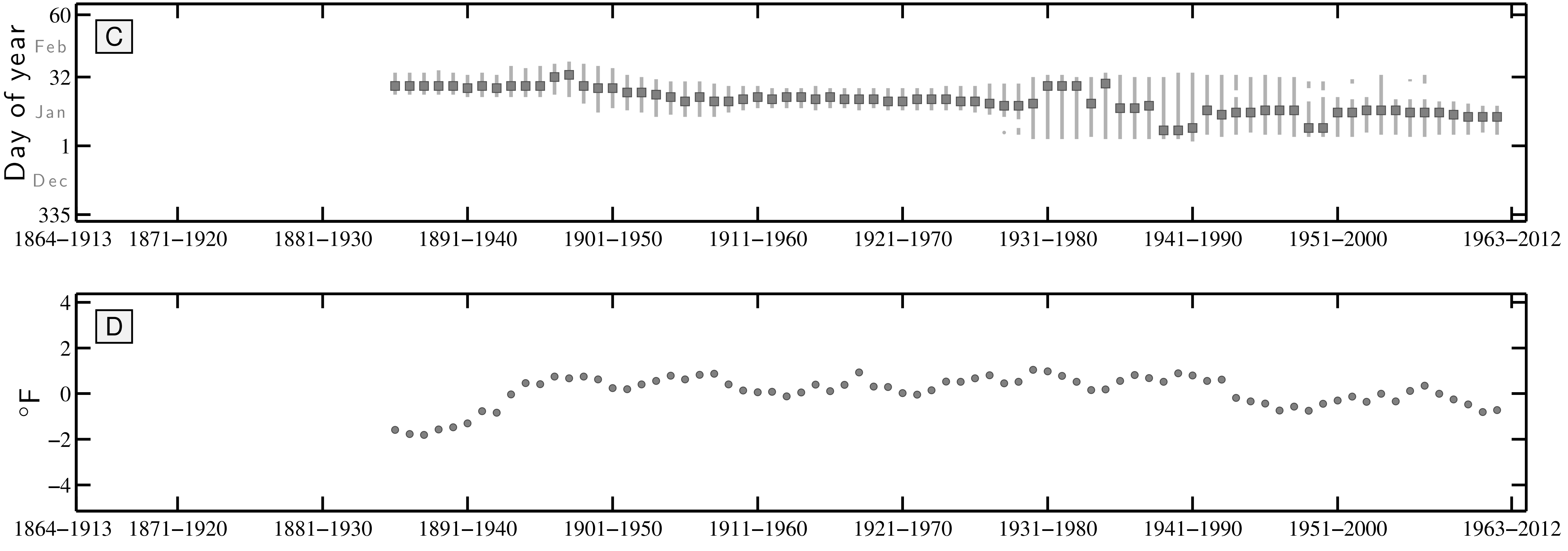

50 year Summer Teletherm dynamics for MURDO, SD:

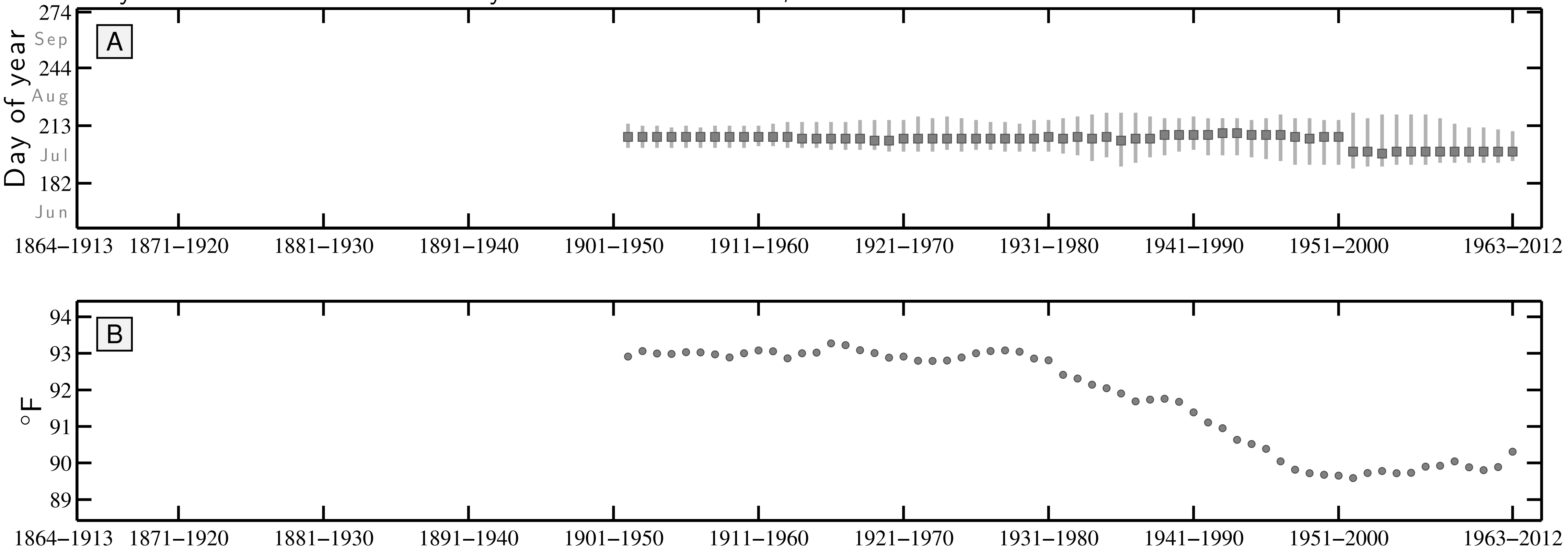

50 year Winter Teletherm dynamics for MURDO, SD:

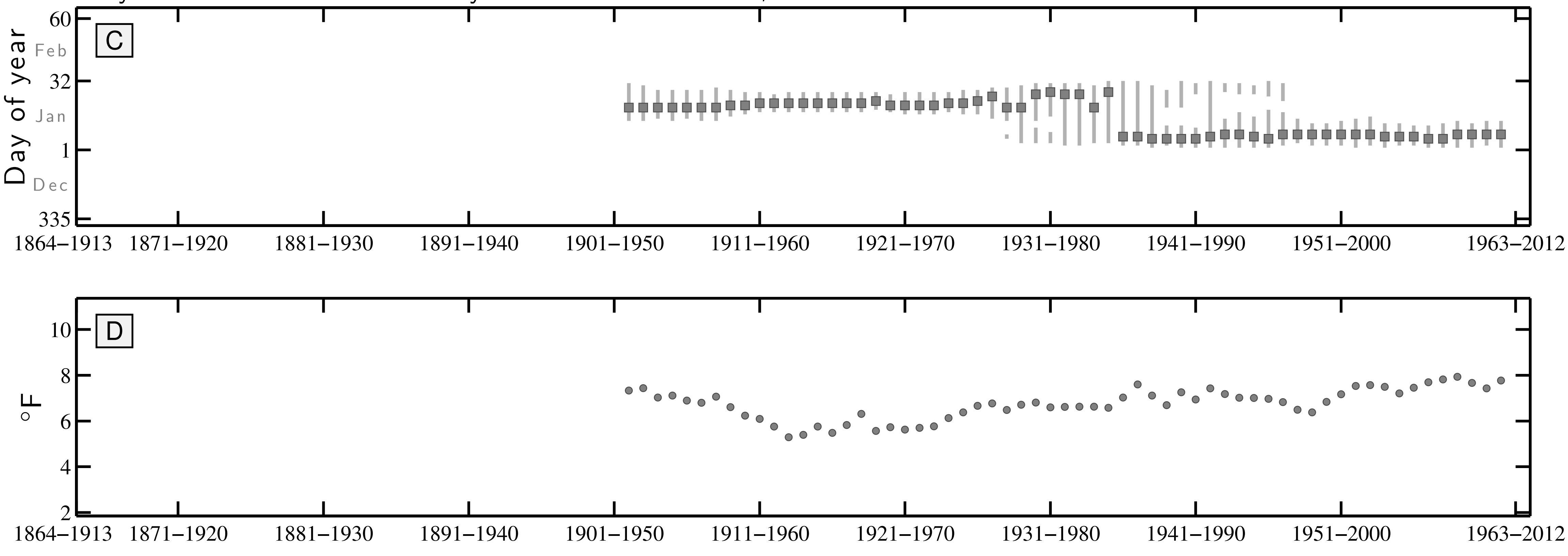

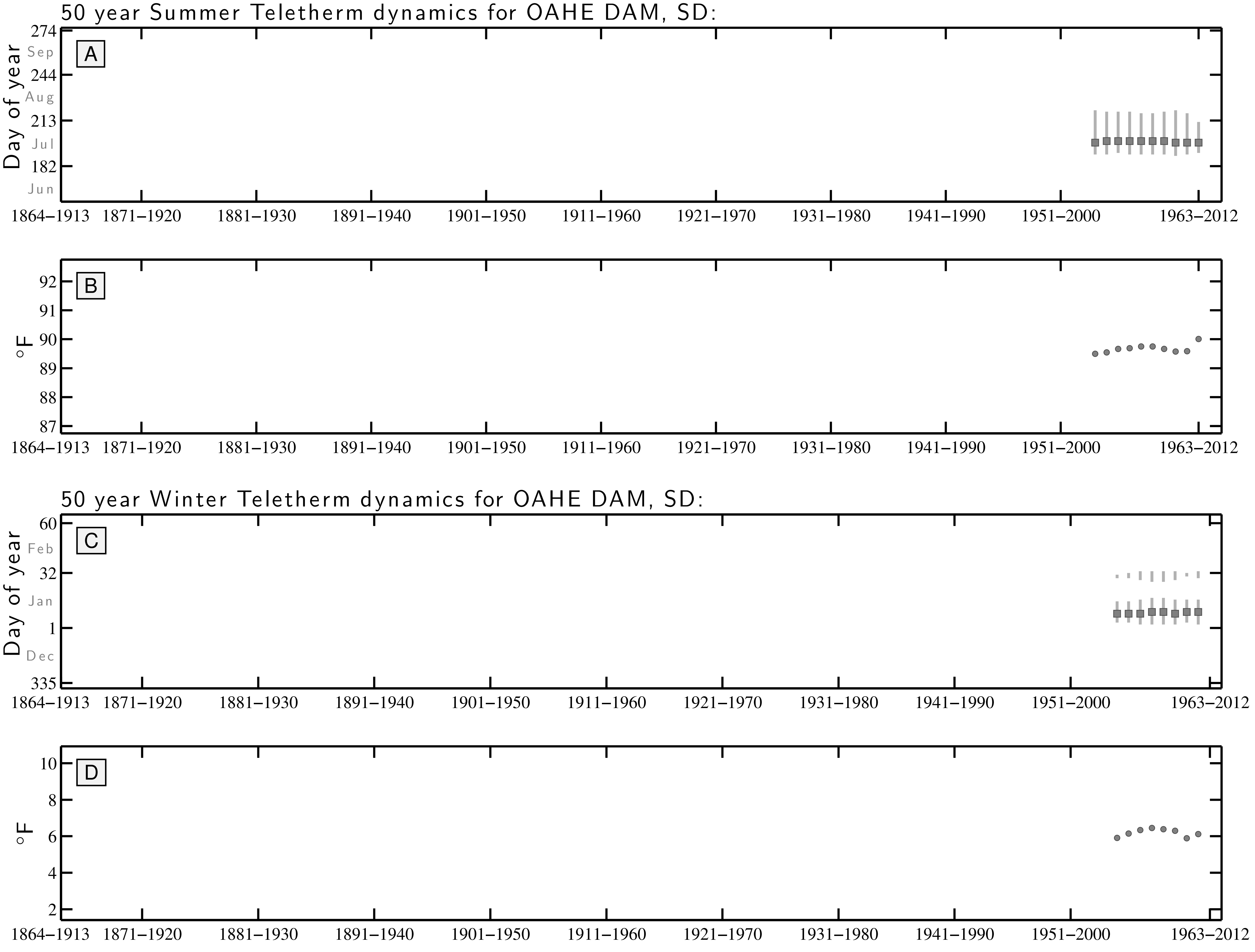
50 year Summer Teletherm dynamics for OAHE DAM, SD:
A
B
Day of year
°F
274
Sep
244
Aug
213
Jul
182
Jun
92
91
90
89
88
87
1864–1913
1871–1920
1881–1930
1891–1940
1901–1950
1911–1960
1921–1970
1931–1980
1941–1990
1951–2000
1963–2012
50 year Winter Teletherm dynamics for OAHE DAM, SD:
C
D
60
Feb
32
Jan
1
Dec
335
10
8
6
4
2

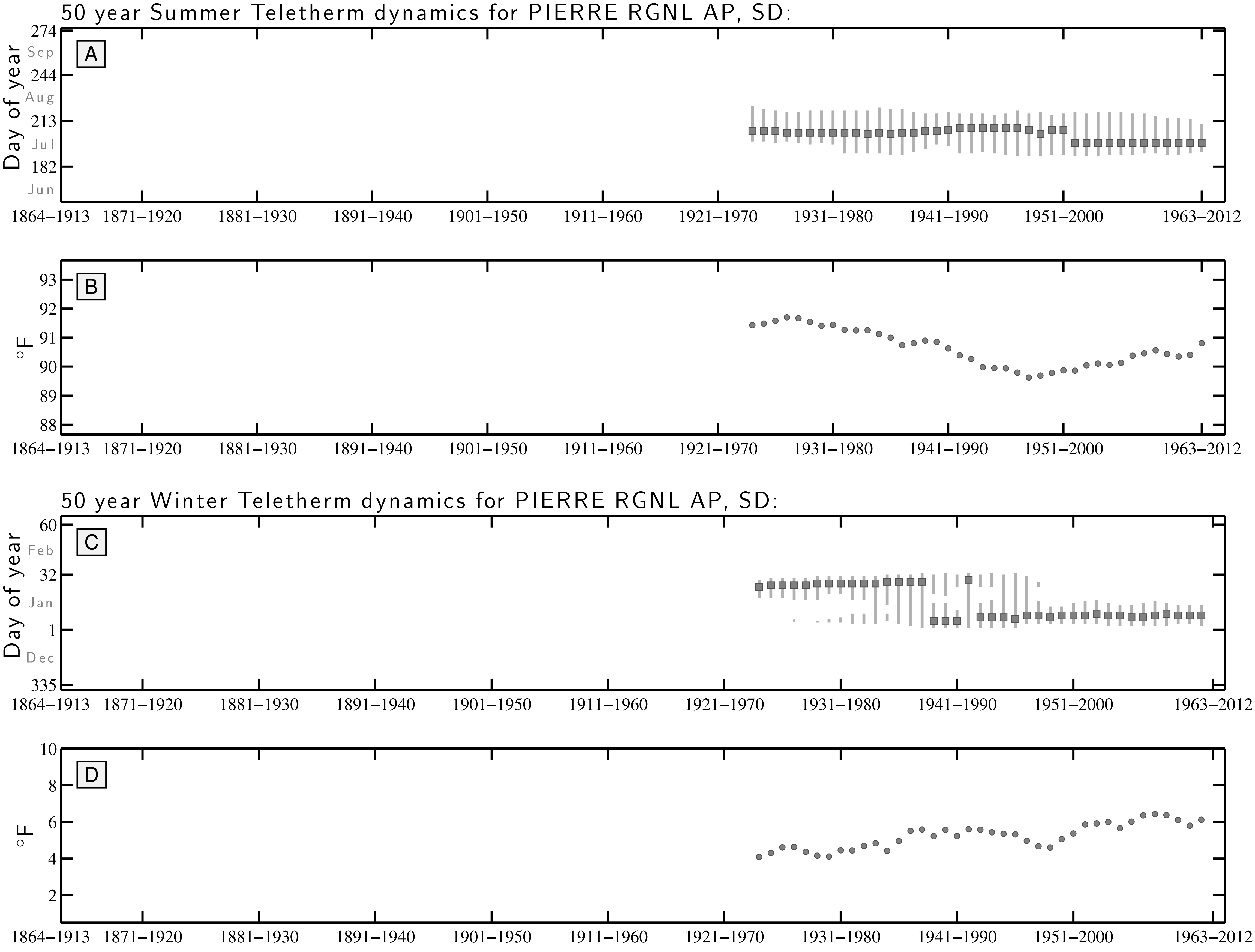
50 year Summer Teletherm dynamics for PIERRE RGNL AP, SD:
A
B
50 year Winter Teletherm dynamics for PIERRE RGNL AP, SD:
C
D
Day of year
°F
274
Sep
244
Aug
213
Jul
182
Jun
93
92
91
90
89
88
60
Feb
32
Jan
1
Dec
335
10
8
6
4
2
1864–1913
1871–1920
1881–1930
1891–1940
1901–1950
1911–1960
1921–1970
1931–1980
1941–1990
1951–2000
1963–2012

50 year Summer Teletherm dynamics for RAPID CITY 4NW, SD:

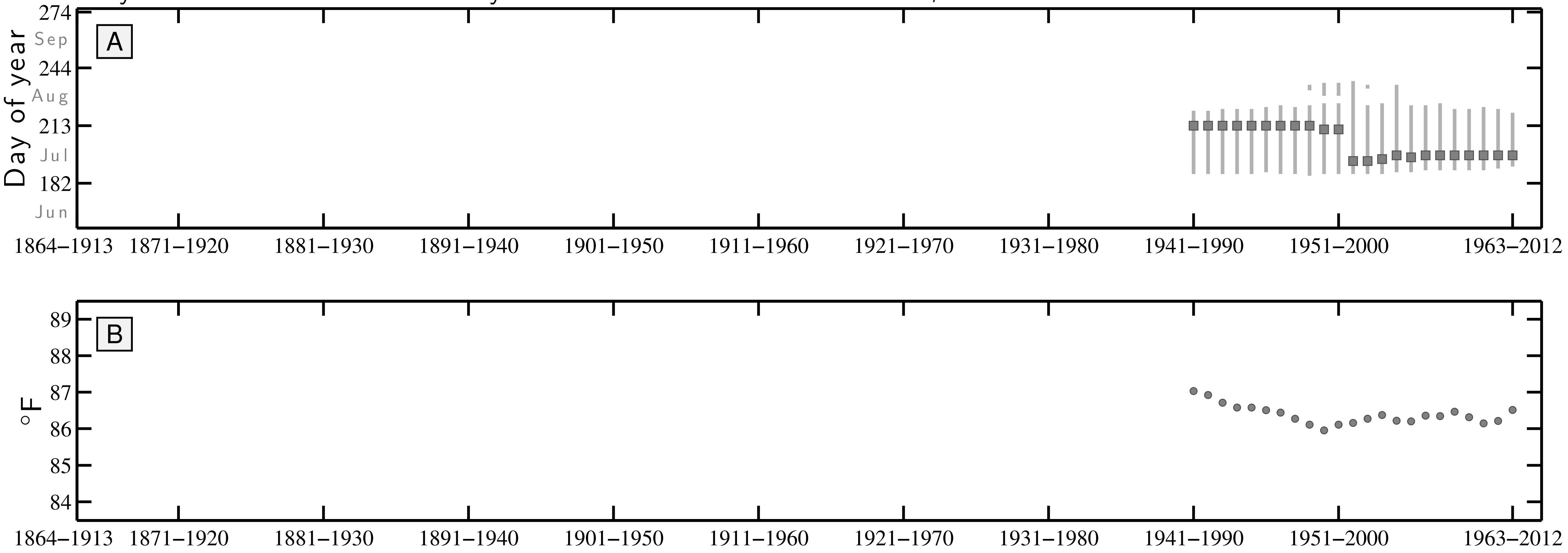

50 year Winter Teletherm dynamics for RAPID CITY 4NW, SD:

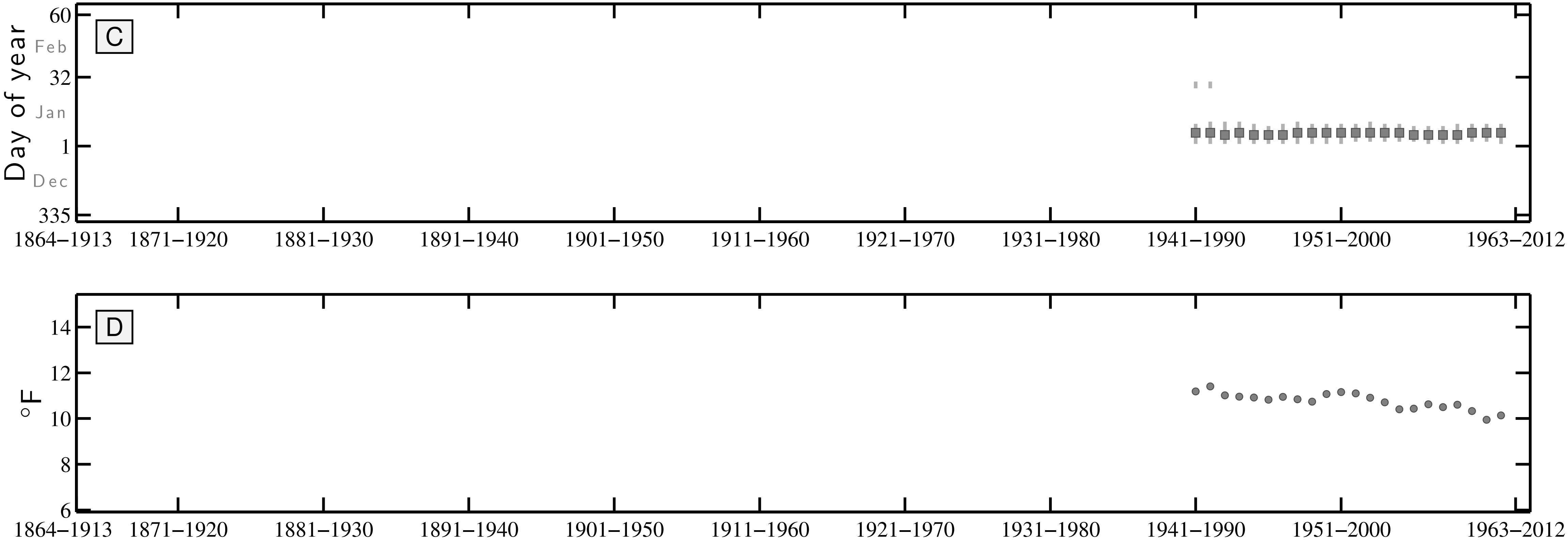

50 year Summer Teletherm dynamics for VERMILLION 2 SE, SD:

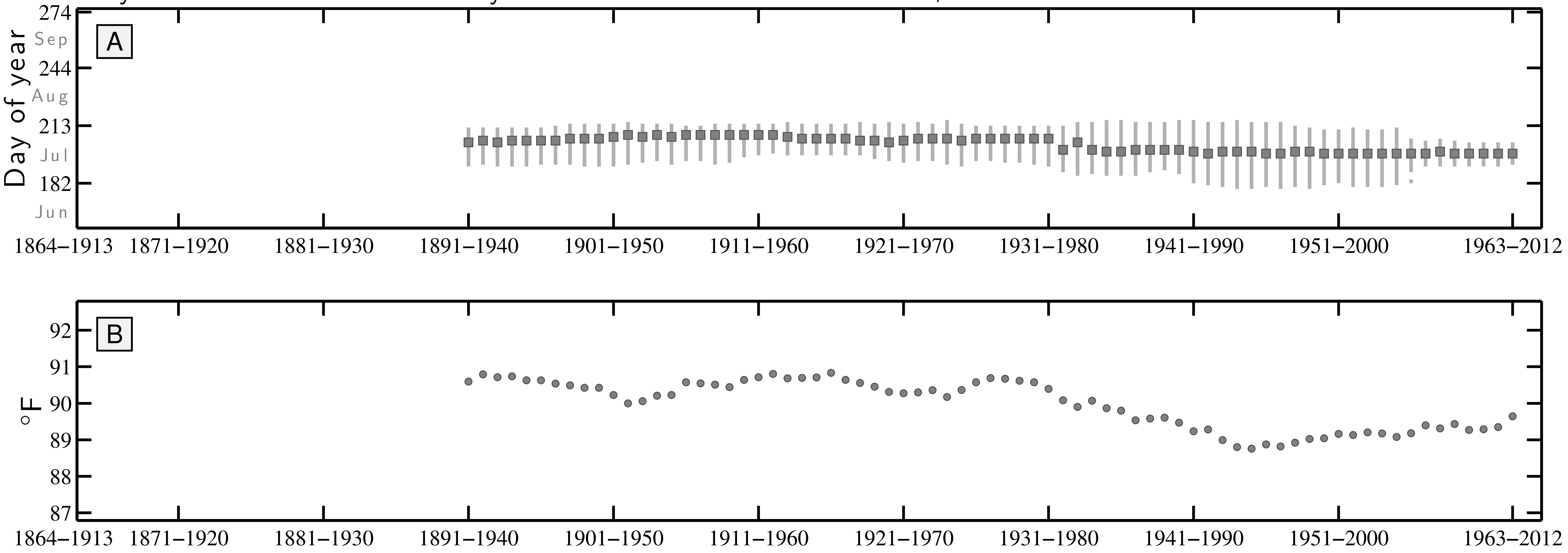

50 year Winter Teletherm dynamics for VERMILLION 2 SE, SD:

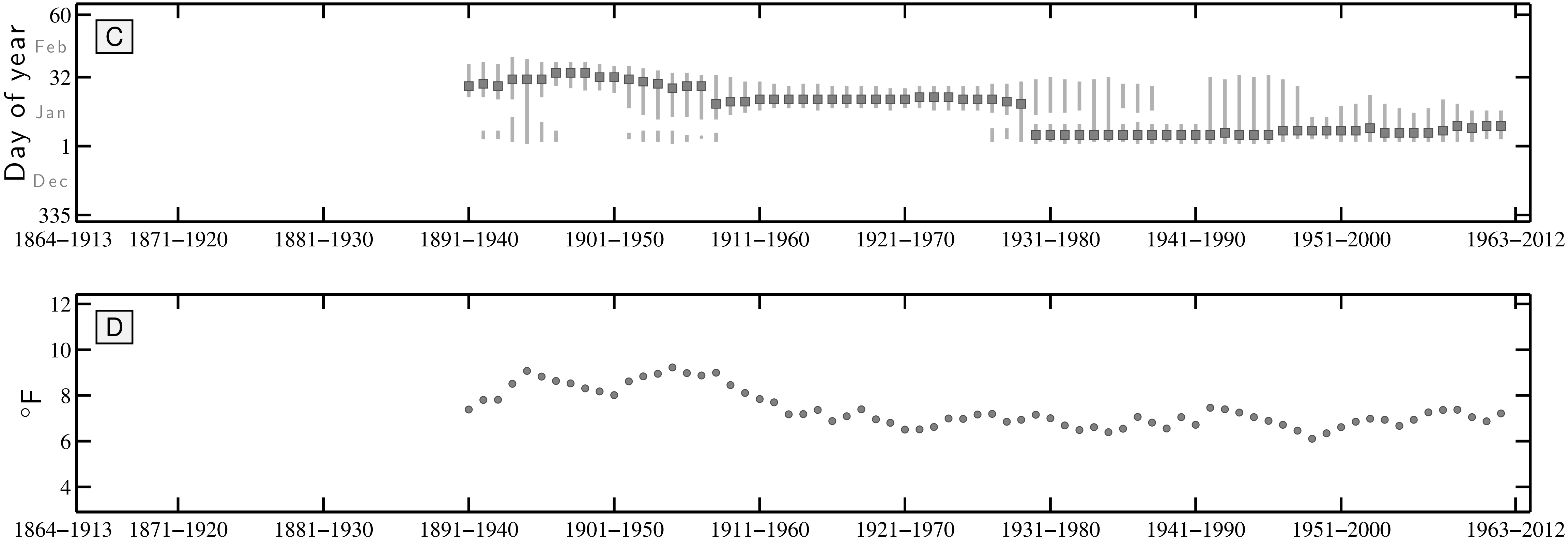

50 year Summer Teletherm dynamics for WATERTOWN RGNL AP, SD:

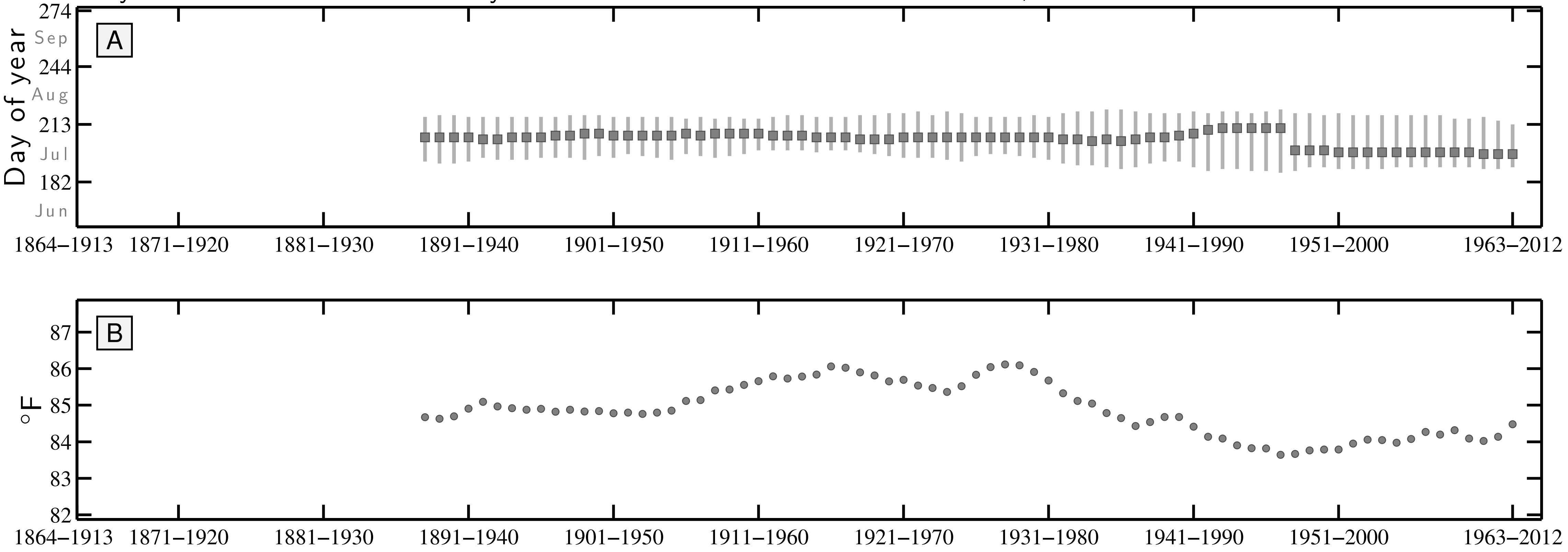

50 year Winter Teletherm dynamics for WATERTOWN RGNL AP, SD:

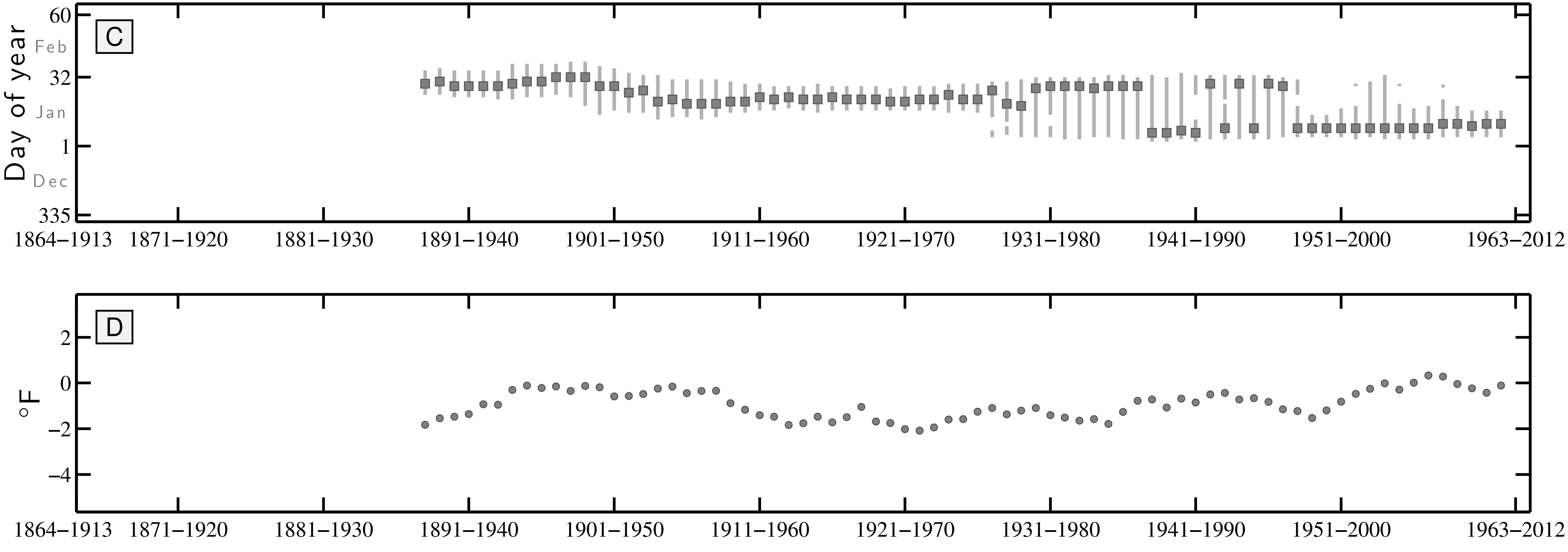

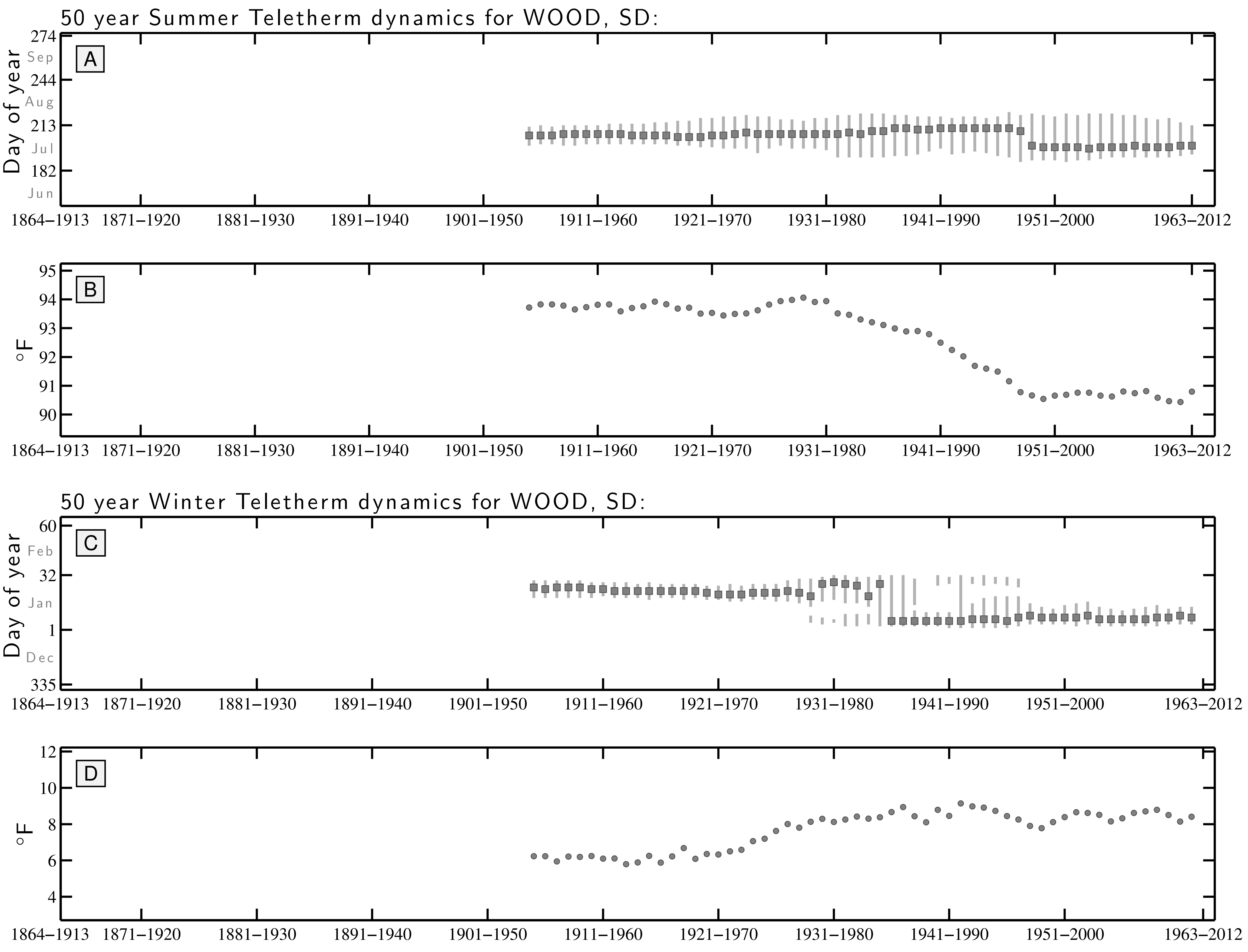
50 year Summer Teletherm dynamics for WOOD, SD:
A
B
50 year Winter Teletherm dynamics for WOOD, SD:
C
D
Day of year
°F
Sep
Aug
Jul
Jun
Feb
Jan
Dec
274
244
213
182
95
94
93
92
91
90
60
32
1
335
12
10
8
6
4
1864–1913
1871–1920
1881–1930
1891–1940
1901–1950
1911–1960
1921–1970
1931–1980
1941–1990
1951–2000
1963–2012

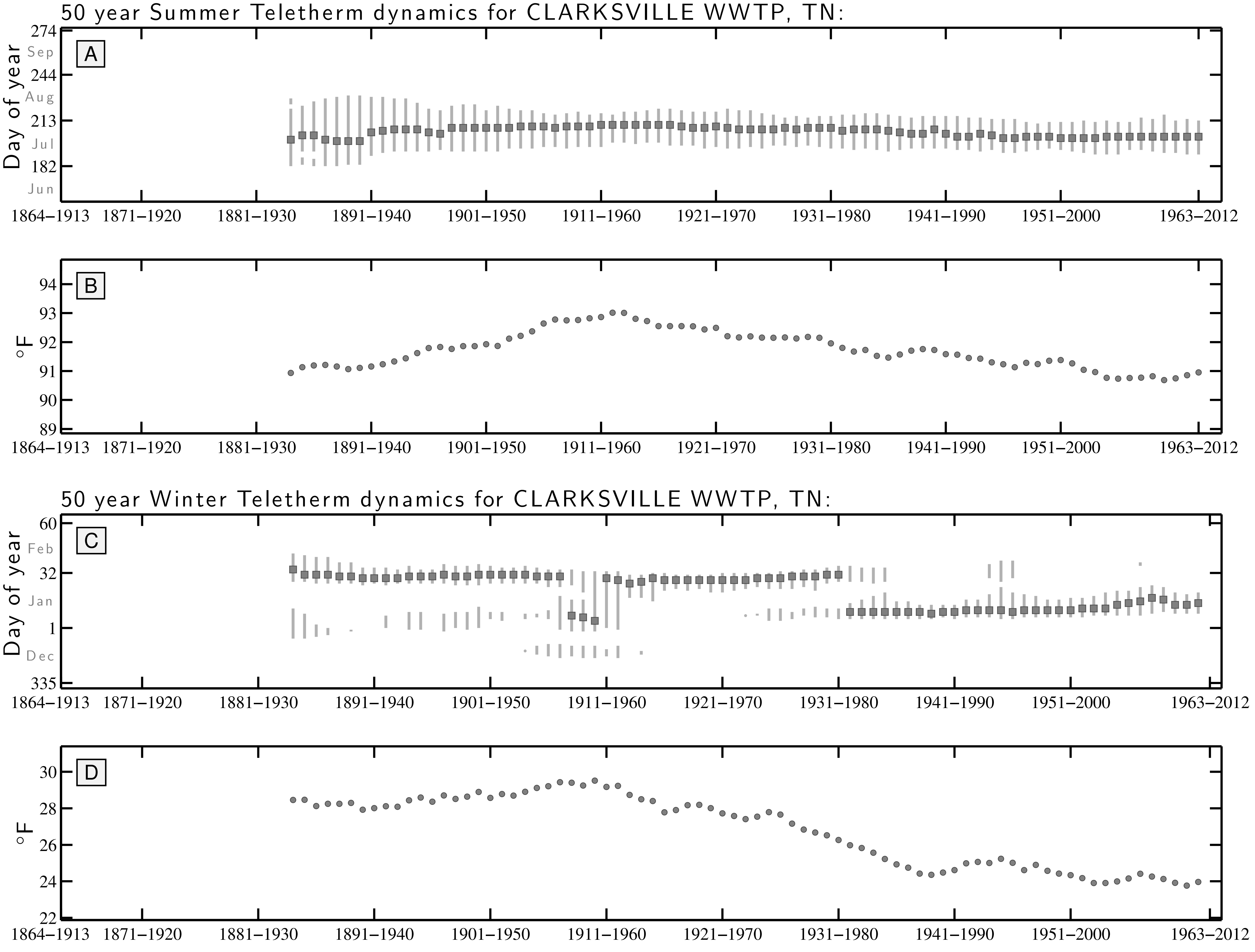

50 year Summer Teletherm dynamics for CLARKSVILLE WWTP, TN:
A
B
Day of year
°F
274
244
213
182
Sep
Aug
Jul
Jun
94
93
92
91
90
89
1864–1913
1871–1920
1881–1930
1891–1940
1901–1950
1911–1960
1921–1970
1931–1980
1941–1990
1951–2000
1963–2012
50 year Winter Teletherm dynamics for CLARKSVILLE WWTP, TN:
C
D
60
32
1
335
Feb
Jan
Dec
30
28
26
24
22

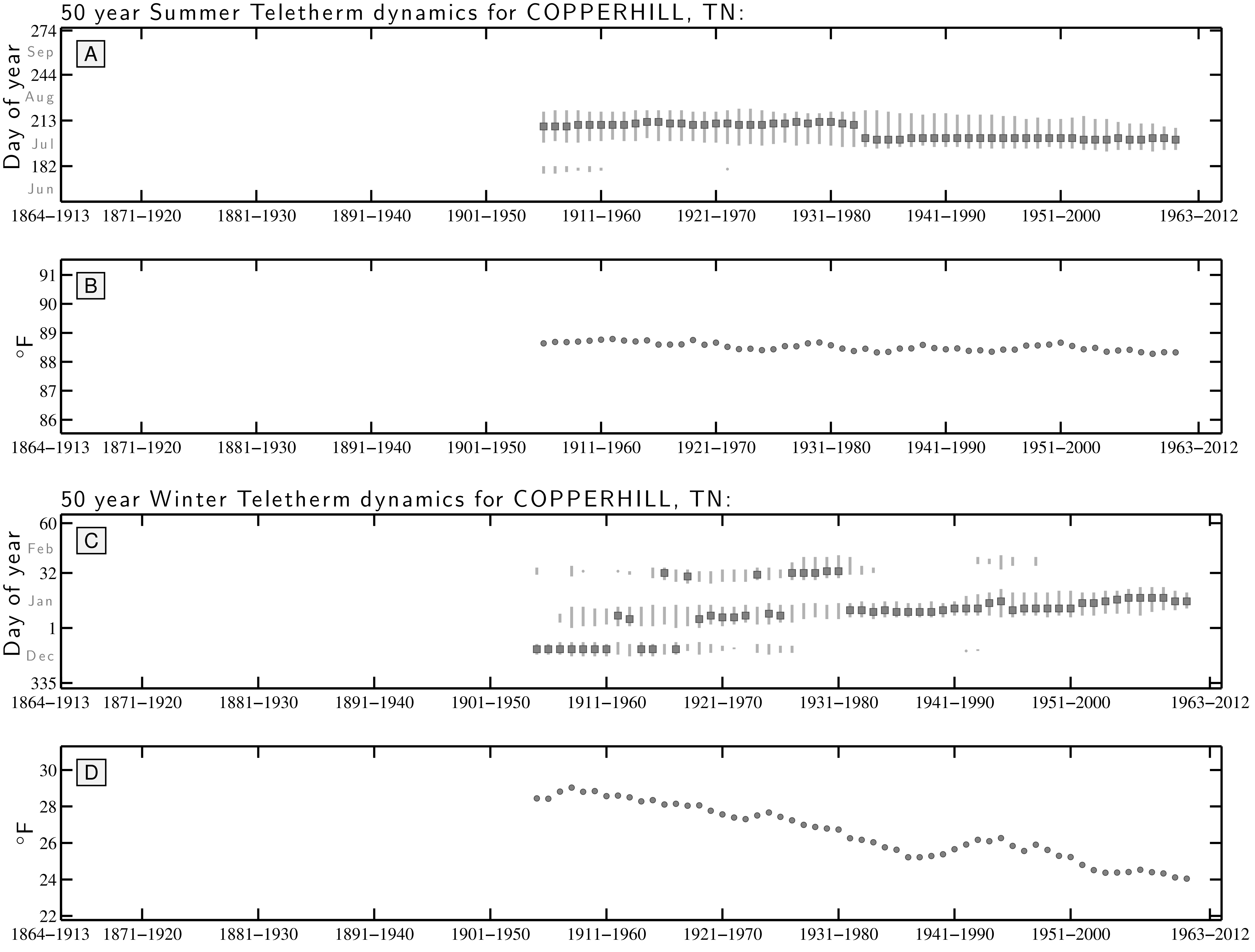
50 year Summer Teletherm dynamics for COPPERHILL, TN:
A
Day of year
274
Sep
244
Aug
213
Jul
182
Jun
B
°F
91
90
89
88
87
86
1864–1913
1871–1920
1881–1930
1891–1940
1901–1950
1911–1960
1921–1970
1931–1980
1941–1990
1951–2000
1963–2012
50 year Winter Teletherm dynamics for COPPERHILL, TN:
C
Day of year
60
Feb
32
Jan
1
Dec
335
D
°F
30
28
26
24
22

50 year Summer Teletherm dynamics for COVINGTON 3 SW, TN:

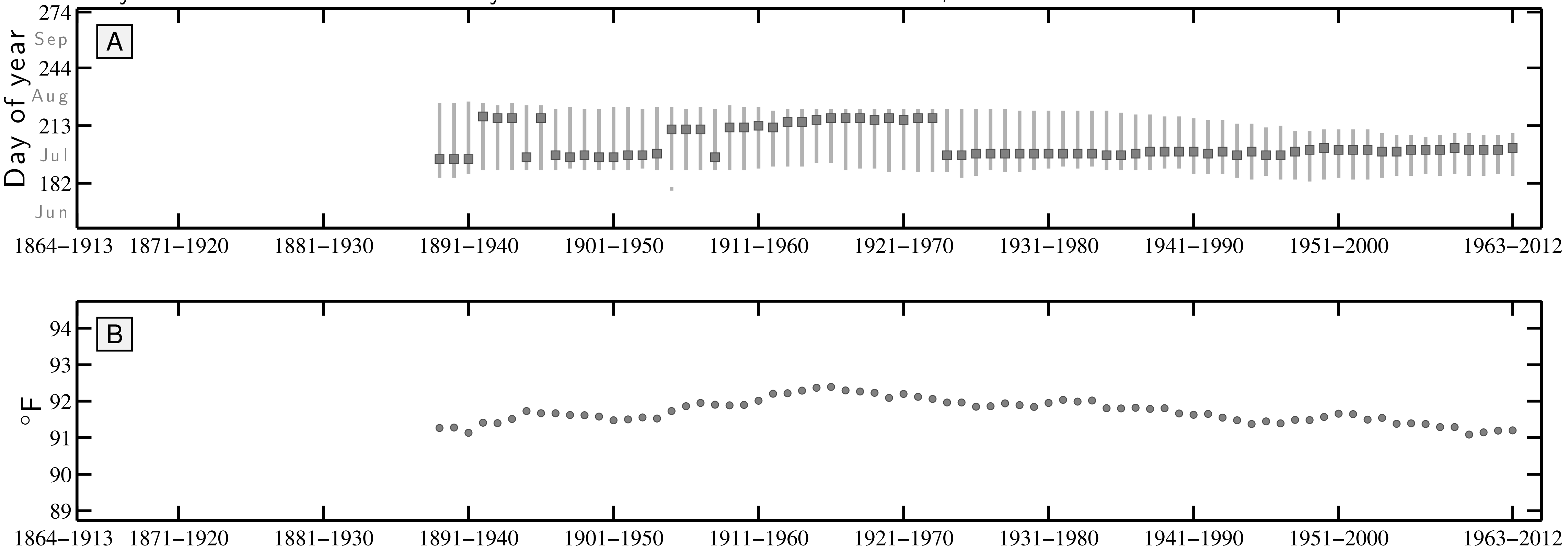

50 year Winter Teletherm dynamics for COVINGTON 3 SW, TN:

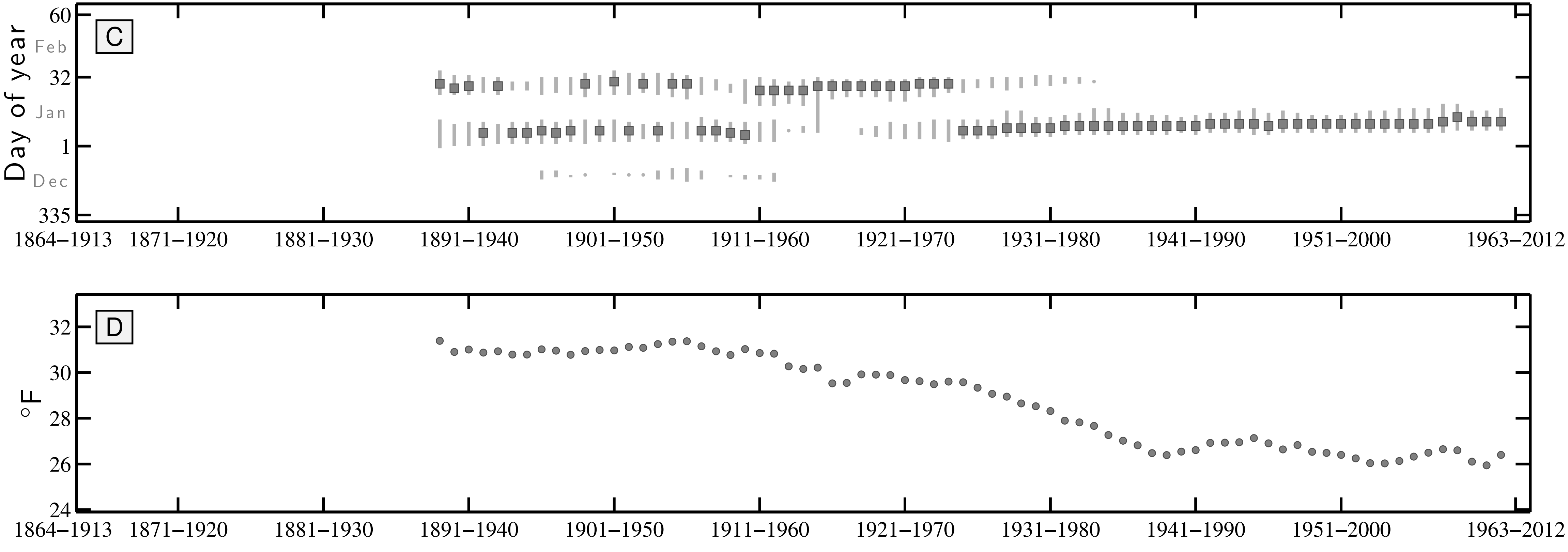

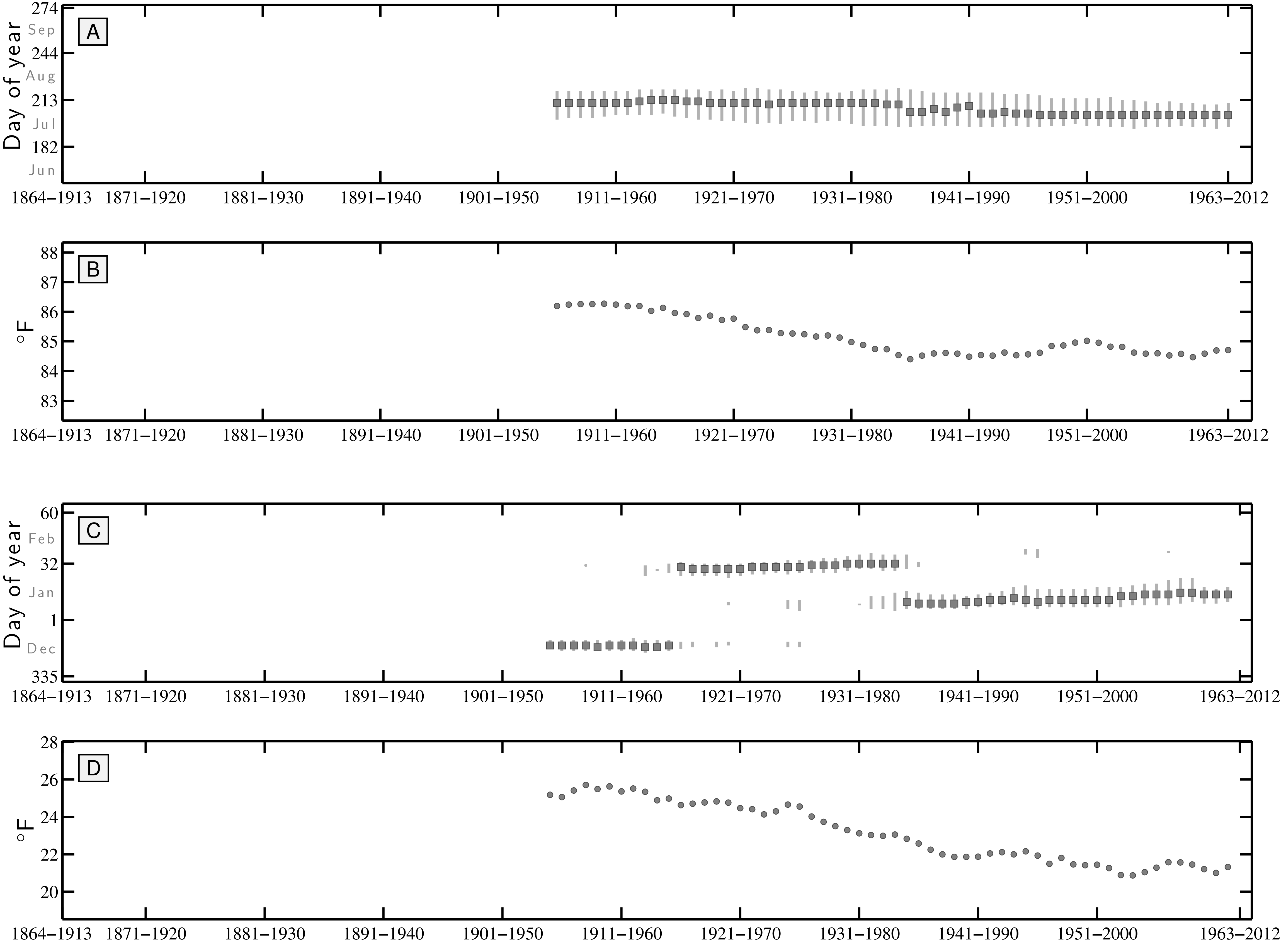
A
Day of year
274
Sep
244
Aug
213
Jul
182
Jun
B
°F
88
87
86
85
84
83
C
Day of year
60
Feb
32
Jan
1
Dec
335
D
°F
28
26
24
22
20
1864–1913
1871–1920
1881–1930
1891–1940
1901–1950
1911–1960
1921–1970
1931–1980
1941–1990
1951–2000
1963–2012

50 year Summer Teletherm dynamics for DICKSON, TN:

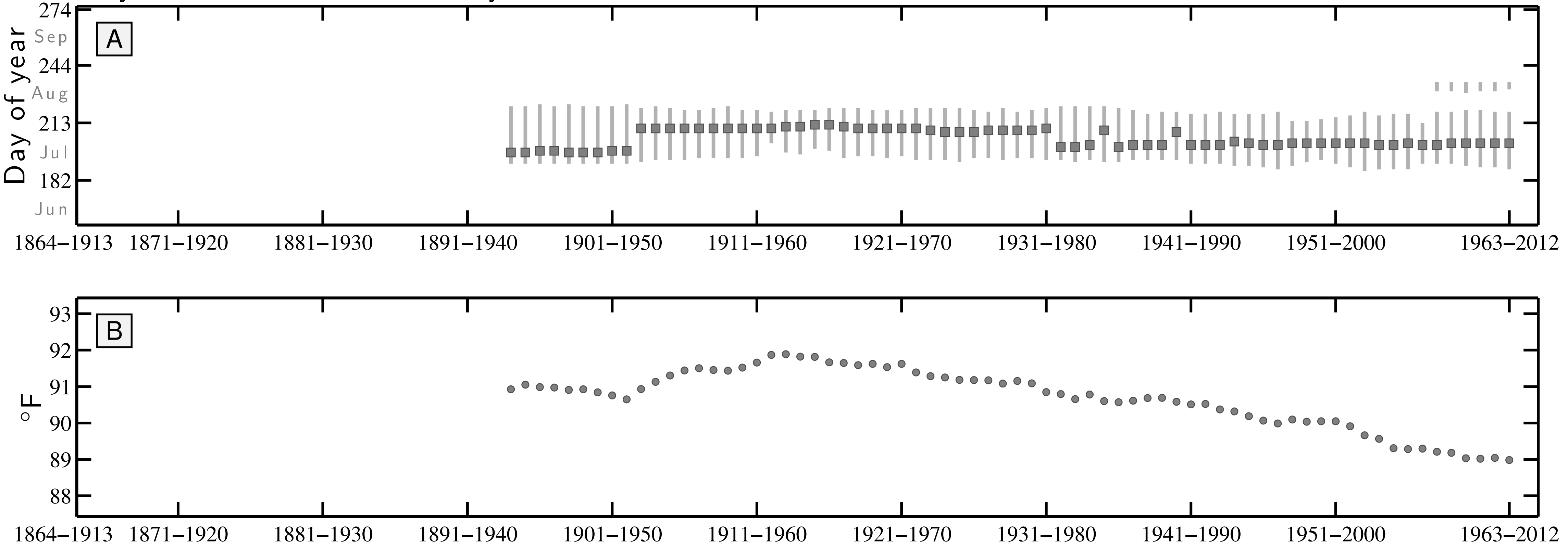

50 year Winter Teletherm dynamics for DICKSON, TN:

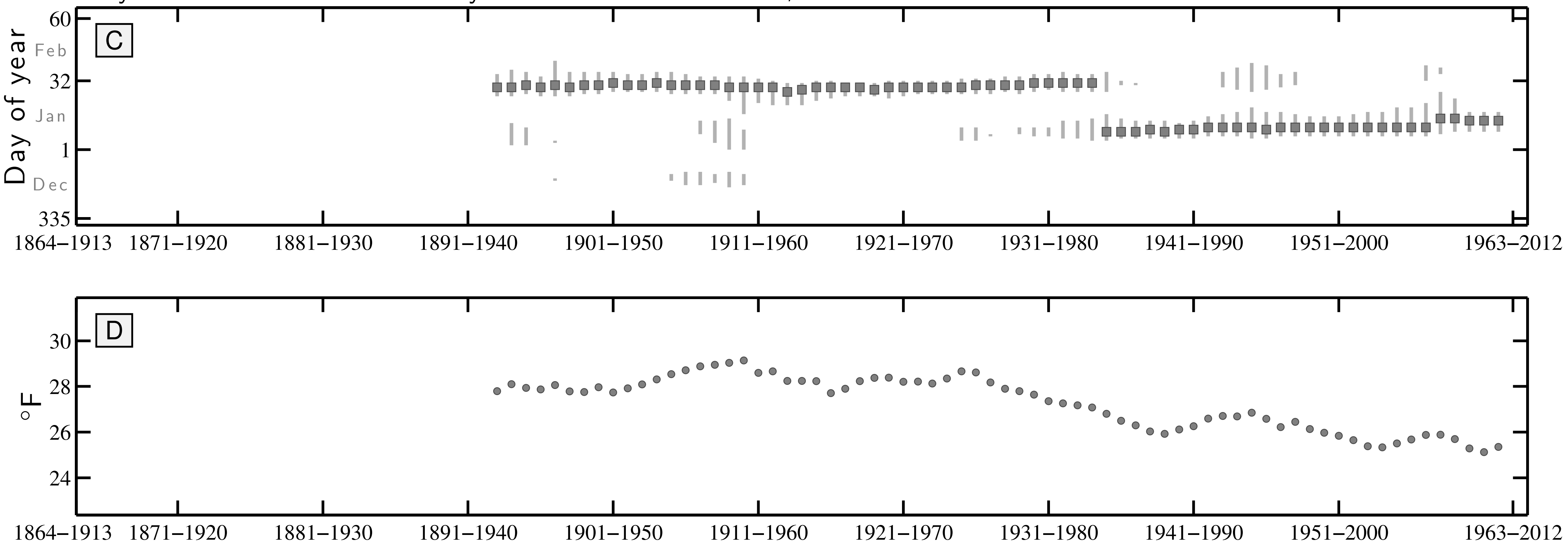

50 year Summer Teletherm dynamics for DOVER 1 W, TN:

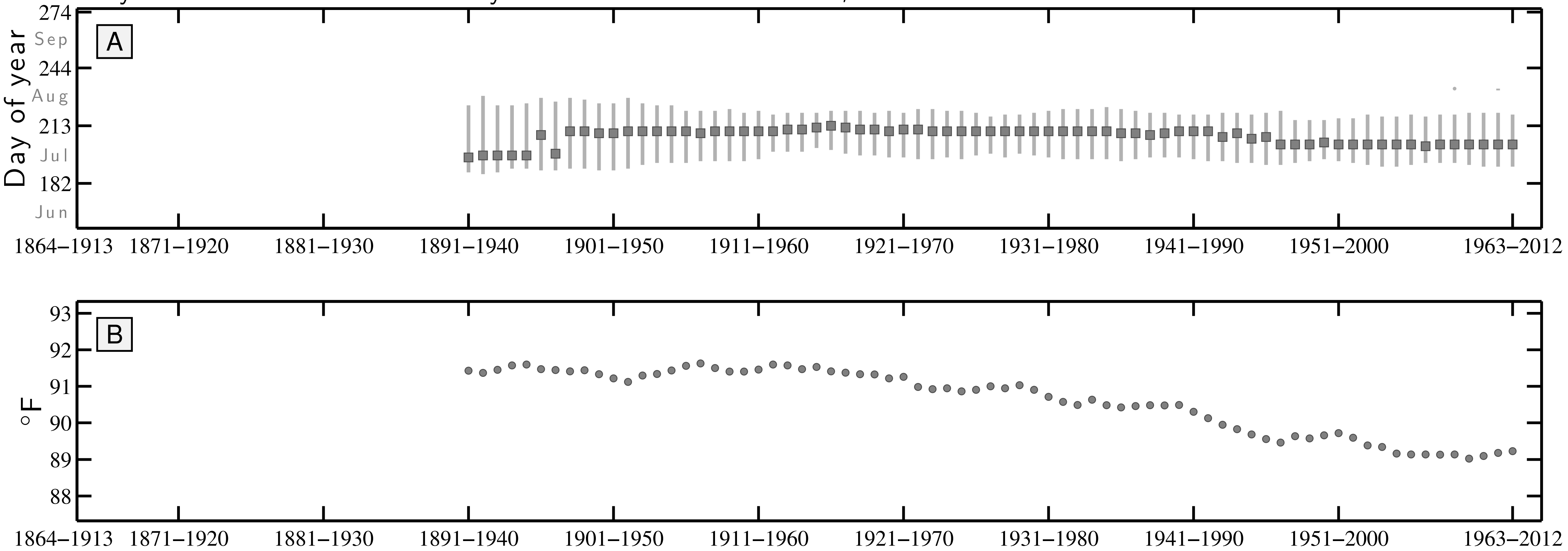

50 year Winter Teletherm dynamics for DOVER 1 W, TN:

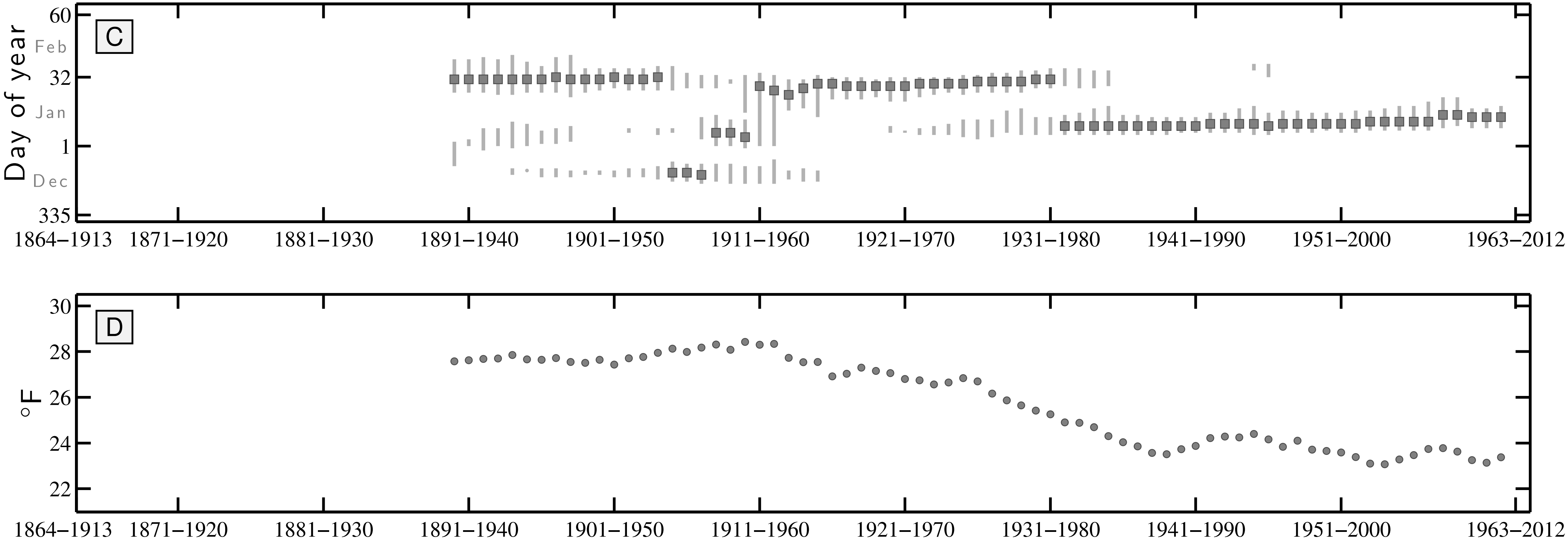

50 year Summer Teletherm dynamics for JACKSON EXP STN, TN:

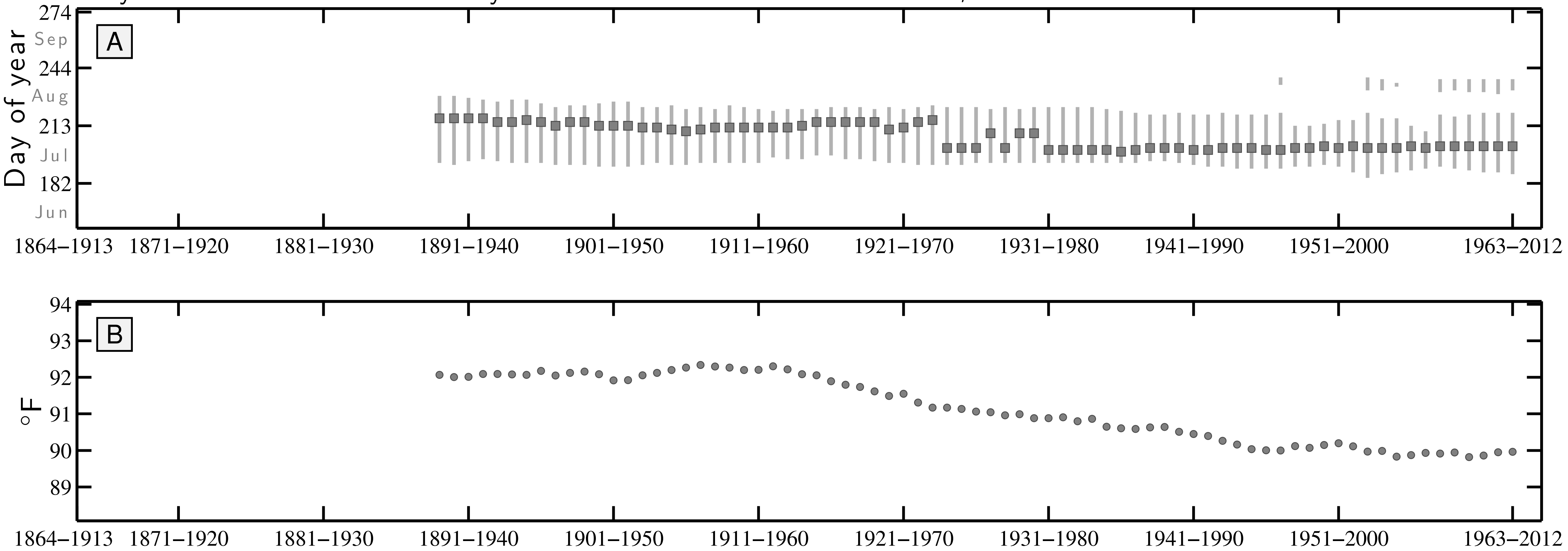

50 year Winter Teletherm dynamics for JACKSON EXP STN, TN:

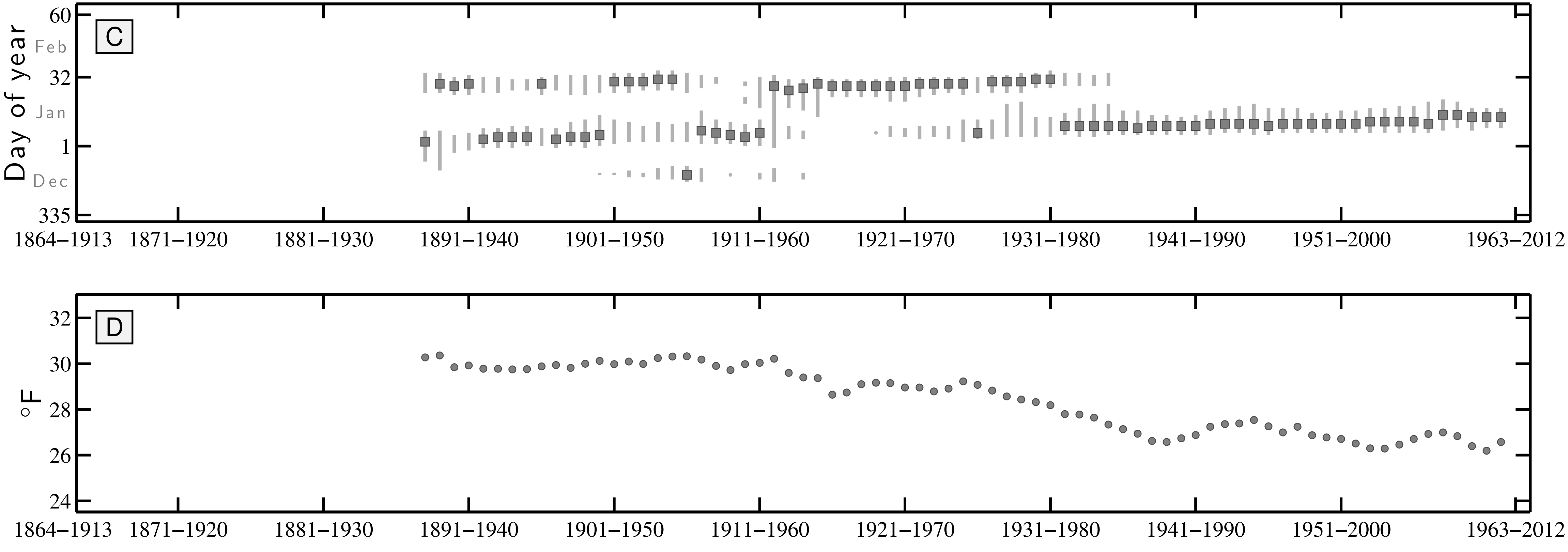

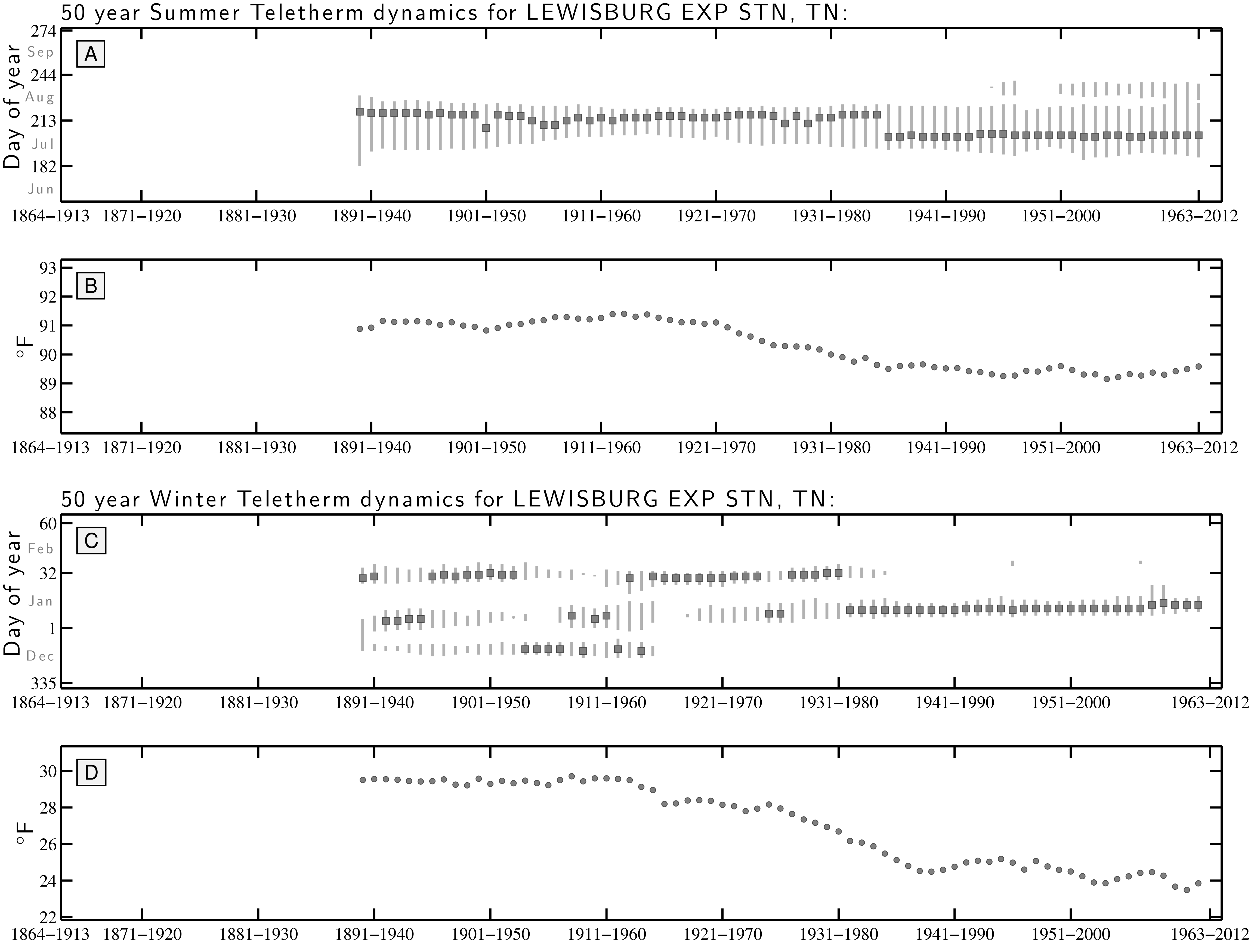

50 year Summer Teletherm dynamics for LEWISBURG EXP STN, TN:
A
Day of year
274
Sep
244
Aug
213
Jul
182
Jun
1864–1913
1871–1920
1881–1930
1891–1940
1901–1950
1911–1960
1921–1970
1931–1980
1941–1990
1951–2000
1963–2012
B
°F
93
92
91
90
89
88
50 year Winter Teletherm dynamics for LEWISBURG EXP STN, TN:
C
Day of year
60
Feb
32
Jan
1
Dec
335
D
°F
30
28
26
24
22

50 year Summer Teletherm dynamics for MC MINNVILLE, TN:

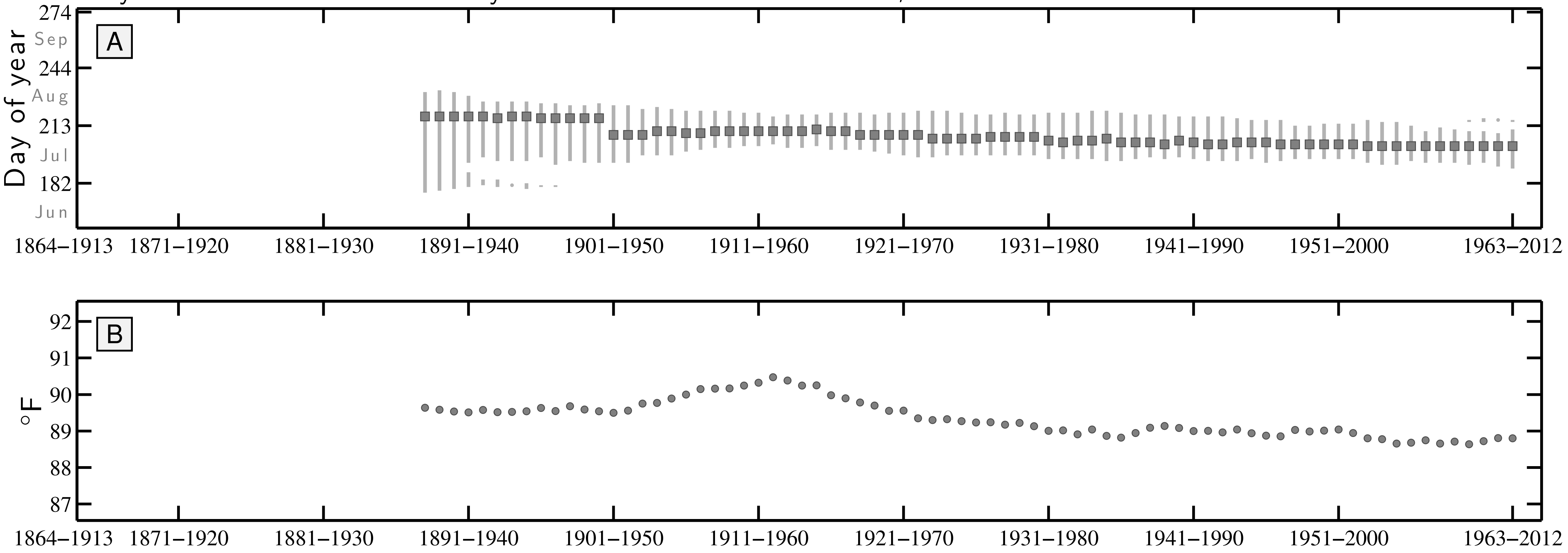

50 year Winter Teletherm dynamics for MC MINNVILLE, TN:

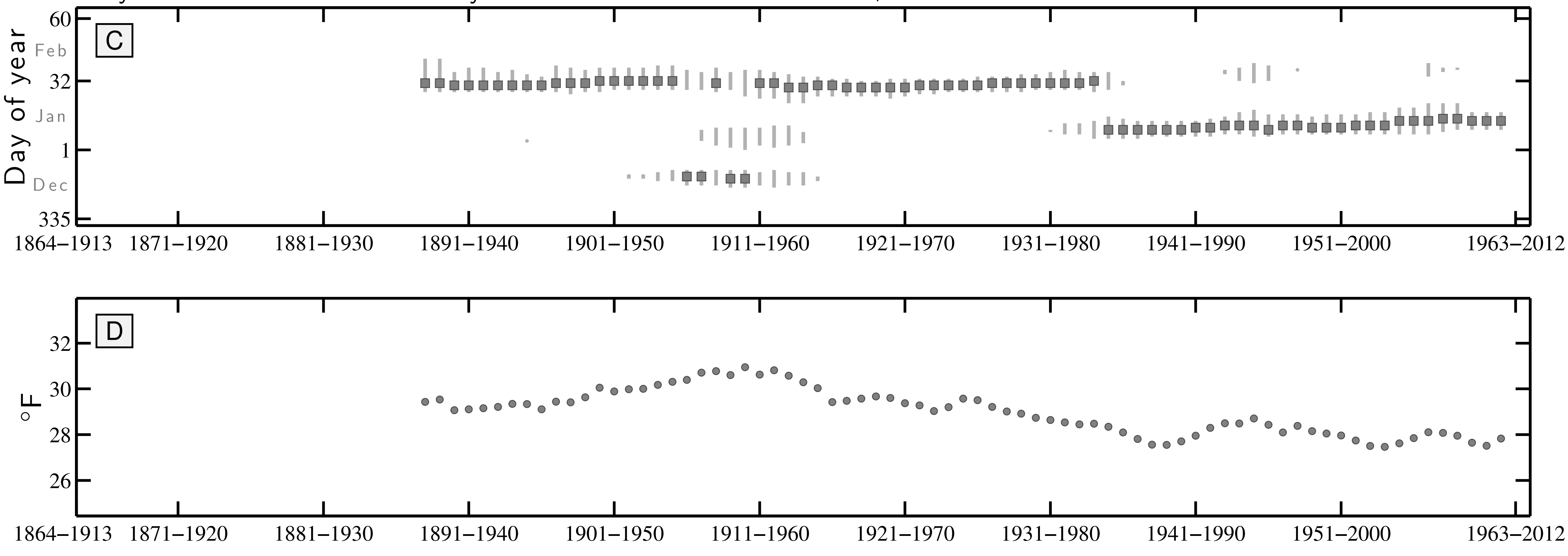

50 year Summer Teletherm dynamics for MURFREESBORO 5 N, TN:

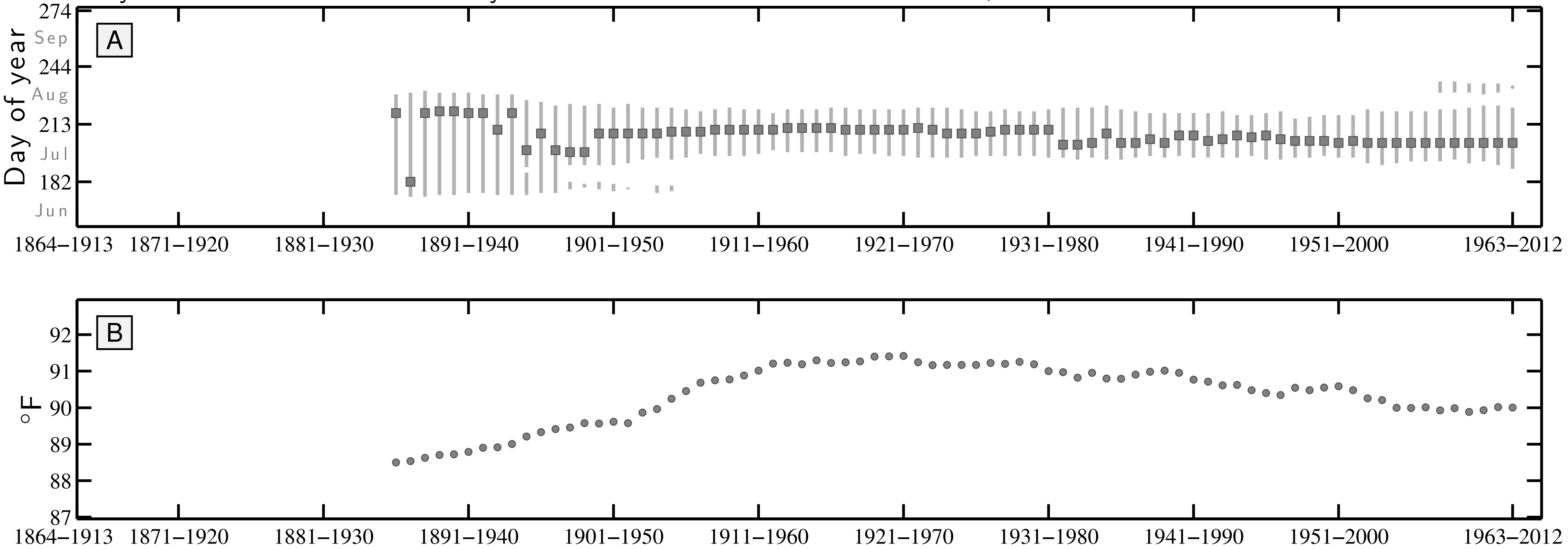

50 year Winter Teletherm dynamics for MURFREESBORO 5 N, TN:

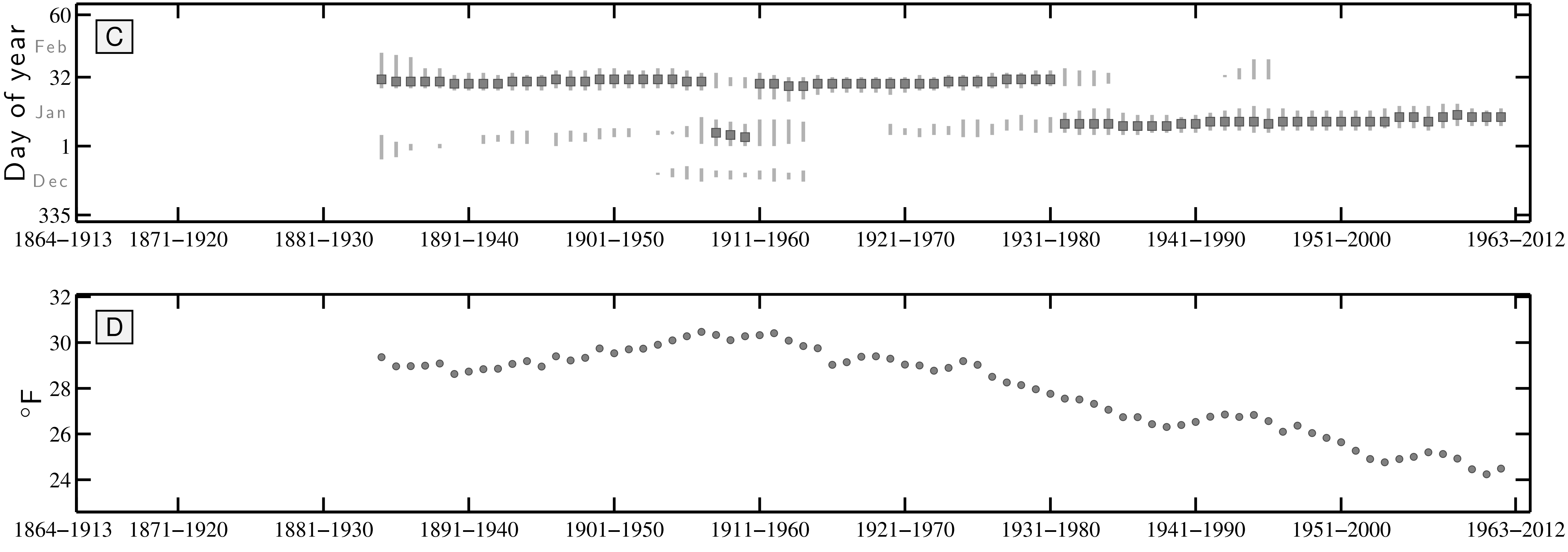

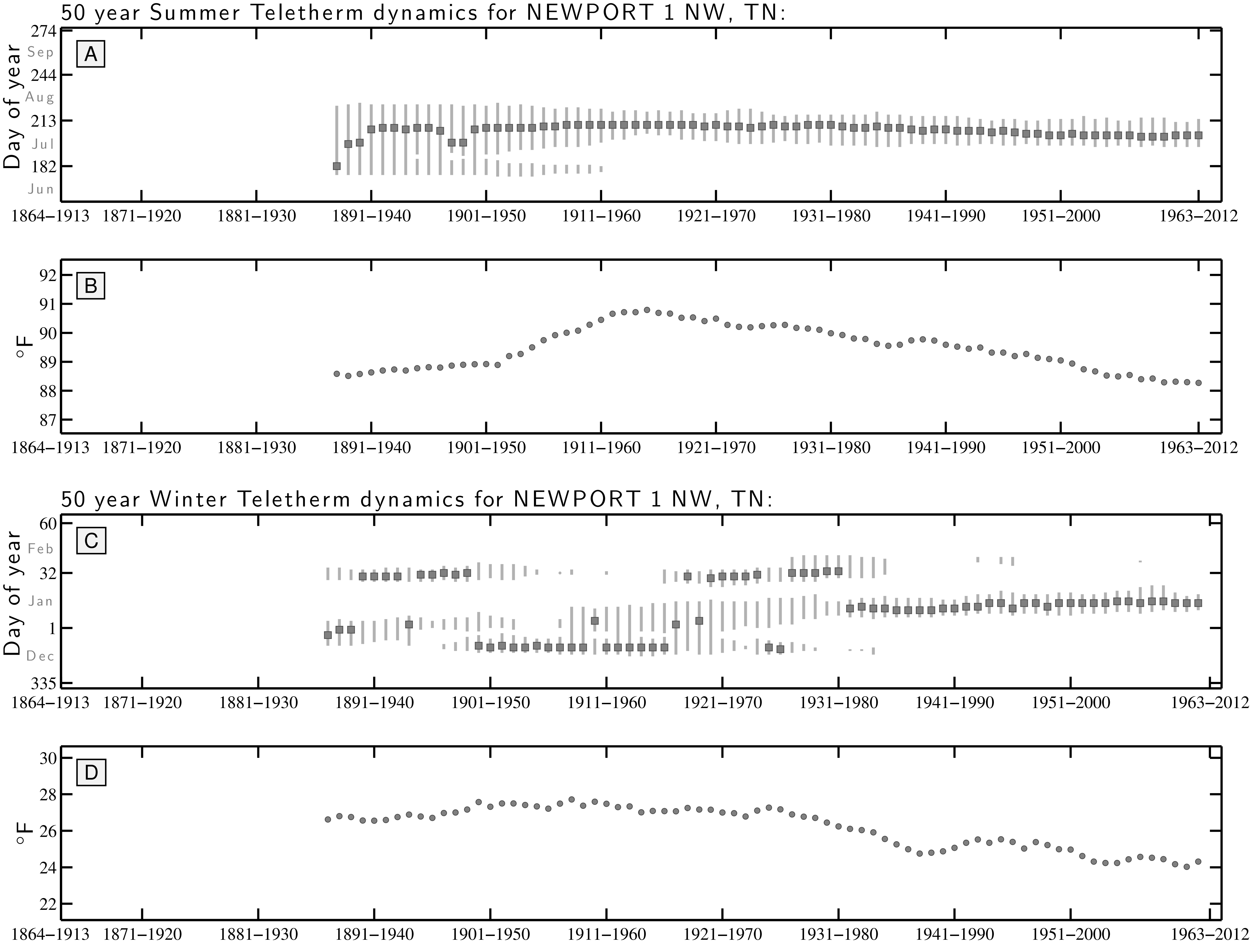
50 year Summer Teletherm dynamics for NEWPORT 1 NW, TN:
A
Day of year
274
Sep
244
Aug
213
Jul
182
Jun
1864–1913
1871–1920
1881–1930
1891–1940
1901–1950
1911–1960
1921–1970
1931–1980
1941–1990
1951–2000
1963–2012
B
°F
92
91
90
89
88
87
50 year Winter Teletherm dynamics for NEWPORT 1 NW, TN:
C
Day of year
60
Feb
32
Jan
1
Dec
335
D
°F
30
28
26
24
22

50 year Summer Teletherm dynamics for ROGERSVILLE 1 NE, TN:

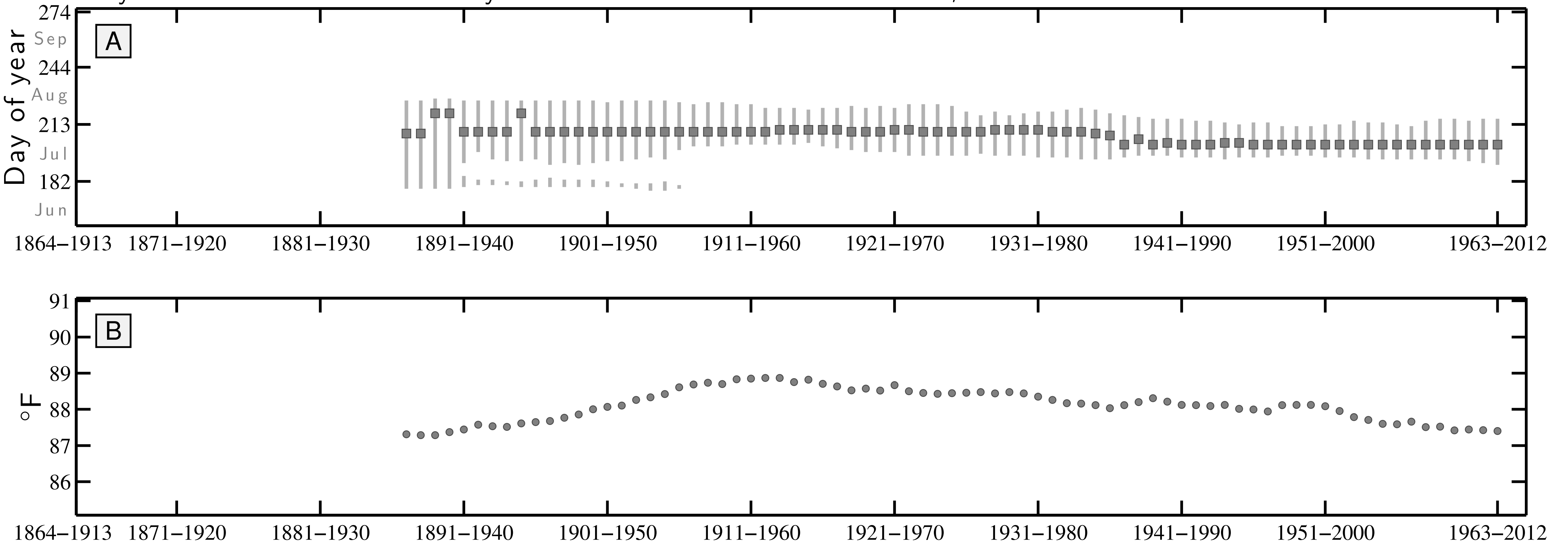

50 year Winter Teletherm dynamics for ROGERSVILLE 1 NE, TN:

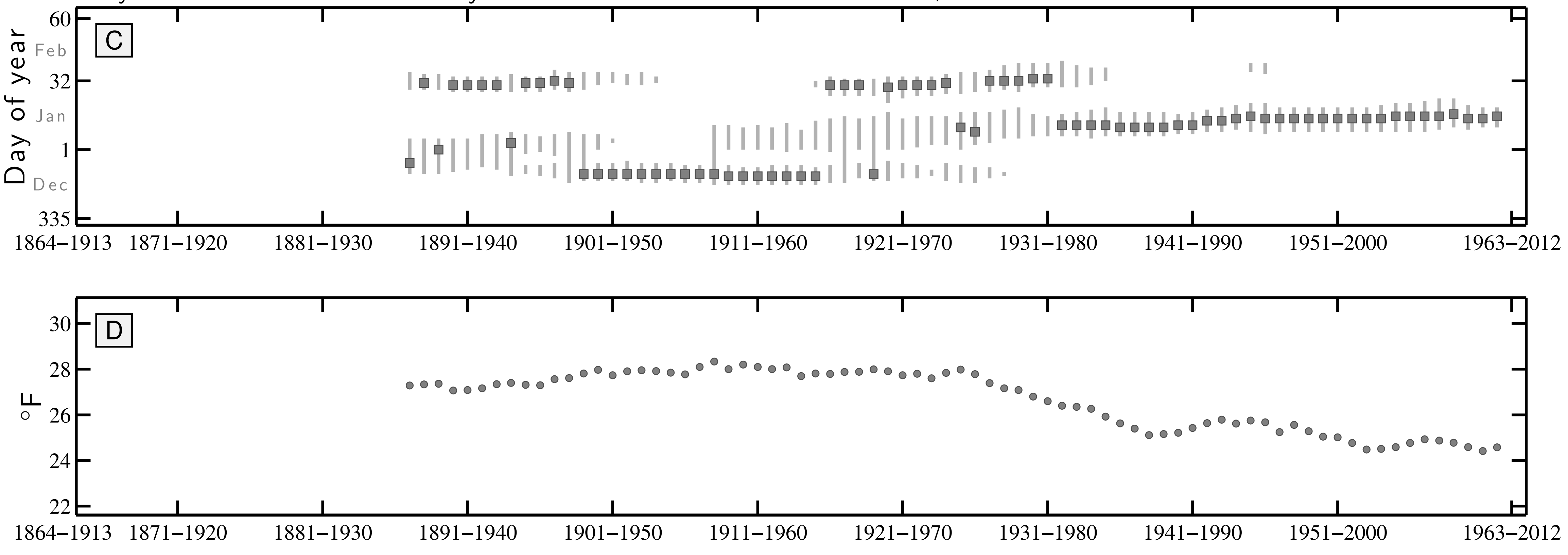

50 year Summer Teletherm dynamics for TULLAHOMA, TN:

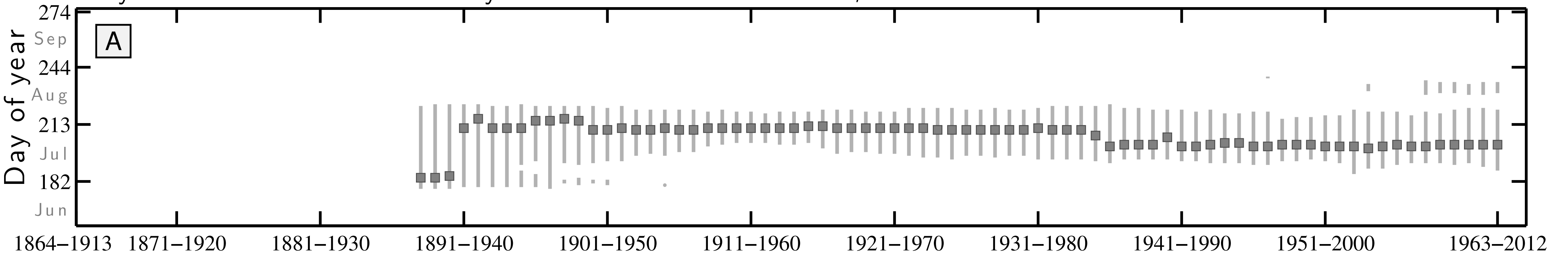

50 year Winter Teletherm dynamics for TULLAHOMA, TN:

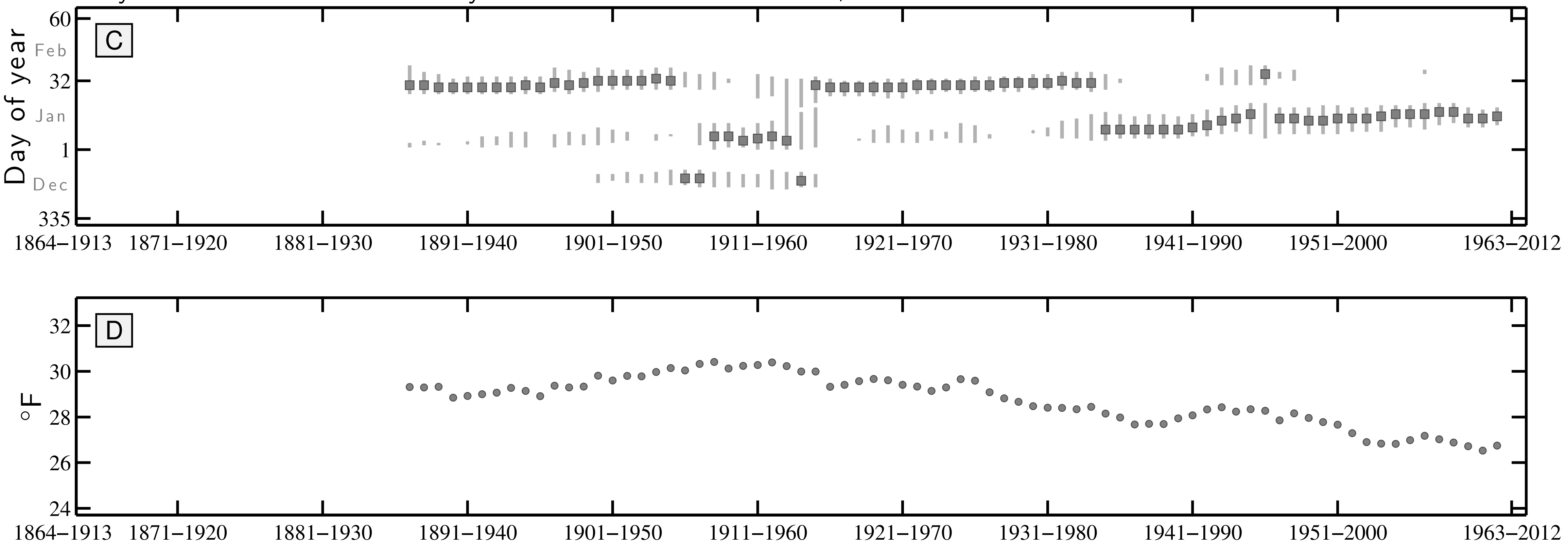

50 year Summer Teletherm dynamics for UNION CITY, TN:

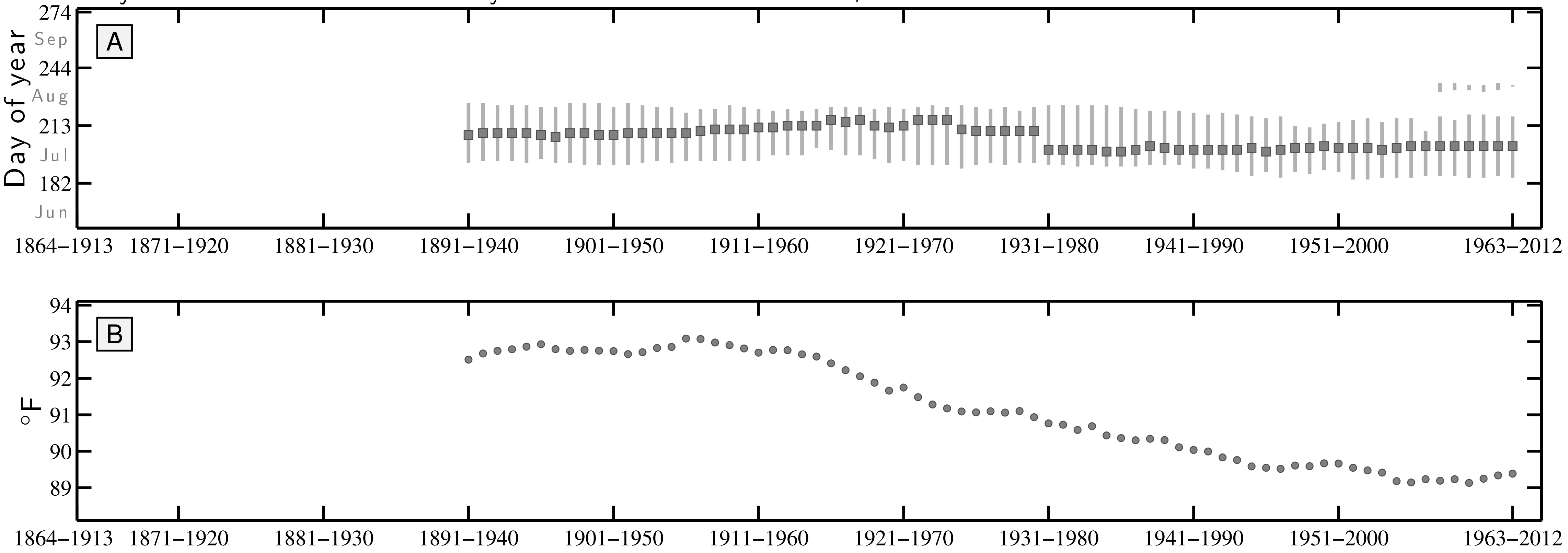

50 year Winter Teletherm dynamics for UNION CITY, TN:

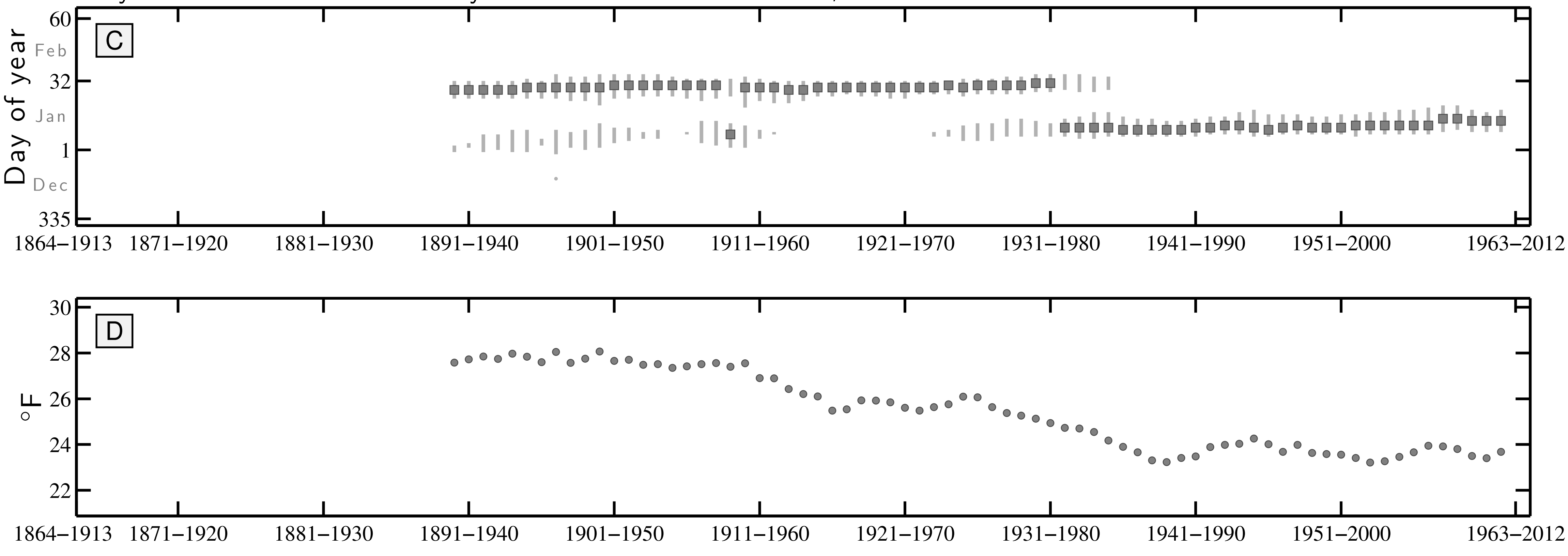

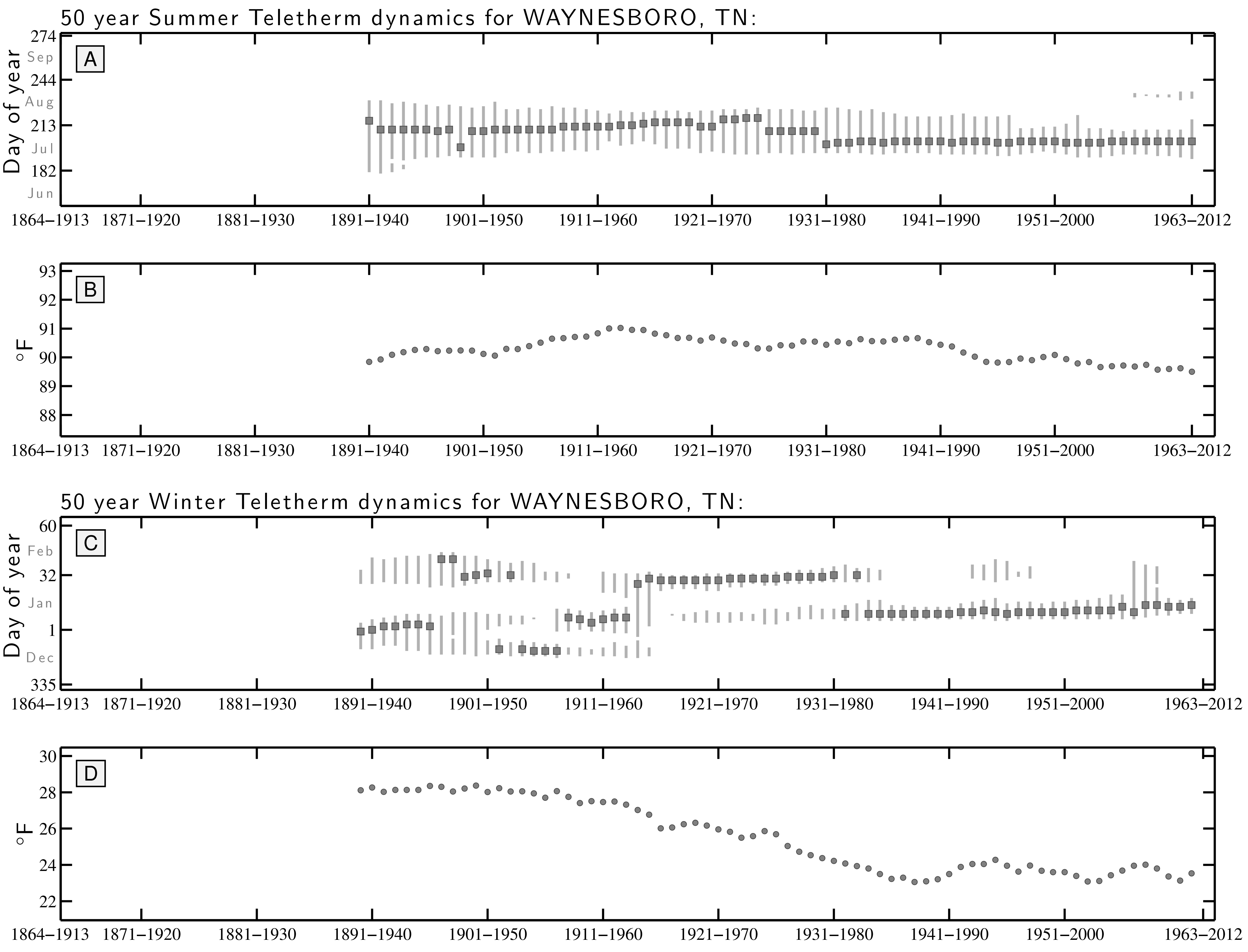
50 year Summer Teletherm dynamics for WAYNESBORO, TN:
A
Day of year
274
Sep
244
Aug
213
Jul
182
Jun
1864–1913
1871–1920
1881–1930
1891–1940
1901–1950
1911–1960
1921–1970
1931–1980
1941–1990
1951–2000
1963–2012
B
°F
93
92
91
90
89
88
50 year Winter Teletherm dynamics for WAYNESBORO, TN:
C
Day of year
60
Feb
32
Jan
1
Dec
335
D
°F
30
28
26
24
22

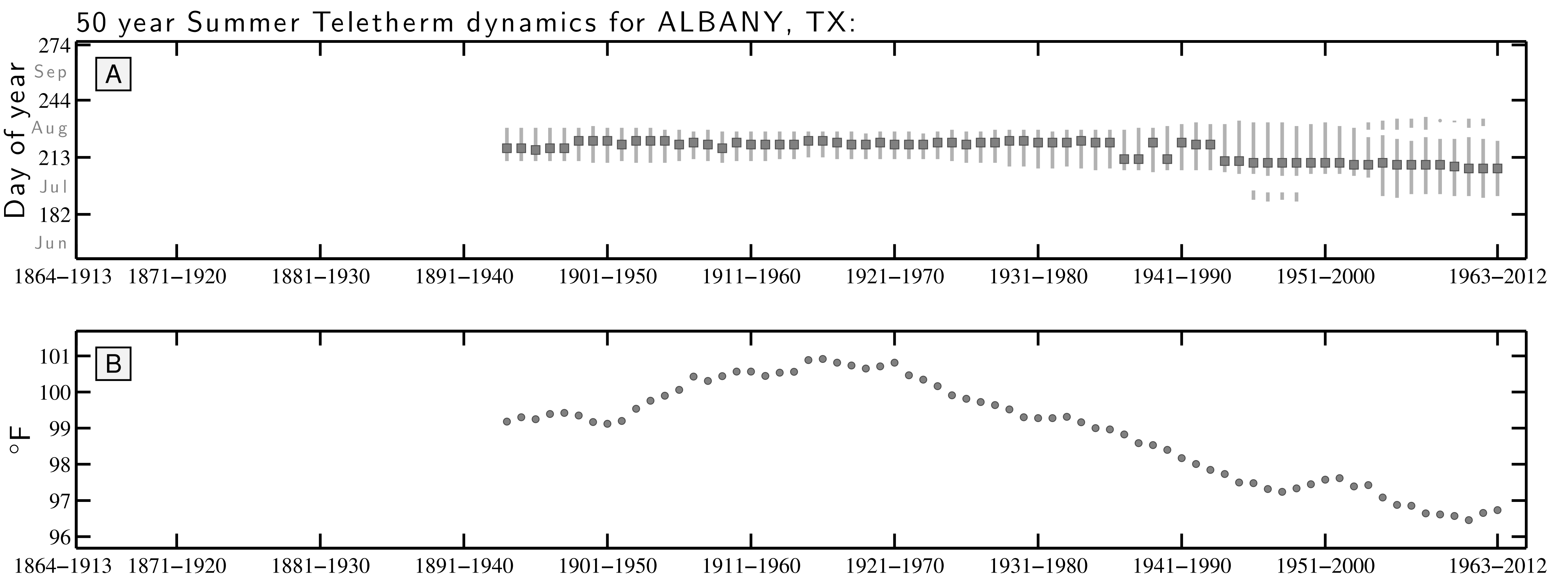
50 year Summer Teletherm dynamics for ALBANY, TX:
A
B
Day of year
°F
274
Sep
244
Aug
213
Jul
182
Jun
101
100
99
98
97
96
1864–1913
1871–1920
1881–1930
1891–1940
1901–1950
1911–1960
1921–1970
1931–1980
1941–1990
1951–2000
1963–2012

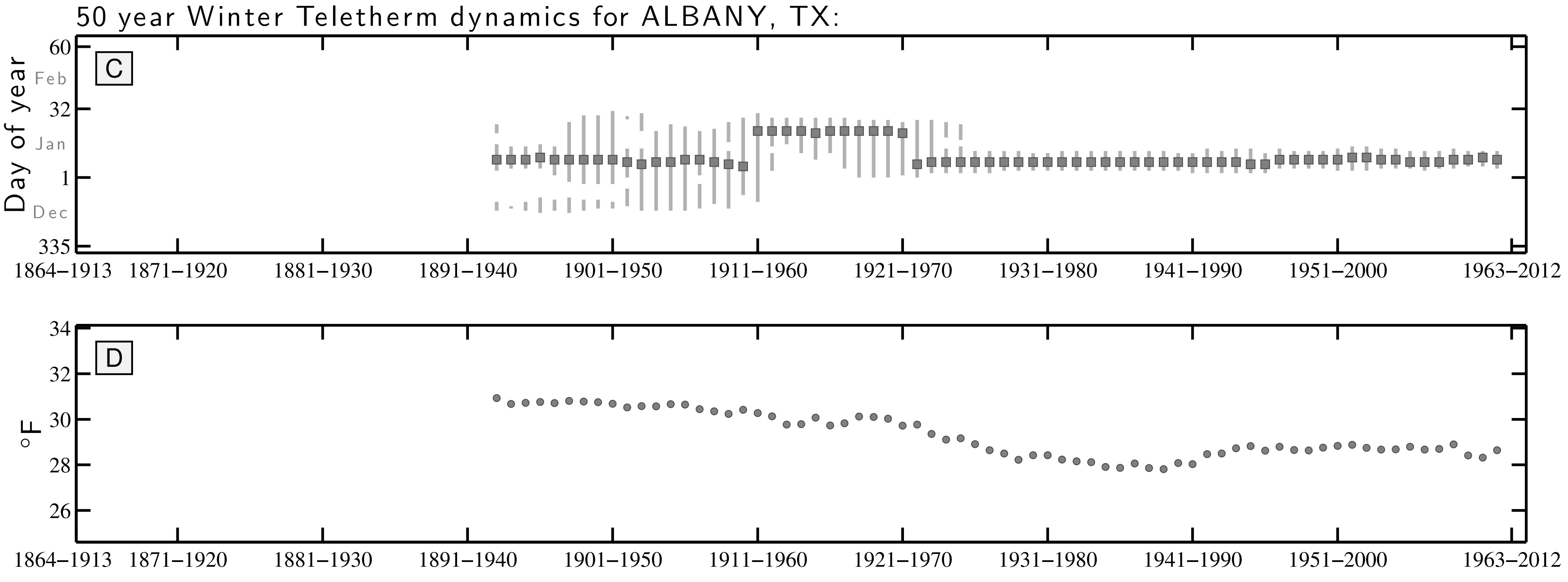
50 year Winter Teletherm dynamics for ALBANY, TX:
C
D
Day of year
°F
60
Feb
32
Jan
1
Dec
335
34
32
30
28
26
1864–1913
1871–1920
1881–1930
1891–1940
1901–1950
1911–1960
1921–1970
1931–1980
1941–1990
1951–2000
1963–2012

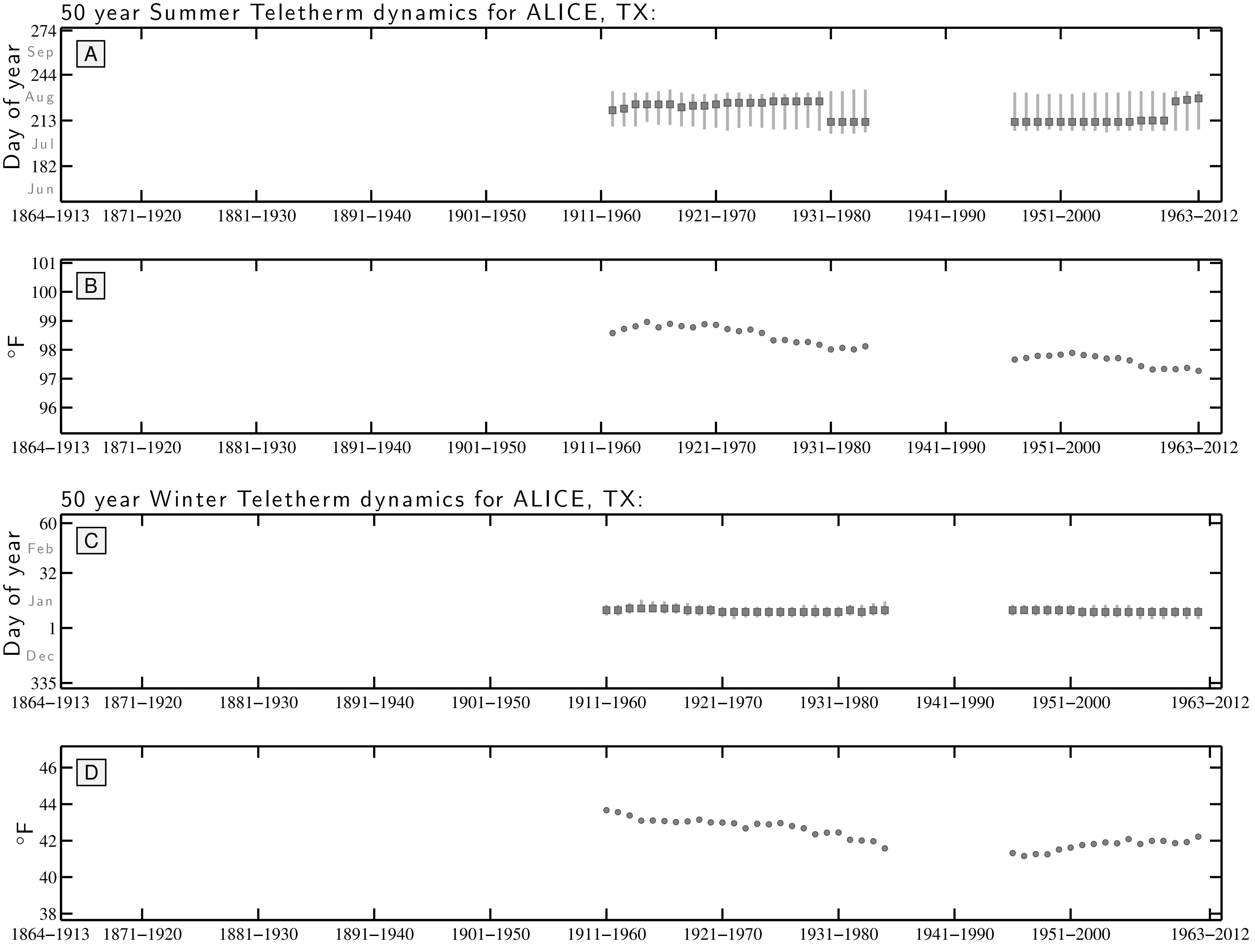
50 year Summer Teletherm dynamics for ALICE, TX:
A
Day of year
274
Sep
244
Aug
213
Jul
182
Jun
1864–1913
1871–1920
1881–1930
1891–1940
1901–1950
1911–1960
1921–1970
1931–1980
1941–1990
1951–2000
1963–2012
B
°F
101
100
99
98
97
96
50 year Winter Teletherm dynamics for ALICE, TX:
C
Day of year
60
Feb
32
Jan
1
Dec
335
D
°F
46
44
42
40
38

50 year Summer Teletherm dynamics for ALPINE, TX:

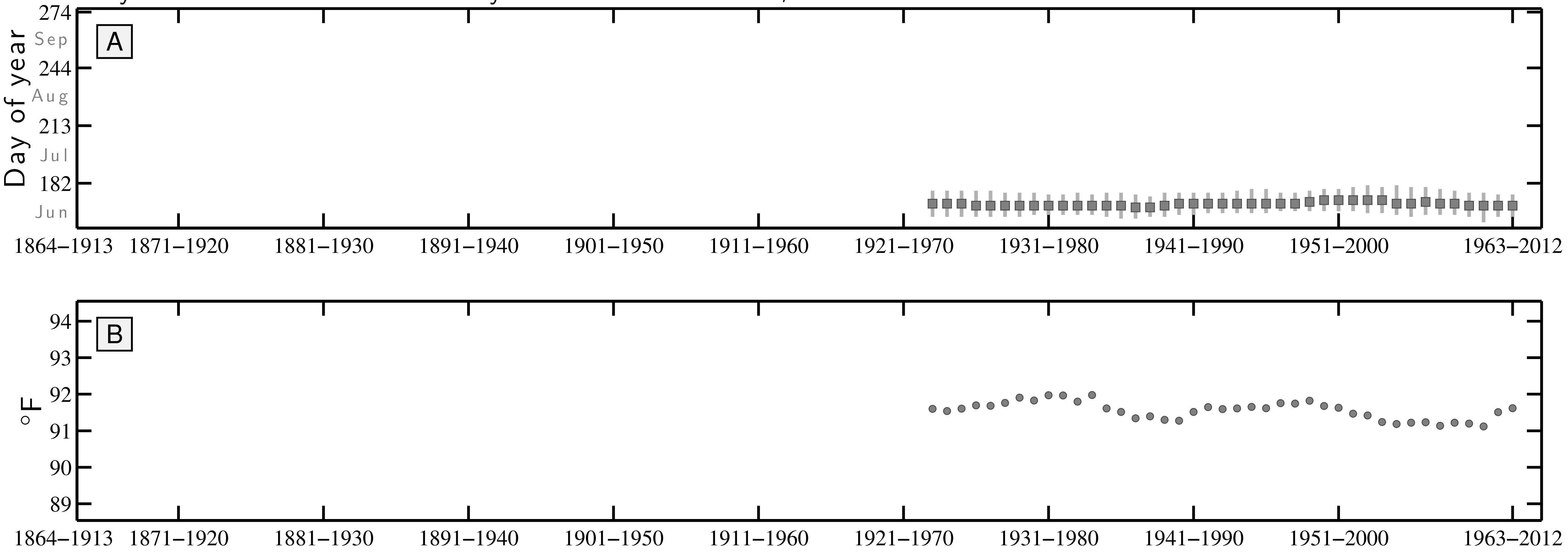

50 year Winter Teletherm dynamics for ALPINE, TX:

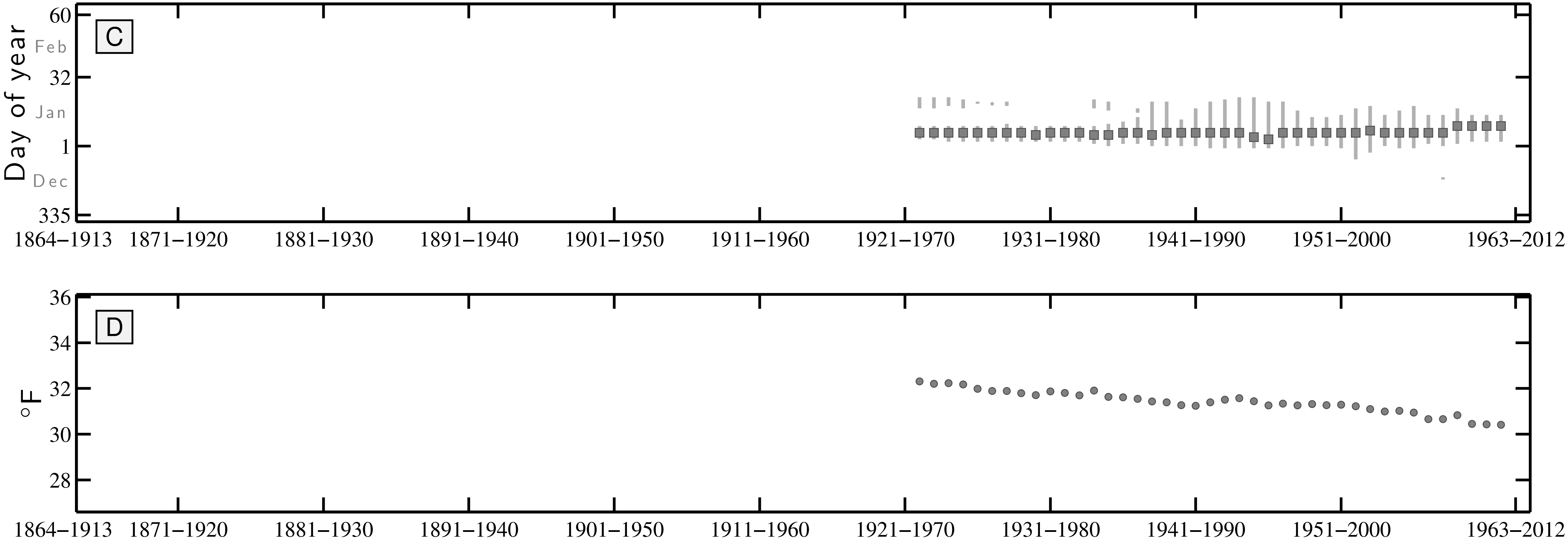

50 year Summer Teletherm dynamics for BALLINGER 2 NW, TX:

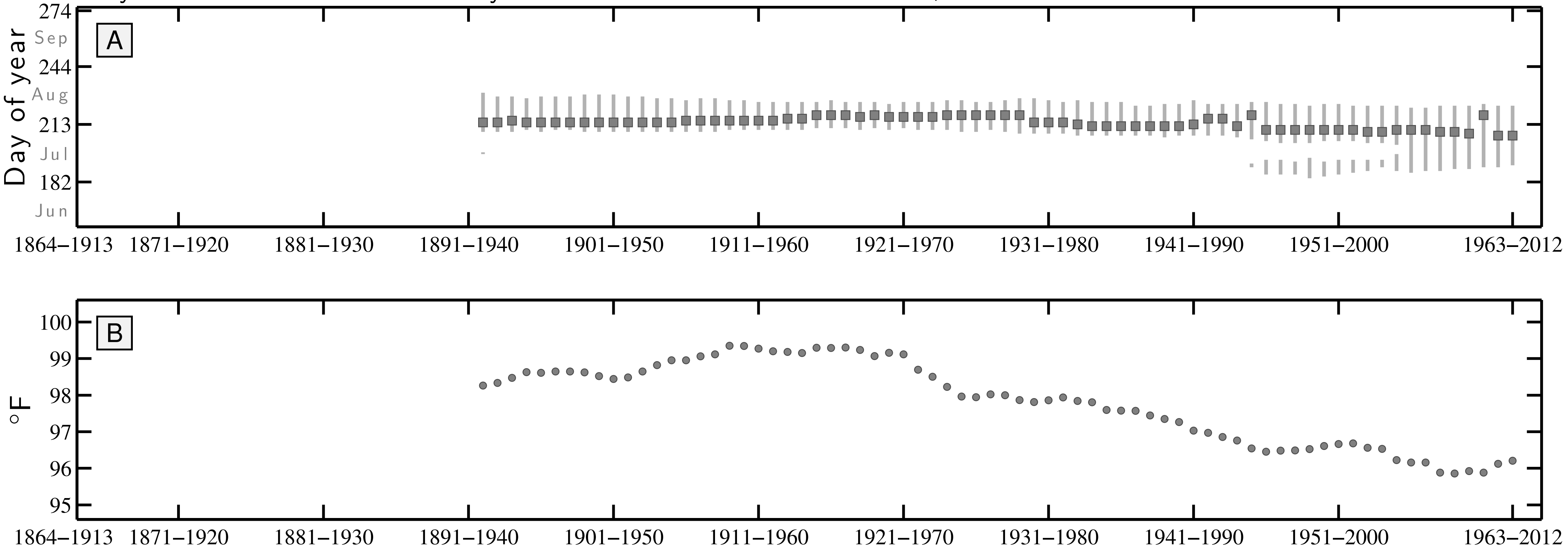

50 year Winter Teletherm dynamics for BALLINGER 2 NW, TX:

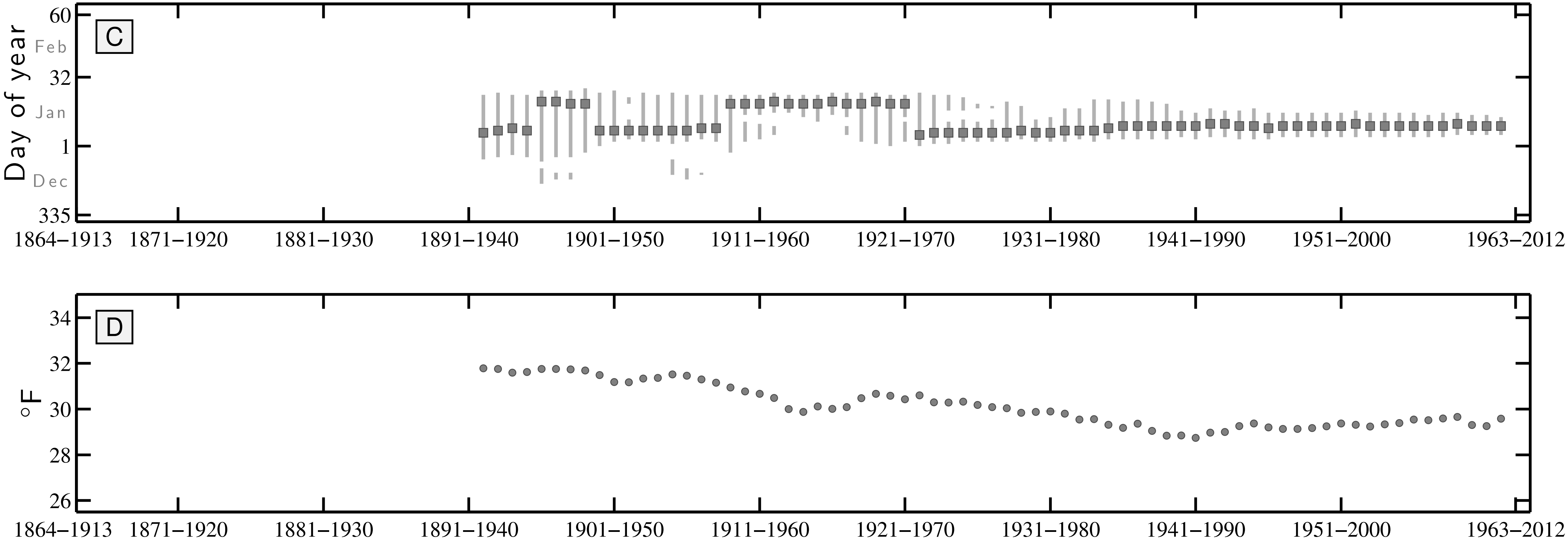

50 year Summer Teletherm dynamics for BALMORHEA, TX:

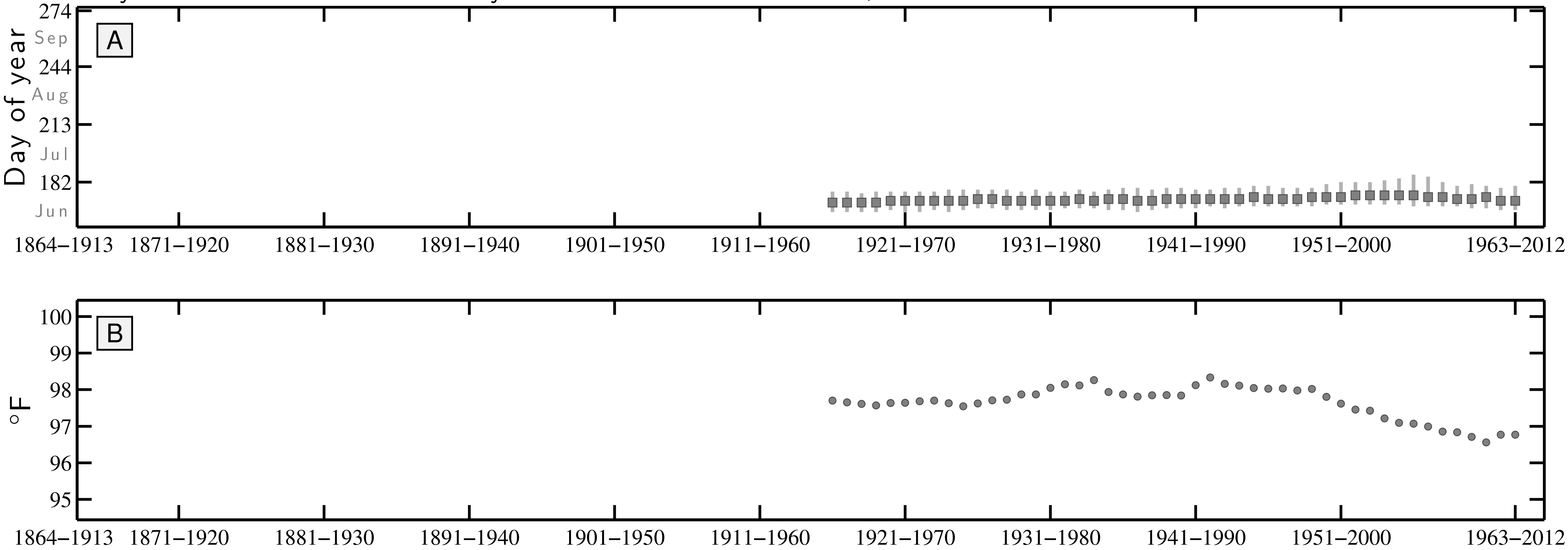

50 year Winter Teletherm dynamics for BALMORHEA, TX:

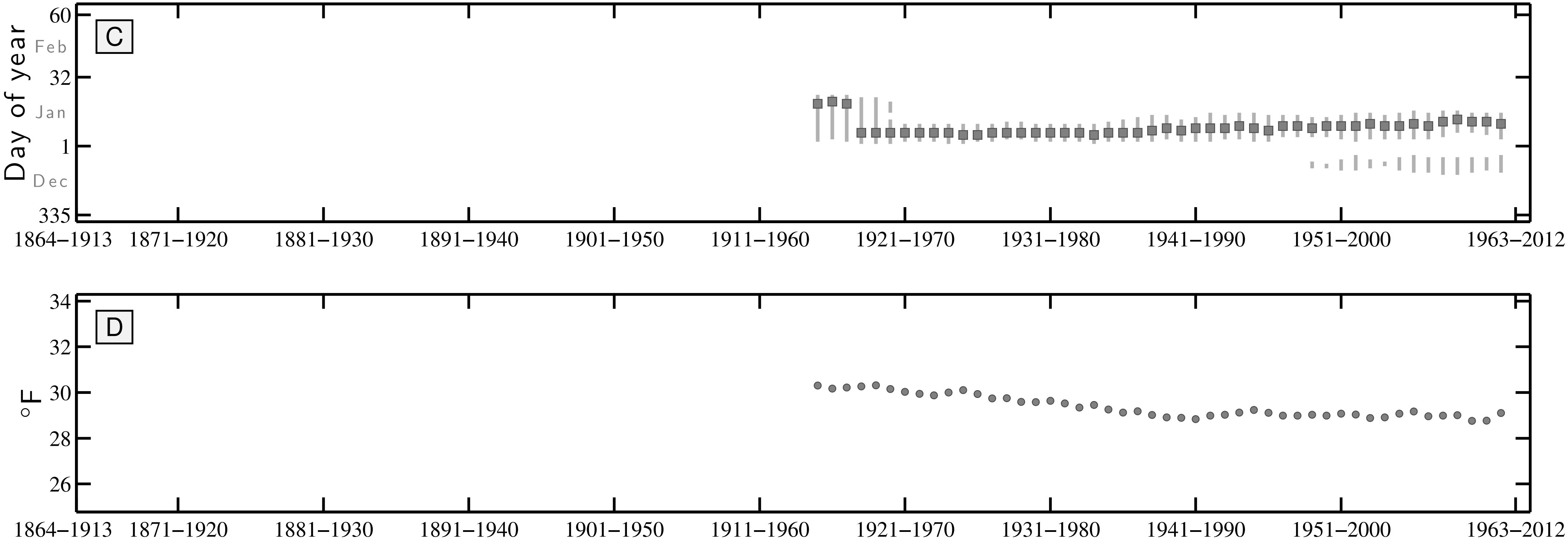

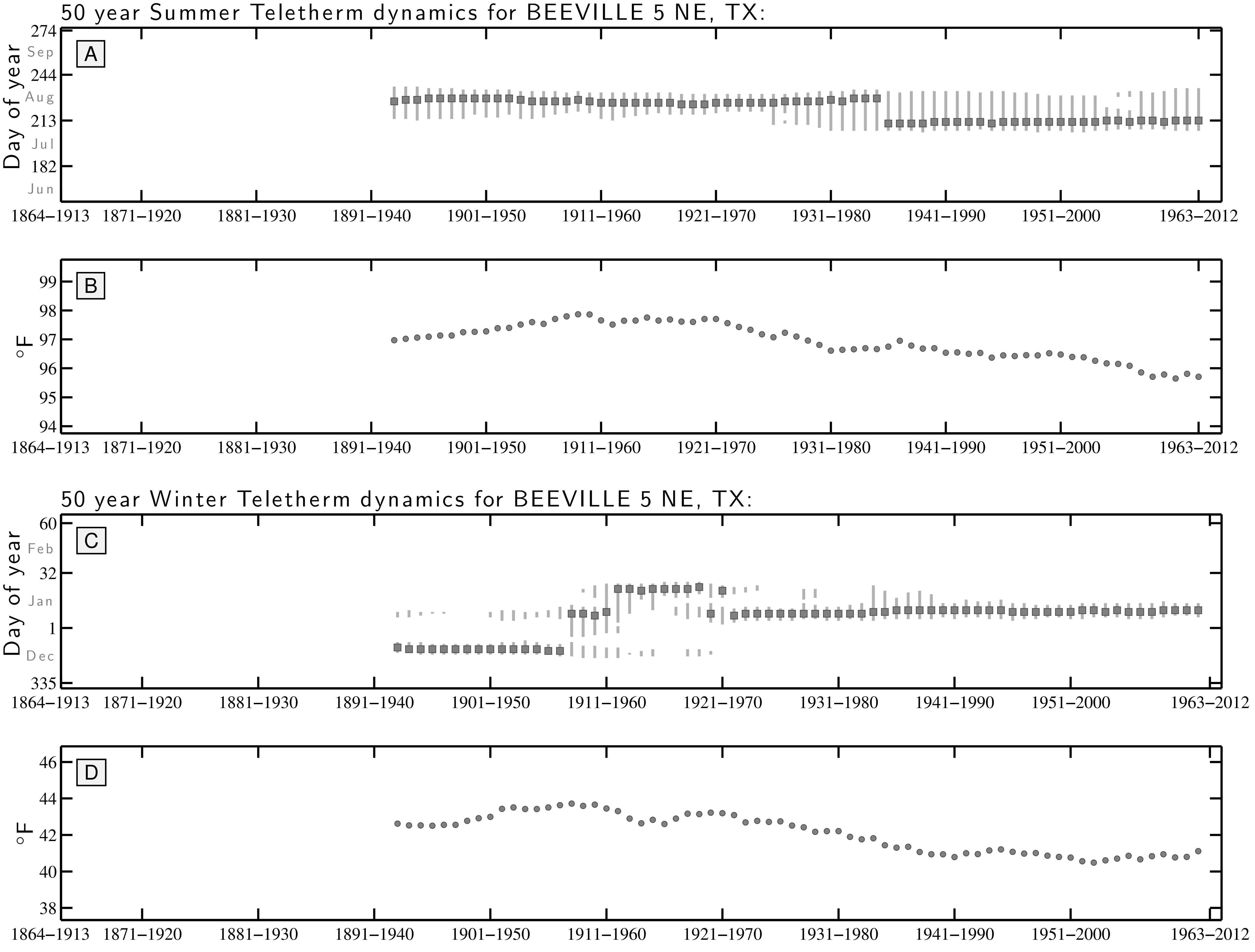

50 year Summer Teletherm dynamics for BEEVILLE 5 NE, TX:
A
Day of year
274
Sep
244
Aug
213
Jul
182
Jun
1864–1913
1871–1920
1881–1930
1891–1940
1901–1950
1911–1960
1921–1970
1931–1980
1941–1990
1951–2000
1963–2012
B
°F
99
98
97
96
95
94
50 year Winter Teletherm dynamics for BEEVILLE 5 NE, TX:
C
Day of year
60
Feb
32
Jan
1
Dec
335
D
°F
46
44
42
40
38

50 year Summer Teletherm dynamics for BLANCO, TX:

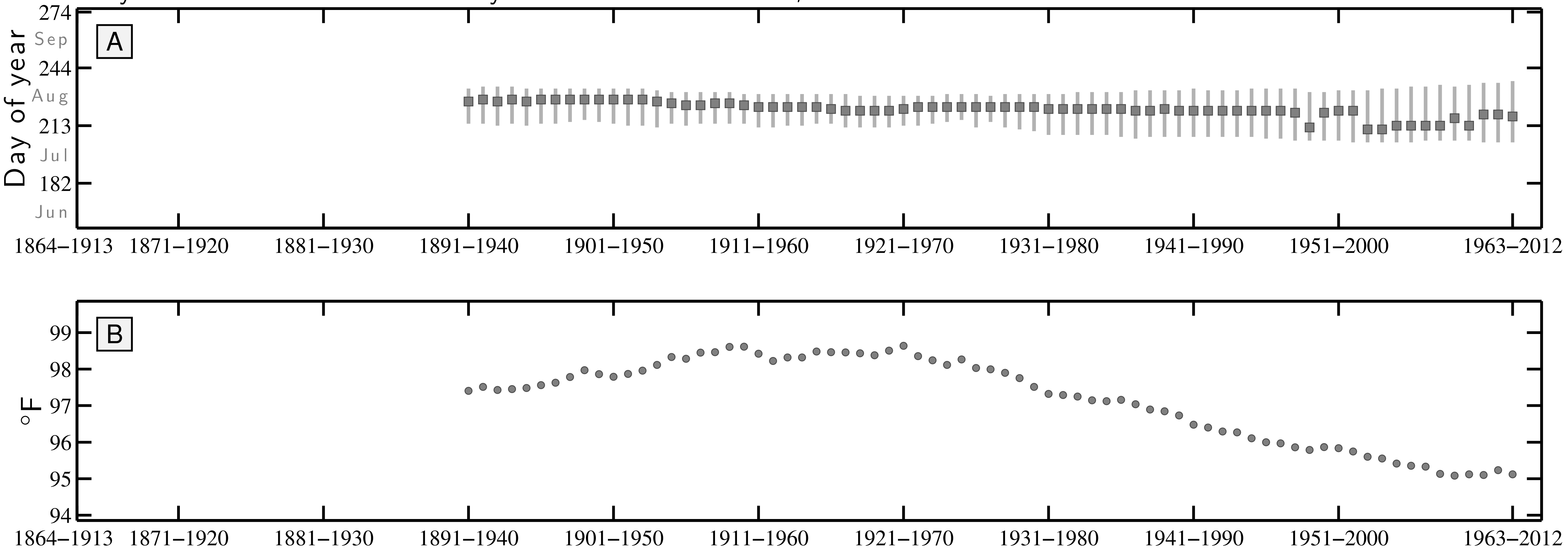

50 year Winter Teletherm dynamics for BLANCO, TX:

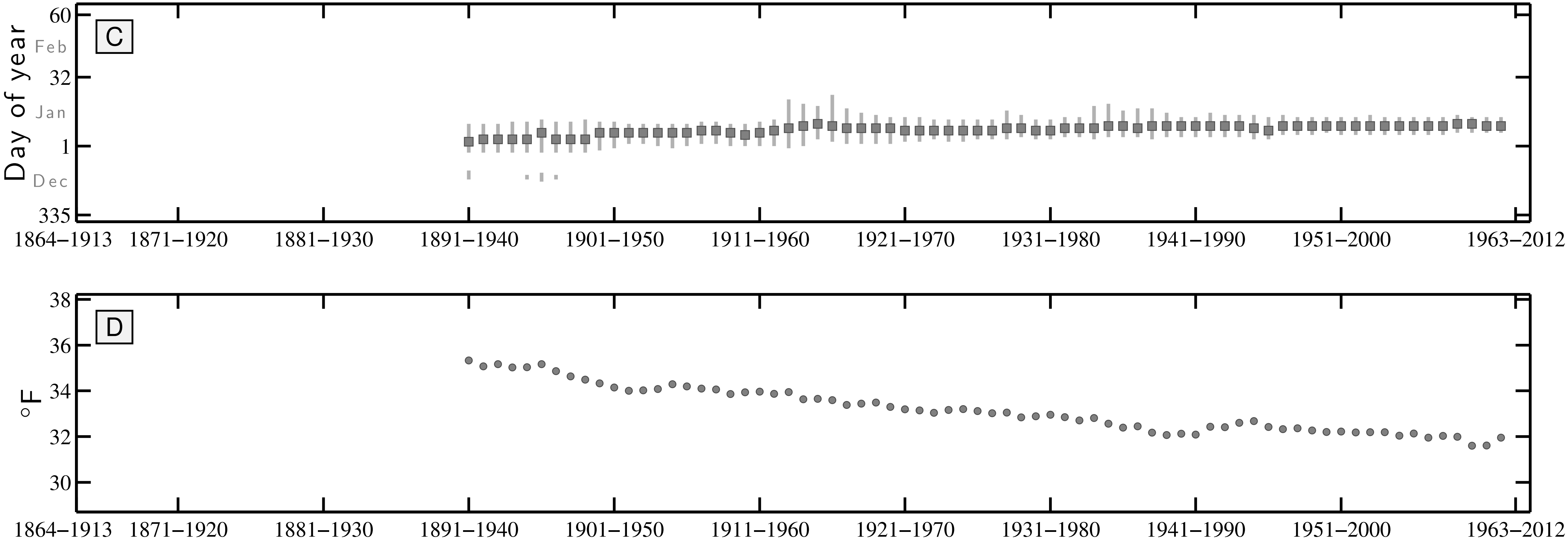

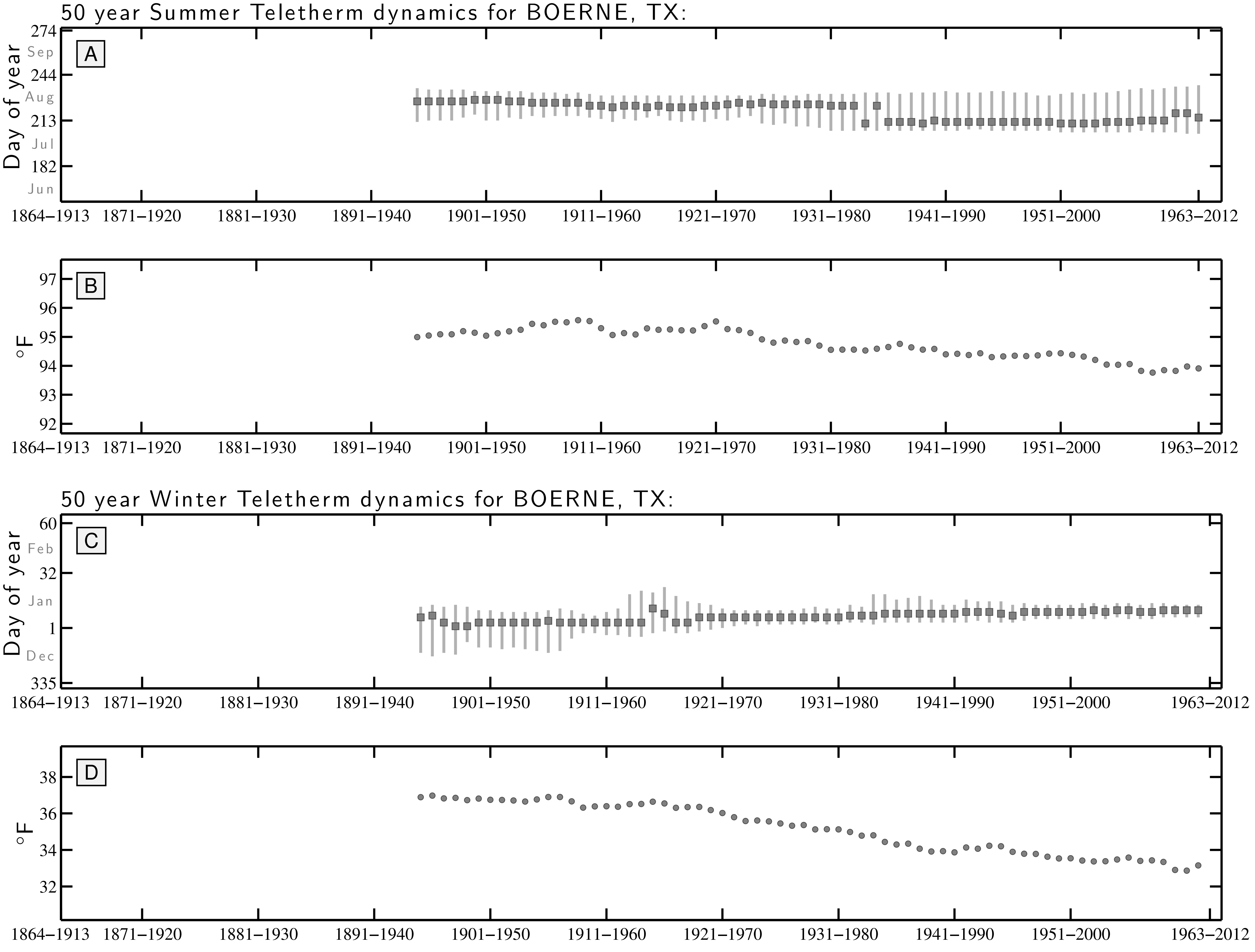
50 year Summer Teletherm dynamics for BOERNE, TX:
A
Day of year
274
Sep
244
Aug
213
Jul
182
Jun
1864–1913
1871–1920
1881–1930
1891–1940
1901–1950
1911–1960
1921–1970
1931–1980
1941–1990
1951–2000
1963–2012
B
°F
97
96
95
94
93
92
50 year Winter Teletherm dynamics for BOERNE, TX:
C
Day of year
60
Feb
32
Jan
1
Dec
335
D
°F
38
36
34
32

50 year Summer Teletherm dynamics for BRENHAM, TX:

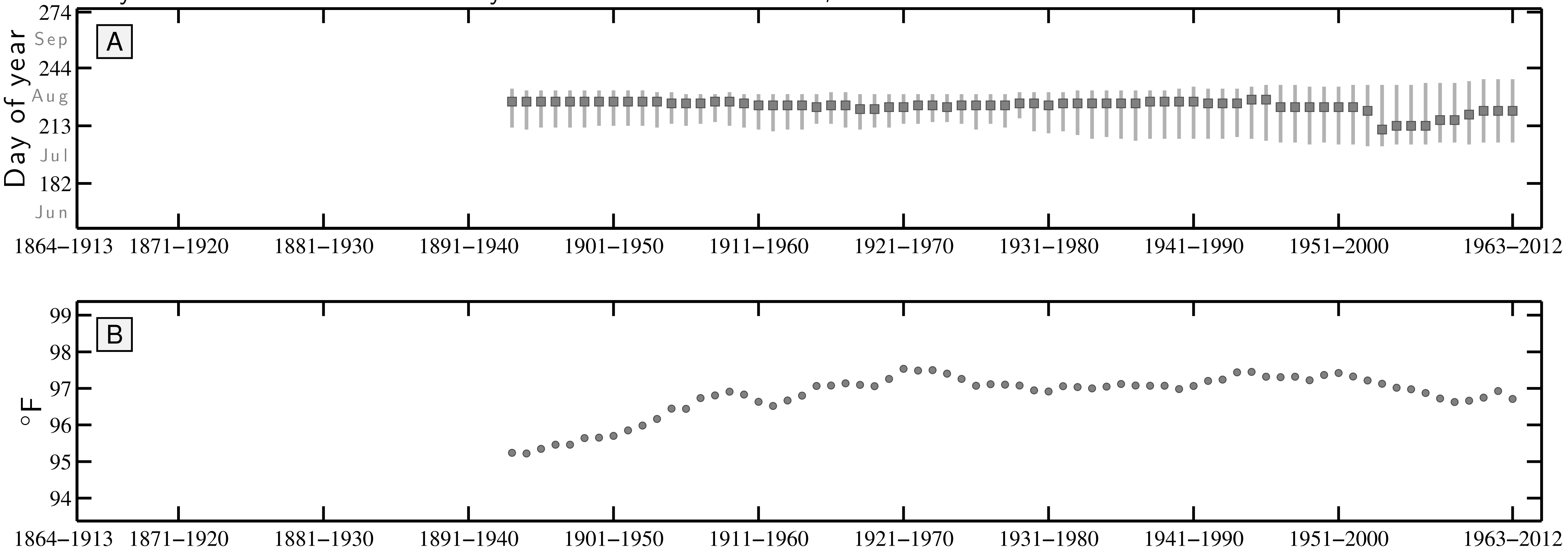

50 year Winter Teletherm dynamics for BRENHAM, TX:

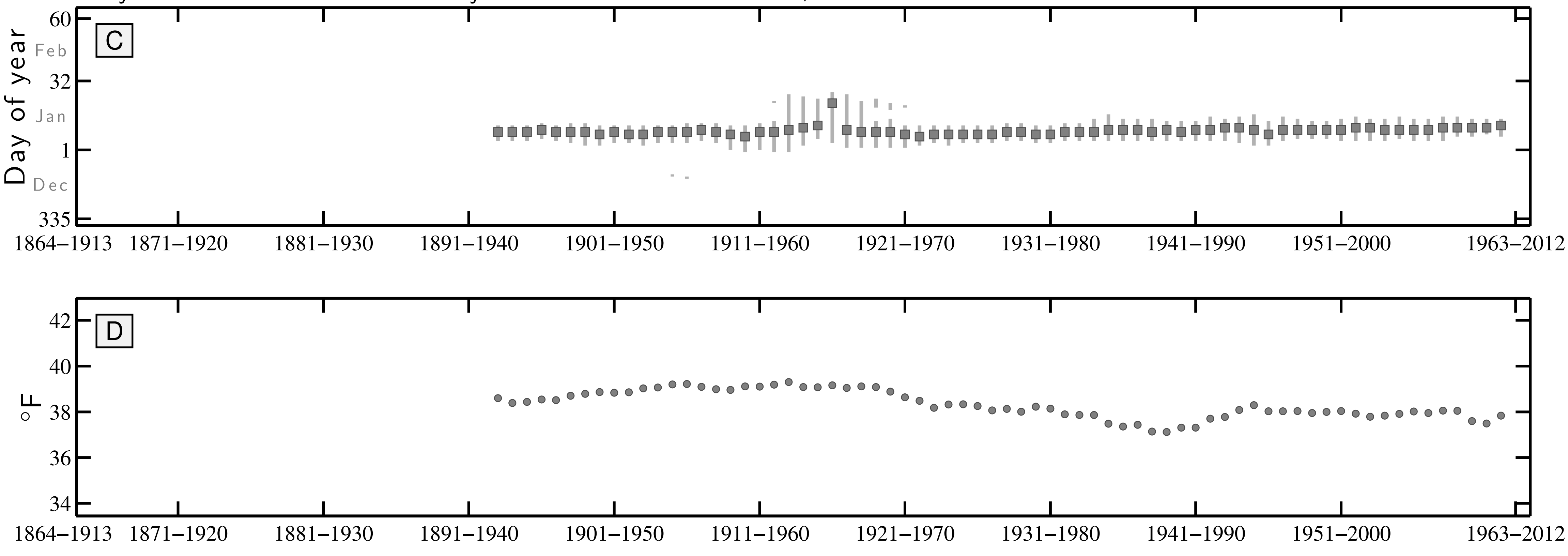

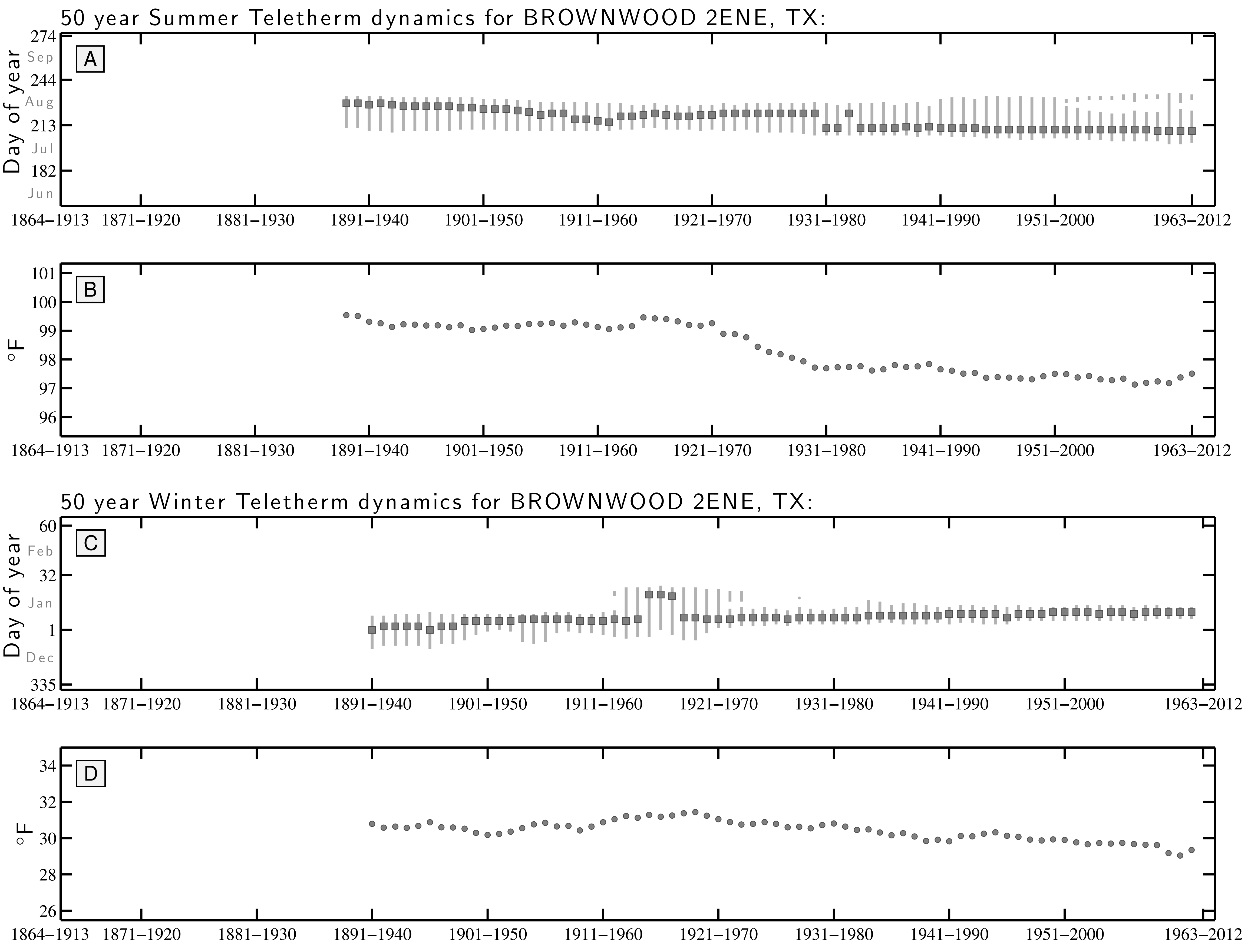

50 year Summer Teletherm dynamics for BROWNWOOD 2ENE, TX:
A
B
50 year Winter Teletherm dynamics for BROWNWOOD 2ENE, TX:
C
D
Day of year
°F
Jun
Jul
Aug
Sep
Dec
Jan
Feb
1864–1913
1871–1920
1881–1930
1891–1940
1901–1950
1911–1960
1921–1970
1931–1980
1941–1990
1951–2000
1963–2012

50 year Summer Teletherm dynamics for CLARKSVILLE 2NE, TX:

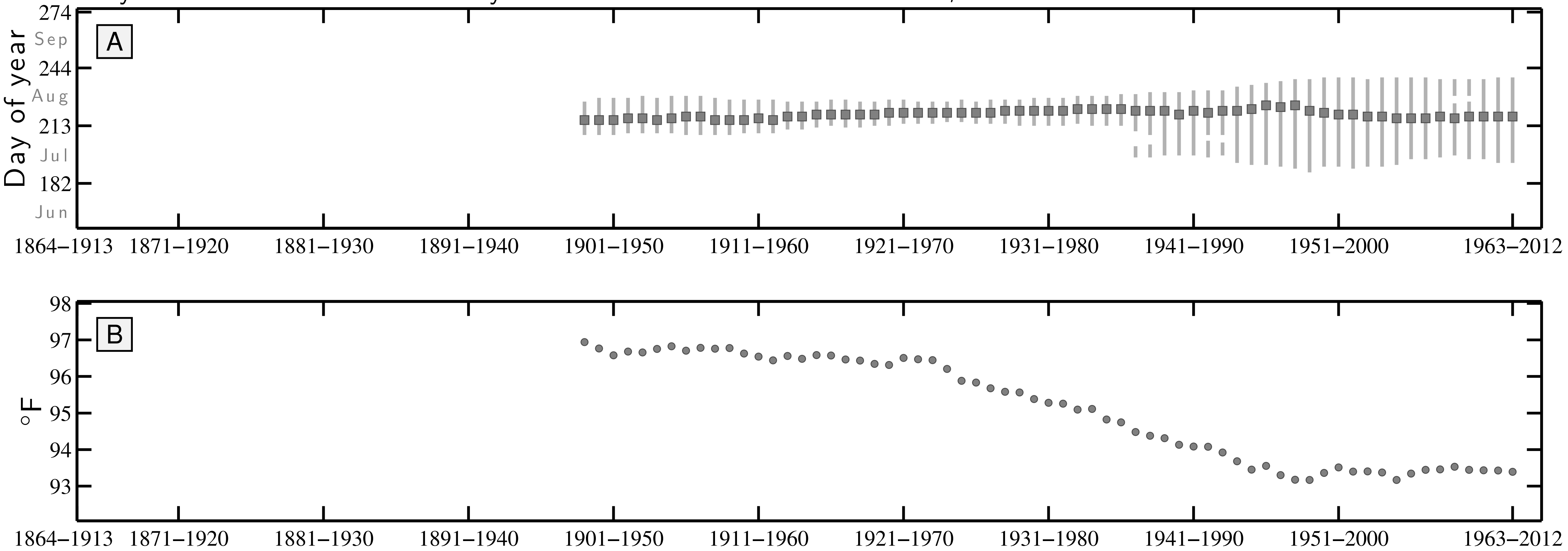

50 year Winter Teletherm dynamics for CLARKSVILLE 2NE, TX:

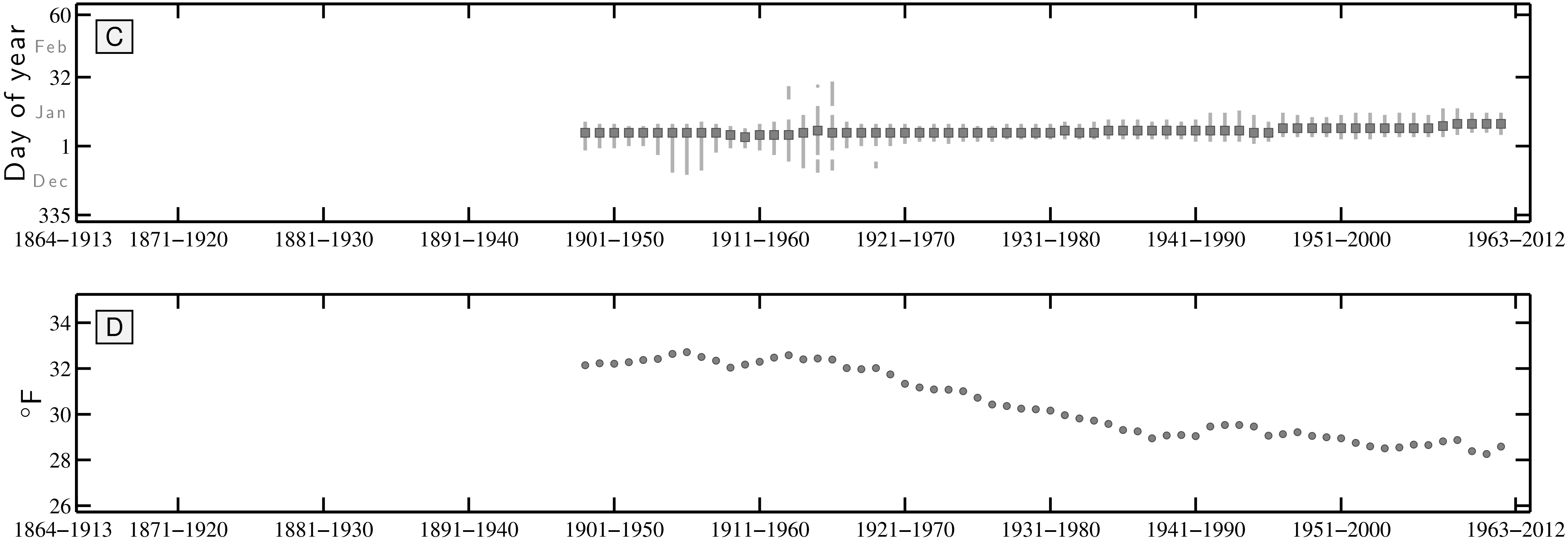

50 year Summer Teletherm dynamics for CORPUS CHRISTI AP, TX:

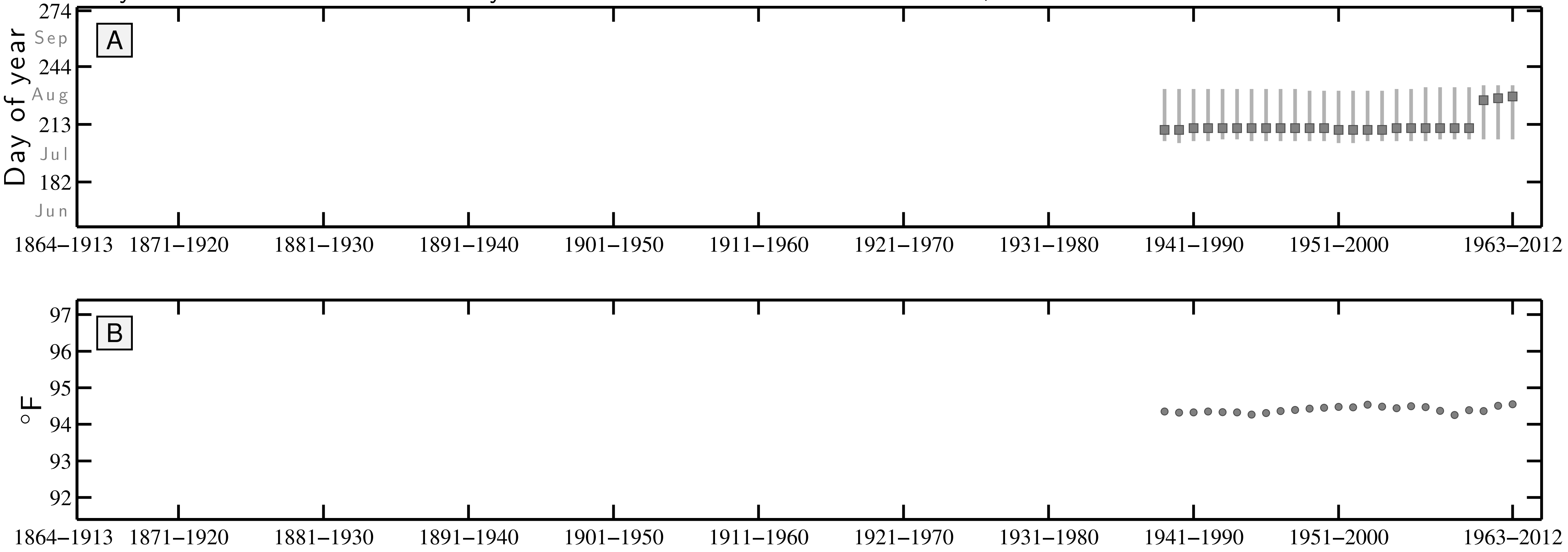

50 year Winter Teletherm dynamics for CORPUS CHRISTI AP, TX:

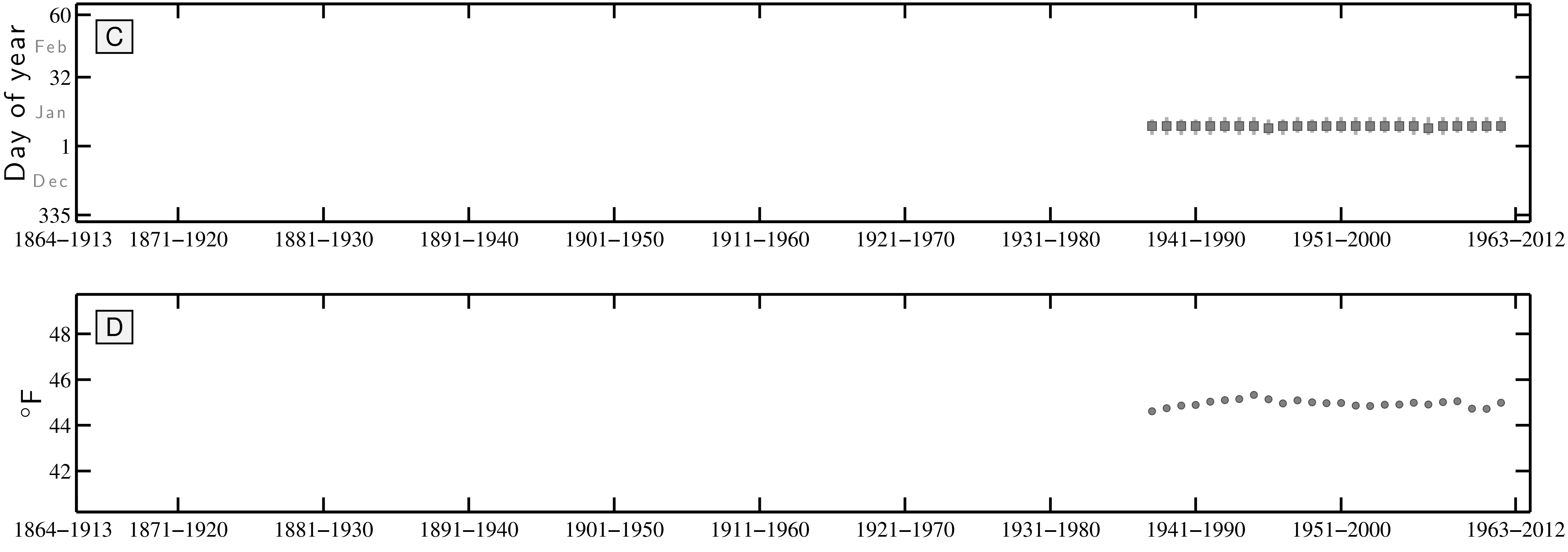

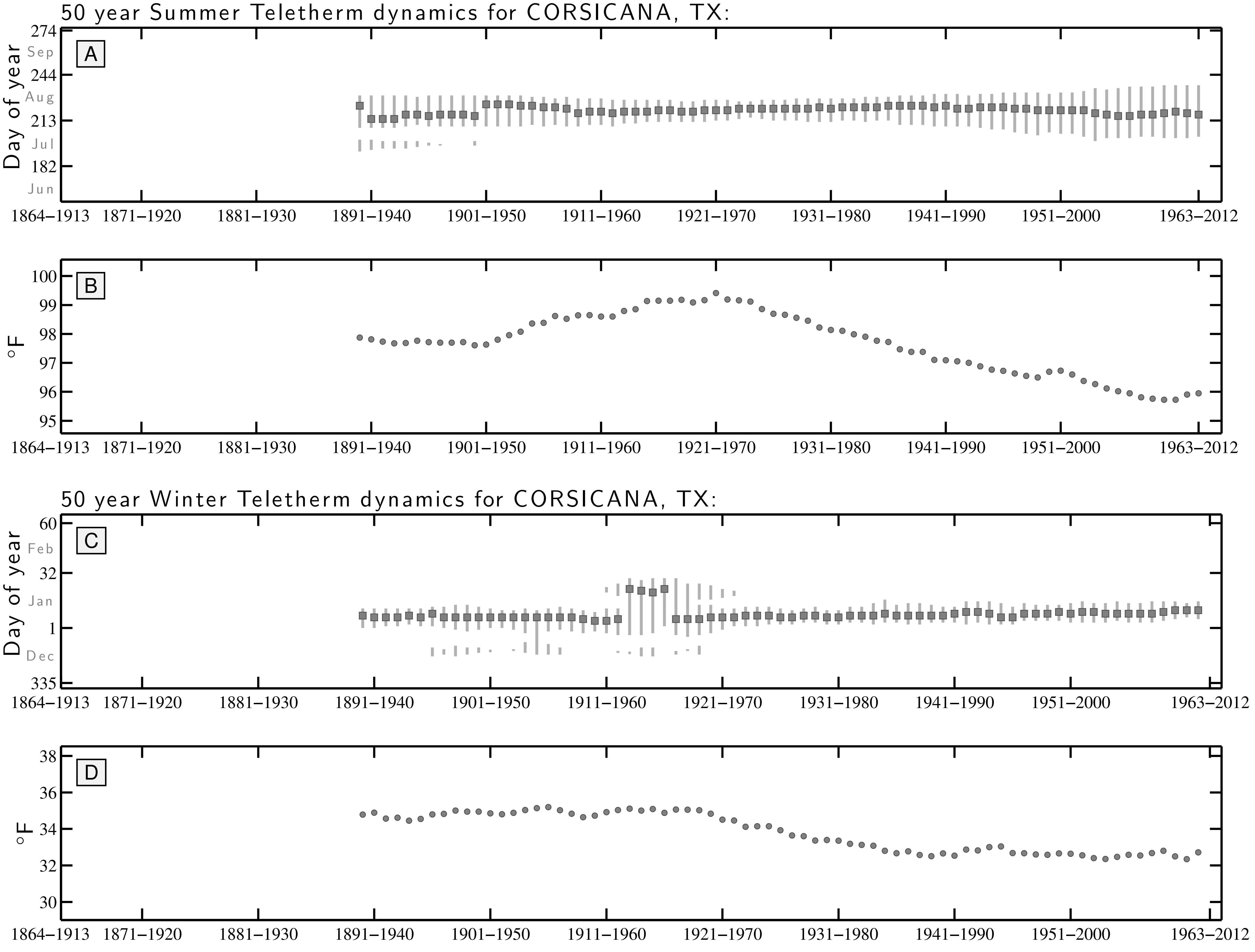
50 year Summer Teletherm dynamics for CORSICANA, TX:
A
Day of year
274
Sep
244
Aug
213
Jul
182
Jun
1864–1913
1871–1920
1881–1930
1891–1940
1901–1950
1911–1960
1921–1970
1931–1980
1941–1990
1951–2000
1963–2012
B
°F
100
99
98
97
96
95
50 year Winter Teletherm dynamics for CORSICANA, TX:
C
Day of year
60
Feb
32
Jan
1
Dec
335
D
°F
38
36
34
32
30

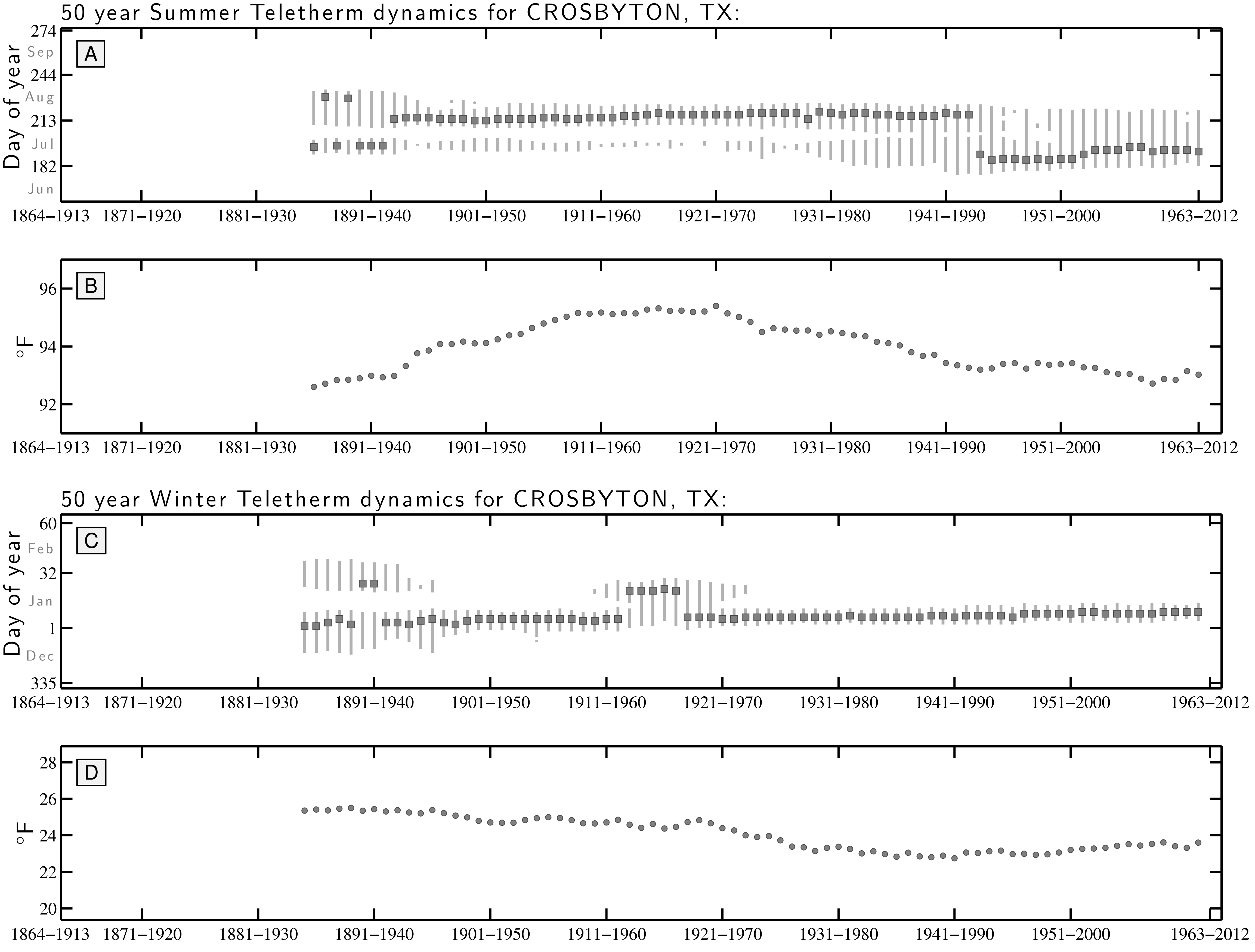
50 year Summer Teletherm dynamics for CROSBYTON, TX:
A
B
Day of year
°F
274
Sep
244
Aug
213
Jul
182
Jun
96
94
92
1864–1913
1871–1920
1881–1930
1891–1940
1901–1950
1911–1960
1921–1970
1931–1980
1941–1990
1951–2000
1963–2012
50 year Winter Teletherm dynamics for CROSBYTON, TX:
C
D
60
Feb
32
Jan
1
Dec
335
28
26
24
22
20

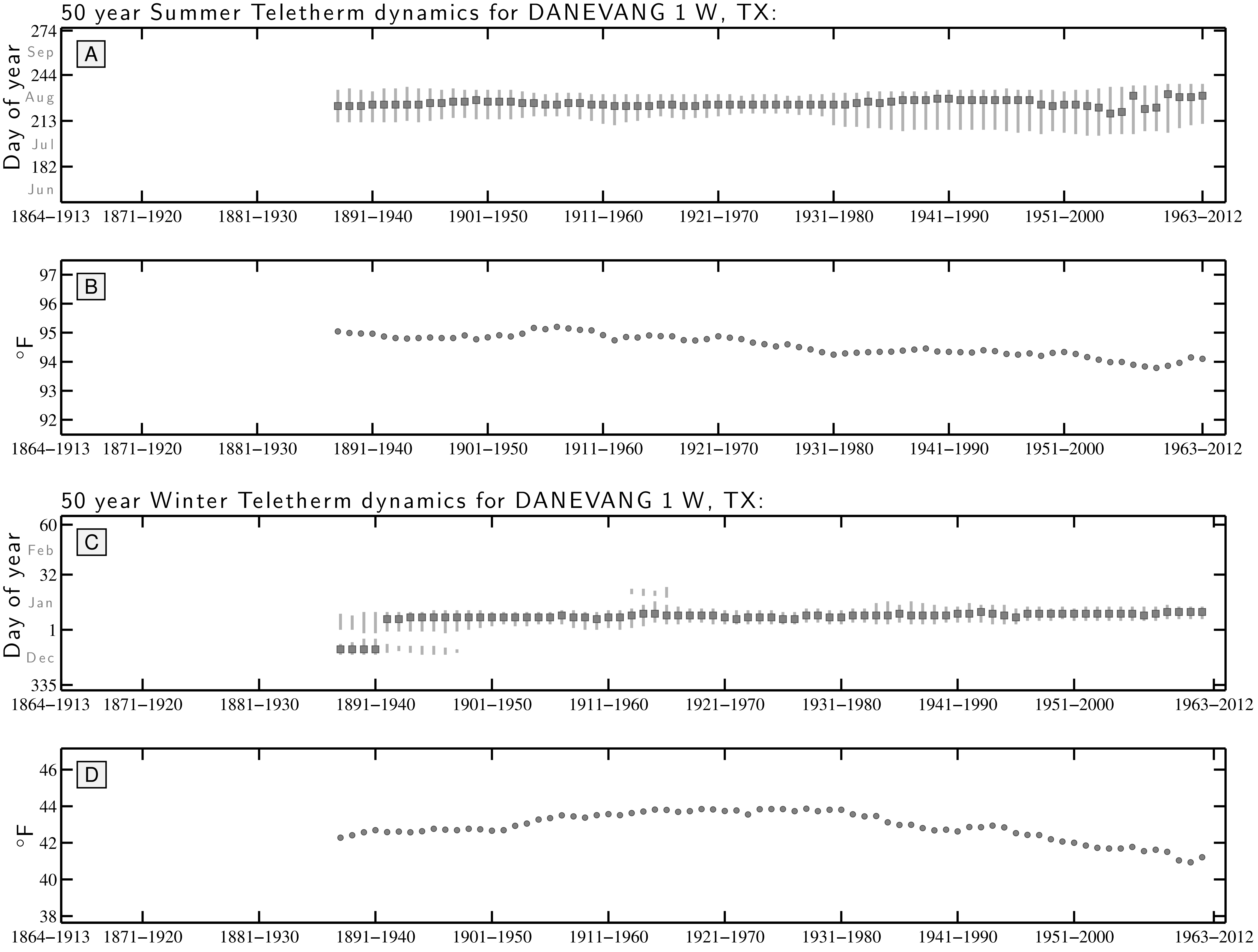
50 year Summer Teletherm dynamics for DANEVANG 1 W, TX:
A
B
Day of year
°F
Sep
Aug
Jul
Jun
274
244
213
182
97
96
95
94
93
92
50 year Winter Teletherm dynamics for DANEVANG 1 W, TX:
C
D
Feb
Jan
Dec
60
32
1
335
46
44
42
40
38
1864–1913
1871–1920
1881–1930
1891–1940
1901–1950
1911–1960
1921–1970
1931–1980
1941–1990
1951–2000
1963–2012

50 year Summer Teletherm dynamics for DUBLIN 2SE, TX:

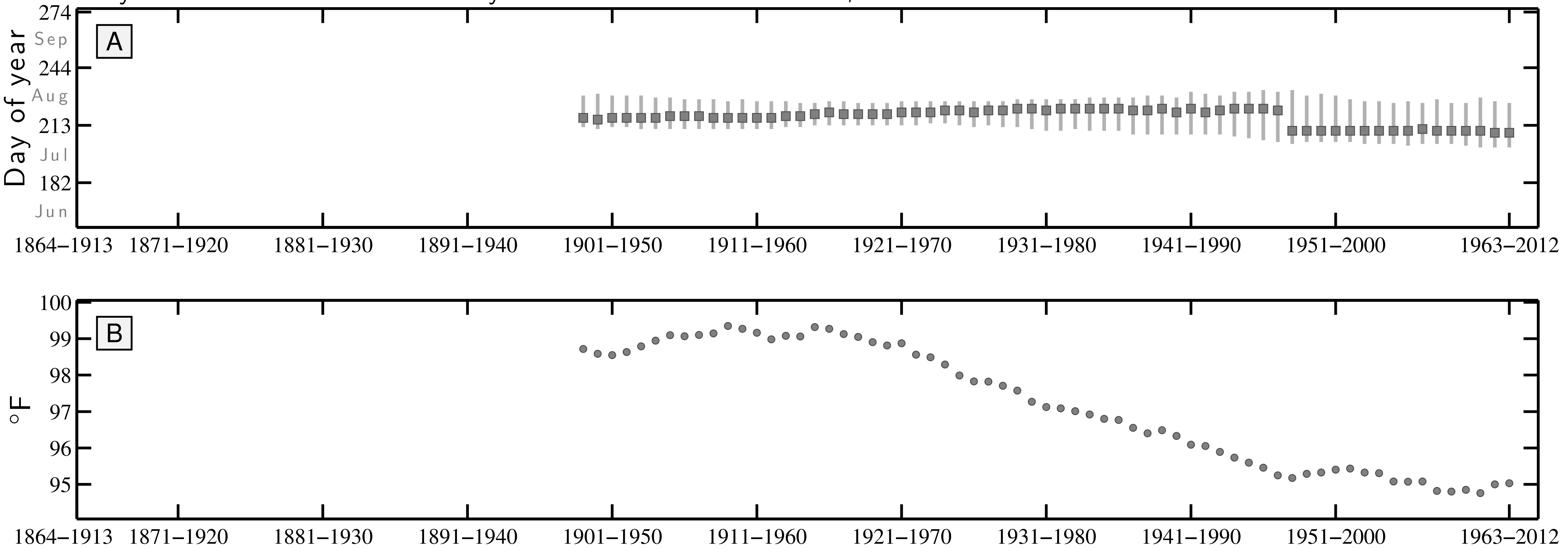

50 year Winter Teletherm dynamics for DUBLIN 2SE, TX:

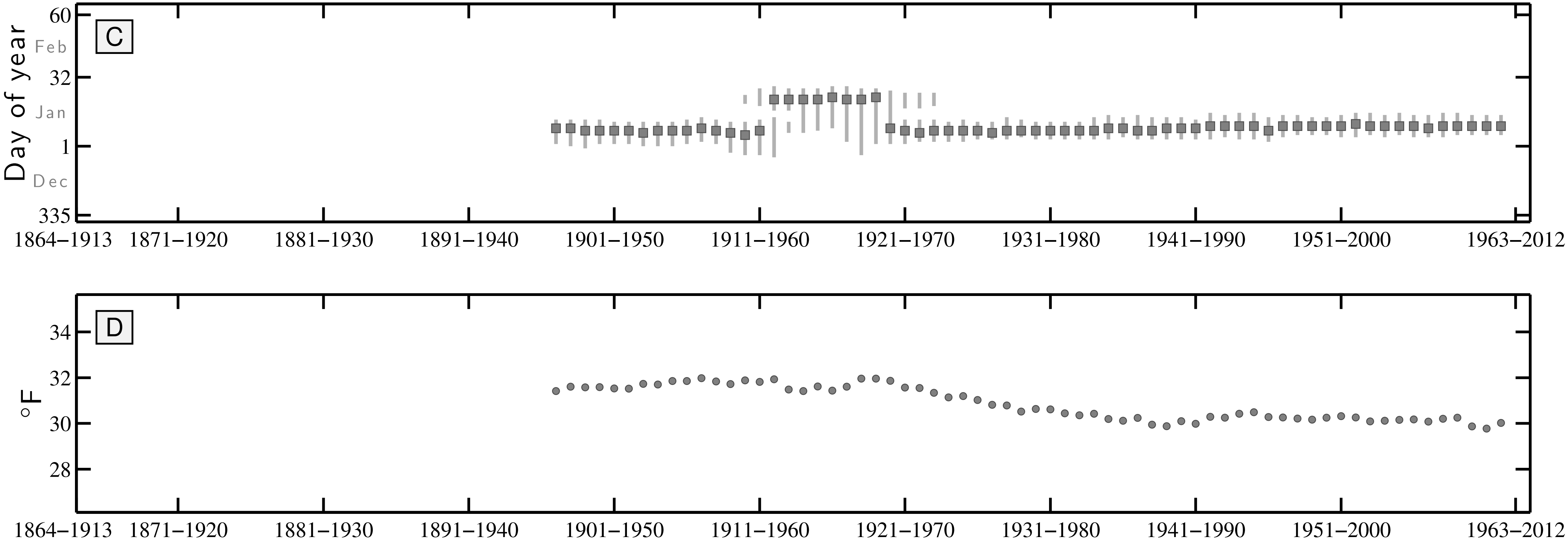

50 year Summer Teletherm dynamics for EAGLE PASS 3N, TX:

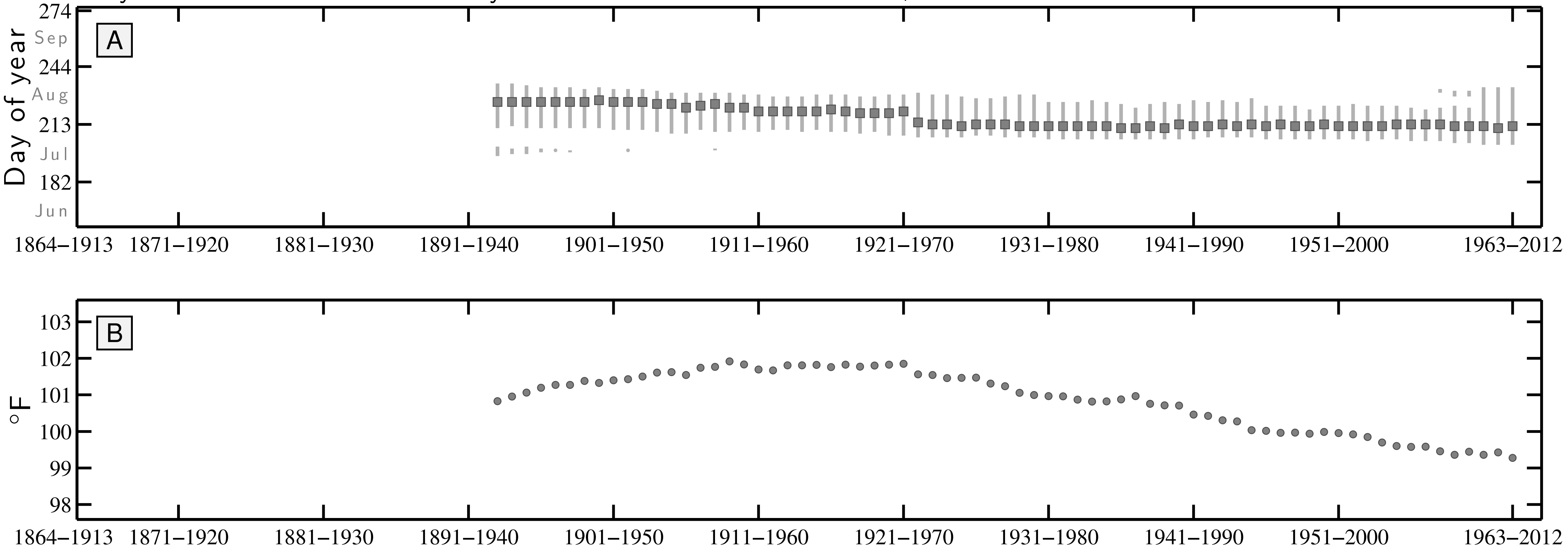

50 year Winter Teletherm dynamics for EAGLE PASS 3N, TX:

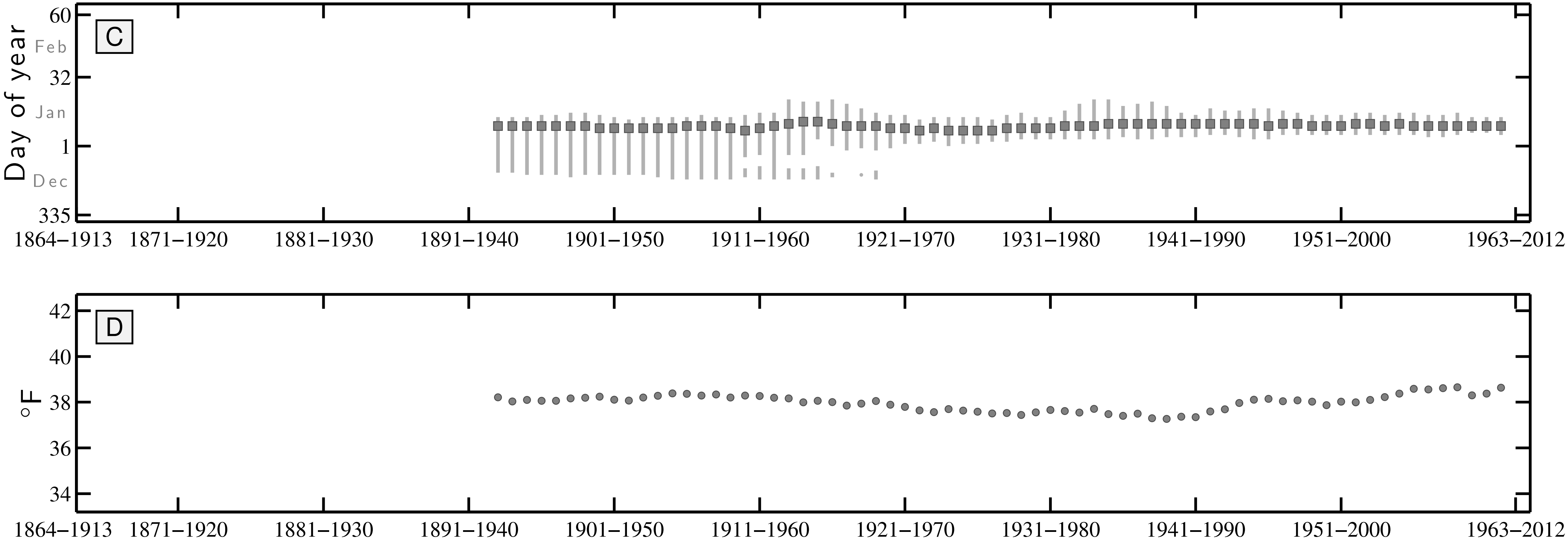

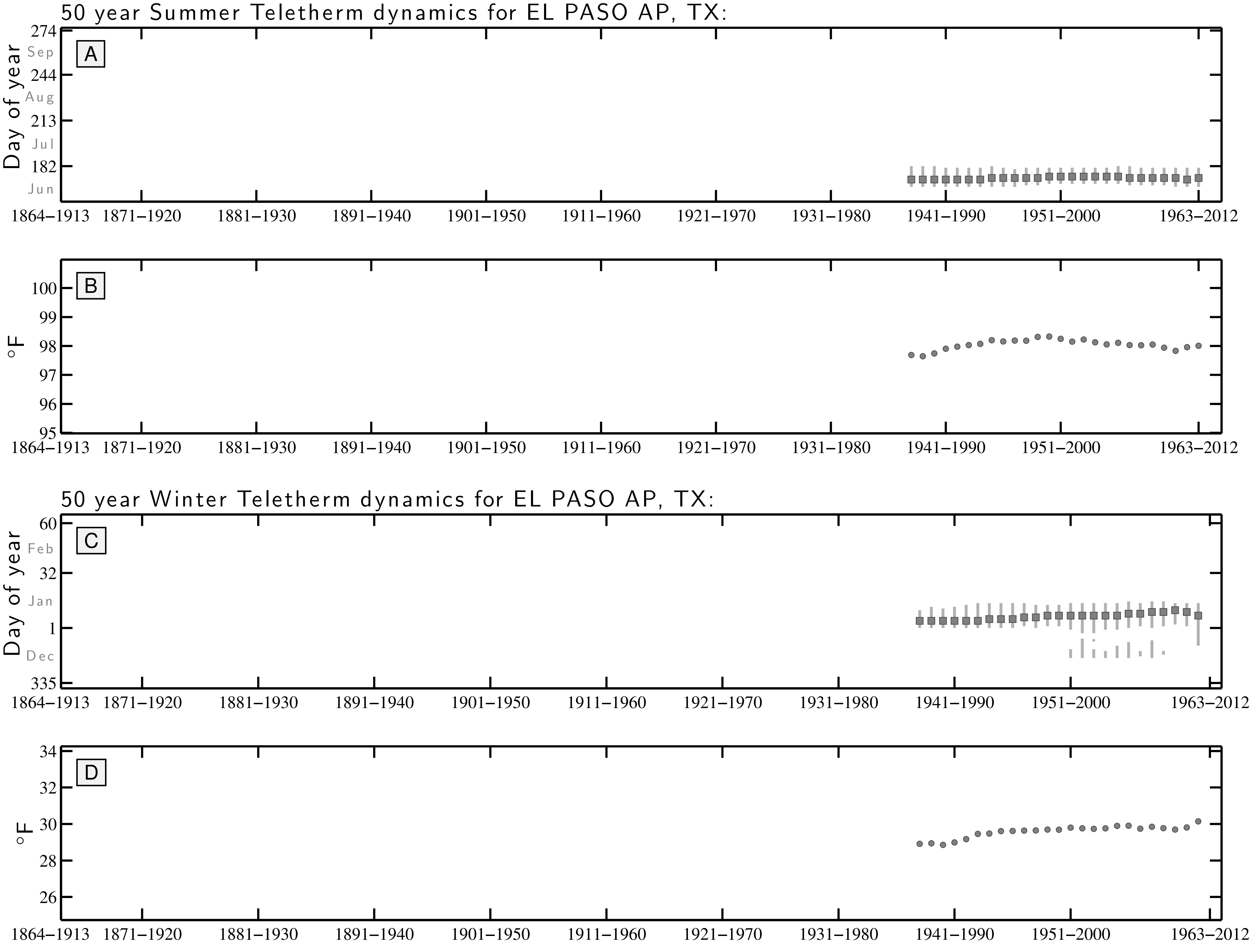
50 year Summer Teletherm dynamics for EL PASO AP, TX:
A
Day of year
274
Sep
244
Aug
213
Jul
182
Jun
B
°F
100
99
98
97
96
95
1864–1913
1871–1920
1881–1930
1891–1940
1901–1950
1911–1960
1921–1970
1931–1980
1941–1990
1951–2000
1963–2012
50 year Winter Teletherm dynamics for EL PASO AP, TX:
C
Day of year
60
Feb
32
Jan
1
Dec
335
D
°F
34
32
30
28
26

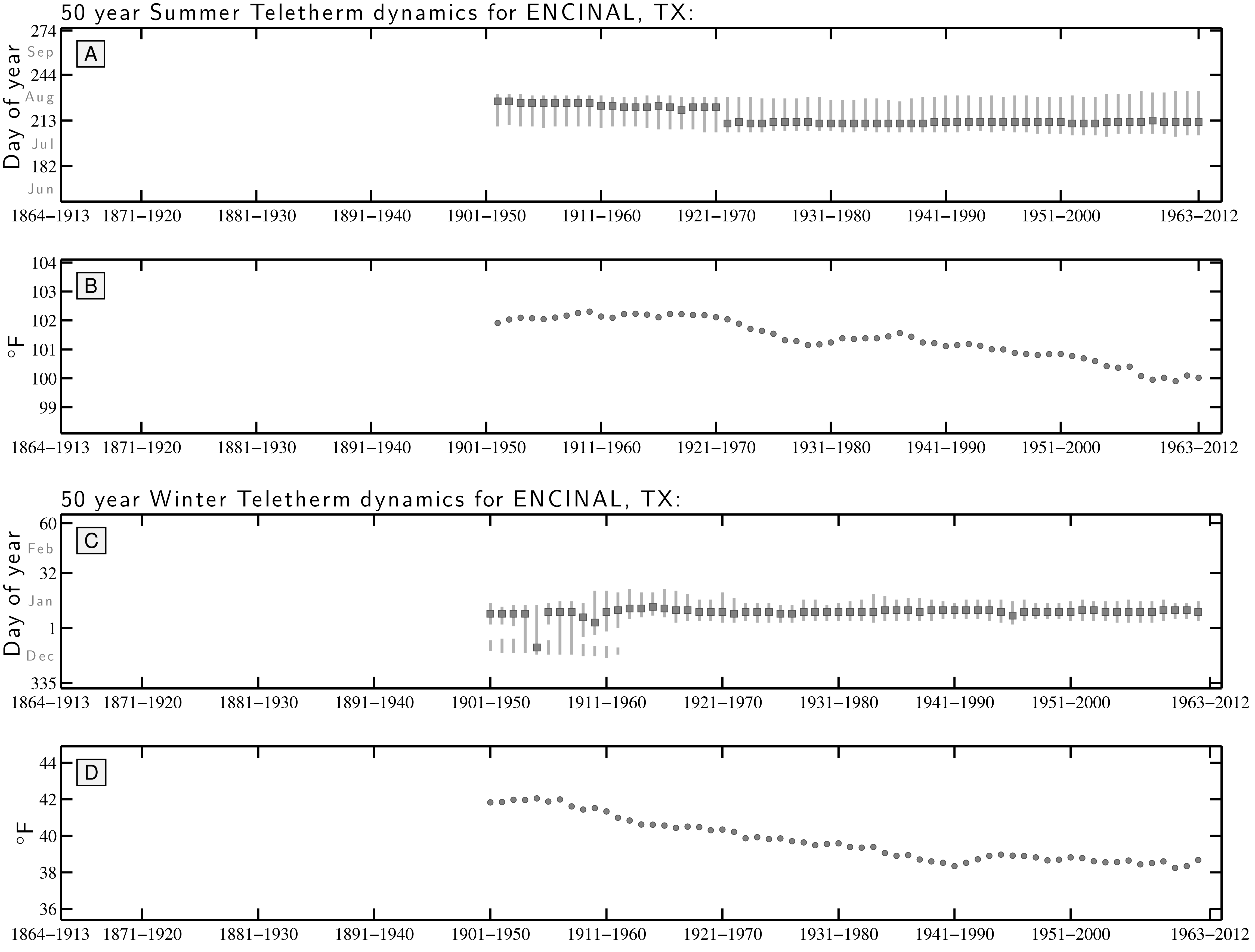
50 year Summer Teletherm dynamics for ENCINAL, TX:
A
Day of year
274
Sep
244
Aug
213
Jul
182
Jun
1864–1913
1871–1920
1881–1930
1891–1940
1901–1950
1911–1960
1921–1970
1931–1980
1941–1990
1951–2000
1963–2012
B
°F
104
103
102
101
100
99
50 year Winter Teletherm dynamics for ENCINAL, TX:
C
Day of year
60
Feb
32
Jan
1
Dec
335
D
°F
44
42
40
38
36

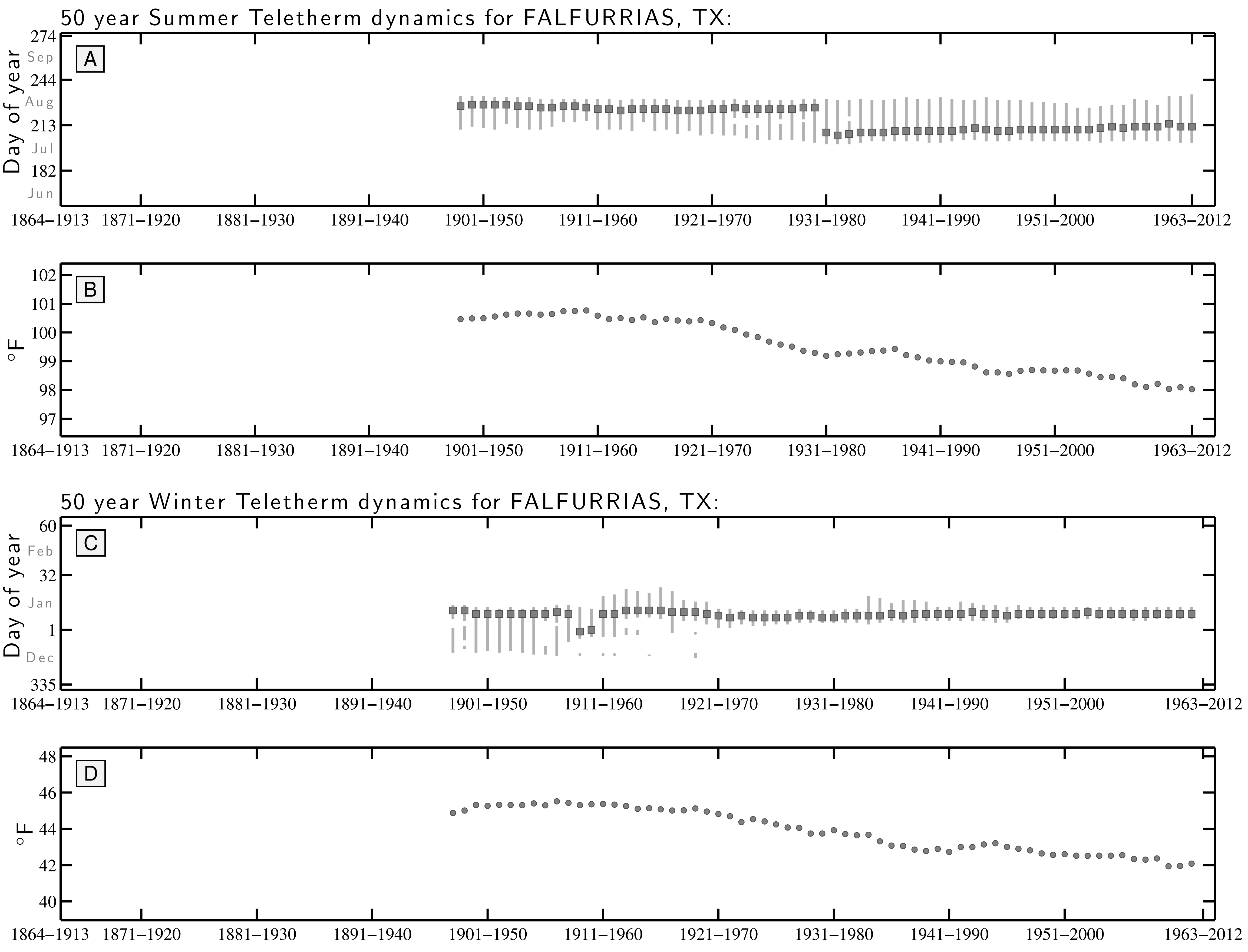

50 year Summer Teletherm dynamics for FALFURRIAS, TX:
A
Day of year
274
Sep
244
Aug
213
Jul
182
Jun
1864–1913
1871–1920
1881–1930
1891–1940
1901–1950
1911–1960
1921–1970
1931–1980
1941–1990
1951–2000
1963–2012
B
°F
102
101
100
99
98
97
50 year Winter Teletherm dynamics for FALFURRIAS, TX:
C
Day of year
60
Feb
32
Jan
1
Dec
335
D
°F
48
46
44
42
40

50 year Summer Teletherm dynamics for FLATONIA, TX:

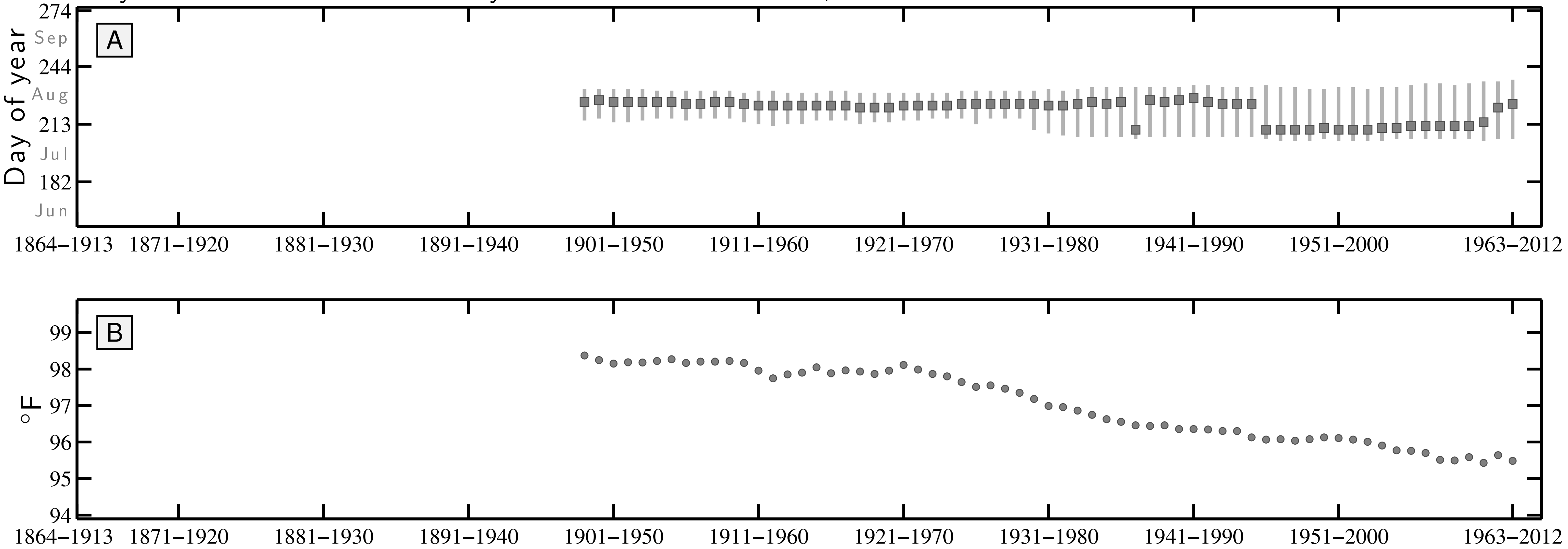

50 year Winter Teletherm dynamics for FLATONIA, TX:

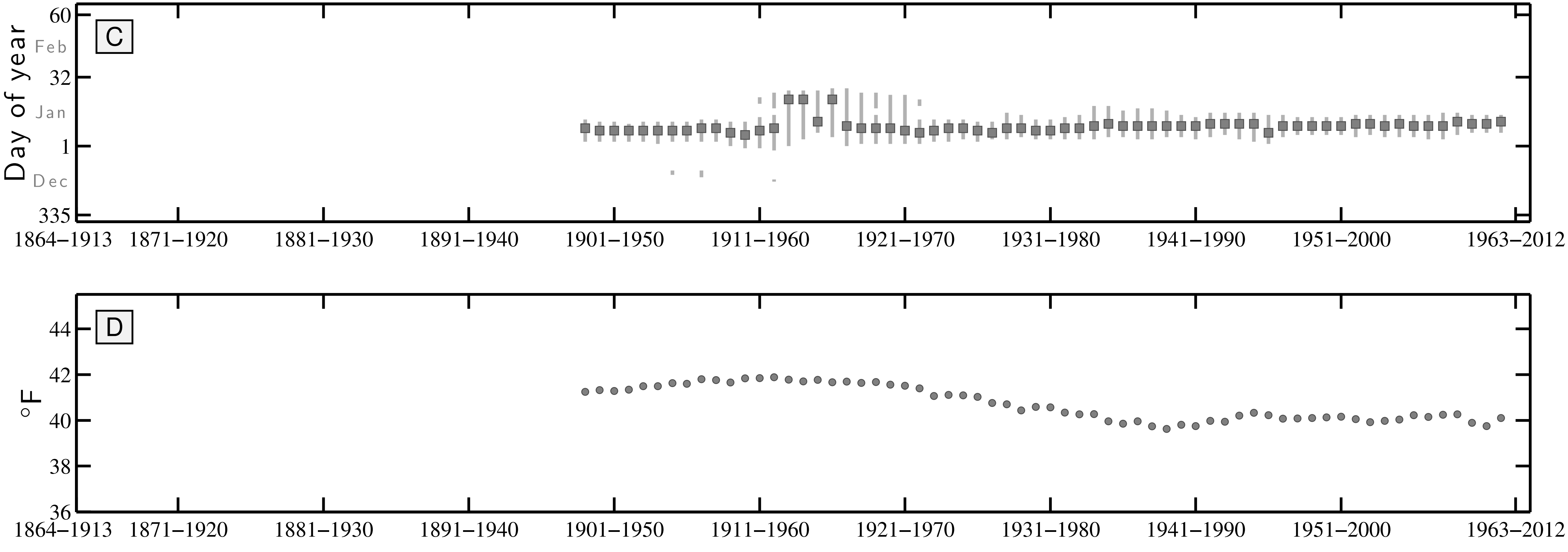

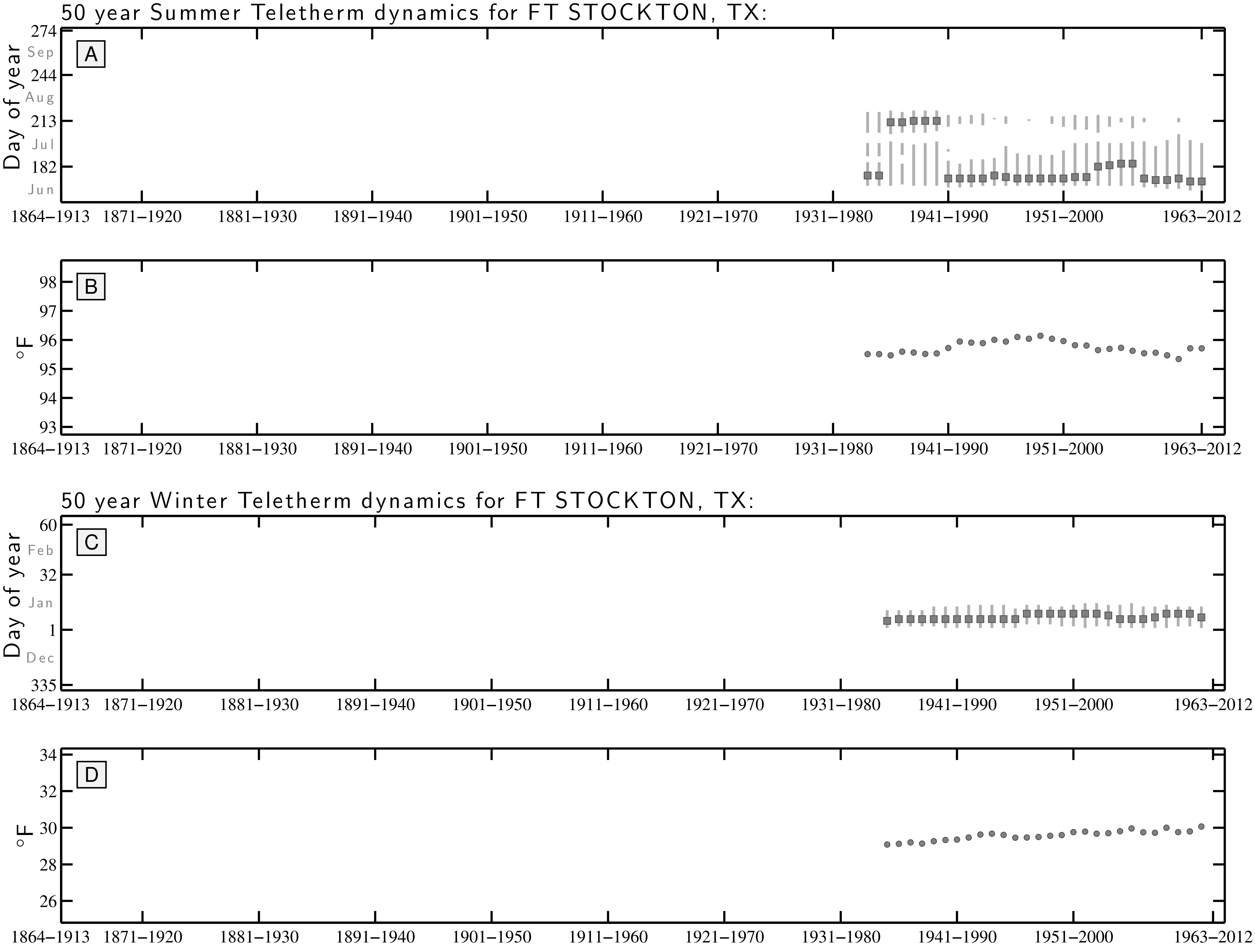
50 year Summer Teletherm dynamics for FT STOCKTON, TX:
A
Day of year
274
Sep
244
Aug
213
Jul
182
Jun
B
°F
98
97
96
95
94
93
50 year Winter Teletherm dynamics for FT STOCKTON, TX:
C
Day of year
60
Feb
32
Jan
1
Dec
335
D
°F
34
32
30
28
26
1864–1913
1871–1920
1881–1930
1891–1940
1901–1950
1911–1960
1921–1970
1931–1980
1941–1990
1951–2000
1963–2012

50 year Summer Teletherm dynamics for GREENVILLE KGVL RADIO, TX:

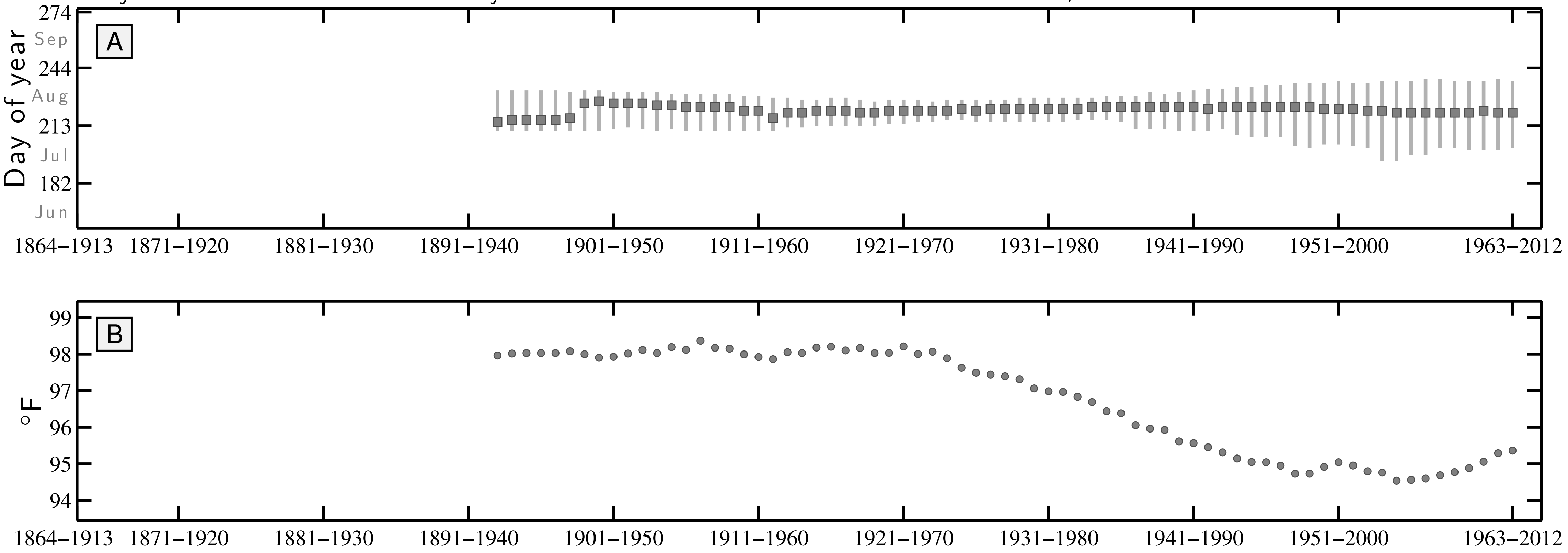

50 year Winter Teletherm dynamics for GREENVILLE KGVL RADIO, TX:

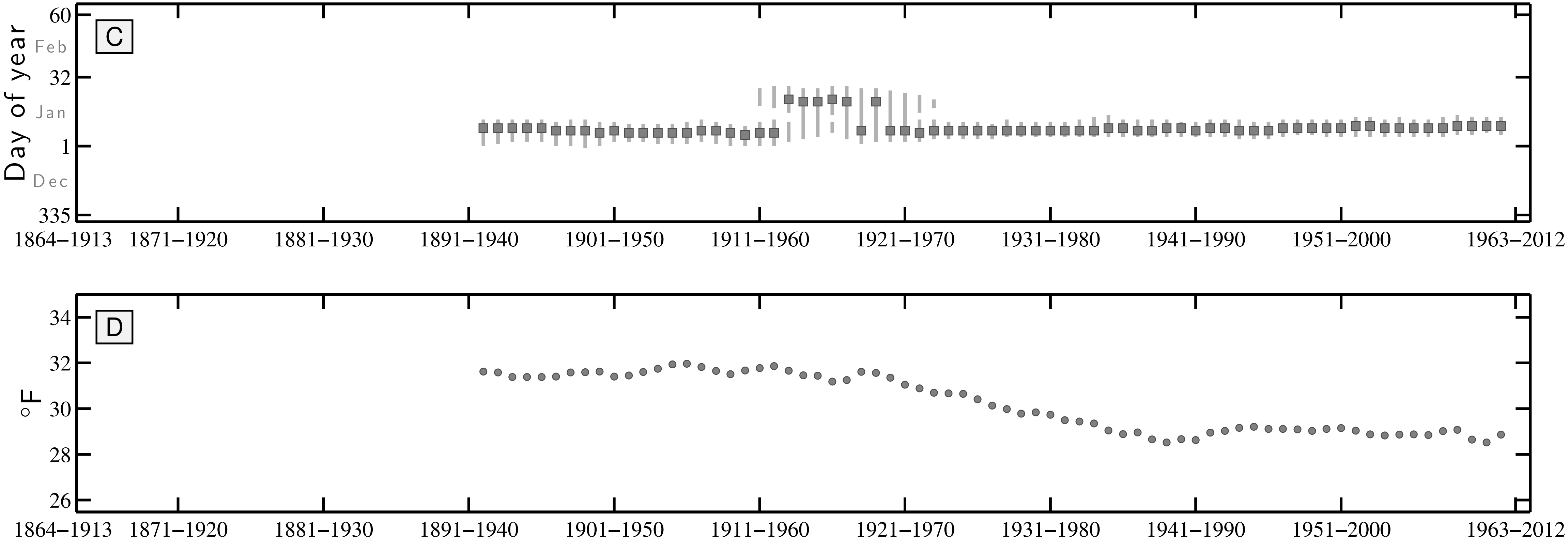

50 year Summer Teletherm dynamics for HALLETTSVILLE 2 N, TX:

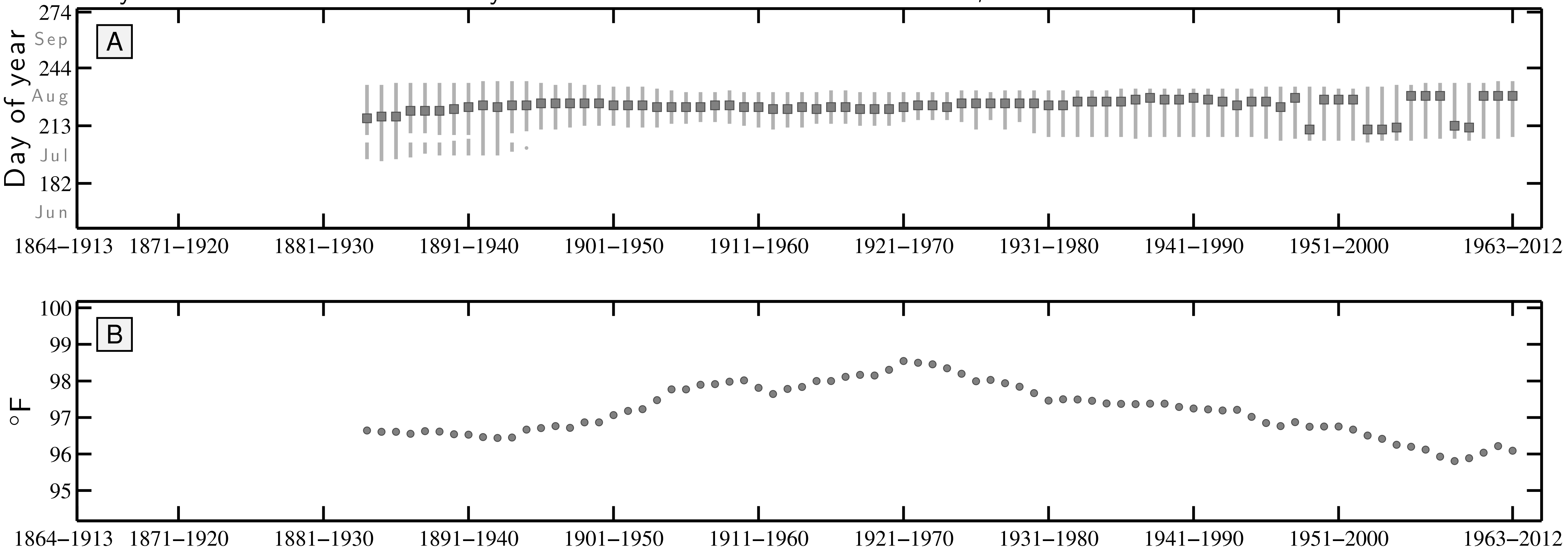

50 year Winter Teletherm dynamics for HALLETTSVILLE 2 N, TX:

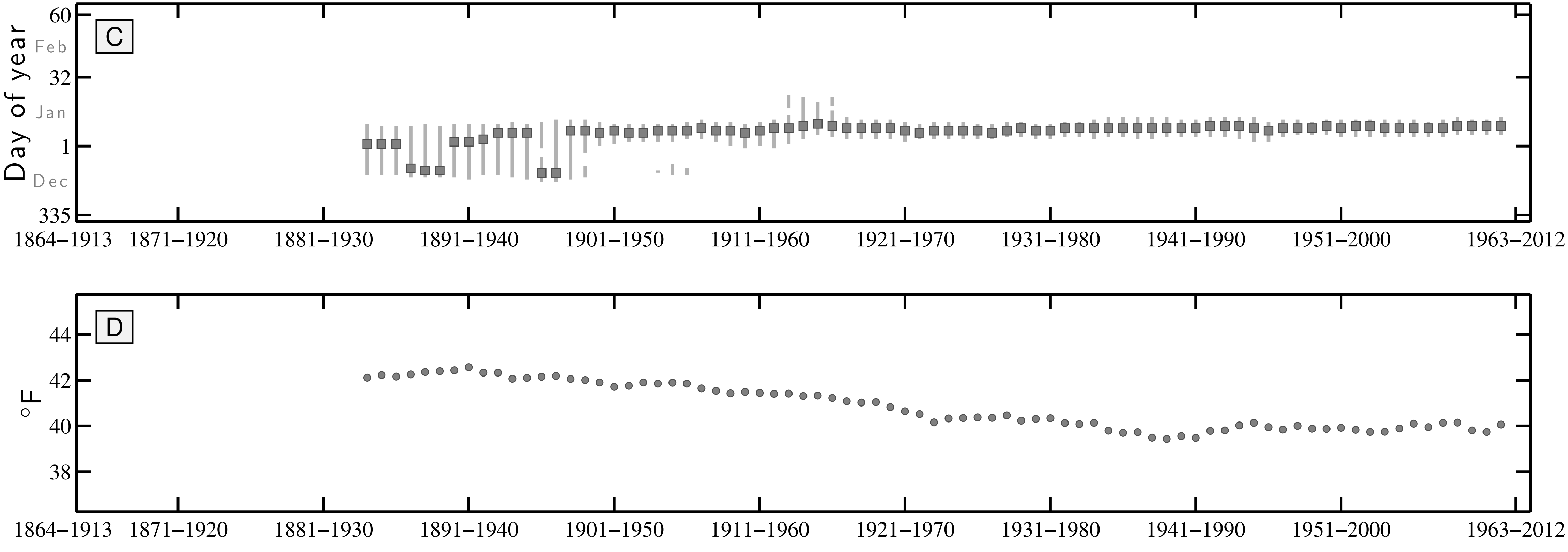

50 year Summer Teletherm dynamics for HASKELL, TX:

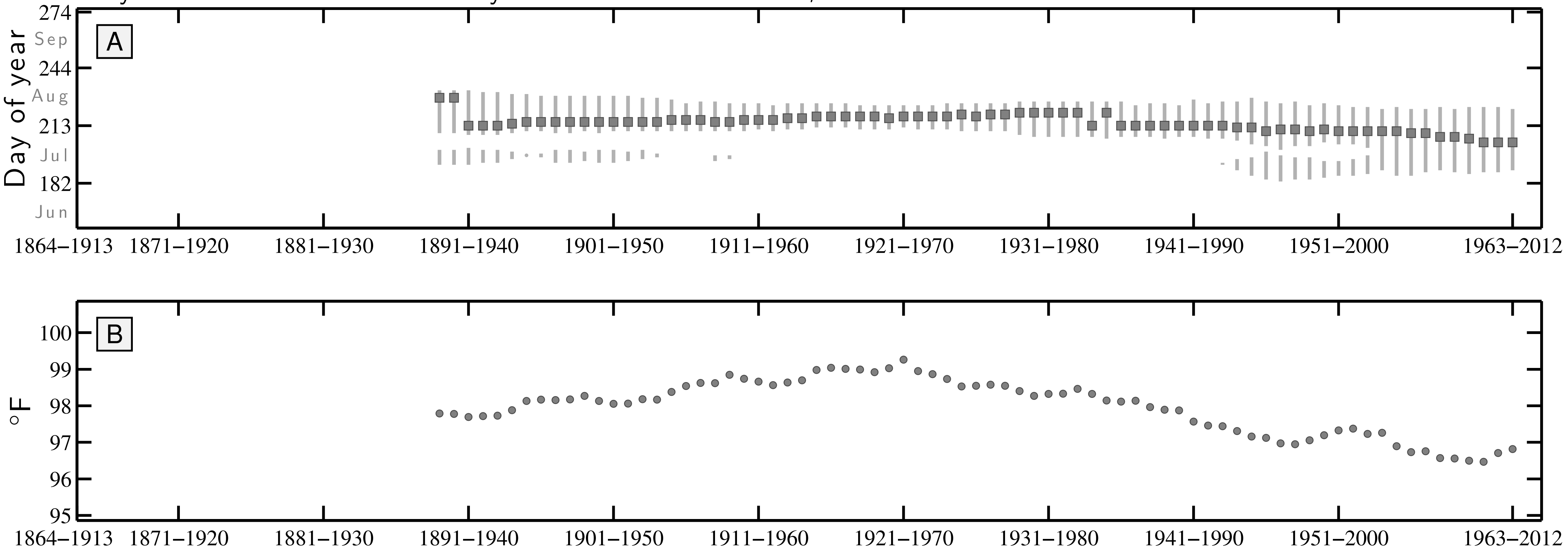

50 year Winter Teletherm dynamics for HASKELL, TX:

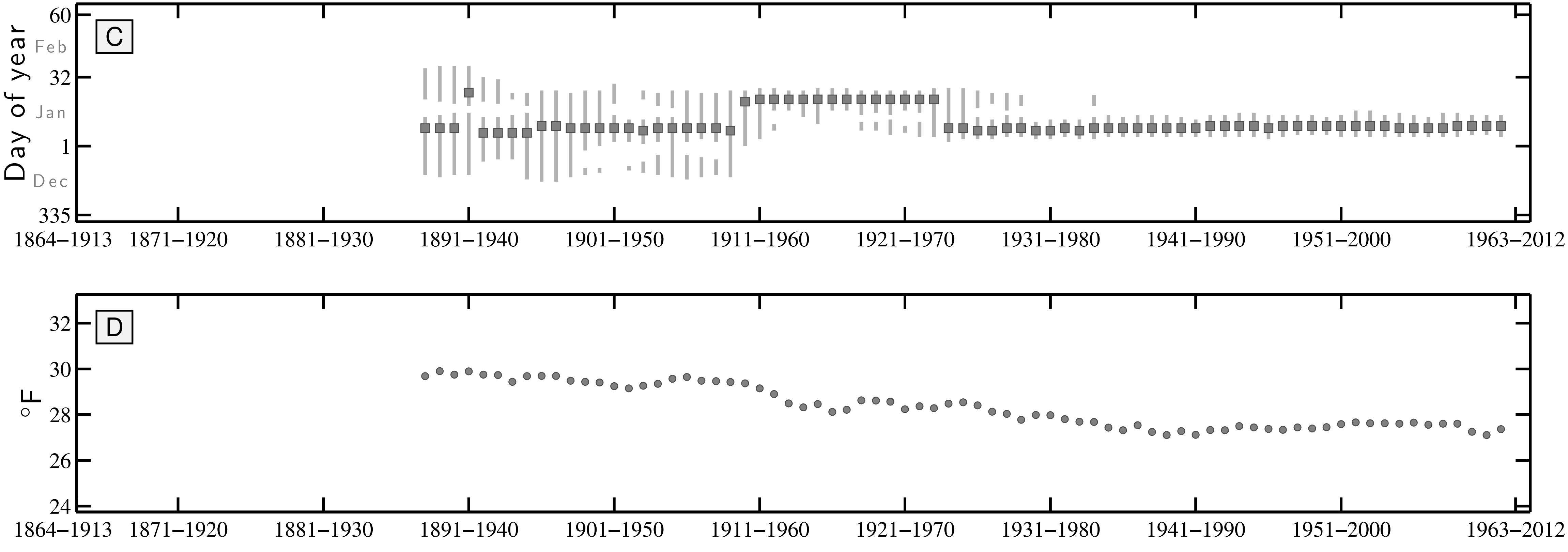

50 year Summer Teletherm dynamics for LAMPASAS, TX:

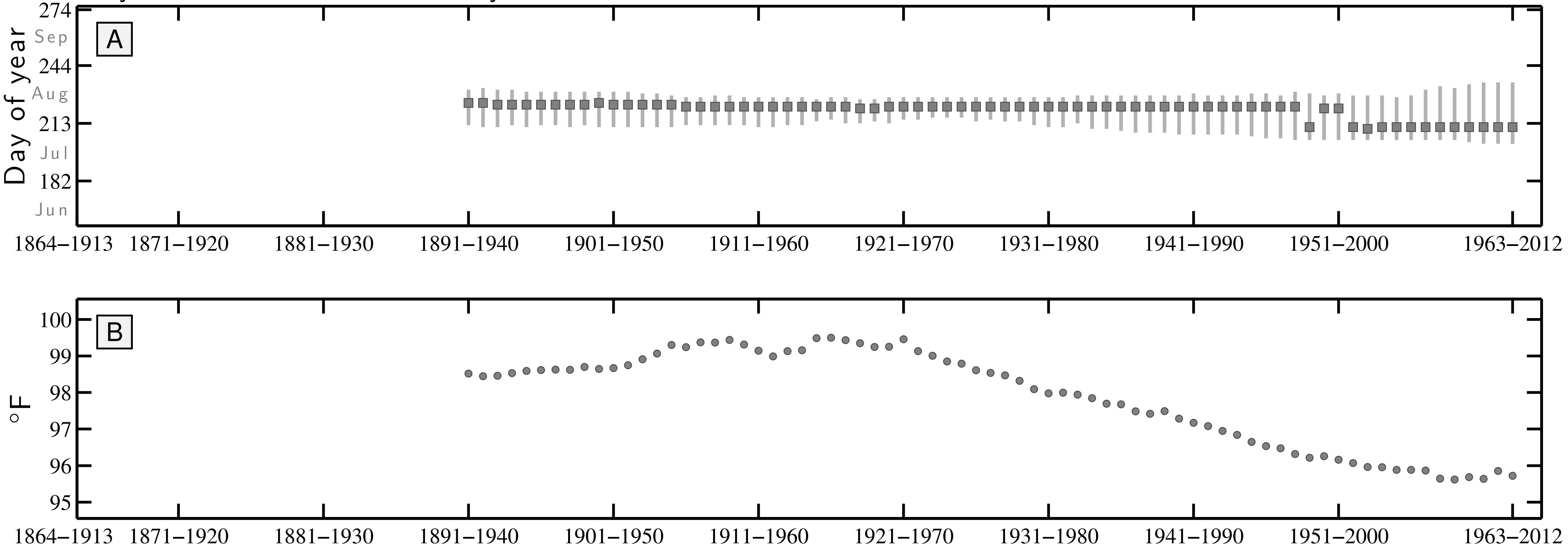

50 year Winter Teletherm dynamics for LAMPASAS, TX:

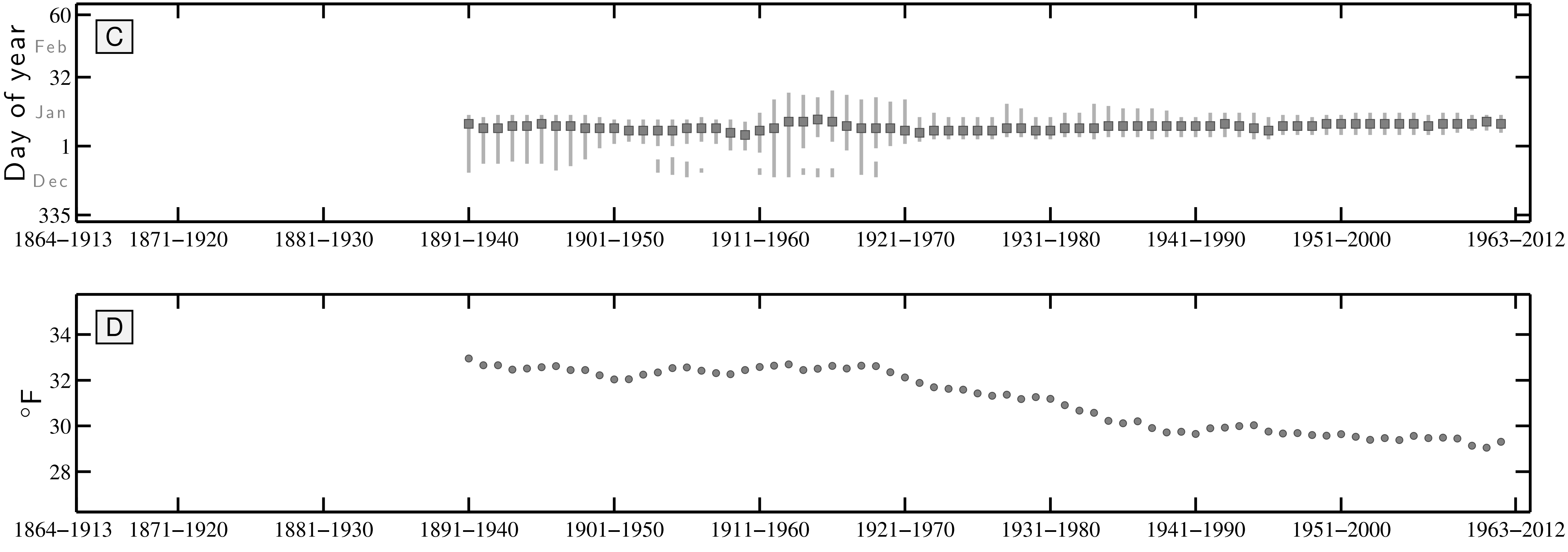

50 year Summer Teletherm dynamics for LIBERTY, TX:

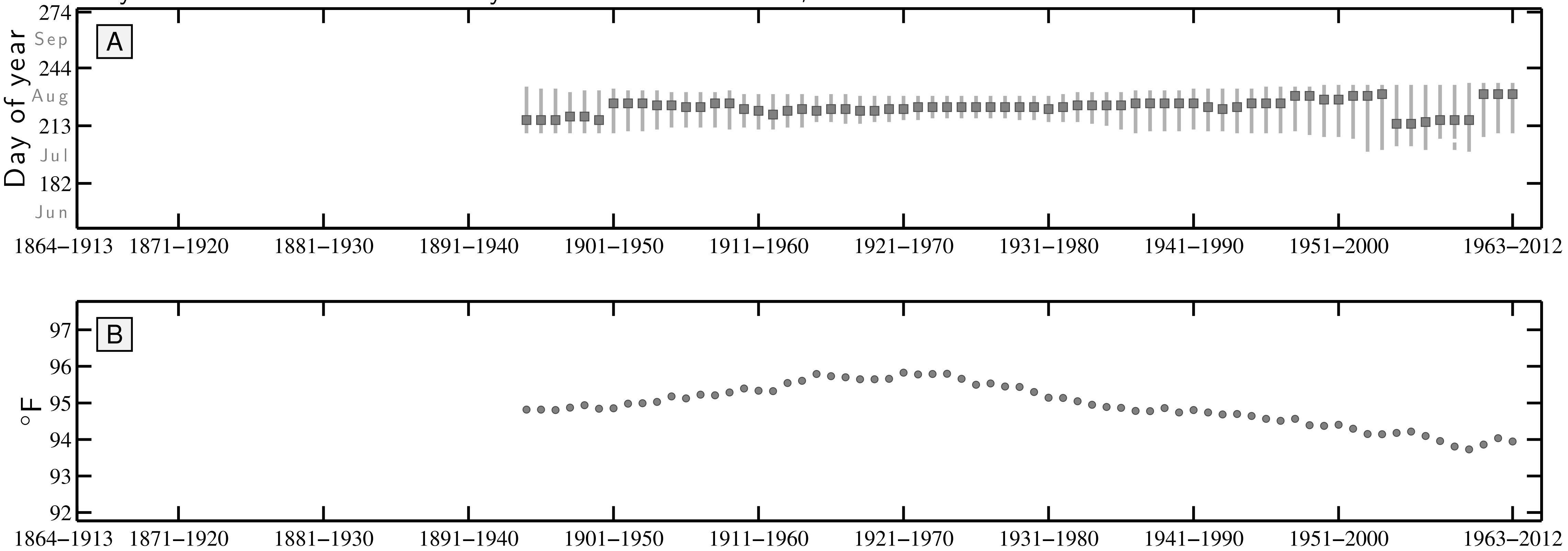

50 year Winter Teletherm dynamics for LIBERTY, TX:

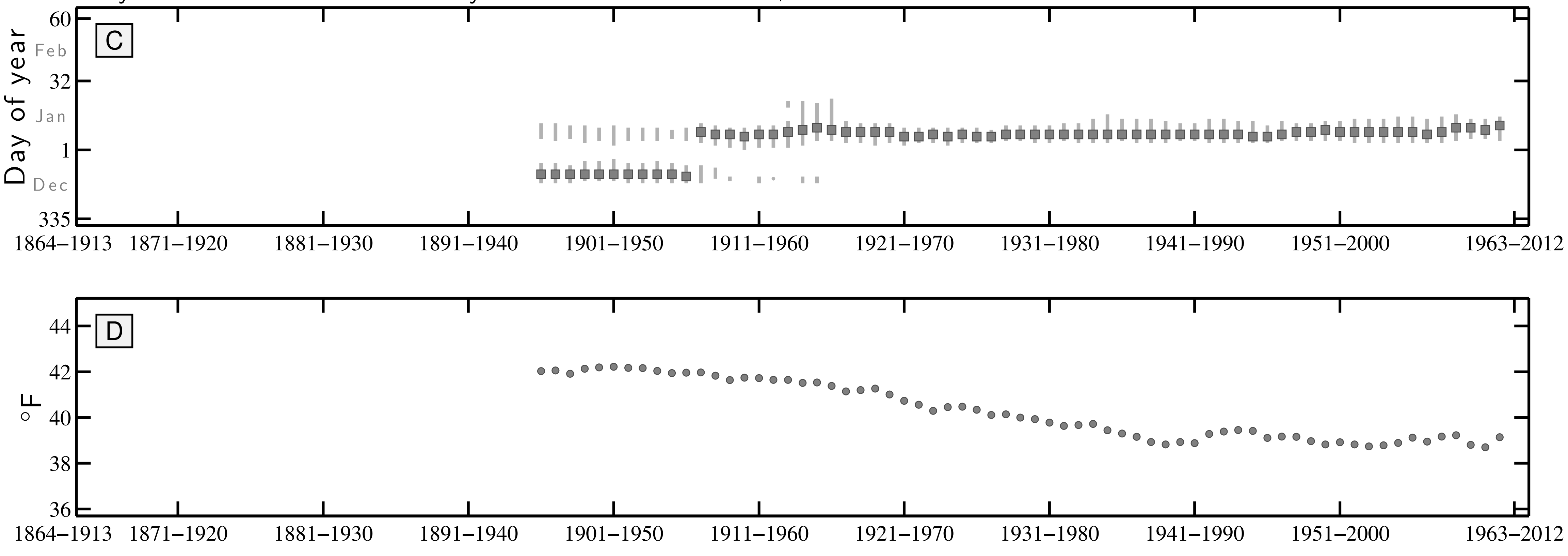

50 year Summer Teletherm dynamics for LLANO, TX:

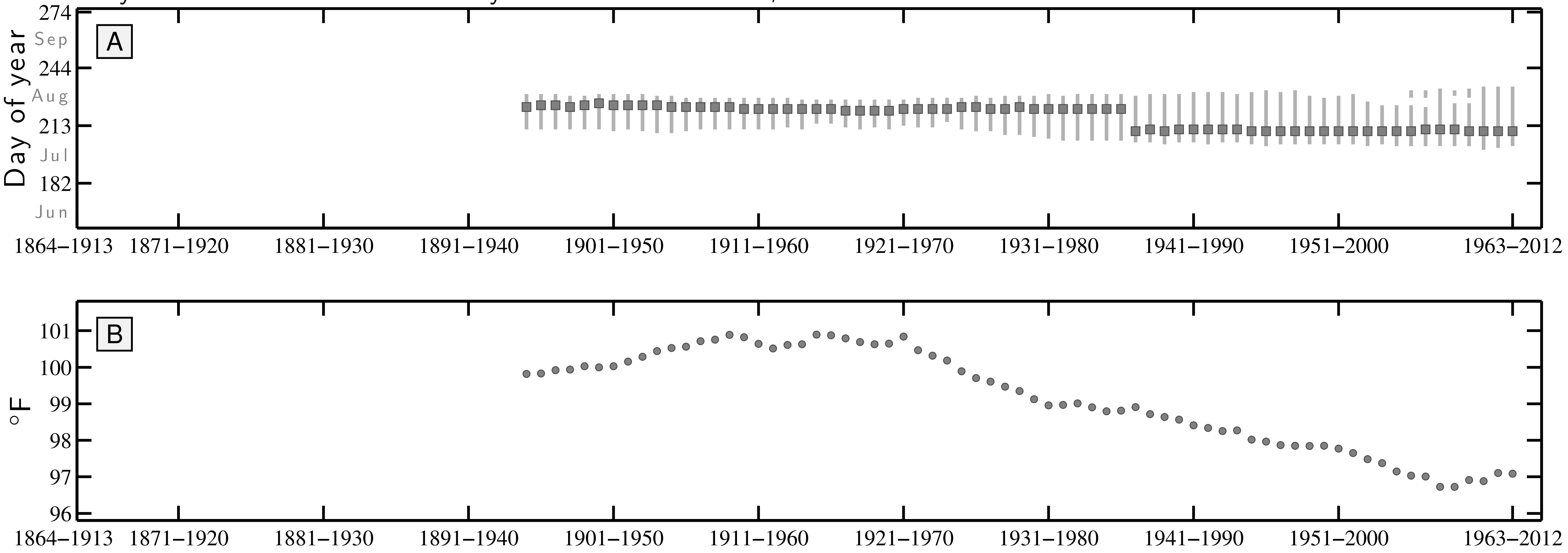

50 year Winter Teletherm dynamics for LLANO, TX:

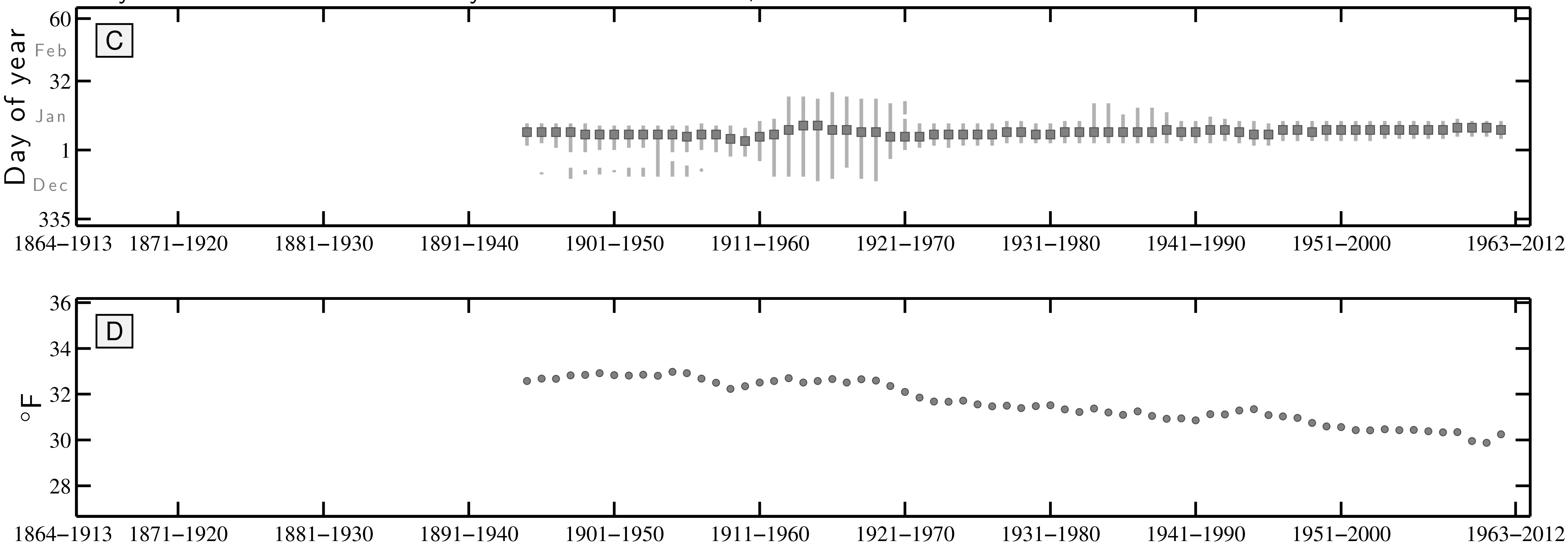

50 year Summer Teletherm dynamics for LULING, TX:

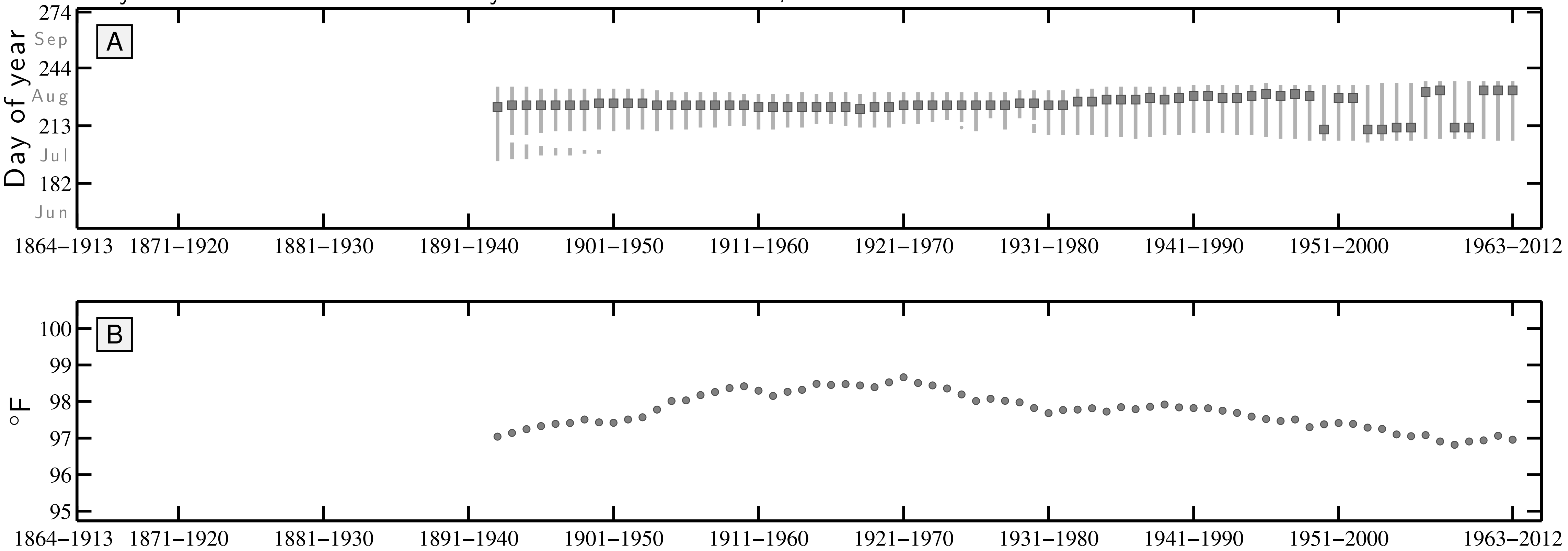

50 year Winter Teletherm dynamics for LULING, TX:

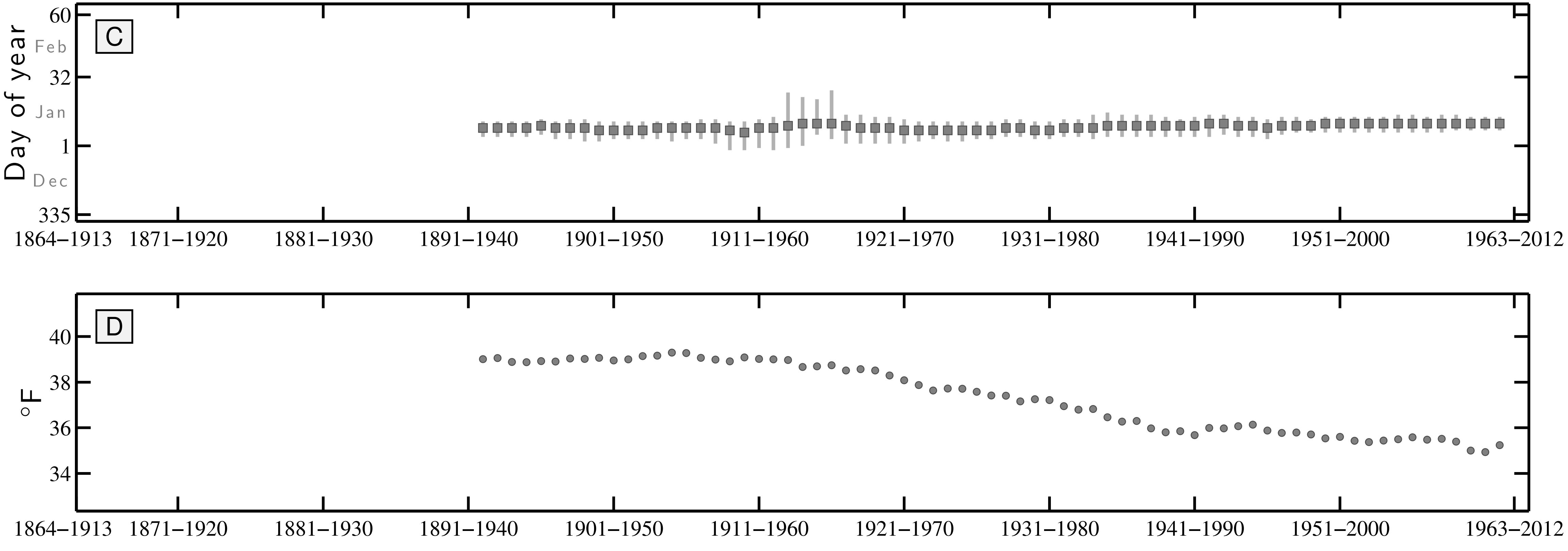

50 year Summer Teletherm dynamics for MARSHALL, TX:

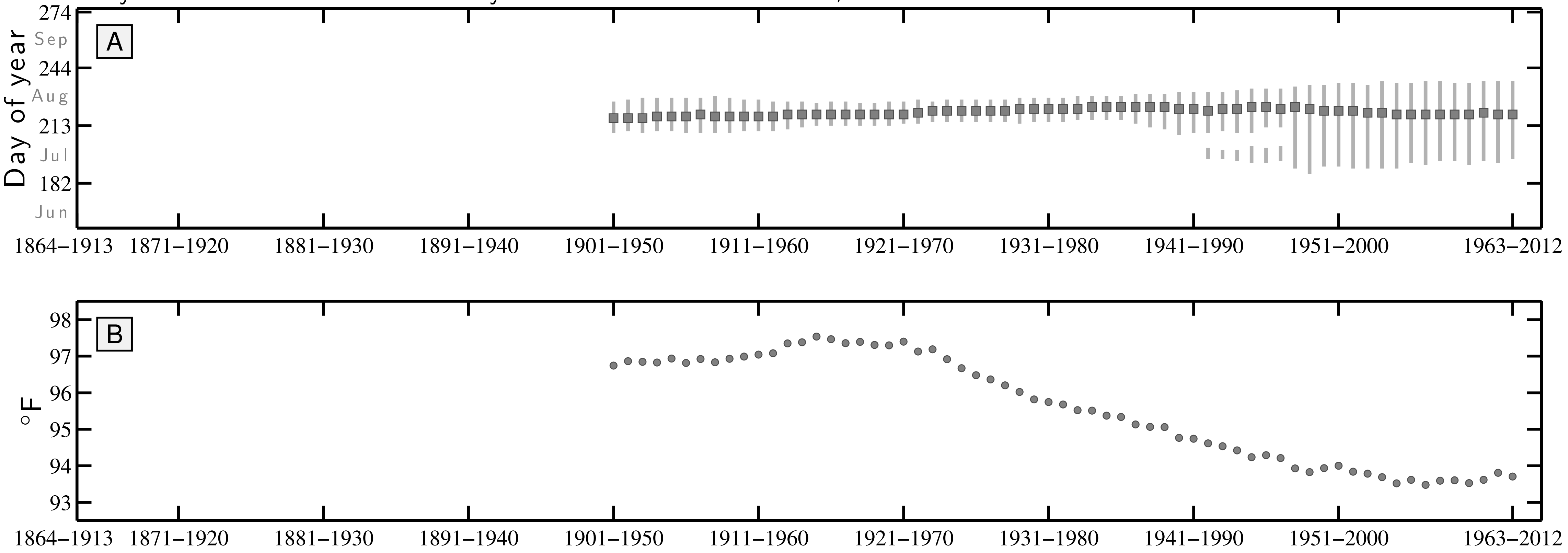

50 year Winter Teletherm dynamics for MARSHALL, TX:

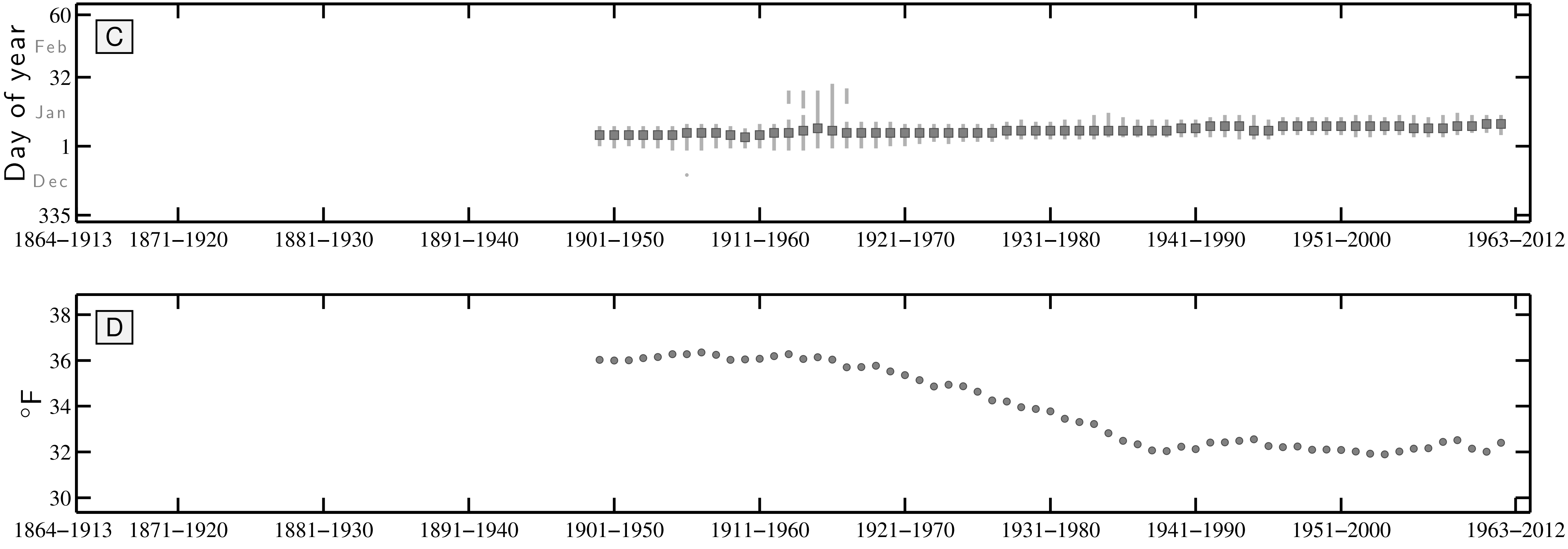

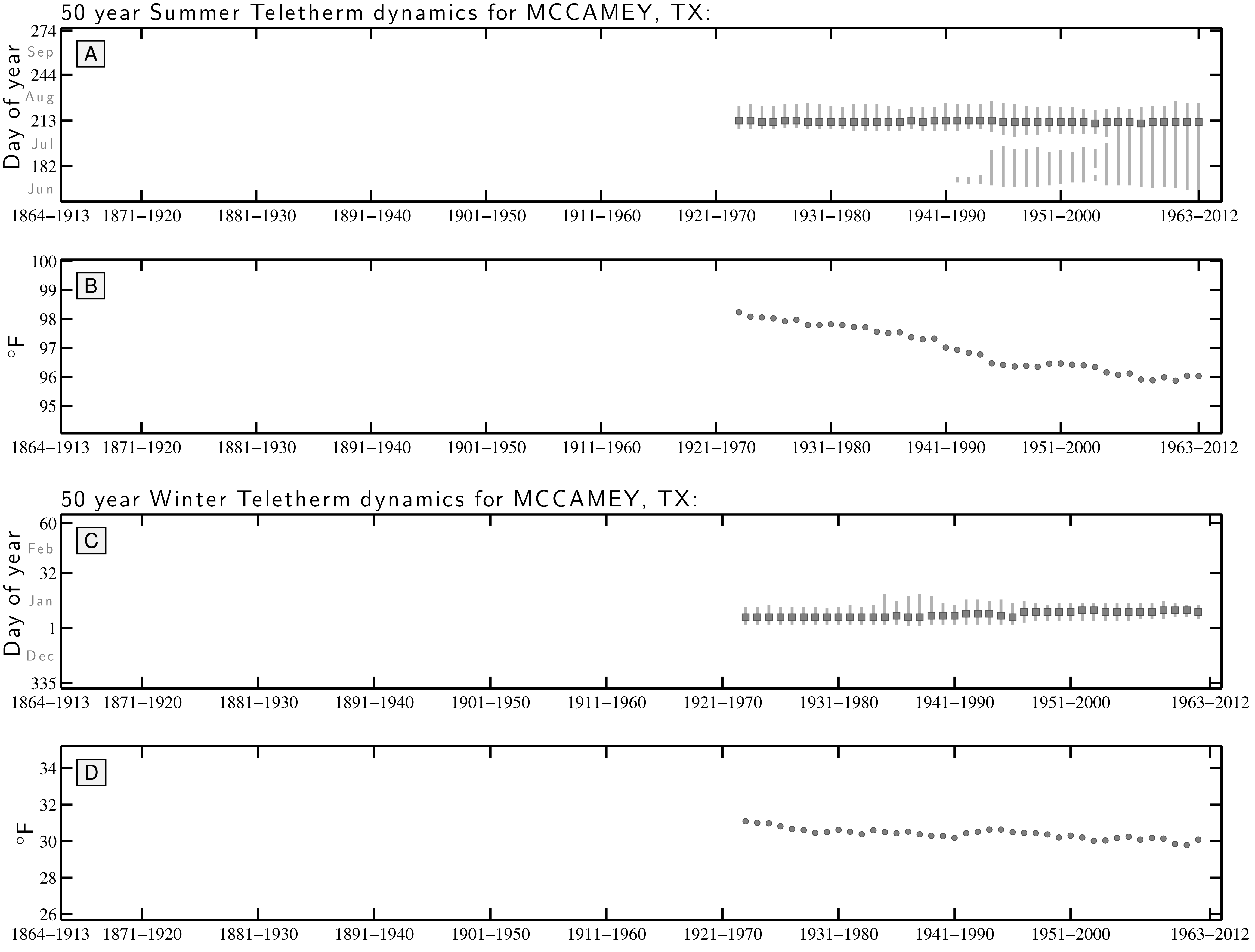
50 year Summer Teletherm dynamics for MCCAMEY, TX:
A
B
Day of year
°F
Sep
Aug
Jul
Jun
274
244
213
182
100
99
98
97
96
95
1864–1913
1871–1920
1881–1930
1891–1940
1901–1950
1911–1960
1921–1970
1931–1980
1941–1990
1951–2000
1963–2012
50 year Winter Teletherm dynamics for MCCAMEY, TX:
C
D
Feb
Jan
Dec
60
32
1
335
34
32
30
28
26

50 year Summer Teletherm dynamics for MEXIA, TX:

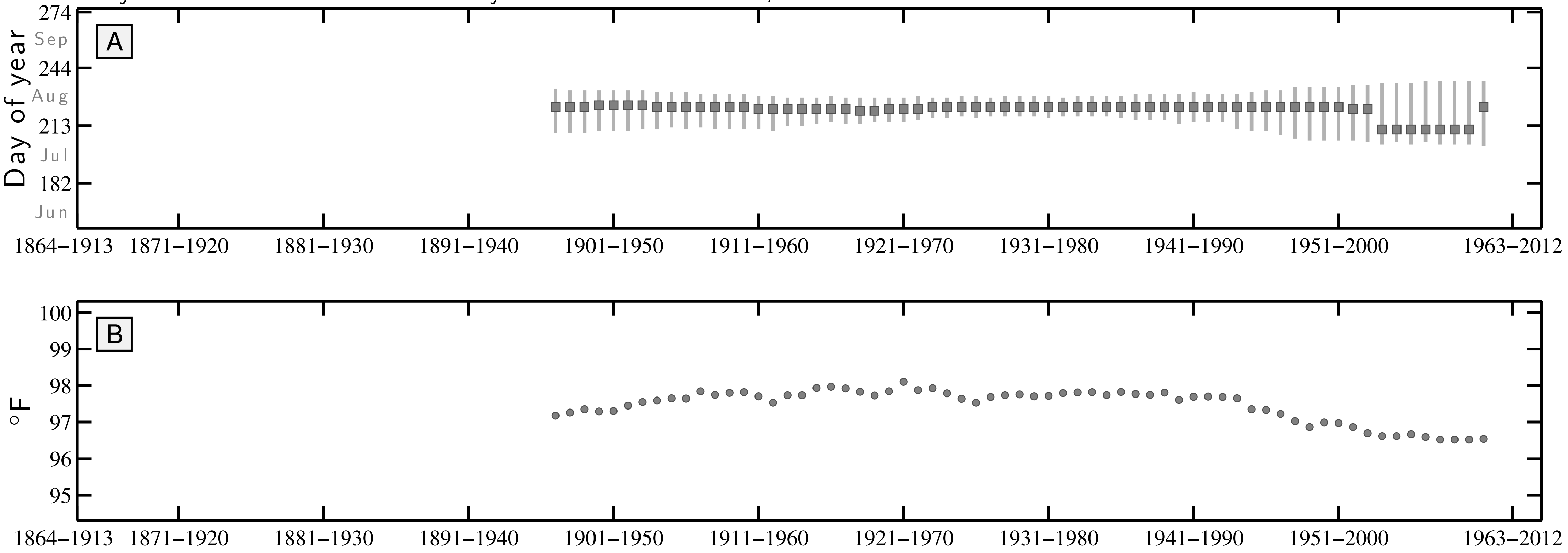

50 year Winter Teletherm dynamics for MEXIA, TX:

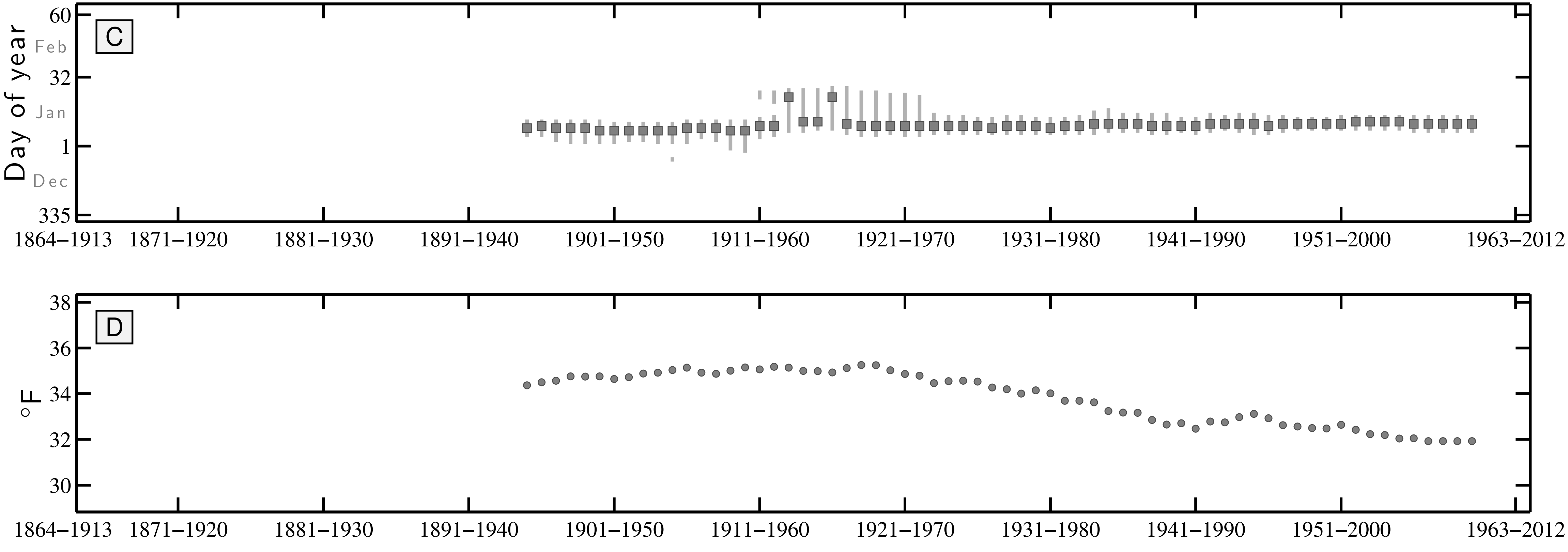

50 year Summer Teletherm dynamics for MIAMI, TX:

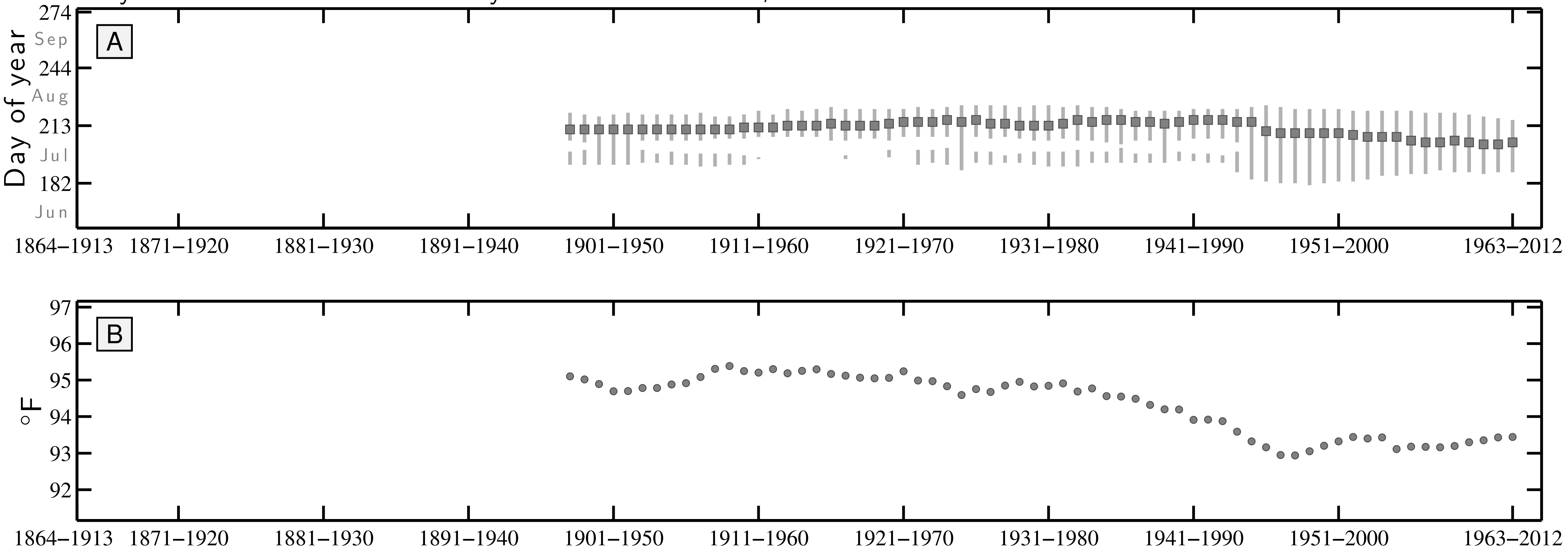

50 year Winter Teletherm dynamics for MIAMI, TX:

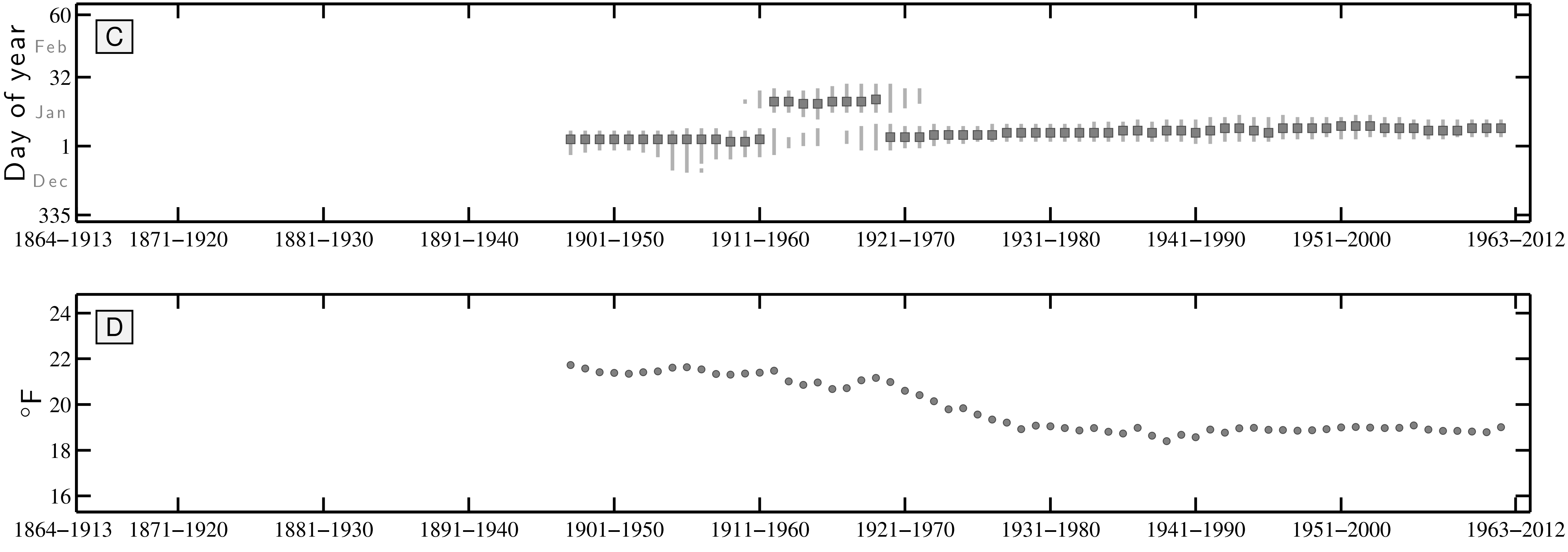

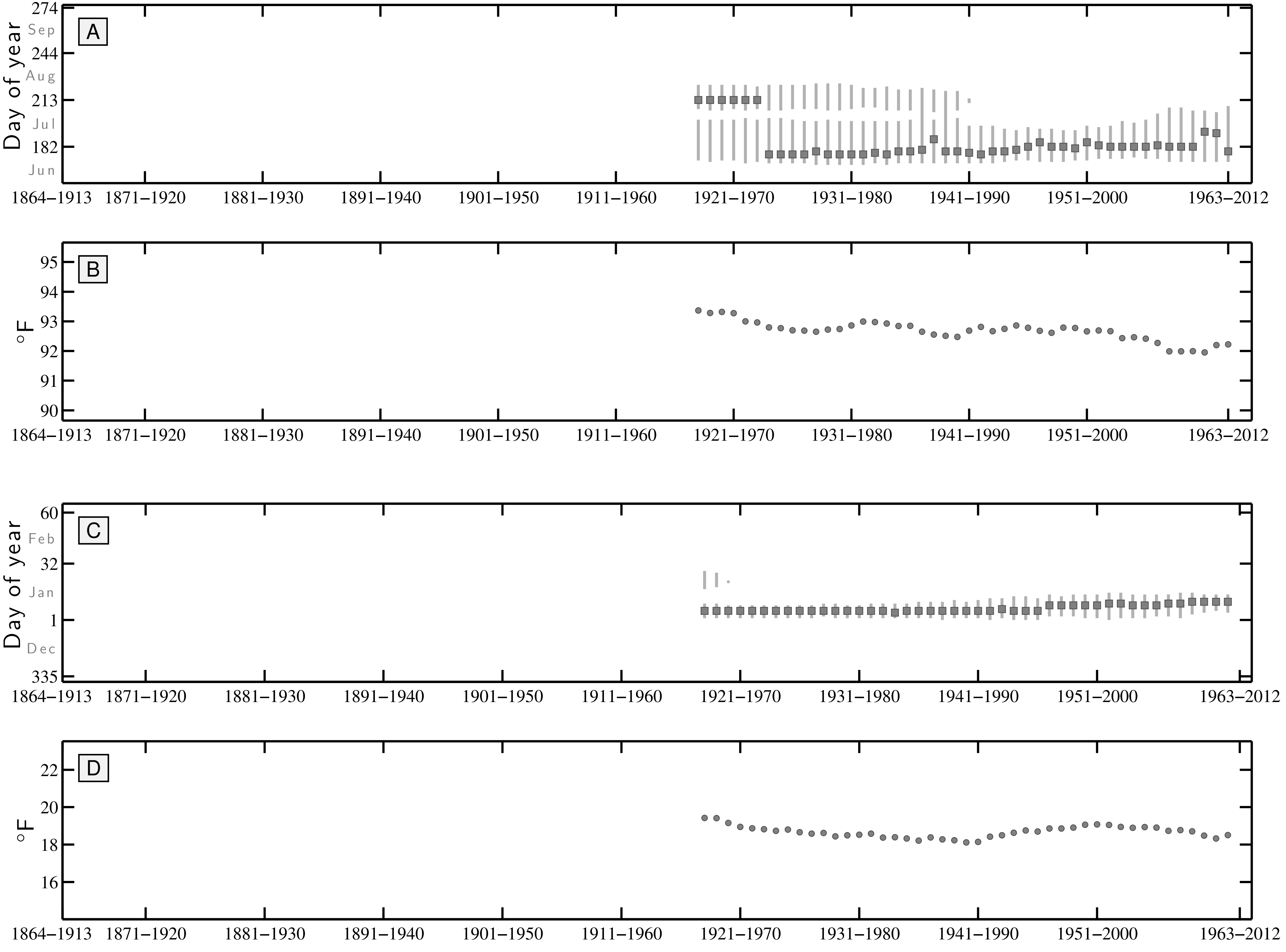
A
Day of year
274
Sep
244
Aug
213
Jul
182
Jun
B
°F
95
94
93
92
91
90
C
Day of year
60
Feb
32
Jan
1
Dec
335
D
°F
22
20
18
16
1864–1913
1871–1920
1881–1930
1891–1940
1901–1950
1911–1960
1921–1970
1931–1980
1941–1990
1951–2000
1963–2012

50 year Summer Teletherm dynamics for NEW BRAUNFELS, TX:

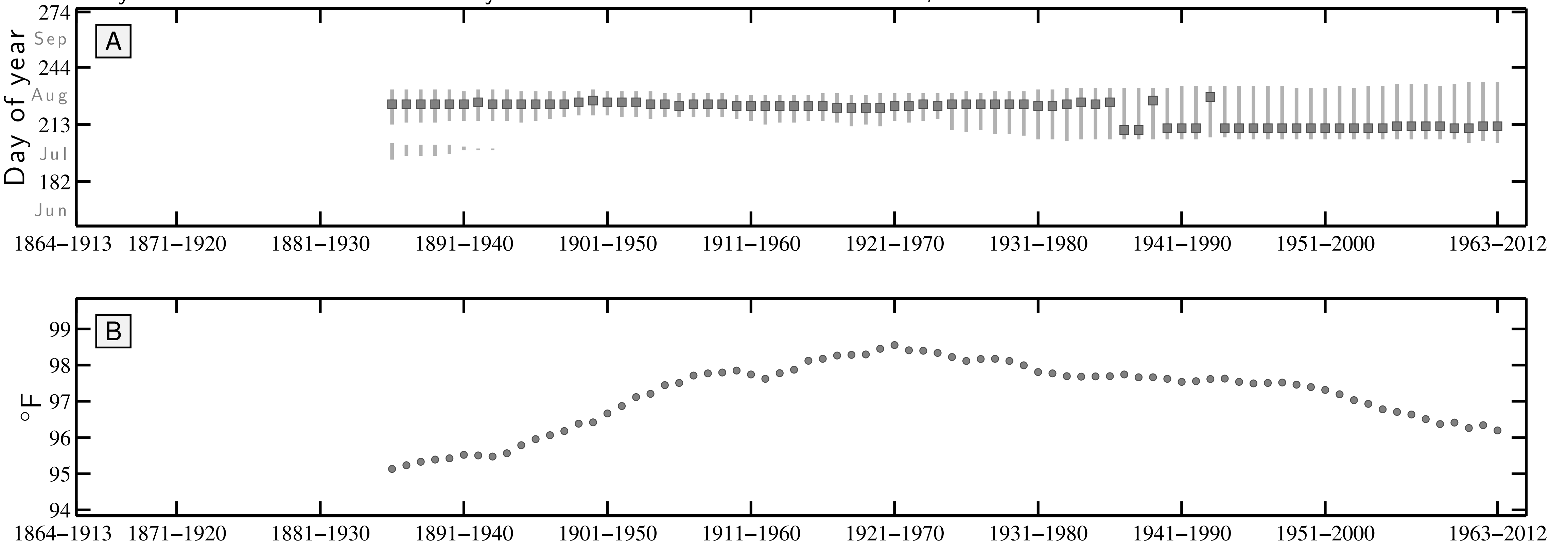

50 year Winter Teletherm dynamics for NEW BRAUNFELS, TX:

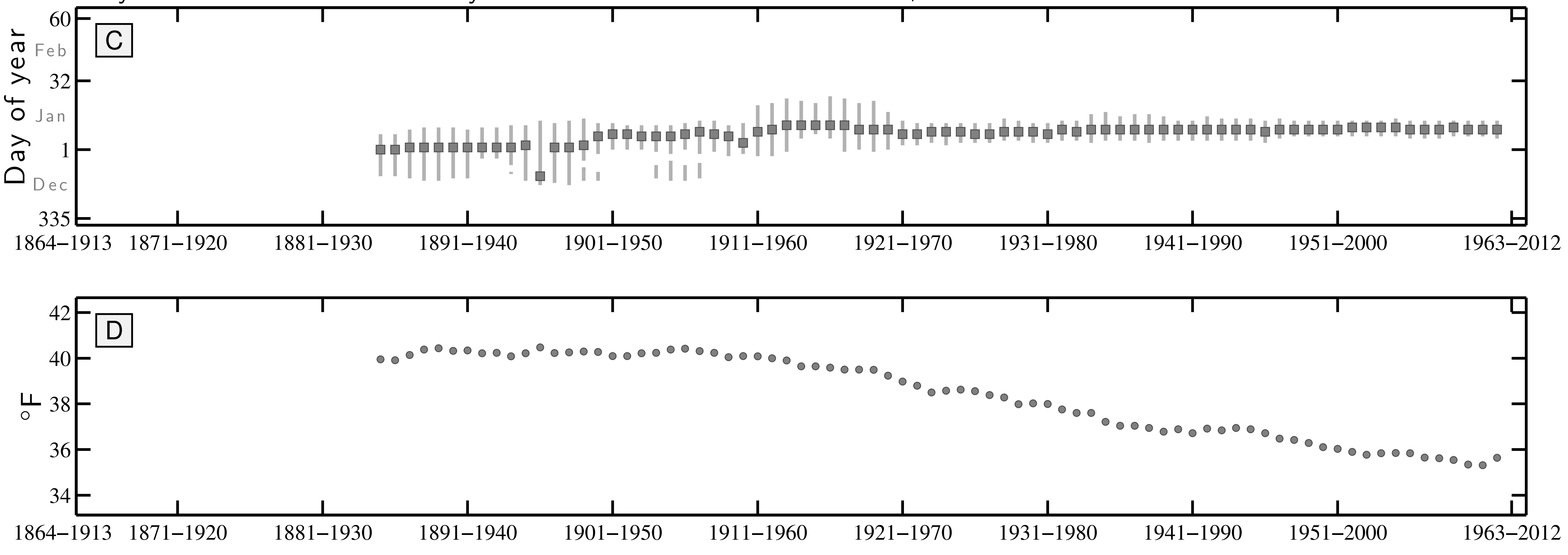

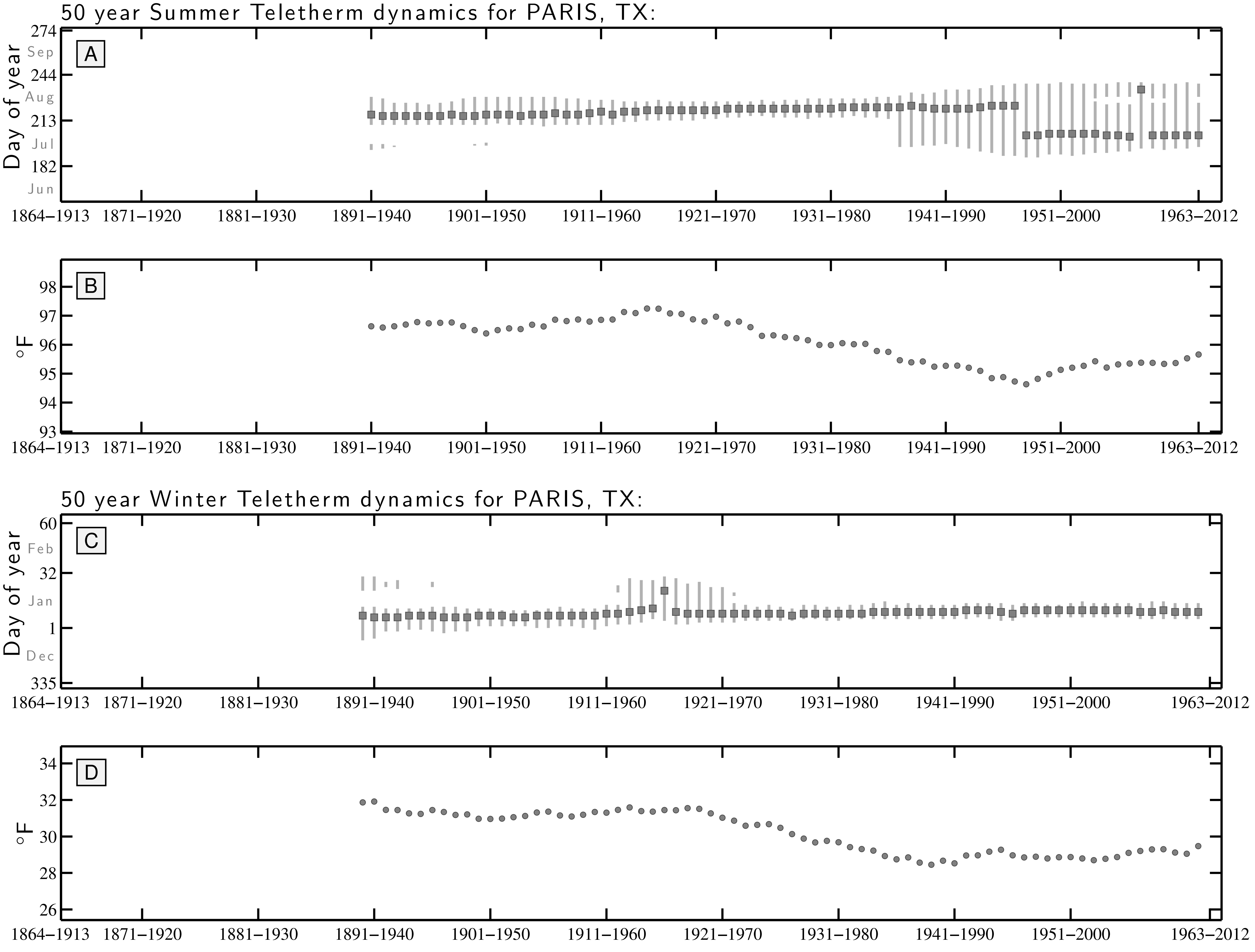

50 year Summer Teletherm dynamics for PARIS, TX:
A
B
Day of year
°F
274
Sep
244
Aug
213
Jul
182
Jun
98
97
96
95
94
93
1864–1913
1871–1920
1881–1930
1891–1940
1901–1950
1911–1960
1921–1970
1931–1980
1941–1990
1951–2000
1963–2012
50 year Winter Teletherm dynamics for PARIS, TX:
C
D
60
Feb
32
Jan
1
Dec
335
34
32
30
28
26

50 year Summer Teletherm dynamics for PECOS, TX:

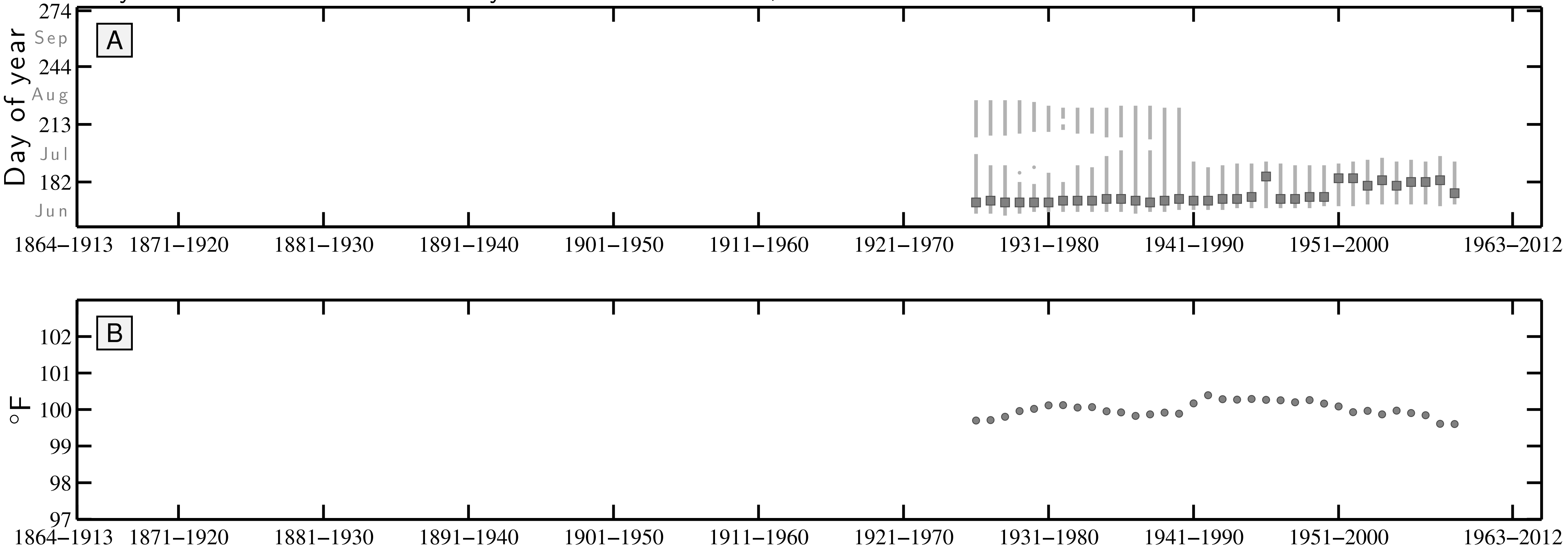

50 year Winter Teletherm dynamics for PECOS, TX:

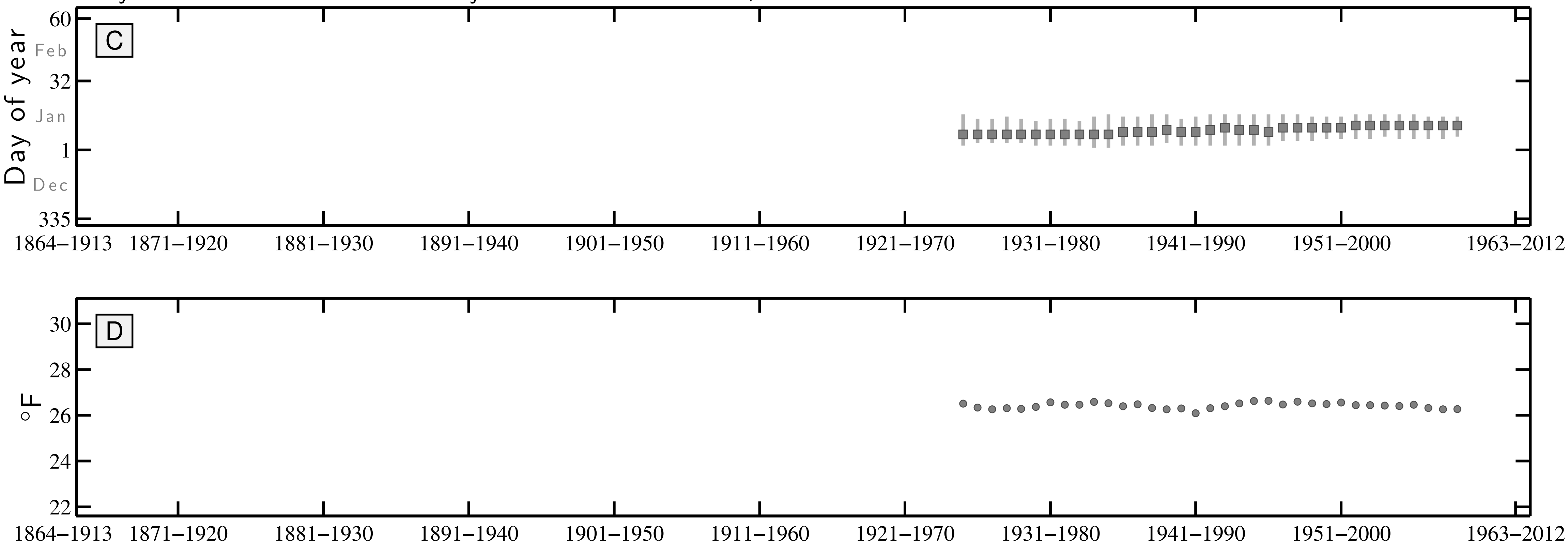

50 year Summer Teletherm dynamics for PLAINVIEW, TX:

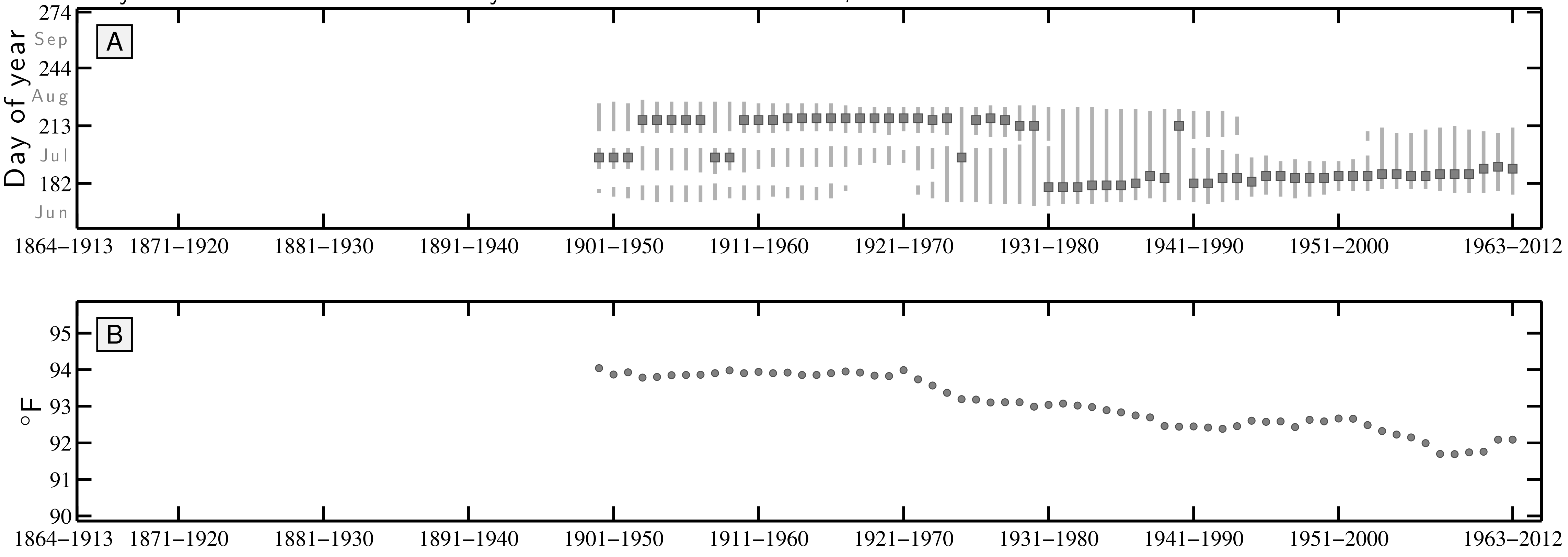

50 year Winter Teletherm dynamics for PLAINVIEW, TX:

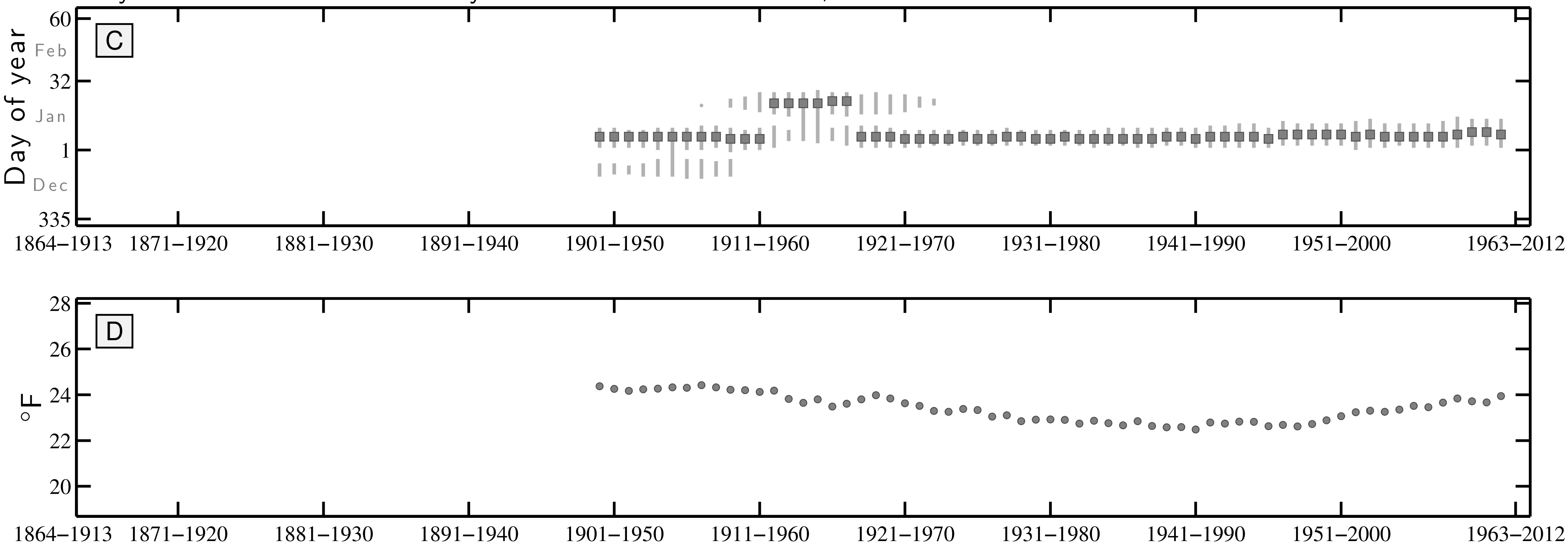

50 year Summer Teletherm dynamics for QUANAH 2 SW, TX:

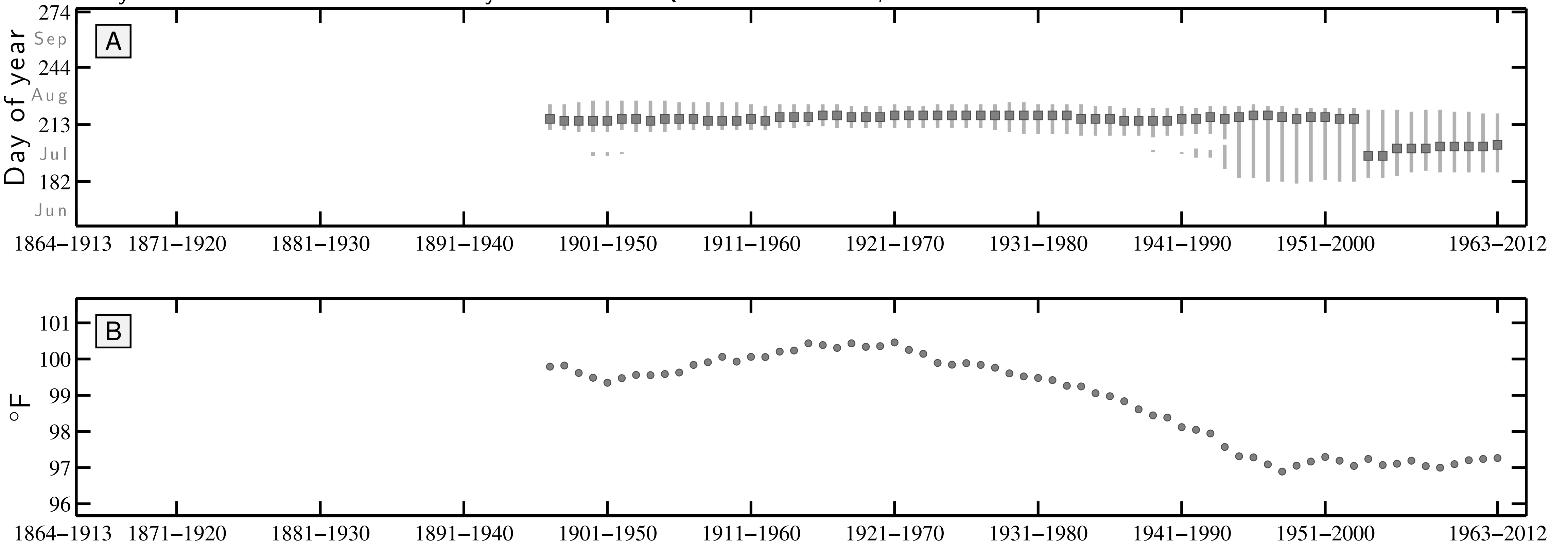

50 year Winter Teletherm dynamics for QUANAH 2 SW, TX:

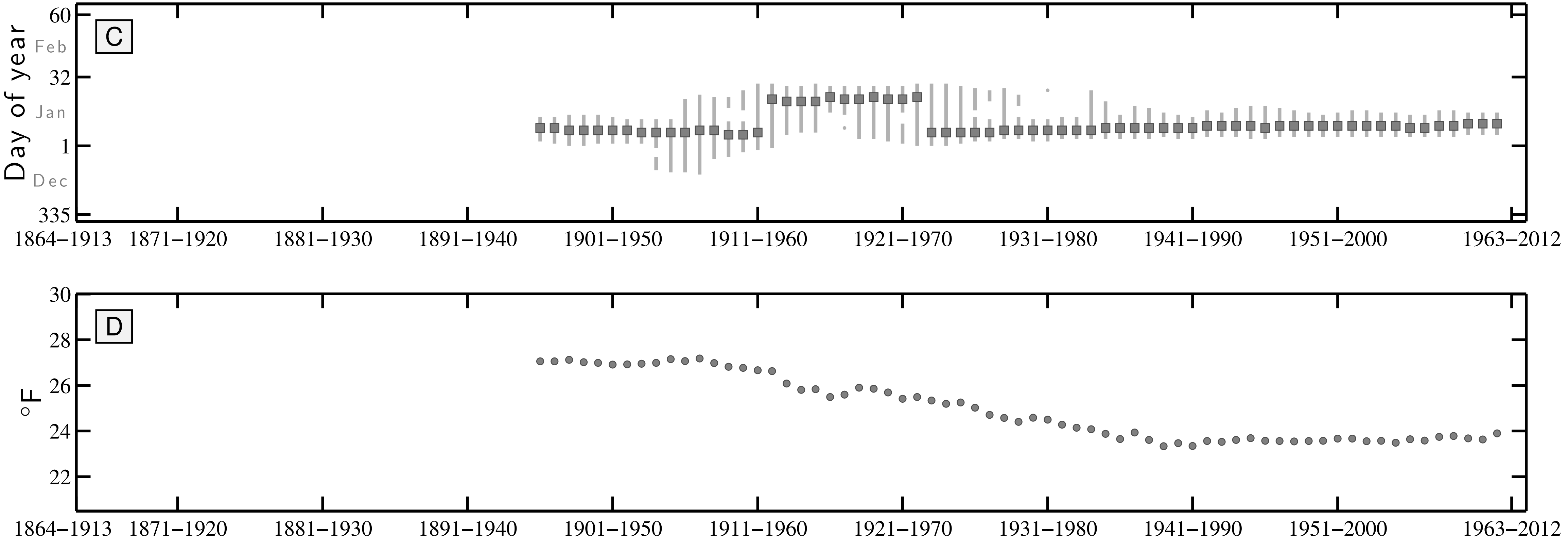

50 year Summer Teletherm dynamics for RIO GRANDE CITY, TX:

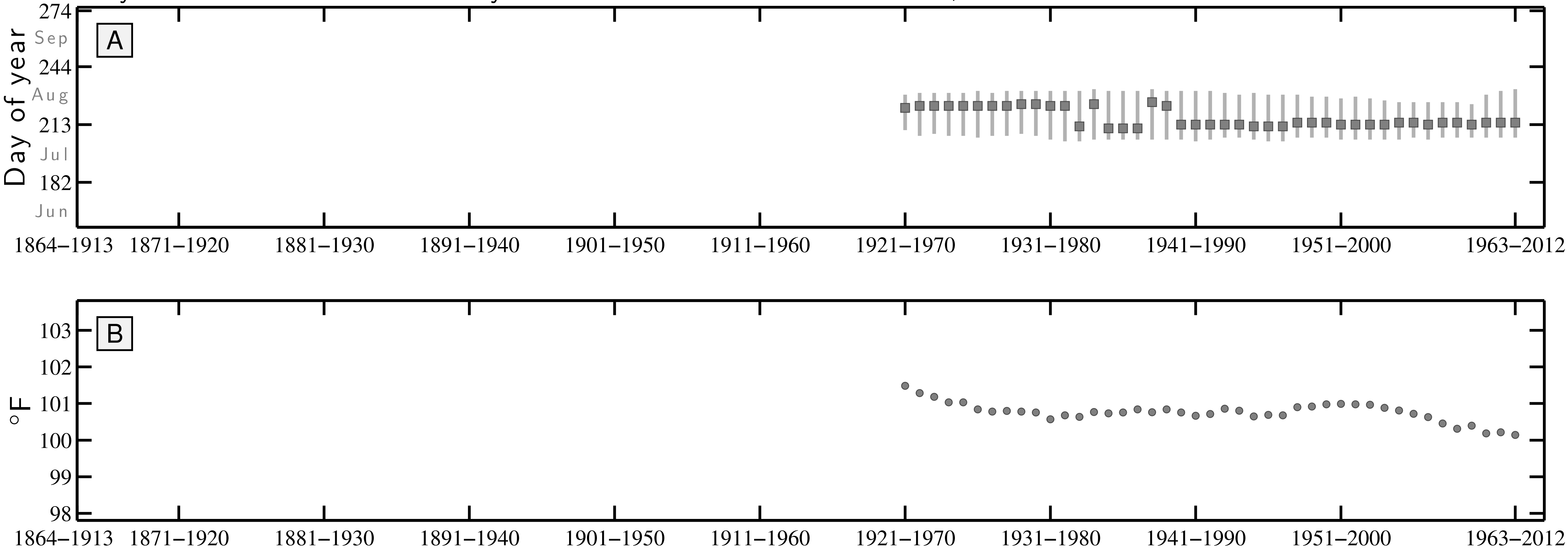

50 year Winter Teletherm dynamics for RIO GRANDE CITY, TX:

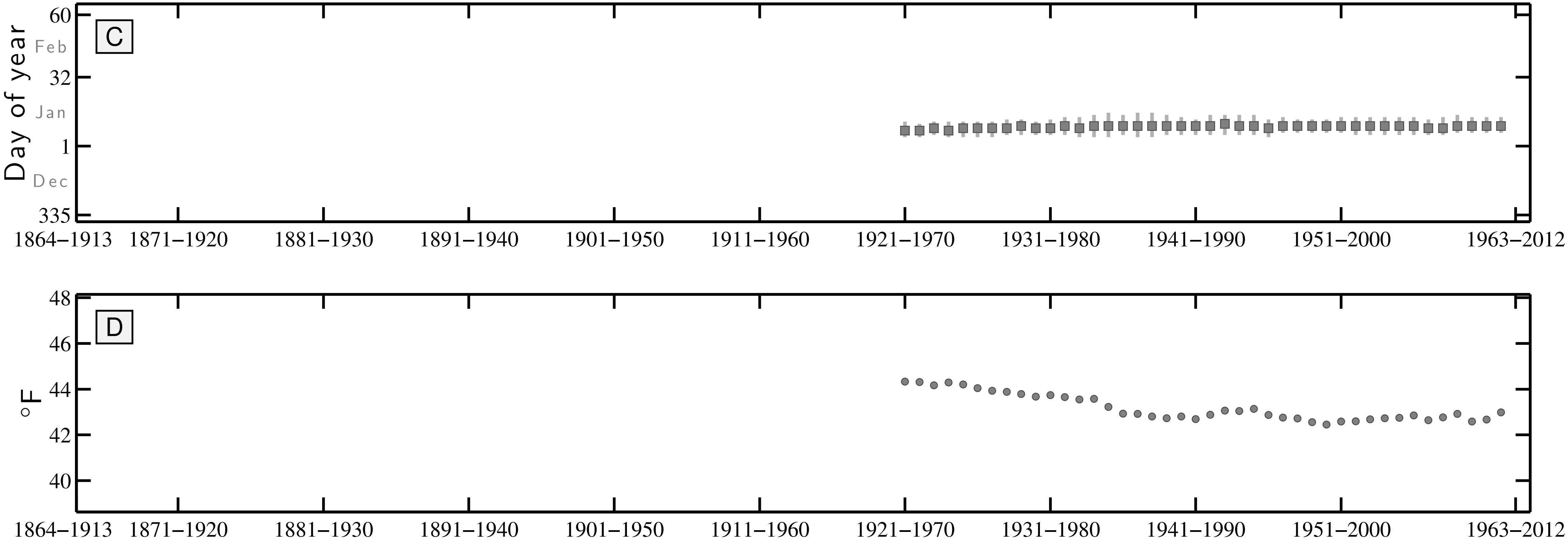

50 year Summer Teletherm dynamics for SAN ANTONIO INTL AP, TX:

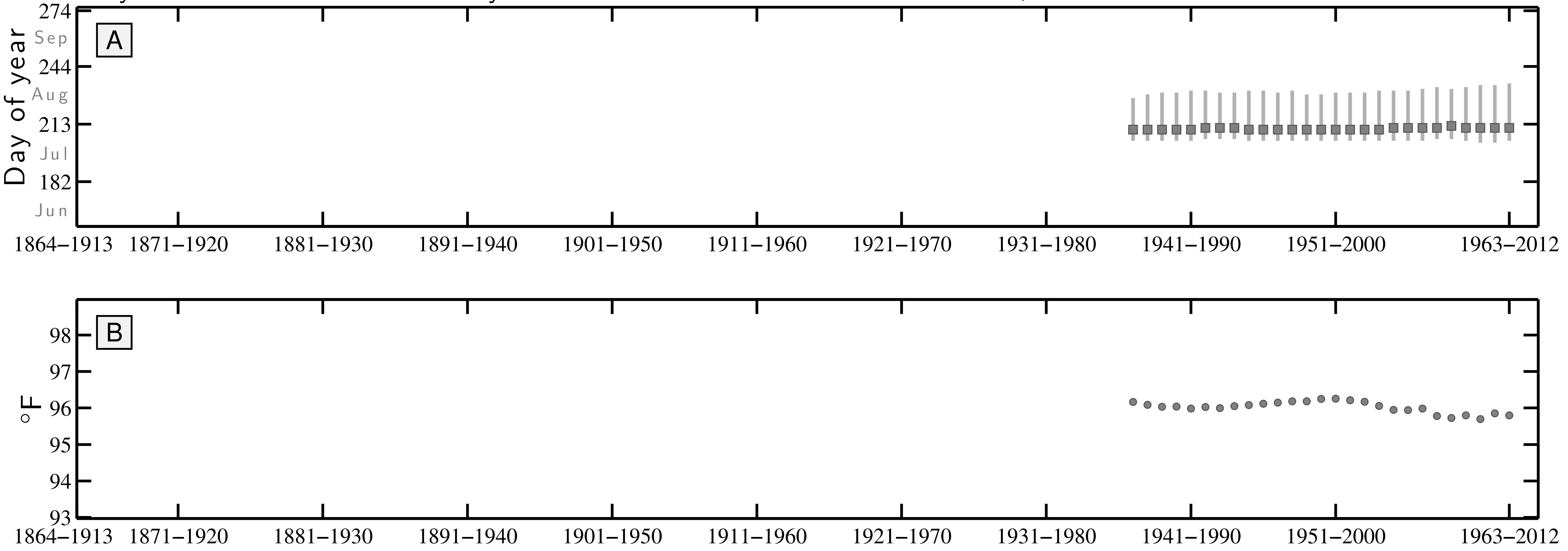

50 year Winter Teletherm dynamics for SAN ANTONIO INTL AP, TX:

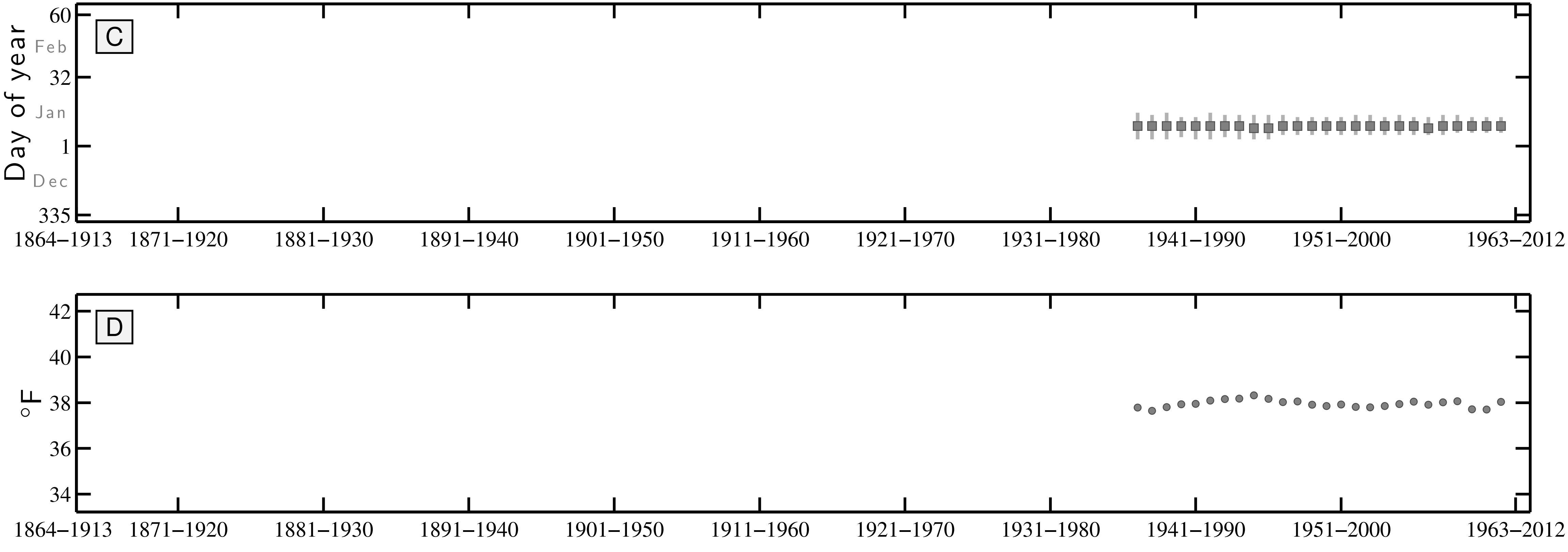

50 year Summer Teletherm dynamics for SEMINOLE, TX:

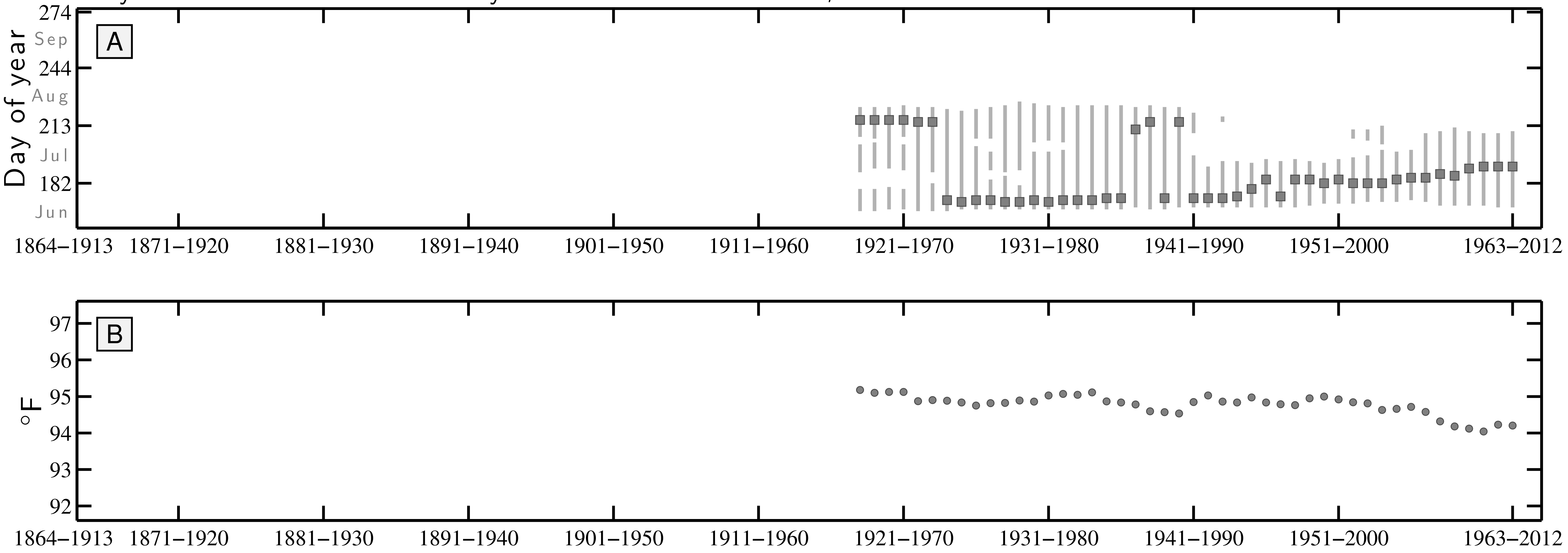

50 year Winter Teletherm dynamics for SEMINOLE, TX:

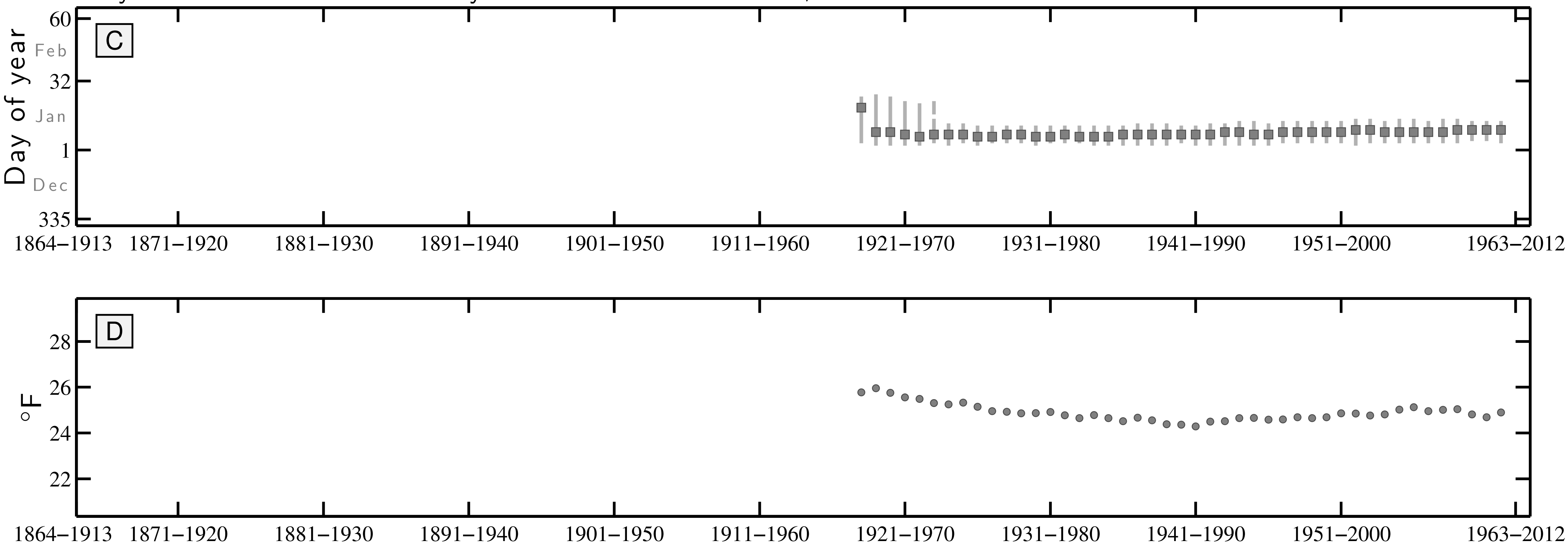

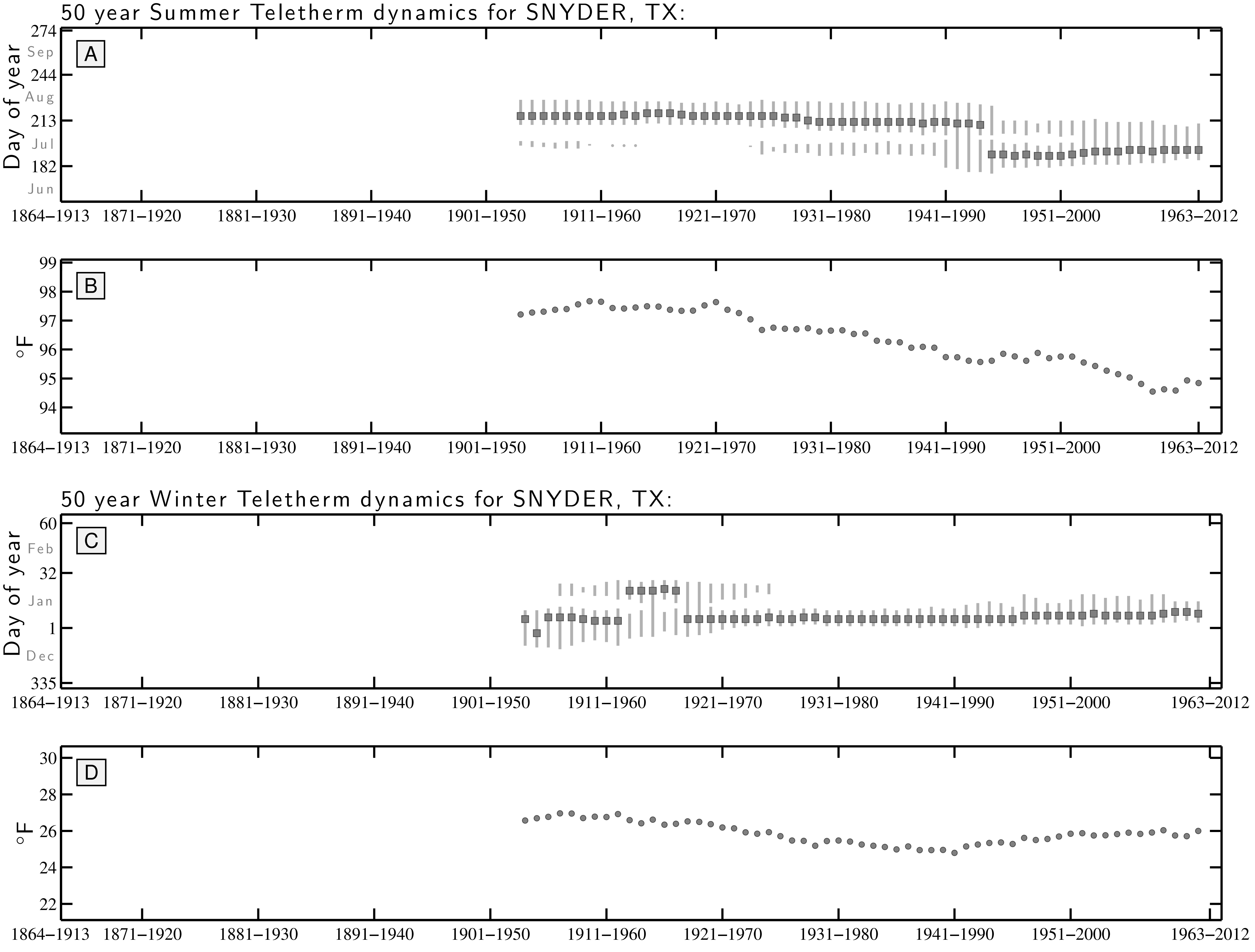

50 year Summer Teletherm dynamics for SNYDER, TX:
A
Day of year
274
Sep
244
Aug
213
Jul
182
Jun
B
°F
99
98
97
96
95
94
1864–1913
1871–1920
1881–1930
1891–1940
1901–1950
1911–1960
1921–1970
1931–1980
1941–1990
1951–2000
1963–2012
50 year Winter Teletherm dynamics for SNYDER, TX:
C
Day of year
60
Feb
32
Jan
1
Dec
335
D
°F
30
28
26
24
22

50 year Summer Teletherm dynamics for STRATFORD, TX:

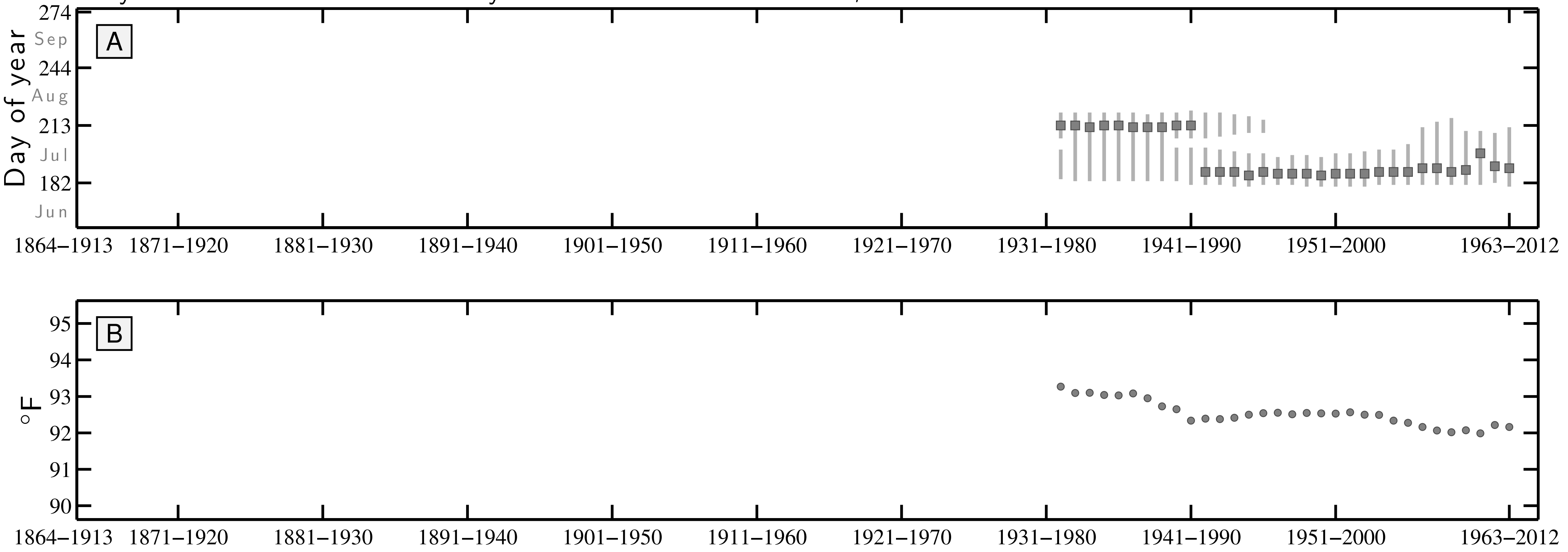

50 year Winter Teletherm dynamics for STRATFORD, TX:

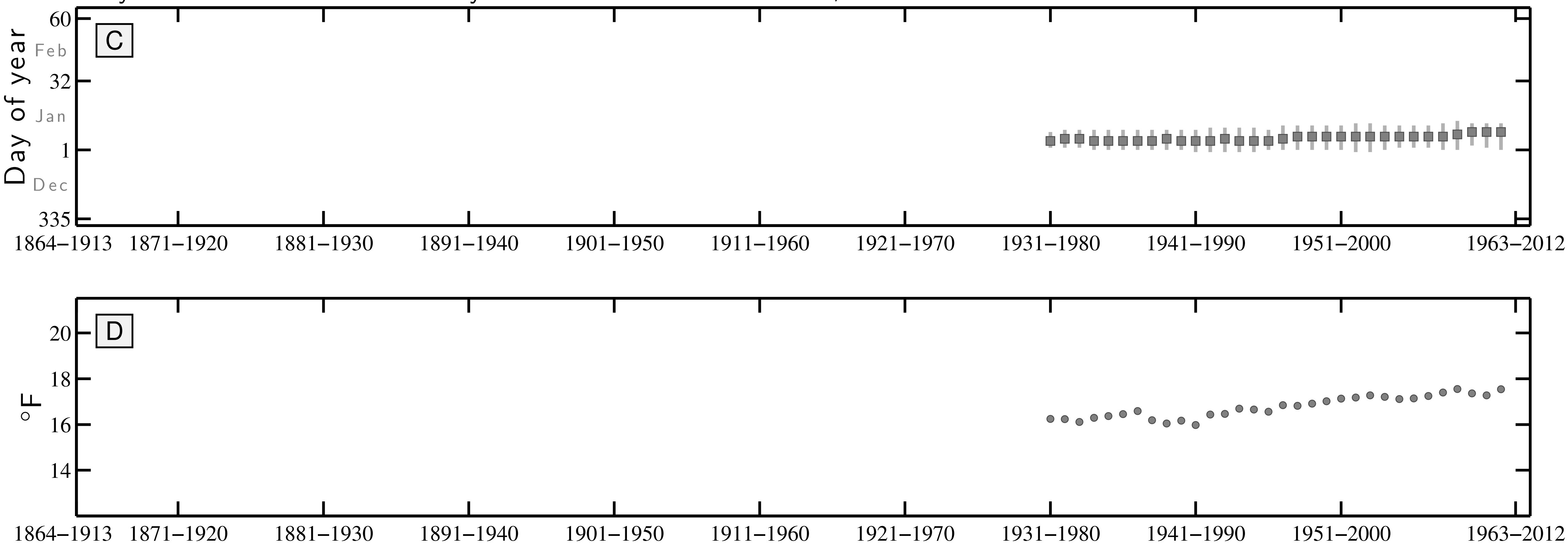

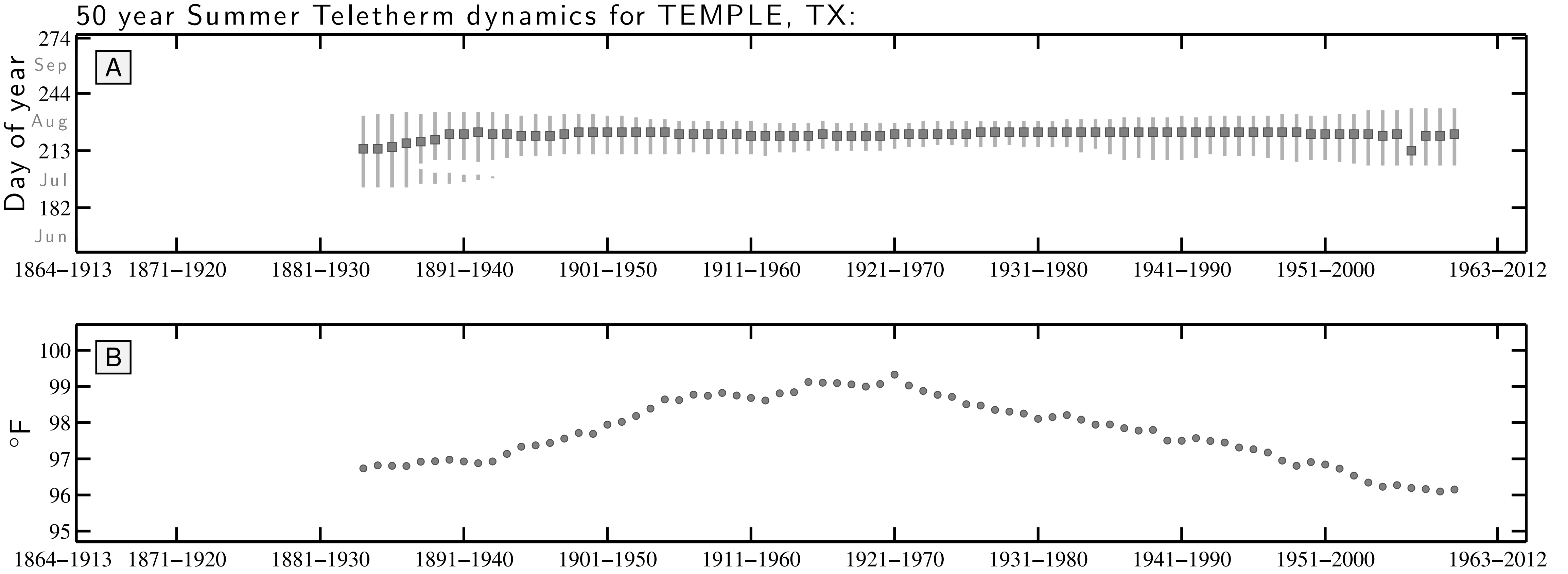
50 year Summer Teletherm dynamics for TEMPLE, TX:
A
B
Day of year
Sep
Aug
Jul
Jun
274
244
213
182
°F
100
99
98
97
96
95
1864–1913
1871–1920
1881–1930
1891–1940
1901–1950
1911–1960
1921–1970
1931–1980
1941–1990
1951–2000
1963–2012

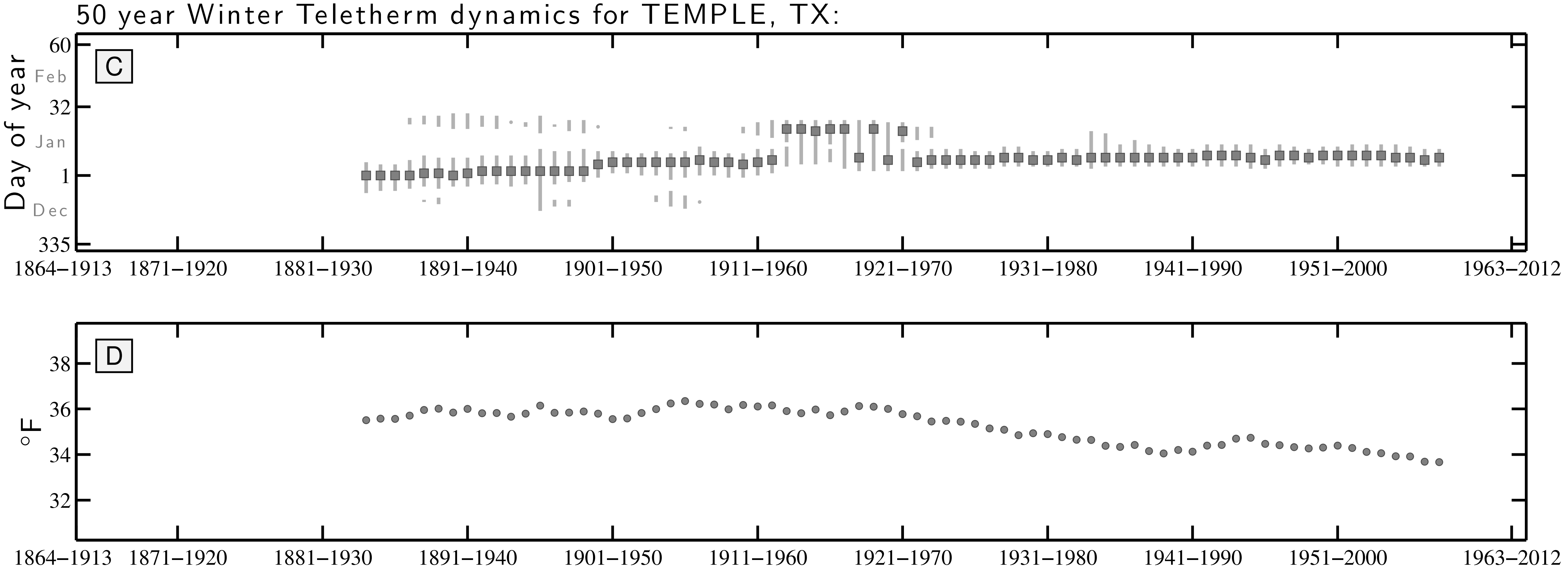
50 year Winter Teletherm dynamics for TEMPLE, TX:
C
D
Day of year
Feb
Jan
Dec
60
32
1
335
°F
38
36
34
32
1864–1913
1871–1920
1881–1930
1891–1940
1901–1950
1911–1960
1921–1970
1931–1980
1941–1990
1951–2000
1963–2012

50 year Summer Teletherm dynamics for WEATHERFORD, TX:

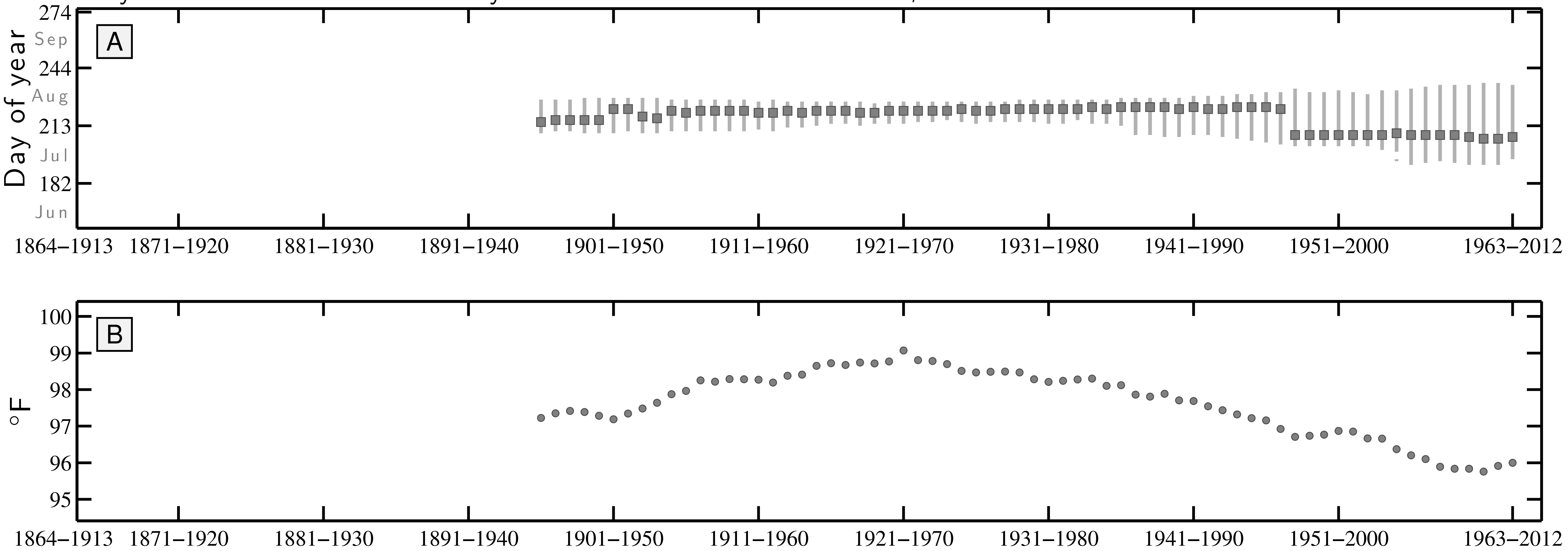

50 year Winter Teletherm dynamics for WEATHERFORD, TX:

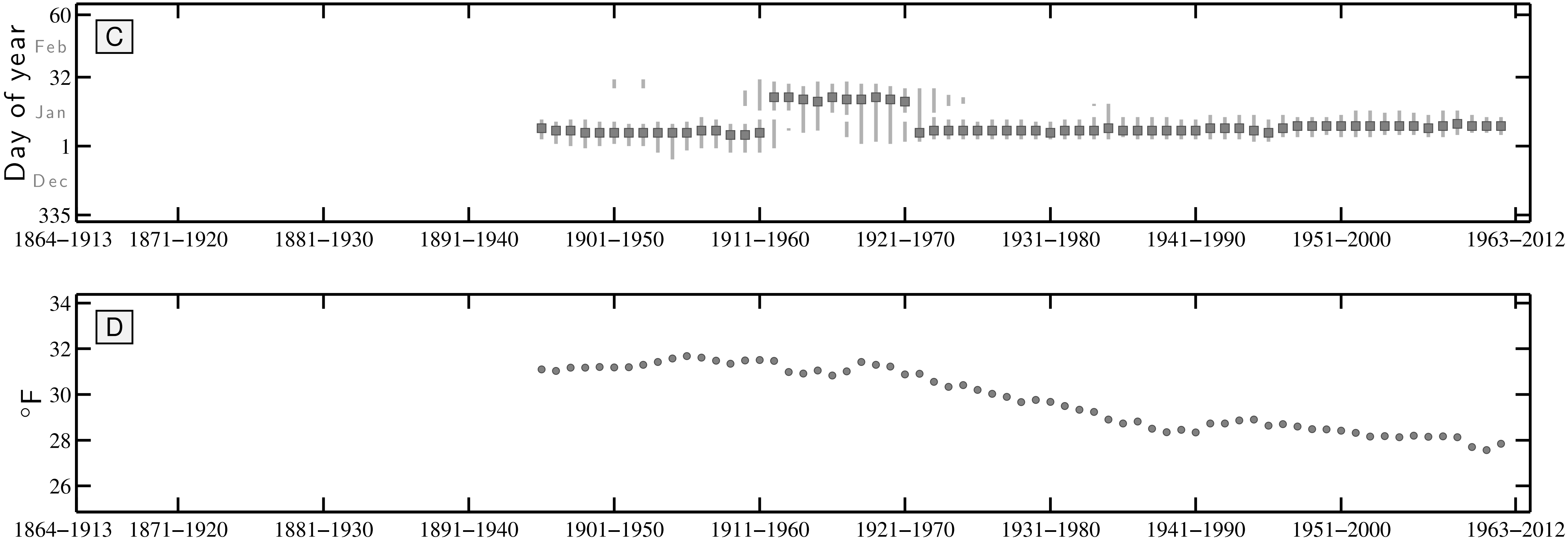

50 year Summer Teletherm dynamics for ALTON, UT:

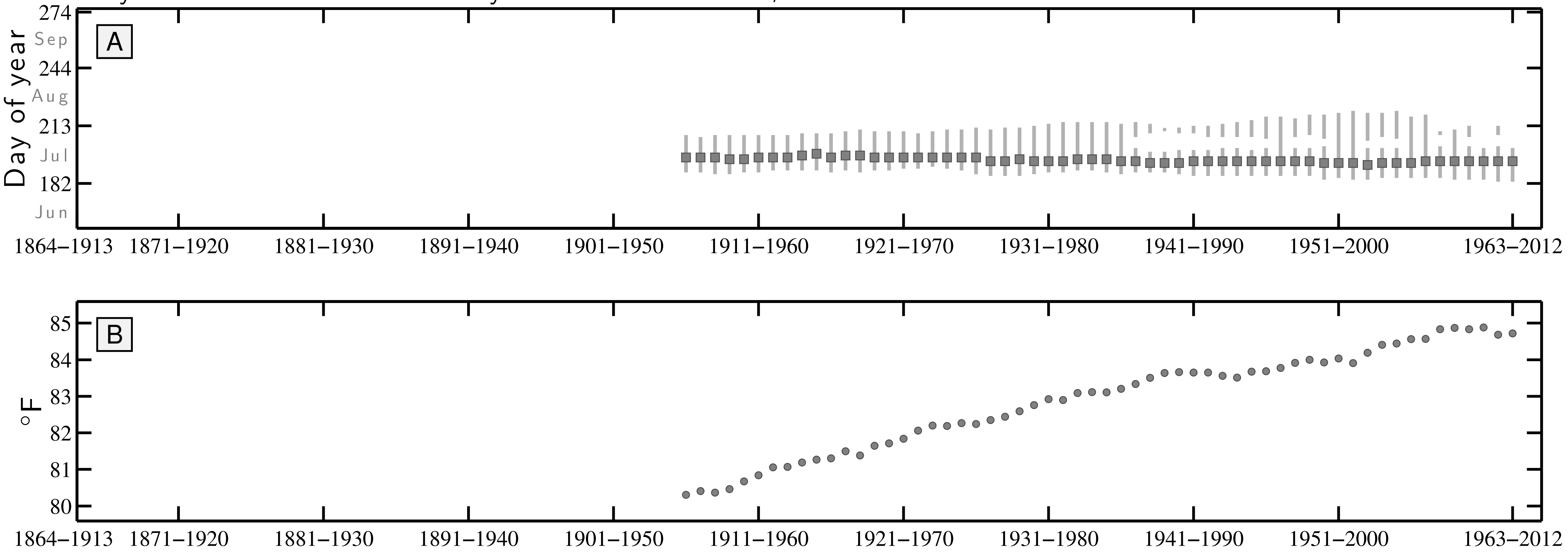

50 year Winter Teletherm dynamics for ALTON, UT:

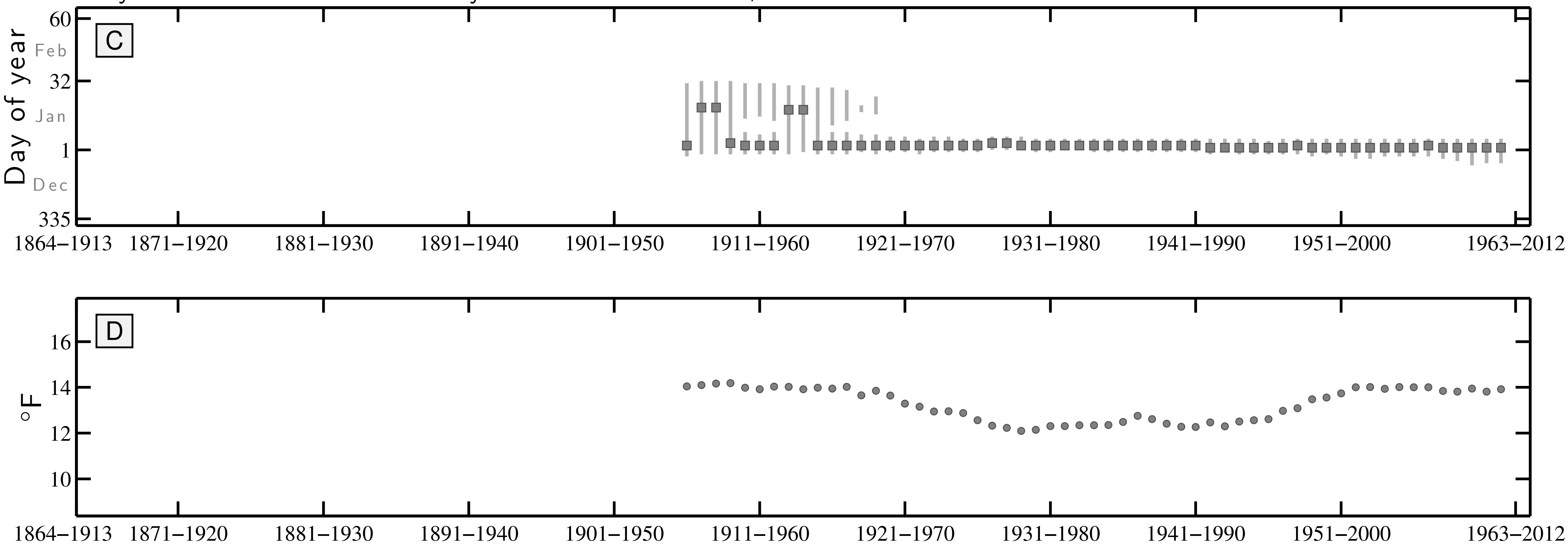

50 year Summer Teletherm dynamics for BLANDING, UT:

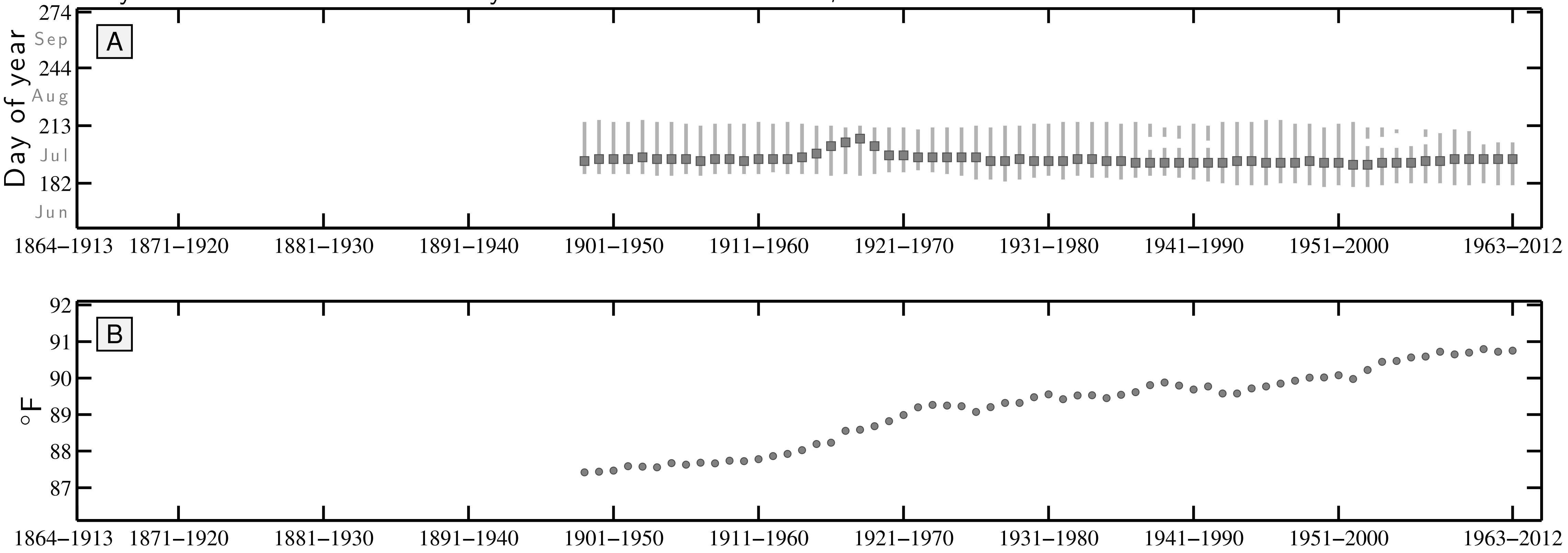

50 year Winter Teletherm dynamics for BLANDING, UT:

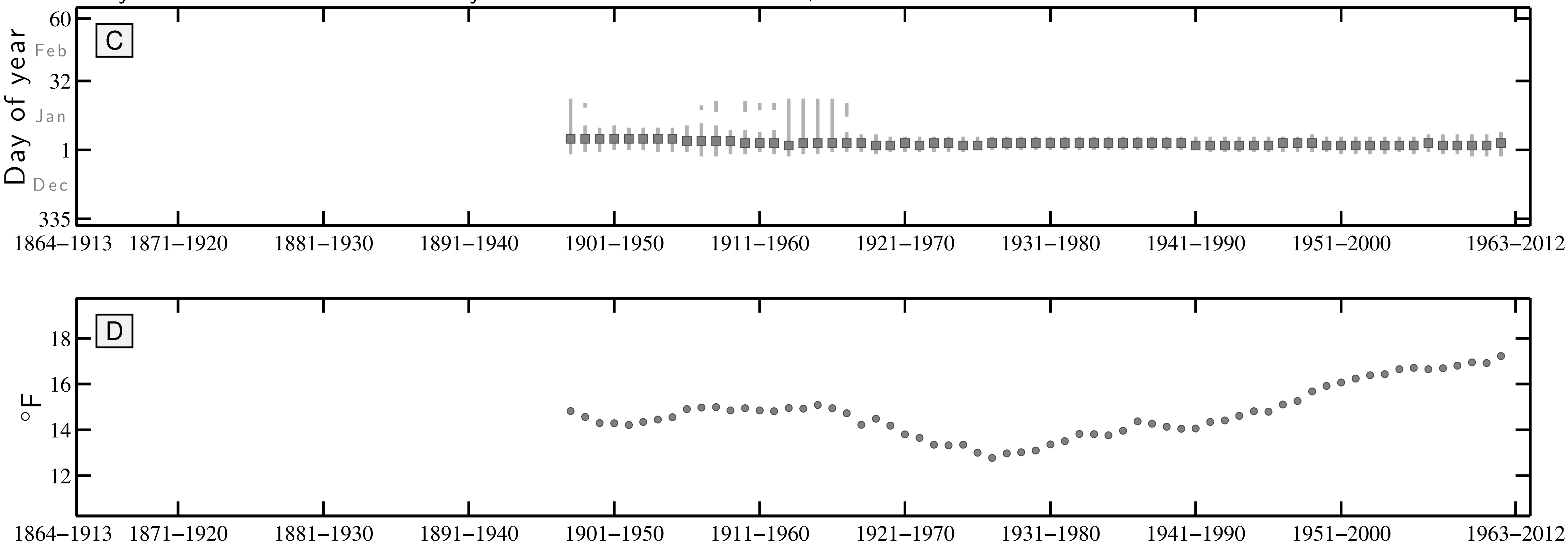

50 year Summer Teletherm dynamics for BLUFF, UT:

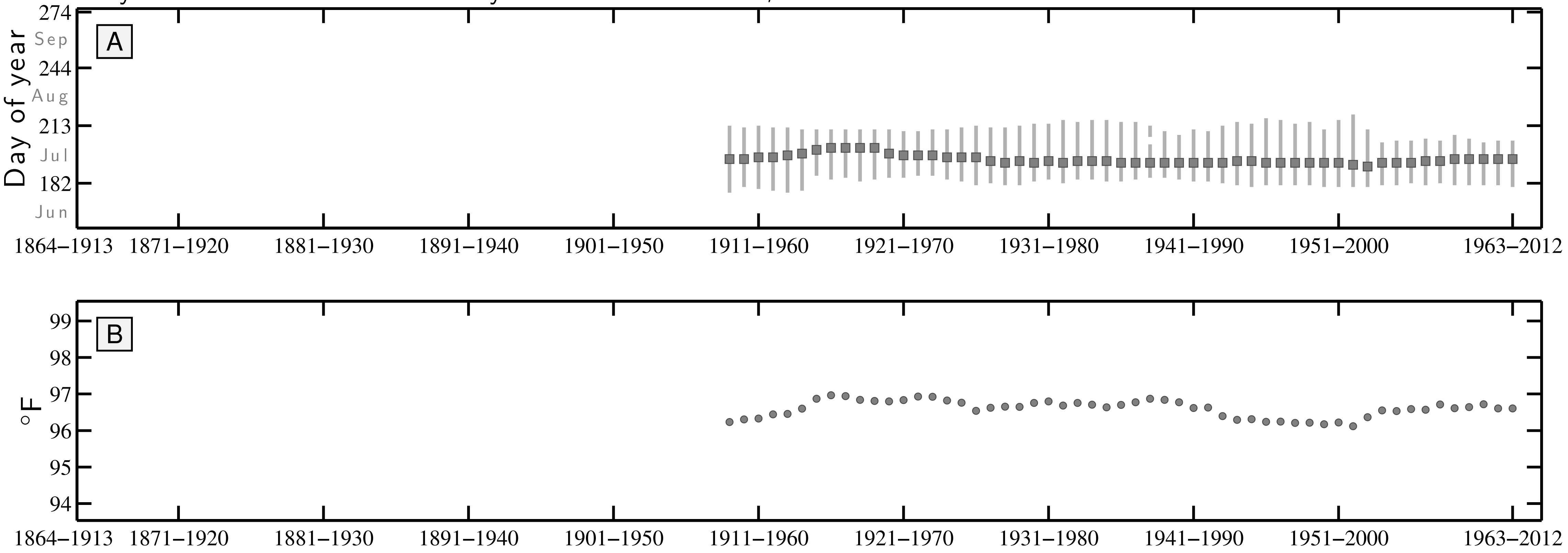

50 year Winter Teletherm dynamics for BLUFF, UT:

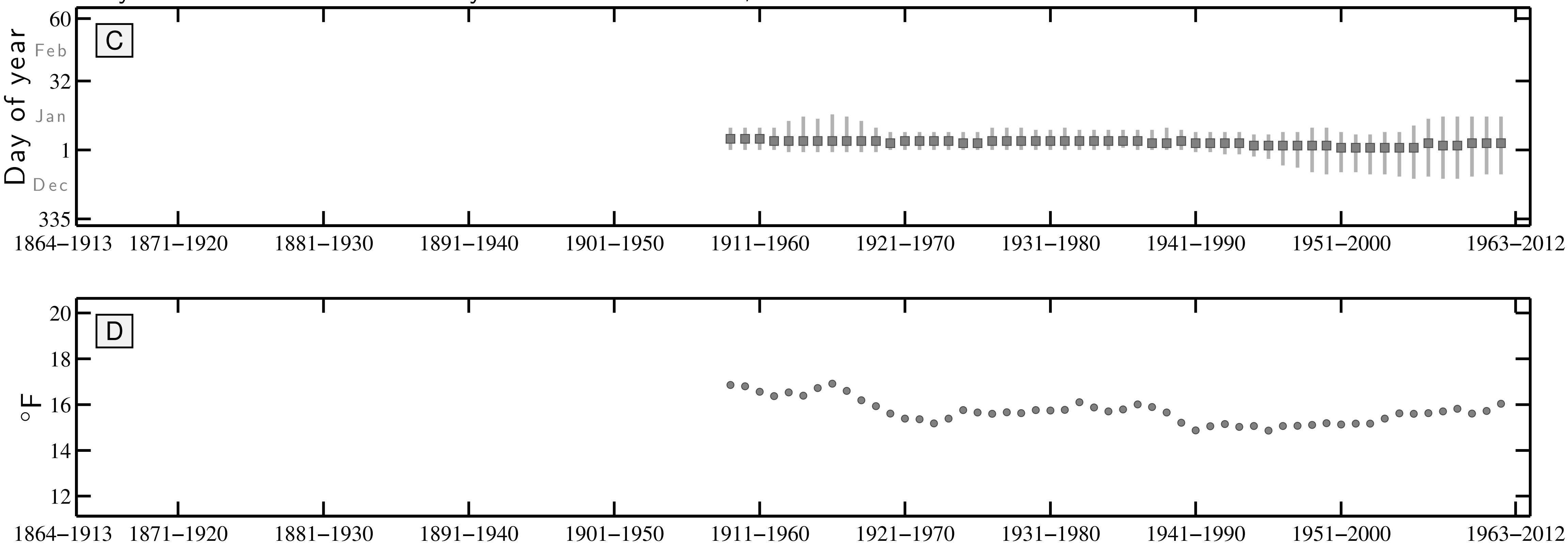

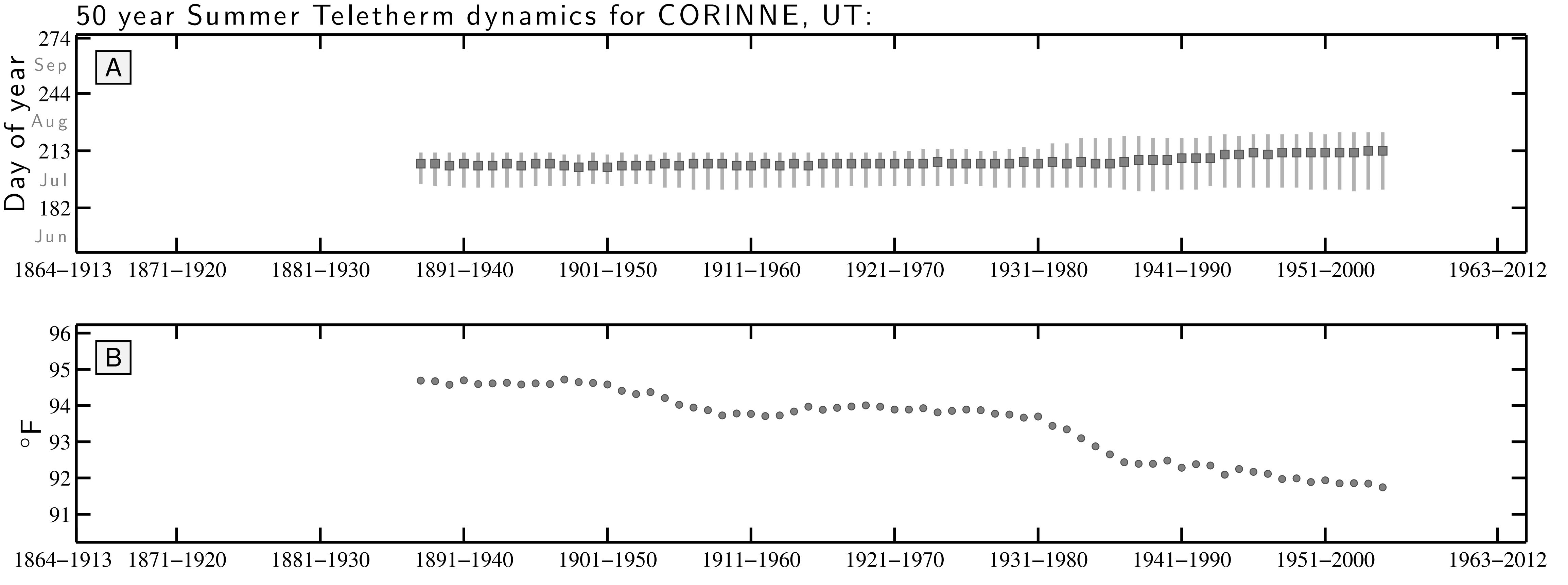
50 year Summer Teletherm dynamics for CORINNE, UT:
A
B
Day of year
°F
274
244
213
182
Sep
Aug
Jul
Jun
96
95
94
93
92
91
1864–1913
1871–1920
1881–1930
1891–1940
1901–1950
1911–1960
1921–1970
1931–1980
1941–1990
1951–2000
1963–2012

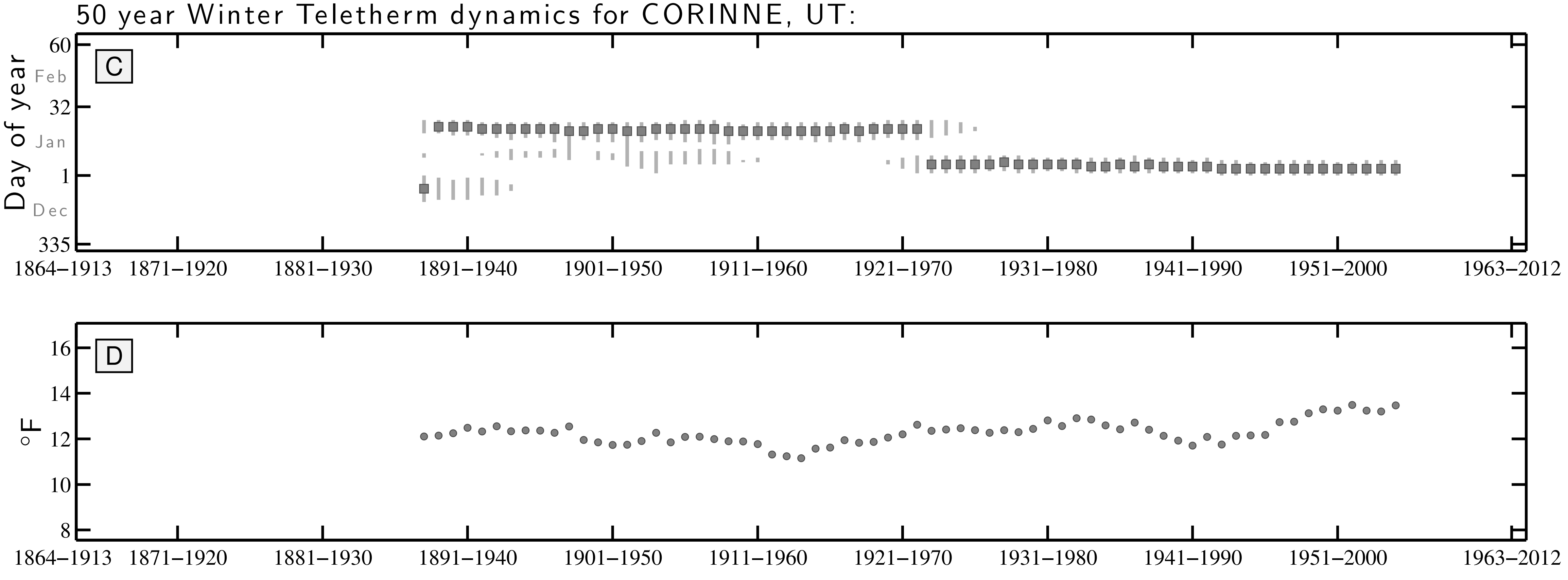
50 year Winter Teletherm dynamics for CORINNE, UT:
C
D
Day of year
°F
60
32
1
335
Feb
Jan
Dec
16
14
12
10
8
1864–1913
1871–1920
1881–1930
1891–1940
1901–1950
1911–1960
1921–1970
1931–1980
1941–1990
1951–2000
1963–2012

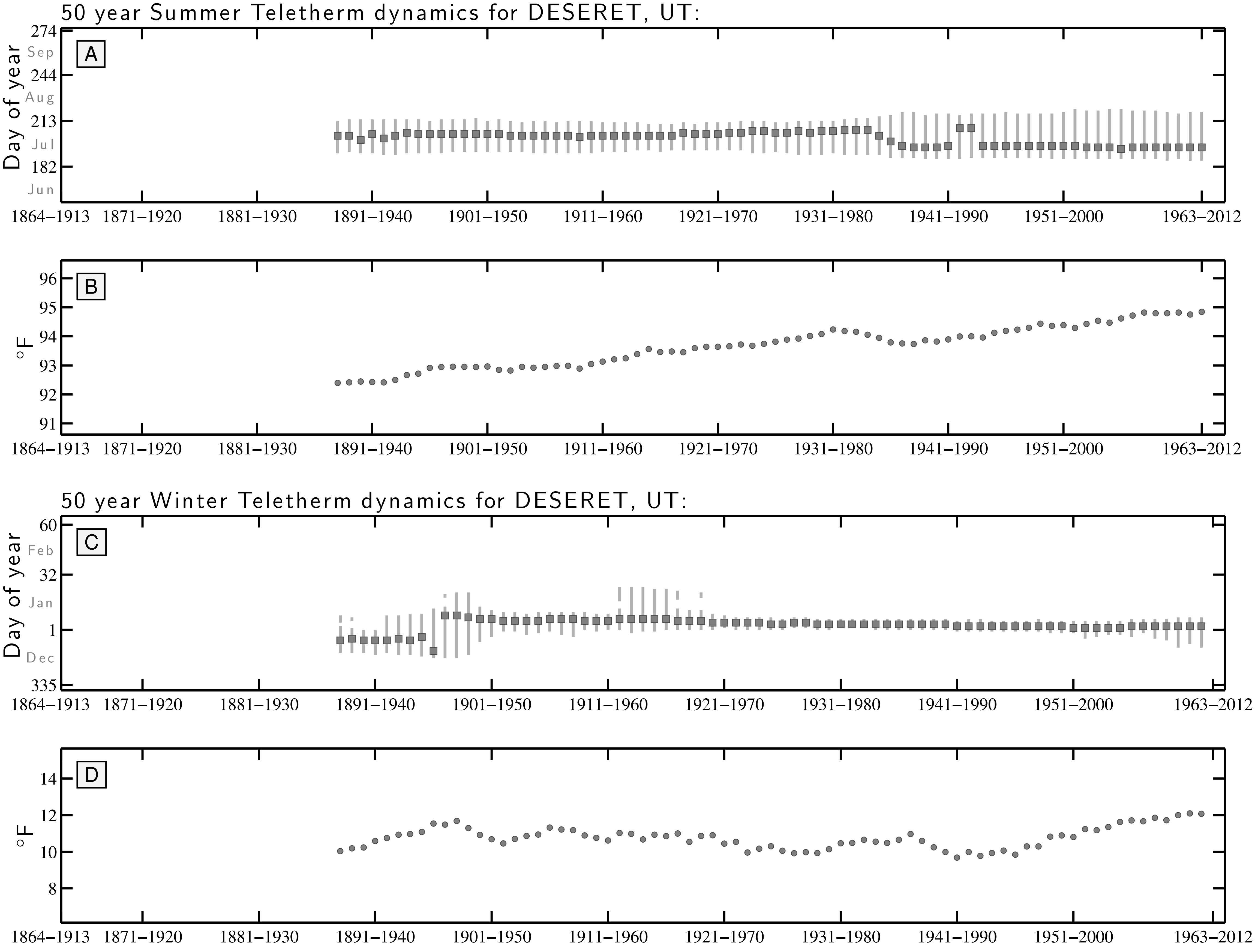
50 year Summer Teletherm dynamics for DESERET, UT:
A
Day of year
274
Sep
244
Aug
213
Jul
182
Jun
1864–1913
1871–1920
1881–1930
1891–1940
1901–1950
1911–1960
1921–1970
1931–1980
1941–1990
1951–2000
1963–2012
B
°F
96
95
94
93
92
91
50 year Winter Teletherm dynamics for DESERET, UT:
C
Day of year
60
Feb
32
Jan
1
Dec
335
D
°F
14
12
10
8

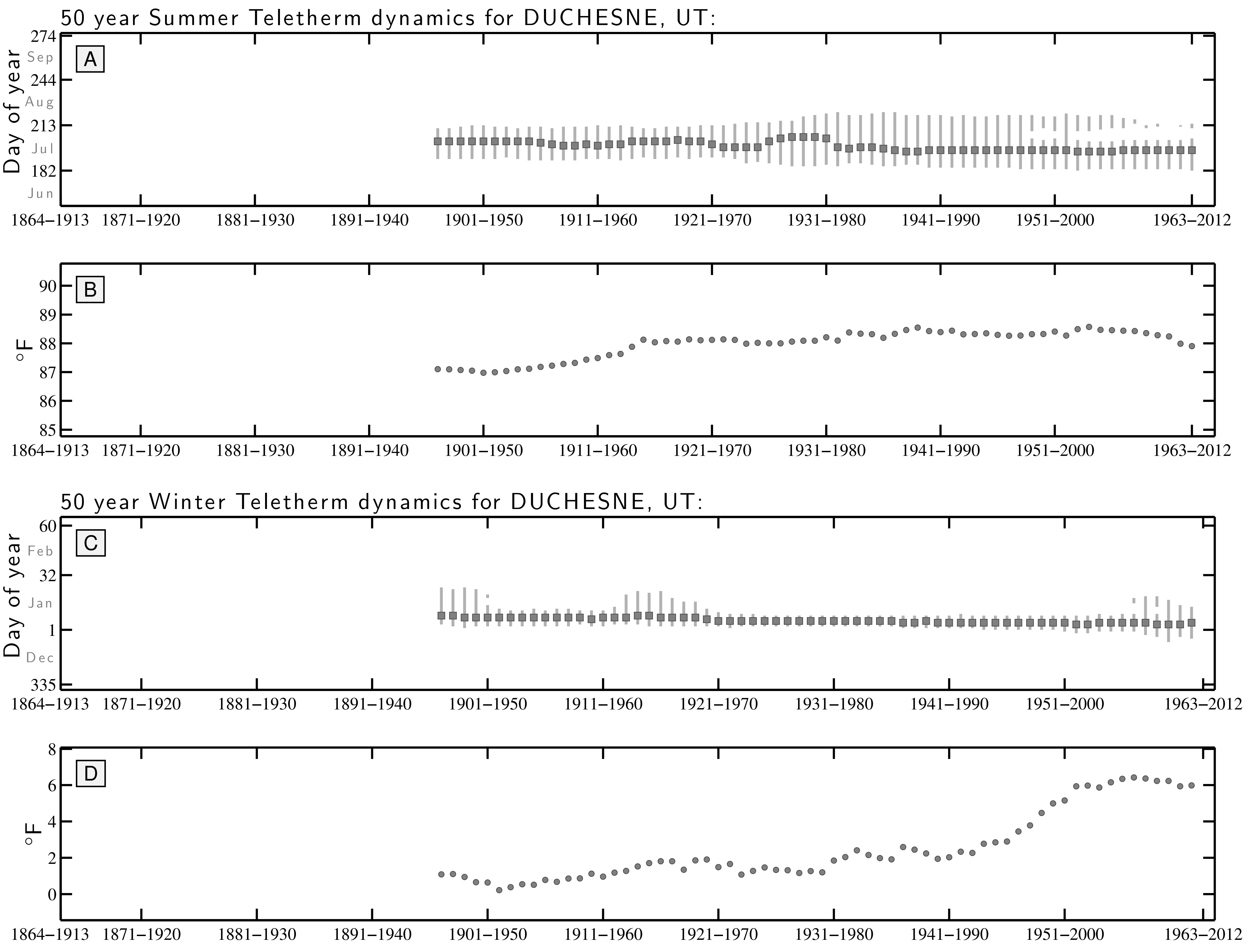
50 year Summer Teletherm dynamics for DUCHESNE, UT:
A
B
Day of year
°F
274
Sep
244
Aug
213
Jul
182
Jun
90
89
88
87
86
85
1864–1913
1871–1920
1881–1930
1891–1940
1901–1950
1911–1960
1921–1970
1931–1980
1941–1990
1951–2000
1963–2012
50 year Winter Teletherm dynamics for DUCHESNE, UT:
C
D
60
Feb
32
Jan
1
Dec
335
8
6
4
2
0

50 year Summer Teletherm dynamics for ESCALANTE, UT:

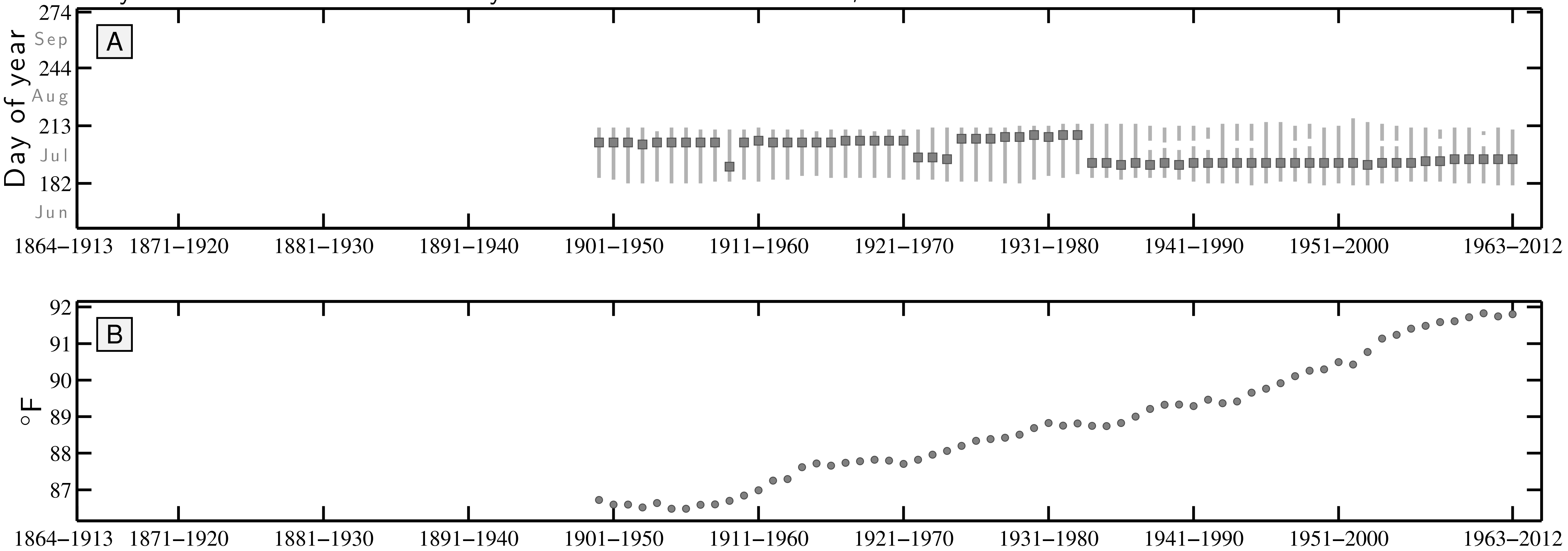

50 year Winter Teletherm dynamics for ESCALANTE, UT:

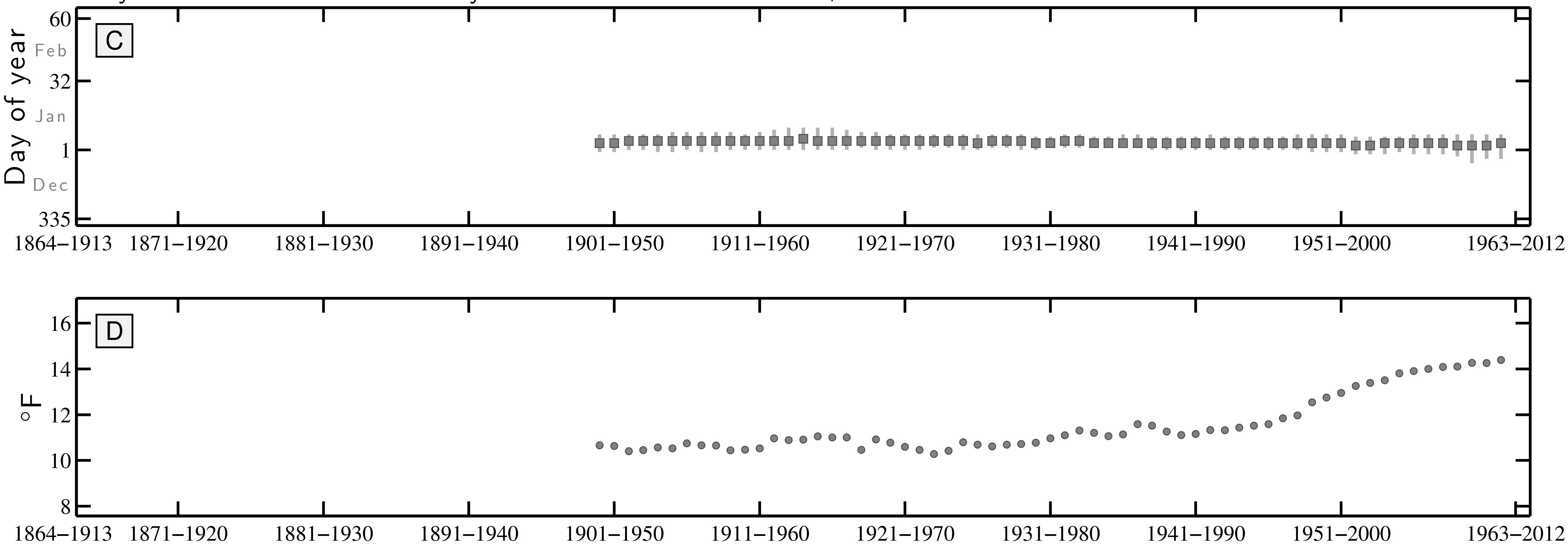

50 year Summer Teletherm dynamics for FARMINGTON 3 NW, UT:

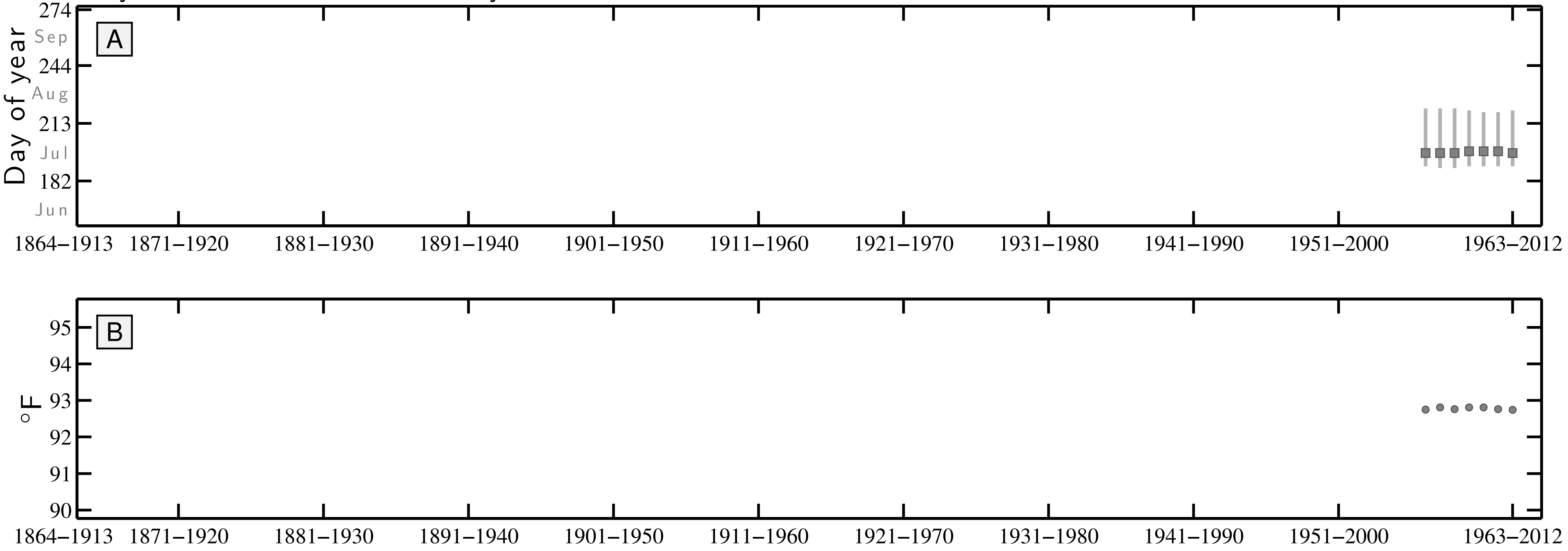

50 year Winter Teletherm dynamics for FARMINGTON 3 NW, UT:

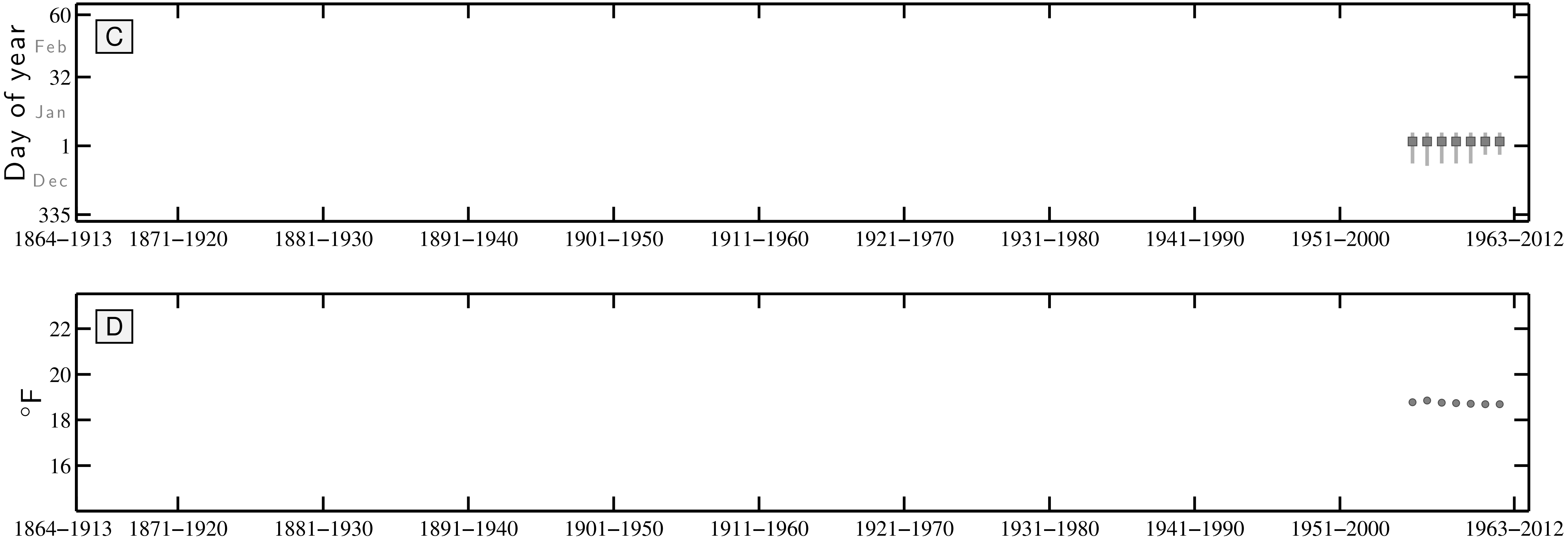

50 year Summer Teletherm dynamics for FILLMORE, UT:

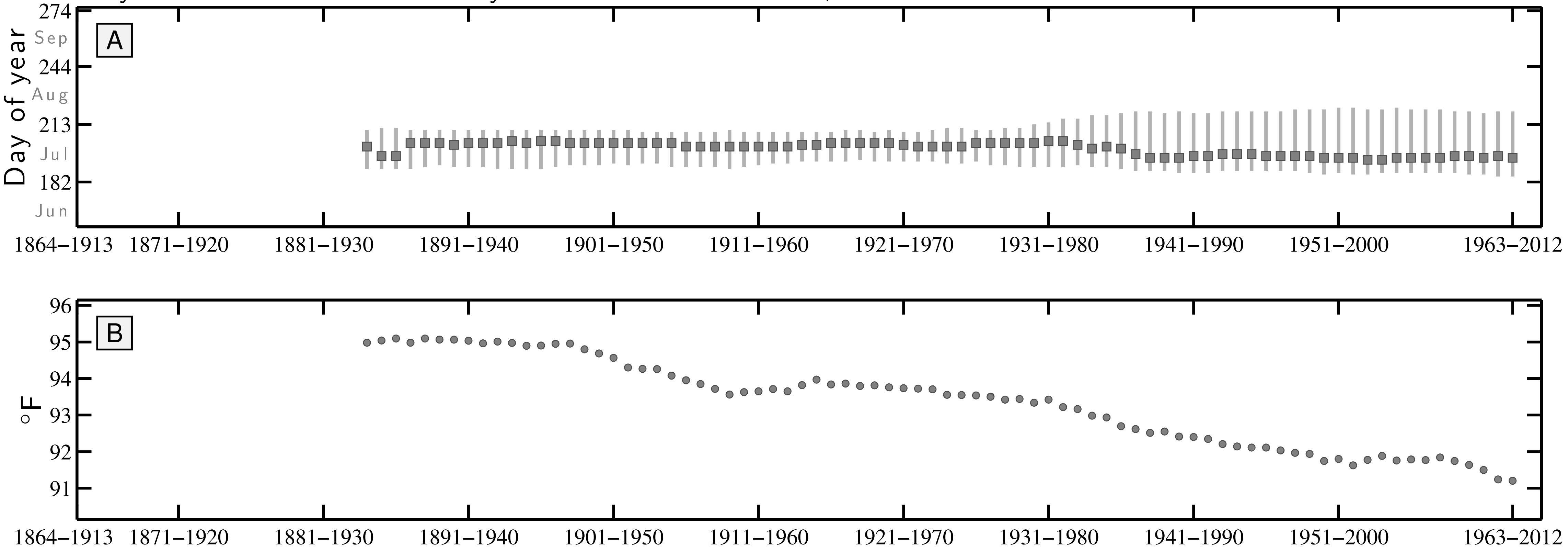

50 year Winter Teletherm dynamics for FILLMORE, UT:

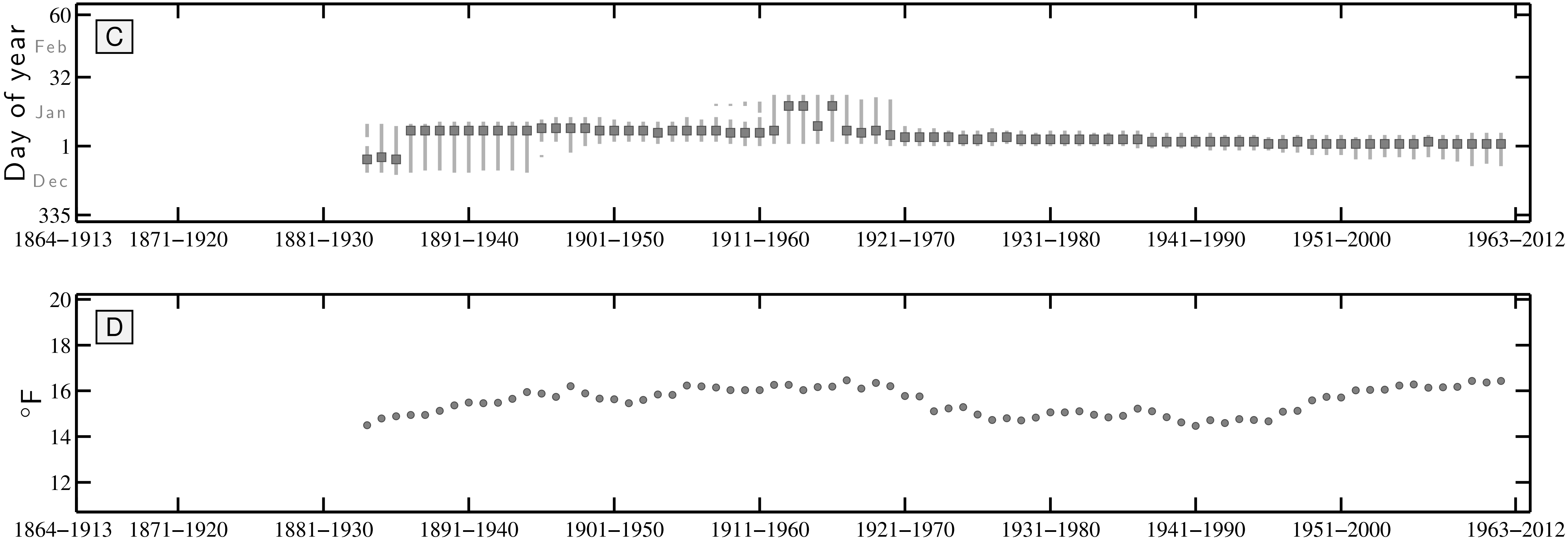

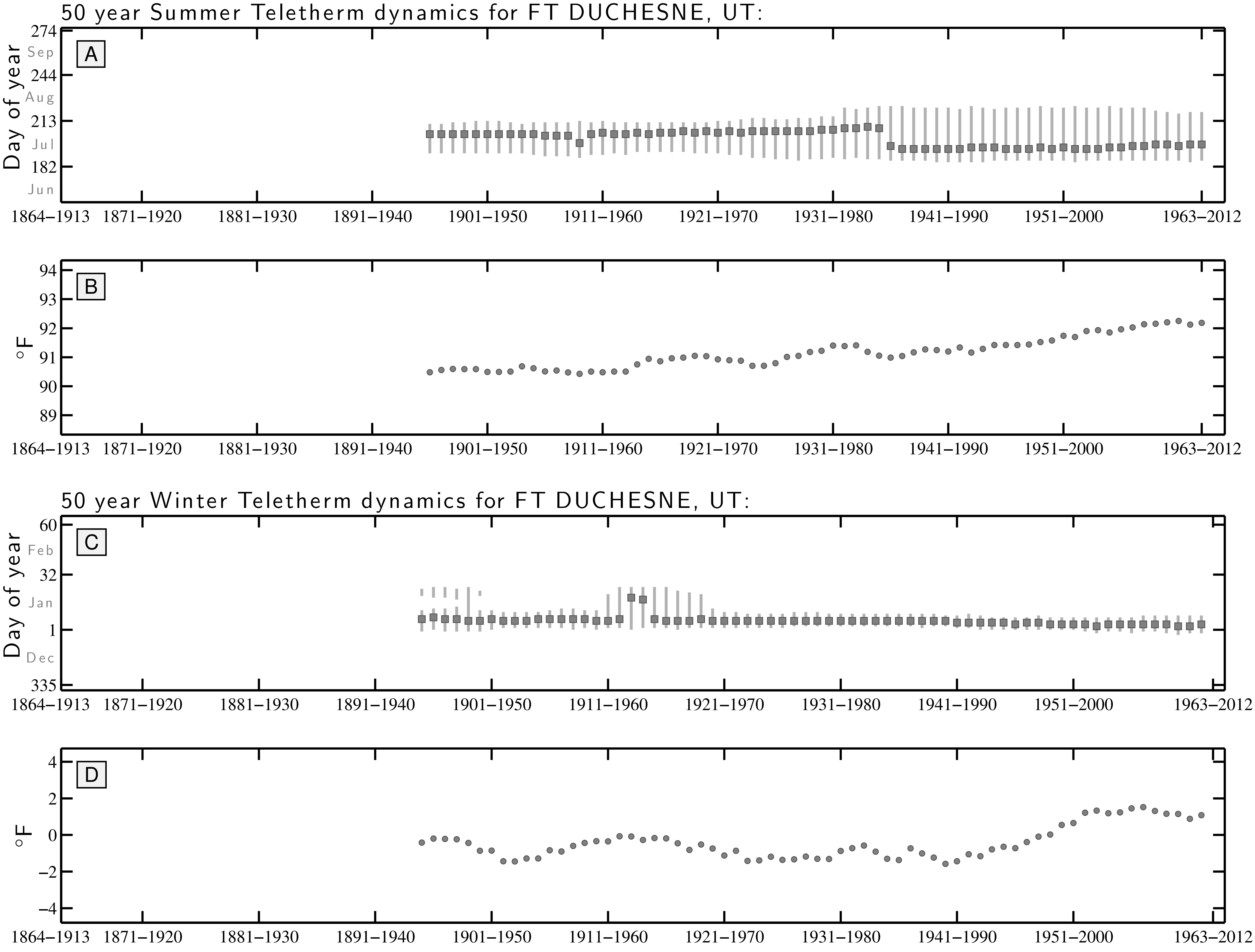
50 year Summer Teletherm dynamics for FT DUCHESNE, UT:
A
Day of year
274
Sep
244
Aug
213
Jul
182
Jun
B
°F
94
93
92
91
90
89
1864–1913
1871–1920
1881–1930
1891–1940
1901–1950
1911–1960
1921–1970
1931–1980
1941–1990
1951–2000
1963–2012
50 year Winter Teletherm dynamics for FT DUCHESNE, UT:
C
Day of year
60
Feb
32
Jan
1
Dec
335
D
°F
4
2
0
−2
−4

50 year Summer Teletherm dynamics for GREEN RIVER AVIATION, UT:

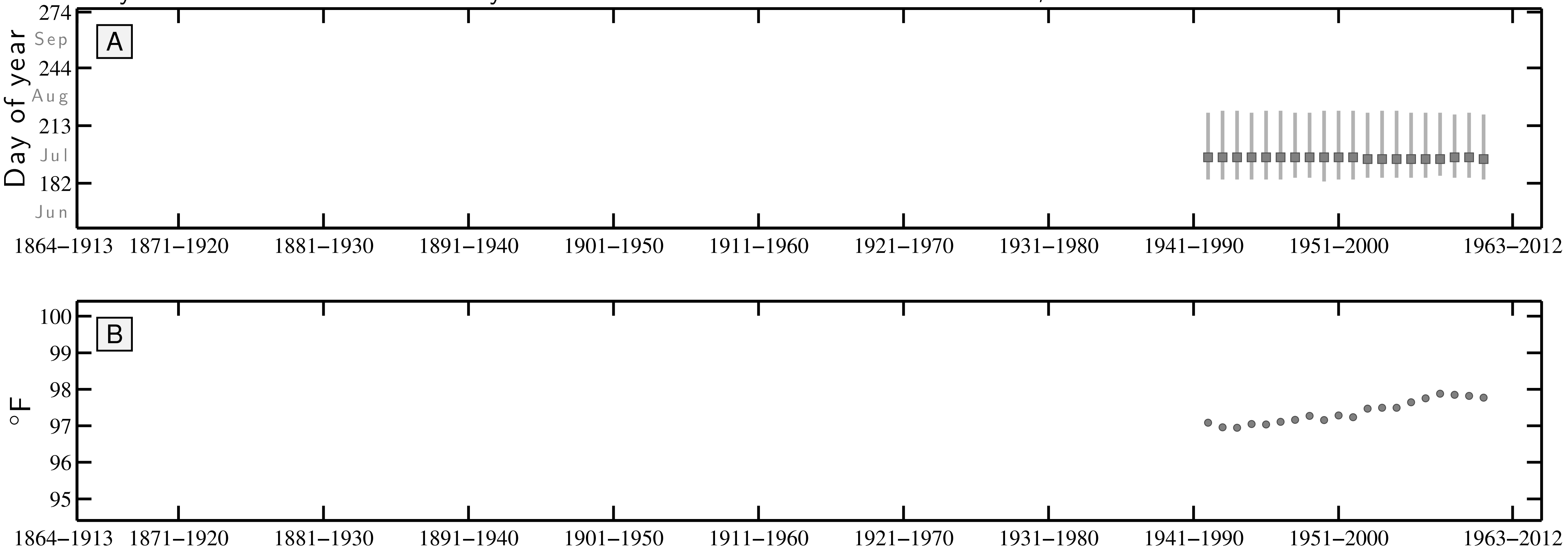

50 year Winter Teletherm dynamics for GREEN RIVER AVIATION, UT:

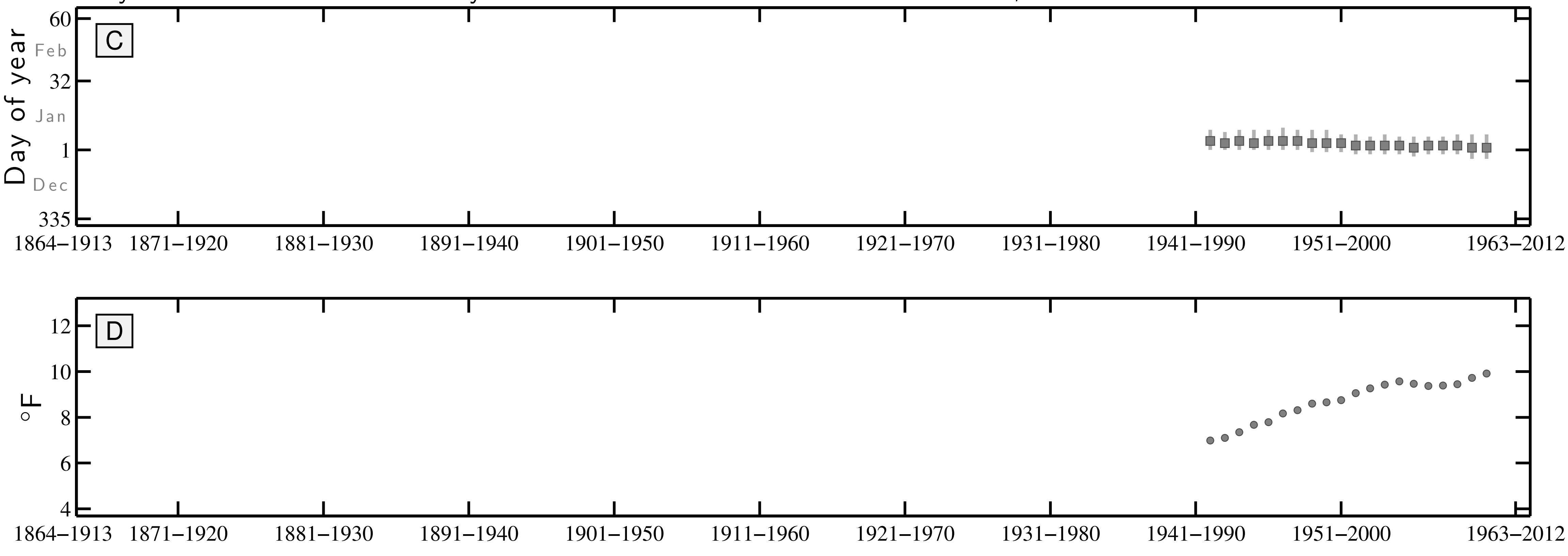

50 year Summer Teletherm dynamics for HANKSVILLE, UT:

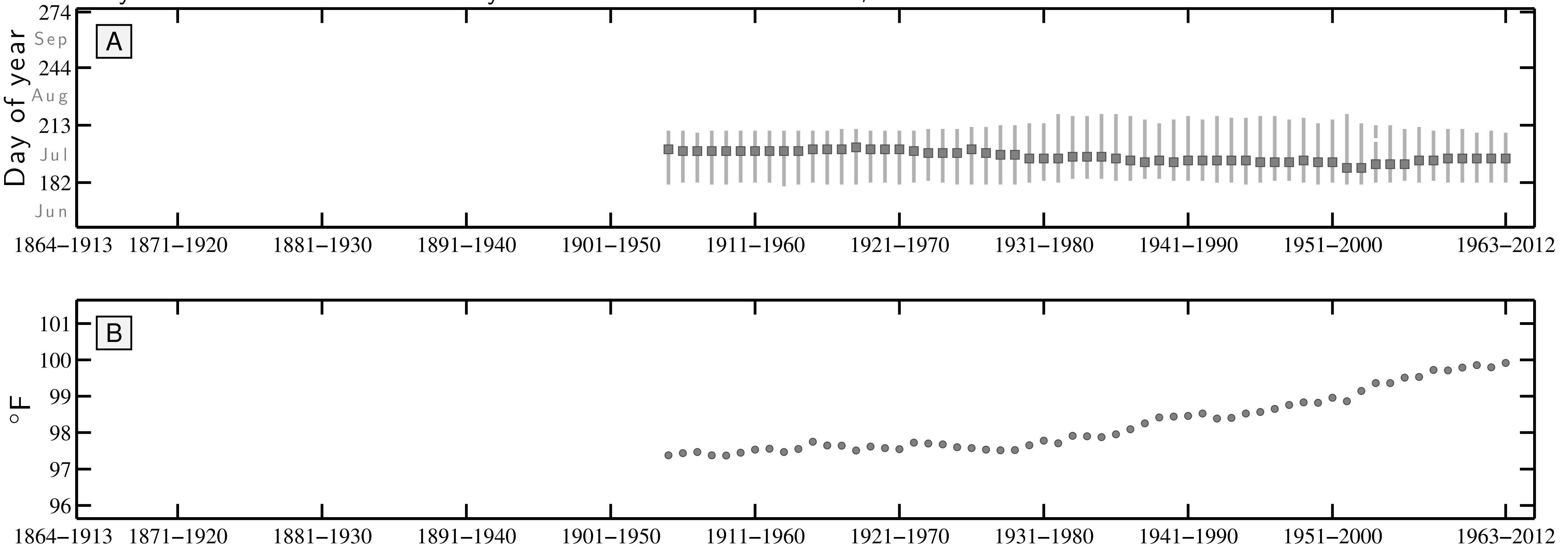

50 year Winter Teletherm dynamics for HANKSVILLE, UT:

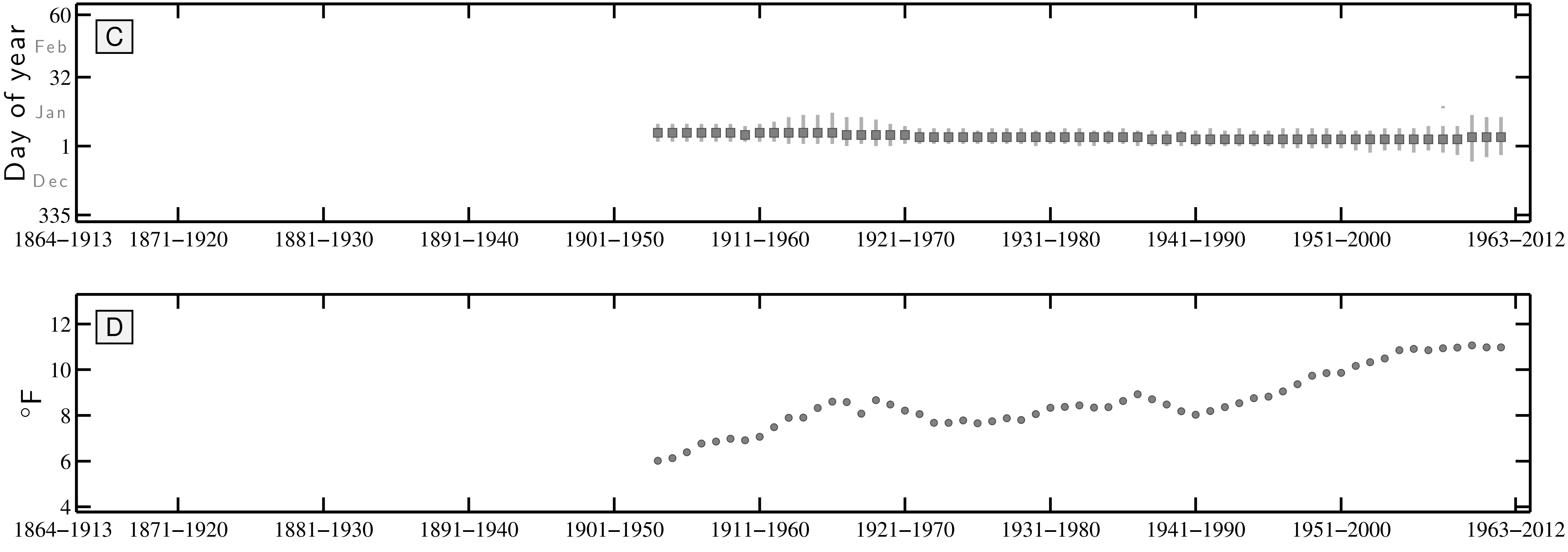

50 year Summer Teletherm dynamics for HEBER, UT:

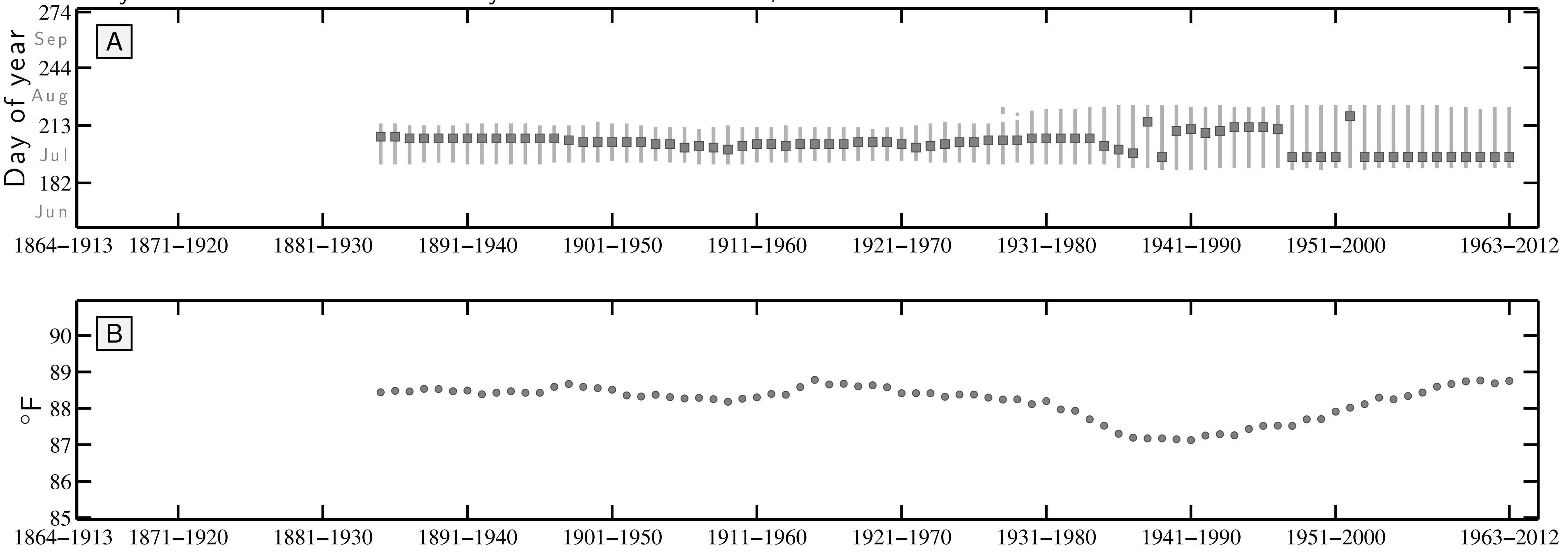

50 year Winter Teletherm dynamics for HEBER, UT:

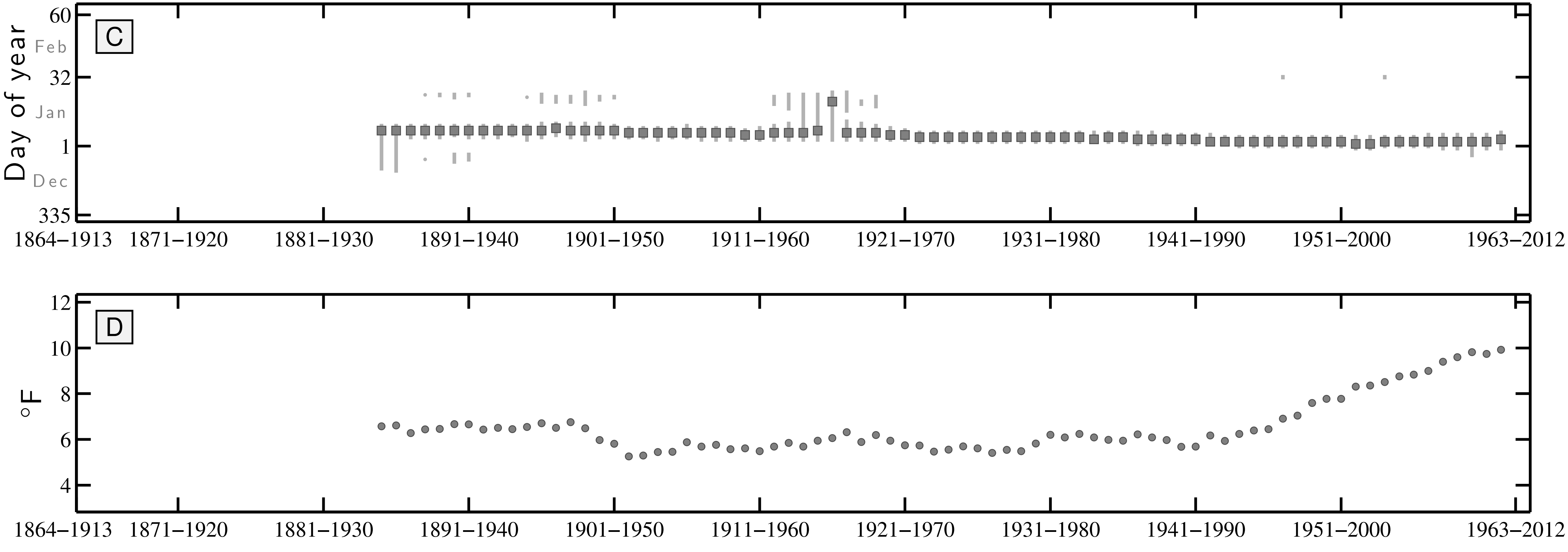

50 year Summer Teletherm dynamics for KANAB, UT:

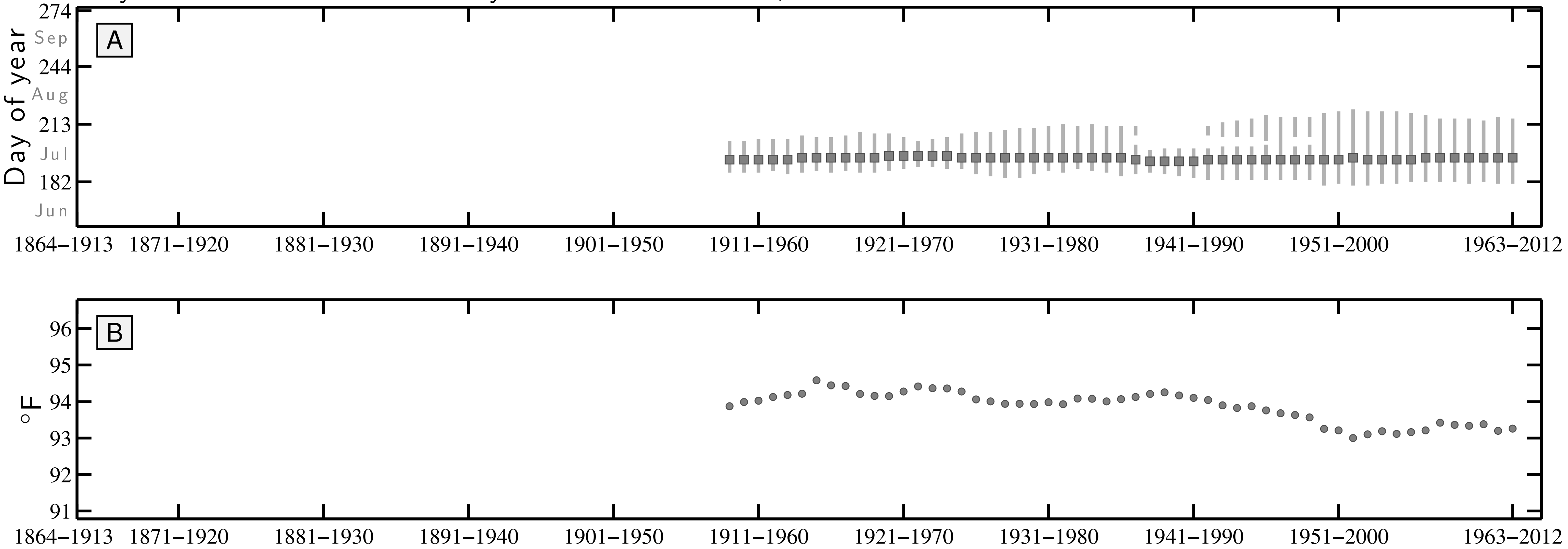

50 year Winter Teletherm dynamics for KANAB, UT:

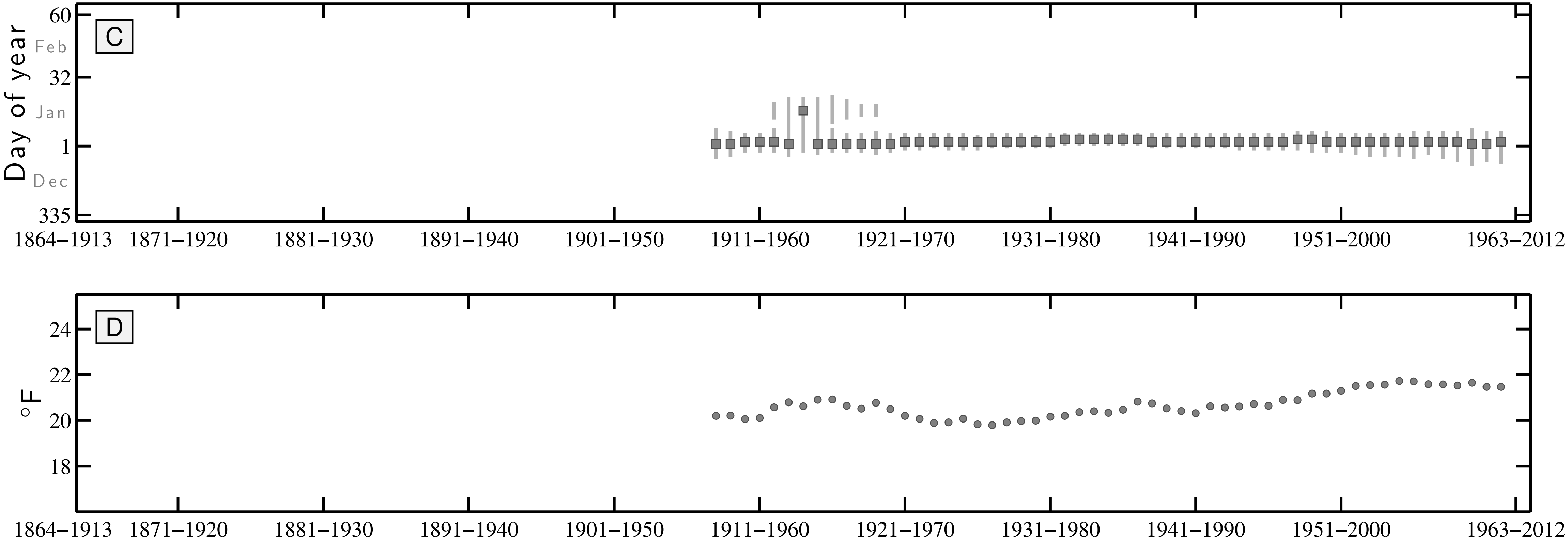

50 year Summer Teletherm dynamics for LAKETOWN, UT:

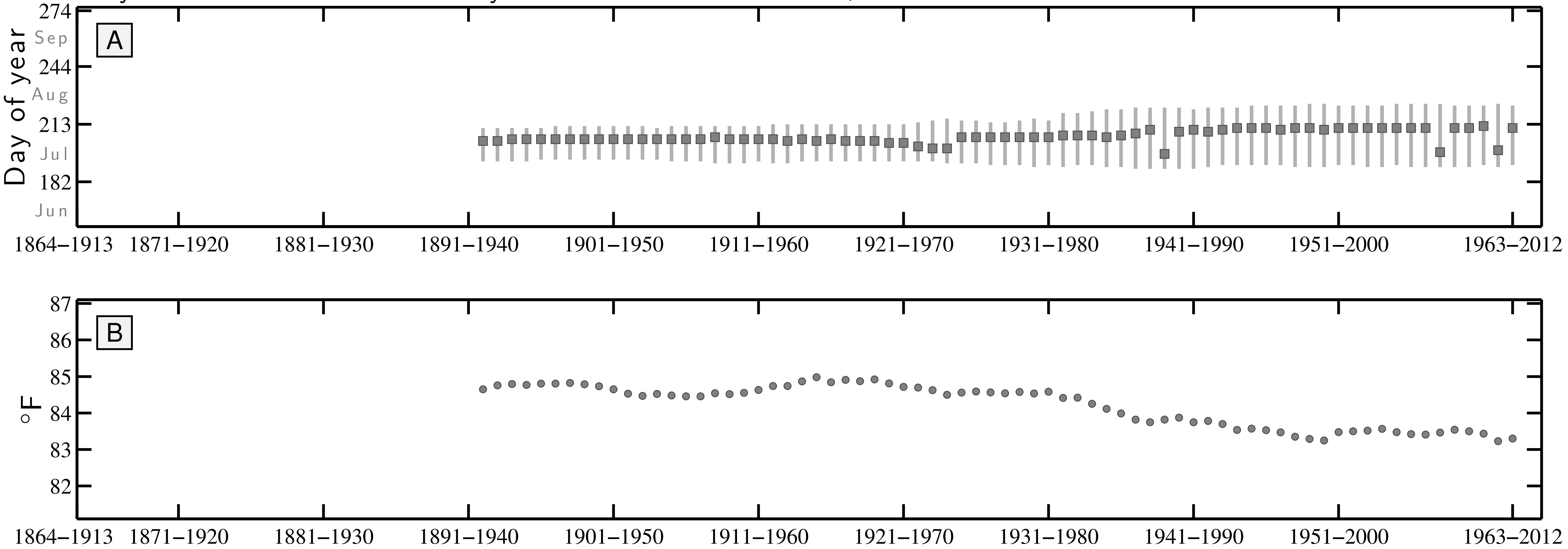

50 year Winter Teletherm dynamics for LAKETOWN, UT:

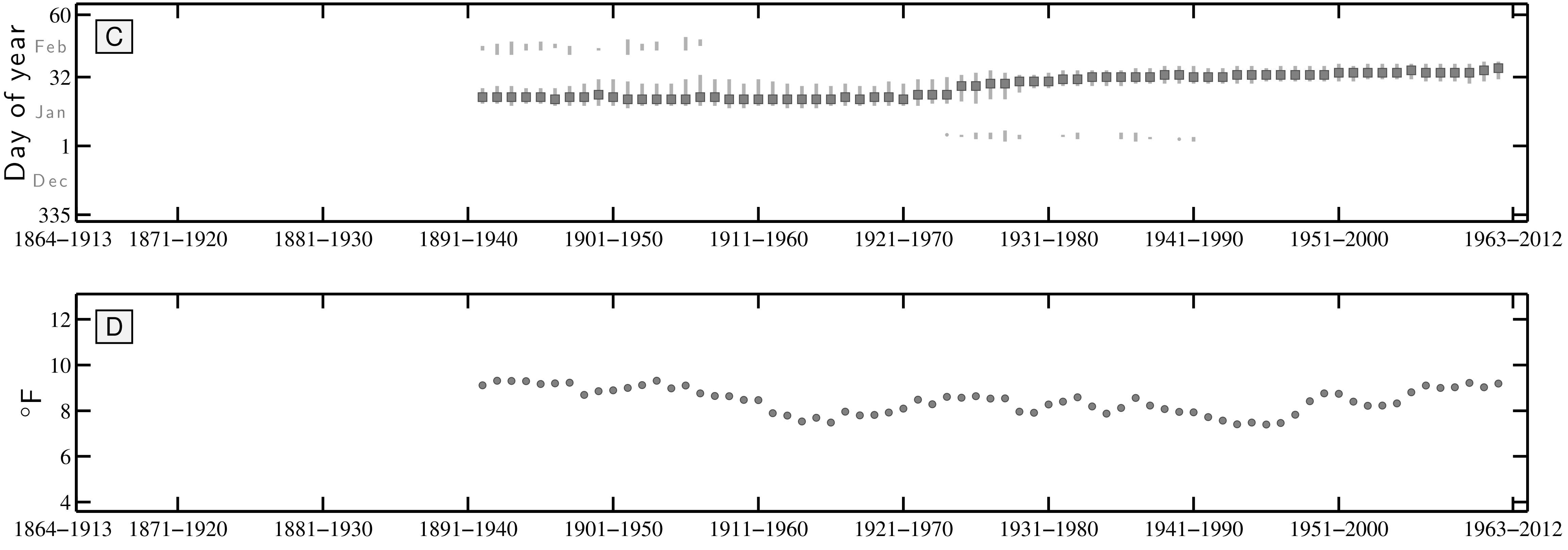

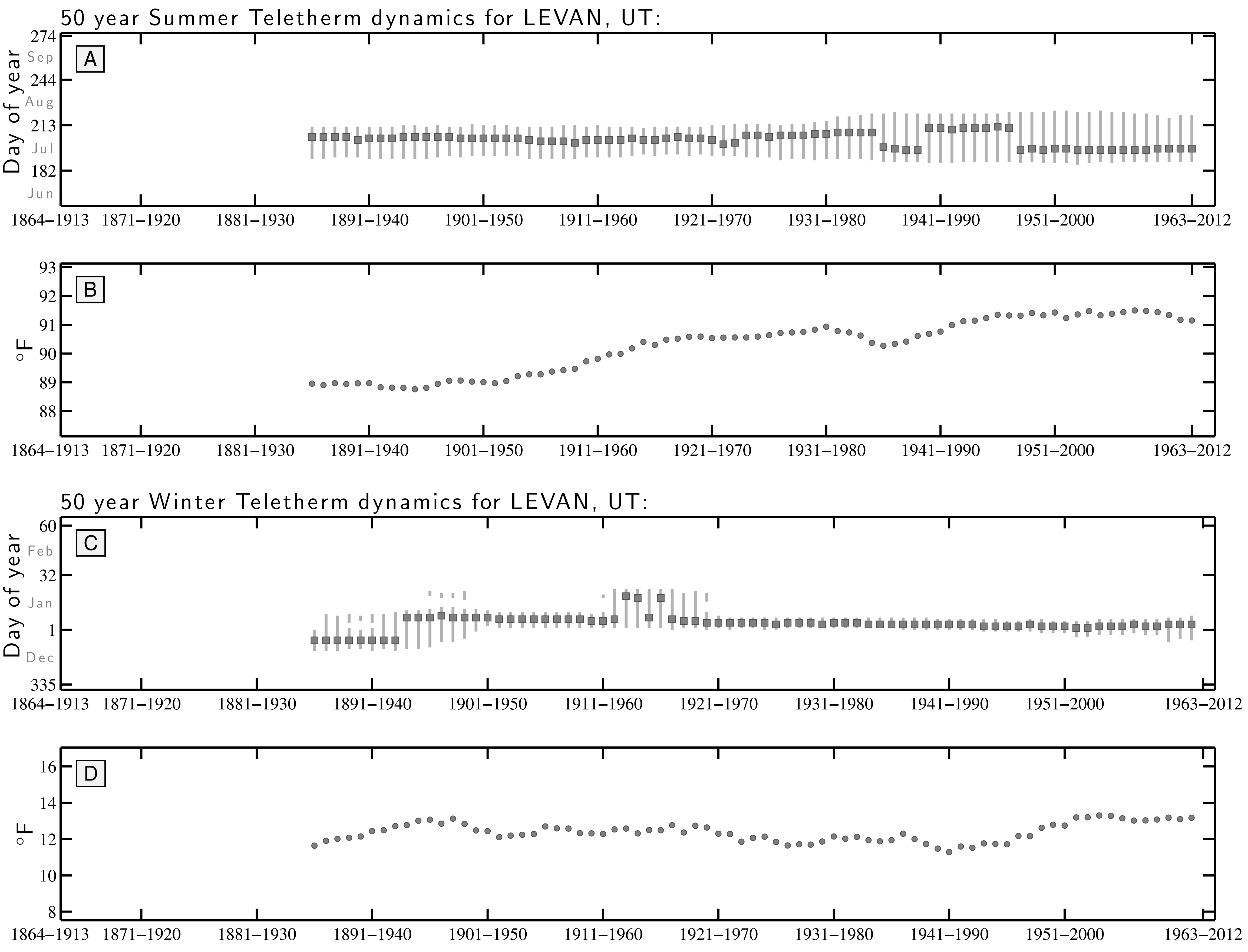
50 year Summer Teletherm dynamics for LEVAN, UT:
A
Day of year
274
Sep
244
Aug
213
Jul
182
Jun
1864–1913
1871–1920
1881–1930
1891–1940
1901–1950
1911–1960
1921–1970
1931–1980
1941–1990
1951–2000
1963–2012
B
°F
93
92
91
90
89
88
50 year Winter Teletherm dynamics for LEVAN, UT:
C
60
Feb
32
Jan
1
Dec
335
D
16
14
12
10
8

50 year Summer Teletherm dynamics for LOGAN UTAH ST UNIV, UT:

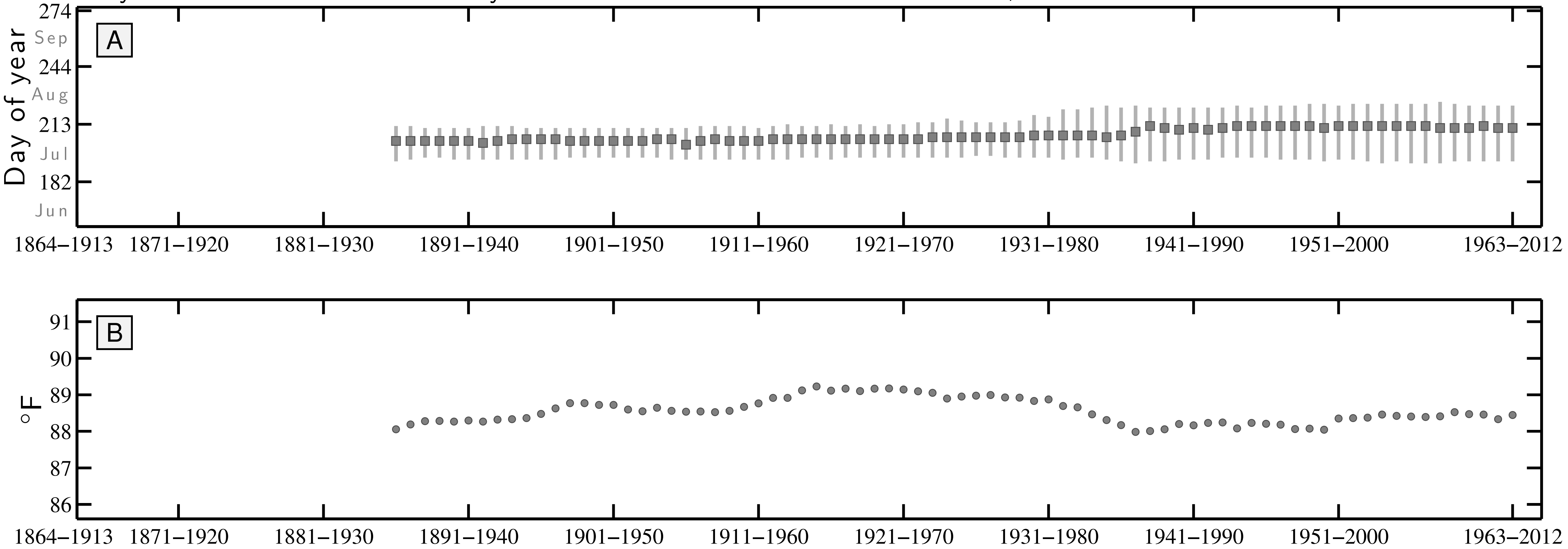

50 year Winter Teletherm dynamics for LOGAN UTAH ST UNIV, UT:

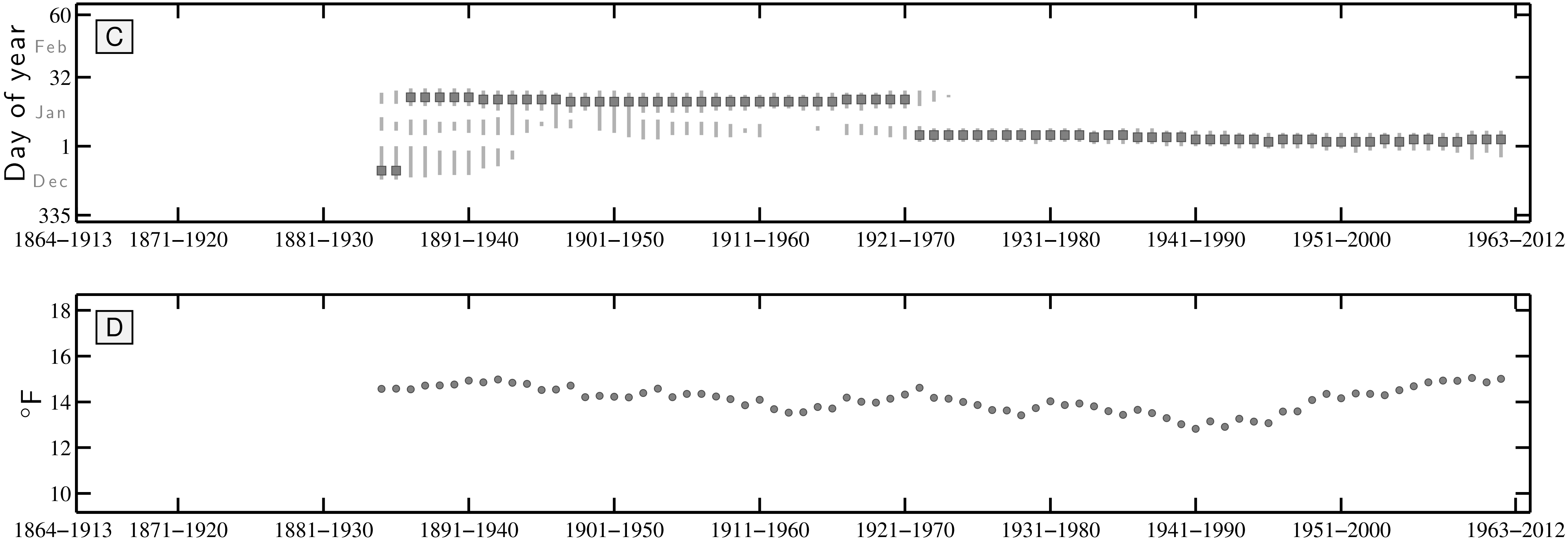

50 year Summer Teletherm dynamics for MANTI, UT:

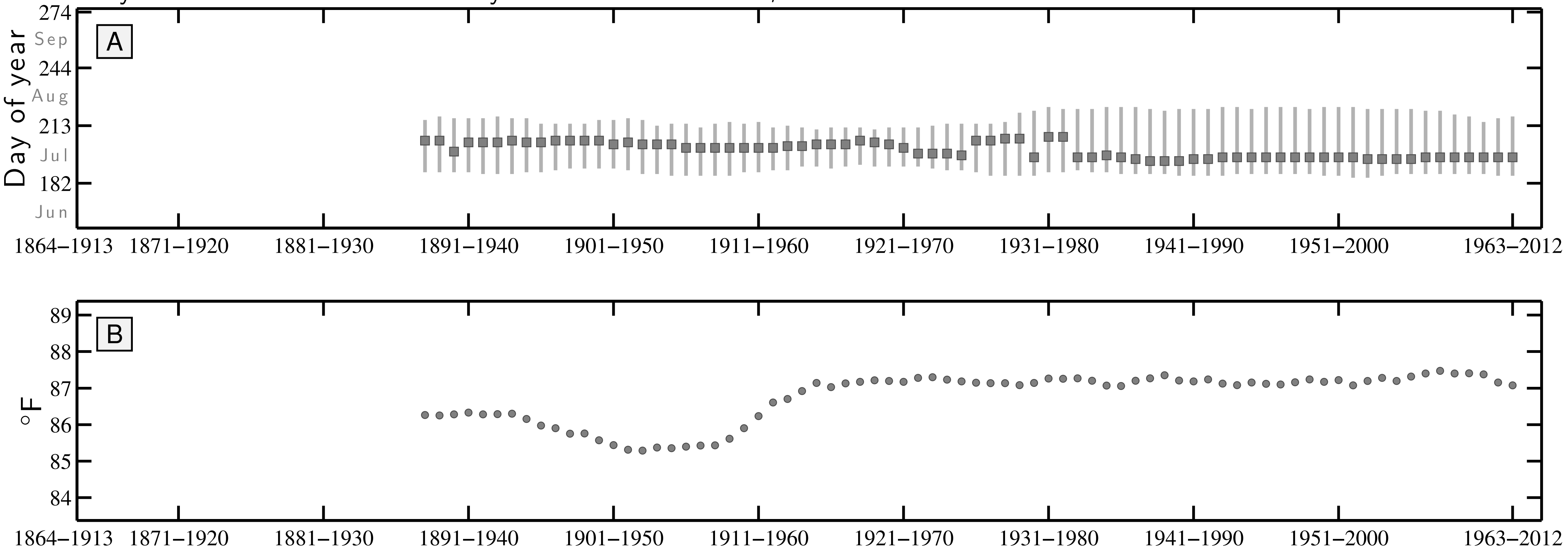

50 year Winter Teletherm dynamics for MANTI, UT:

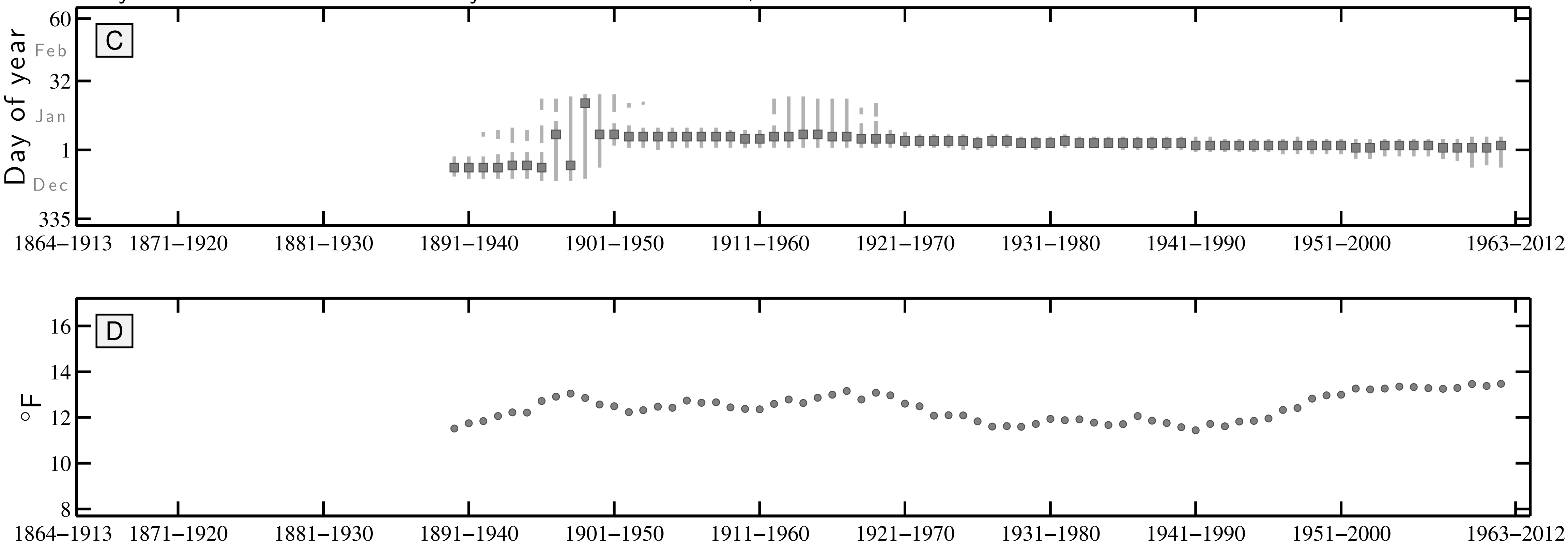

50 year Summer Teletherm dynamics for MARYSVALE, UT:

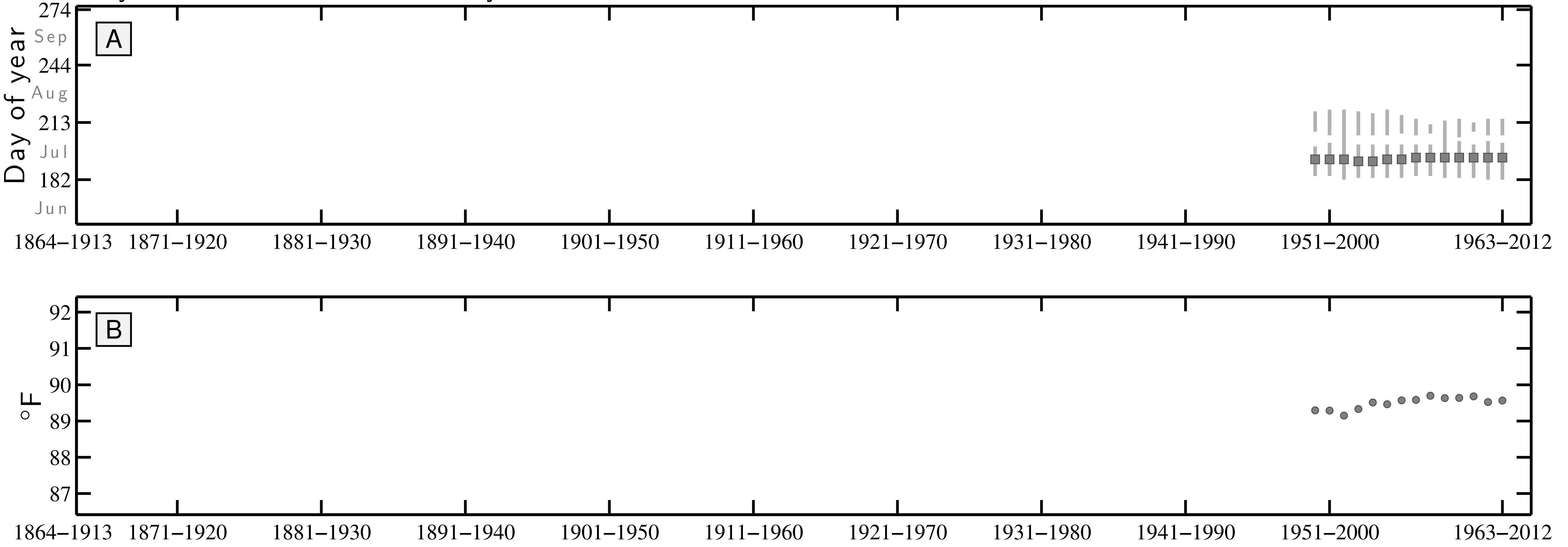

50 year Winter Teletherm dynamics for MARYSVALE, UT:

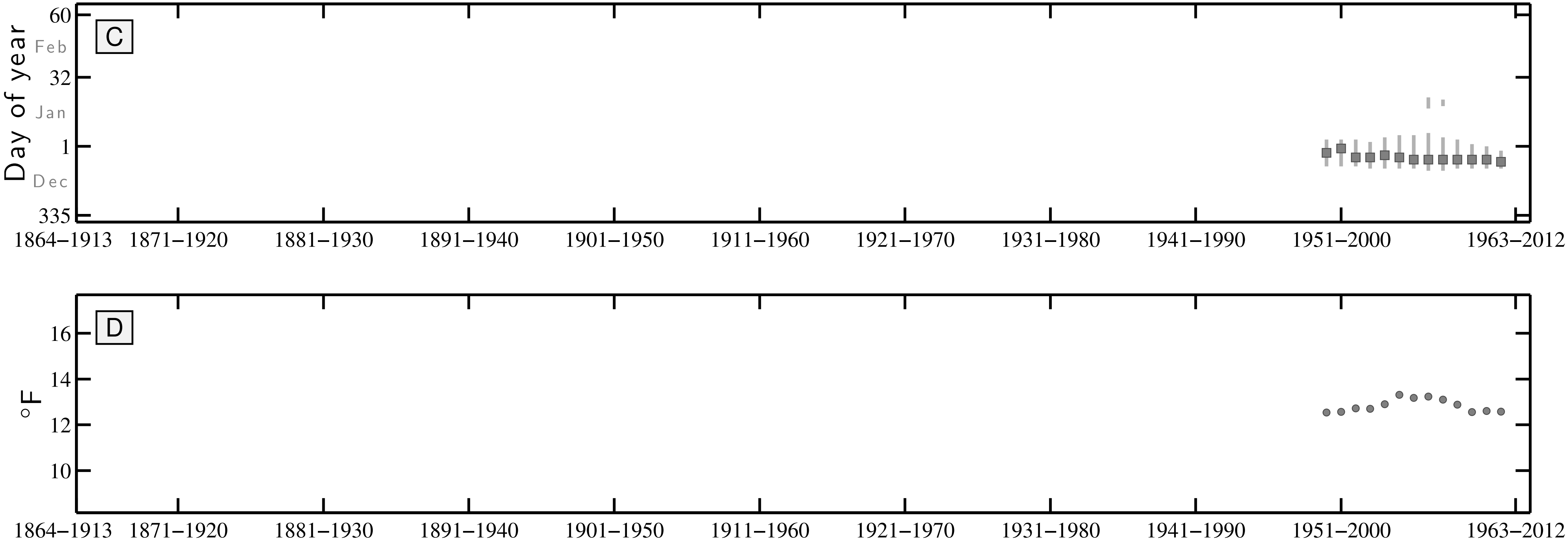

50 year Summer Teletherm dynamics for MOAB, UT:

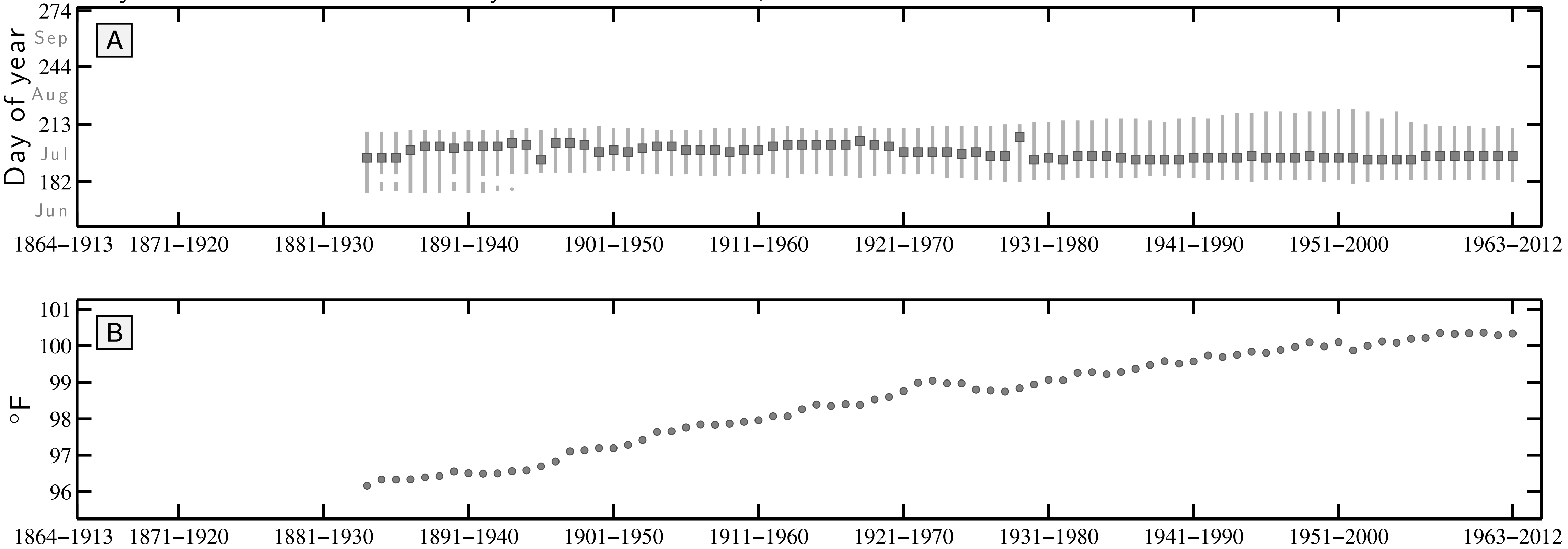

50 year Winter Teletherm dynamics for MOAB, UT:

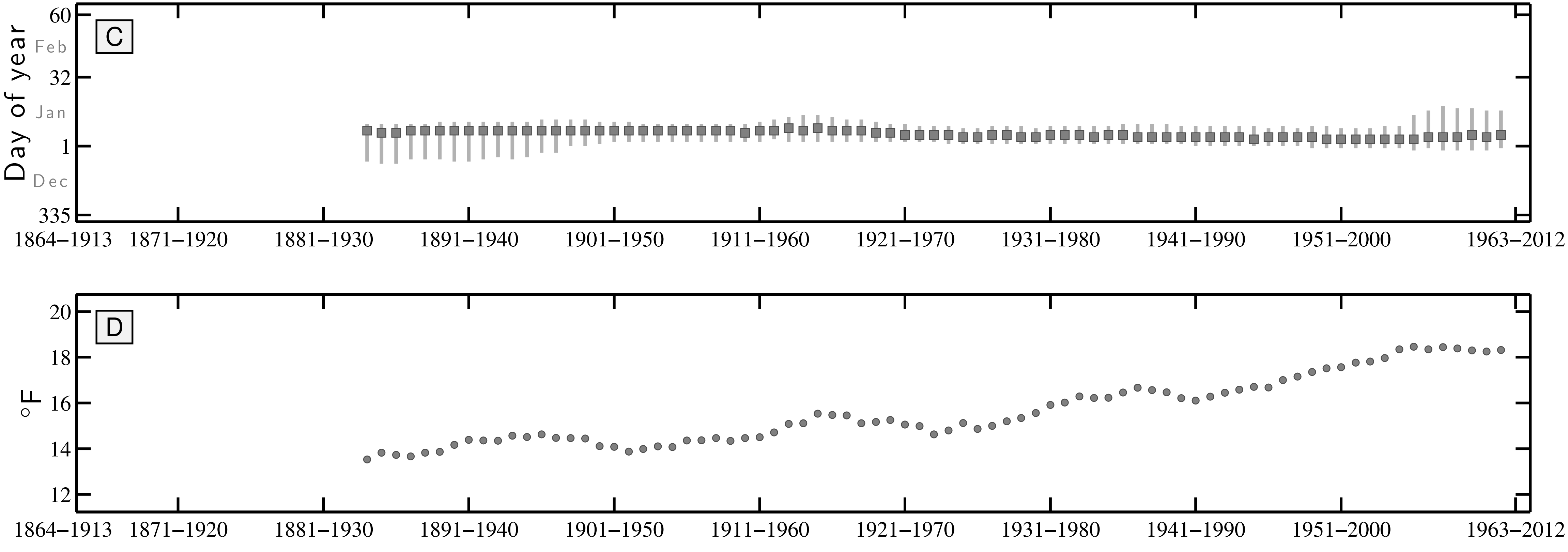

50 year Summer Teletherm dynamics for MODENA, UT:

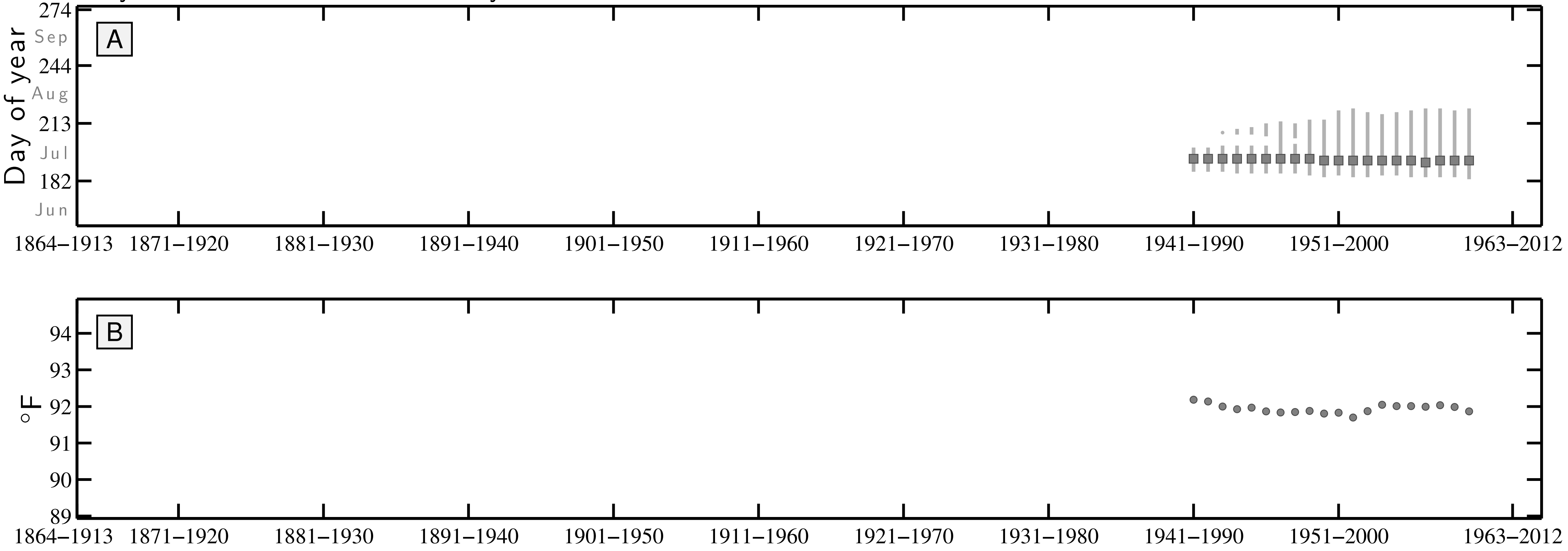

50 year Winter Teletherm dynamics for MODENA, UT:

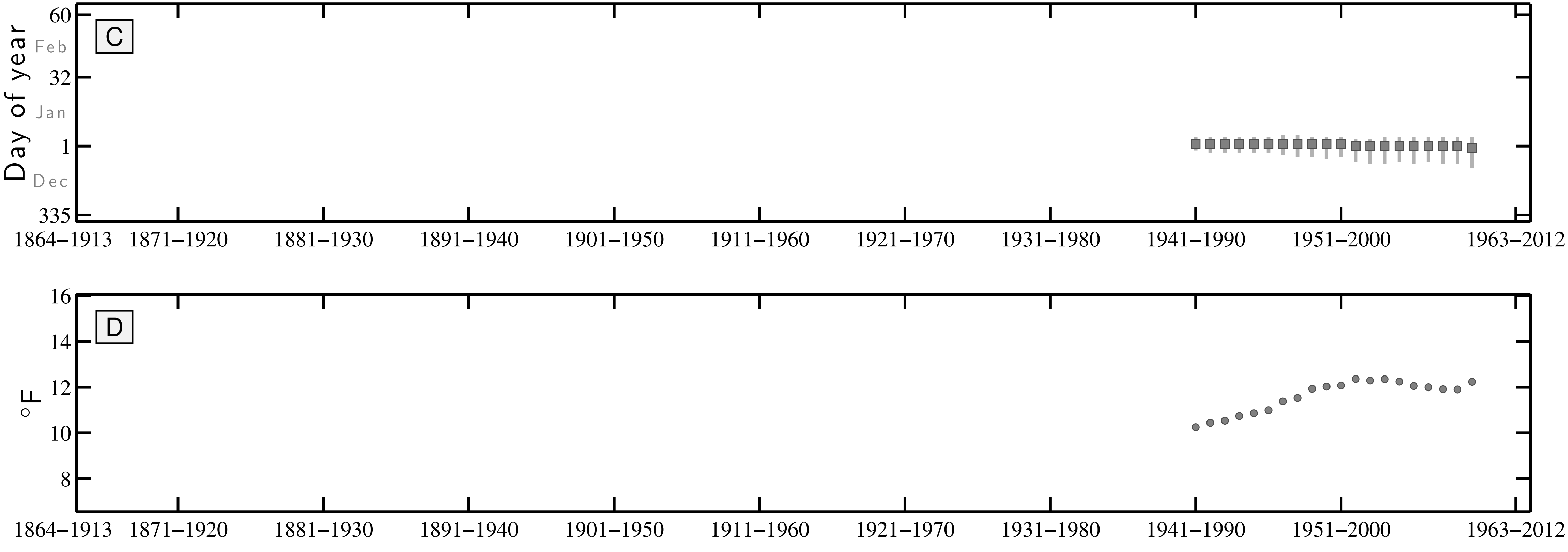

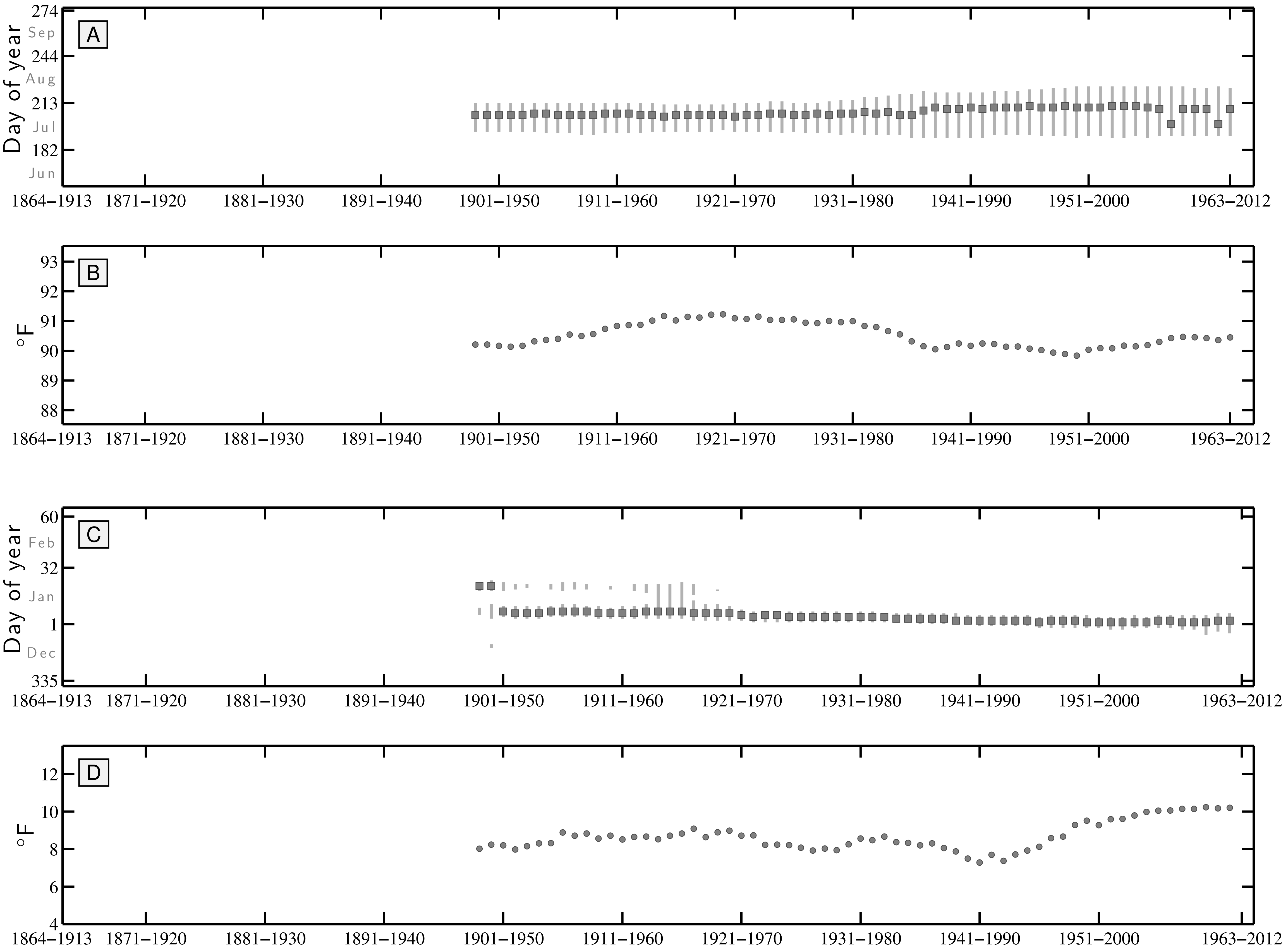

A
Day of year
274
Sep
244
Aug
213
Jul
182
Jun
1864–1913
1871–1920
1881–1930
1891–1940
1901–1950
1911–1960
1921–1970
1931–1980
1941–1990
1951–2000
1963–2012
B
°F
93
92
91
90
89
88
C
Day of year
60
Feb
32
Jan
1
Dec
335
D
°F
12
10
8
6
4

50 year Summer Teletherm dynamics for NEPHI, UT:

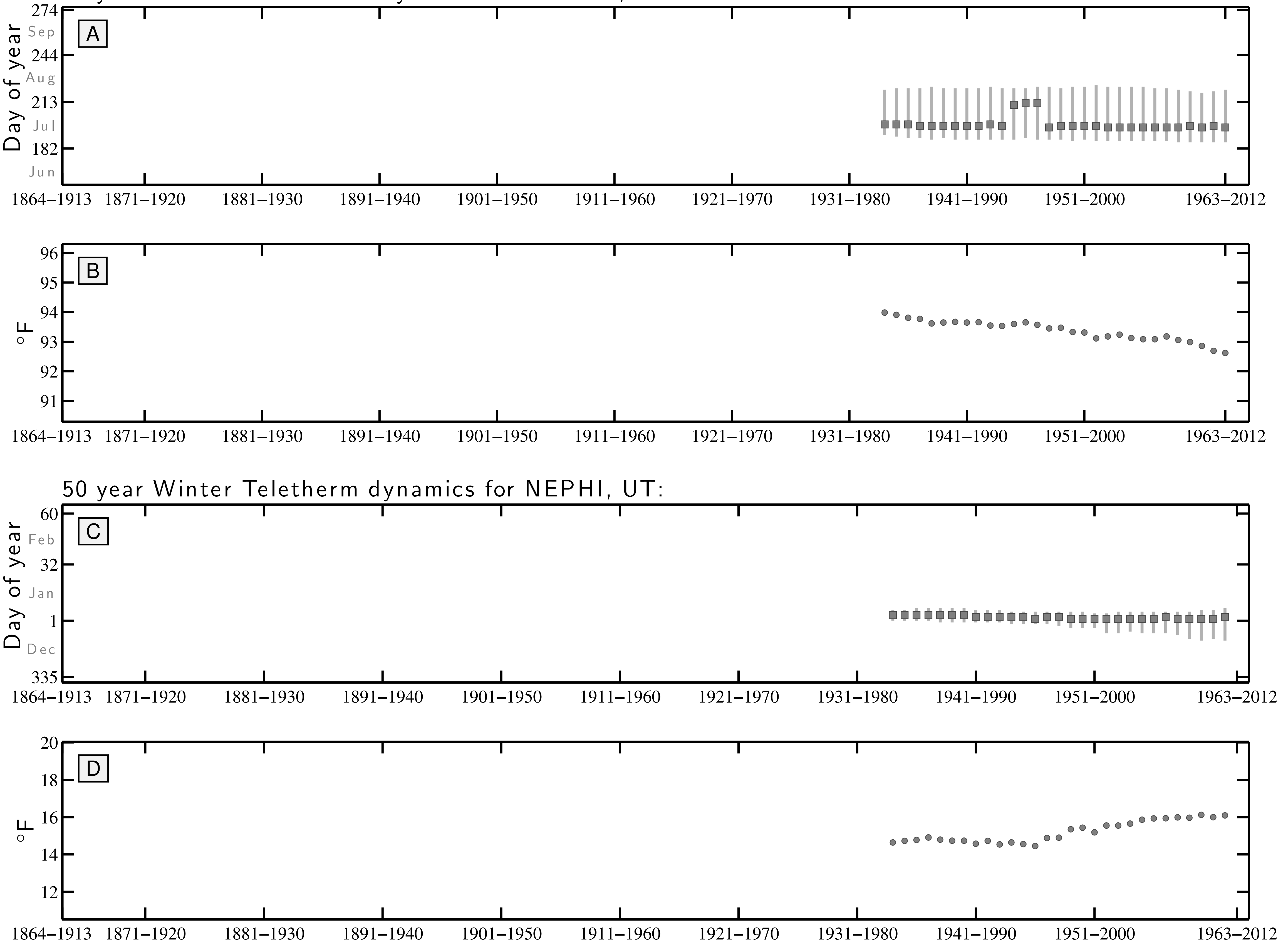

50 year Winter Teletherm dynamics for NEPHI, UT:

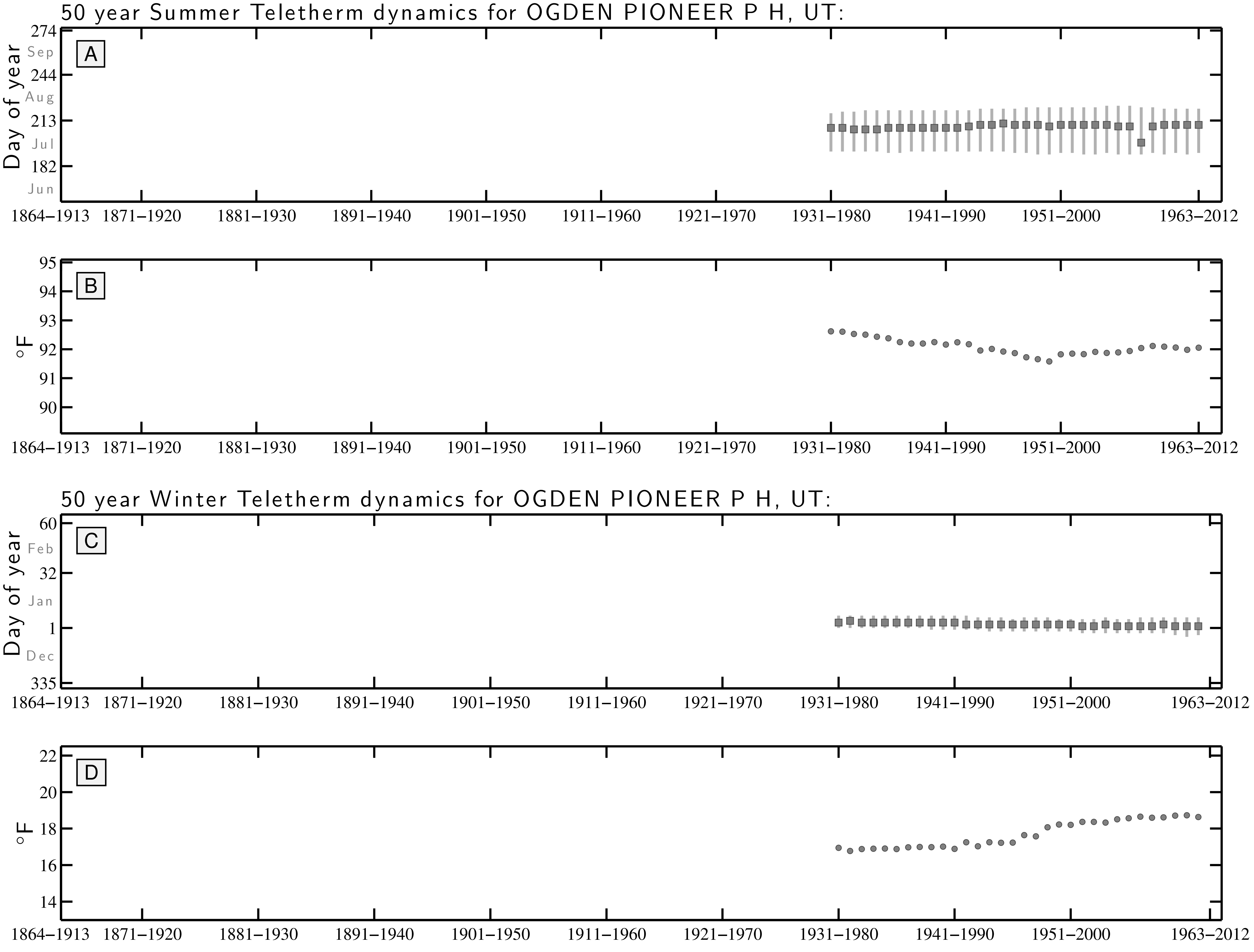
50 year Summer Teletherm dynamics for OGDEN PIONEER P H, UT:
A
Day of year
274
Sep
244
Aug
213
Jul
182
Jun
B
°F
95
94
93
92
91
90
50 year Winter Teletherm dynamics for OGDEN PIONEER P H, UT:
C
Day of year
60
Feb
32
Jan
1
Dec
335
D
°F
22
20
18
16
14
1864–1913
1871–1920
1881–1930
1891–1940
1901–1950
1911–1960
1921–1970
1931–1980
1941–1990
1951–2000
1963–2012

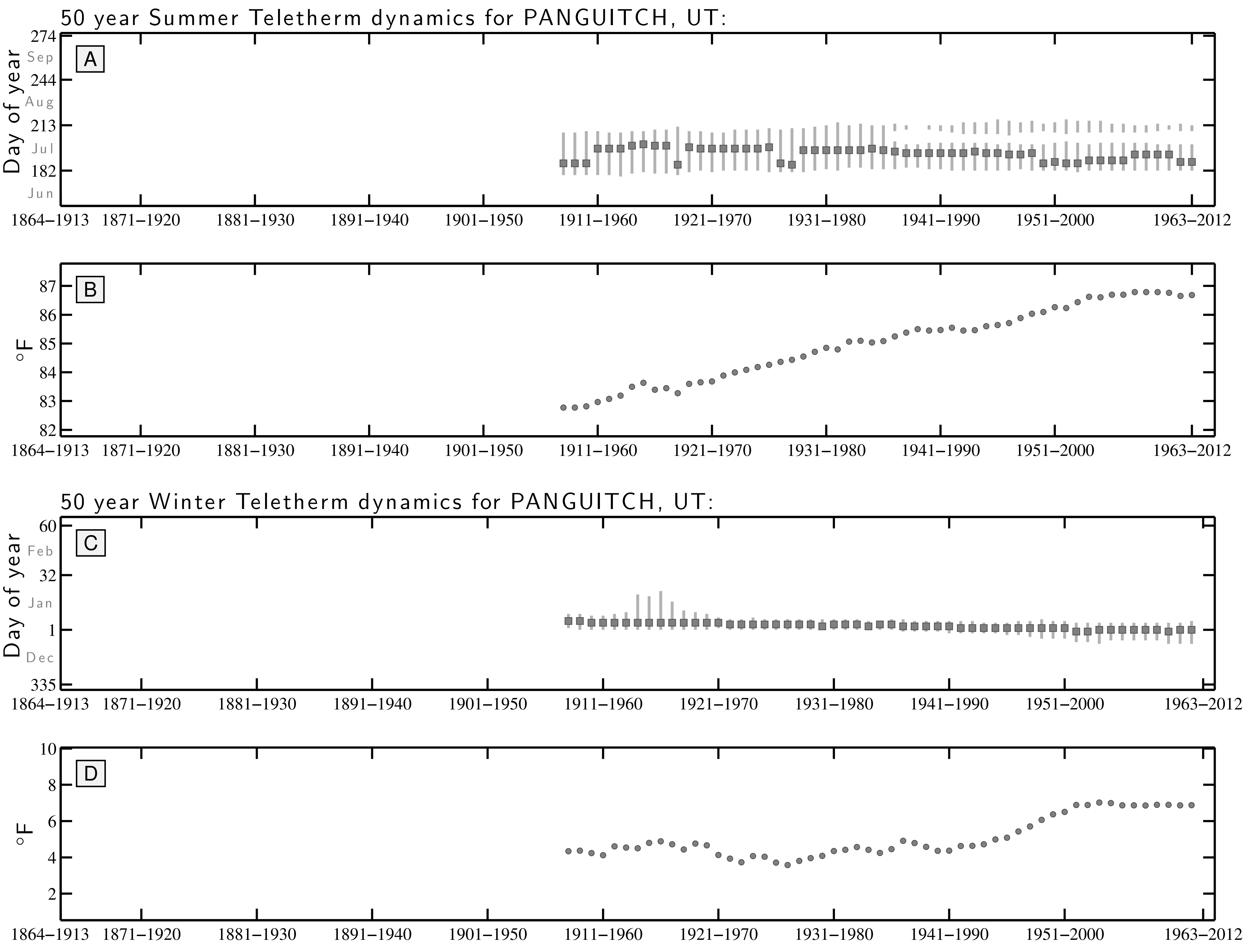
50 year Summer Teletherm dynamics for PANGUITCH, UT:
A
B
Day of year
°F
Sep
Aug
Jul
Jun
274
244
213
182
87
86
85
84
83
82
1864–1913
1871–1920
1881–1930
1891–1940
1901–1950
1911–1960
1921–1970
1931–1980
1941–1990
1951–2000
1963–2012
50 year Winter Teletherm dynamics for PANGUITCH, UT:
C
D
Feb
Jan
Dec
60
32
1
335
10
8
6
4
2

50 year Summer Teletherm dynamics for PAROWAN PWR, UT:

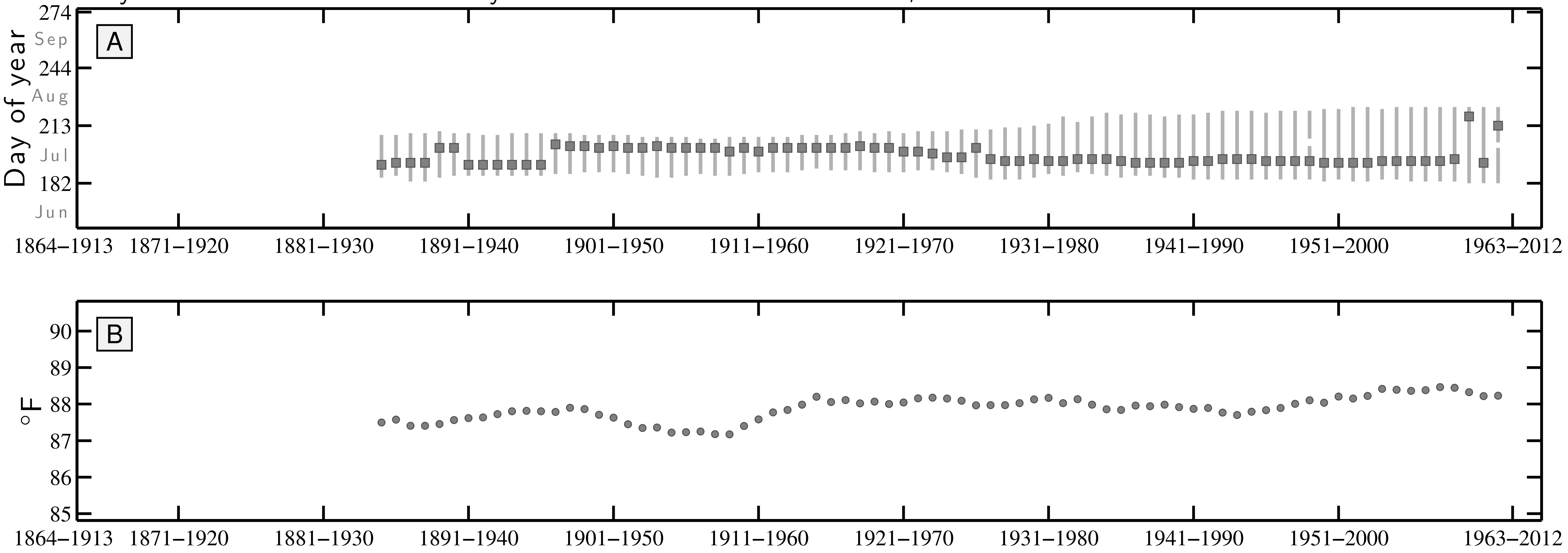

50 year Winter Teletherm dynamics for PAROWAN PWR, UT:

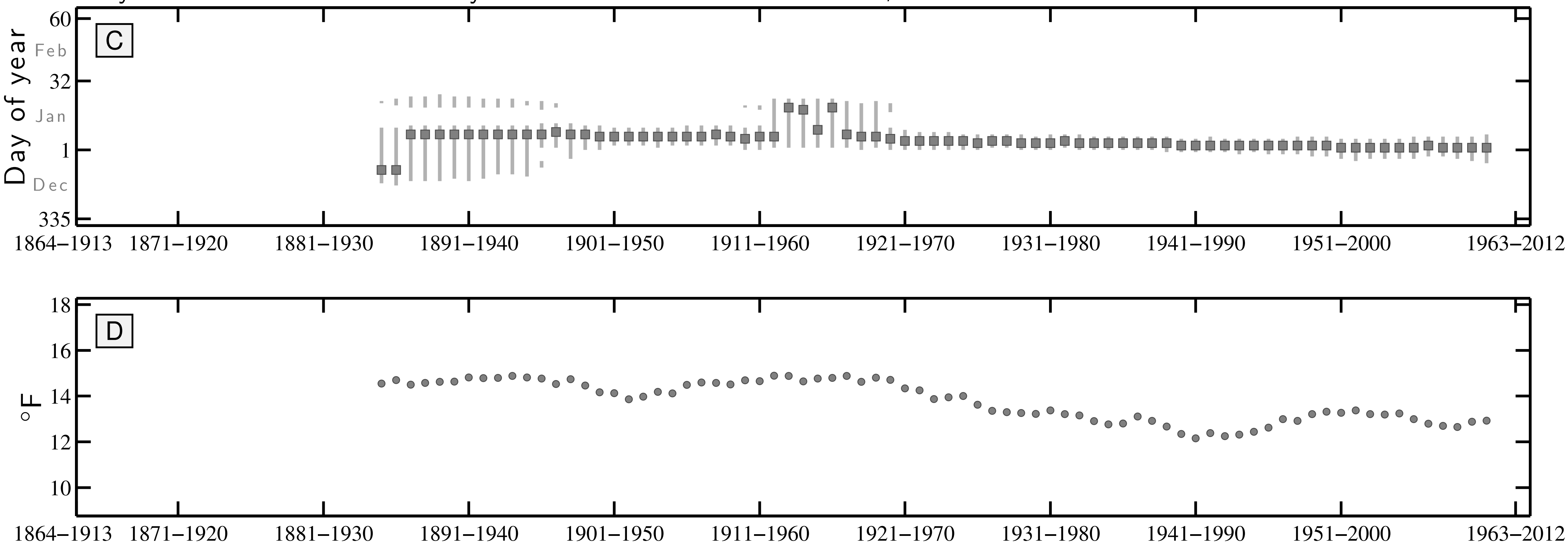

50 year Summer Teletherm dynamics for RICHFIELD RADIO KSVC, UT:

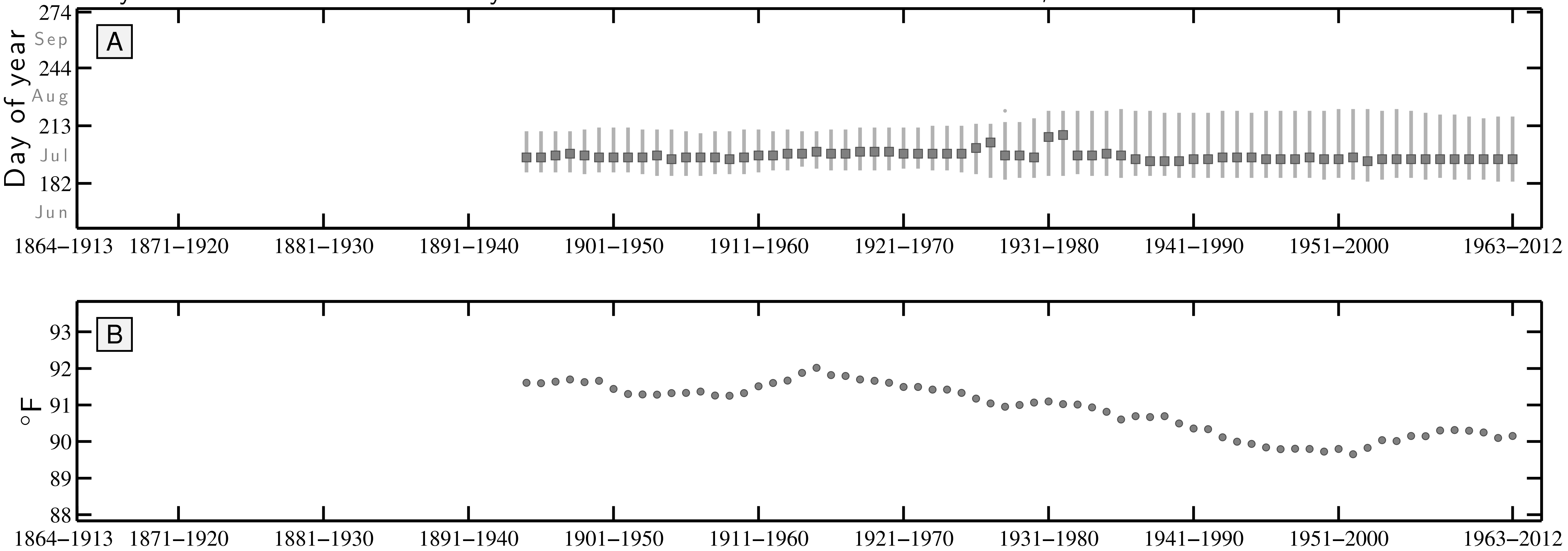

50 year Winter Teletherm dynamics for RICHFIELD RADIO KSVC, UT:

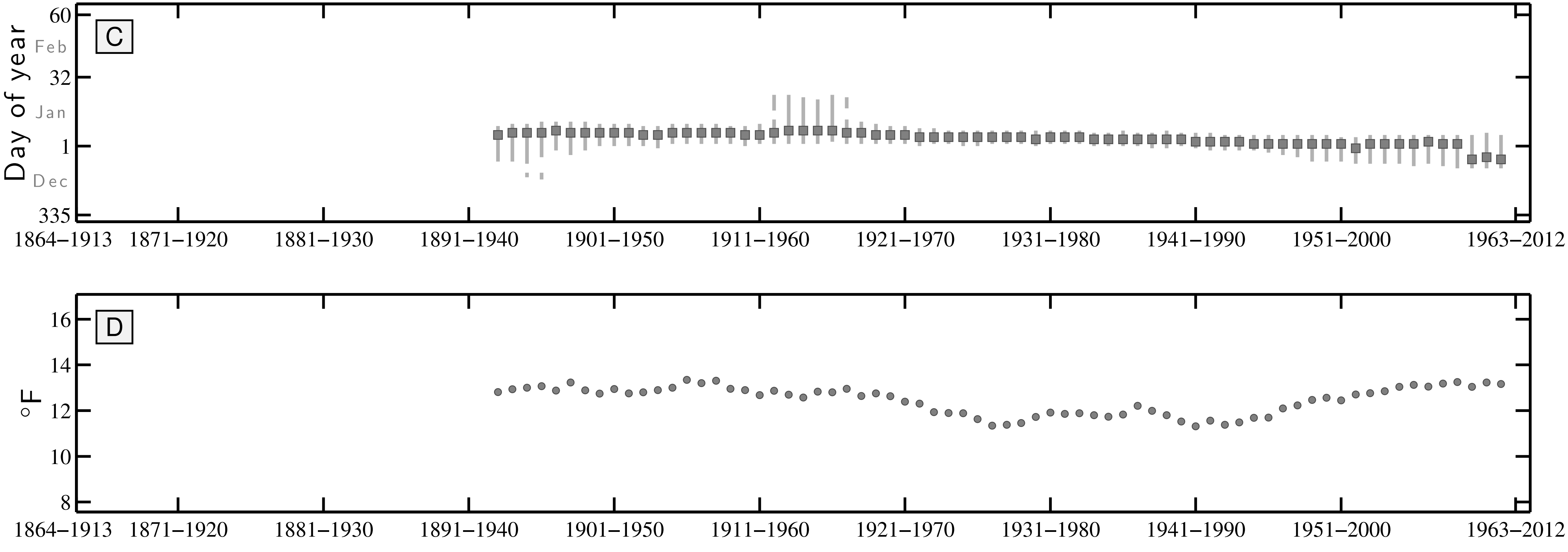

50 year Summer Teletherm dynamics for ST GEORGE, UT:

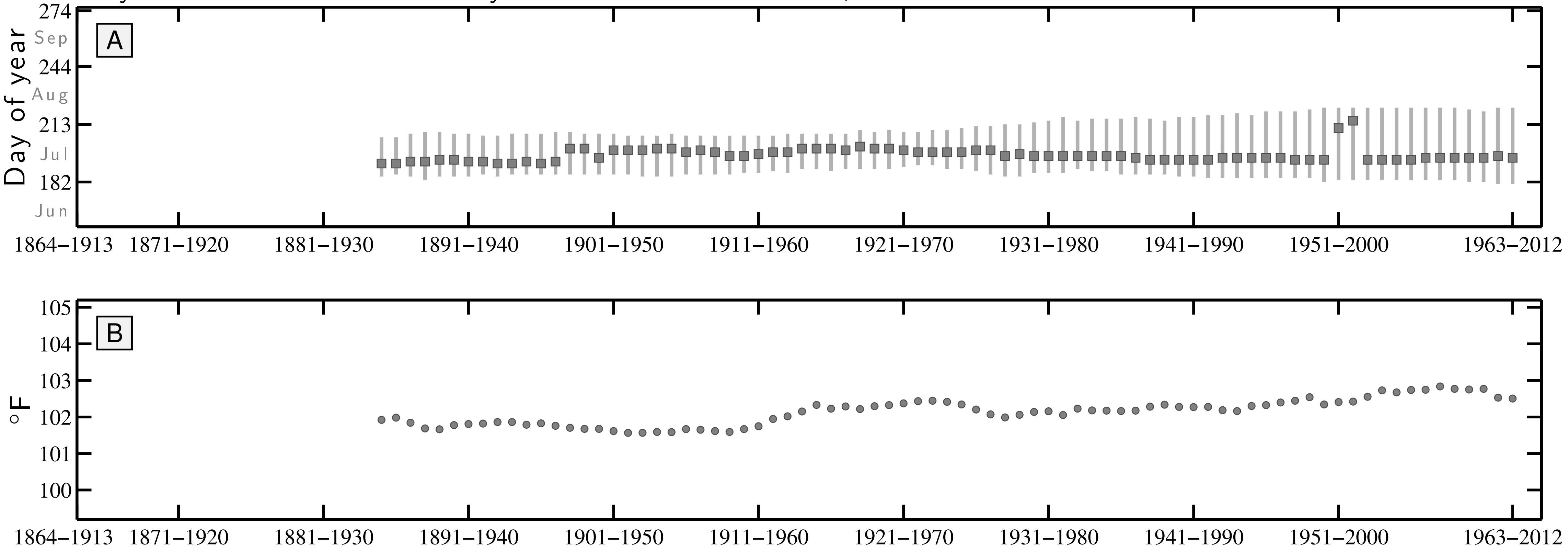

50 year Winter Teletherm dynamics for ST GEORGE, UT:

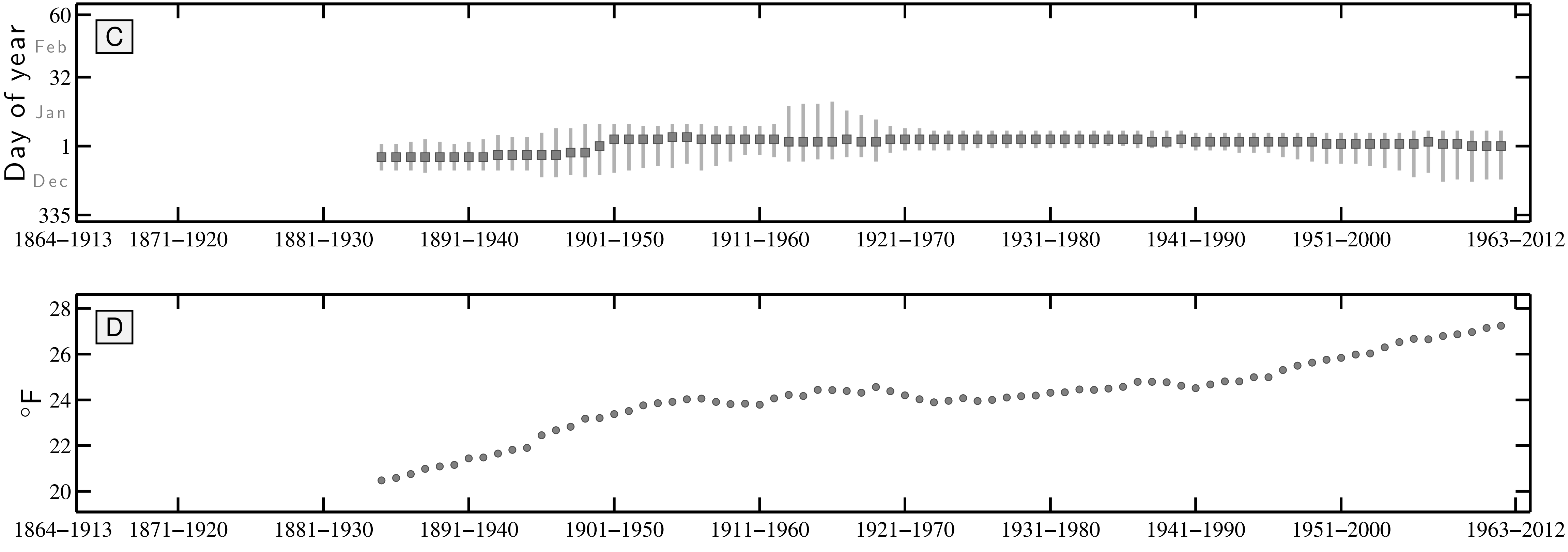

50 year Summer Teletherm dynamics for SCIPIO, UT:

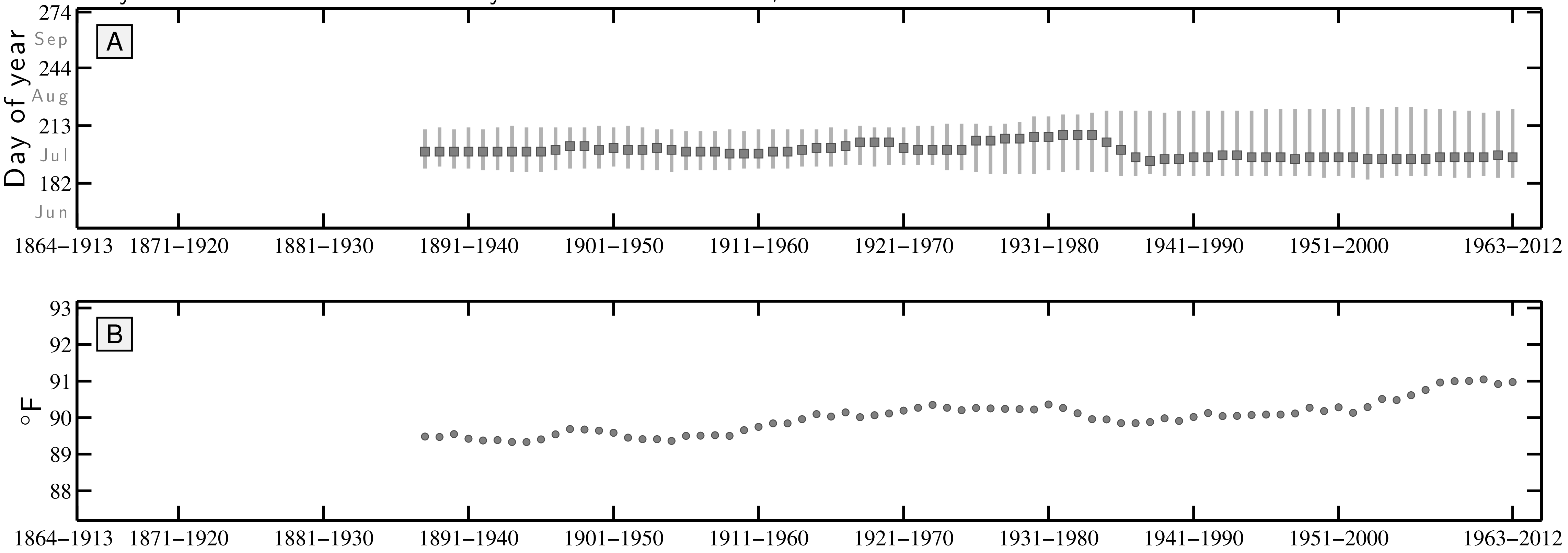

50 year Winter Teletherm dynamics for SCIPIO, UT:

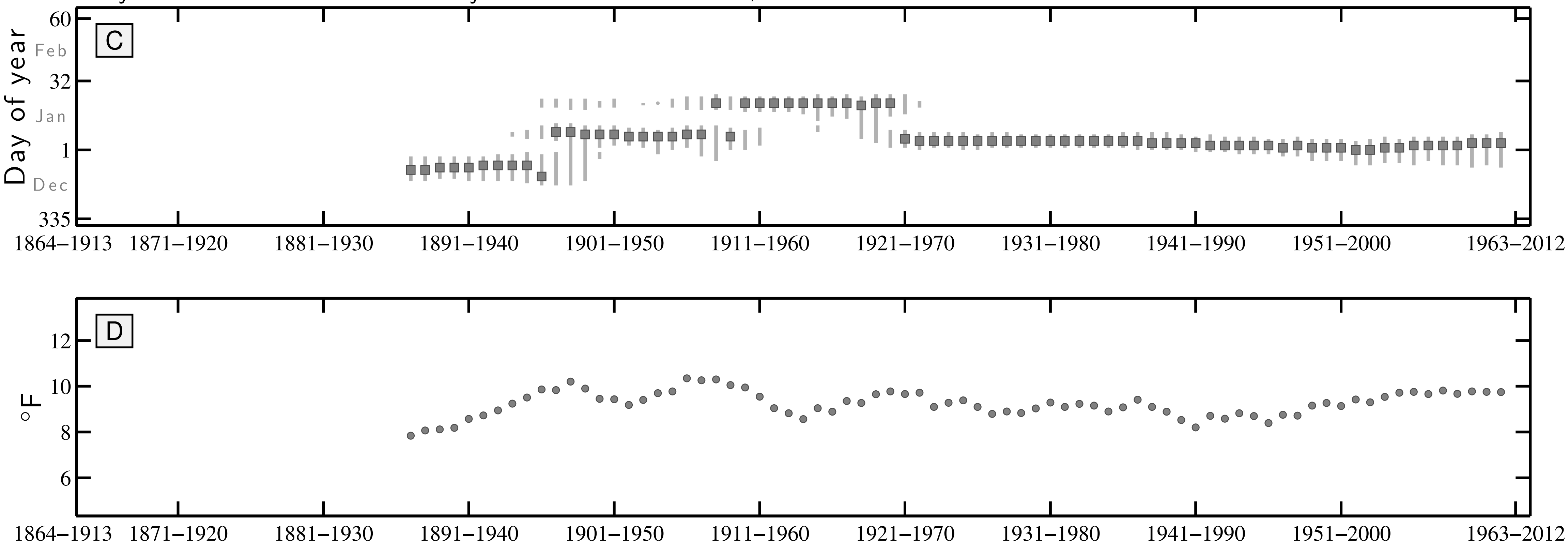

50 year Summer Teletherm dynamics for SNAKE CREEK POWERHOUSE, UT:

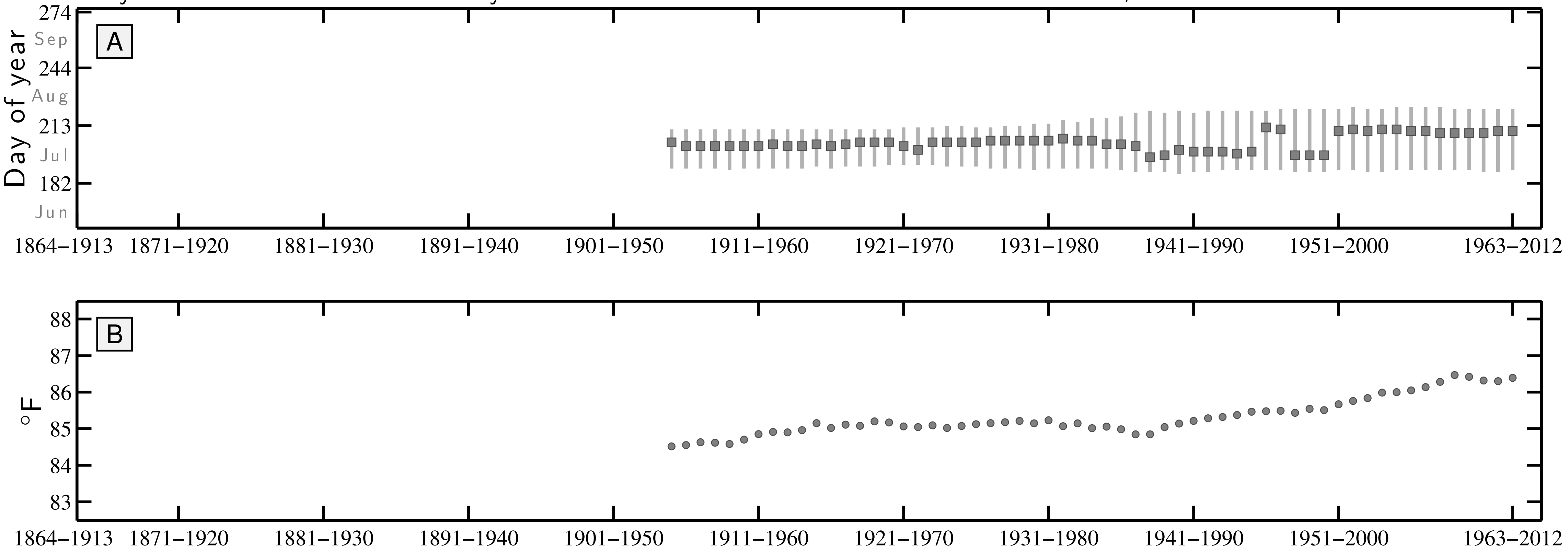

50 year Winter Teletherm dynamics for SNAKE CREEK POWERHOUSE, UT:

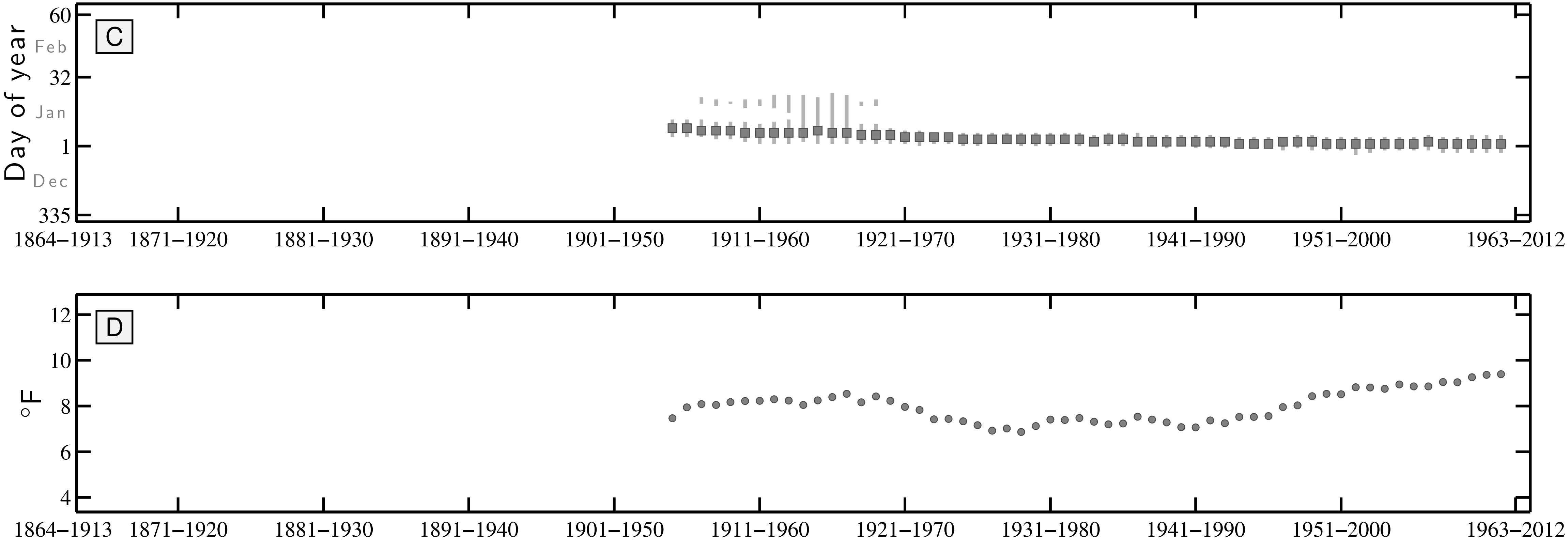

50 year Summer Teletherm dynamics for SPANISH FORK PWR HOUSE, UT:

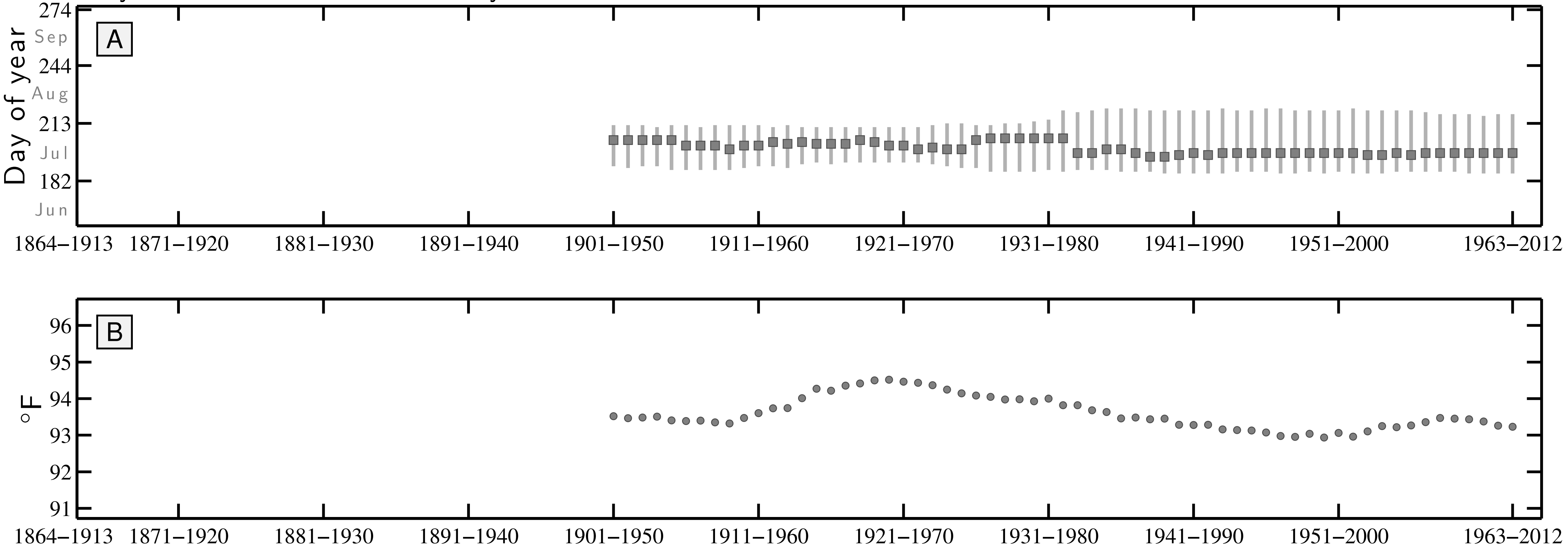

50 year Winter Teletherm dynamics for SPANISH FORK PWR HOUSE, UT:

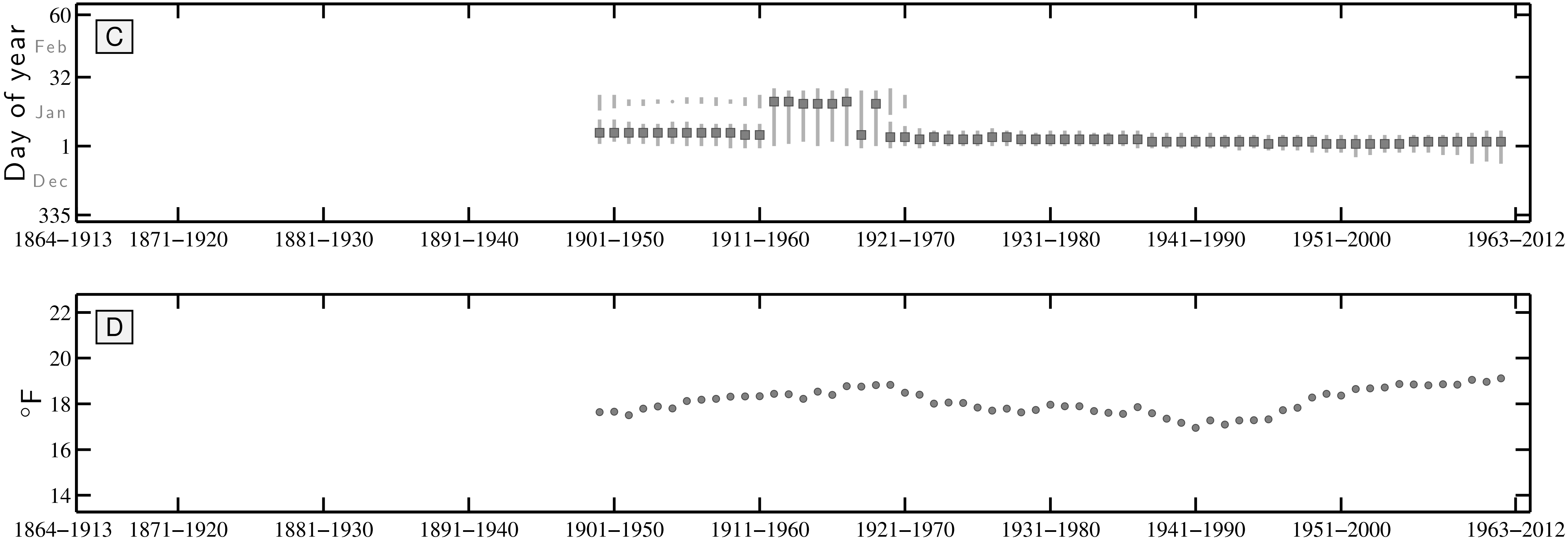

50 year Summer Teletherm dynamics for THOMPSON, UT:

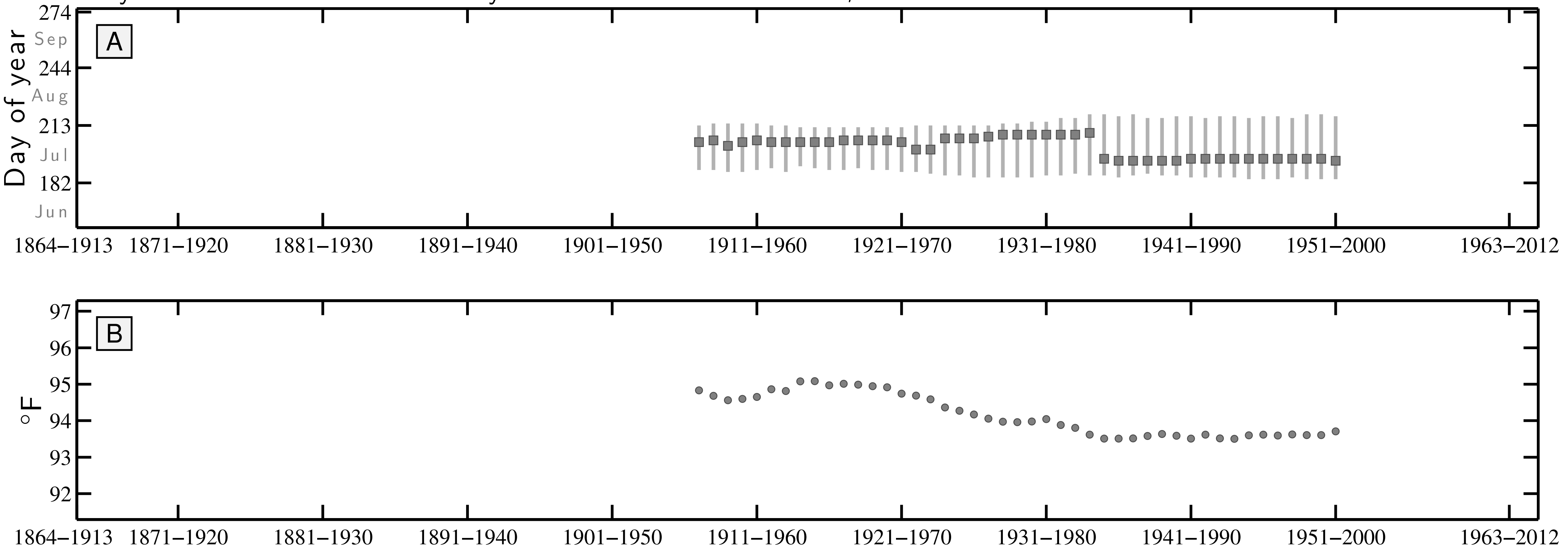

50 year Winter Teletherm dynamics for THOMPSON, UT:

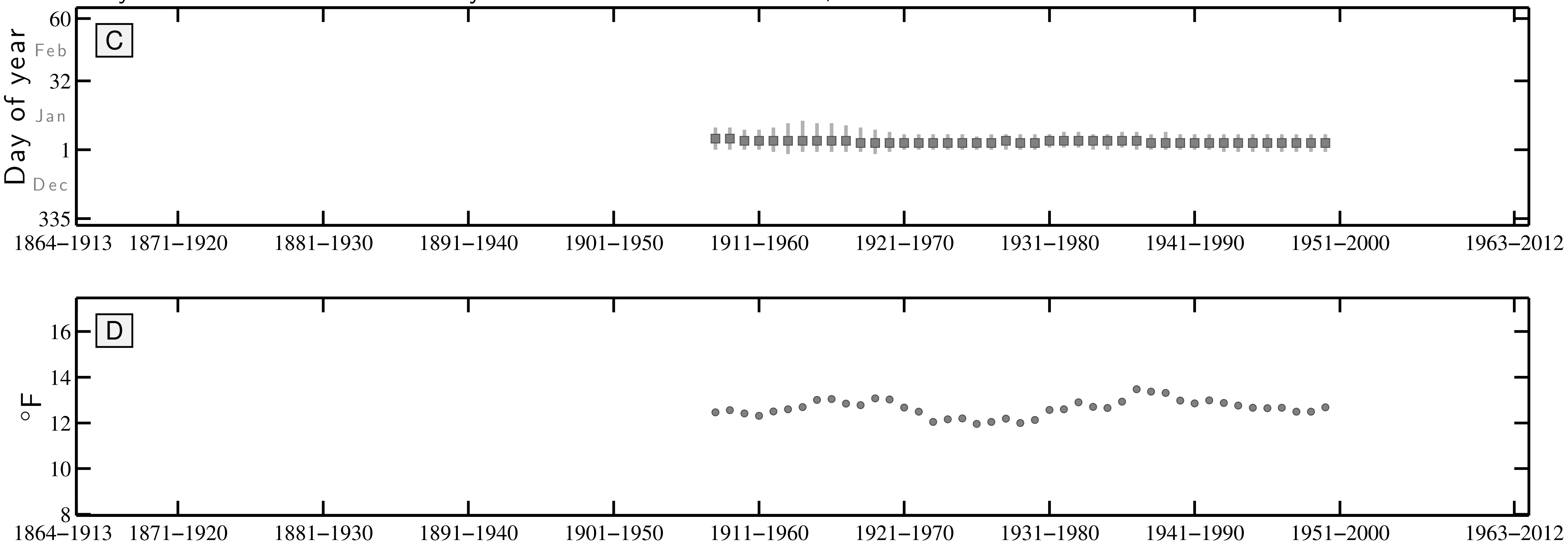

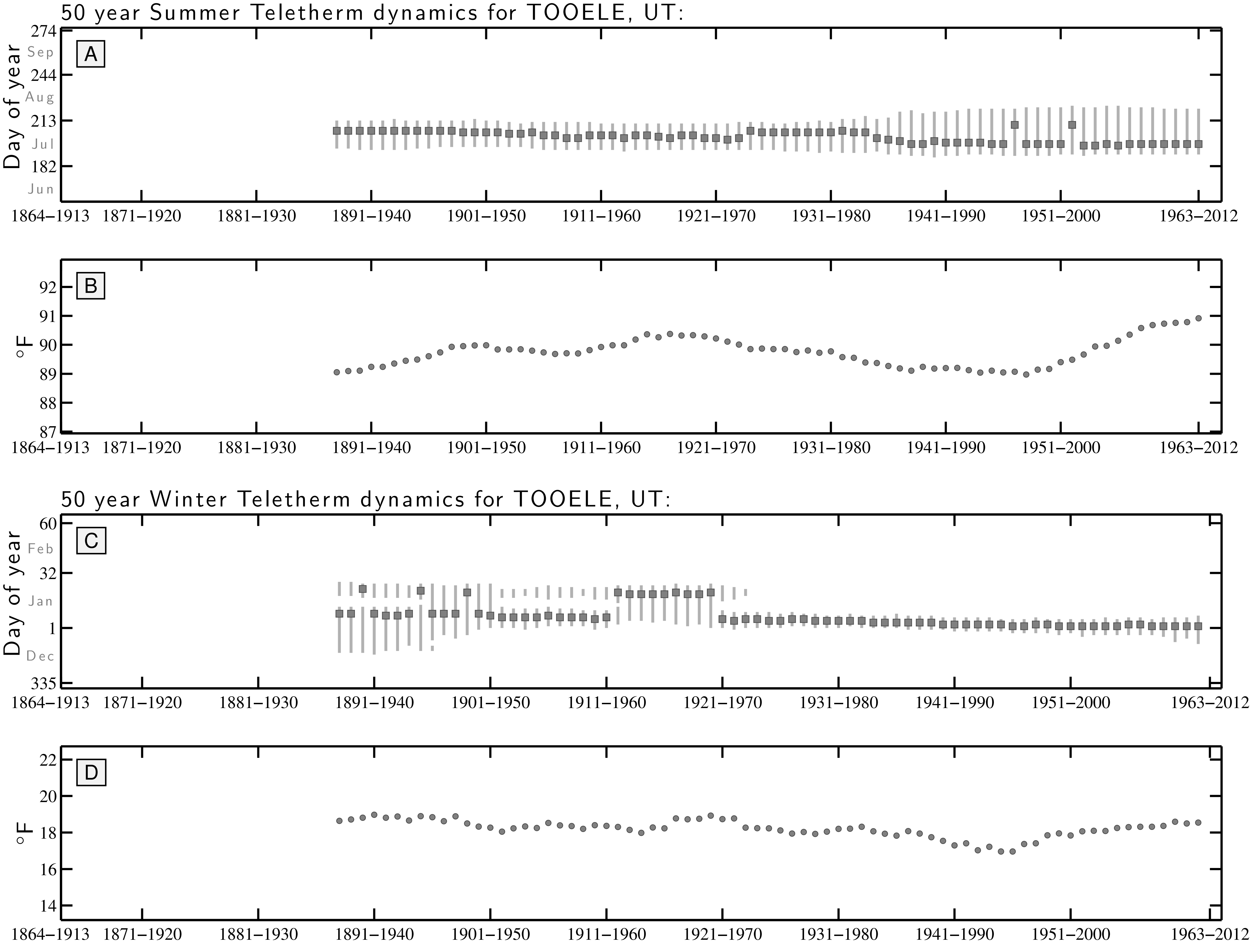

50 year Summer Teletherm dynamics for TOOELE, UT:
A
Day of year
274
Sep
244
Aug
213
Jul
182
Jun
1864–1913
1871–1920
1881–1930
1891–1940
1901–1950
1911–1960
1921–1970
1931–1980
1941–1990
1951–2000
1963–2012
B
°F
92
91
90
89
88
87
50 year Winter Teletherm dynamics for TOOELE, UT:
C
60
Feb
32
Jan
1
Dec
335
D
22
20
18
16
14

50 year Summer Teletherm dynamics for UTAH LAKE LEHI, UT:

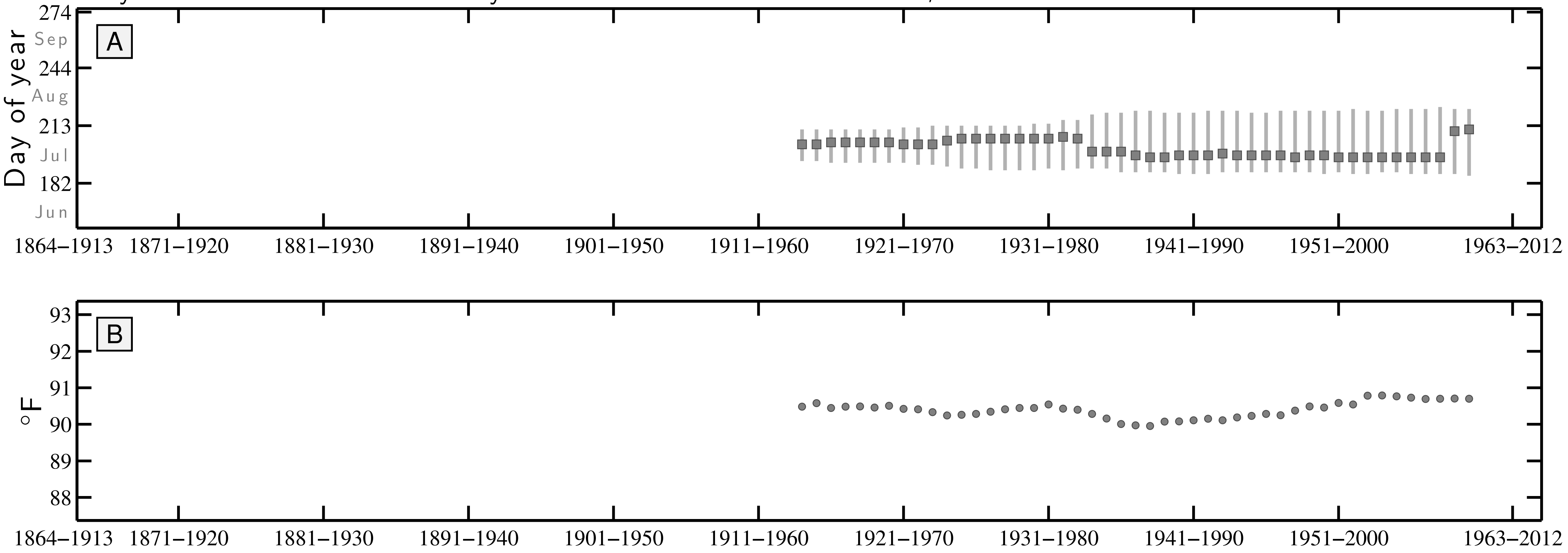

50 year Winter Teletherm dynamics for UTAH LAKE LEHI, UT:

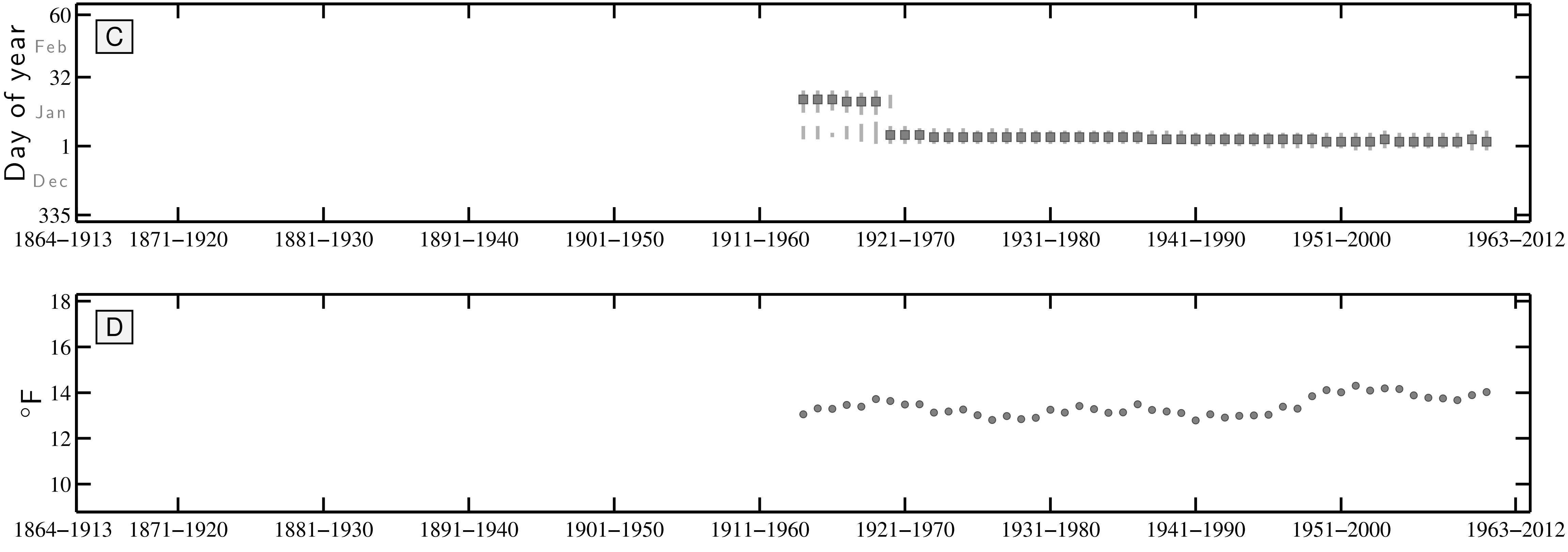

50 year Summer Teletherm dynamics for VERNAL 2SW, UT:

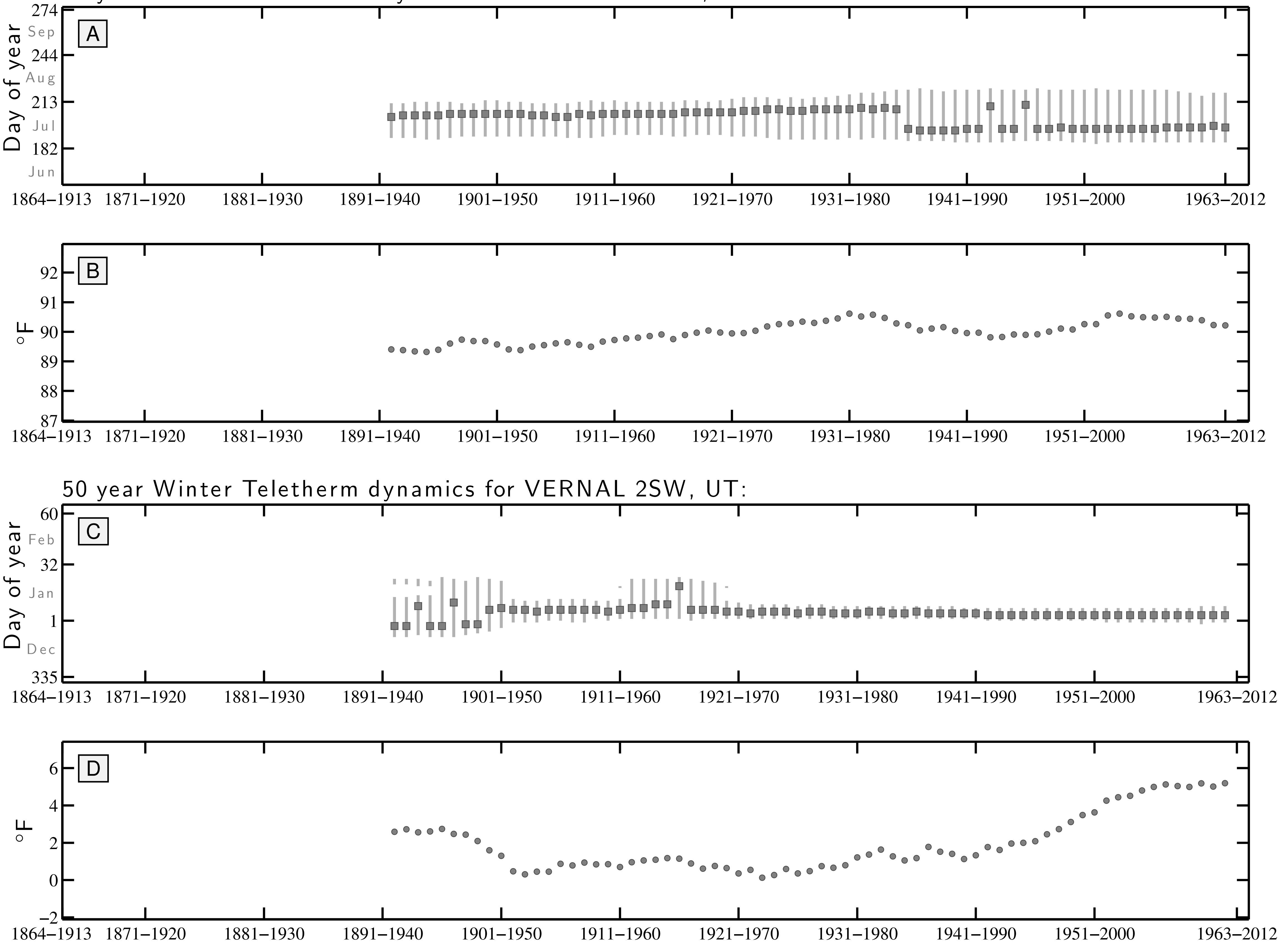

50 year Winter Teletherm dynamics for VERNAL 2SW, UT:

50 year Summer Teletherm dynamics for WENDOVER AP AWOS, UT:

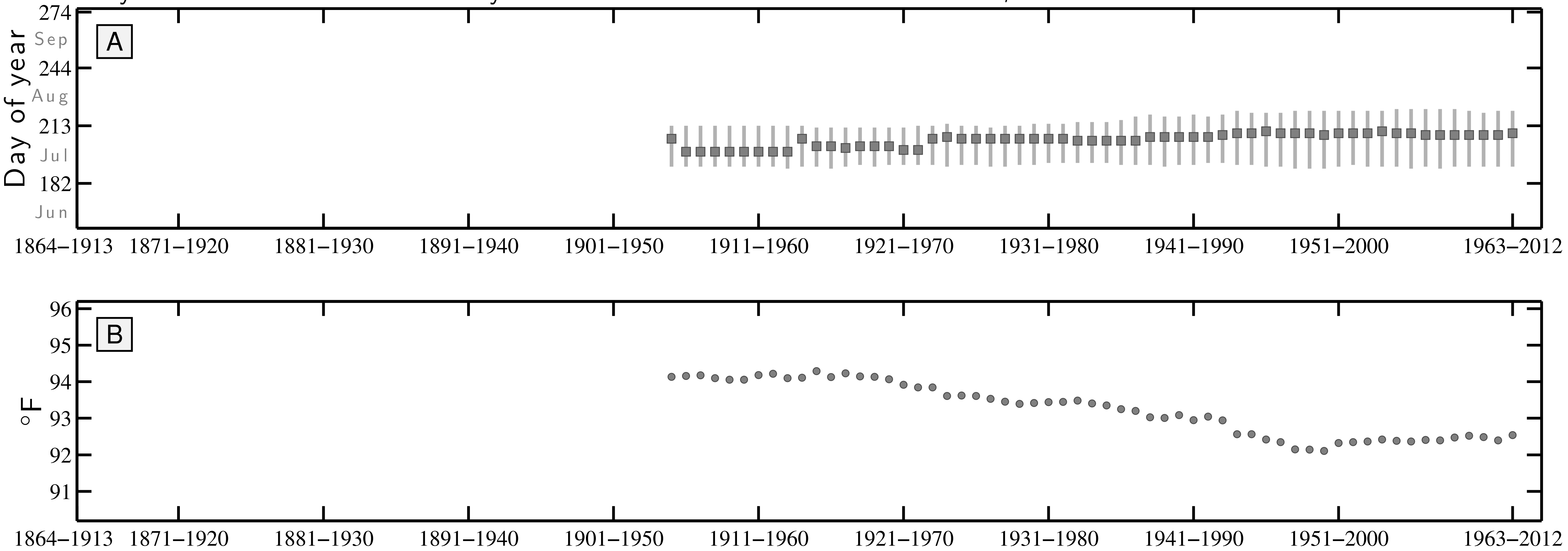

50 year Winter Teletherm dynamics for WENDOVER AP AWOS, UT:

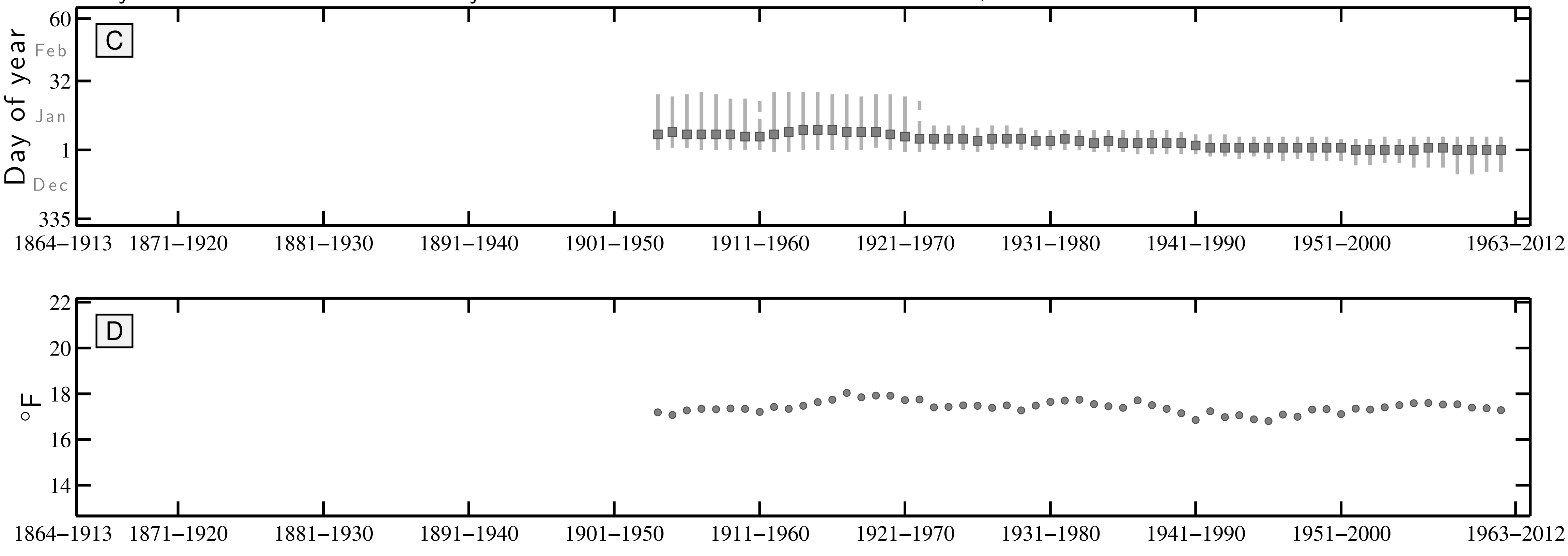

50 year Summer Teletherm dynamics for WOODRUFF, UT:

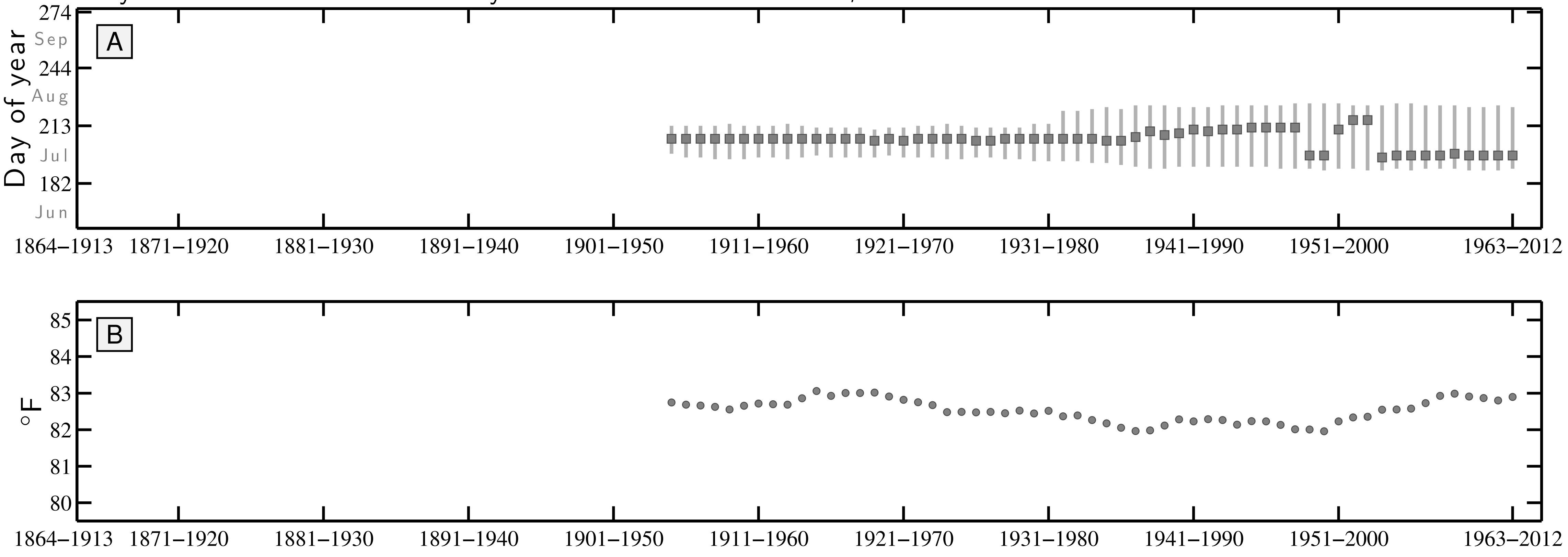

50 year Winter Teletherm dynamics for WOODRUFF, UT:

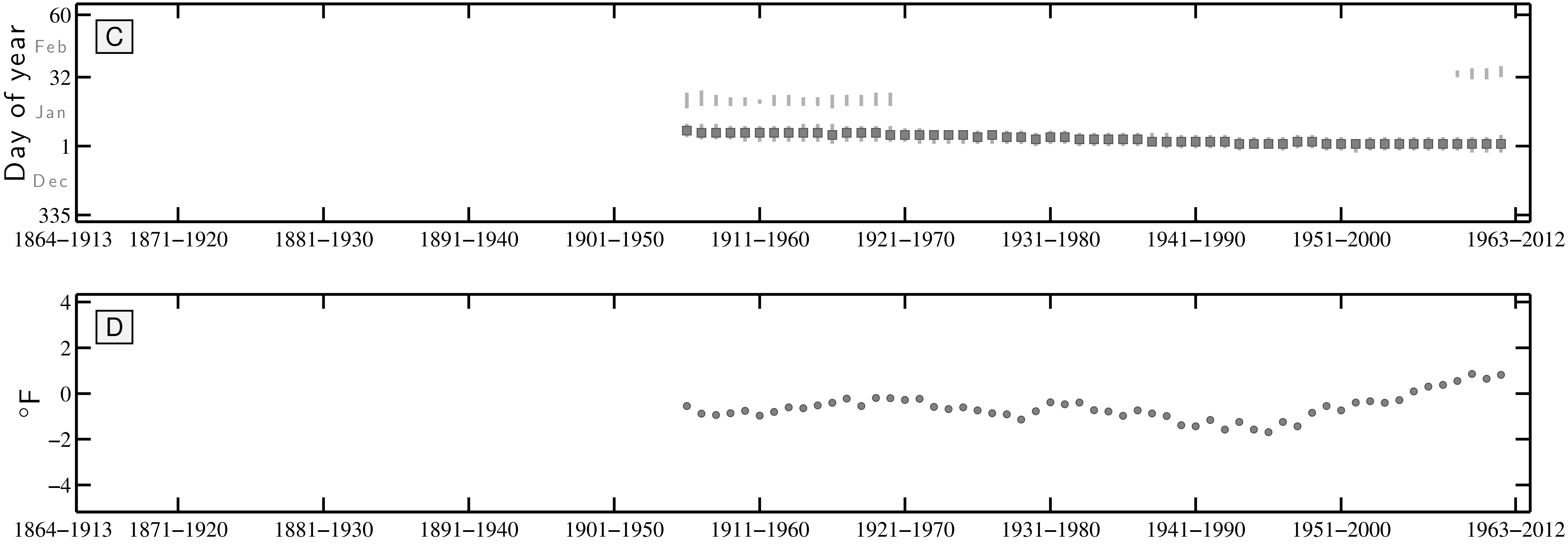

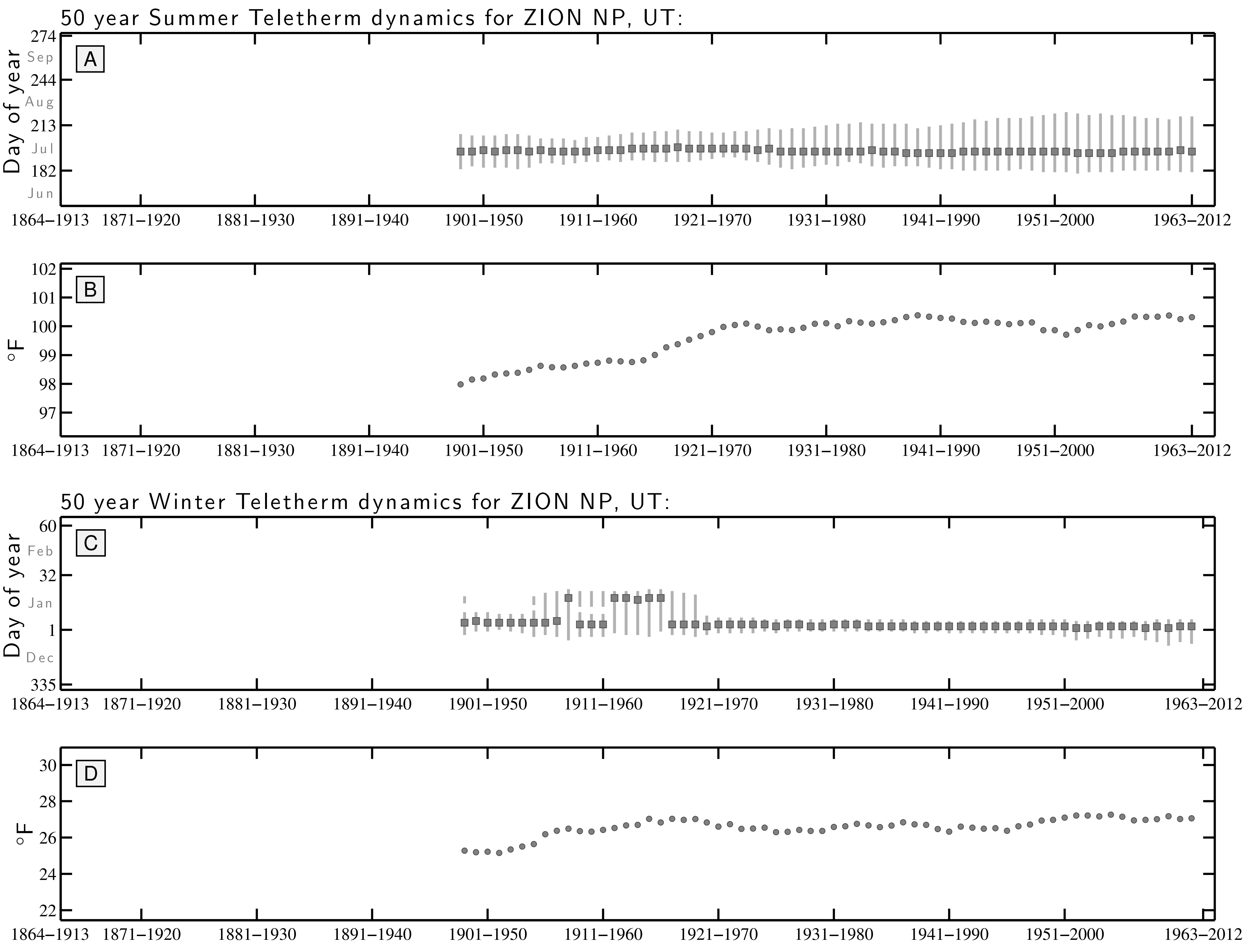
50 year Summer Teletherm dynamics for ZION NP, UT:
A
Day of year
274
Sep
244
Aug
213
Jul
182
Jun
1864–1913
1871–1920
1881–1930
1891–1940
1901–1950
1911–1960
1921–1970
1931–1980
1941–1990
1951–2000
1963–2012
B
°F
102
101
100
99
98
97
50 year Winter Teletherm dynamics for ZION NP, UT:
C
Day of year
60
Feb
32
Jan
1
Dec
335
D
°F
30
28
26
24
22

50 year Summer Teletherm dynamics for BURLINGTON WSO AP, VT:

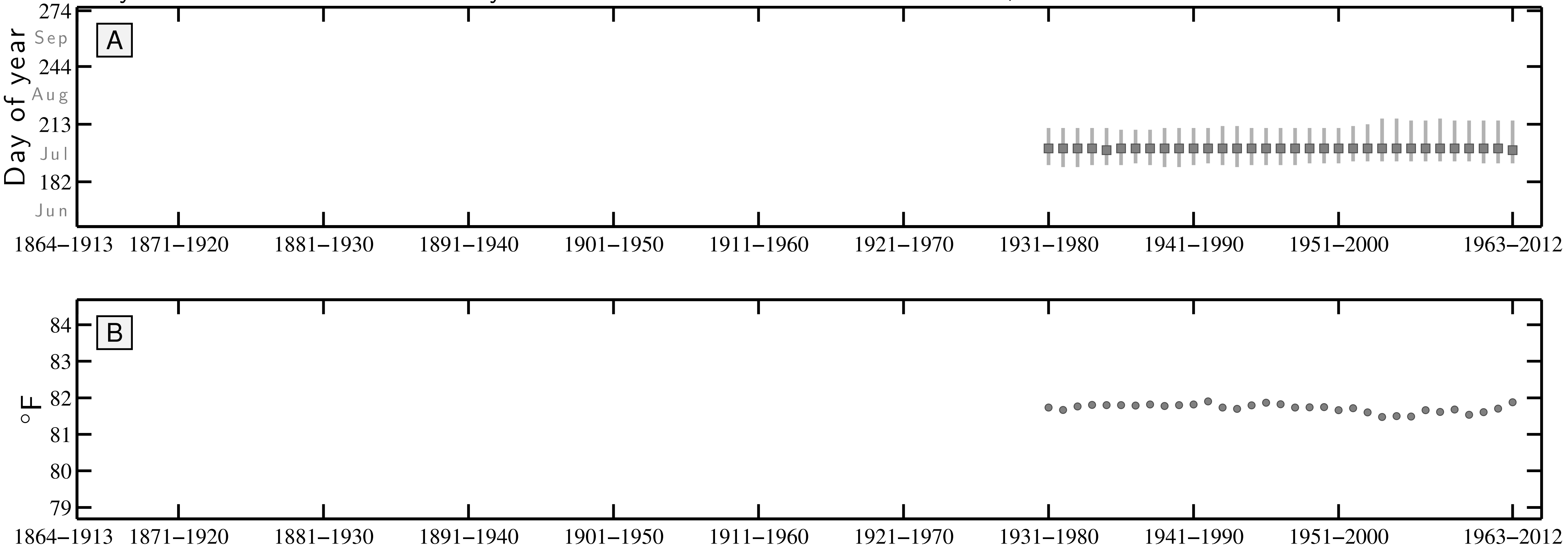

50 year Winter Teletherm dynamics for BURLINGTON WSO AP, VT:

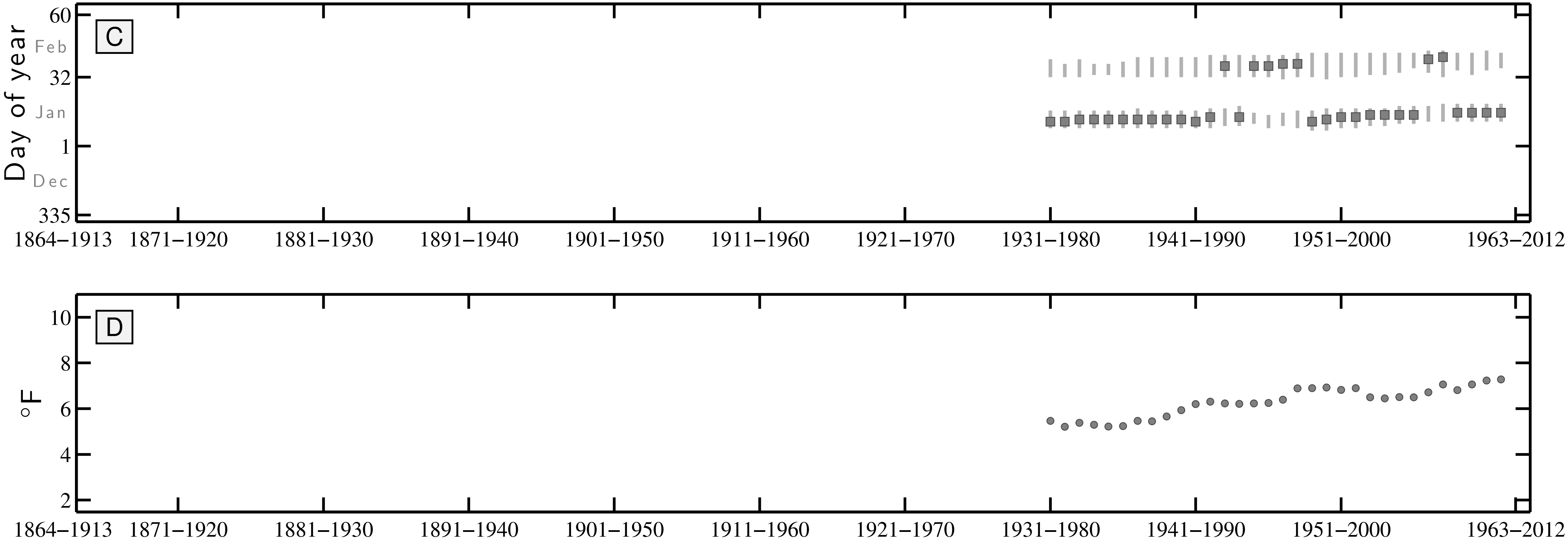

50 year Summer Teletherm dynamics for CAVENDISH, VT:

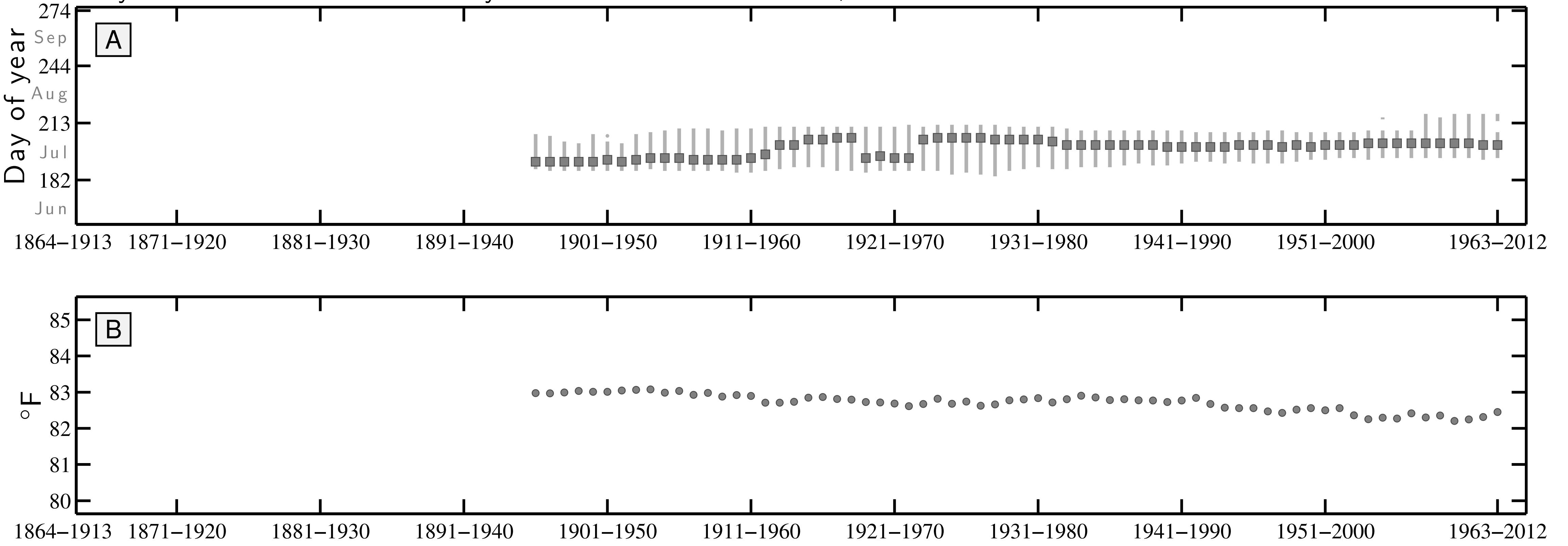

50 year Winter Teletherm dynamics for CAVENDISH, VT:

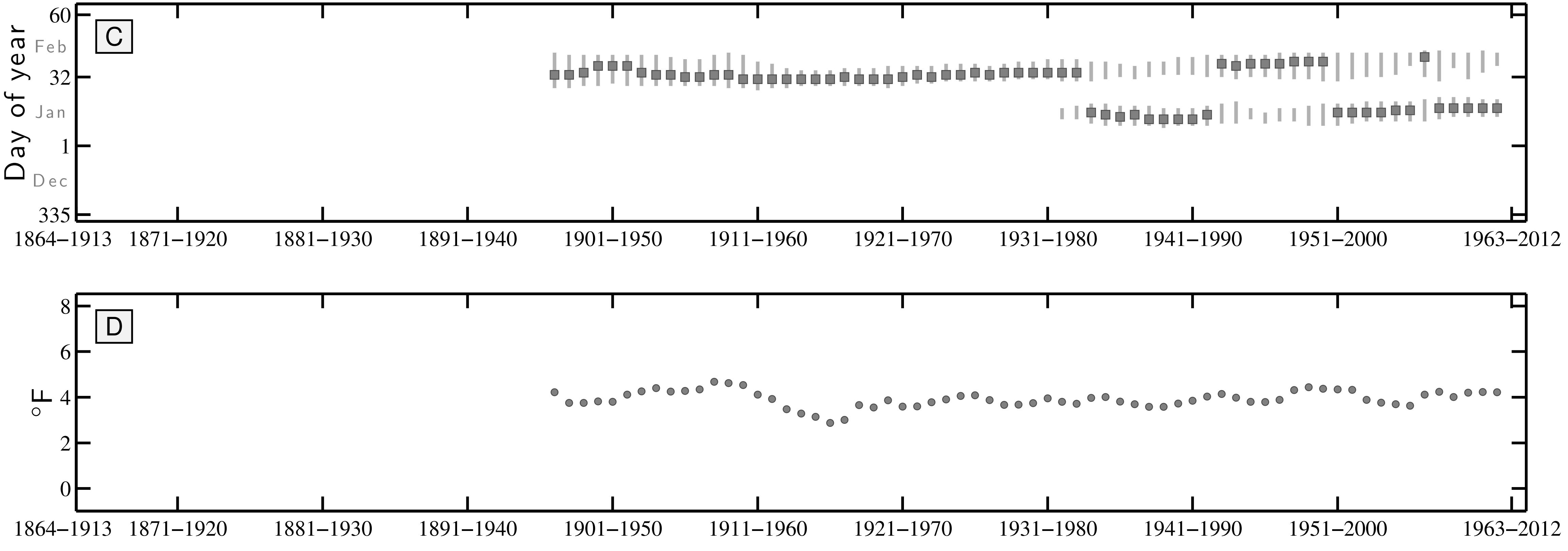

50 year Summer Teletherm dynamics for CHELSEA, VT:

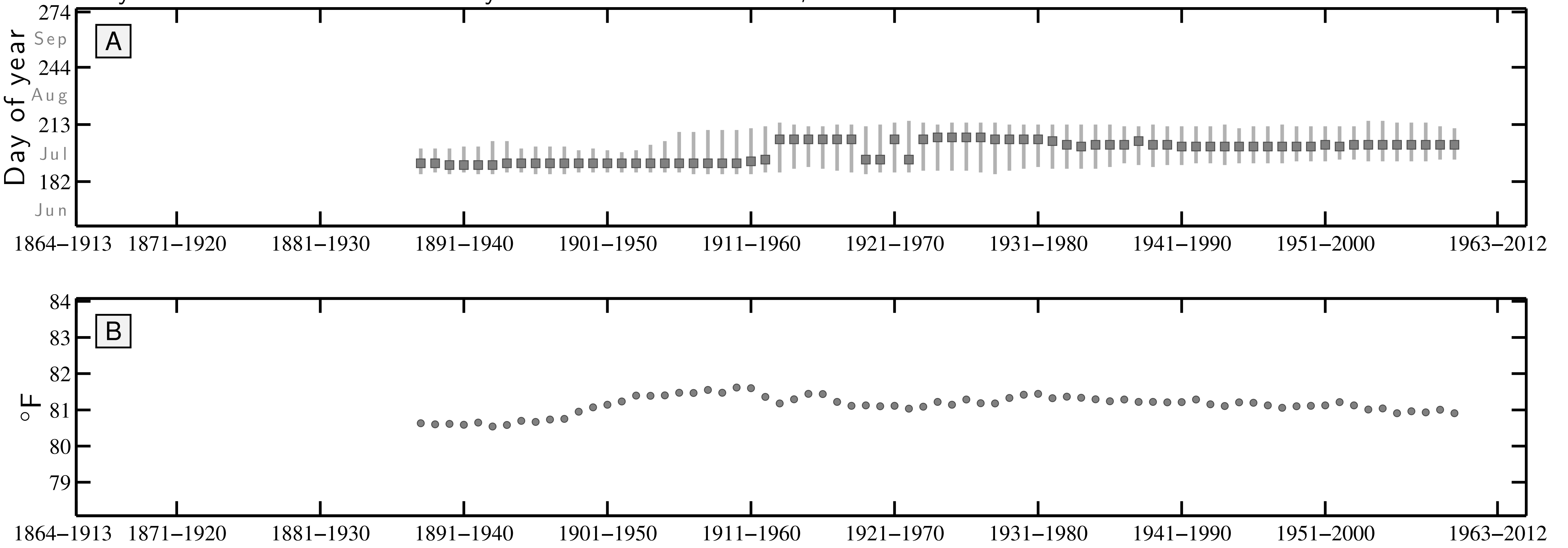

50 year Winter Teletherm dynamics for CHELSEA, VT:

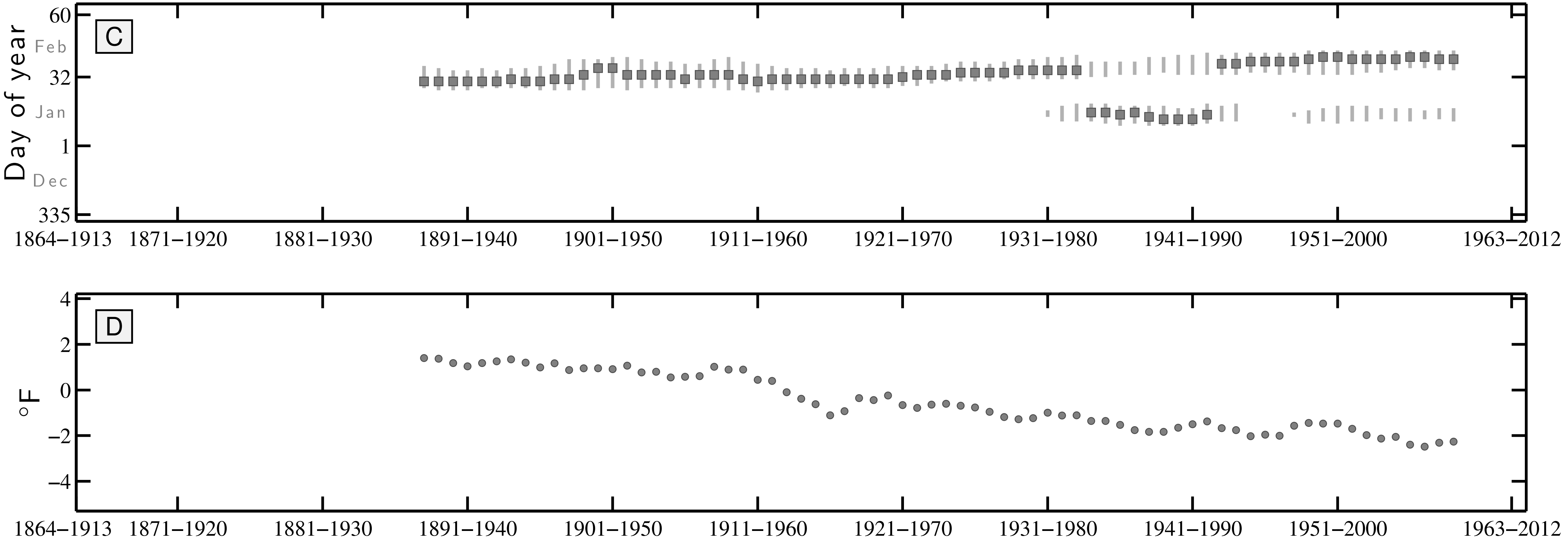

50 year Summer Teletherm dynamics for CORNWALL, VT:

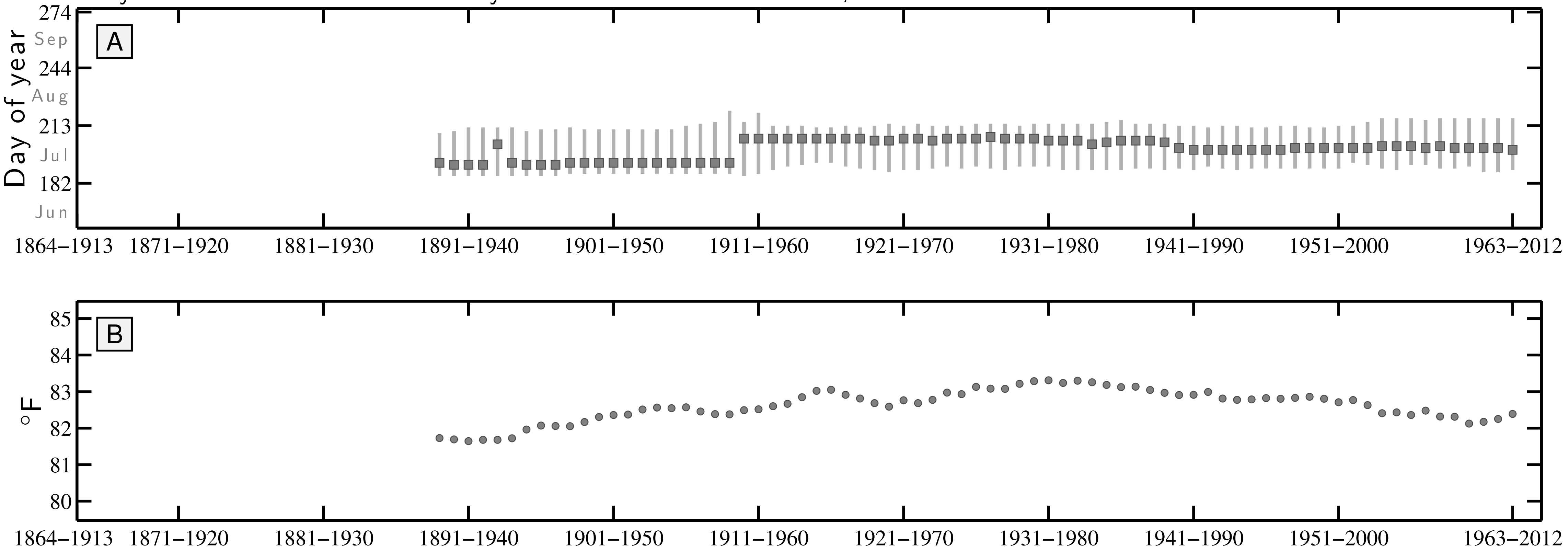

50 year Winter Teletherm dynamics for CORNWALL, VT:

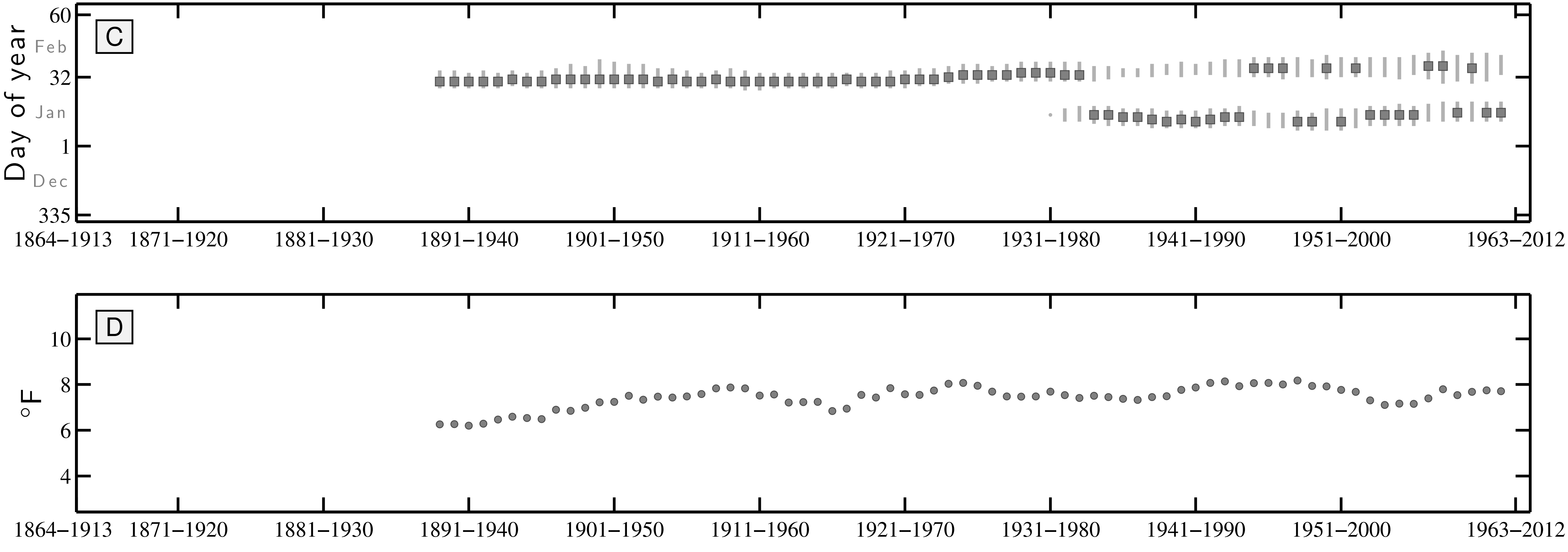

50 year Summer Teletherm dynamics for ENOSBURG FALLS, VT:

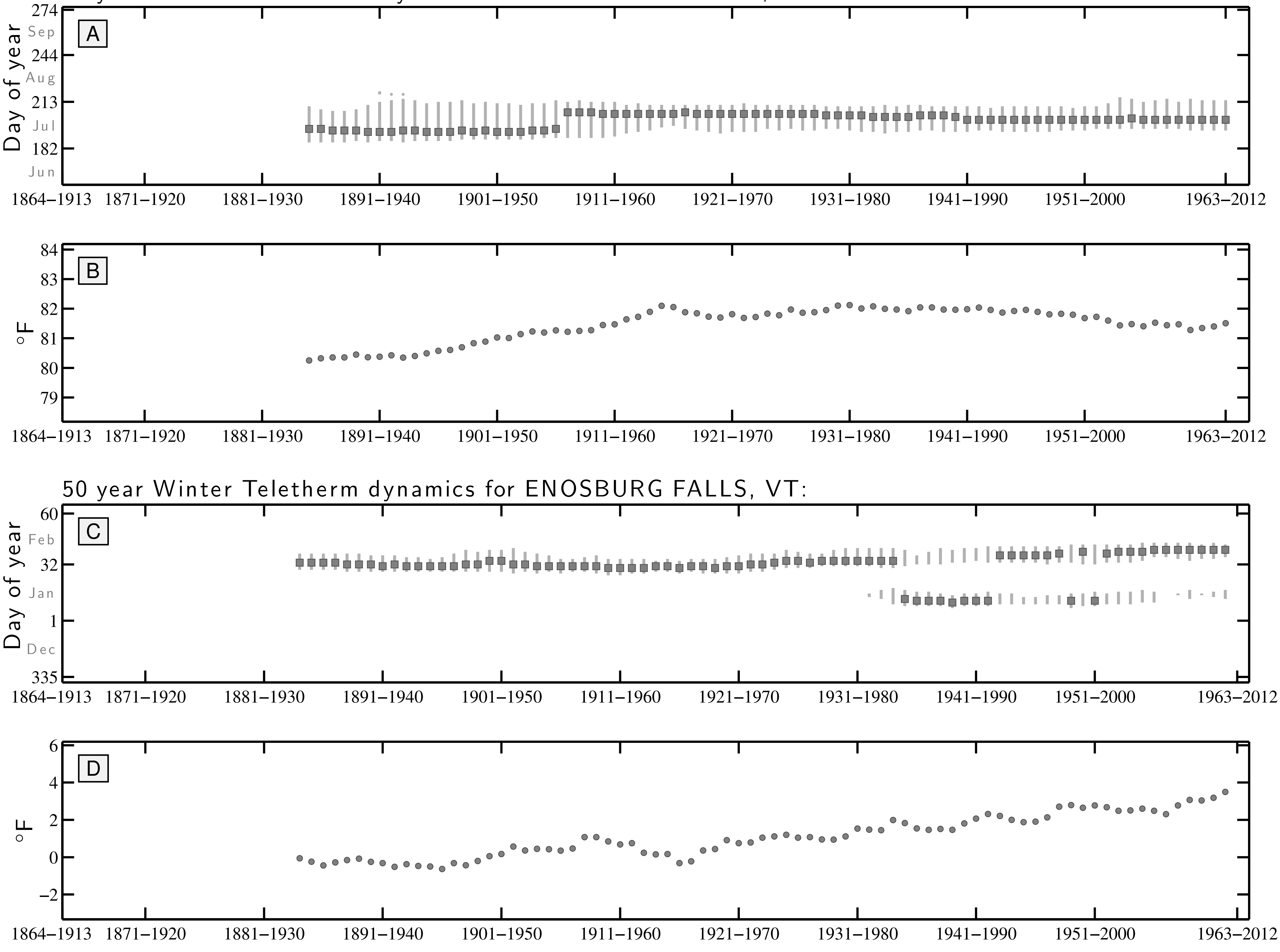

50 year Winter Teletherm dynamics for ENOSBURG FALLS, VT:

50 year Summer Teletherm dynamics for SAINT JOHNSBURY, VT:

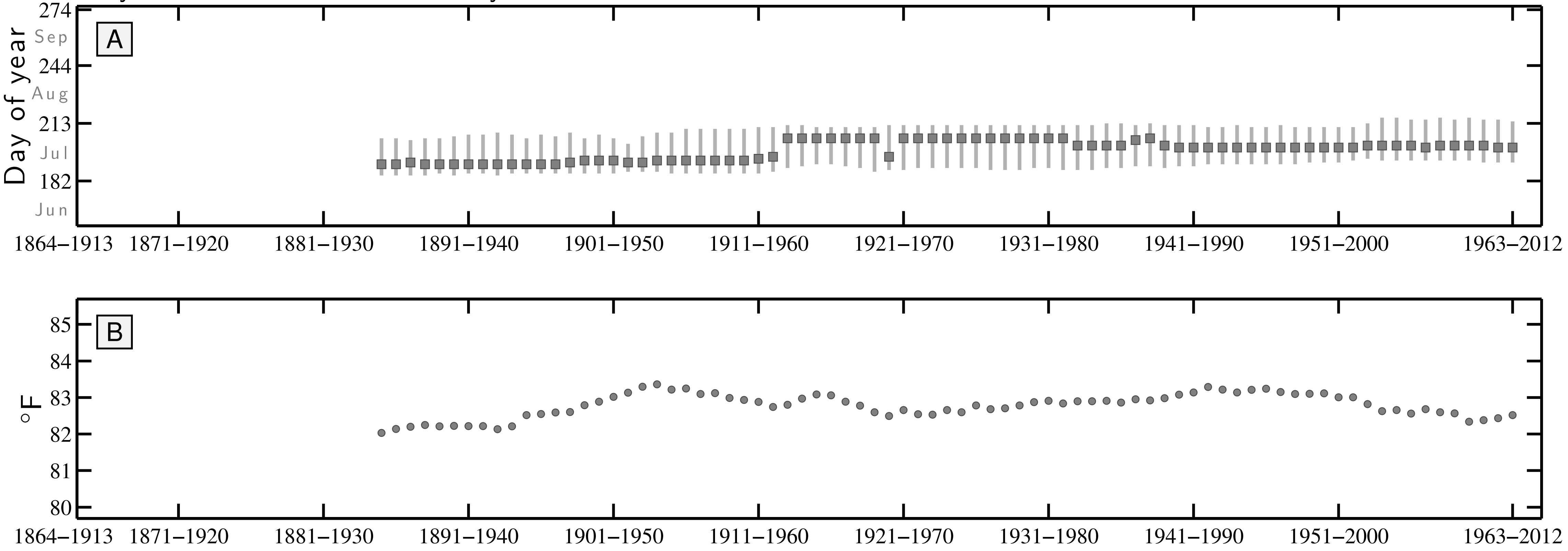

50 year Winter Teletherm dynamics for SAINT JOHNSBURY, VT:

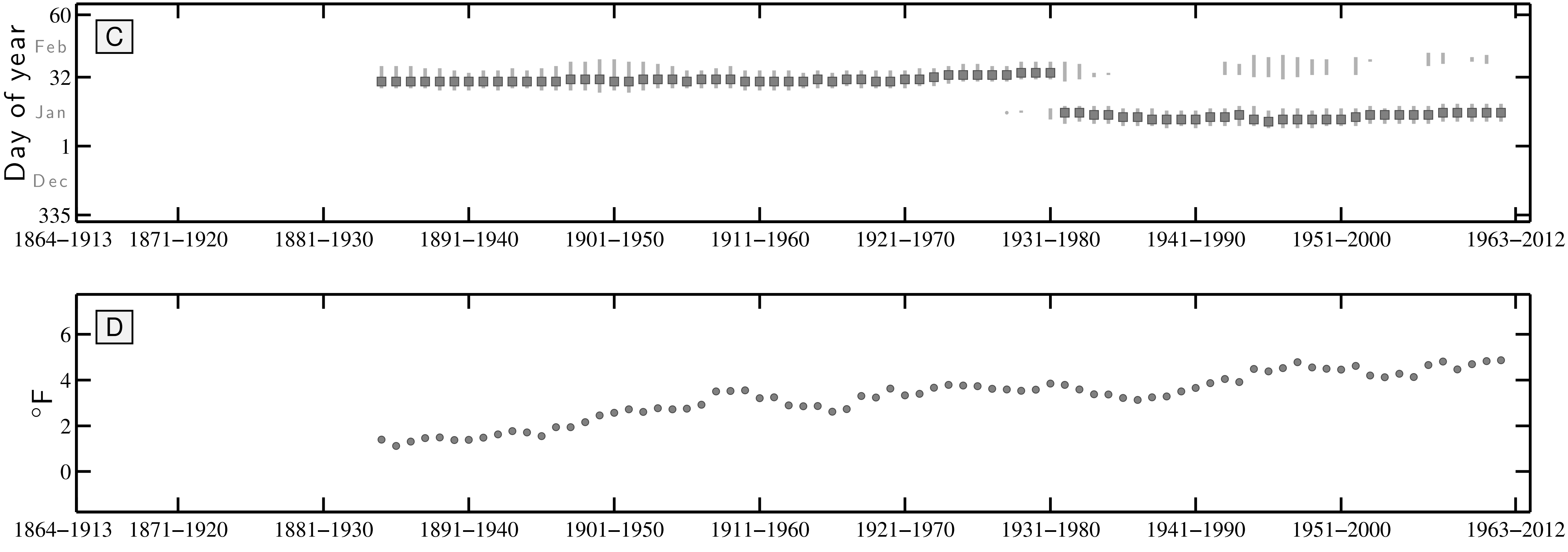

50 year Summer Teletherm dynamics for SOUTH HERO, VT:

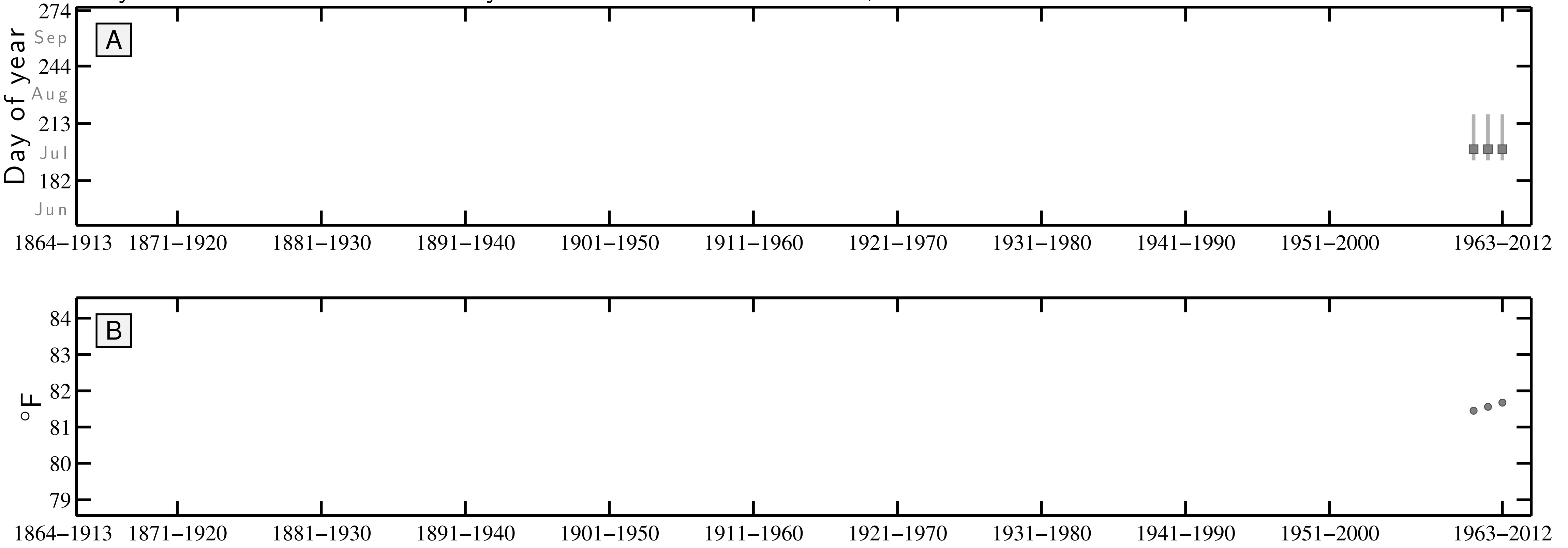

50 year Winter Teletherm dynamics for SOUTH HERO, VT:

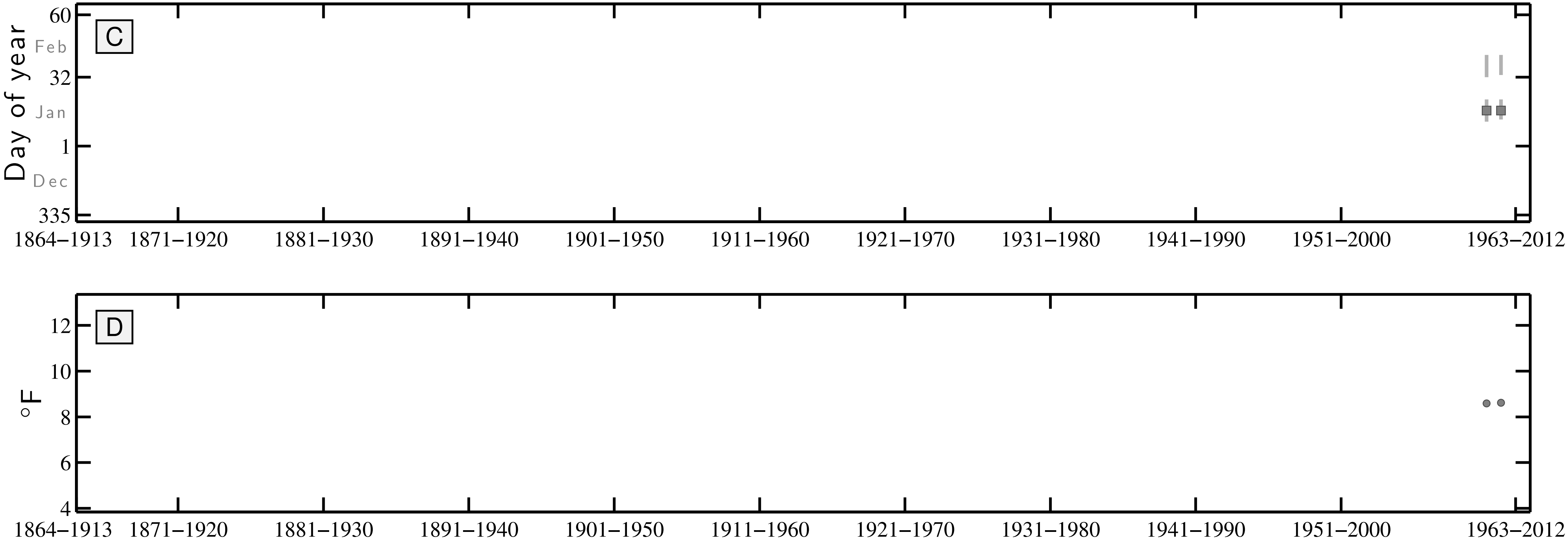

50 year Summer Teletherm dynamics for BLACKSBURG NWSO, VA:

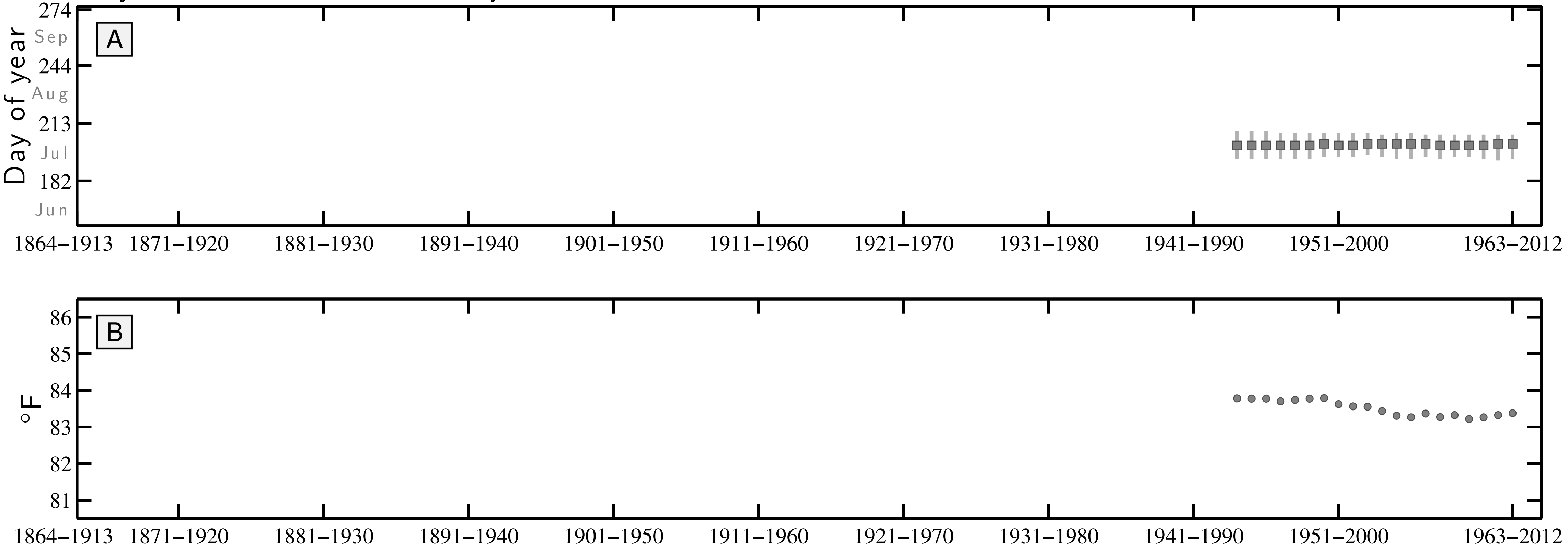

50 year Winter Teletherm dynamics for BLACKSBURG NWSO, VA:

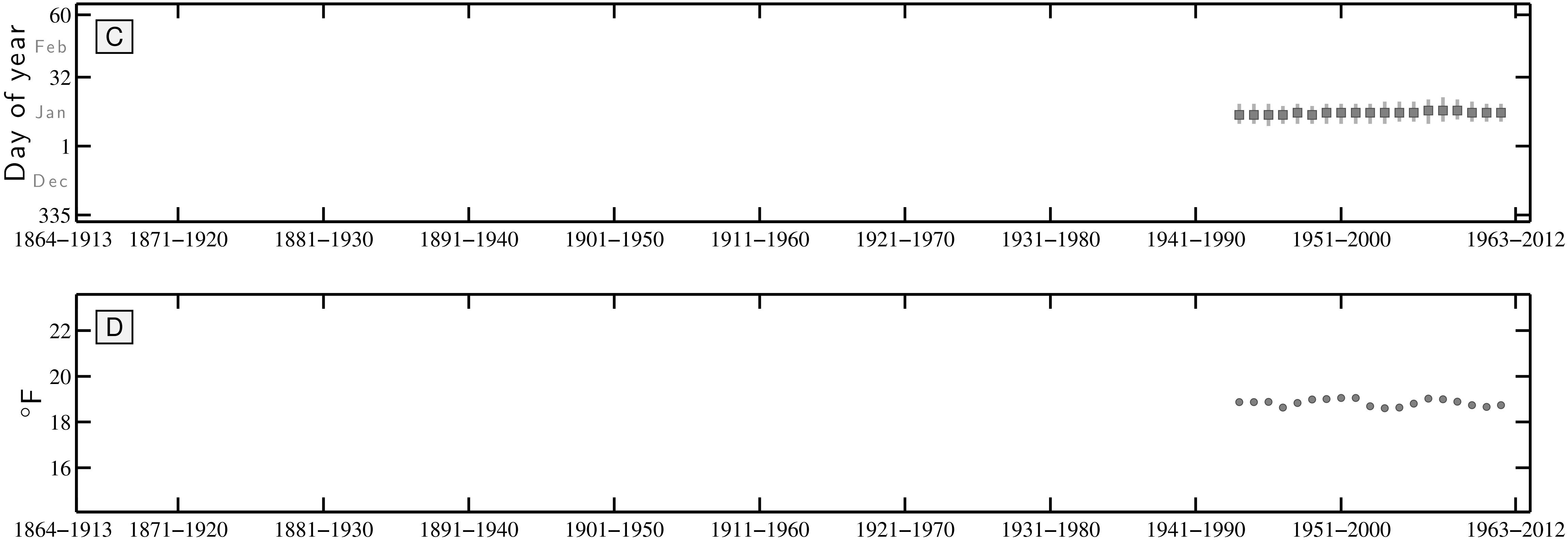

50 year Summer Teletherm dynamics for BURKES GARDEN, VA:

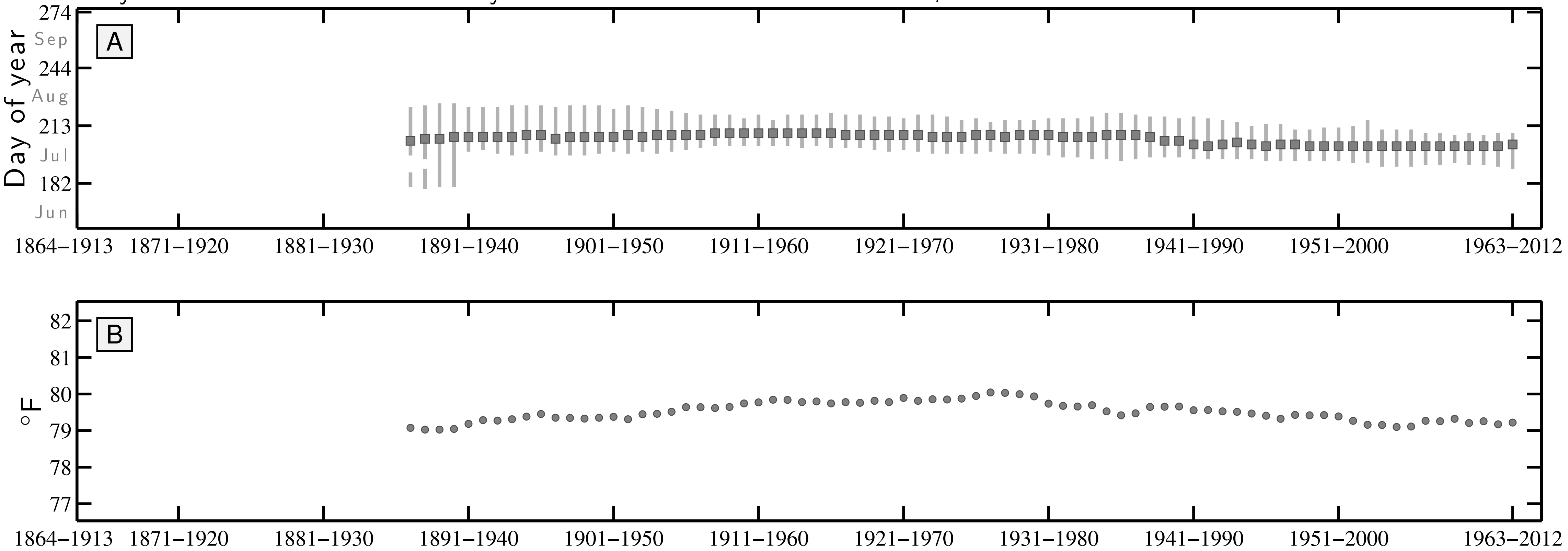

50 year Winter Teletherm dynamics for BURKES GARDEN, VA:

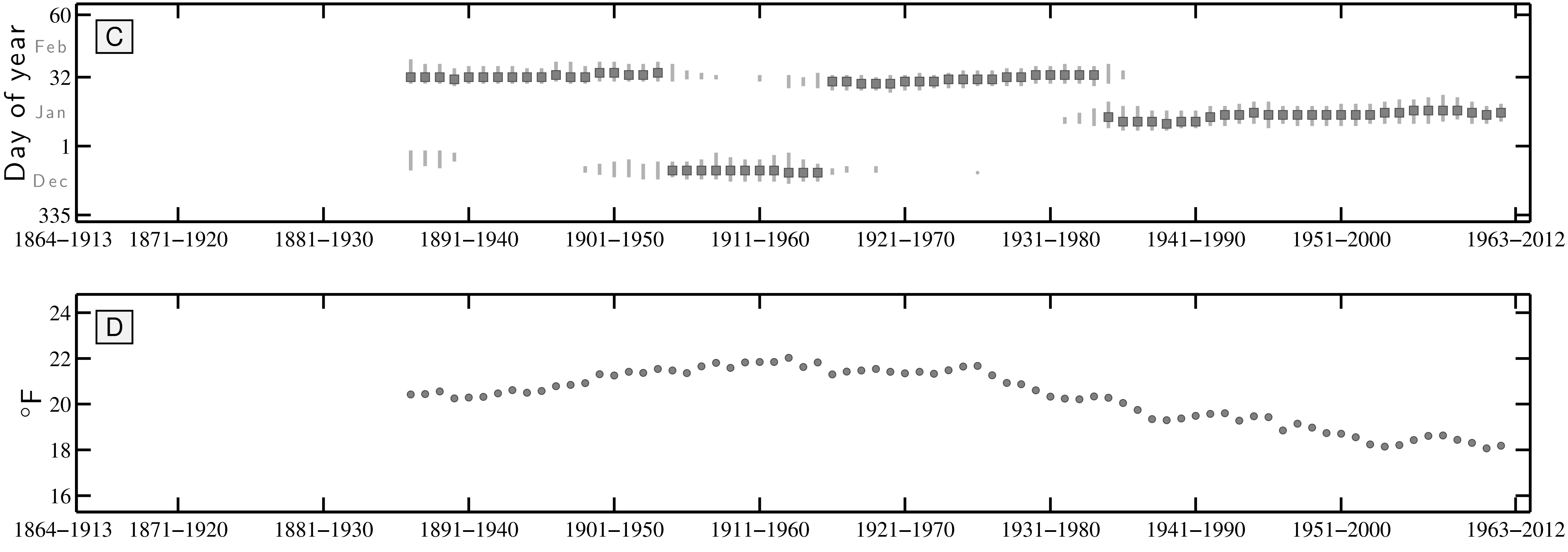

50 year Summer Teletherm dynamics for CHARLOTTESVILLE 2W, VA:

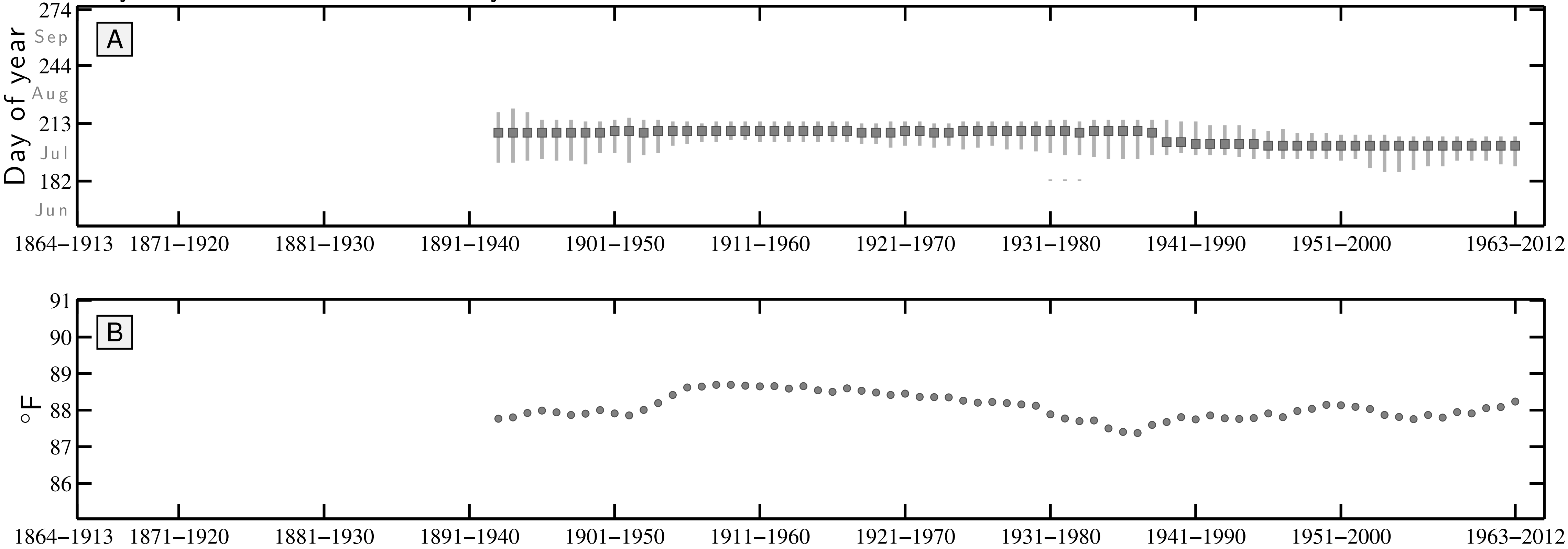

50 year Winter Teletherm dynamics for CHARLOTTESVILLE 2W, VA:

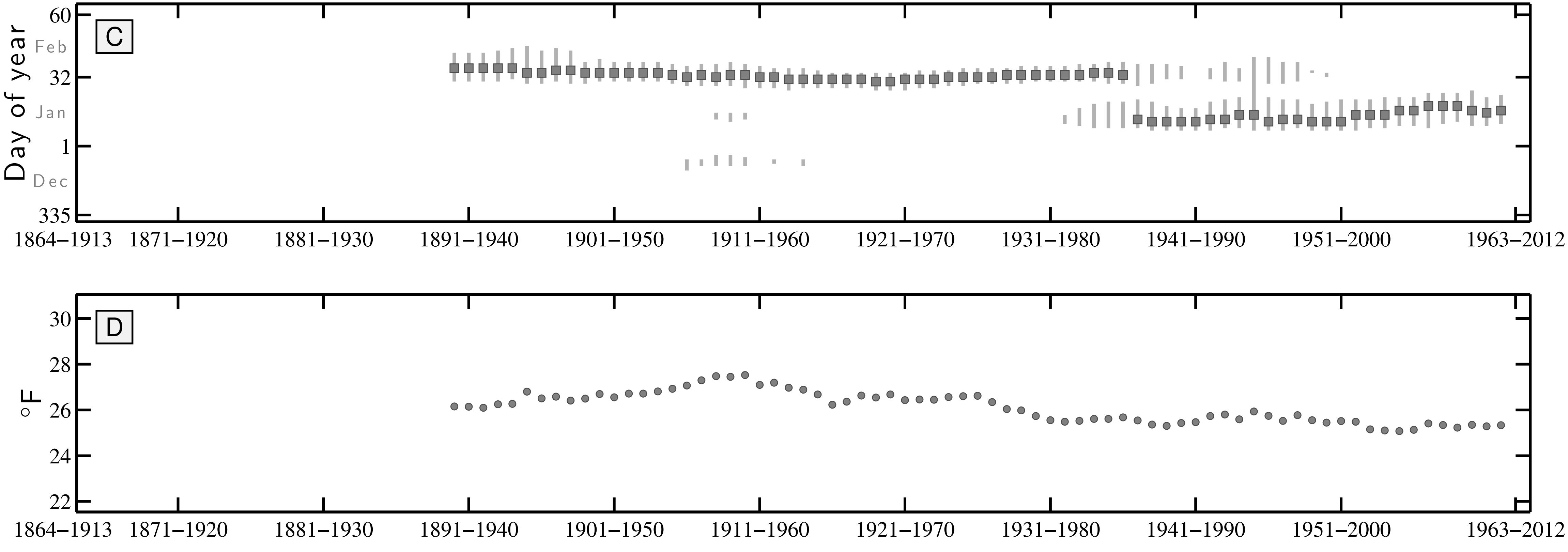

50 year Summer Teletherm dynamics for DALE ENTERPRISE, VA:

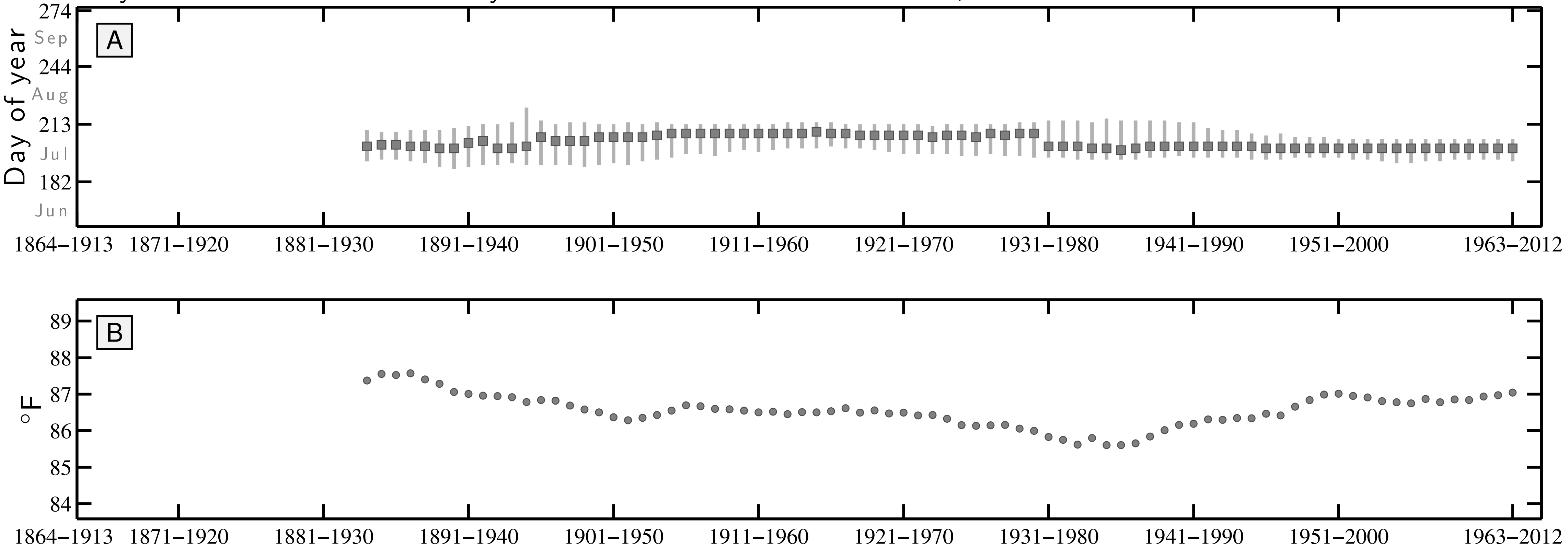

50 year Winter Teletherm dynamics for DALE ENTERPRISE, VA:

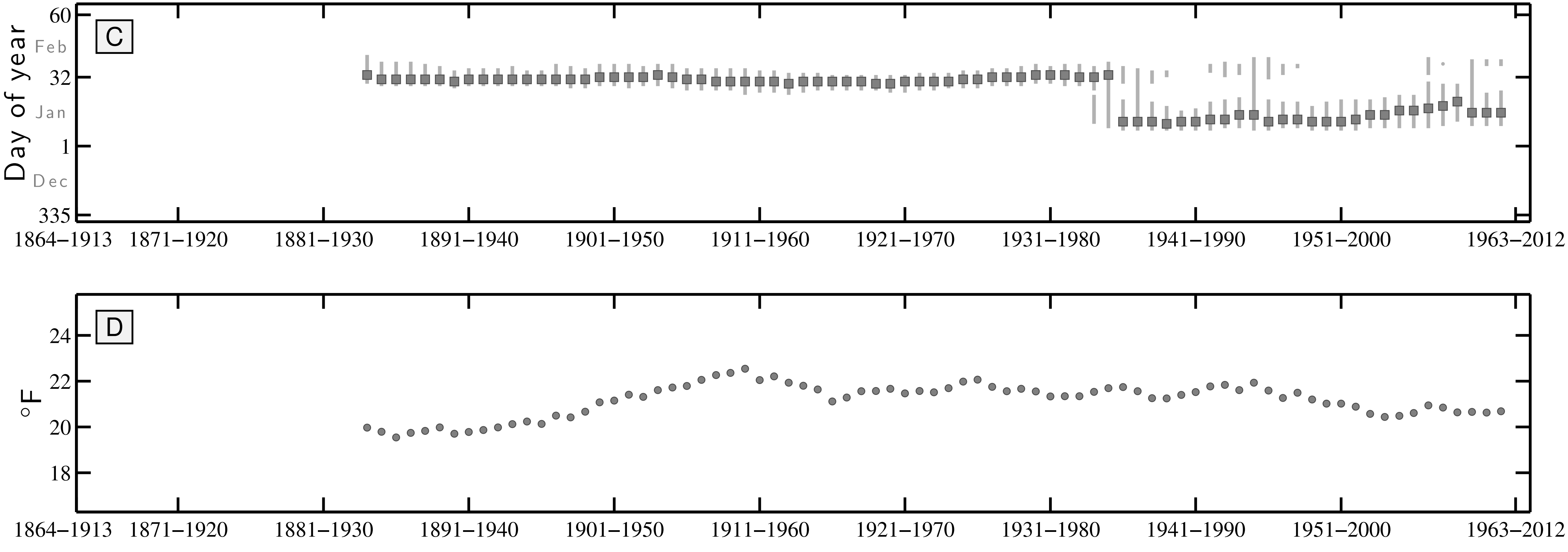

50 year Summer Teletherm dynamics for DANVILLE, VA:

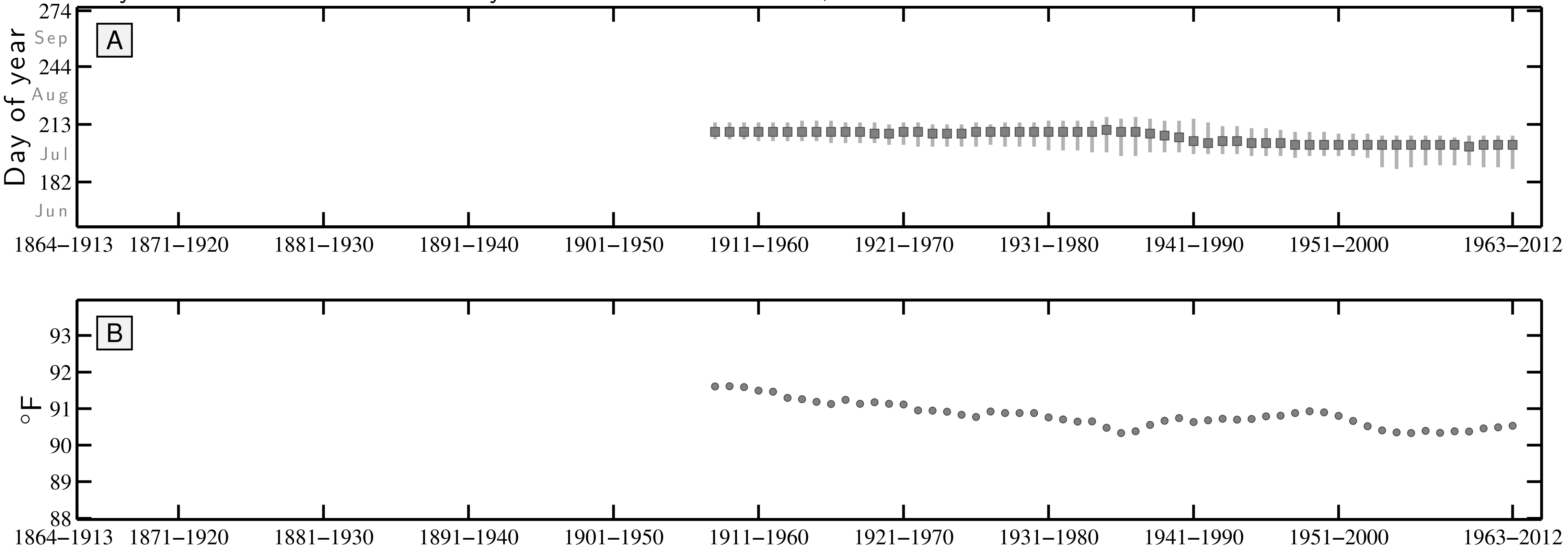

50 year Winter Teletherm dynamics for DANVILLE, VA:

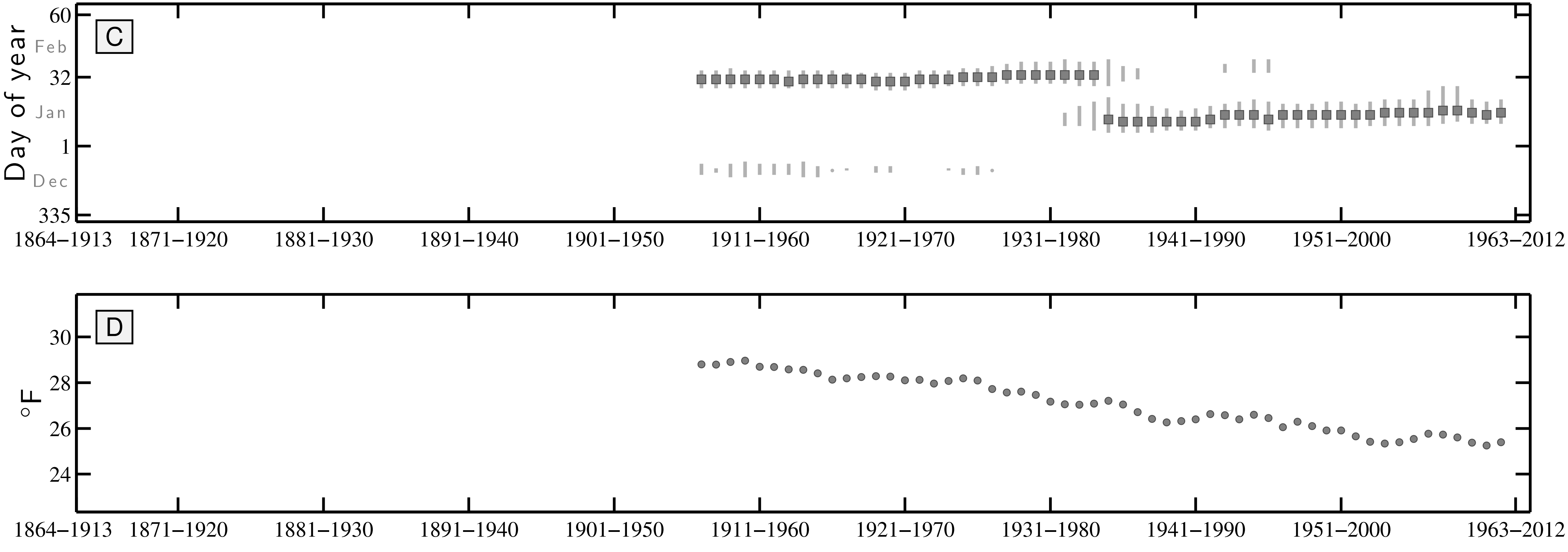

50 year Summer Teletherm dynamics for FARMVILLE 2 N, VA:

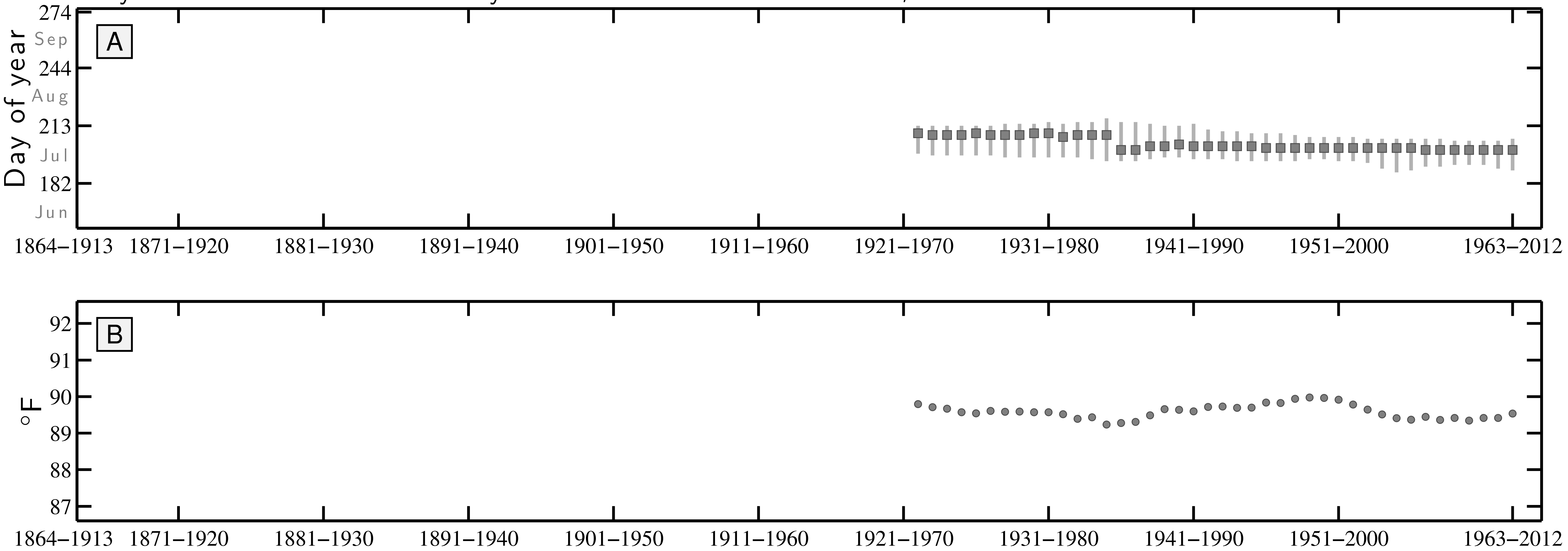

50 year Winter Teletherm dynamics for FARMVILLE 2 N, VA:

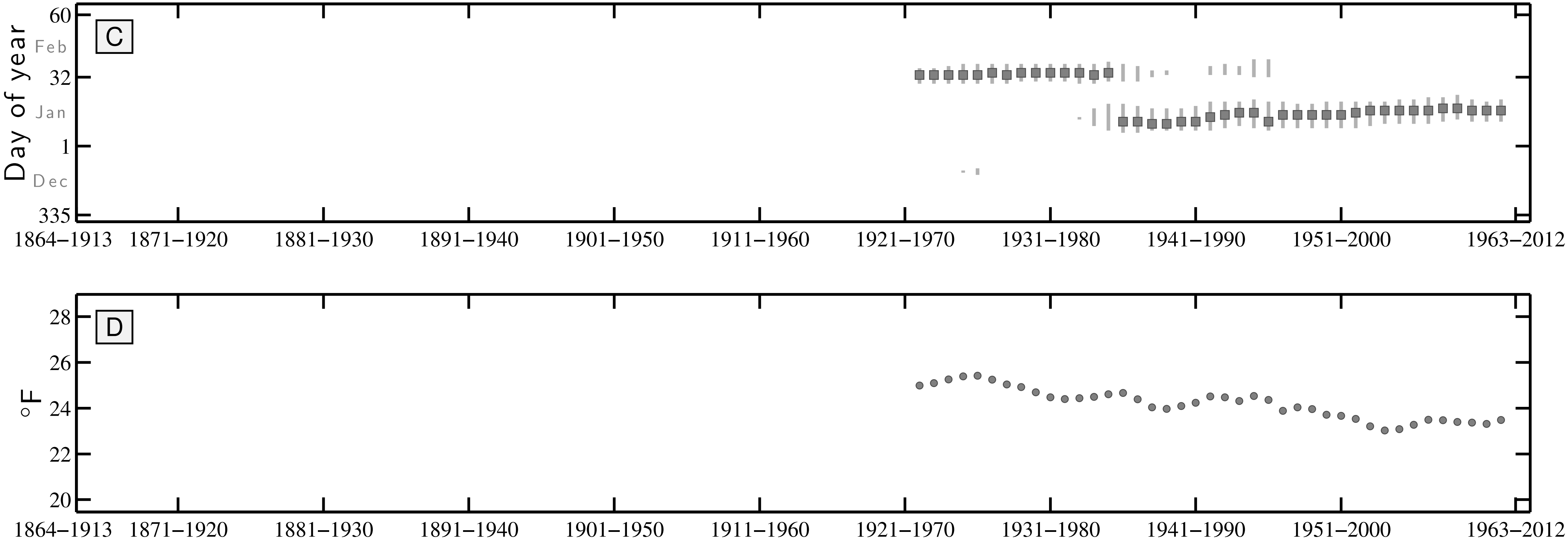

50 year Summer Teletherm dynamics for FREDERICKSBURG NP, VA:

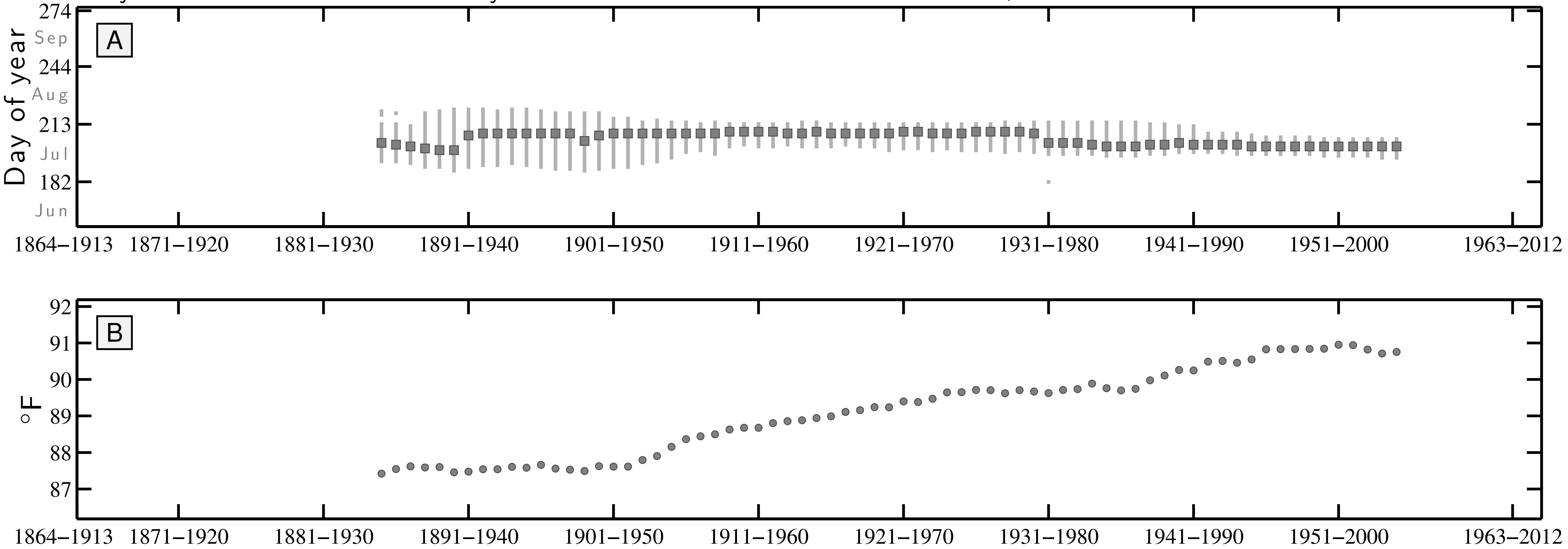

50 year Winter Teletherm dynamics for FREDERICKSBURG NP, VA:

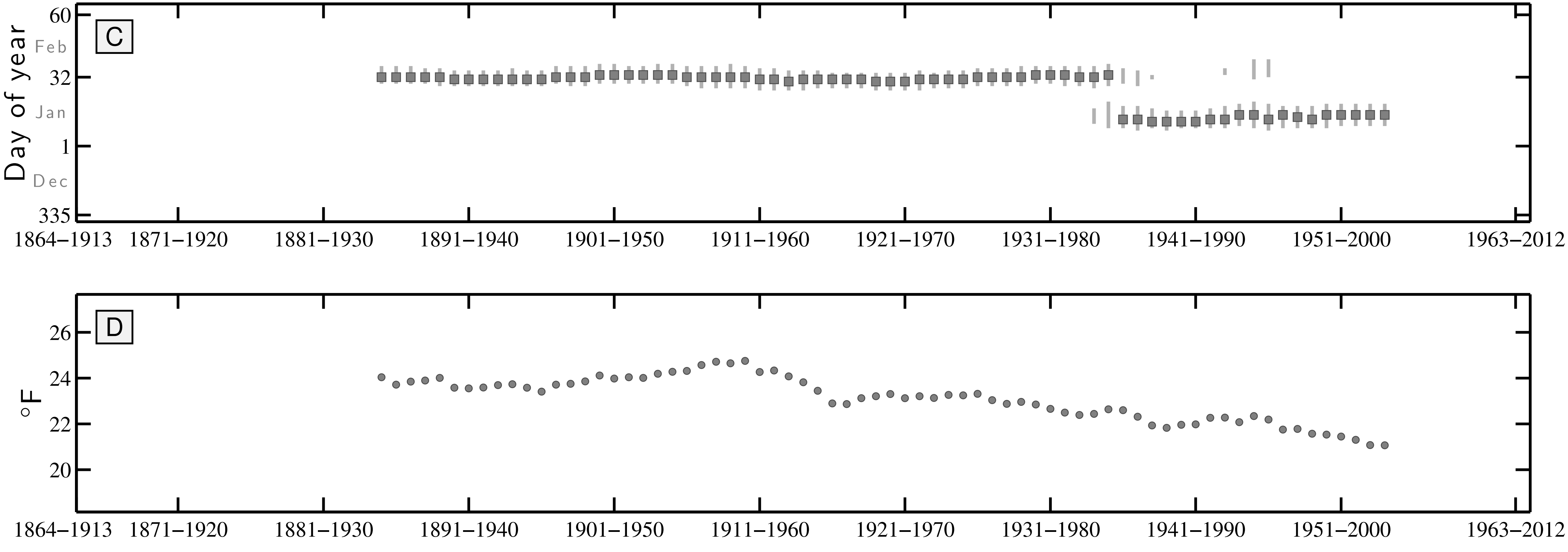

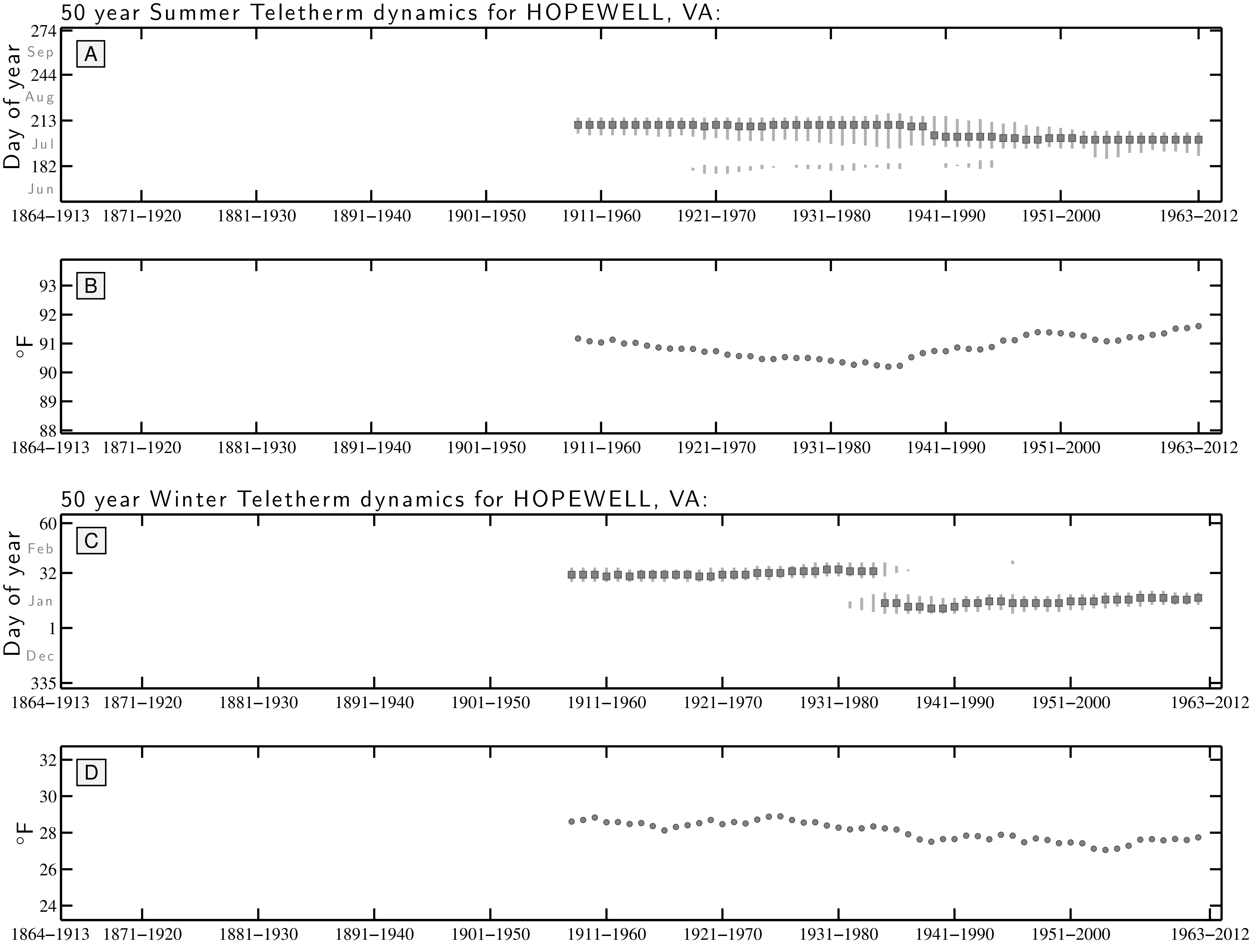

50 year Summer Teletherm dynamics for HOPEWELL, VA:
A
Day of year
274
Sep
244
Aug
213
Jul
182
Jun
1864–1913
1871–1920
1881–1930
1891–1940
1901–1950
1911–1960
1921–1970
1931–1980
1941–1990
1951–2000
1963–2012
B
°F
93
92
91
90
89
88
50 year Winter Teletherm dynamics for HOPEWELL, VA:
C
Day of year
60
Feb
32
Jan
1
Dec
335
D
°F
32
30
28
26
24

50 year Summer Teletherm dynamics for HOT SPRINGS, VA:

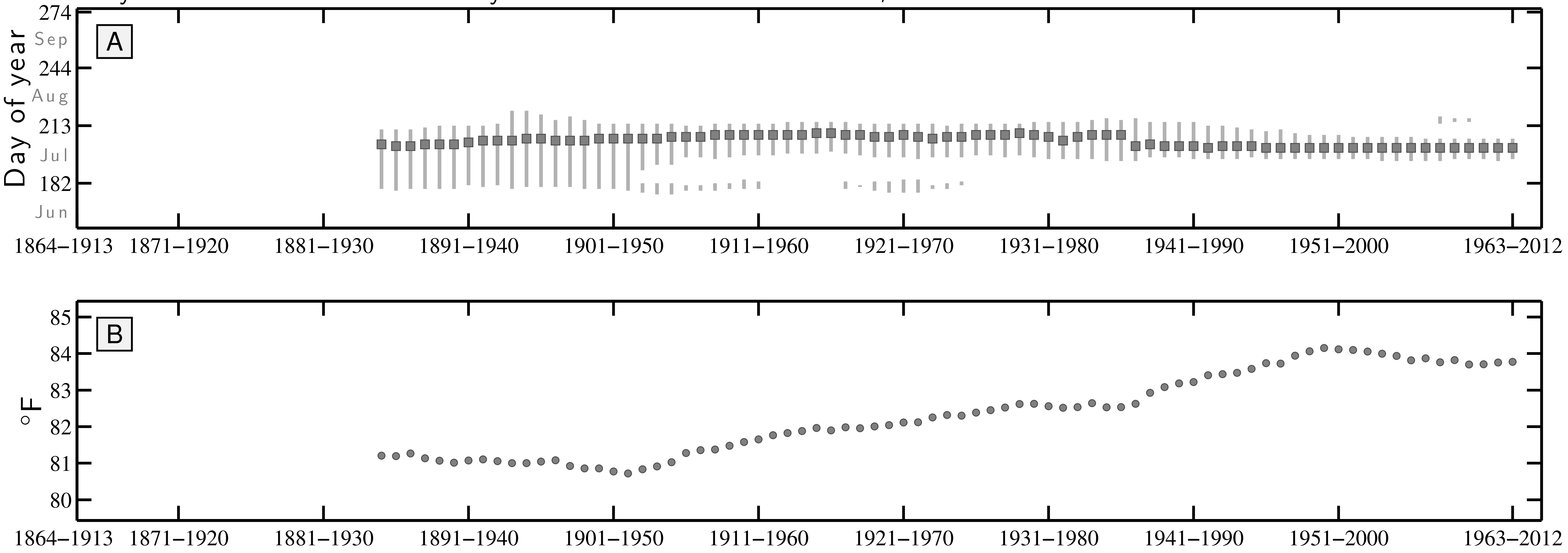

50 year Winter Teletherm dynamics for HOT SPRINGS, VA:

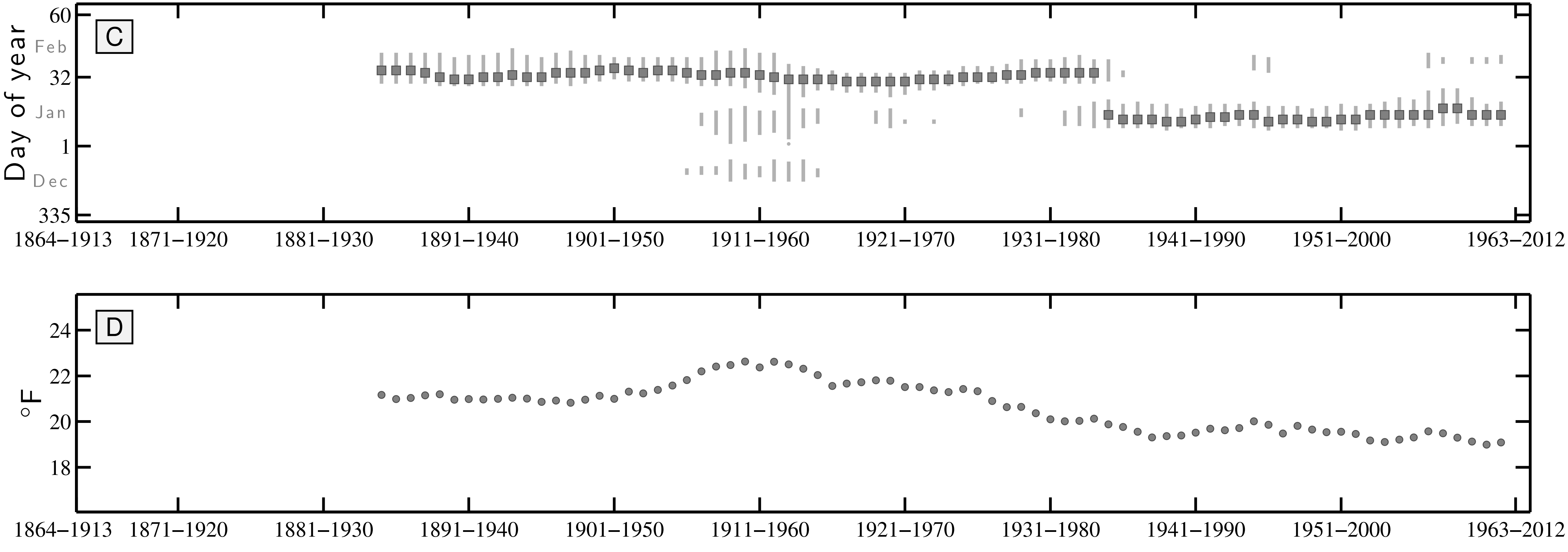

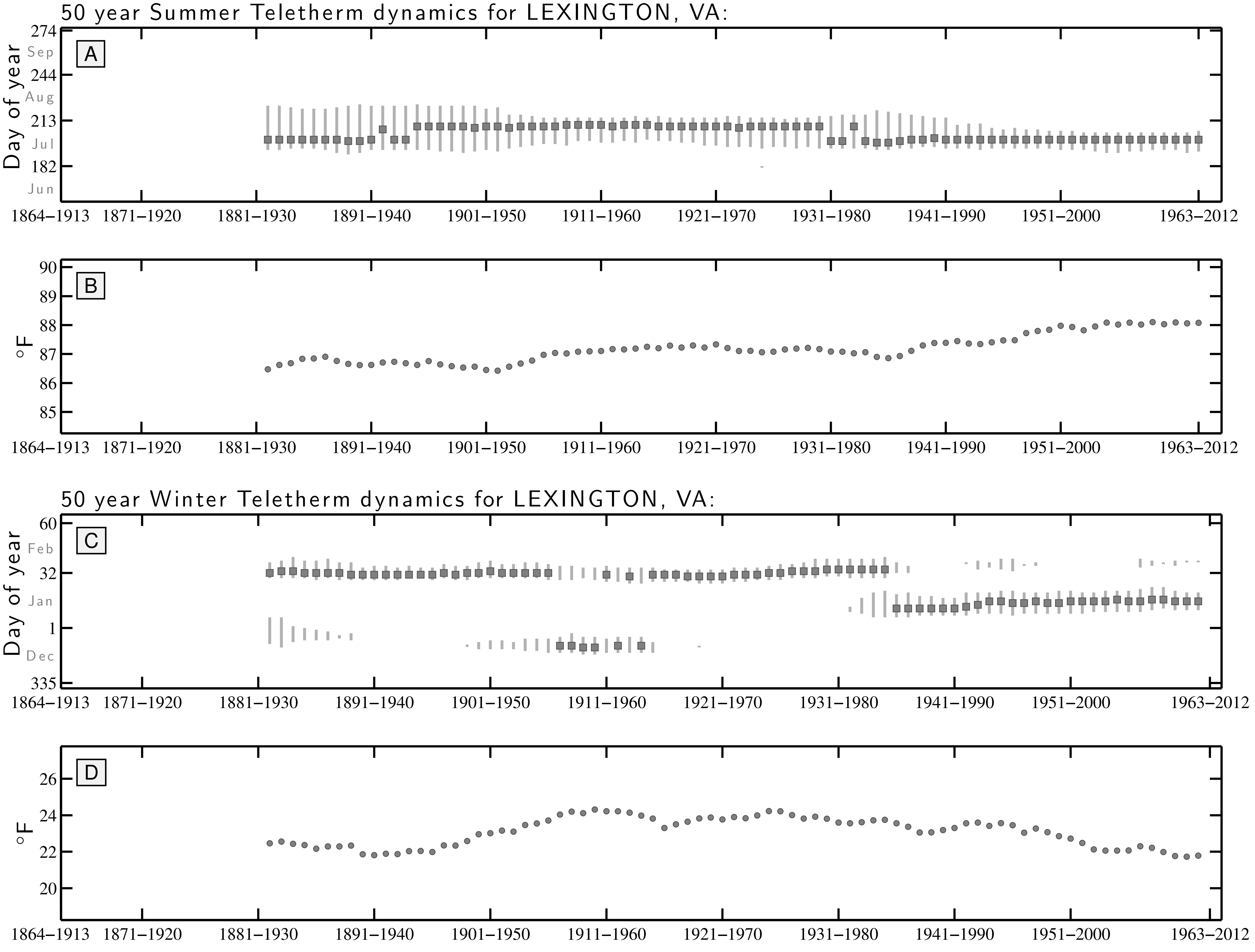

50 year Summer Teletherm dynamics for LEXINGTON, VA:
A
Day of year
274
Sep
244
Aug
213
Jul
182
Jun
B
°F
90
89
88
87
86
85
50 year Winter Teletherm dynamics for LEXINGTON, VA:
C
60
Feb
32
Jan
1
Dec
335
D
26
24
22
20
1864–1913
1871–1920
1881–1930
1891–1940
1901–1950
1911–1960
1921–1970
1931–1980
1941–1990
1951–2000
1963–2012

50 year Summer Teletherm dynamics for LINCOLN, VA:

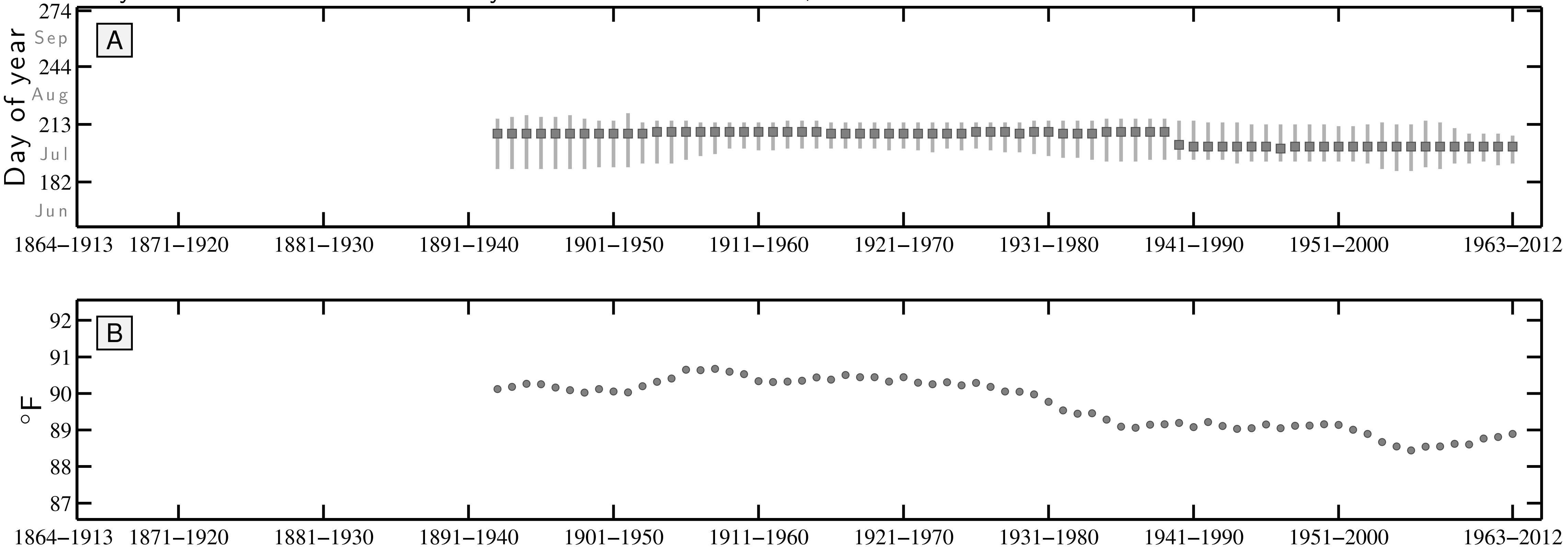

50 year Winter Teletherm dynamics for LINCOLN, VA:

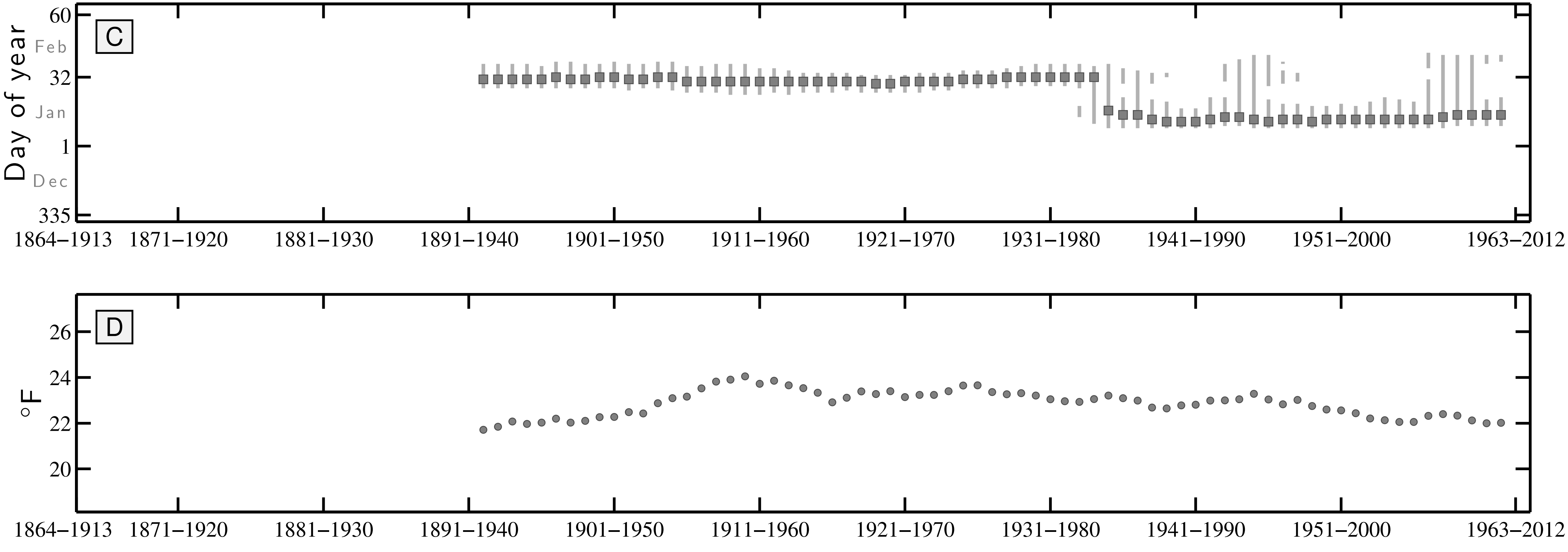

50 year Summer Teletherm dynamics for NORFOLK INTL AP, VA:

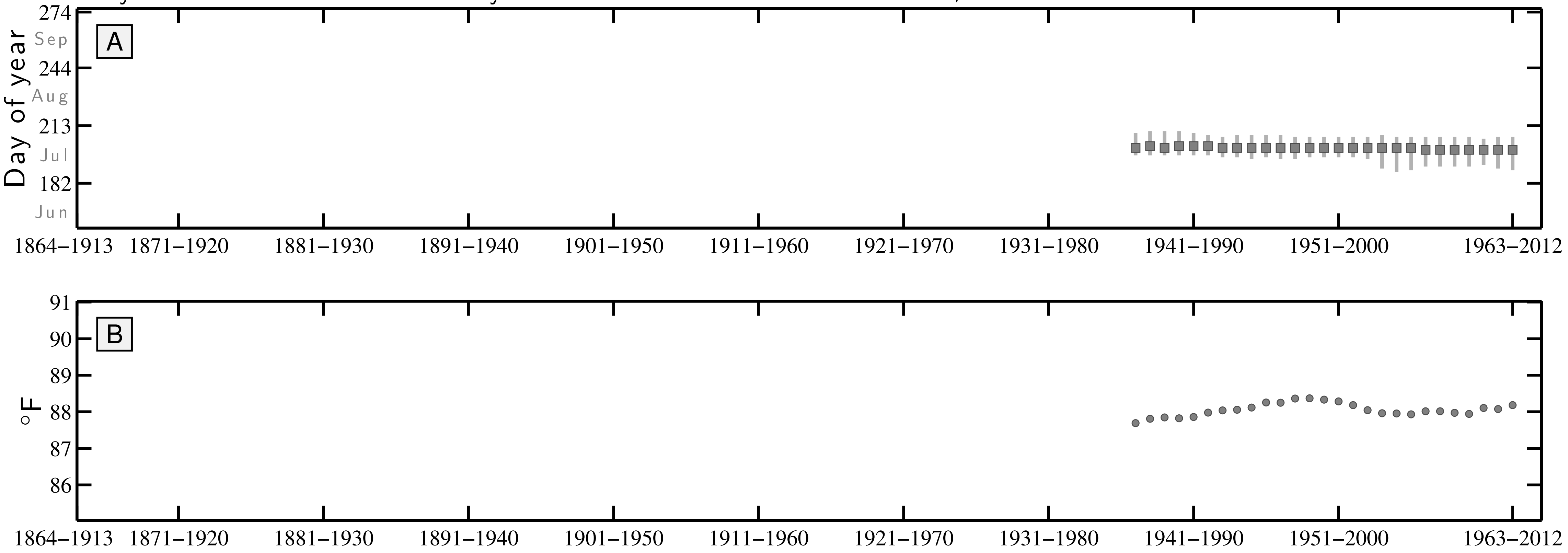

50 year Winter Teletherm dynamics for NORFOLK INTL AP, VA:

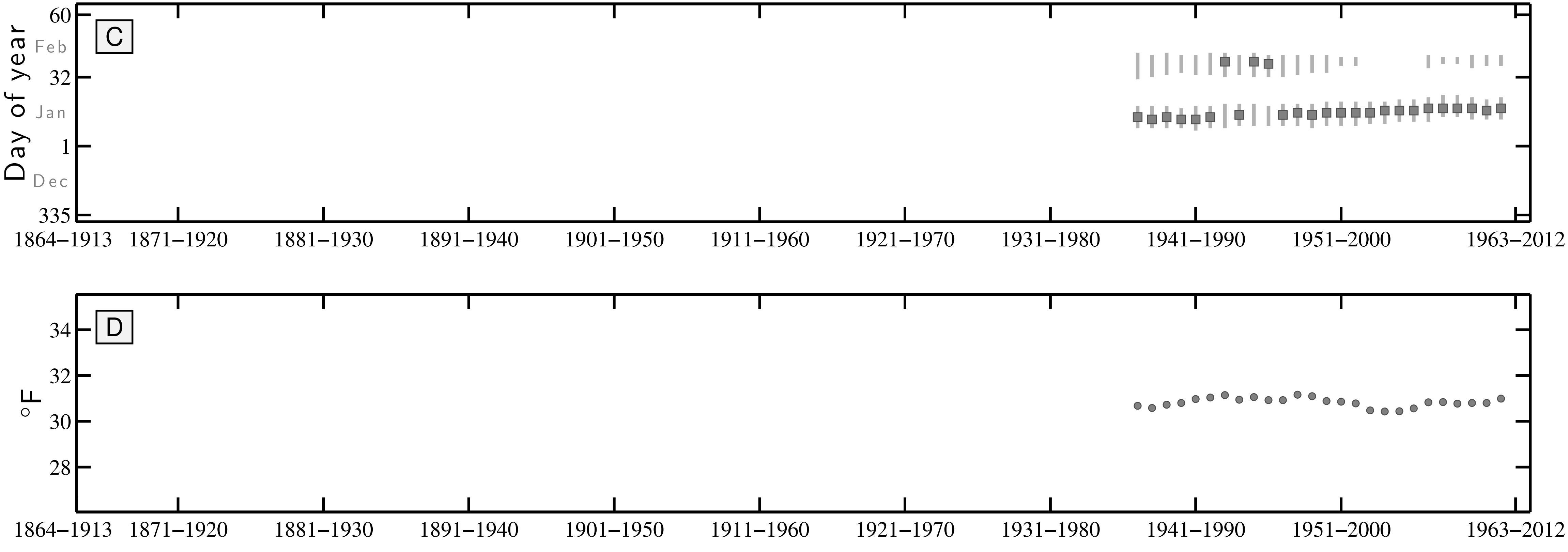

50 year Summer Teletherm dynamics for PENNINGTON GAP, VA:

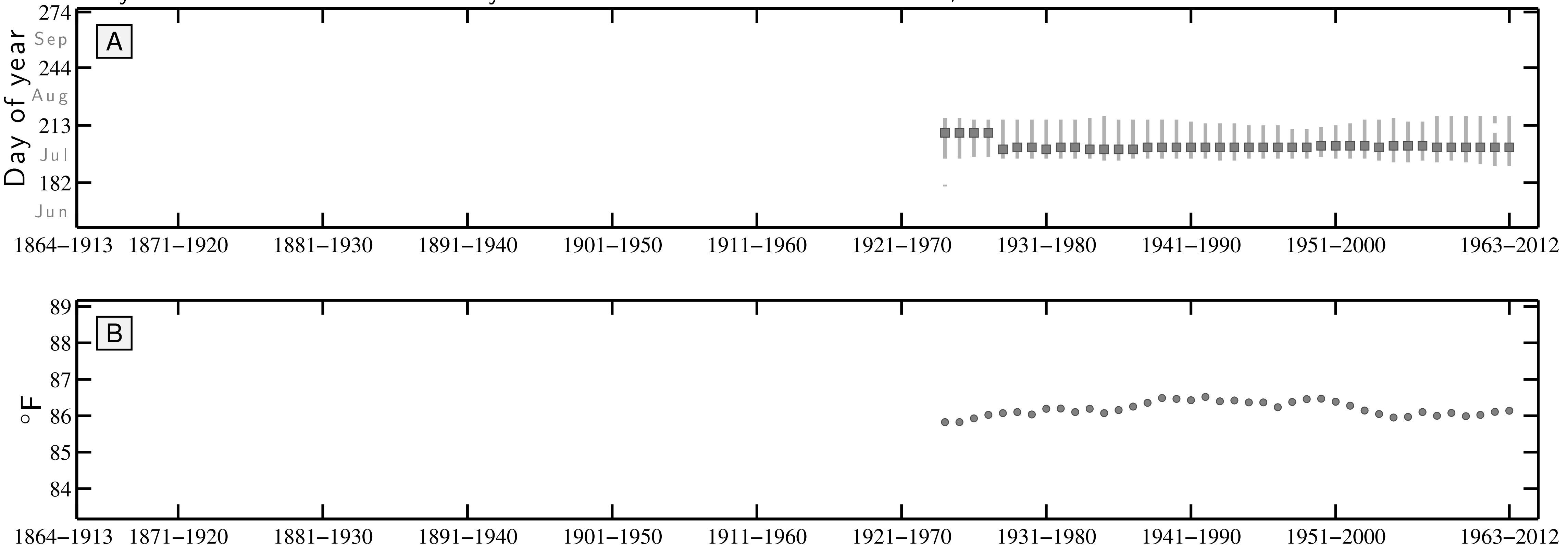

50 year Winter Teletherm dynamics for PENNINGTON GAP, VA:

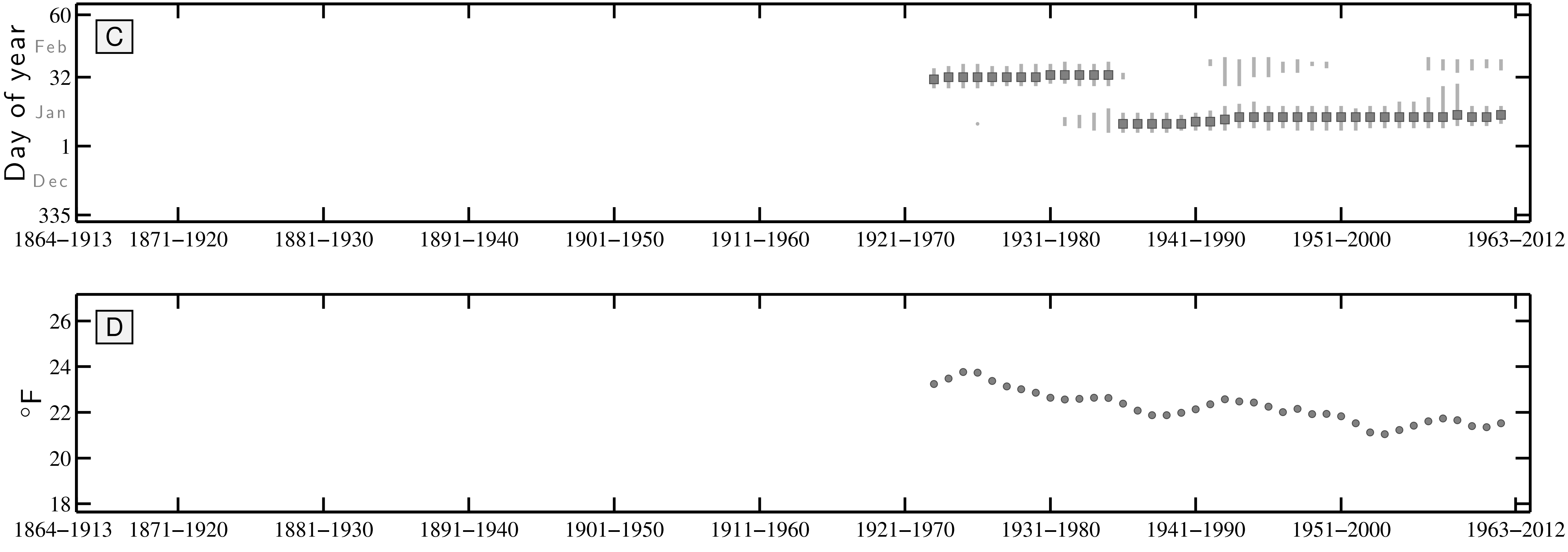

50 year Summer Teletherm dynamics for PIEDMONT RSCH STN, VA:

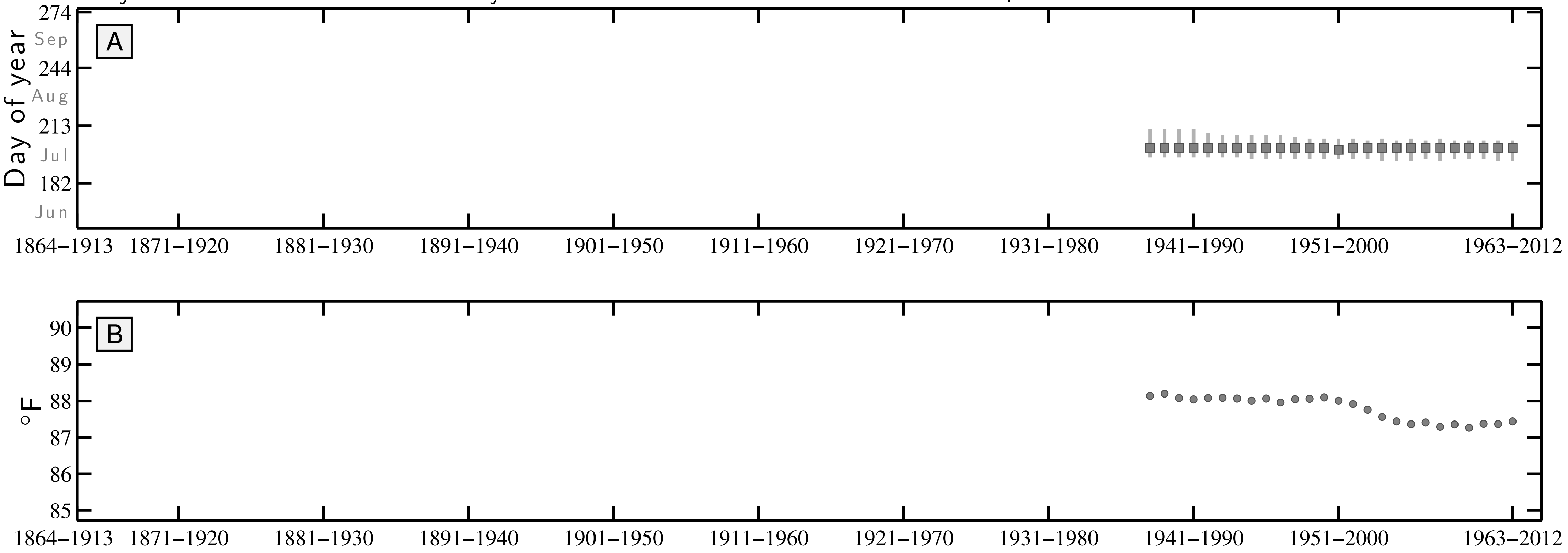

50 year Winter Teletherm dynamics for PIEDMONT RSCH STN, VA:

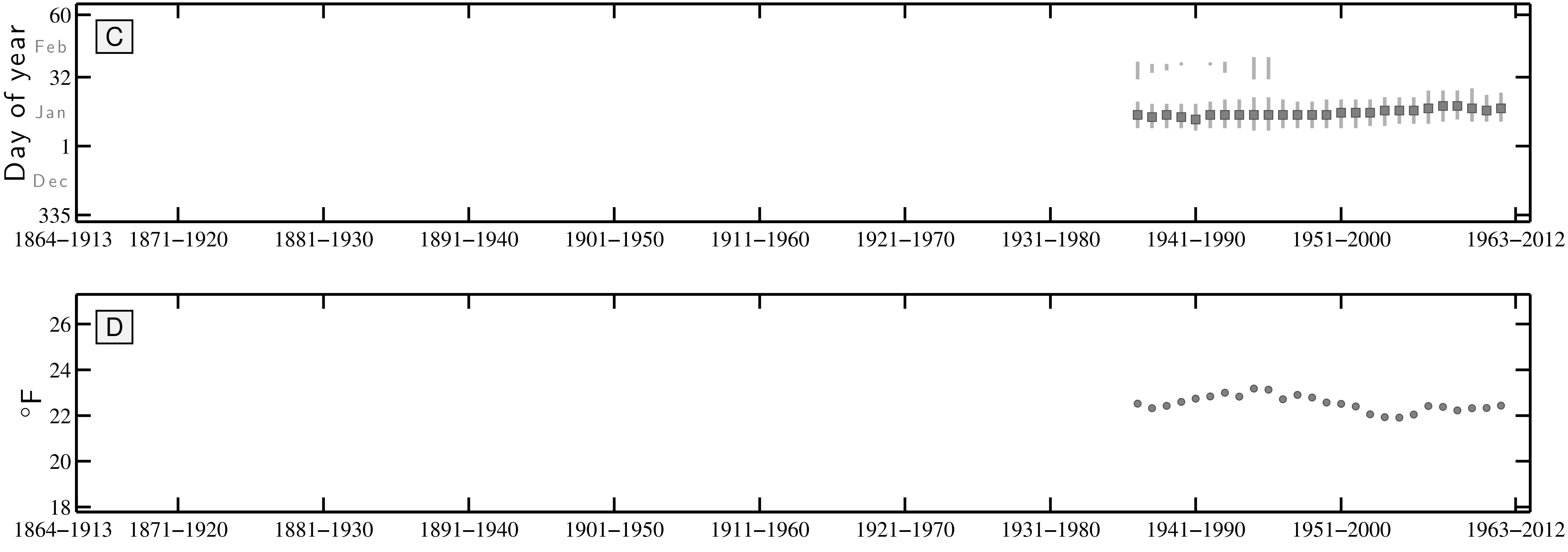

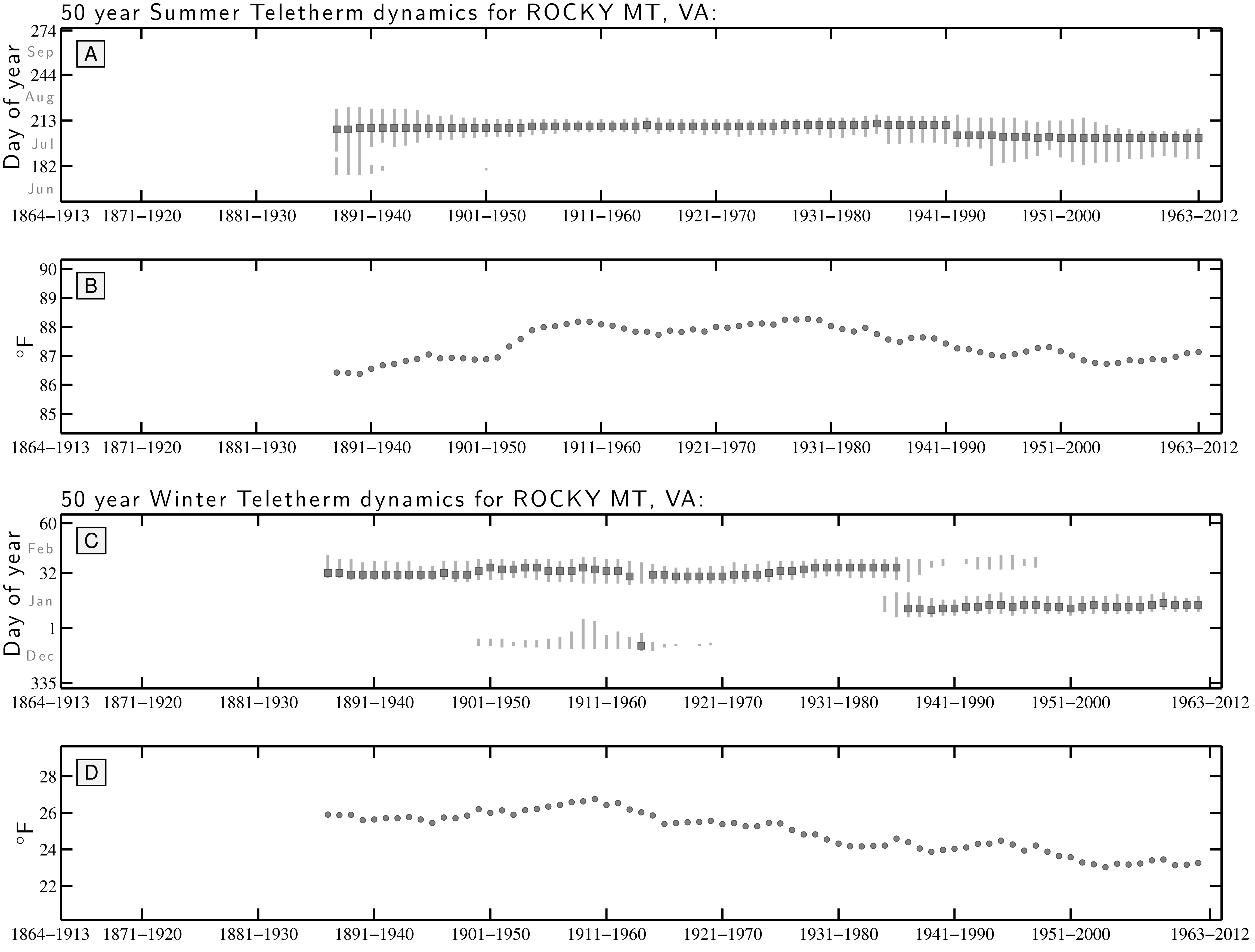
50 year Summer Teletherm dynamics for ROCKY MT, VA:
A
B
Day of year
°F
Sep
Aug
Jul
Jun
274
244
213
182
90
89
88
87
86
85
50 year Winter Teletherm dynamics for ROCKY MT, VA:
C
D
Feb
Jan
Dec
60
32
1
335
28
26
24
22
1864–1913
1871–1920
1881–1930
1891–1940
1901–1950
1911–1960
1921–1970
1931–1980
1941–1990
1951–2000
1963–2012

50 year Summer Teletherm dynamics for STAUNTON WATER TRMTMT PLT, VA:

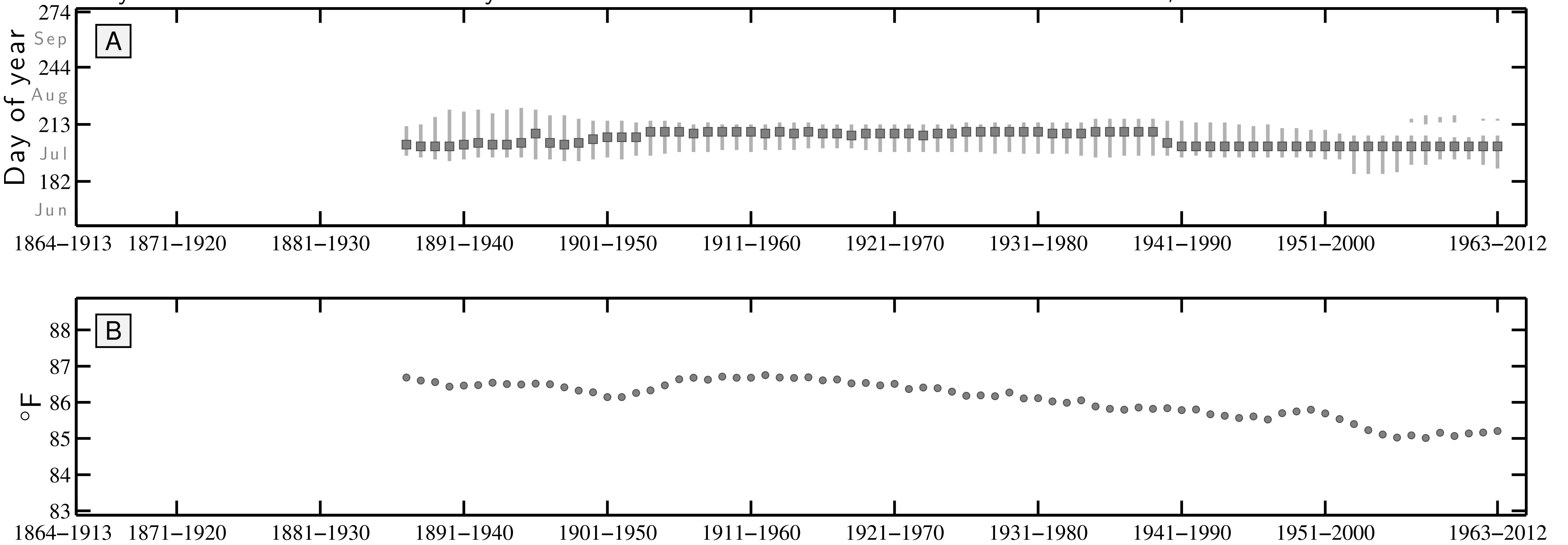

50 year Winter Teletherm dynamics for STAUNTON WATER TRMTMT PLT, VA:

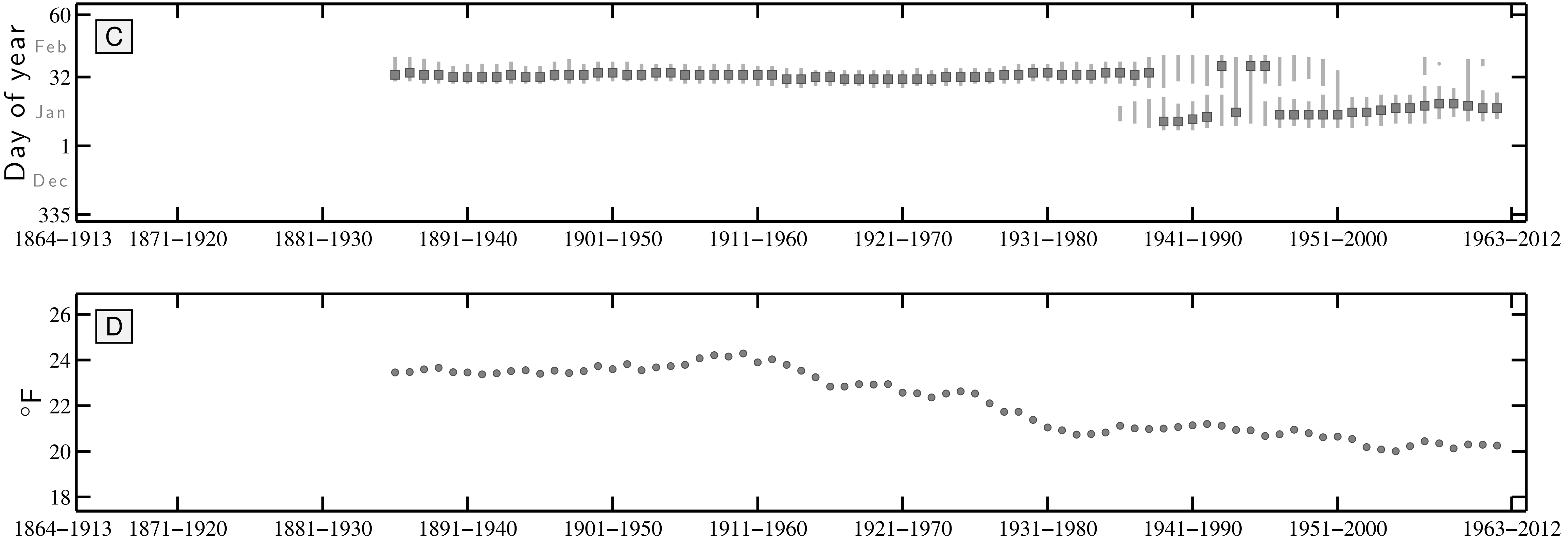

50 year Summer Teletherm dynamics for WILLIAMSBURG 2 N, VA:

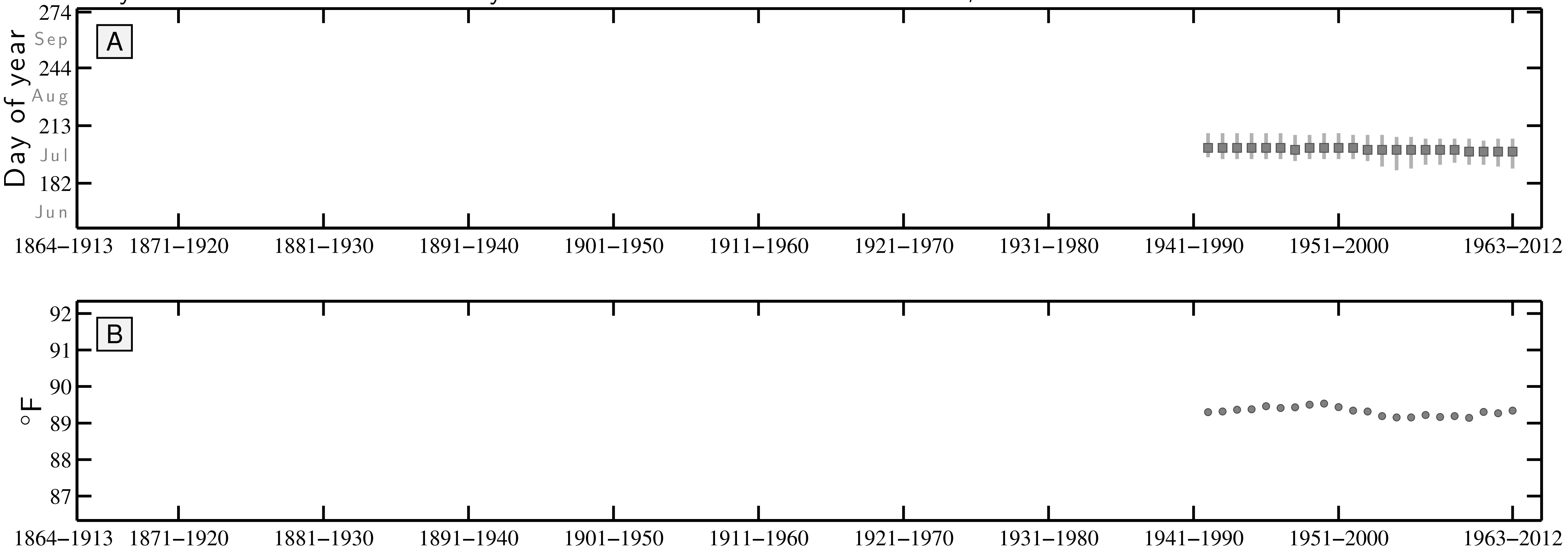

50 year Winter Teletherm dynamics for WILLIAMSBURG 2 N, VA:

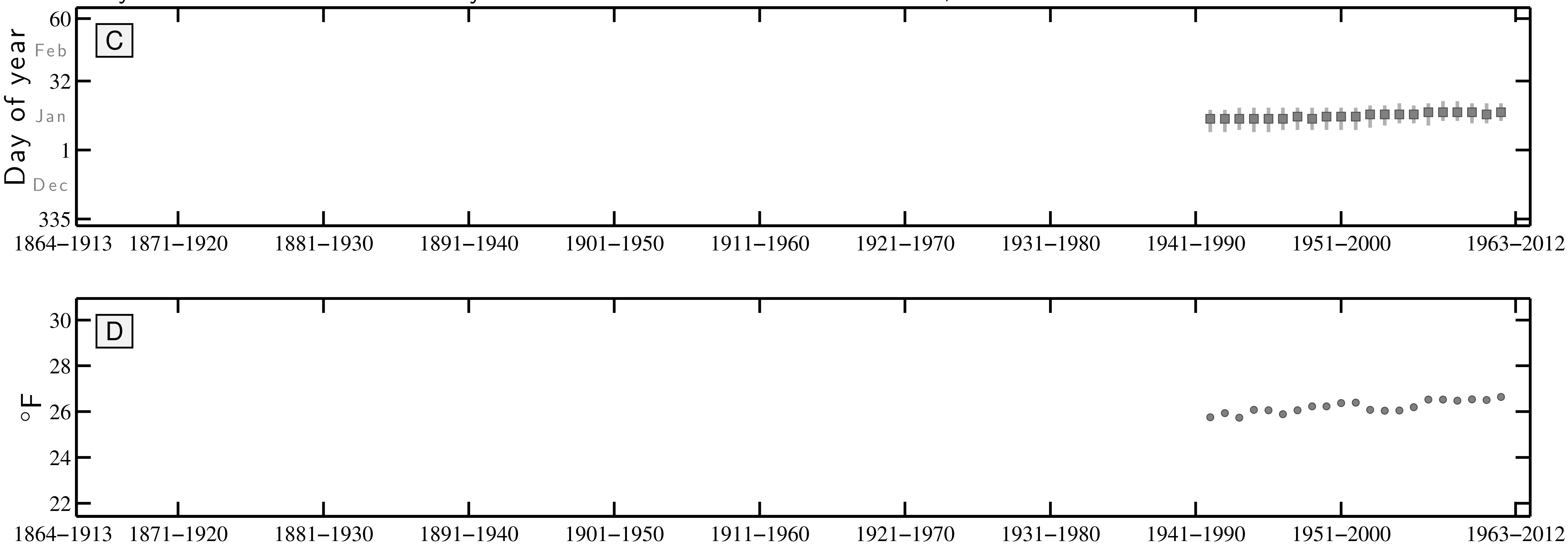

50 year Summer Teletherm dynamics for WOODSTOCK 2 NE, VA:

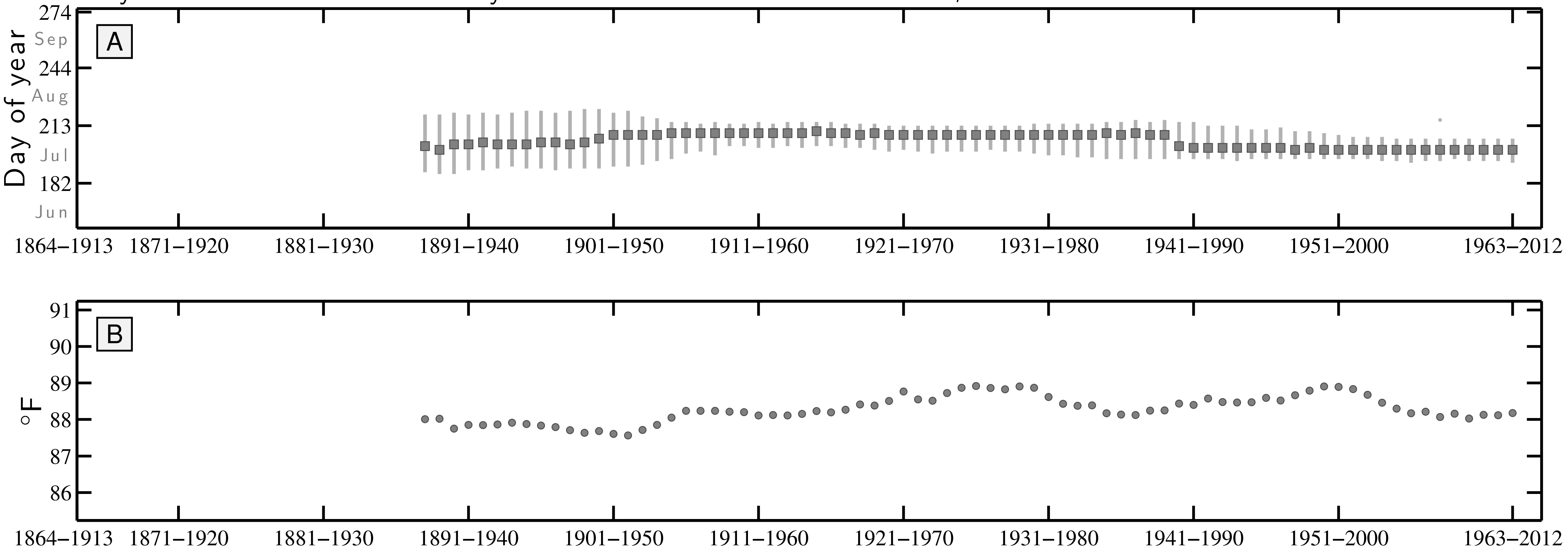

50 year Winter Teletherm dynamics for WOODSTOCK 2 NE, VA:

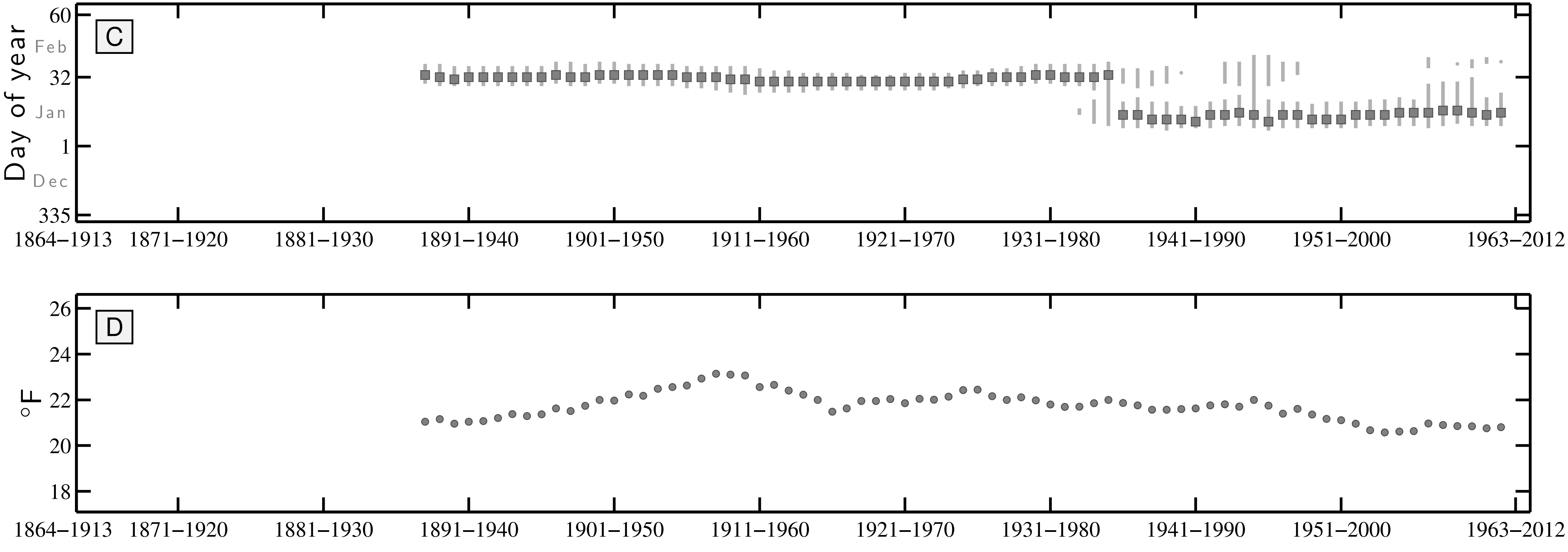

50 year Summer Teletherm dynamics for ABERDEEN, WA:

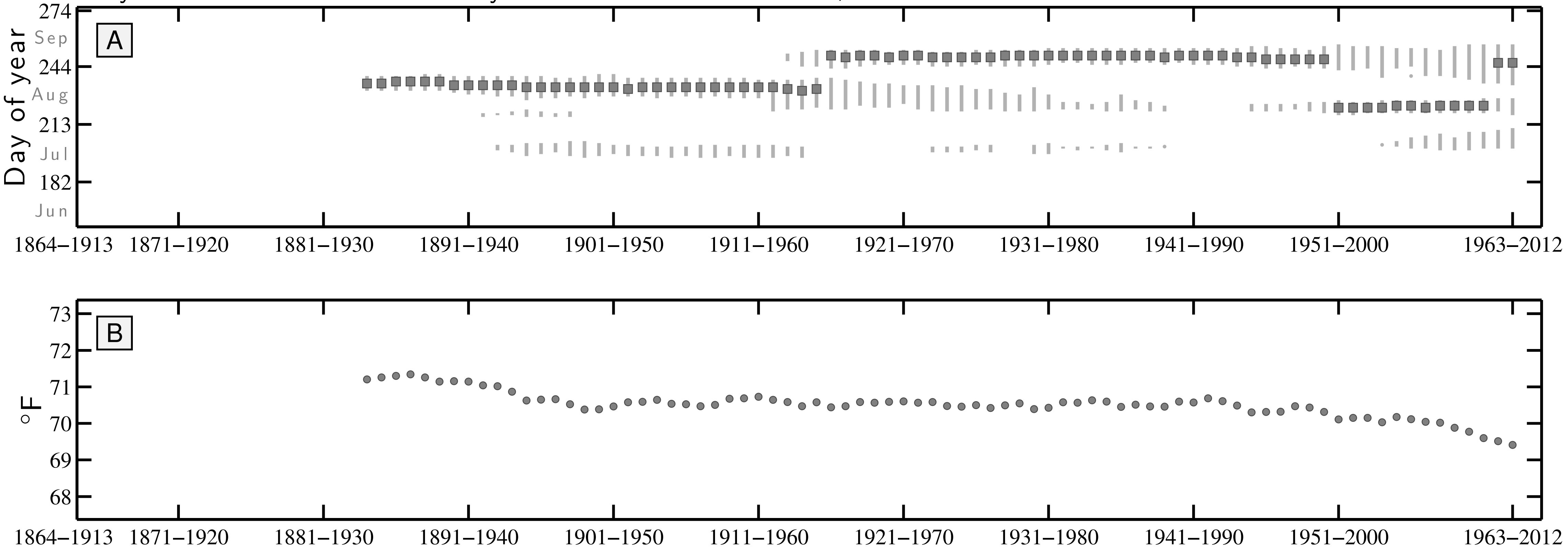

50 year Winter Teletherm dynamics for ABERDEEN, WA:

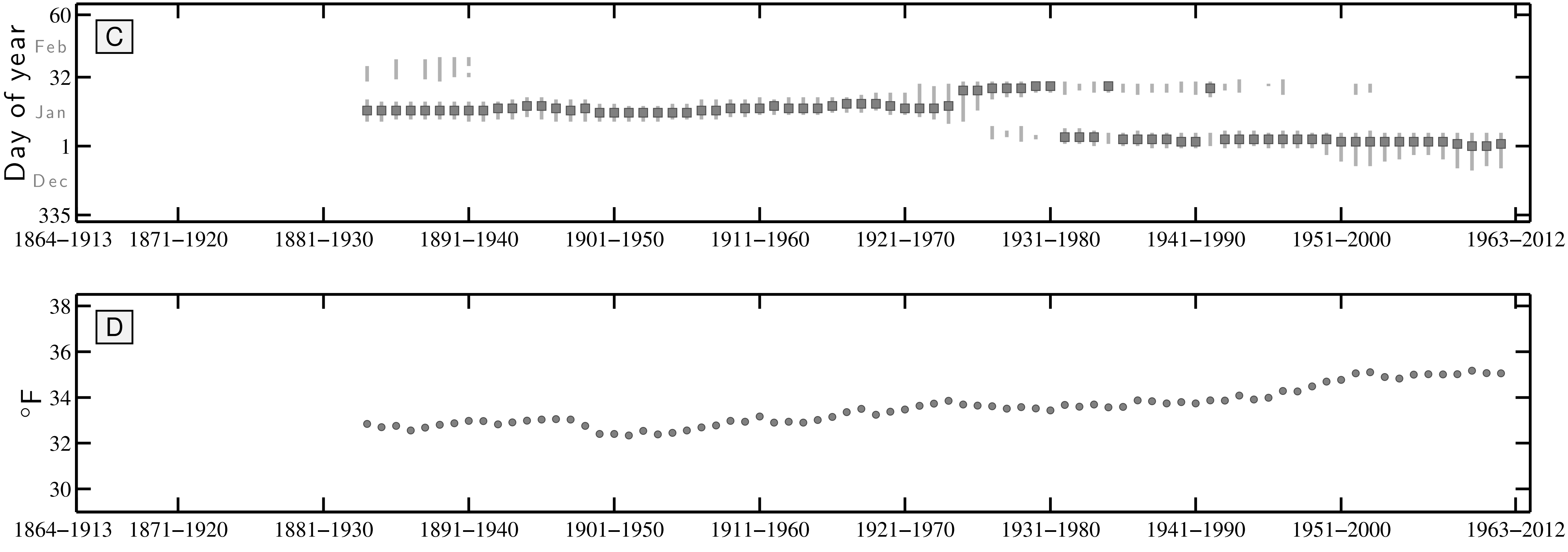

50 year Summer Teletherm dynamics for BLAINE, WA:

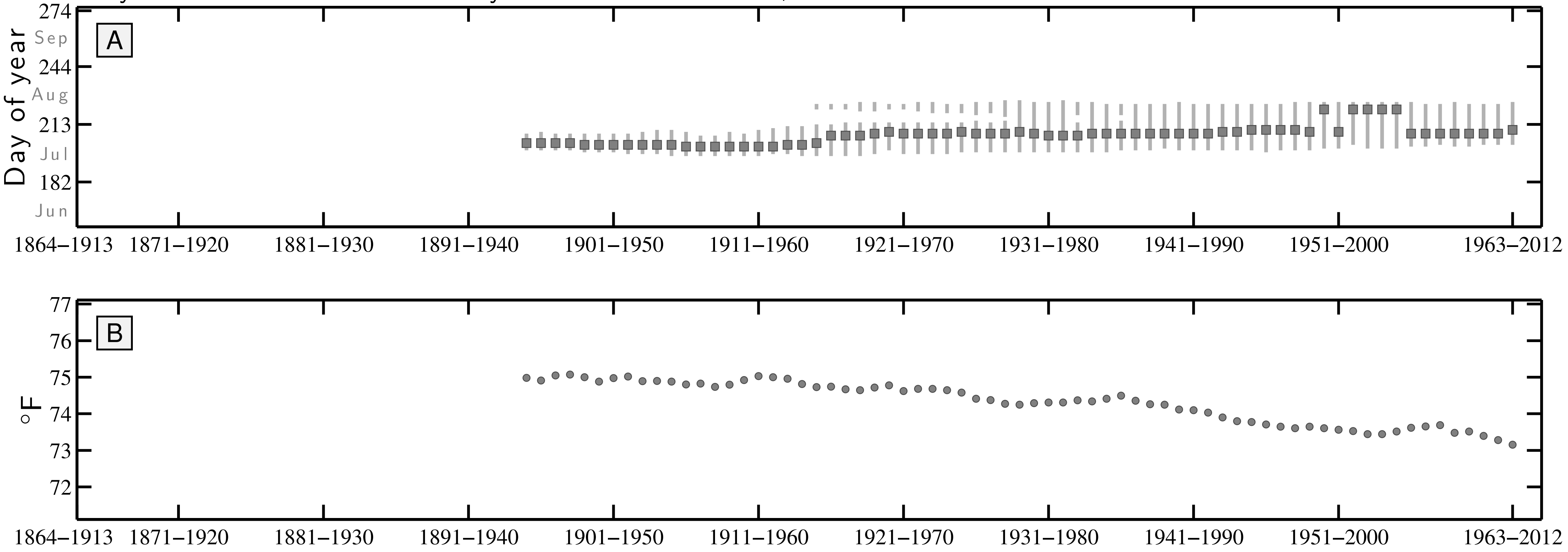

50 year Winter Teletherm dynamics for BLAINE, WA:

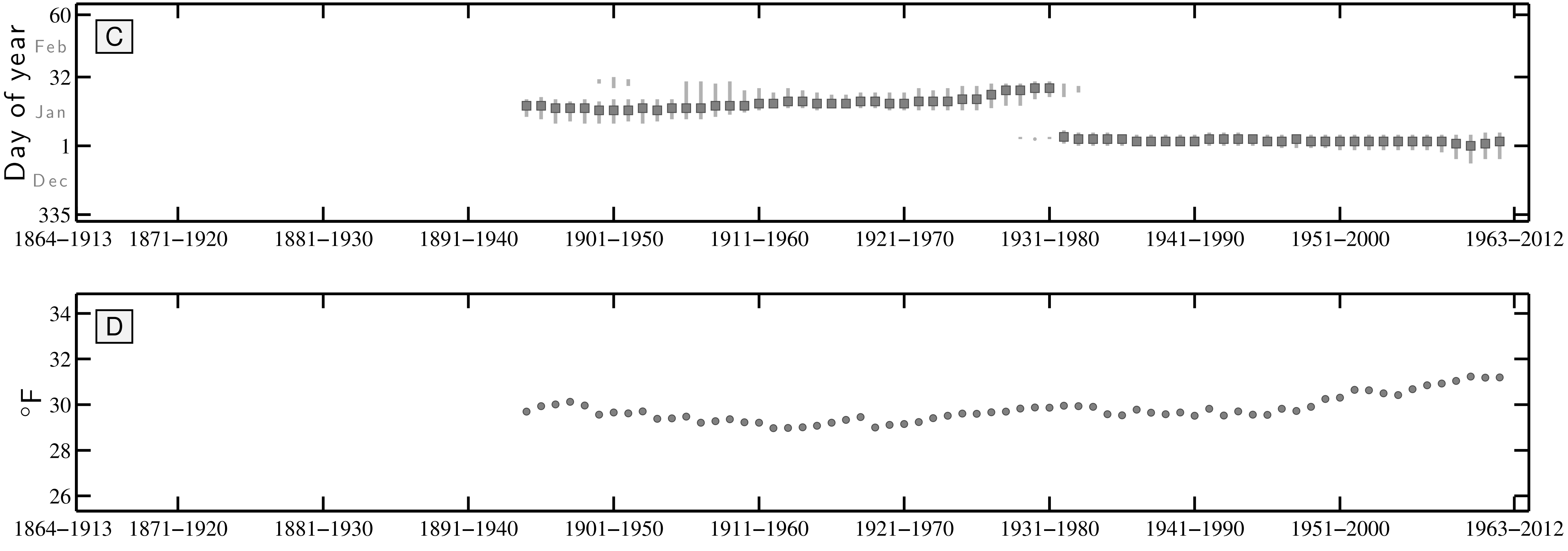

50 year Summer Teletherm dynamics for BUCKLEY 1 NE, WA:

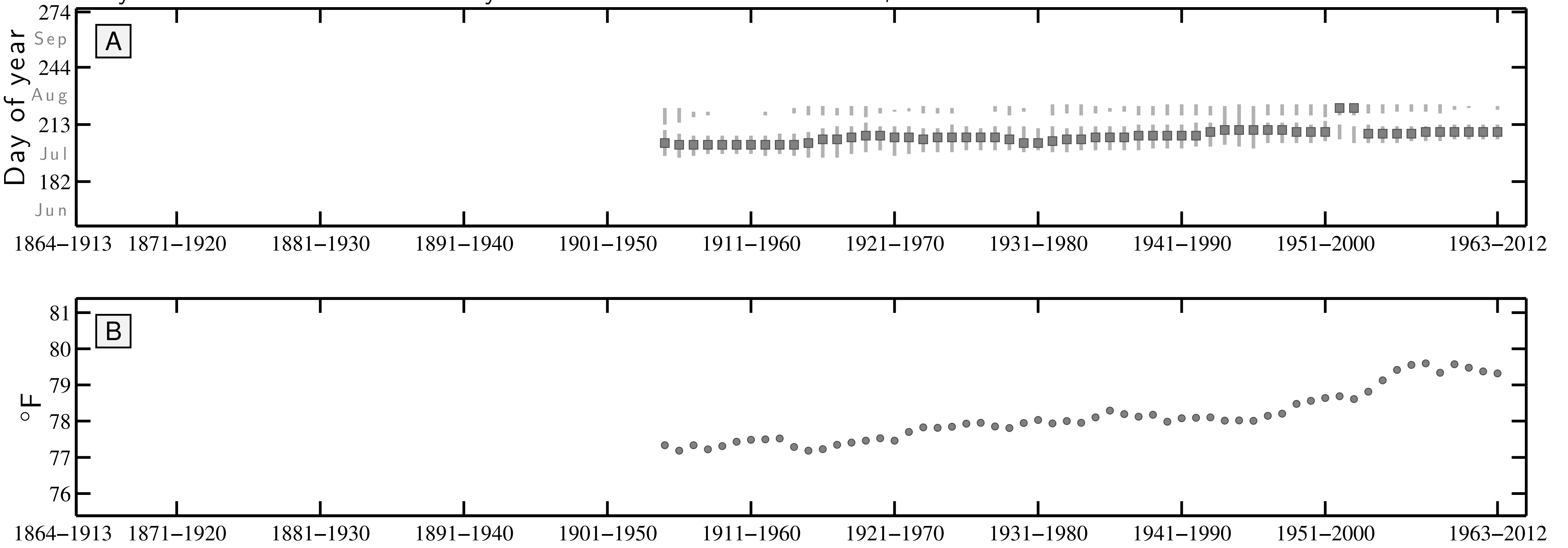

50 year Winter Teletherm dynamics for BUCKLEY 1 NE, WA:

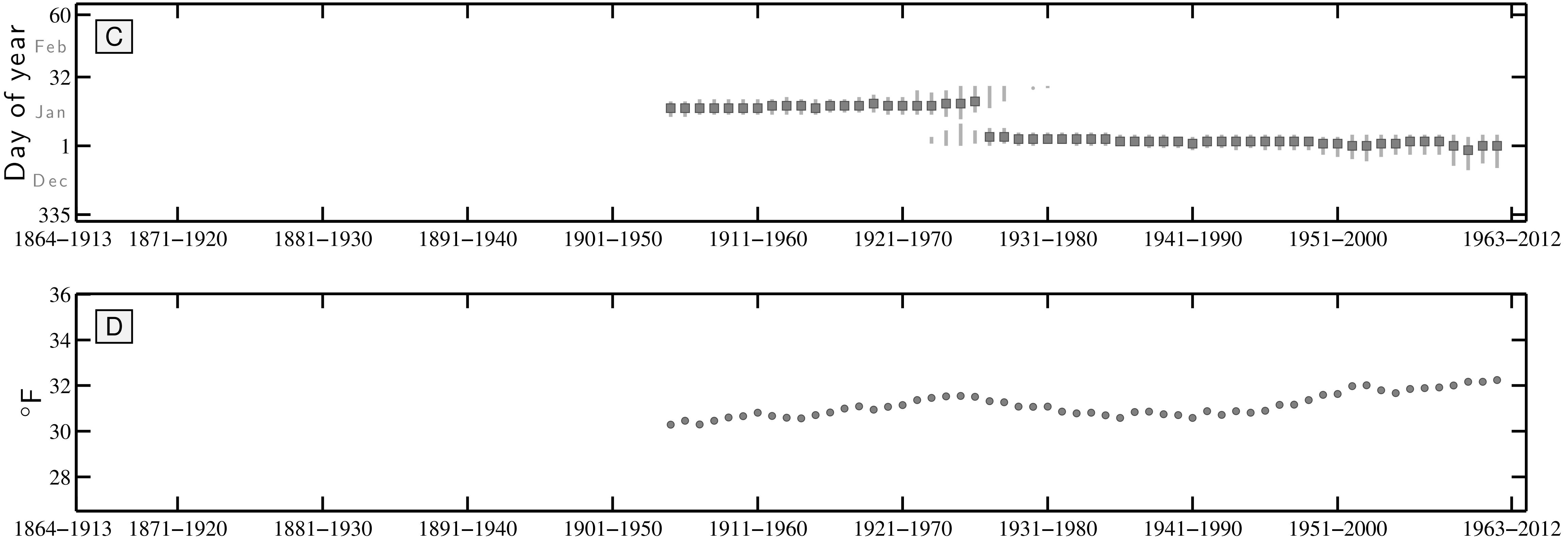

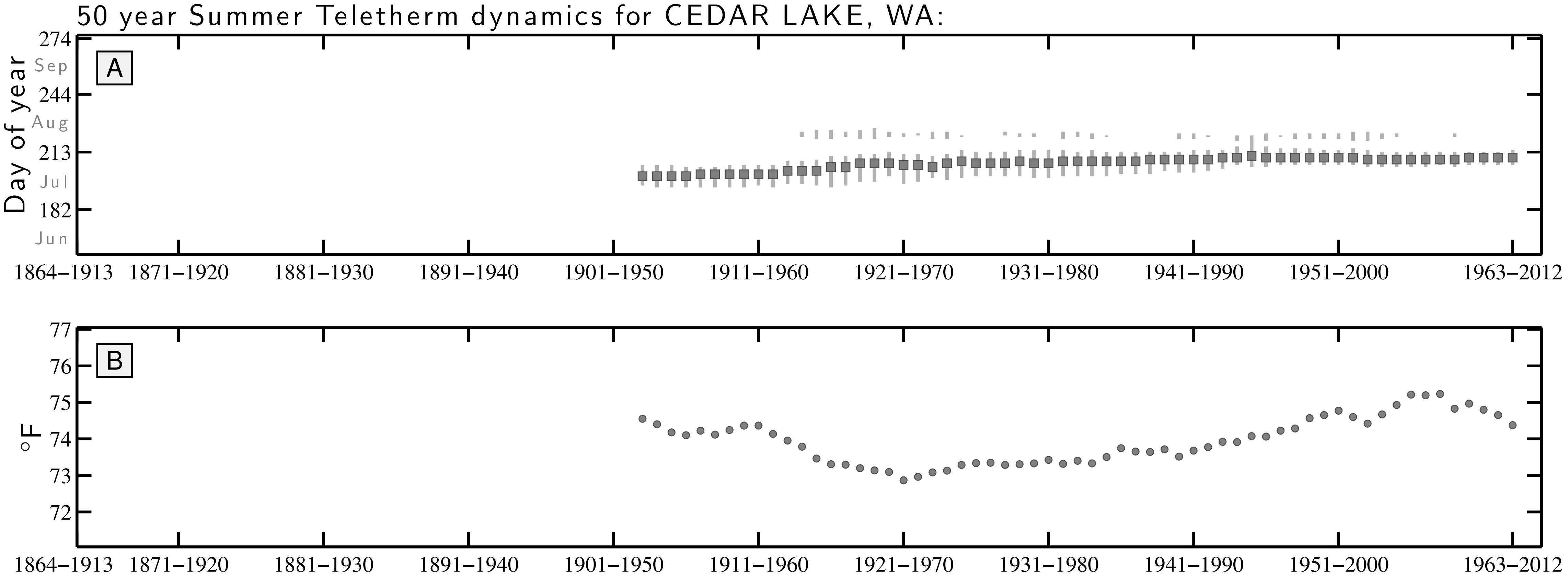
50 year Summer Teletherm dynamics for CEDAR LAKE, WA:
A
B
Day of year
°F
274
244
213
182
Sep
Aug
Jul
Jun
77
76
75
74
73
72
1864–1913
1871–1920
1881–1930
1891–1940
1901–1950
1911–1960
1921–1970
1931–1980
1941–1990
1951–2000
1963–2012

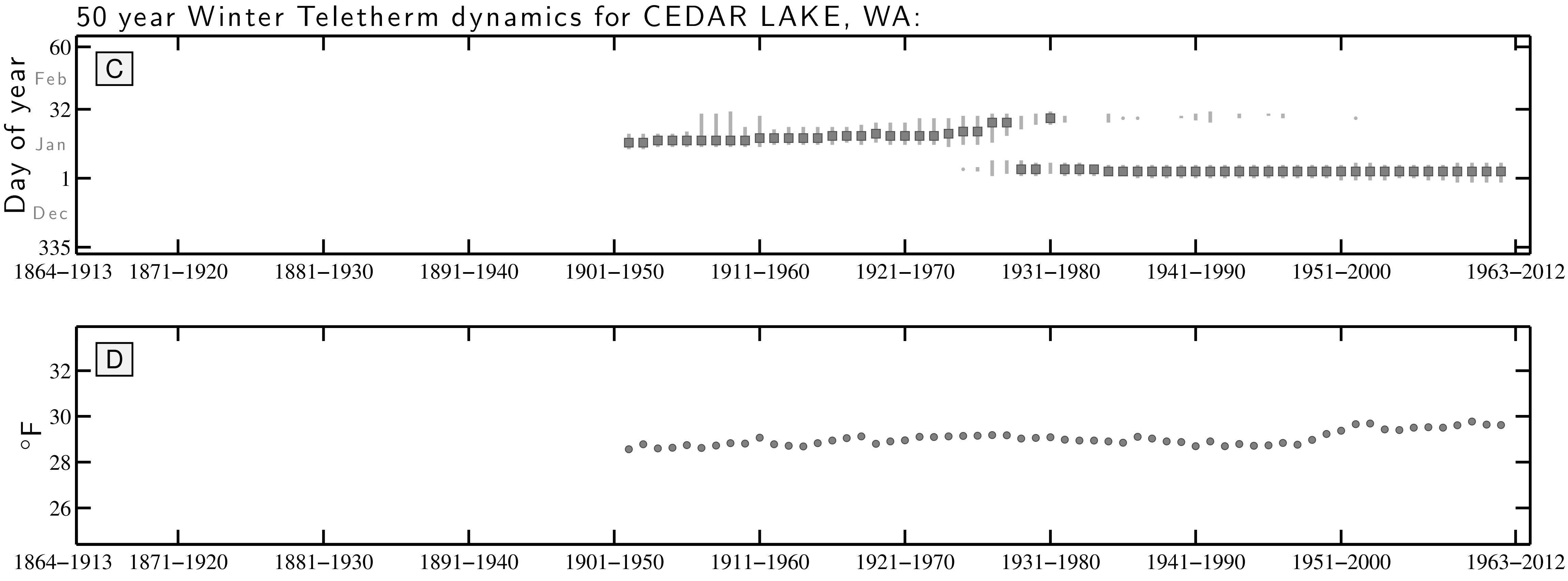
50 year Winter Teletherm dynamics for CEDAR LAKE, WA:
C
D
Day of year
°F
60
32
1
335
Feb
Jan
Dec
32
30
28
26
1864–1913
1871–1920
1881–1930
1891–1940
1901–1950
1911–1960
1921–1970
1931–1980
1941–1990
1951–2000
1963–2012

50 year Summer Teletherm dynamics for CENTRALIA, WA:

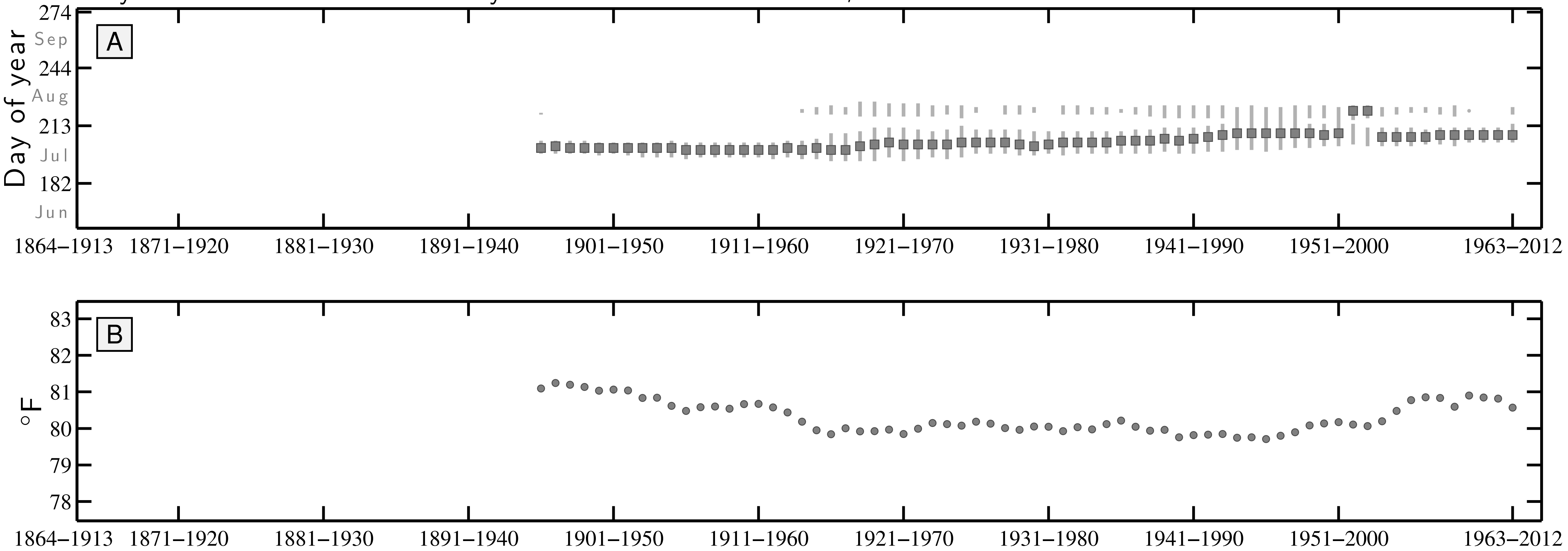

50 year Winter Teletherm dynamics for CENTRALIA, WA:

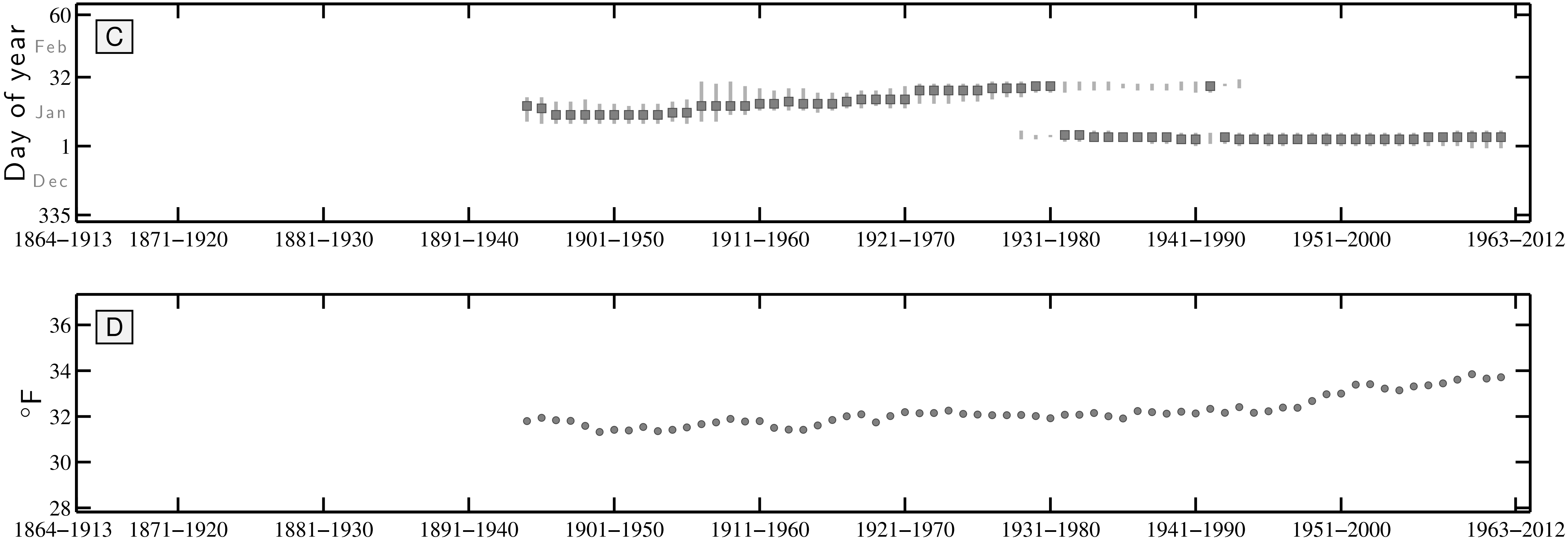

50 year Summer Teletherm dynamics for CLEARBROOK, WA:

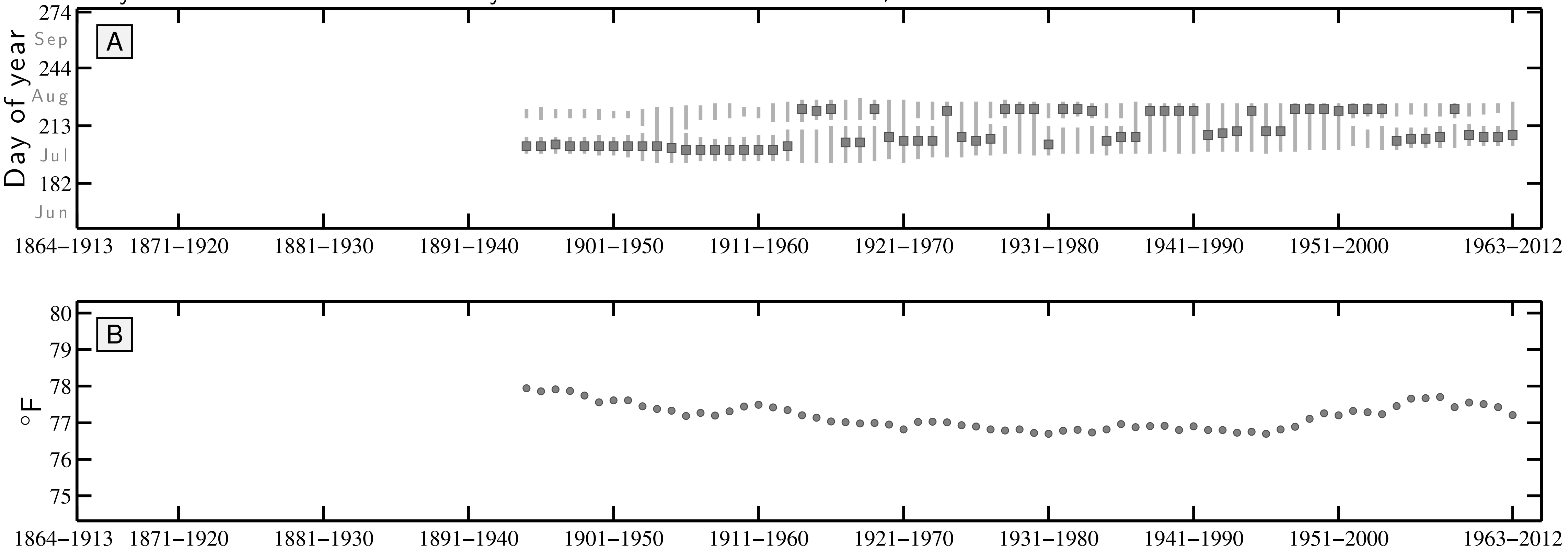

50 year Winter Teletherm dynamics for CLEARBROOK, WA:

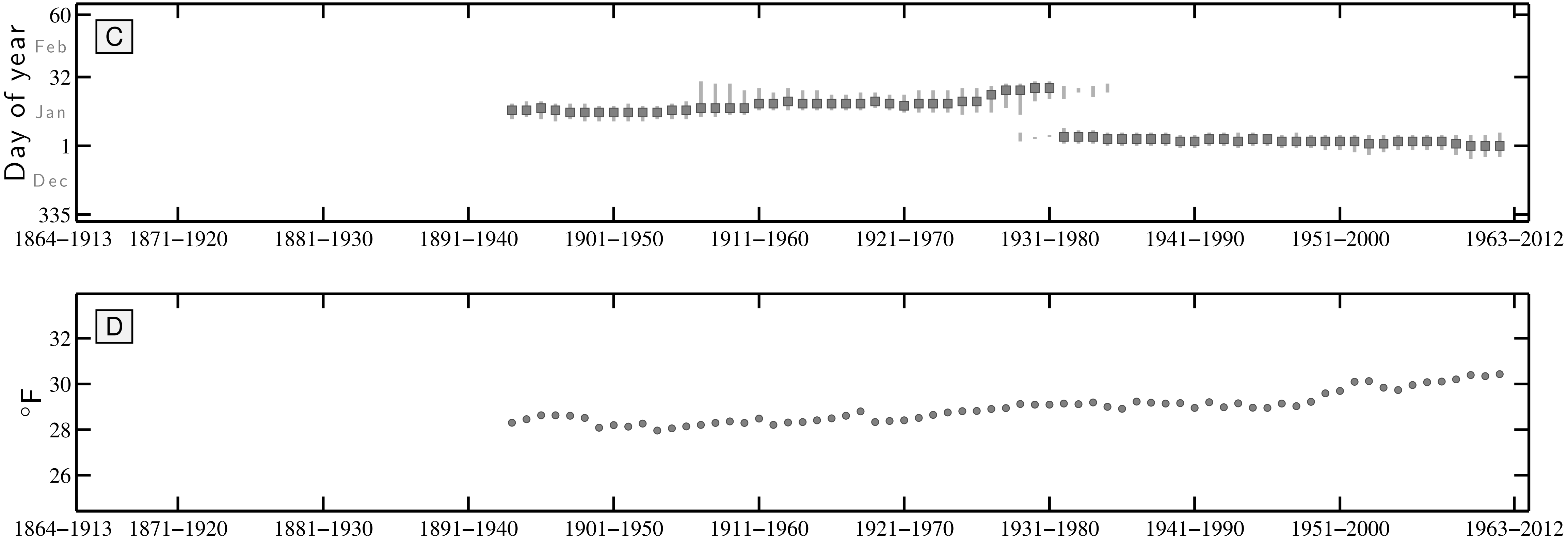

50 year Summer Teletherm dynamics for CLE ELUM, WA:

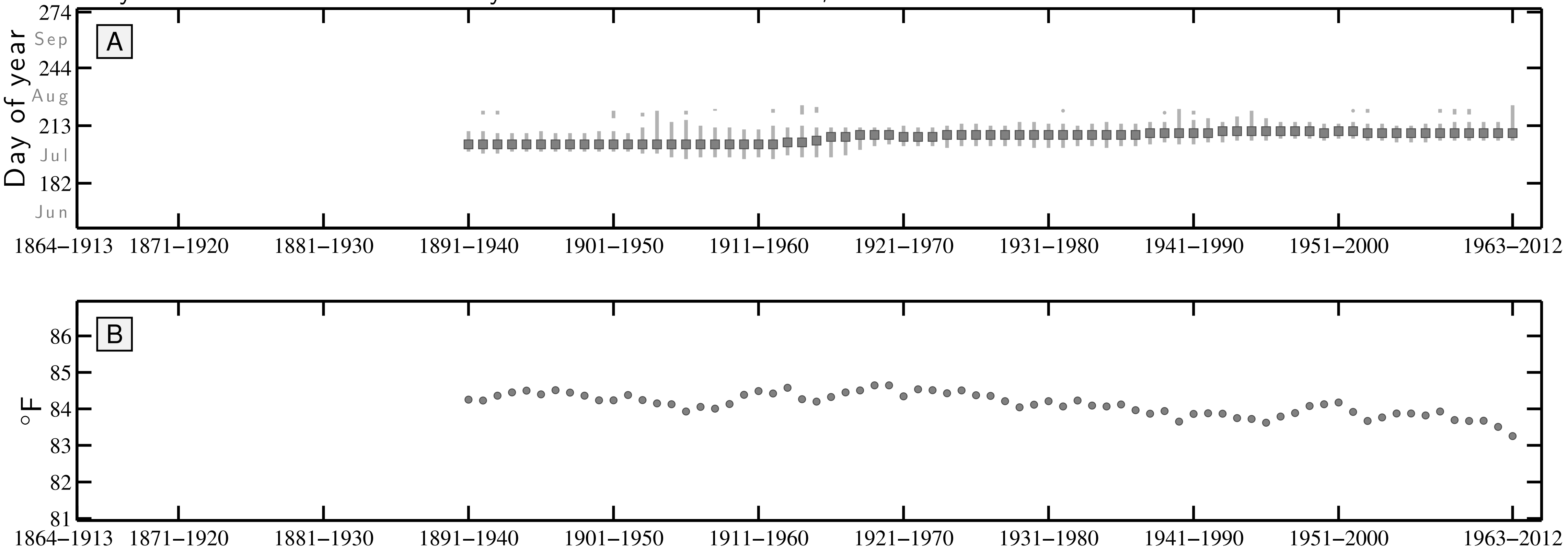

50 year Winter Teletherm dynamics for CLE ELUM, WA:

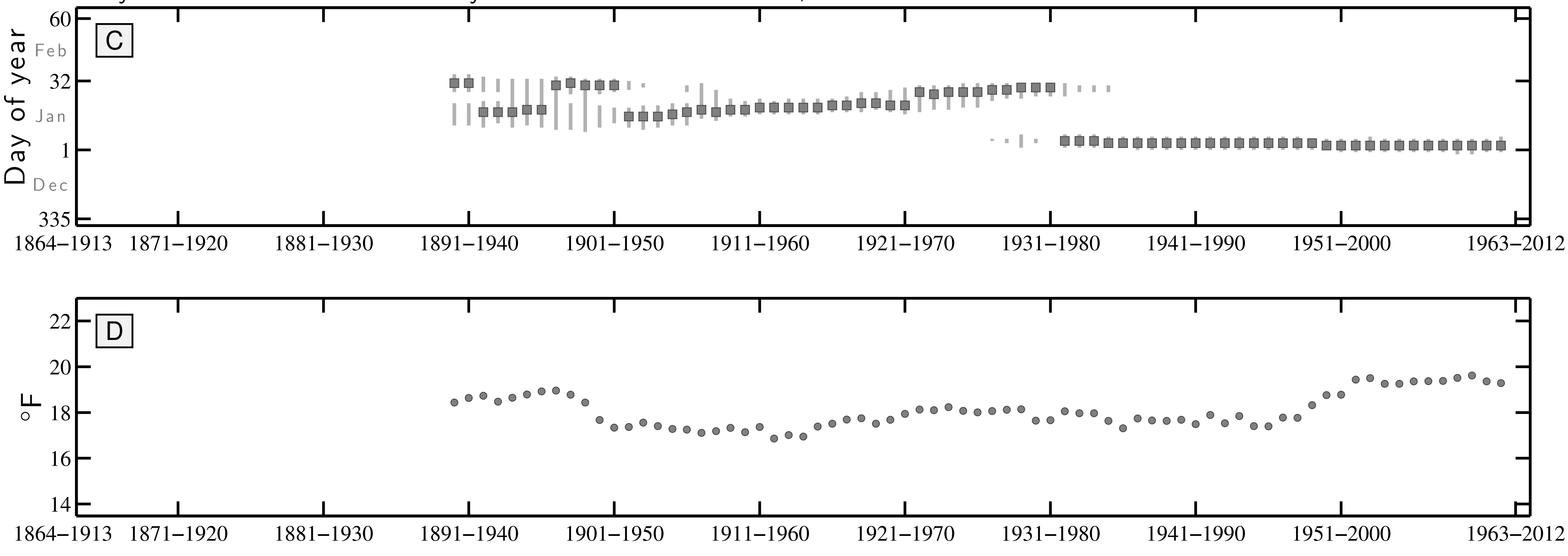

50 year Summer Teletherm dynamics for COLVILLE, WA:

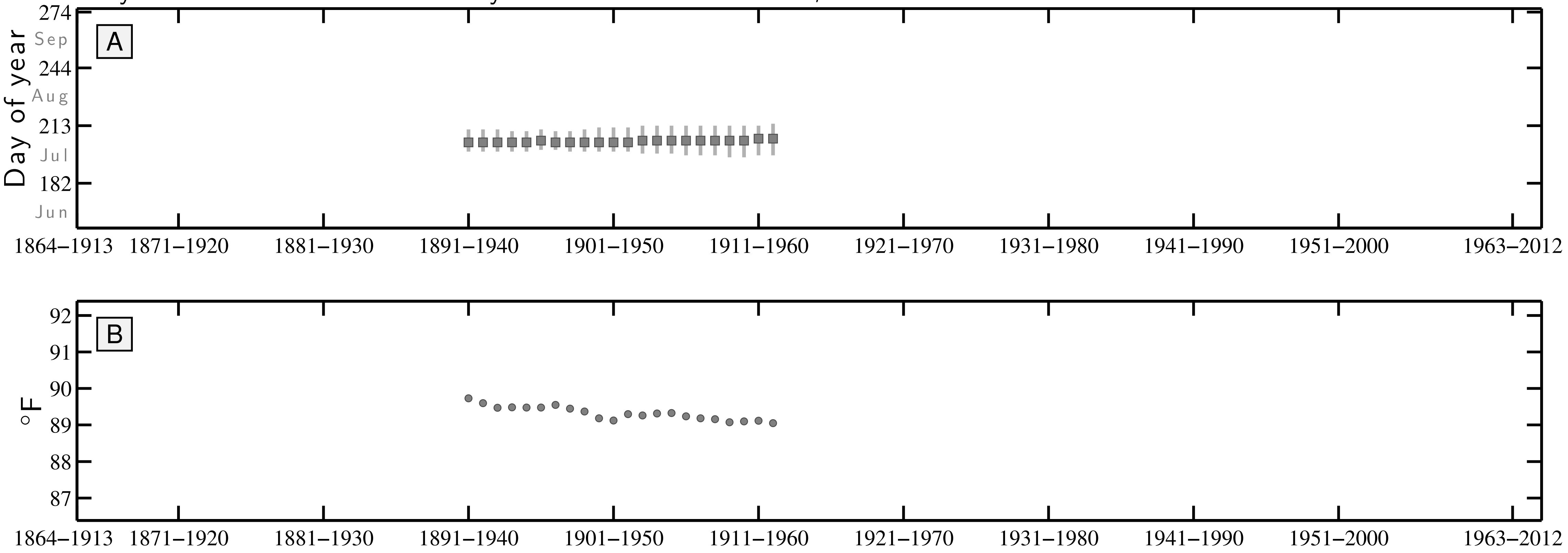

50 year Winter Teletherm dynamics for COLVILLE, WA:

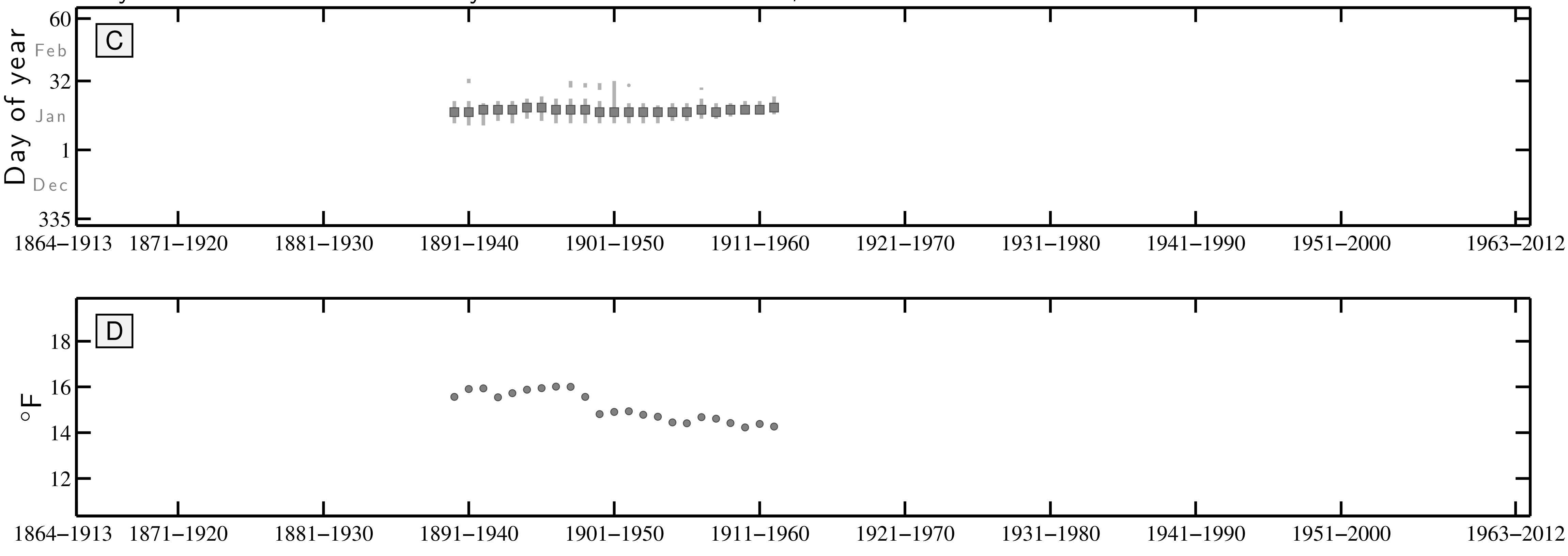

50 year Summer Teletherm dynamics for CONCONULLY, WA:

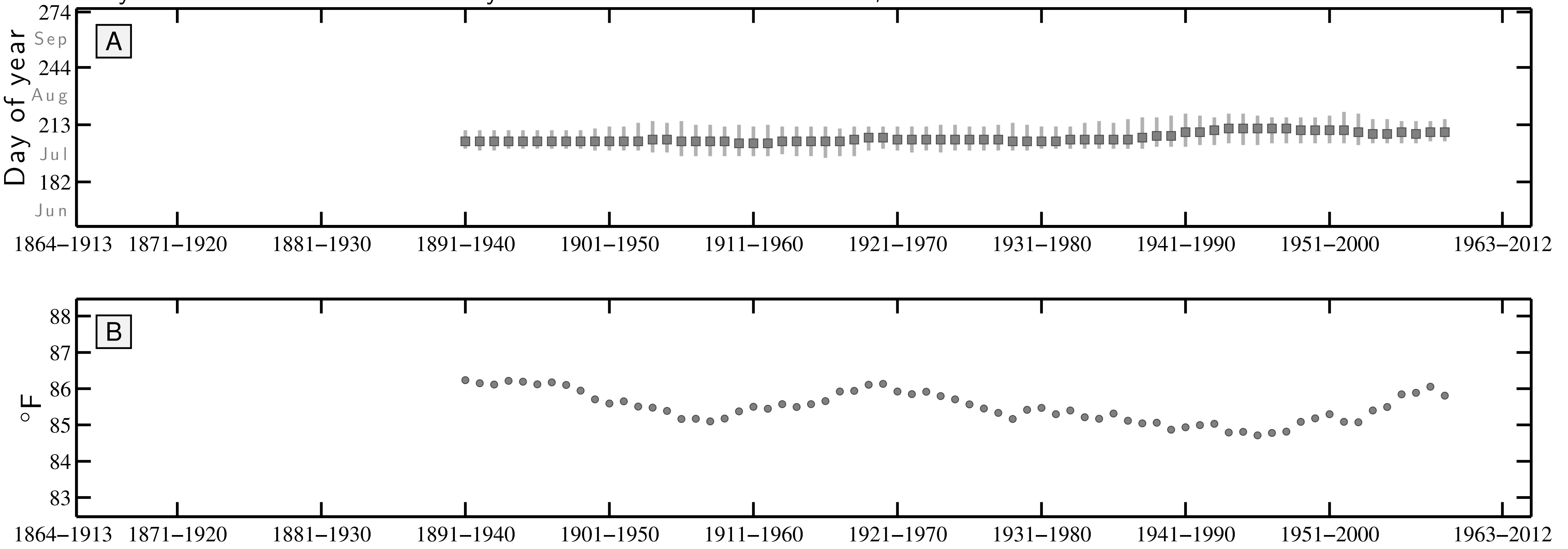

50 year Winter Teletherm dynamics for CONCONULLY, WA:

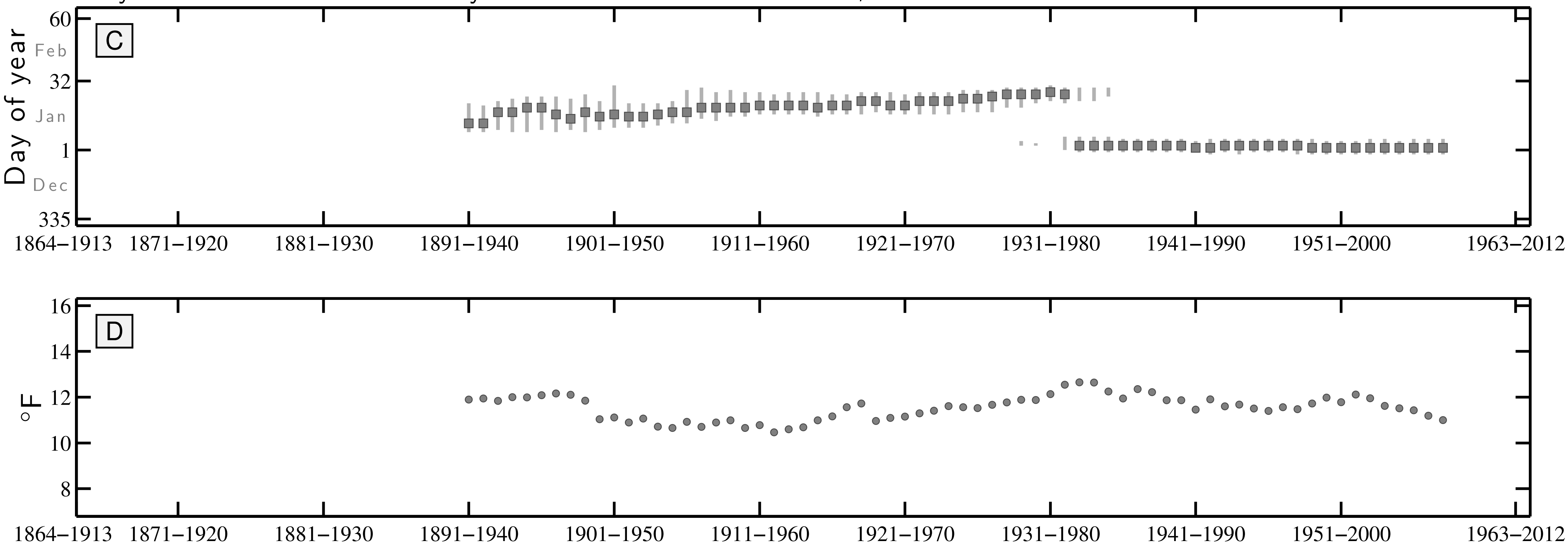

50 year Summer Teletherm dynamics for DAVENPORT, WA:

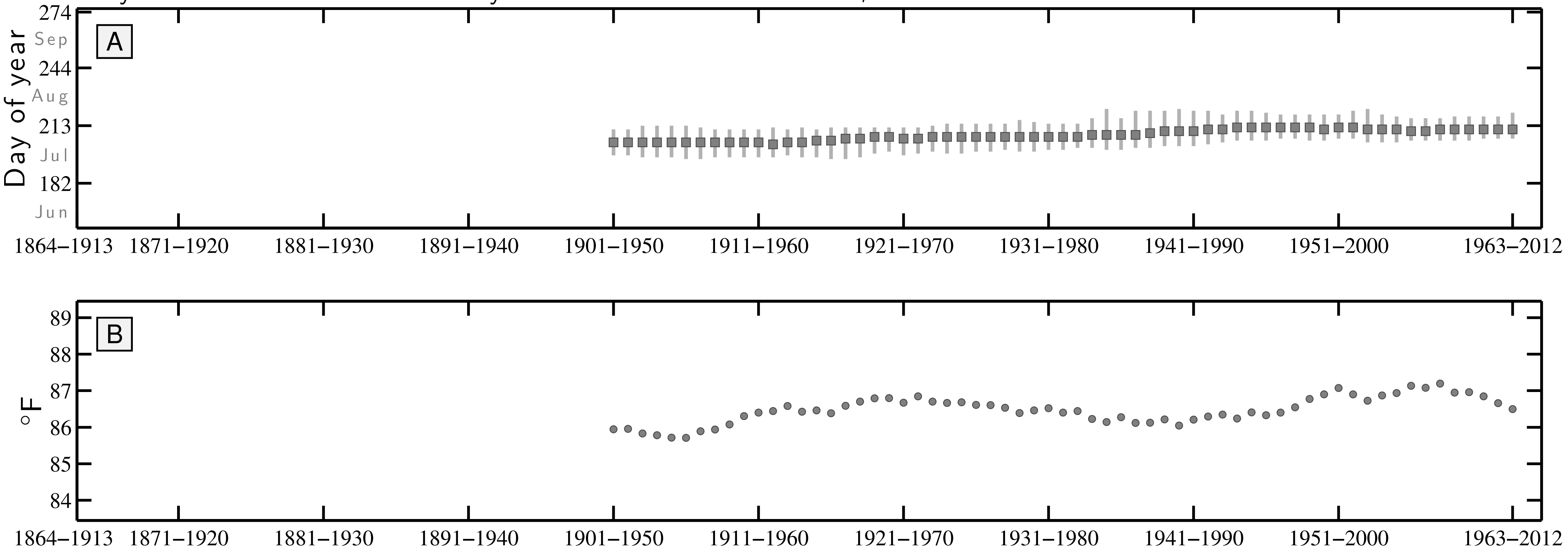

50 year Winter Teletherm dynamics for DAVENPORT, WA:

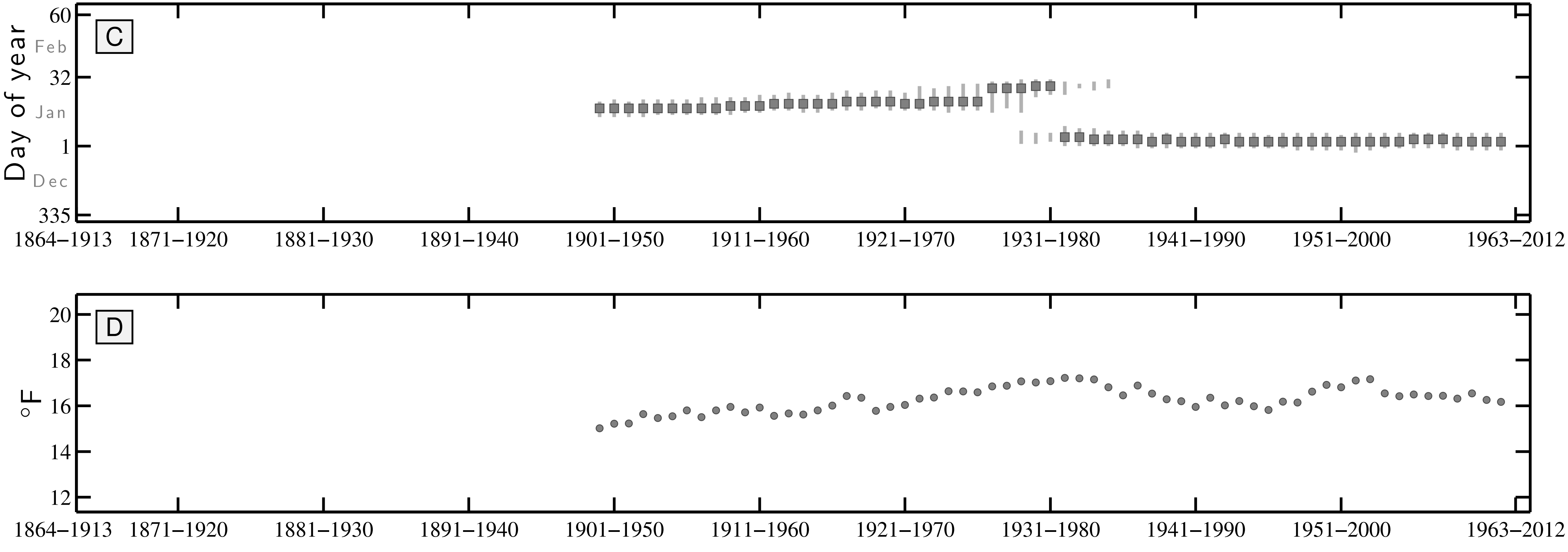

50 year Summer Teletherm dynamics for DAYTON 1 WSW, WA:

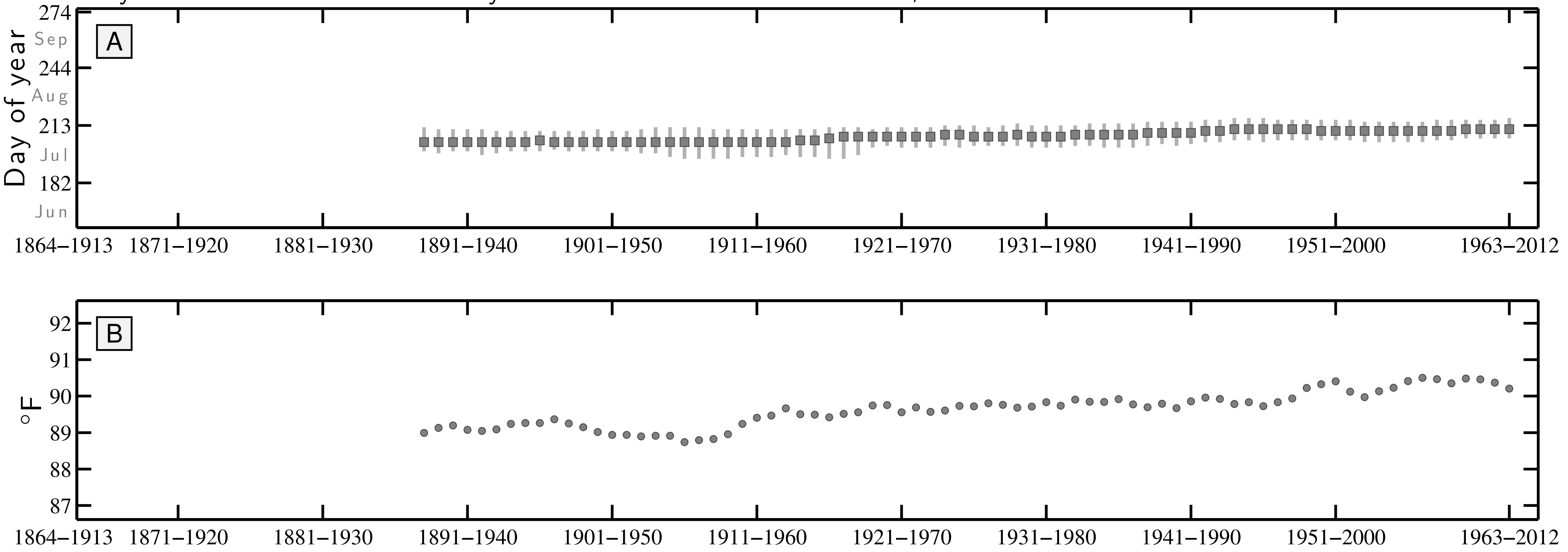

50 year Winter Teletherm dynamics for DAYTON 1 WSW, WA:

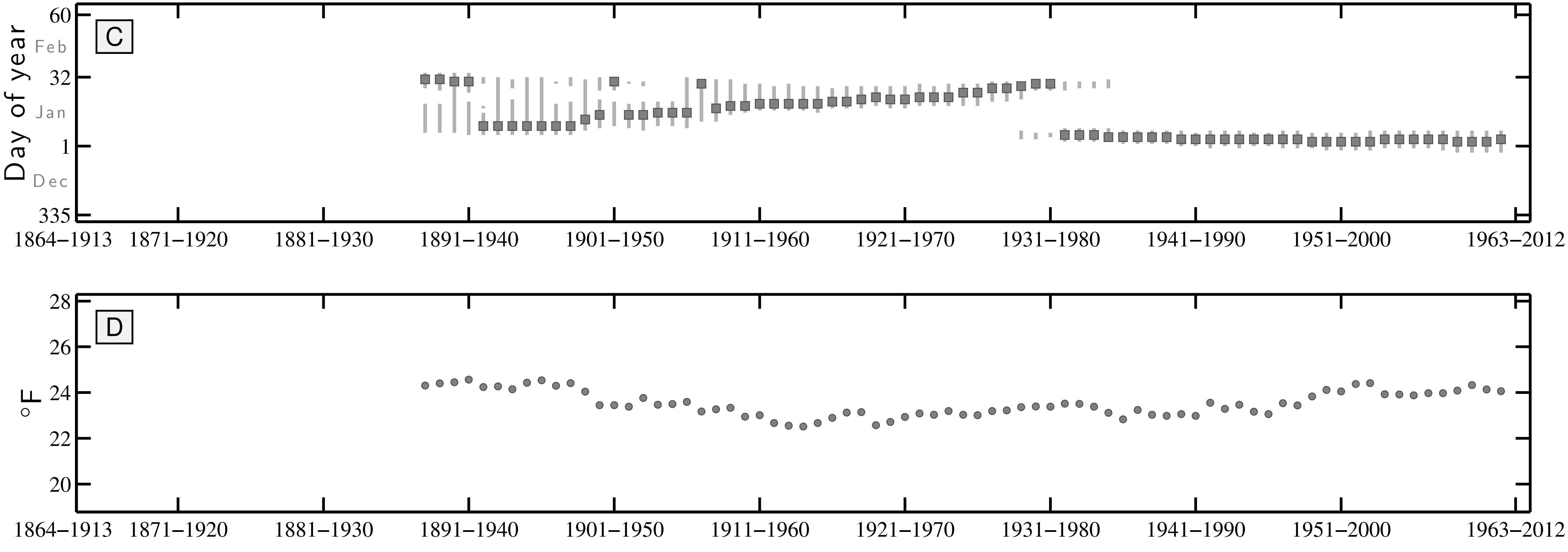

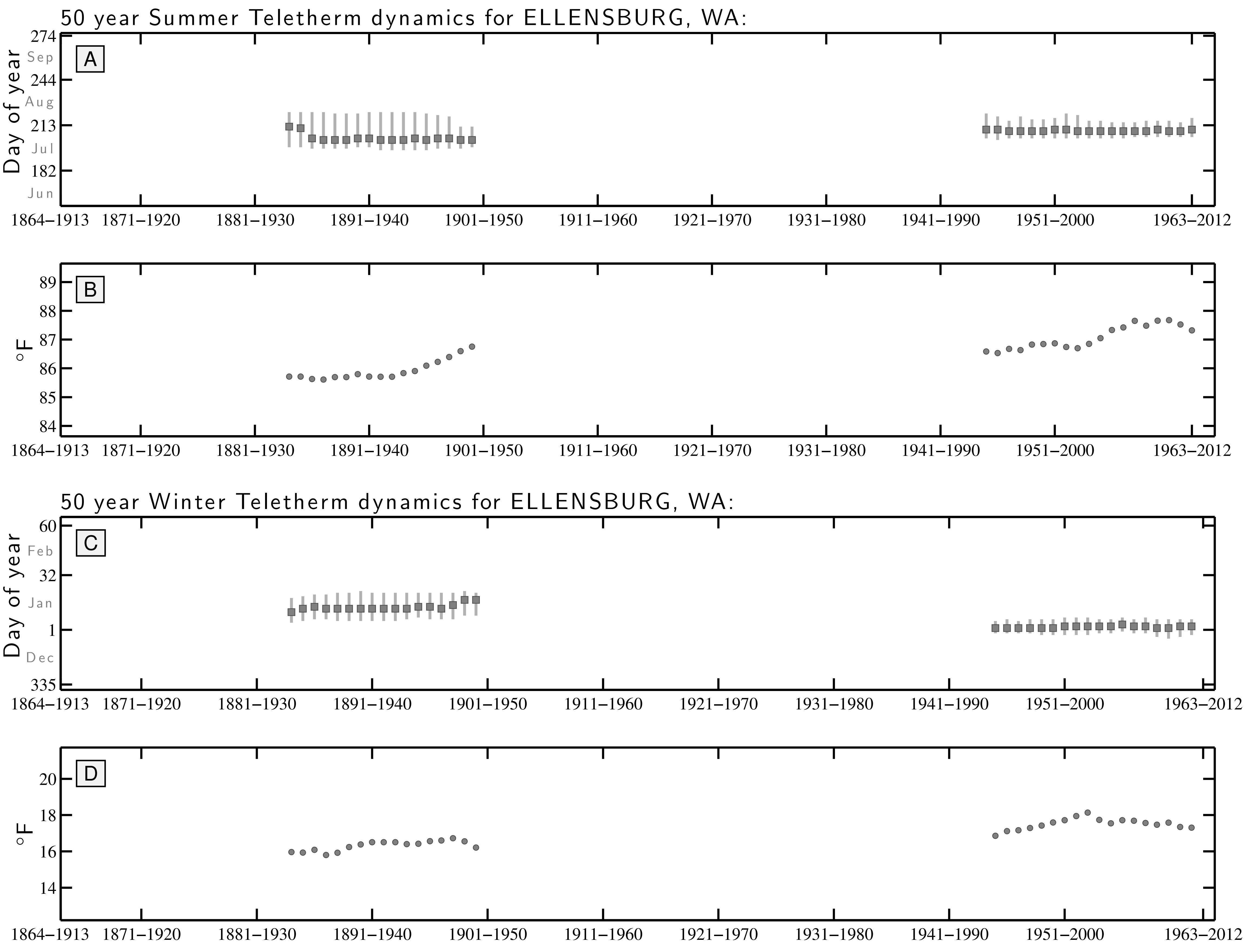
50 year Summer Teletherm dynamics for ELLENSBURG, WA:
A
Day of year
274
Sep
244
Aug
213
Jul
182
Jun
1864–1913
1871–1920
1881–1930
1891–1940
1901–1950
1911–1960
1921–1970
1931–1980
1941–1990
1951–2000
1963–2012
B
°F
89
88
87
86
85
84
50 year Winter Teletherm dynamics for ELLENSBURG, WA:
C
60
Feb
32
Jan
1
Dec
335
D
20
18
16
14

50 year Summer Teletherm dynamics for EVERETT, WA:

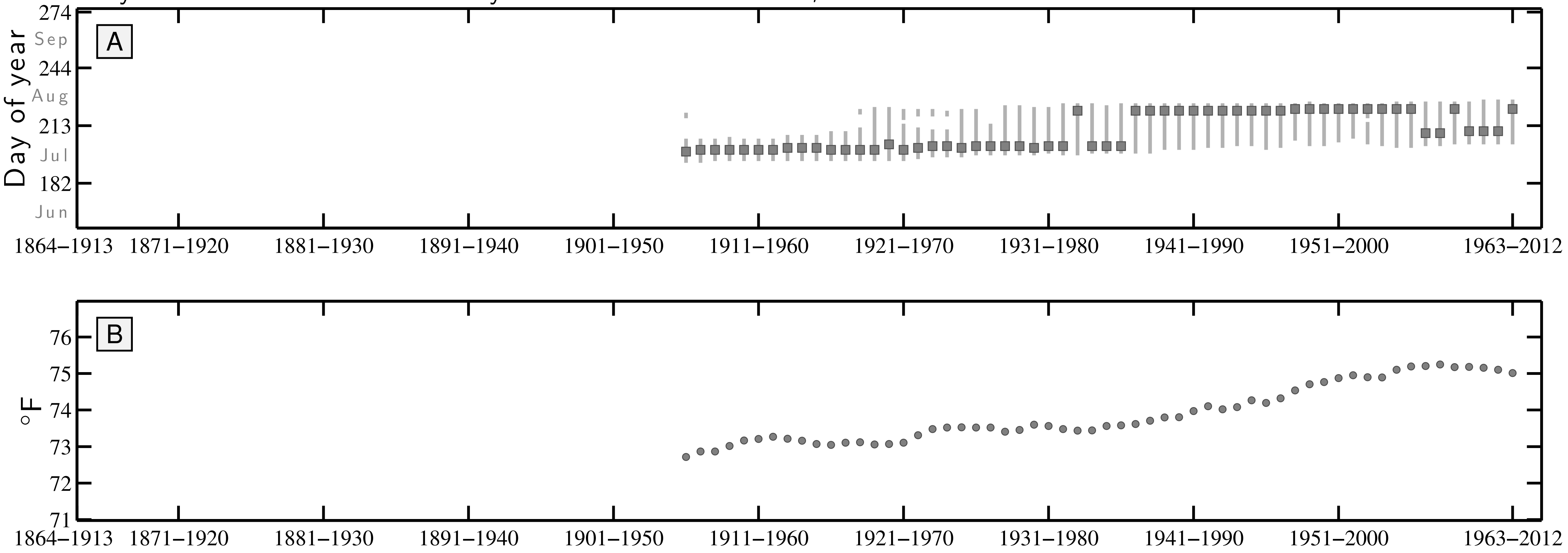

50 year Winter Teletherm dynamics for EVERETT, WA:

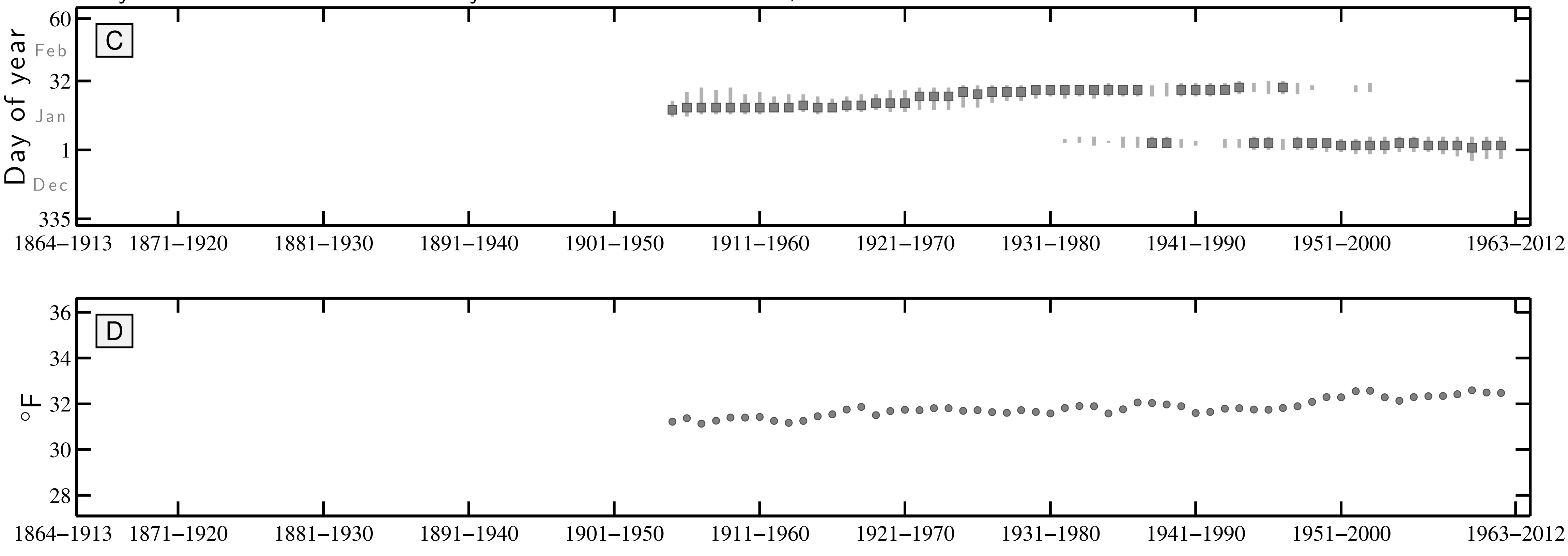

50 year Summer Teletherm dynamics for FORKS 1 E, WA:

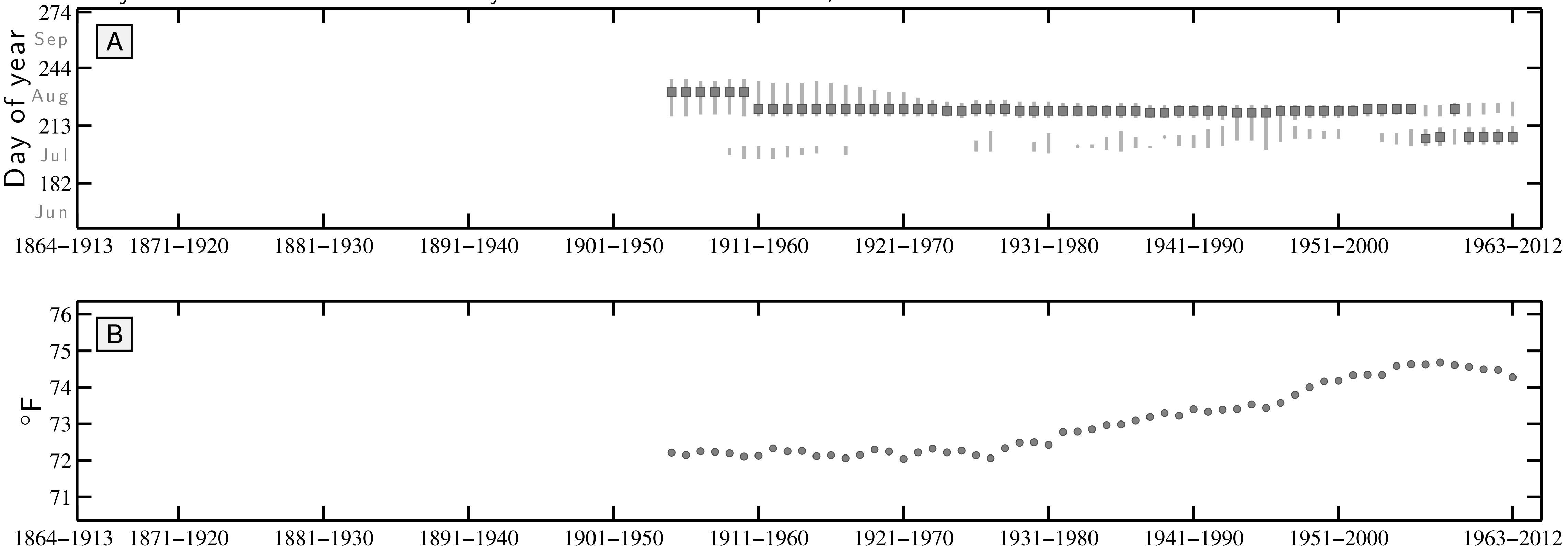

50 year Winter Teletherm dynamics for FORKS 1 E, WA:

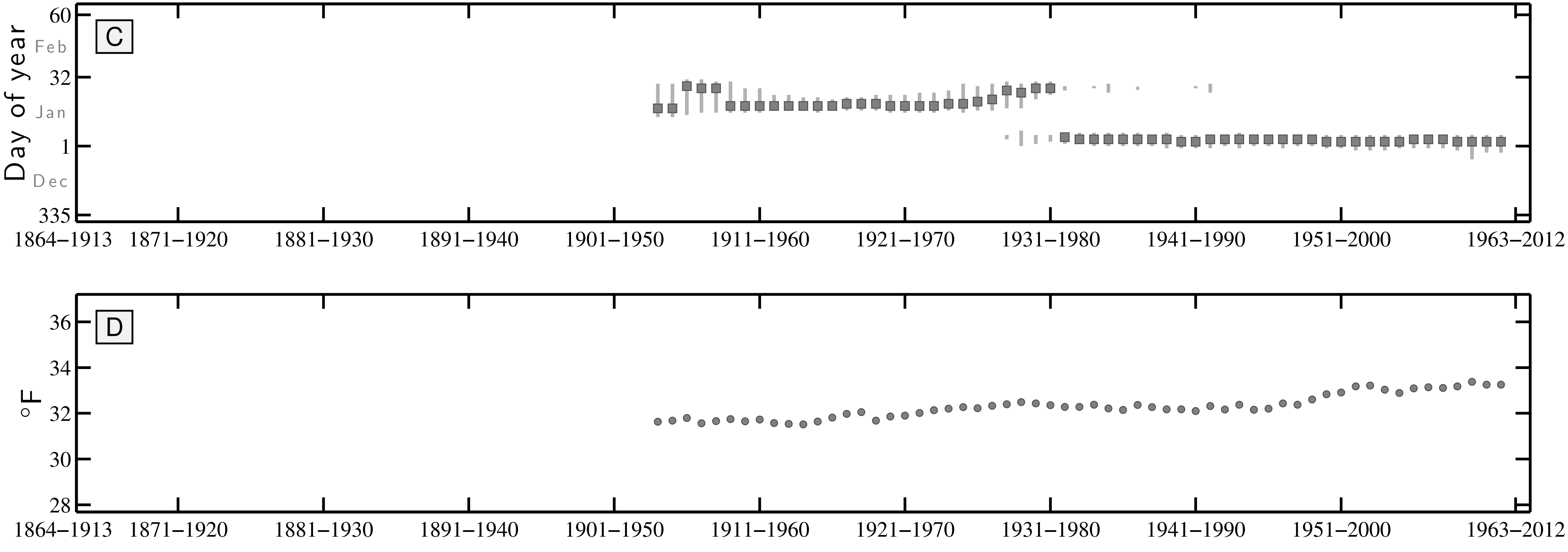

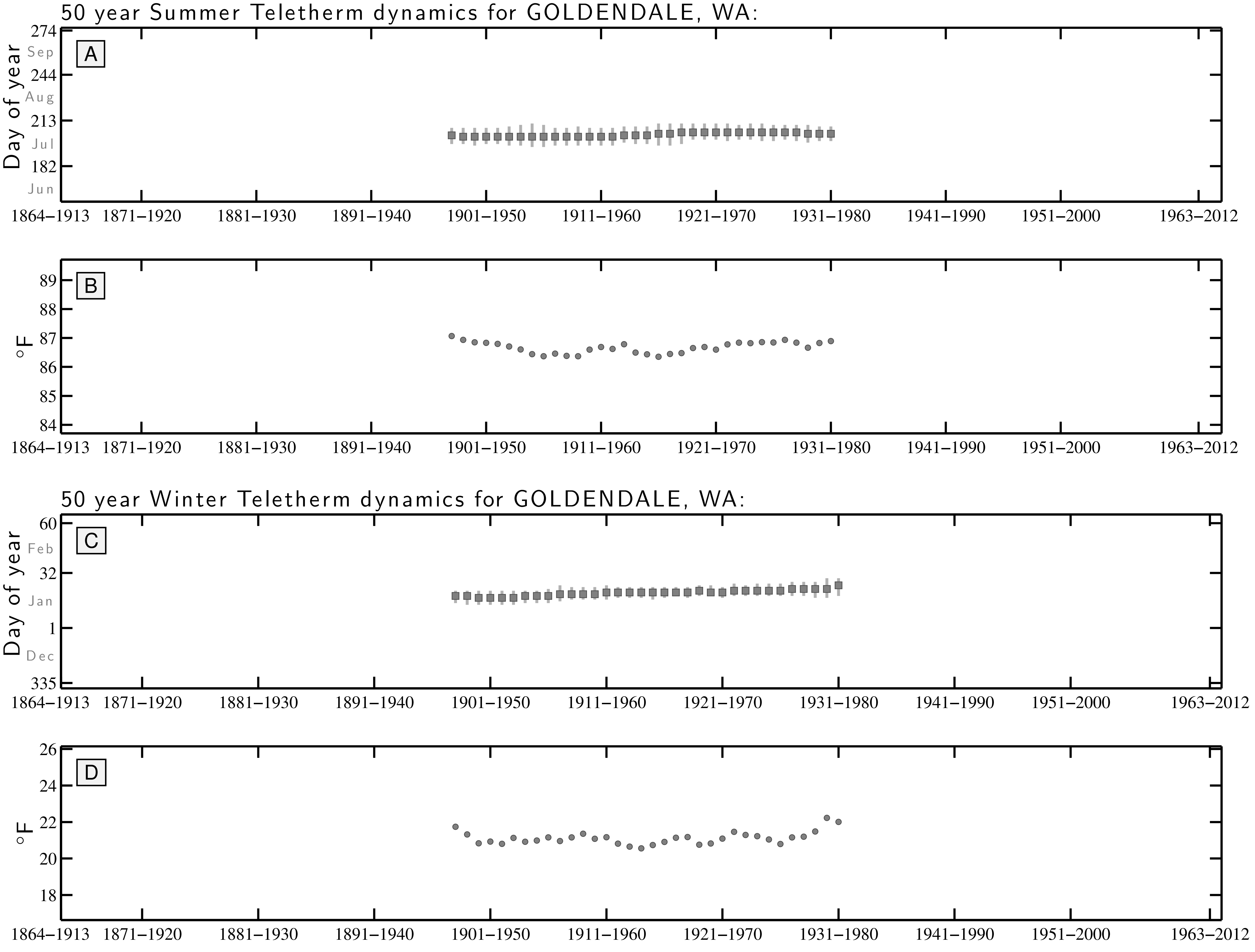

50 year Summer Teletherm dynamics for GOLDENDALE, WA:
A
B
Day of year
274
Sep
244
Aug
213
Jul
182
Jun
°F
89
88
87
86
85
84
1864–1913
1871–1920
1881–1930
1891–1940
1901–1950
1911–1960
1921–1970
1931–1980
1941–1990
1951–2000
1963–2012
50 year Winter Teletherm dynamics for GOLDENDALE, WA:
C
D
60
Feb
32
Jan
1
Dec
335
26
24
22
20
18

50 year Summer Teletherm dynamics for KENNEWICK, WA:

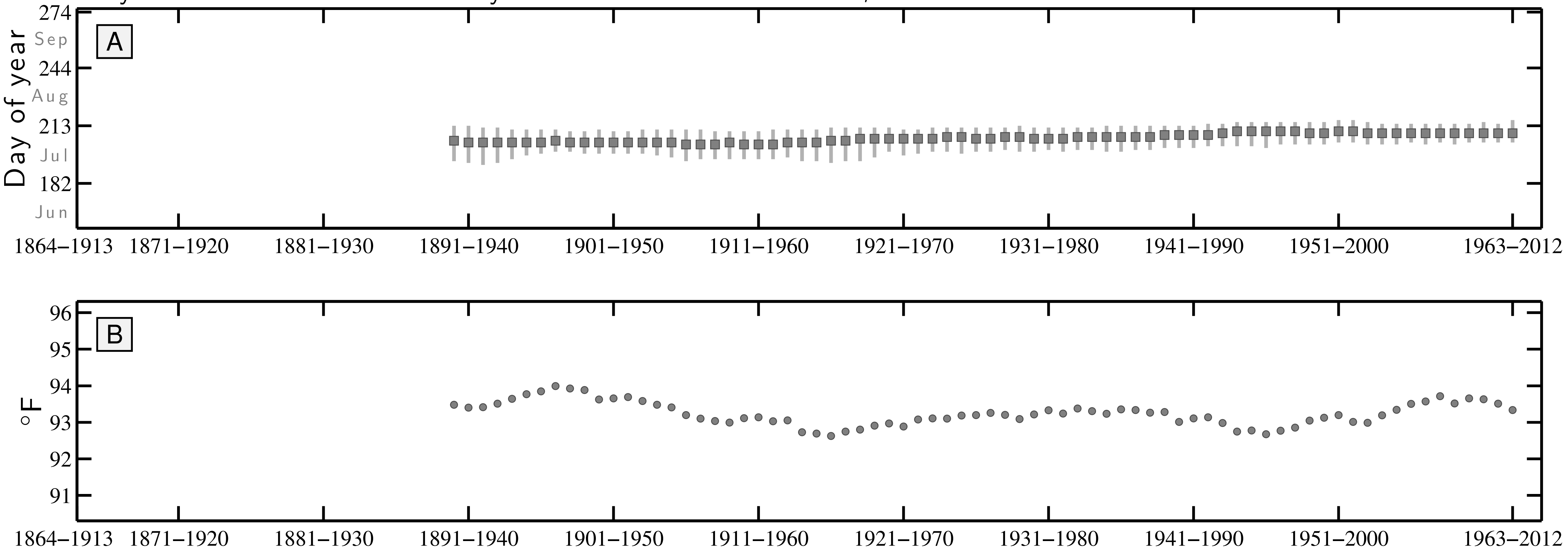

50 year Winter Teletherm dynamics for KENNEWICK, WA:

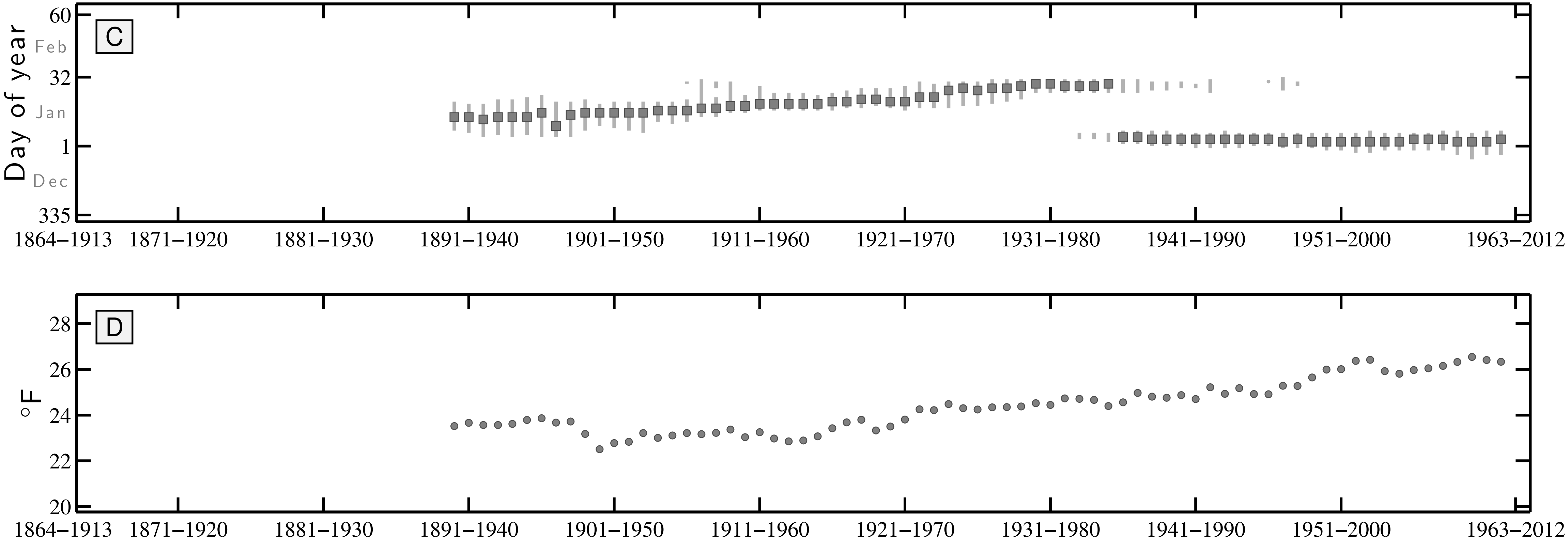

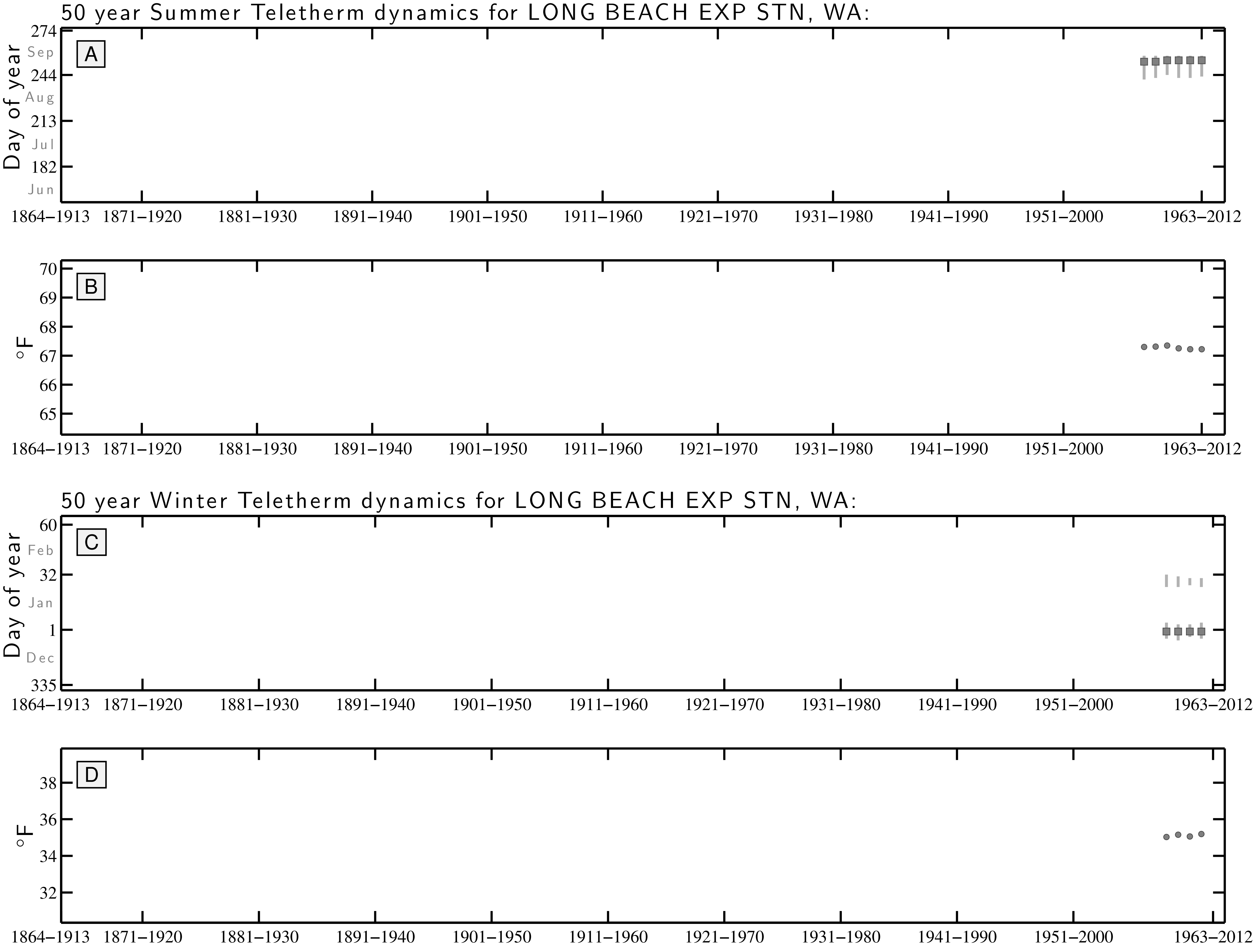
50 year Summer Teletherm dynamics for LONG BEACH EXP STN, WA:
A
Day of year
274
Sep
244
Aug
213
Jul
182
Jun
B
°F
70
69
68
67
66
65
50 year Winter Teletherm dynamics for LONG BEACH EXP STN, WA:
C
Day of year
60
Feb
32
Jan
1
Dec
335
D
°F
38
36
34
32
1864–1913
1871–1920
1881–1930
1891–1940
1901–1950
1911–1960
1921–1970
1931–1980
1941–1990
1951–2000
1963–2012

50 year Summer Teletherm dynamics for LONGVIEW, WA:

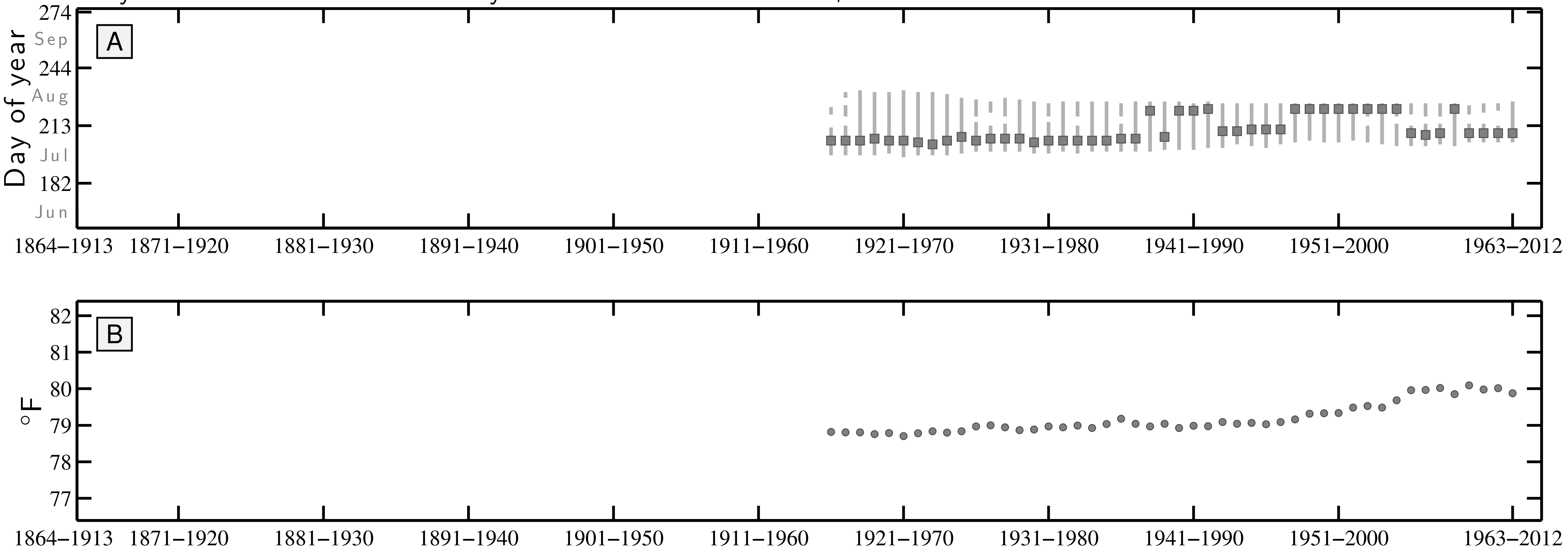

50 year Winter Teletherm dynamics for LONGVIEW, WA:

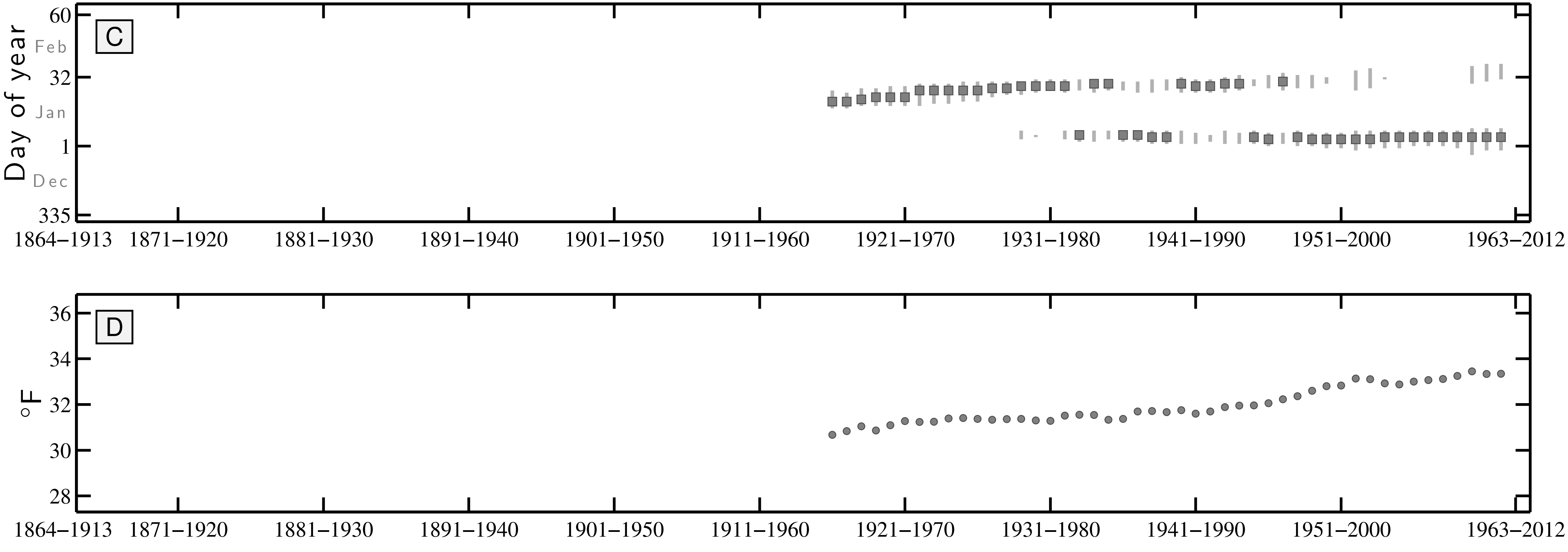

50 year Summer Teletherm dynamics for MC MILLIN RSVR, WA:

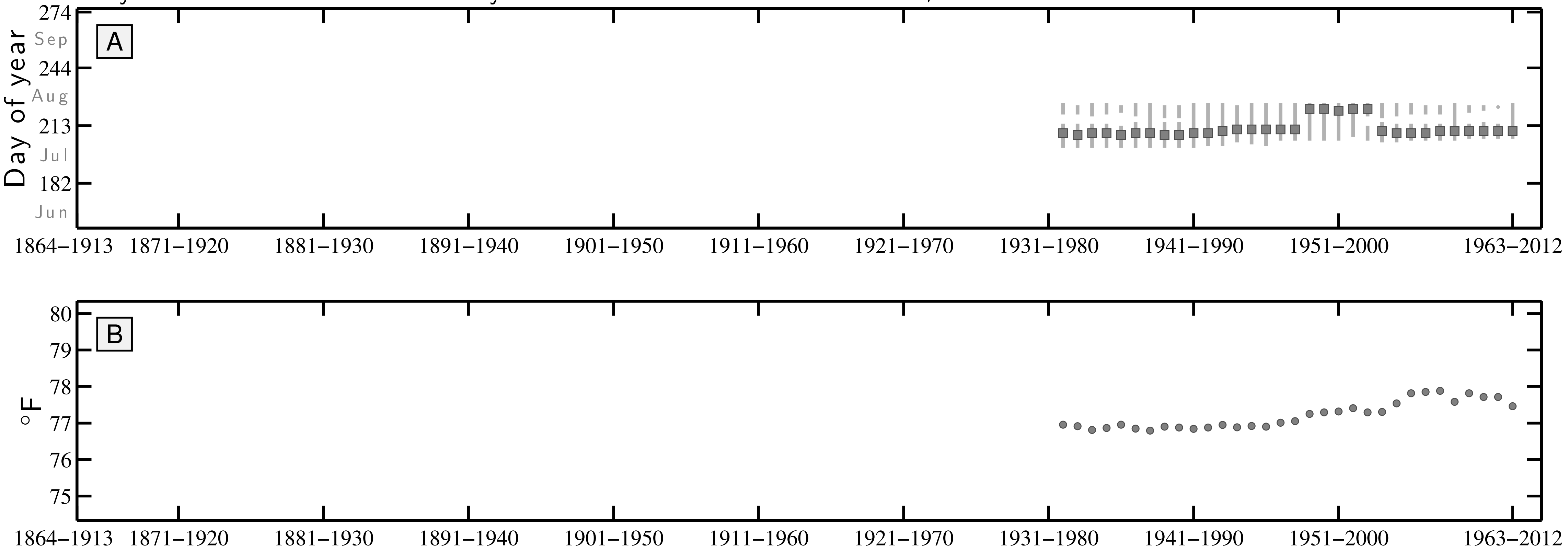

50 year Winter Teletherm dynamics for MC MILLIN RSVR, WA:

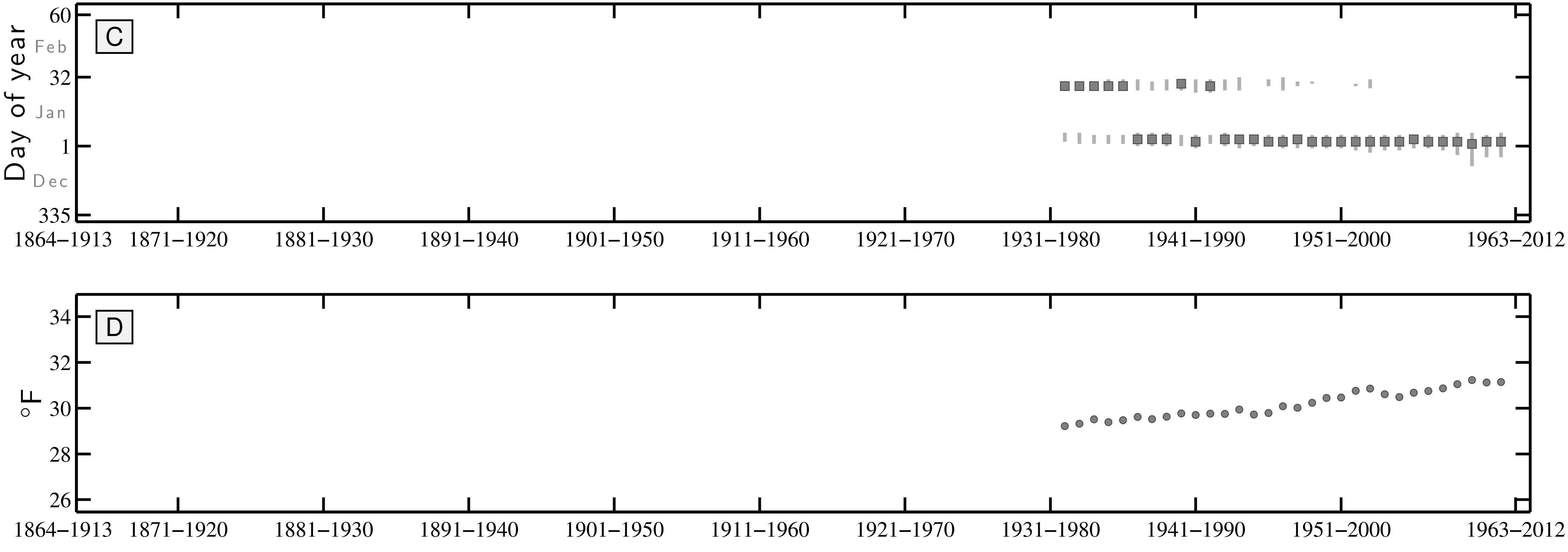

50 year Summer Teletherm dynamics for NORTHPORT, WA:

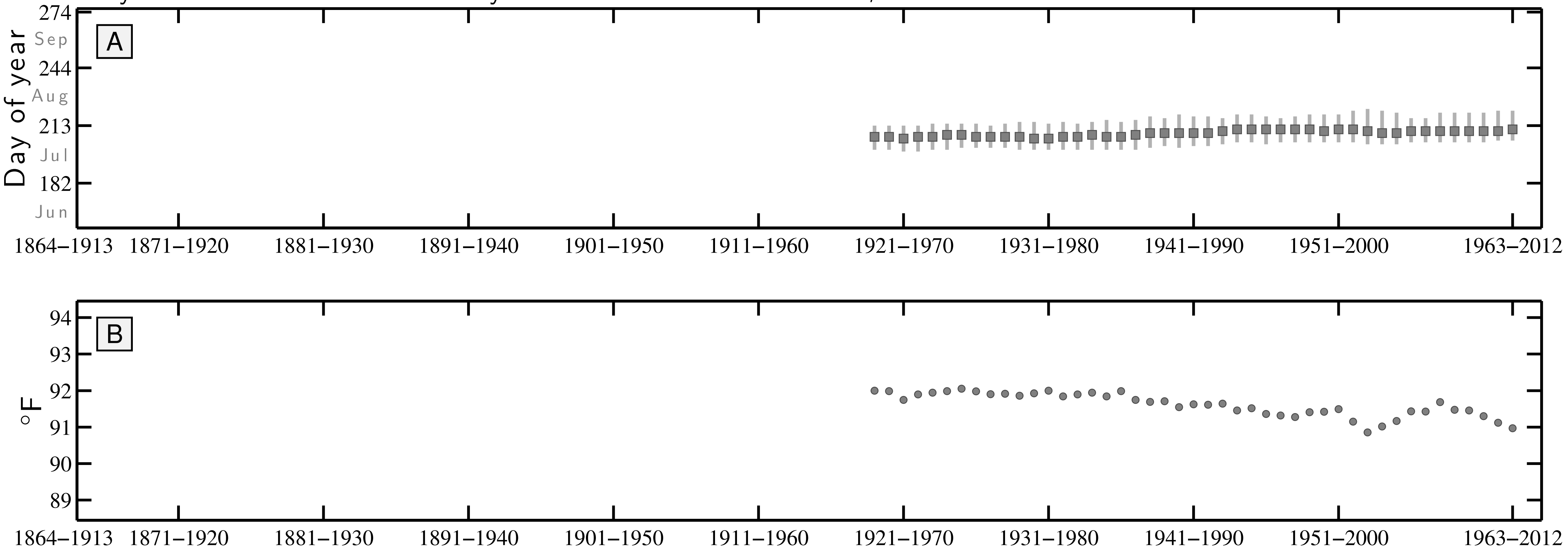

50 year Winter Teletherm dynamics for NORTHPORT, WA:

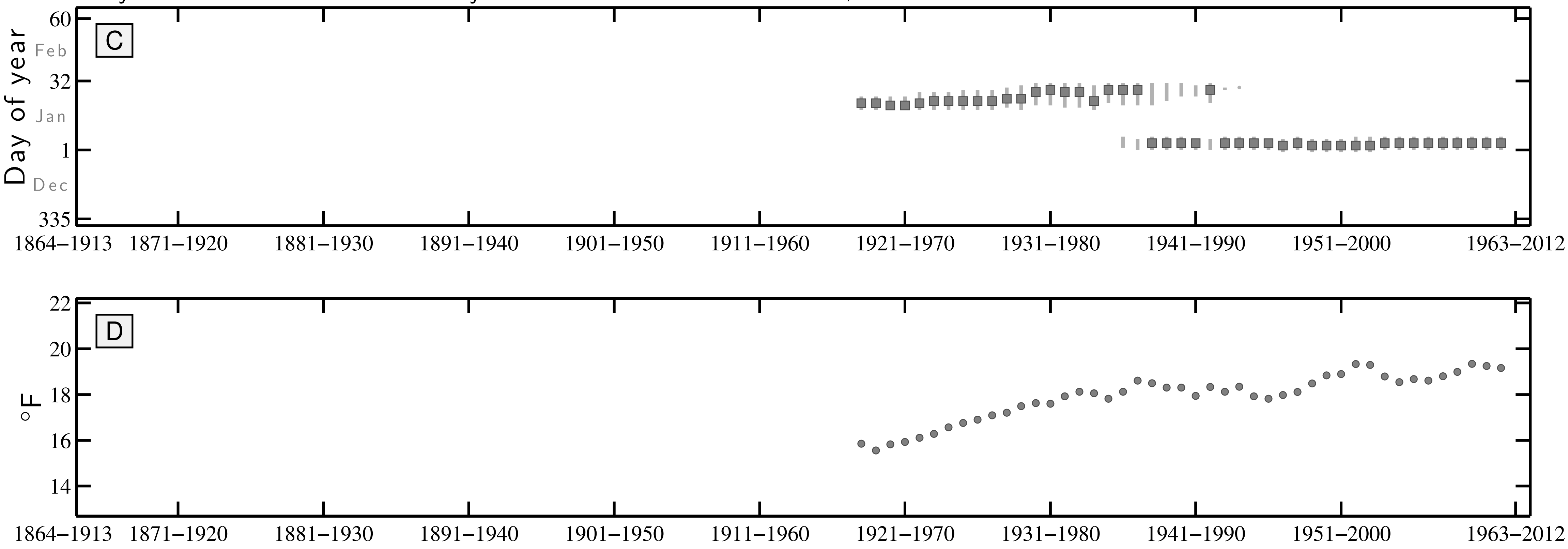

50 year Summer Teletherm dynamics for ODESSA, WA:

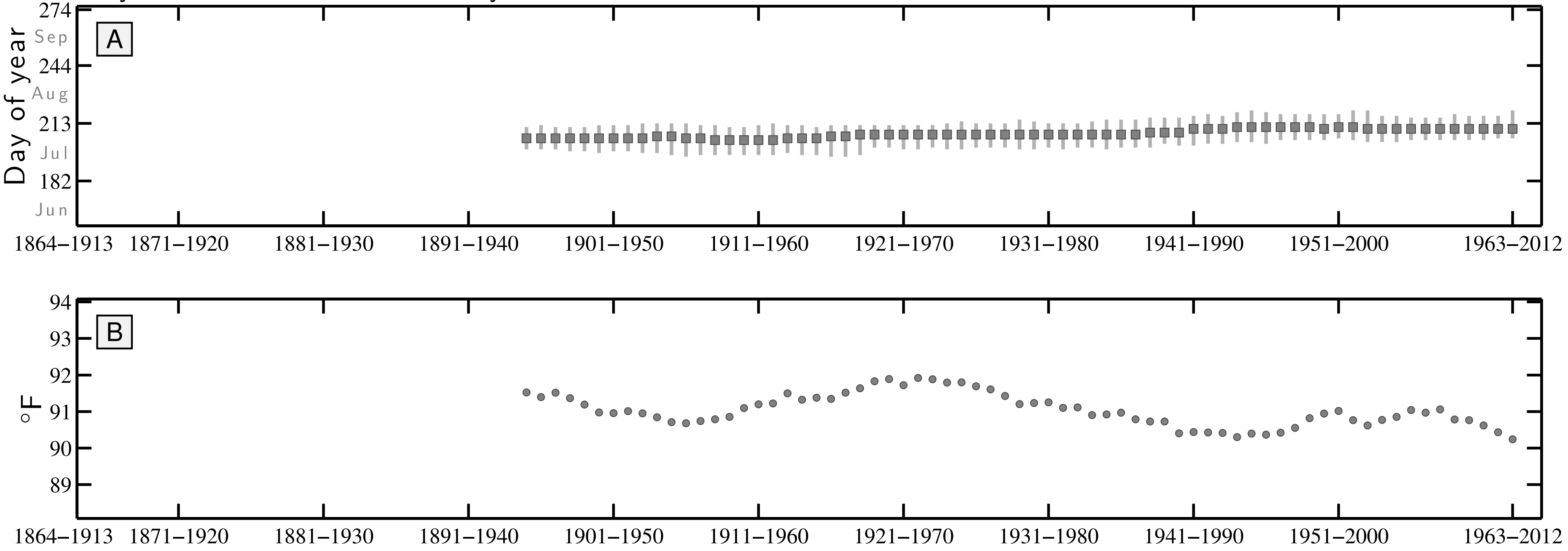

50 year Winter Teletherm dynamics for ODESSA, WA:

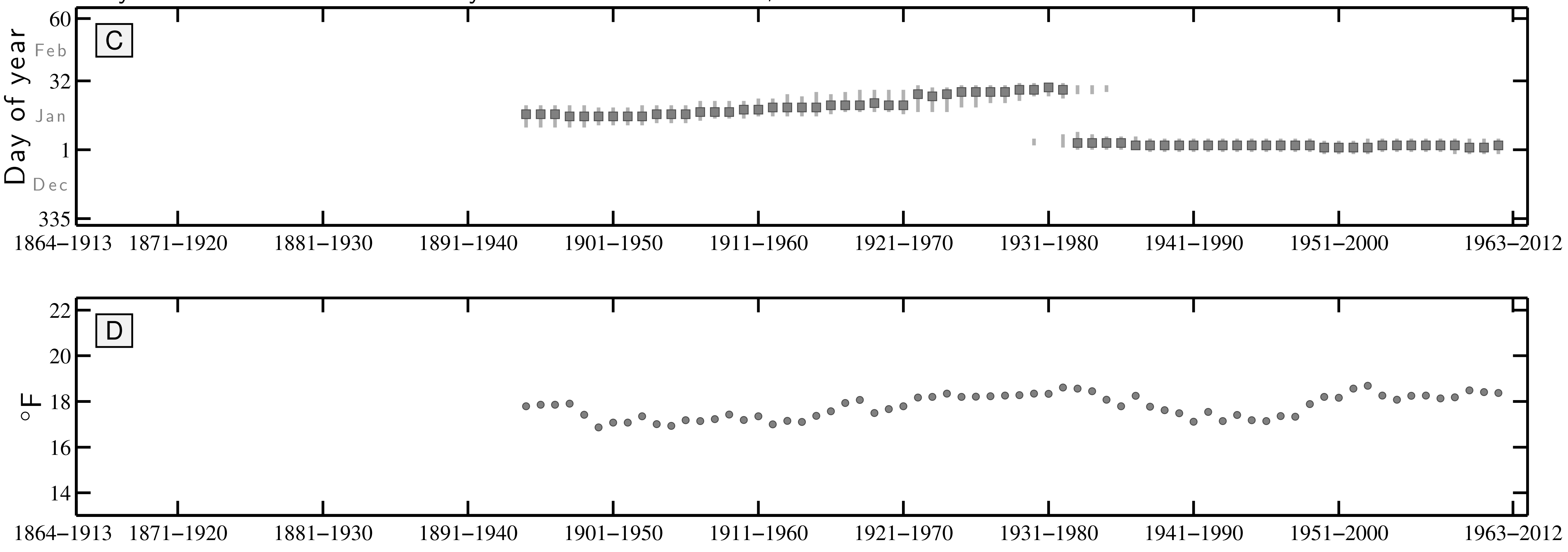

50 year Summer Teletherm dynamics for OLGA 2 SE, WA:

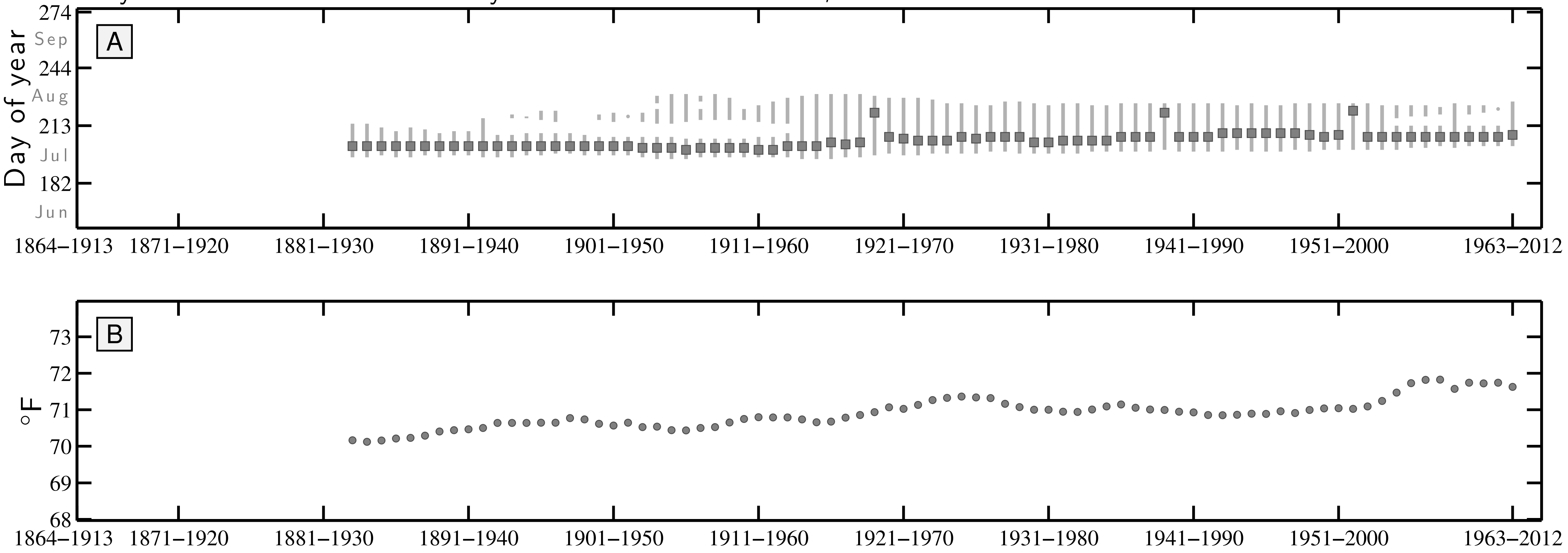

50 year Winter Teletherm dynamics for OLGA 2 SE, WA:

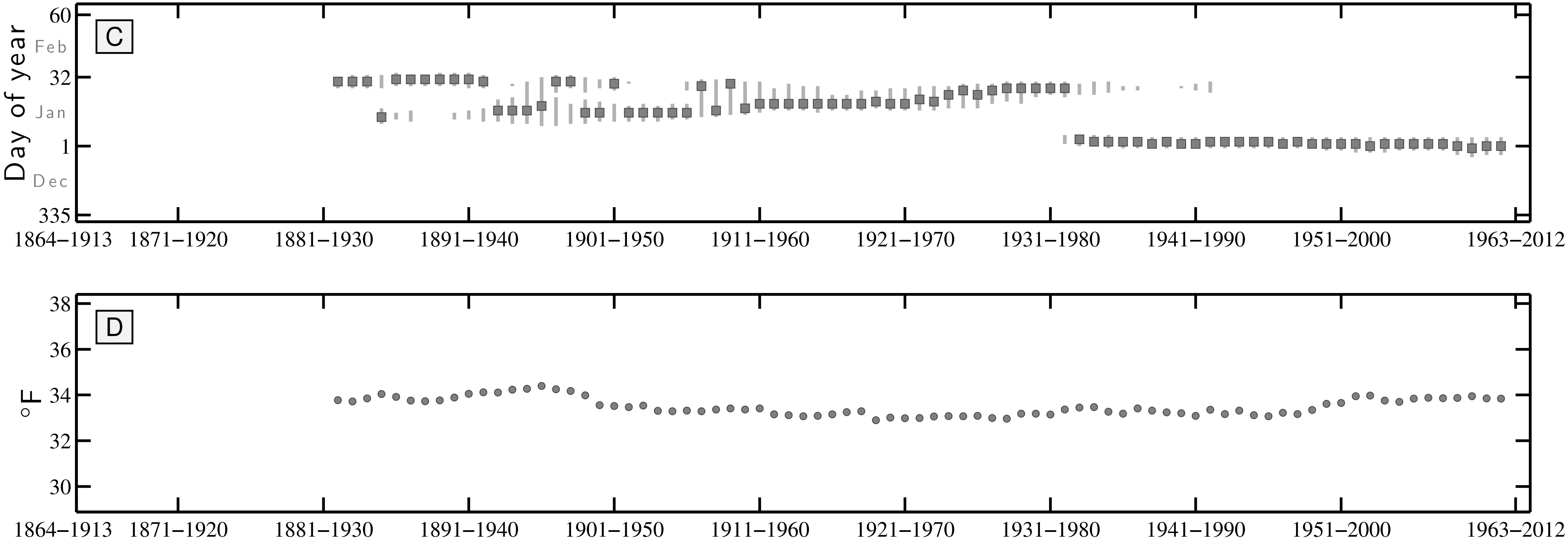

50 year Summer Teletherm dynamics for POMEROY, WA:

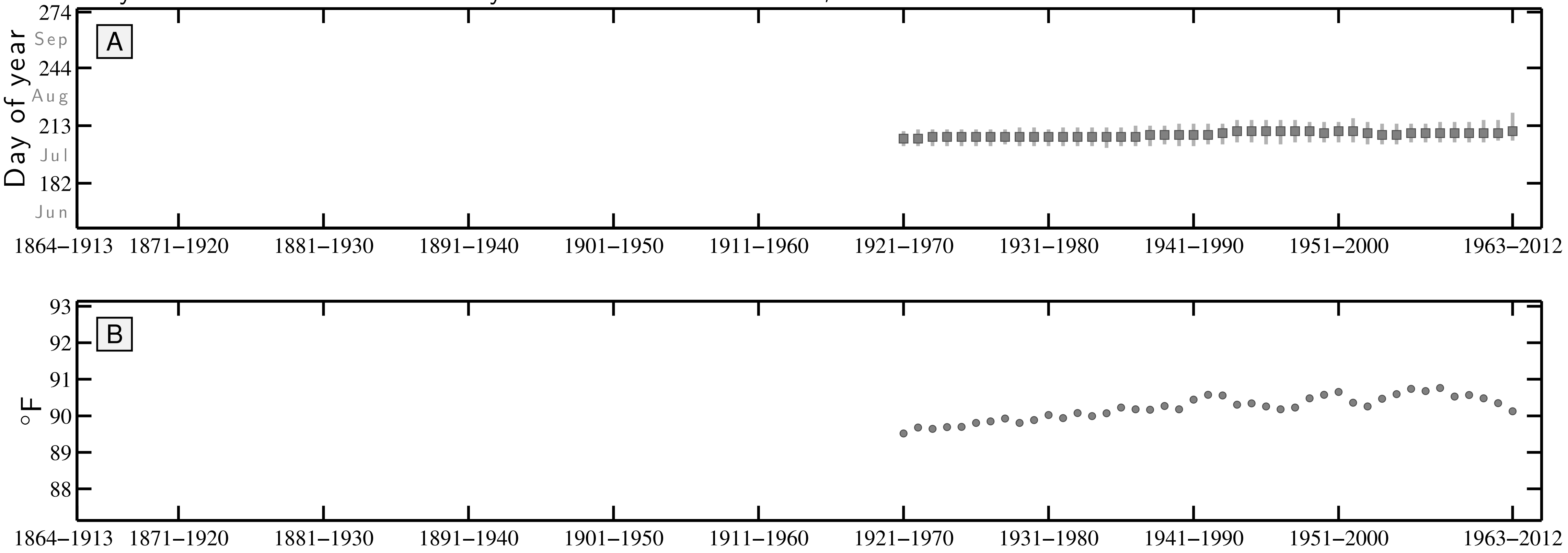

50 year Winter Teletherm dynamics for POMEROY, WA:

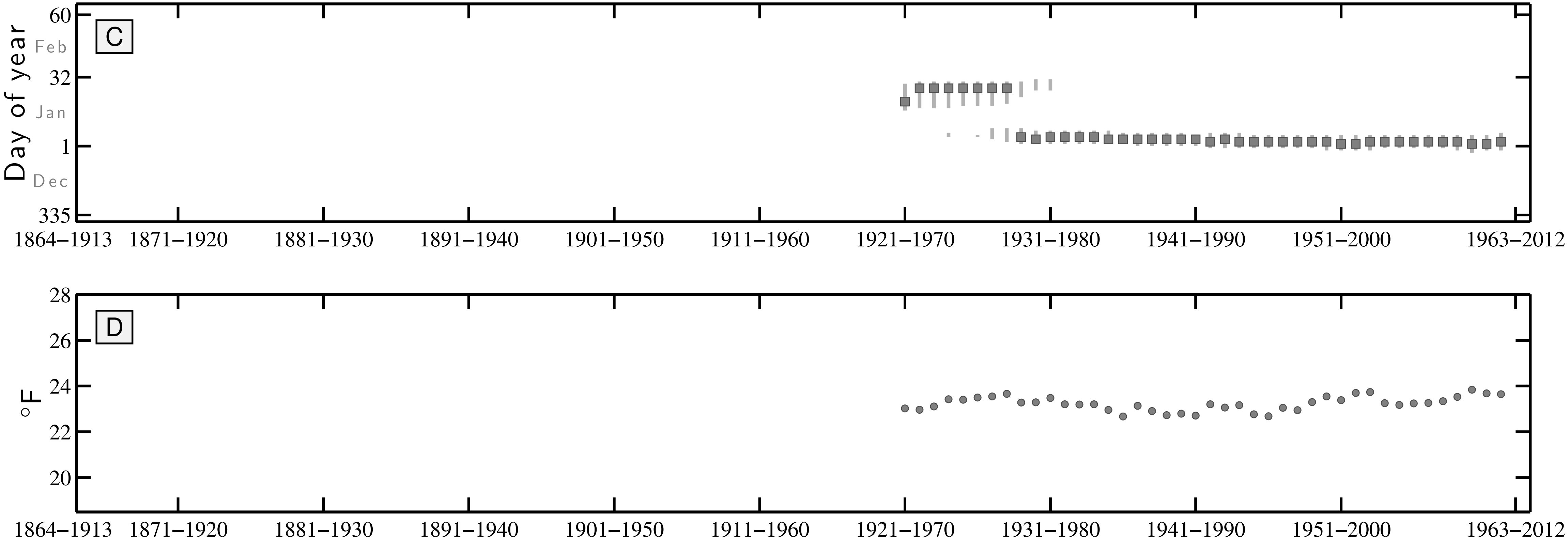

50 year Summer Teletherm dynamics for PORT ANGELES, WA:

50 year Winter Teletherm dynamics for PORT ANGELES, WA:

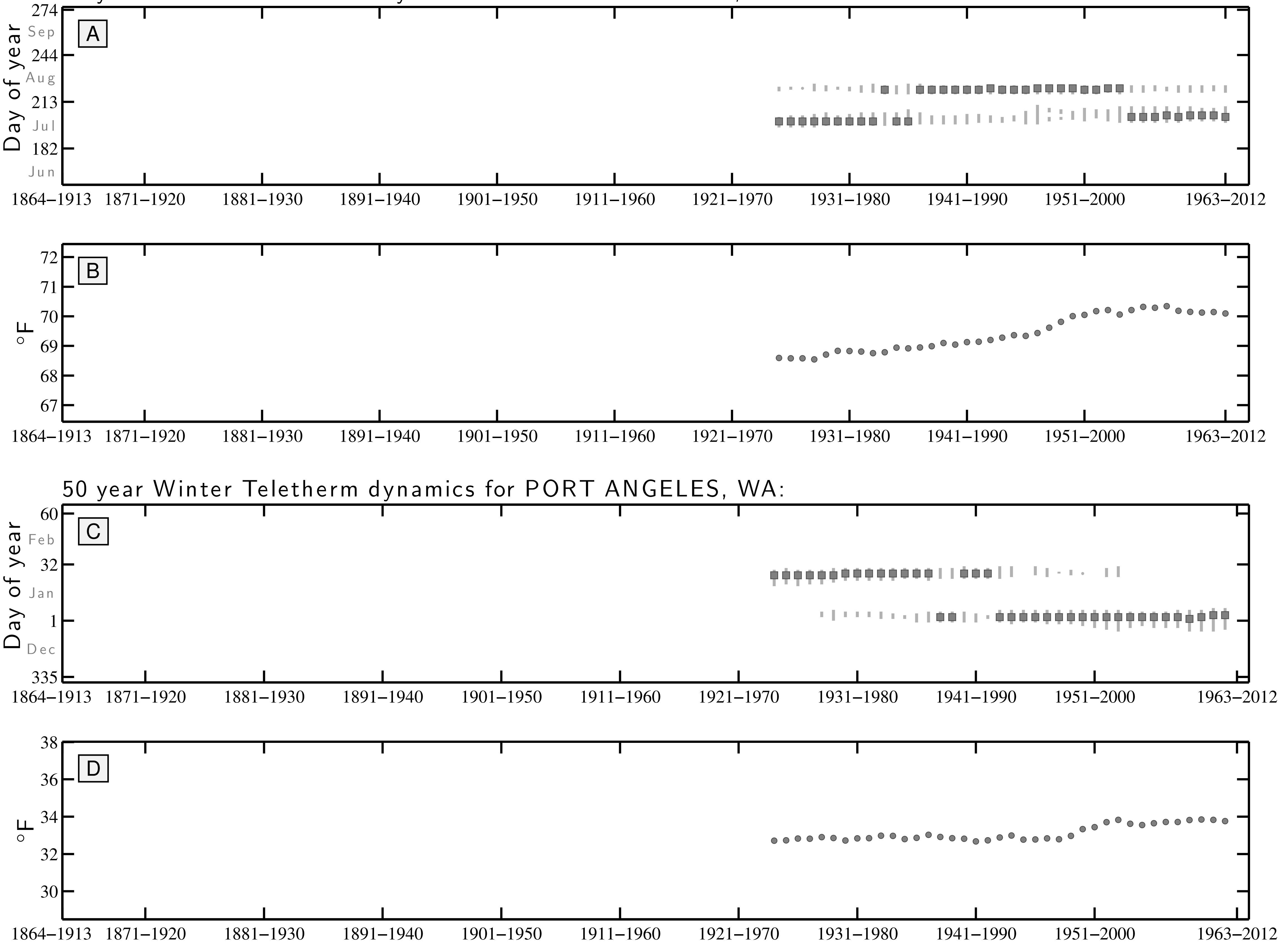

50 year Summer Teletherm dynamics for PORT TOWNSEND, WA:

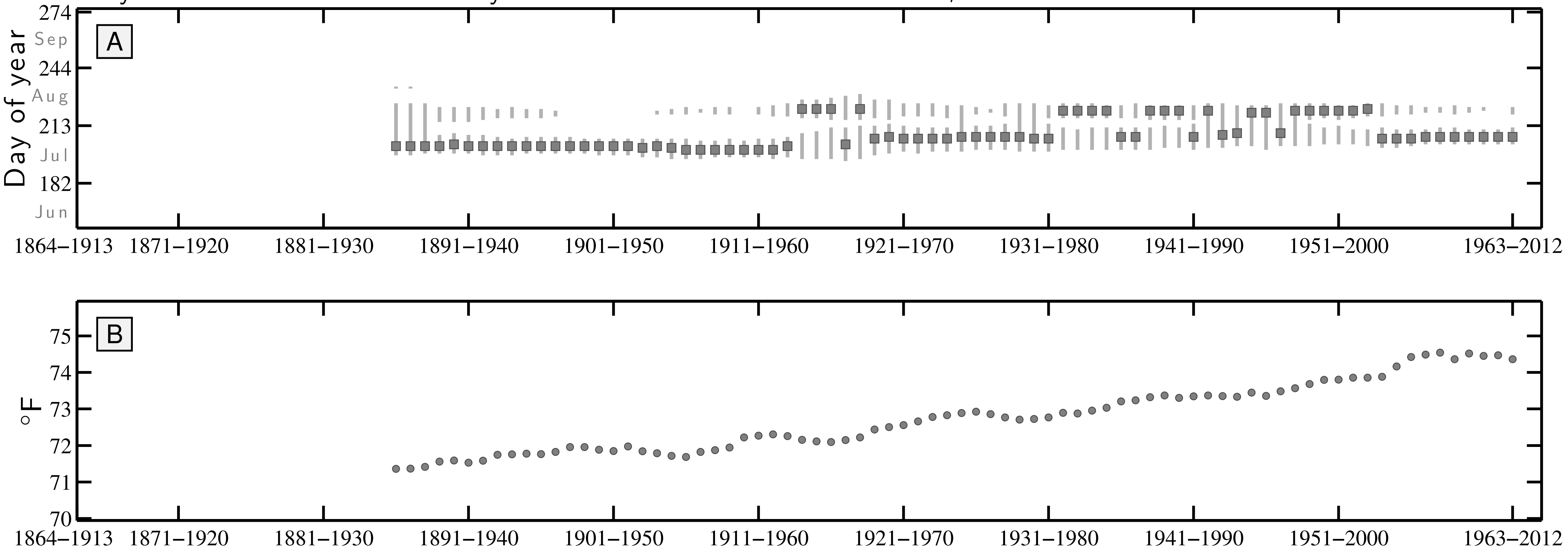

50 year Winter Teletherm dynamics for PORT TOWNSEND, WA:

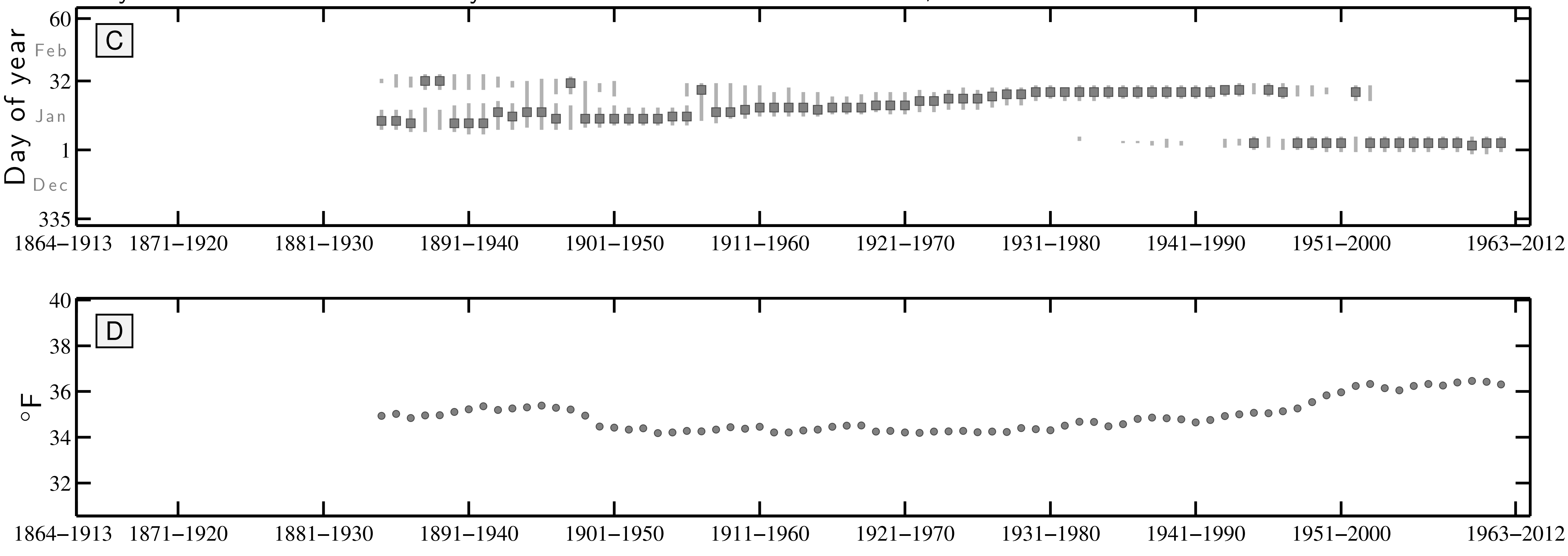

50 year Summer Teletherm dynamics for PULLMAN 2 NW, WA:

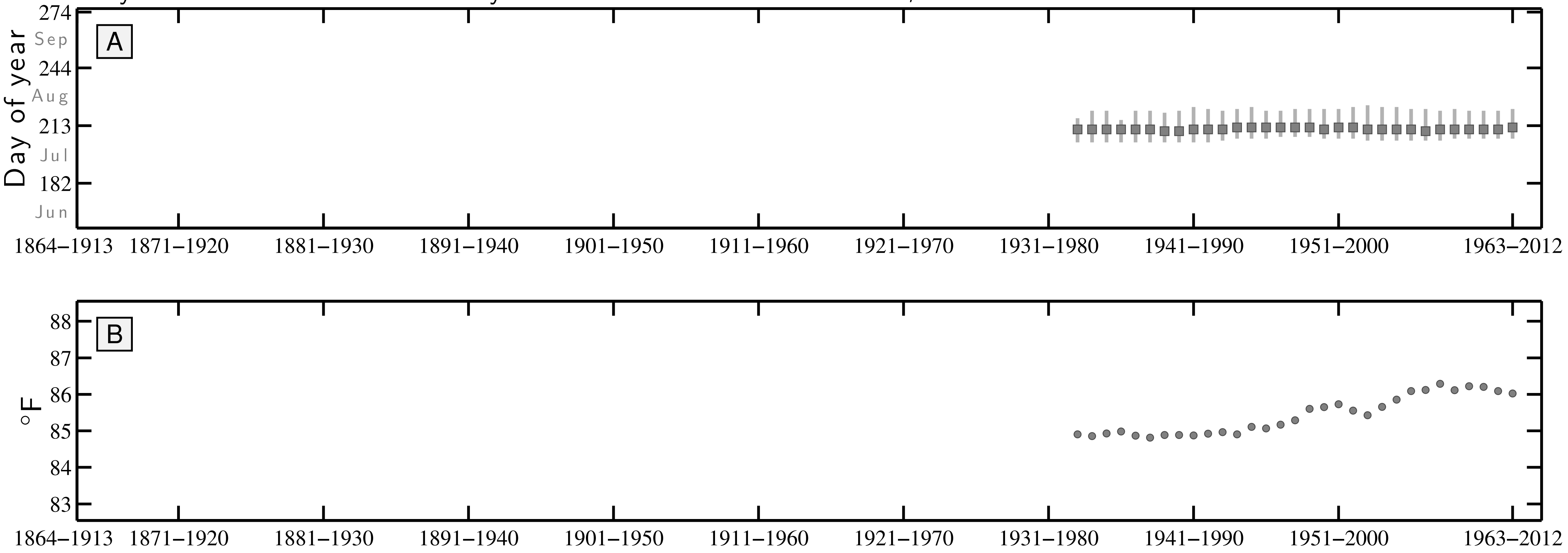

50 year Winter Teletherm dynamics for PULLMAN 2 NW, WA:

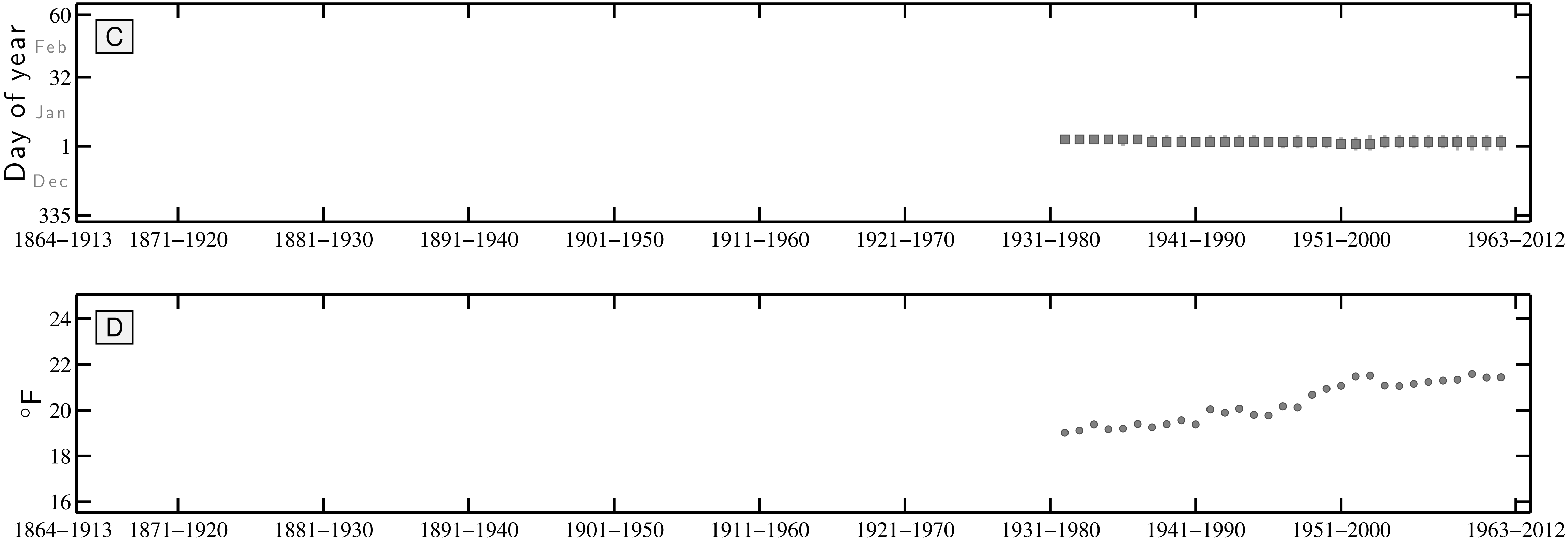

50 year Summer Teletherm dynamics for RITZVILLE 1 SSE, WA:

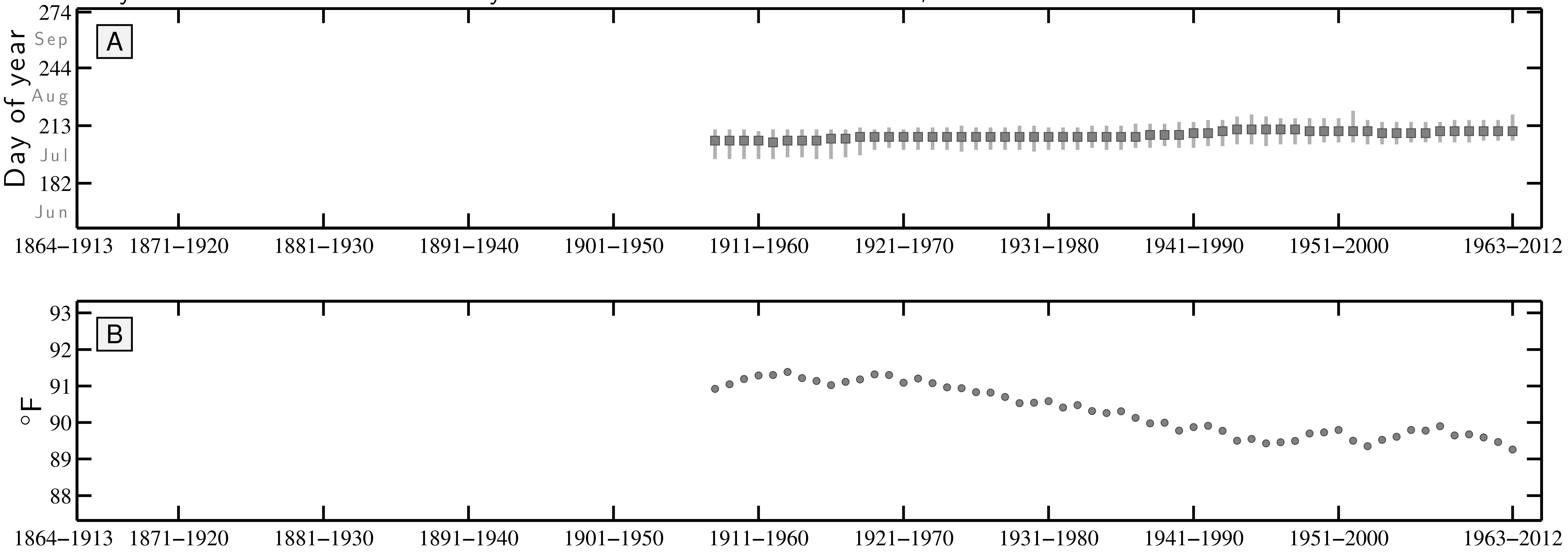

50 year Winter Teletherm dynamics for RITZVILLE 1 SSE, WA:

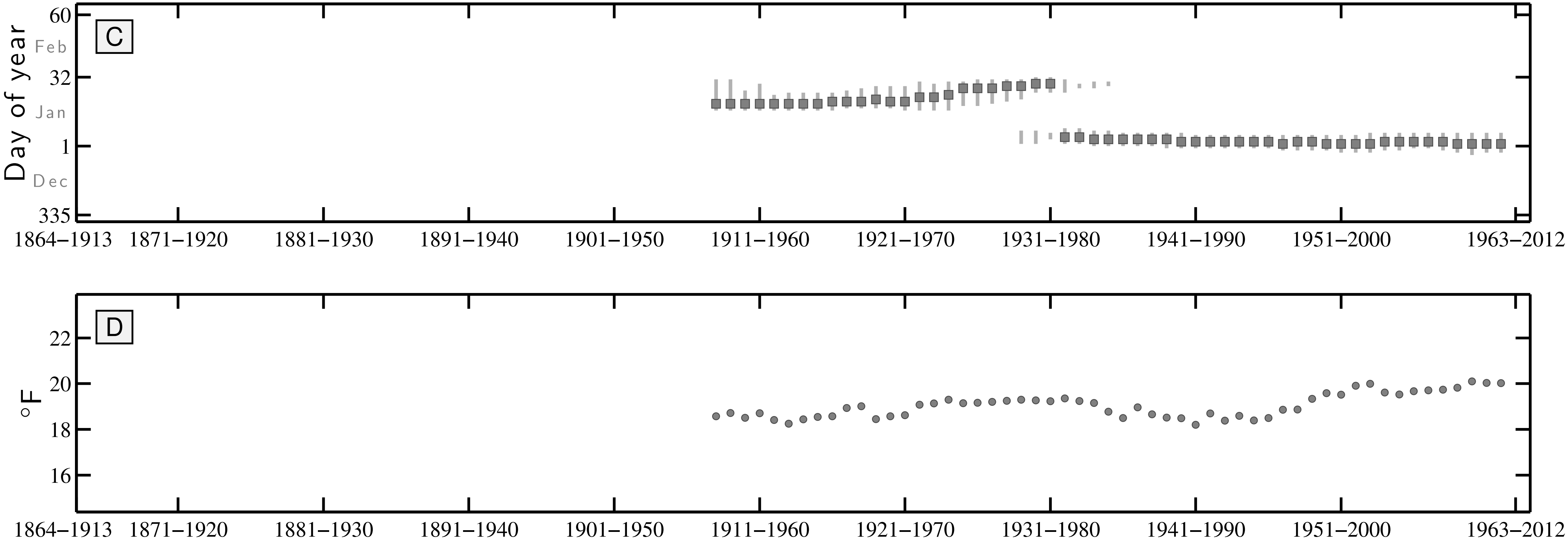

50 year Summer Teletherm dynamics for SAINT JOHN, WA:

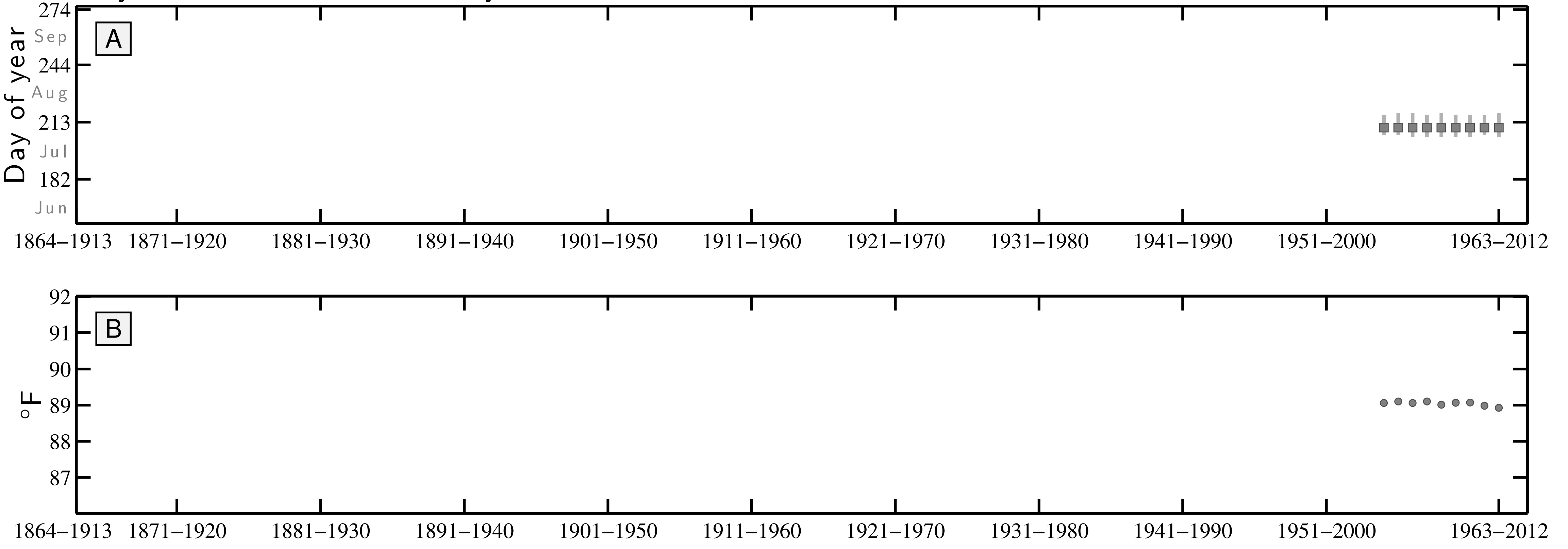

50 year Winter Teletherm dynamics for SAINT JOHN, WA:

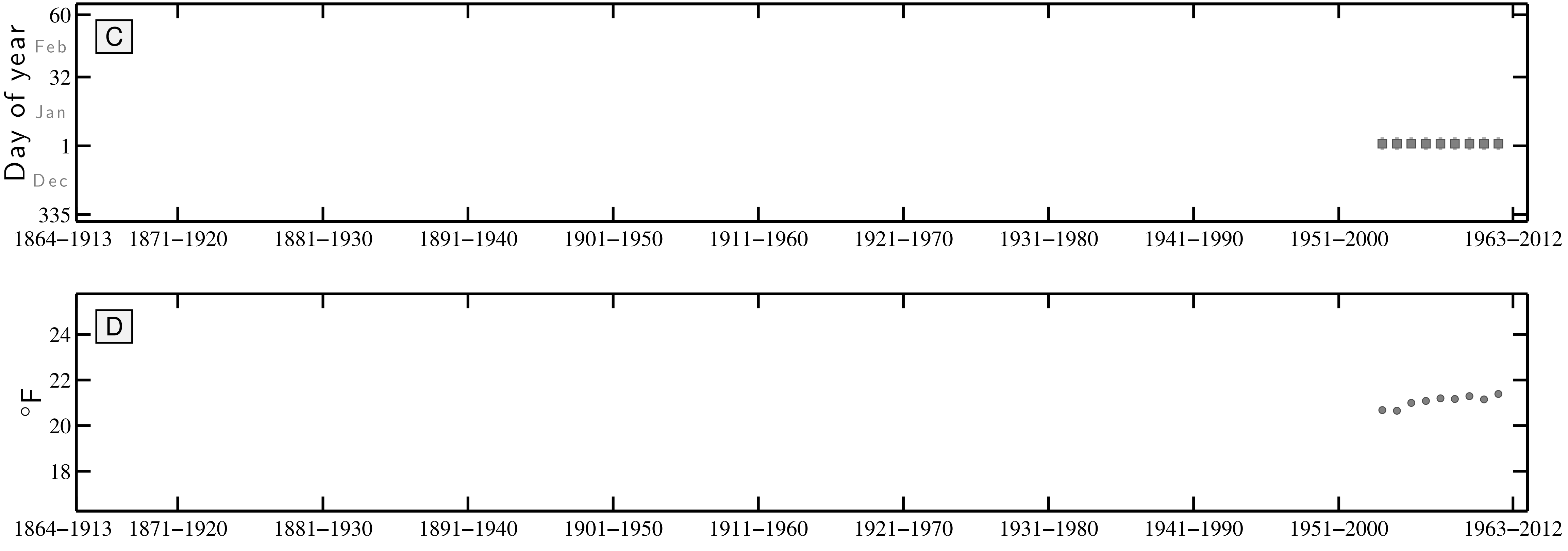

50 year Summer Teletherm dynamics for SEATTLE URBAN SITE, WA:

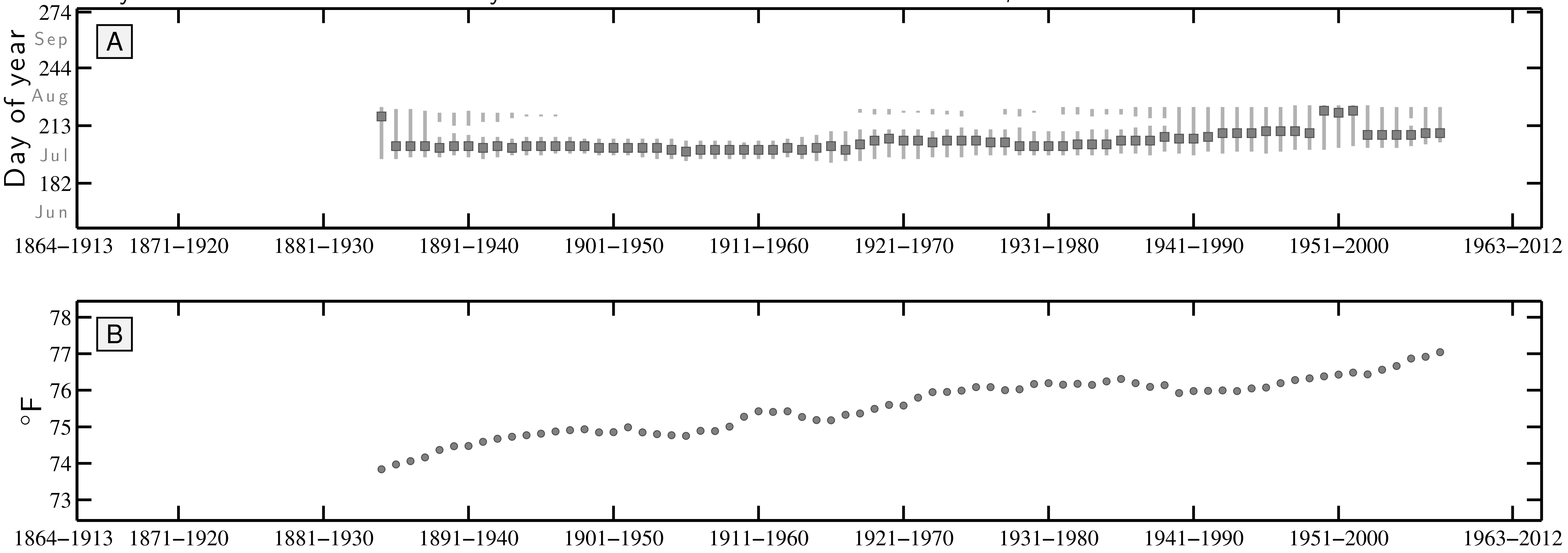

50 year Winter Teletherm dynamics for SEATTLE URBAN SITE, WA:

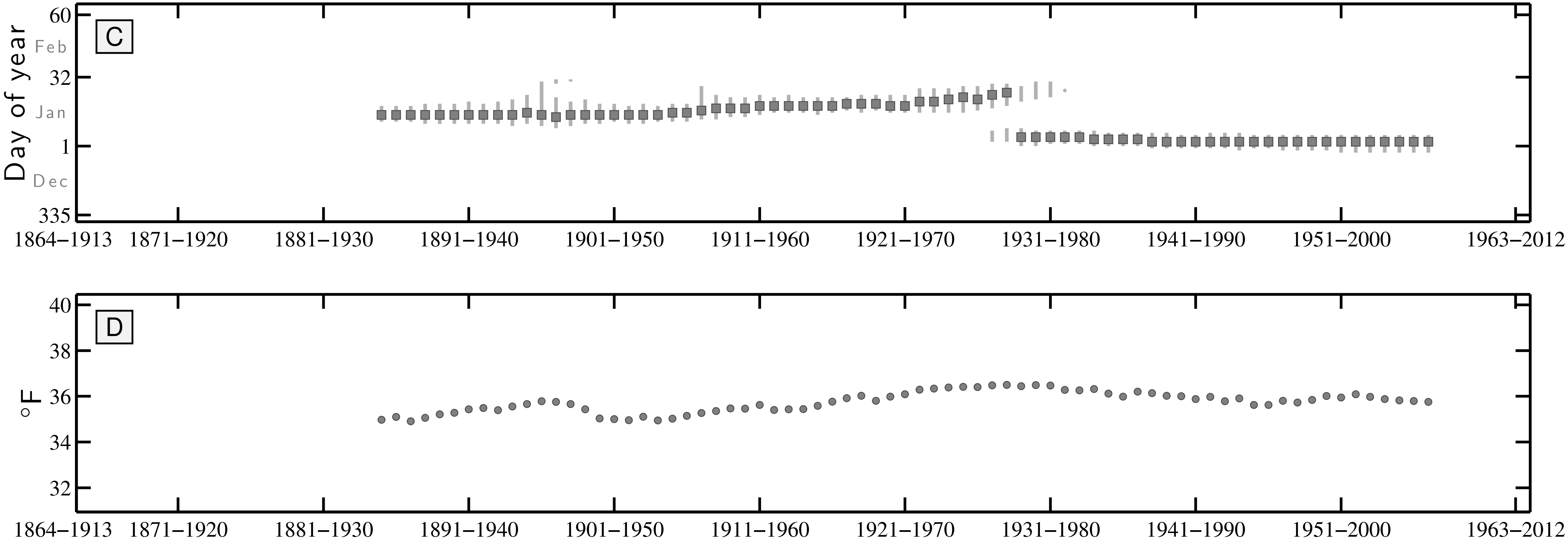

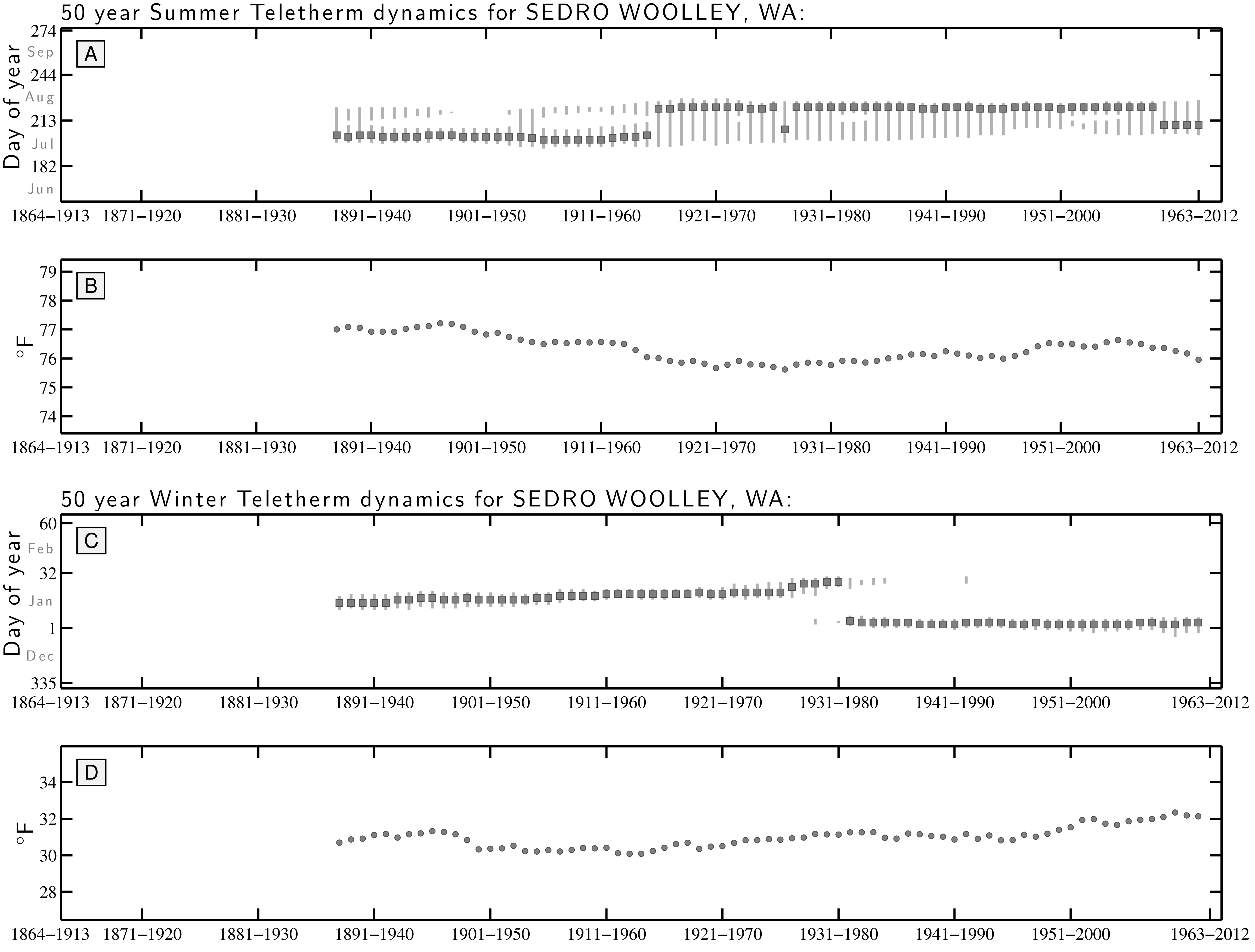
50 year Summer Teletherm dynamics for SEDRO WOOLLEY, WA:
A
B
Day of year
°F
274
Sep
244
Aug
213
Jul
182
Jun
79
78
77
76
75
74
1864–1913
1871–1920
1881–1930
1891–1940
1901–1950
1911–1960
1921–1970
1931–1980
1941–1990
1951–2000
1963–2012
50 year Winter Teletherm dynamics for SEDRO WOOLLEY, WA:
C
D
60
Feb
32
Jan
1
Dec
335
34
32
30
28

50 year Summer Teletherm dynamics for SNOQUALMIE FALLS, WA:

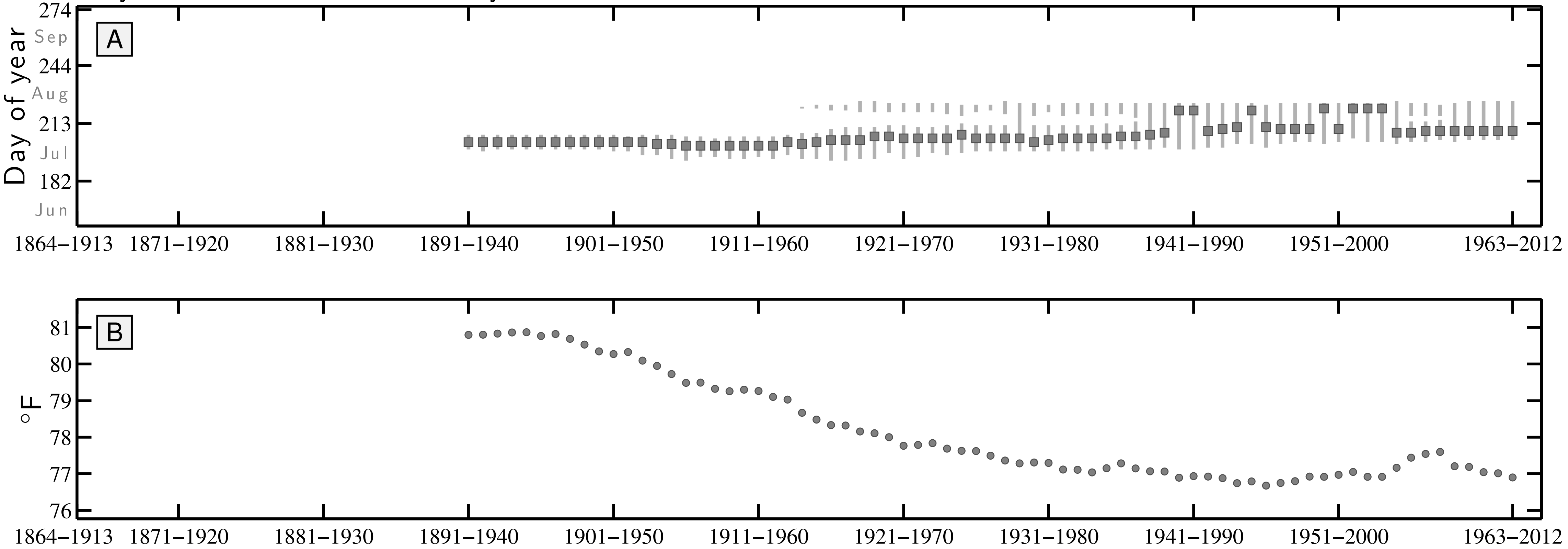

50 year Winter Teletherm dynamics for SNOQUALMIE FALLS, WA:

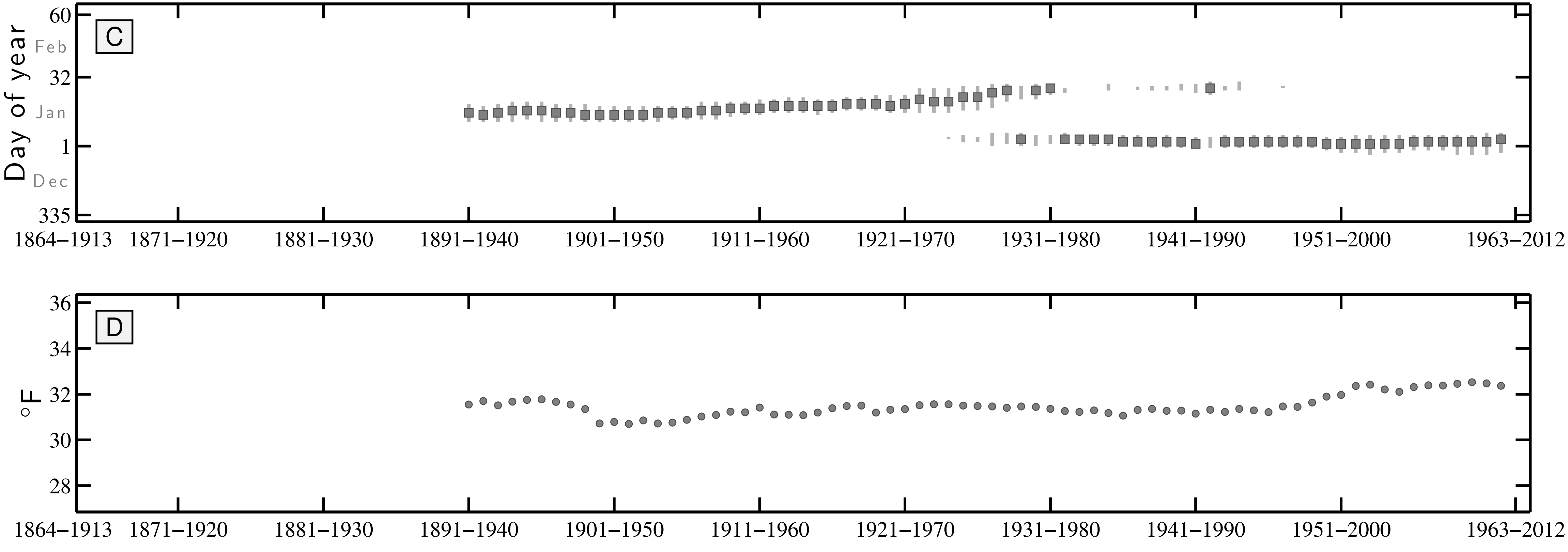

50 year Summer Teletherm dynamics for SPOKANE INTL AP, WA:

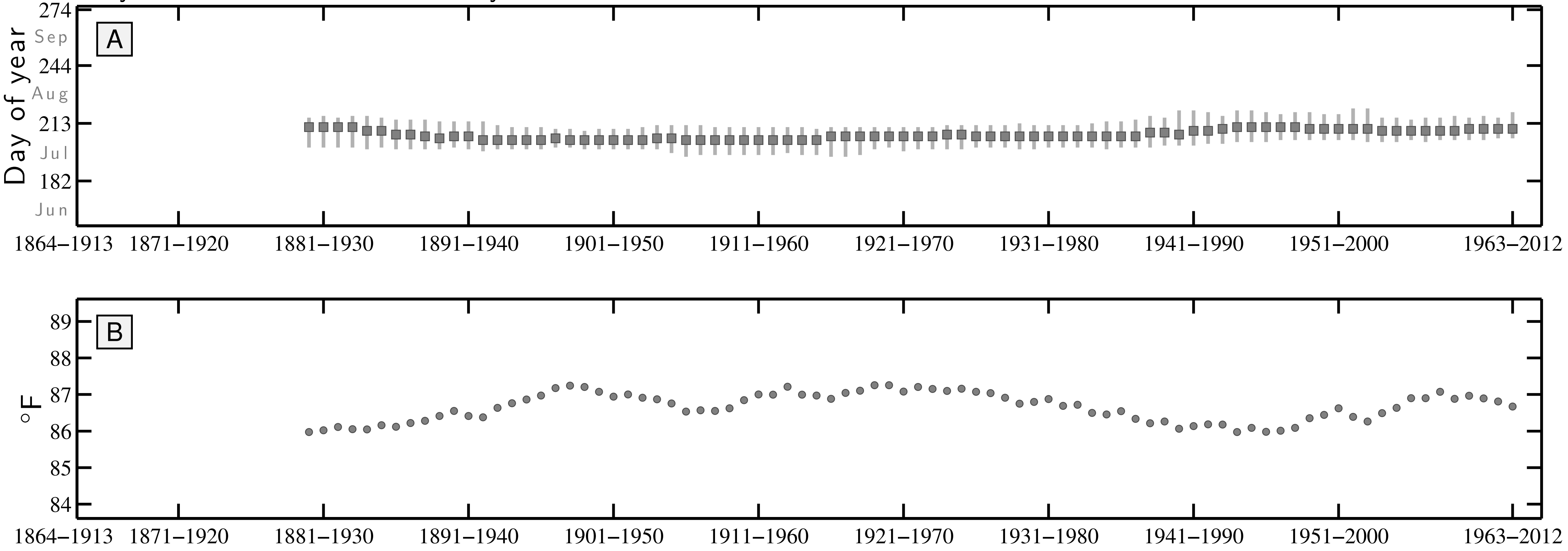

50 year Winter Teletherm dynamics for SPOKANE INTL AP, WA:

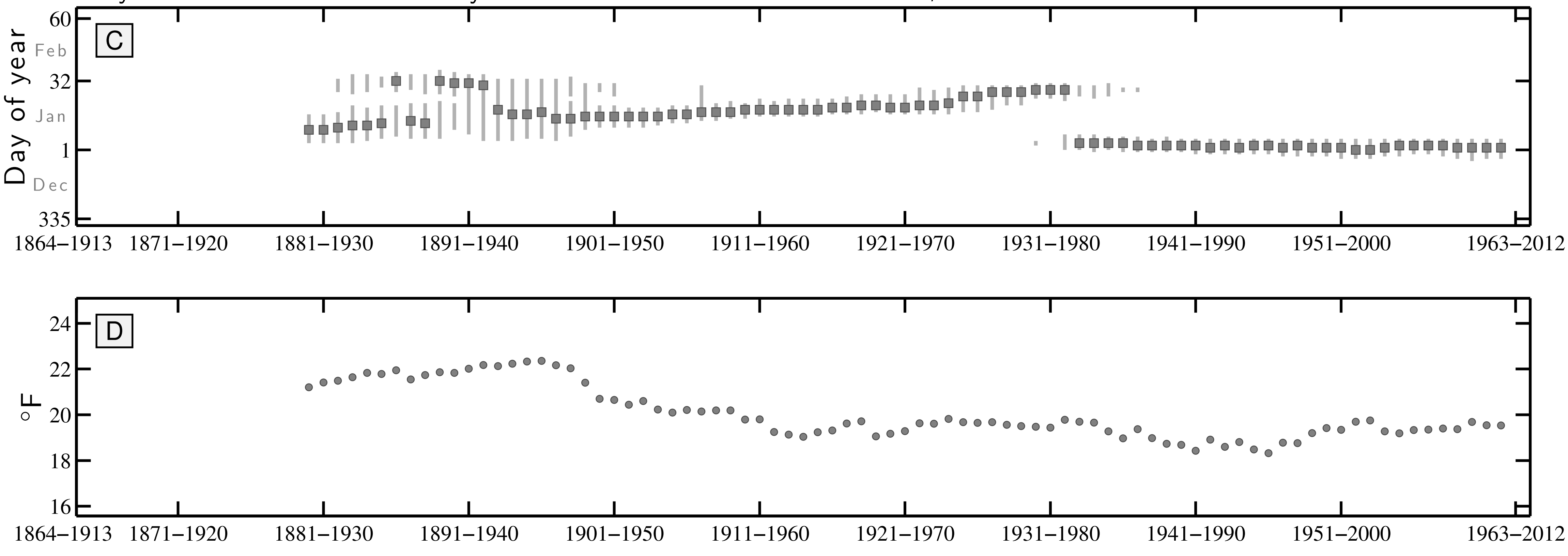

50 year Summer Teletherm dynamics for STEHEKIN 4 NW, WA:

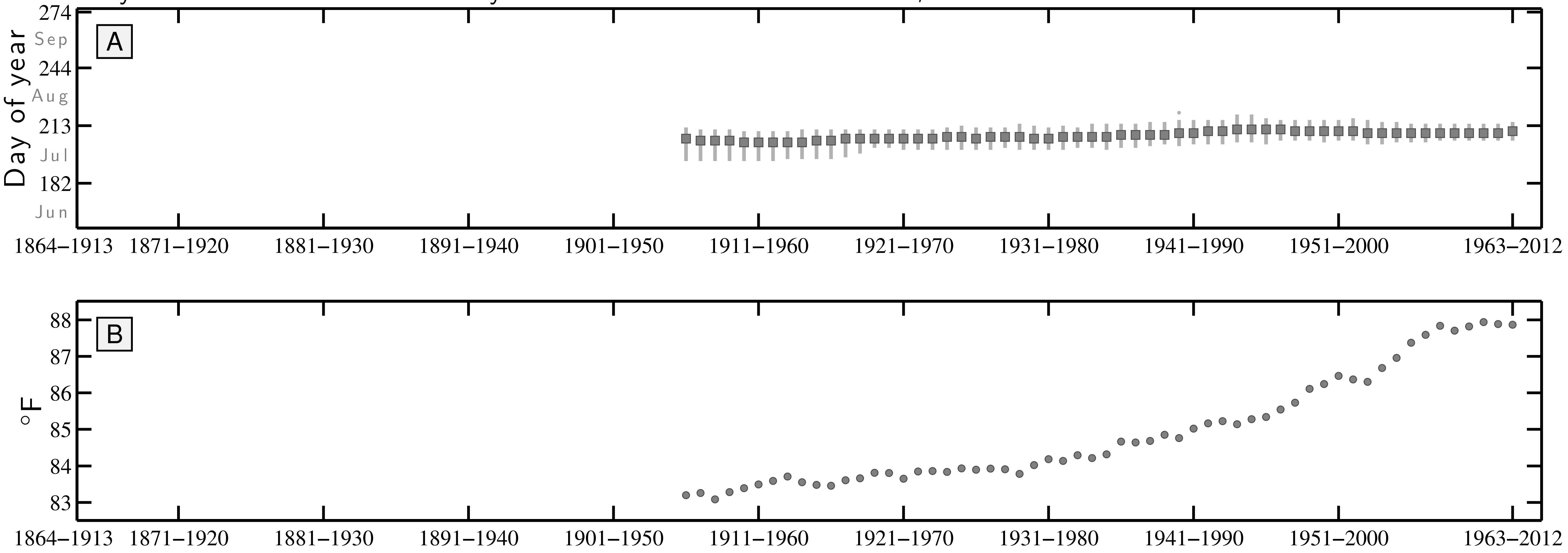

50 year Winter Teletherm dynamics for STEHEKIN 4 NW, WA:

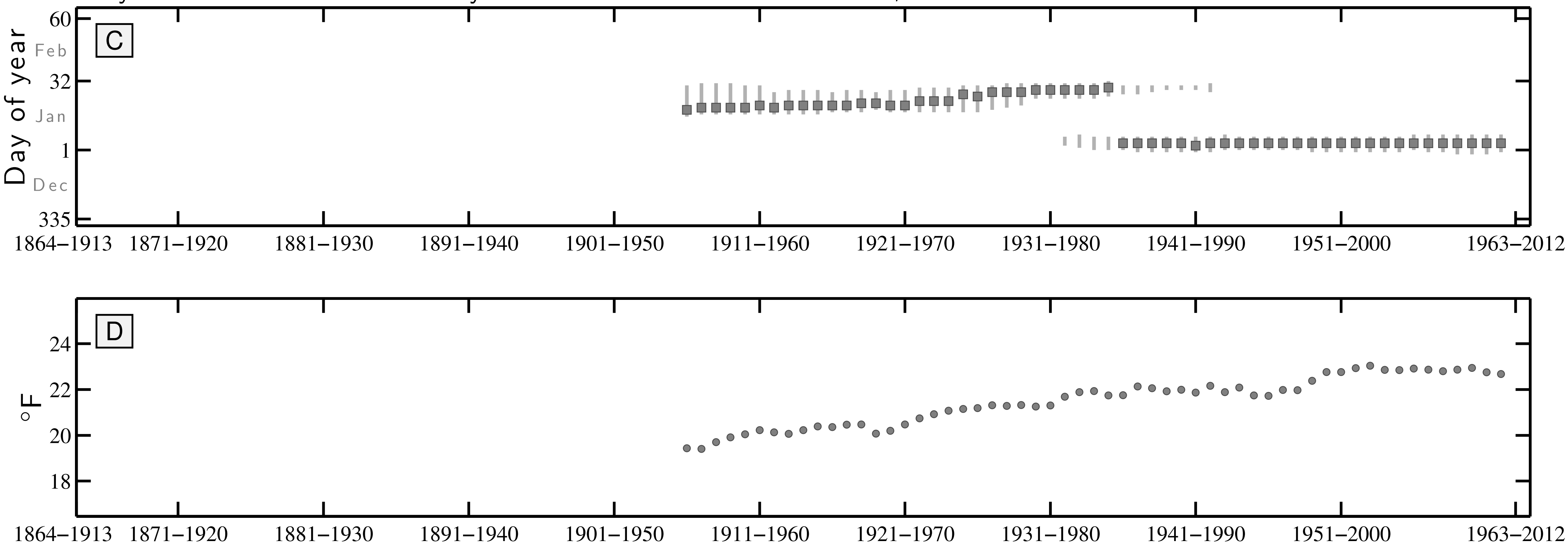

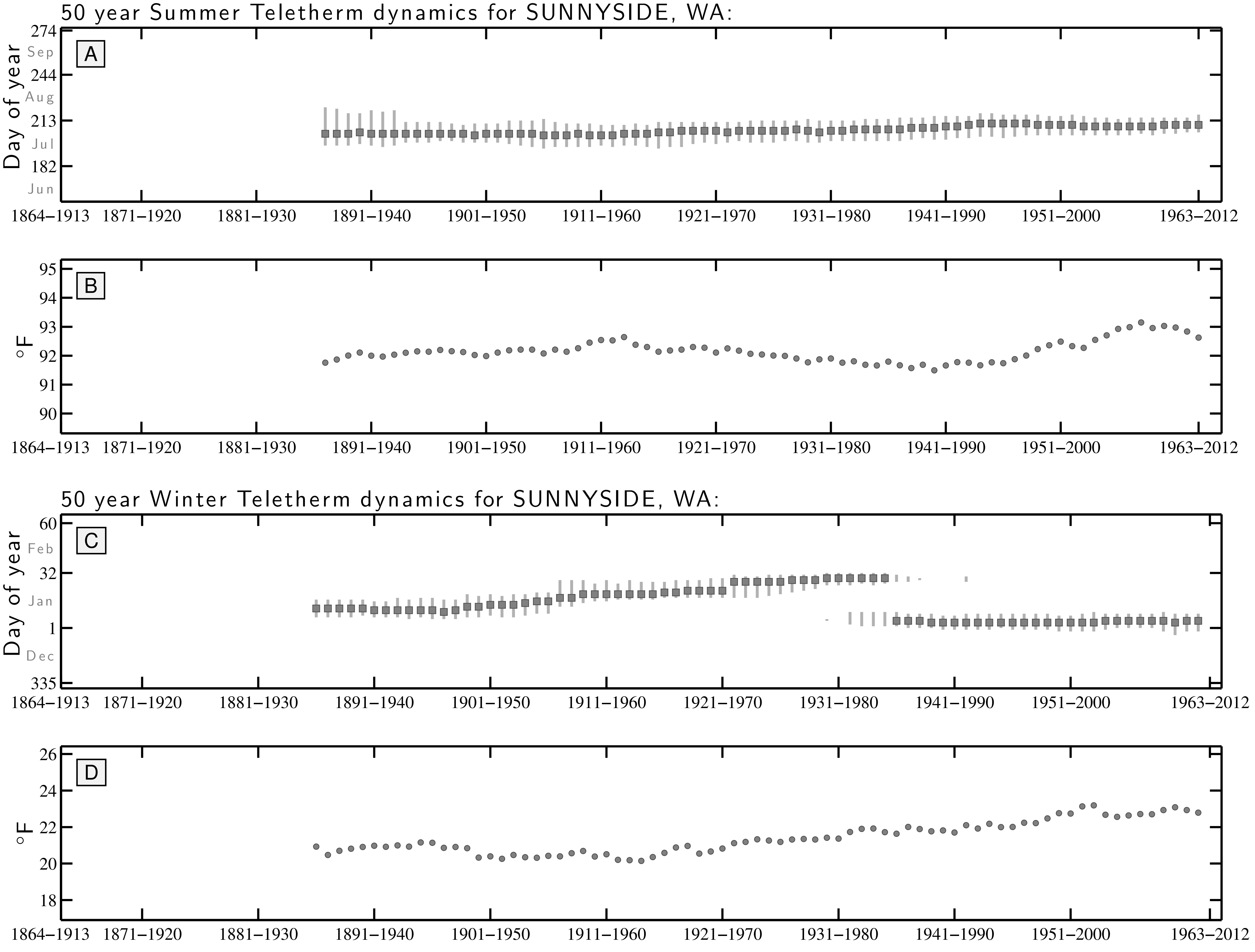

50 year Summer Teletherm dynamics for SUNNYSIDE, WA:
A
Day of year
274
Sep
244
Aug
213
Jul
182
Jun
1864–1913
1871–1920
1881–1930
1891–1940
1901–1950
1911–1960
1921–1970
1931–1980
1941–1990
1951–2000
1963–2012
B
°F
95
94
93
92
91
90
50 year Winter Teletherm dynamics for SUNNYSIDE, WA:
C
Day of year
60
Feb
32
Jan
1
Dec
335
D
°F
26
24
22
20
18

50 year Summer Teletherm dynamics for VANCOUVER 4 NNE, WA:

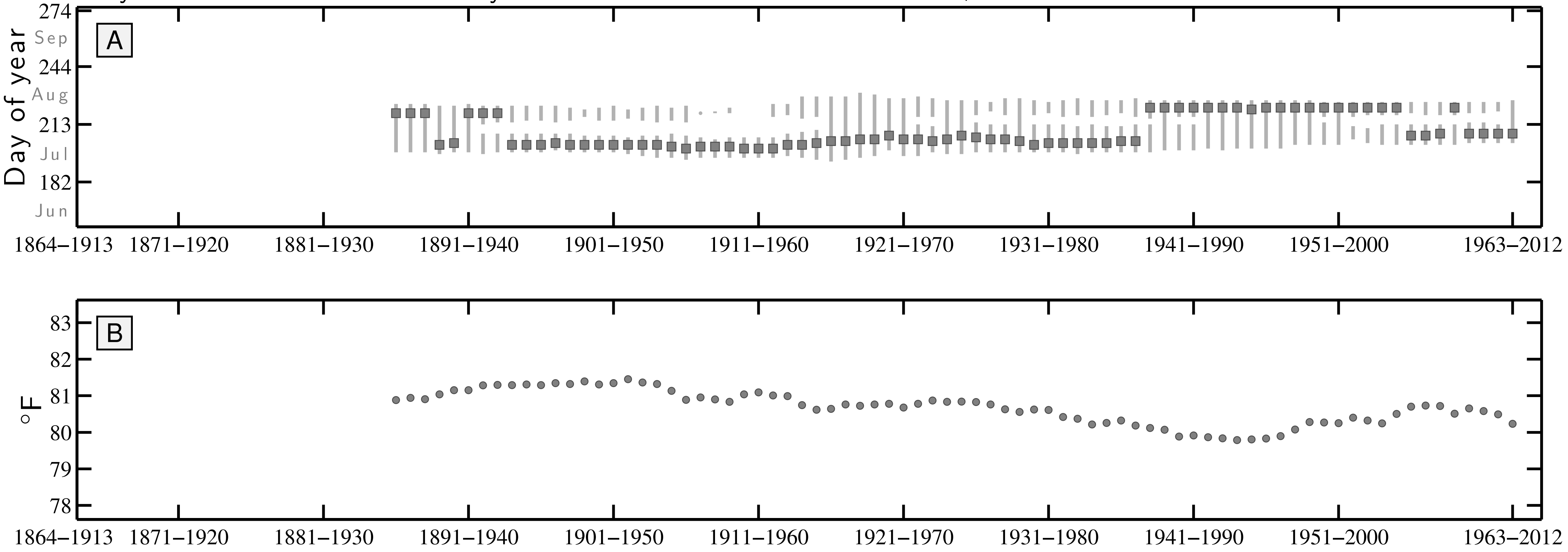

50 year Winter Teletherm dynamics for VANCOUVER 4 NNE, WA:

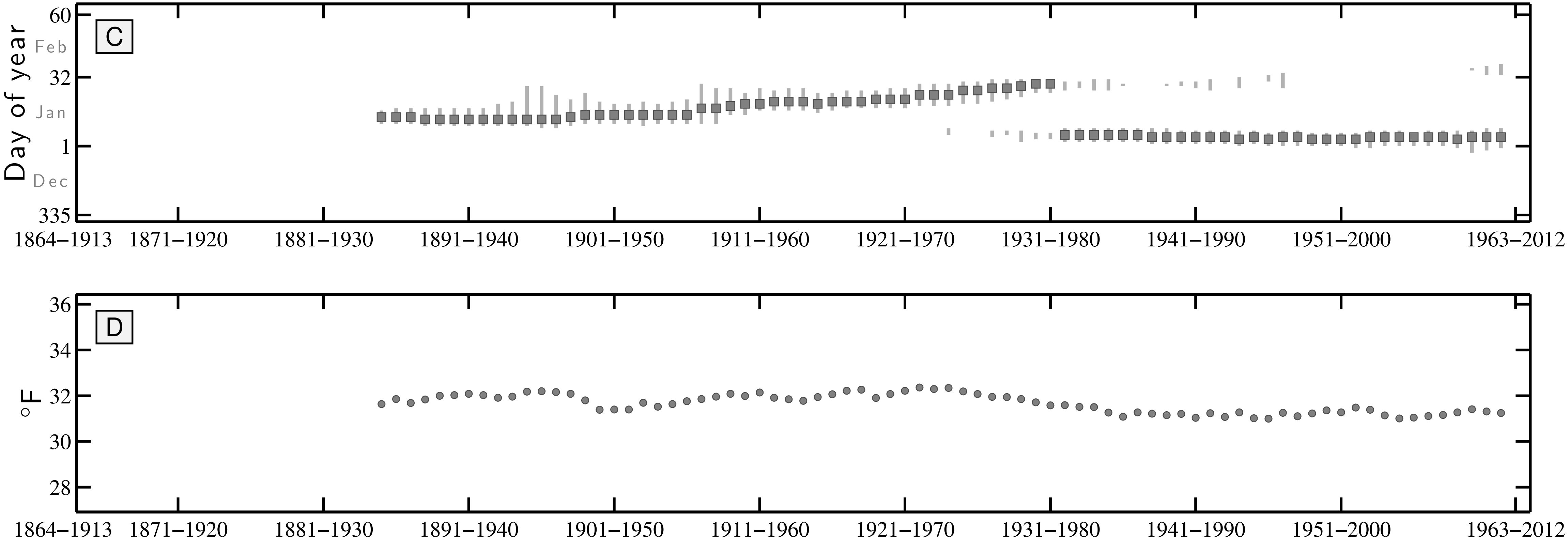

50 year Summer Teletherm dynamics for WALLA WALLA FAA AP, WA:

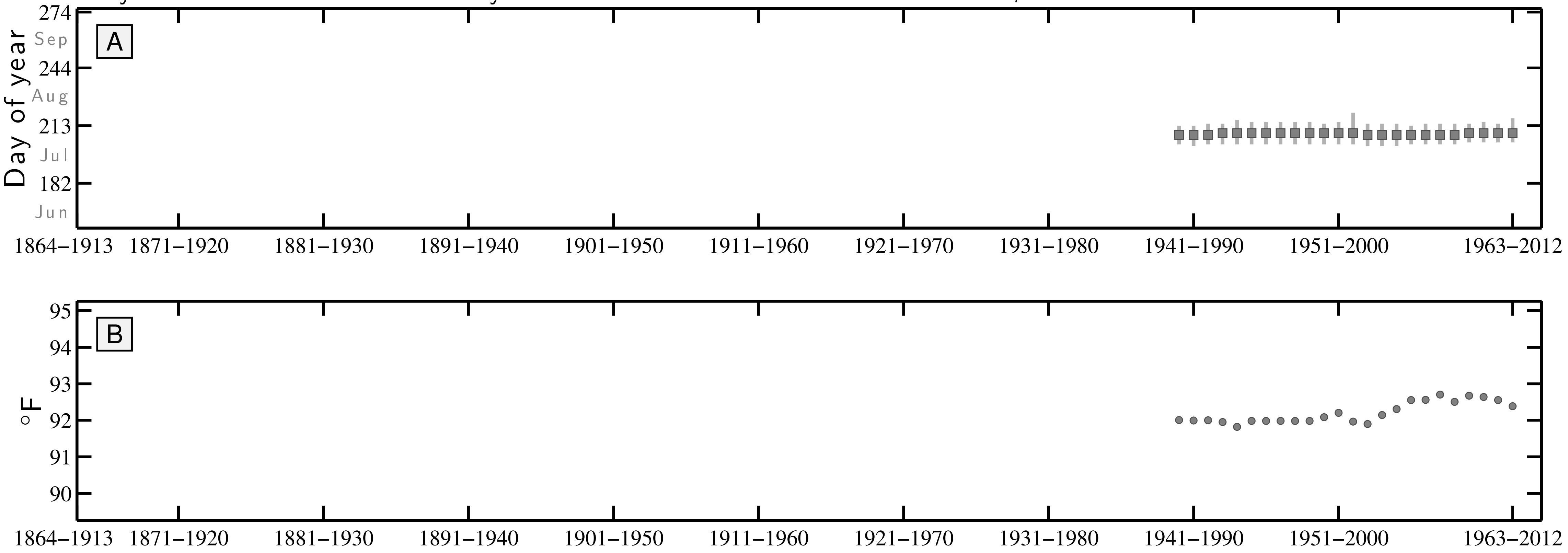

50 year Winter Teletherm dynamics for WALLA WALLA FAA AP, WA:

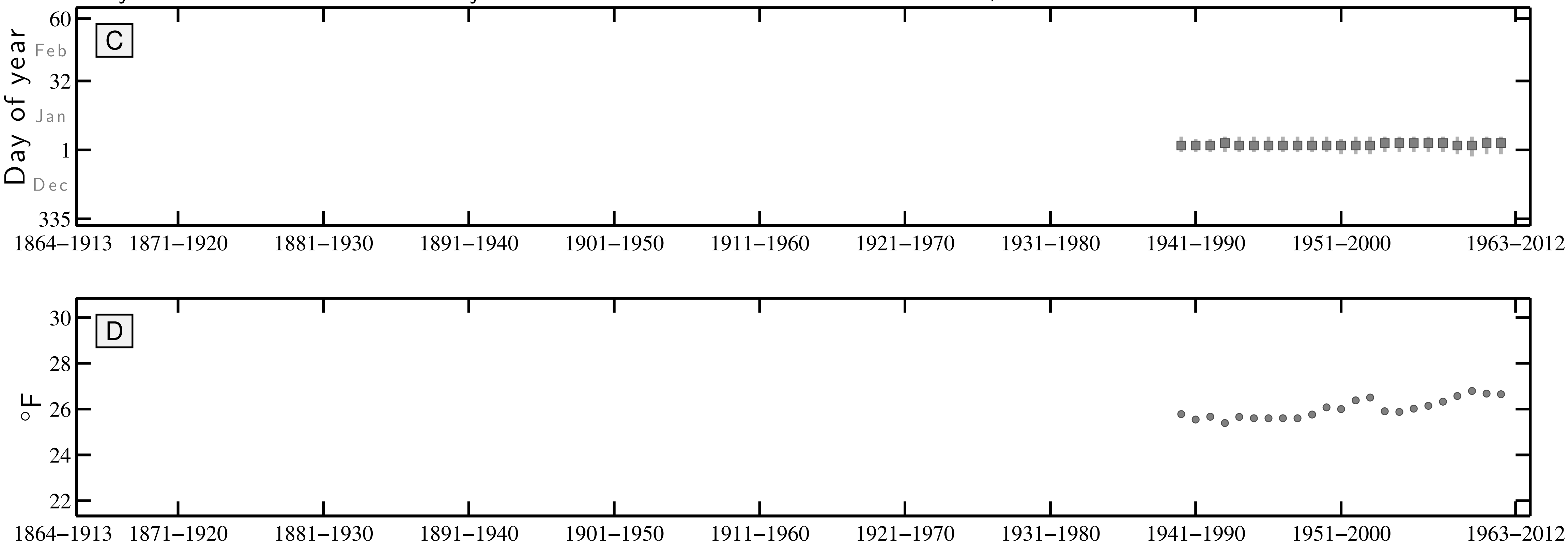

50 year Summer Teletherm dynamics for WATERVILLE, WA:

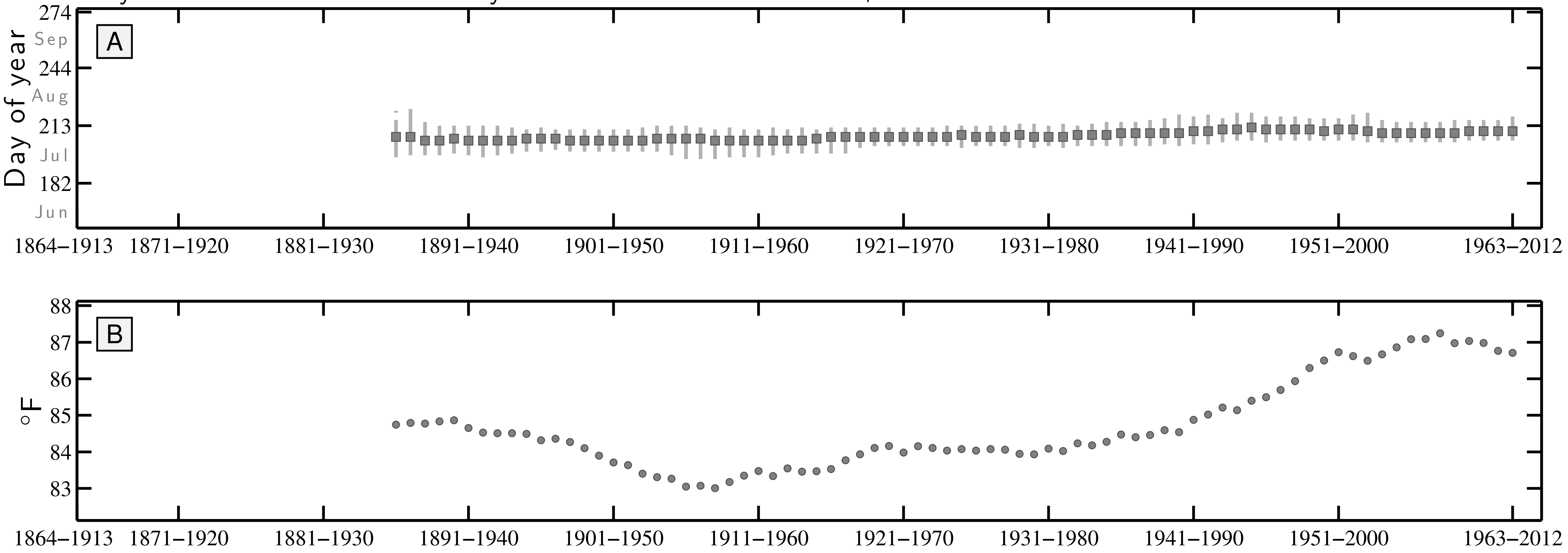

50 year Winter Teletherm dynamics for WATERVILLE, WA:

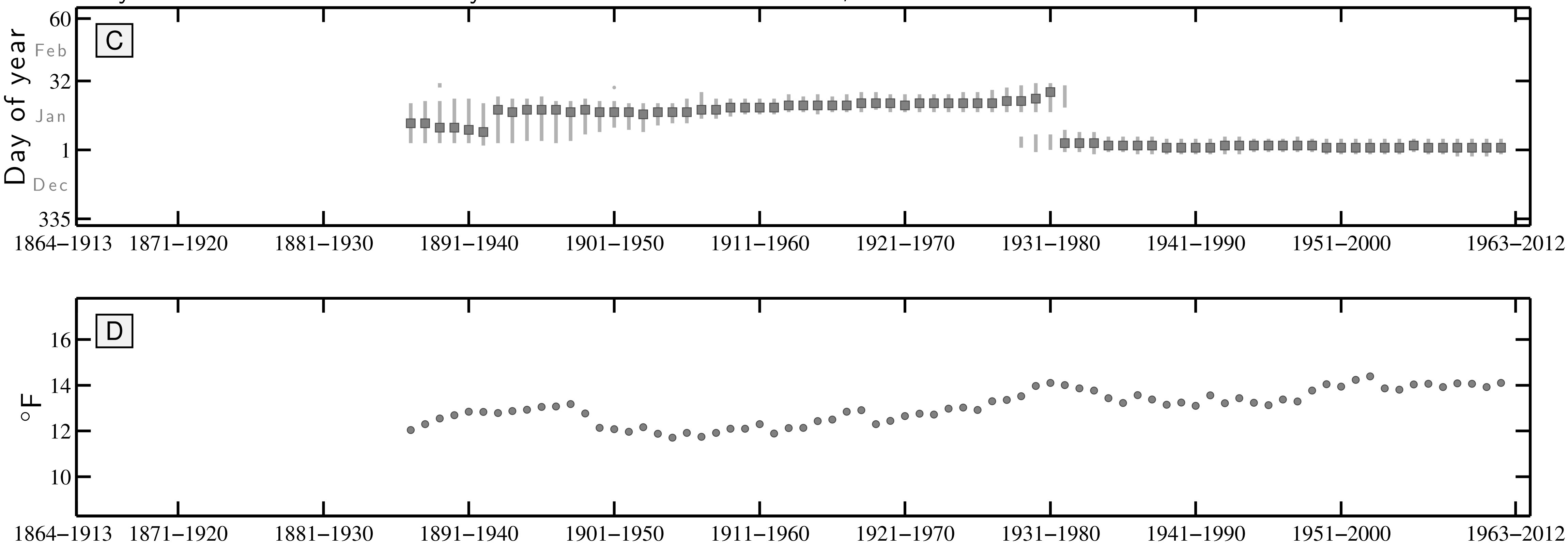

50 year Summer Teletherm dynamics for WENATCHEE, WA:

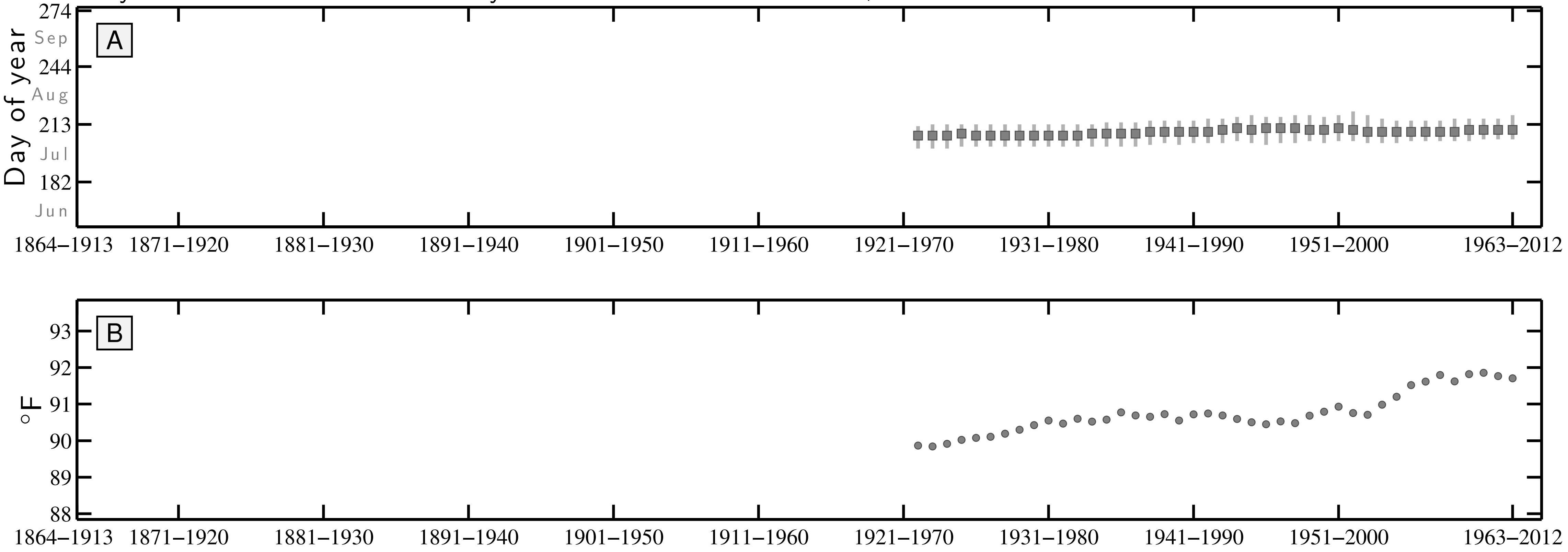

50 year Winter Teletherm dynamics for WENATCHEE, WA:

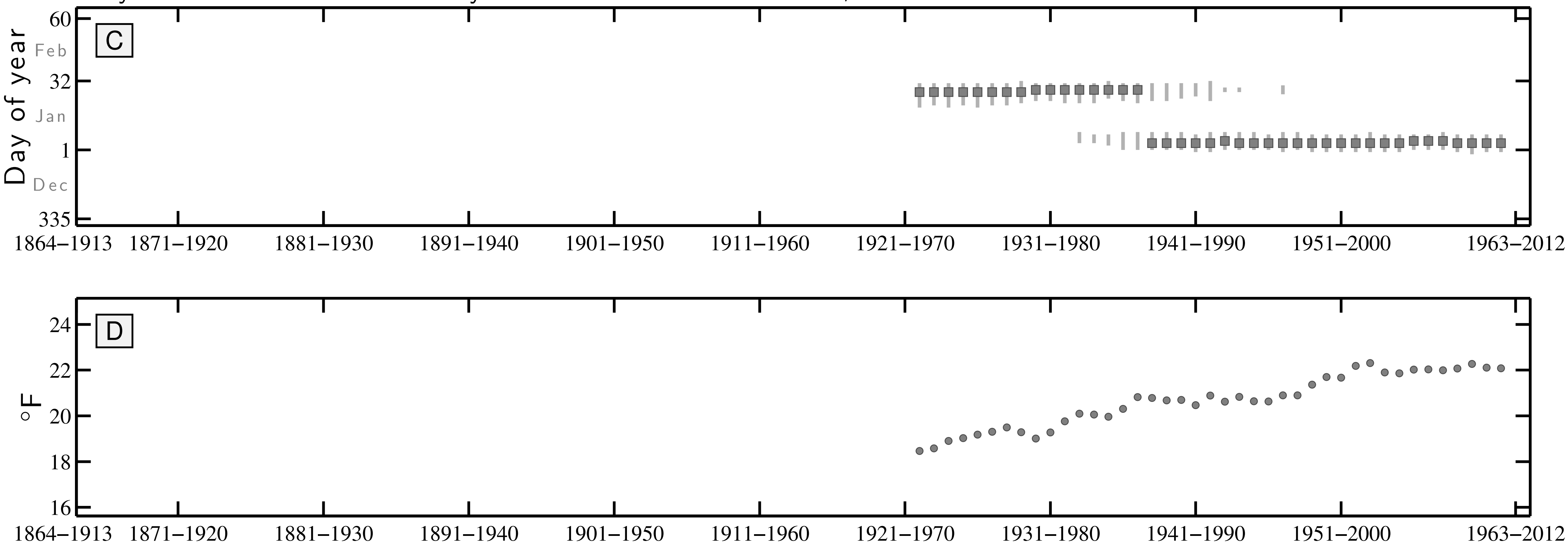

50 year Summer Teletherm dynamics for WILBUR, WA:

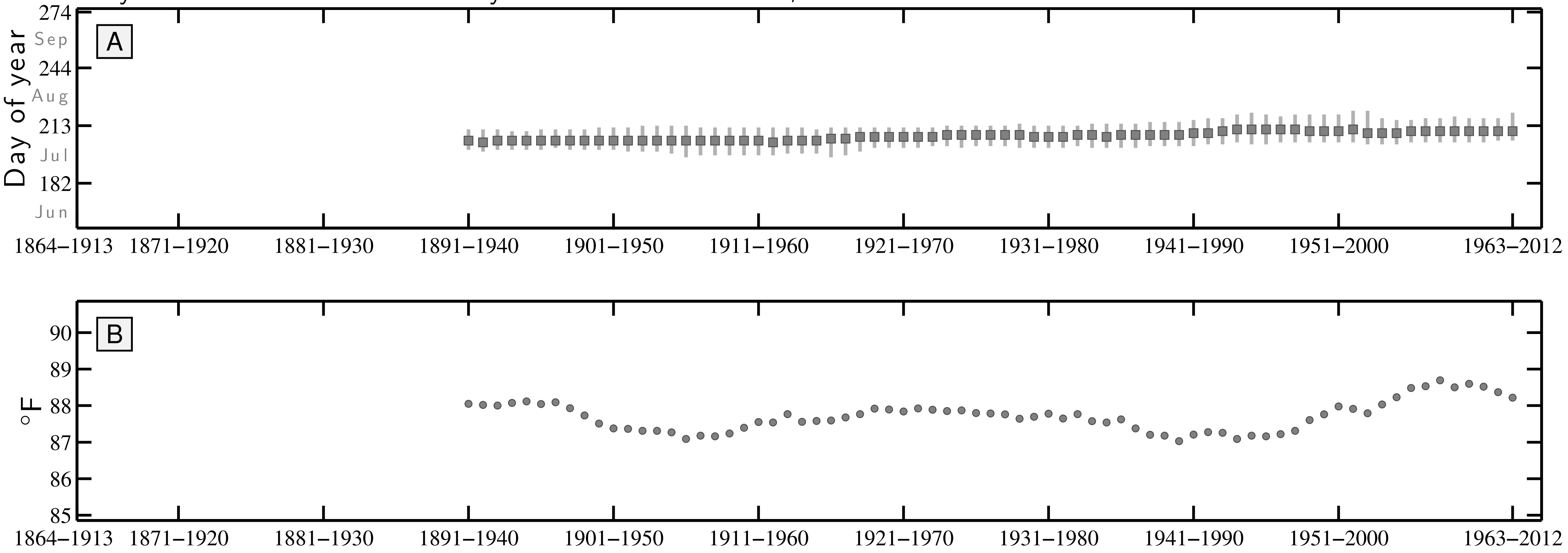

50 year Winter Teletherm dynamics for WILBUR, WA:

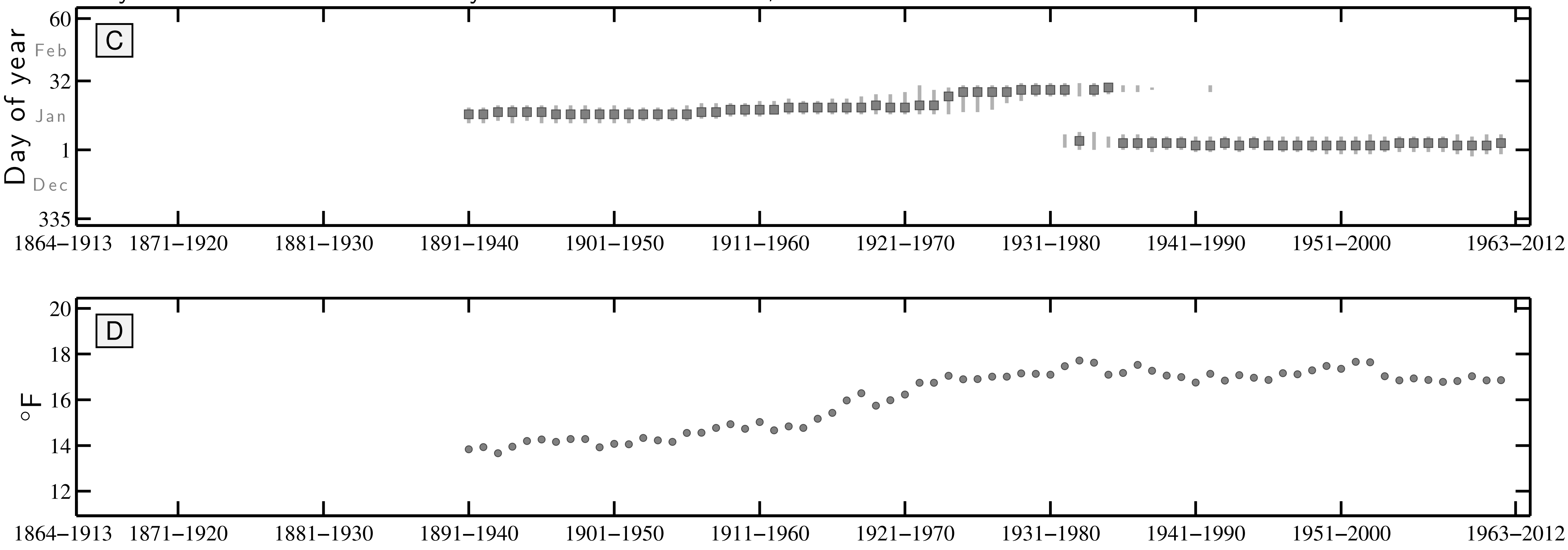

50 year Summer Teletherm dynamics for WINTHROP 1 WSW, WA:

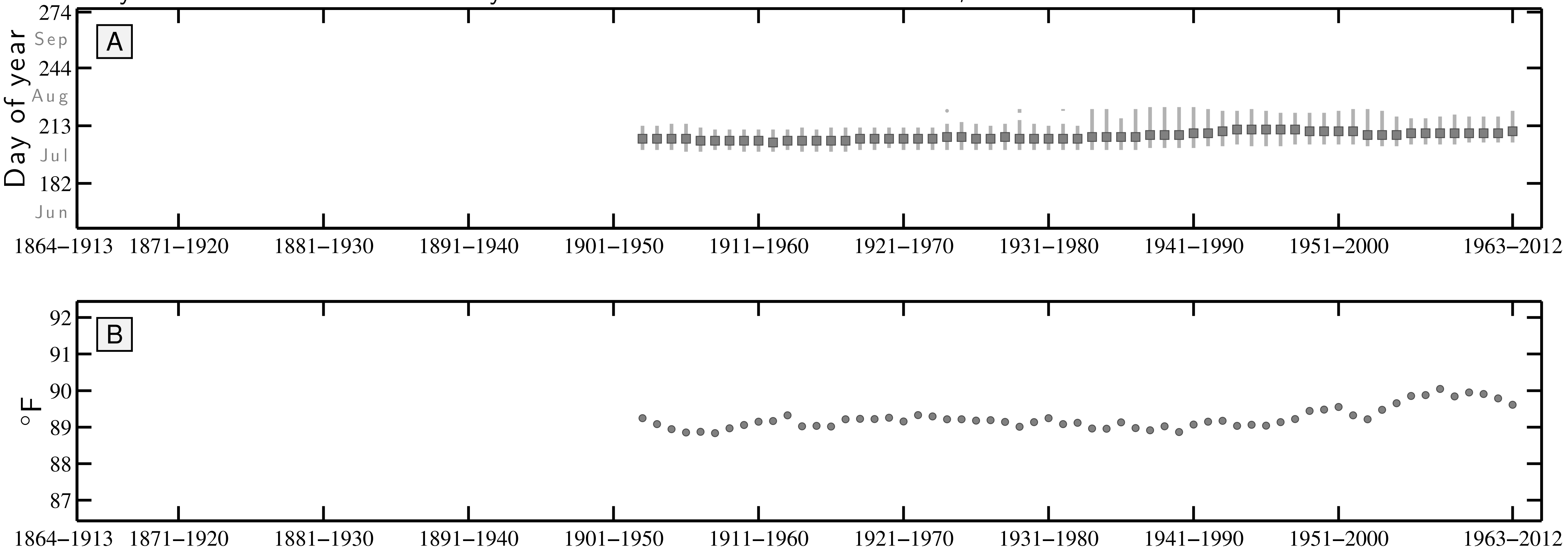

50 year Winter Teletherm dynamics for WINTHROP 1 WSW, WA:

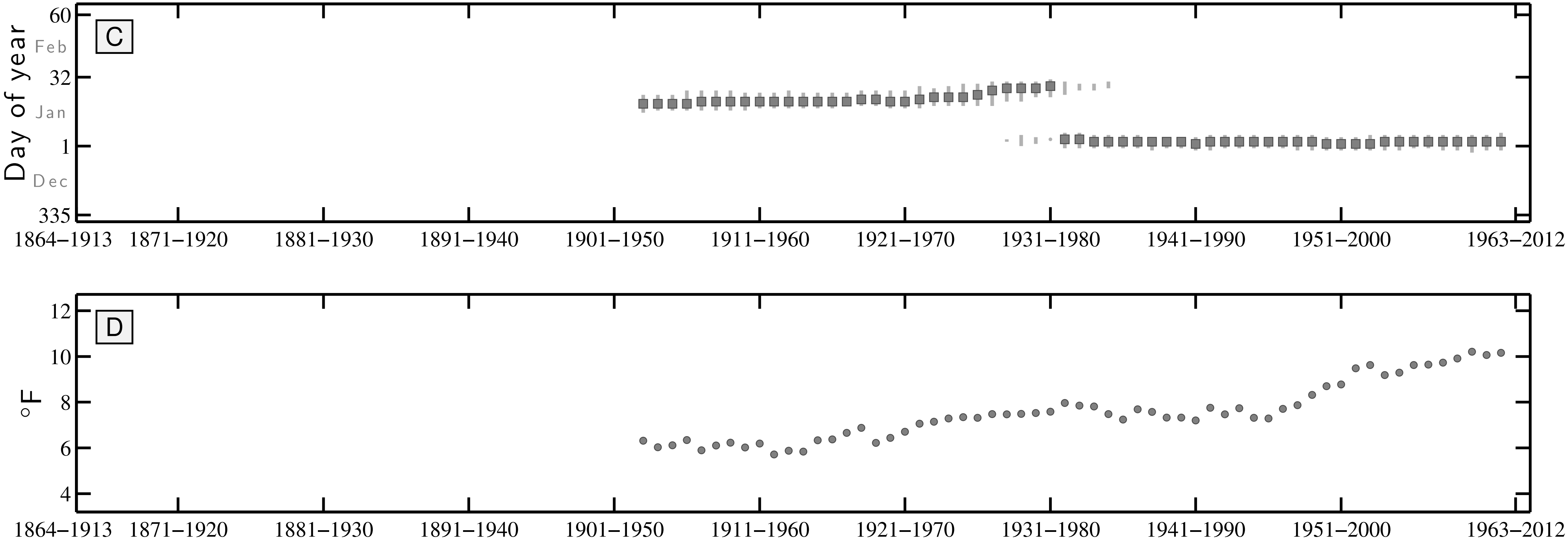

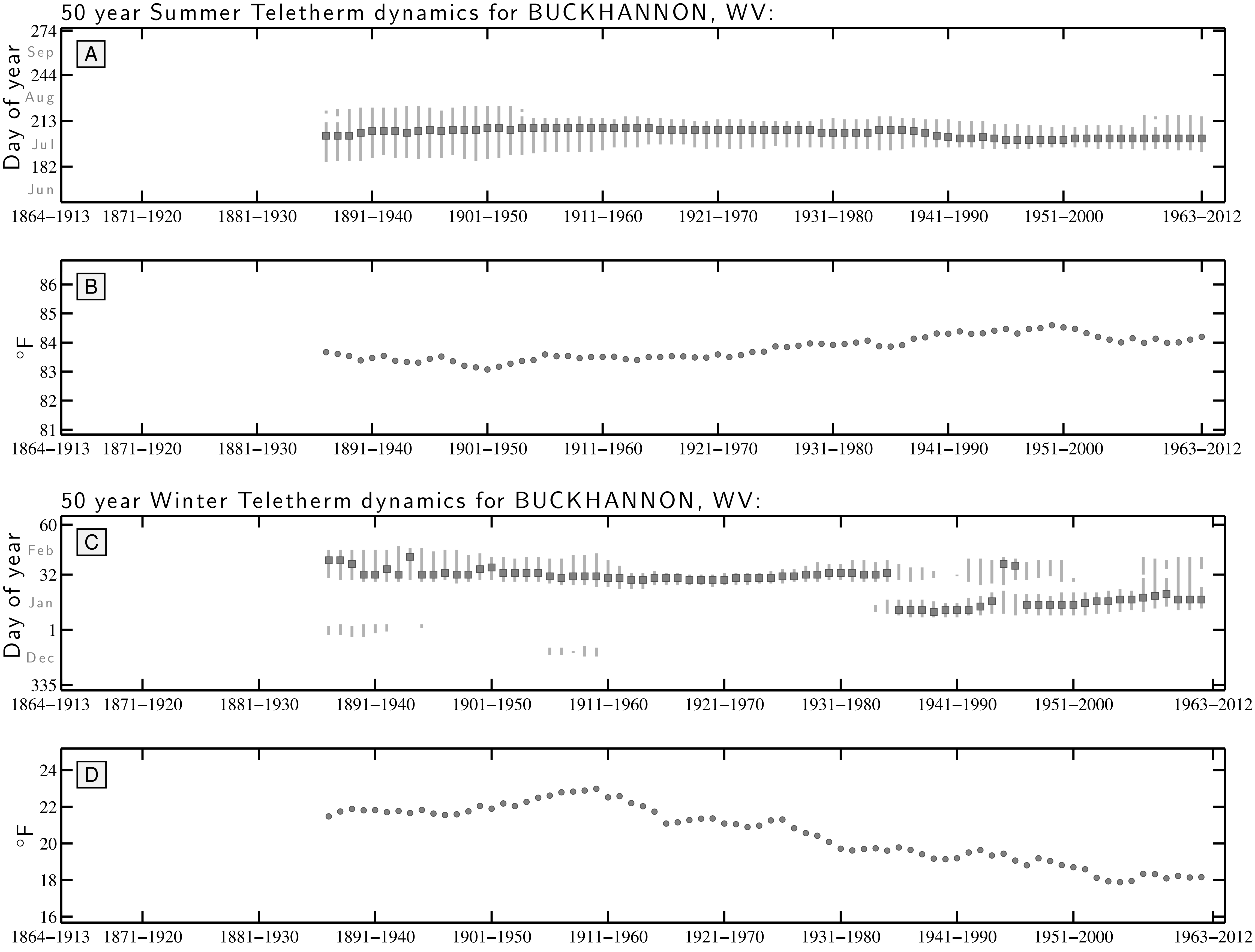
50 year Summer Teletherm dynamics for BUCKHANNON, WV:
A
B
Day of year
°F
Sep
Aug
Jul
Jun
274
244
213
182
86
85
84
83
82
81
50 year Winter Teletherm dynamics for BUCKHANNON, WV:
C
D
Feb
Jan
Dec
60
32
1
335
24
22
20
18
16
1864–1913
1871–1920
1881–1930
1891–1940
1901–1950
1911–1960
1921–1970
1931–1980
1941–1990
1951–2000
1963–2012

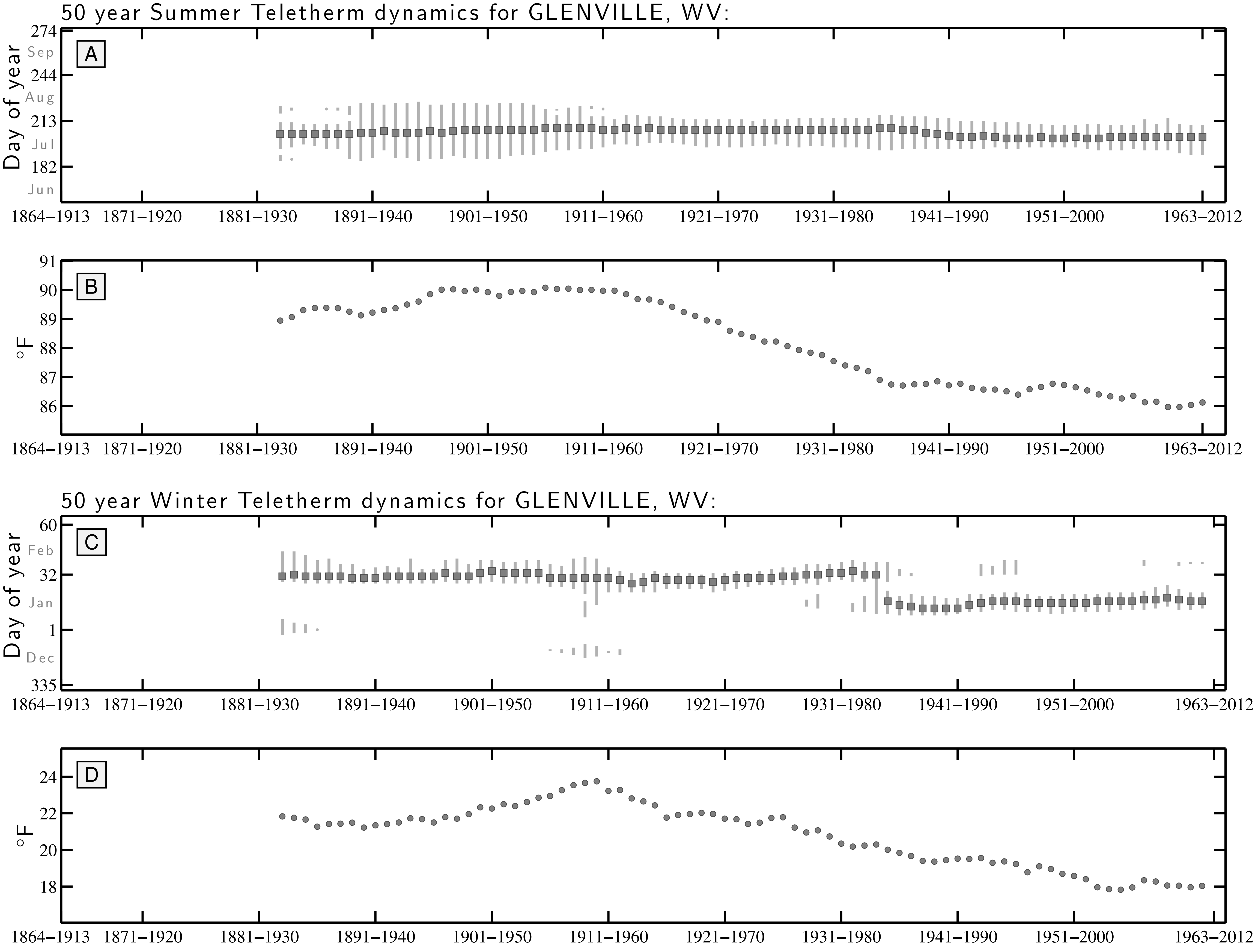
50 year Summer Teletherm dynamics for GLENVILLE, WV:
A
B
Day of year
°F
274
244
213
182
Sep
Aug
Jul
Jun
91
90
89
88
87
86
1864–1913
1871–1920
1881–1930
1891–1940
1901–1950
1911–1960
1921–1970
1931–1980
1941–1990
1951–2000
1963–2012
50 year Winter Teletherm dynamics for GLENVILLE, WV:
C
D
60
32
1
335
Feb
Jan
Dec
24
22
20
18

50 year Summer Teletherm dynamics for LEWISBURG 3 N, WV:

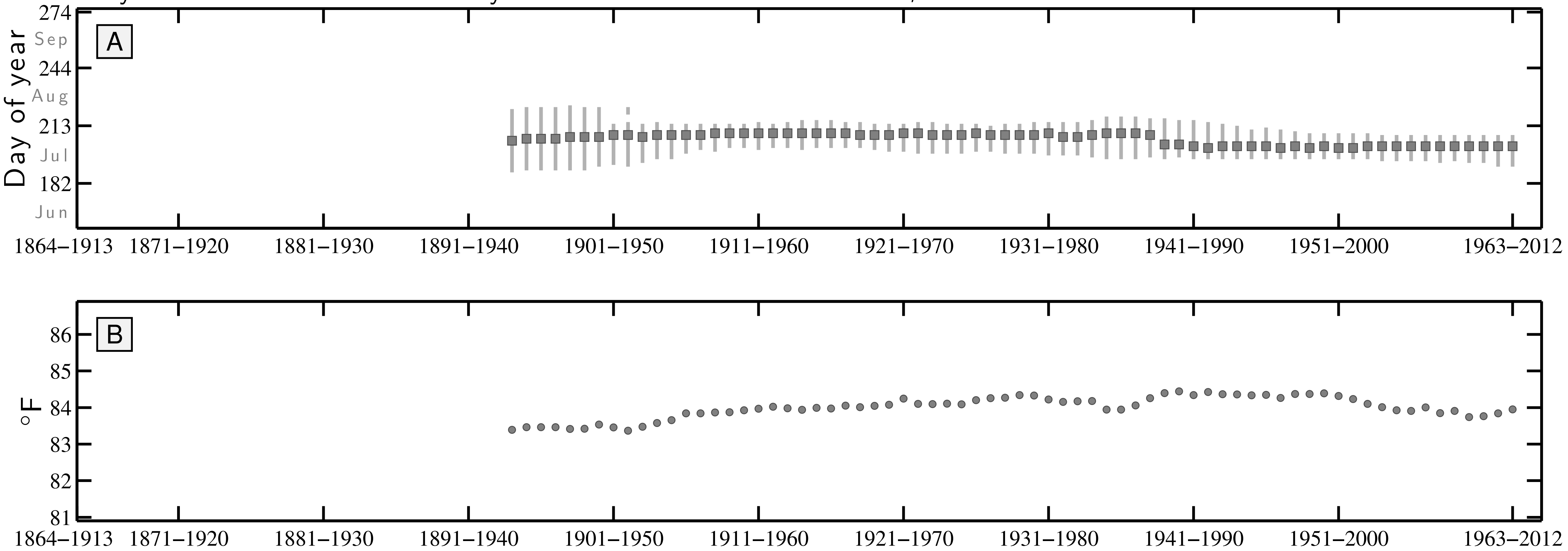

50 year Winter Teletherm dynamics for LEWISBURG 3 N, WV:

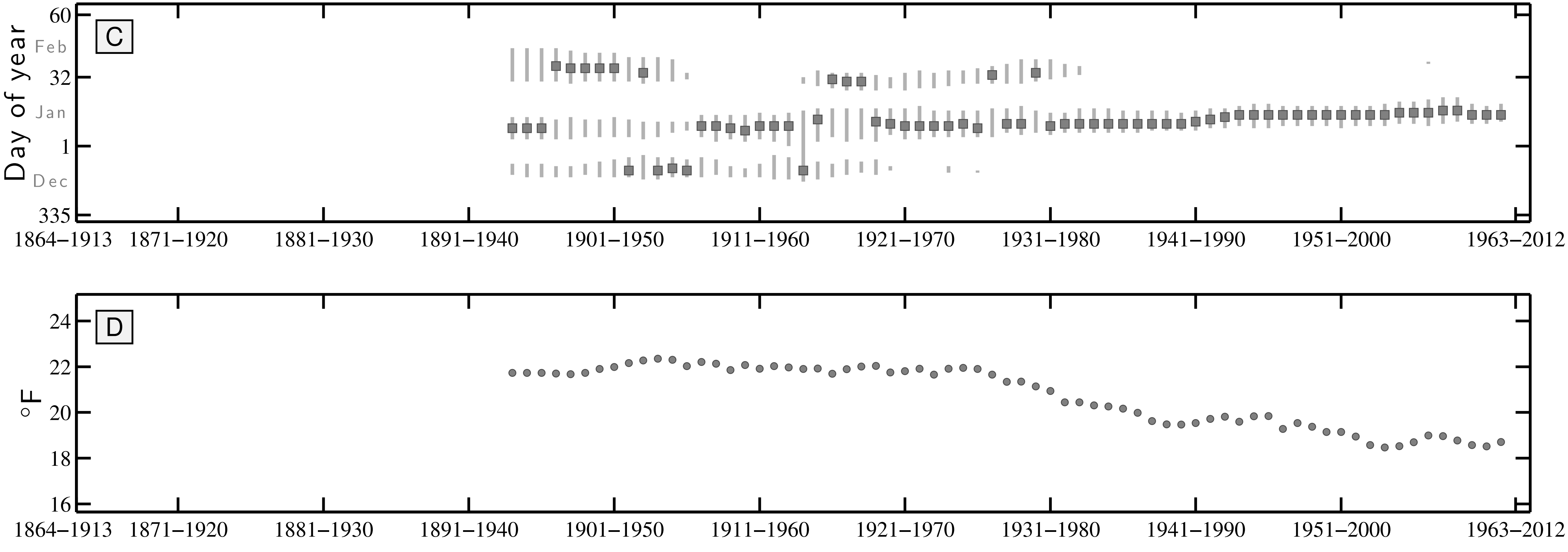

50 year Summer Teletherm dynamics for MARTINSBURG E WV RGNL, WV:

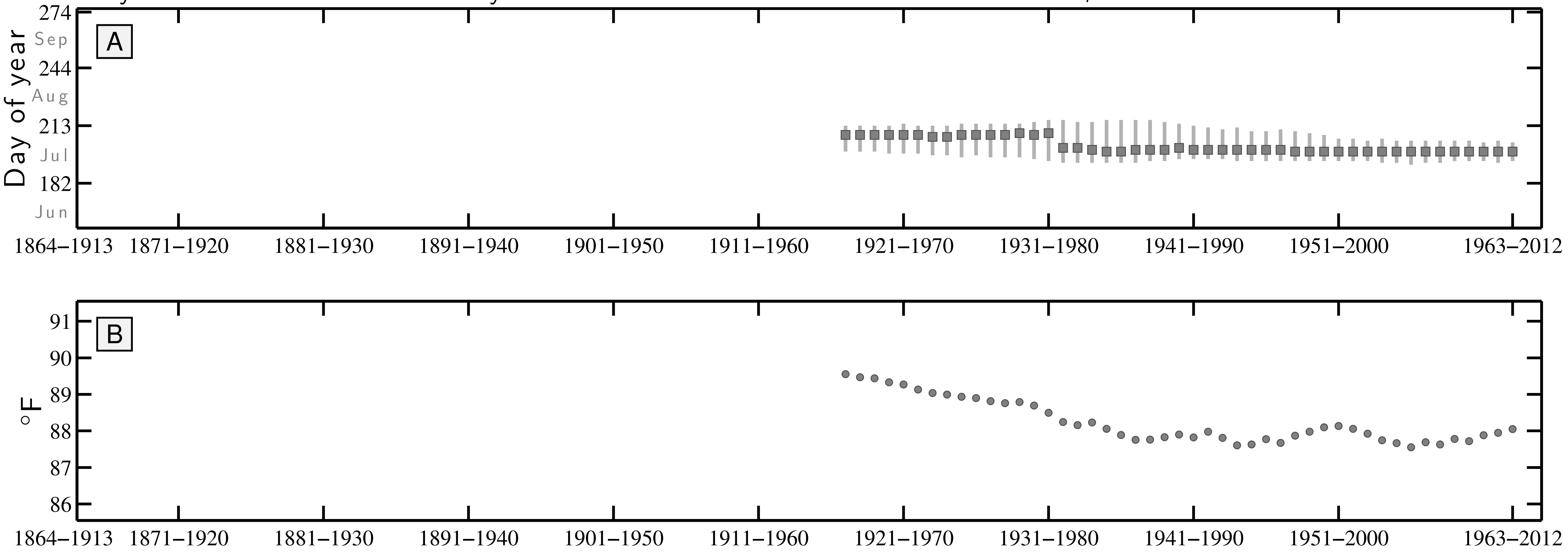

50 year Winter Teletherm dynamics for MARTINSBURG E WV RGNL, WV:

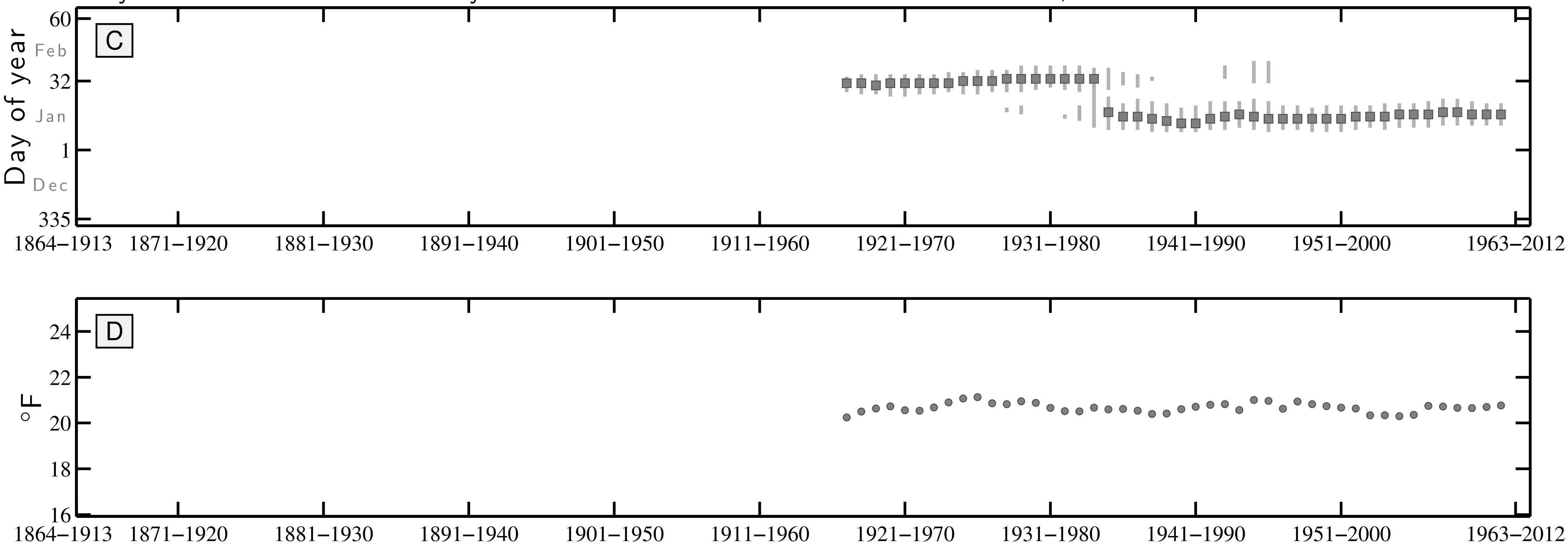

50 year Summer Teletherm dynamics for PARSONS 1 NE, WV:

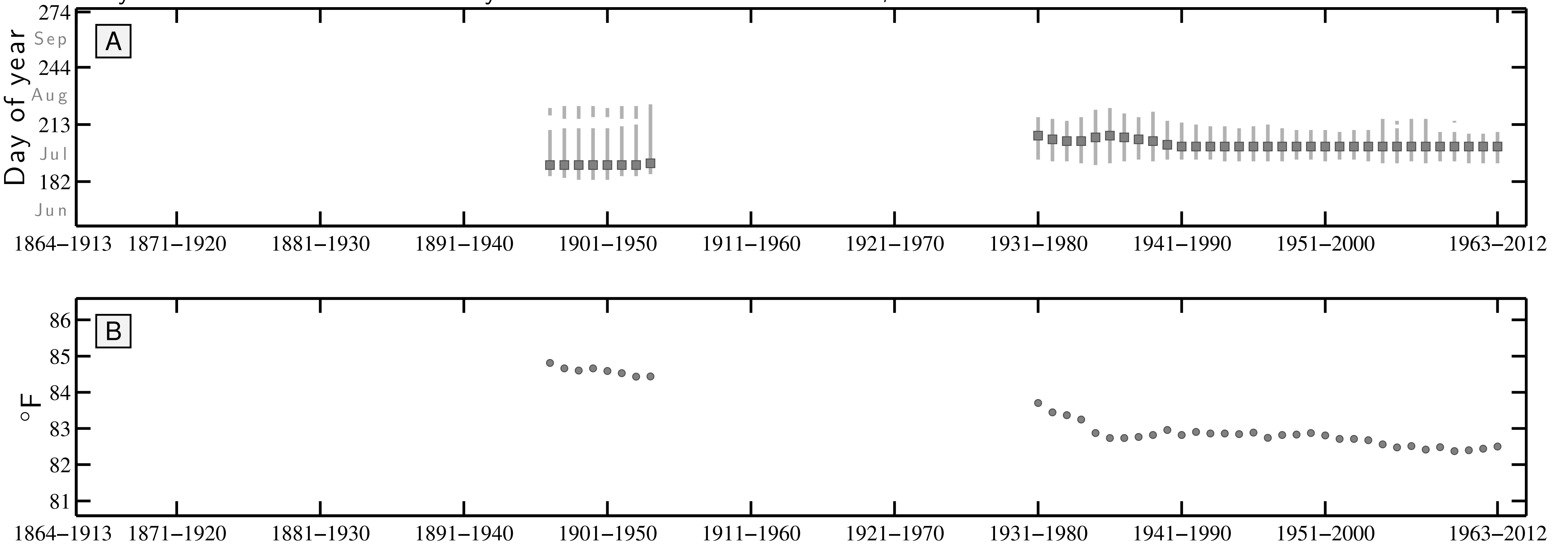

50 year Winter Teletherm dynamics for PARSONS 1 NE, WV:

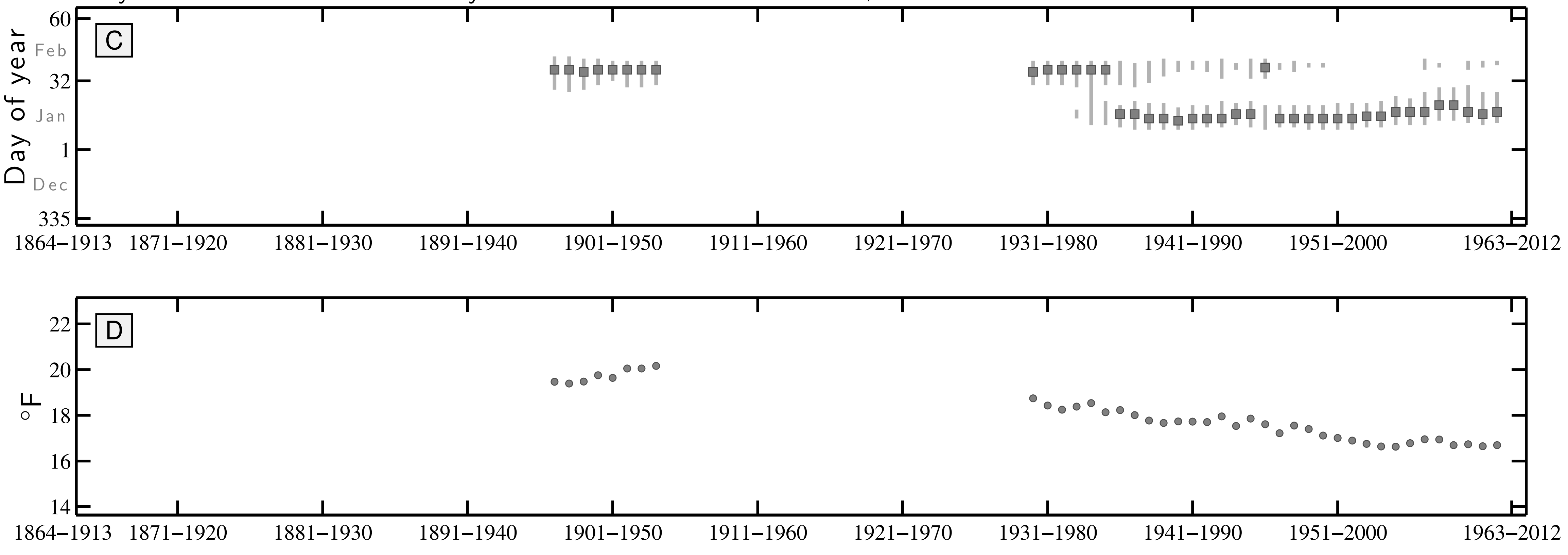

50 year Summer Teletherm dynamics for PINEVILLE, WV:

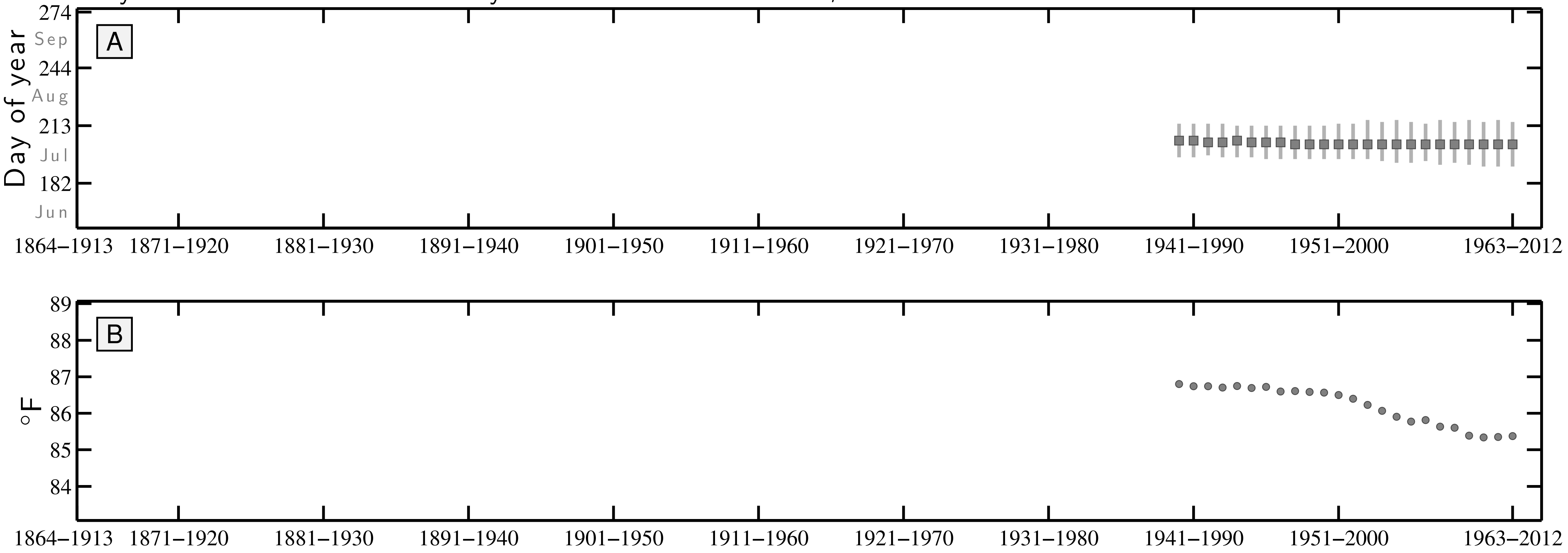

50 year Winter Teletherm dynamics for PINEVILLE, WV:

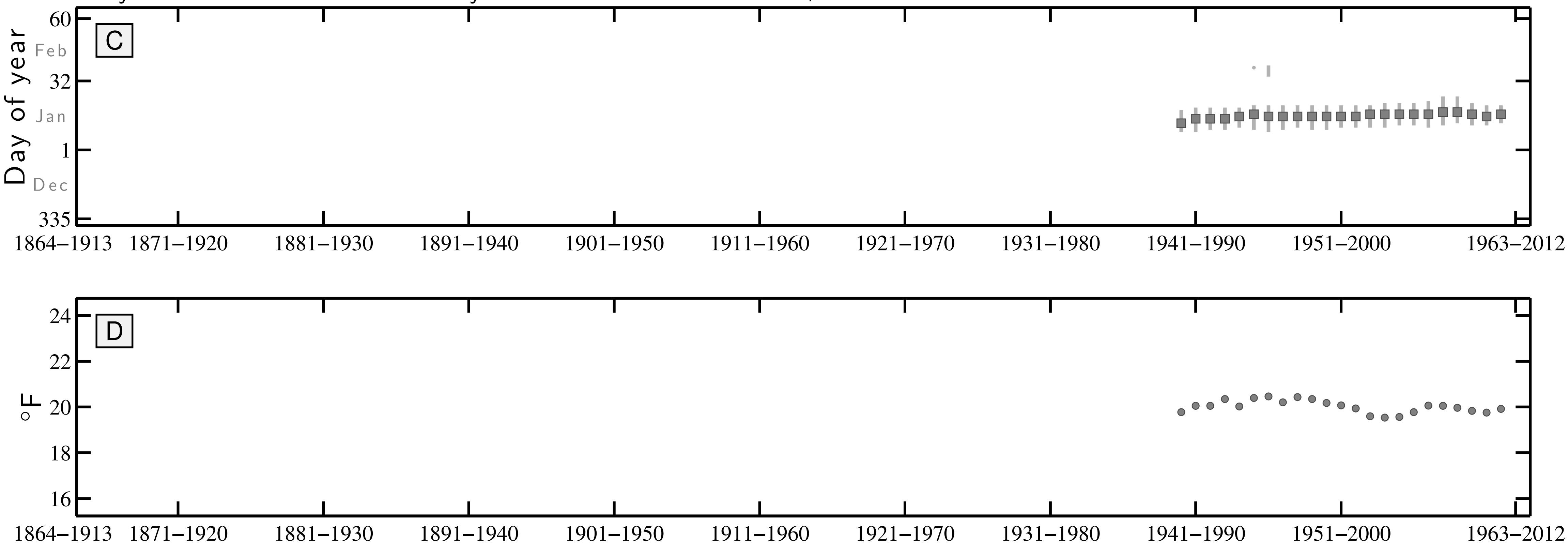

50 year Summer Teletherm dynamics for SPENCER, WV:

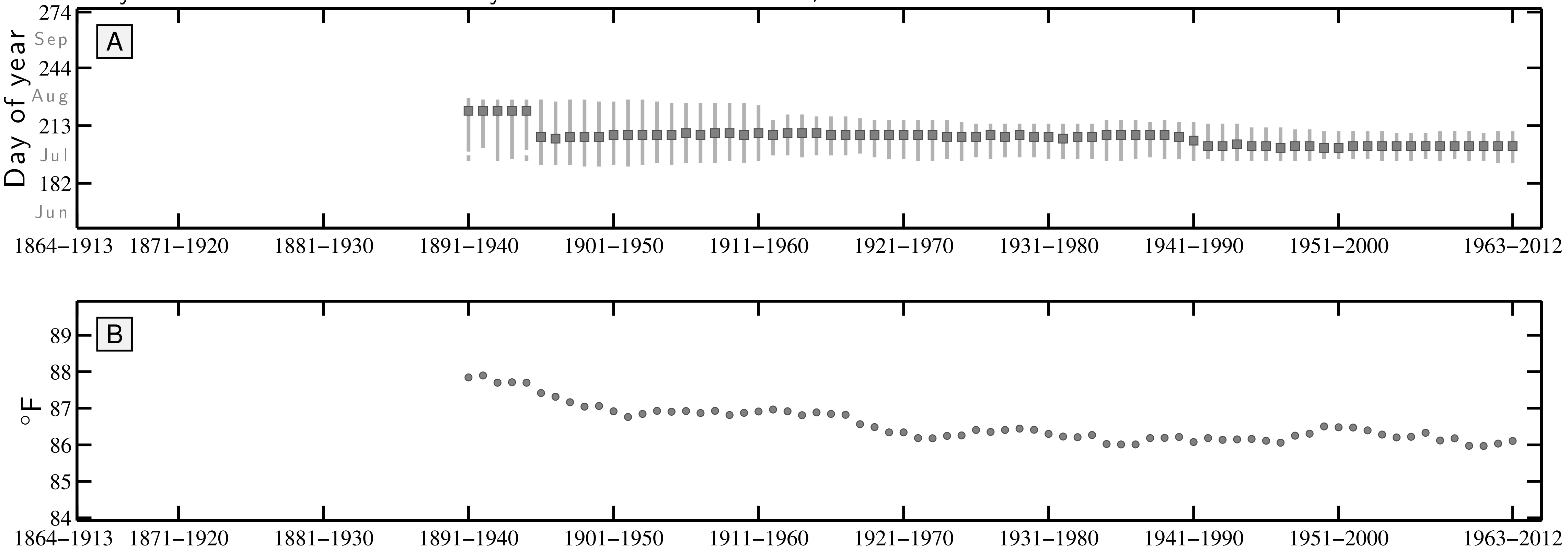

50 year Winter Teletherm dynamics for SPENCER, WV:

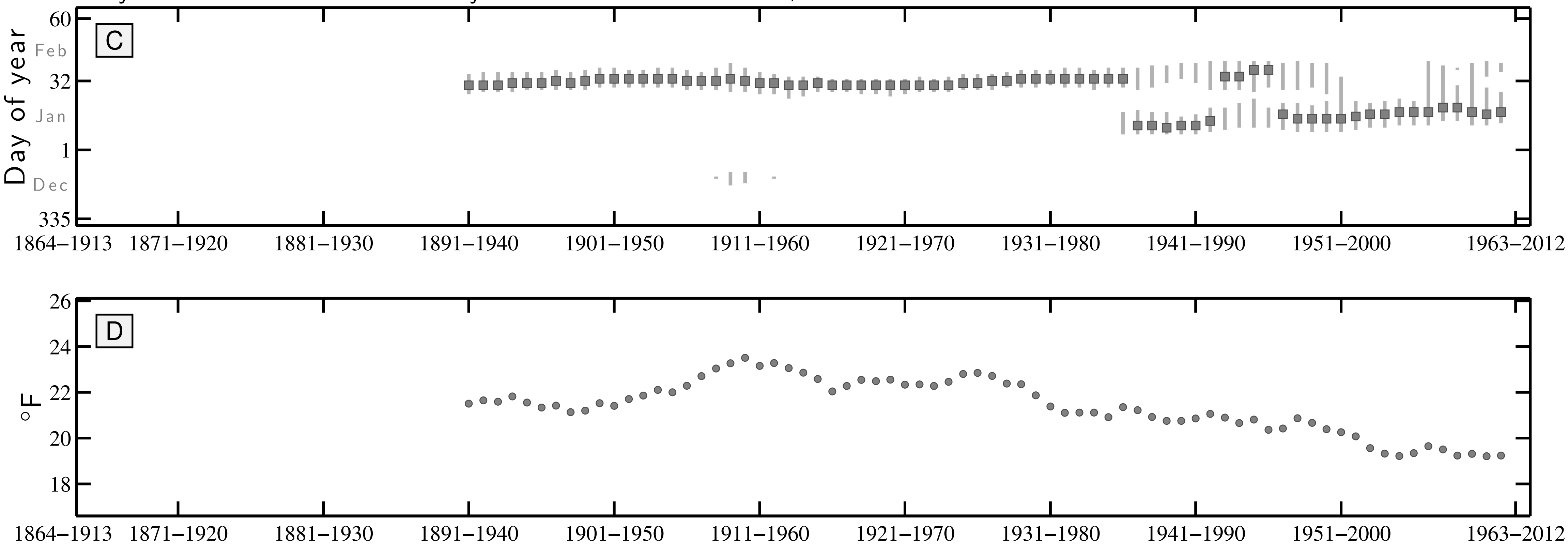

50 year Summer Teletherm dynamics for WELLSBURG WTR TRMT PL, WV:

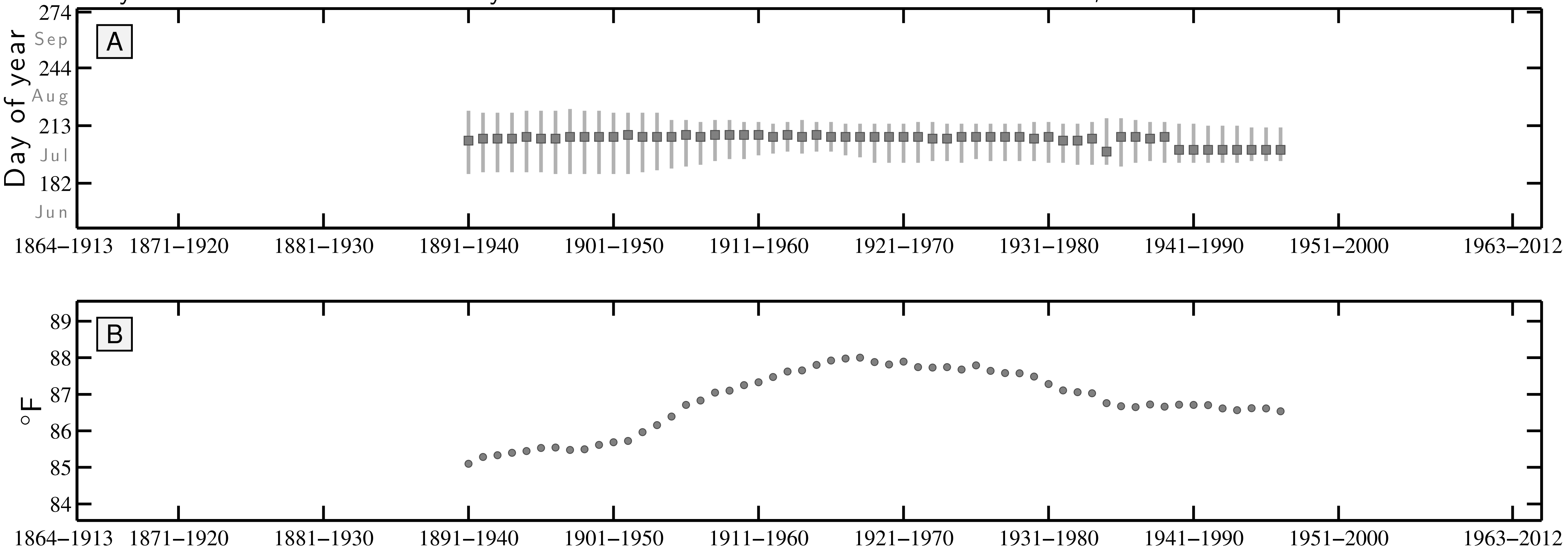

50 year Winter Teletherm dynamics for WELLSBURG WTR TRMT PL, WV:

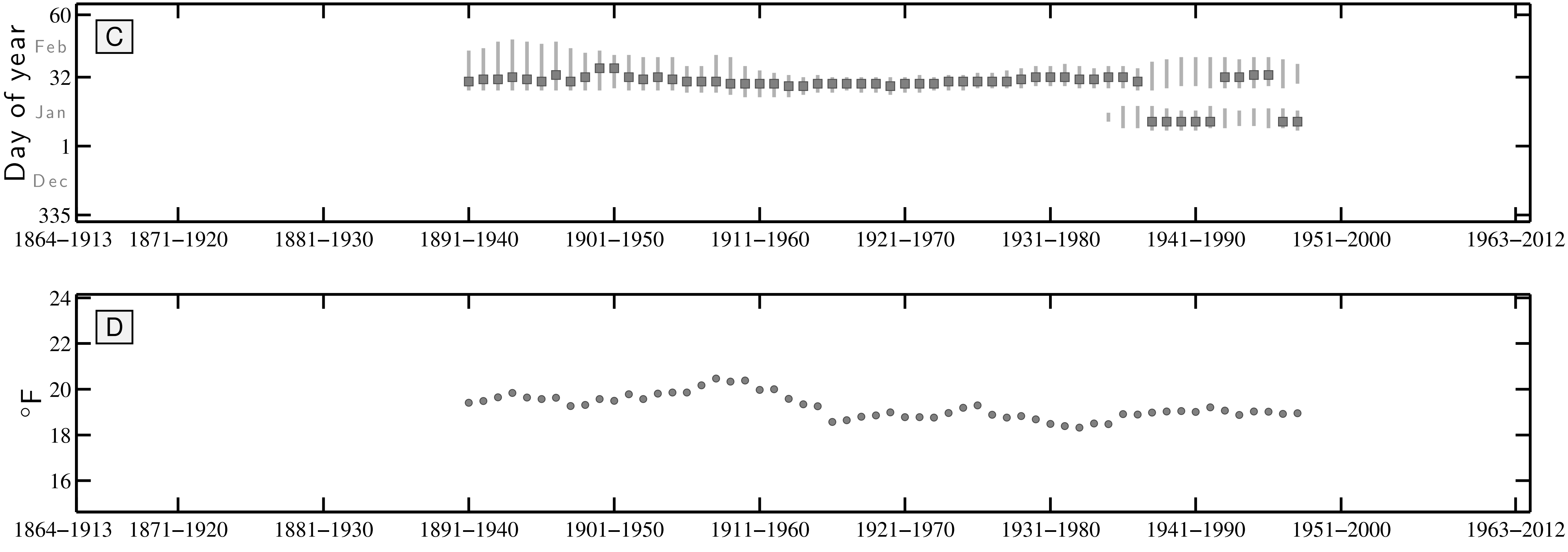

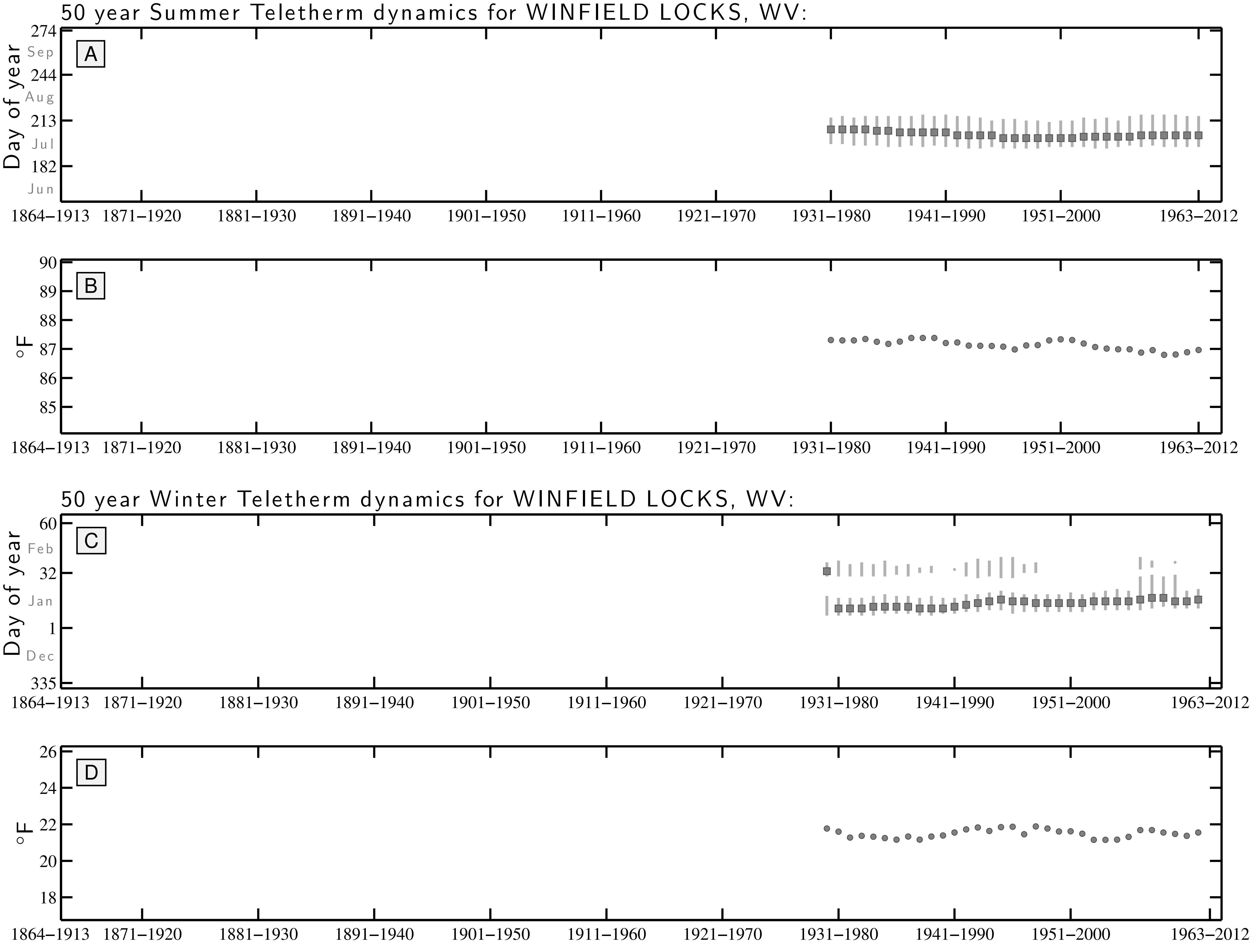
50 year Summer Teletherm dynamics for WINFIELD LOCKS, WV:
A
B
Day of year
°F
Jun
Jul
Aug
Sep
182
213
244
274
85
86
87
88
89
90
50 year Winter Teletherm dynamics for WINFIELD LOCKS, WV:
C
D
Dec
Jan
Feb
335
1
32
60
18
20
22
24
26
1864–1913
1871–1920
1881–1930
1891–1940
1901–1950
1911–1960
1921–1970
1931–1980
1941–1990
1951–2000
1963–2012

50 year Summer Teletherm dynamics for ASHLAND EXP FARM, WI:

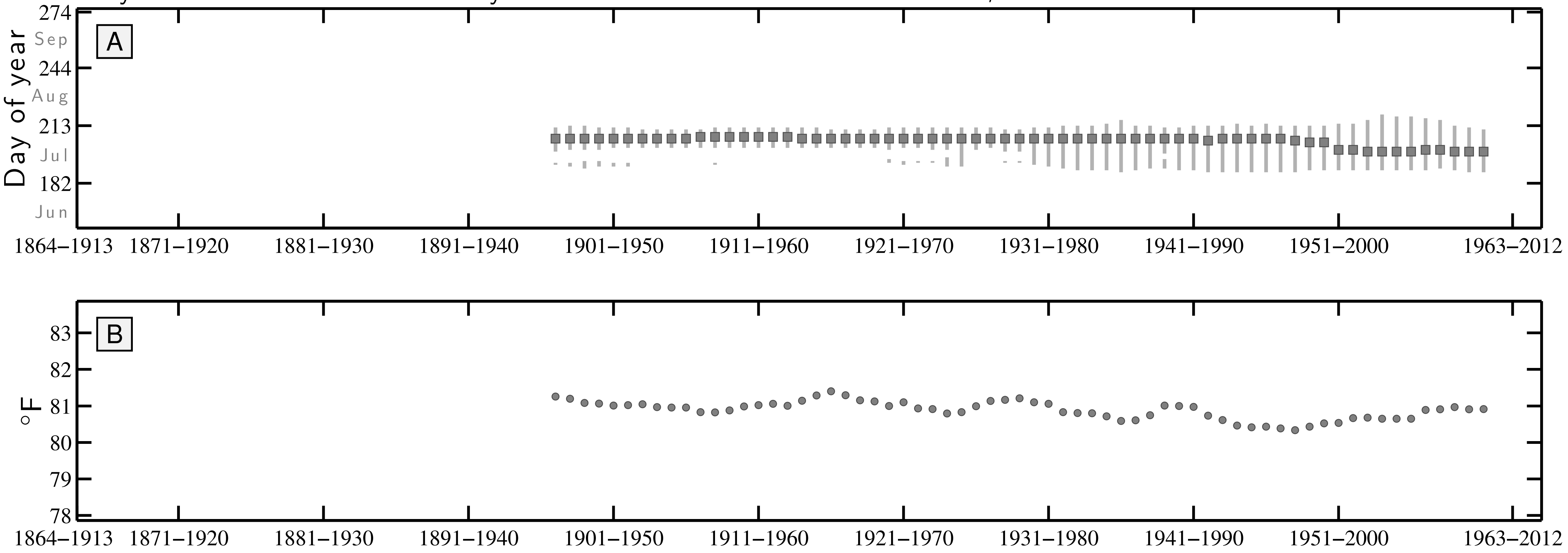

50 year Winter Teletherm dynamics for ASHLAND EXP FARM, WI:

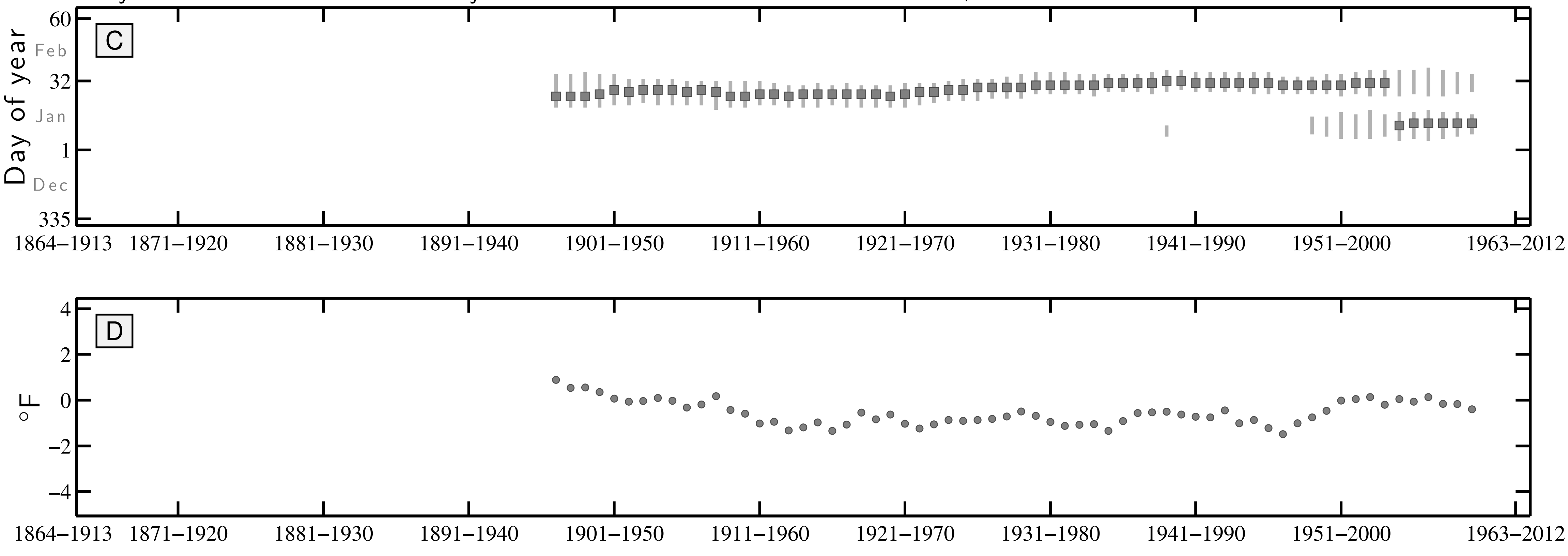

50 year Summer Teletherm dynamics for BRODHEAD, WI:

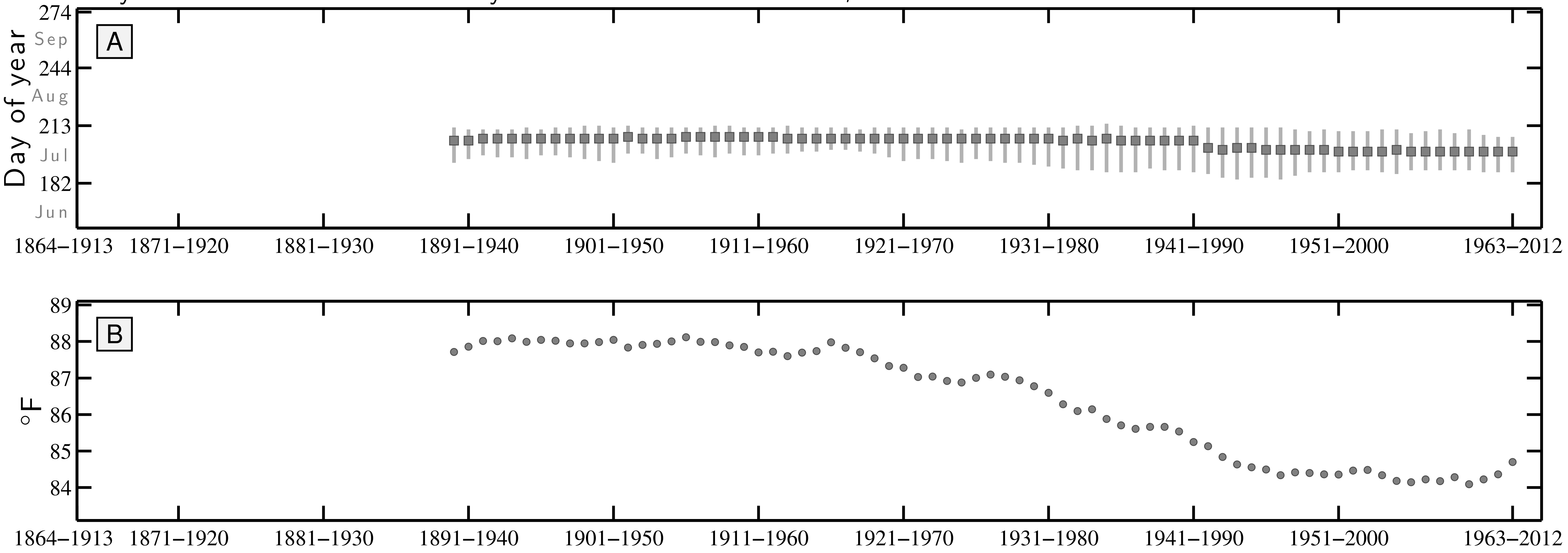

50 year Winter Teletherm dynamics for BRODHEAD, WI:

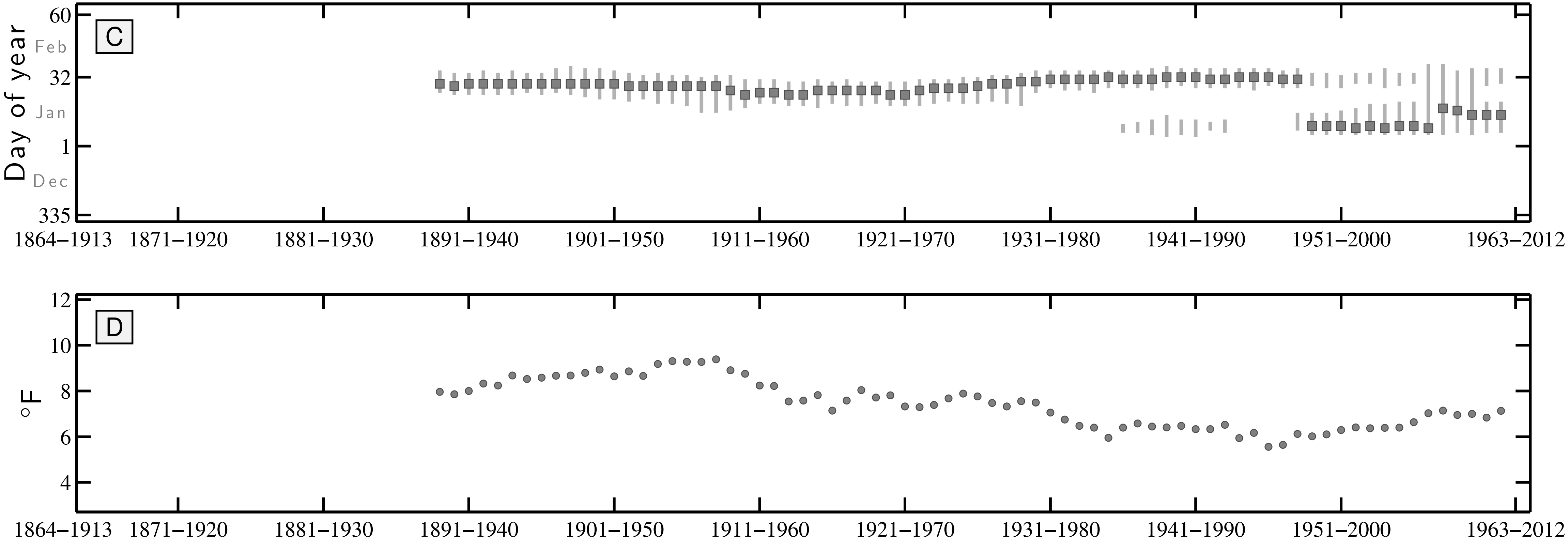

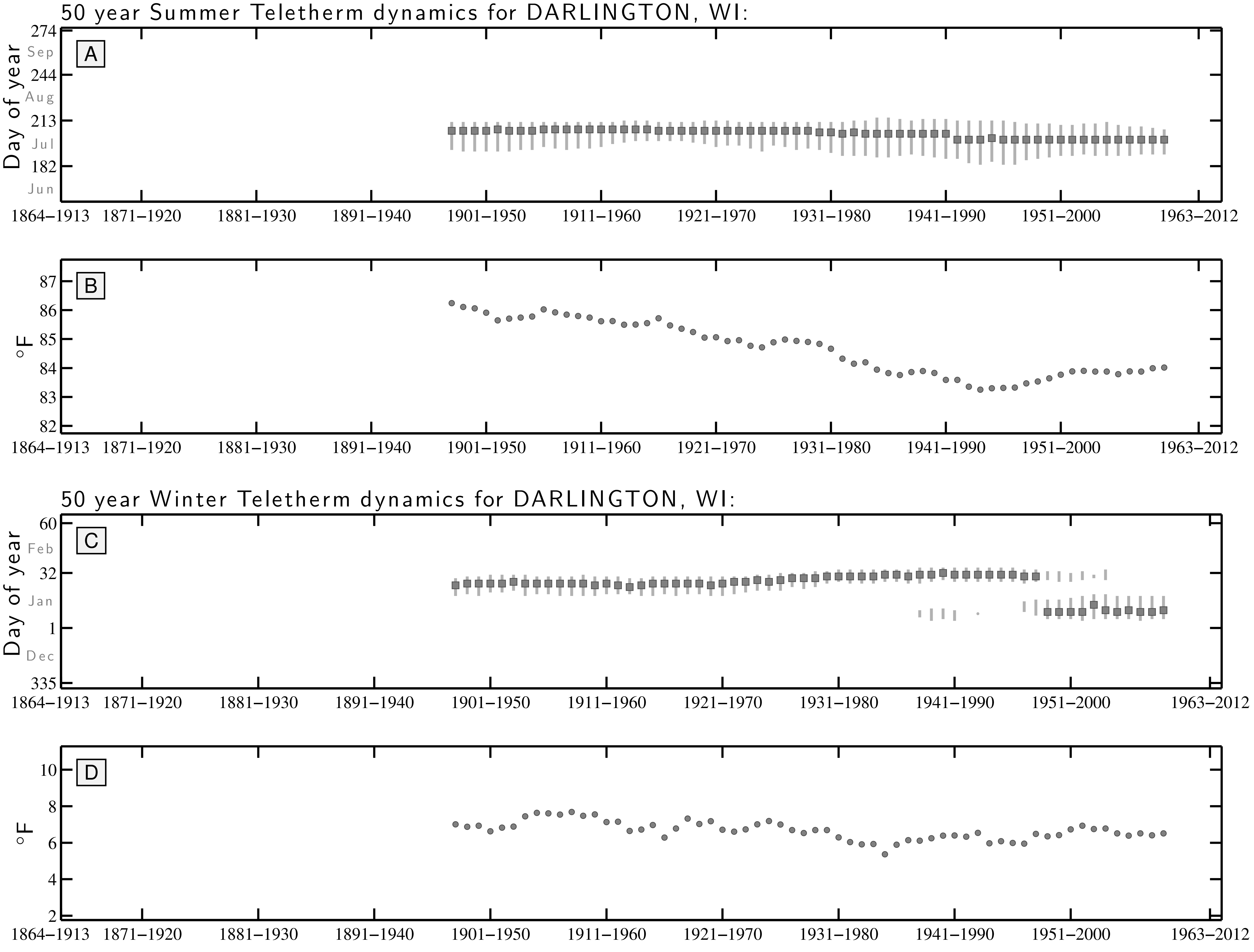
50 year Summer Teletherm dynamics for DARLINGTON, WI:
A
B
Day of year
°F
274
Sep
244
Aug
213
Jul
182
Jun
87
86
85
84
83
82
1864–1913
1871–1920
1881–1930
1891–1940
1901–1950
1911–1960
1921–1970
1931–1980
1941–1990
1951–2000
1963–2012
50 year Winter Teletherm dynamics for DARLINGTON, WI:
C
D
60
Feb
32
Jan
1
Dec
335
10
8
6
4
2

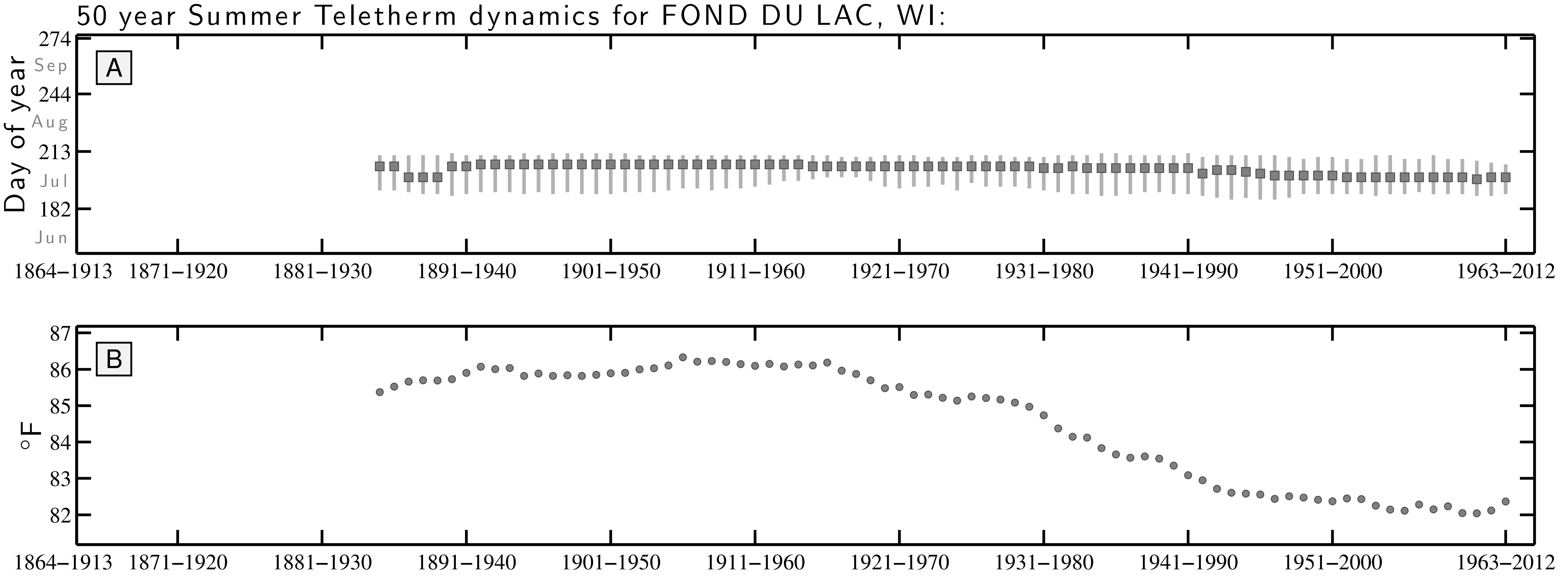
50 year Summer Teletherm dynamics for FOND DU LAC, WI:
A
B
Day of year
°F
274
244
213
182
Sep
Aug
Jul
Jun
87
86
85
84
83
82
1864–1913
1871–1920
1881–1930
1891–1940
1901–1950
1911–1960
1921–1970
1931–1980
1941–1990
1951–2000
1963–2012

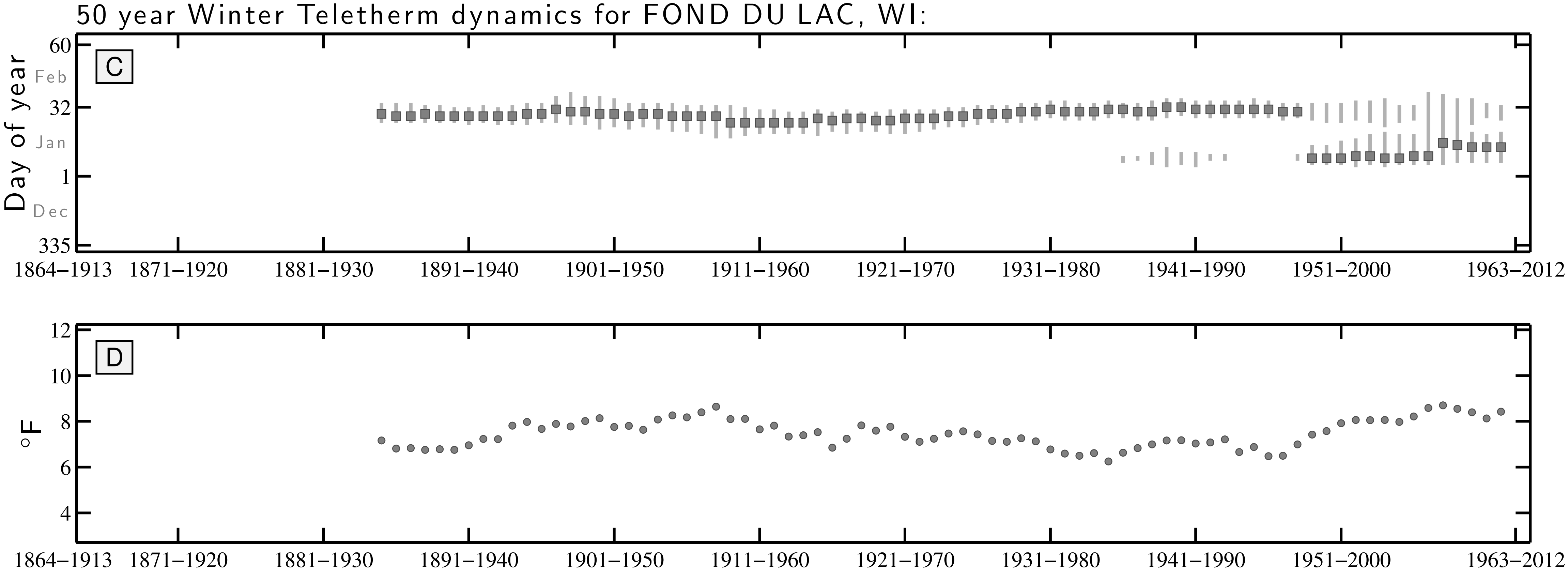
50 year Winter Teletherm dynamics for FOND DU LAC, WI:
C
D
Day of year
°F
60
32
1
335
Feb
Jan
Dec
12
10
8
6
4
1864–1913
1871–1920
1881–1930
1891–1940
1901–1950
1911–1960
1921–1970
1931–1980
1941–1990
1951–2000
1963–2012

50 year Summer Teletherm dynamics for HANCOCK EXP FARM, WI:

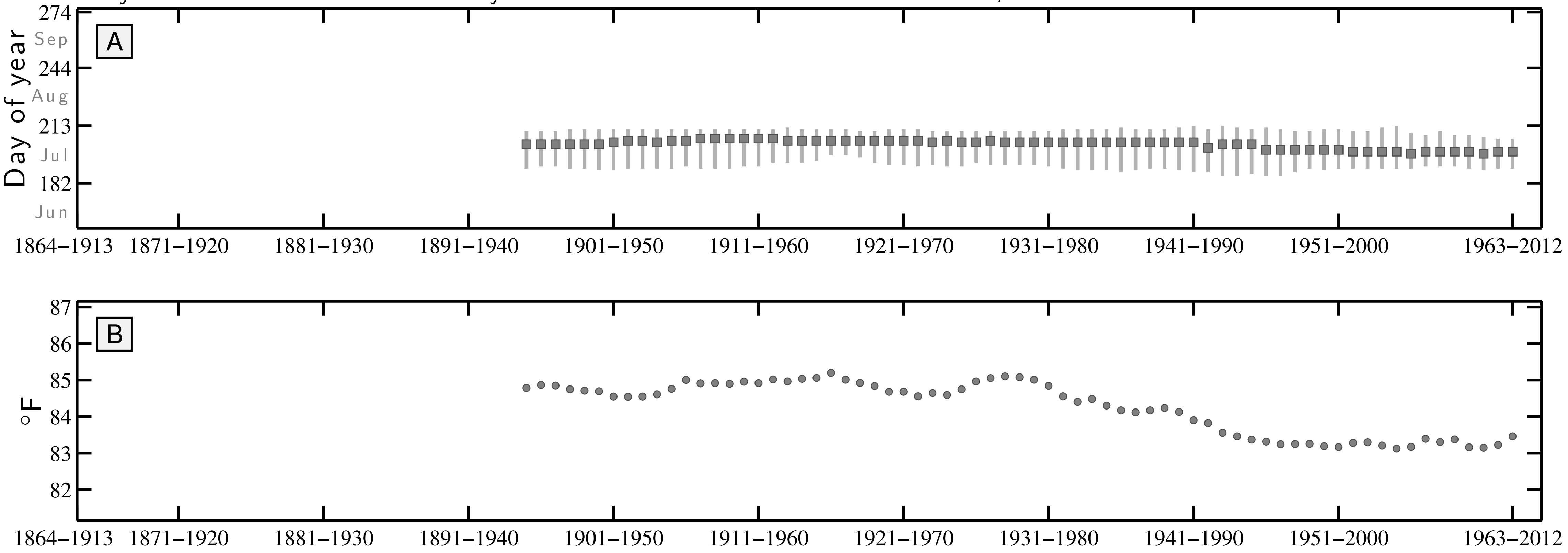

50 year Winter Teletherm dynamics for HANCOCK EXP FARM, WI:

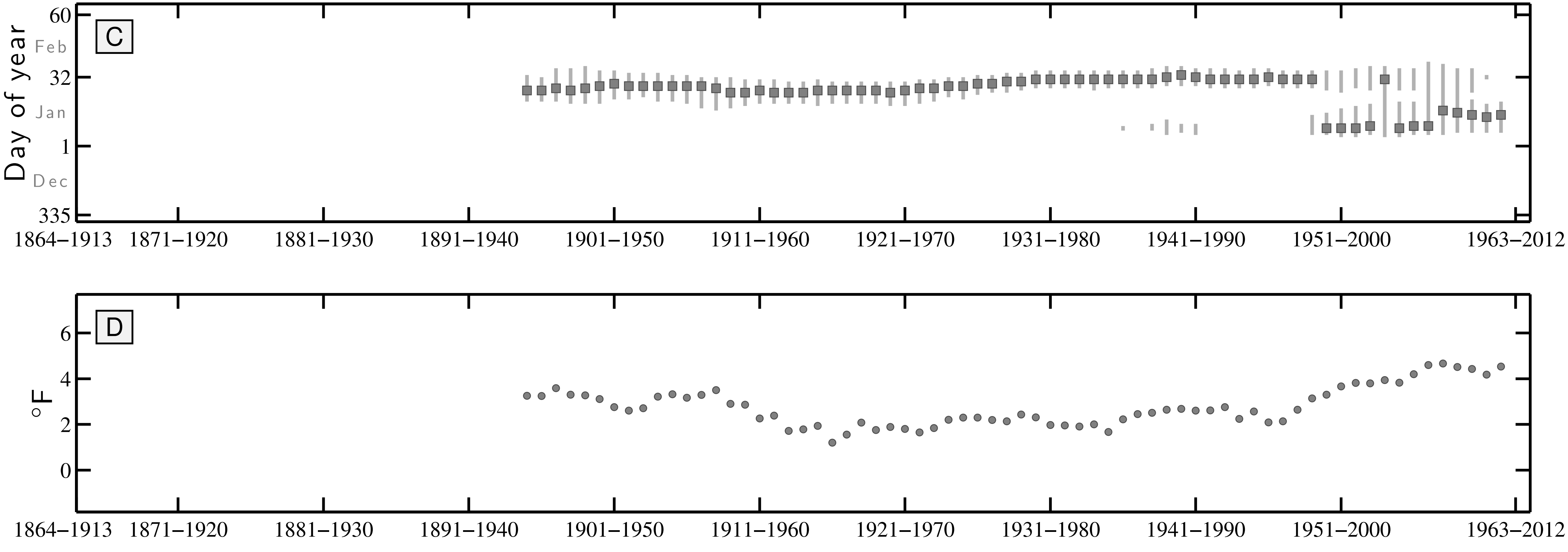

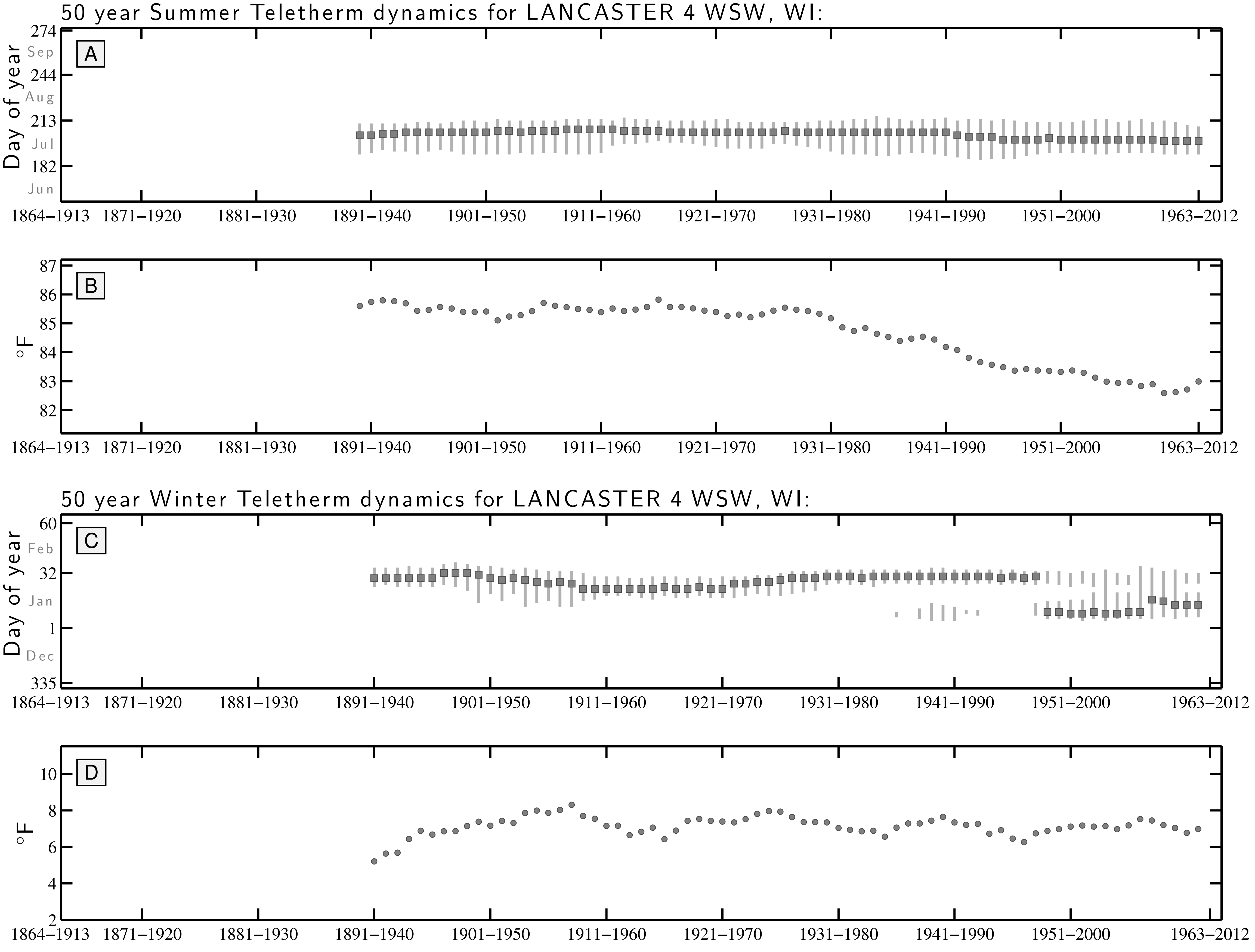

50 year Summer Teletherm dynamics for LANCASTER 4 WSW, WI:
A
Day of year
274
Sep
244
Aug
213
Jul
182
Jun
1864–1913
1871–1920
1881–1930
1891–1940
1901–1950
1911–1960
1921–1970
1931–1980
1941–1990
1951–2000
1963–2012
B
°F
87
86
85
84
83
82
50 year Winter Teletherm dynamics for LANCASTER 4 WSW, WI:
C
60
Feb
32
Jan
1
Dec
335
D
10
8
6
4
2

50 year Summer Teletherm dynamics for MANITOWOC, WI:

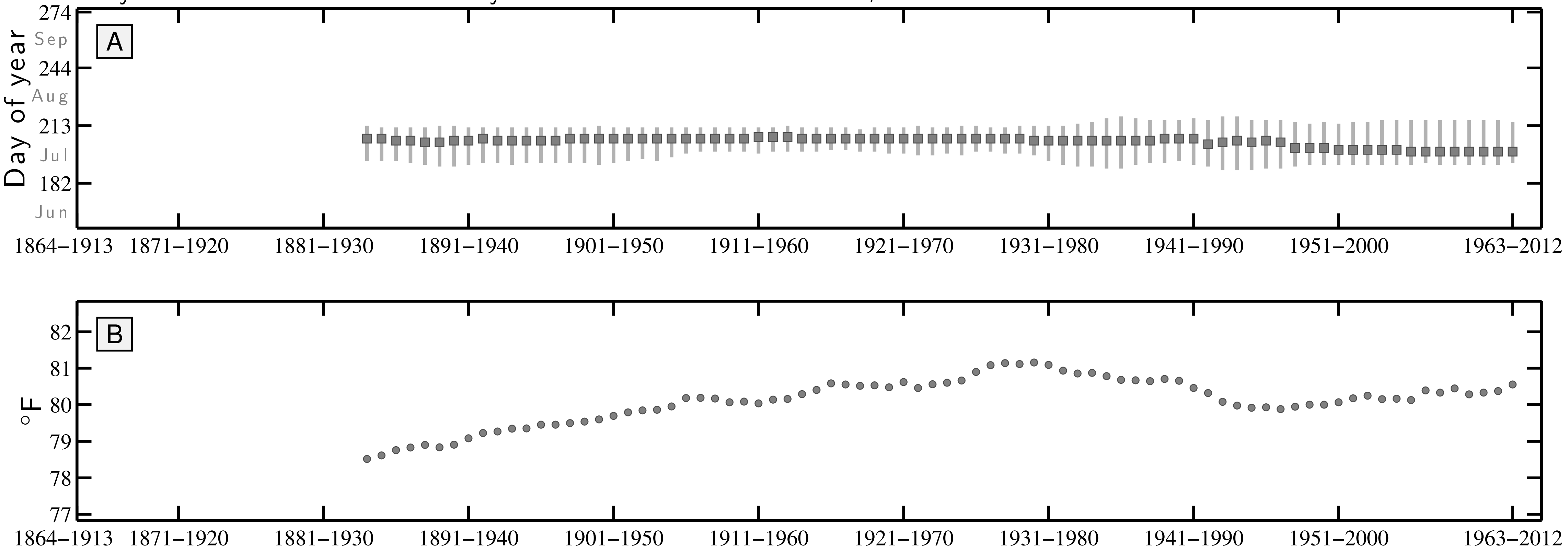

50 year Winter Teletherm dynamics for MANITOWOC, WI:

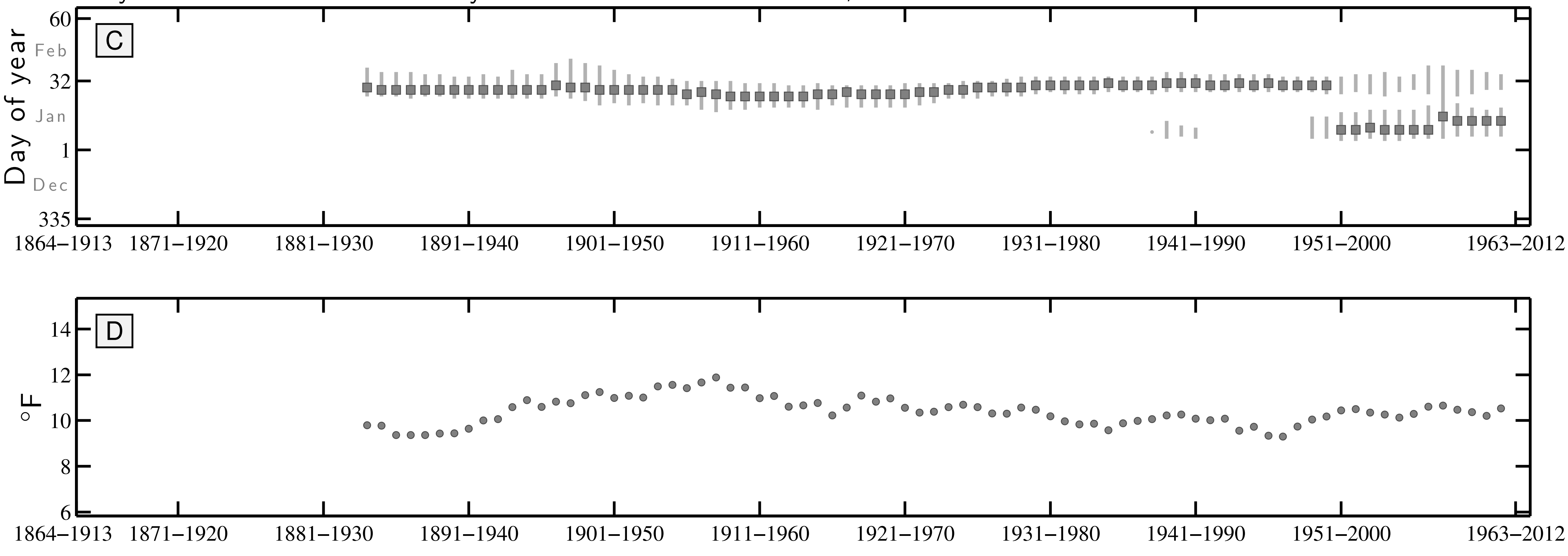

50 year Summer Teletherm dynamics for MARSHFIELD EXP FARM, WI:

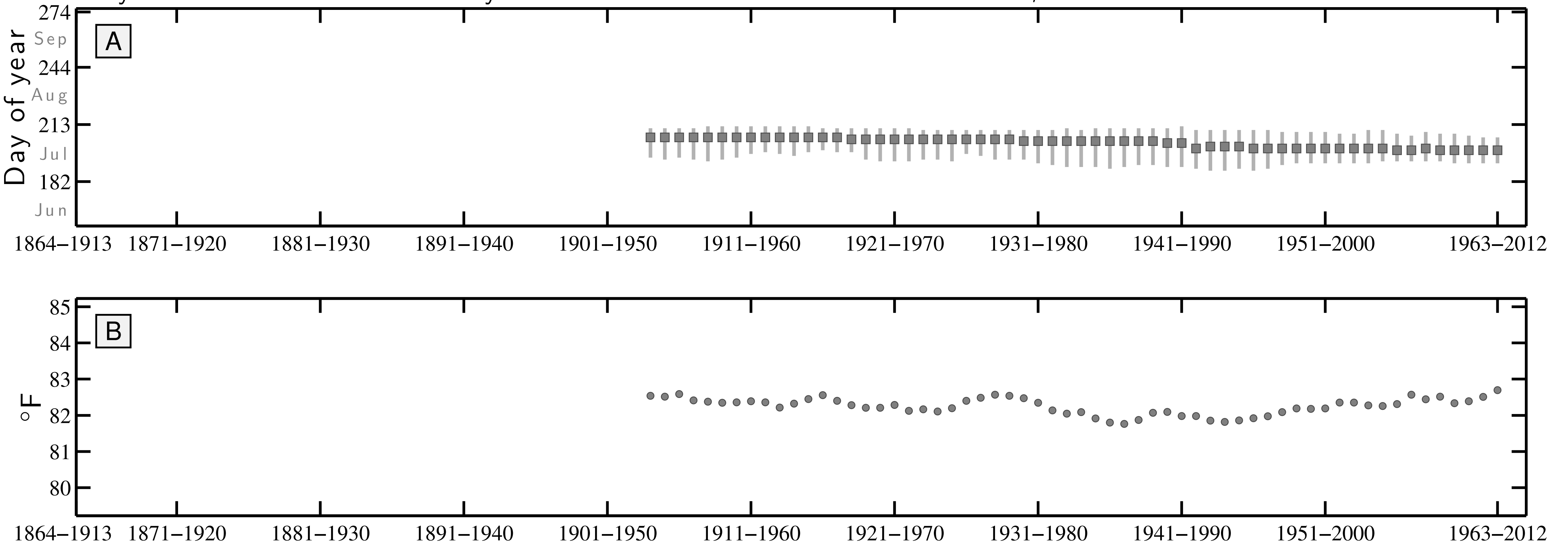

50 year Winter Teletherm dynamics for MARSHFIELD EXP FARM, WI:

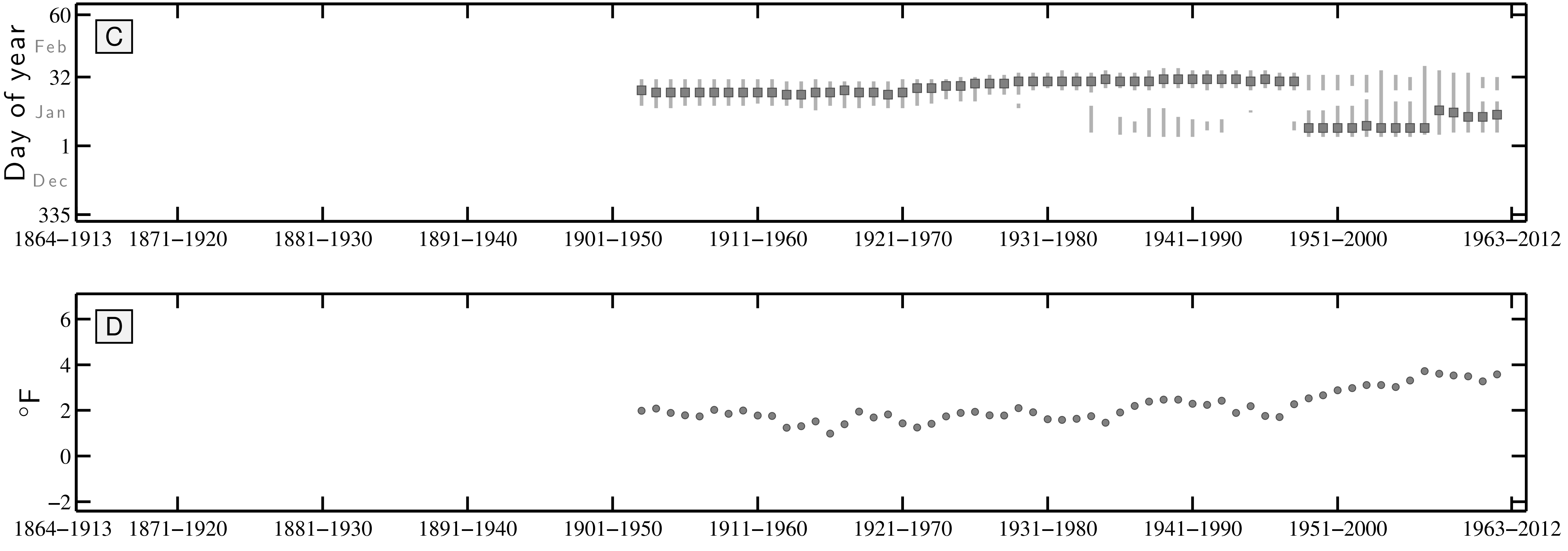

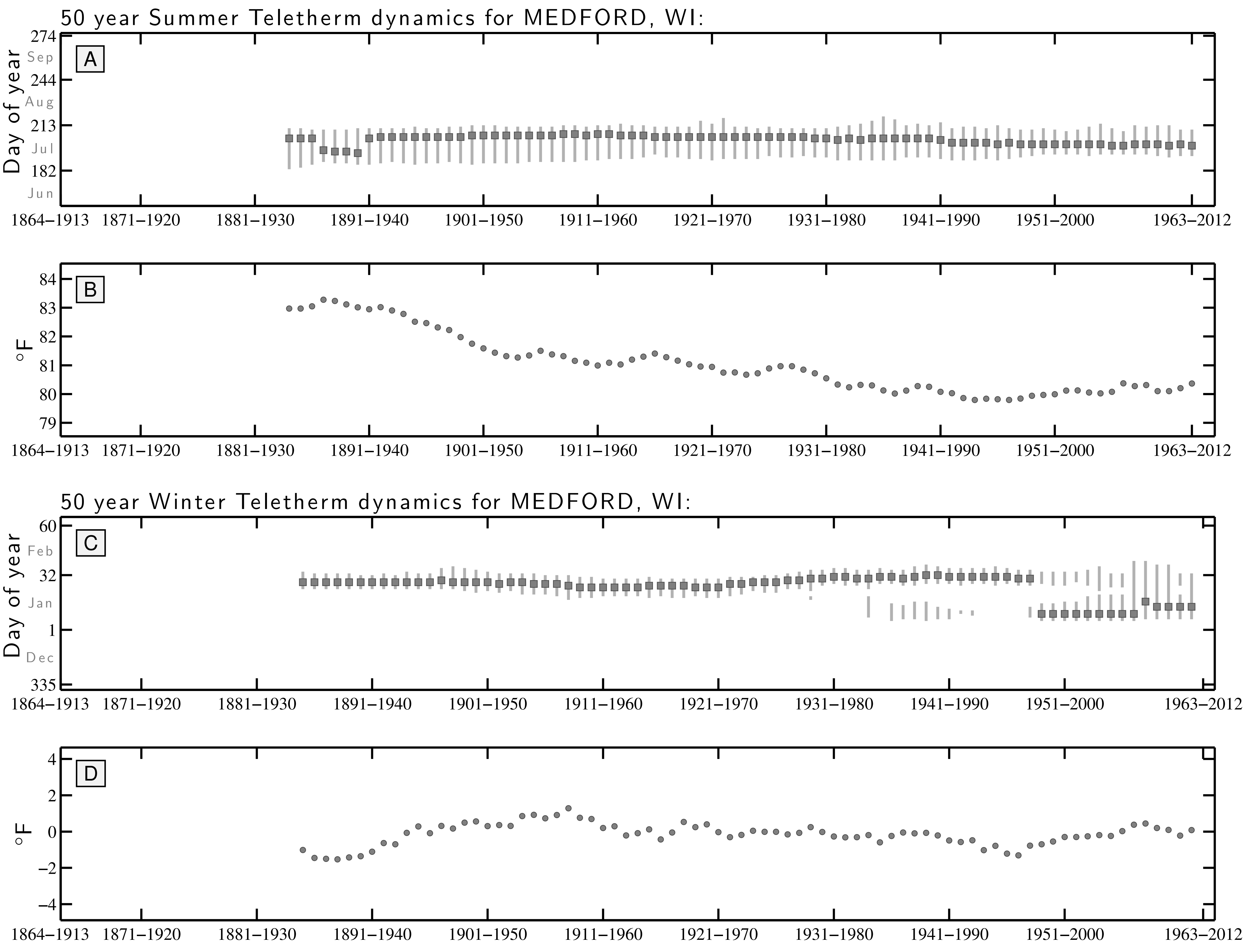
50 year Summer Teletherm dynamics for MEDFORD, WI:
A
Day of year
274
Sep
244
Aug
213
Jul
182
Jun
B
°F
84
83
82
81
80
79
1864–1913
1871–1920
1881–1930
1891–1940
1901–1950
1911–1960
1921–1970
1931–1980
1941–1990
1951–2000
1963–2012
50 year Winter Teletherm dynamics for MEDFORD, WI:
C
Day of year
60
Feb
32
Jan
1
Dec
335
D
°F
4
2
0
−2
−4

50 year Summer Teletherm dynamics for MILWAUKEE MT MARY COL, WI:

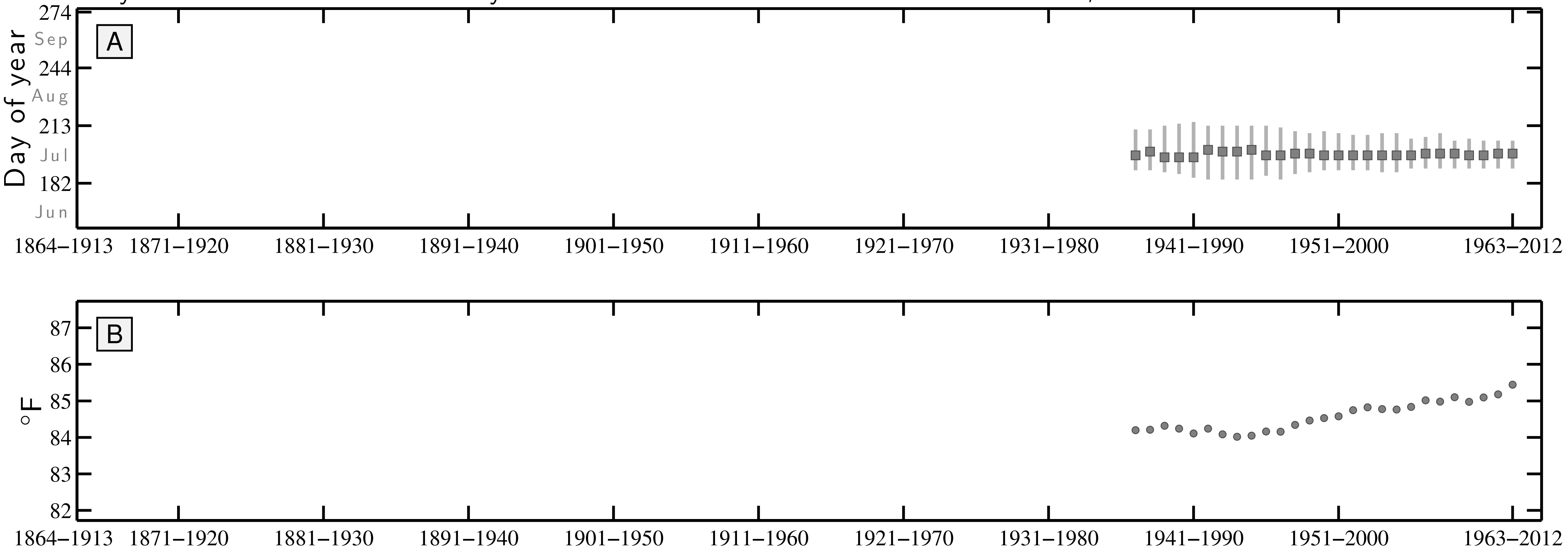

50 year Winter Teletherm dynamics for MILWAUKEE MT MARY COL, WI:

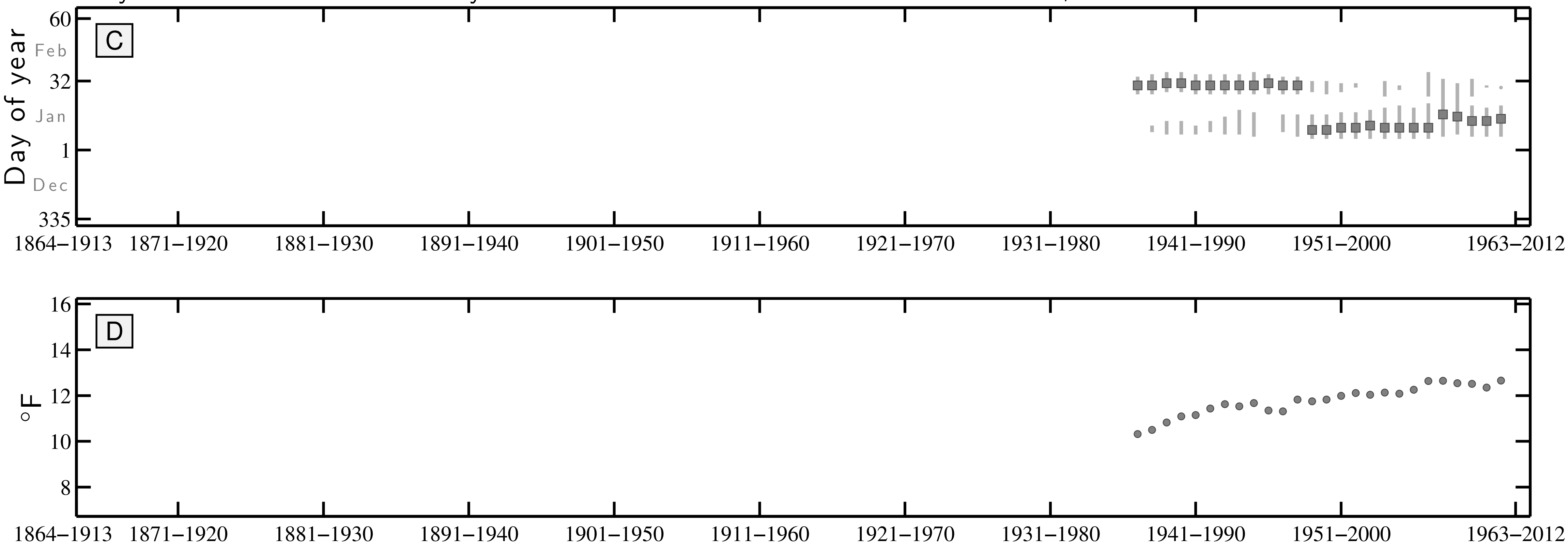

50 year Summer Teletherm dynamics for MINOCQUA, WI:

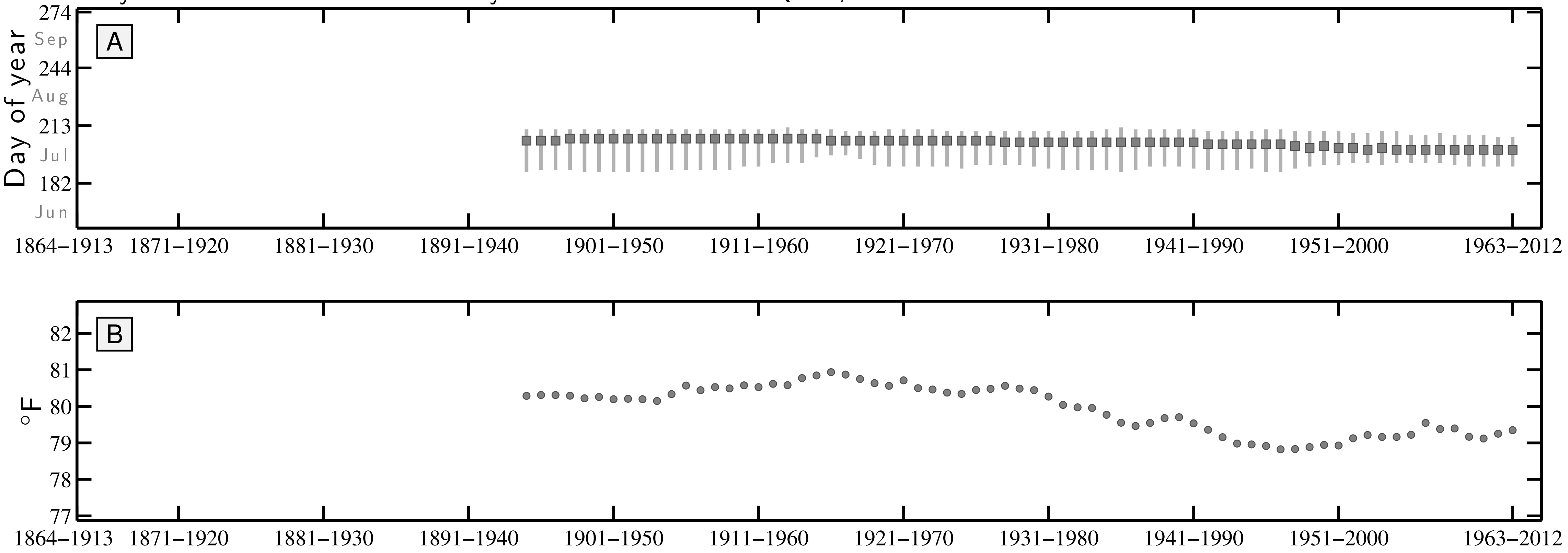

50 year Winter Teletherm dynamics for MINOCQUA, WI:

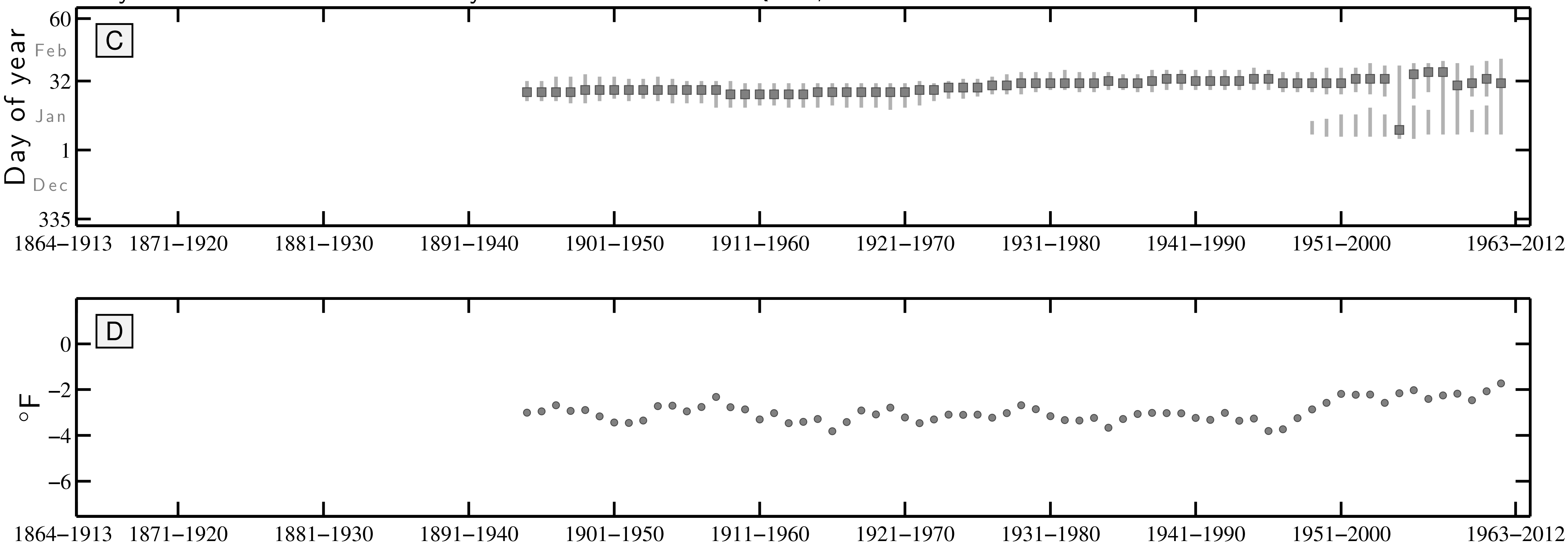

50 year Summer Teletherm dynamics for NEILLSVILLE 3 SW, WI:

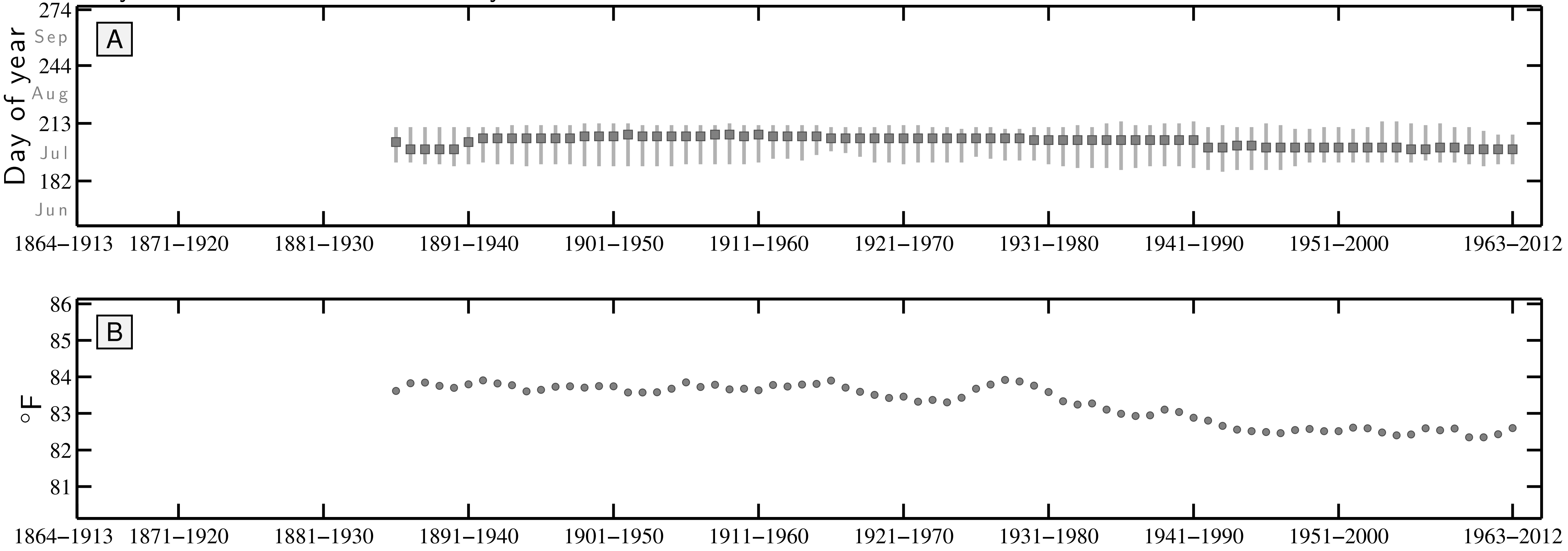

50 year Winter Teletherm dynamics for NEILLSVILLE 3 SW, WI:

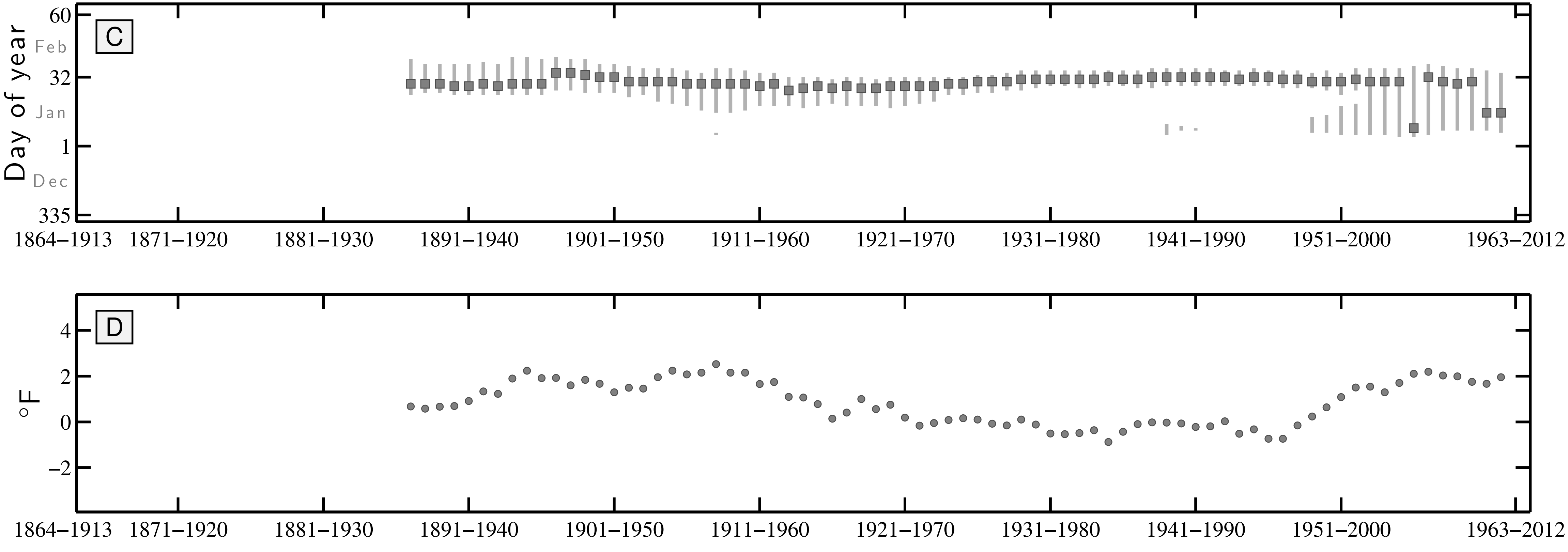

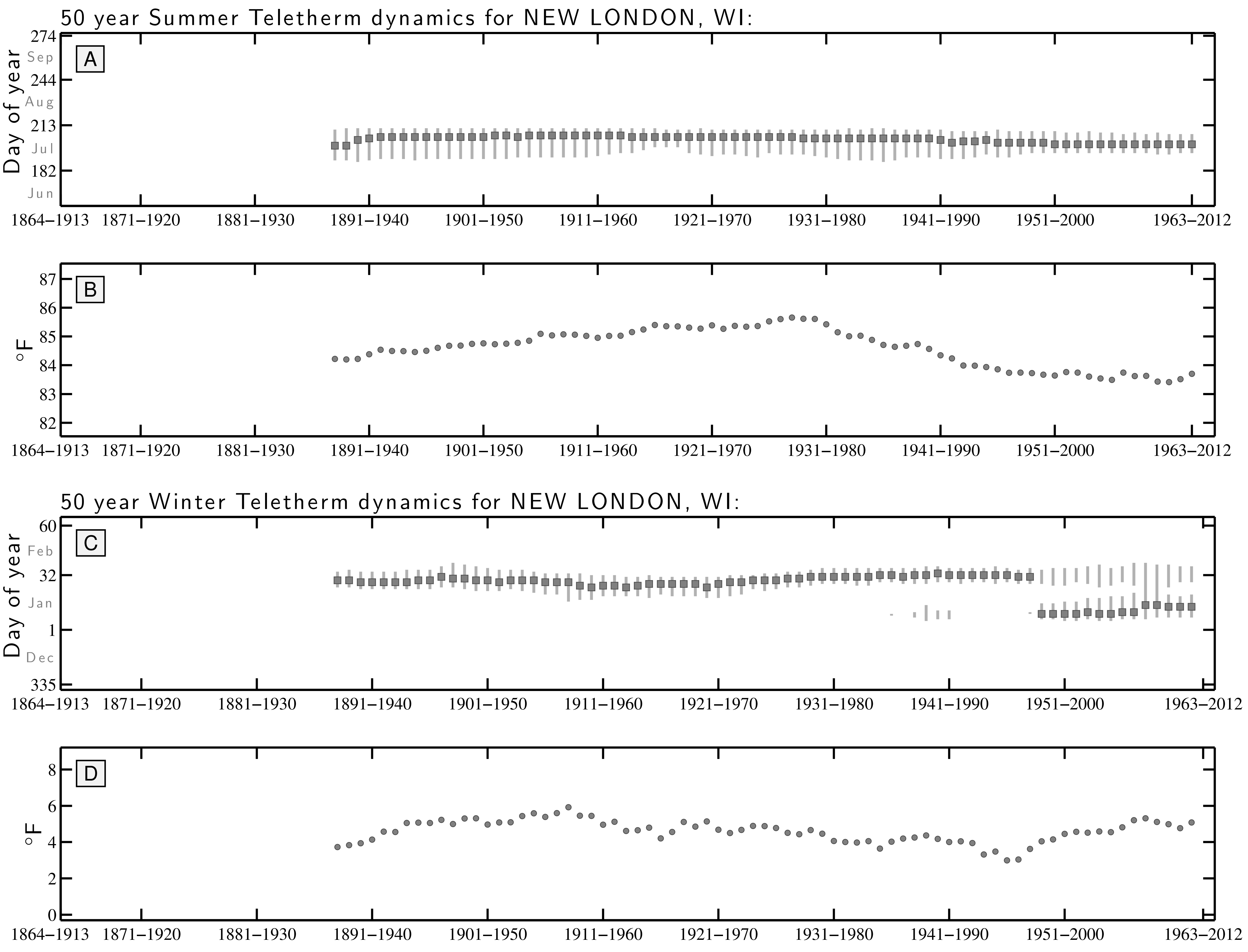

50 year Summer Teletherm dynamics for NEW LONDON, WI:
A
Day of year
274
Sep
244
Aug
213
Jul
182
Jun
1864–1913
1871–1920
1881–1930
1891–1940
1901–1950
1911–1960
1921–1970
1931–1980
1941–1990
1951–2000
1963–2012
B
°F
87
86
85
84
83
82
50 year Winter Teletherm dynamics for NEW LONDON, WI:
C
60
Feb
32
Jan
1
Dec
335
D
8
6
4
2
0

50 year Summer Teletherm dynamics for OCONTO 4 W, WI:

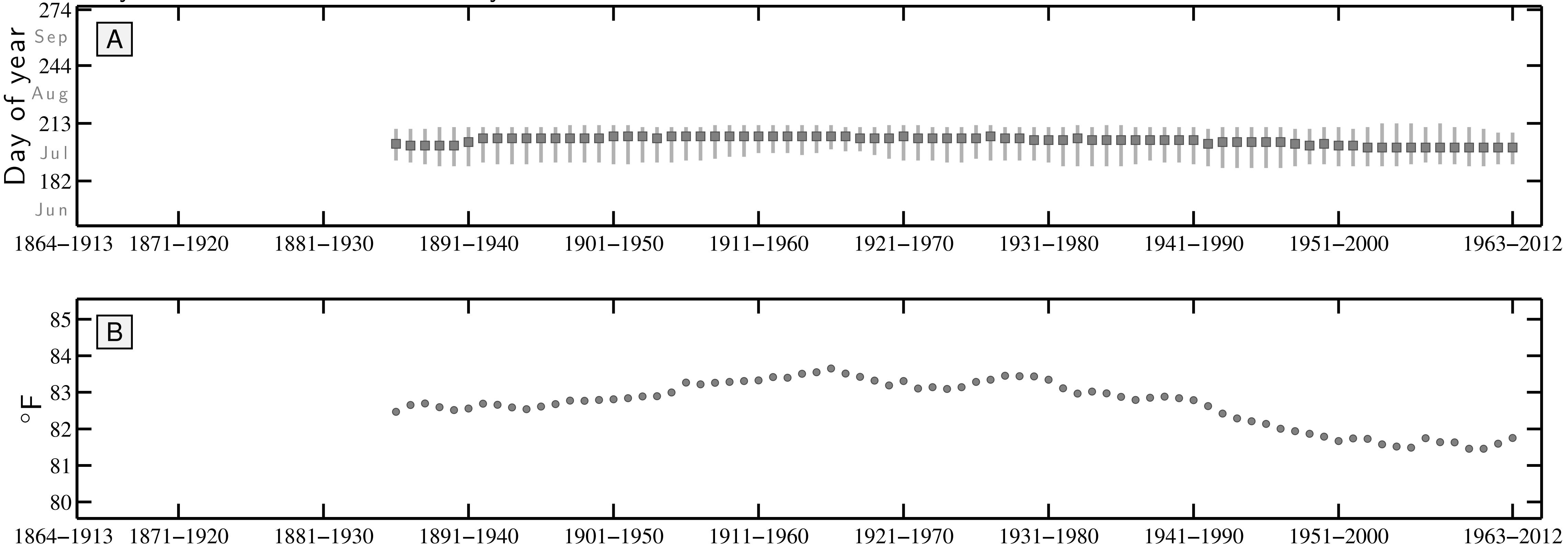

50 year Winter Teletherm dynamics for OCONTO 4 W, WI:

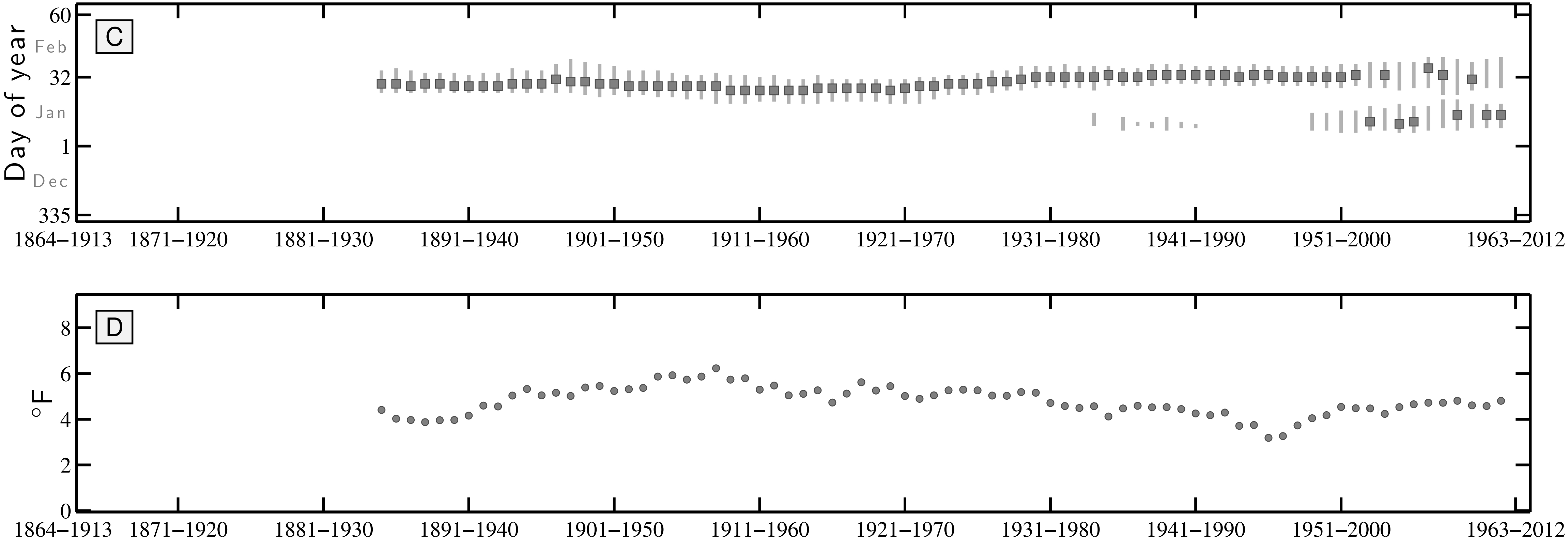

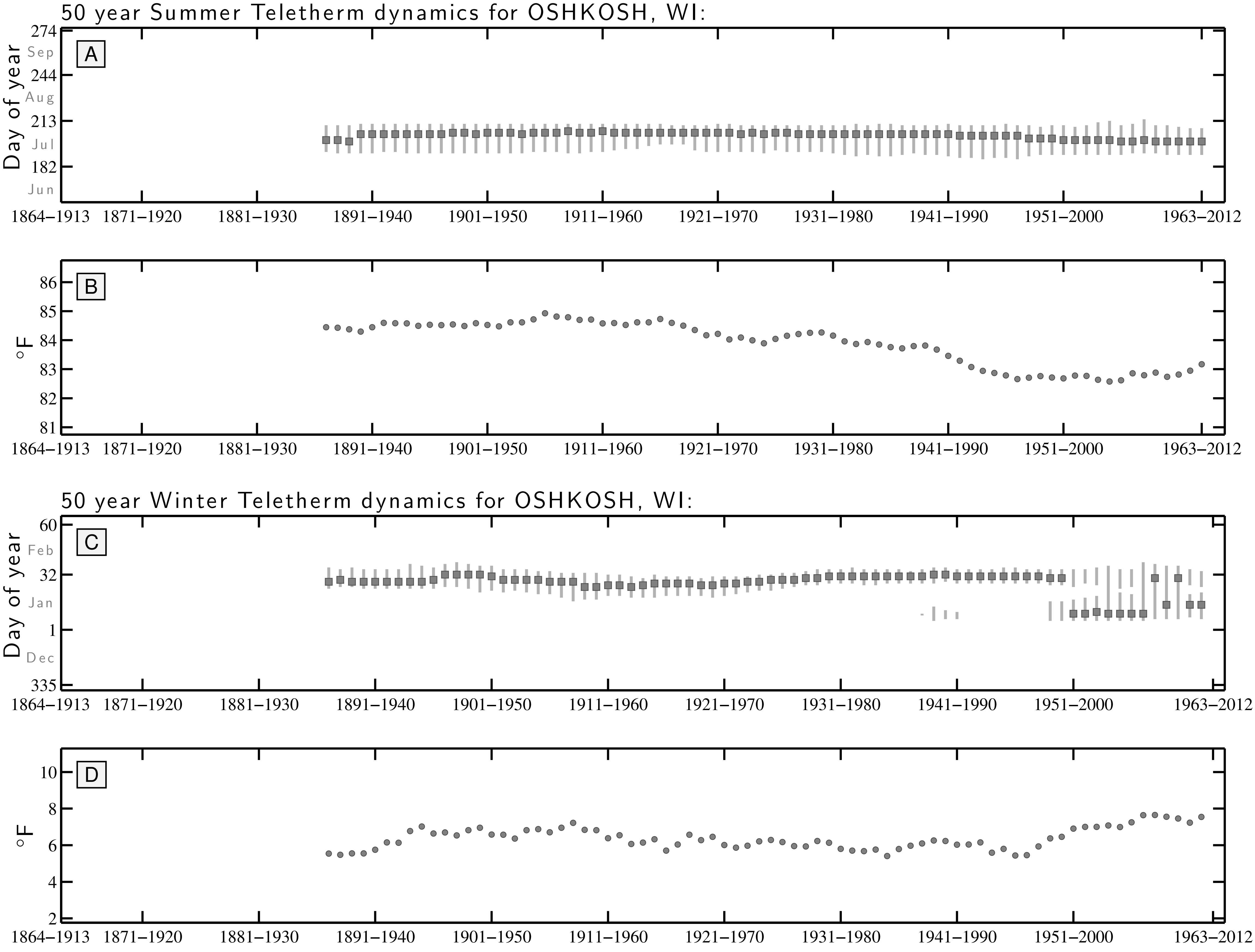

50 year Summer Teletherm dynamics for OSHKOSH, WI:
A
Day of year
274
Sep
244
Aug
213
Jul
182
Jun
1864–1913
1871–1920
1881–1930
1891–1940
1901–1950
1911–1960
1921–1970
1931–1980
1941–1990
1951–2000
1963–2012
B
°F
86
85
84
83
82
81
50 year Winter Teletherm dynamics for OSHKOSH, WI:
C
Day of year
60
Feb
32
Jan
1
Dec
335
D
°F
10
8
6
4
2

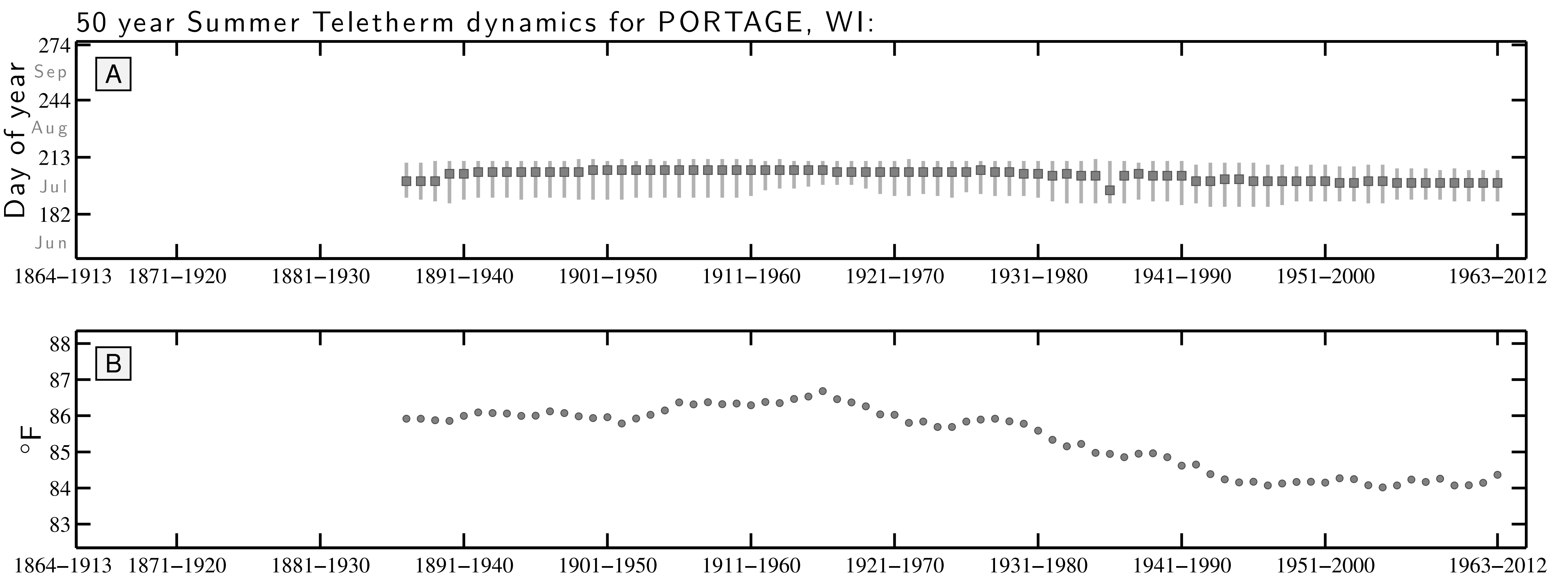
50 year Summer Teletherm dynamics for PORTAGE, WI:
A
B
Day of year
°F
274
Sep
244
Aug
213
Jul
182
Jun
88
87
86
85
84
83
1864–1913
1871–1920
1881–1930
1891–1940
1901–1950
1911–1960
1921–1970
1931–1980
1941–1990
1951–2000
1963–2012

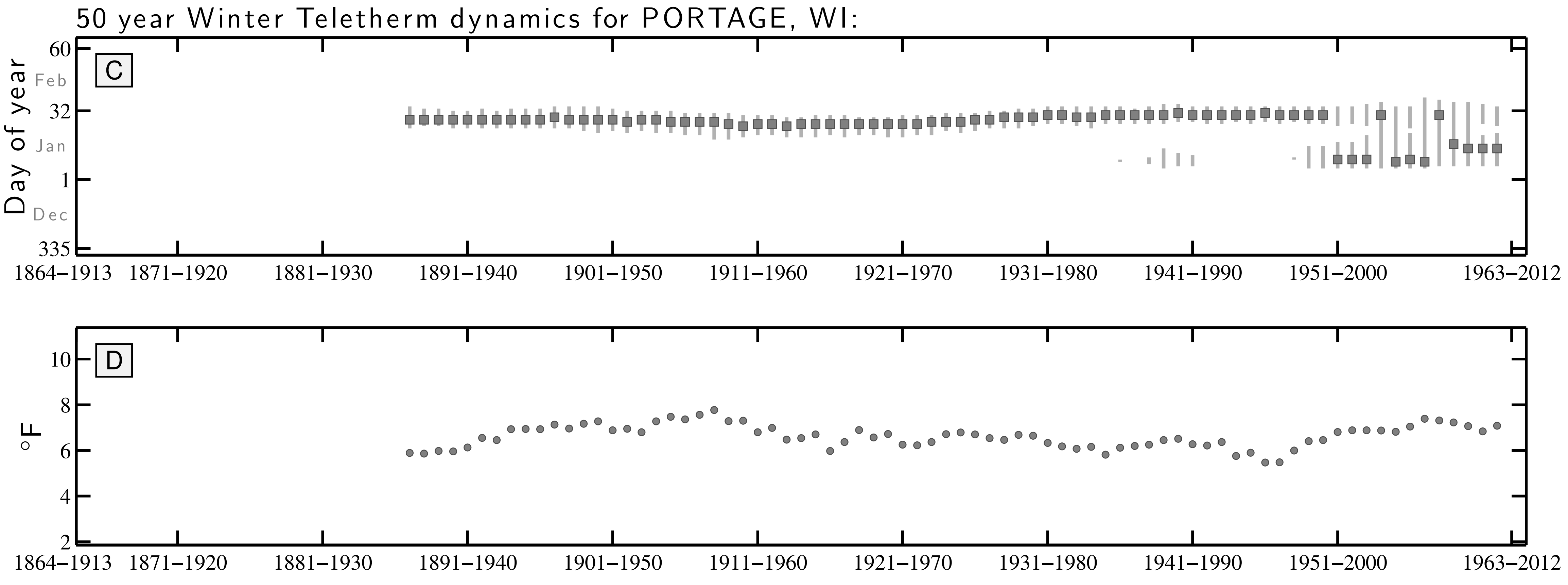
50 year Winter Teletherm dynamics for PORTAGE, WI:
C
D
Day of year
°F
60
Feb
32
Jan
1
Dec
335
10
8
6
4
2
1864–1913
1871–1920
1881–1930
1891–1940
1901–1950
1911–1960
1921–1970
1931–1980
1941–1990
1951–2000
1963–2012

50 year Summer Teletherm dynamics for PRAIRIE DU CHIEN, WI:

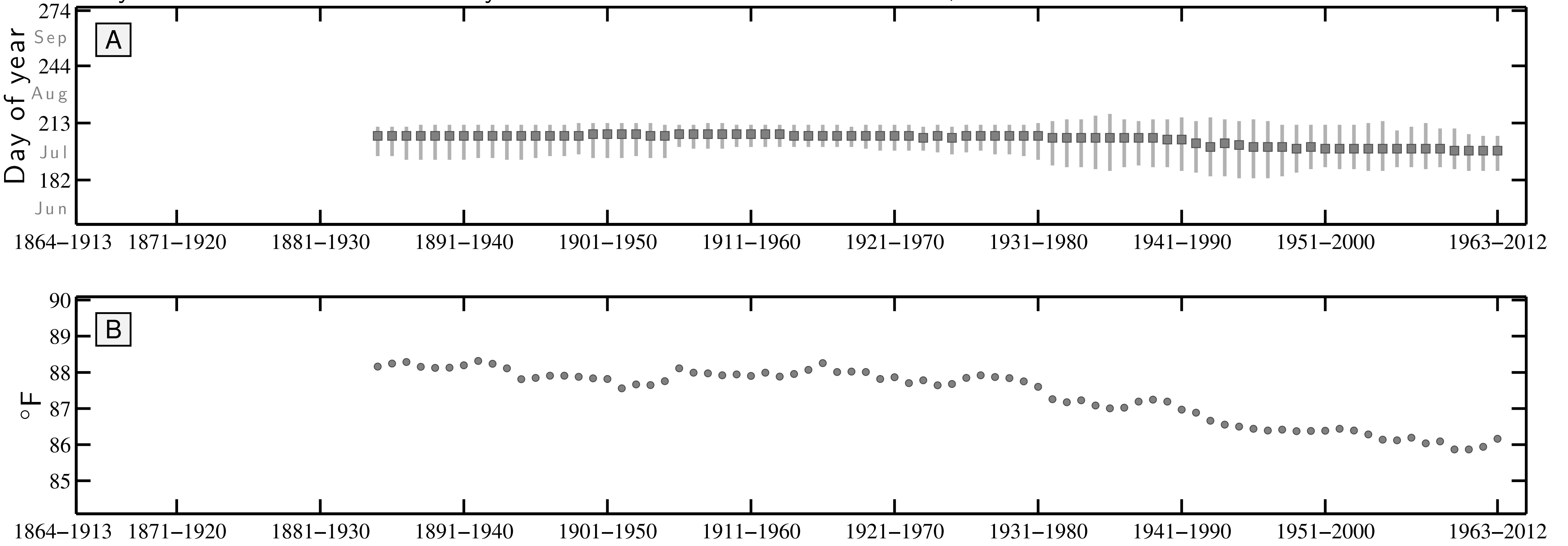

50 year Winter Teletherm dynamics for PRAIRIE DU CHIEN, WI:

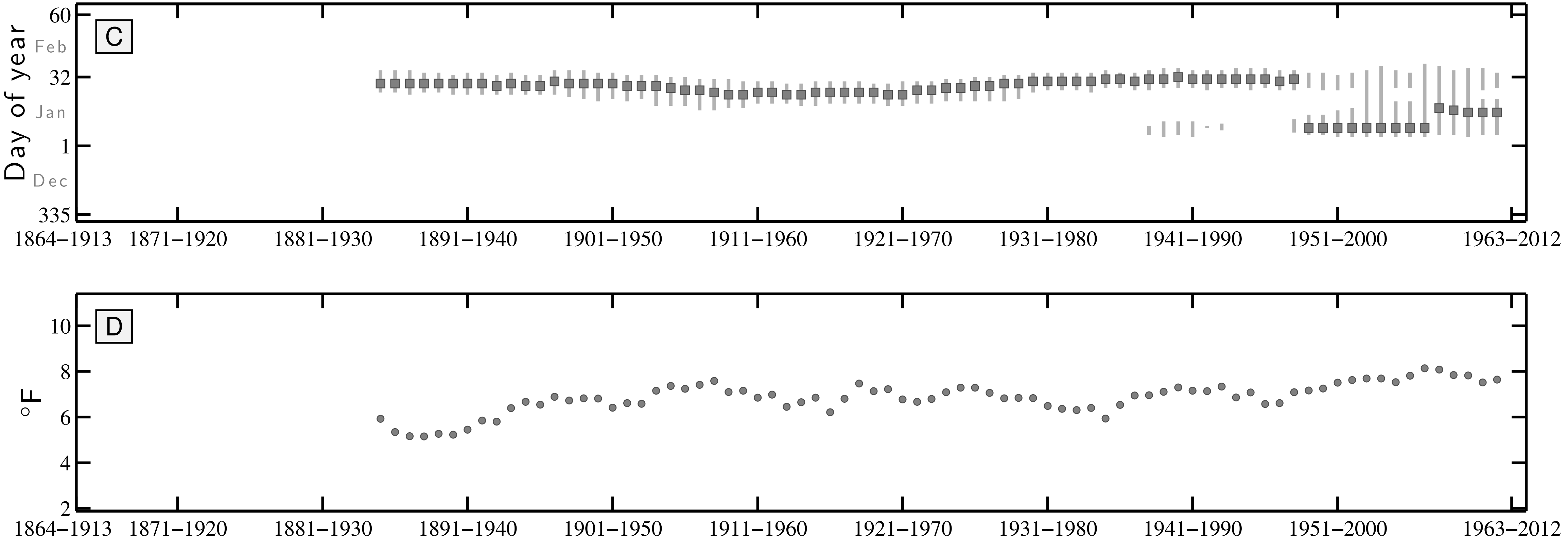

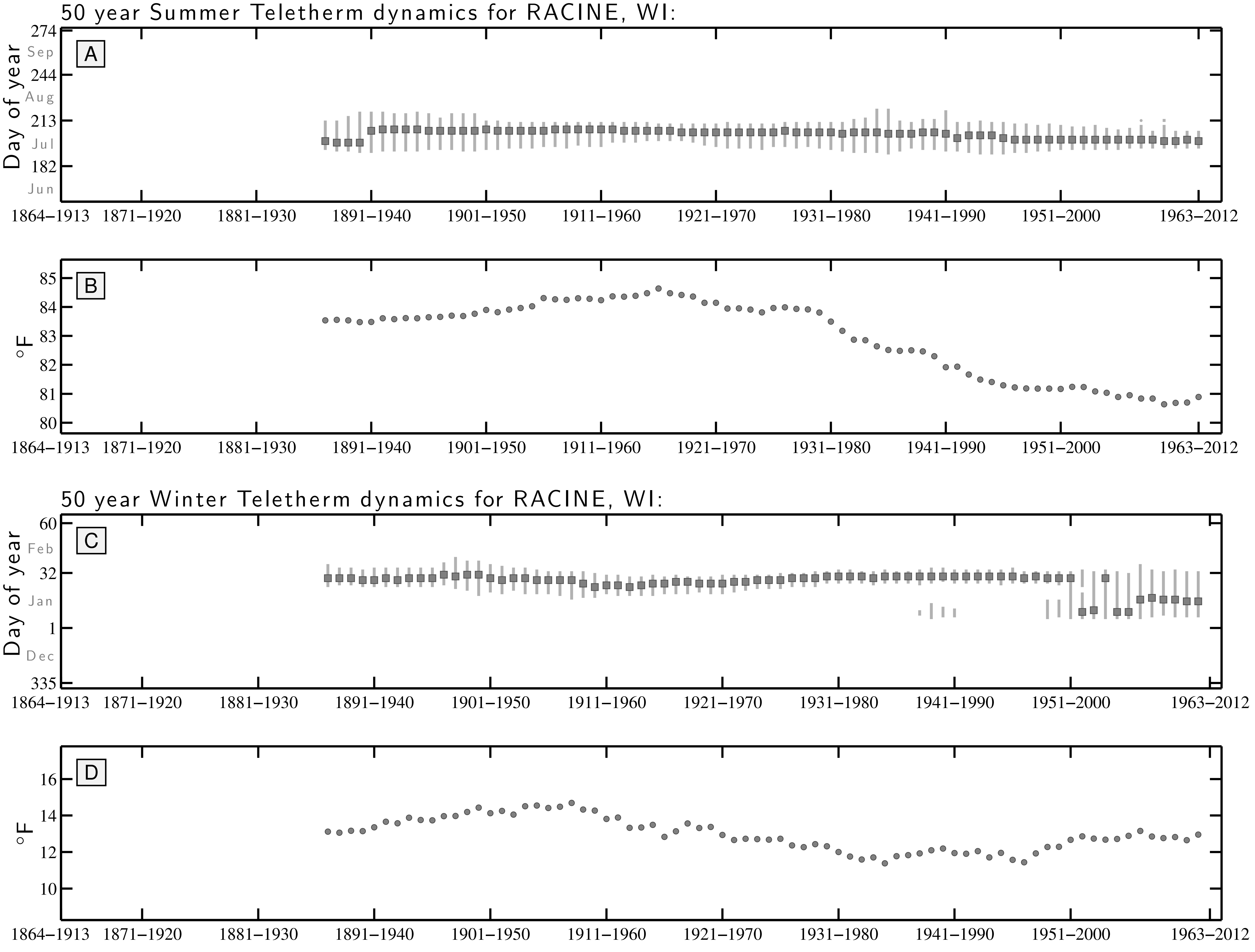

50 year Summer Teletherm dynamics for RACINE, WI:
A
Day of year
274
Sep
244
Aug
213
Jul
182
Jun
B
°F
85
84
83
82
81
80
1864–1913
1871–1920
1881–1930
1891–1940
1901–1950
1911–1960
1921–1970
1931–1980
1941–1990
1951–2000
1963–2012
50 year Winter Teletherm dynamics for RACINE, WI:
C
Day of year
60
Feb
32
Jan
1
Dec
335
D
°F
16
14
12
10

50 year Summer Teletherm dynamics for SPOONER AG RES STN, WI:

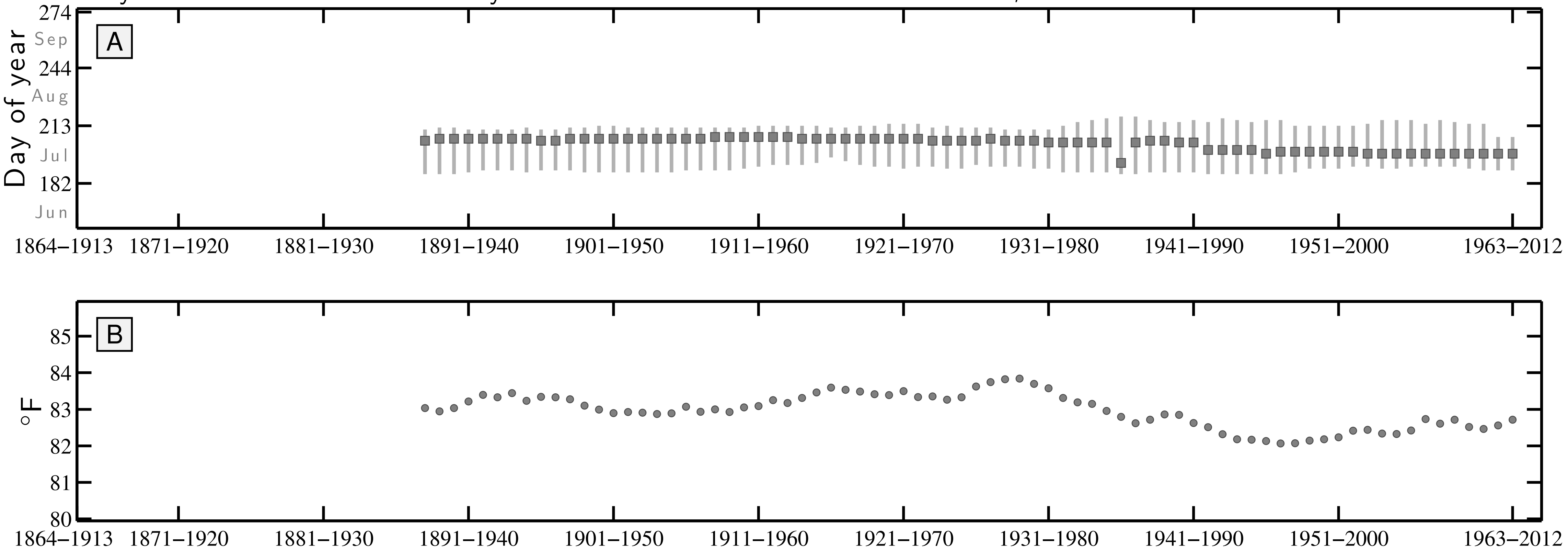

50 year Winter Teletherm dynamics for SPOONER AG RES STN, WI:

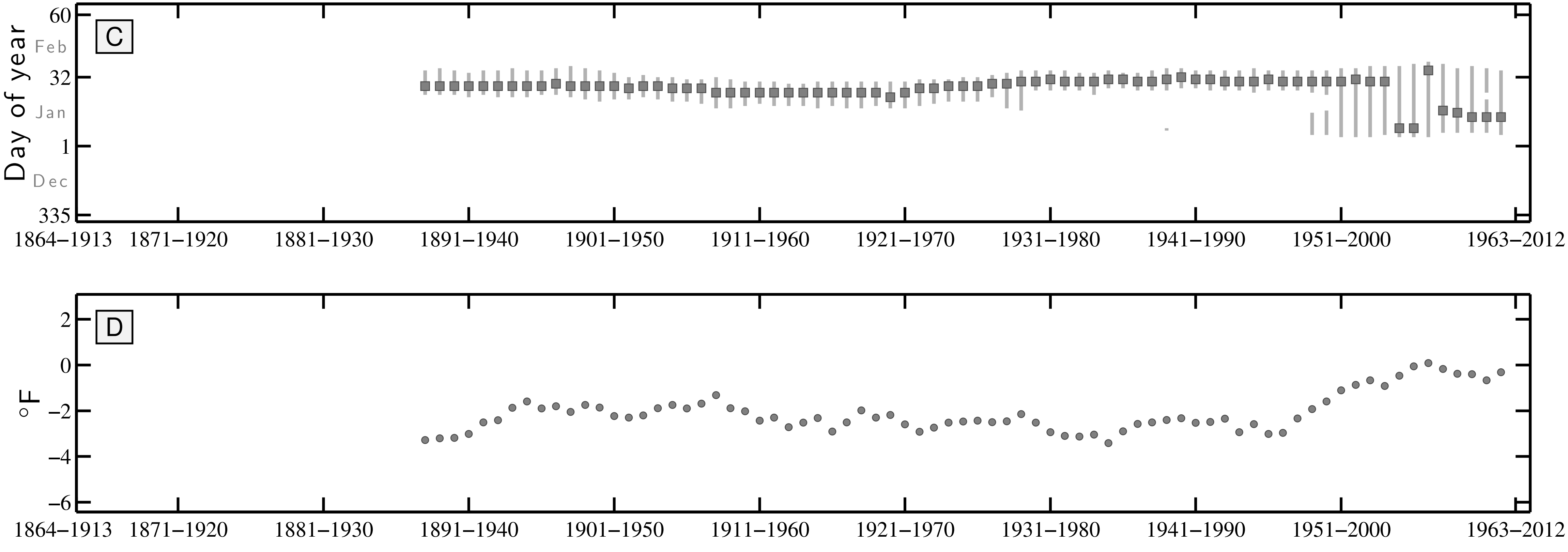

50 year Summer Teletherm dynamics for STANLEY, WI:

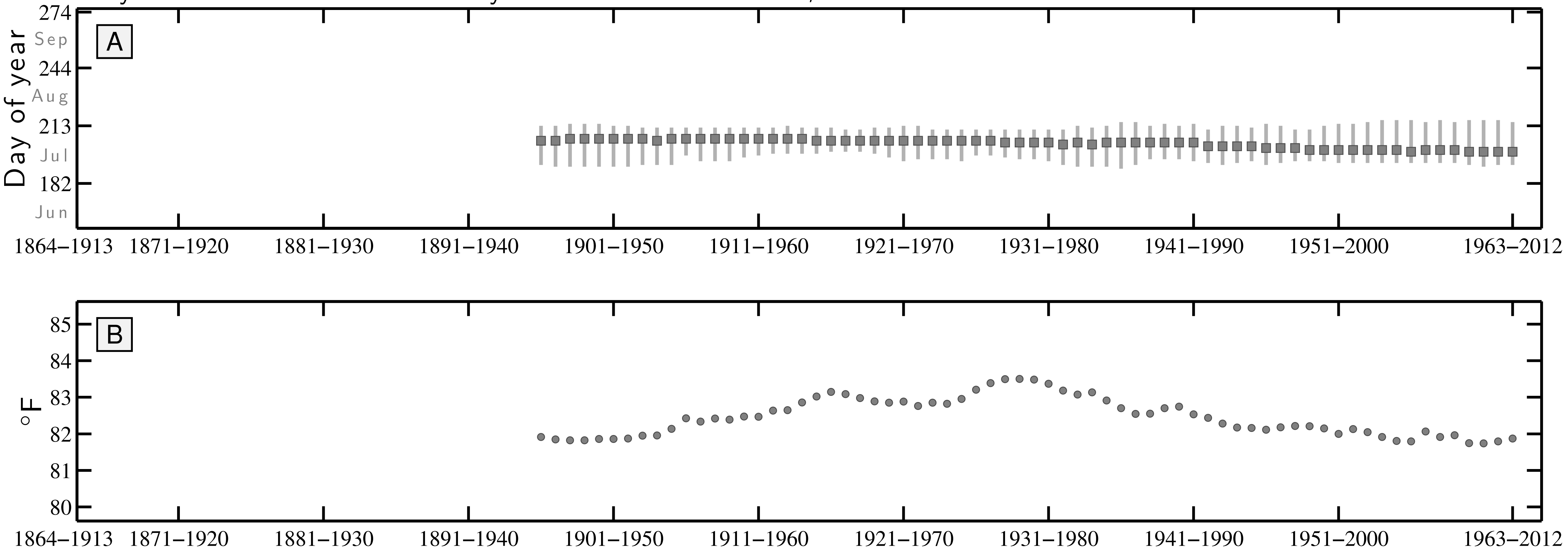

50 year Winter Teletherm dynamics for STANLEY, WI:

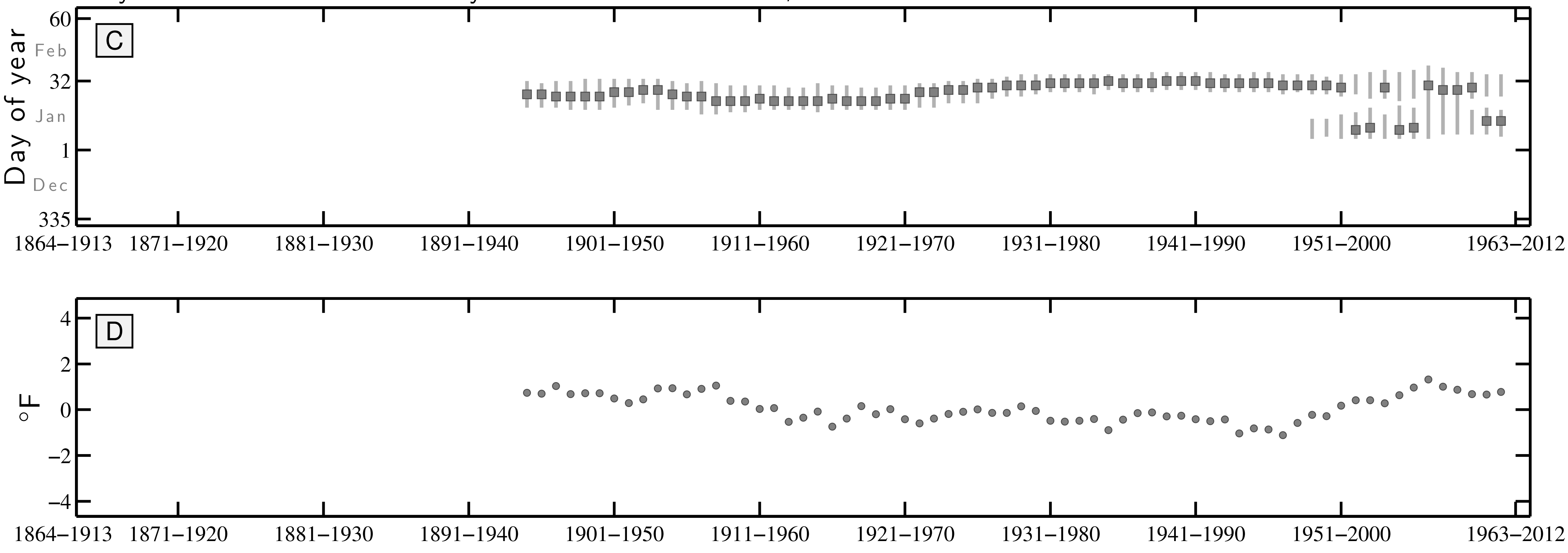

50 year Summer Teletherm dynamics for VIROQUA, WI:

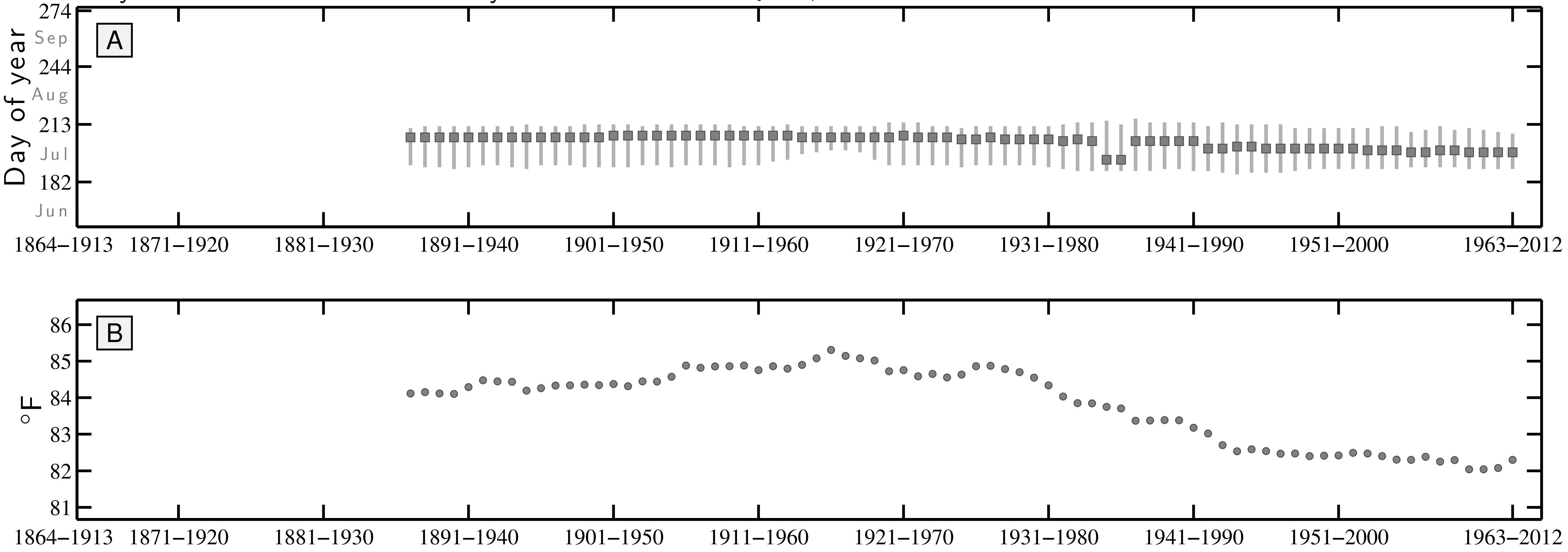

50 year Winter Teletherm dynamics for VIROQUA, WI:

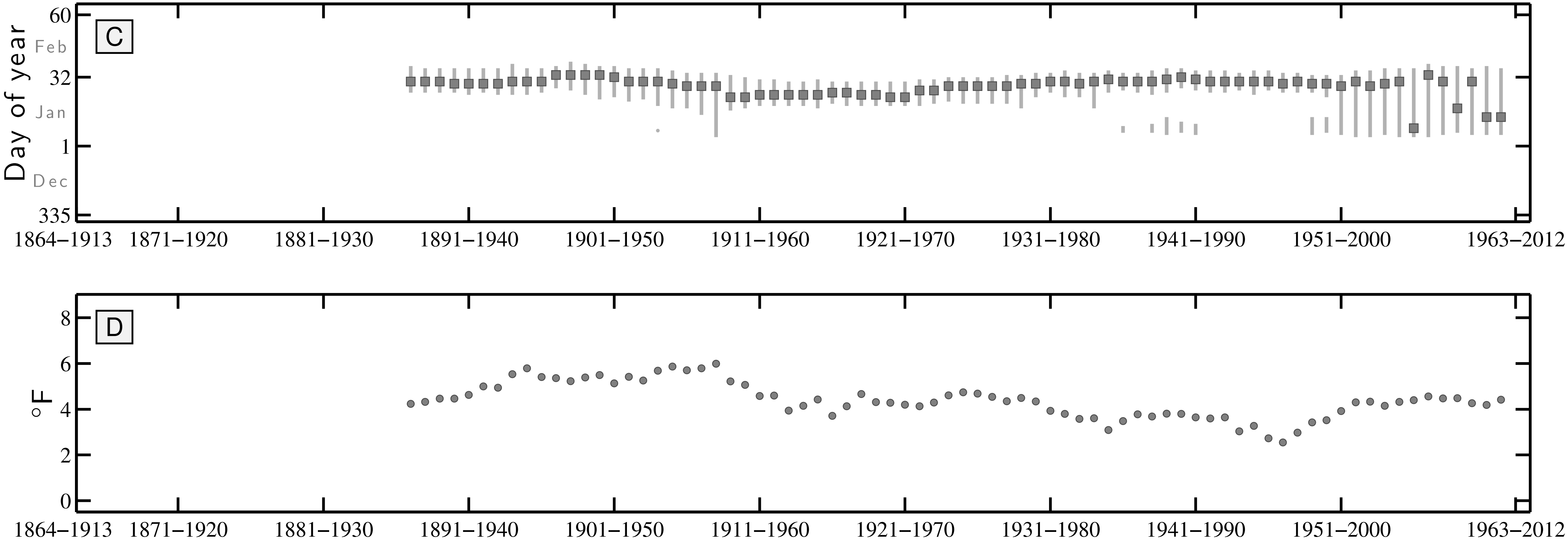

50 year Summer Teletherm dynamics for WATERTOWN, WI:

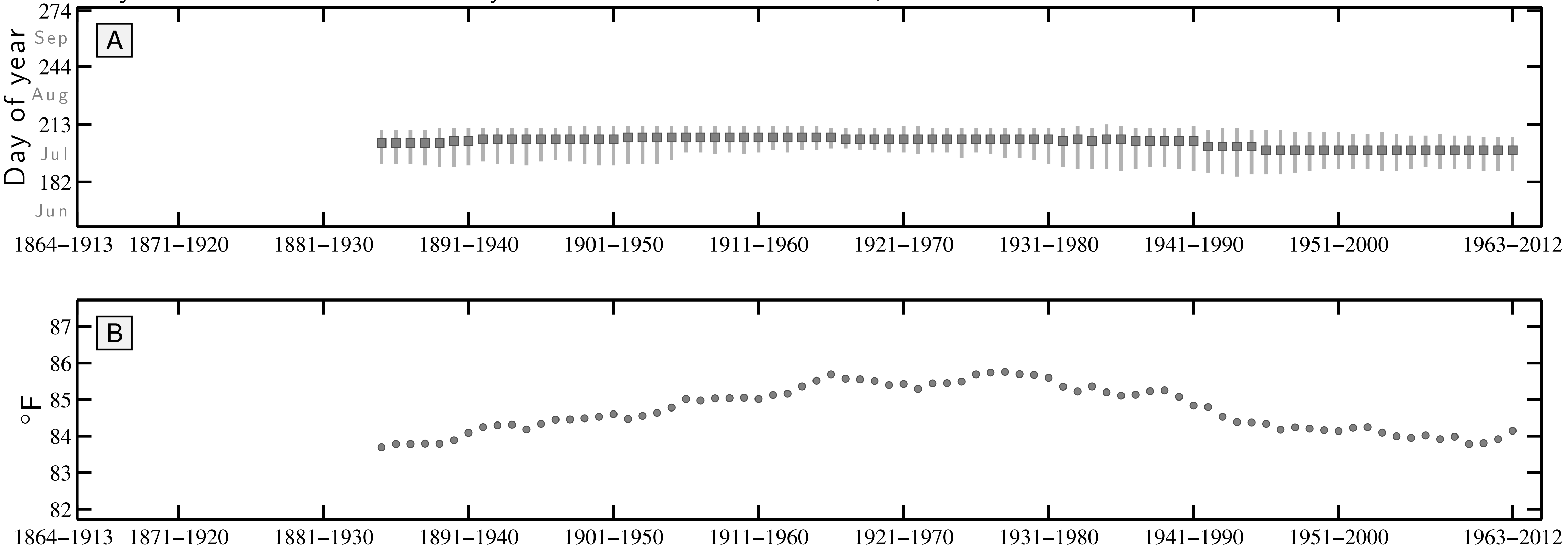

50 year Winter Teletherm dynamics for WATERTOWN, WI:

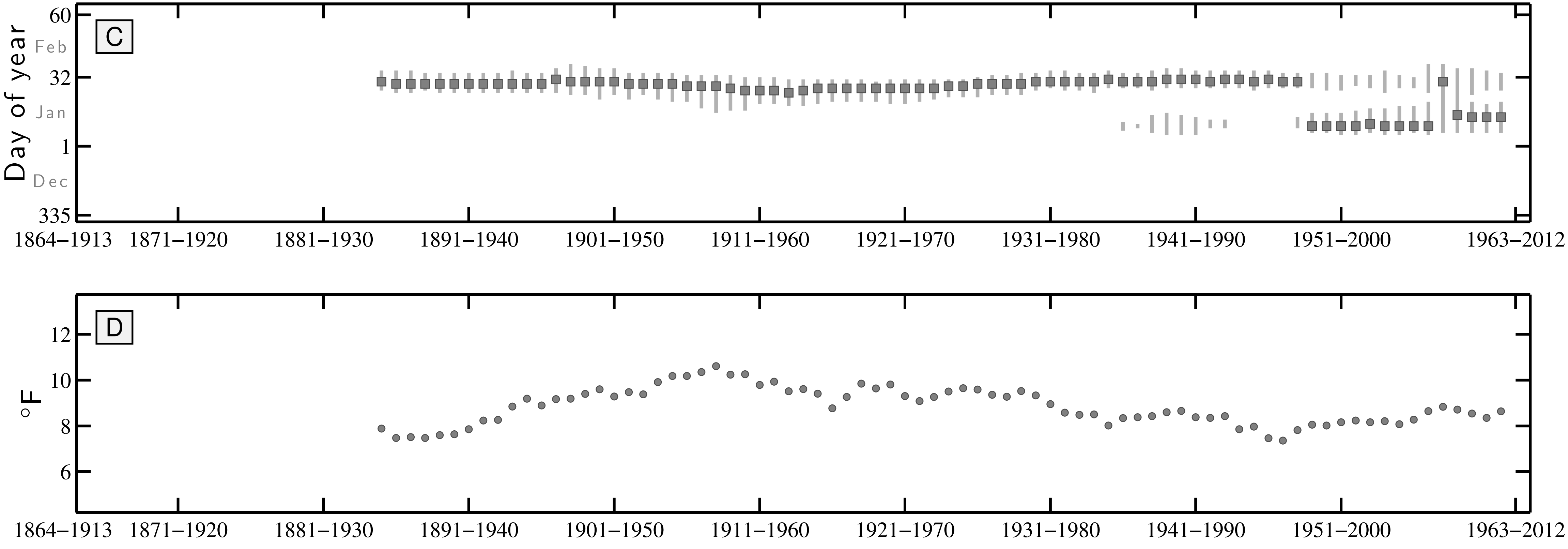

50 year Summer Teletherm dynamics for ALTA 1 NNW, WY:

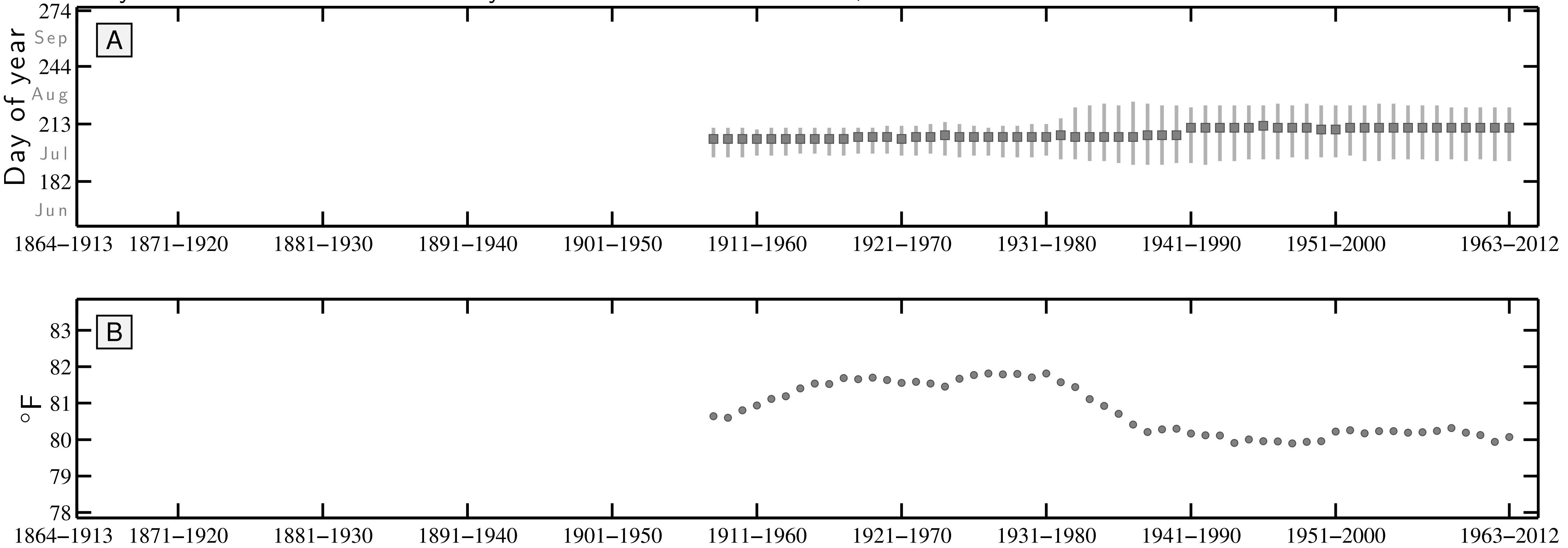

50 year Winter Teletherm dynamics for ALTA 1 NNW, WY:

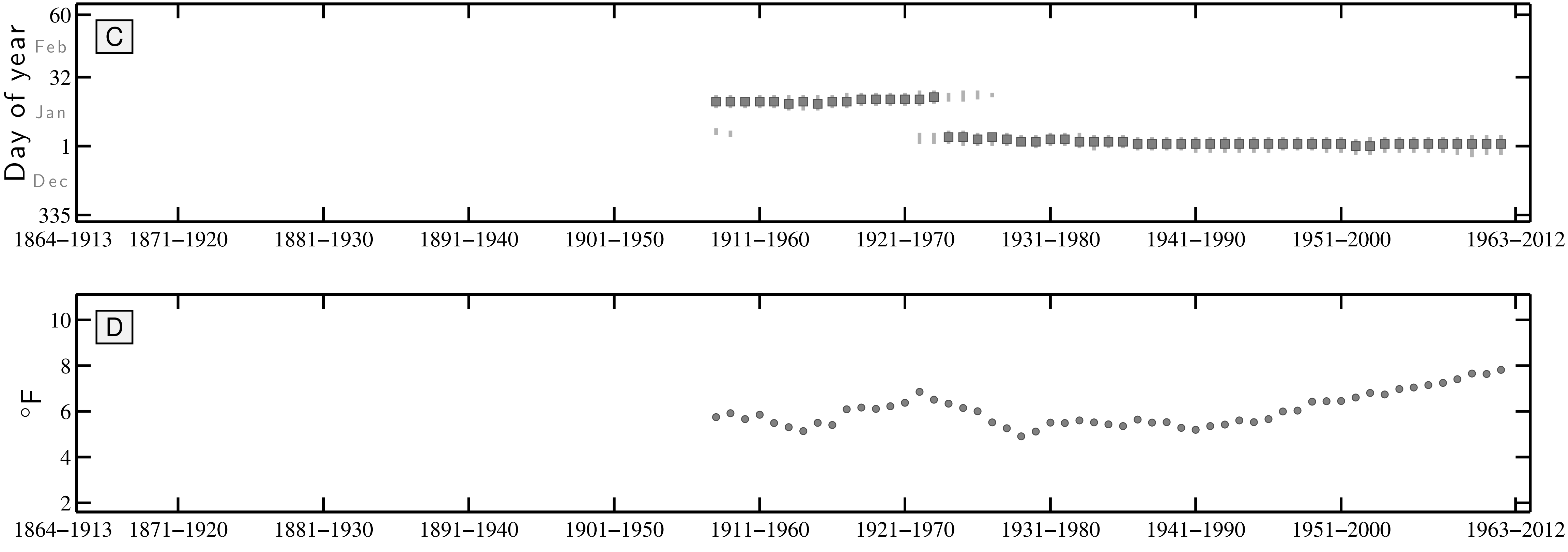

50 year Summer Teletherm dynamics for BASIN, WY:

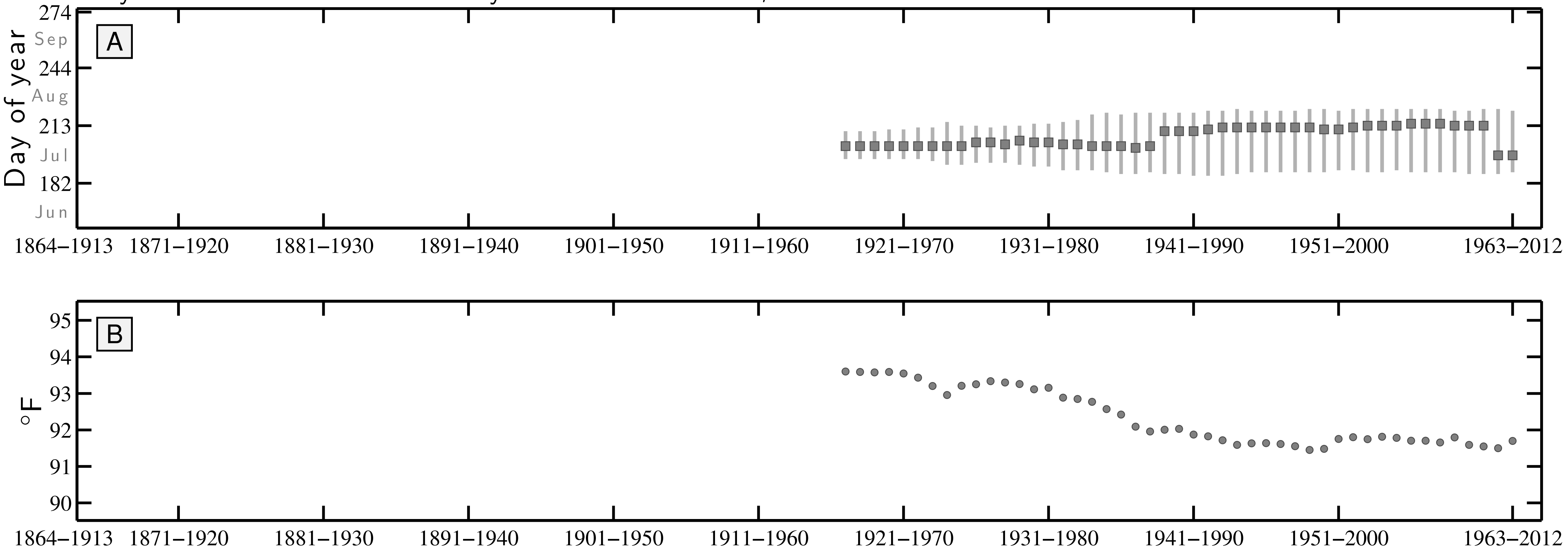

50 year Winter Teletherm dynamics for BASIN, WY:

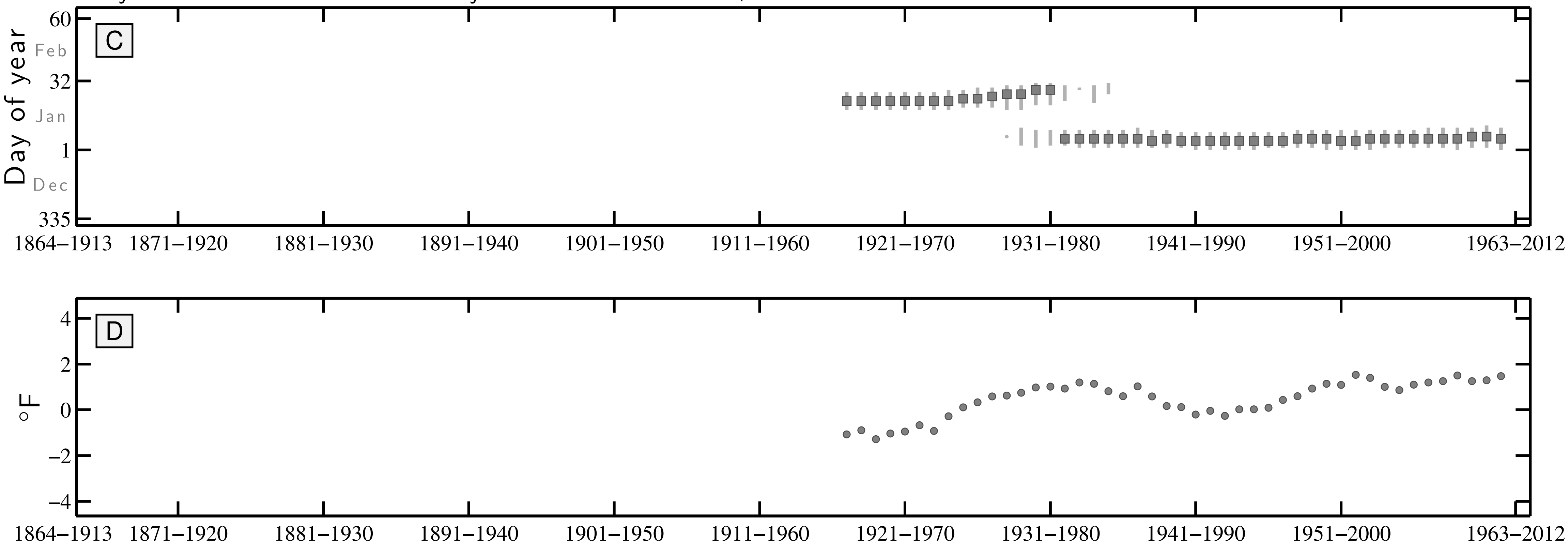

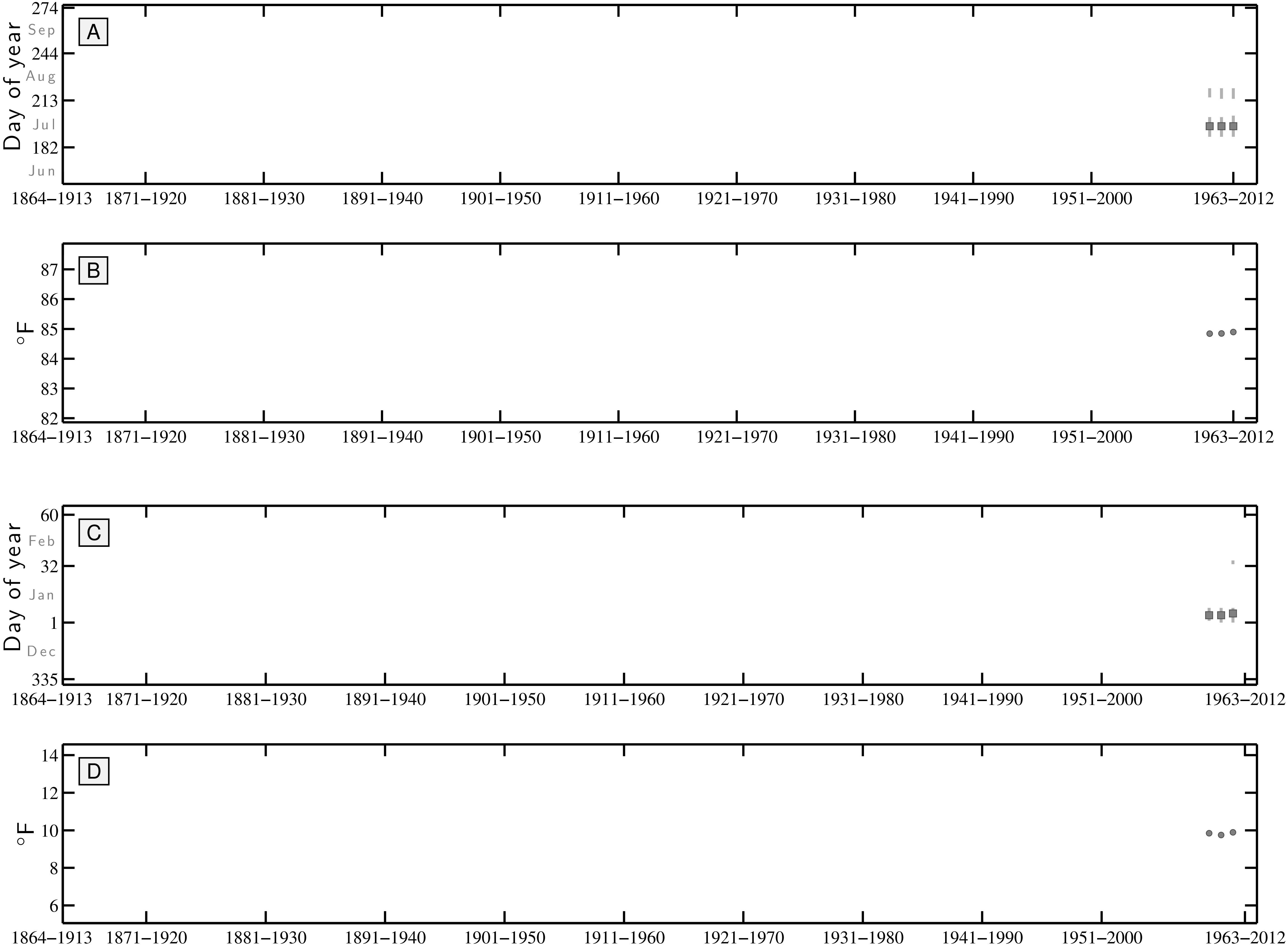
A
Day of year
274
Sep
244
Aug
213
Jul
182
Jun
1864–1913
1871–1920
1881–1930
1891–1940
1901–1950
1911–1960
1921–1970
1931–1980
1941–1990
1951–2000
1963–2012
B
°F
87
86
85
84
83
82
C
Day of year
60
Feb
32
Jan
1
Dec
335
D
°F
14
12
10
8
6

50 year Summer Teletherm dynamics for CHEYENNE WSFO AP, WY:

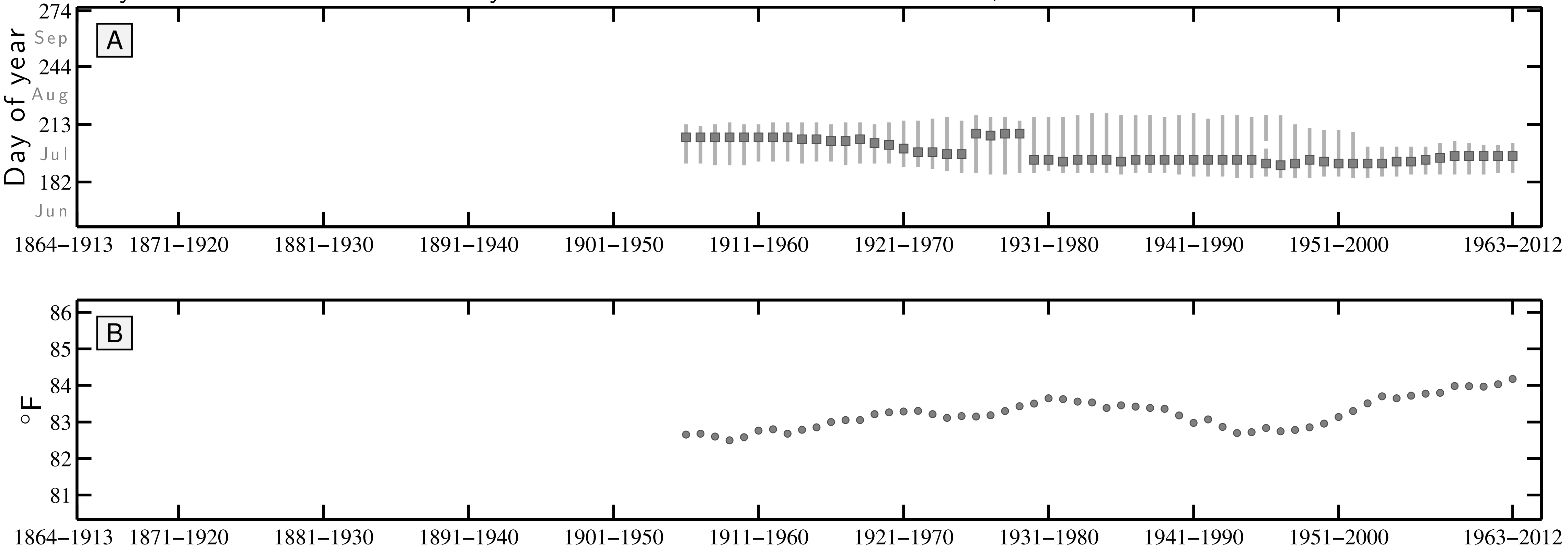

50 year Winter Teletherm dynamics for CHEYENNE WSFO AP, WY:

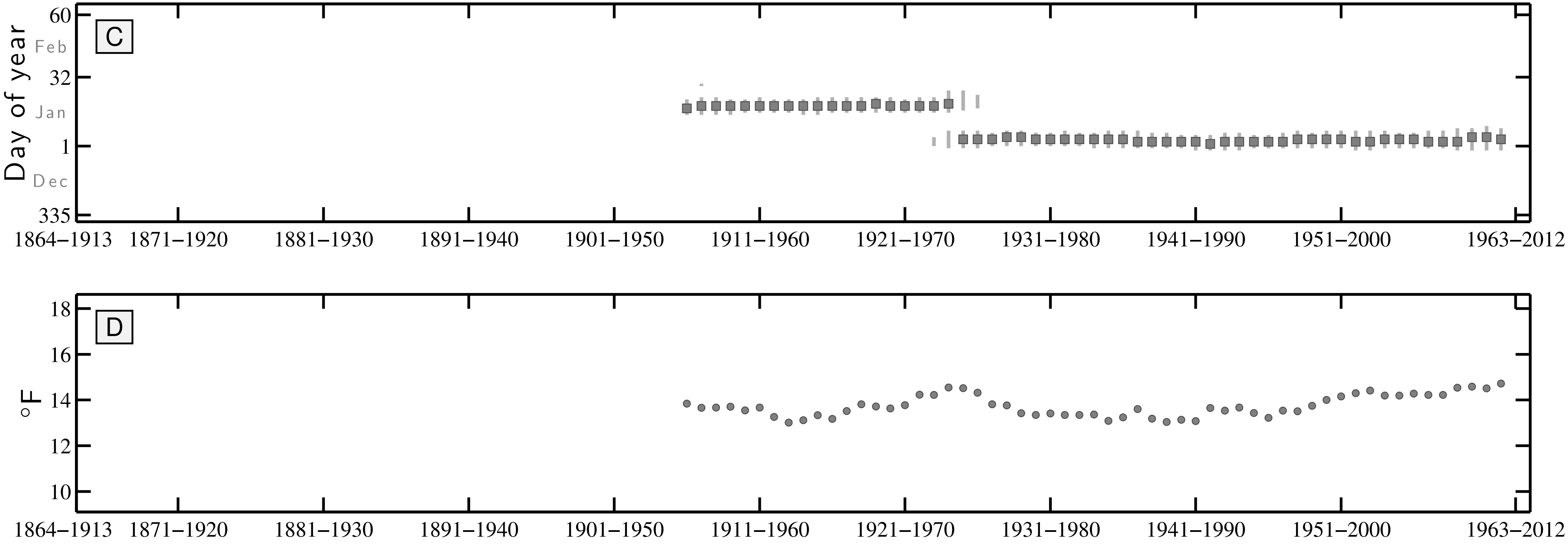

50 year Summer Teletherm dynamics for CHUGWATER, WY:

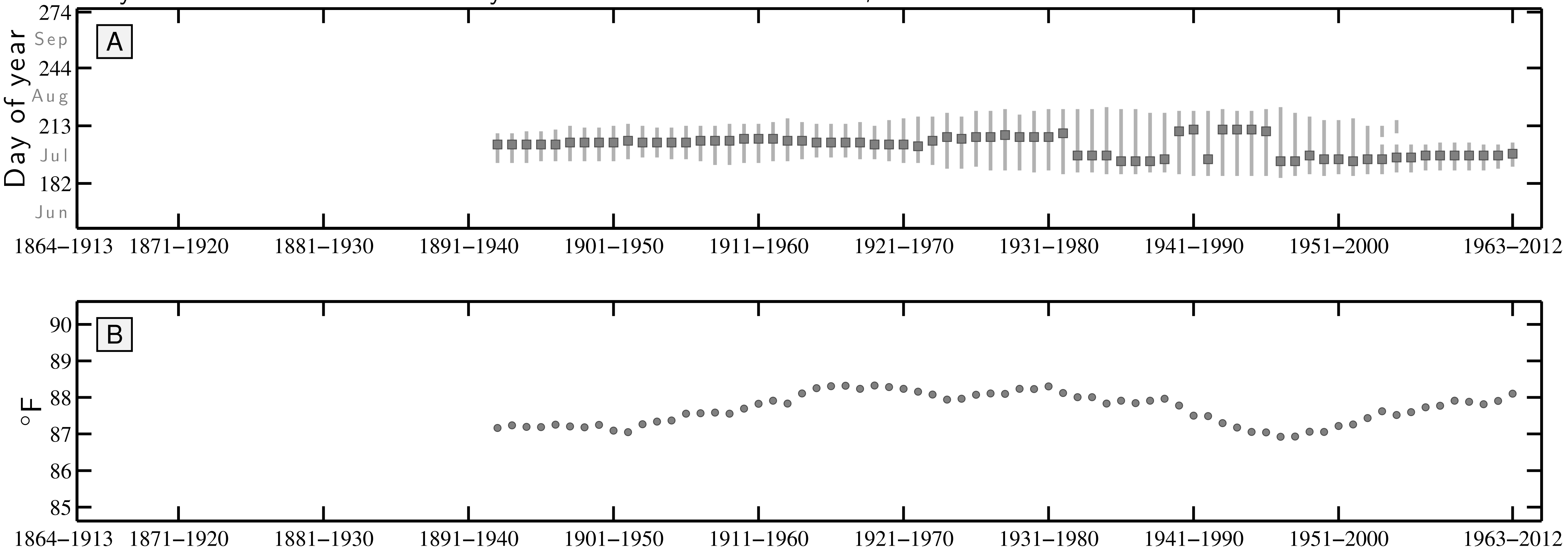

50 year Winter Teletherm dynamics for CHUGWATER, WY:

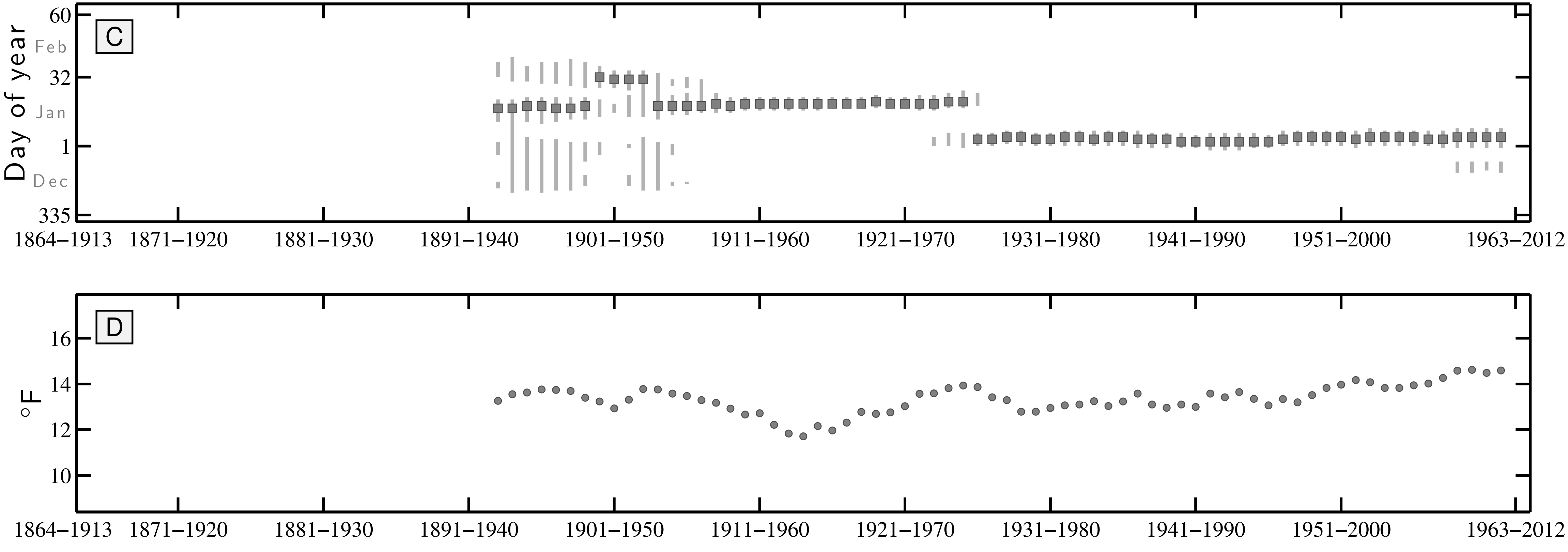

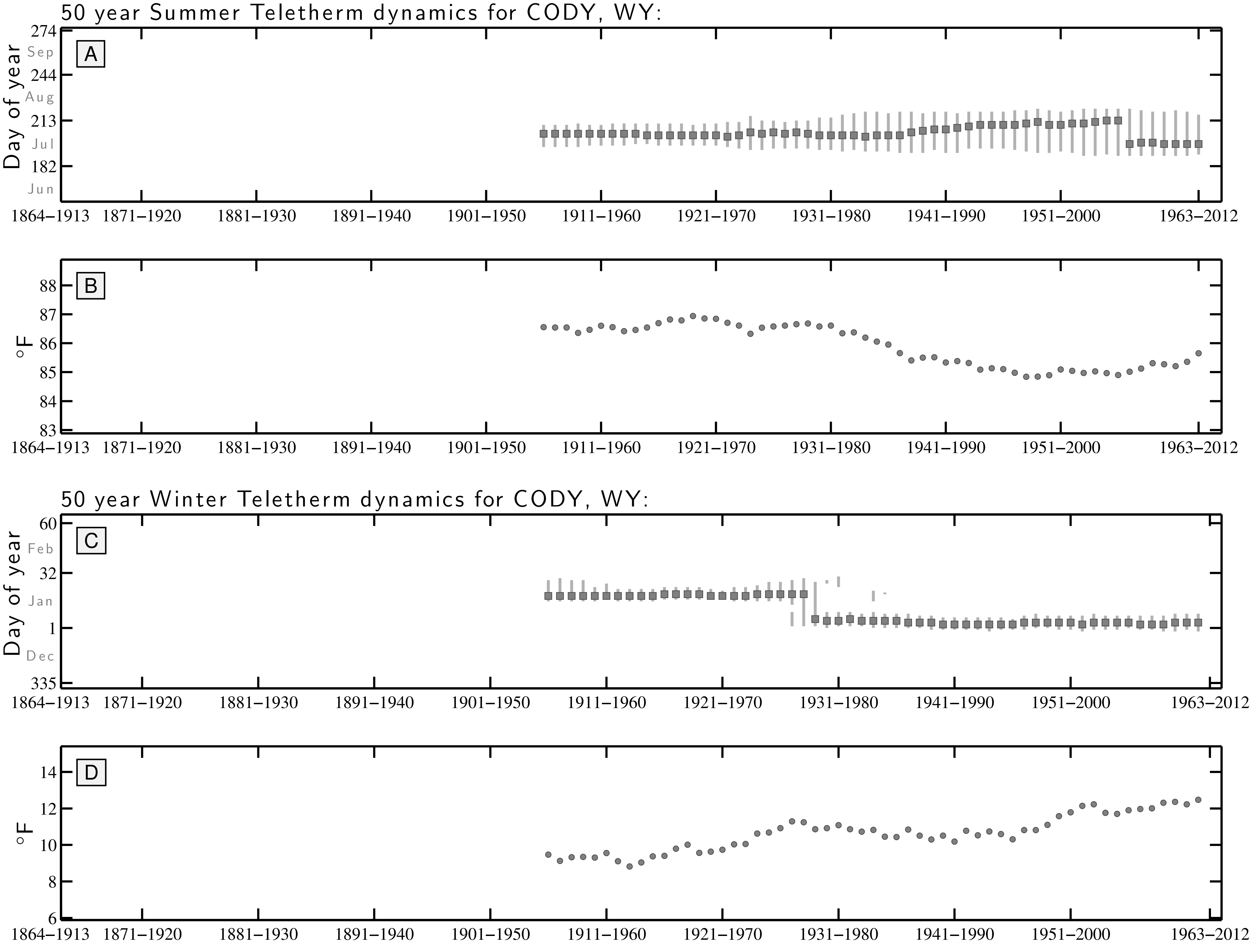

50 year Summer Teletherm dynamics for CODY, WY:
A
B
Day of year
°F
274
Sep
244
Aug
213
Jul
182
Jun
88
87
86
85
84
83
50 year Winter Teletherm dynamics for CODY, WY:
C
D
60
Feb
32
Jan
1
Dec
335
14
12
10
8
6
1864–1913
1871–1920
1881–1930
1891–1940
1901–1950
1911–1960
1921–1970
1931–1980
1941–1990
1951–2000
1963–2012

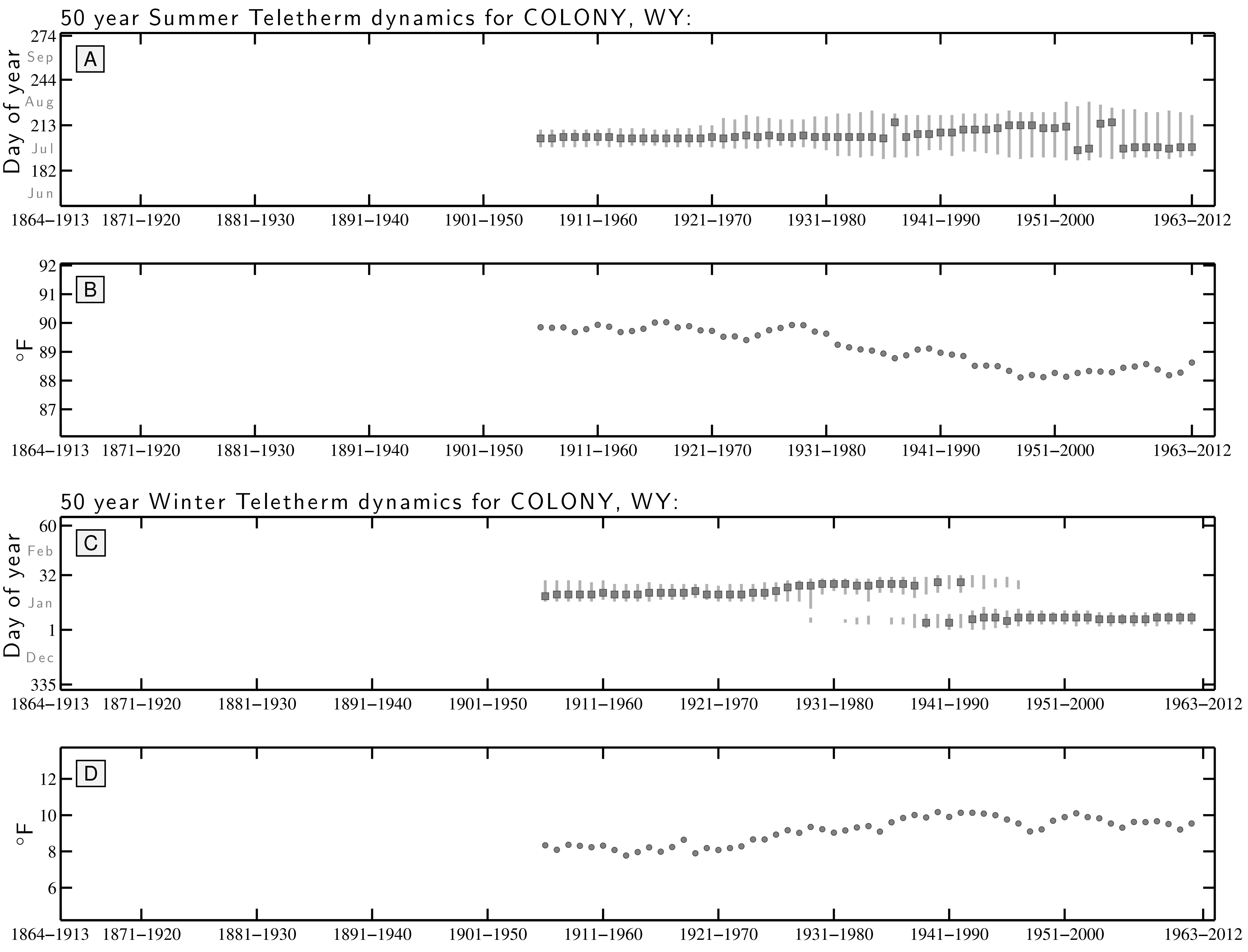
50 year Summer Teletherm dynamics for COLONY, WY:
A
Day of year
274
Sep
244
Aug
213
Jul
182
Jun
B
°F
92
91
90
89
88
87
50 year Winter Teletherm dynamics for COLONY, WY:
C
Day of year
60
Feb
32
Jan
1
Dec
335
D
°F
12
10
8
6
1864–1913
1871–1920
1881–1930
1891–1940
1901–1950
1911–1960
1921–1970
1931–1980
1941–1990
1951–2000
1963–2012

50 year Summer Teletherm dynamics for DIVERSION DAM, WY:

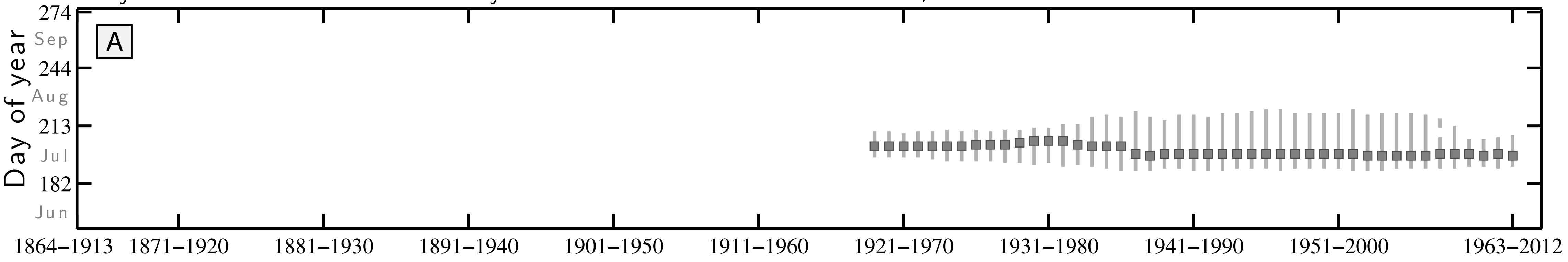

50 year Winter Teletherm dynamics for DIVERSION DAM, WY:

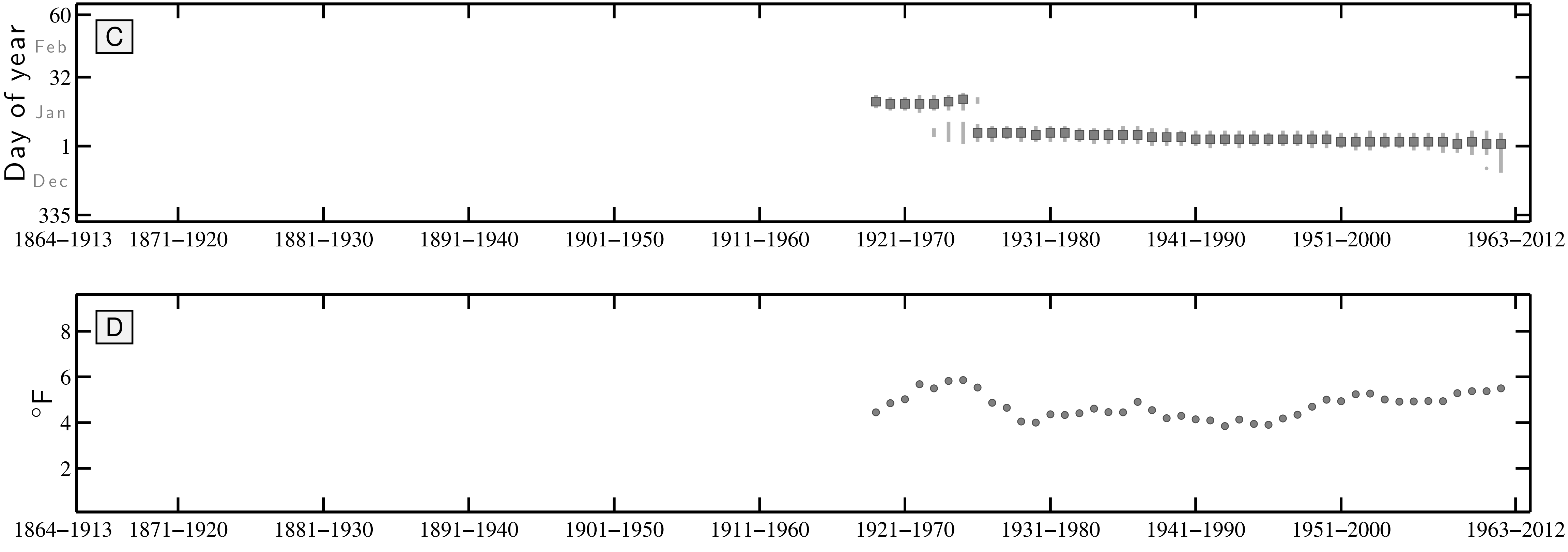

50 year Summer Teletherm dynamics for DUBOIS, WY:

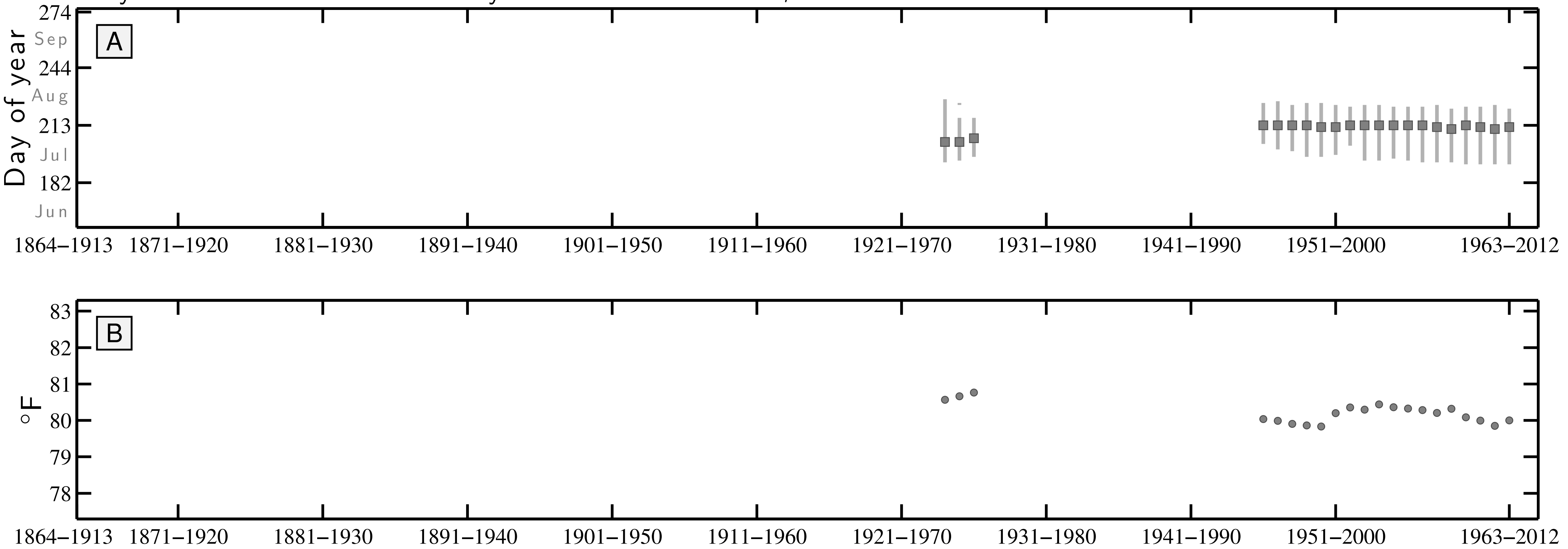

50 year Winter Teletherm dynamics for DUBOIS, WY:

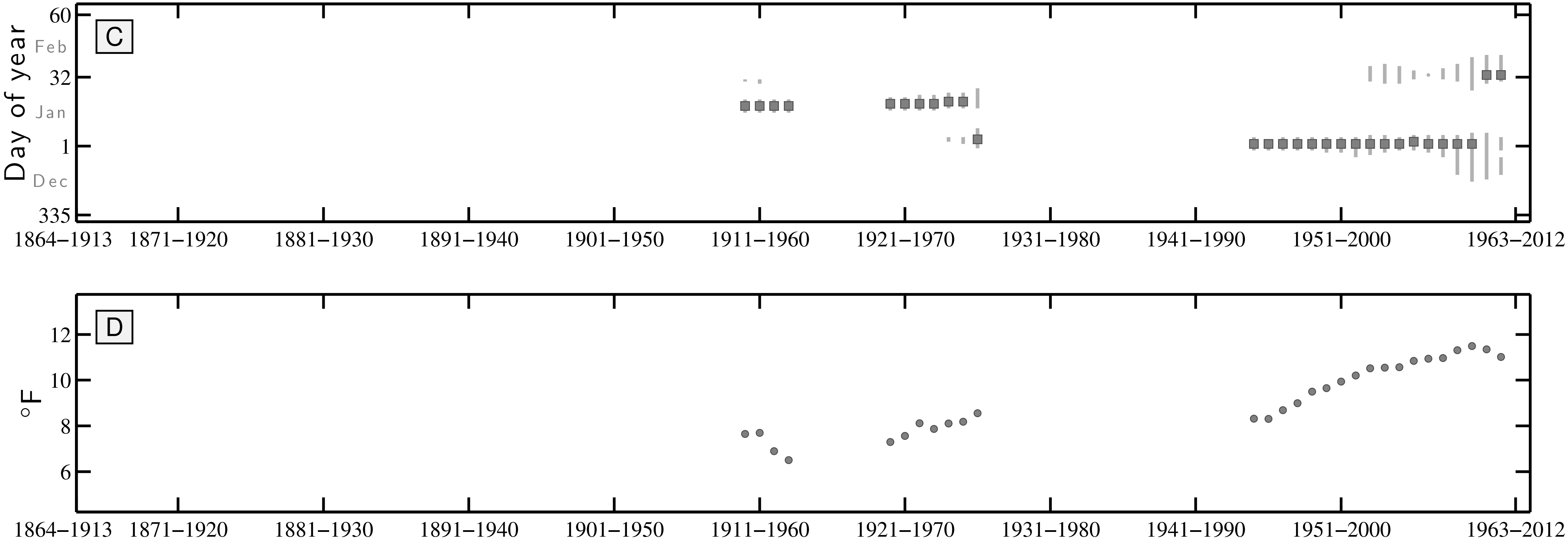

50 year Summer Teletherm dynamics for EVANSTON 1 E, WY:

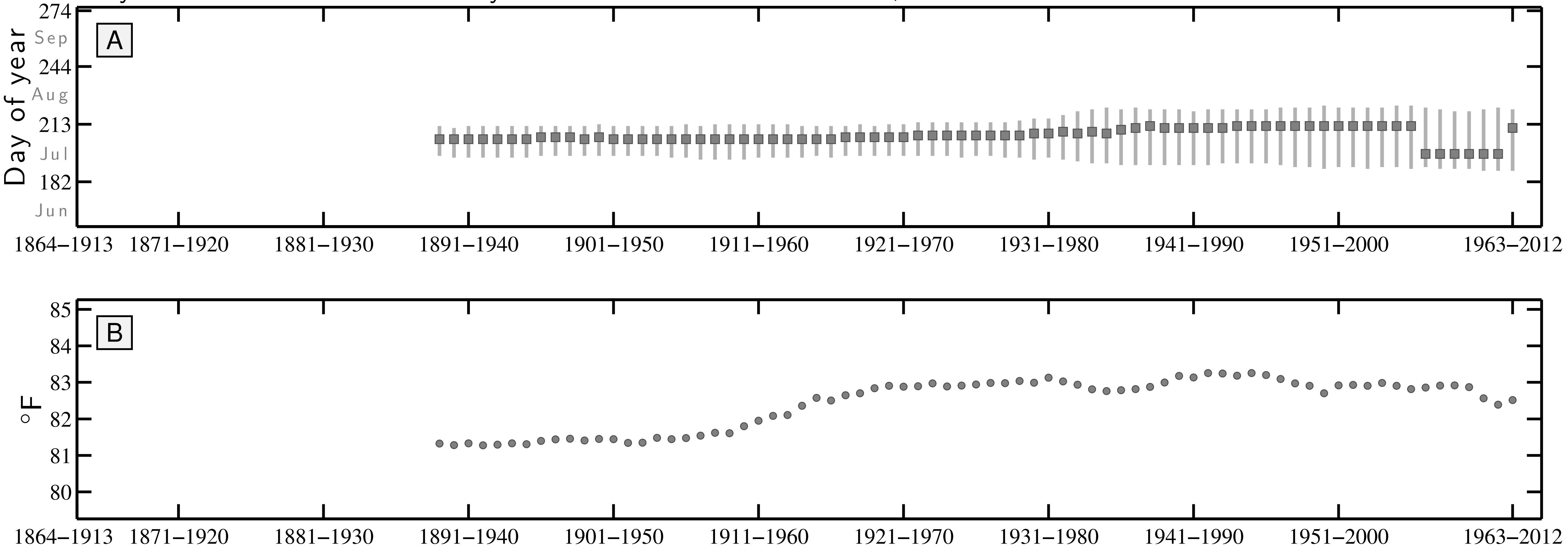

50 year Winter Teletherm dynamics for EVANSTON 1 E, WY:

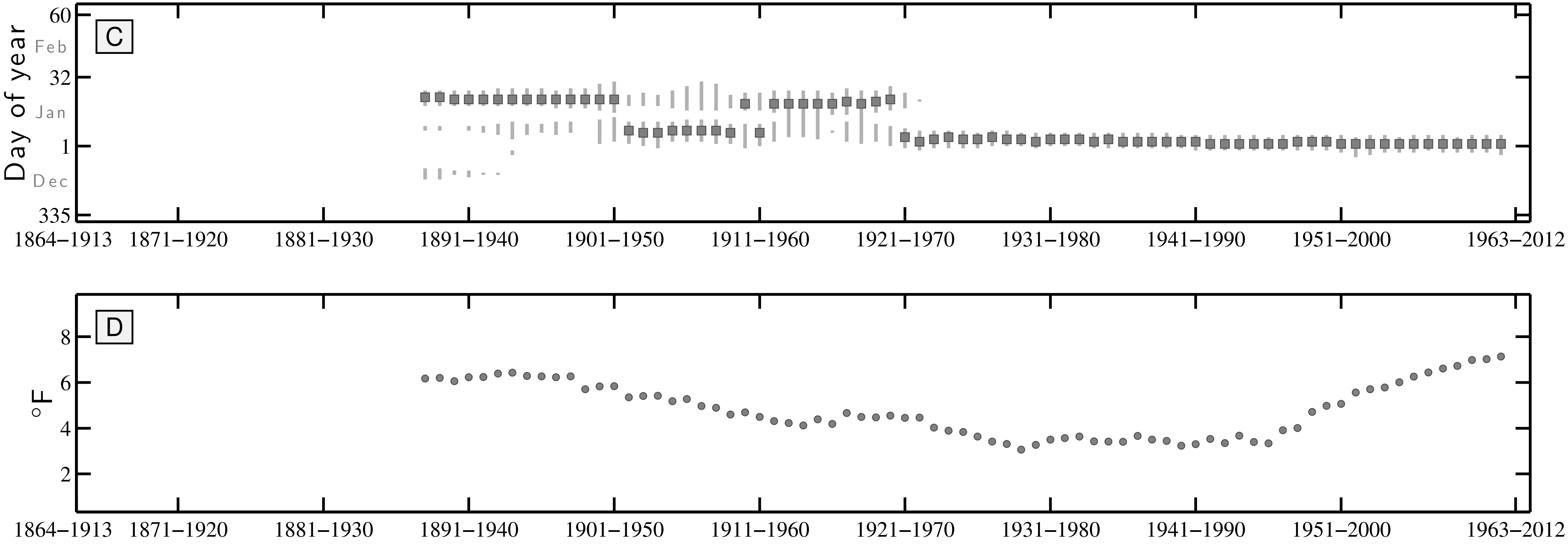

50 year Summer Teletherm dynamics for GREEN RIVER, WY:

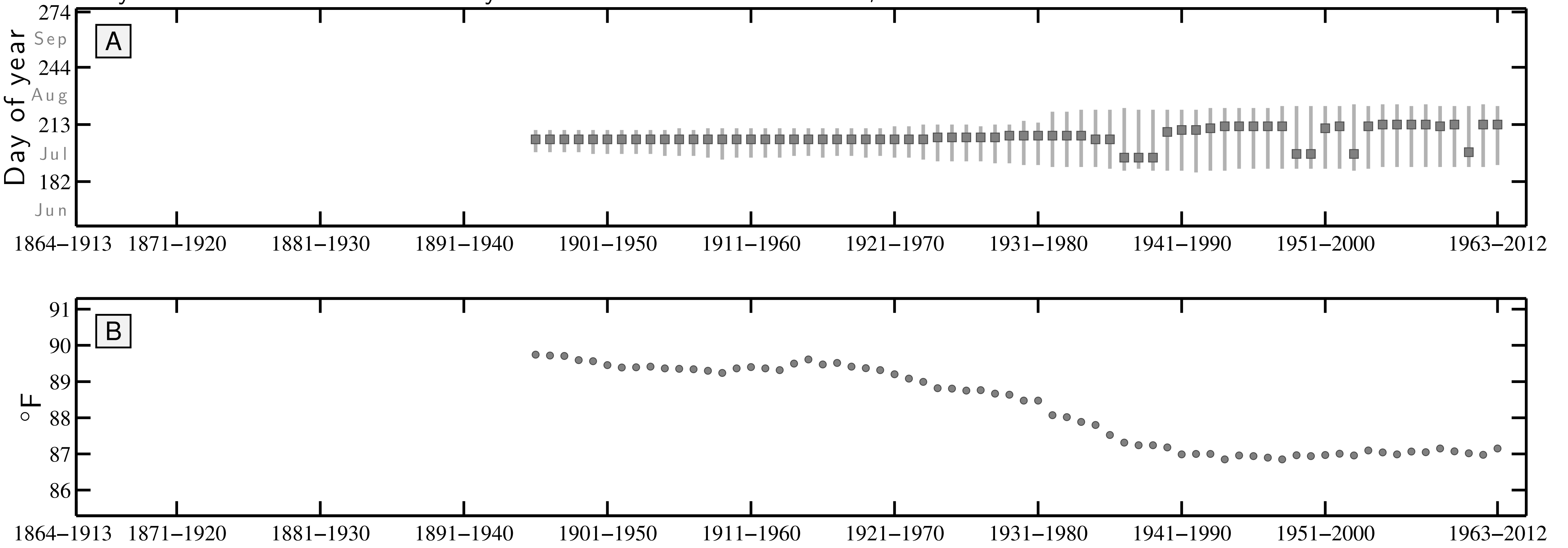

50 year Winter Teletherm dynamics for GREEN RIVER, WY:

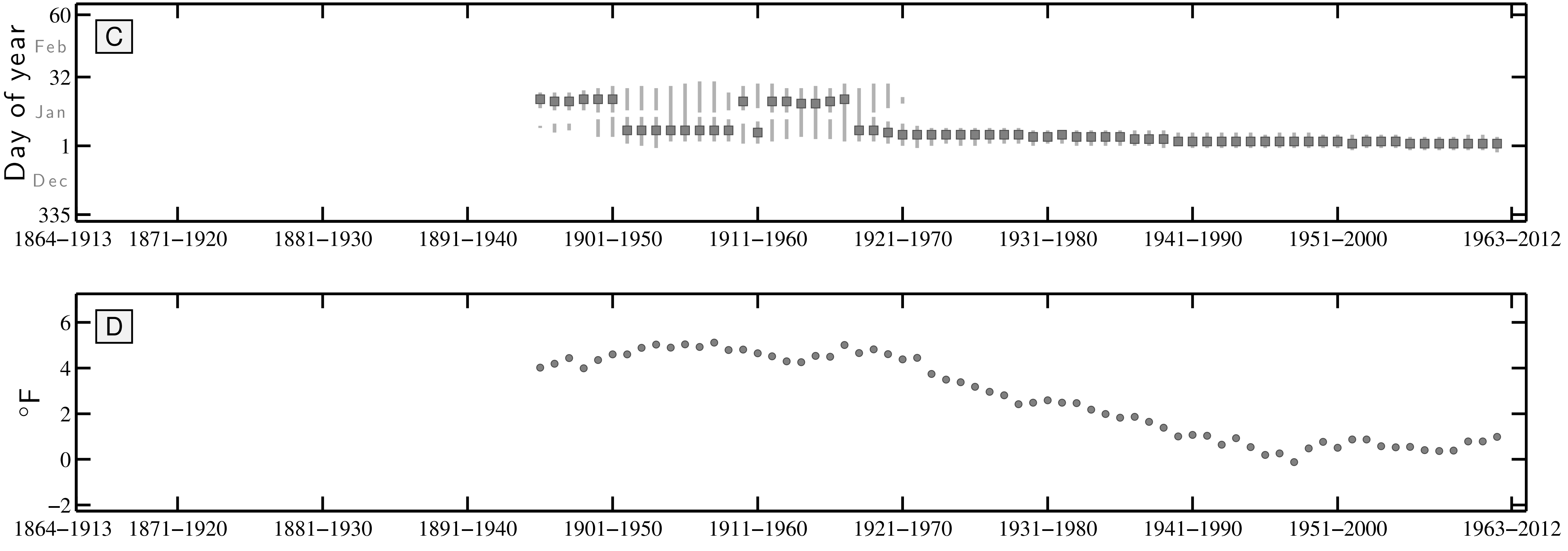

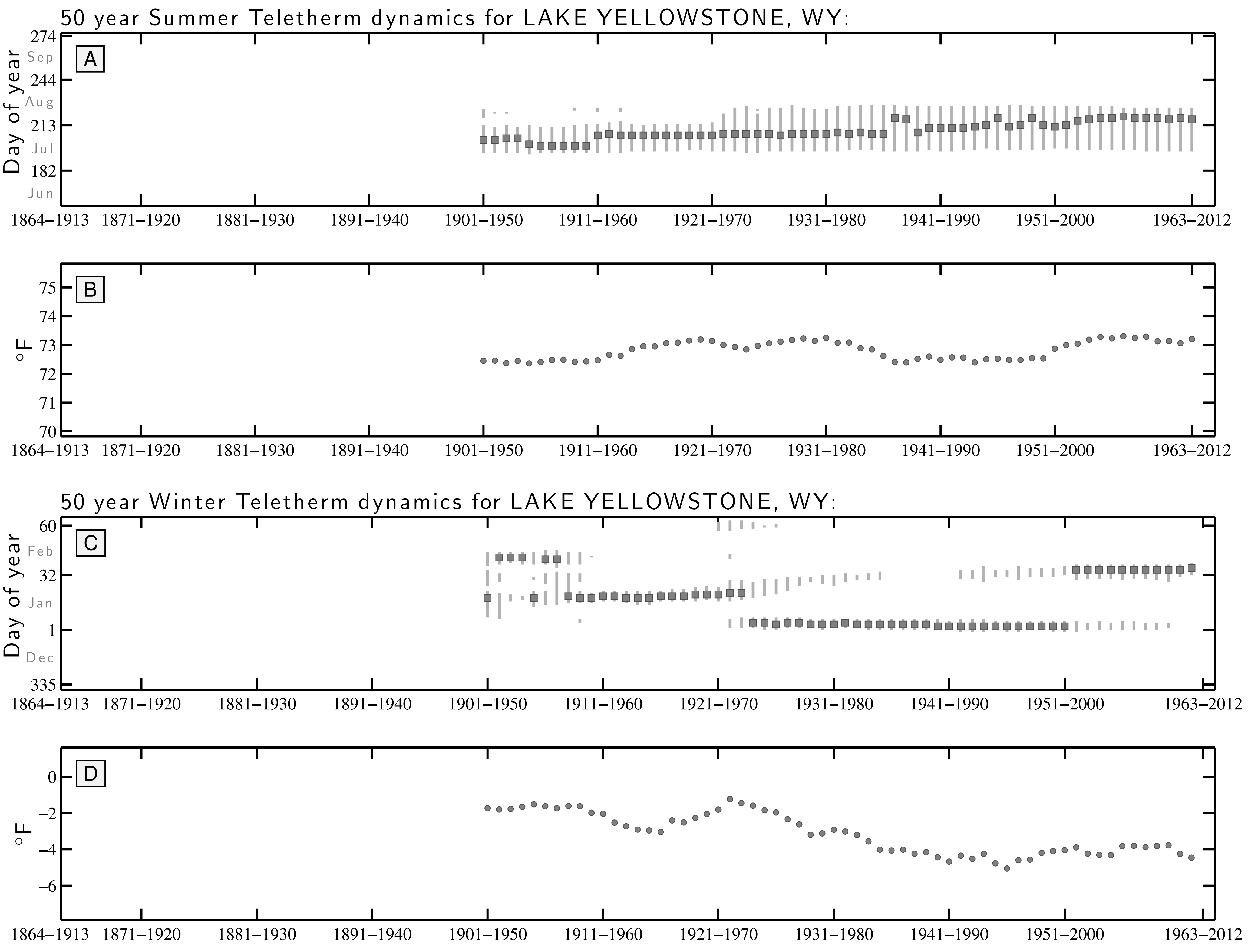
50 year Summer Teletherm dynamics for LAKE YELLOWSTONE, WY:
A
Day of year
274
Sep
244
Aug
213
Jul
182
Jun
1864–1913
1871–1920
1881–1930
1891–1940
1901–1950
1911–1960
1921–1970
1931–1980
1941–1990
1951–2000
1963–2012
B
°F
75
74
73
72
71
70
50 year Winter Teletherm dynamics for LAKE YELLOWSTONE, WY:
C
Day of year
60
Feb
32
Jan
1
Dec
335
D
°F
0
−2
−4
−6

50 year Summer Teletherm dynamics for LARAMIE RGNL AP, WY:

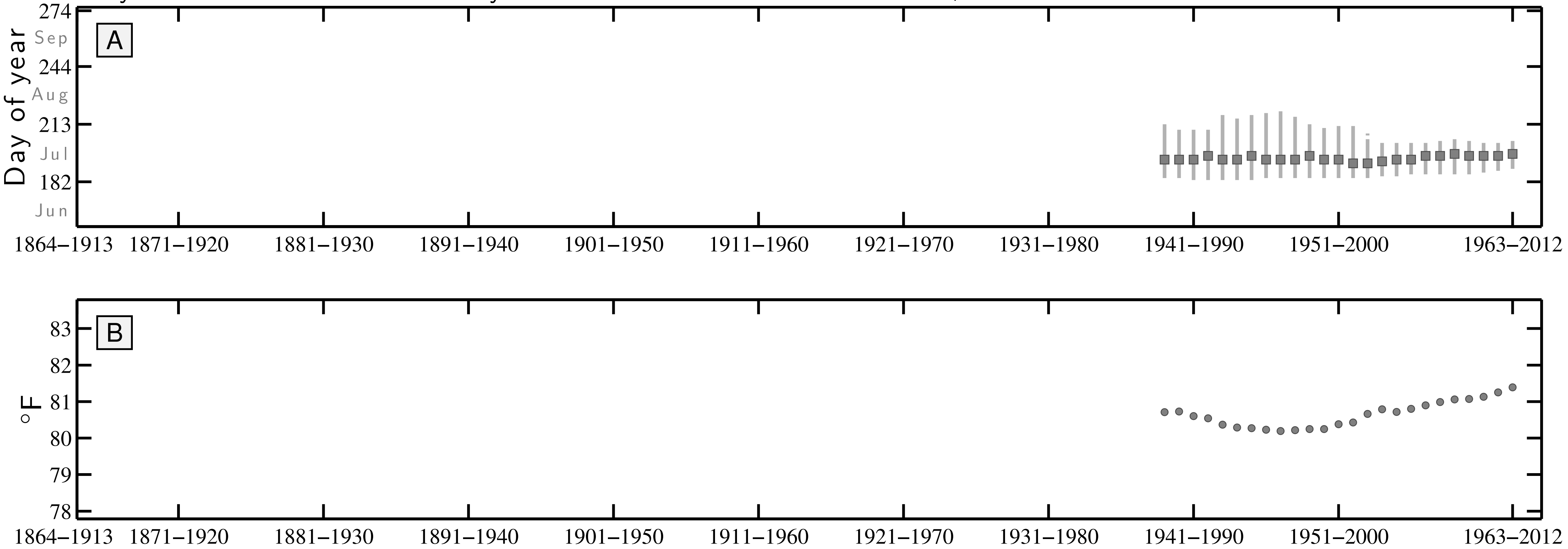

50 year Winter Teletherm dynamics for LARAMIE RGNL AP, WY:

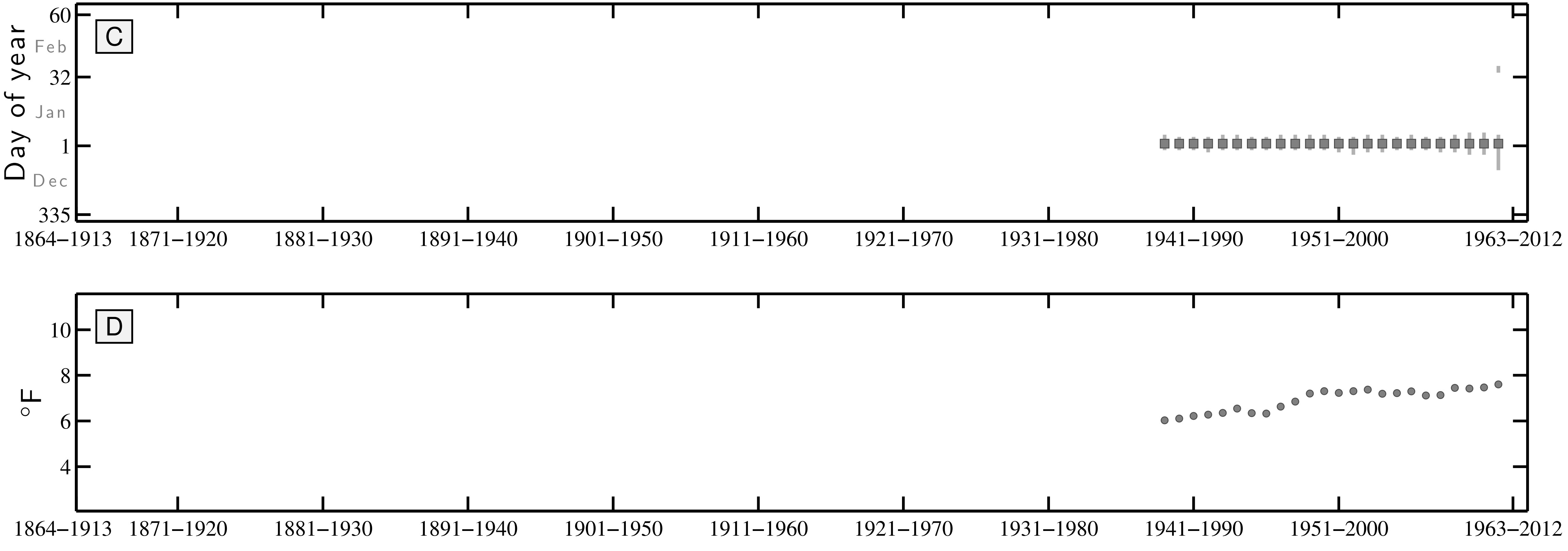

50 year Summer Teletherm dynamics for LUSK 2 SW, WY:

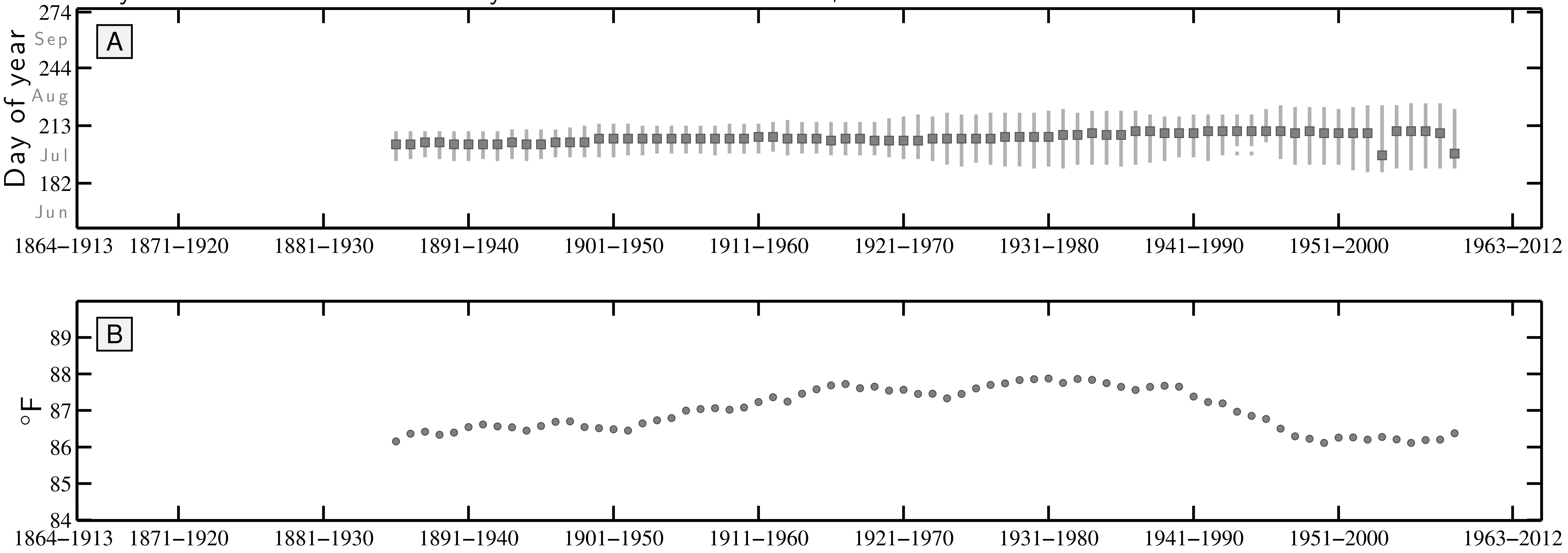

50 year Winter Teletherm dynamics for LUSK 2 SW, WY:

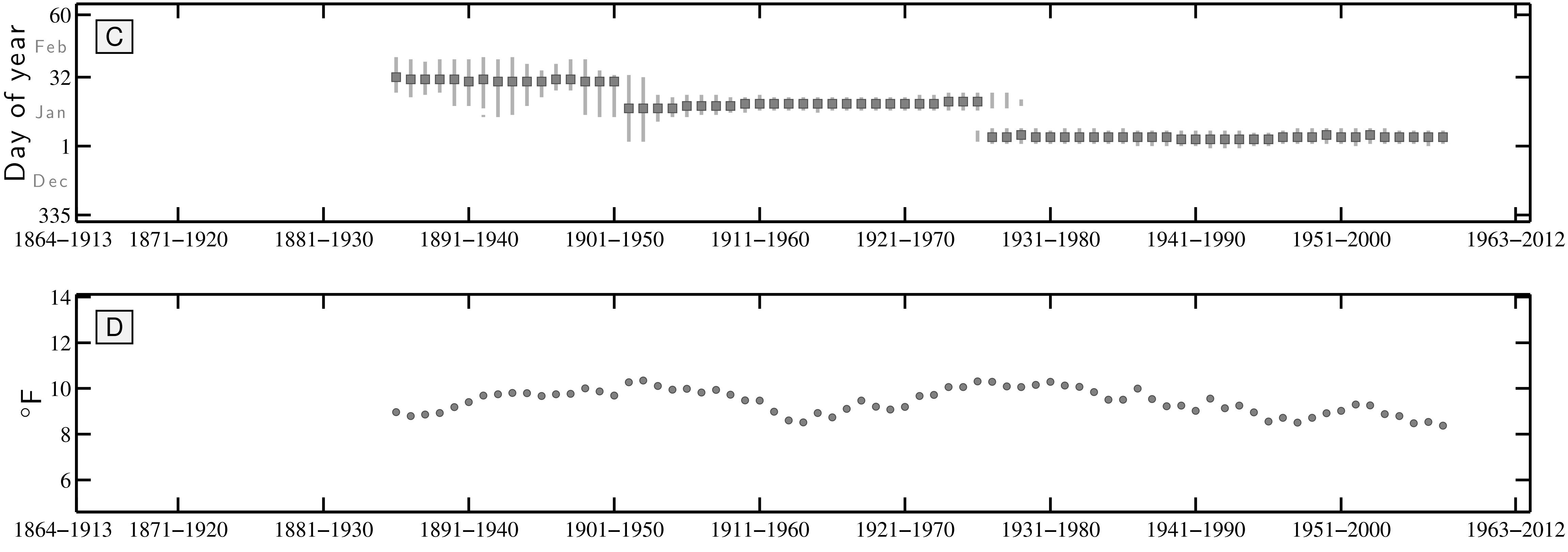

50 year Summer Teletherm dynamics for MIDWEST, WY:

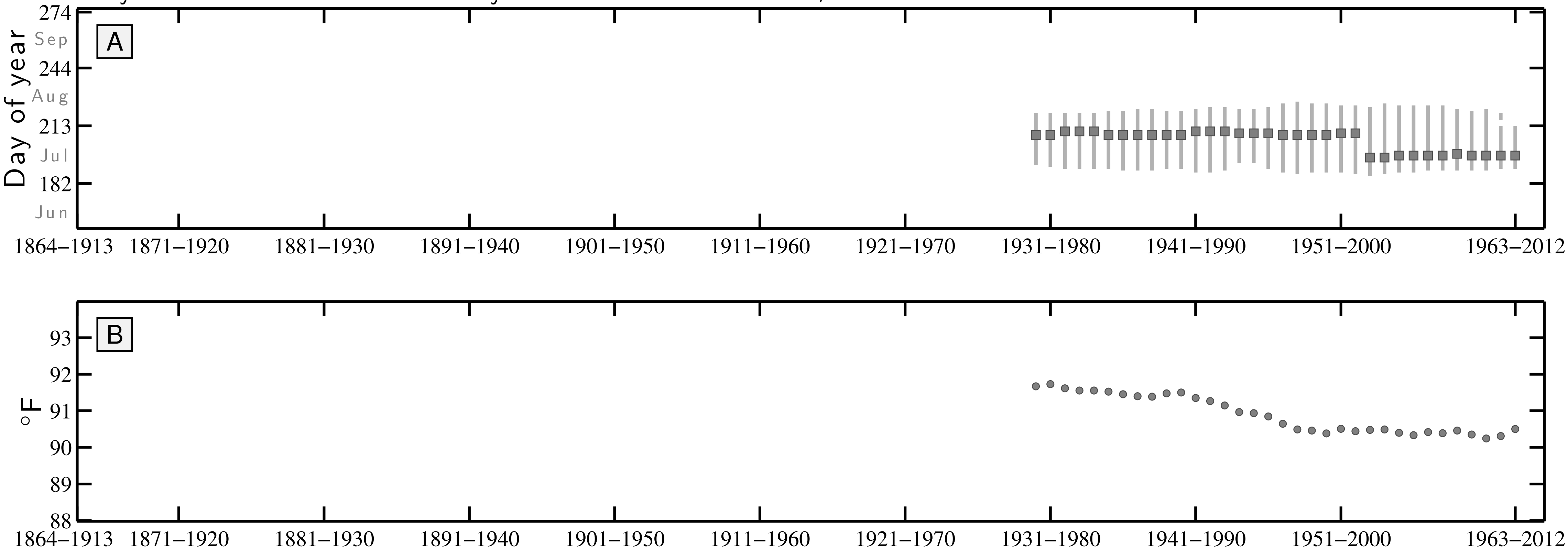

50 year Winter Teletherm dynamics for MIDWEST, WY:

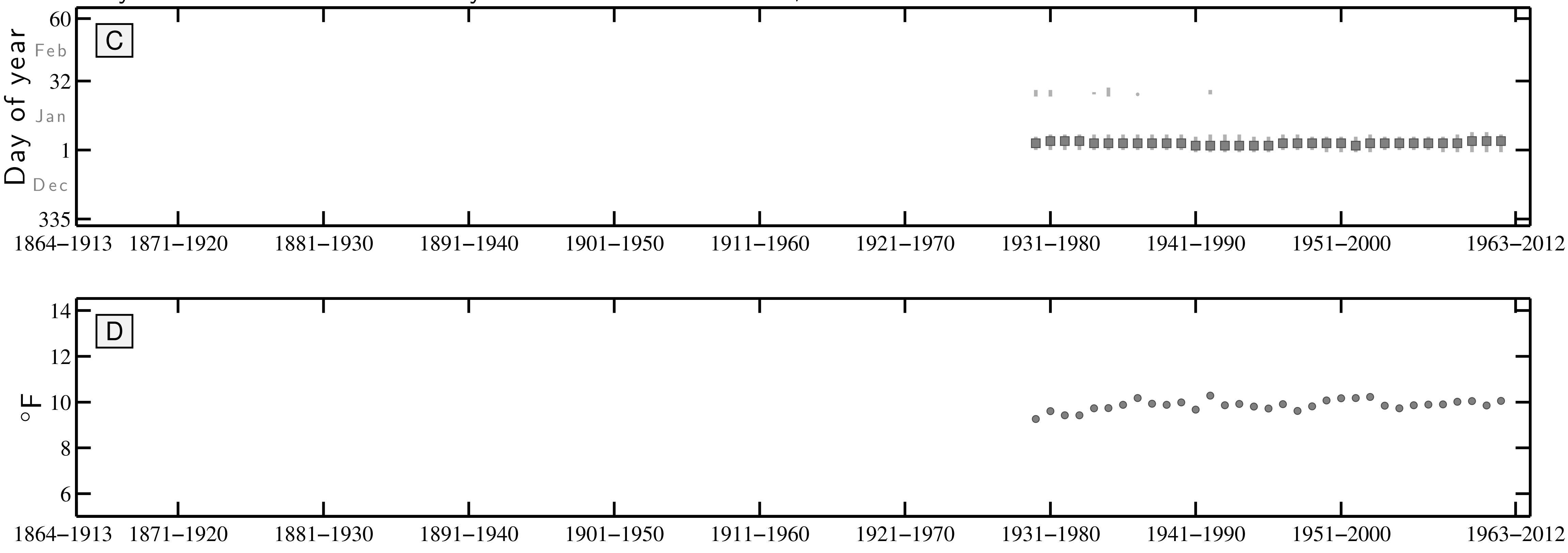

50 year Summer Teletherm dynamics for MORAN 5 WNW, WY:

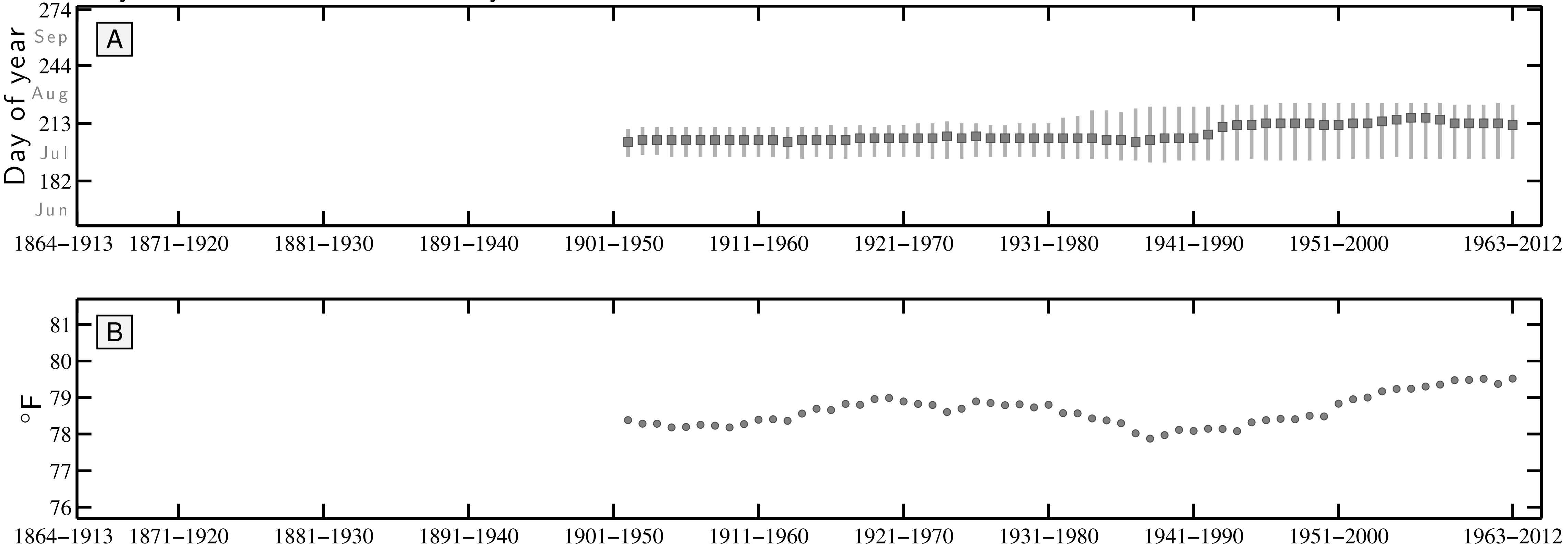

50 year Winter Teletherm dynamics for MORAN 5 WNW, WY:

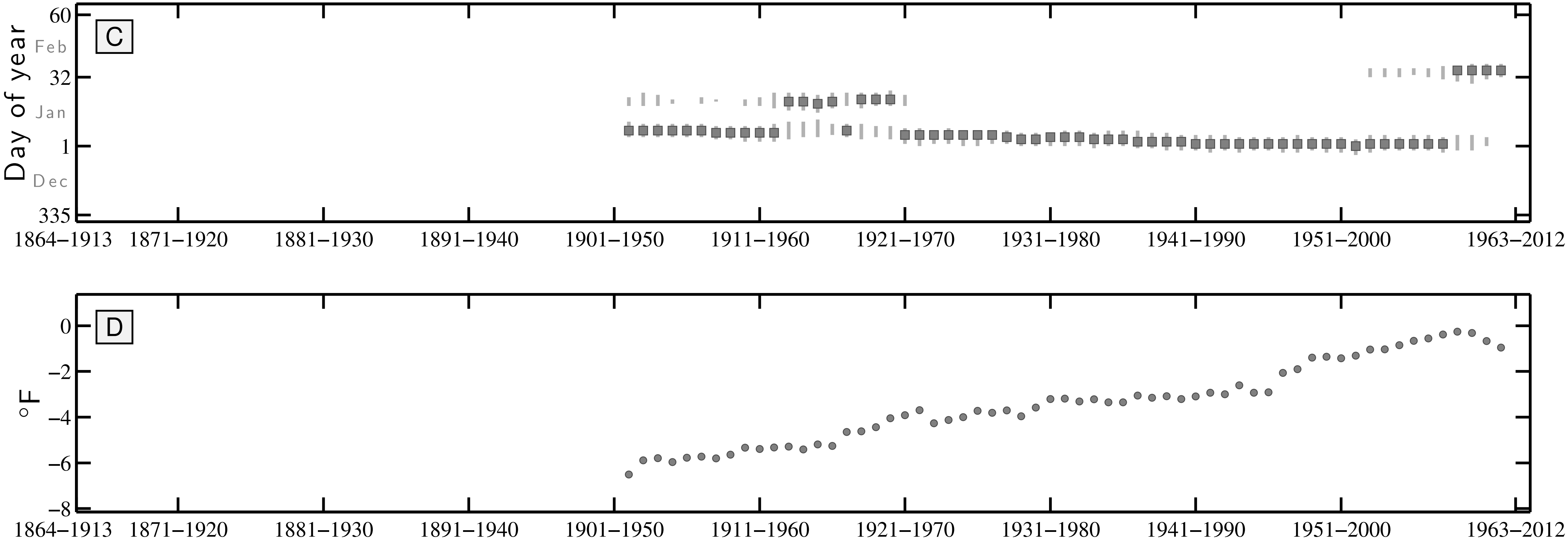

50 year Summer Teletherm dynamics for NEWCASTLE, WY:

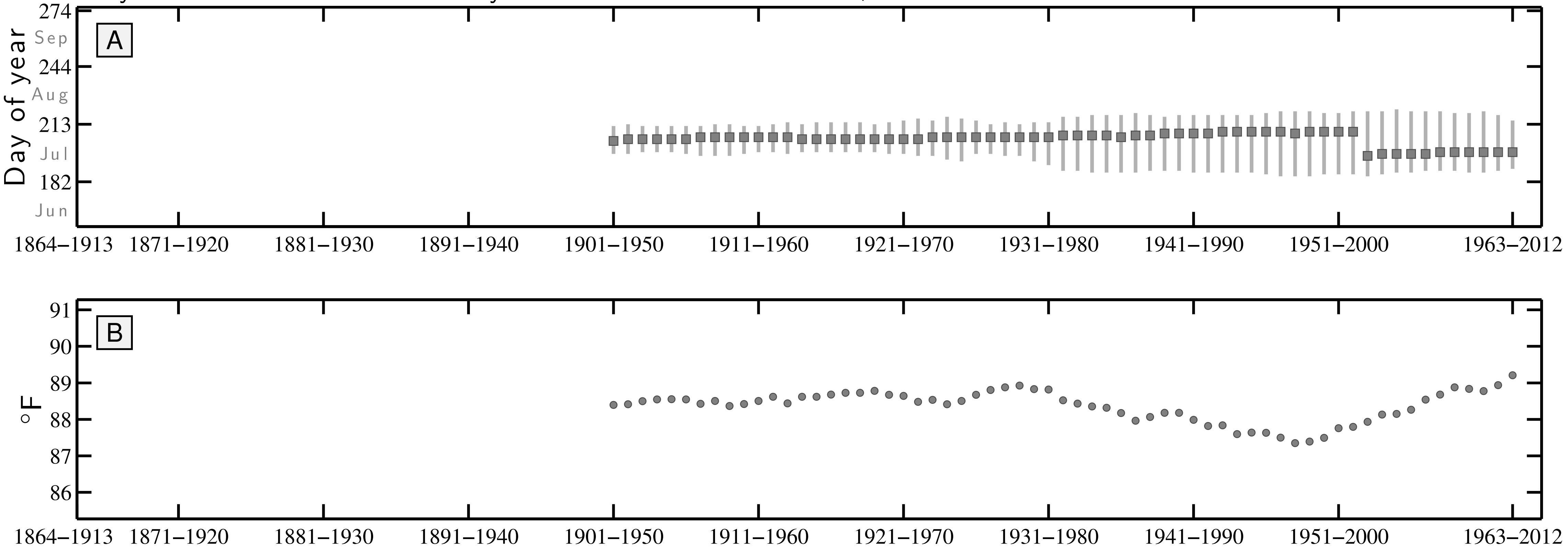

50 year Winter Teletherm dynamics for NEWCASTLE, WY:

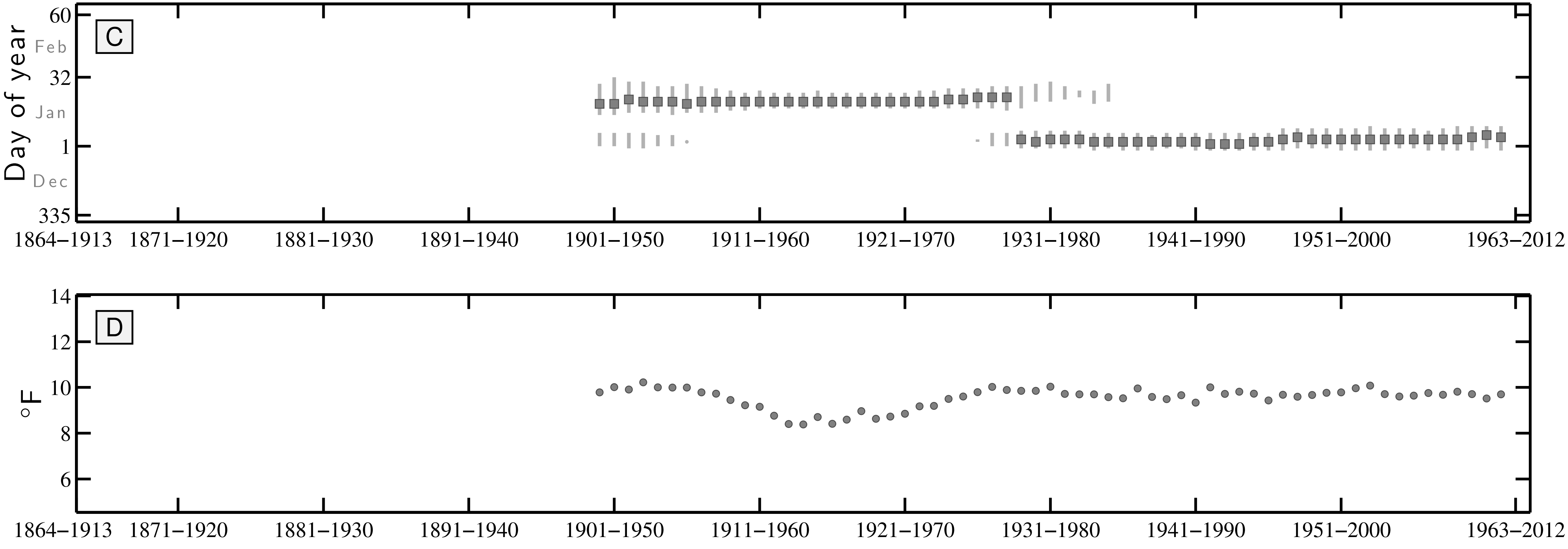

50 year Summer Teletherm dynamics for PAVILLION, WY:

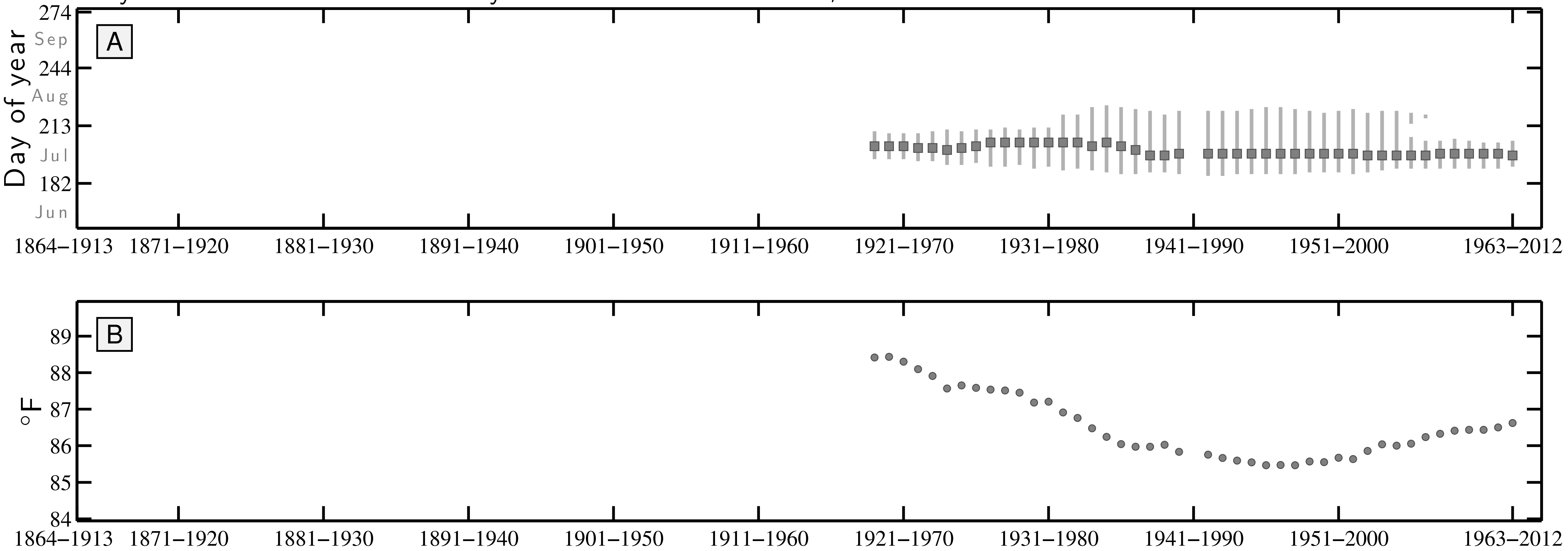

50 year Winter Teletherm dynamics for PAVILLION, WY:

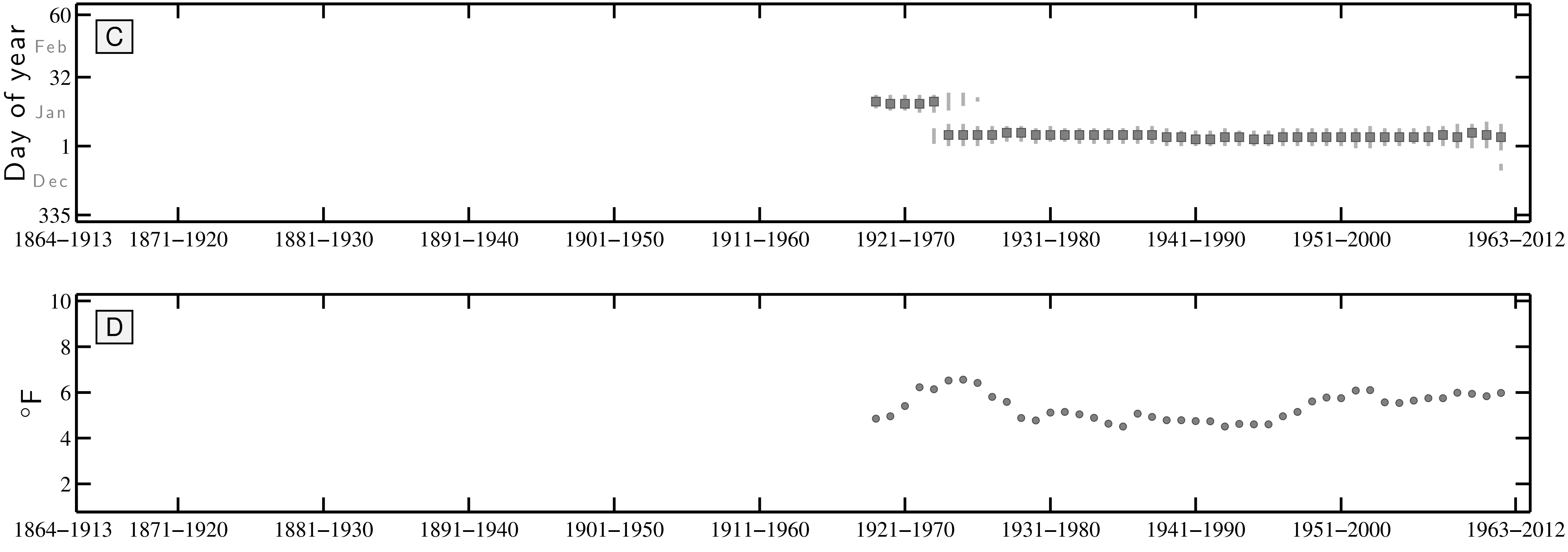

50 year Summer Teletherm dynamics for PINEDALE, WY:

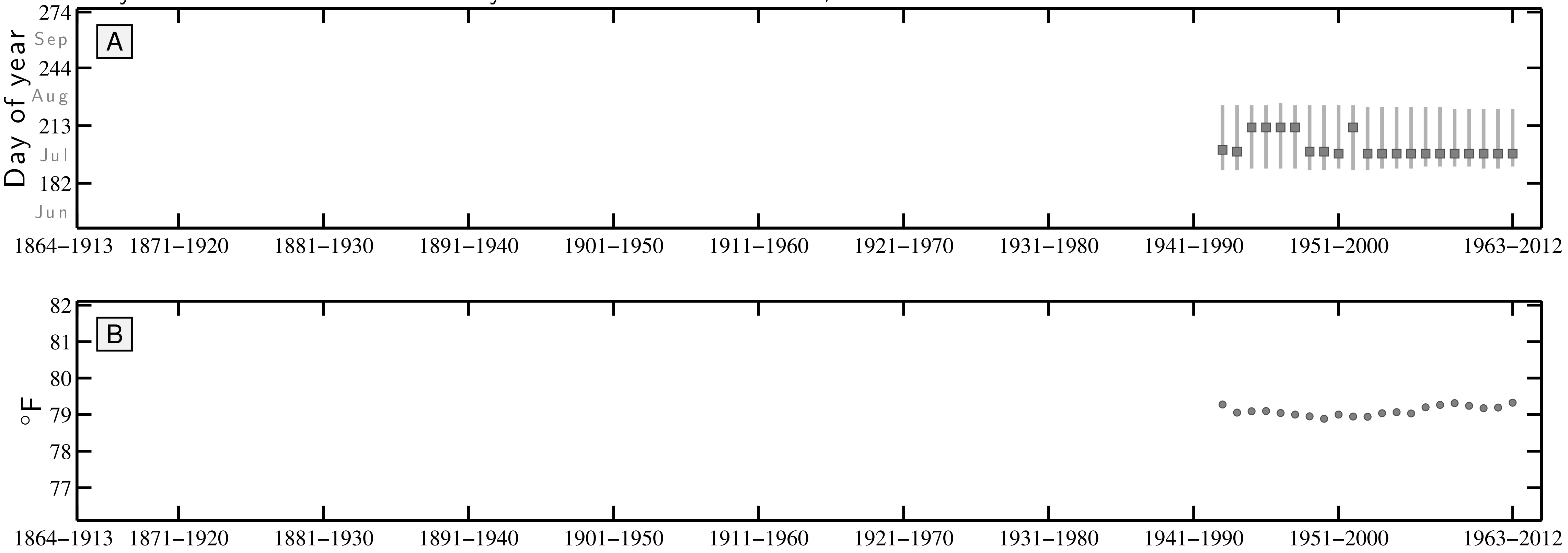

50 year Winter Teletherm dynamics for PINEDALE, WY:

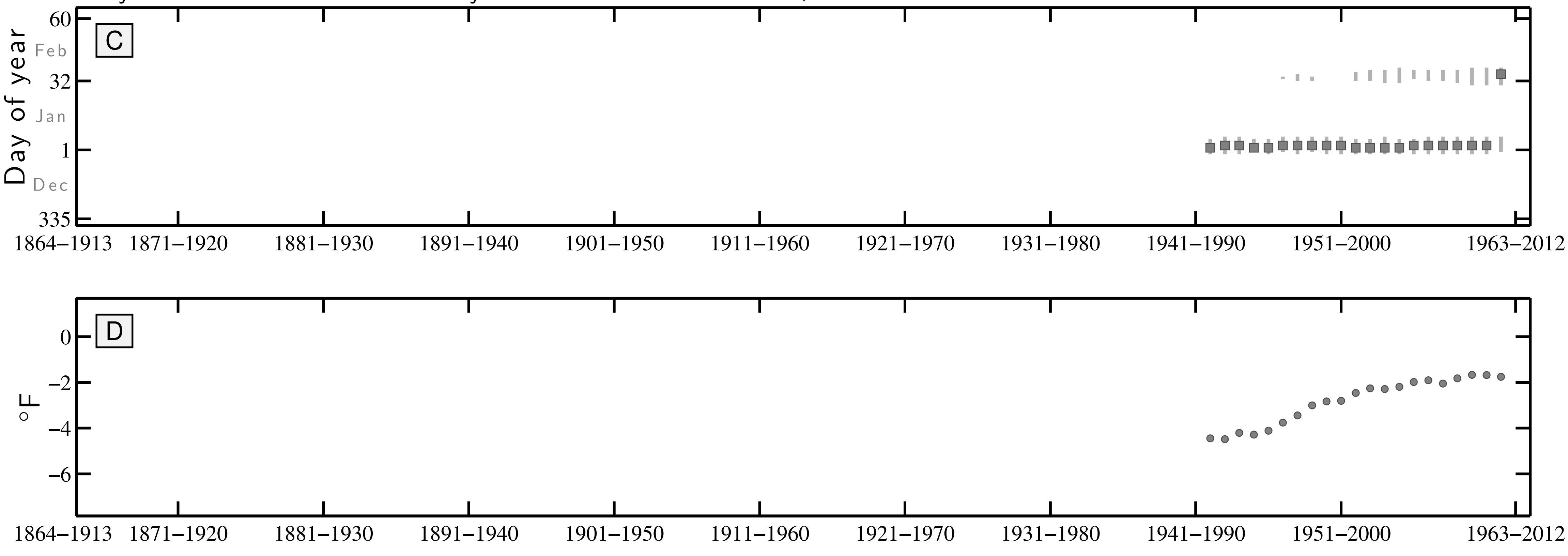

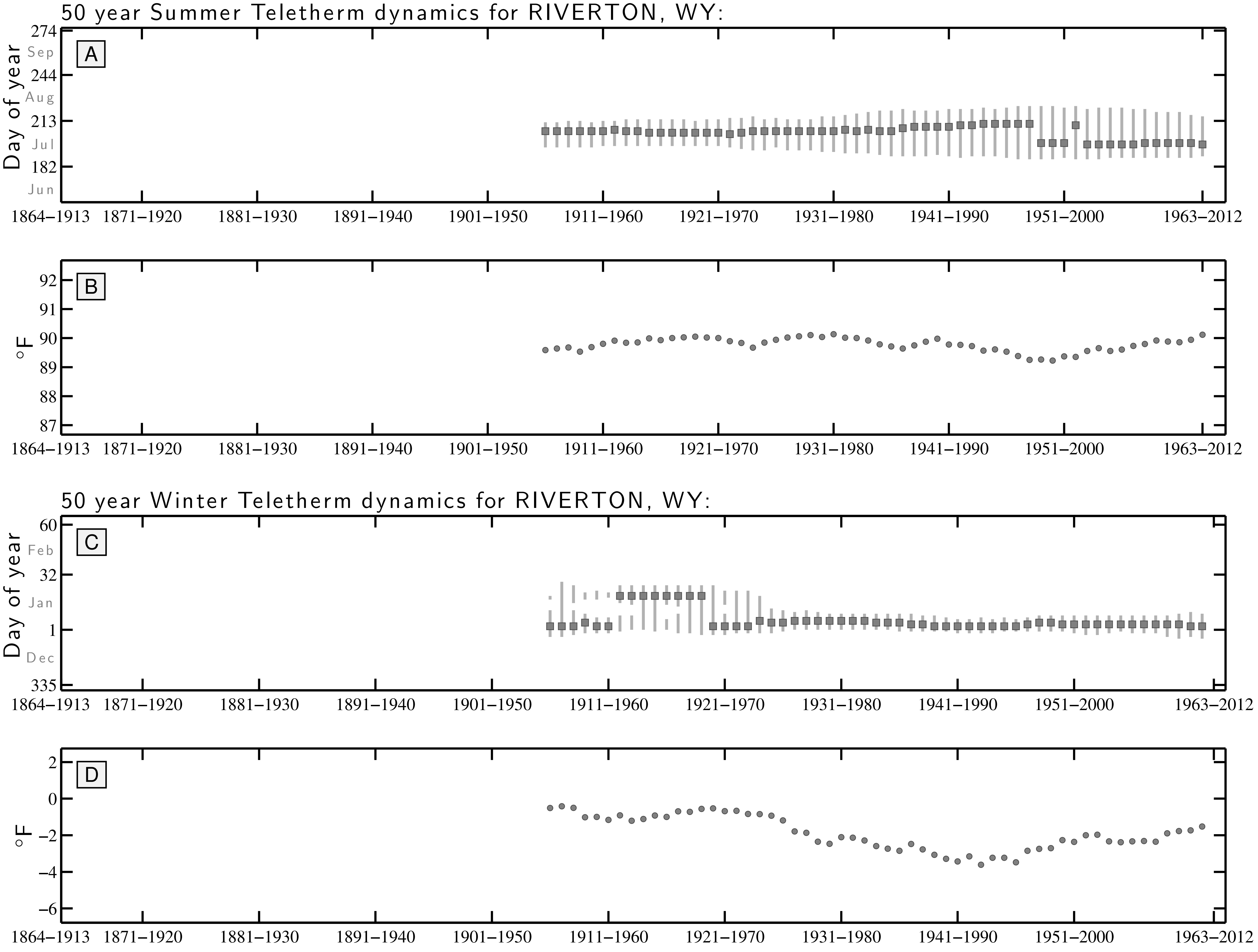

50 year Summer Teletherm dynamics for RIVERTON, WY:
A
Day of year
274
Sep
244
Aug
213
Jul
182
Jun
B
°F
92
91
90
89
88
87
50 year Winter Teletherm dynamics for RIVERTON, WY:
C
Day of year
60
Feb
32
Jan
1
Dec
335
D
°F
2
0
−2
−4
−6
1864–1913
1871–1920
1881–1930
1891–1940
1901–1950
1911–1960
1921–1970
1931–1980
1941–1990
1951–2000
1963–2012

50 year Summer Teletherm dynamics for ROCK SPRINGS AP, WY:

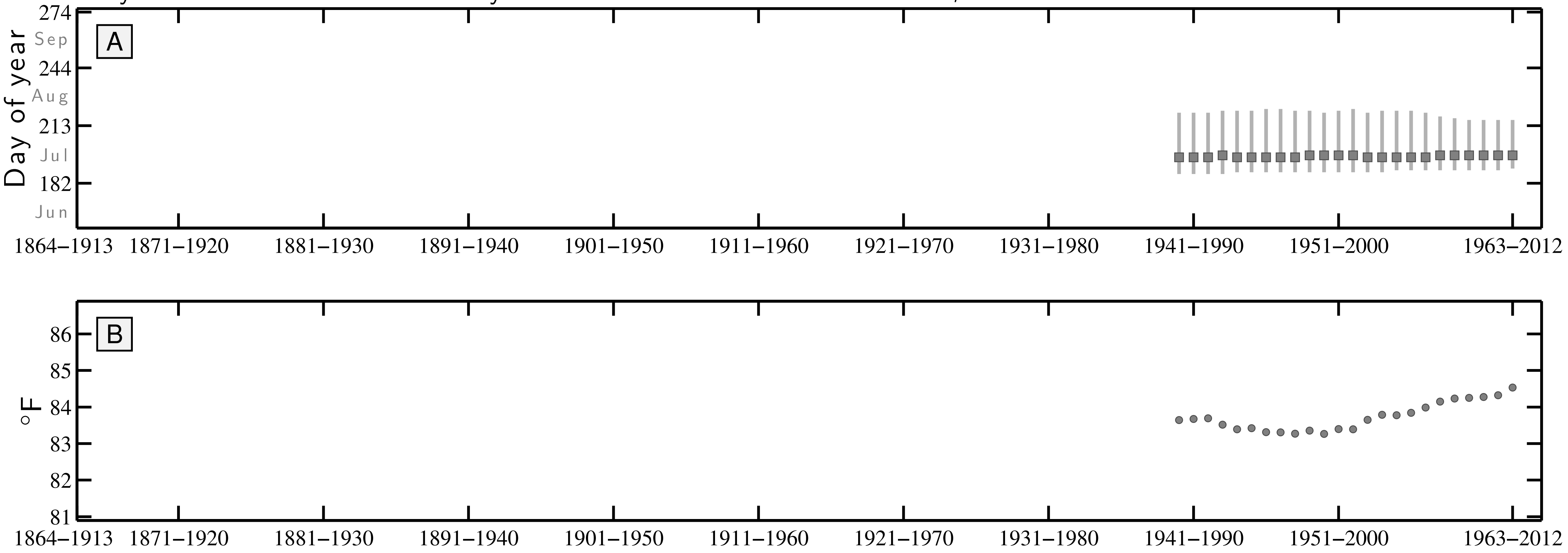

50 year Winter Teletherm dynamics for ROCK SPRINGS AP, WY:

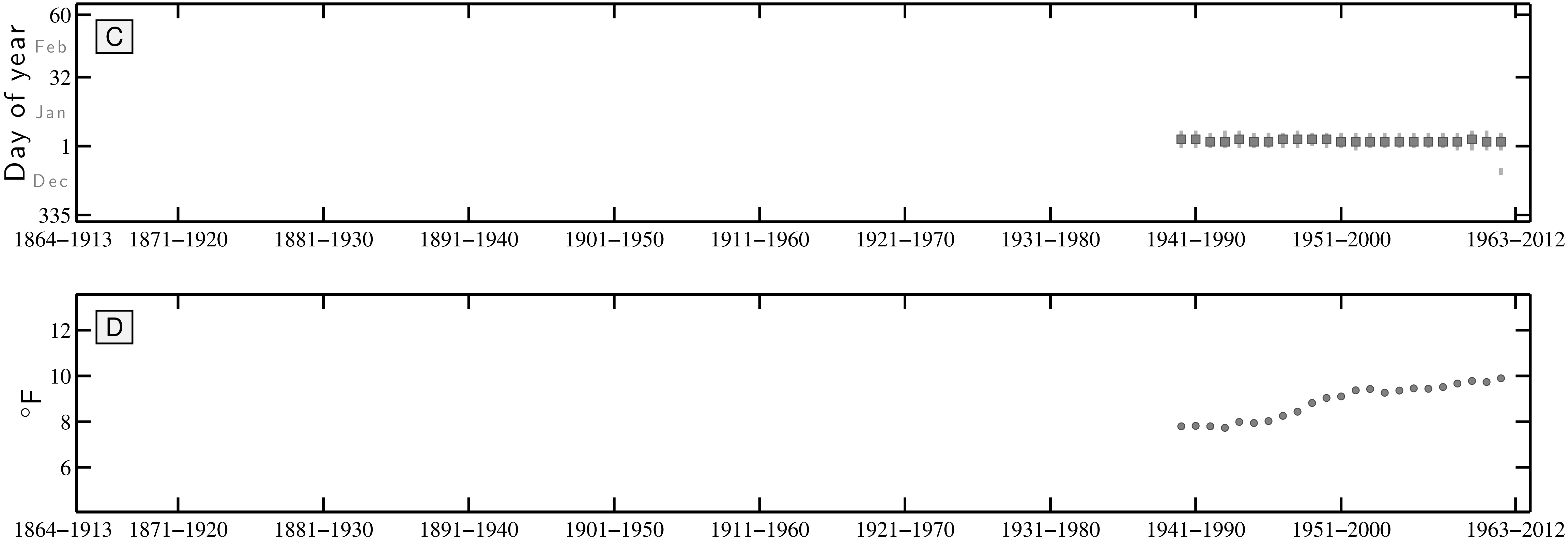

50 year Summer Teletherm dynamics for SARATOGA, WY:

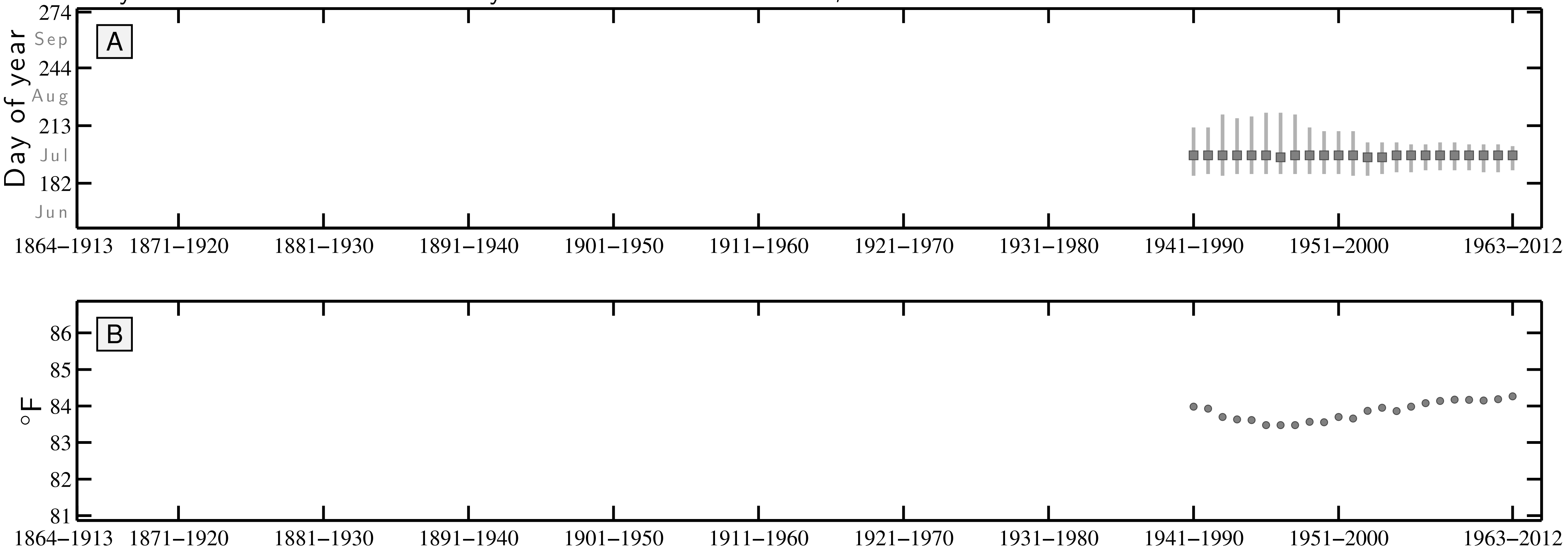

50 year Winter Teletherm dynamics for SARATOGA, WY:

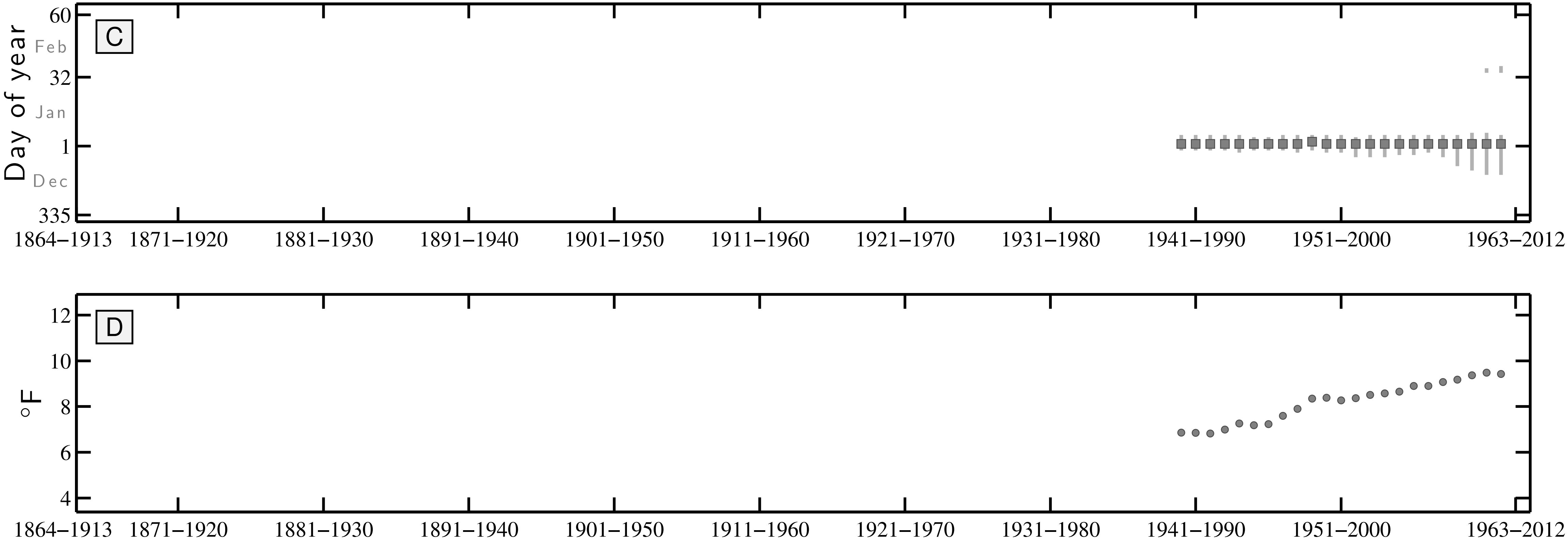

50 year Summer Teletherm dynamics for SHERIDAN FLD STN, WY:

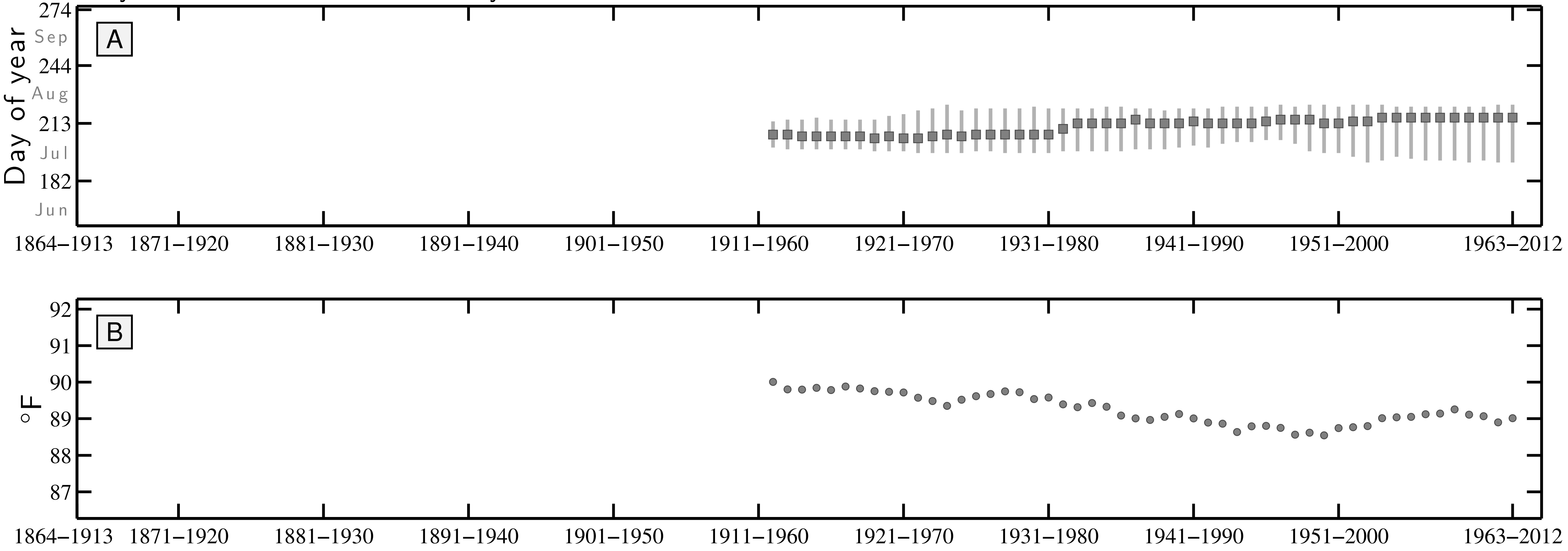

50 year Winter Teletherm dynamics for SHERIDAN FLD STN, WY:

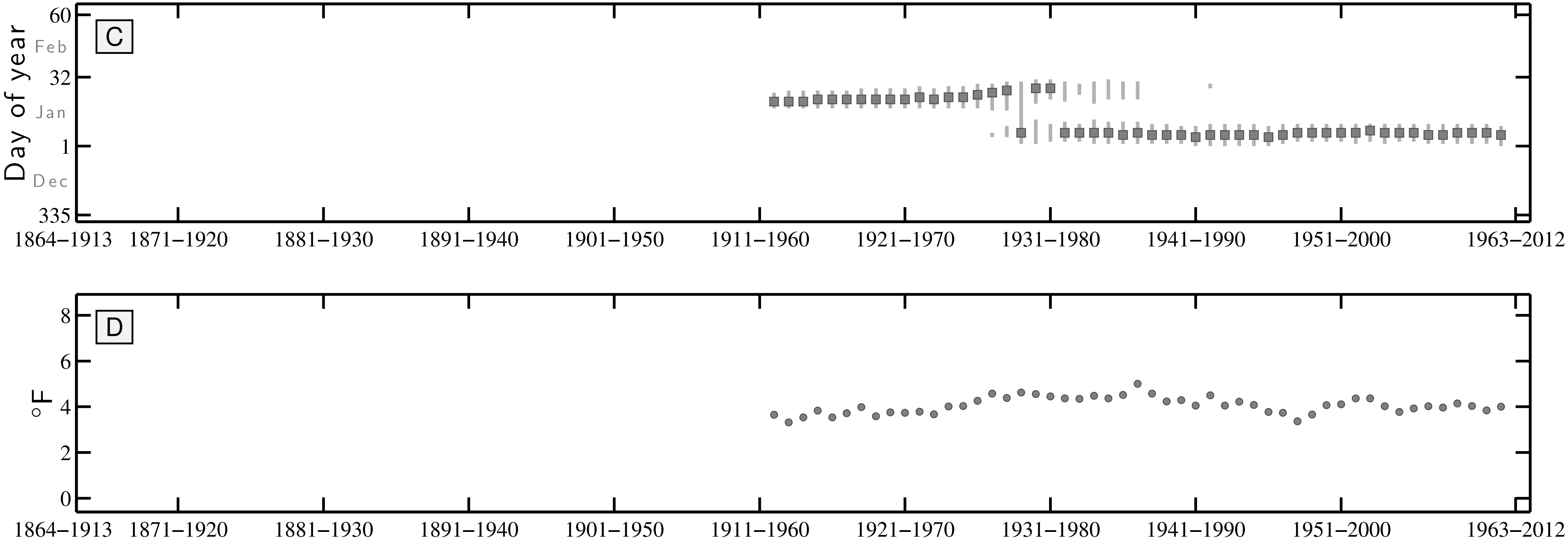

50 year Summer Teletherm dynamics for TORRINGTON EXP FARM, WY:

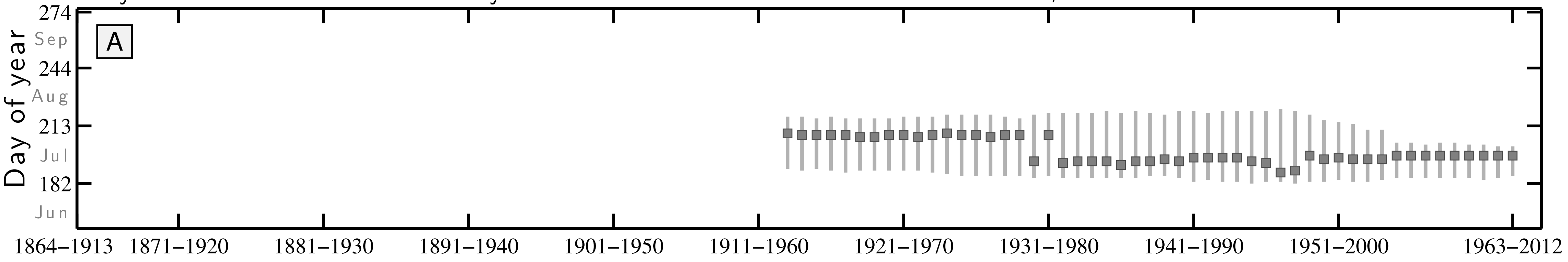

50 year Winter Teletherm dynamics for TORRINGTON EXP FARM, WY:

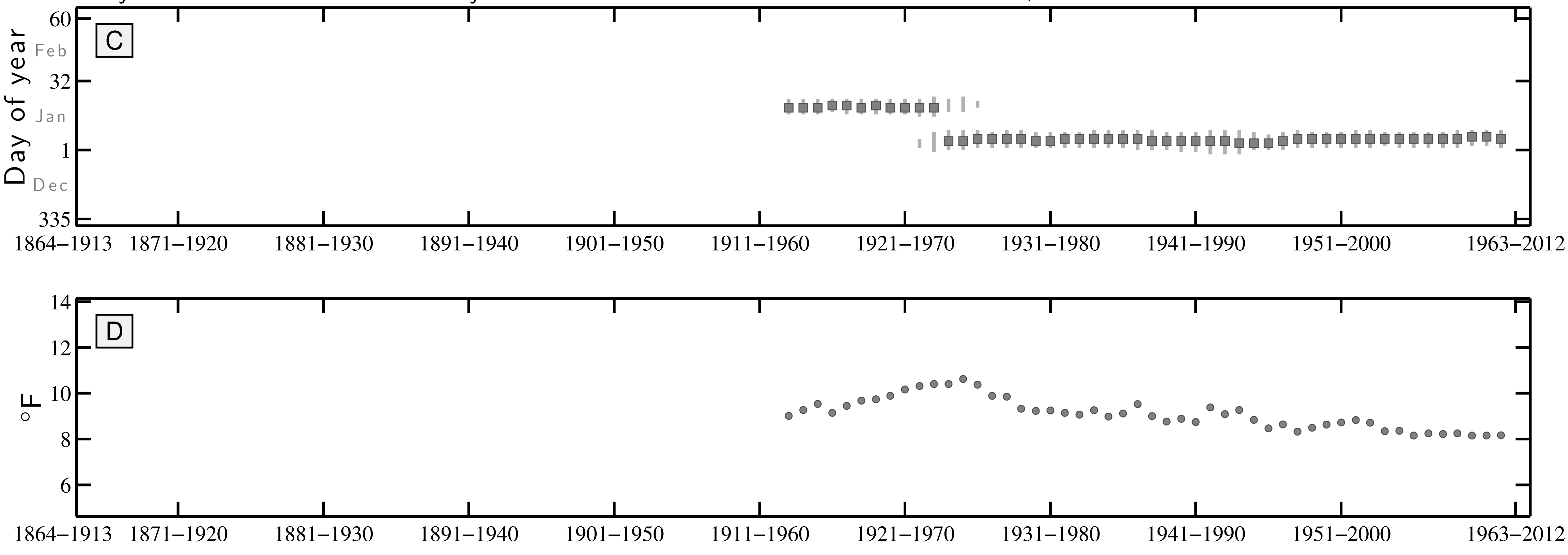

50 year Summer Teletherm dynamics for WHEATLAND 4 N, WY:

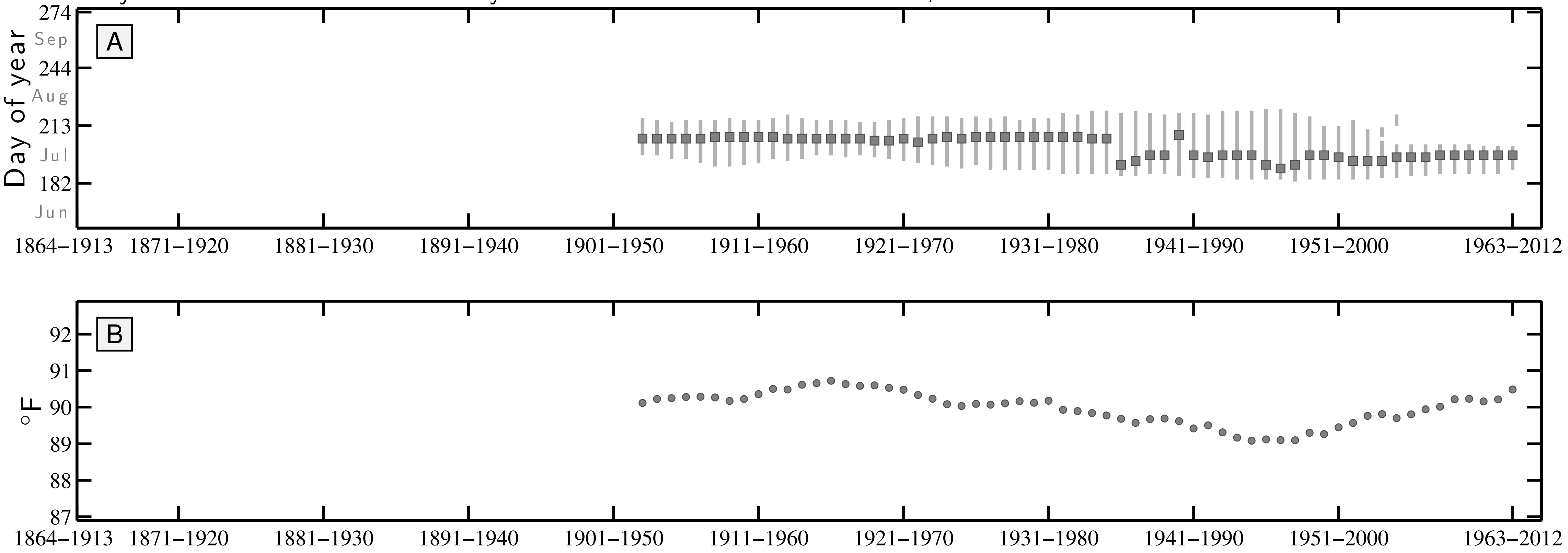

50 year Winter Teletherm dynamics for WHEATLAND 4 N, WY:

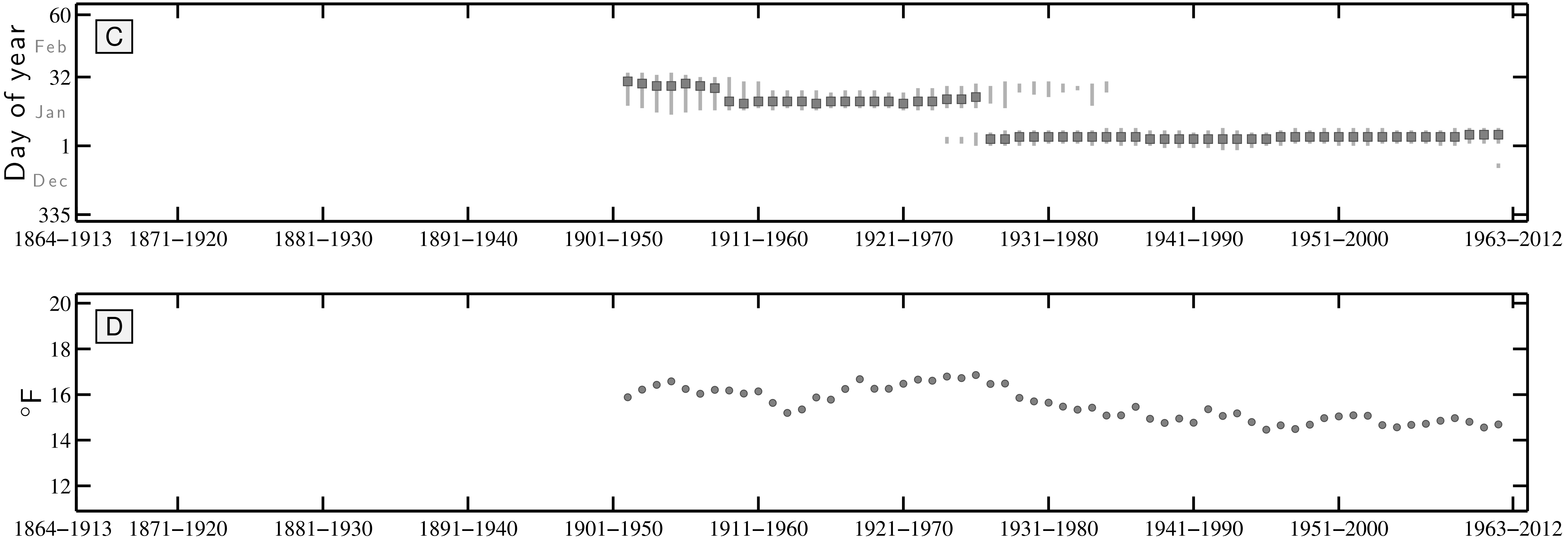

50 year Summer Teletherm dynamics for WORLAND, WY:

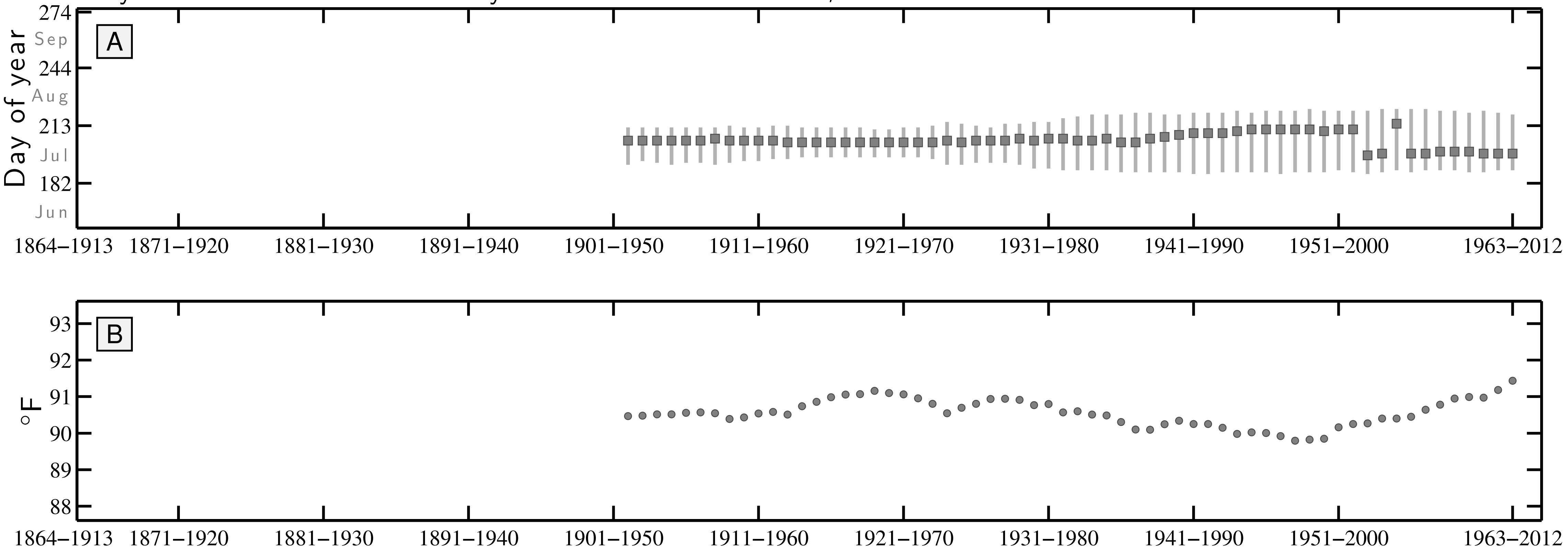

50 year Winter Teletherm dynamics for WORLAND, WY:

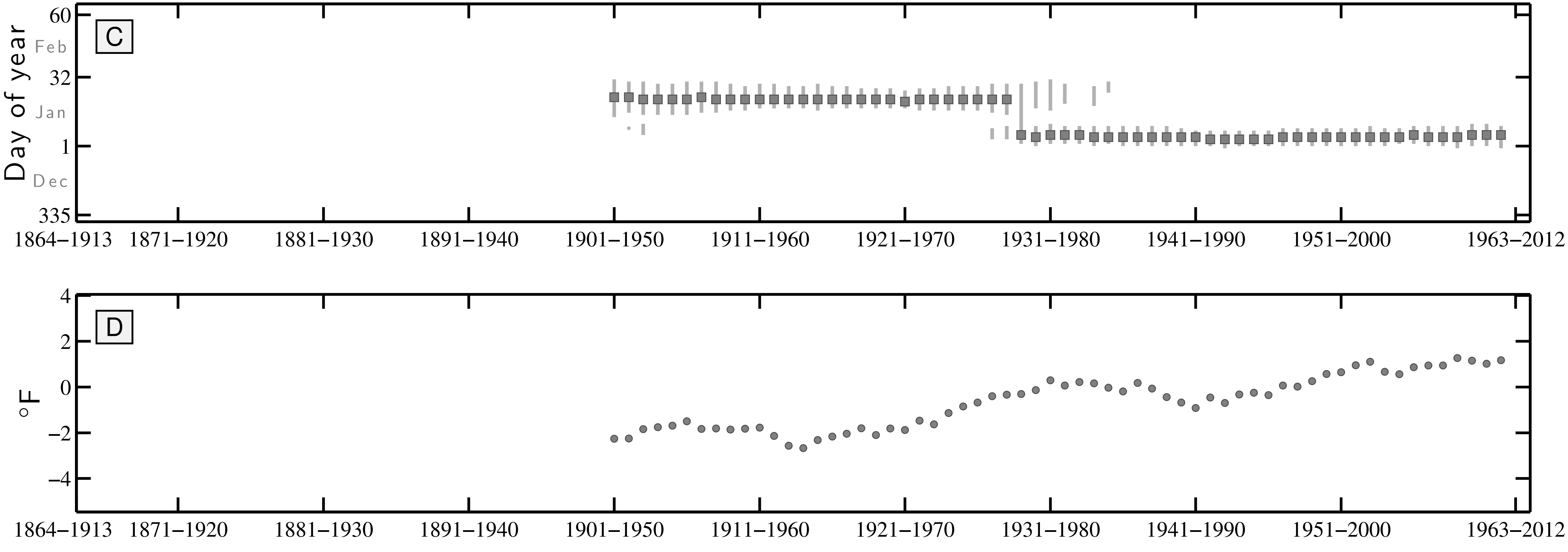

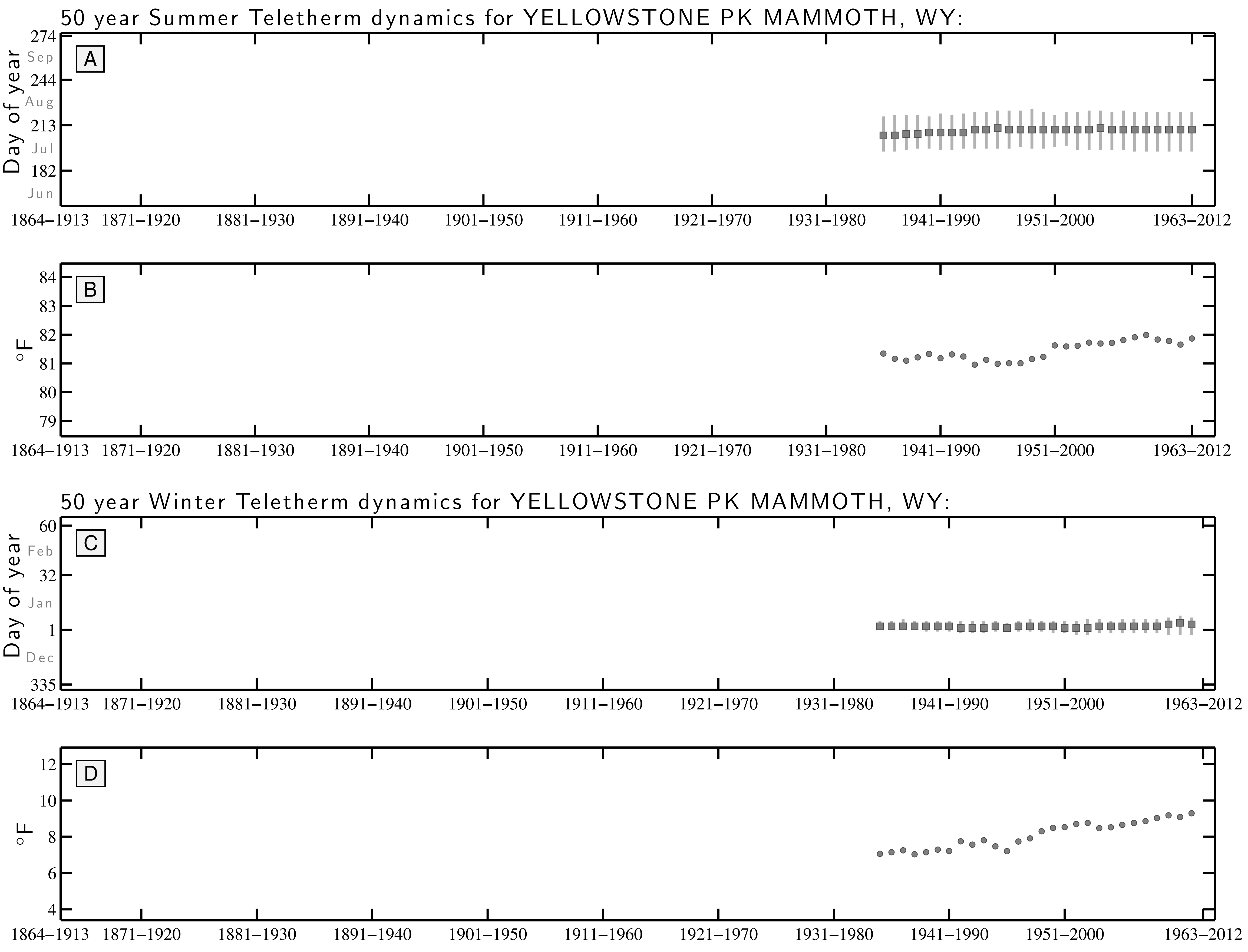
50 year Summer Teletherm dynamics for YELLOWSTONE PK MAMMOTH, WY:
A
Day of year
274
Sep
244
Aug
213
Jul
182
Jun
B
°F
84
83
82
81
80
79
50 year Winter Teletherm dynamics for YELLOWSTONE PK MAMMOTH, WY:
C
Day of year
60
Feb
32
Jan
1
Dec
335
D
°F
12
10
8
6
4
1864–1913
1871–1920
1881–1930
1891–1940
1901–1950
1911–1960
1921–1970
1931–1980
1941–1990
1951–2000
1963–2012
