# Supplementary material for: Tracking Climate Change through the Spatiotemporal Dynamics of the Teletherms, the Statistically Hottest and Coldest Days of the Year
Source: PLoS One. 2016 May 11;11(5):e0154184. doi: 10.1371/journal.pone.0154184 (PMC4864332; doi:10.1371/journal.pone.0154184)

10 year Summer Teletherm dynamics for BREWTON 3 SSE, AL:

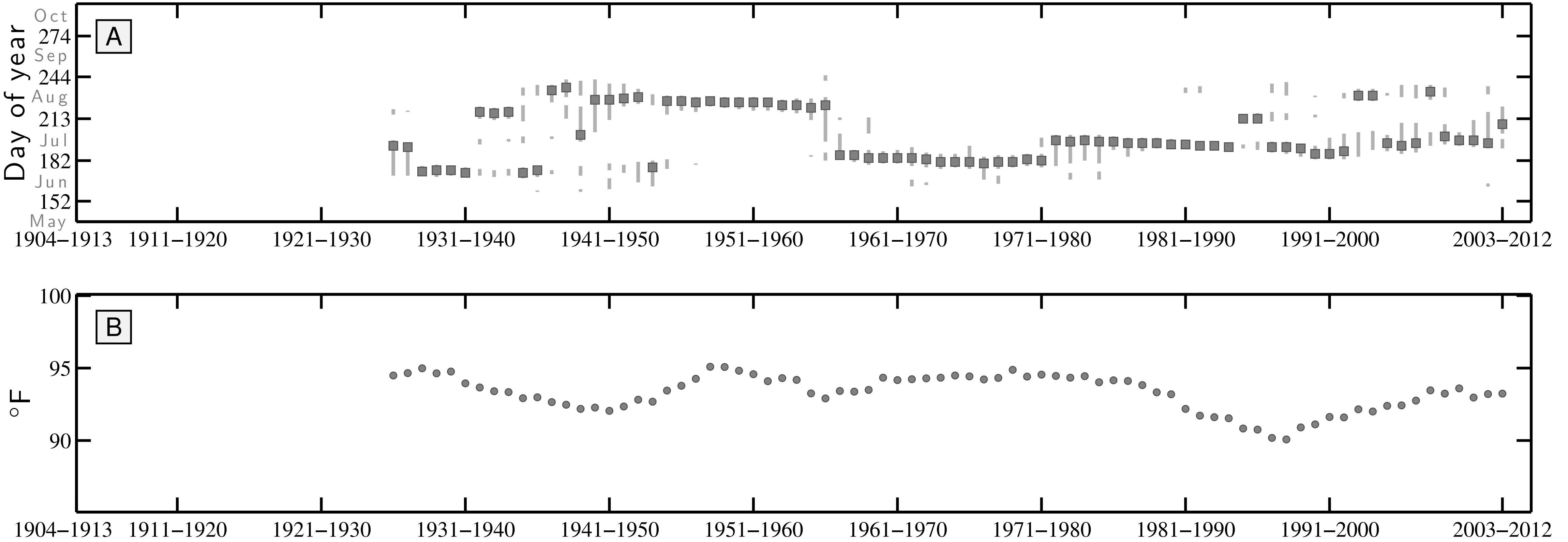

10 year Winter Teletherm dynamics for BREWTON 3 SSE, AL:

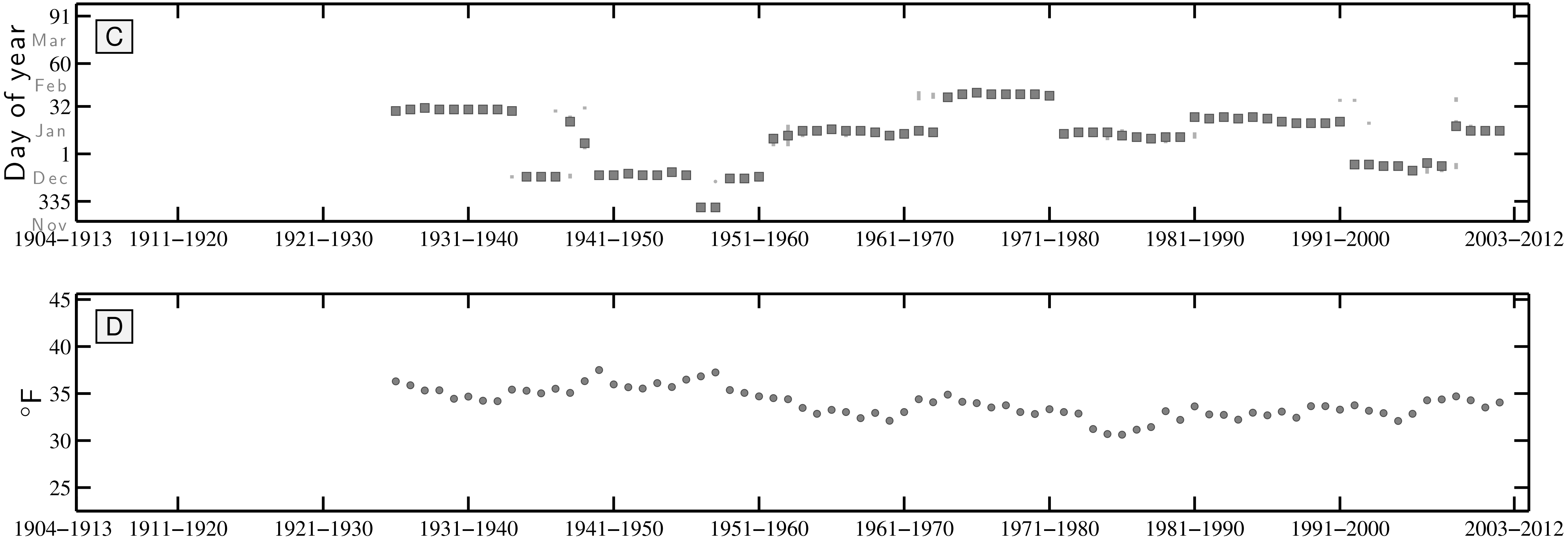

10 year Summer Teletherm dynamics for FAIRHOPE 2 NE, AL:

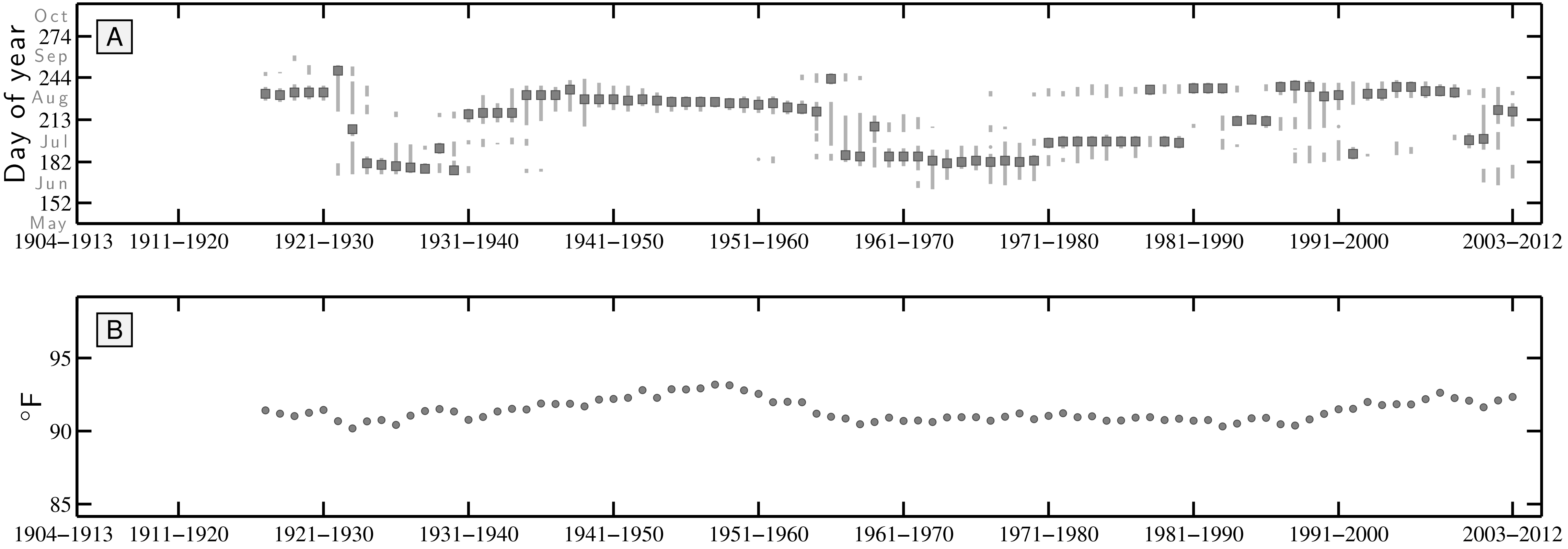

10 year Winter Teletherm dynamics for FAIRHOPE 2 NE, AL:

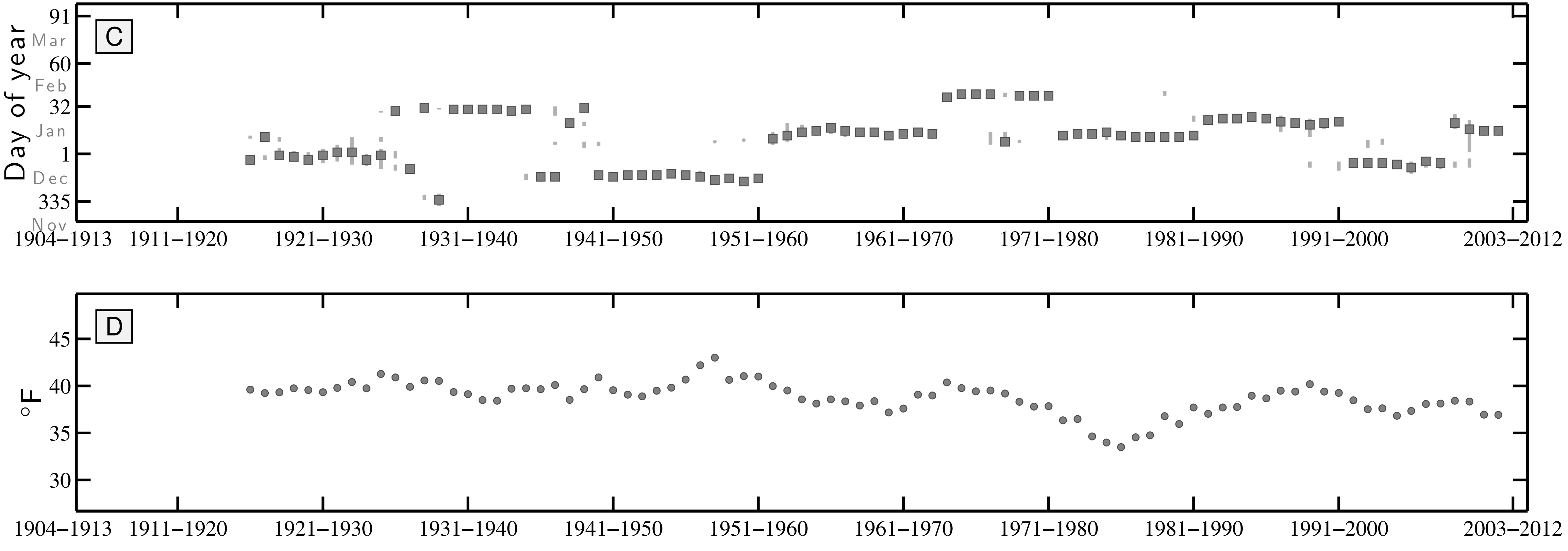

10 year Summer Teletherm dynamics for GAINESVILLE LOCK, AL:

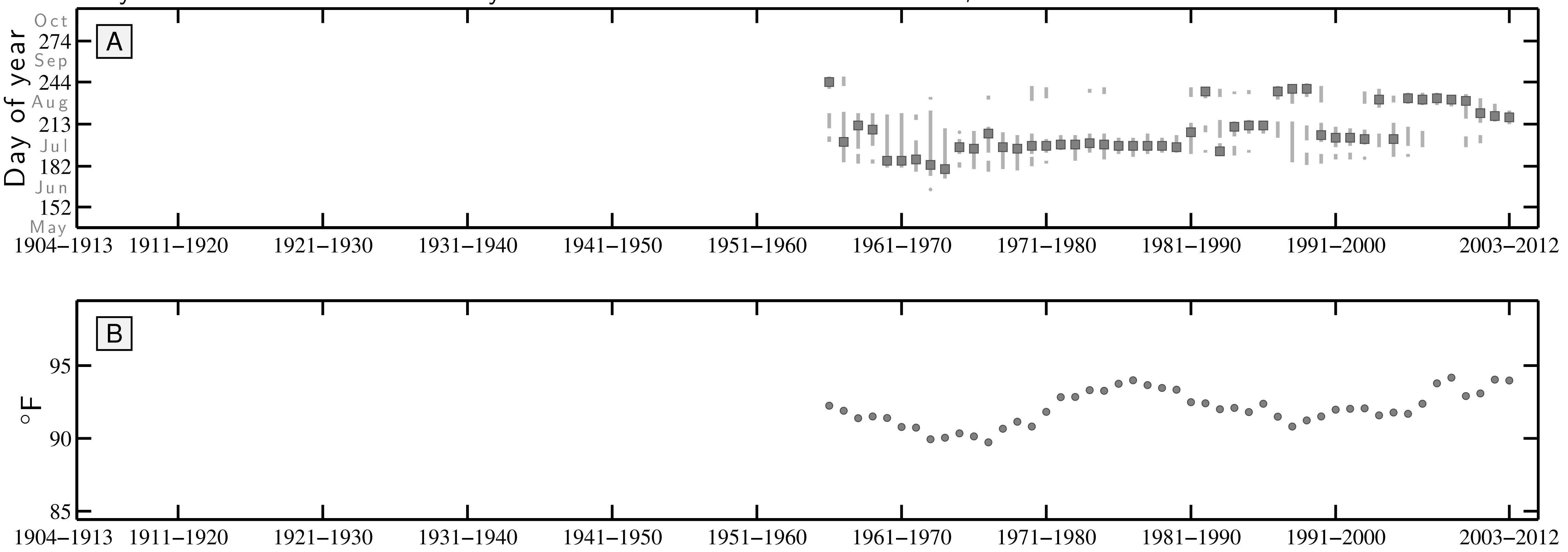

10 year Winter Teletherm dynamics for GAINESVILLE LOCK, AL:

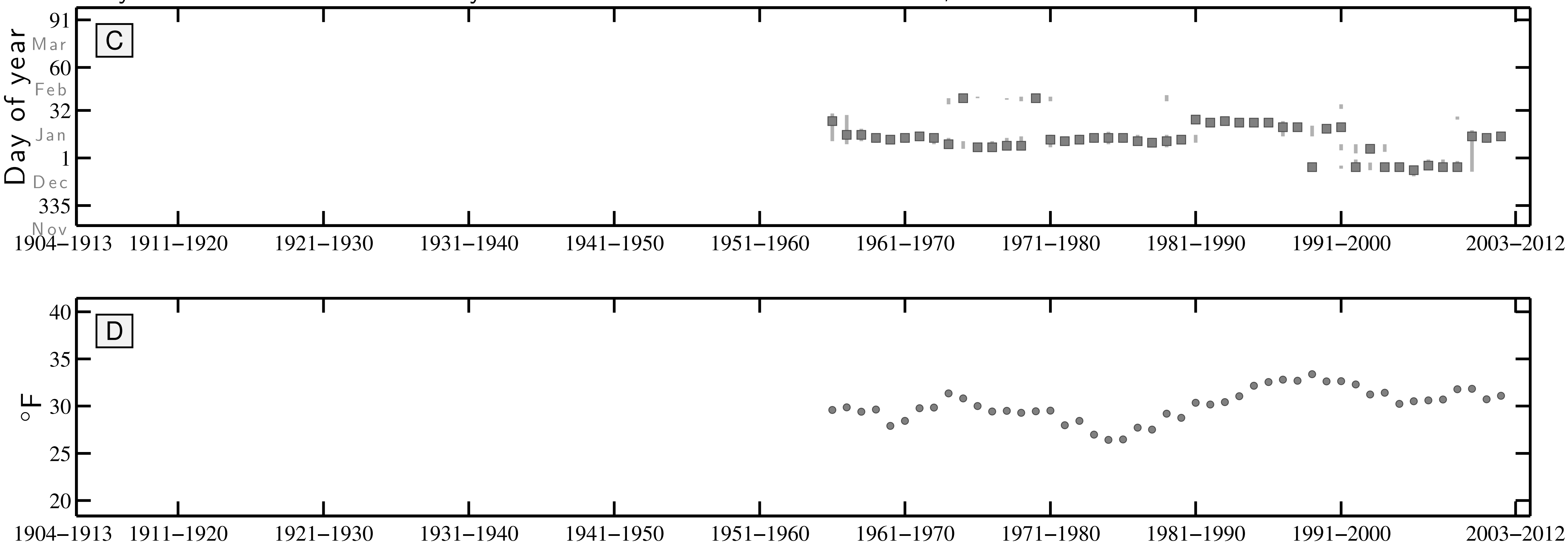

10 year Summer Teletherm dynamics for GREENSBORO, AL:

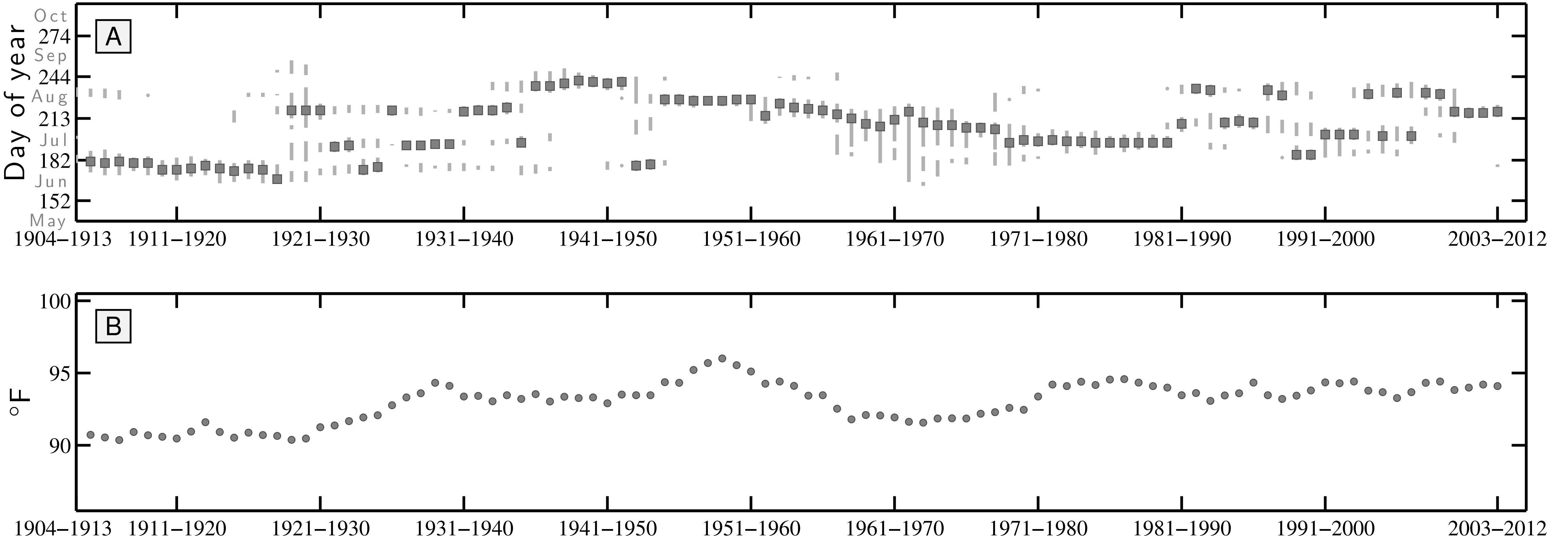

10 year Winter Teletherm dynamics for GREENSBORO, AL:

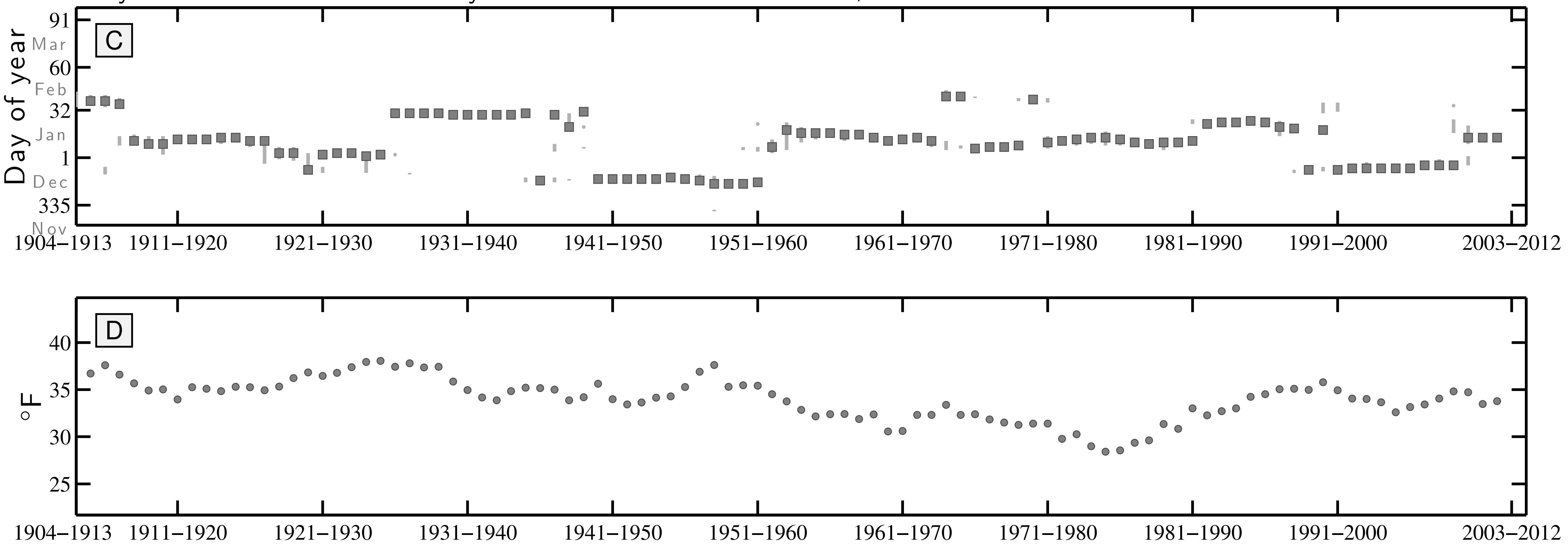

10 year Summer Teletherm dynamics for HIGHLAND HOME, AL:

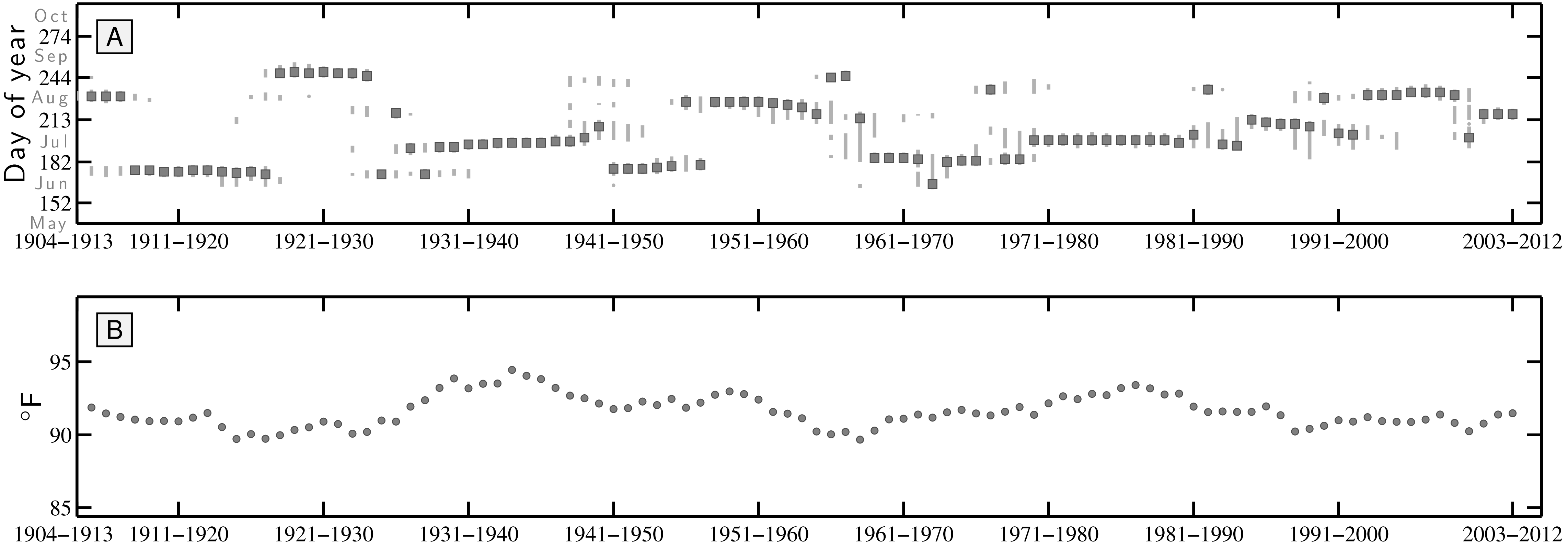

10 year Winter Teletherm dynamics for HIGHLAND HOME, AL:

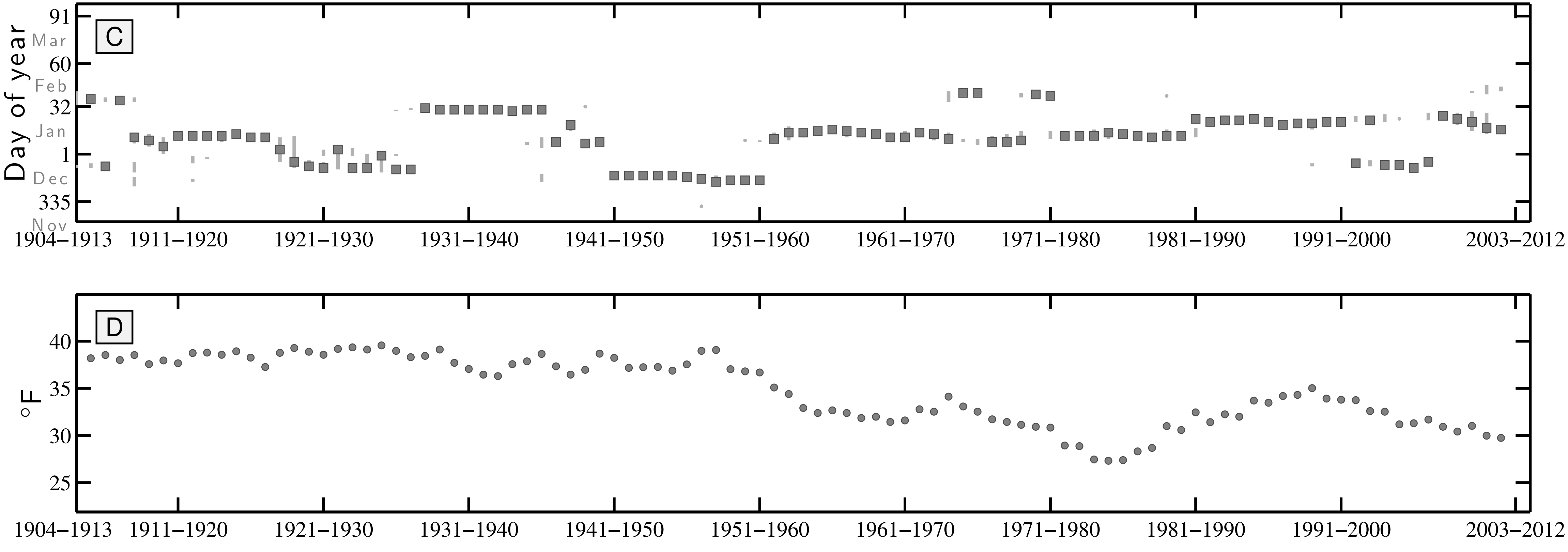

10 year Summer Teletherm dynamics for MUSCLE SHOALS AP, AL:

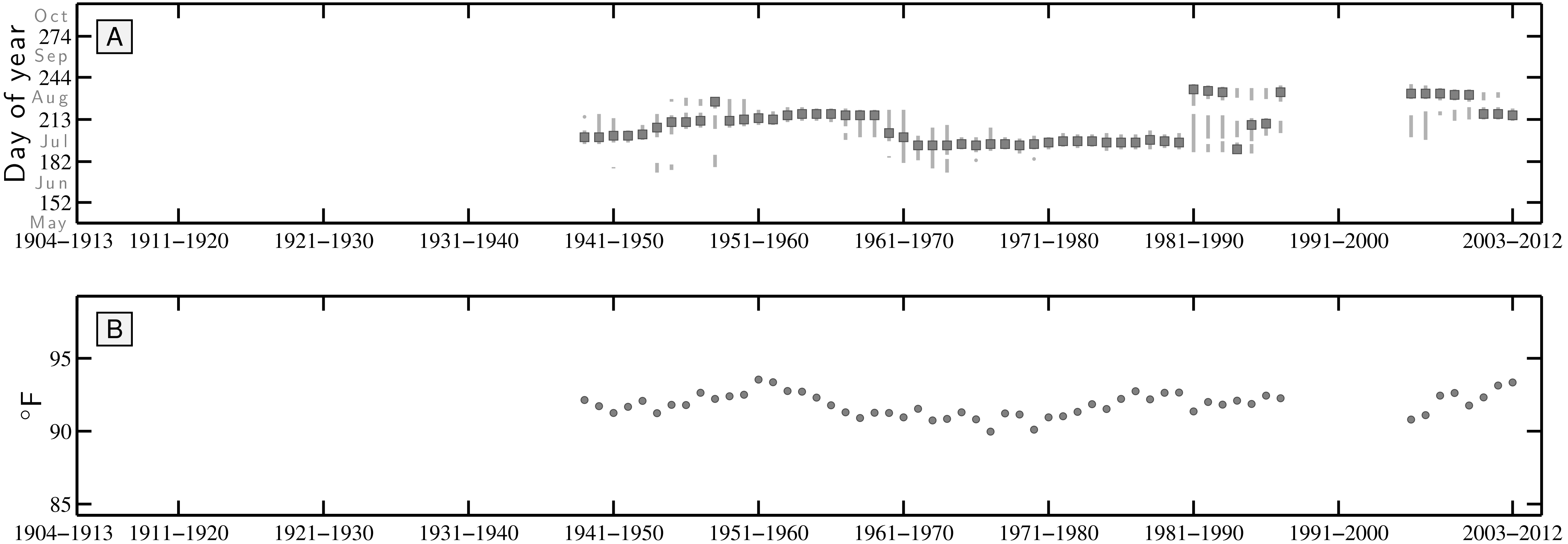

10 year Winter Teletherm dynamics for MUSCLE SHOALS AP, AL:

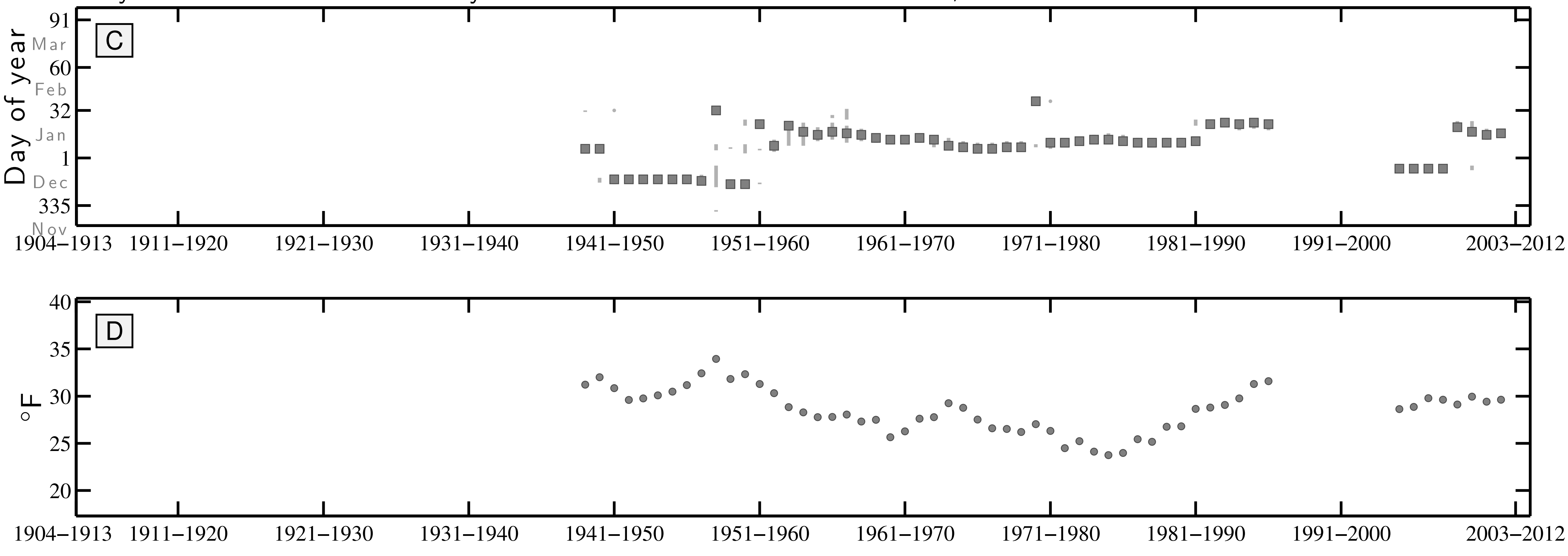

10 year Summer Teletherm dynamics for SAINT BERNARD, AL:

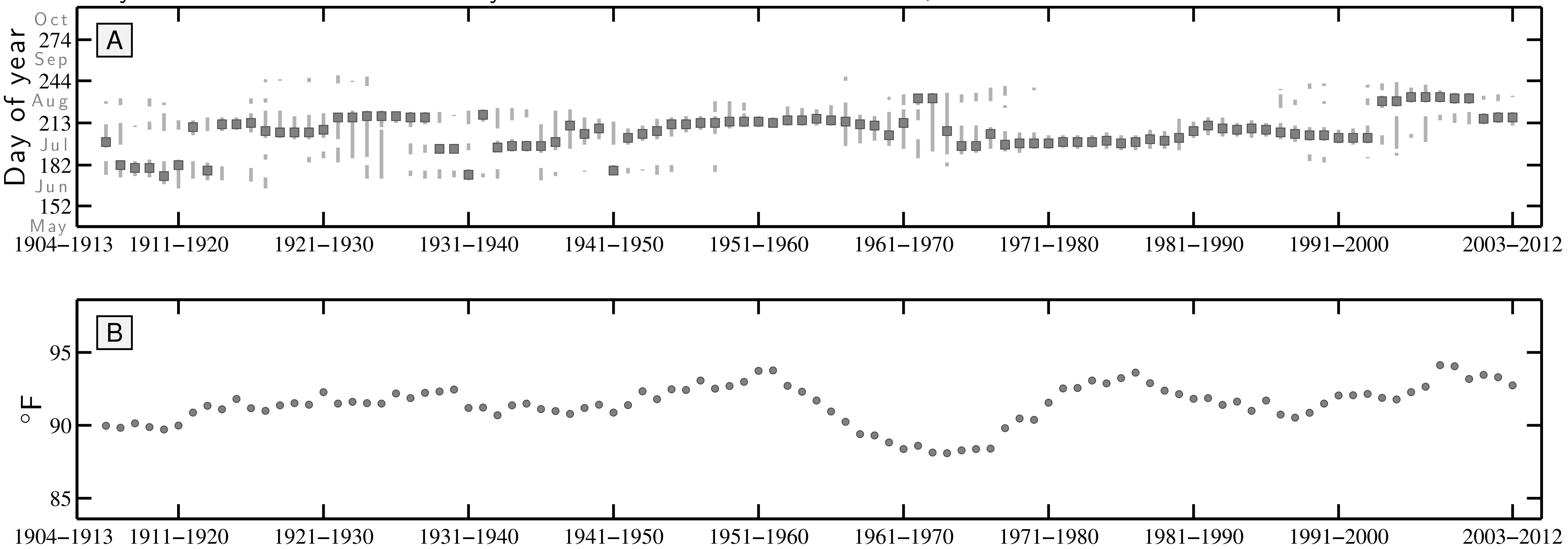

10 year Winter Teletherm dynamics for SAINT BERNARD, AL:

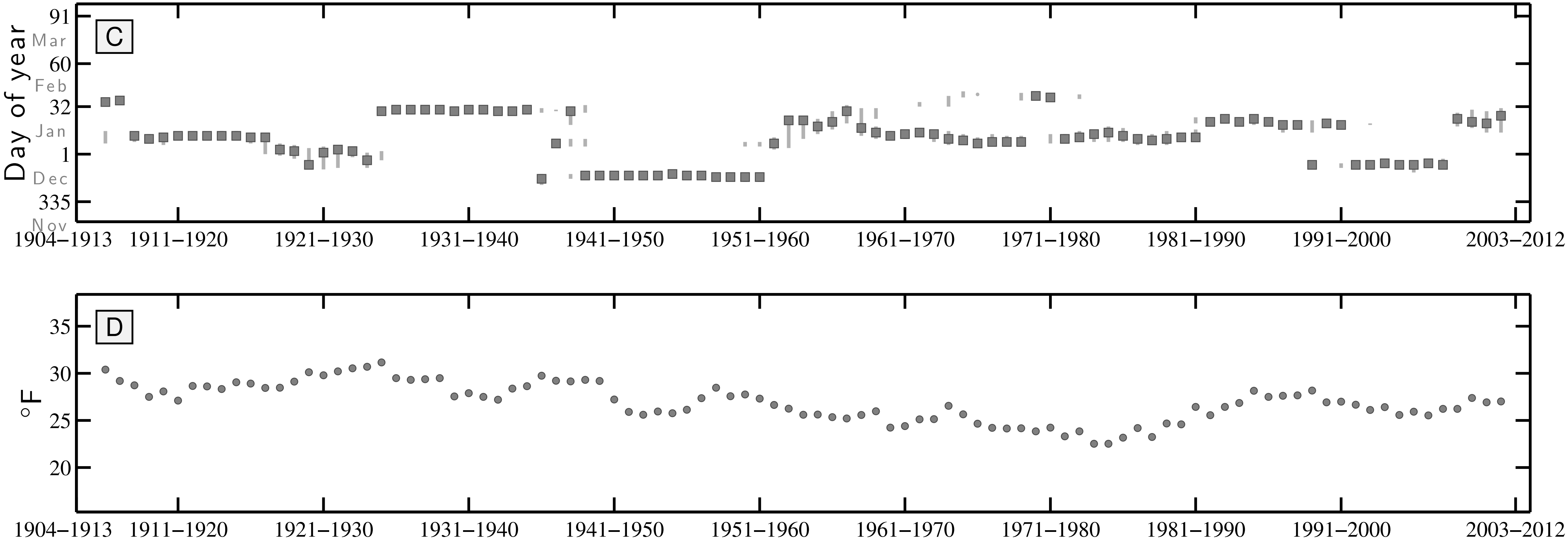

10 year Summer Teletherm dynamics for SCOTTSBORO, AL:

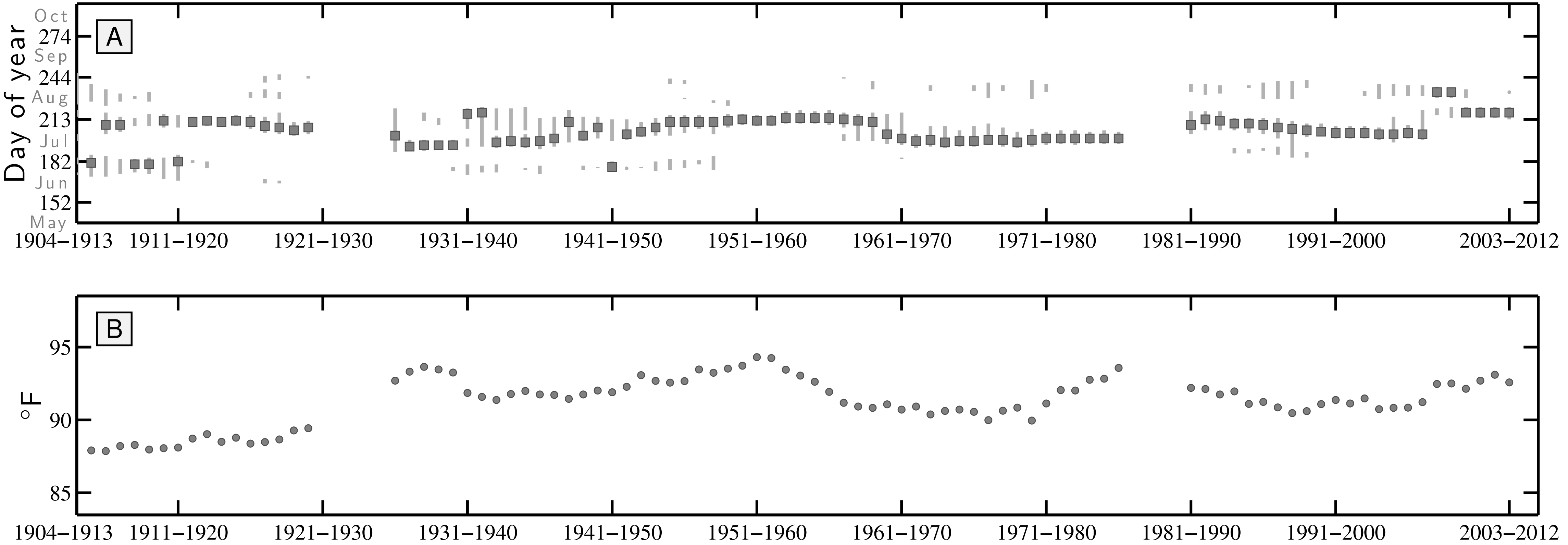

10 year Winter Teletherm dynamics for SCOTTSBORO, AL:

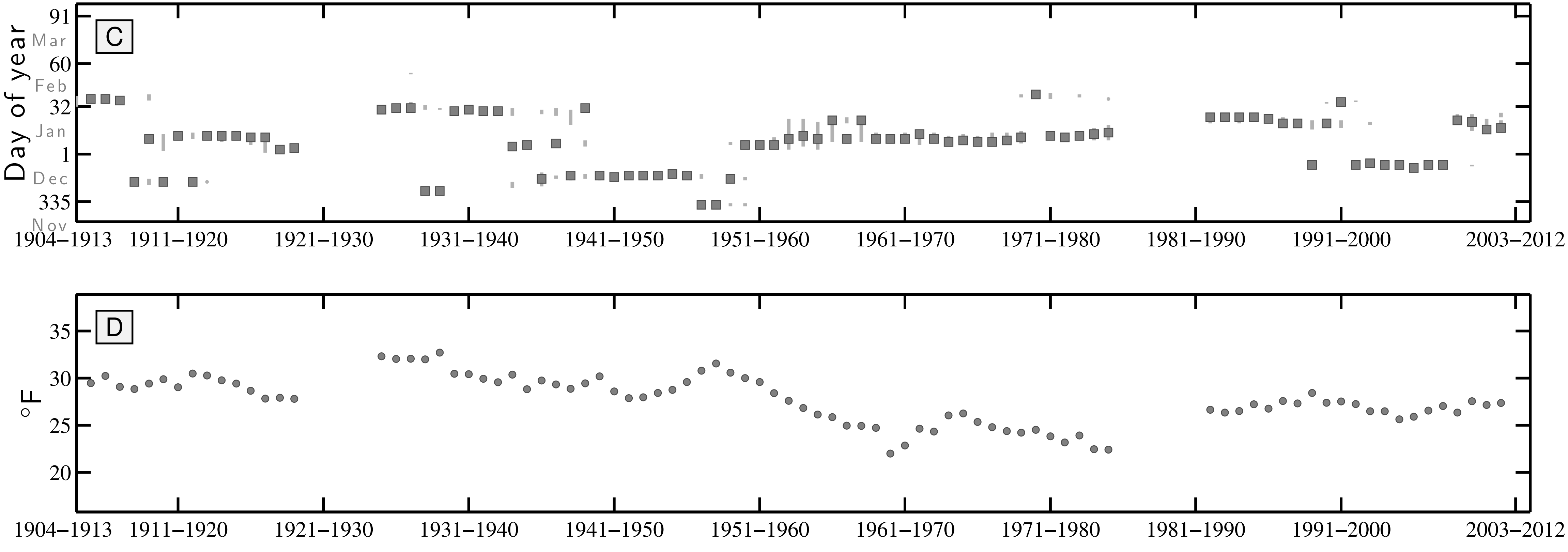

10 year Summer Teletherm dynamics for SELMA, AL:

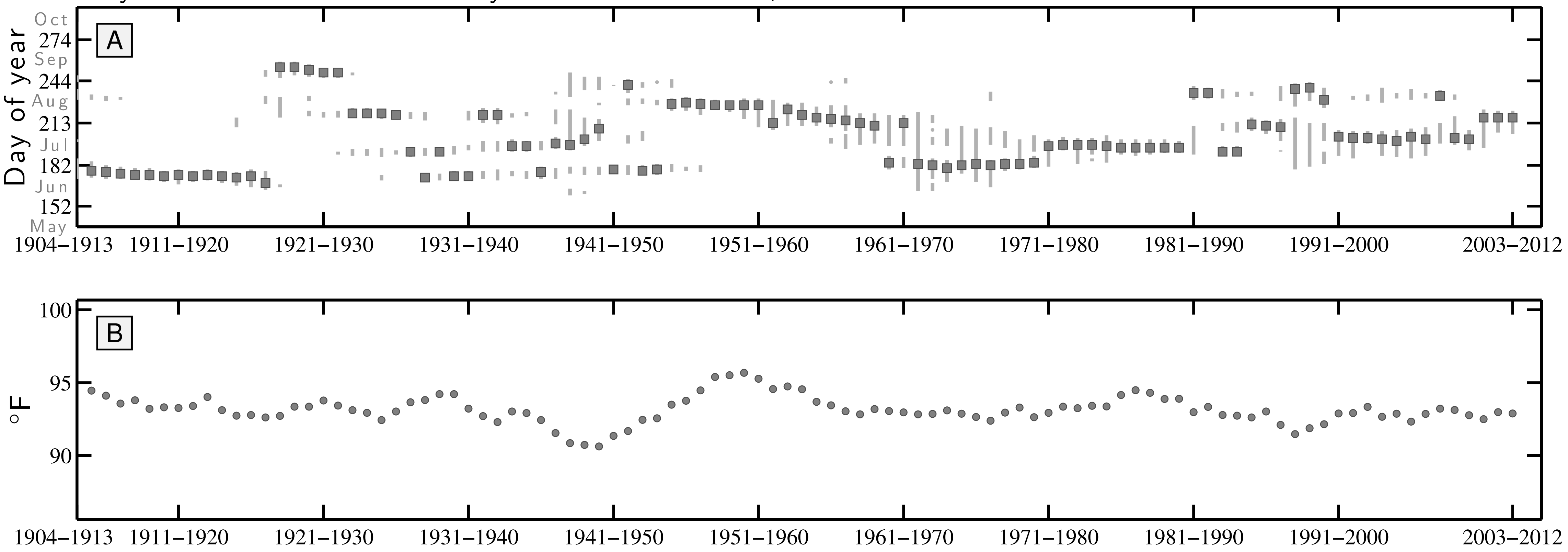

10 year Winter Teletherm dynamics for SELMA, AL:

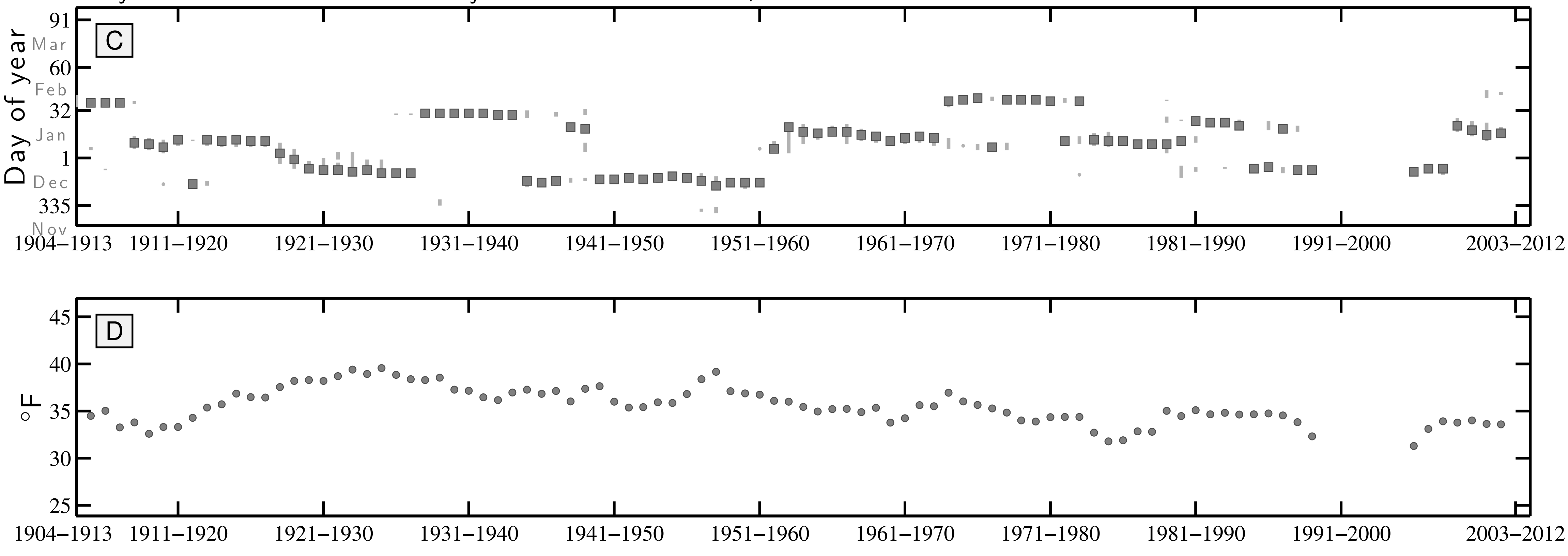

10 year Summer Teletherm dynamics for TALLADEGA, AL:

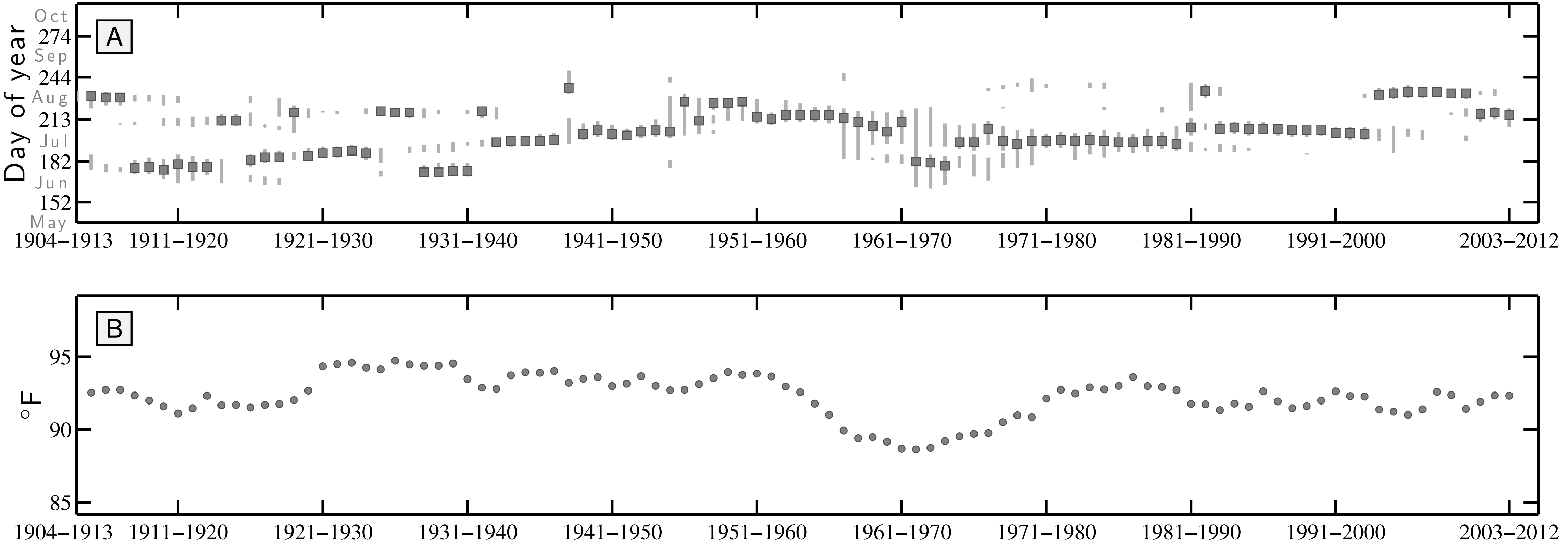

10 year Winter Teletherm dynamics for TALLADEGA, AL:

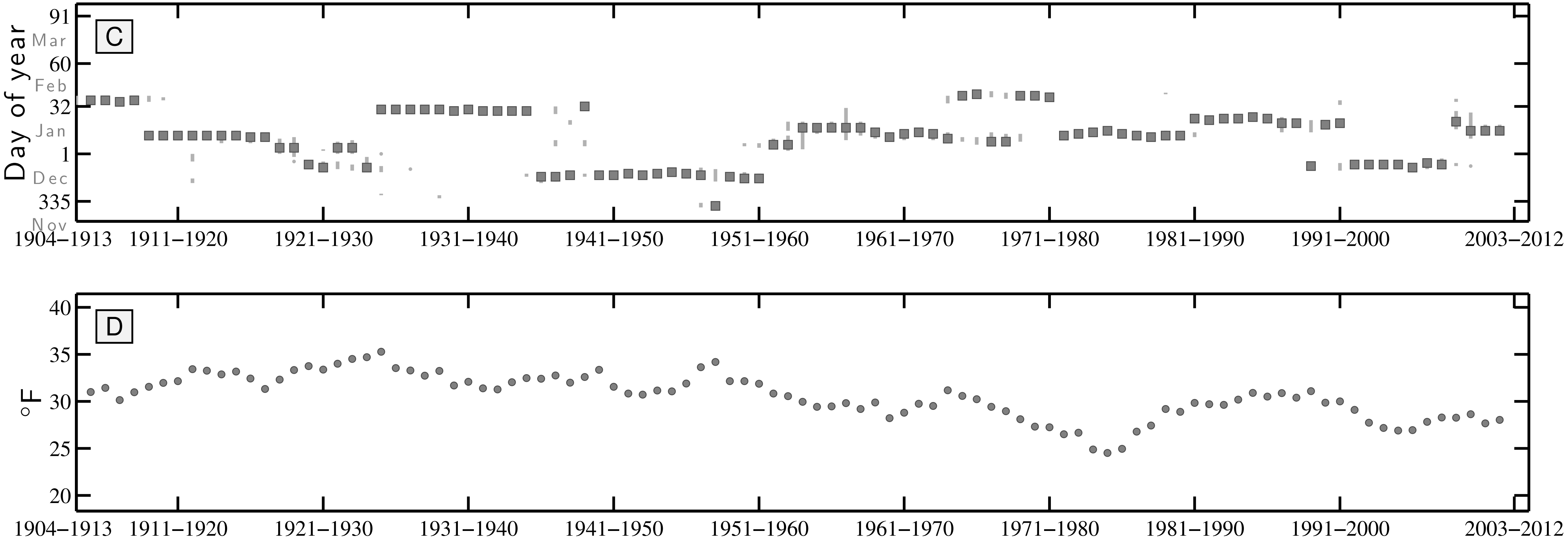

10 year Summer Teletherm dynamics for THOMASVILLE, AL:

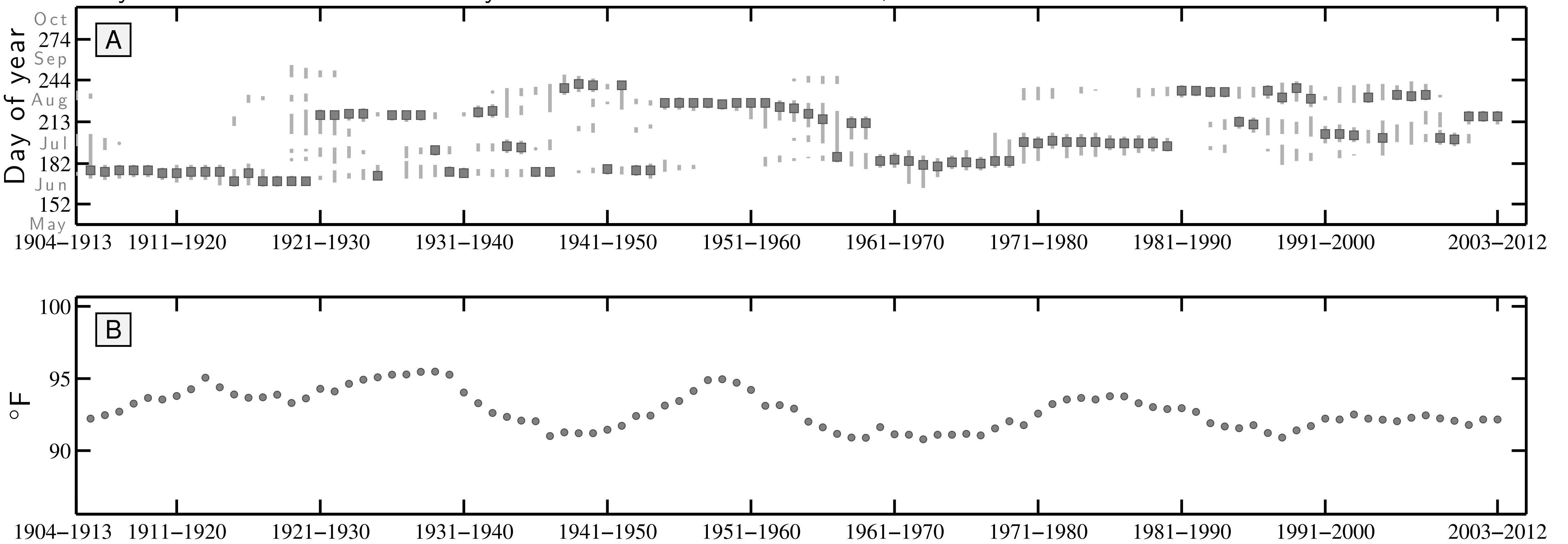

10 year Winter Teletherm dynamics for THOMASVILLE, AL:

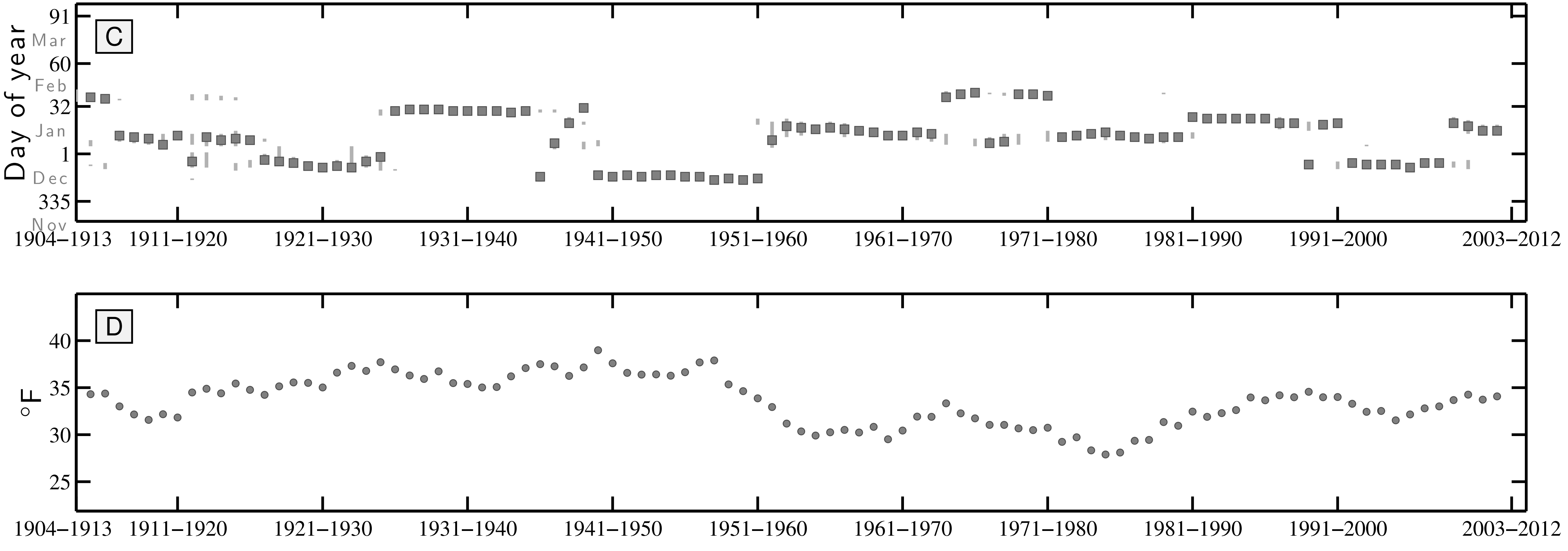

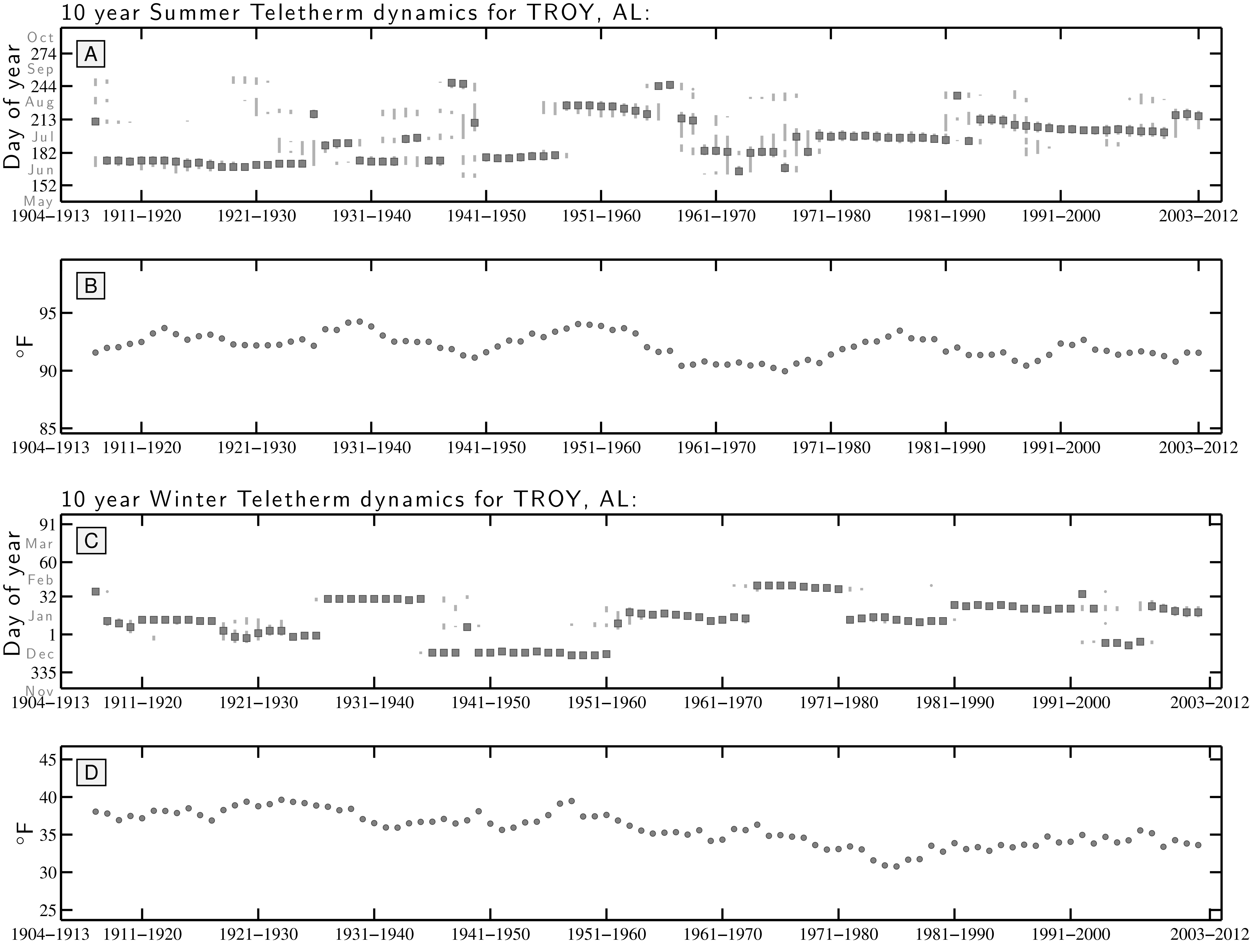
10 year Summer Teletherm dynamics for TROY, AL:
A
Day of year
Oct
274
Sep
244
Aug
213
Jul
182
Jun
152
May
1904–1913
1911–1920
1921–1930
1931–1940
1941–1950
1951–1960
1961–1970
1971–1980
1981–1990
1991–2000
2003–2012
B
°F
95
90
85
10 year Winter Teletherm dynamics for TROY, AL:
C
Day of year
91
Mar
60
Feb
32
Jan
1
Dec
335
Nov
D
°F
45
40
35
30
25

10 year Summer Teletherm dynamics for TUSCALOOSA ACFD, AL:

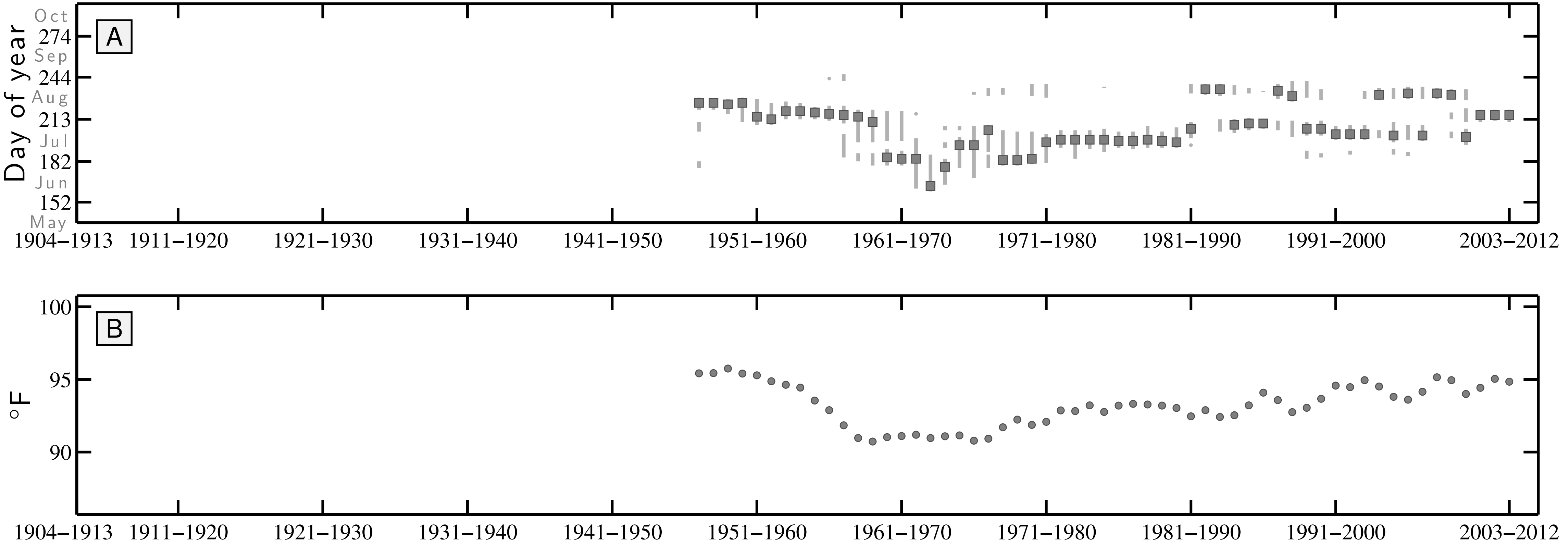

10 year Winter Teletherm dynamics for TUSCALOOSA ACFD, AL:

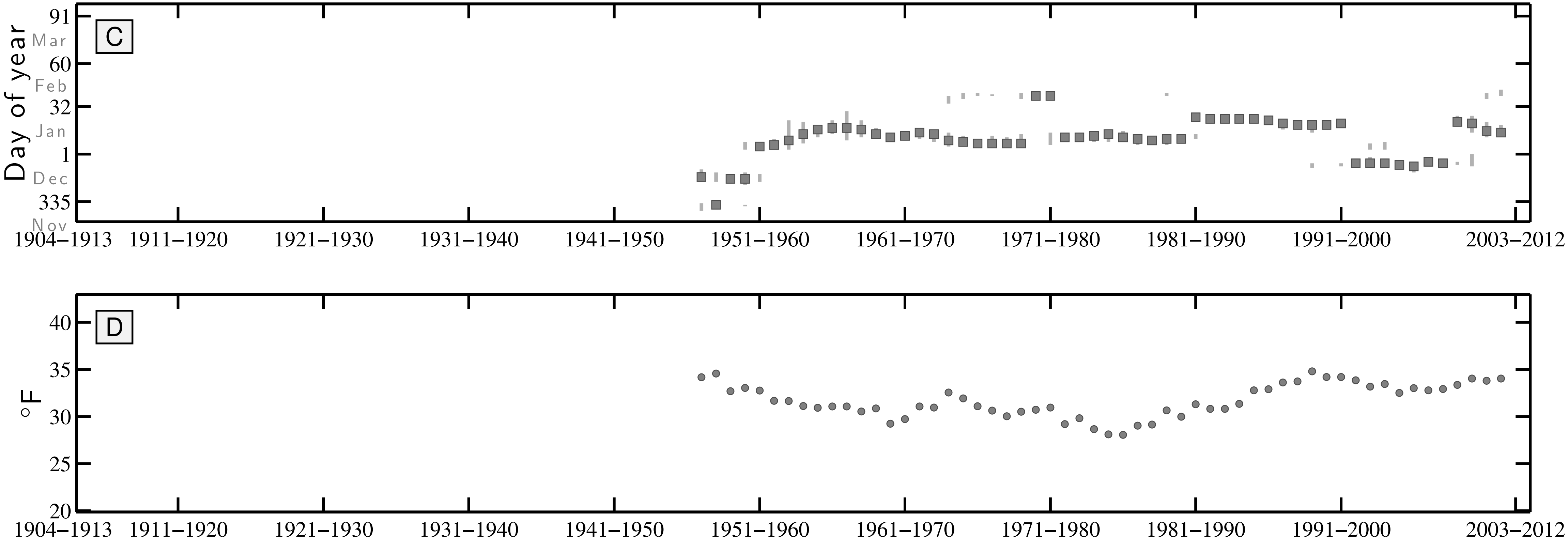

10 year Summer Teletherm dynamics for UNION SPRINGS 9 S, AL:

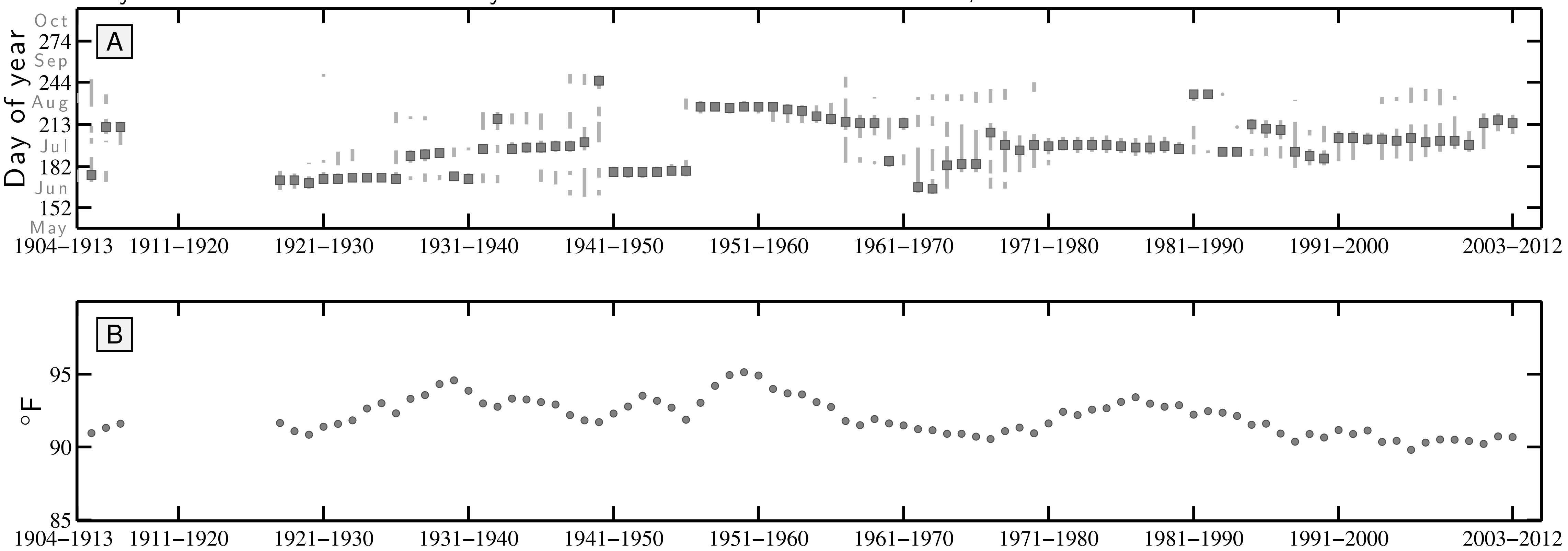

10 year Winter Teletherm dynamics for UNION SPRINGS 9 S, AL:

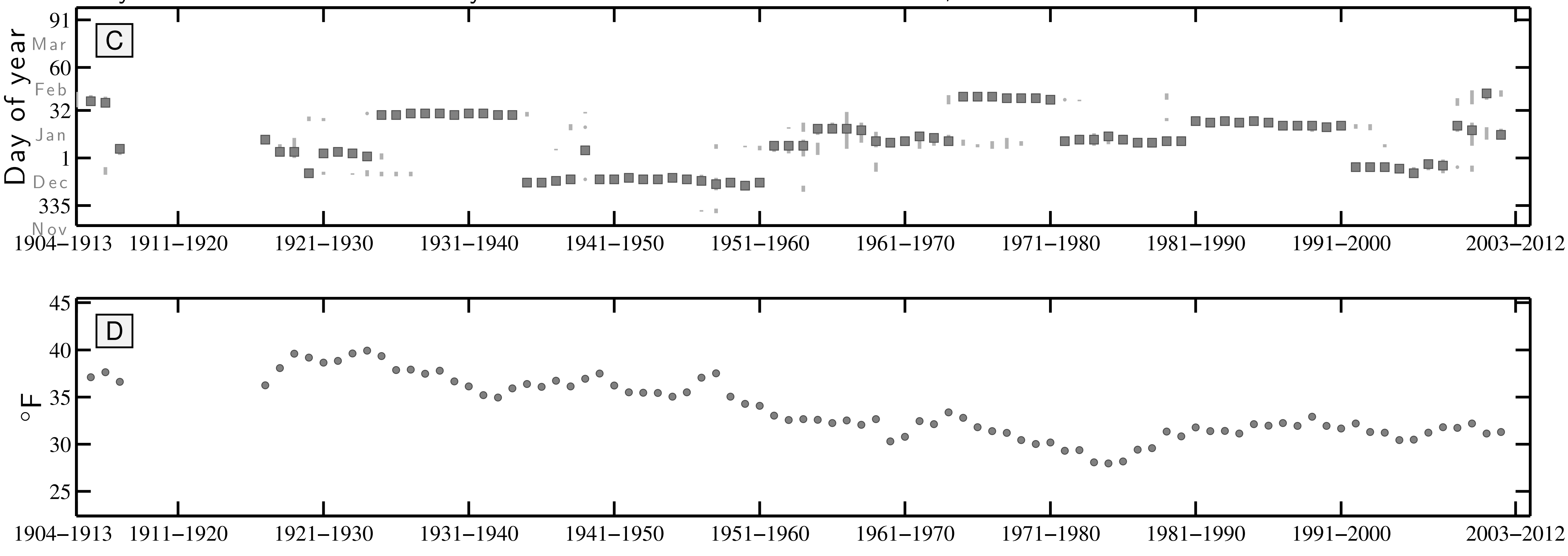

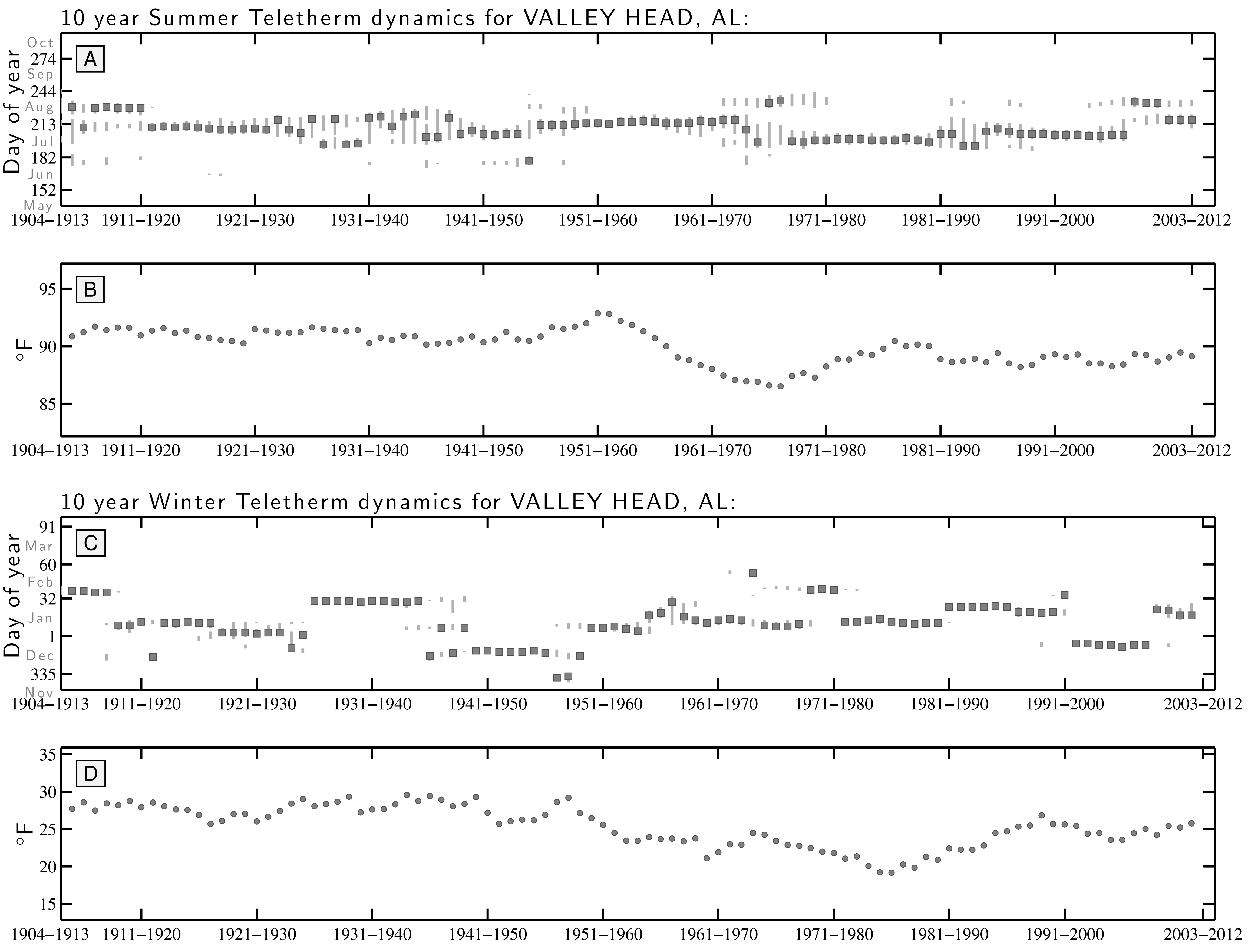
10 year Summer Teletherm dynamics for VALLEY HEAD, AL:
A
Day of year
Oct
274
Sep
244
Aug
213
Jul
182
Jun
152
May
1904–1913
1911–1920
1921–1930
1931–1940
1941–1950
1951–1960
1961–1970
1971–1980
1981–1990
1991–2000
2003–2012
B
°F
95
90
85
10 year Winter Teletherm dynamics for VALLEY HEAD, AL:
C
91
Mar
60
Feb
32
Jan
1
Dec
335
Nov
D
35
30
25
20
15

10 year Summer Teletherm dynamics for AJO, AZ:

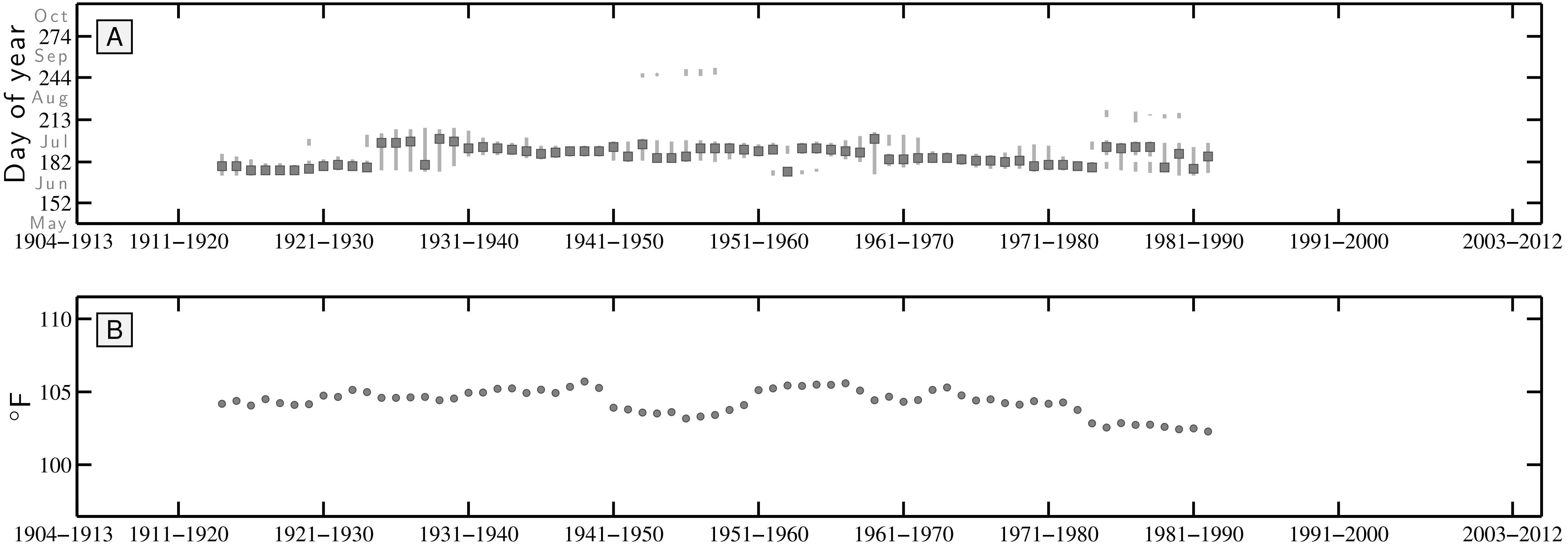

10 year Winter Teletherm dynamics for AJO, AZ:

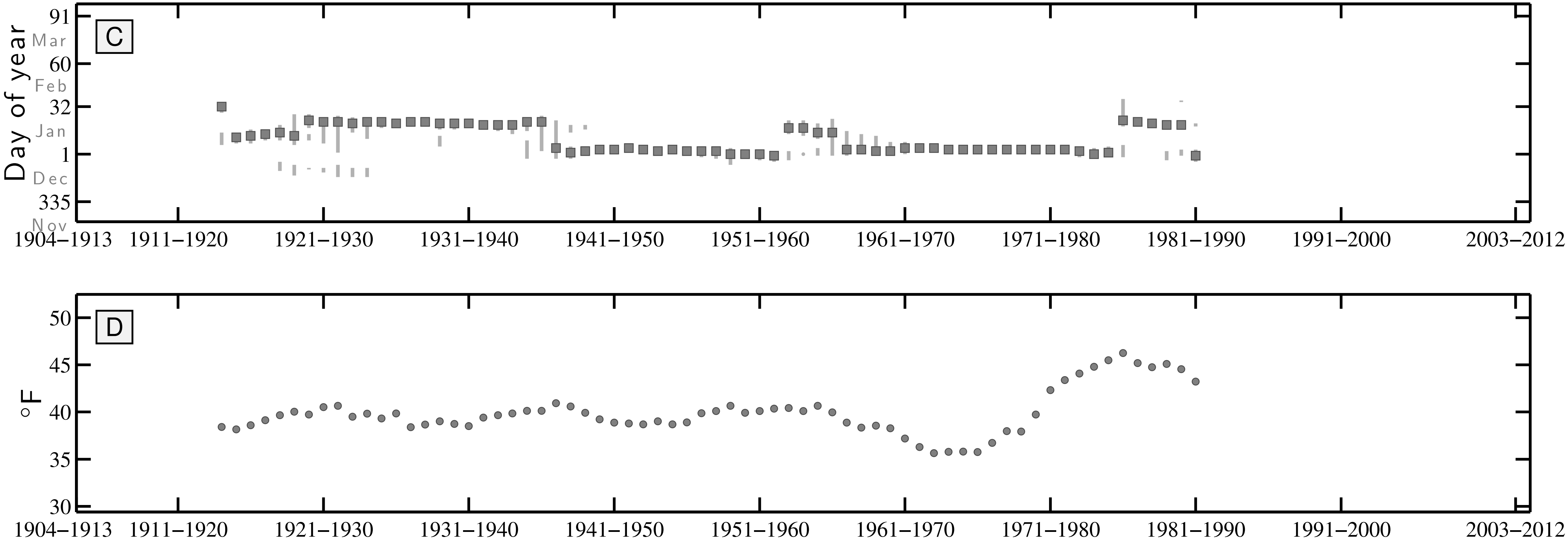

10 year Summer Teletherm dynamics for BUCKEYE, AZ:

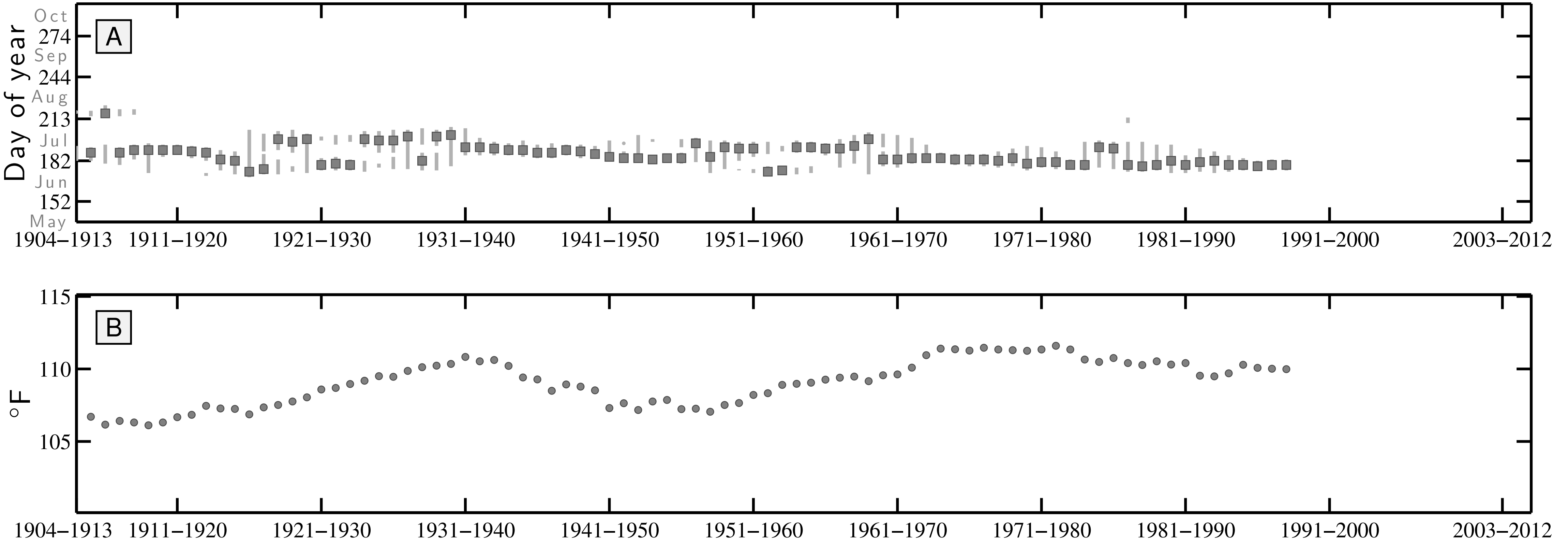

10 year Winter Teletherm dynamics for BUCKEYE, AZ:

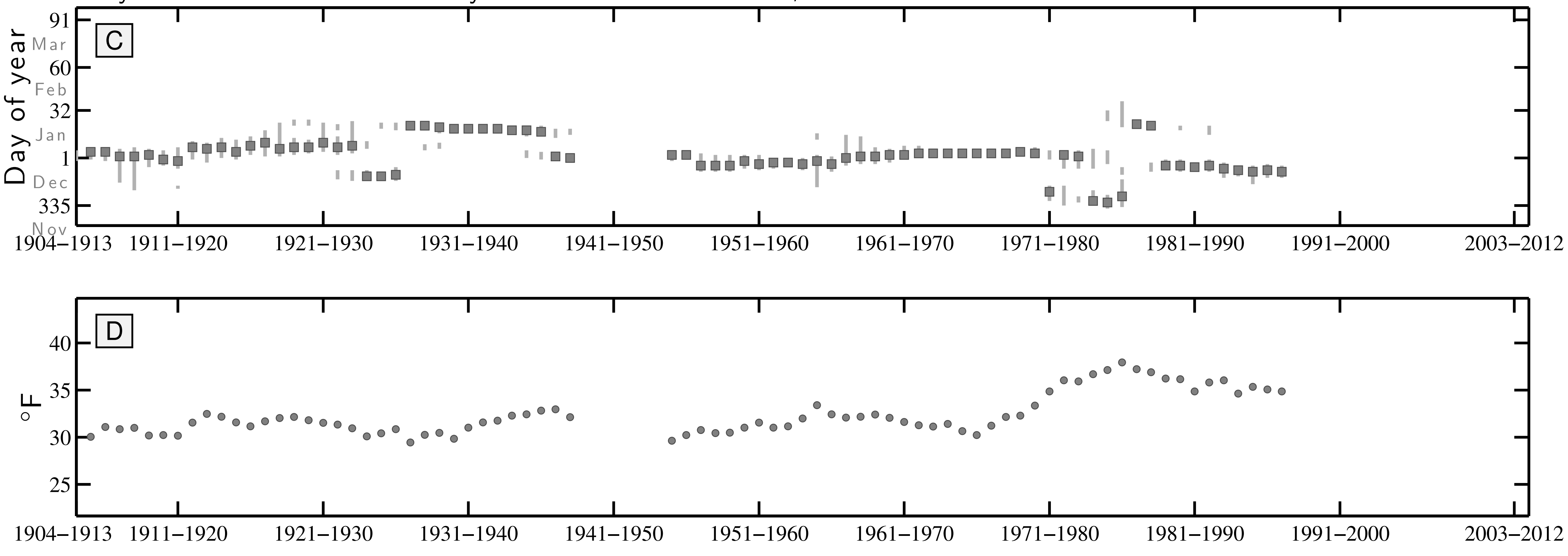

10 year Summer Teletherm dynamics for CANYON DE CHELLY, AZ:

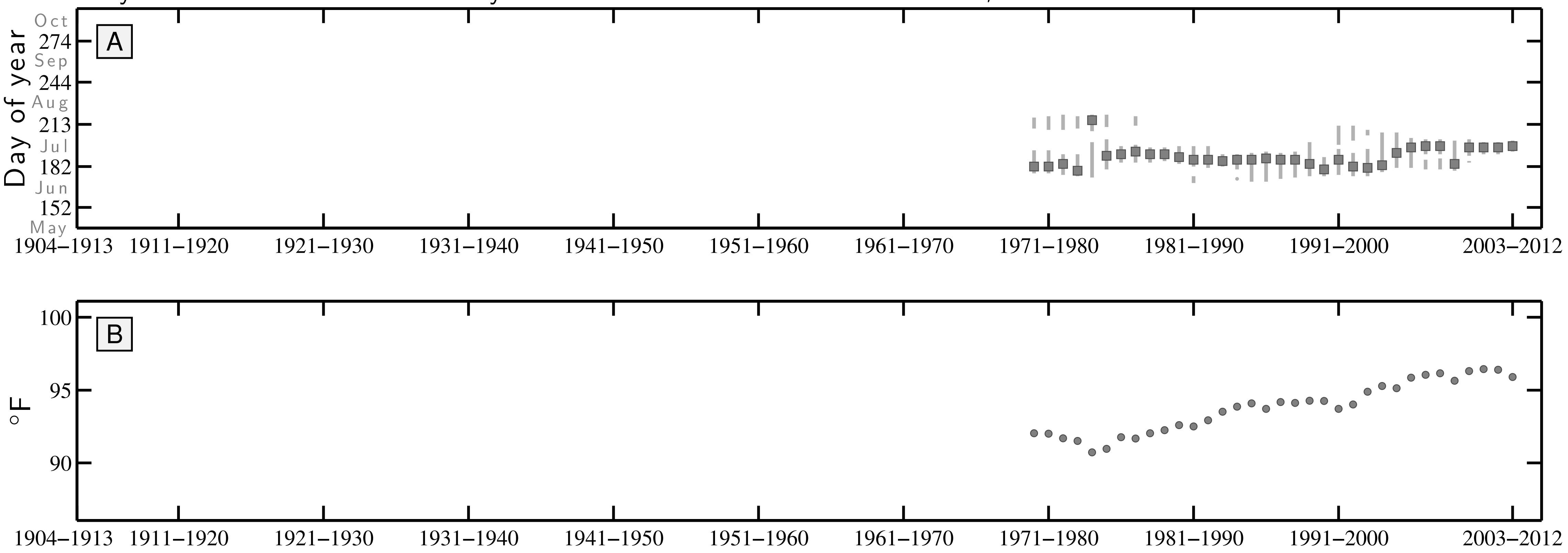

10 year Winter Teletherm dynamics for CANYON DE CHELLY, AZ:

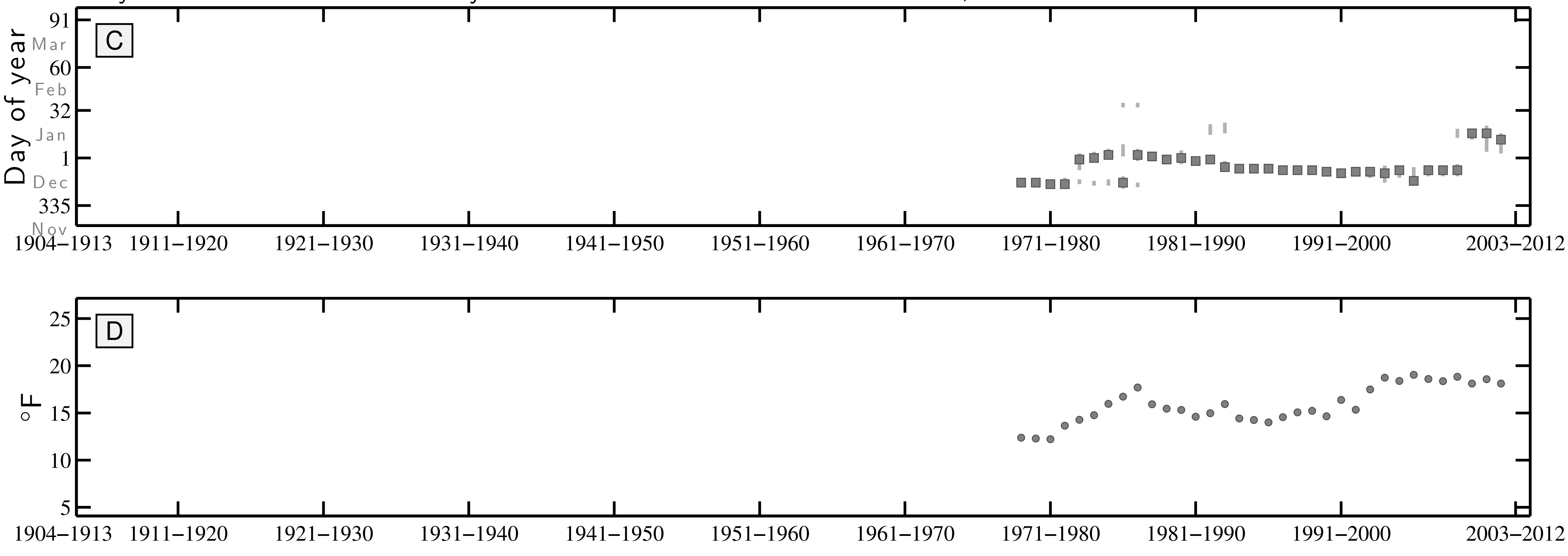

10 year Summer Teletherm dynamics for CHANDLER HEIGHTS, AZ:

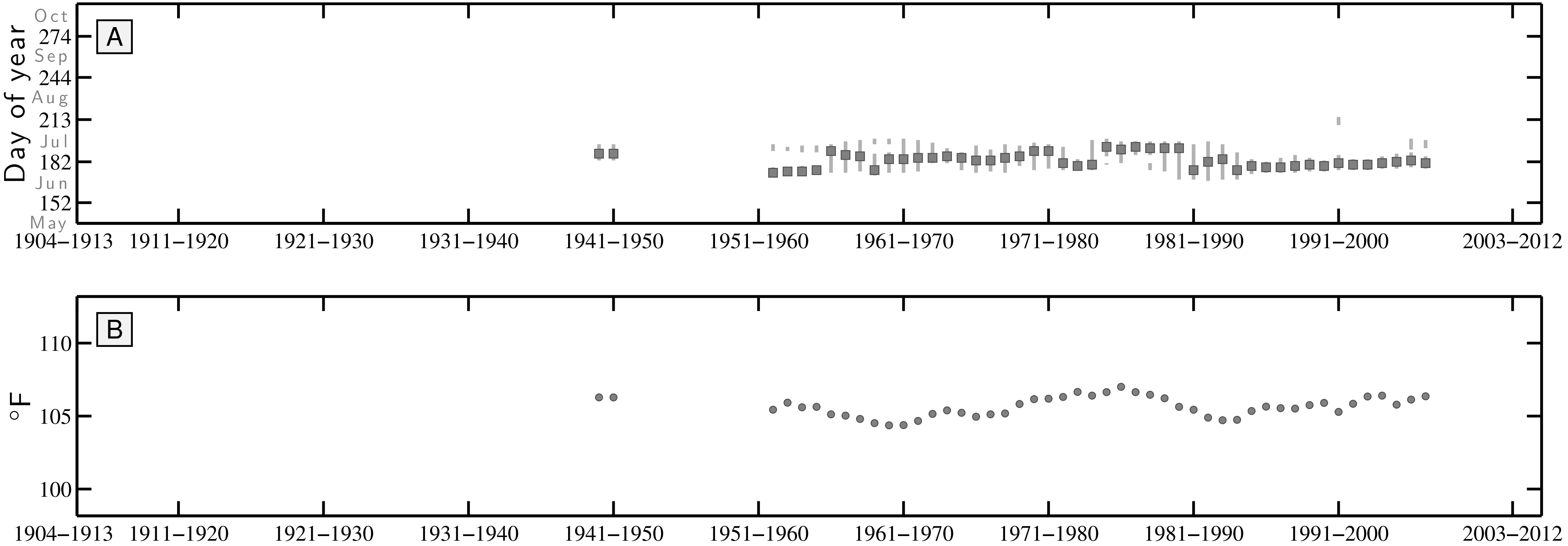

10 year Winter Teletherm dynamics for CHANDLER HEIGHTS, AZ:

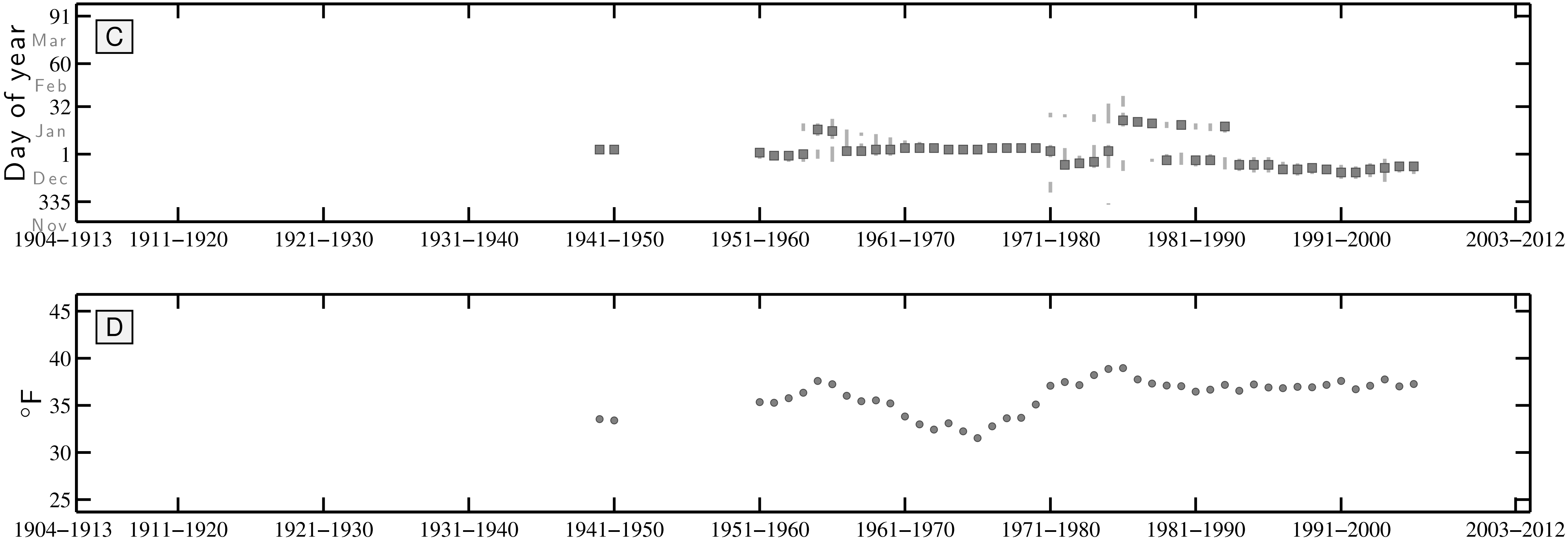

10 year Summer Teletherm dynamics for CHILDS, AZ:

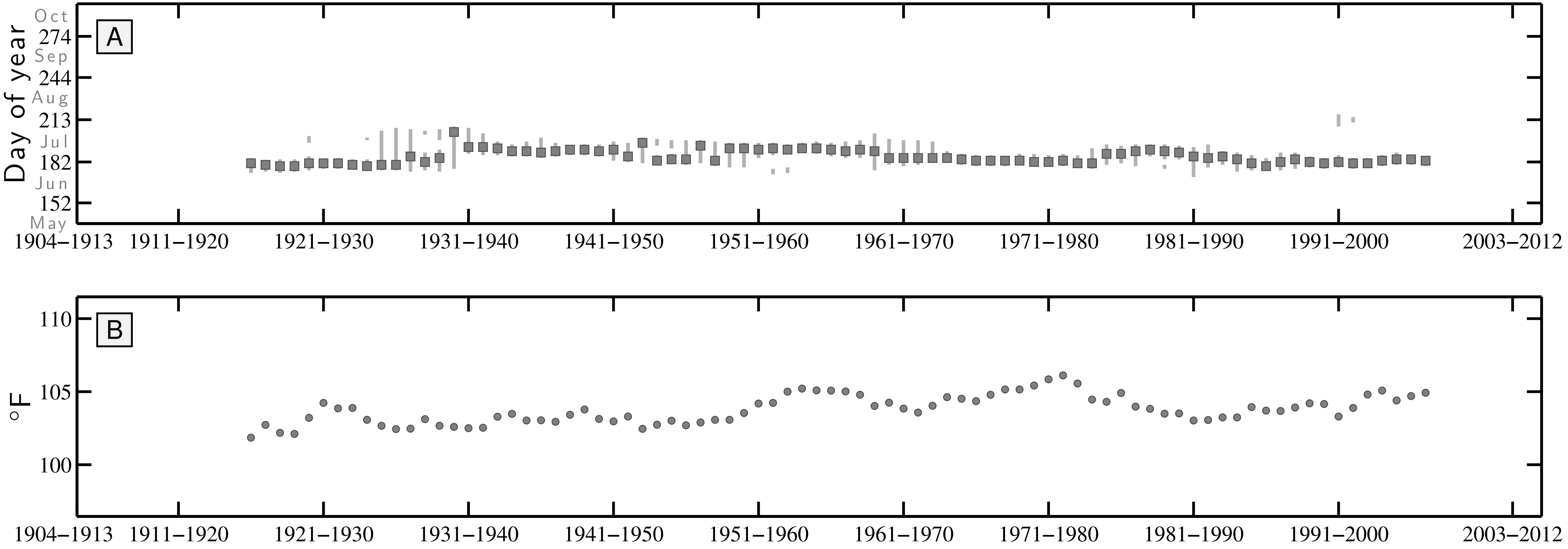

10 year Winter Teletherm dynamics for CHILDS, AZ:

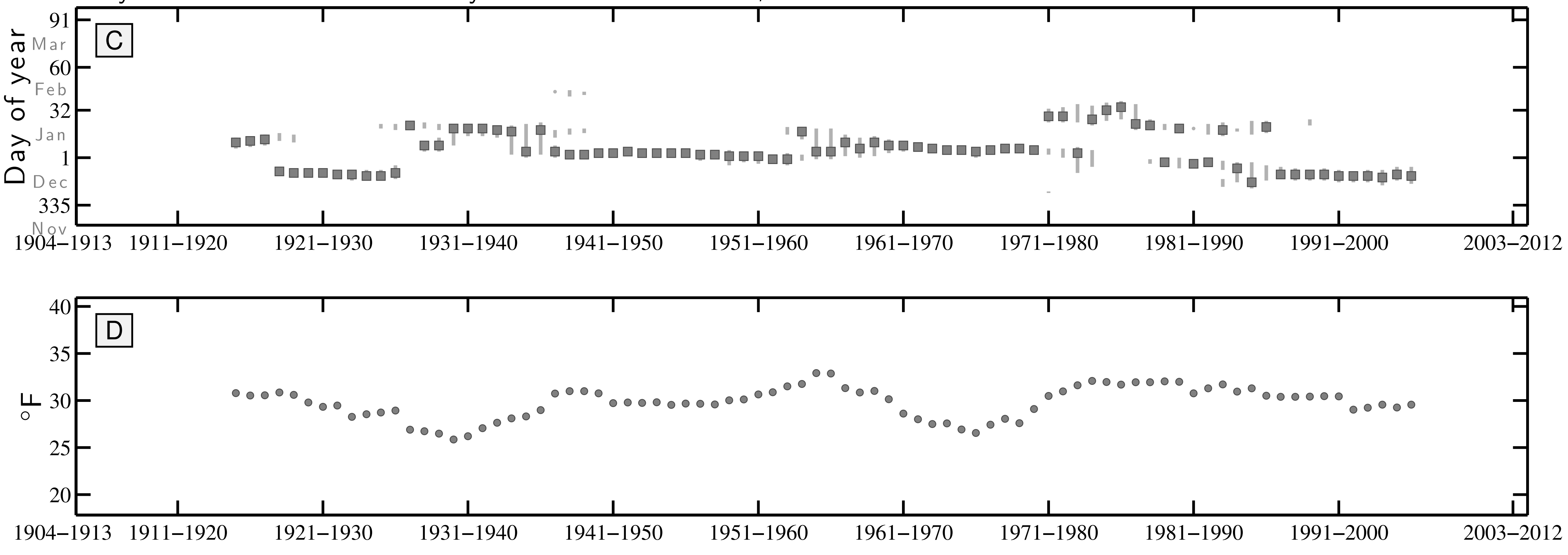

10 year Summer Teletherm dynamics for FT VALLEY, AZ:

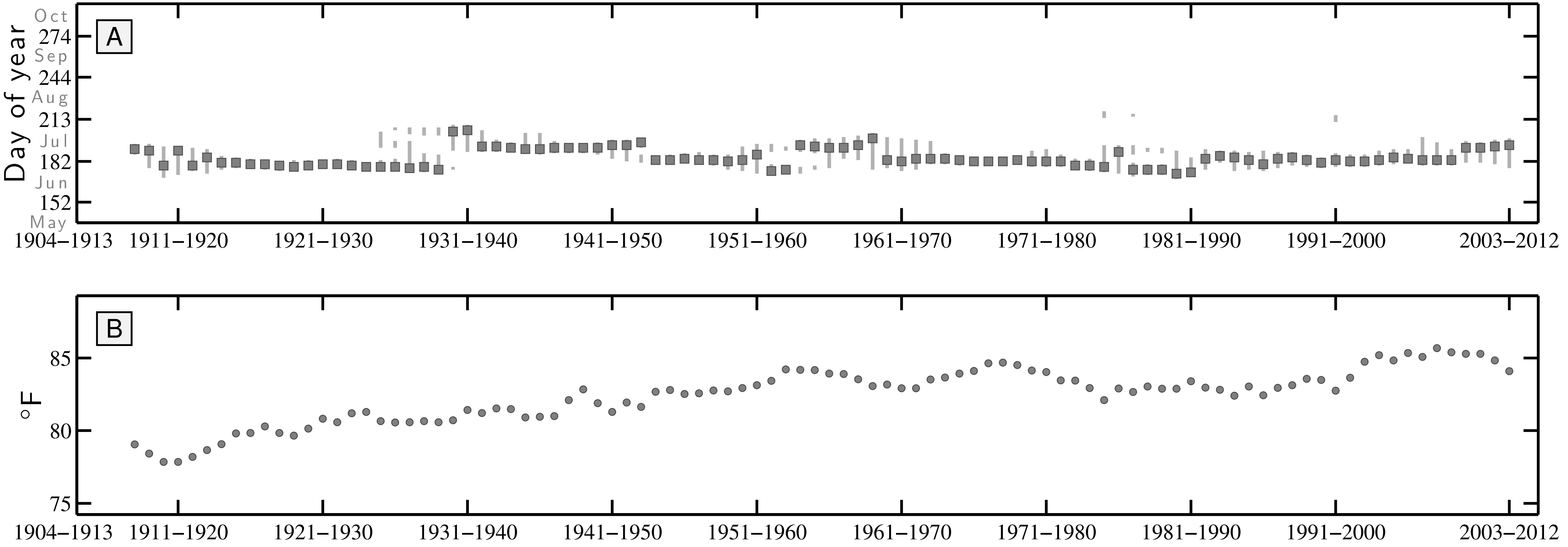

10 year Winter Teletherm dynamics for FT VALLEY, AZ:

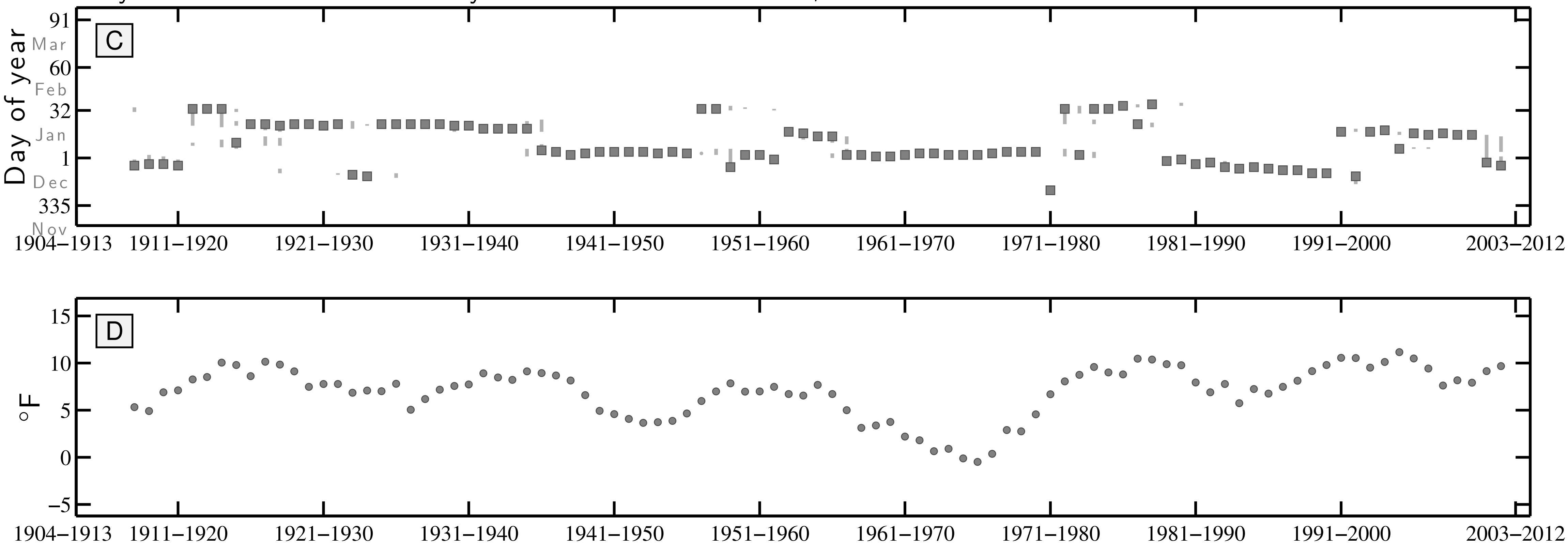

10 year Summer Teletherm dynamics for GRAND CANYON NP 2, AZ:

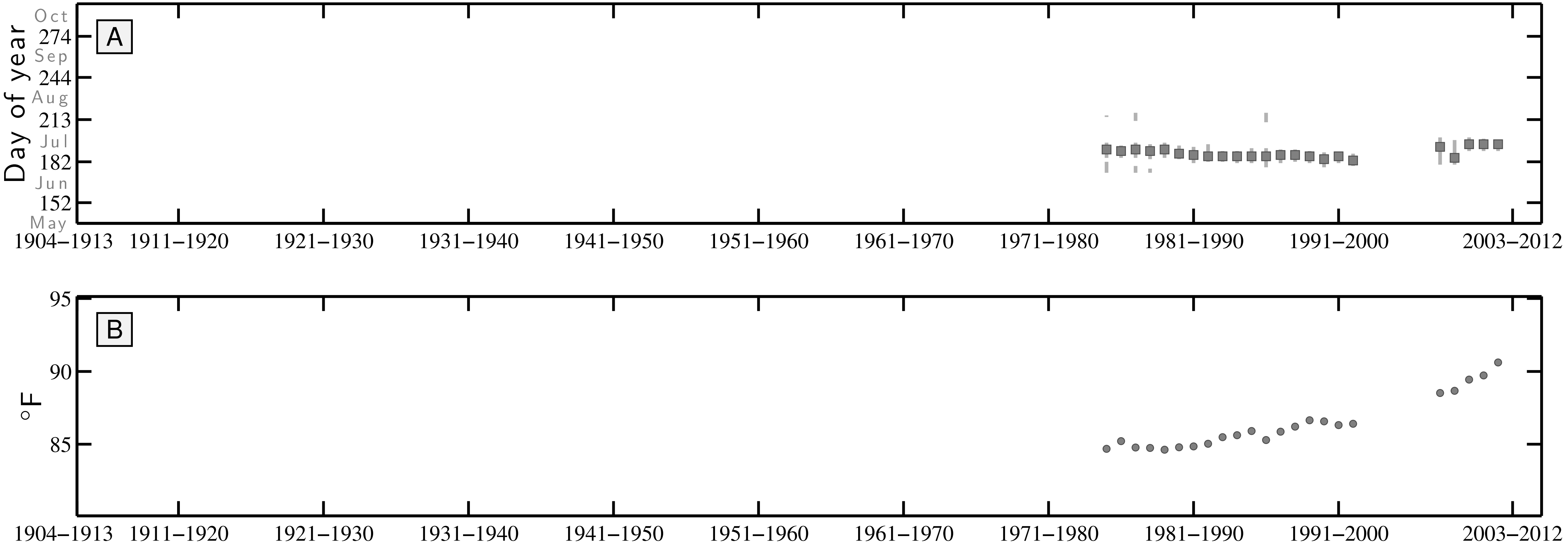

10 year Winter Teletherm dynamics for GRAND CANYON NP 2, AZ:

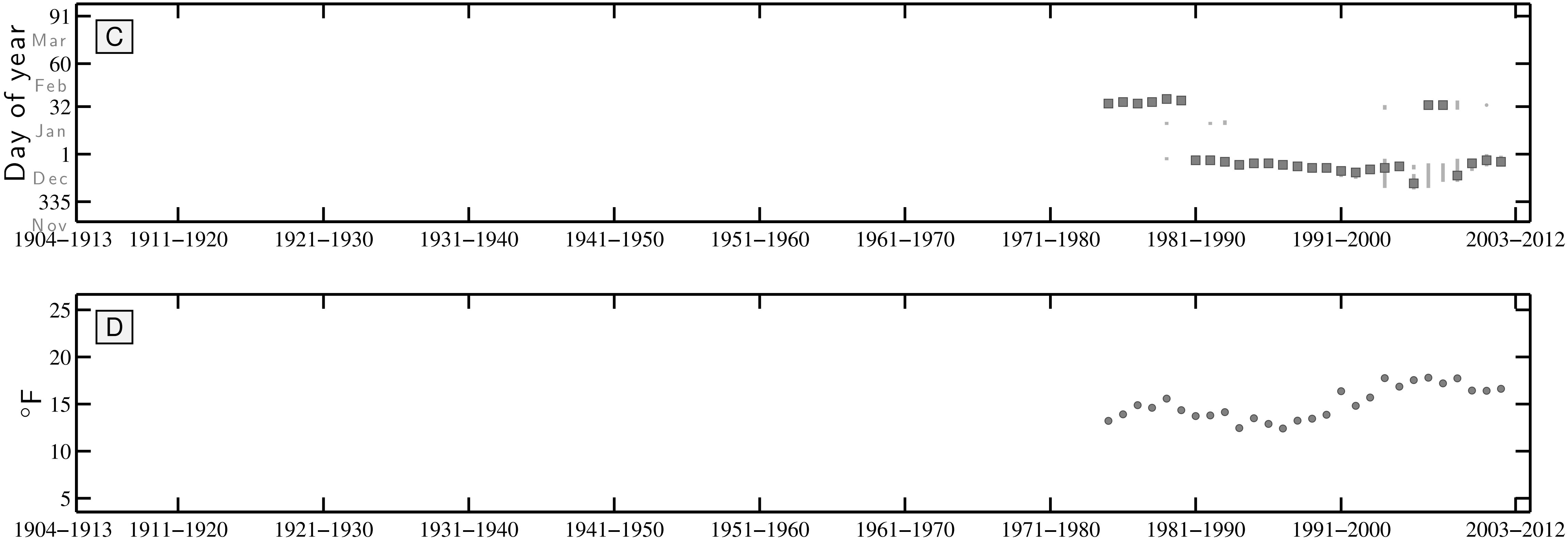

10 year Summer Teletherm dynamics for HOLBROOK, AZ:

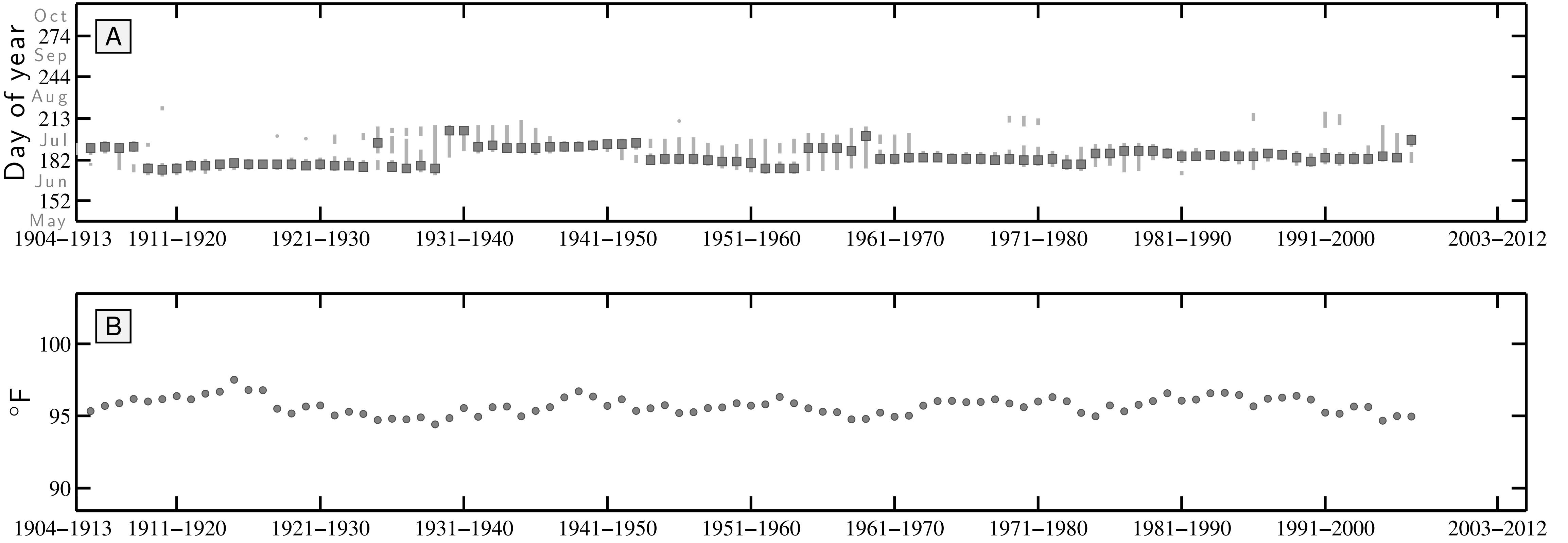

10 year Winter Teletherm dynamics for HOLBROOK, AZ:

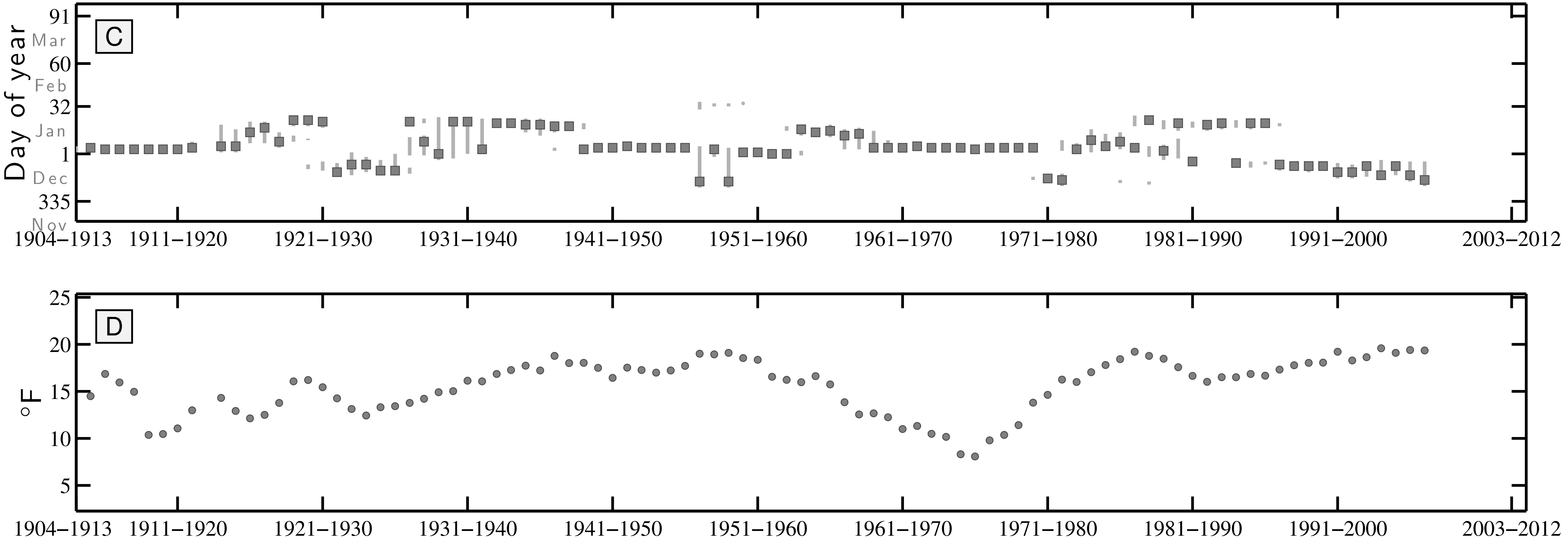

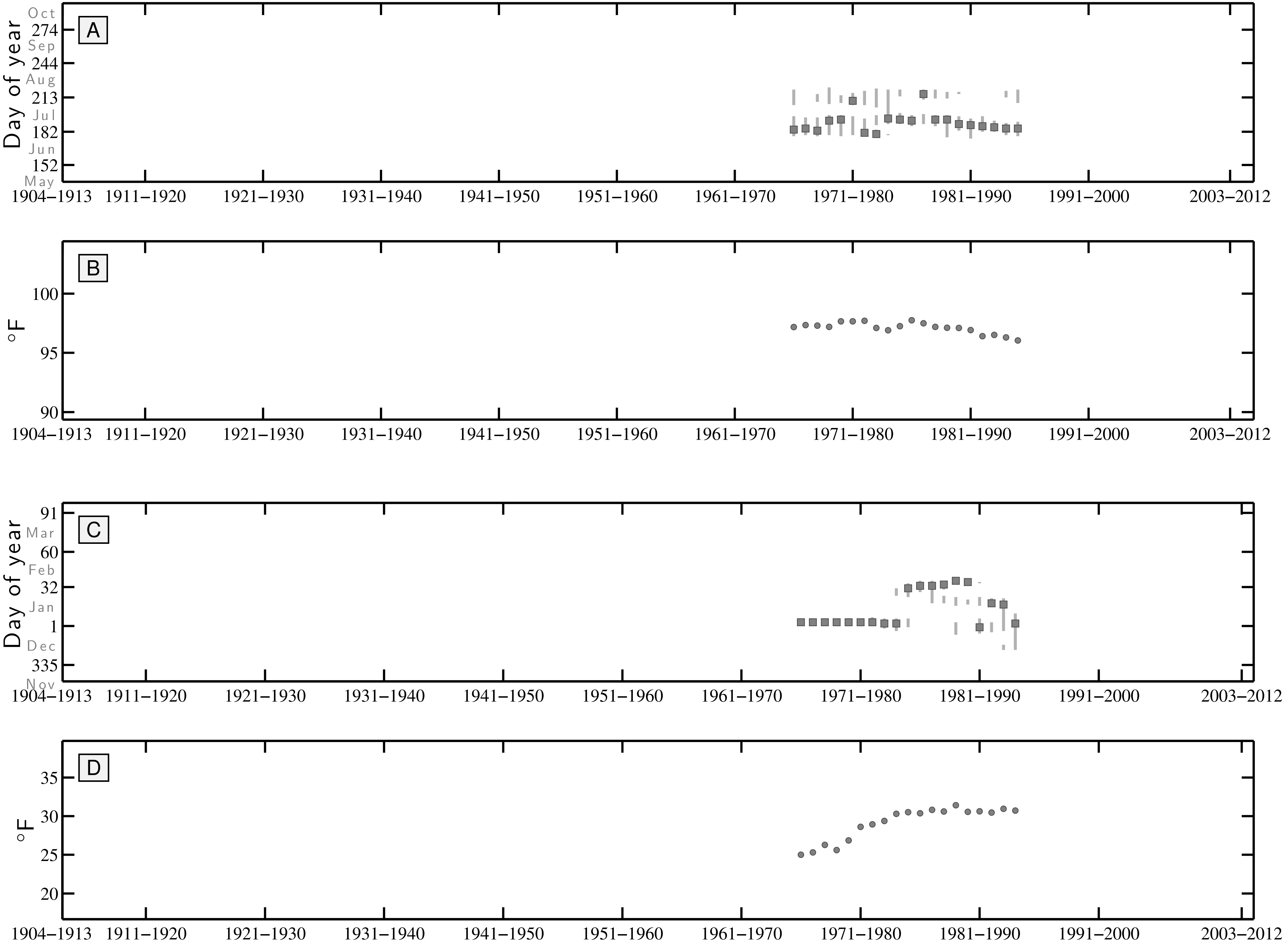
A
Day of year
Oct
274
Sep
244
Aug
213
Jul
182
Jun
152
May
1904–1913
1911–1920
1921–1930
1931–1940
1941–1950
1951–1960
1961–1970
1971–1980
1981–1990
1991–2000
2003–2012
B
°F
100
95
90
C
91
Mar
60
Feb
32
Jan
1
Dec
335
Nov
D
35
30
25
20

10 year Summer Teletherm dynamics for LEES FERRY, AZ:

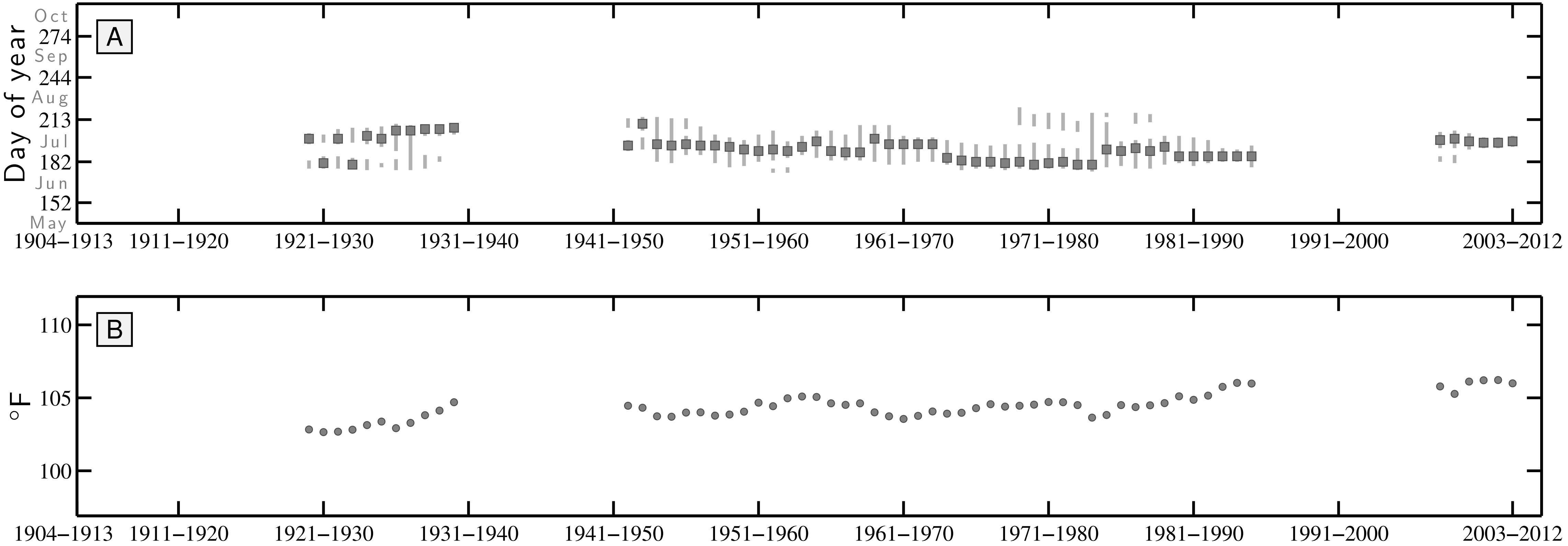

10 year Winter Teletherm dynamics for LEES FERRY, AZ:

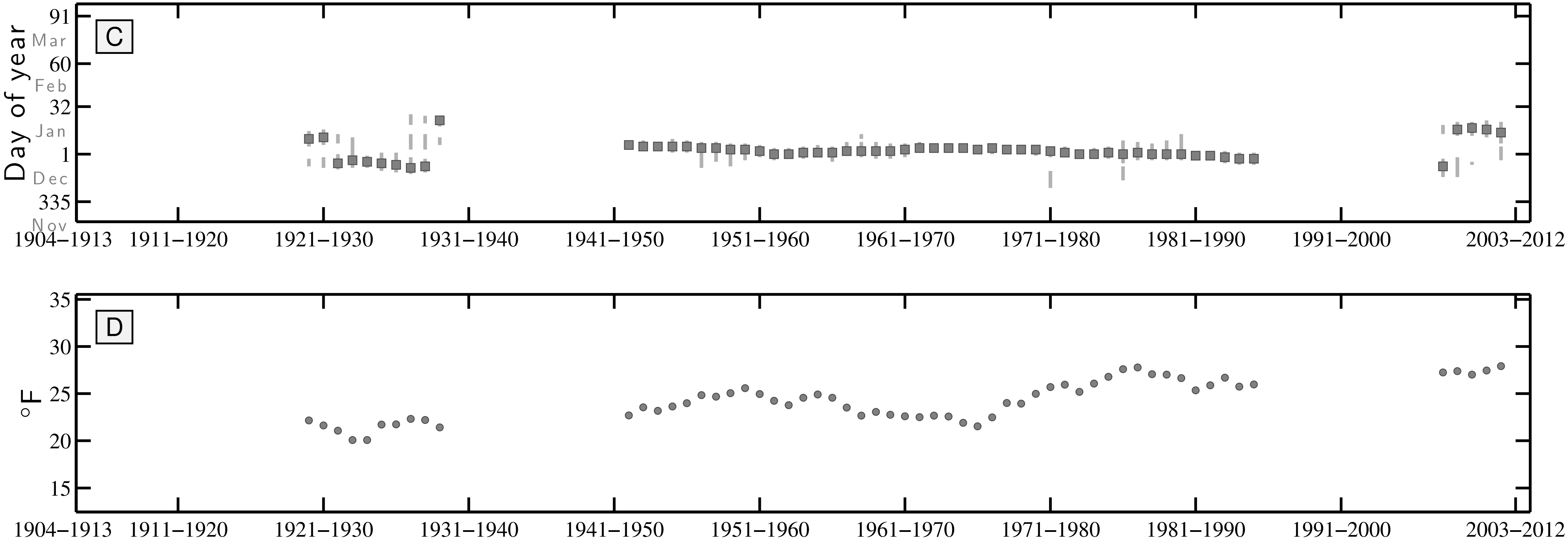

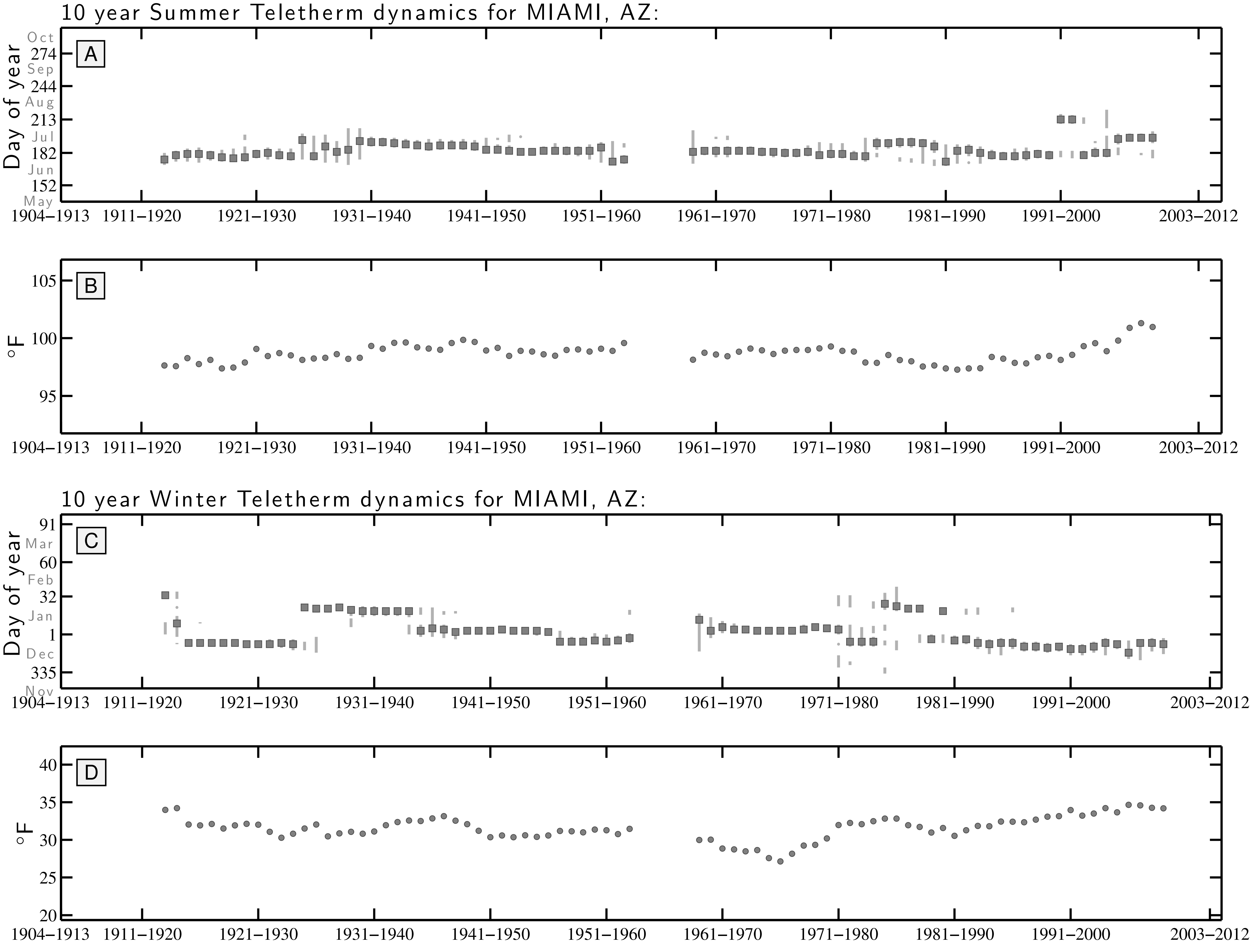
10 year Summer Teletherm dynamics for MIAMI, AZ:
A
Day of year
Oct
274
Sep
244
Aug
213
Jul
182
Jun
152
May
1904–1913
1911–1920
1921–1930
1931–1940
1941–1950
1951–1960
1961–1970
1971–1980
1981–1990
1991–2000
2003–2012
B
°F
105
100
95
10 year Winter Teletherm dynamics for MIAMI, AZ:
C
Day of year
91
Mar
60
Feb
32
Jan
1
Dec
335
Nov
D
°F
40
35
30
25
20

10 year Summer Teletherm dynamics for PARKER, AZ:

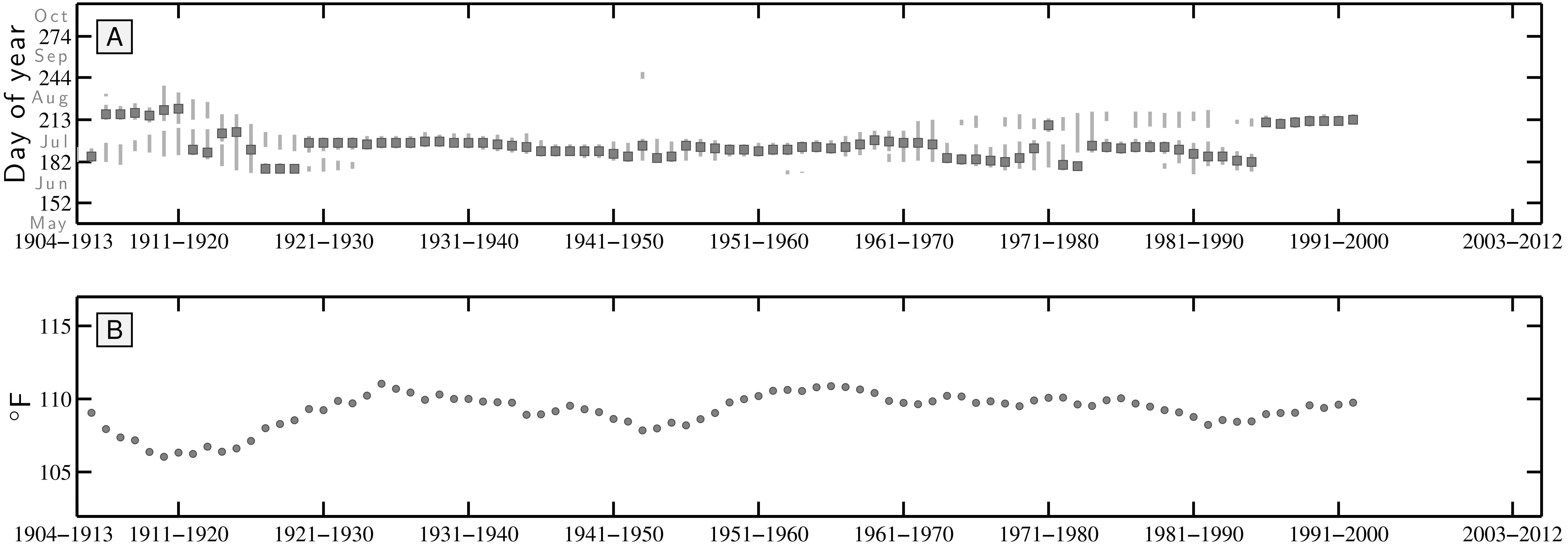

10 year Winter Teletherm dynamics for PARKER, AZ:

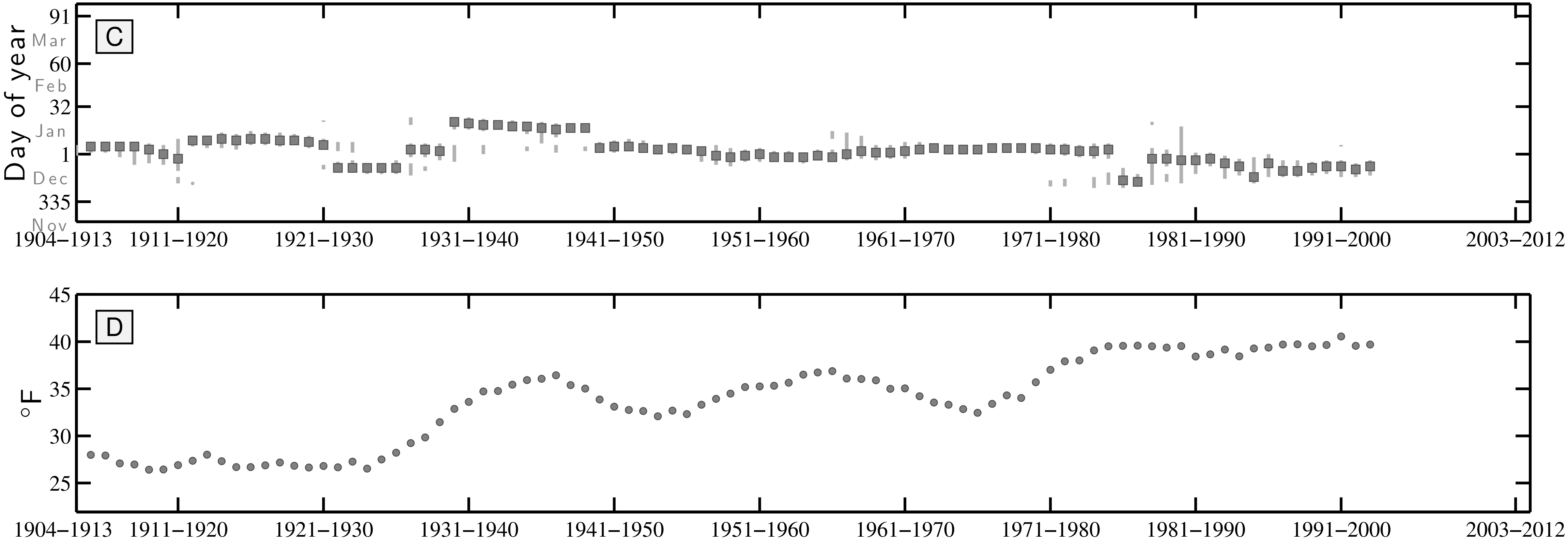

10 year Summer Teletherm dynamics for PEARCE SUNSITES, AZ:

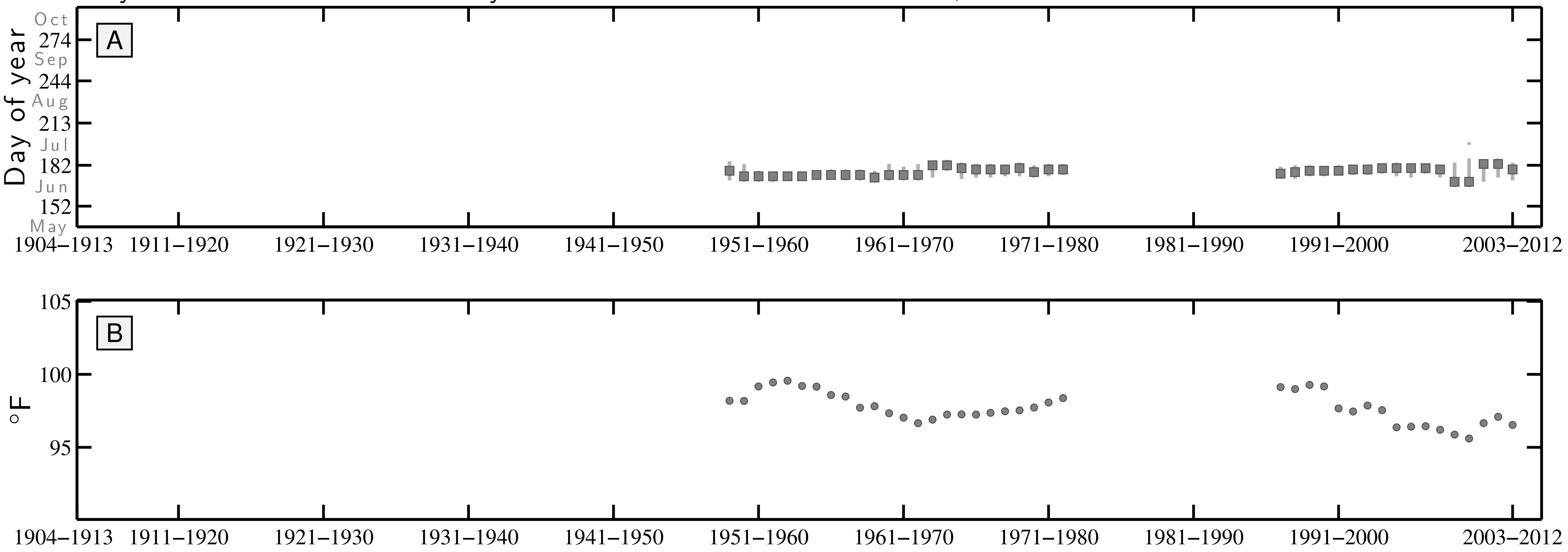

10 year Winter Teletherm dynamics for PEARCE SUNSITES, AZ:

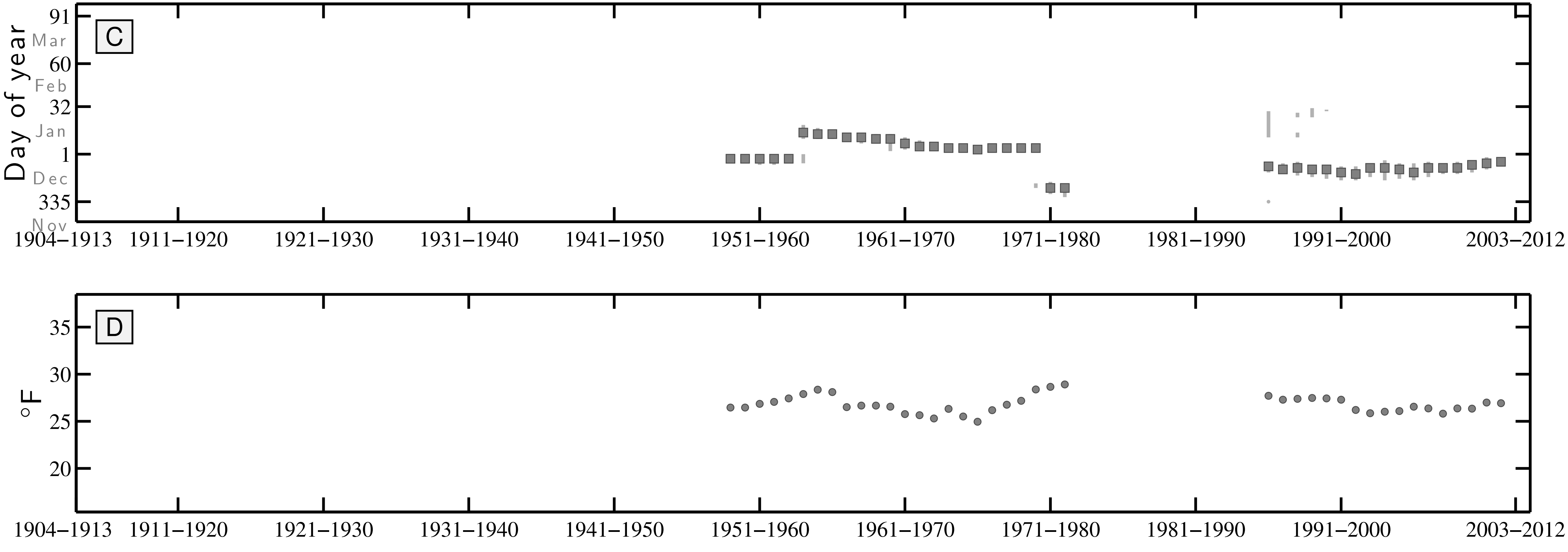

10 year Summer Teletherm dynamics for PRESCOTT, AZ:

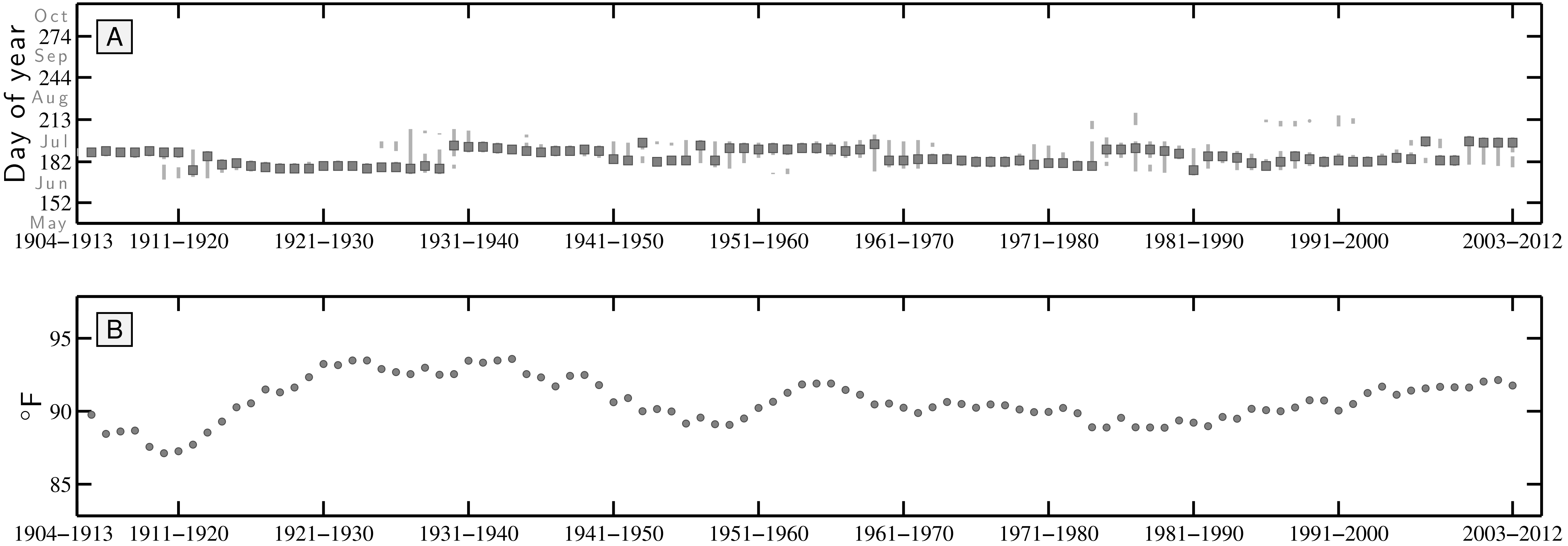

10 year Winter Teletherm dynamics for PRESCOTT, AZ:

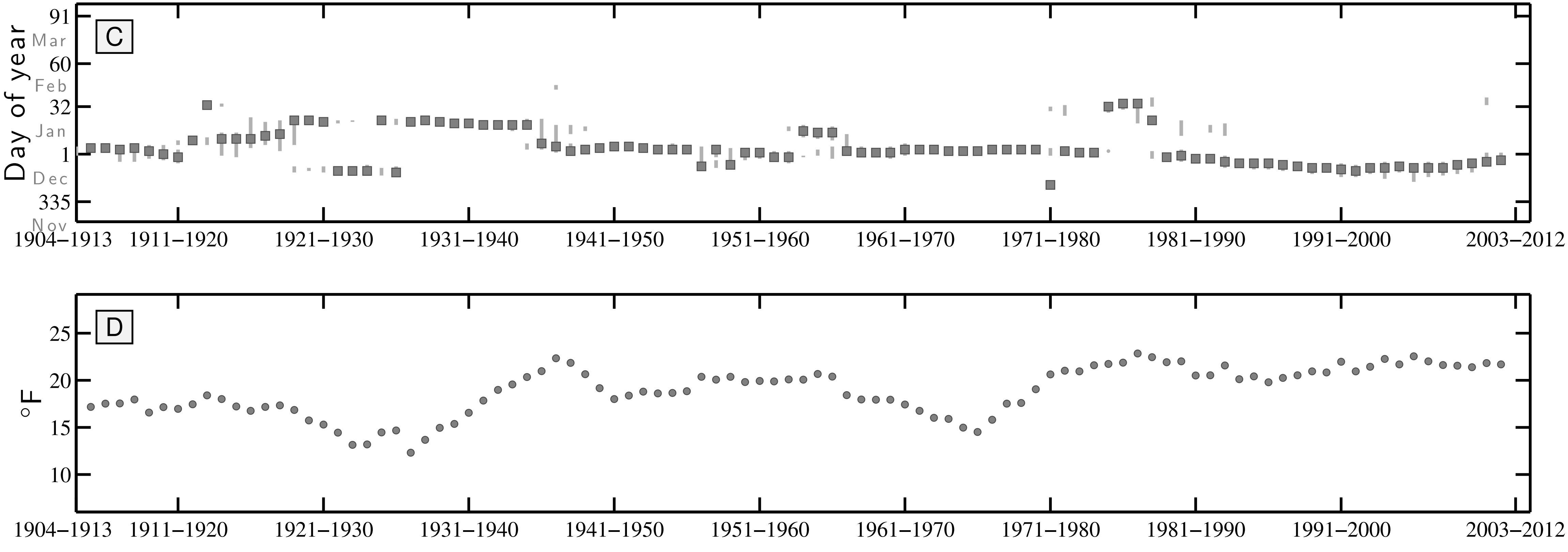

10 year Summer Teletherm dynamics for ROOSEVELT 1 WNW, AZ:

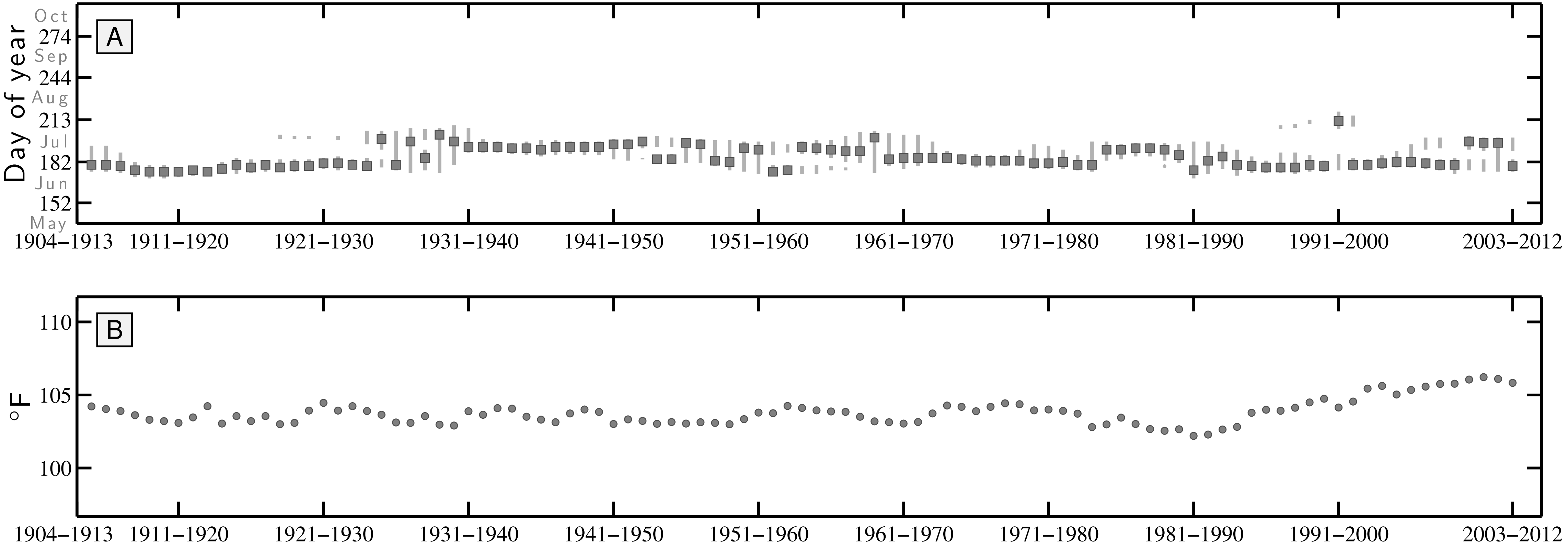

10 year Winter Teletherm dynamics for ROOSEVELT 1 WNW, AZ:

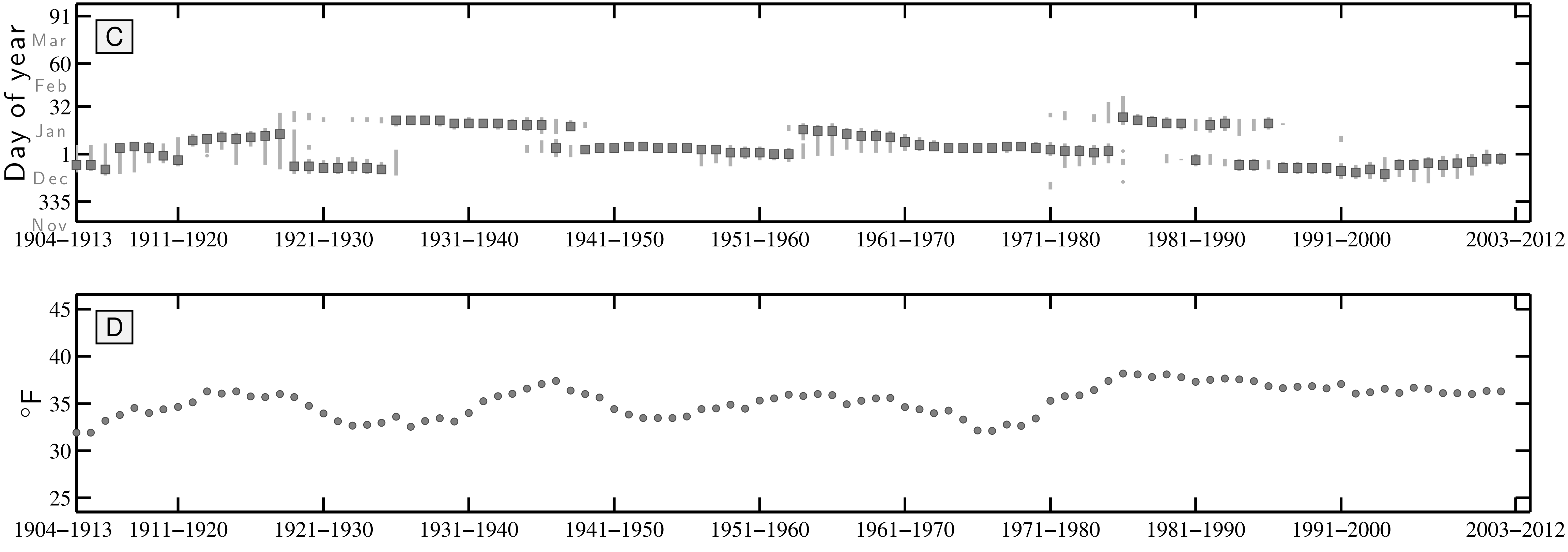

10 year Summer Teletherm dynamics for SACATON, AZ:

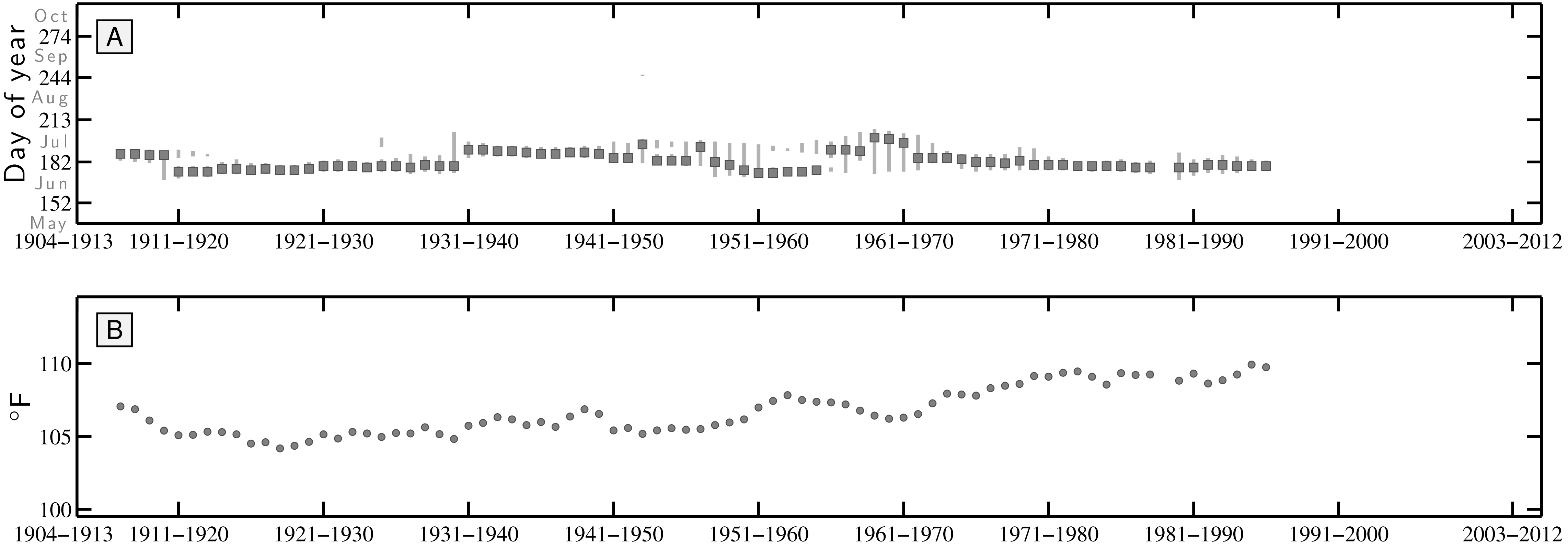

10 year Winter Teletherm dynamics for SACATON, AZ:

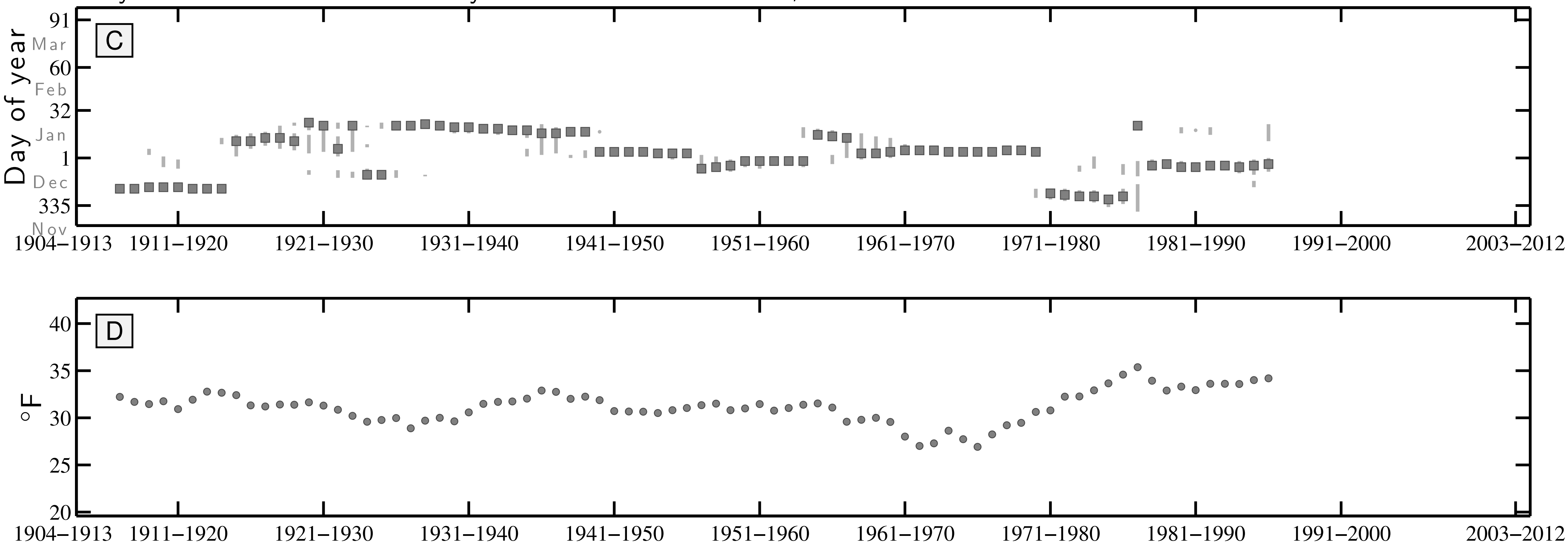

10 year Summer Teletherm dynamics for SAFFORD AGRICULTRL CTR, AZ:

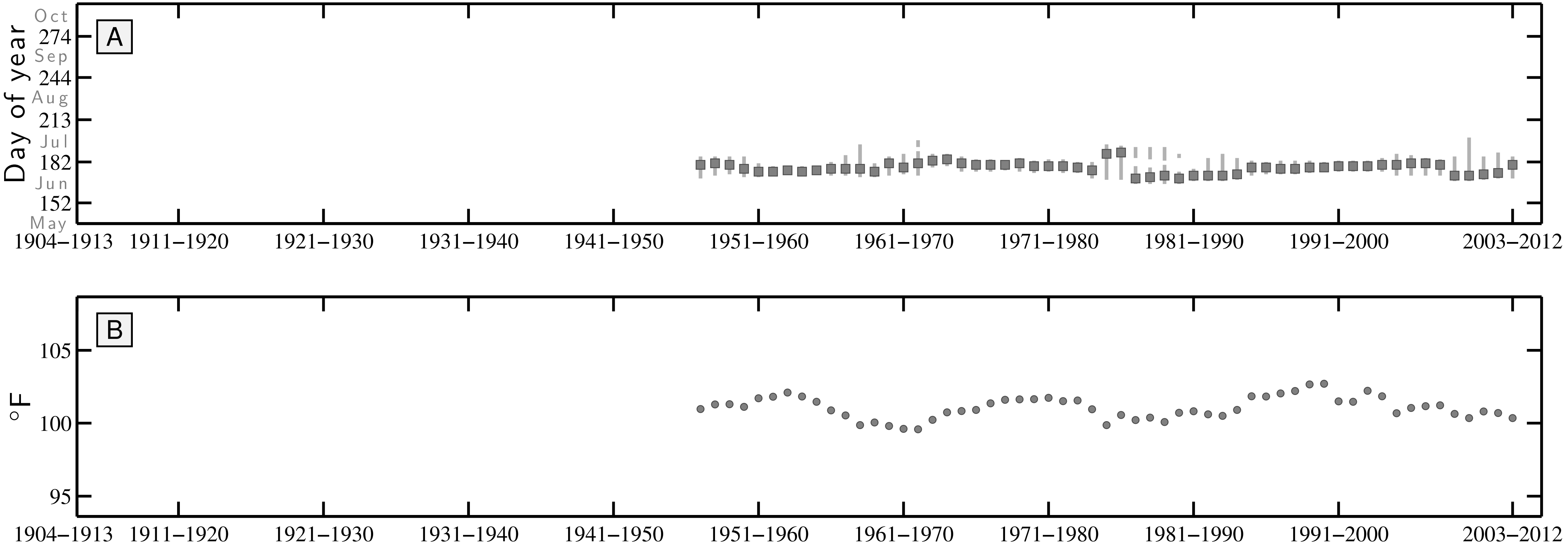

10 year Winter Teletherm dynamics for SAFFORD AGRICULTRL CTR, AZ:

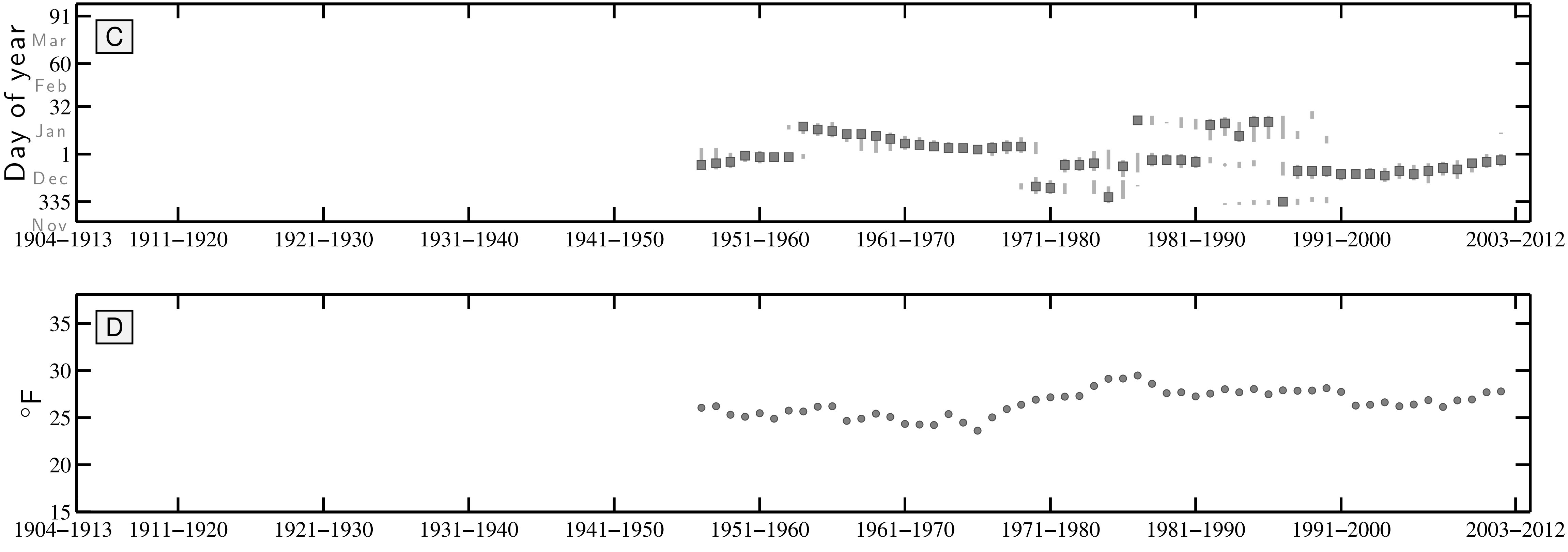

10 year Summer Teletherm dynamics for SAINT JOHNS, AZ:

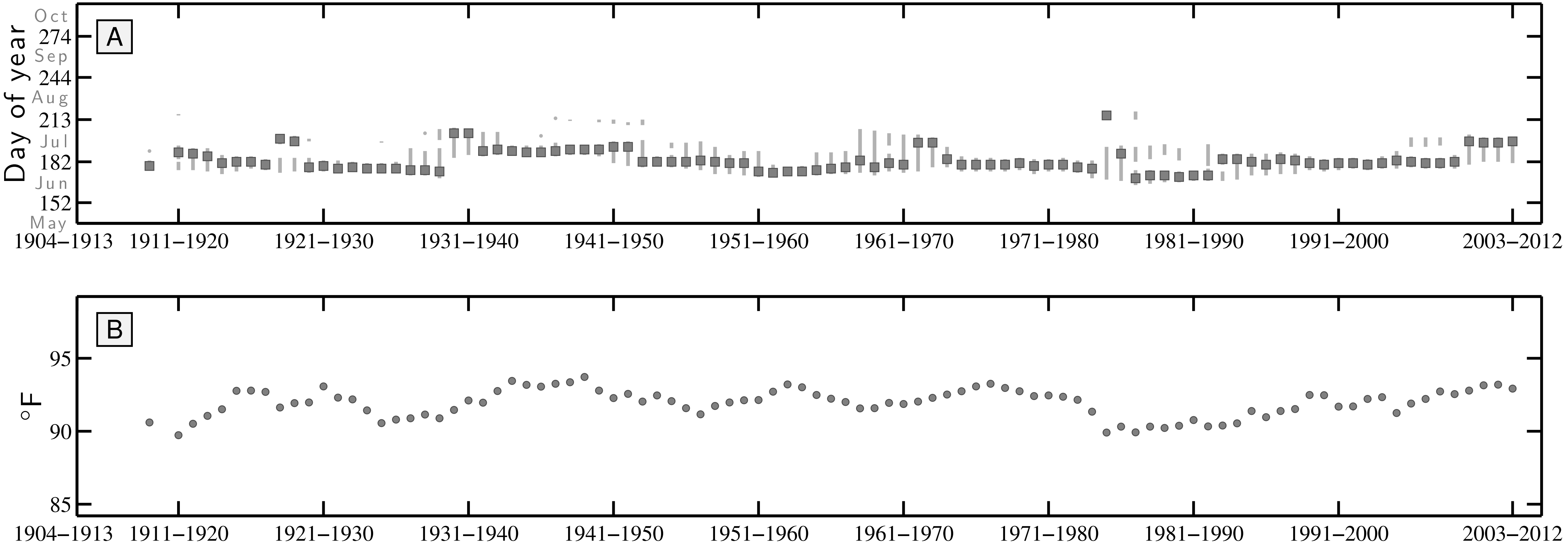

10 year Winter Teletherm dynamics for SAINT JOHNS, AZ:

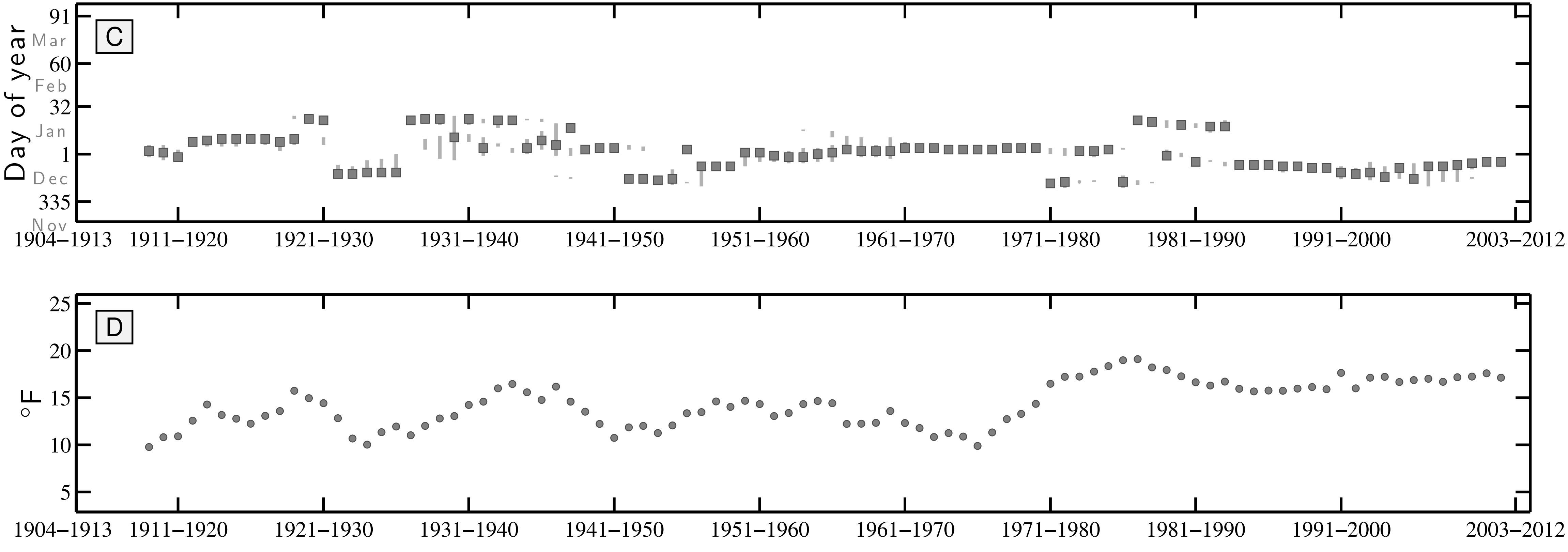

10 year Summer Teletherm dynamics for SELIGMAN, AZ:

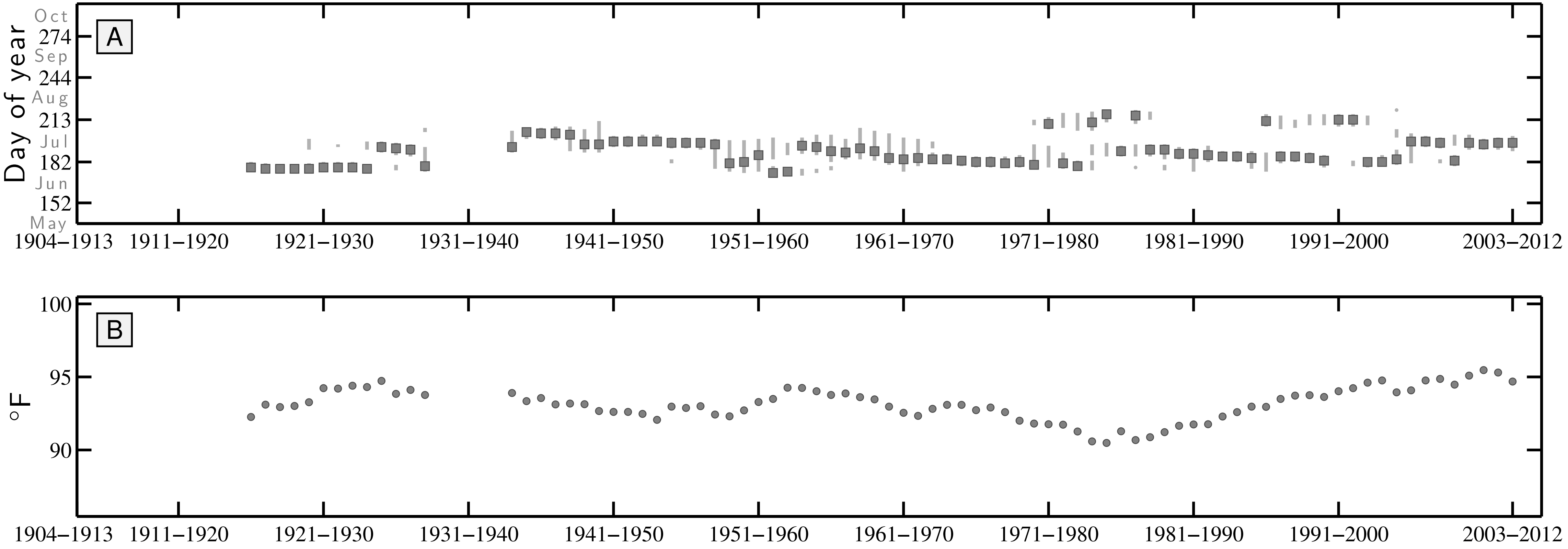

10 year Winter Teletherm dynamics for SELIGMAN, AZ:

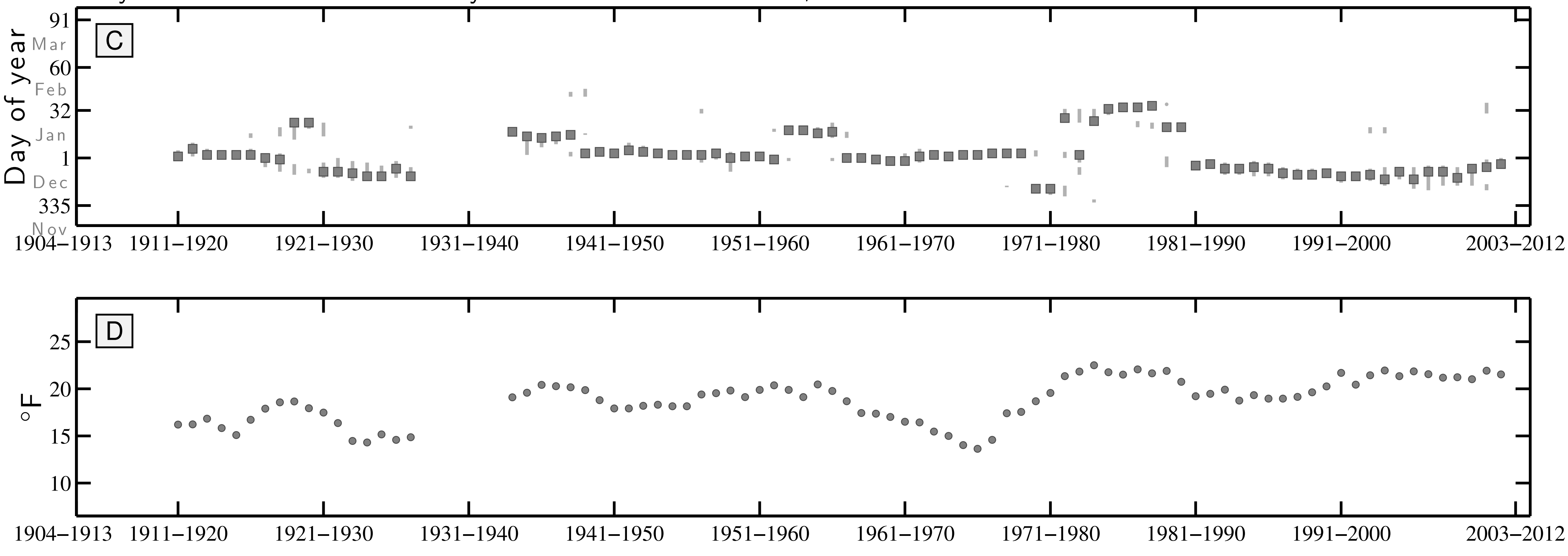

10 year Summer Teletherm dynamics for TOMBSTONE, AZ:

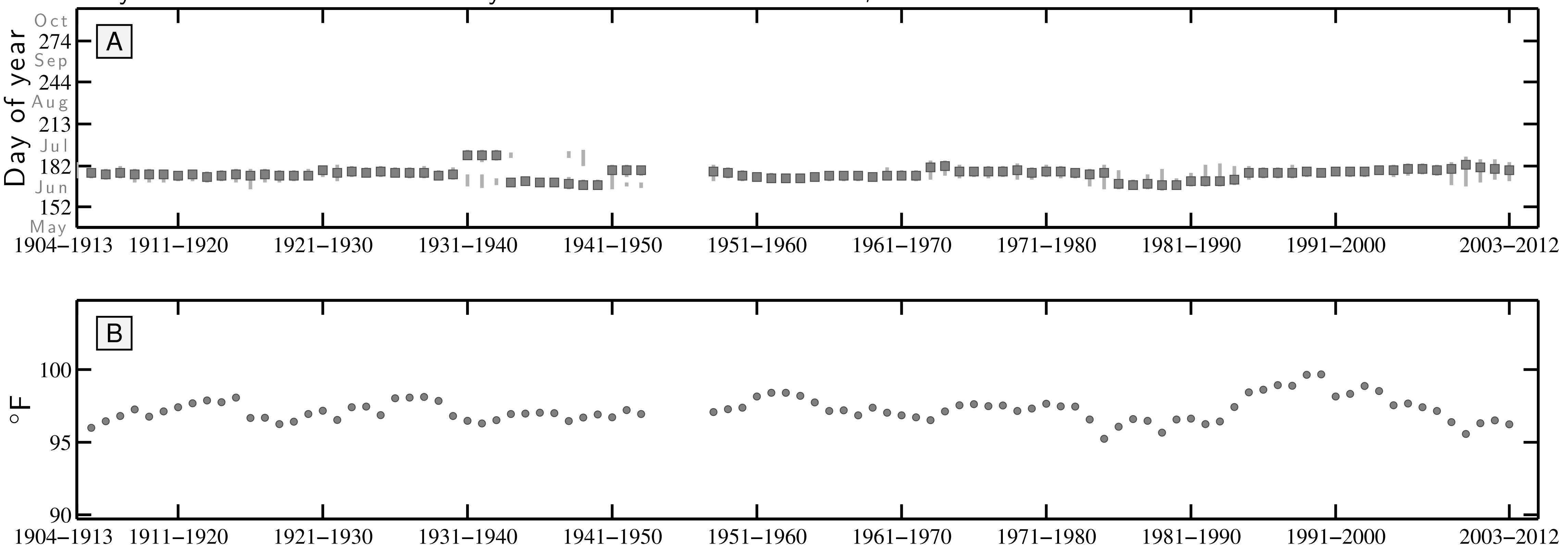

10 year Winter Teletherm dynamics for TOMBSTONE, AZ:

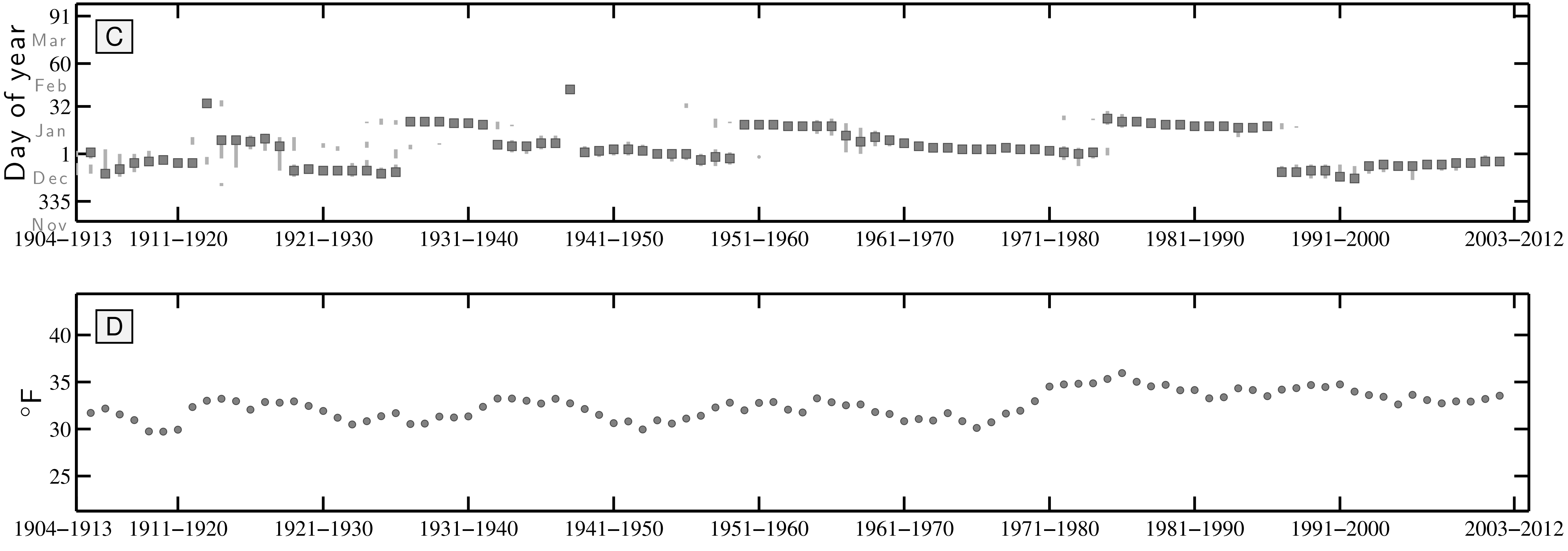

10 year Summer Teletherm dynamics for TUCSON WFO, AZ:

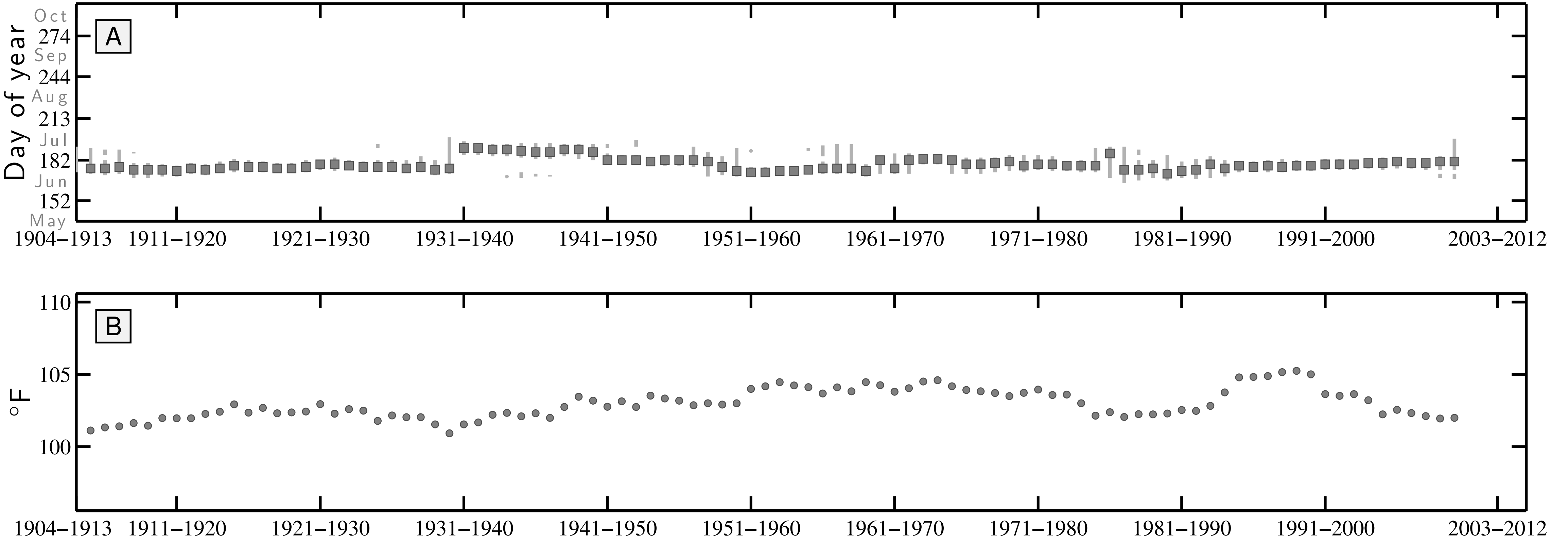

10 year Winter Teletherm dynamics for TUCSON WFO, AZ:

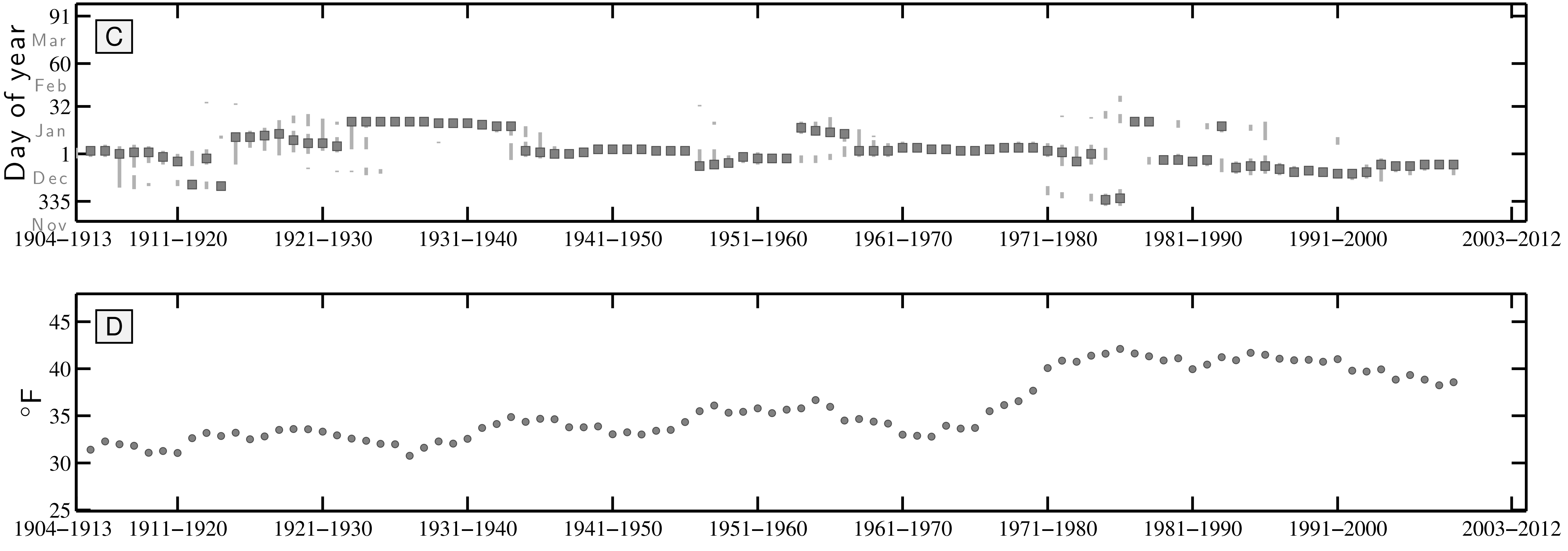

10 year Summer Teletherm dynamics for WHITERIVER 1 SW, AZ:

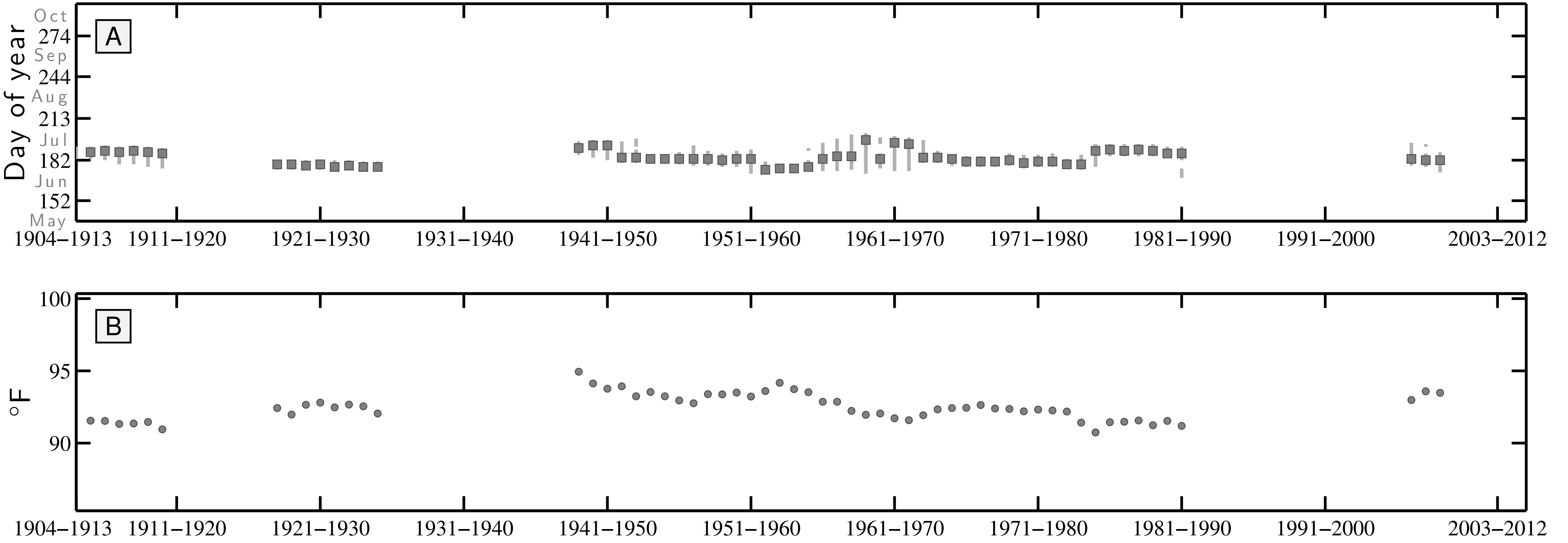

10 year Winter Teletherm dynamics for WHITERIVER 1 SW, AZ:

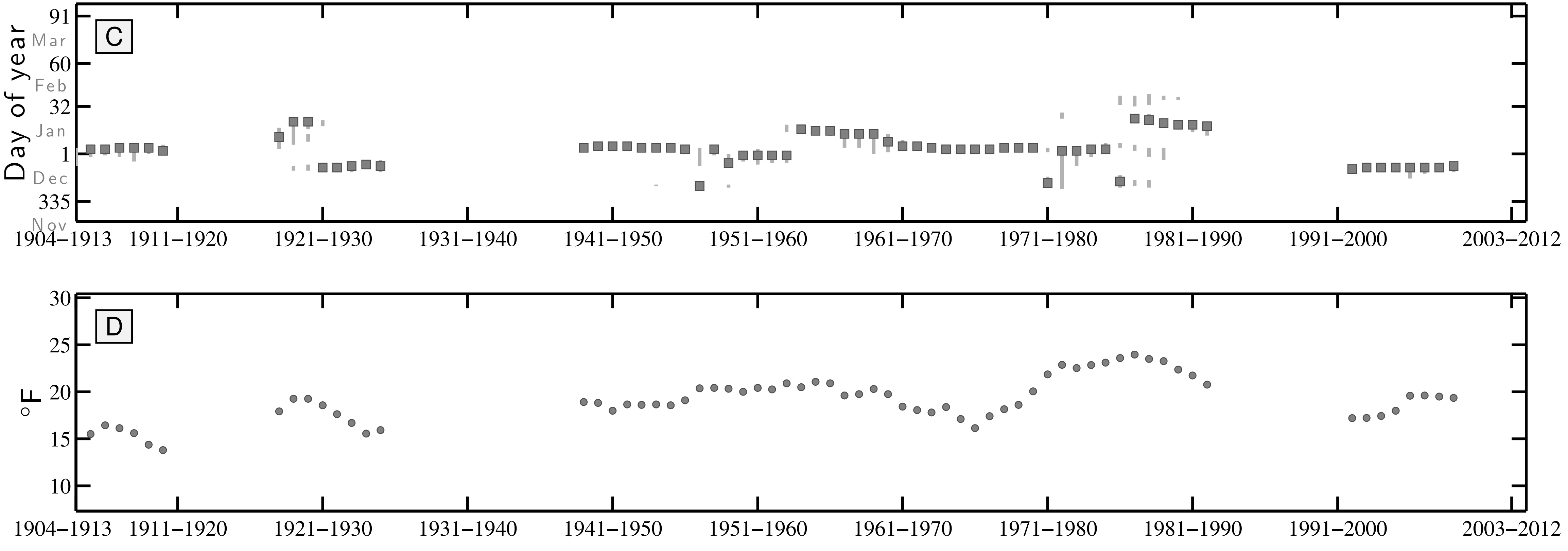

10 year Summer Teletherm dynamics for WICKENBURG, AZ:

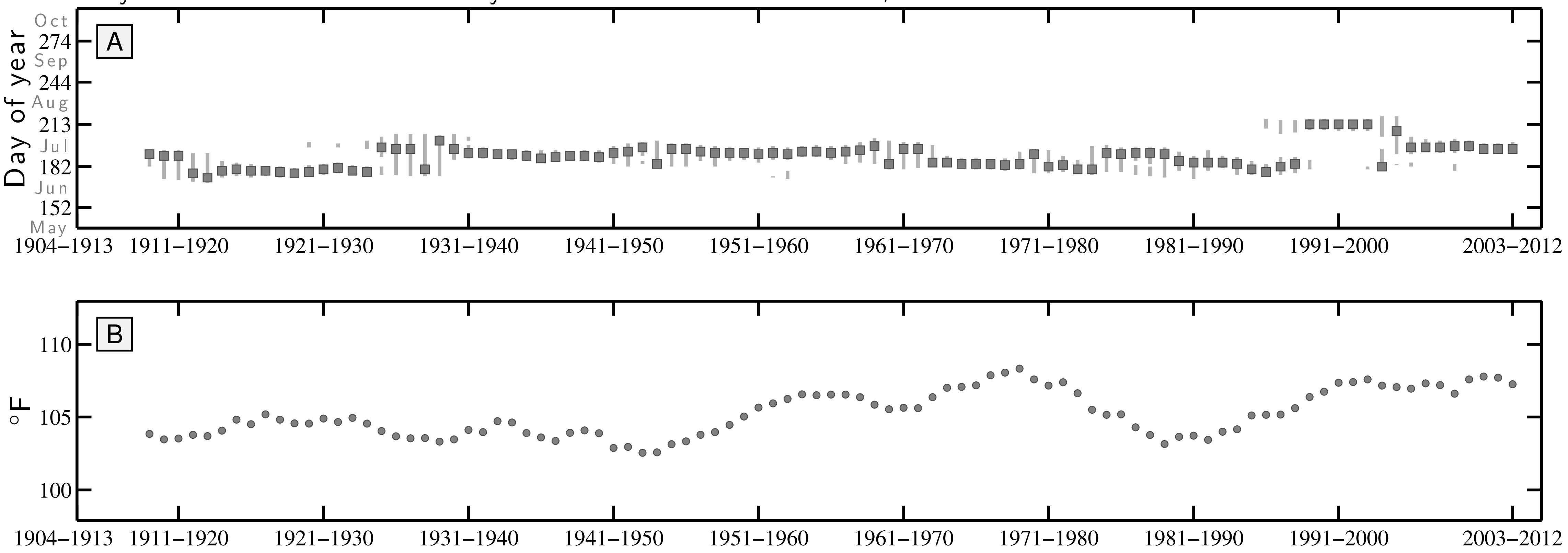

10 year Winter Teletherm dynamics for WICKENBURG, AZ:

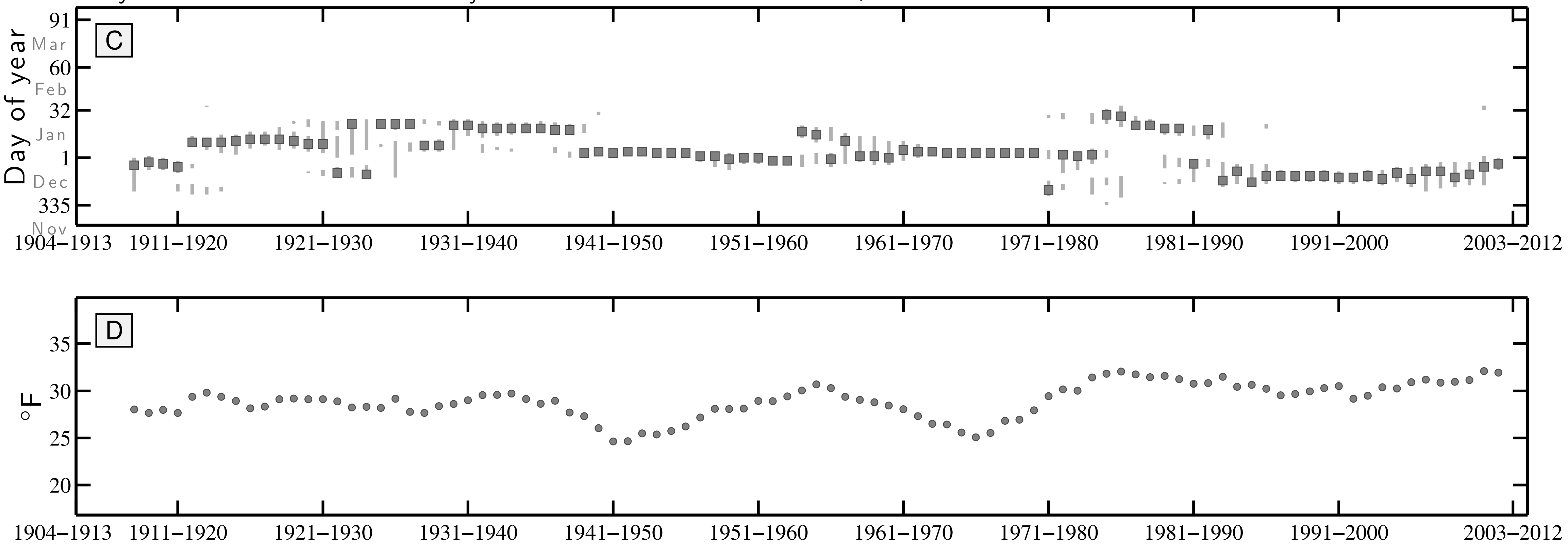

10 year Summer Teletherm dynamics for WILLIAMS, AZ:

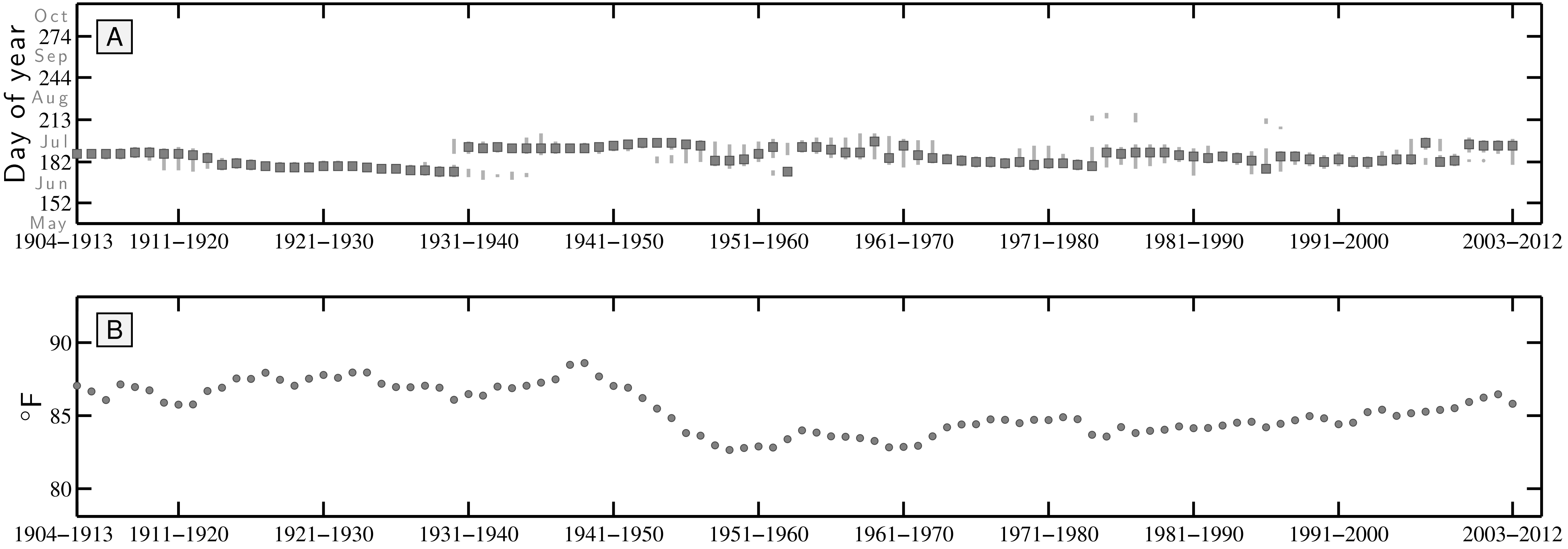

10 year Winter Teletherm dynamics for WILLIAMS, AZ:

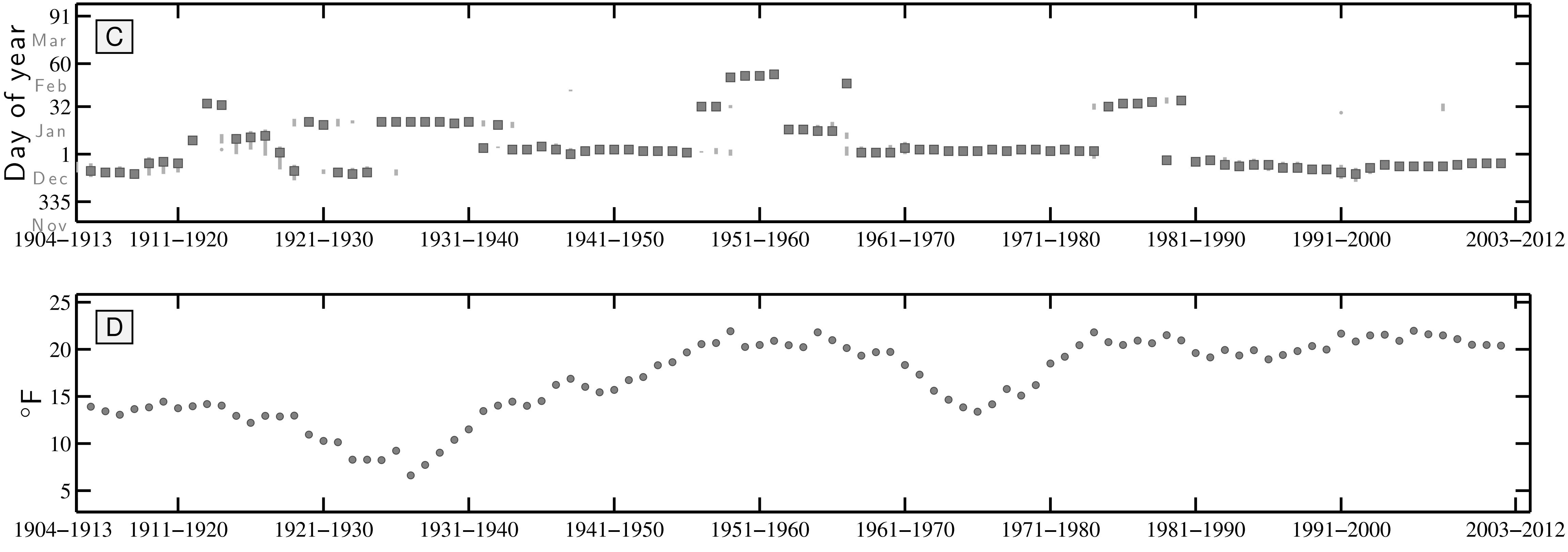

10 year Summer Teletherm dynamics for YUMA CITRUS STN, AZ:

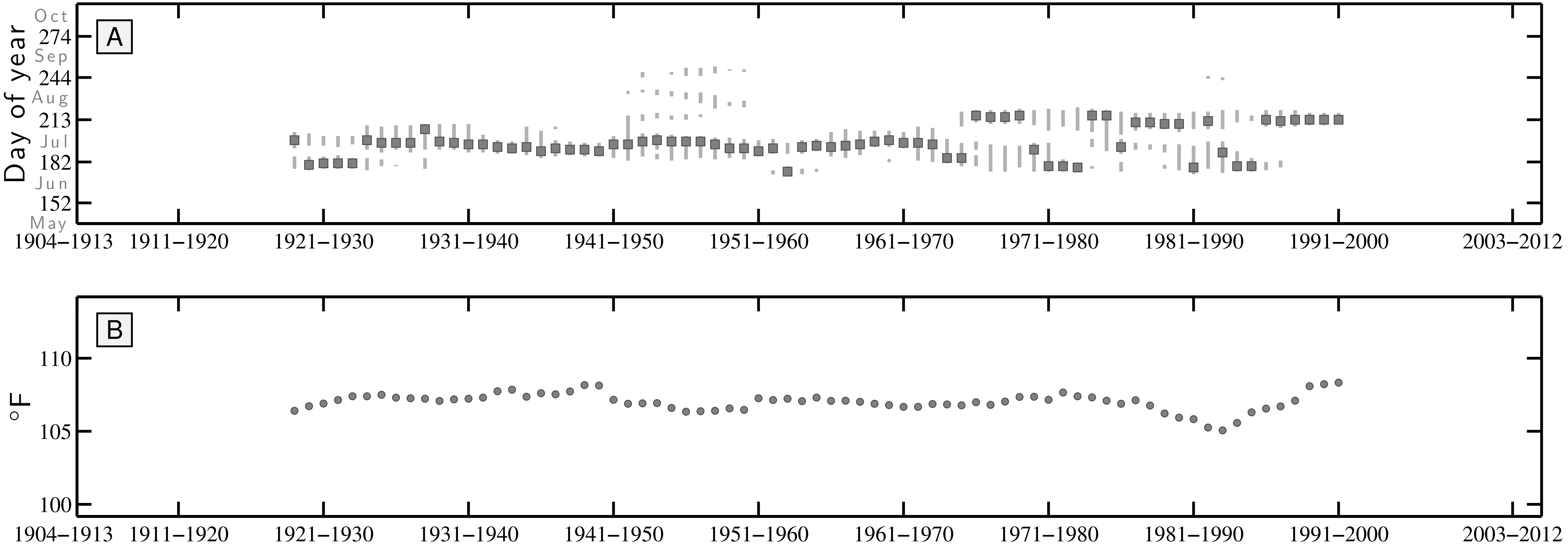

10 year Winter Teletherm dynamics for YUMA CITRUS STN, AZ:

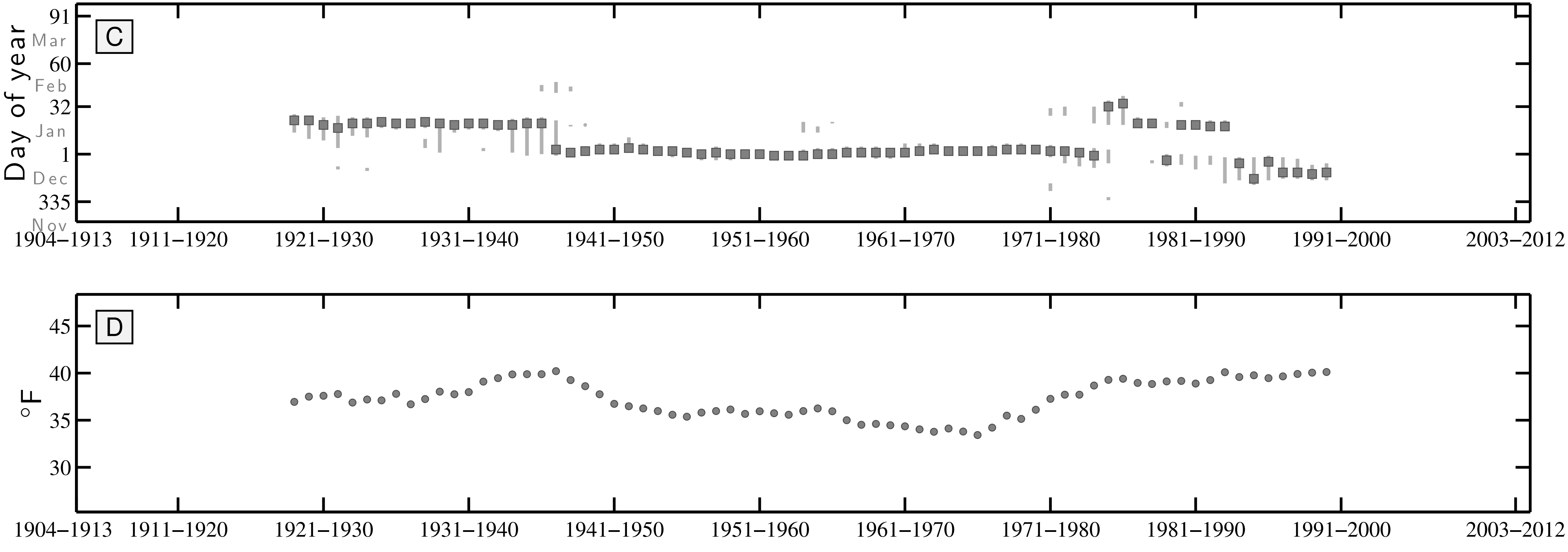

10 year Summer Teletherm dynamics for BRINKLEY, AR:

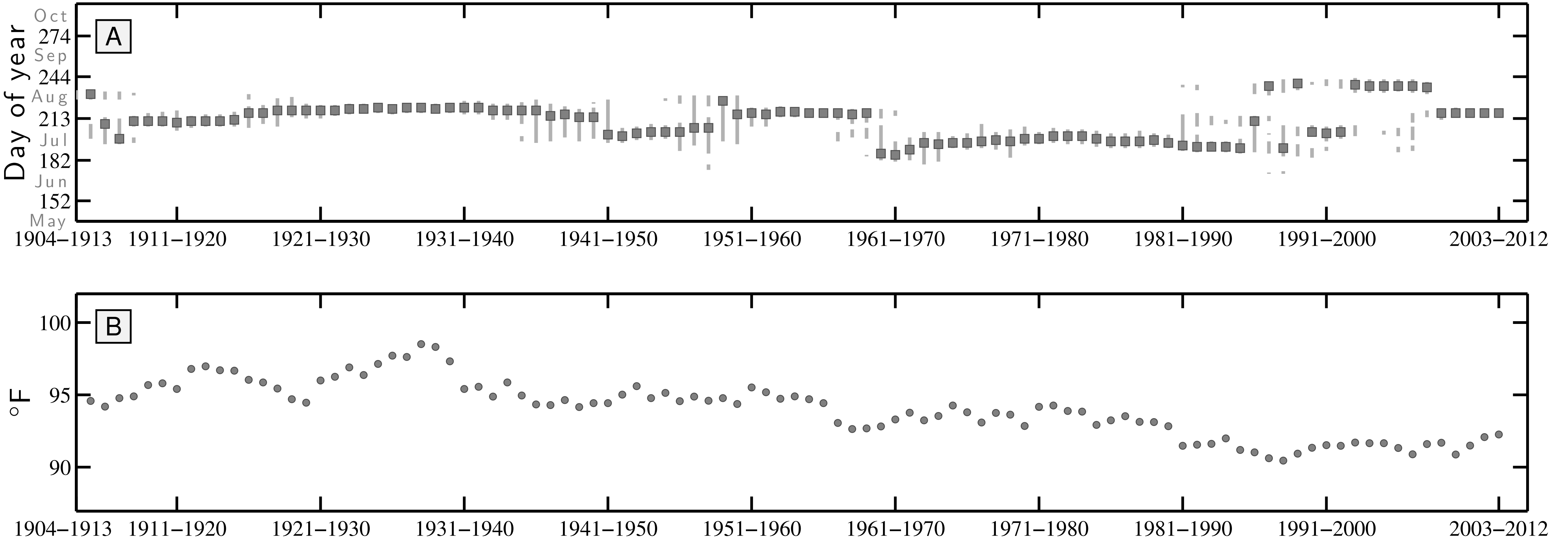

10 year Winter Teletherm dynamics for BRINKLEY, AR:

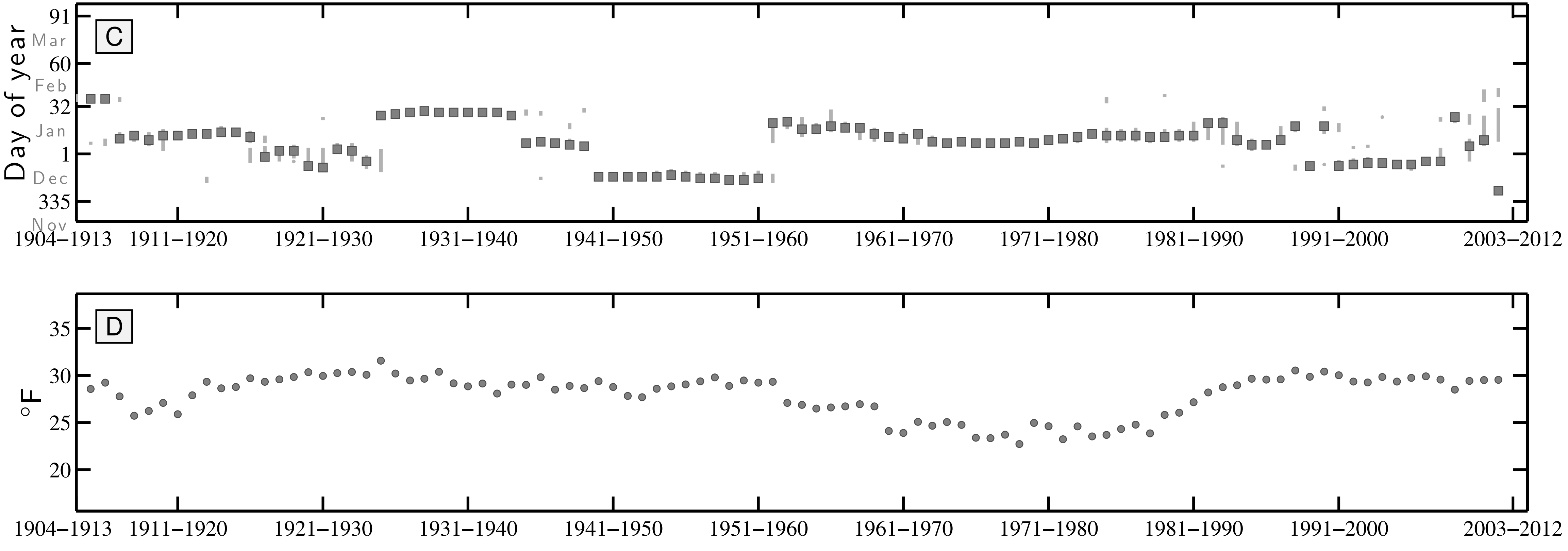

10 year Summer Teletherm dynamics for CONWAY, AR:

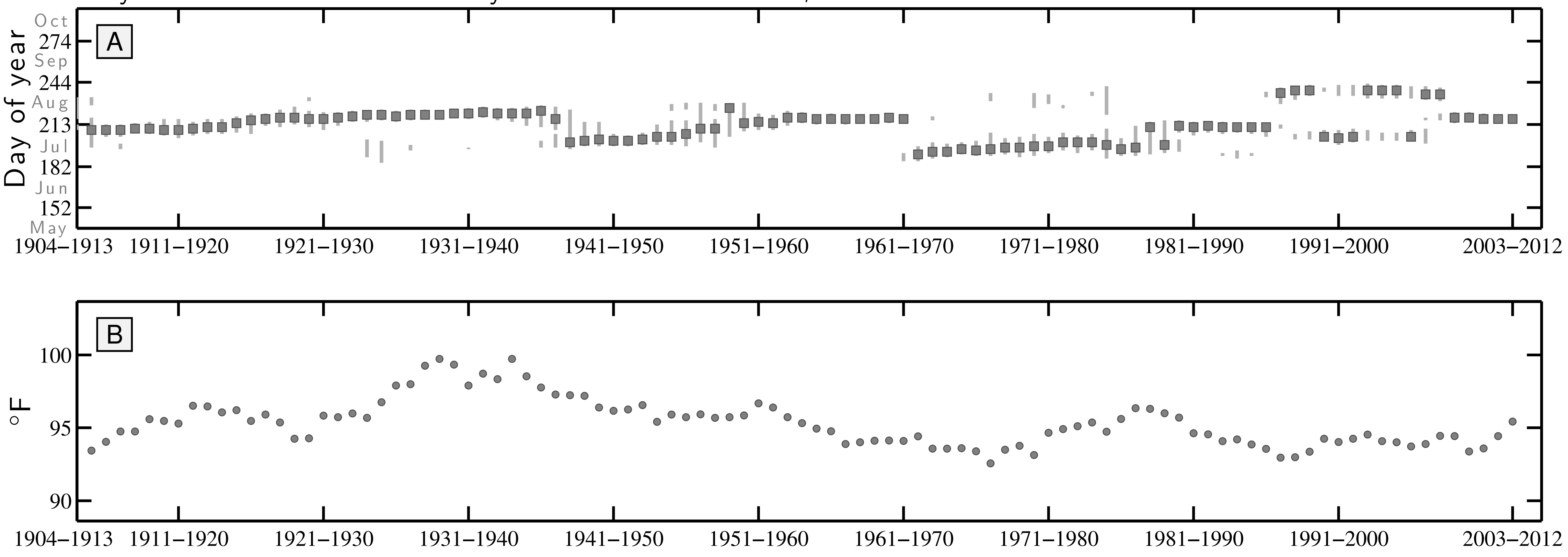

10 year Winter Teletherm dynamics for CONWAY, AR:

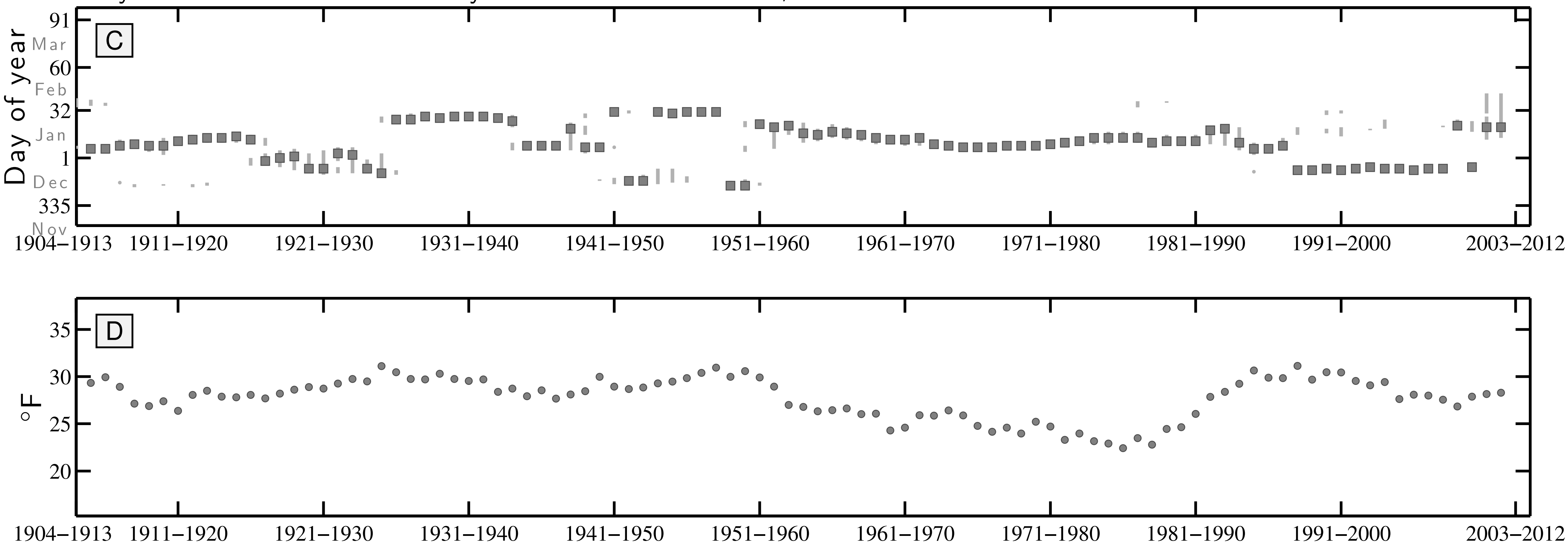

10 year Summer Teletherm dynamics for CORNING, AR:

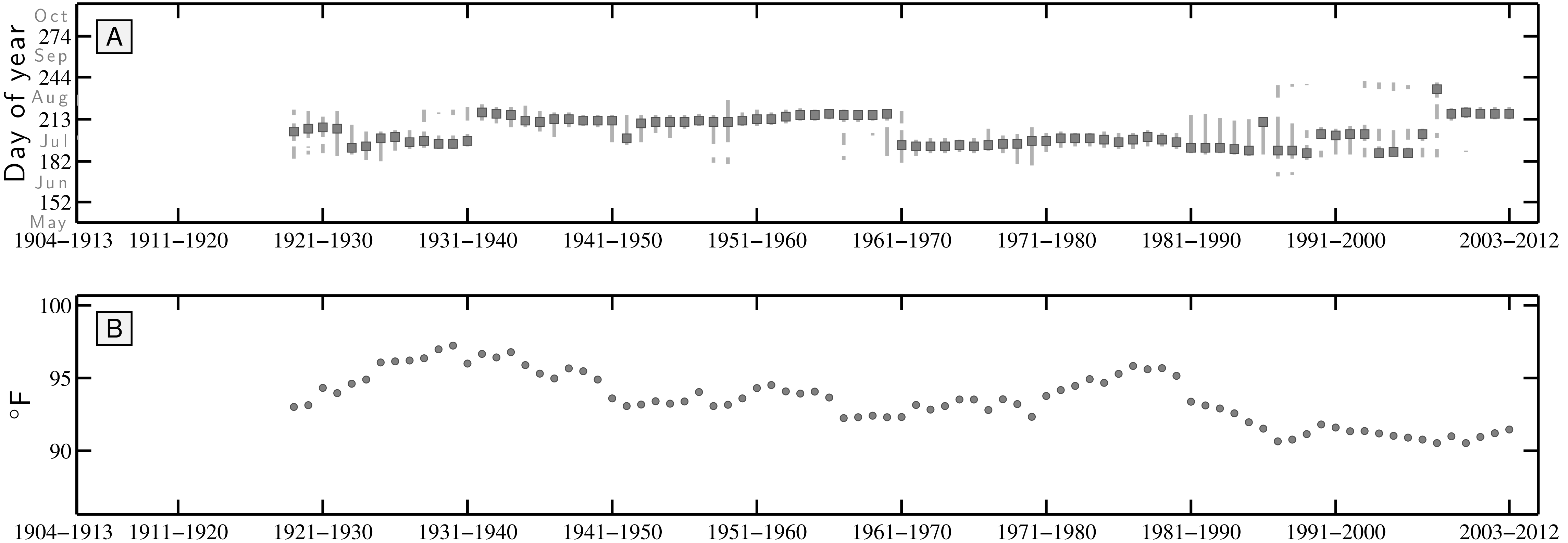

10 year Winter Teletherm dynamics for CORNING, AR:

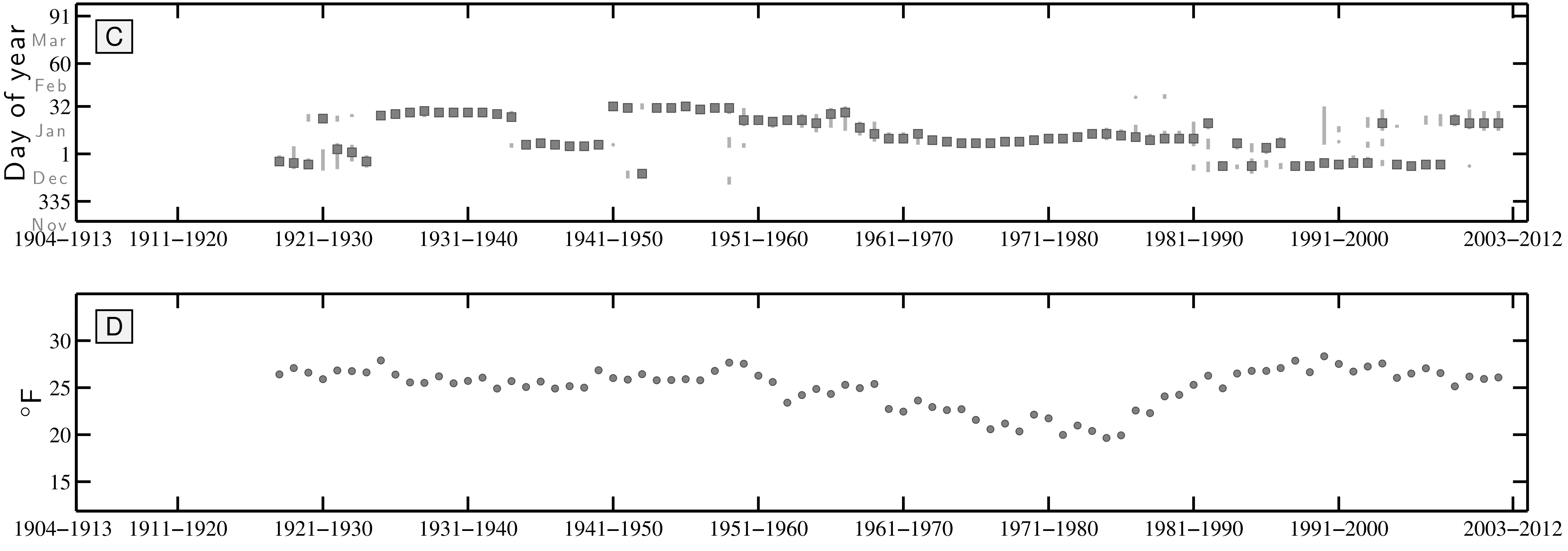

10 year Summer Teletherm dynamics for EUREKA SPRINGS 3 WNW, AR:

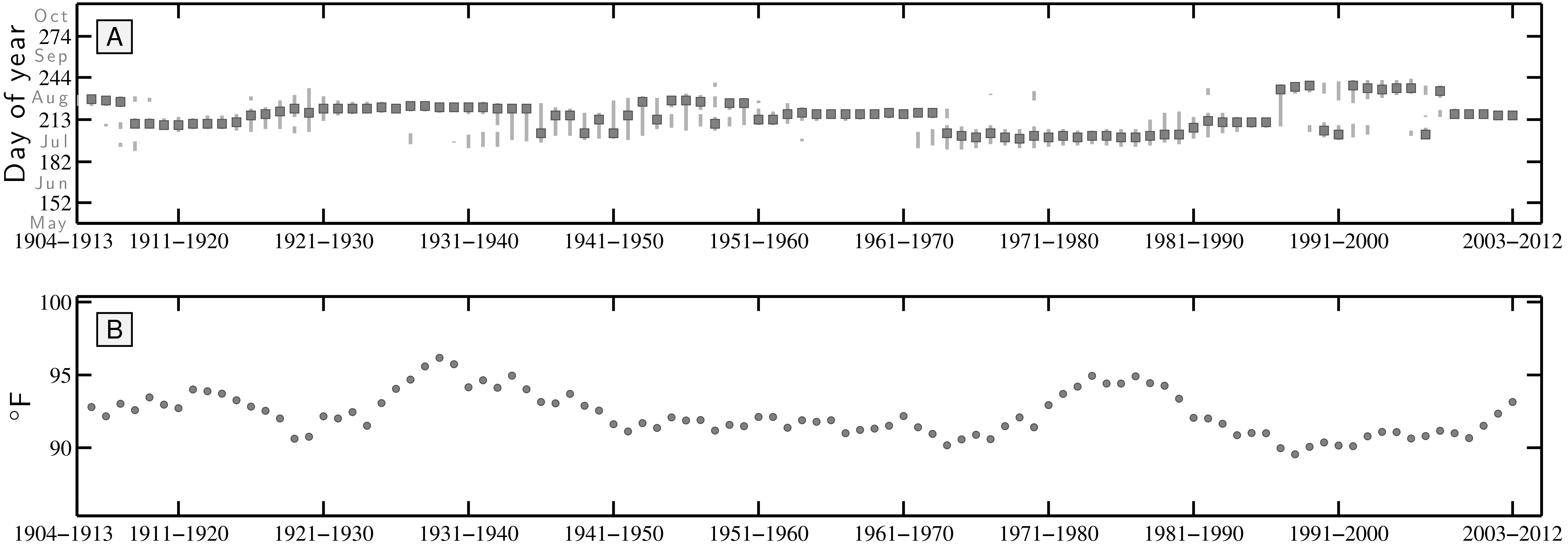

10 year Winter Teletherm dynamics for EUREKA SPRINGS 3 WNW, AR:

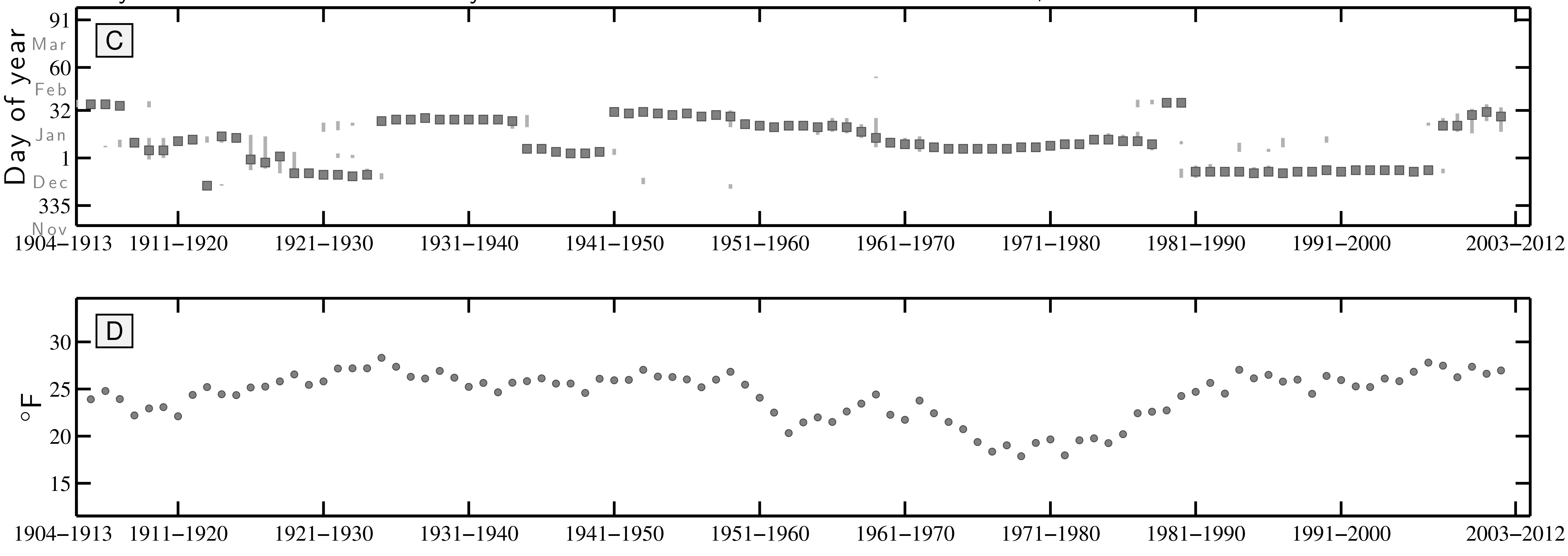

10 year Summer Teletherm dynamics for FAYETTEVILLE EXP STN, AR:

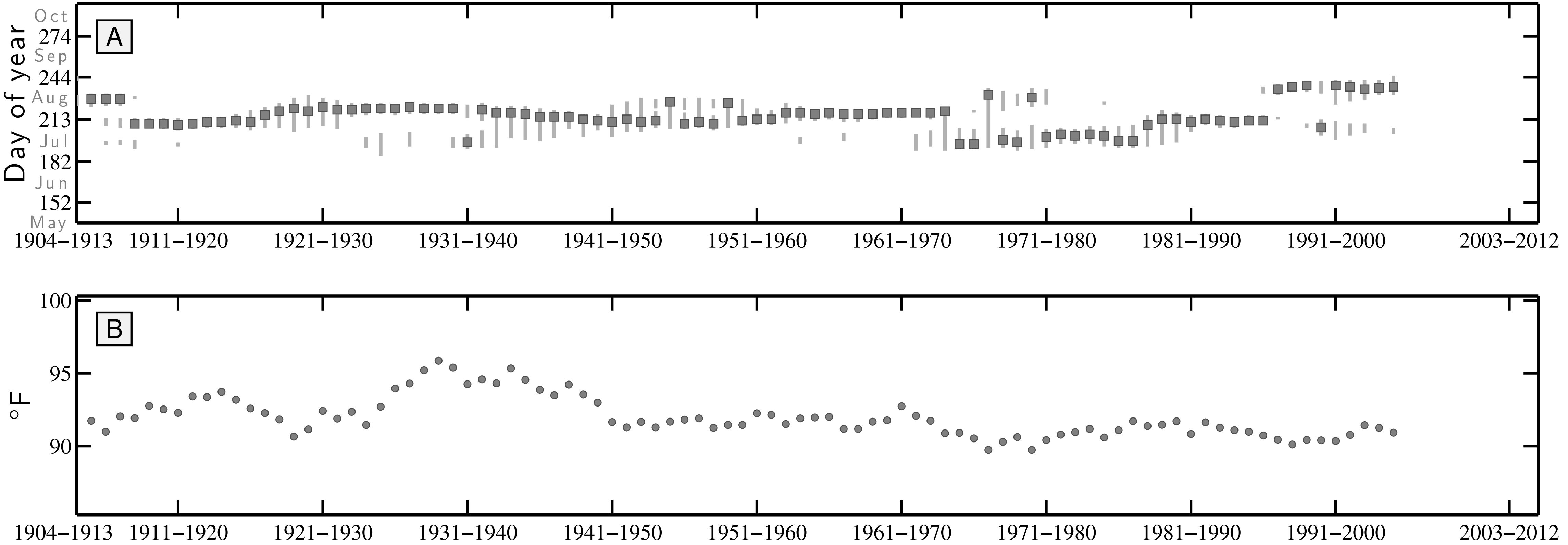

10 year Winter Teletherm dynamics for FAYETTEVILLE EXP STN, AR:

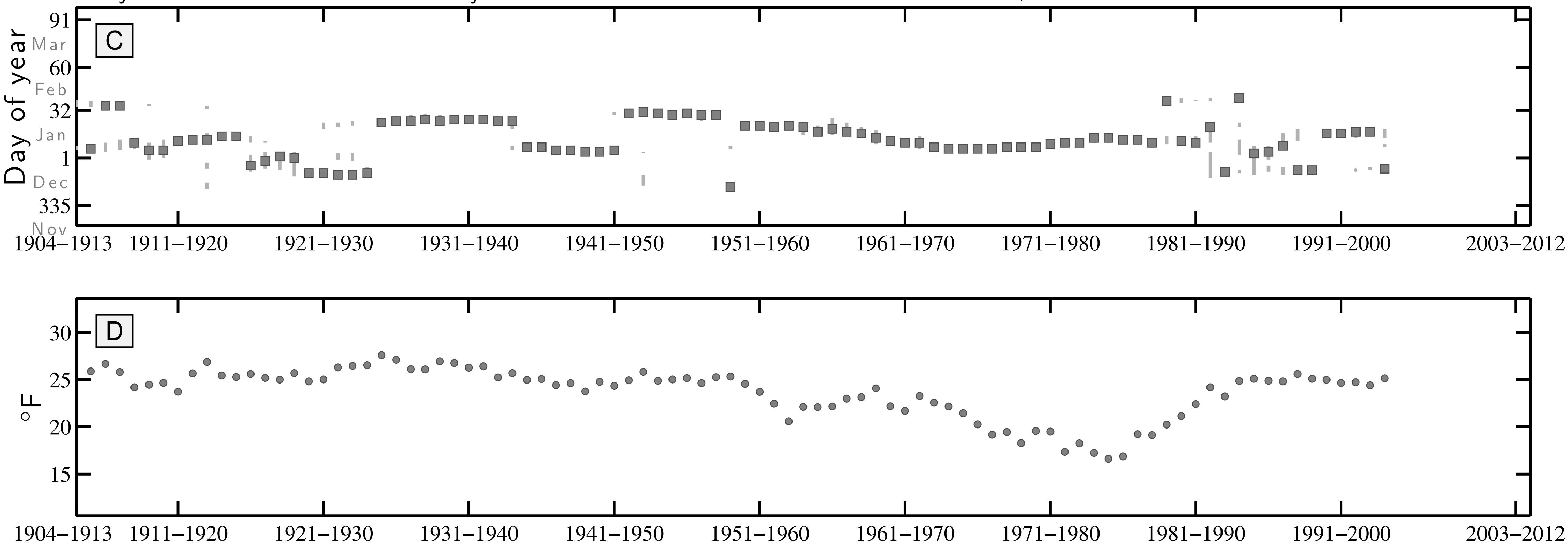

10 year Summer Teletherm dynamics for GRAVETTE, AR:

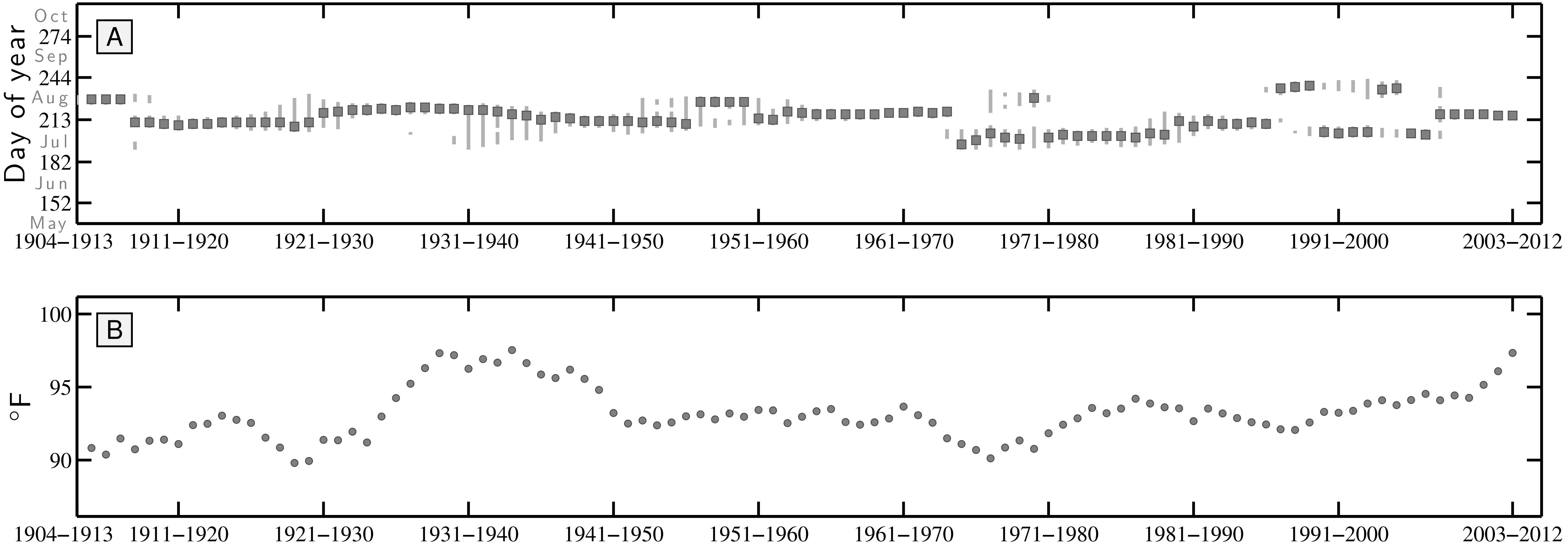

10 year Winter Teletherm dynamics for GRAVETTE, AR:

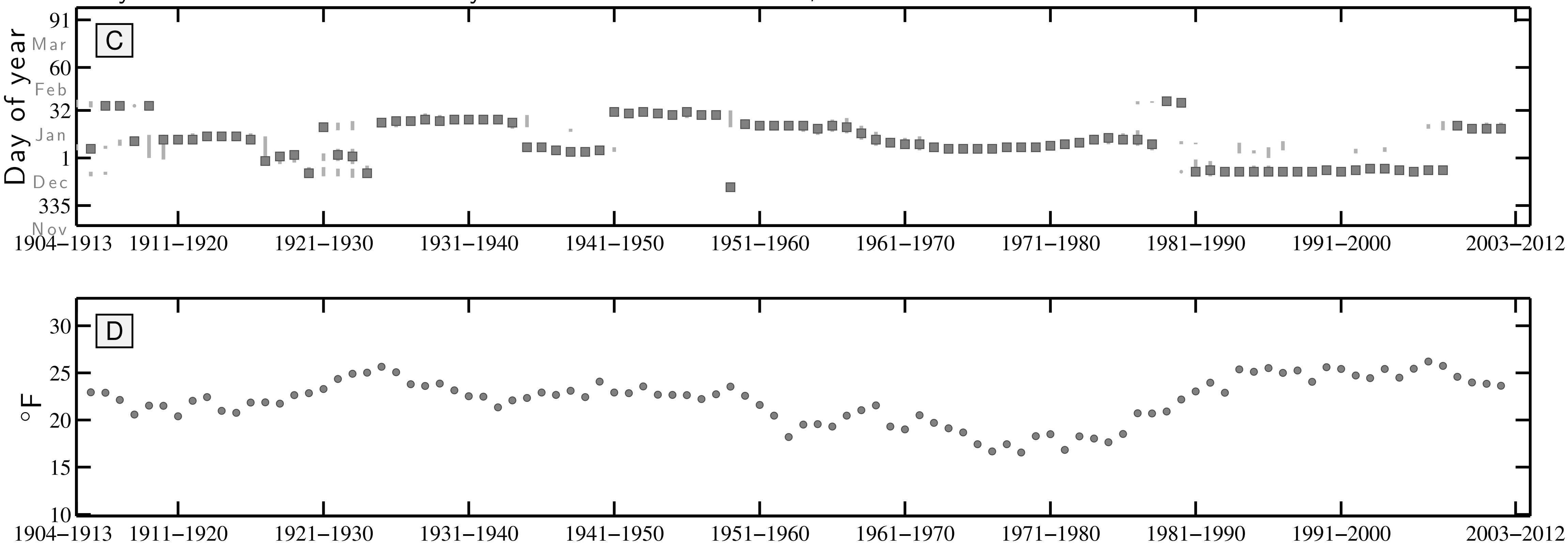

10 year Summer Teletherm dynamics for MAMMOTH SPRING, AR:

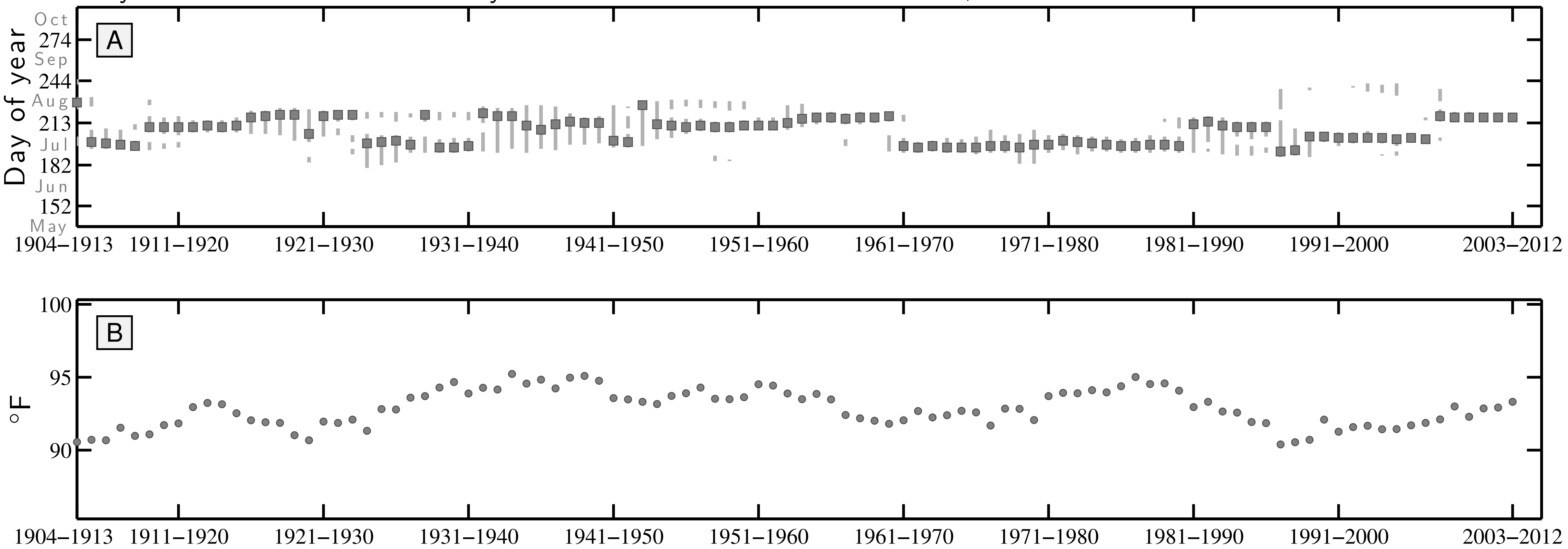

10 year Winter Teletherm dynamics for MAMMOTH SPRING, AR:

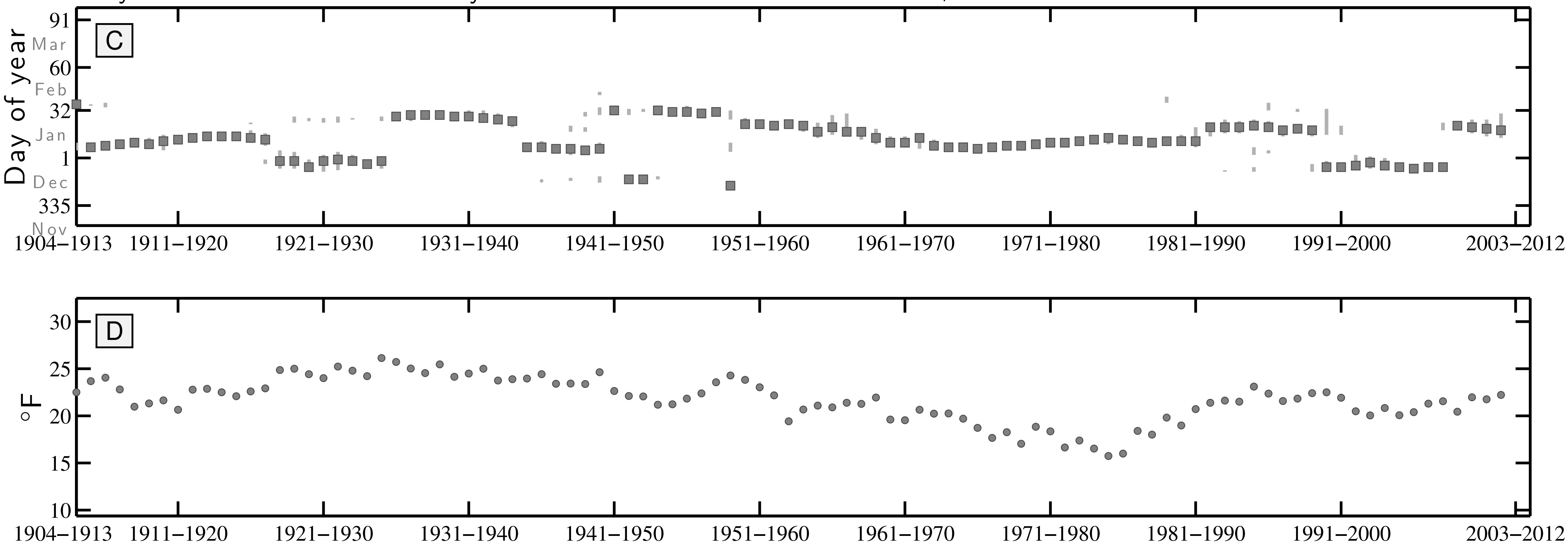

10 year Summer Teletherm dynamics for MENA, AR:

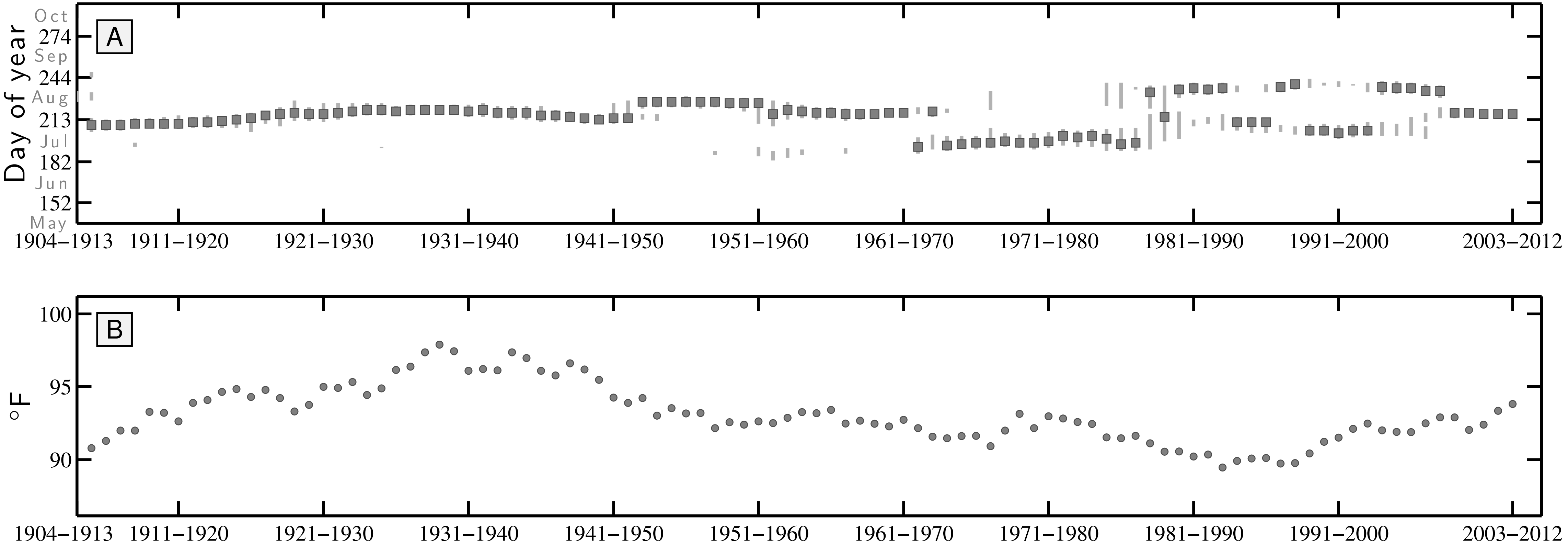

10 year Winter Teletherm dynamics for MENA, AR:

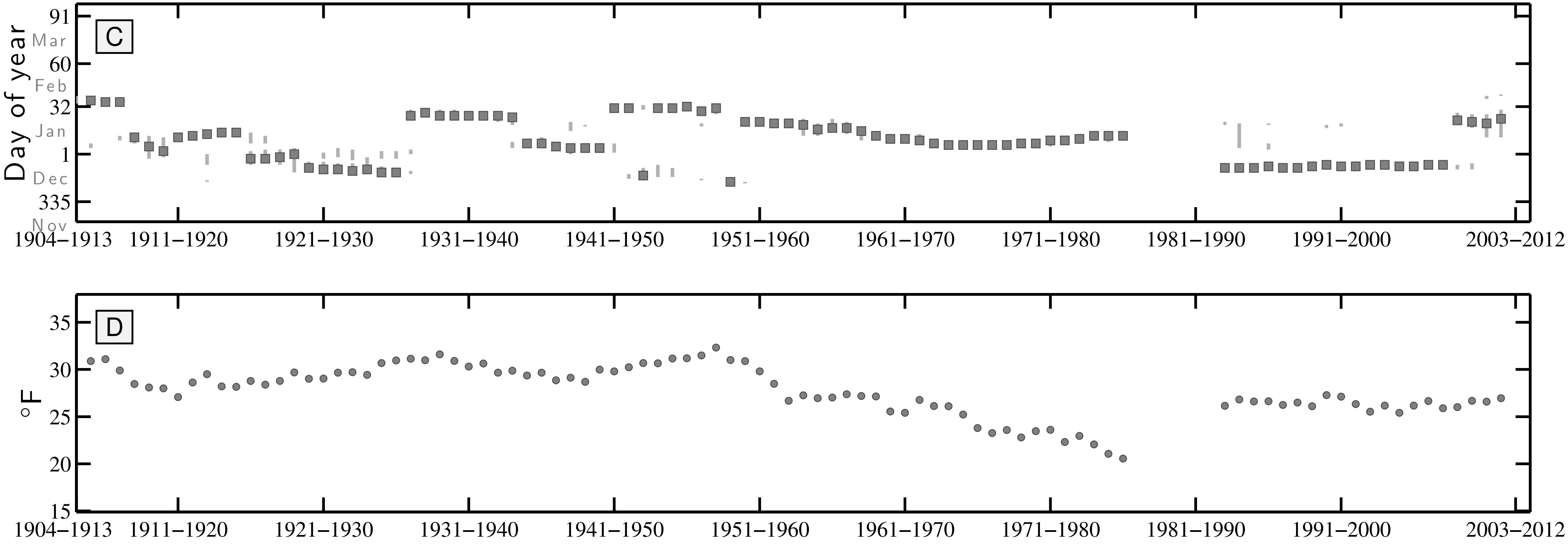

10 year Summer Teletherm dynamics for NEWPORT, AR:

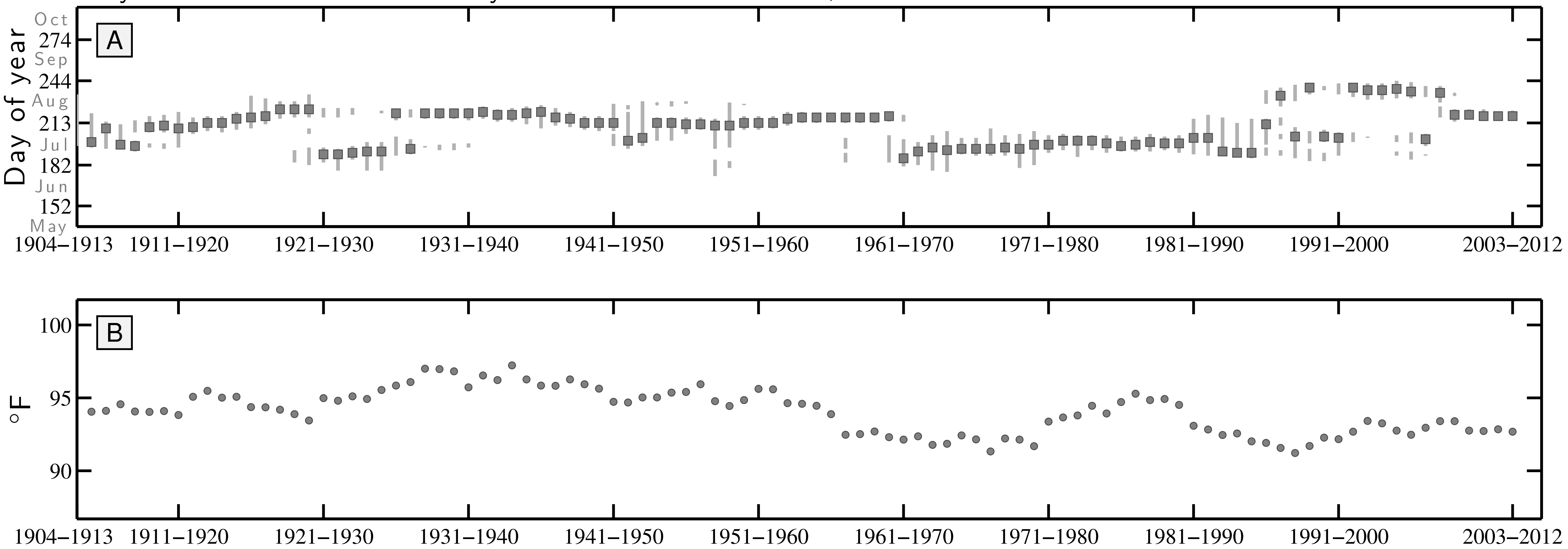

10 year Winter Teletherm dynamics for NEWPORT, AR:

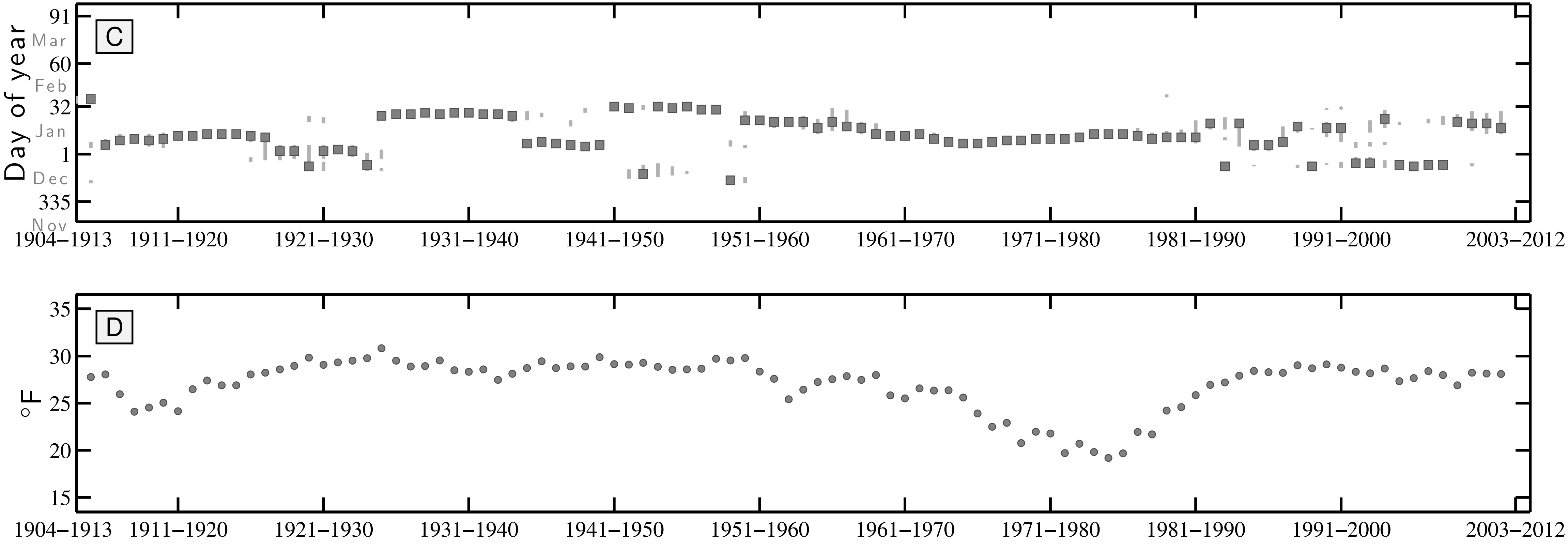

10 year Summer Teletherm dynamics for OZARK 2, AR:

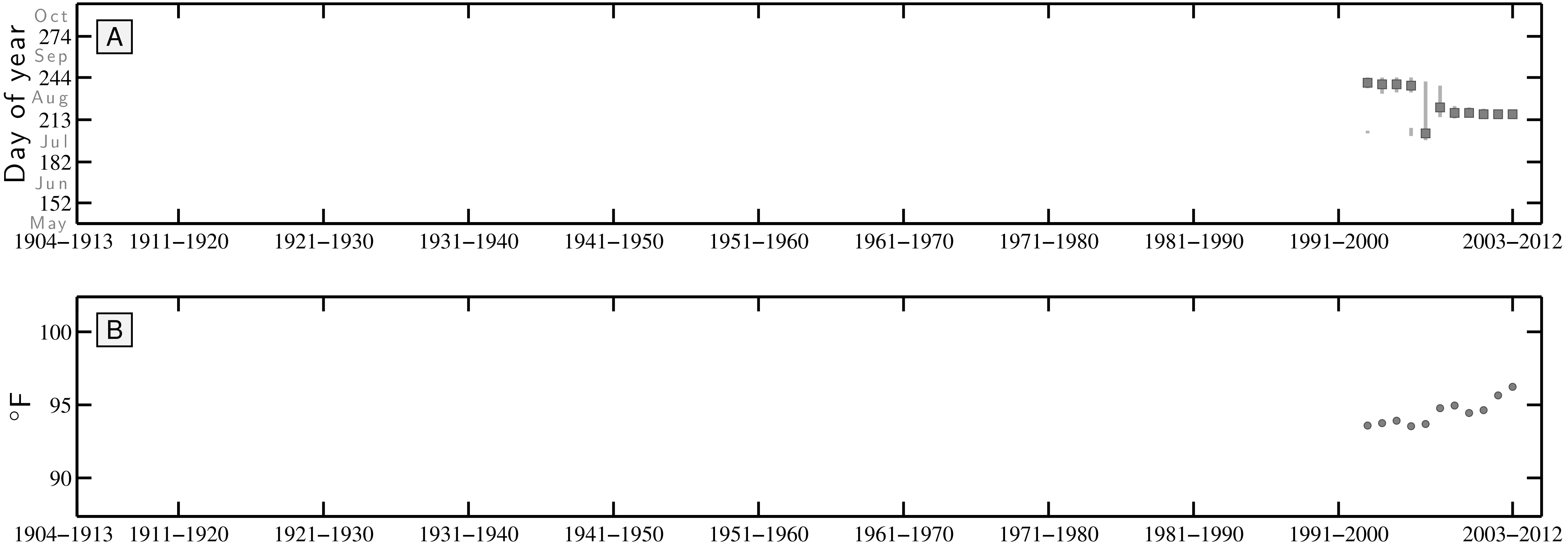

10 year Winter Teletherm dynamics for OZARK 2, AR:

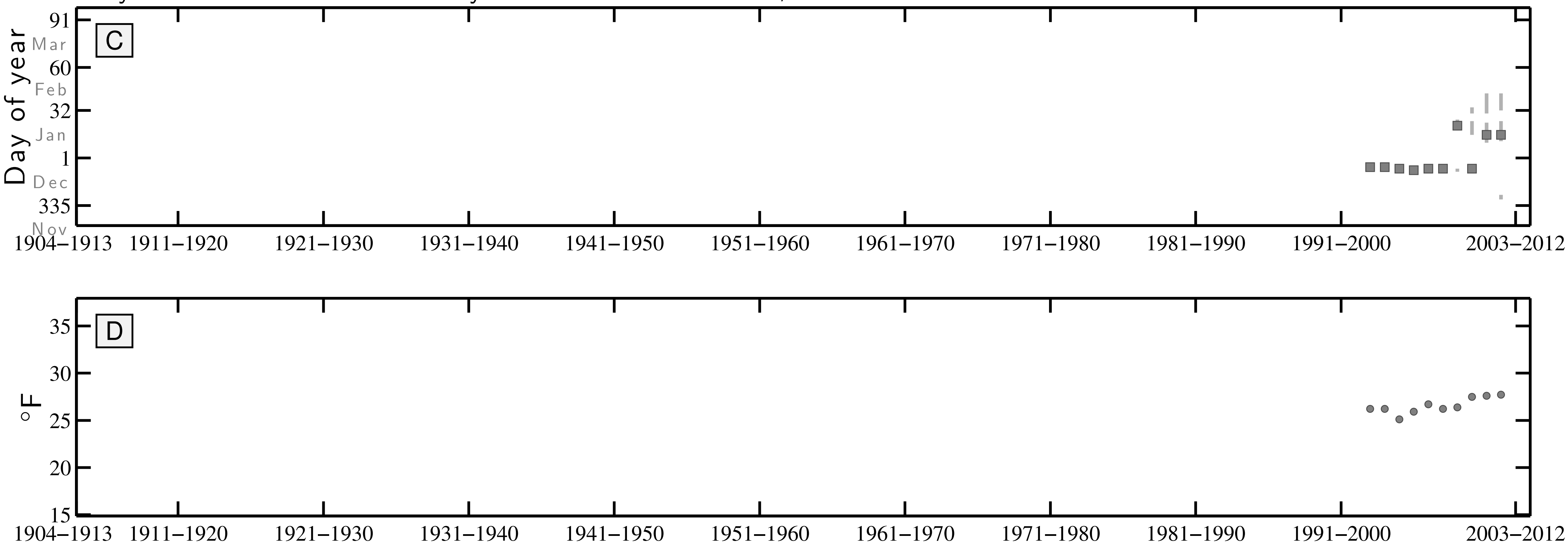

10 year Summer Teletherm dynamics for PINE BLUFF, AR:

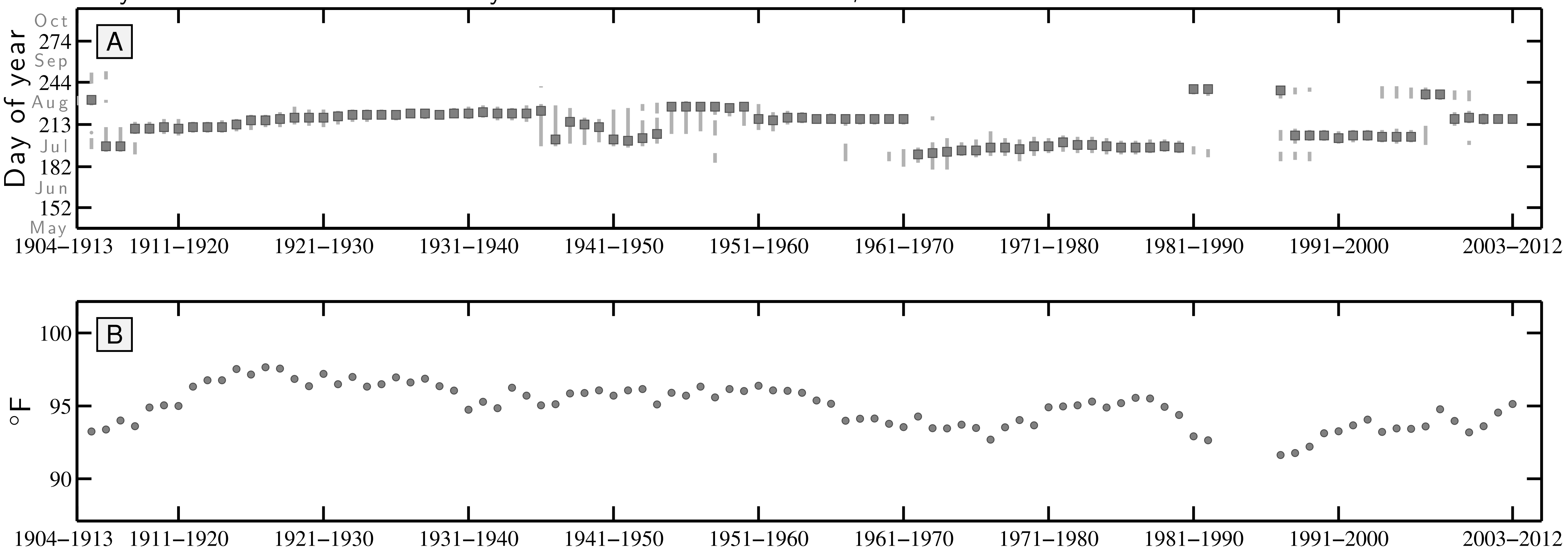

10 year Winter Teletherm dynamics for PINE BLUFF, AR:

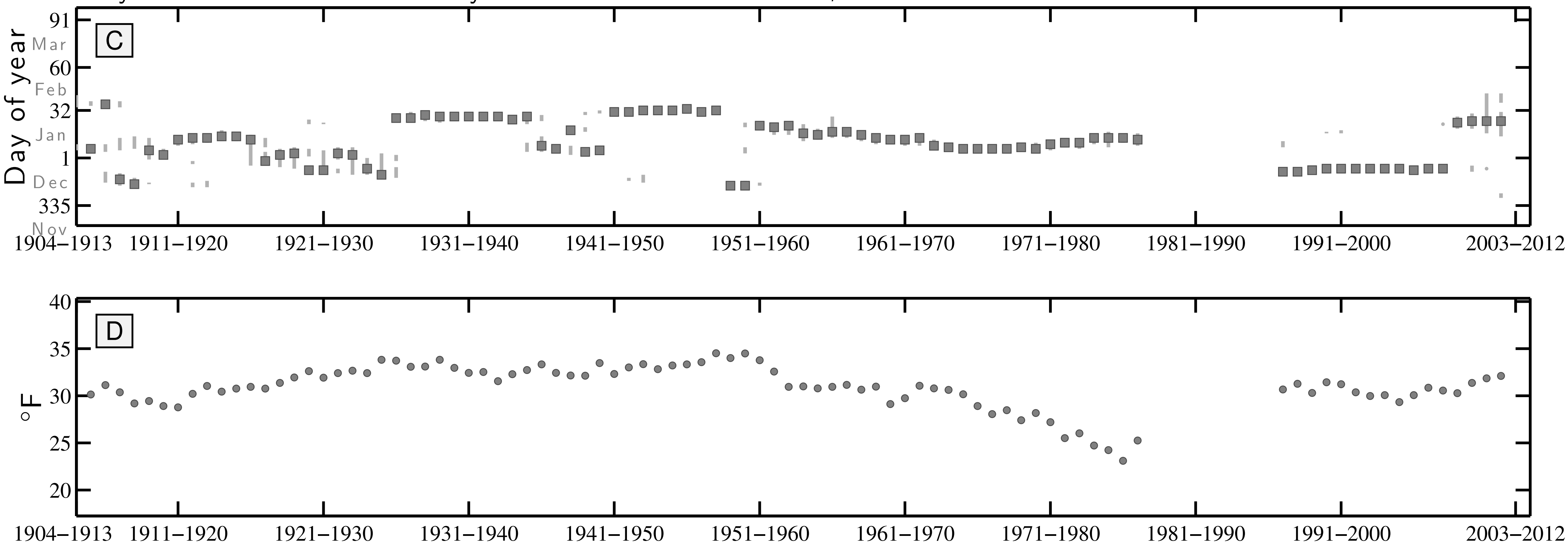

10 year Summer Teletherm dynamics for POCAHONTAS 1, AR:

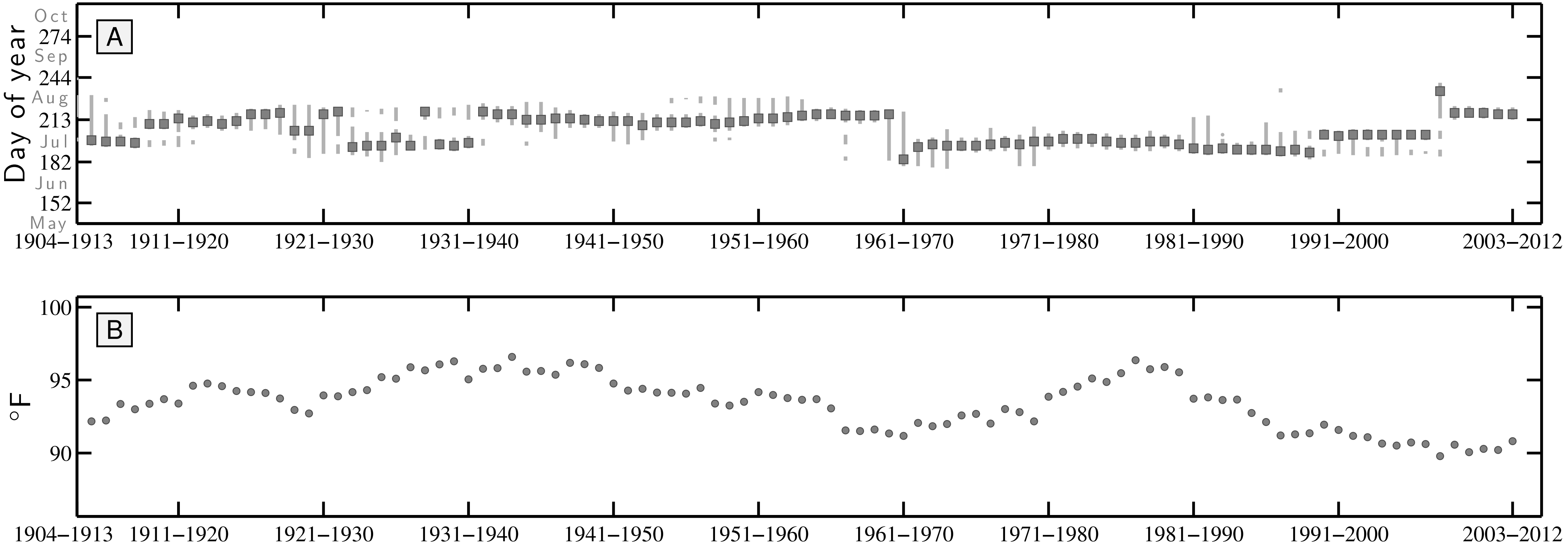

10 year Winter Teletherm dynamics for POCAHONTAS 1, AR:

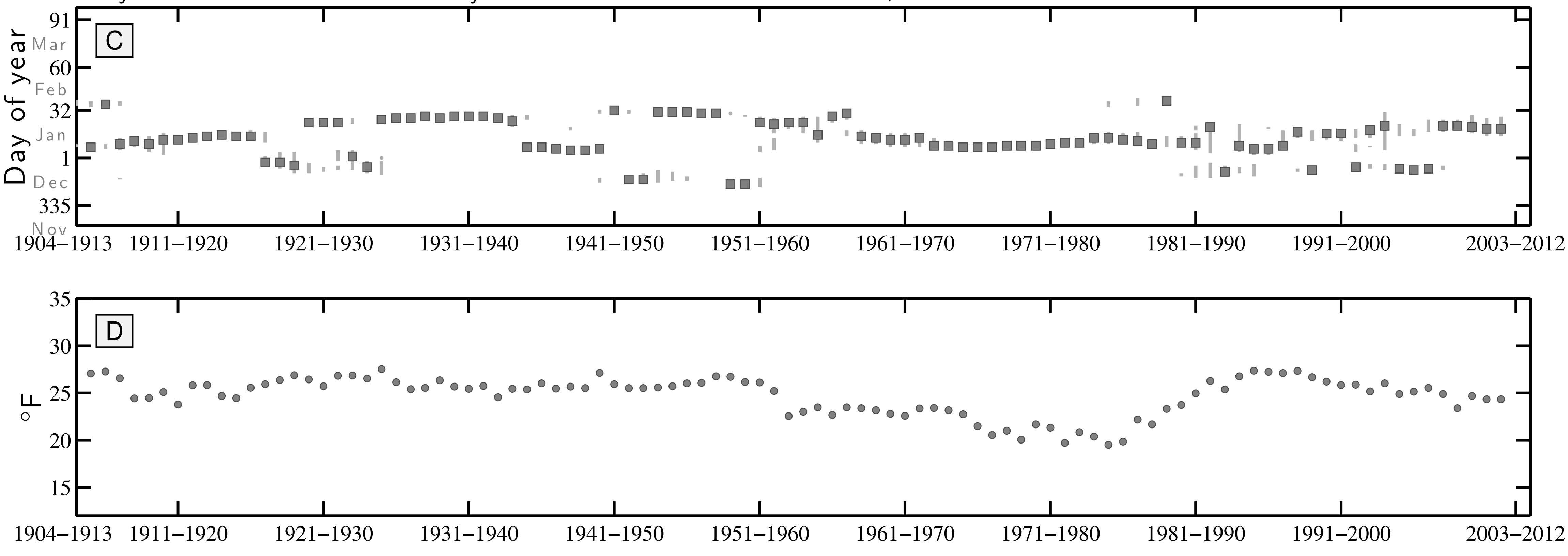

10 year Summer Teletherm dynamics for PRESCOTT 2 NNW, AR:

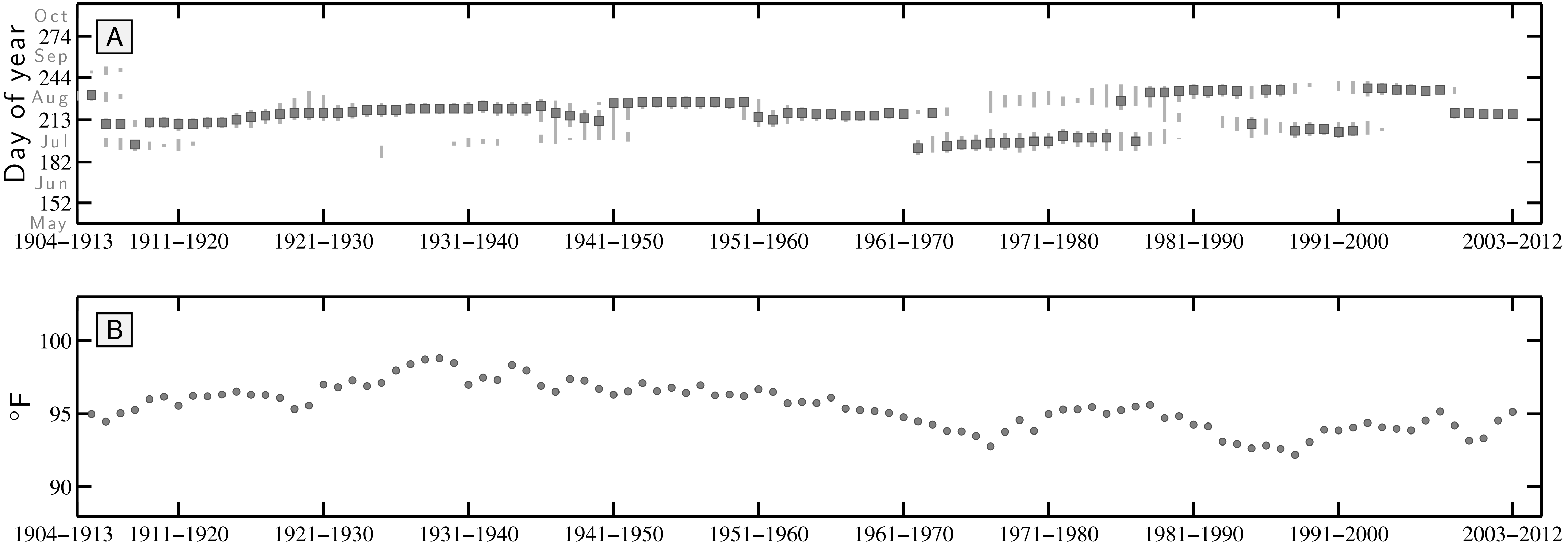

10 year Winter Teletherm dynamics for PRESCOTT 2 NNW, AR:

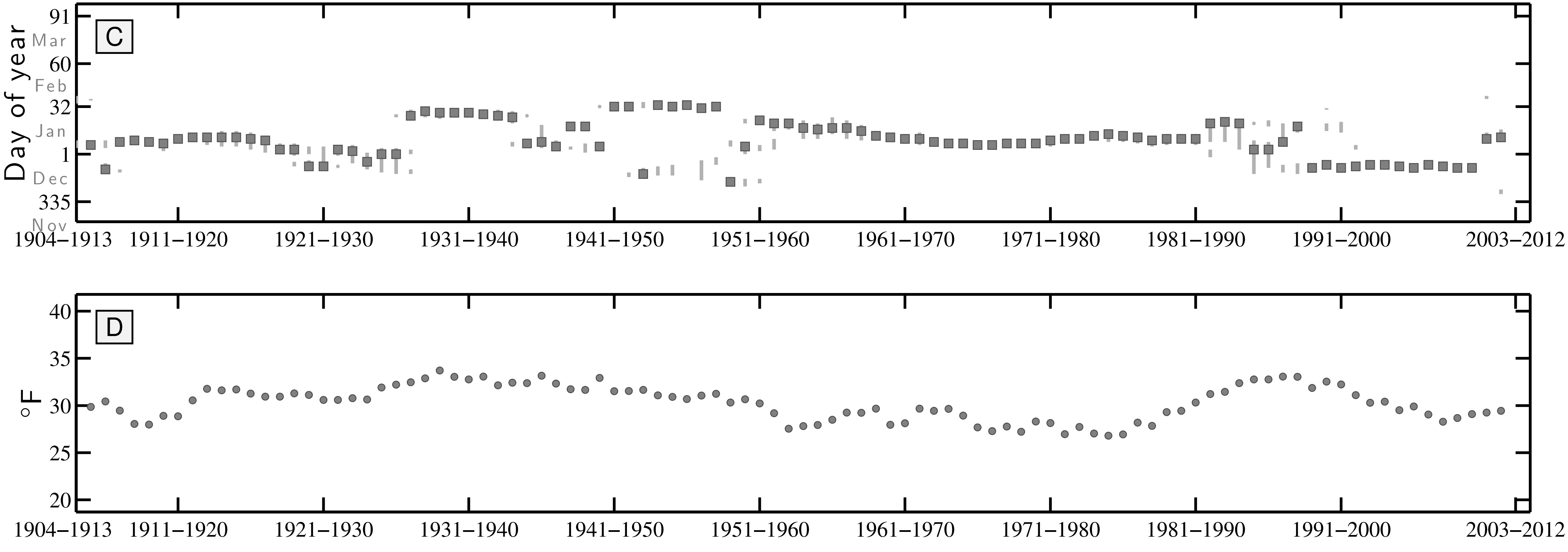

10 year Summer Teletherm dynamics for ROHWER 2 NNE, AR:

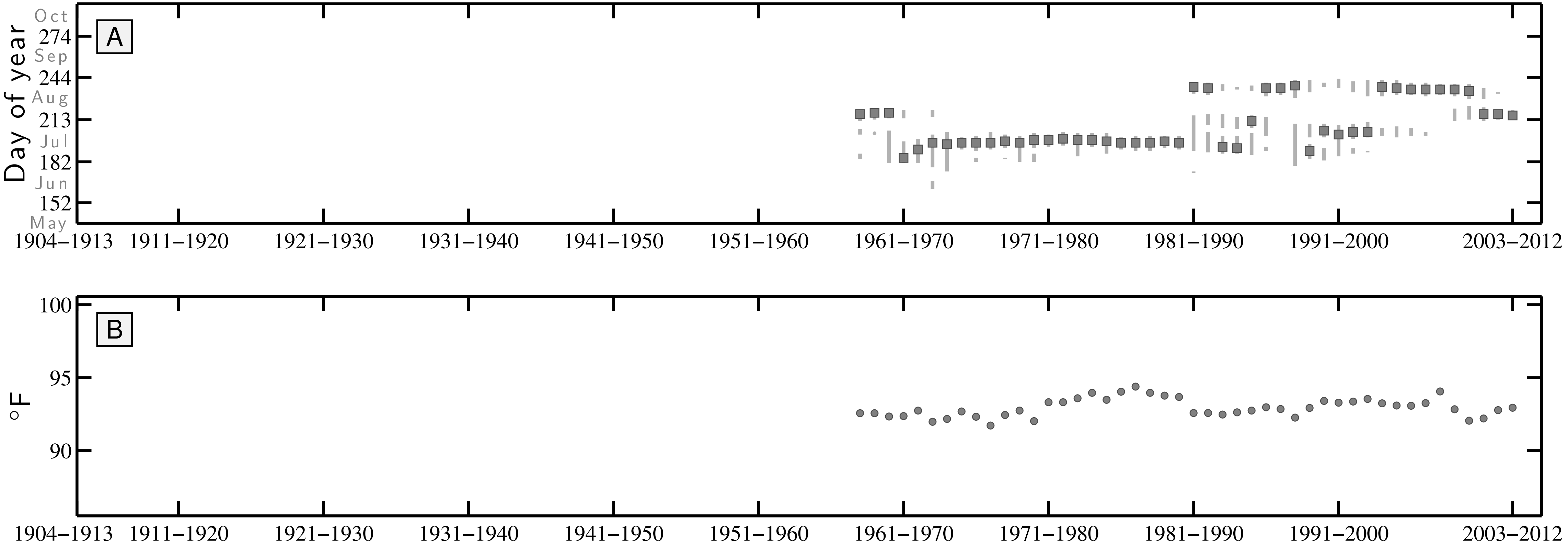

10 year Winter Teletherm dynamics for ROHWER 2 NNE, AR:

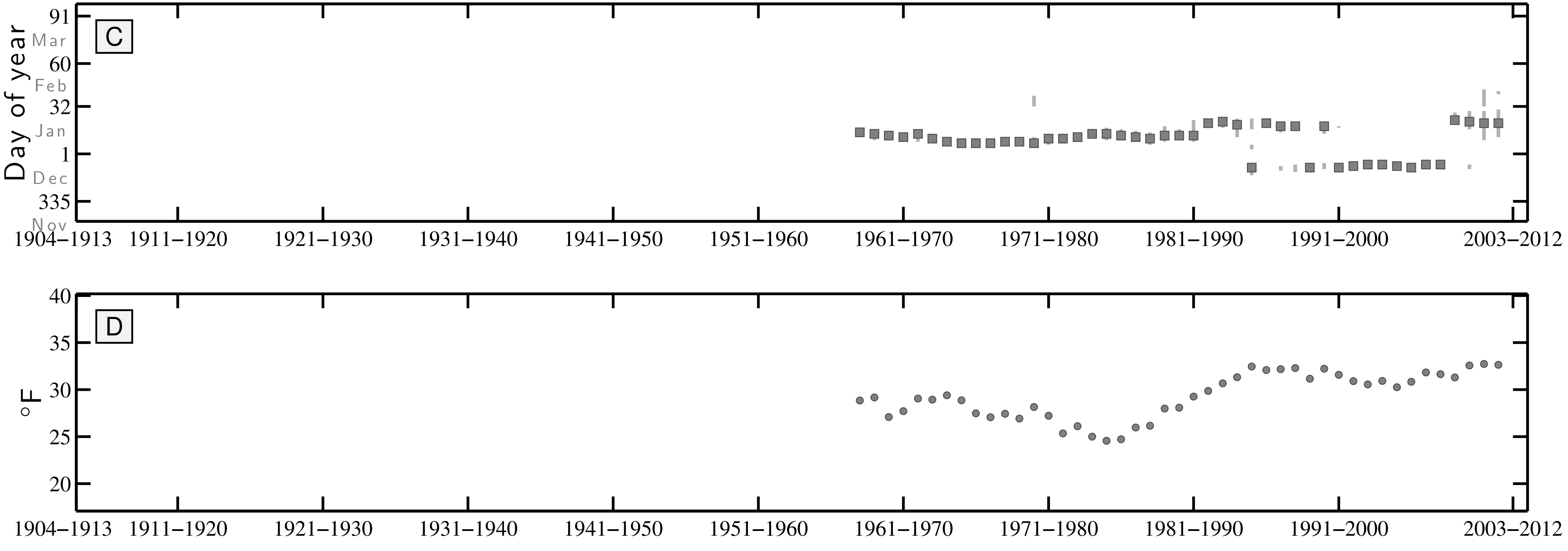

10 year Summer Teletherm dynamics for SUBIACO, AR:

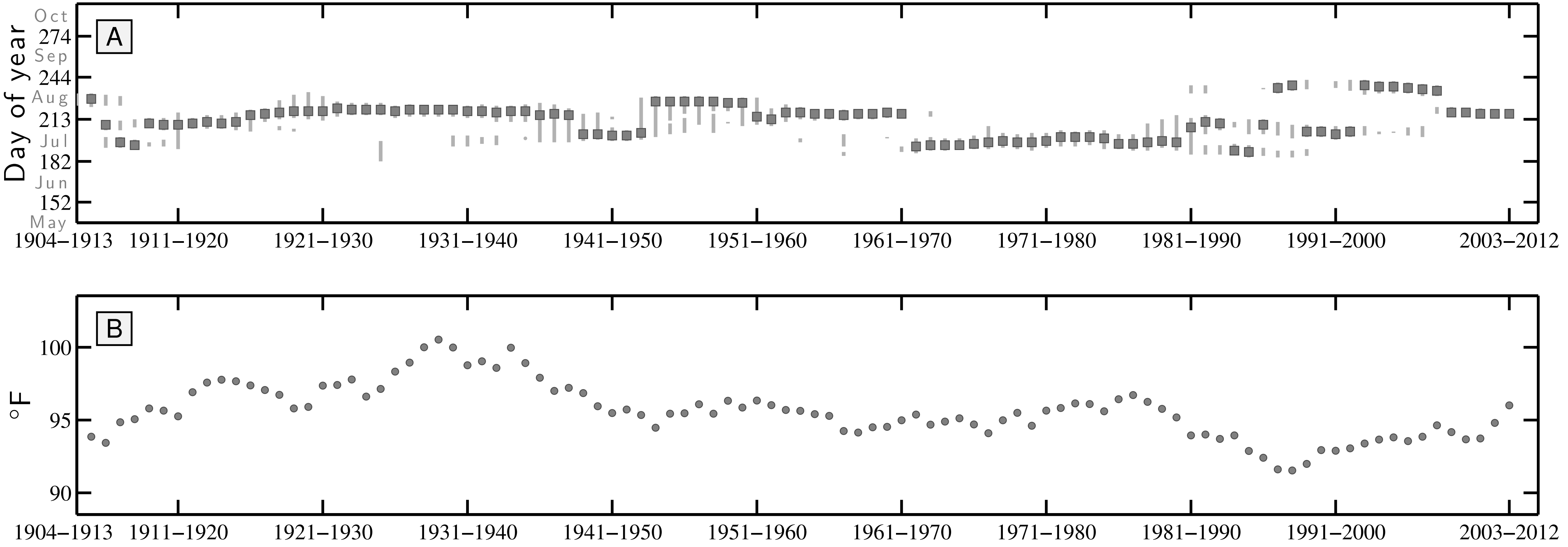

10 year Winter Teletherm dynamics for SUBIACO, AR:

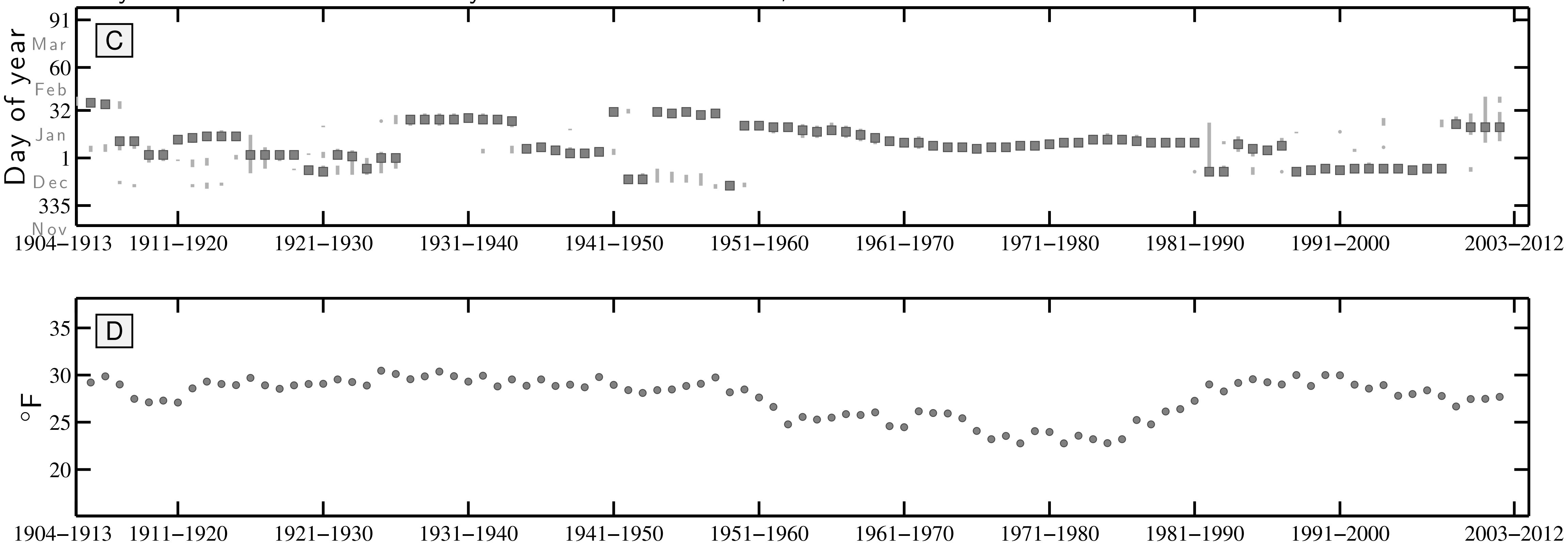

10 year Summer Teletherm dynamics for BERKELEY, CA:

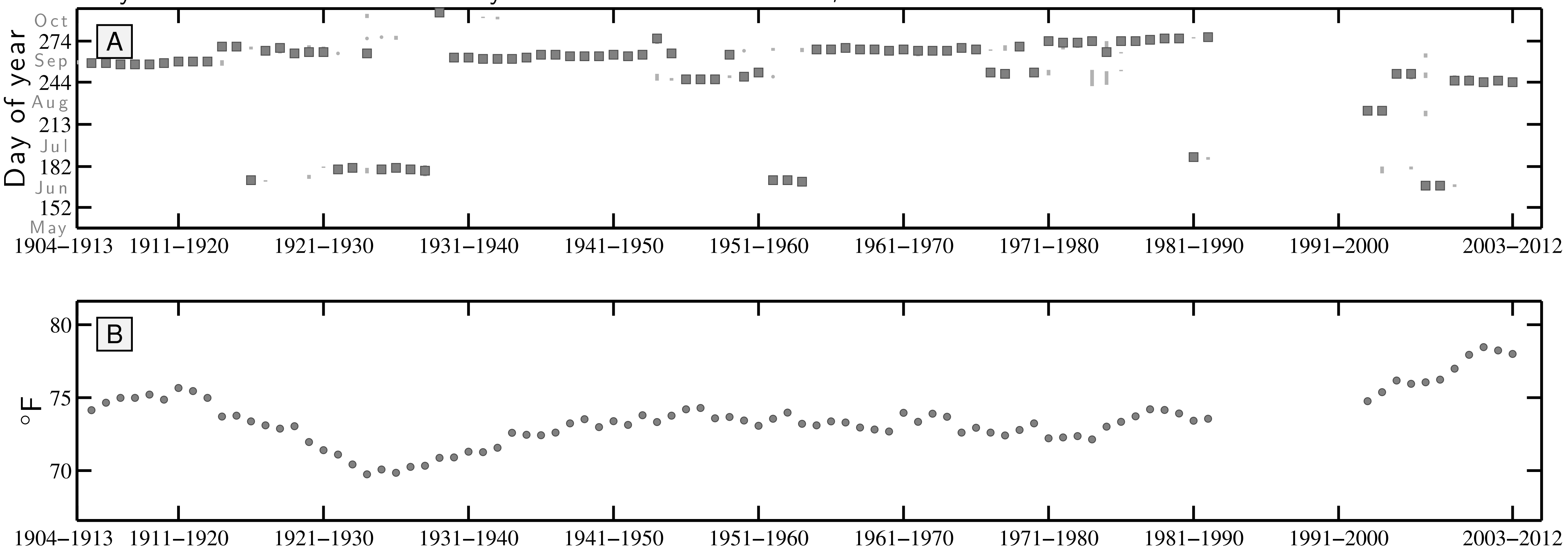

10 year Winter Teletherm dynamics for BERKELEY, CA:

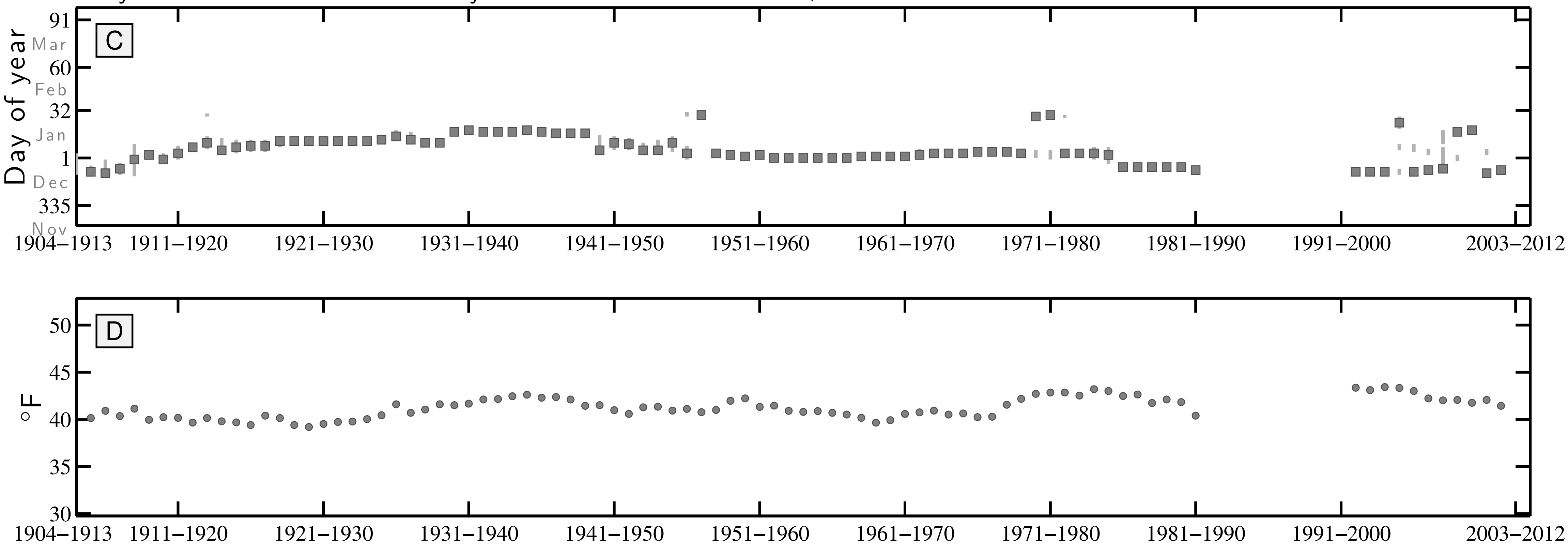

10 year Summer Teletherm dynamics for BLYTHE, CA:

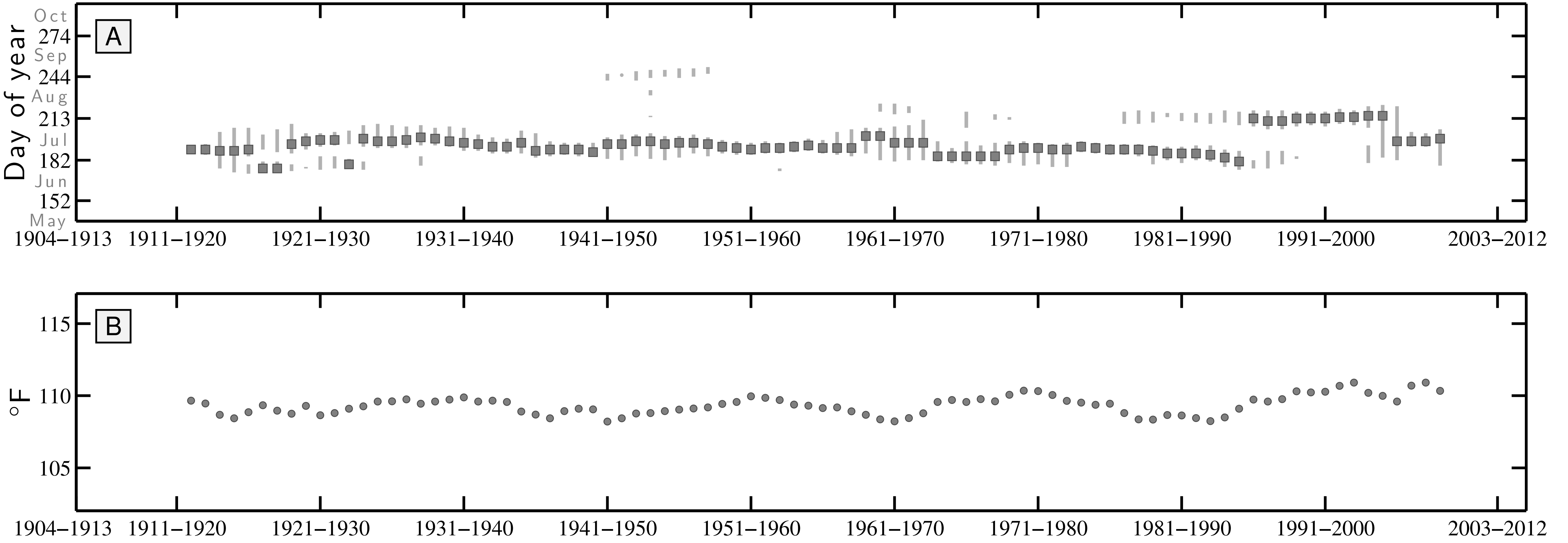

10 year Winter Teletherm dynamics for BLYTHE, CA:

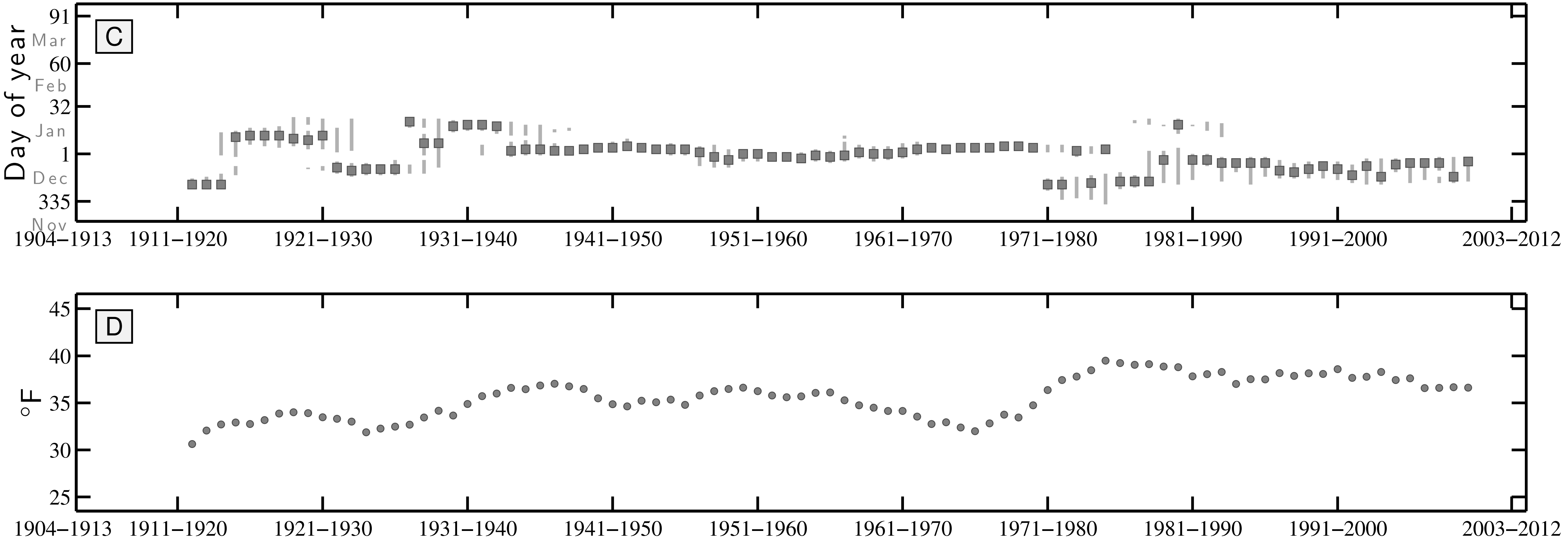

10 year Summer Teletherm dynamics for BRAWLEY 2 SW, CA:

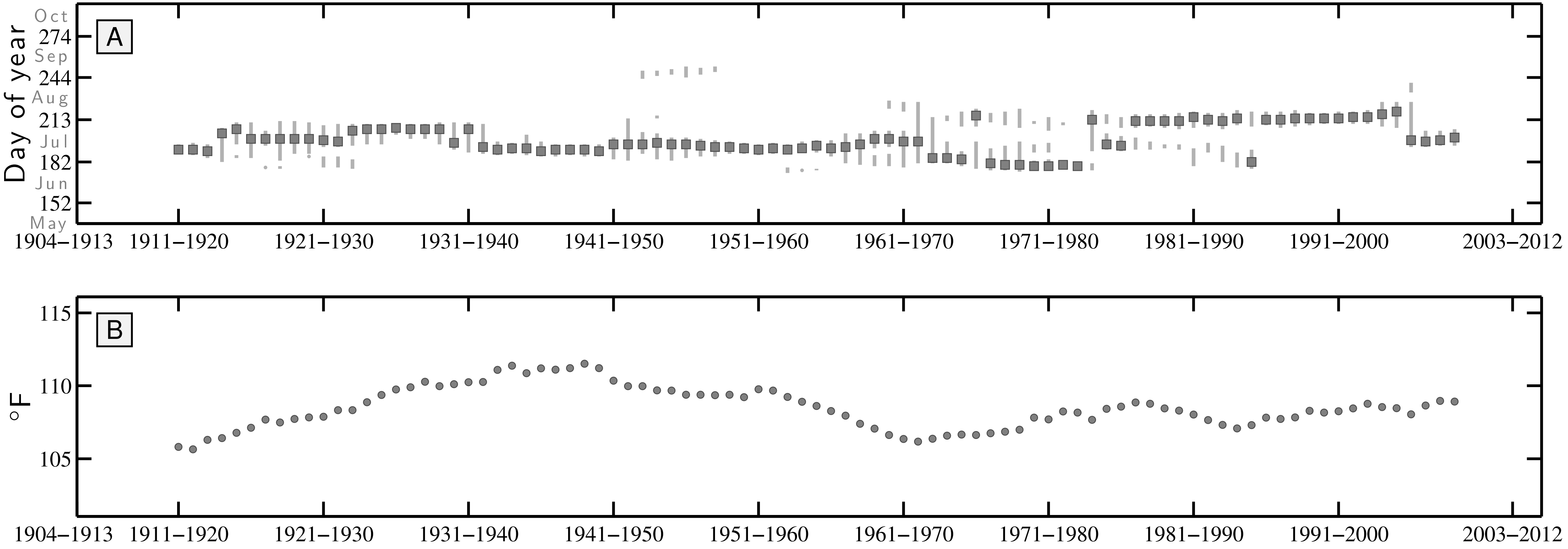

10 year Winter Teletherm dynamics for BRAWLEY 2 SW, CA:

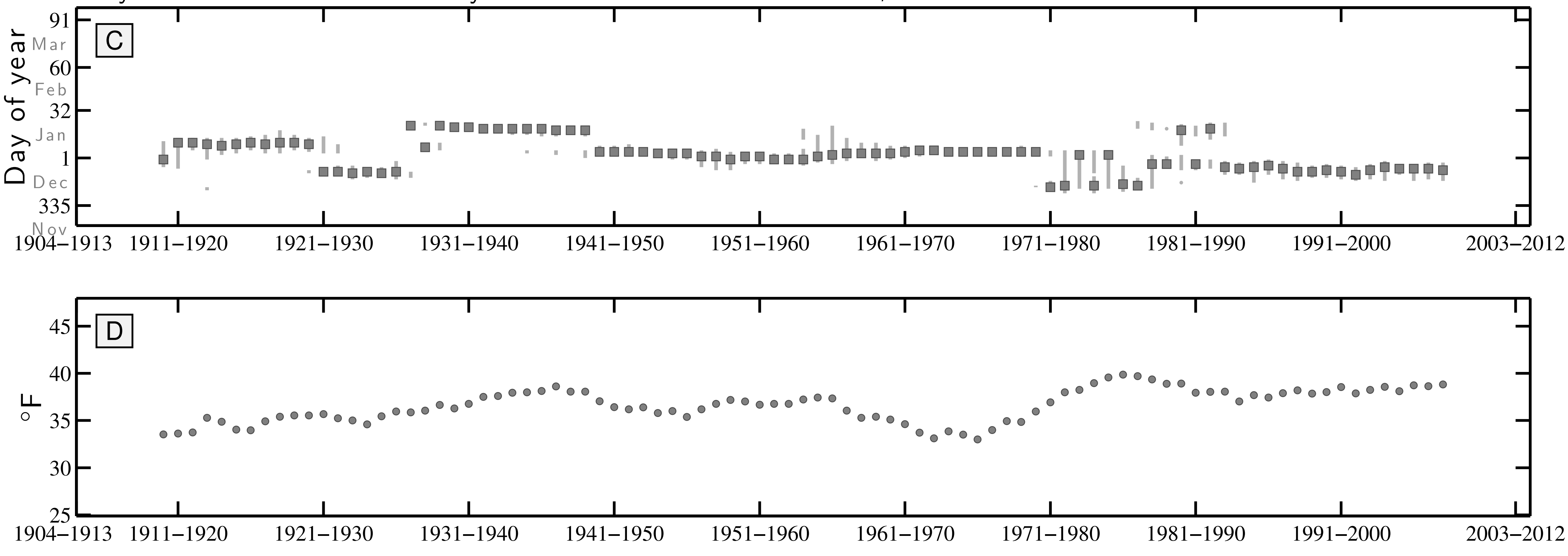

10 year Summer Teletherm dynamics for CEDARVILLE, CA:

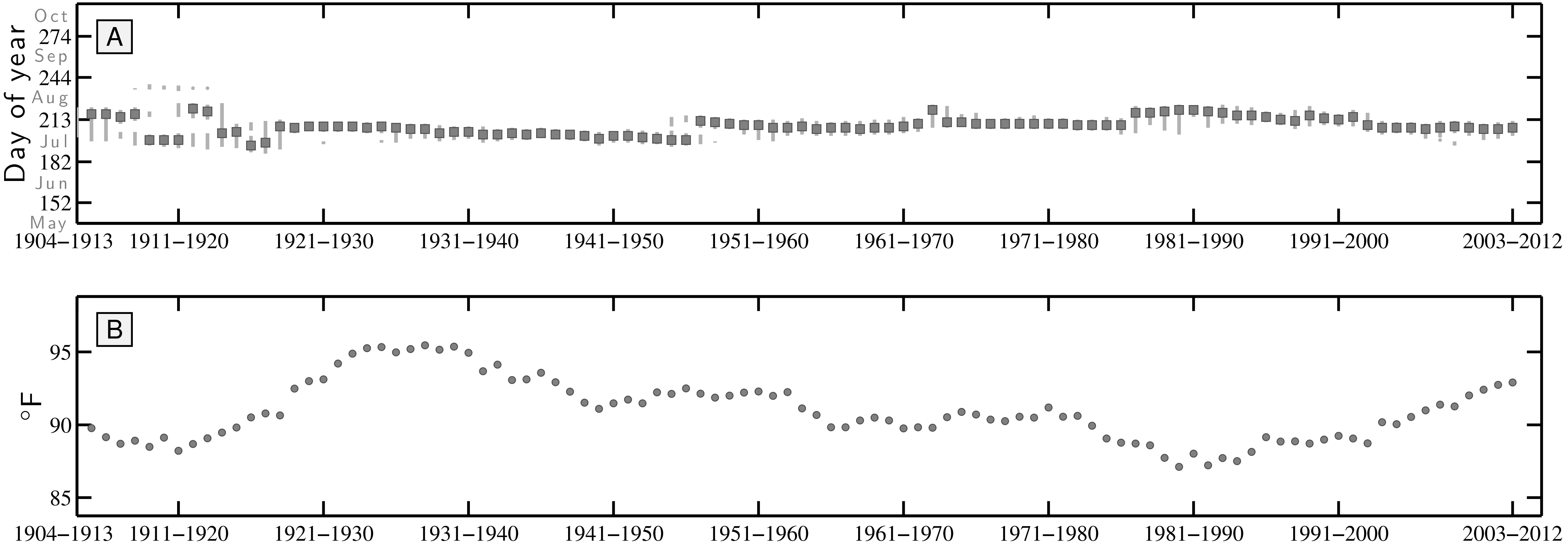

10 year Winter Teletherm dynamics for CEDARVILLE, CA:

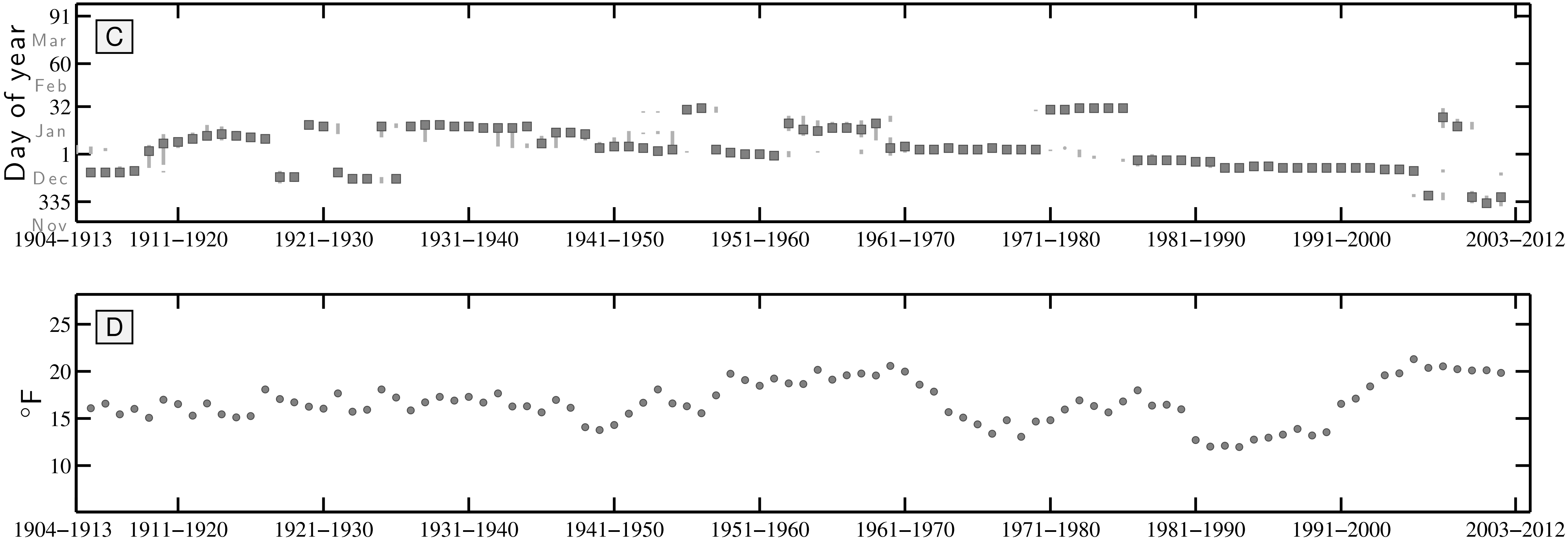

10 year Summer Teletherm dynamics for CHICO UNIV FARM, CA:

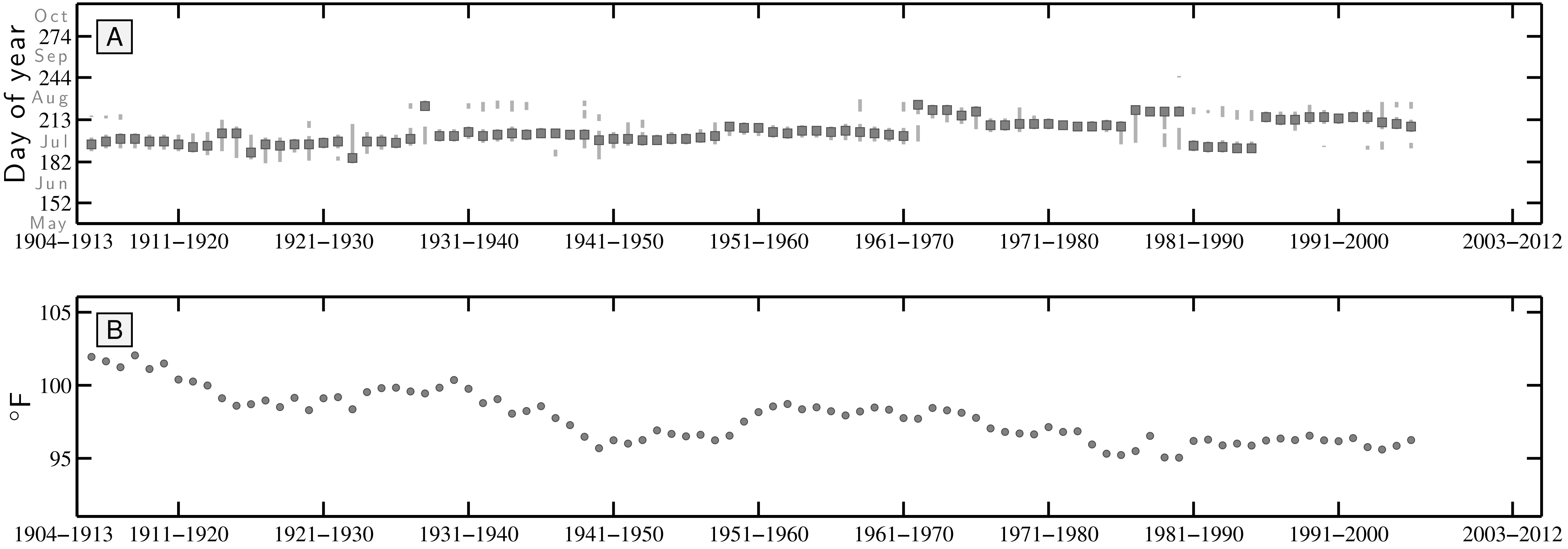

10 year Winter Teletherm dynamics for CHICO UNIV FARM, CA:

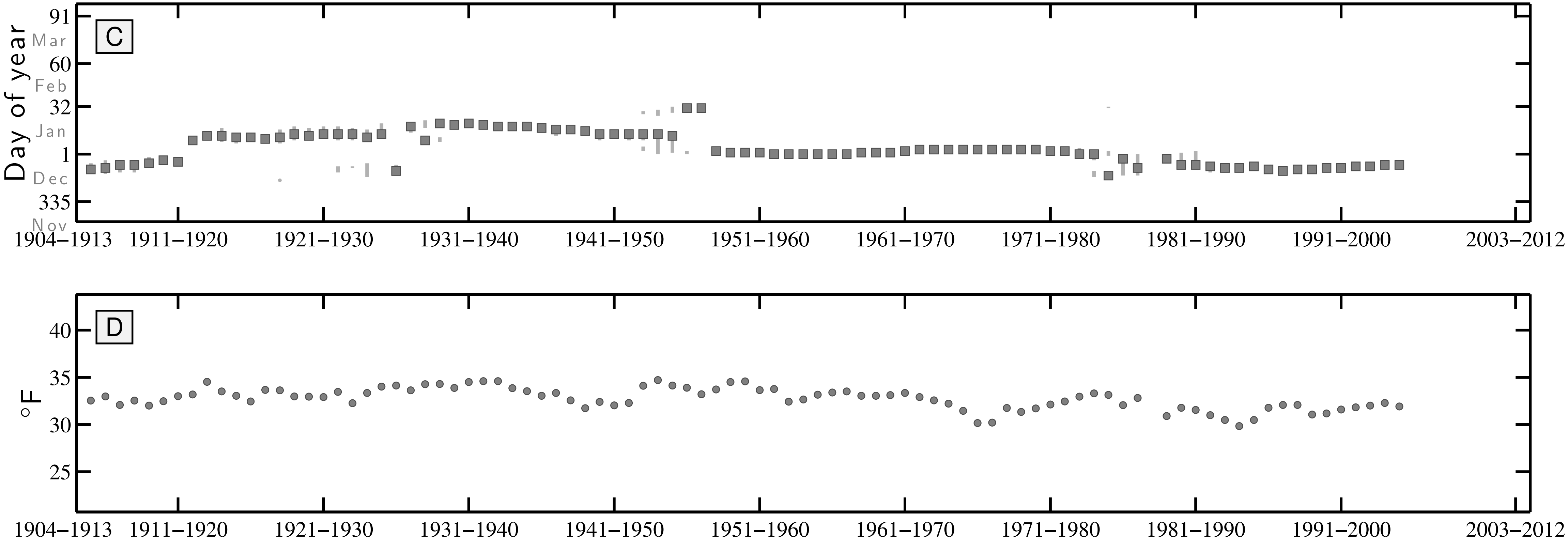

10 year Summer Teletherm dynamics for CHULA VISTA, CA:

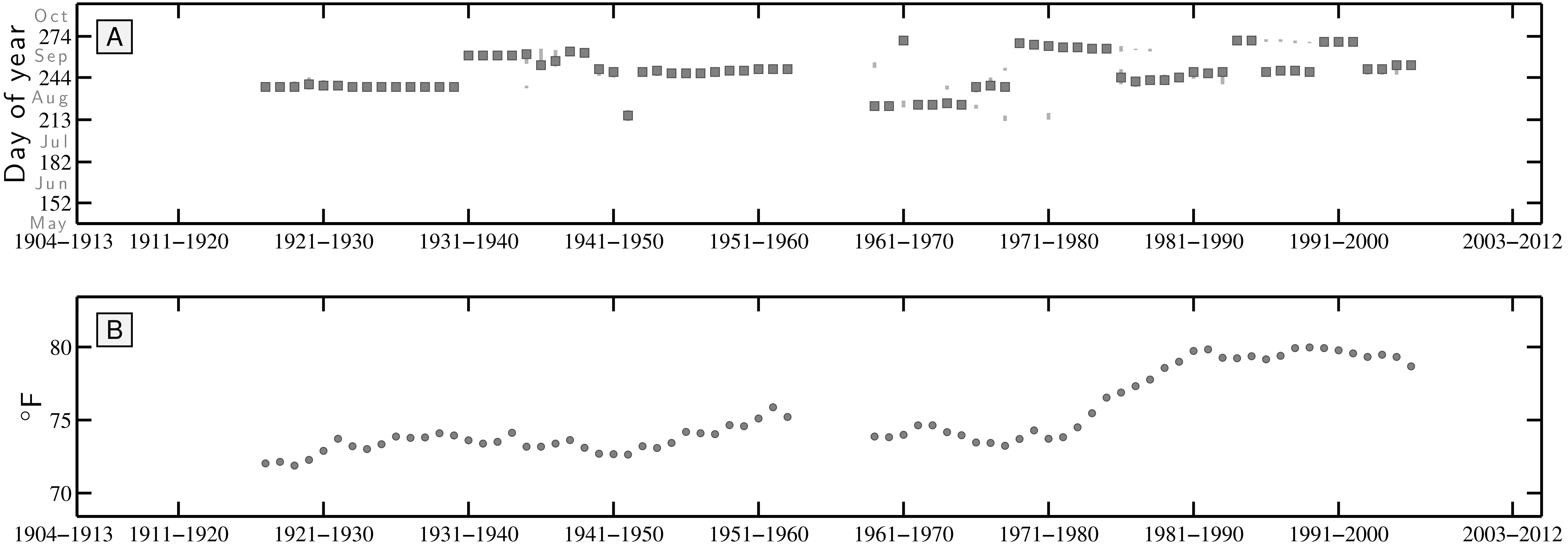

10 year Winter Teletherm dynamics for CHULA VISTA, CA:

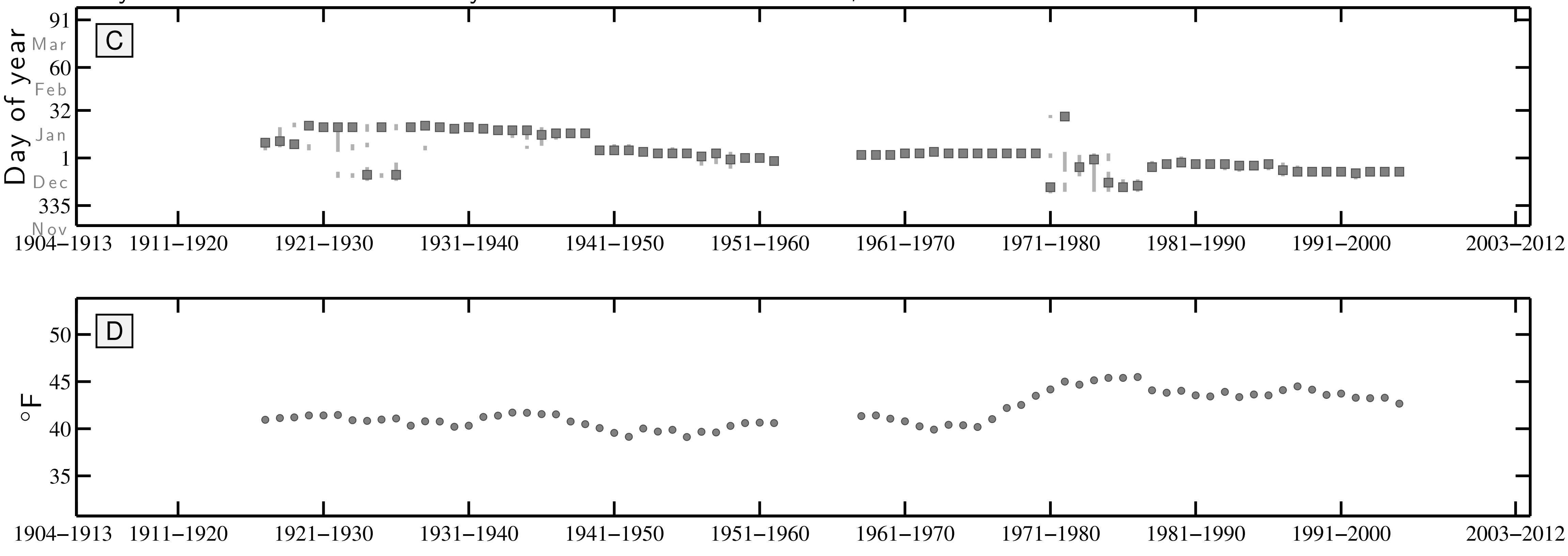

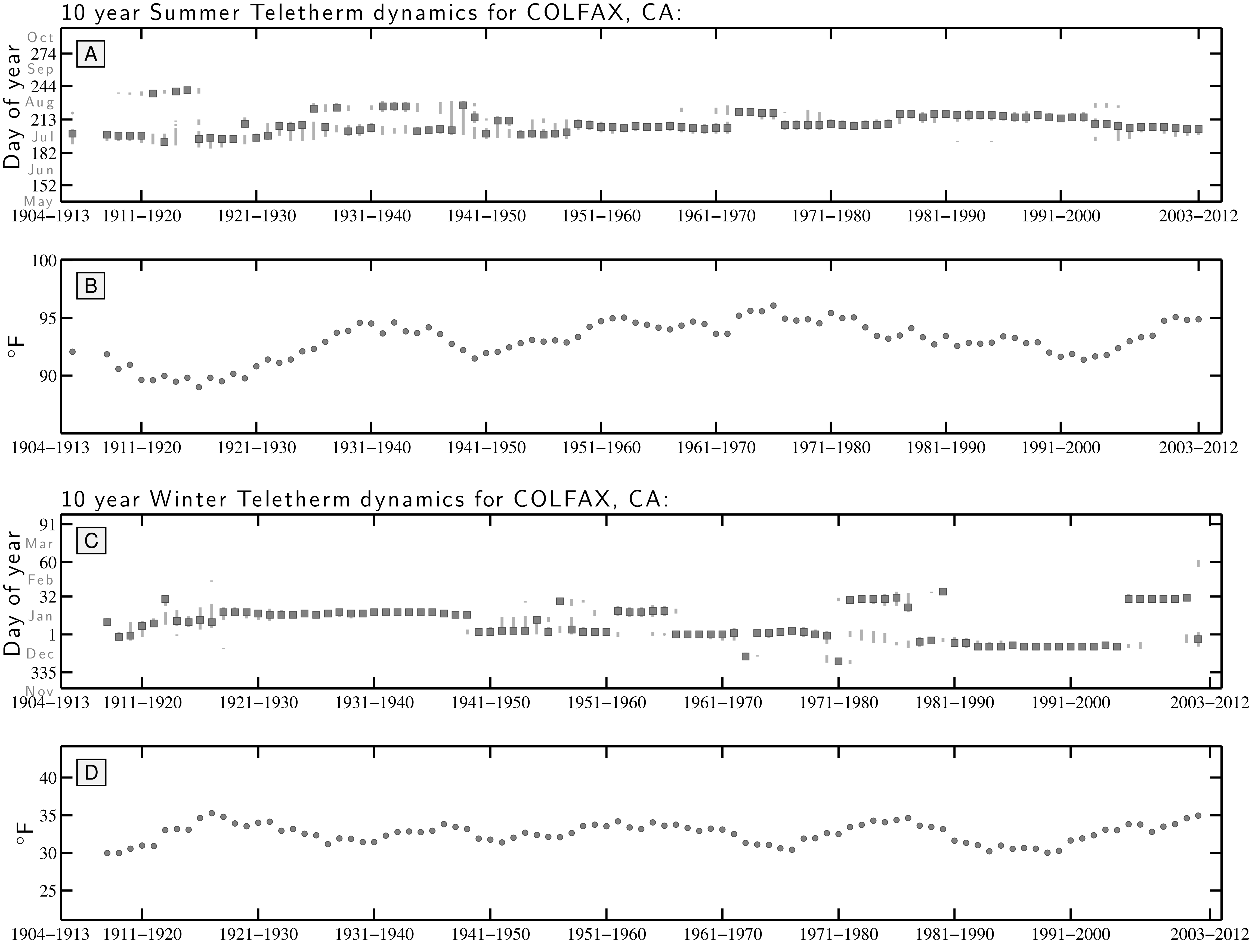
10 year Summer Teletherm dynamics for COLFAX, CA:
A
B
Day of year
°F
Oct
274
Sep
244
Aug
213
Jul
182
Jun
152
May
100
95
90
1904–1913
1911–1920
1921–1930
1931–1940
1941–1950
1951–1960
1961–1970
1971–1980
1981–1990
1991–2000
2003–2012
10 year Winter Teletherm dynamics for COLFAX, CA:
C
D
91
Mar
60
Feb
32
Jan
1
Dec
335
Nov
40
35
30
25

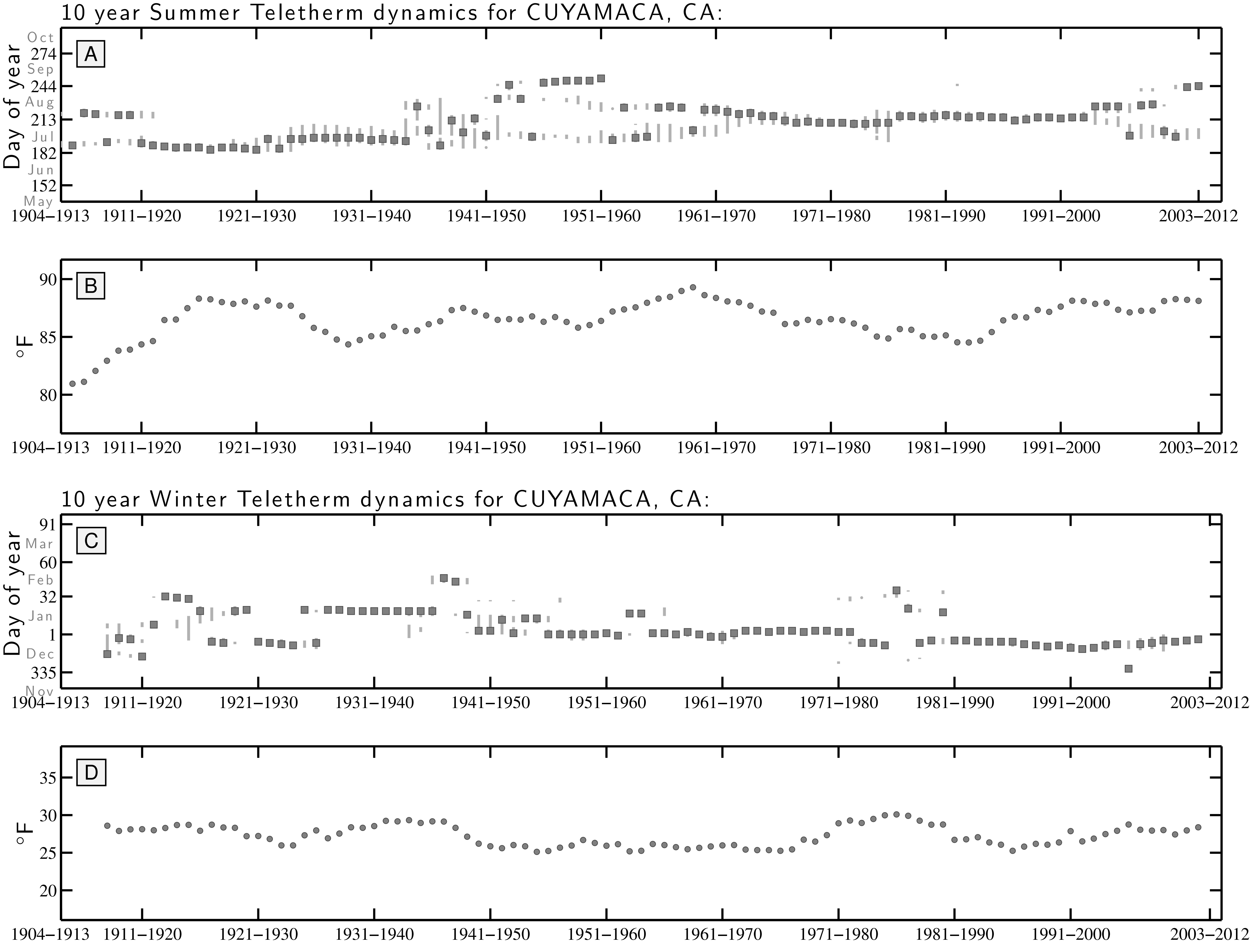
10 year Summer Teletherm dynamics for CUYAMACA, CA:
A
B
Day of year
°F
Oct
274
Sep
244
Aug
213
Jul
182
Jun
152
May
90
85
80
1904–1913
1911–1920
1921–1930
1931–1940
1941–1950
1951–1960
1961–1970
1971–1980
1981–1990
1991–2000
2003–2012
10 year Winter Teletherm dynamics for CUYAMACA, CA:
C
D
91
Mar
60
Feb
32
Jan
1
Dec
335
Nov
35
30
25
20

10 year Summer Teletherm dynamics for DAVIS 2 WSW EXP, CA:

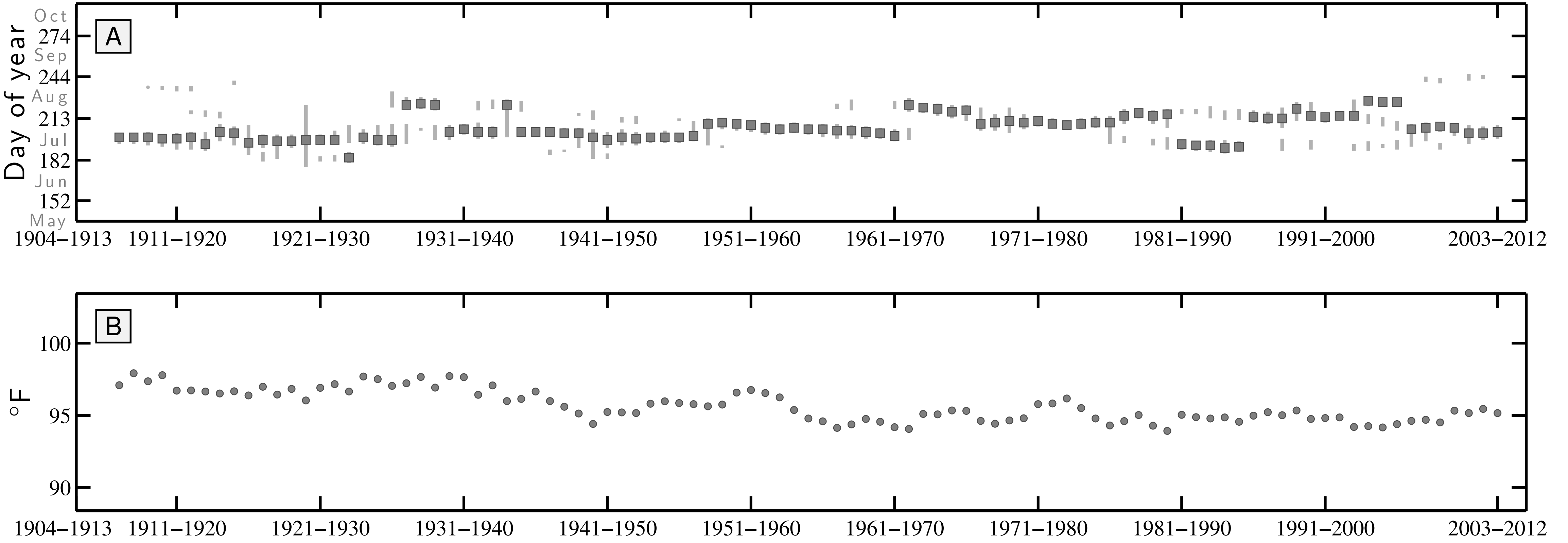

10 year Winter Teletherm dynamics for DAVIS 2 WSW EXP, CA:

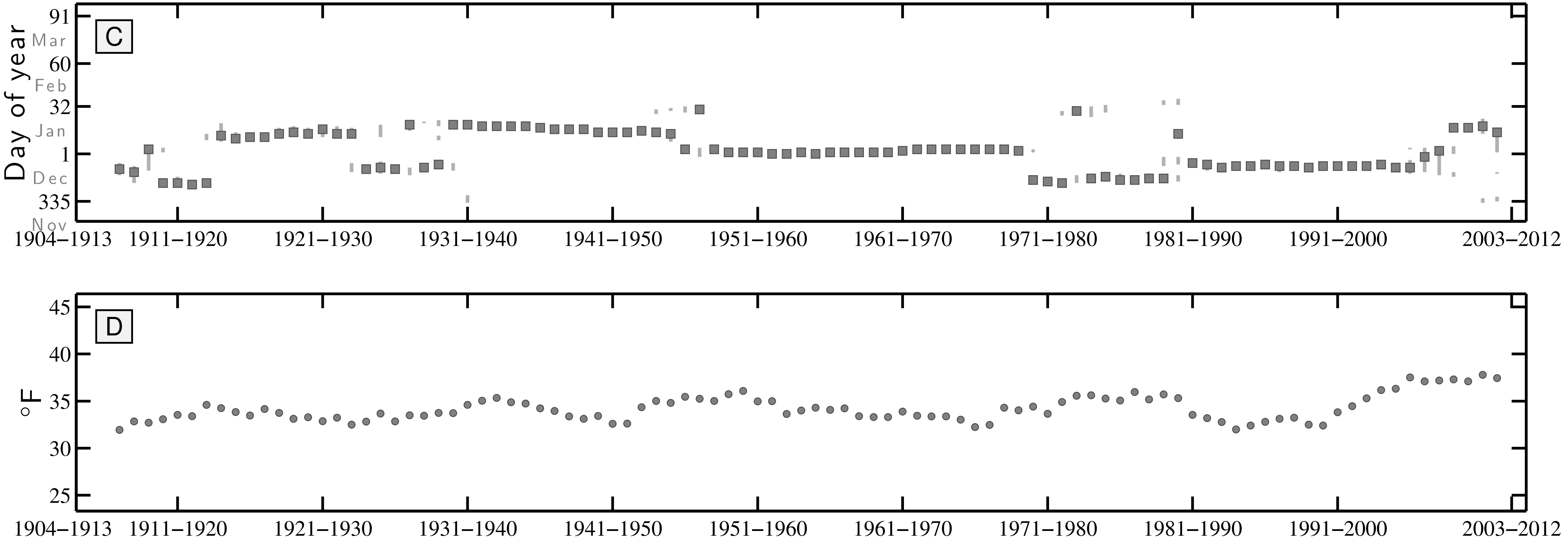

10 year Summer Teletherm dynamics for DEATH VALLEY, CA:

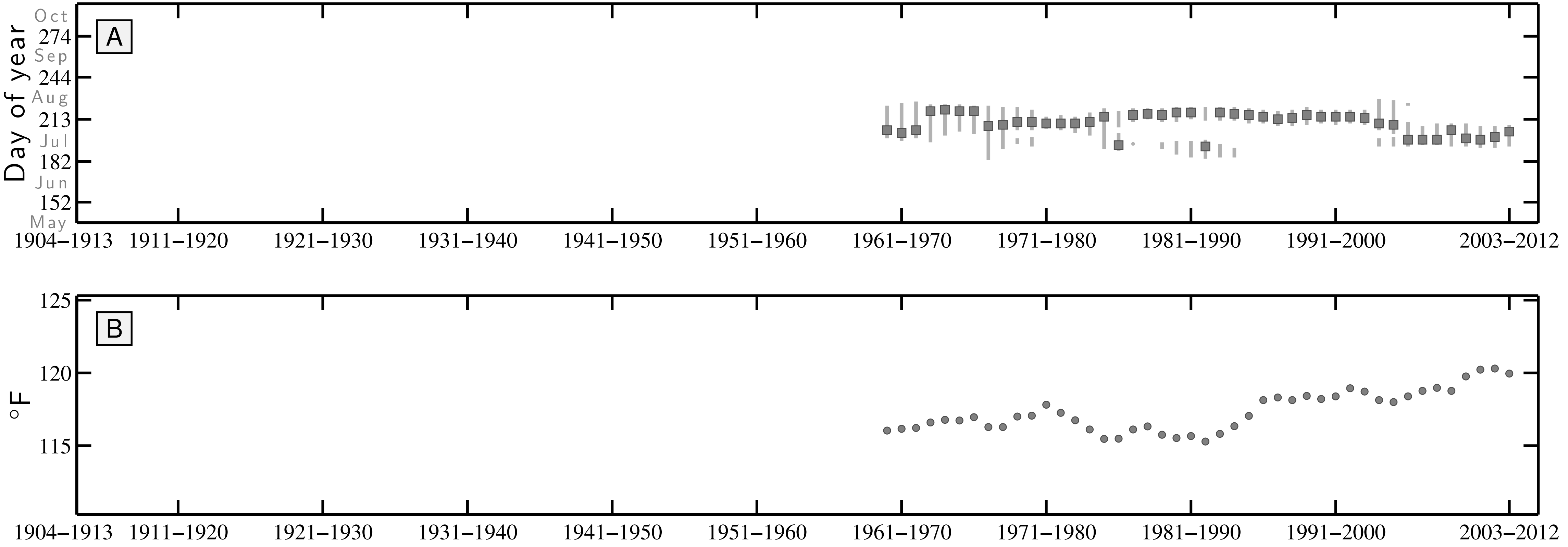

10 year Winter Teletherm dynamics for DEATH VALLEY, CA:

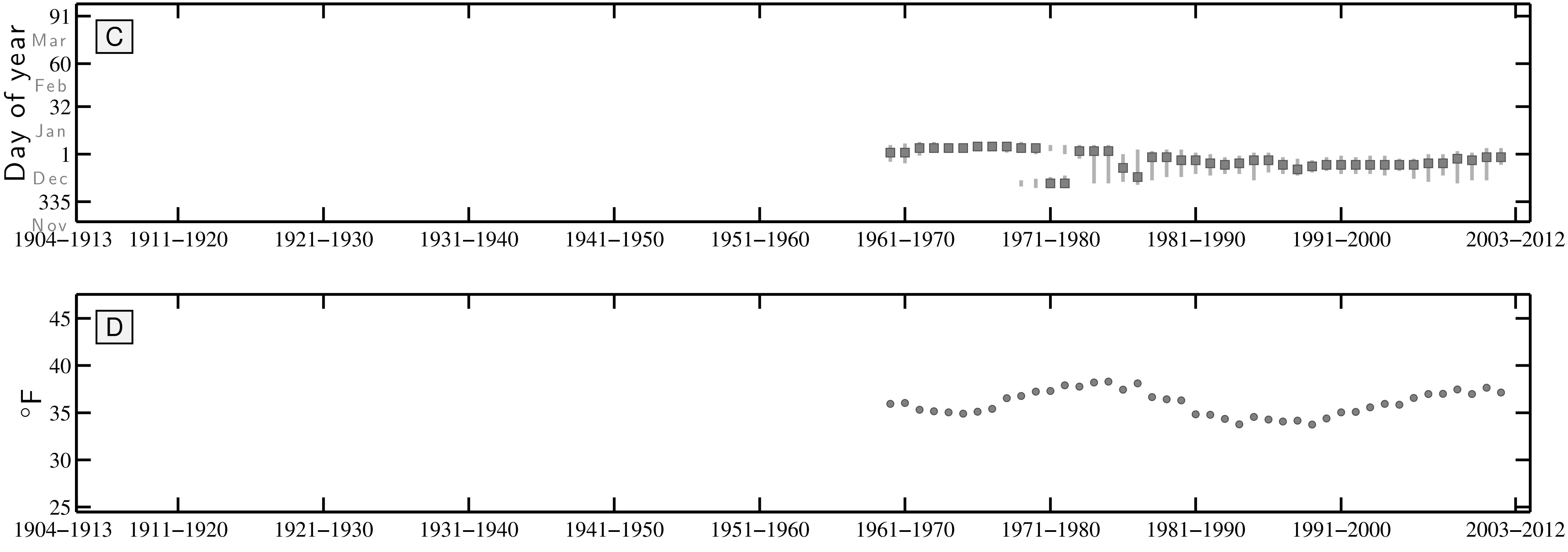

10 year Summer Teletherm dynamics for ELECTRA P H, CA:

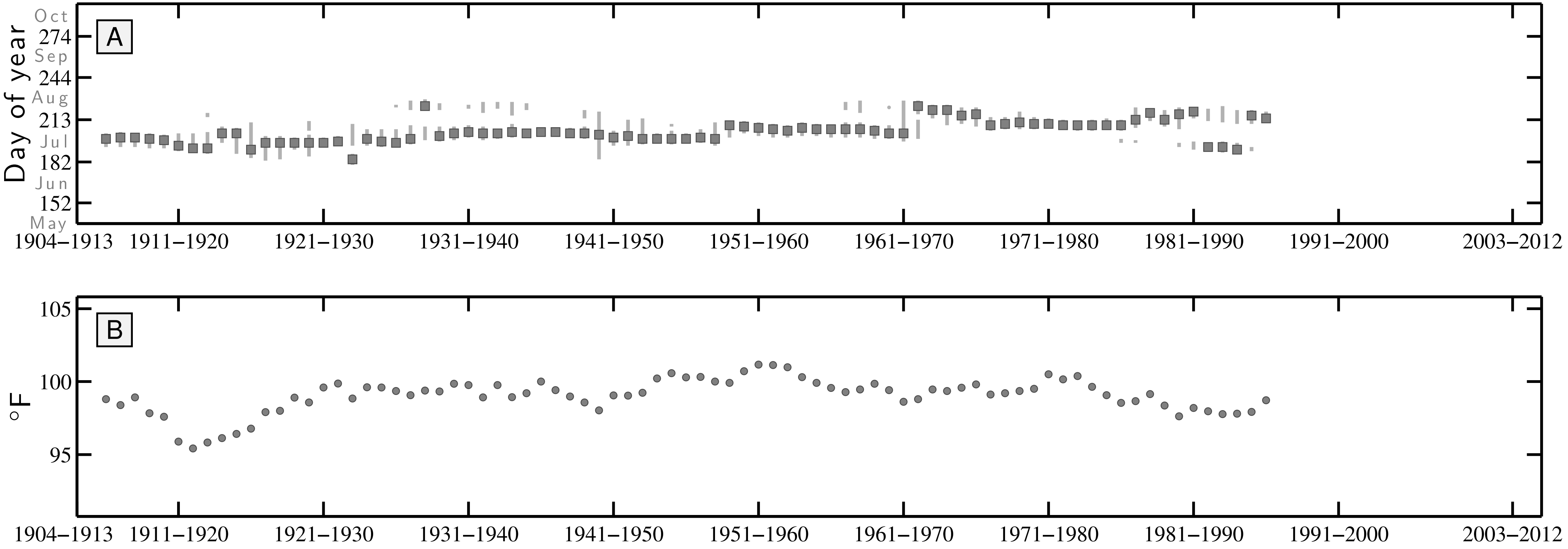

10 year Winter Teletherm dynamics for ELECTRA P H, CA:

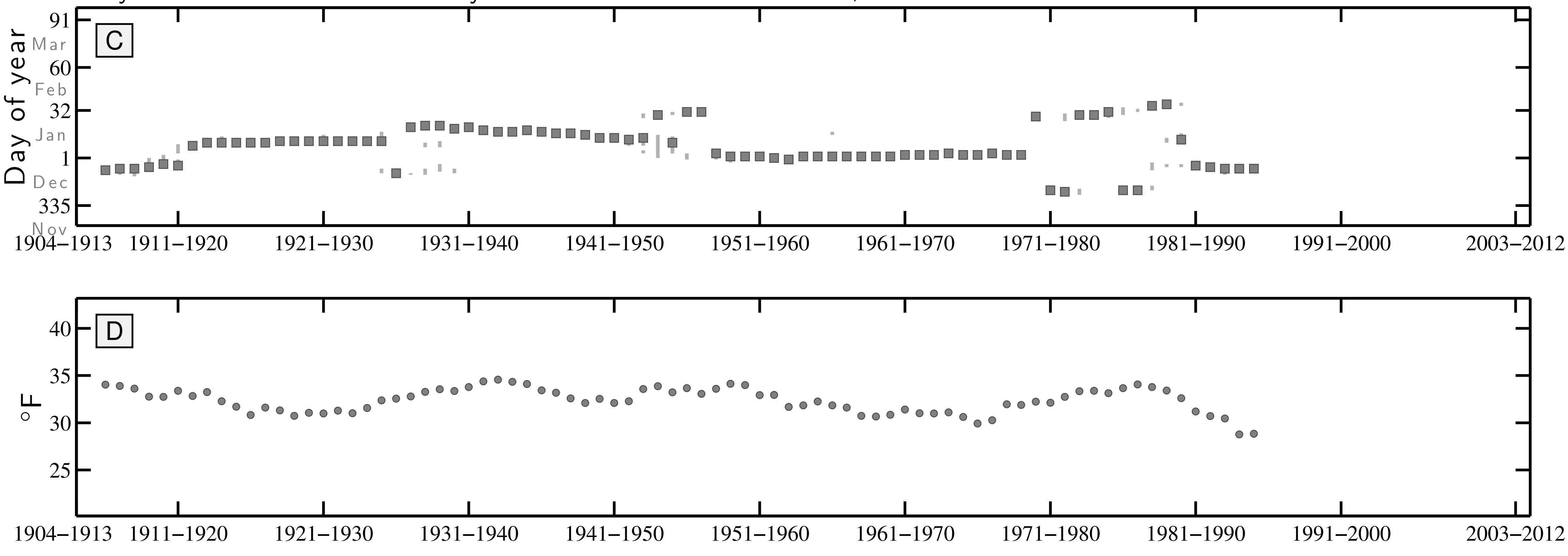

10 year Summer Teletherm dynamics for EUREKA WFO WOODLEY IS, CA:

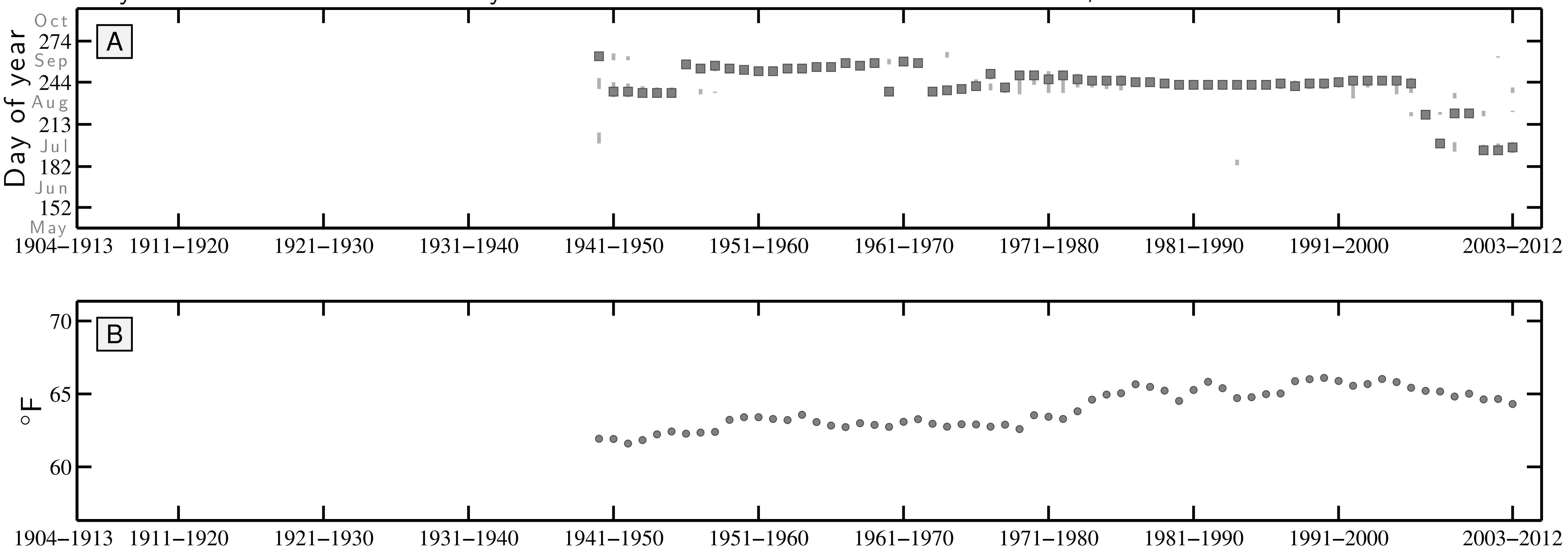

10 year Winter Teletherm dynamics for EUREKA WFO WOODLEY IS, CA:

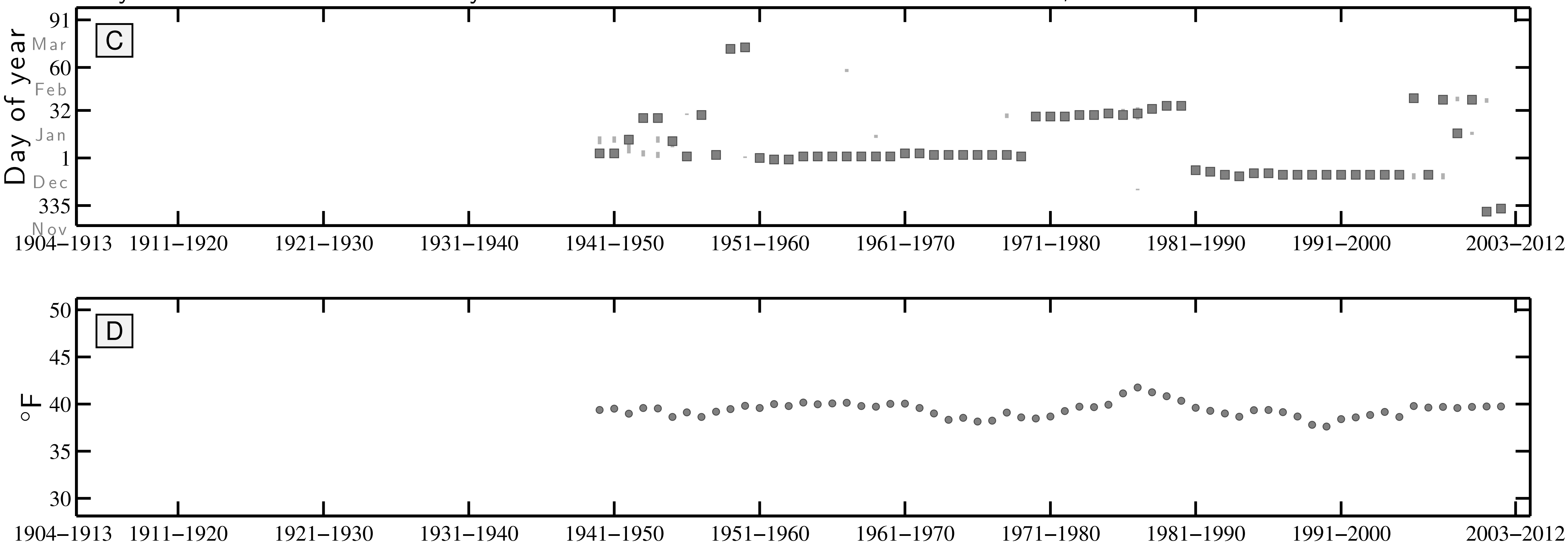

10 year Summer Teletherm dynamics for FAIRMONT, CA:

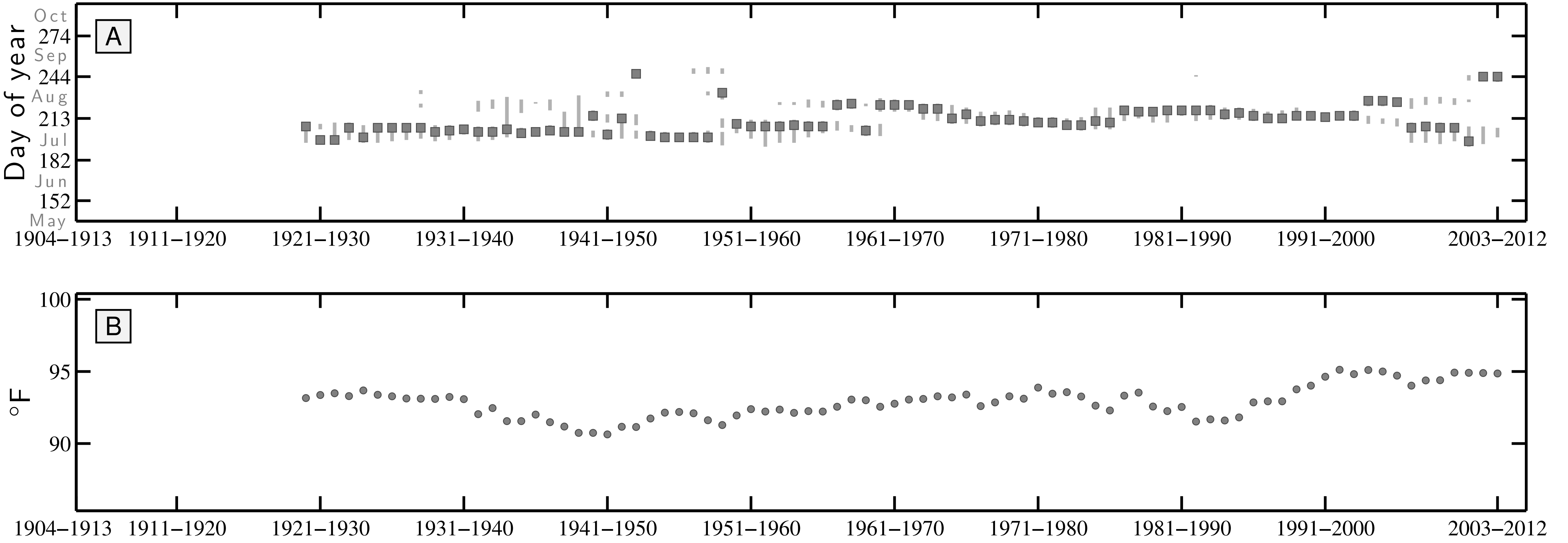

10 year Winter Teletherm dynamics for FAIRMONT, CA:

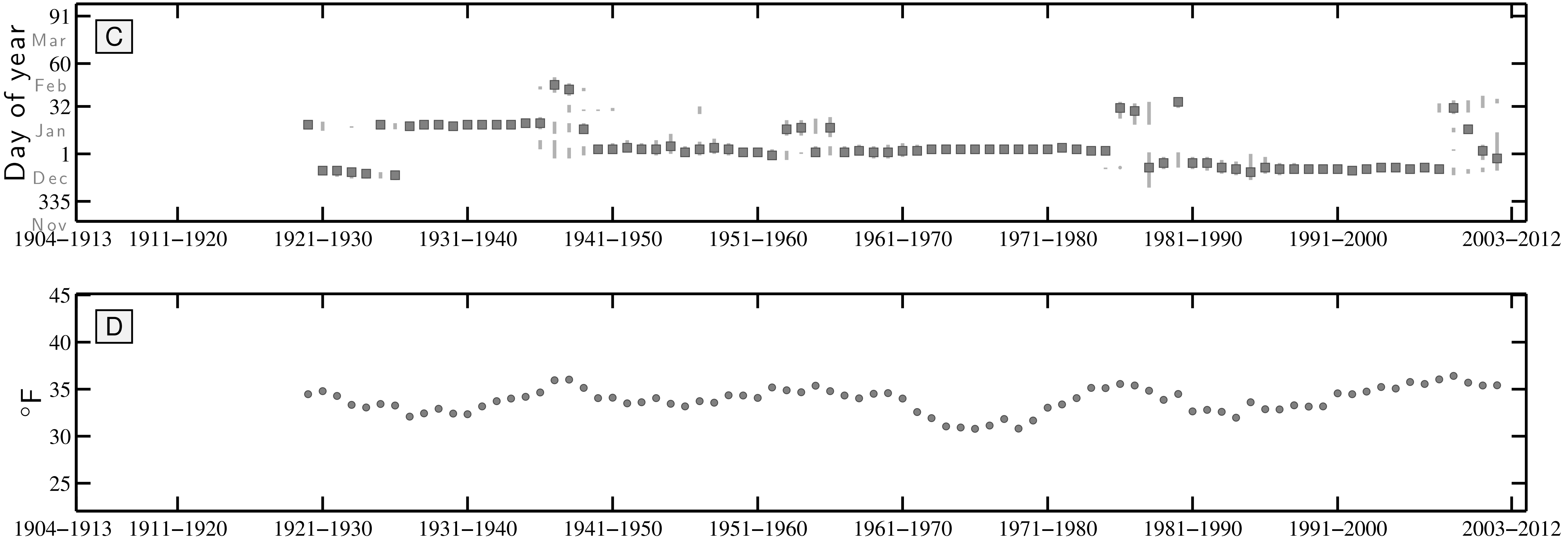

10 year Summer Teletherm dynamics for FT BRAGG 5 N, CA:

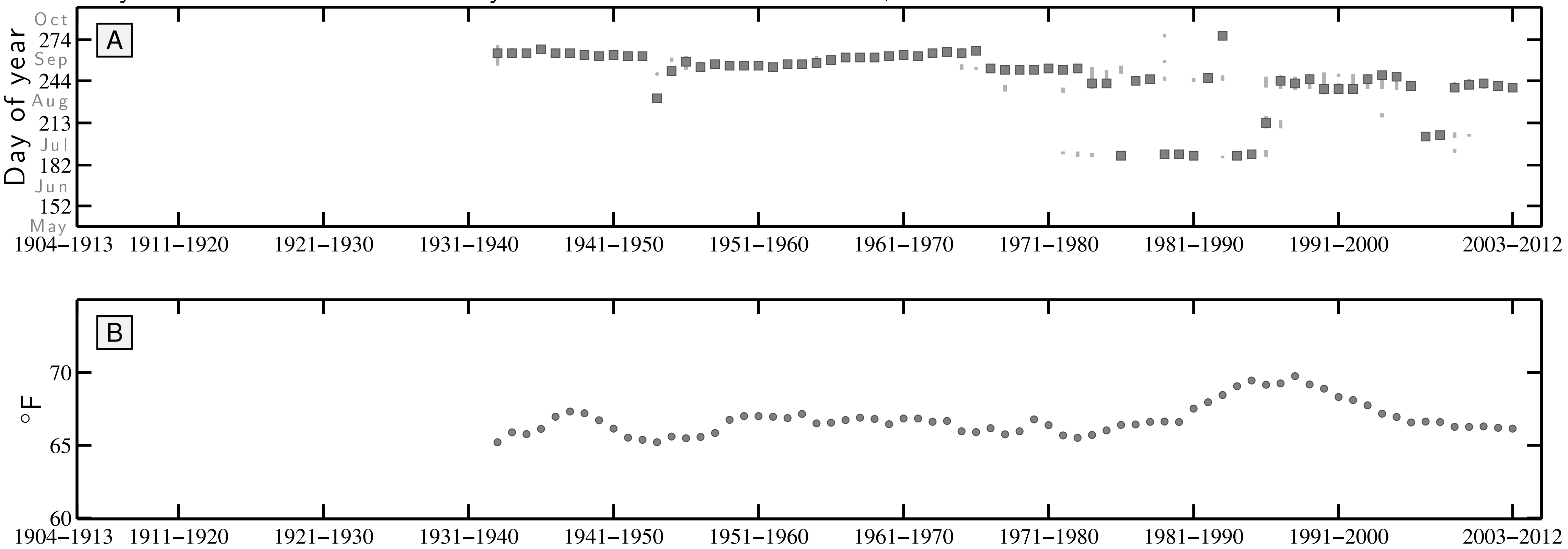

10 year Winter Teletherm dynamics for FT BRAGG 5 N, CA:

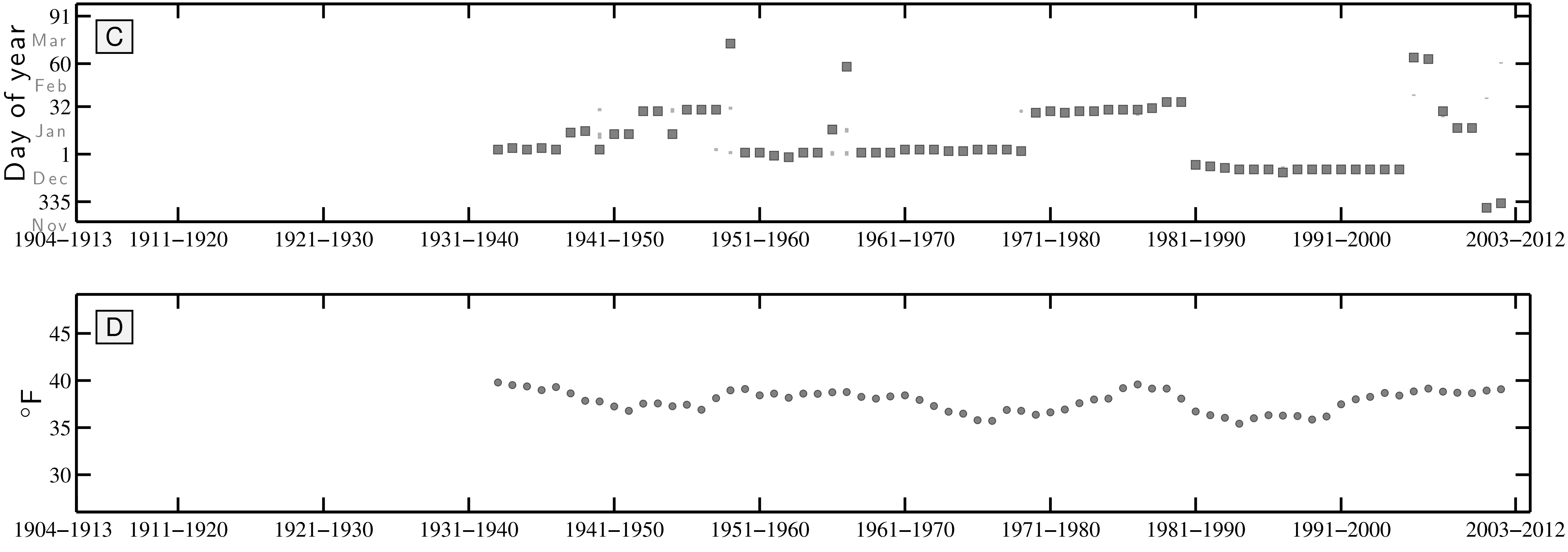

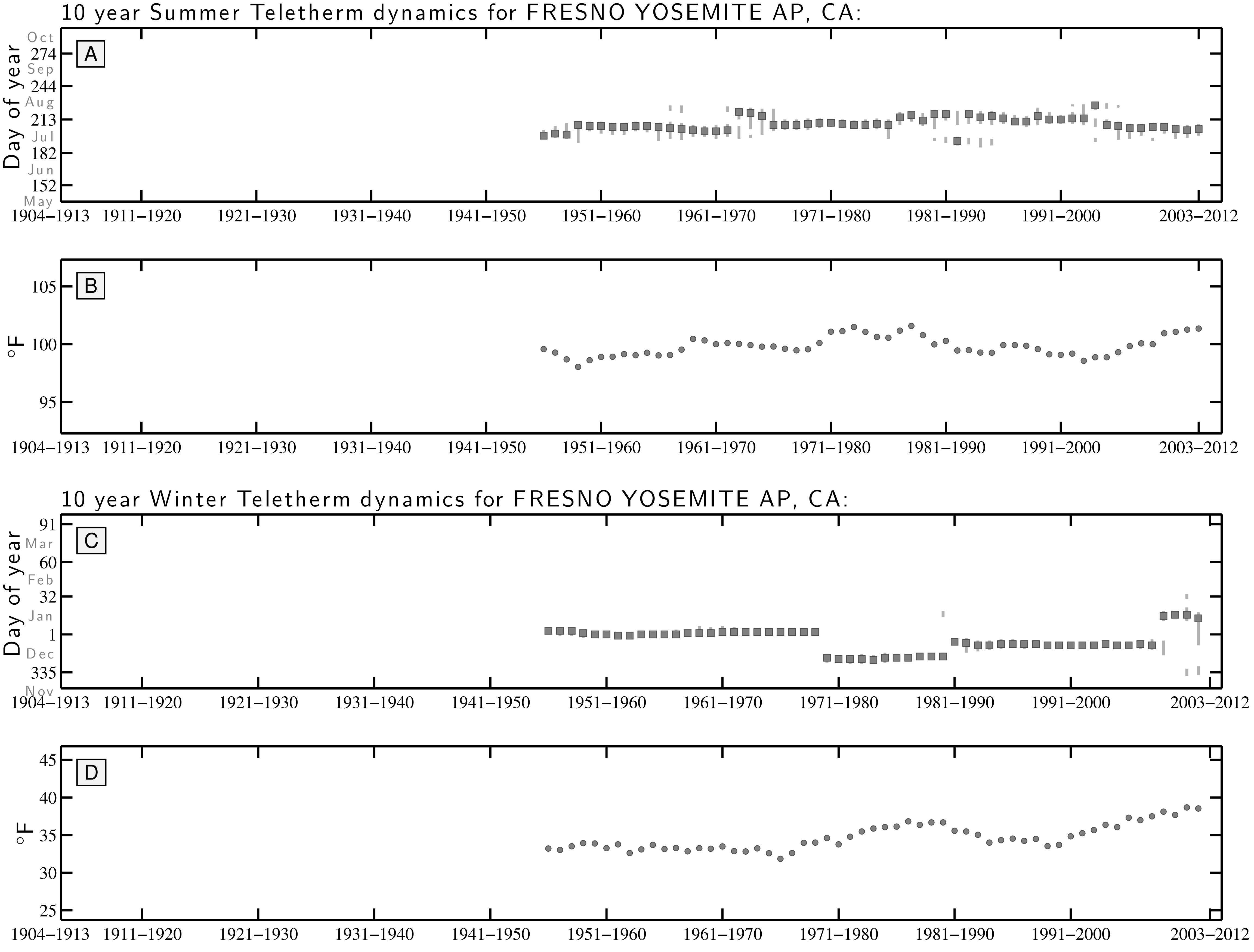
10 year Summer Teletherm dynamics for FRESNO YOSEMITE AP, CA:
A
Day of year
Oct
274
Sep
244
Aug
213
Jul
182
Jun
152
May
B
°F
105
100
95
10 year Winter Teletherm dynamics for FRESNO YOSEMITE AP, CA:
C
Day of year
91
Mar
60
Feb
32
Jan
1
Dec
335
Nov
D
°F
45
40
35
30
25
1904–1913
1911–1920
1921–1930
1931–1940
1941–1950
1951–1960
1961–1970
1971–1980
1981–1990
1991–2000
2003–2012

10 year Summer Teletherm dynamics for HANFORD 1 S, CA:

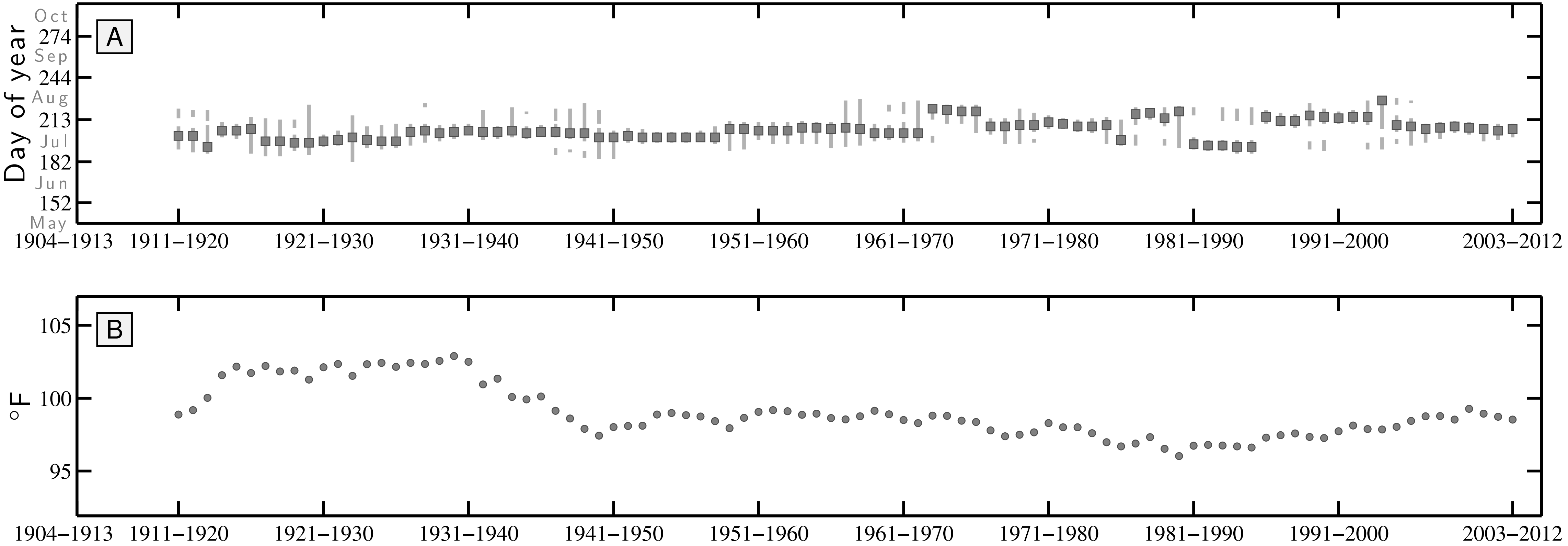

10 year Winter Teletherm dynamics for HANFORD 1 S, CA:

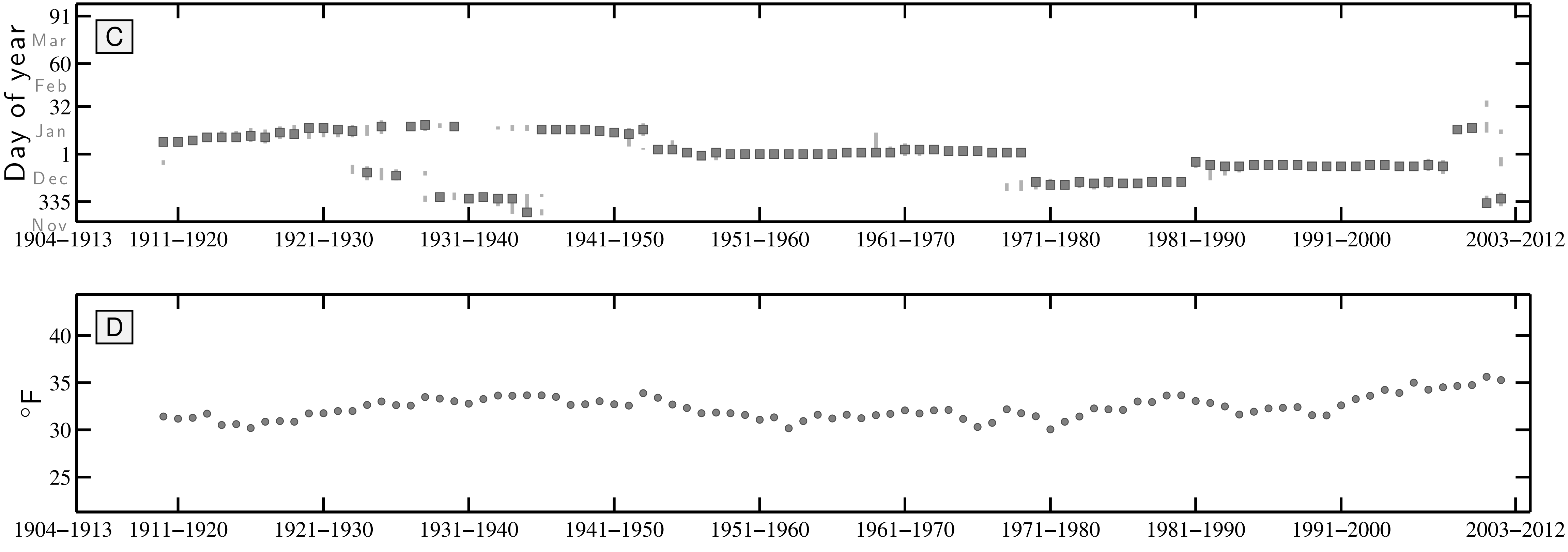

10 year Summer Teletherm dynamics for HAPPY CAMP RS, CA:

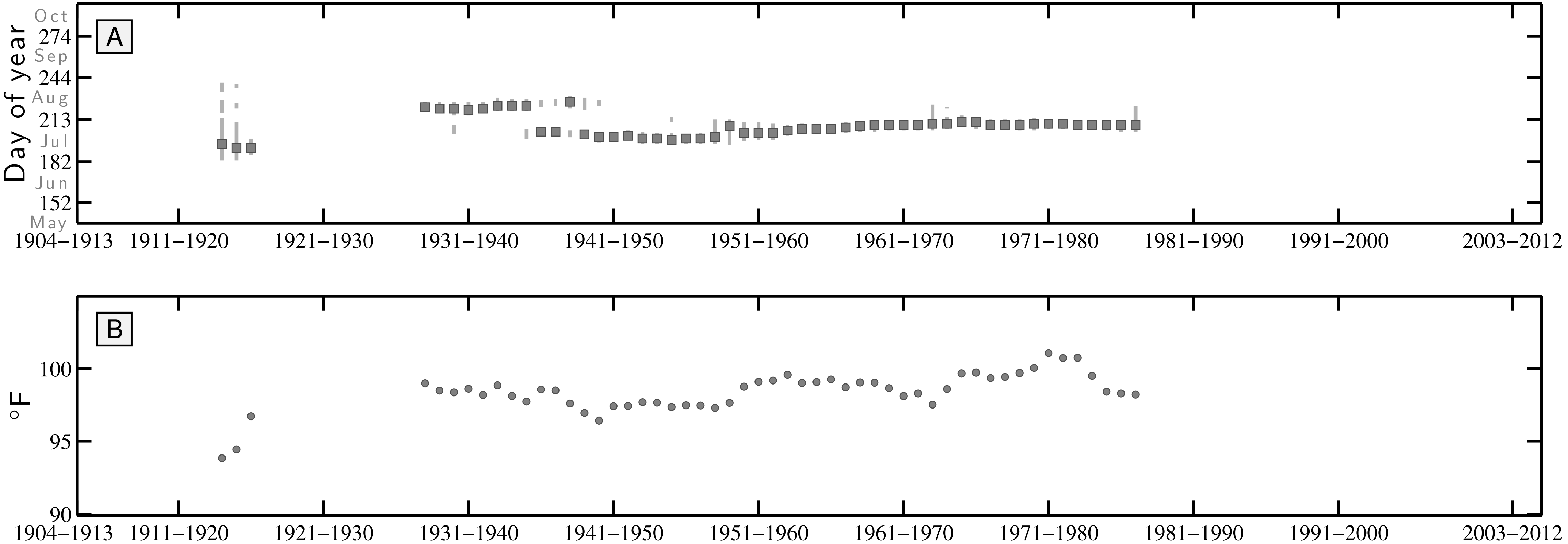

10 year Winter Teletherm dynamics for HAPPY CAMP RS, CA:

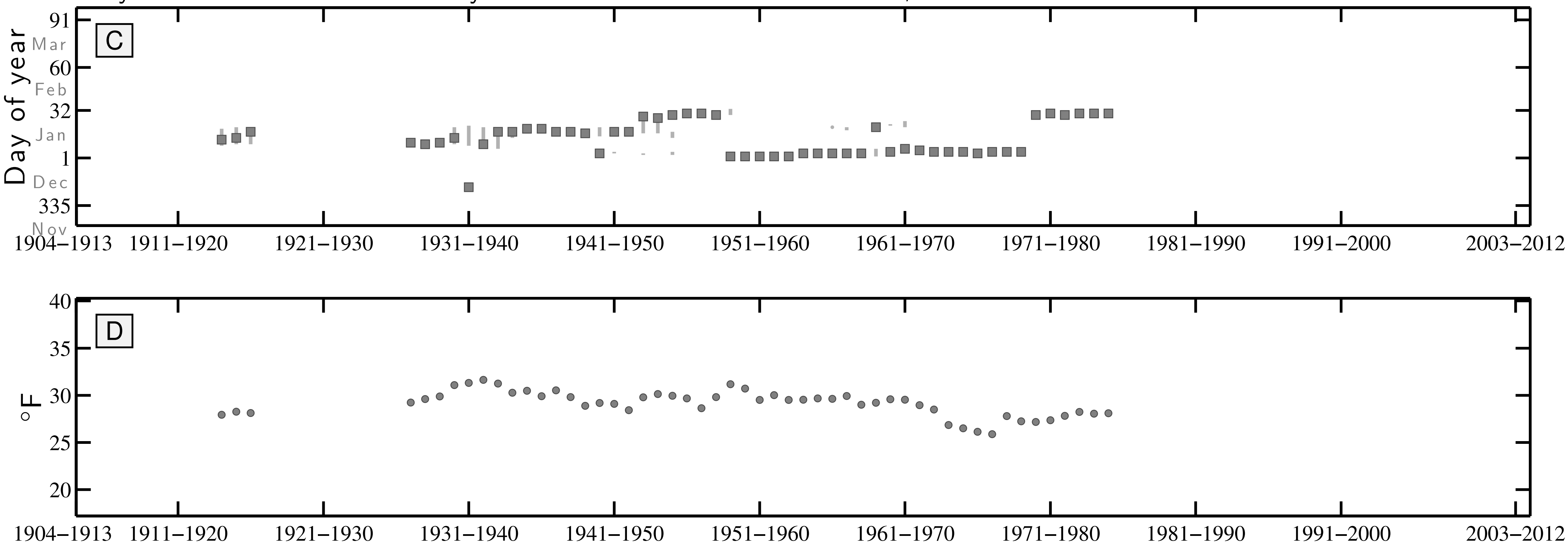

10 year Summer Teletherm dynamics for HEALDSBURG, CA:

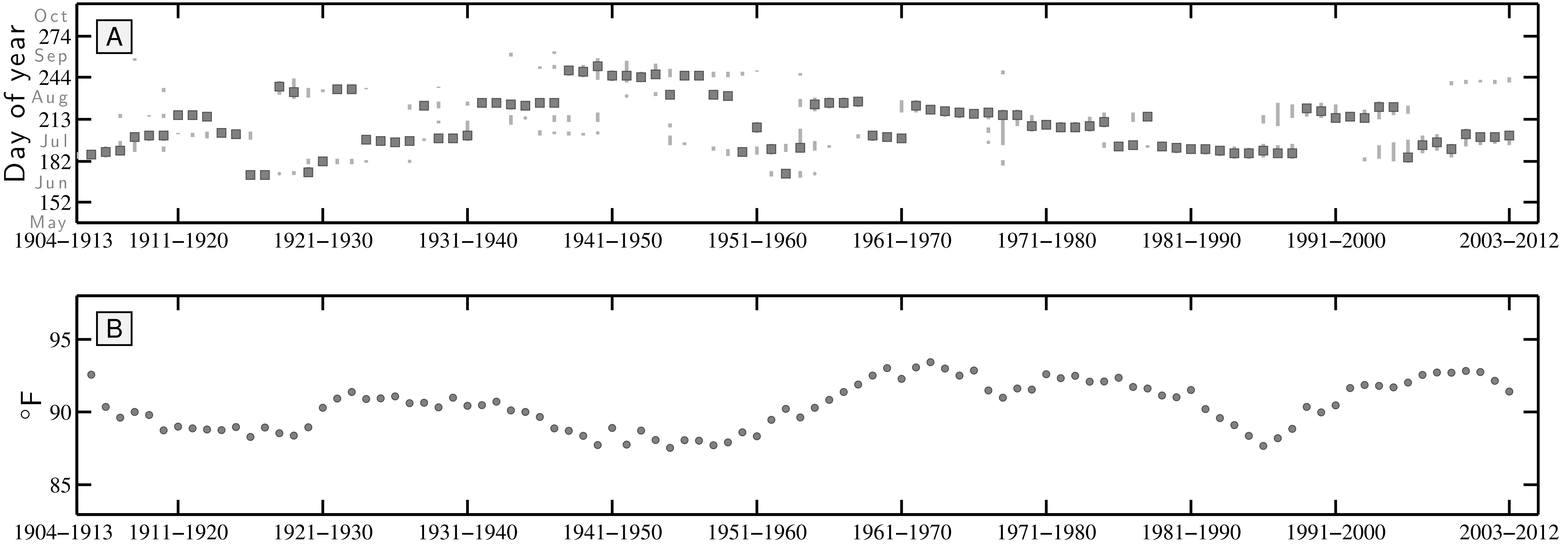

10 year Winter Teletherm dynamics for HEALDSBURG, CA:

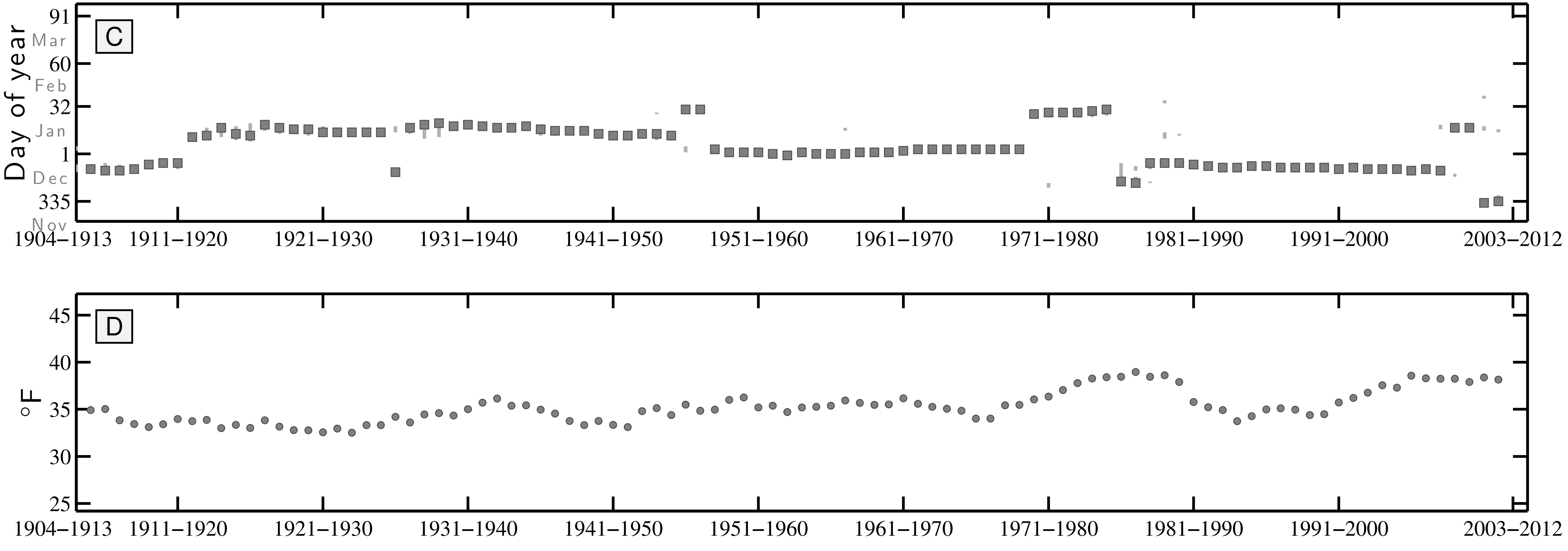

10 year Summer Teletherm dynamics for INDEPENDENCE, CA:

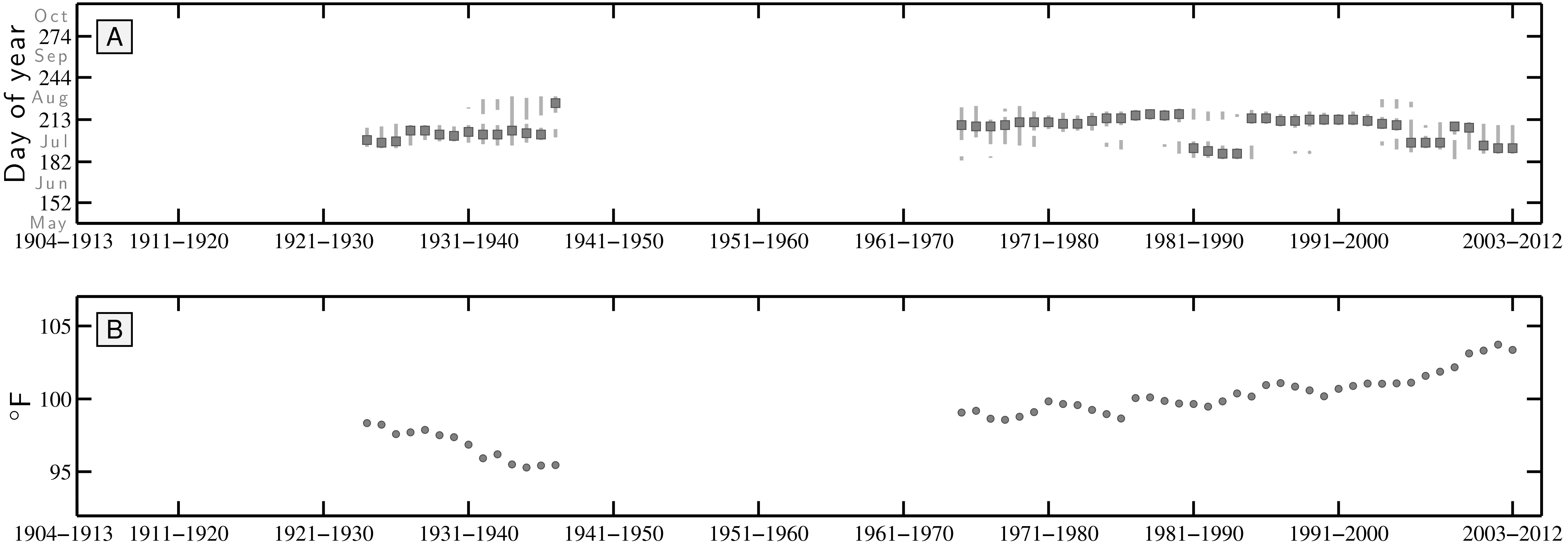

10 year Winter Teletherm dynamics for INDEPENDENCE, CA:

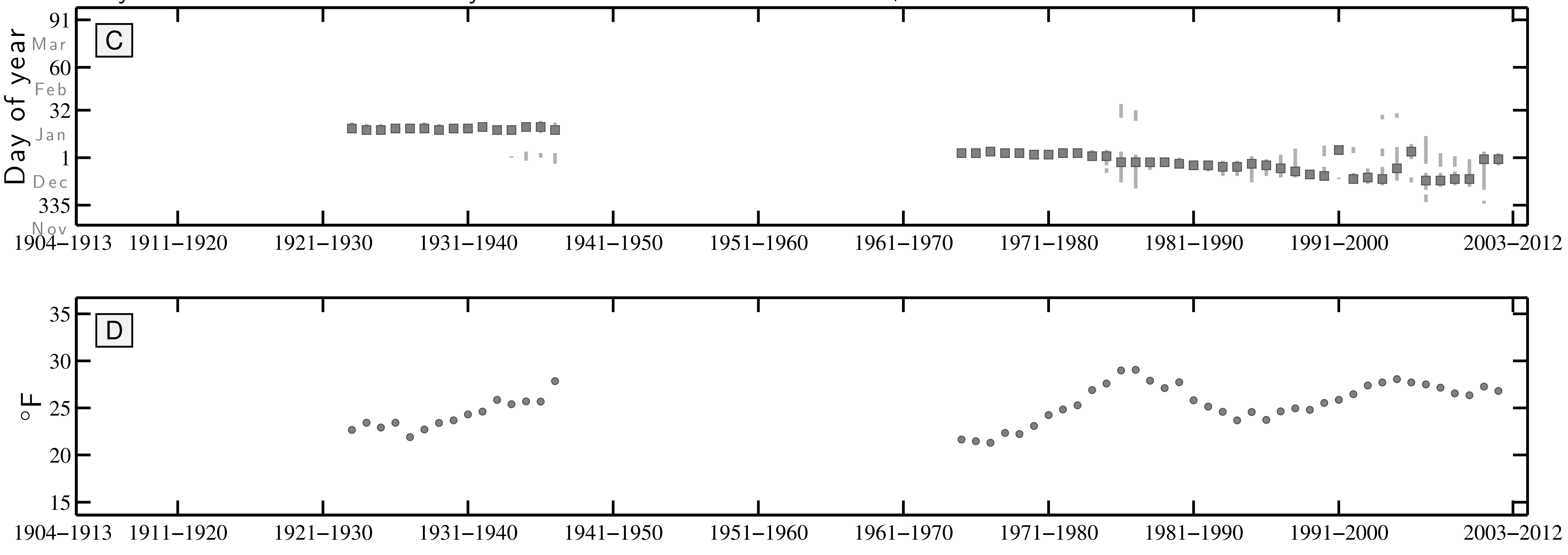

10 year Summer Teletherm dynamics for INDIO FIRE STN, CA:

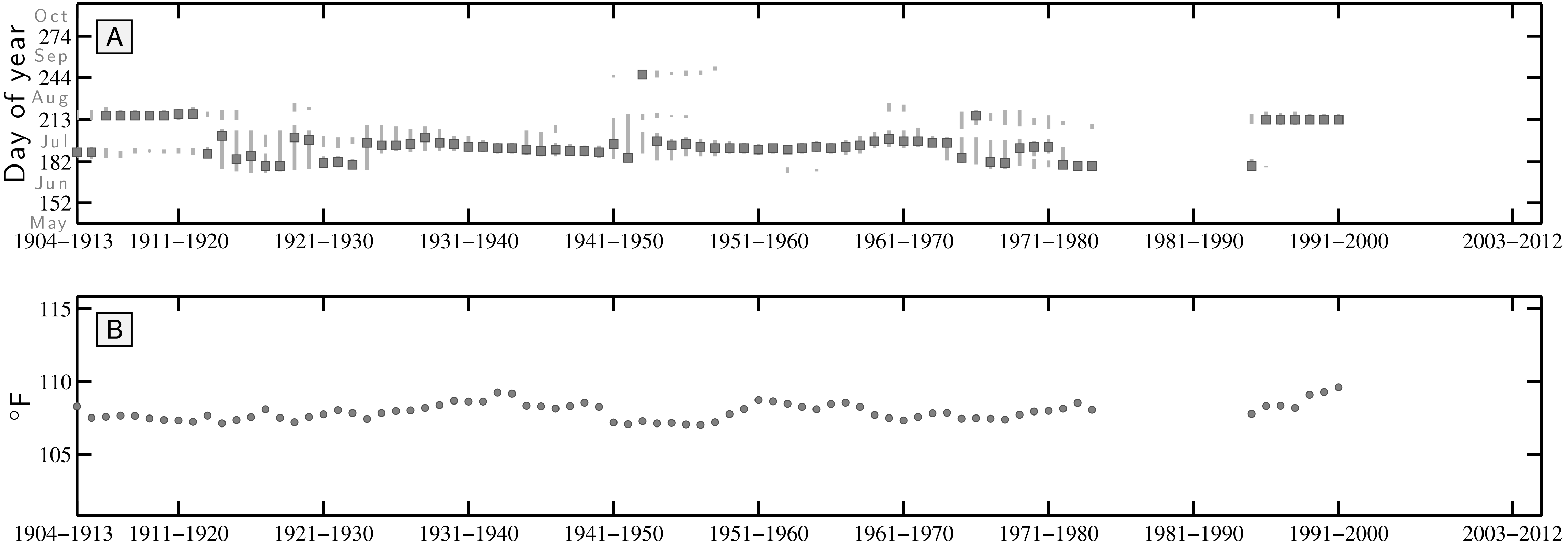

10 year Winter Teletherm dynamics for INDIO FIRE STN, CA:

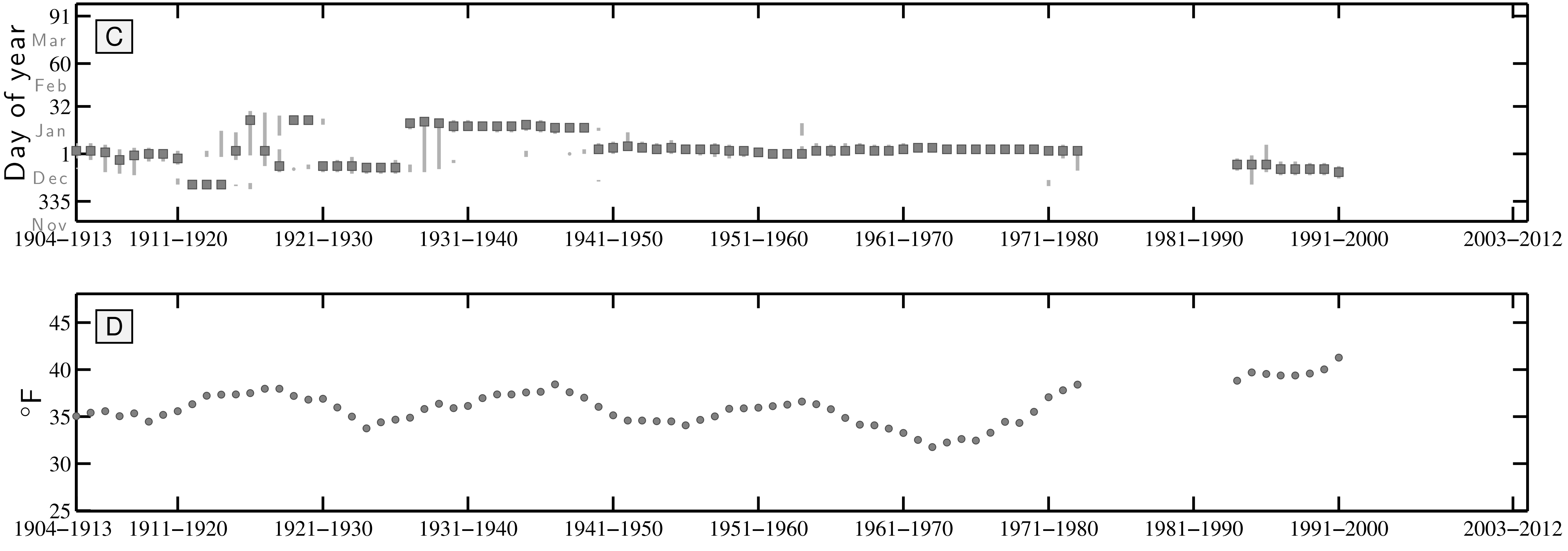

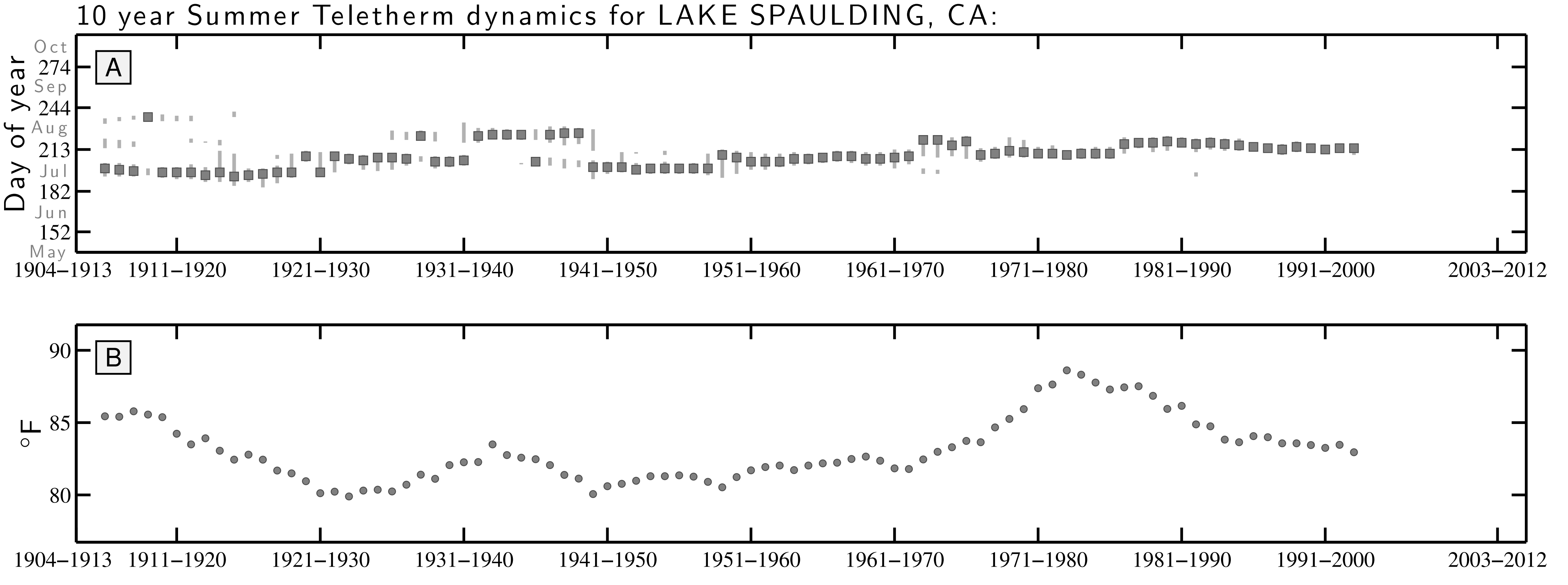
10 year Summer Teletherm dynamics for LAKE SPAULDING, CA:
A
Day of year
Oct
274
Sep
244
Aug
213
Jul
182
Jun
152
May
B
°F
90
85
80
1904–1913
1911–1920
1921–1930
1931–1940
1941–1950
1951–1960
1961–1970
1971–1980
1981–1990
1991–2000
2003–2012

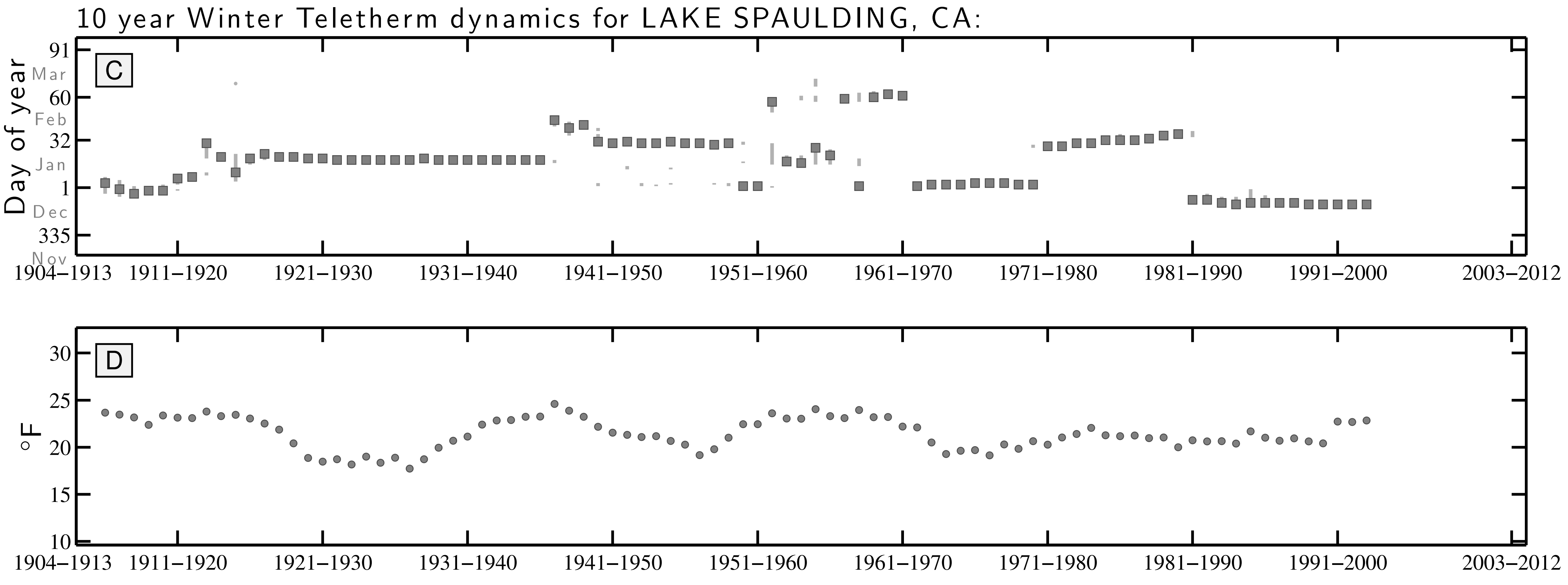
10 year Winter Teletherm dynamics for LAKE SPAULDING, CA:
C
Day of year
91
Mar
60
Feb
32
Jan
1
Dec
335
Nov
D
°F
30
25
20
15
10
1904–1913
1911–1920
1921–1930
1931–1940
1941–1950
1951–1960
1961–1970
1971–1980
1981–1990
1991–2000
2003–2012

10 year Summer Teletherm dynamics for LEMON COVE, CA:

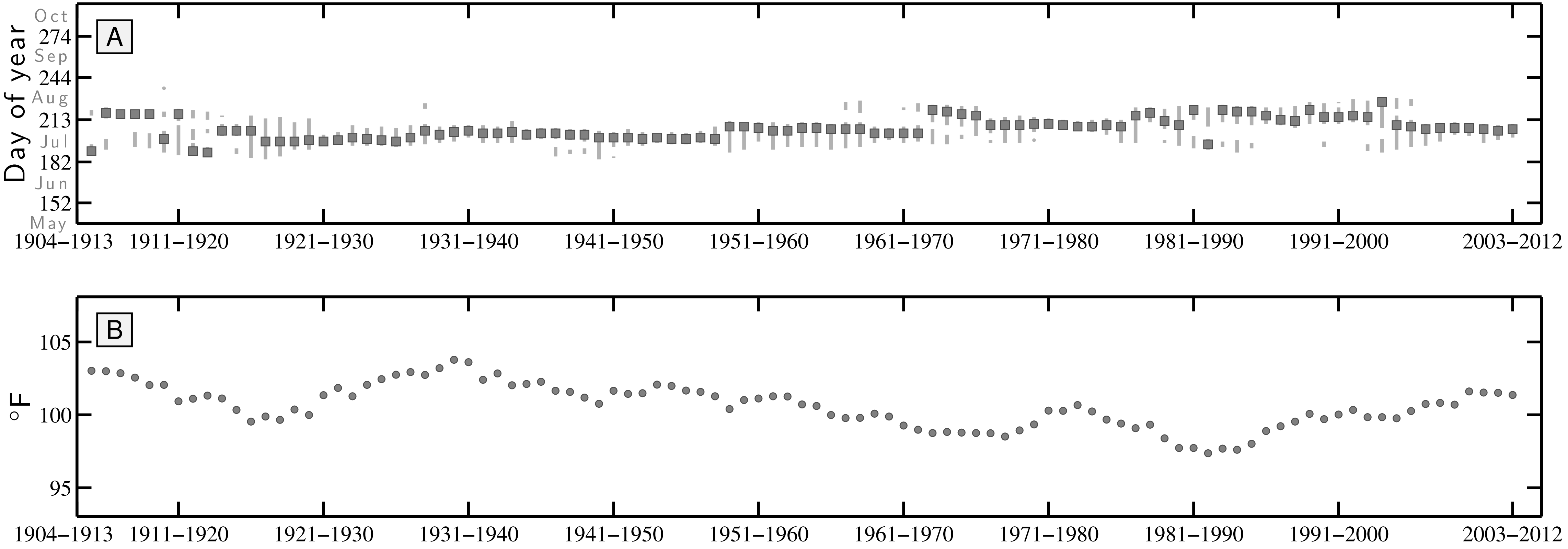

10 year Winter Teletherm dynamics for LEMON COVE, CA:

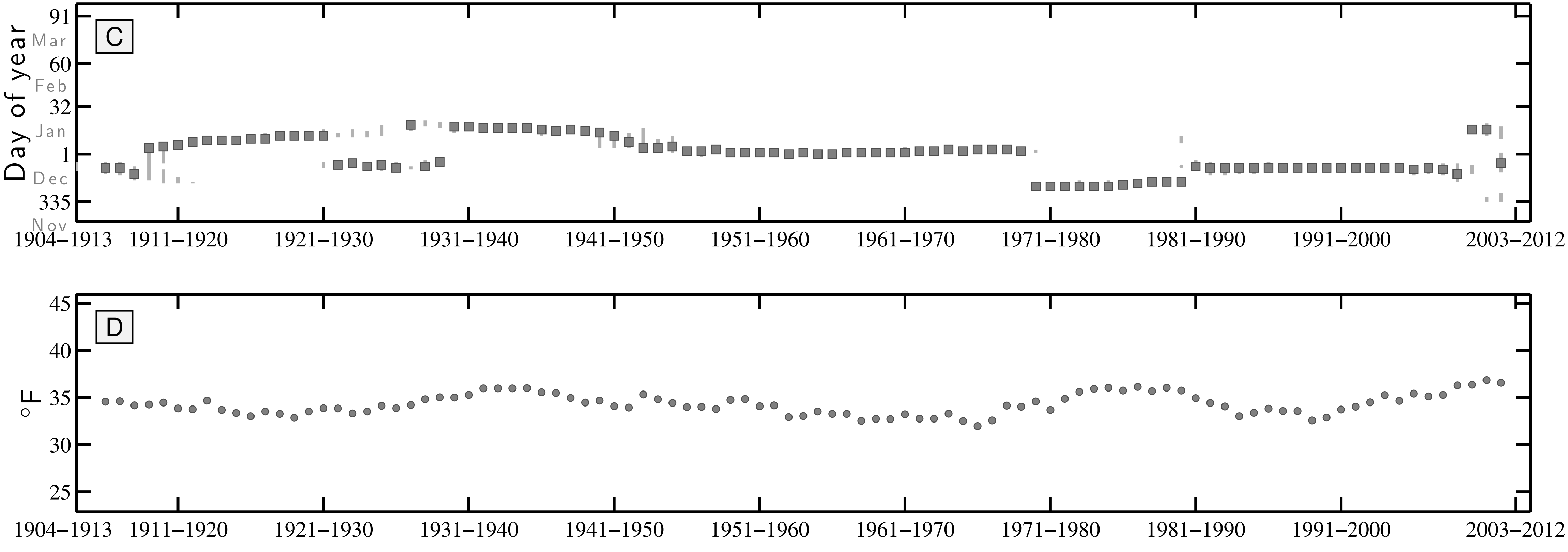

10 year Summer Teletherm dynamics for LIVERMORE, CA:

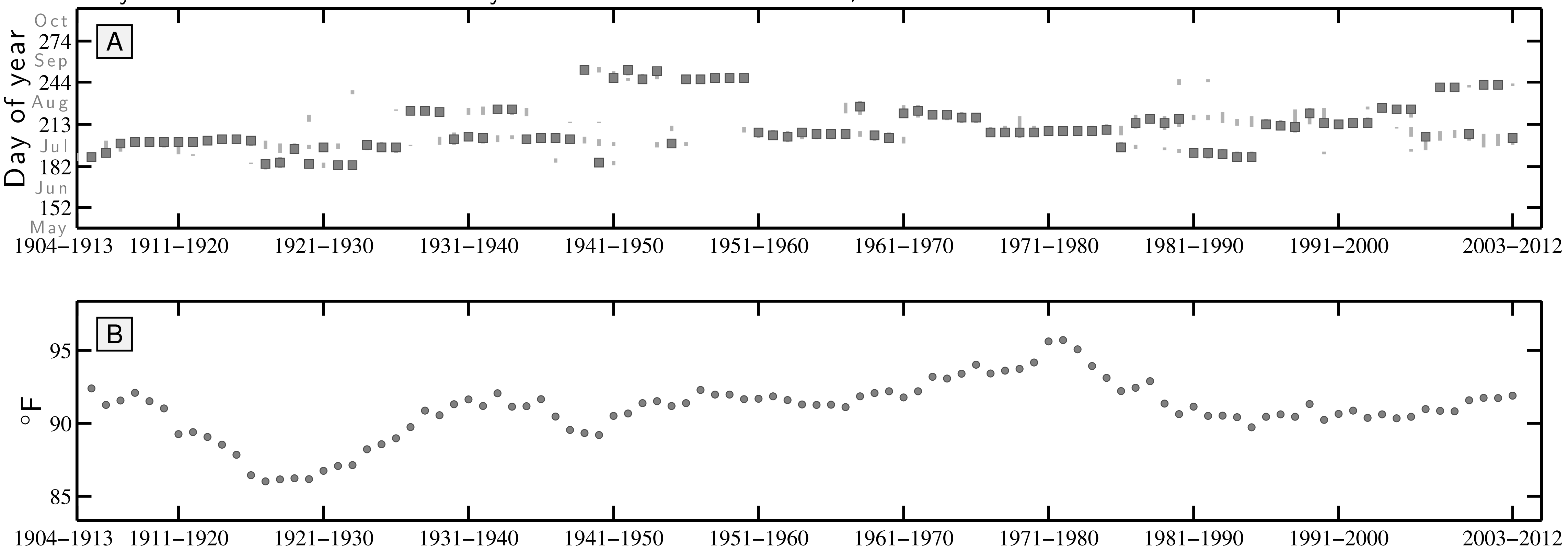

10 year Winter Teletherm dynamics for LIVERMORE, CA:

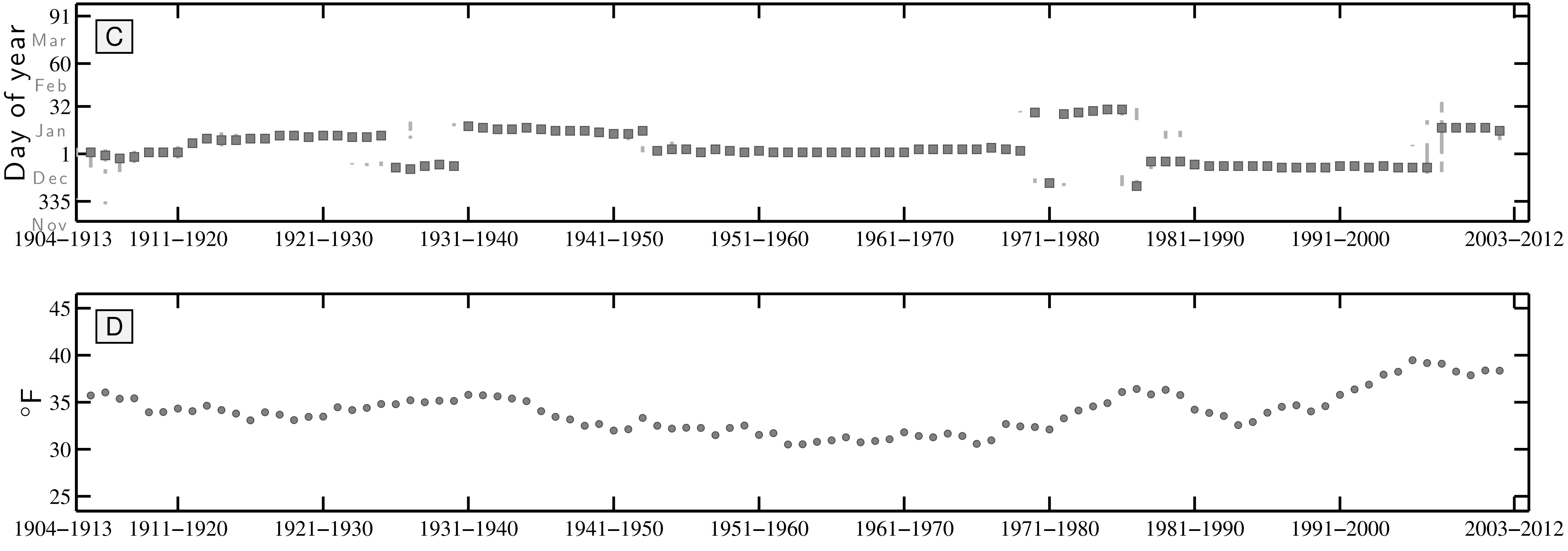

10 year Summer Teletherm dynamics for LODI, CA:

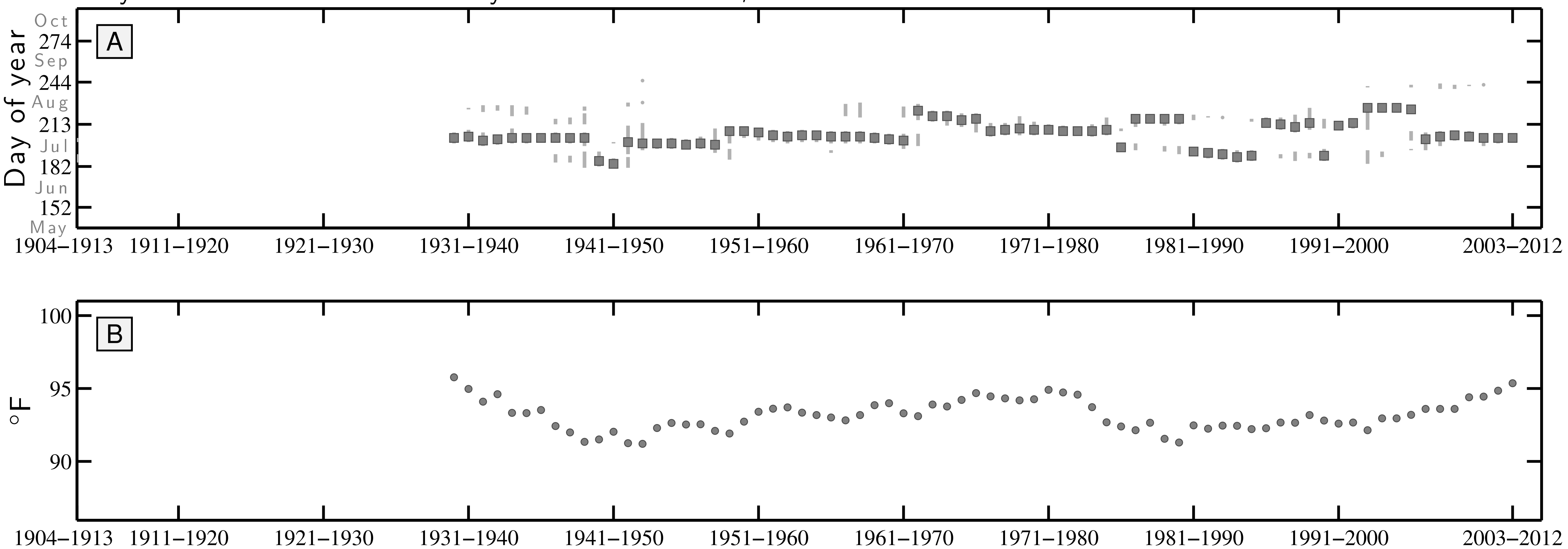

10 year Winter Teletherm dynamics for LODI, CA:

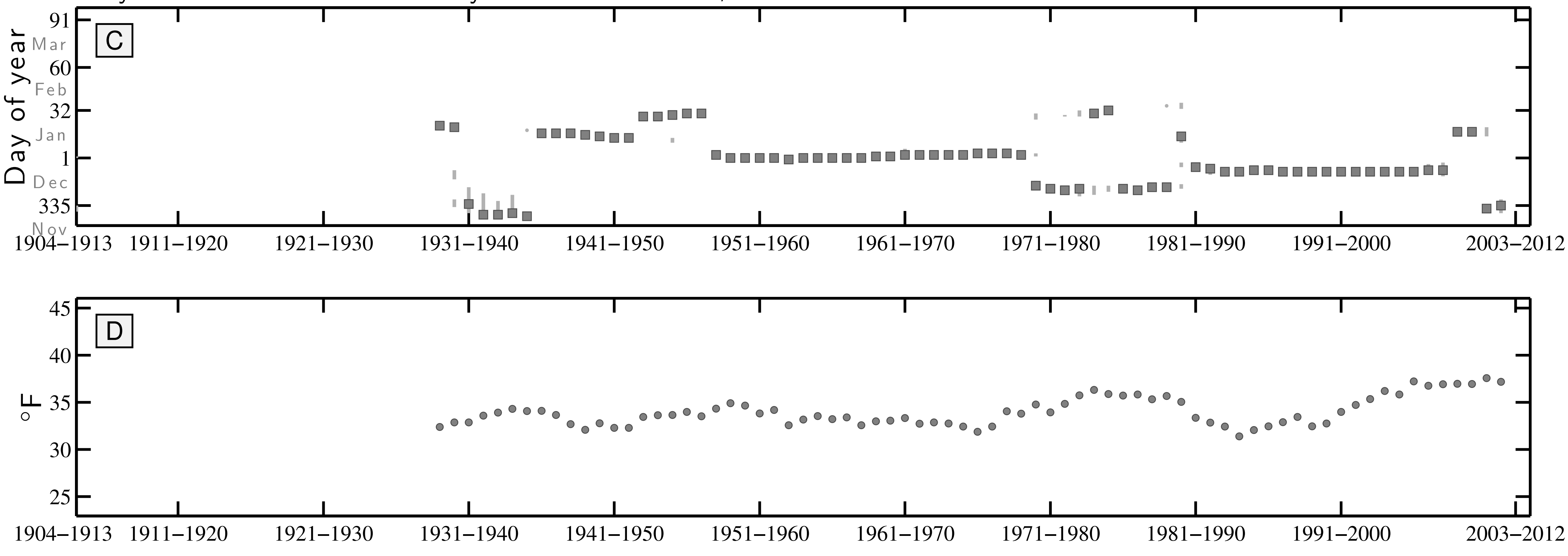

10 year Summer Teletherm dynamics for MARYSVILLE, CA:

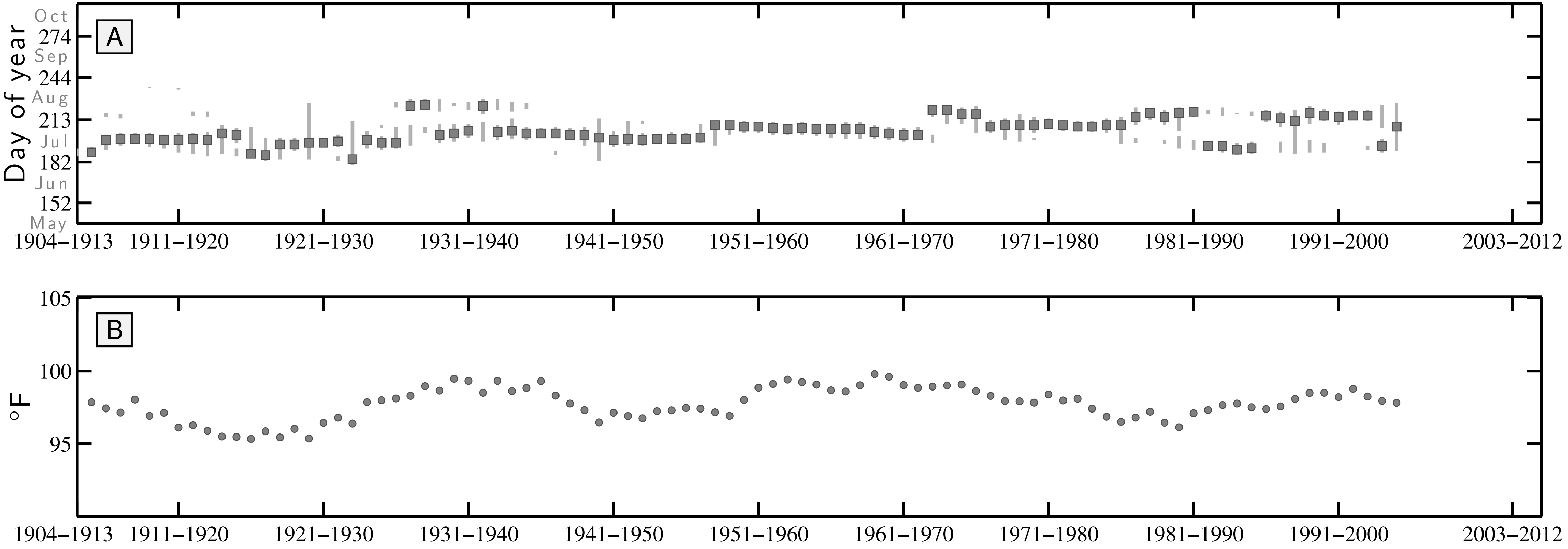

10 year Winter Teletherm dynamics for MARYSVILLE, CA:

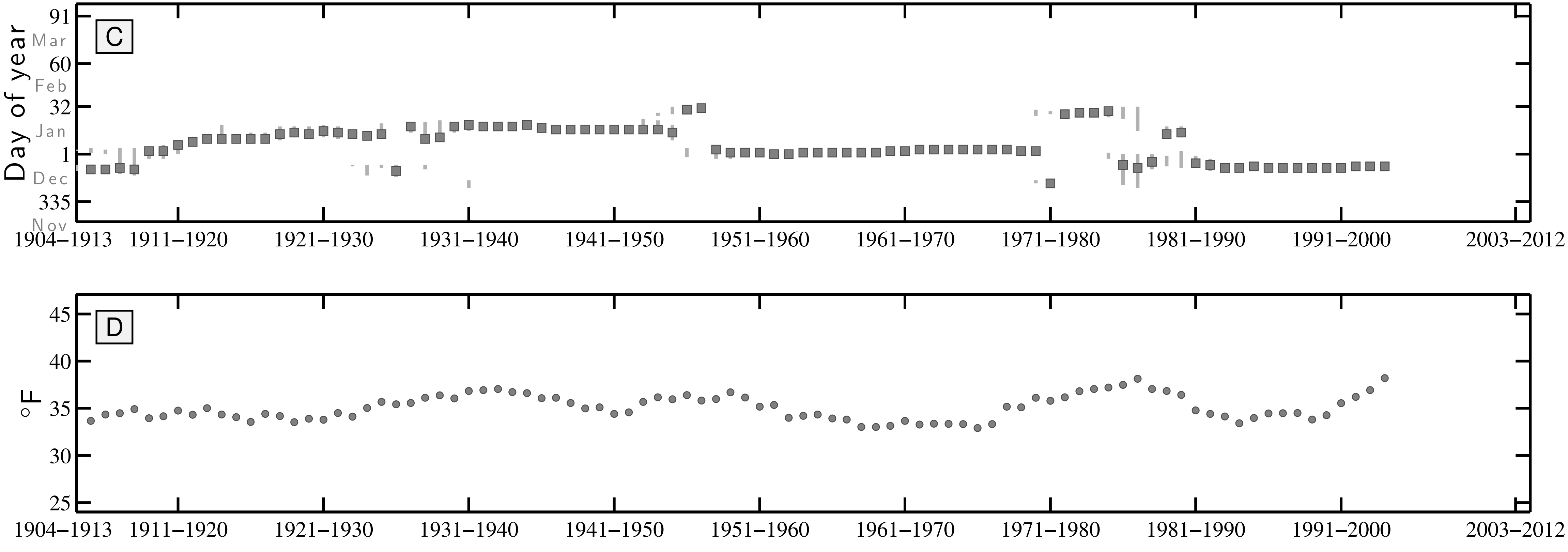

10 year Summer Teletherm dynamics for MERCED, CA:

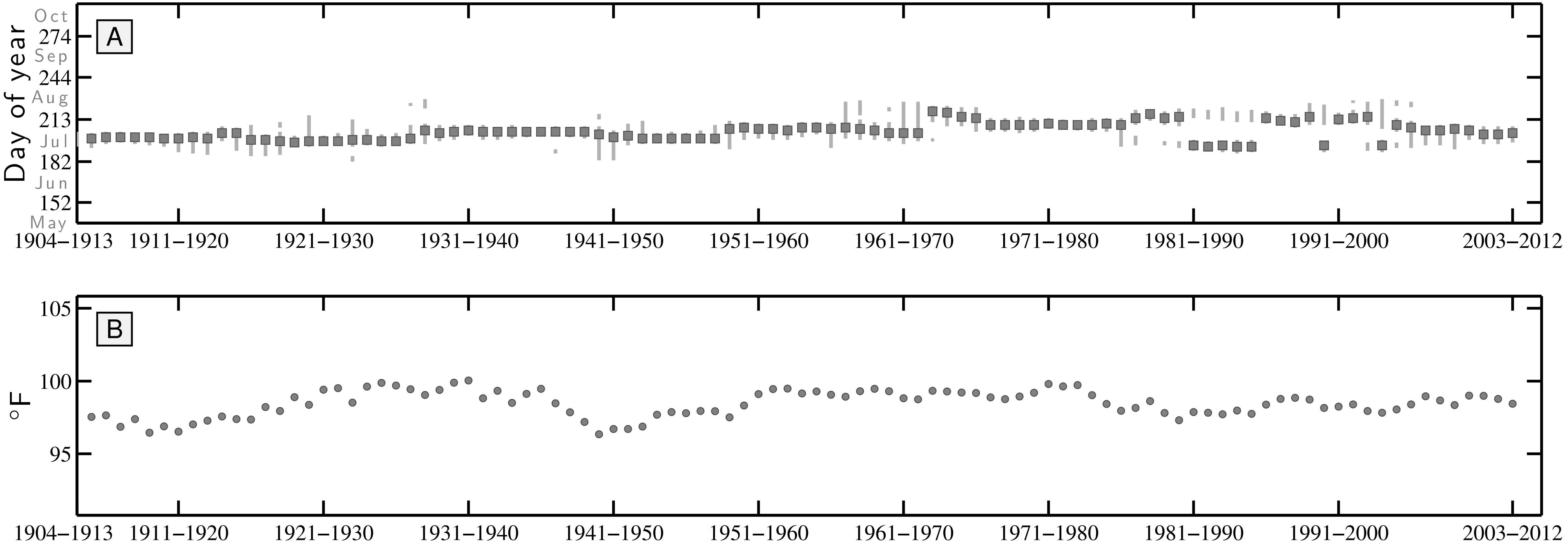

10 year Winter Teletherm dynamics for MERCED, CA:

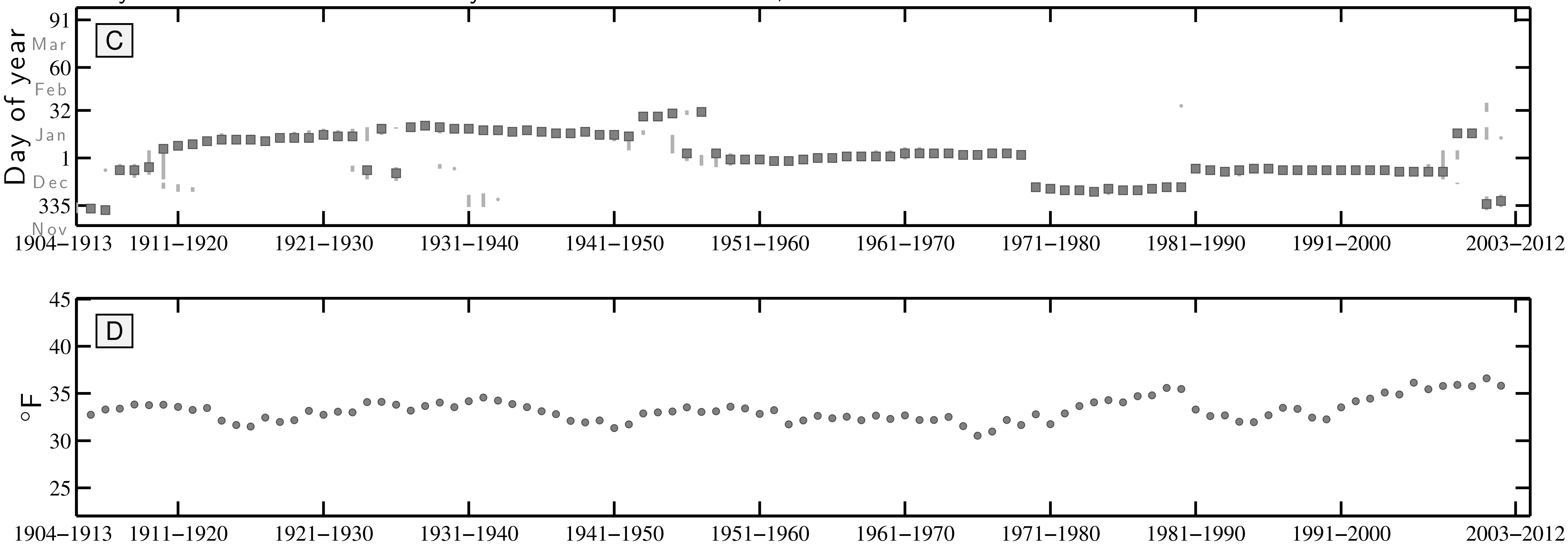

10 year Summer Teletherm dynamics for MT SHASTA, CA:

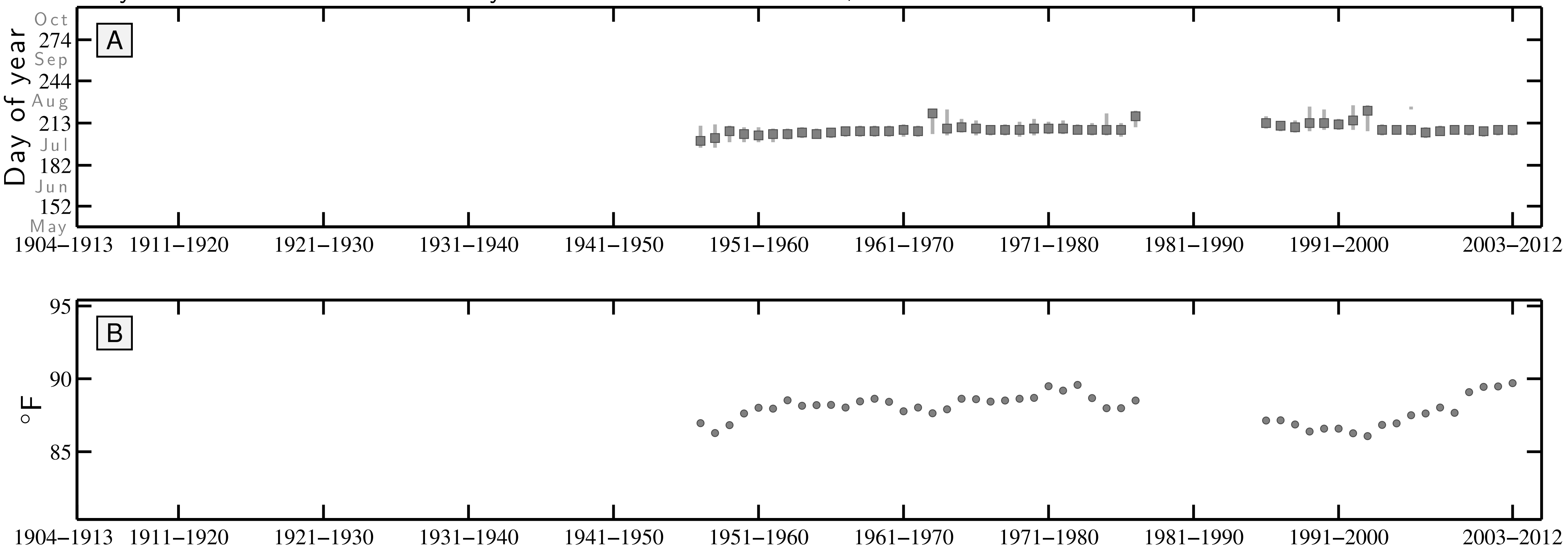

10 year Winter Teletherm dynamics for MT SHASTA, CA:

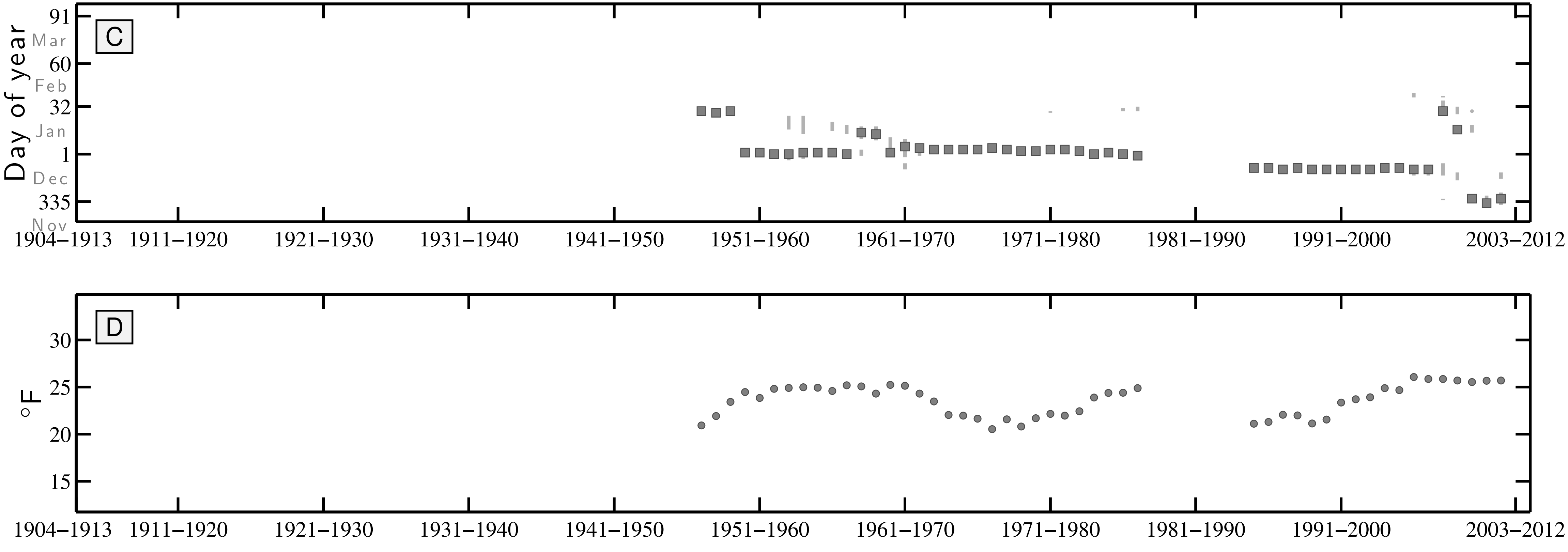

10 year Summer Teletherm dynamics for NAPA STATE HOSPITAL, CA:

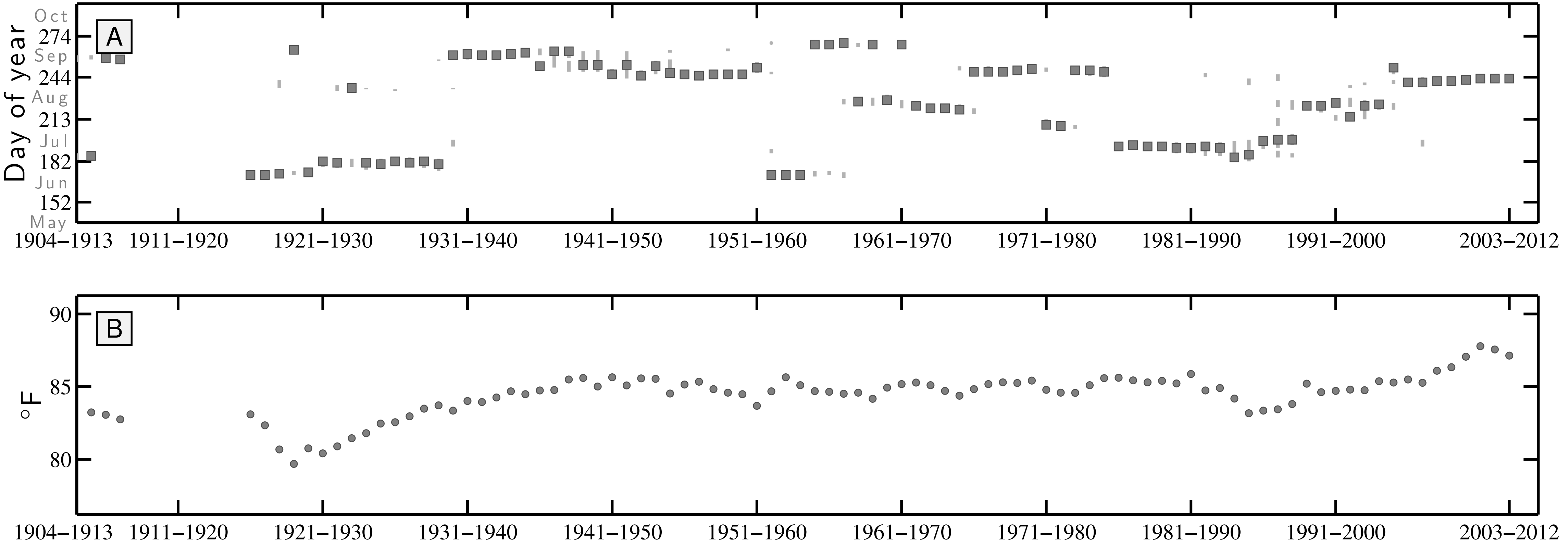

10 year Winter Teletherm dynamics for NAPA STATE HOSPITAL, CA:

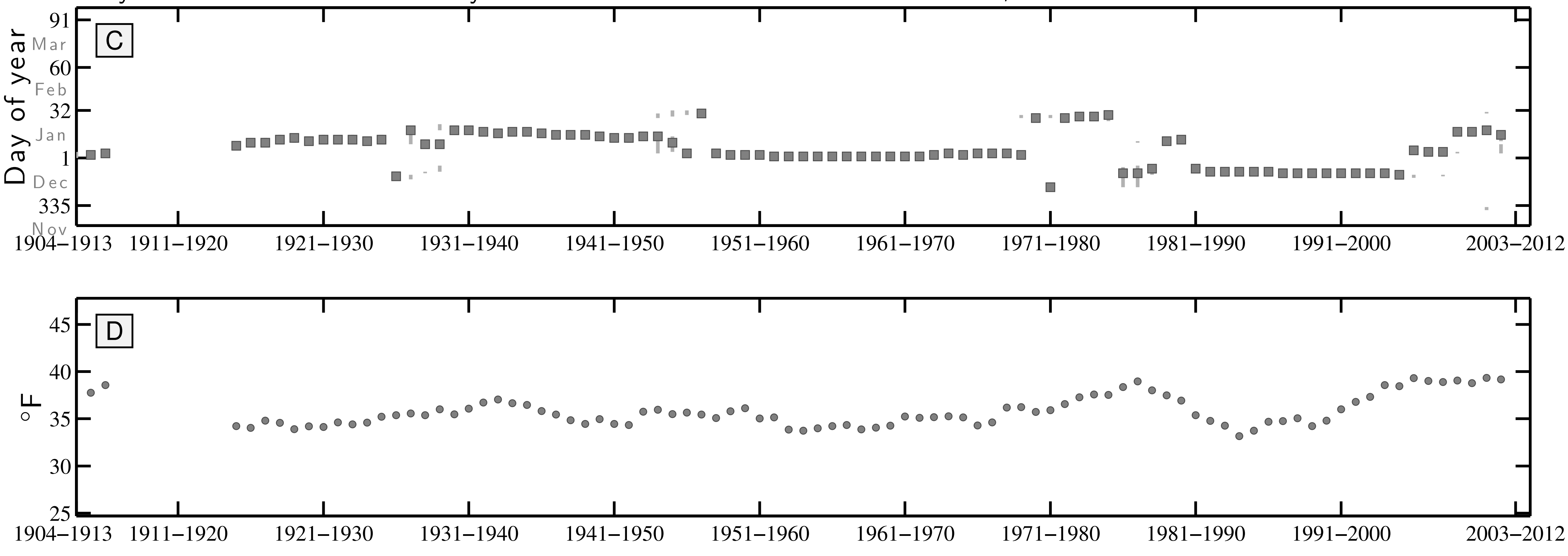

10 year Summer Teletherm dynamics for NEEDLES AP, CA:

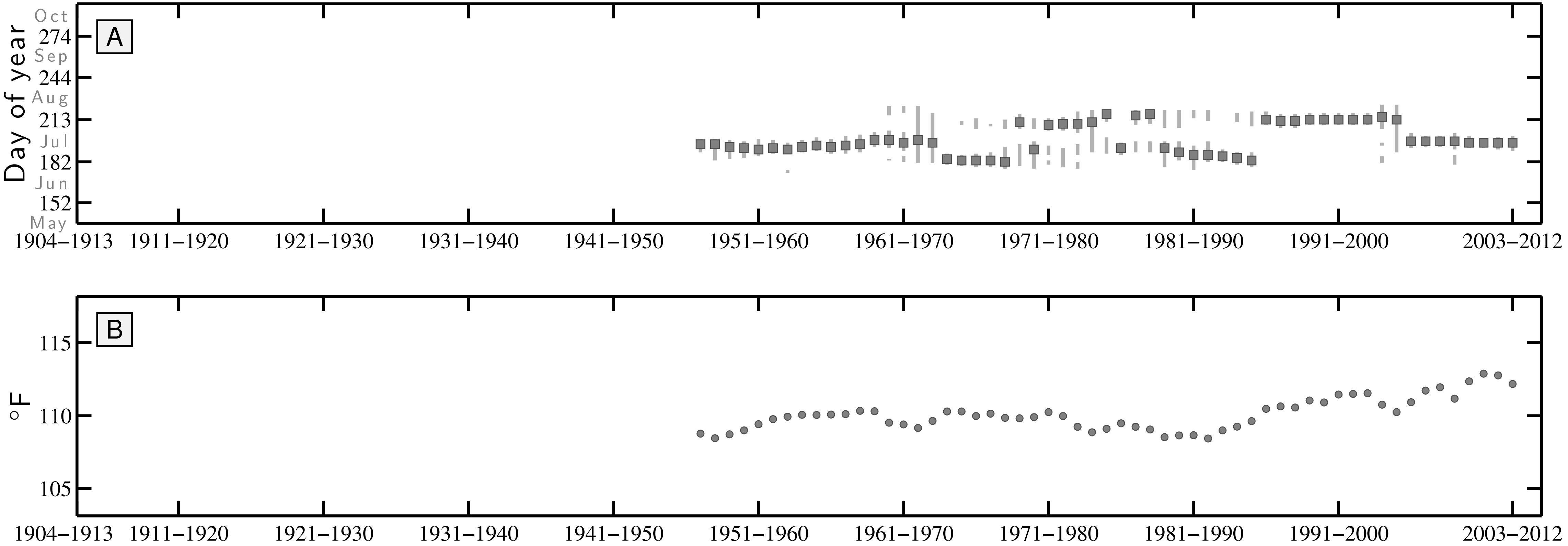

10 year Winter Teletherm dynamics for NEEDLES AP, CA:

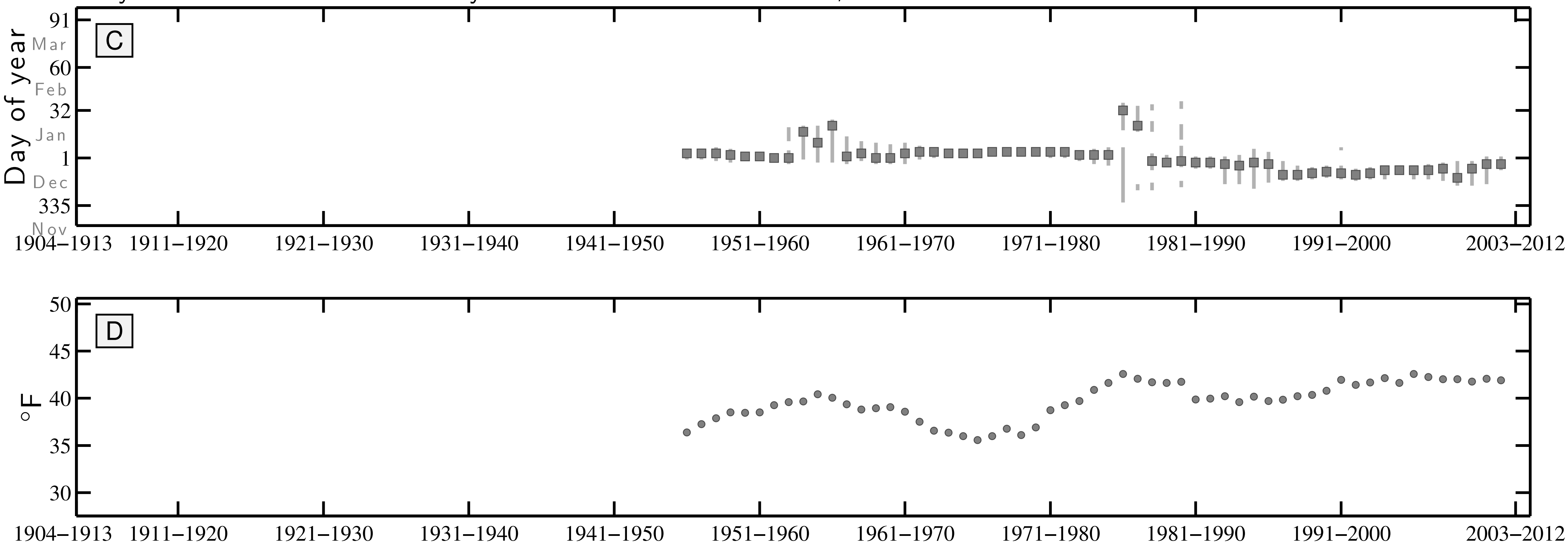

10 year Summer Teletherm dynamics for NEWPORT BEACH HARBOR, CA:

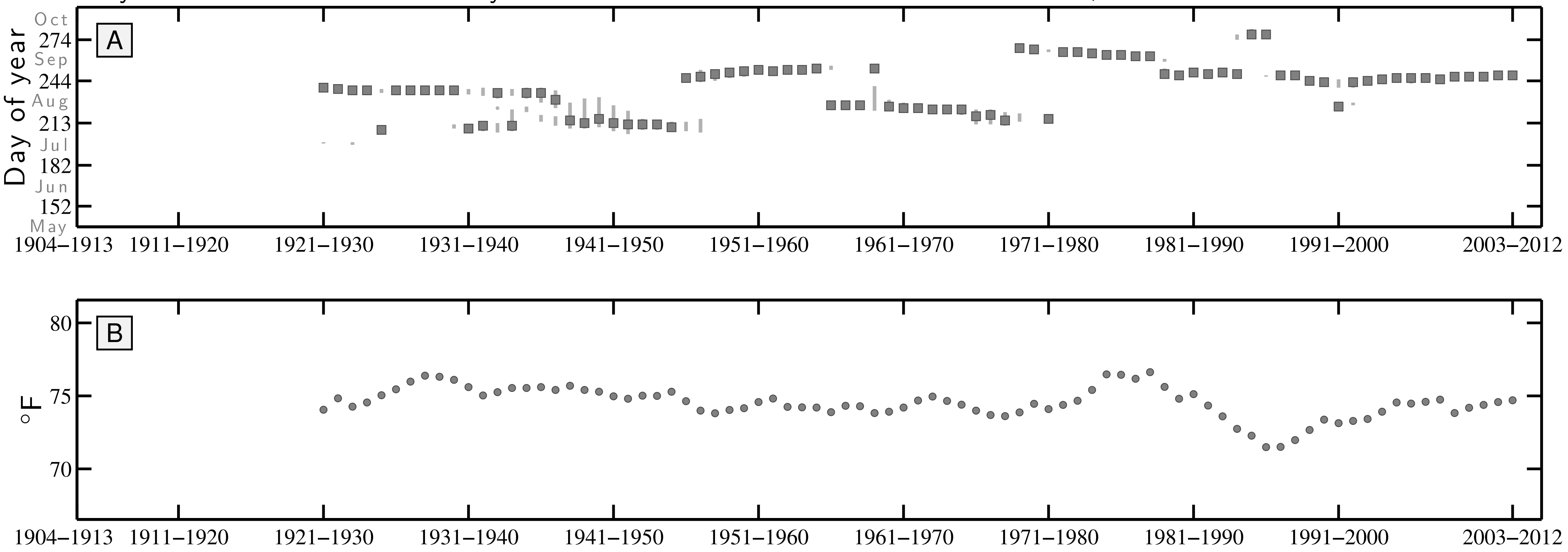

10 year Winter Teletherm dynamics for NEWPORT BEACH HARBOR, CA:

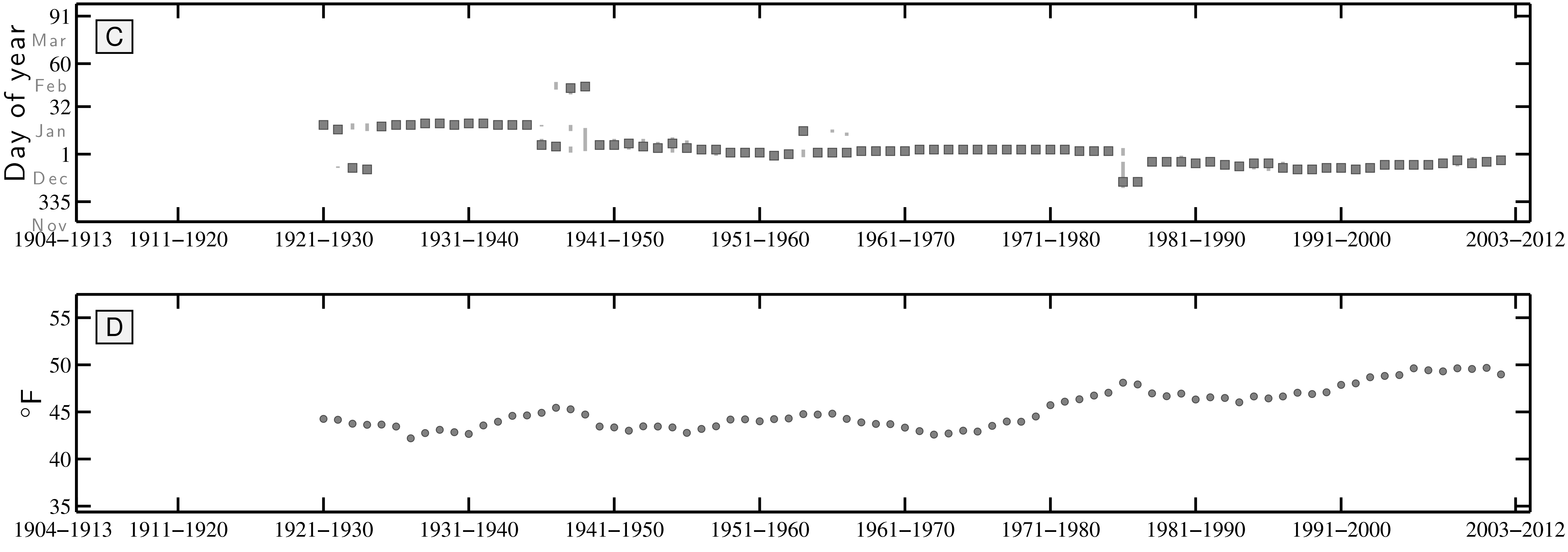

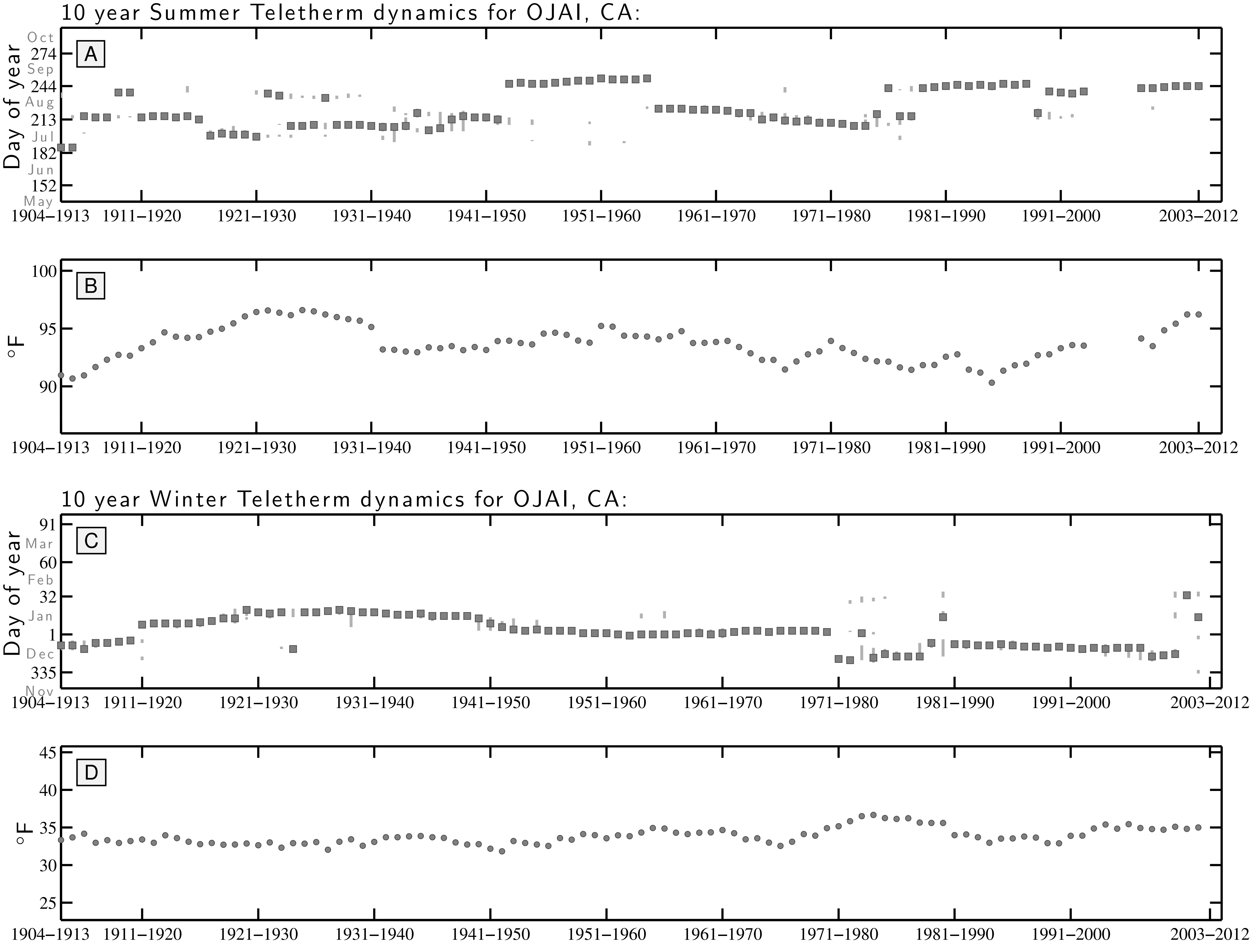

10 year Summer Teletherm dynamics for OJAI, CA:
A
Day of year
Oct
274
Sep
244
Aug
213
Jul
182
Jun
152
May
1904–1913
1911–1920
1921–1930
1931–1940
1941–1950
1951–1960
1961–1970
1971–1980
1981–1990
1991–2000
2003–2012
B
°F
100
95
90
10 year Winter Teletherm dynamics for OJAI, CA:
C
Day of year
91
Mar
60
Feb
32
Jan
1
Dec
335
Nov
D
°F
45
40
35
30
25

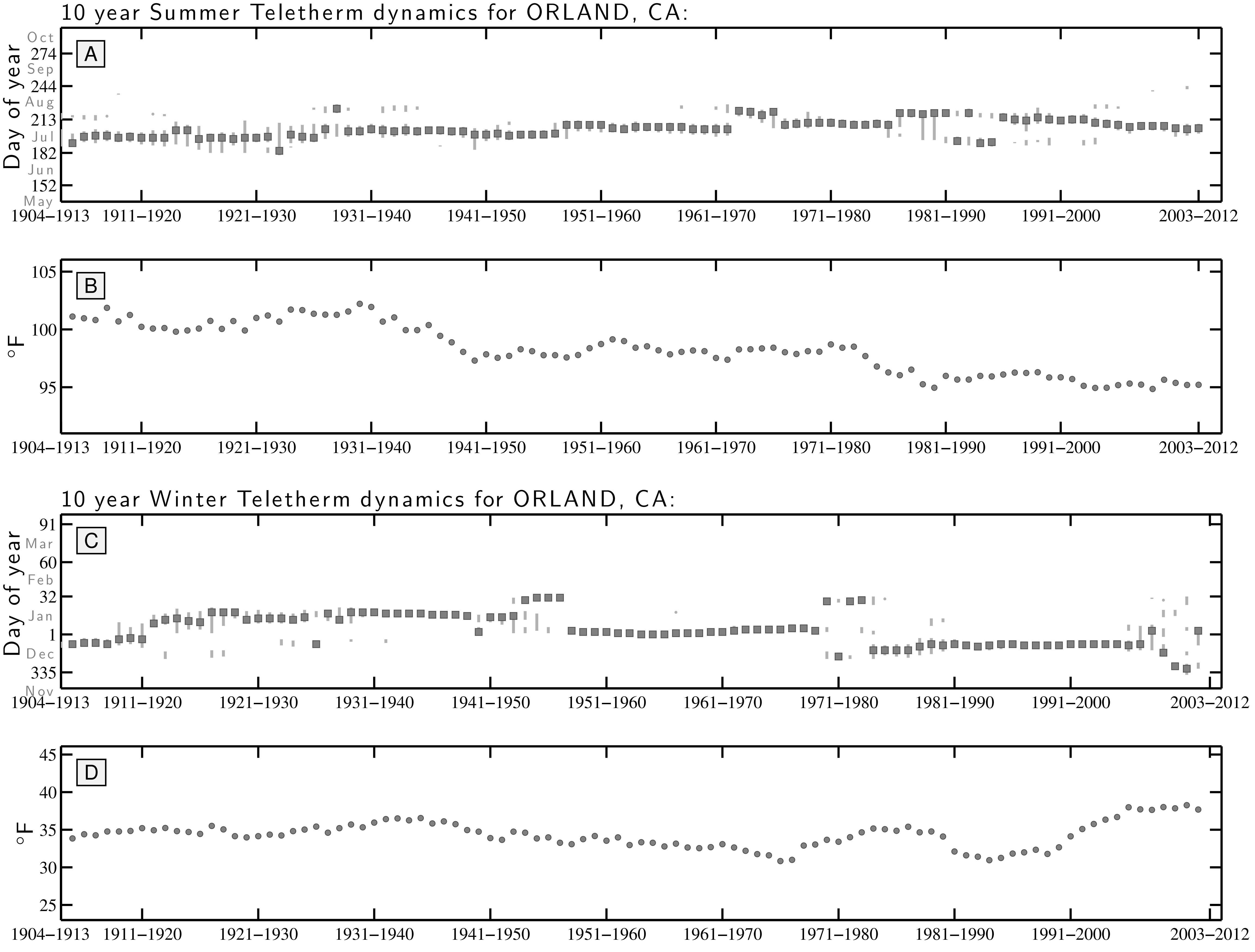
10 year Summer Teletherm dynamics for ORLAND, CA:
A
B
Day of year
°F
Oct
274
Sep
244
Aug
213
Jul
182
Jun
152
May
105
100
95
1904–1913
1911–1920
1921–1930
1931–1940
1941–1950
1951–1960
1961–1970
1971–1980
1981–1990
1991–2000
2003–2012
10 year Winter Teletherm dynamics for ORLAND, CA:
C
D
91
Mar
60
Feb
32
Jan
1
Dec
335
Nov
45
40
35
30
25

10 year Summer Teletherm dynamics for ORLEANS, CA:

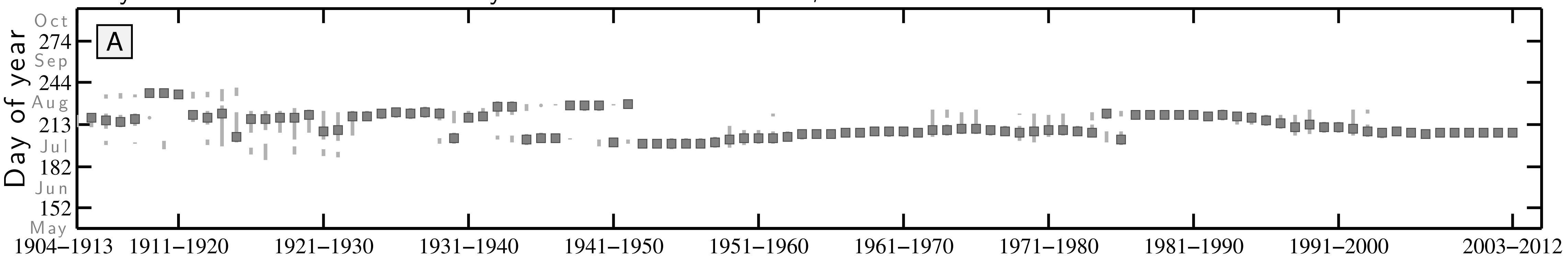

10 year Winter Teletherm dynamics for ORLEANS, CA:

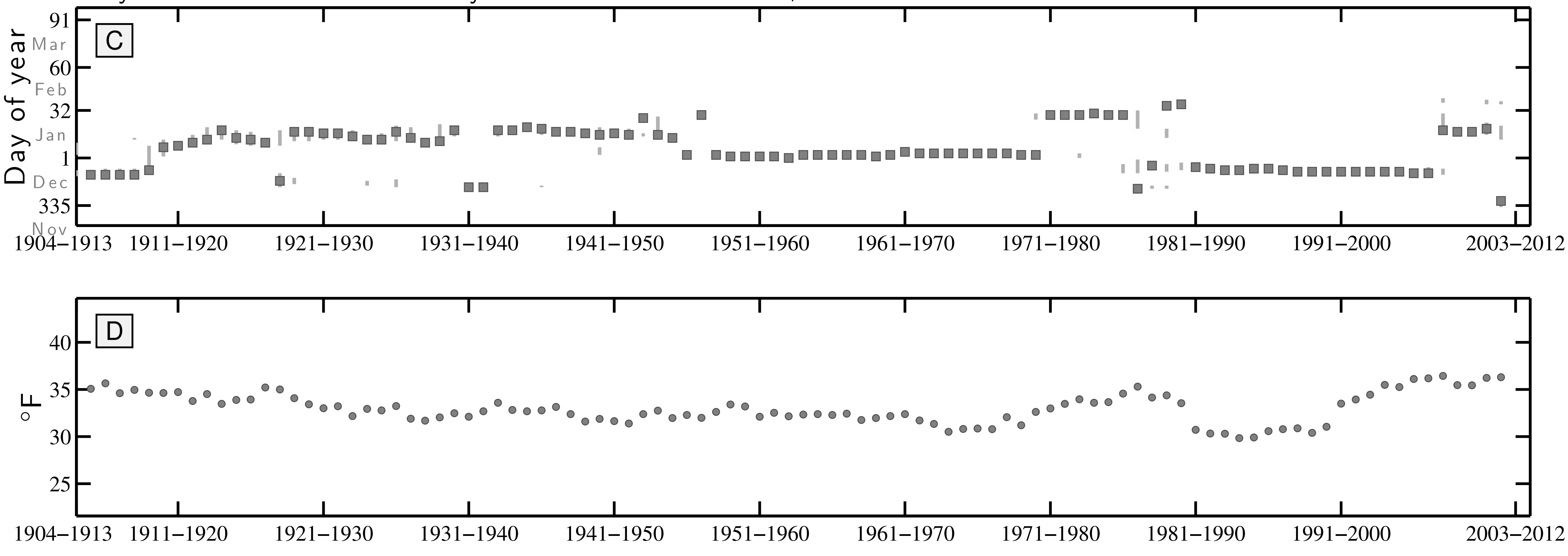

10 year Summer Teletherm dynamics for PASADENA, CA:

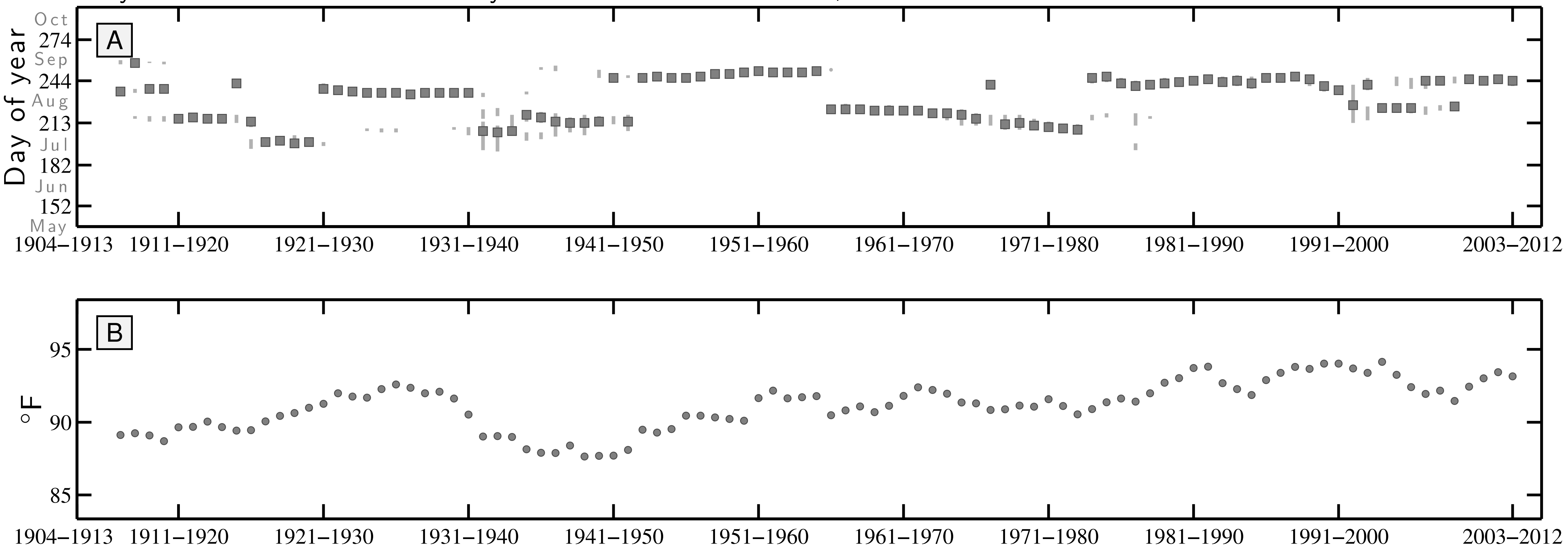

10 year Winter Teletherm dynamics for PASADENA, CA:

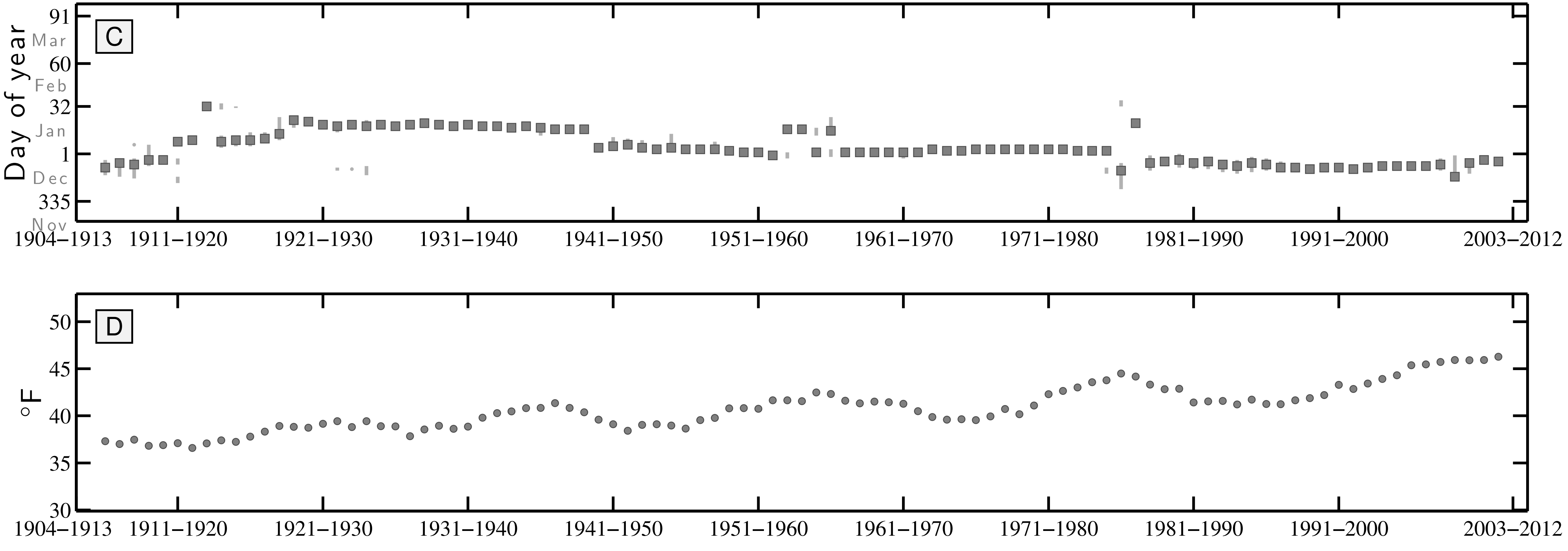

10 year Summer Teletherm dynamics for PASO ROBLES, CA:

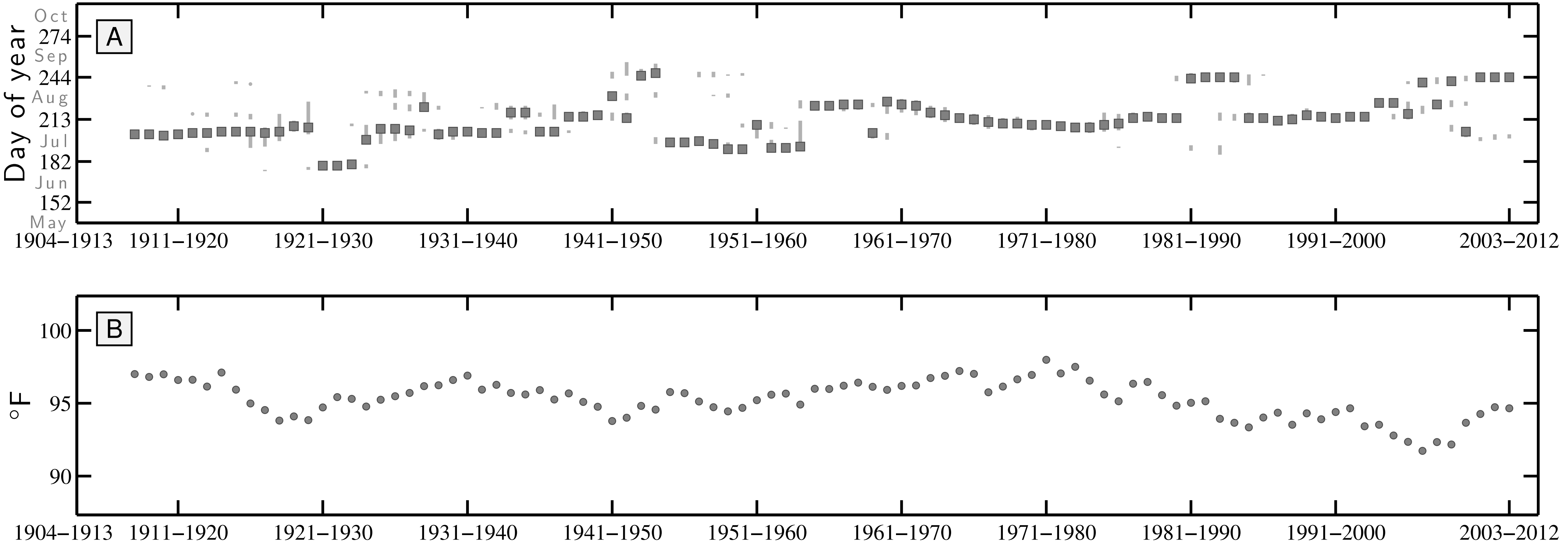

10 year Winter Teletherm dynamics for PASO ROBLES, CA:

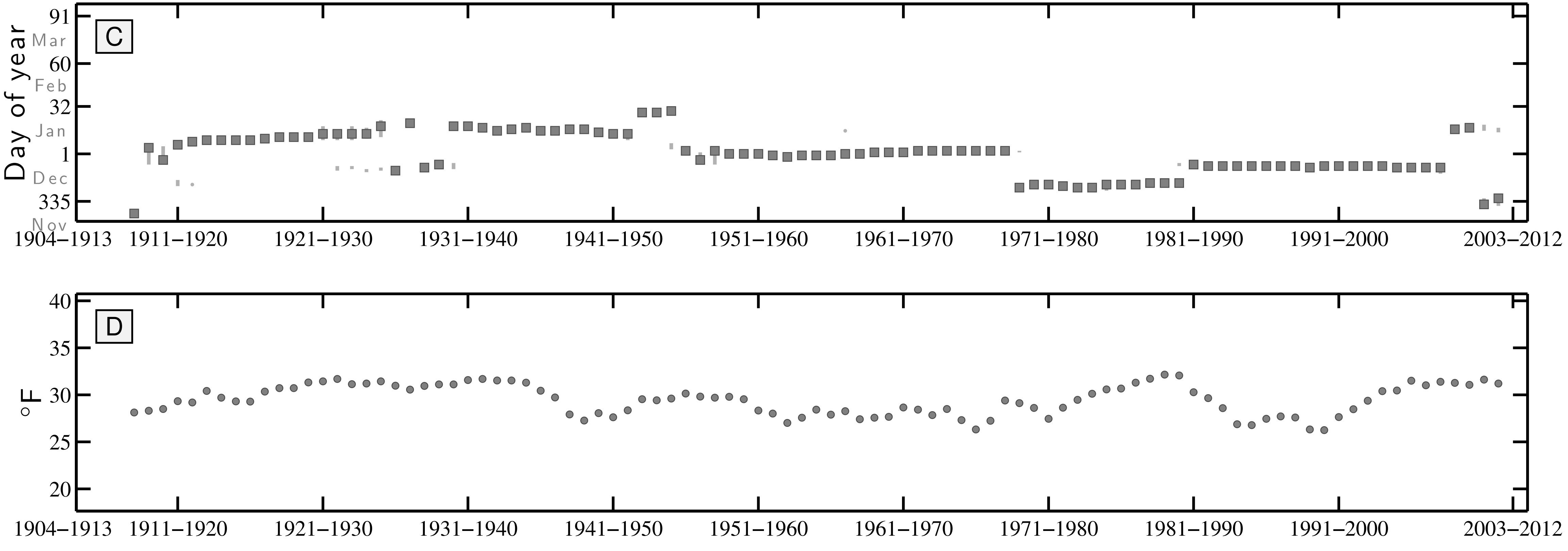

10 year Summer Teletherm dynamics for PETALUMA AP, CA:

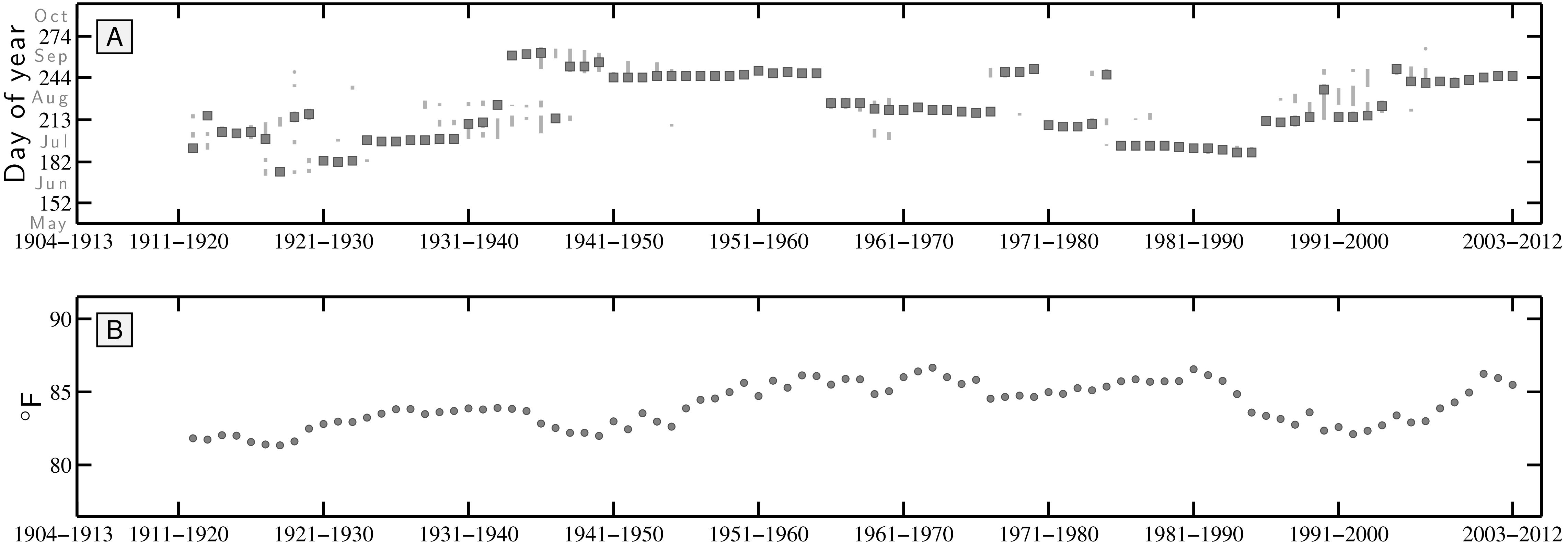

10 year Winter Teletherm dynamics for PETALUMA AP, CA:

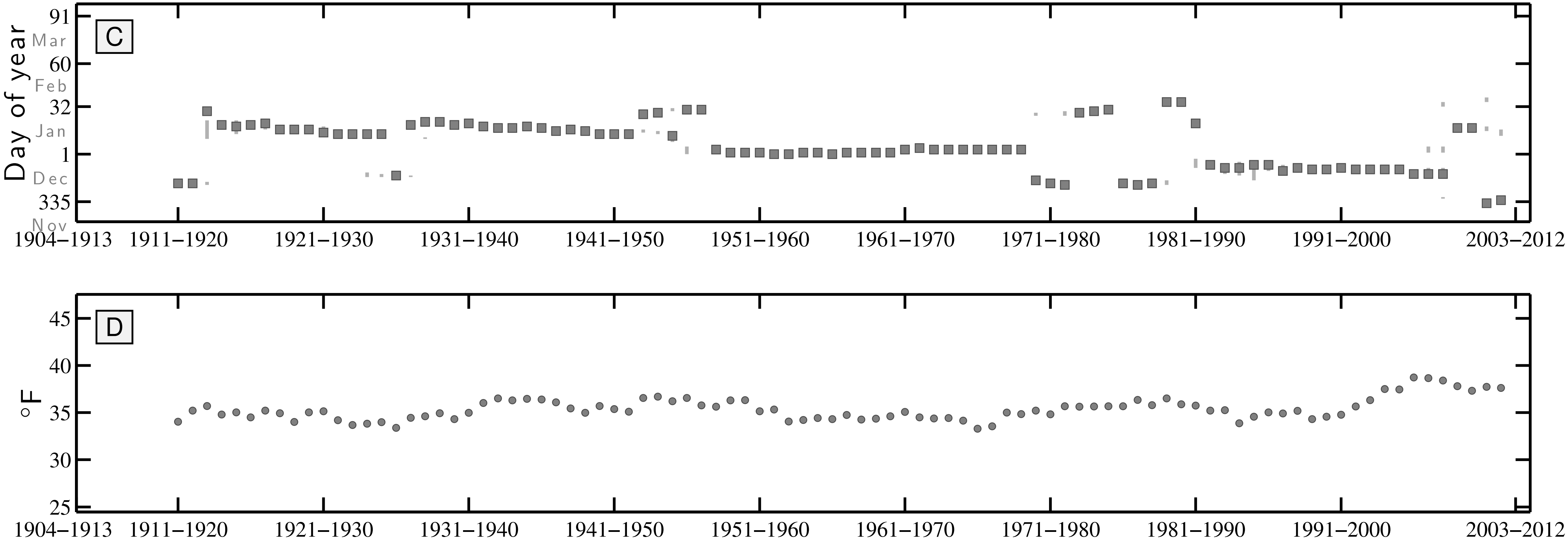

10 year Summer Teletherm dynamics for QUINCY, CA:

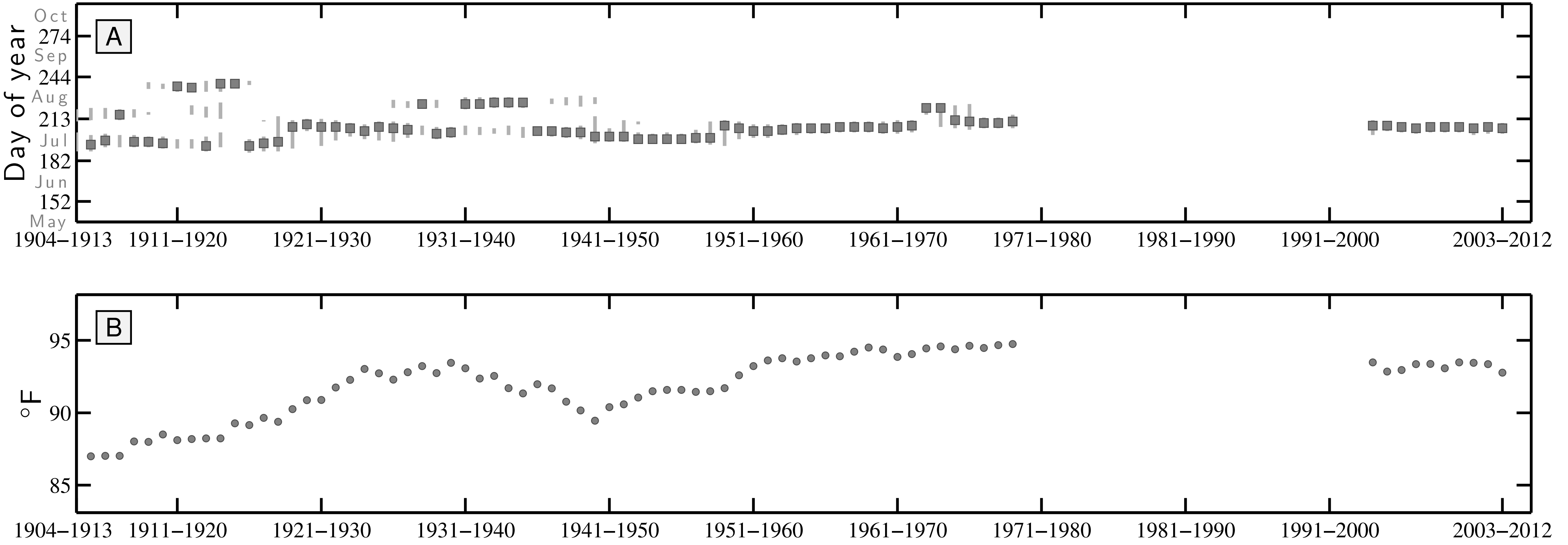

10 year Winter Teletherm dynamics for QUINCY, CA:

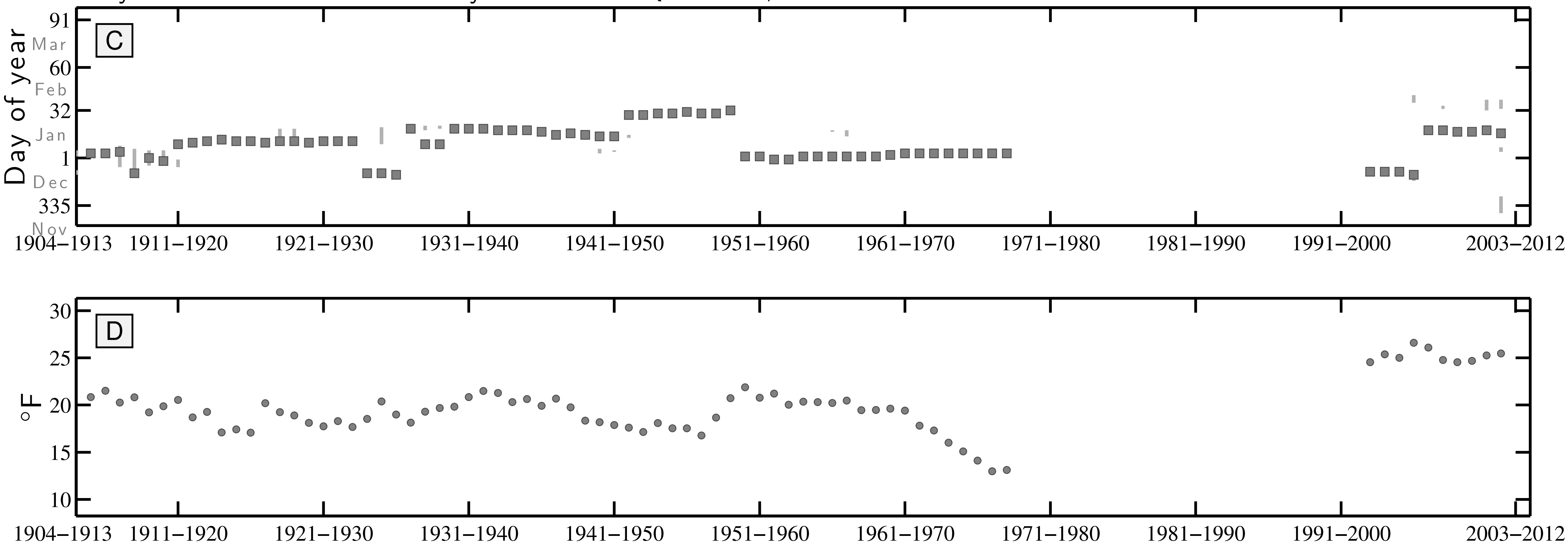

10 year Summer Teletherm dynamics for REDDING MUNI AP, CA:

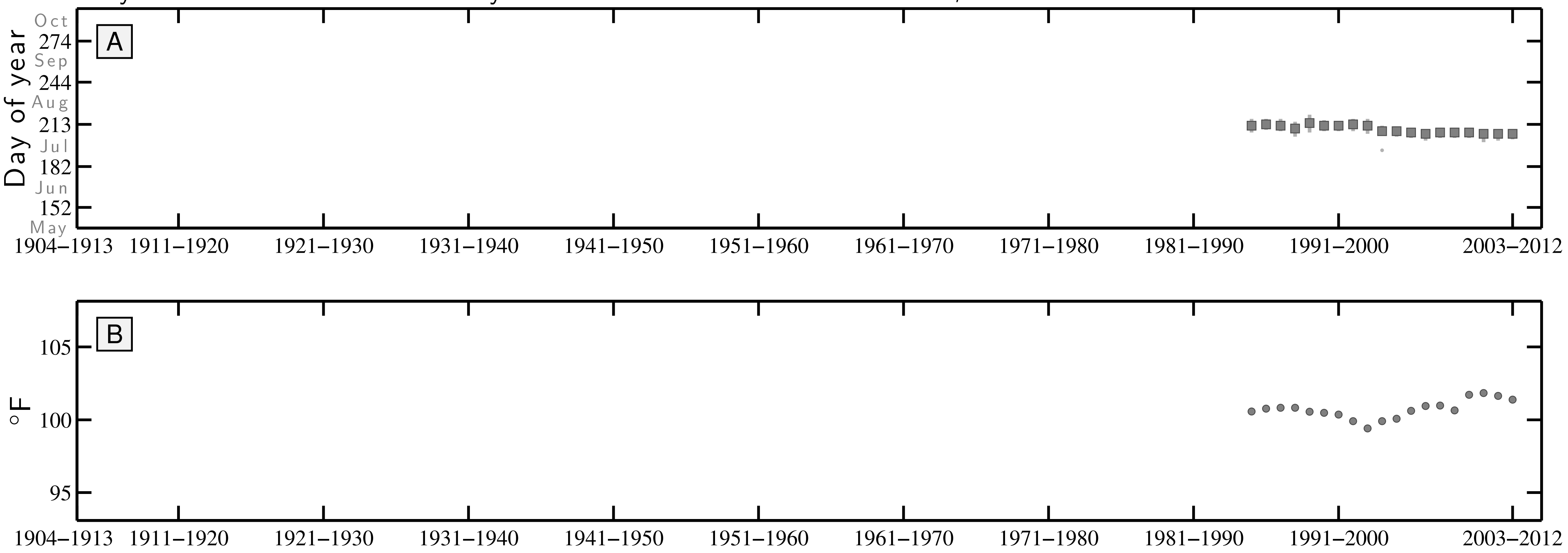

10 year Winter Teletherm dynamics for REDDING MUNI AP, CA:

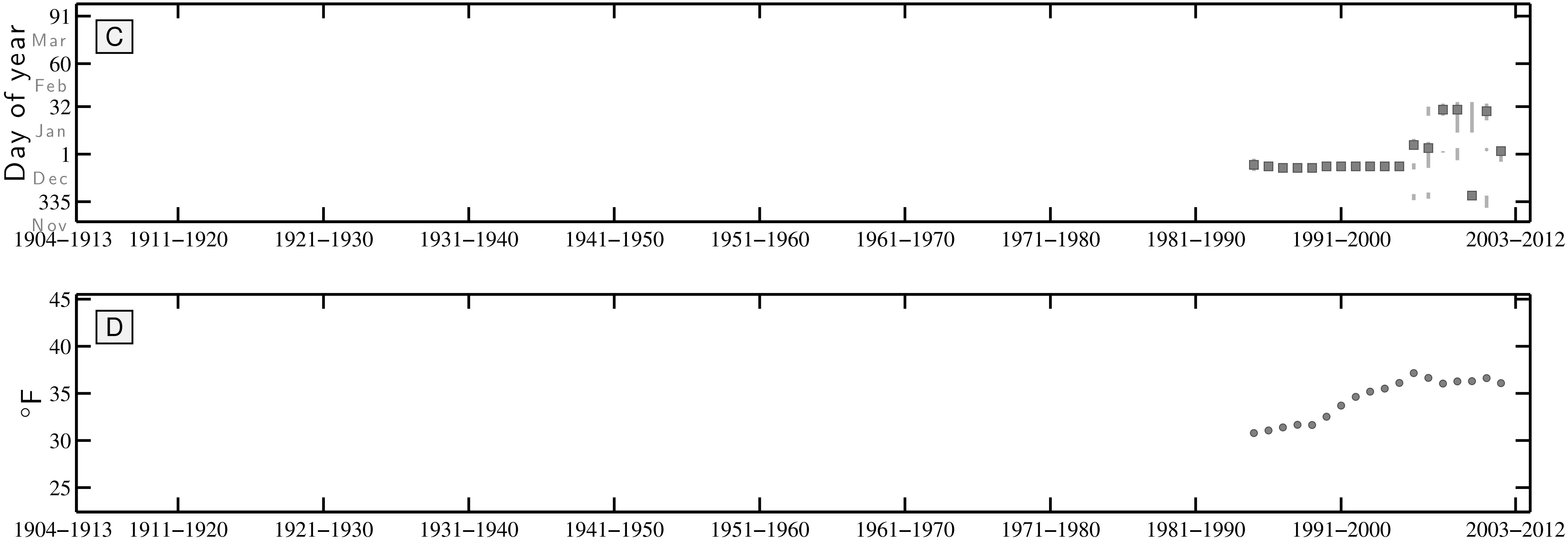

10 year Summer Teletherm dynamics for REDLANDS, CA:

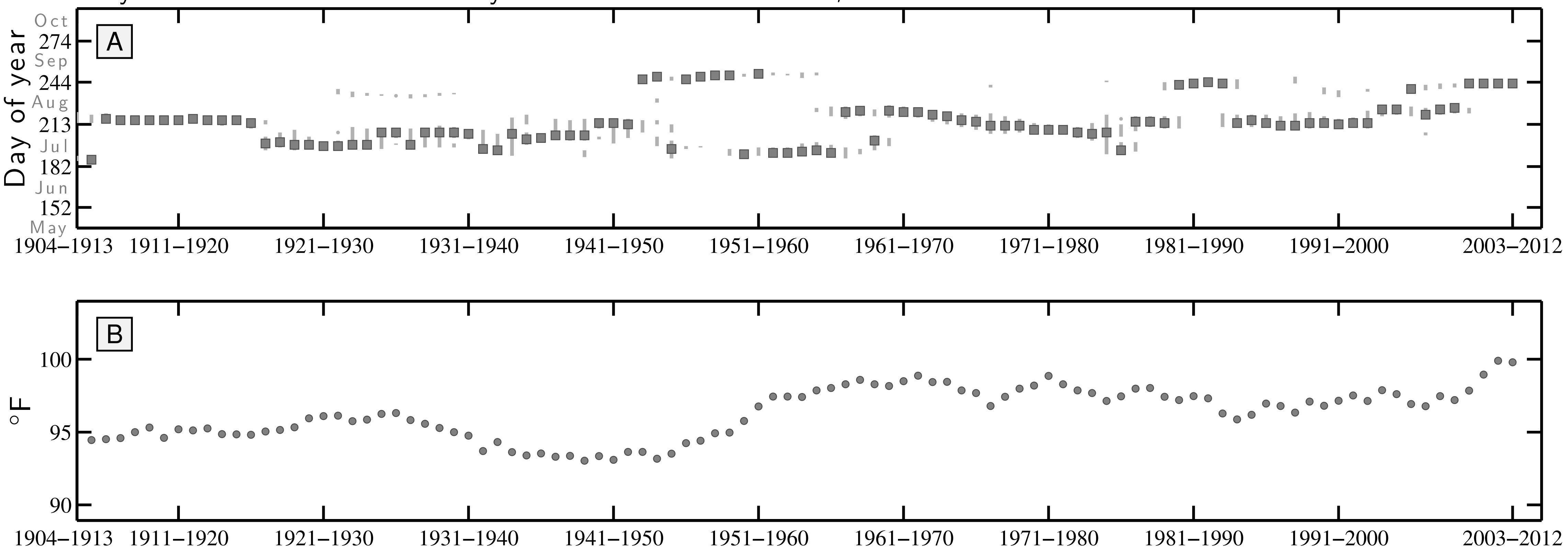

10 year Winter Teletherm dynamics for REDLANDS, CA:

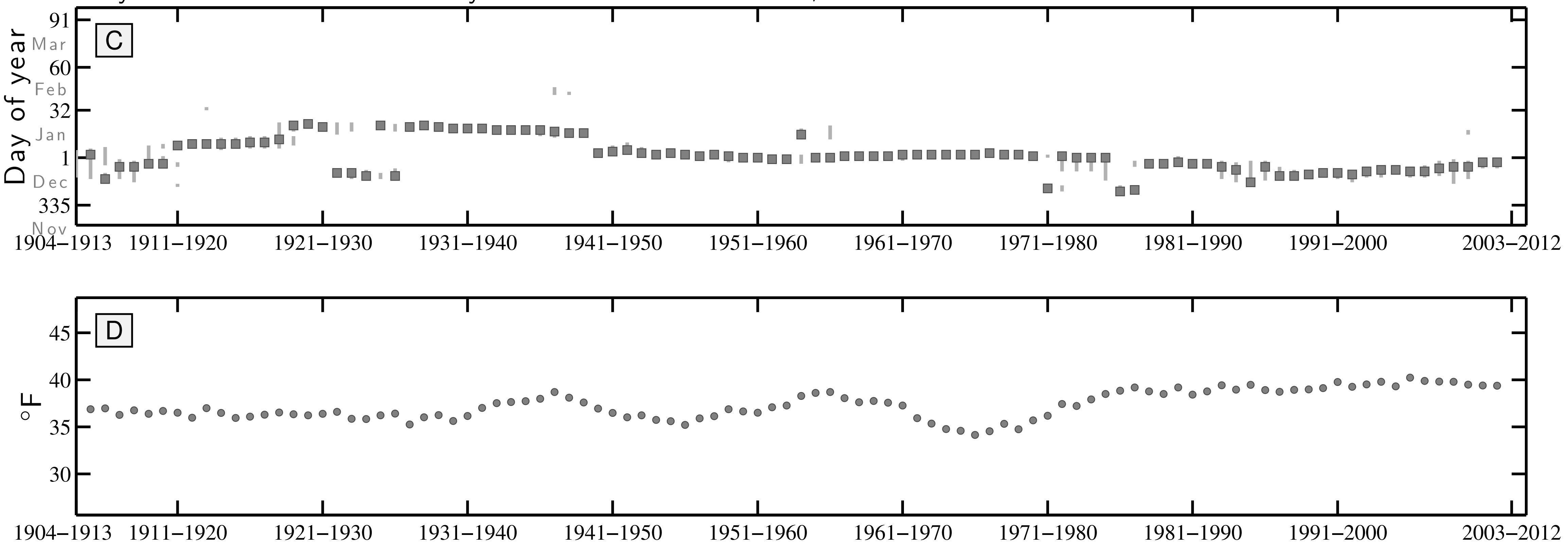

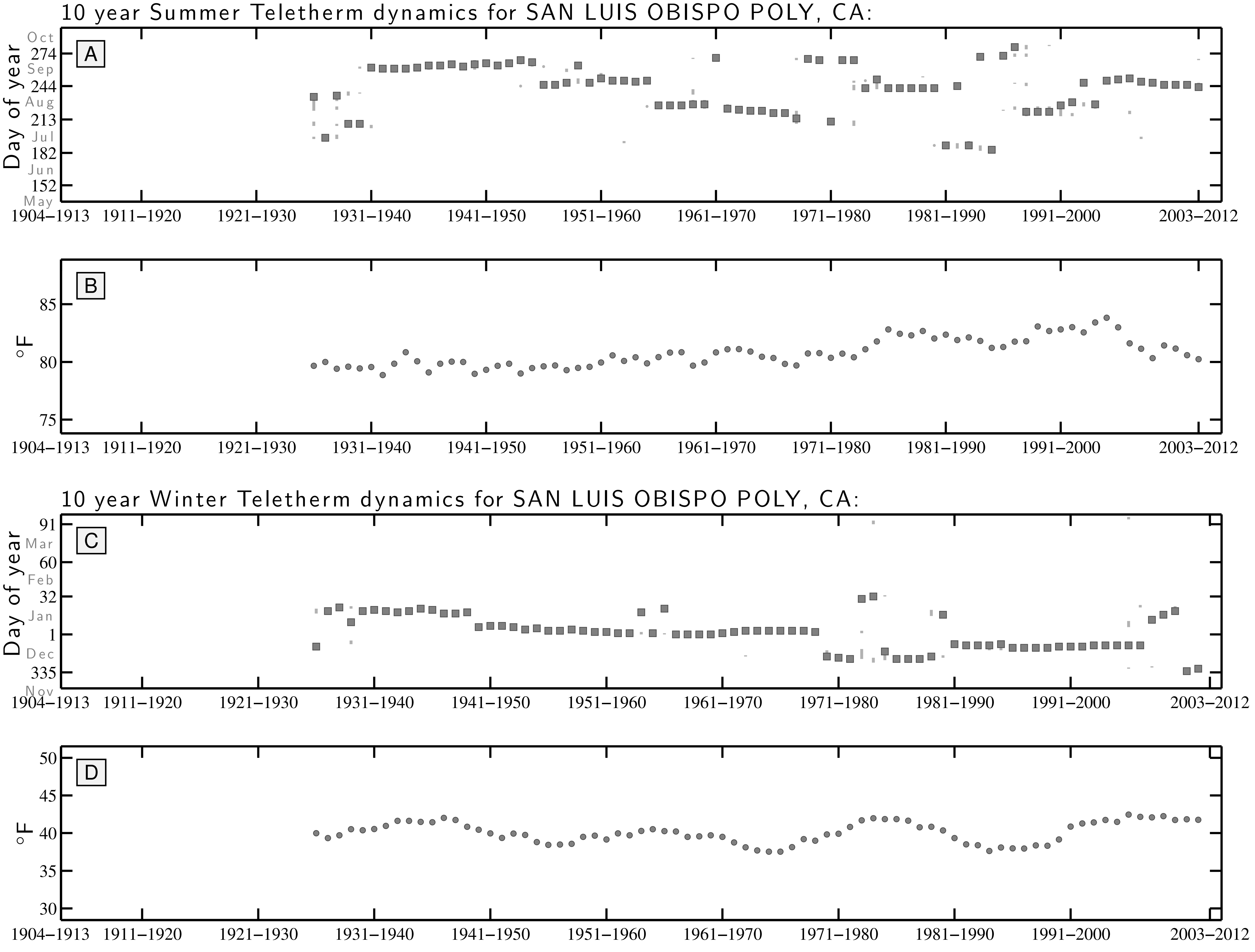

10 year Summer Teletherm dynamics for SAN LUIS OBISPO POLY, CA:
A
B
Day of year
°F
Oct
274
Sep
244
Aug
213
Jul
182
Jun
152
May
85
80
75
10 year Winter Teletherm dynamics for SAN LUIS OBISPO POLY, CA:
C
D
91
Mar
60
Feb
32
Jan
1
Dec
335
Nov
50
45
40
35
30
1904–1913
1911–1920
1921–1930
1931–1940
1941–1950
1951–1960
1961–1970
1971–1980
1981–1990
1991–2000
2003–2012

10 year Summer Teletherm dynamics for SANTA BARBARA, CA:

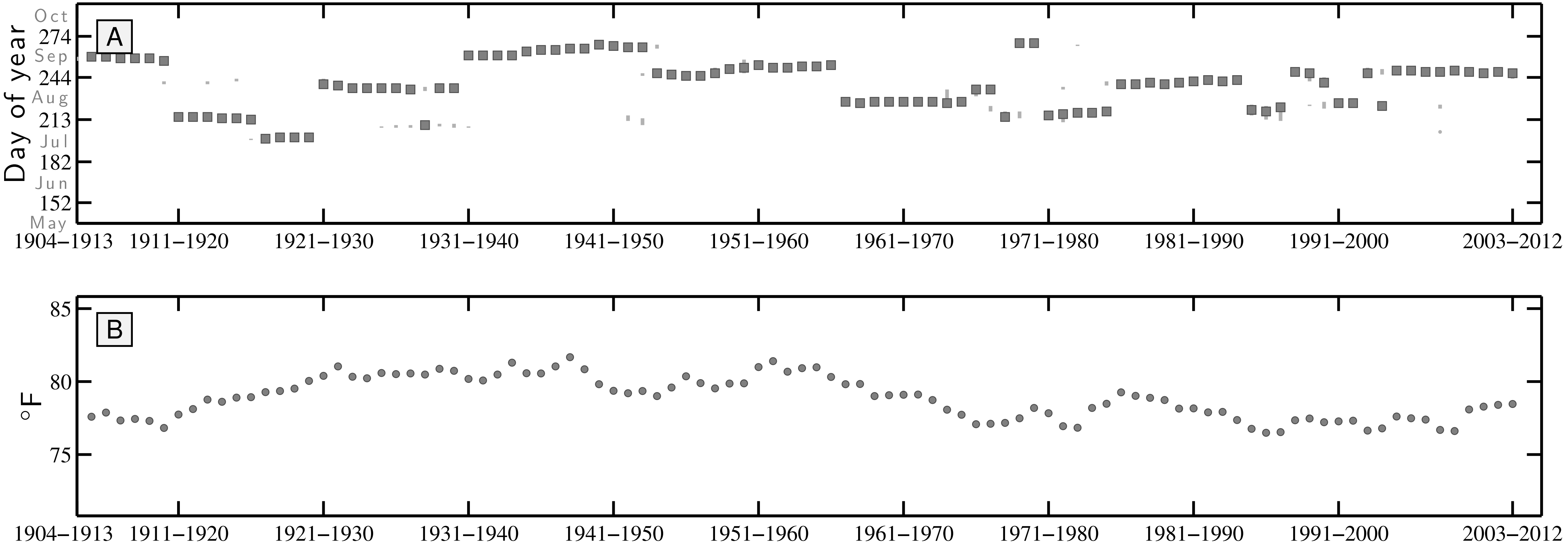

10 year Winter Teletherm dynamics for SANTA BARBARA, CA:

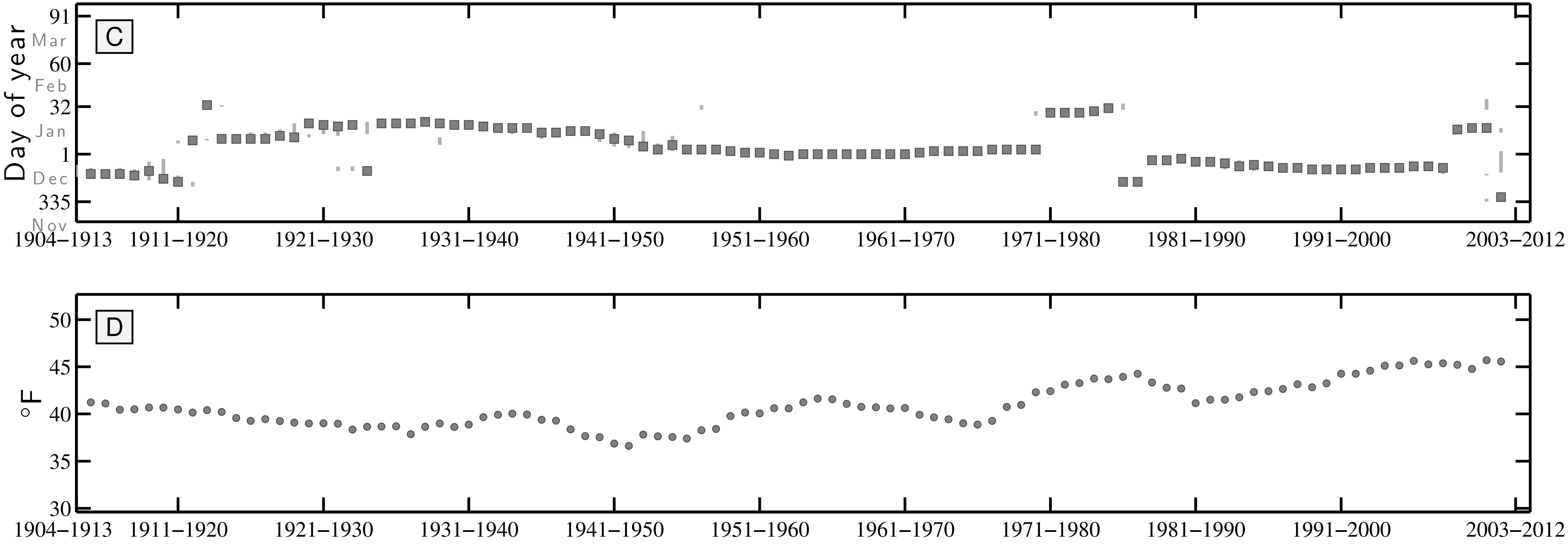

10 year Summer Teletherm dynamics for SANTA CRUZ, CA:

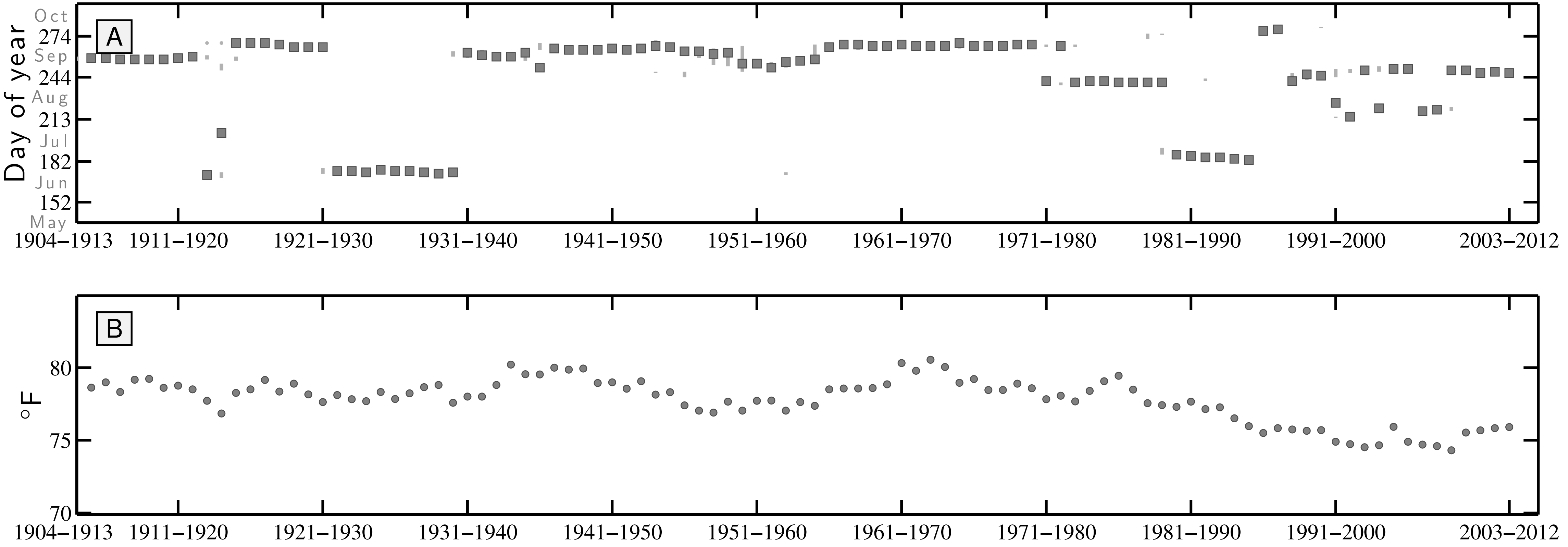

10 year Winter Teletherm dynamics for SANTA CRUZ, CA:

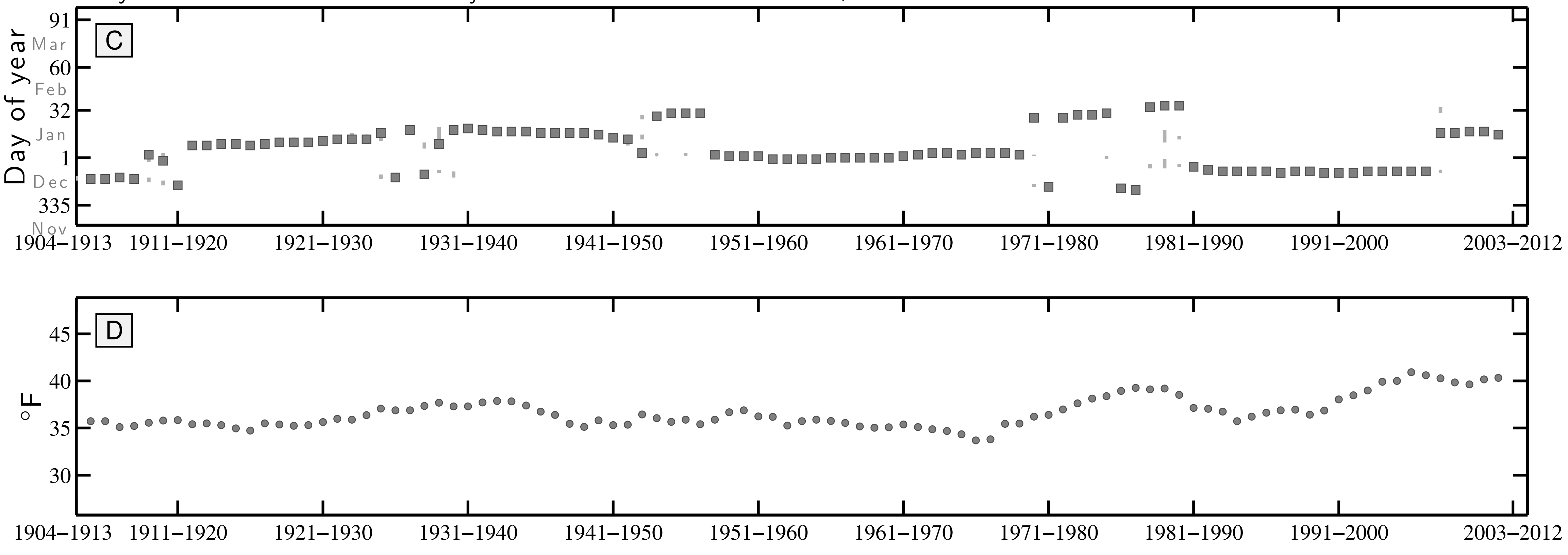

10 year Summer Teletherm dynamics for SANTA ROSA, CA:

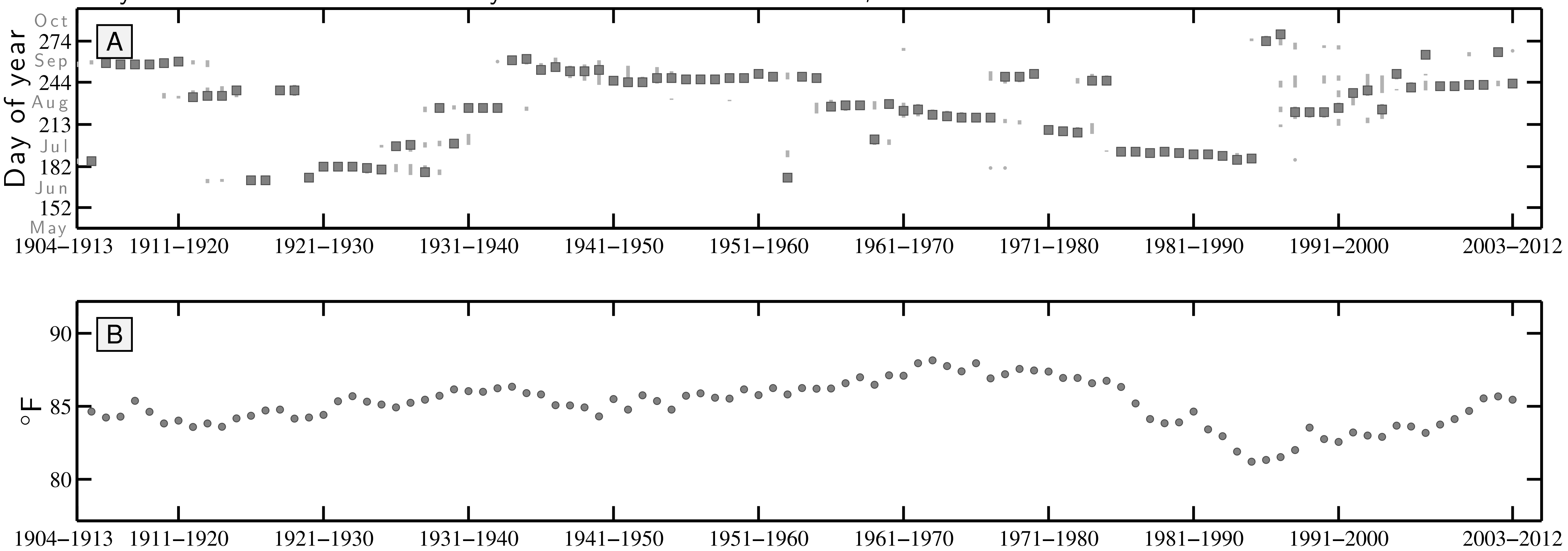

10 year Winter Teletherm dynamics for SANTA ROSA, CA:

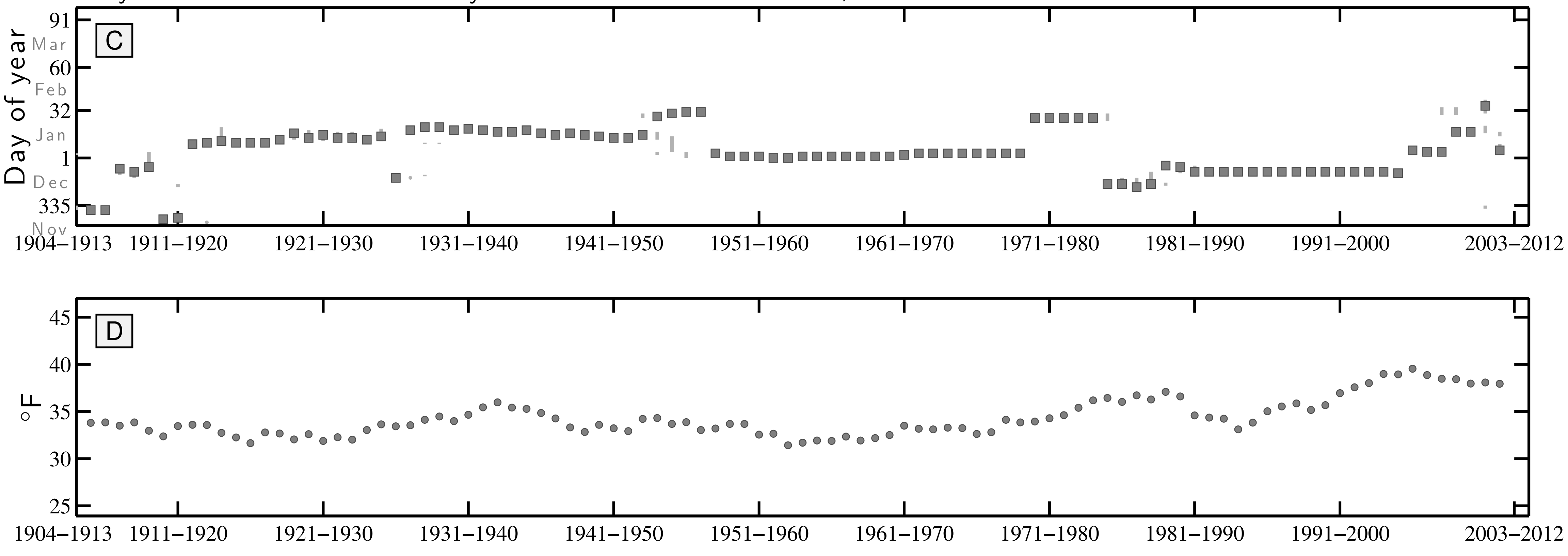

10 year Summer Teletherm dynamics for SUSANVILLE 2SW, CA:

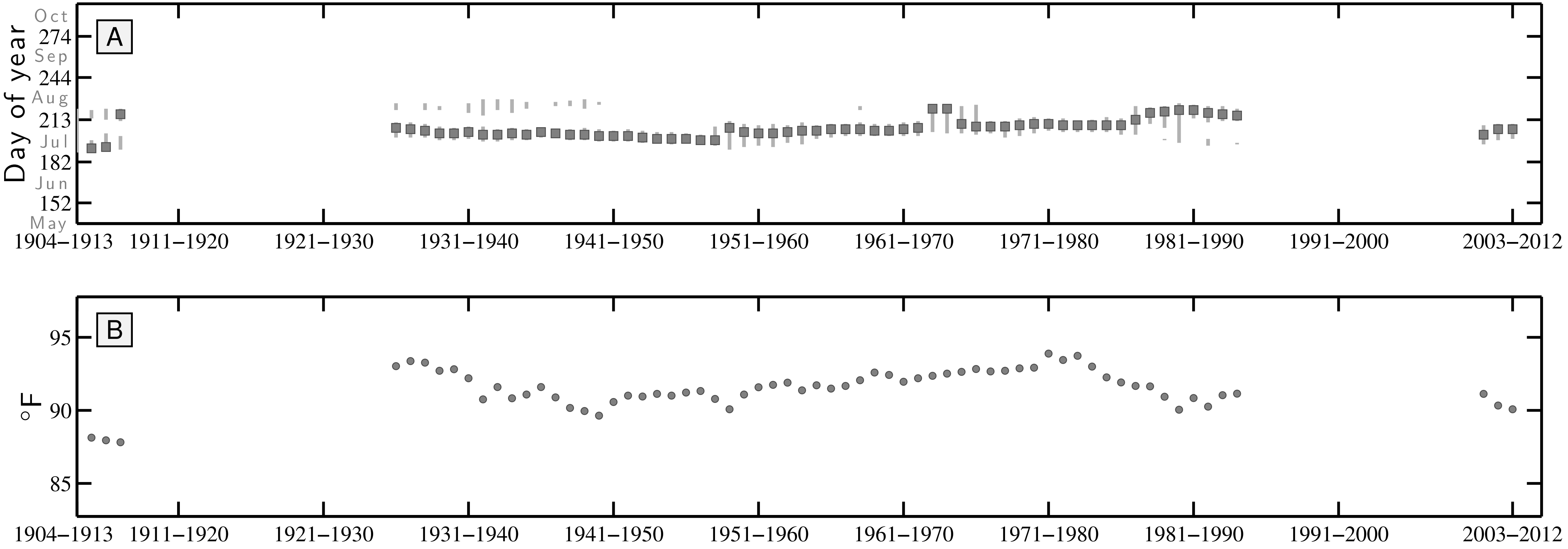

10 year Winter Teletherm dynamics for SUSANVILLE 2SW, CA:

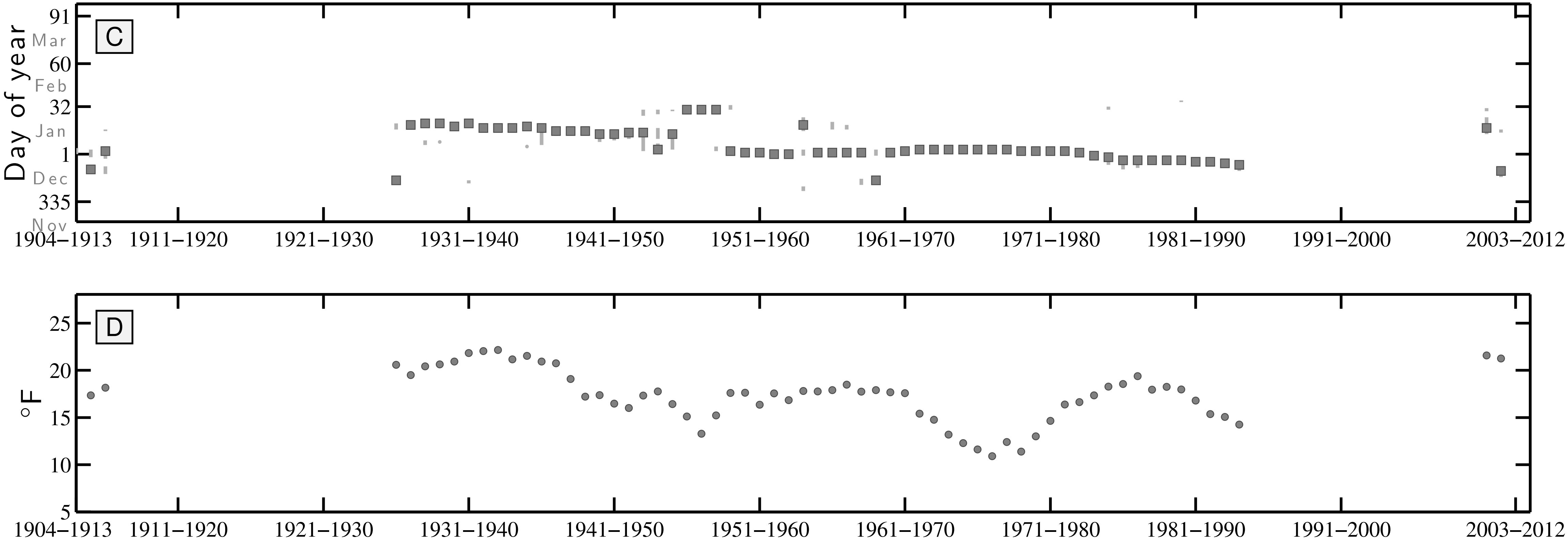

10 year Summer Teletherm dynamics for TAHOE CITY, CA:

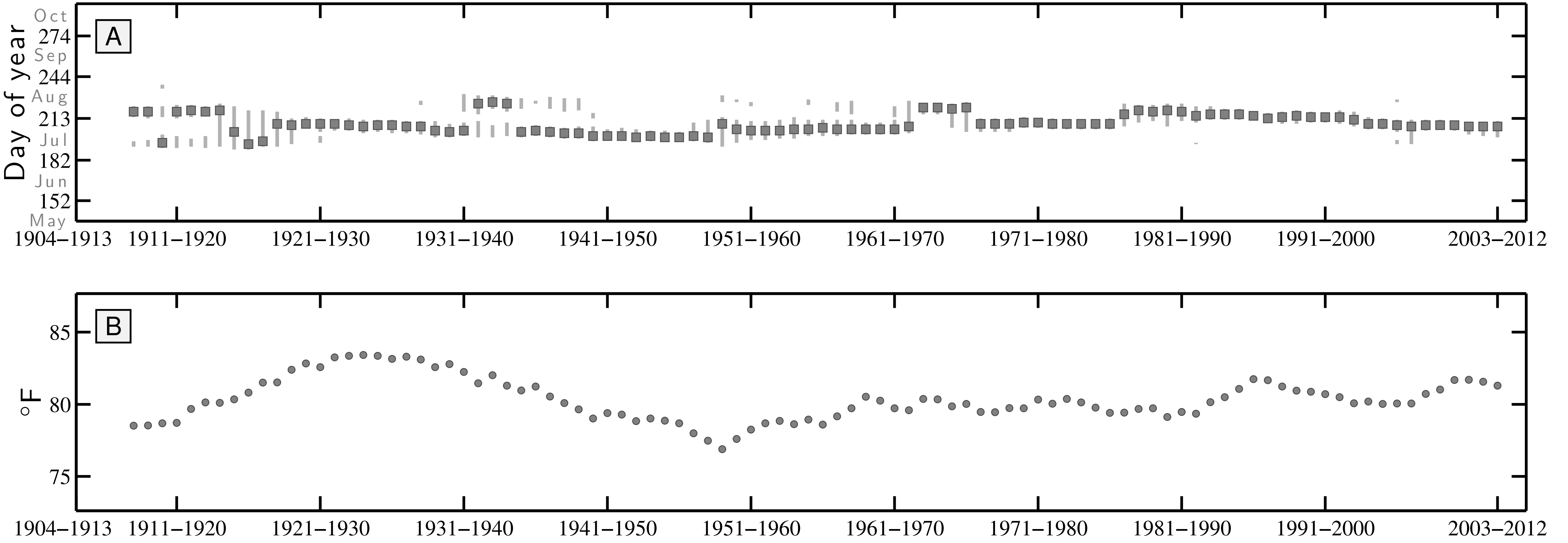

10 year Winter Teletherm dynamics for TAHOE CITY, CA:

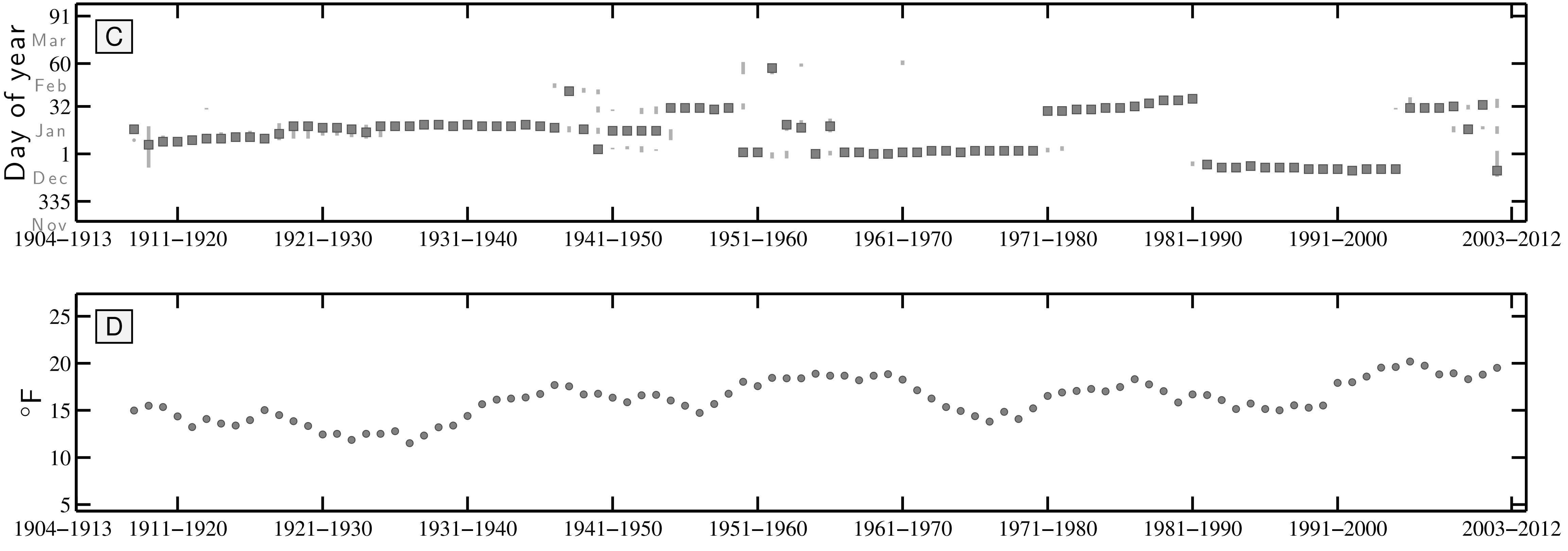

10 year Summer Teletherm dynamics for TEJON RANCHO, CA:

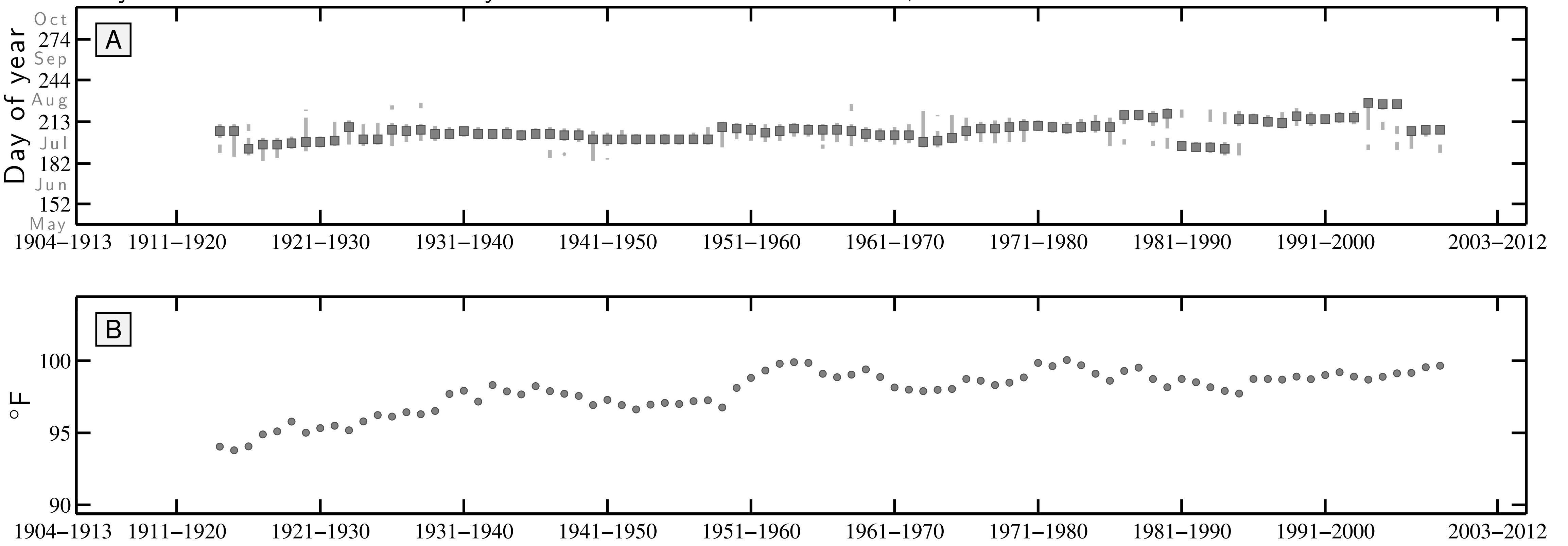

10 year Winter Teletherm dynamics for TEJON RANCHO, CA:

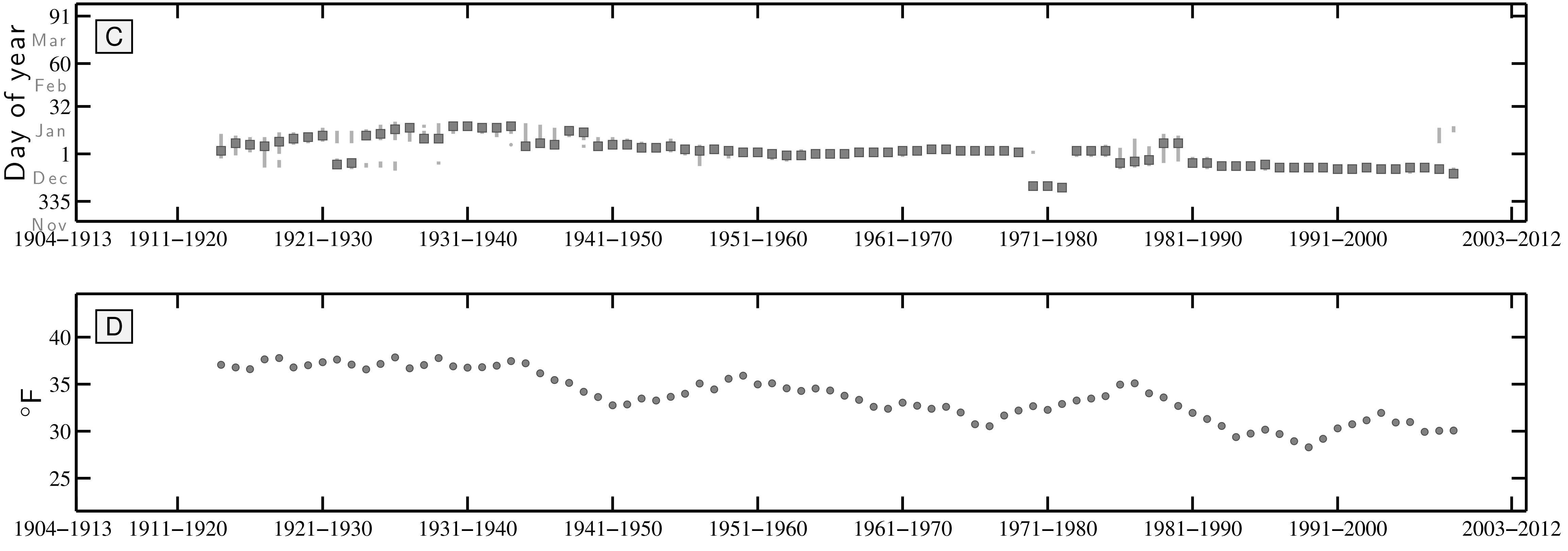

10 year Summer Teletherm dynamics for TUSTIN IRVINE RCH, CA:

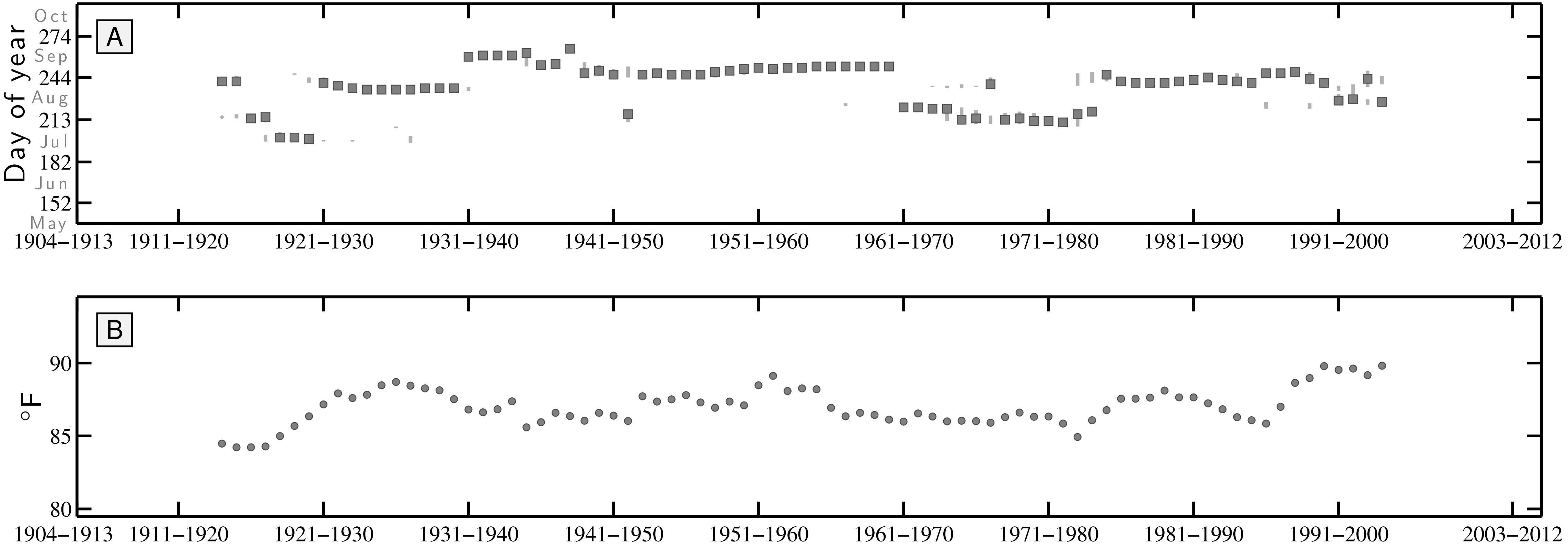

10 year Winter Teletherm dynamics for TUSTIN IRVINE RCH, CA:

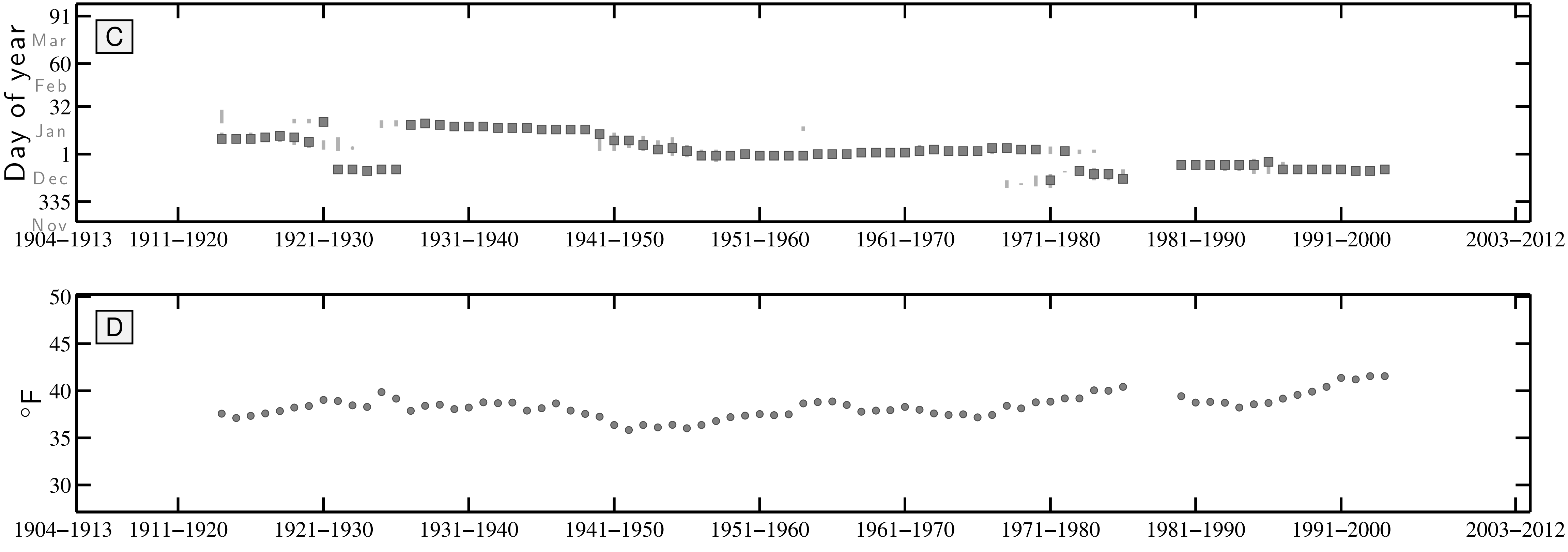

10 year Summer Teletherm dynamics for UKIAH, CA:

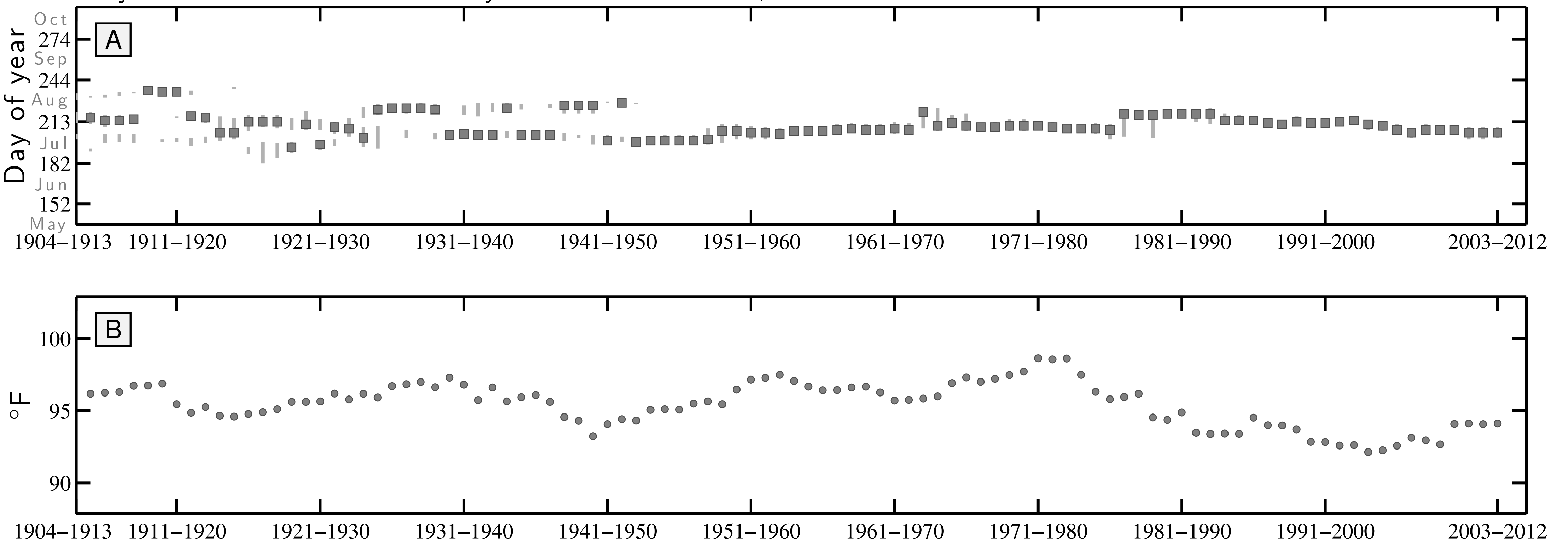

10 year Winter Teletherm dynamics for UKIAH, CA:

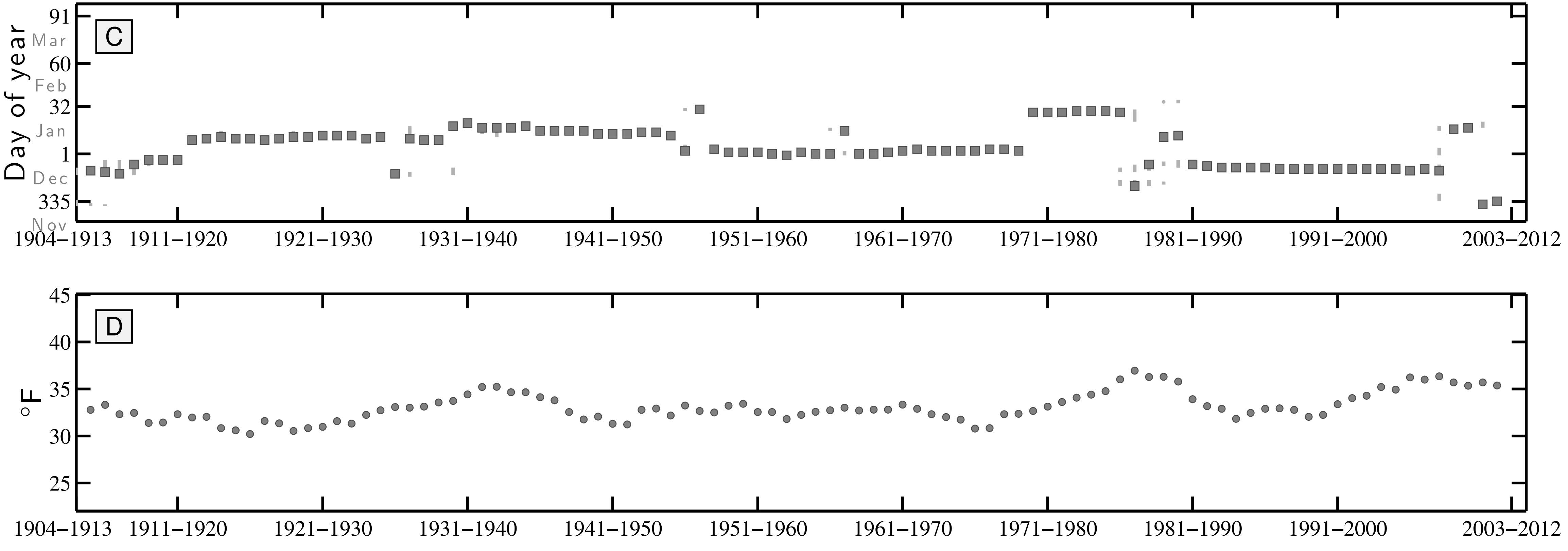

10 year Summer Teletherm dynamics for VACAVILLE, CA:

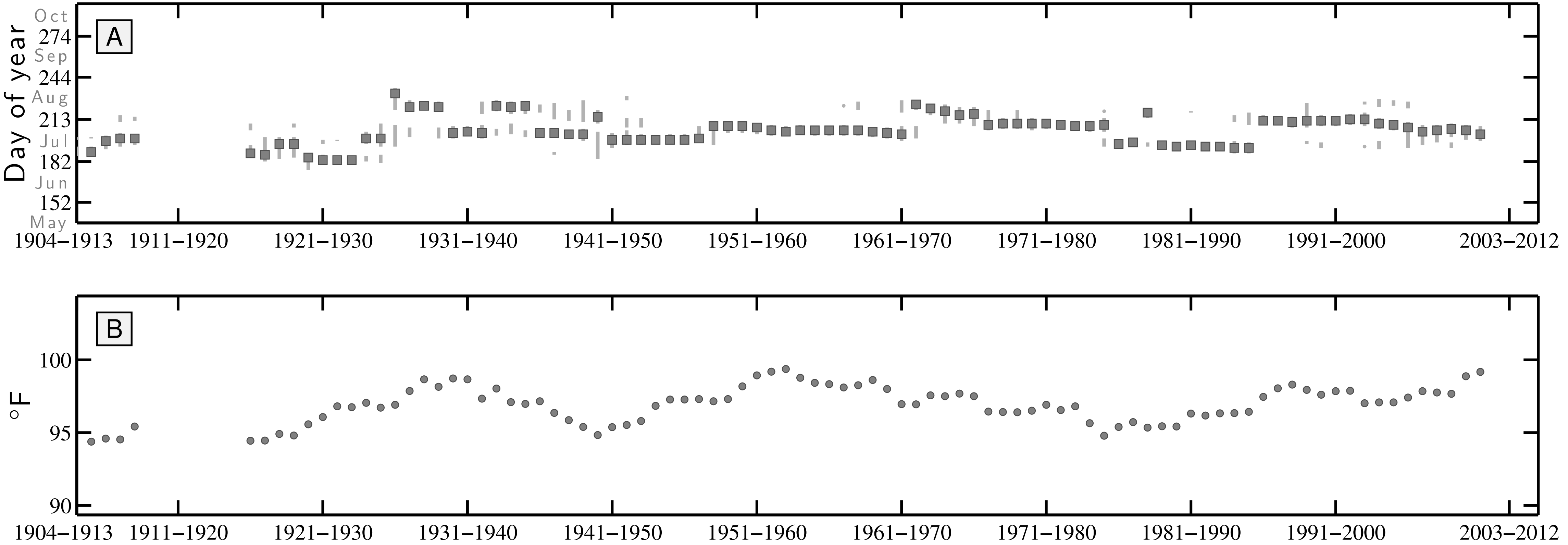

10 year Winter Teletherm dynamics for VACAVILLE, CA:

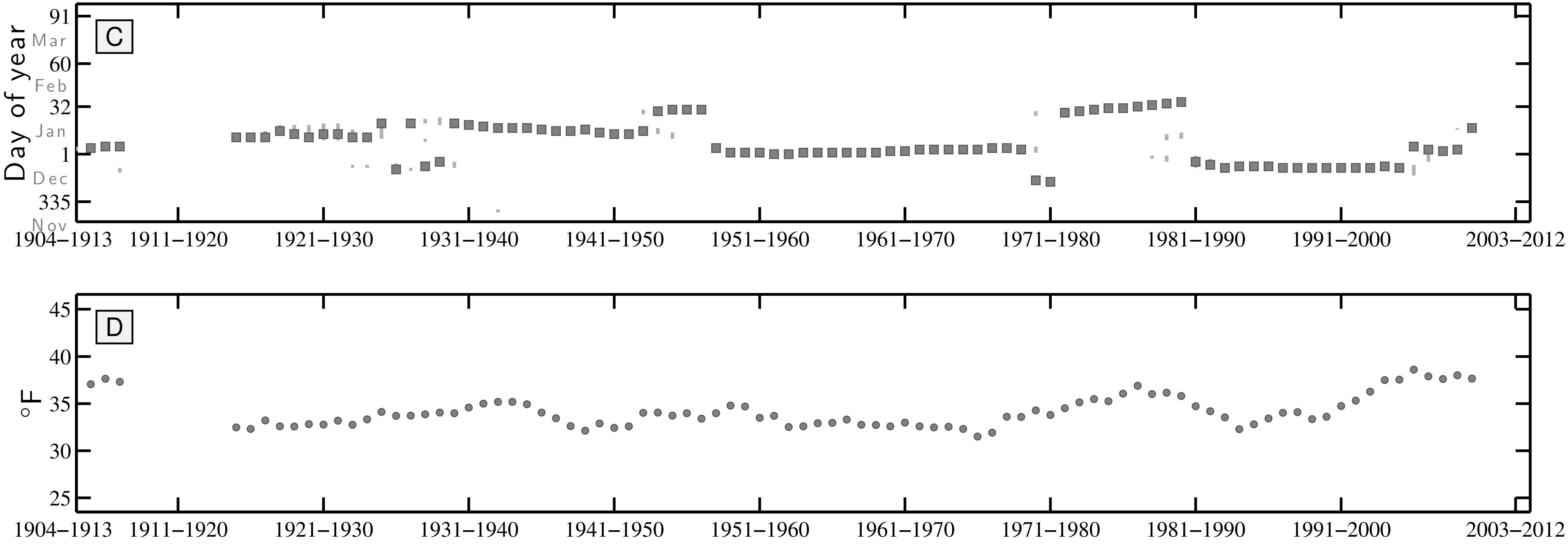

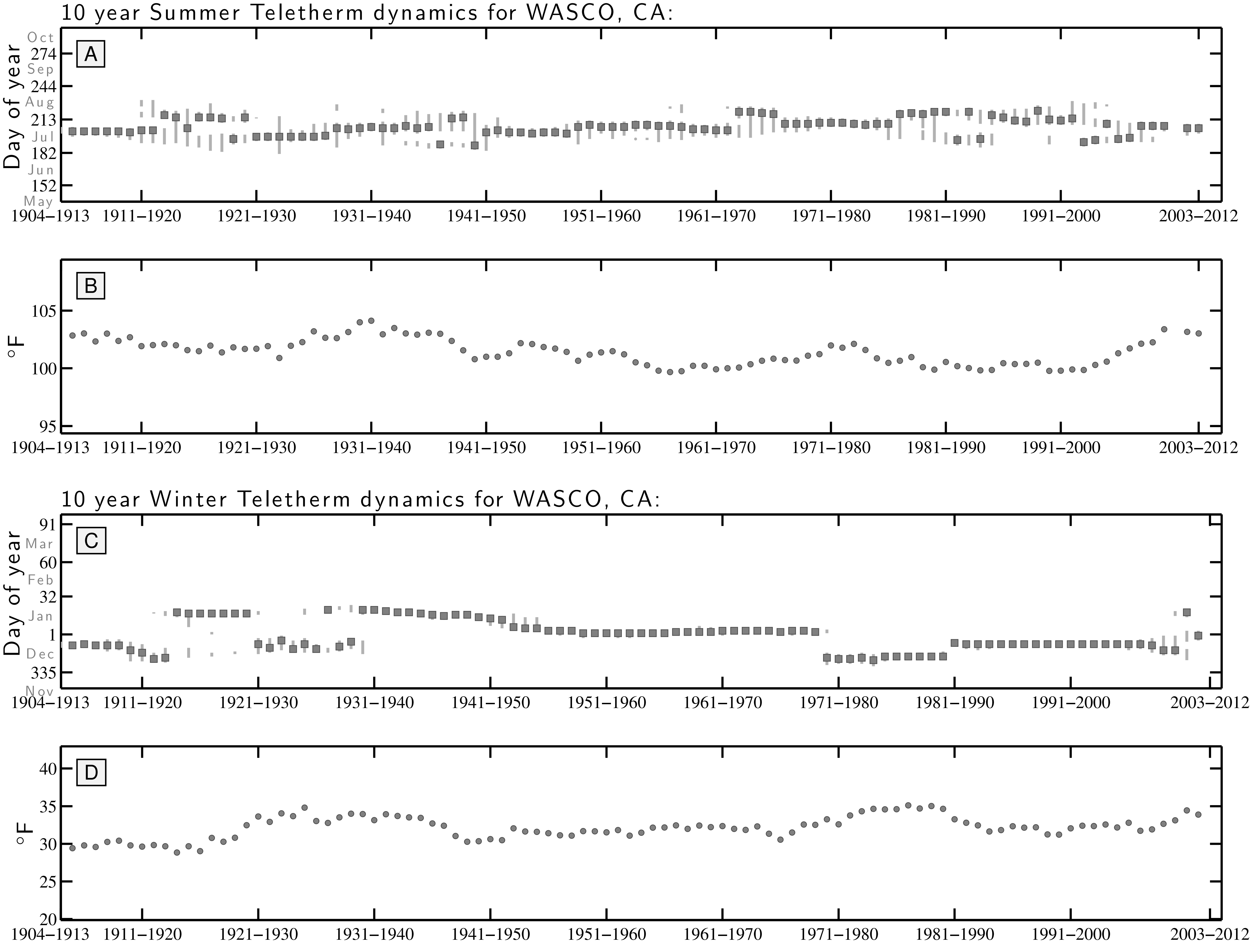
10 year Summer Teletherm dynamics for WASCO, CA:
A
B
Day of year
°F
Oct
274
Sep
244
Aug
213
Jul
182
Jun
152
May
105
100
95
1904–1913
1911–1920
1921–1930
1931–1940
1941–1950
1951–1960
1961–1970
1971–1980
1981–1990
1991–2000
2003–2012
10 year Winter Teletherm dynamics for WASCO, CA:
C
D
91
Mar
60
Feb
32
Jan
1
Dec
335
Nov
40
35
30
25
20

10 year Summer Teletherm dynamics for WEAVERVILLE, CA:

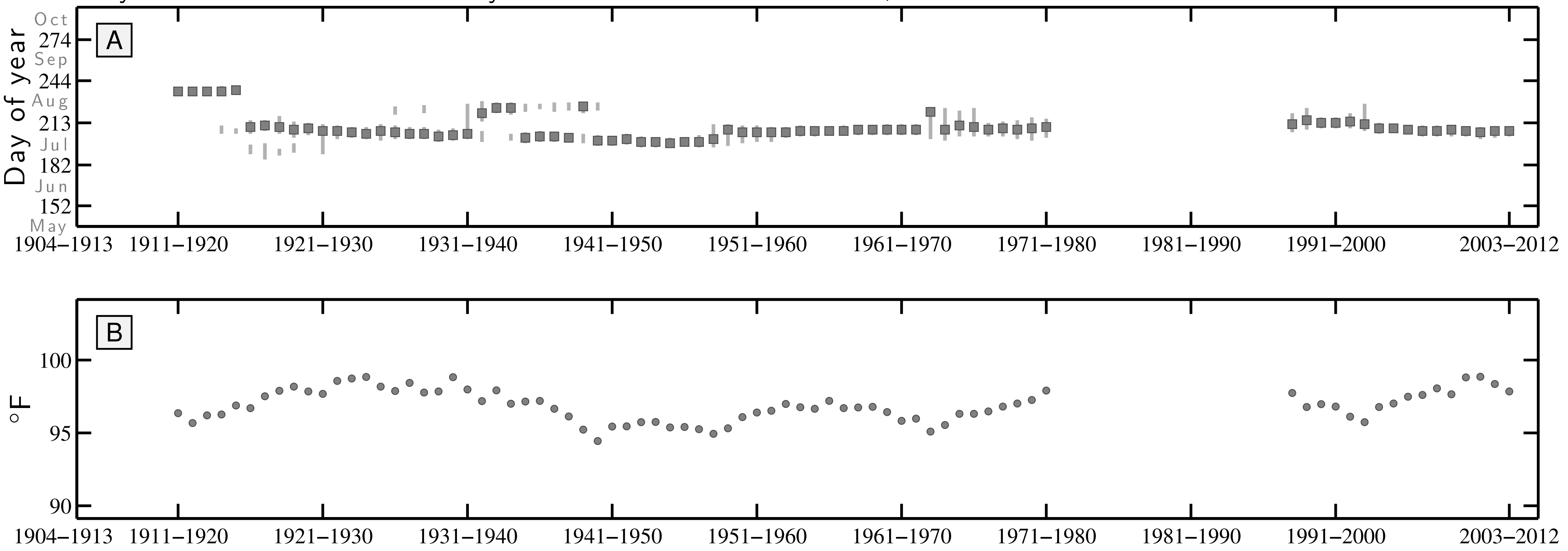

10 year Winter Teletherm dynamics for WEAVERVILLE, CA:

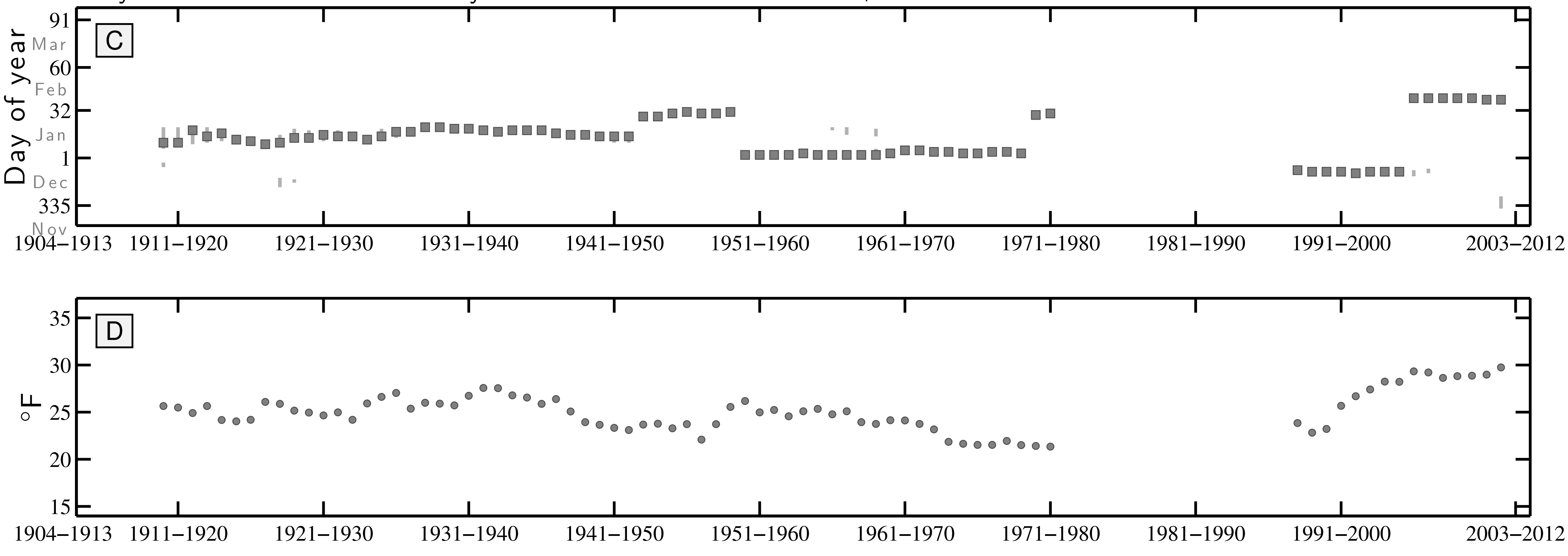

10 year Summer Teletherm dynamics for WILLOWS 6 W, CA:

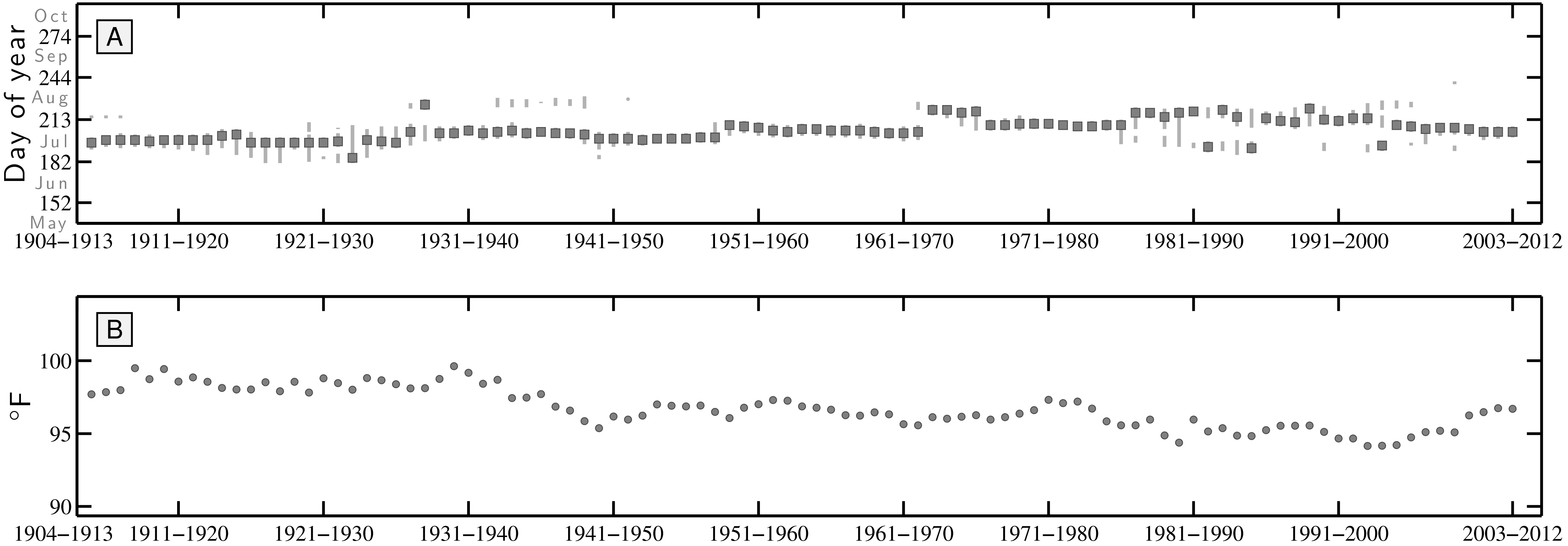

10 year Winter Teletherm dynamics for WILLOWS 6 W, CA:

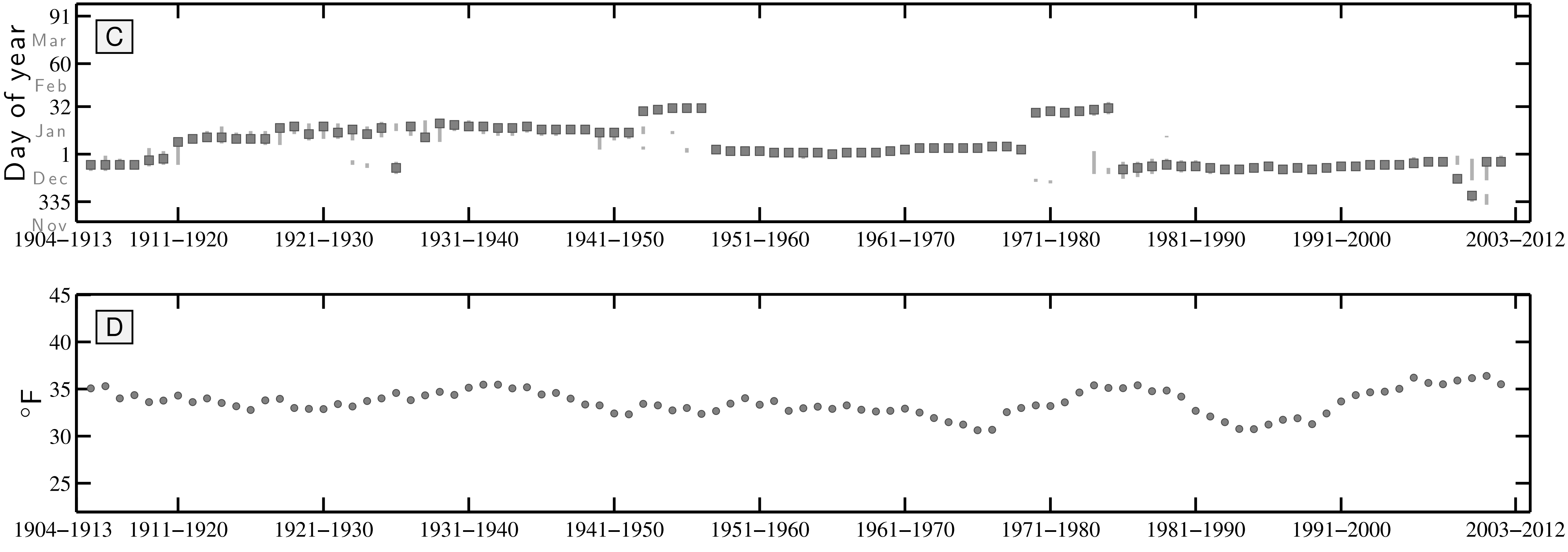

10 year Summer Teletherm dynamics for YOSEMITE PARK HQ, CA:

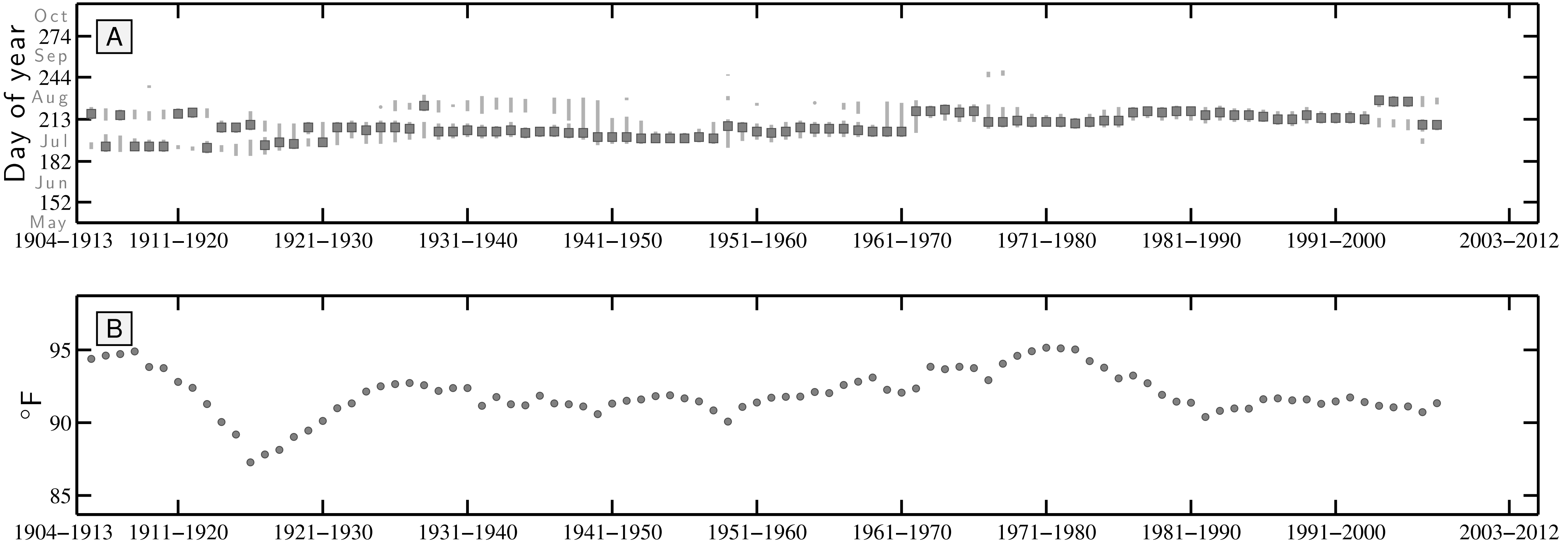

10 year Winter Teletherm dynamics for YOSEMITE PARK HQ, CA:

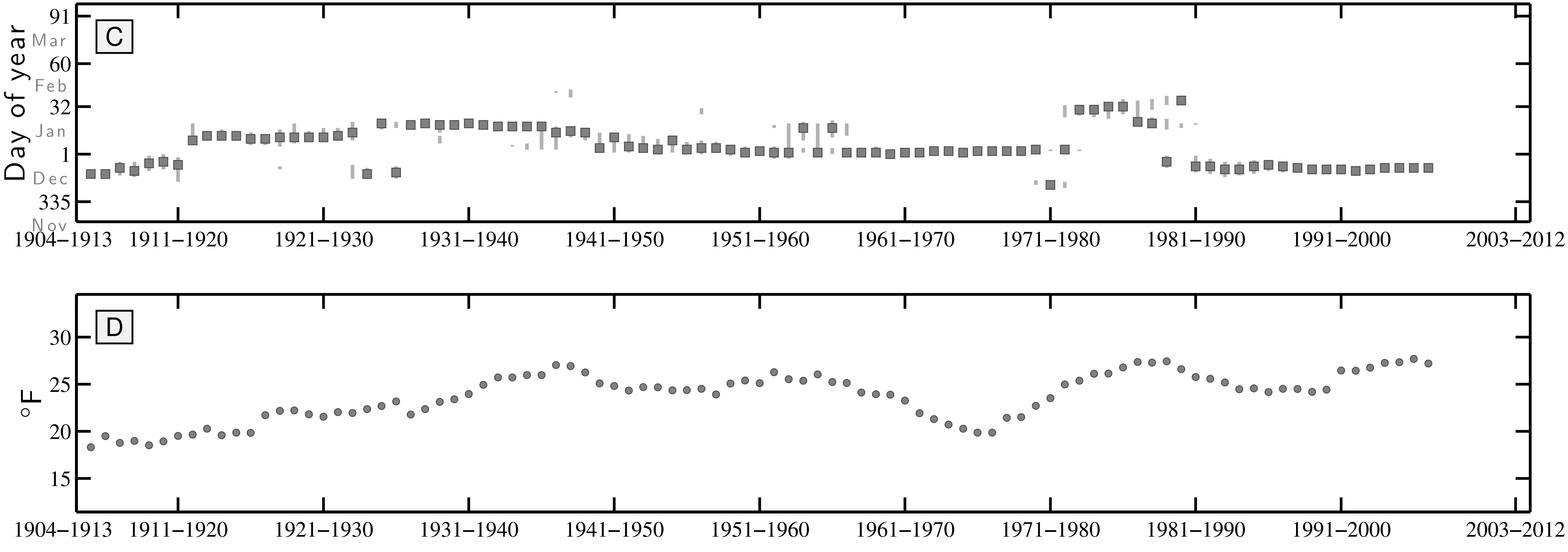

10 year Summer Teletherm dynamics for YREKA, CA:

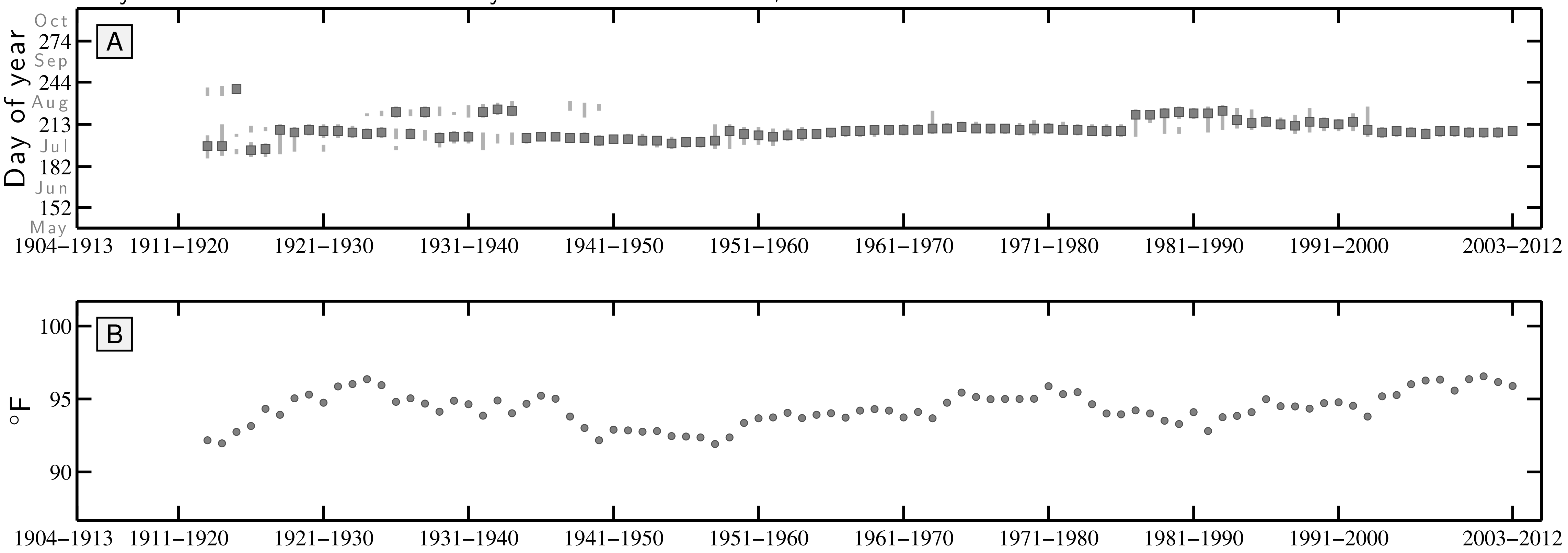

10 year Winter Teletherm dynamics for YREKA, CA:

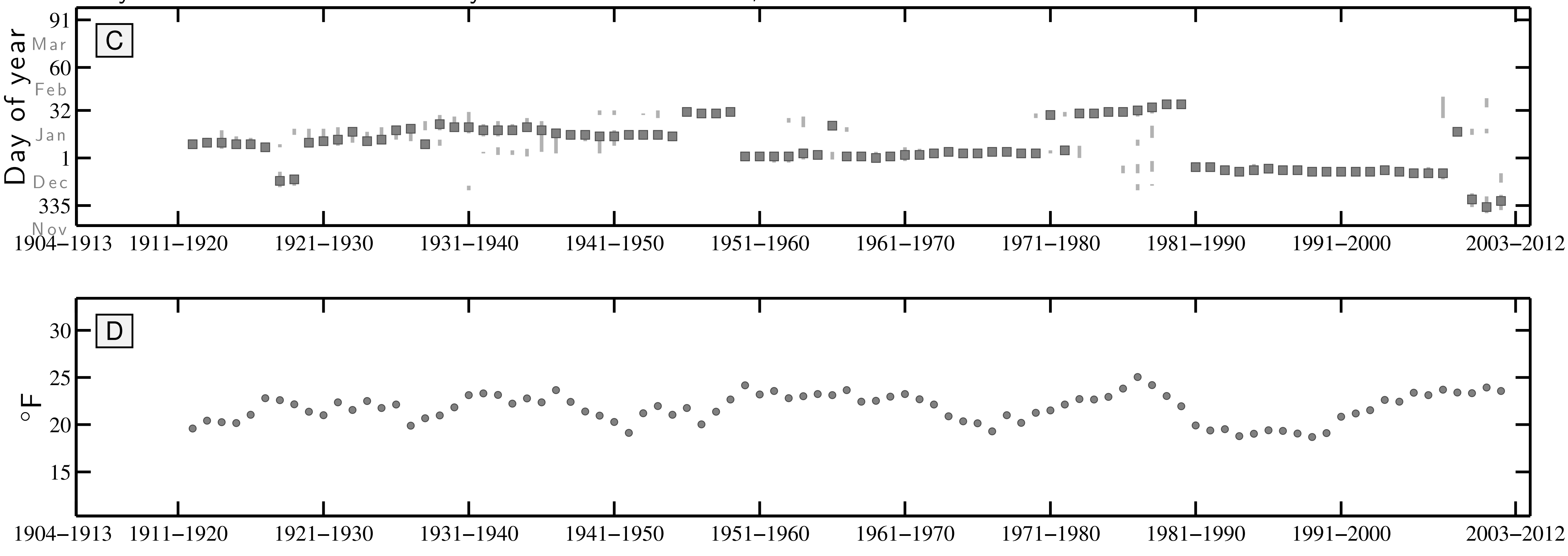

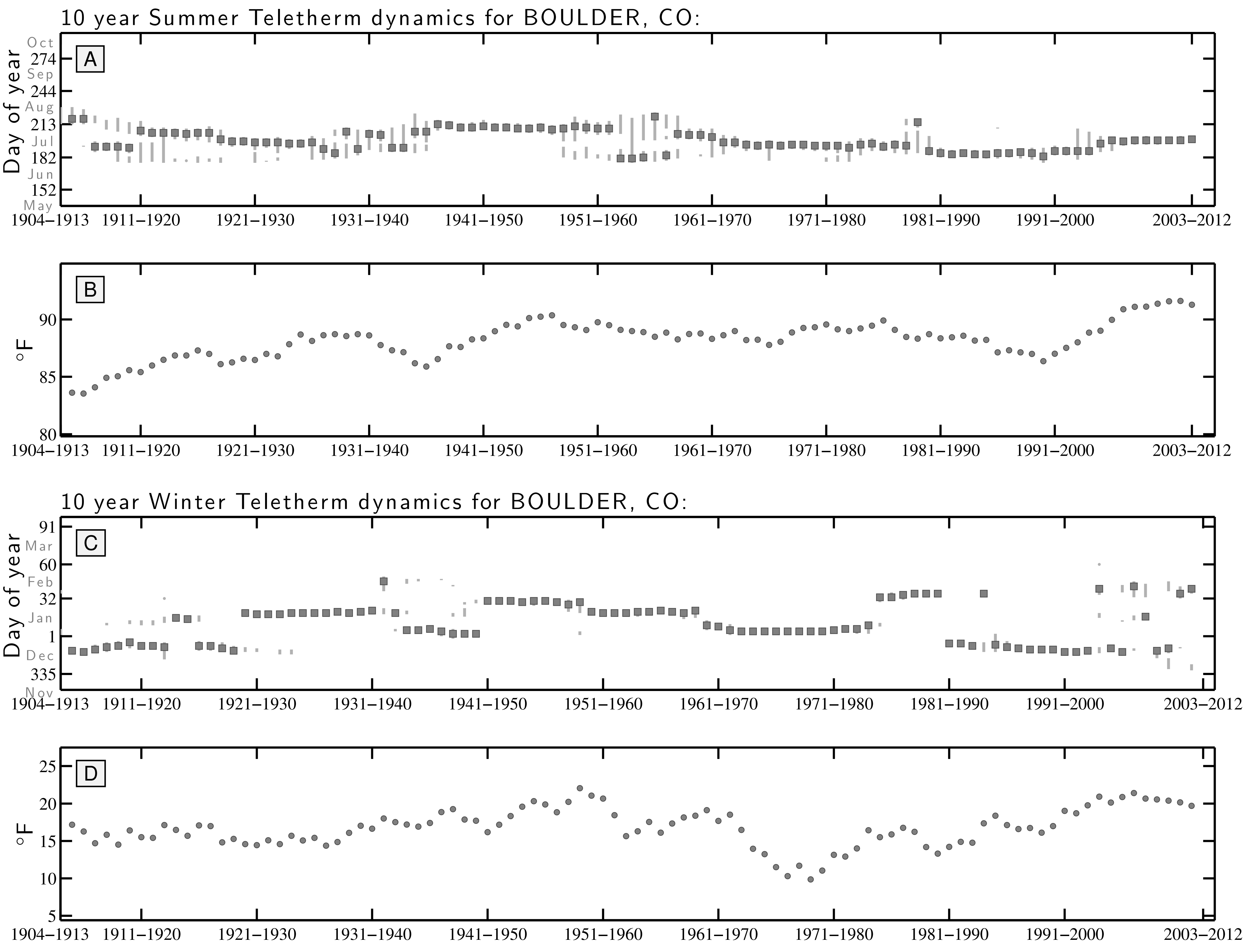
10 year Summer Teletherm dynamics for BOULDER, CO:
A
Day of year
Oct 274 Sep 244 Aug 213 Jul 182 Jun 152 May
B
°F
80 85 90
10 year Winter Teletherm dynamics for BOULDER, CO:
C
Day of year
91 Mar 60 Feb 32 Jan 1 Dec 335 Nov
D
°F
5 10 15 20 25
1904–1913 1911–1920 1921–1930 1931–1940 1941–1950 1951–1960 1961–1970 1971–1980 1981–1990 1991–2000 2003–2012

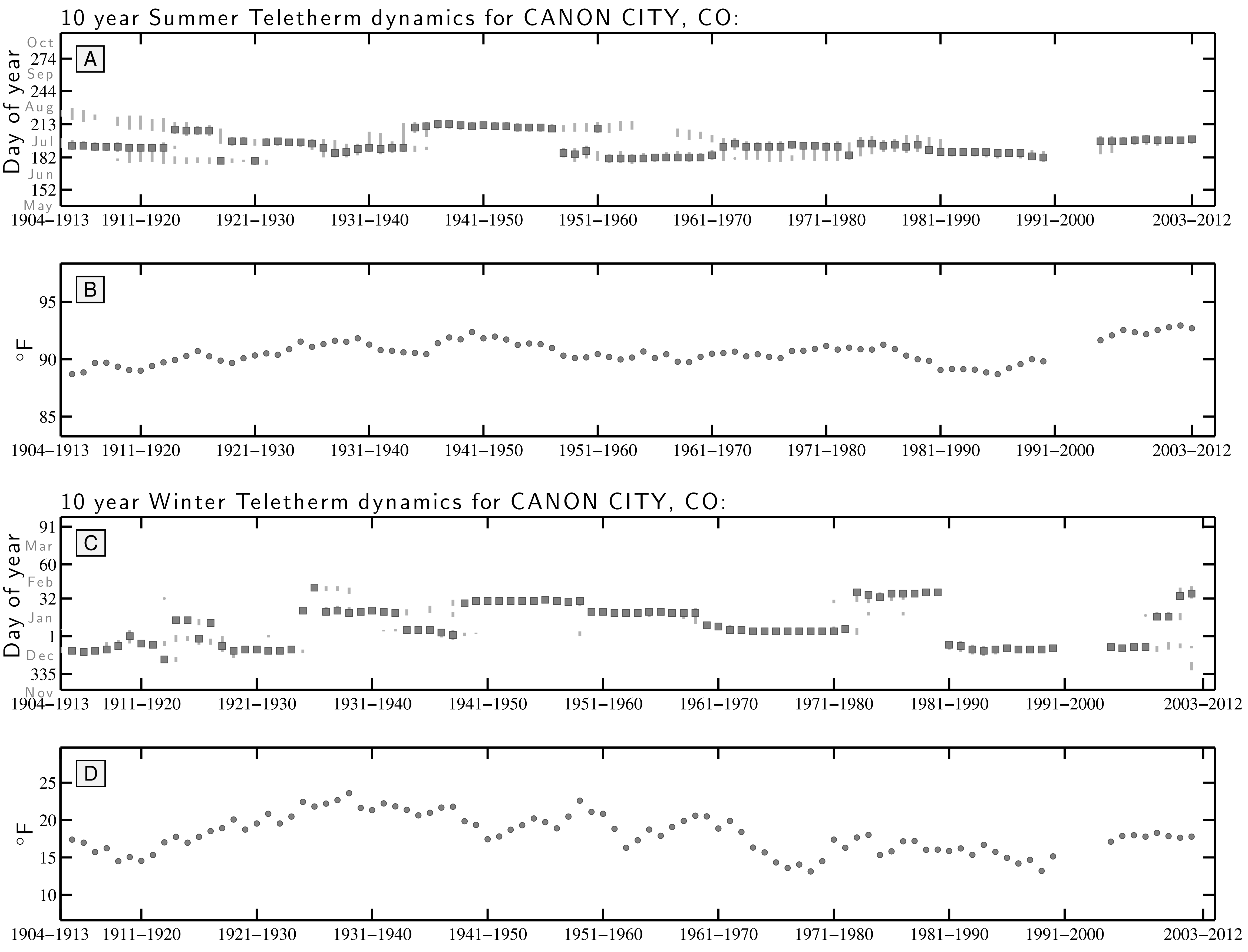
10 year Summer Teletherm dynamics for CANON CITY, CO:
A
Day of year
Oct
274
Sep
244
Aug
213
Jul
182
Jun
152
May
1904–1913
1911–1920
1921–1930
1931–1940
1941–1950
1951–1960
1961–1970
1971–1980
1981–1990
1991–2000
2003–2012
B
°F
95
90
85
10 year Winter Teletherm dynamics for CANON CITY, CO:
C
Day of year
91
Mar
60
Feb
32
Jan
1
Dec
335
Nov
D
°F
25
20
15
10

10 year Summer Teletherm dynamics for CHEESMAN, CO:

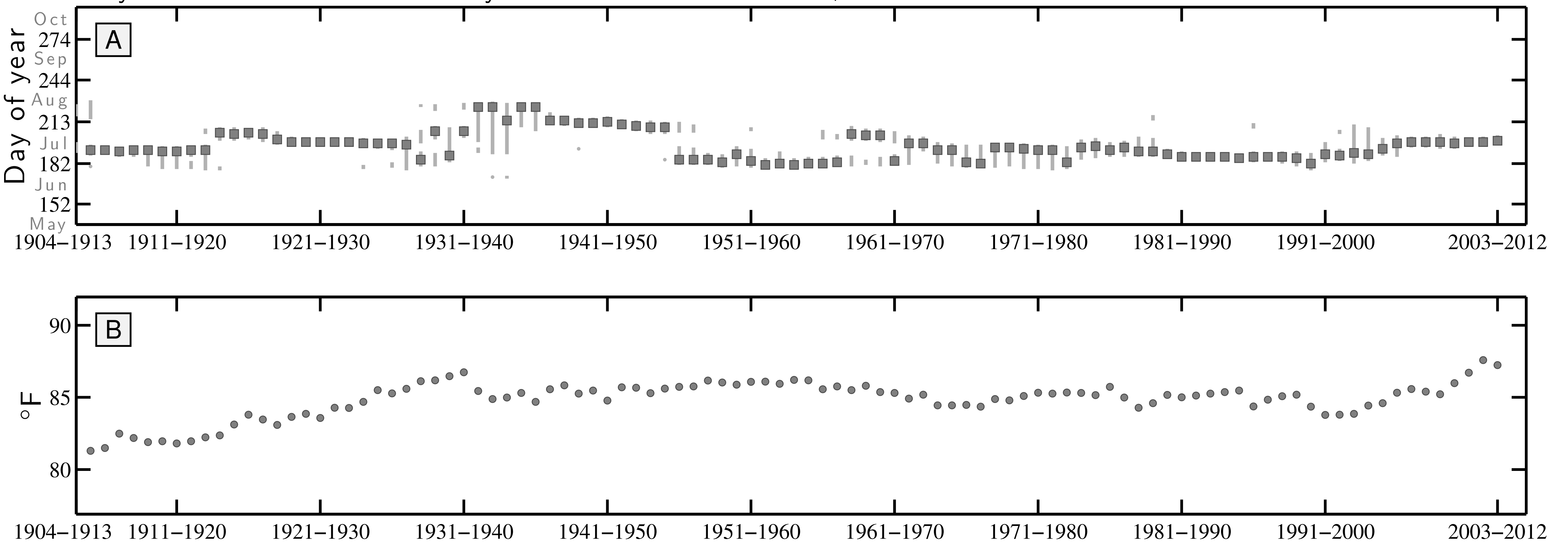

10 year Winter Teletherm dynamics for CHEESMAN, CO:

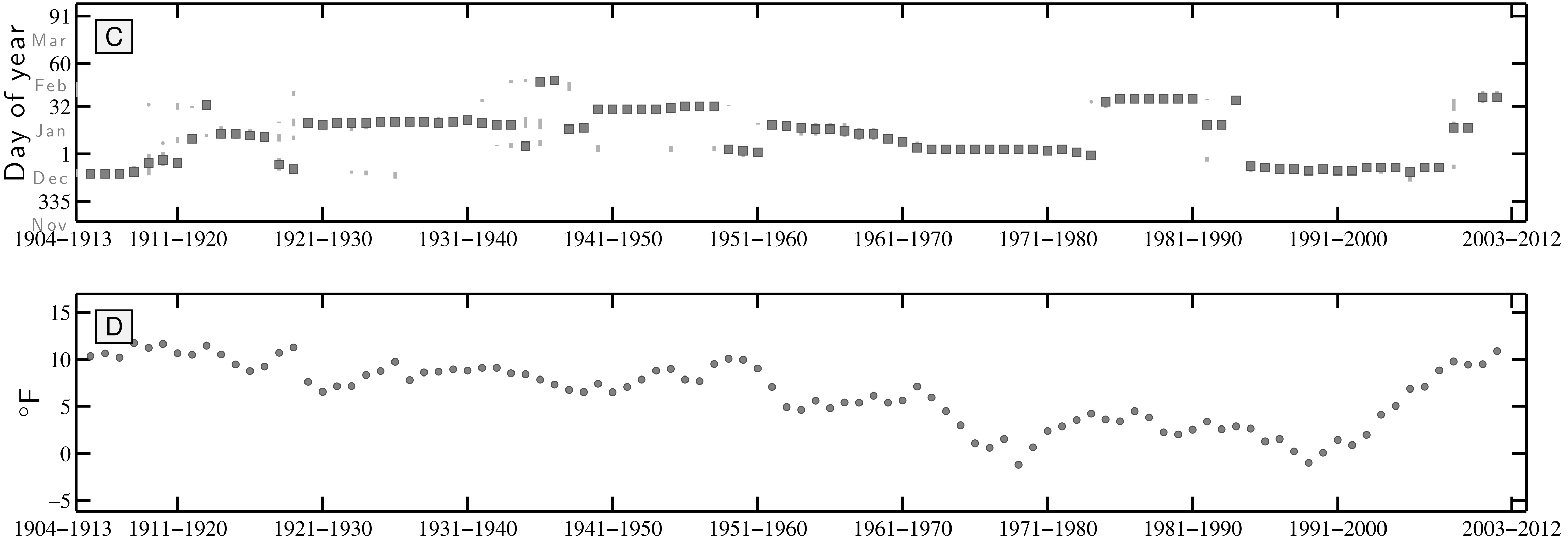

10 year Summer Teletherm dynamics for CHEYENNE WELLS, CO:

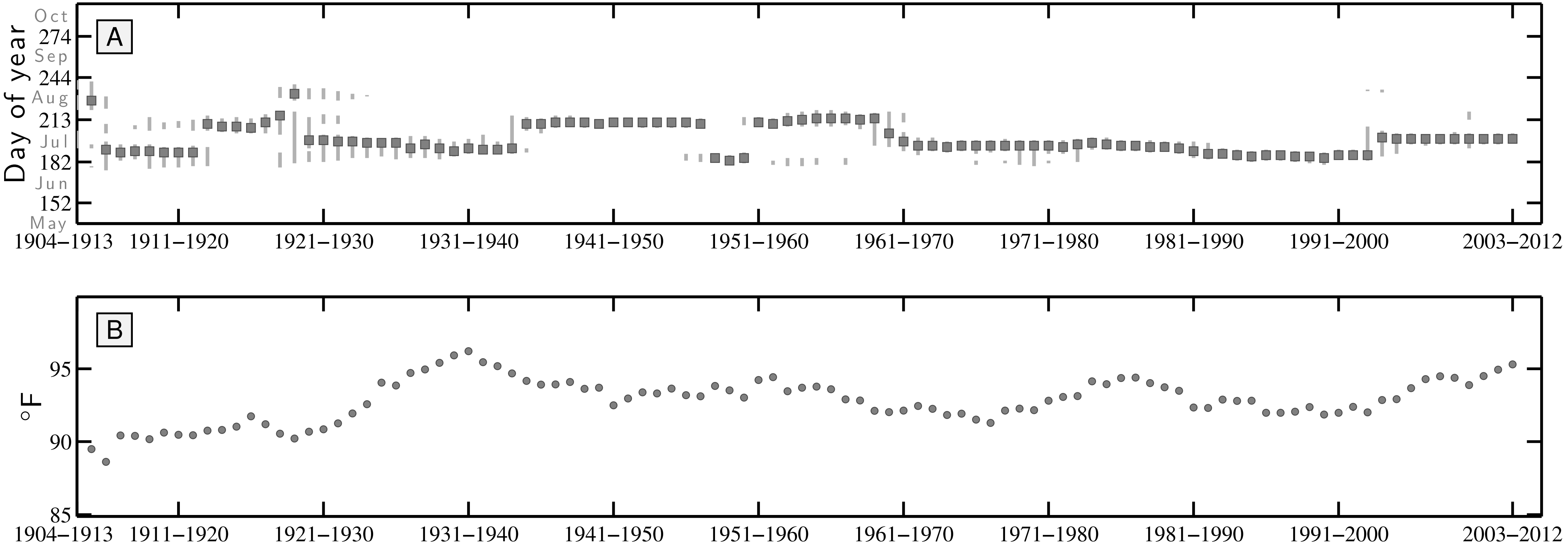

10 year Winter Teletherm dynamics for CHEYENNE WELLS, CO:

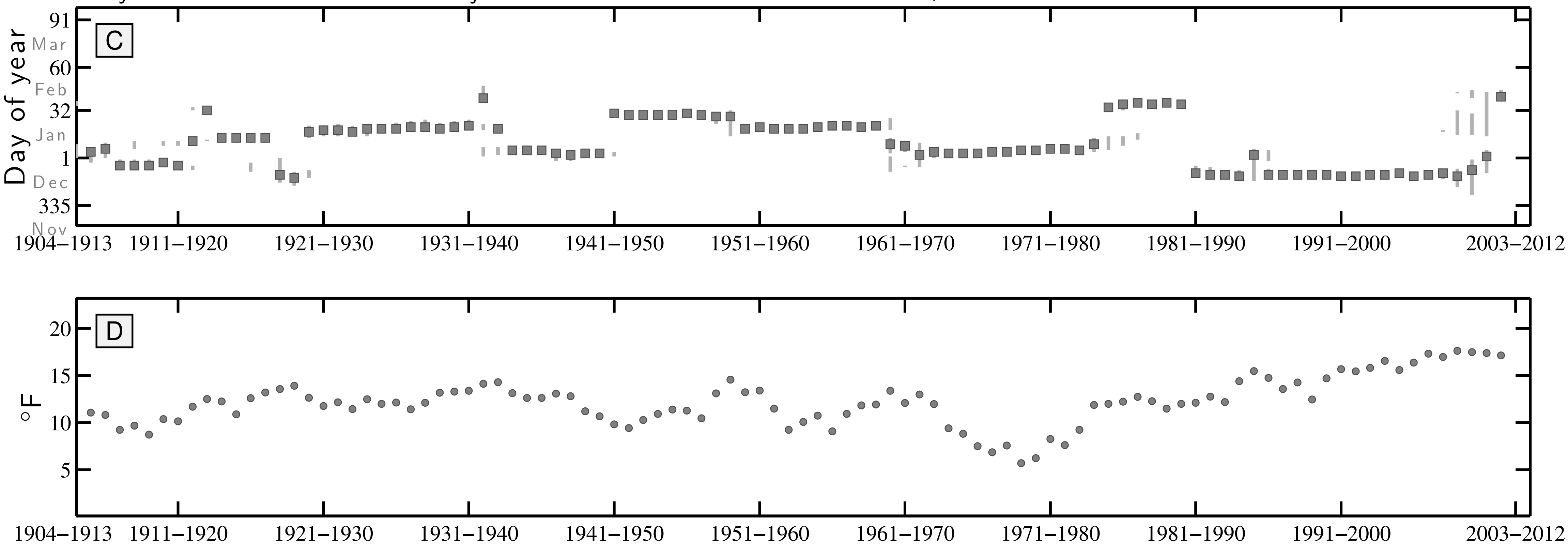

10 year Summer Teletherm dynamics for COLLBRAN, CO:

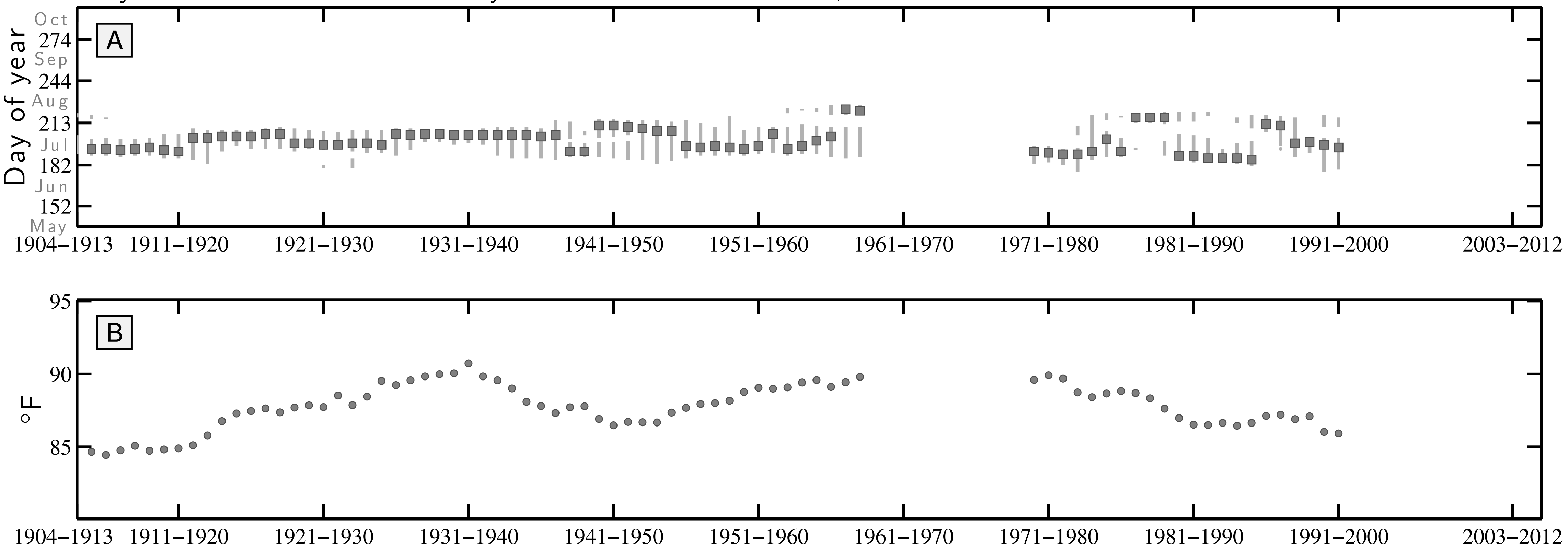

10 year Winter Teletherm dynamics for COLLBRAN, CO:

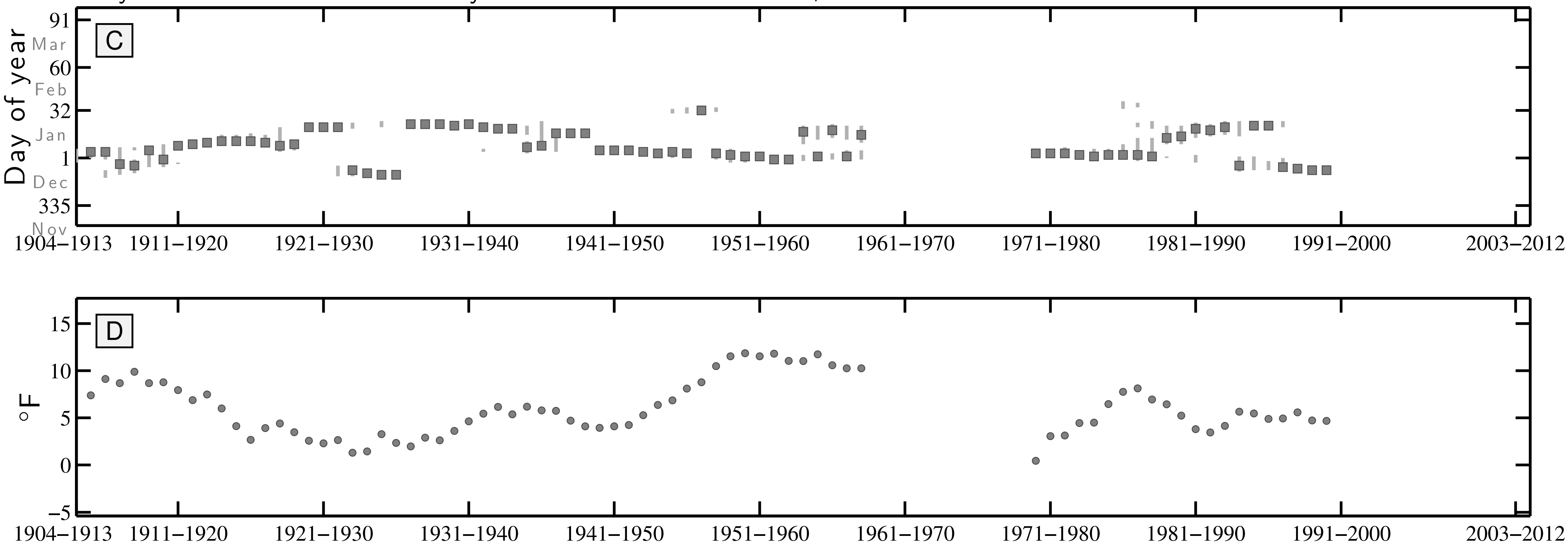

10 year Summer Teletherm dynamics for DEL NORTE 2E, CO:

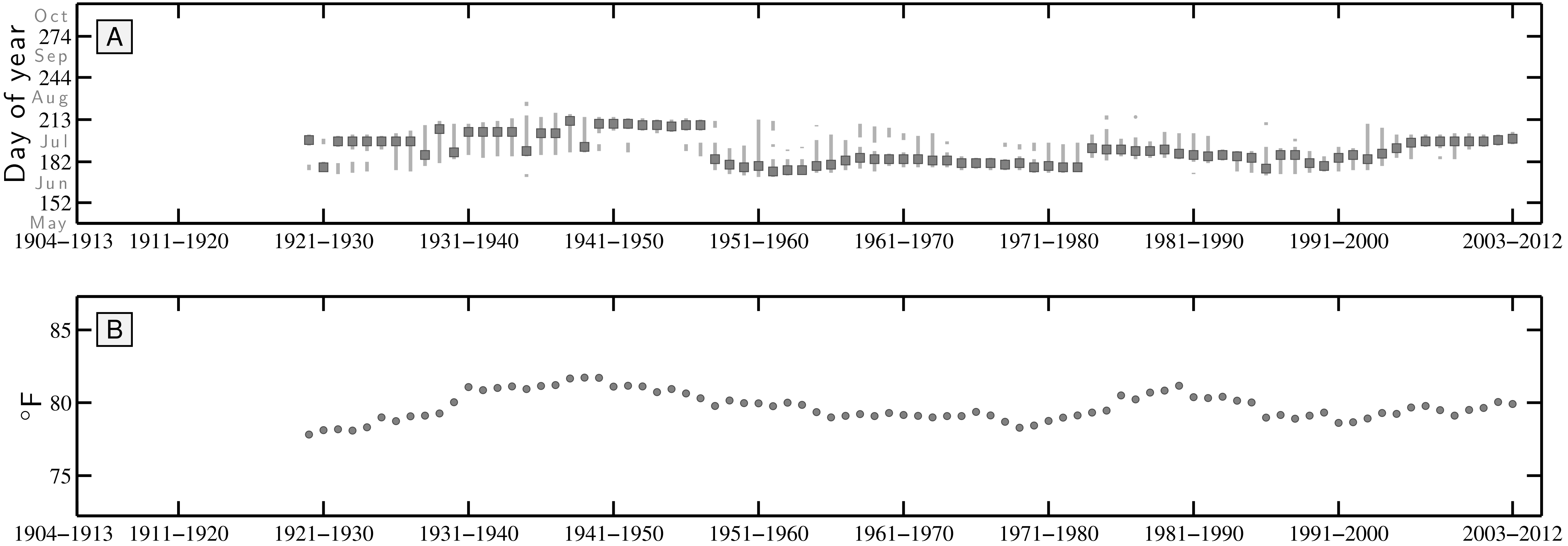

10 year Winter Teletherm dynamics for DEL NORTE 2E, CO:

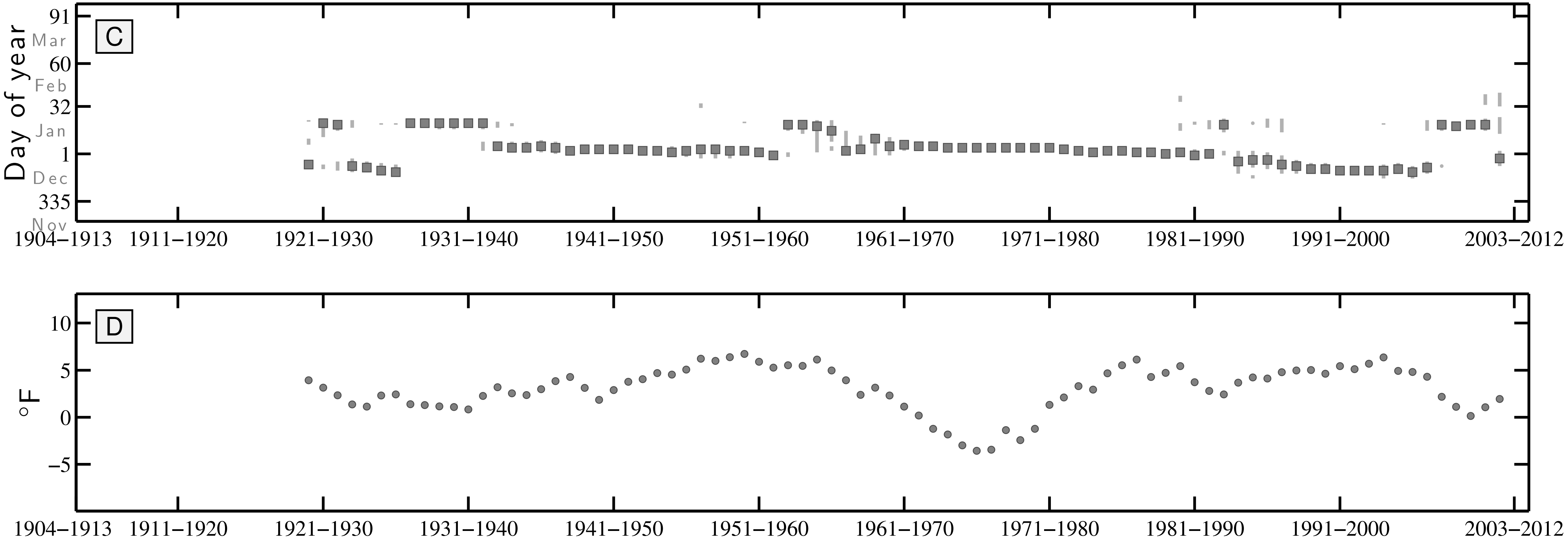

10 year Summer Teletherm dynamics for DILLON 1 E, CO:

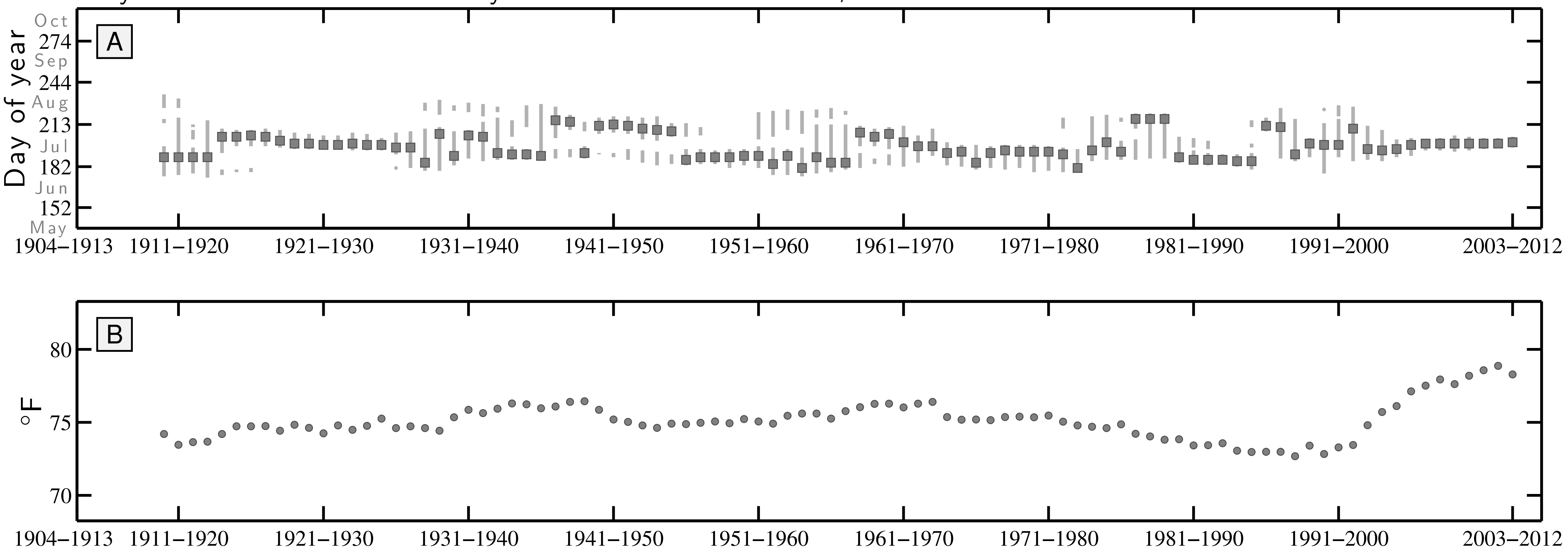

10 year Winter Teletherm dynamics for DILLON 1 E, CO:

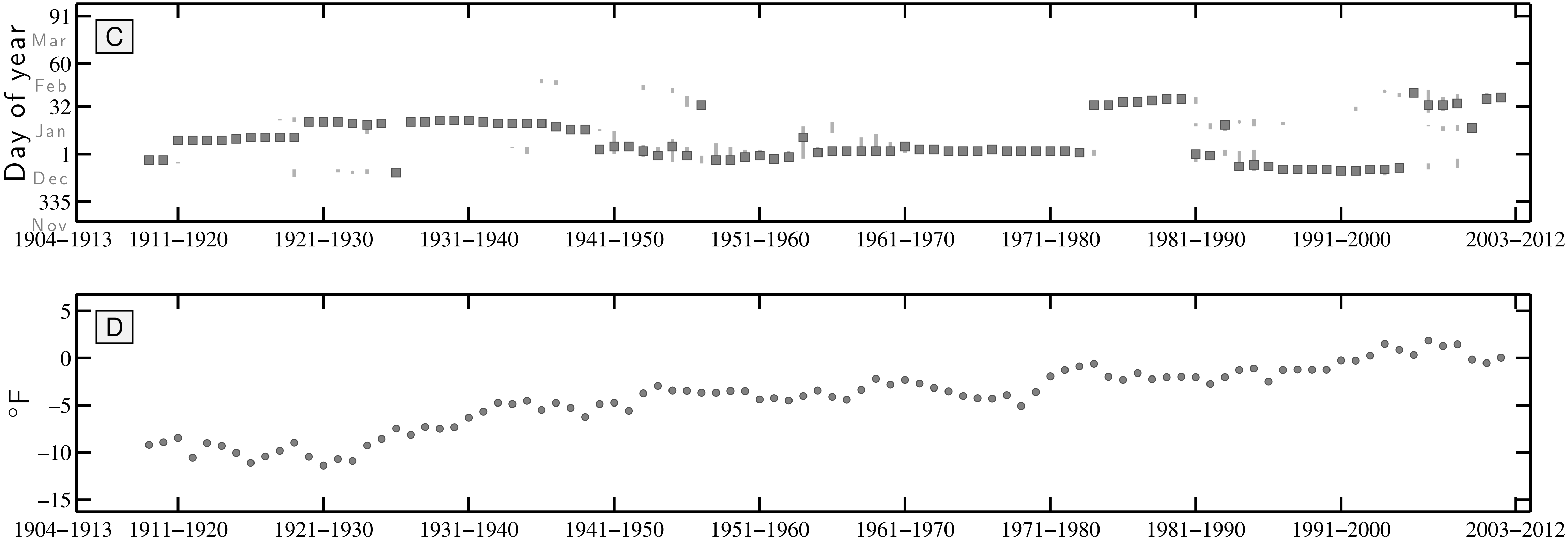

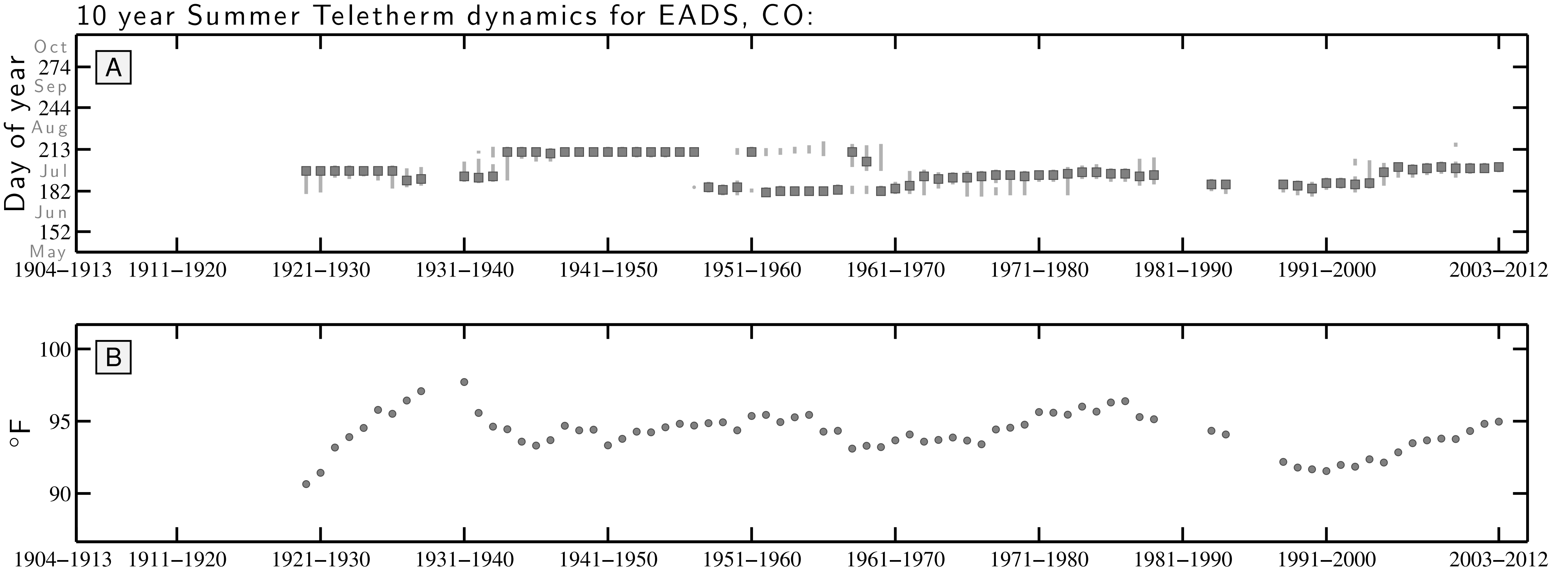
10 year Summer Teletherm dynamics for EADS, CO:
A
B
Day of year
°F
Oct
274
Sep
244
Aug
213
Jul
182
Jun
152
May
100
95
90
1904–1913
1911–1920
1921–1930
1931–1940
1941–1950
1951–1960
1961–1970
1971–1980
1981–1990
1991–2000
2003–2012

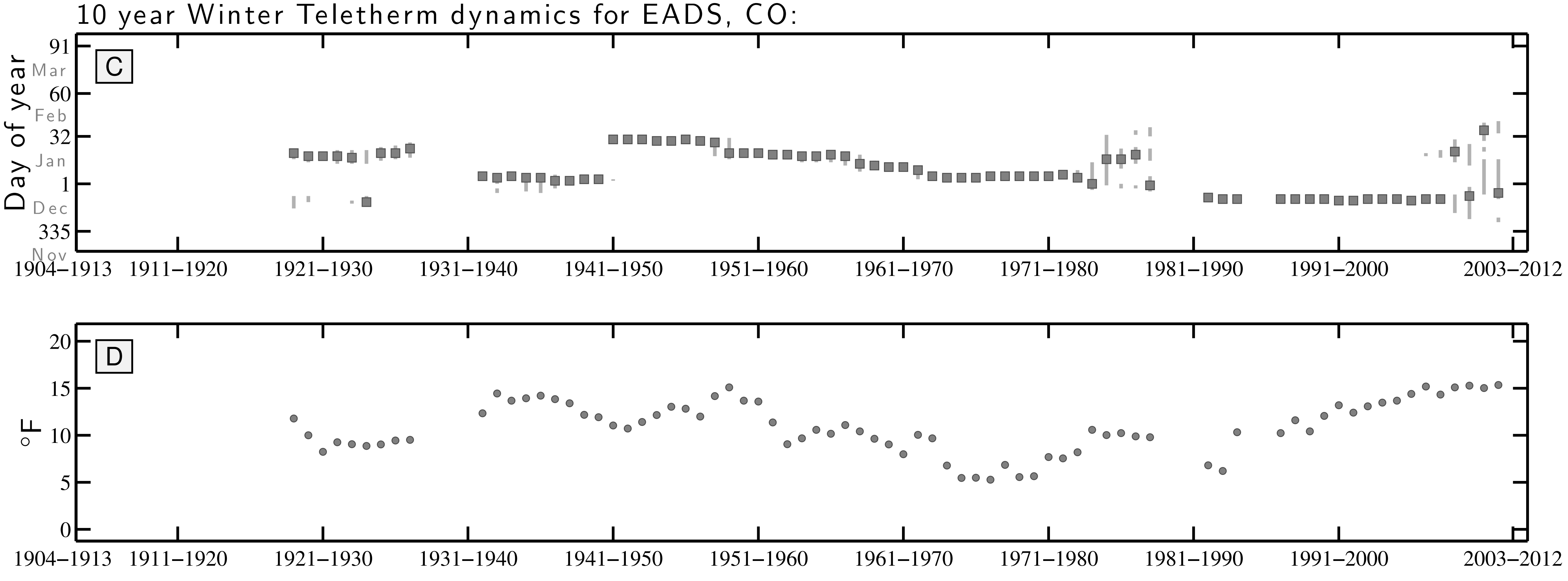
10 year Winter Teletherm dynamics for EADS, CO:
C
D
Day of year
°F
91
Mar
60
Feb
32
Jan
1
Dec
335
Nov
20
15
10
5
0
1904–1913
1911–1920
1921–1930
1931–1940
1941–1950
1951–1960
1961–1970
1971–1980
1981–1990
1991–2000
2003–2012

10 year Summer Teletherm dynamics for FT COLLINS, CO:

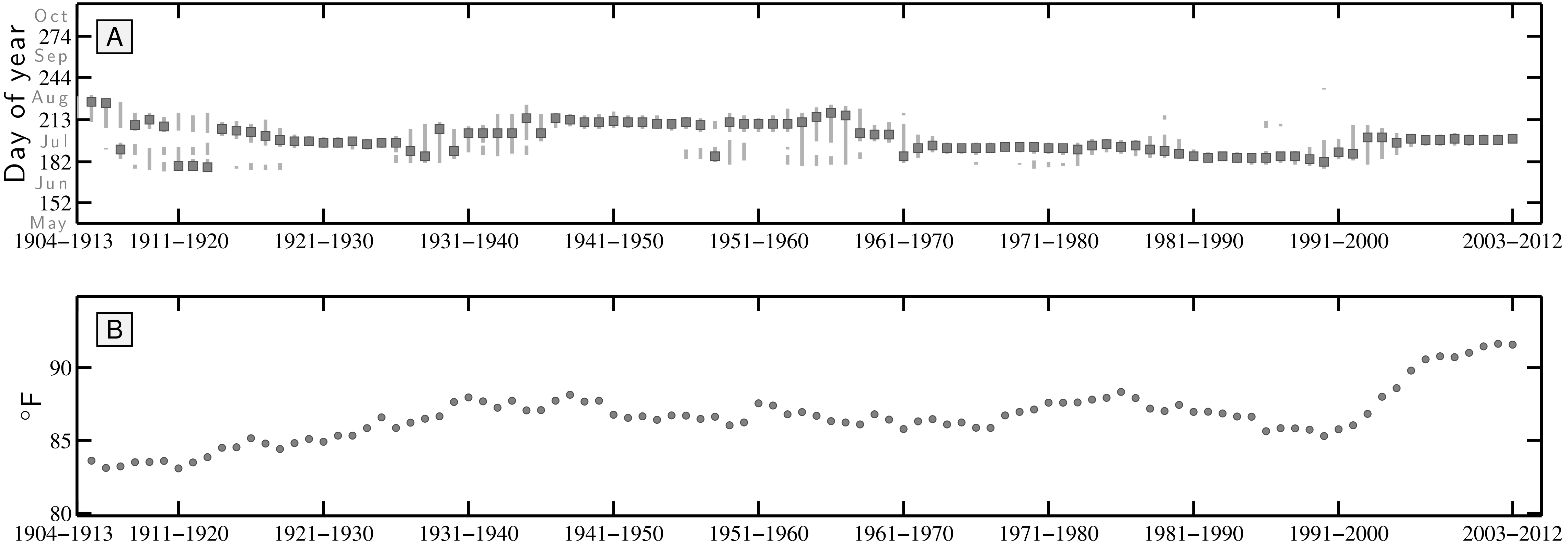

10 year Winter Teletherm dynamics for FT COLLINS, CO:

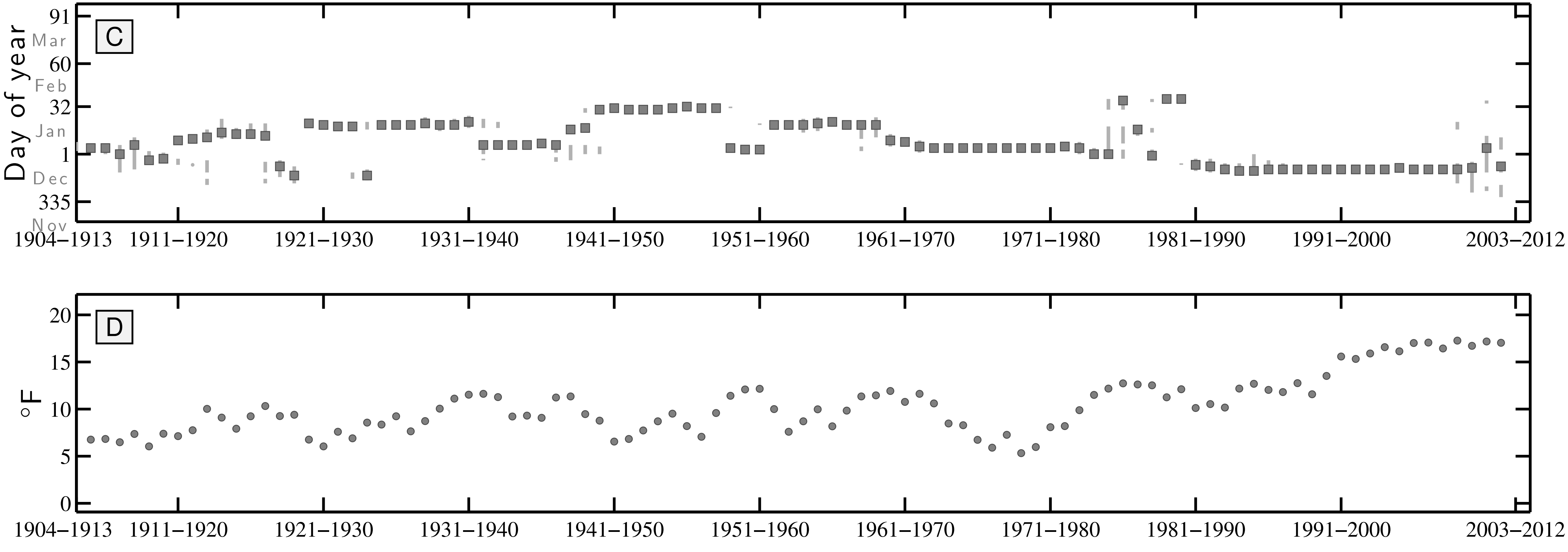

10 year Summer Teletherm dynamics for FT MORGAN, CO:

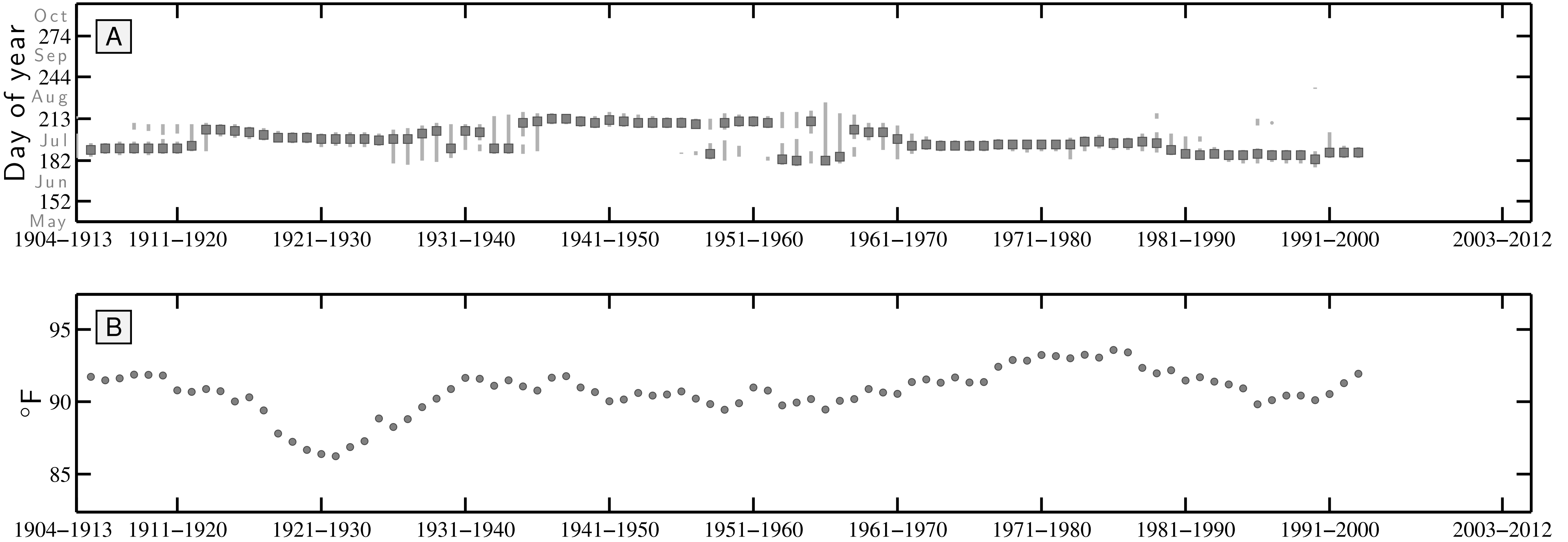

10 year Winter Teletherm dynamics for FT MORGAN, CO:

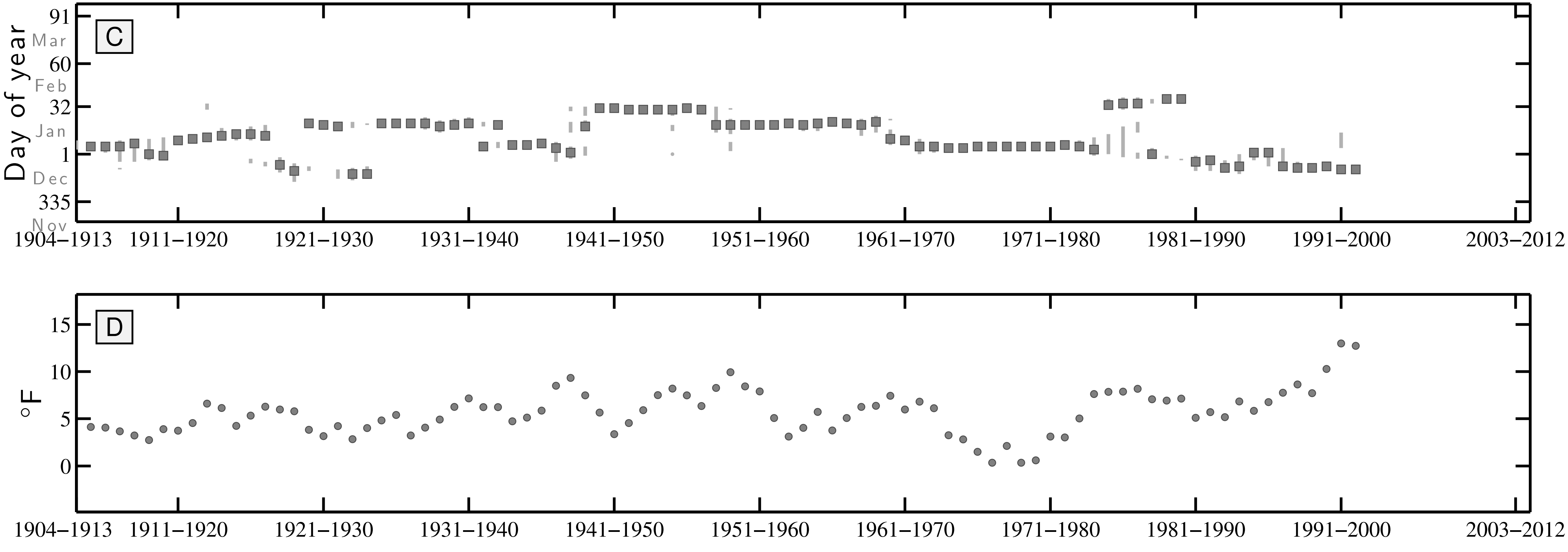

10 year Summer Teletherm dynamics for FRUITA, CO:

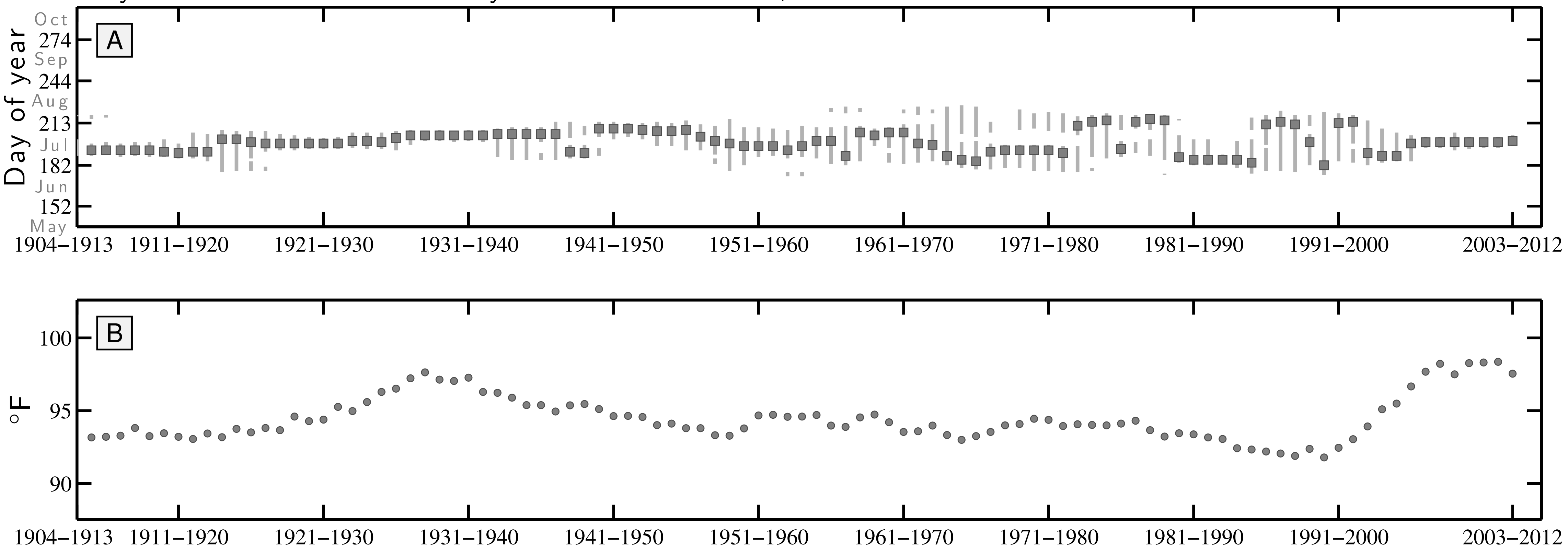

10 year Winter Teletherm dynamics for FRUITA, CO:

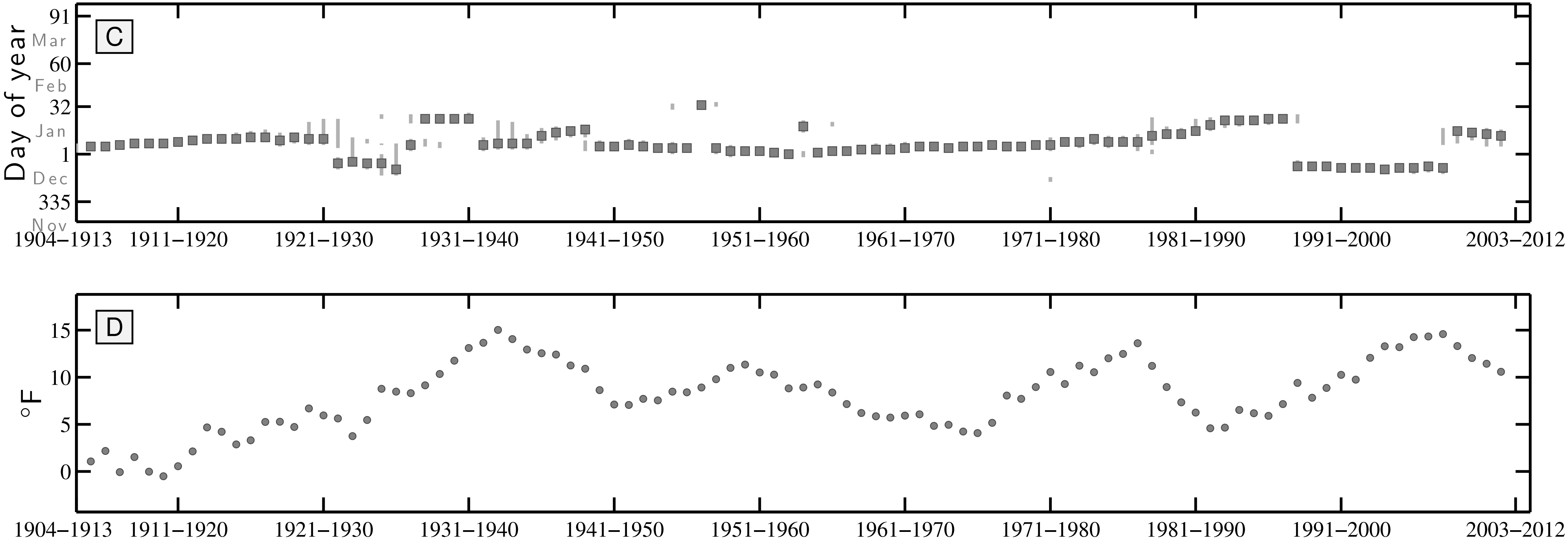

10 year Summer Teletherm dynamics for GUNNISON 3SW, CO:

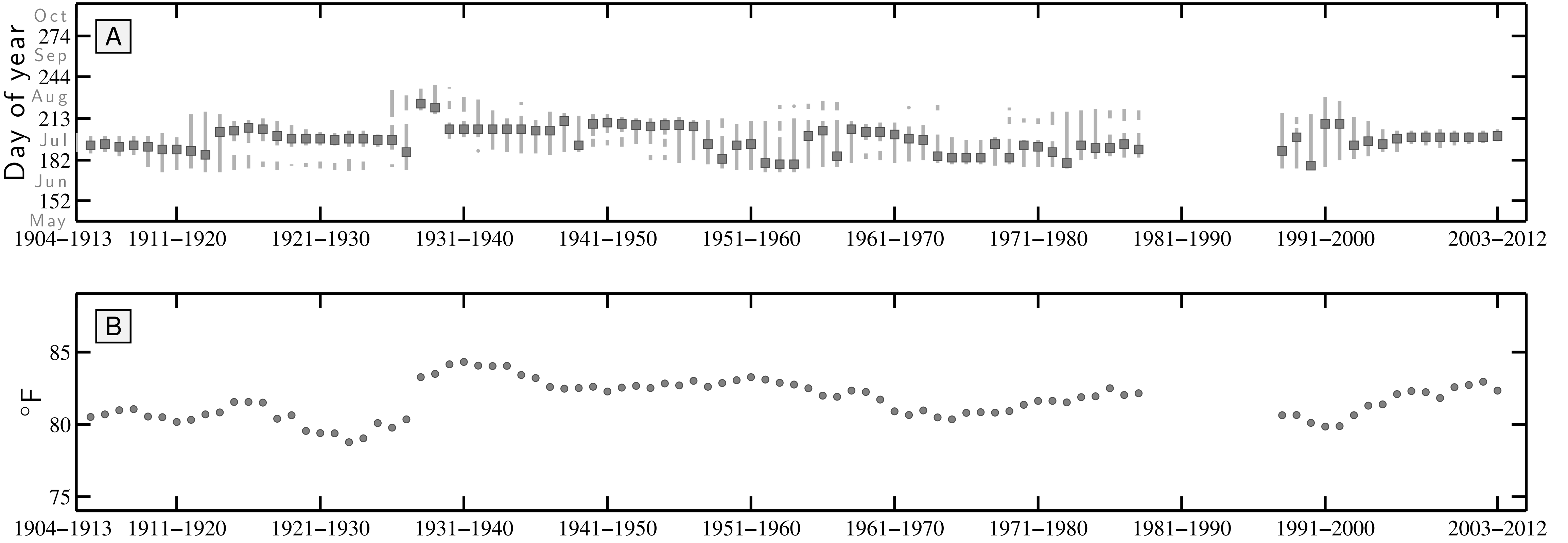

10 year Winter Teletherm dynamics for GUNNISON 3SW, CO:

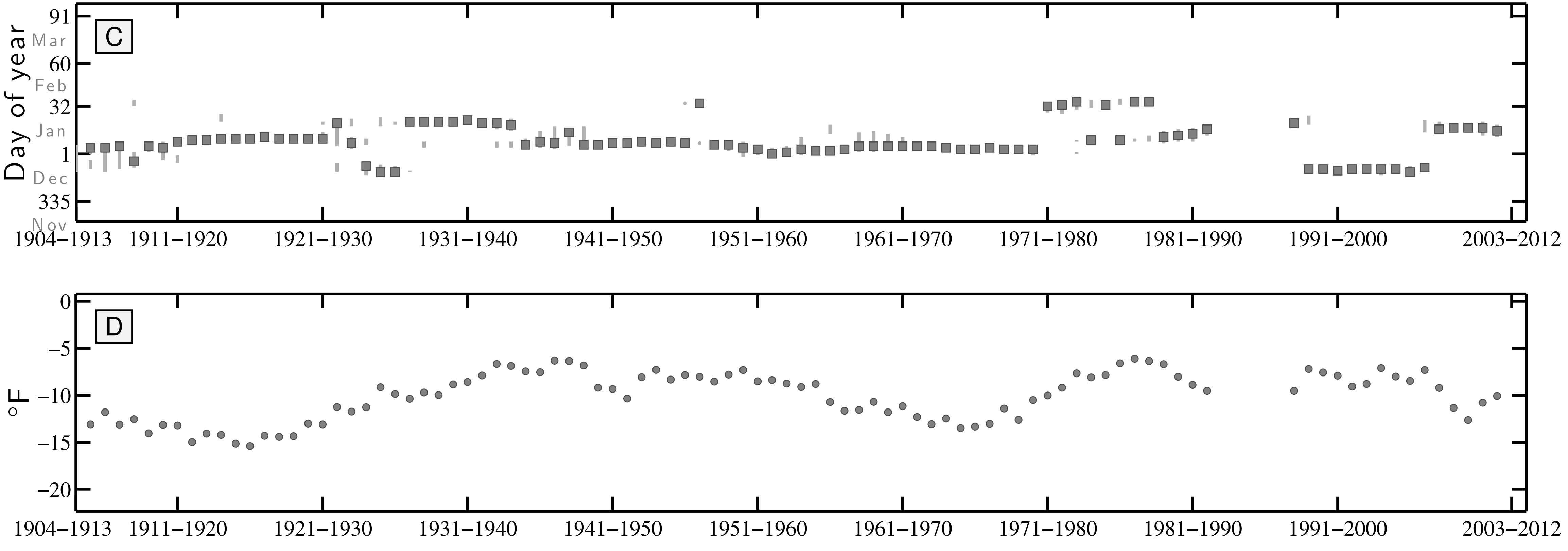

10 year Summer Teletherm dynamics for HERMIT 7 ESE, CO:

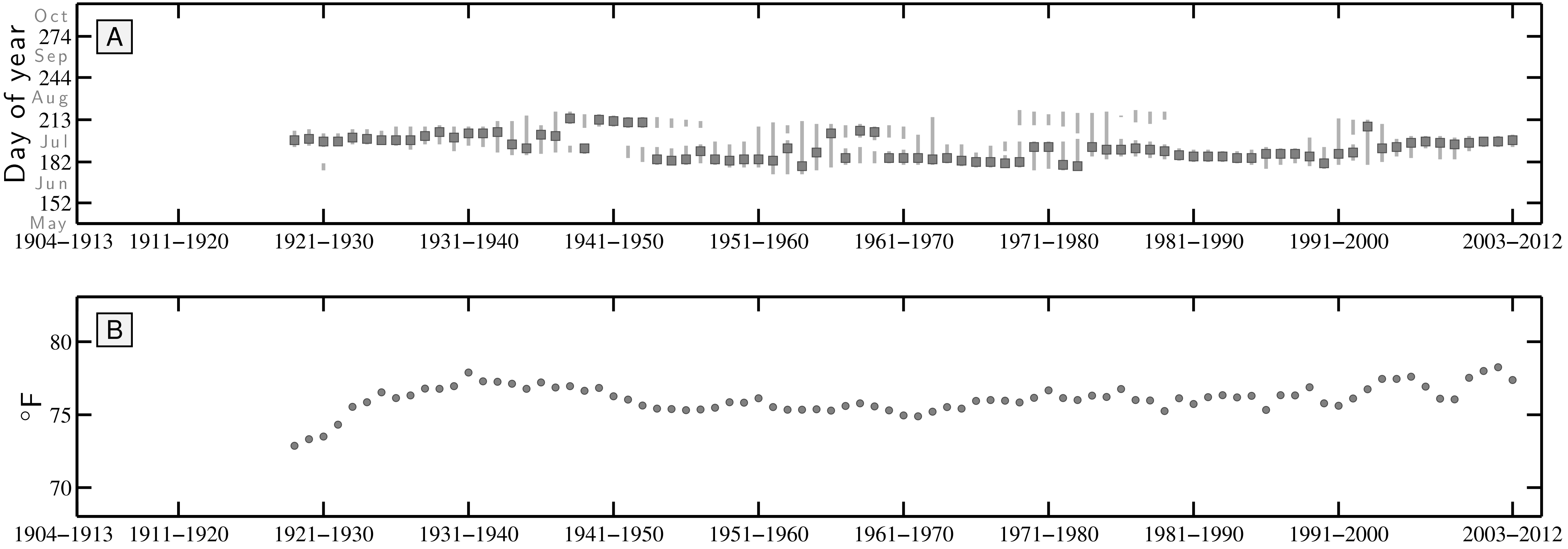

10 year Winter Teletherm dynamics for HERMIT 7 ESE, CO:

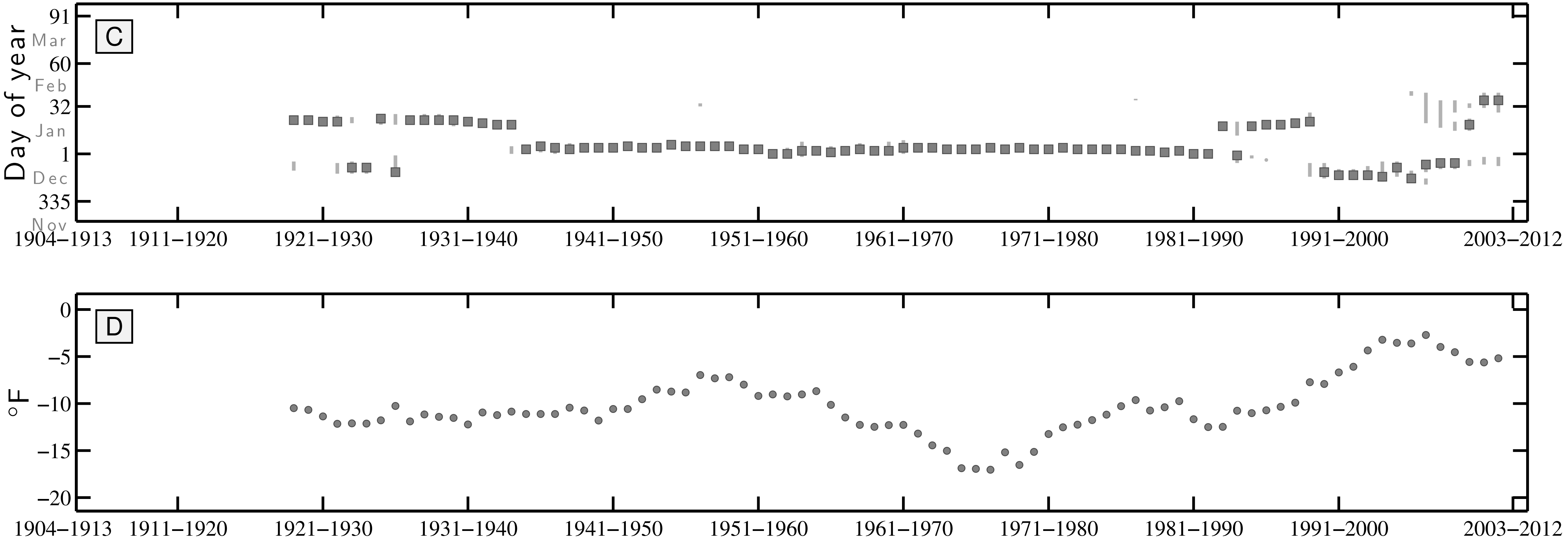

10 year Summer Teletherm dynamics for HOLLY, CO:

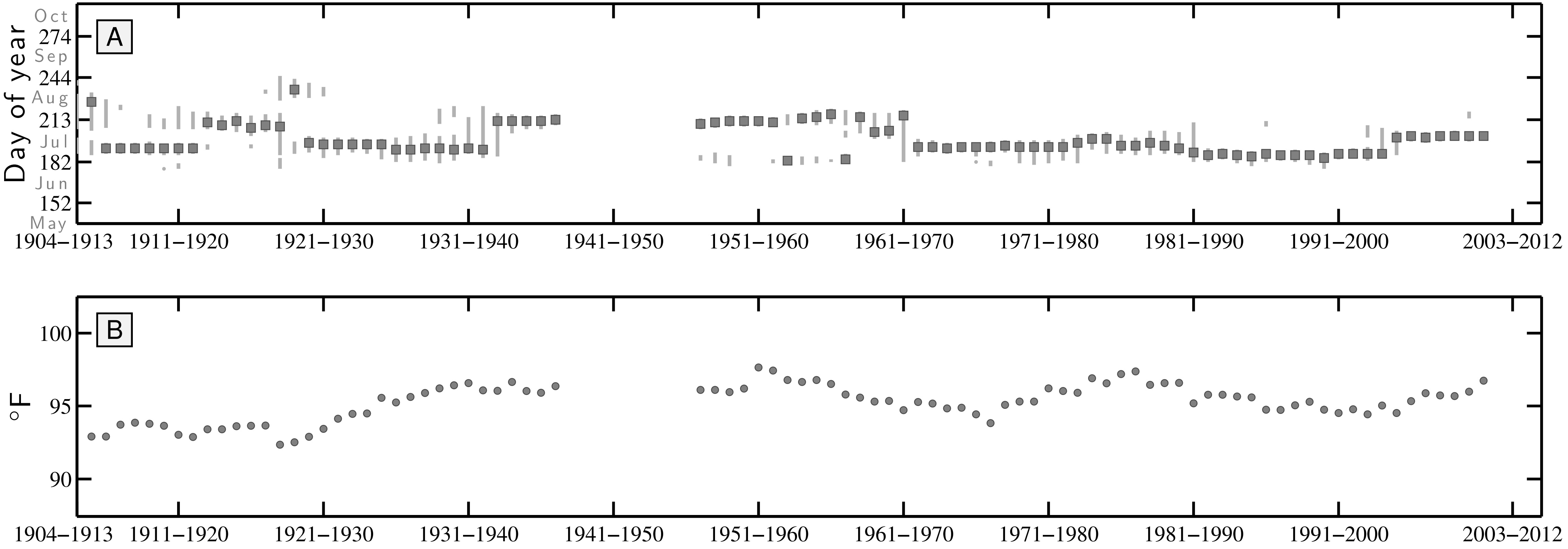

10 year Winter Teletherm dynamics for HOLLY, CO:

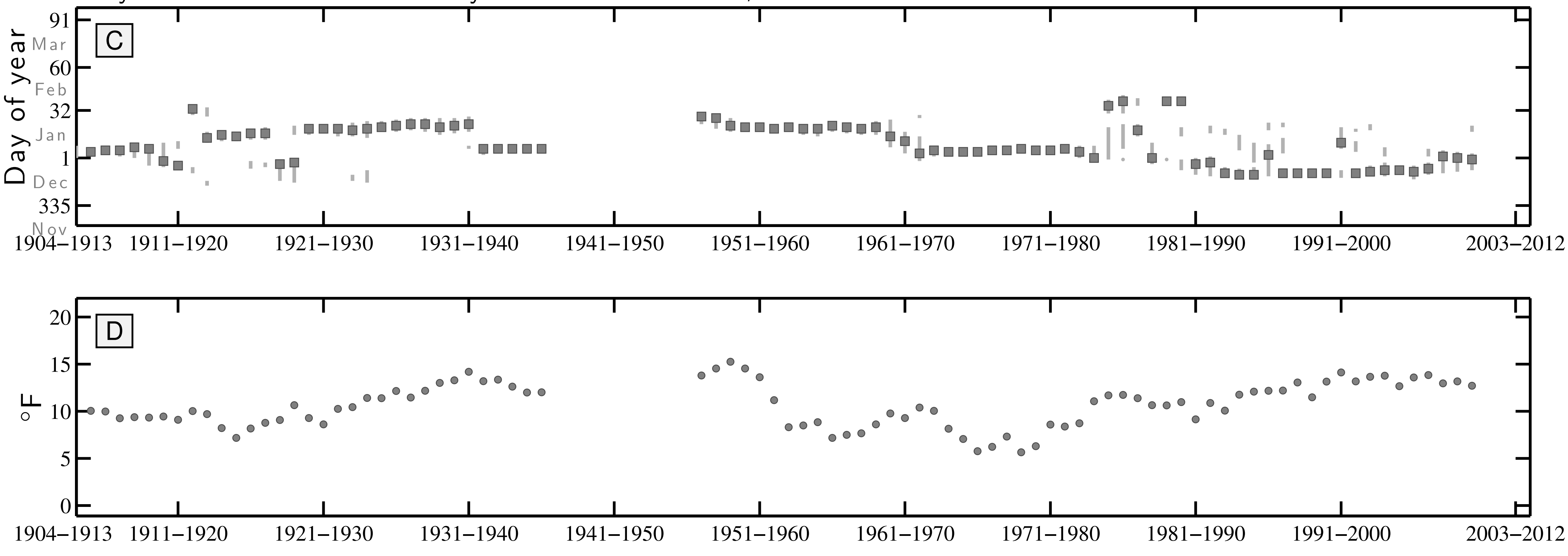

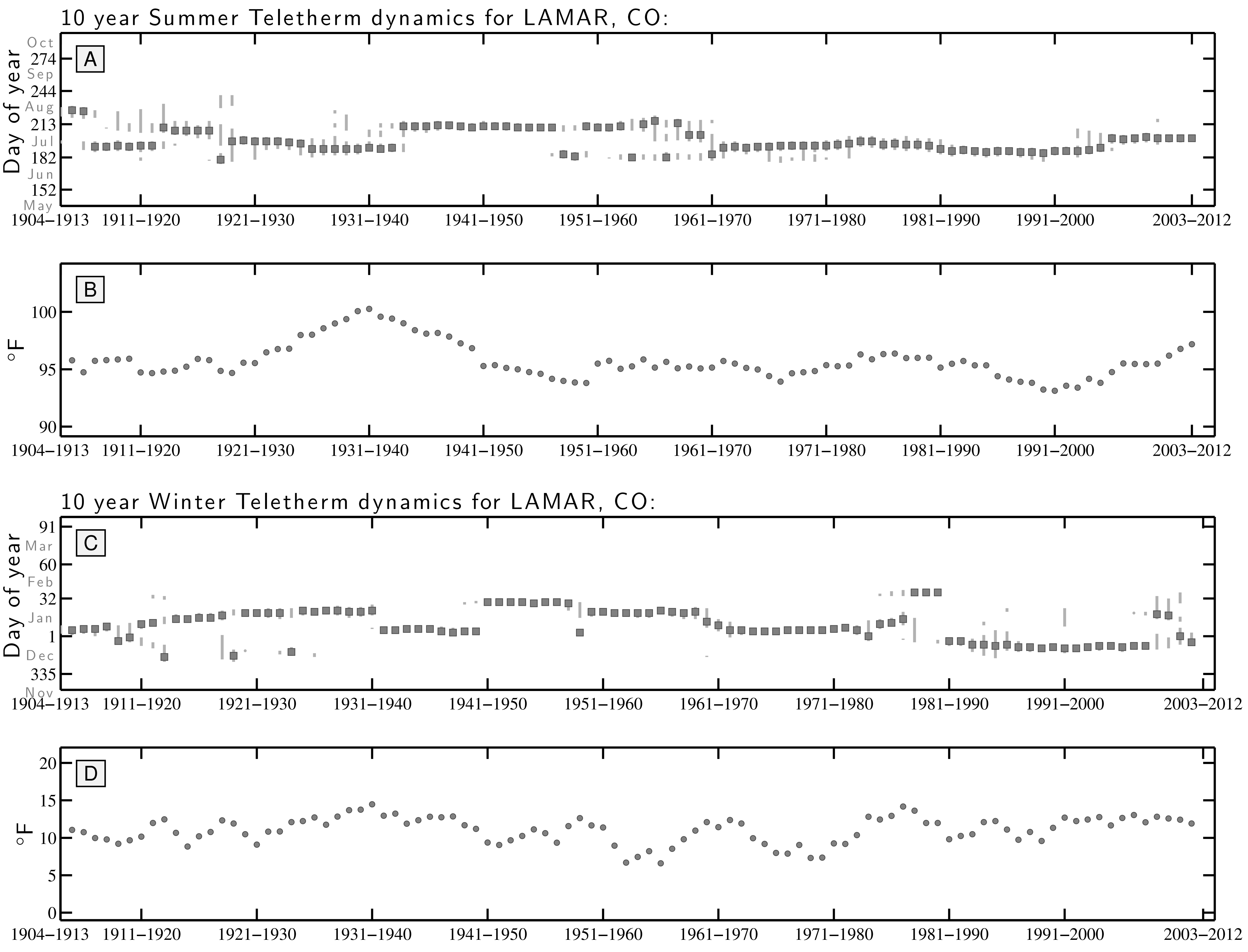
10 year Summer Teletherm dynamics for LAMAR, CO:
A
B
Day of year
°F
Oct
274
Sep
244
Aug
213
Jul
182
Jun
152
May
100
95
90
1904–1913
1911–1920
1921–1930
1931–1940
1941–1950
1951–1960
1961–1970
1971–1980
1981–1990
1991–2000
2003–2012
10 year Winter Teletherm dynamics for LAMAR, CO:
C
D
91
Mar
60
Feb
32
Jan
1
Dec
335
Nov
20
15
10
5
0

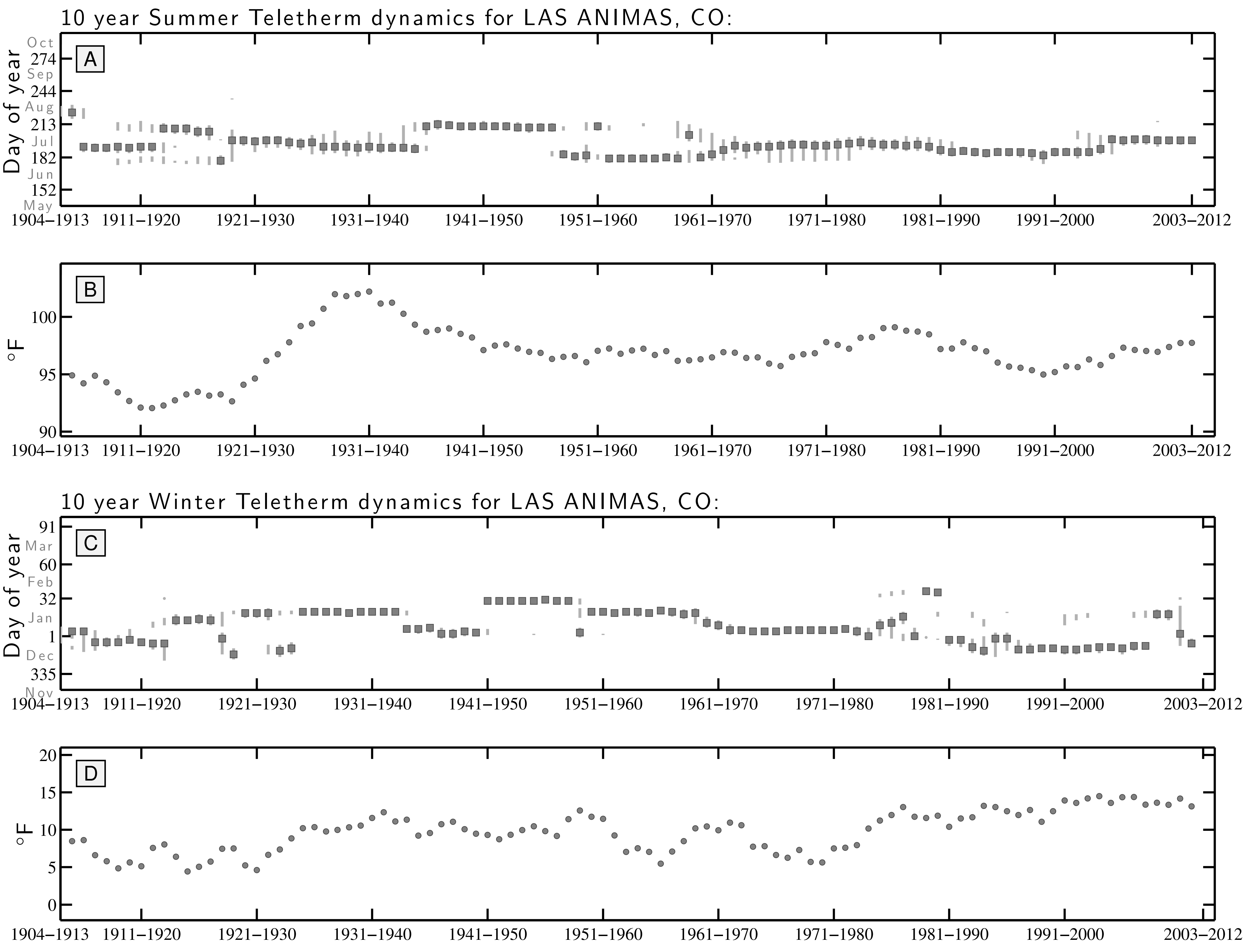

10 year Summer Teletherm dynamics for LAS ANIMAS, CO:
A
Day of year
Oct
274
Sep
244
Aug
213
Jul
182
Jun
152
May
1904–1913
1911–1920
1921–1930
1931–1940
1941–1950
1951–1960
1961–1970
1971–1980
1981–1990
1991–2000
2003–2012
B
°F
100
95
90
10 year Winter Teletherm dynamics for LAS ANIMAS, CO:
C
91
Mar
60
Feb
32
Jan
1
Dec
335
Nov
D
20
15
10
5
0

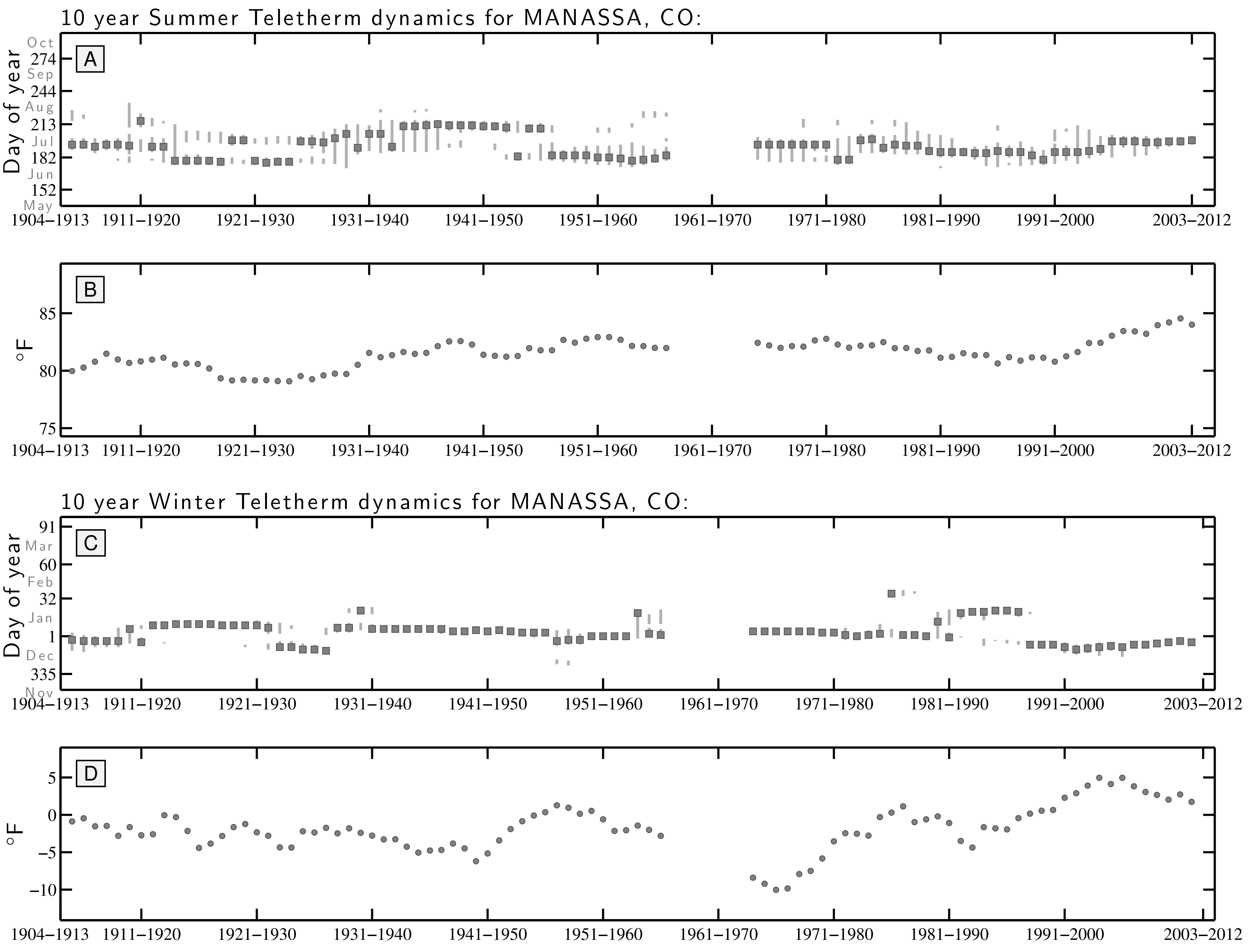

10 year Summer Teletherm dynamics for MANASSA, CO:
A
B
Day of year
°F
Oct
274
Sep
244
Aug
213
Jul
182
Jun
152
May
85
80
75
10 year Winter Teletherm dynamics for MANASSA, CO:
C
D
91
Mar
60
Feb
32
Jan
1
Dec
335
Nov
5
0
−5
−10
1904–1913
1911–1920
1921–1930
1931–1940
1941–1950
1951–1960
1961–1970
1971–1980
1981–1990
1991–2000
2003–2012

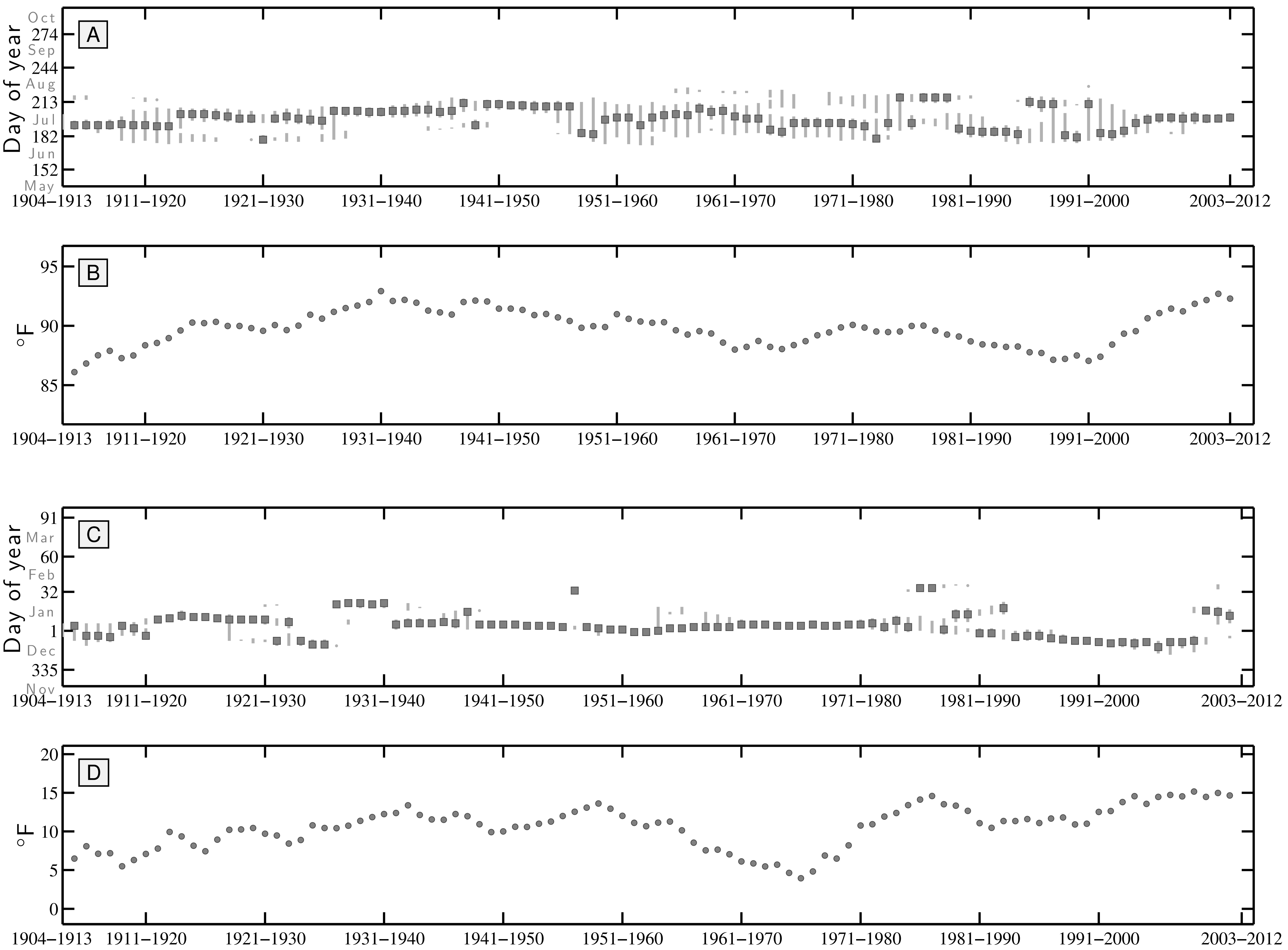
A
B
C
D
Day of year
°F
Oct
274
Sep
244
Aug
213
Jul
182
Jun
152
May
95
90
85
91
Mar
60
Feb
32
Jan
1
Dec
335
Nov
20
15
10
5
0
1904–1913
1911–1920
1921–1930
1931–1940
1941–1950
1951–1960
1961–1970
1971–1980
1981–1990
1991–2000
2003–2012

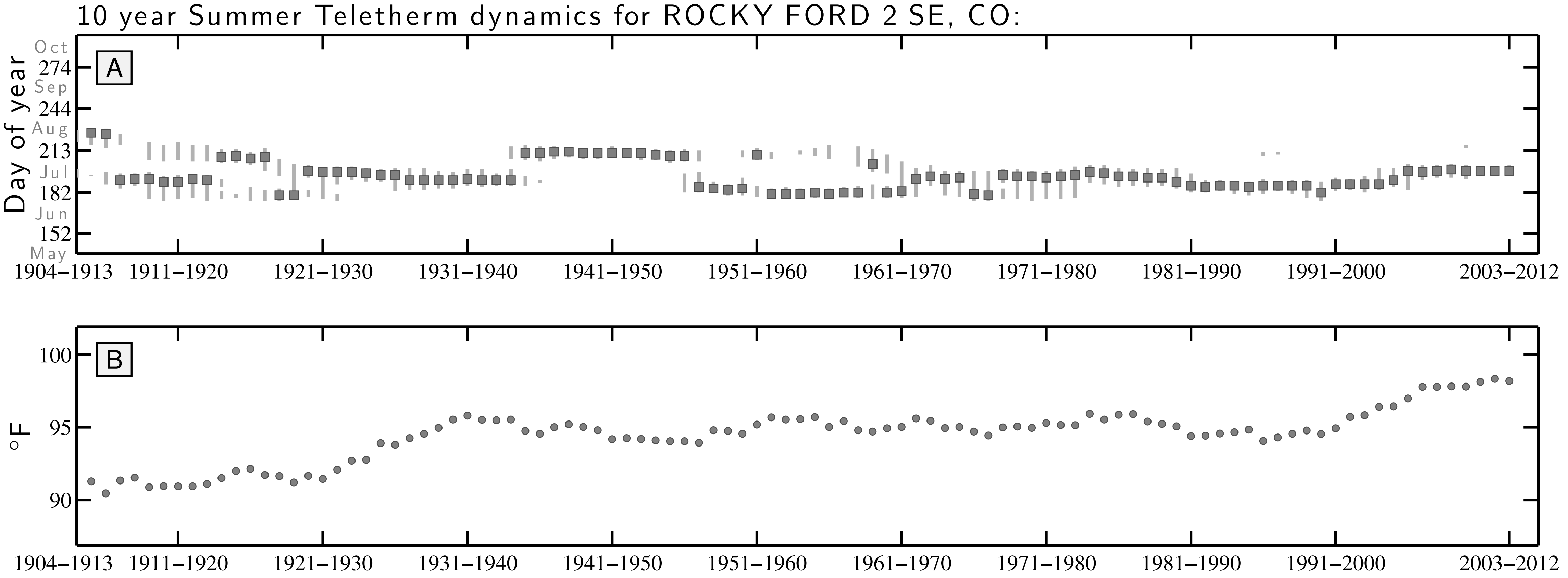
10 year Summer Teletherm dynamics for ROCKY FORD 2 SE, CO:
A
B
Day of year
°F
Oct
274
Sep
244
Aug
213
Jul
182
Jun
152
May
100
95
90
1904–1913
1911–1920
1921–1930
1931–1940
1941–1950
1951–1960
1961–1970
1971–1980
1981–1990
1991–2000
2003–2012

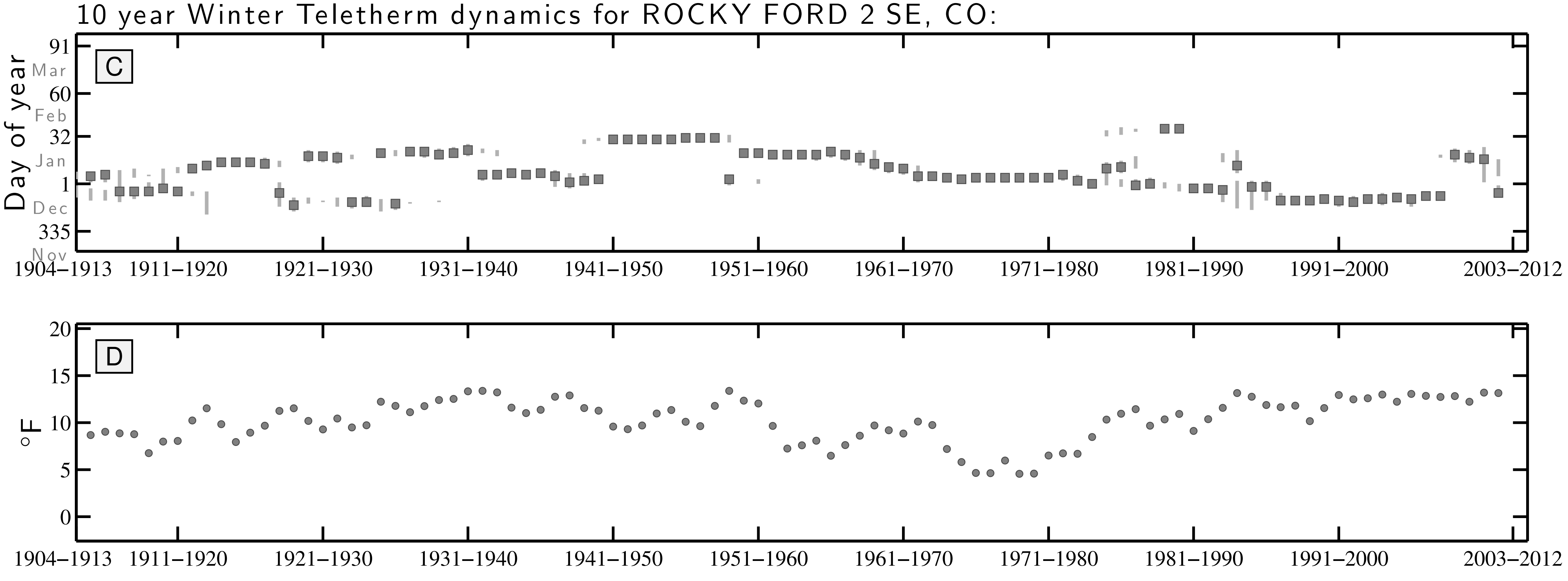
10 year Winter Teletherm dynamics for ROCKY FORD 2 SE, CO:
C
D
Day of year
°F
91
Mar
60
Feb
32
Jan
1
Dec
335
Nov
20
15
10
5
0
1904–1913
1911–1920
1921–1930
1931–1940
1941–1950
1951–1960
1961–1970
1971–1980
1981–1990
1991–2000
2003–2012

10 year Summer Teletherm dynamics for SAGUACHE, CO:

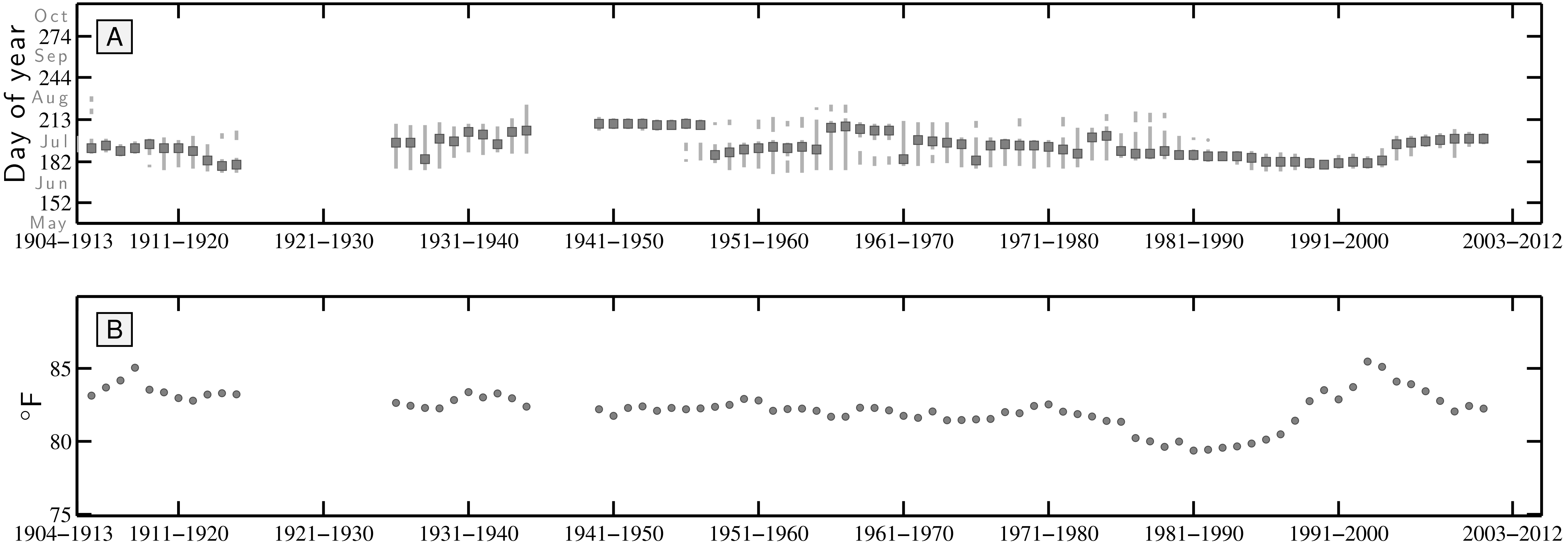

10 year Winter Teletherm dynamics for SAGUACHE, CO:

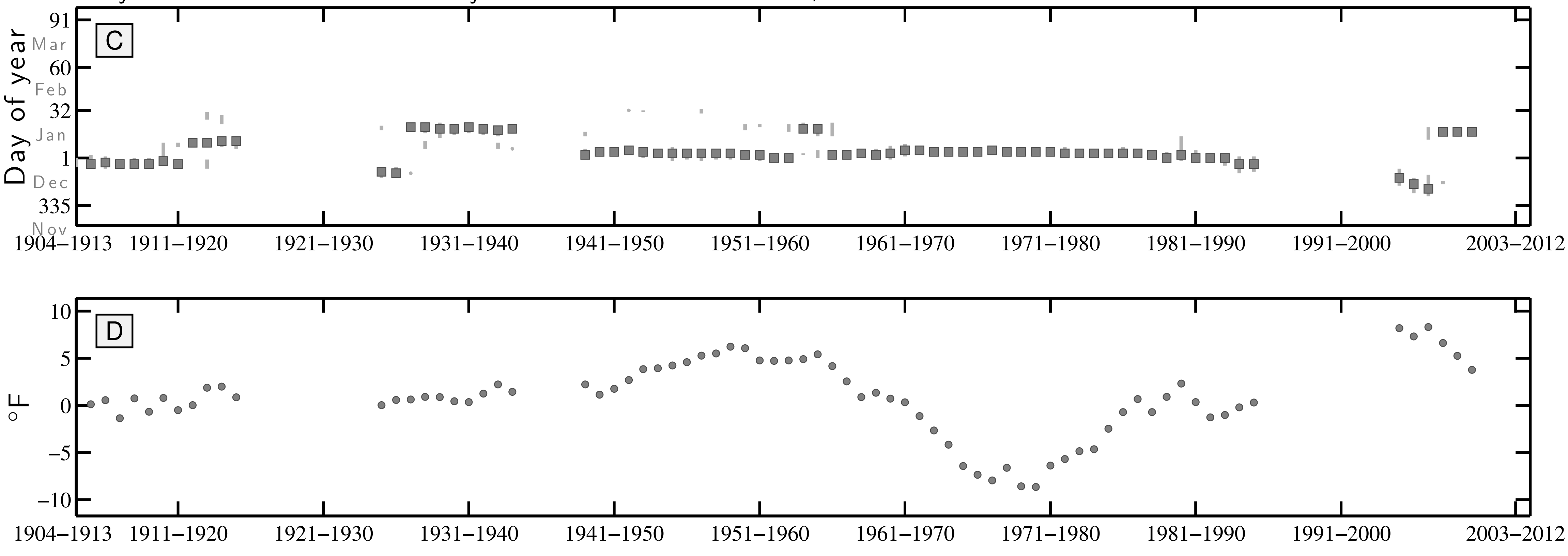

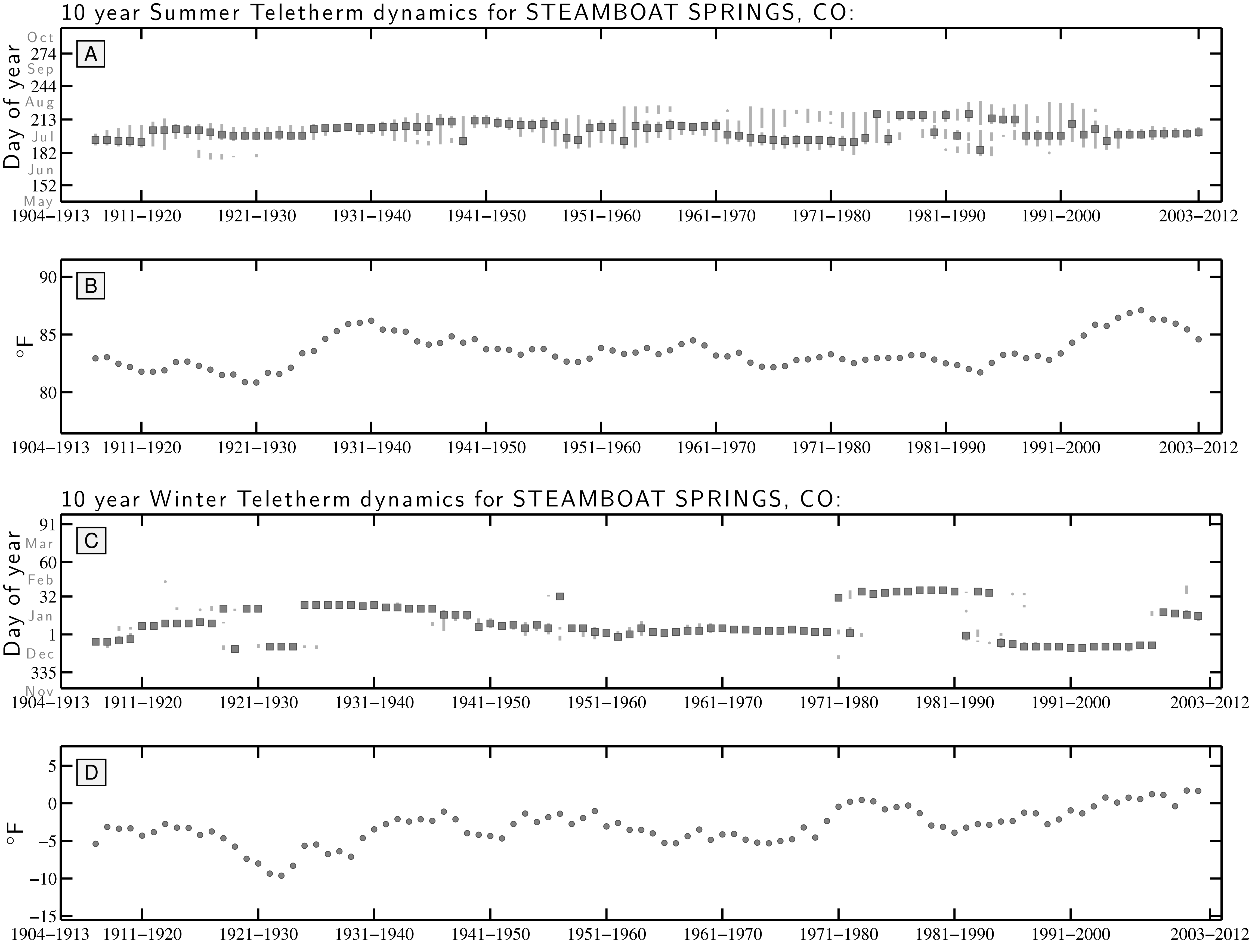
10 year Summer Teletherm dynamics for STEAMBOAT SPRINGS, CO:
A
B
Day of year
°F
10 year Winter Teletherm dynamics for STEAMBOAT SPRINGS, CO:
C
D
1904–1913
1911–1920
1921–1930
1931–1940
1941–1950
1951–1960
1961–1970
1971–1980
1981–1990
1991–2000
2003–2012

10 year Summer Teletherm dynamics for TELLURIDE 4WNW, CO:

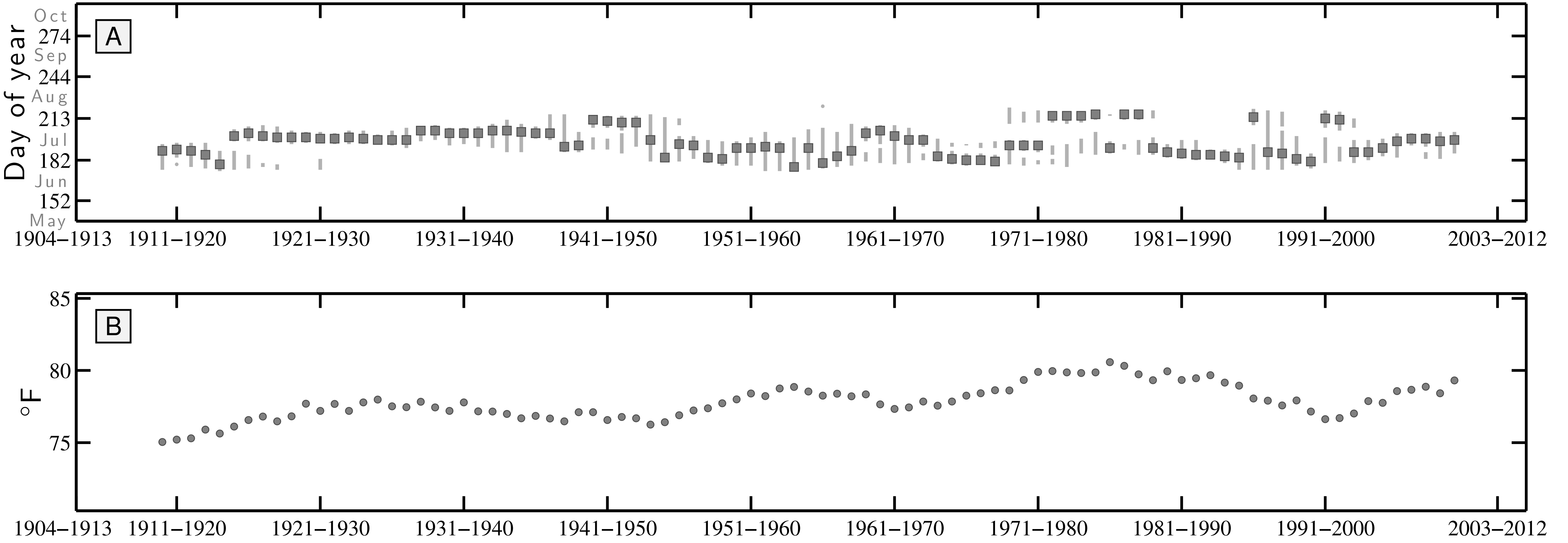

10 year Winter Teletherm dynamics for TELLURIDE 4WNW, CO:

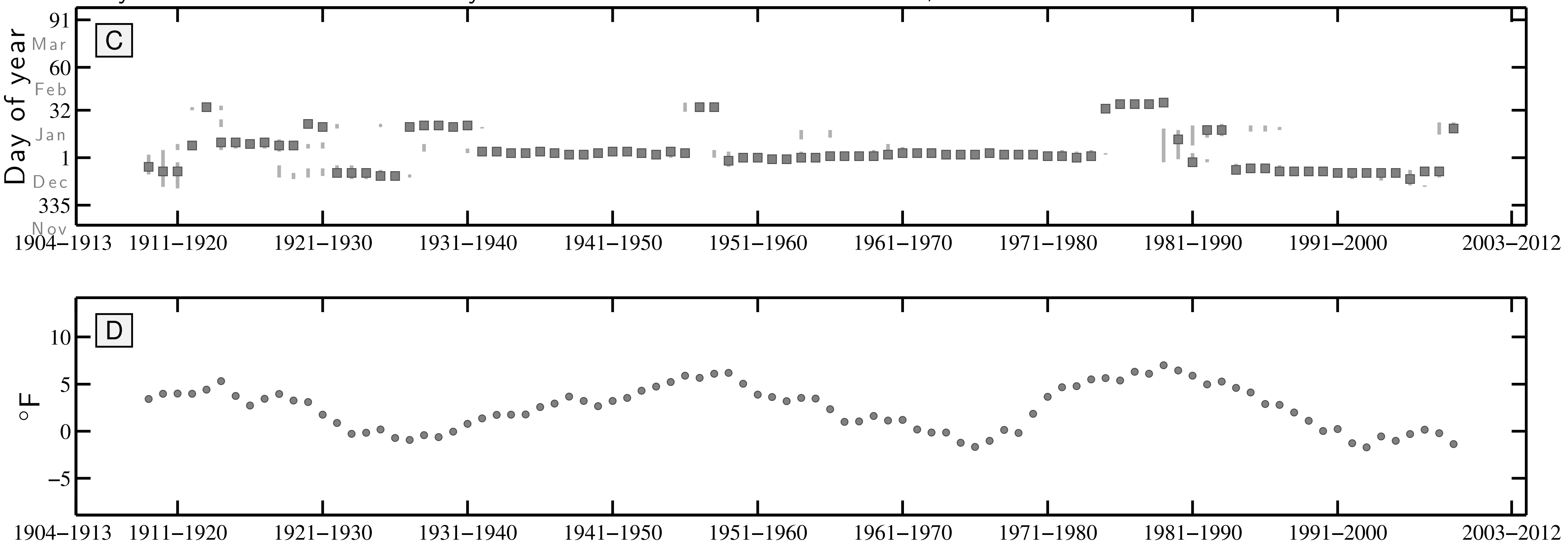

10 year Summer Teletherm dynamics for TRINIDAD, CO:

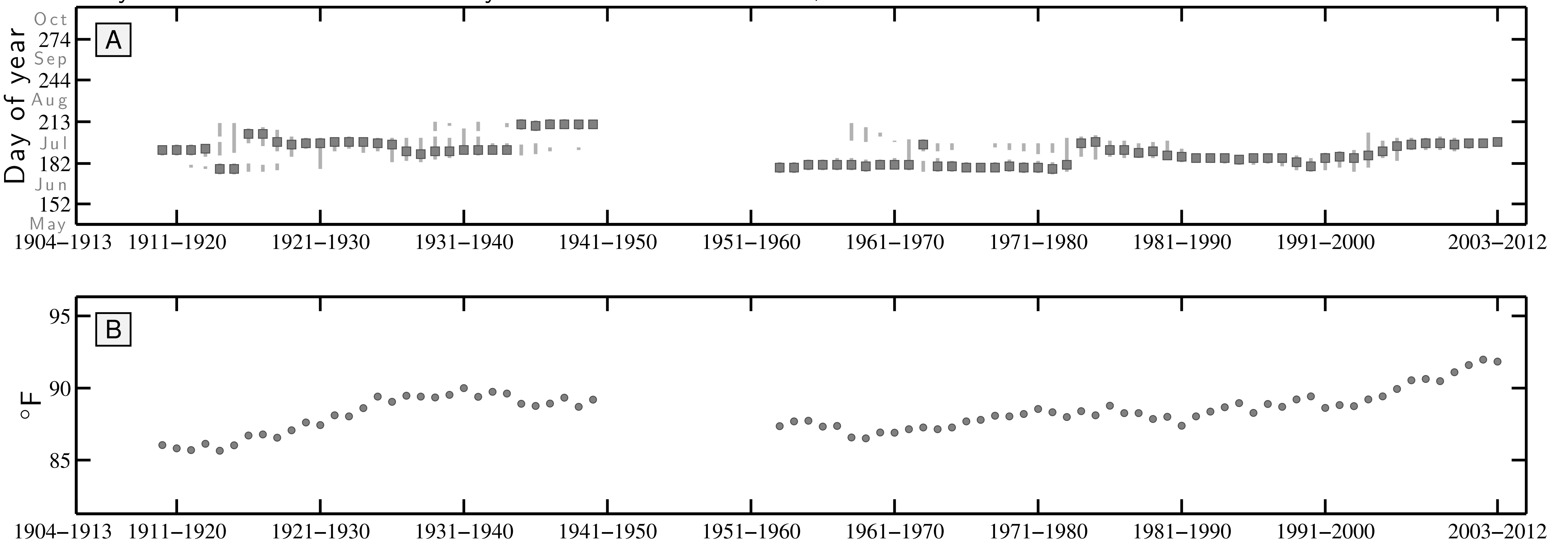

10 year Winter Teletherm dynamics for TRINIDAD, CO:

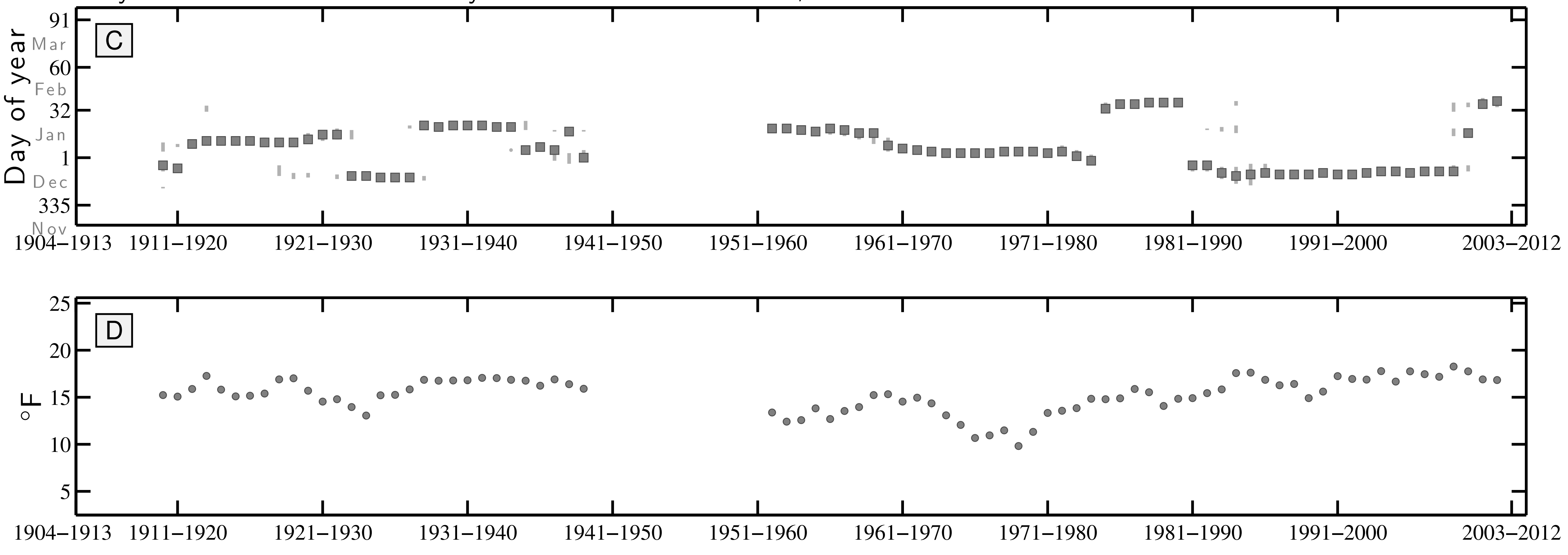

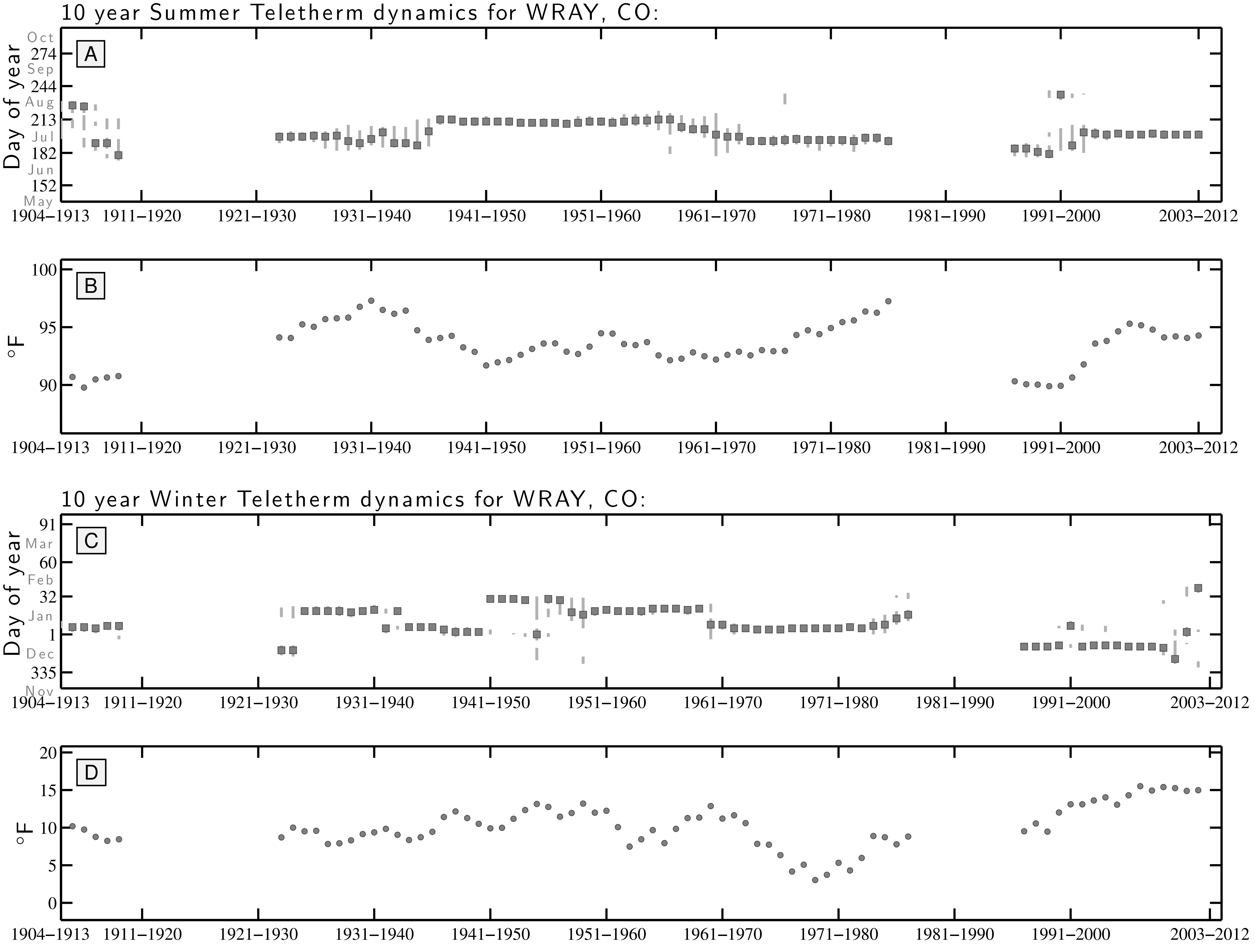
10 year Summer Teletherm dynamics for WRAY, CO:
A
B
Day of year
°F
Oct
274
Sep
244
Aug
213
Jul
182
Jun
152
May
100
95
90
10 year Winter Teletherm dynamics for WRAY, CO:
C
D
91
Mar
60
Feb
32
Jan
1
Dec
335
Nov
20
15
10
5
0
1904–1913
1911–1920
1921–1930
1931–1940
1941–1950
1951–1960
1961–1970
1971–1980
1981–1990
1991–2000
2003–2012

10 year Summer Teletherm dynamics for FALLS VILLAGE, CT:

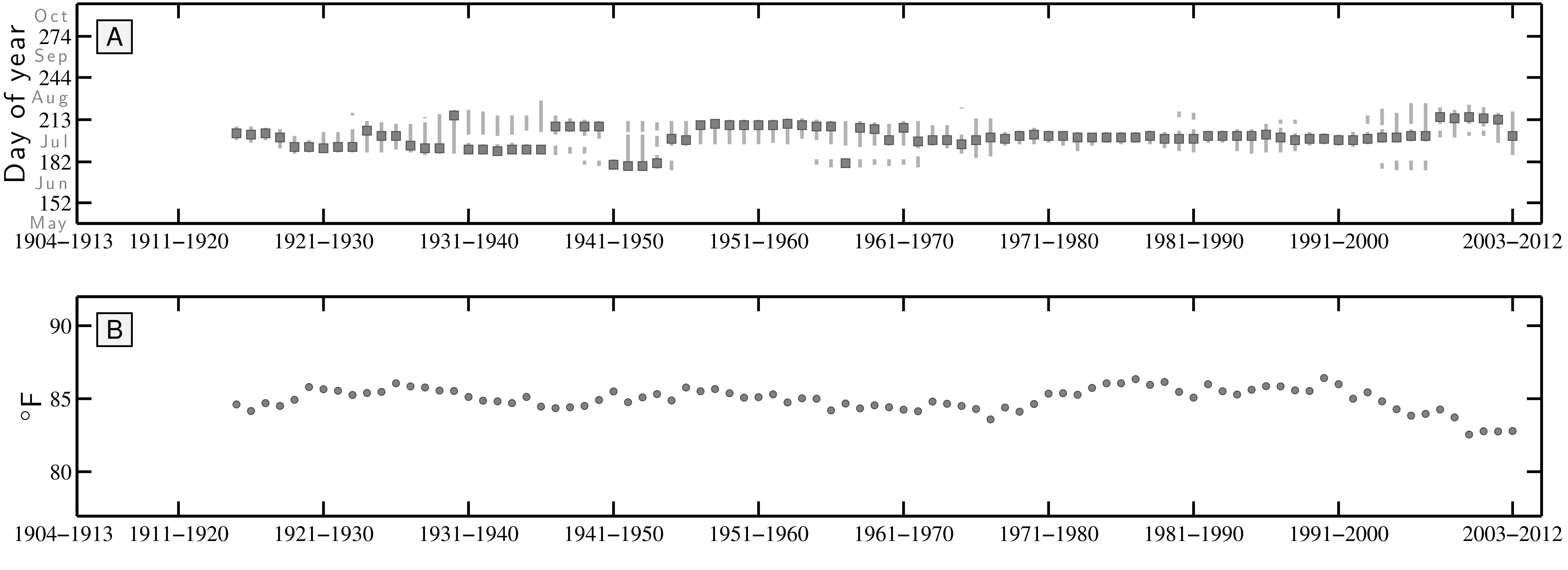

10 year Winter Teletherm dynamics for FALLS VILLAGE, CT:

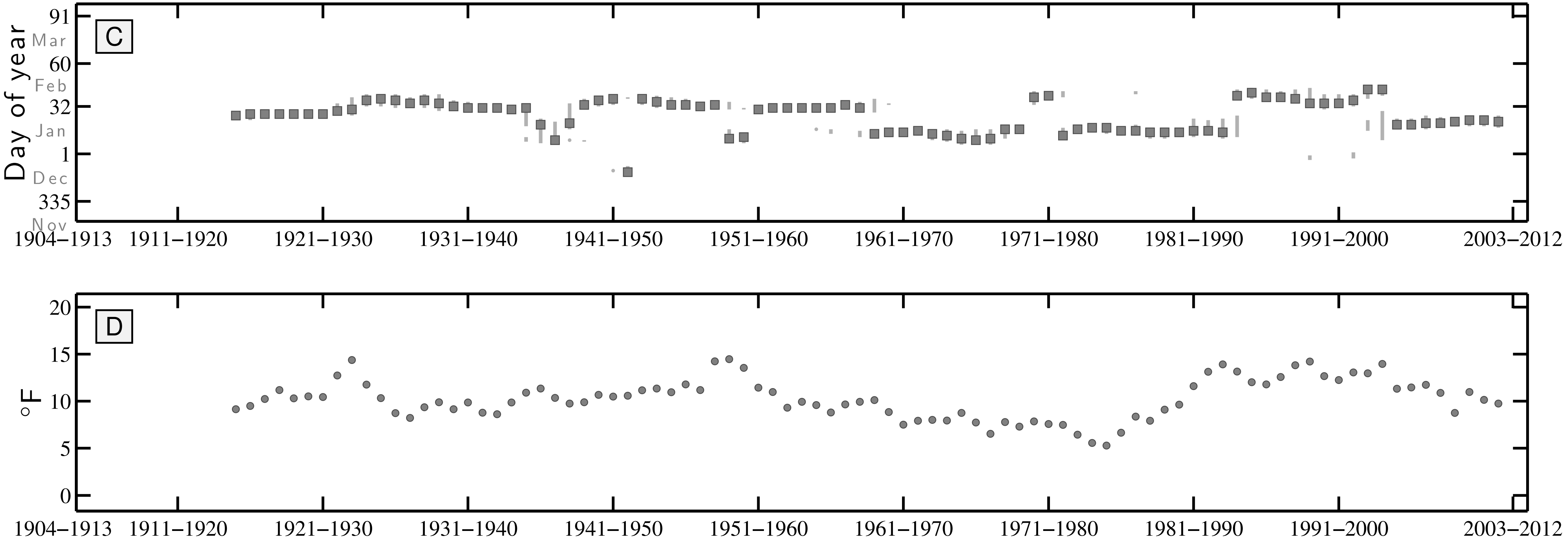

10 year Summer Teletherm dynamics for GROTON, CT:

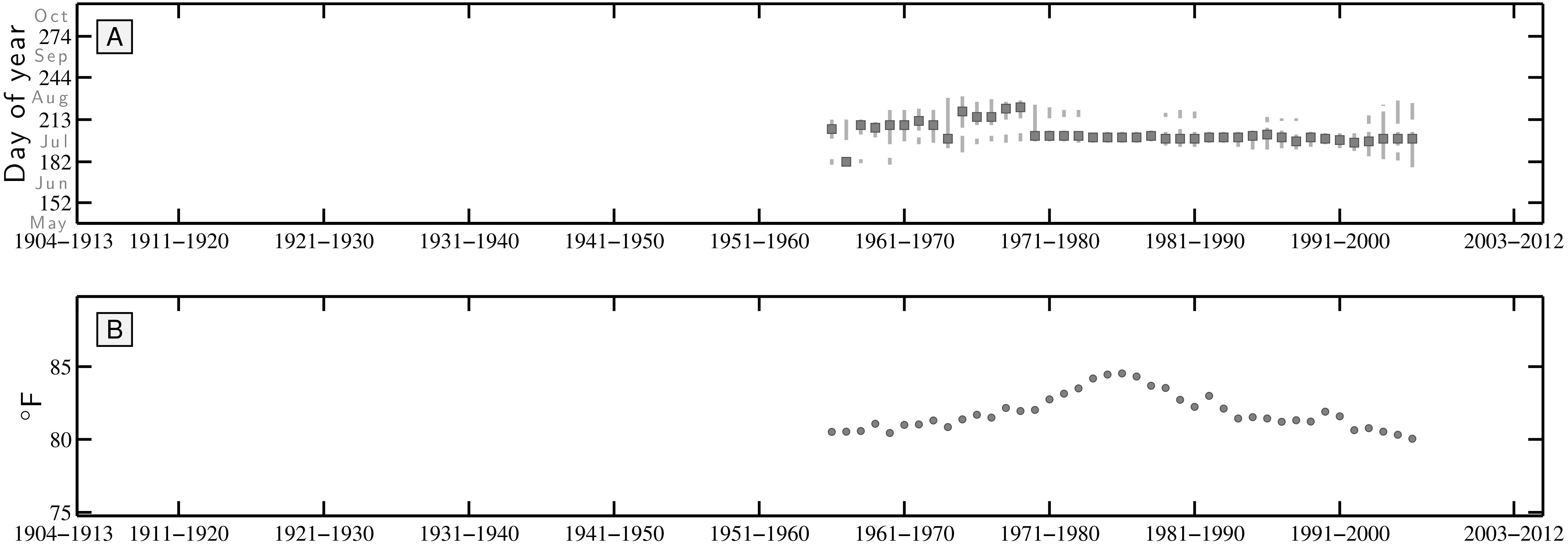

10 year Winter Teletherm dynamics for GROTON, CT:

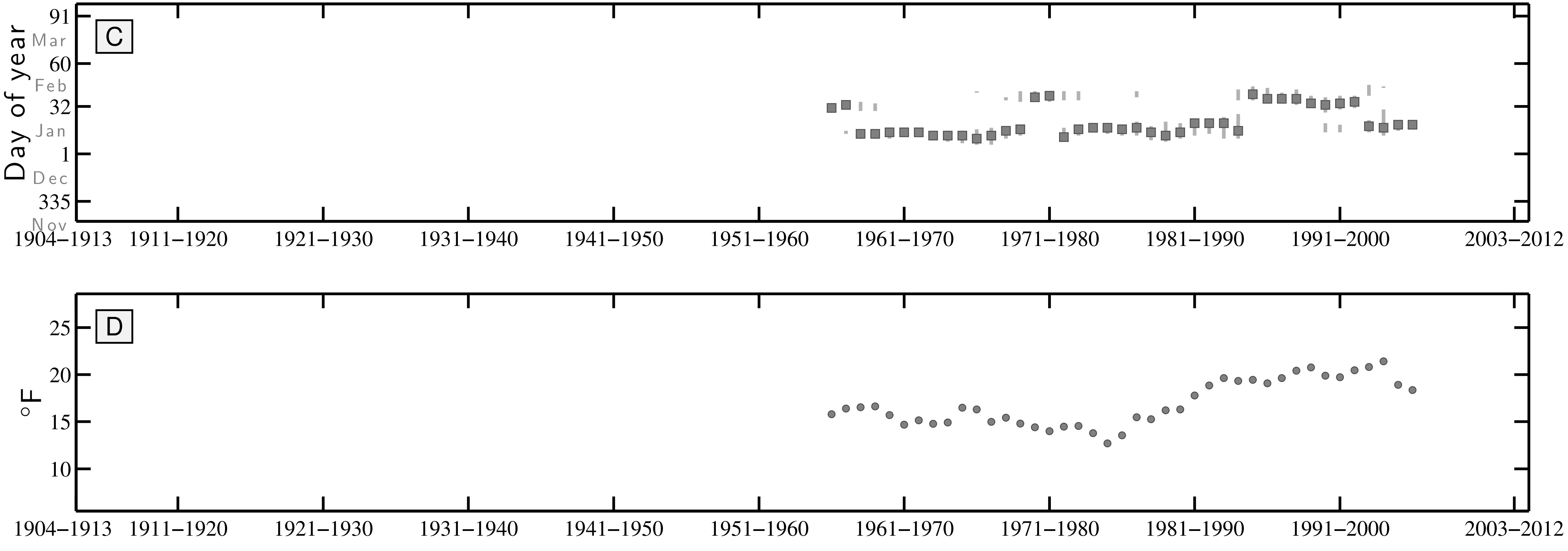

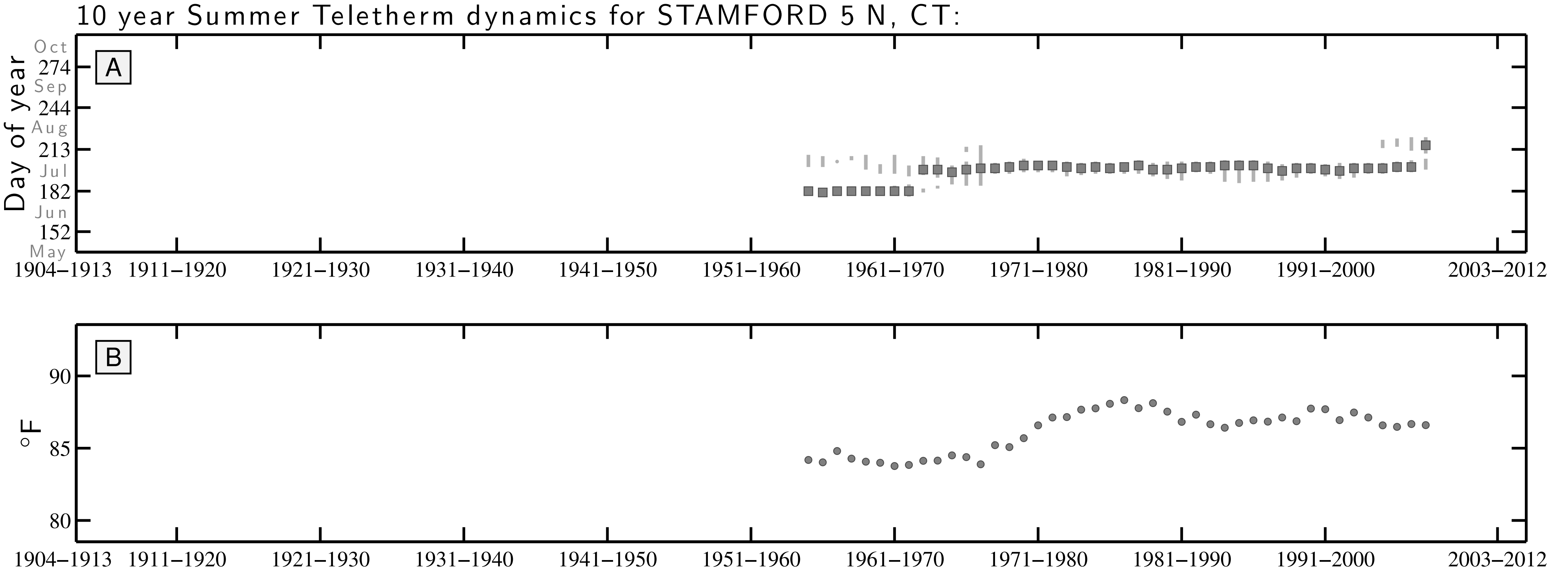
10 year Summer Teletherm dynamics for STAMFORD 5 N, CT:
A
B
Day of year
°F
Oct
274
Sep
244
Aug
213
Jul
182
Jun
152
May
90
85
80
1904–1913
1911–1920
1921–1930
1931–1940
1941–1950
1951–1960
1961–1970
1971–1980
1981–1990
1991–2000
2003–2012

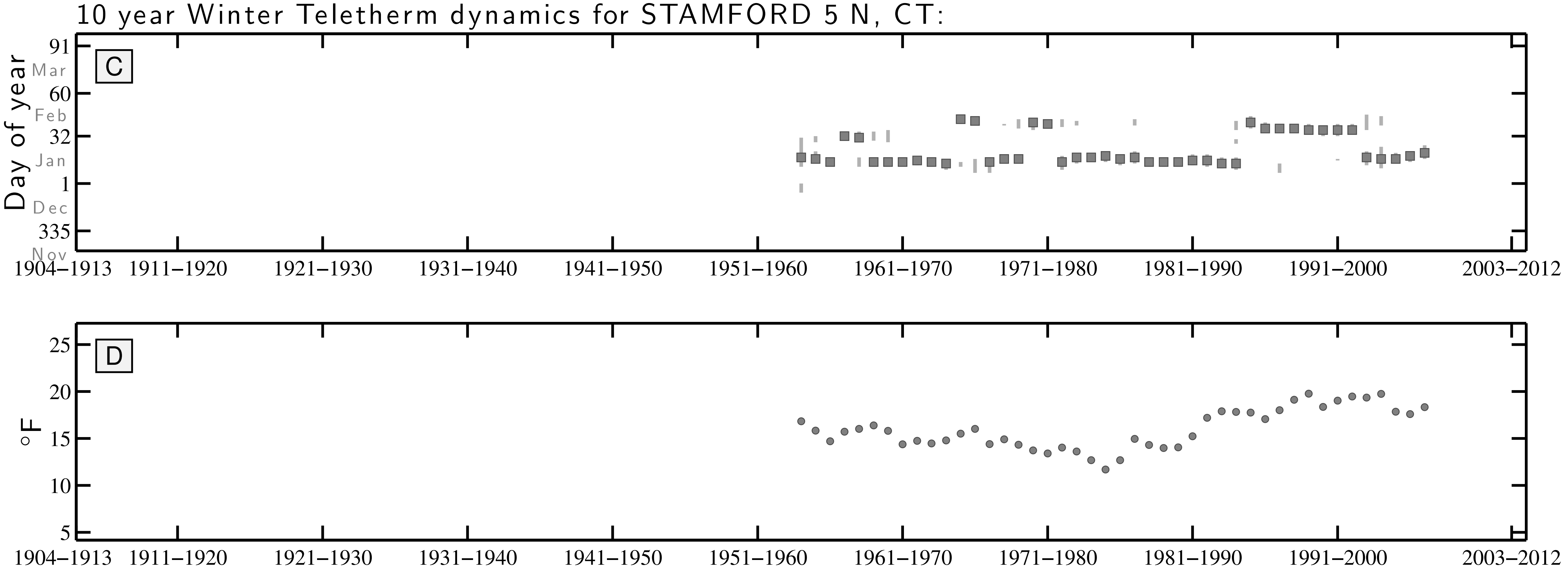
10 year Winter Teletherm dynamics for STAMFORD 5 N, CT:
C
D
Day of year
°F
91
Mar
60
Feb
32
Jan
1
Dec
335
Nov
25
20
15
10
5
1904–1913
1911–1920
1921–1930
1931–1940
1941–1950
1951–1960
1961–1970
1971–1980
1981–1990
1991–2000
2003–2012

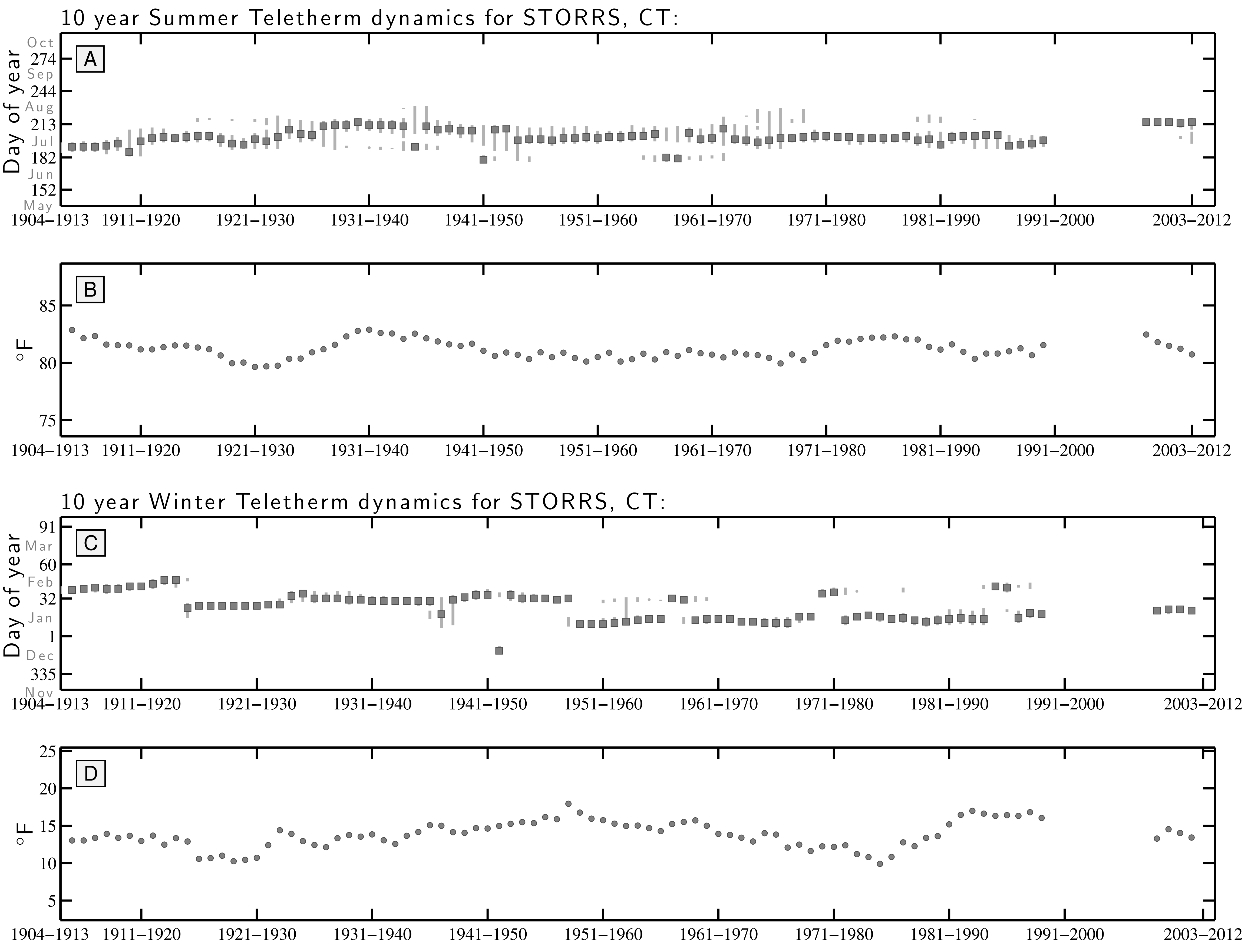

10 year Summer Teletherm dynamics for STORRS, CT:
A
Day of year
Oct
274
Sep
244
Aug
213
Jul
182
Jun
152
May
1904–1913
1911–1920
1921–1930
1931–1940
1941–1950
1951–1960
1961–1970
1971–1980
1981–1990
1991–2000
2003–2012
B
°F
85
80
75
10 year Winter Teletherm dynamics for STORRS, CT:
C
Day of year
91
Mar
60
Feb
32
Jan
1
Dec
335
Nov
D
°F
25
20
15
10
5

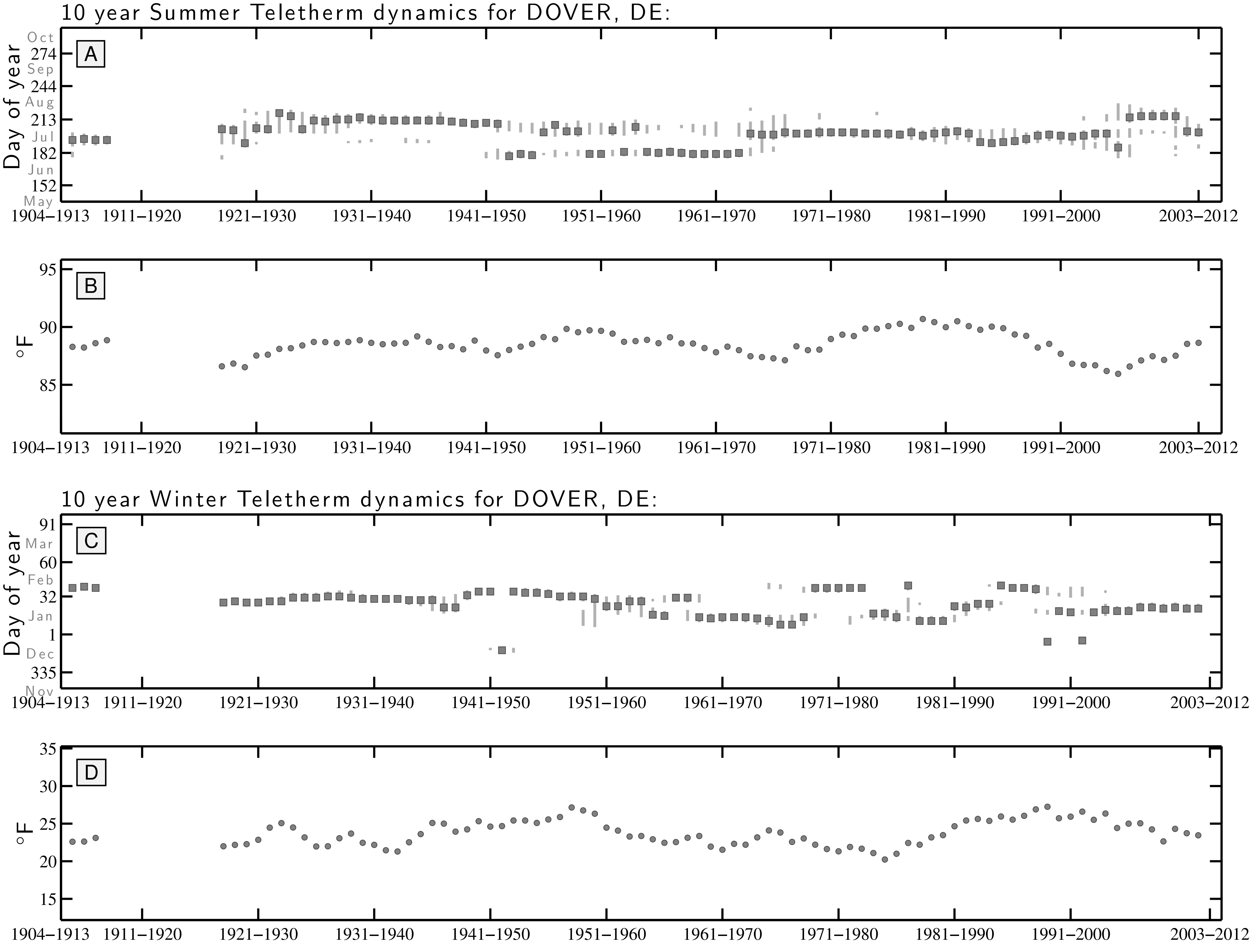
10 year Summer Teletherm dynamics for DOVER, DE:
A
Day of year
Oct
274
Sep
244
Aug
213
Jul
182
Jun
152
May
B
°F
95
90
85
10 year Winter Teletherm dynamics for DOVER, DE:
C
Day of year
91
Mar
60
Feb
32
Jan
1
Dec
335
Nov
D
°F
35
30
25
20
15
1904–1913
1911–1920
1921–1930
1931–1940
1941–1950
1951–1960
1961–1970
1971–1980
1981–1990
1991–2000
2003–2012

10 year Summer Teletherm dynamics for GREENWOOD 2NE, DE:

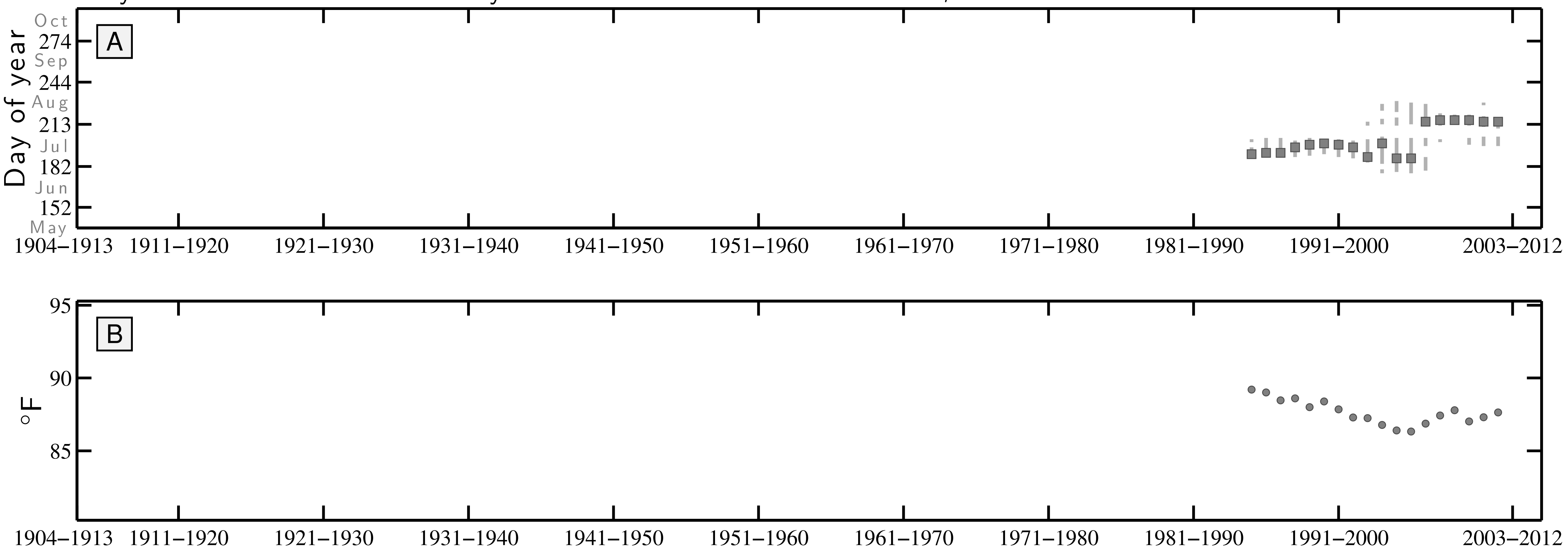

10 year Winter Teletherm dynamics for GREENWOOD 2NE, DE:

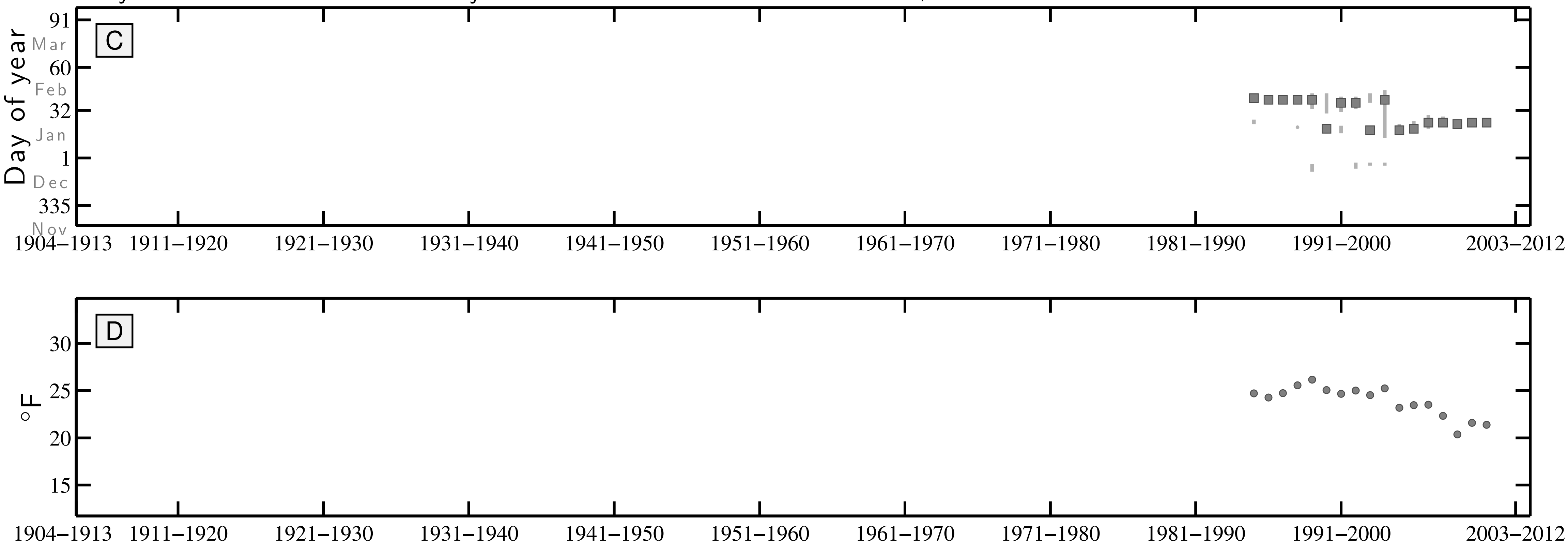

10 year Summer Teletherm dynamics for MILFORD 2 SE, DE:

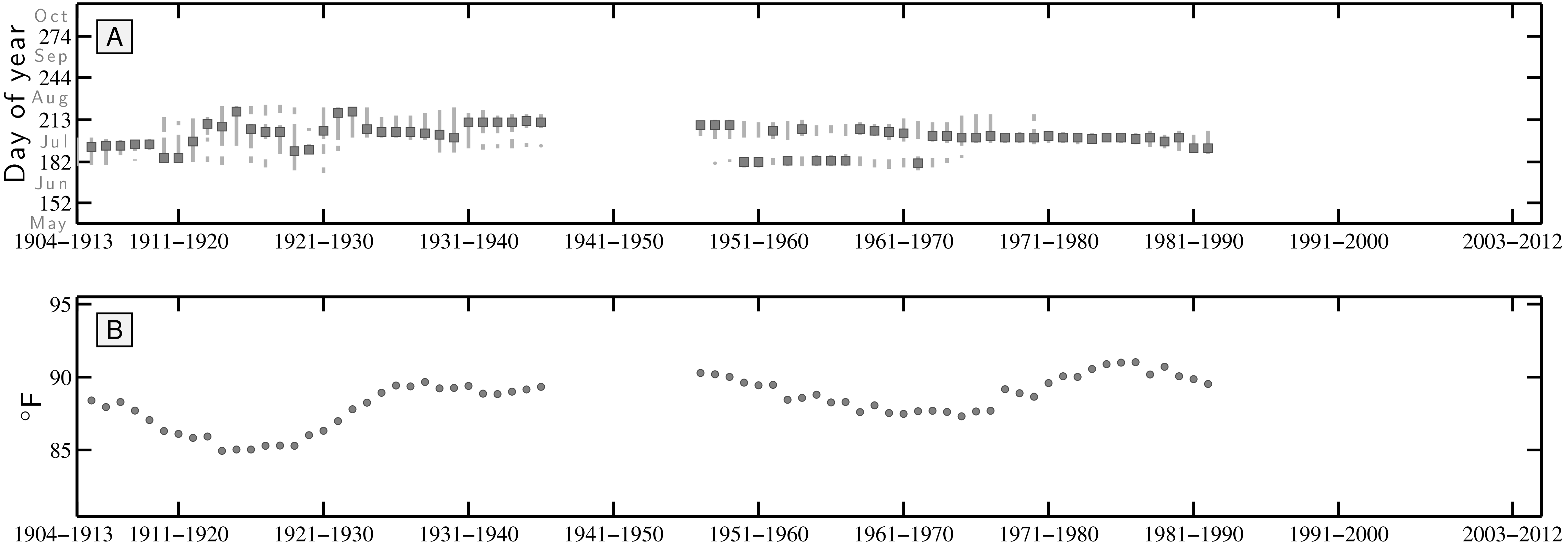

10 year Winter Teletherm dynamics for MILFORD 2 SE, DE:

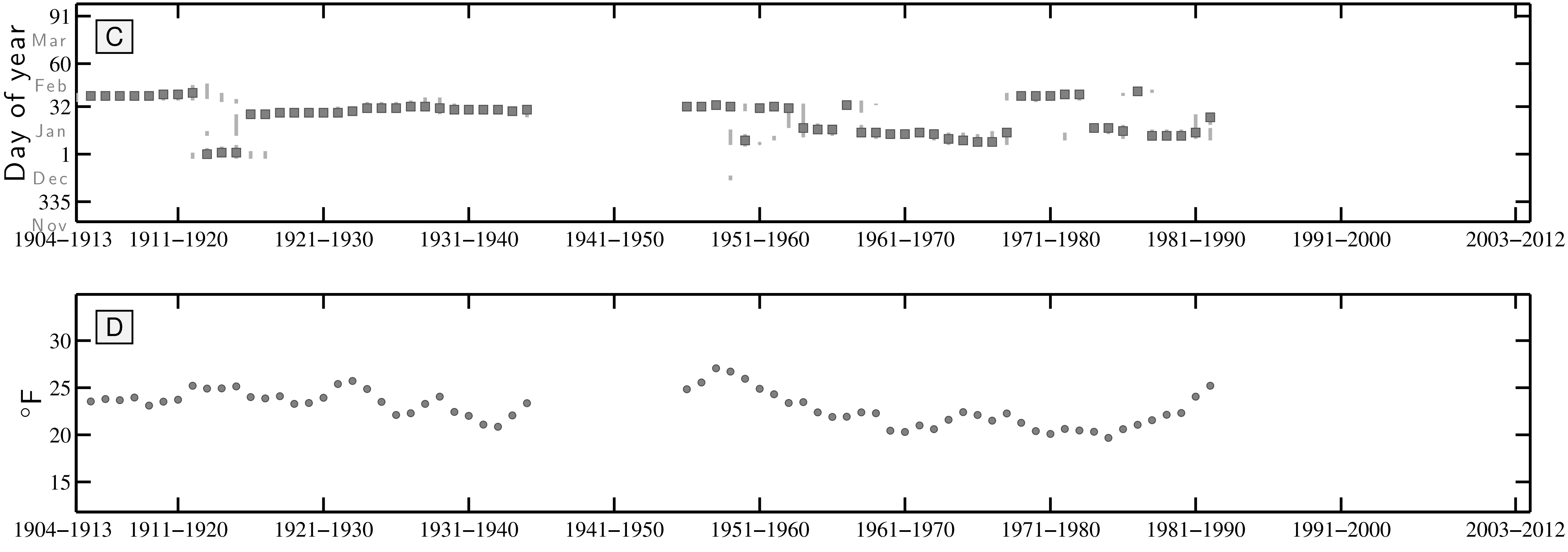

10 year Summer Teletherm dynamics for NEWARK UNIV FARM, DE:

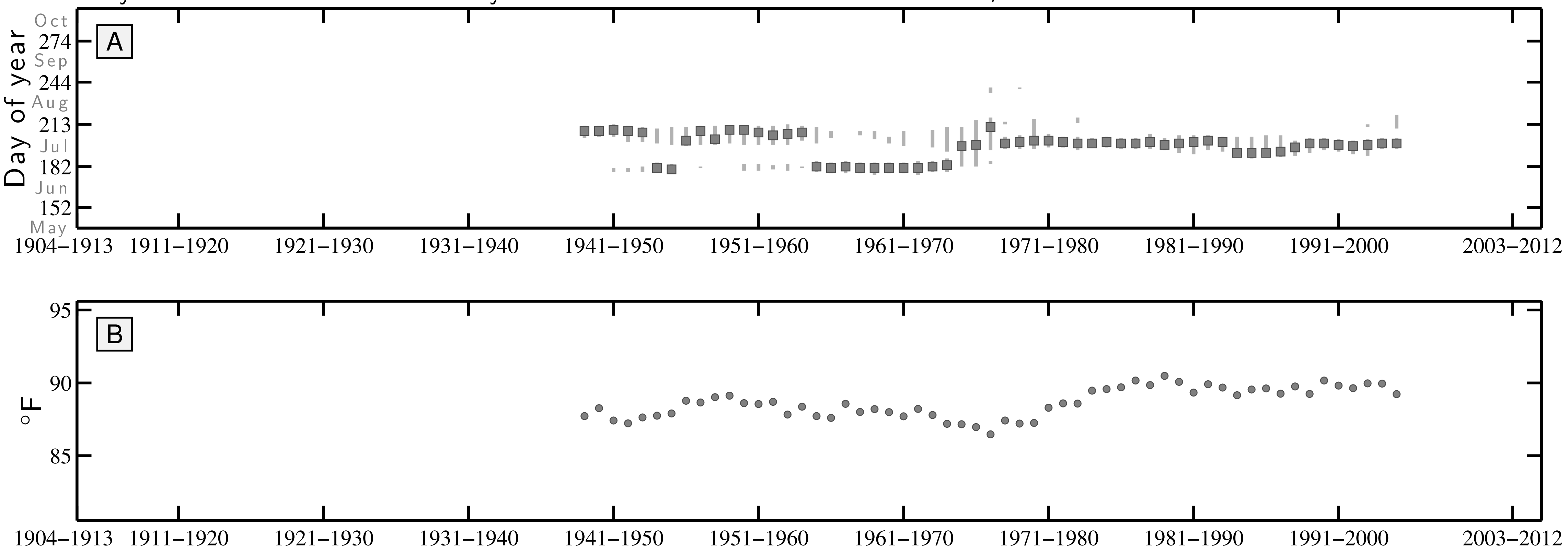

10 year Winter Teletherm dynamics for NEWARK UNIV FARM, DE:

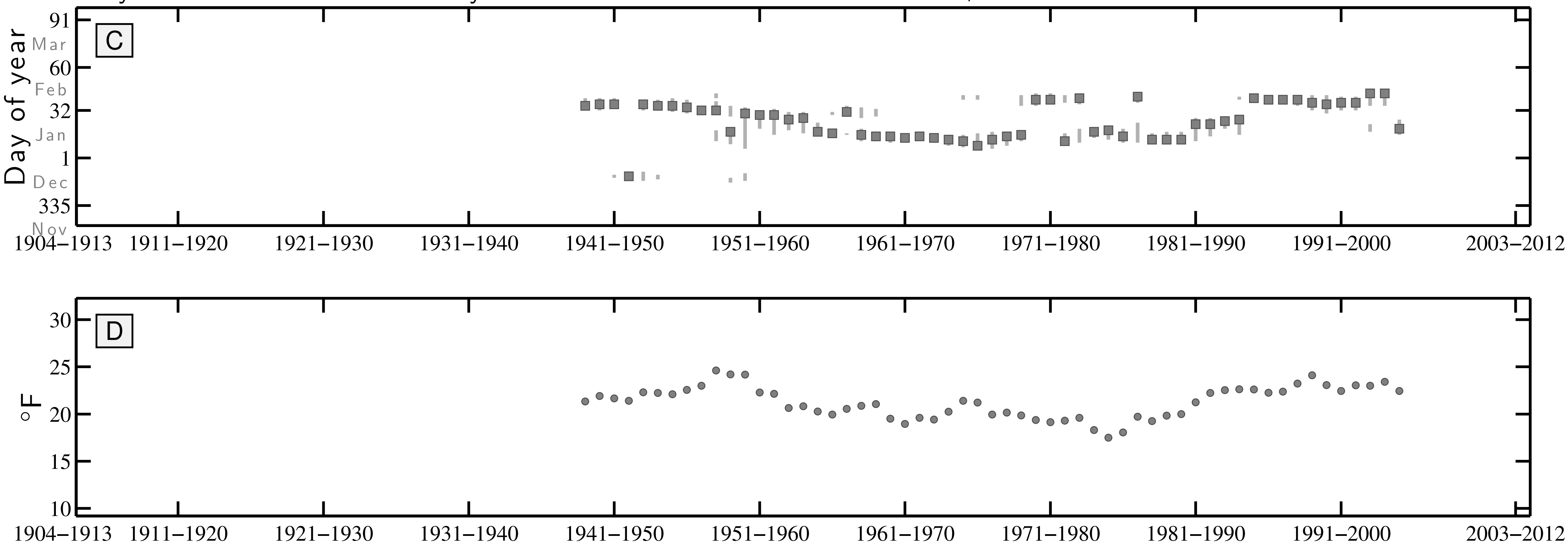

10 year Summer Teletherm dynamics for WILMINGTON PORTER RES, DE:

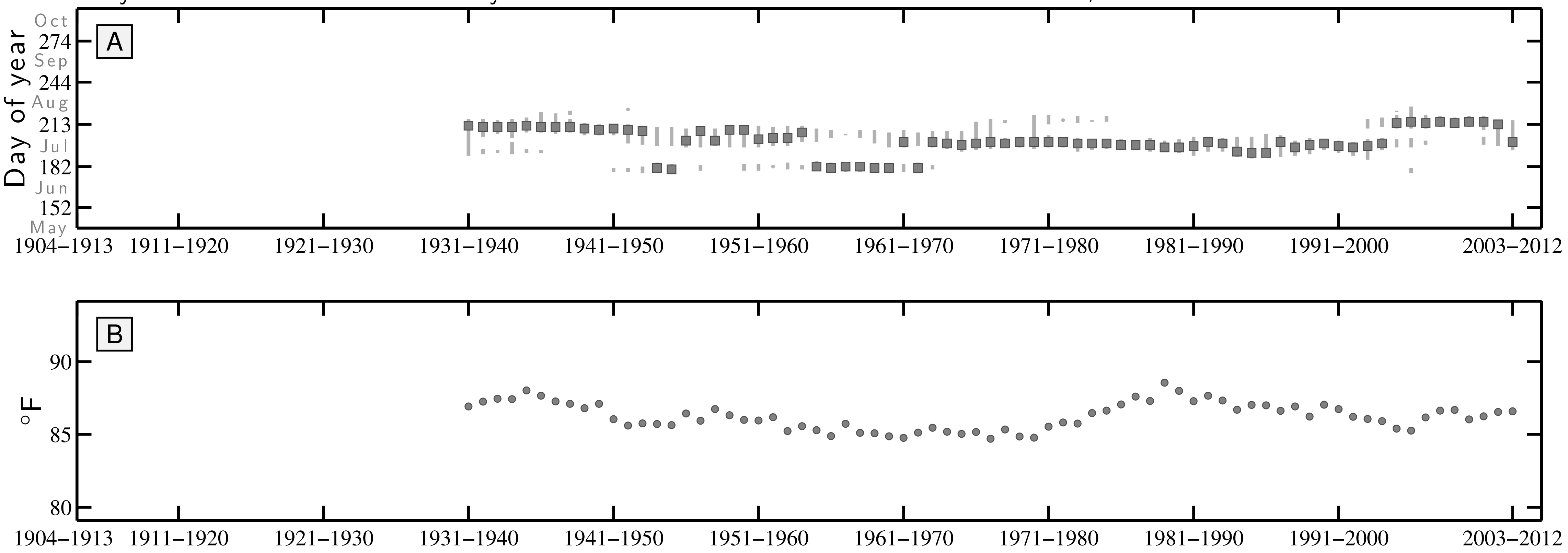

10 year Winter Teletherm dynamics for WILMINGTON PORTER RES, DE:

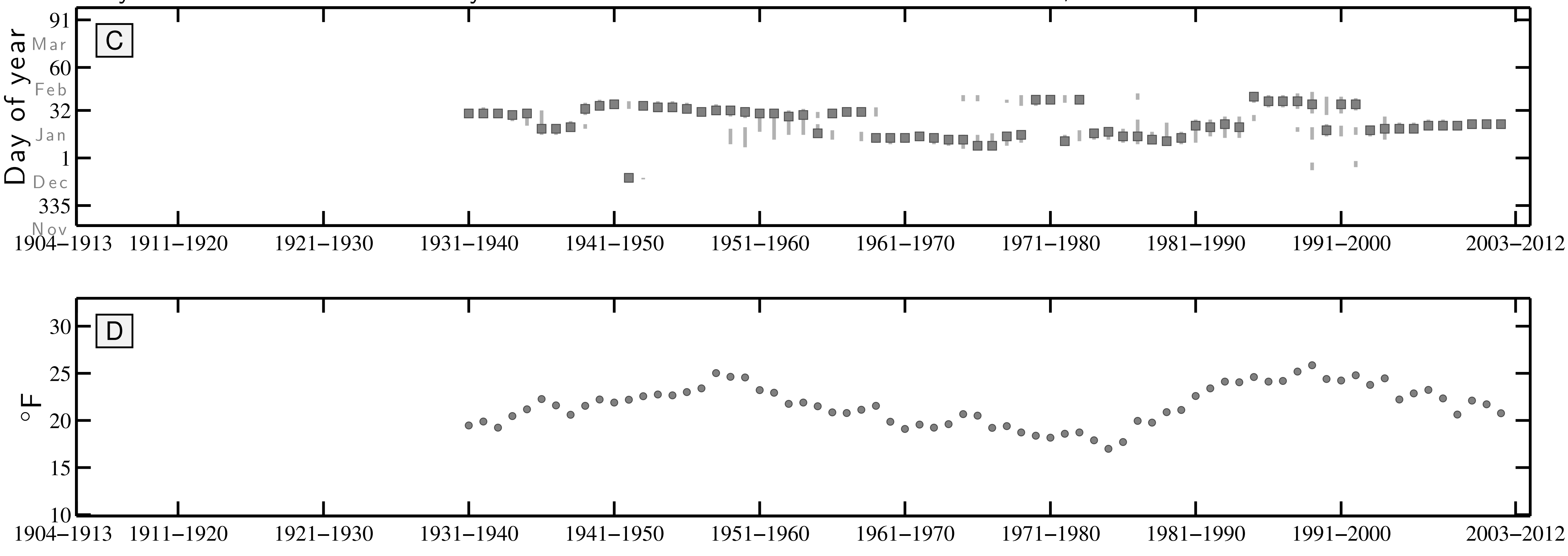

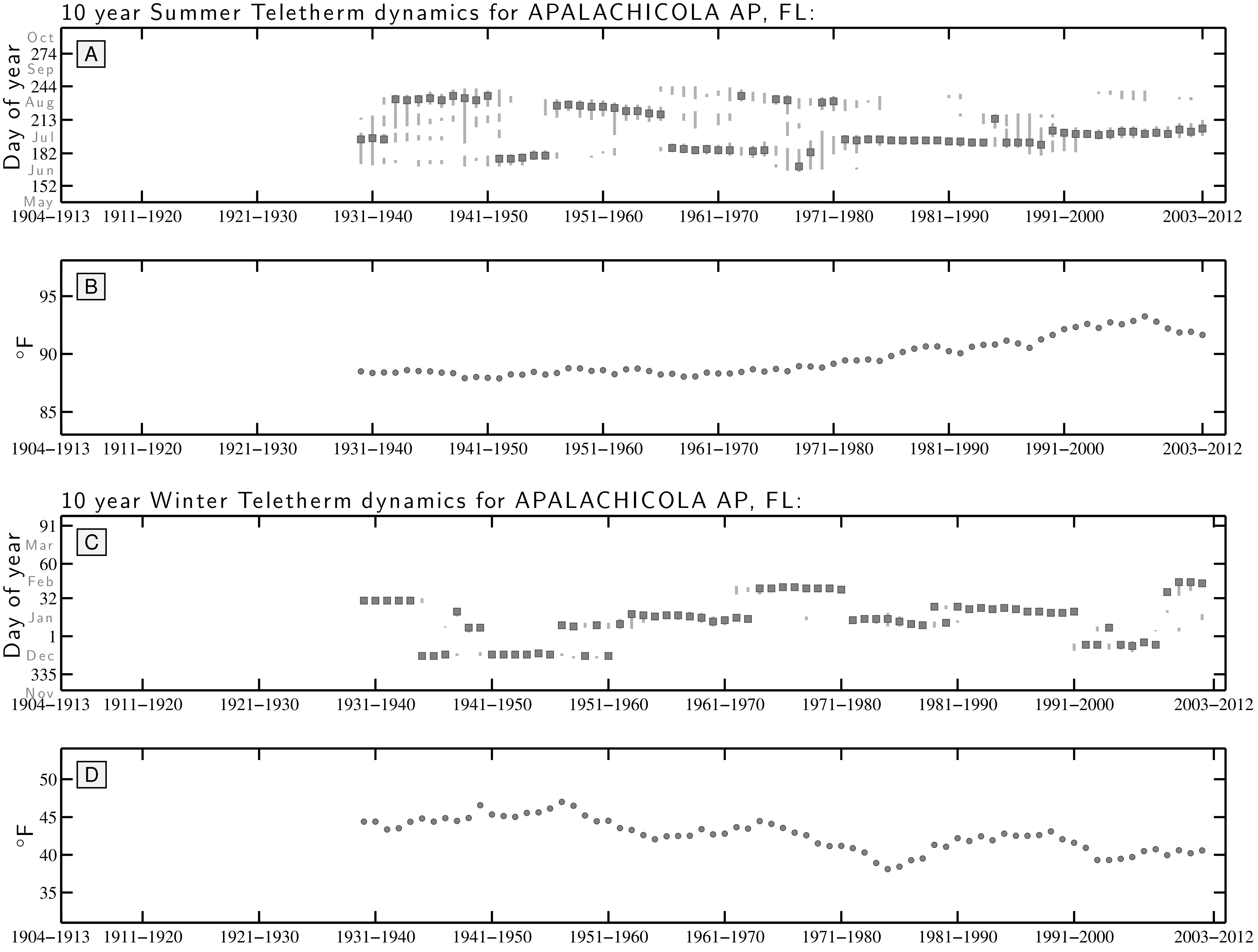

10 year Summer Teletherm dynamics for APALACHICOLA AP, FL:
A
Day of year
Oct
274
Sep
244
Aug
213
Jul
182
Jun
152
May
1904–1913
1911–1920
1921–1930
1931–1940
1941–1950
1951–1960
1961–1970
1971–1980
1981–1990
1991–2000
2003–2012
B
°F
95
90
85
10 year Winter Teletherm dynamics for APALACHICOLA AP, FL:
C
91
Mar
60
Feb
32
Jan
1
Dec
335
Nov
D
50
45
40
35

10 year Summer Teletherm dynamics for ARCADIA, FL:

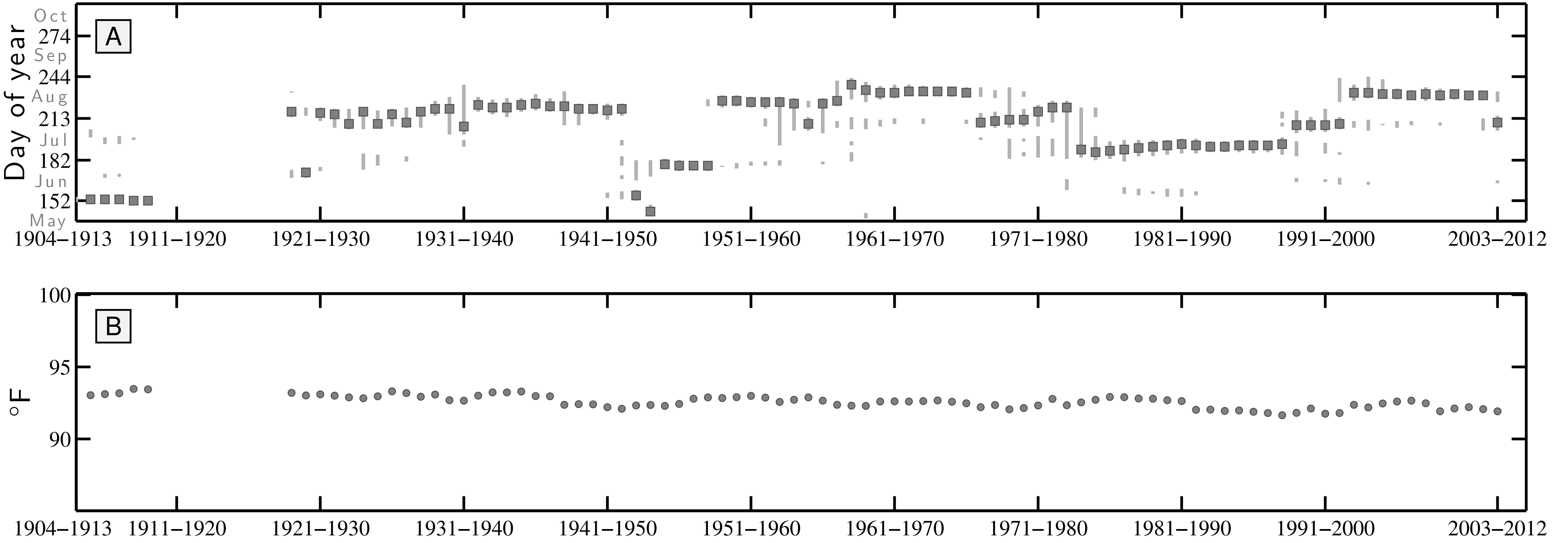

10 year Winter Teletherm dynamics for ARCADIA, FL:

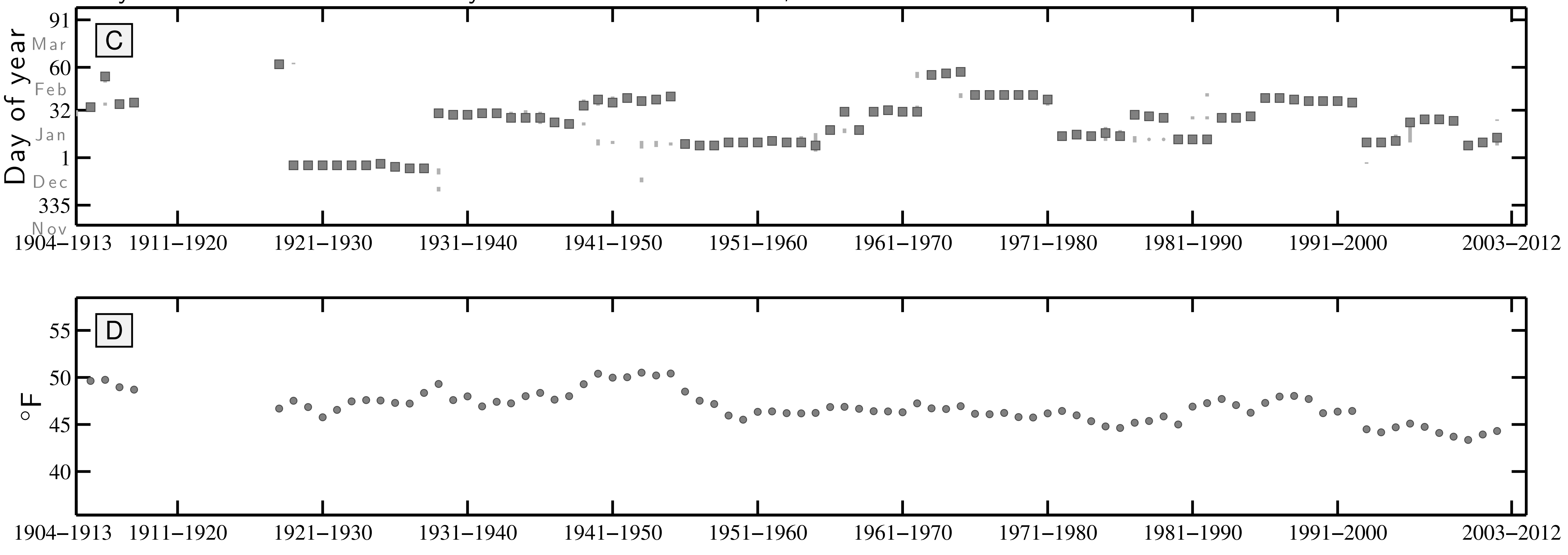

10 year Summer Teletherm dynamics for BARTOW, FL:

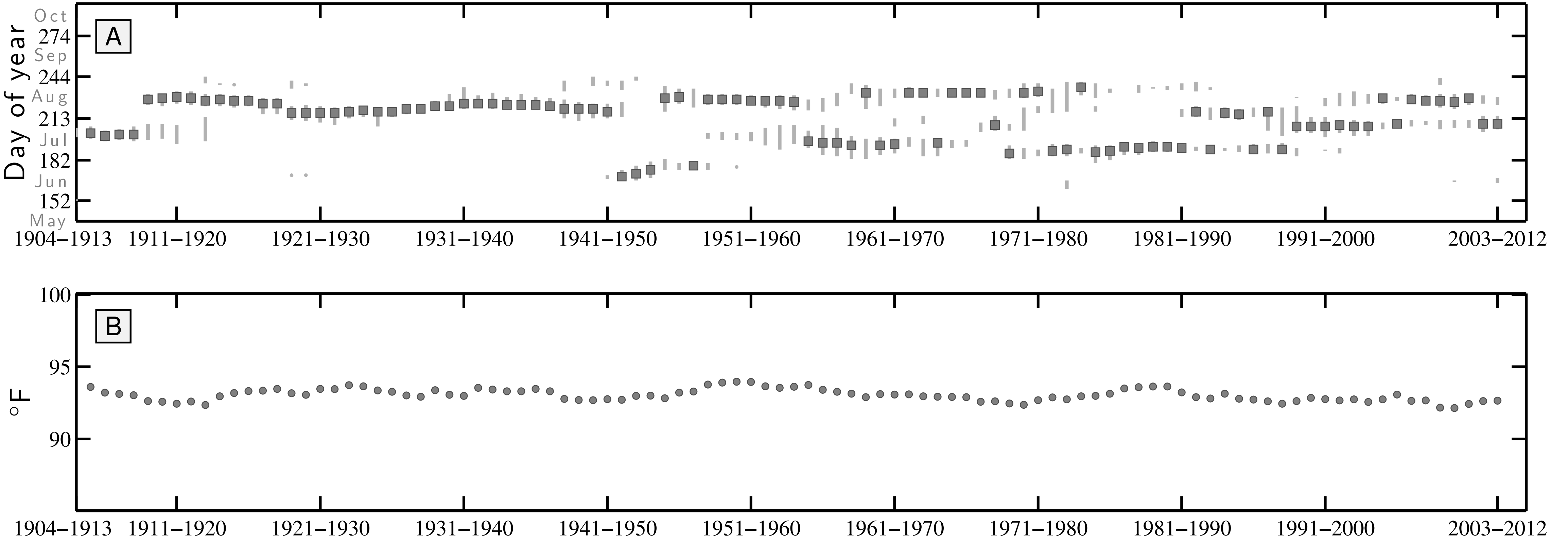

10 year Winter Teletherm dynamics for BARTOW, FL:

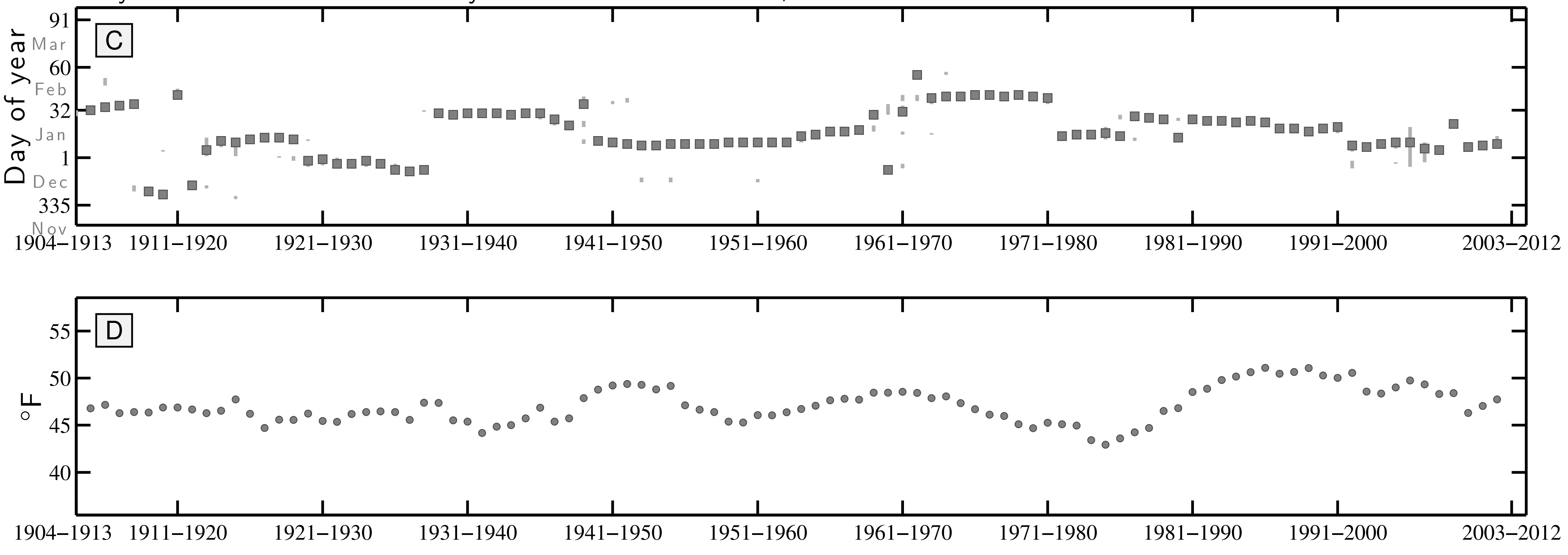

10 year Summer Teletherm dynamics for BELLE GLADE, FL:

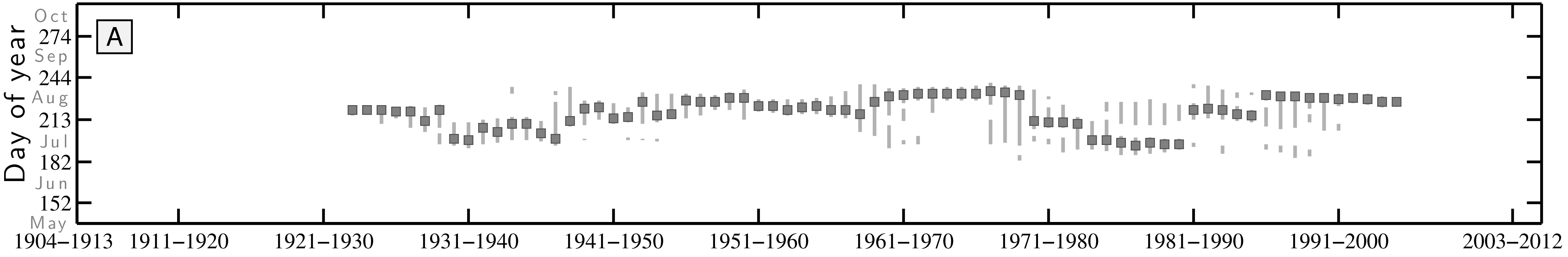

10 year Winter Teletherm dynamics for BELLE GLADE, FL:

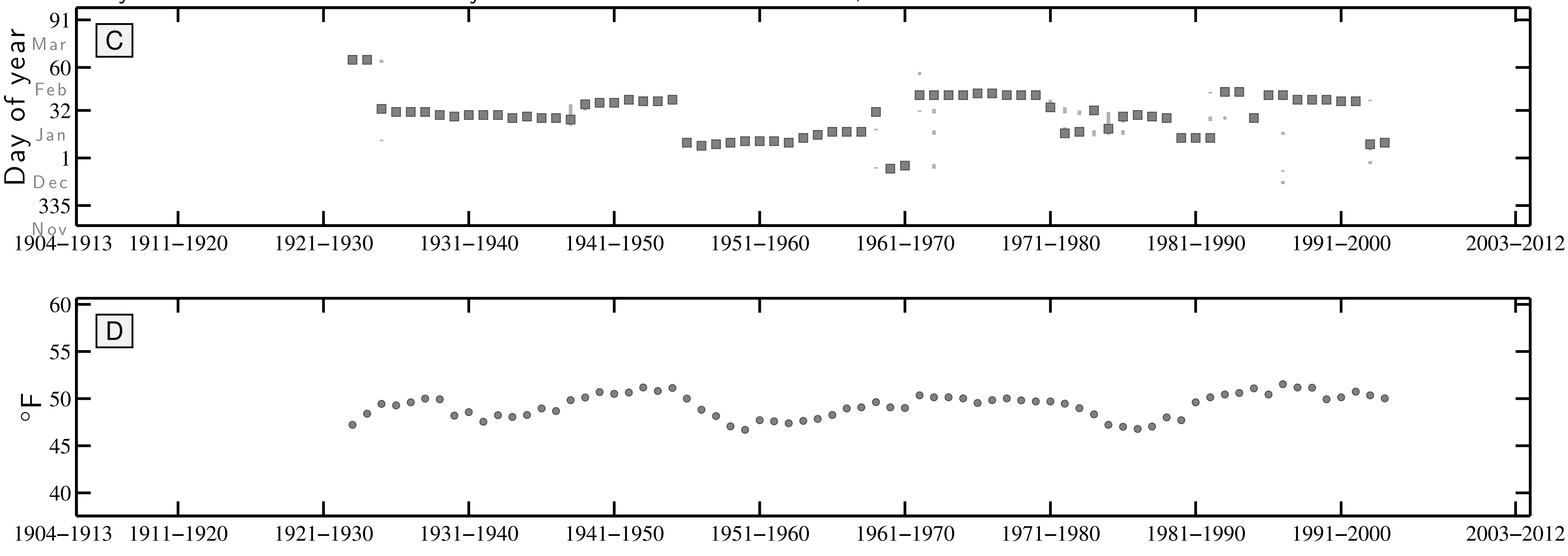

10 year Summer Teletherm dynamics for DE FUNIAK SPRINGS 1, FL:

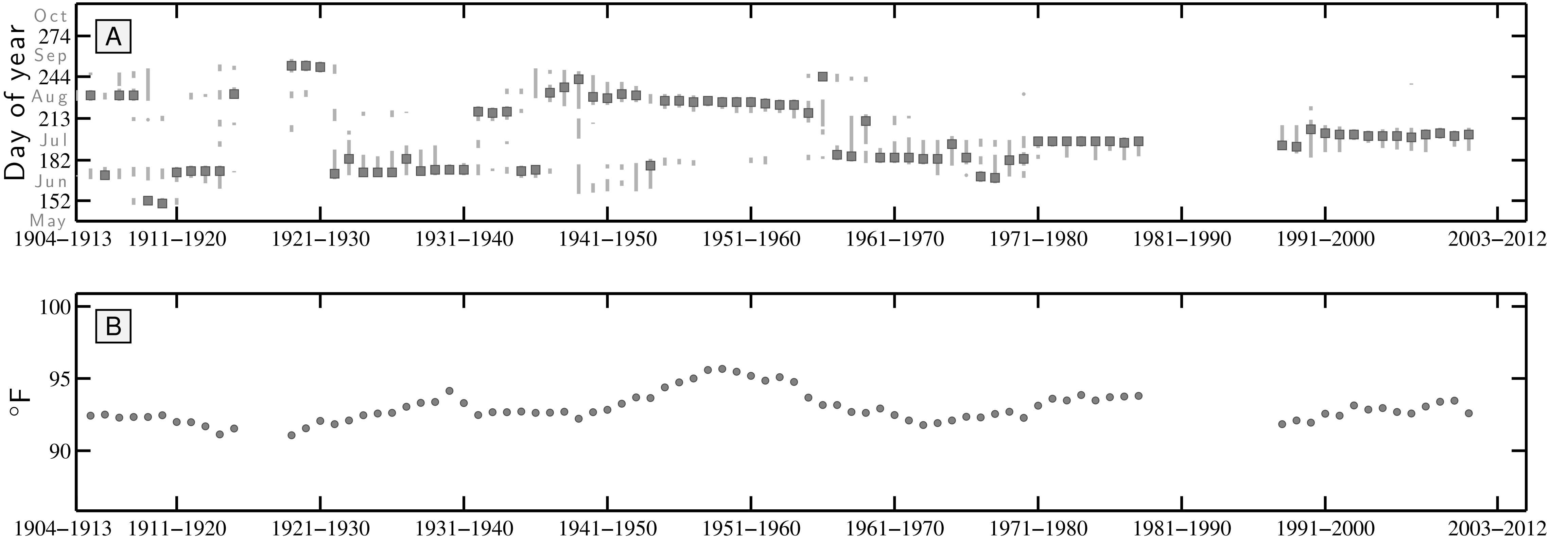

10 year Winter Teletherm dynamics for DE FUNIAK SPRINGS 1, FL:

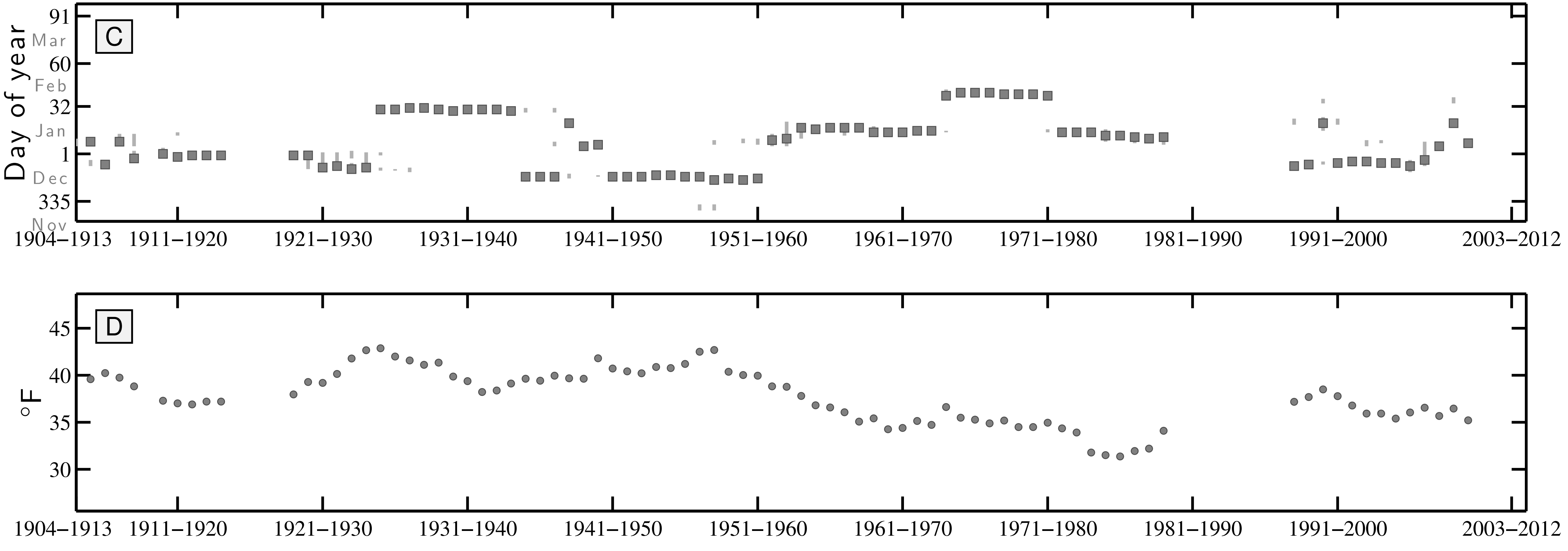

10 year Summer Teletherm dynamics for EVERGLADES, FL:

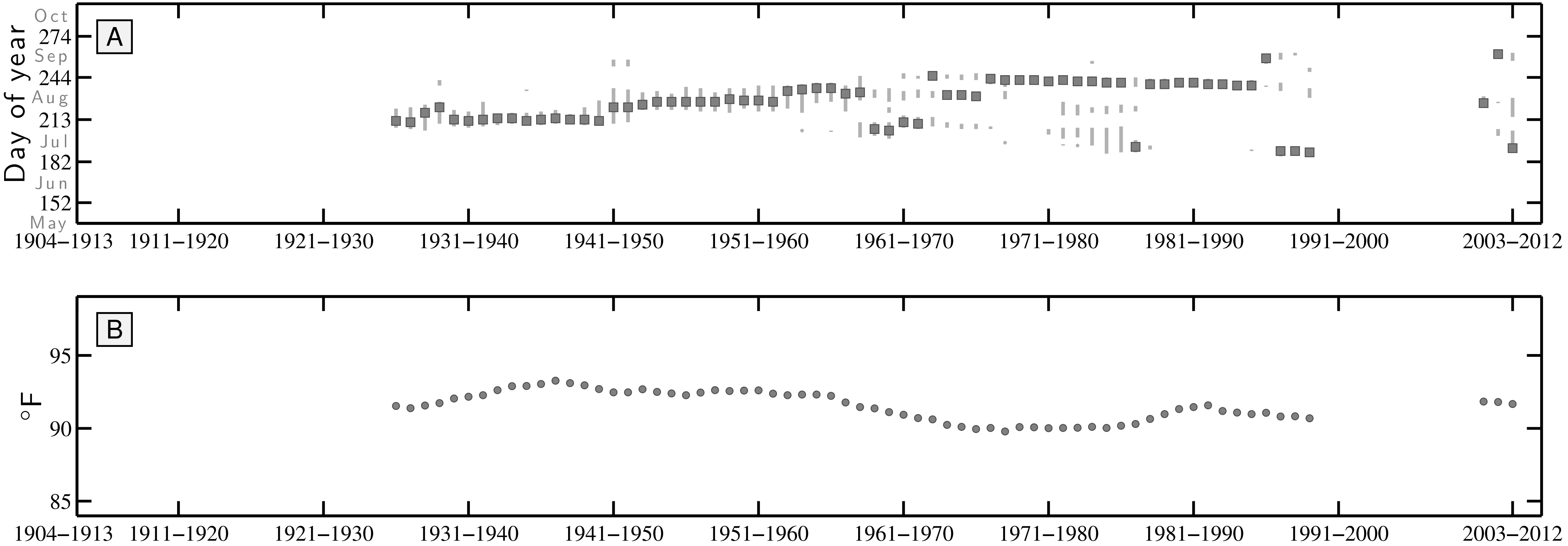

10 year Winter Teletherm dynamics for EVERGLADES, FL:

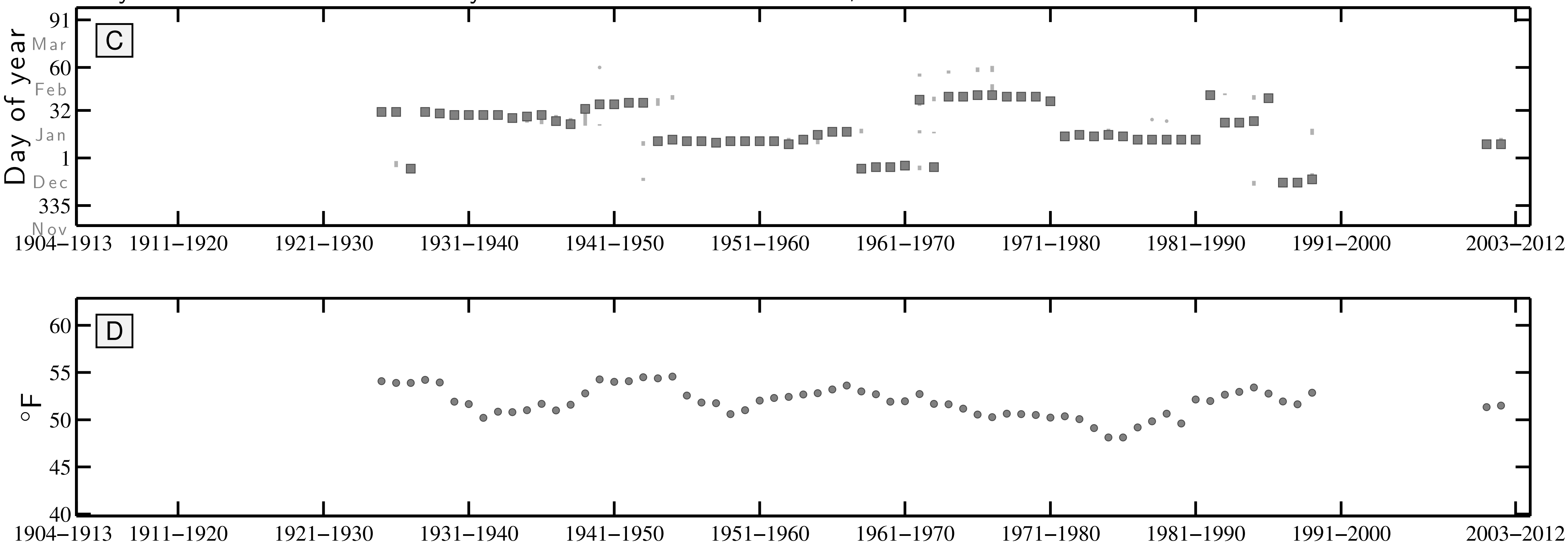

10 year Summer Teletherm dynamics for FEDERAL POINT, FL:

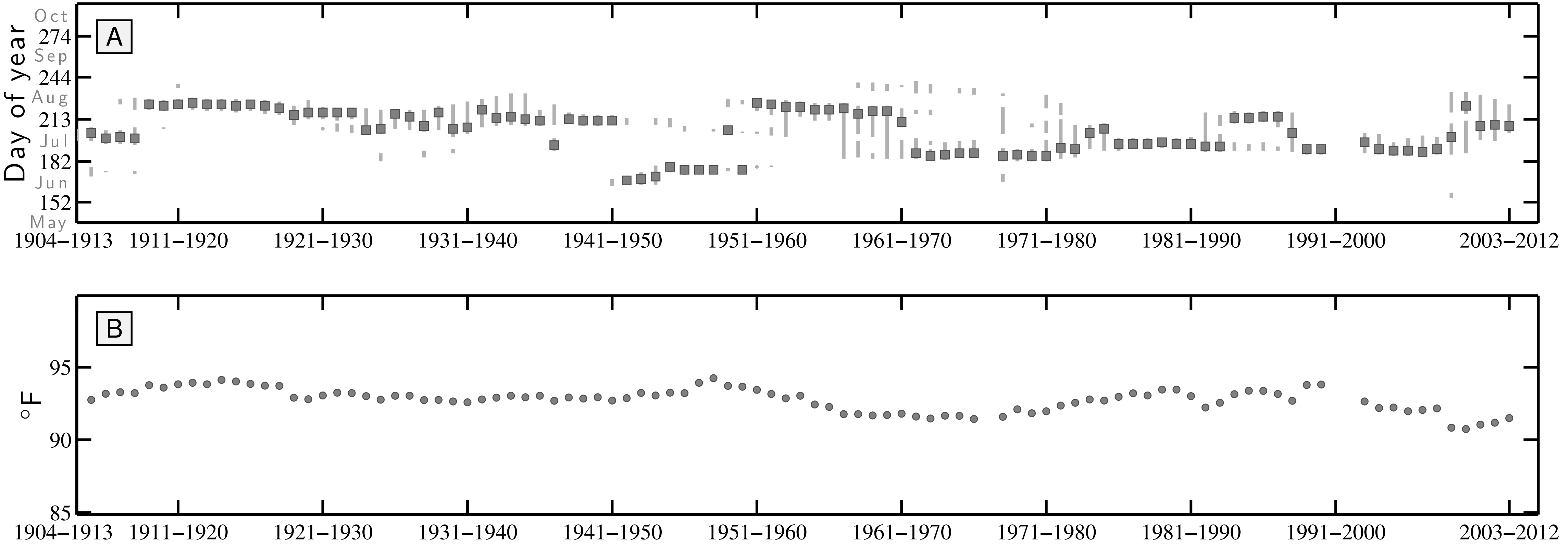

10 year Winter Teletherm dynamics for FEDERAL POINT, FL:

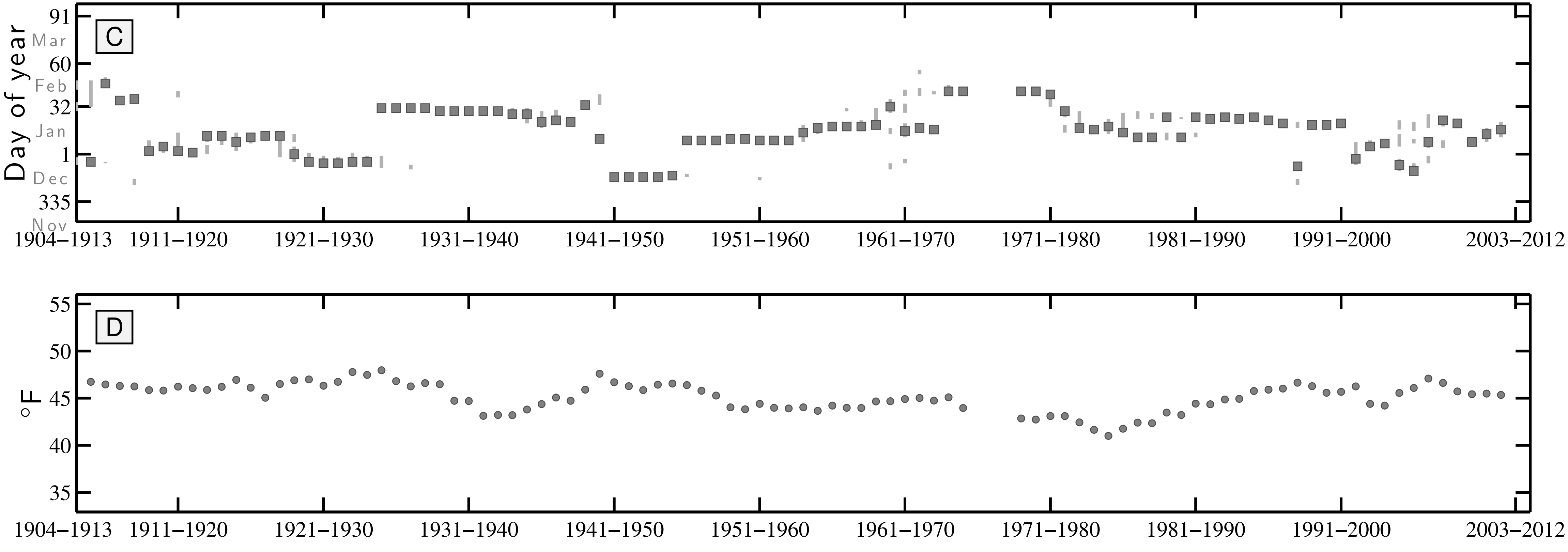

10 year Summer Teletherm dynamics for FERNANDINA BEACH, FL:

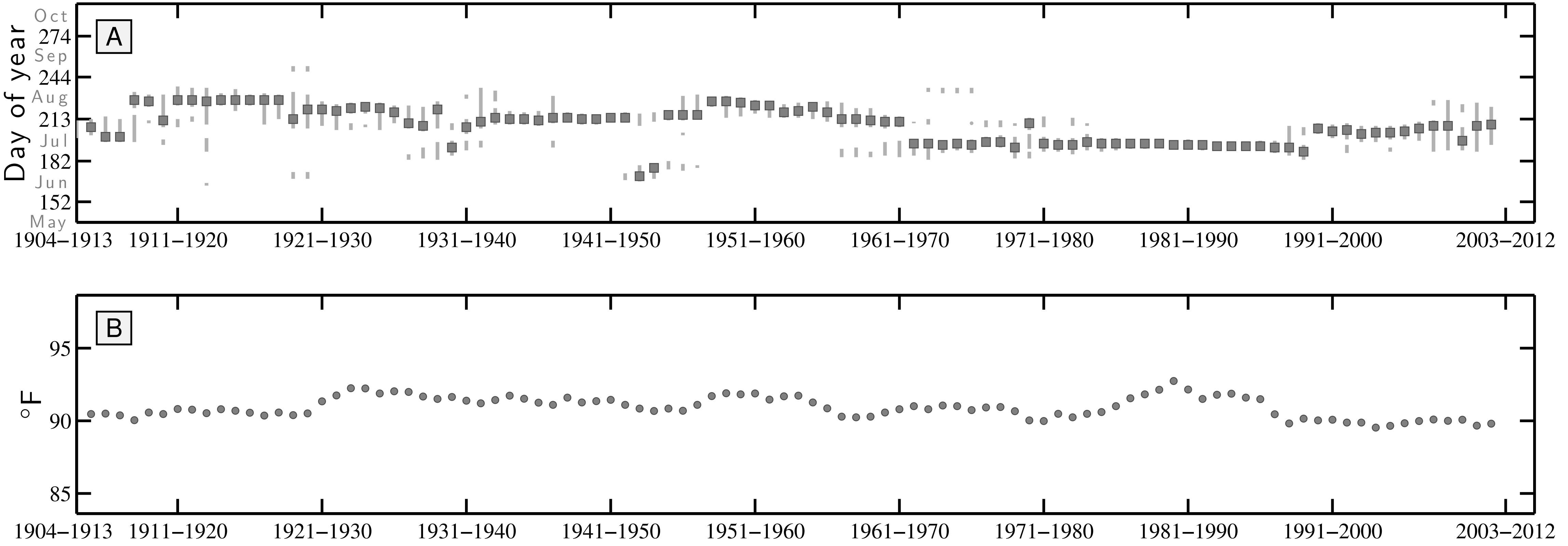

10 year Winter Teletherm dynamics for FERNANDINA BEACH, FL:

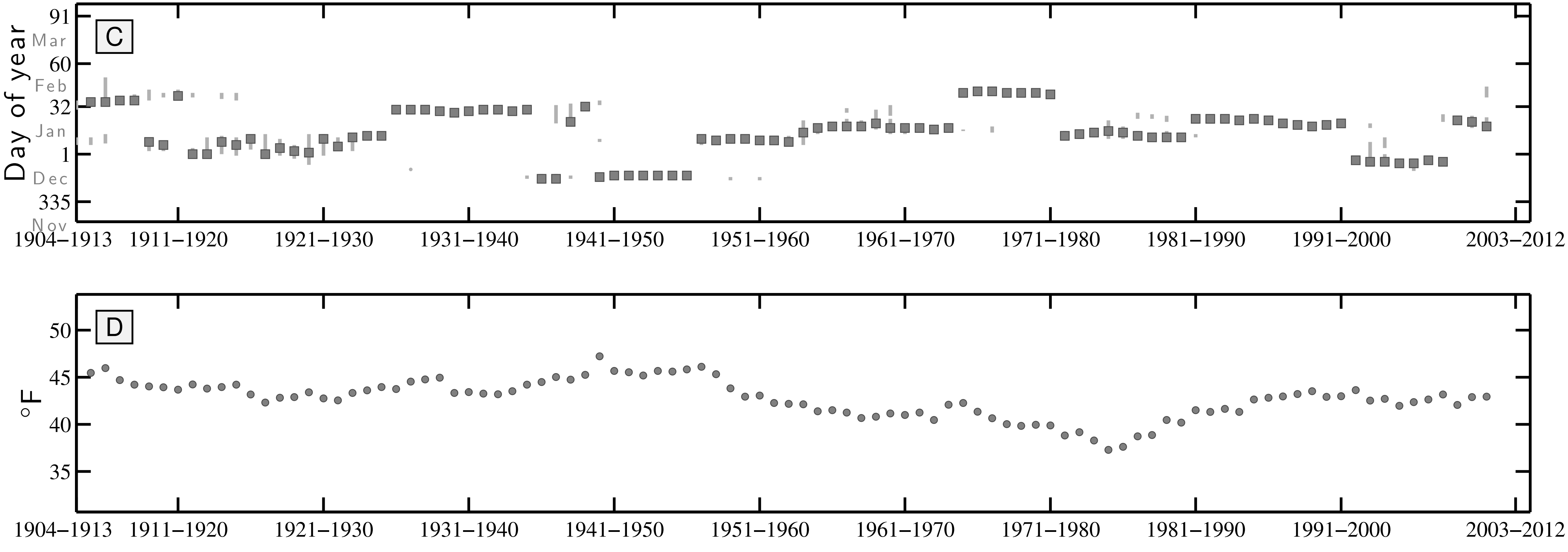

10 year Summer Teletherm dynamics for FT LAUDERDALE, FL:

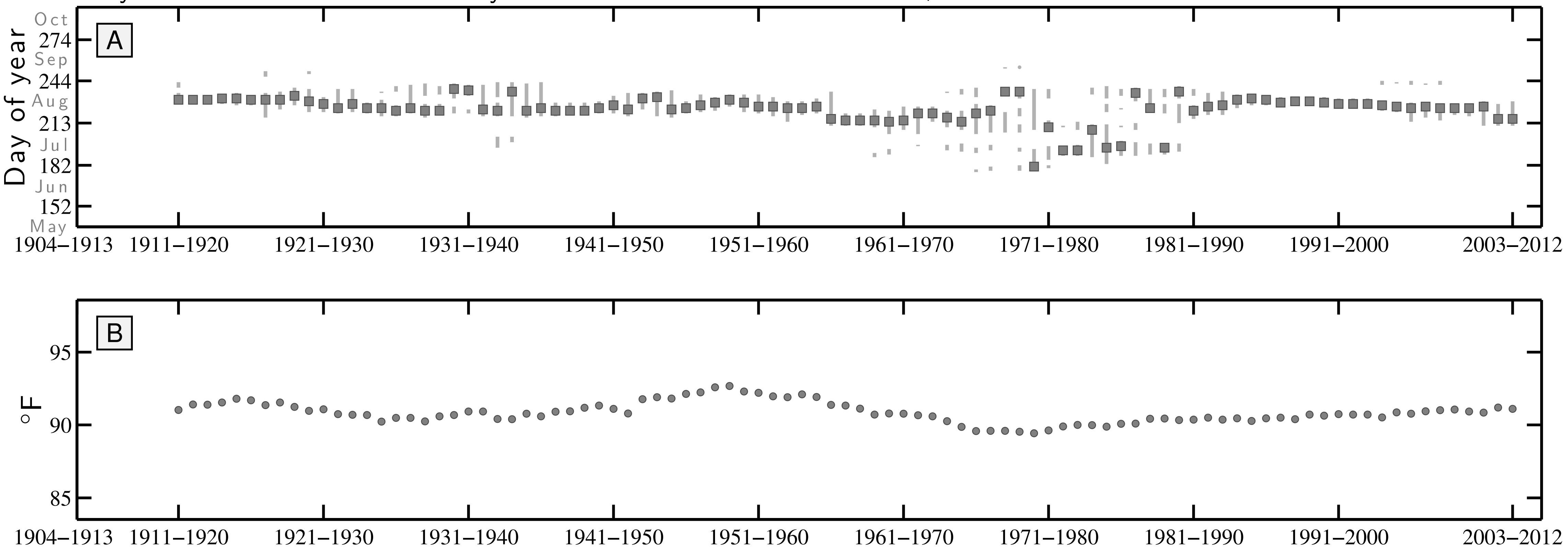

10 year Winter Teletherm dynamics for FT LAUDERDALE, FL:

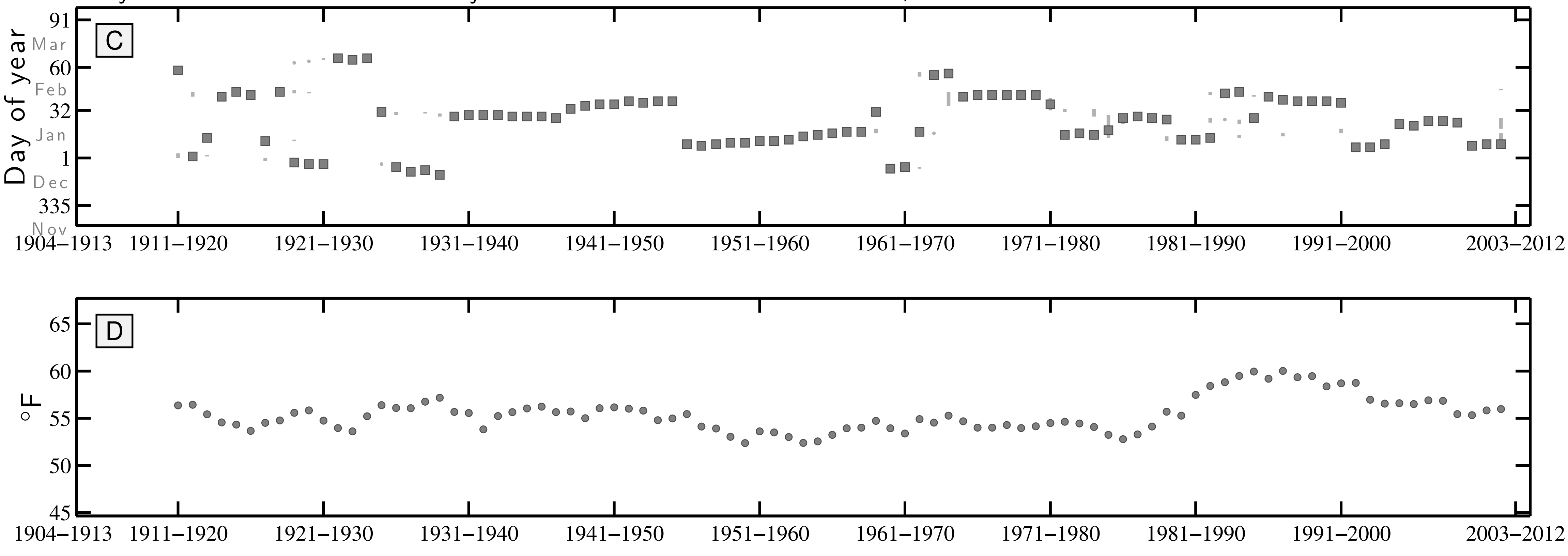

10 year Summer Teletherm dynamics for FT MYERS PAGE FLD, FL:

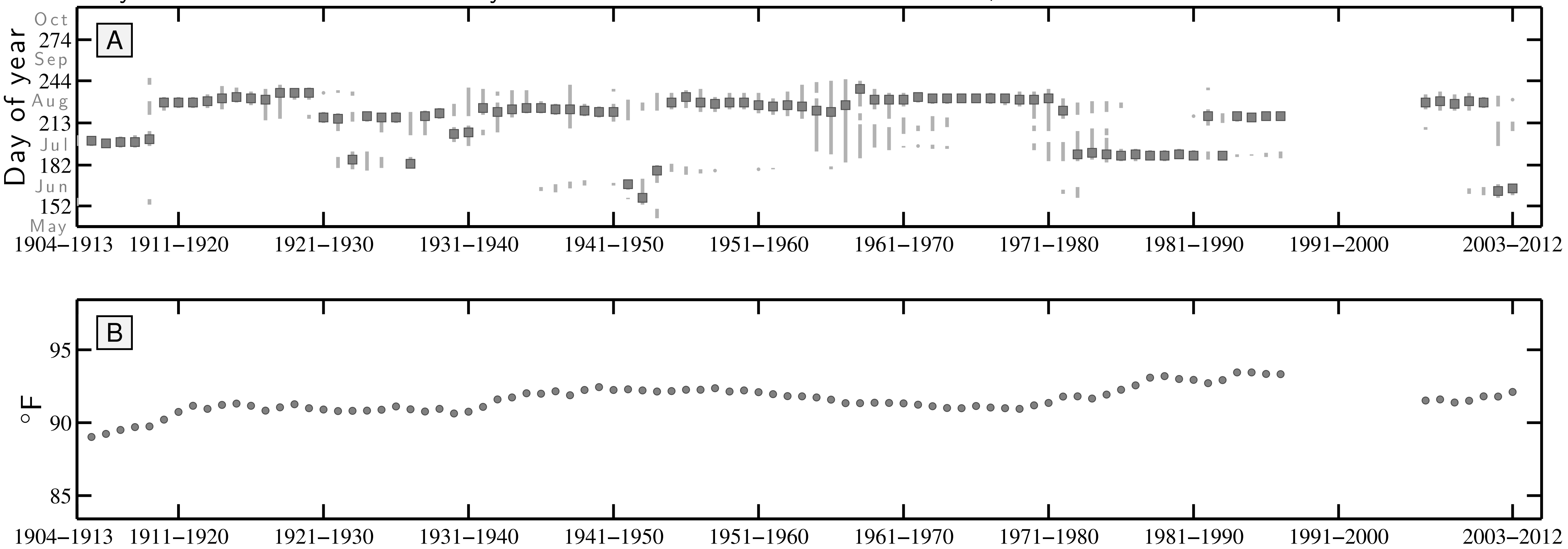

10 year Winter Teletherm dynamics for FT MYERS PAGE FLD, FL:

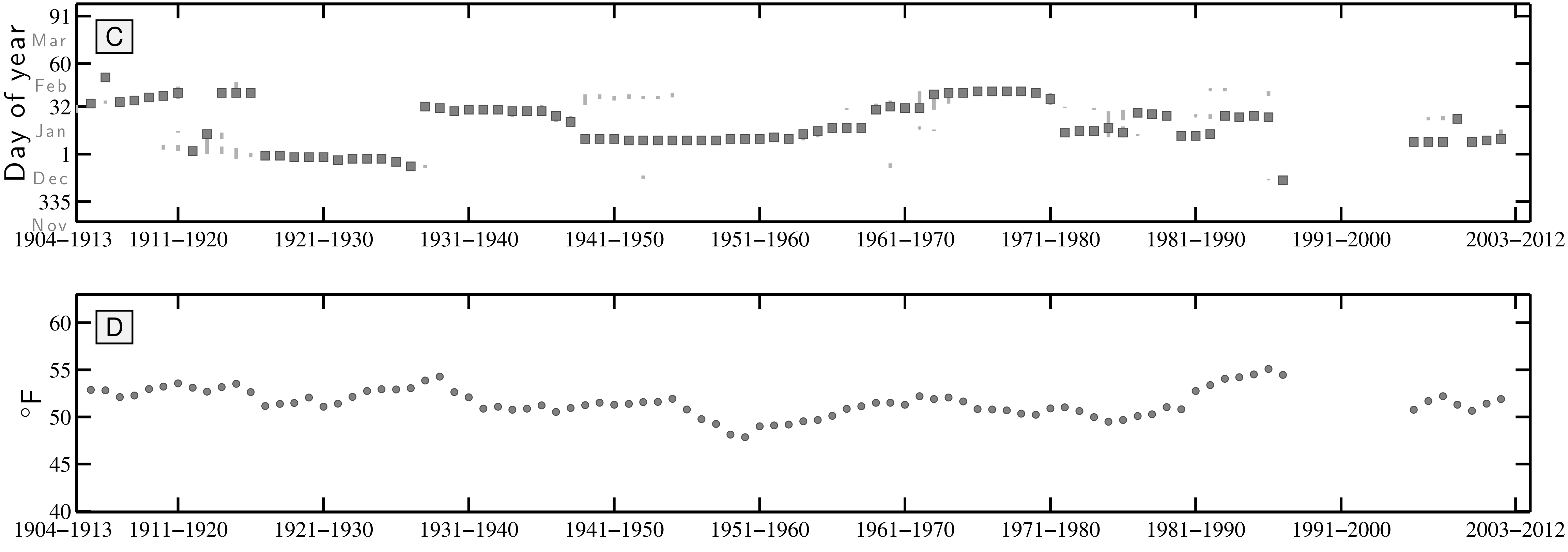

10 year Summer Teletherm dynamics for FT PIERCE, FL:

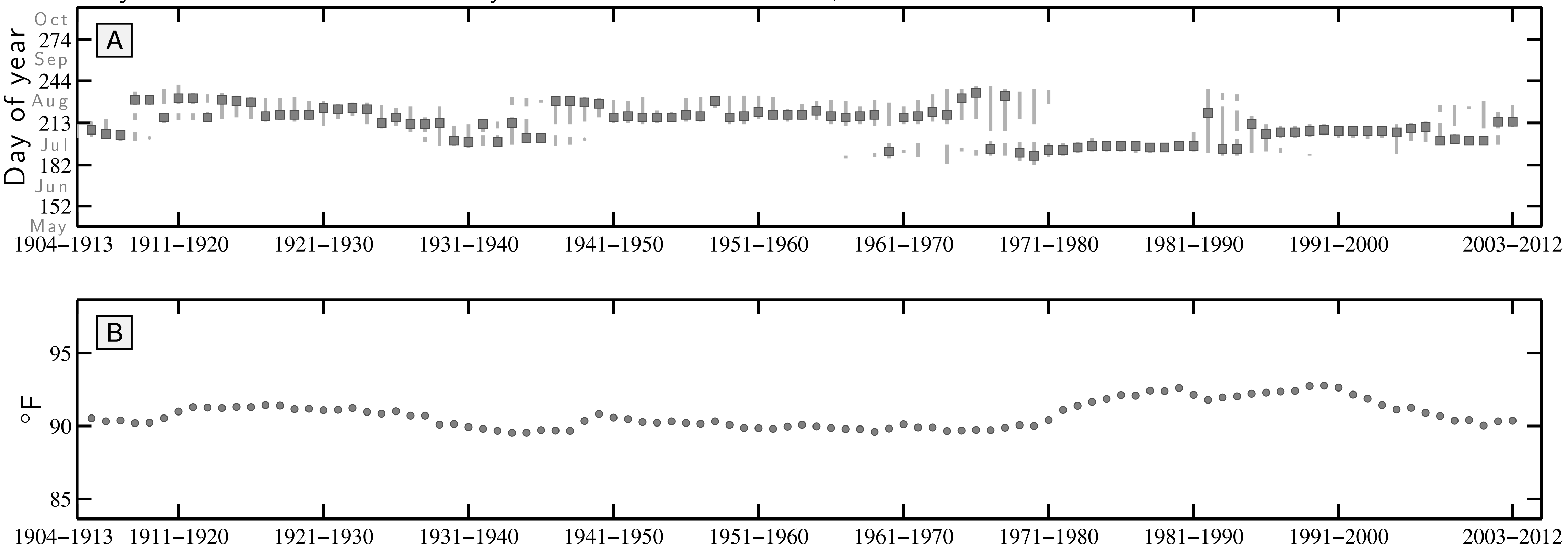

10 year Winter Teletherm dynamics for FT PIERCE, FL:

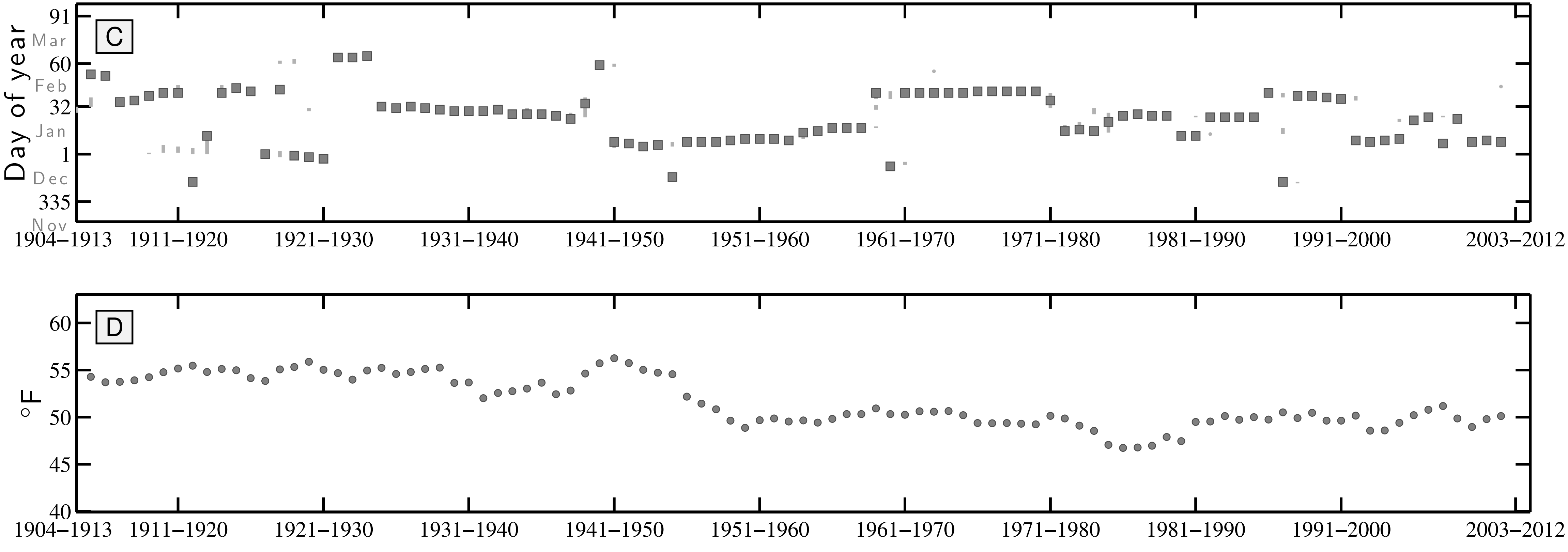

10 year Summer Teletherm dynamics for INVERNESS 3 SE, FL:

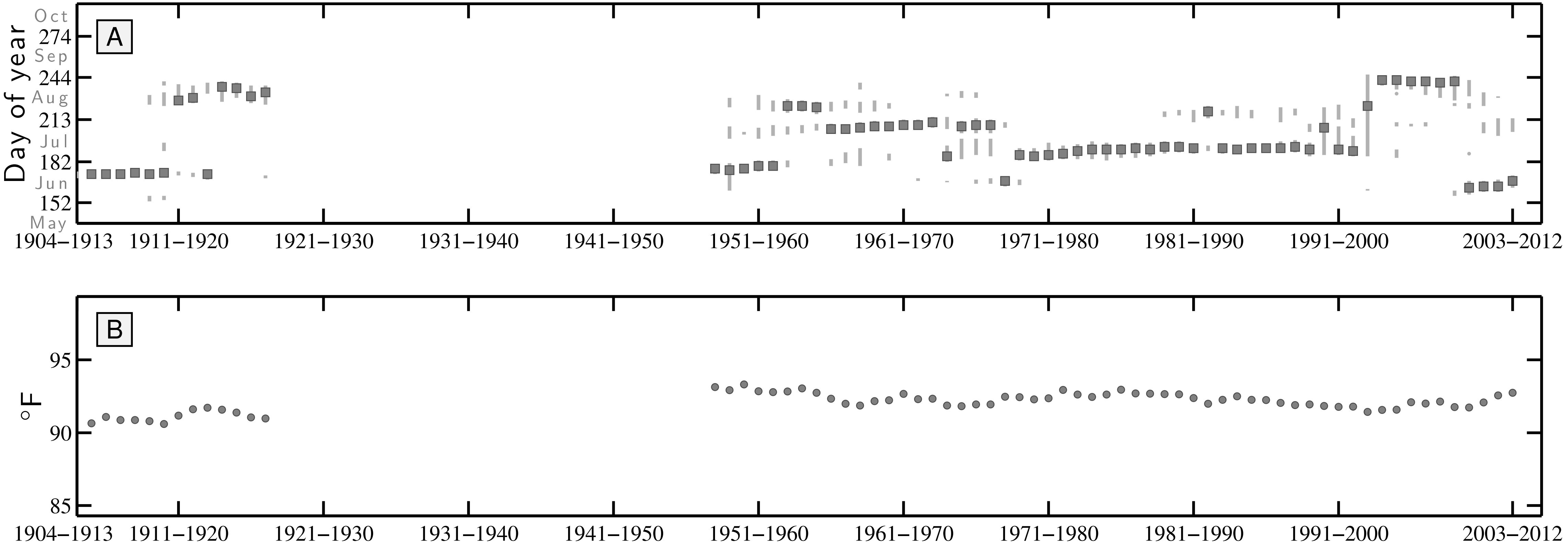

10 year Winter Teletherm dynamics for INVERNESS 3 SE, FL:

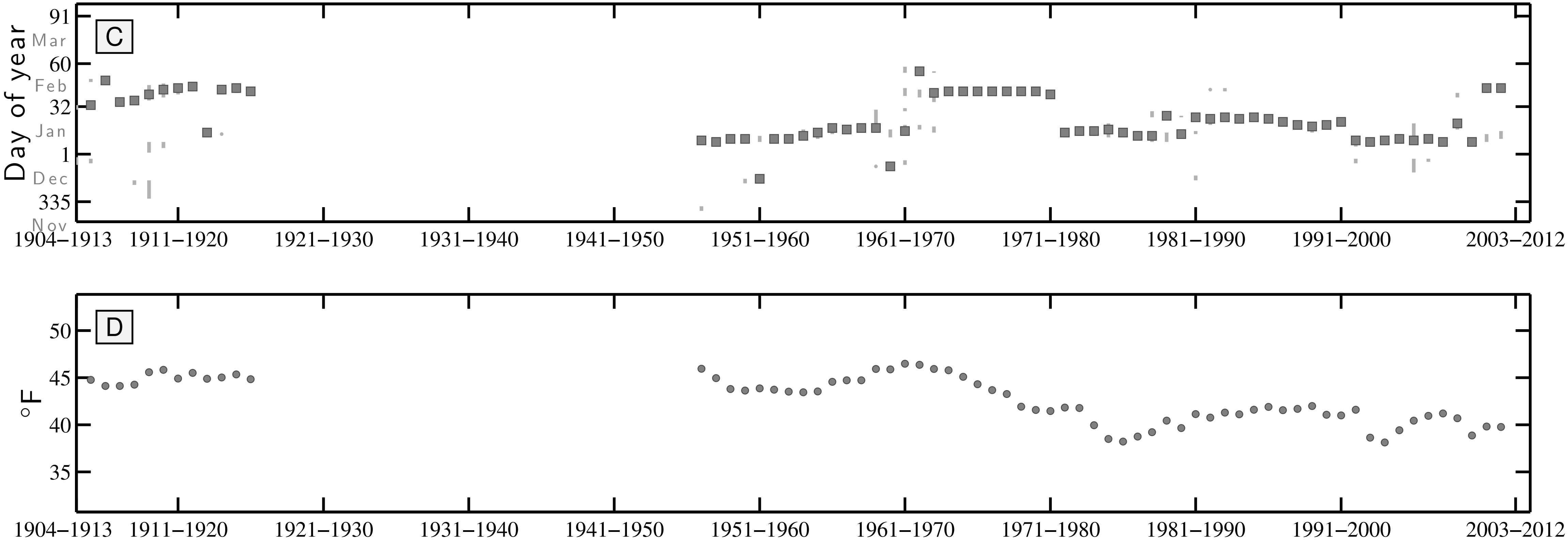

10 year Summer Teletherm dynamics for KEY WEST INTL AP, FL:

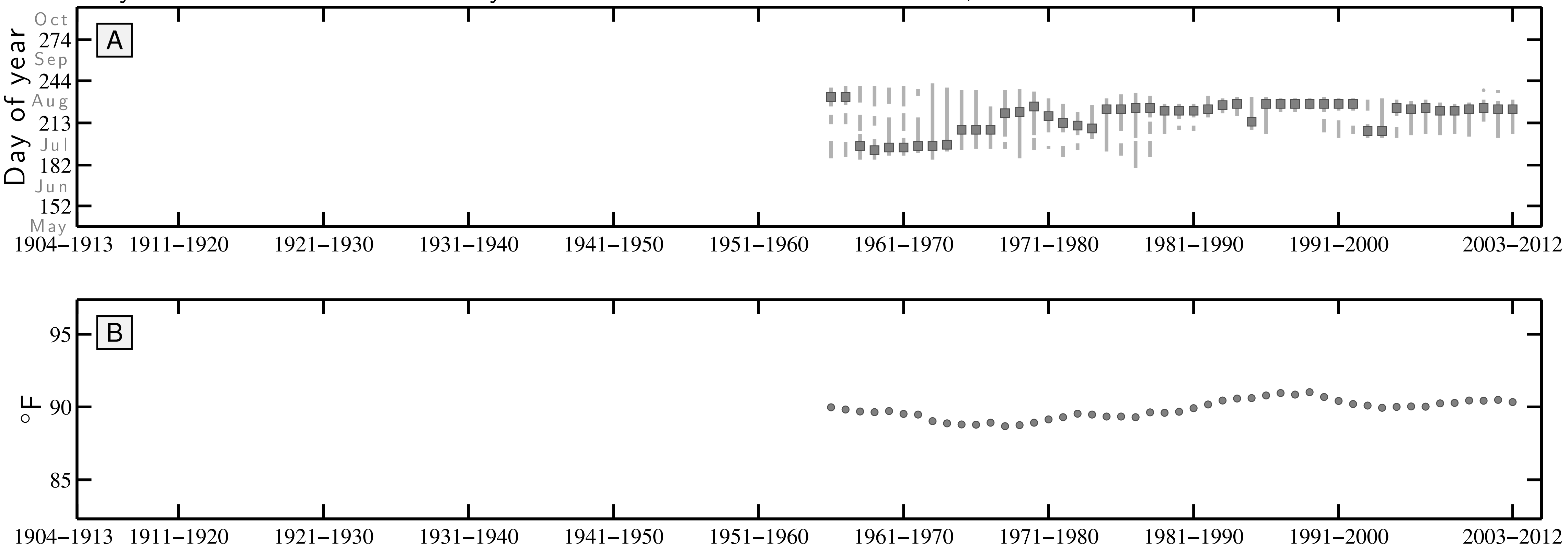

10 year Winter Teletherm dynamics for KEY WEST INTL AP, FL:

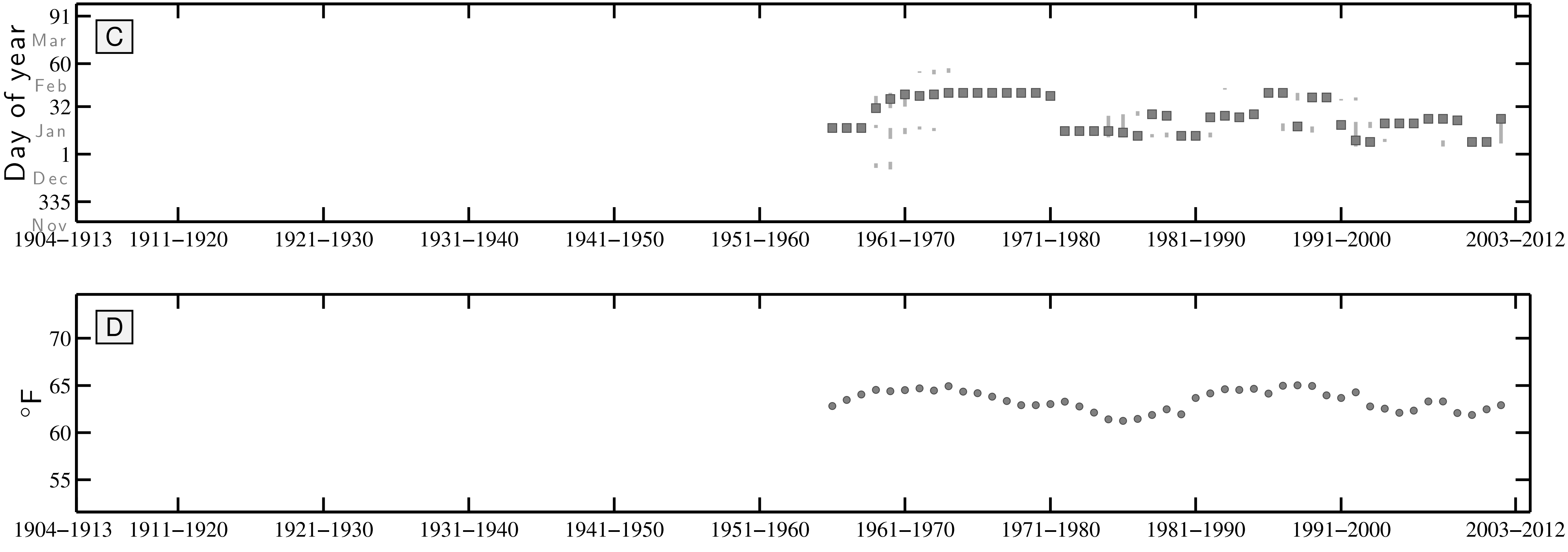

10 year Summer Teletherm dynamics for LAKE CITY 2 E, FL:

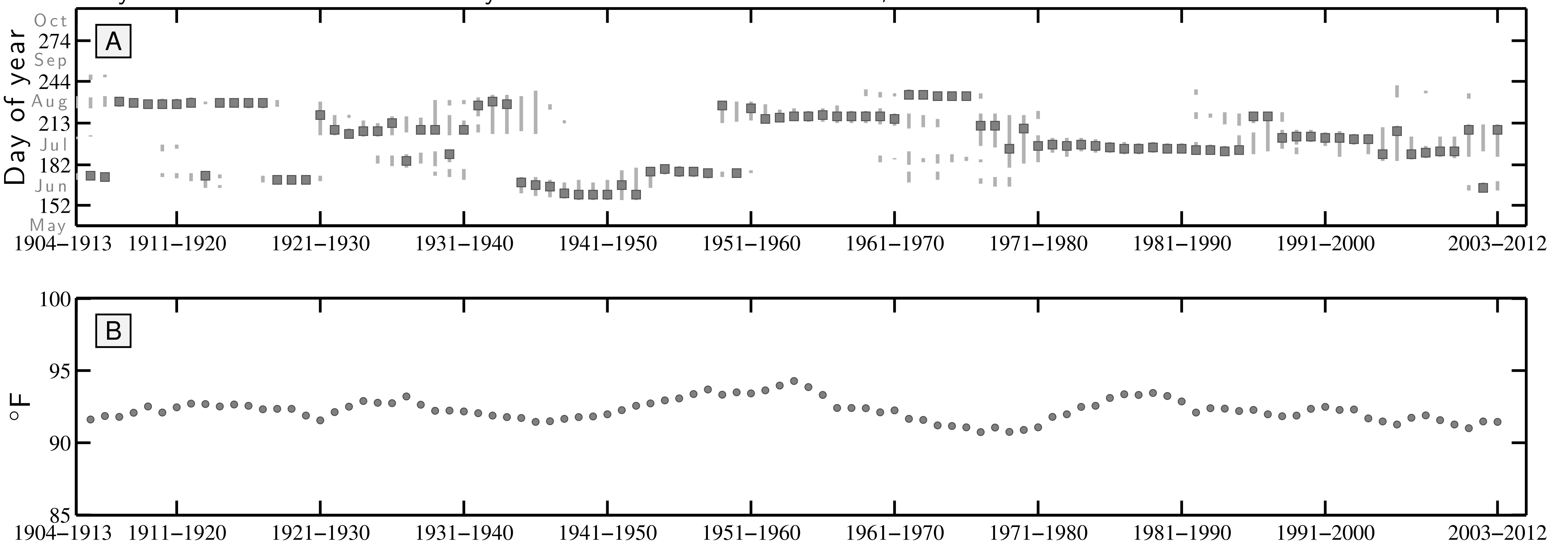

10 year Winter Teletherm dynamics for LAKE CITY 2 E, FL:

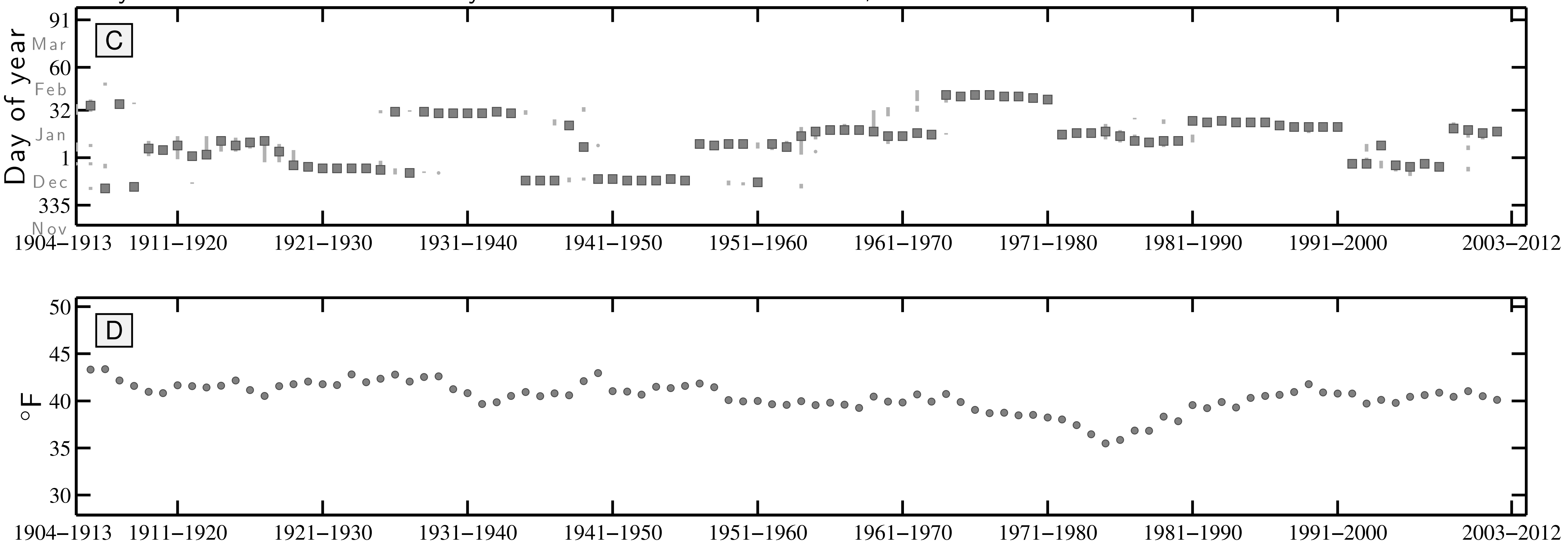

10 year Summer Teletherm dynamics for MADISON, FL:

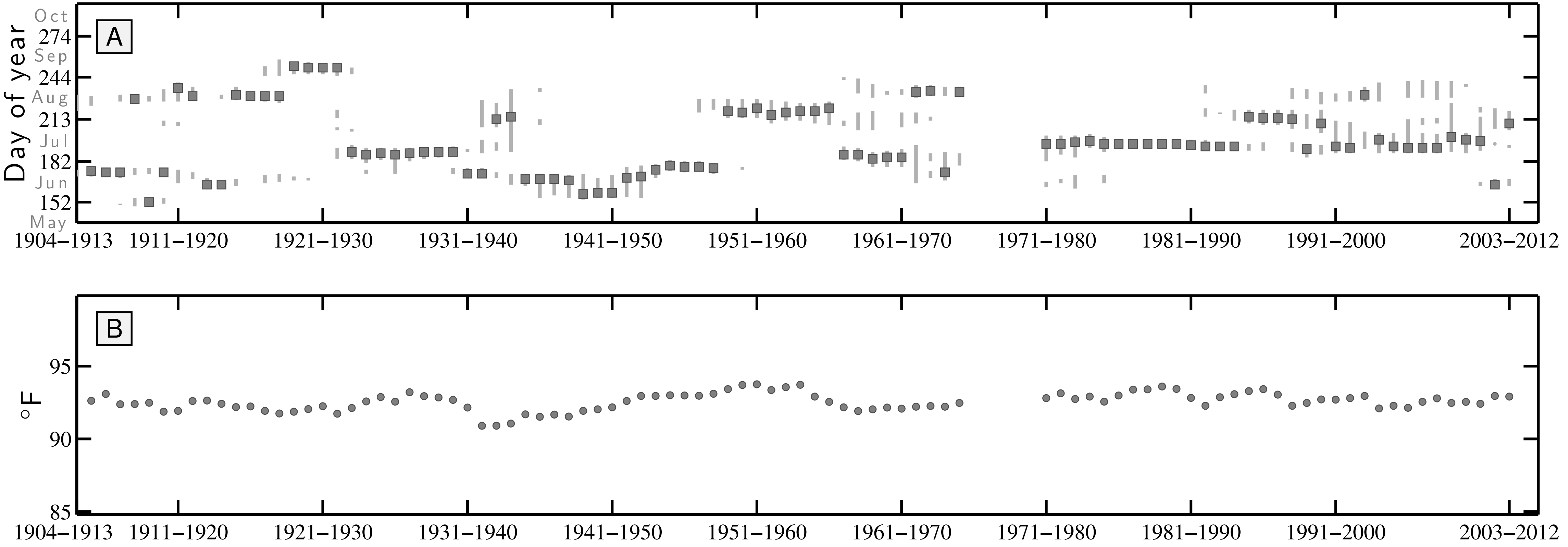

10 year Winter Teletherm dynamics for MADISON, FL:

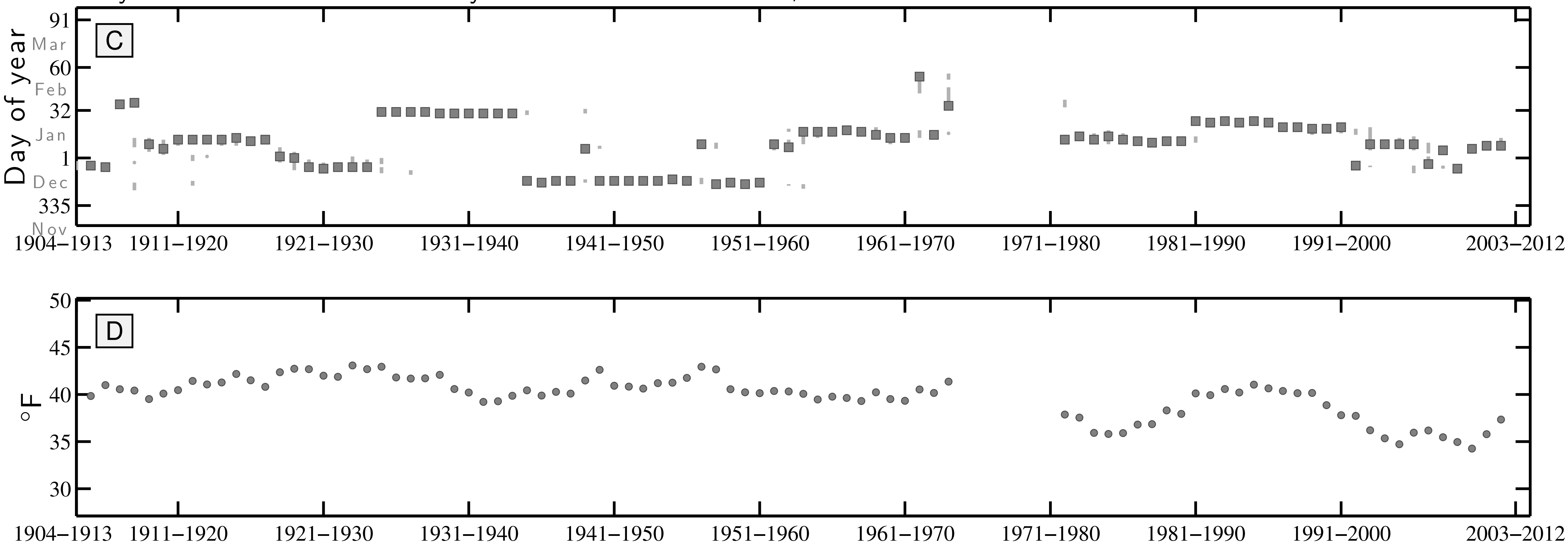

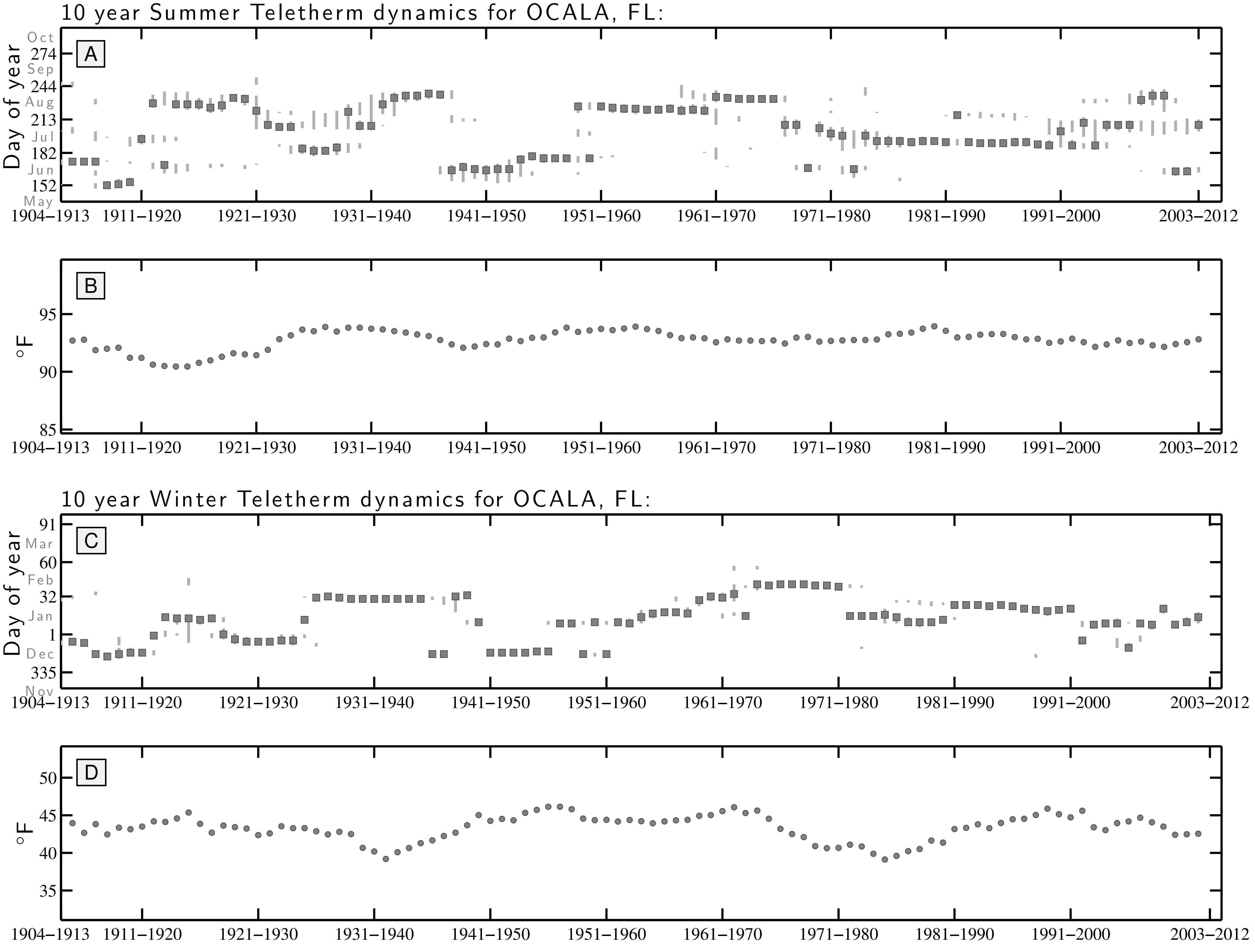
10 year Summer Teletherm dynamics for OCALA, FL:
A
Day of year
Oct
274
Sep
244
Aug
213
Jul
182
Jun
152
May
B
°F
95
90
85
10 year Winter Teletherm dynamics for OCALA, FL:
C
Day of year
91
Mar
60
Feb
32
Jan
1
Dec
335
Nov
D
°F
50
45
40
35
1904–1913
1911–1920
1921–1930
1931–1940
1941–1950
1951–1960
1961–1970
1971–1980
1981–1990
1991–2000
2003–2012

10 year Summer Teletherm dynamics for PENSACOLA RGNL AP, FL:

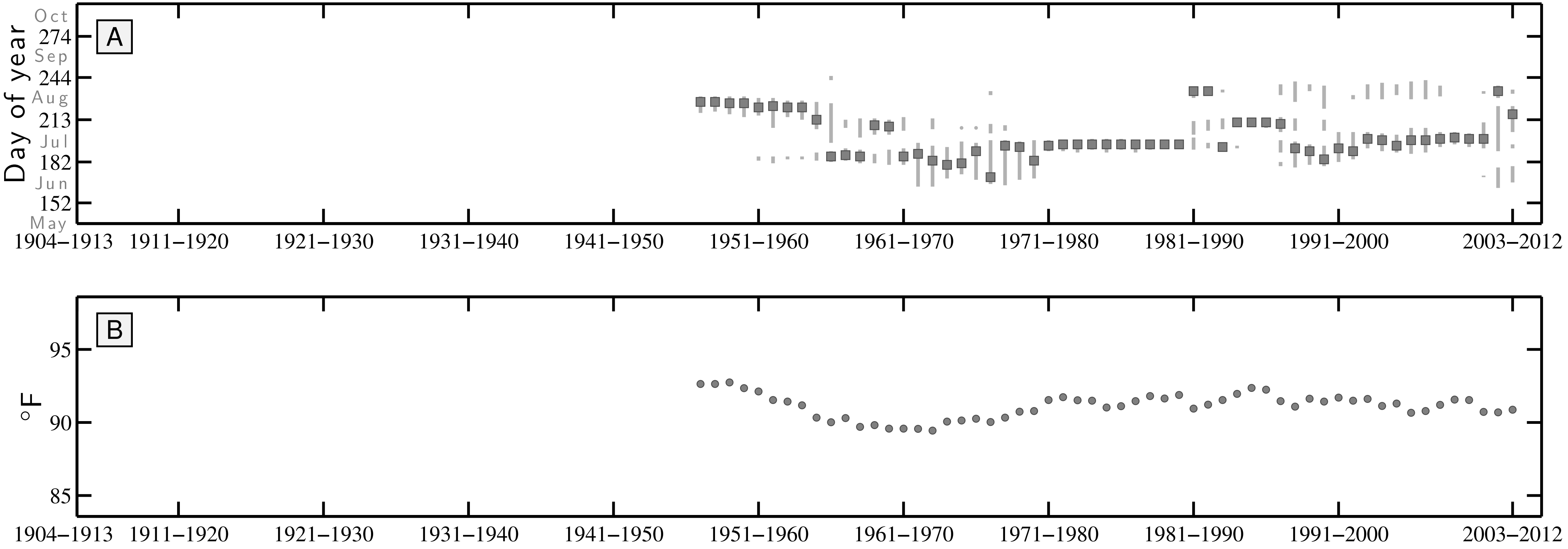

10 year Winter Teletherm dynamics for PENSACOLA RGNL AP, FL:

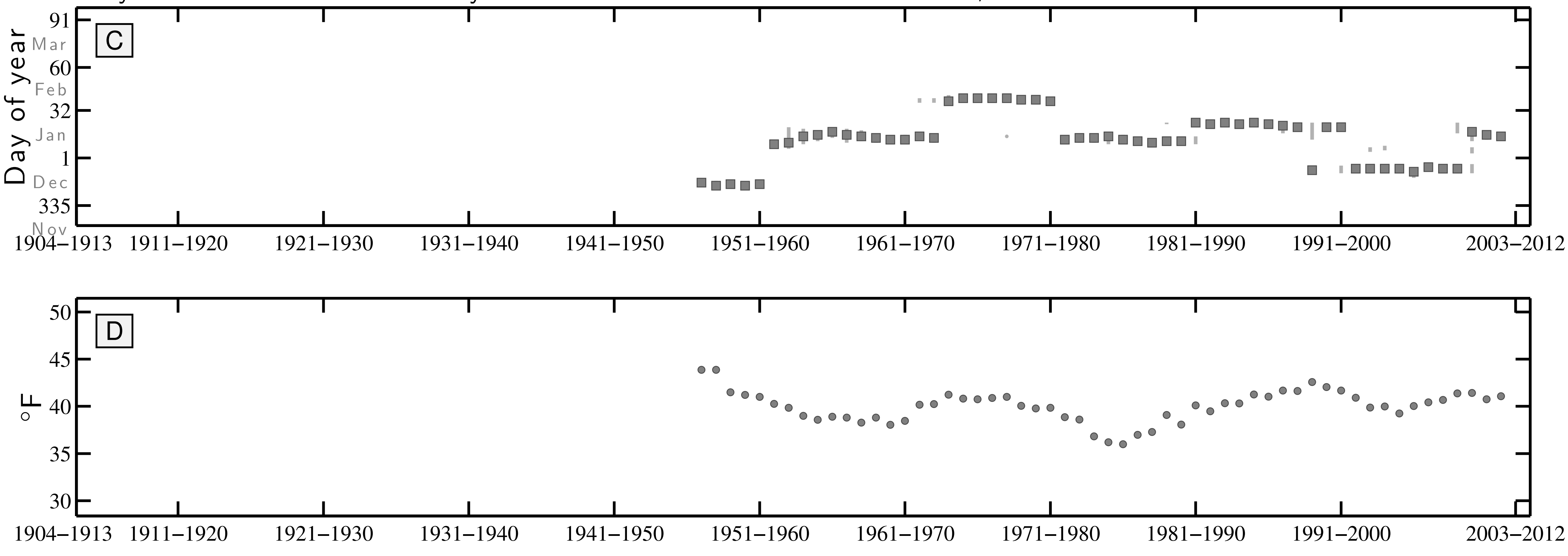

10 year Summer Teletherm dynamics for PERRINE 4W, FL:

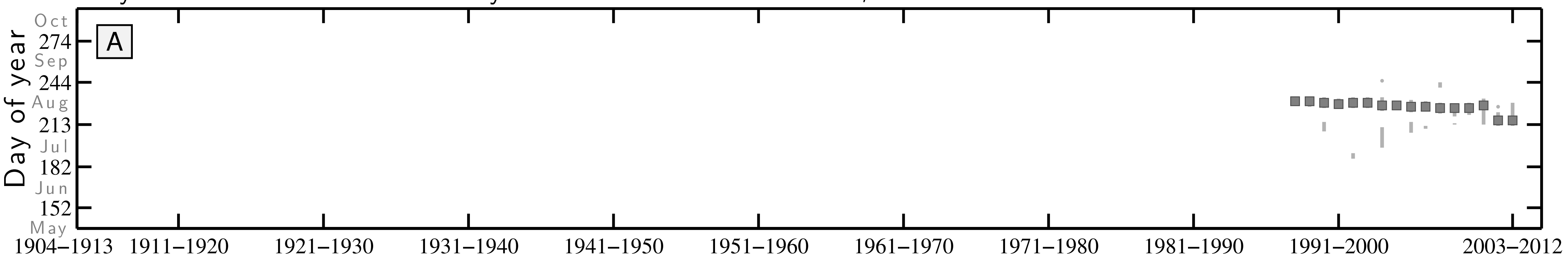

10 year Winter Teletherm dynamics for PERRINE 4W, FL:

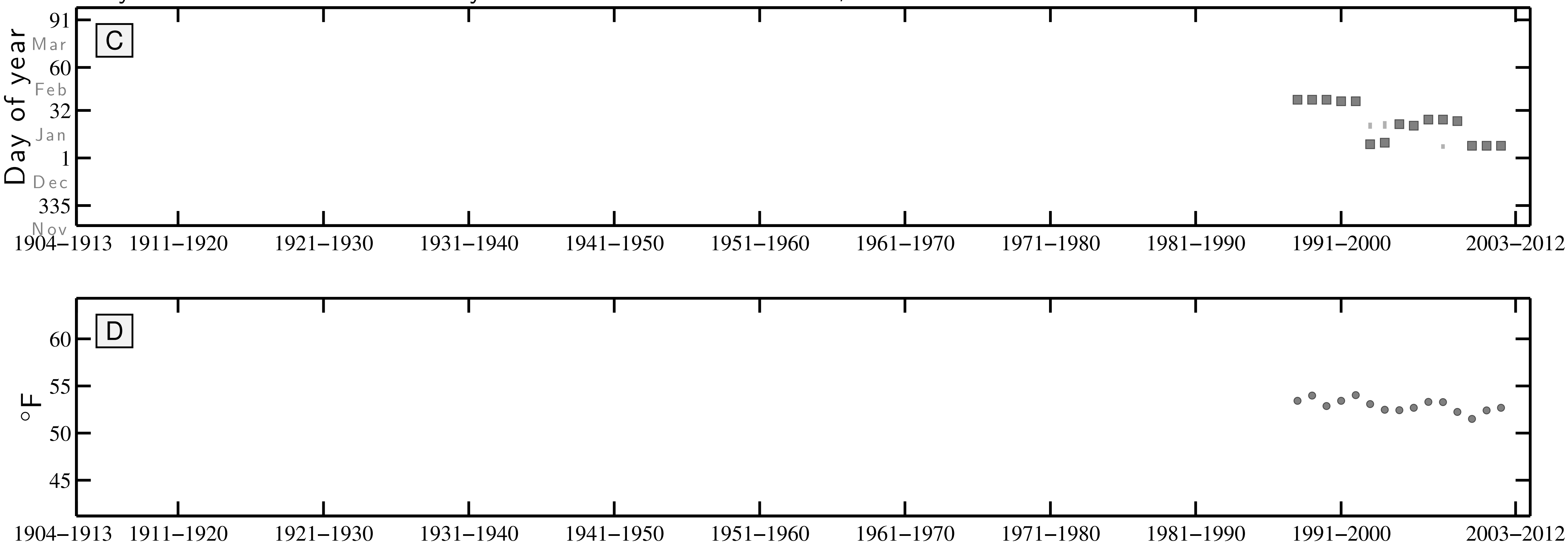

10 year Summer Teletherm dynamics for SAINT LEO, FL:

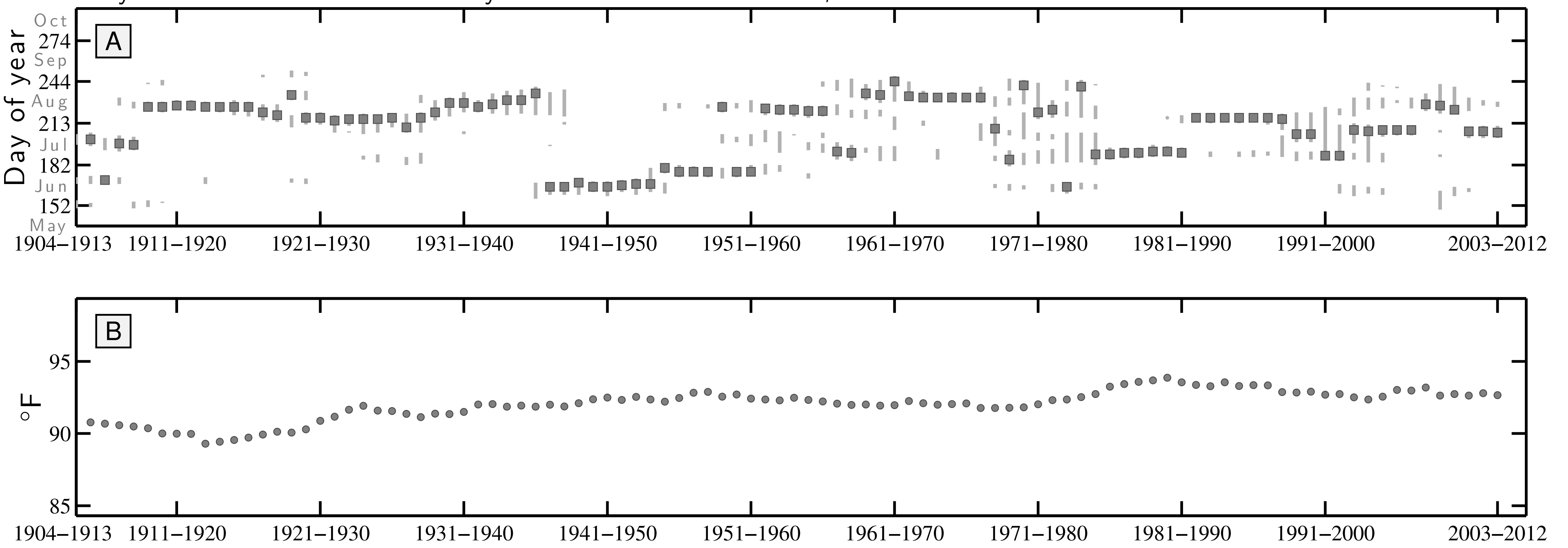

10 year Winter Teletherm dynamics for SAINT LEO, FL:

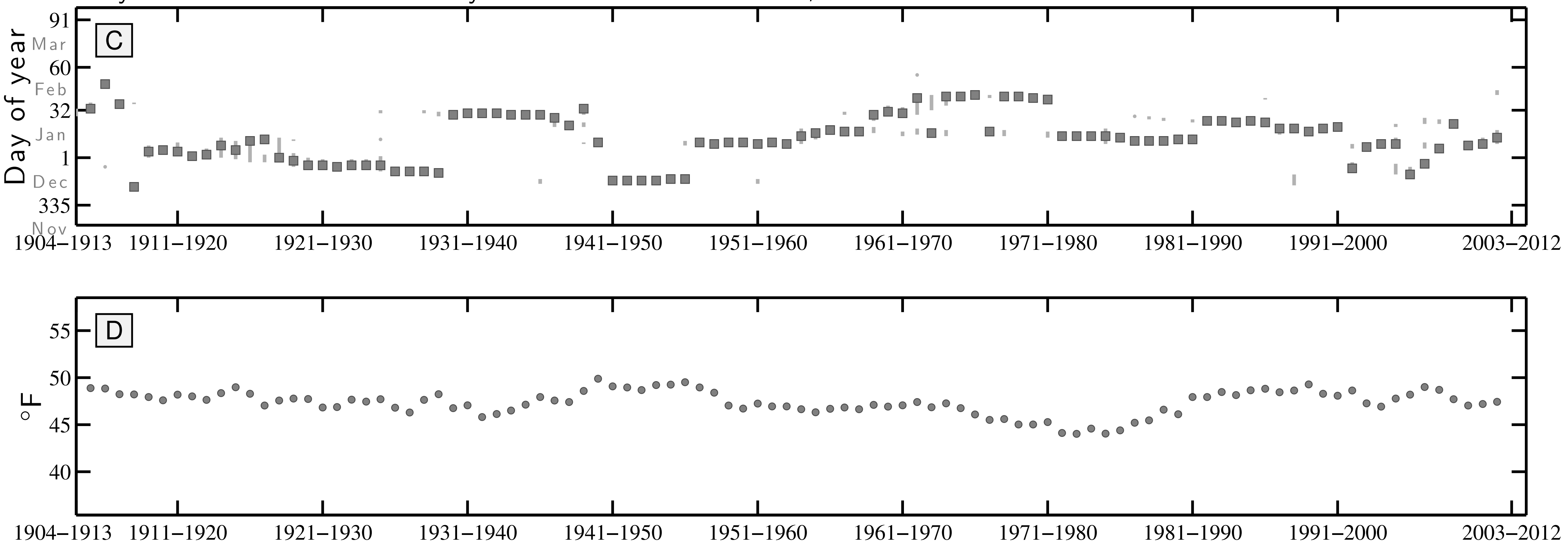

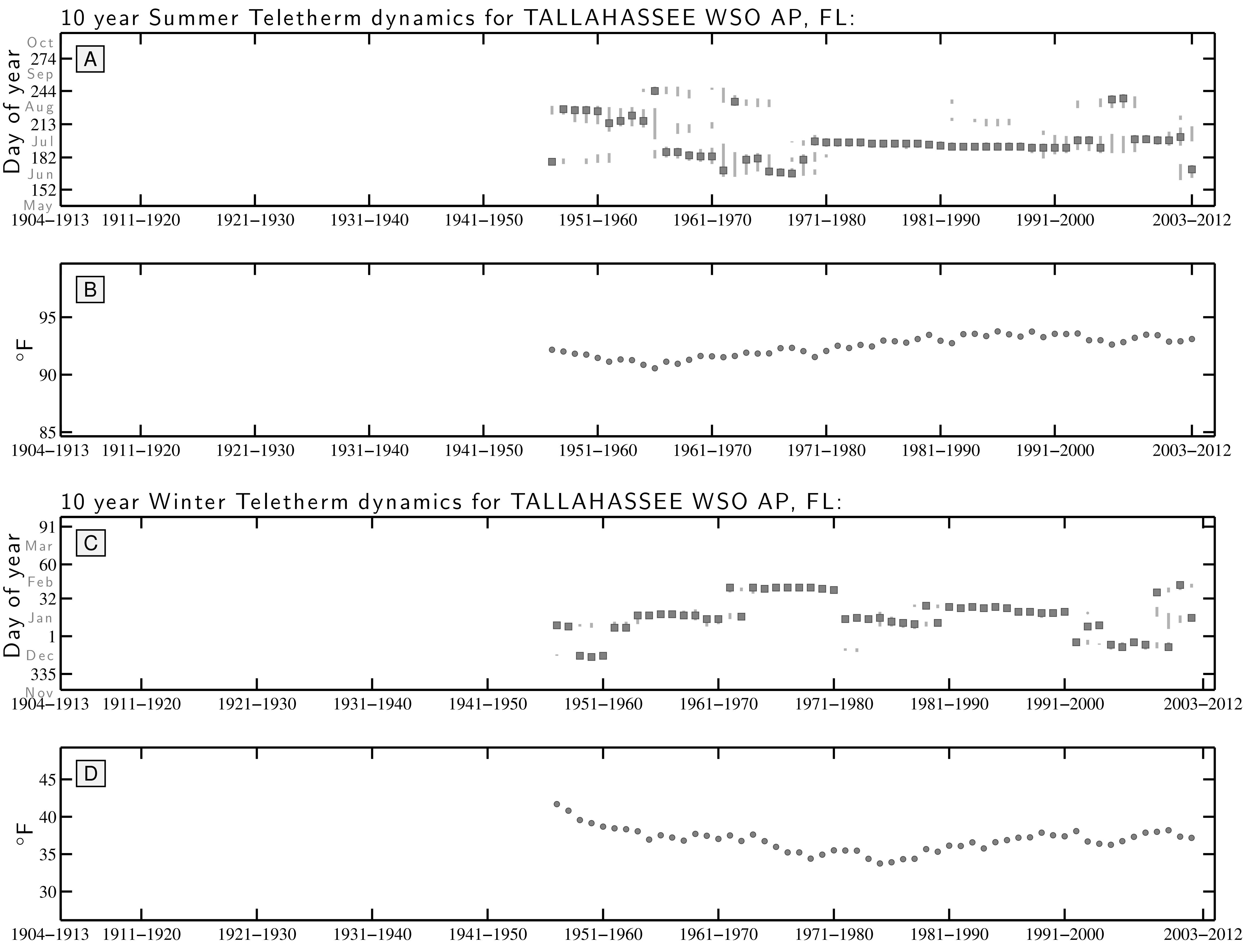
10 year Summer Teletherm dynamics for TALLAHASSEE WSO AP, FL:
A
B
Day of year
°F
10 year Winter Teletherm dynamics for TALLAHASSEE WSO AP, FL:
C
D
1904–1913
1911–1920
1921–1930
1931–1940
1941–1950
1951–1960
1961–1970
1971–1980
1981–1990
1991–2000
2003–2012

10 year Summer Teletherm dynamics for TARPON SPGS SEWAGE PL, FL:

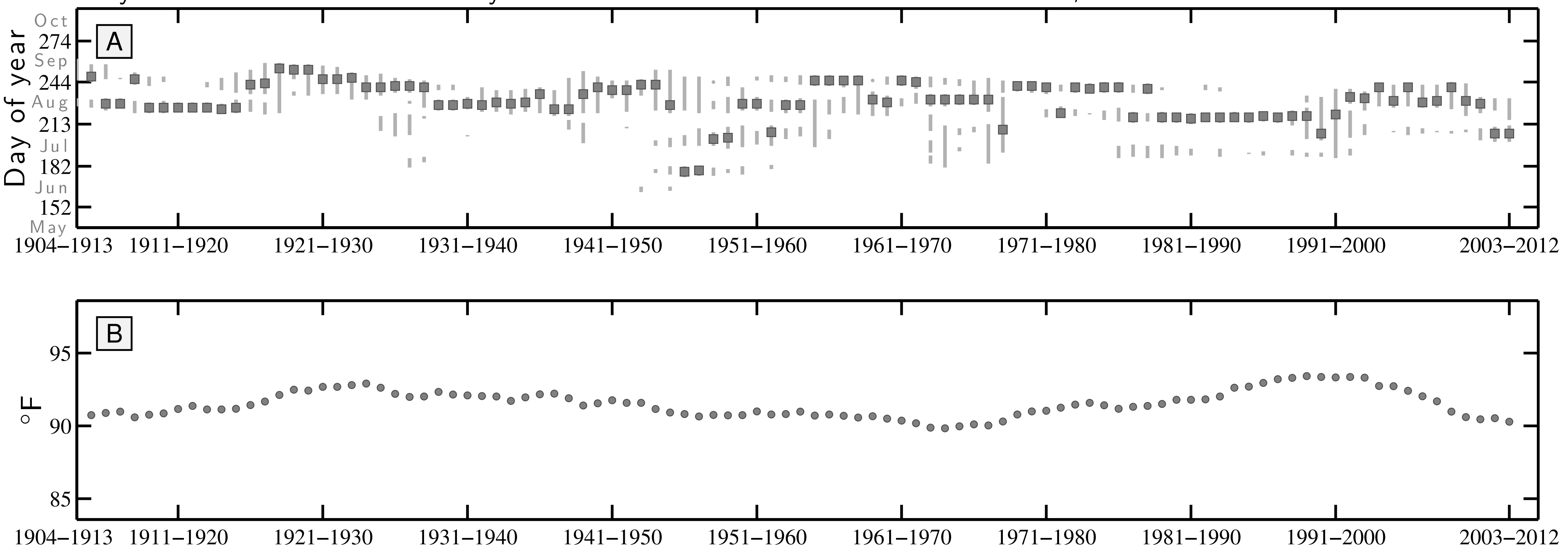

10 year Winter Teletherm dynamics for TARPON SPGS SEWAGE PL, FL:

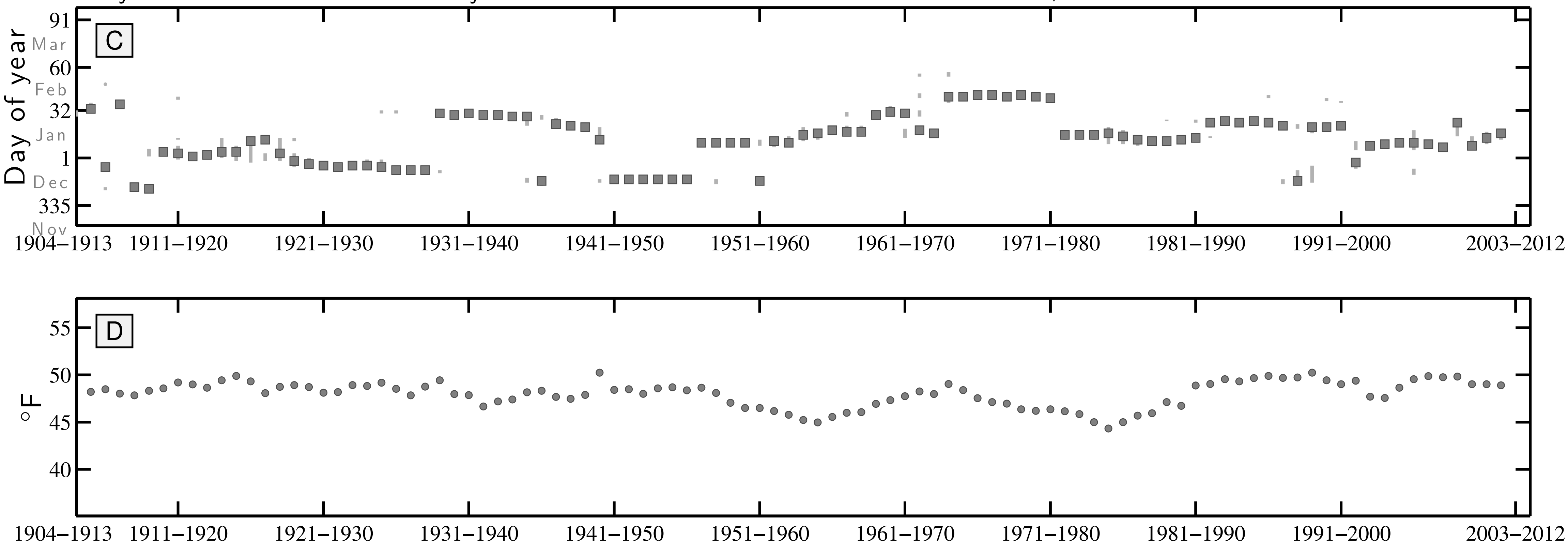

10 year Summer Teletherm dynamics for TITUSVILLE, FL:

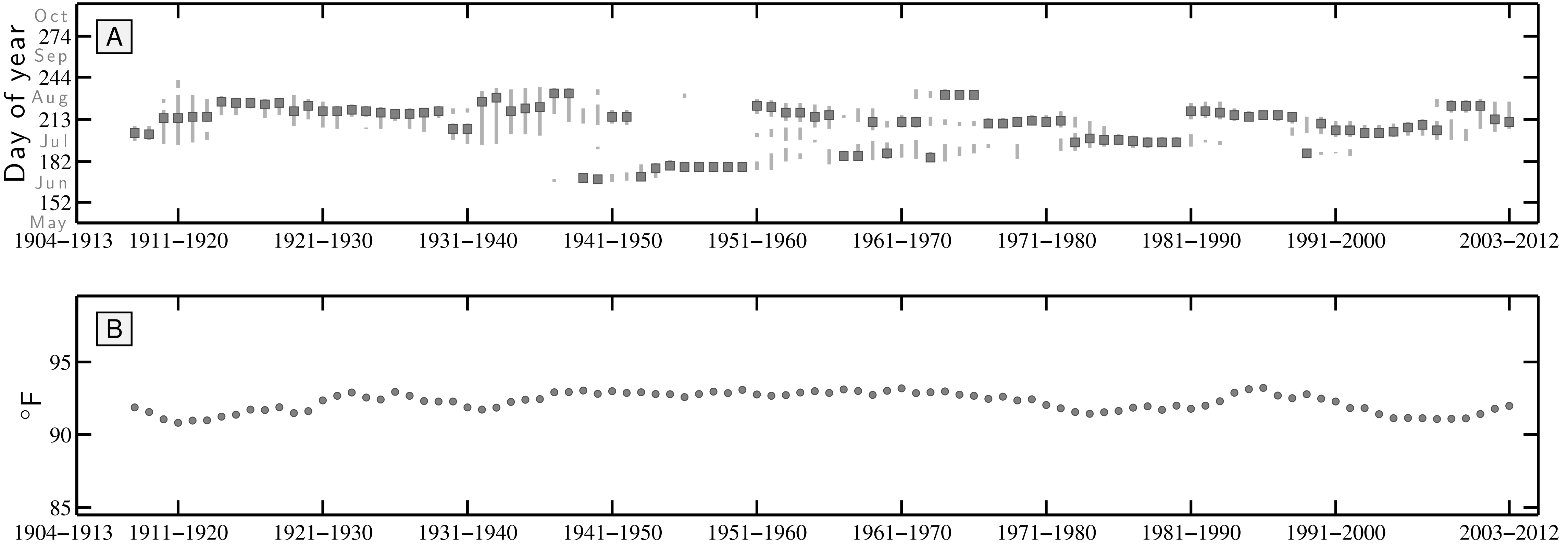

10 year Winter Teletherm dynamics for TITUSVILLE, FL:

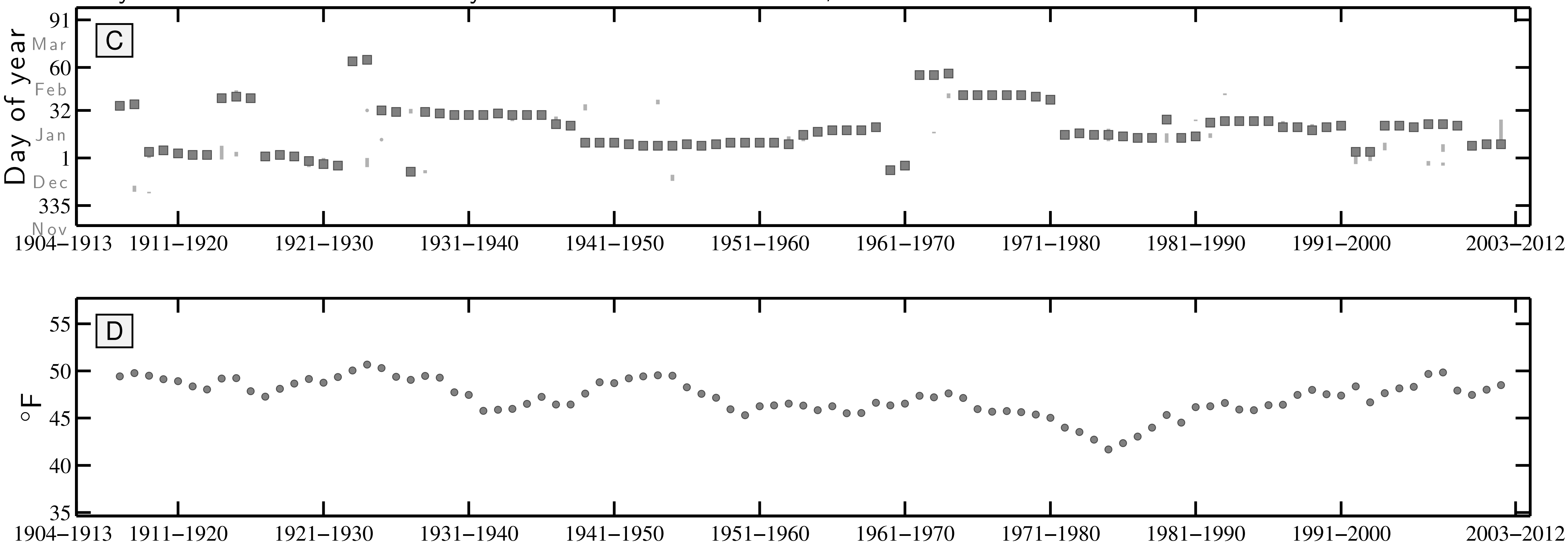

10 year Summer Teletherm dynamics for ALBANY 3 SE, GA:

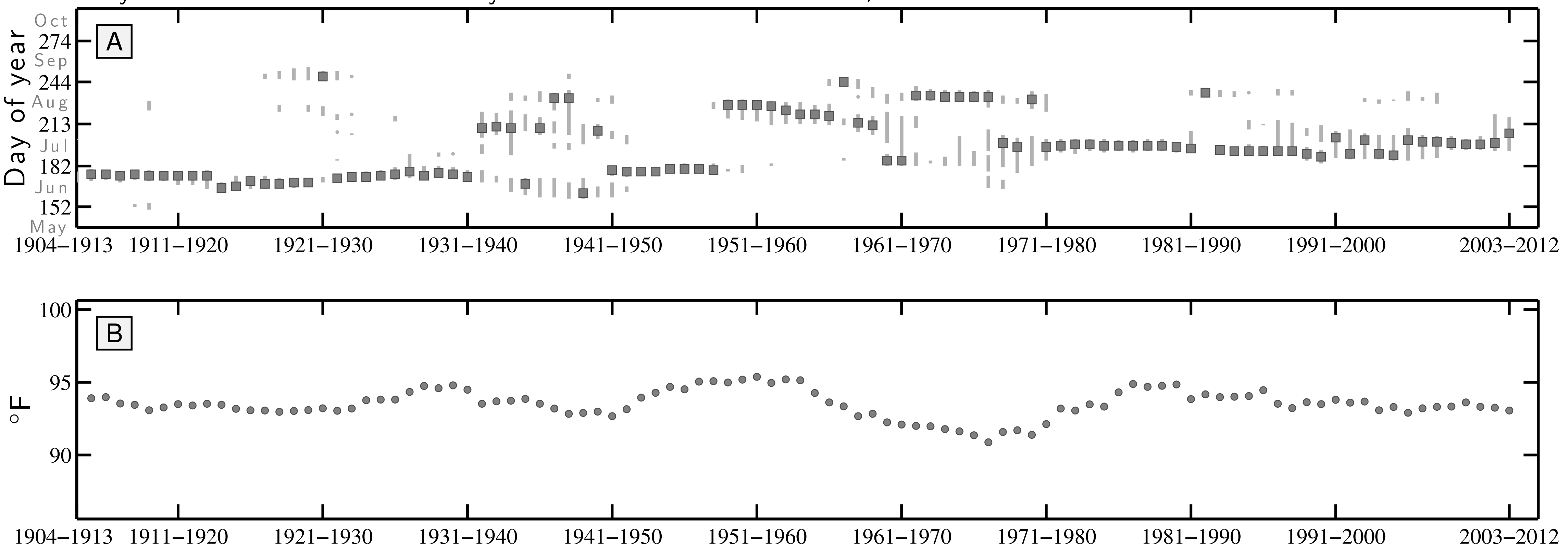

10 year Winter Teletherm dynamics for ALBANY 3 SE, GA:

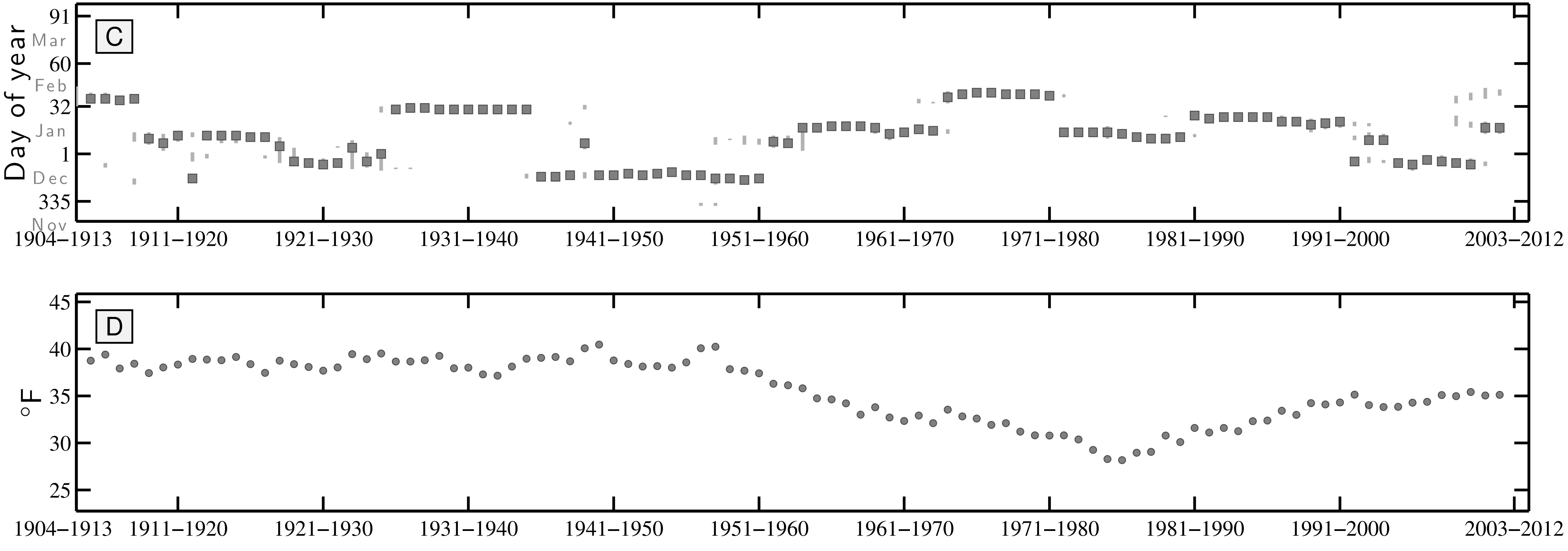

10 year Summer Teletherm dynamics for BAINBRIDGE INTL PAPER, GA:

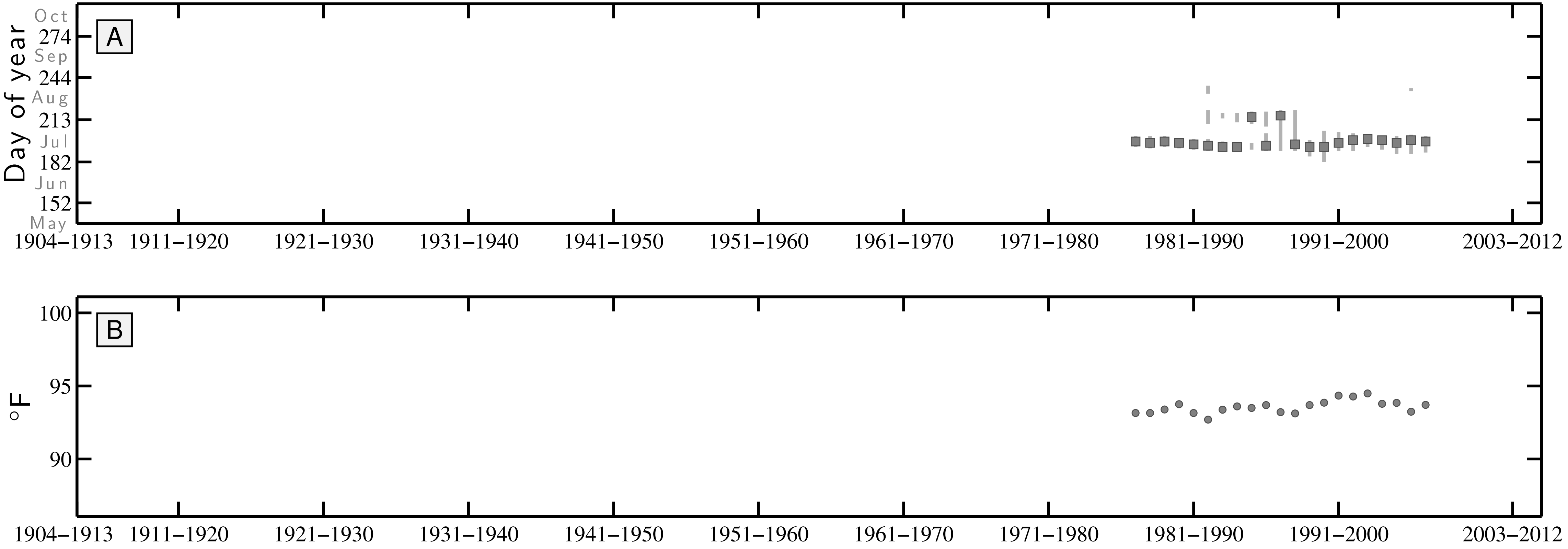

10 year Winter Teletherm dynamics for BAINBRIDGE INTL PAPER, GA:

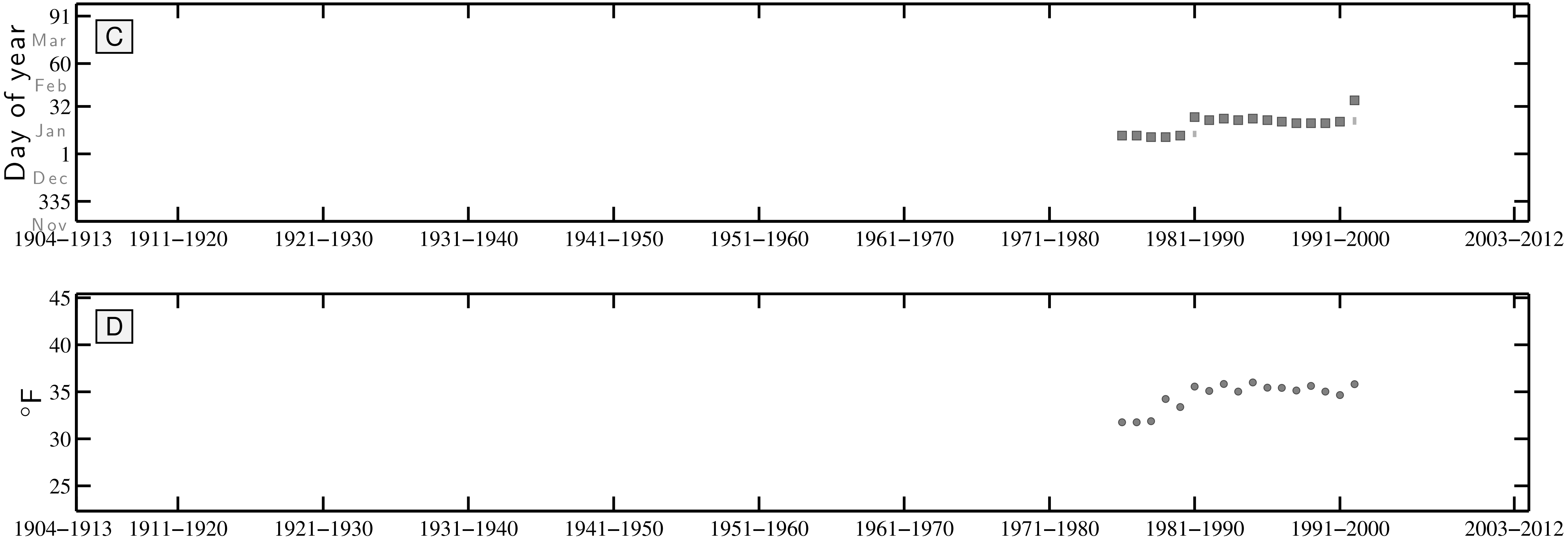

10 year Summer Teletherm dynamics for BRUNSWICK, GA:

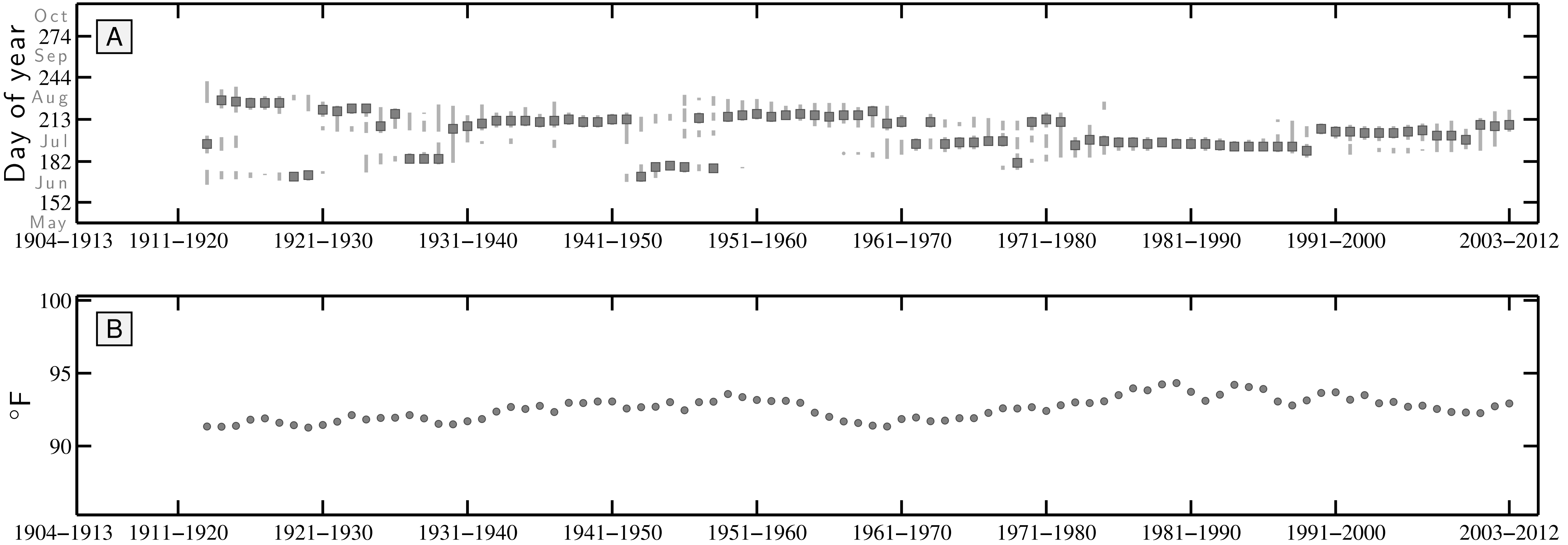

10 year Winter Teletherm dynamics for BRUNSWICK, GA:

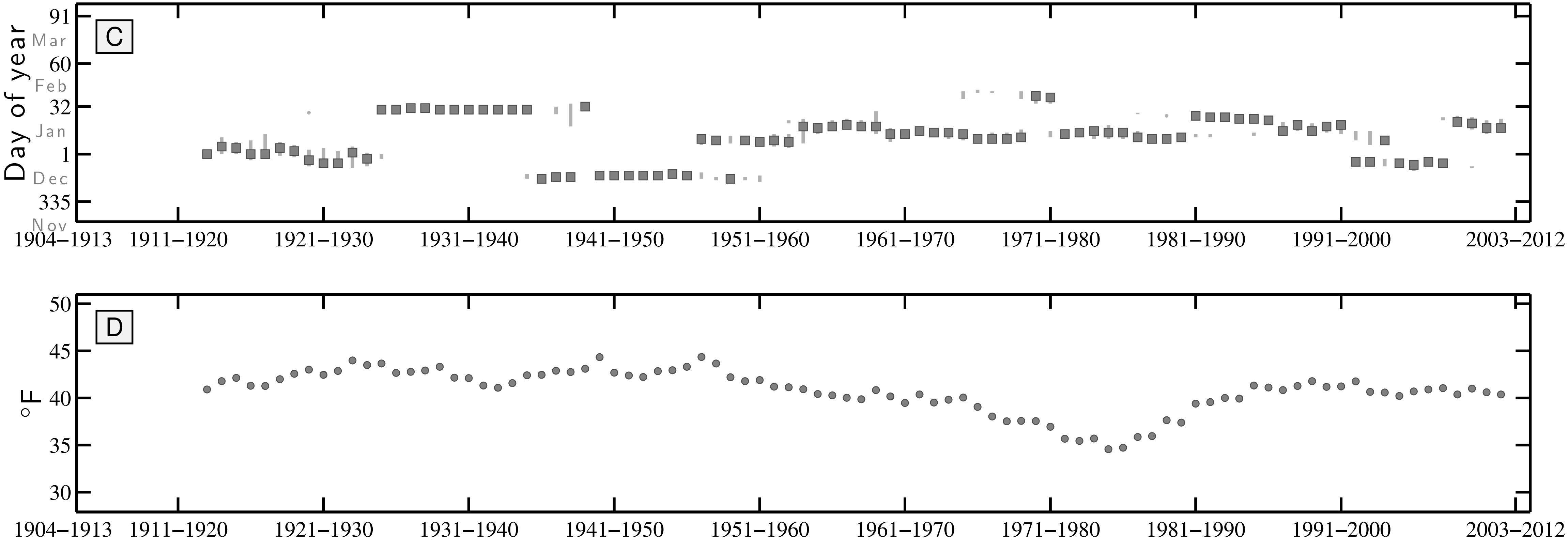

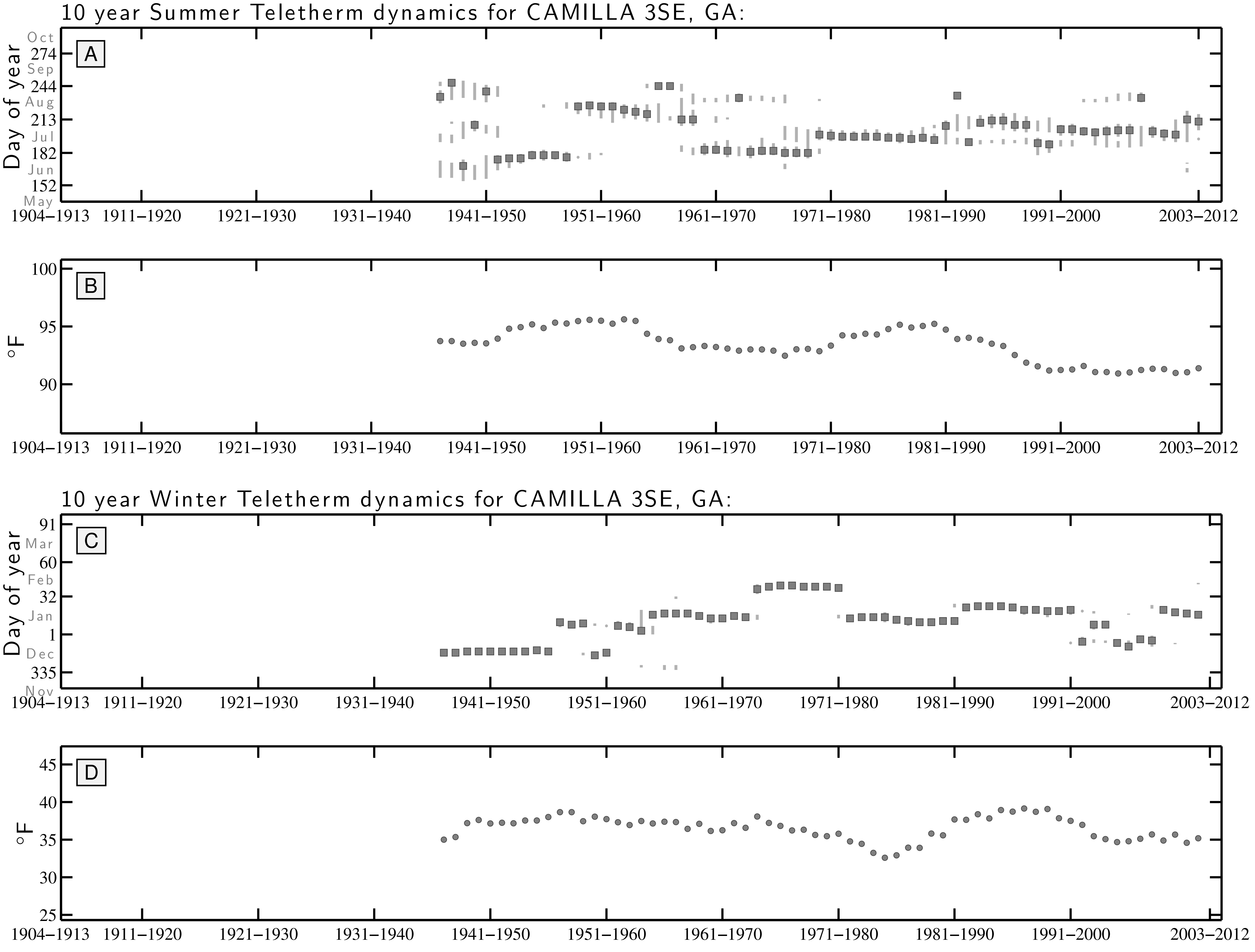
10 year Summer Teletherm dynamics for CAMILLA 3SE, GA:
A
B
Day of year
°F
Oct
274
Sep
244
Aug
213
Jul
182
Jun
152
May
100
95
90
10 year Winter Teletherm dynamics for CAMILLA 3SE, GA:
C
D
Day of year
°F
91
Mar
60
Feb
32
Jan
1
Dec
335
Nov
45
40
35
30
25
1904–1913
1911–1920
1921–1930
1931–1940
1941–1950
1951–1960
1961–1970
1971–1980
1981–1990
1991–2000
2003–2012

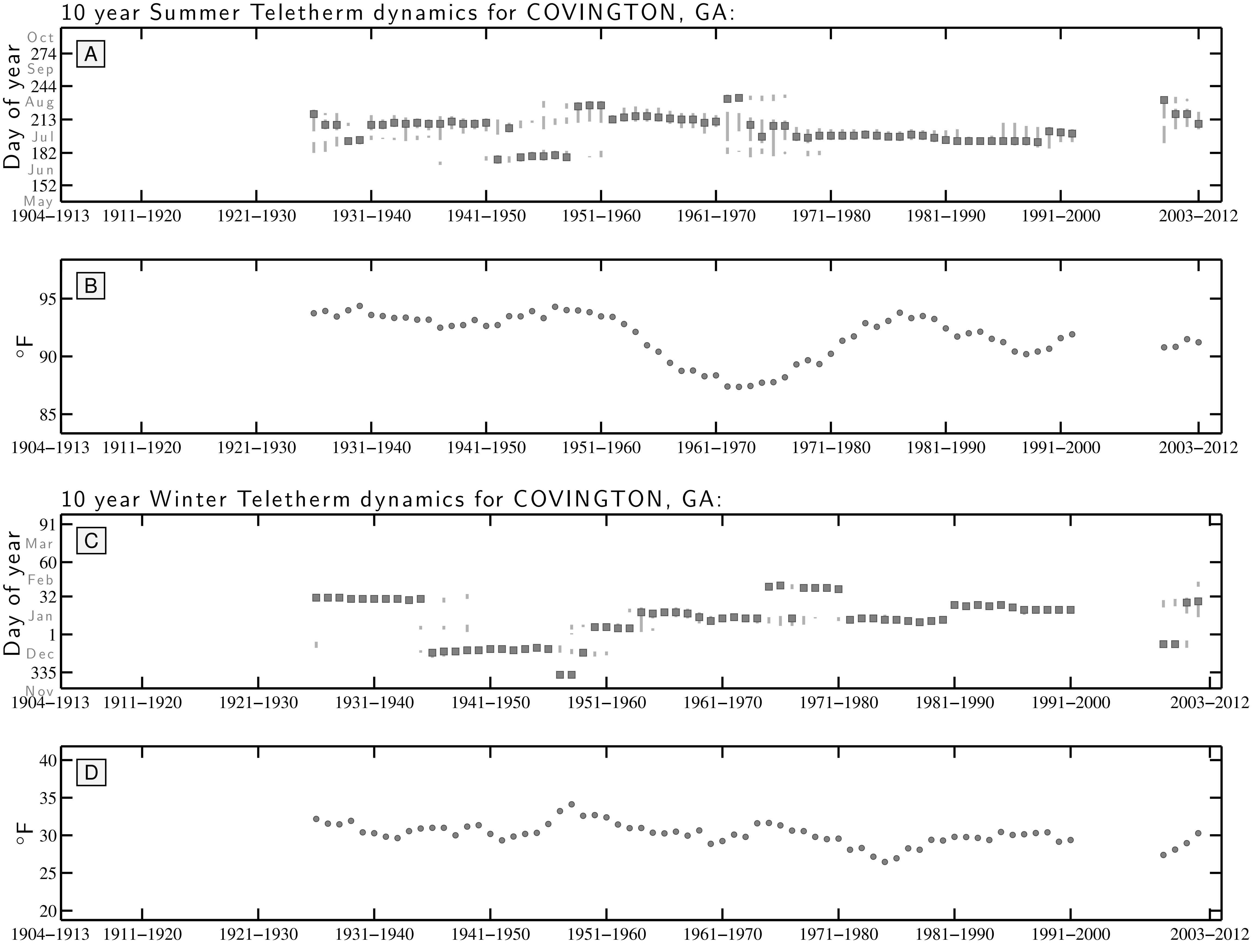
10 year Summer Teletherm dynamics for COVINGTON, GA:
A
Day of year
Oct
274
Sep
244
Aug
213
Jul
182
Jun
152
May
B
°F
95
90
85
10 year Winter Teletherm dynamics for COVINGTON, GA:
C
Day of year
91
Mar
60
Feb
32
Jan
1
Dec
335
Nov
D
°F
40
35
30
25
20
1904–1913
1911–1920
1921–1930
1931–1940
1941–1950
1951–1960
1961–1970
1971–1980
1981–1990
1991–2000
2003–2012

10 year Summer Teletherm dynamics for DAHLONEGA, GA:

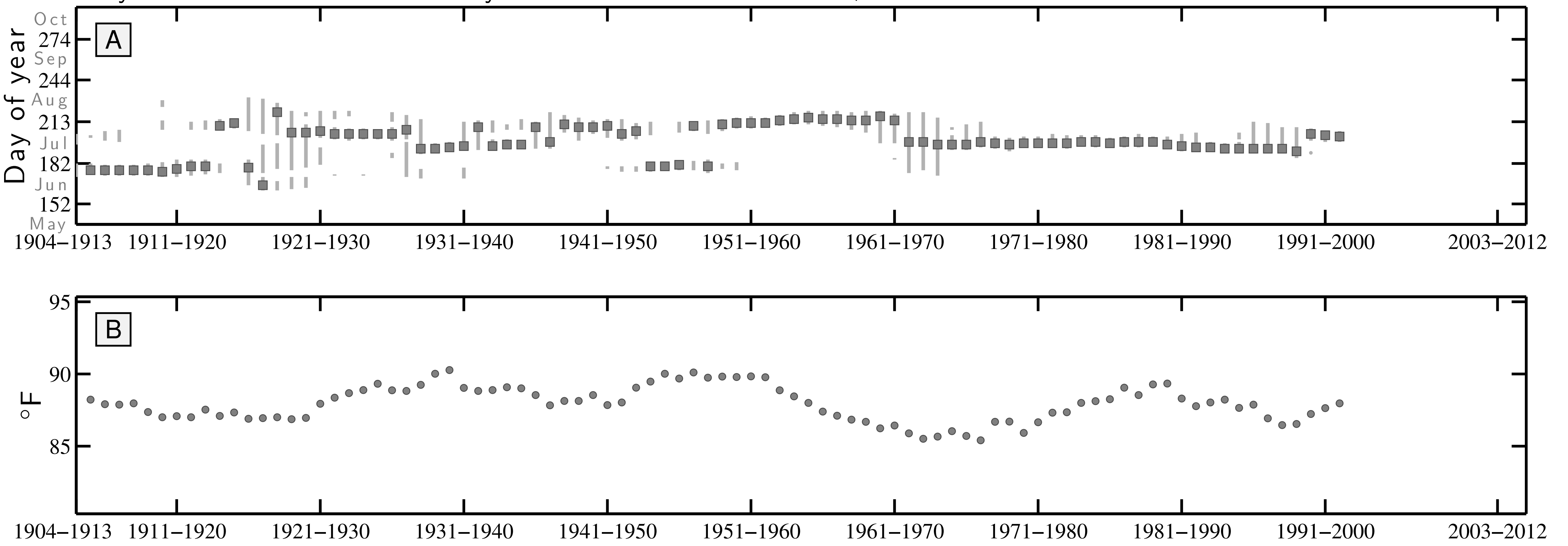

10 year Winter Teletherm dynamics for DAHLONEGA, GA:

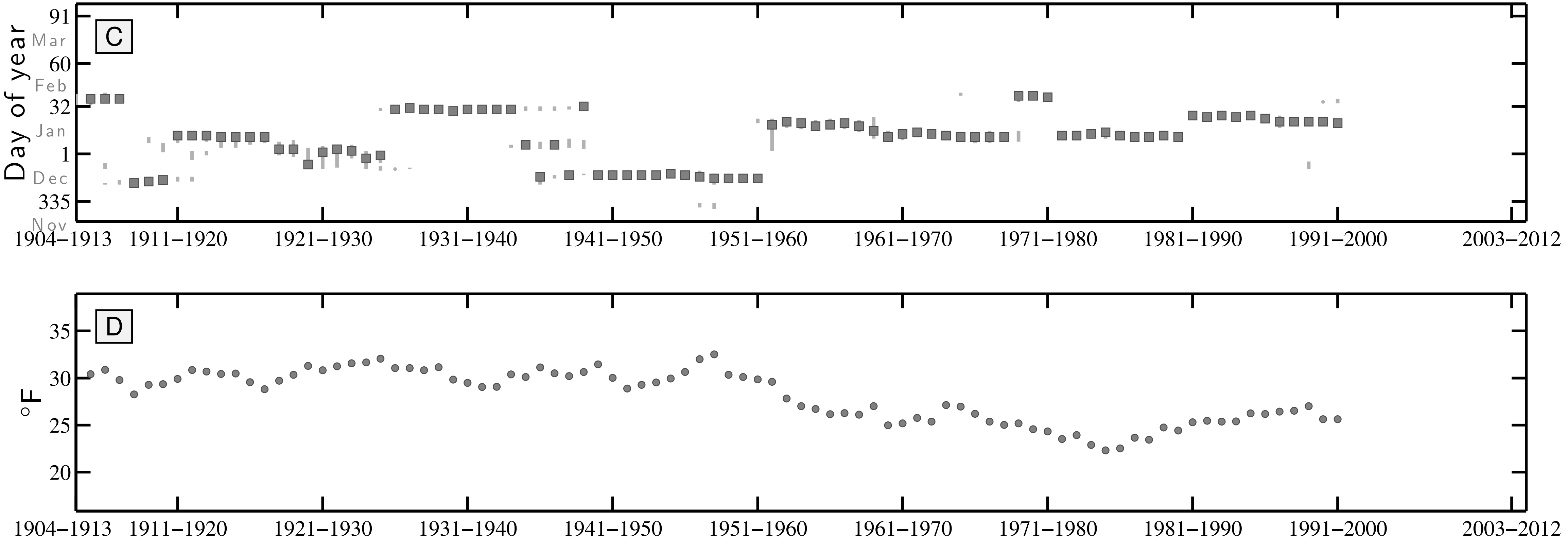

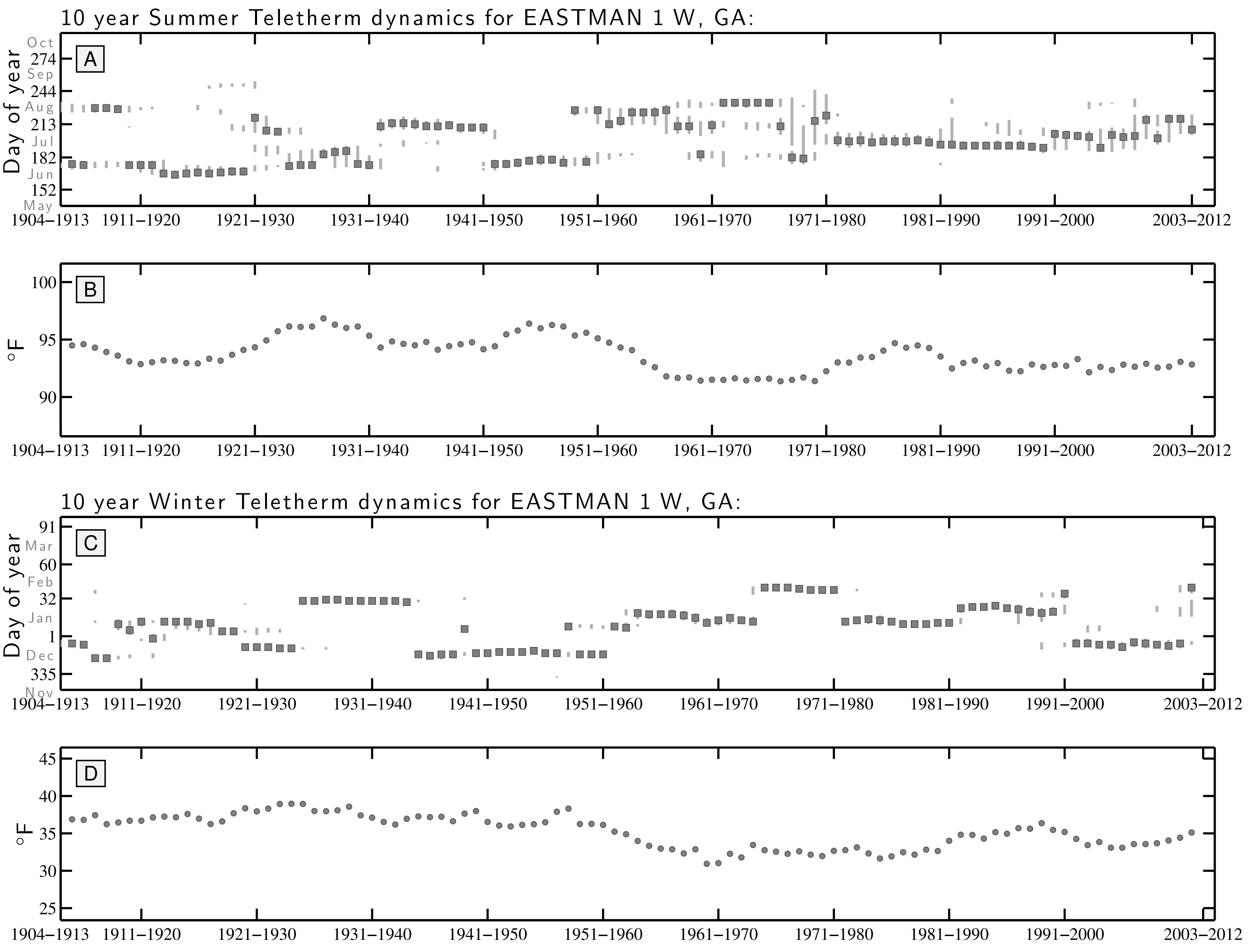
10 year Summer Teletherm dynamics for EASTMAN 1 W, GA:
A
B
Day of year
°F
Oct
274
Sep
244
Aug
213
Jul
182
Jun
152
May
100
95
90
1904–1913
1911–1920
1921–1930
1931–1940
1941–1950
1951–1960
1961–1970
1971–1980
1981–1990
1991–2000
2003–2012
10 year Winter Teletherm dynamics for EASTMAN 1 W, GA:
C
D
91
Mar
60
Feb
32
Jan
1
Dec
335
Nov
45
40
35
30
25

10 year Summer Teletherm dynamics for GAINESVILLE, GA:

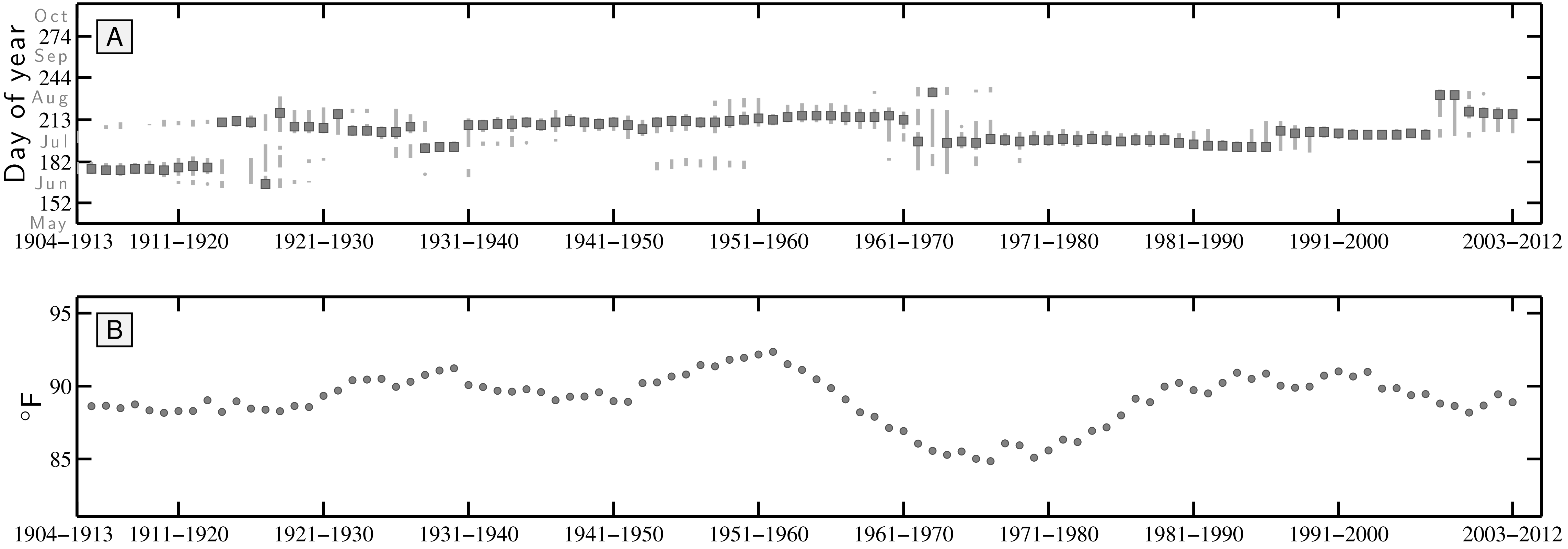

10 year Winter Teletherm dynamics for GAINESVILLE, GA:

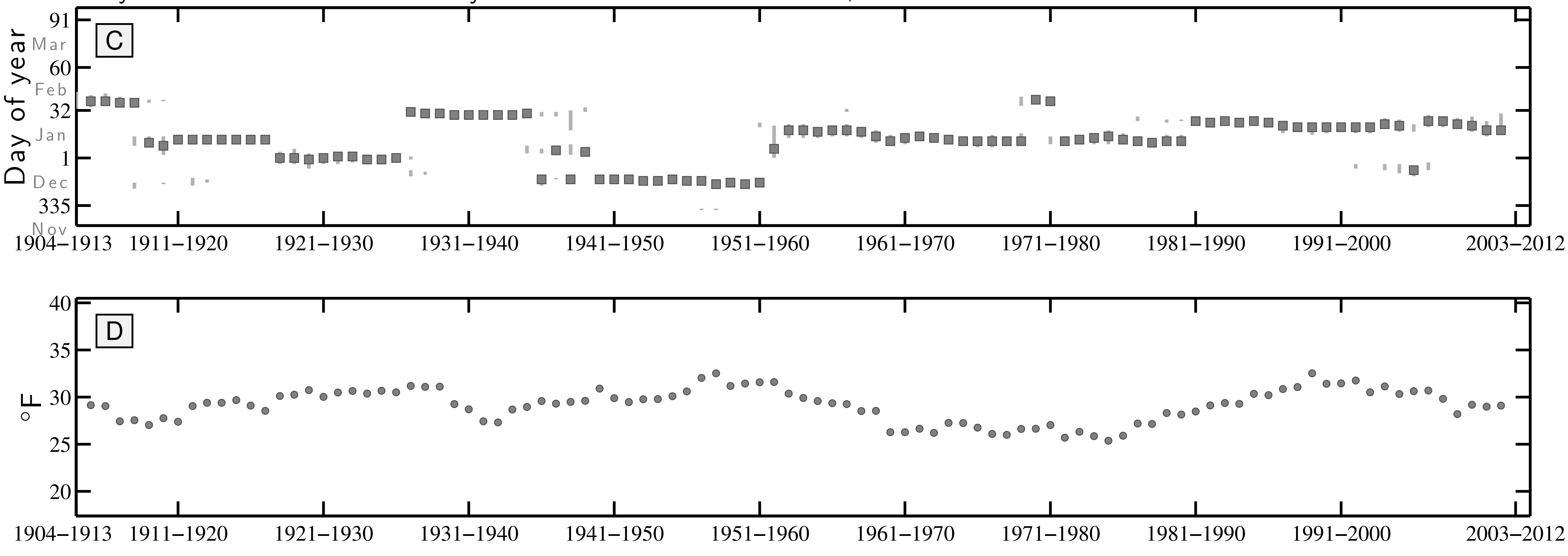

10 year Summer Teletherm dynamics for GLENNVILLE 3NW, GA:

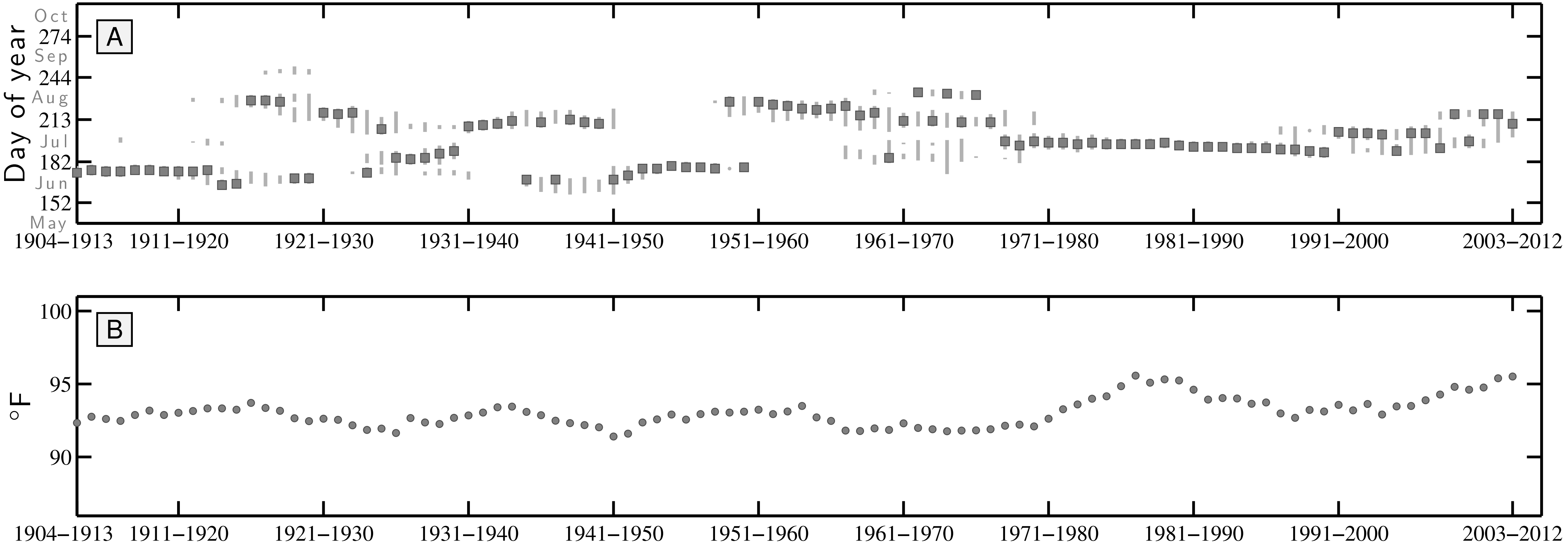

10 year Winter Teletherm dynamics for GLENNVILLE 3NW, GA:

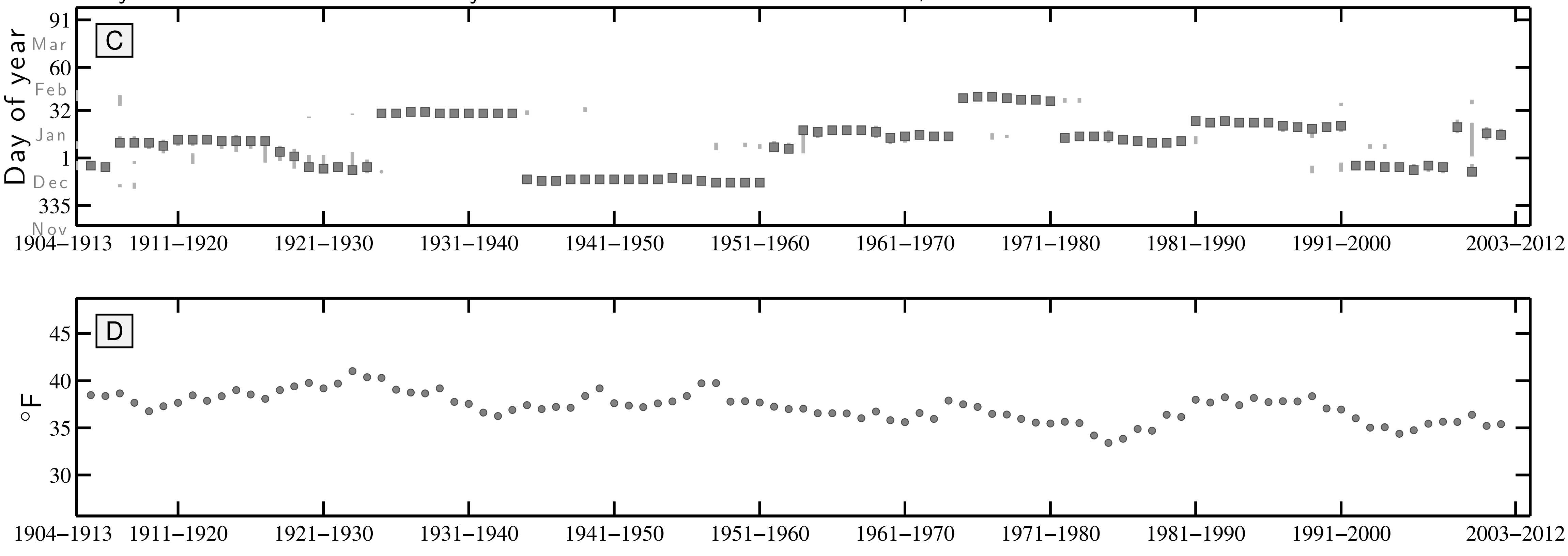

10 year Summer Teletherm dynamics for HAWKINSVILLE, GA:

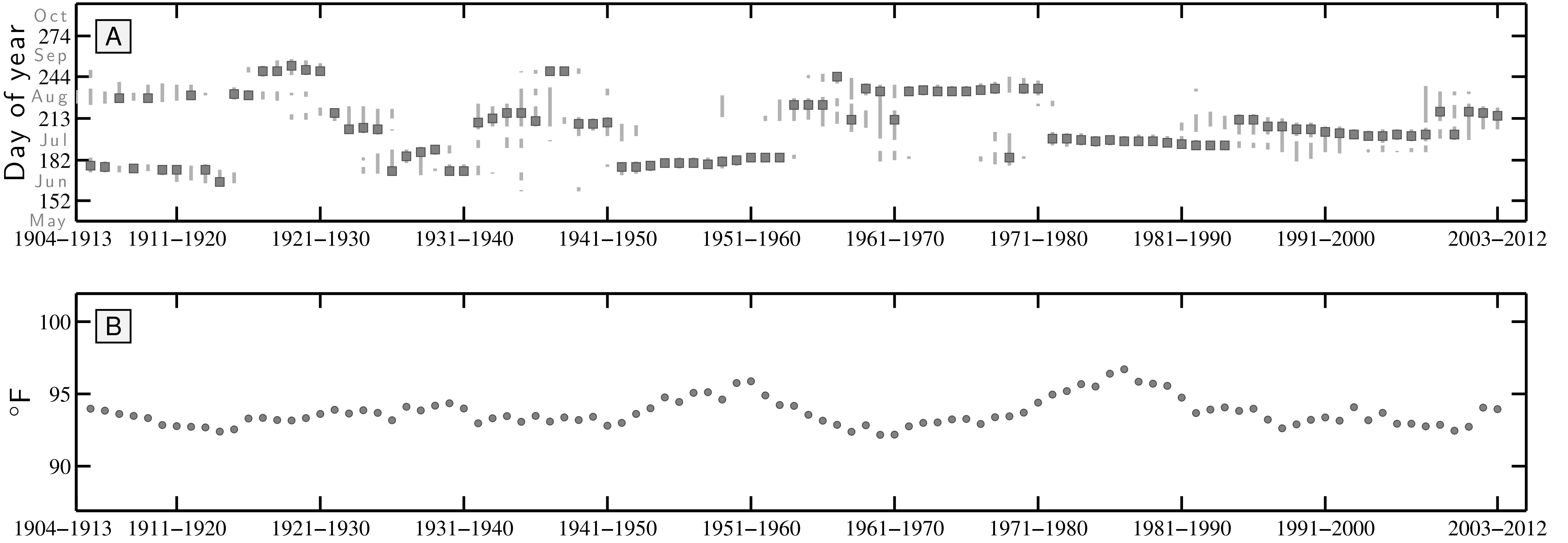

10 year Winter Teletherm dynamics for HAWKINSVILLE, GA:

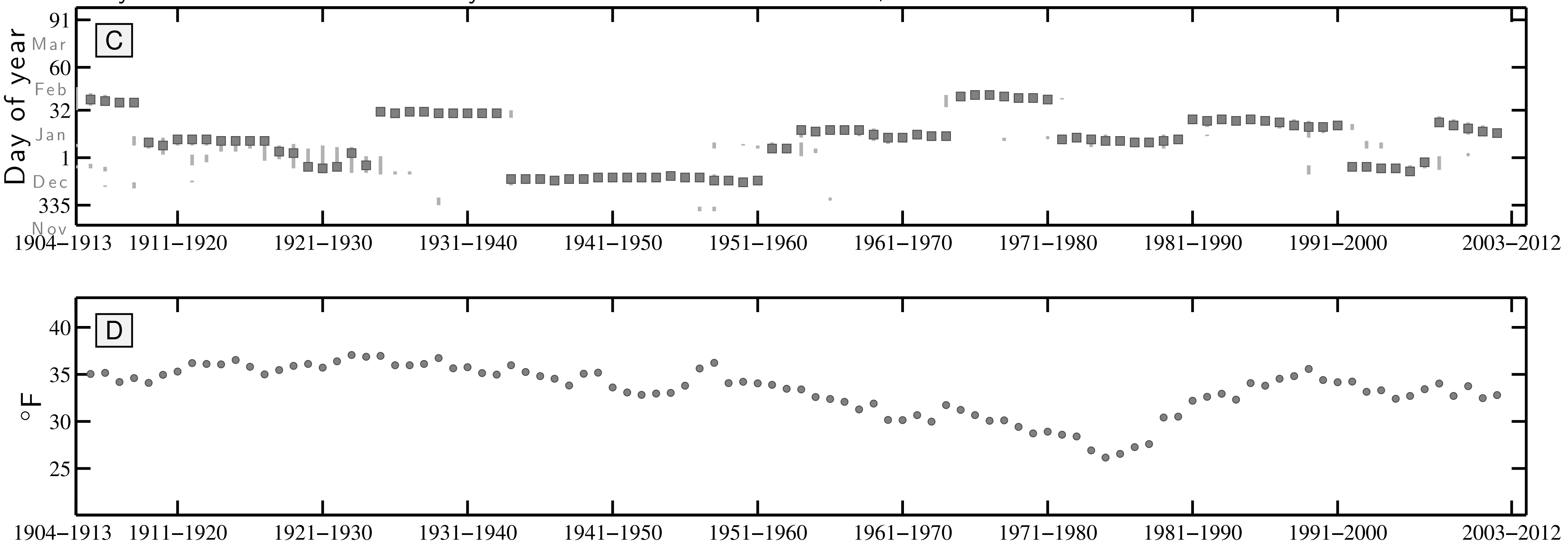

10 year Summer Teletherm dynamics for MILLEDGEVILLE, GA:

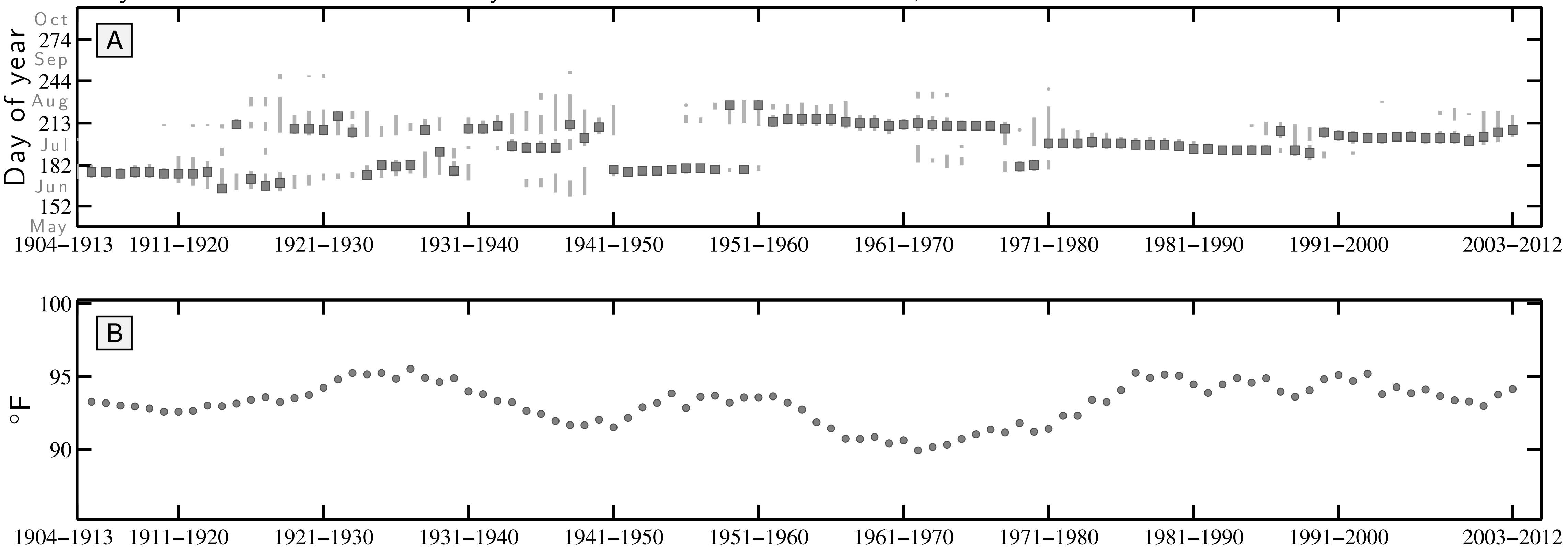

10 year Winter Teletherm dynamics for MILLEDGEVILLE, GA:

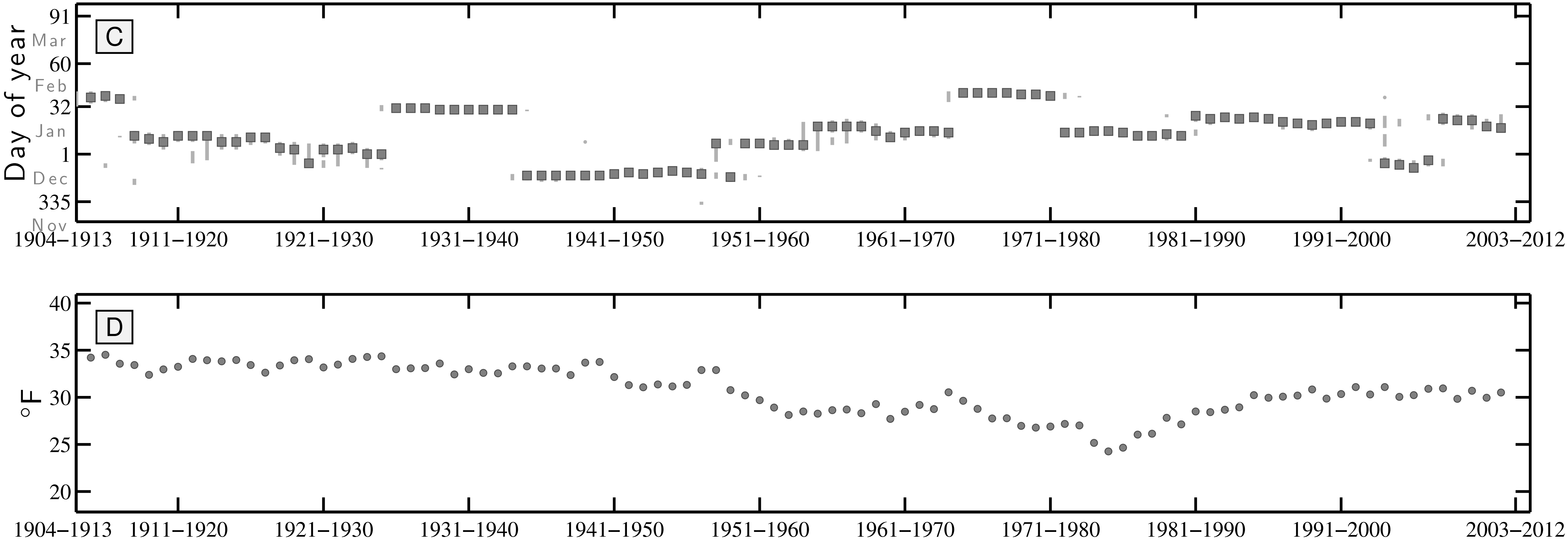

10 year Summer Teletherm dynamics for MILLEN 4 N, GA:

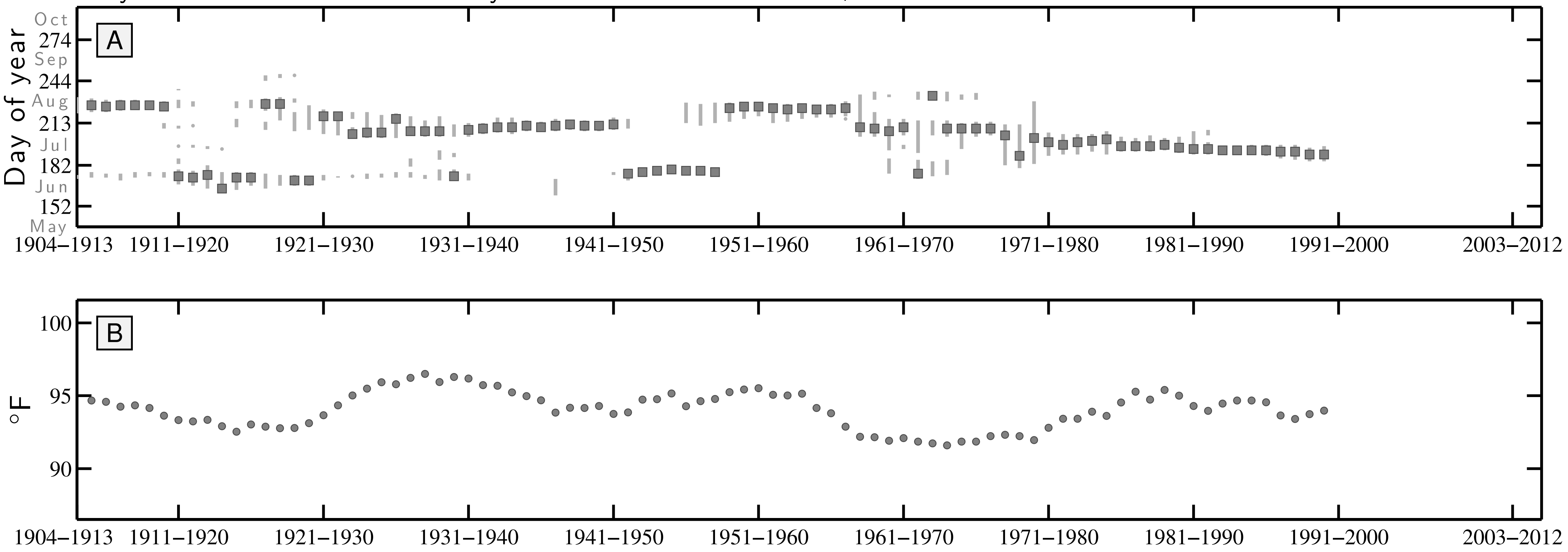

10 year Winter Teletherm dynamics for MILLEN 4 N, GA:

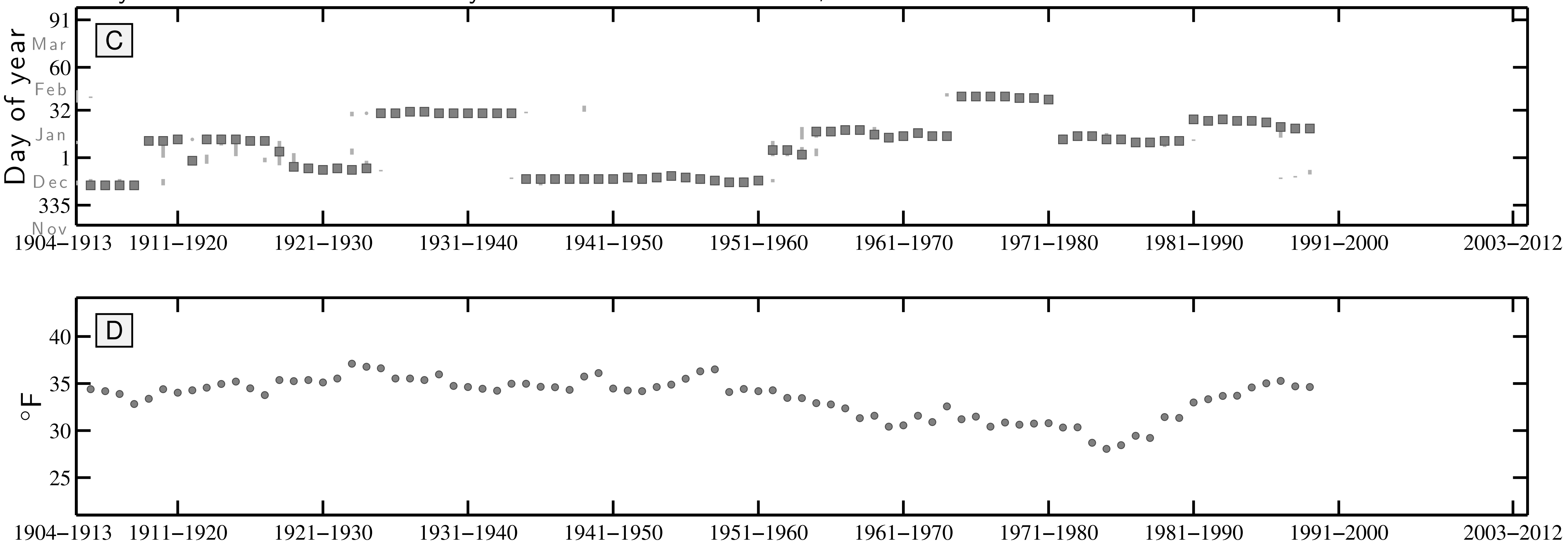

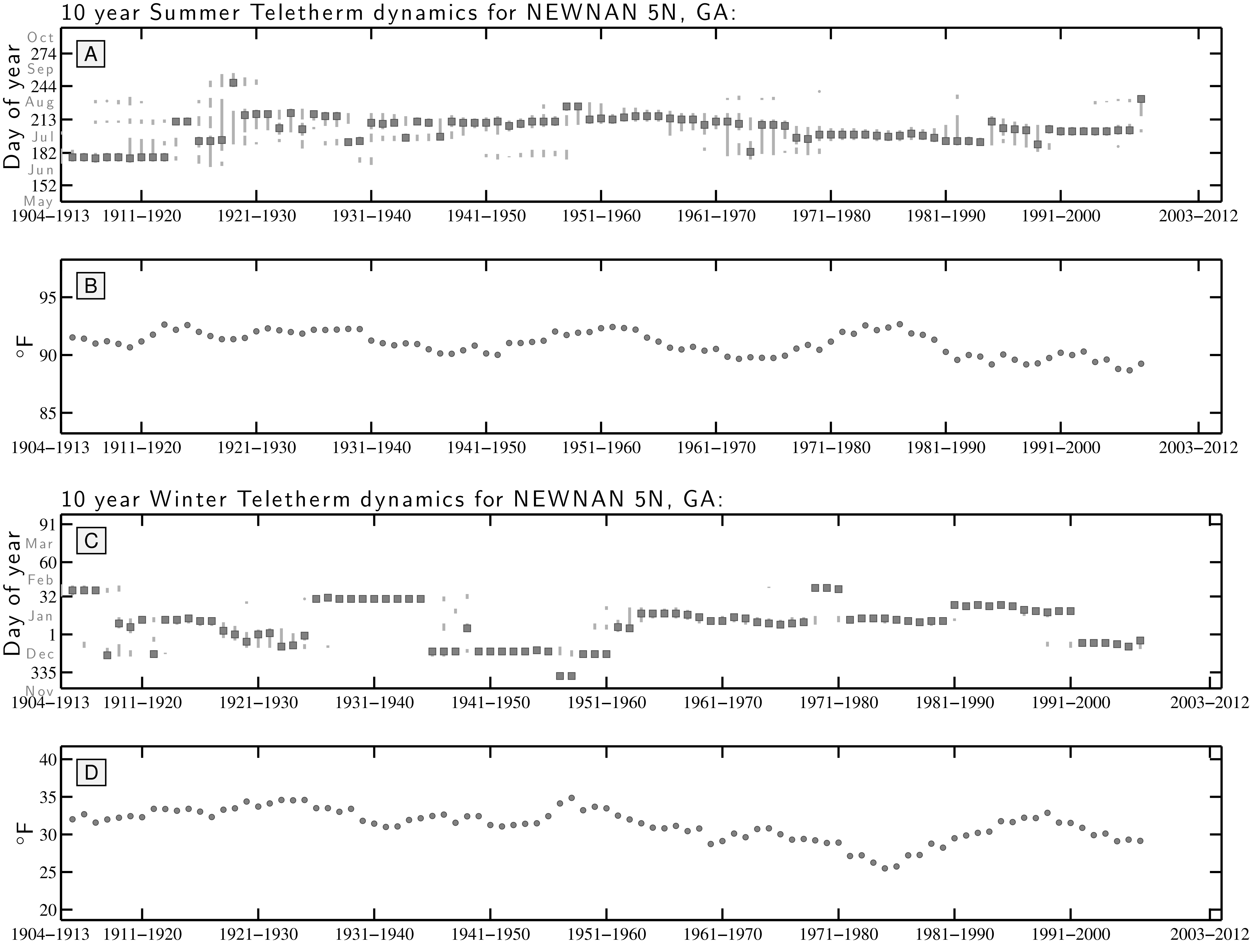
10 year Summer Teletherm dynamics for NEWNAN 5N, GA:
A
Day of year
Oct
274
Sep
244
Aug
213
Jul
182
Jun
152
May
B
°F
95
90
85
10 year Winter Teletherm dynamics for NEWNAN 5N, GA:
C
Day of year
91
Mar
60
Feb
32
Jan
1
Dec
335
Nov
D
°F
40
35
30
25
20
1904–1913
1911–1920
1921–1930
1931–1940
1941–1950
1951–1960
1961–1970
1971–1980
1981–1990
1991–2000
2003–2012

10 year Summer Teletherm dynamics for QUITMAN 2 NW, GA:

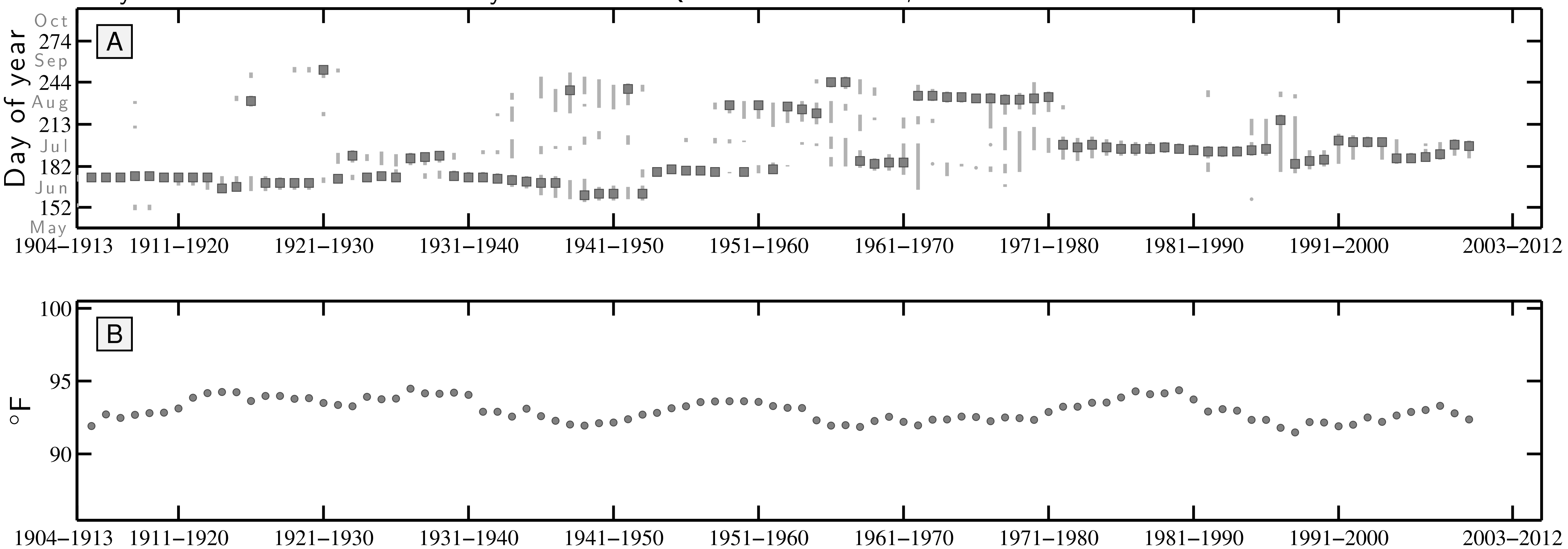

10 year Winter Teletherm dynamics for QUITMAN 2 NW, GA:

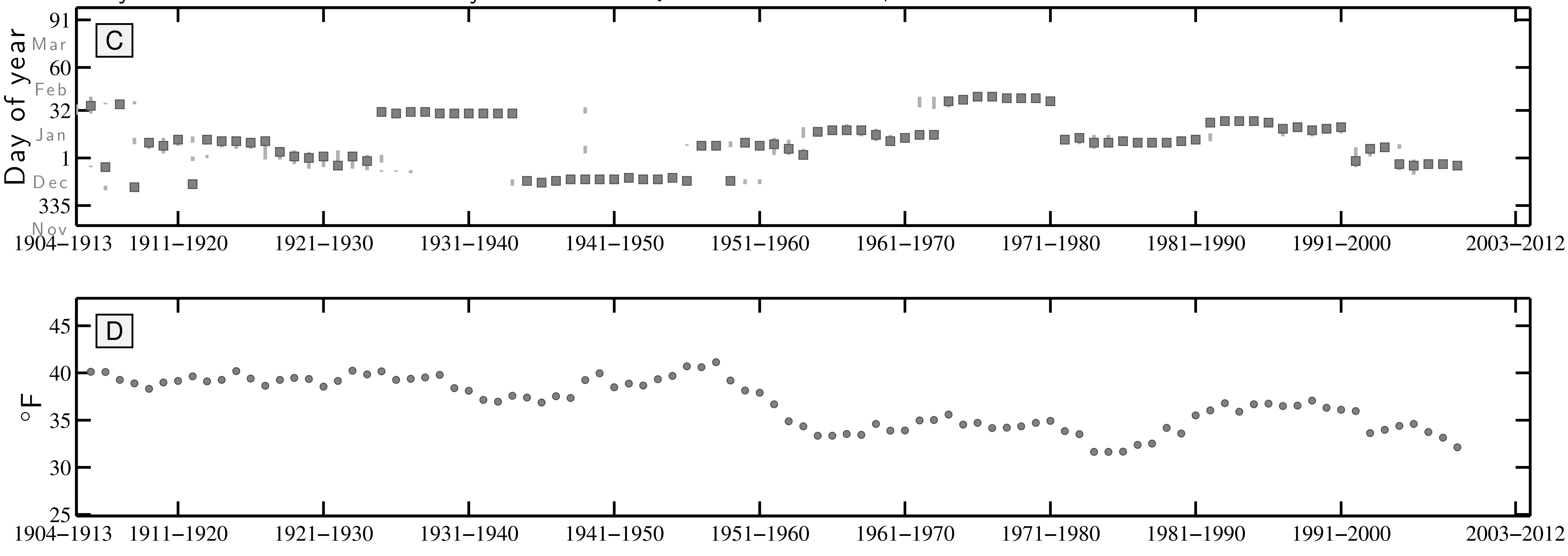

10 year Summer Teletherm dynamics for ROME, GA:

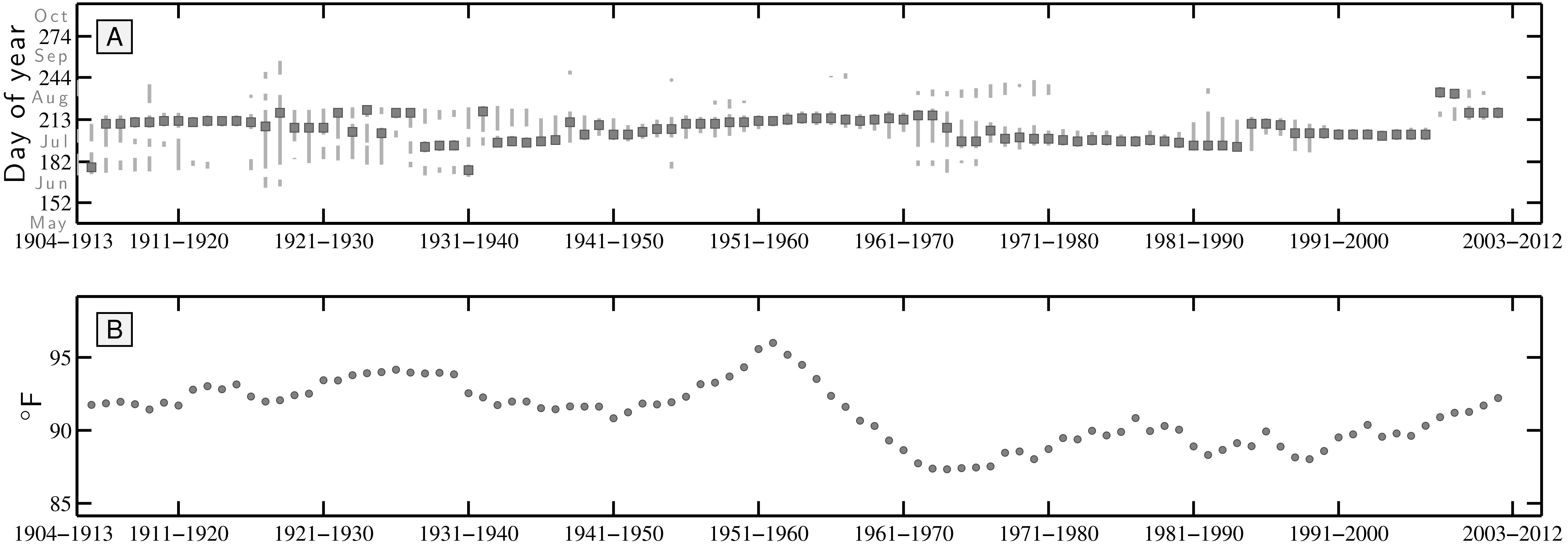

10 year Winter Teletherm dynamics for ROME, GA:

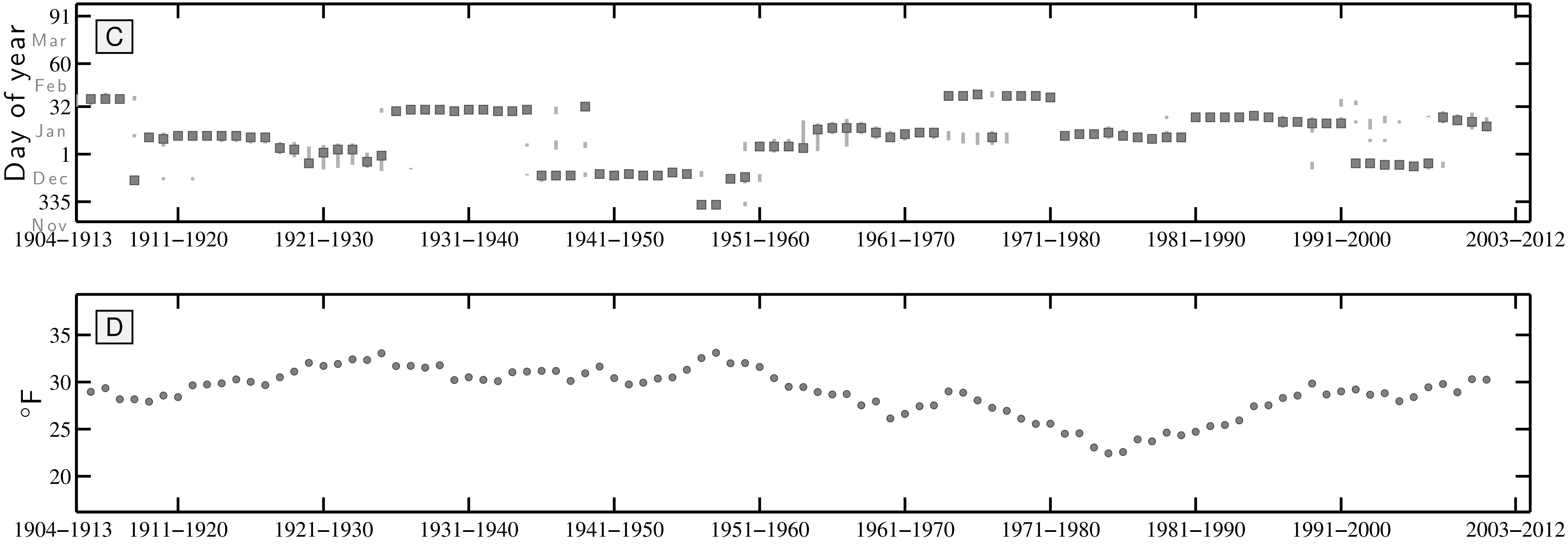

10 year Summer Teletherm dynamics for SAVANNAH INTL AP, GA:

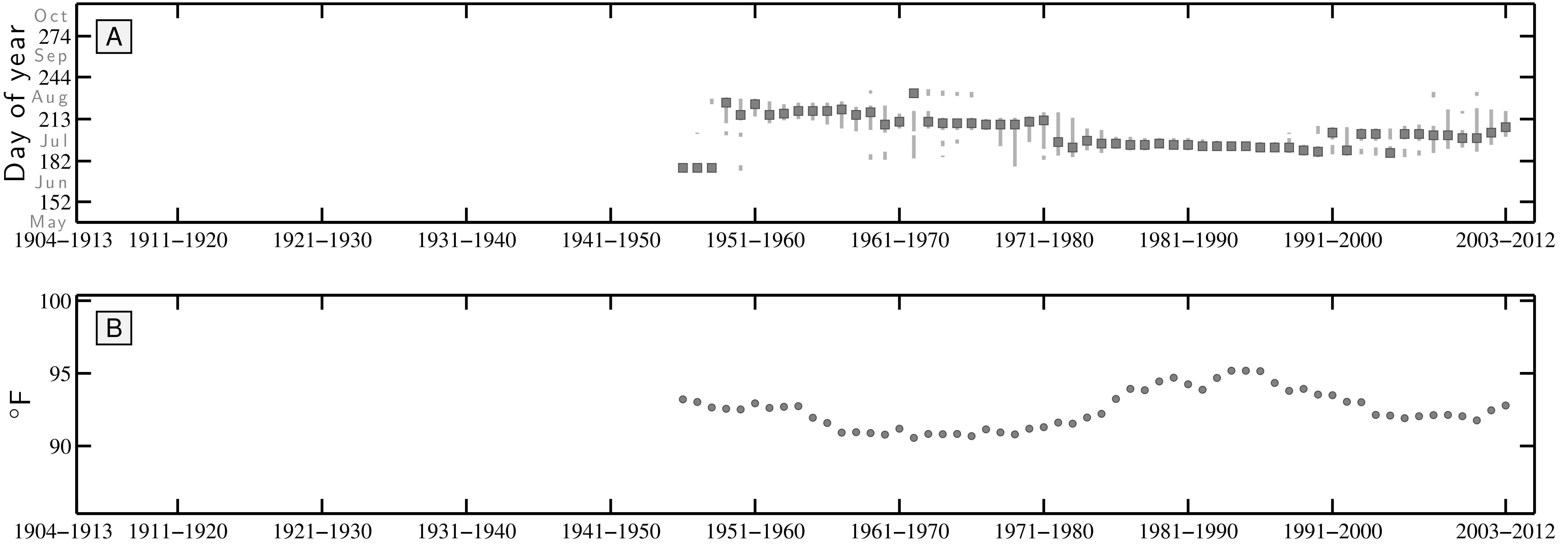

10 year Winter Teletherm dynamics for SAVANNAH INTL AP, GA:

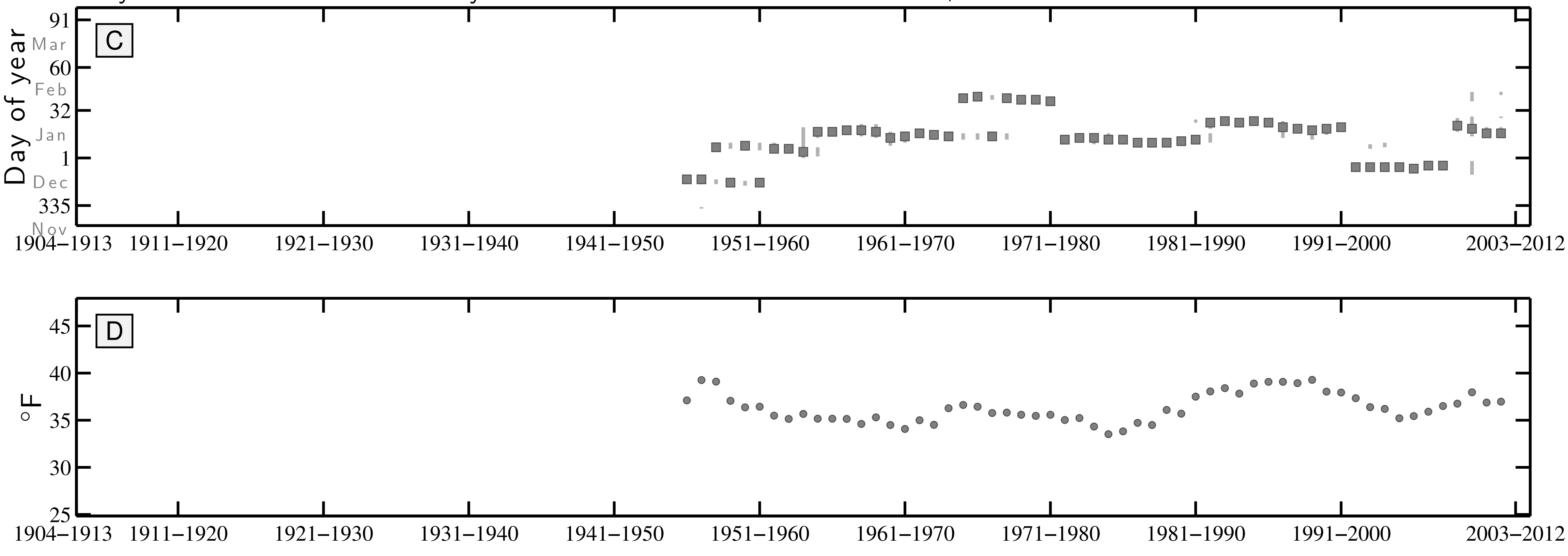

10 year Summer Teletherm dynamics for TALBOTTON, GA:

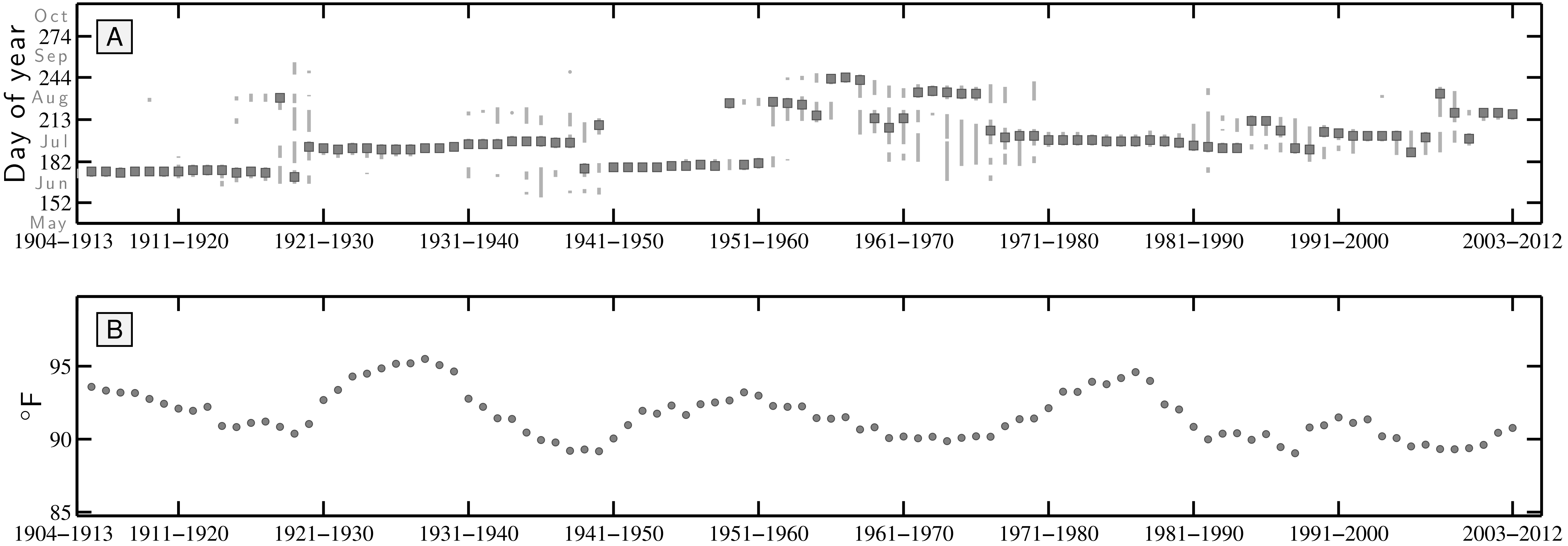

10 year Winter Teletherm dynamics for TALBOTTON, GA:

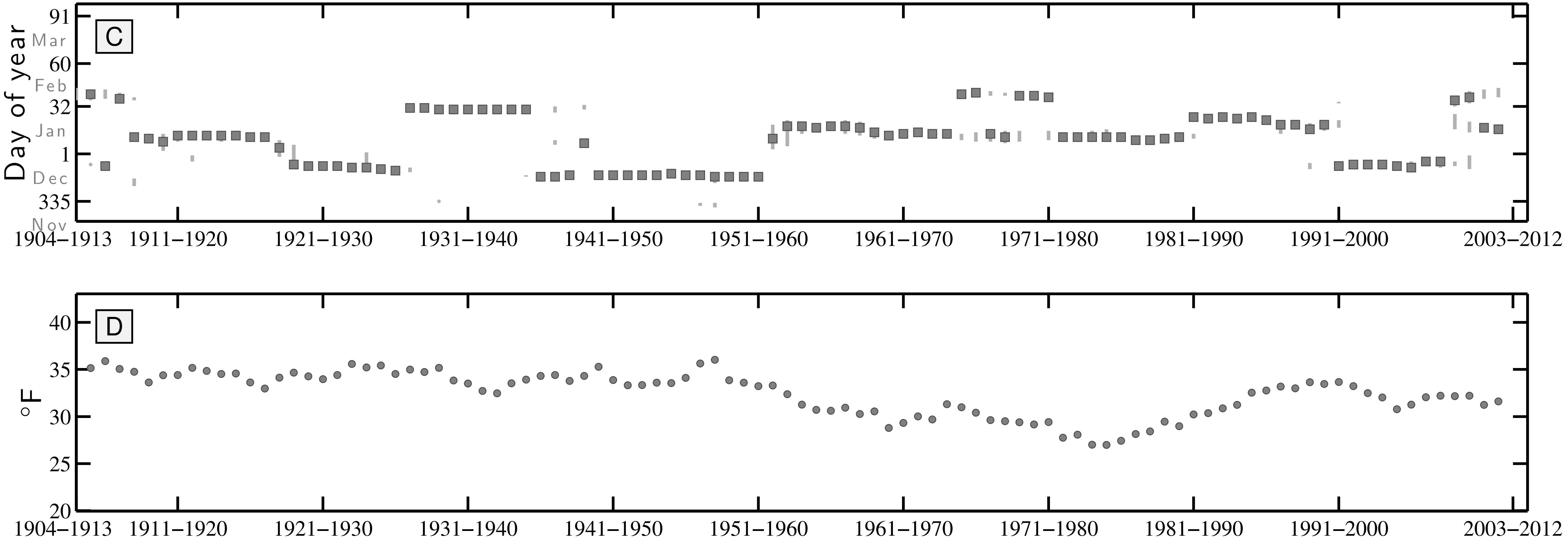

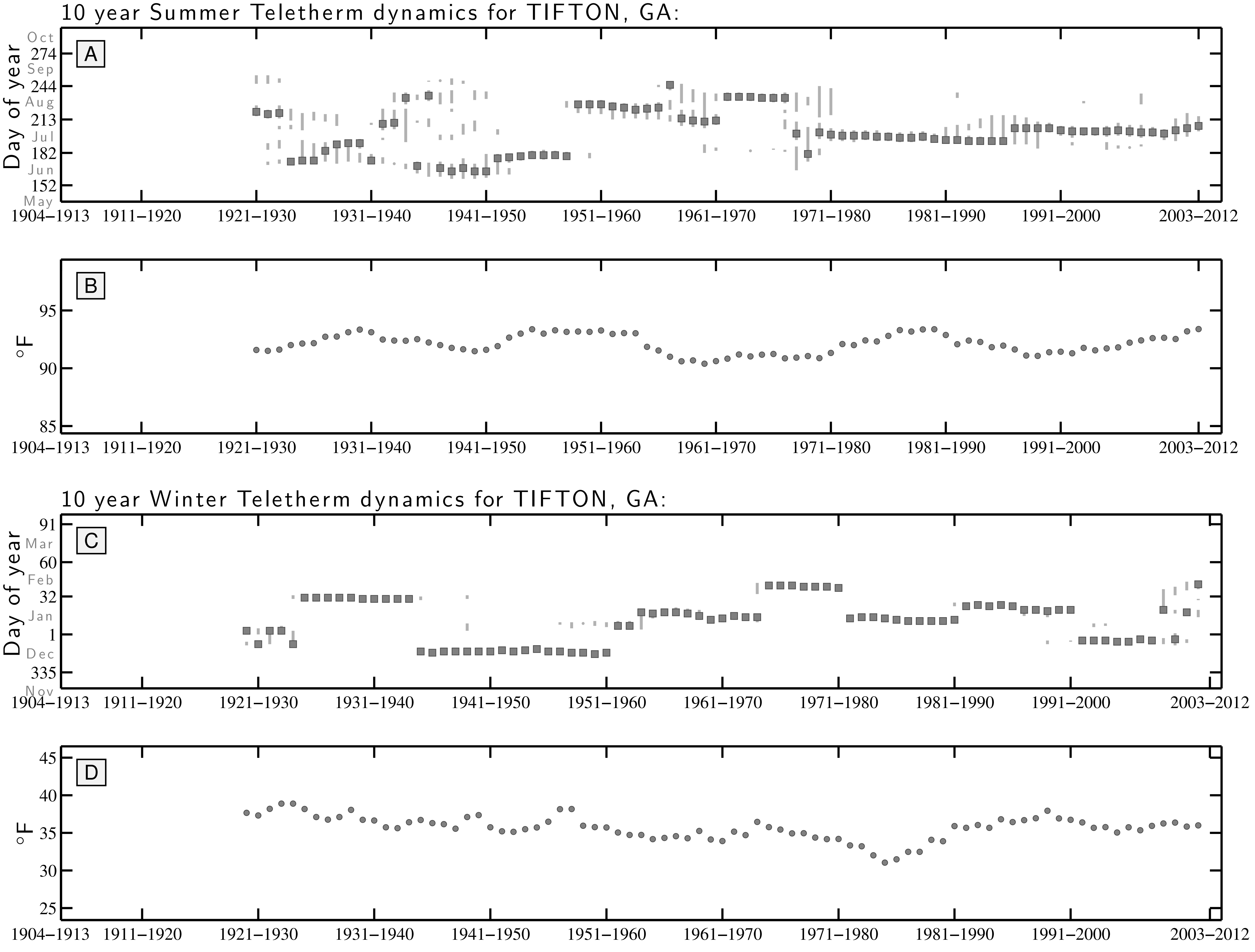
10 year Summer Teletherm dynamics for TIFTON, GA:
A
Day of year
Oct
274
Sep
244
Aug
213
Jul
182
Jun
152
May
1904–1913
1911–1920
1921–1930
1931–1940
1941–1950
1951–1960
1961–1970
1971–1980
1981–1990
1991–2000
2003–2012
B
°F
95
90
85
10 year Winter Teletherm dynamics for TIFTON, GA:
C
Day of year
91
Mar
60
Feb
32
Jan
1
Dec
335
Nov
D
°F
45
40
35
30
25

10 year Summer Teletherm dynamics for TOCCOA, GA:

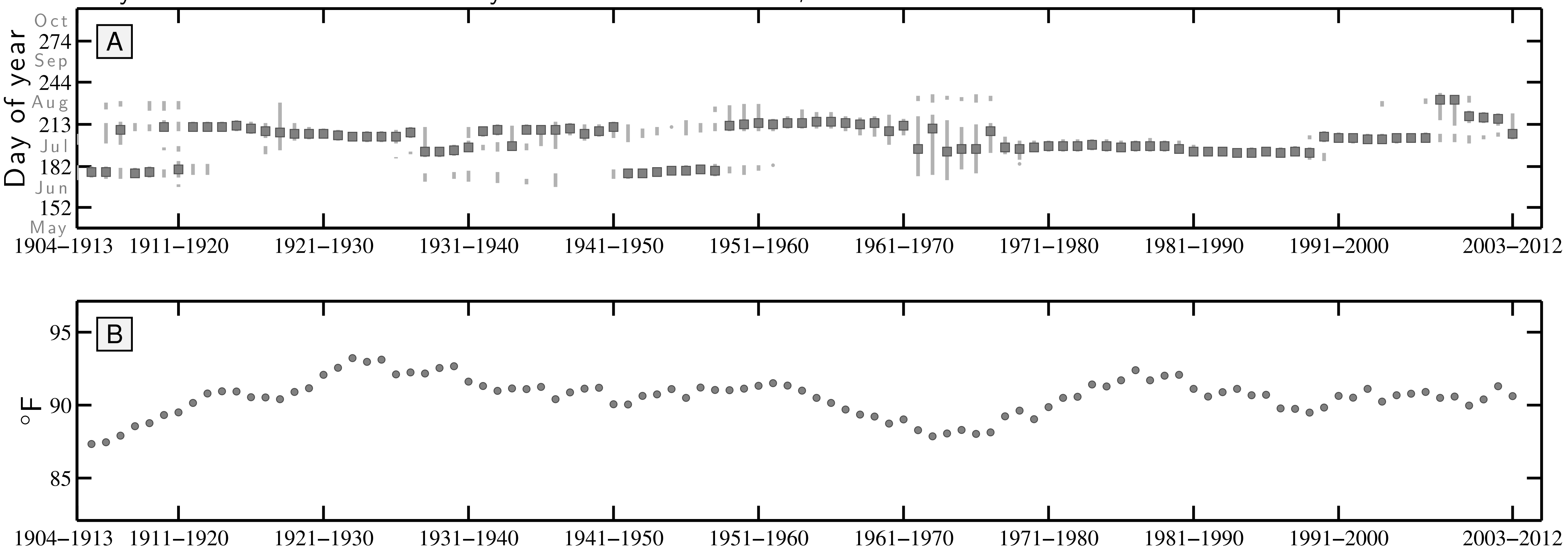

10 year Winter Teletherm dynamics for TOCCOA, GA:

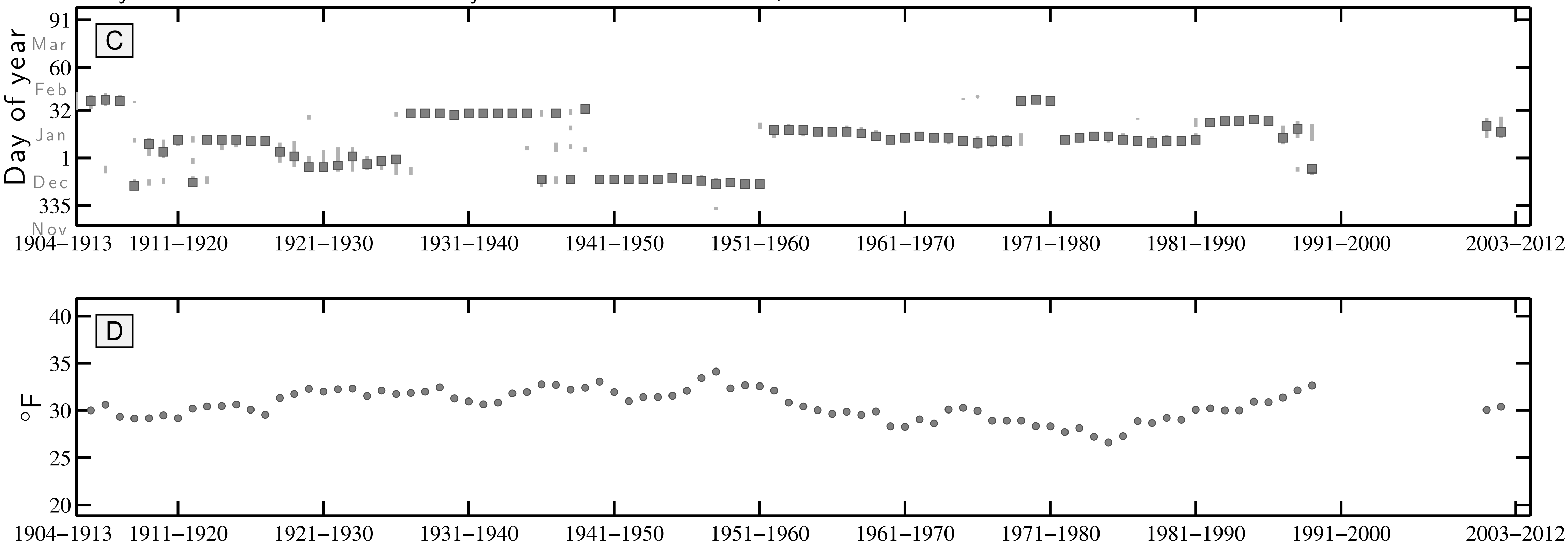

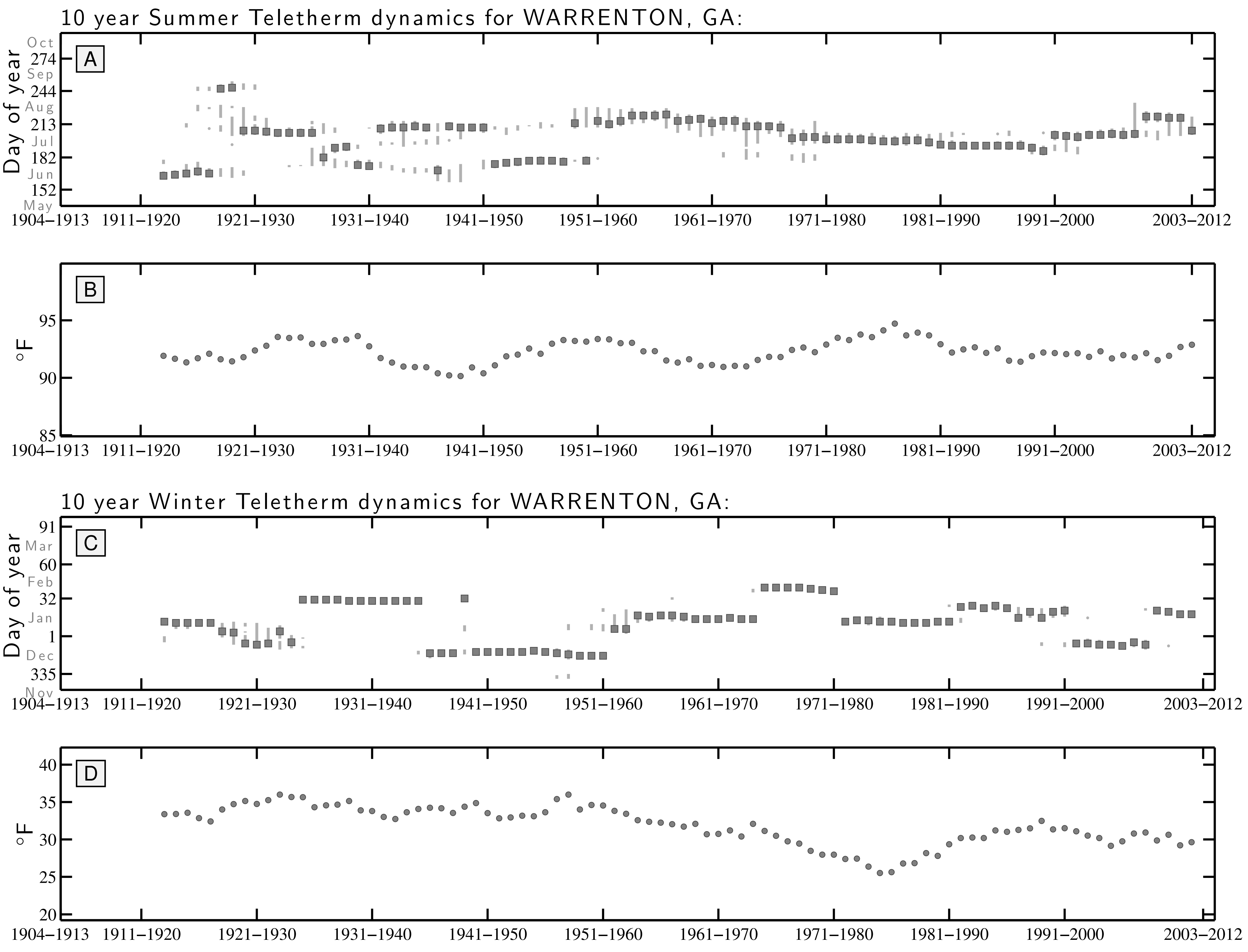

10 year Summer Teletherm dynamics for WARRENTON, GA:
A
B
Day of year
°F
May
Jun
Jul
Aug
Sep
Oct
152
182
213
244
274
85
90
95
1904–1913
1911–1920
1921–1930
1931–1940
1941–1950
1951–1960
1961–1970
1971–1980
1981–1990
1991–2000
2003–2012
10 year Winter Teletherm dynamics for WARRENTON, GA:
C
D
Nov
Dec
Jan
Feb
Mar
335
1
32
60
91
20
25
30
35
40

10 year Summer Teletherm dynamics for WASHINGTON 2 ESE, GA:

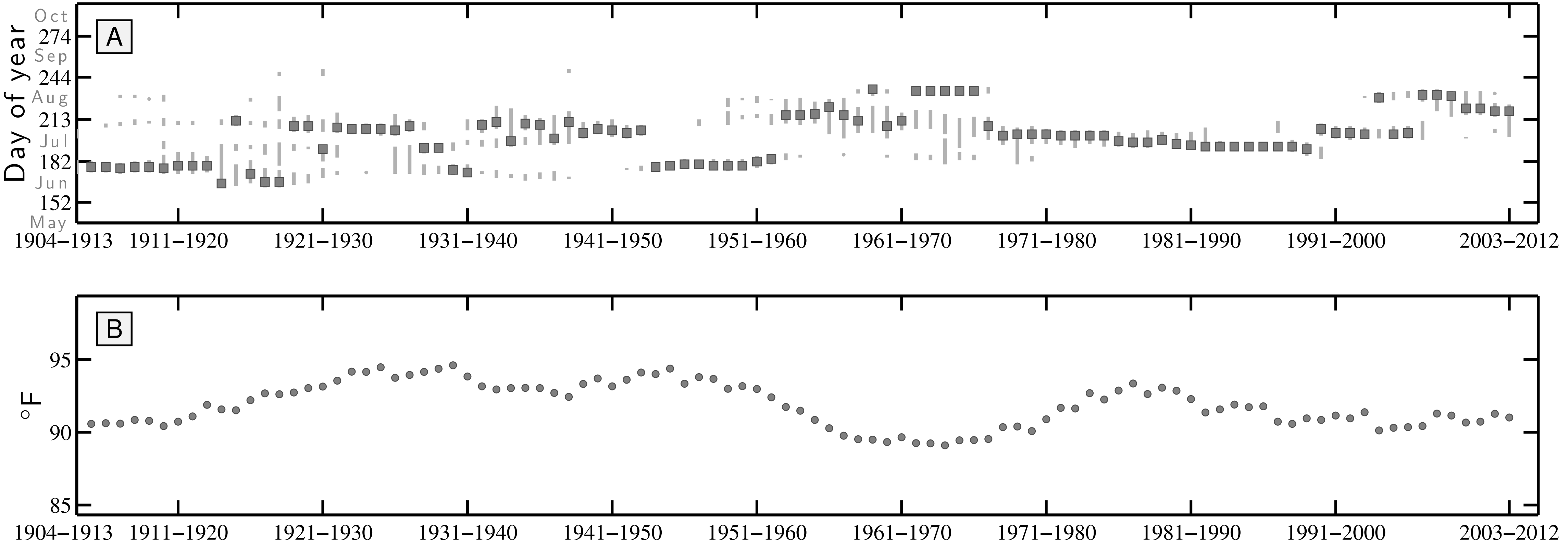

10 year Winter Teletherm dynamics for WASHINGTON 2 ESE, GA:

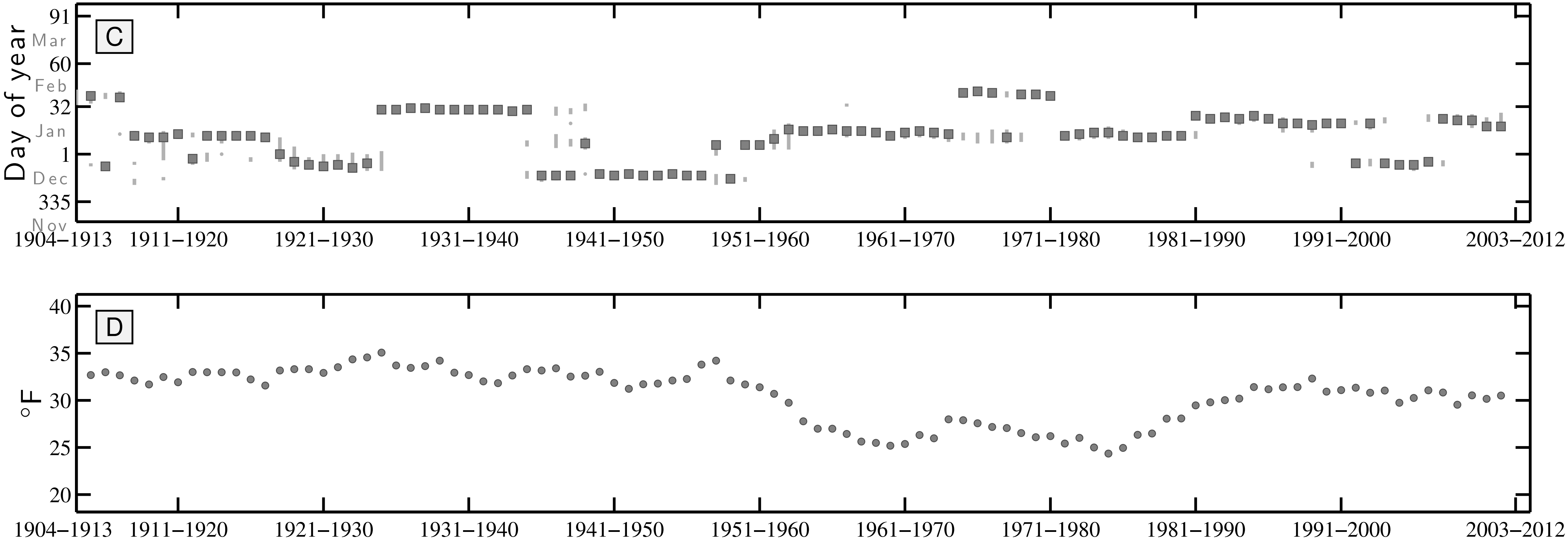

10 year Summer Teletherm dynamics for WAYCROSS 4 NE, GA:

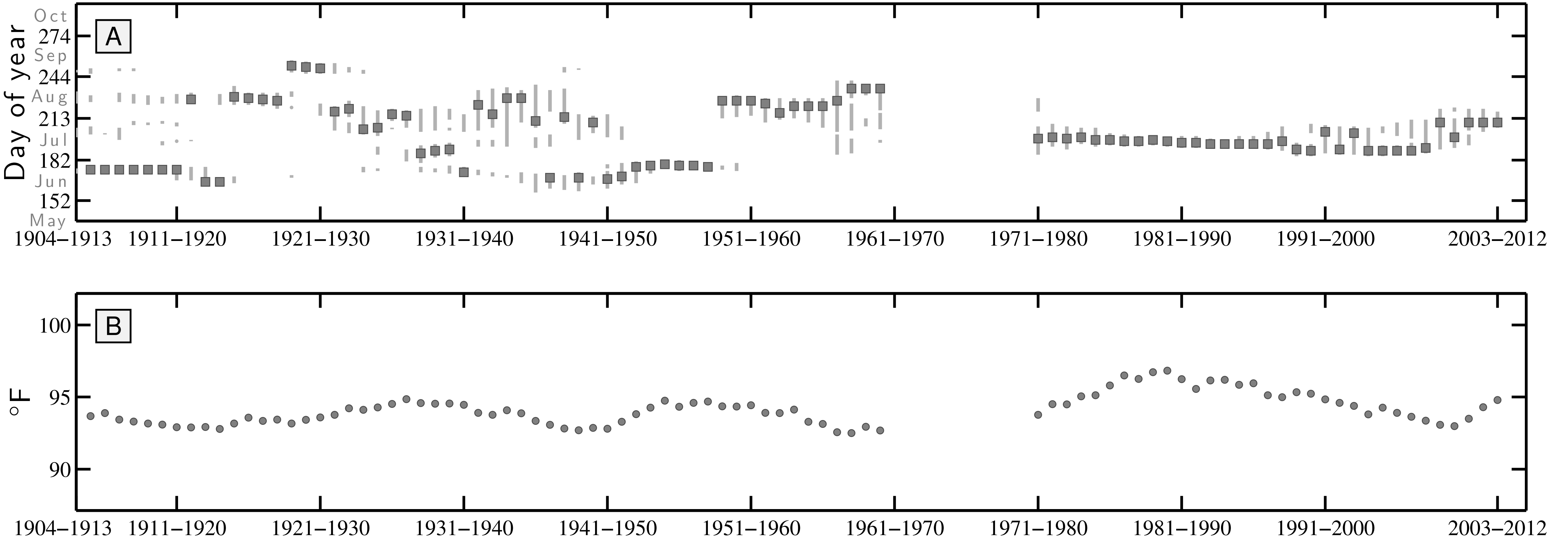

10 year Winter Teletherm dynamics for WAYCROSS 4 NE, GA:

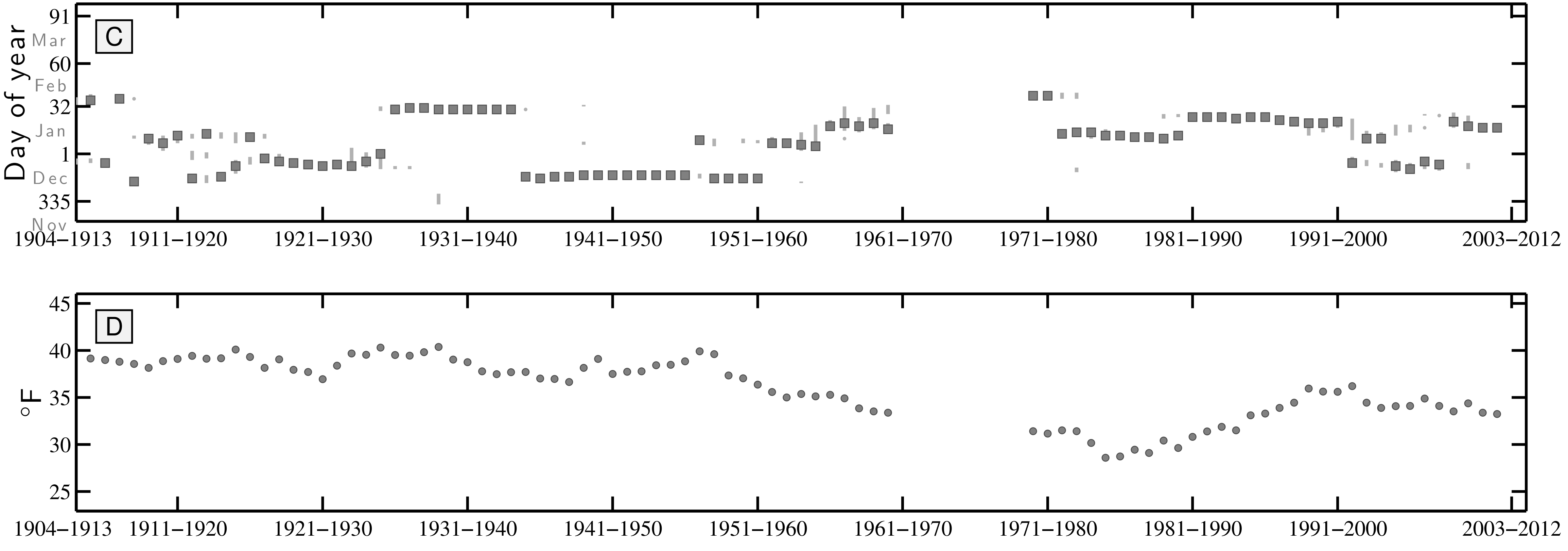

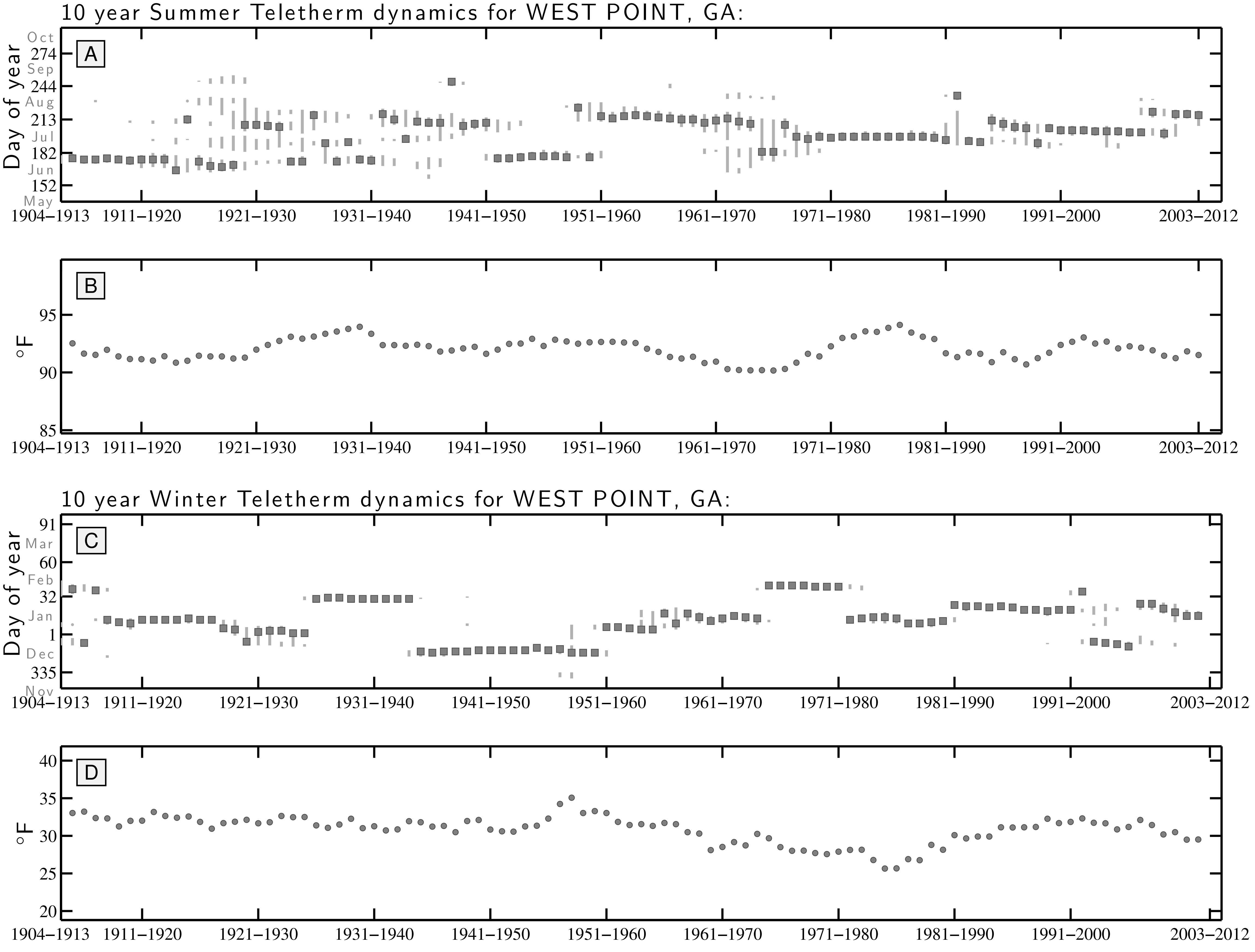
10 year Summer Teletherm dynamics for WEST POINT, GA:
A
Day of year
Oct
274
Sep
244
Aug
213
Jul
182
Jun
152
May
1904–1913
1911–1920
1921–1930
1931–1940
1941–1950
1951–1960
1961–1970
1971–1980
1981–1990
1991–2000
2003–2012
B
°F
95
90
85
10 year Winter Teletherm dynamics for WEST POINT, GA:
C
91
Mar
60
Feb
32
Jan
1
Dec
335
Nov
D
40
35
30
25
20

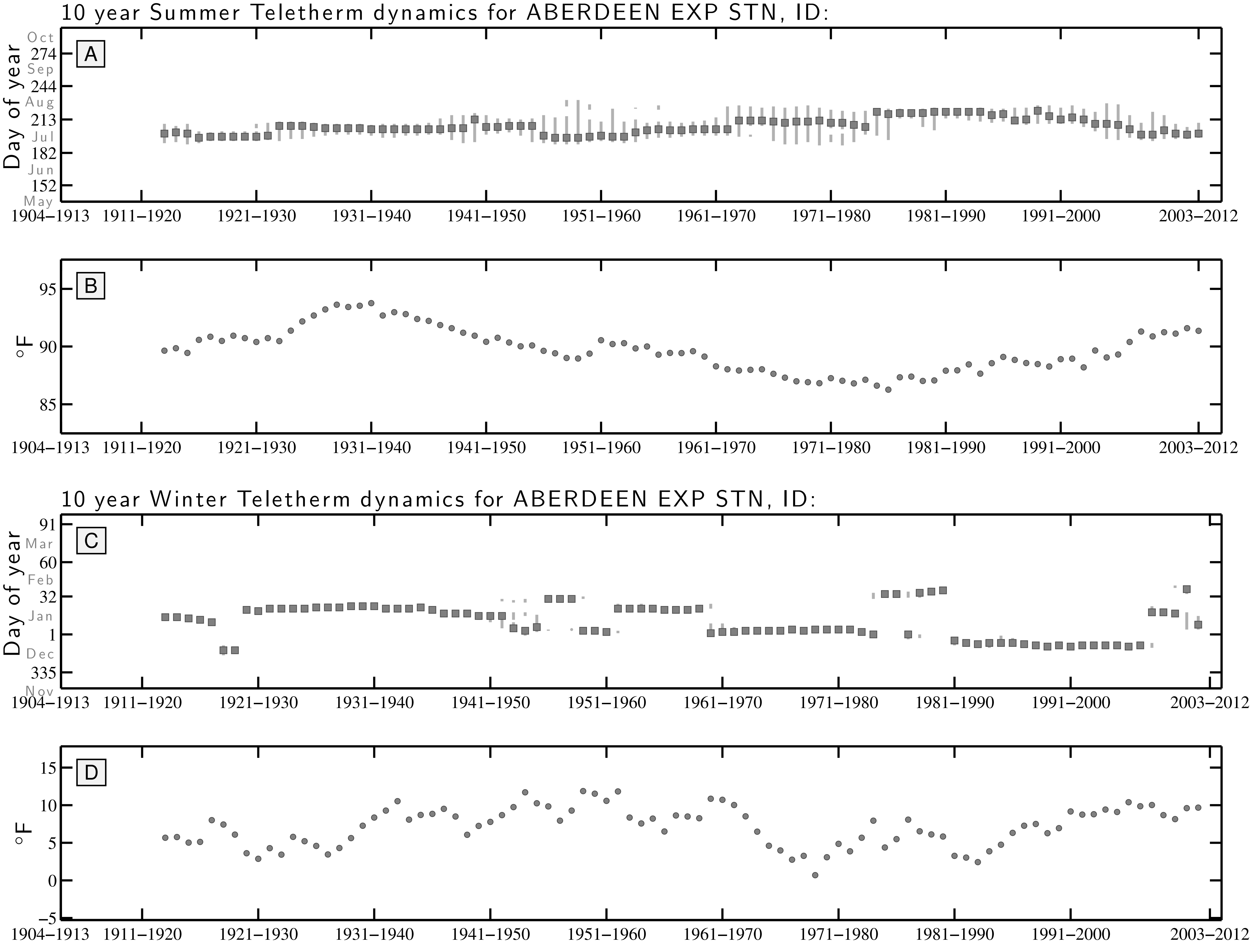

10 year Summer Teletherm dynamics for ABERDEEN EXP STN, ID:
A
Day of year
Oct
274
Sep
244
Aug
213
Jul
182
Jun
152
May
B
°F
95
90
85
1904–1913
1911–1920
1921–1930
1931–1940
1941–1950
1951–1960
1961–1970
1971–1980
1981–1990
1991–2000
2003–2012
10 year Winter Teletherm dynamics for ABERDEEN EXP STN, ID:
C
Day of year
91
Mar
60
Feb
32
Jan
1
Dec
335
Nov
D
°F
15
10
5
0
−5

10 year Summer Teletherm dynamics for ARROWROCK DAM, ID:

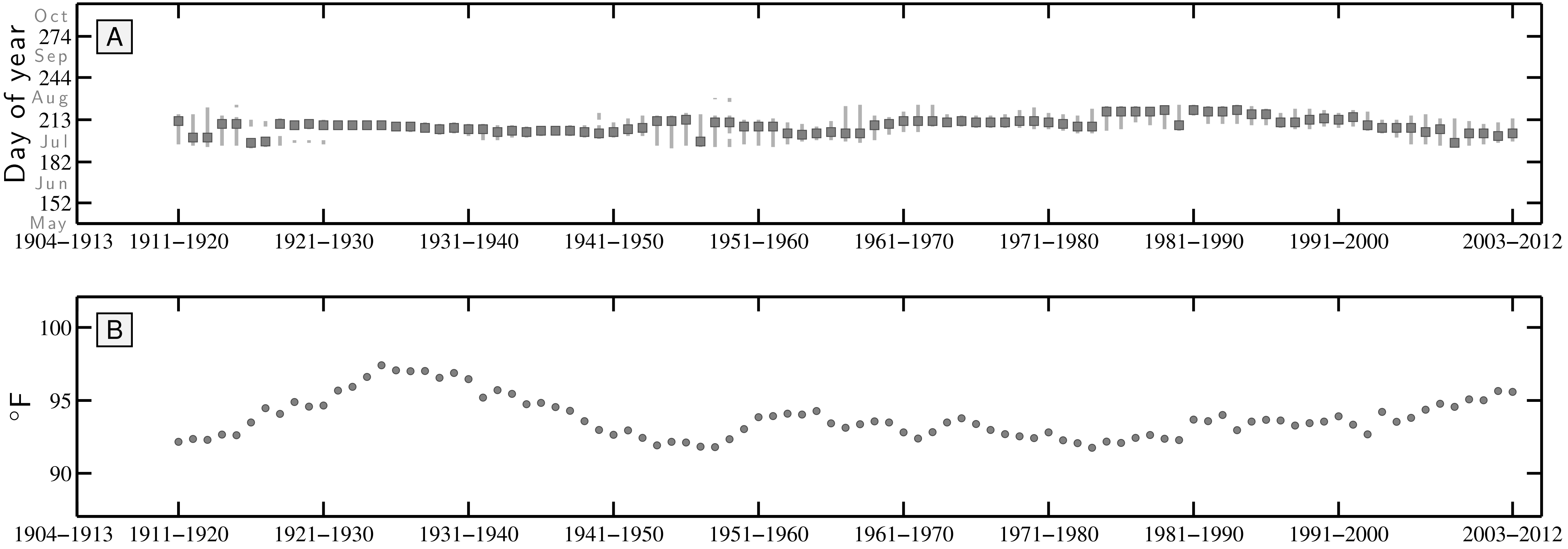

10 year Winter Teletherm dynamics for ARROWROCK DAM, ID:

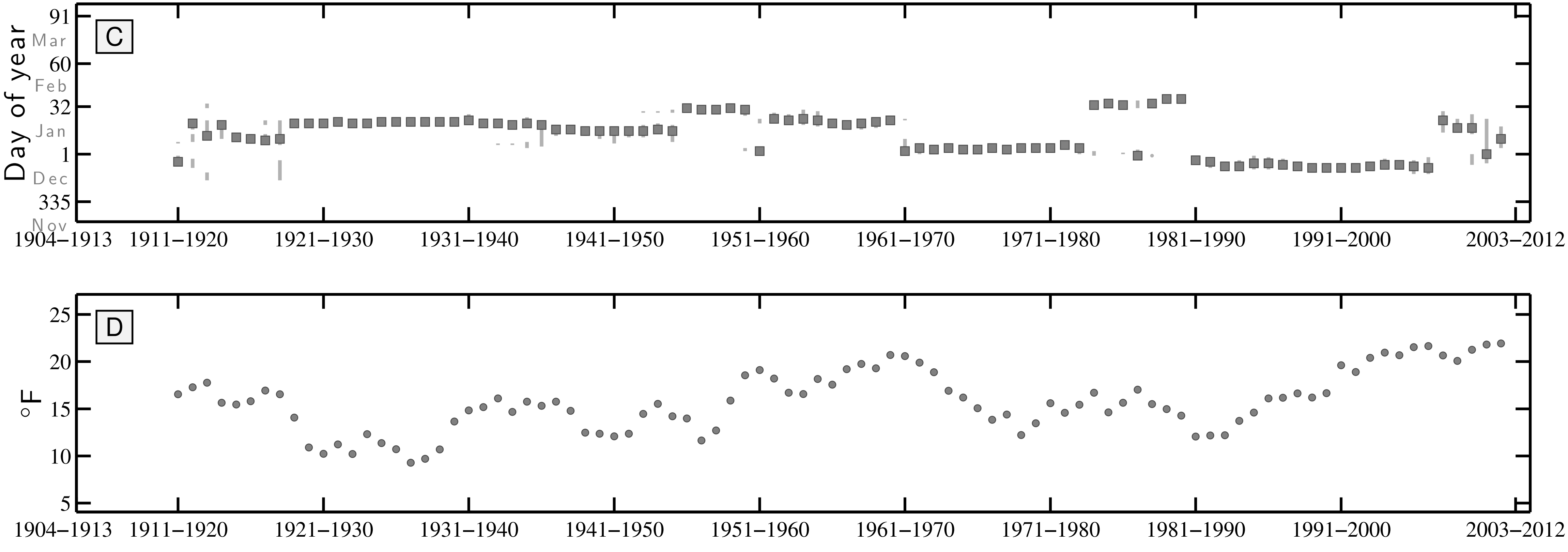

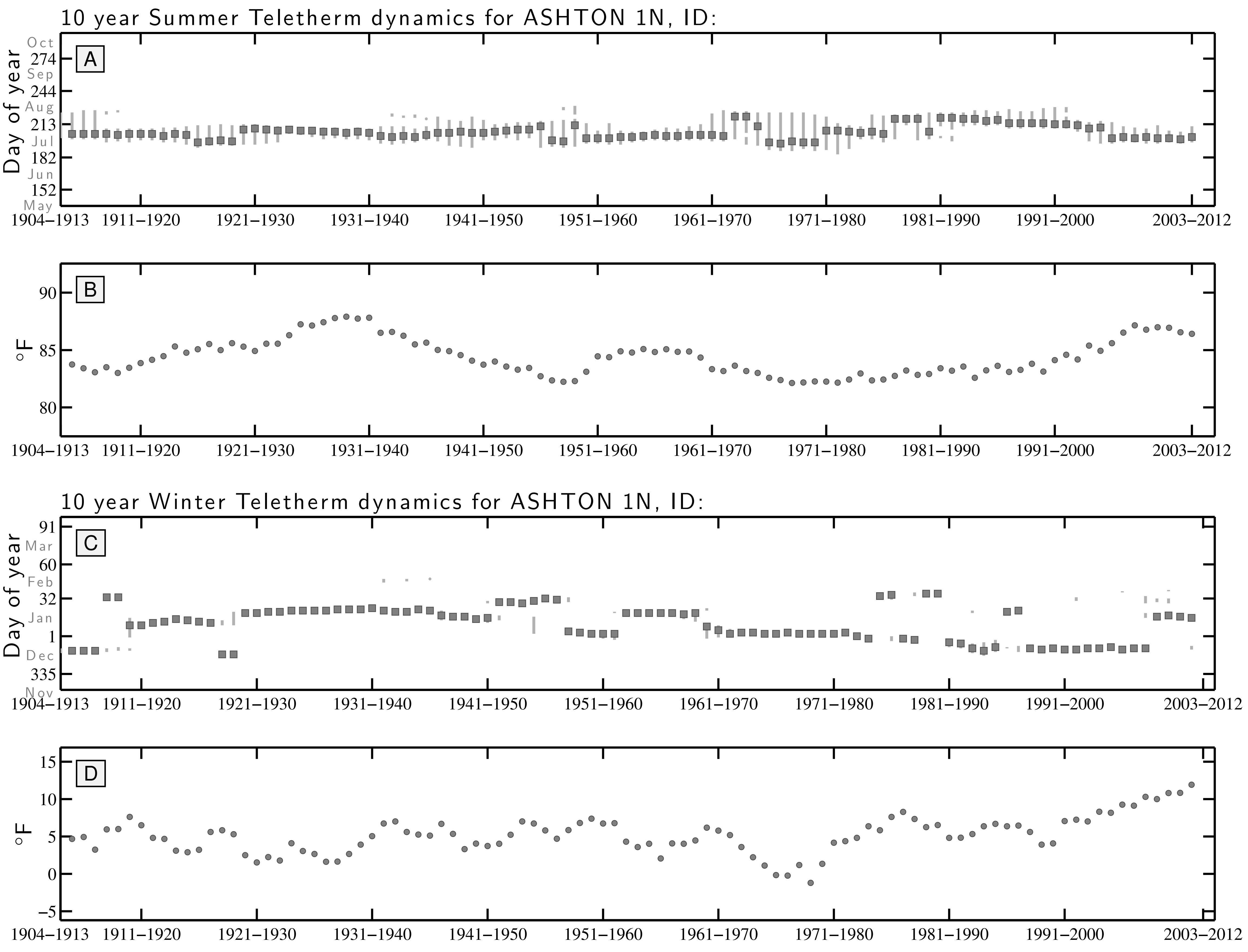
10 year Summer Teletherm dynamics for ASHTON 1N, ID:
A
B
Day of year
°F
Oct
274
Sep
244
Aug
213
Jul
182
Jun
152
May
90
85
80
10 year Winter Teletherm dynamics for ASHTON 1N, ID:
C
D
91
Mar
60
Feb
32
Jan
1
Dec
335
Nov
15
10
5
0
−5
1904–1913
1911–1920
1921–1930
1931–1940
1941–1950
1951–1960
1961–1970
1971–1980
1981–1990
1991–2000
2003–2012

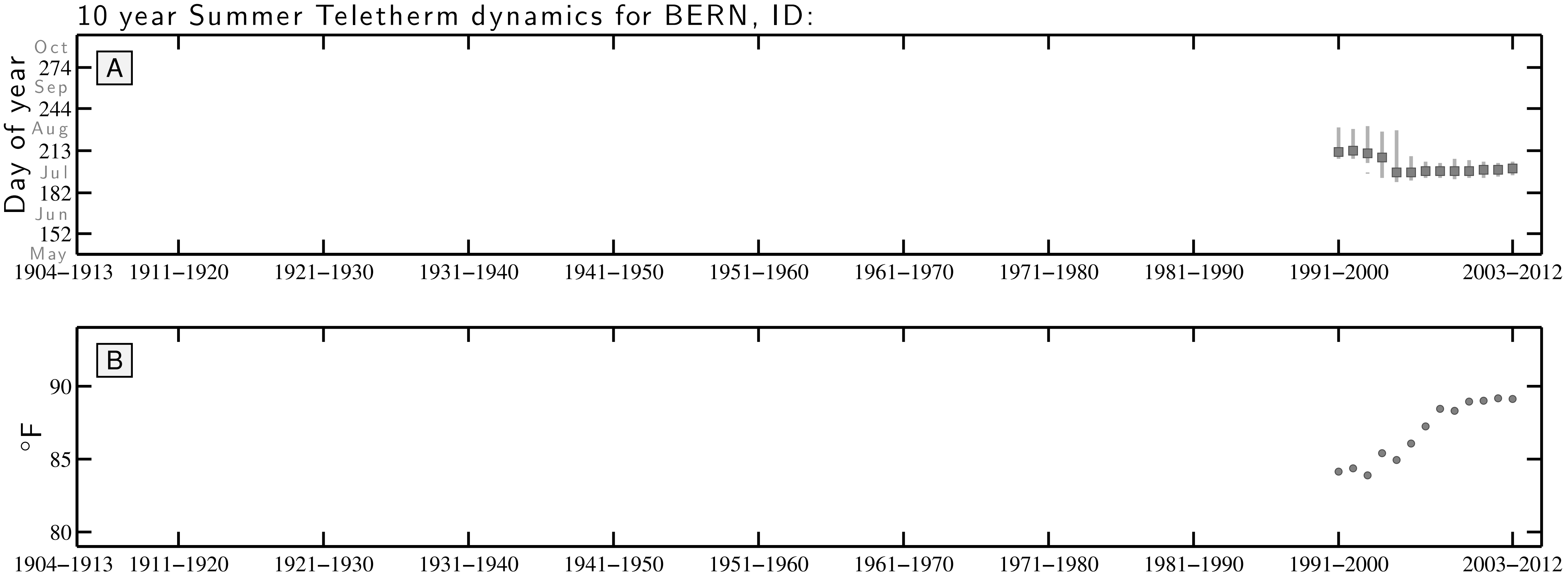
10 year Summer Teletherm dynamics for BERN, ID:
A
B
Day of year
°F
Oct
274
Sep
244
Aug
213
Jul
182
Jun
152
May
90
85
80
1904–1913
1911–1920
1921–1930
1931–1940
1941–1950
1951–1960
1961–1970
1971–1980
1981–1990
1991–2000
2003–2012

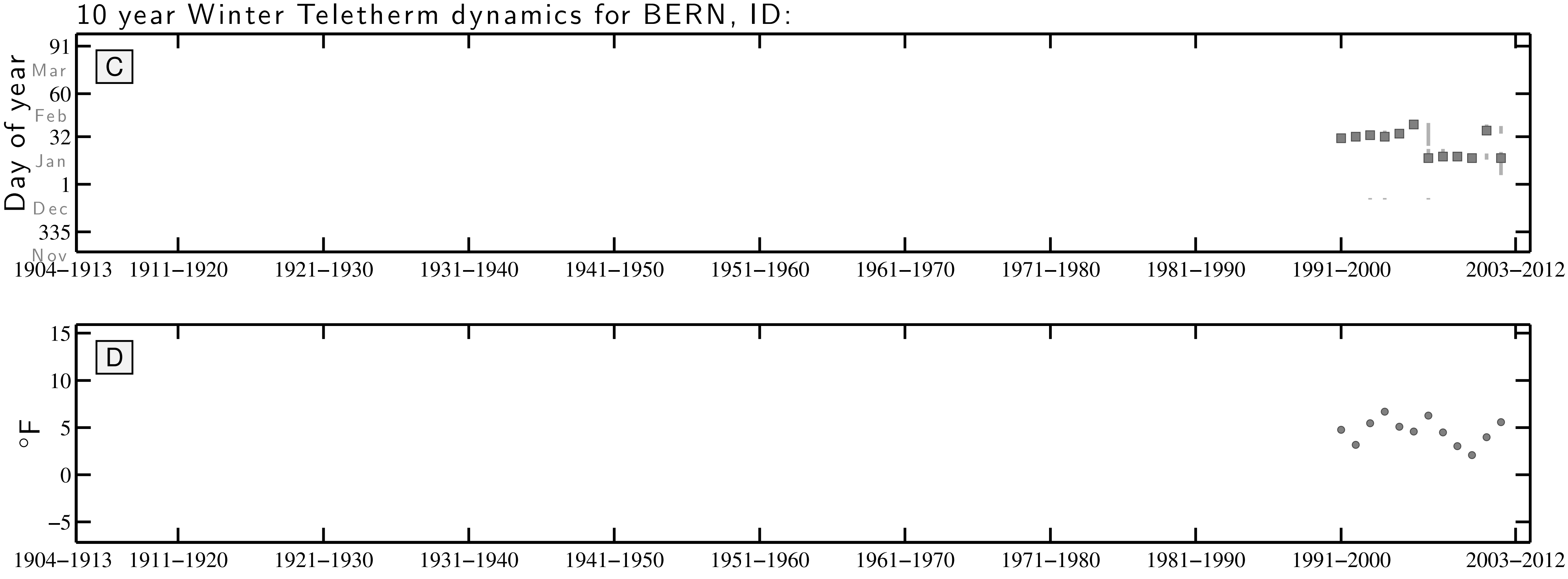
10 year Winter Teletherm dynamics for BERN, ID:
C
D
Day of year
°F
91
Mar
60
Feb
32
Jan
1
Dec
335
Nov
15
10
5
0
−5
1904–1913
1911–1920
1921–1930
1931–1940
1941–1950
1951–1960
1961–1970
1971–1980
1981–1990
1991–2000
2003–2012

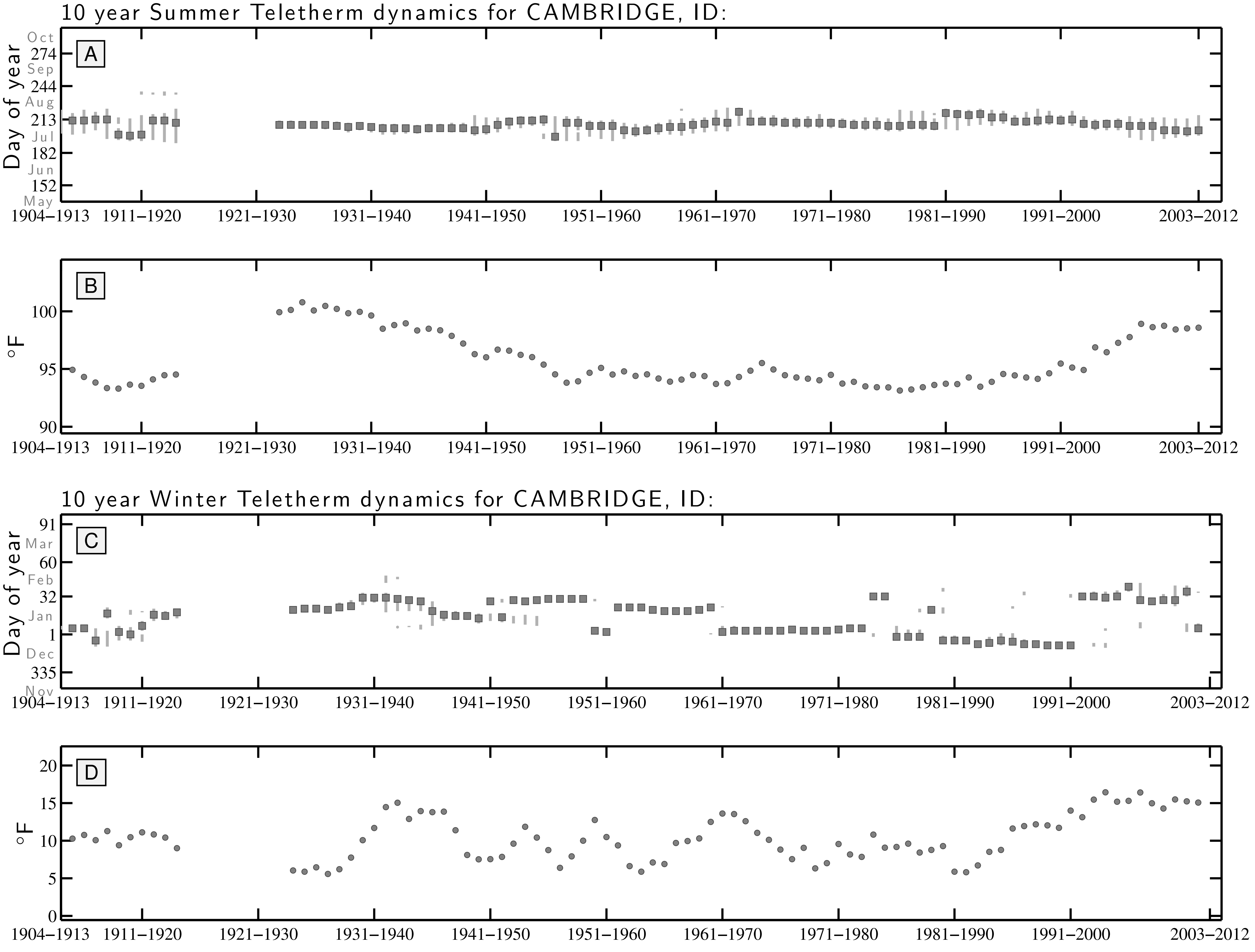
10 year Summer Teletherm dynamics for CAMBRIDGE, ID:
A
Day of year
Oct
274
Sep
244
Aug
213
Jul
182
Jun
152
May
1904–1913
1911–1920
1921–1930
1931–1940
1941–1950
1951–1960
1961–1970
1971–1980
1981–1990
1991–2000
2003–2012
B
°F
100
95
90
10 year Winter Teletherm dynamics for CAMBRIDGE, ID:
C
Day of year
91
Mar
60
Feb
32
Jan
1
Dec
335
Nov
D
°F
20
15
10
5
0

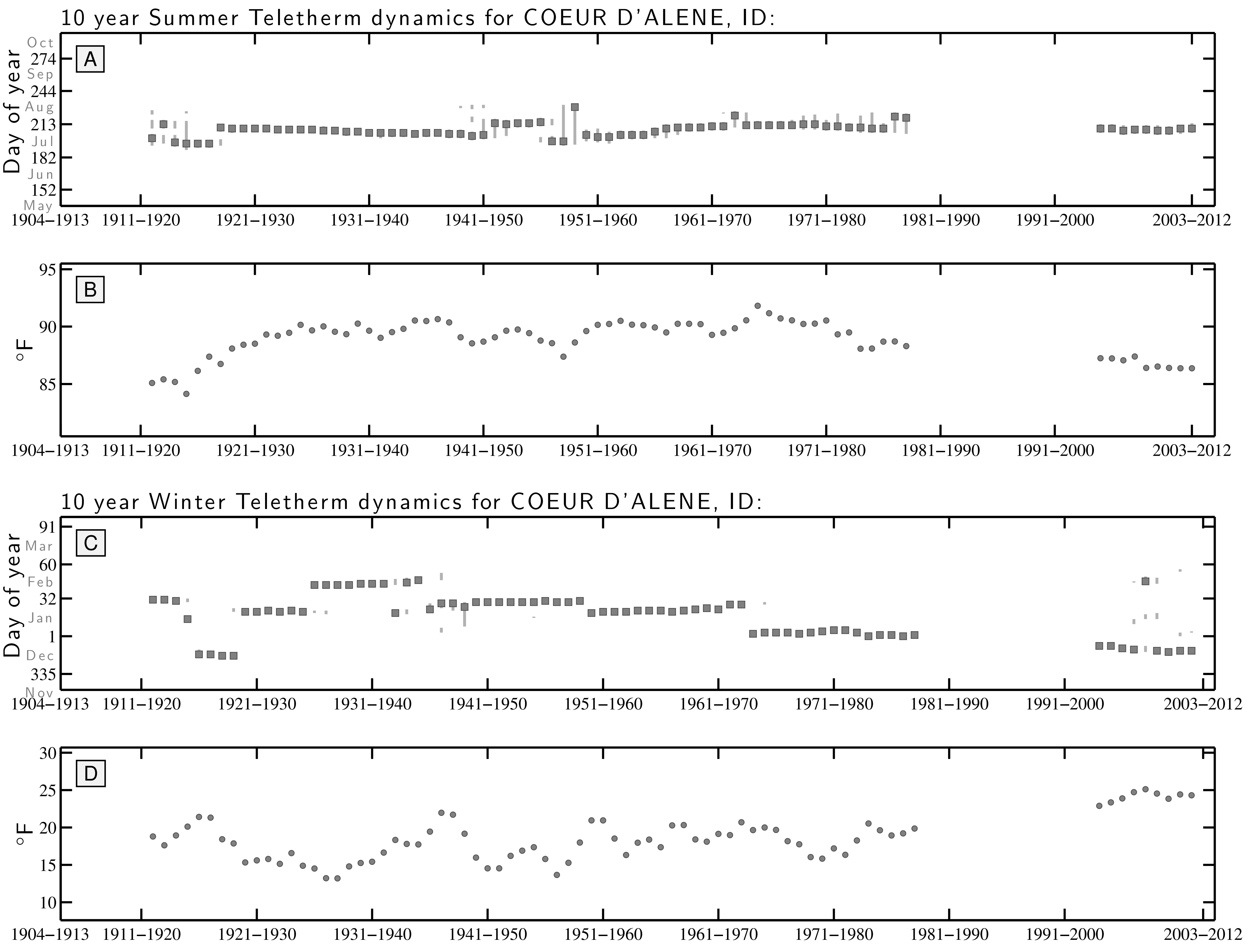

10 year Summer Teletherm dynamics for COEUR D'ALENE, ID:
A
Day of year
Oct
274
Sep
244
Aug
213
Jul
182
Jun
152
May
B
°F
95
90
85
10 year Winter Teletherm dynamics for COEUR D'ALENE, ID:
C
Day of year
91
Mar
60
Feb
32
Jan
1
Dec
335
Nov
D
°F
30
25
20
15
10
1904–1913
1911–1920
1921–1930
1931–1940
1941–1950
1951–1960
1961–1970
1971–1980
1981–1990
1991–2000
2003–2012

10 year Summer Teletherm dynamics for DWORSHAK FISH HATCHERY, ID:

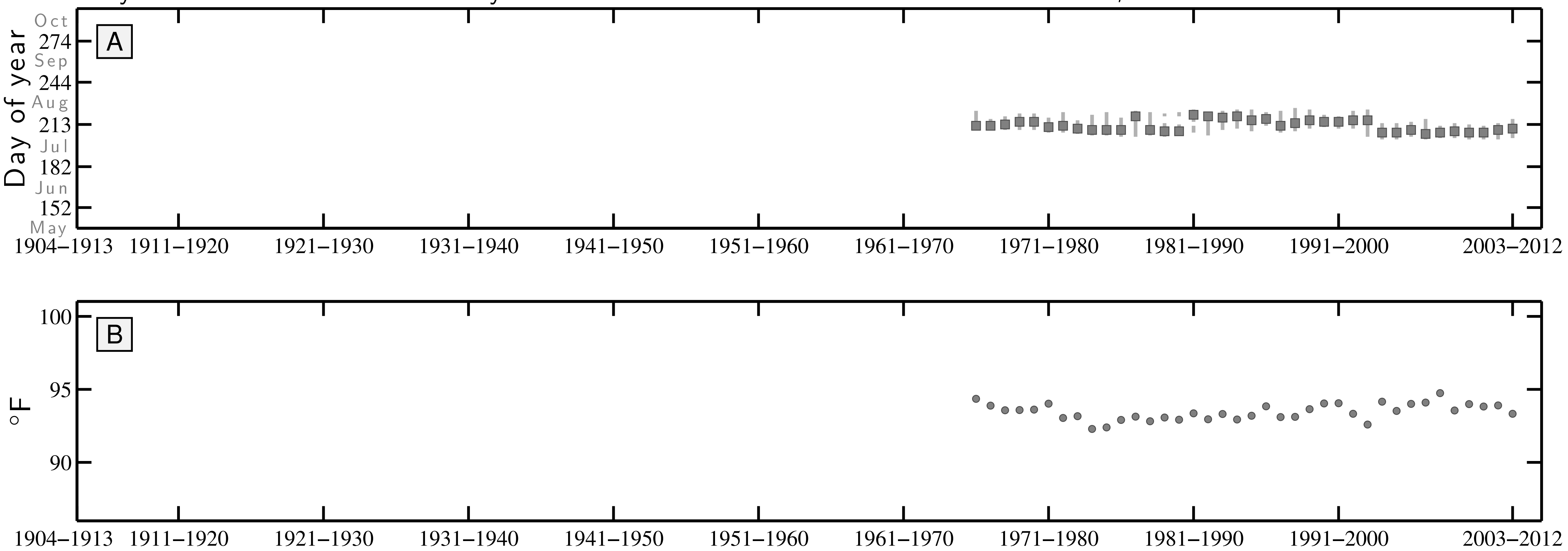

10 year Winter Teletherm dynamics for DWORSHAK FISH HATCHERY, ID:

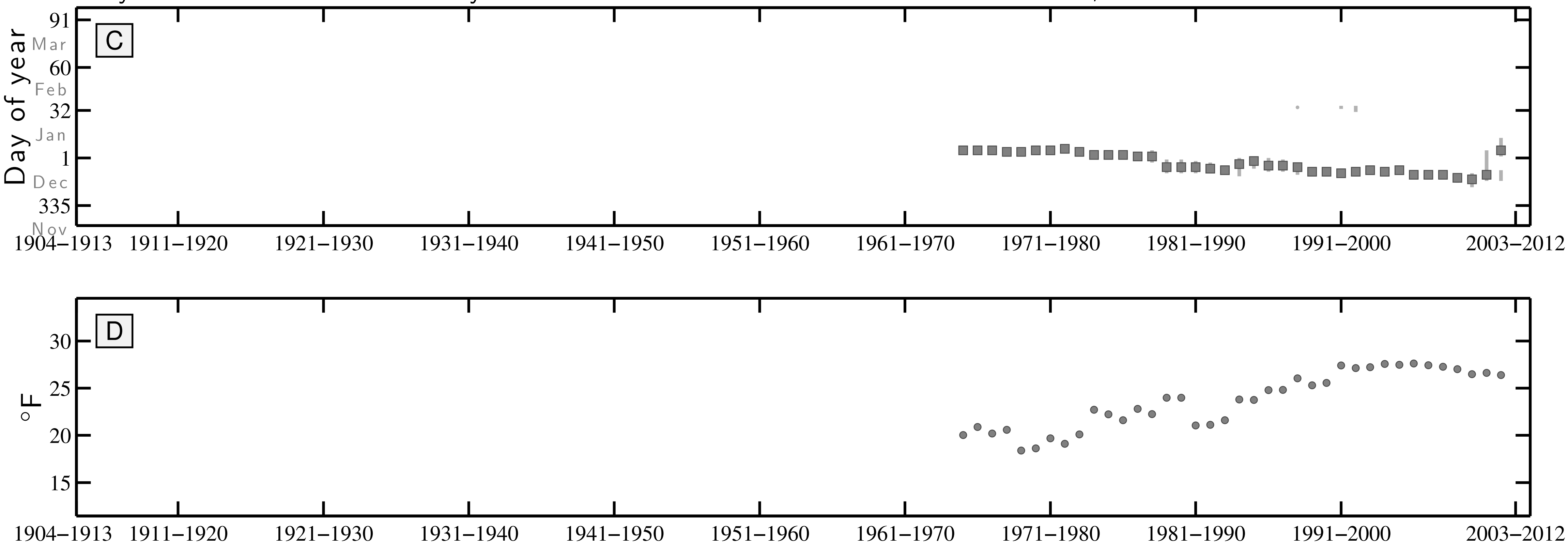

10 year Summer Teletherm dynamics for FENN RS, ID:

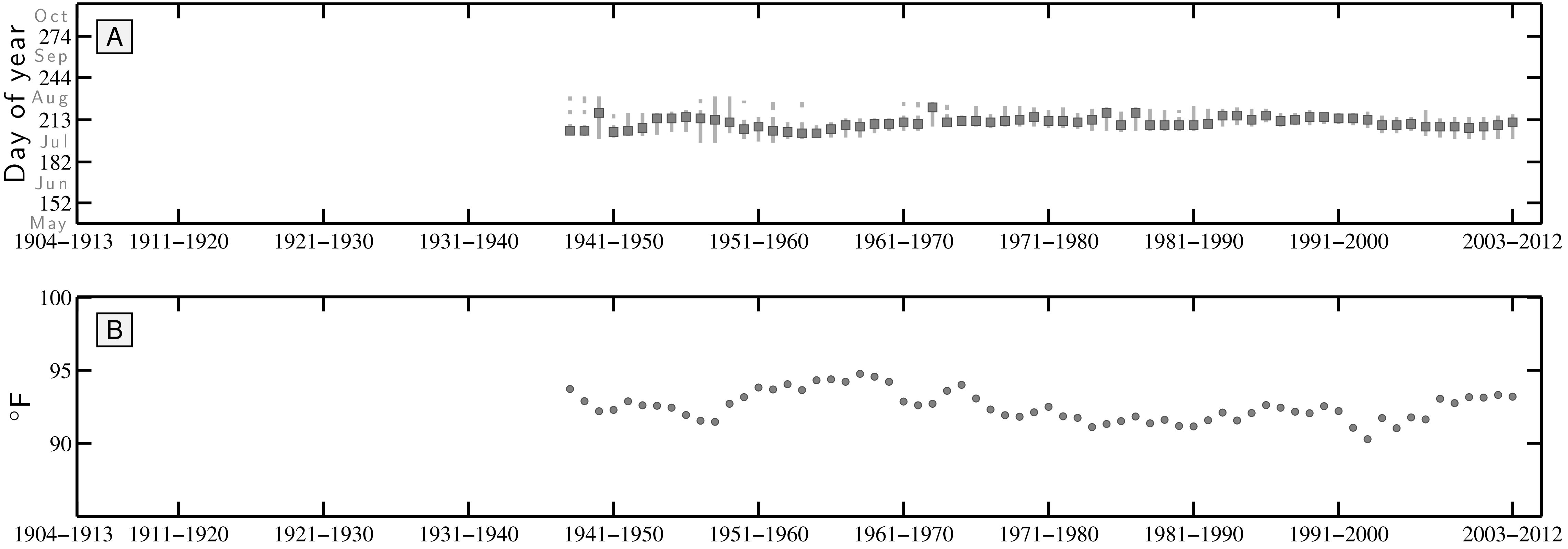

10 year Winter Teletherm dynamics for FENN RS, ID:

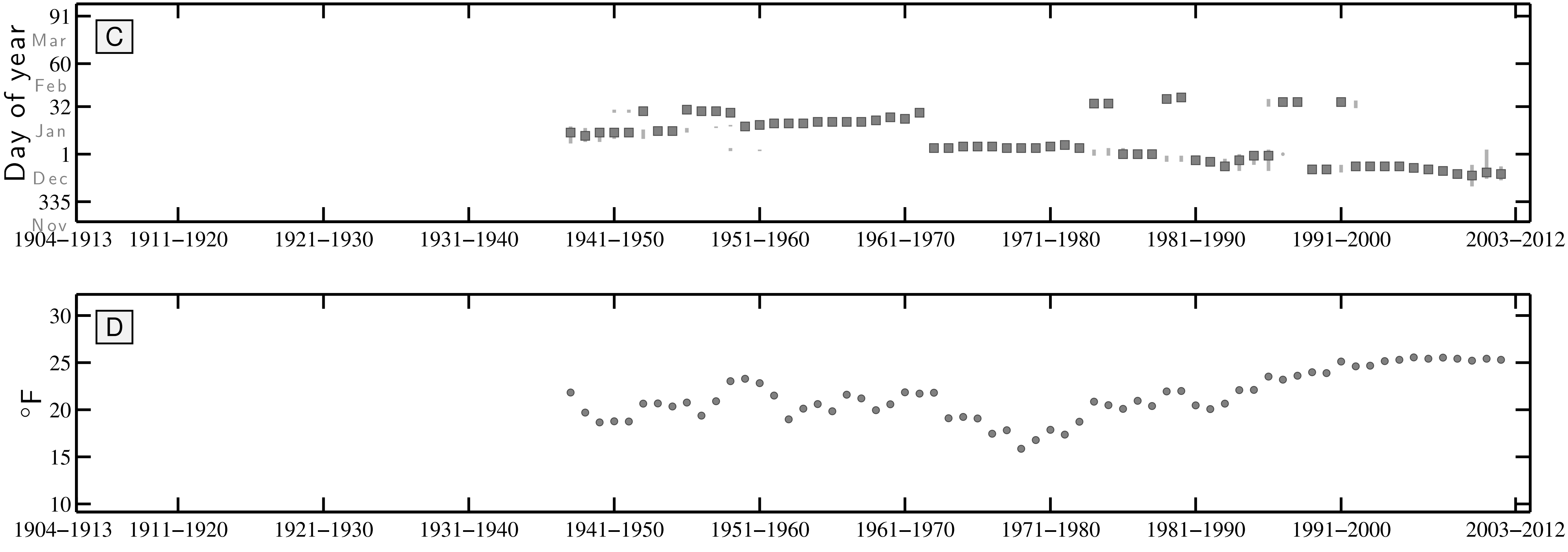

10 year Summer Teletherm dynamics for GLENNS FERRY, ID:

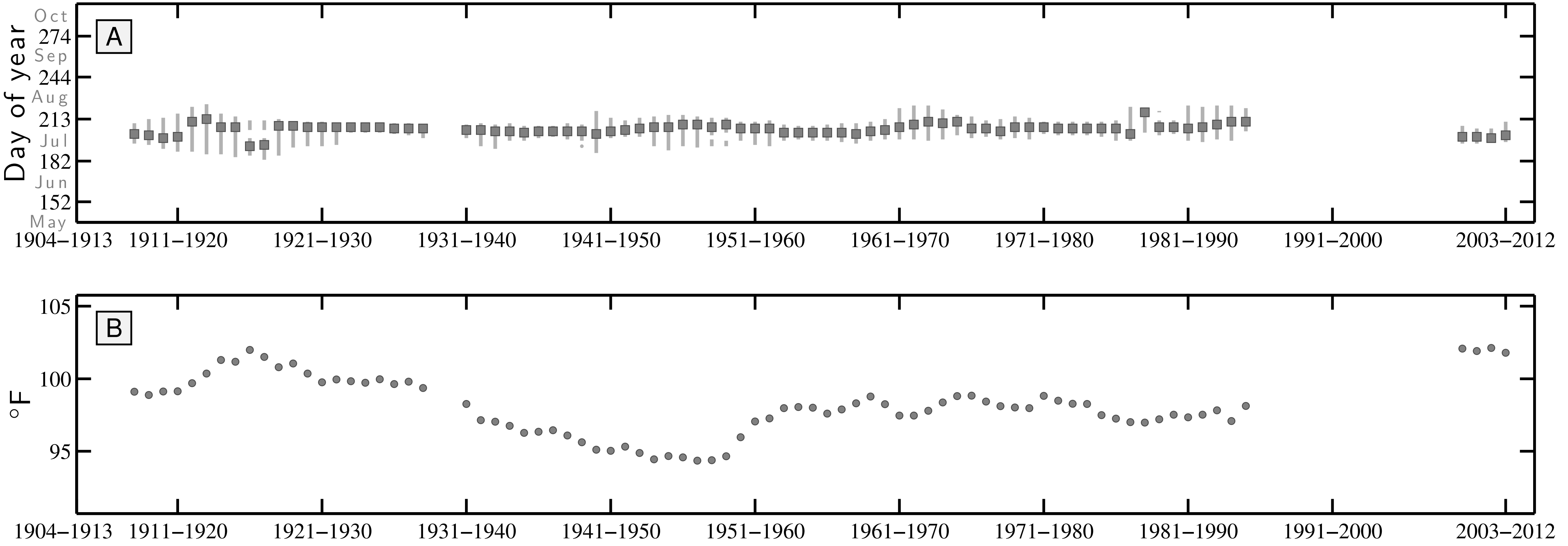

10 year Winter Teletherm dynamics for GLENNS FERRY, ID:

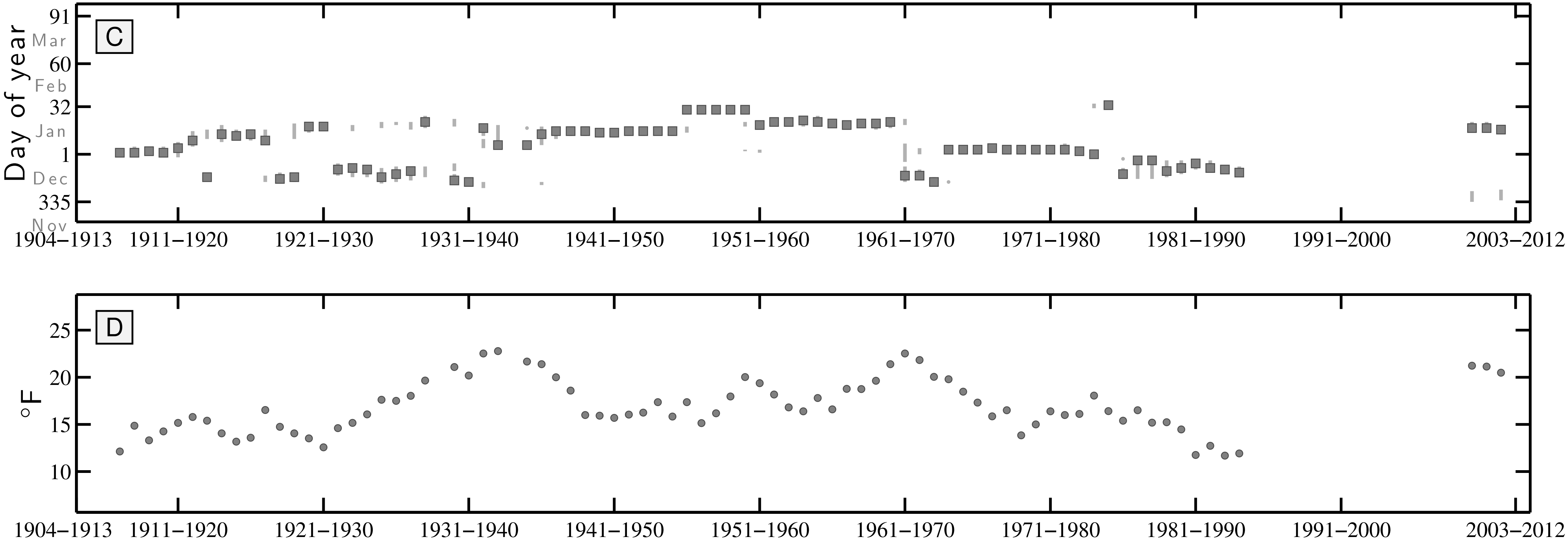

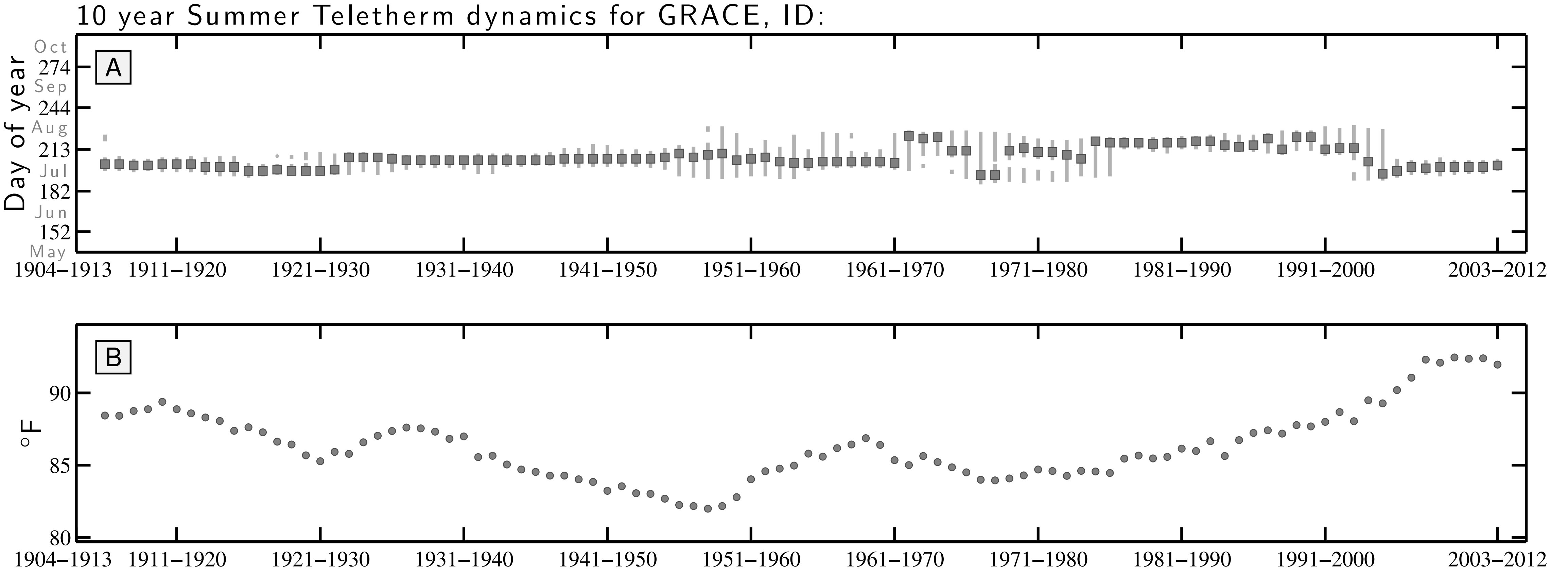
10 year Summer Teletherm dynamics for GRACE, ID:
A
B
Day of year
Oct
274
Sep
244
Aug
213
Jul
182
Jun
152
May
°F
90
85
80
1904–1913
1911–1920
1921–1930
1931–1940
1941–1950
1951–1960
1961–1970
1971–1980
1981–1990
1991–2000
2003–2012

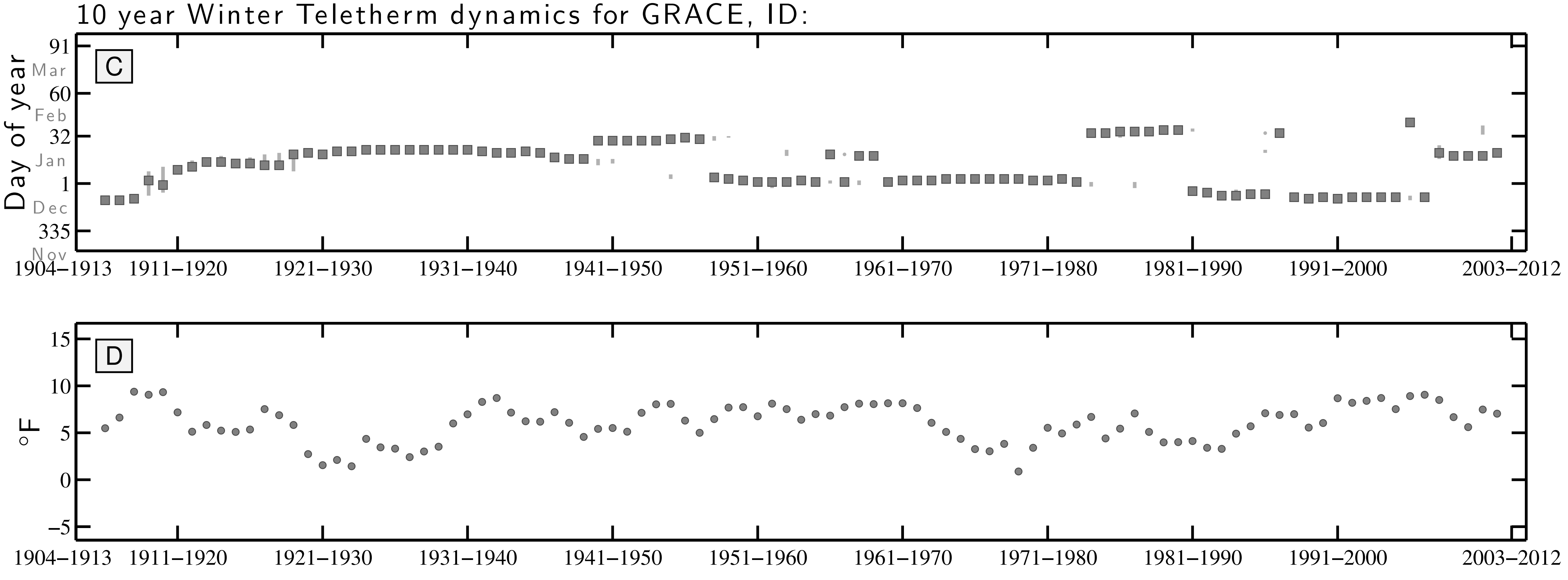
10 year Winter Teletherm dynamics for GRACE, ID:
C
D
Day of year
91
Mar
60
Feb
32
Jan
1
Dec
335
Nov
°F
15
10
5
0
−5
1904–1913
1911–1920
1921–1930
1931–1940
1941–1950
1951–1960
1961–1970
1971–1980
1981–1990
1991–2000
2003–2012

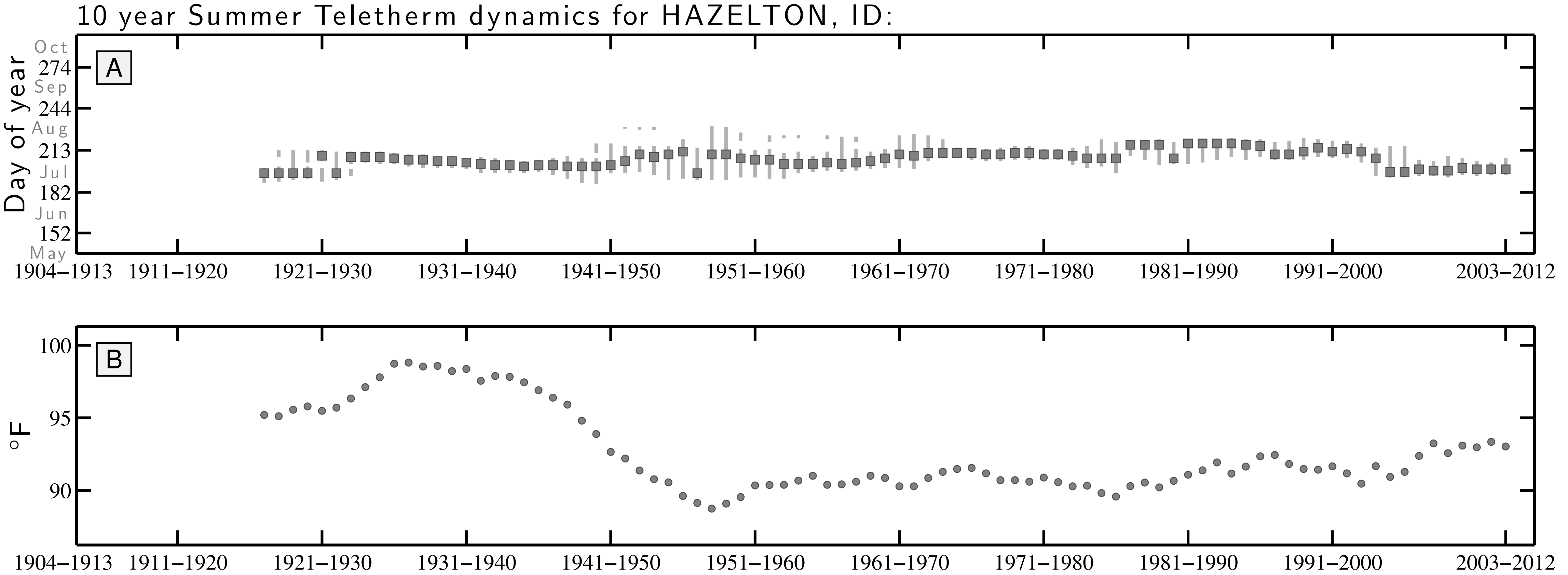
10 year Summer Teletherm dynamics for HAZELTON, ID:
A
Day of year
Oct
274
Sep
244
Aug
213
Jul
182
Jun
152
May
B
°F
100
95
90
1904–1913
1911–1920
1921–1930
1931–1940
1941–1950
1951–1960
1961–1970
1971–1980
1981–1990
1991–2000
2003–2012

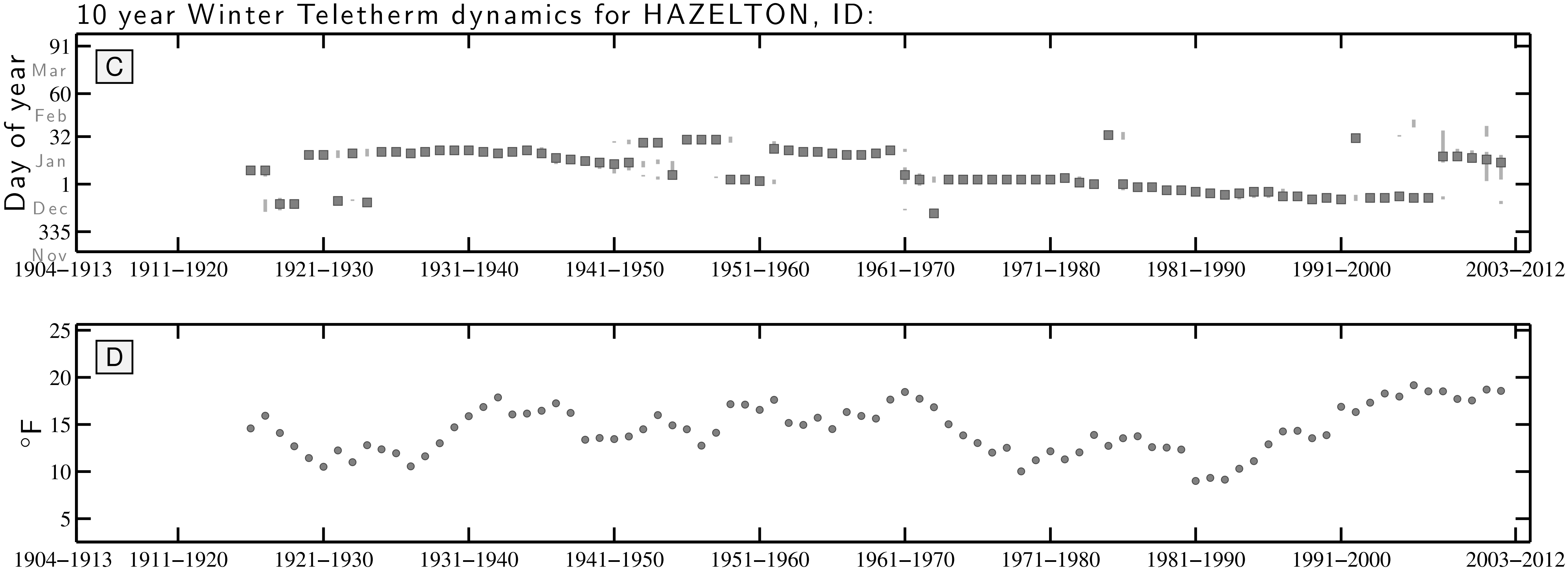
10 year Winter Teletherm dynamics for HAZELTON, ID:
C
Day of year
91
Mar
60
Feb
32
Jan
1
Dec
335
Nov
D
°F
25
20
15
10
5
1904–1913
1911–1920
1921–1930
1931–1940
1941–1950
1951–1960
1961–1970
1971–1980
1981–1990
1991–2000
2003–2012

10 year Summer Teletherm dynamics for HOLLISTER, ID:

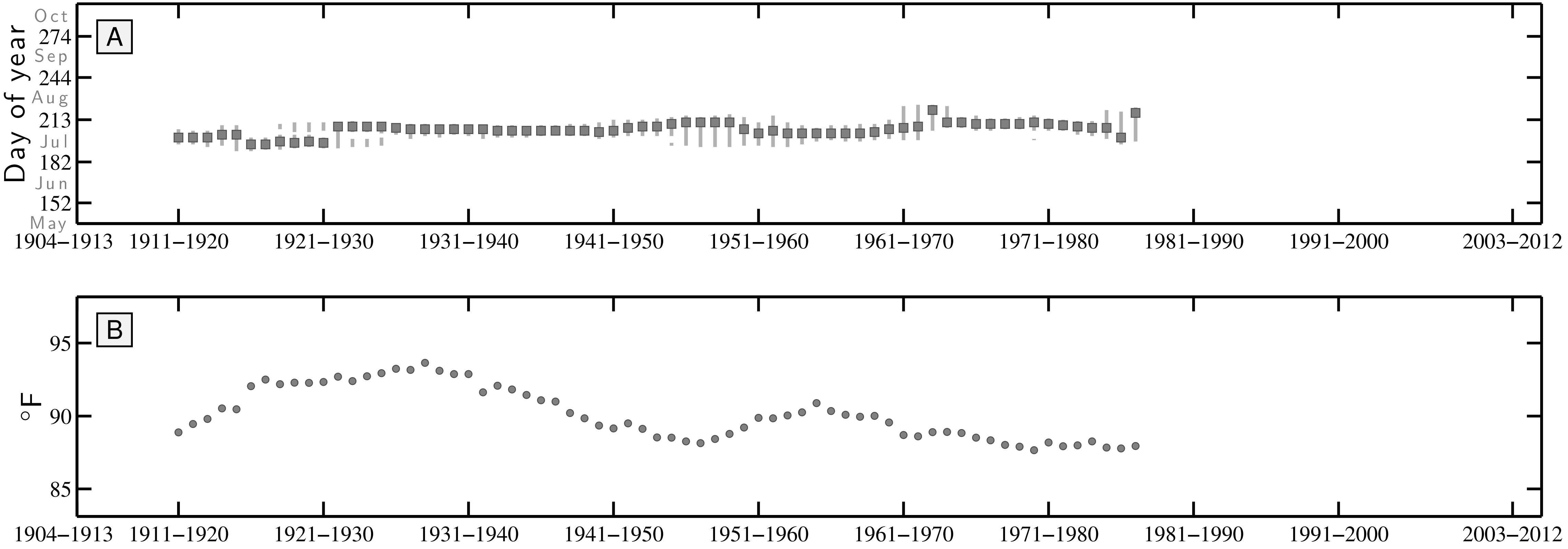

10 year Winter Teletherm dynamics for HOLLISTER, ID:

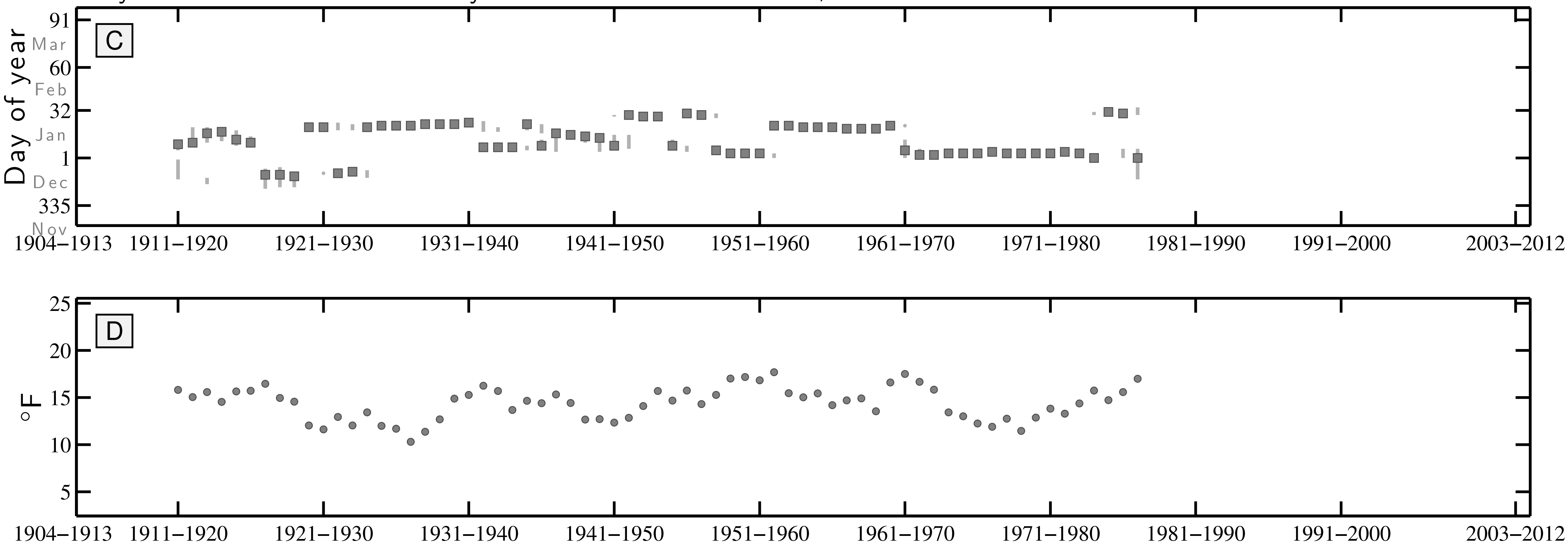

10 year Summer Teletherm dynamics for JEROME, ID:

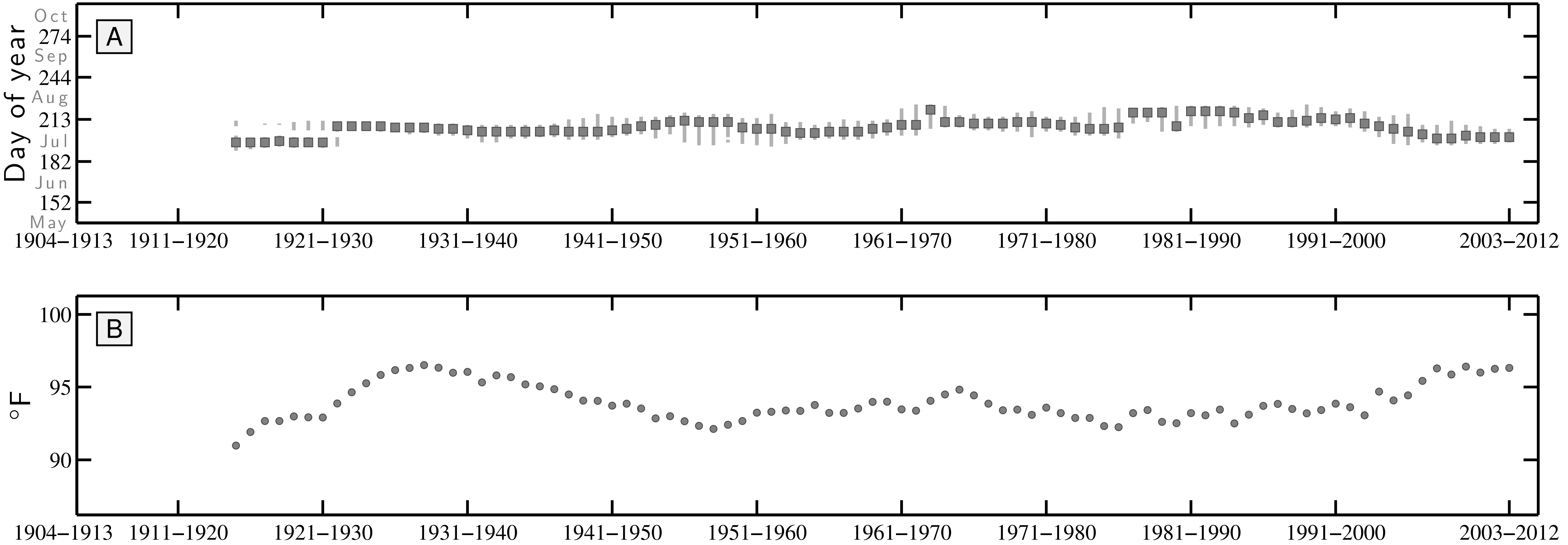

10 year Winter Teletherm dynamics for JEROME, ID:

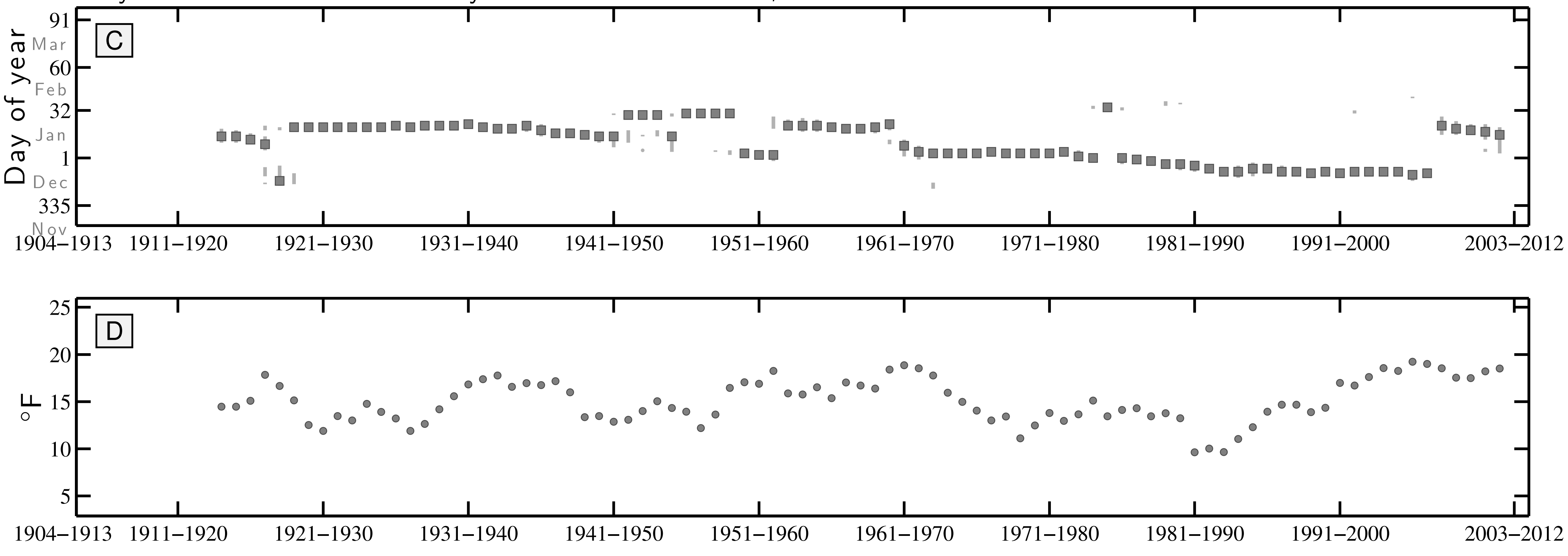

10 year Summer Teletherm dynamics for KELLOGG, ID:

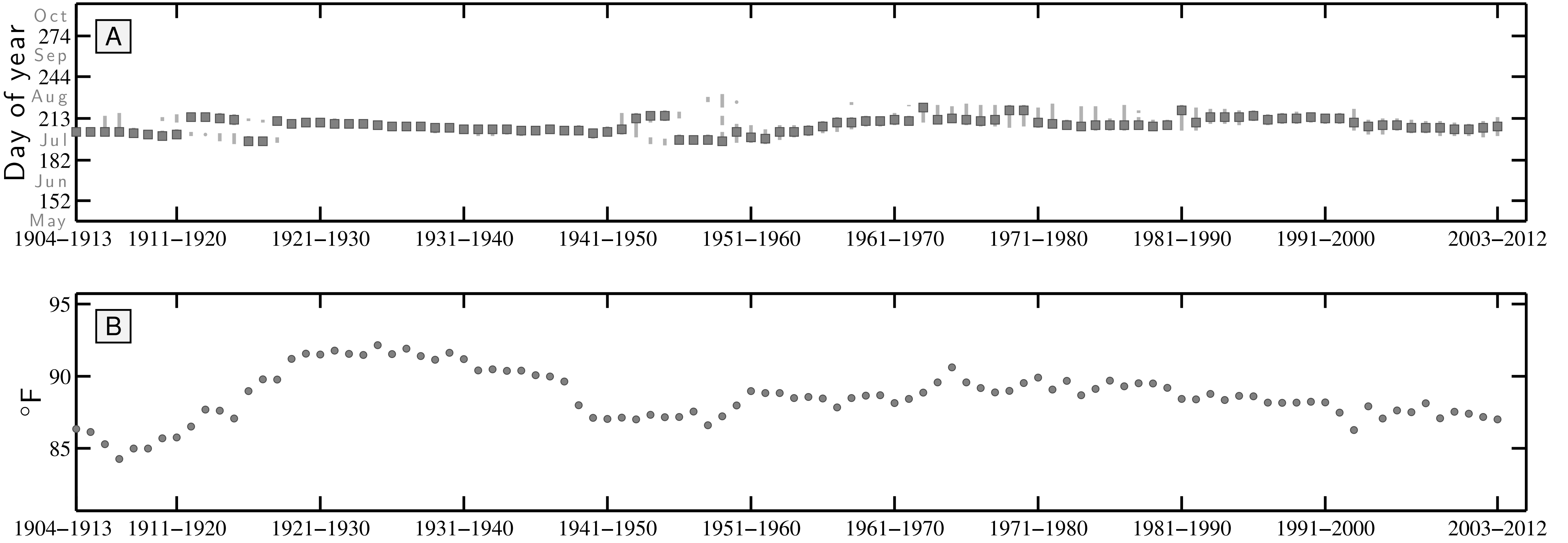

10 year Winter Teletherm dynamics for KELLOGG, ID:

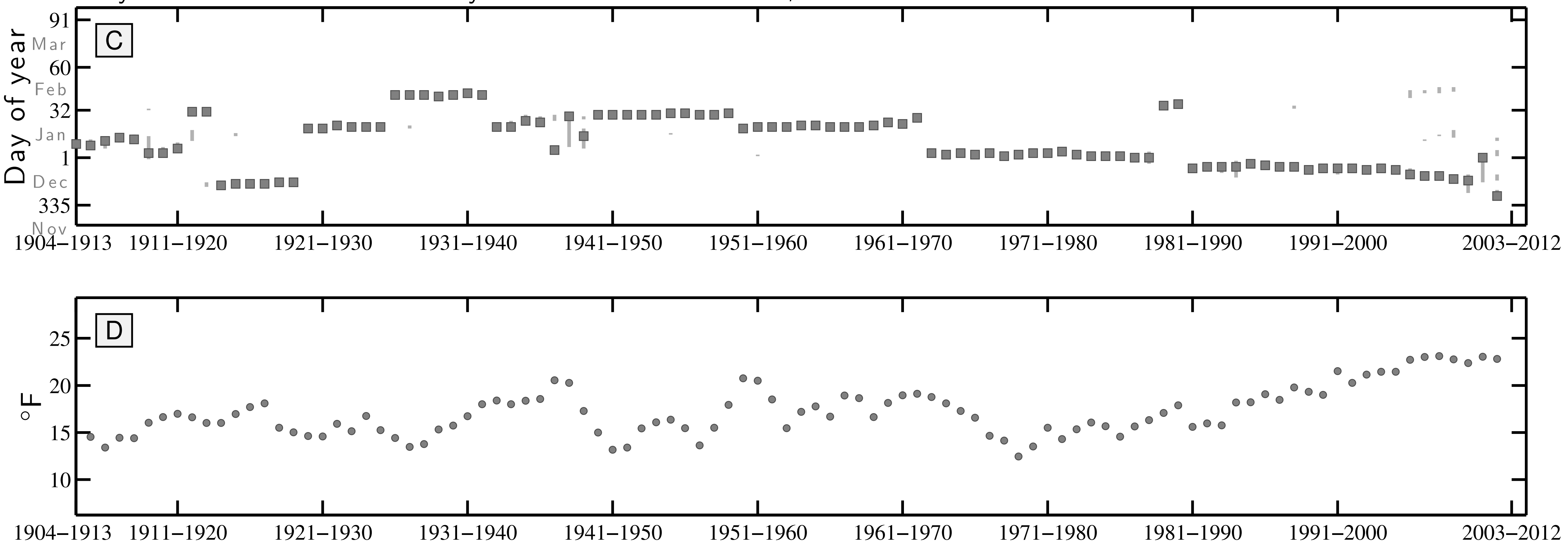

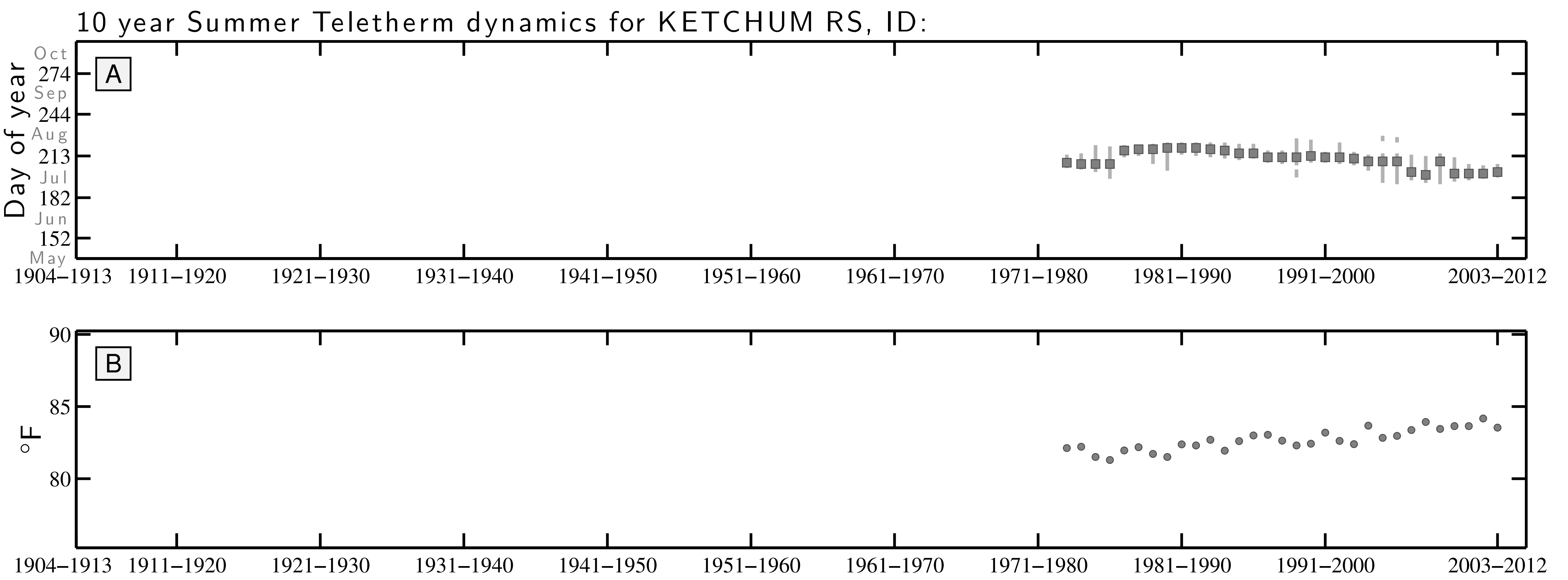
10 year Summer Teletherm dynamics for KETCHUM RS, ID:
A
B
Day of year
°F
Oct
274
Sep
244
Aug
213
Jul
182
Jun
152
May
90
85
80
1904–1913
1911–1920
1921–1930
1931–1940
1941–1950
1951–1960
1961–1970
1971–1980
1981–1990
1991–2000
2003–2012

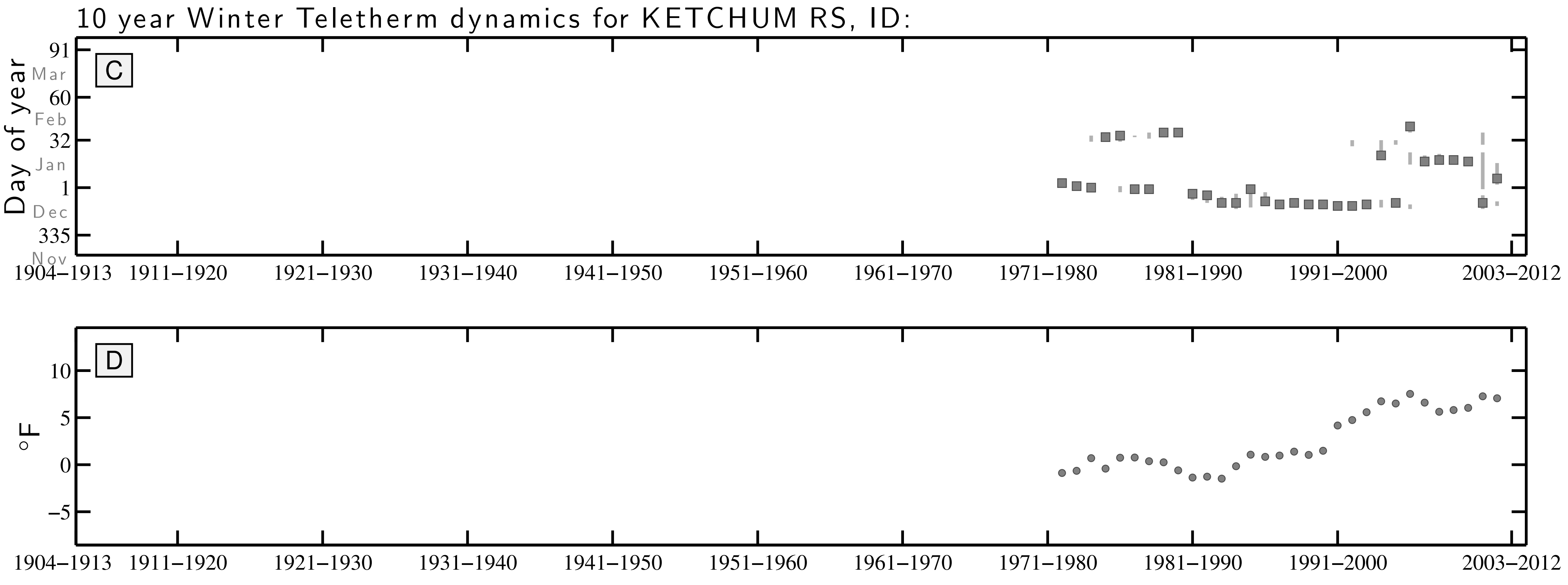
10 year Winter Teletherm dynamics for KETCHUM RS, ID:
C
D
Day of year
°F
91
Mar
60
Feb
32
Jan
1
Dec
335
Nov
10
5
0
−5
1904–1913
1911–1920
1921–1930
1931–1940
1941–1950
1951–1960
1961–1970
1971–1980
1981–1990
1991–2000
2003–2012

10 year Summer Teletherm dynamics for LEWISTON AP, ID:

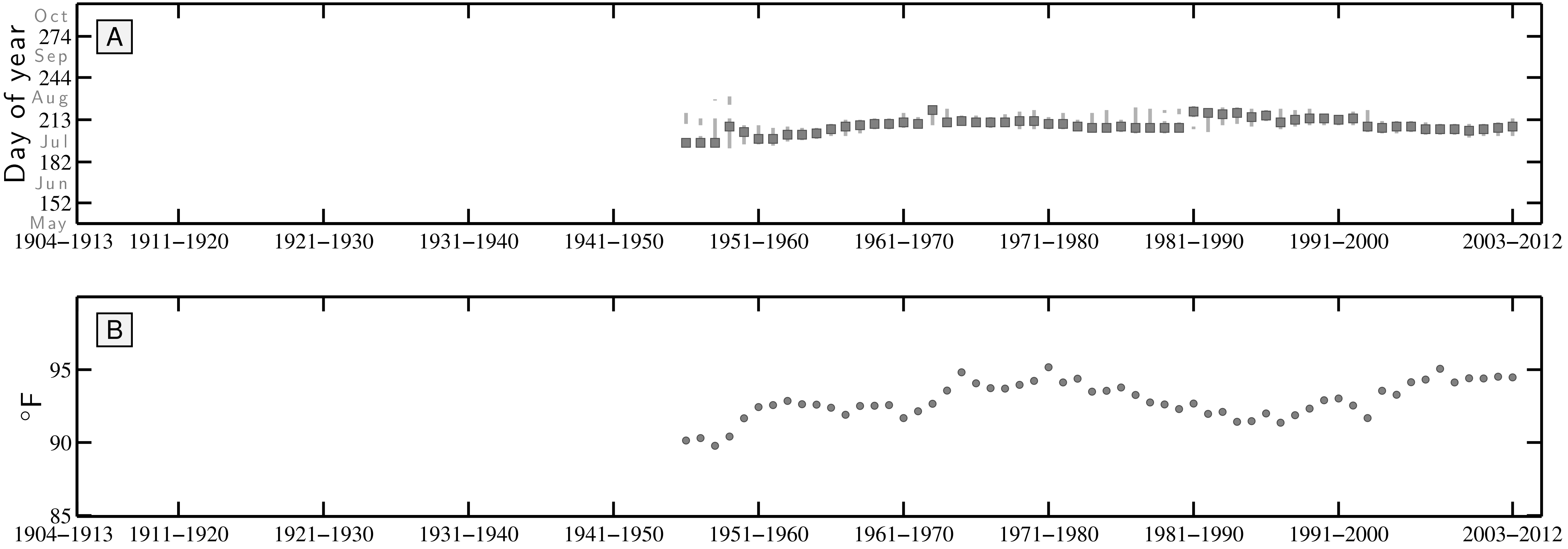

10 year Winter Teletherm dynamics for LEWISTON AP, ID:

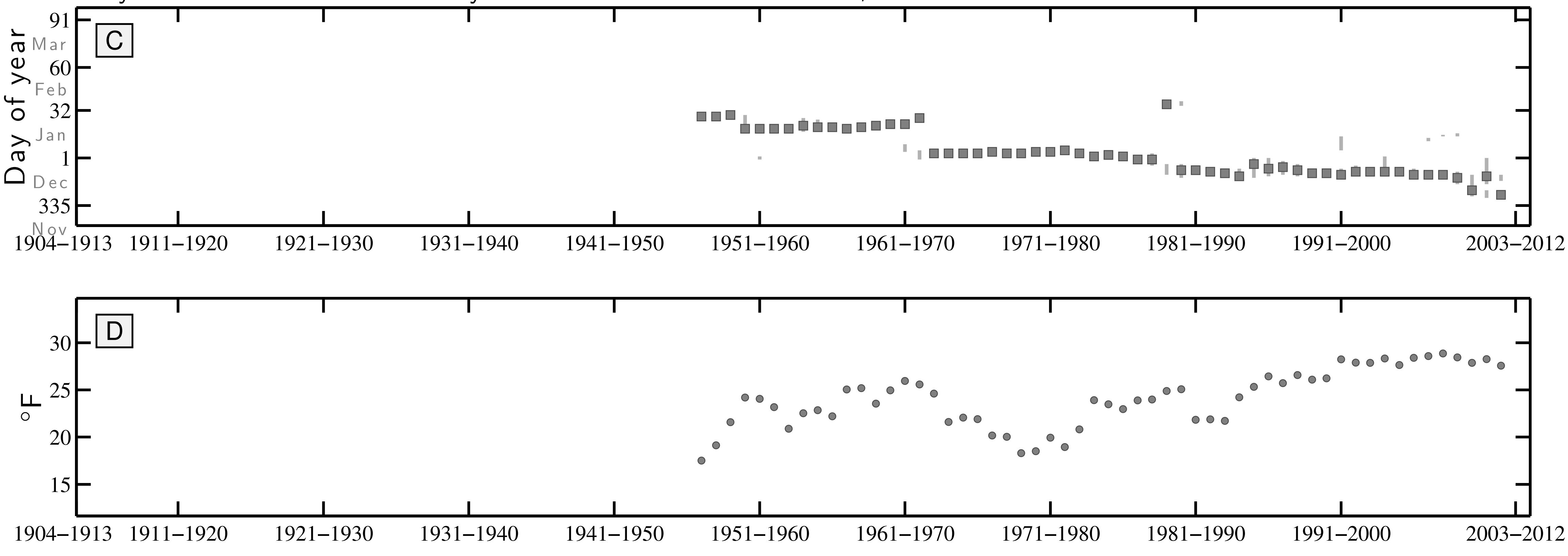

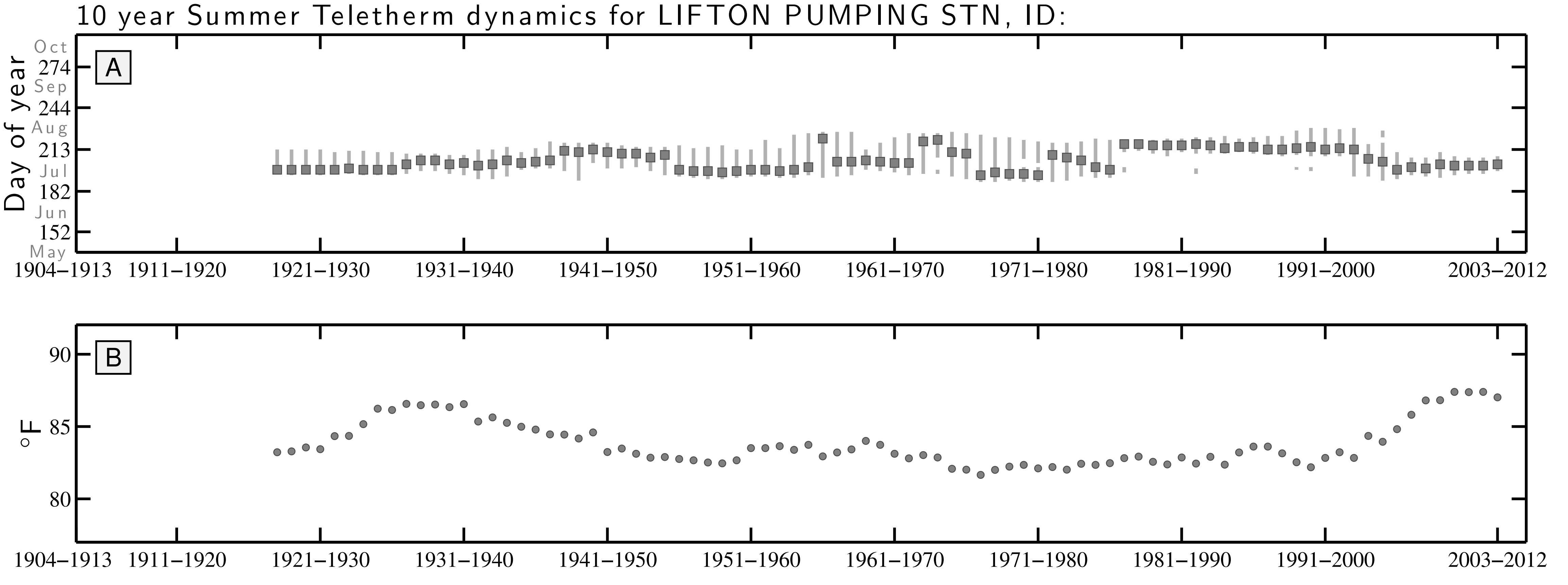
10 year Summer Teletherm dynamics for LIFTON PUMPING STN, ID:
A
B
Day of year
°F
Oct
274
Sep
244
Aug
213
Jul
182
Jun
152
May
90
85
80
1904–1913
1911–1920
1921–1930
1931–1940
1941–1950
1951–1960
1961–1970
1971–1980
1981–1990
1991–2000
2003–2012

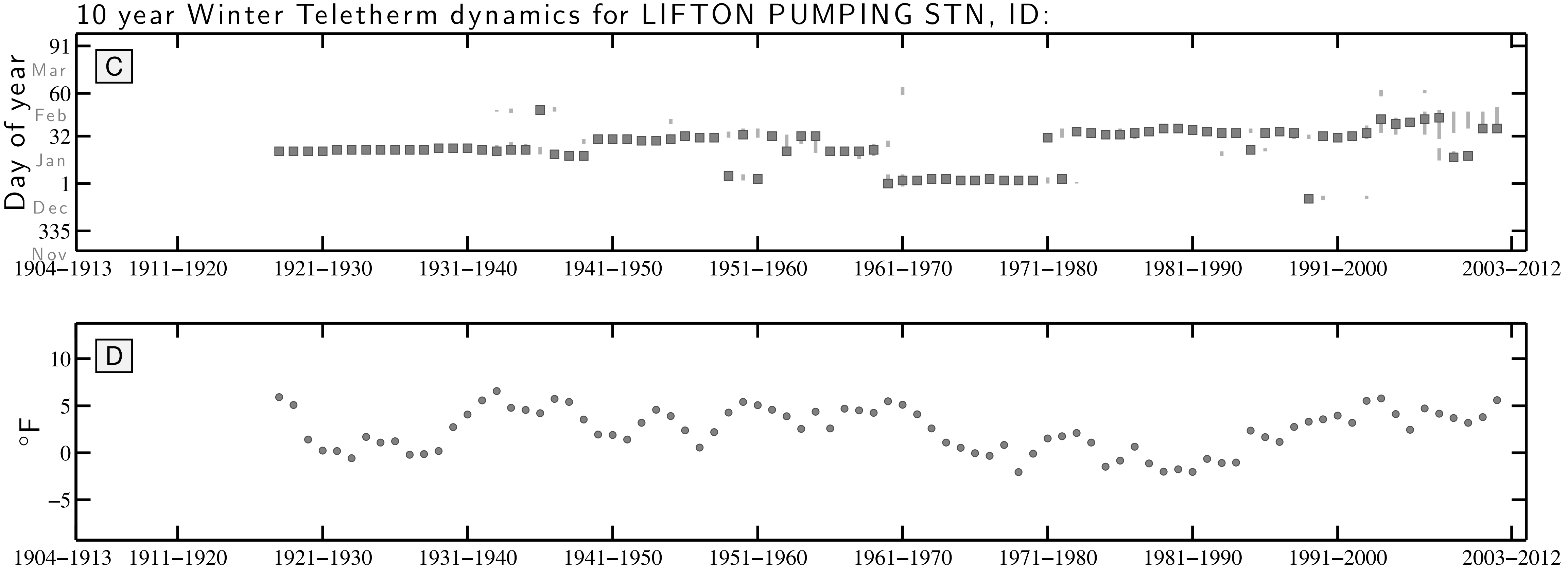
10 year Winter Teletherm dynamics for LIFTON PUMPING STN, ID:
C
D
Day of year
°F
91
Mar
60
Feb
32
Jan
1
Dec
335
Nov
10
5
0
−5
1904–1913
1911–1920
1921–1930
1931–1940
1941–1950
1951–1960
1961–1970
1971–1980
1981–1990
1991–2000
2003–2012

10 year Summer Teletherm dynamics for MACKAY LOST RIVER RS, ID:

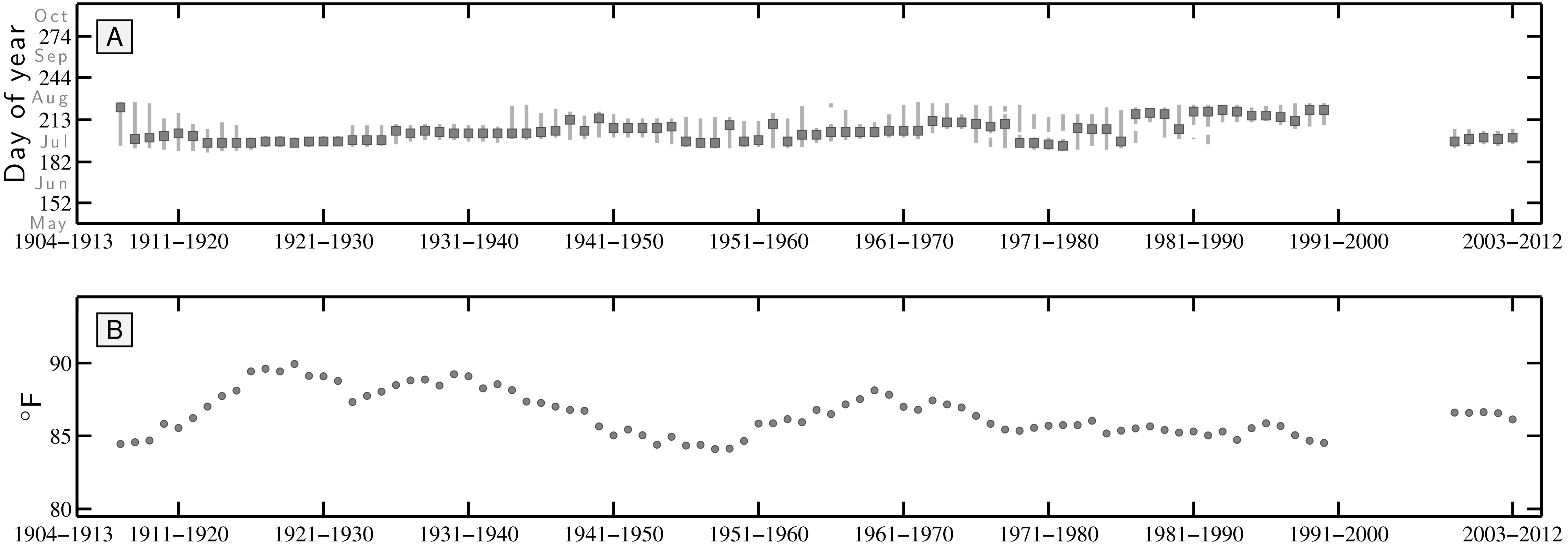

10 year Winter Teletherm dynamics for MACKAY LOST RIVER RS, ID:

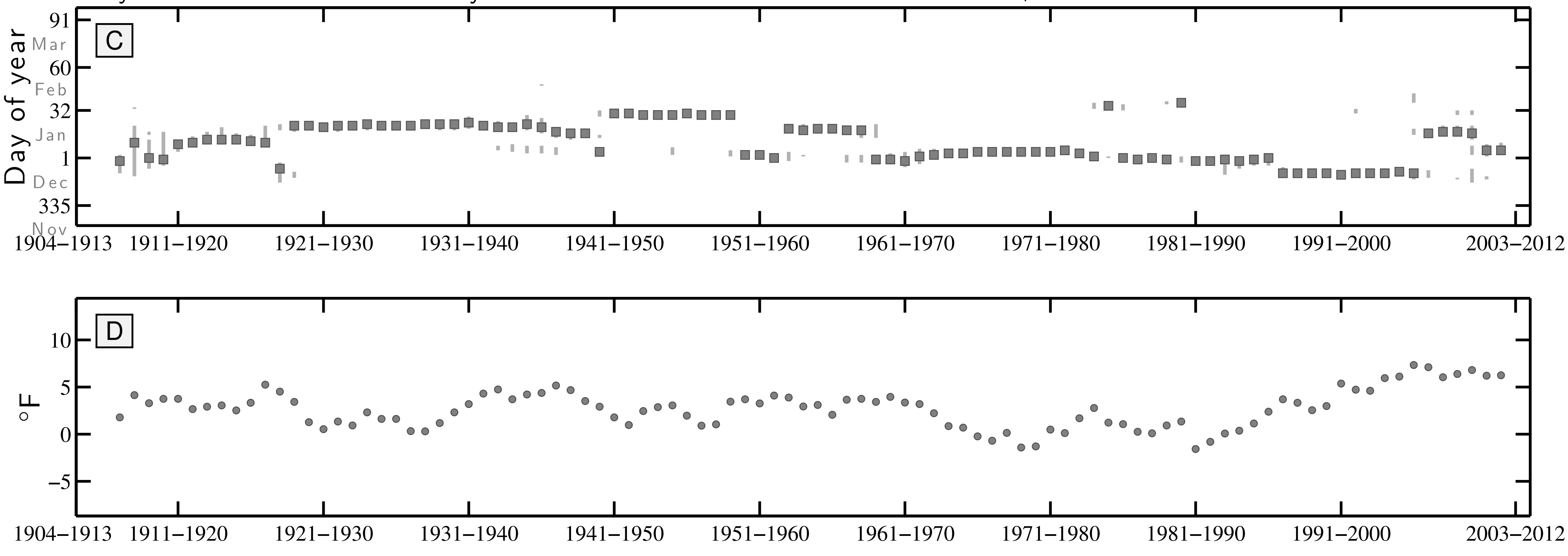

10 year Summer Teletherm dynamics for MALAD CITY, ID:

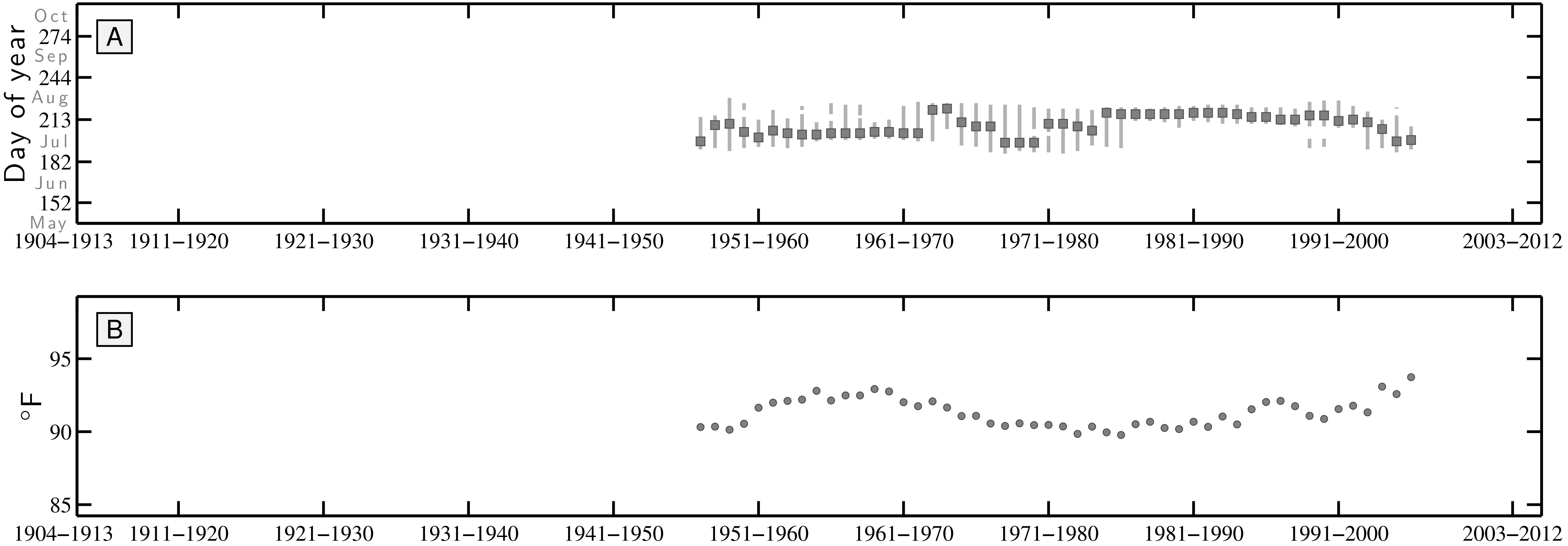

10 year Winter Teletherm dynamics for MALAD CITY, ID:

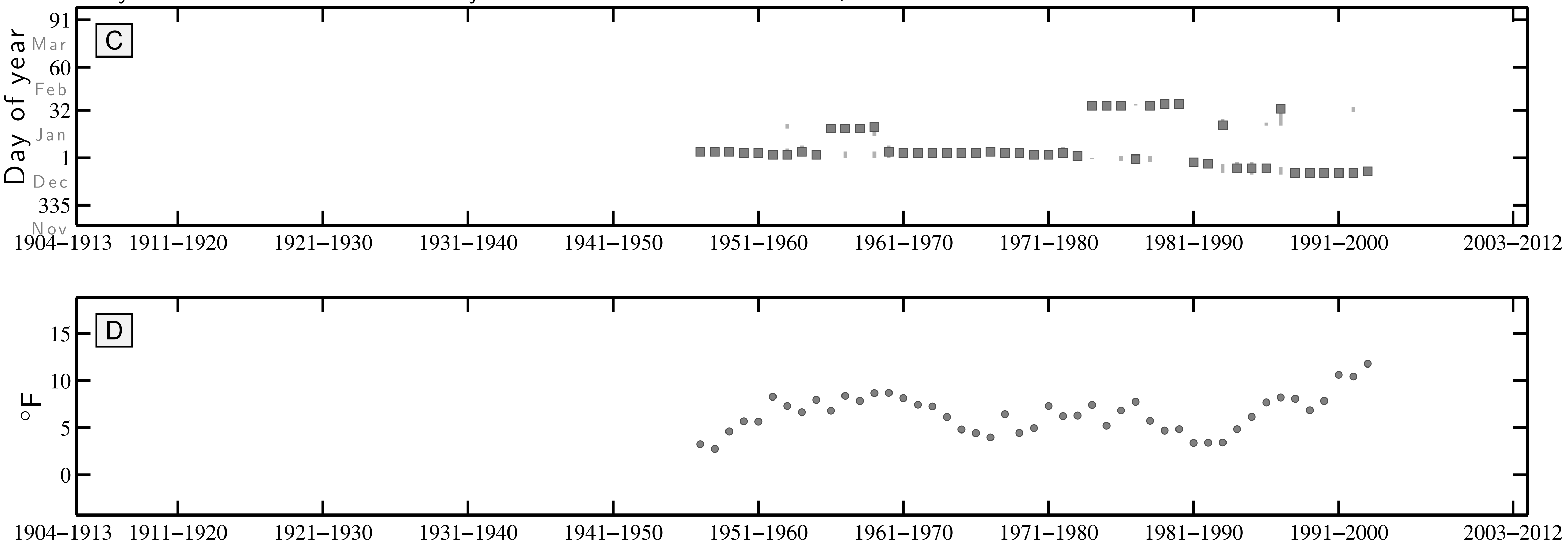

10 year Summer Teletherm dynamics for MAY 2SSE, ID:

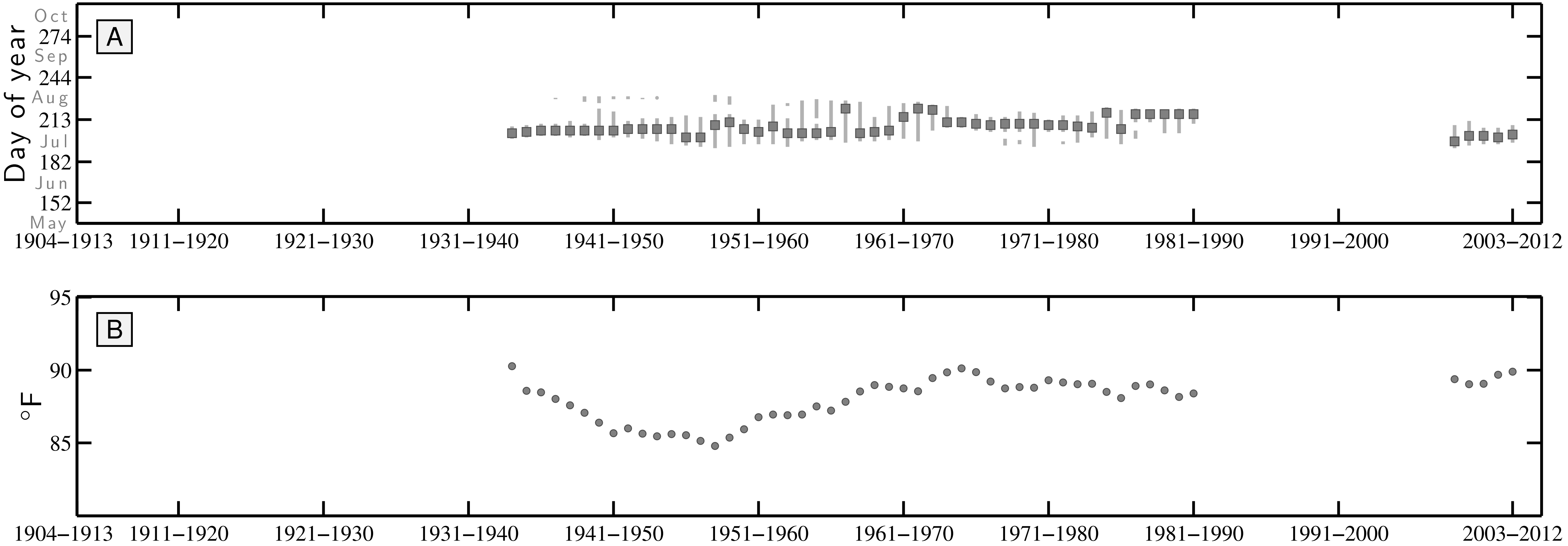

10 year Winter Teletherm dynamics for MAY 2SSE, ID:

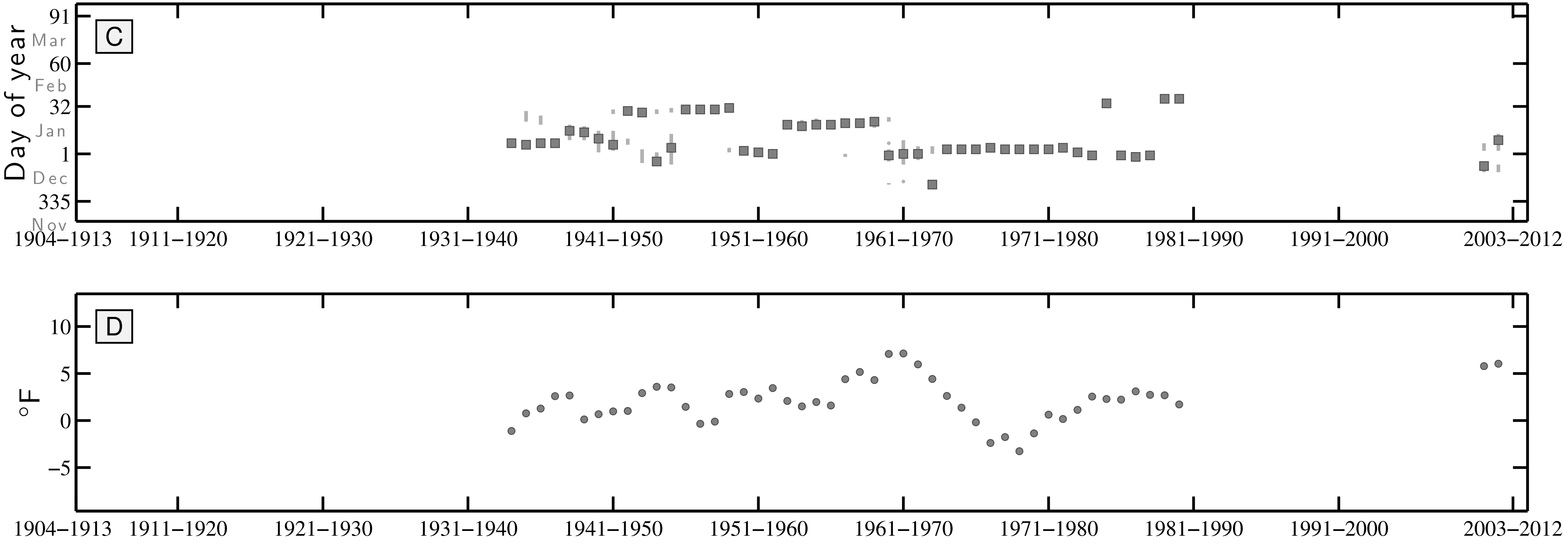

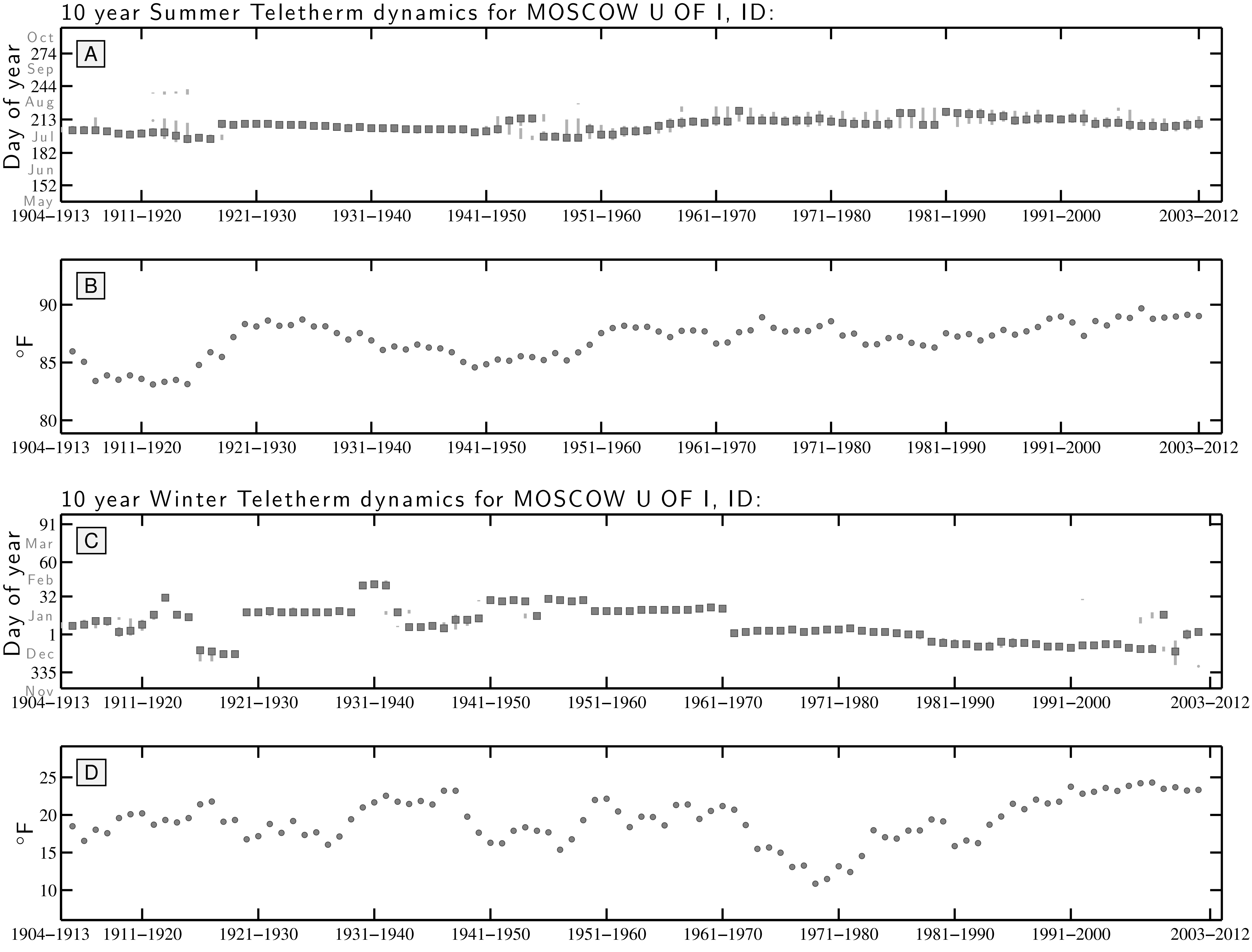
10 year Summer Teletherm dynamics for MOSCOW U OF I, ID:
A
Day of year
Oct
274
Sep
244
Aug
213
Jul
182
Jun
152
May
B
°F
90
85
80
10 year Winter Teletherm dynamics for MOSCOW U OF I, ID:
C
91
Mar
60
Feb
32
Jan
1
Dec
335
Nov
D
25
20
15
10
1904–1913
1911–1920
1921–1930
1931–1940
1941–1950
1951–1960
1961–1970
1971–1980
1981–1990
1991–2000
2003–2012

10 year Summer Teletherm dynamics for NAMPA SUGAR FACTORY, ID:

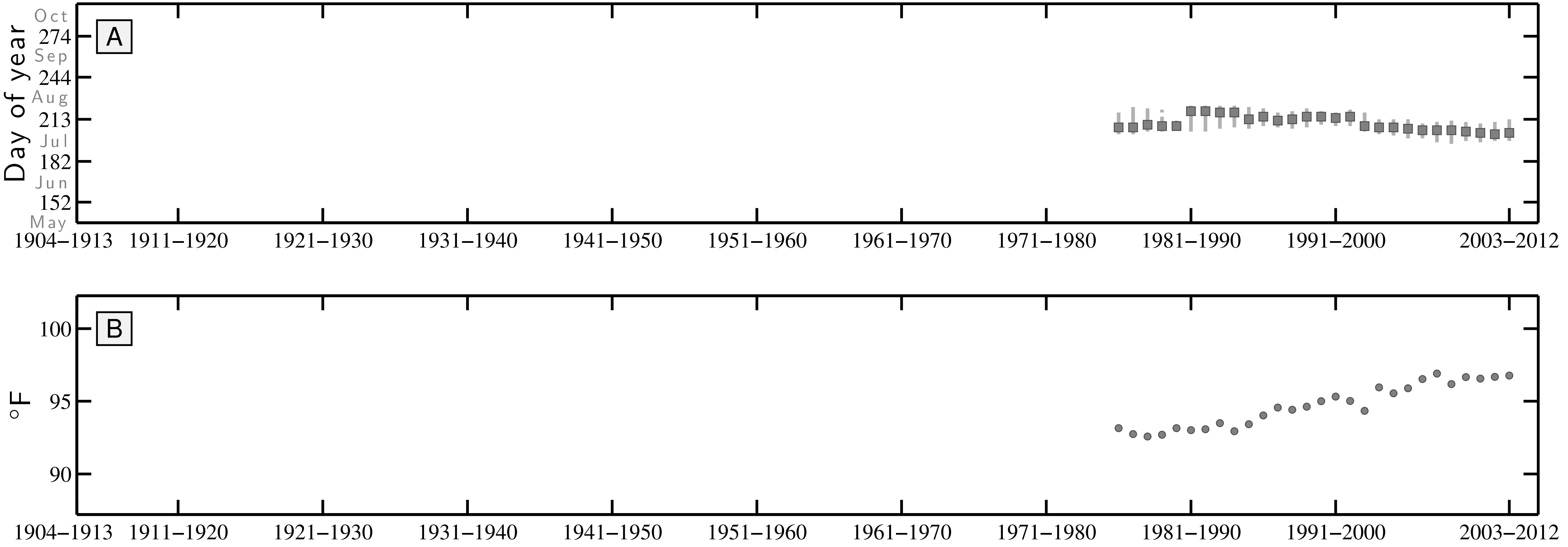

10 year Winter Teletherm dynamics for NAMPA SUGAR FACTORY, ID:

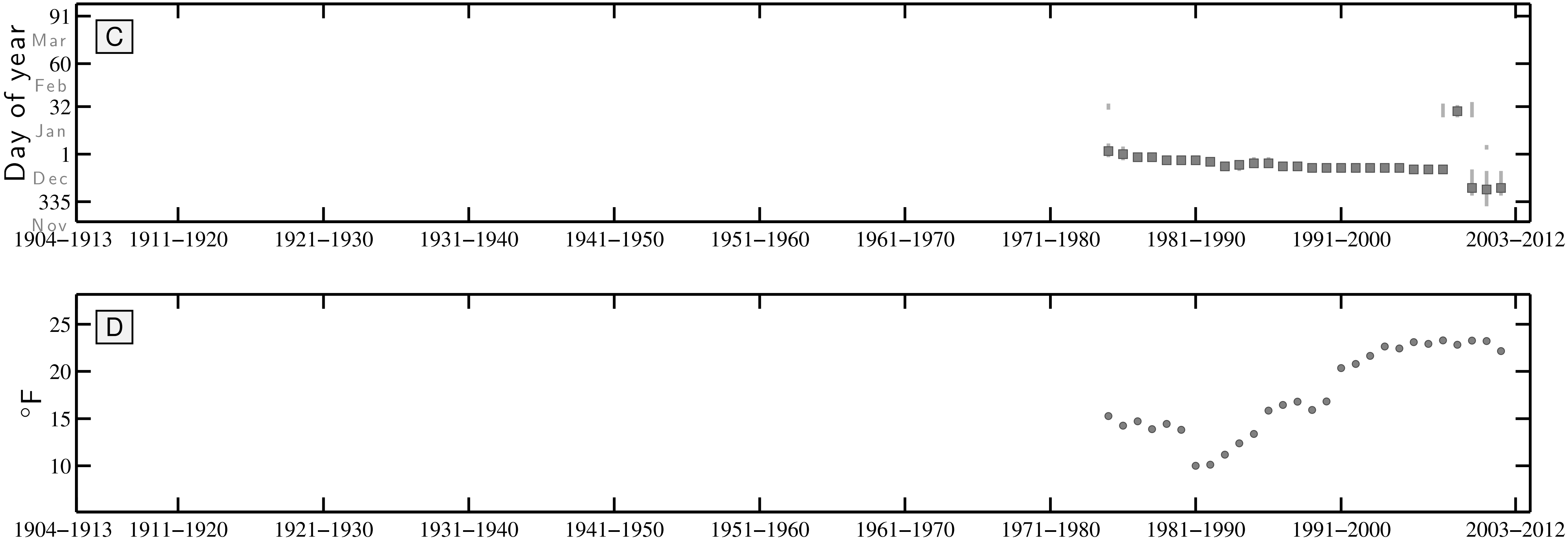

10 year Summer Teletherm dynamics for NEW MEADOWS RS, ID:

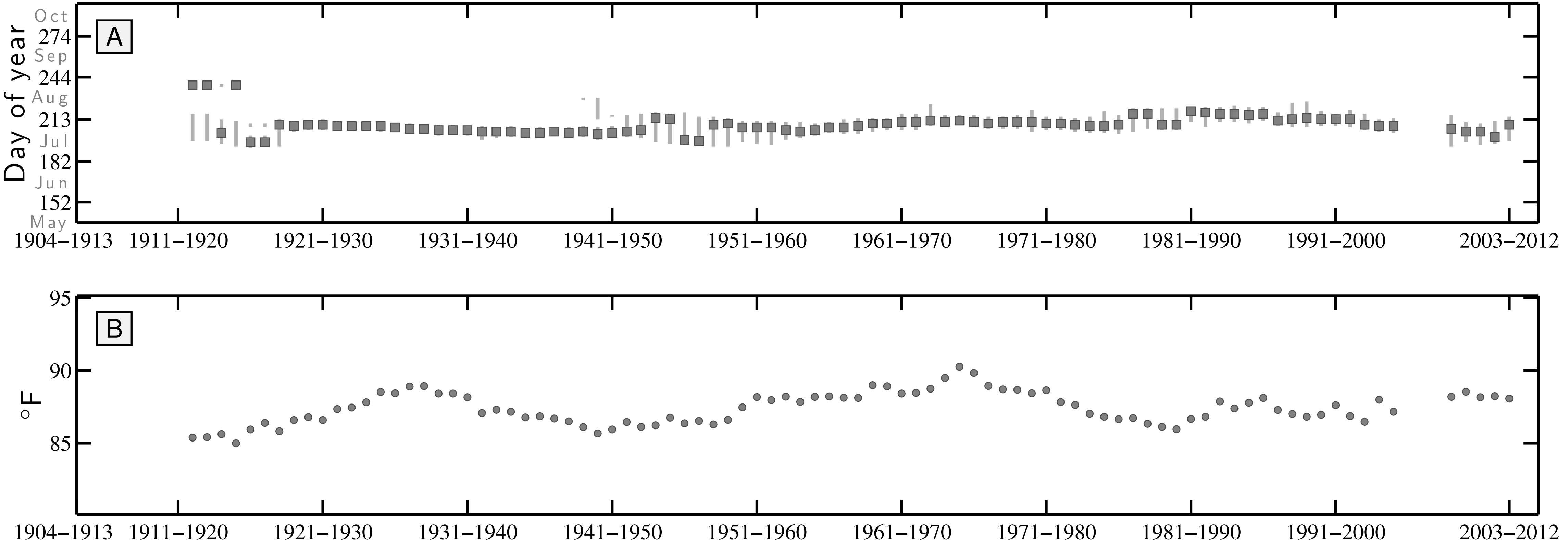

10 year Winter Teletherm dynamics for NEW MEADOWS RS, ID:

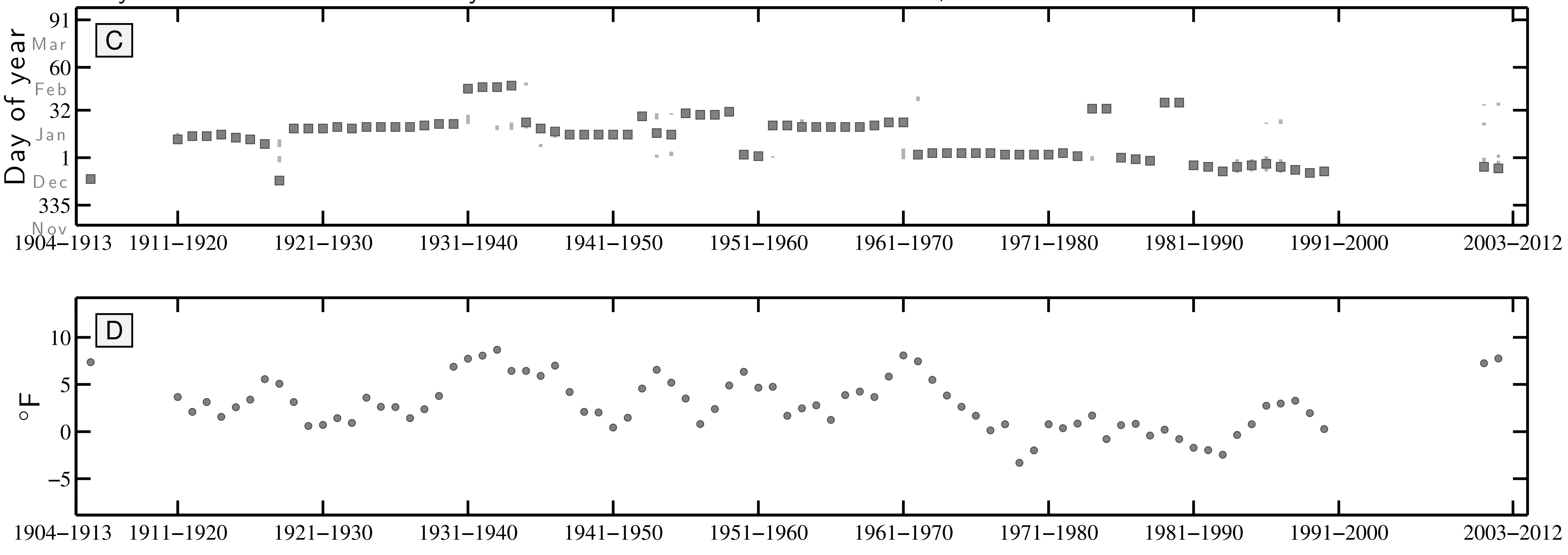

10 year Summer Teletherm dynamics for OAKLEY, ID:

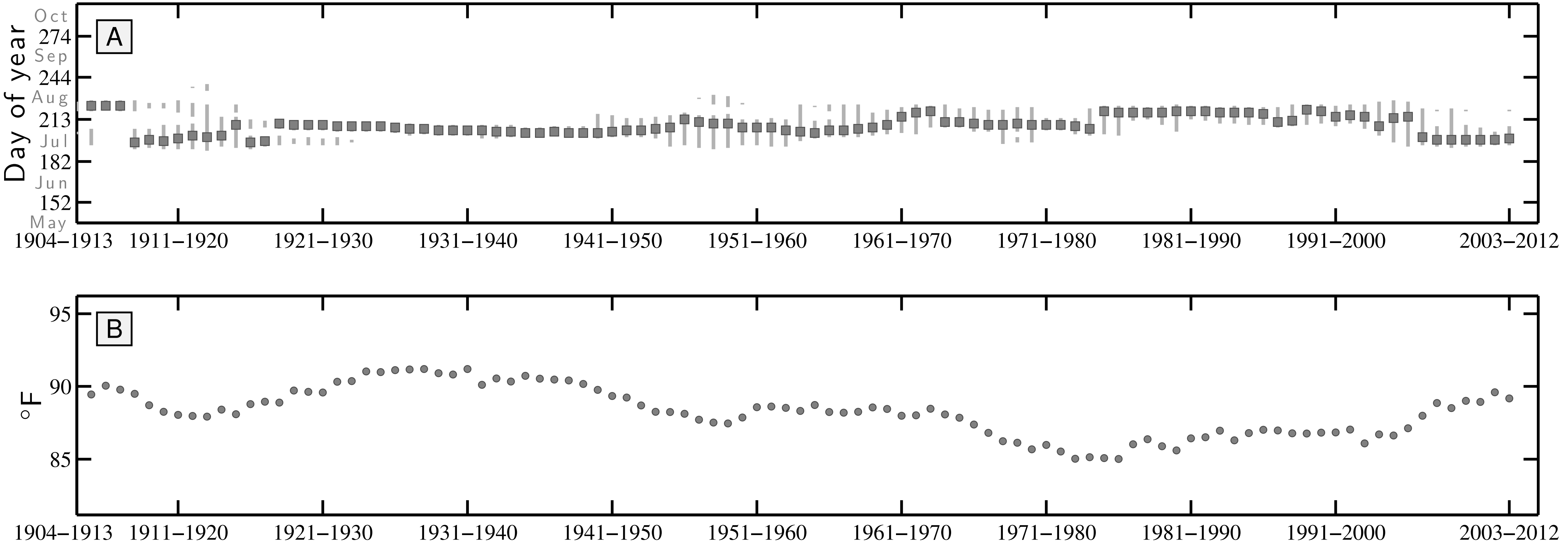

10 year Winter Teletherm dynamics for OAKLEY, ID:

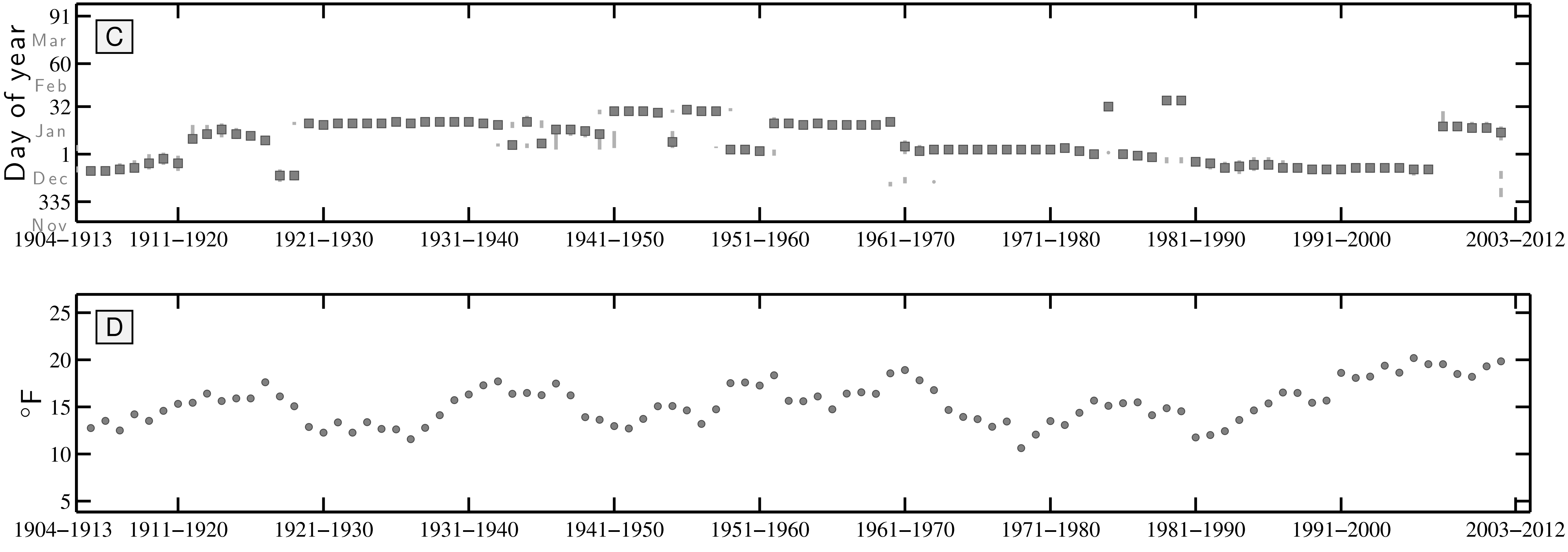

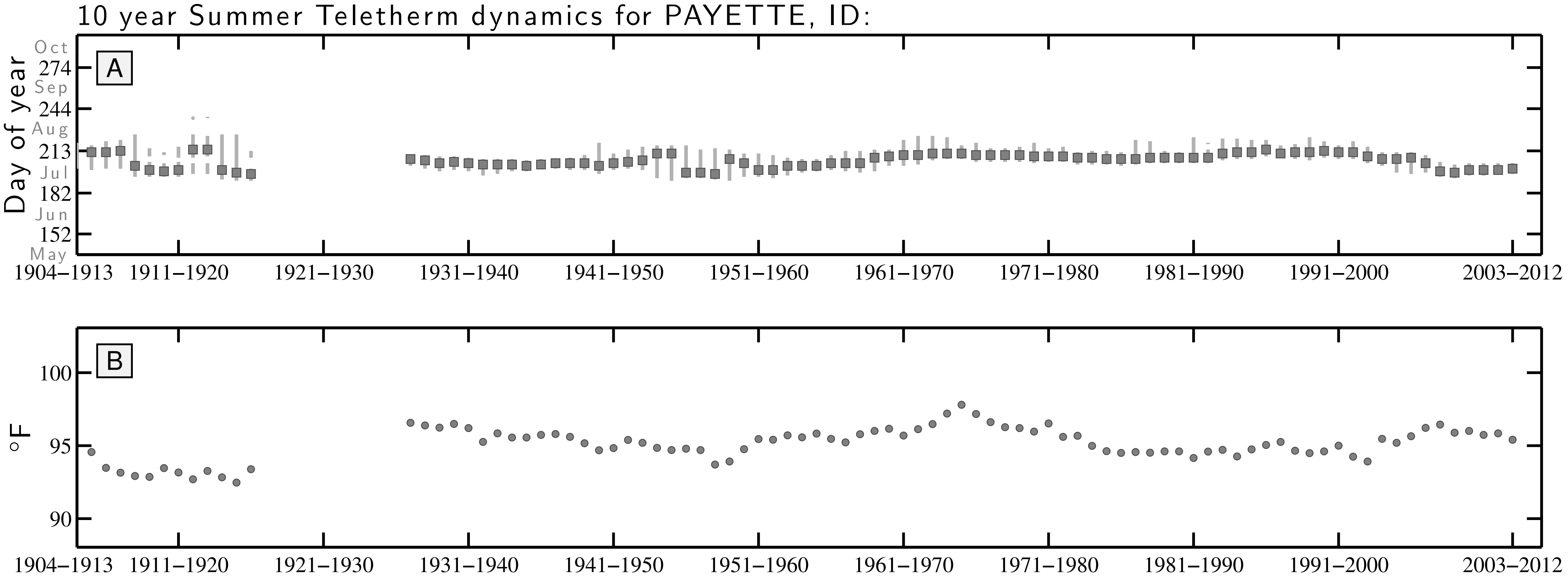
10 year Summer Teletherm dynamics for PAYETTE, ID:
A
B
Day of year
°F
Oct
274
Sep
244
Aug
213
Jul
182
Jun
152
May
100
95
90
1904–1913
1911–1920
1921–1930
1931–1940
1941–1950
1951–1960
1961–1970
1971–1980
1981–1990
1991–2000
2003–2012

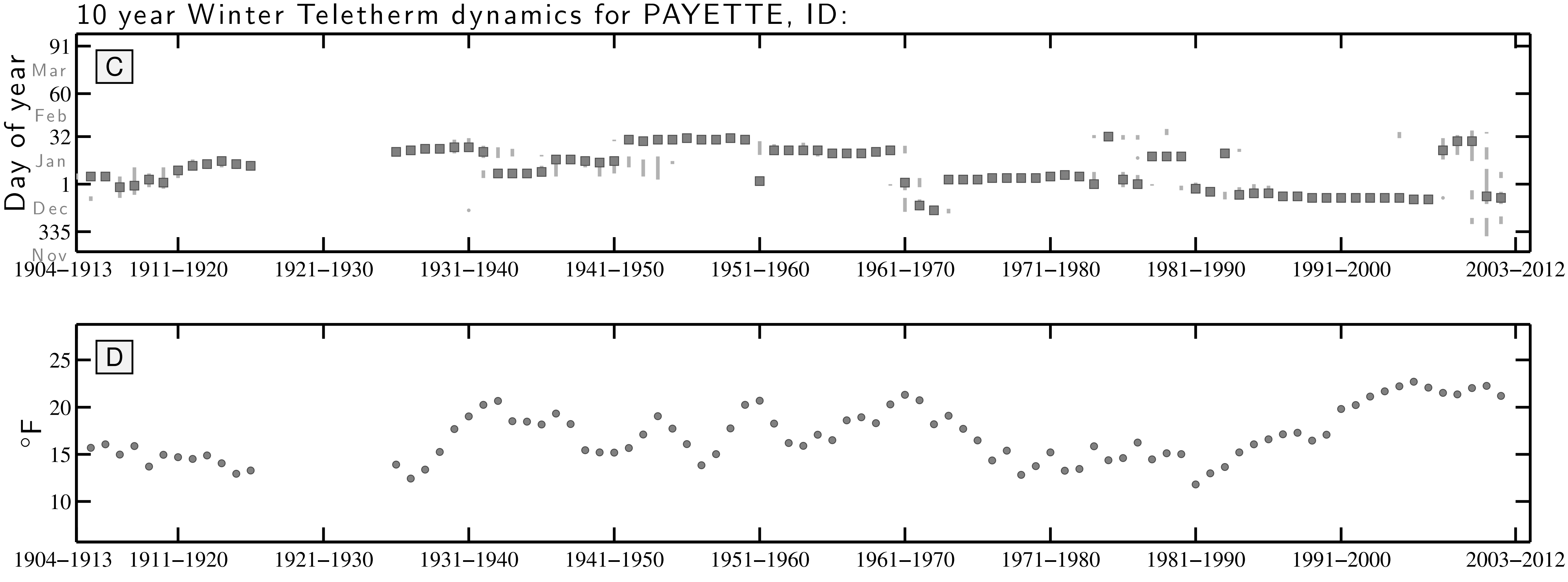
10 year Winter Teletherm dynamics for PAYETTE, ID:
C
D
Day of year
°F
91
Mar
60
Feb
32
Jan
1
Dec
335
Nov
25
20
15
10
1904–1913
1911–1920
1921–1930
1931–1940
1941–1950
1951–1960
1961–1970
1971–1980
1981–1990
1991–2000
2003–2012

10 year Summer Teletherm dynamics for PORTHILL, ID:

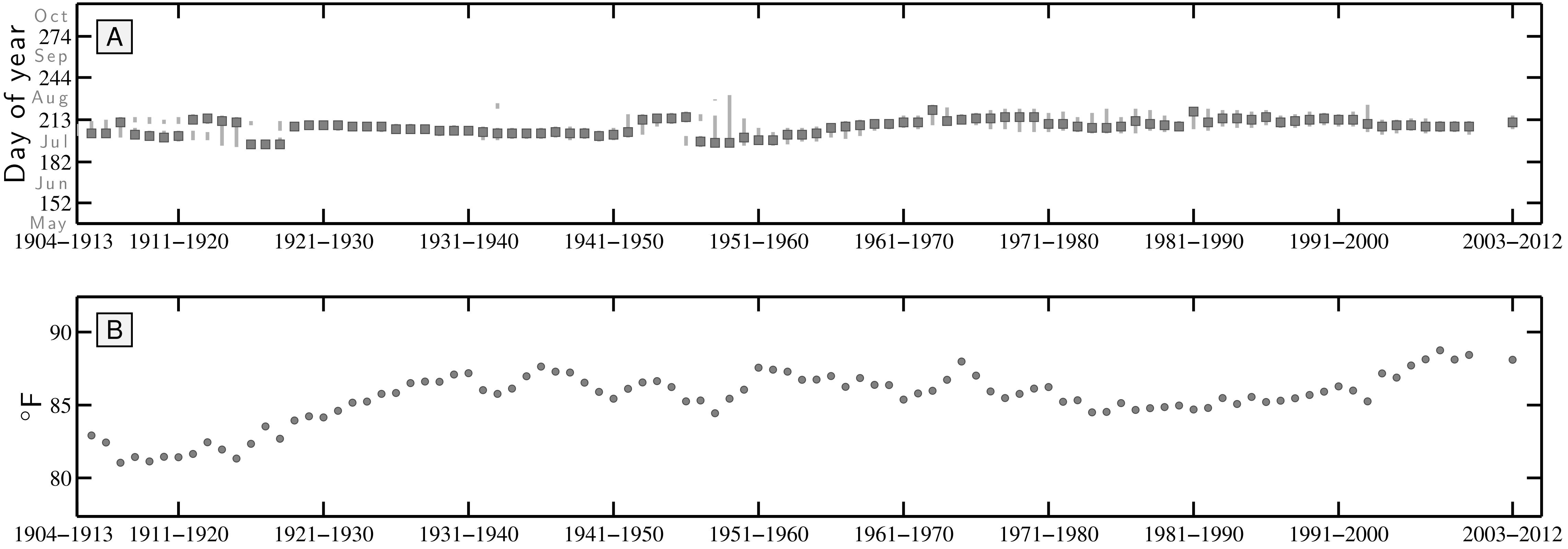

10 year Winter Teletherm dynamics for PORTHILL, ID:

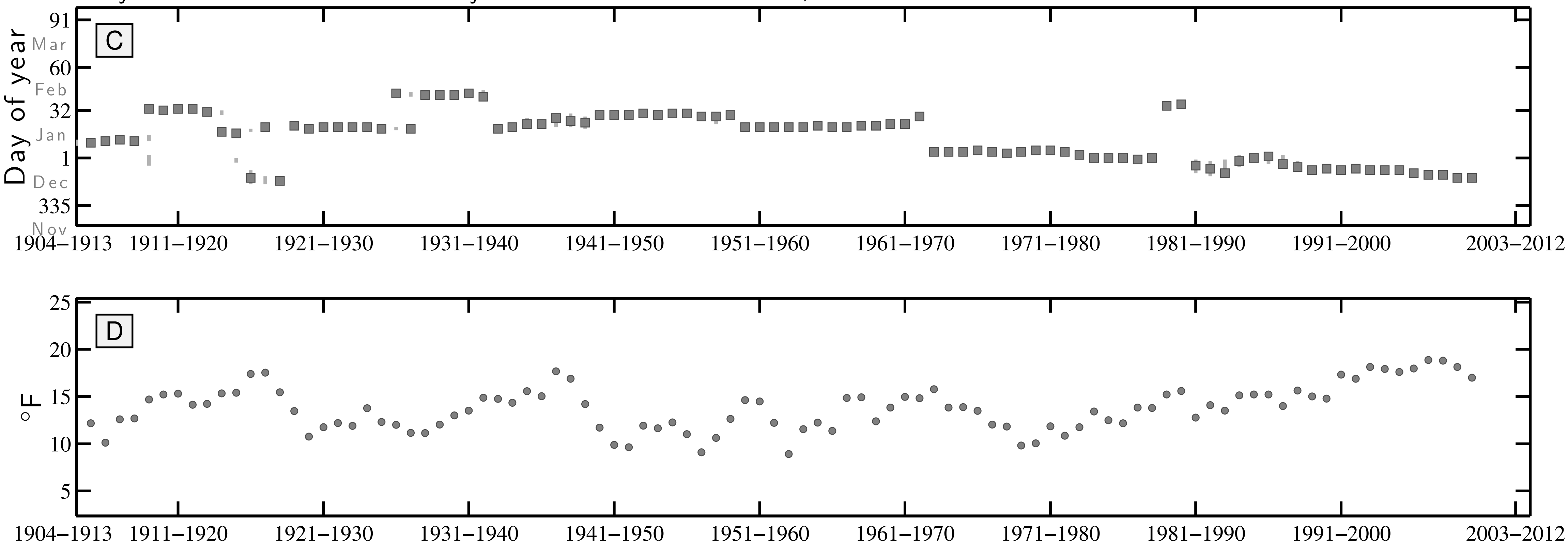

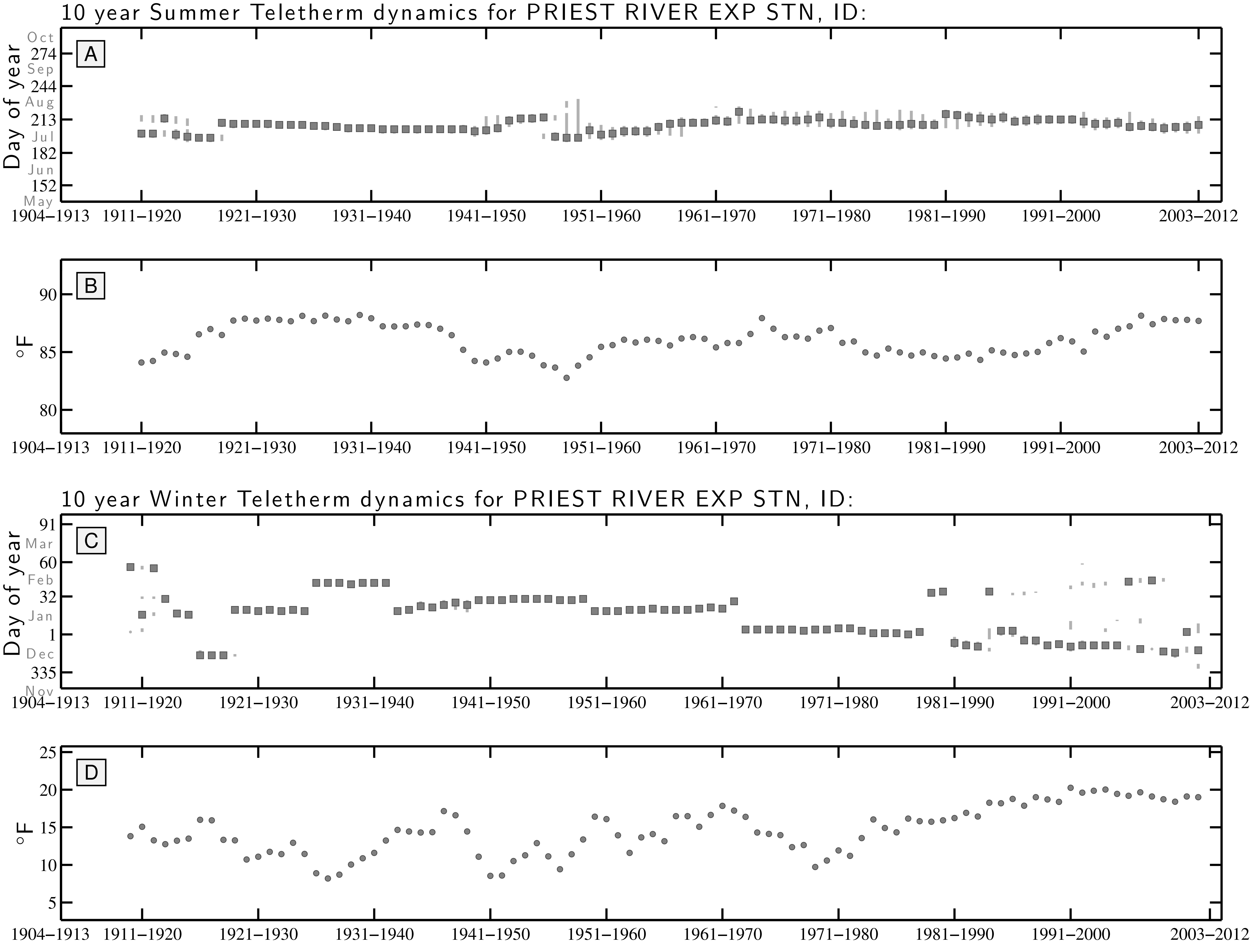
10 year Summer Teletherm dynamics for PRIEST RIVER EXP STN, ID:
A
Day of year
Oct
274
Sep
244
Aug
213
Jul
182
Jun
152
May
B
°F
90
85
80
10 year Winter Teletherm dynamics for PRIEST RIVER EXP STN, ID:
C
Day of year
91
Mar
60
Feb
32
Jan
1
Dec
335
Nov
D
°F
25
20
15
10
5
1904–1913
1911–1920
1921–1930
1931–1940
1941–1950
1951–1960
1961–1970
1971–1980
1981–1990
1991–2000
2003–2012

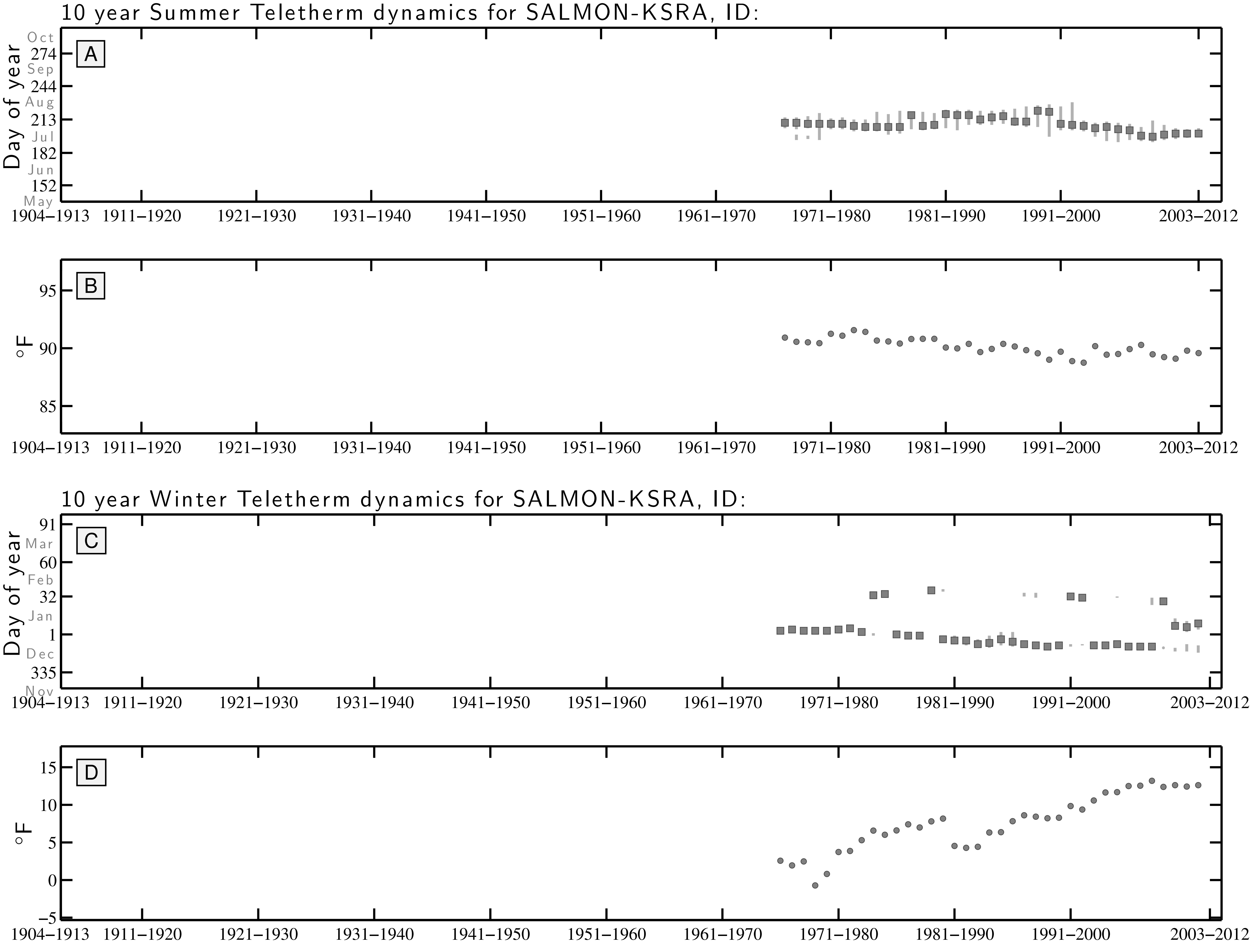
10 year Summer Teletherm dynamics for SALMON-KSRA, ID:
A
Day of year
Oct
274
Sep
244
Aug
213
Jul
182
Jun
152
May
B
°F
95
90
85
10 year Winter Teletherm dynamics for SALMON-KSRA, ID:
C
Day of year
91
Mar
60
Feb
32
Jan
1
Dec
335
Nov
D
°F
15
10
5
0
−5
1904–1913
1911–1920
1921–1930
1931–1940
1941–1950
1951–1960
1961–1970
1971–1980
1981–1990
1991–2000
2003–2012

10 year Summer Teletherm dynamics for SANDPOINT EXP STN, ID:

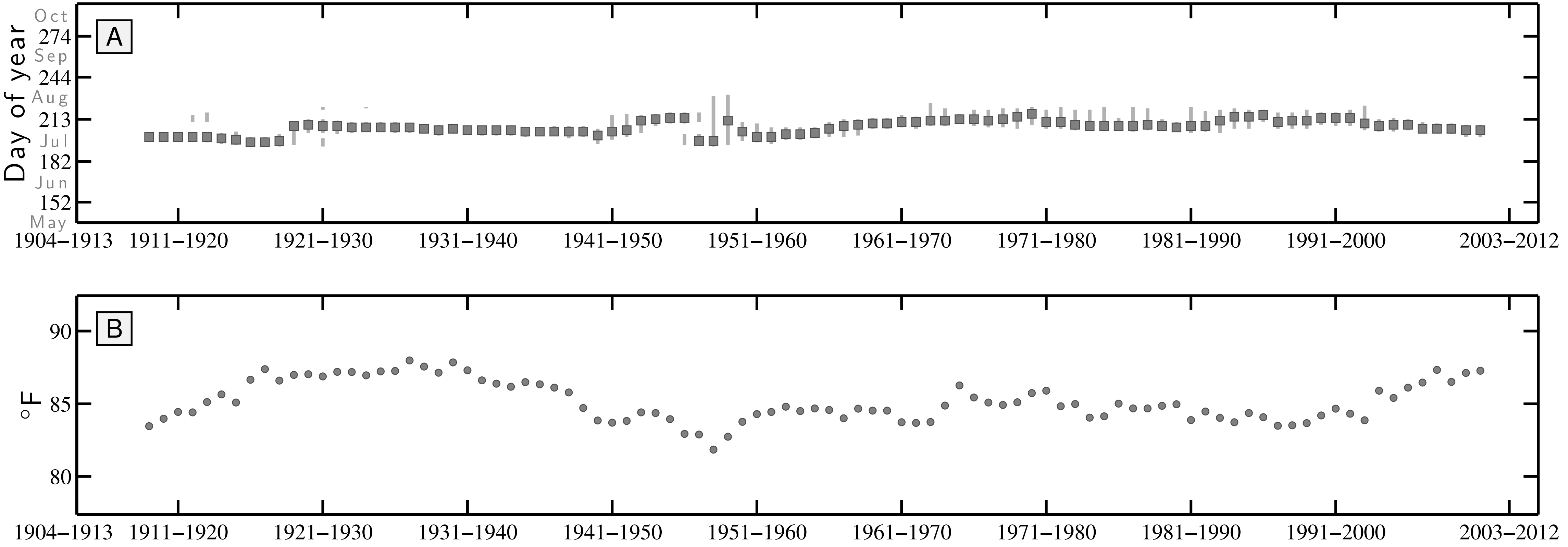

10 year Winter Teletherm dynamics for SANDPOINT EXP STN, ID:

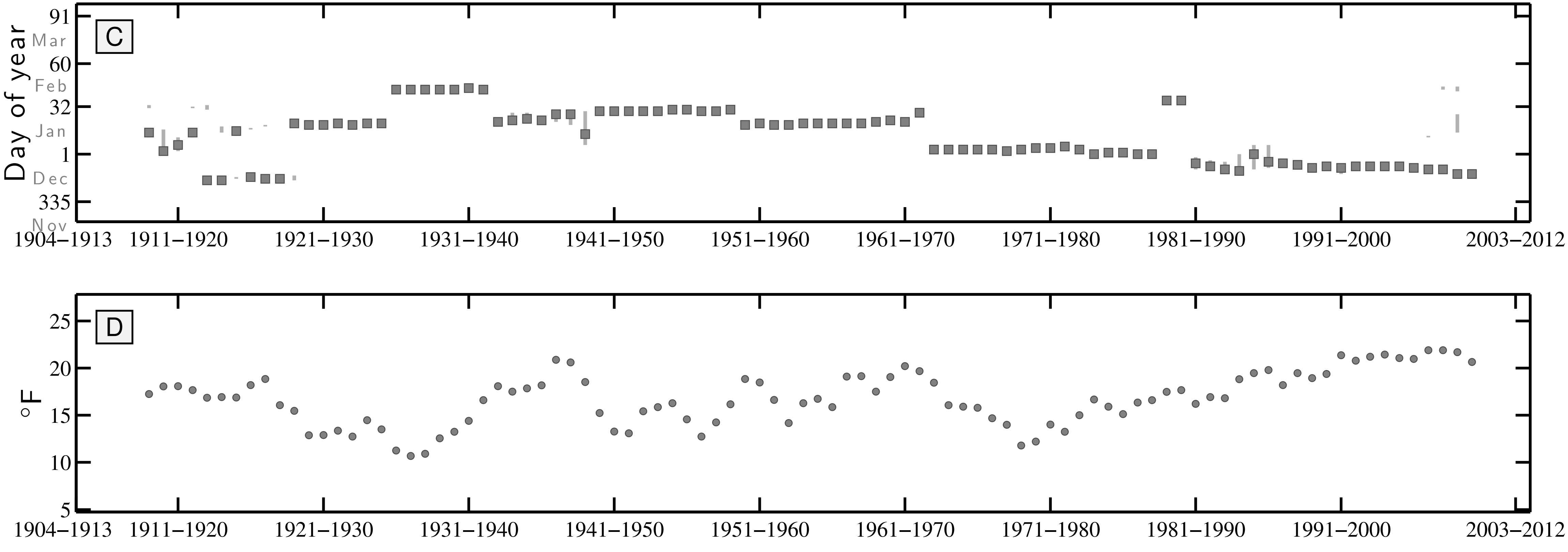

10 year Summer Teletherm dynamics for ALEDO, IL:

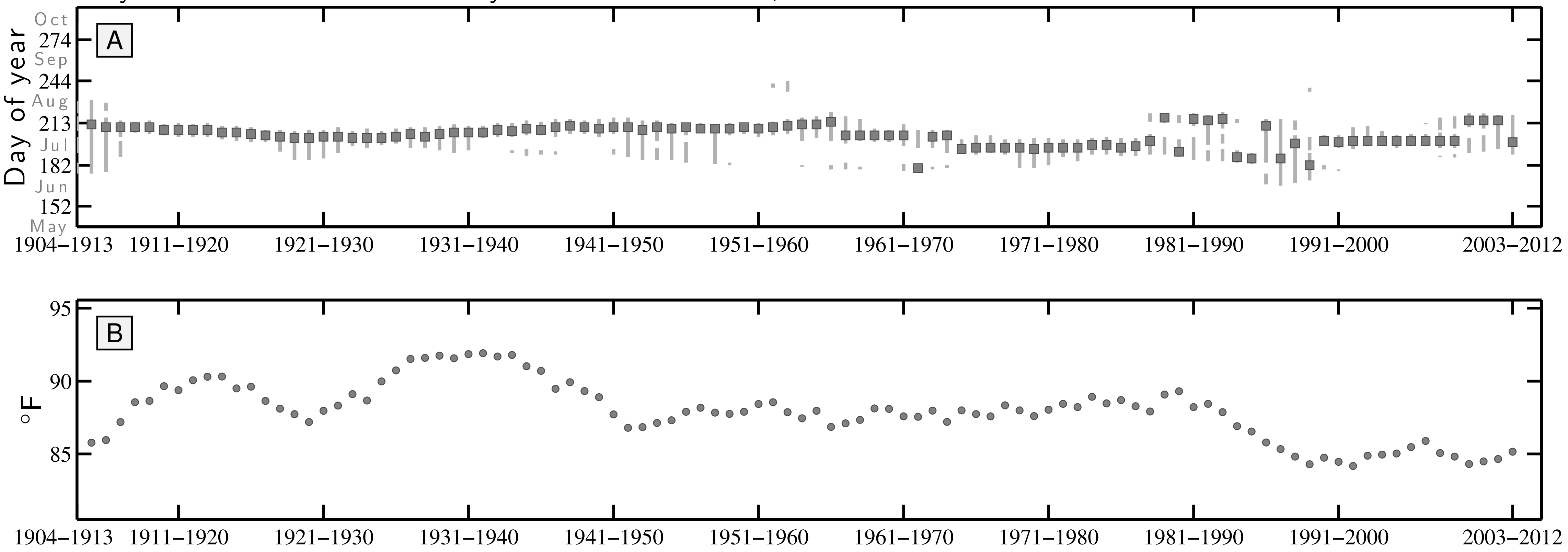

10 year Winter Teletherm dynamics for ALEDO, IL:

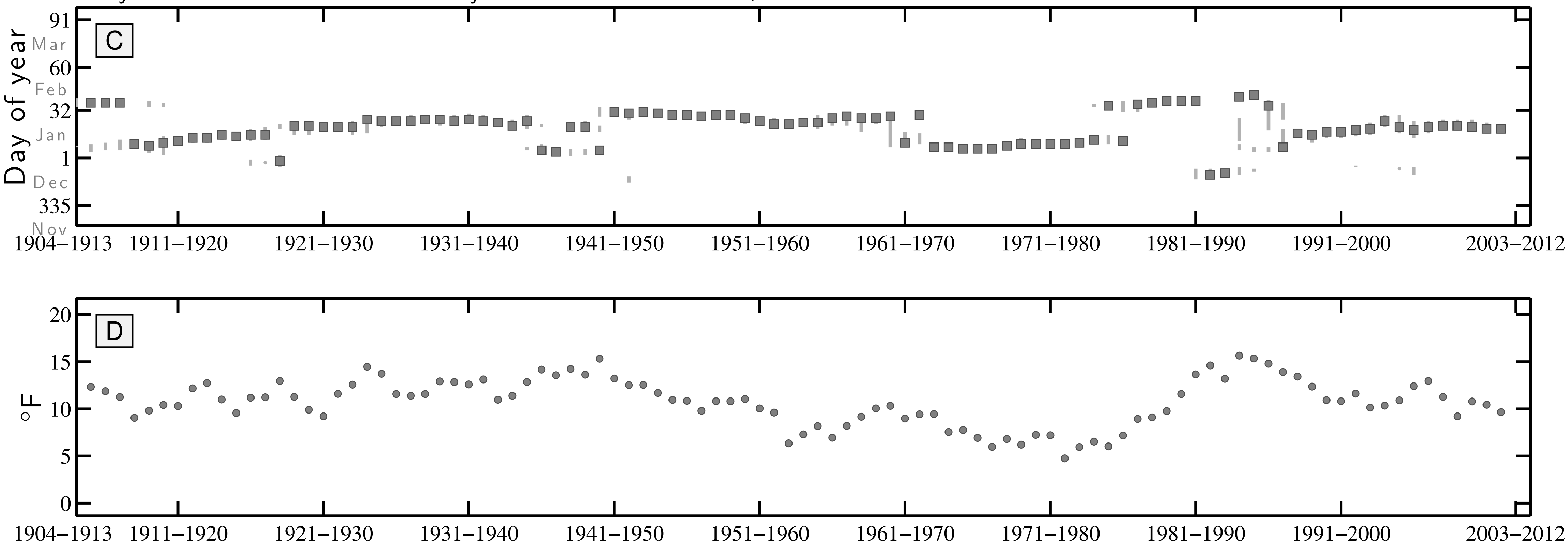

10 year Summer Teletherm dynamics for ANNA 2 NNE, IL:

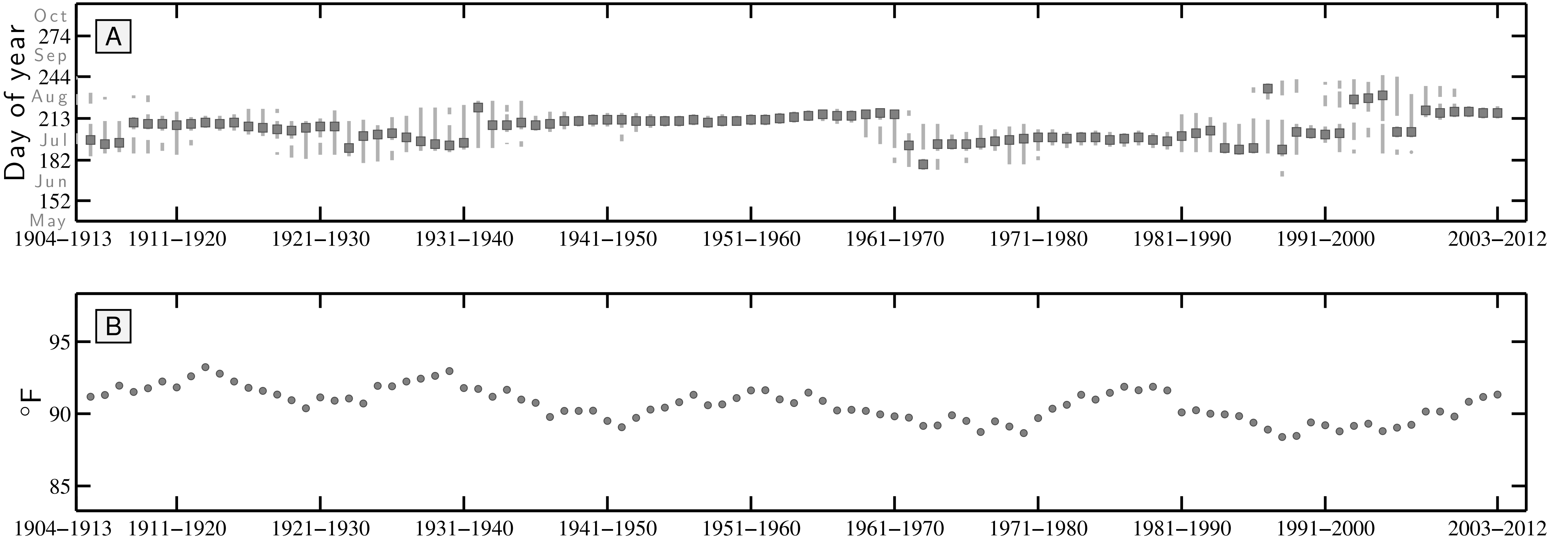

10 year Winter Teletherm dynamics for ANNA 2 NNE, IL:

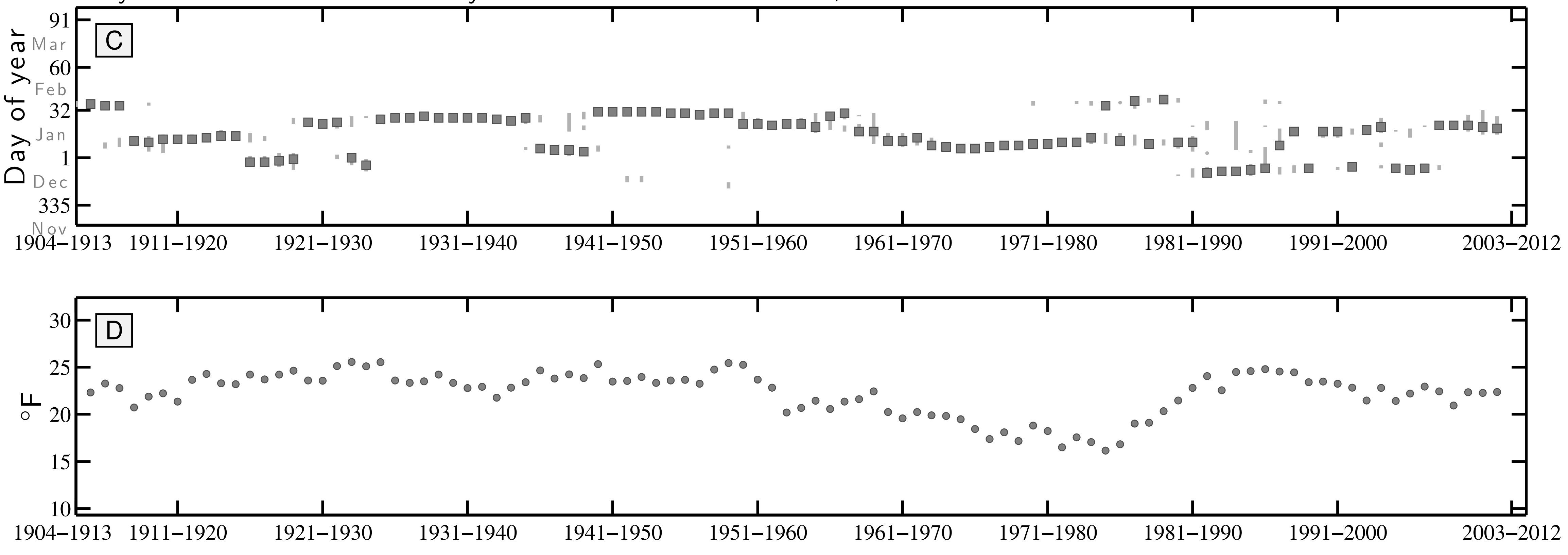

10 year Summer Teletherm dynamics for AURORA, IL:

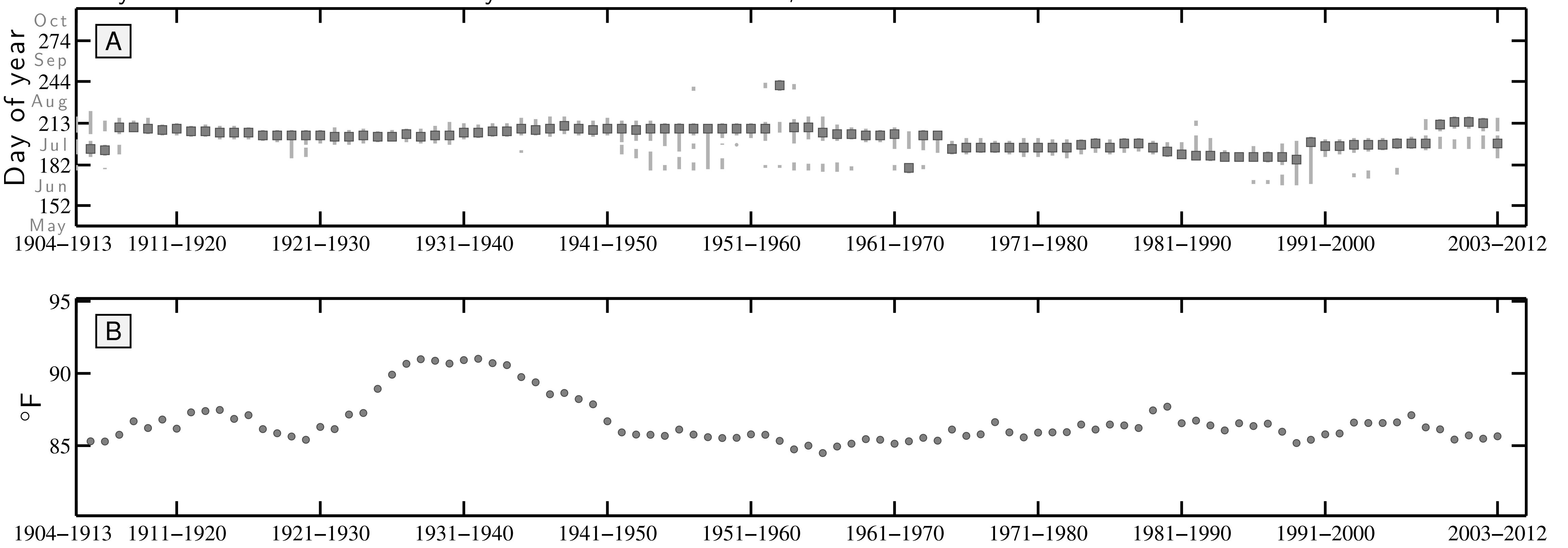

10 year Winter Teletherm dynamics for AURORA, IL:

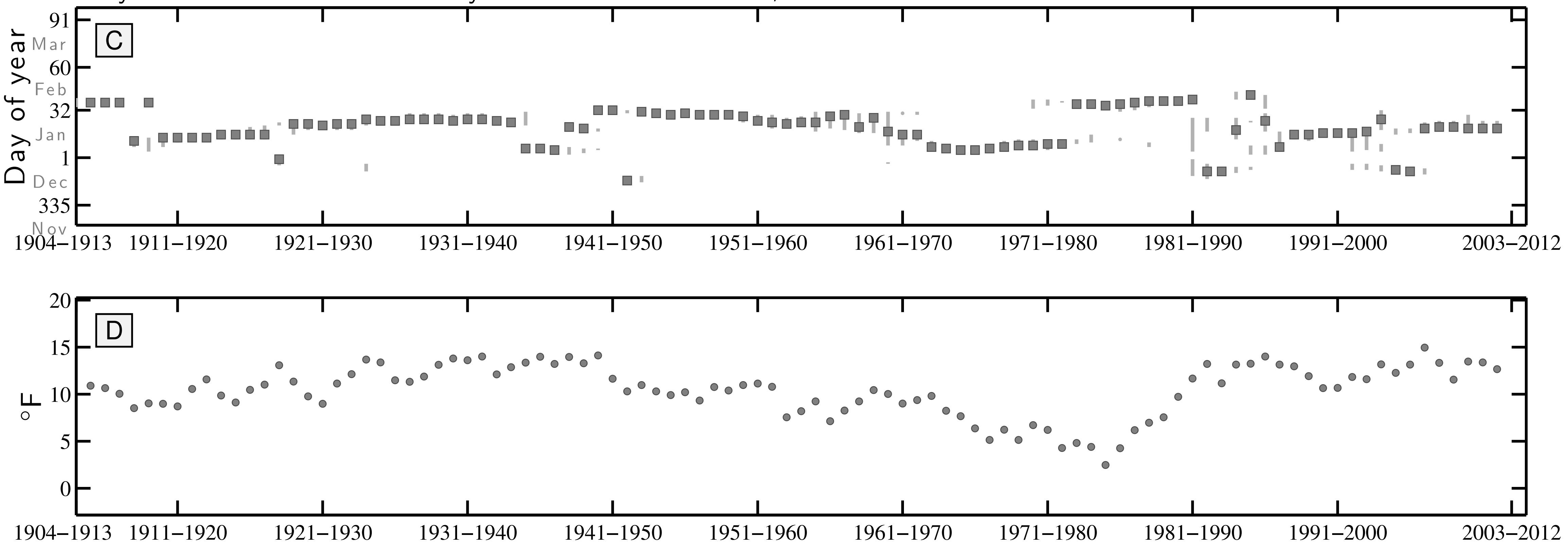

10 year Summer Teletherm dynamics for CARLINVILLE, IL:

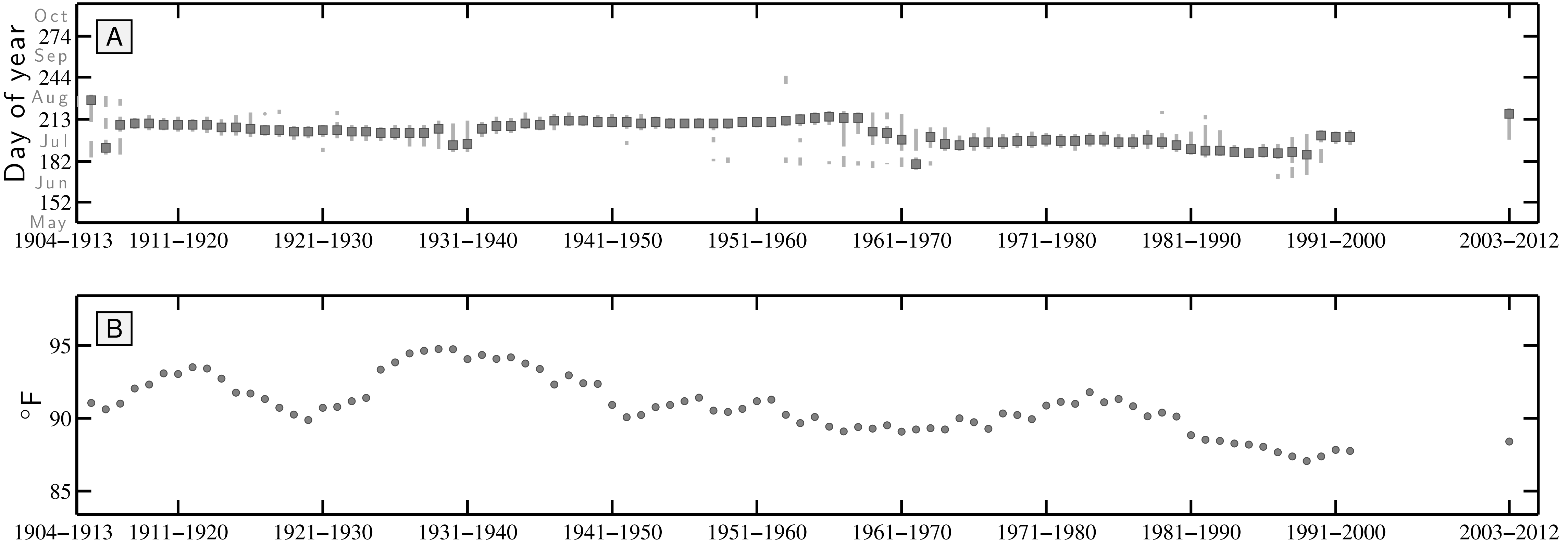

10 year Winter Teletherm dynamics for CARLINVILLE, IL:

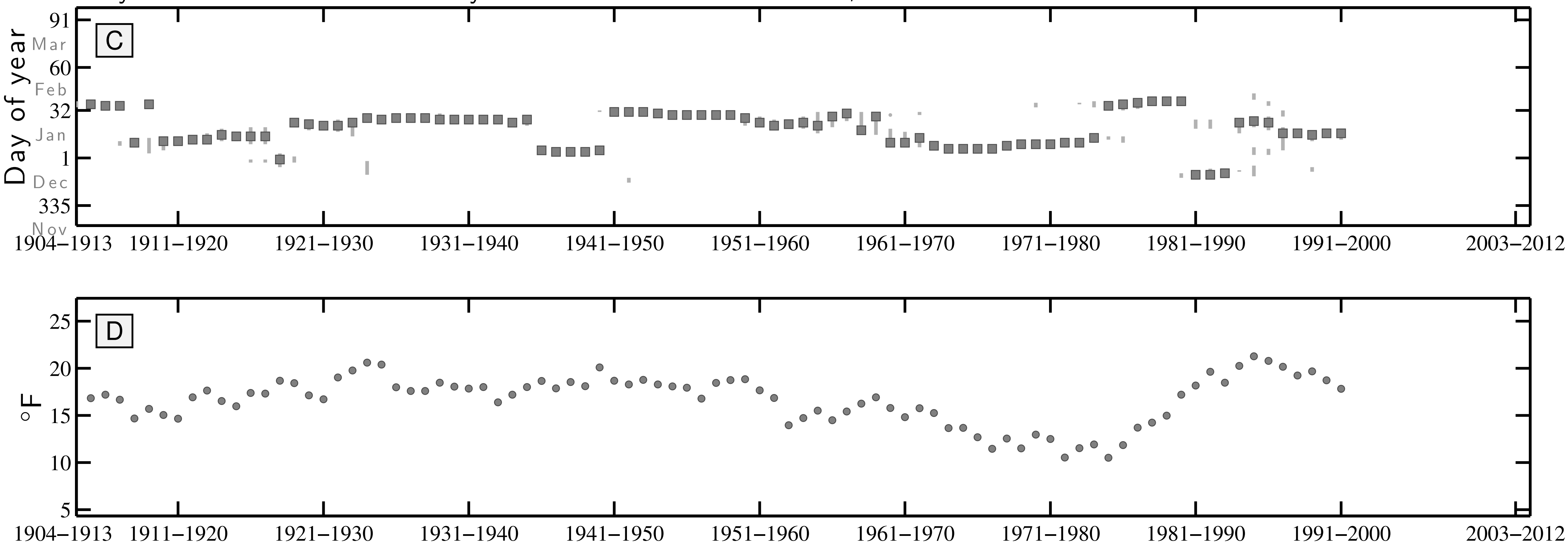

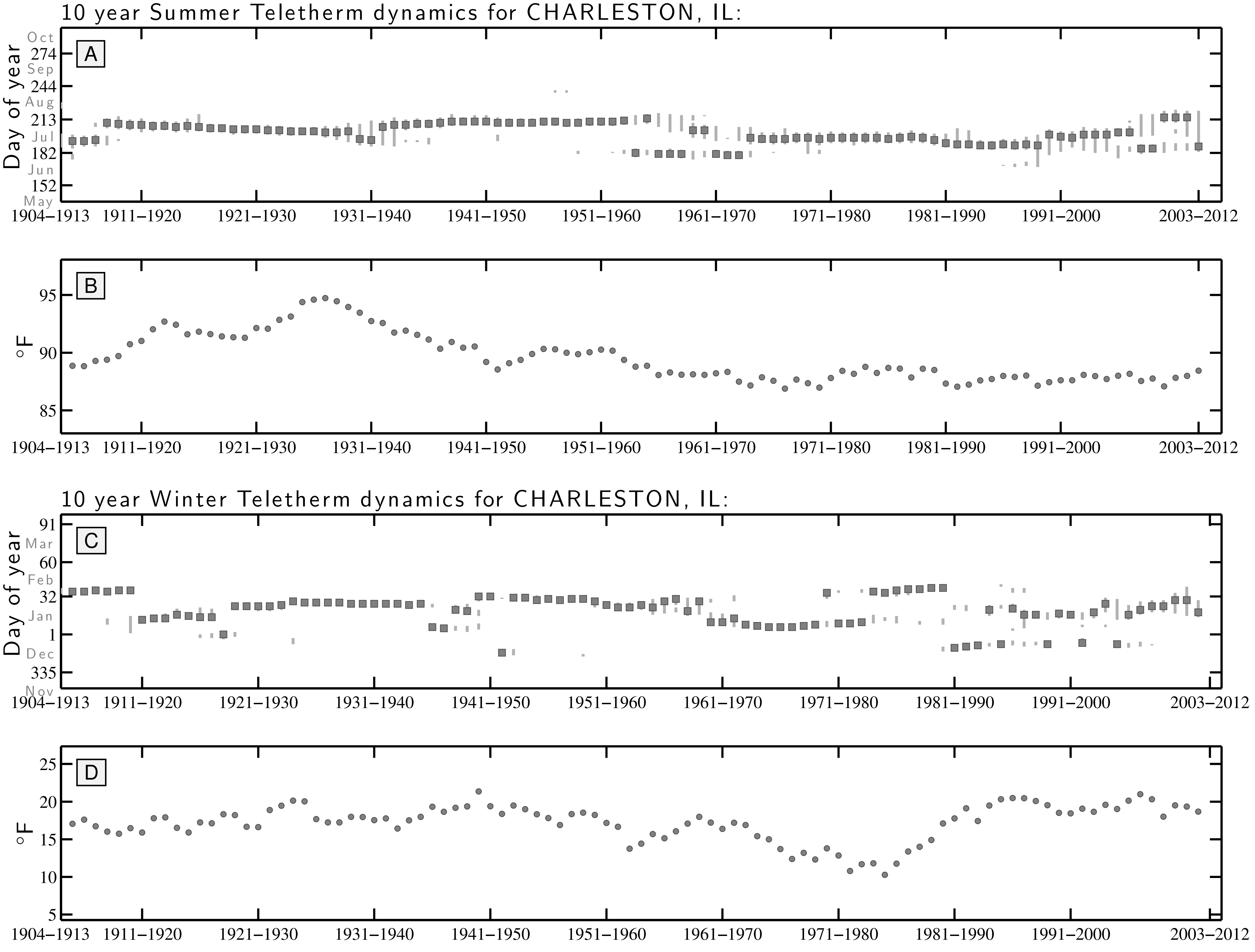
10 year Summer Teletherm dynamics for CHARLESTON, IL:
A
Day of year
Oct
274
Sep
244
Aug
213
Jul
182
Jun
152
May
B
°F
95
90
85
10 year Winter Teletherm dynamics for CHARLESTON, IL:
C
Day of year
91
Mar
60
Feb
32
Jan
1
Dec
335
Nov
D
°F
25
20
15
10
5
1904–1913
1911–1920
1921–1930
1931–1940
1941–1950
1951–1960
1961–1970
1971–1980
1981–1990
1991–2000
2003–2012

10 year Summer Teletherm dynamics for DANVILLE, IL:

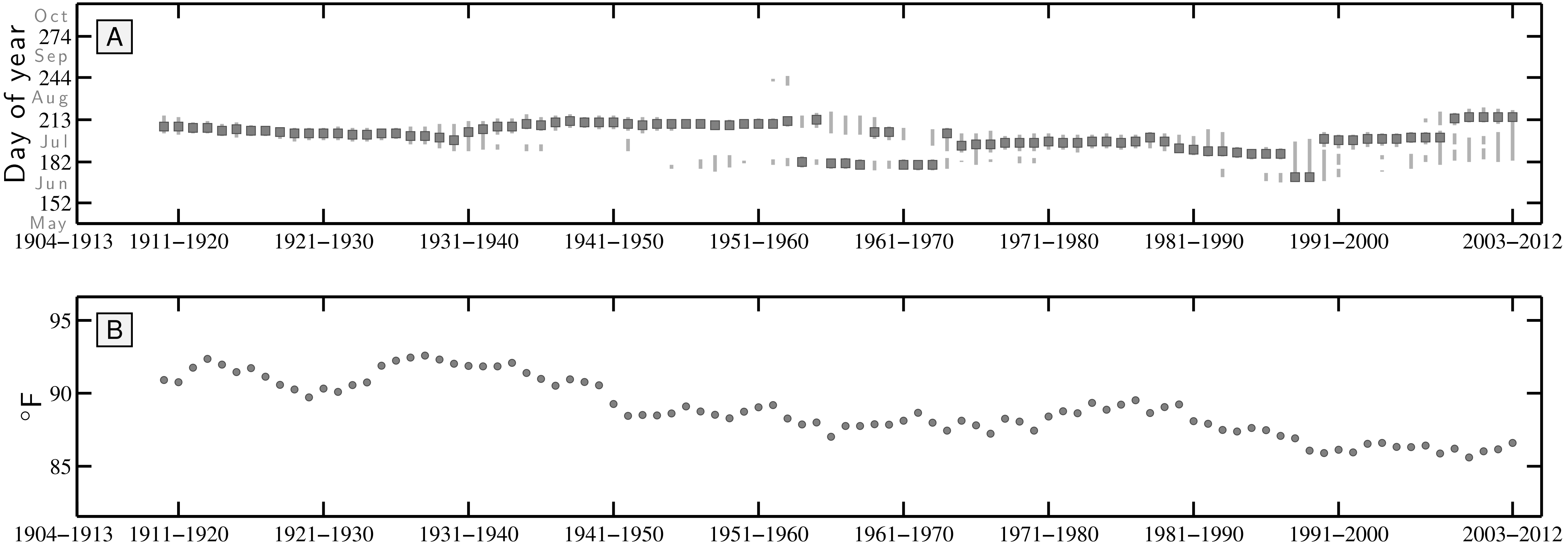

10 year Winter Teletherm dynamics for DANVILLE, IL:

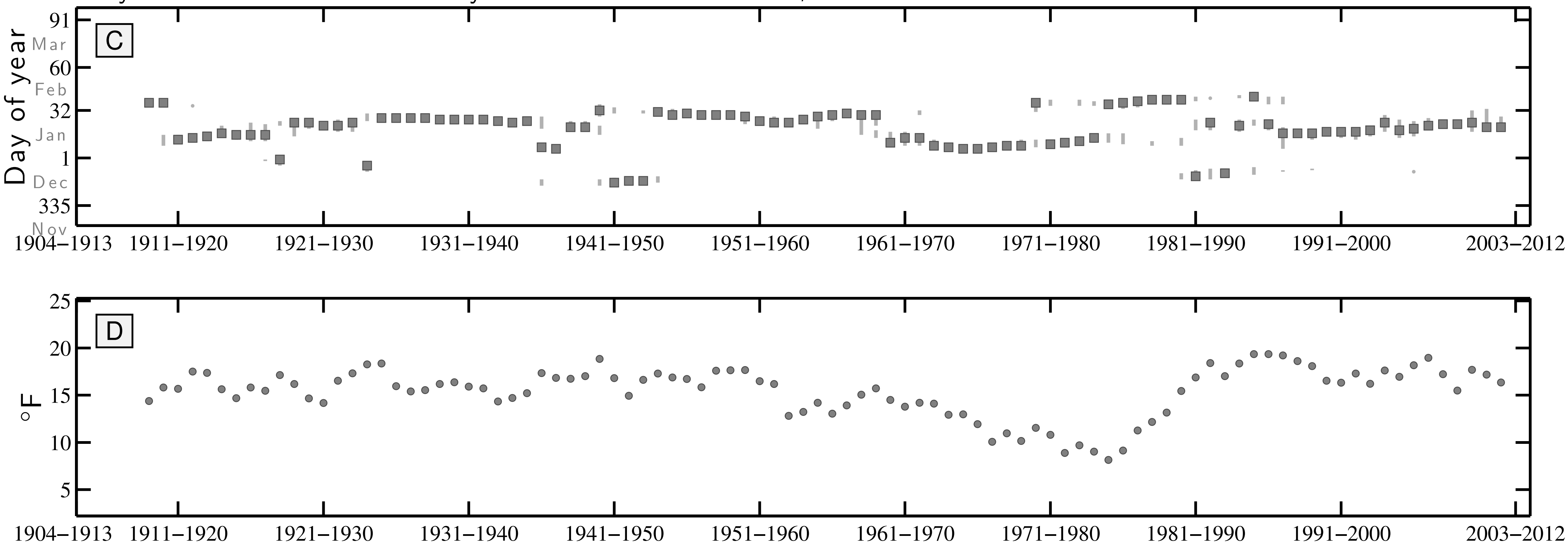

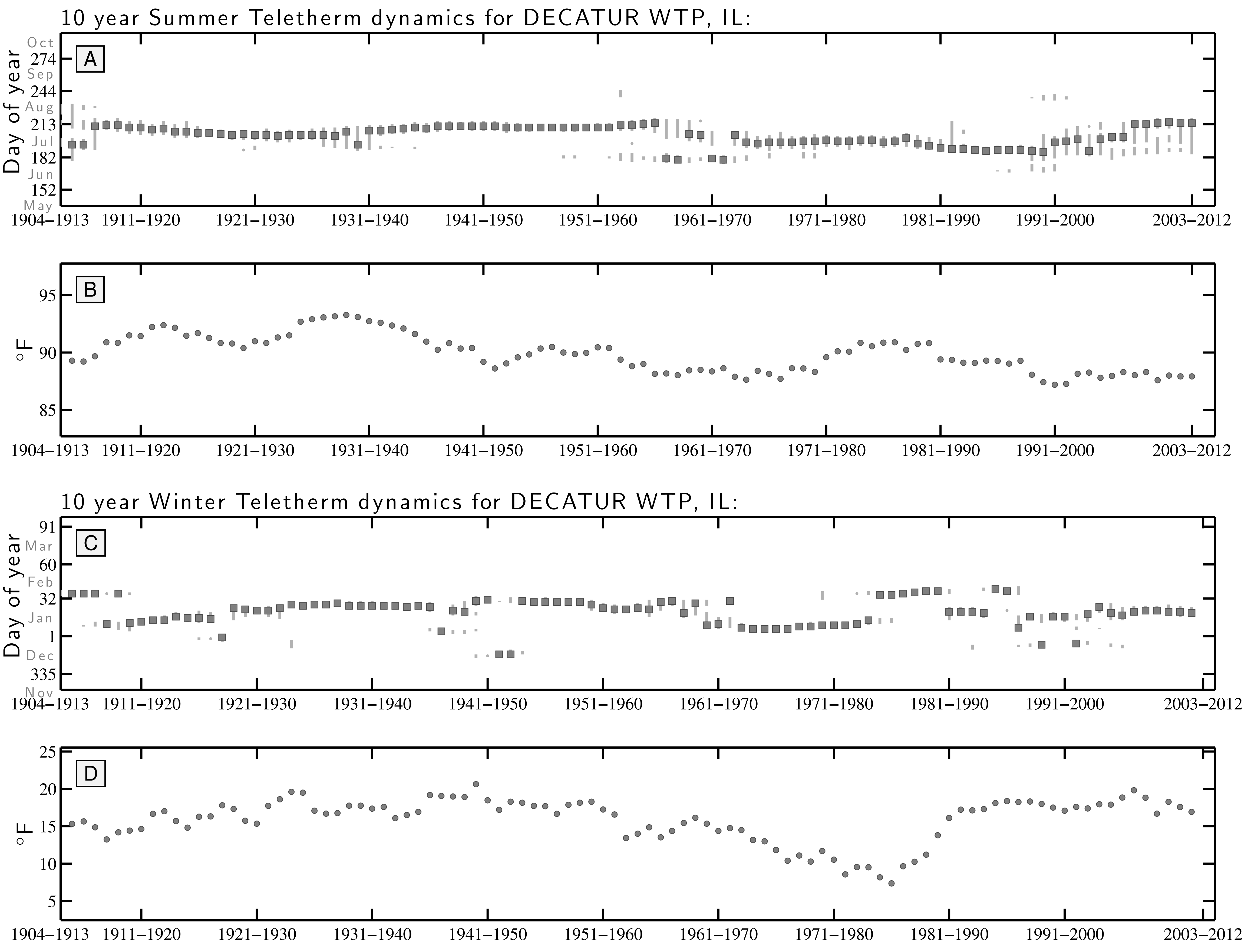
10 year Summer Teletherm dynamics for DECATUR WTP, IL:
A
Day of year
Oct
274
Sep
244
Aug
213
Jul
182
Jun
152
May
1904–1913
1911–1920
1921–1930
1931–1940
1941–1950
1951–1960
1961–1970
1971–1980
1981–1990
1991–2000
2003–2012
B
°F
95
90
85
10 year Winter Teletherm dynamics for DECATUR WTP, IL:
C
Day of year
91
Mar
60
Feb
32
Jan
1
Dec
335
Nov
D
°F
25
20
15
10
5

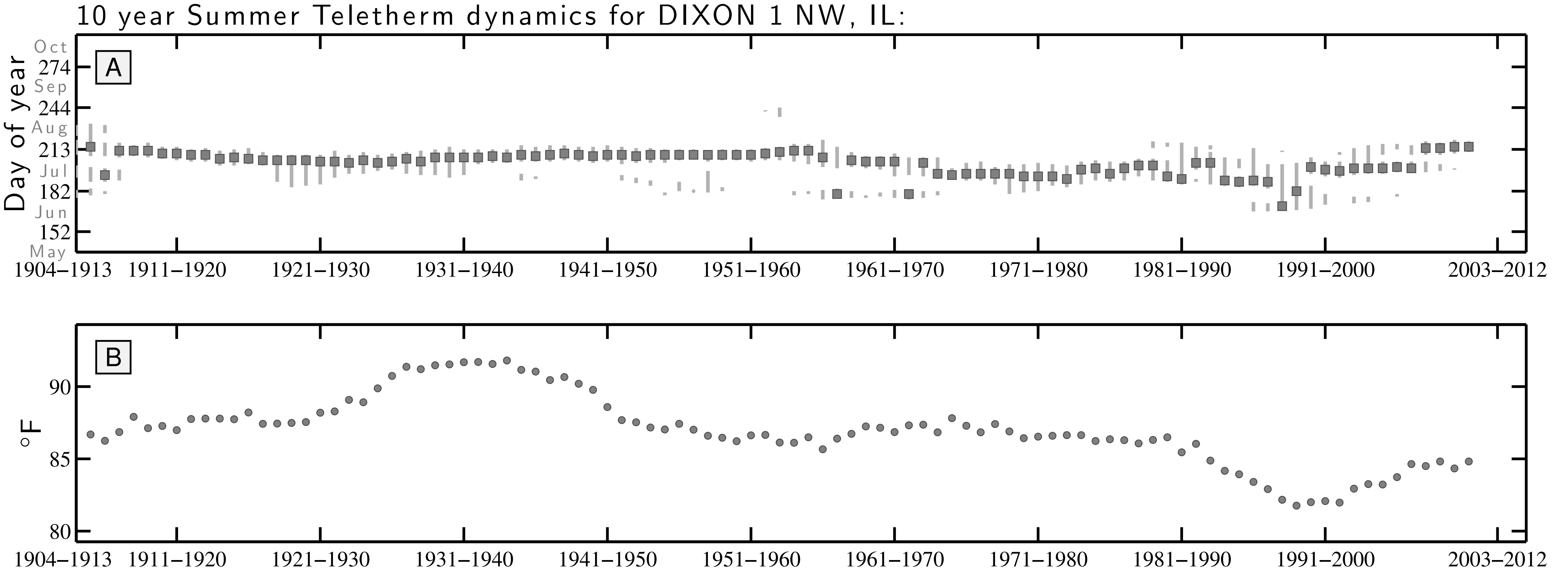
10 year Summer Teletherm dynamics for DIXON 1 NW, IL:
A
B
Day of year
°F
May
Jun
Jul
Aug
Sep
Oct
152
182
213
244
274
80
85
90
1904–1913
1911–1920
1921–1930
1931–1940
1941–1950
1951–1960
1961–1970
1971–1980
1981–1990
1991–2000
2003–2012

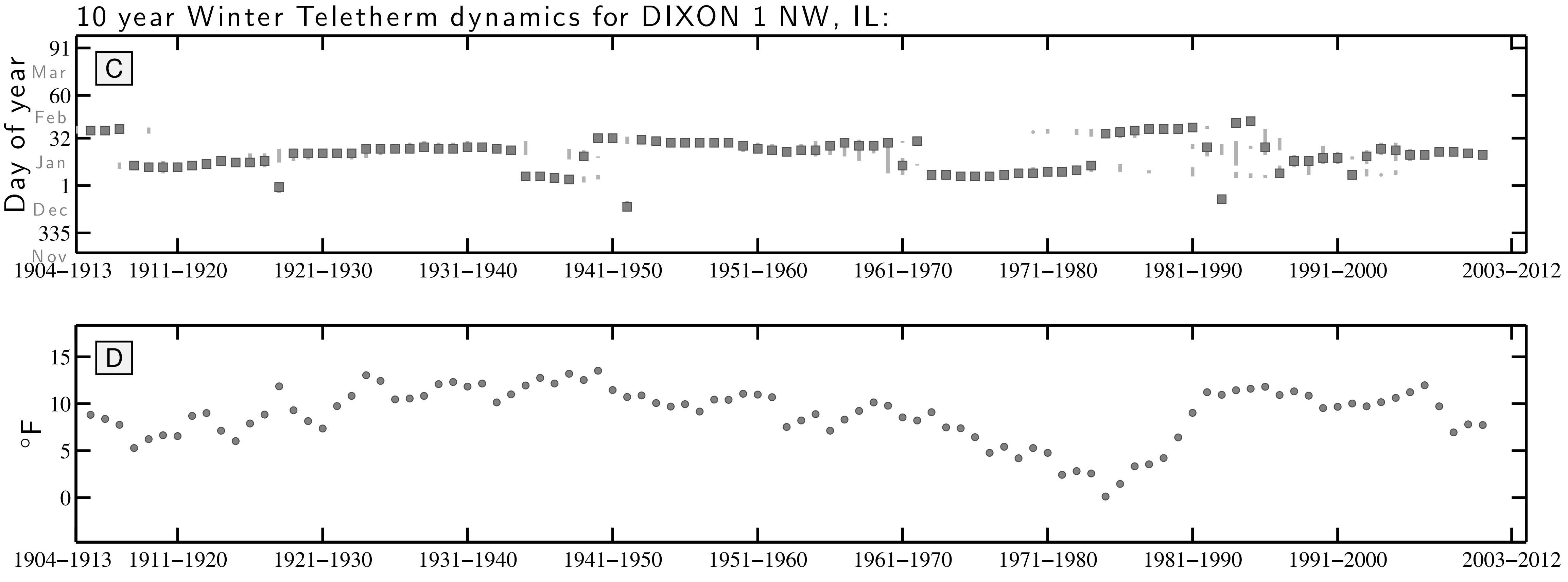
10 year Winter Teletherm dynamics for DIXON 1 NW, IL:
C
D
Day of year
°F
Nov
Dec
Jan
Feb
Mar
335
1
32
60
91
0
5
10
15
1904–1913
1911–1920
1921–1930
1931–1940
1941–1950
1951–1960
1961–1970
1971–1980
1981–1990
1991–2000
2003–2012

10 year Summer Teletherm dynamics for DU QUOIN 4 SE, IL:

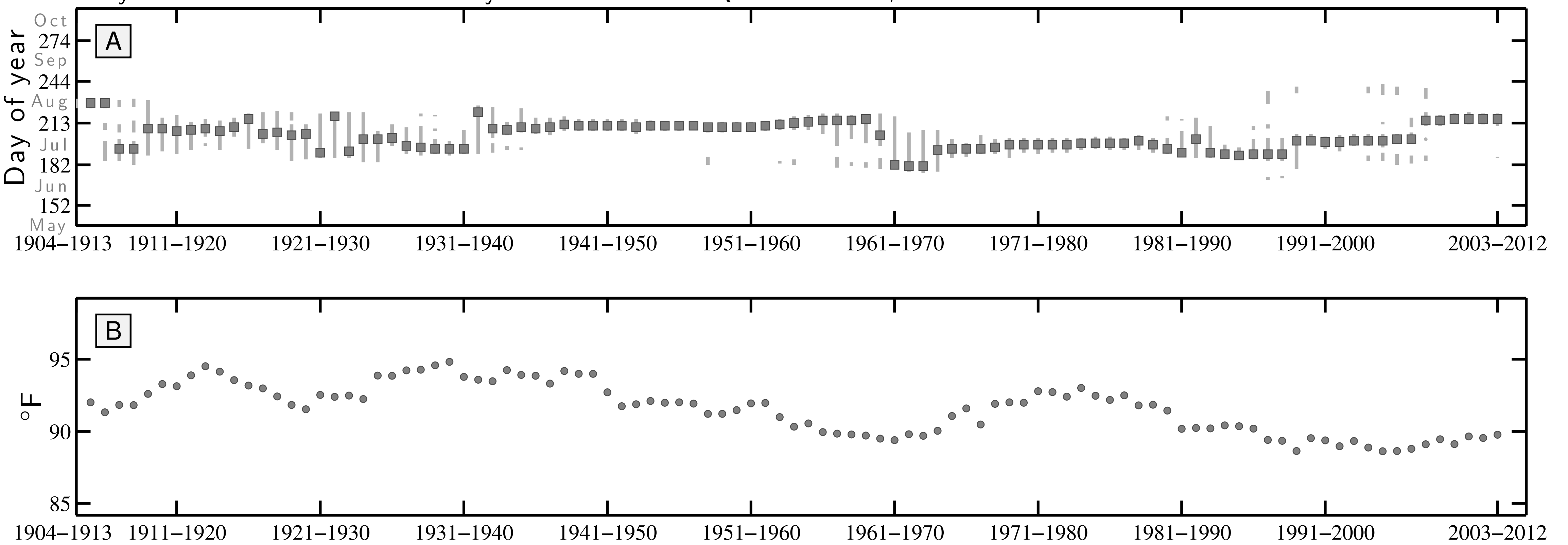

10 year Winter Teletherm dynamics for DU QUOIN 4 SE, IL:

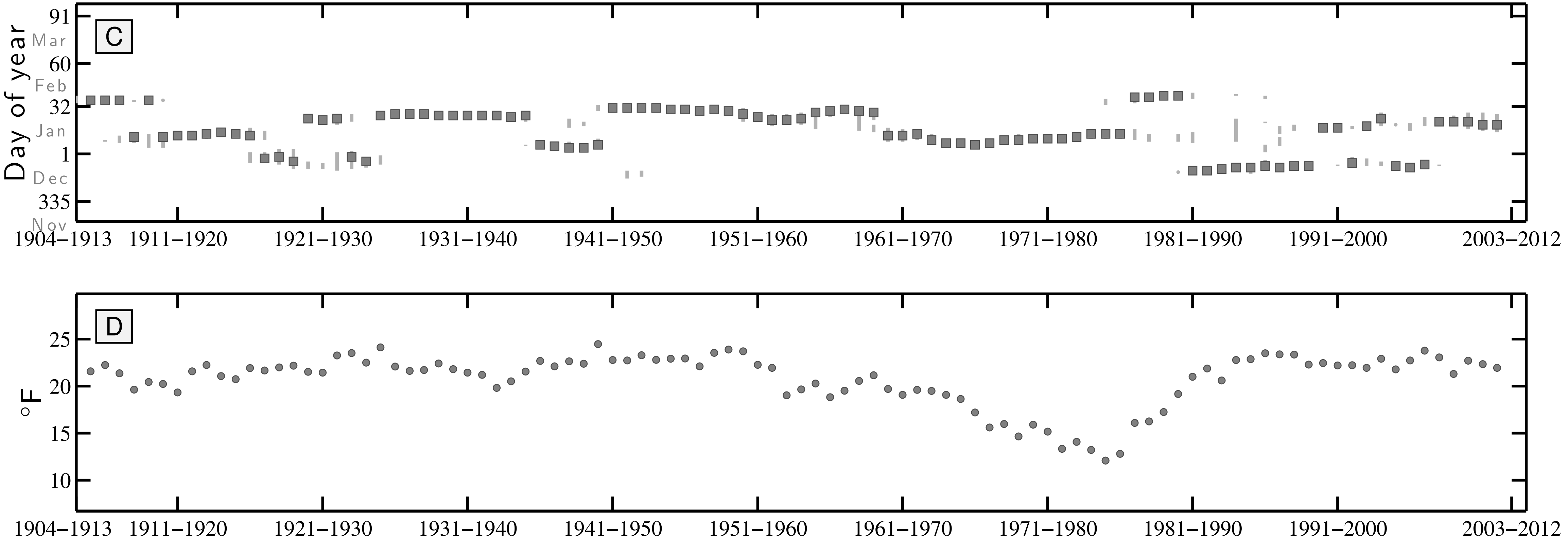

10 year Summer Teletherm dynamics for GALVA, IL:

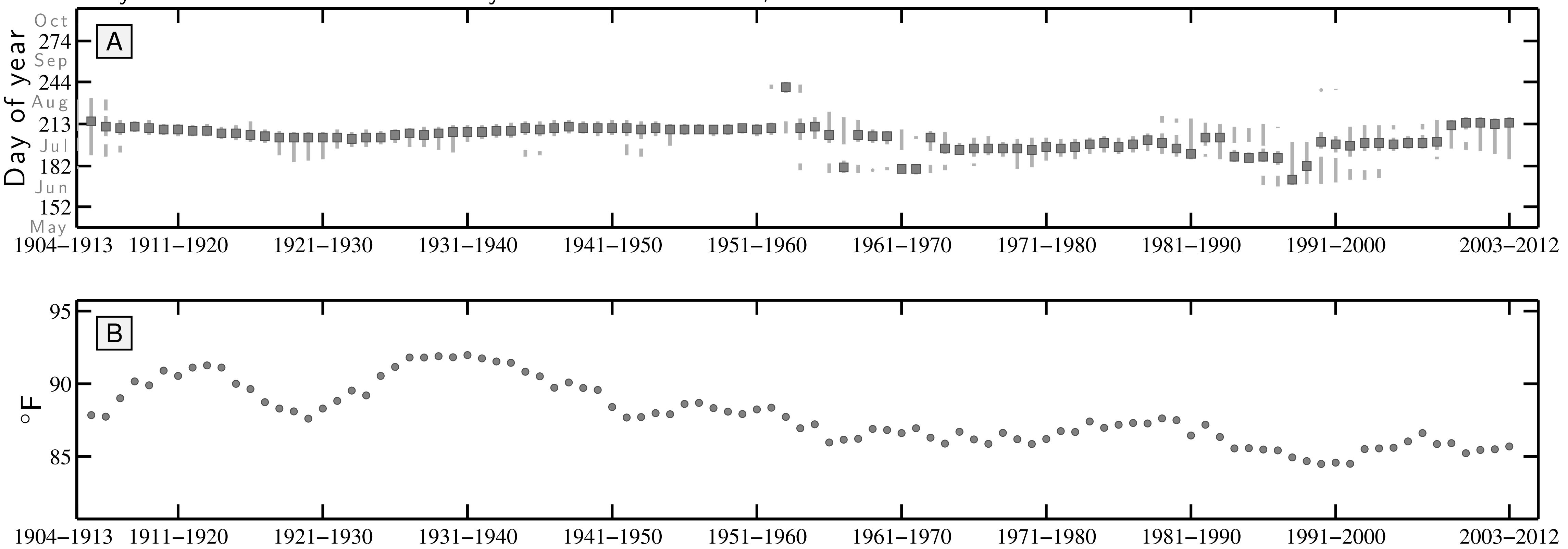

10 year Winter Teletherm dynamics for GALVA, IL:

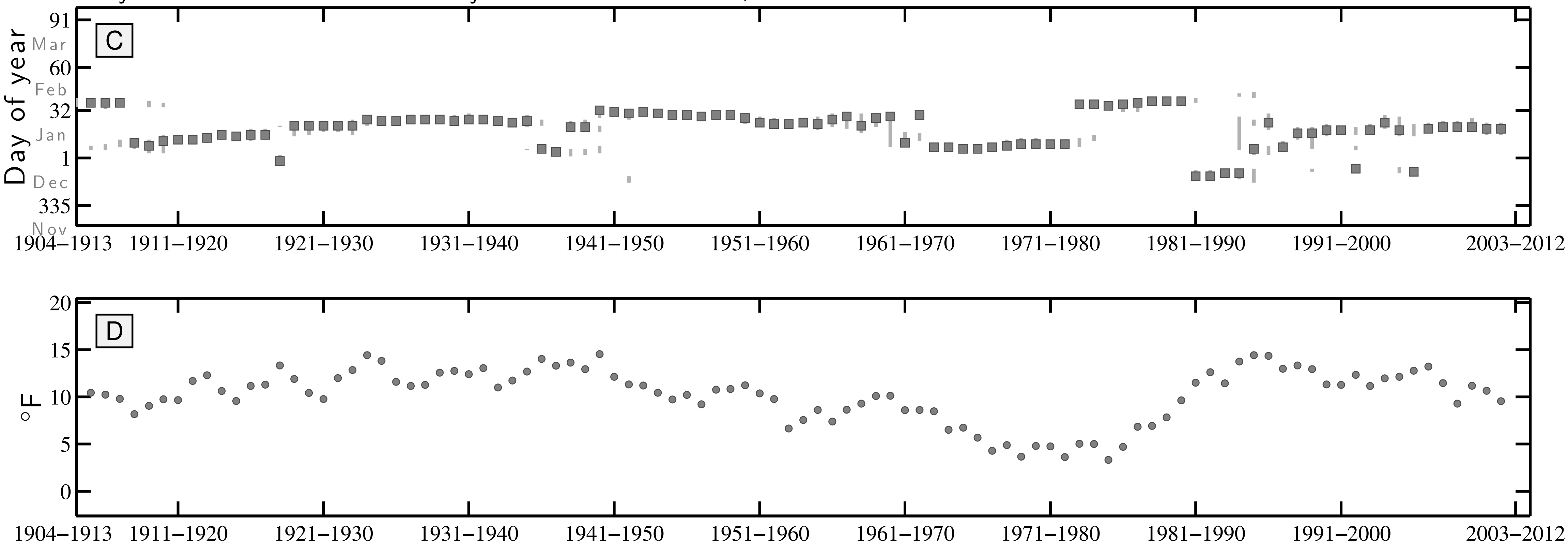

10 year Summer Teletherm dynamics for HARRISBURG, IL:

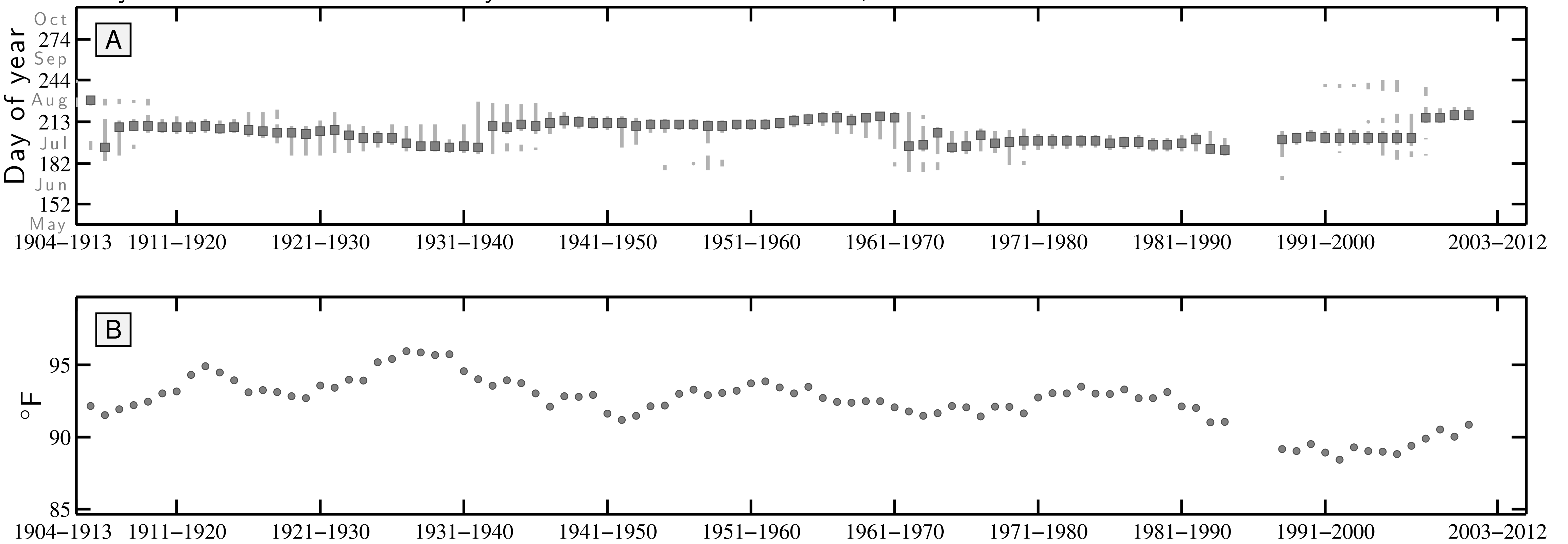

10 year Winter Teletherm dynamics for HARRISBURG, IL:

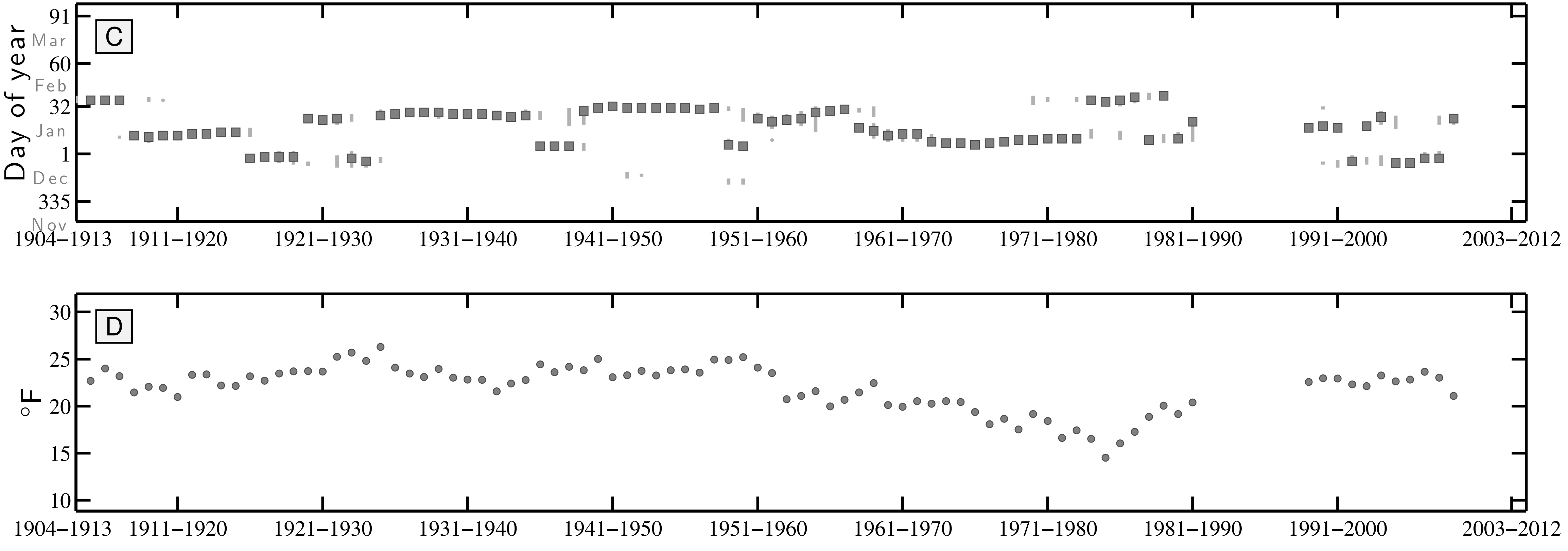

10 year Summer Teletherm dynamics for HILLSBORO, IL:

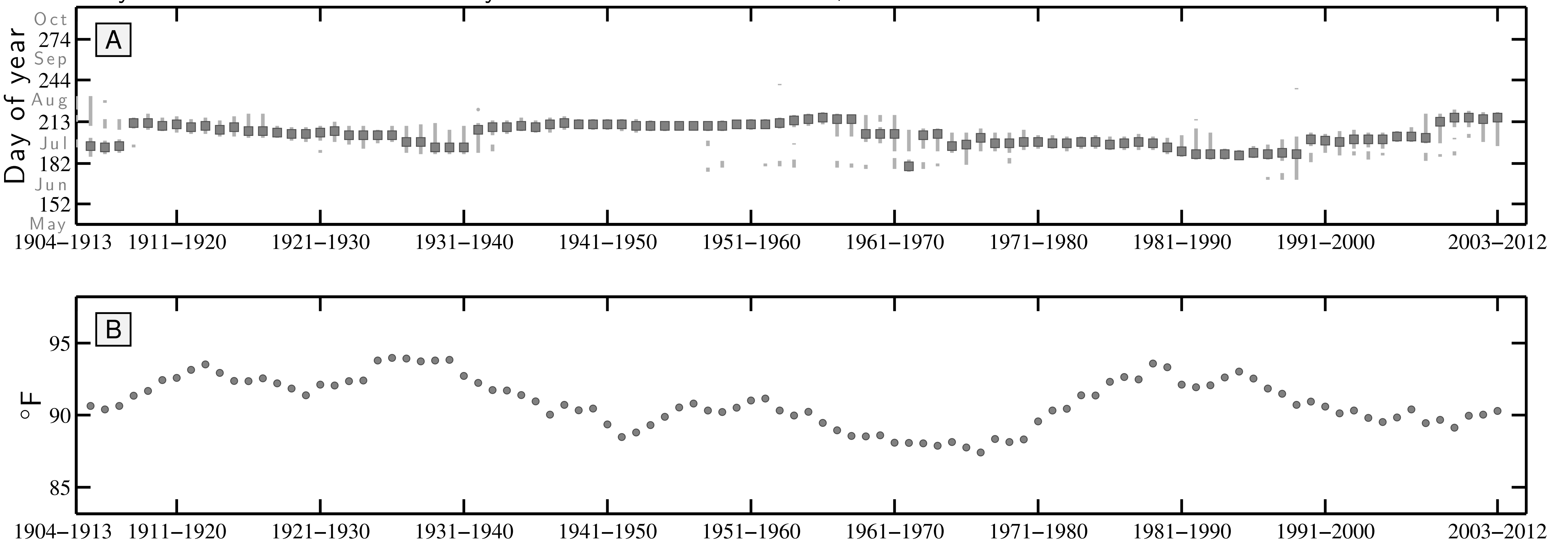

10 year Winter Teletherm dynamics for HILLSBORO, IL:

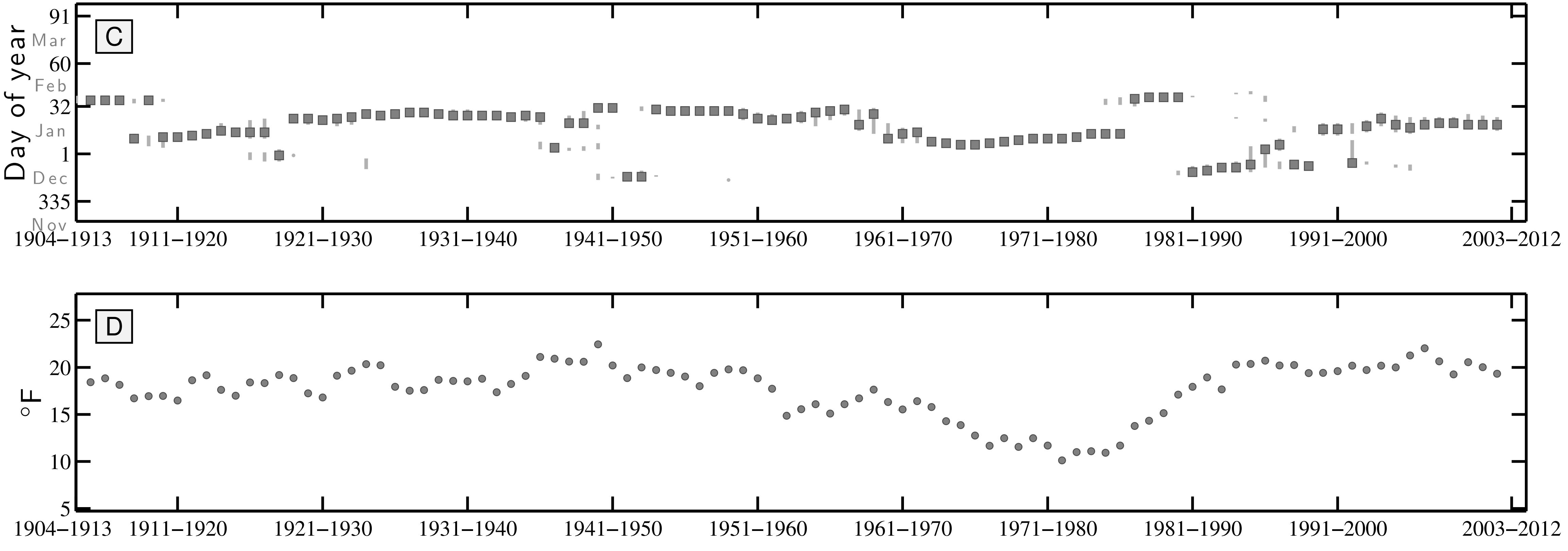

10 year Summer Teletherm dynamics for HOOPESTON 1 NE, IL:

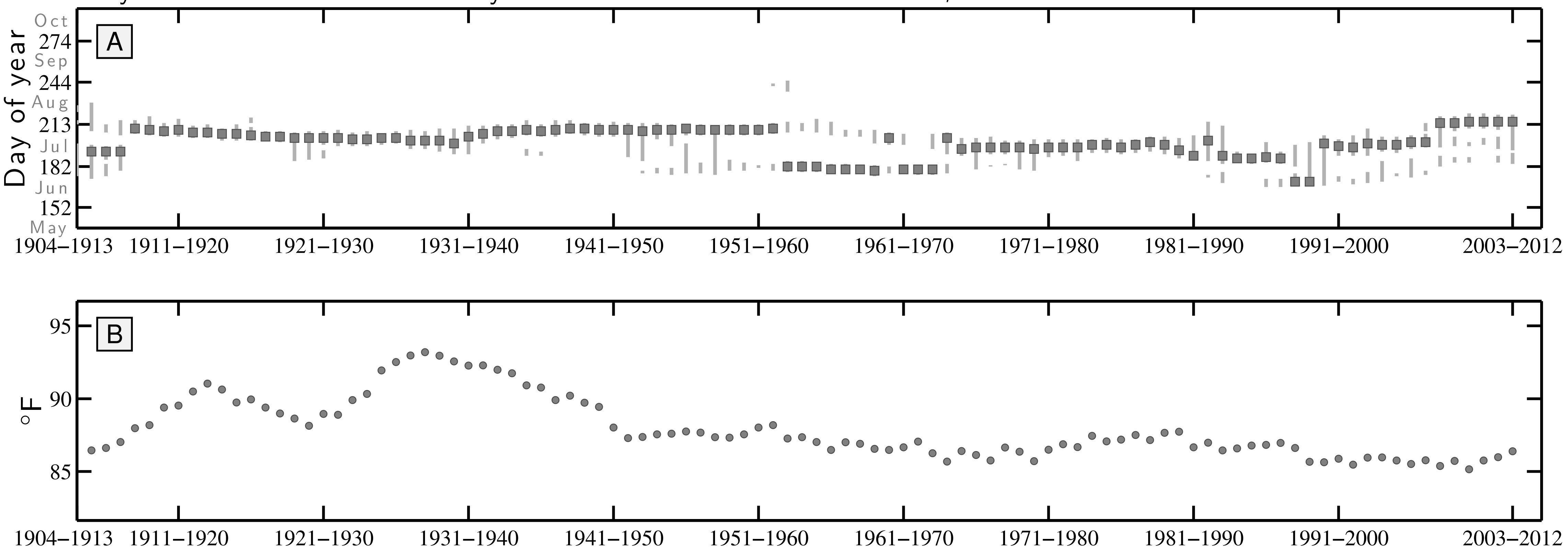

10 year Winter Teletherm dynamics for HOOPESTON 1 NE, IL:

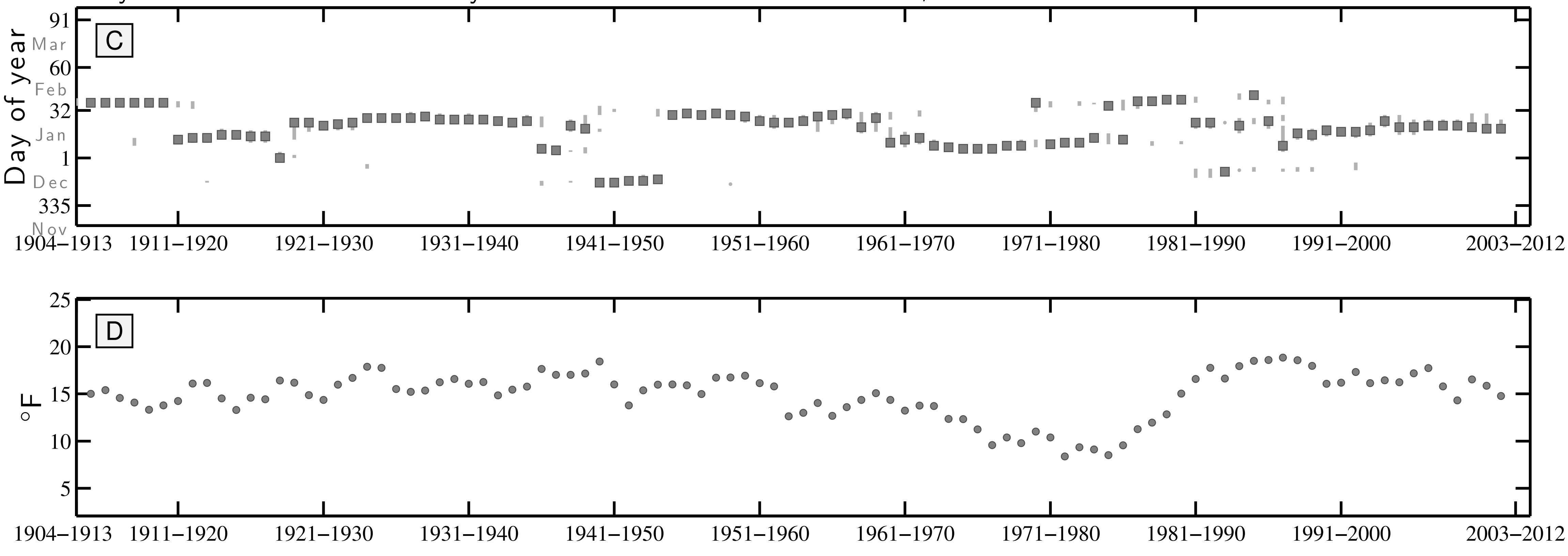

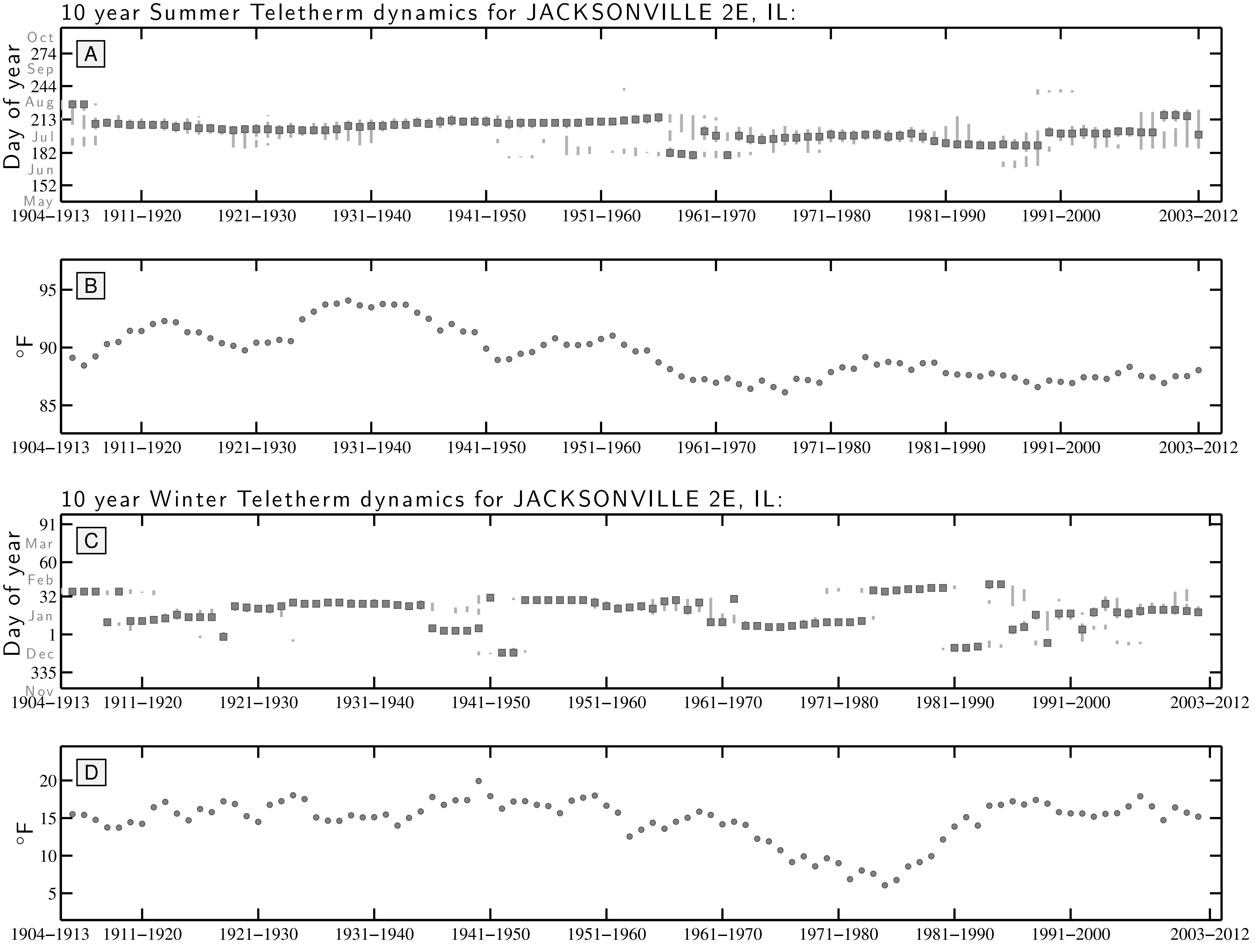
10 year Summer Teletherm dynamics for JACKSONVILLE 2E, IL:
A
Day of year
Oct
274
Sep
244
Aug
213
Jul
182
Jun
152
May
B
°F
95
90
85
10 year Winter Teletherm dynamics for JACKSONVILLE 2E, IL:
C
Day of year
91
Mar
60
Feb
32
Jan
1
Dec
335
Nov
D
°F
20
15
10
5
1904–1913
1911–1920
1921–1930
1931–1940
1941–1950
1951–1960
1961–1970
1971–1980
1981–1990
1991–2000
2003–2012

10 year Summer Teletherm dynamics for LA HARPE, IL:

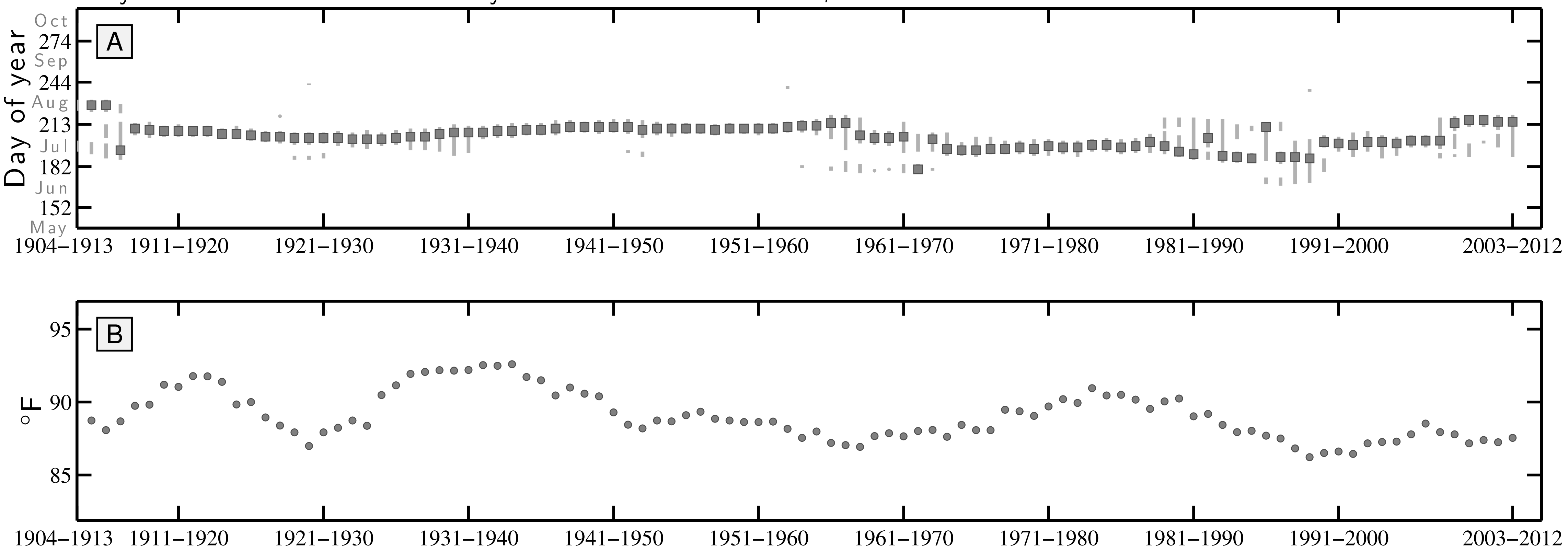

10 year Winter Teletherm dynamics for LA HARPE, IL:

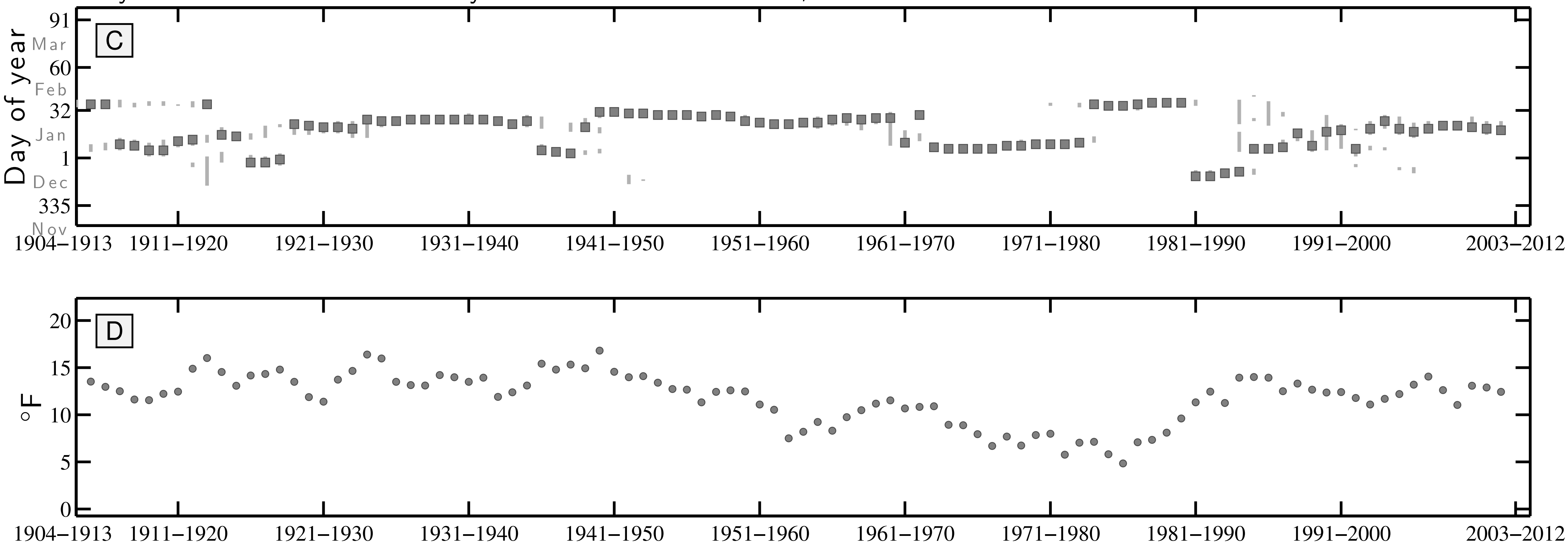

10 year Summer Teletherm dynamics for LINCOLN, IL:

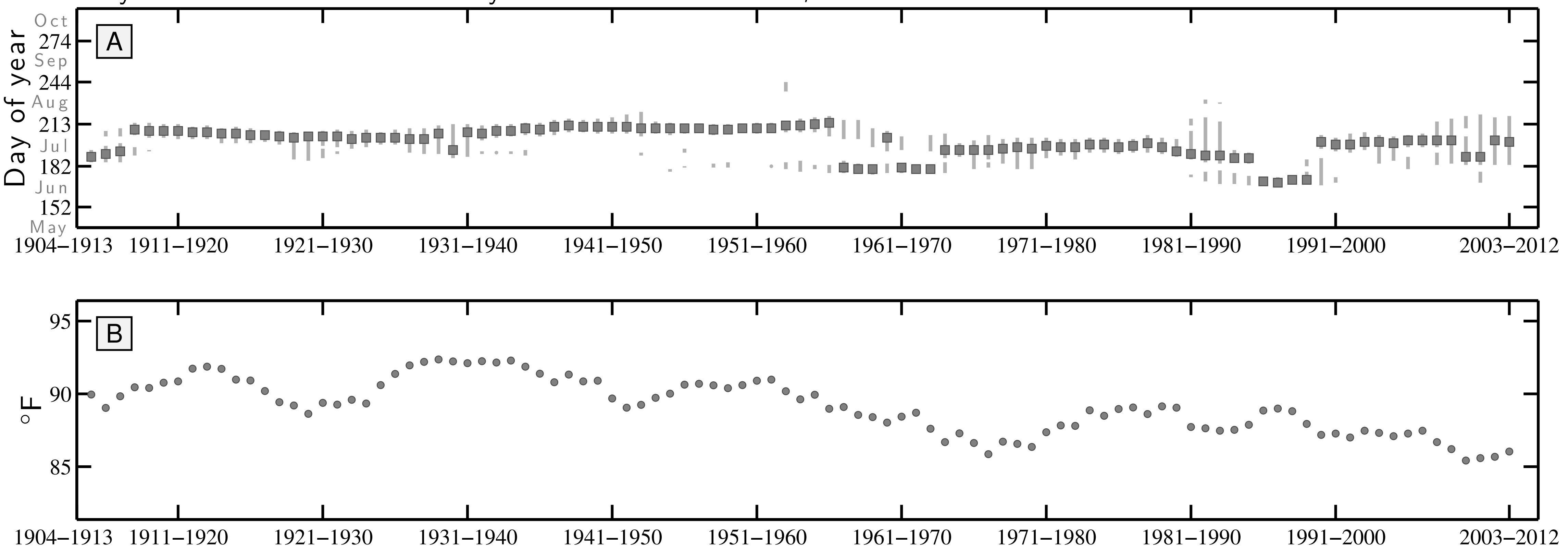

10 year Winter Teletherm dynamics for LINCOLN, IL:

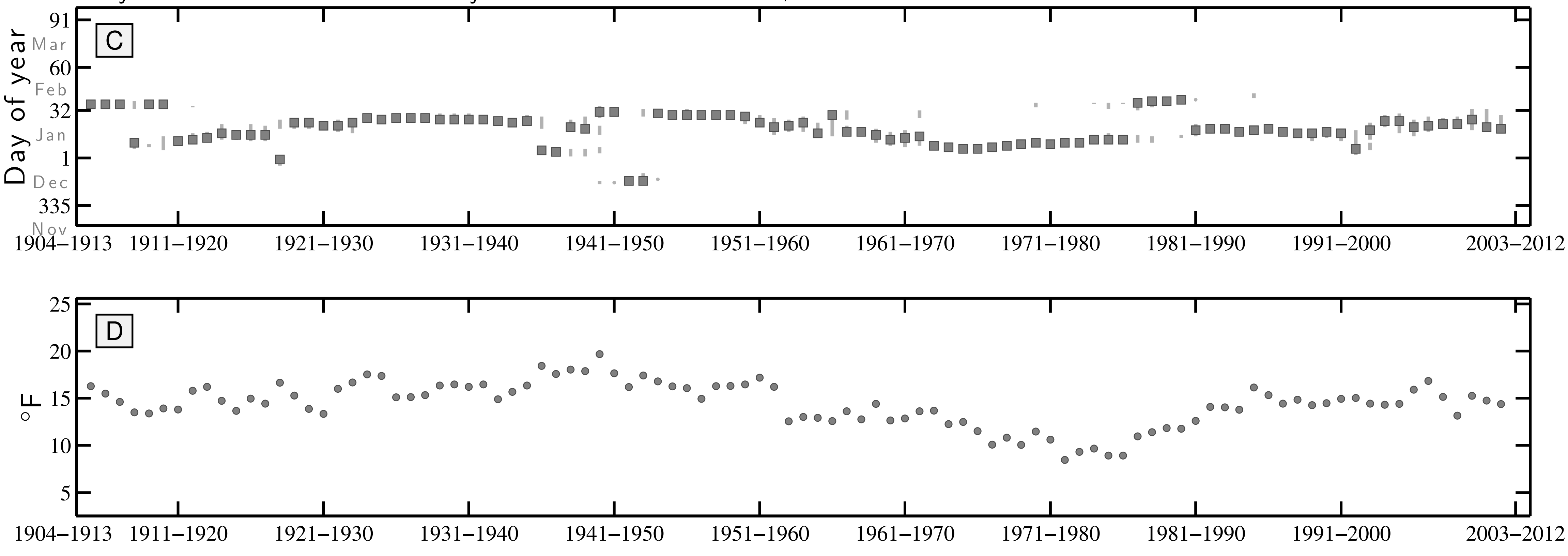

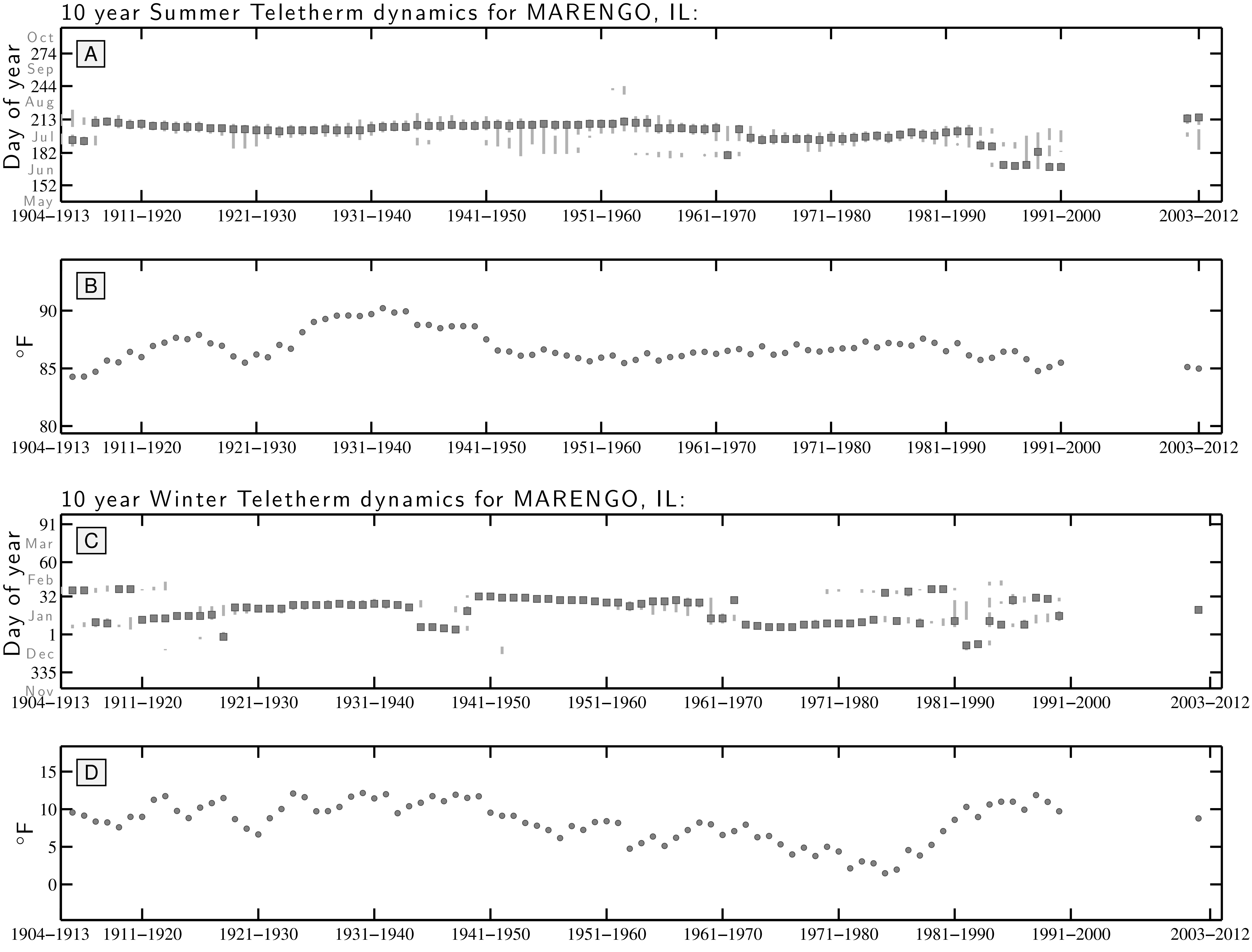

10 year Summer Teletherm dynamics for MARENGO, IL:
A
Day of year
Oct
274
Sep
244
Aug
213
Jul
182
Jun
152
May
B
°F
90
85
80
10 year Winter Teletherm dynamics for MARENGO, IL:
C
Day of year
91
Mar
60
Feb
32
Jan
1
Dec
335
Nov
D
°F
15
10
5
0
1904–1913
1911–1920
1921–1930
1931–1940
1941–1950
1951–1960
1961–1970
1971–1980
1981–1990
1991–2000
2003–2012

10 year Summer Teletherm dynamics for MCLEANSBORO, IL:

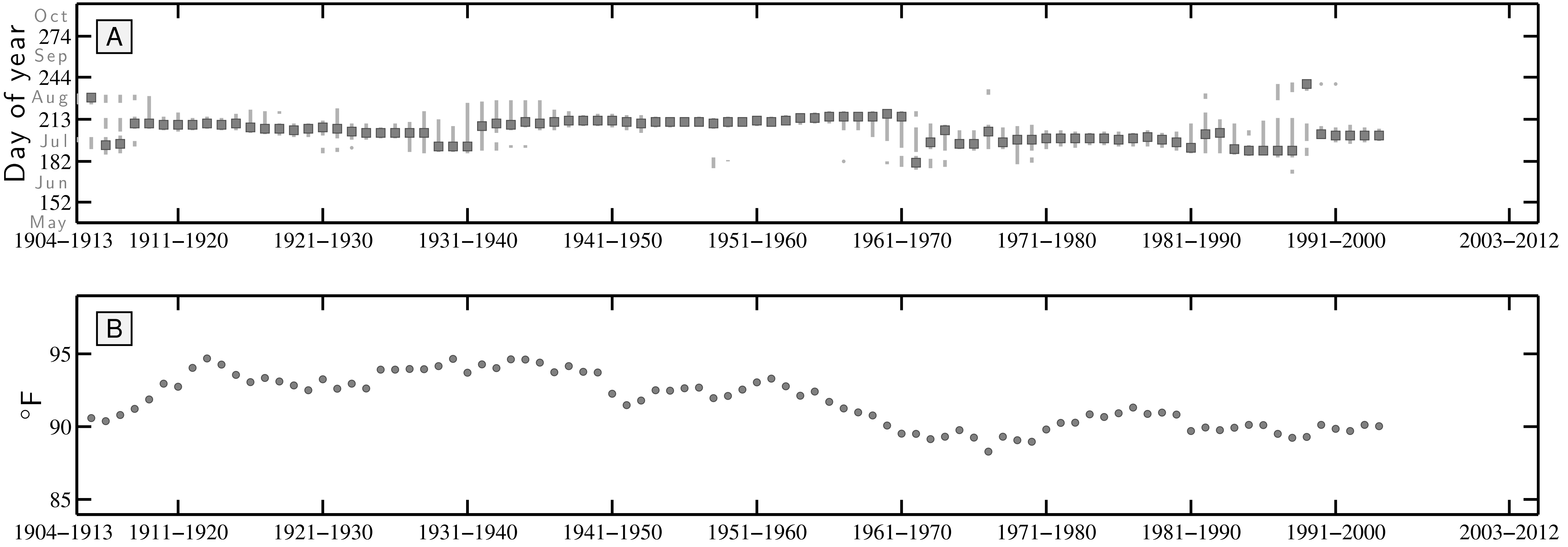

10 year Winter Teletherm dynamics for MCLEANSBORO, IL:

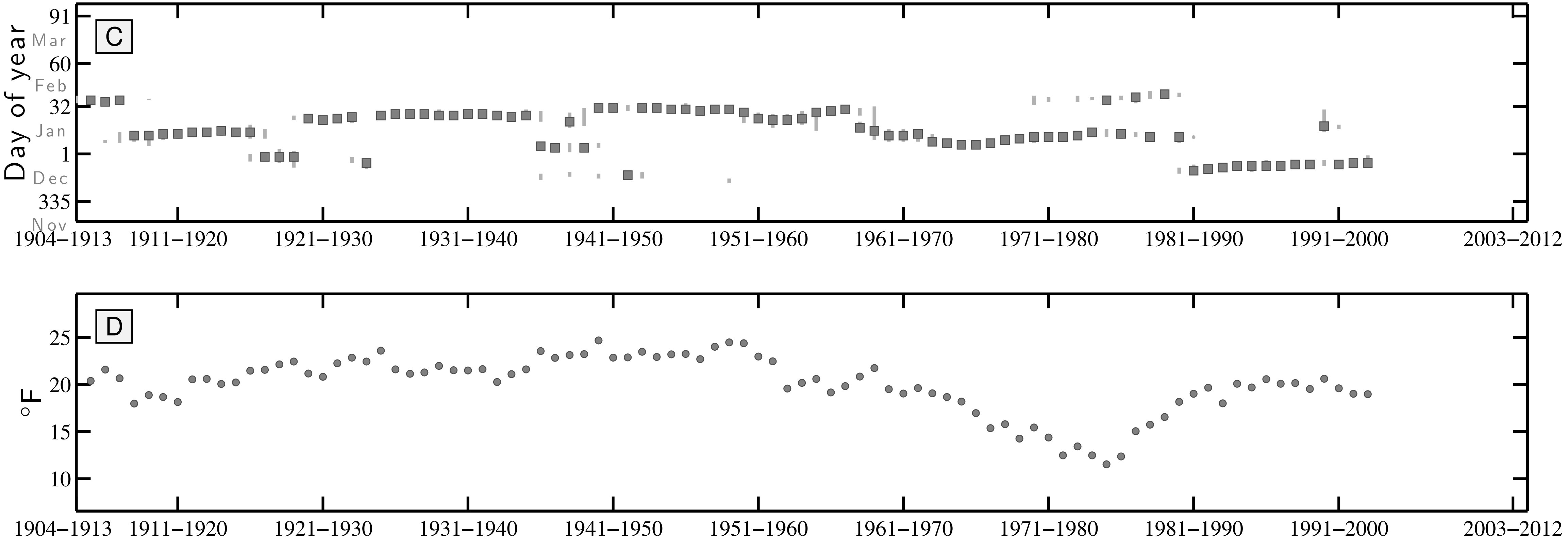

10 year Summer Teletherm dynamics for MINONK, IL:

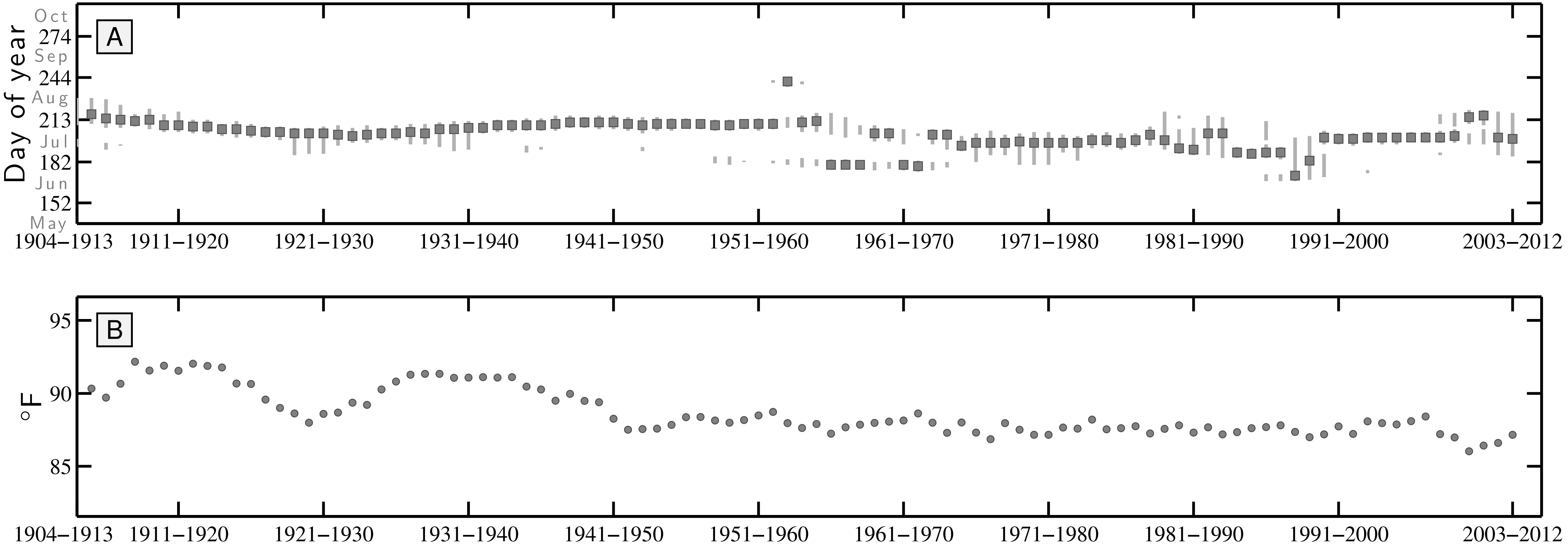

10 year Winter Teletherm dynamics for MINONK, IL:

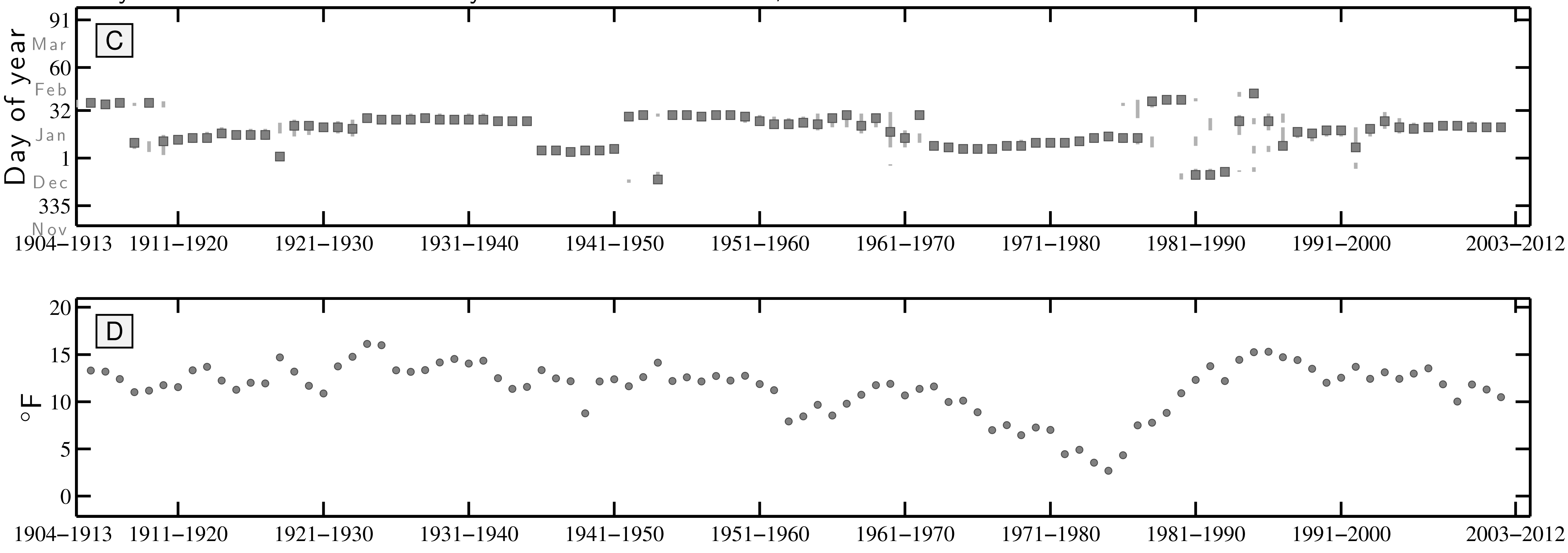

10 year Summer Teletherm dynamics for MONMOUTH, IL:

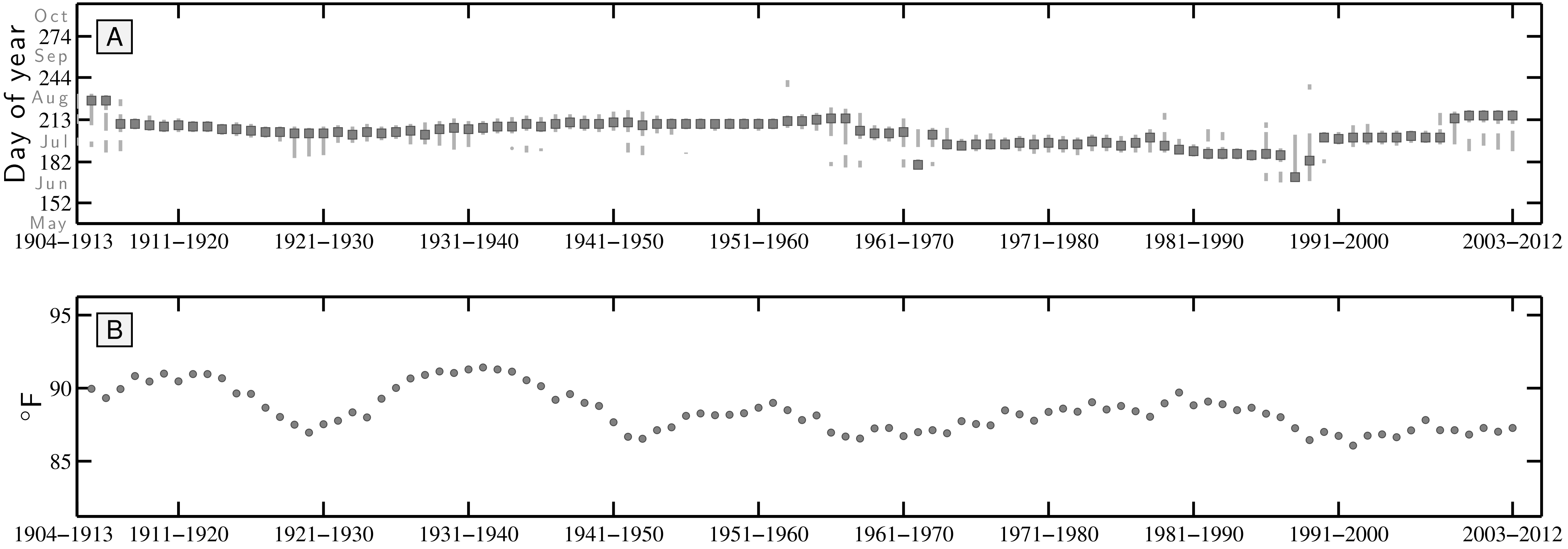

10 year Winter Teletherm dynamics for MONMOUTH, IL:

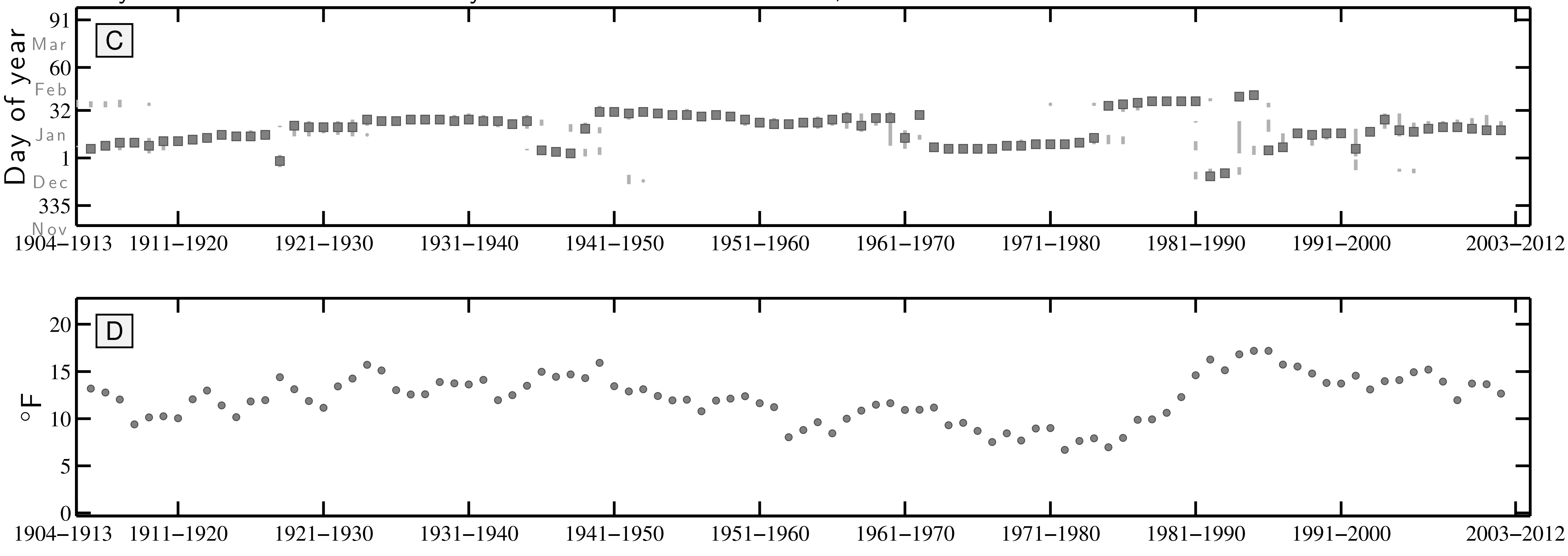

10 year Summer Teletherm dynamics for MORRISON, IL:

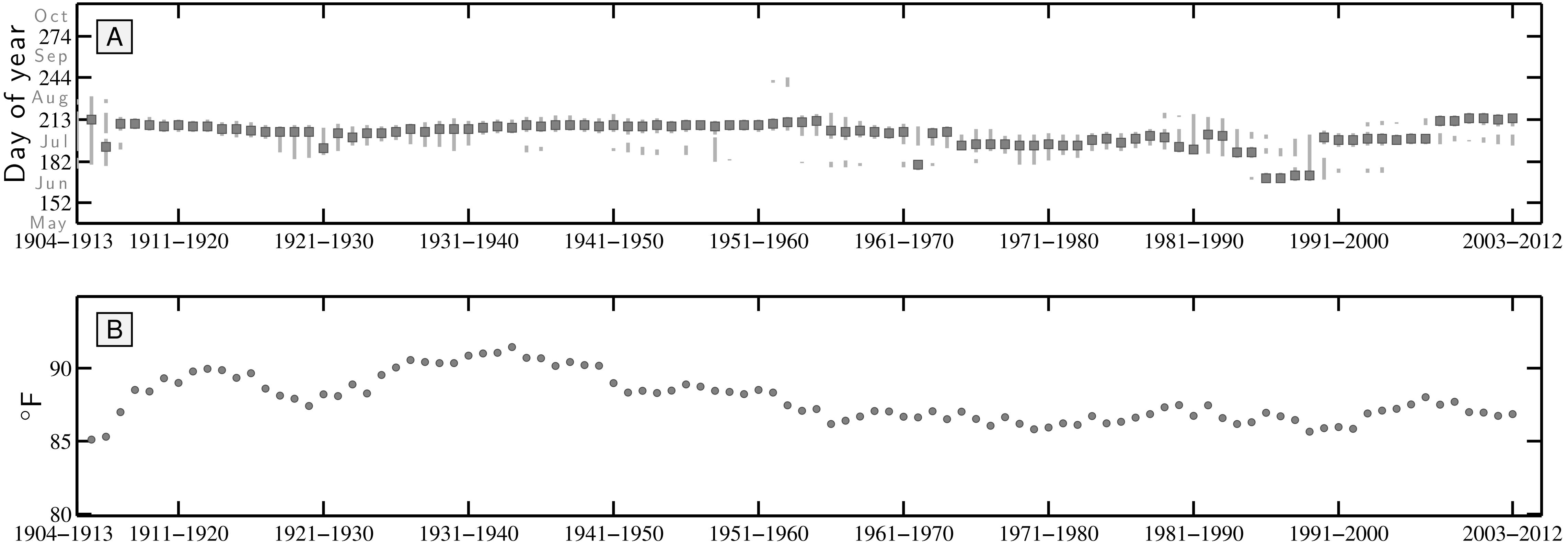

10 year Winter Teletherm dynamics for MORRISON, IL:

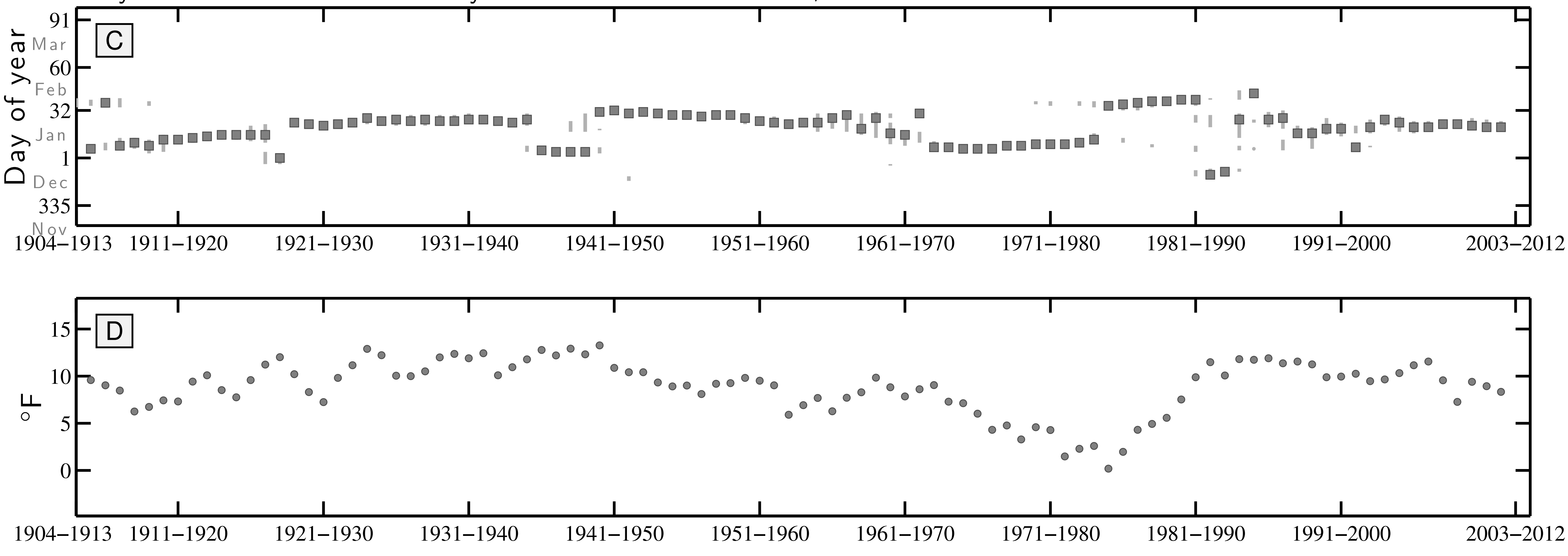

10 year Summer Teletherm dynamics for MT CARROLL, IL:

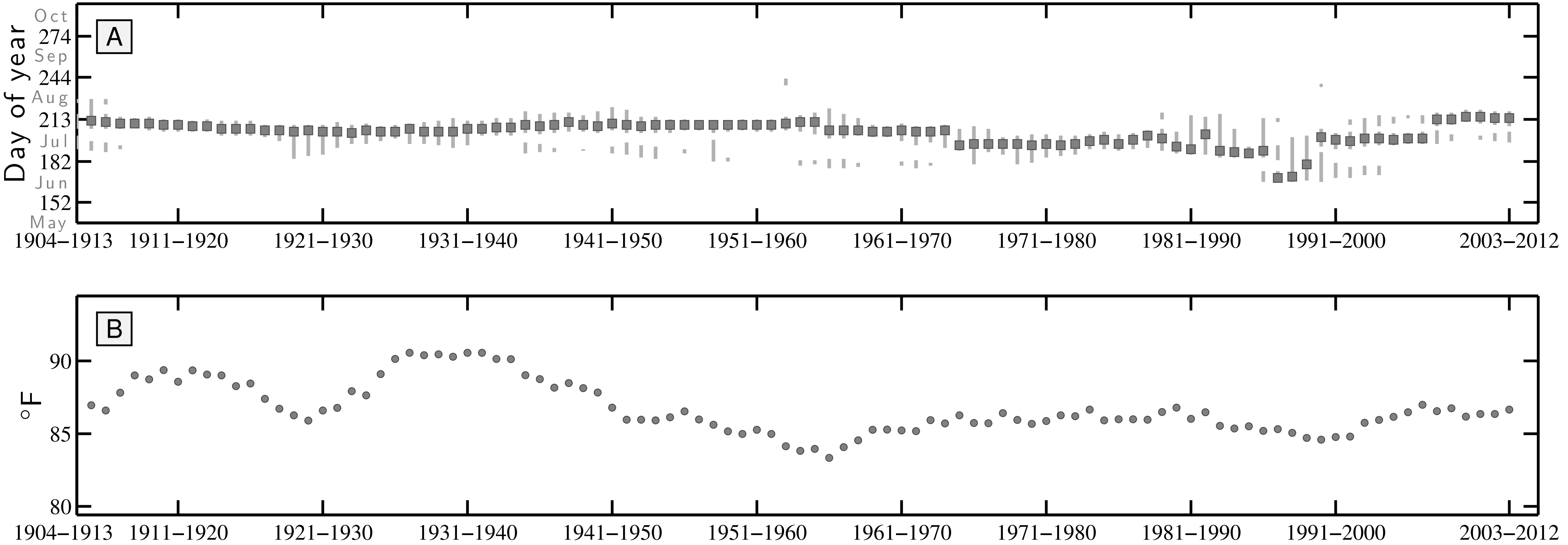

10 year Winter Teletherm dynamics for MT CARROLL, IL:

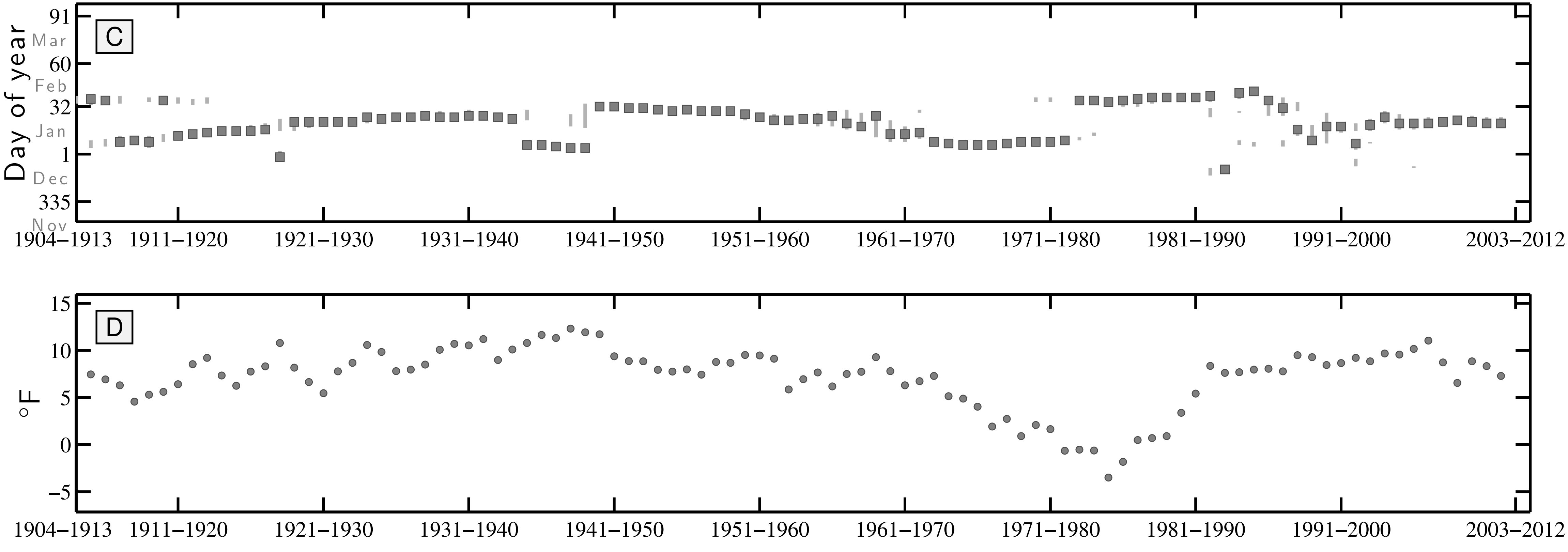

10 year Summer Teletherm dynamics for MT VERNON 3 NE, IL:

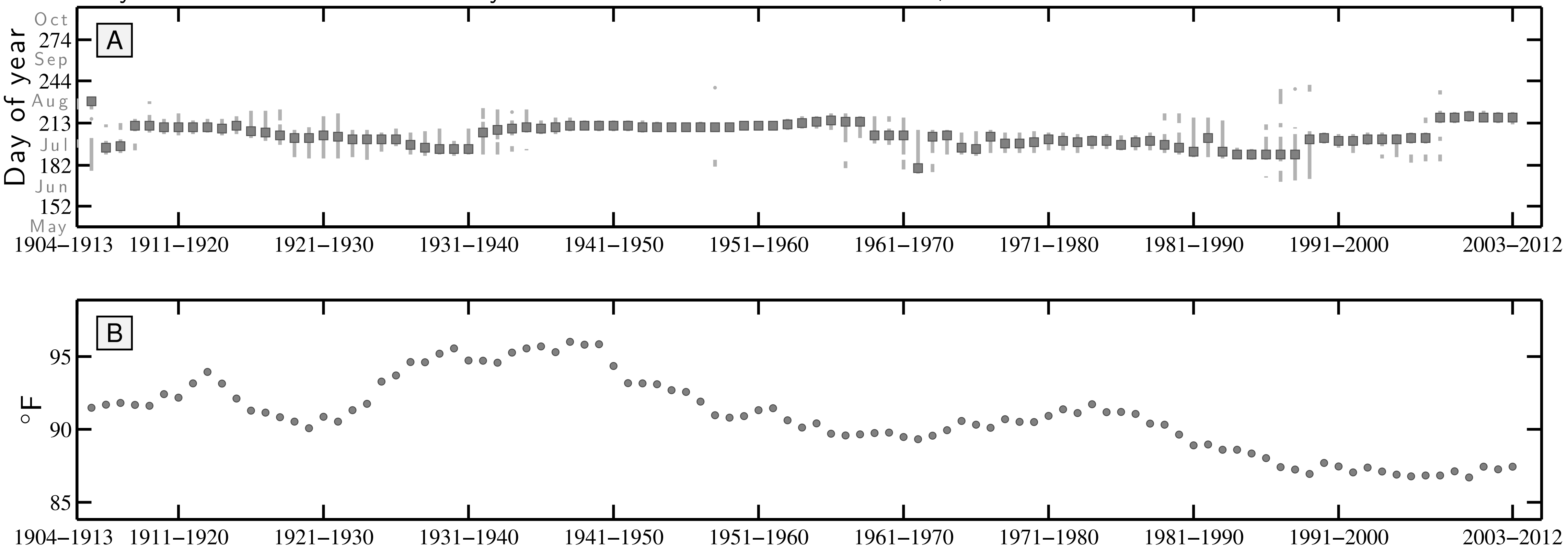

10 year Winter Teletherm dynamics for MT VERNON 3 NE, IL:

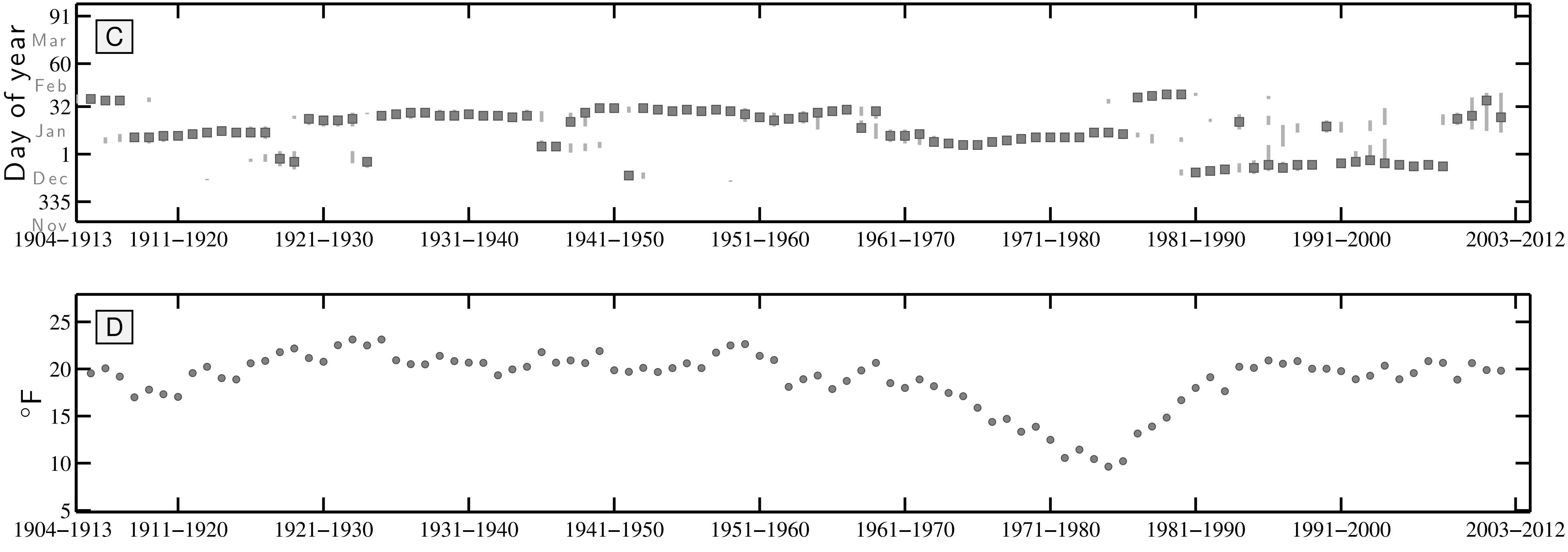

10 year Summer Teletherm dynamics for OLNEY 2S, IL:

10 year Winter Teletherm dynamics for OLNEY 2S, IL:

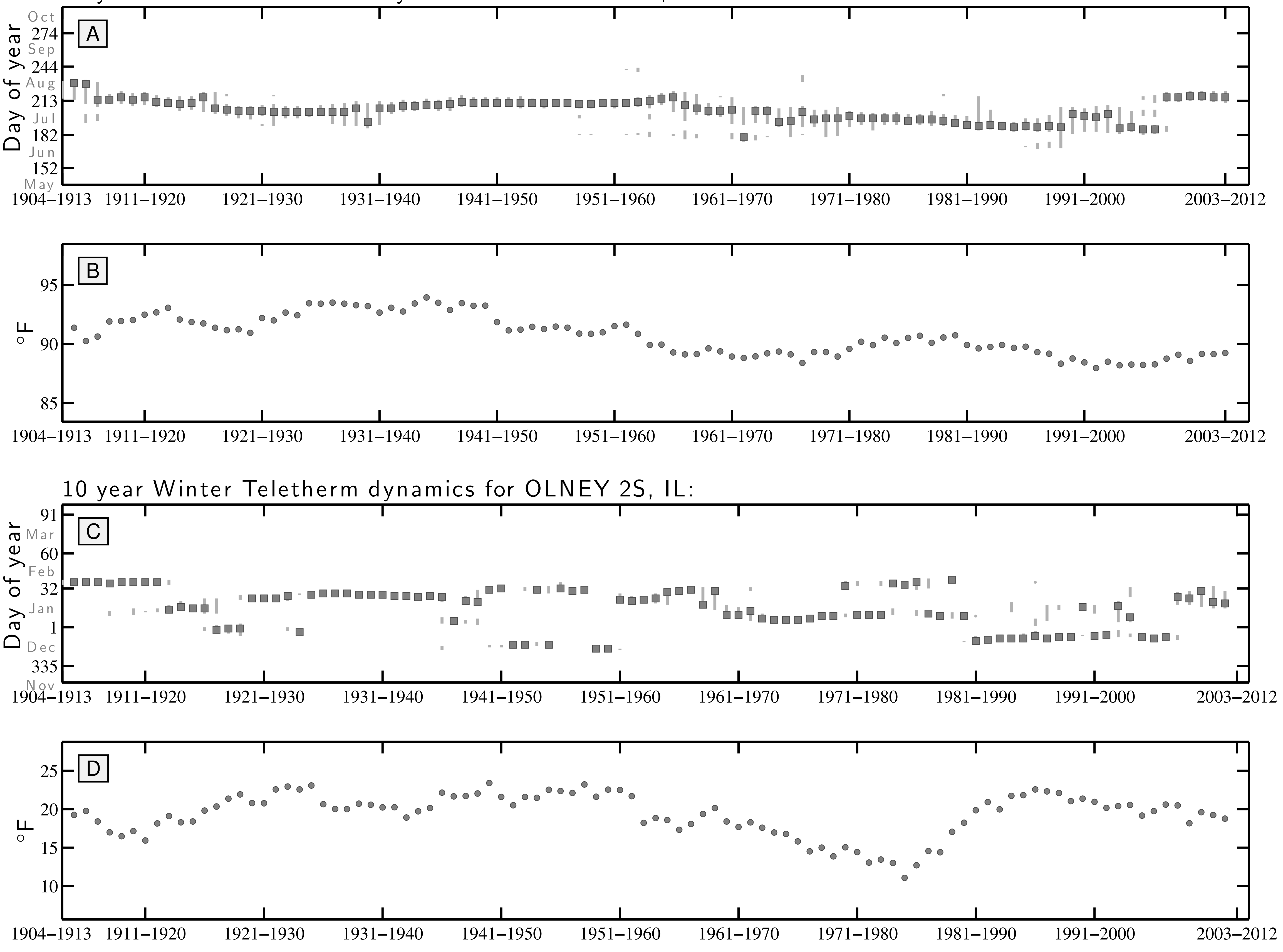

10 year Summer Teletherm dynamics for OTTAWA 5SW, IL:

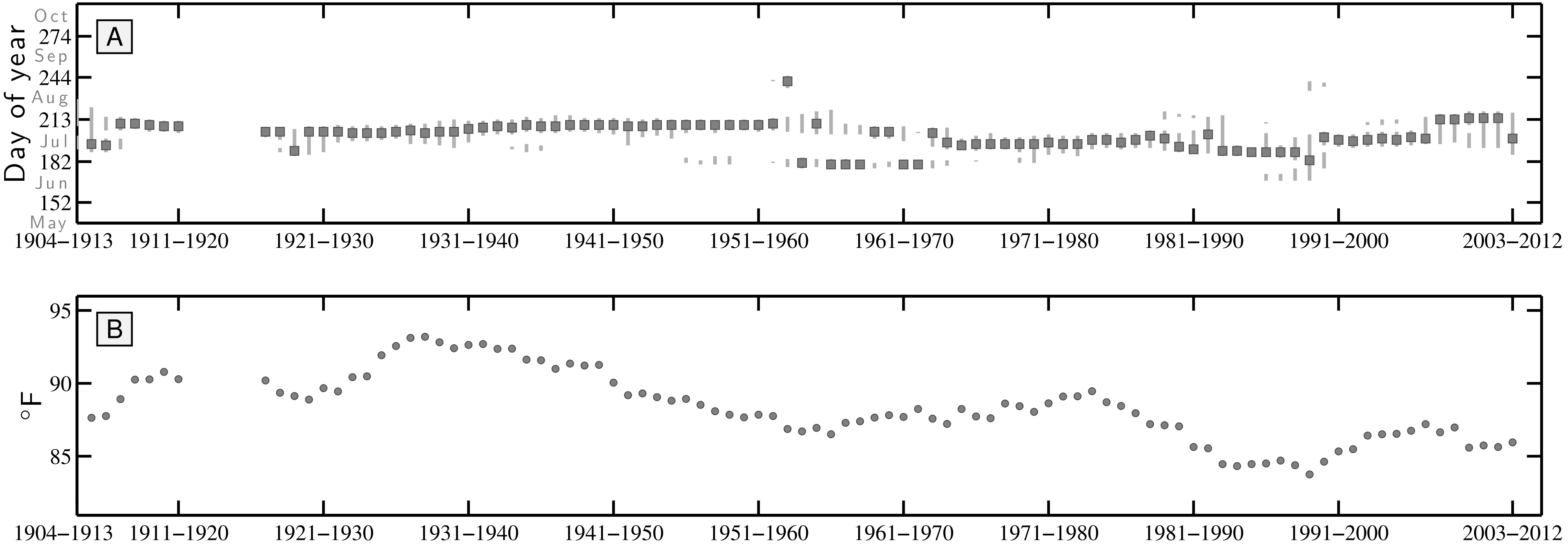

10 year Winter Teletherm dynamics for OTTAWA 5SW, IL:

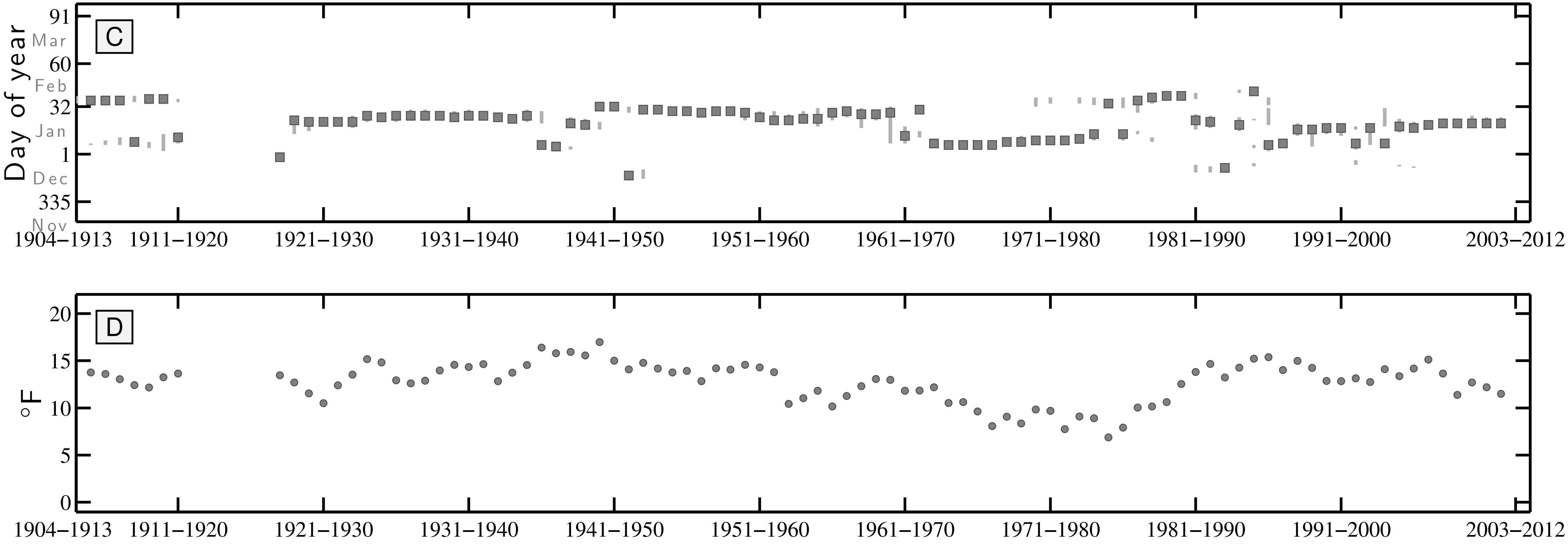

10 year Summer Teletherm dynamics for PALESTINE, IL:

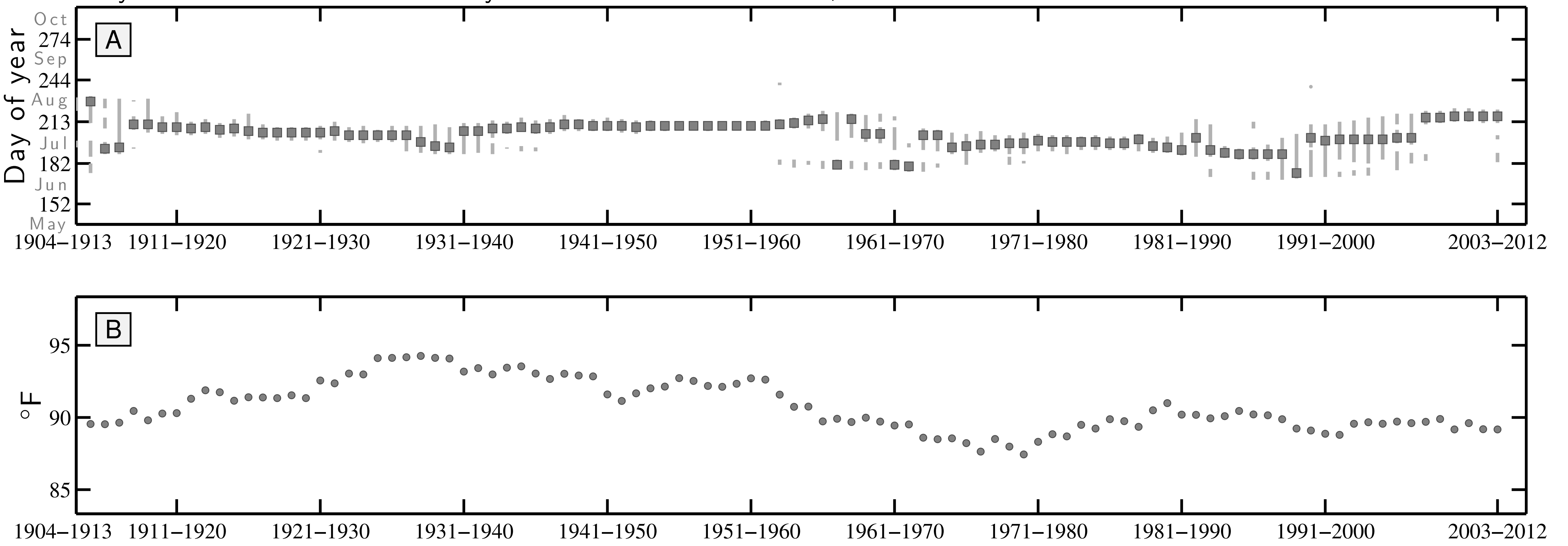

10 year Winter Teletherm dynamics for PALESTINE, IL:

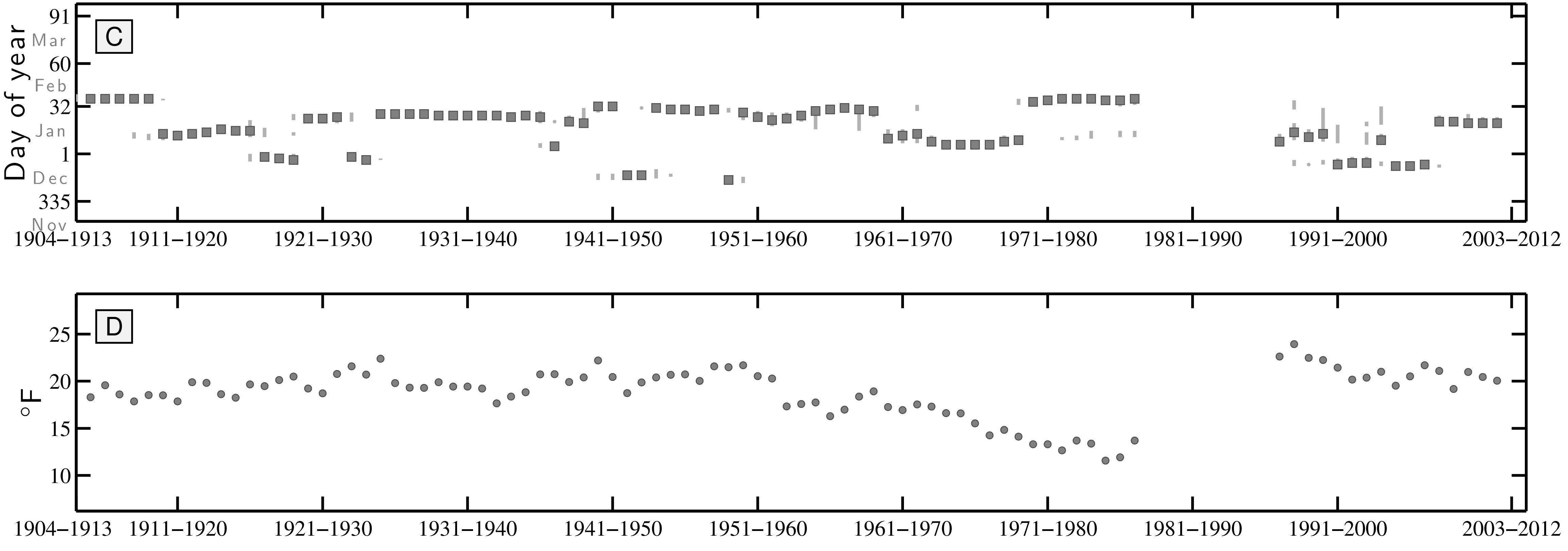

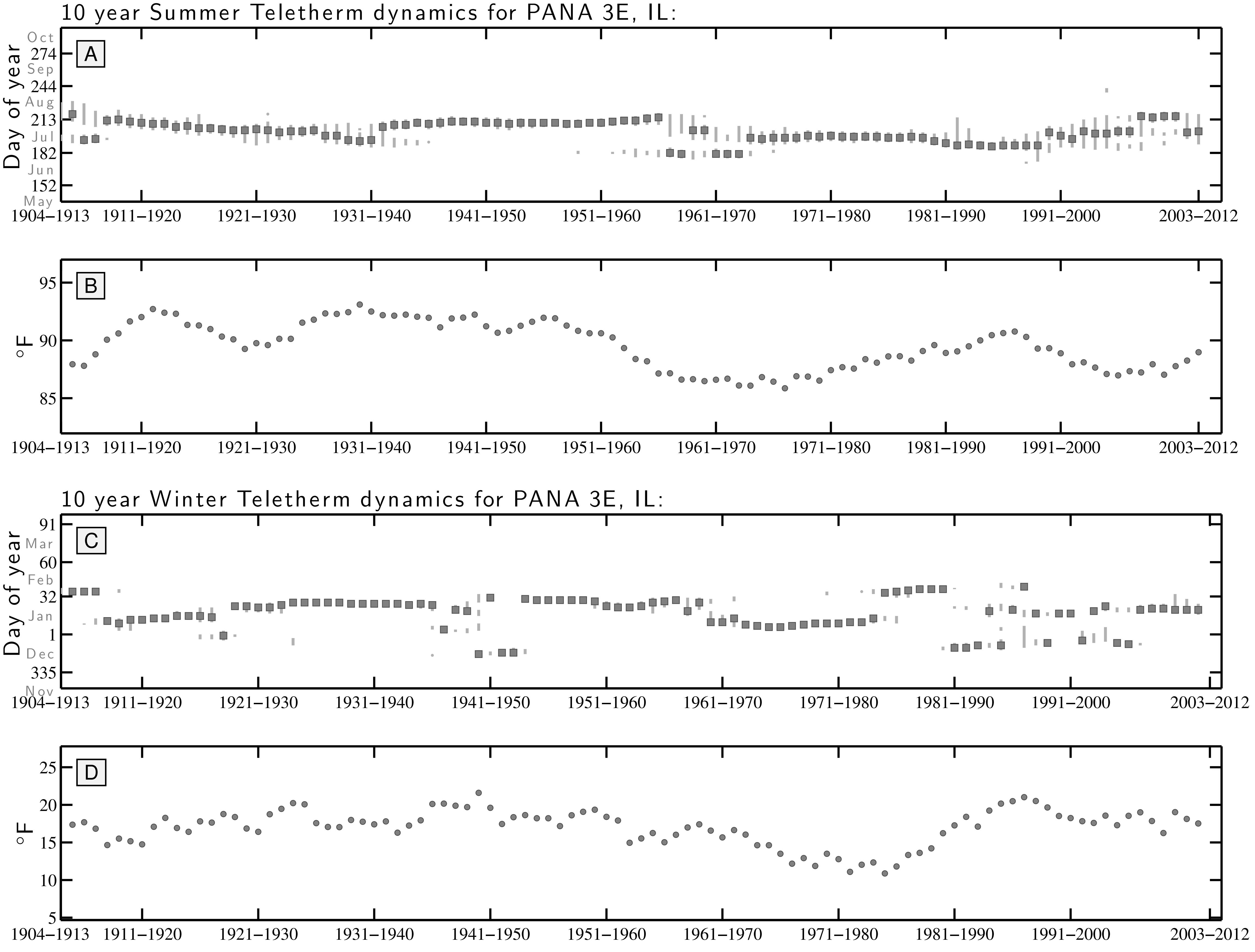
10 year Summer Teletherm dynamics for PANA 3E, IL:
A
Day of year
Oct
274
Sep
244
Aug
213
Jul
182
Jun
152
May
1904–1913
1911–1920
1921–1930
1931–1940
1941–1950
1951–1960
1961–1970
1971–1980
1981–1990
1991–2000
2003–2012
B
°F
95
90
85
10 year Winter Teletherm dynamics for PANA 3E, IL:
C
Day of year
91
Mar
60
Feb
32
Jan
1
Dec
335
Nov
D
°F
25
20
15
10
5

10 year Summer Teletherm dynamics for PARIS WTR WKS, IL:

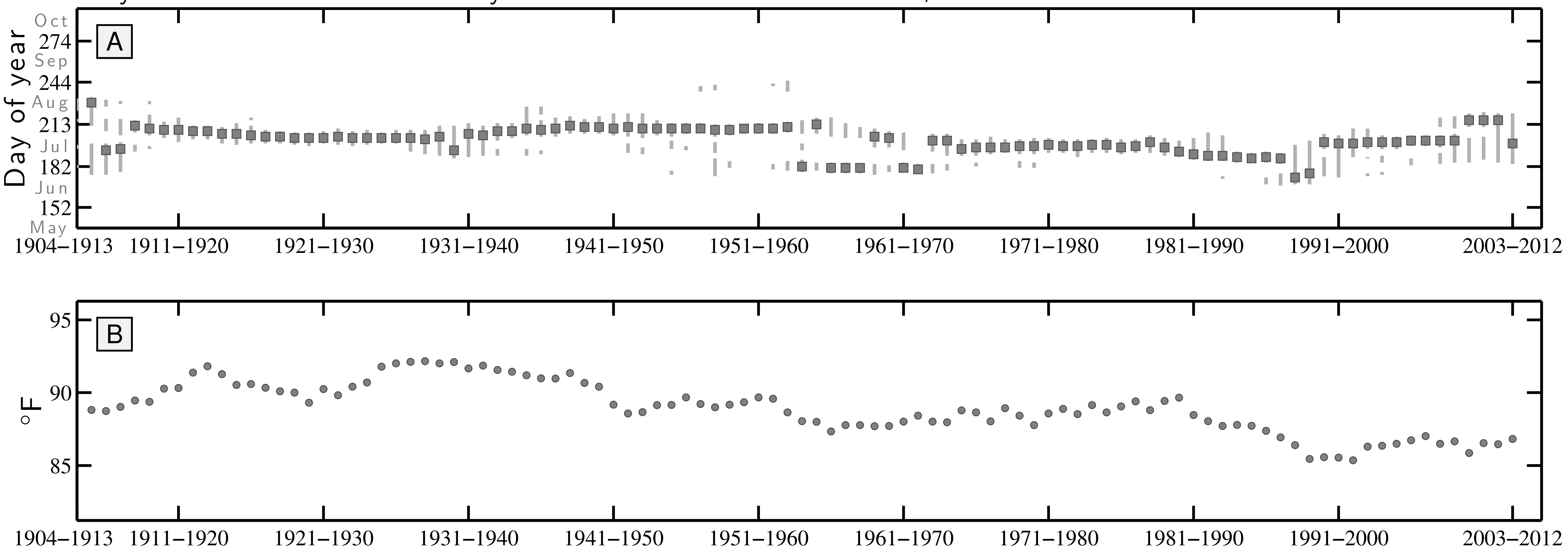

10 year Winter Teletherm dynamics for PARIS WTR WKS, IL:

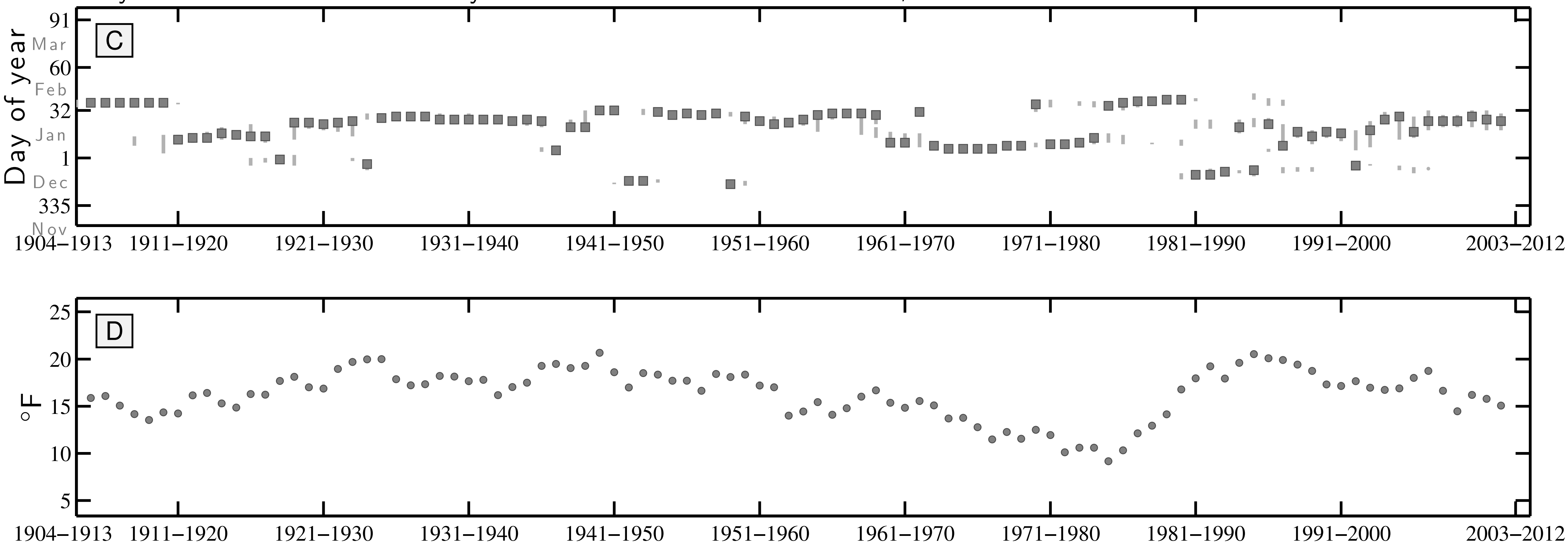

10 year Summer Teletherm dynamics for PERRY 6 NW, IL:

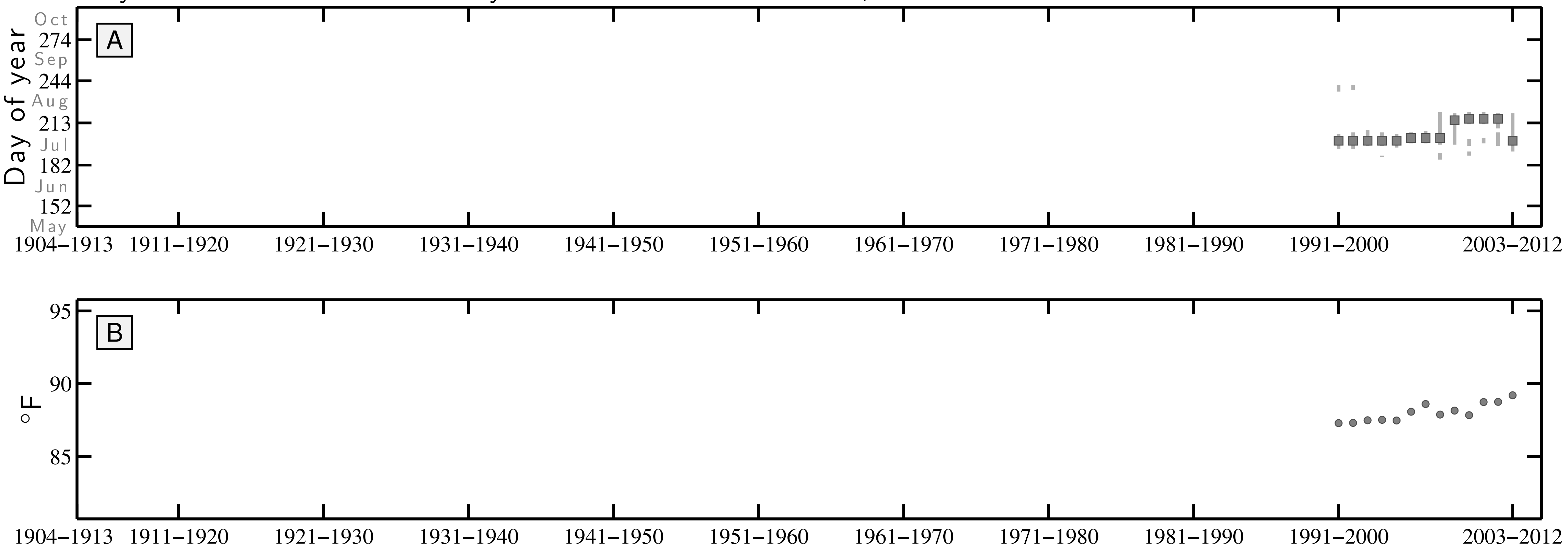

10 year Winter Teletherm dynamics for PERRY 6 NW, IL:

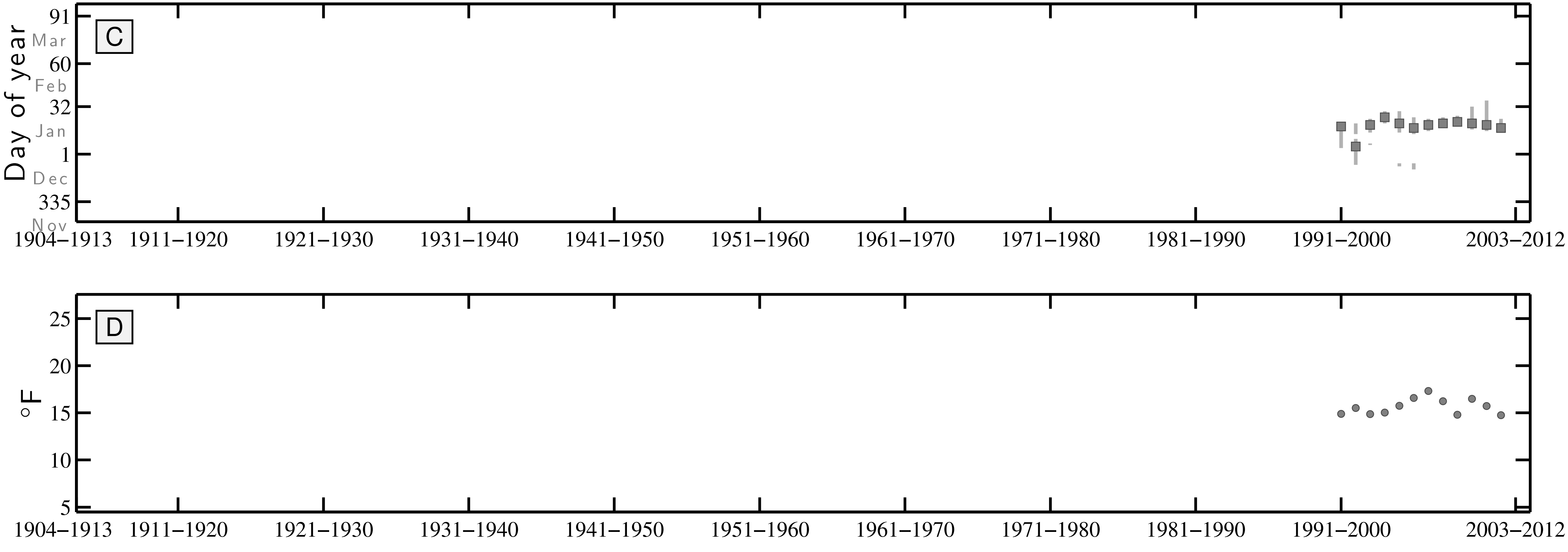

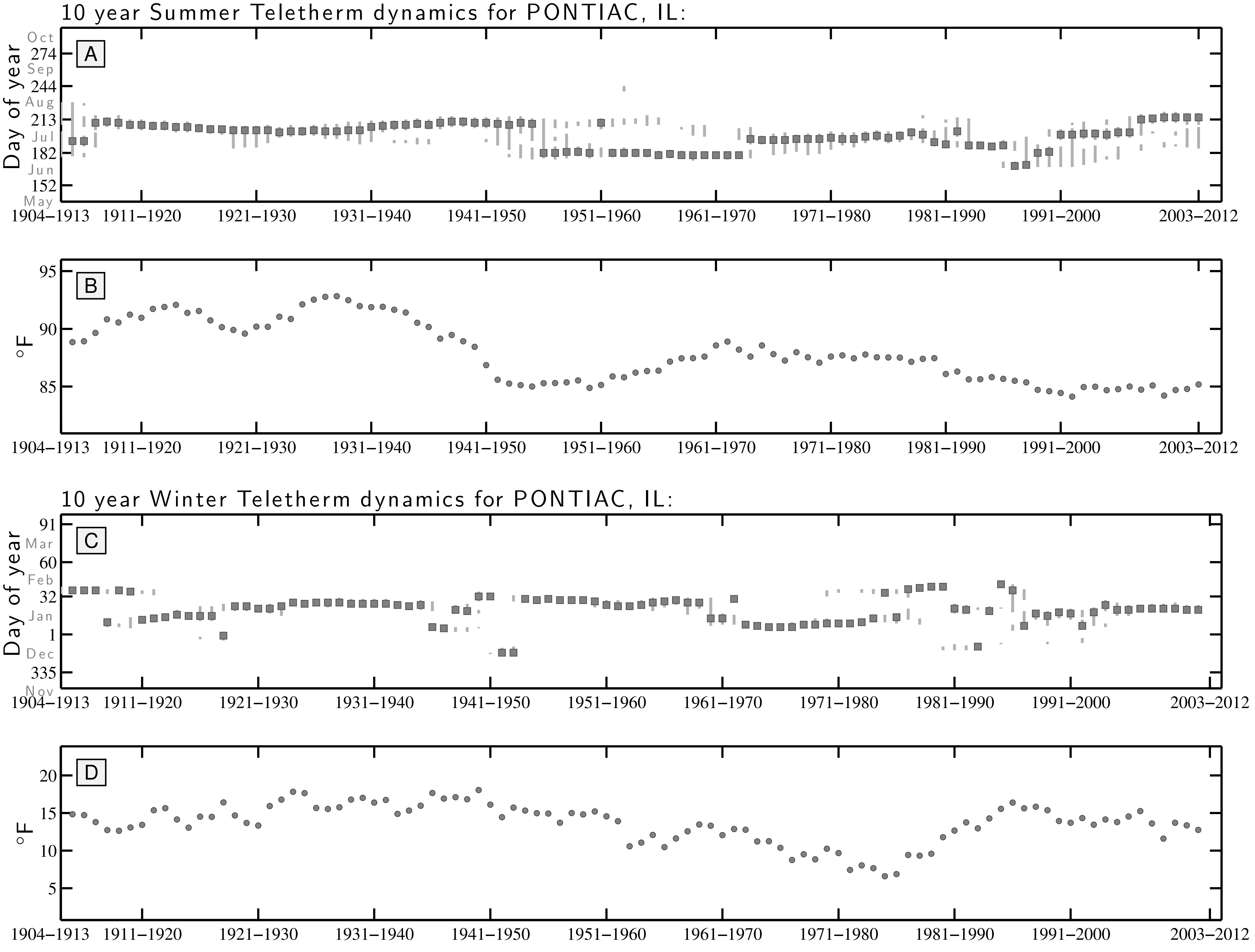
10 year Summer Teletherm dynamics for PONTIAC, IL:
A
Day of year
Oct
274
Sep
244
Aug
213
Jul
182
Jun
152
May
1904–1913
1911–1920
1921–1930
1931–1940
1941–1950
1951–1960
1961–1970
1971–1980
1981–1990
1991–2000
2003–2012
B
°F
95
90
85
10 year Winter Teletherm dynamics for PONTIAC, IL:
C
91
Mar
60
Feb
32
Jan
1
Dec
335
Nov
D
20
15
10
5

10 year Summer Teletherm dynamics for RUSHVILLE, IL:

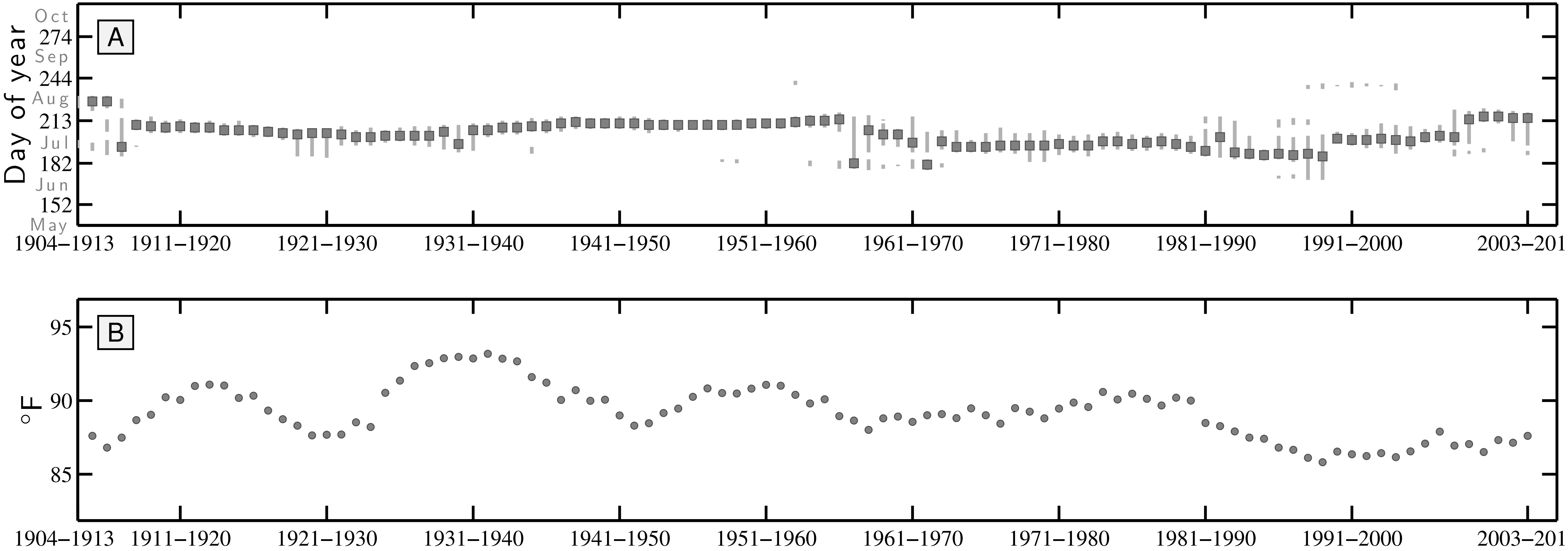

10 year Winter Teletherm dynamics for RUSHVILLE, IL:

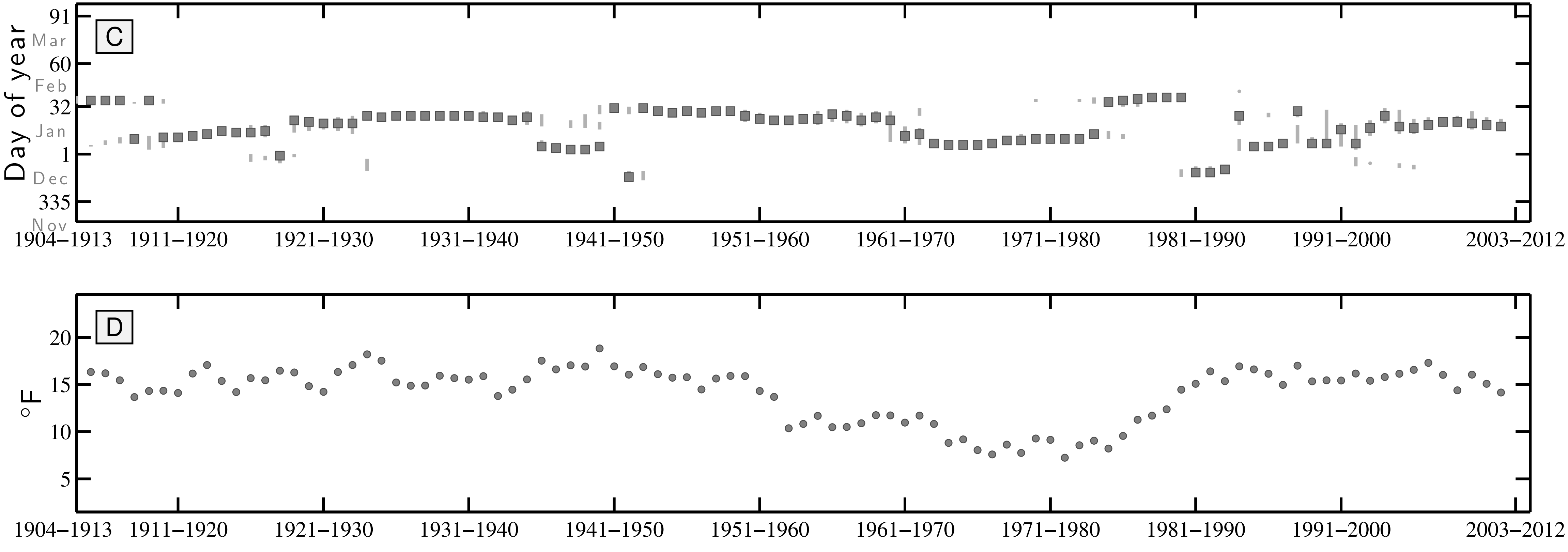

10 year Summer Teletherm dynamics for SPARTA 1 W, IL:

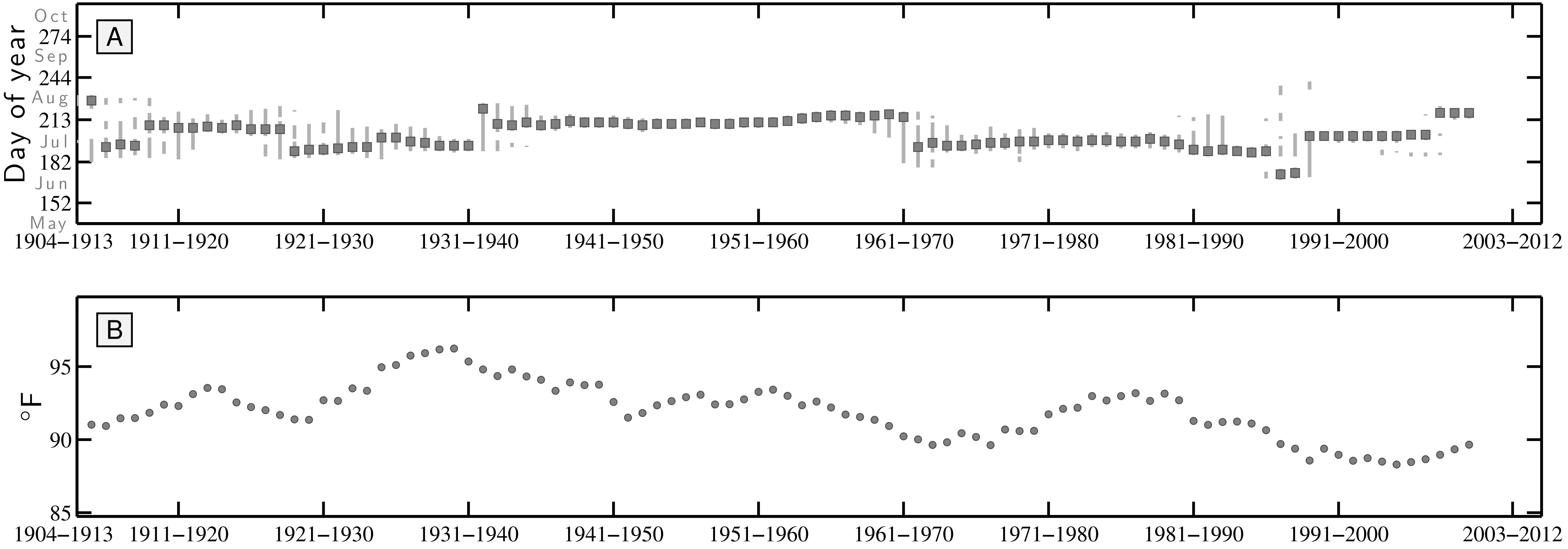

10 year Winter Teletherm dynamics for SPARTA 1 W, IL:

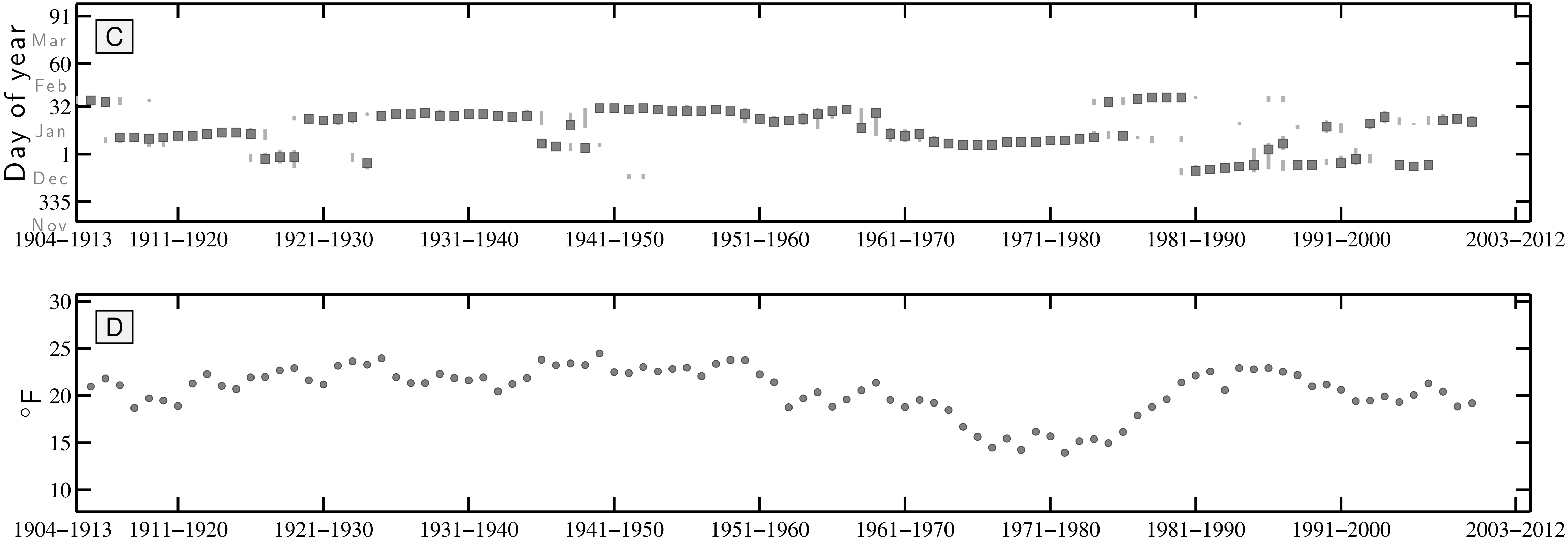

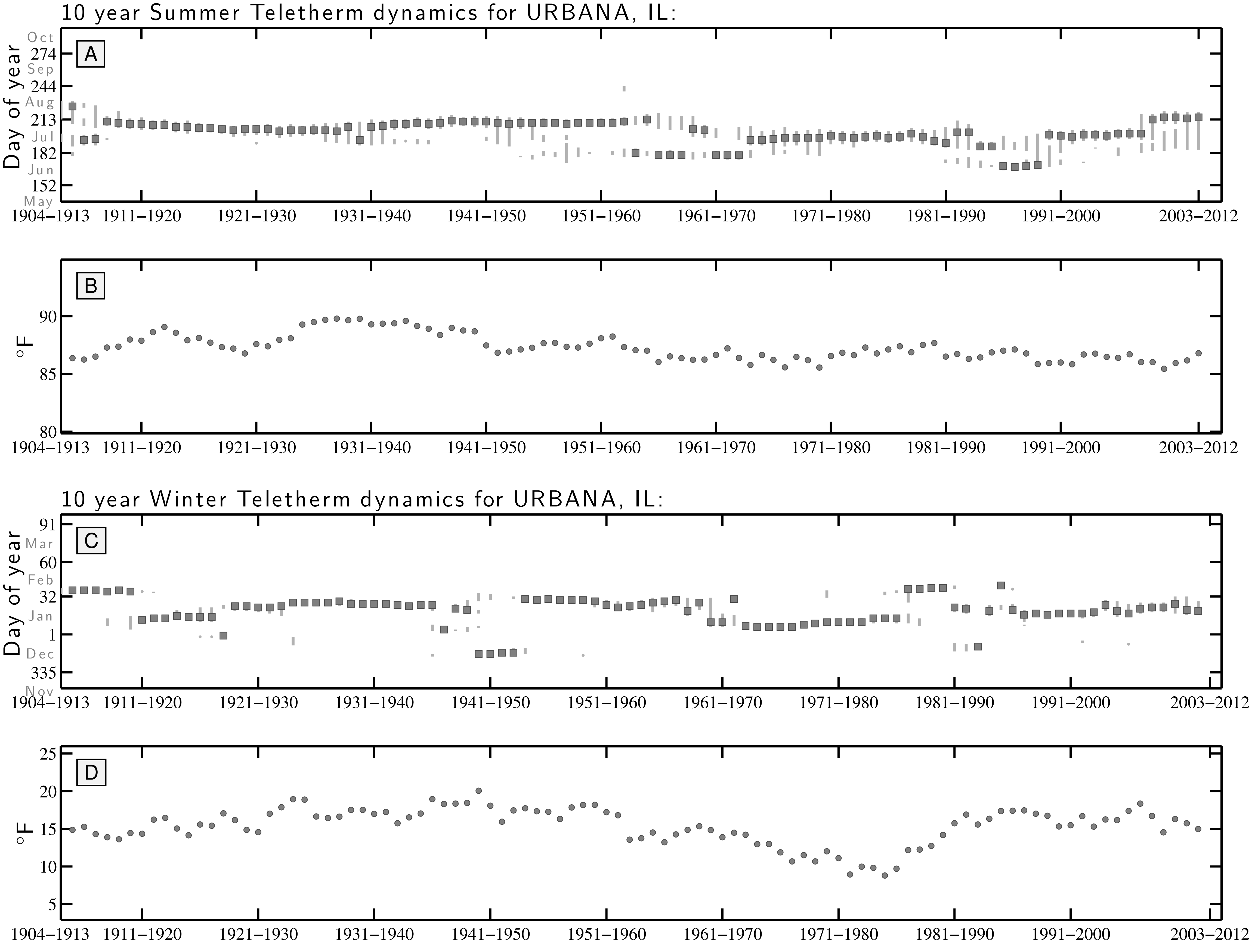
10 year Summer Teletherm dynamics for URBANA, IL:
A
B
Day of year
°F
Oct
274
Sep
244
Aug
213
Jul
182
Jun
152
May
90
85
80
1904–1913
1911–1920
1921–1930
1931–1940
1941–1950
1951–1960
1961–1970
1971–1980
1981–1990
1991–2000
2003–2012
10 year Winter Teletherm dynamics for URBANA, IL:
C
D
91
Mar
60
Feb
32
Jan
1
Dec
335
Nov
25
20
15
10
5

10 year Summer Teletherm dynamics for WALNUT, IL:

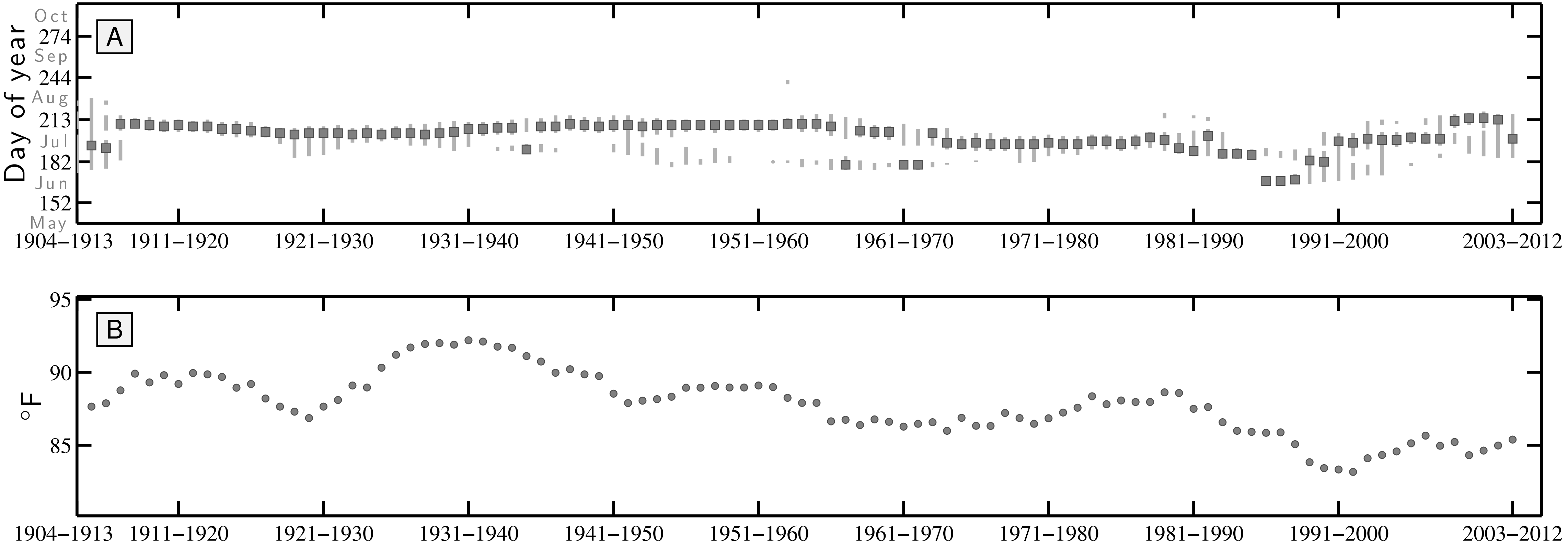

10 year Winter Teletherm dynamics for WALNUT, IL:

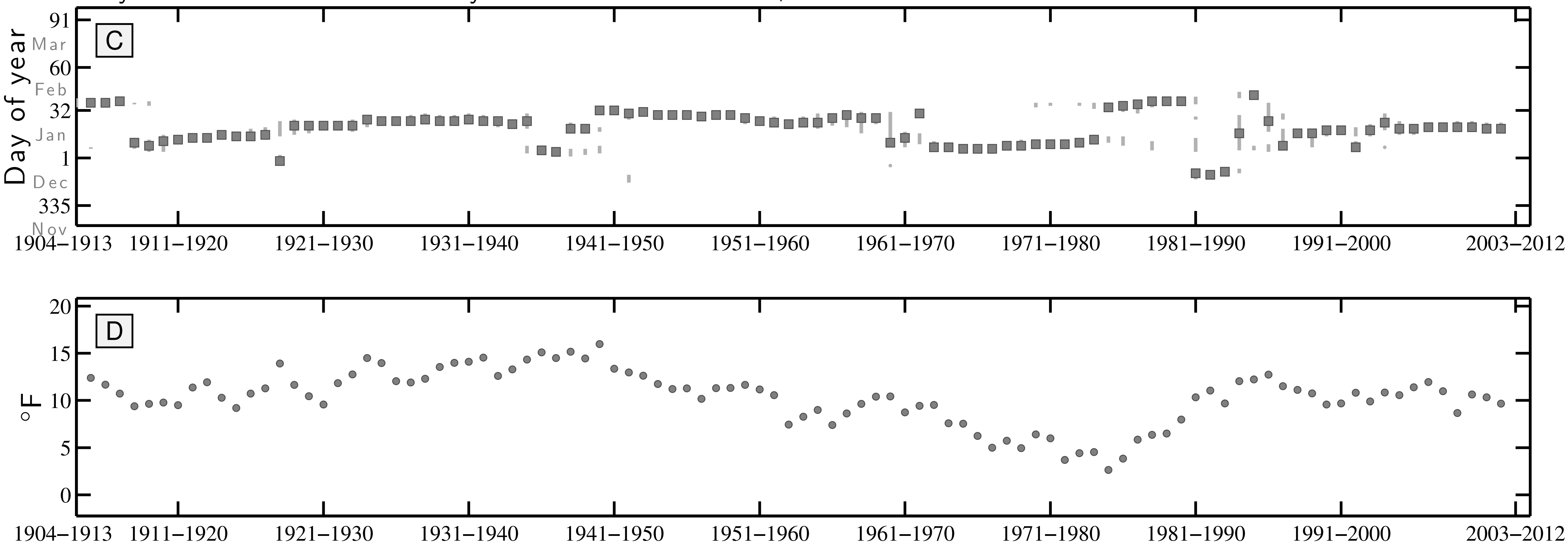

10 year Summer Teletherm dynamics for WHITE HALL 1 E, IL:

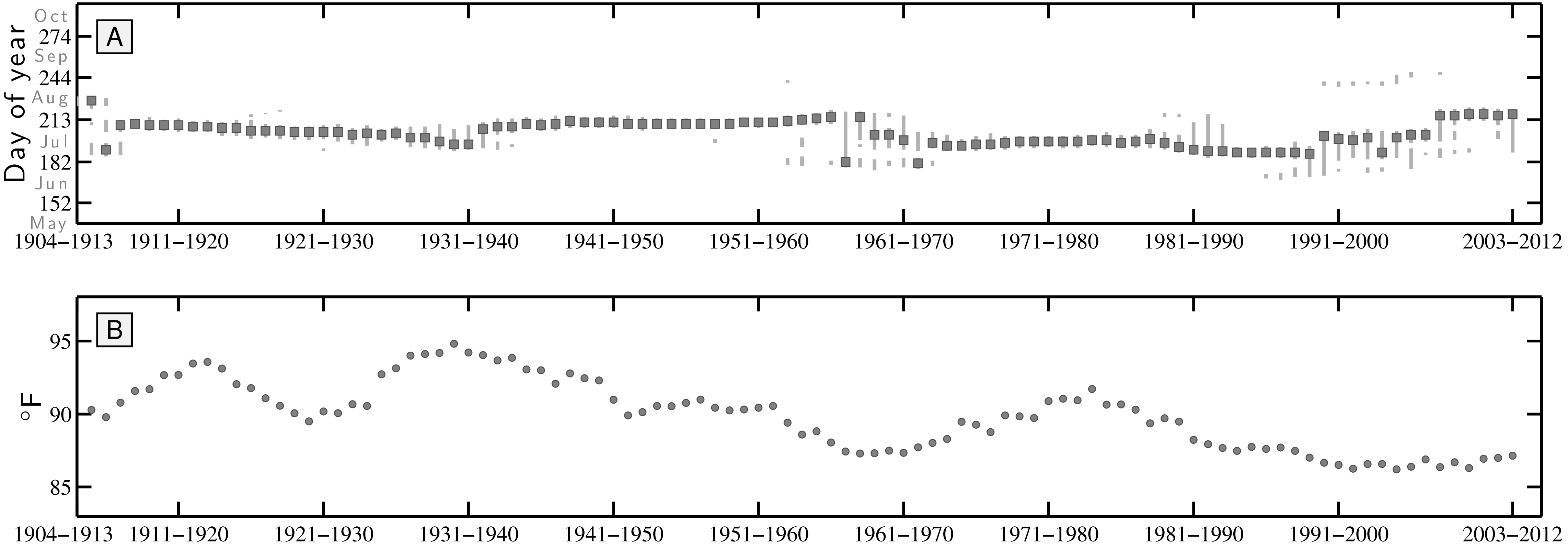

10 year Winter Teletherm dynamics for WHITE HALL 1 E, IL:

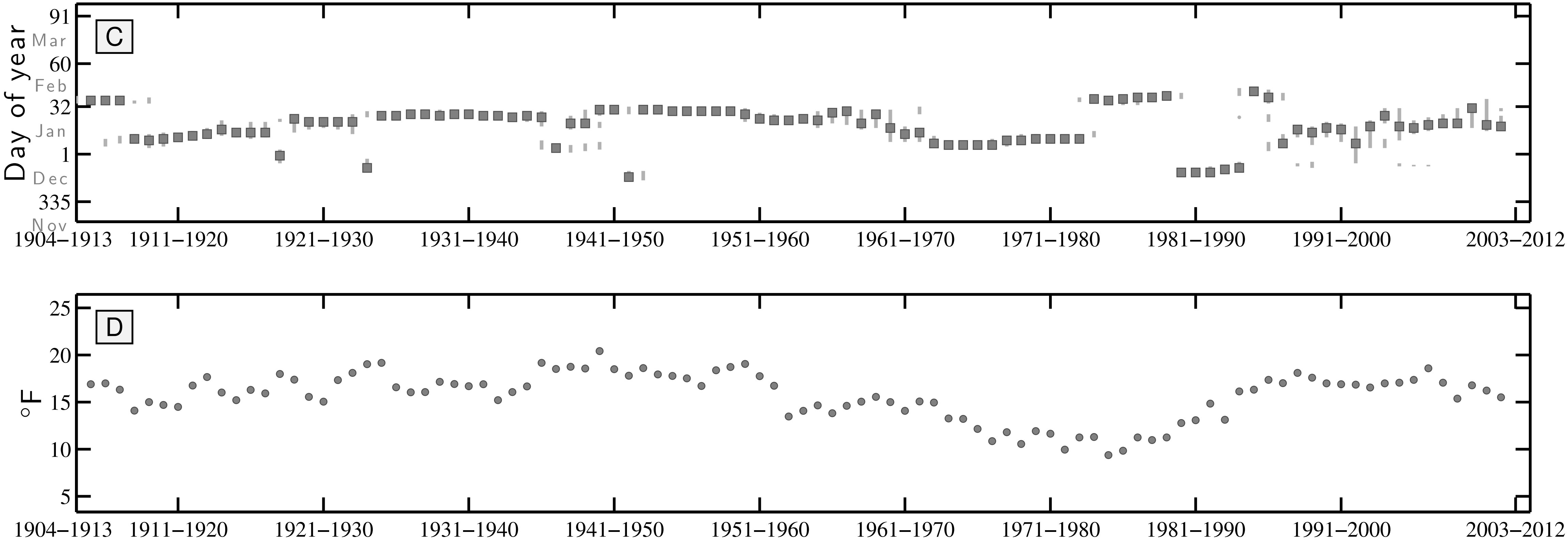

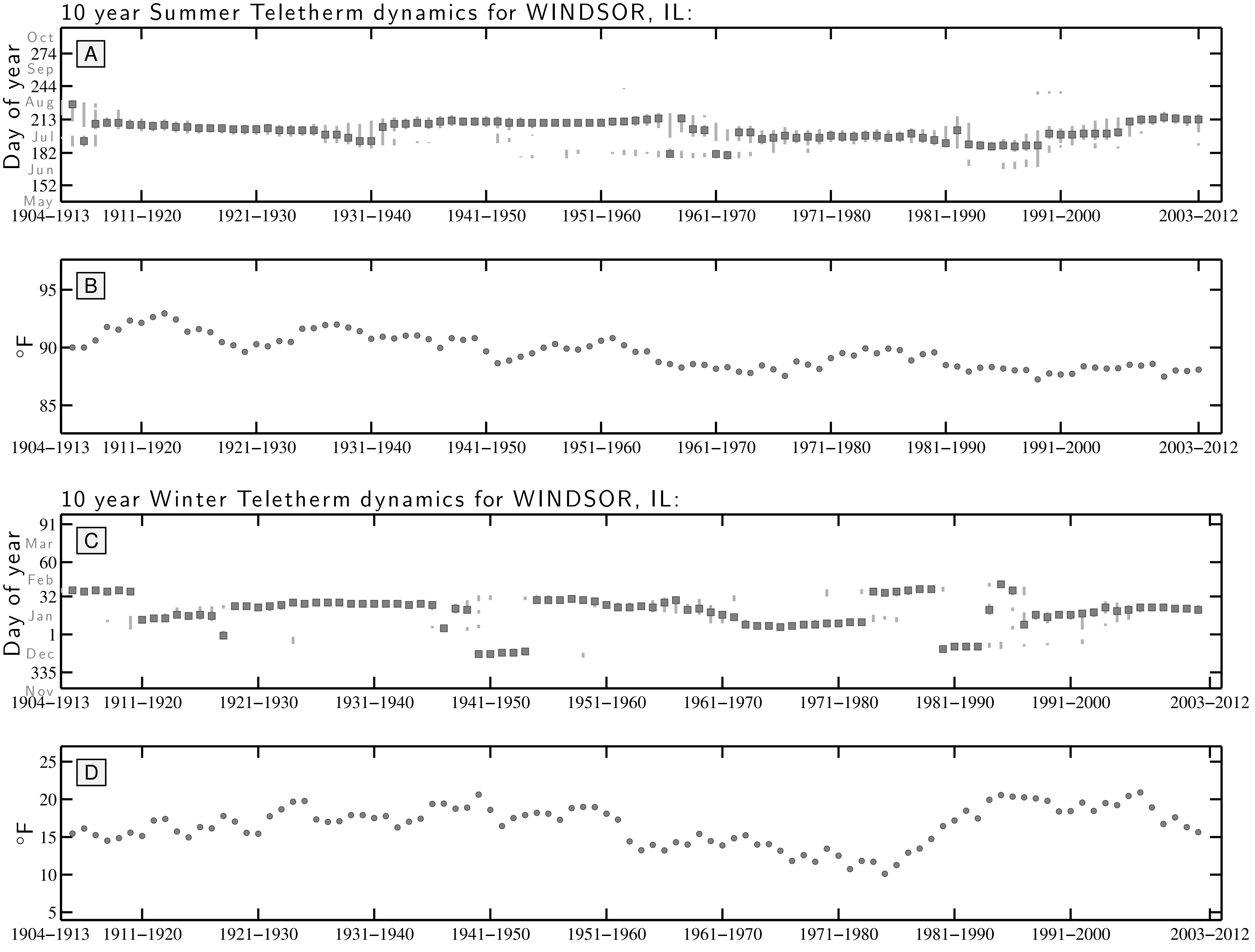
10 year Summer Teletherm dynamics for WINDSOR, IL:
A
Day of year
Oct
274
Sep
244
Aug
213
Jul
182
Jun
152
May
1904–1913
1911–1920
1921–1930
1931–1940
1941–1950
1951–1960
1961–1970
1971–1980
1981–1990
1991–2000
2003–2012
B
°F
95
90
85
10 year Winter Teletherm dynamics for WINDSOR, IL:
C
Day of year
91
Mar
60
Feb
32
Jan
1
Dec
335
Nov
D
°F
25
20
15
10
5

10 year Summer Teletherm dynamics for ANDERSON SEWAGE PLT, IN:

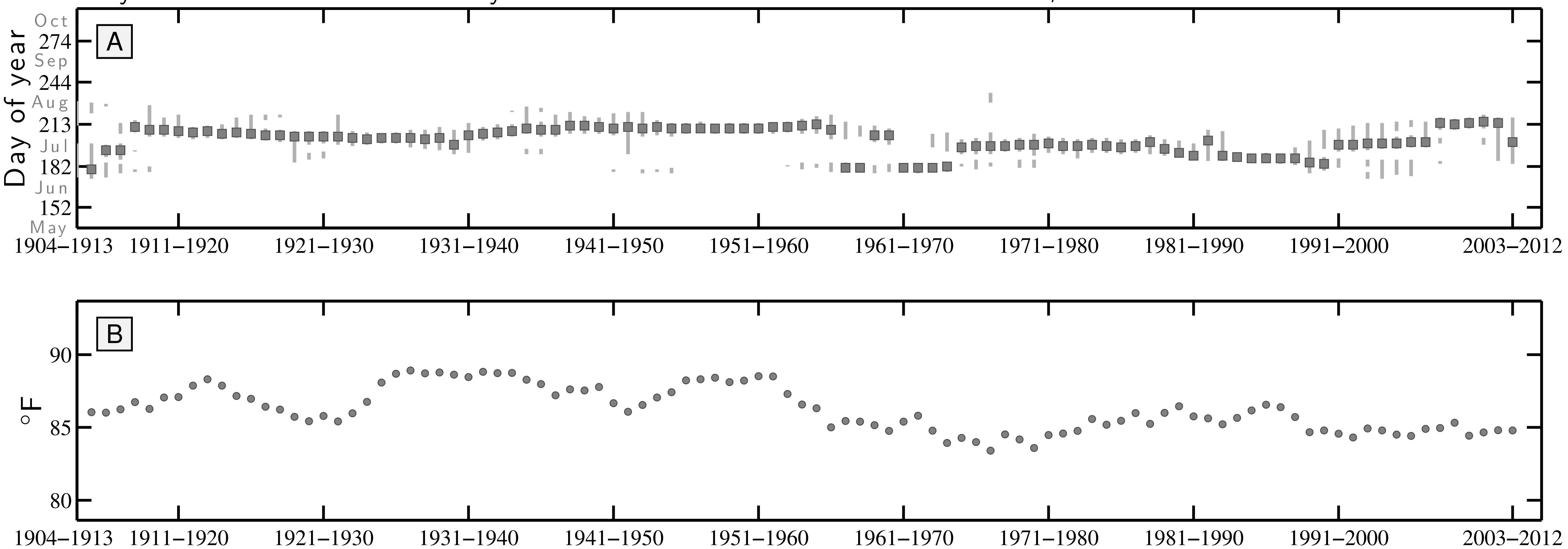

10 year Winter Teletherm dynamics for ANDERSON SEWAGE PLT, IN:

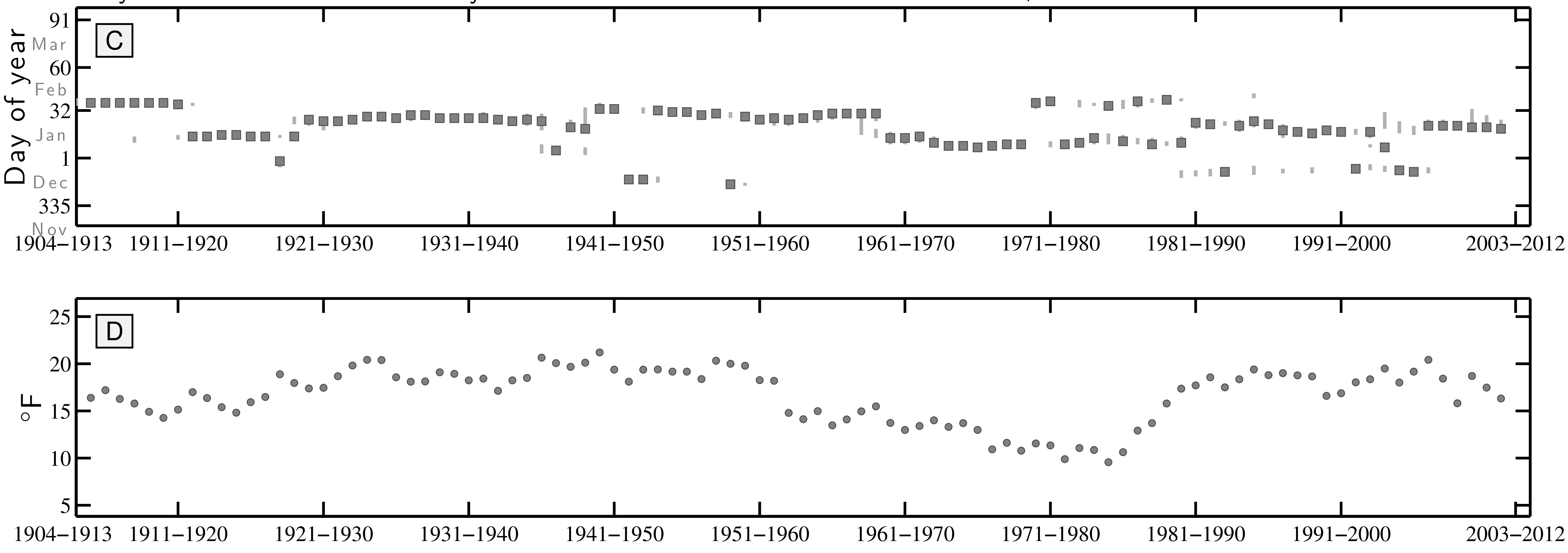

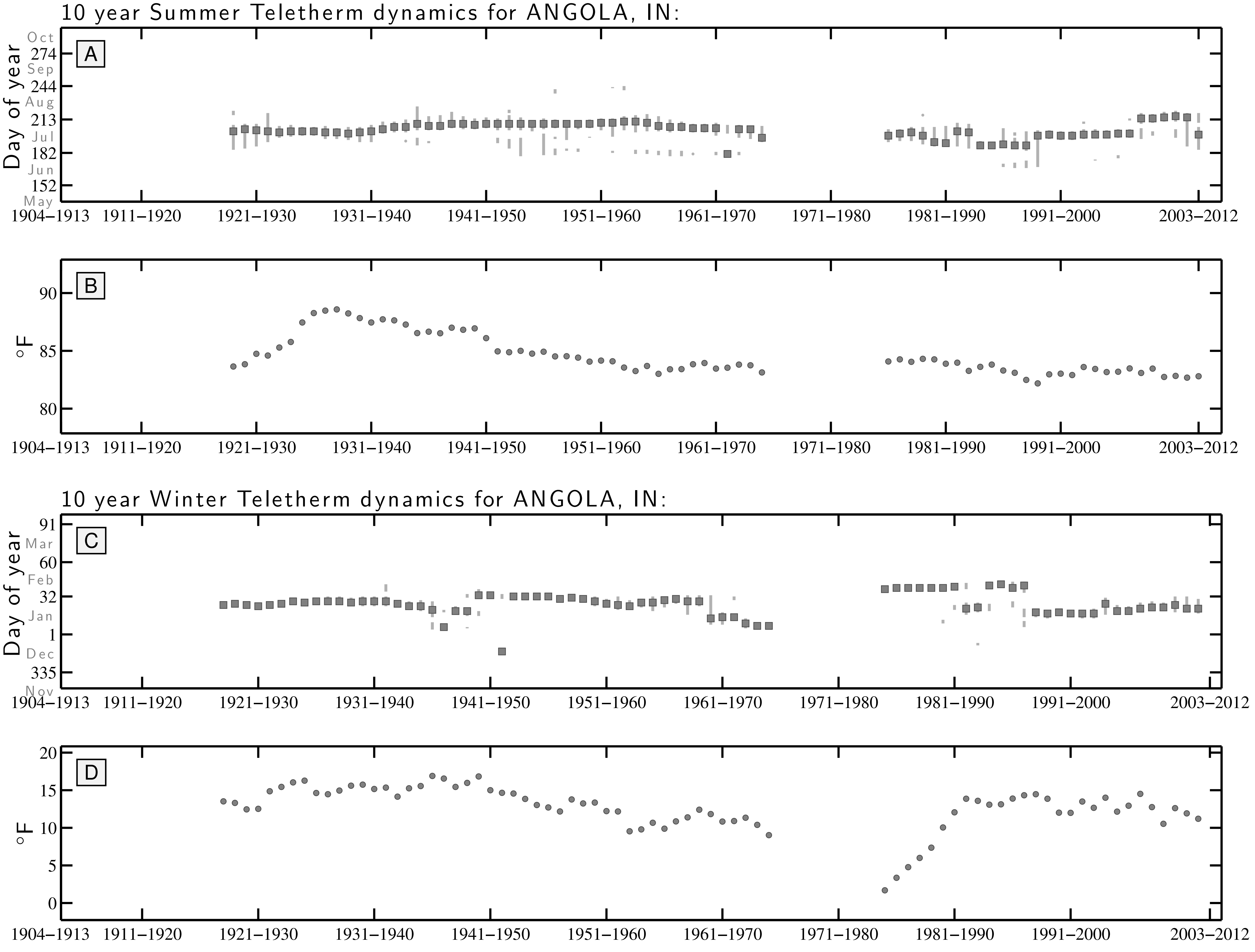
10 year Summer Teletherm dynamics for ANGOLA, IN:
A
Day of year
Oct
274
Sep
244
Aug
213
Jul
182
Jun
152
May
1904–1913
1911–1920
1921–1930
1931–1940
1941–1950
1951–1960
1961–1970
1971–1980
1981–1990
1991–2000
2003–2012
B
°F
90
85
80
10 year Winter Teletherm dynamics for ANGOLA, IN:
C
91
Mar
60
Feb
32
Jan
1
Dec
335
Nov
D
20
15
10
5
0

10 year Summer Teletherm dynamics for BERNE WWTP, IN:

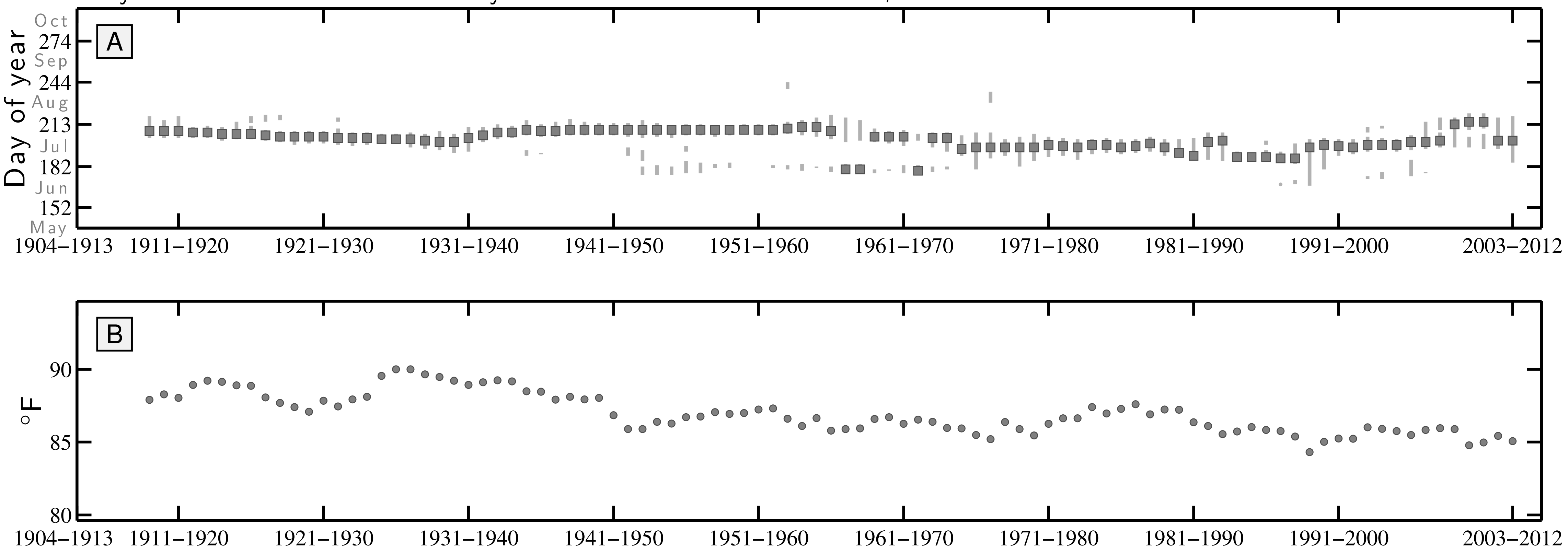

10 year Winter Teletherm dynamics for BERNE WWTP, IN:

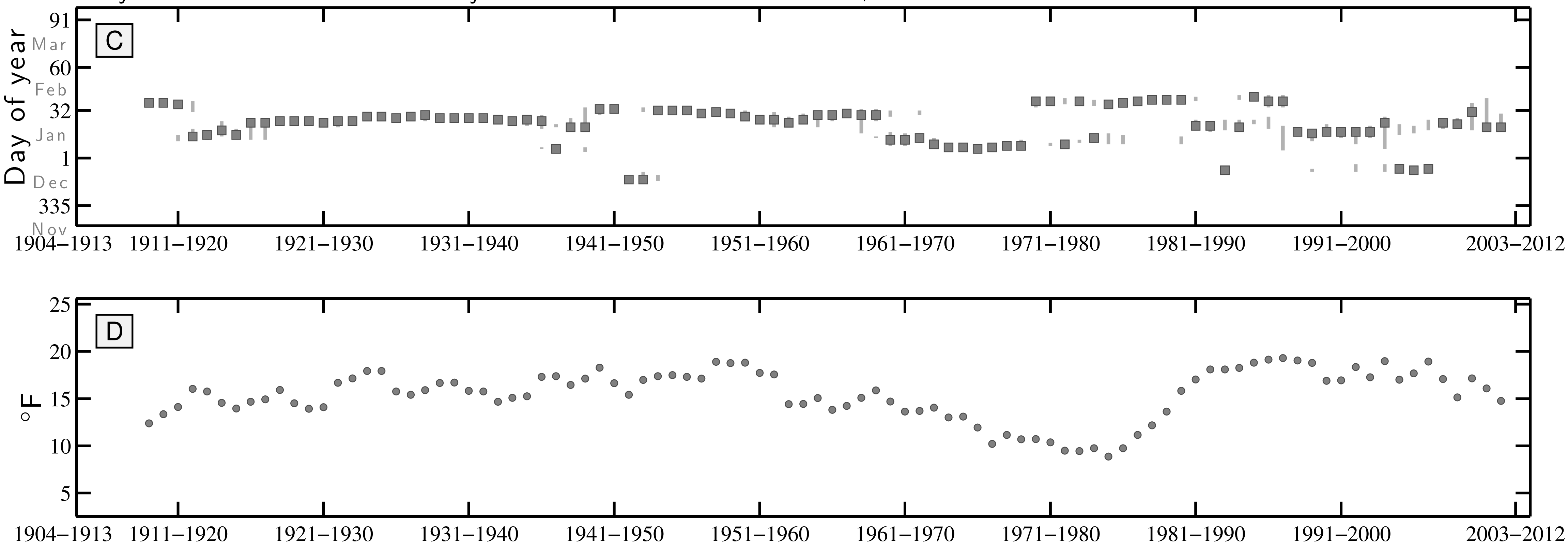

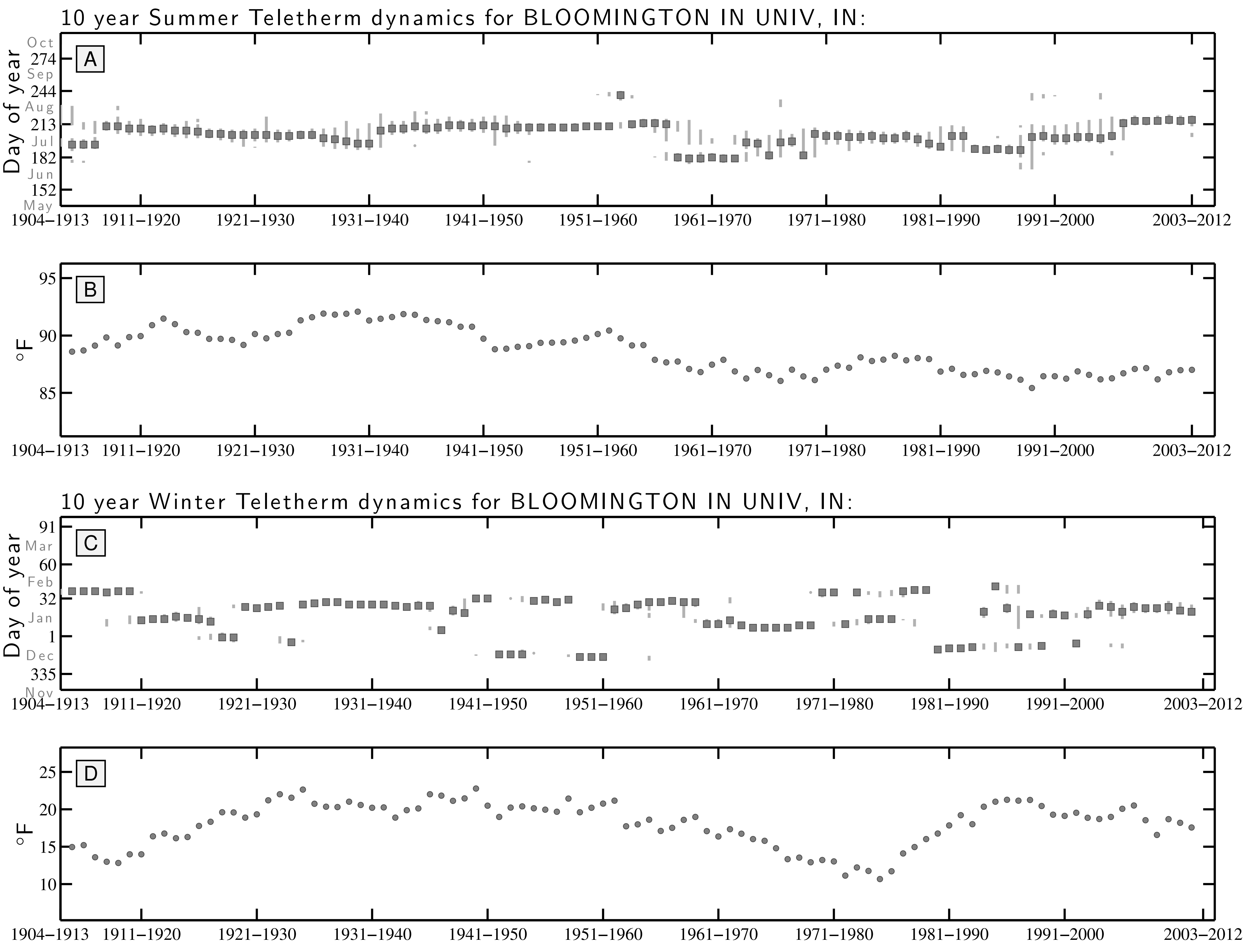
10 year Summer Teletherm dynamics for BLOOMINGTON IN UNIV, IN:
A
Day of year
Oct
274
Sep
244
Aug
213
Jul
182
Jun
152
May
B
°F
95
90
85
1904–1913
1911–1920
1921–1930
1931–1940
1941–1950
1951–1960
1961–1970
1971–1980
1981–1990
1991–2000
2003–2012
10 year Winter Teletherm dynamics for BLOOMINGTON IN UNIV, IN:
C
Day of year
91
Mar
60
Feb
32
Jan
1
Dec
335
Nov
D
°F
25
20
15
10

10 year Summer Teletherm dynamics for BROOKVILLE, IN:

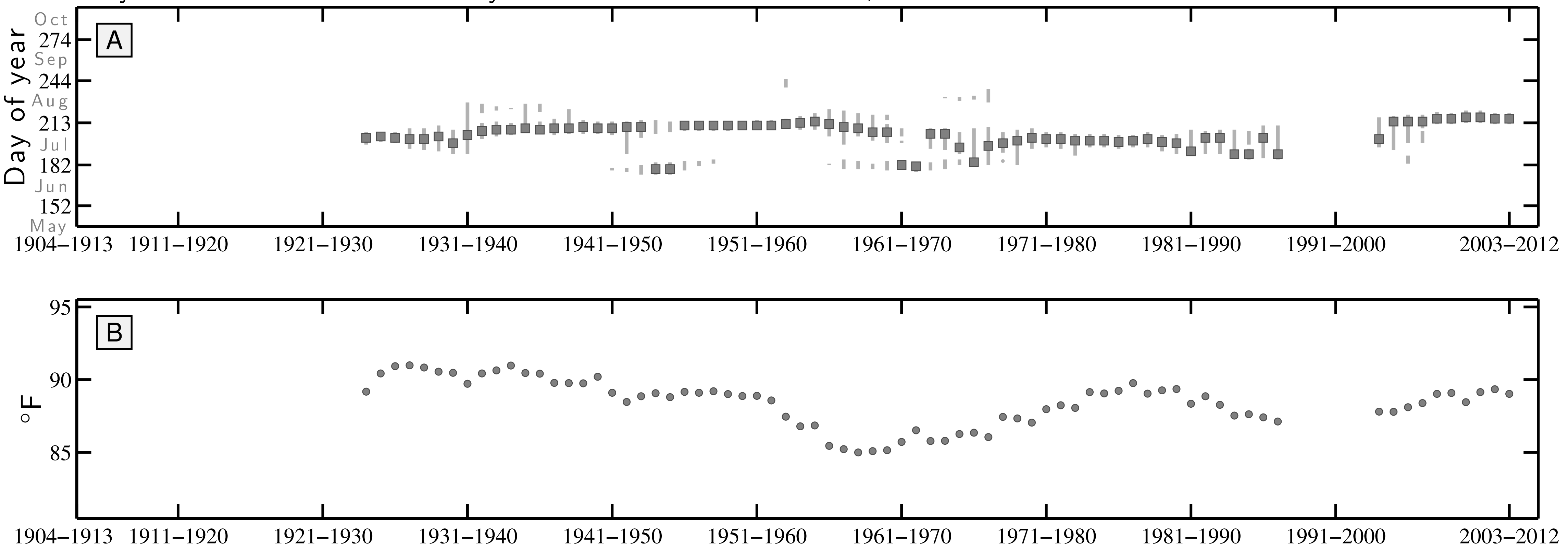

10 year Winter Teletherm dynamics for BROOKVILLE, IN:

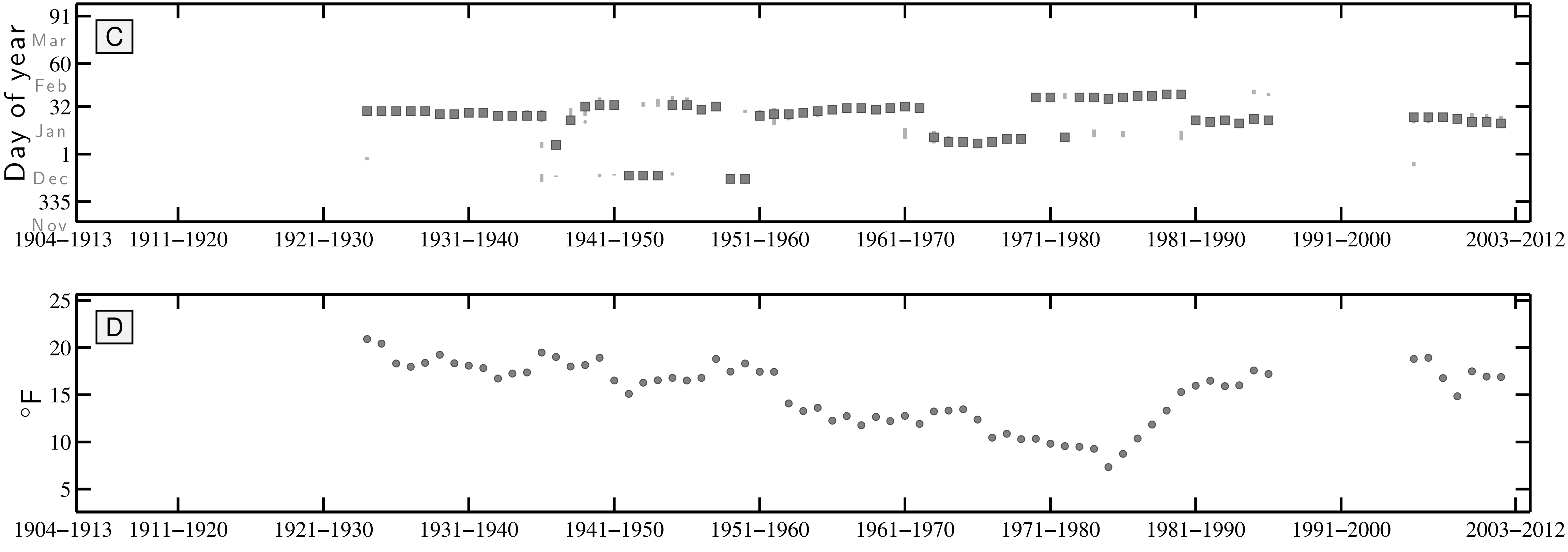

10 year Summer Teletherm dynamics for CAMBRIDGE CITY 3 N, IN:

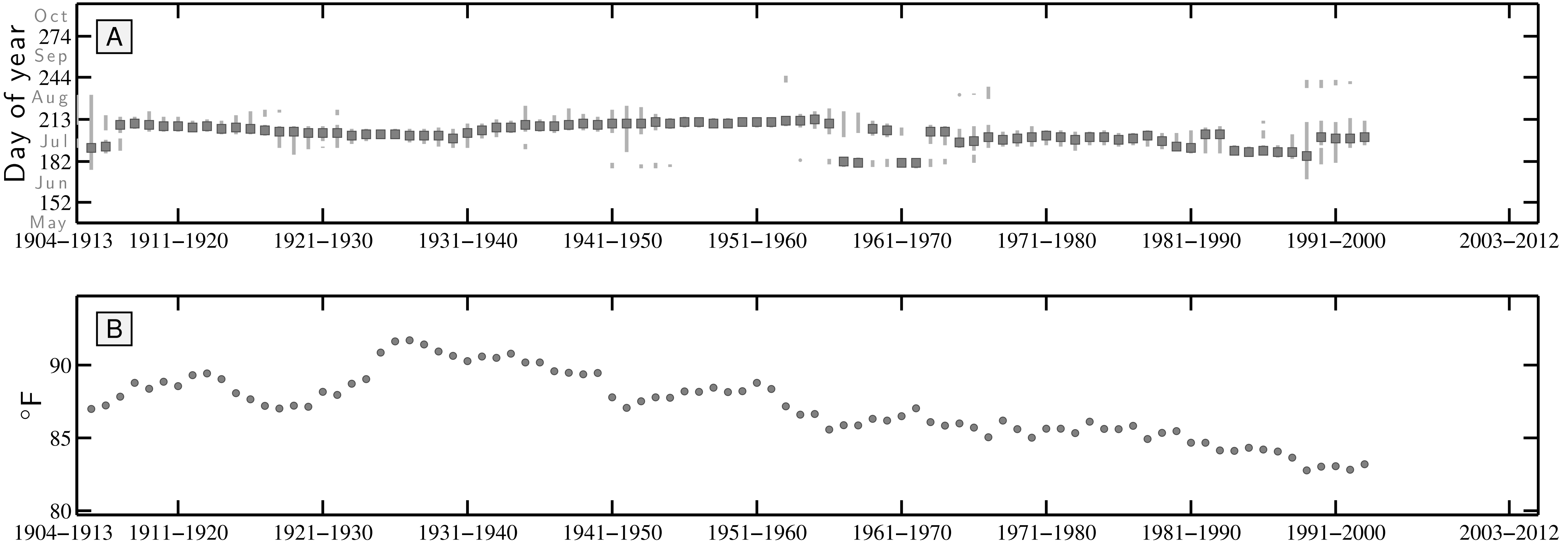

10 year Winter Teletherm dynamics for CAMBRIDGE CITY 3 N, IN:

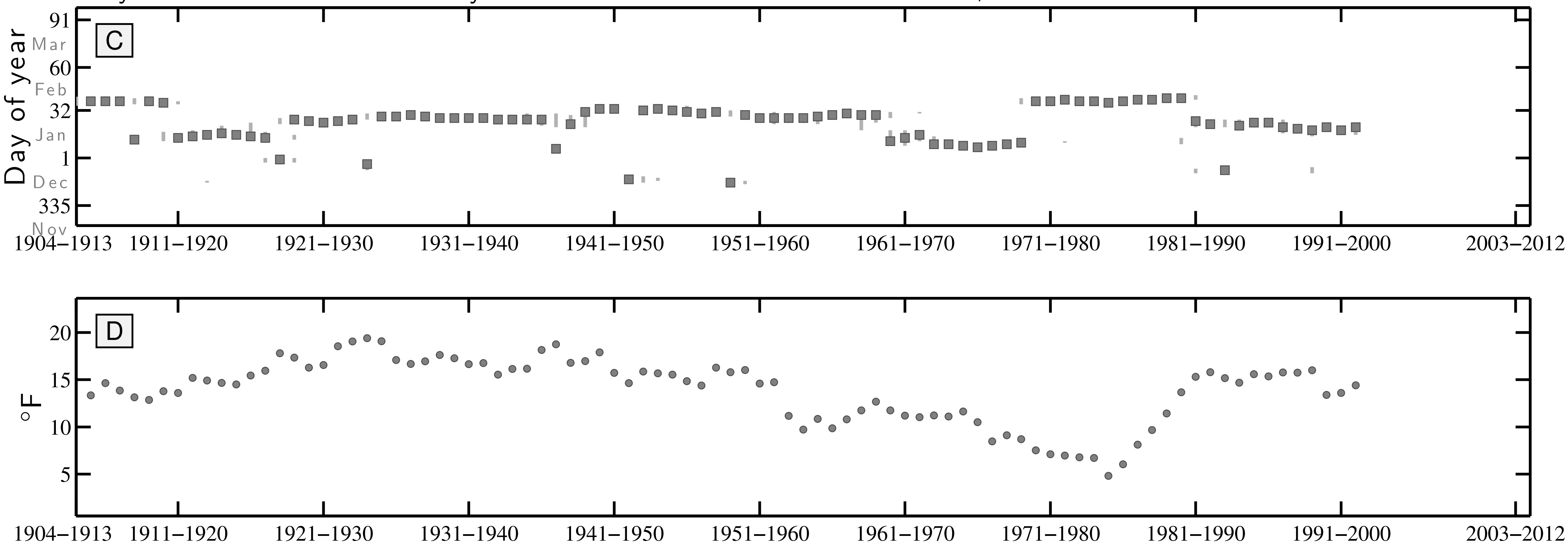

10 year Summer Teletherm dynamics for CHARLESTOWN 5 NNW, IN:

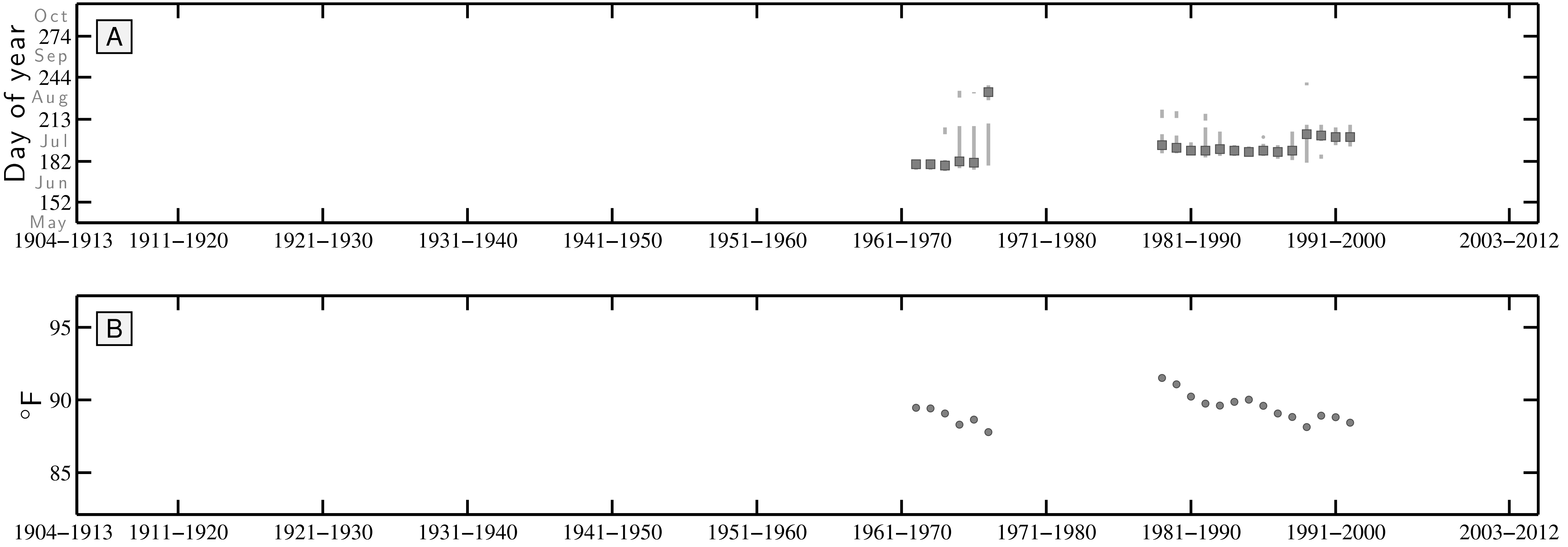

10 year Winter Teletherm dynamics for CHARLESTOWN 5 NNW, IN:

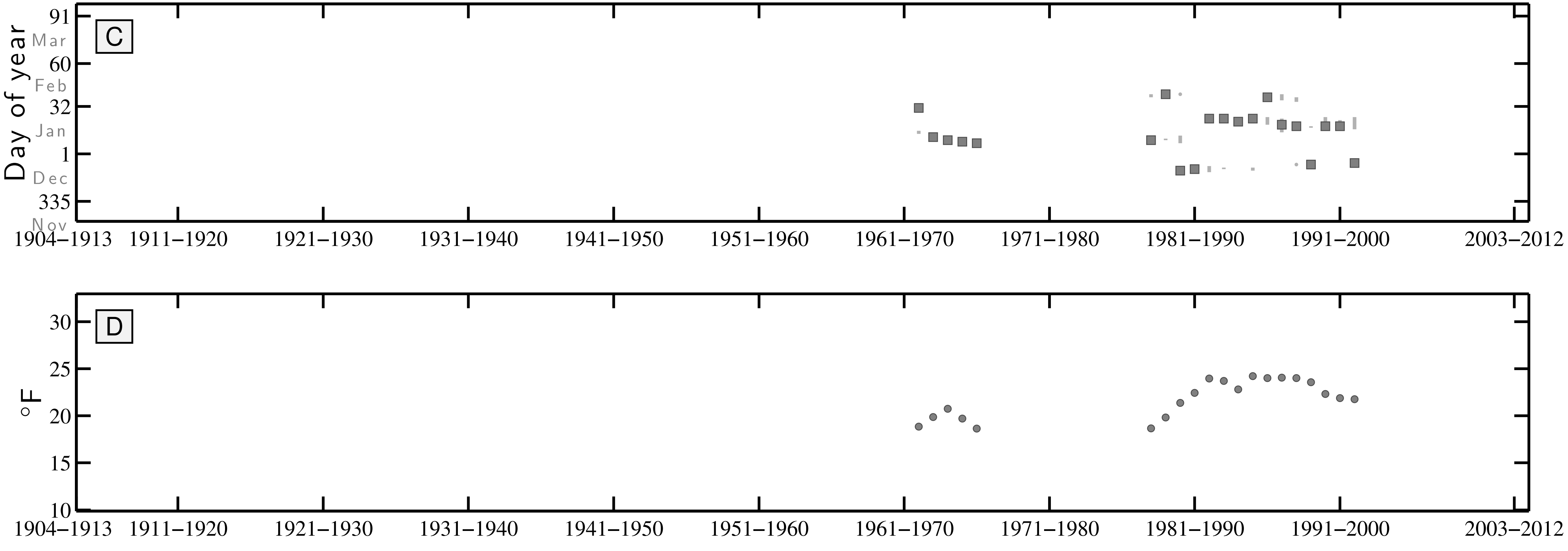

10 year Summer Teletherm dynamics for COLUMBUS, IN:

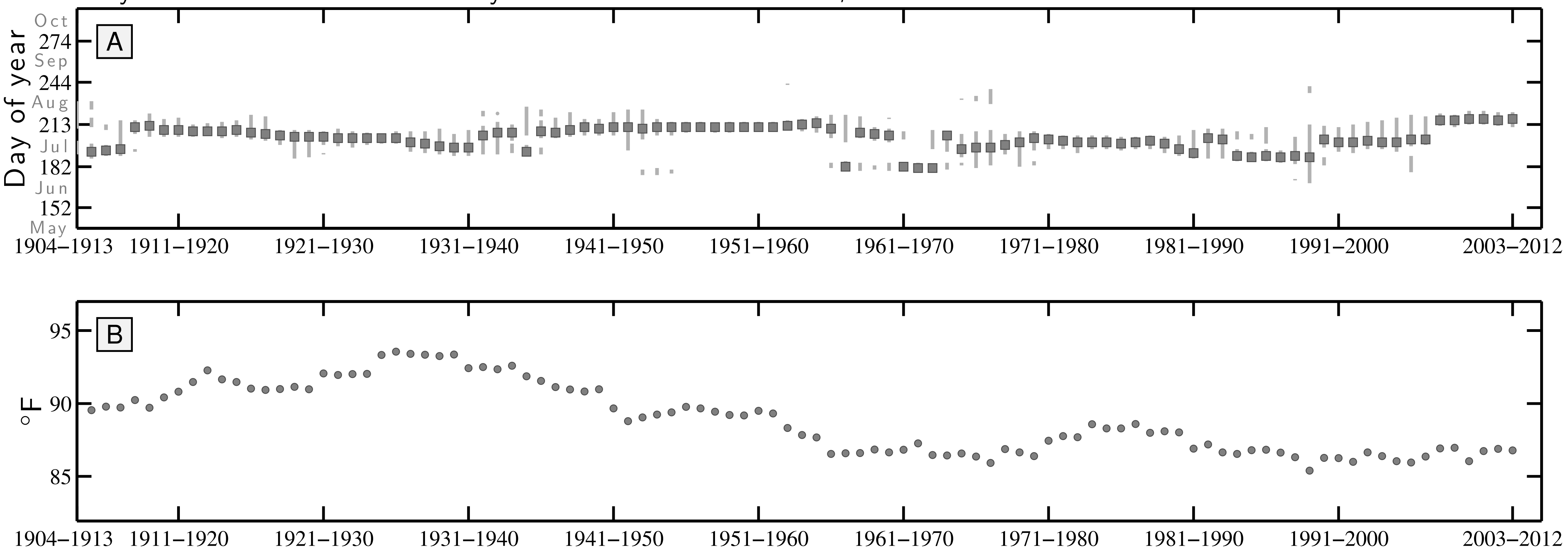

10 year Winter Teletherm dynamics for COLUMBUS, IN:

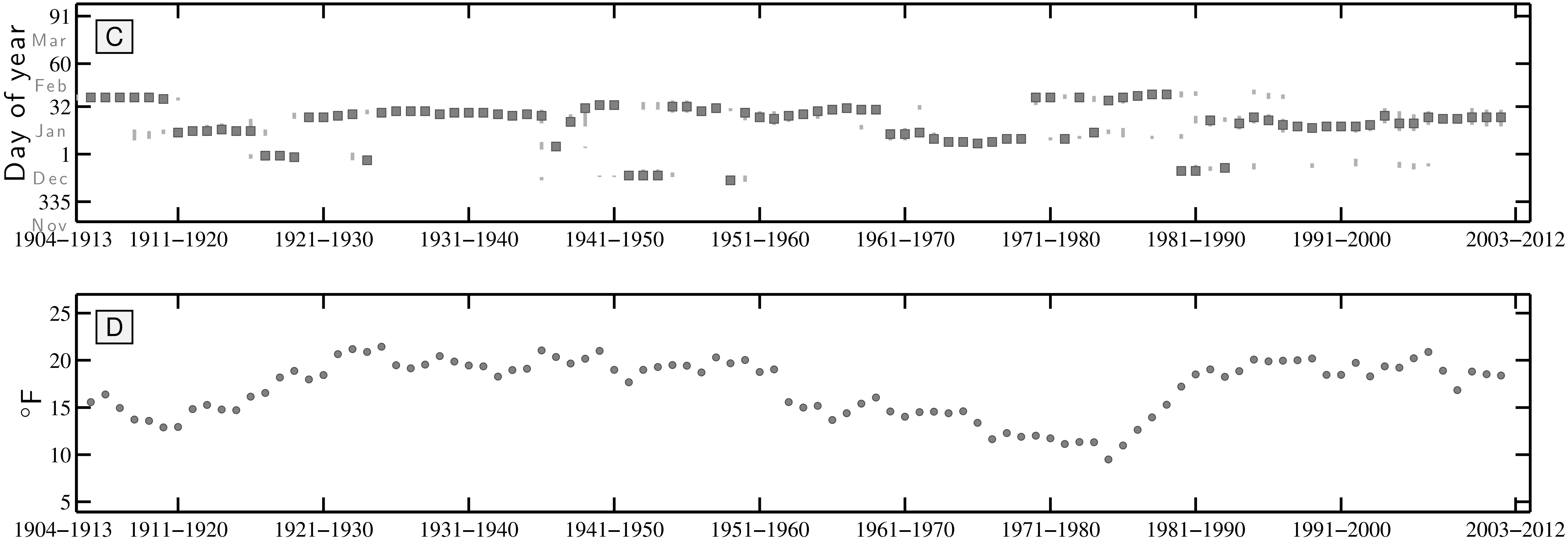

10 year Summer Teletherm dynamics for CRAWFORDSVILLE 6 SE, IN:

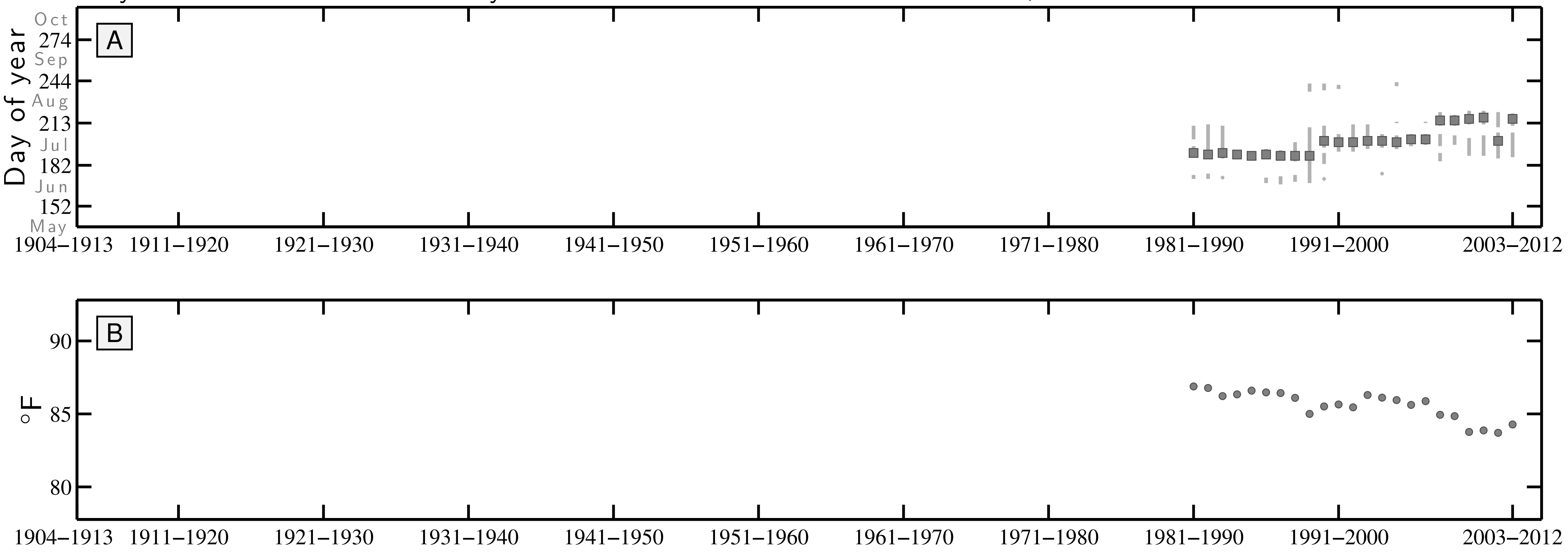

10 year Winter Teletherm dynamics for CRAWFORDSVILLE 6 SE, IN:

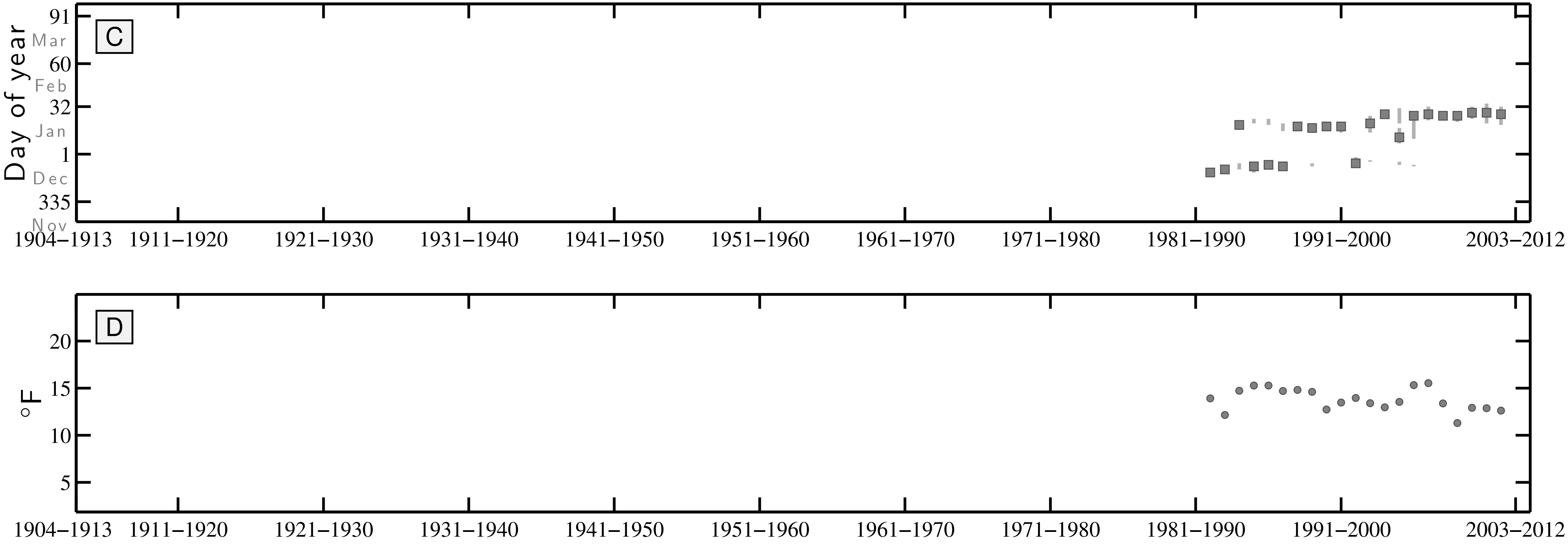

10 year Summer Teletherm dynamics for DELPHI 2 N, IN:

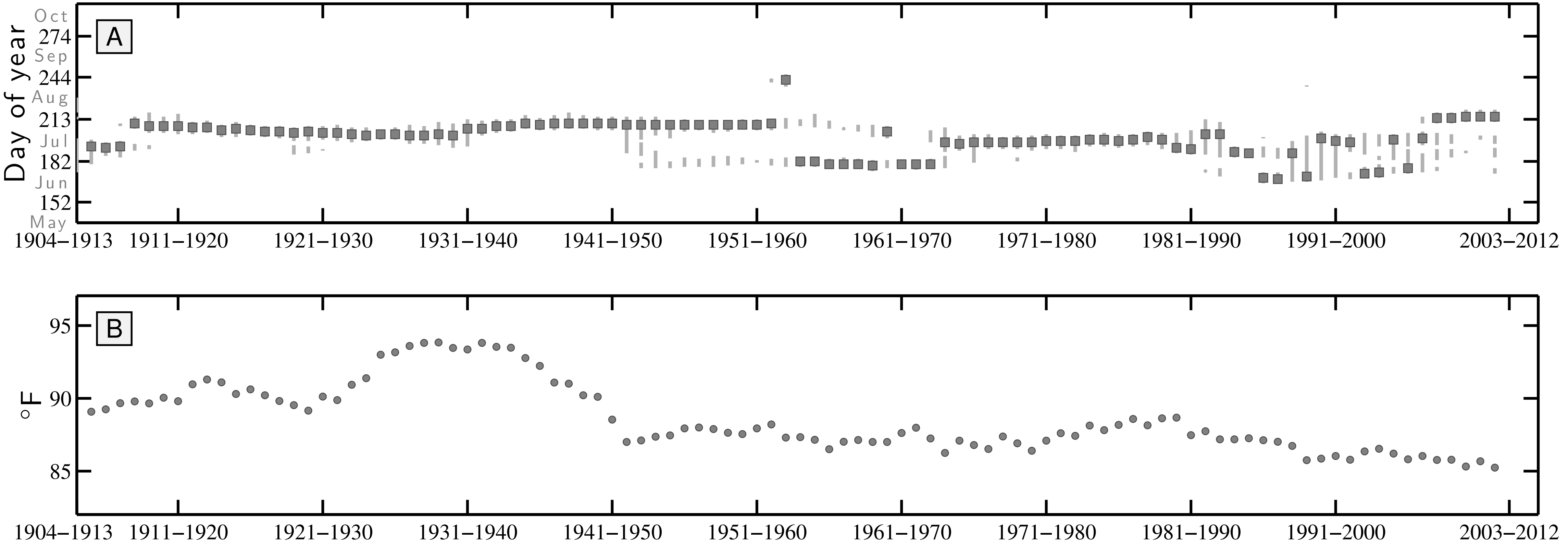

10 year Winter Teletherm dynamics for DELPHI 2 N, IN:

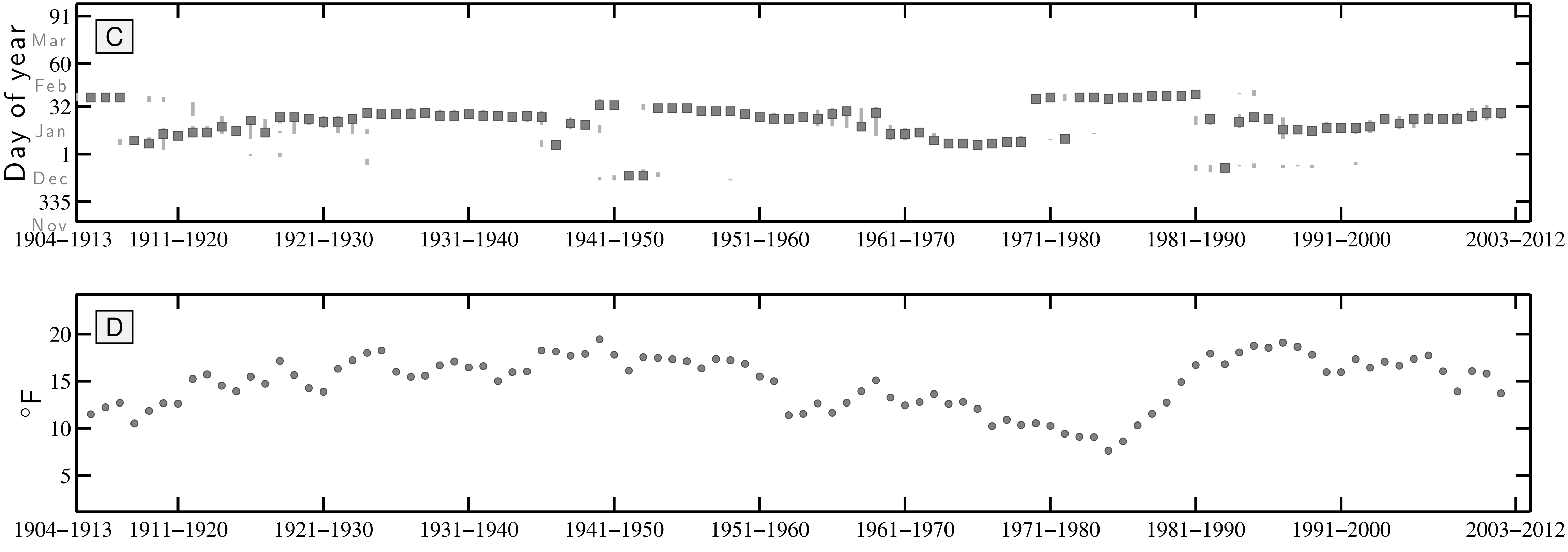

10 year Summer Teletherm dynamics for GOSHEN 3SW, IN:

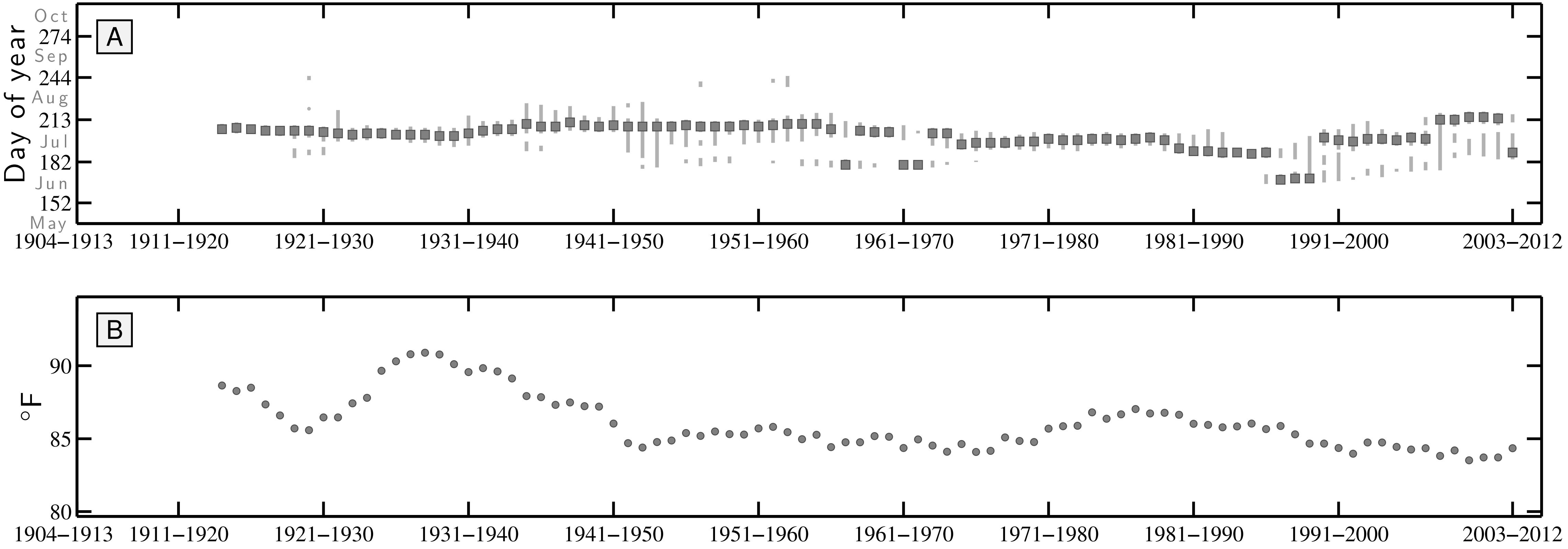

10 year Winter Teletherm dynamics for GOSHEN 3SW, IN:

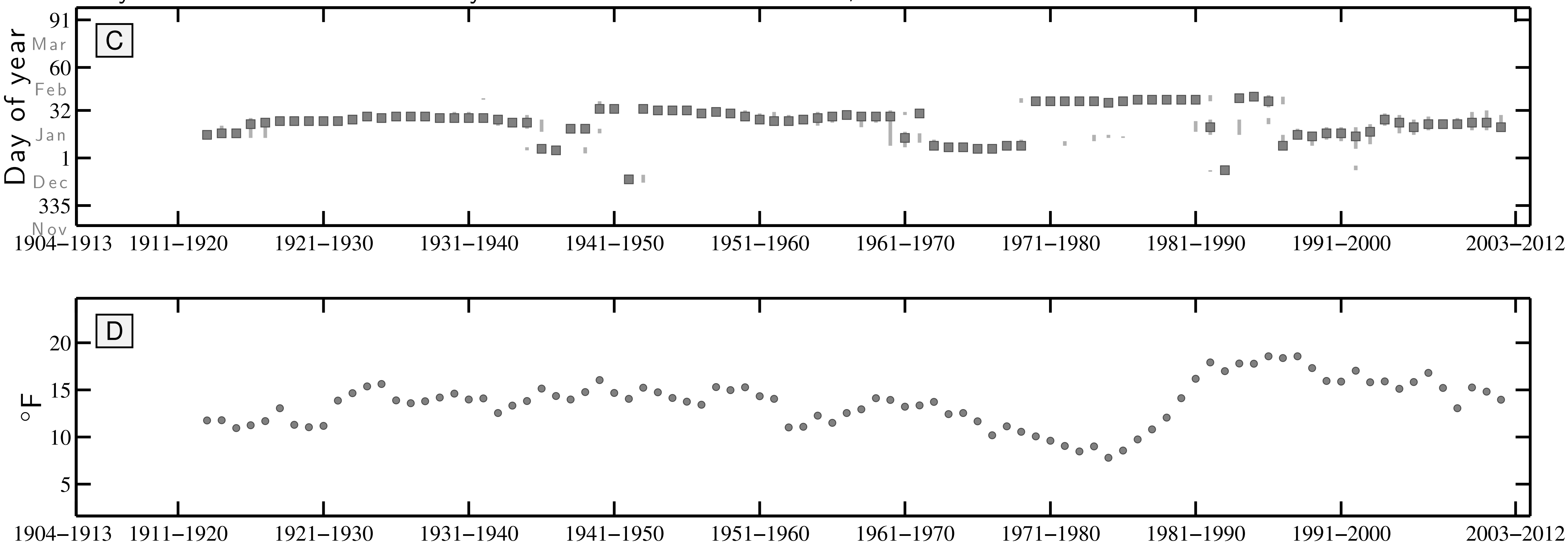

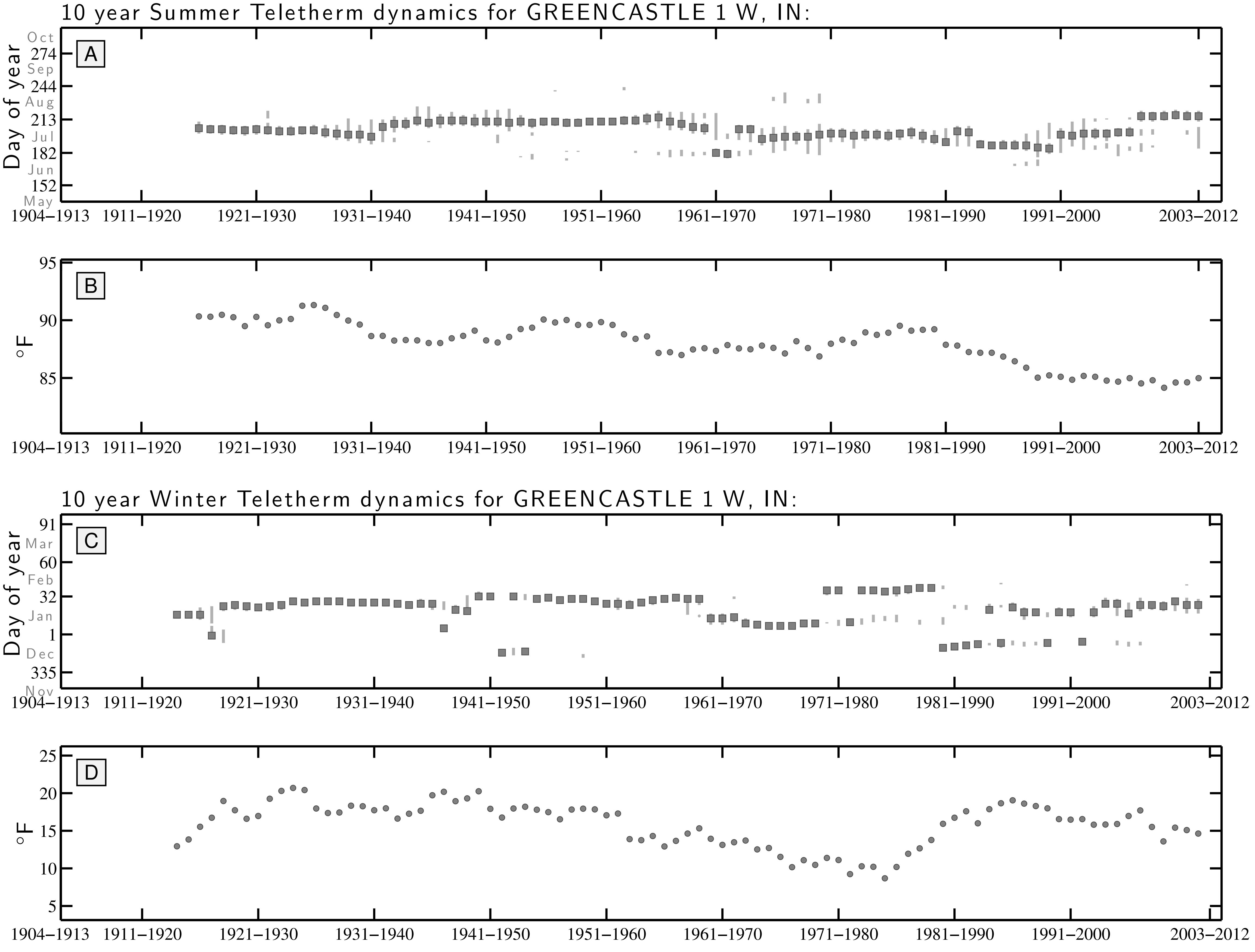

10 year Summer Teletherm dynamics for GREENCASTLE 1 W, IN:
A
Day of year
Oct
274
Sep
244
Aug
213
Jul
182
Jun
152
May
B
°F
95
90
85
1904–1913
1911–1920
1921–1930
1931–1940
1941–1950
1951–1960
1961–1970
1971–1980
1981–1990
1991–2000
2003–2012
10 year Winter Teletherm dynamics for GREENCASTLE 1 W, IN:
C
Day of year
91
Mar
60
Feb
32
Jan
1
Dec
335
Nov
D
°F
25
20
15
10
5

10 year Summer Teletherm dynamics for GREENFIELD, IN:

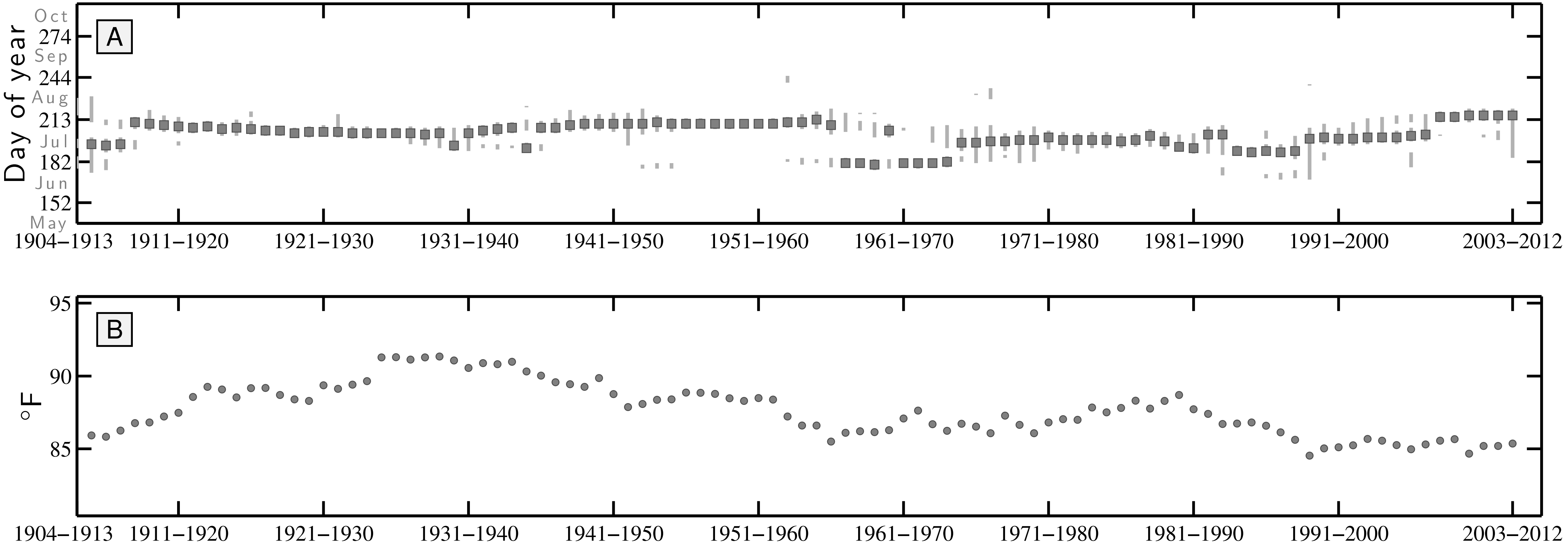

10 year Winter Teletherm dynamics for GREENFIELD, IN:

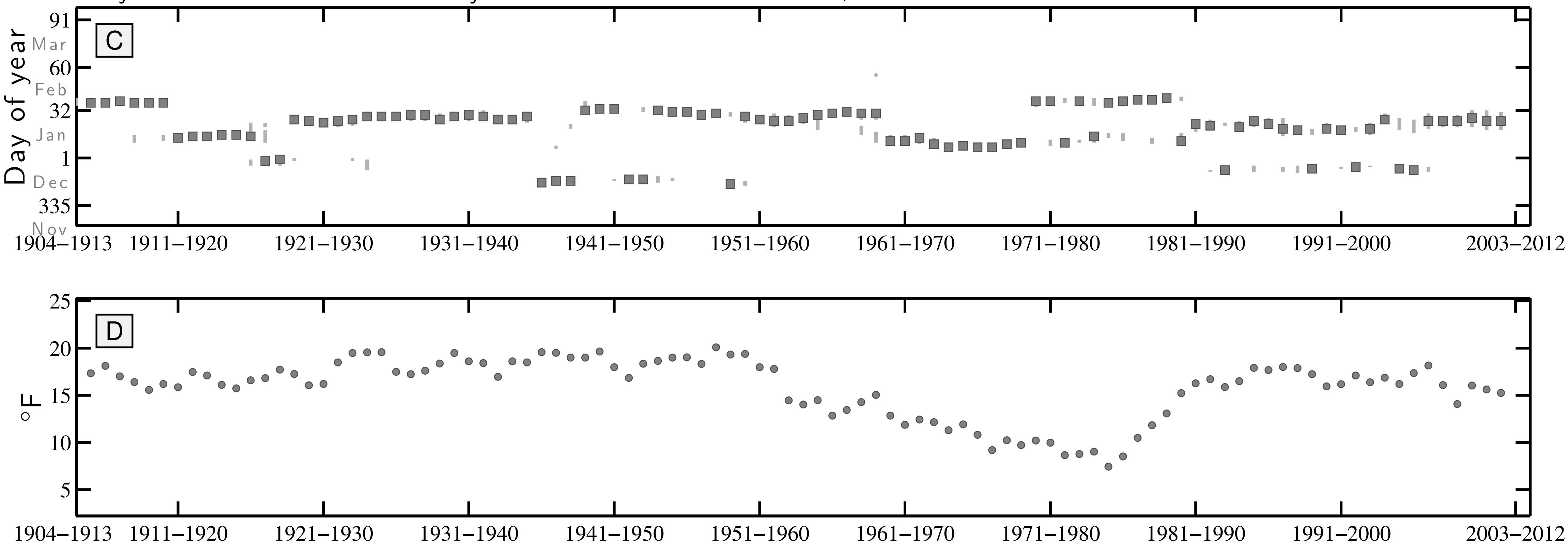

10 year Summer Teletherm dynamics for HOBART 2 WNW, IN:

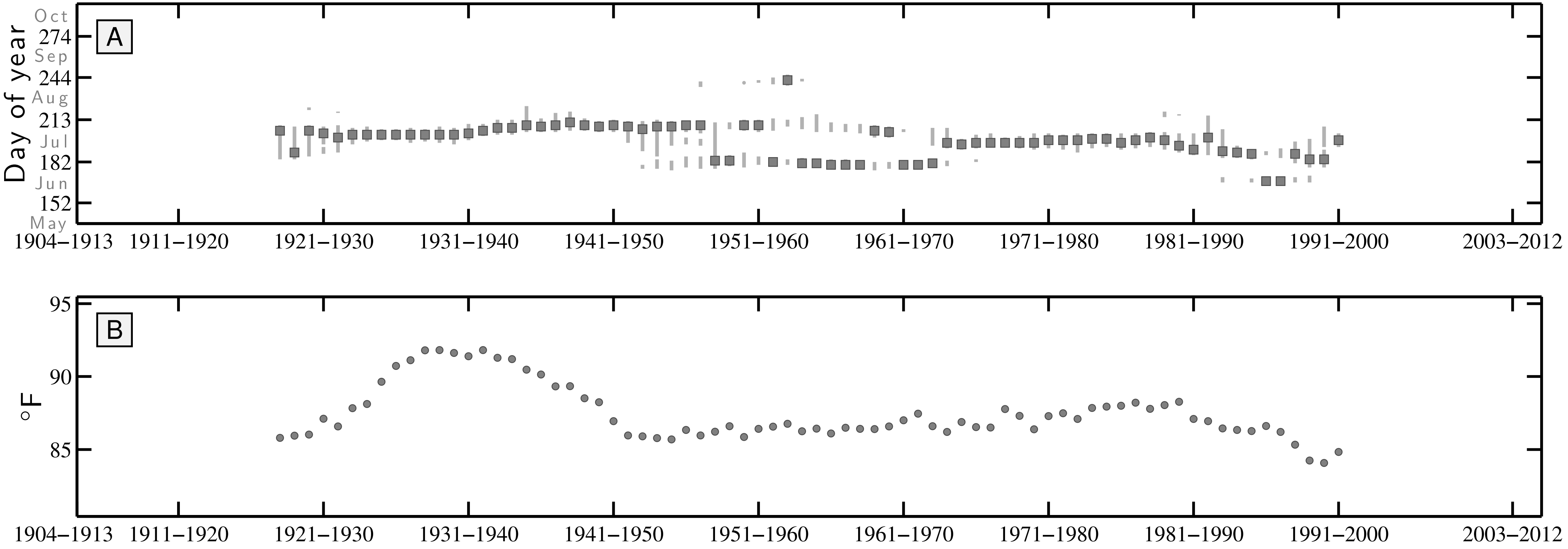

10 year Winter Teletherm dynamics for HOBART 2 WNW, IN:

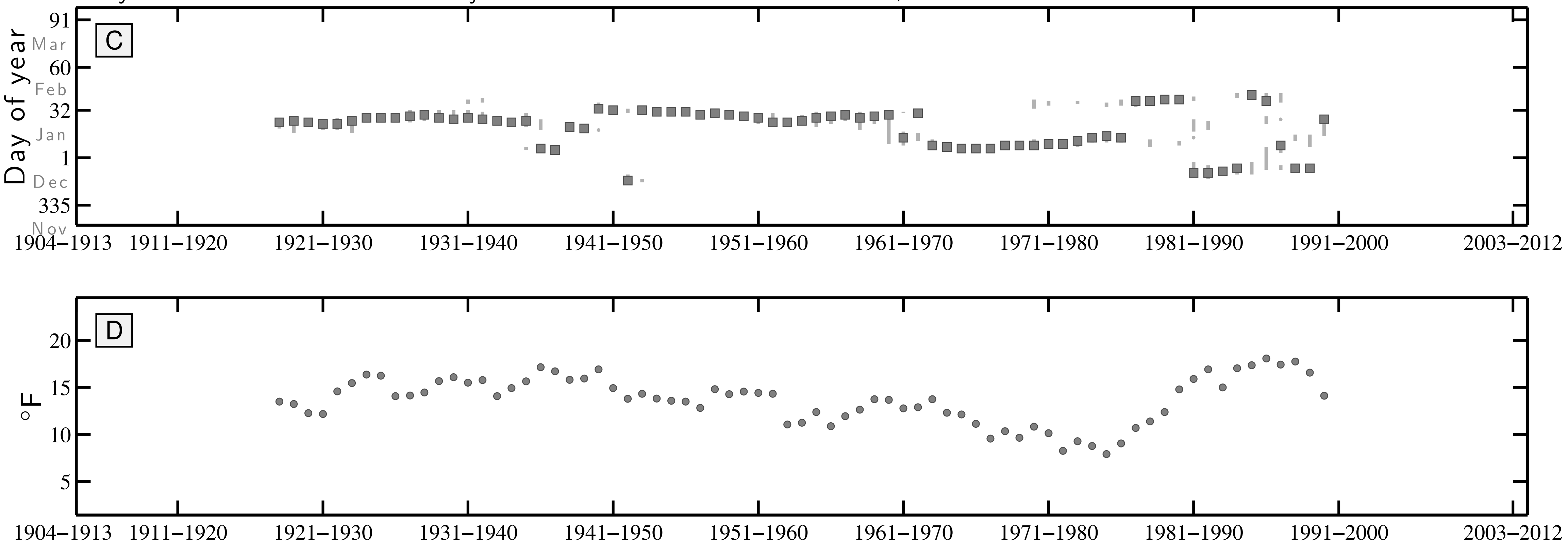

10 year Summer Teletherm dynamics for HUNTINGTON, IN:

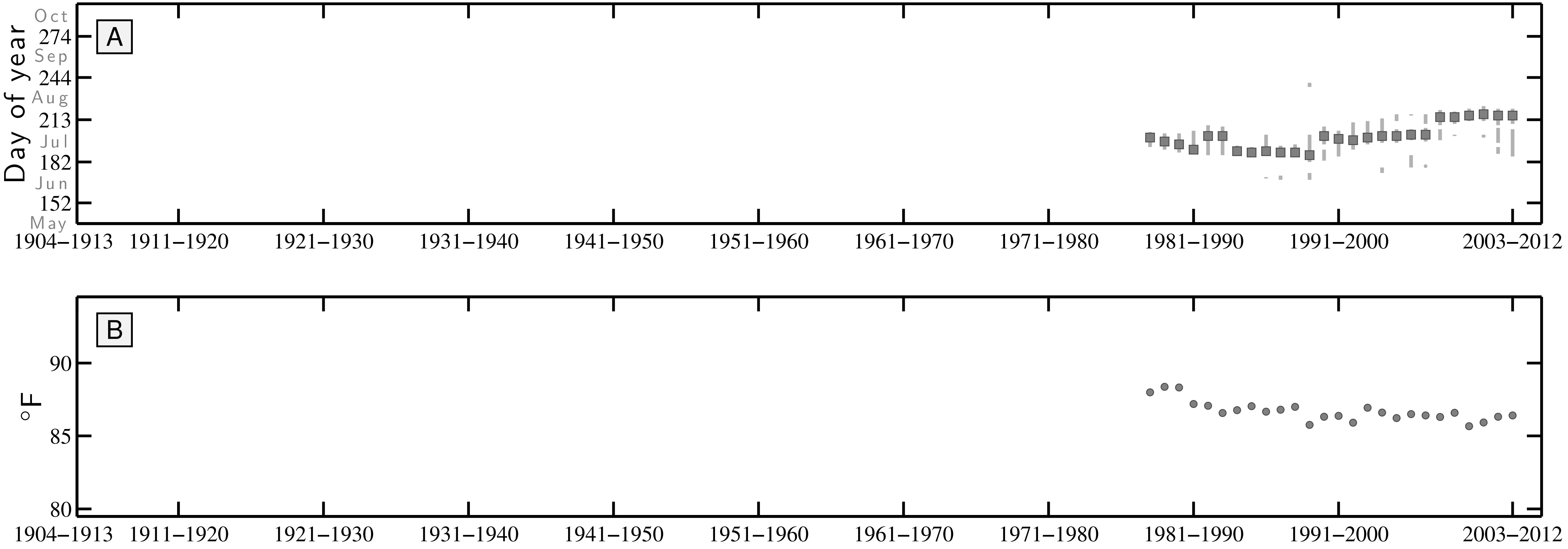

10 year Winter Teletherm dynamics for HUNTINGTON, IN:

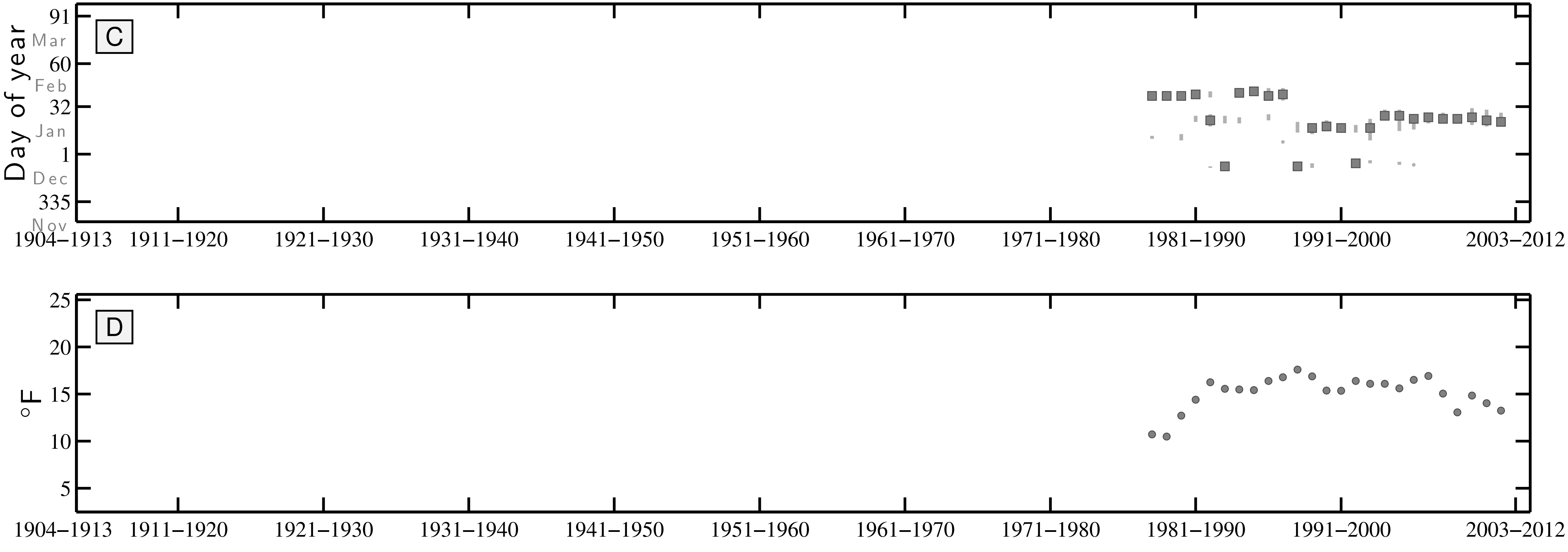

10 year Summer Teletherm dynamics for LAPORTE, IN:

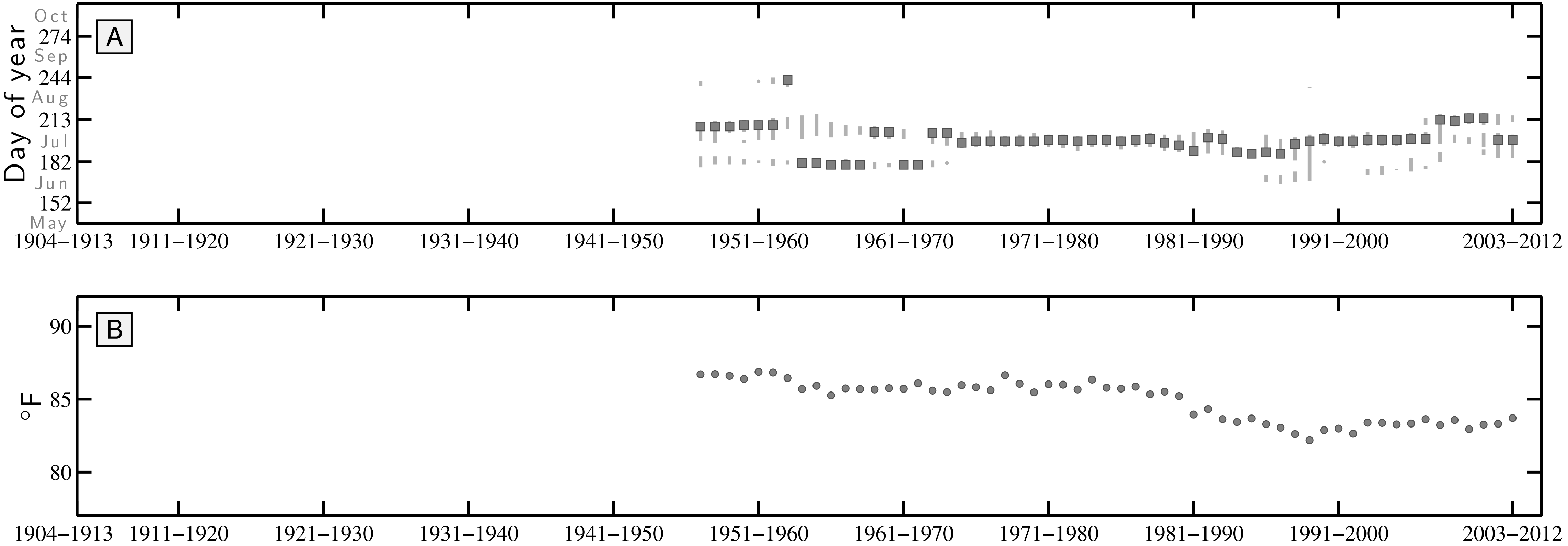

10 year Winter Teletherm dynamics for LAPORTE, IN:

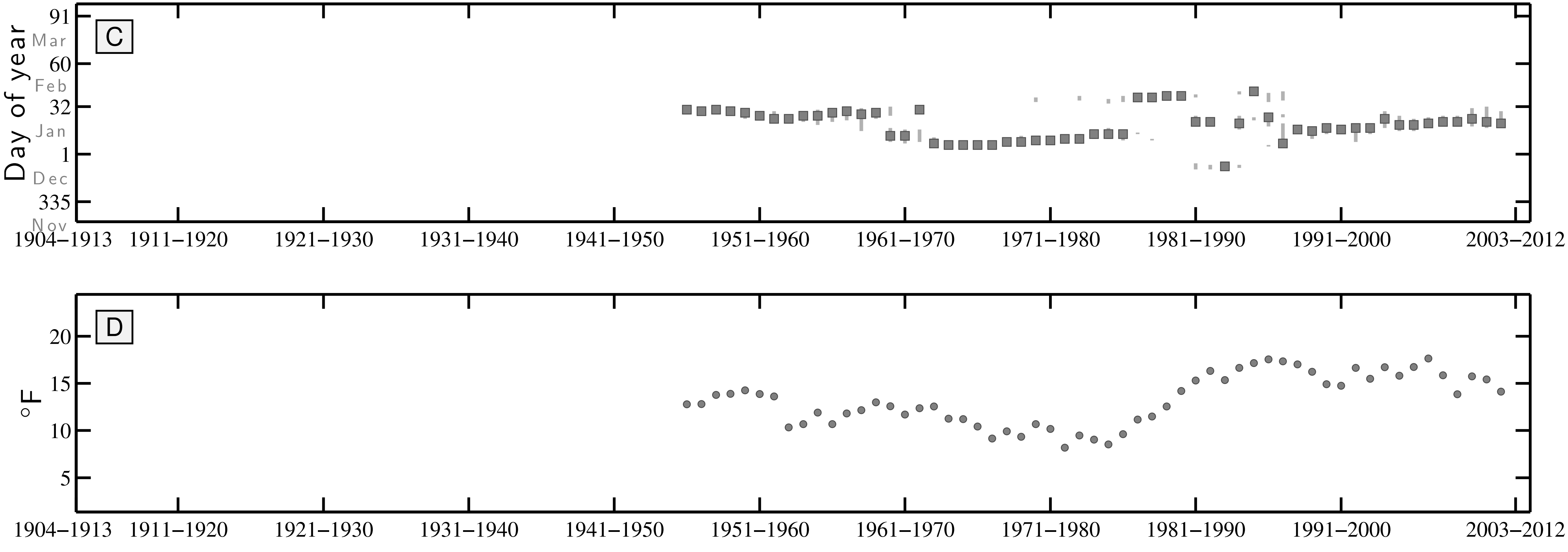

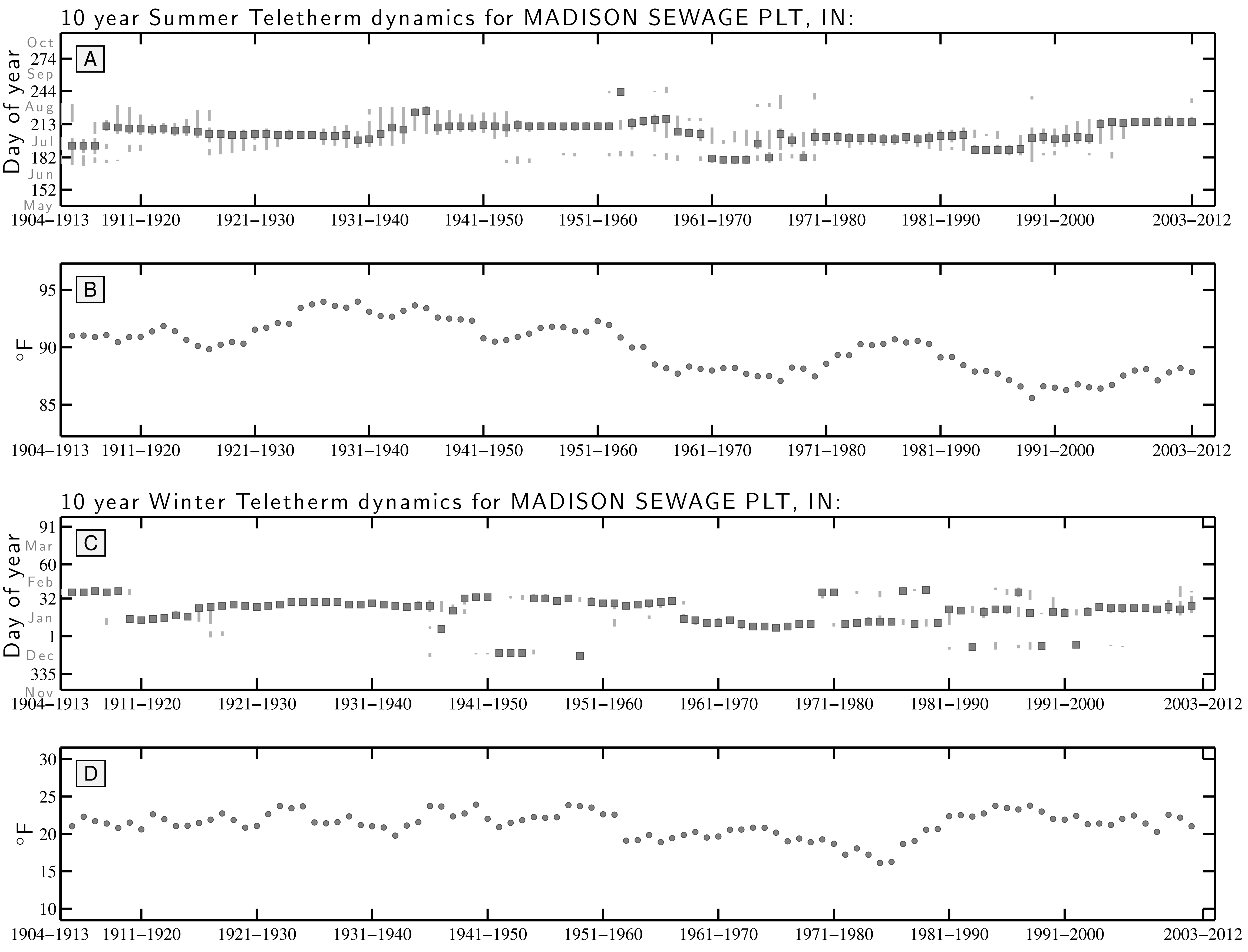
10 year Summer Teletherm dynamics for MADISON SEWAGE PLT, IN:
A
B
10 year Winter Teletherm dynamics for MADISON SEWAGE PLT, IN:
C
D
Day of year
°F
1904–1913
1911–1920
1921–1930
1931–1940
1941–1950
1951–1960
1961–1970
1971–1980
1981–1990
1991–2000
2003–2012

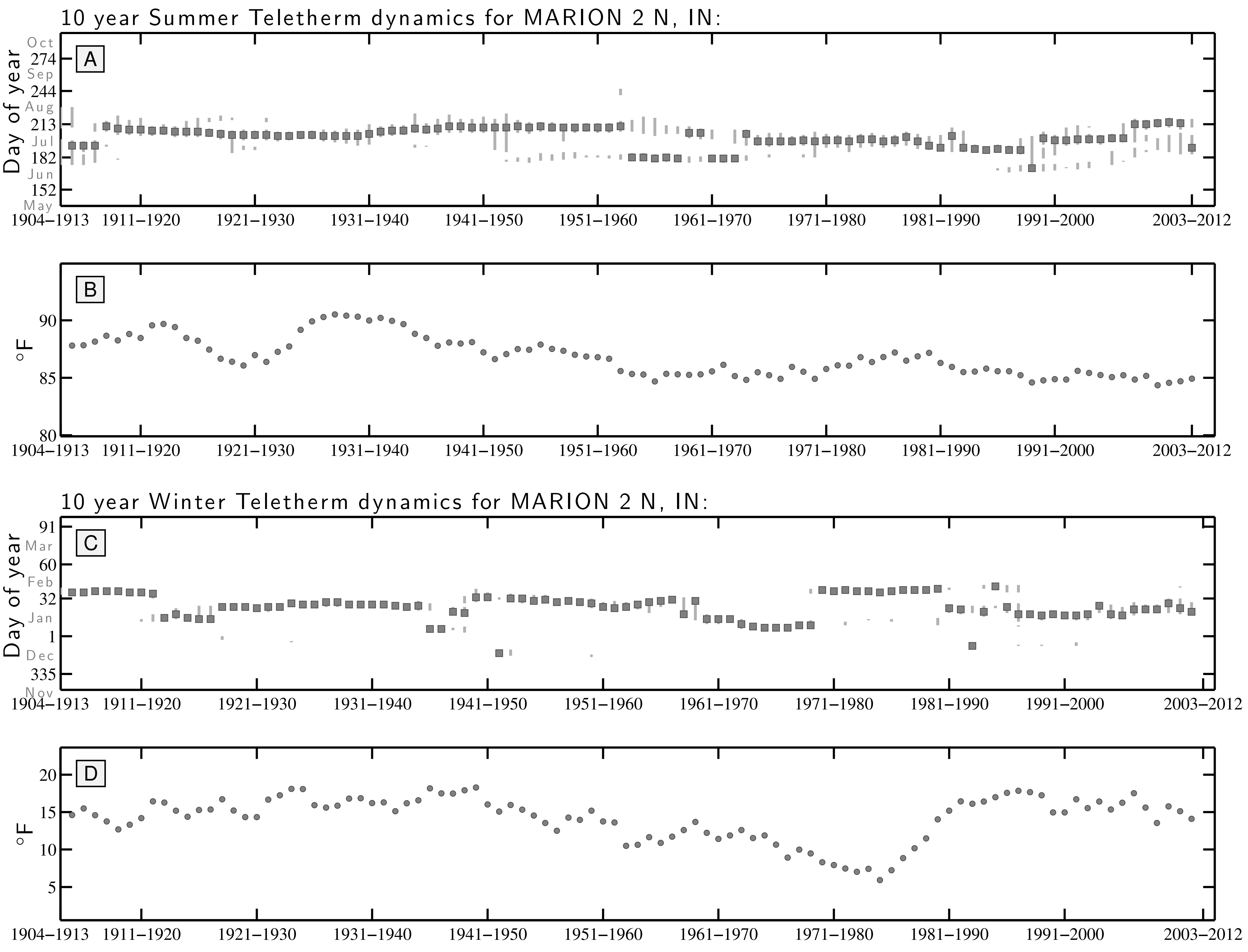
10 year Summer Teletherm dynamics for MARION 2 N, IN:
A
B
10 year Winter Teletherm dynamics for MARION 2 N, IN:
C
D
Day of year
°F
Oct
274
Sep
244
Aug
213
Jul
182
Jun
152
May
91
Mar
60
Feb
32
Jan
1
Dec
335
Nov
90
85
80
20
15
10
5
1904–1913
1911–1920
1921–1930
1931–1940
1941–1950
1951–1960
1961–1970
1971–1980
1981–1990
1991–2000
2003–2012

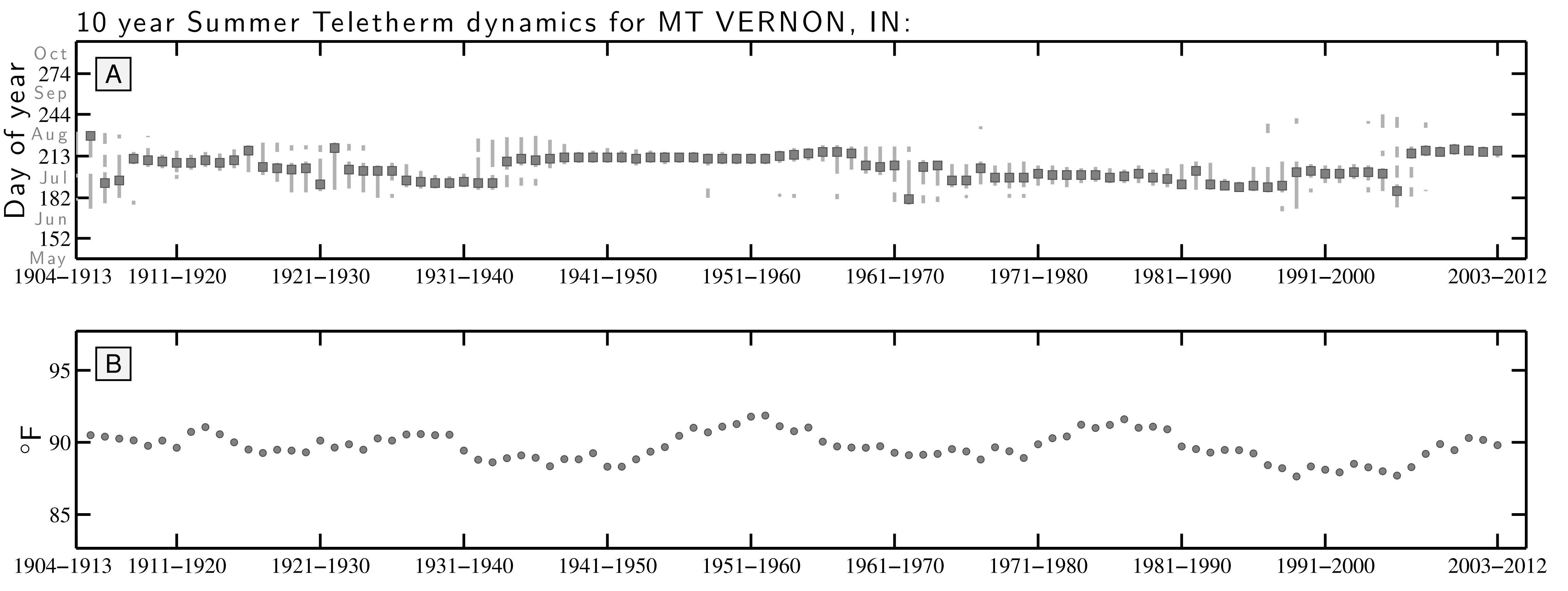
10 year Summer Teletherm dynamics for MT VERNON, IN:
A
B
Day of year
°F
Oct
274
Sep
244
Aug
213
Jul
182
Jun
152
May
95
90
85
1904–1913
1911–1920
1921–1930
1931–1940
1941–1950
1951–1960
1961–1970
1971–1980
1981–1990
1991–2000
2003–2012

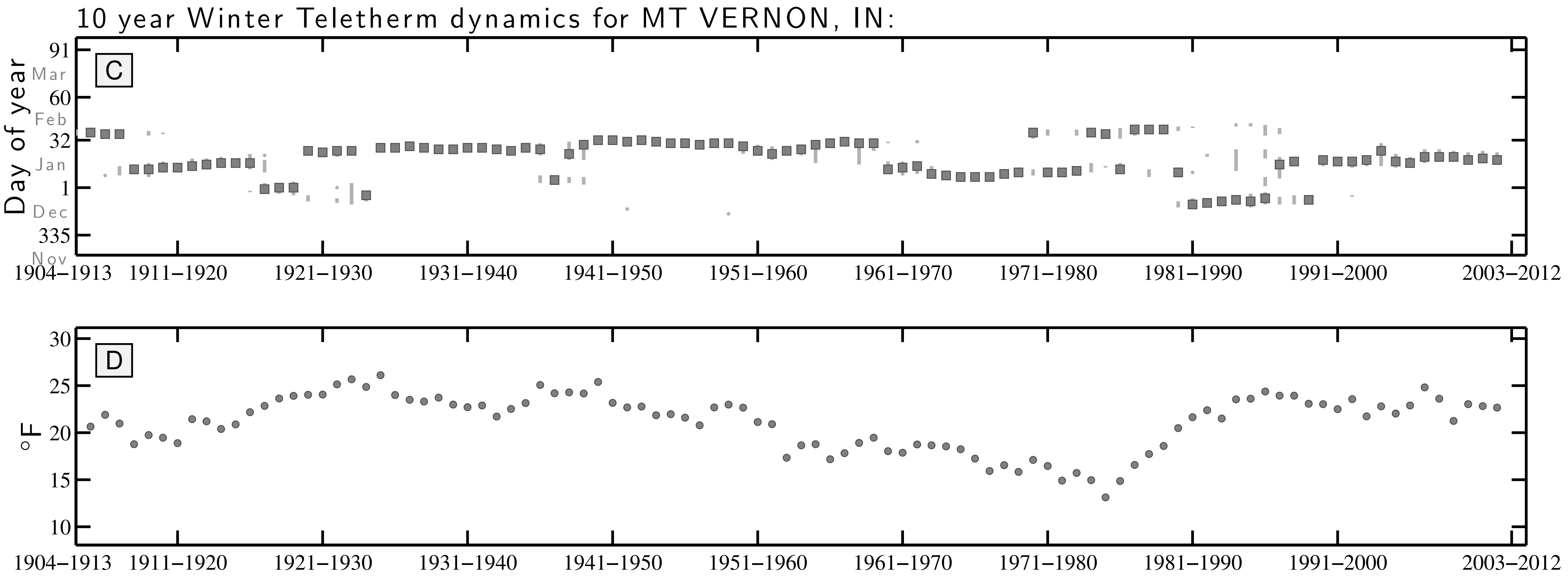
10 year Winter Teletherm dynamics for MT VERNON, IN:
C
D
Day of year
°F
91
Mar
60
Feb
32
Jan
1
Dec
335
Nov
30
25
20
15
10
1904–1913
1911–1920
1921–1930
1931–1940
1941–1950
1951–1960
1961–1970
1971–1980
1981–1990
1991–2000
2003–2012

10 year Summer Teletherm dynamics for OOLITIC PURDUE EX FRM, IN:

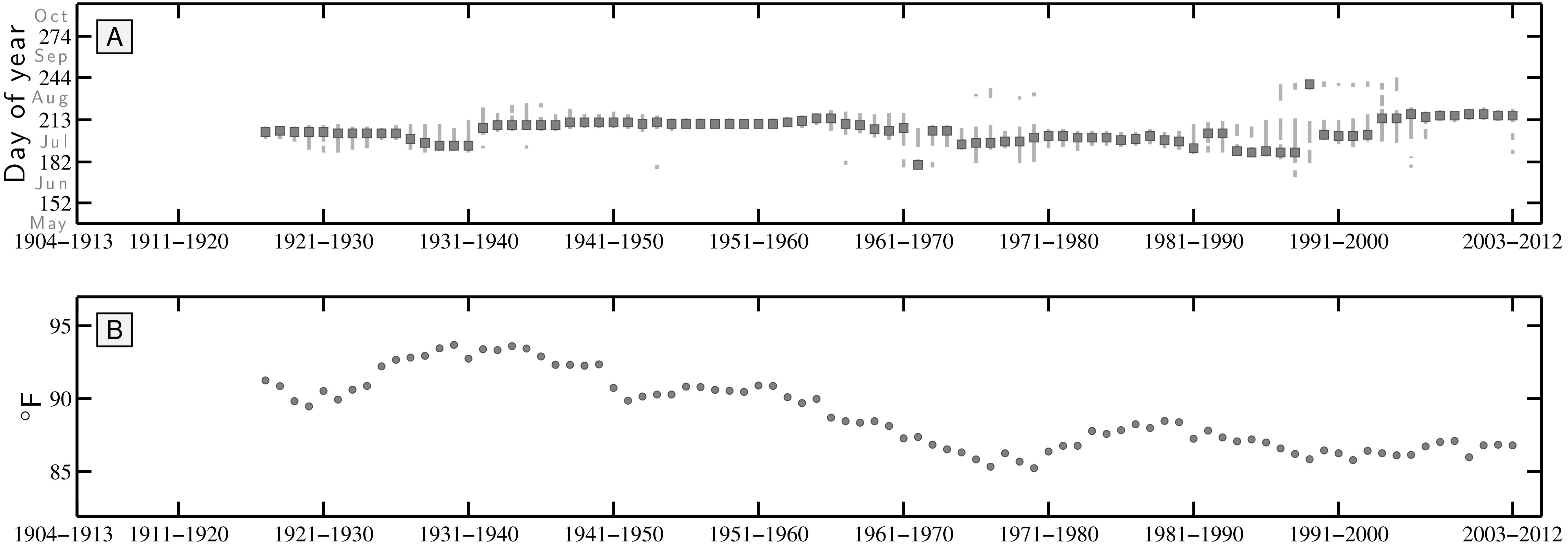

10 year Winter Teletherm dynamics for OOLITIC PURDUE EX FRM, IN:

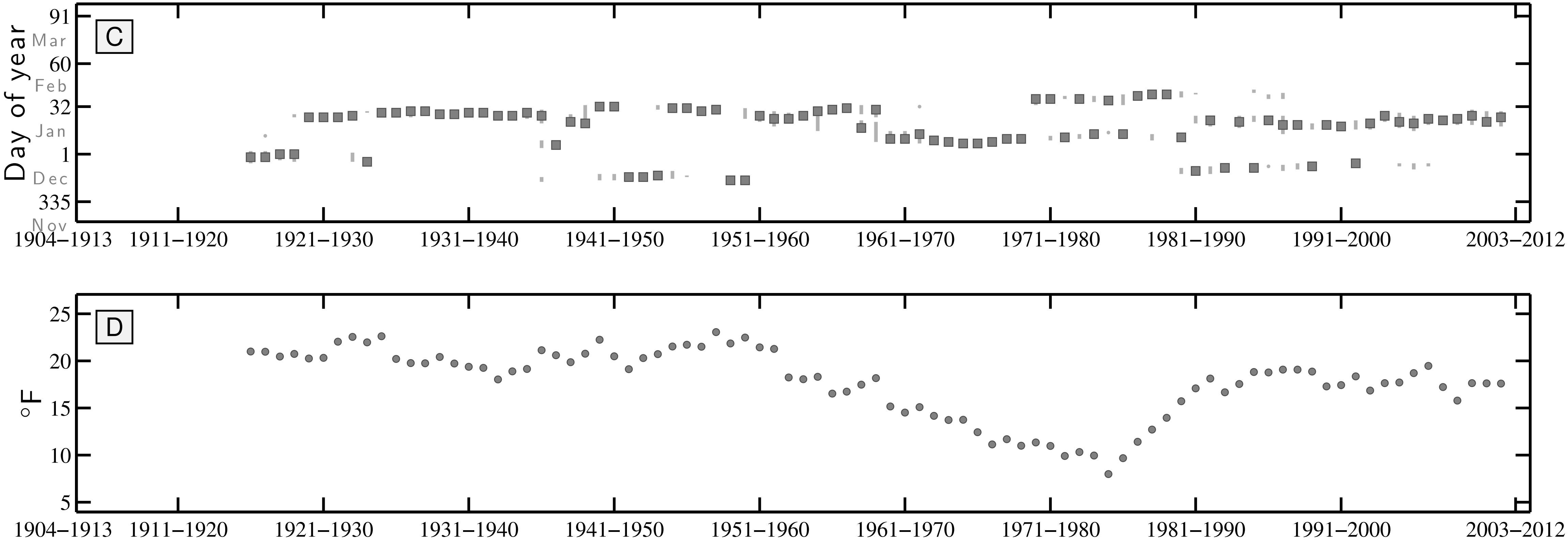

10 year Summer Teletherm dynamics for PAOLI, IN:

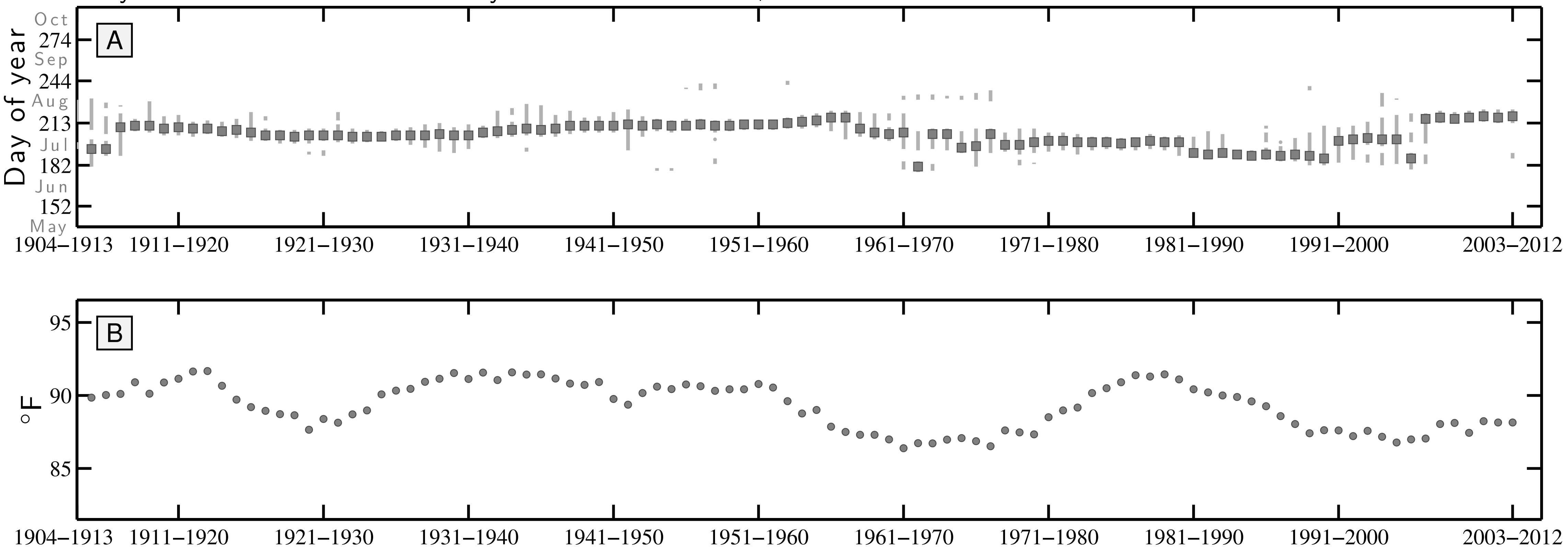

10 year Winter Teletherm dynamics for PAOLI, IN:

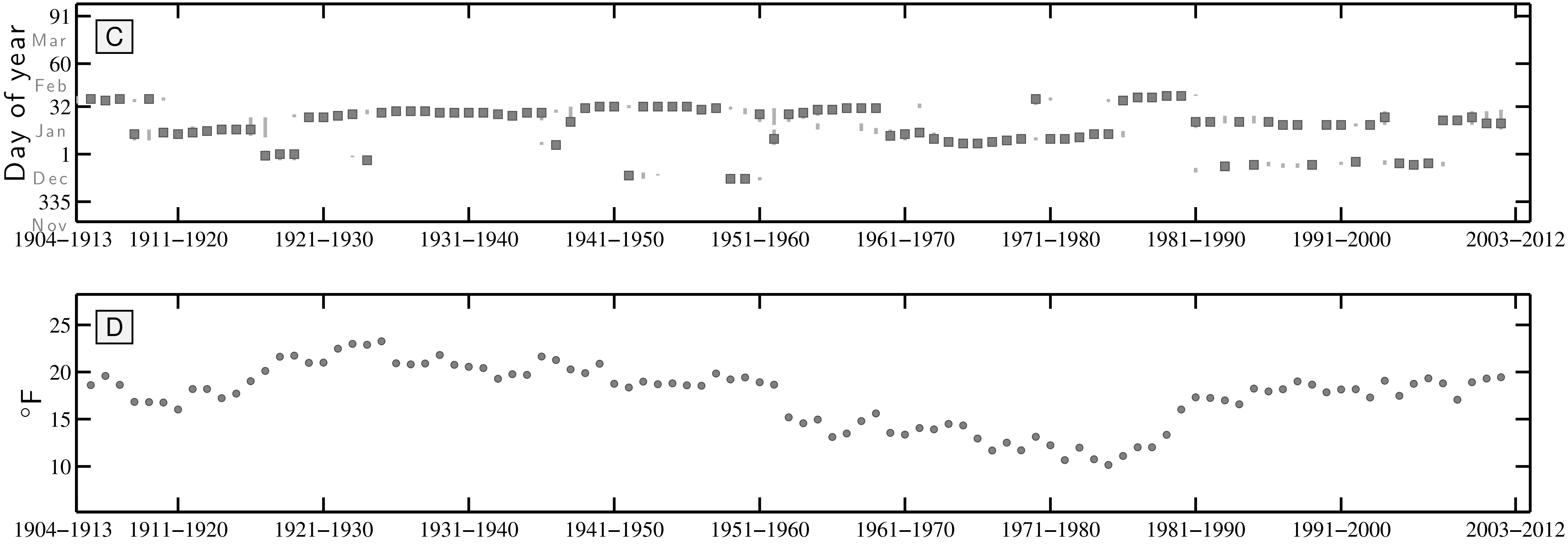

10 year Summer Teletherm dynamics for PRINCETON 1 W, IN:

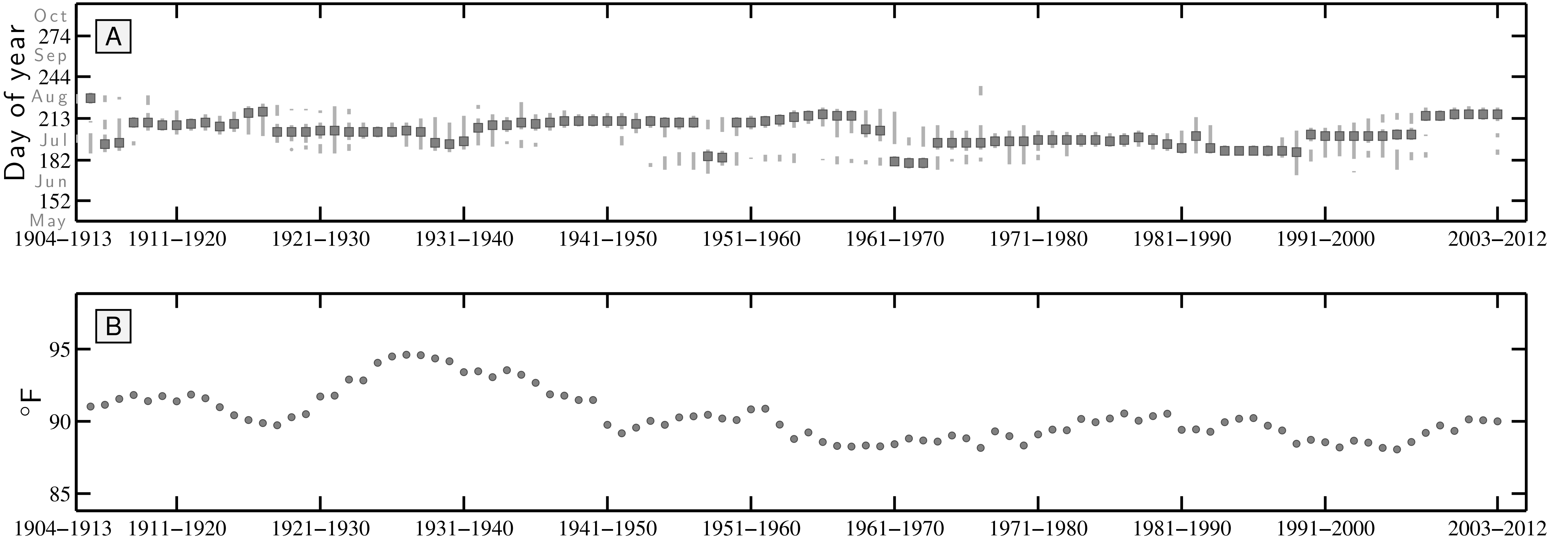

10 year Winter Teletherm dynamics for PRINCETON 1 W, IN:

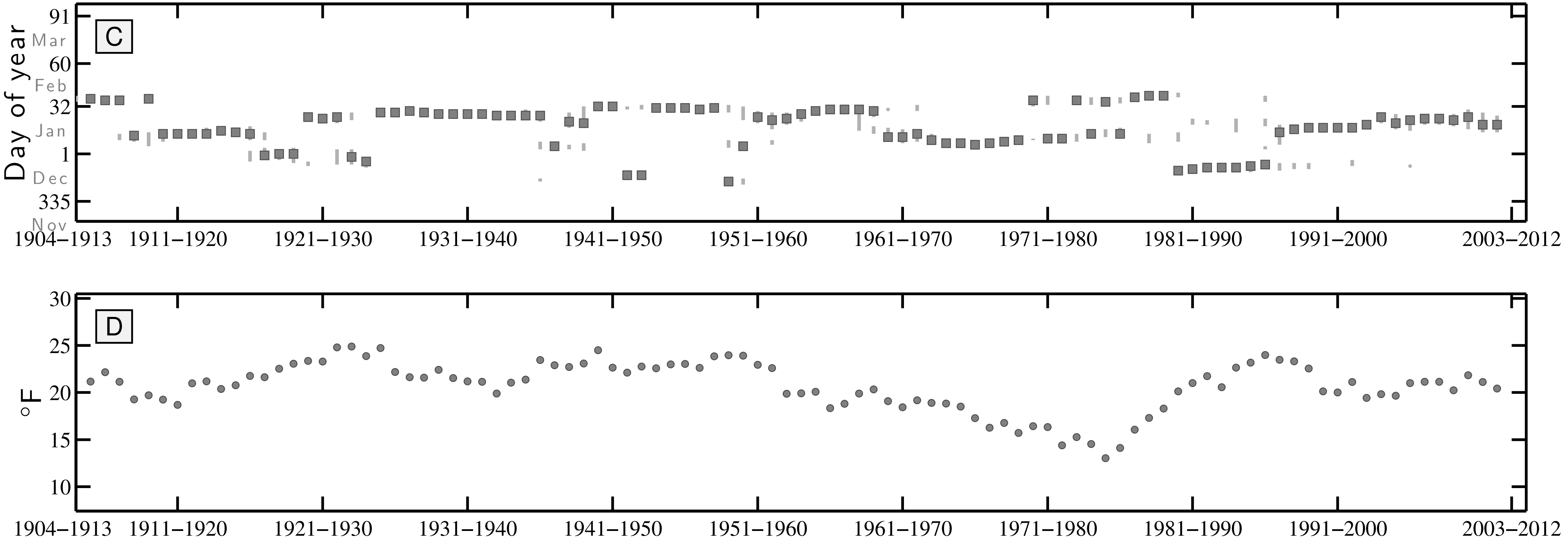

10 year Summer Teletherm dynamics for RENSSELAER, IN:

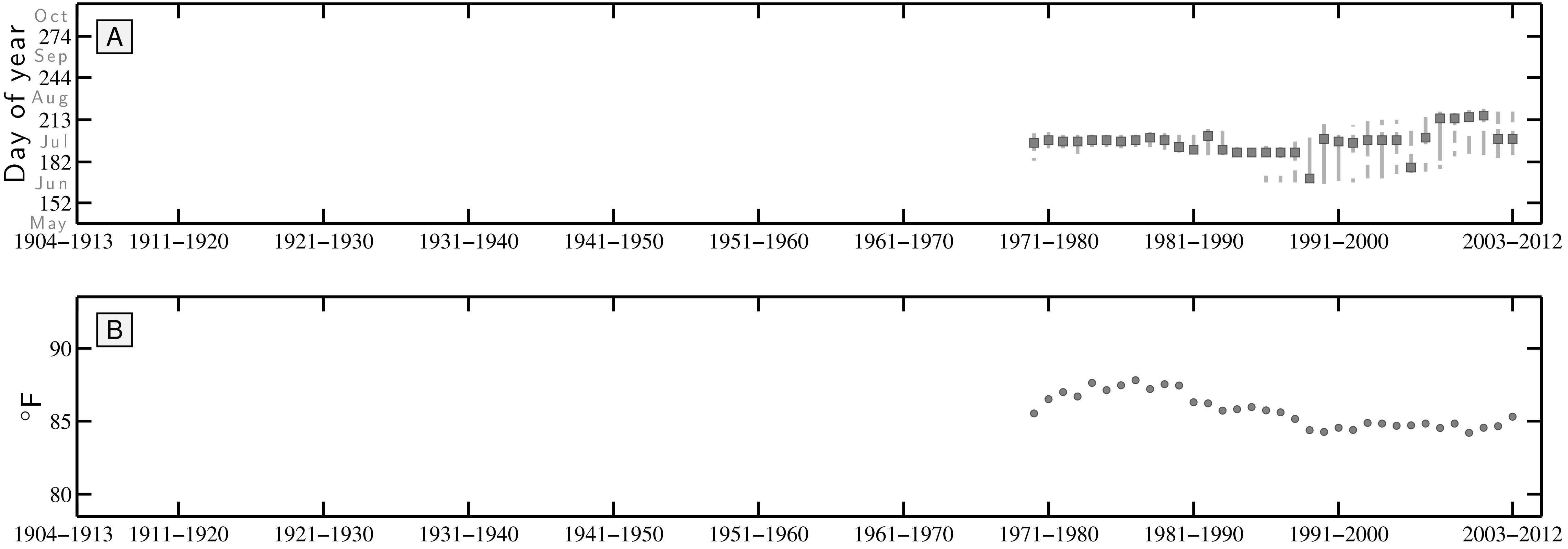

10 year Winter Teletherm dynamics for RENSSELAER, IN:

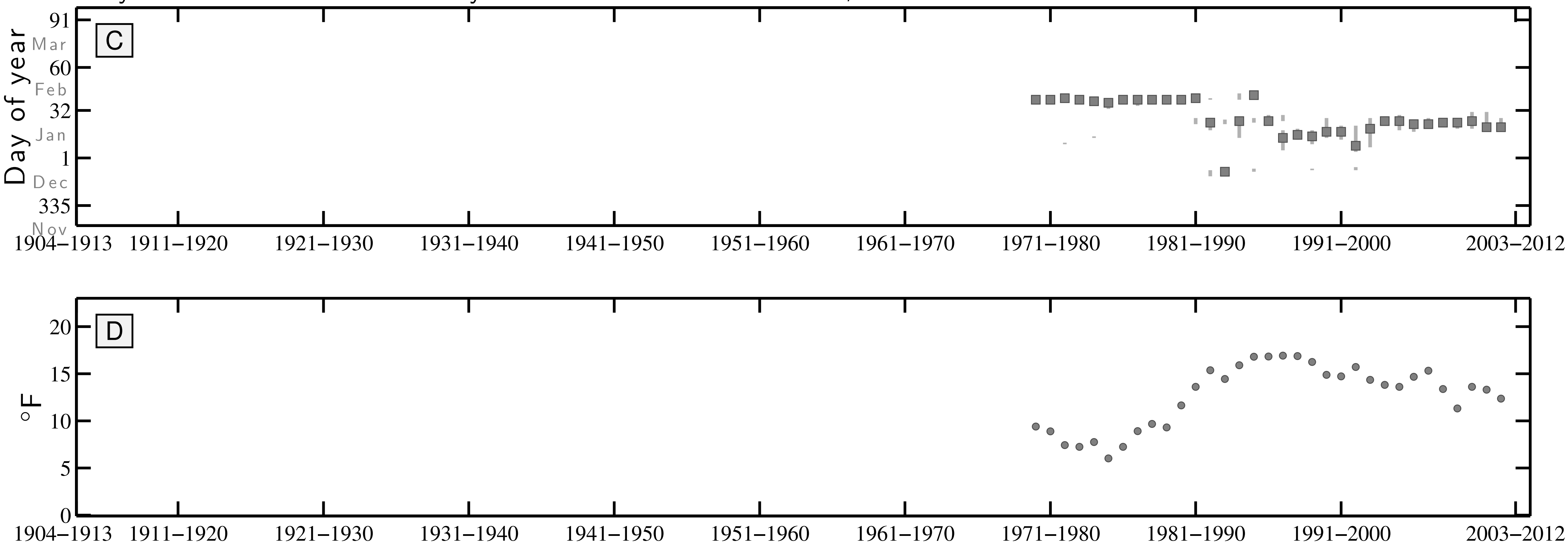

10 year Summer Teletherm dynamics for ROCHESTER, IN:

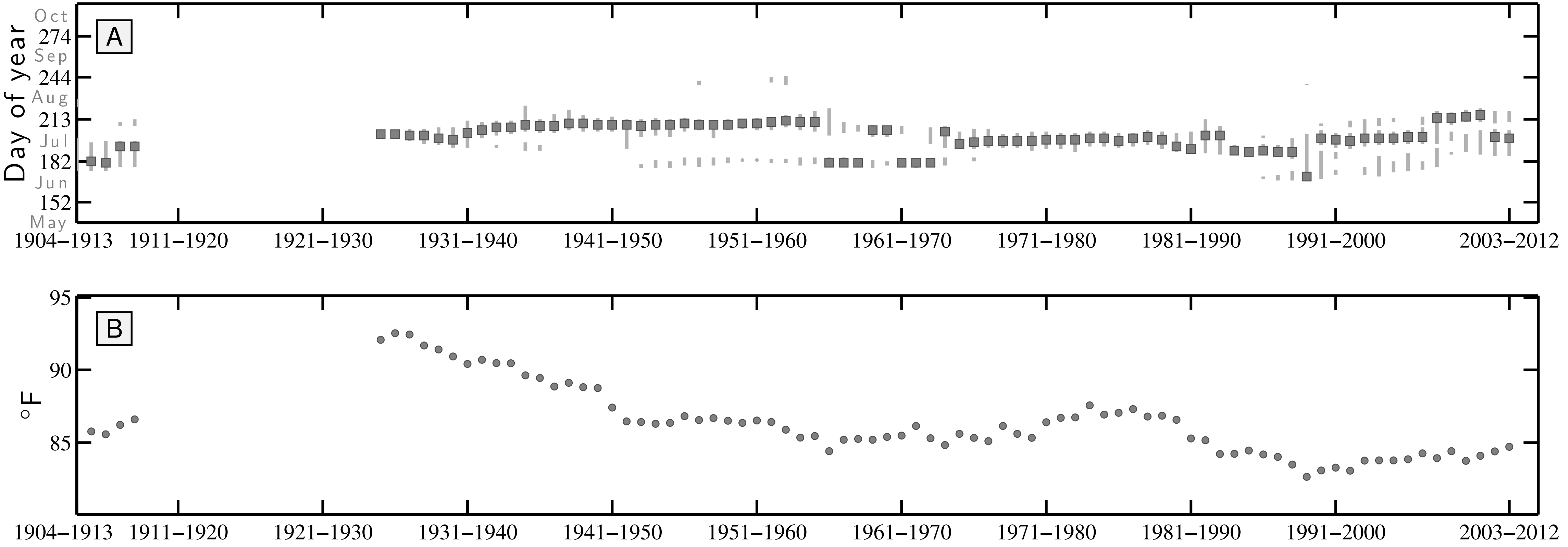

10 year Winter Teletherm dynamics for ROCHESTER, IN:

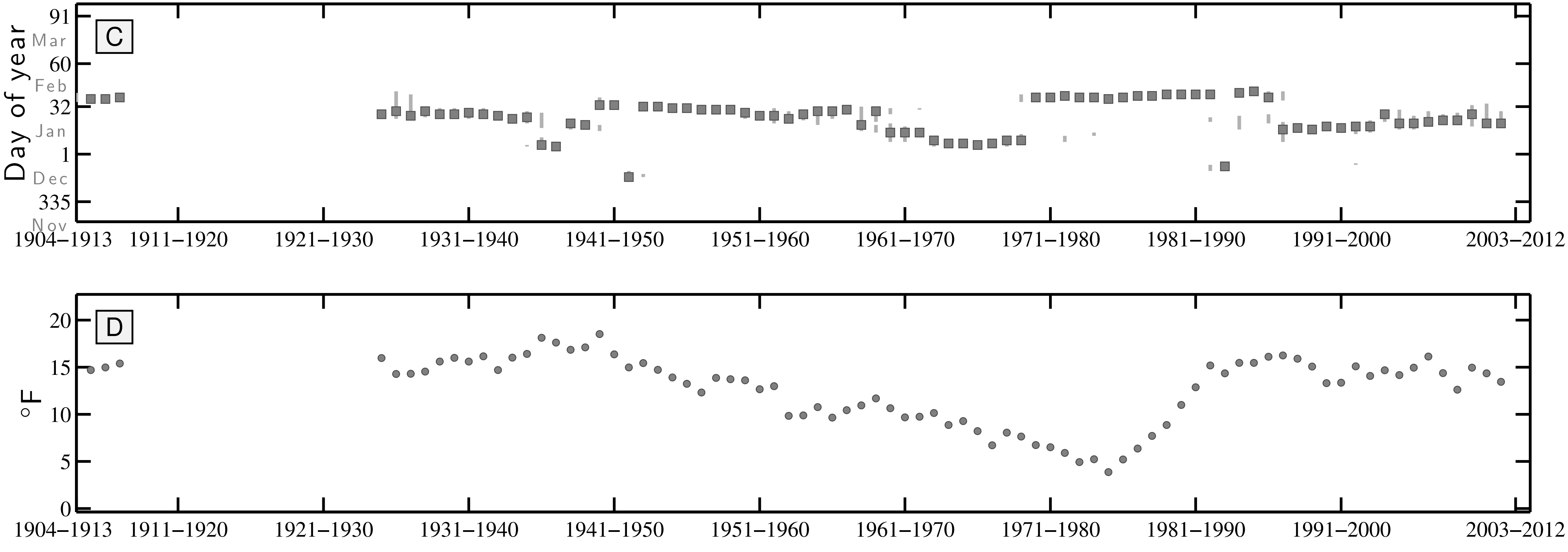

10 year Summer Teletherm dynamics for ROCKVILLE, IN:

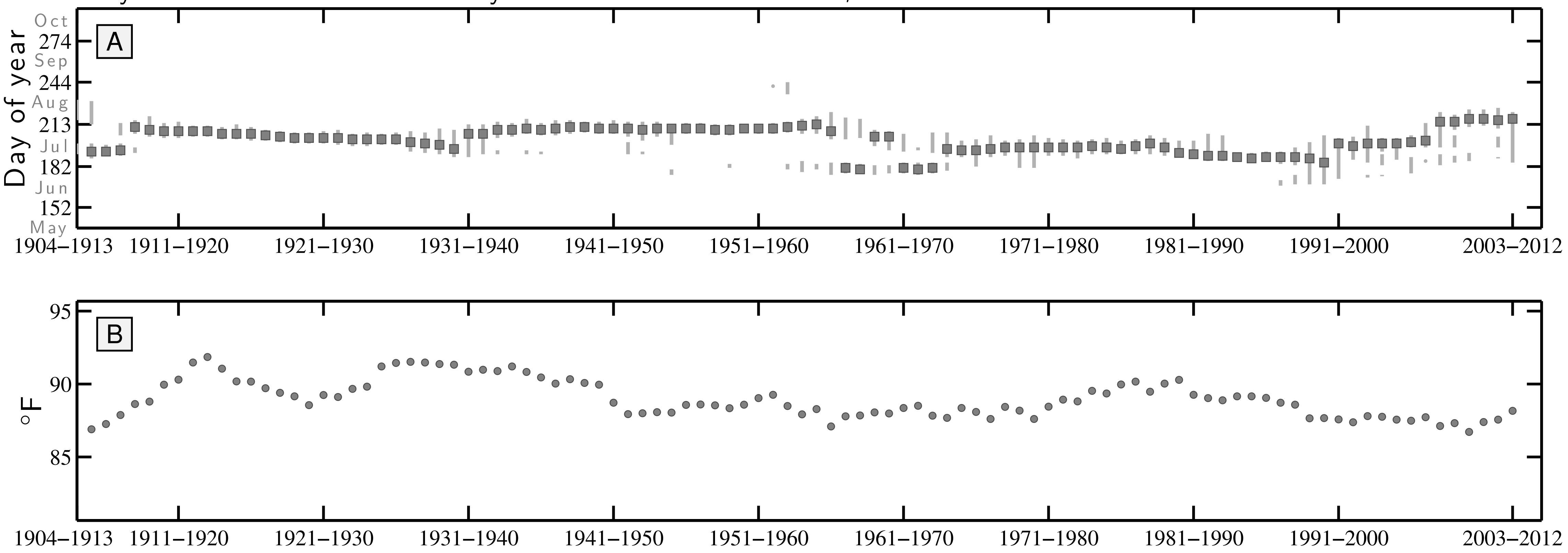

10 year Winter Teletherm dynamics for ROCKVILLE, IN:

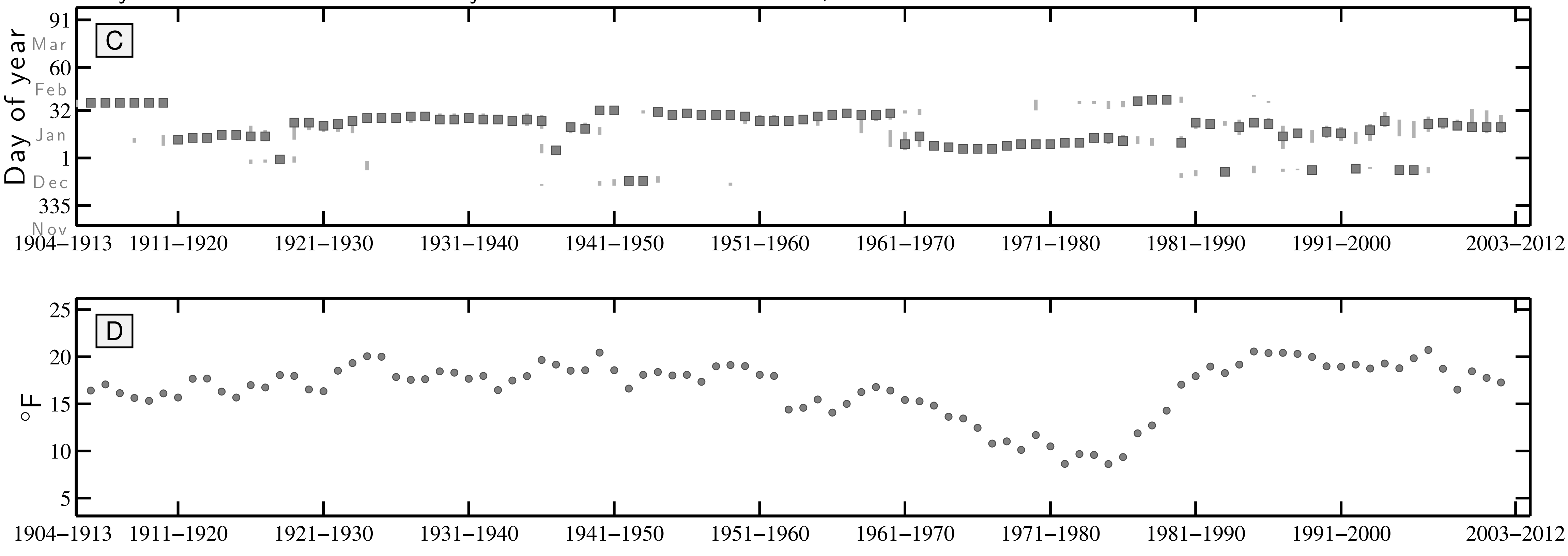

10 year Summer Teletherm dynamics for RUSHVILLE, IN:

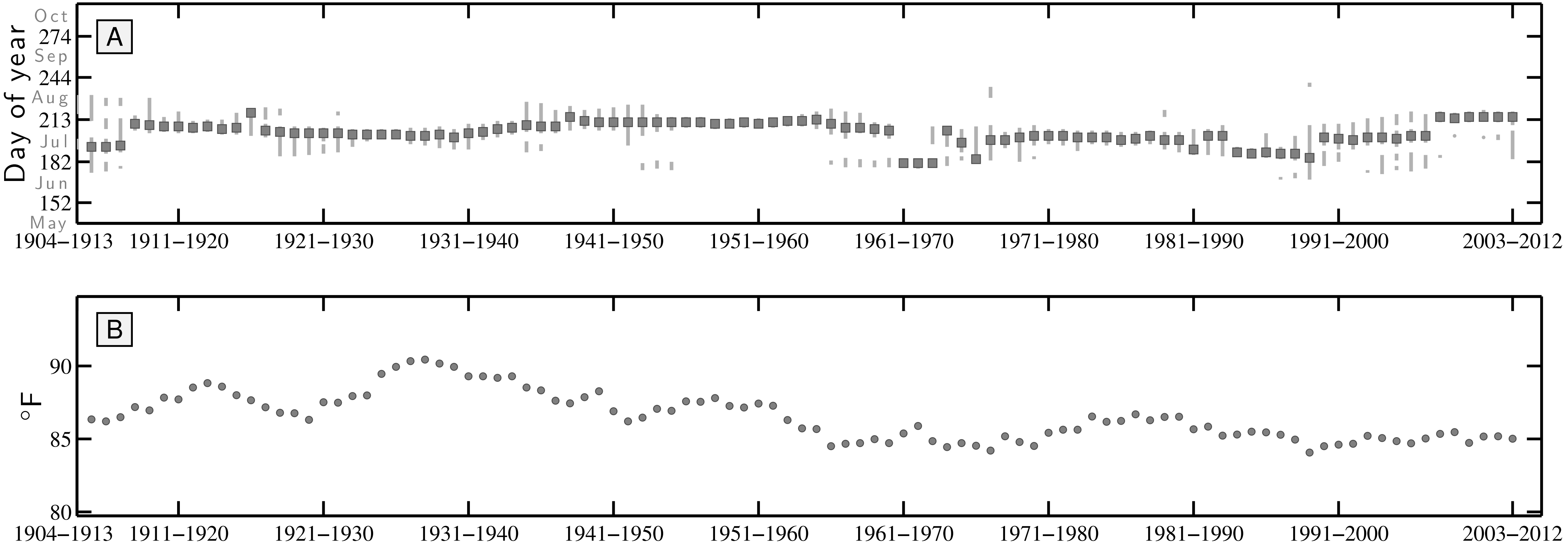

10 year Winter Teletherm dynamics for RUSHVILLE, IN:

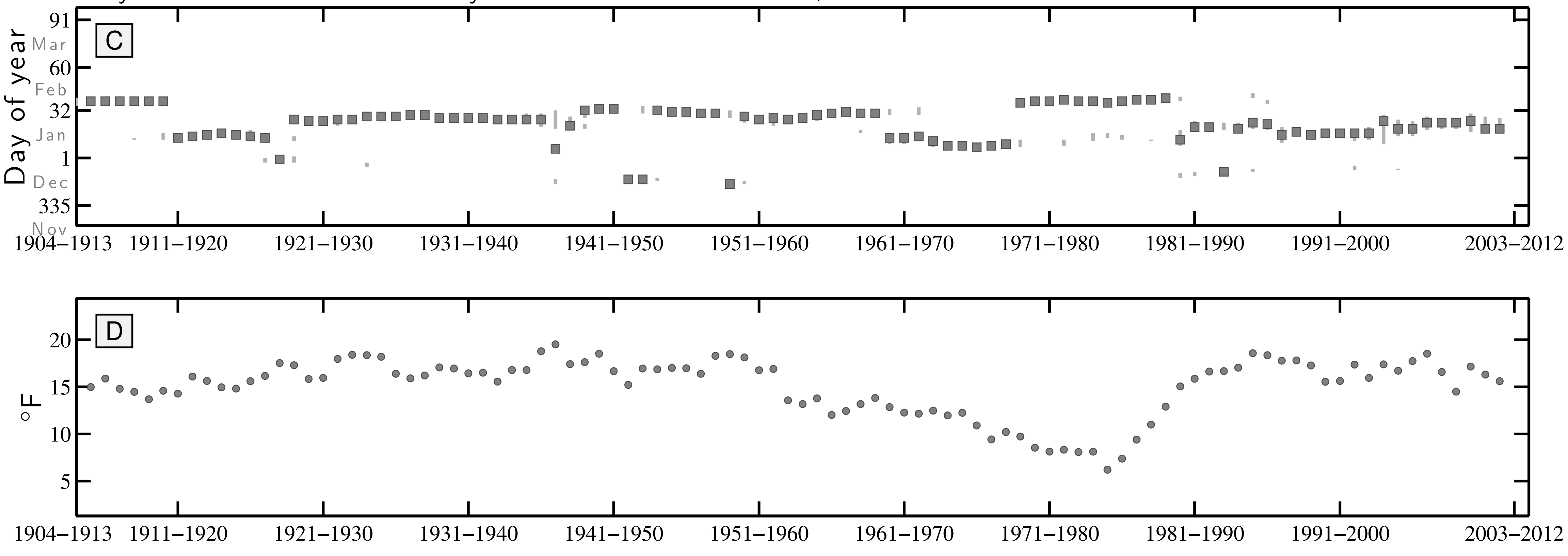

10 year Summer Teletherm dynamics for SALEM, IN:

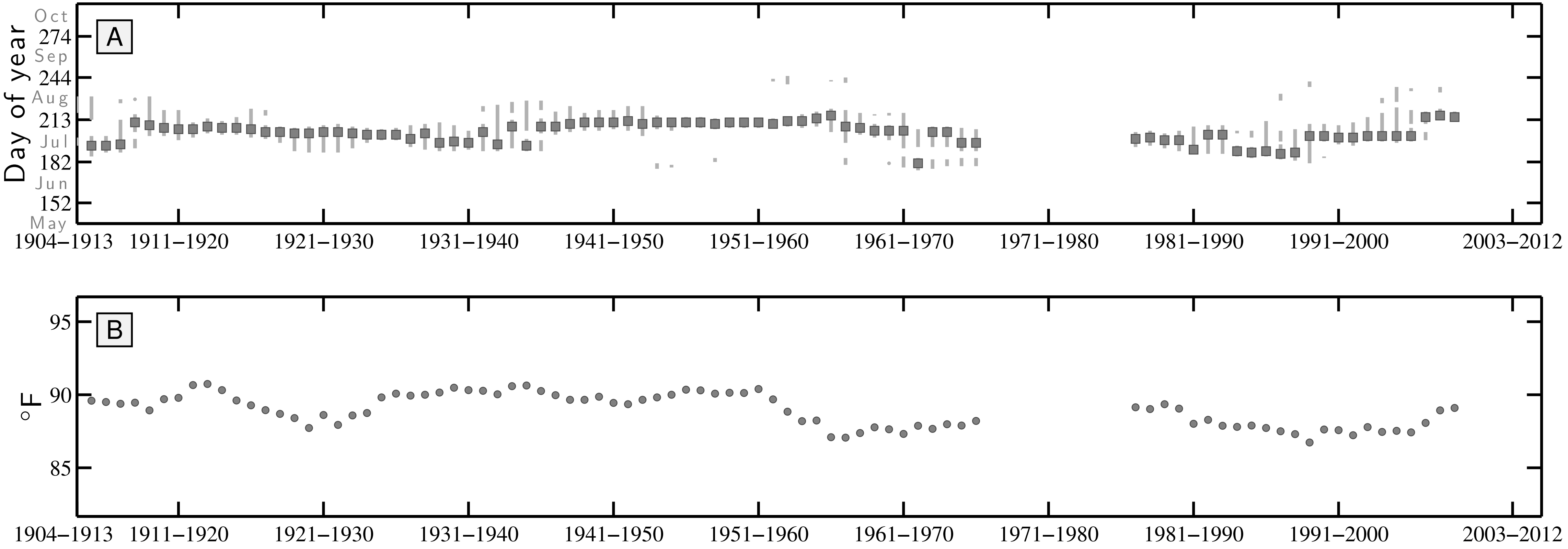

10 year Winter Teletherm dynamics for SALEM, IN:

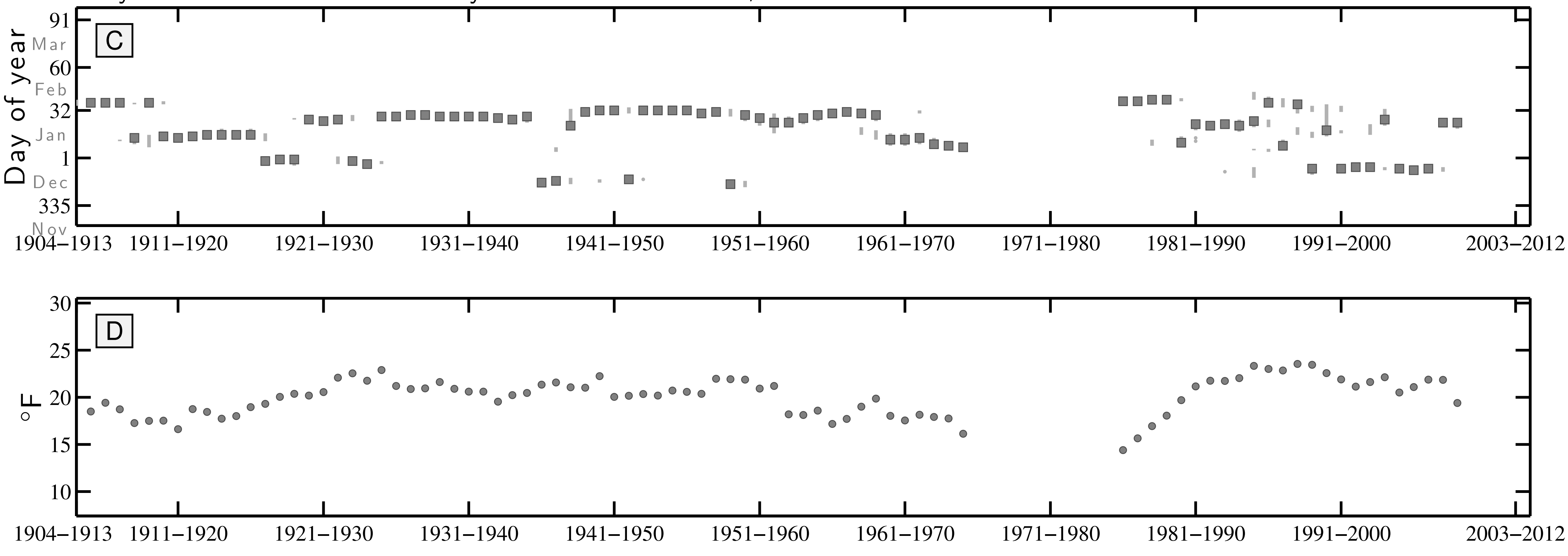

10 year Summer Teletherm dynamics for SCOTTSBURG, IN:

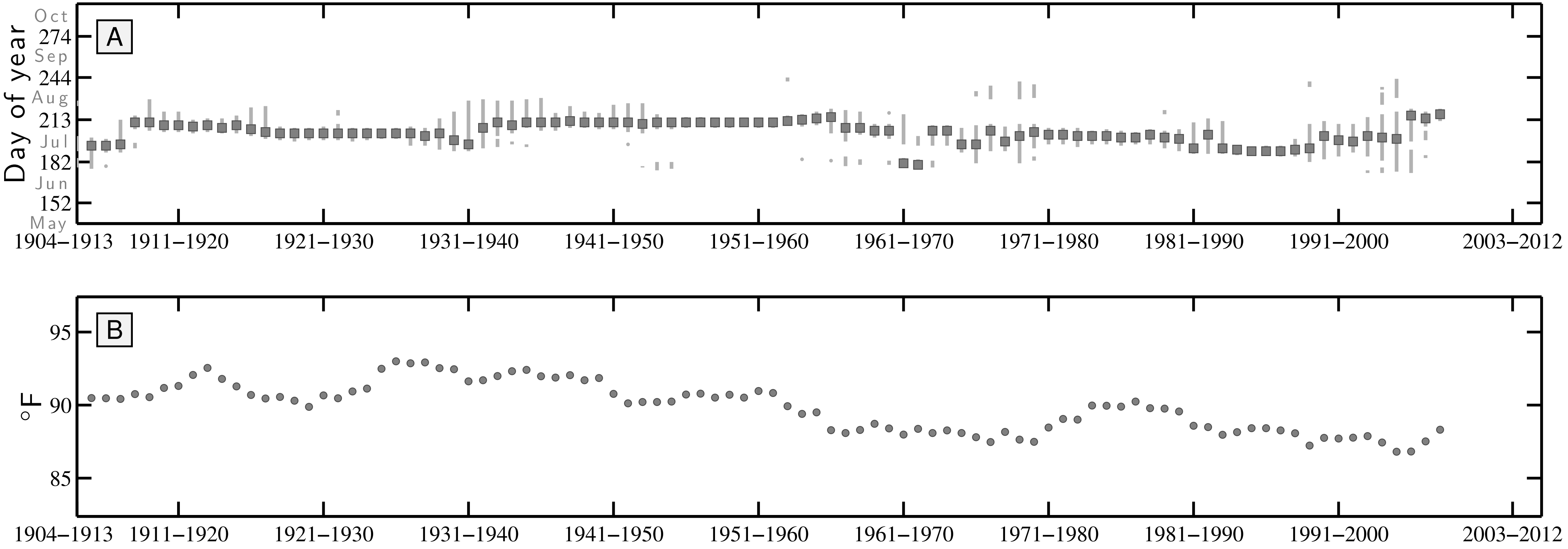

10 year Winter Teletherm dynamics for SCOTTSBURG, IN:

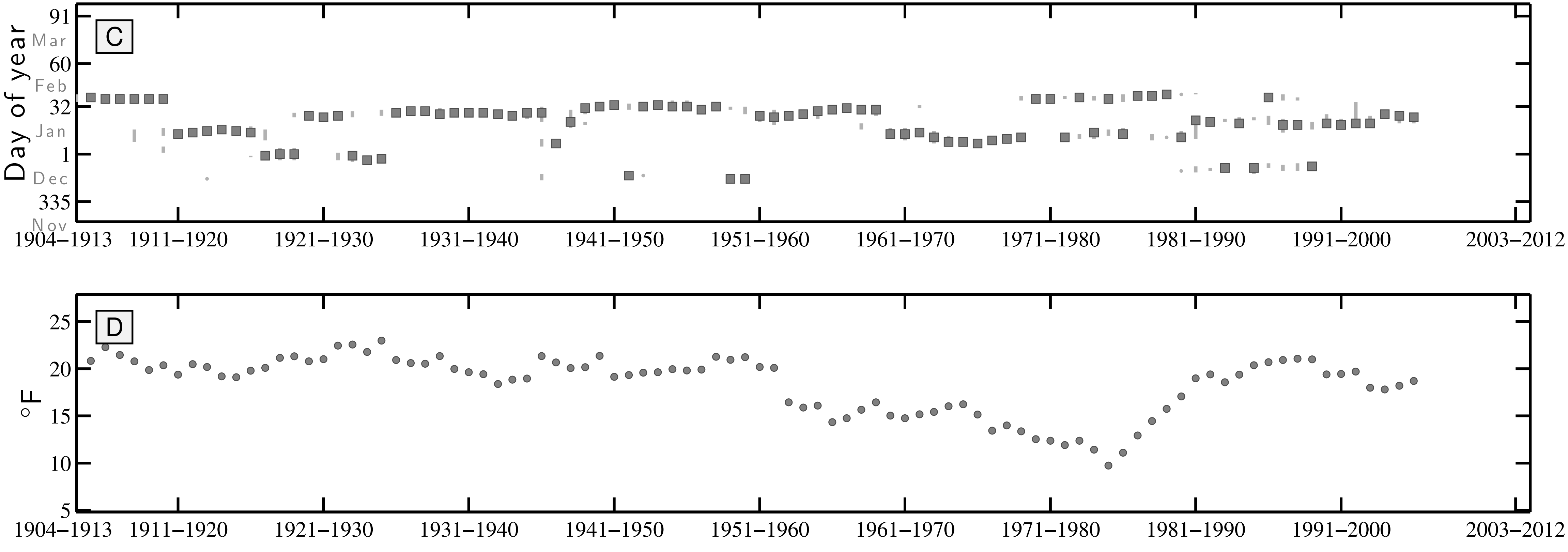

10 year Summer Teletherm dynamics for SEYMOUR 2 N, IN:

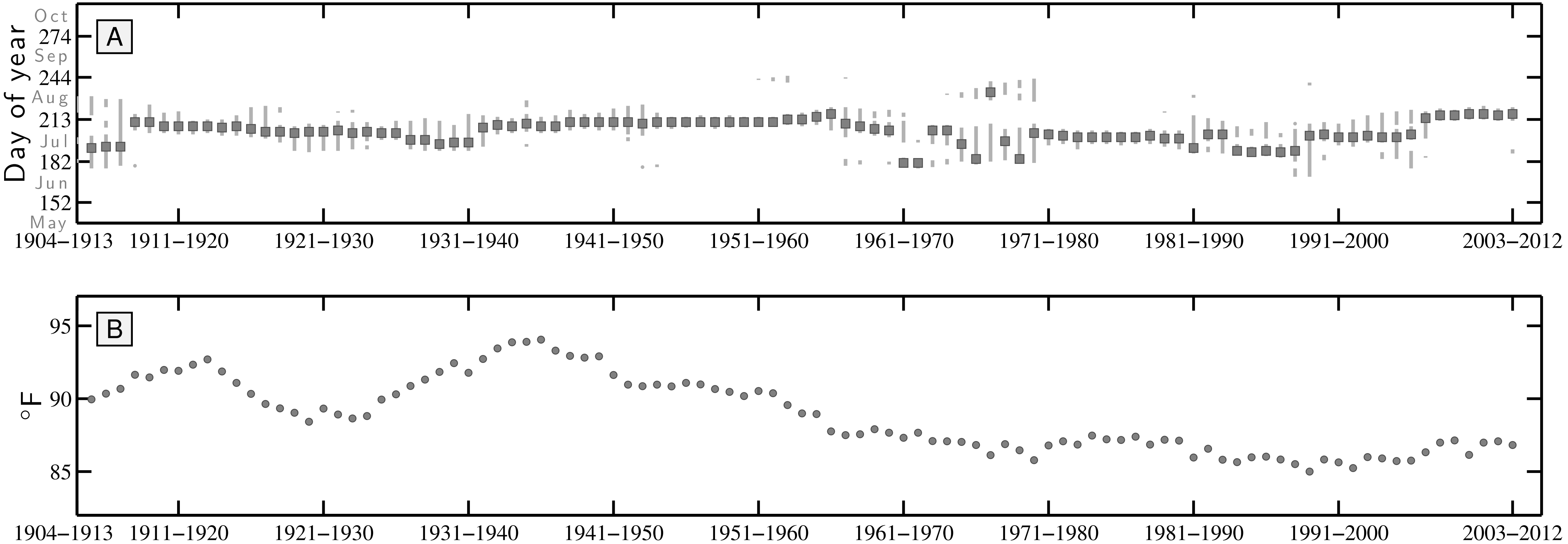

10 year Winter Teletherm dynamics for SEYMOUR 2 N, IN:

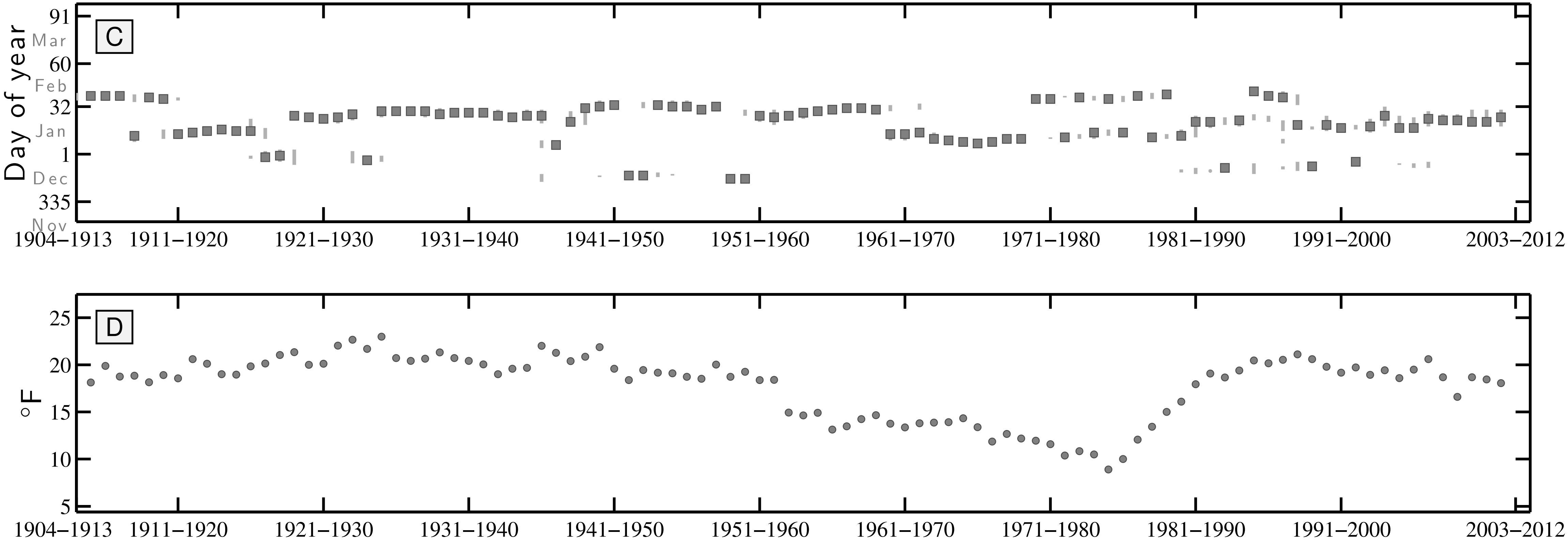

10 year Summer Teletherm dynamics for SHOALS 8 S, IN:

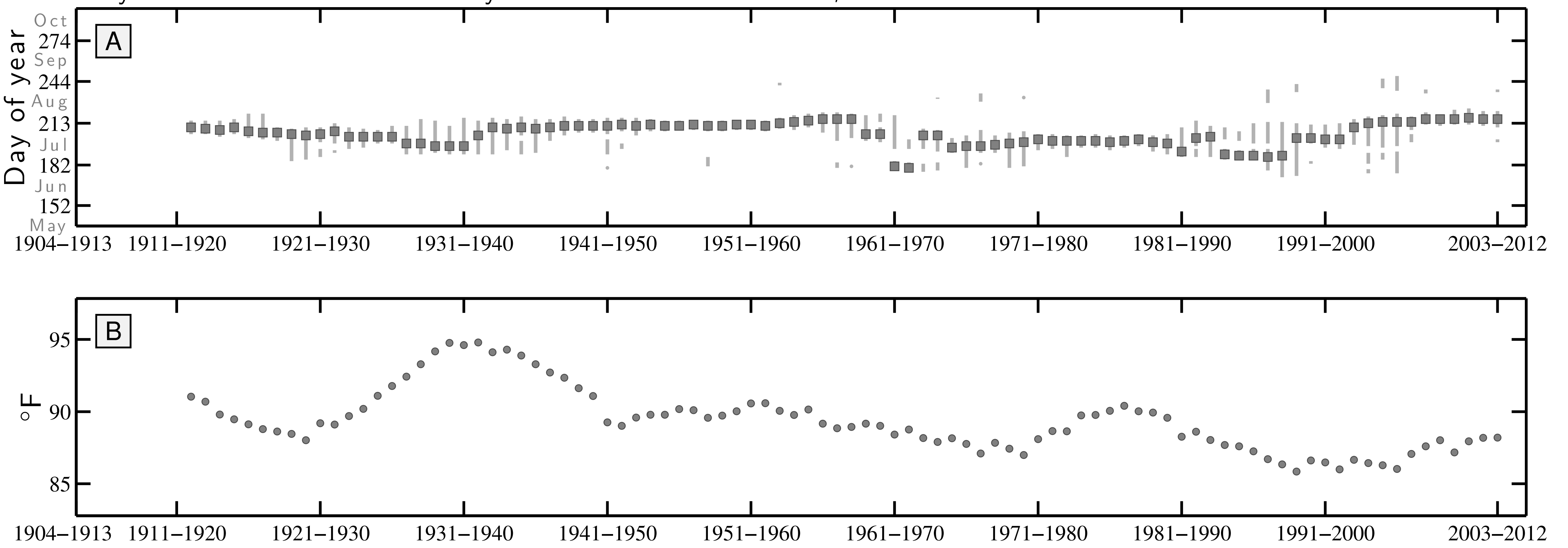

10 year Winter Teletherm dynamics for SHOALS 8 S, IN:

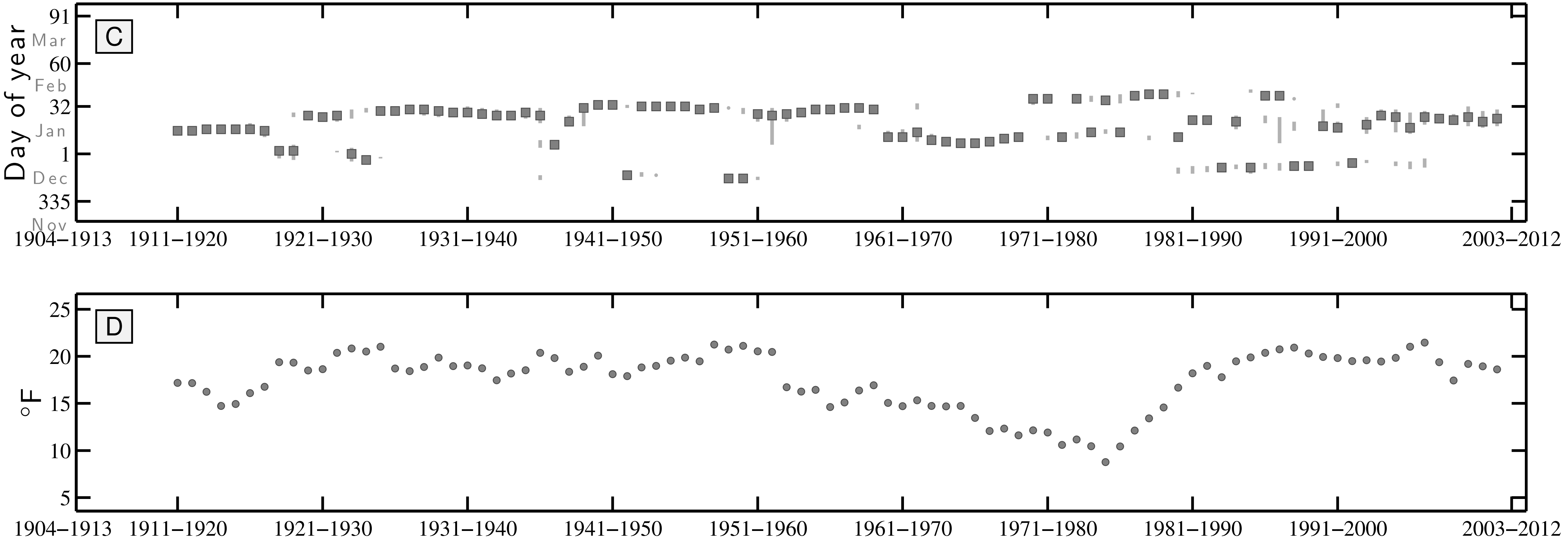

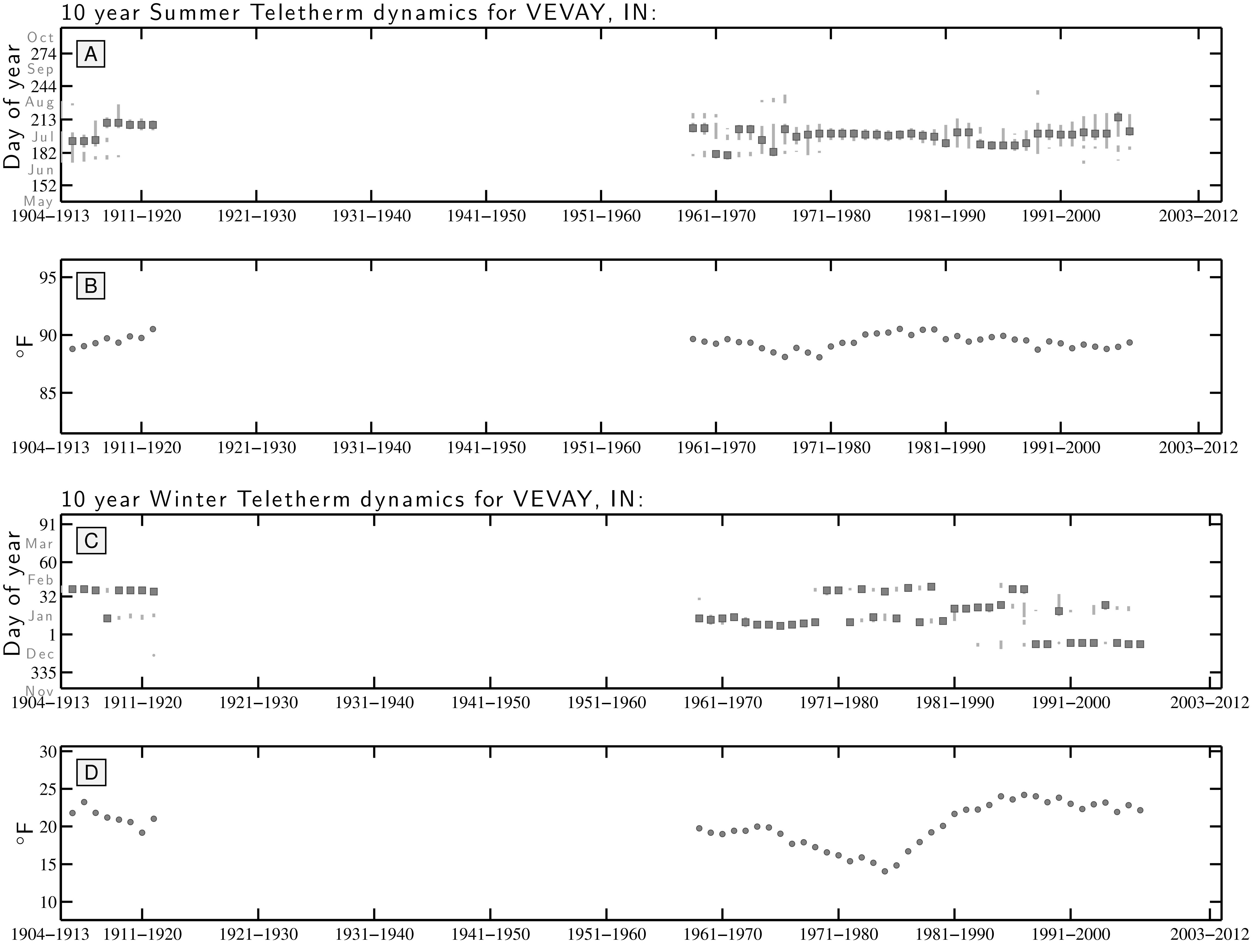
10 year Summer Teletherm dynamics for VEVAY, IN:
A
Day of year
Oct
274
Sep
244
Aug
213
Jul
182
Jun
152
May
1904–1913
1911–1920
1921–1930
1931–1940
1941–1950
1951–1960
1961–1970
1971–1980
1981–1990
1991–2000
2003–2012
B
°F
95
90
85
10 year Winter Teletherm dynamics for VEVAY, IN:
C
91
Mar
60
Feb
32
Jan
1
Dec
335
Nov
D
30
25
20
15
10

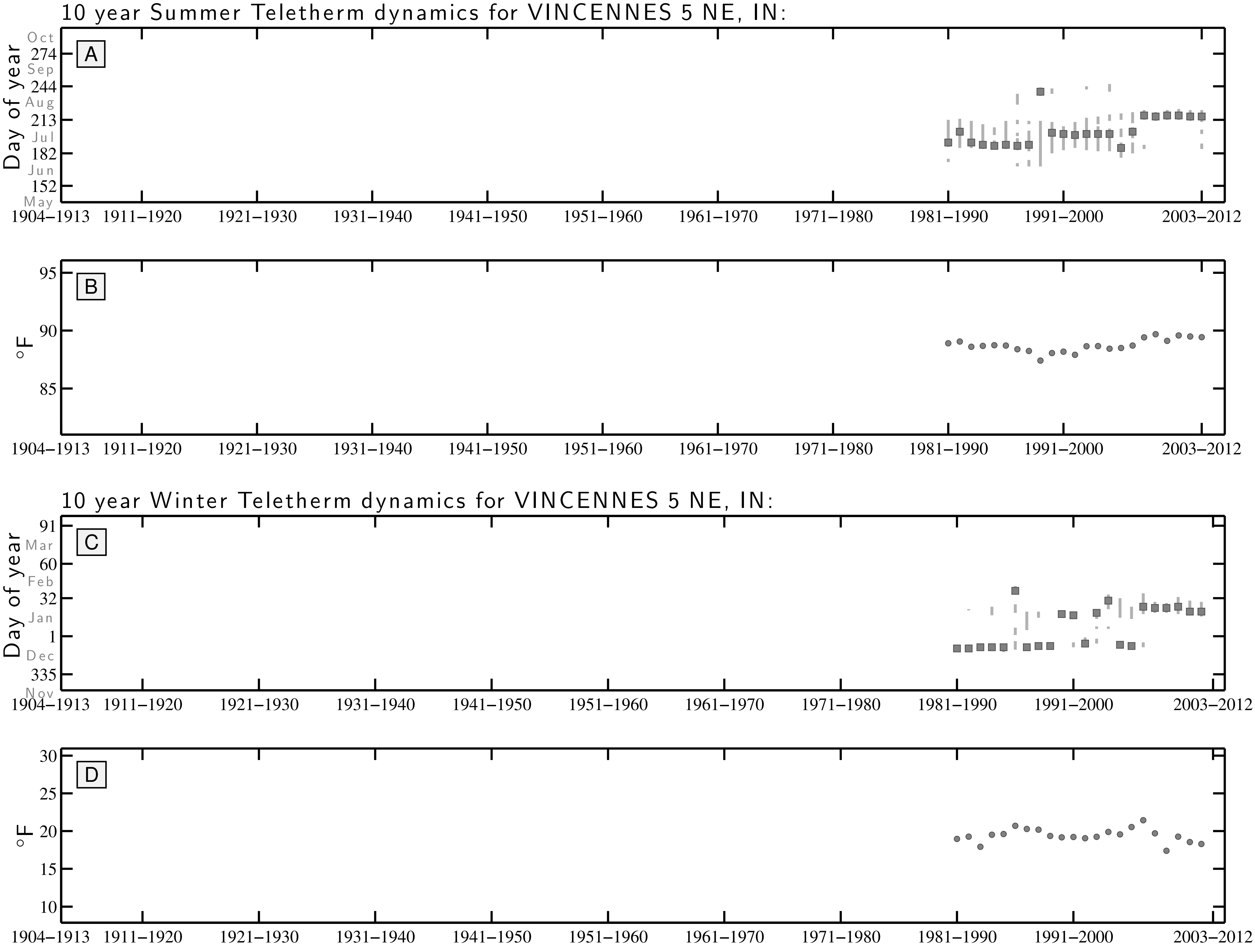
10 year Summer Teletherm dynamics for VINCENNES 5 NE, IN:
A
B
Day of year
°F
Oct
274
Sep
244
Aug
213
Jul
182
Jun
152
May
95
90
85
1904–1913
1911–1920
1921–1930
1931–1940
1941–1950
1951–1960
1961–1970
1971–1980
1981–1990
1991–2000
2003–2012
10 year Winter Teletherm dynamics for VINCENNES 5 NE, IN:
C
D
91
Mar
60
Feb
32
Jan
1
Dec
335
Nov
30
25
20
15
10

10 year Summer Teletherm dynamics for WASHINGTON 1 W, IN:

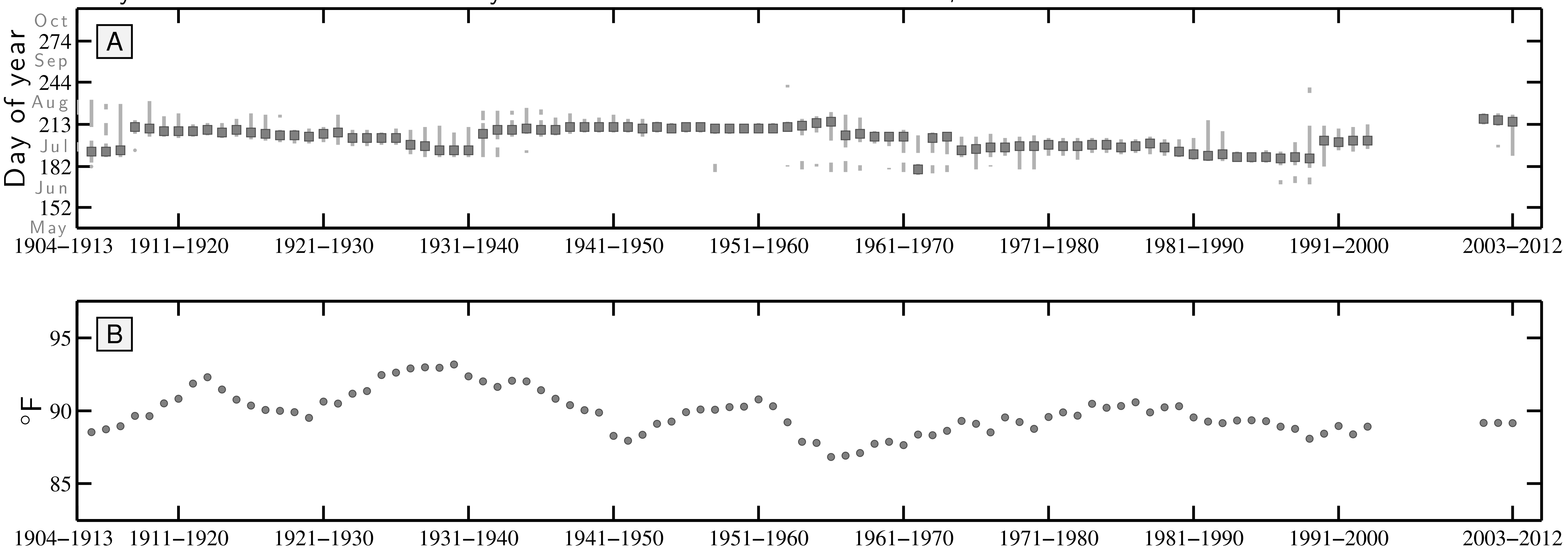

10 year Winter Teletherm dynamics for WASHINGTON 1 W, IN:

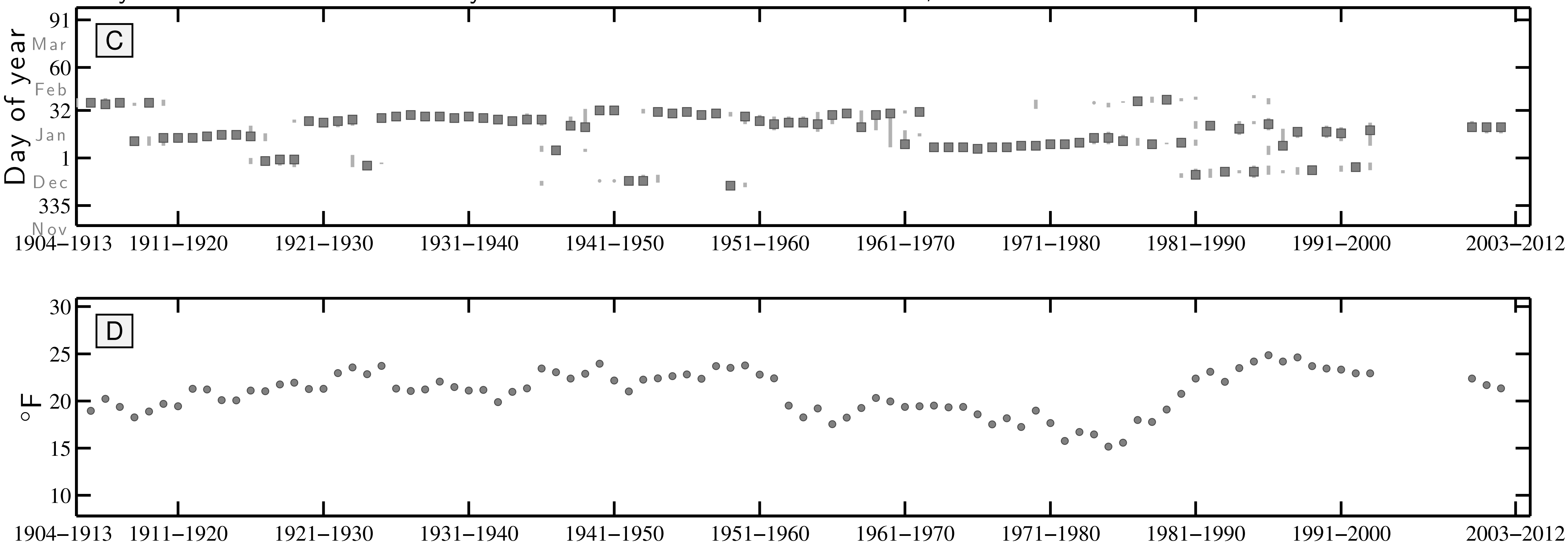

10 year Summer Teletherm dynamics for WHEATFIELD, IN:

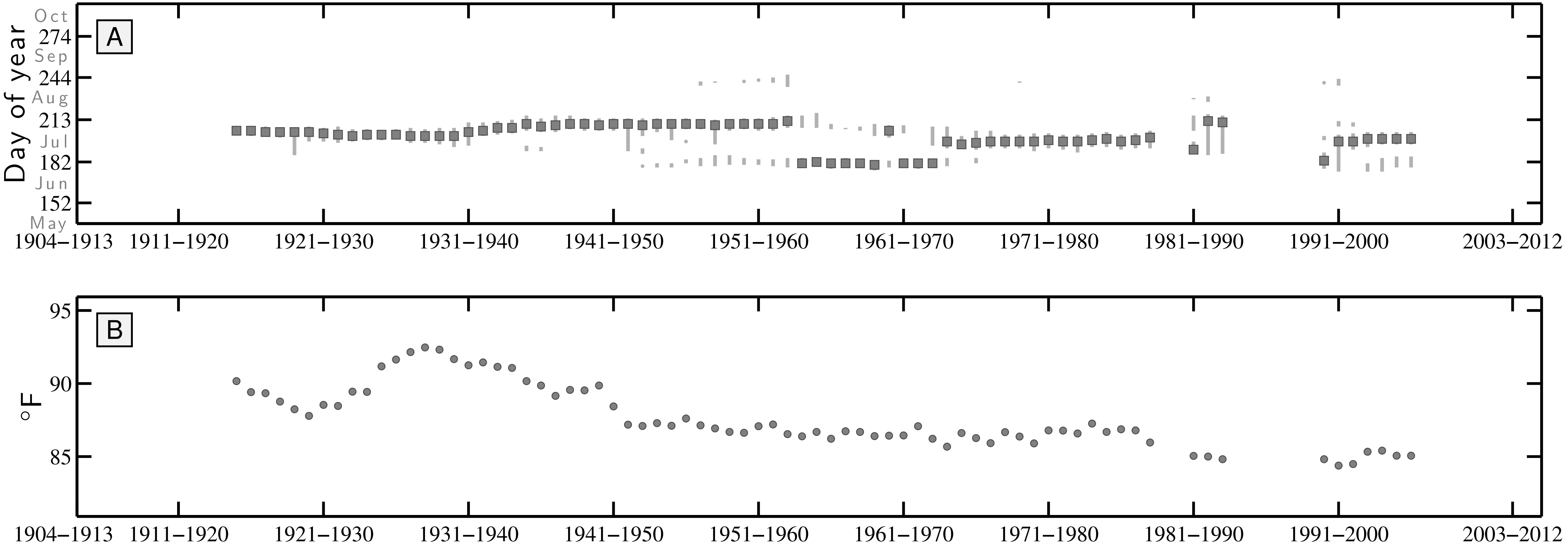

10 year Winter Teletherm dynamics for WHEATFIELD, IN:

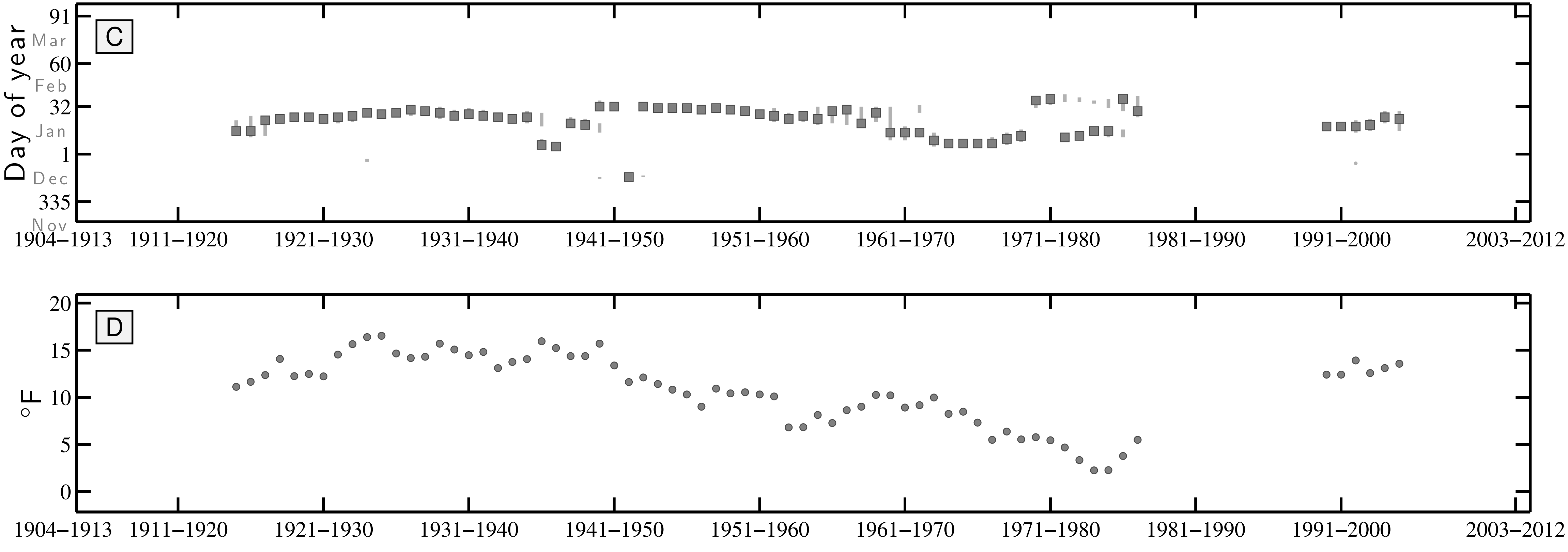

10 year Summer Teletherm dynamics for WHITESTOWN, IN:

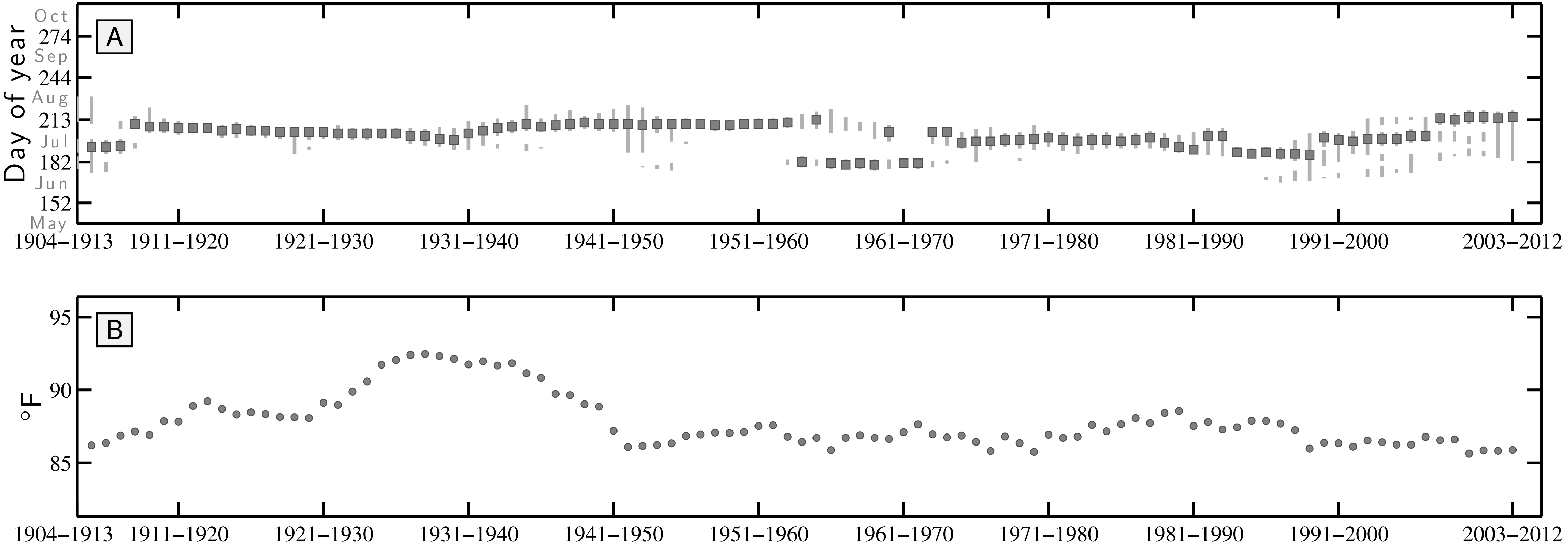

10 year Winter Teletherm dynamics for WHITESTOWN, IN:

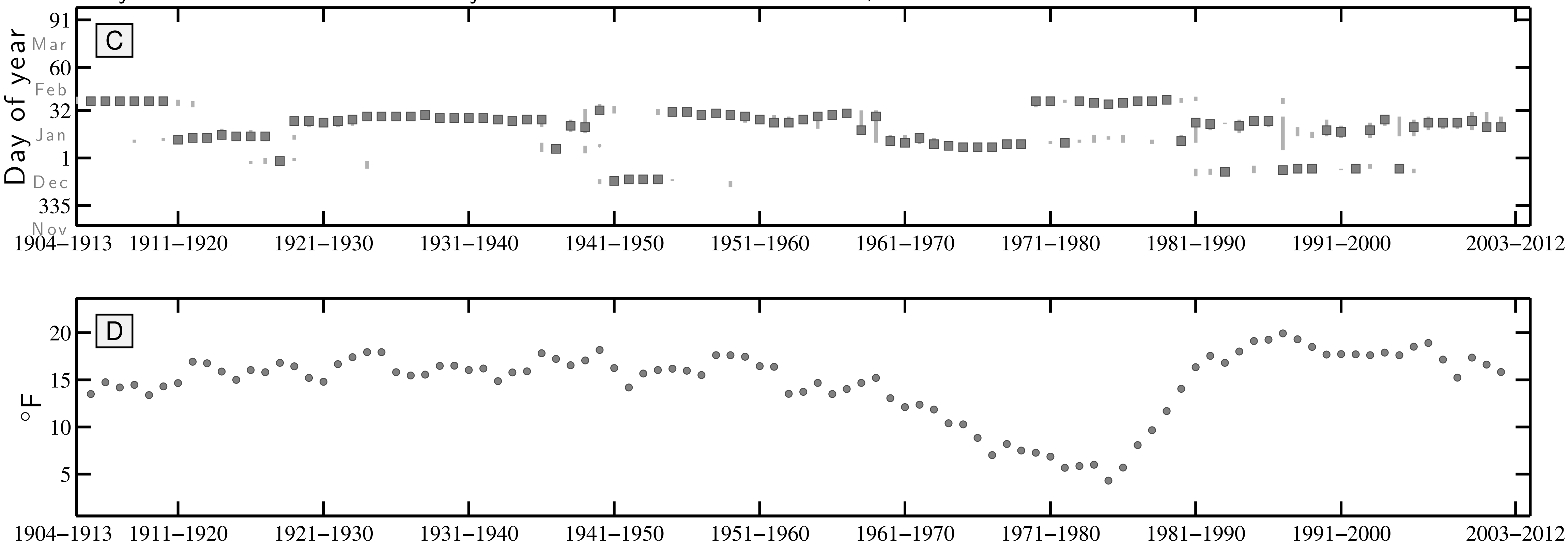

10 year Summer Teletherm dynamics for WINAMAC 2SSE, IN:

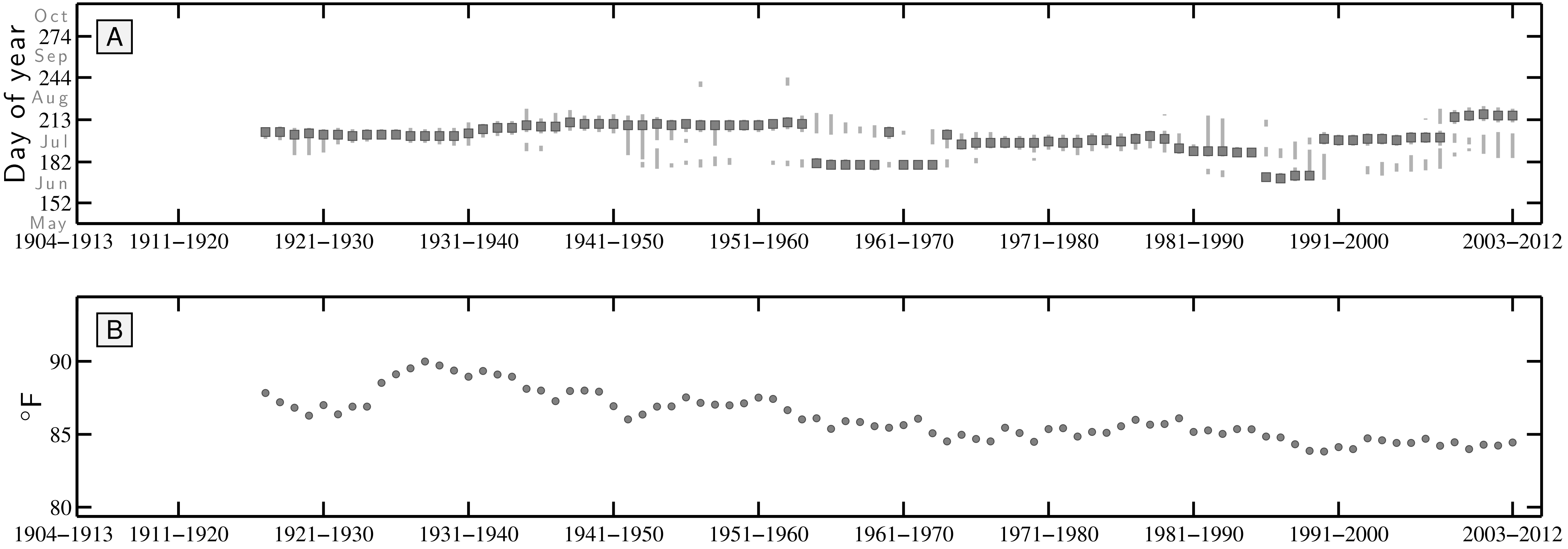

10 year Winter Teletherm dynamics for WINAMAC 2SSE, IN:

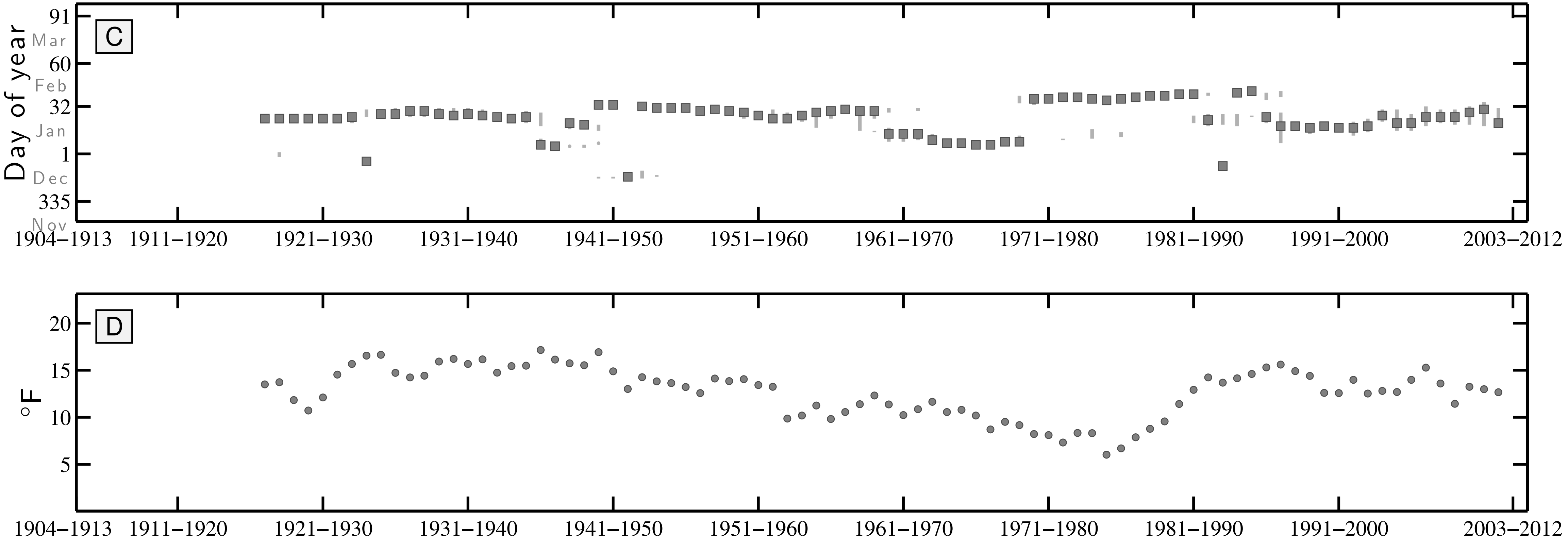

10 year Summer Teletherm dynamics for ALBIA 3 NNE, IA:

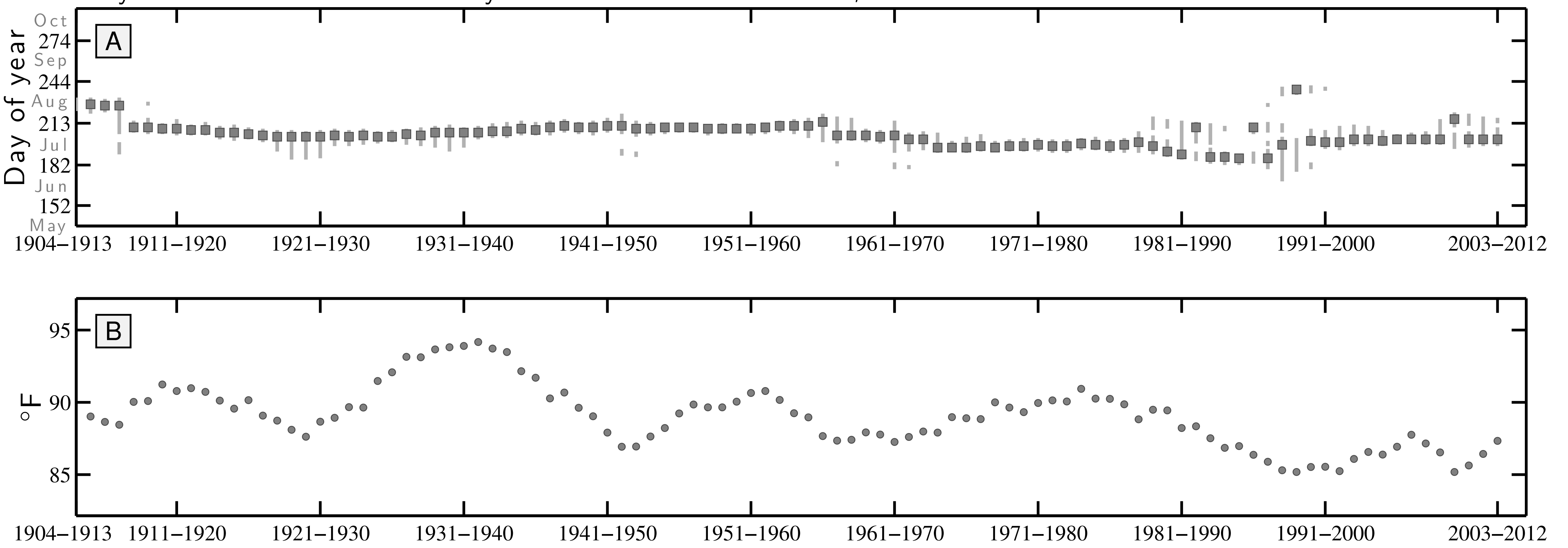

10 year Winter Teletherm dynamics for ALBIA 3 NNE, IA:

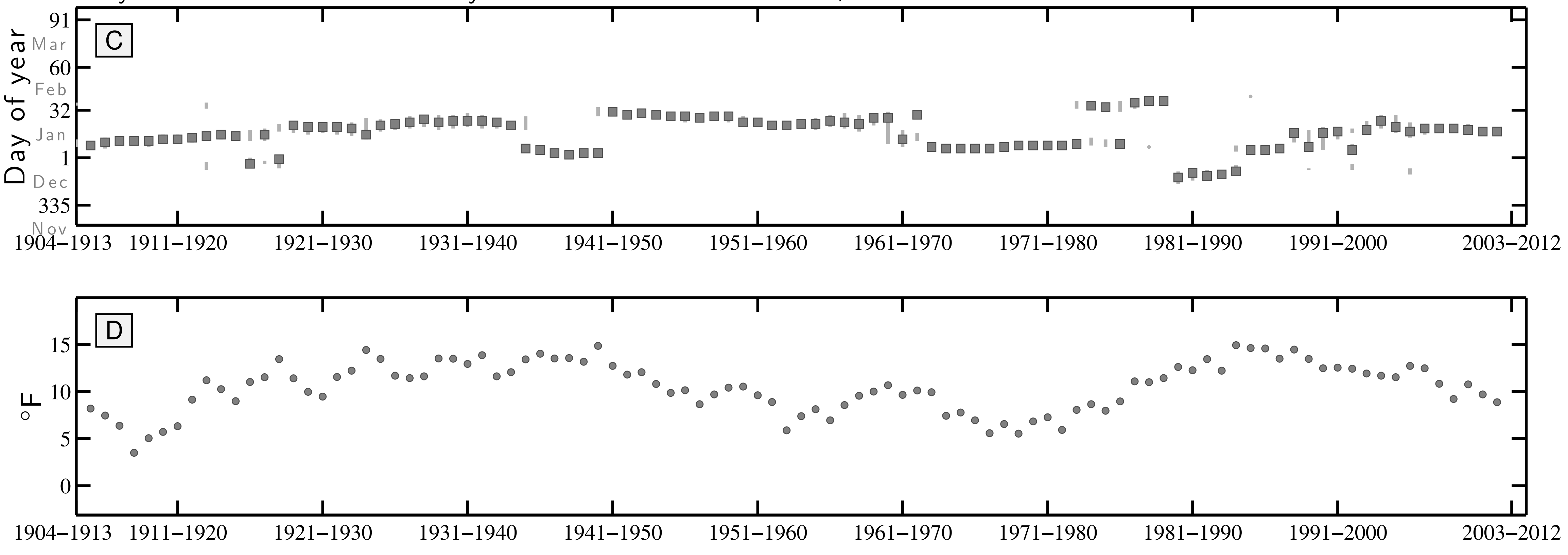

10 year Summer Teletherm dynamics for ALGONA 3 W, IA:

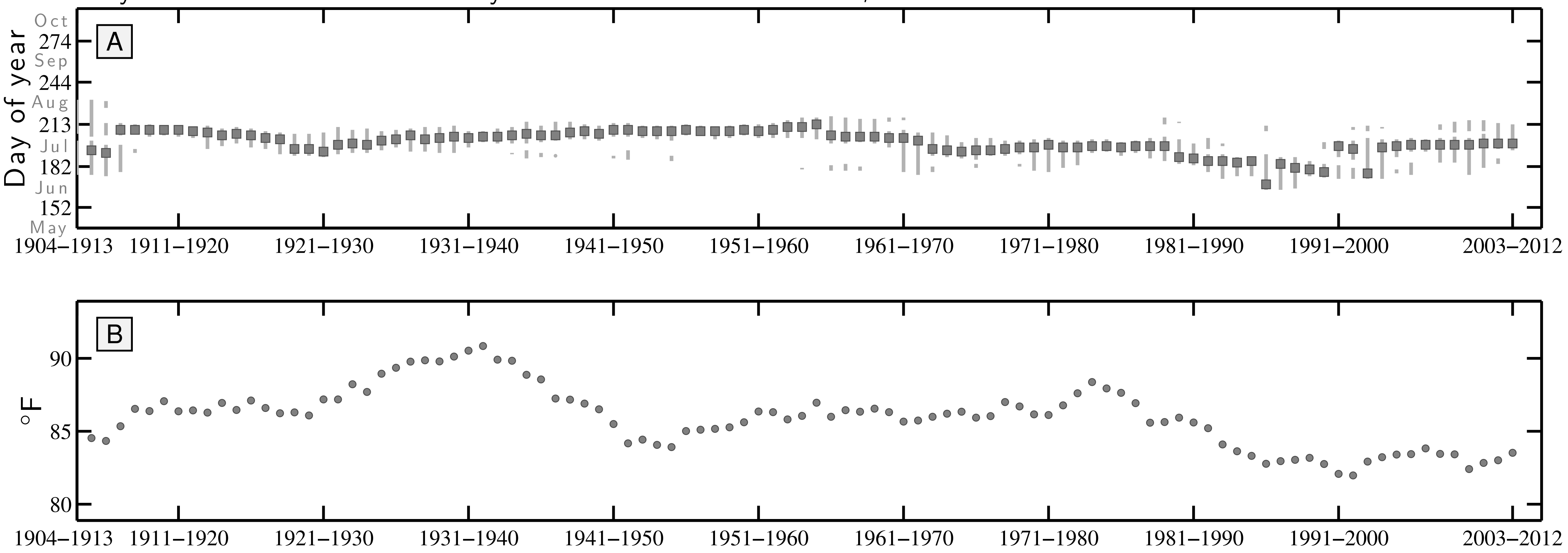

10 year Winter Teletherm dynamics for ALGONA 3 W, IA:

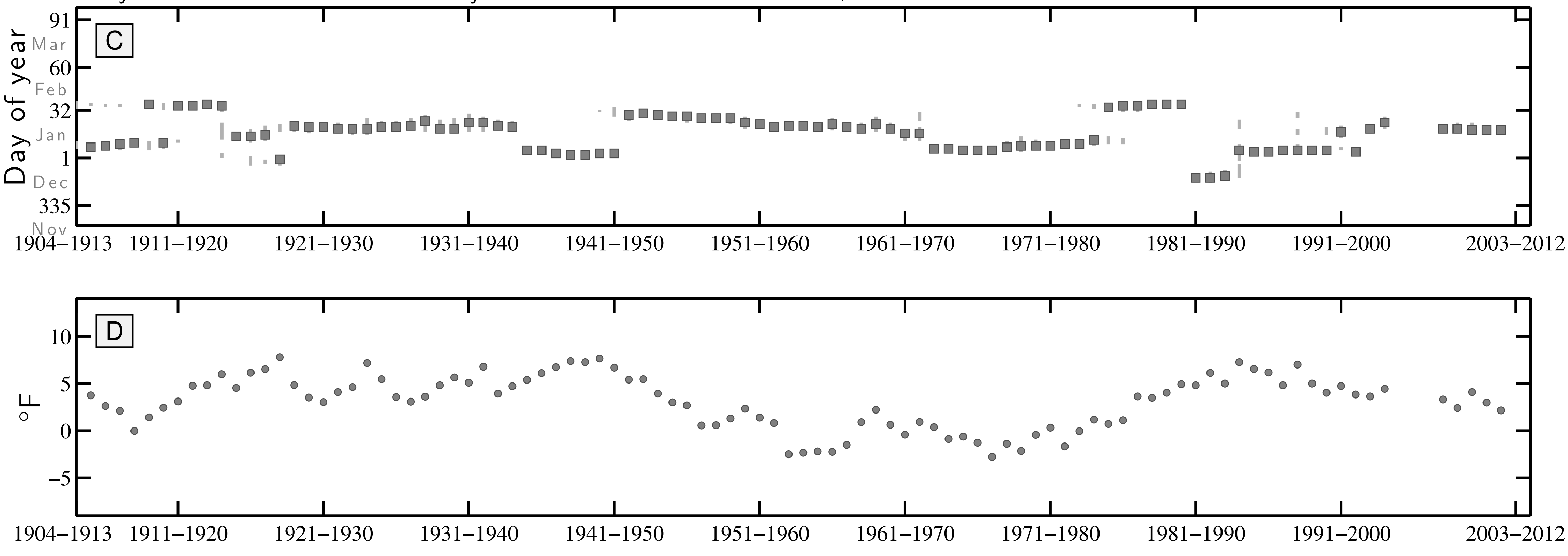

10 year Summer Teletherm dynamics for BELLE PLAINE, IA:

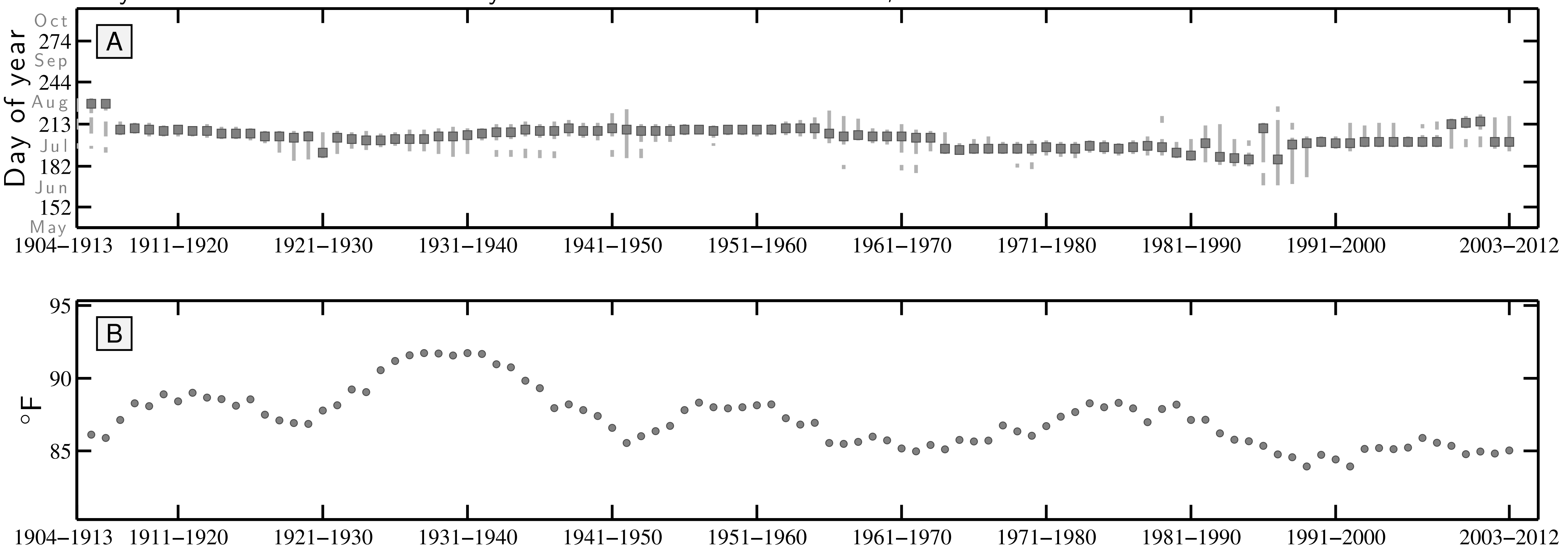

10 year Winter Teletherm dynamics for BELLE PLAINE, IA:

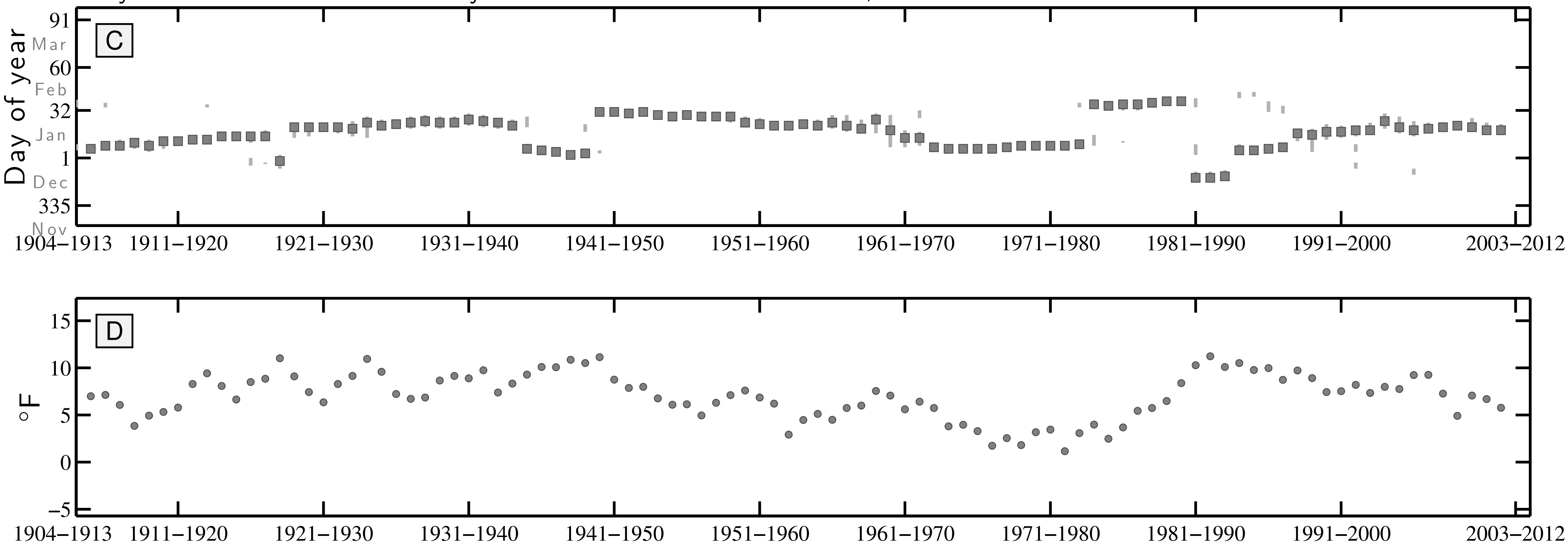

10 year Summer Teletherm dynamics for CHARLES CITY, IA:

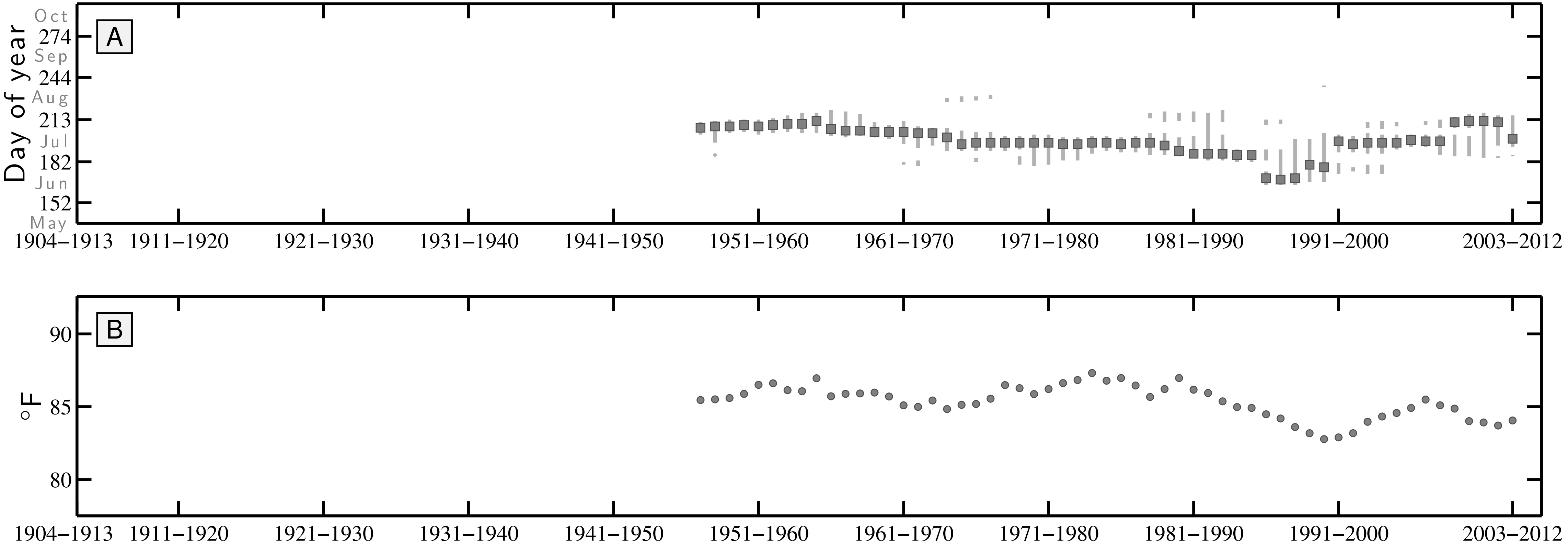

10 year Winter Teletherm dynamics for CHARLES CITY, IA:

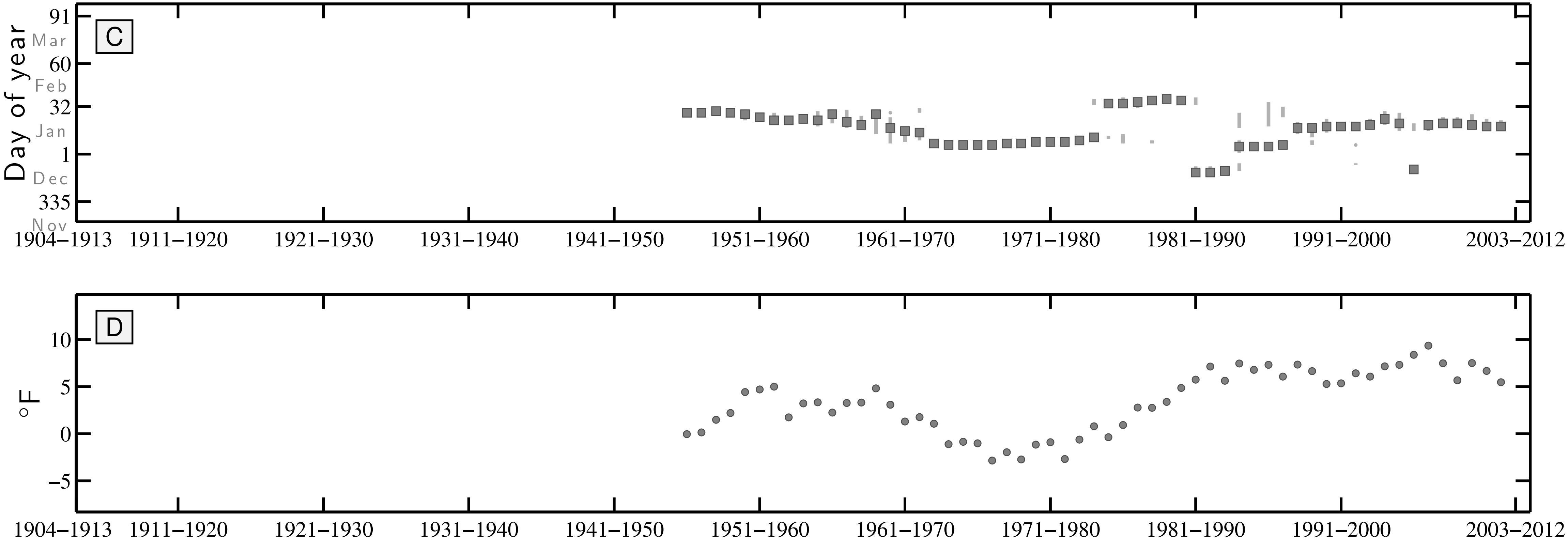

10 year Summer Teletherm dynamics for CLARINDA, IA:

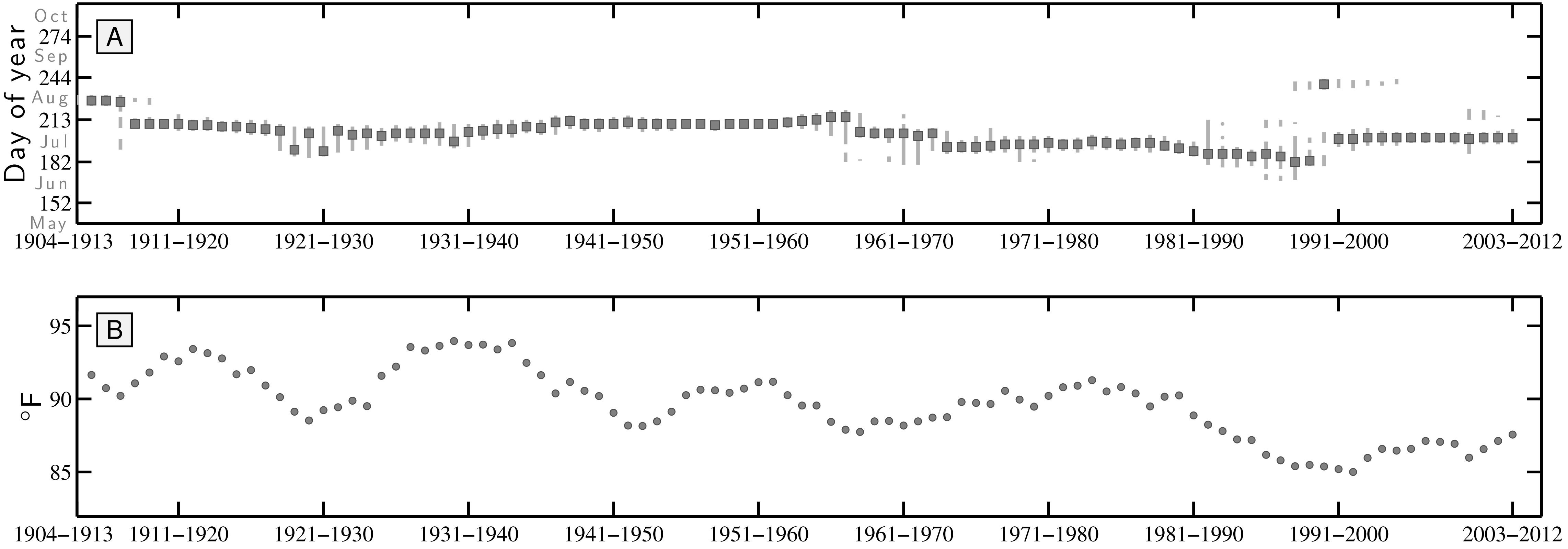

10 year Winter Teletherm dynamics for CLARINDA, IA:

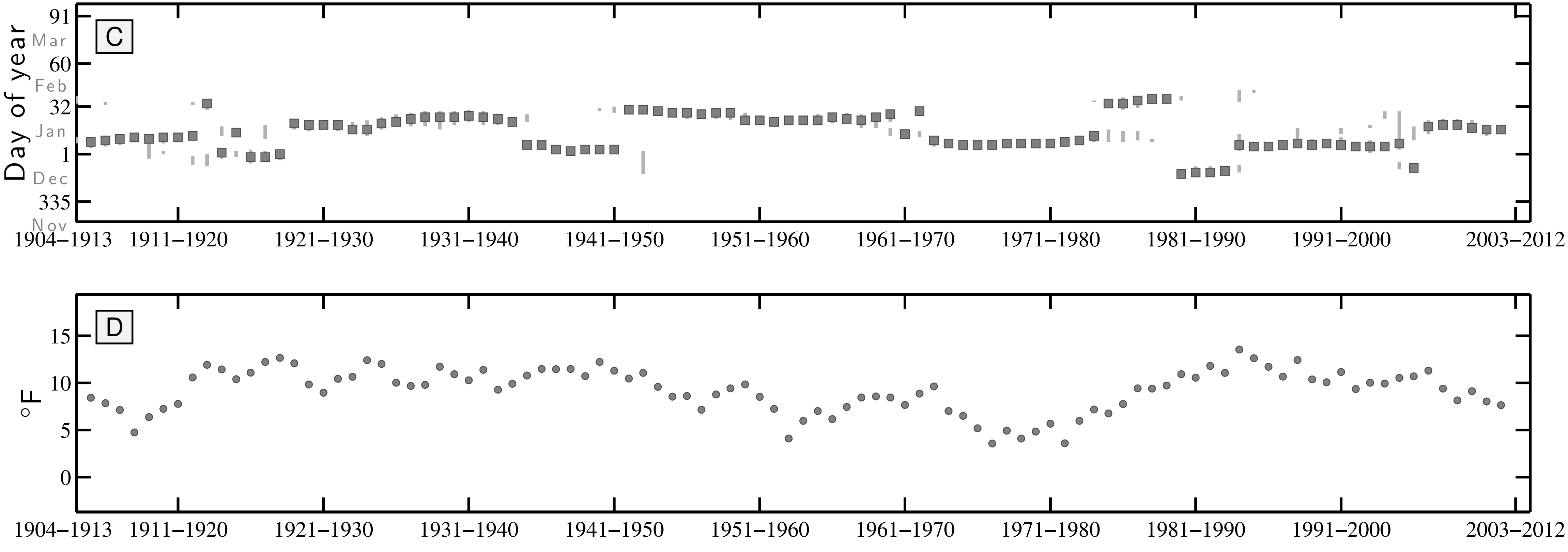

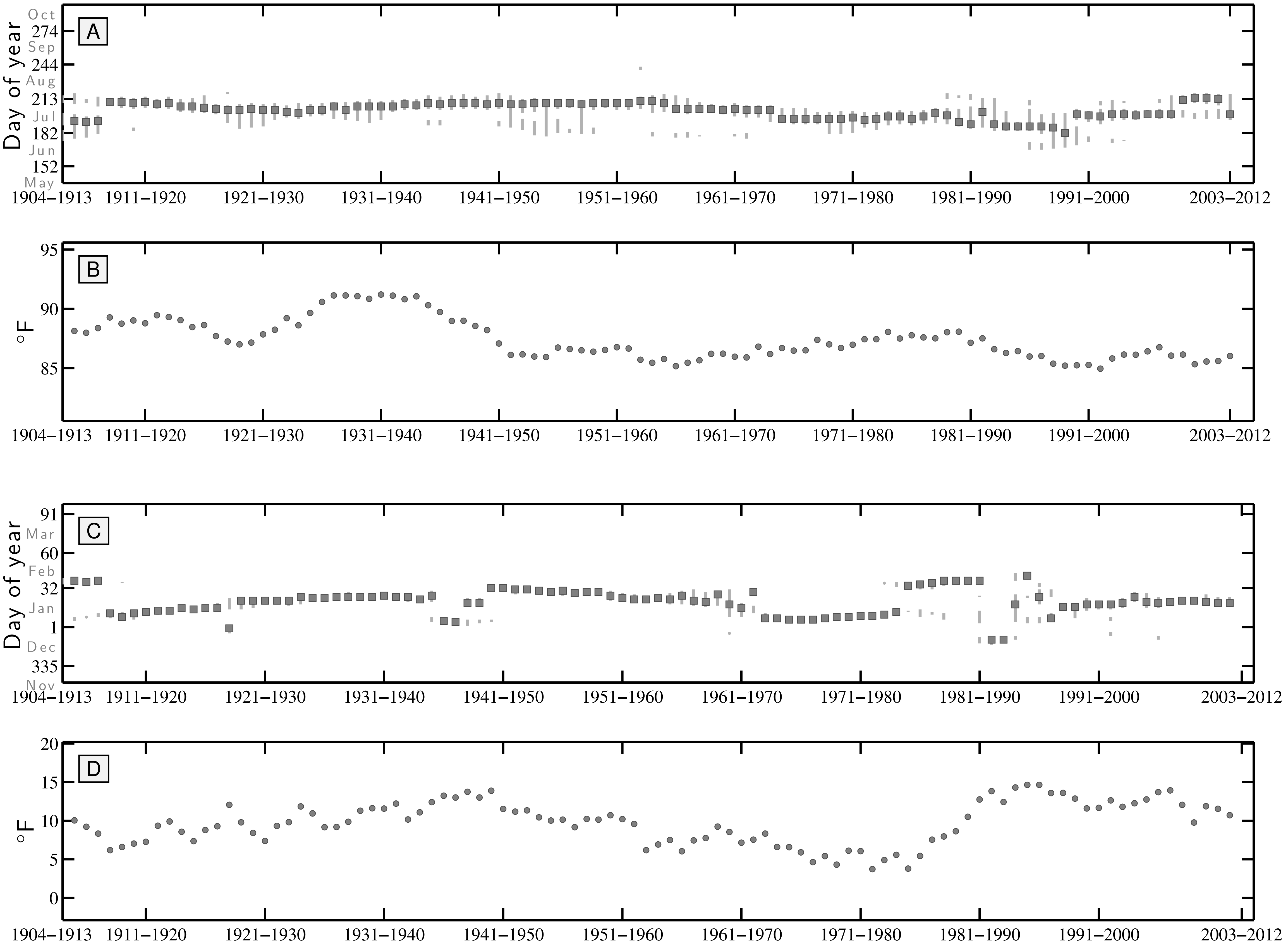

A
B
C
D
Day of year
Oct
274
Sep
244
Aug
213
Jul
182
Jun
152
May
°F
95
90
85
Mar
91
60
Feb
32
Jan
1
Dec
335
Nov
20
15
10
5
0
1904–1913
1911–1920
1921–1930
1931–1940
1941–1950
1951–1960
1961–1970
1971–1980
1981–1990
1991–2000
2003–2012

10 year Summer Teletherm dynamics for ESTHERVILLE 2 N, IA:

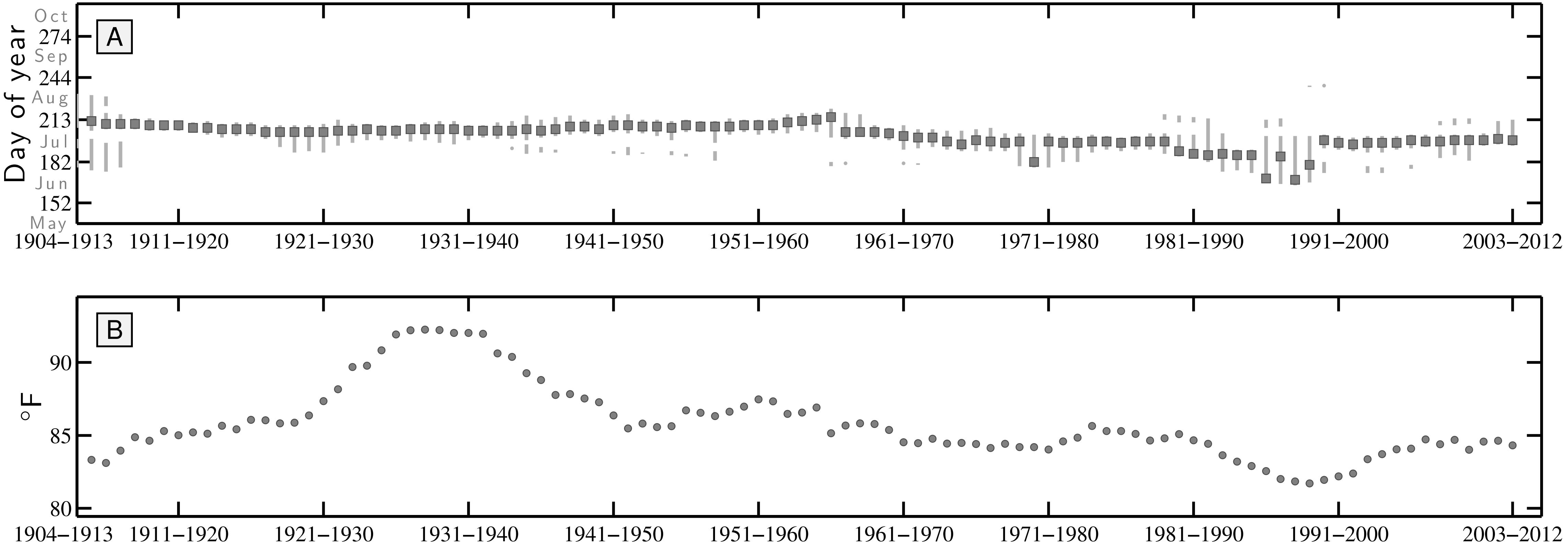

10 year Winter Teletherm dynamics for ESTHERVILLE 2 N, IA:

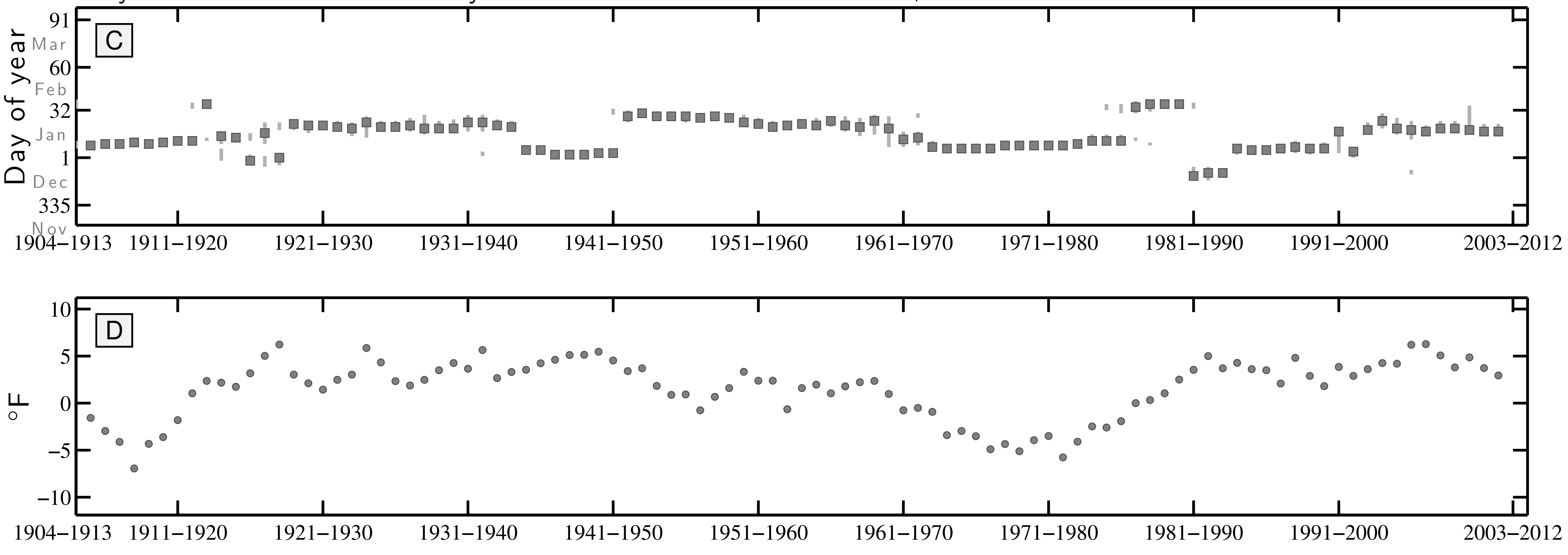

10 year Summer Teletherm dynamics for FAIRFIELD, IA:

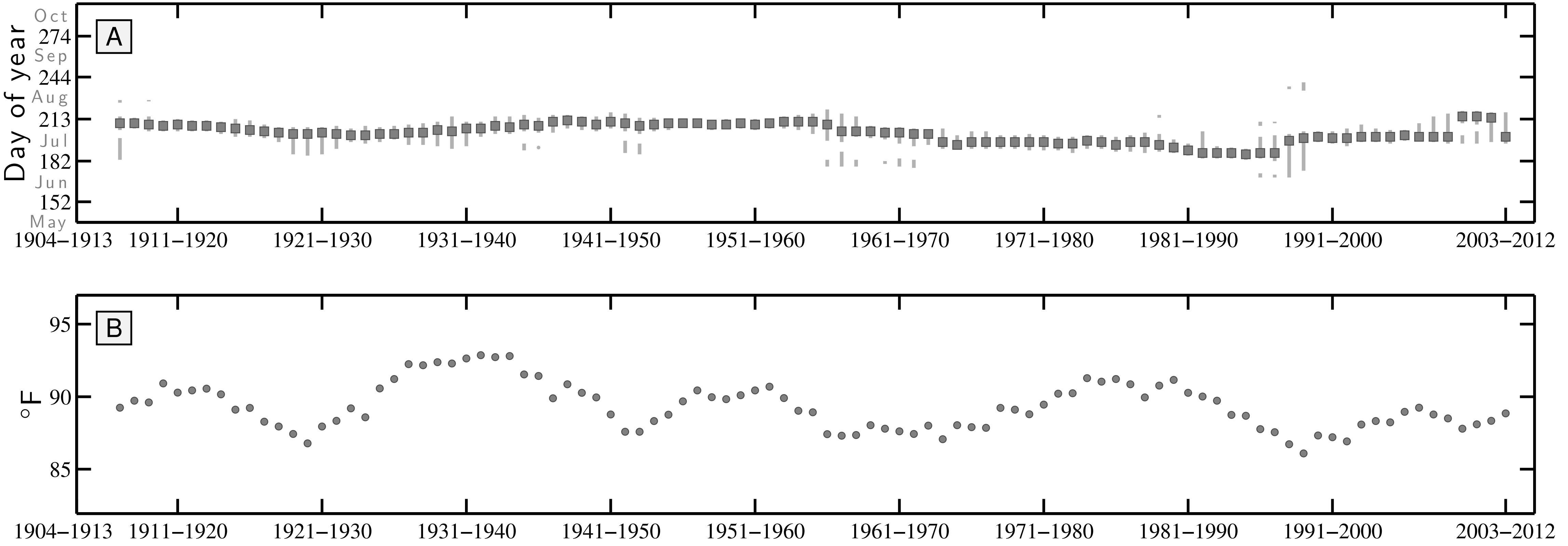

10 year Winter Teletherm dynamics for FAIRFIELD, IA:

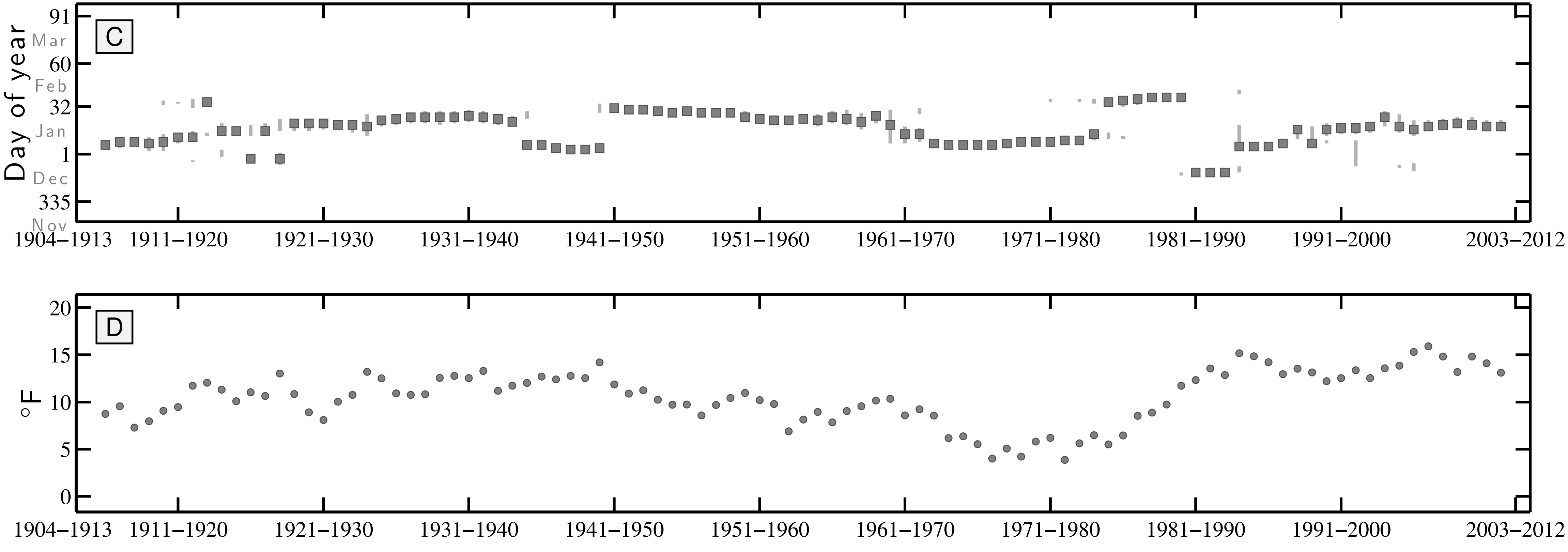

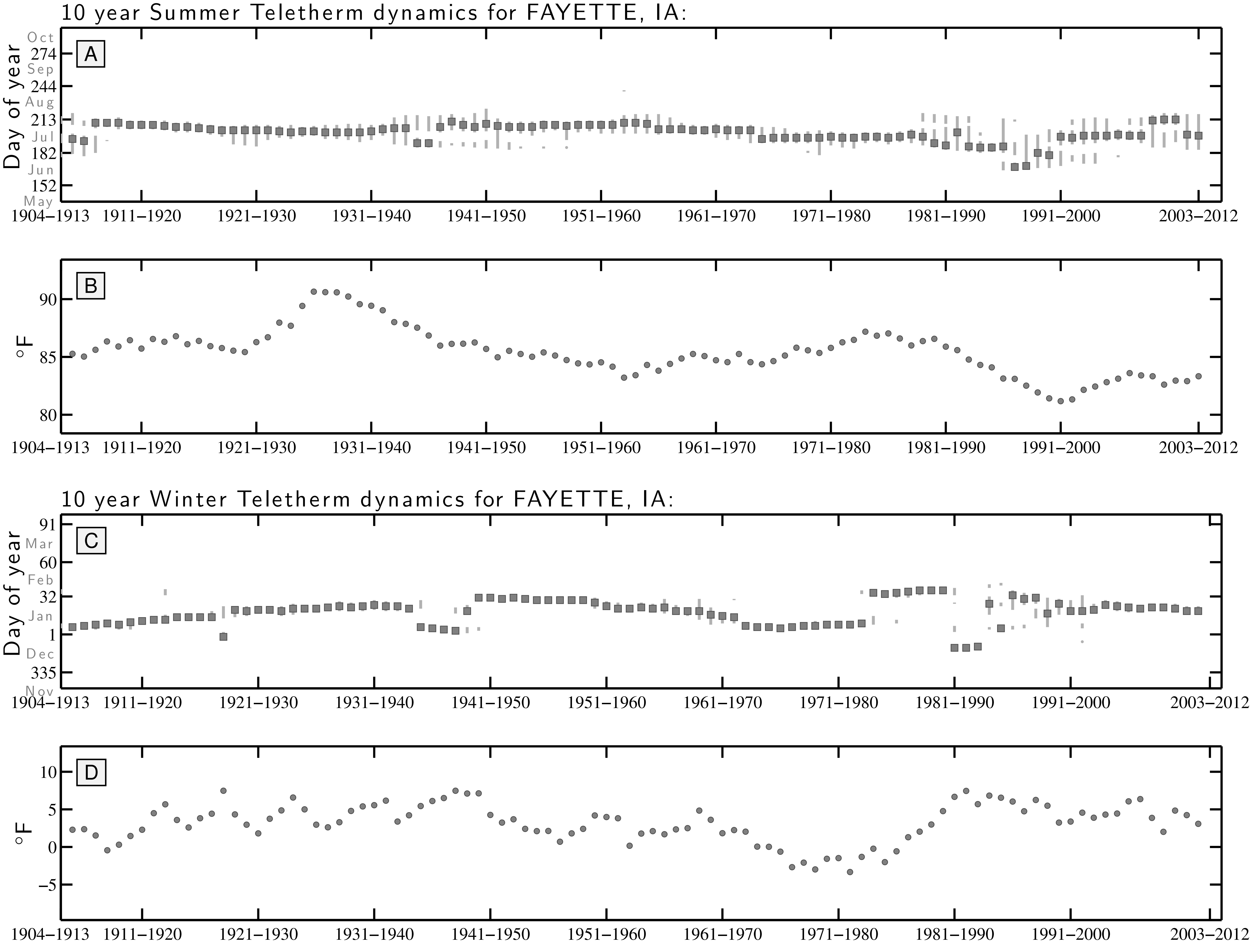
10 year Summer Teletherm dynamics for FAYETTE, IA:
A
B
Day of year
°F
Oct
274
Sep
244
Aug
213
Jul
182
Jun
152
May
90
85
80
1904–1913
1911–1920
1921–1930
1931–1940
1941–1950
1951–1960
1961–1970
1971–1980
1981–1990
1991–2000
2003–2012
10 year Winter Teletherm dynamics for FAYETTE, IA:
C
D
91
Mar
60
Feb
32
Jan
1
Dec
335
Nov
10
5
0
−5

10 year Summer Teletherm dynamics for FOREST CITY 2 NNE, IA:

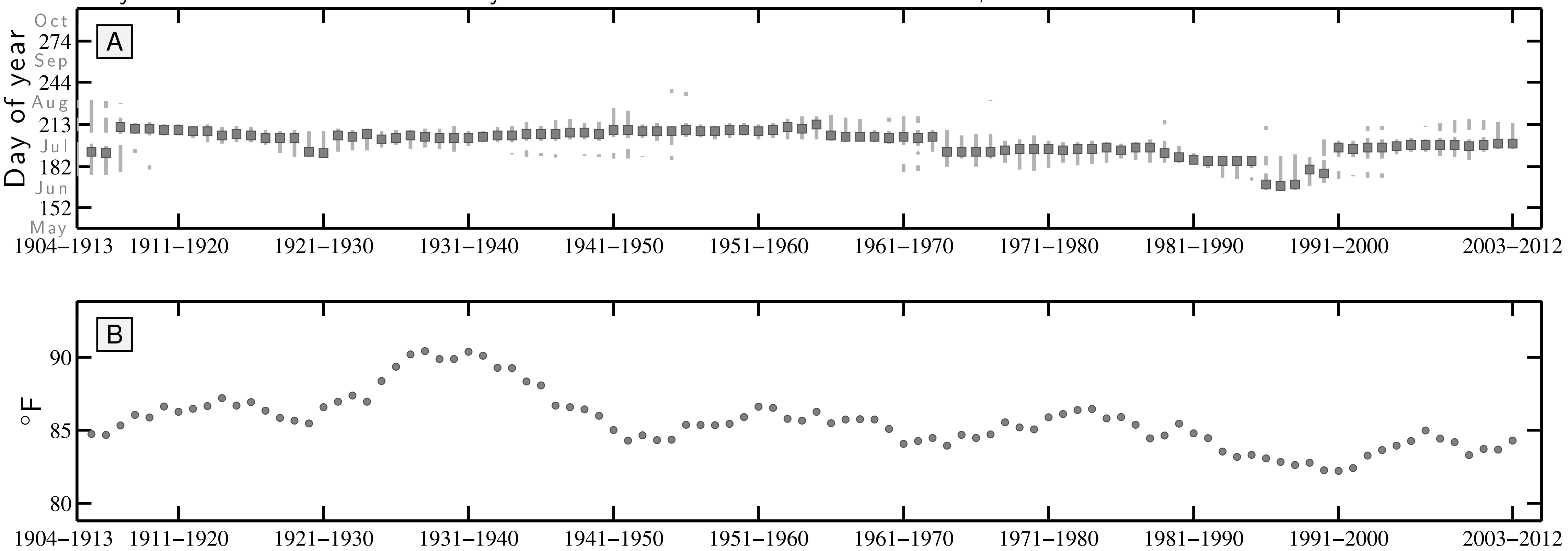

10 year Winter Teletherm dynamics for FOREST CITY 2 NNE, IA:

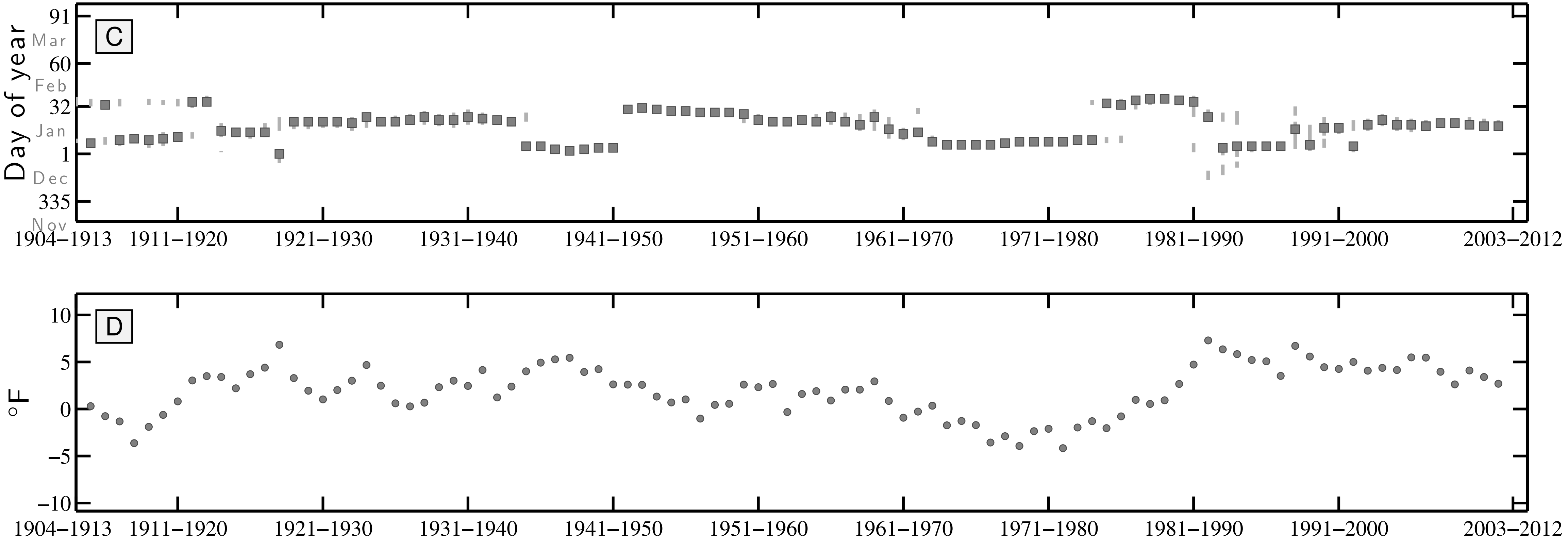

10 year Summer Teletherm dynamics for FORT DODGE 5NNW, IA:

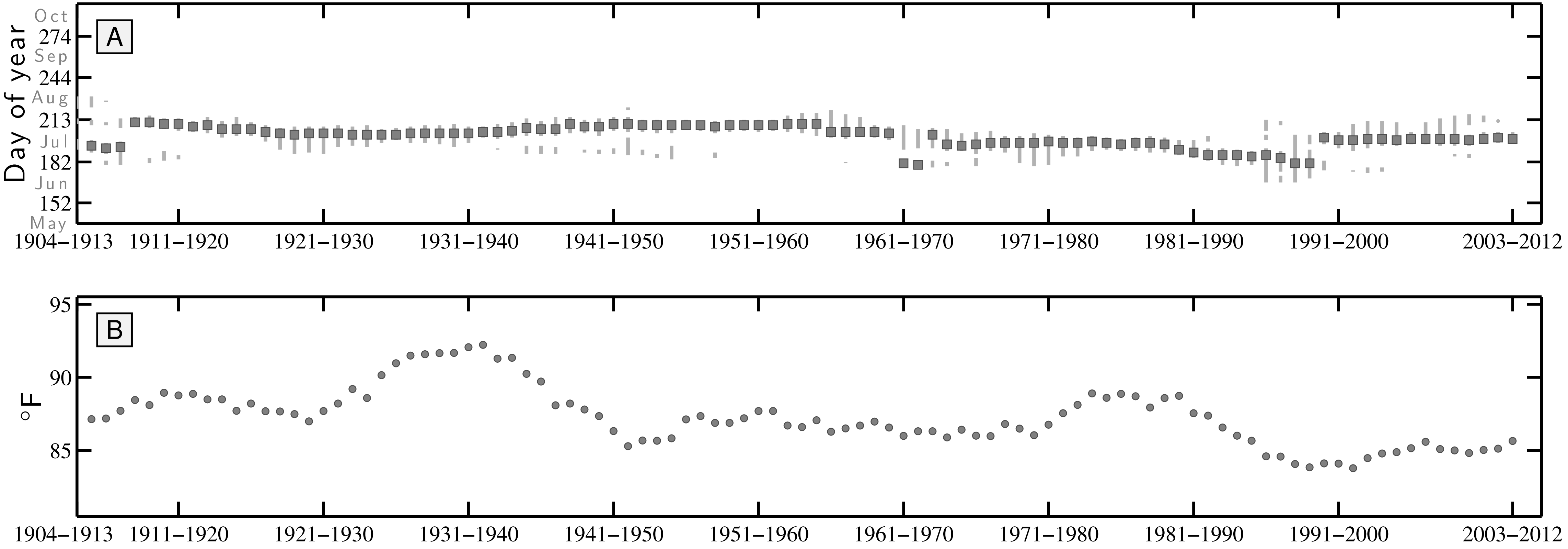

10 year Winter Teletherm dynamics for FORT DODGE 5NNW, IA:

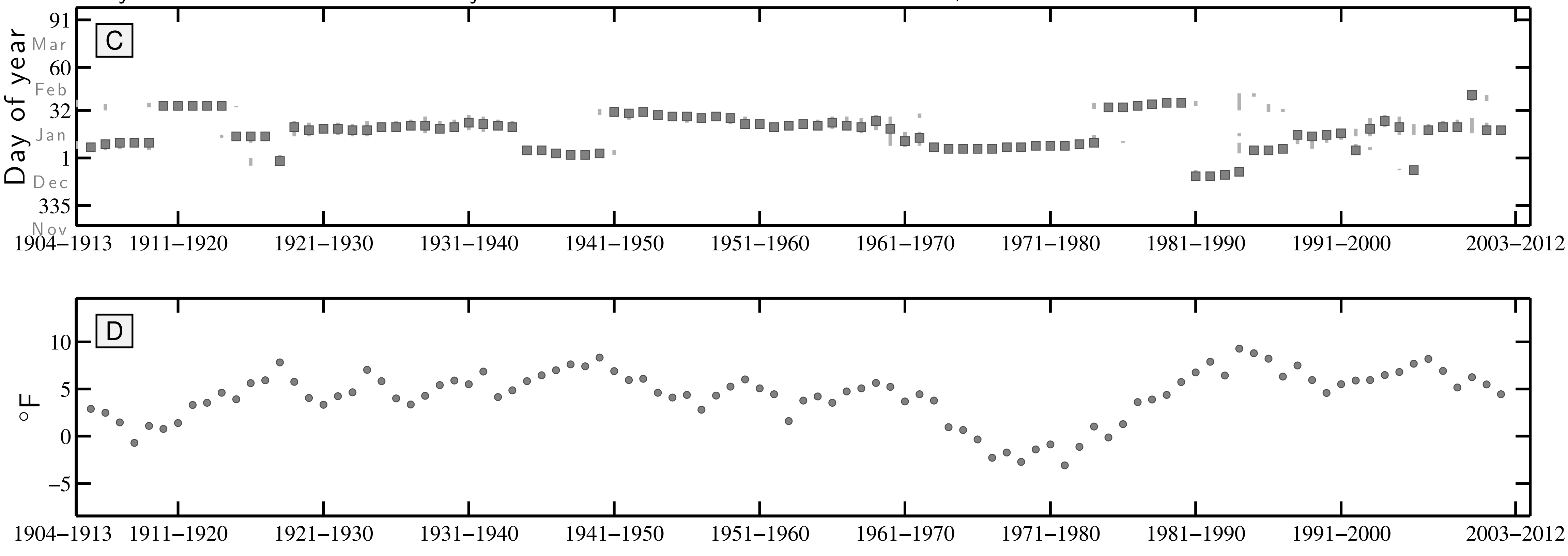

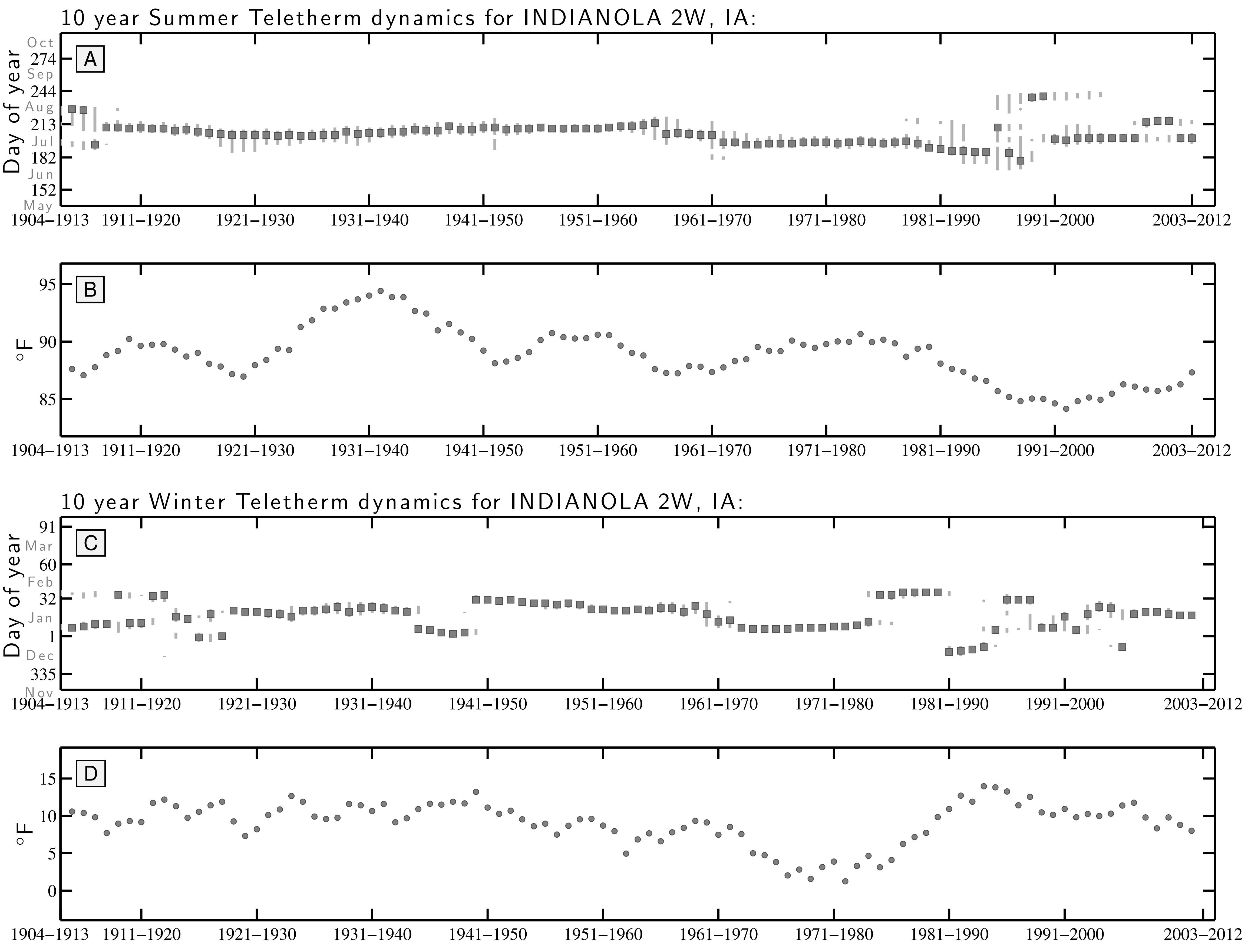
10 year Summer Teletherm dynamics for INDIANOLA 2W, IA:
A
Day of year
Oct
274
Sep
244
Aug
213
Jul
182
Jun
152
May
1904–1913
1911–1920
1921–1930
1931–1940
1941–1950
1951–1960
1961–1970
1971–1980
1981–1990
1991–2000
2003–2012
B
°F
95
90
85
10 year Winter Teletherm dynamics for INDIANOLA 2W, IA:
C
91
Mar
60
Feb
32
Jan
1
Dec
335
Nov
D
15
10
5
0

10 year Summer Teletherm dynamics for IOWA FALLS, IA:

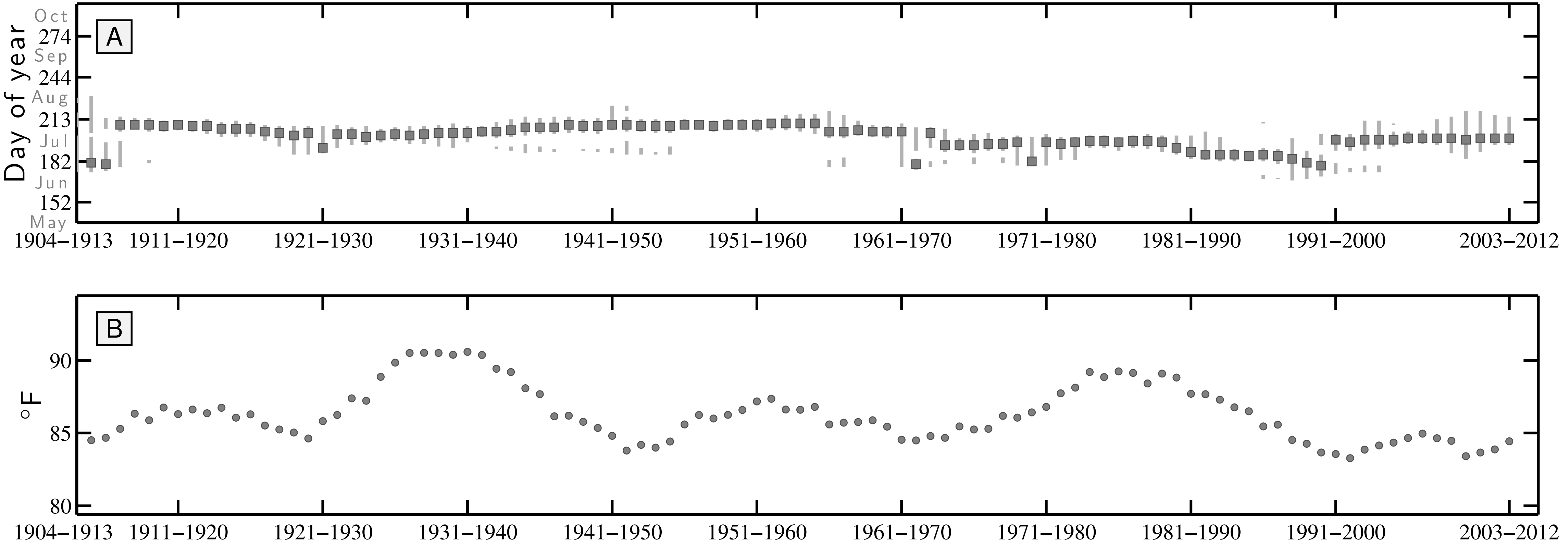

10 year Winter Teletherm dynamics for IOWA FALLS, IA:

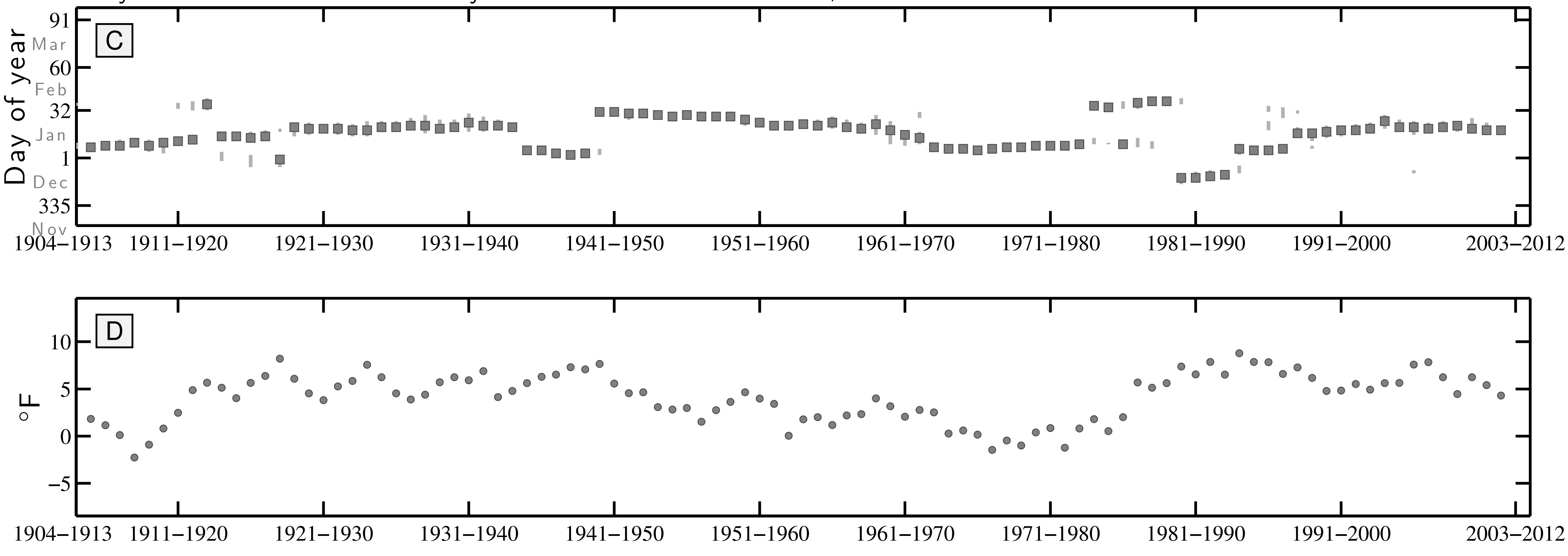

10 year Summer Teletherm dynamics for LE MARS, IA:

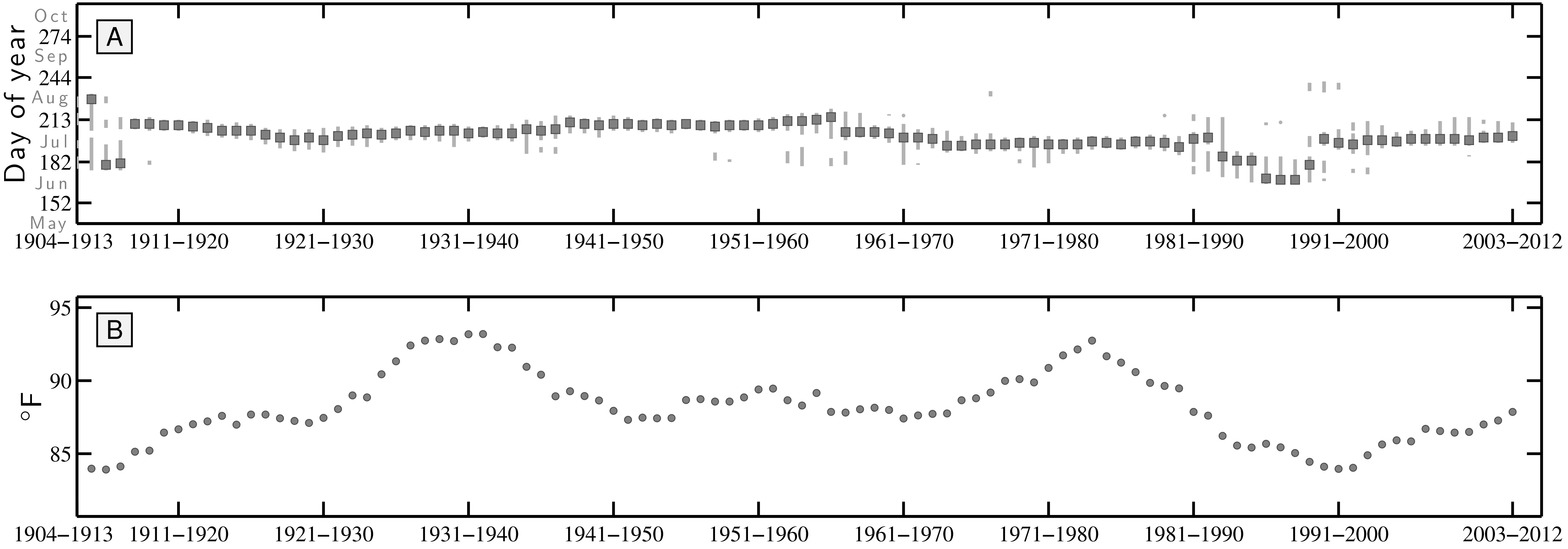

10 year Winter Teletherm dynamics for LE MARS, IA:

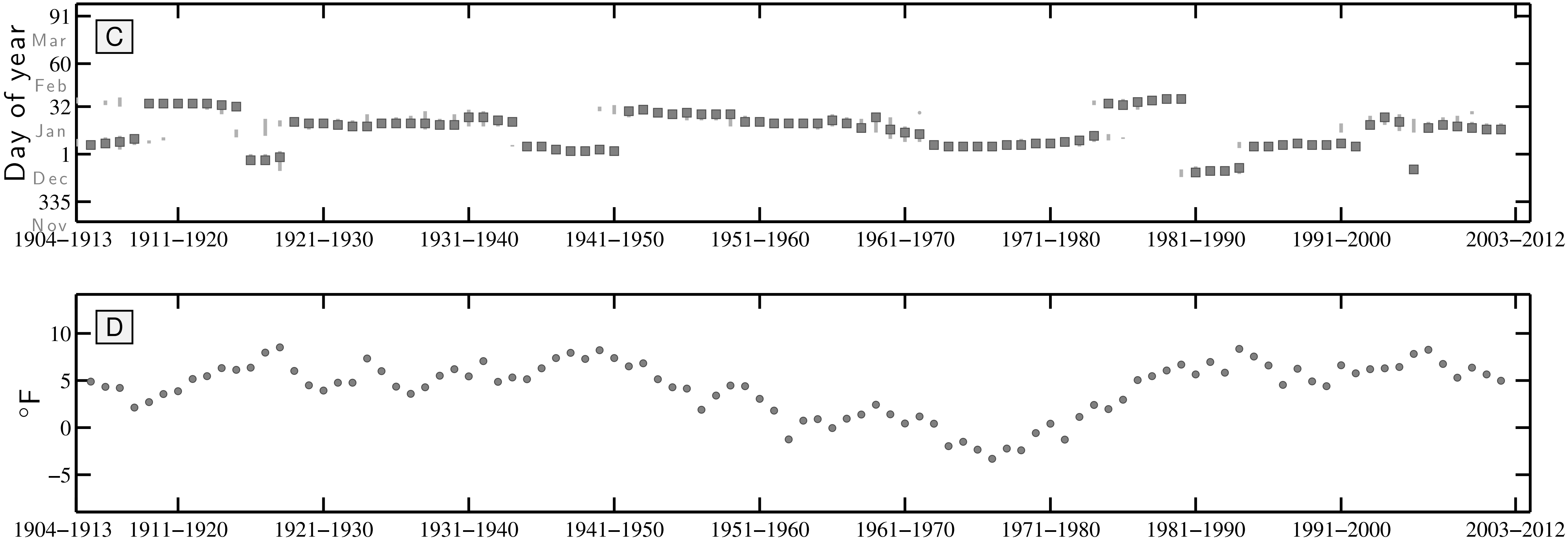

10 year Summer Teletherm dynamics for LOGAN, IA:

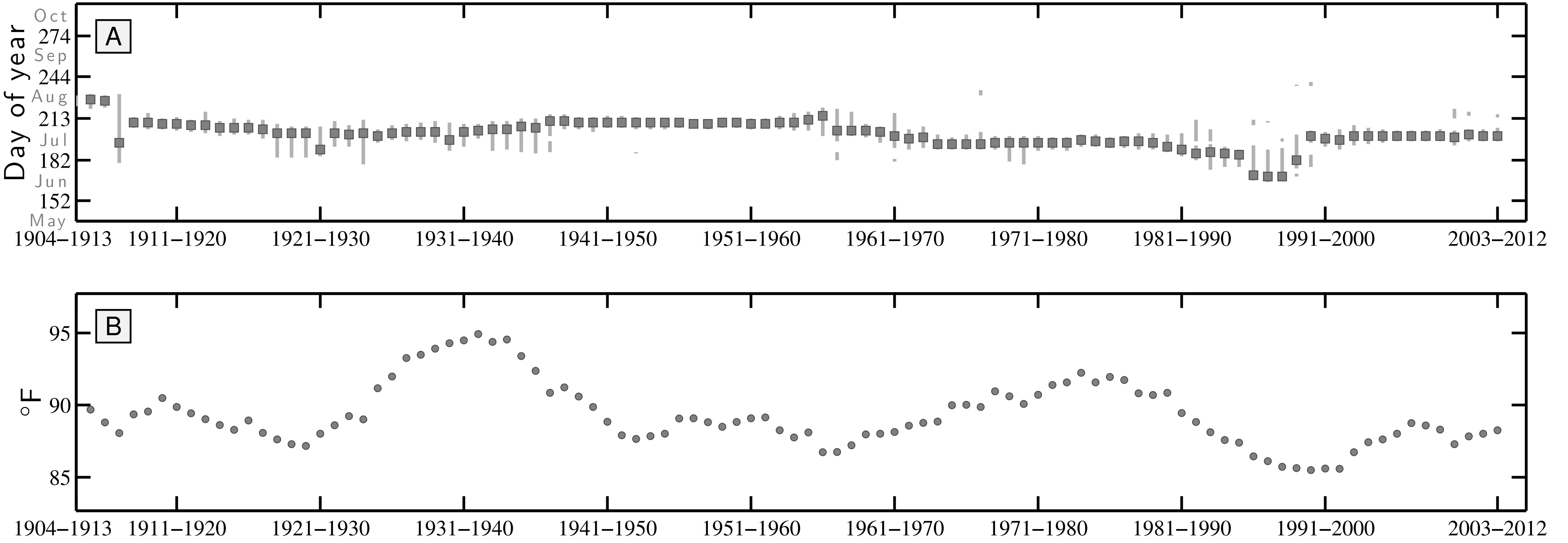

10 year Winter Teletherm dynamics for LOGAN, IA:

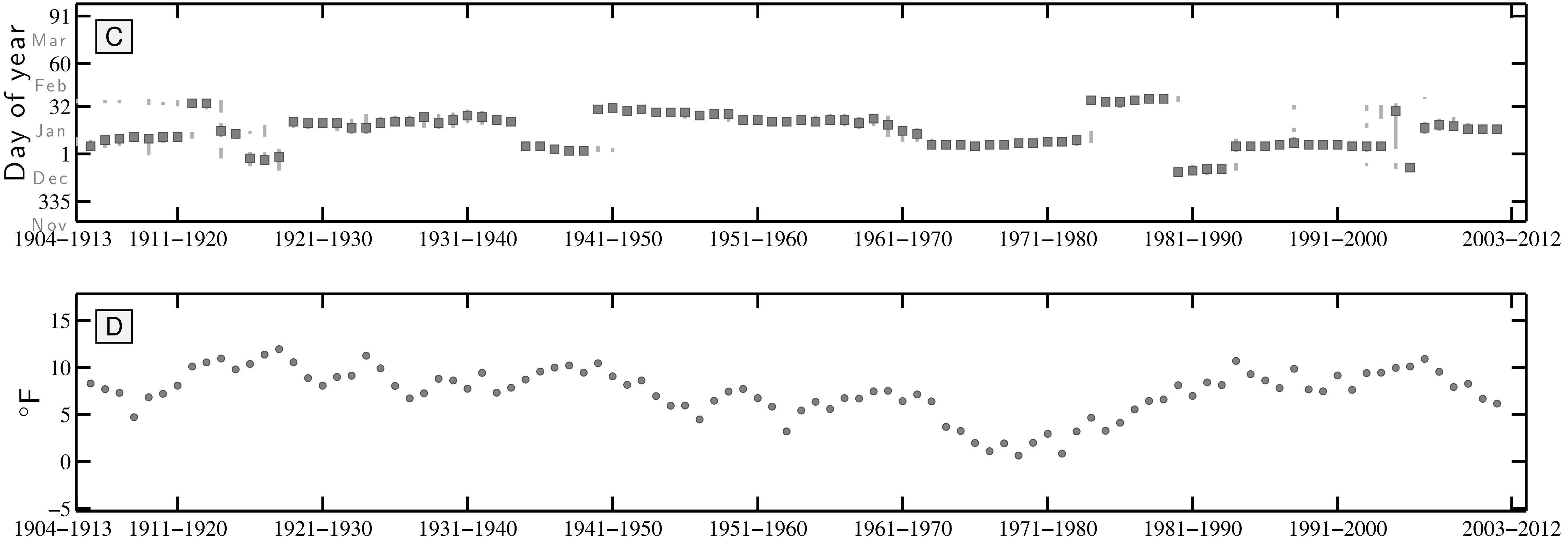

10 year Summer Teletherm dynamics for MT AYR, IA:

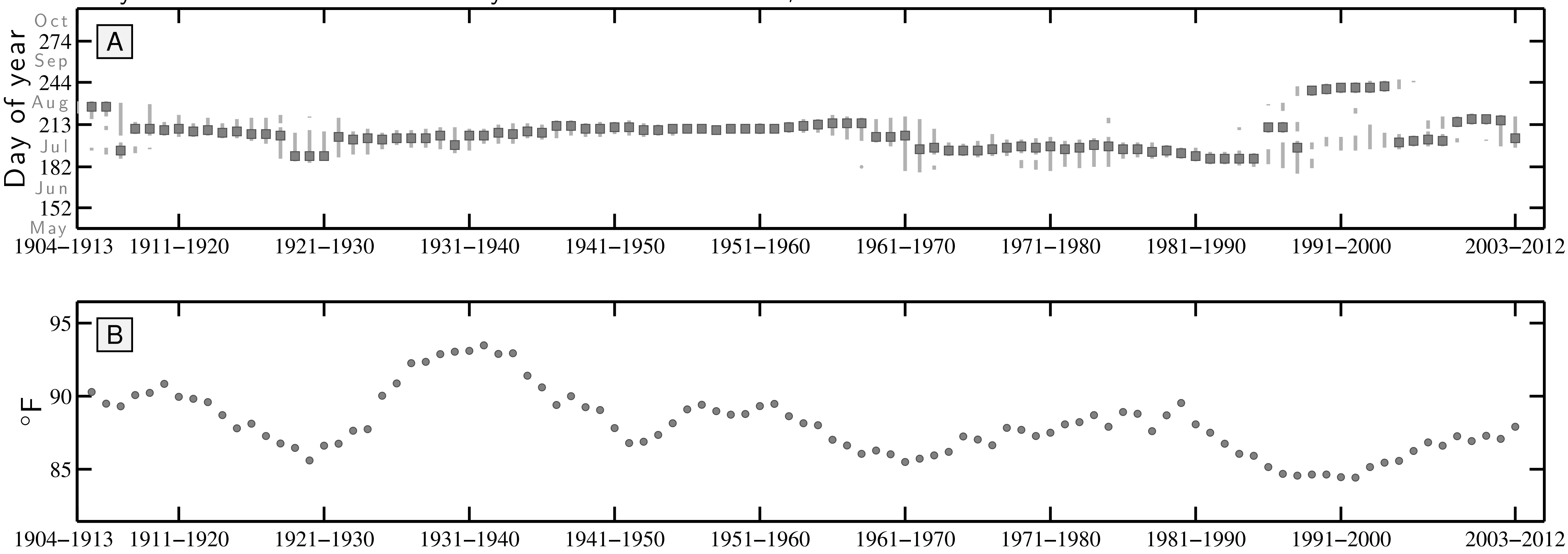

10 year Winter Teletherm dynamics for MT AYR, IA:

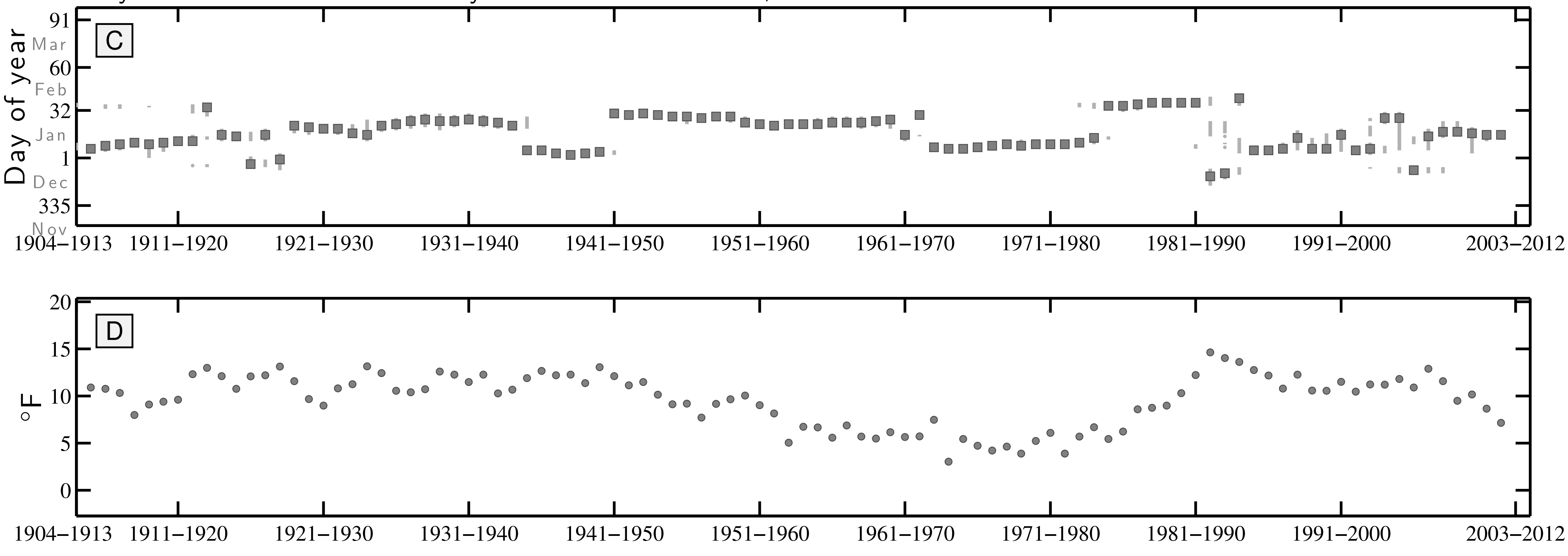

10 year Summer Teletherm dynamics for MT PLEASANT 1 SSW, IA:

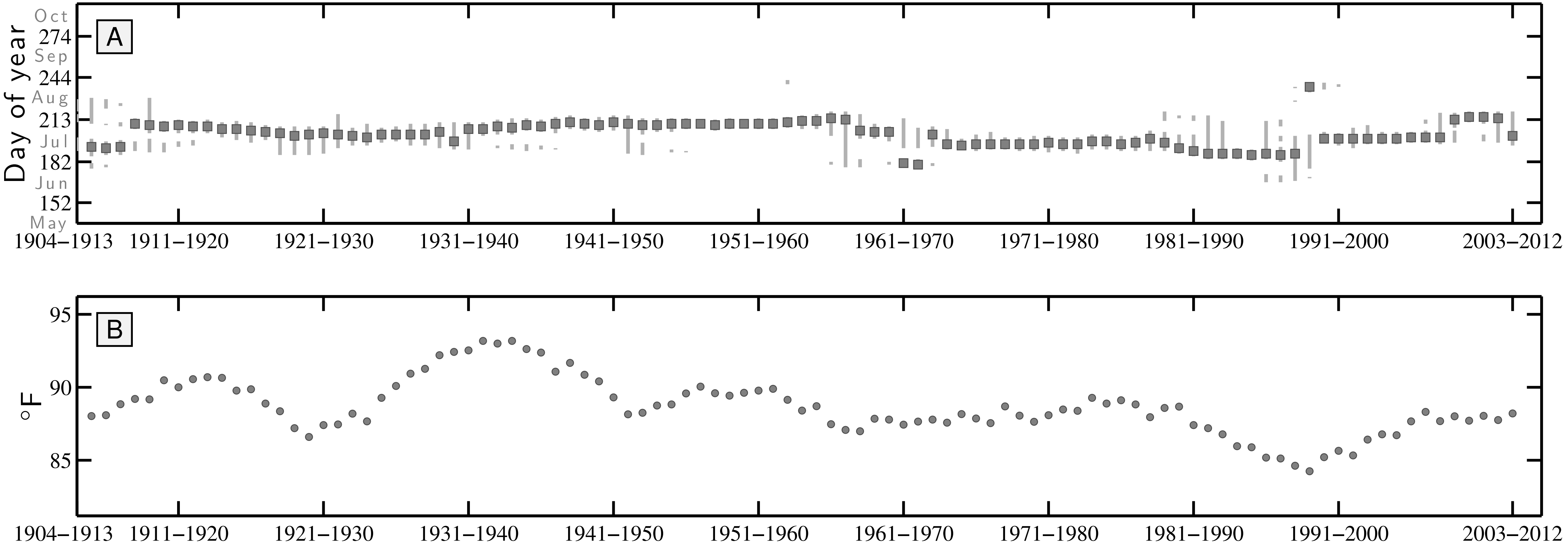

10 year Winter Teletherm dynamics for MT PLEASANT 1 SSW, IA:

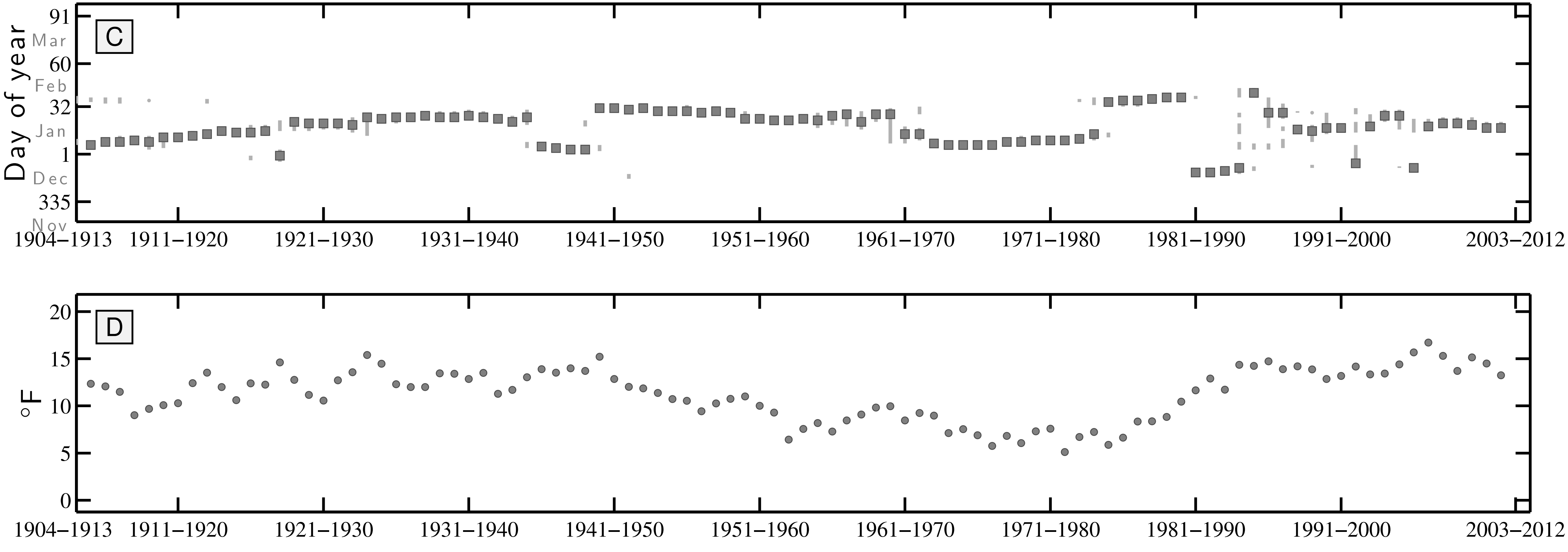

10 year Summer Teletherm dynamics for NEW HAMPTON, IA:

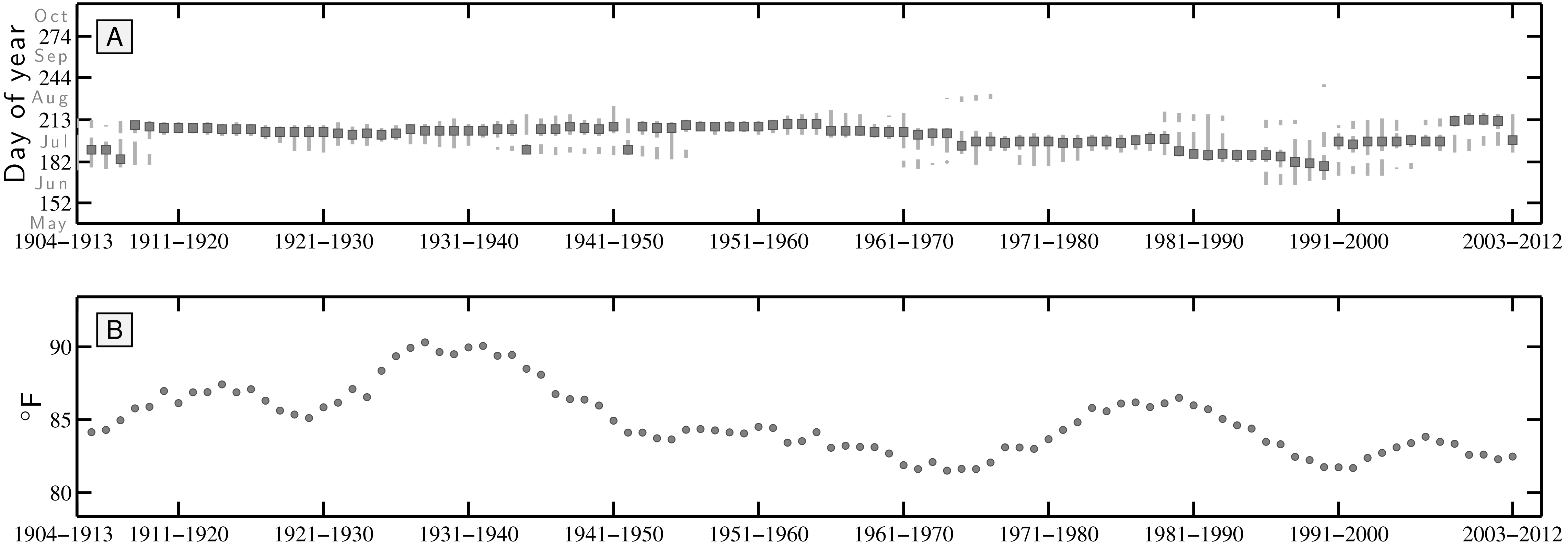

10 year Winter Teletherm dynamics for NEW HAMPTON, IA:

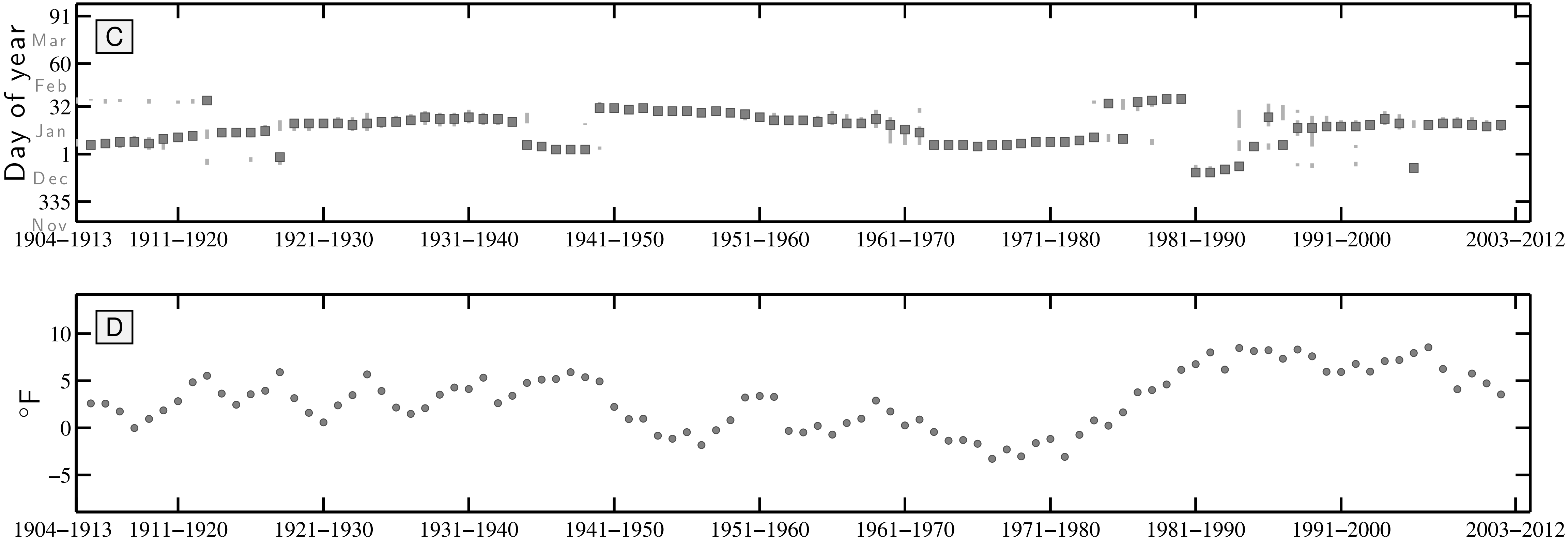

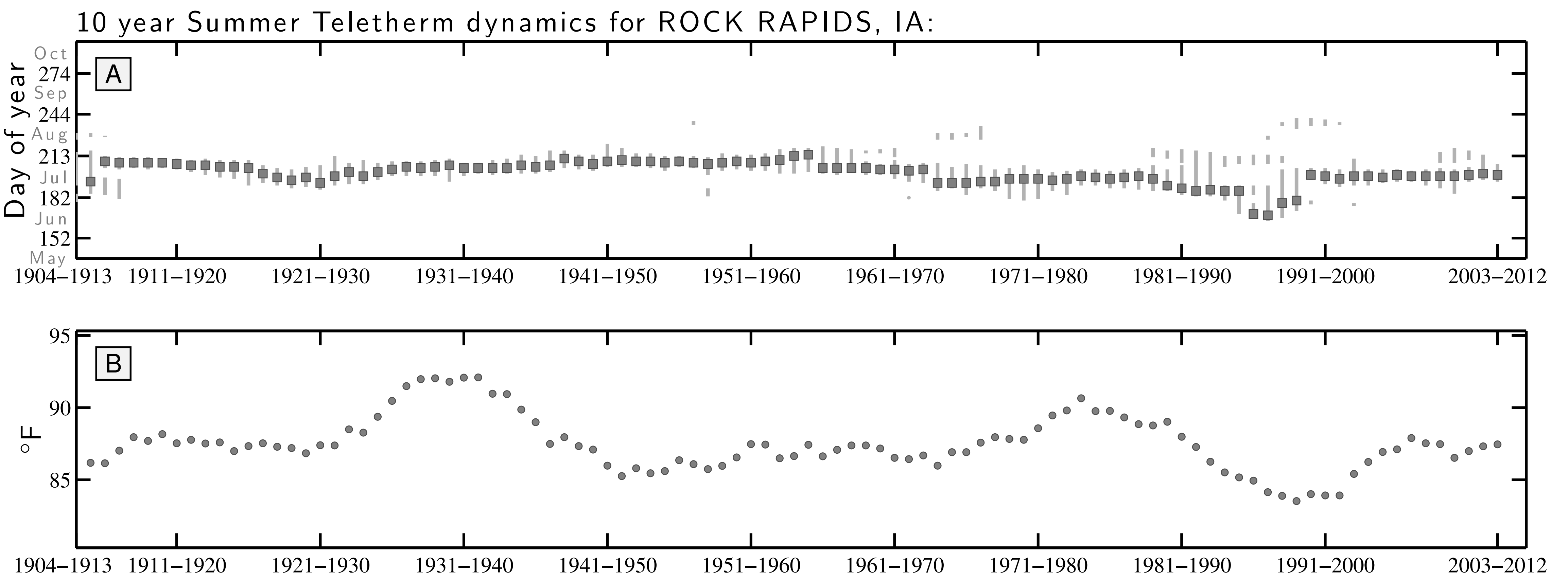
10 year Summer Teletherm dynamics for ROCK RAPIDS, IA:
A
B
Day of year
°F
Oct
274
Sep
244
Aug
213
Jul
182
Jun
152
May
95
90
85
1904–1913
1911–1920
1921–1930
1931–1940
1941–1950
1951–1960
1961–1970
1971–1980
1981–1990
1991–2000
2003–2012

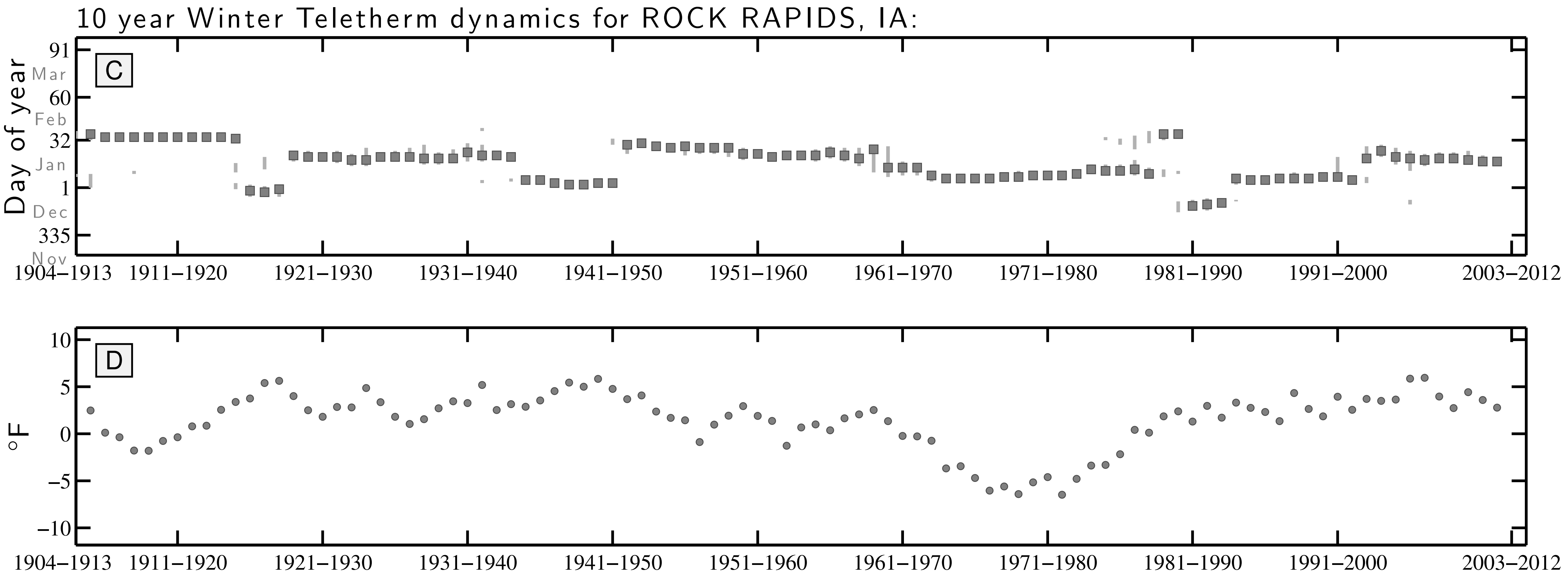
10 year Winter Teletherm dynamics for ROCK RAPIDS, IA:
C
D
Day of year
°F
91
Mar
60
Feb
32
Jan
1
Dec
335
Nov
10
5
0
−5
−10
1904–1913
1911–1920
1921–1930
1931–1940
1941–1950
1951–1960
1961–1970
1971–1980
1981–1990
1991–2000
2003–2012

10 year Summer Teletherm dynamics for ROCKWELL CITY, IA:

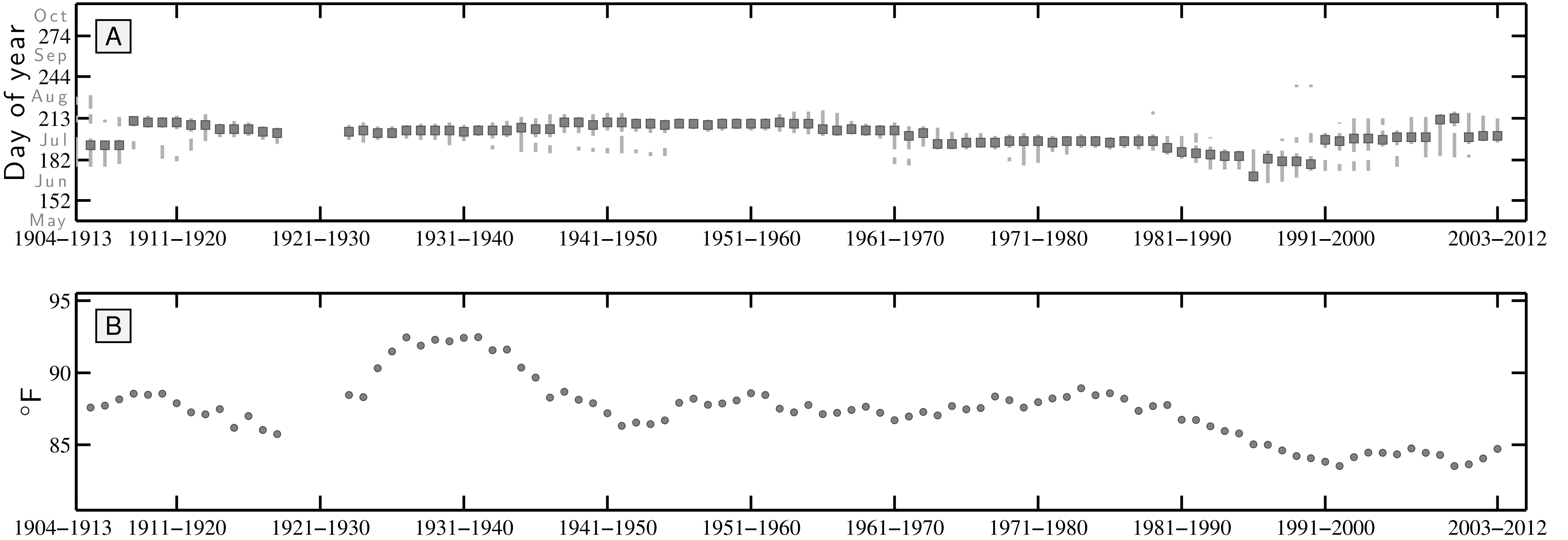

10 year Winter Teletherm dynamics for ROCKWELL CITY, IA:

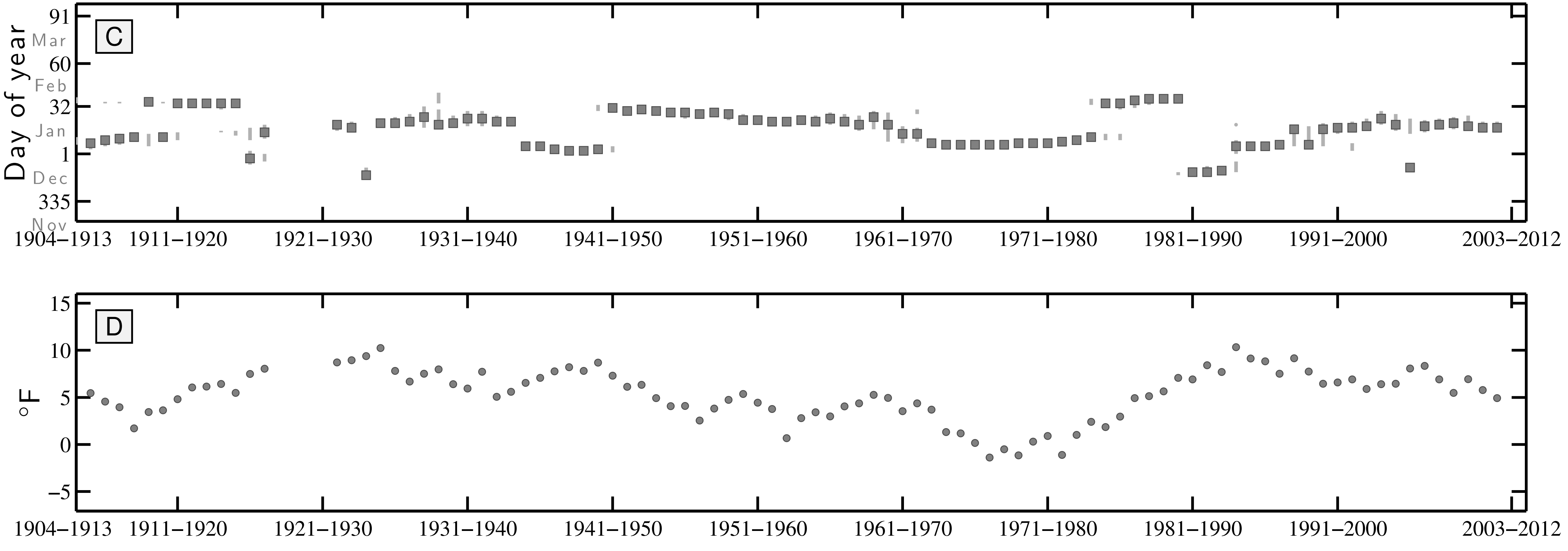

10 year Summer Teletherm dynamics for STORM LAKE 2 E, IA:

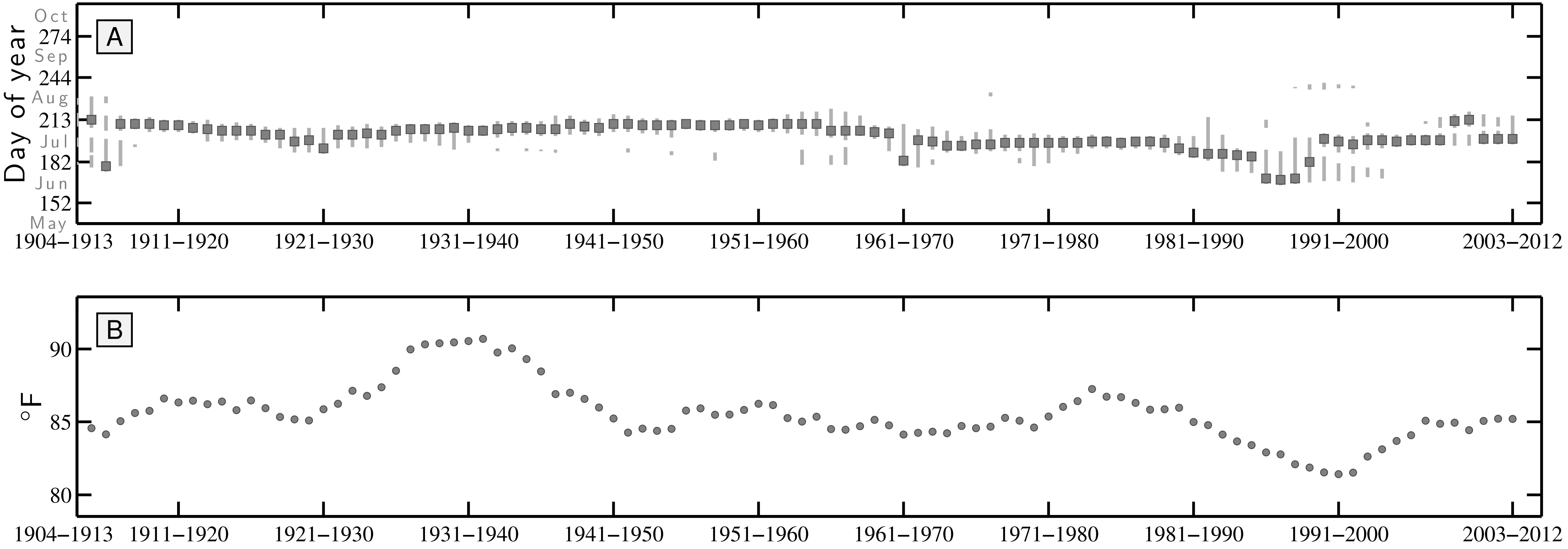

10 year Winter Teletherm dynamics for STORM LAKE 2 E, IA:

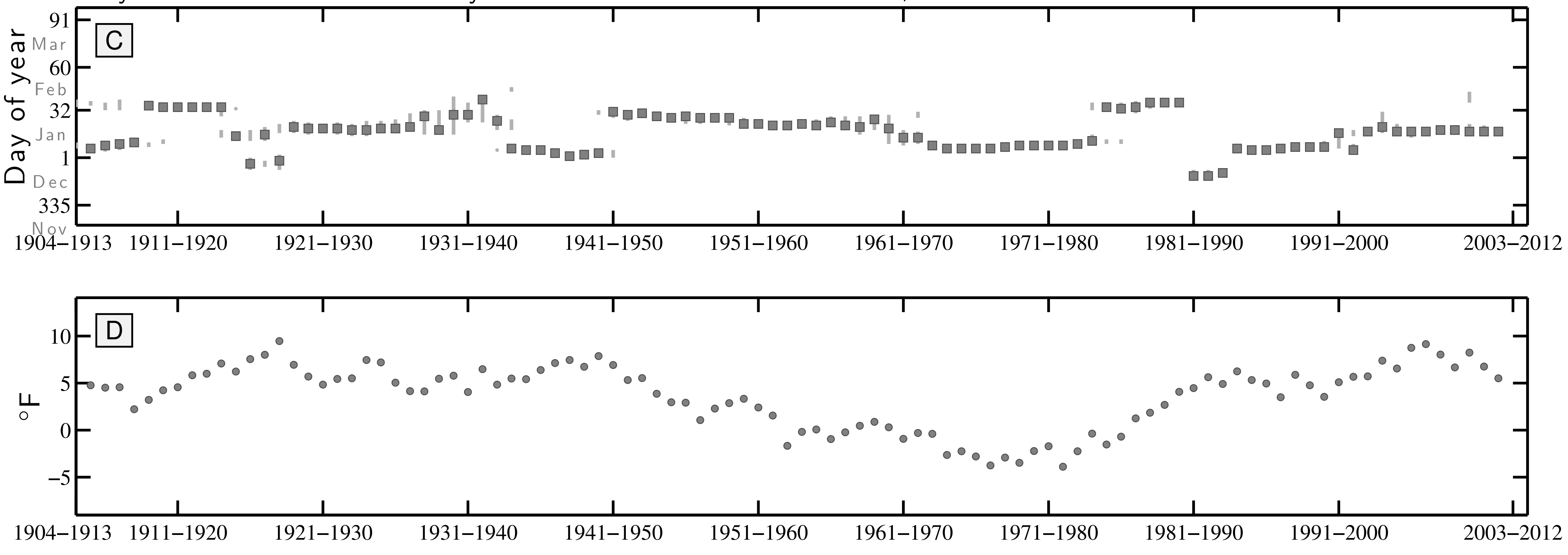

10 year Summer Teletherm dynamics for TOLEDO 3N, IA:

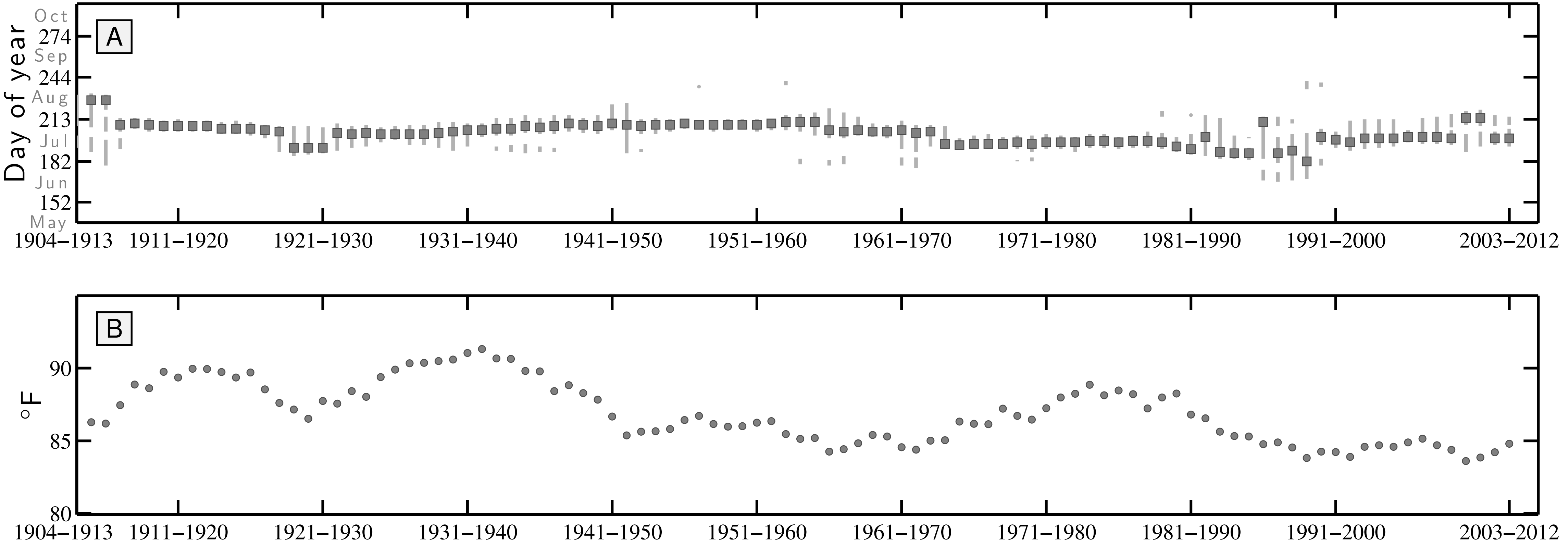

10 year Winter Teletherm dynamics for TOLEDO 3N, IA:

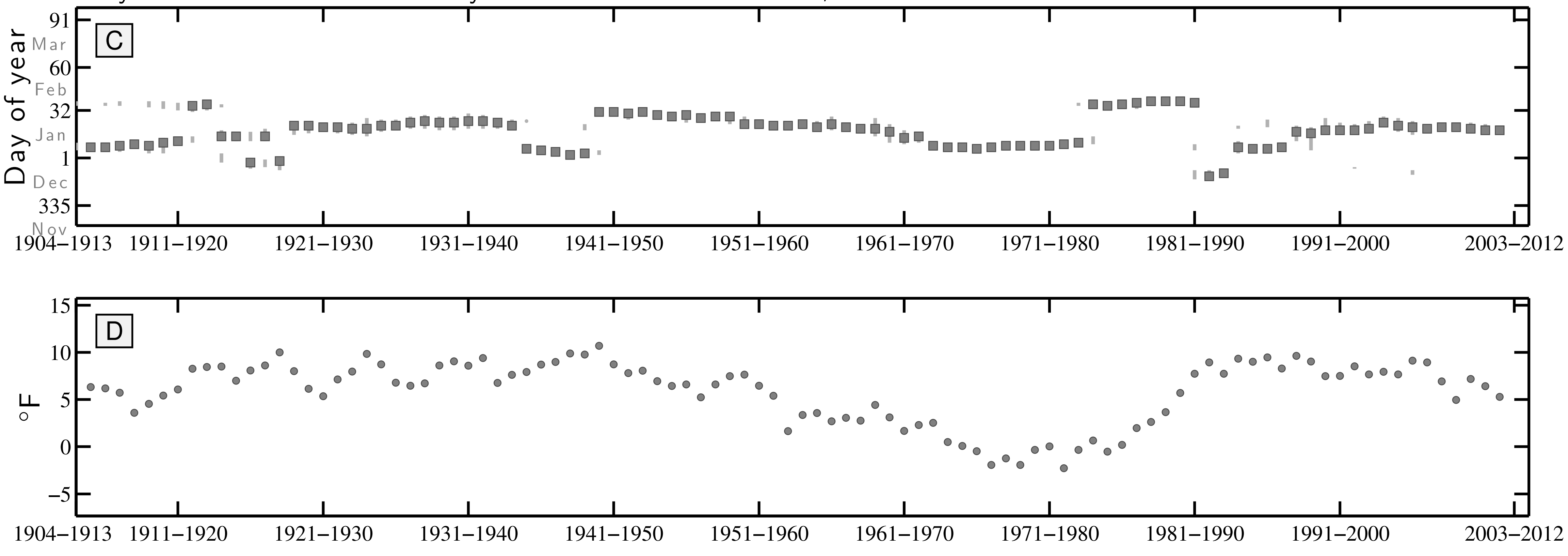

10 year Summer Teletherm dynamics for WASHINGTON, IA:

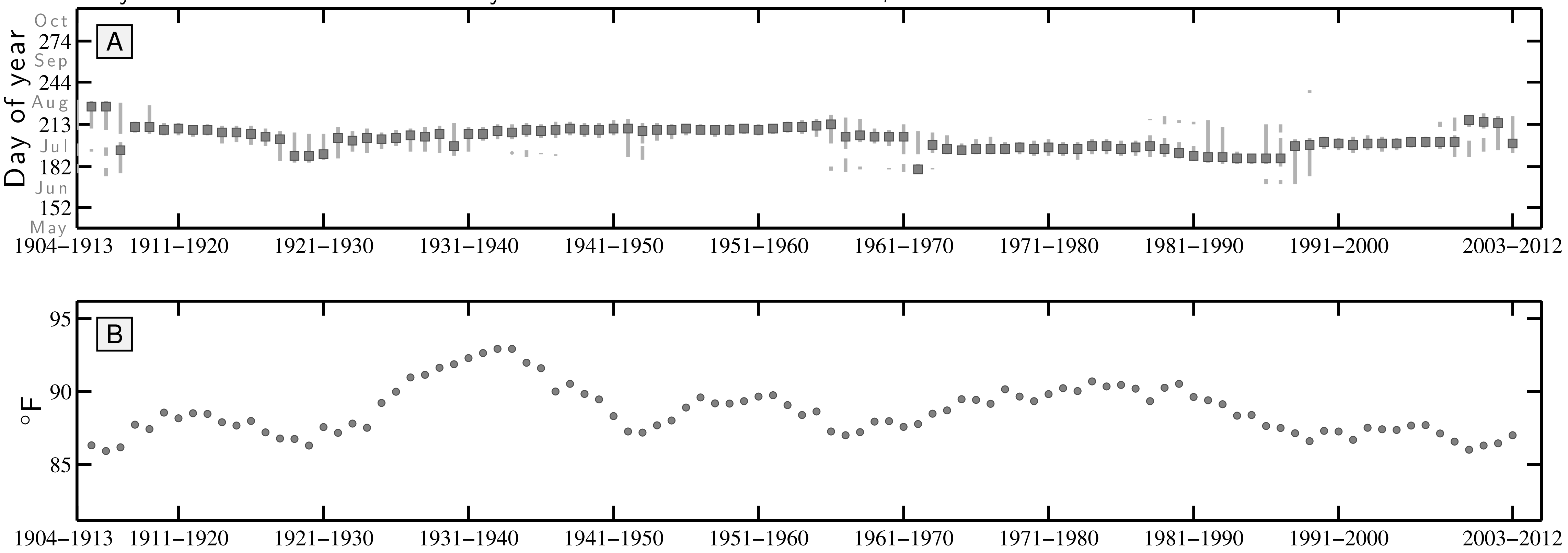

10 year Winter Teletherm dynamics for WASHINGTON, IA:

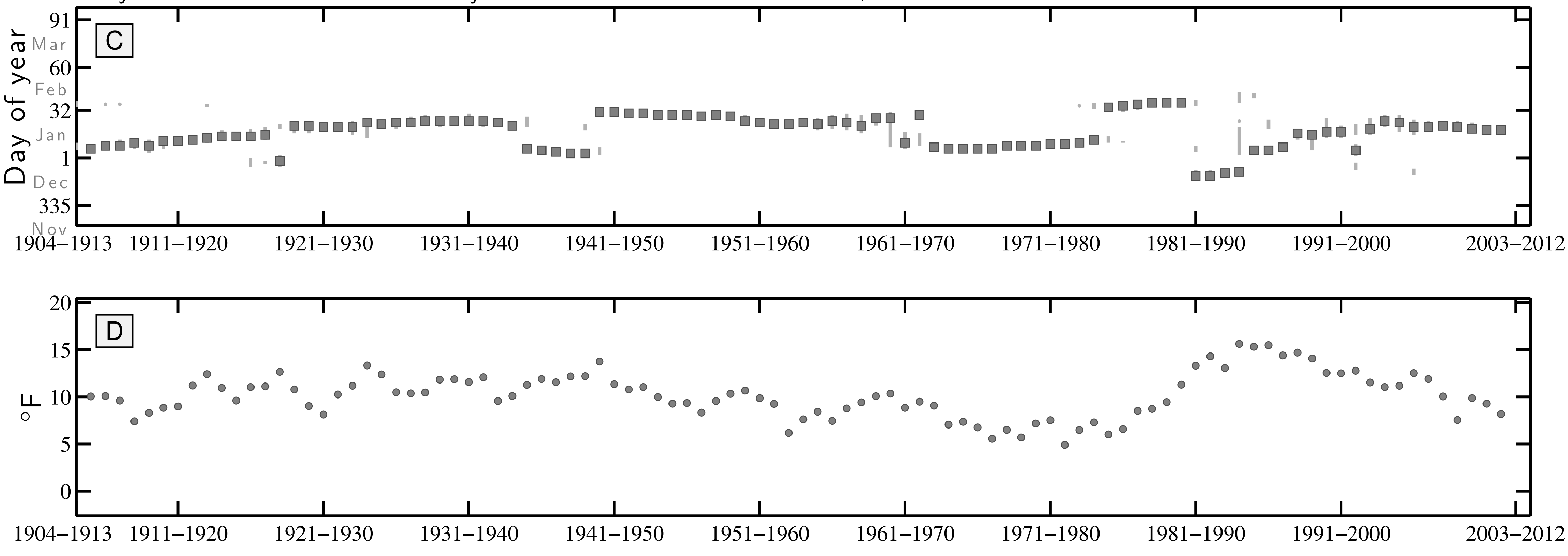

10 year Summer Teletherm dynamics for ANTHONY, KS:

10 year Winter Teletherm dynamics for ANTHONY, KS:

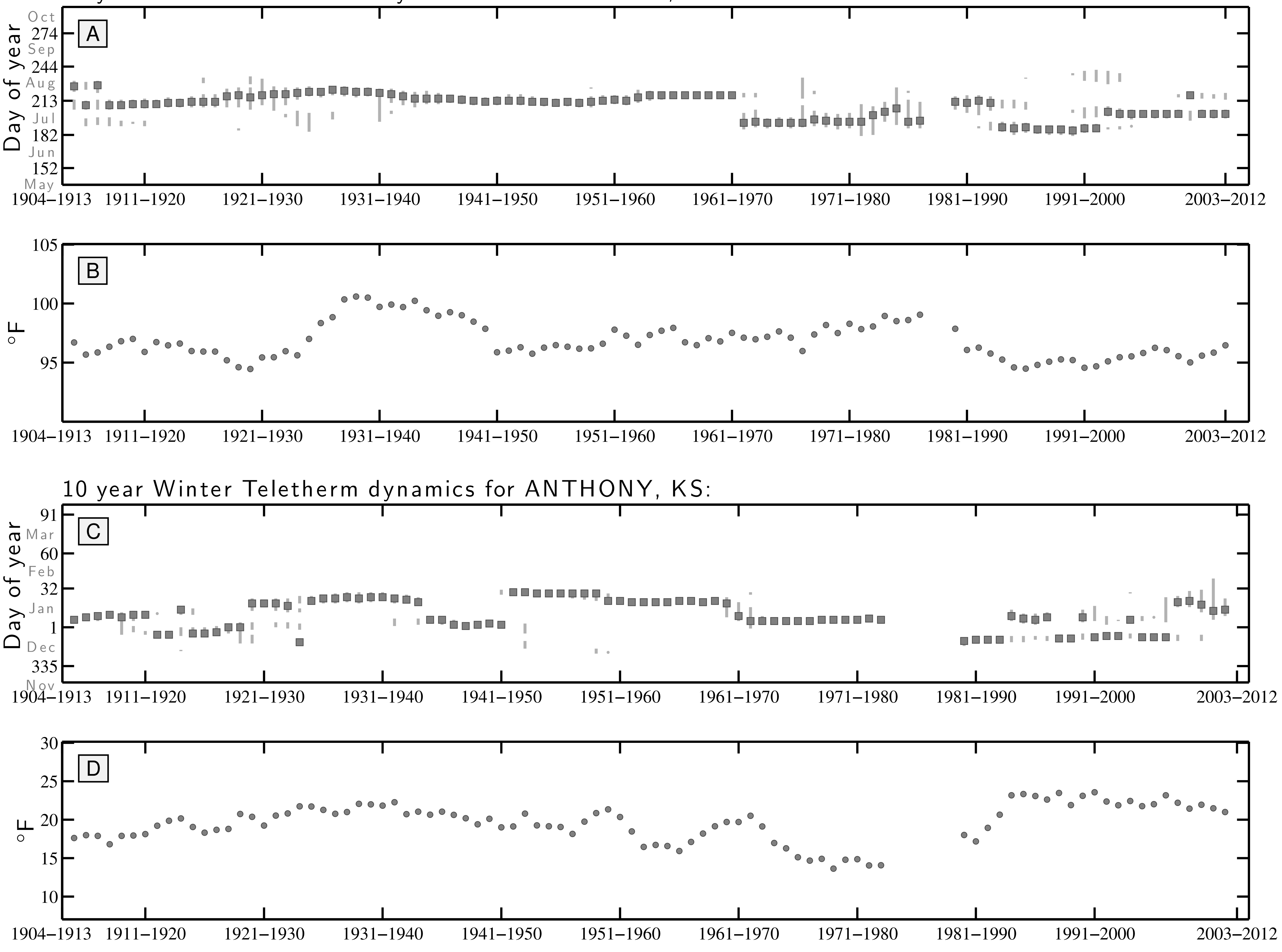

10 year Summer Teletherm dynamics for ASHLAND, KS:

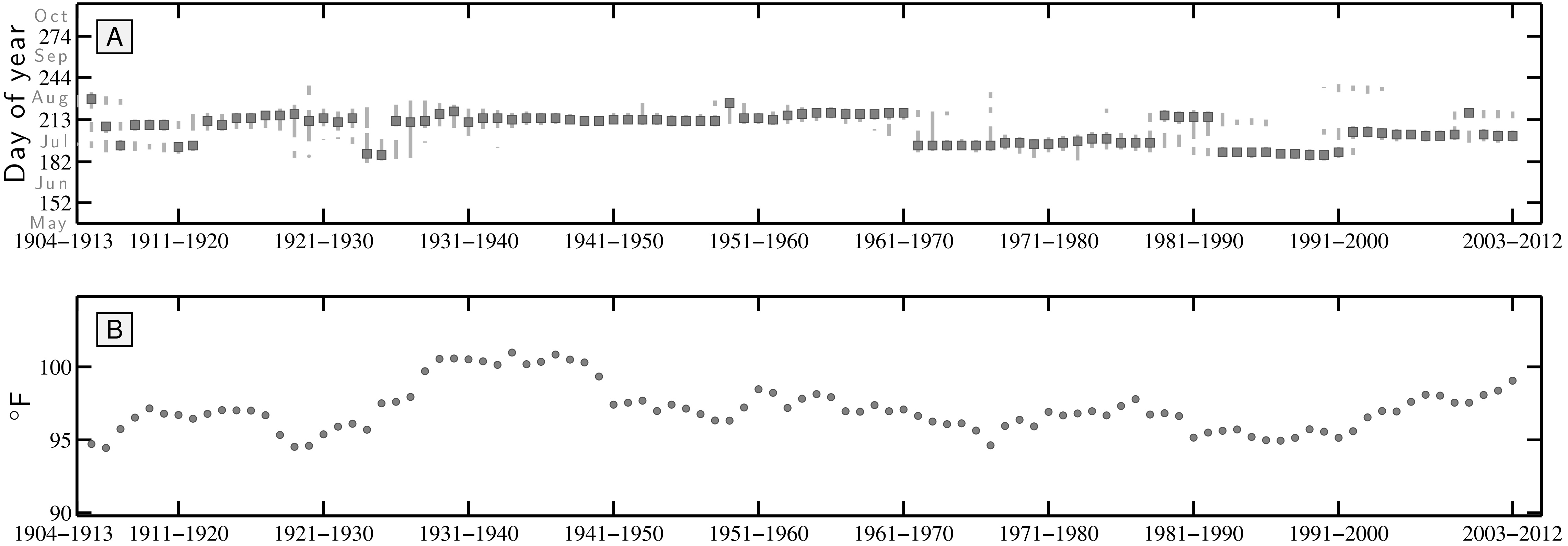

10 year Winter Teletherm dynamics for ASHLAND, KS:

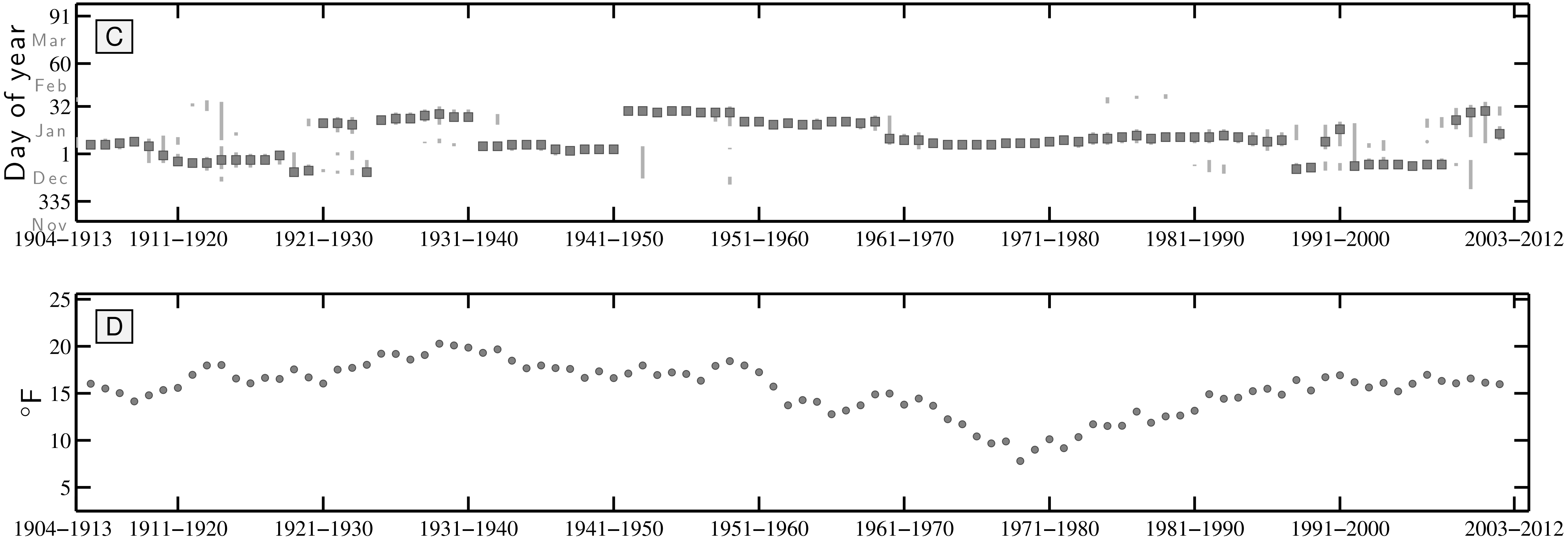

10 year Summer Teletherm dynamics for ATCHISON, KS:

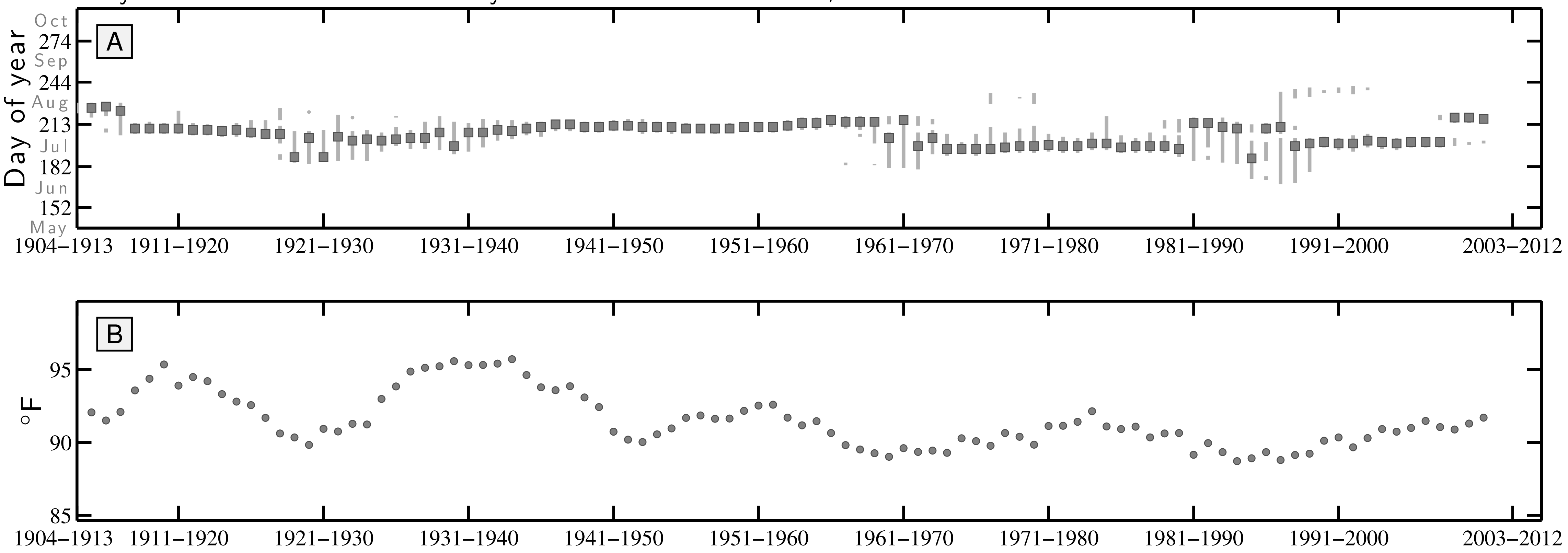

10 year Winter Teletherm dynamics for ATCHISON, KS:

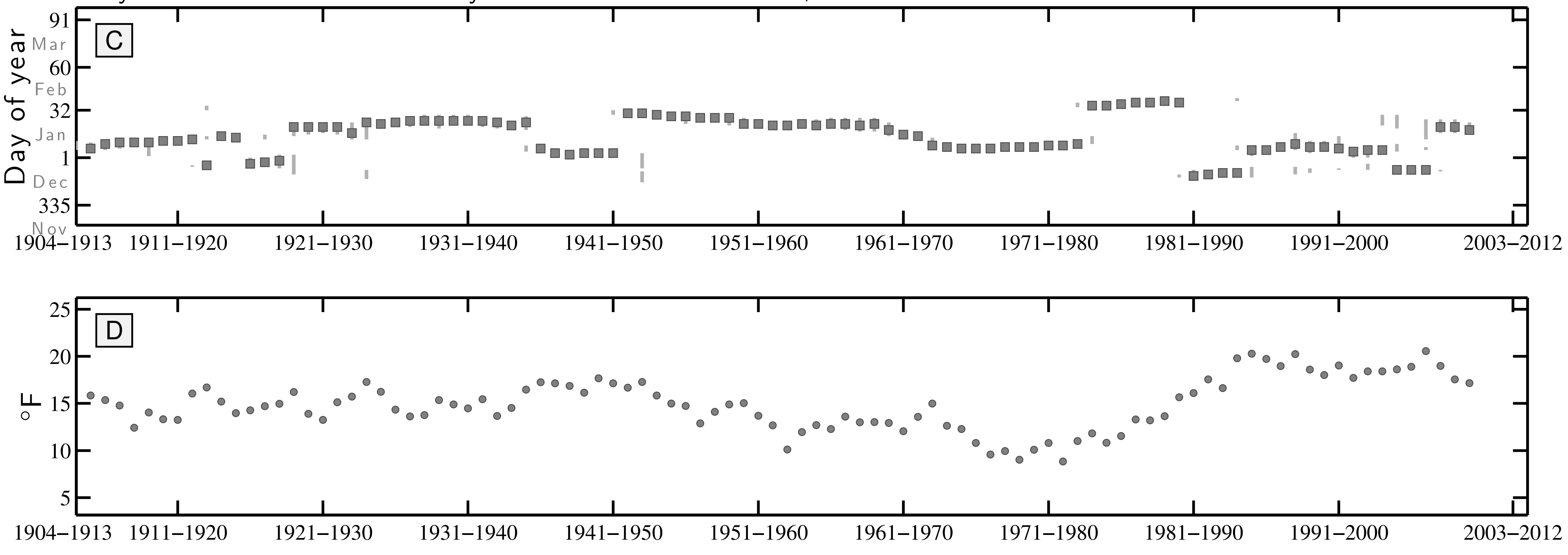

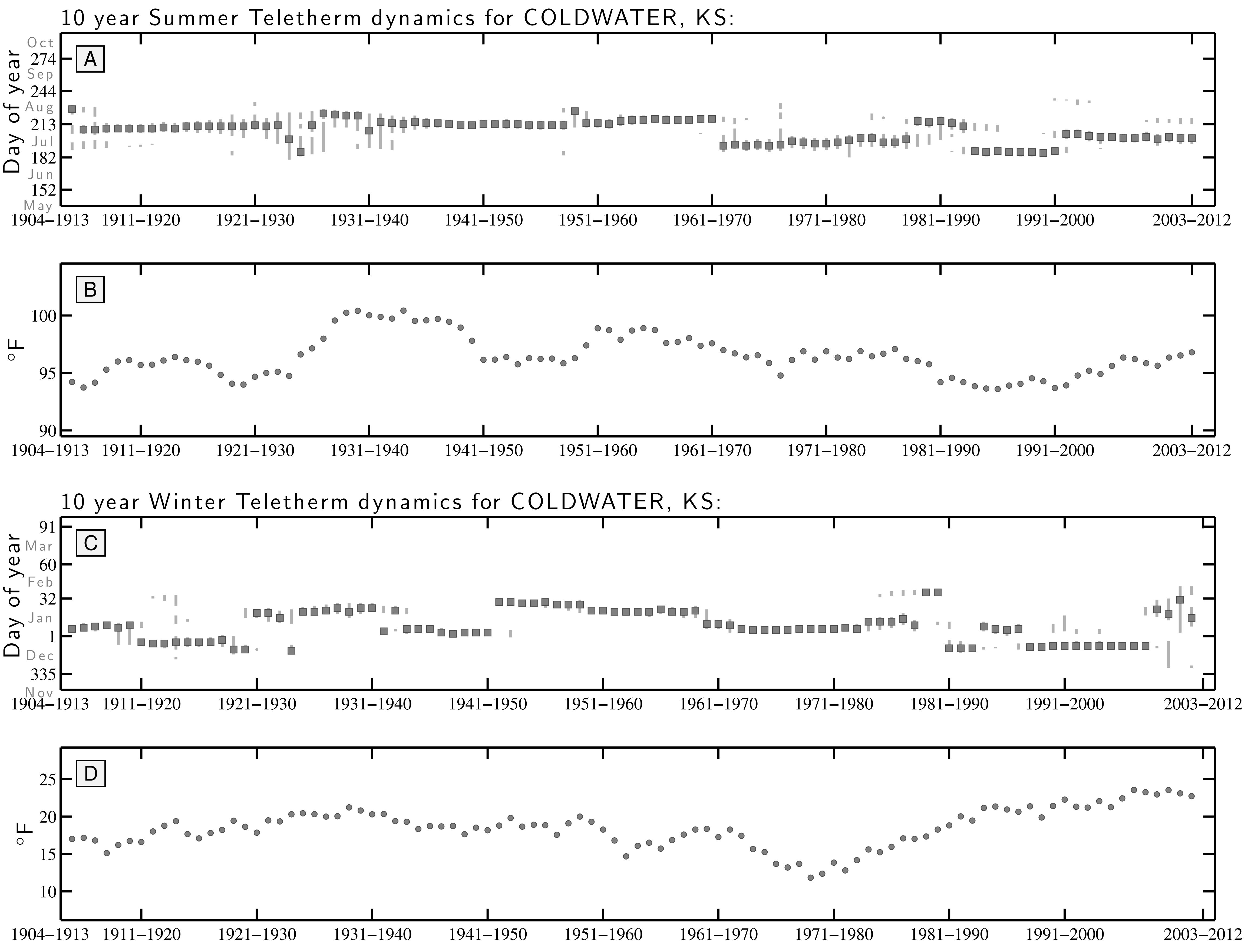
10 year Summer Teletherm dynamics for COLDWATER, KS:
A
B
Day of year
°F
10 year Winter Teletherm dynamics for COLDWATER, KS:
C
D
1904–1913
1911–1920
1921–1930
1931–1940
1941–1950
1951–1960
1961–1970
1971–1980
1981–1990
1991–2000
2003–2012

10 year Summer Teletherm dynamics for COLUMBUS, KS:

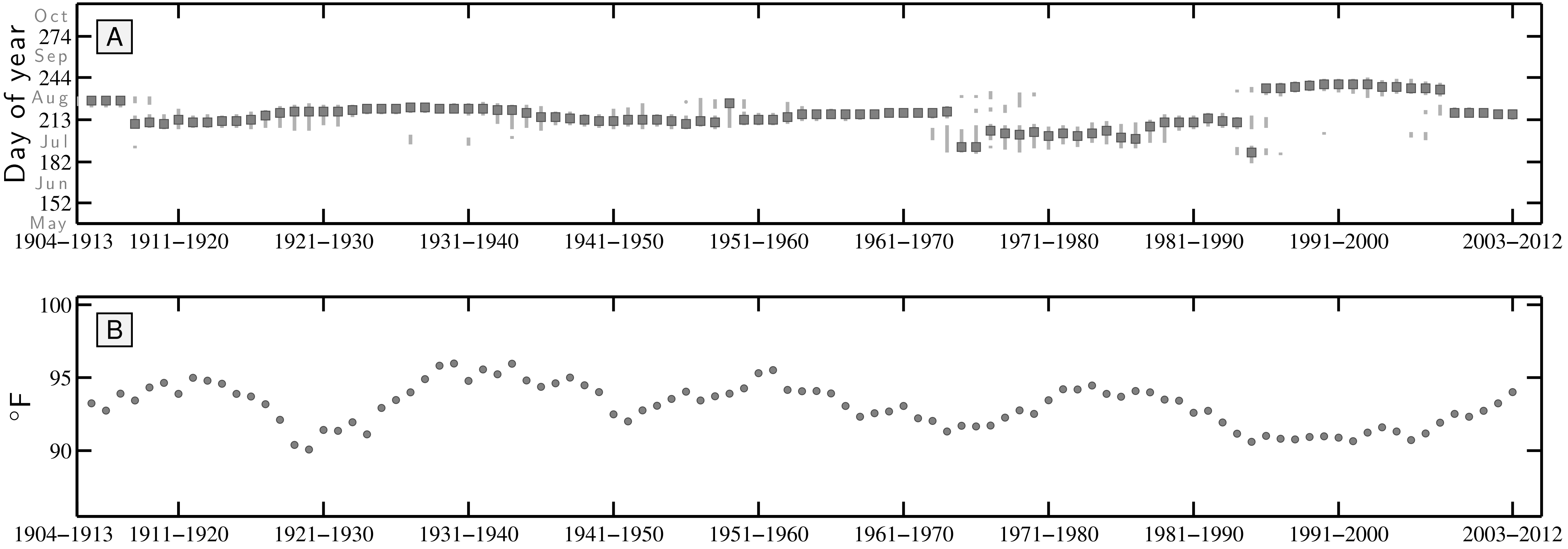

10 year Winter Teletherm dynamics for COLUMBUS, KS:

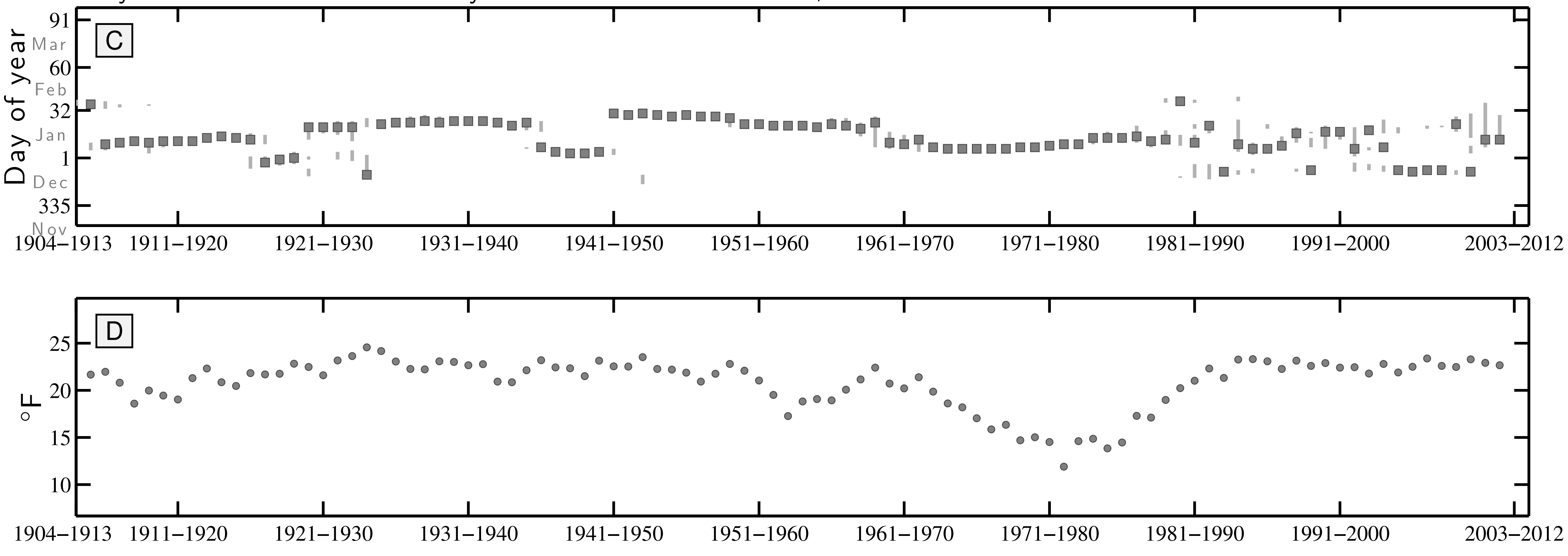

10 year Summer Teletherm dynamics for COUNCIL GROVE LAKE, KS:

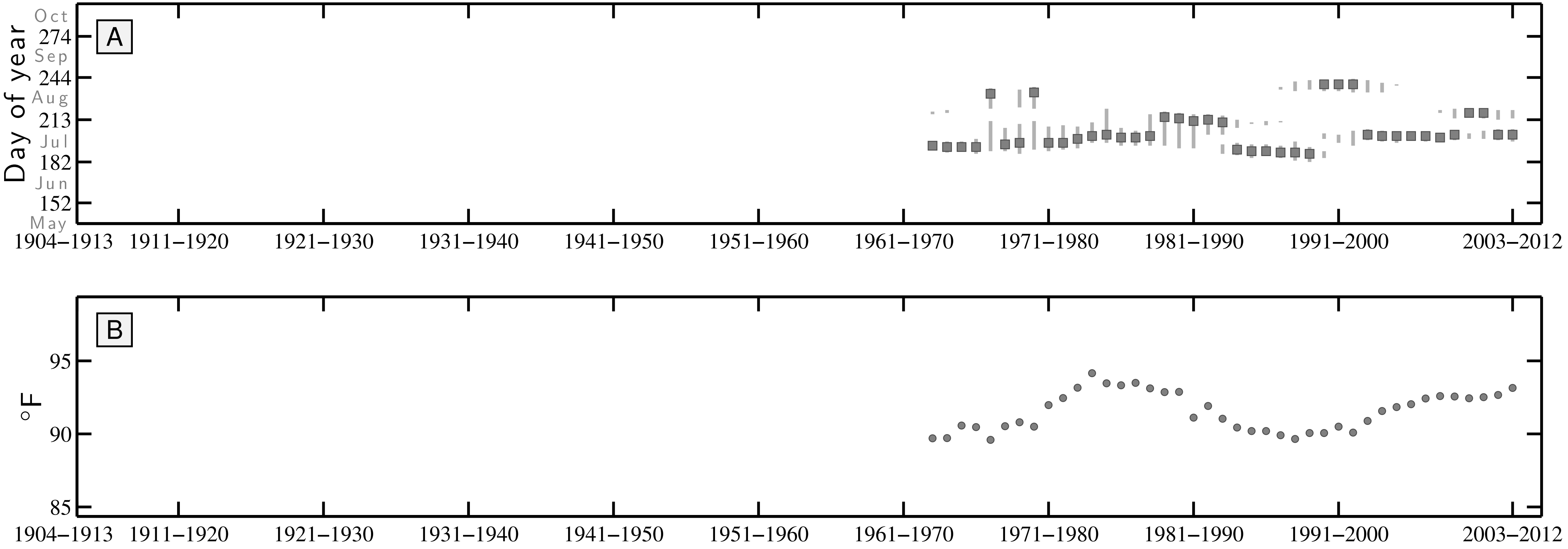

10 year Winter Teletherm dynamics for COUNCIL GROVE LAKE, KS:

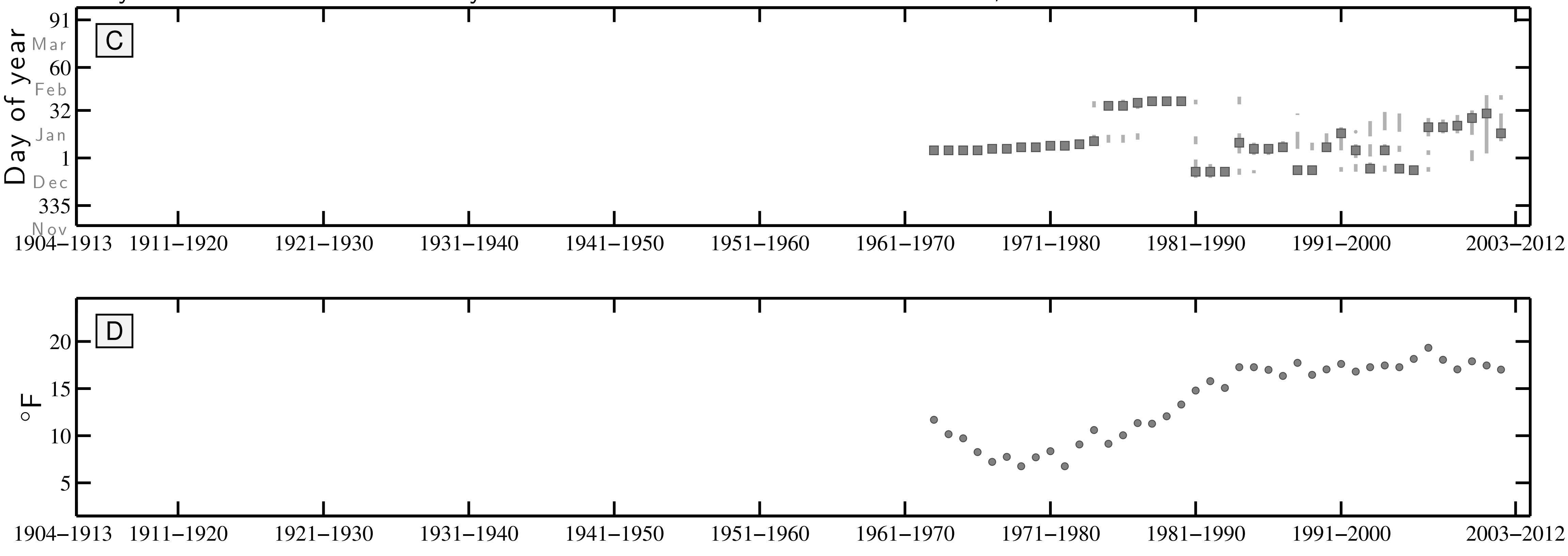

10 year Summer Teletherm dynamics for EL DORADO, KS:

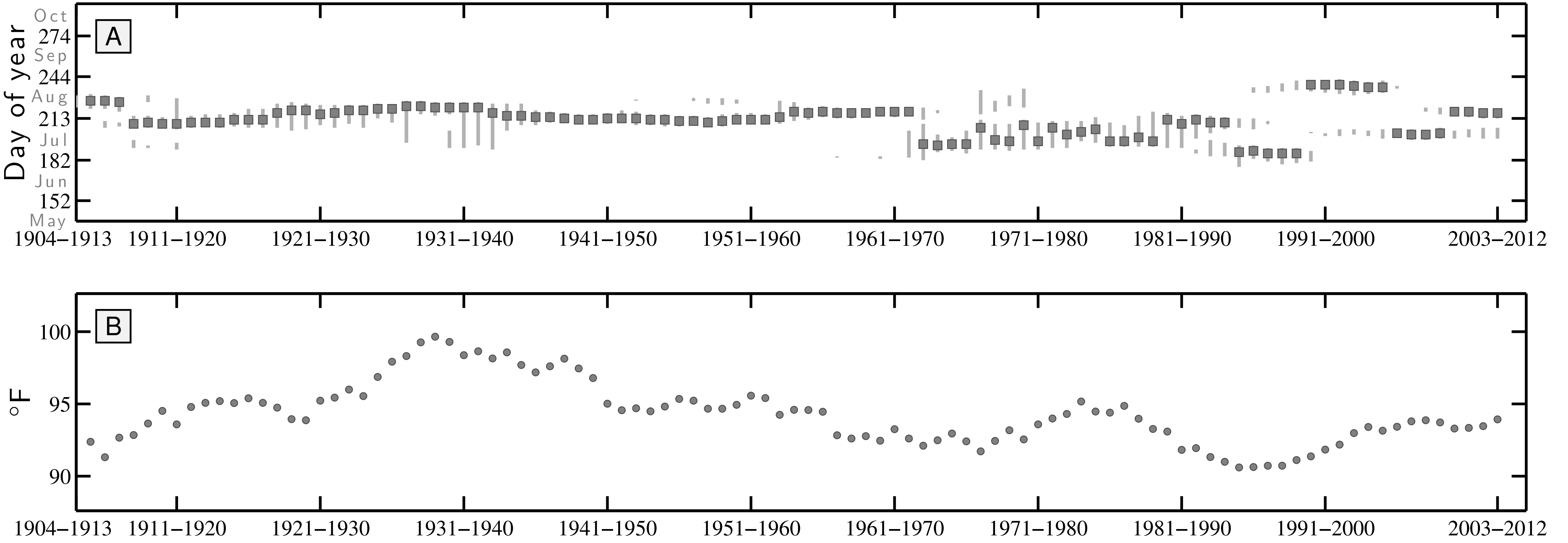

10 year Winter Teletherm dynamics for EL DORADO, KS:

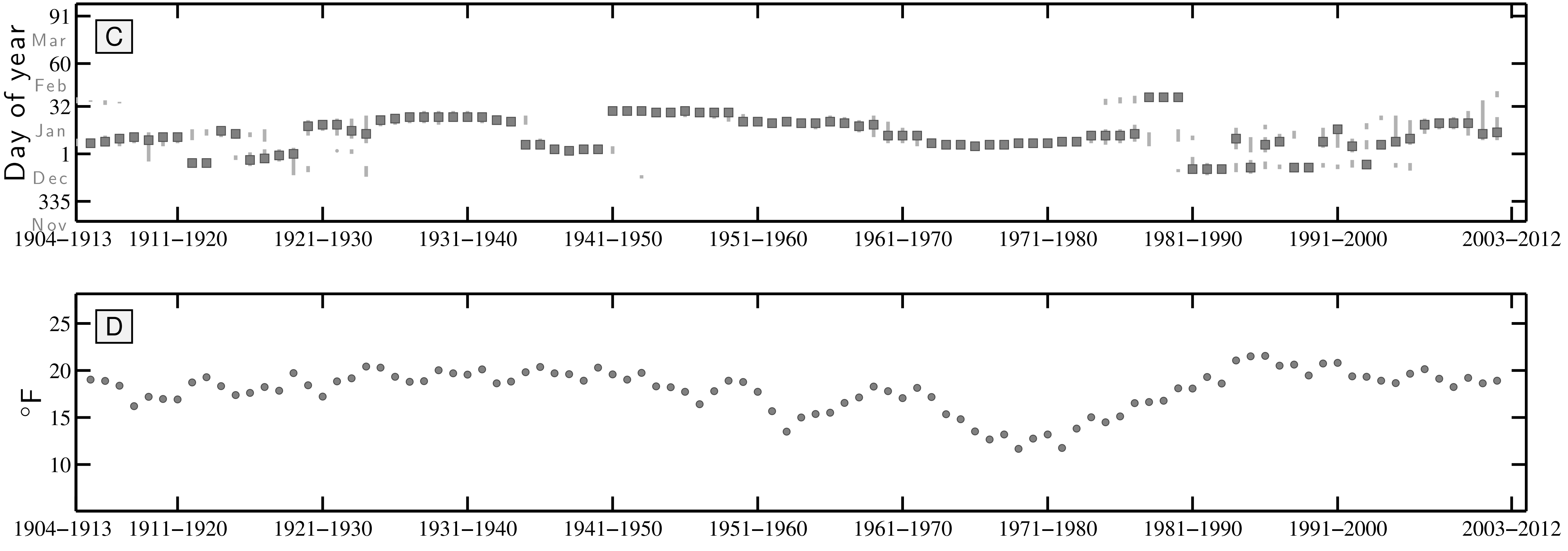

10 year Summer Teletherm dynamics for ELLSWORTH, KS:

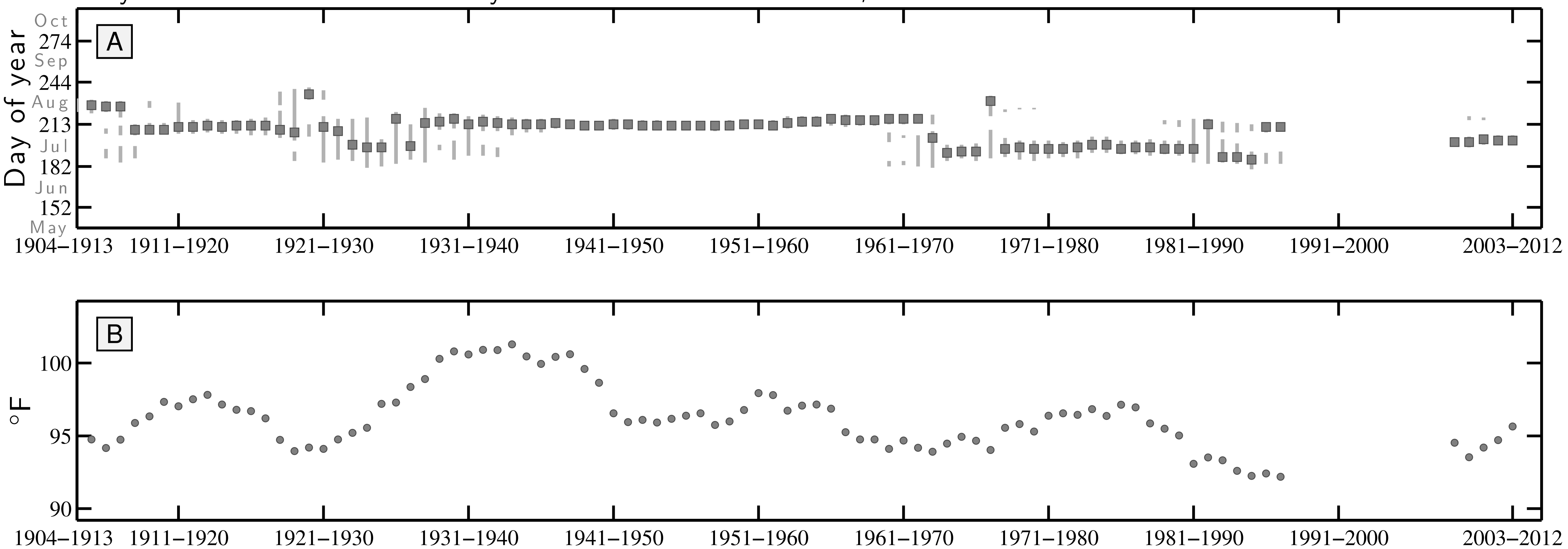

10 year Winter Teletherm dynamics for ELLSWORTH, KS:

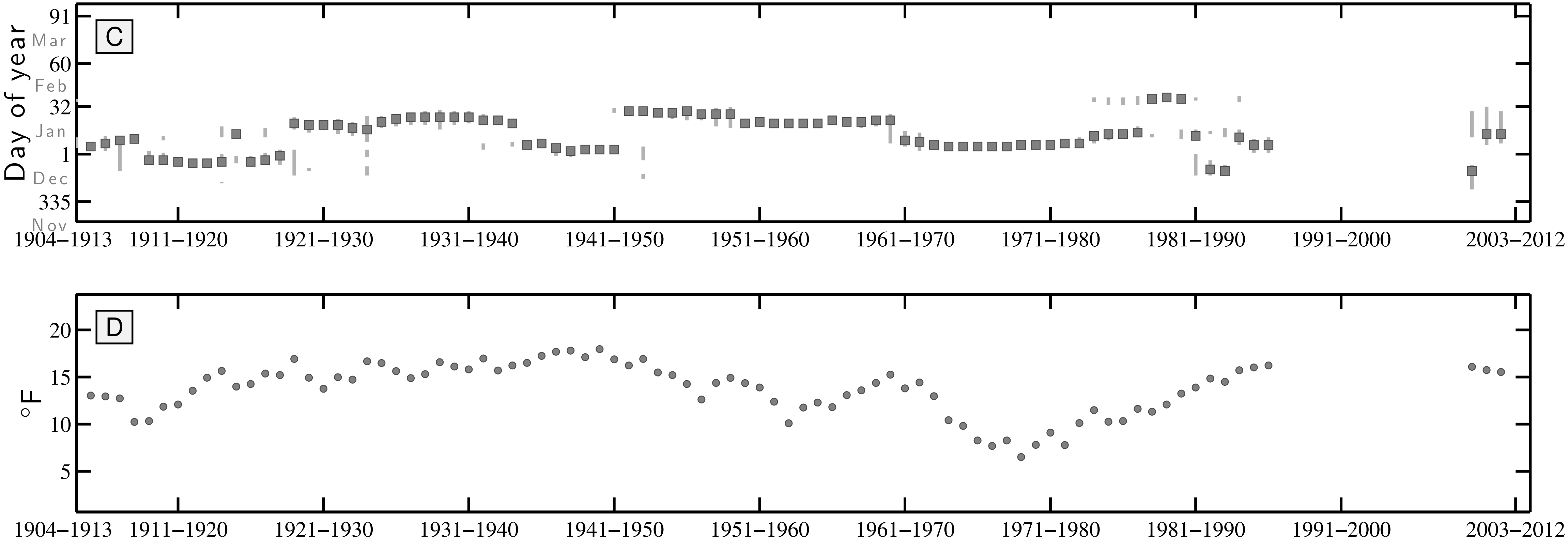

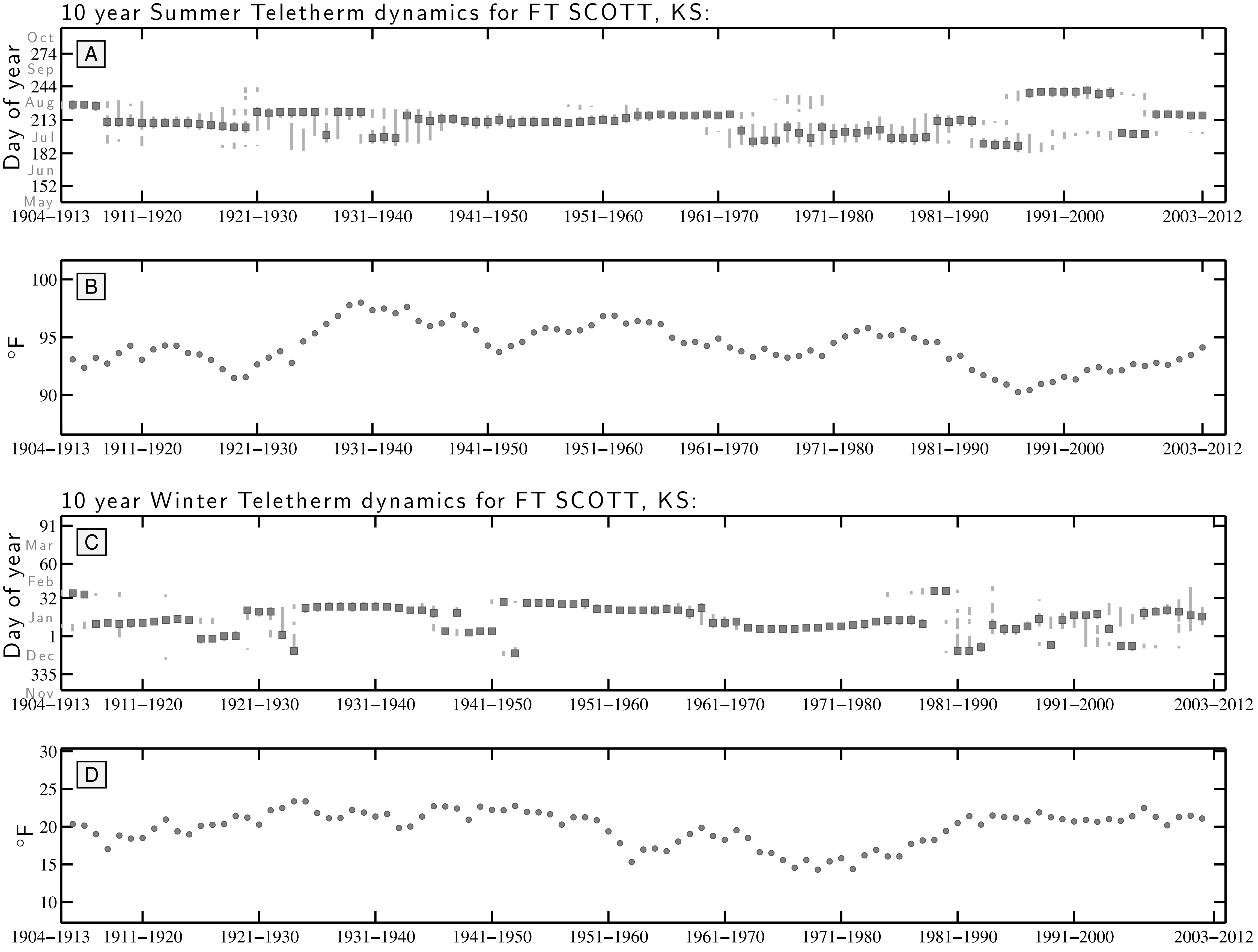
10 year Summer Teletherm dynamics for FT SCOTT, KS:
A
Day of year
Oct
274
Sep
244
Aug
213
Jul
182
Jun
152
May
1904–1913
1911–1920
1921–1930
1931–1940
1941–1950
1951–1960
1961–1970
1971–1980
1981–1990
1991–2000
2003–2012
B
°F
100
95
90
10 year Winter Teletherm dynamics for FT SCOTT, KS:
C
Day of year
91
Mar
60
Feb
32
Jan
1
Dec
335
Nov
D
°F
30
25
20
15
10

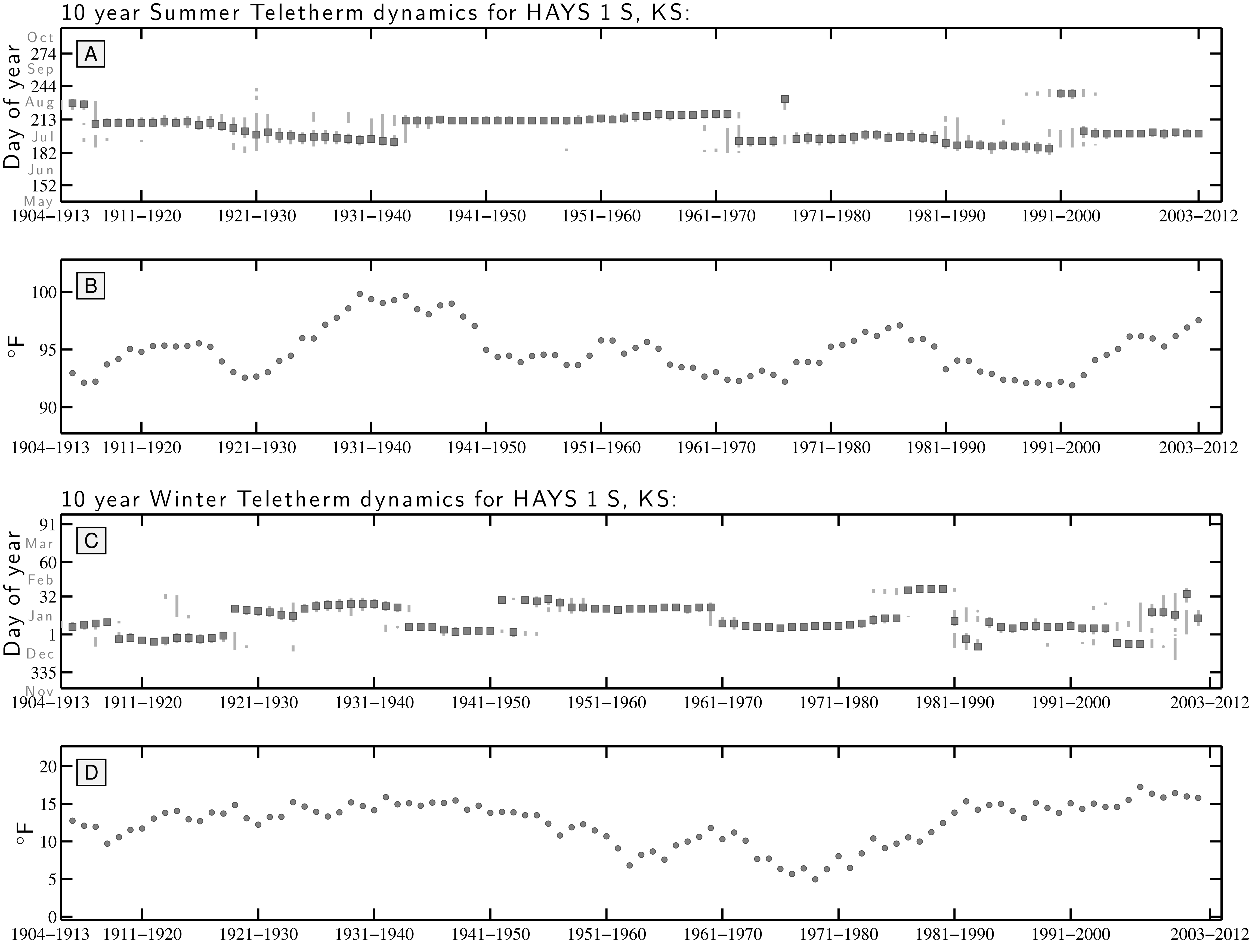
10 year Summer Teletherm dynamics for HAYS 1 S, KS:
A
Day of year
Oct
274
Sep
244
Aug
213
Jul
182
Jun
152
May
1904–1913
1911–1920
1921–1930
1931–1940
1941–1950
1951–1960
1961–1970
1971–1980
1981–1990
1991–2000
2003–2012
B
°F
100
95
90
10 year Winter Teletherm dynamics for HAYS 1 S, KS:
C
Day of year
91
Mar
60
Feb
32
Jan
1
Dec
335
Nov
D
°F
20
15
10
5
0

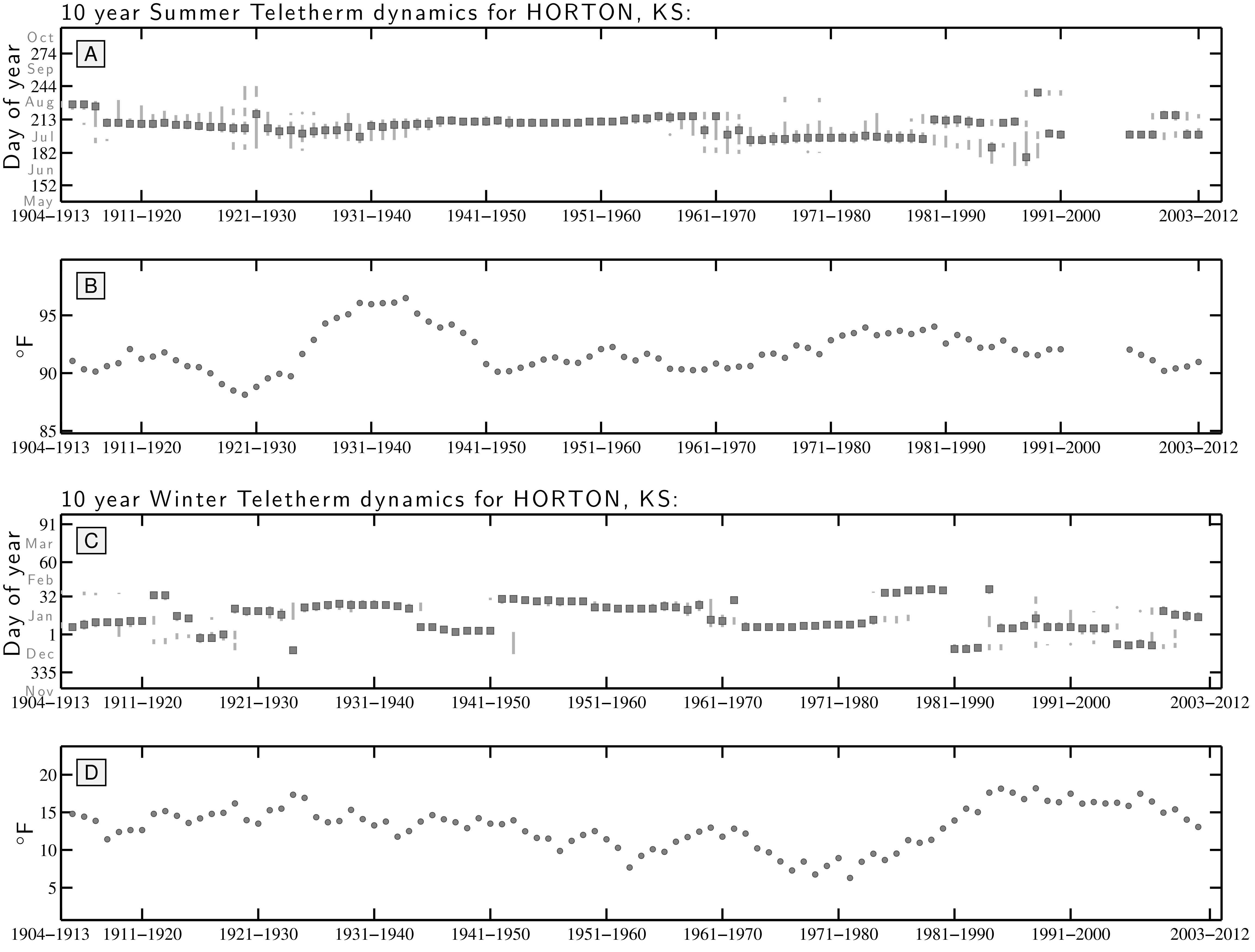
10 year Summer Teletherm dynamics for HORTON, KS:
A
Day of year
Oct
274
Sep
244
Aug
213
Jul
182
Jun
152
May
1904–1913
1911–1920
1921–1930
1931–1940
1941–1950
1951–1960
1961–1970
1971–1980
1981–1990
1991–2000
2003–2012
B
°F
95
90
85
10 year Winter Teletherm dynamics for HORTON, KS:
C
91
Mar
60
Feb
32
Jan
1
Dec
335
Nov
D
20
15
10
5

10 year Summer Teletherm dynamics for INDEPENDENCE, KS:

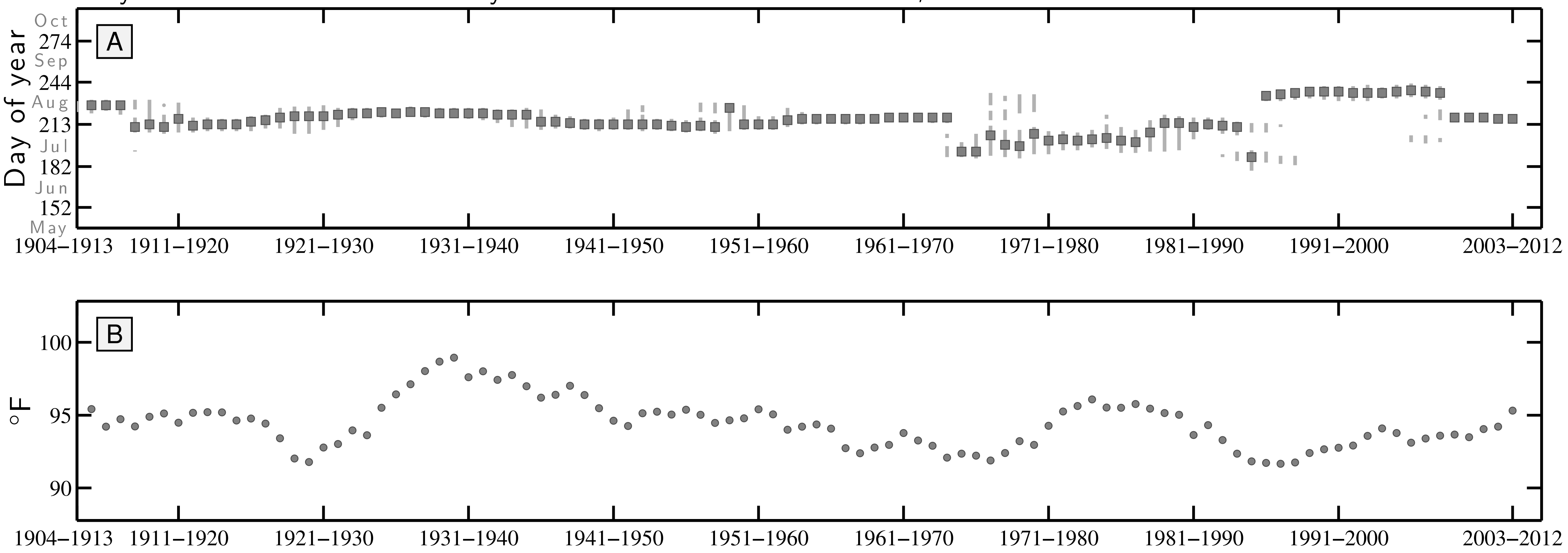

10 year Winter Teletherm dynamics for INDEPENDENCE, KS:

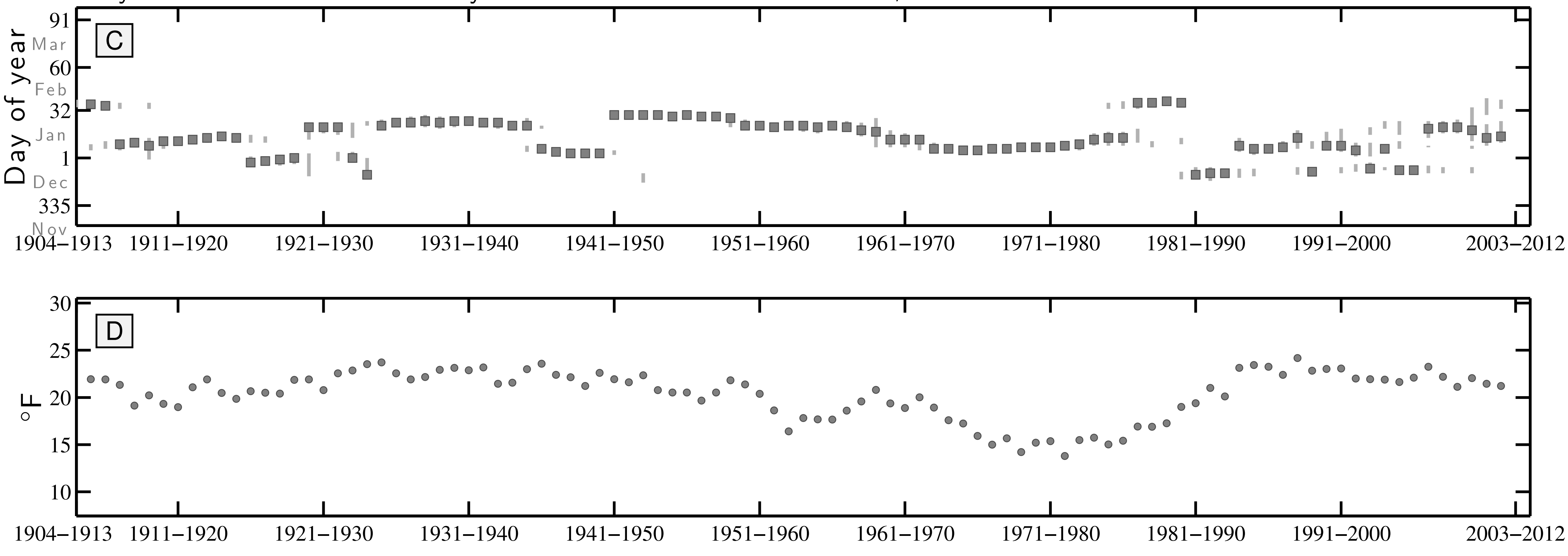

10 year Summer Teletherm dynamics for JETMORE 8NNW, KS:

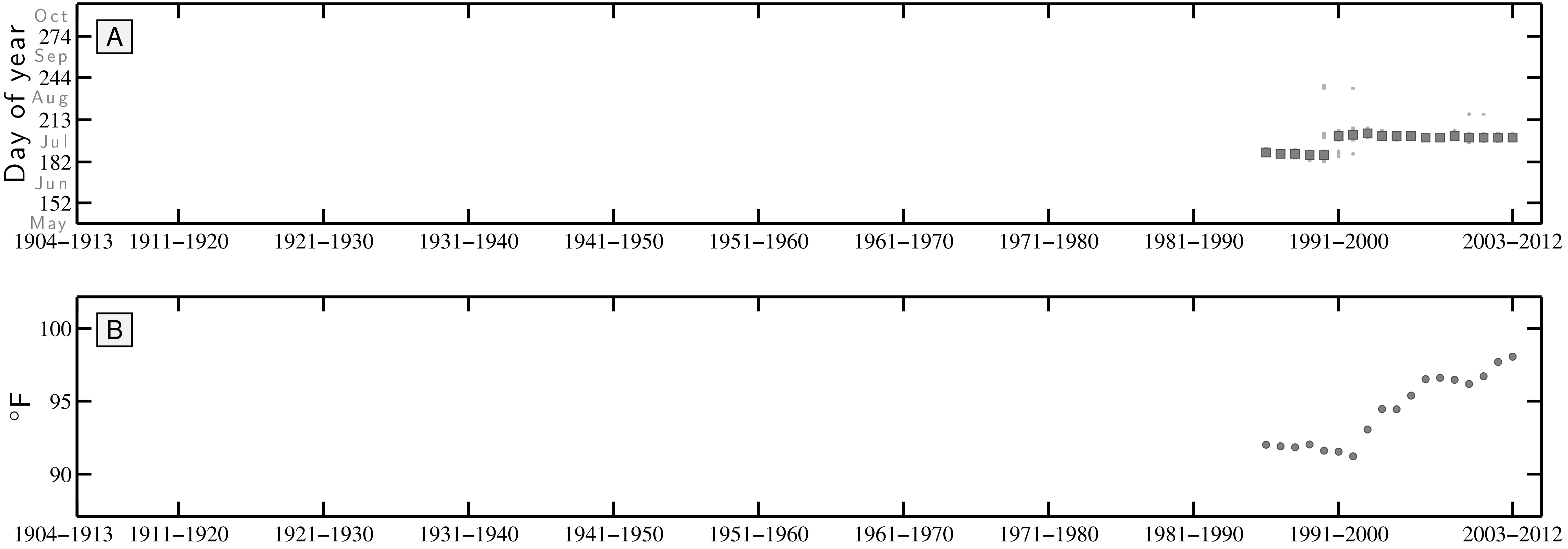

10 year Winter Teletherm dynamics for JETMORE 8NNW, KS:

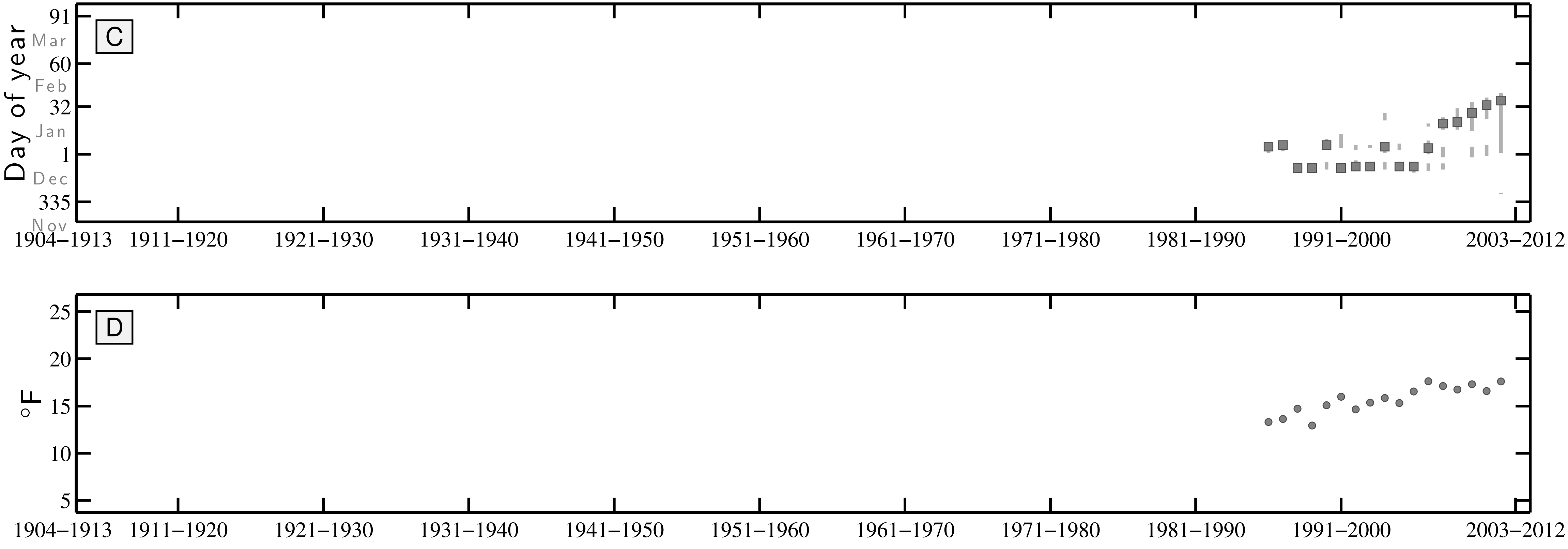

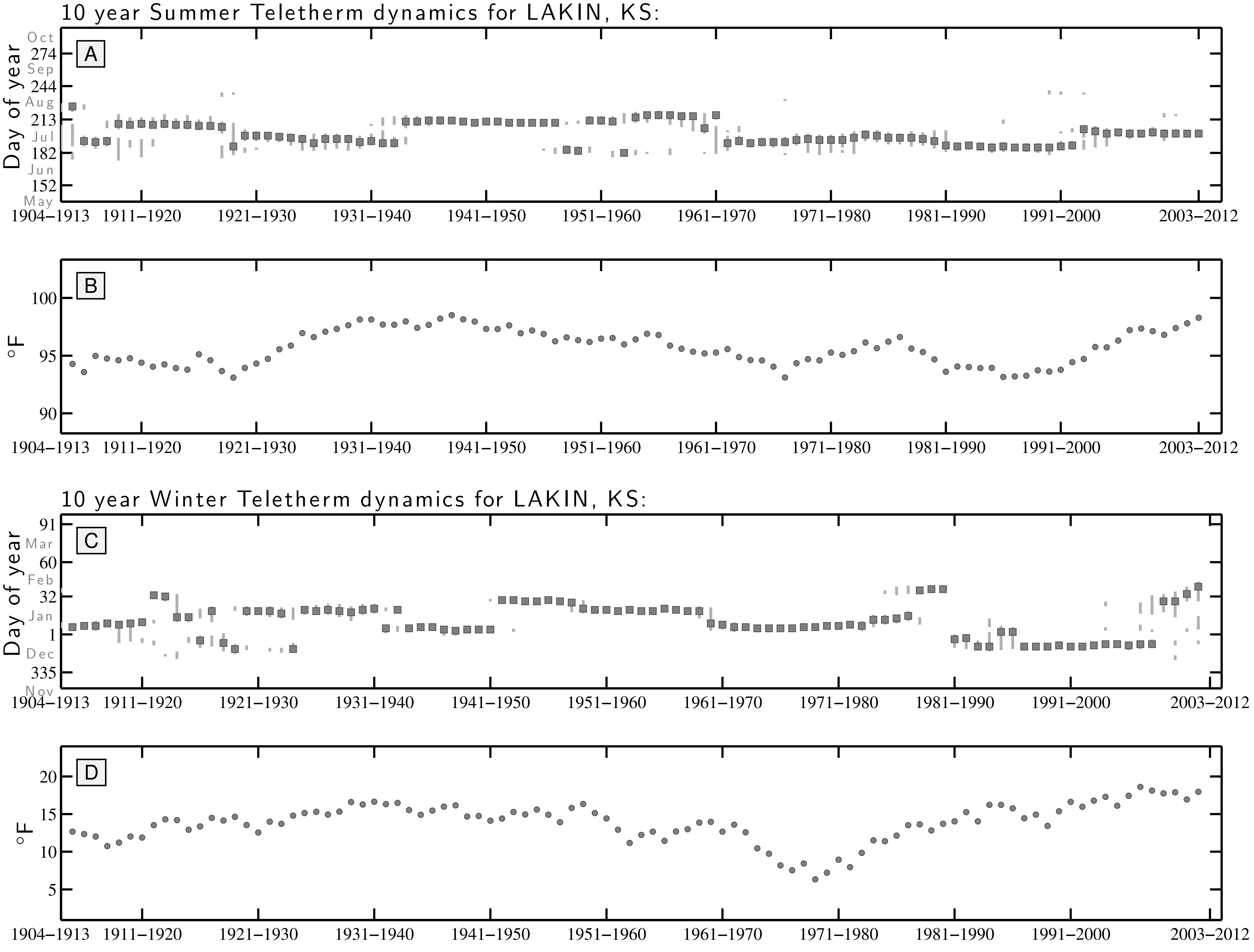

10 year Summer Teletherm dynamics for LAKIN, KS:
A
Day of year
Oct
274
Sep
244
Aug
213
Jul
182
Jun
152
May
1904–1913
1911–1920
1921–1930
1931–1940
1941–1950
1951–1960
1961–1970
1971–1980
1981–1990
1991–2000
2003–2012
B
°F
100
95
90
10 year Winter Teletherm dynamics for LAKIN, KS:
C
Day of year
91
Mar
60
Feb
32
Jan
1
Dec
335
Nov
D
°F
20
15
10
5

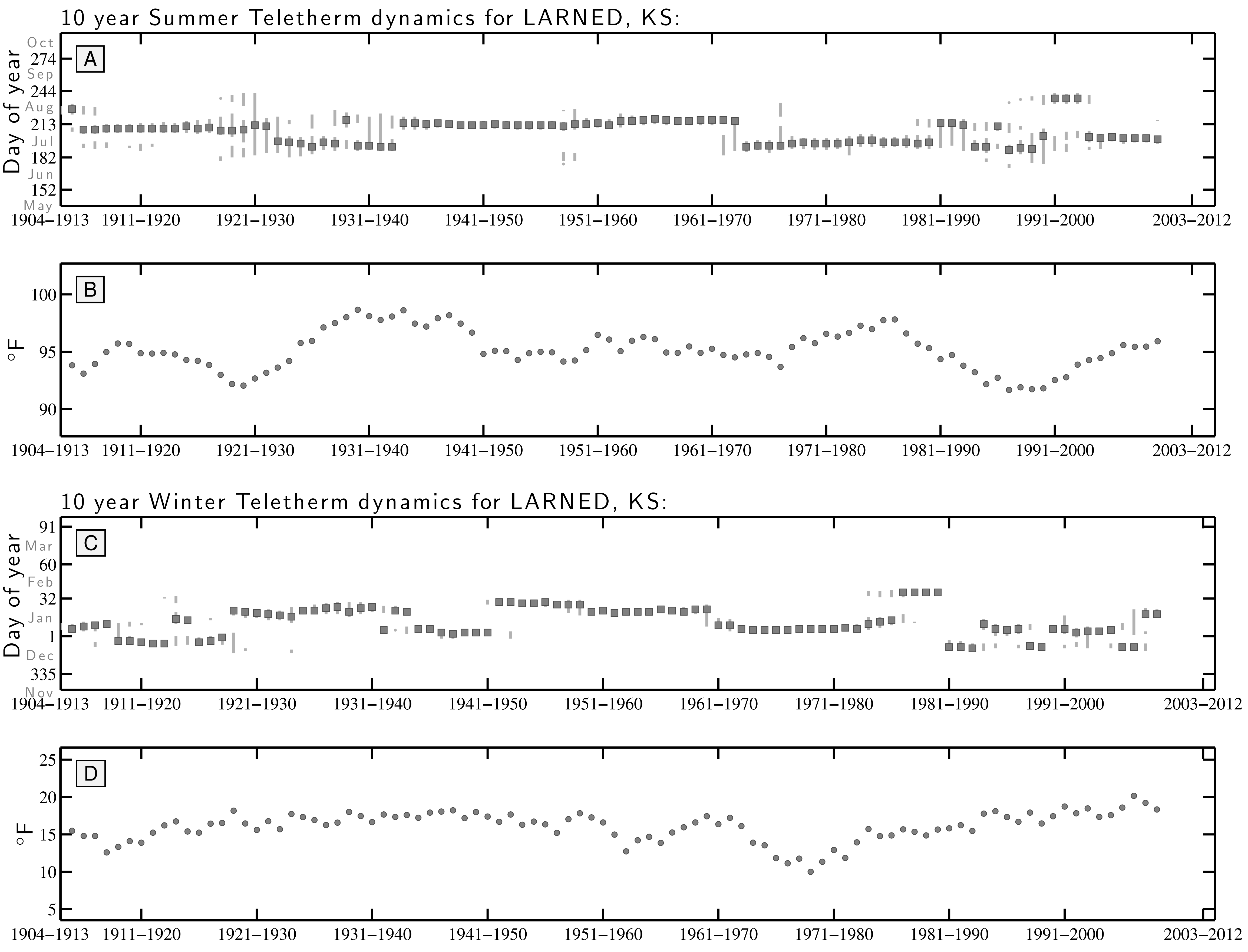
10 year Summer Teletherm dynamics for LARNED, KS:
A
Day of year
Oct
274
Sep
244
Aug
213
Jul
182
Jun
152
May
B
°F
100
95
90
10 year Winter Teletherm dynamics for LARNED, KS:
C
Day of year
91
Mar
60
Feb
32
Jan
1
Dec
335
Nov
D
°F
25
20
15
10
5
1904–1913
1911–1920
1921–1930
1931–1940
1941–1950
1951–1960
1961–1970
1971–1980
1981–1990
1991–2000
2003–2012

10 year Summer Teletherm dynamics for LAWRENCE, KS:

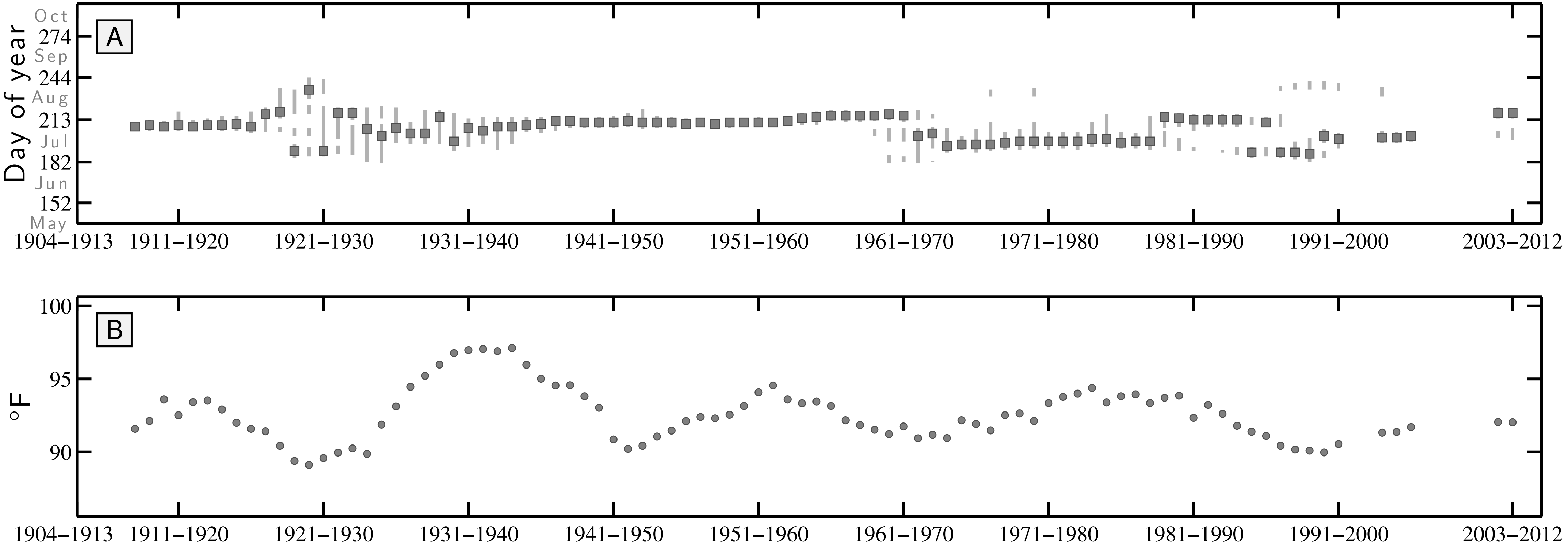

10 year Winter Teletherm dynamics for LAWRENCE, KS:

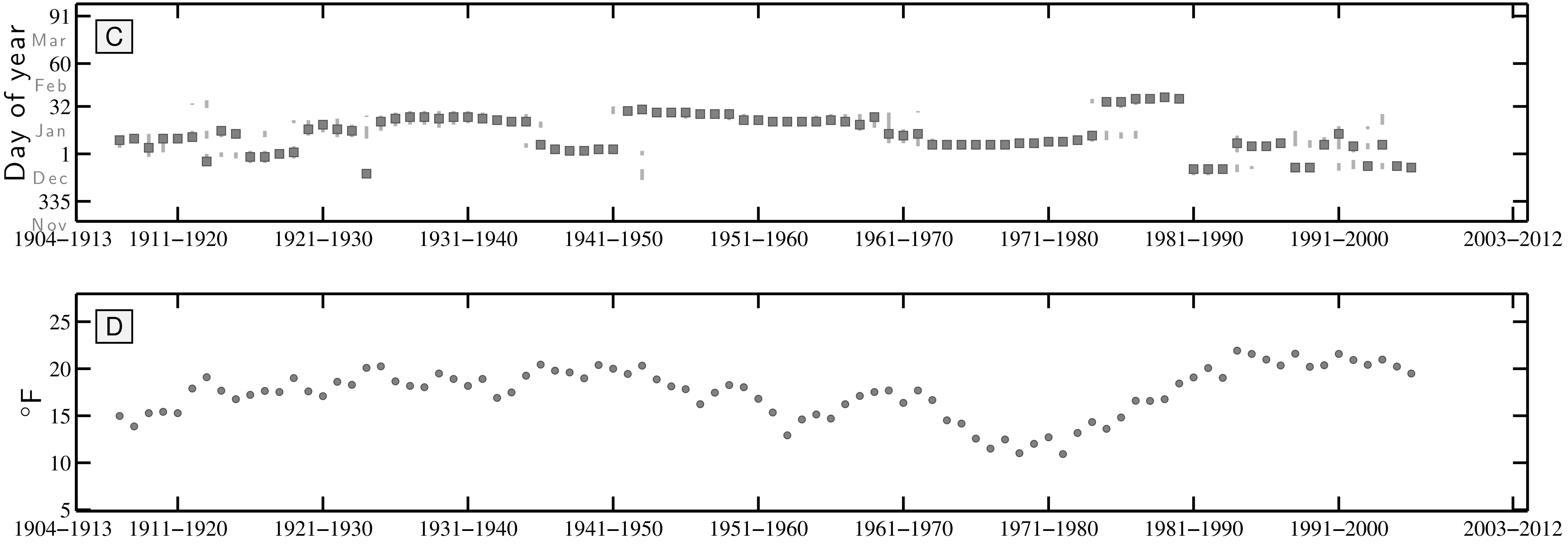

10 year Summer Teletherm dynamics for LEAVENWORTH, KS:

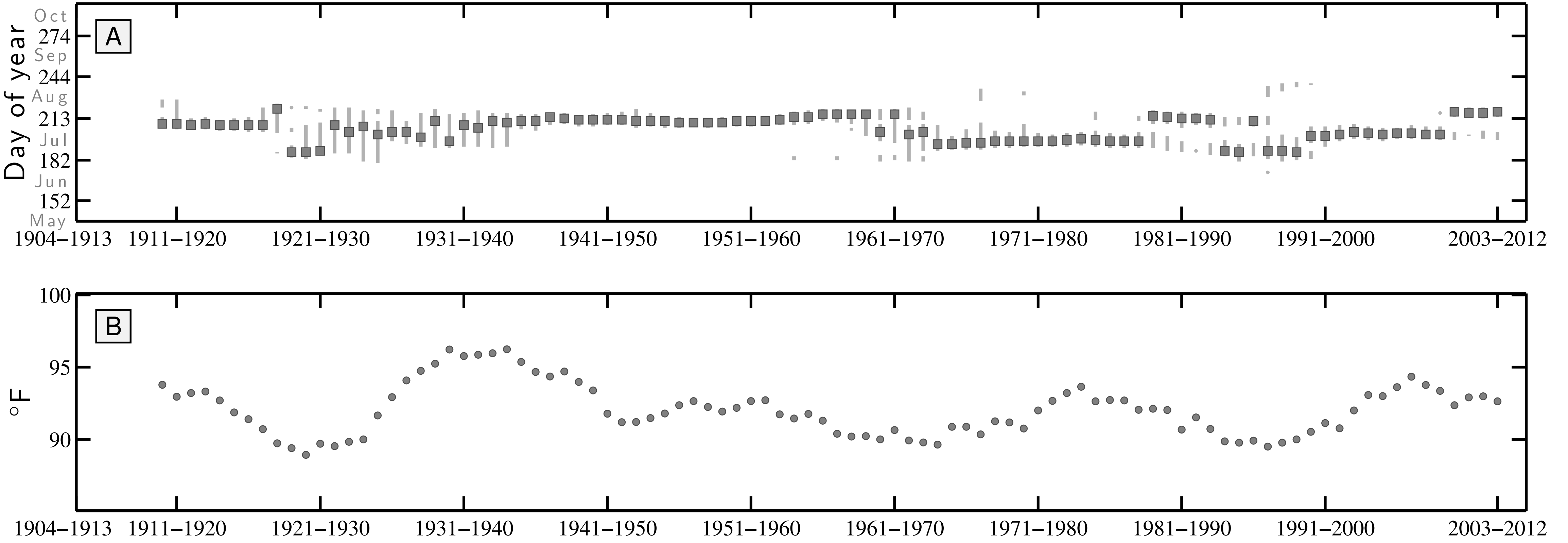

10 year Winter Teletherm dynamics for LEAVENWORTH, KS:

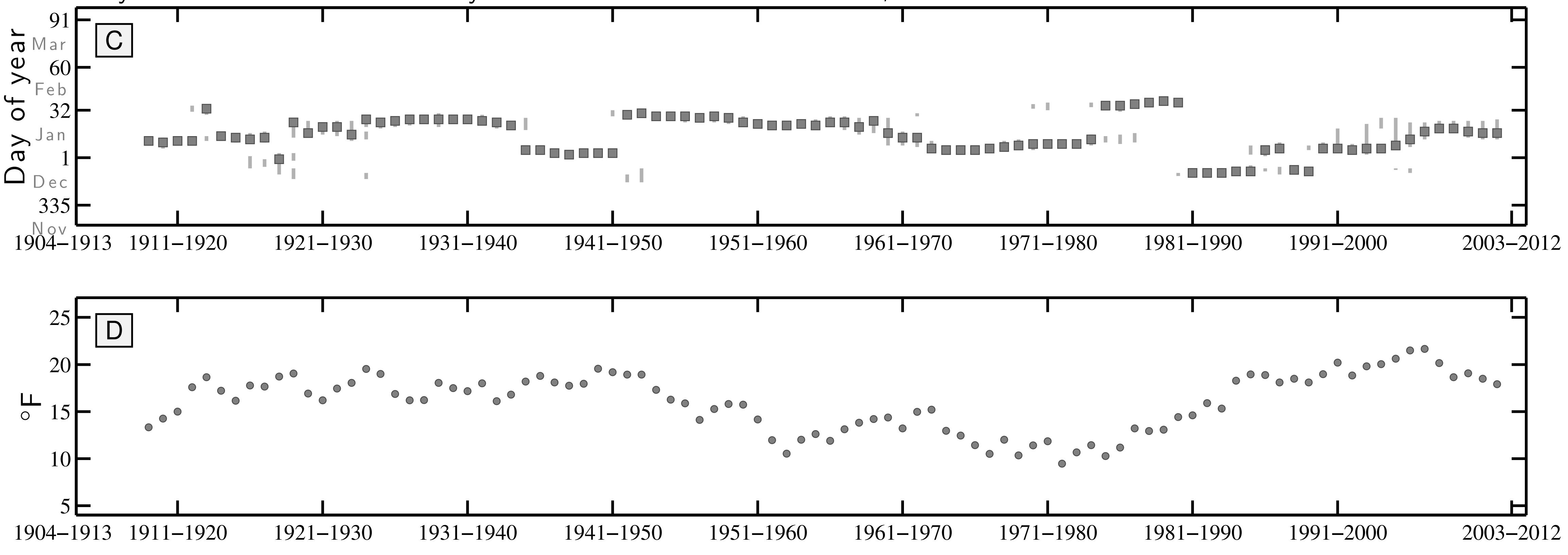

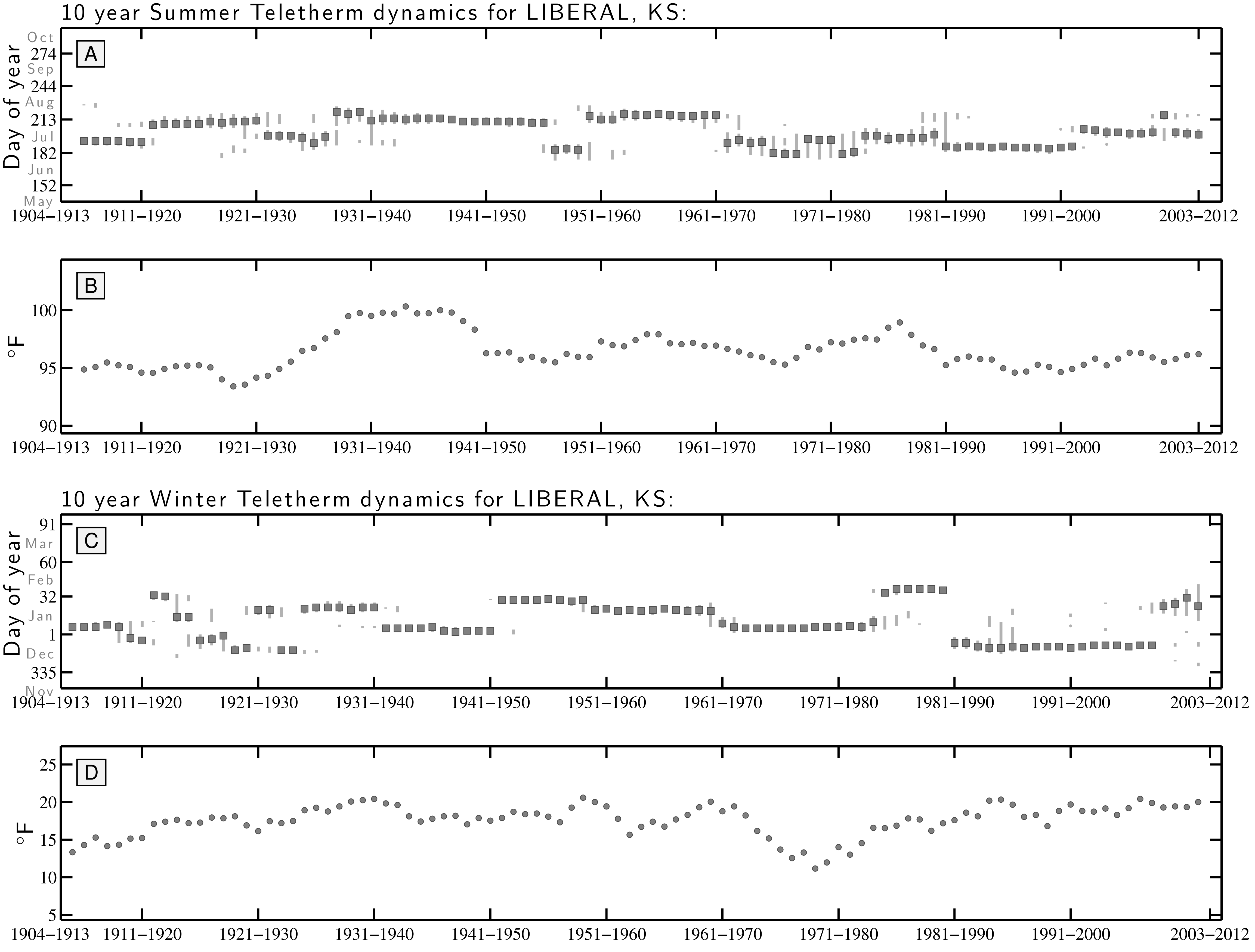
10 year Summer Teletherm dynamics for LIBERAL, KS:
A
Day of year
Oct
274
Sep
244
Aug
213
Jul
182
Jun
152
May
1904–1913
1911–1920
1921–1930
1931–1940
1941–1950
1951–1960
1961–1970
1971–1980
1981–1990
1991–2000
2003–2012
B
°F
100
95
90
10 year Winter Teletherm dynamics for LIBERAL, KS:
C
91
Mar
60
Feb
32
Jan
1
Dec
335
Nov
D
25
20
15
10
5

10 year Summer Teletherm dynamics for MANHATTAN, KS:

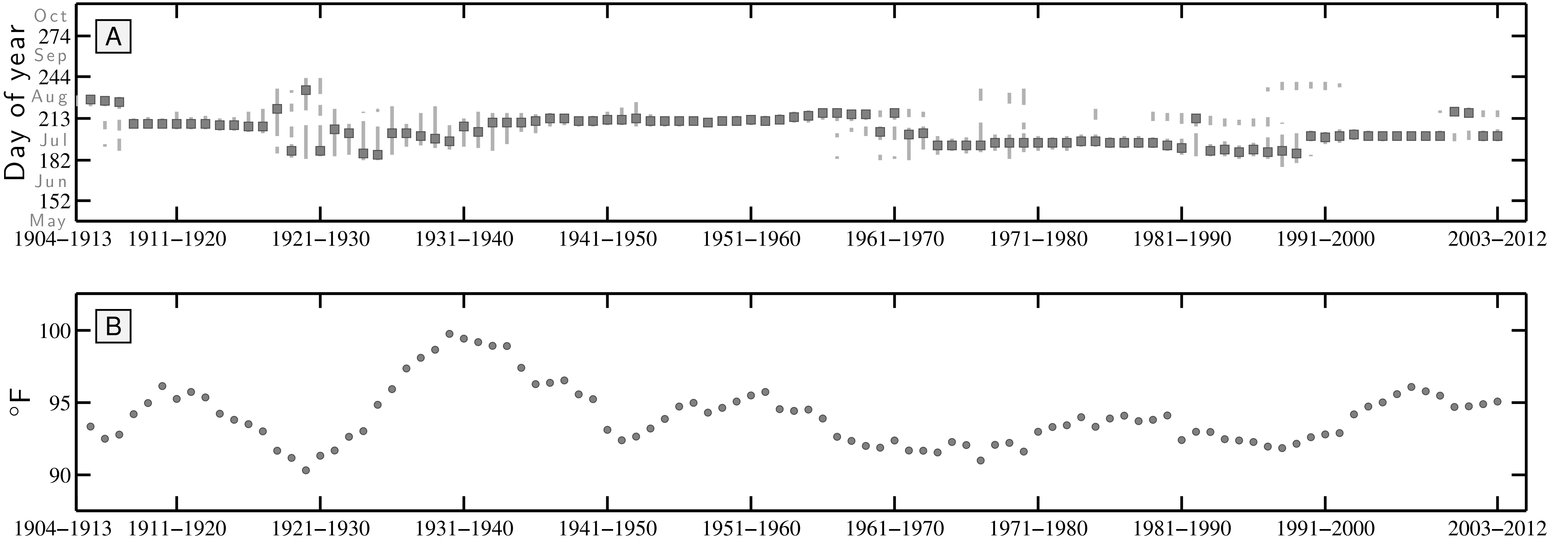

10 year Winter Teletherm dynamics for MANHATTAN, KS:

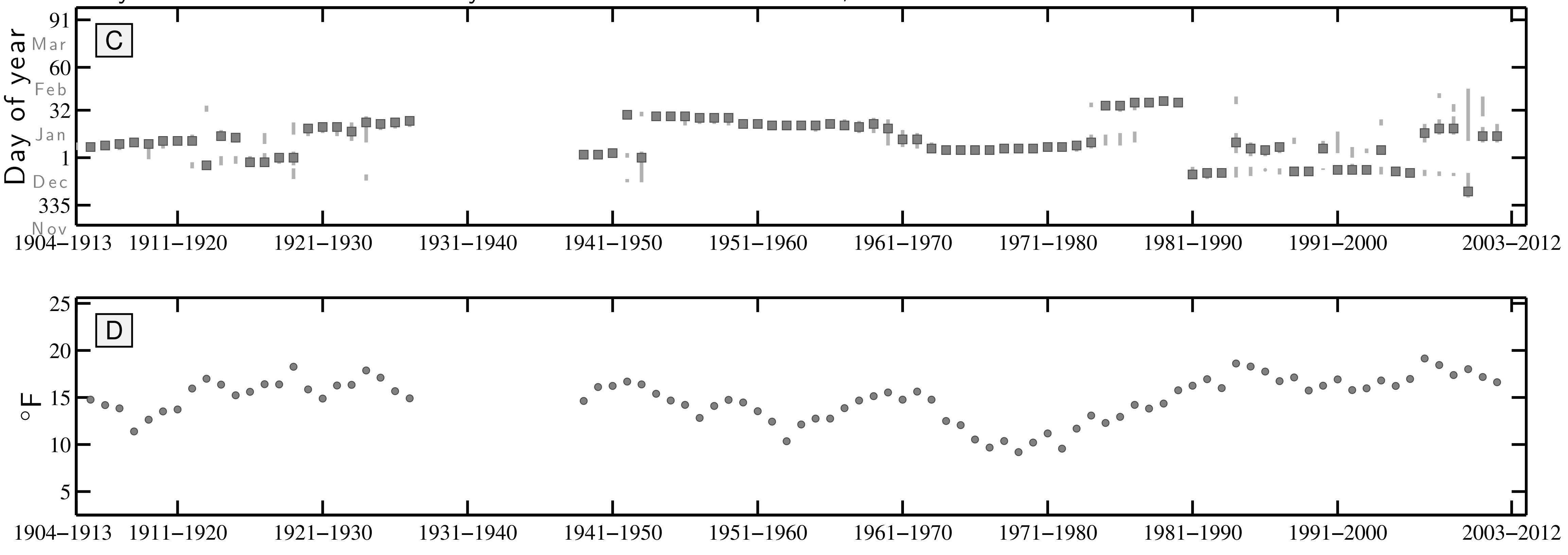

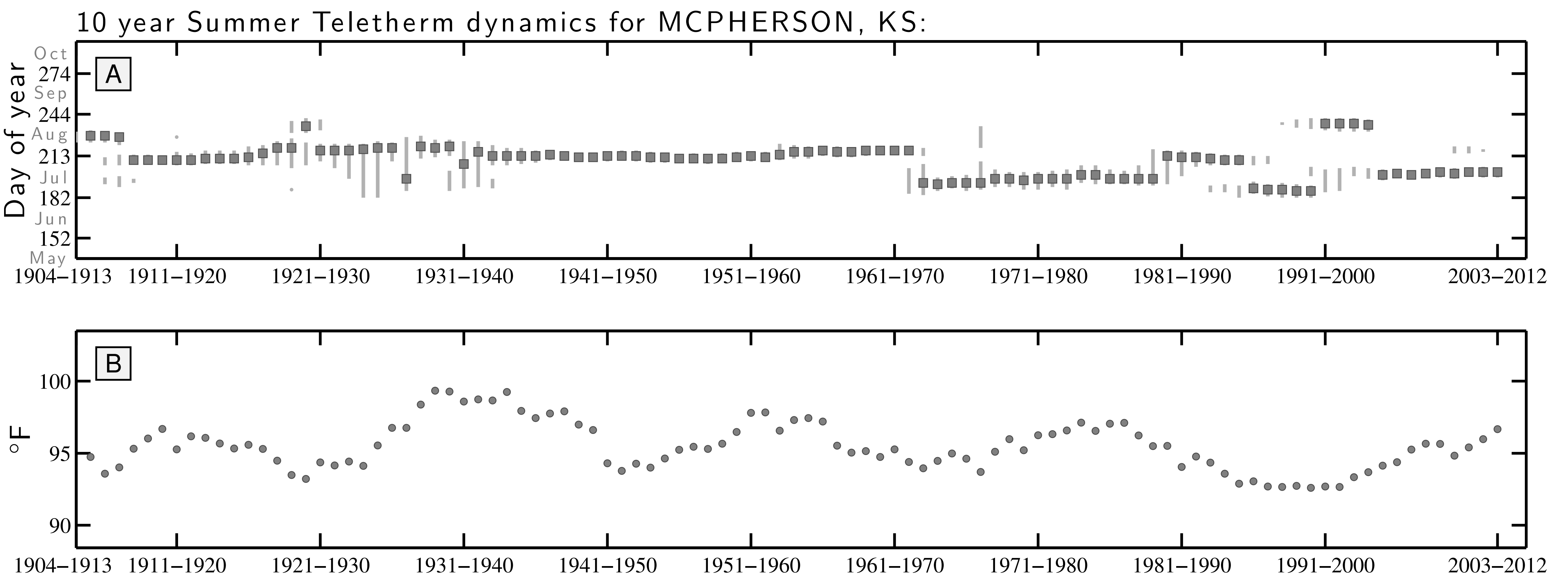
10 year Summer Teletherm dynamics for MCPHERSON, KS:
A
B
Day of year
°F
Oct
274
Sep
244
Aug
213
Jul
182
Jun
152
May
100
95
90
1904–1913
1911–1920
1921–1930
1931–1940
1941–1950
1951–1960
1961–1970
1971–1980
1981–1990
1991–2000
2003–2012

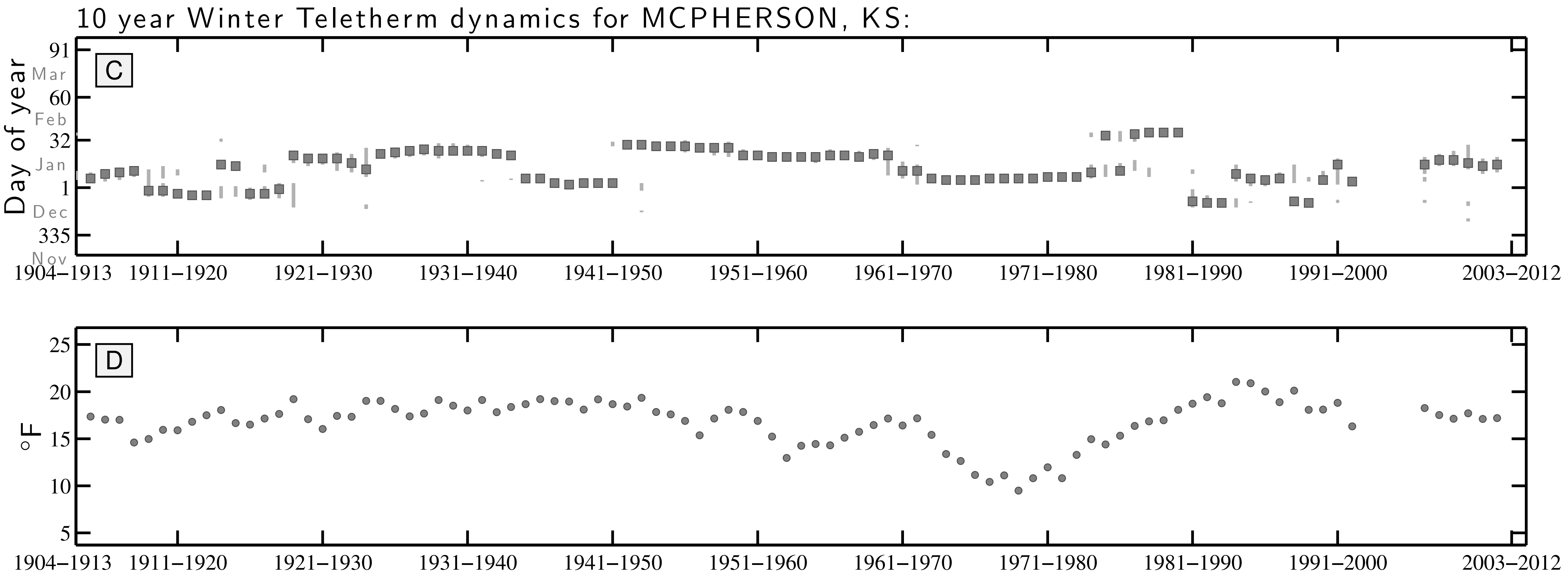
10 year Winter Teletherm dynamics for MCPHERSON, KS:
C
D
Day of year
°F
91
Mar
60
Feb
32
Jan
1
Dec
335
Nov
25
20
15
10
5
1904–1913
1911–1920
1921–1930
1931–1940
1941–1950
1951–1960
1961–1970
1971–1980
1981–1990
1991–2000
2003–2012

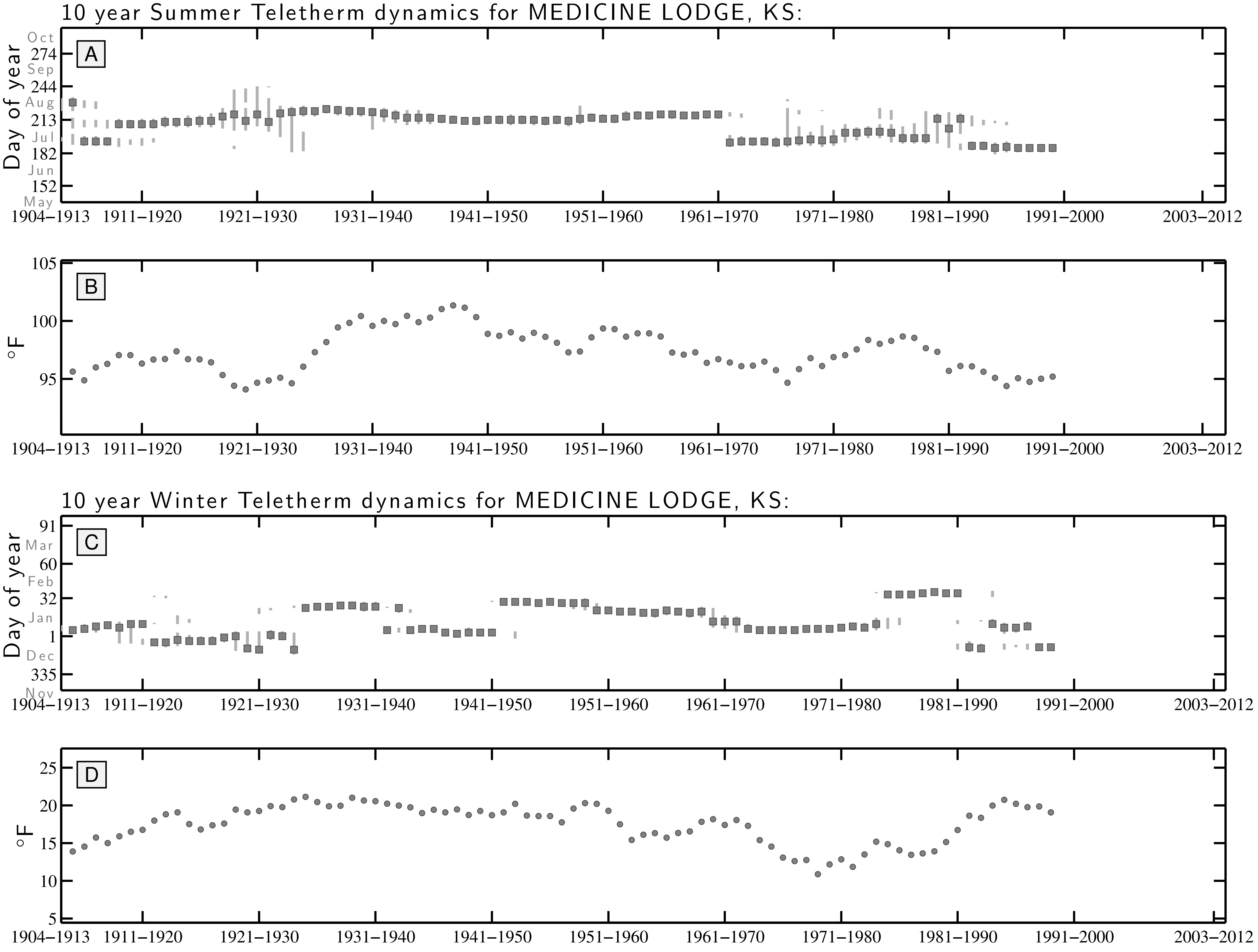
10 year Summer Teletherm dynamics for MEDICINE LODGE, KS:
A
Day of year
Oct
274
Sep
244
Aug
213
Jul
182
Jun
152
May
B
°F
105
100
95
10 year Winter Teletherm dynamics for MEDICINE LODGE, KS:
C
91
Mar
60
Feb
32
Jan
1
Dec
335
Nov
D
25
20
15
10
5
1904–1913
1911–1920
1921–1930
1931–1940
1941–1950
1951–1960
1961–1970
1971–1980
1981–1990
1991–2000
2003–2012

10 year Summer Teletherm dynamics for MINNEAPOLIS, KS:

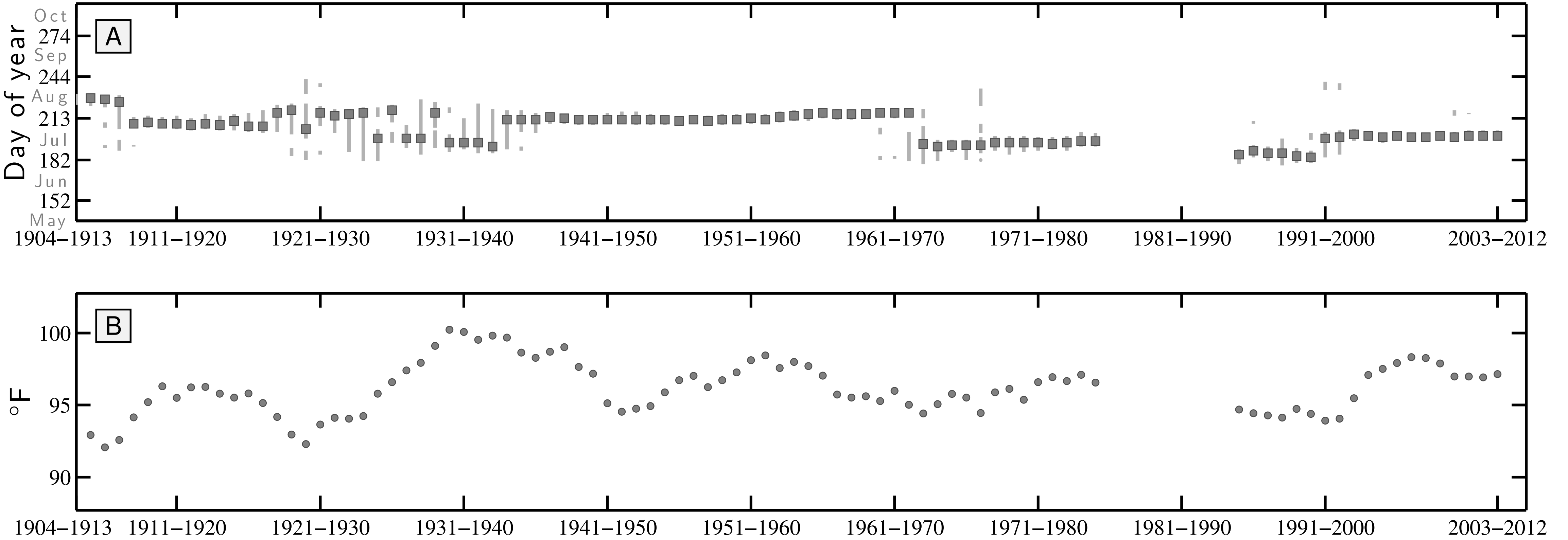

10 year Winter Teletherm dynamics for MINNEAPOLIS, KS:

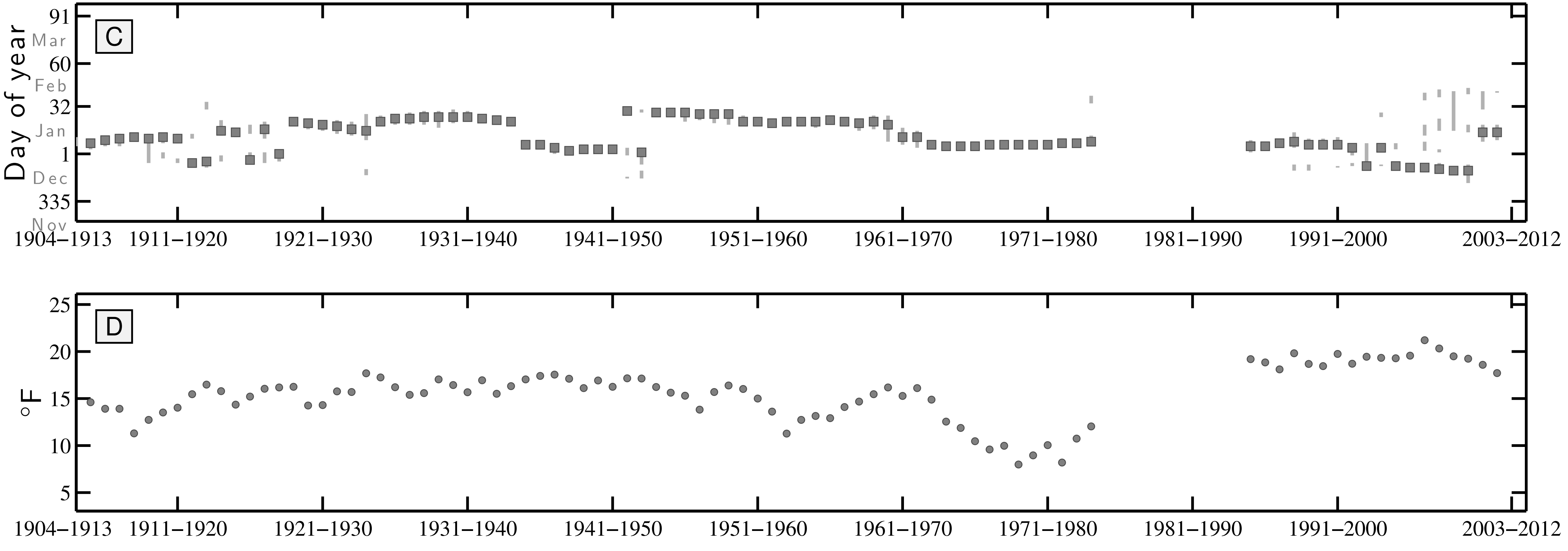

10 year Summer Teletherm dynamics for NORTON 9SSE, KS:

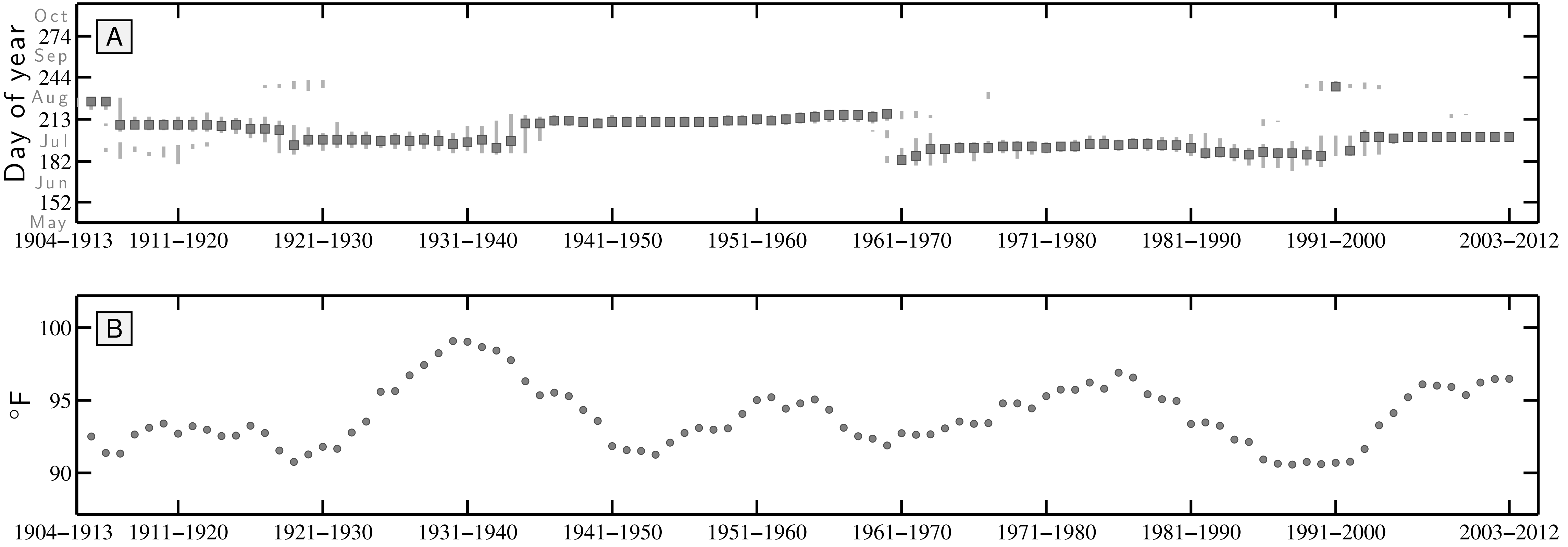

10 year Winter Teletherm dynamics for NORTON 9SSE, KS:

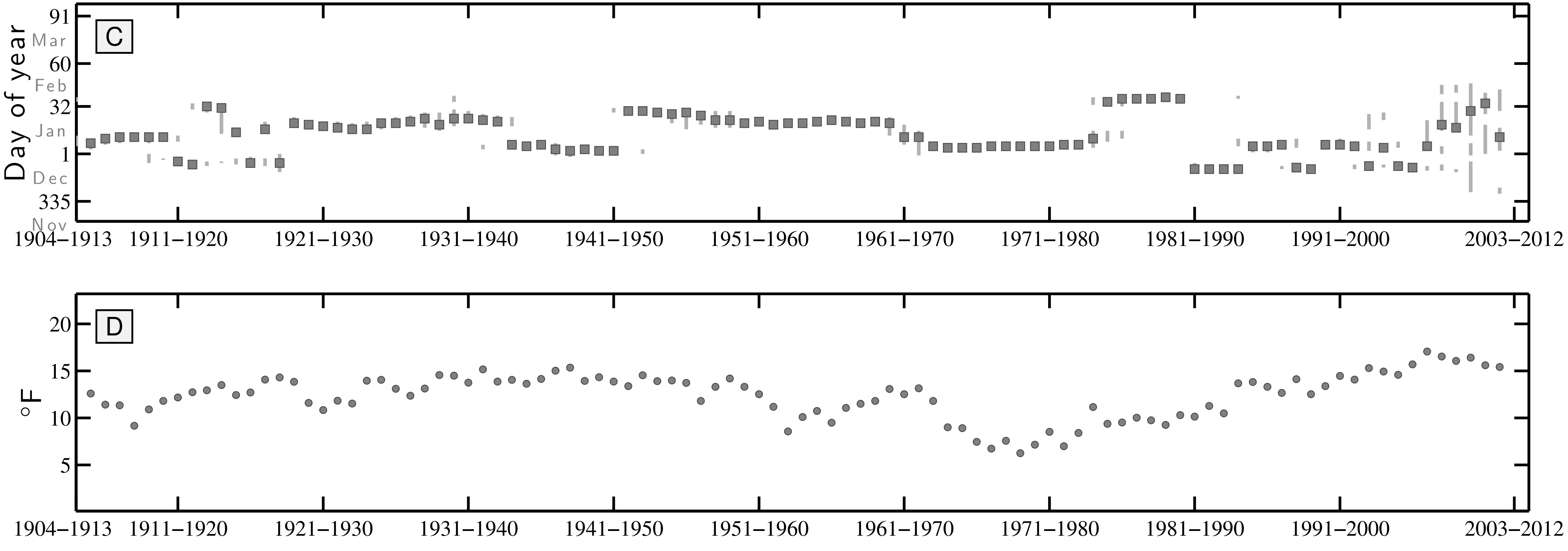

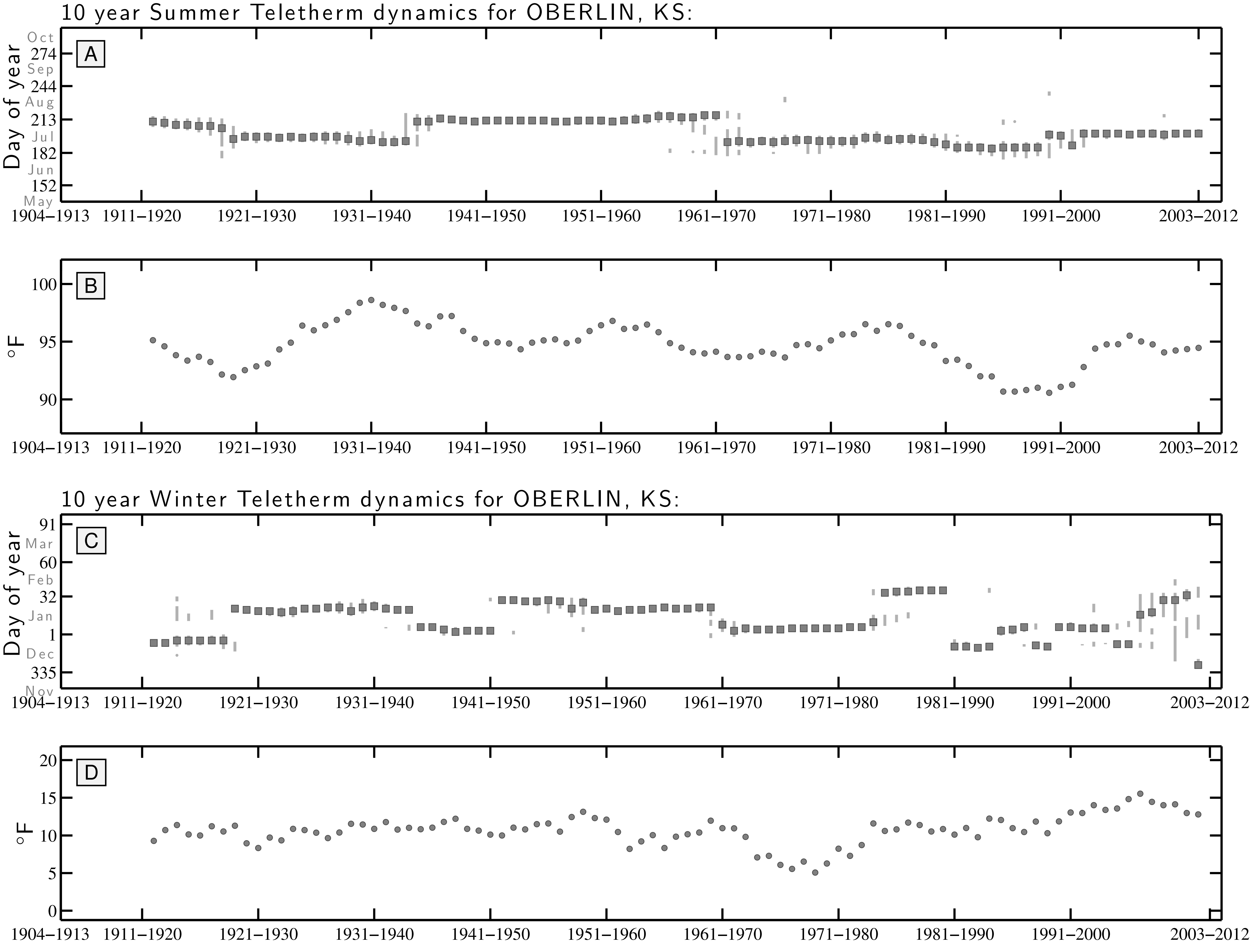
10 year Summer Teletherm dynamics for OBERLIN, KS:
A
Day of year
Oct
274
Sep
244
Aug
213
Jul
182
Jun
152
May
B
°F
100
95
90
10 year Winter Teletherm dynamics for OBERLIN, KS:
C
Day of year
91
Mar
60
Feb
32
Jan
1
Dec
335
Nov
D
°F
20
15
10
5
0
1904–1913
1911–1920
1921–1930
1931–1940
1941–1950
1951–1960
1961–1970
1971–1980
1981–1990
1991–2000
2003–2012

10 year Summer Teletherm dynamics for OLATHE 3E, KS:

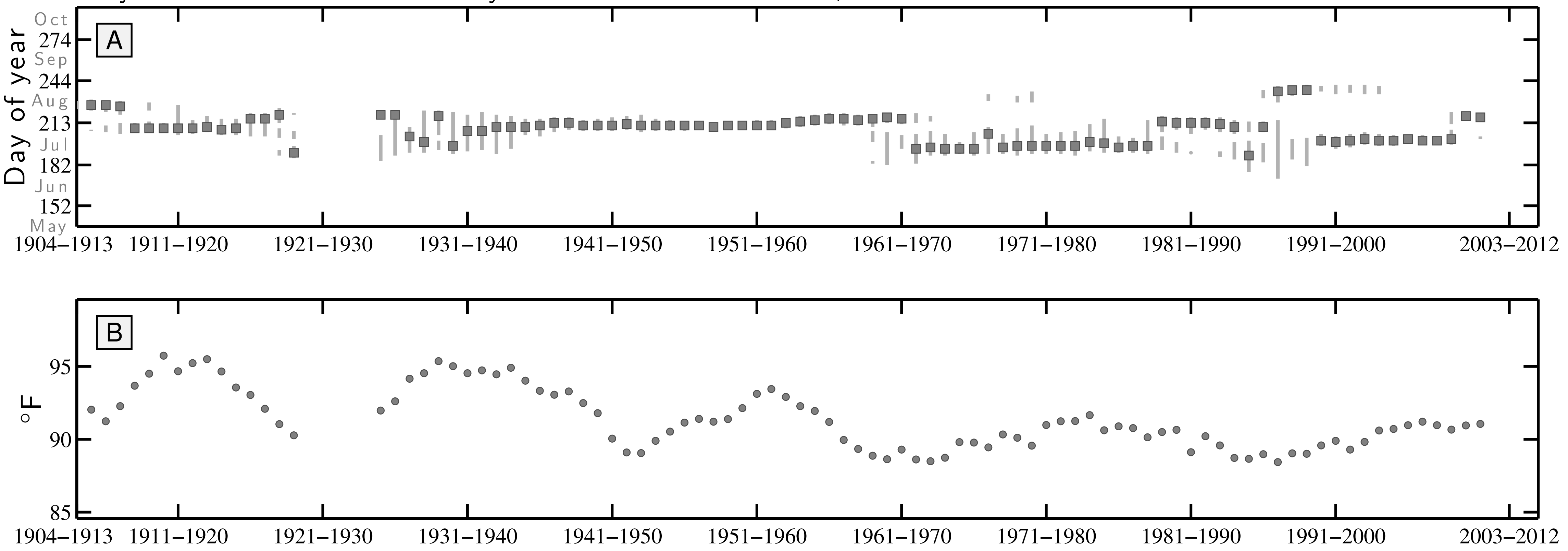

10 year Winter Teletherm dynamics for OLATHE 3E, KS:

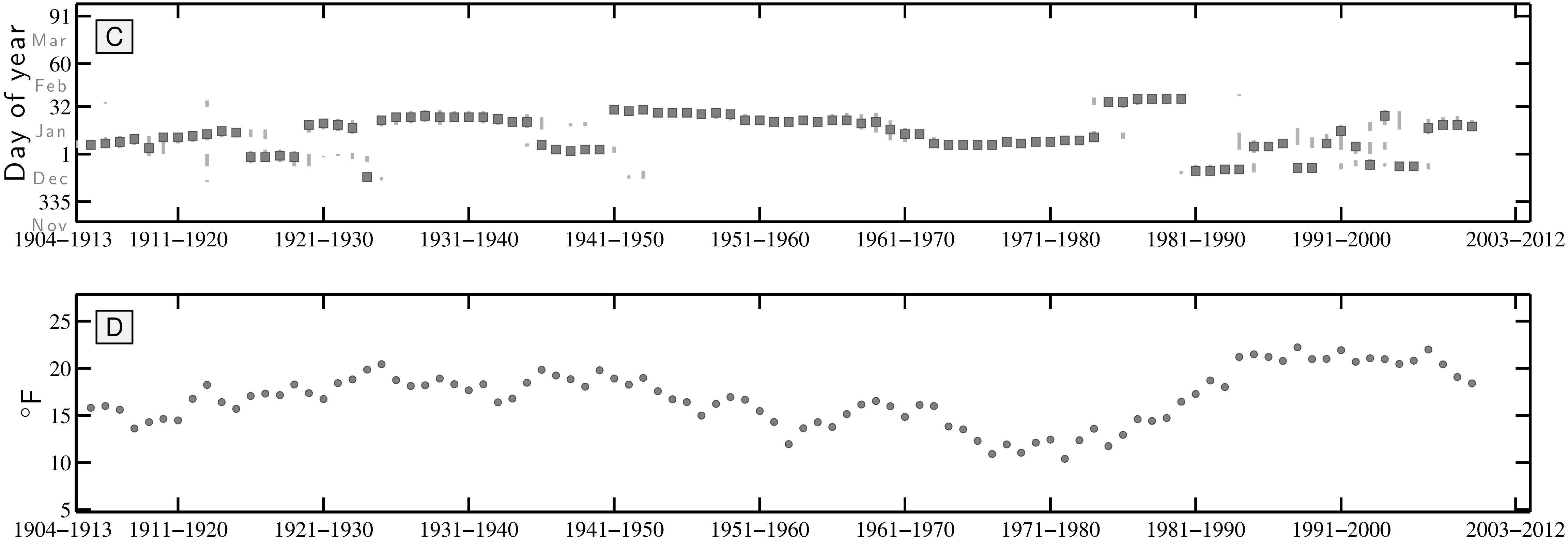

10 year Summer Teletherm dynamics for OTTAWA, KS:

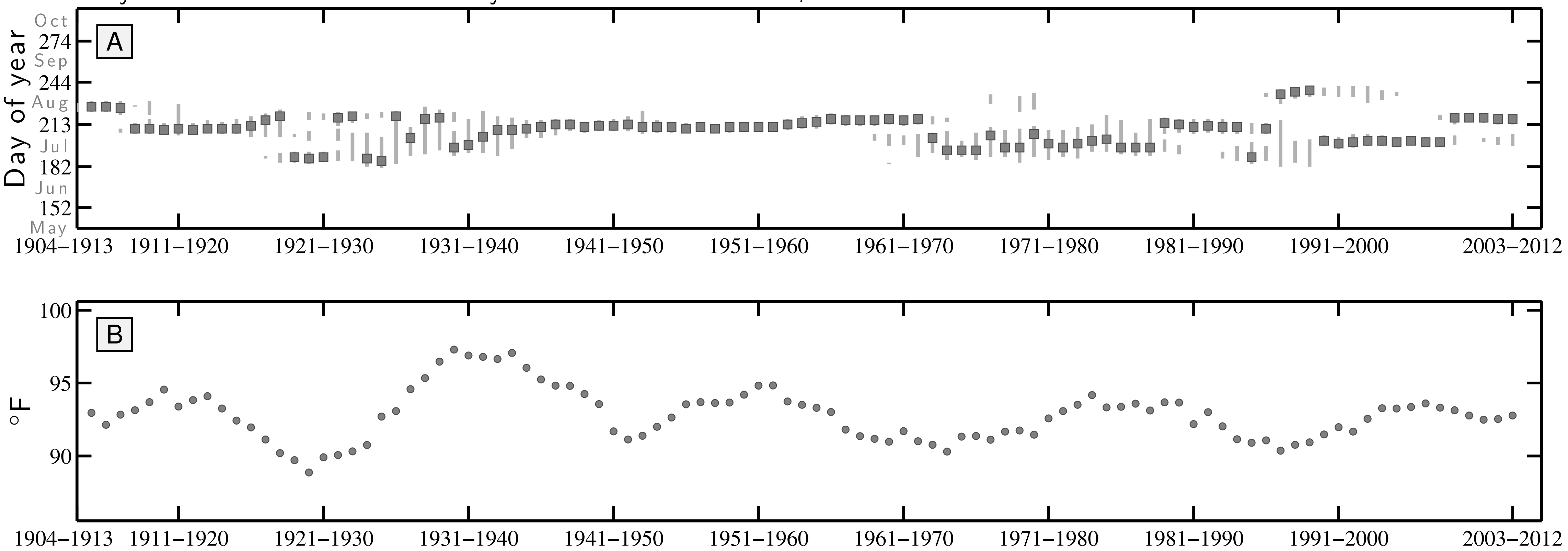

10 year Winter Teletherm dynamics for OTTAWA, KS:

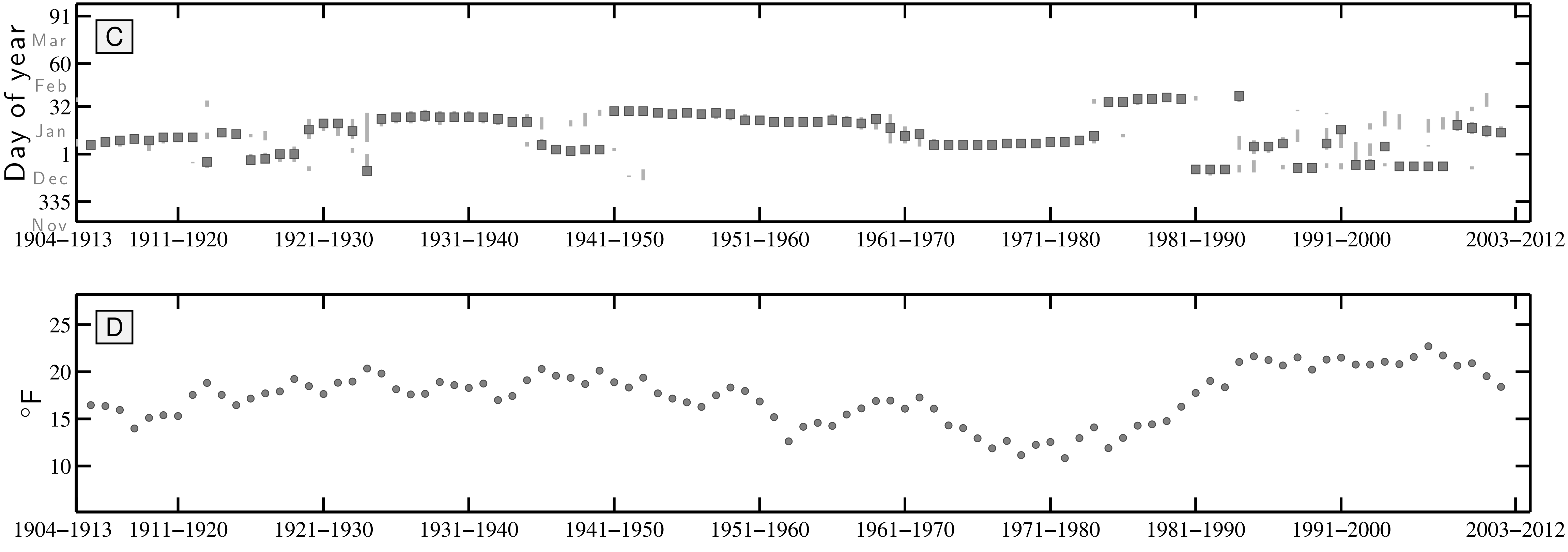

10 year Summer Teletherm dynamics for SAINT FRANCIS, KS:

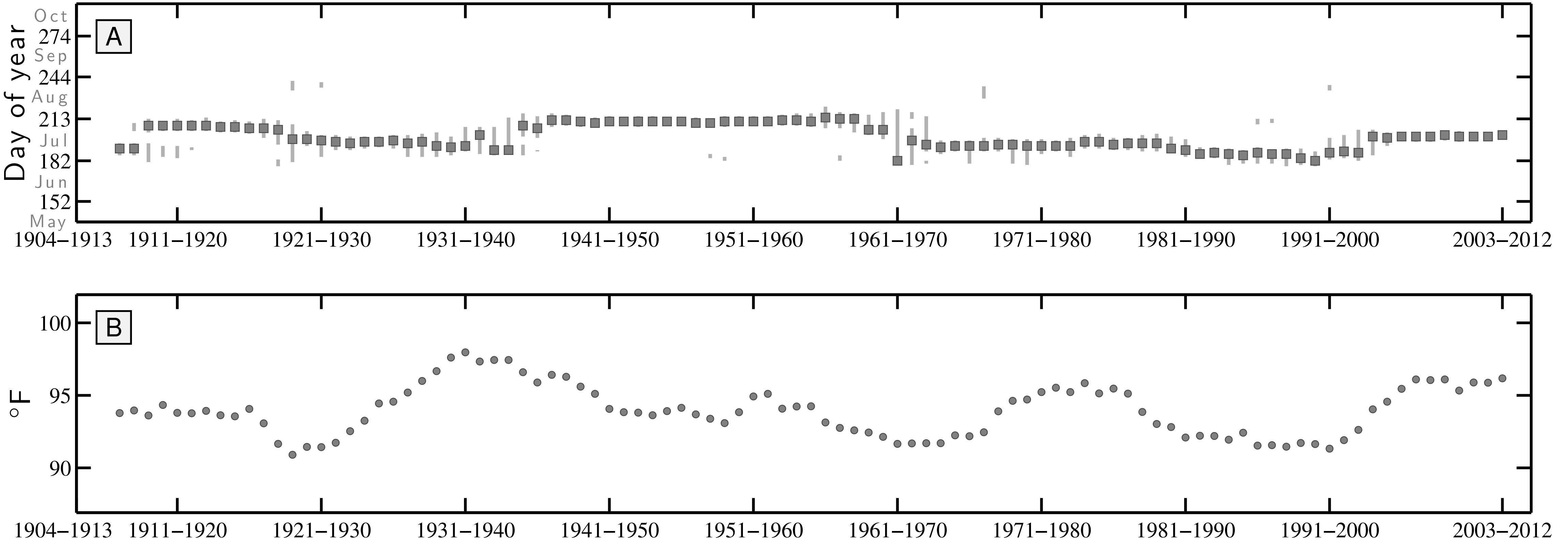

10 year Winter Teletherm dynamics for SAINT FRANCIS, KS:

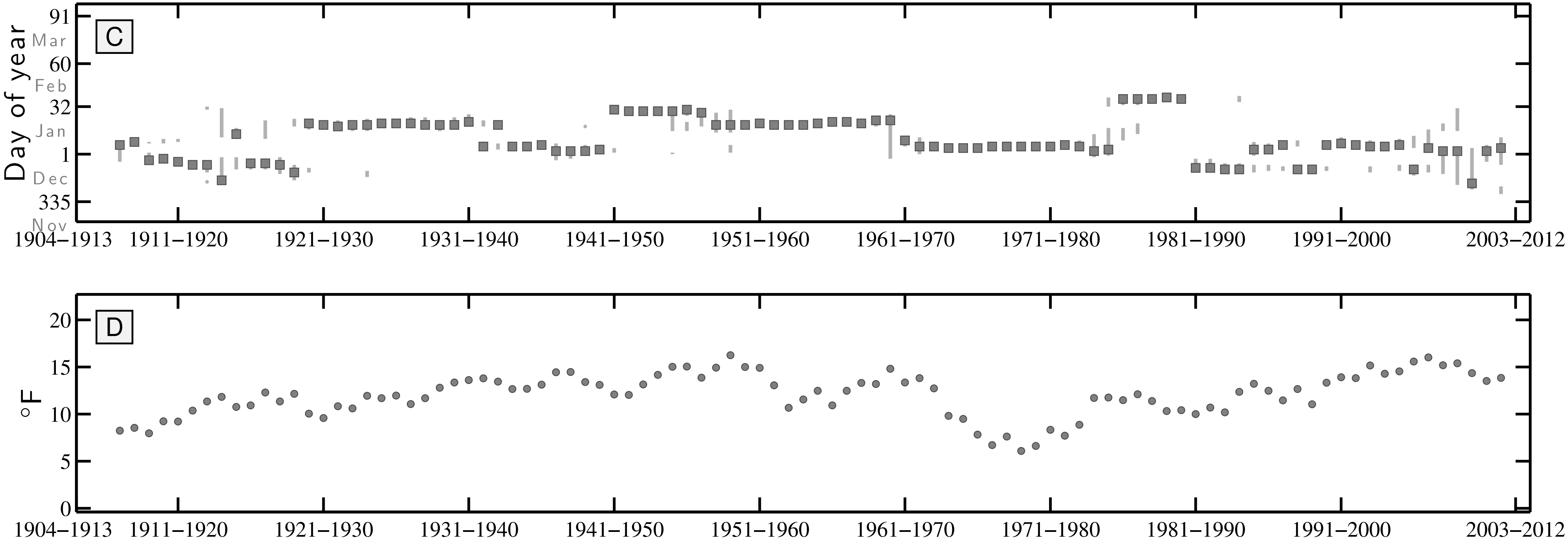

10 year Summer Teletherm dynamics for SCOTT CITY, KS:

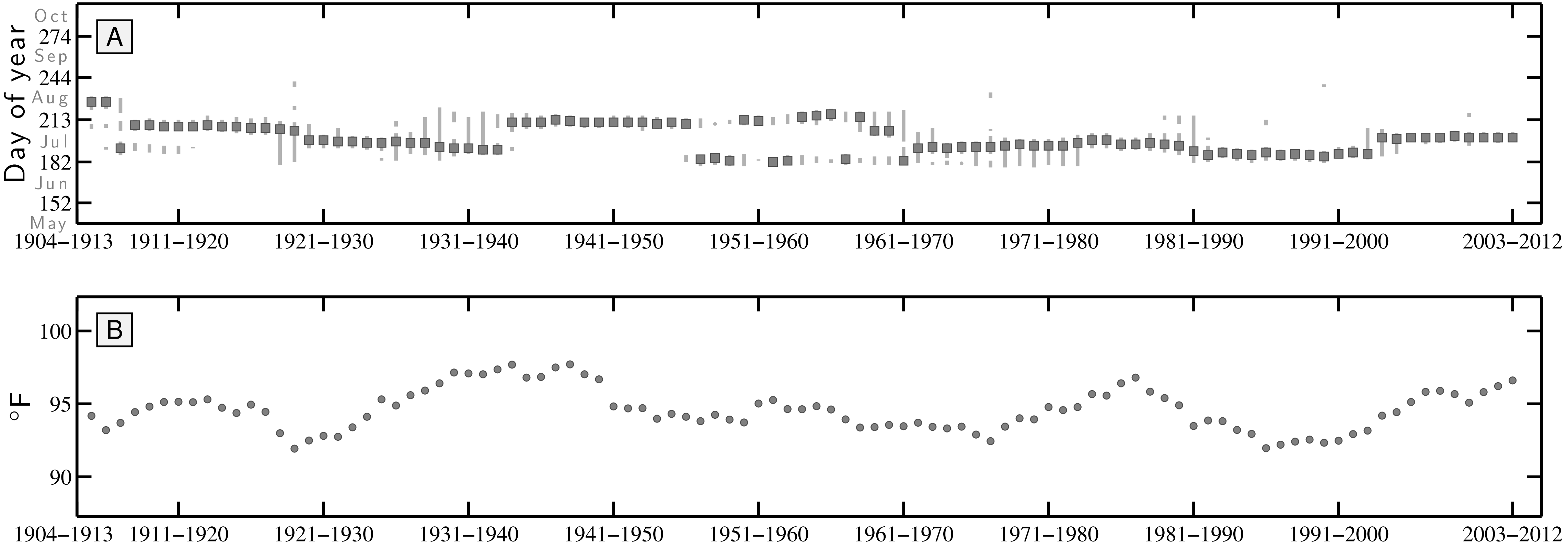

10 year Winter Teletherm dynamics for SCOTT CITY, KS:

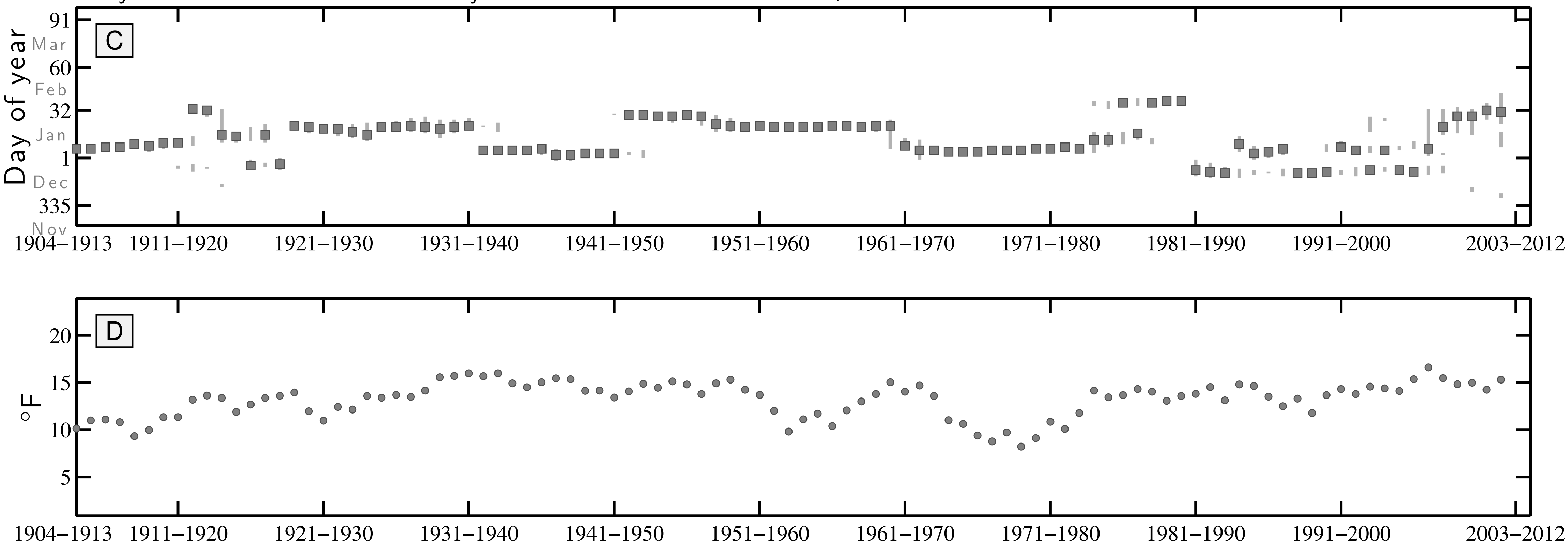

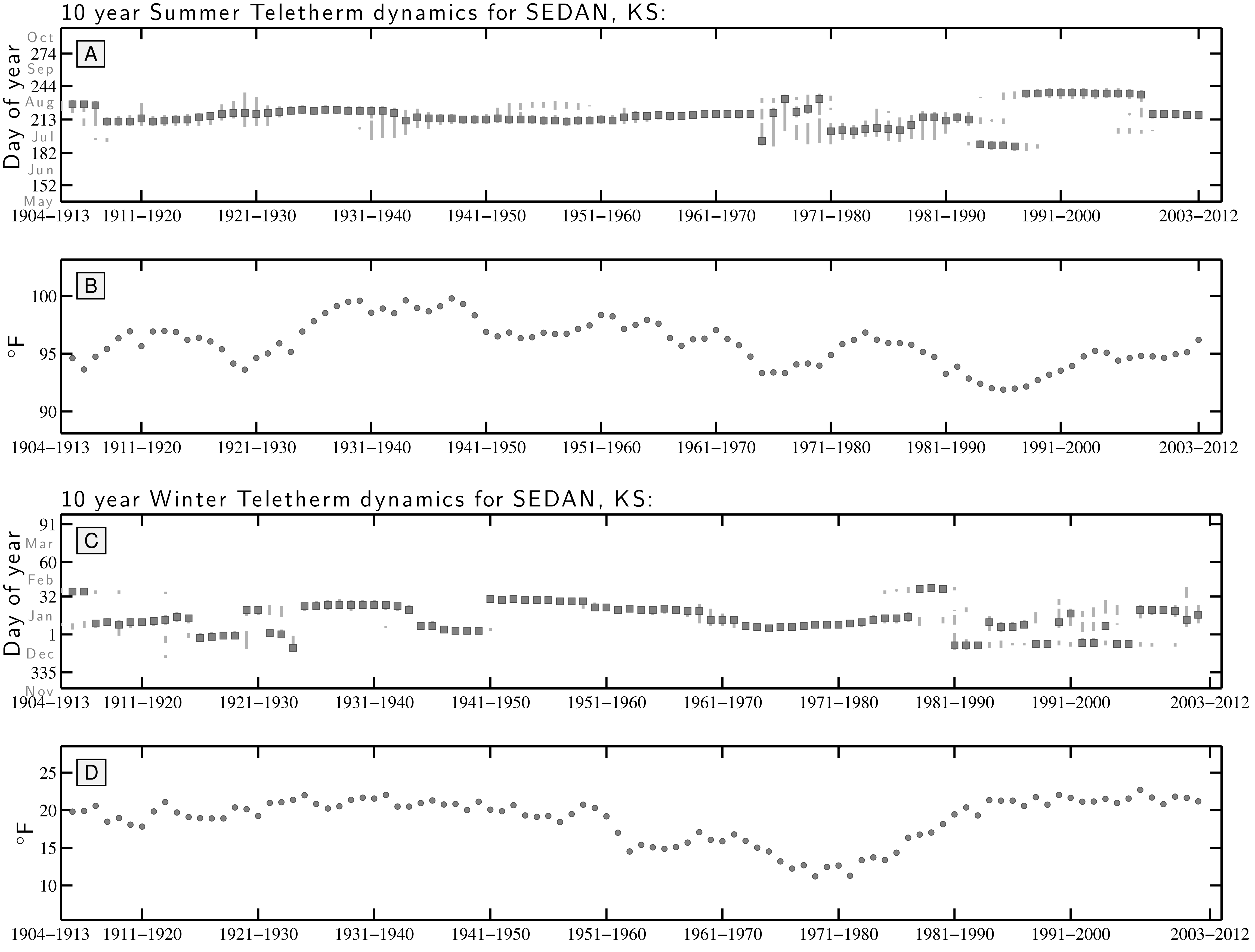
10 year Summer Teletherm dynamics for SEDAN, KS:
A
Day of year
Oct
274
Sep
244
Aug
213
Jul
182
Jun
152
May
B
°F
100
95
90
10 year Winter Teletherm dynamics for SEDAN, KS:
C
Day of year
91
Mar
60
Feb
32
Jan
1
Dec
335
Nov
D
°F
25
20
15
10
1904–1913
1911–1920
1921–1930
1931–1940
1941–1950
1951–1960
1961–1970
1971–1980
1981–1990
1991–2000
2003–2012

10 year Summer Teletherm dynamics for SMITH CTR, KS:

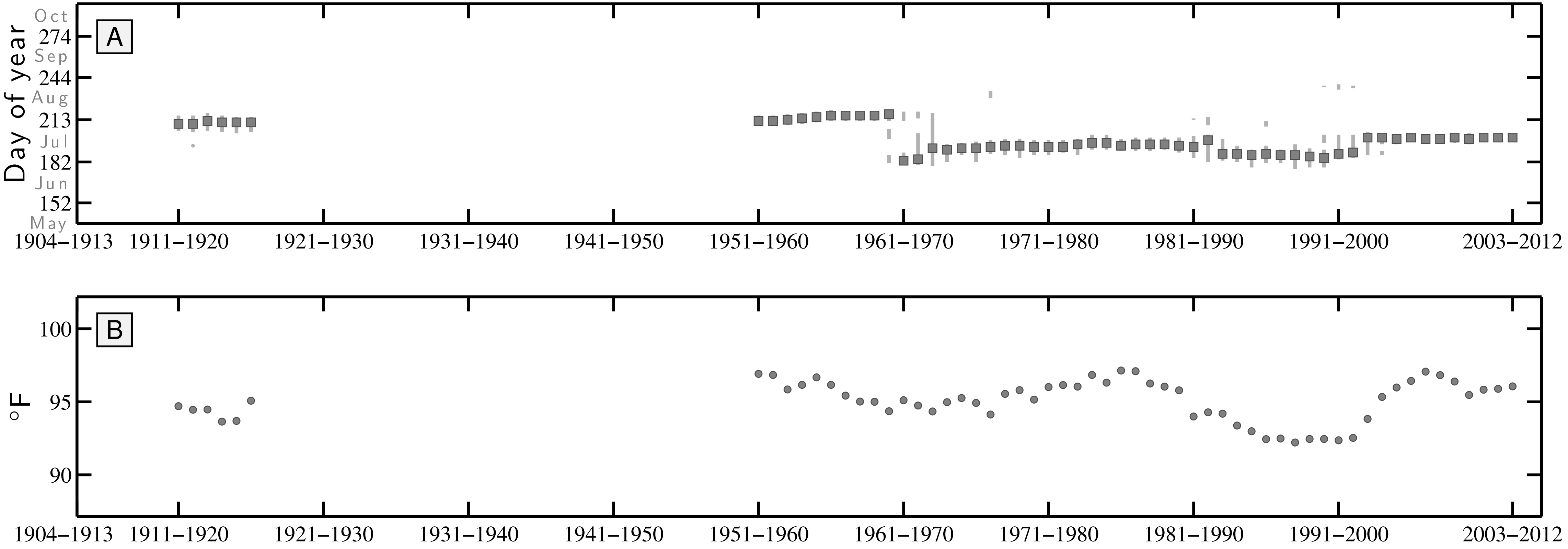

10 year Winter Teletherm dynamics for SMITH CTR, KS:

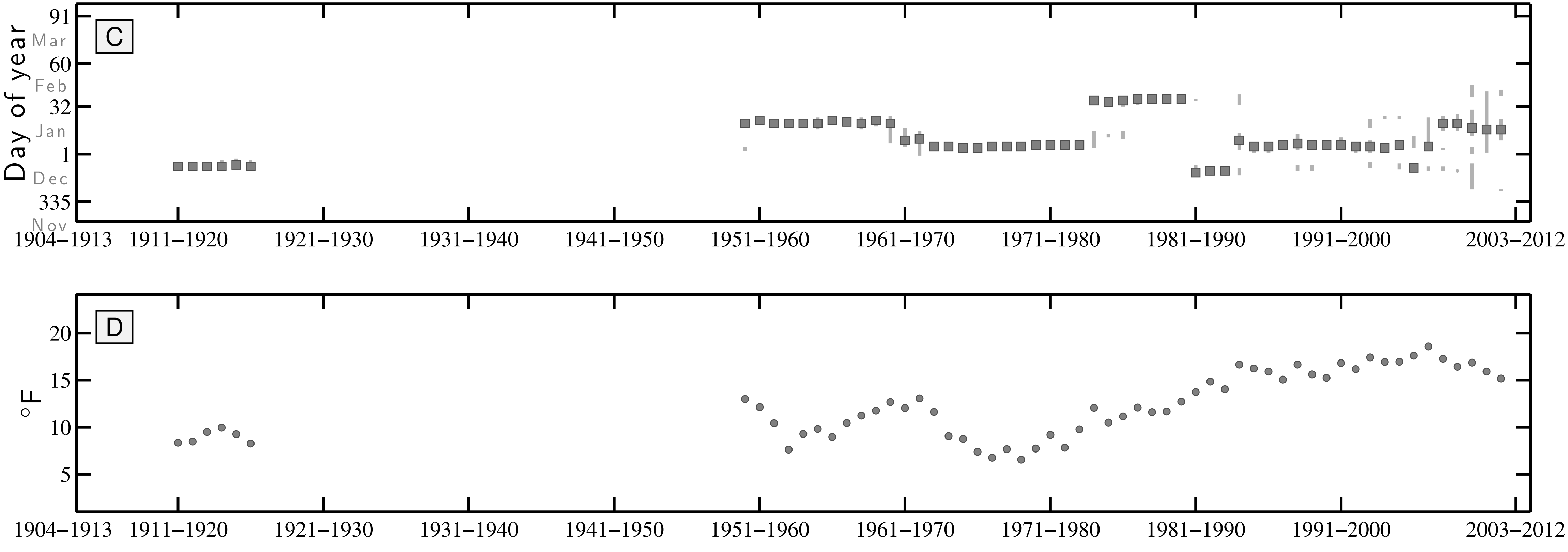

10 year Summer Teletherm dynamics for WAKEENEY, KS:

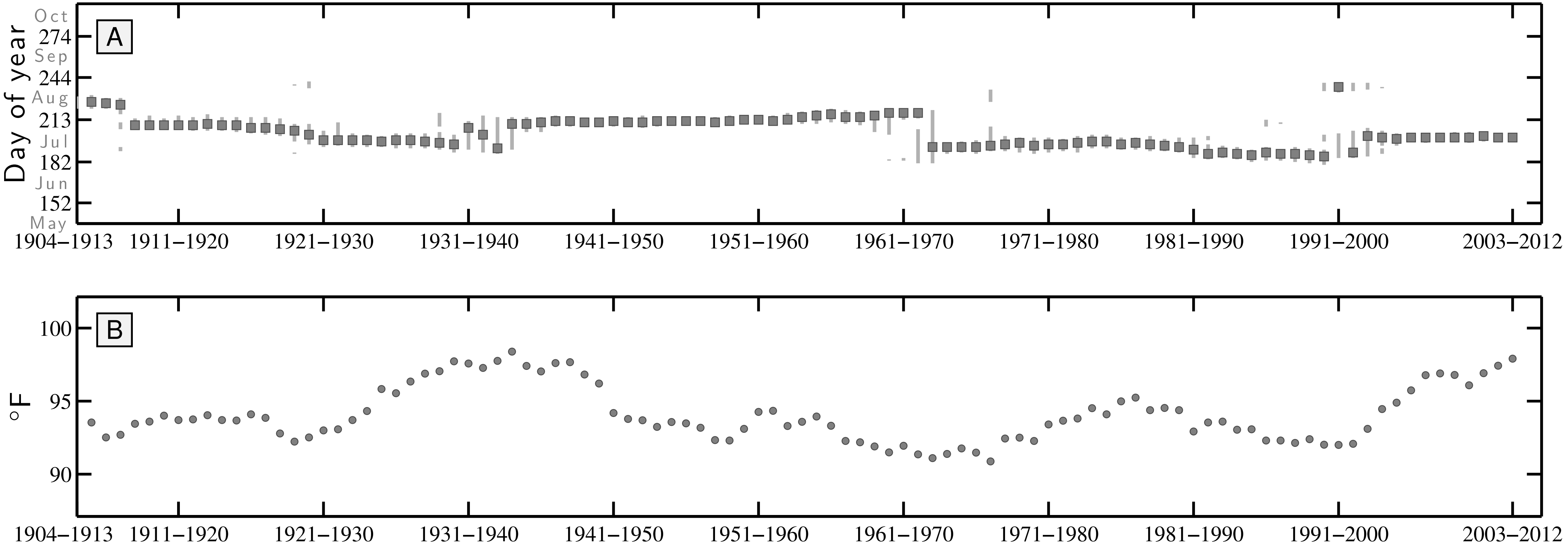

10 year Winter Teletherm dynamics for WAKEENEY, KS:

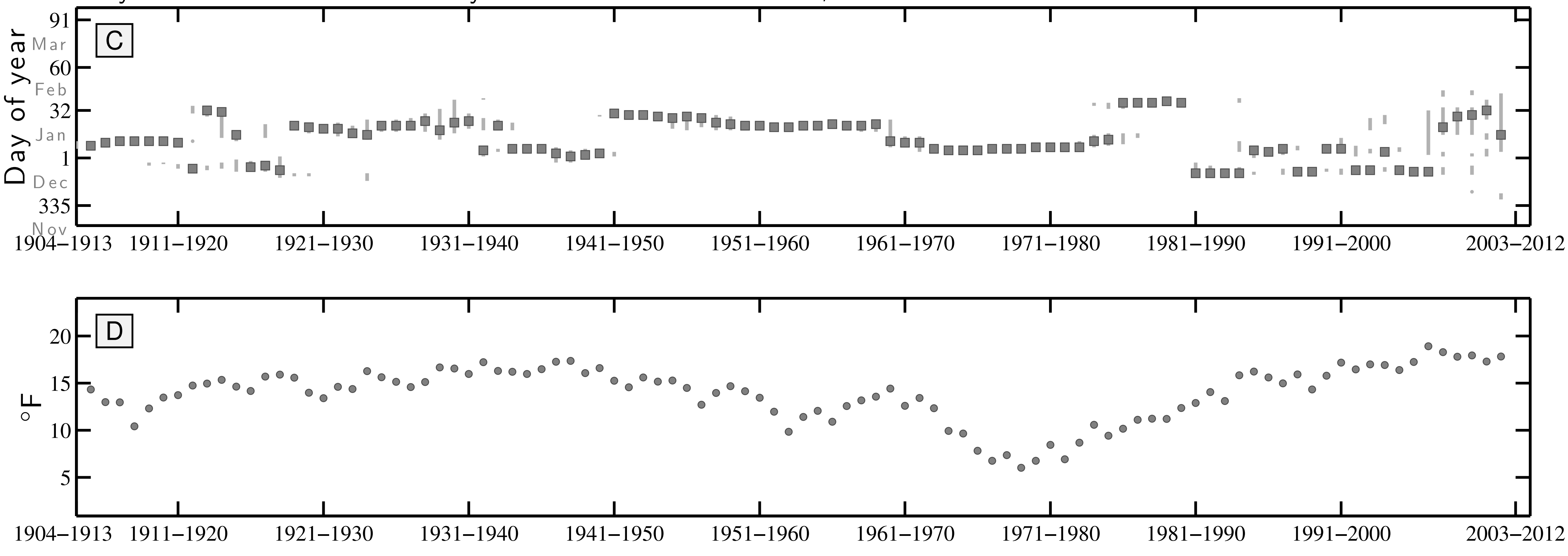

10 year Summer Teletherm dynamics for ASHLAND, KY:

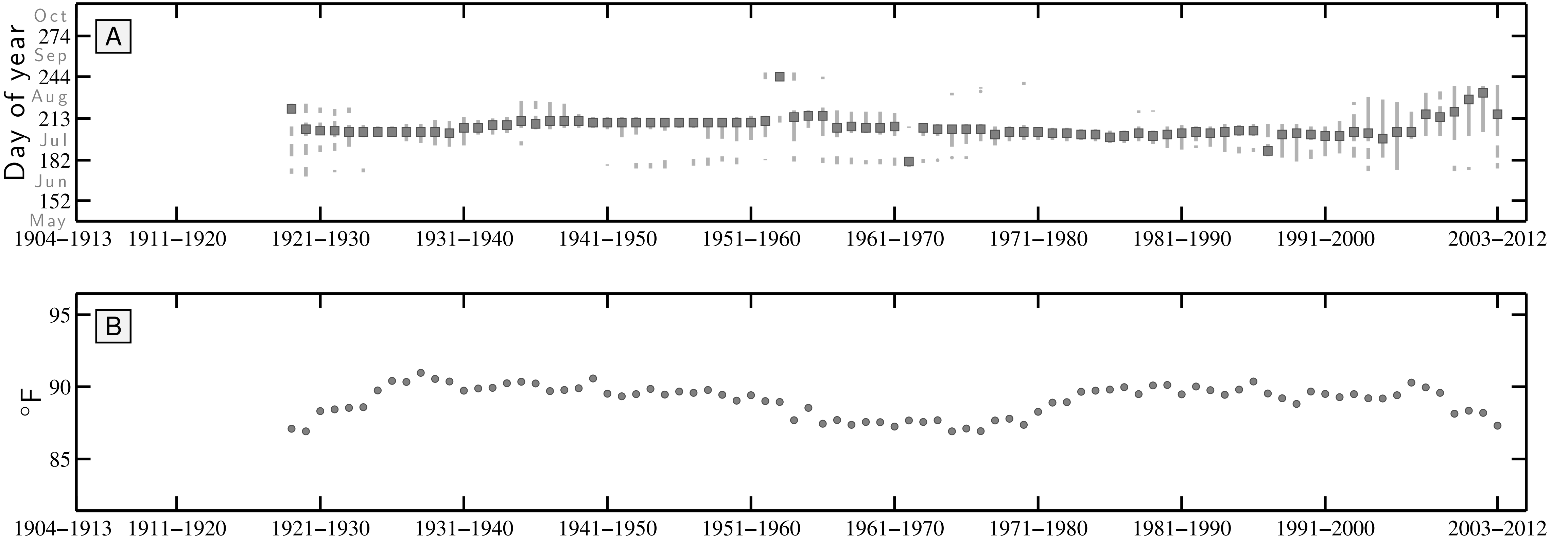

10 year Winter Teletherm dynamics for ASHLAND, KY:

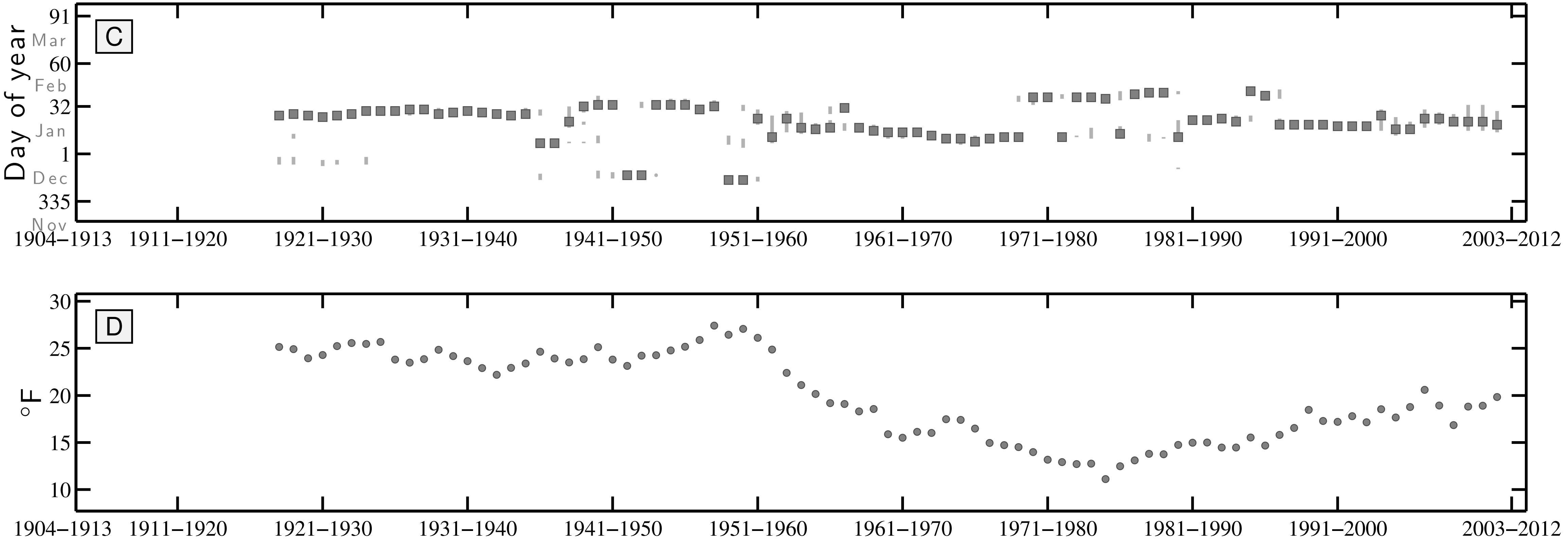

10 year Summer Teletherm dynamics for BARBOURVILLE, KY:

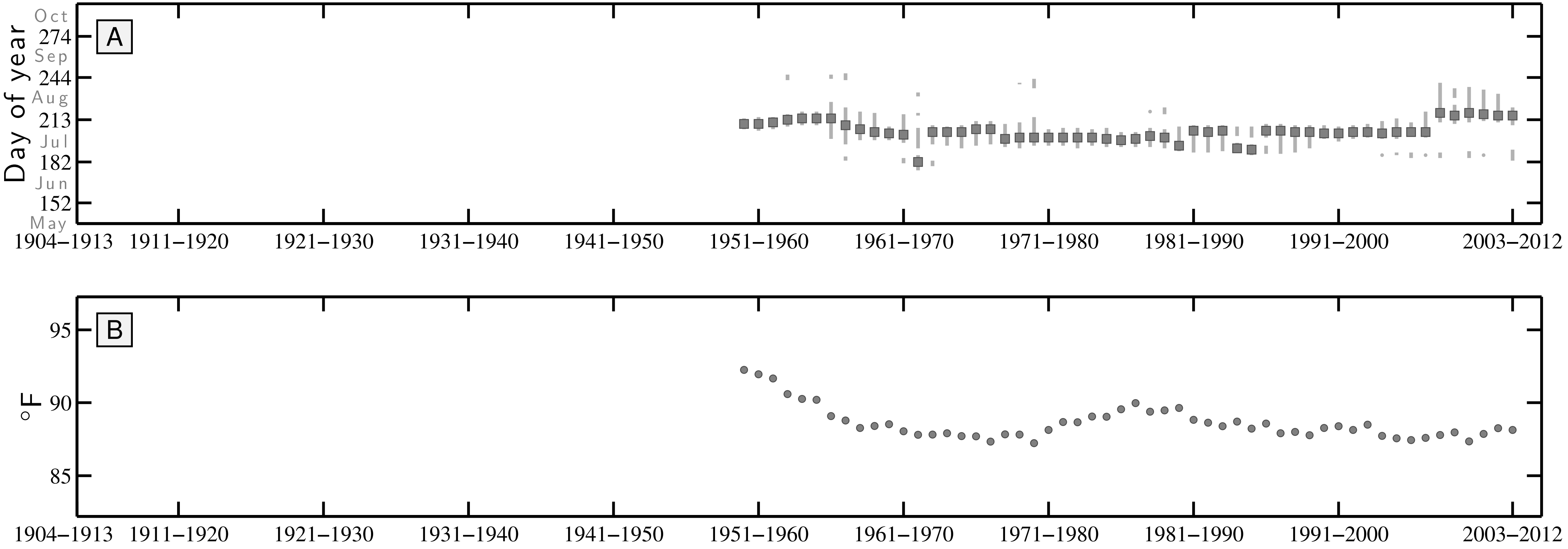

10 year Winter Teletherm dynamics for BARBOURVILLE, KY:

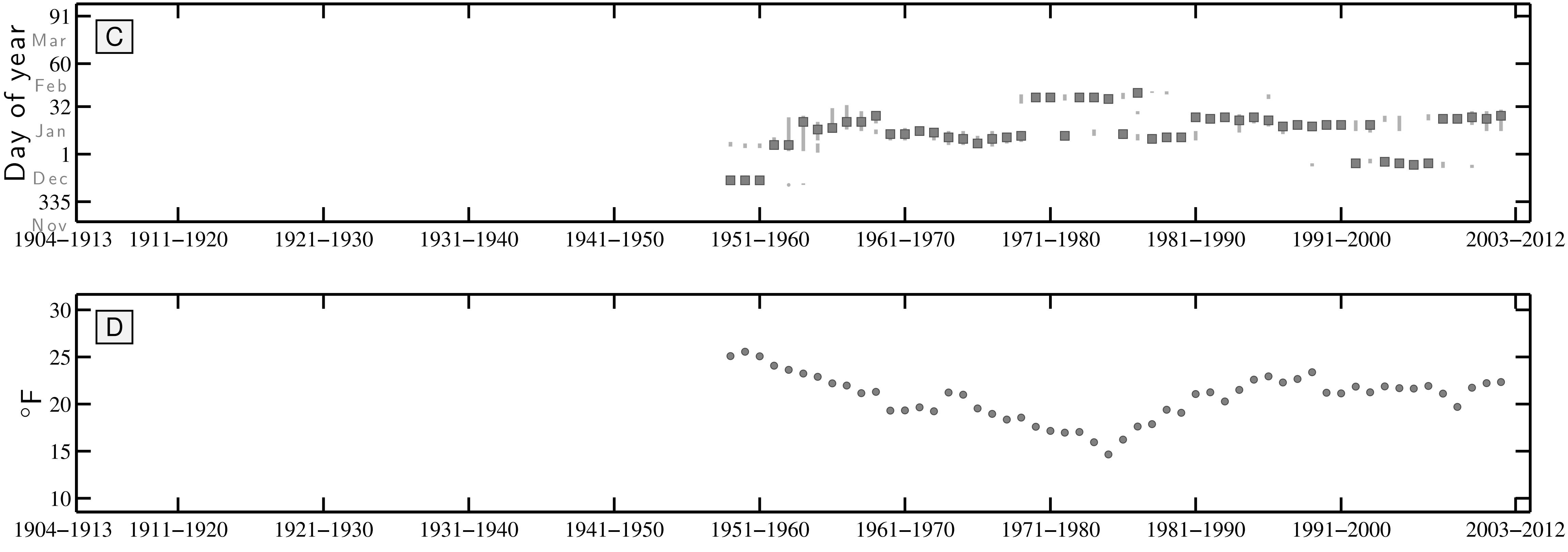

10 year Summer Teletherm dynamics for BEREA COLLEGE, KY:

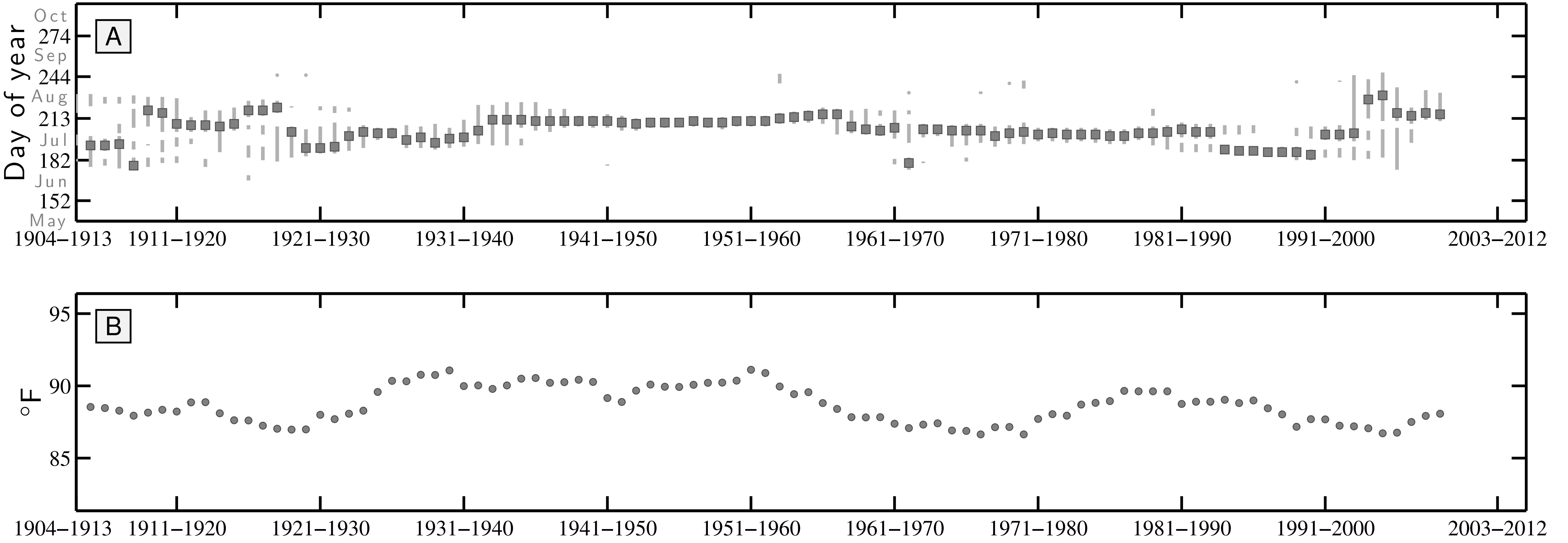

10 year Winter Teletherm dynamics for BEREA COLLEGE, KY:

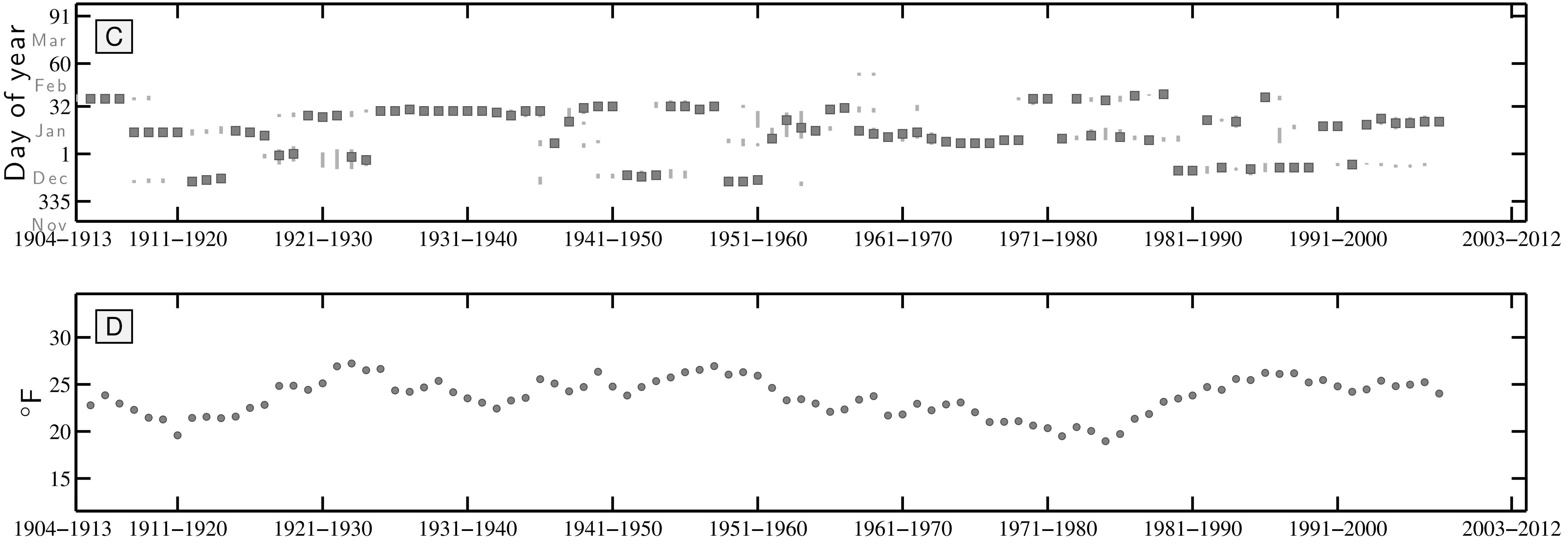

10 year Summer Teletherm dynamics for BOWLING GREEN RGNL AP, KY:

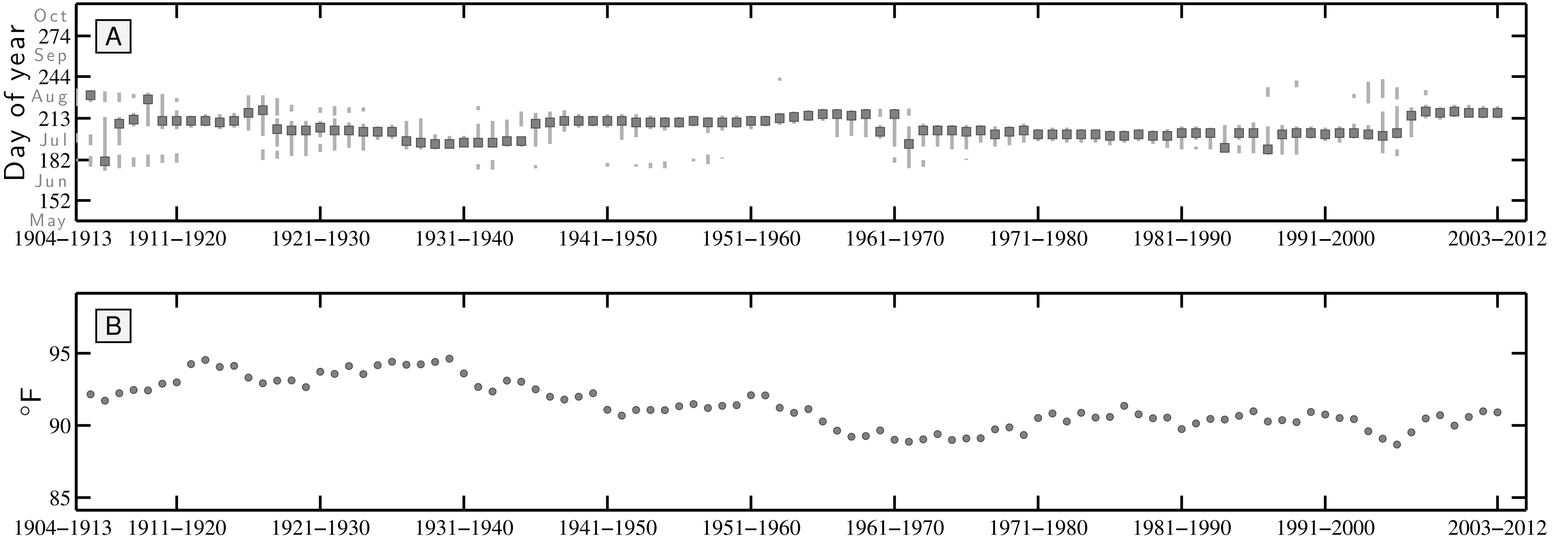

10 year Winter Teletherm dynamics for BOWLING GREEN RGNL AP, KY:

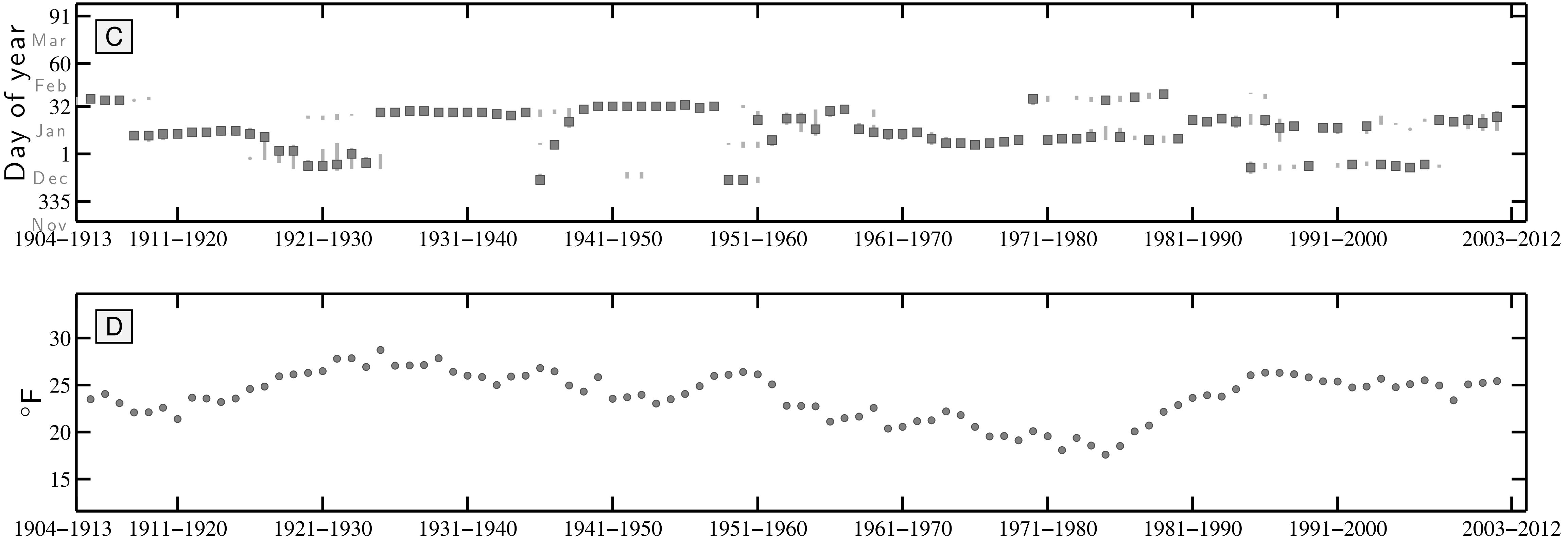

10 year Summer Teletherm dynamics for FARMERS 2 S, KY:

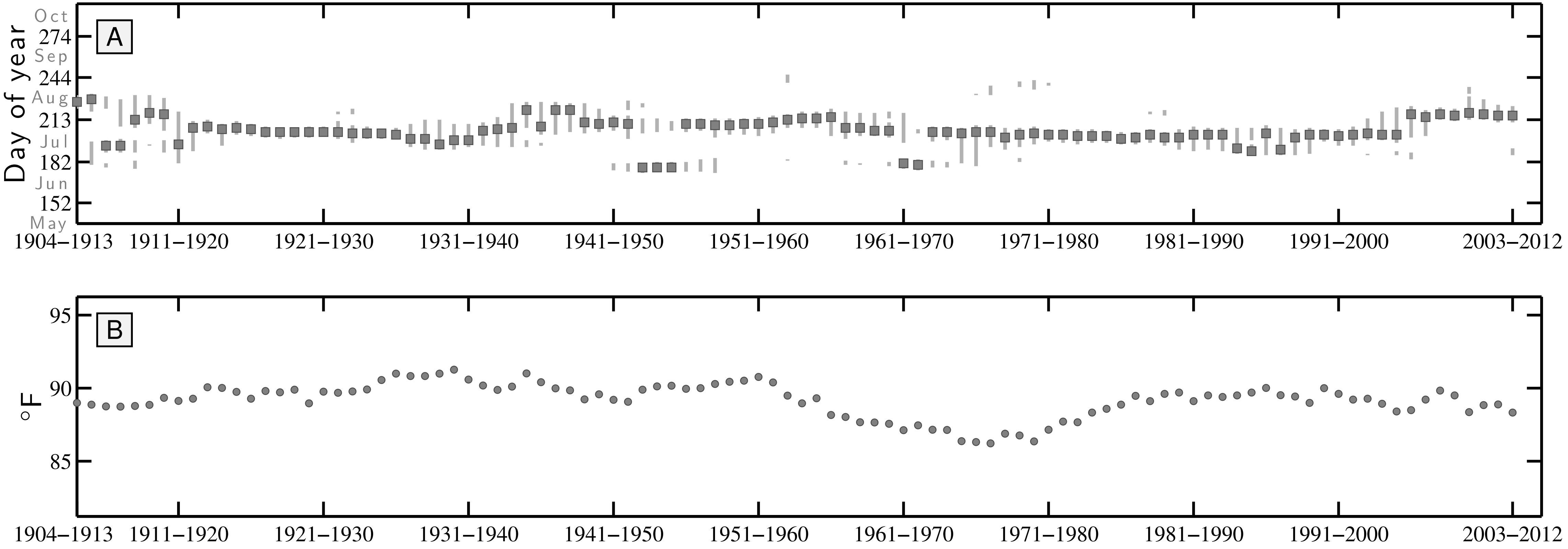

10 year Winter Teletherm dynamics for FARMERS 2 S, KY:

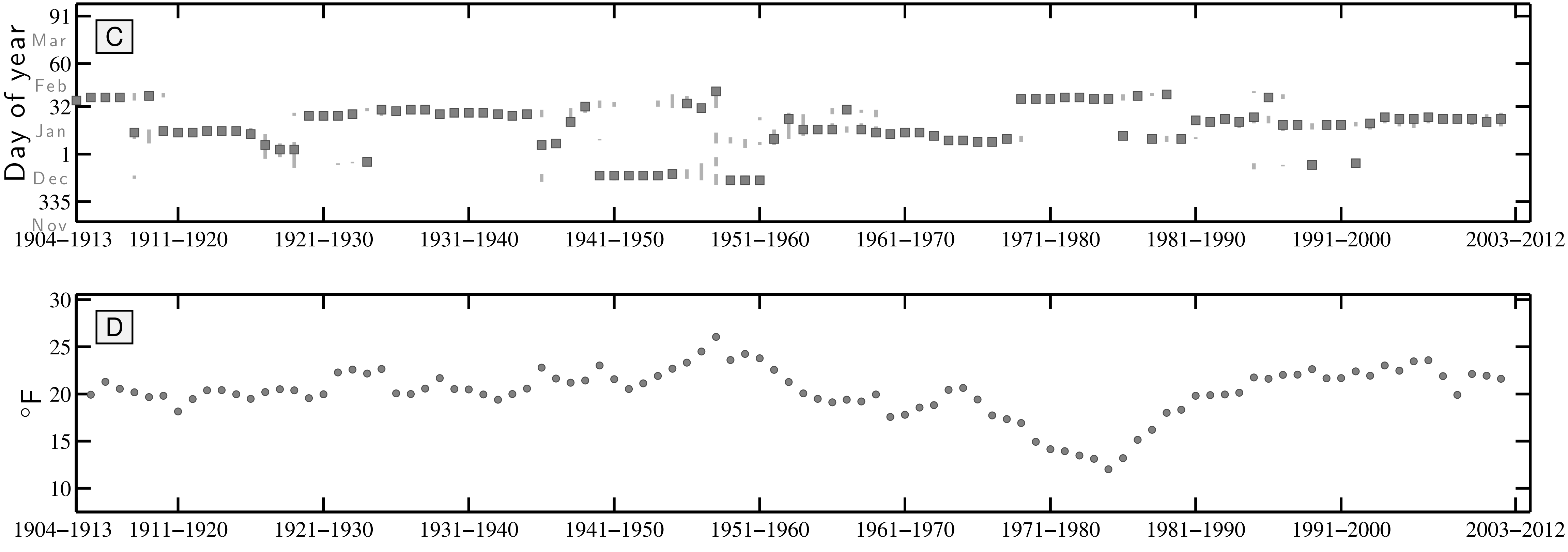

10 year Summer Teletherm dynamics for FRANKFORT DOWNTOWN, KY:

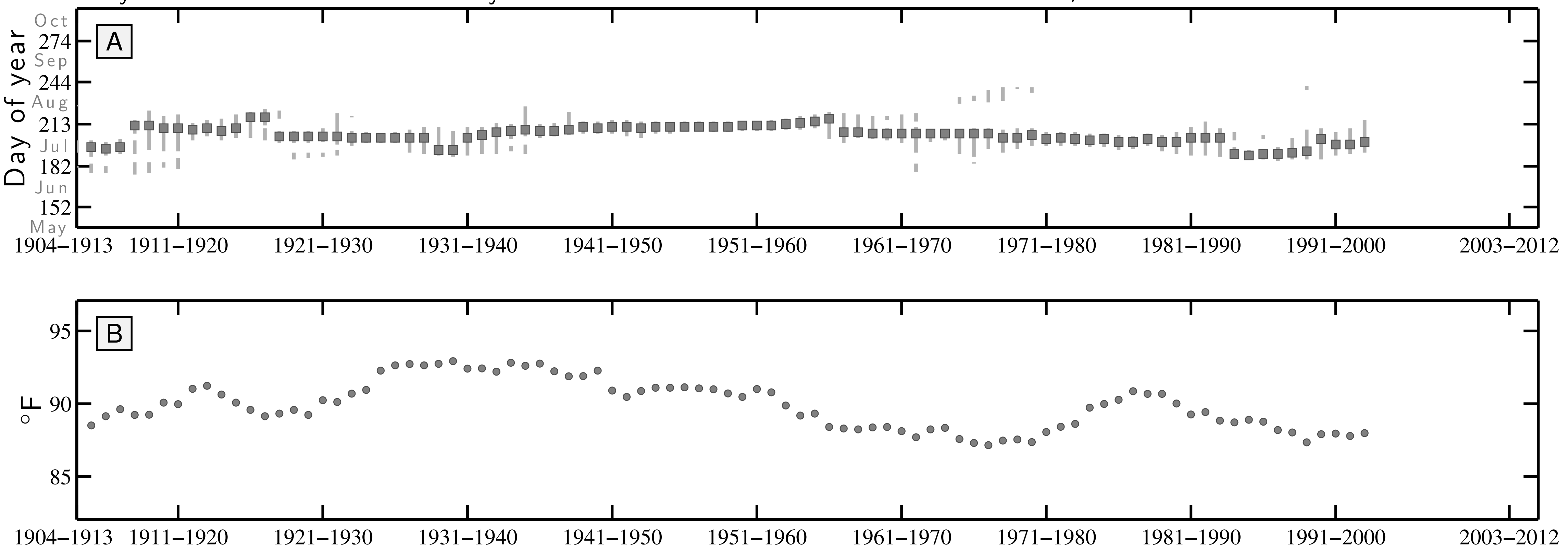

10 year Winter Teletherm dynamics for FRANKFORT DOWNTOWN, KY:

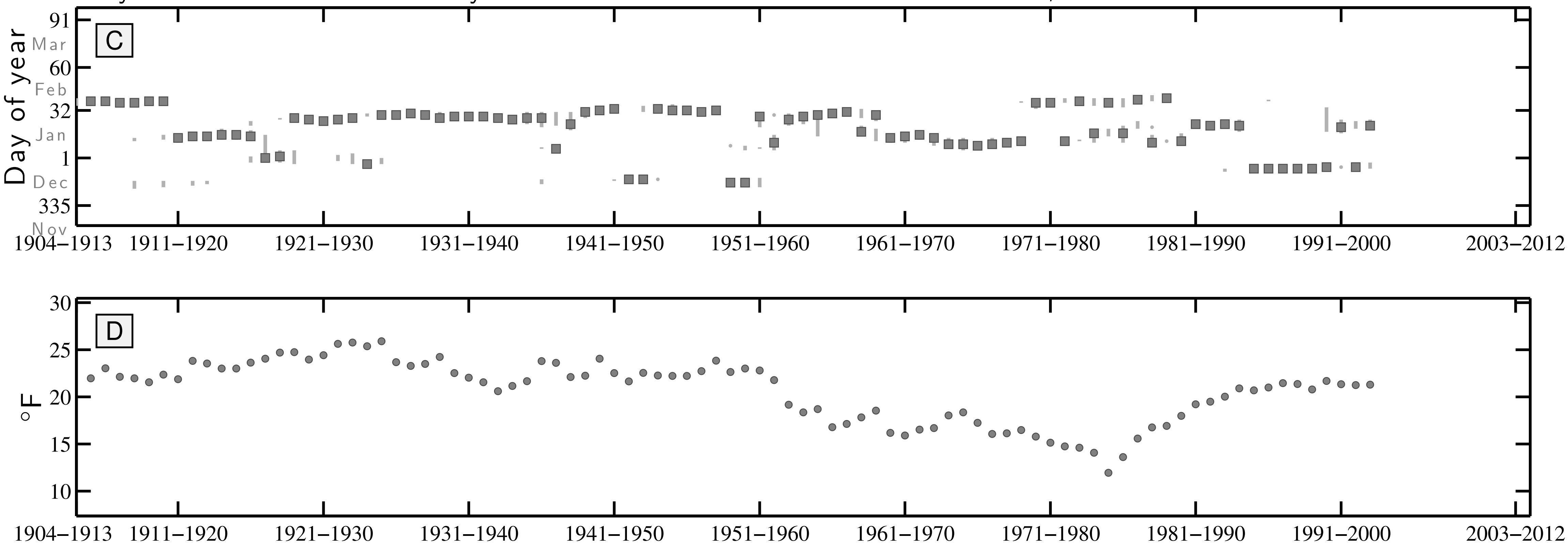

10 year Summer Teletherm dynamics for GREENSBURG, KY:

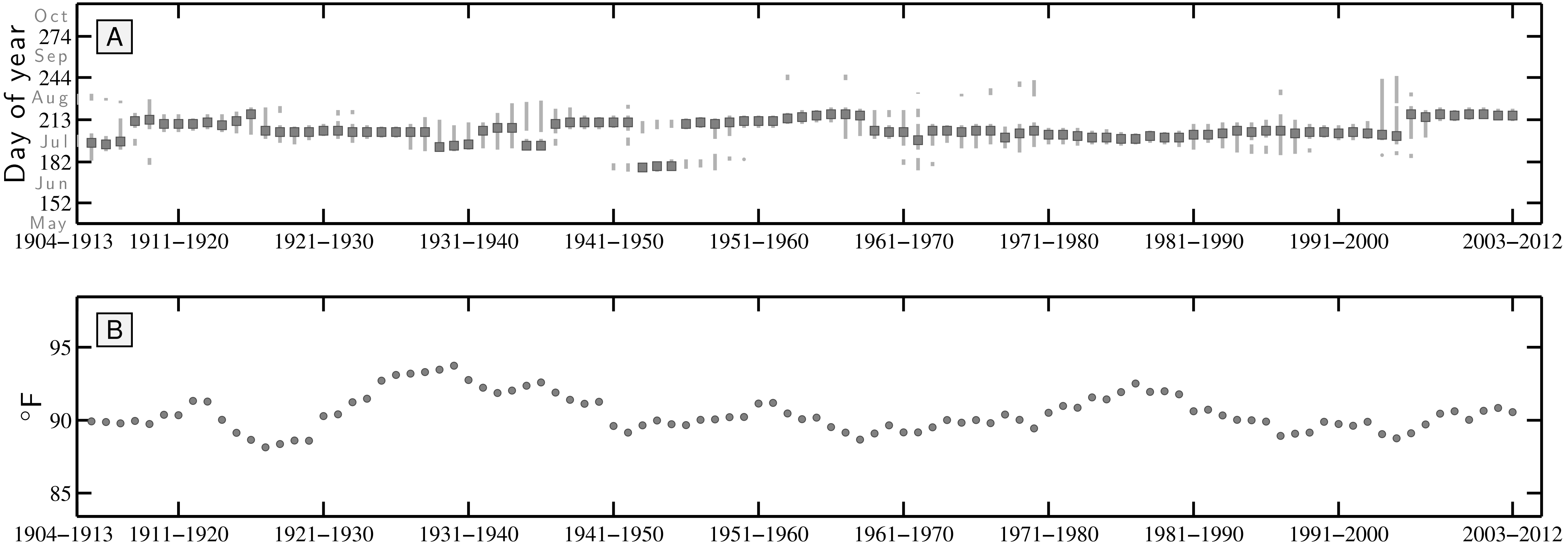

10 year Winter Teletherm dynamics for GREENSBURG, KY:

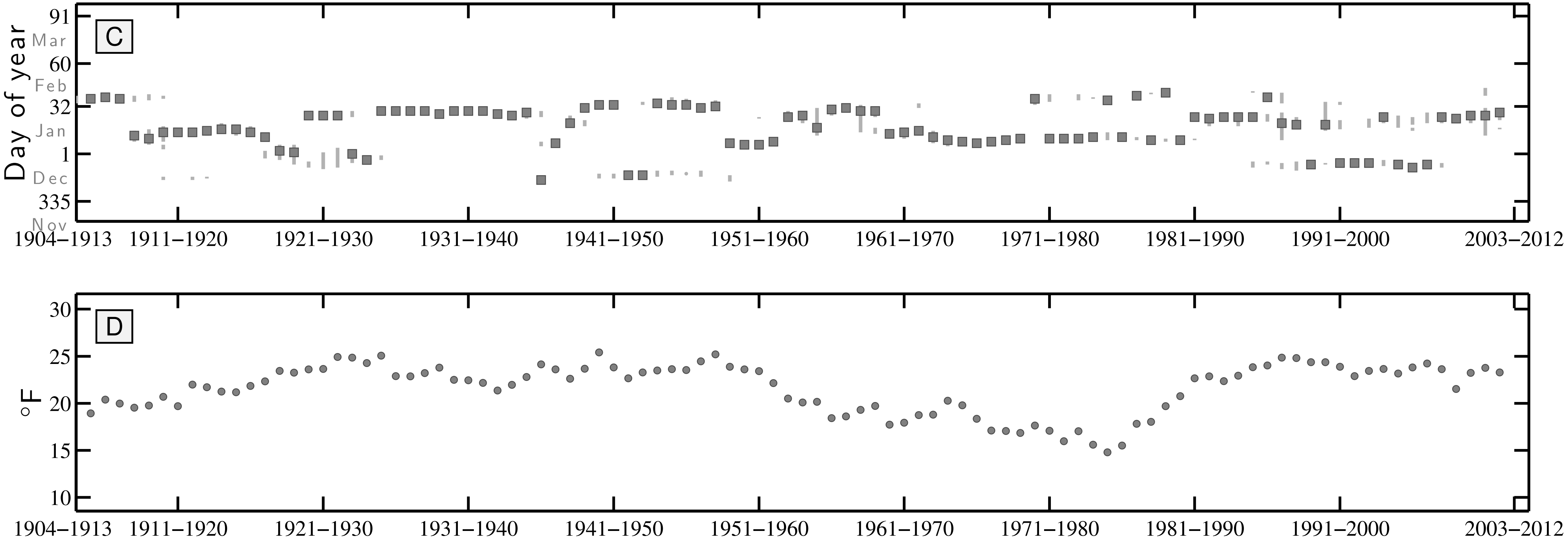

10 year Summer Teletherm dynamics for HENDERSON 8 SSW, KY:

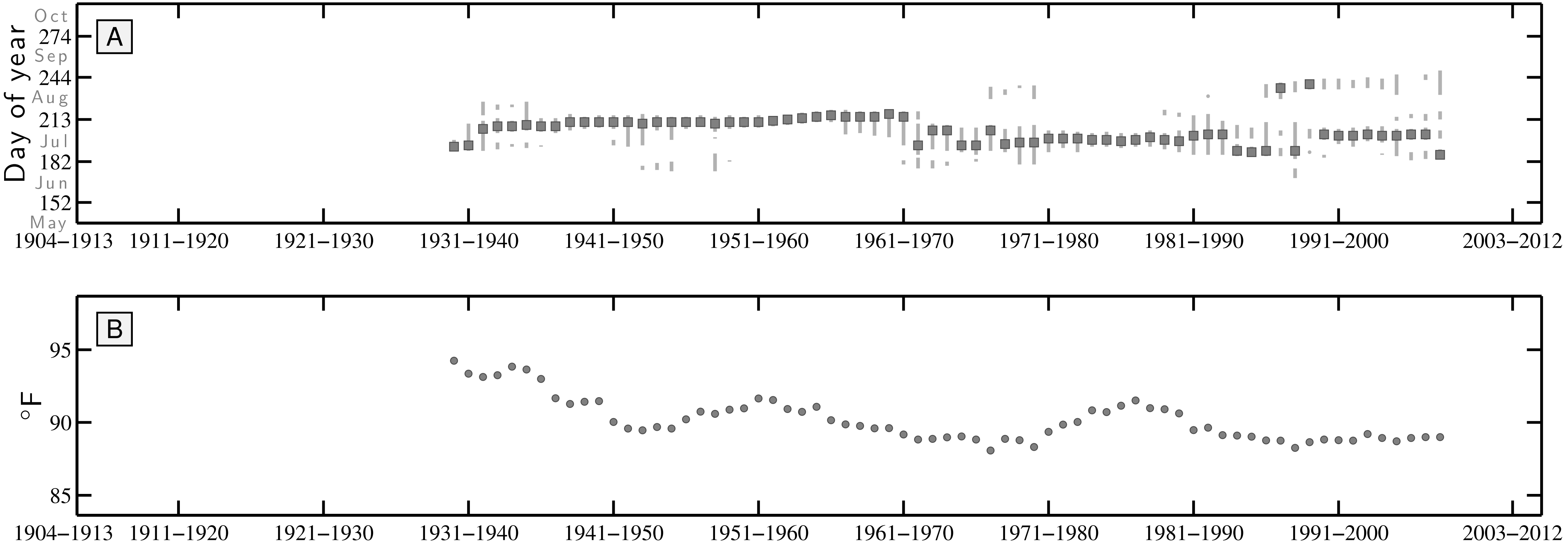

10 year Winter Teletherm dynamics for HENDERSON 8 SSW, KY:

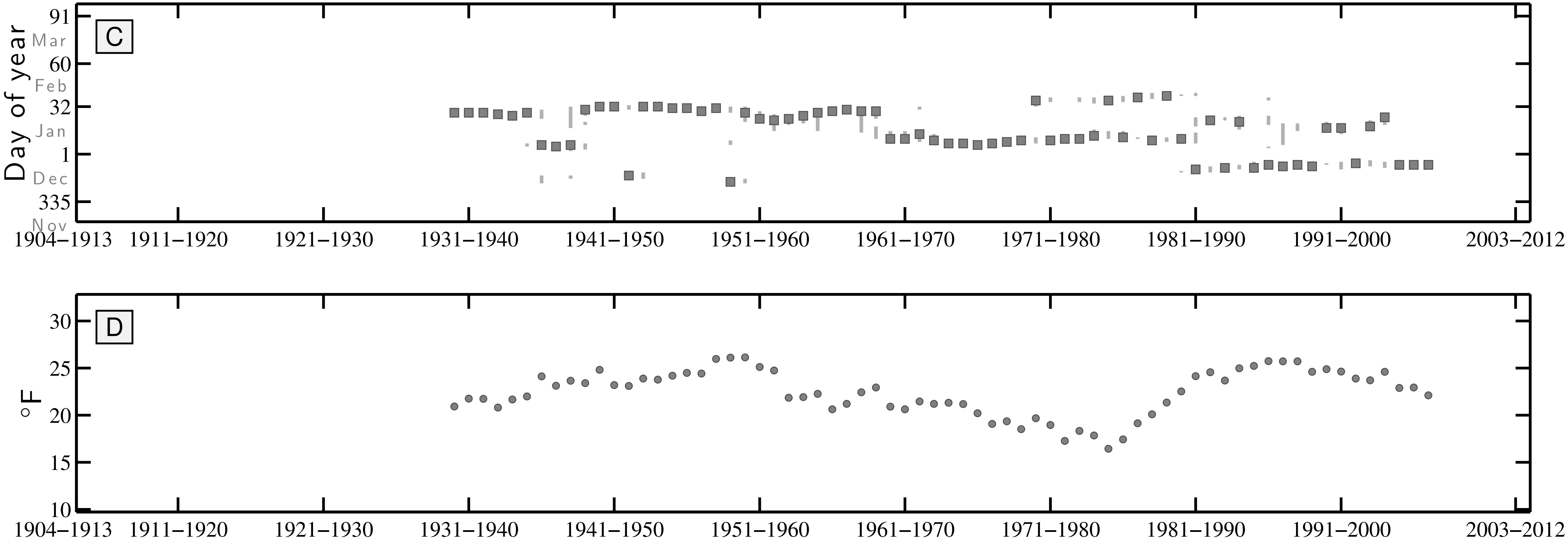

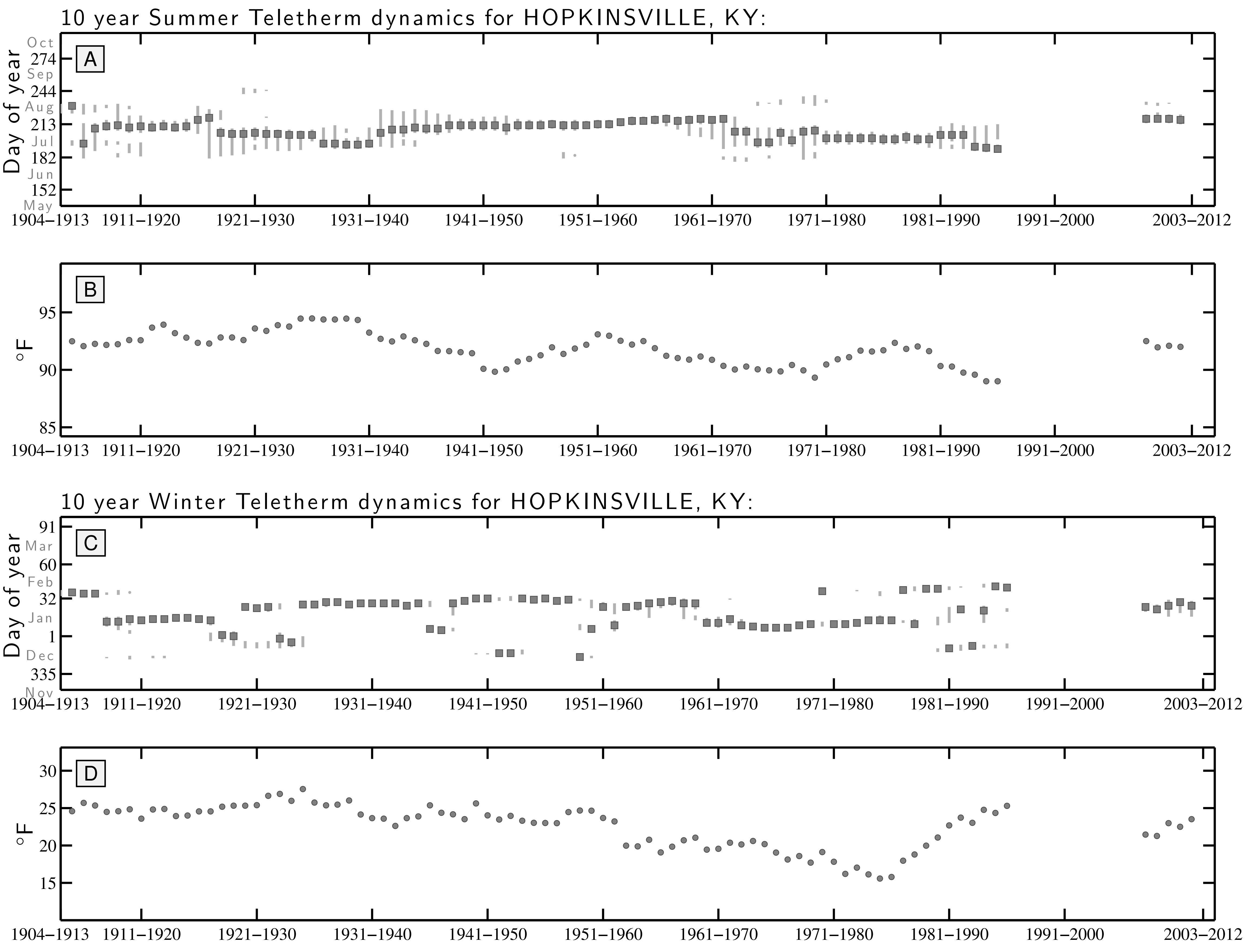
10 year Summer Teletherm dynamics for HOPKINSVILLE, KY:
A
Day of year
Oct
274
Sep
244
Aug
213
Jul
182
Jun
152
May
1904–1913
1911–1920
1921–1930
1931–1940
1941–1950
1951–1960
1961–1970
1971–1980
1981–1990
1991–2000
2003–2012
B
°F
95
90
85
10 year Winter Teletherm dynamics for HOPKINSVILLE, KY:
C
91
Mar
60
Feb
32
Jan
1
Dec
335
Nov
D
30
25
20
15

10 year Summer Teletherm dynamics for LEITCHFIELD 2 N, KY:

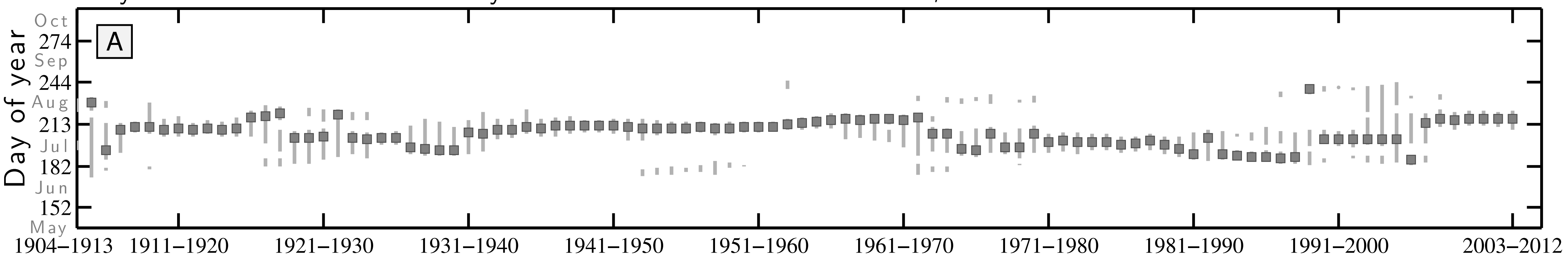

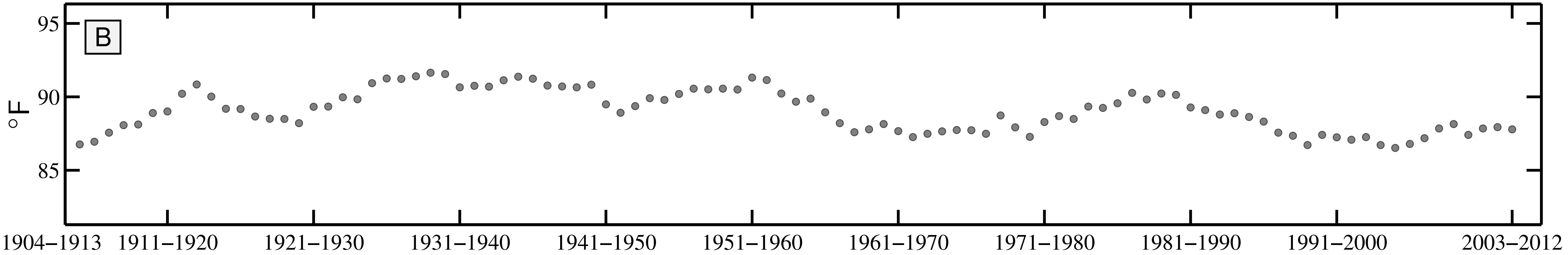

10 year Winter Teletherm dynamics for LEITCHFIELD 2 N, KY:

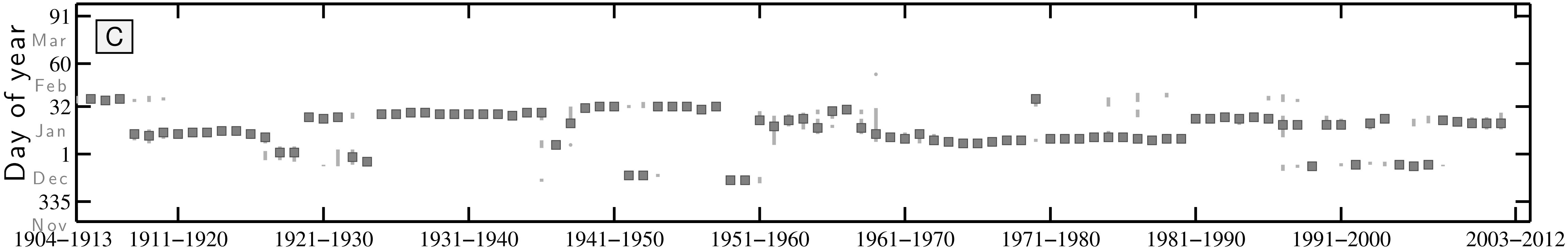

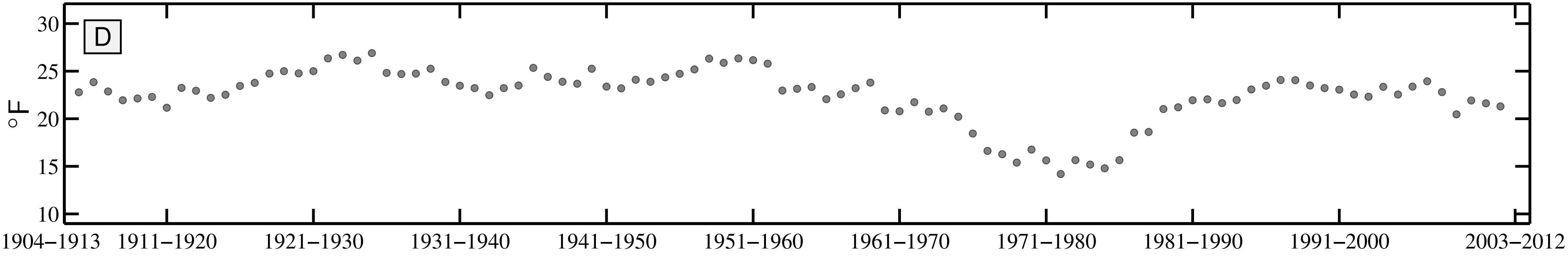

10 year Summer Teletherm dynamics for SHELBYVILLE 1 E, KY:

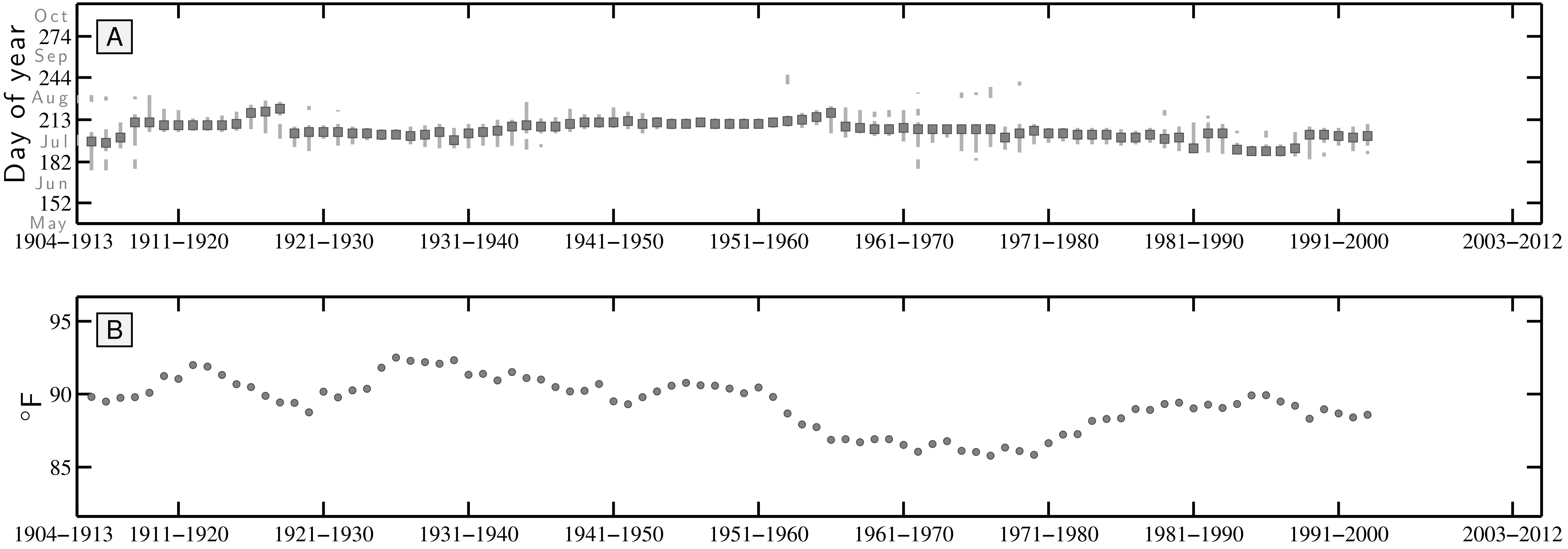

10 year Winter Teletherm dynamics for SHELBYVILLE 1 E, KY:

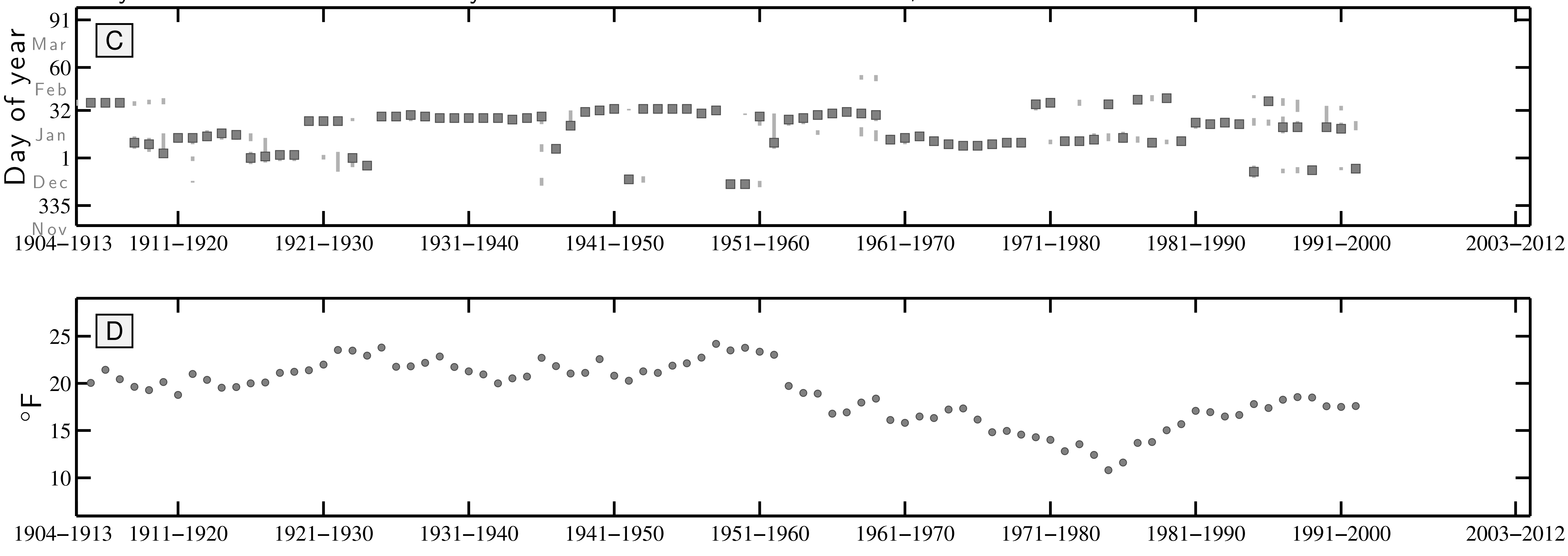

10 year Summer Teletherm dynamics for WILLIAMSBURG, KY:

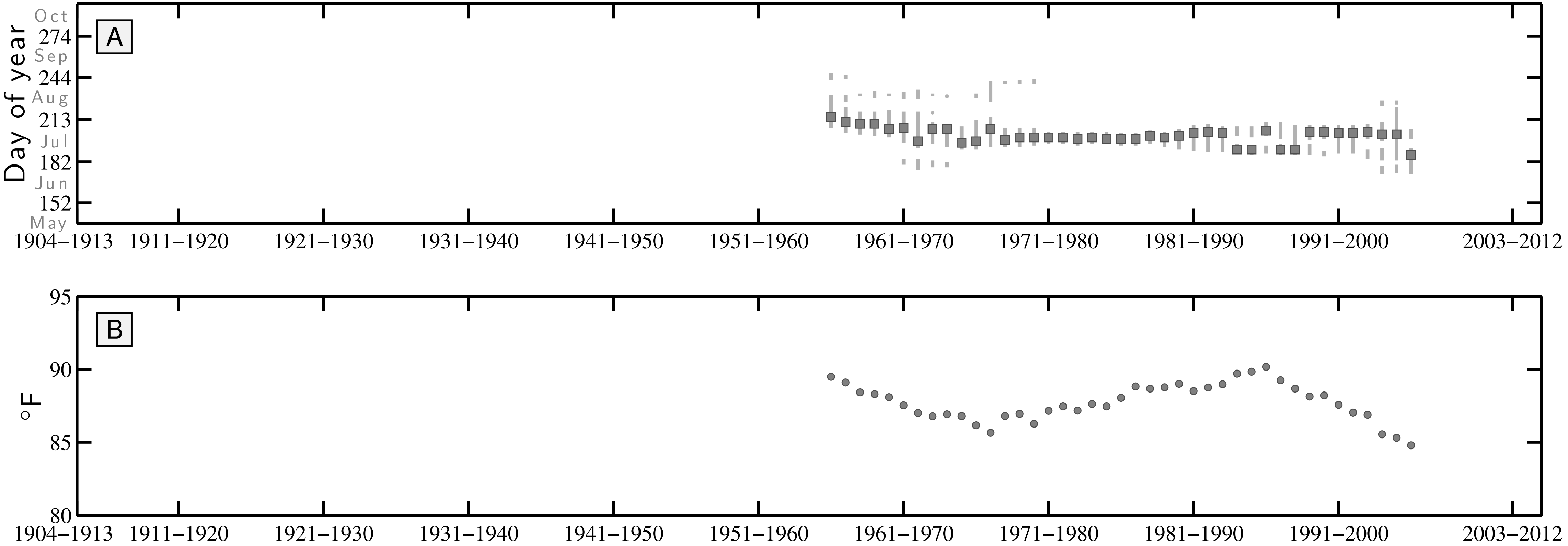

10 year Winter Teletherm dynamics for WILLIAMSBURG, KY:

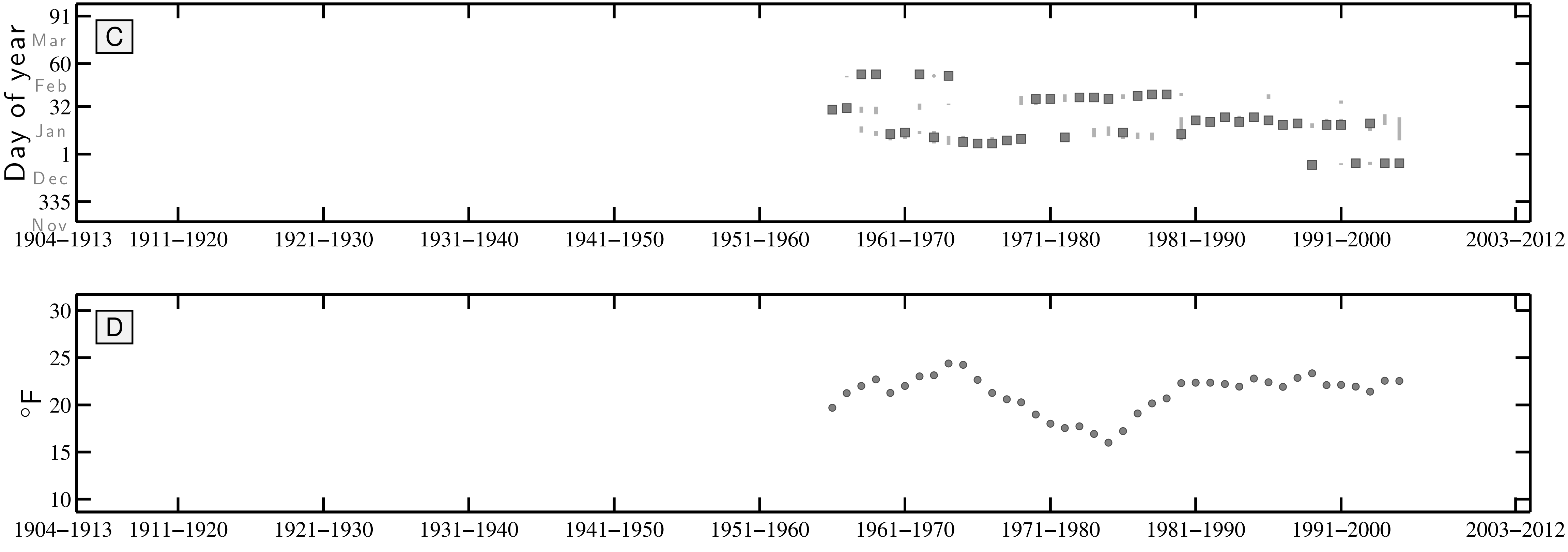

10 year Summer Teletherm dynamics for WILLIAMSTOWN 3 W, KY:

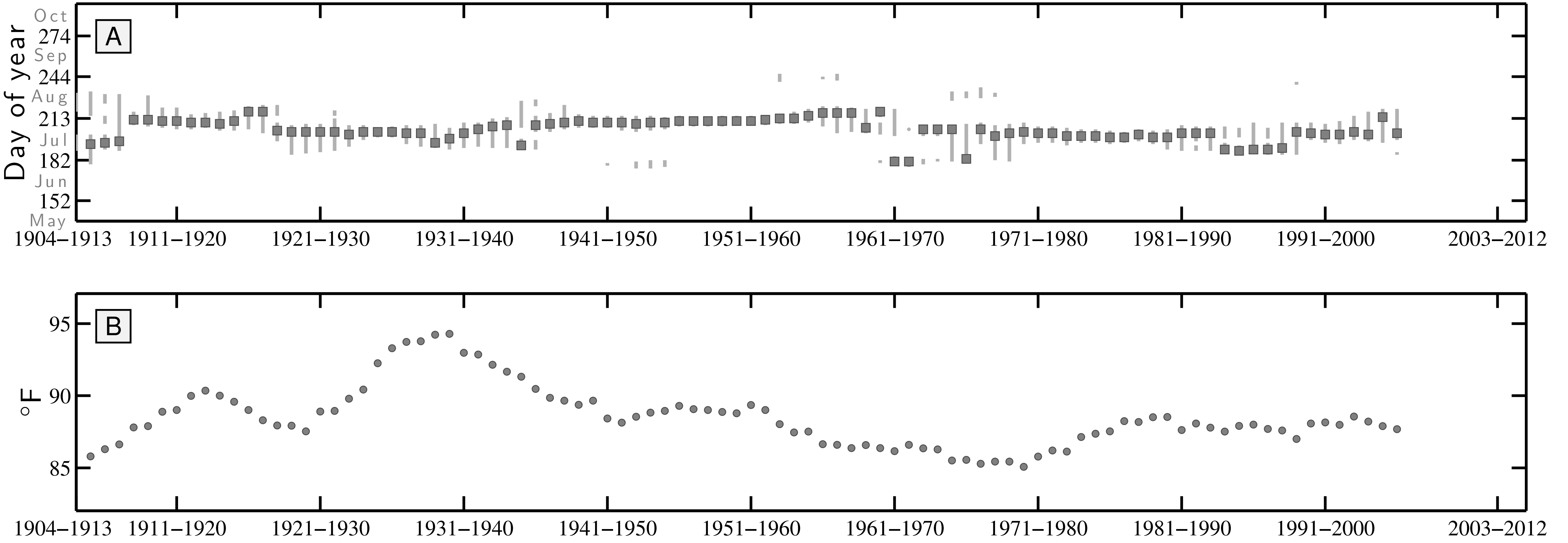

10 year Winter Teletherm dynamics for WILLIAMSTOWN 3 W, KY:

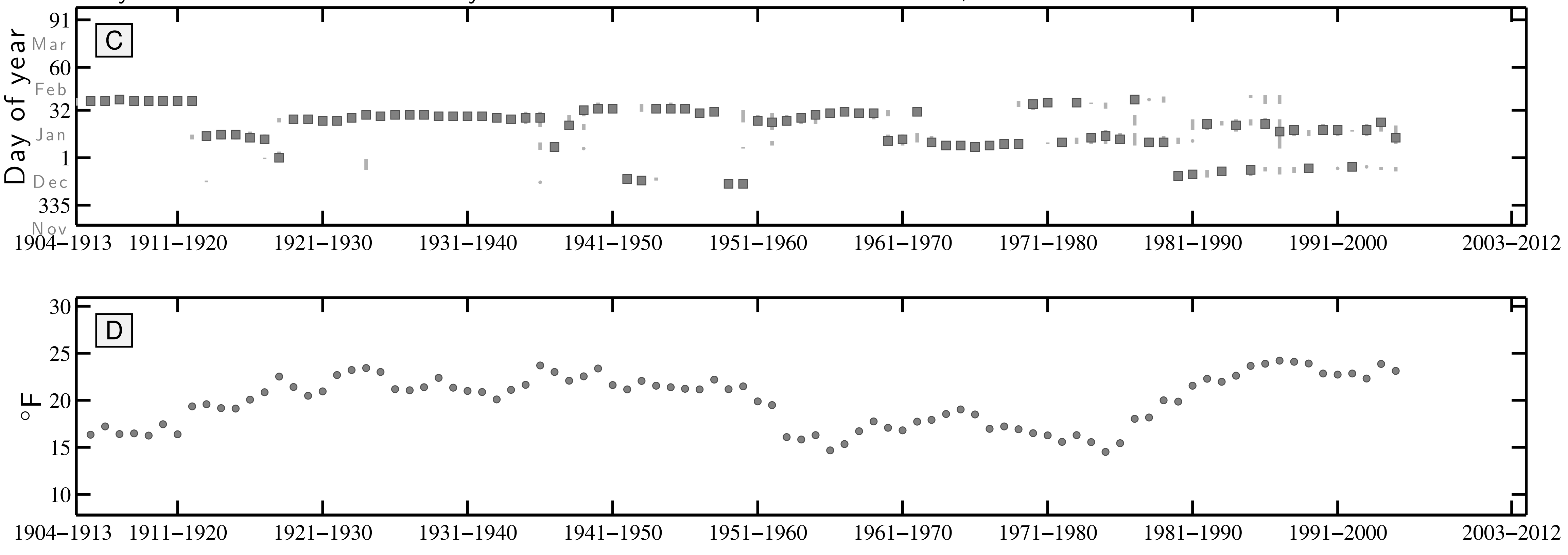

10 year Summer Teletherm dynamics for ALEXANDRIA, LA:

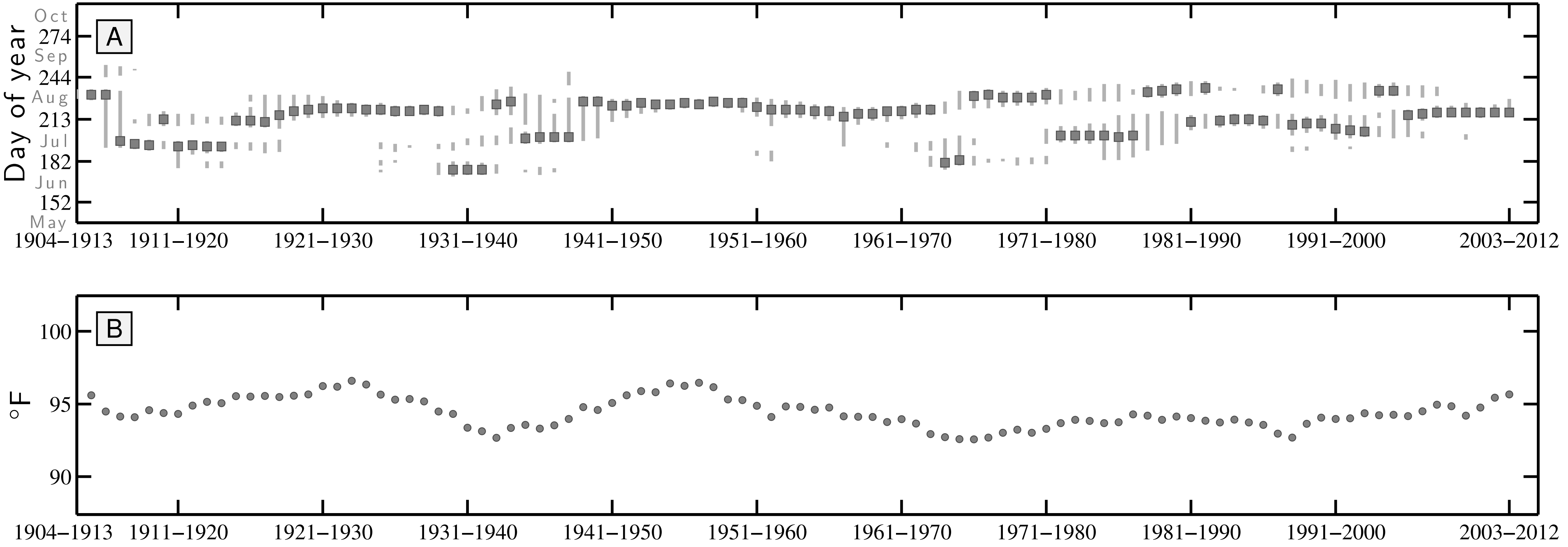

10 year Winter Teletherm dynamics for ALEXANDRIA, LA:

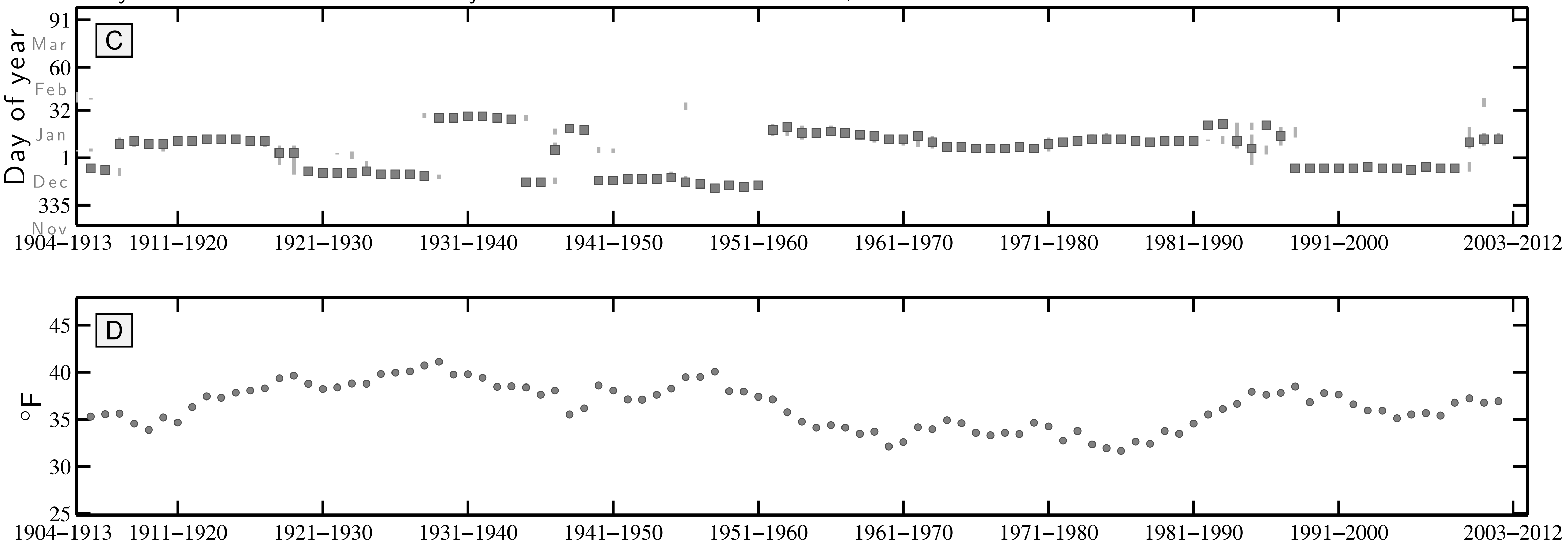

10 year Summer Teletherm dynamics for AMITE, LA:

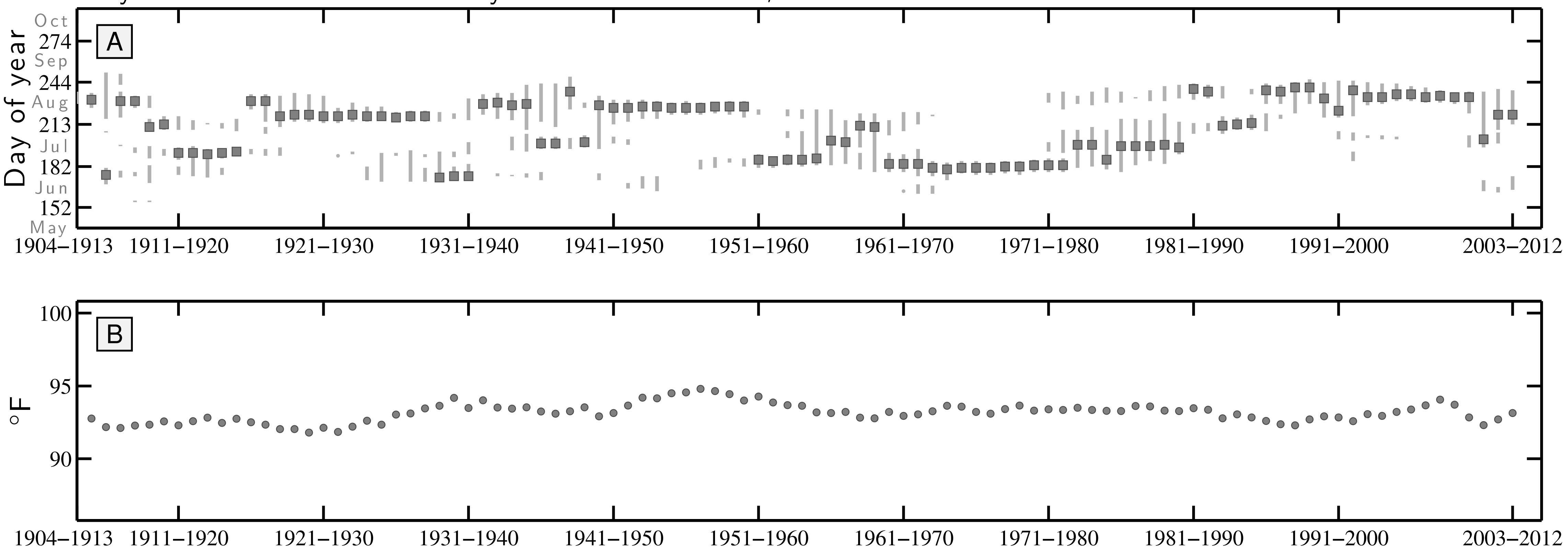

10 year Winter Teletherm dynamics for AMITE, LA:

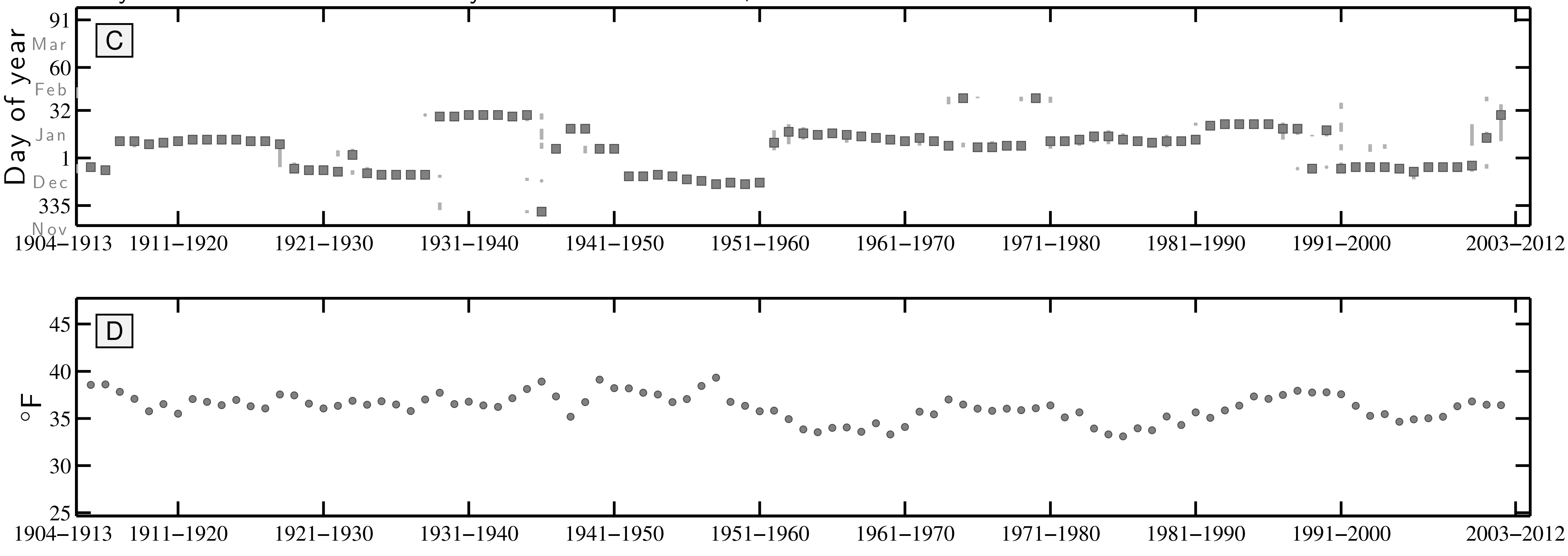

10 year Summer Teletherm dynamics for BASTROP, LA:

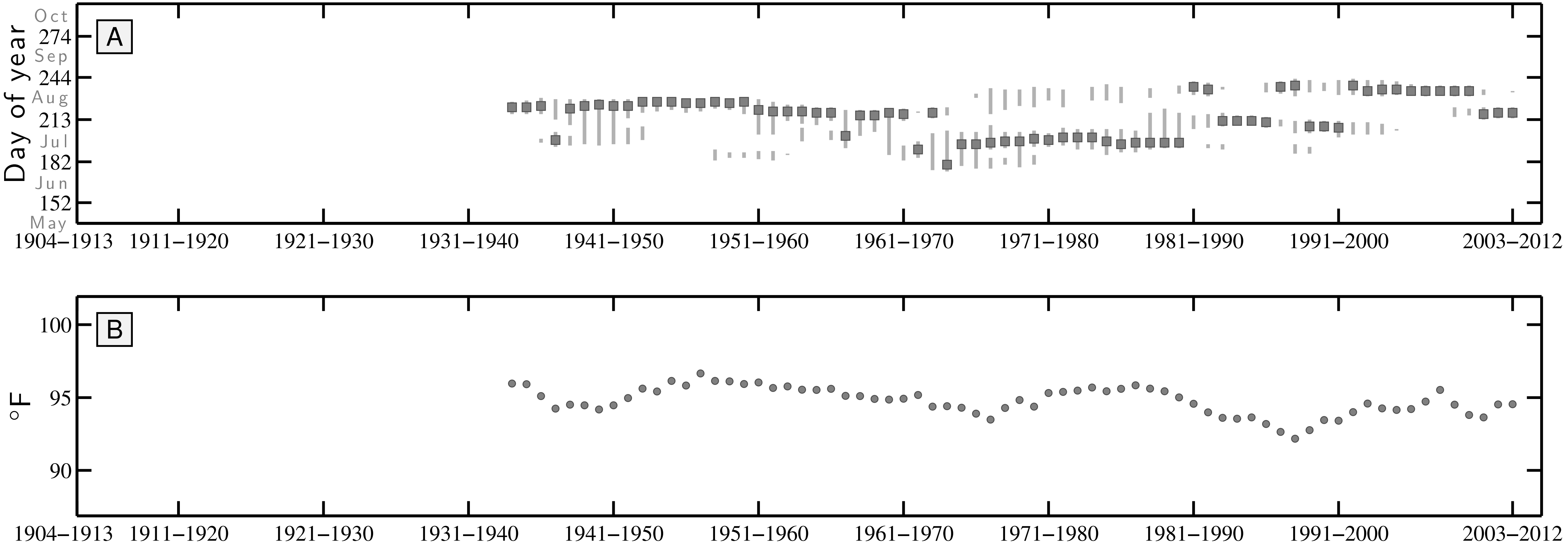

10 year Winter Teletherm dynamics for BASTROP, LA:

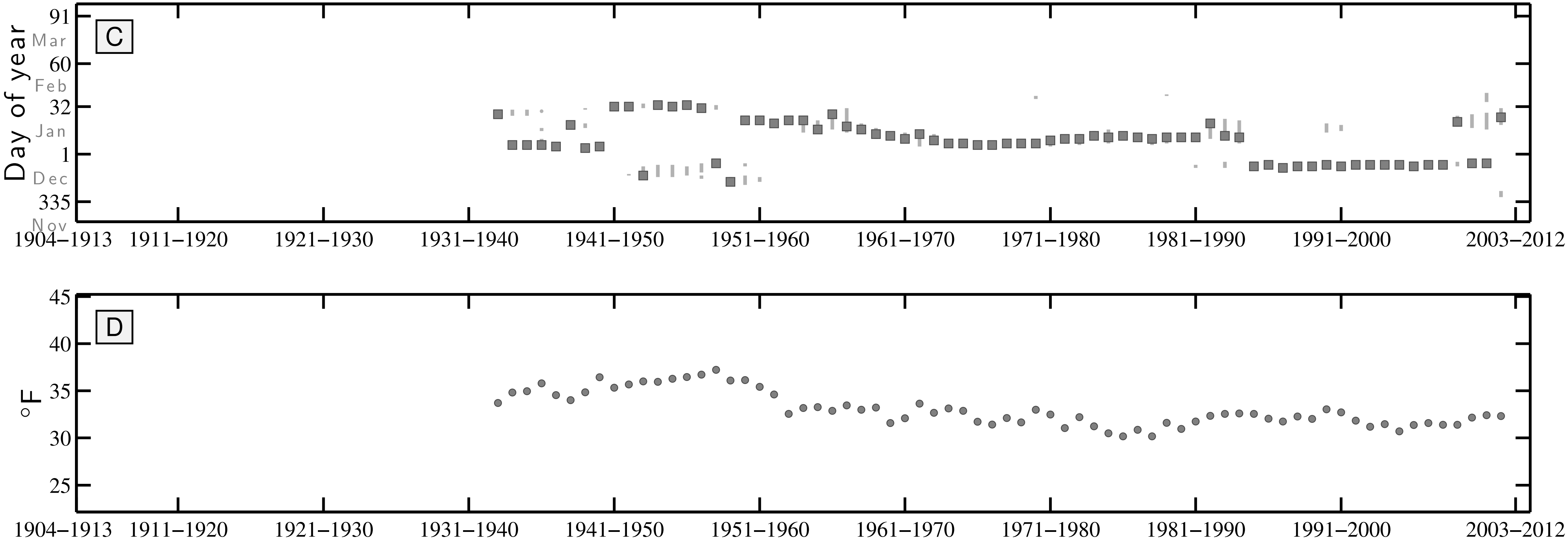

10 year Summer Teletherm dynamics for BATON ROUGE METRO AP, LA:

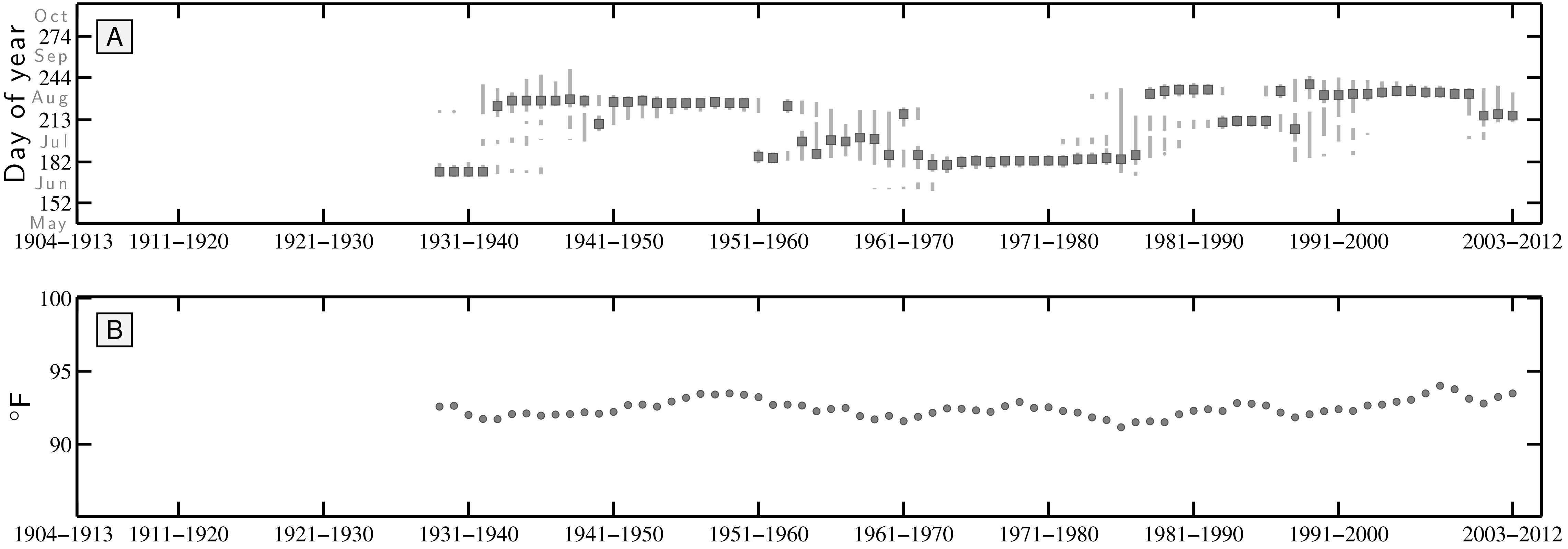

10 year Winter Teletherm dynamics for BATON ROUGE METRO AP, LA:

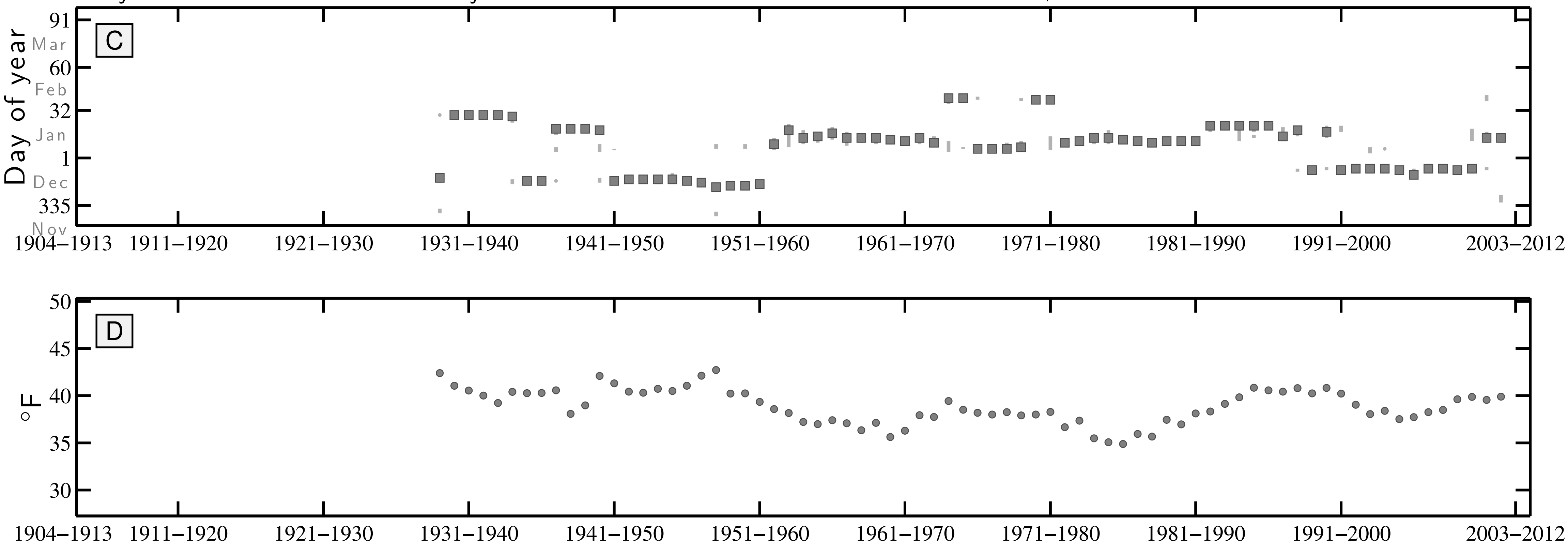

10 year Summer Teletherm dynamics for BUNKIE, LA:

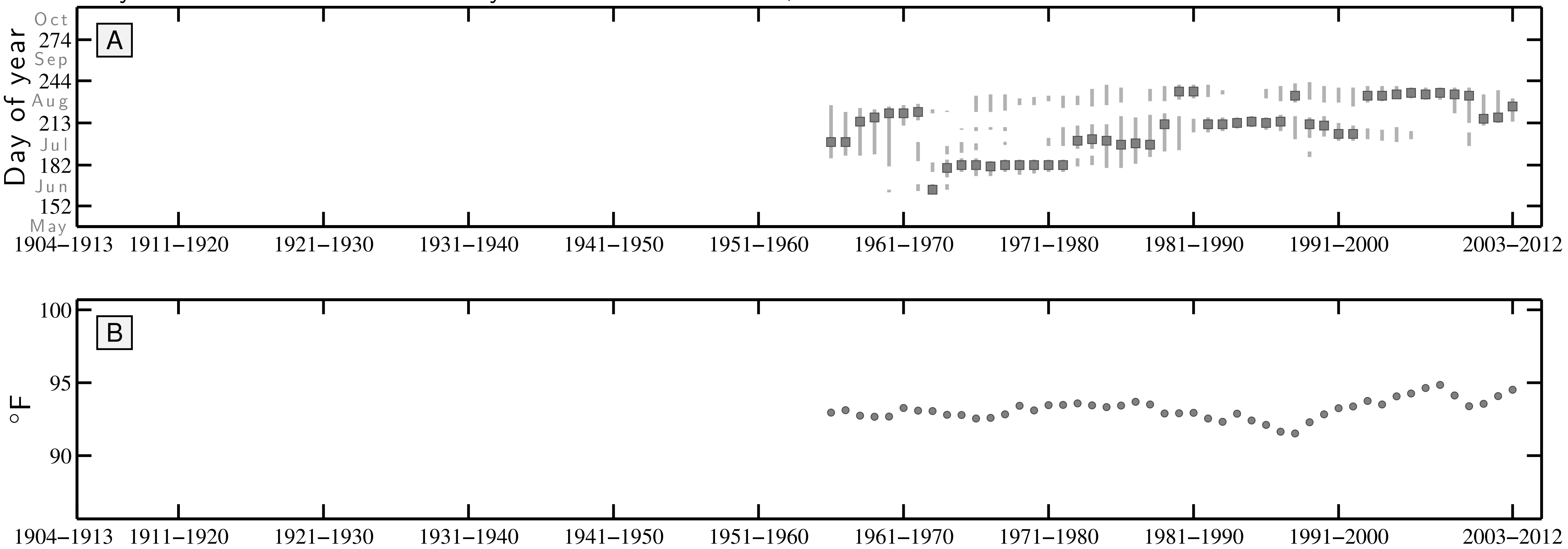

10 year Winter Teletherm dynamics for BUNKIE, LA:

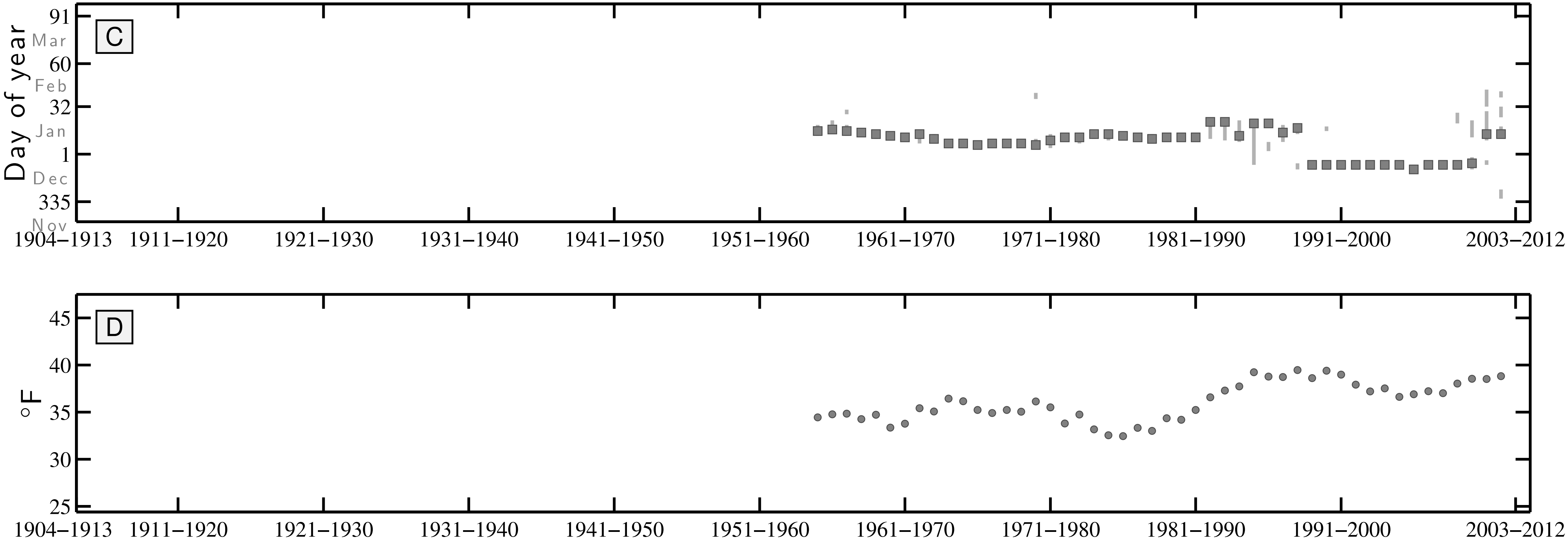

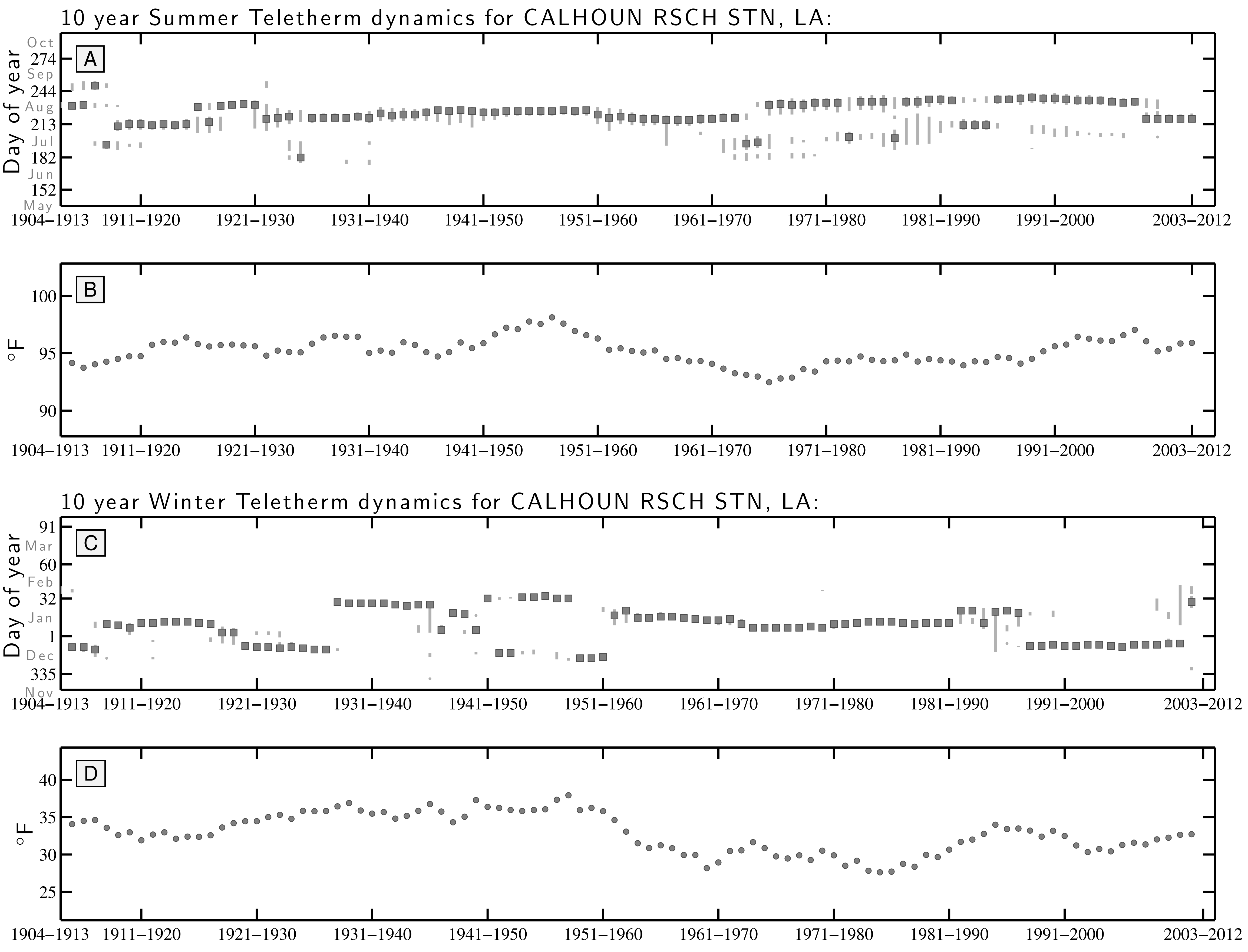
10 year Summer Teletherm dynamics for CALHOUN RSCH STN, LA:
A
Day of year
Oct
274
Sep
244
Aug
213
Jul
182
Jun
152
May
1904–1913
1911–1920
1921–1930
1931–1940
1941–1950
1951–1960
1961–1970
1971–1980
1981–1990
1991–2000
2003–2012
B
°F
100
95
90
10 year Winter Teletherm dynamics for CALHOUN RSCH STN, LA:
C
91
Mar
60
Feb
32
Jan
1
Dec
335
Nov
D
40
35
30
25

10 year Summer Teletherm dynamics for COVINGTON 4 NNW, LA:

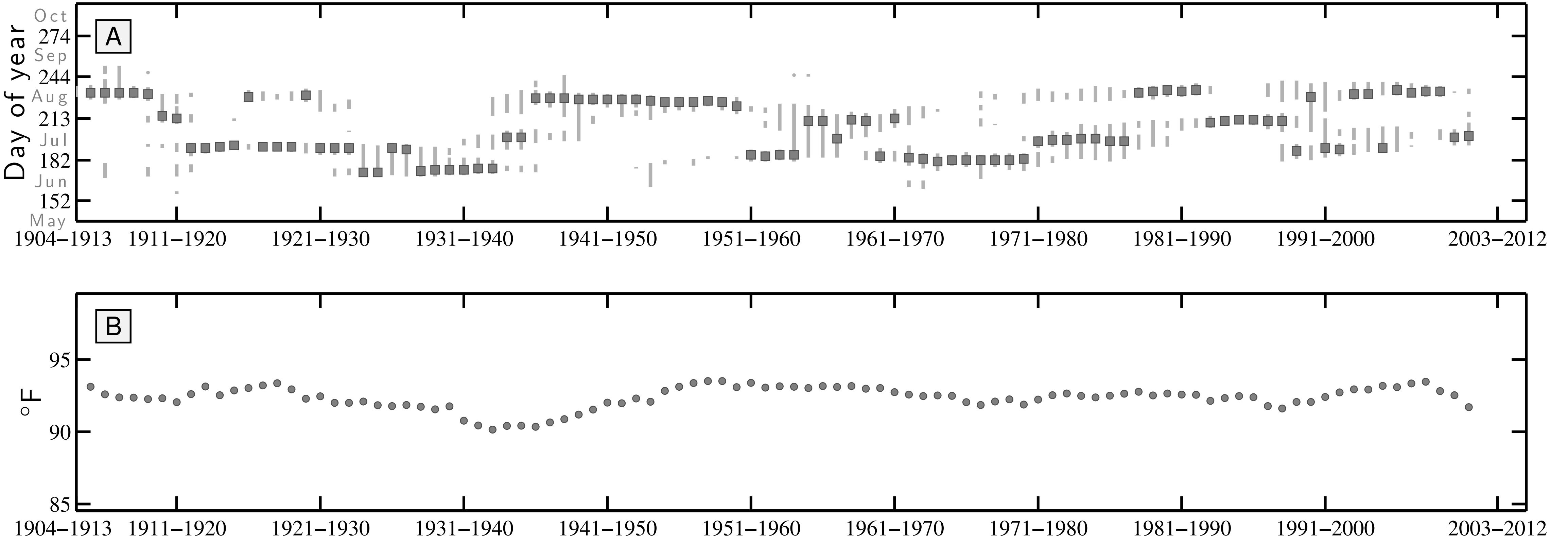

10 year Winter Teletherm dynamics for COVINGTON 4 NNW, LA:

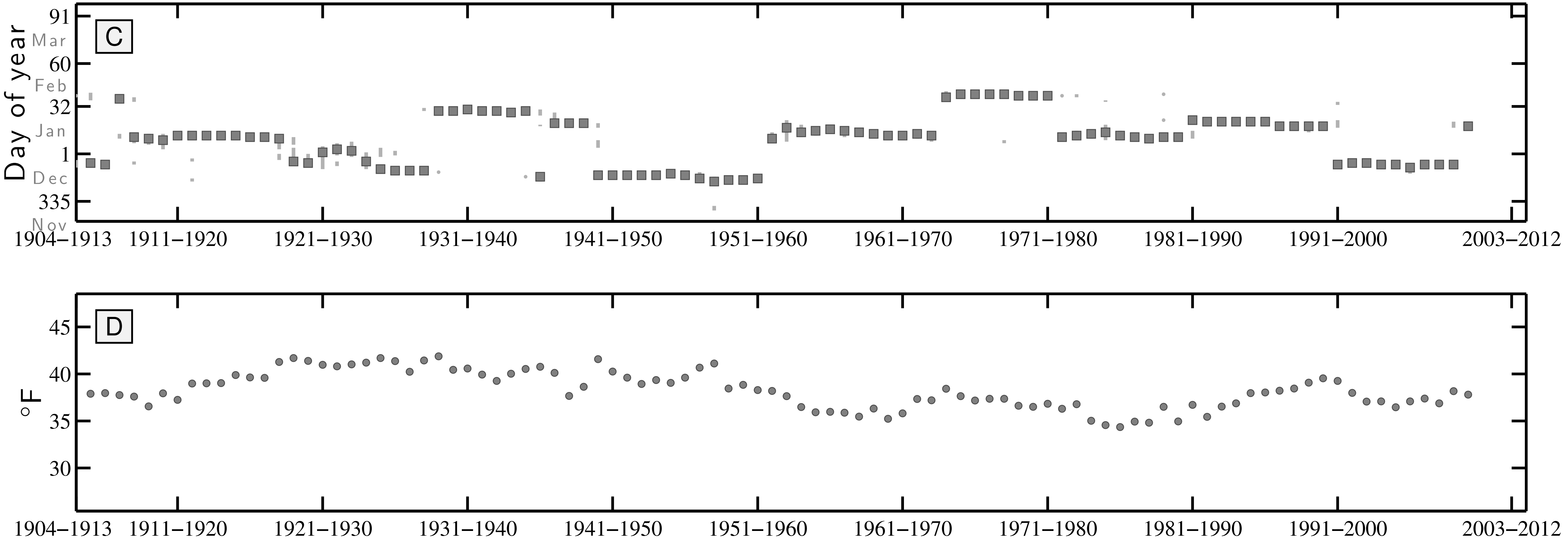

10 year Summer Teletherm dynamics for DONALDSONVILLE 4 SW, LA:

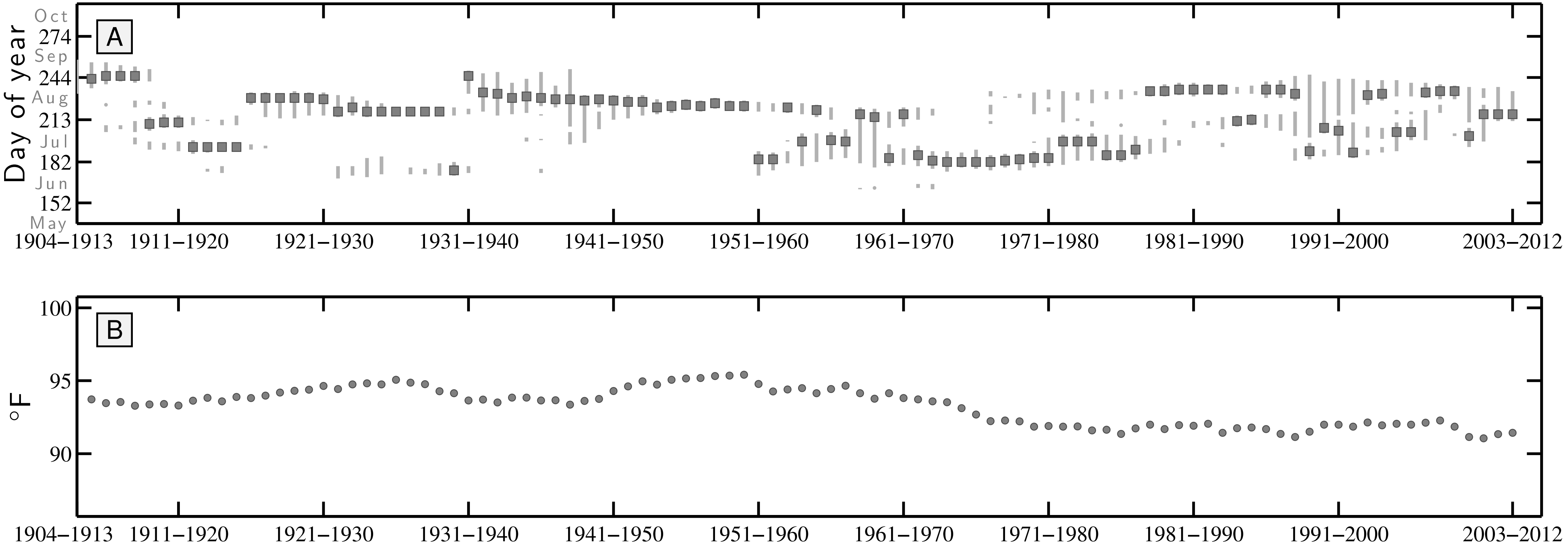

10 year Winter Teletherm dynamics for DONALDSONVILLE 4 SW, LA:

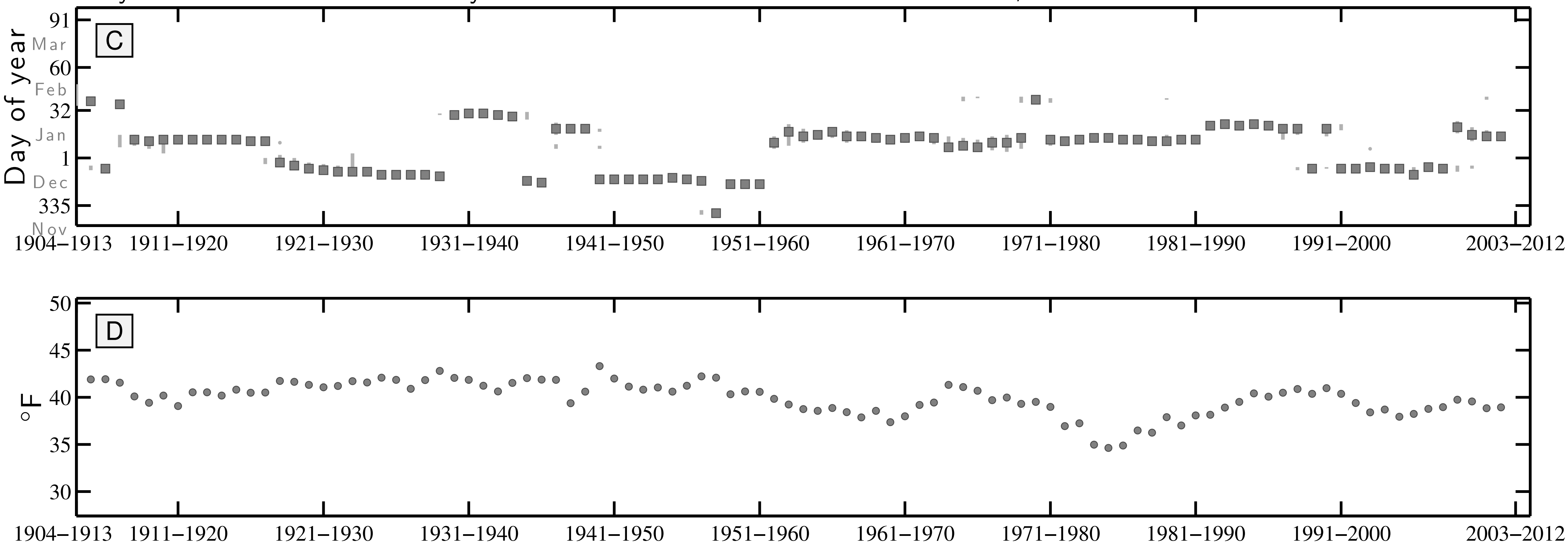

10 year Summer Teletherm dynamics for FRANKLIN 3 NW, LA:

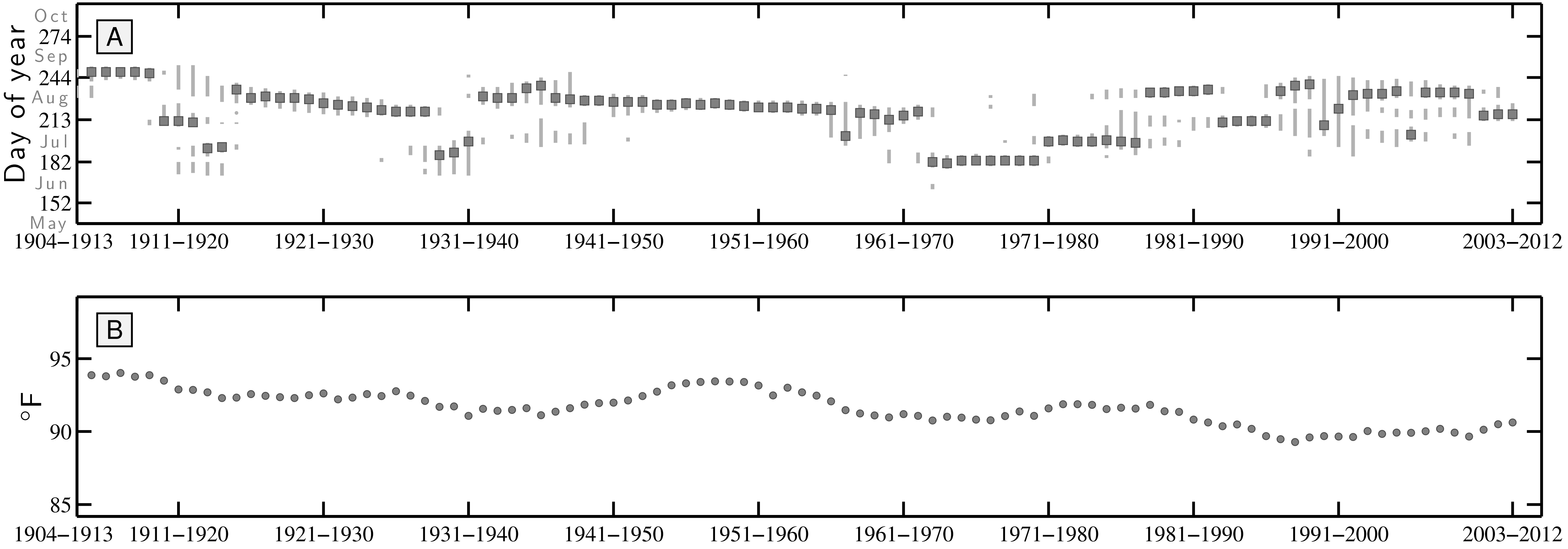

10 year Winter Teletherm dynamics for FRANKLIN 3 NW, LA:

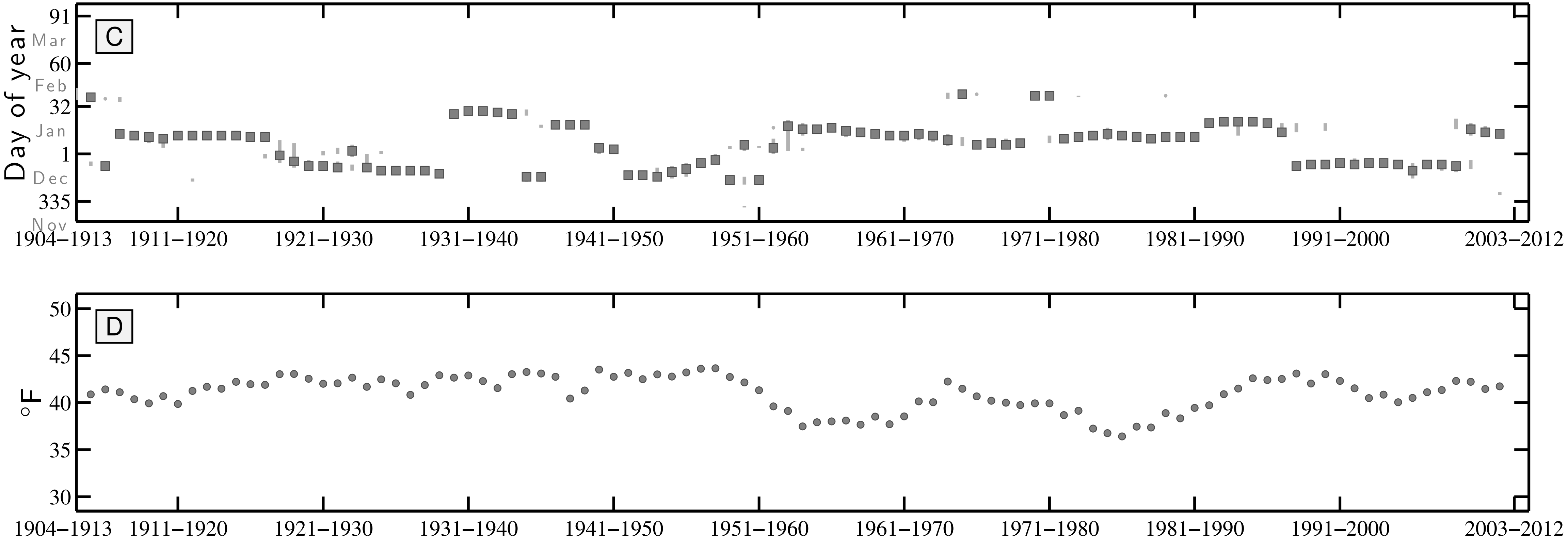

10 year Summer Teletherm dynamics for GRAND COTEAU, LA:

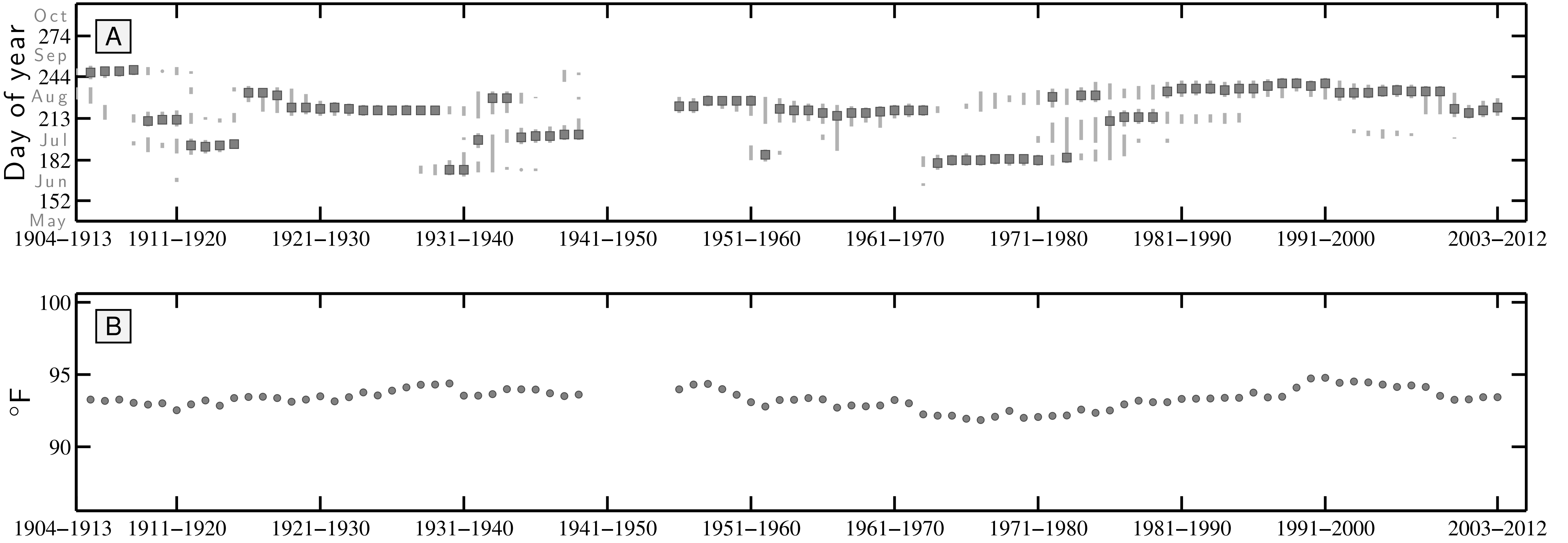

10 year Winter Teletherm dynamics for GRAND COTEAU, LA:

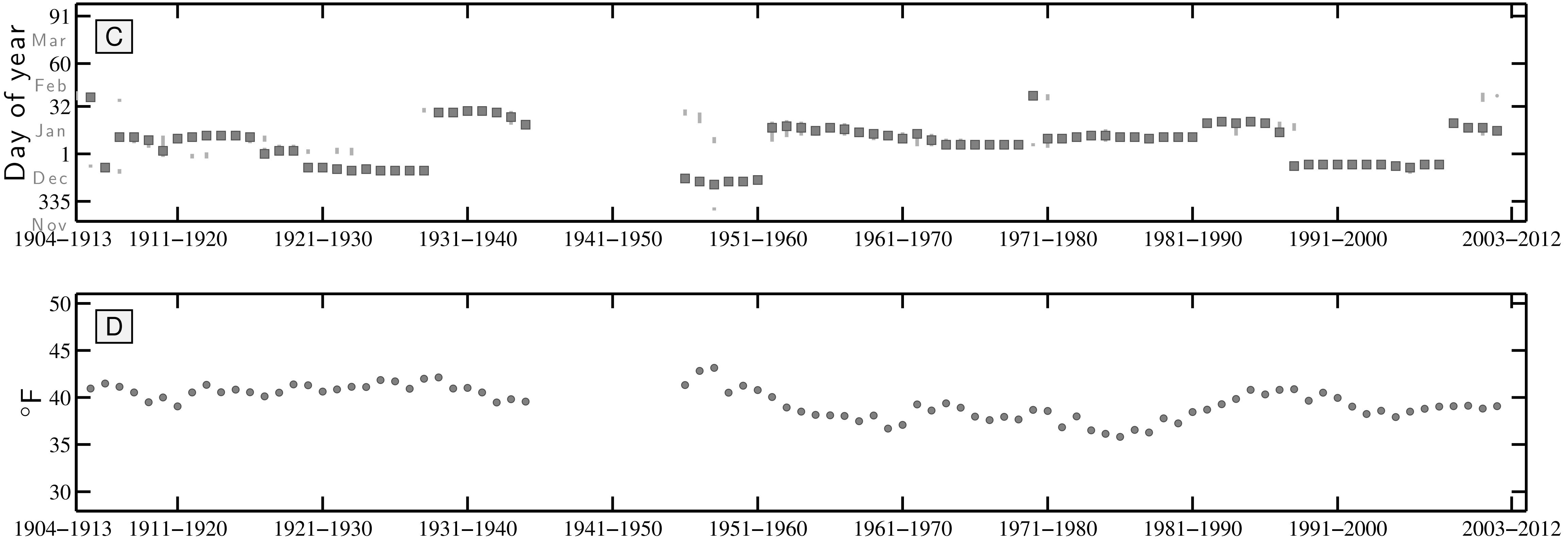

10 year Summer Teletherm dynamics for HOUMA, LA:

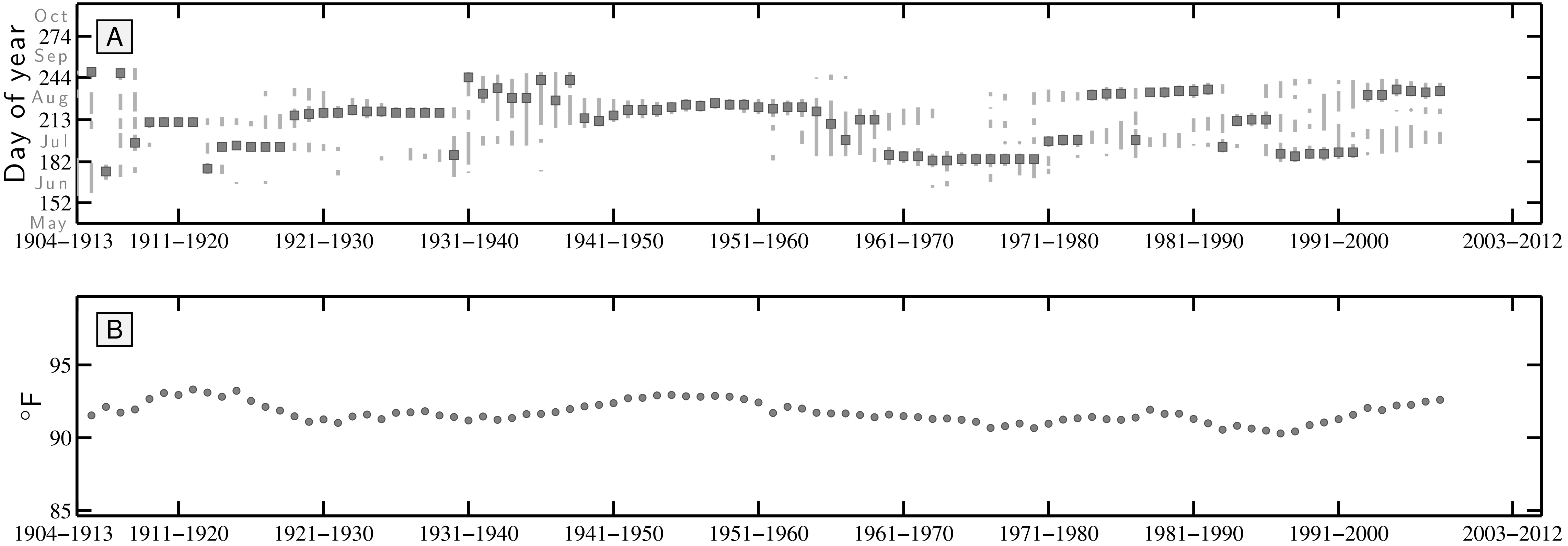

10 year Winter Teletherm dynamics for HOUMA, LA:

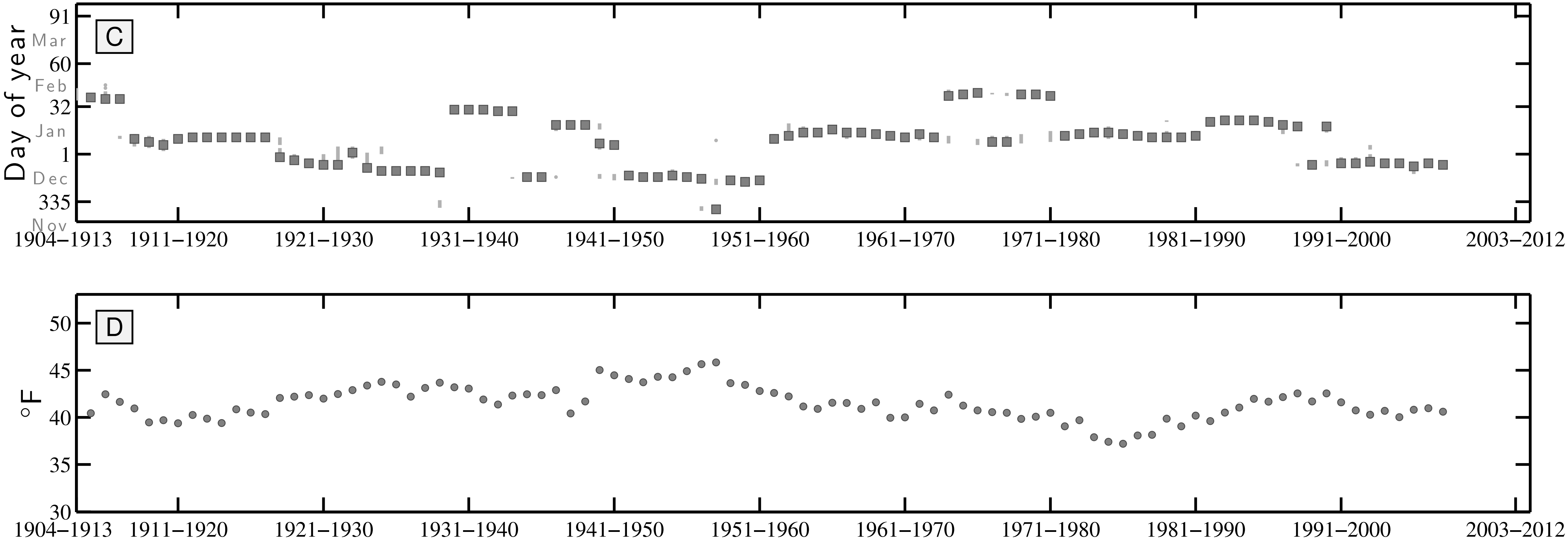

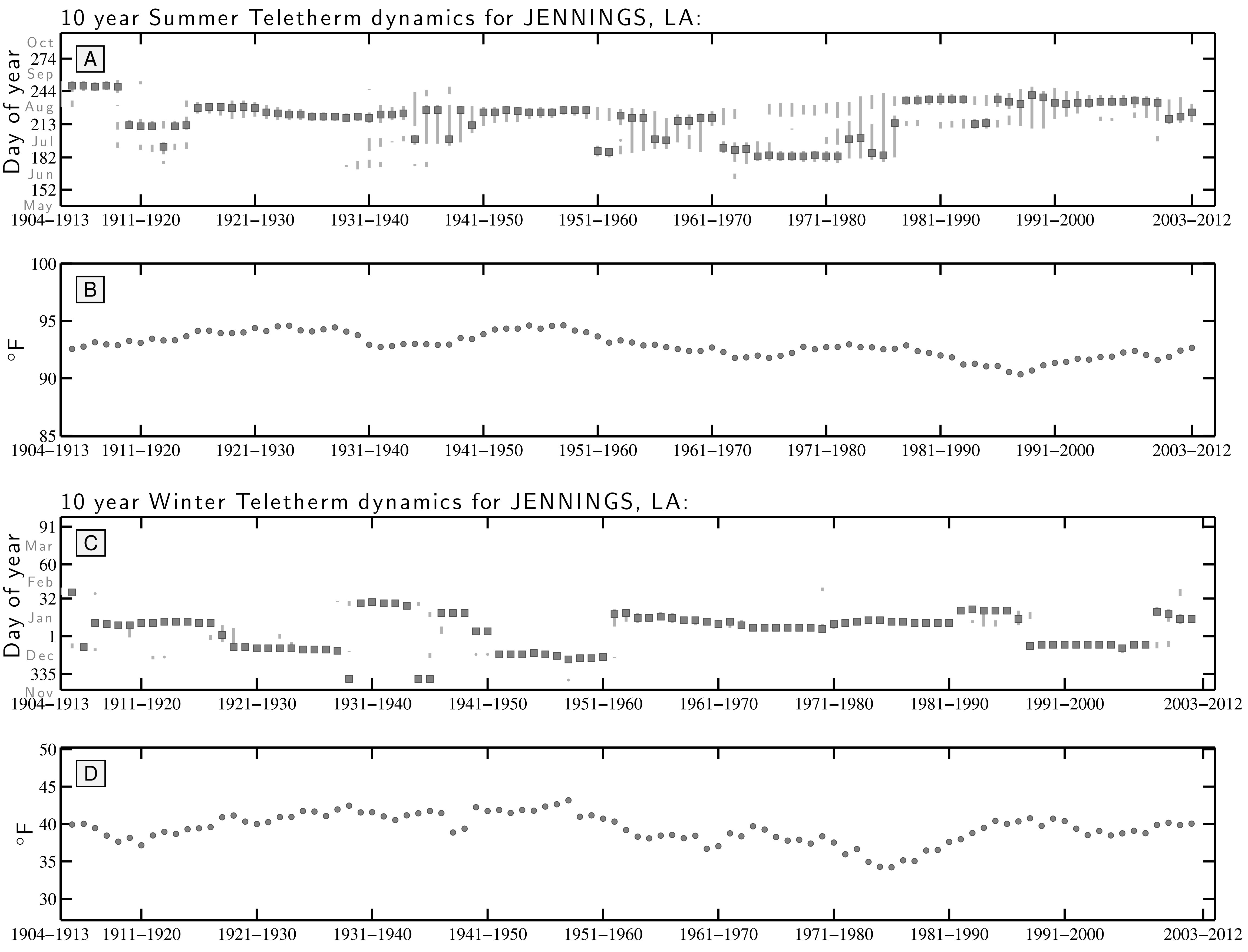
10 year Summer Teletherm dynamics for JENNINGS, LA:
A
B
Day of year
°F
Oct
Sep
Aug
Jul
Jun
May
274
244
213
182
152
100
95
90
85
10 year Winter Teletherm dynamics for JENNINGS, LA:
C
D
Mar
Feb
Jan
Dec
Nov
91
60
32
1
335
50
45
40
35
30
1904–1913
1911–1920
1921–1930
1931–1940
1941–1950
1951–1960
1961–1970
1971–1980
1981–1990
1991–2000
2003–2012

10 year Summer Teletherm dynamics for LAFAYETTE FCWOS, LA:

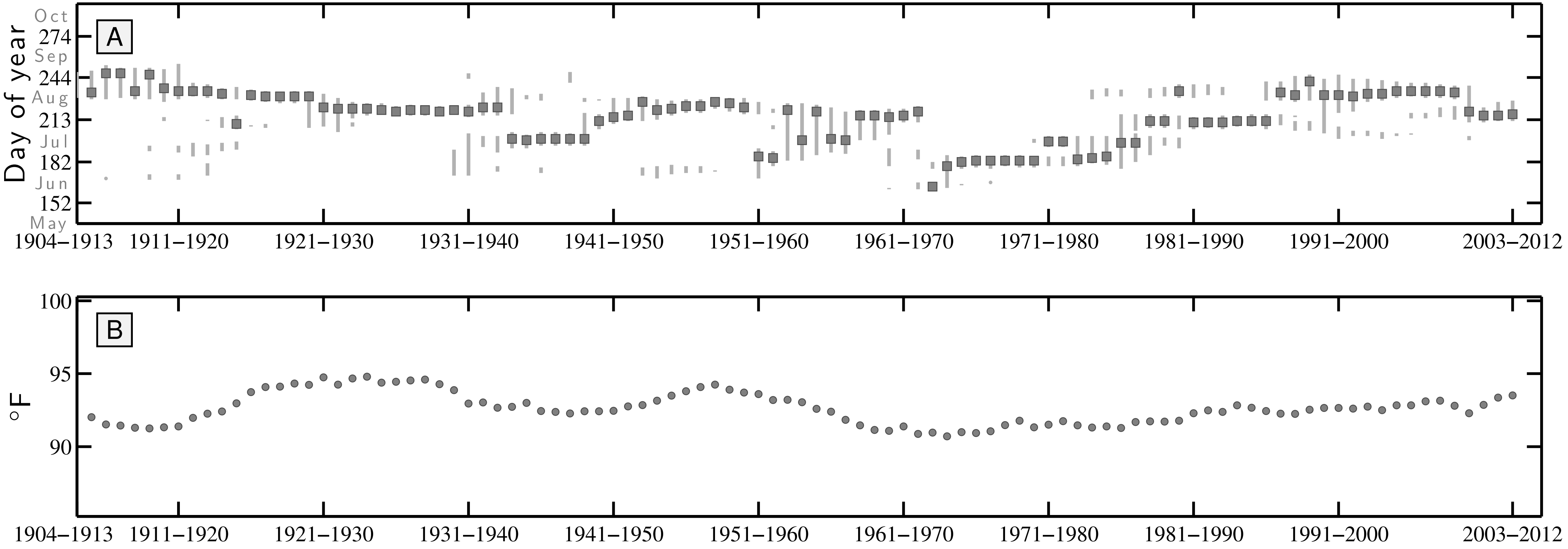

10 year Winter Teletherm dynamics for LAFAYETTE FCWOS, LA:

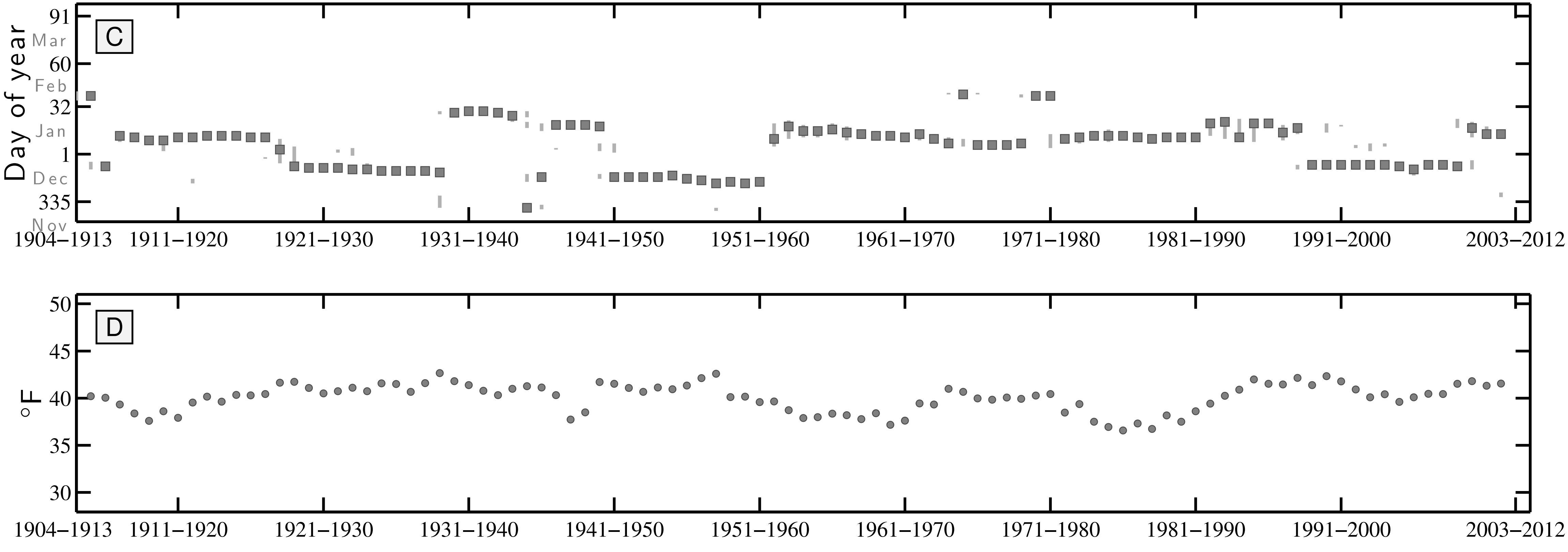

10 year Summer Teletherm dynamics for NEW ORLEANS AUDUBON, LA:

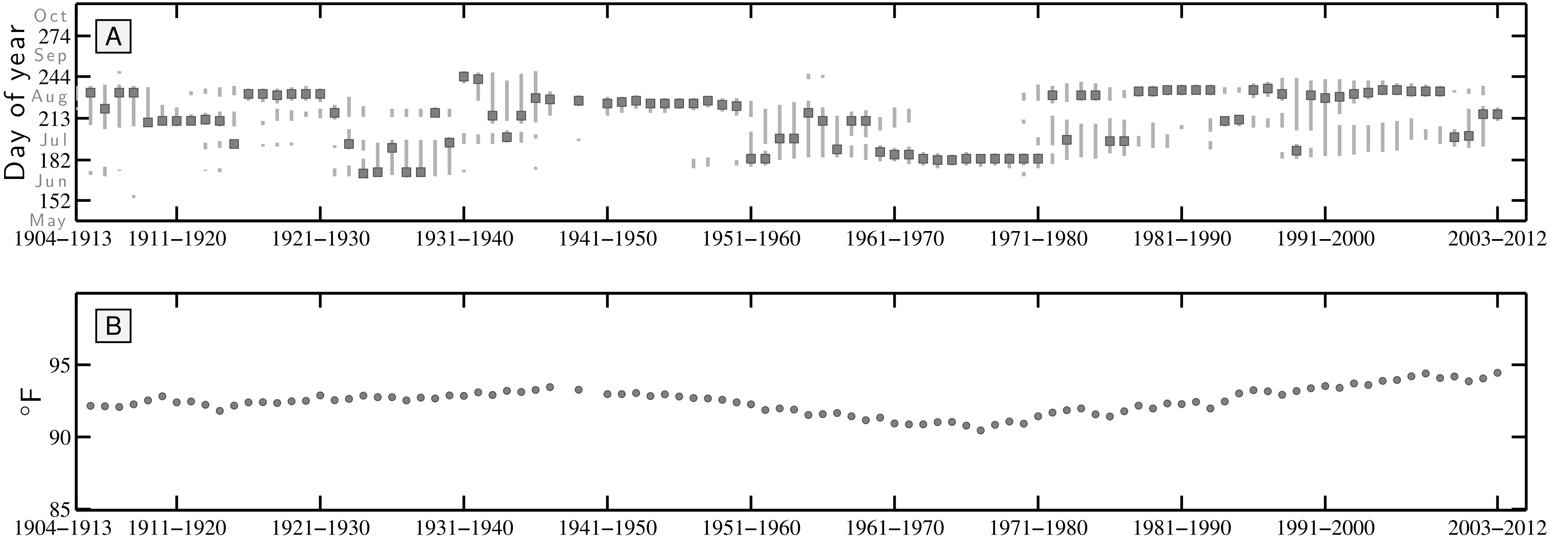

10 year Winter Teletherm dynamics for NEW ORLEANS AUDUBON, LA:

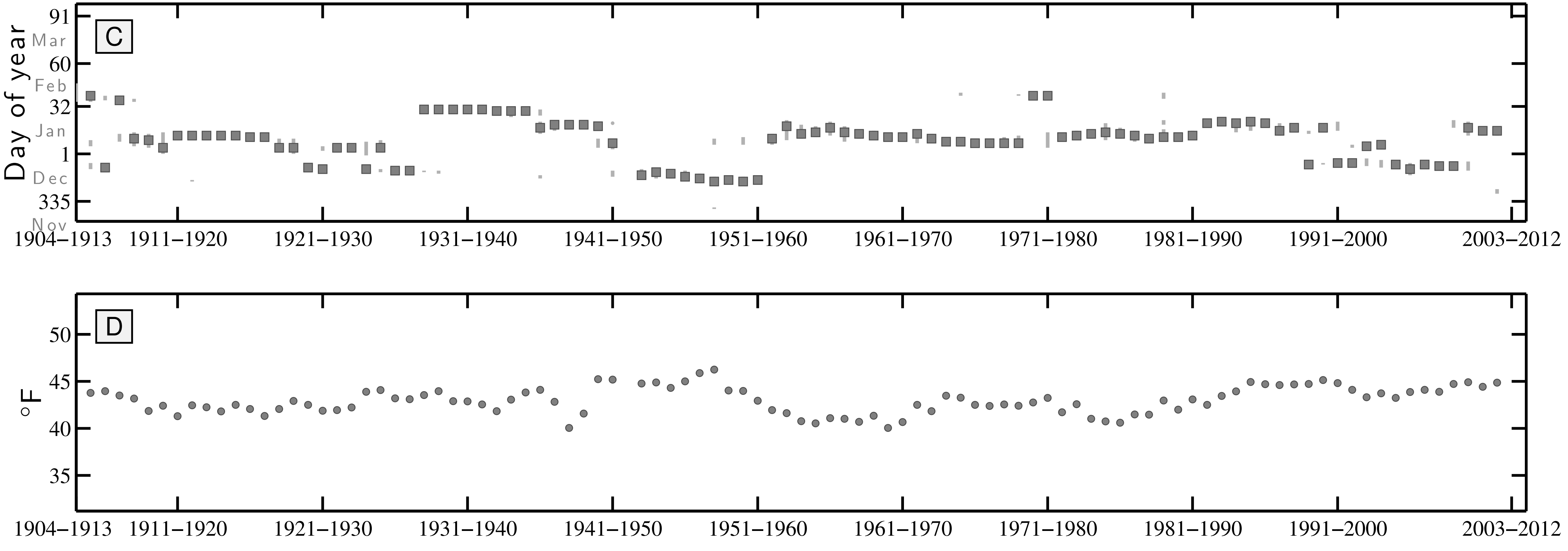

10 year Summer Teletherm dynamics for PLAIN DEALING 4 W, LA:

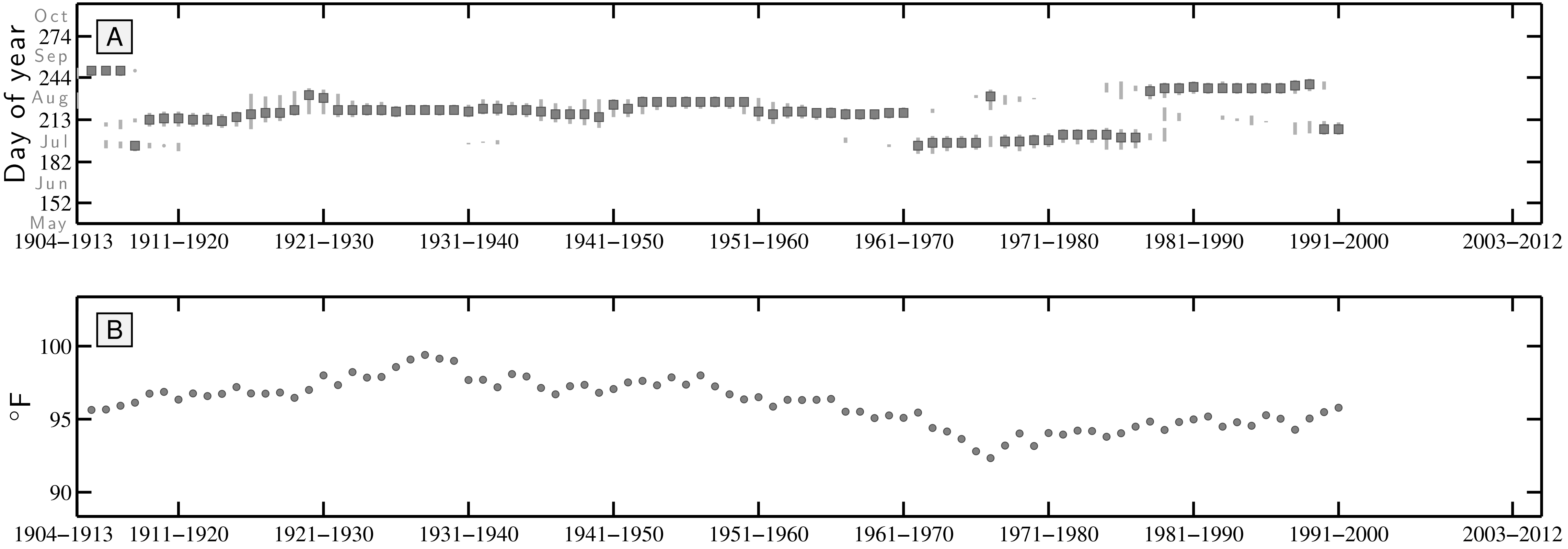

10 year Winter Teletherm dynamics for PLAIN DEALING 4 W, LA:

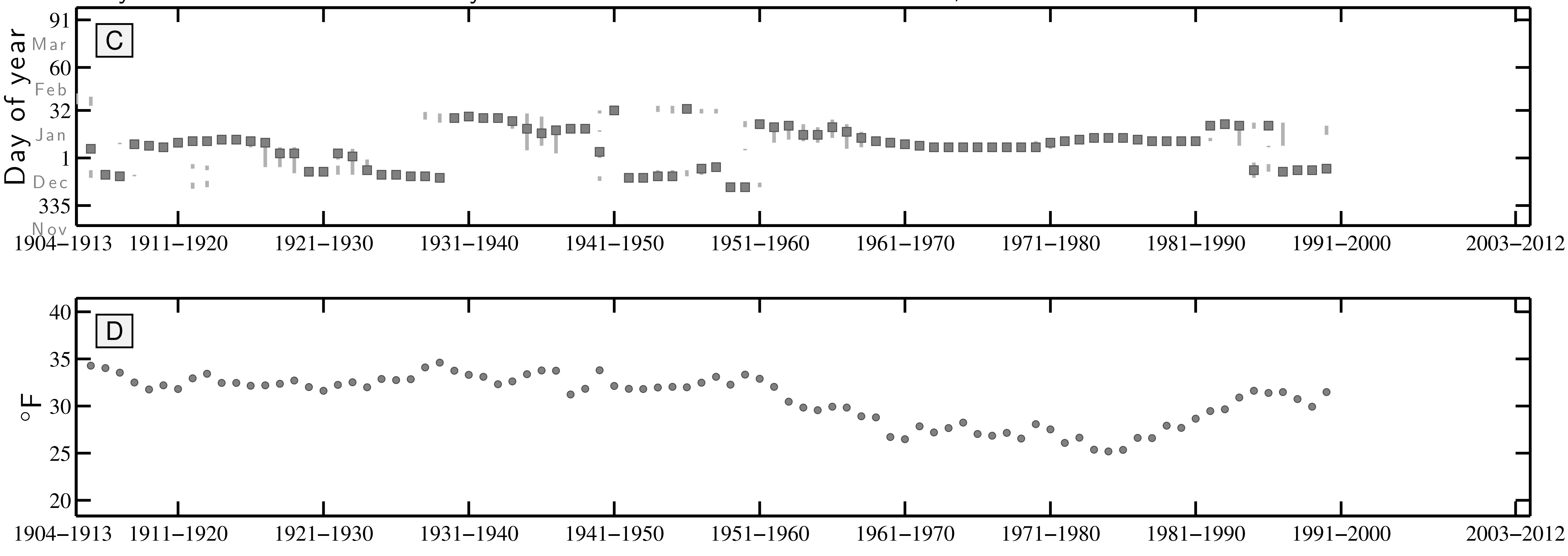

10 year Summer Teletherm dynamics for ST JOSEPH 3 N, LA:

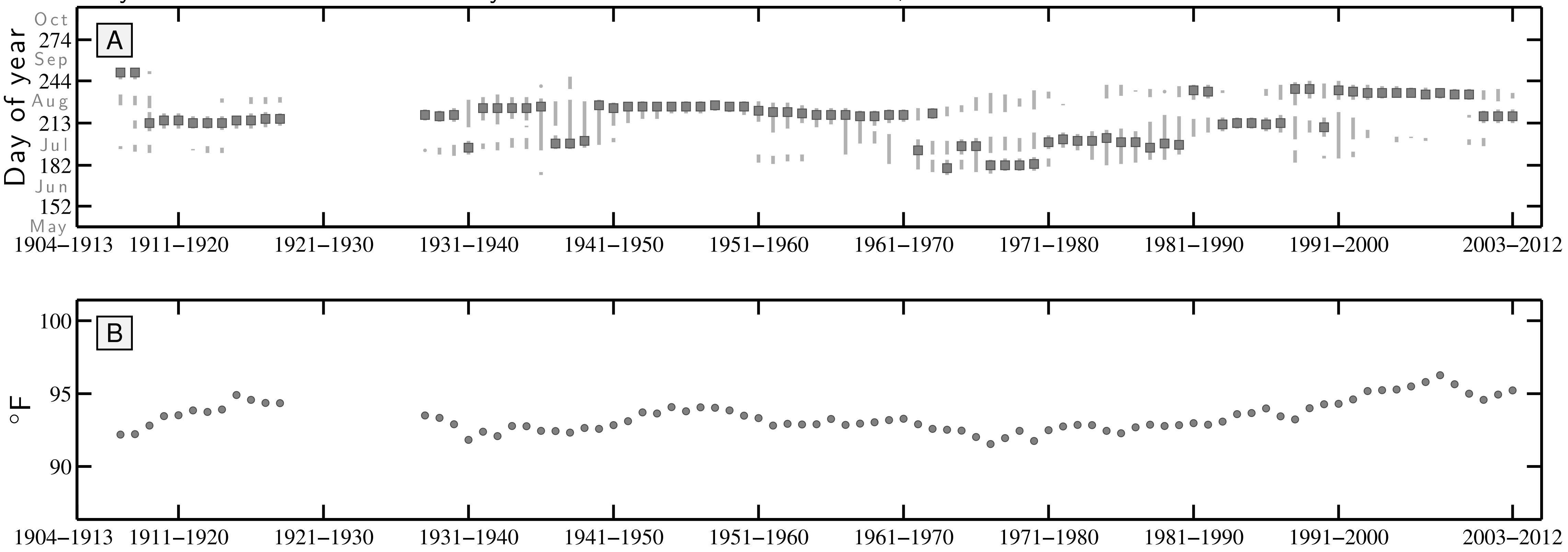

10 year Winter Teletherm dynamics for ST JOSEPH 3 N, LA:

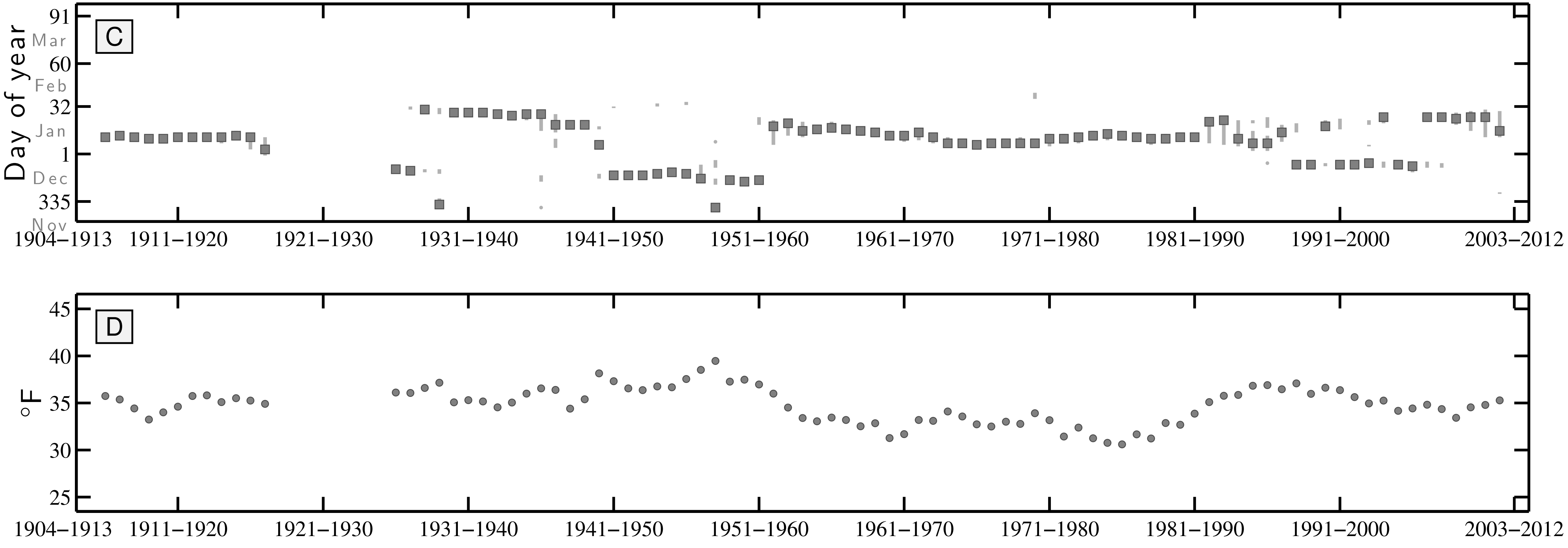

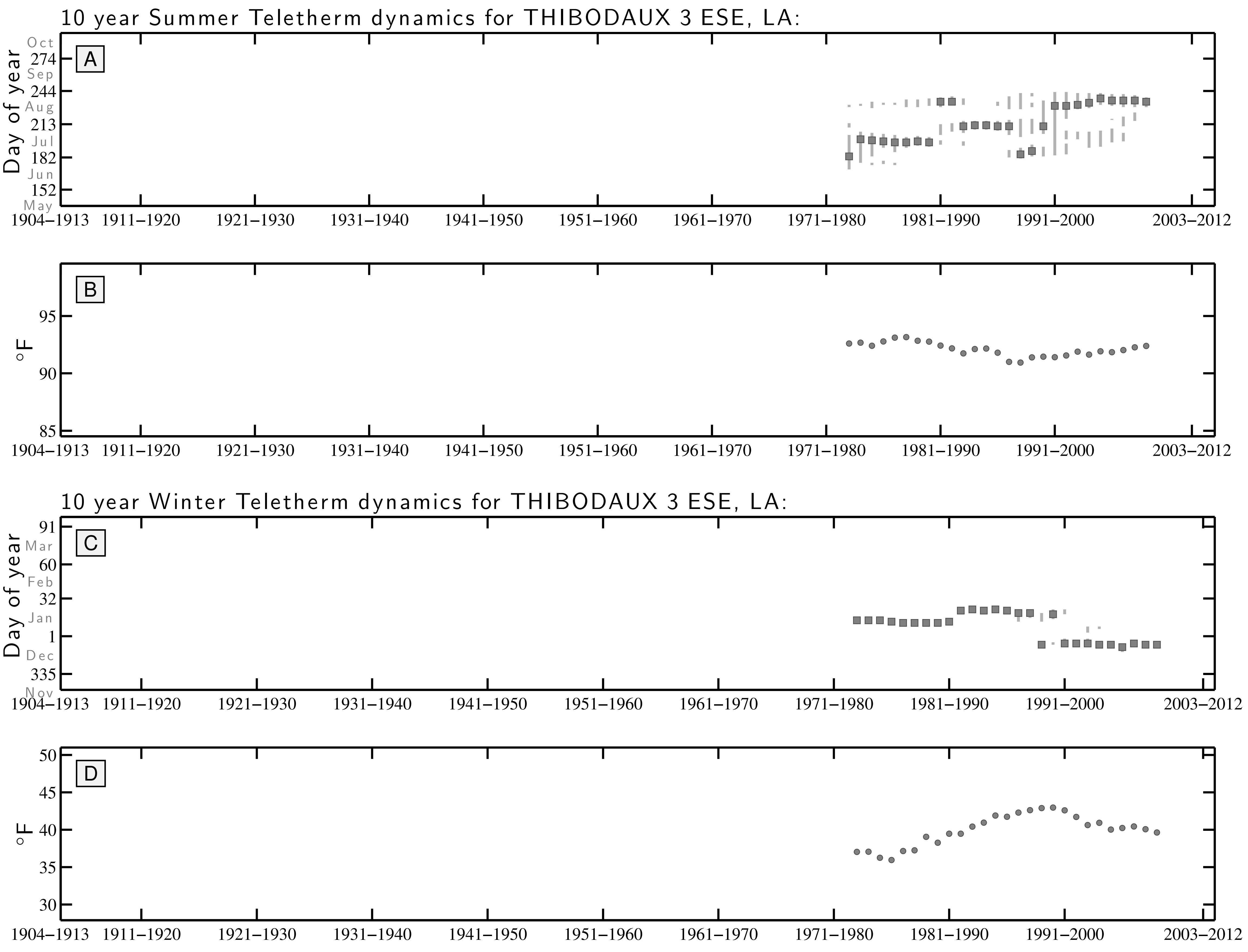
10 year Summer Teletherm dynamics for THIBODAUX 3 ESE, LA:
A
Day of year
Oct
274
Sep
244
Aug
213
Jul
182
Jun
152
May
B
°F
95
90
85
10 year Winter Teletherm dynamics for THIBODAUX 3 ESE, LA:
C
Day of year
91
Mar
60
Feb
32
Jan
1
Dec
335
Nov
D
°F
50
45
40
35
30
1904–1913
1911–1920
1921–1930
1931–1940
1941–1950
1951–1960
1961–1970
1971–1980
1981–1990
1991–2000
2003–2012

10 year Summer Teletherm dynamics for WINNSBORO 5 SSE, LA:

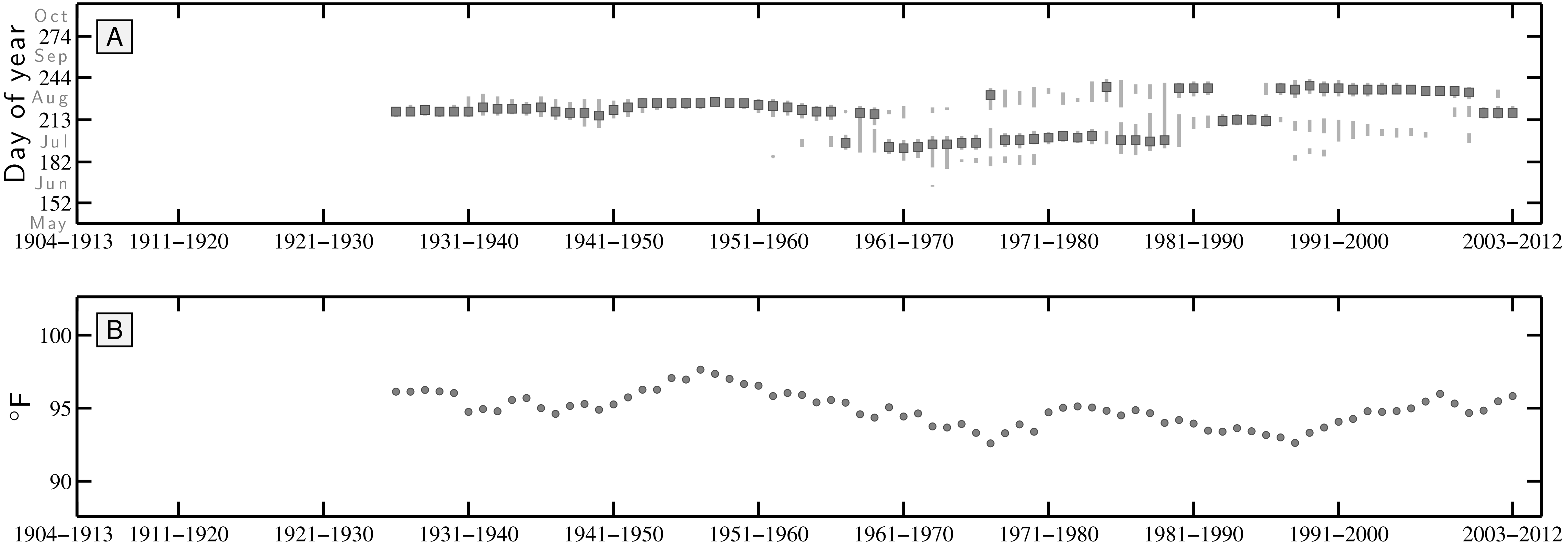

10 year Winter Teletherm dynamics for WINNSBORO 5 SSE, LA:

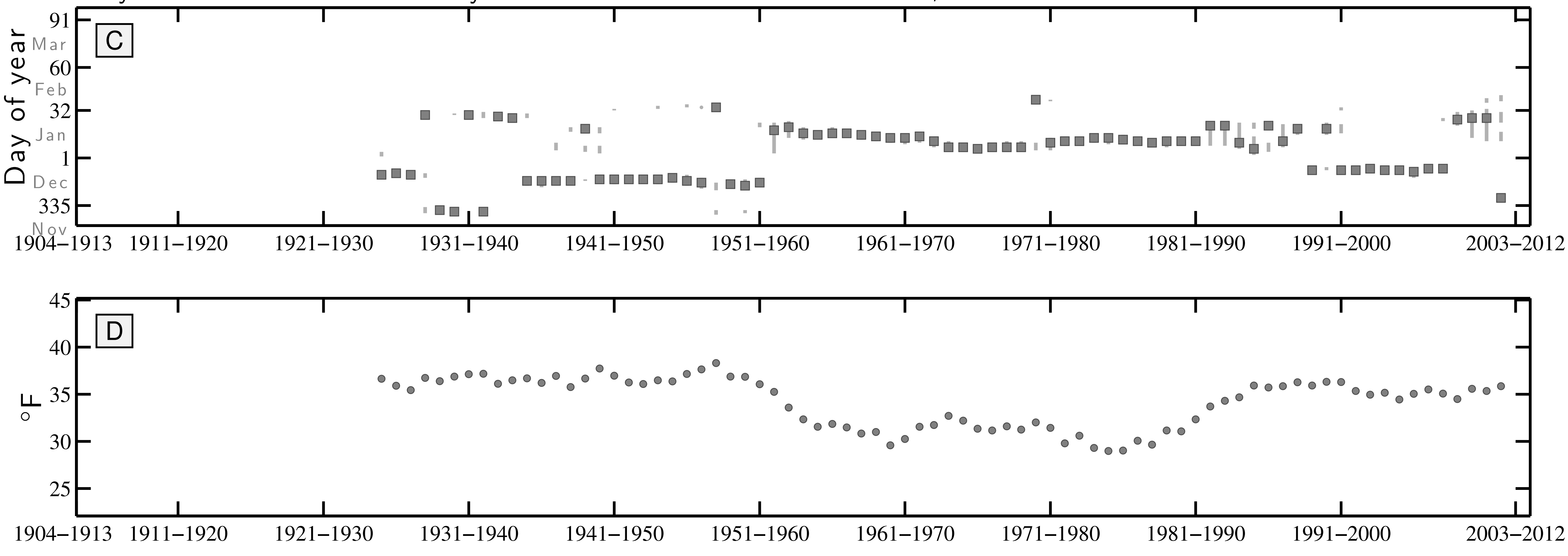

10 year Summer Teletherm dynamics for ACADIA NP, ME:

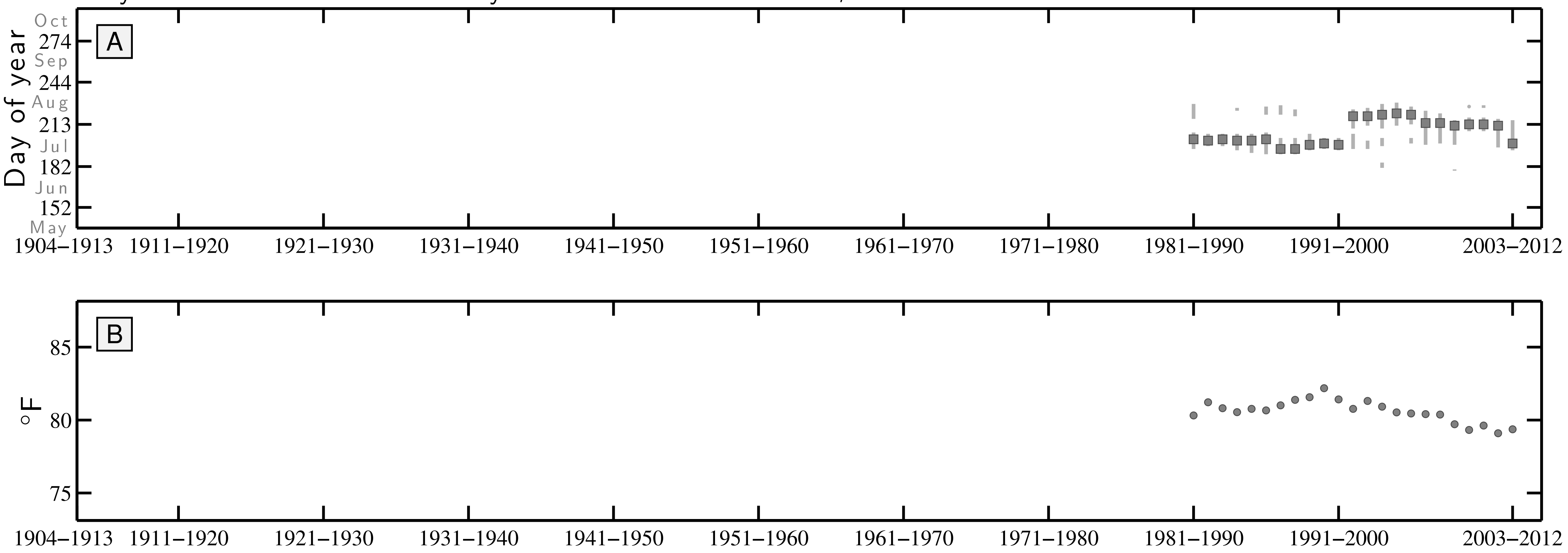

10 year Winter Teletherm dynamics for ACADIA NP, ME:

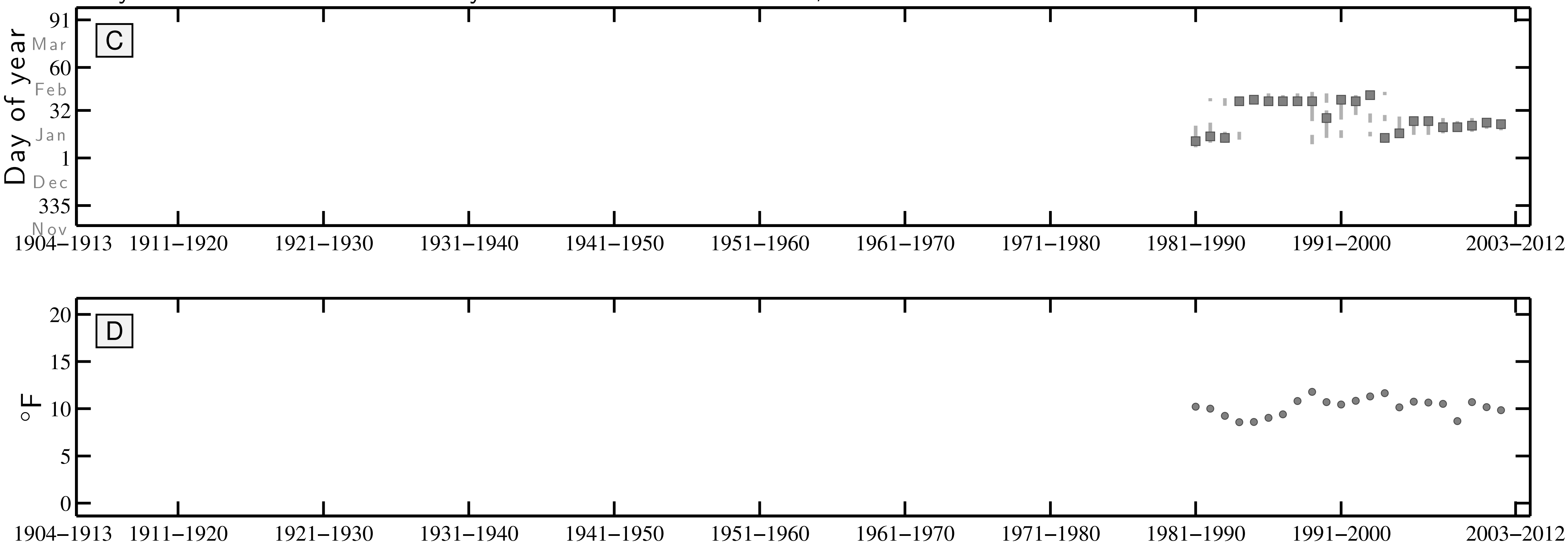

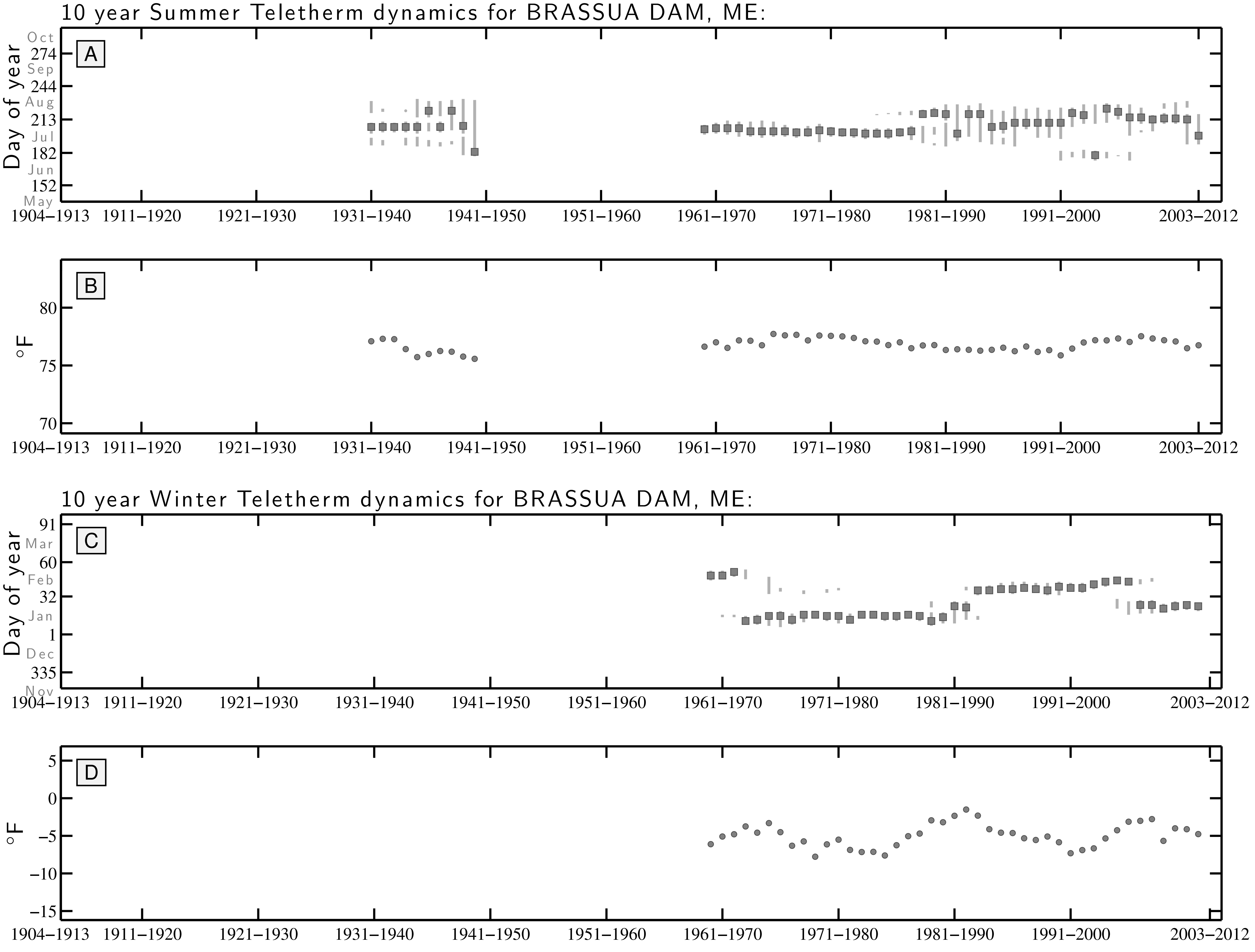
10 year Summer Teletherm dynamics for BRASSUA DAM, ME:
A
B
Day of year
°F
Oct
274
Sep
244
Aug
213
Jul
182
Jun
152
May
80
75
70
10 year Winter Teletherm dynamics for BRASSUA DAM, ME:
C
D
91
Mar
60
Feb
32
Jan
1
Dec
335
Nov
5
0
−5
−10
−15
1904–1913
1911–1920
1921–1930
1931–1940
1941–1950
1951–1960
1961–1970
1971–1980
1981–1990
1991–2000
2003–2012

10 year Summer Teletherm dynamics for CORINNA, ME:

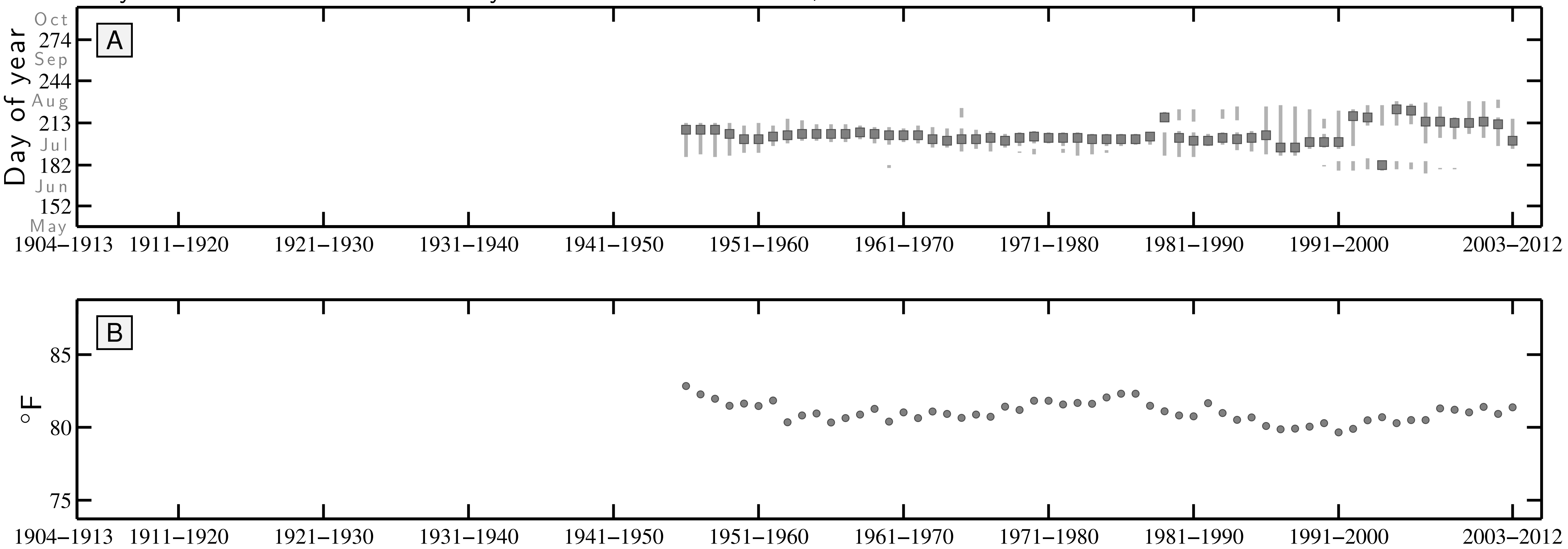

10 year Winter Teletherm dynamics for CORINNA, ME:

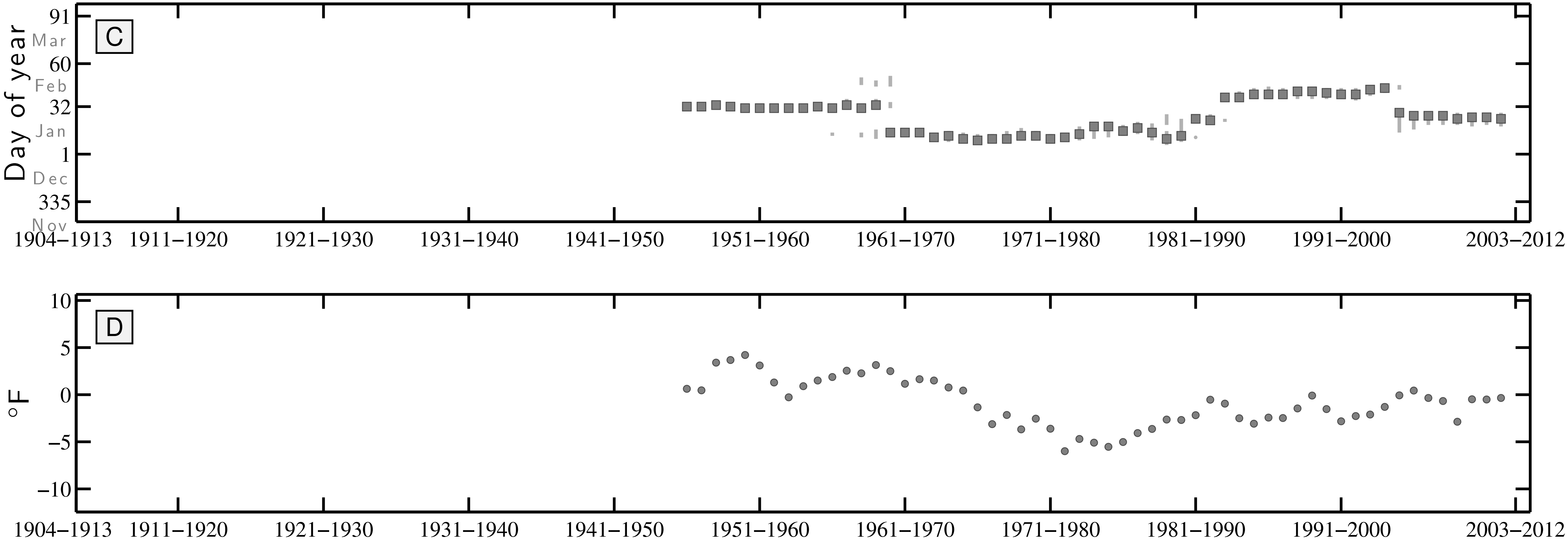

10 year Summer Teletherm dynamics for EASTPORT, ME:

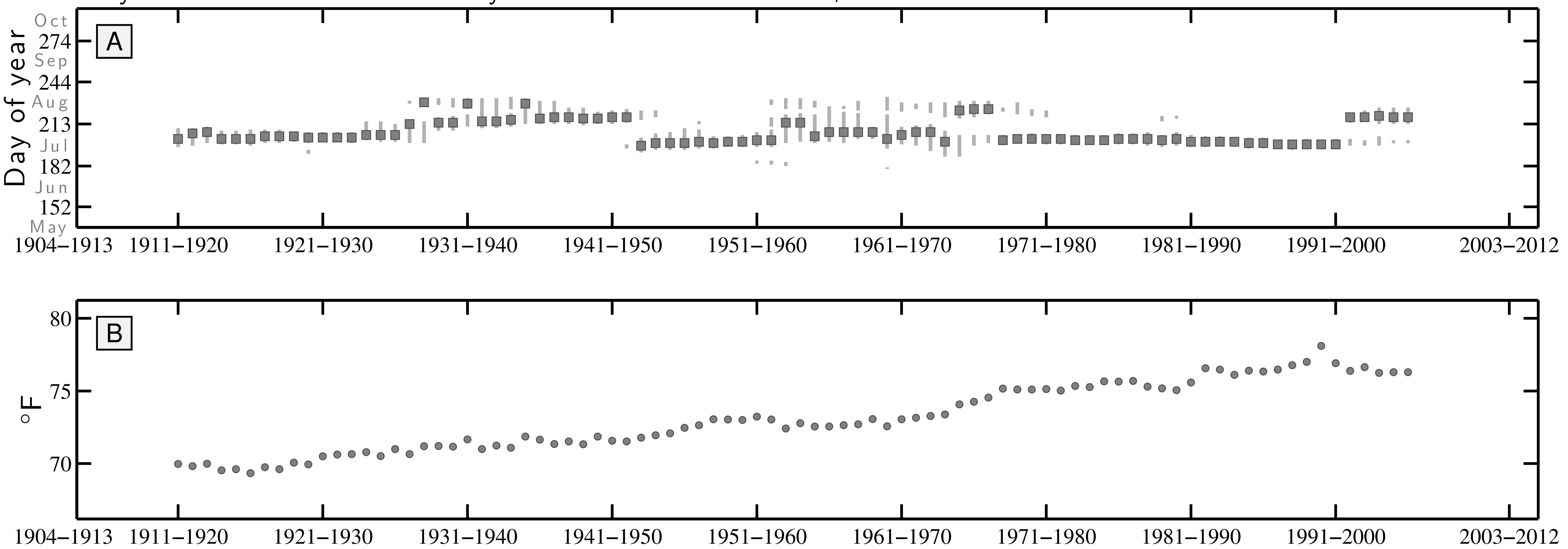

10 year Winter Teletherm dynamics for EASTPORT, ME:

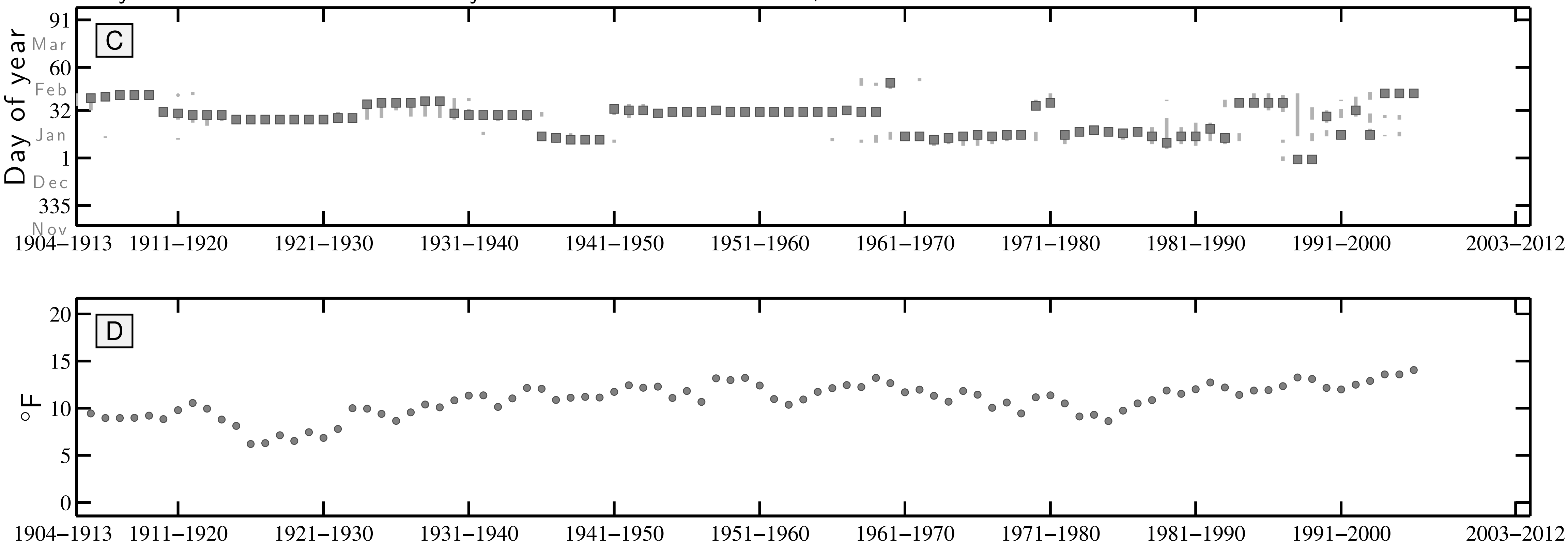

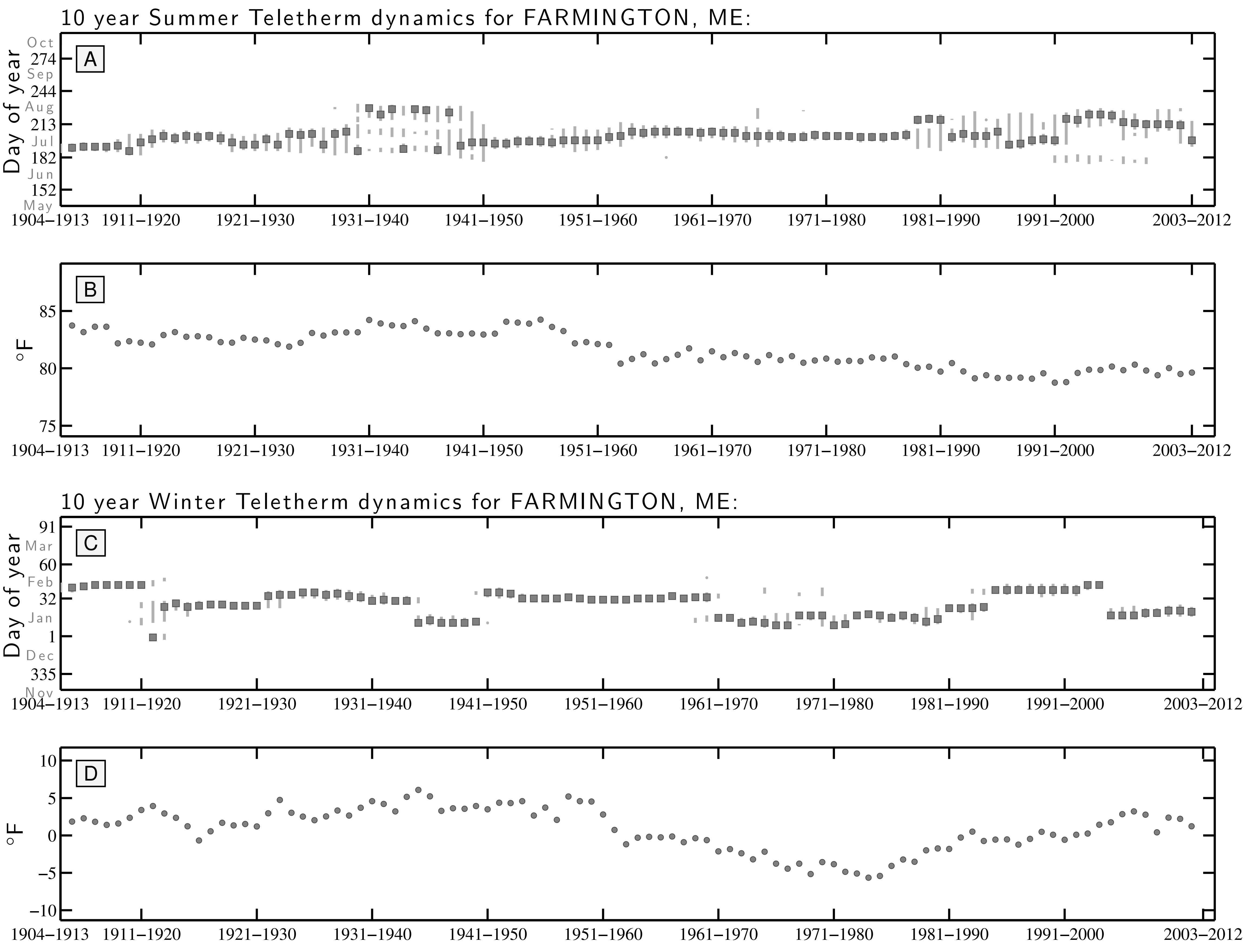
10 year Summer Teletherm dynamics for FARMINGTON, ME:
A
Day of year
Oct
274
Sep
244
Aug
213
Jul
182
Jun
152
May
B
°F
85
80
75
1904–1913
1911–1920
1921–1930
1931–1940
1941–1950
1951–1960
1961–1970
1971–1980
1981–1990
1991–2000
2003–2012
10 year Winter Teletherm dynamics for FARMINGTON, ME:
C
Day of year
91
Mar
60
Feb
32
Jan
1
Dec
335
Nov
D
°F
10
5
0
−5
−10

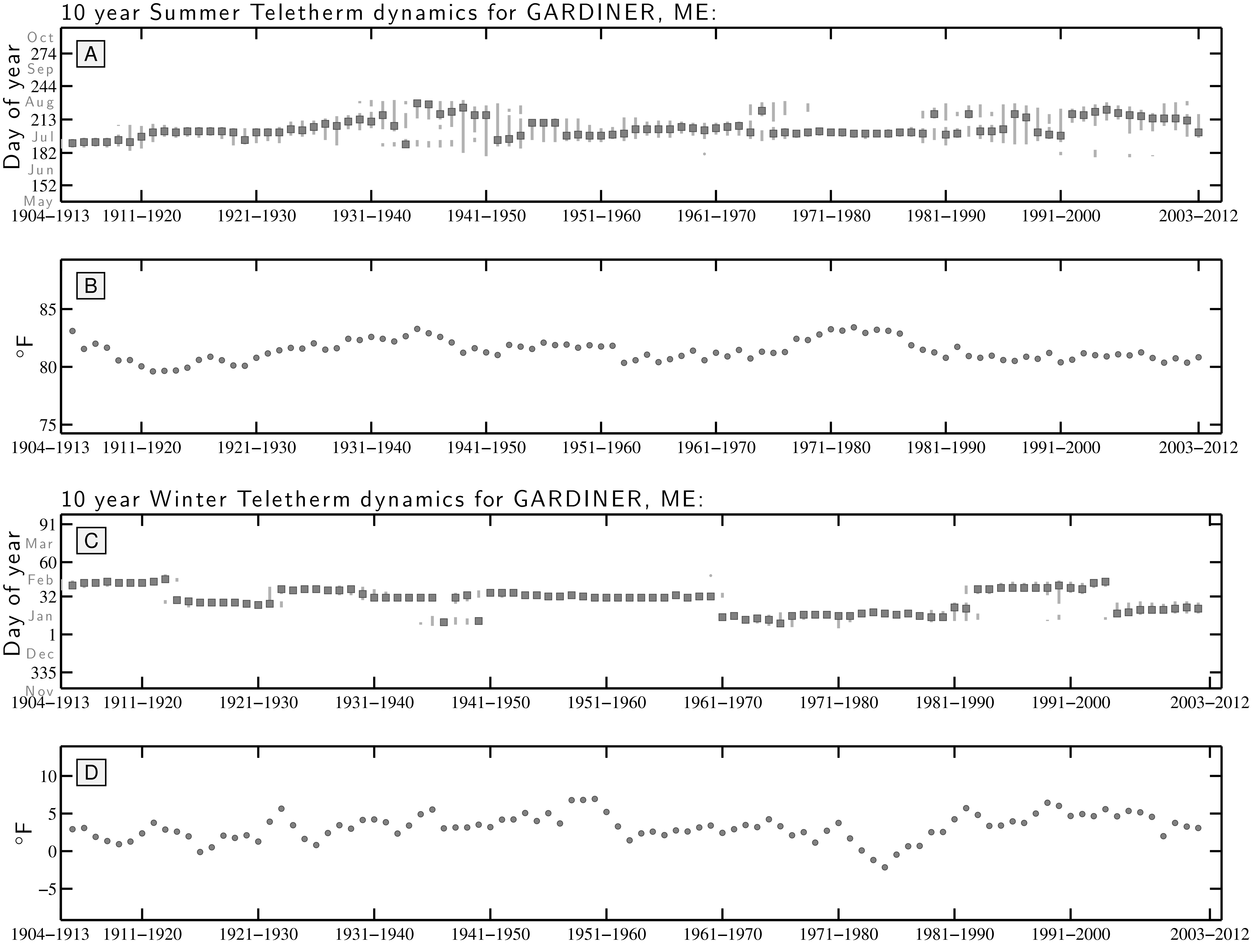
10 year Summer Teletherm dynamics for GARDINER, ME:
A
Day of year
Oct
274
Sep
244
Aug
213
Jul
182
Jun
152
May
B
°F
85
80
75
10 year Winter Teletherm dynamics for GARDINER, ME:
C
Day of year
91
Mar
60
Feb
32
Jan
1
Dec
335
Nov
D
°F
10
5
0
−5
1904–1913
1911–1920
1921–1930
1931–1940
1941–1950
1951–1960
1961–1970
1971–1980
1981–1990
1991–2000
2003–2012

10 year Summer Teletherm dynamics for HOULTON 5N, ME:

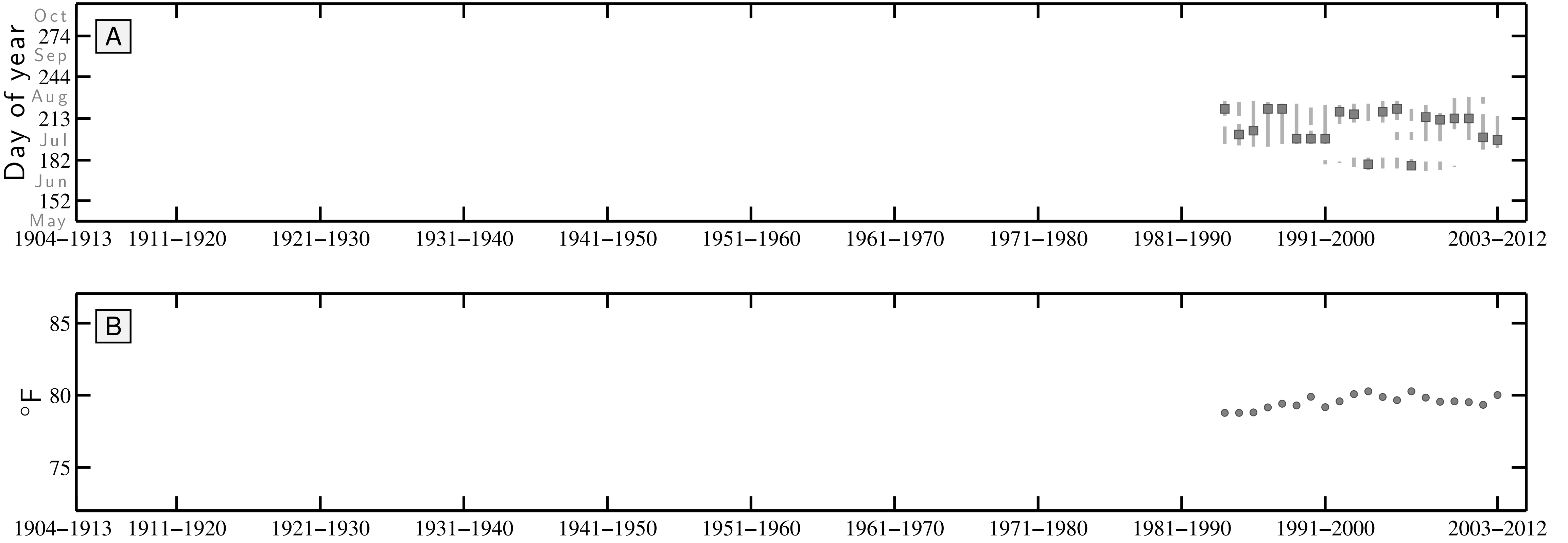

10 year Winter Teletherm dynamics for HOULTON 5N, ME:

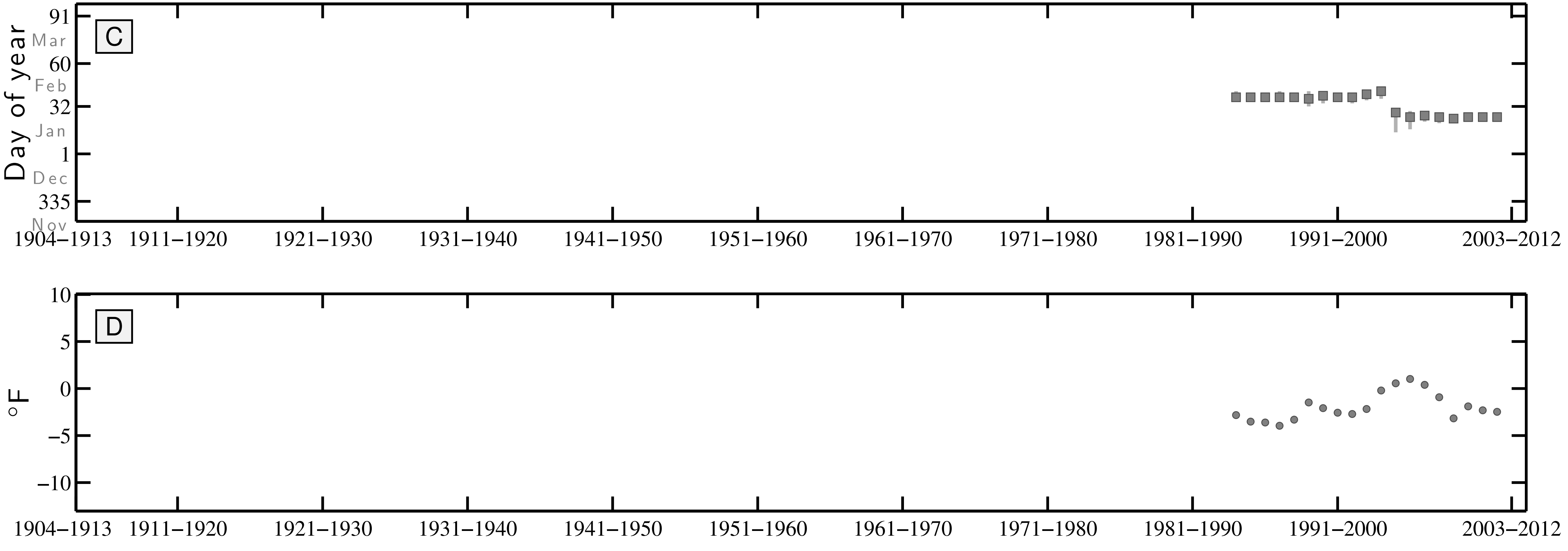

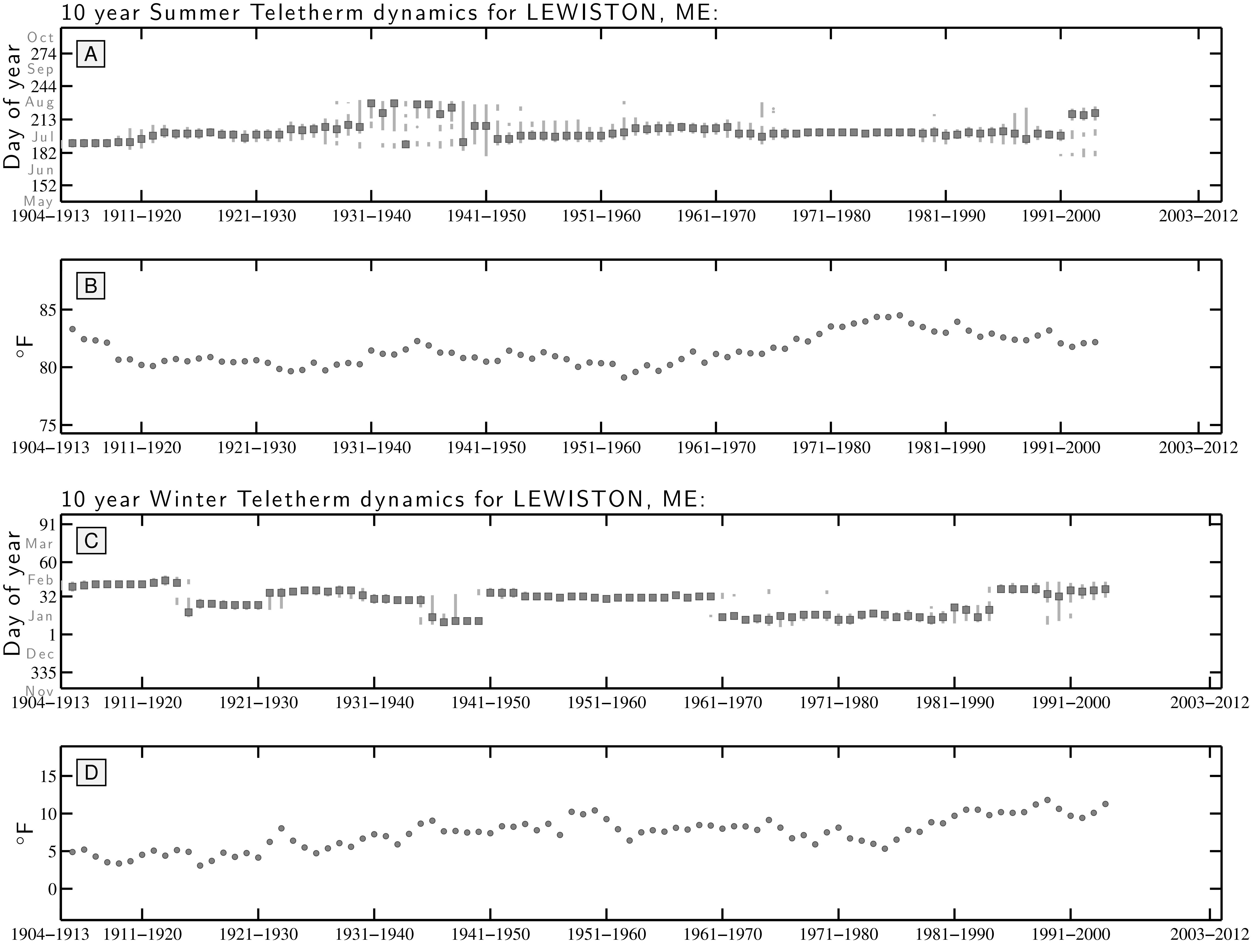
10 year Summer Teletherm dynamics for LEWISTON, ME:
A
B
10 year Winter Teletherm dynamics for LEWISTON, ME:
C
D
Day of year
°F
Oct
274
Sep
244
Aug
213
Jul
182
Jun
152
May
85
80
75
91
Mar
60
Feb
32
Jan
1
Dec
335
Nov
15
10
5
0
1904–1913
1911–1920
1921–1930
1931–1940
1941–1950
1951–1960
1961–1970
1971–1980
1981–1990
1991–2000
2003–2012

10 year Summer Teletherm dynamics for MILLINOCKET, ME:

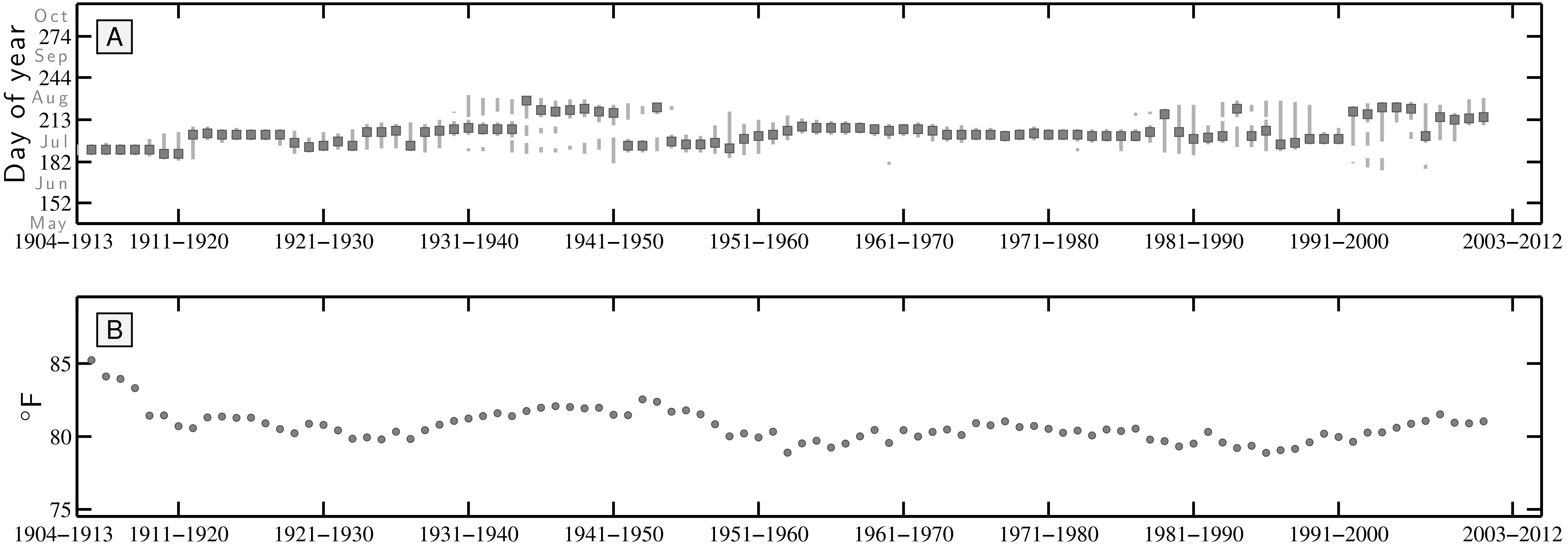

10 year Winter Teletherm dynamics for MILLINOCKET, ME:

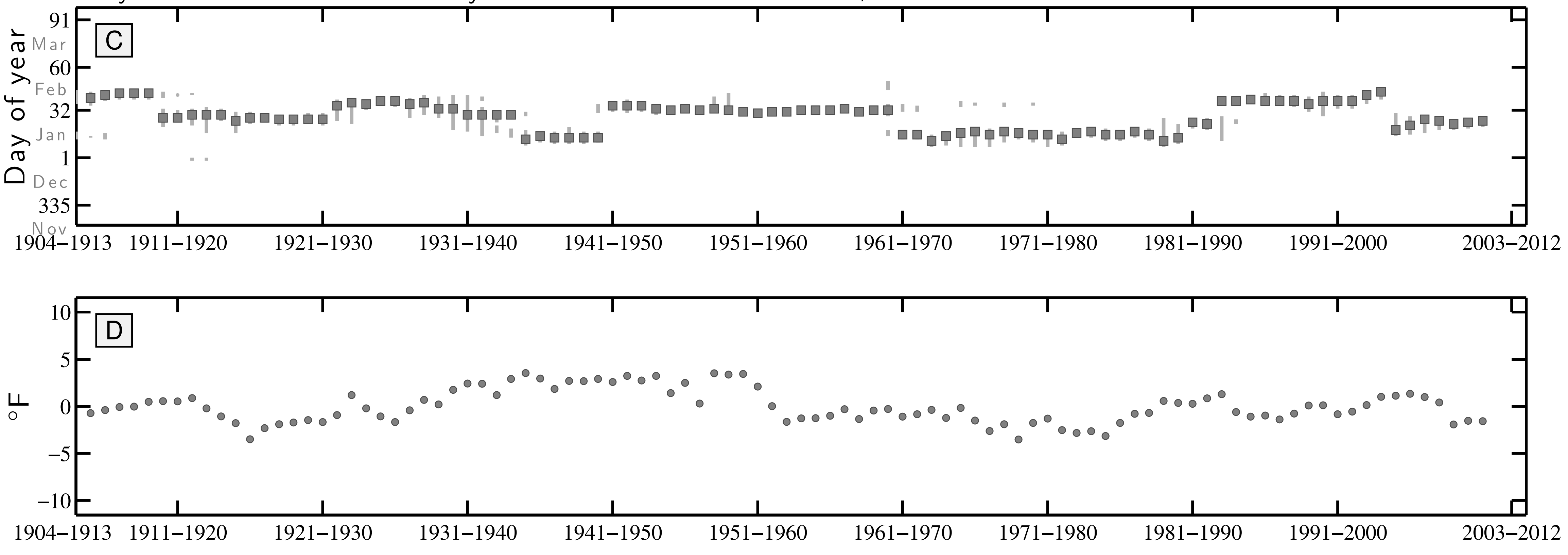

10 year Summer Teletherm dynamics for PORTLAND JETPORT, ME:

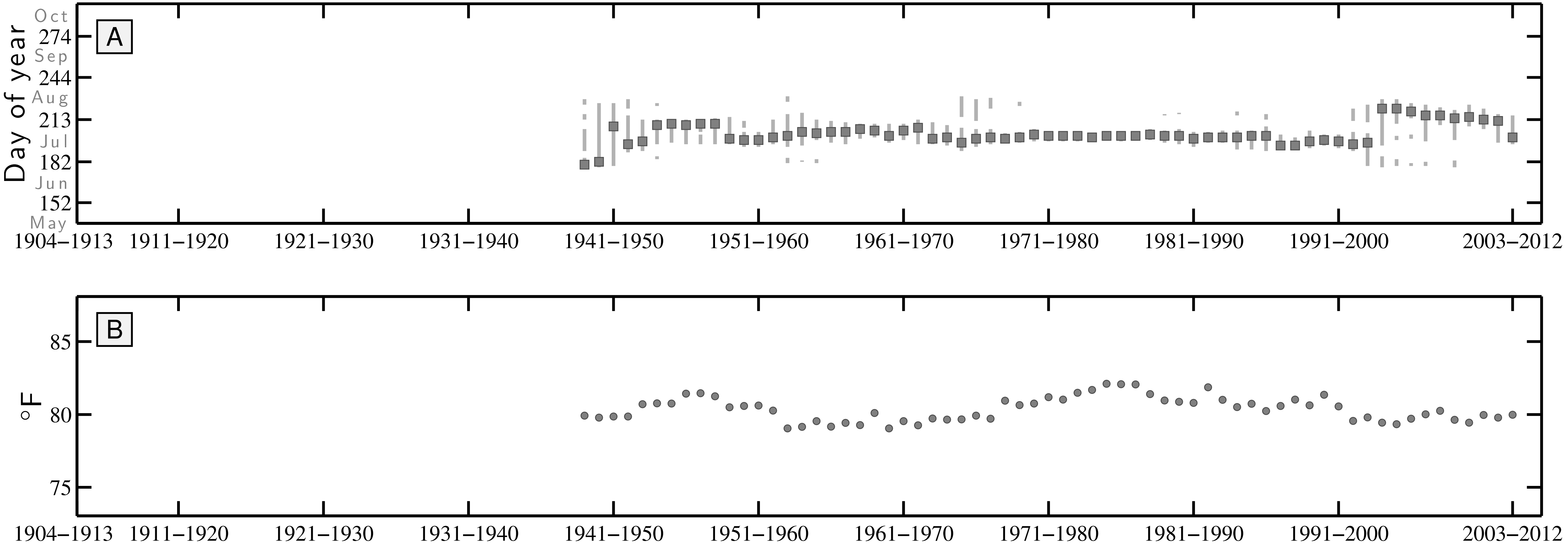

10 year Winter Teletherm dynamics for PORTLAND JETPORT, ME:

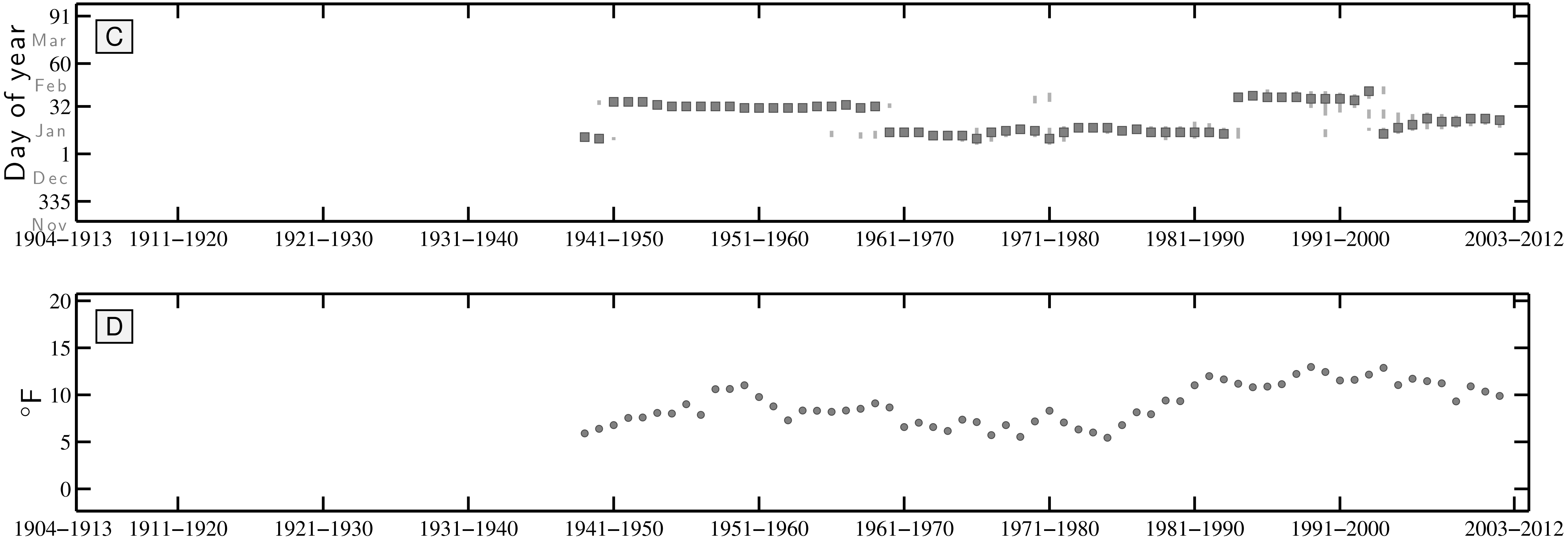

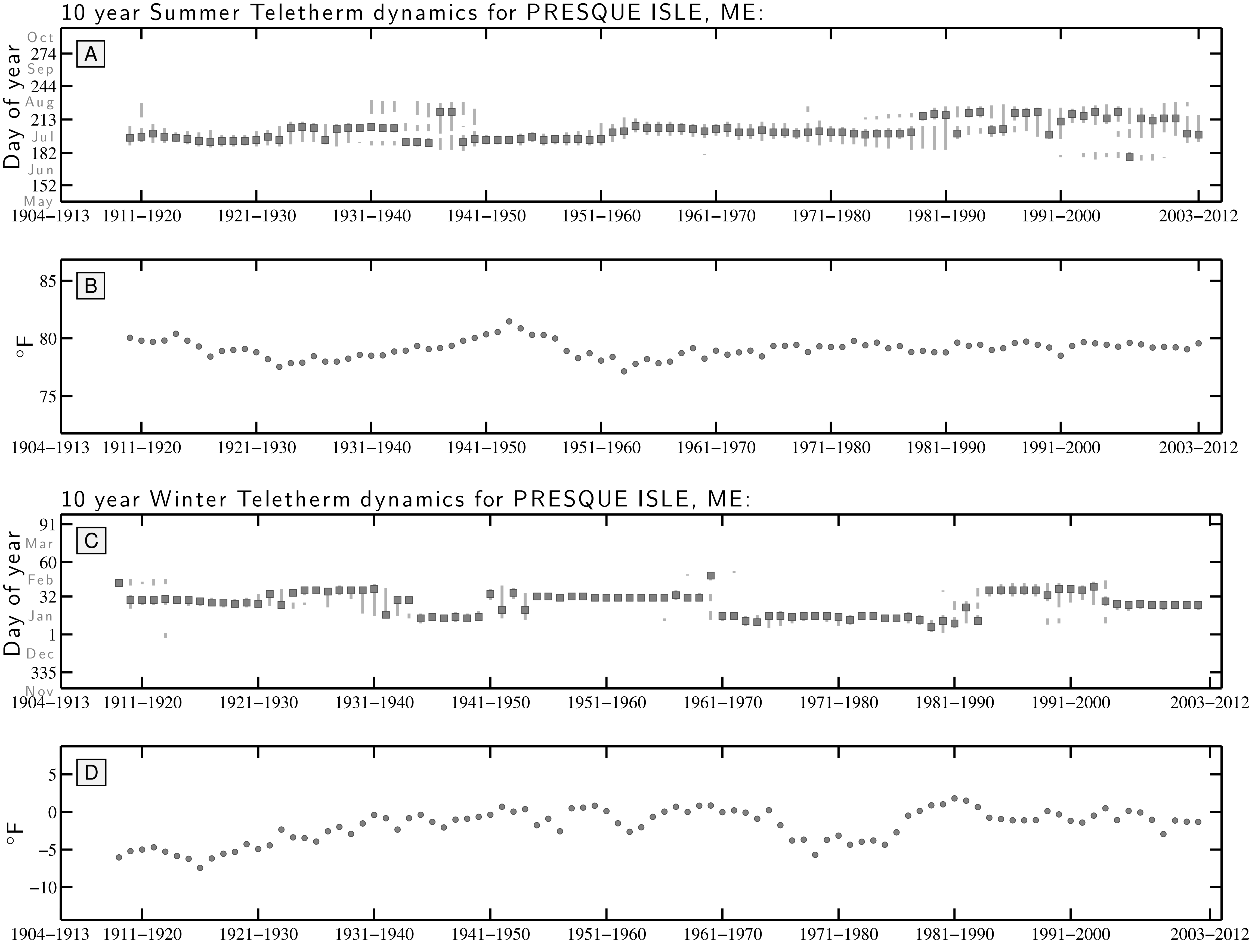
10 year Summer Teletherm dynamics for PRESQUE ISLE, ME:
A
Day of year
Oct
274
Sep
244
Aug
213
Jul
182
Jun
152
May
1904–1913
1911–1920
1921–1930
1931–1940
1941–1950
1951–1960
1961–1970
1971–1980
1981–1990
1991–2000
2003–2012
B
°F
85
80
75
10 year Winter Teletherm dynamics for PRESQUE ISLE, ME:
C
91
Mar
60
Feb
32
Jan
1
Dec
335
Nov
D
5
0
−5
−10

10 year Summer Teletherm dynamics for WOODLAND, ME:

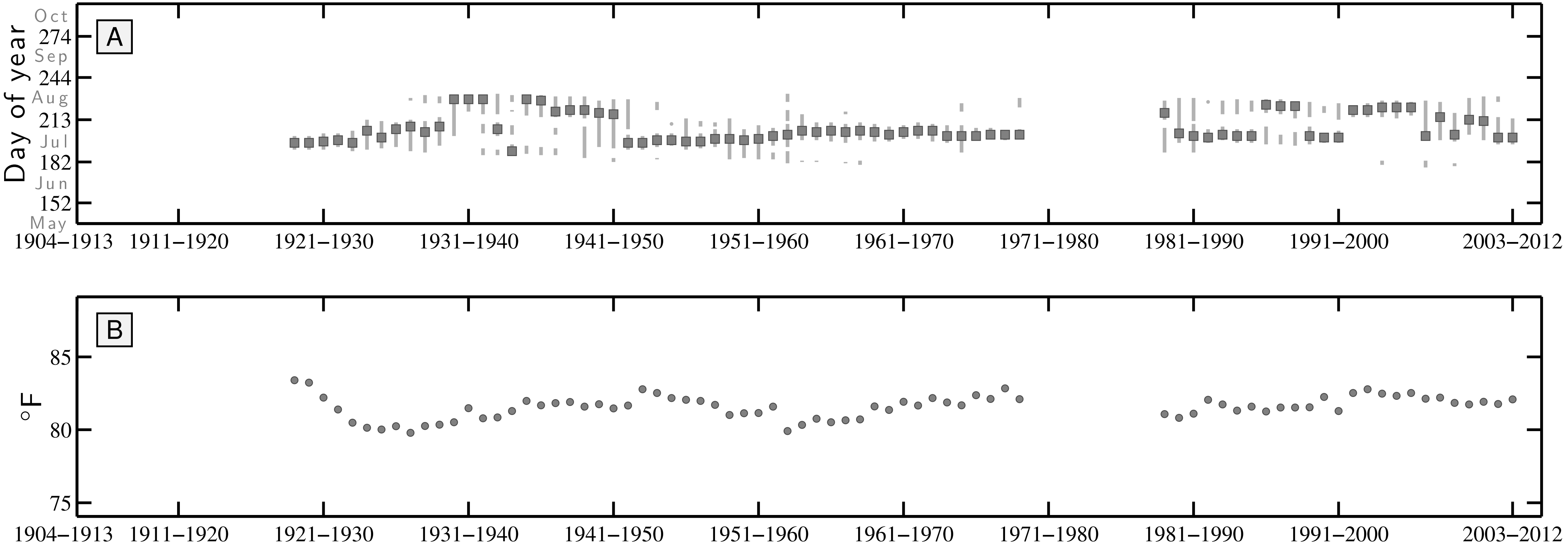

10 year Winter Teletherm dynamics for WOODLAND, ME:

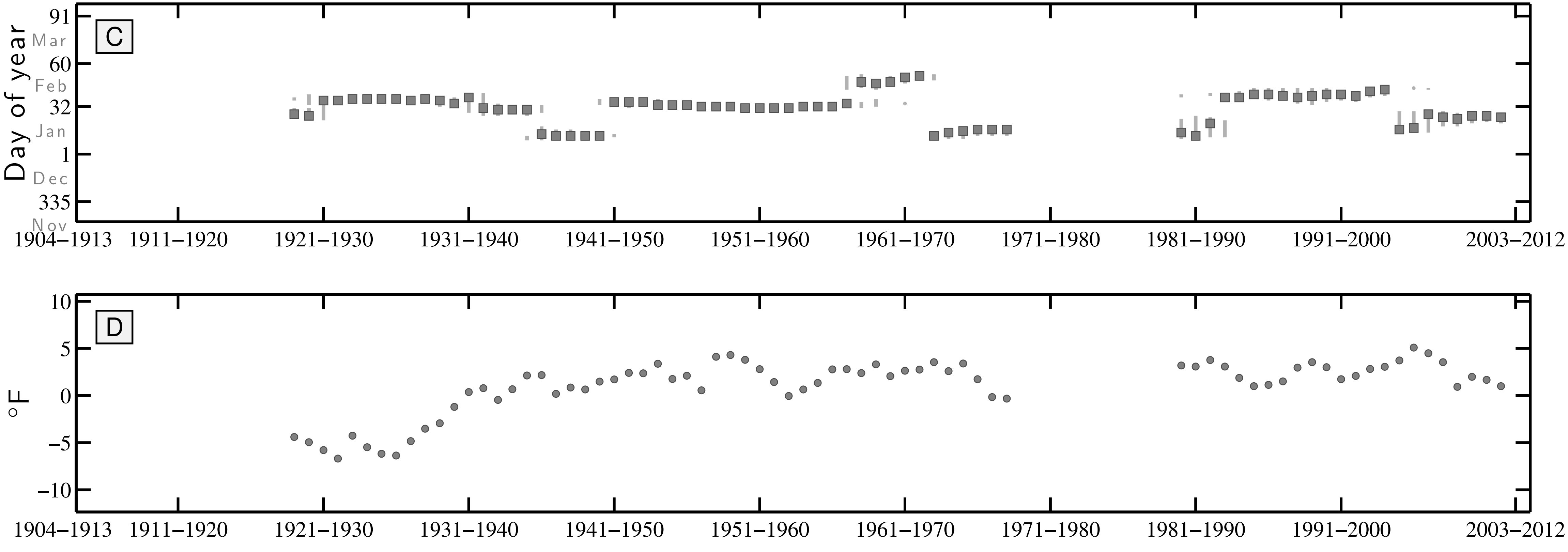

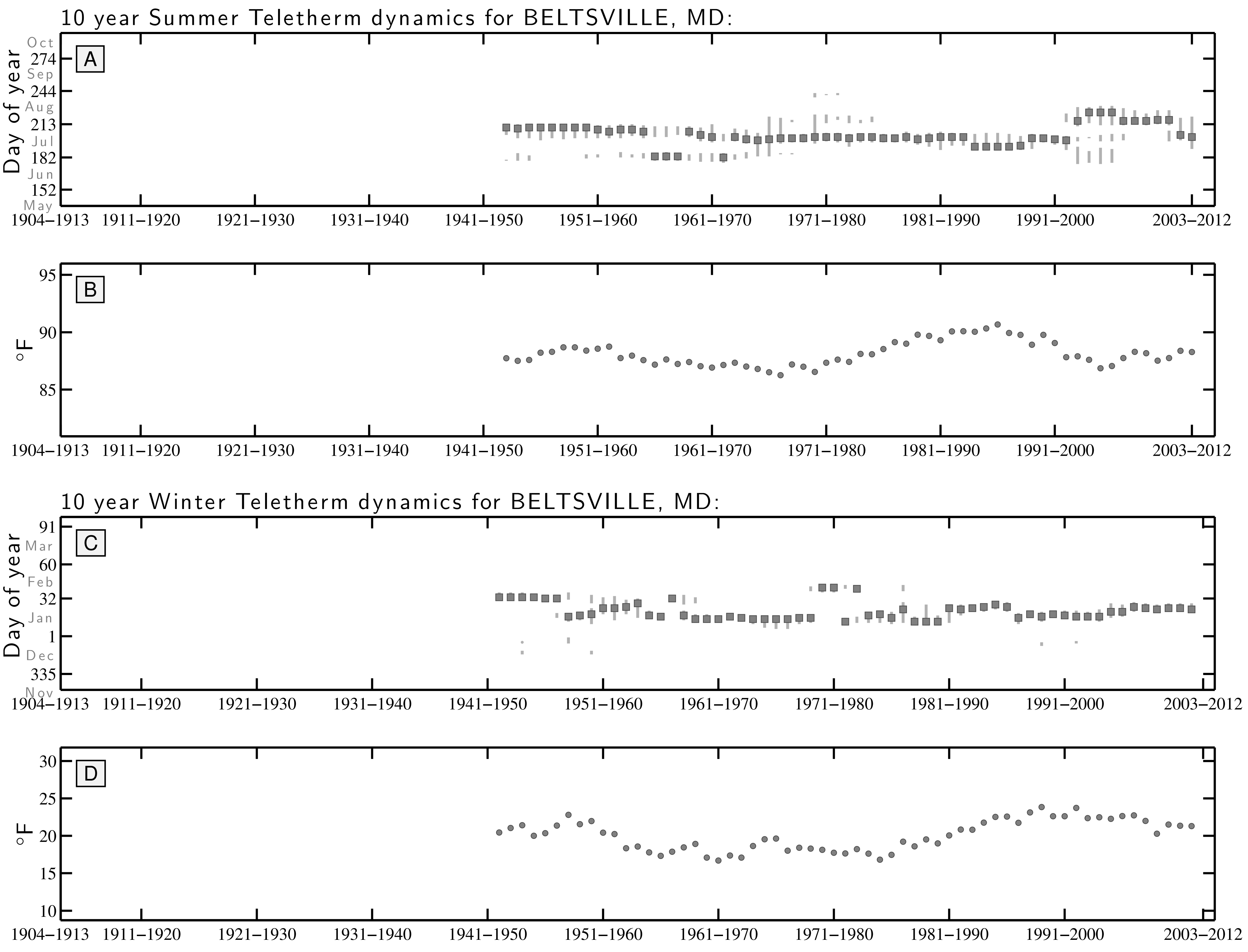

10 year Summer Teletherm dynamics for BELTSVILLE, MD:
A
Day of year
Oct
274
Sep
244
Aug
213
Jul
182
Jun
152
May
1904–1913
1911–1920
1921–1930
1931–1940
1941–1950
1951–1960
1961–1970
1971–1980
1981–1990
1991–2000
2003–2012
B
°F
95
90
85
10 year Winter Teletherm dynamics for BELTSVILLE, MD:
C
91
Mar
60
Feb
32
Jan
1
Dec
335
Nov
D
30
25
20
15
10

10 year Summer Teletherm dynamics for CAMBRIDGE WATER TRMT P, MD:

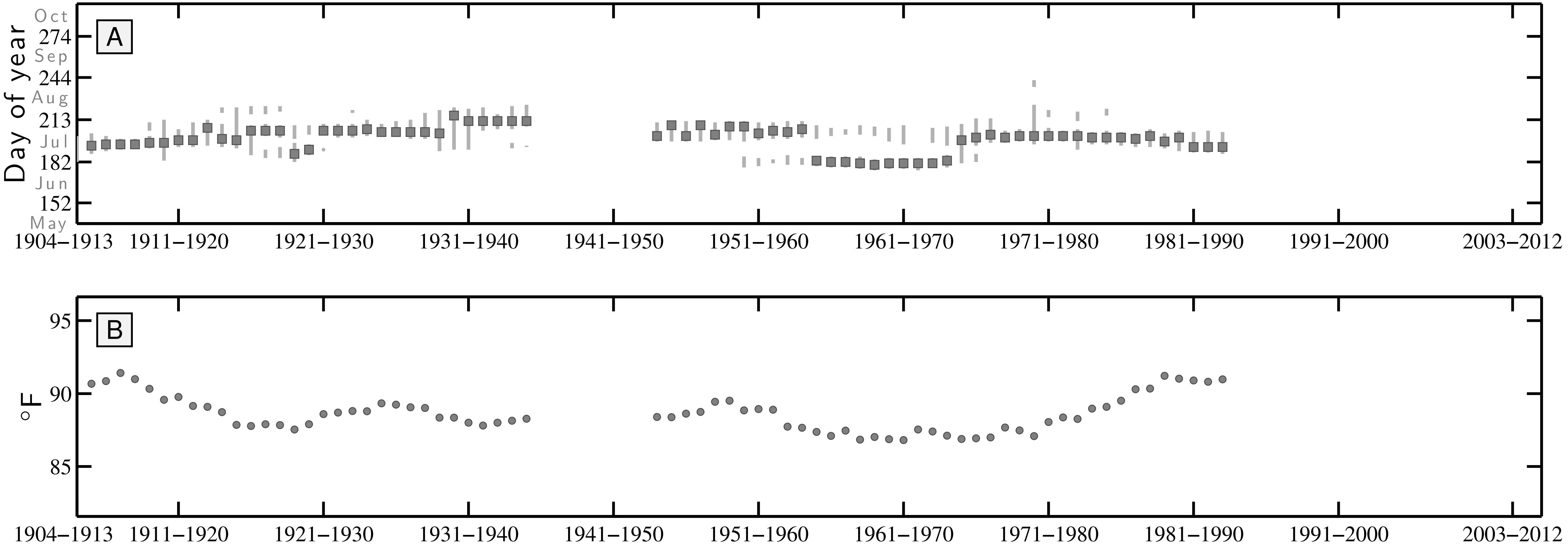

10 year Winter Teletherm dynamics for CAMBRIDGE WATER TRMT P, MD:

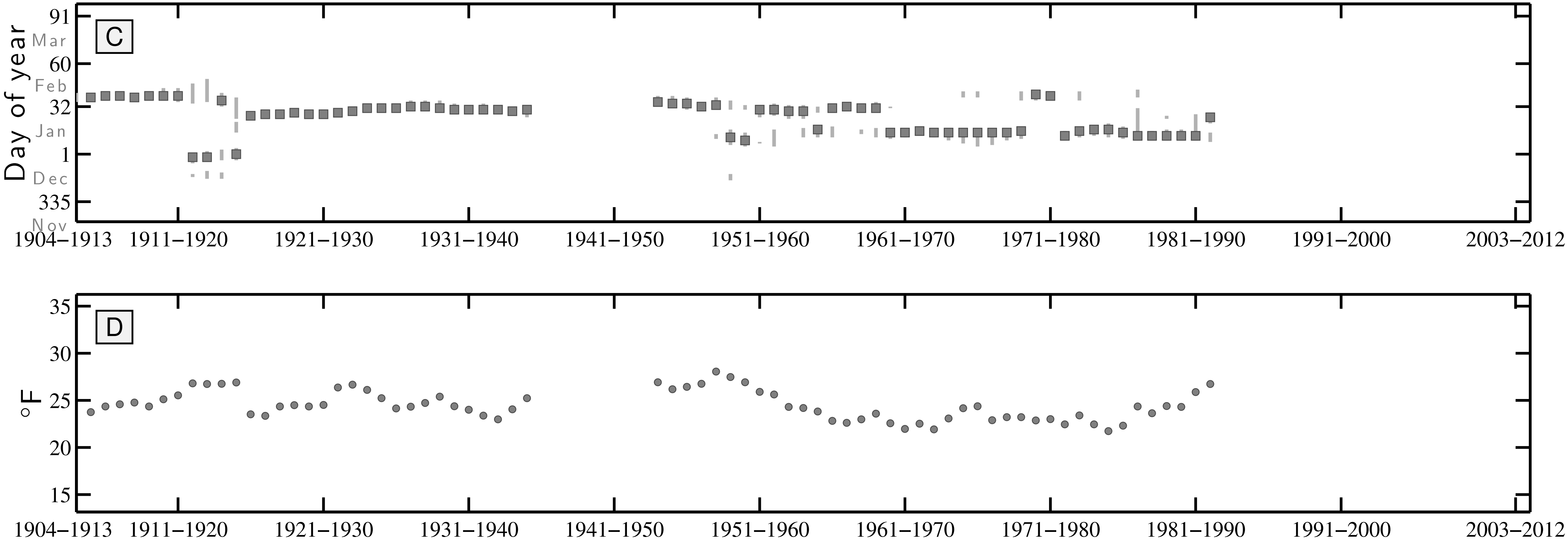

10 year Summer Teletherm dynamics for CHESTERTOWN, MD:

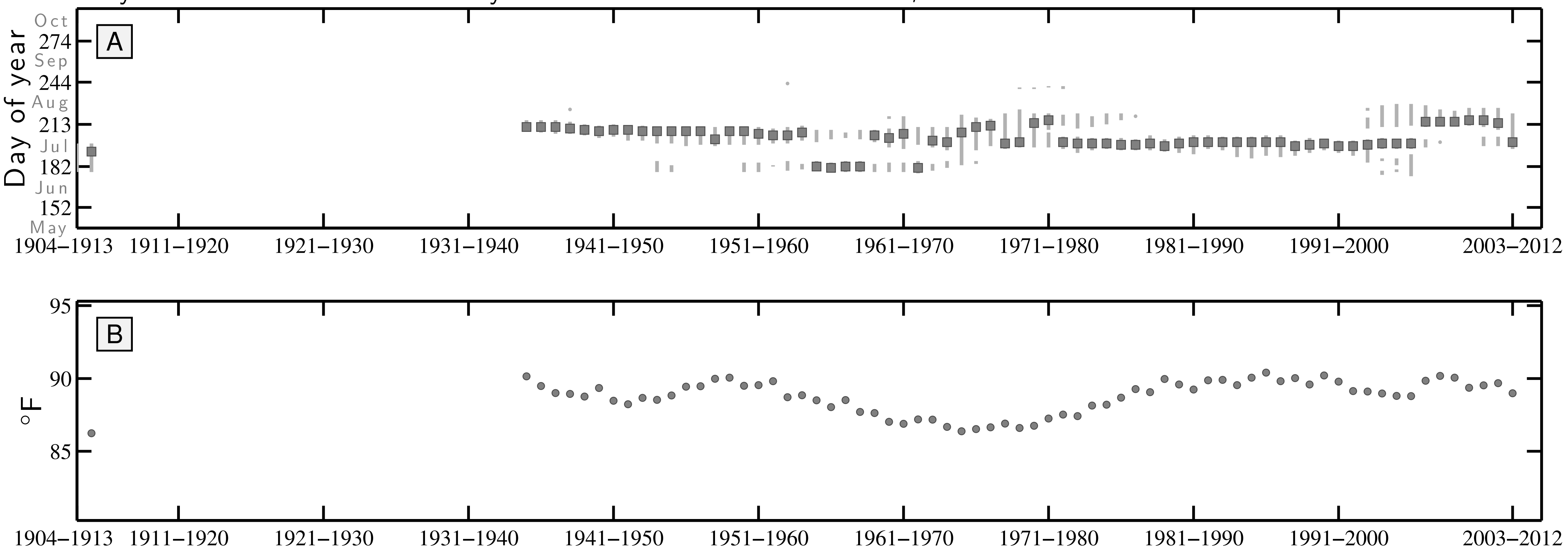

10 year Winter Teletherm dynamics for CHESTERTOWN, MD:

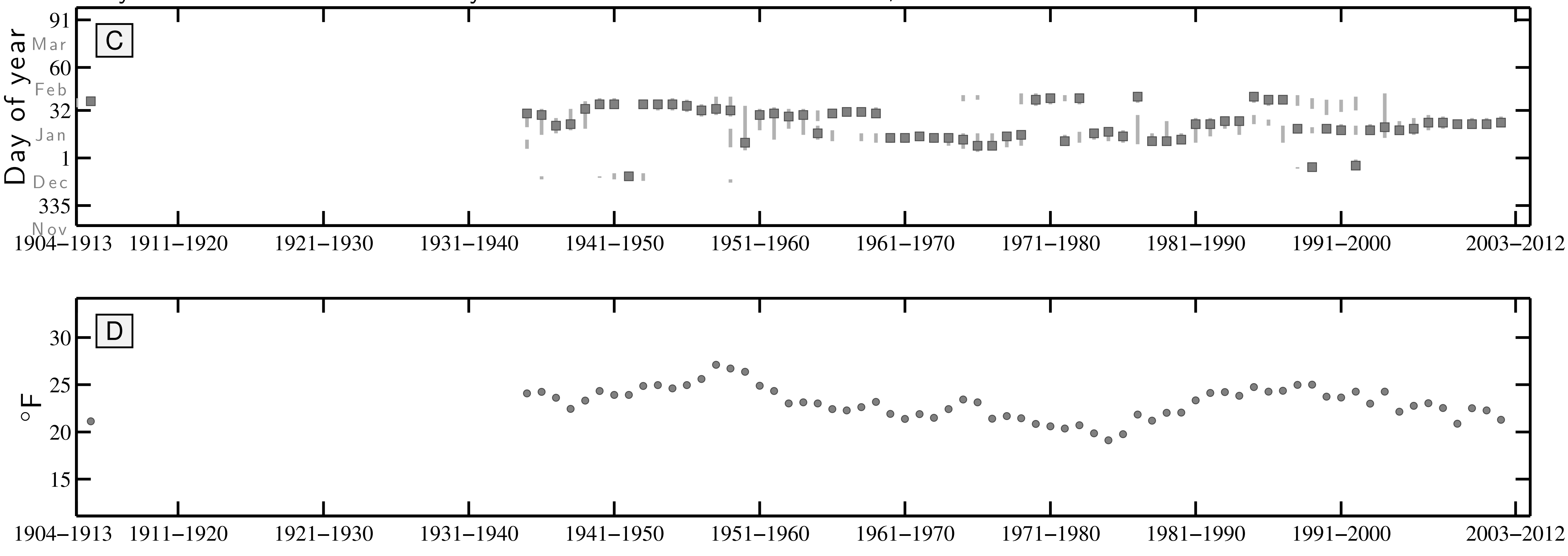

10 year Summer Teletherm dynamics for CUMBERLAND 2, MD:

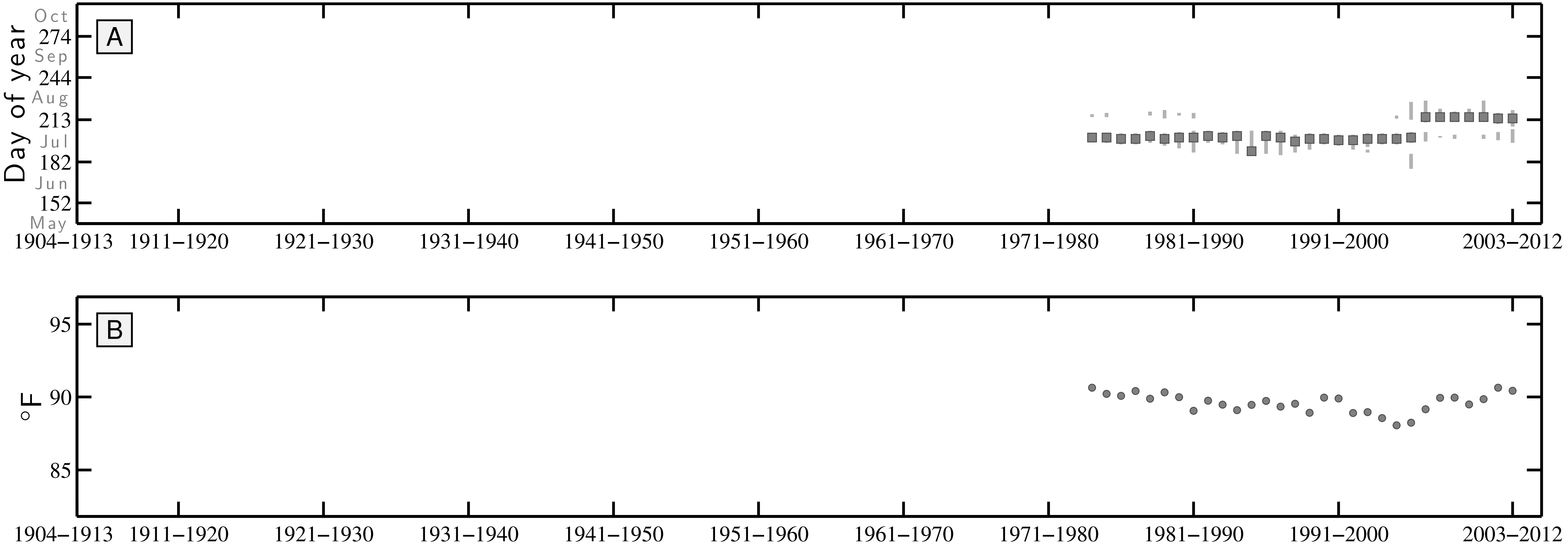

10 year Winter Teletherm dynamics for CUMBERLAND 2, MD:

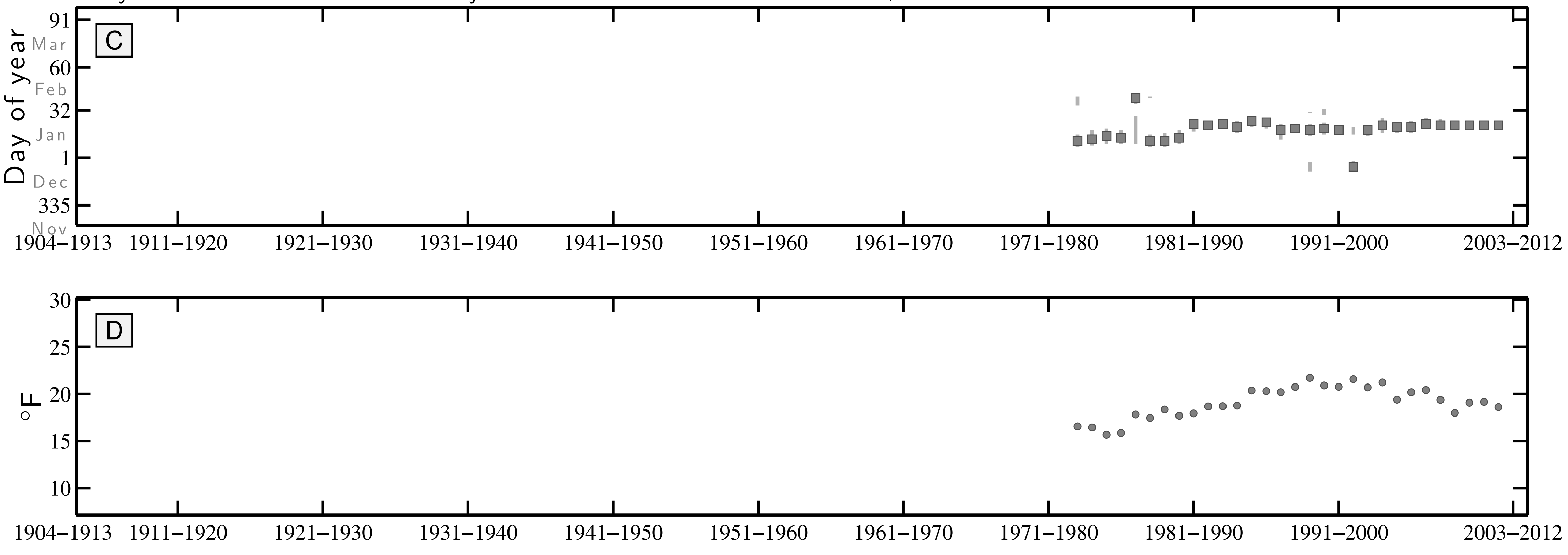

10 year Summer Teletherm dynamics for DENTON 2 E, MD:

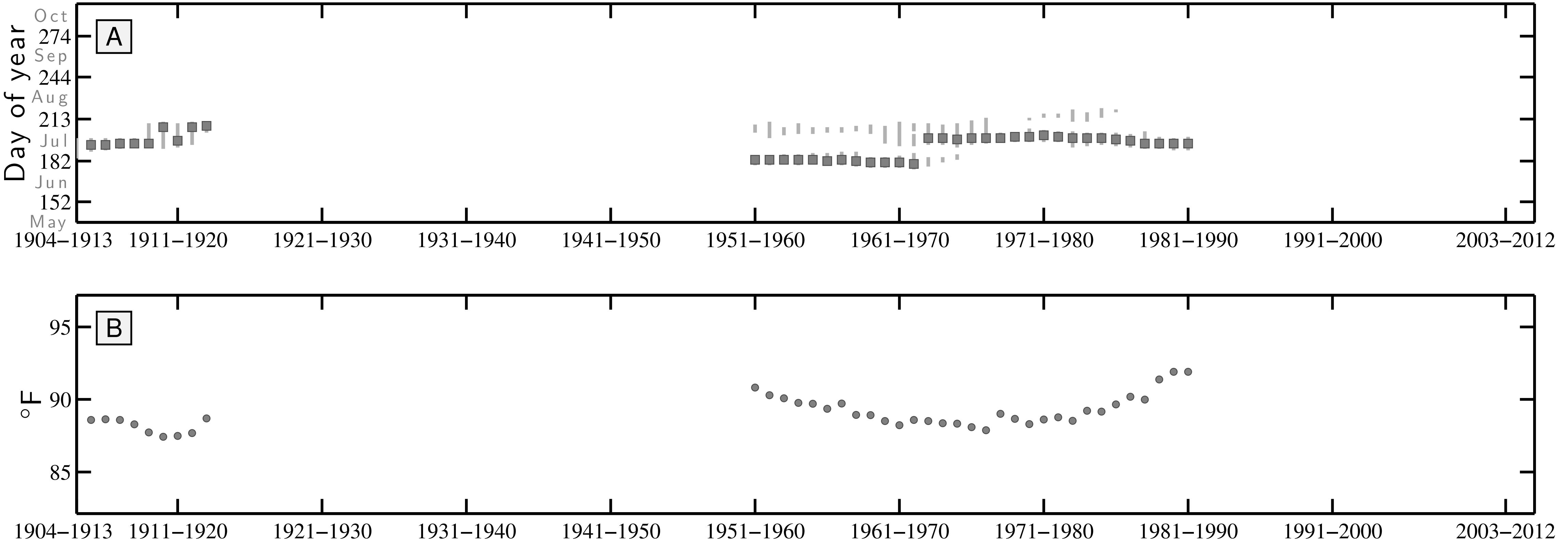

10 year Winter Teletherm dynamics for DENTON 2 E, MD:

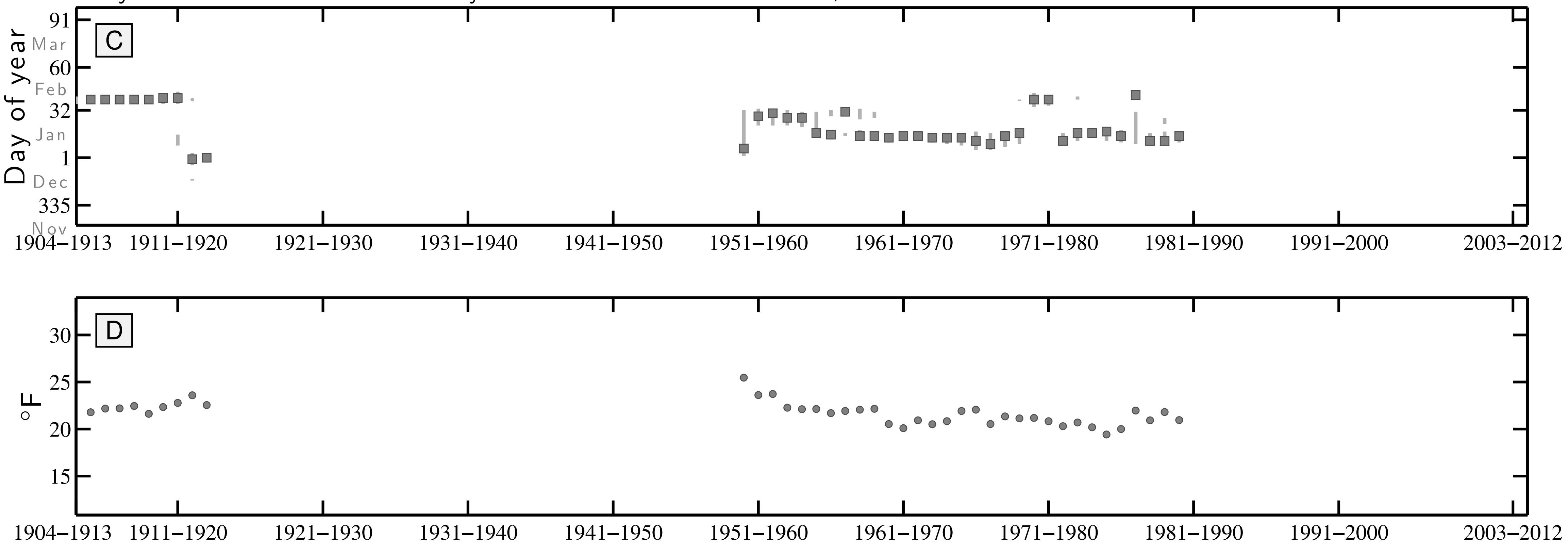

10 year Summer Teletherm dynamics for GLENN DALE BELL STN, MD:

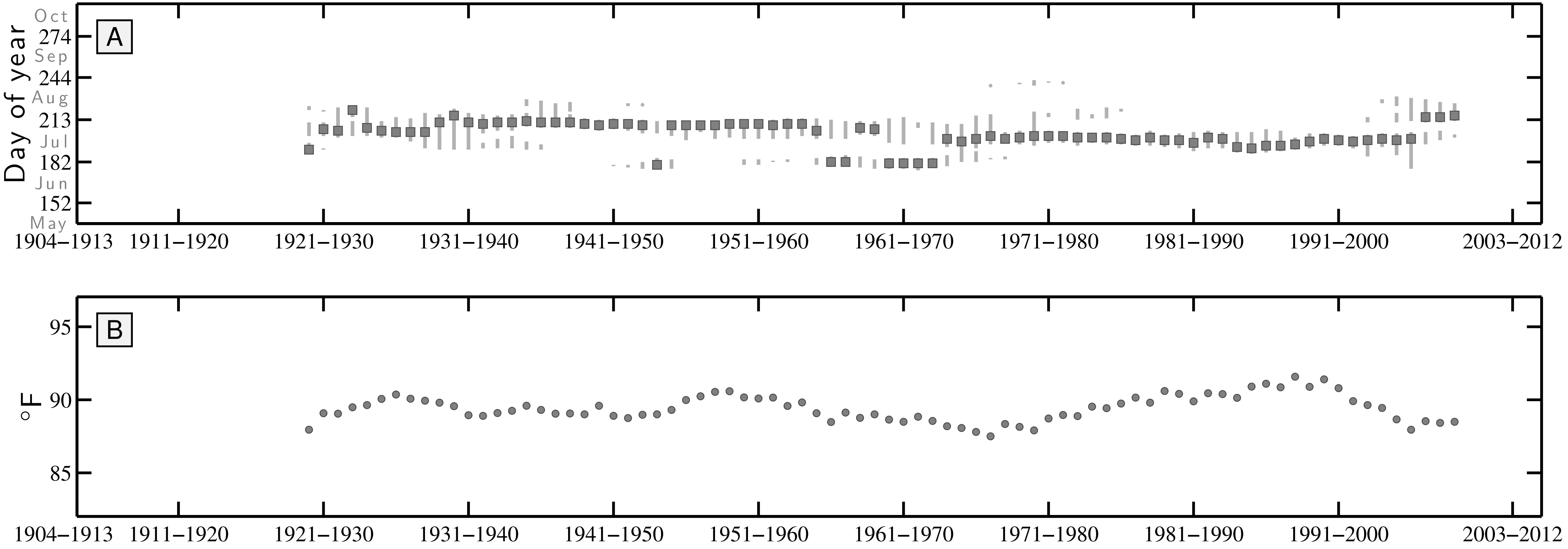

10 year Winter Teletherm dynamics for GLENN DALE BELL STN, MD:

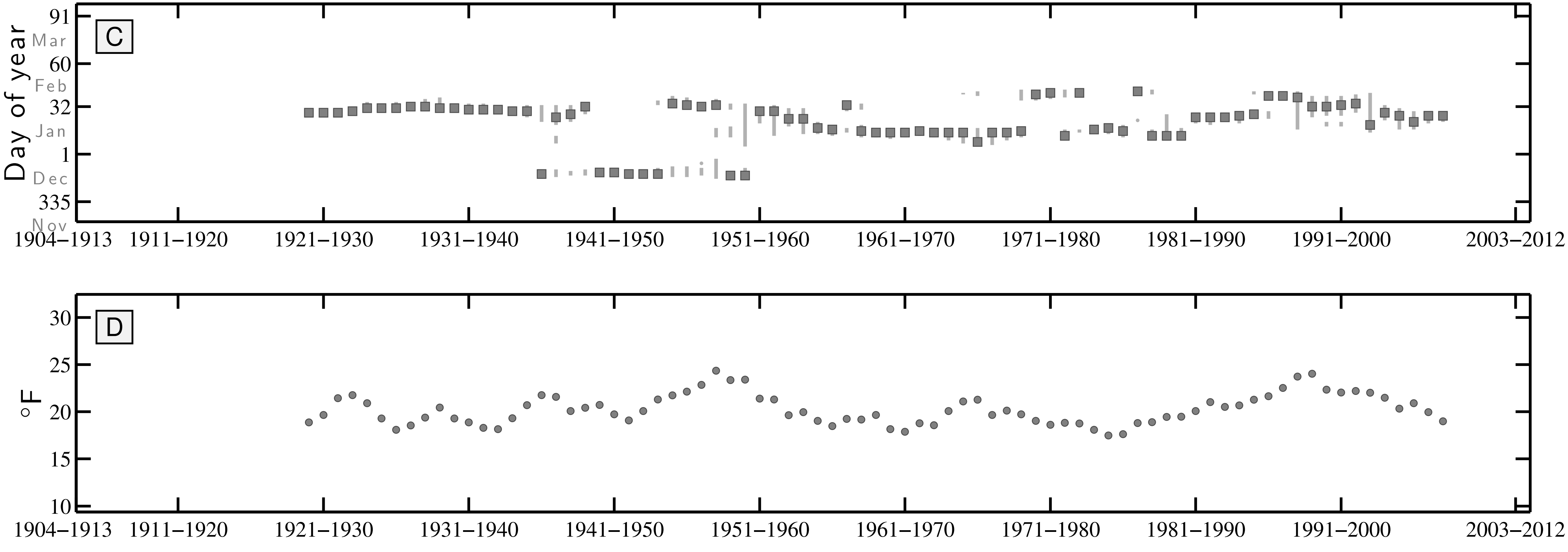

10 year Summer Teletherm dynamics for LAUREL 3 W, MD:

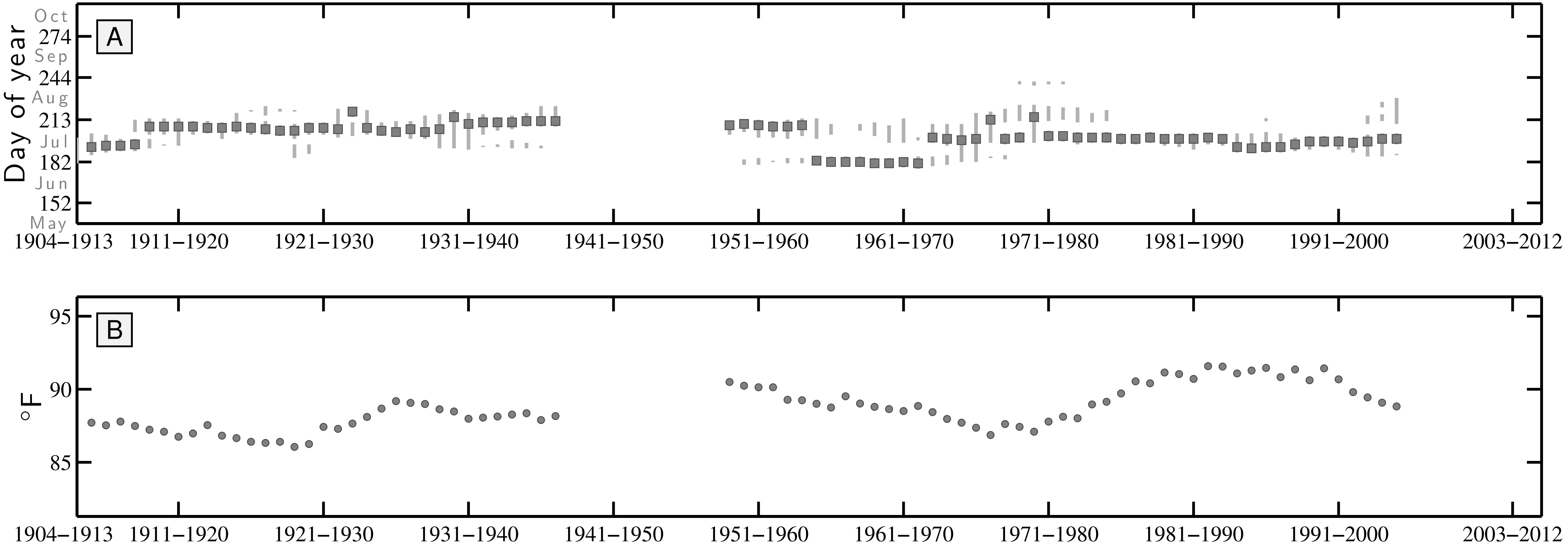

10 year Winter Teletherm dynamics for LAUREL 3 W, MD:

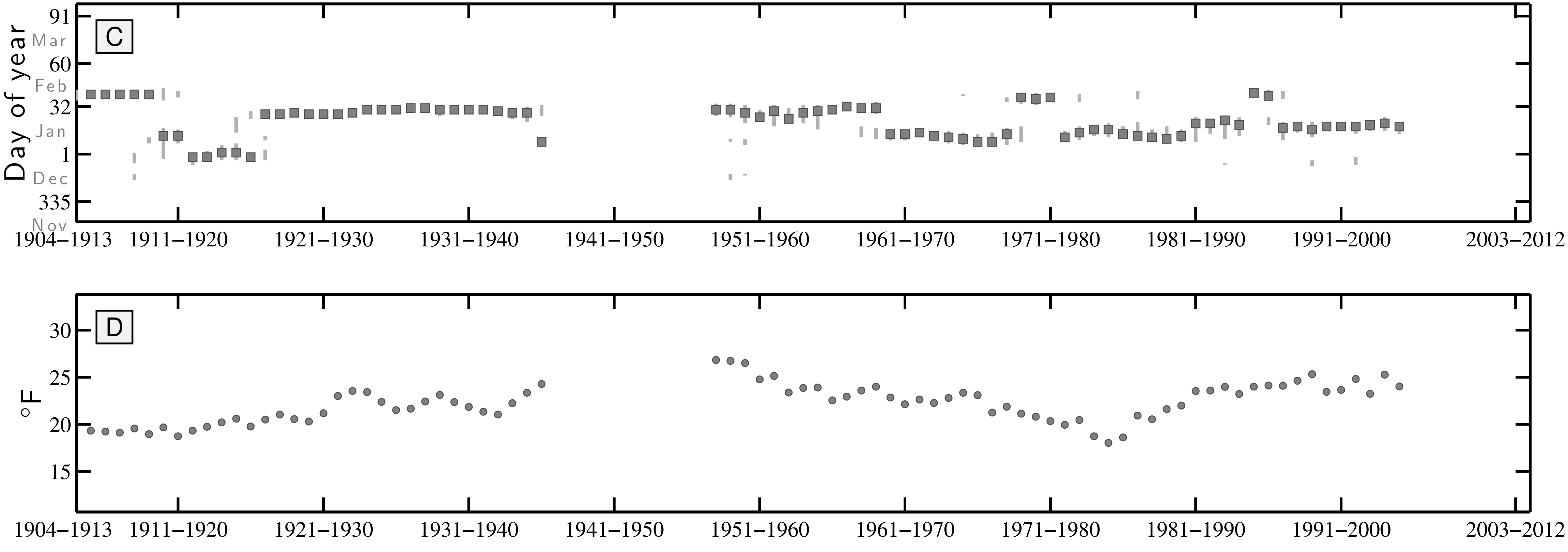

10 year Summer Teletherm dynamics for MD SCI CTR BALTIMORE, MD:

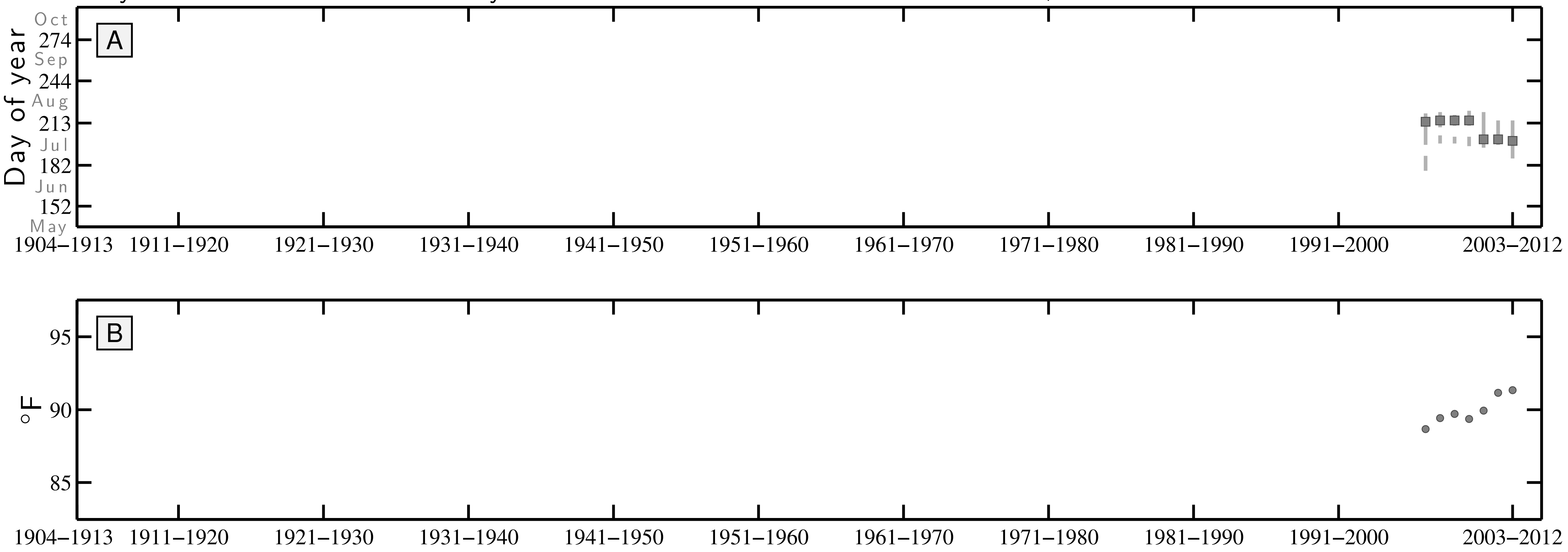

10 year Winter Teletherm dynamics for MD SCI CTR BALTIMORE, MD:

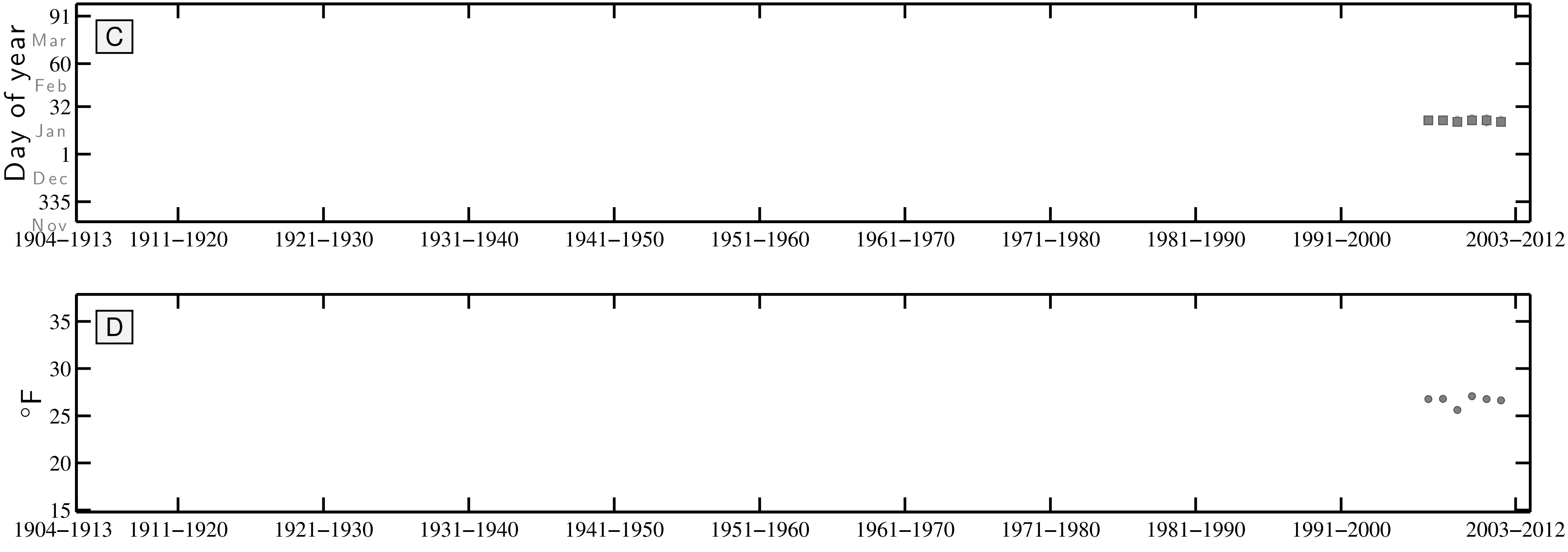

10 year Summer Teletherm dynamics for MILLINGTON 1 SE, MD:

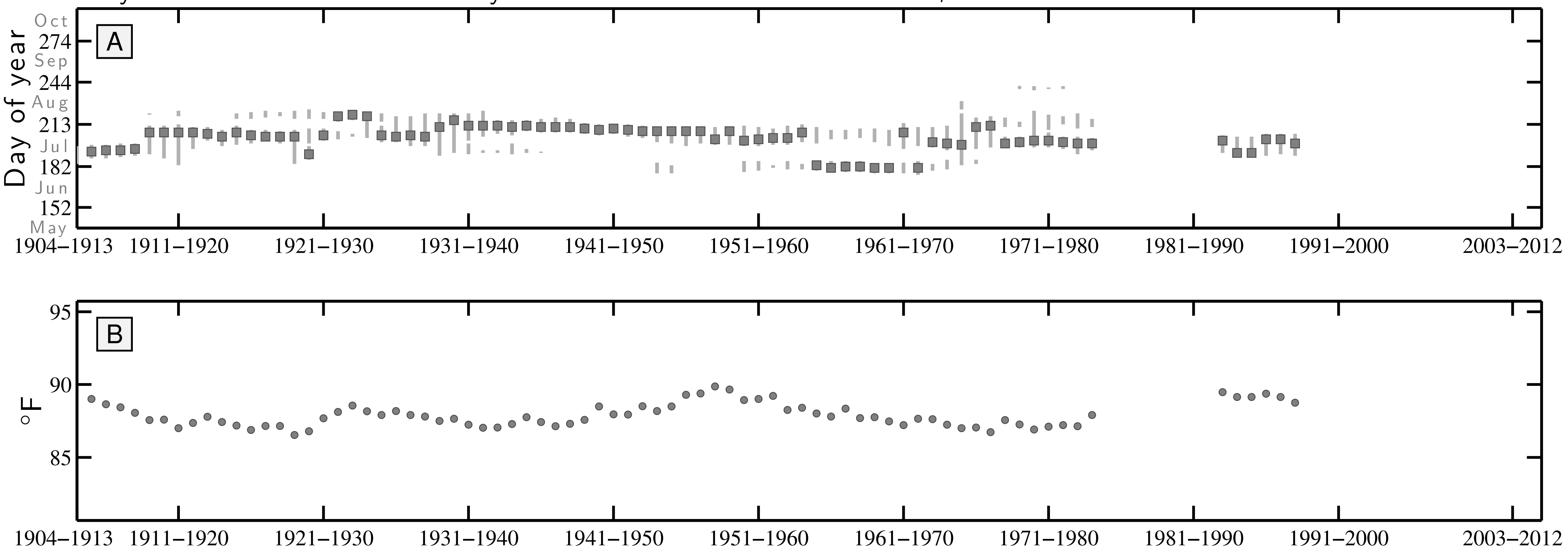

10 year Winter Teletherm dynamics for MILLINGTON 1 SE, MD:

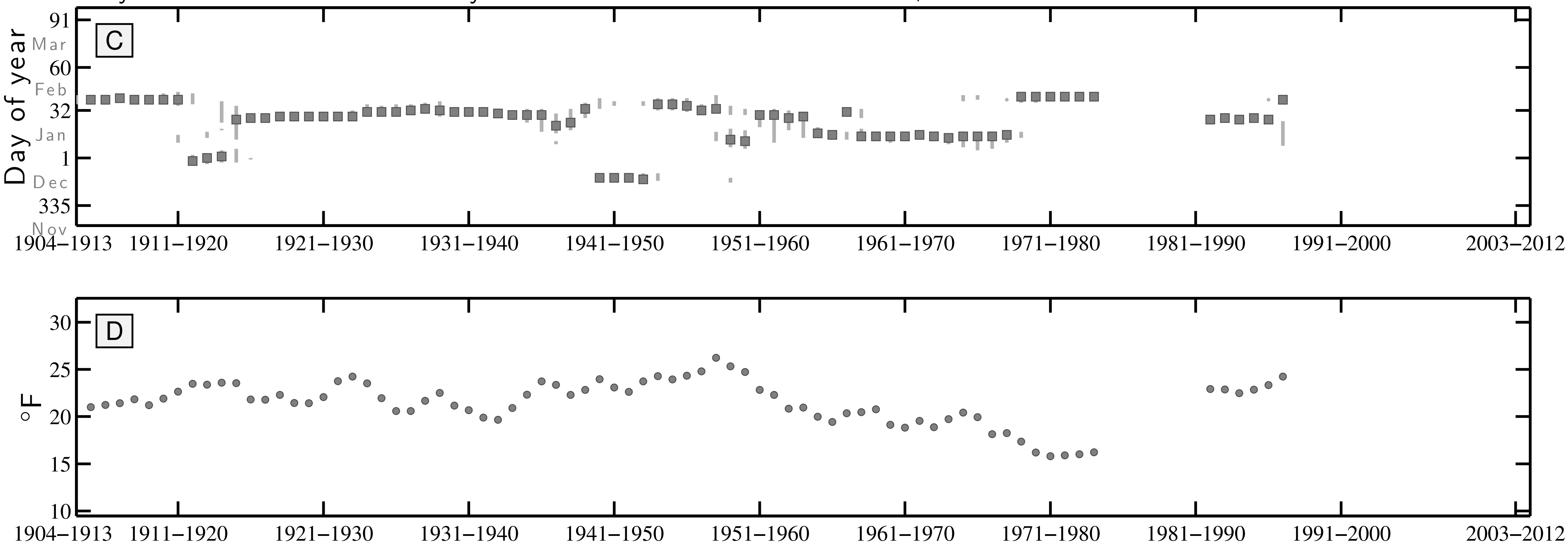

10 year Summer Teletherm dynamics for OAKLAND 1 SE, MD:

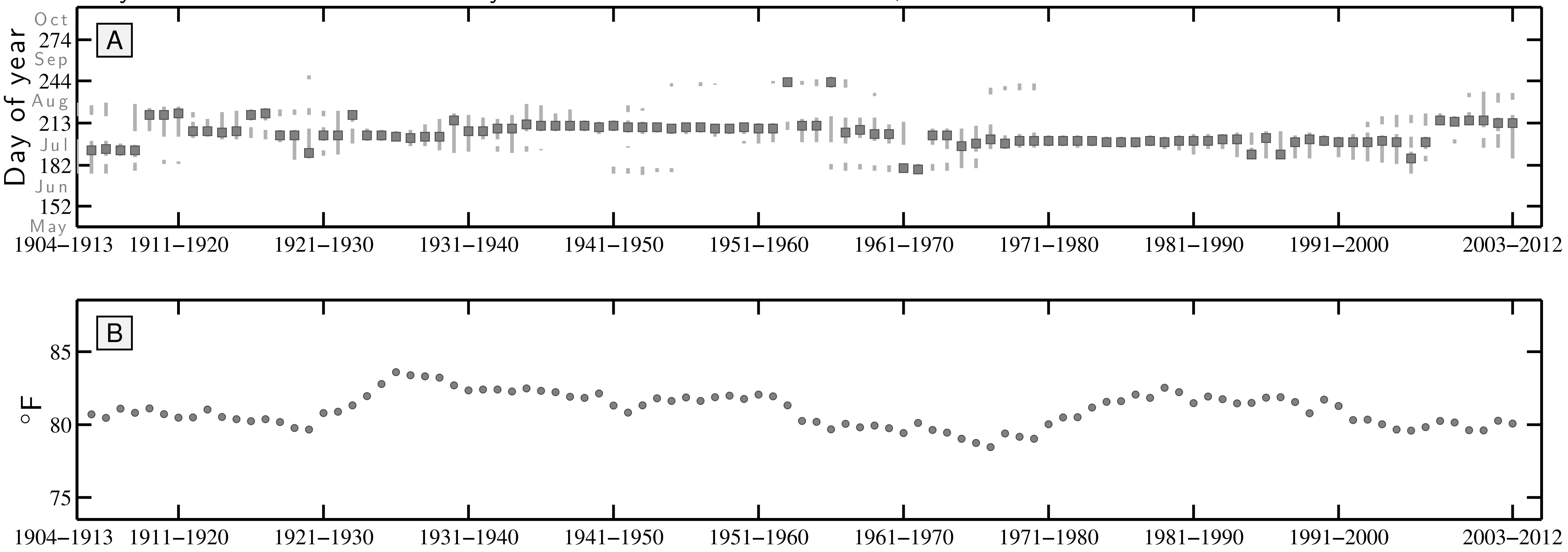

10 year Winter Teletherm dynamics for OAKLAND 1 SE, MD:

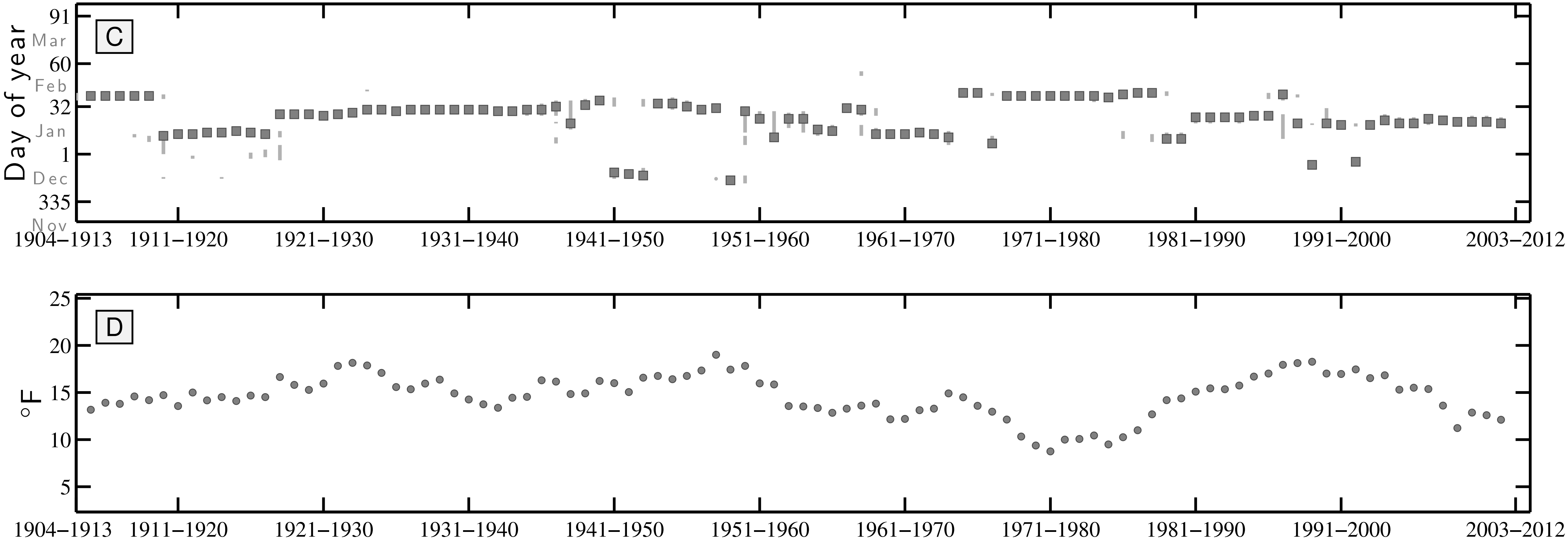

10 year Summer Teletherm dynamics for OWINGS FERRY LANDING, MD:

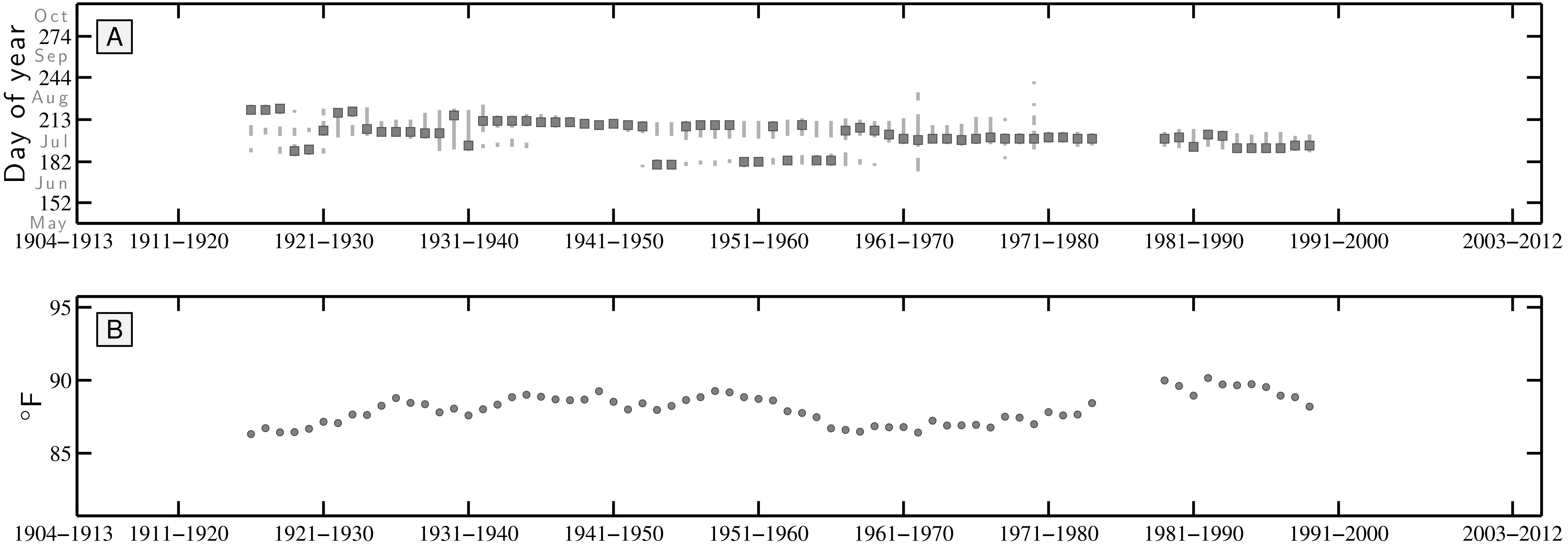

10 year Winter Teletherm dynamics for OWINGS FERRY LANDING, MD:

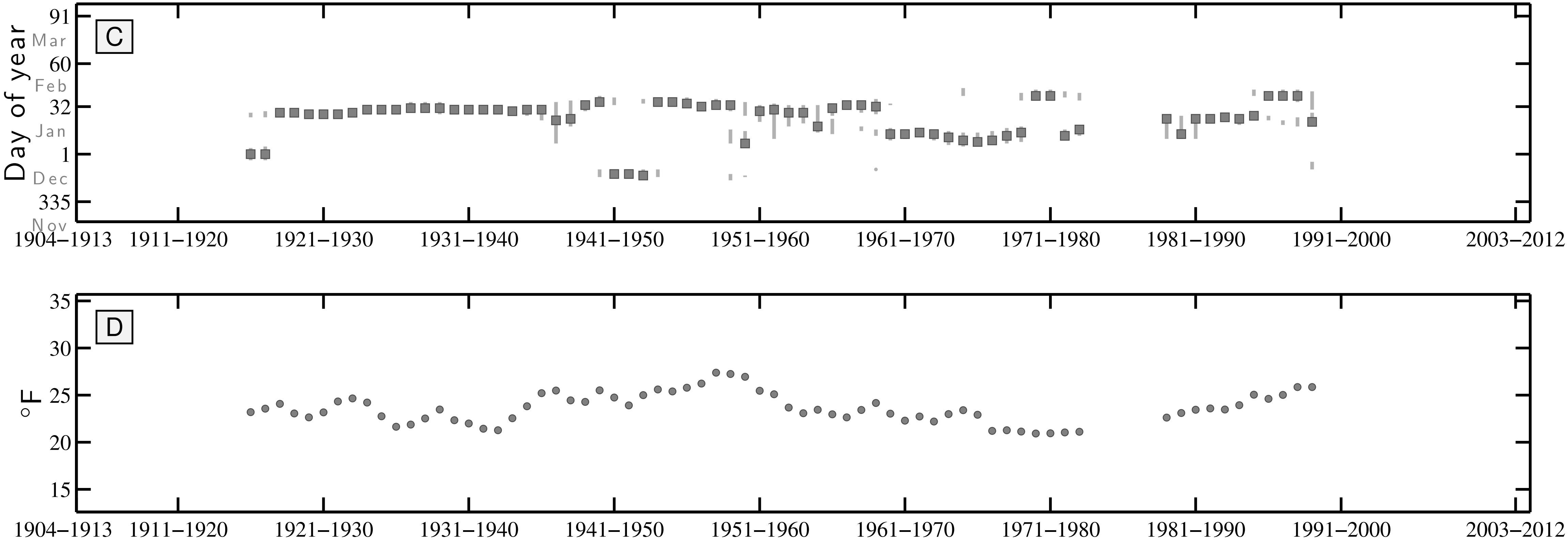

10 year Summer Teletherm dynamics for PRINCESS ANNE, MD:

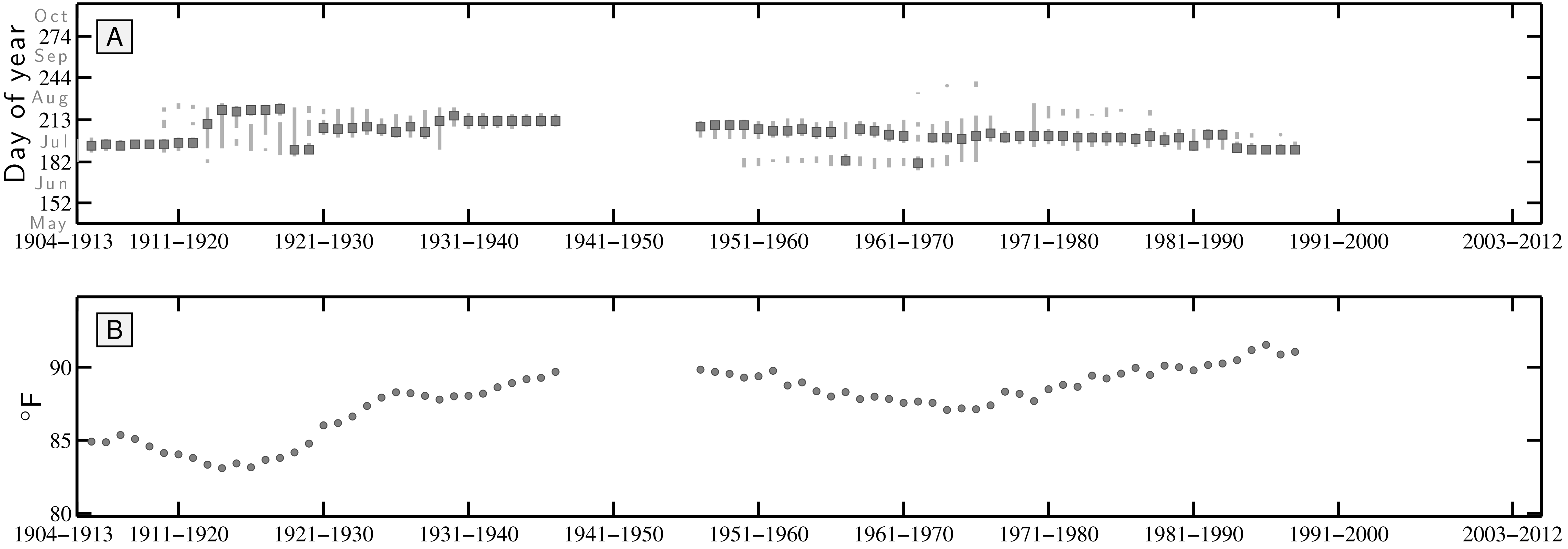

10 year Winter Teletherm dynamics for PRINCESS ANNE, MD:

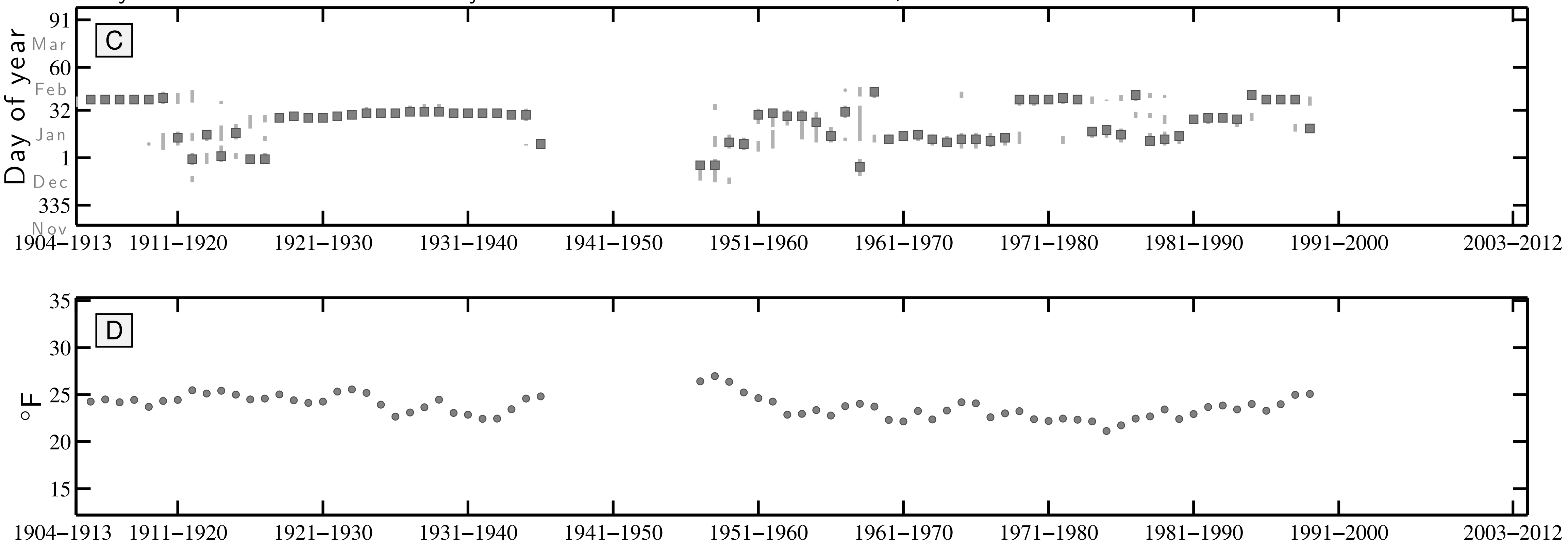

10 year Summer Teletherm dynamics for ROYAL OAK 2 SSW, MD:

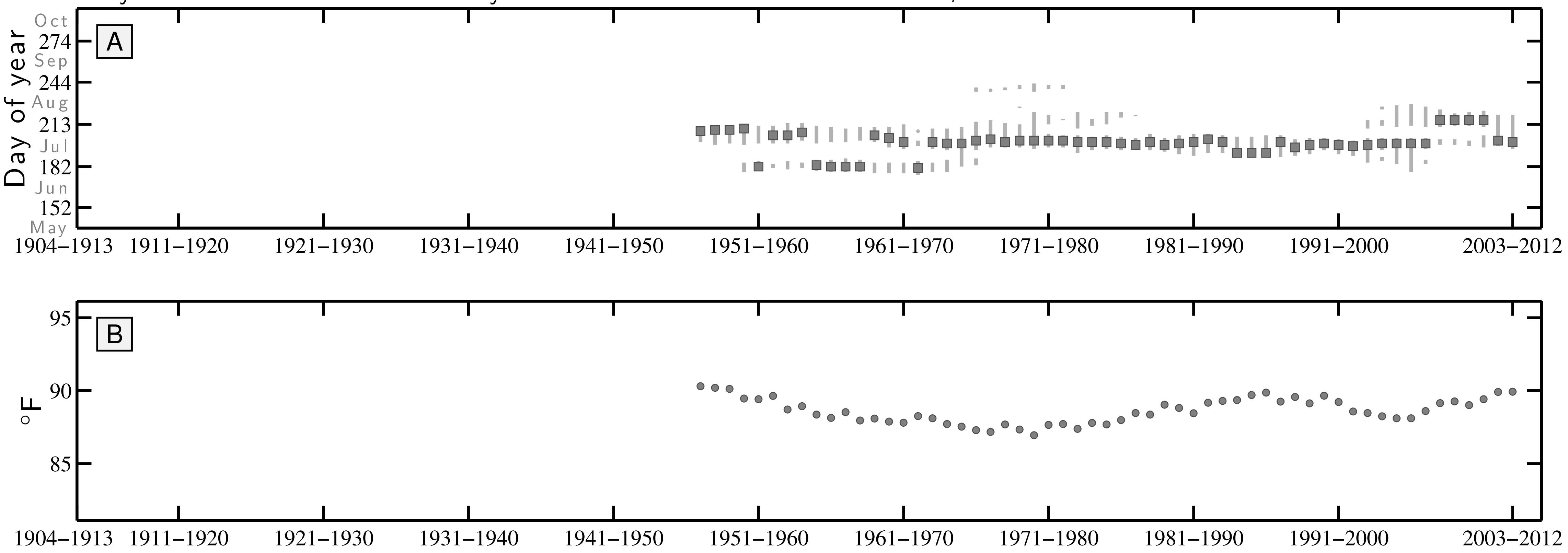

10 year Winter Teletherm dynamics for ROYAL OAK 2 SSW, MD:

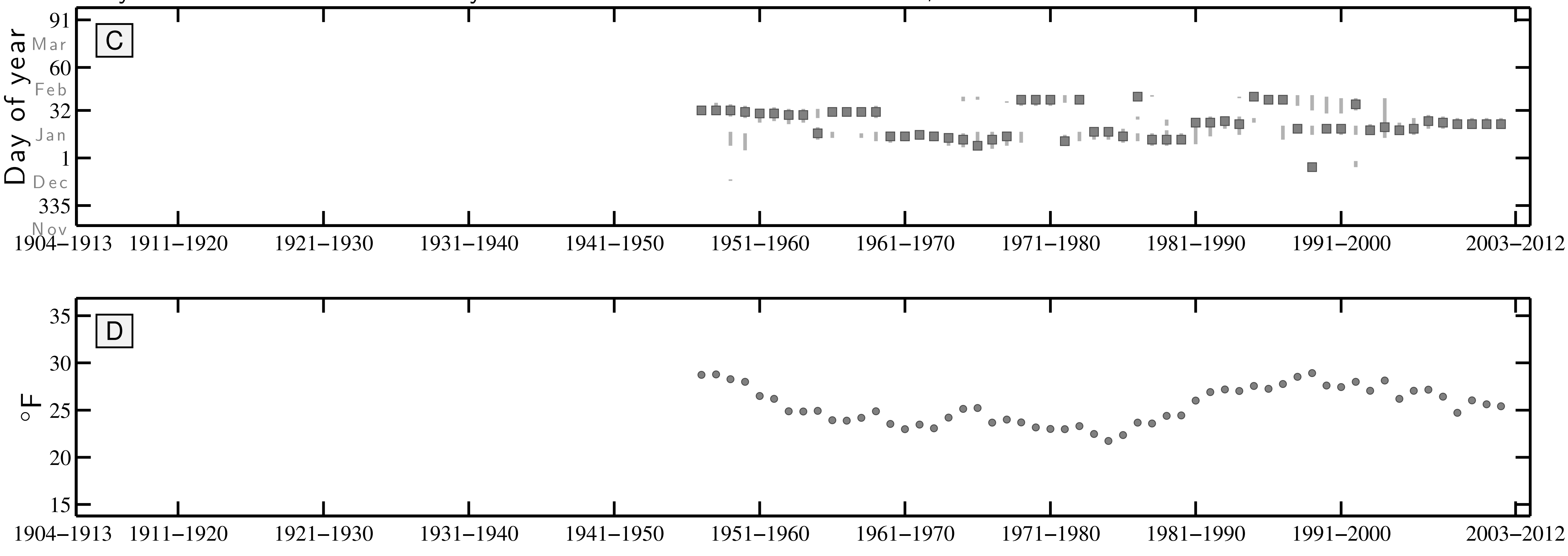

10 year Summer Teletherm dynamics for SALISBURY, MD:

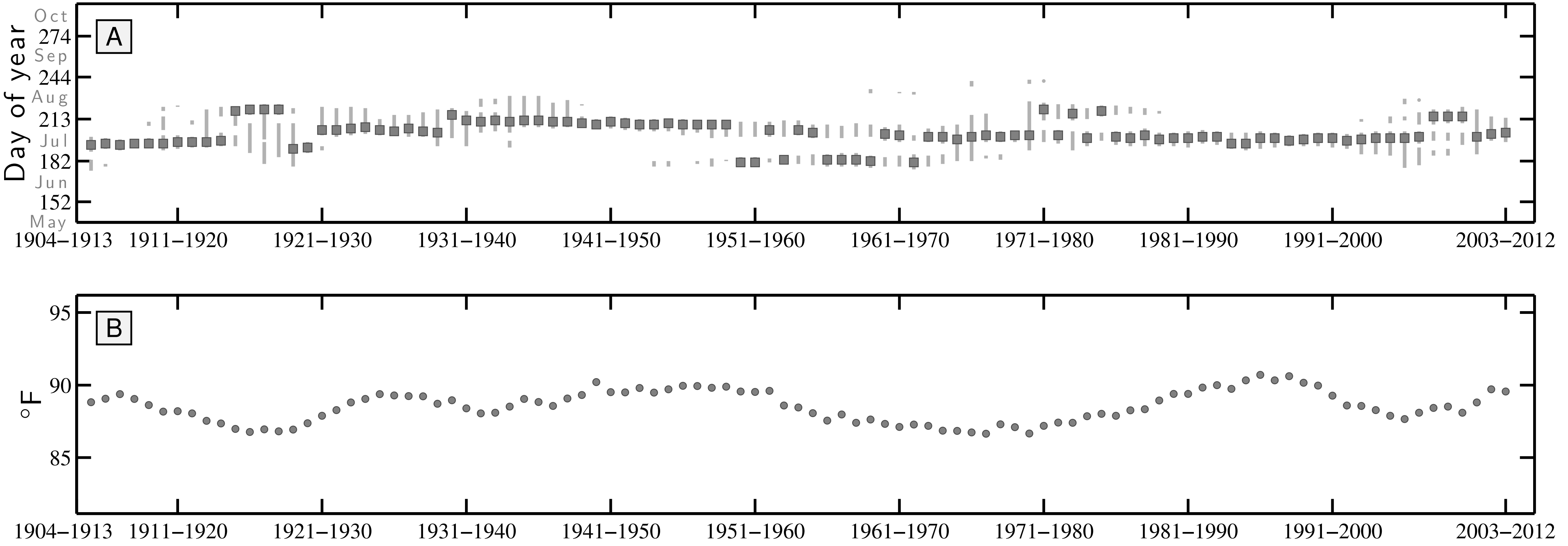

10 year Winter Teletherm dynamics for SALISBURY, MD:

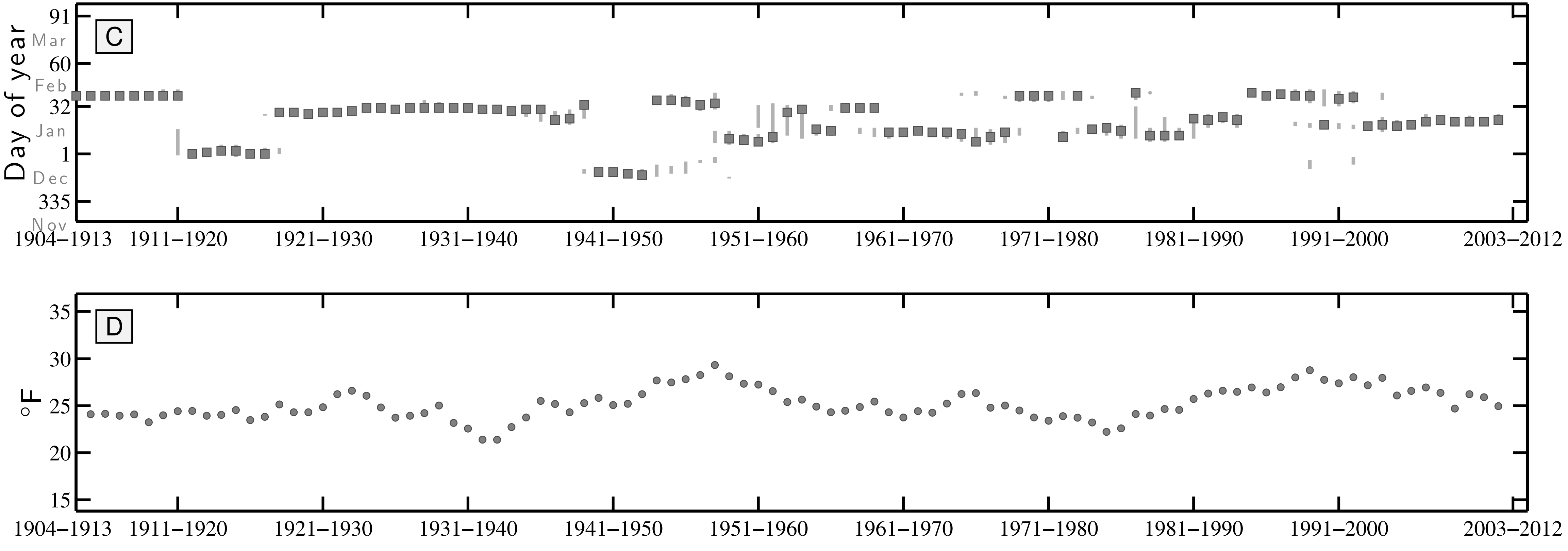

10 year Summer Teletherm dynamics for WESTMINSTER POL BRKS, MD:

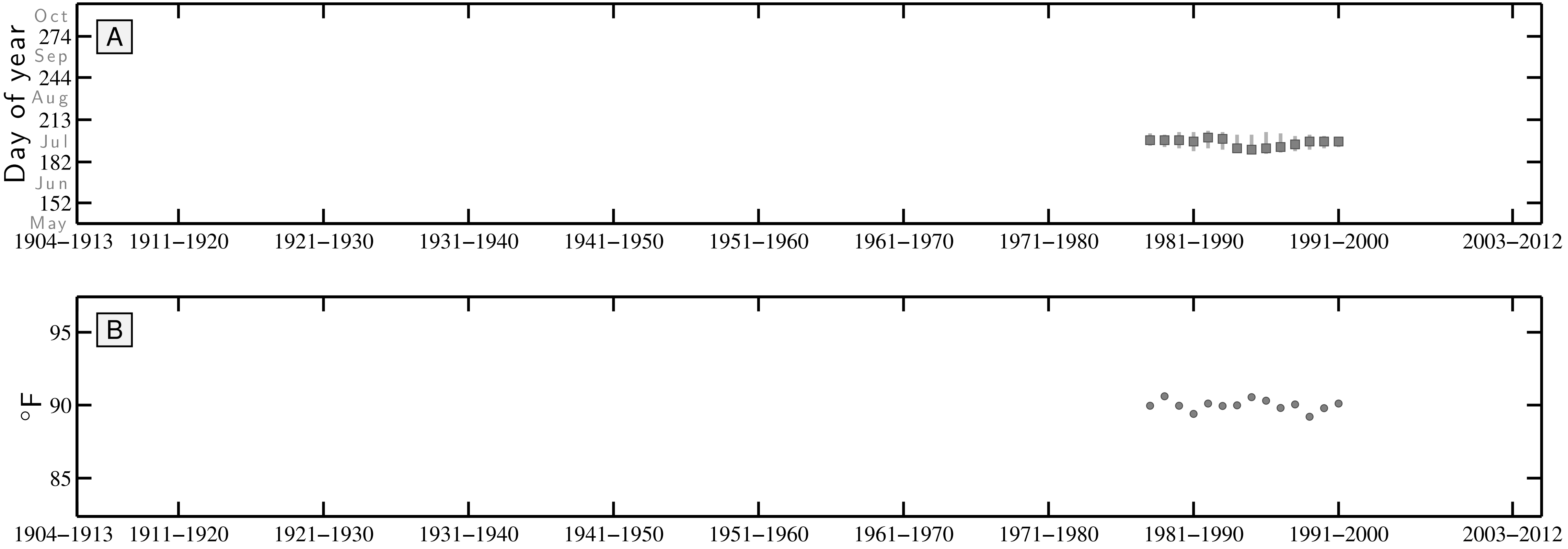

10 year Winter Teletherm dynamics for WESTMINSTER POL BRKS, MD:

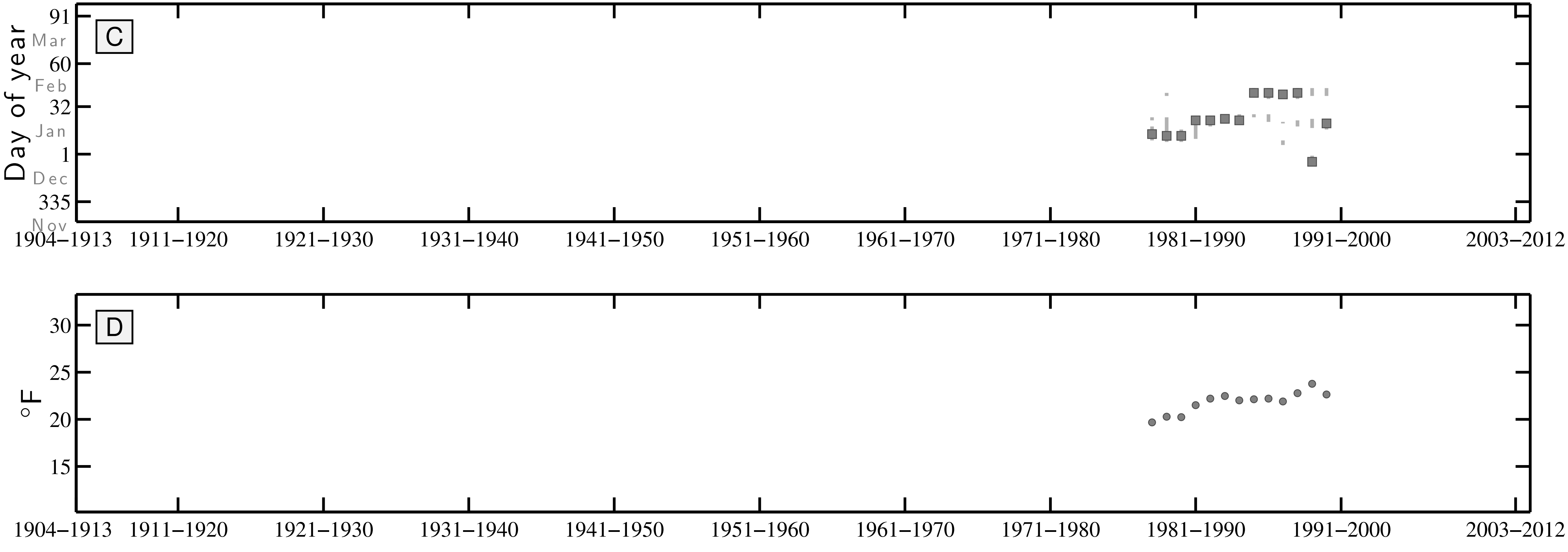

10 year Summer Teletherm dynamics for WOODSTOCK, MD:

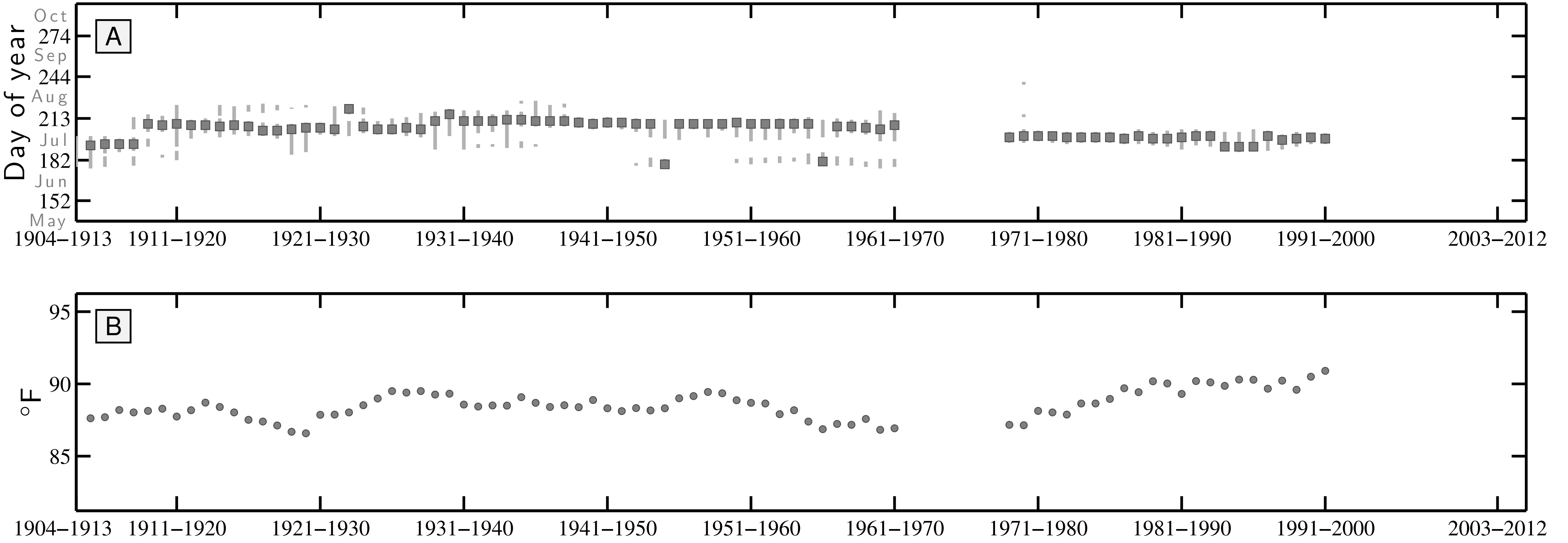

10 year Winter Teletherm dynamics for WOODSTOCK, MD:

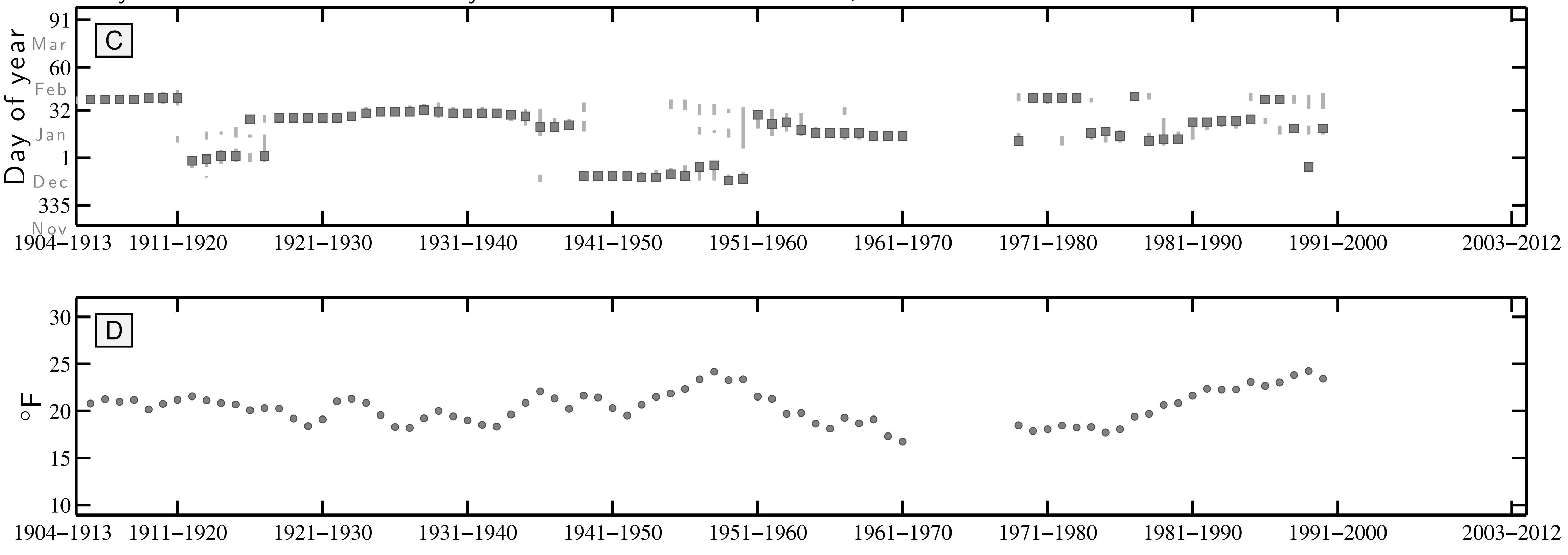

10 year Summer Teletherm dynamics for AMHERST, MA:

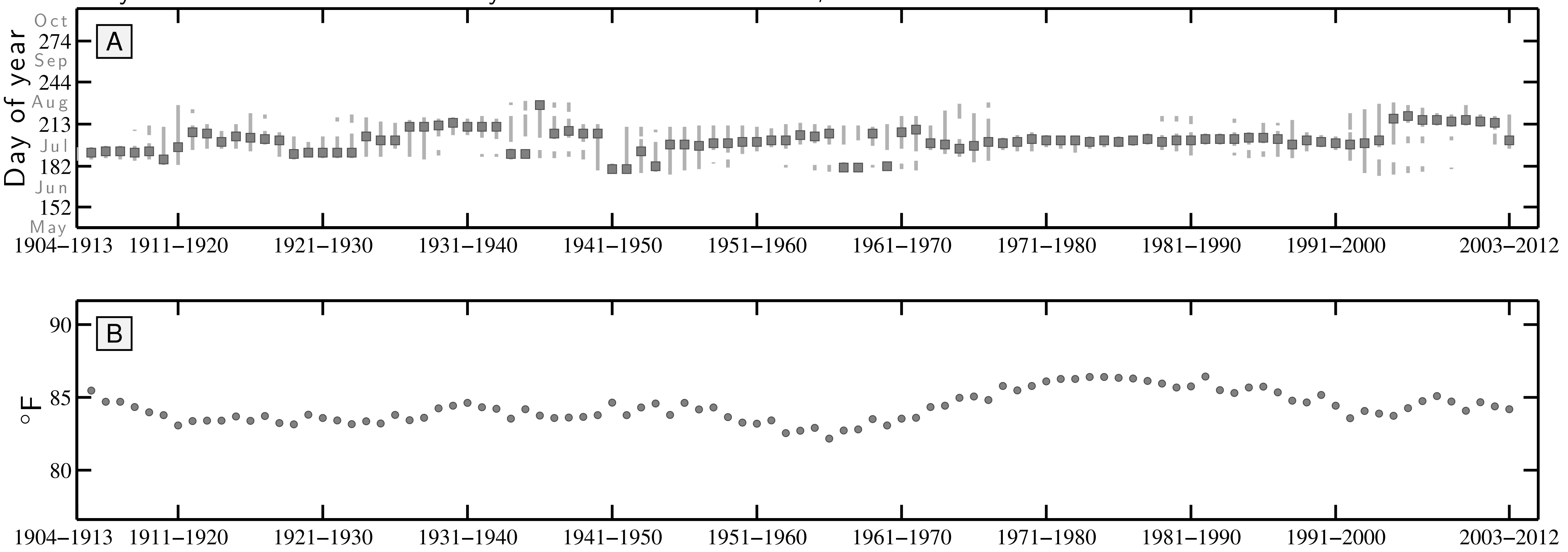

10 year Winter Teletherm dynamics for AMHERST, MA:

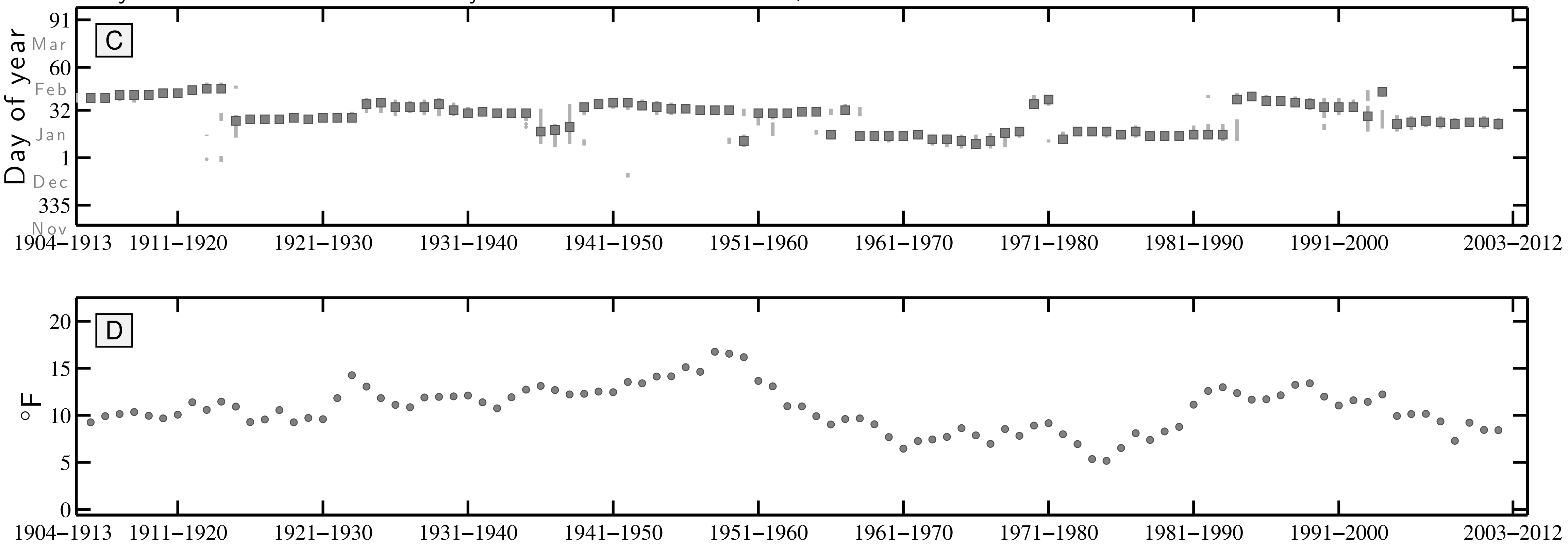

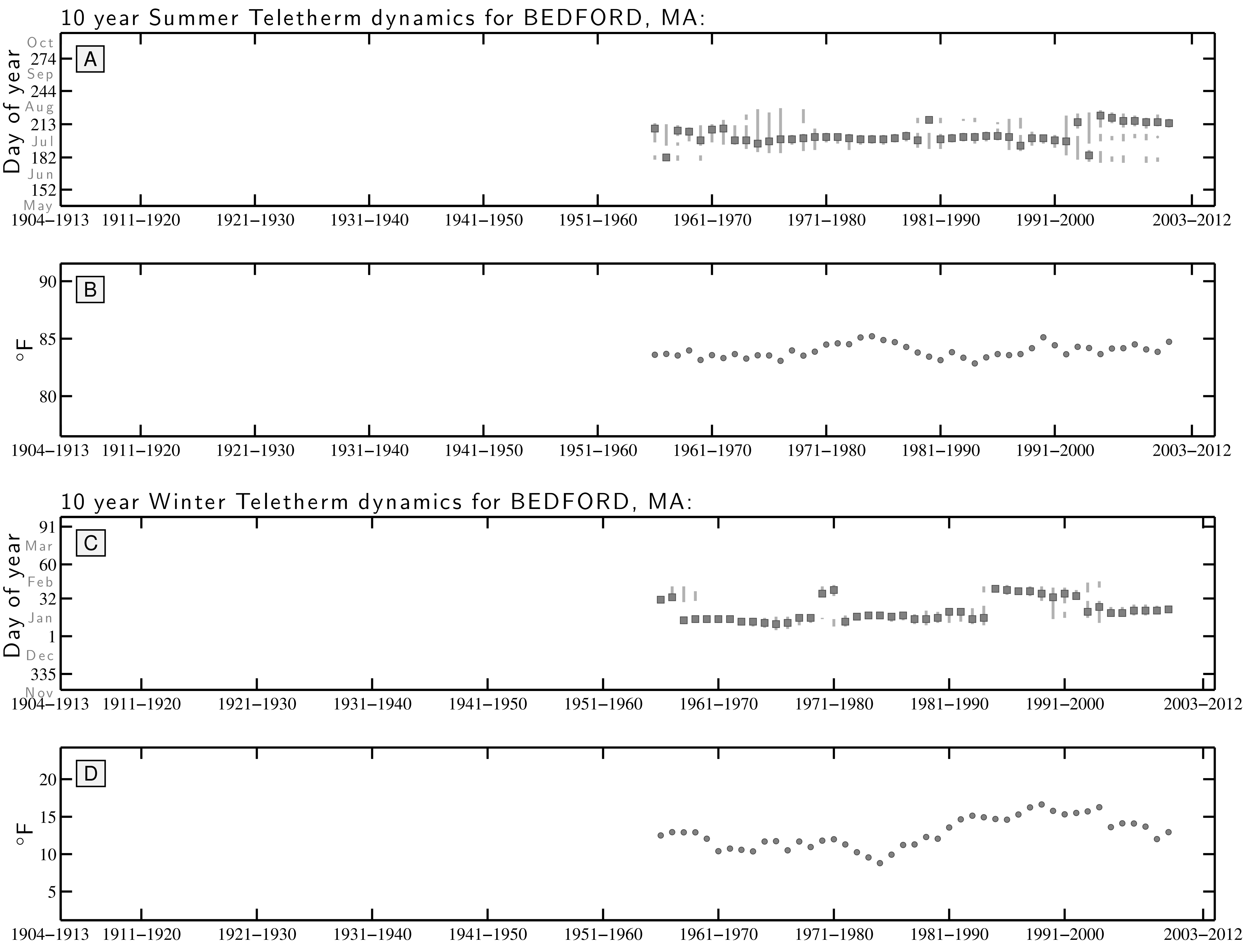
10 year Summer Teletherm dynamics for BEDFORD, MA:
A
Day of year
Oct
274
Sep
244
Aug
213
Jul
182
Jun
152
May
1904–1913
1911–1920
1921–1930
1931–1940
1941–1950
1951–1960
1961–1970
1971–1980
1981–1990
1991–2000
2003–2012
B
°F
90
85
80
10 year Winter Teletherm dynamics for BEDFORD, MA:
C
91
Mar
60
Feb
32
Jan
1
Dec
335
Nov
D
20
15
10
5

10 year Summer Teletherm dynamics for BLUE HILL, MA:

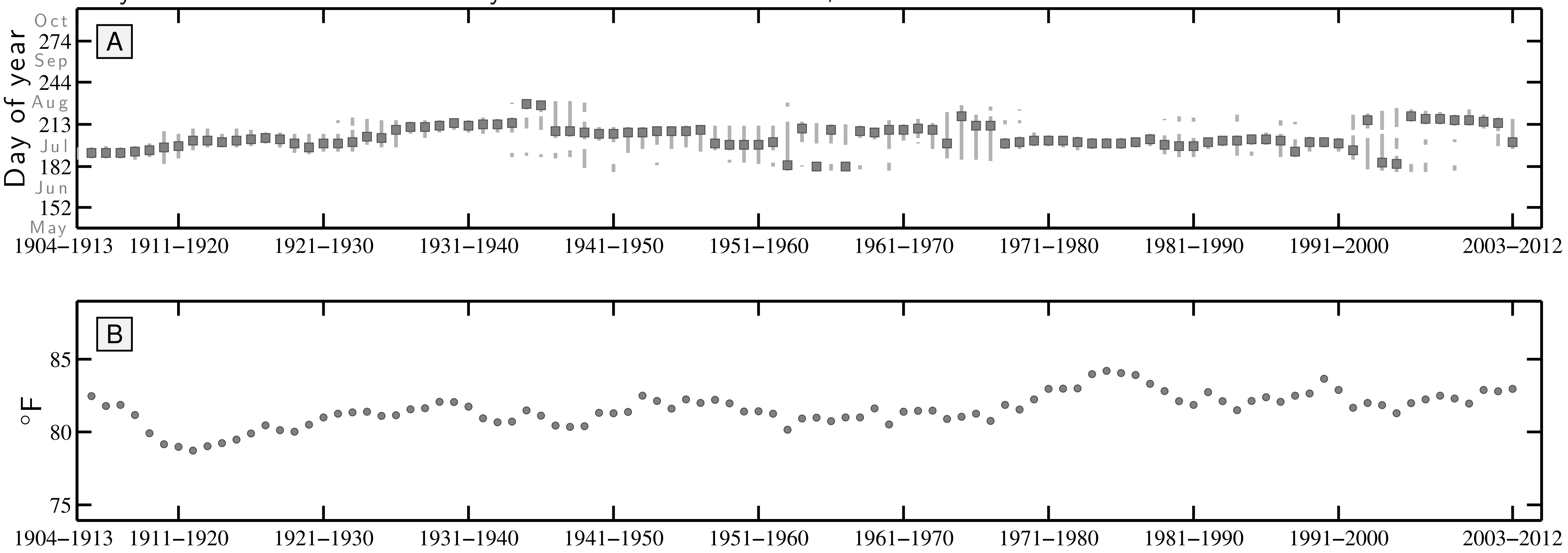

10 year Winter Teletherm dynamics for BLUE HILL, MA:

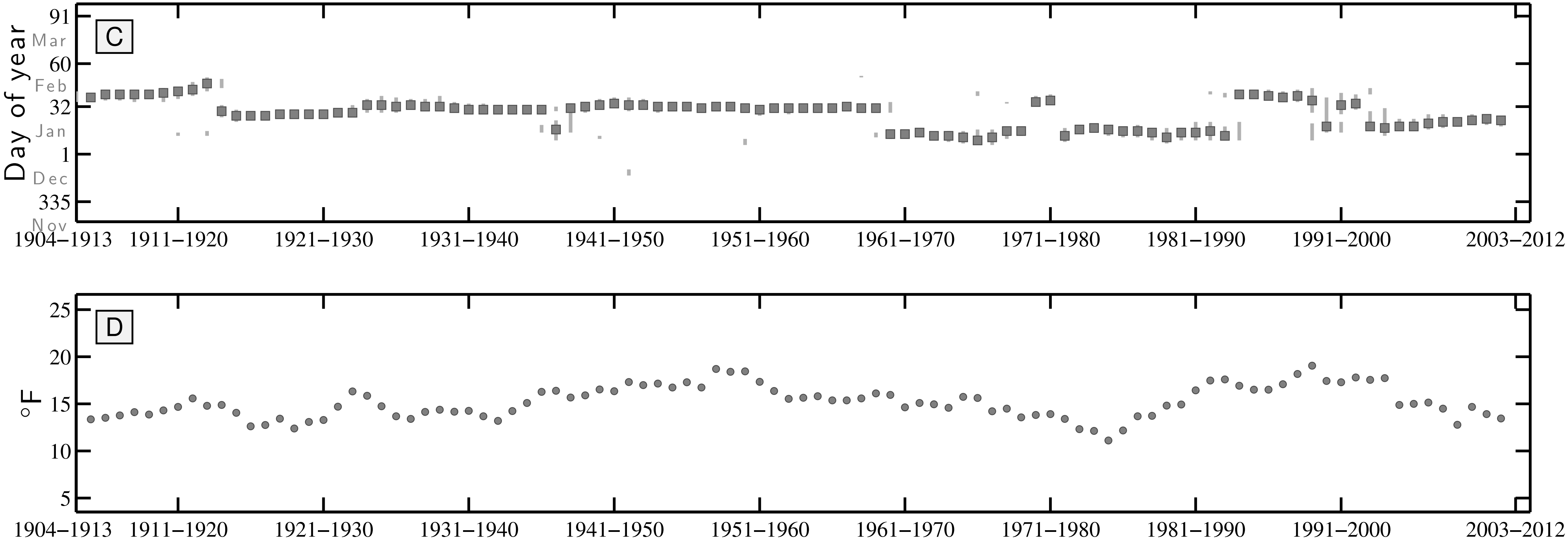

10 year Summer Teletherm dynamics for GREAT BARRINGTON 5 SW, MA:

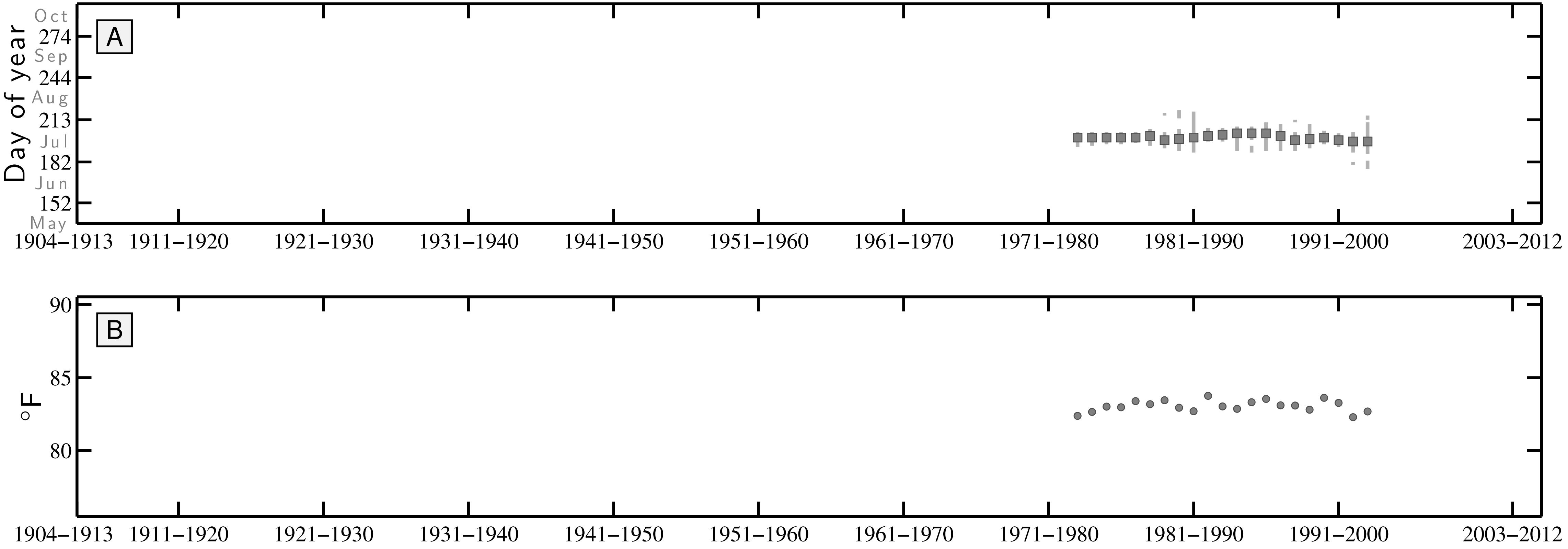

10 year Winter Teletherm dynamics for GREAT BARRINGTON 5 SW, MA:

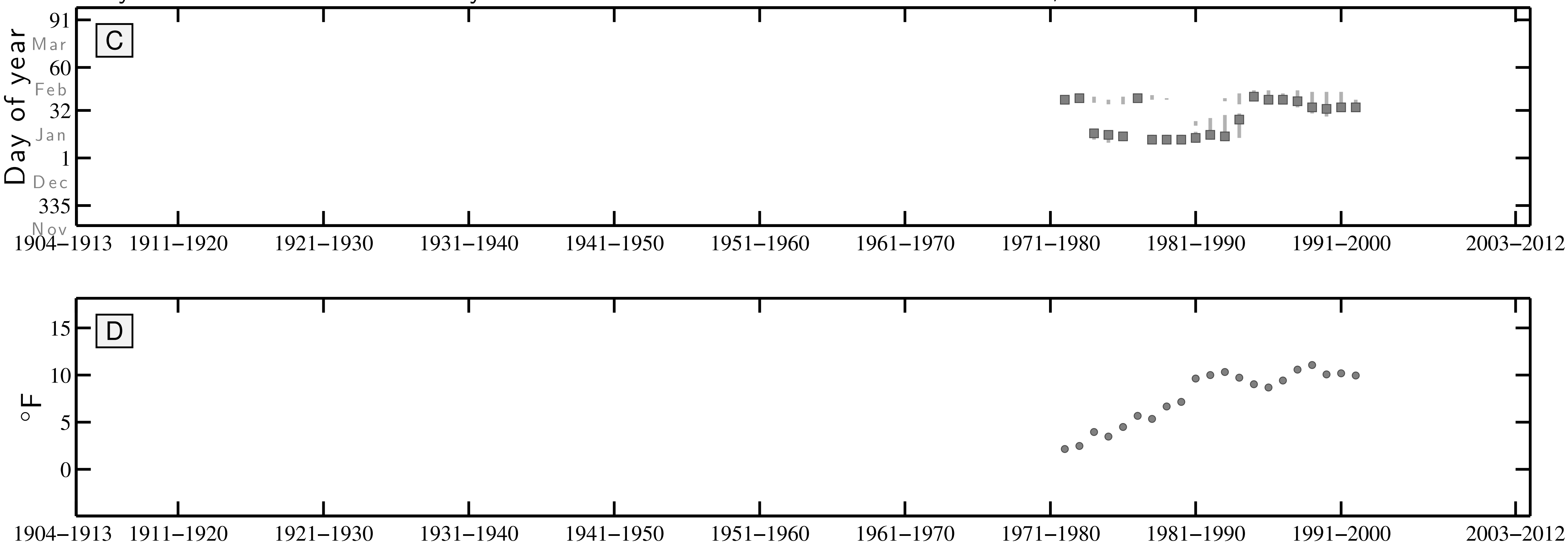

10 year Summer Teletherm dynamics for LAWRENCE, MA:

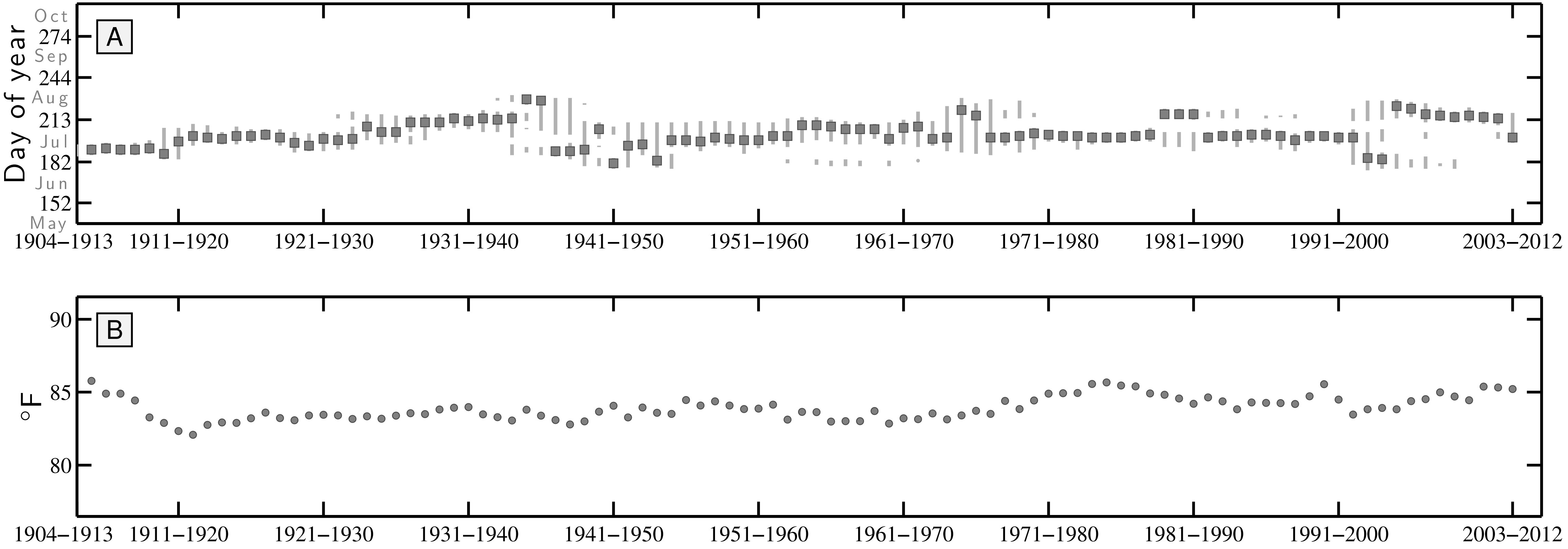

10 year Winter Teletherm dynamics for LAWRENCE, MA:

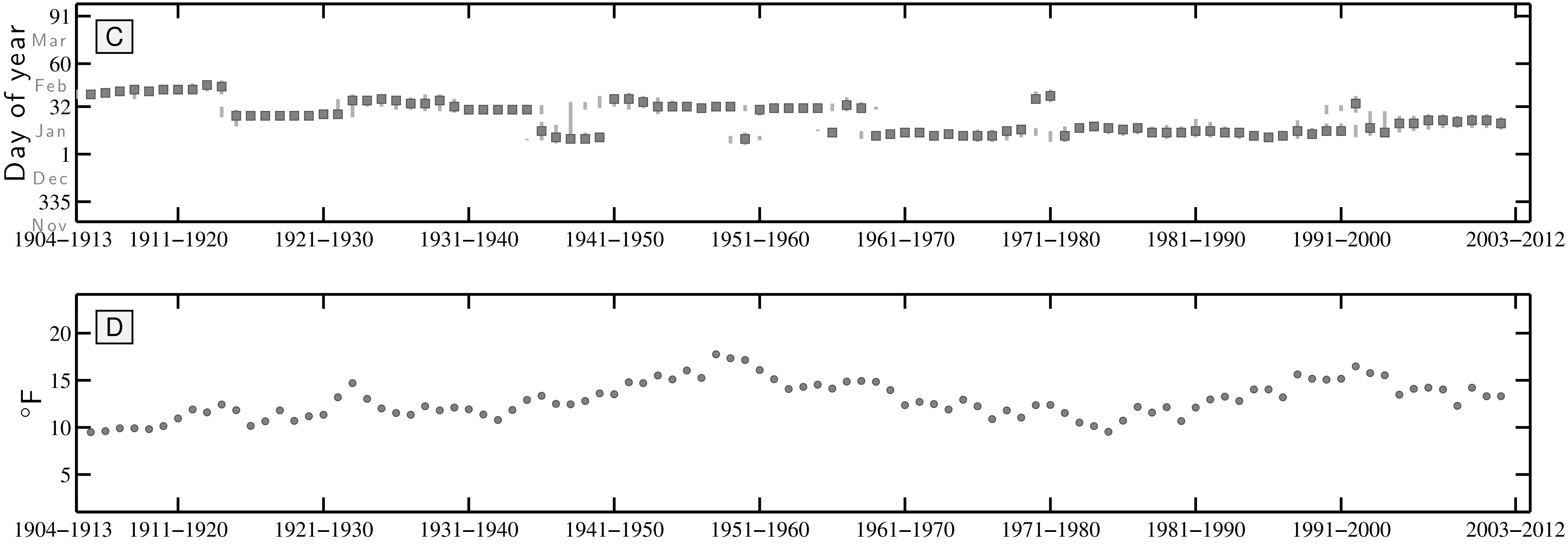

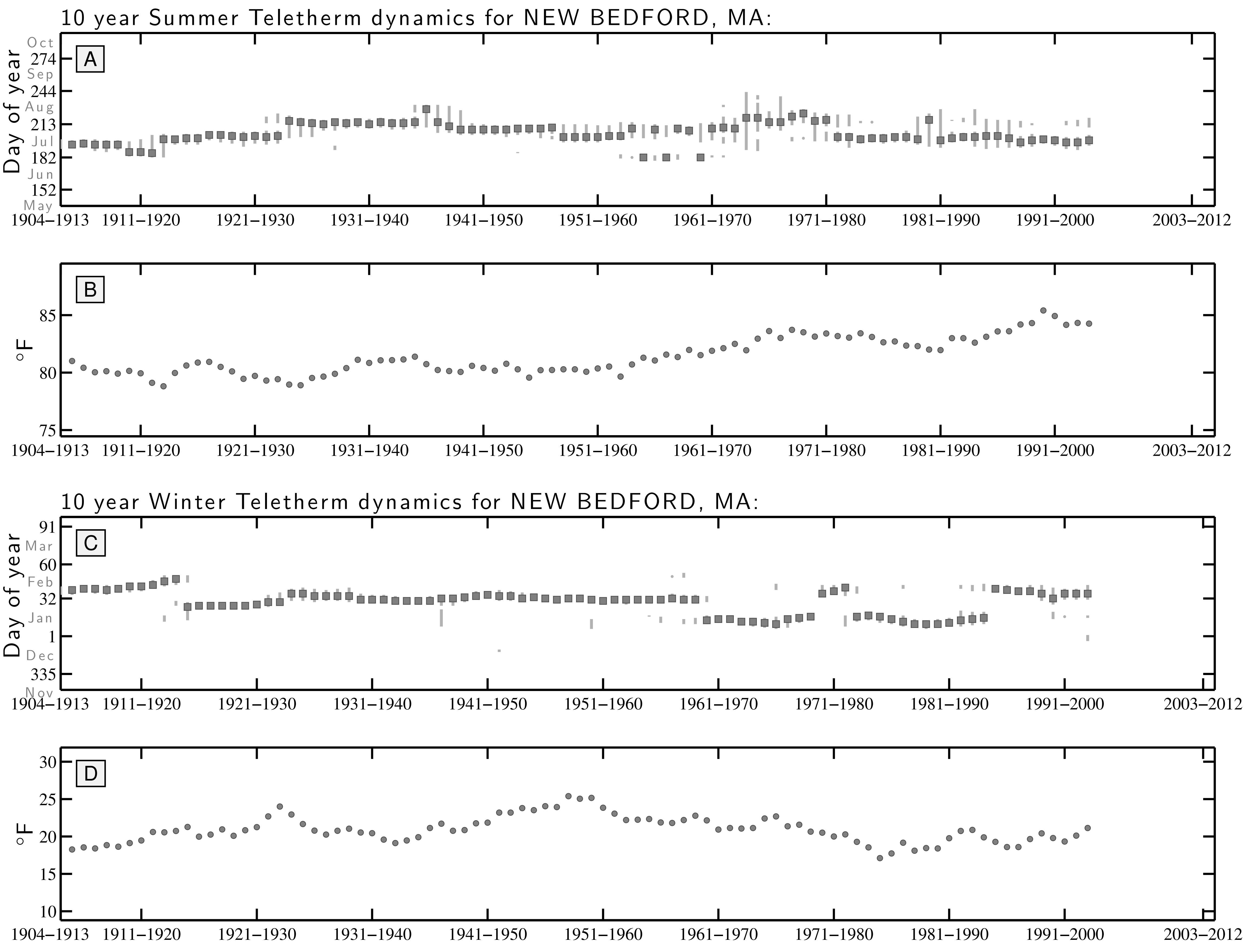
10 year Summer Teletherm dynamics for NEW BEDFORD, MA:
A
Day of year
Oct
274
Sep
244
Aug
213
Jul
182
Jun
152
May
B
°F
85
80
75
1904–1913
1911–1920
1921–1930
1931–1940
1941–1950
1951–1960
1961–1970
1971–1980
1981–1990
1991–2000
2003–2012
10 year Winter Teletherm dynamics for NEW BEDFORD, MA:
C
Day of year
91
Mar
60
Feb
32
Jan
1
Dec
335
Nov
D
°F
30
25
20
15
10

10 year Summer Teletherm dynamics for PLYMOUTH-KINGSTON, MA:

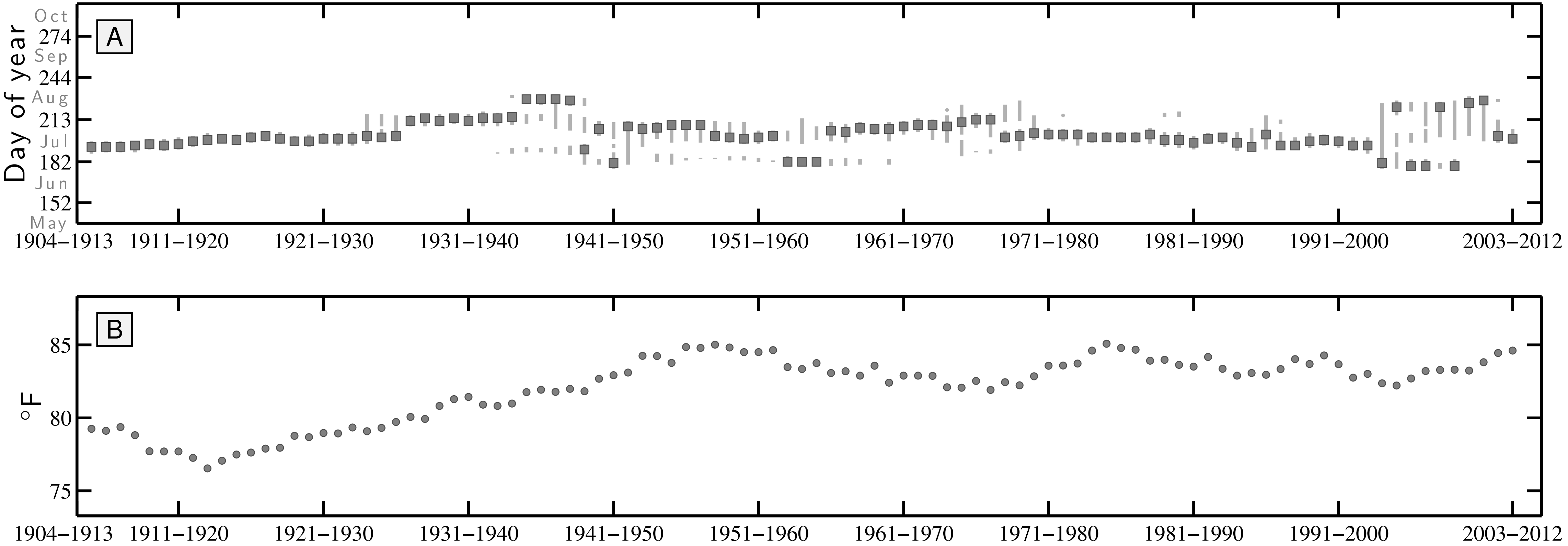

10 year Winter Teletherm dynamics for PLYMOUTH-KINGSTON, MA:

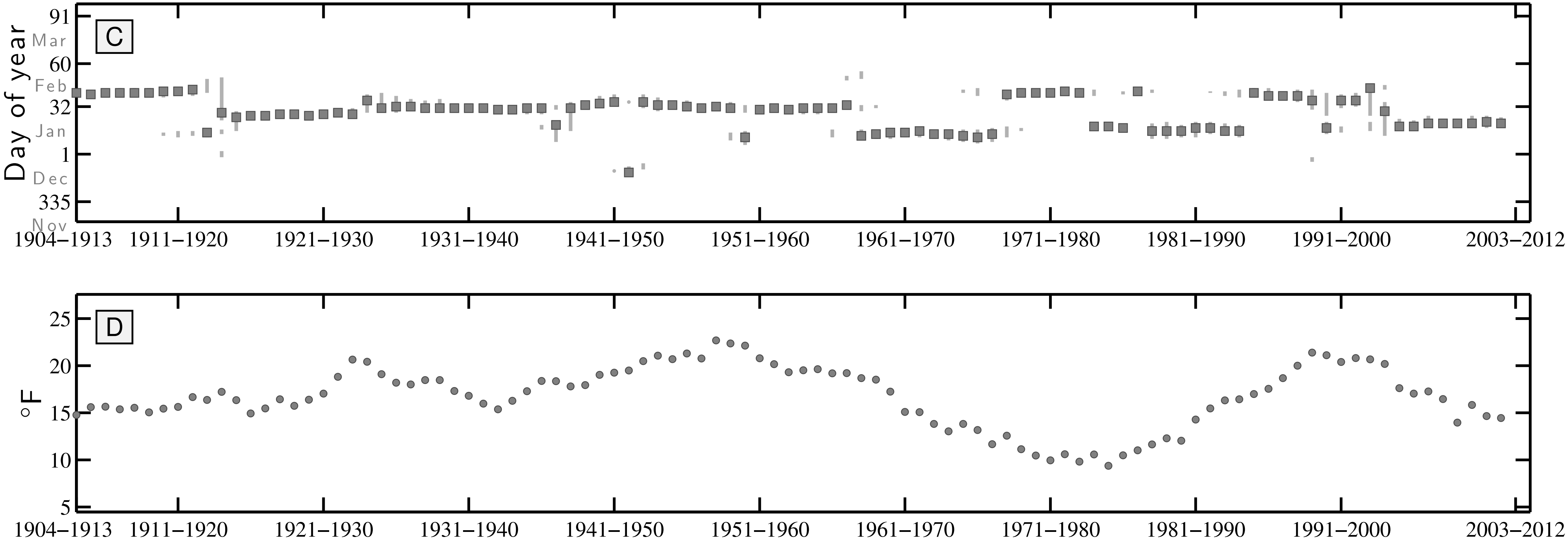

10 year Summer Teletherm dynamics for PROVINCETOWN, MA:

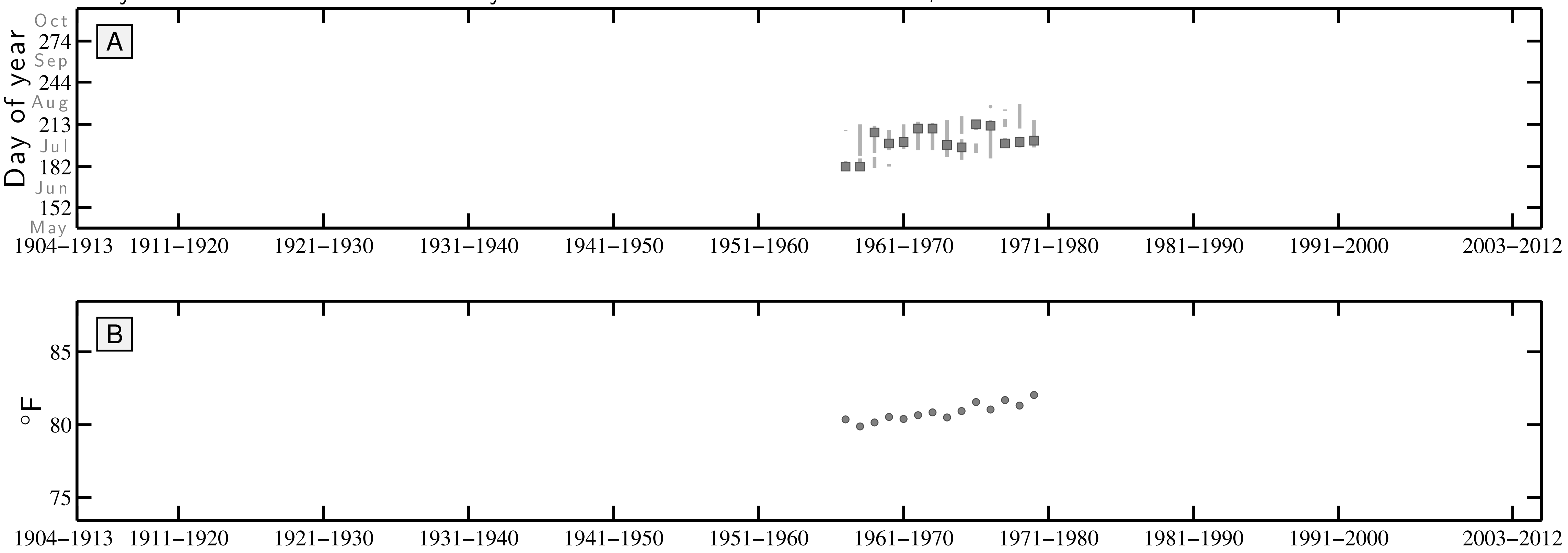

10 year Winter Teletherm dynamics for PROVINCETOWN, MA:

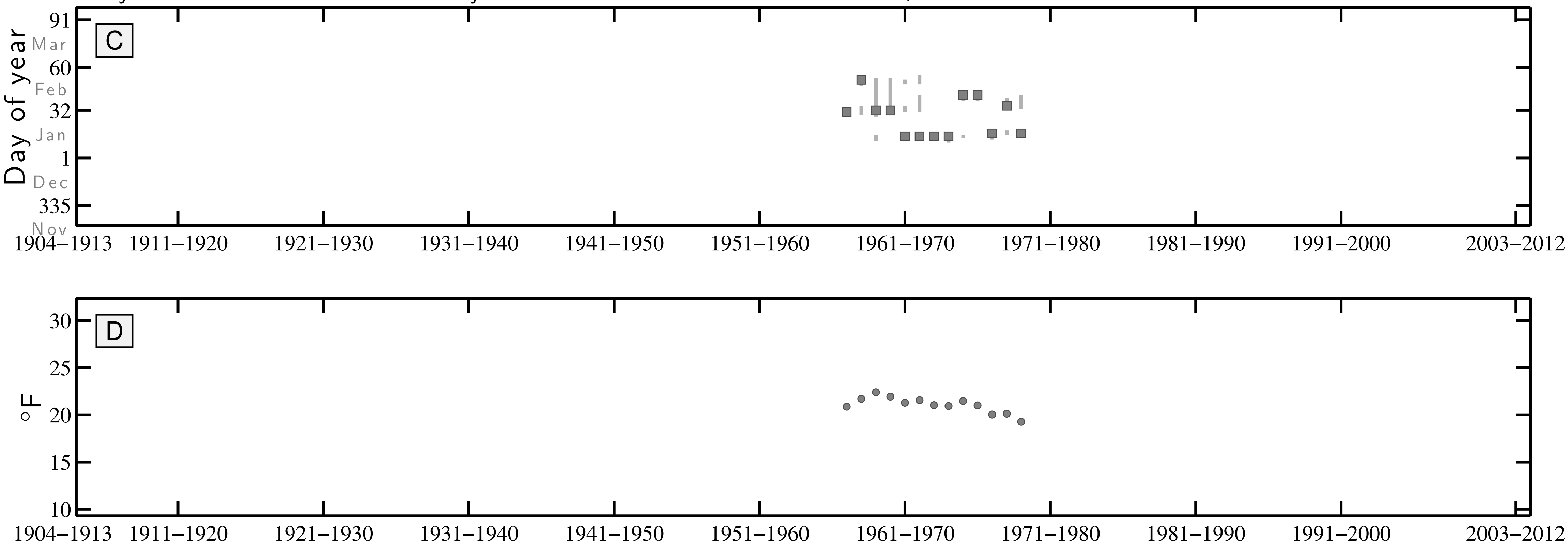

10 year Summer Teletherm dynamics for READING, MA:

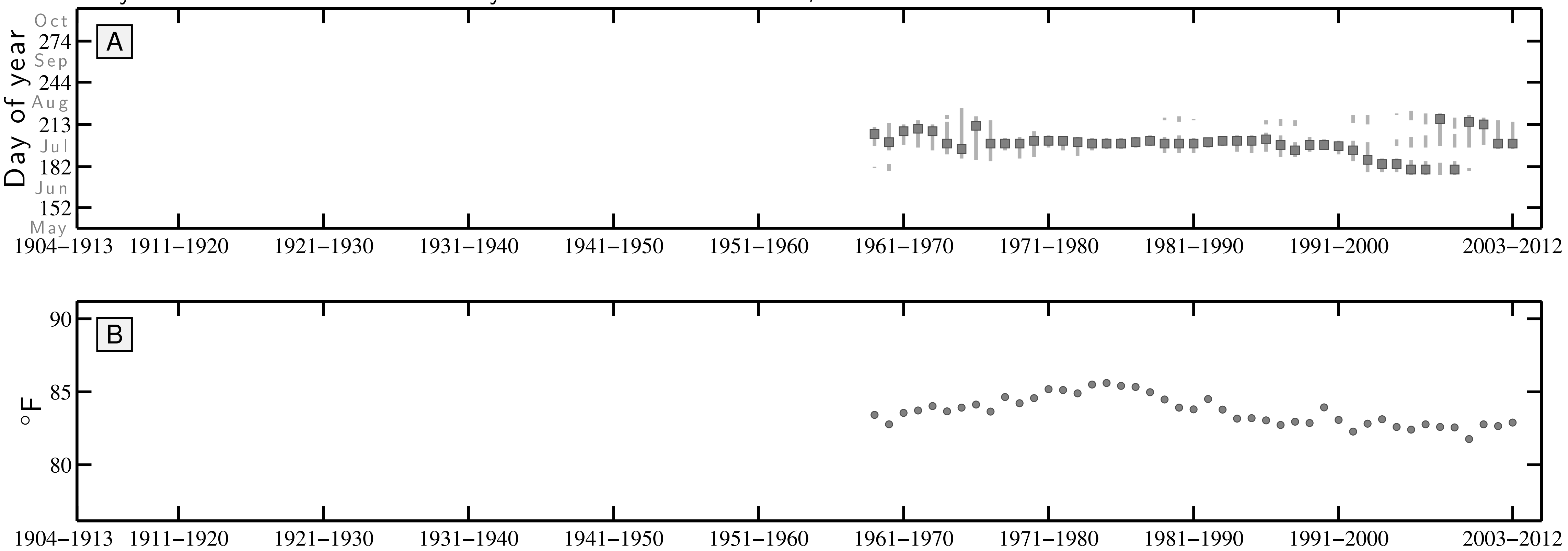

10 year Winter Teletherm dynamics for READING, MA:

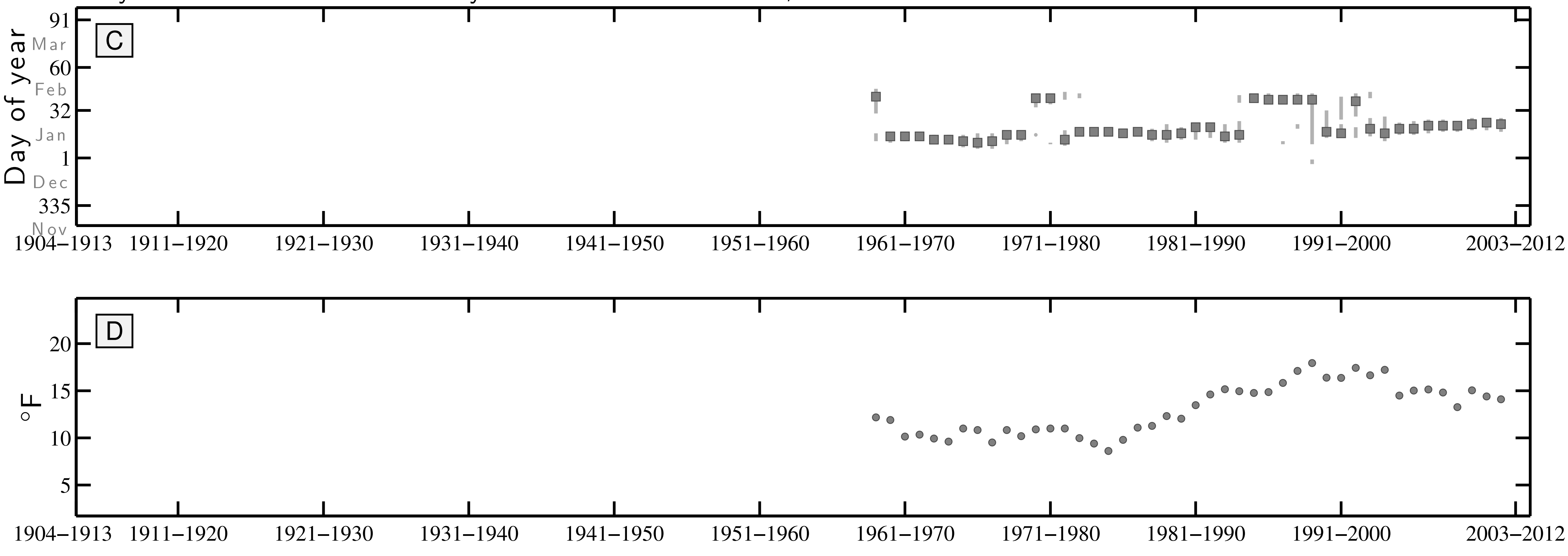

10 year Summer Teletherm dynamics for TAUNTON, MA:

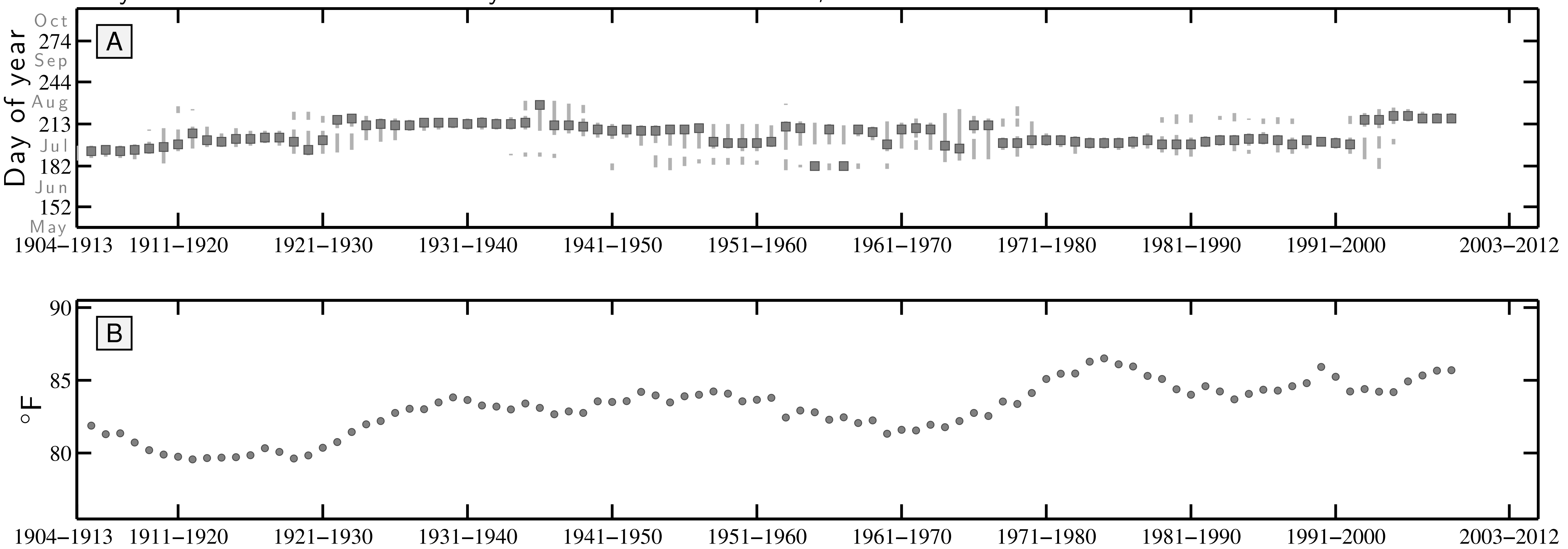

10 year Winter Teletherm dynamics for TAUNTON, MA:

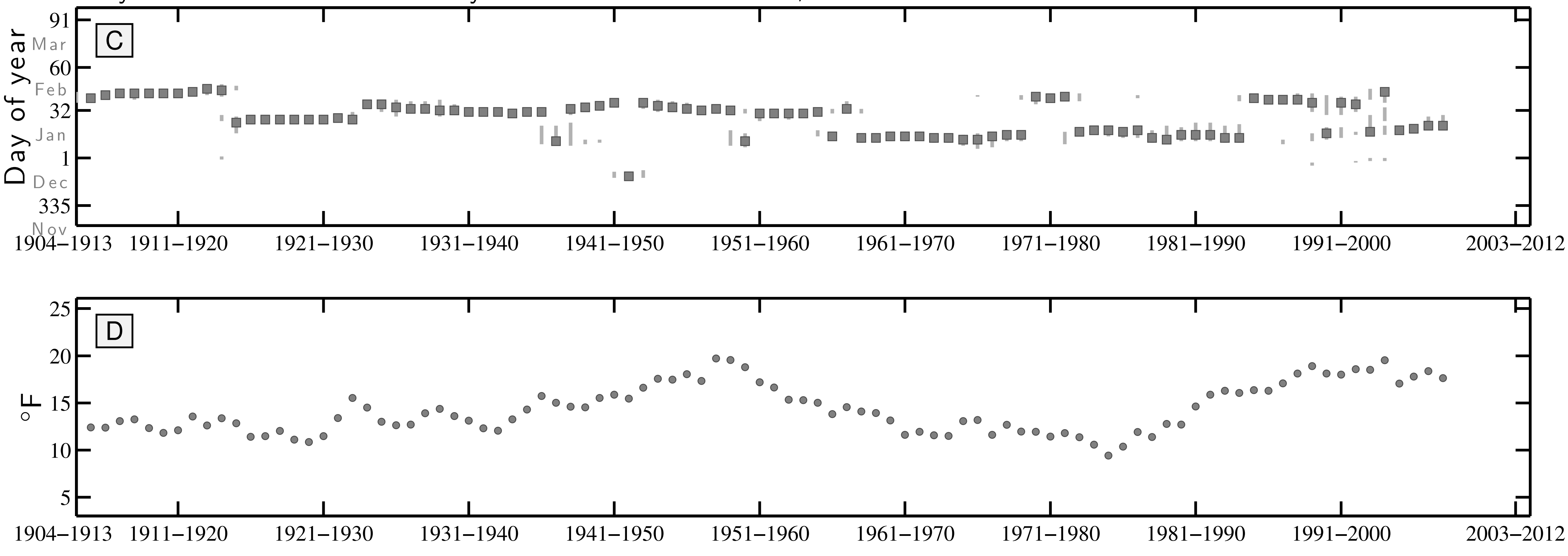

10 year Summer Teletherm dynamics for WALPOLE 2, MA:

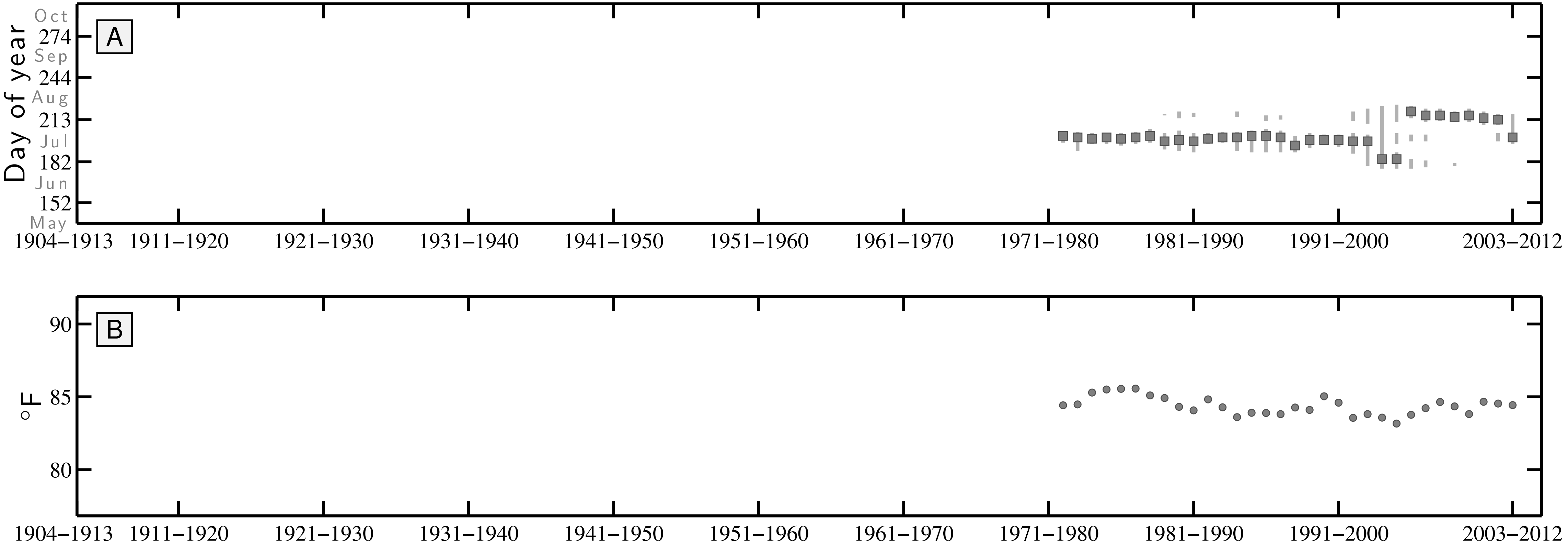

10 year Winter Teletherm dynamics for WALPOLE 2, MA:

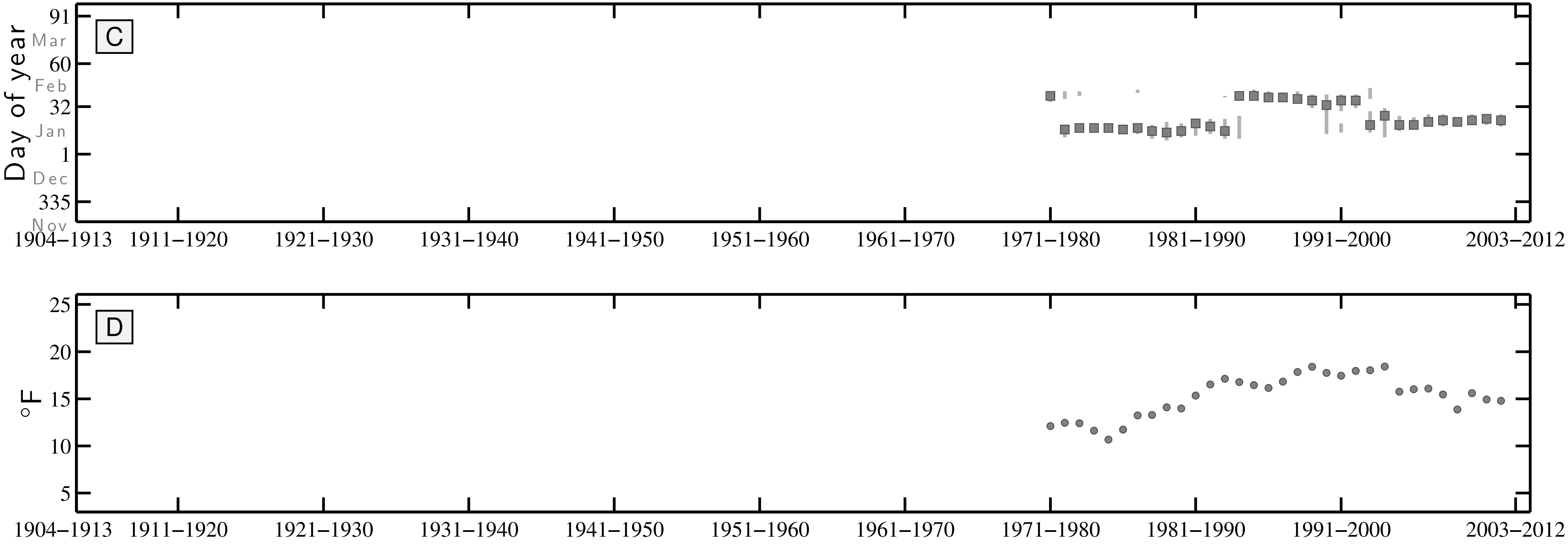

10 year Summer Teletherm dynamics for WEST MEDWAY, MA:

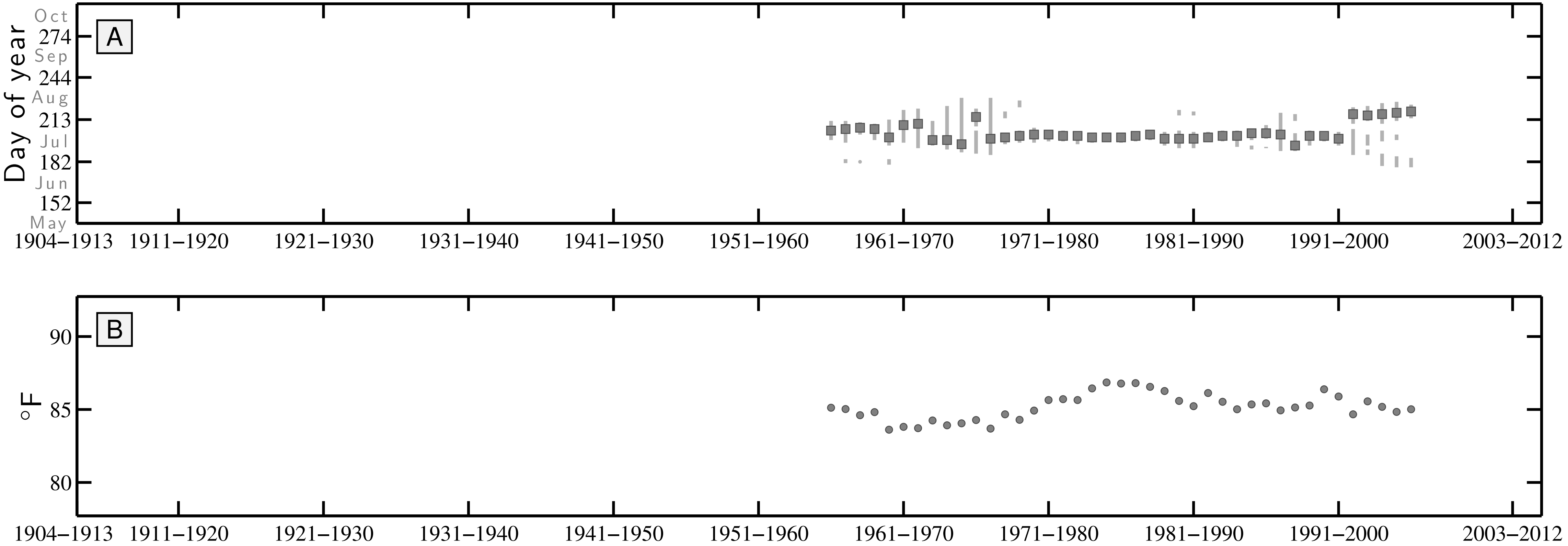

10 year Winter Teletherm dynamics for WEST MEDWAY, MA:

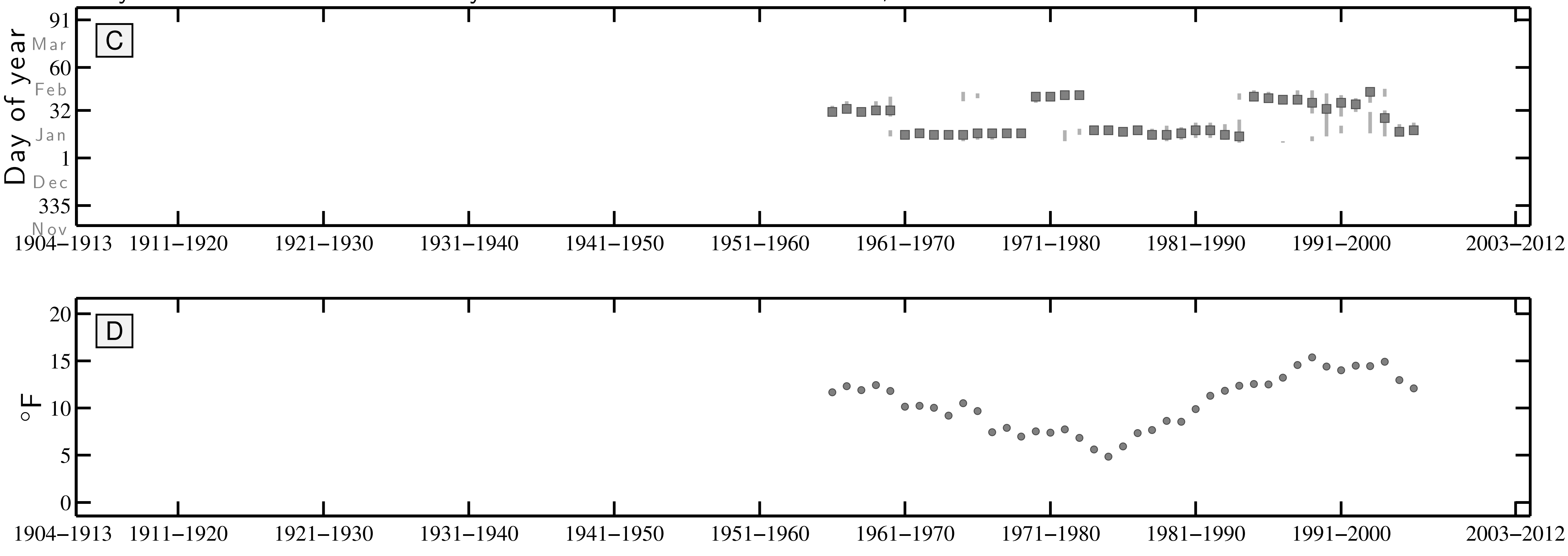

10 year Summer Teletherm dynamics for ADRIAN 2 NNE, MI:

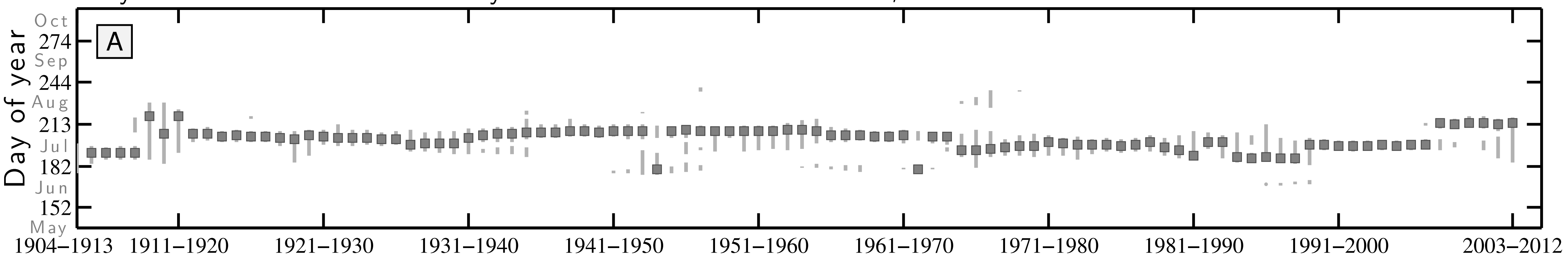

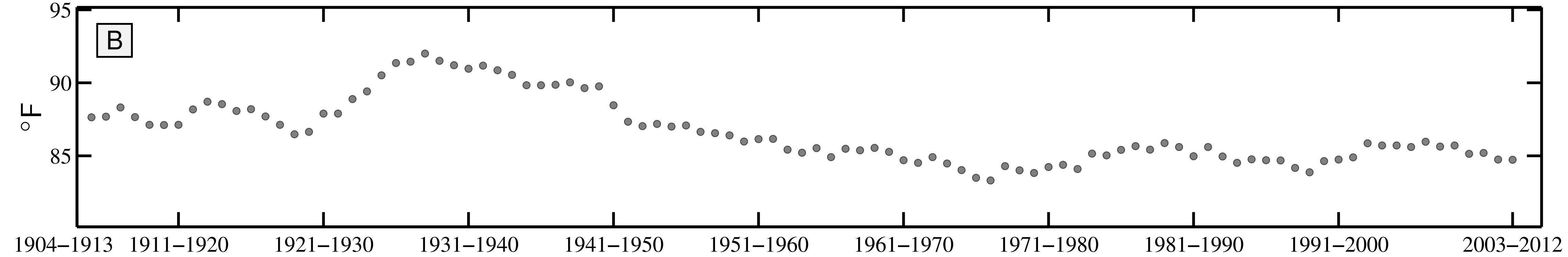

10 year Winter Teletherm dynamics for ADRIAN 2 NNE, MI:

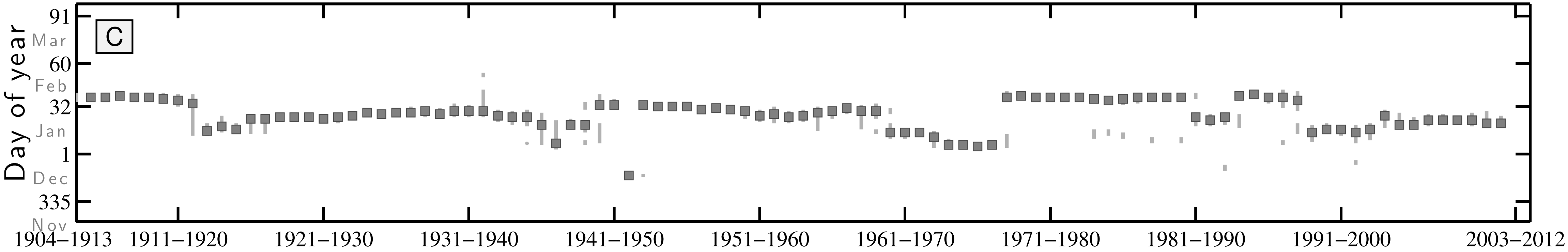

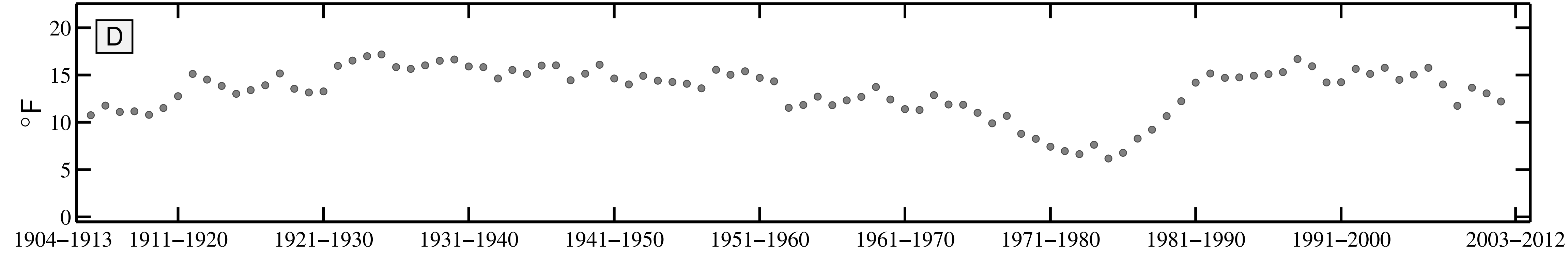

10 year Summer Teletherm dynamics for ALLEGAN 5NE, MI:

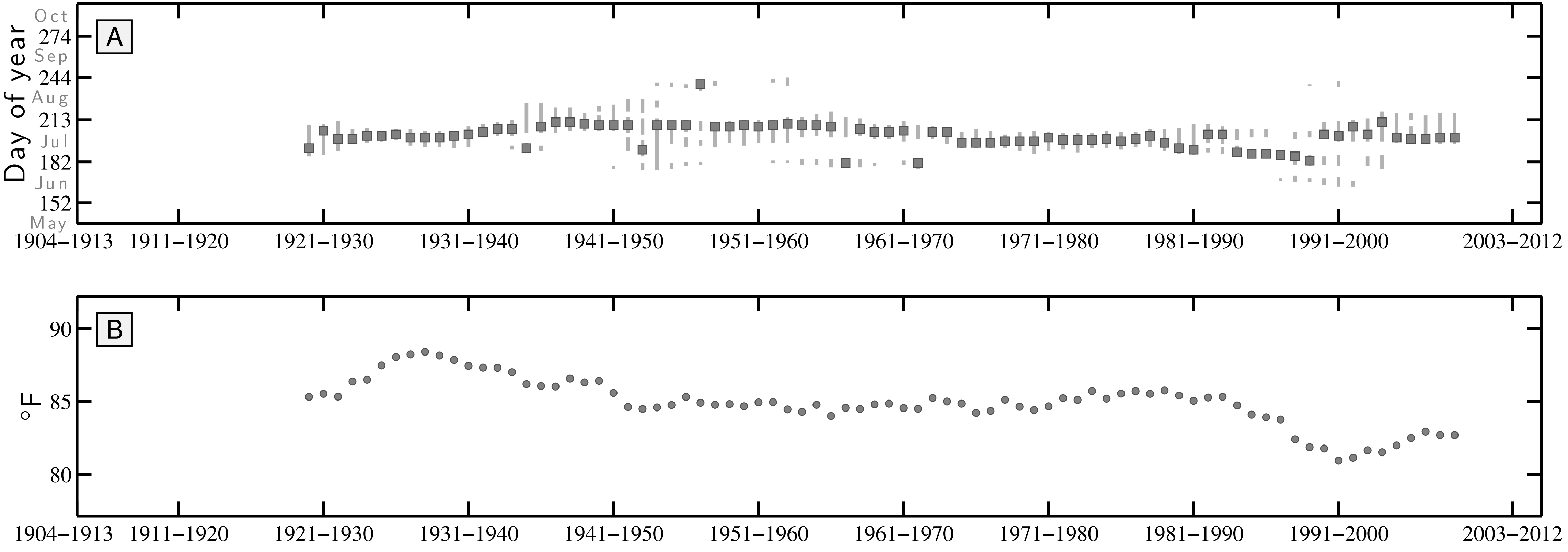

10 year Winter Teletherm dynamics for ALLEGAN 5NE, MI:

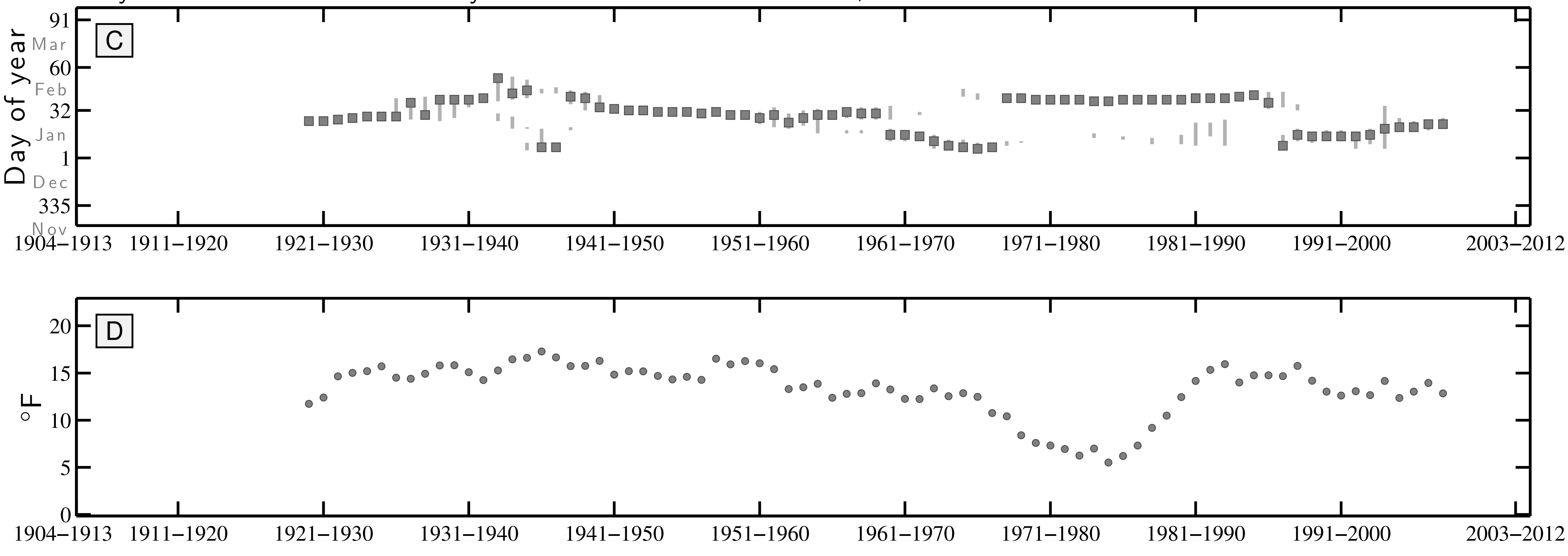

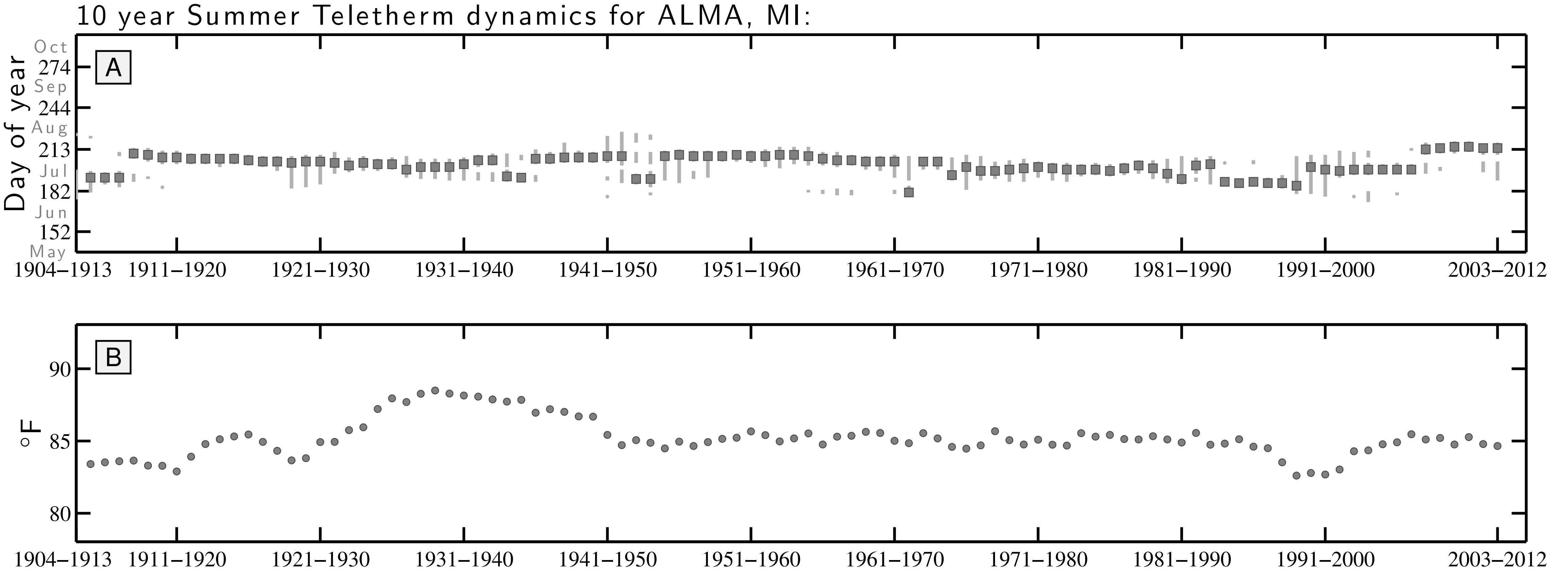
10 year Summer Teletherm dynamics for ALMA, MI:
A
B
Day of year
°F
Oct
274
Sep
244
Aug
213
Jul
182
Jun
152
May
90
85
80
1904–1913
1911–1920
1921–1930
1931–1940
1941–1950
1951–1960
1961–1970
1971–1980
1981–1990
1991–2000
2003–2012

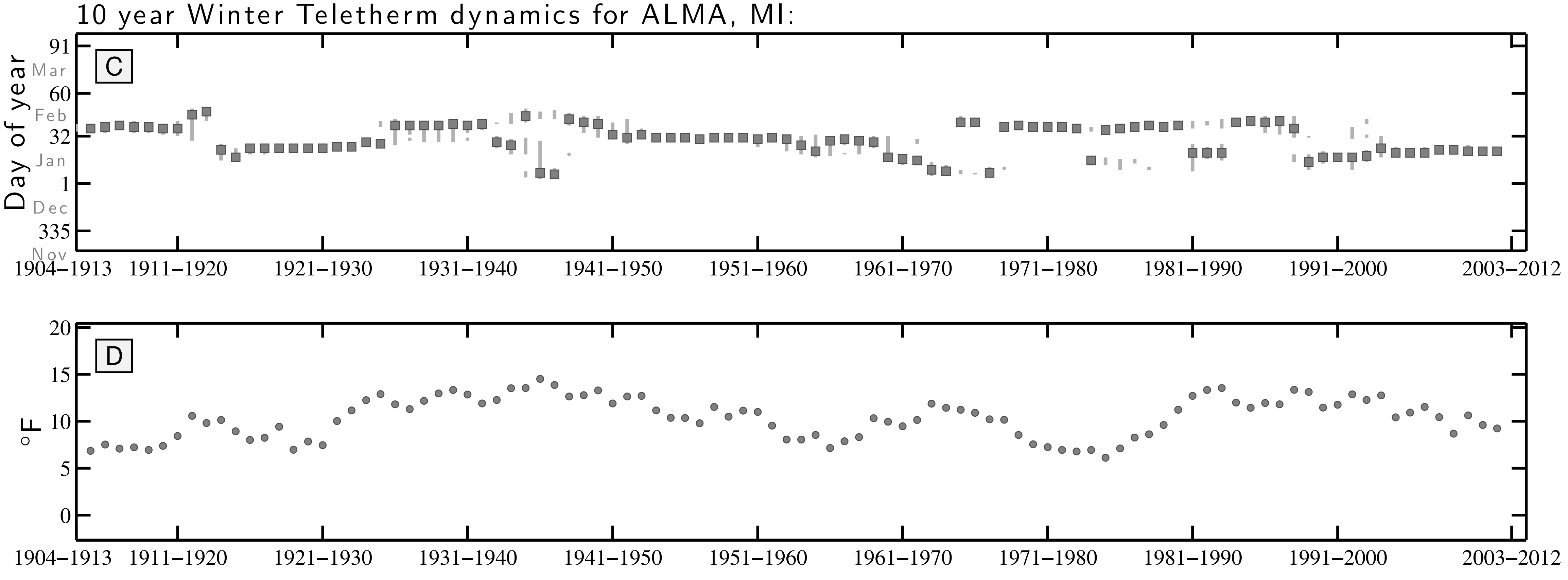
10 year Winter Teletherm dynamics for ALMA, MI:
C
D
Day of year
°F
91
Mar
60
Feb
32
Jan
1
Dec
335
Nov
20
15
10
5
0
1904–1913
1911–1920
1921–1930
1931–1940
1941–1950
1951–1960
1961–1970
1971–1980
1981–1990
1991–2000
2003–2012

10 year Summer Teletherm dynamics for ANN ARBOR U OF, MI:

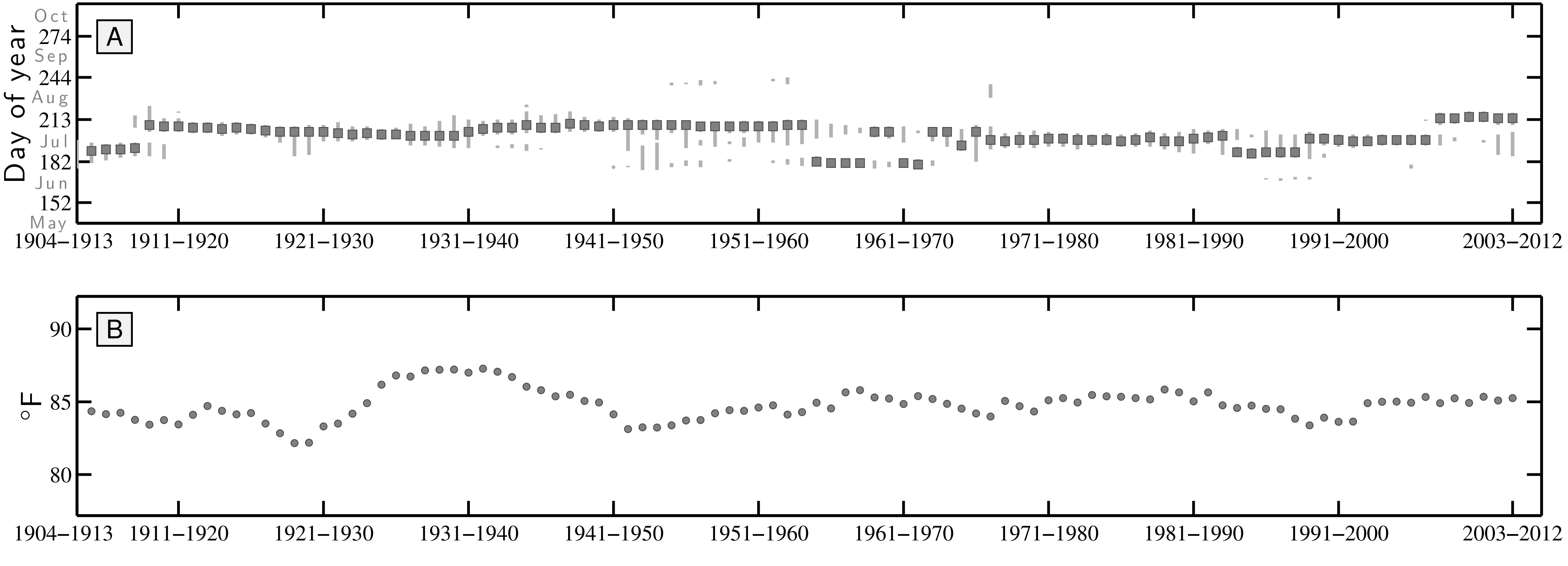

10 year Winter Teletherm dynamics for ANN ARBOR U OF, MI:

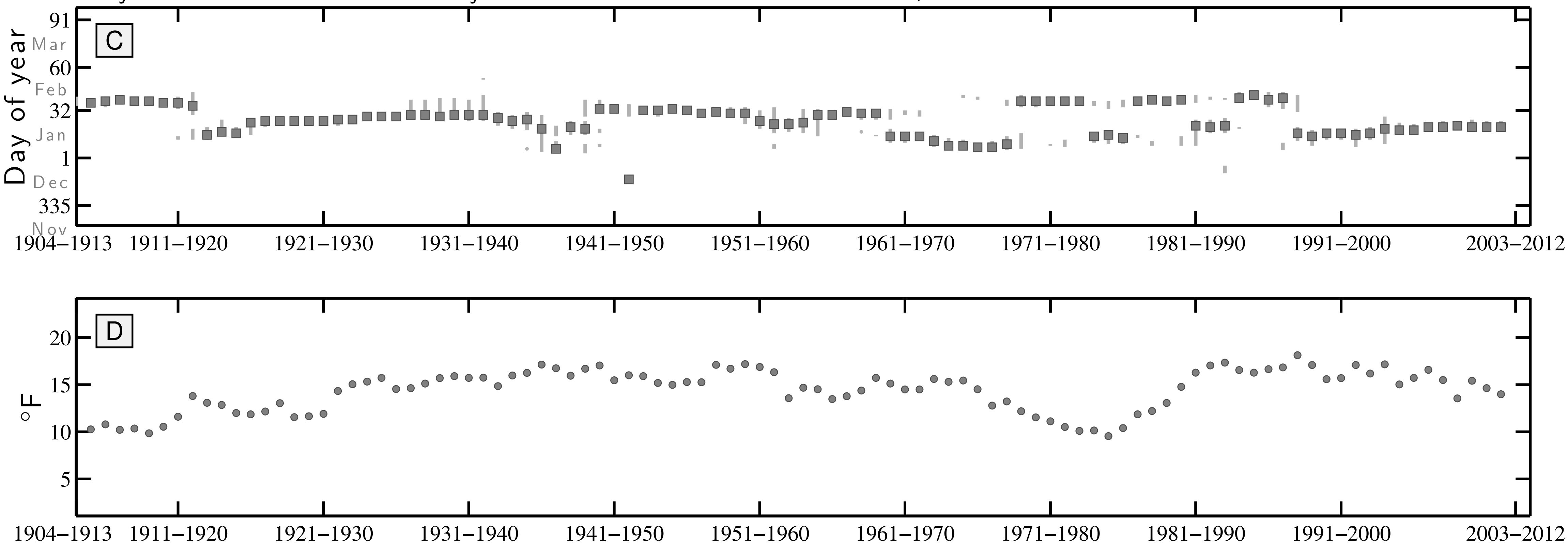

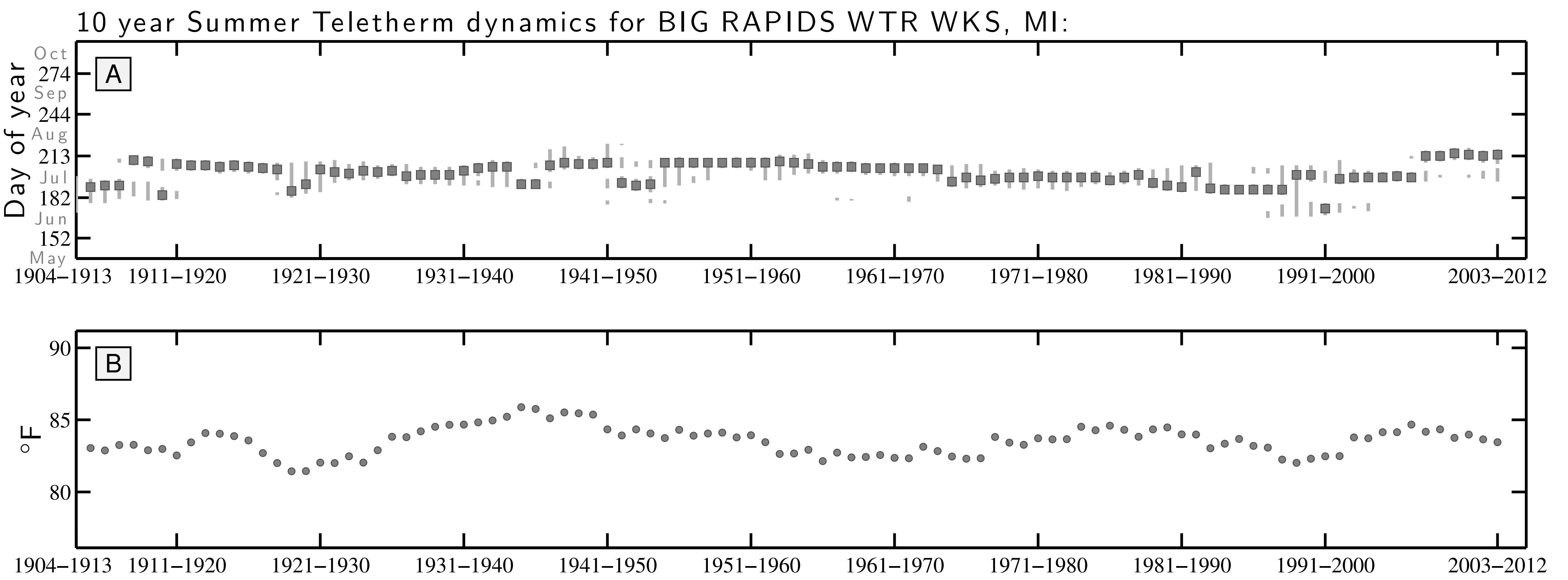
10 year Summer Teletherm dynamics for BIG RAPIDS WTR WKS, MI:
A
B
Day of year
°F
Oct
274
Sep
244
Aug
213
Jul
182
Jun
152
May
90
85
80
1904–1913
1911–1920
1921–1930
1931–1940
1941–1950
1951–1960
1961–1970
1971–1980
1981–1990
1991–2000
2003–2012

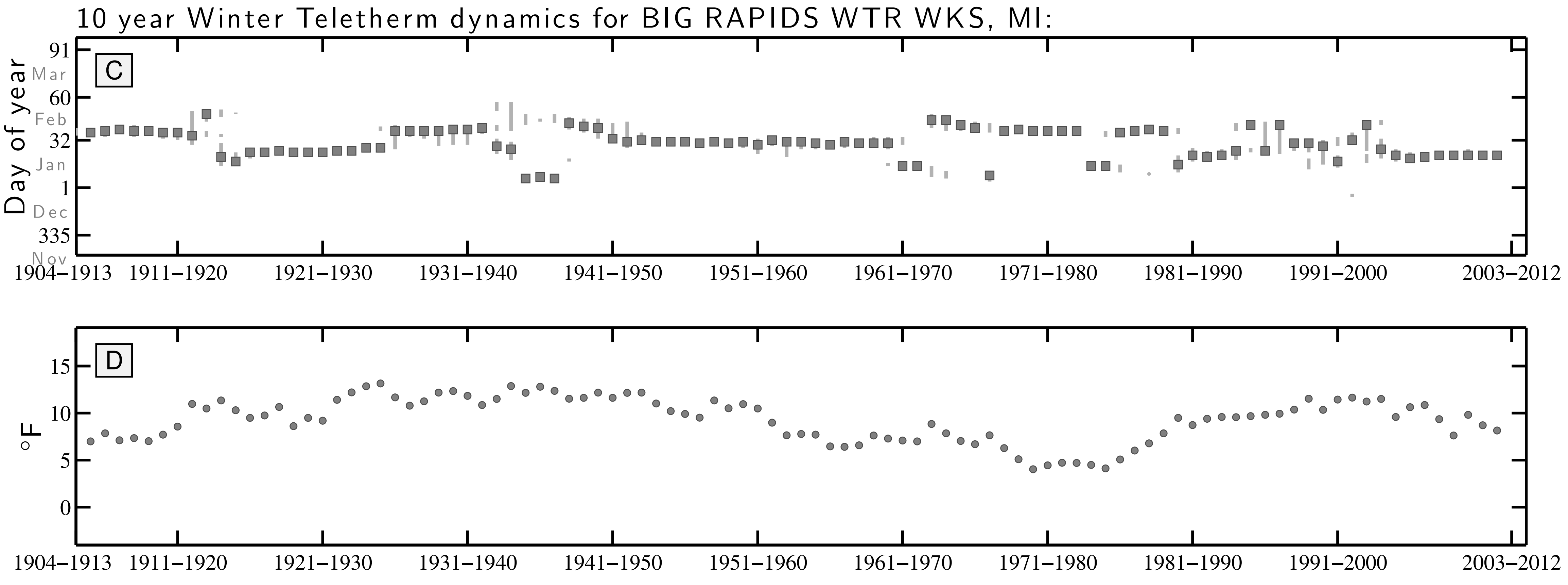
10 year Winter Teletherm dynamics for BIG RAPIDS WTR WKS, MI:
C
D
Day of year
°F
91
Mar
60
Feb
32
Jan
1
Dec
335
Nov
15
10
5
0
1904–1913
1911–1920
1921–1930
1931–1940
1941–1950
1951–1960
1961–1970
1971–1980
1981–1990
1991–2000
2003–2012

10 year Summer Teletherm dynamics for CHAMPION VAN RIPER PK, MI:

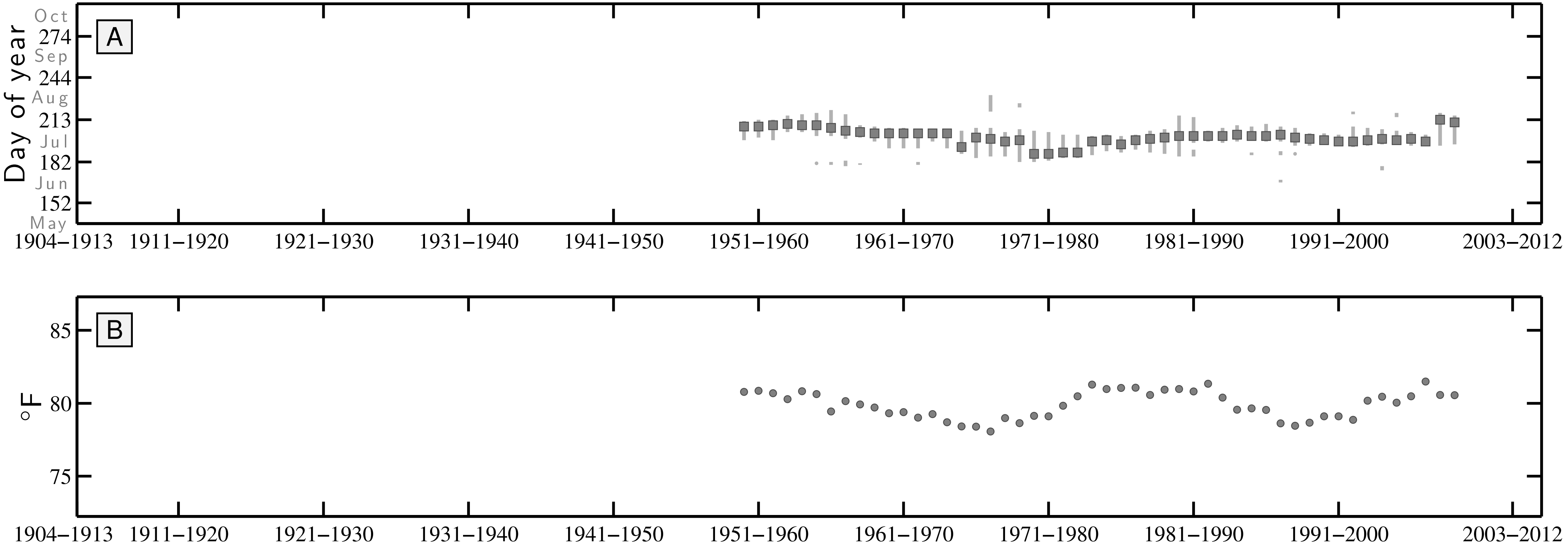

10 year Winter Teletherm dynamics for CHAMPION VAN RIPER PK, MI:

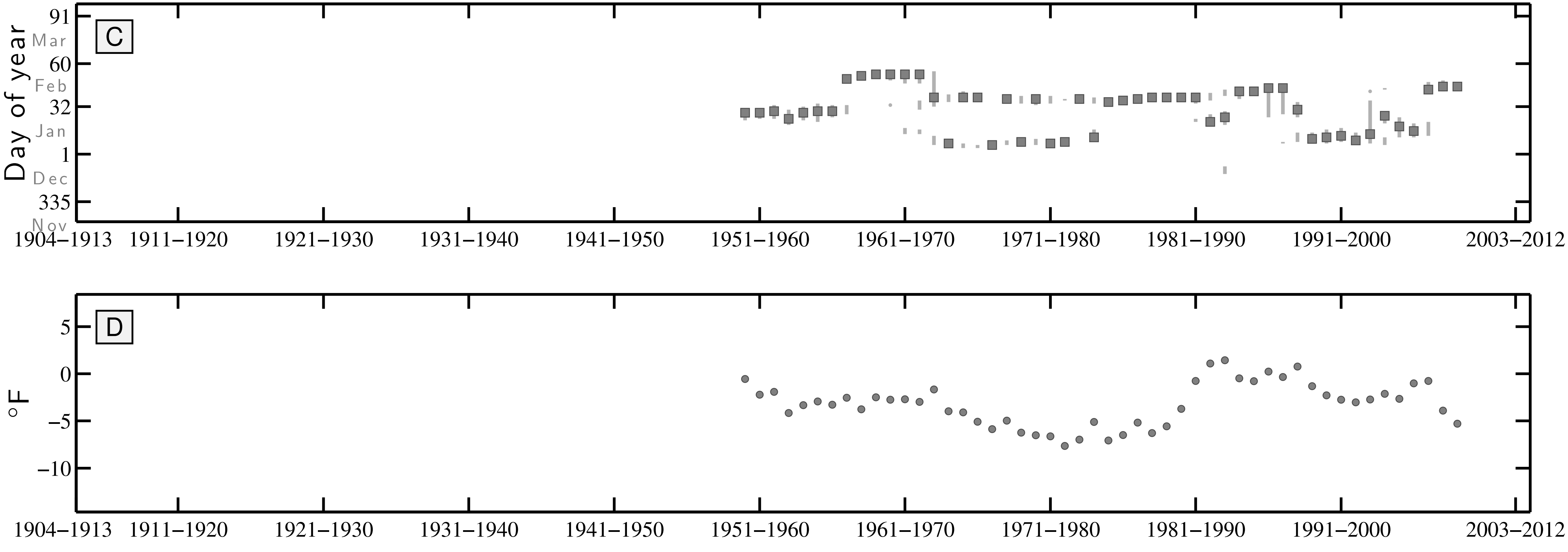

10 year Summer Teletherm dynamics for CHATHAM EXP FARM 2, MI:

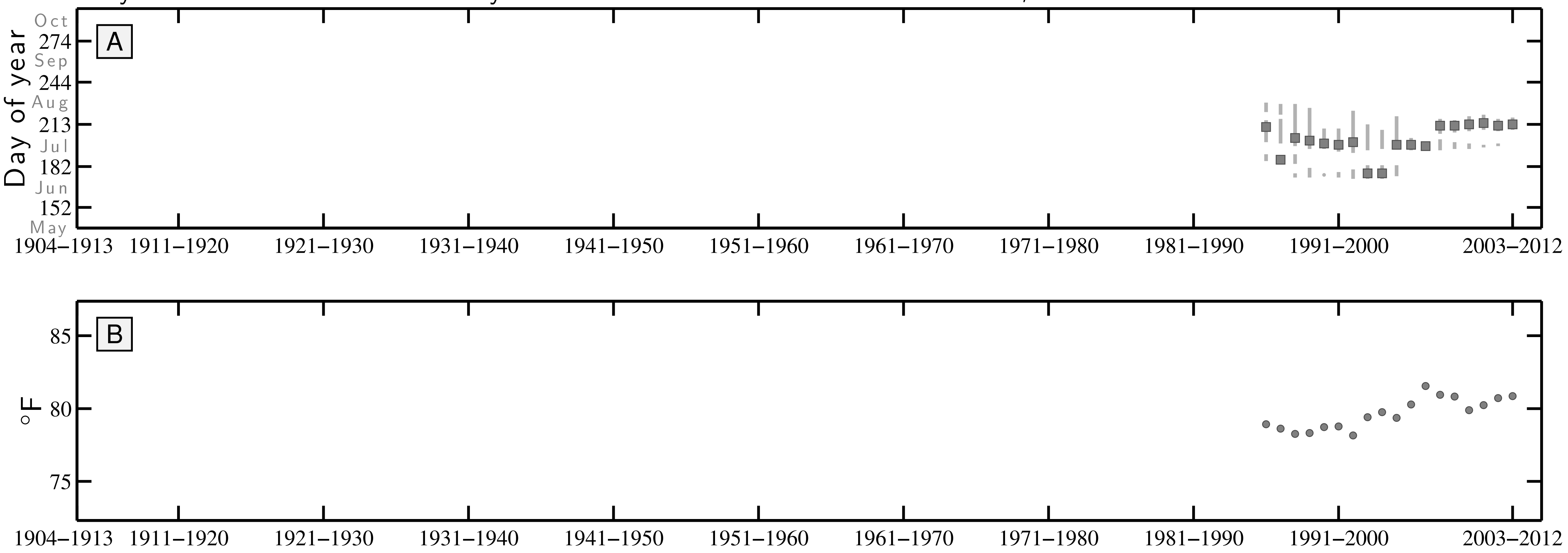

10 year Winter Teletherm dynamics for CHATHAM EXP FARM 2, MI:

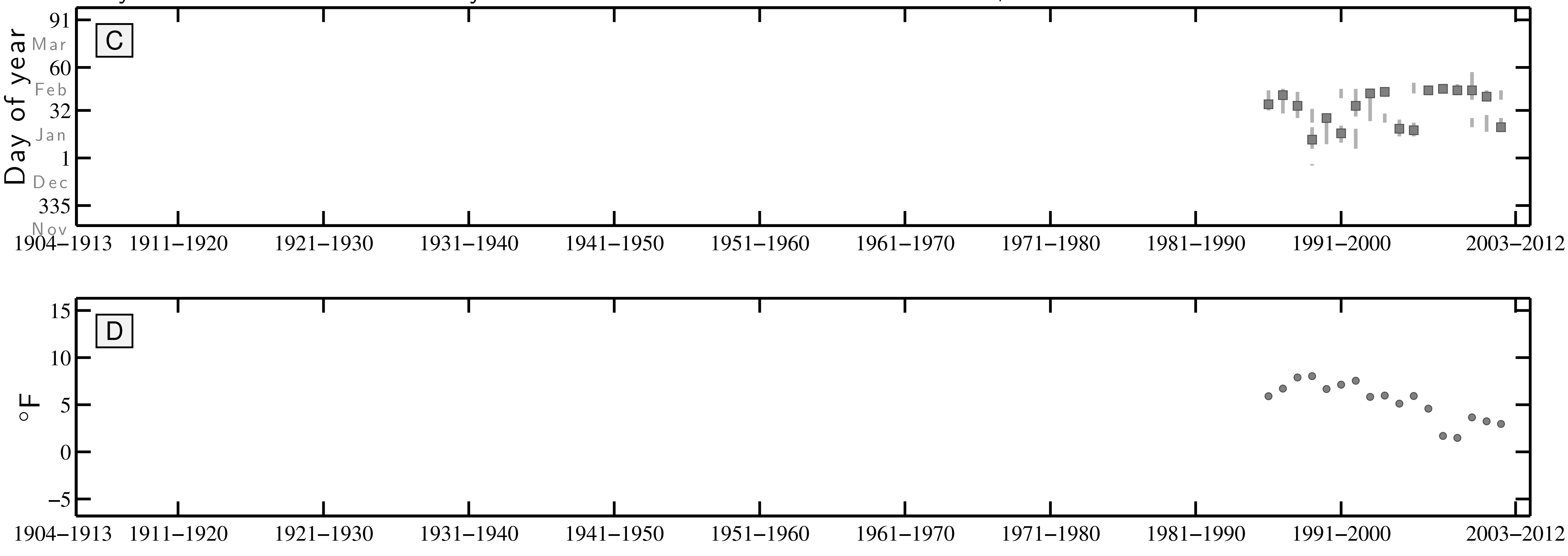

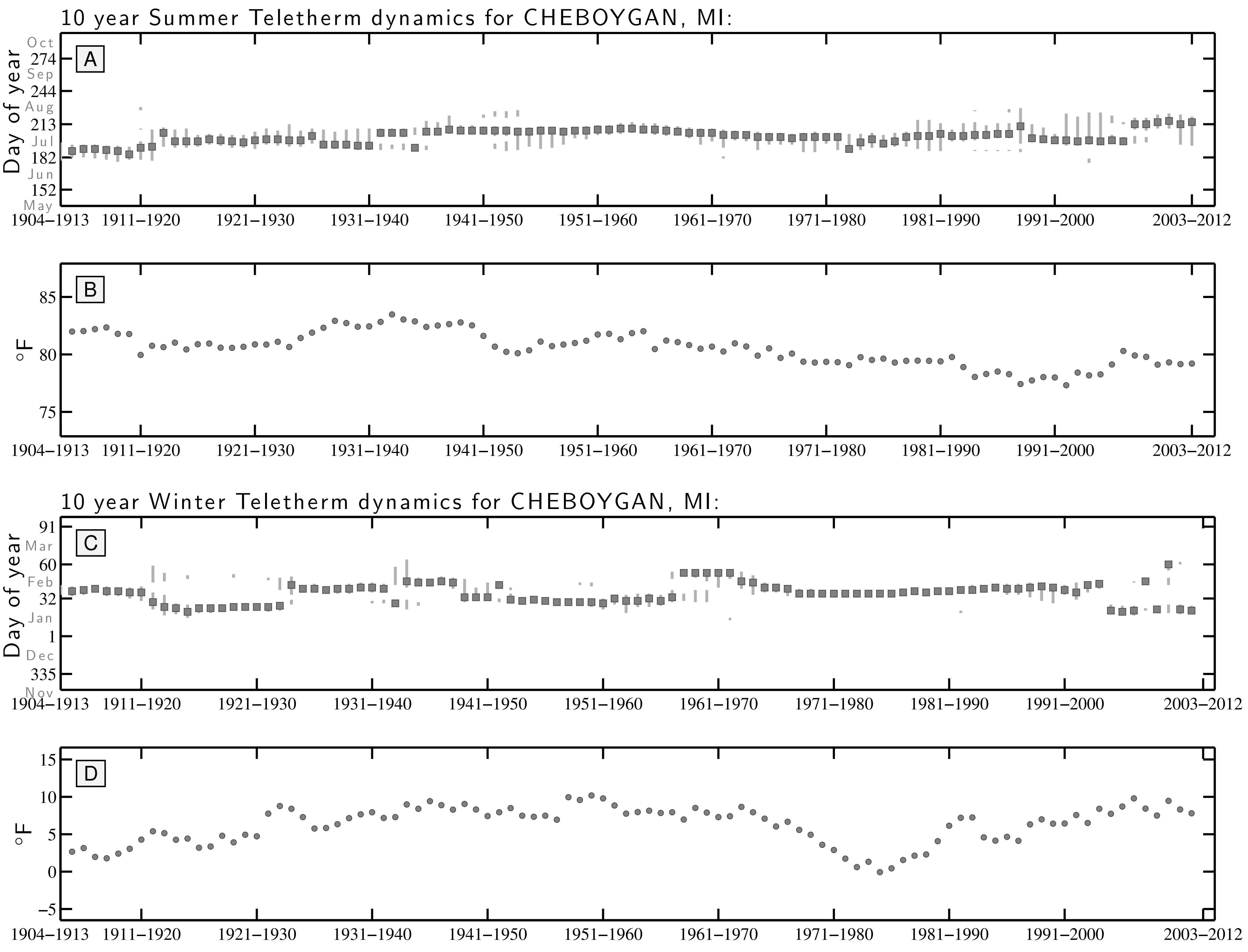
10 year Summer Teletherm dynamics for CHEBOYGAN, MI:
A
Day of year
Oct
274
Sep
244
Aug
213
Jul
182
Jun
152
May
B
°F
85
80
75
1904–1913
1911–1920
1921–1930
1931–1940
1941–1950
1951–1960
1961–1970
1971–1980
1981–1990
1991–2000
2003–2012
10 year Winter Teletherm dynamics for CHEBOYGAN, MI:
C
Day of year
91
Mar
60
Feb
32
Jan
1
Dec
335
Nov
D
°F
15
10
5
0
−5

10 year Summer Teletherm dynamics for COLDWATER ST SCHOOL, MI:

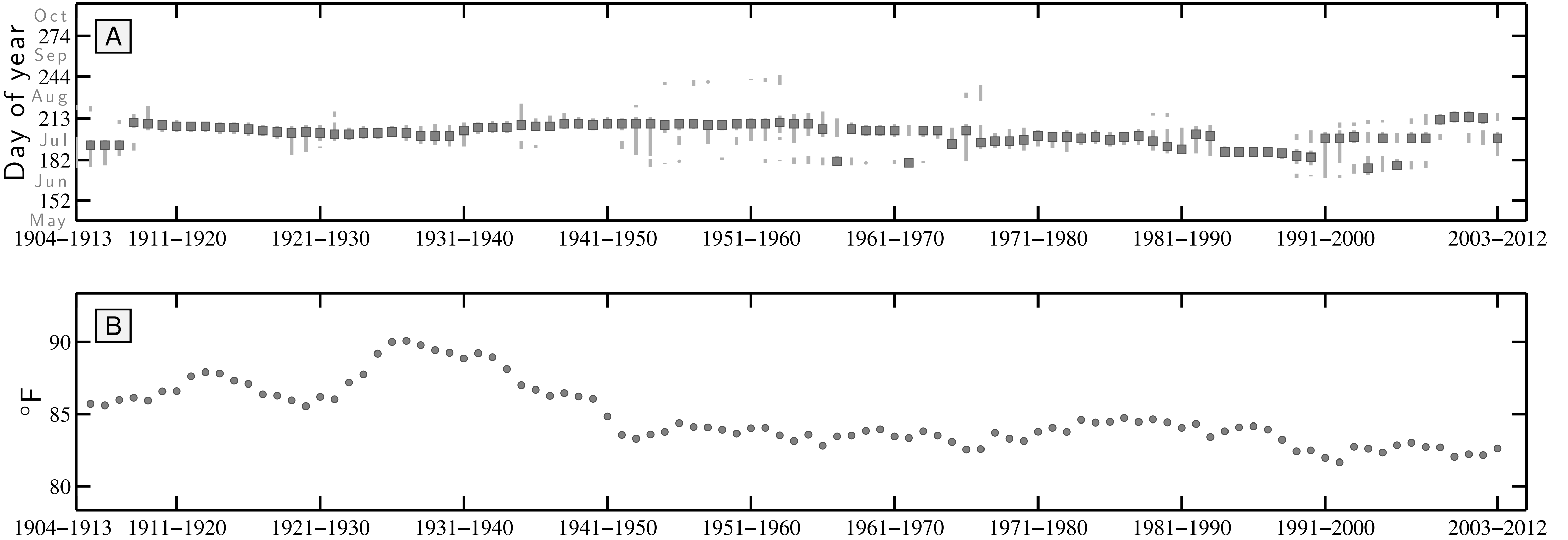

10 year Winter Teletherm dynamics for COLDWATER ST SCHOOL, MI:

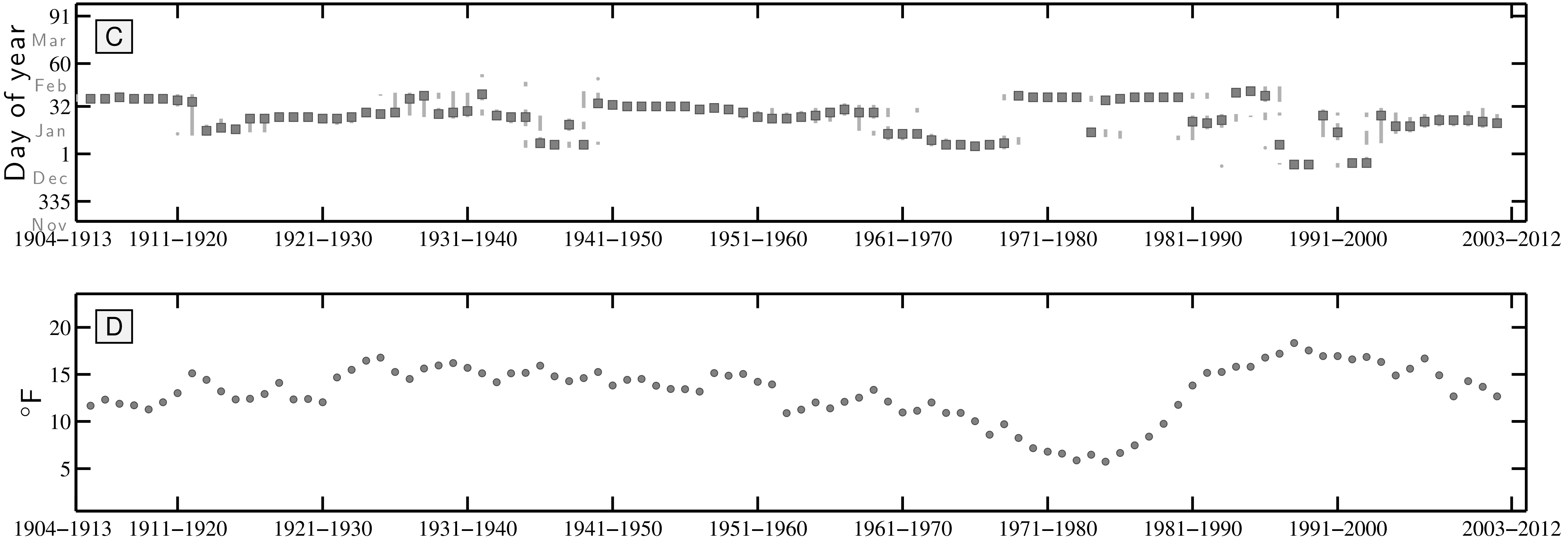

10 year Summer Teletherm dynamics for EAST TAWAS, MI:

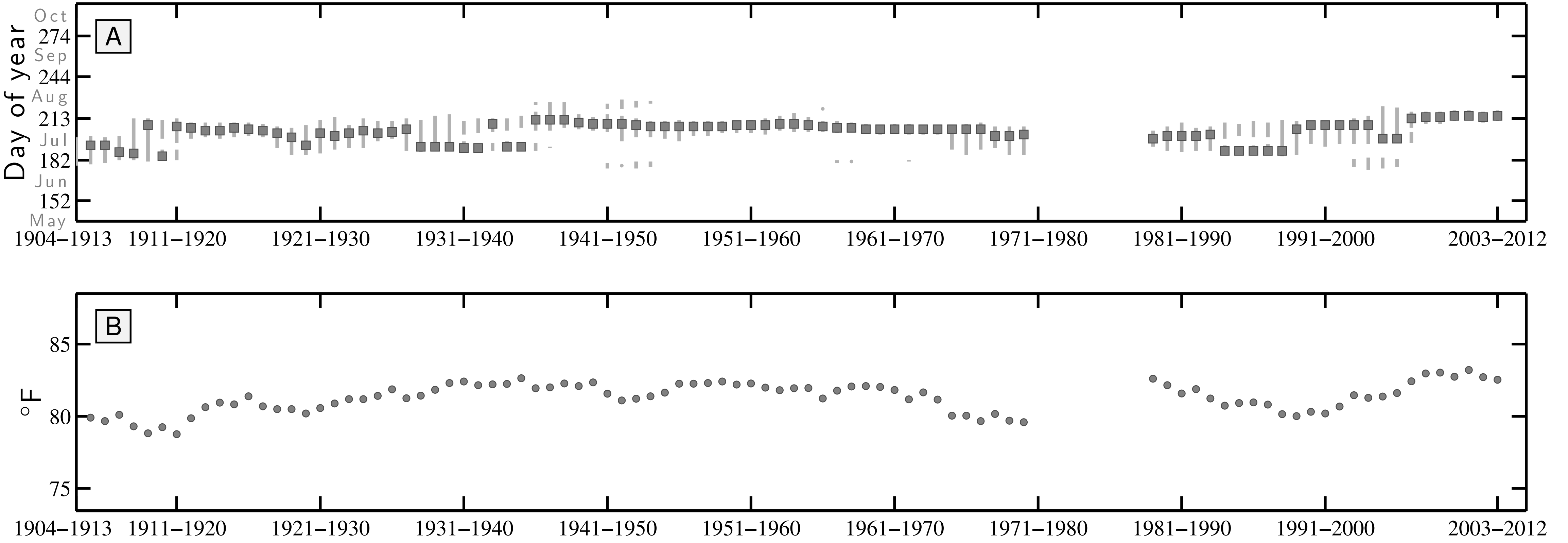

10 year Winter Teletherm dynamics for EAST TAWAS, MI:

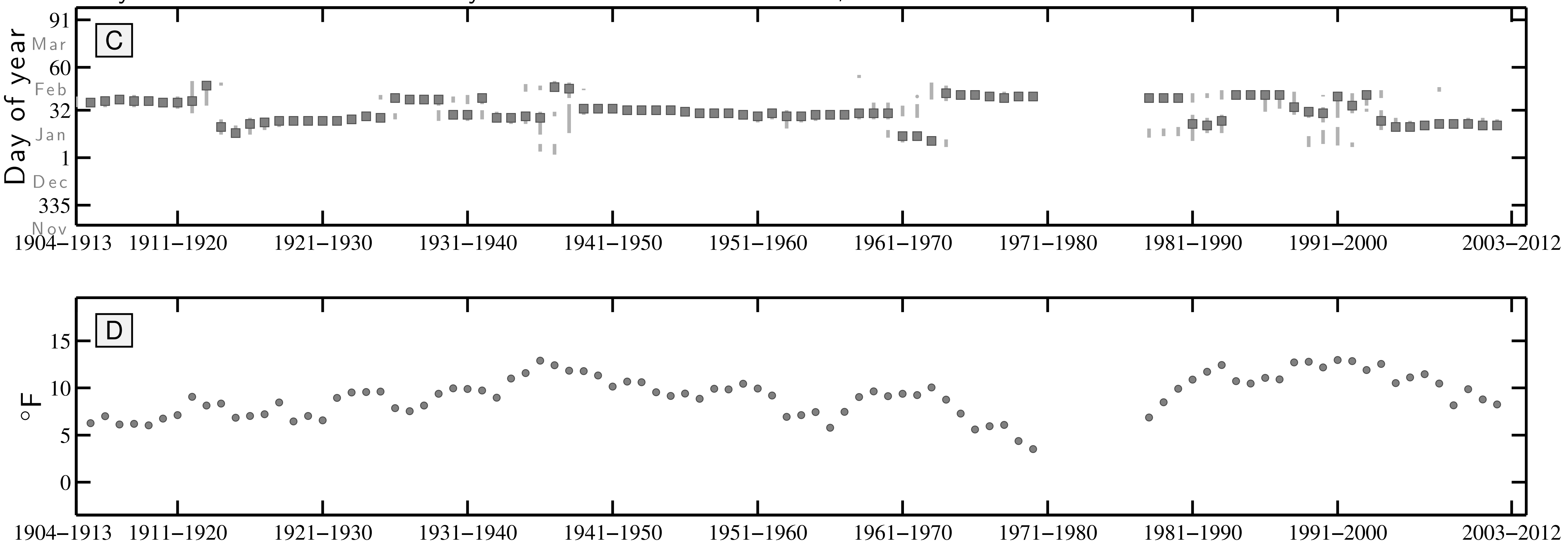

10 year Summer Teletherm dynamics for FAYETTE 4 SW, MI:

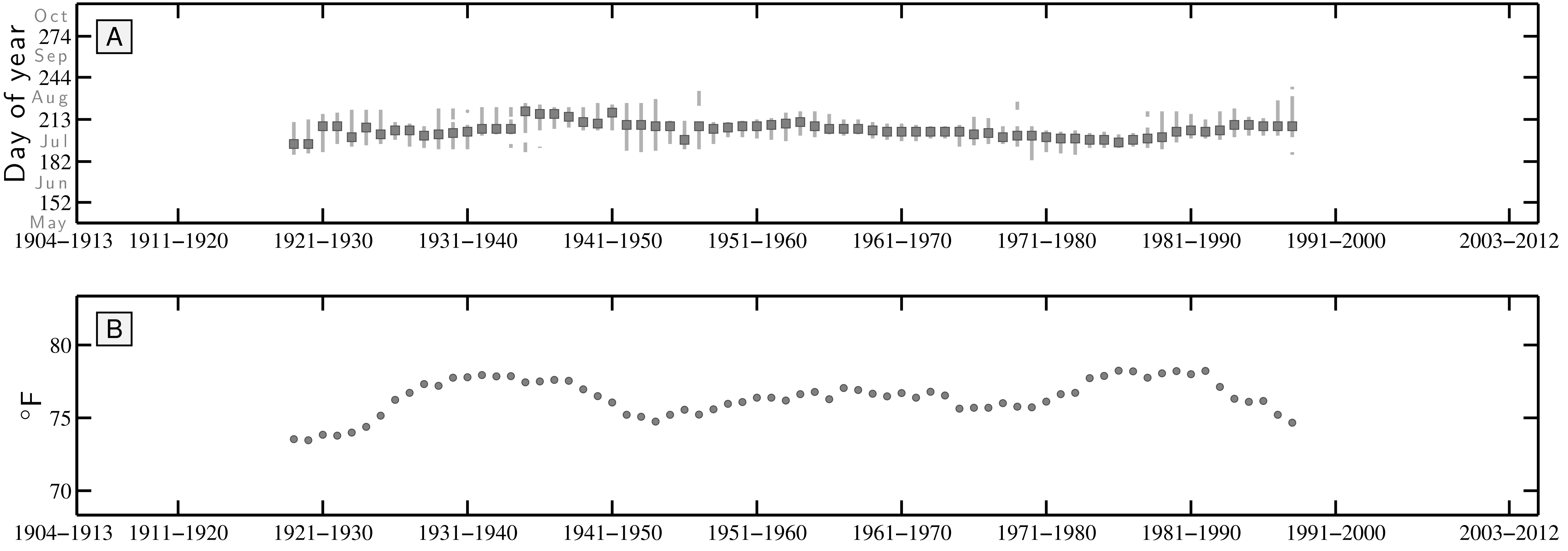

10 year Winter Teletherm dynamics for FAYETTE 4 SW, MI:

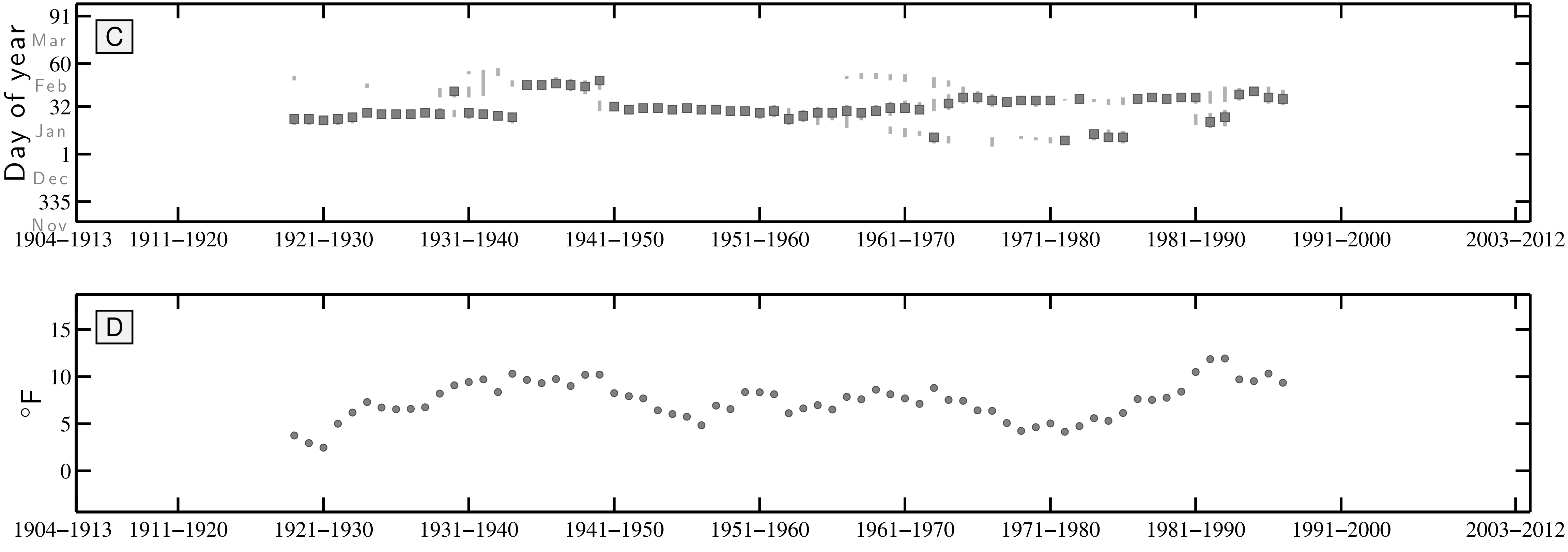

10 year Summer Teletherm dynamics for HART 3 WSW, MI:

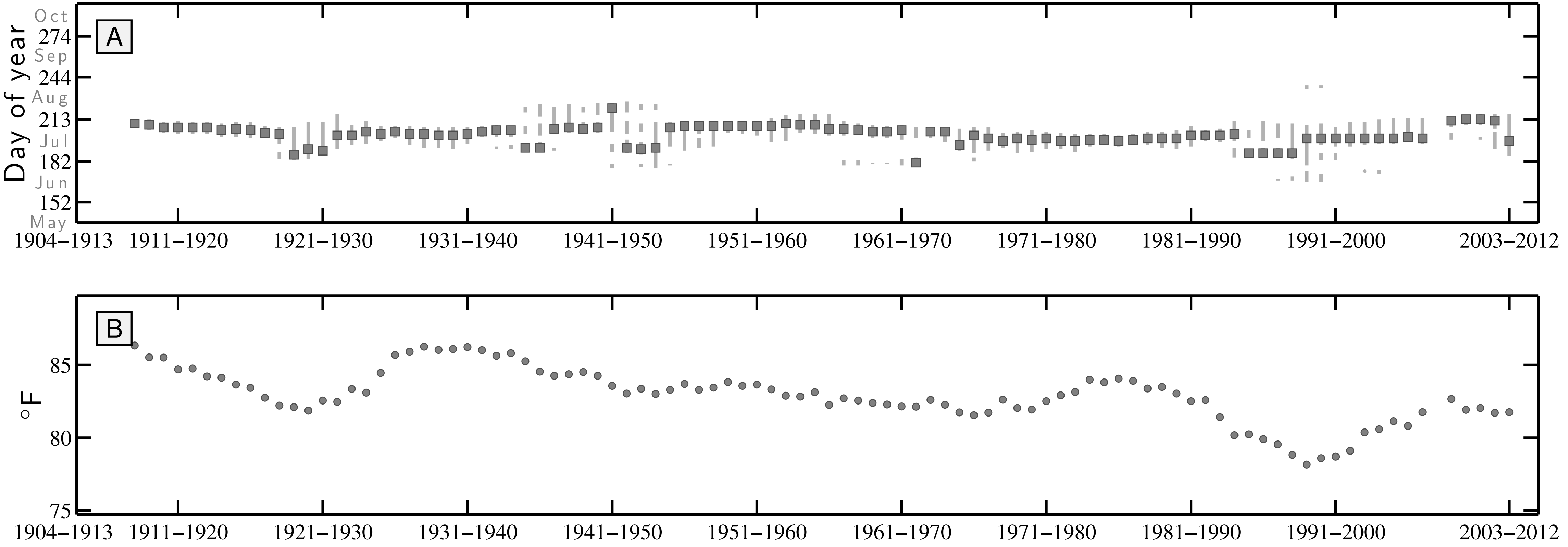

10 year Winter Teletherm dynamics for HART 3 WSW, MI:

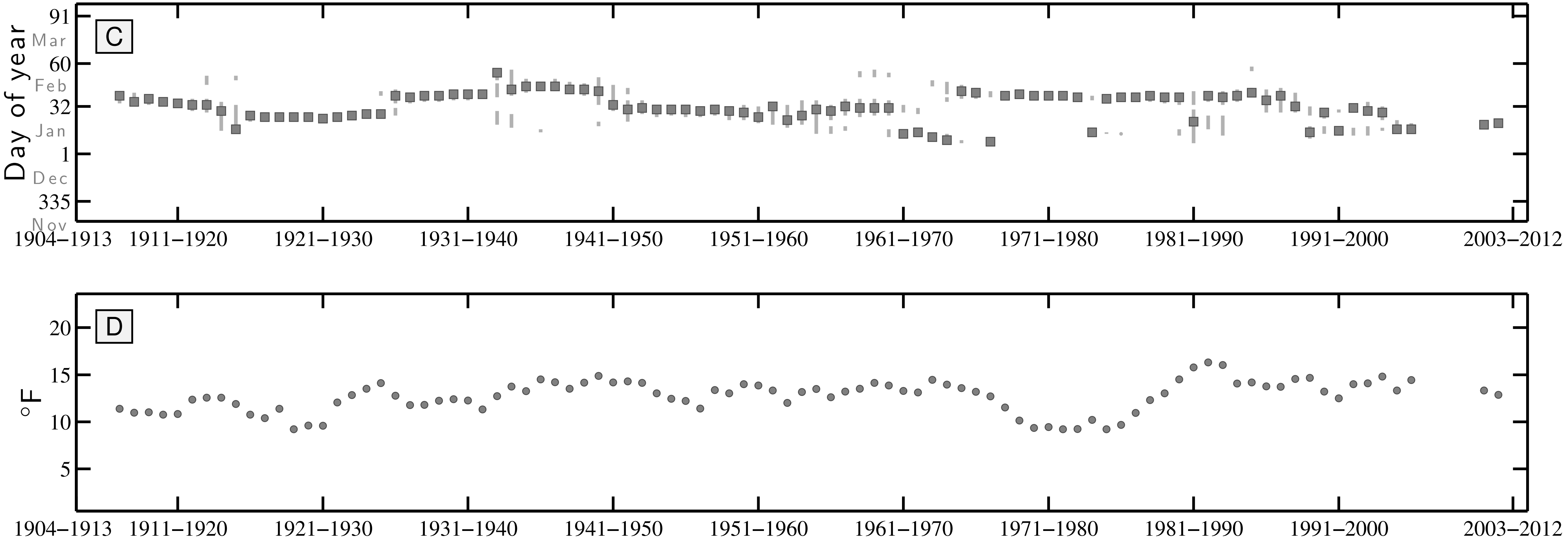

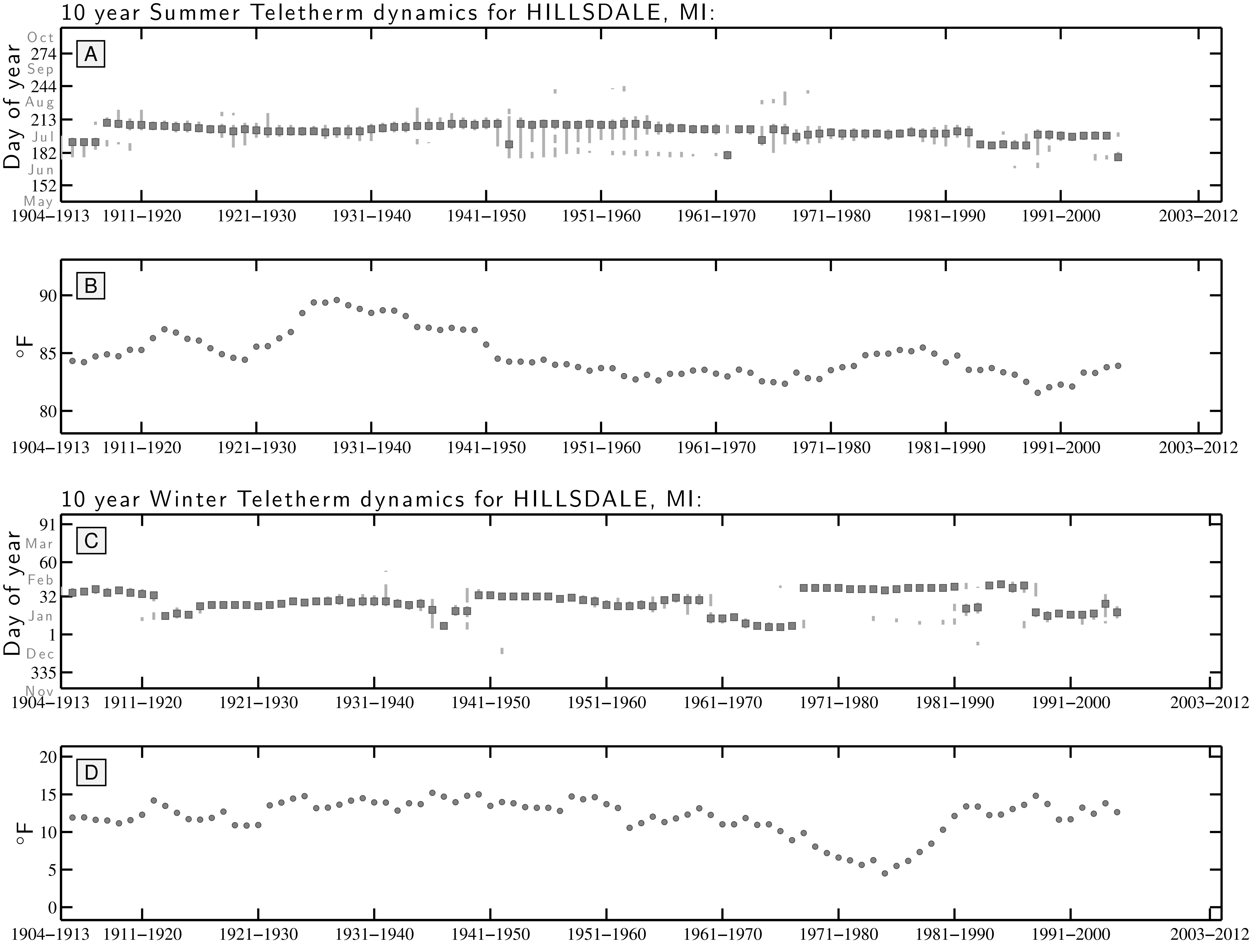
10 year Summer Teletherm dynamics for HILLSDALE, MI:
A
Day of year
Oct
274
Sep
244
Aug
213
Jul
182
Jun
152
May
1904–1913
1911–1920
1921–1930
1931–1940
1941–1950
1951–1960
1961–1970
1971–1980
1981–1990
1991–2000
2003–2012
B
°F
90
85
80
10 year Winter Teletherm dynamics for HILLSDALE, MI:
C
91
Mar
60
Feb
32
Jan
1
Dec
335
Nov
D
20
15
10
5
0

10 year Summer Teletherm dynamics for IRON MT KINGSFORD WWTP, MI:

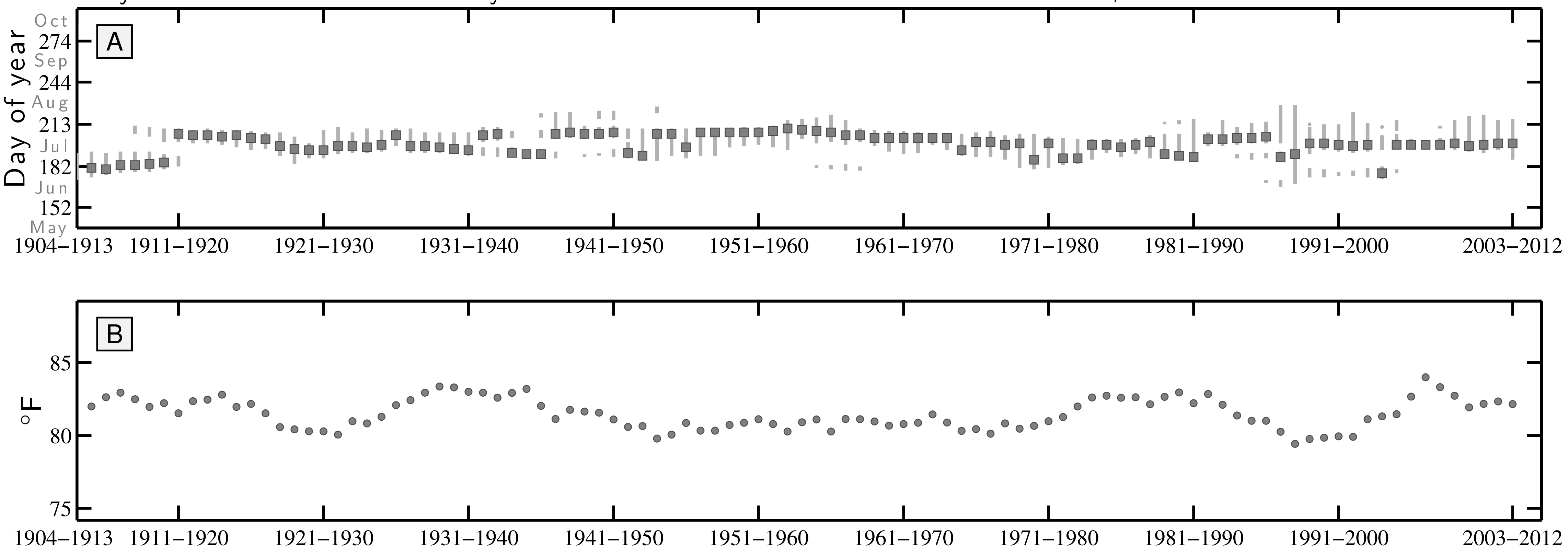

10 year Winter Teletherm dynamics for IRON MT KINGSFORD WWTP, MI:

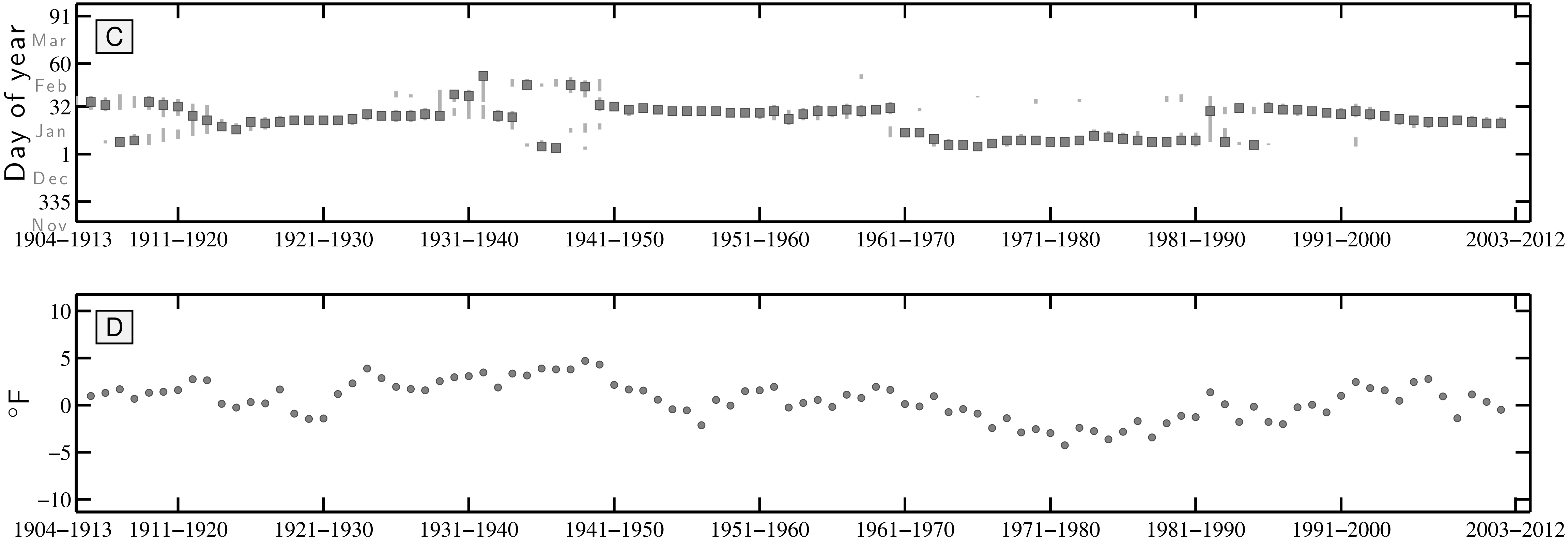

10 year Summer Teletherm dynamics for IRONWOOD, MI:

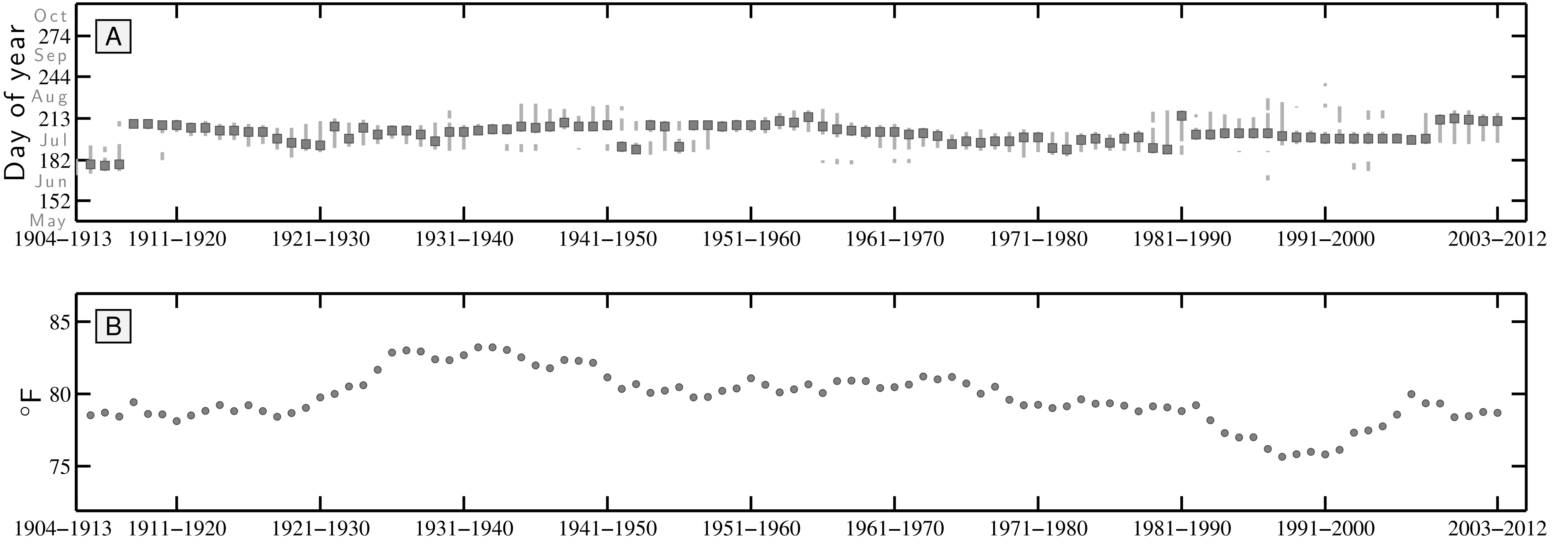

10 year Winter Teletherm dynamics for IRONWOOD, MI:

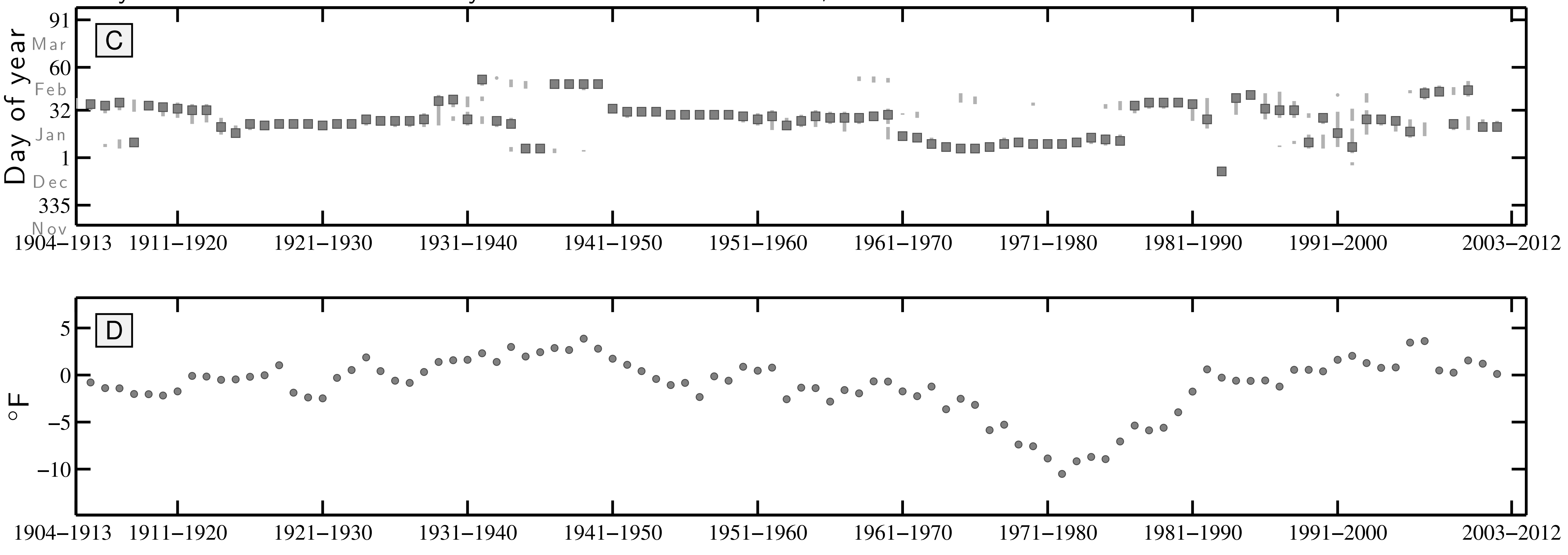

10 year Summer Teletherm dynamics for KALAMAZOO STATE HOSP, MI:

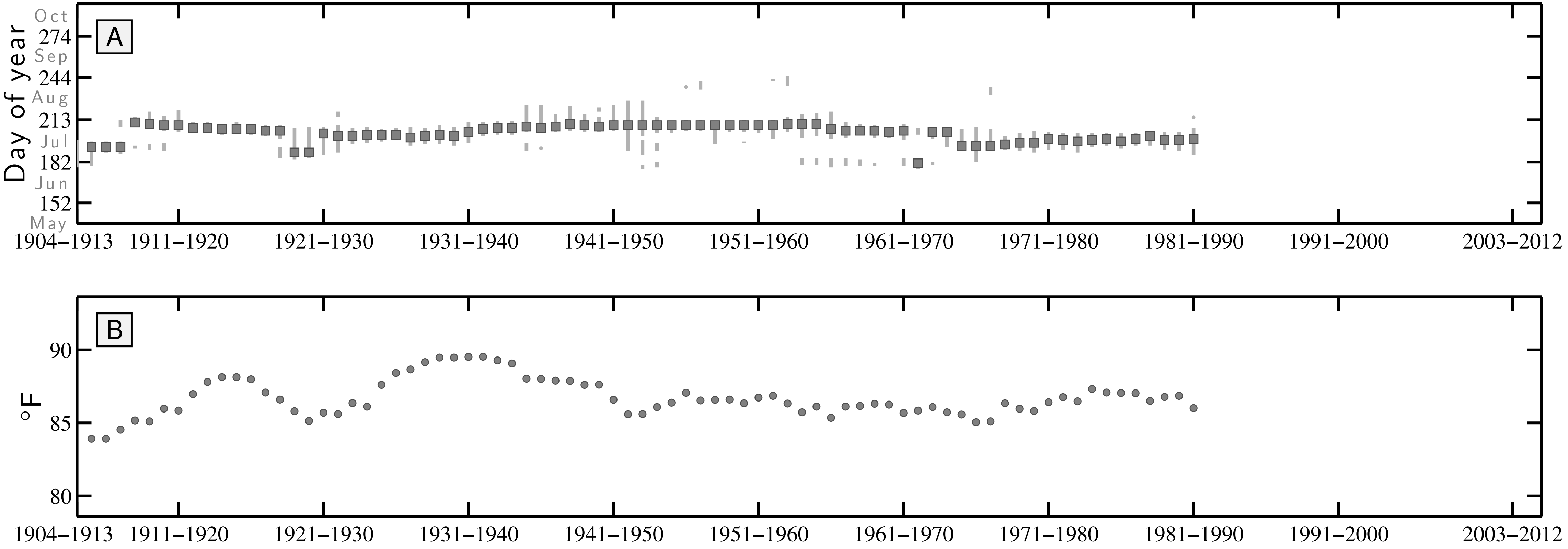

10 year Winter Teletherm dynamics for KALAMAZOO STATE HOSP, MI:

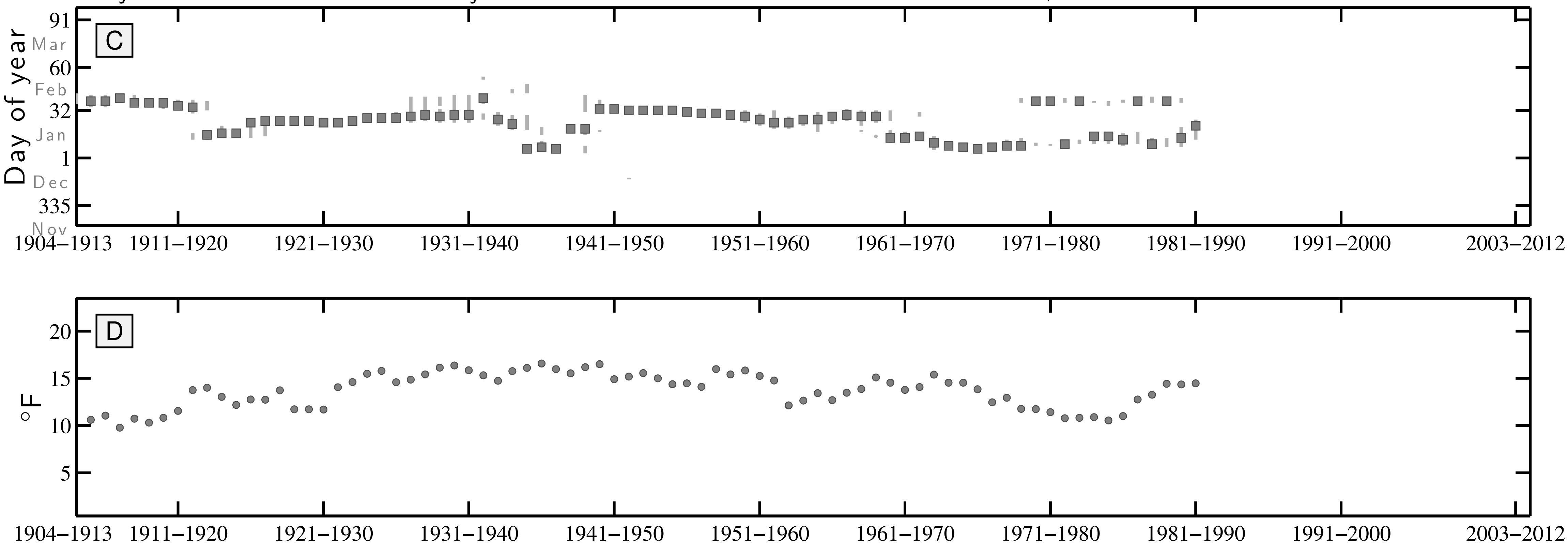

10 year Summer Teletherm dynamics for MIDLAND, MI:

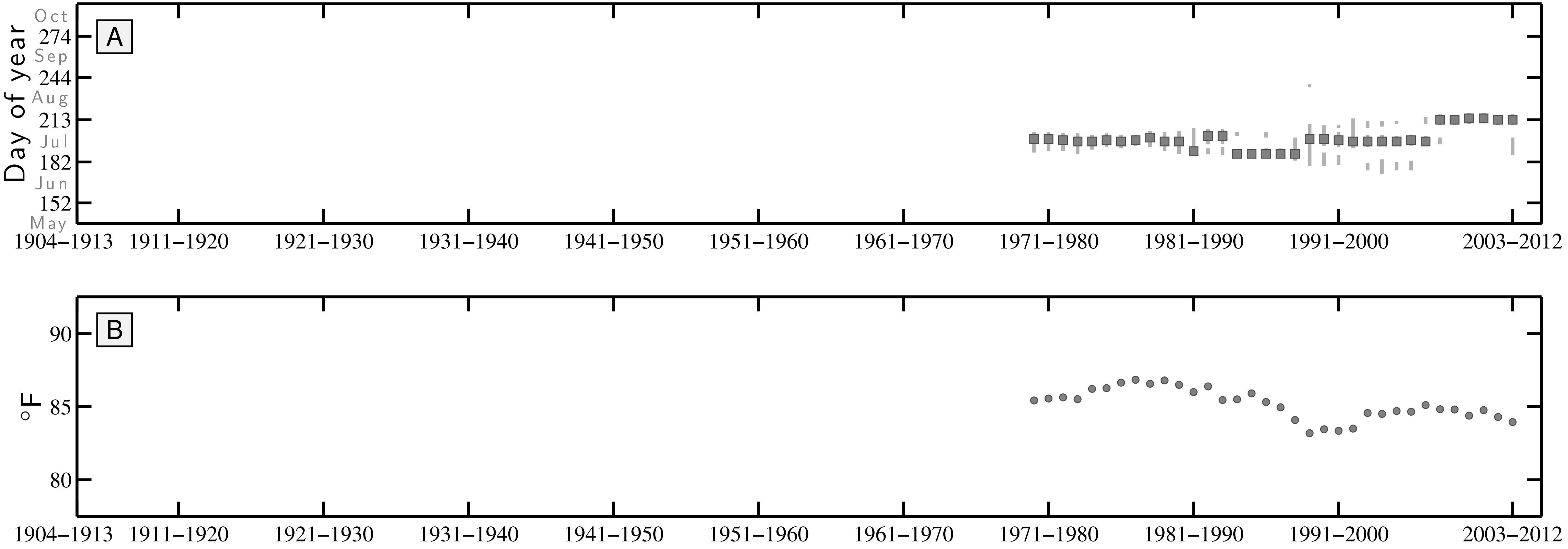

10 year Winter Teletherm dynamics for MIDLAND, MI:

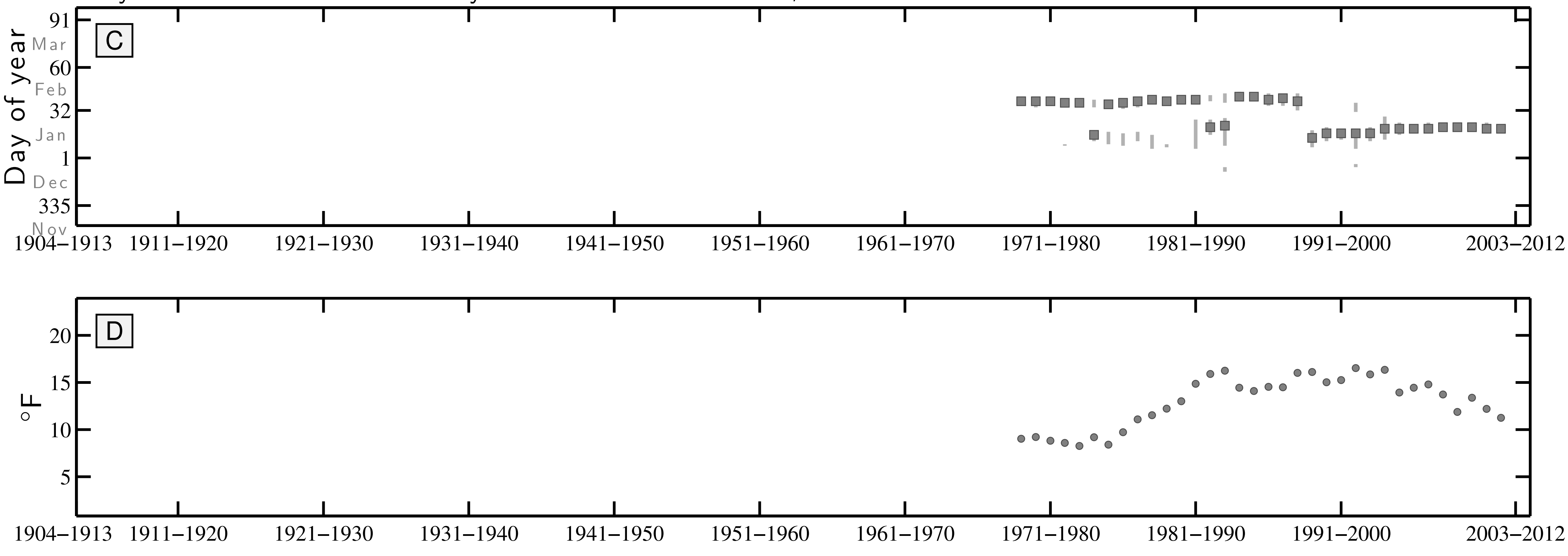

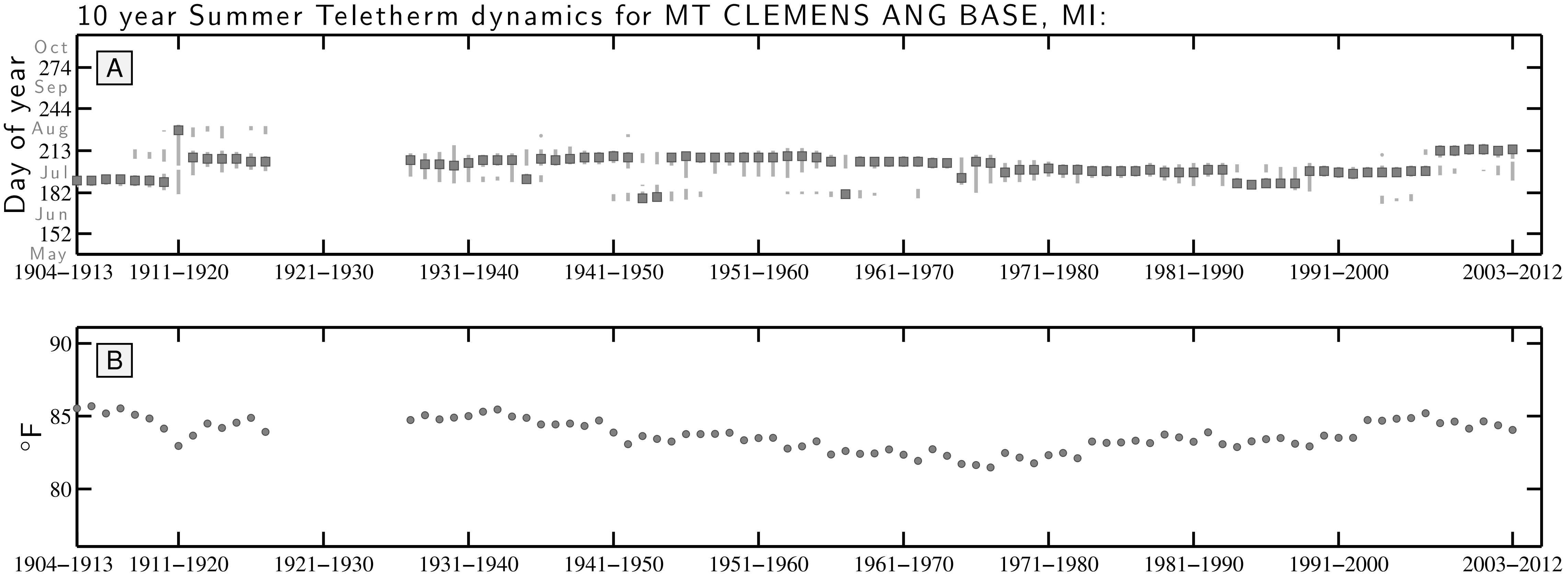
10 year Summer Teletherm dynamics for MT CLEMENS ANG BASE, MI:
A
B
Day of year
°F
Oct
274
Sep
244
Aug
213
Jul
182
Jun
152
May
90
85
80
1904–1913
1911–1920
1921–1930
1931–1940
1941–1950
1951–1960
1961–1970
1971–1980
1981–1990
1991–2000
2003–2012

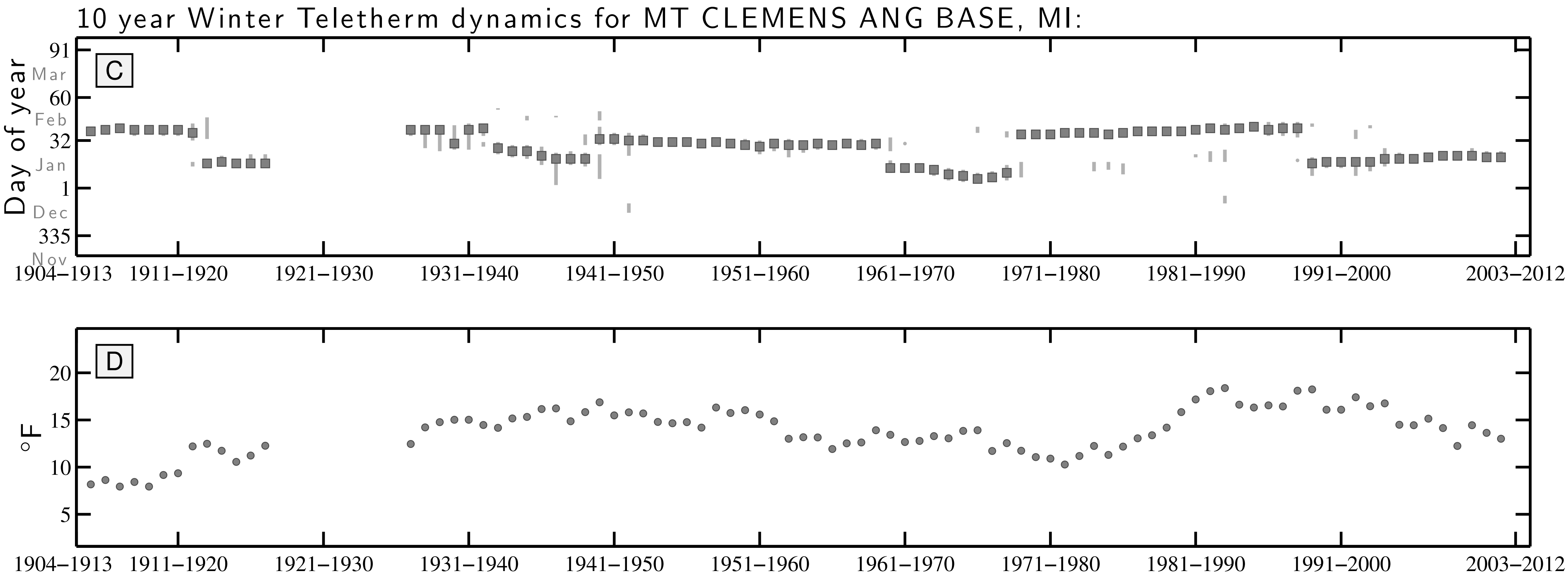
10 year Winter Teletherm dynamics for MT CLEMENS ANG BASE, MI:
C
D
Day of year
°F
91
Mar
60
Feb
32
Jan
1
Dec
335
Nov
20
15
10
5
1904–1913
1911–1920
1921–1930
1931–1940
1941–1950
1951–1960
1961–1970
1971–1980
1981–1990
1991–2000
2003–2012

10 year Summer Teletherm dynamics for MT PLEASANT UNIV, MI:

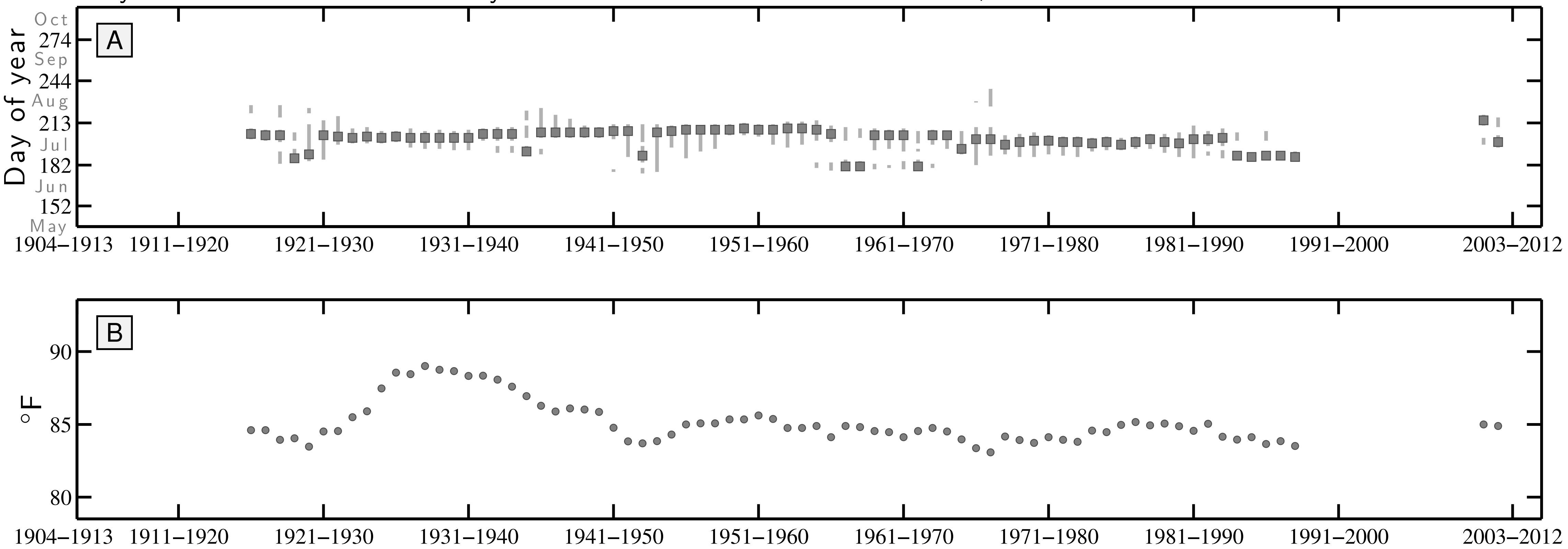

10 year Winter Teletherm dynamics for MT PLEASANT UNIV, MI:

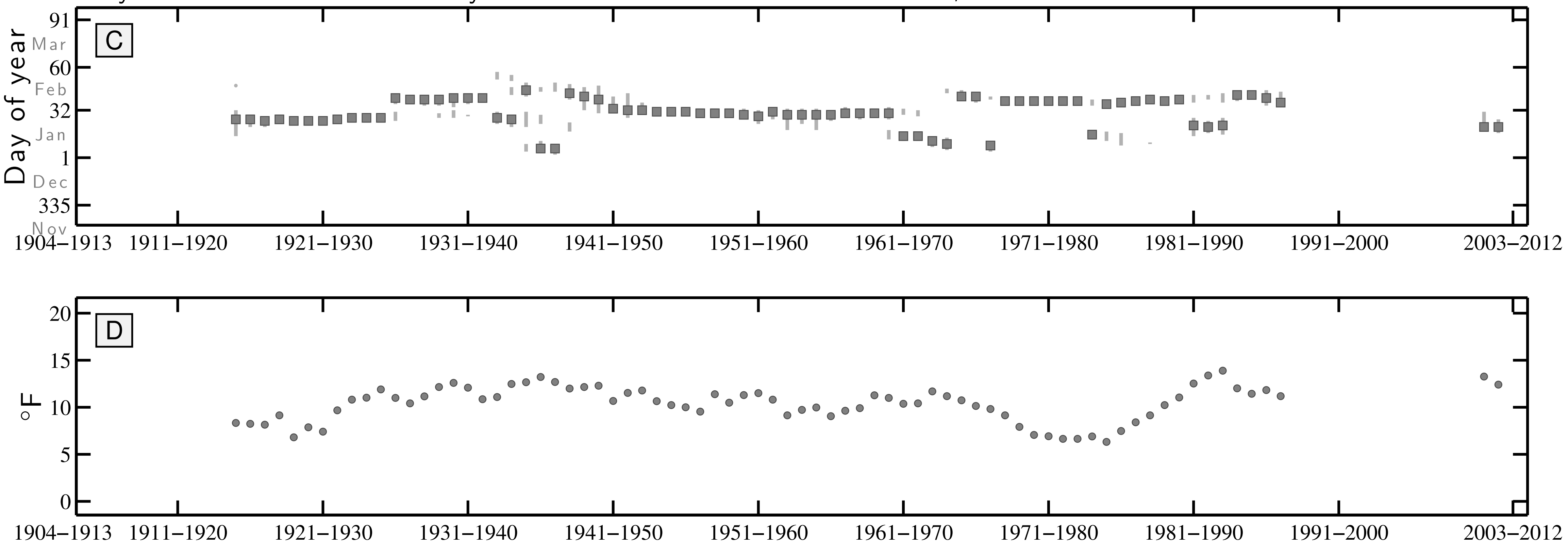

10 year Summer Teletherm dynamics for MUNISING, MI:

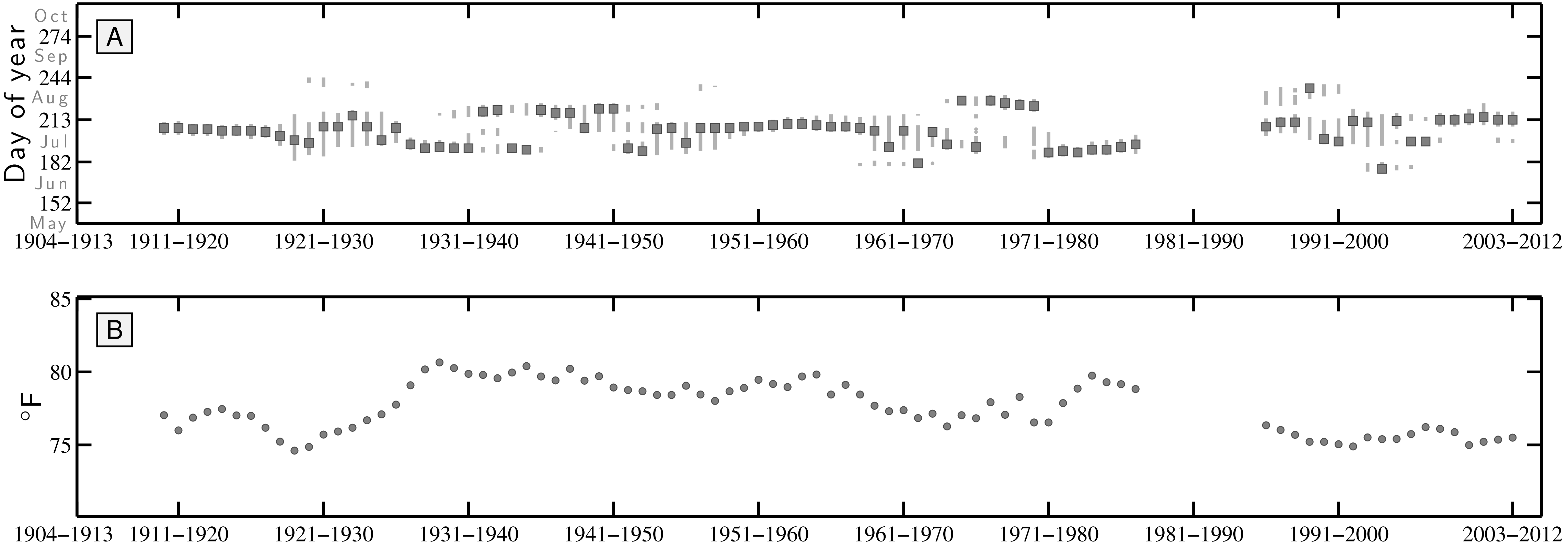

10 year Winter Teletherm dynamics for MUNISING, MI:

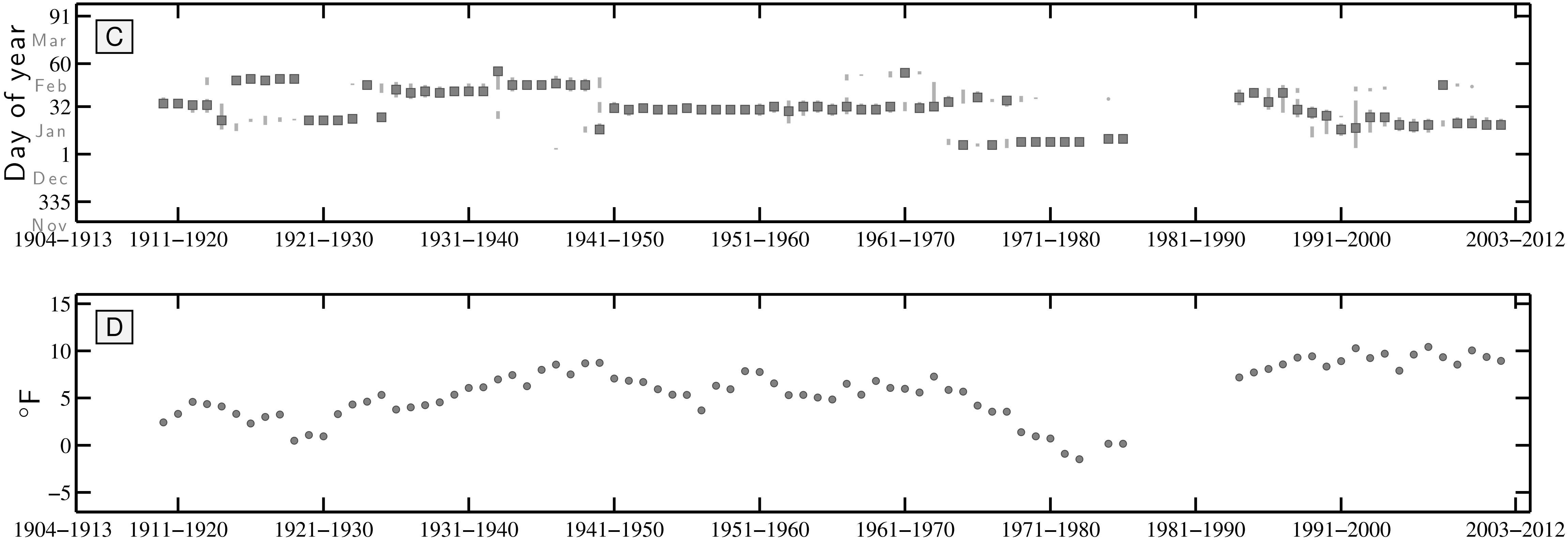

10 year Summer Teletherm dynamics for NEWBERRY 3S, MI:

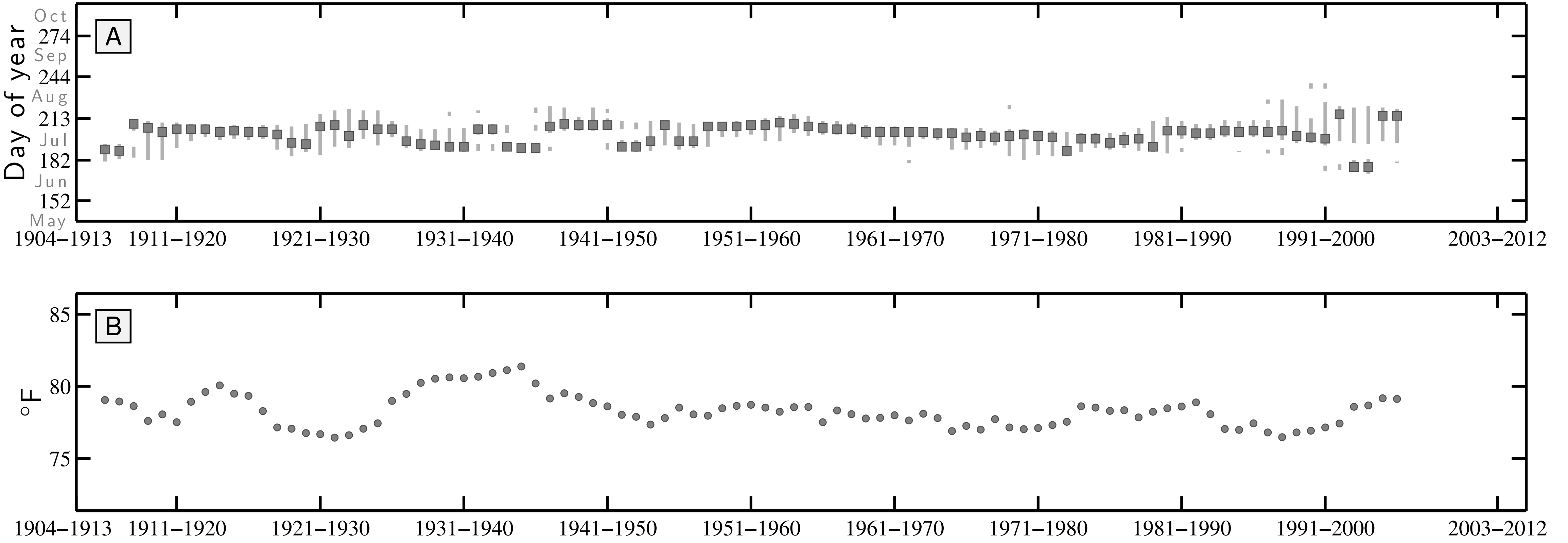

10 year Winter Teletherm dynamics for NEWBERRY 3S, MI:

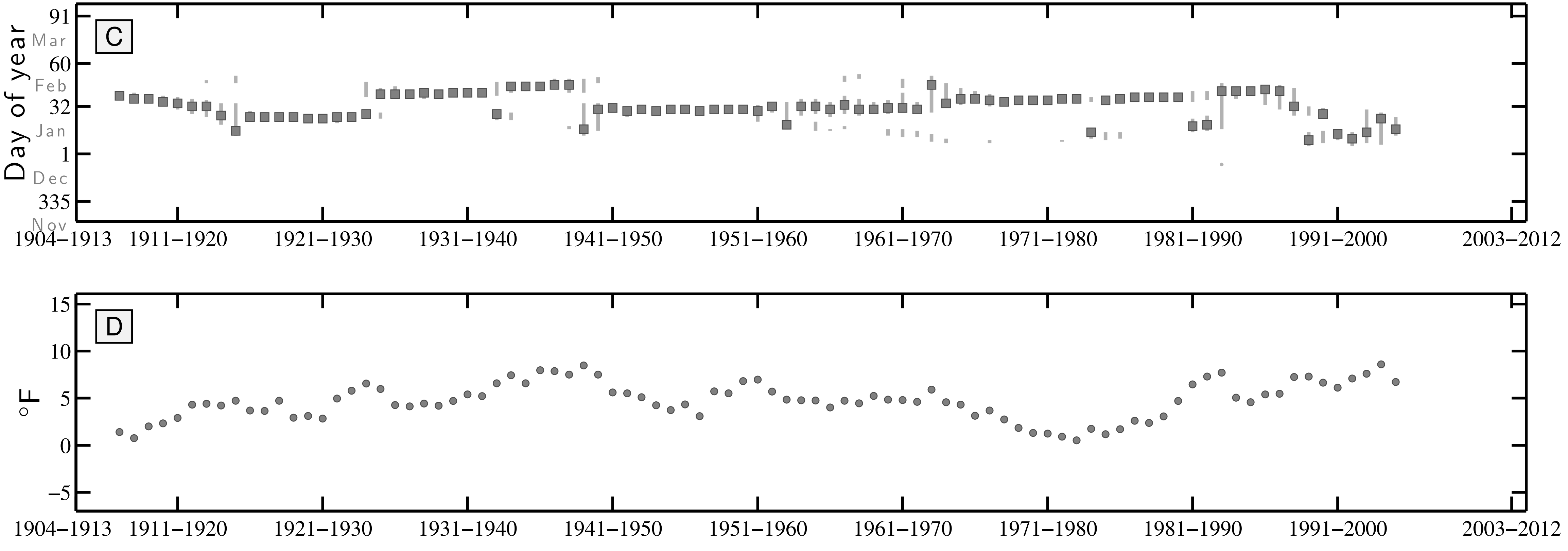

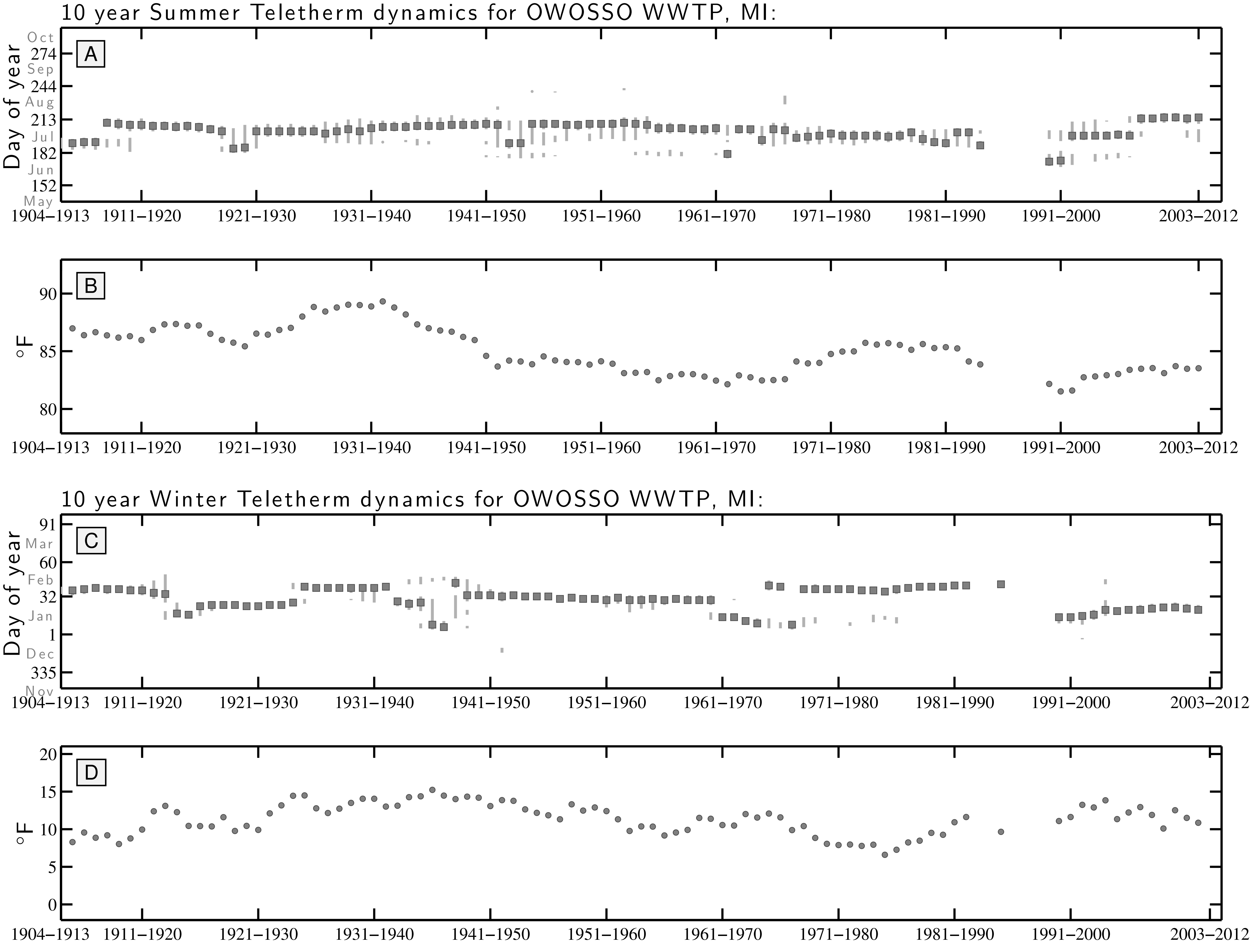
10 year Summer Teletherm dynamics for OWOSSO WWTP, MI:
A
B
Day of year
°F
Oct
274
Sep
244
Aug
213
Jul
182
Jun
152
May
90
85
80
10 year Winter Teletherm dynamics for OWOSSO WWTP, MI:
C
D
91
Mar
60
Feb
32
Jan
1
Dec
335
Nov
20
15
10
5
0
1904–1913
1911–1920
1921–1930
1931–1940
1941–1950
1951–1960
1961–1970
1971–1980
1981–1990
1991–2000
2003–2012

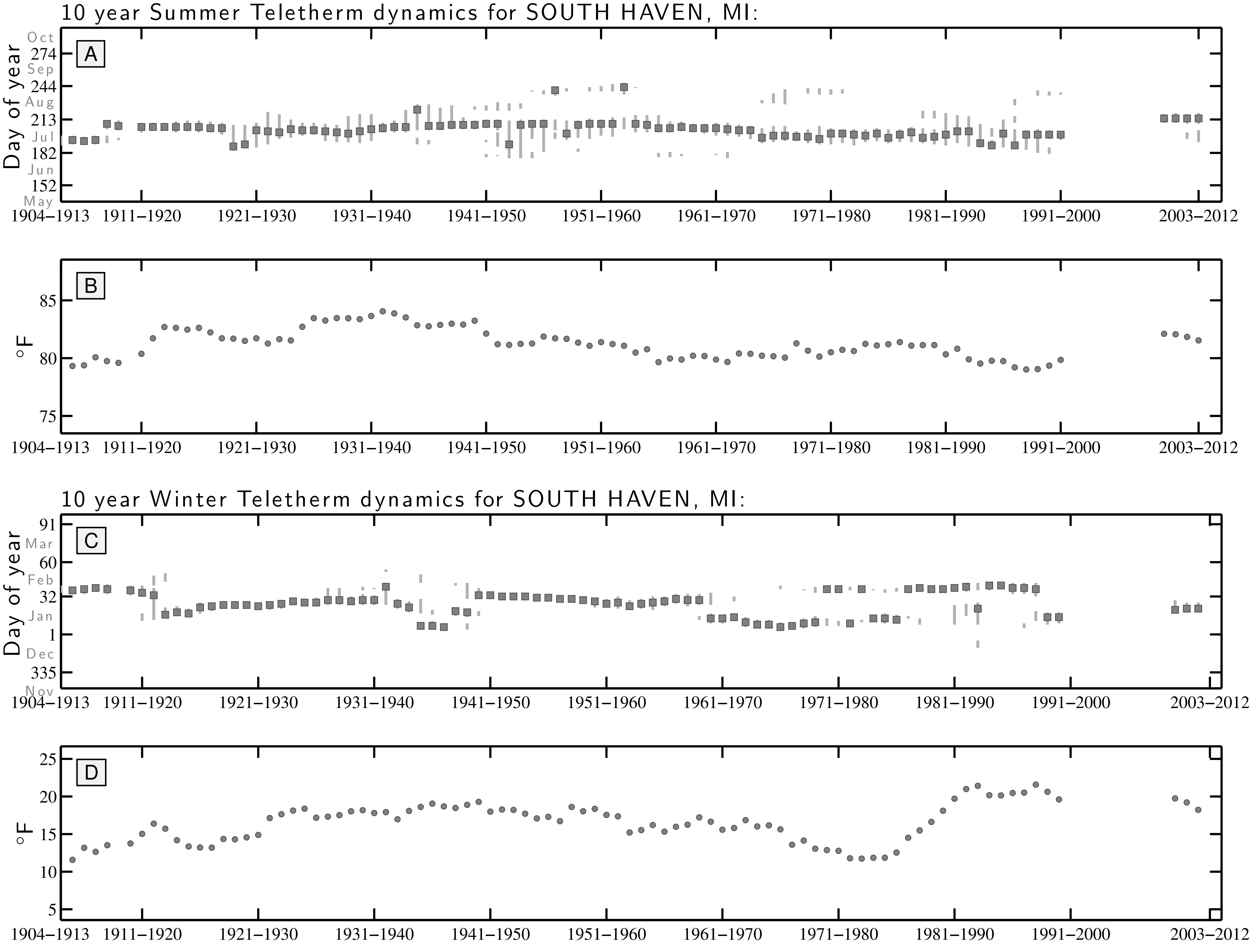
10 year Summer Teletherm dynamics for SOUTH HAVEN, MI:
A
Day of year
Oct
274
Sep
244
Aug
213
Jul
182
Jun
152
May
1904–1913
1911–1920
1921–1930
1931–1940
1941–1950
1951–1960
1961–1970
1971–1980
1981–1990
1991–2000
2003–2012
B
°F
85
80
75
10 year Winter Teletherm dynamics for SOUTH HAVEN, MI:
C
Day of year
91
Mar
60
Feb
32
Jan
1
Dec
335
Nov
D
°F
25
20
15
10
5

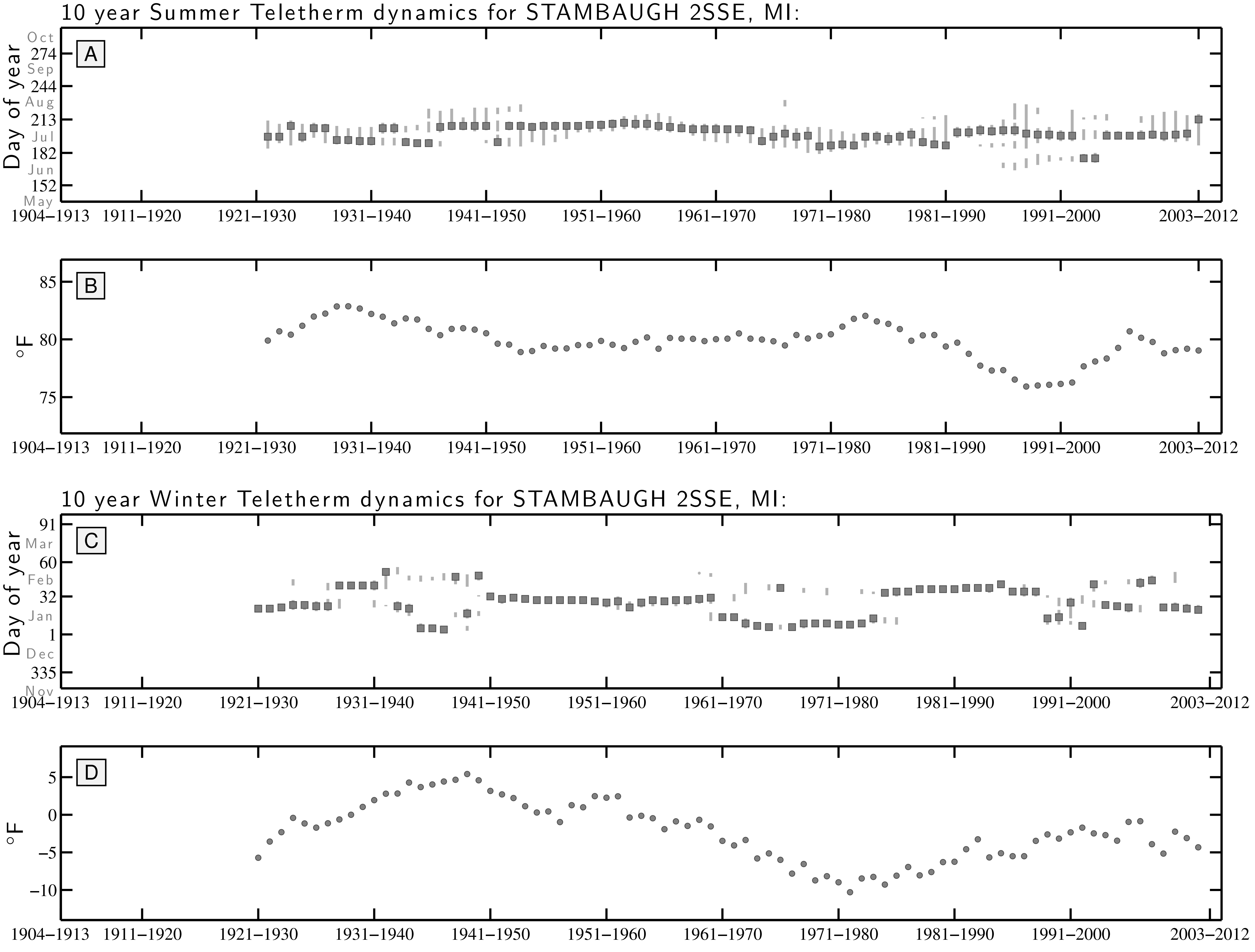
10 year Summer Teletherm dynamics for STAMBAUGH 2SSE, MI:
A
Day of year
Oct
274
Sep
244
Aug
213
Jul
182
Jun
152
May
1904–1913
1911–1920
1921–1930
1931–1940
1941–1950
1951–1960
1961–1970
1971–1980
1981–1990
1991–2000
2003–2012
B
°F
85
80
75
10 year Winter Teletherm dynamics for STAMBAUGH 2SSE, MI:
C
91
Mar
60
Feb
32
Jan
1
Dec
335
Nov
D
5
0
−5
−10

10 year Summer Teletherm dynamics for ADA, MN:

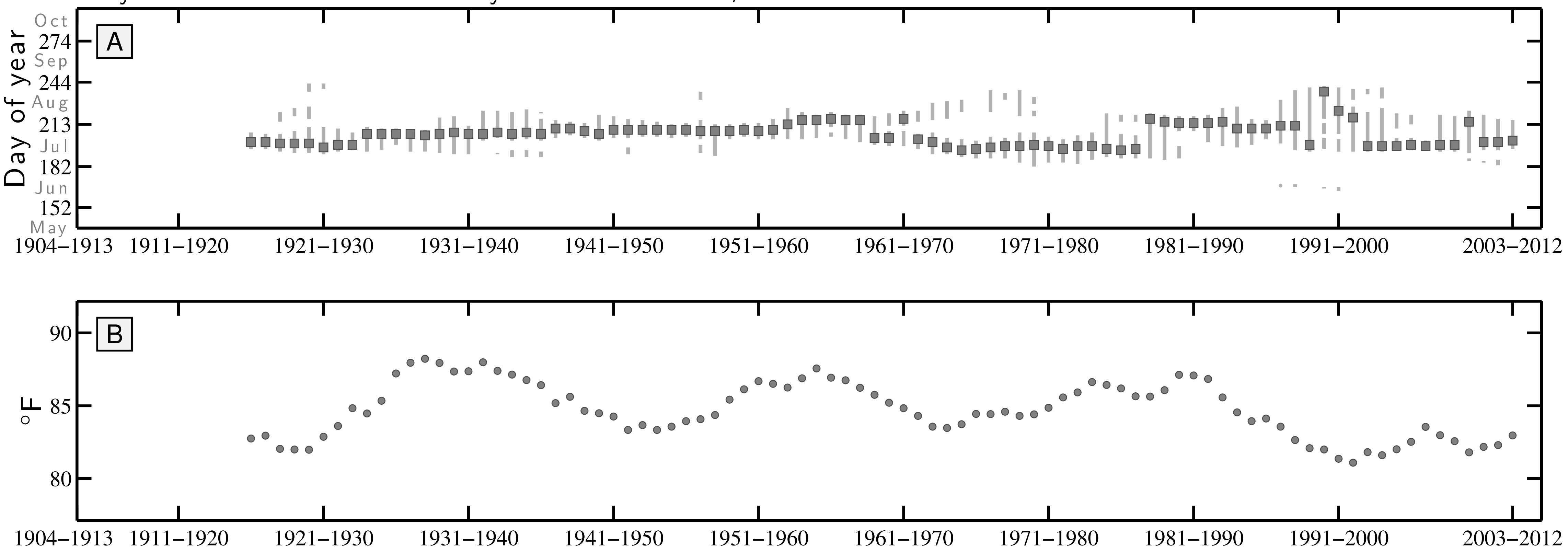

10 year Winter Teletherm dynamics for ADA, MN:

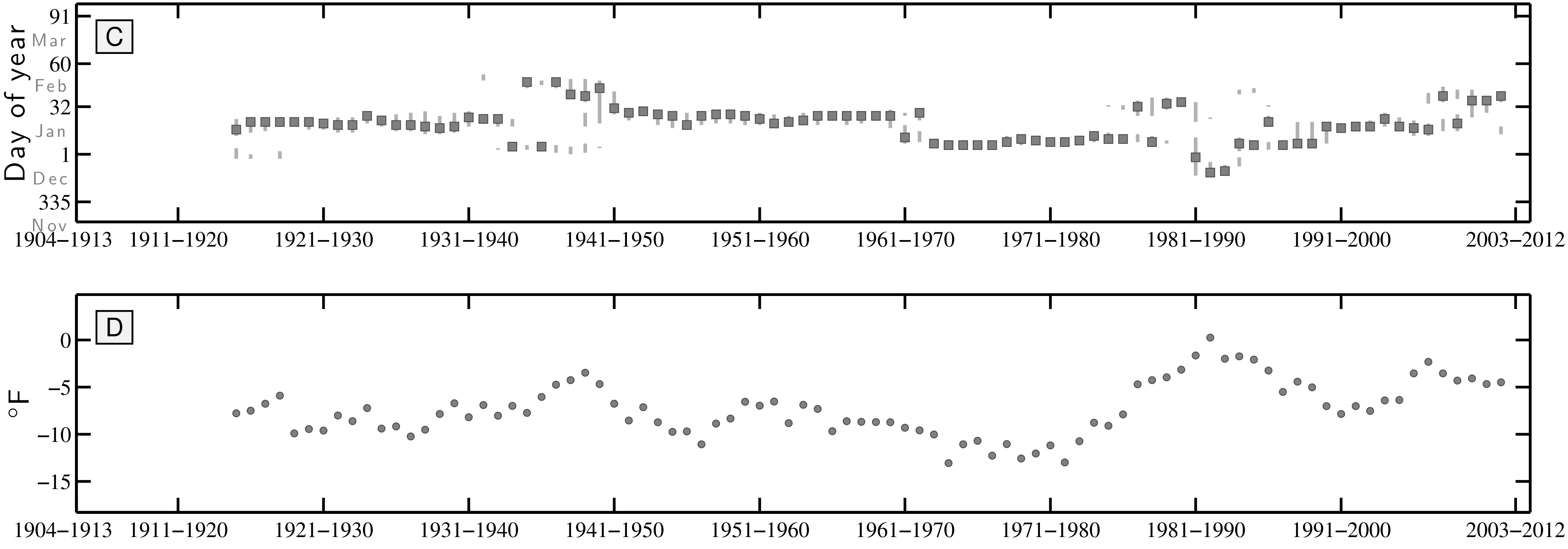

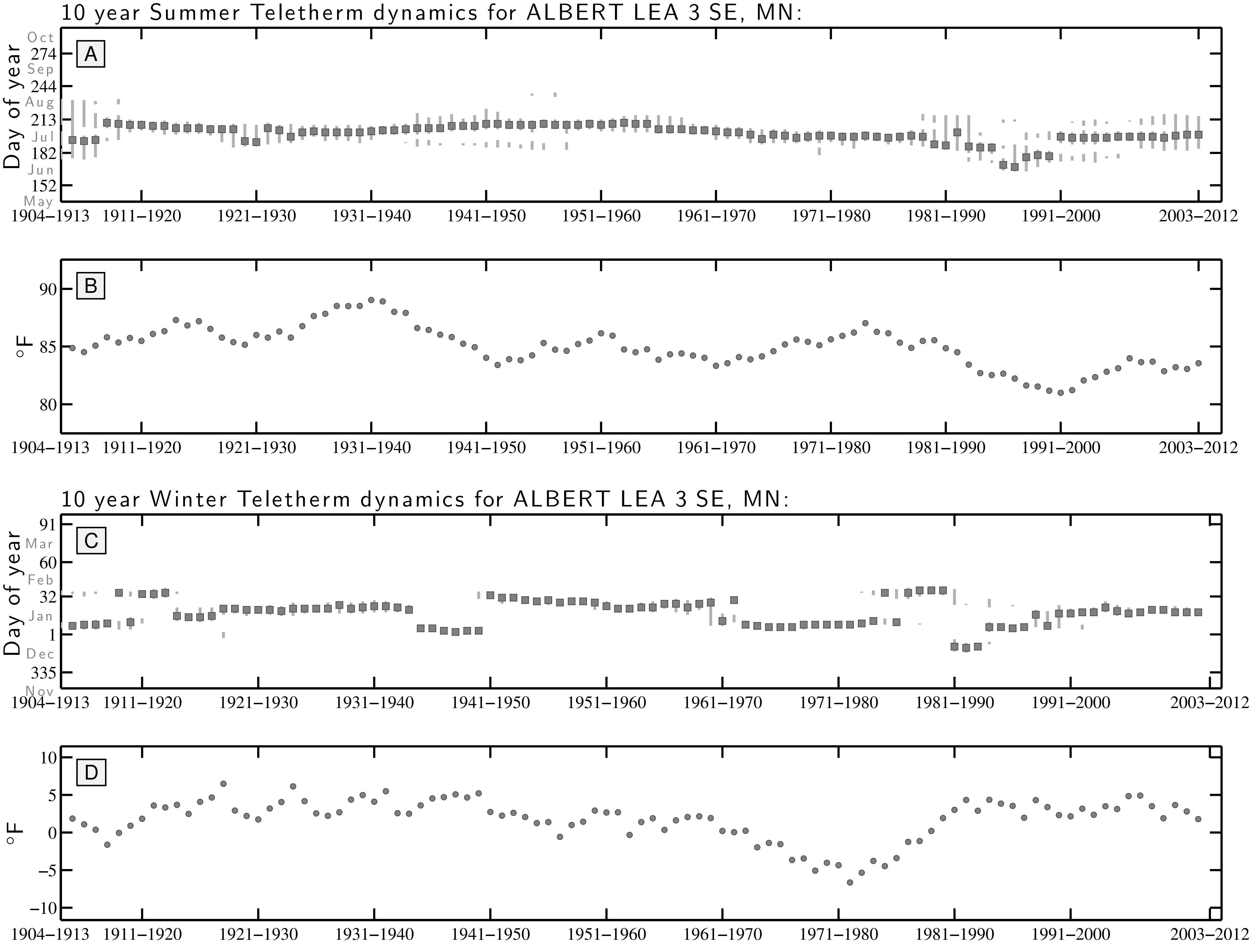
10 year Summer Teletherm dynamics for ALBERT LEA 3 SE, MN:
A
Day of year
Oct
274
Sep
244
Aug
213
Jul
182
Jun
152
May
B
°F
90
85
80
10 year Winter Teletherm dynamics for ALBERT LEA 3 SE, MN:
C
Day of year
91
Mar
60
Feb
32
Jan
1
Dec
335
Nov
D
°F
10
5
0
−5
−10
1904–1913
1911–1920
1921–1930
1931–1940
1941–1950
1951–1960
1961–1970
1971–1980
1981–1990
1991–2000
2003–2012

10 year Summer Teletherm dynamics for ARGYLE, MN:

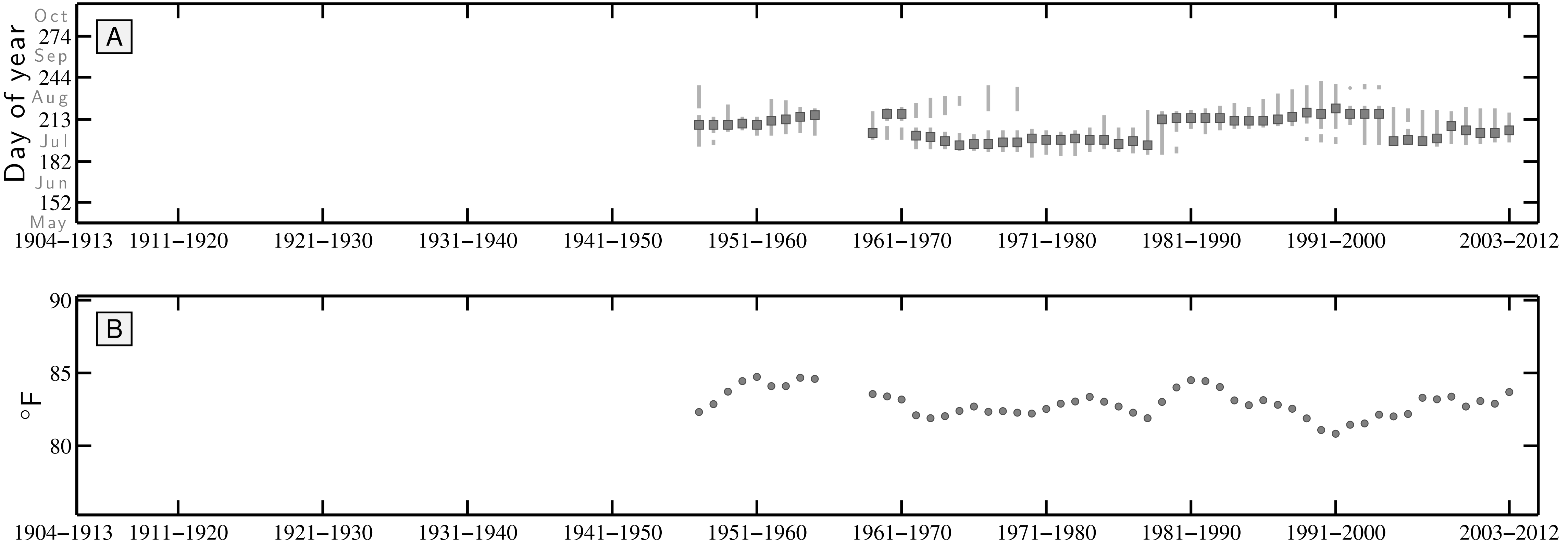

10 year Winter Teletherm dynamics for ARGYLE, MN:

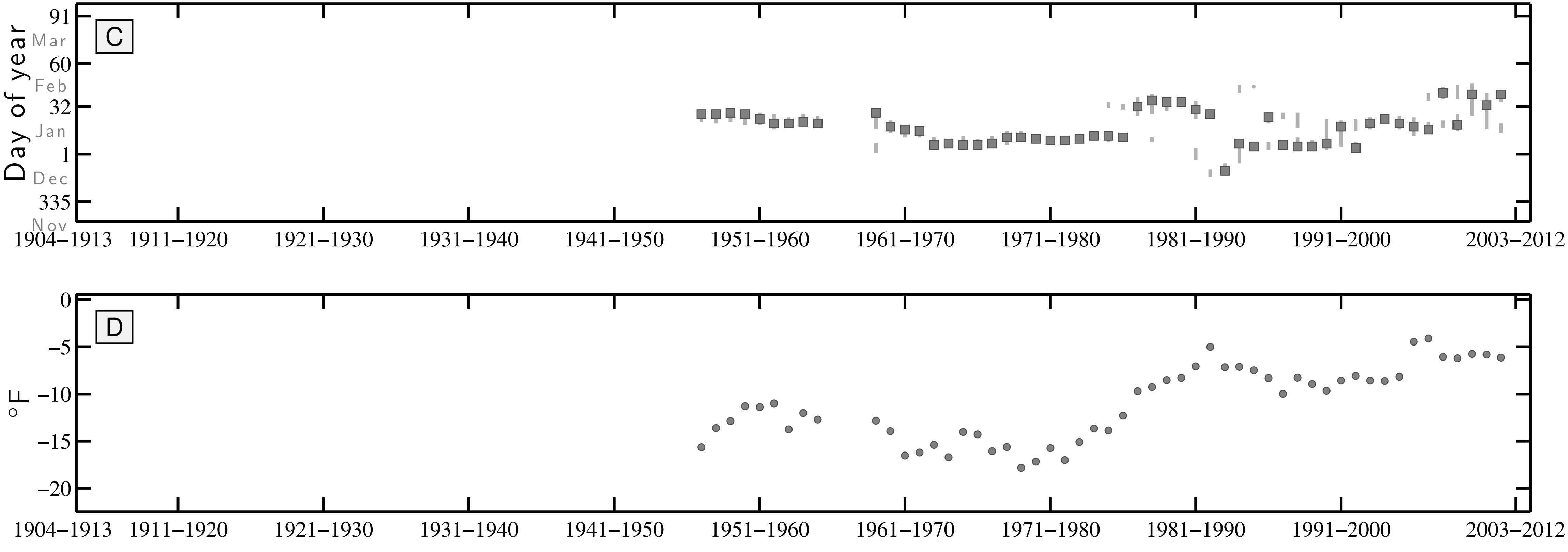

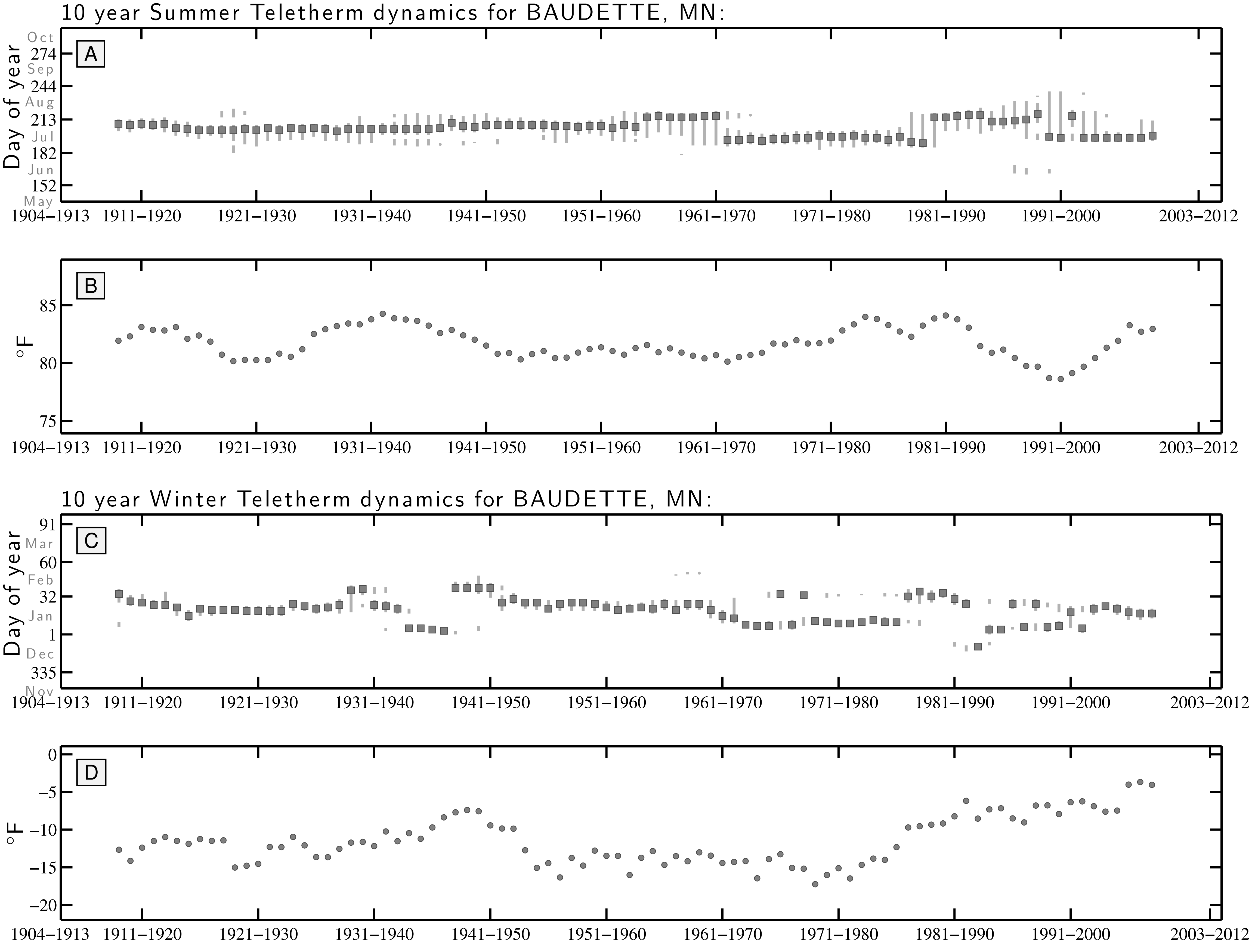

10 year Summer Teletherm dynamics for BAUDETTE, MN:
A
Day of year
Oct
274
Sep
244
Aug
213
Jul
182
Jun
152
May
B
°F
85
80
75
10 year Winter Teletherm dynamics for BAUDETTE, MN:
C
91
Mar
60
Feb
32
Jan
1
Dec
335
Nov
D
0
−5
−10
−15
−20
1904–1913
1911–1920
1921–1930
1931–1940
1941–1950
1951–1960
1961–1970
1971–1980
1981–1990
1991–2000
2003–2012

10 year Summer Teletherm dynamics for CHASKA, MN:

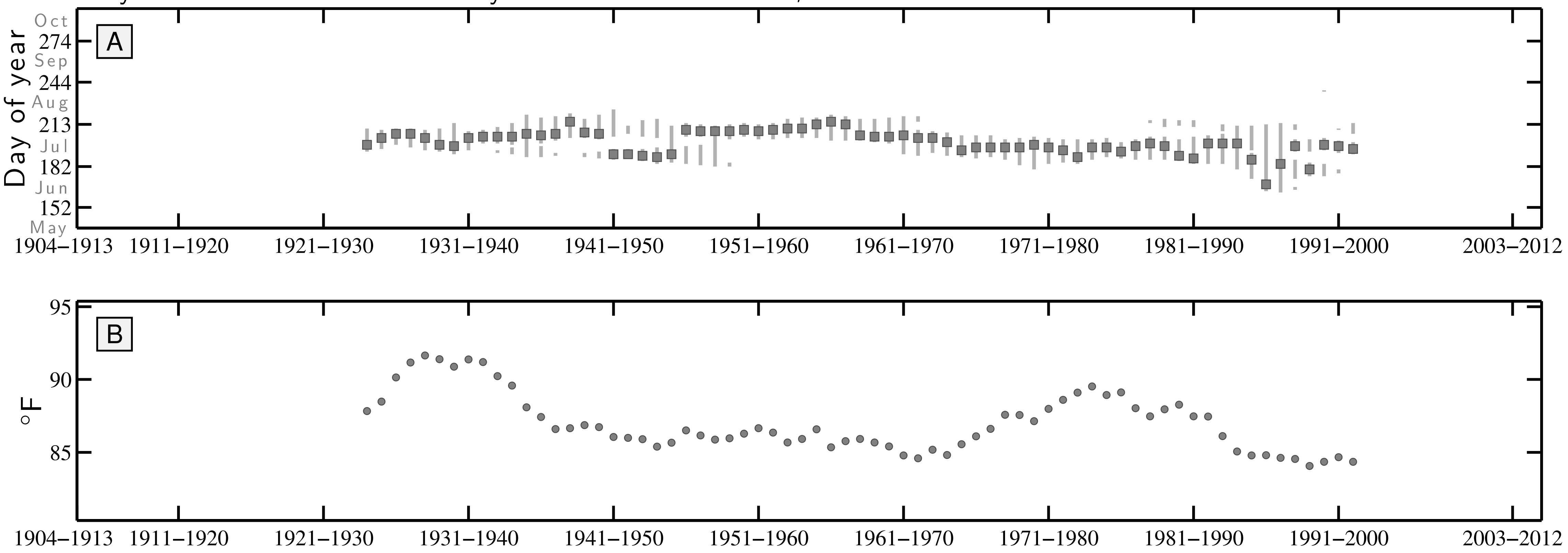

10 year Winter Teletherm dynamics for CHASKA, MN:

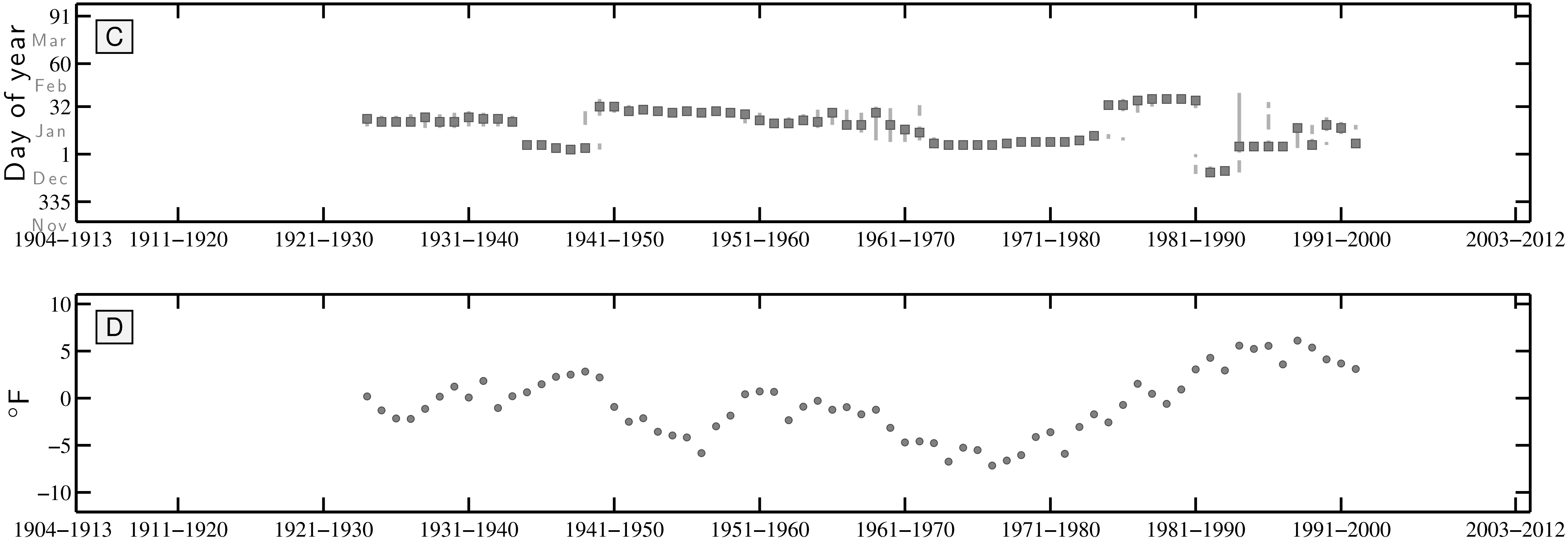

10 year Summer Teletherm dynamics for CLOQUET, MN:

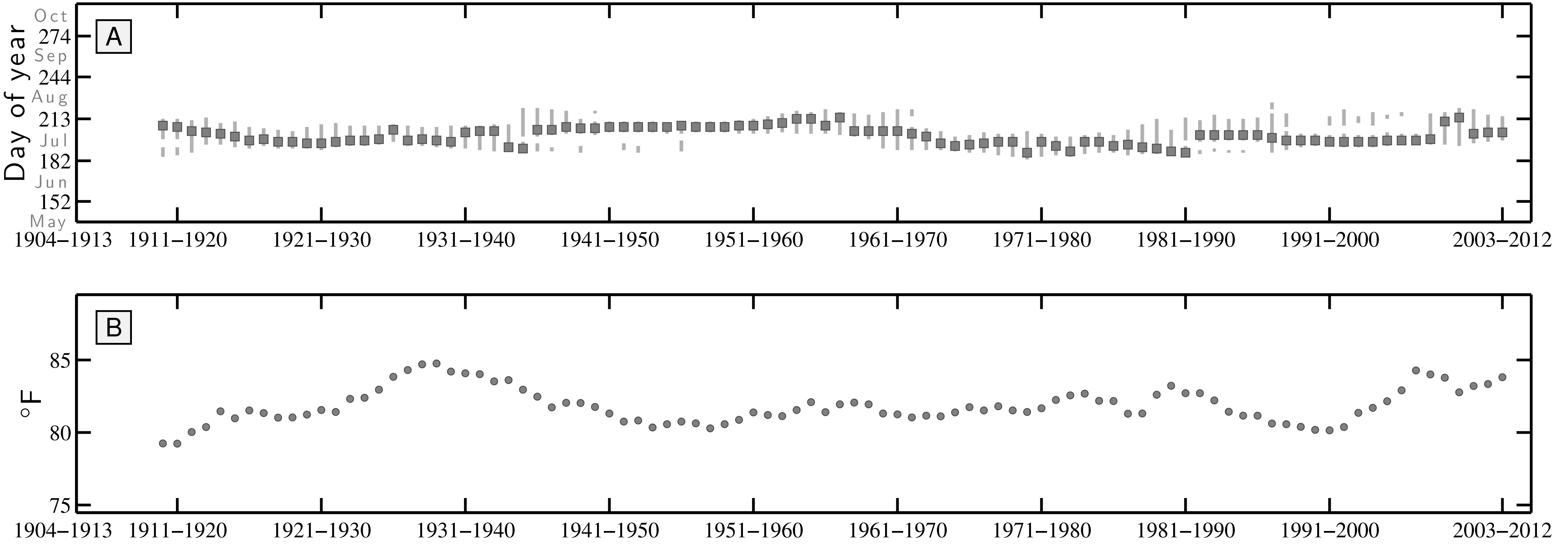

10 year Winter Teletherm dynamics for CLOQUET, MN:

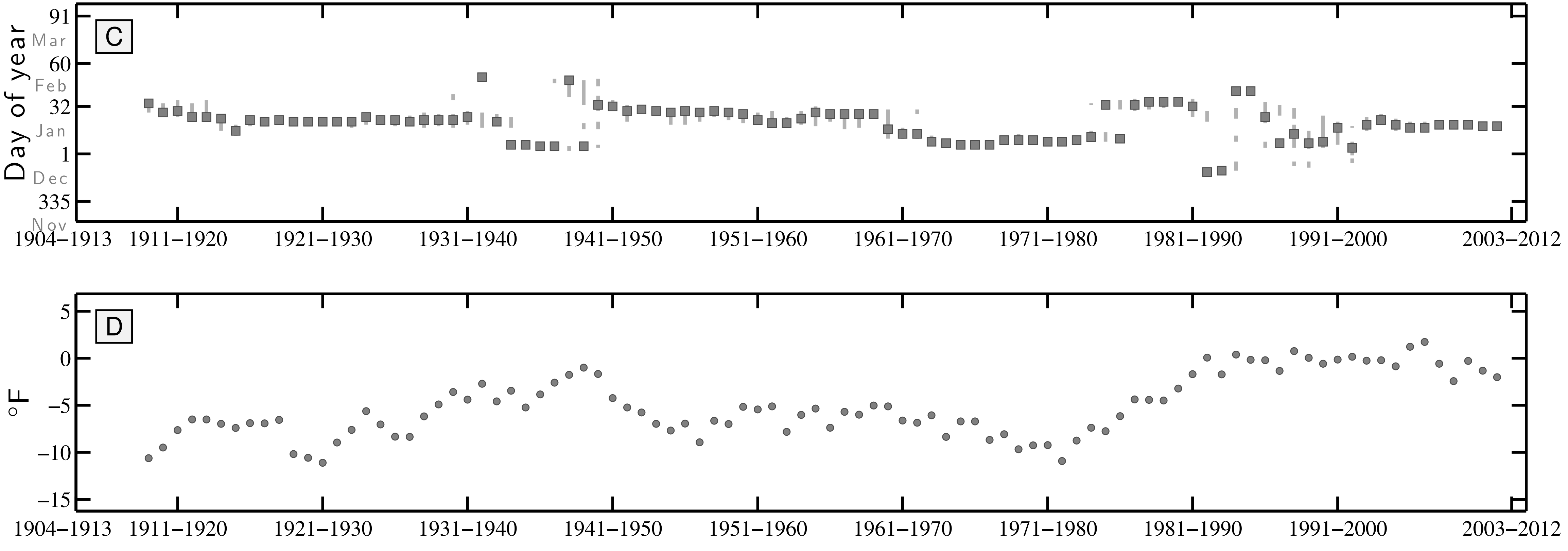

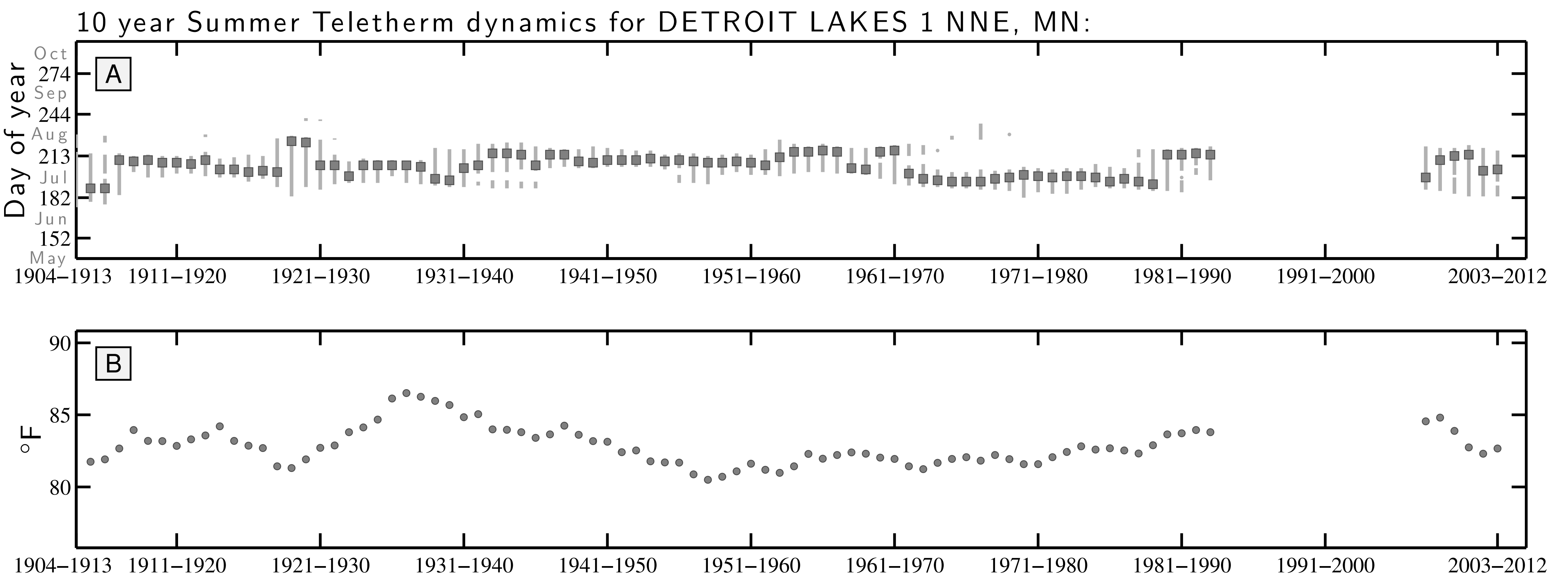
10 year Summer Teletherm dynamics for DETROIT LAKES 1 NNE, MN:
A
B
Day of year
°F
Oct
274
Sep
244
Aug
213
Jul
182
Jun
152
May
90
85
80
1904–1913
1911–1920
1921–1930
1931–1940
1941–1950
1951–1960
1961–1970
1971–1980
1981–1990
1991–2000
2003–2012

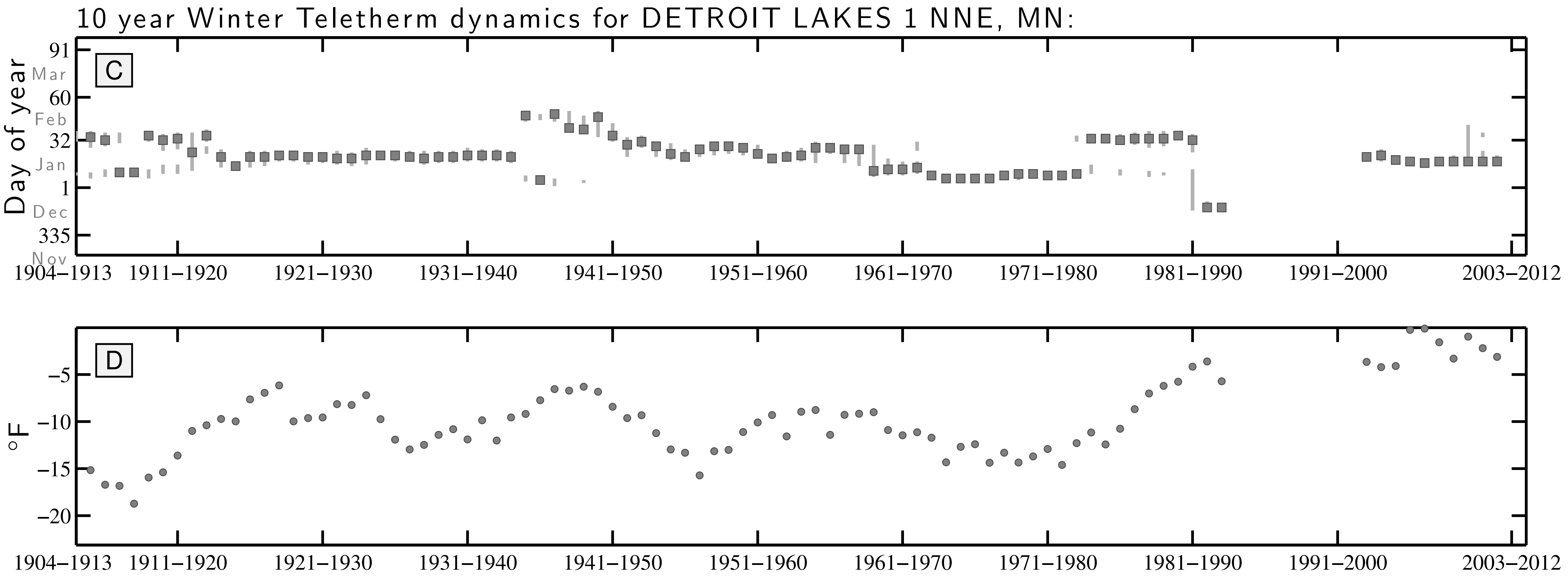
10 year Winter Teletherm dynamics for DETROIT LAKES 1 NNE, MN:
C
D
Day of year
°F
91
Mar
60
Feb
32
Jan
1
Dec
335
Nov
−5
−10
−15
−20
1904–1913
1911–1920
1921–1930
1931–1940
1941–1950
1951–1960
1961–1970
1971–1980
1981–1990
1991–2000
2003–2012

10 year Summer Teletherm dynamics for EVELETH WWTP, MN:

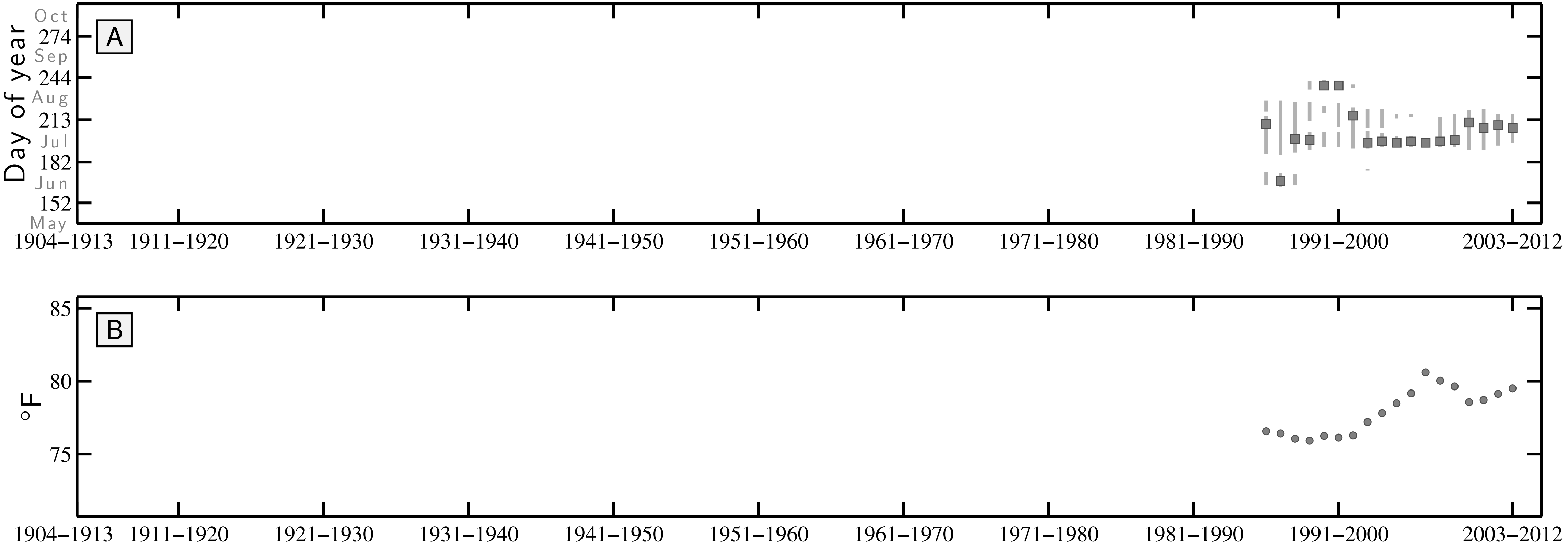

10 year Winter Teletherm dynamics for EVELETH WWTP, MN:

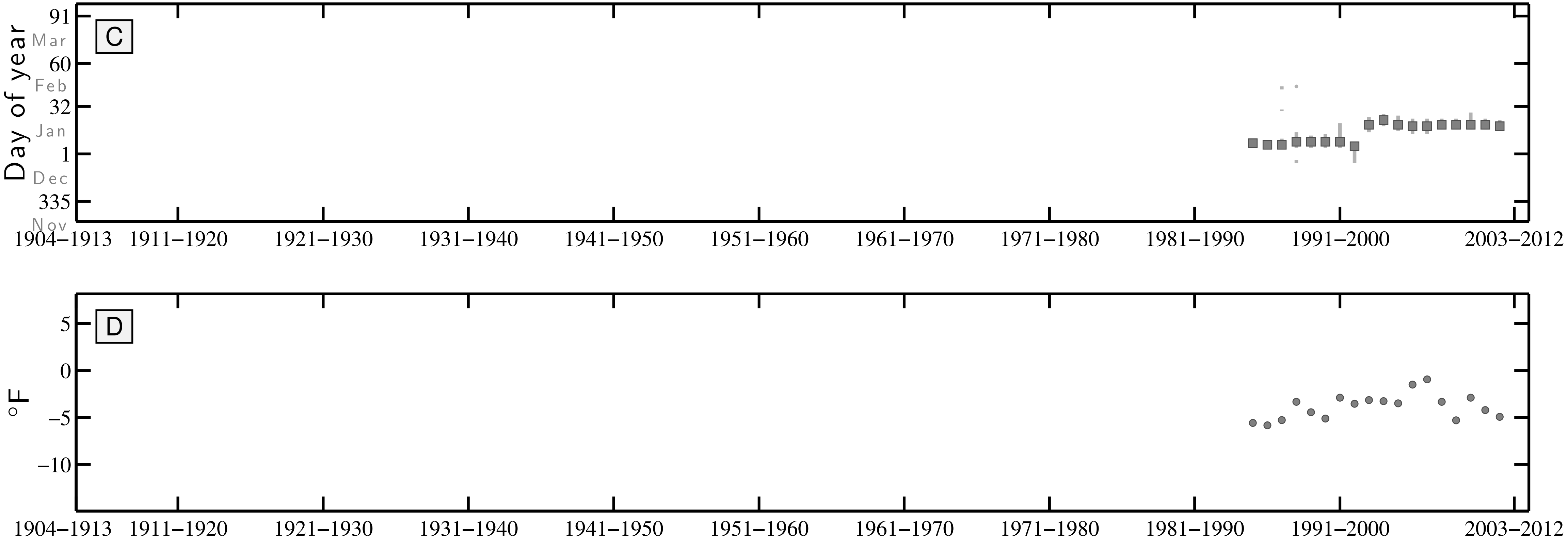

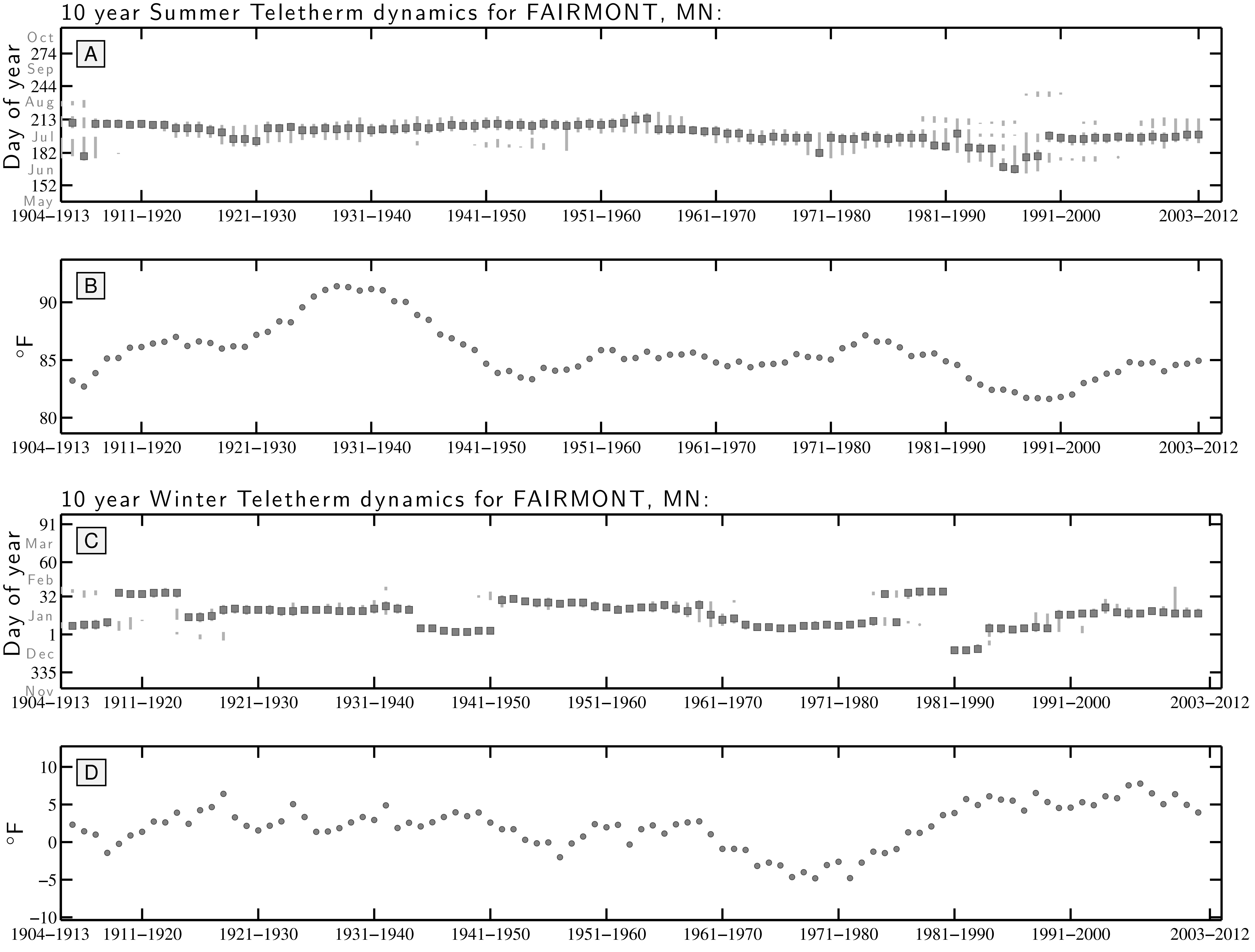
10 year Summer Teletherm dynamics for FAIRMONT, MN:
A
B
Day of year
°F
Oct
274
Sep
244
Aug
213
Jul
182
Jun
152
May
90
85
80
10 year Winter Teletherm dynamics for FAIRMONT, MN:
C
D
91
Mar
60
Feb
32
Jan
1
Dec
335
Nov
10
5
0
−5
−10
1904–1913
1911–1920
1921–1930
1931–1940
1941–1950
1951–1960
1961–1970
1971–1980
1981–1990
1991–2000
2003–2012

10 year Summer Teletherm dynamics for FARMINGTON 3 NW, MN:

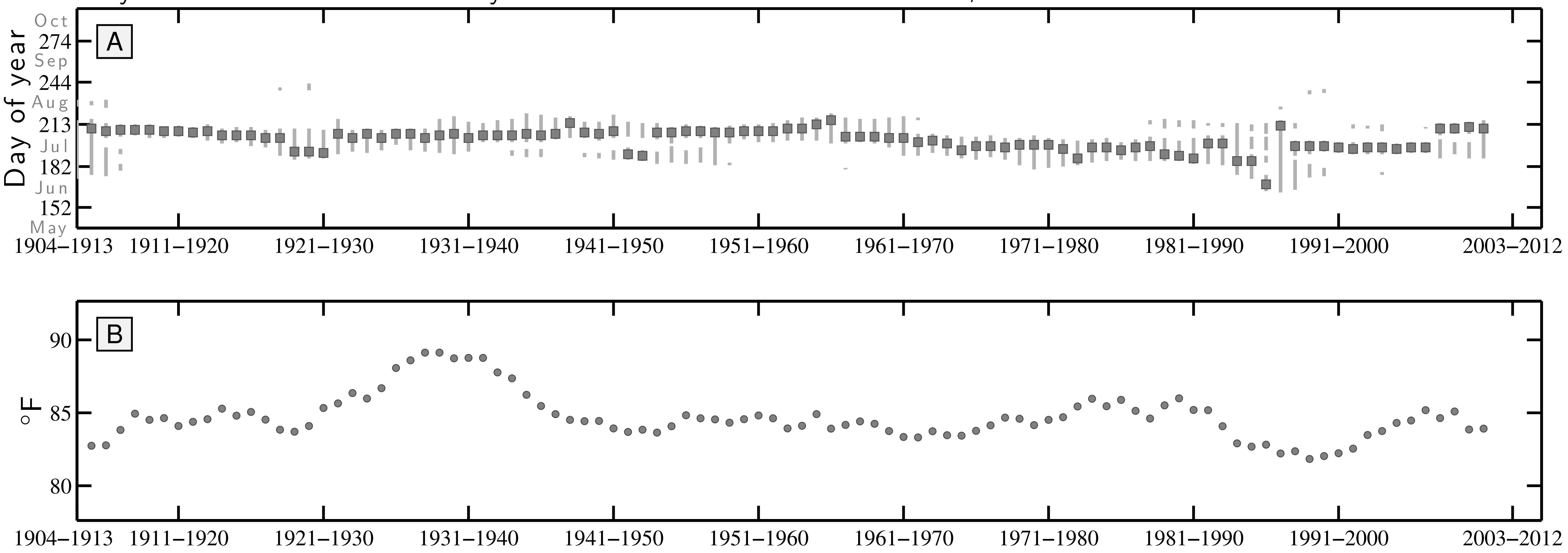

10 year Winter Teletherm dynamics for FARMINGTON 3 NW, MN:

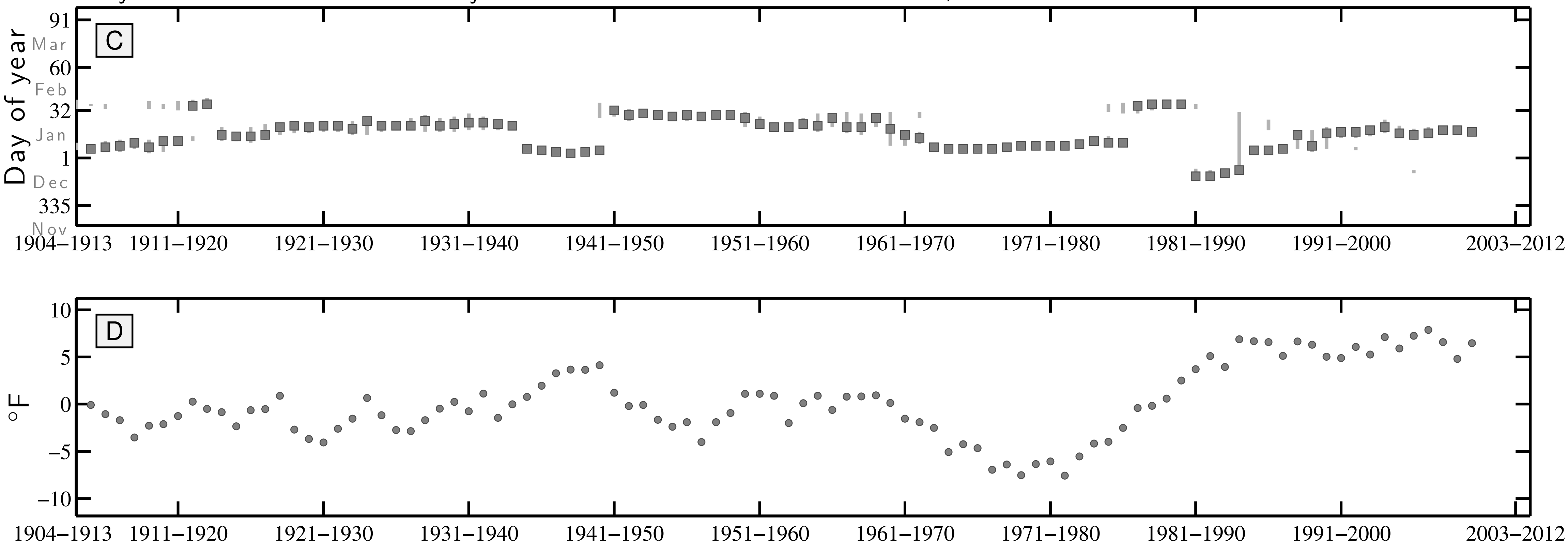

10 year Summer Teletherm dynamics for FOSSTON 1 E, MN:

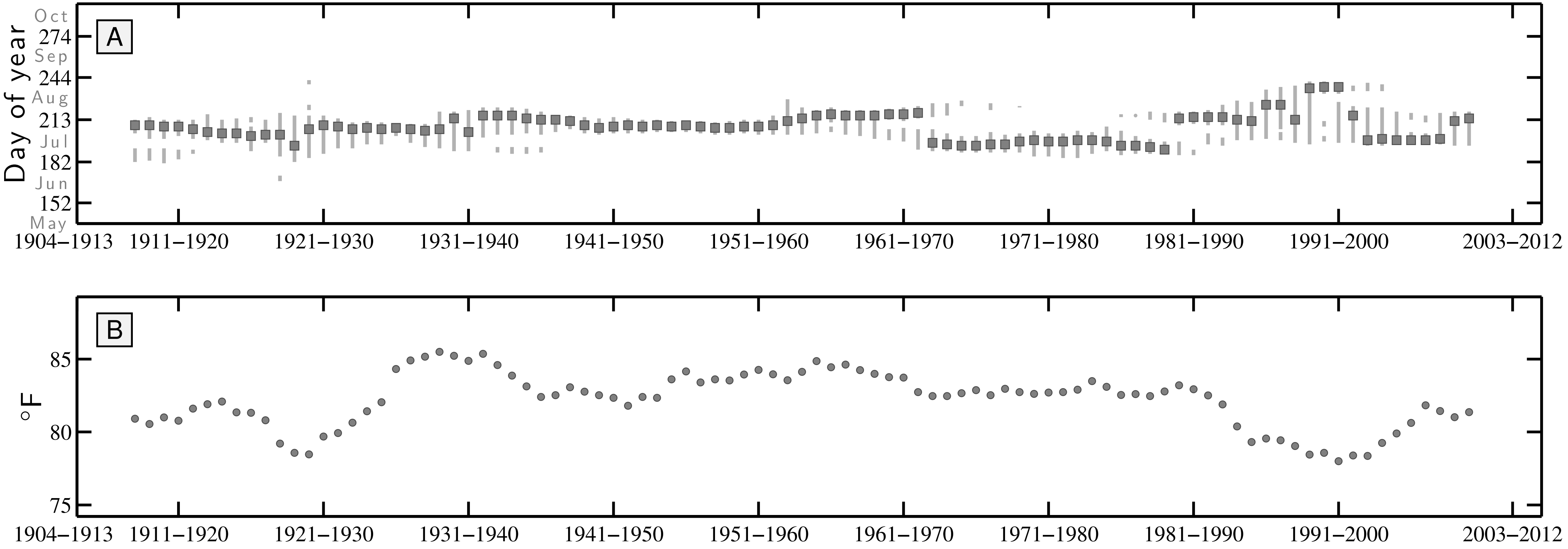

10 year Winter Teletherm dynamics for FOSSTON 1 E, MN:

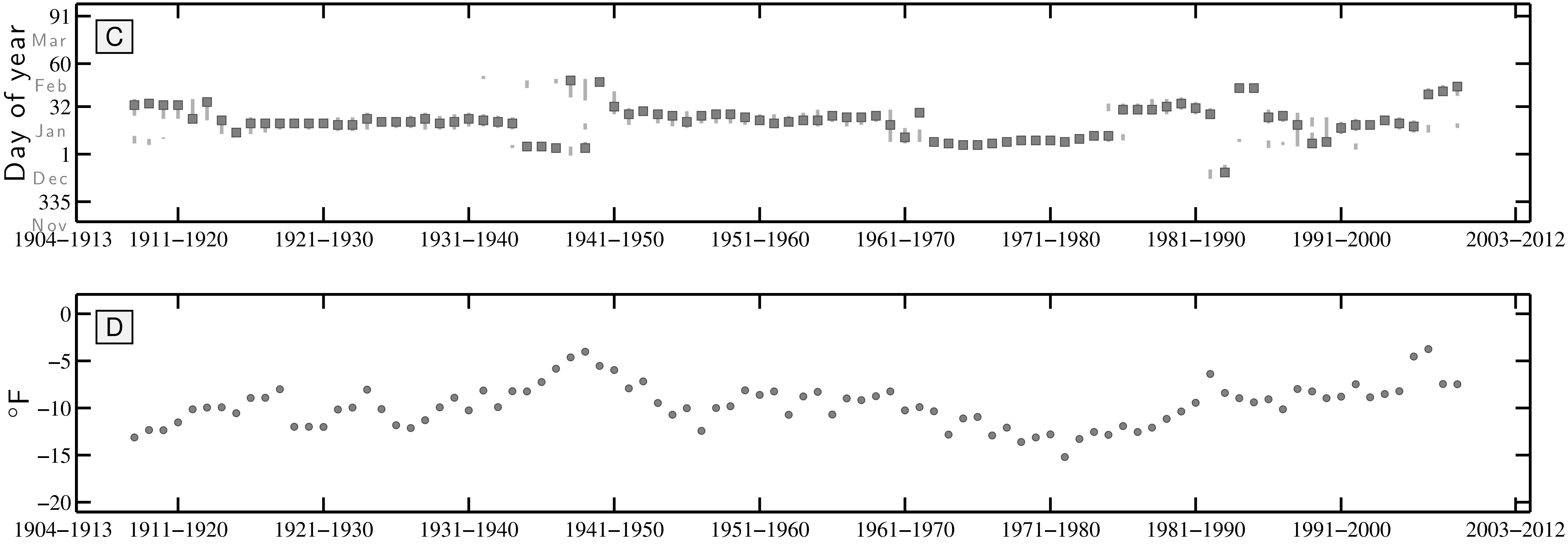

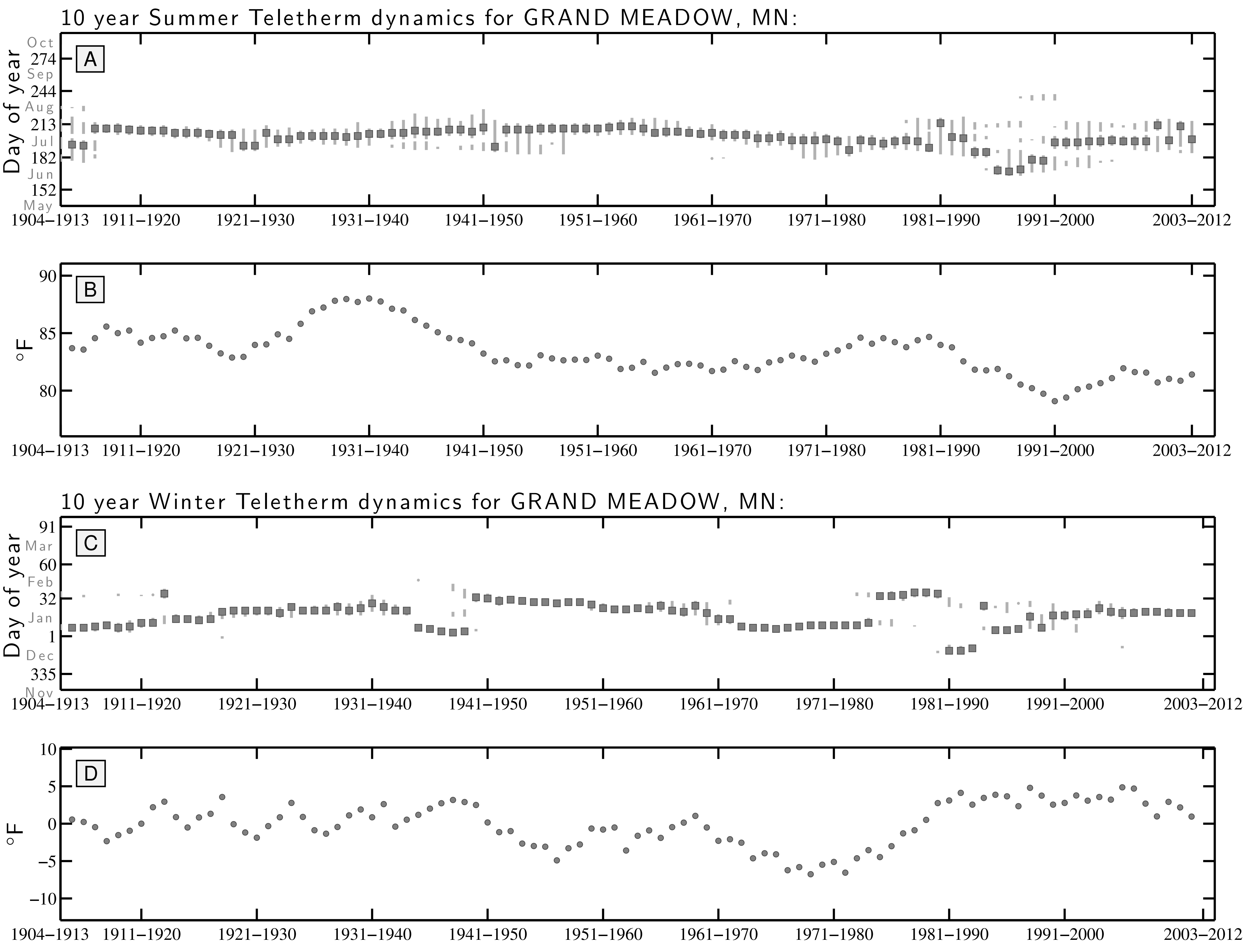

10 year Summer Teletherm dynamics for GRAND MEADOW, MN:
A
Day of year
Oct
274
Sep
244
Aug
213
Jul
182
Jun
152
May
1904–1913
1911–1920
1921–1930
1931–1940
1941–1950
1951–1960
1961–1970
1971–1980
1981–1990
1991–2000
2003–2012
B
°F
90
85
80
10 year Winter Teletherm dynamics for GRAND MEADOW, MN:
C
91
Mar
60
Feb
32
Jan
1
Dec
335
Nov
D
10
5
0
−5
−10

10 year Summer Teletherm dynamics for GRAND RPDS FOREST LAB, MN:

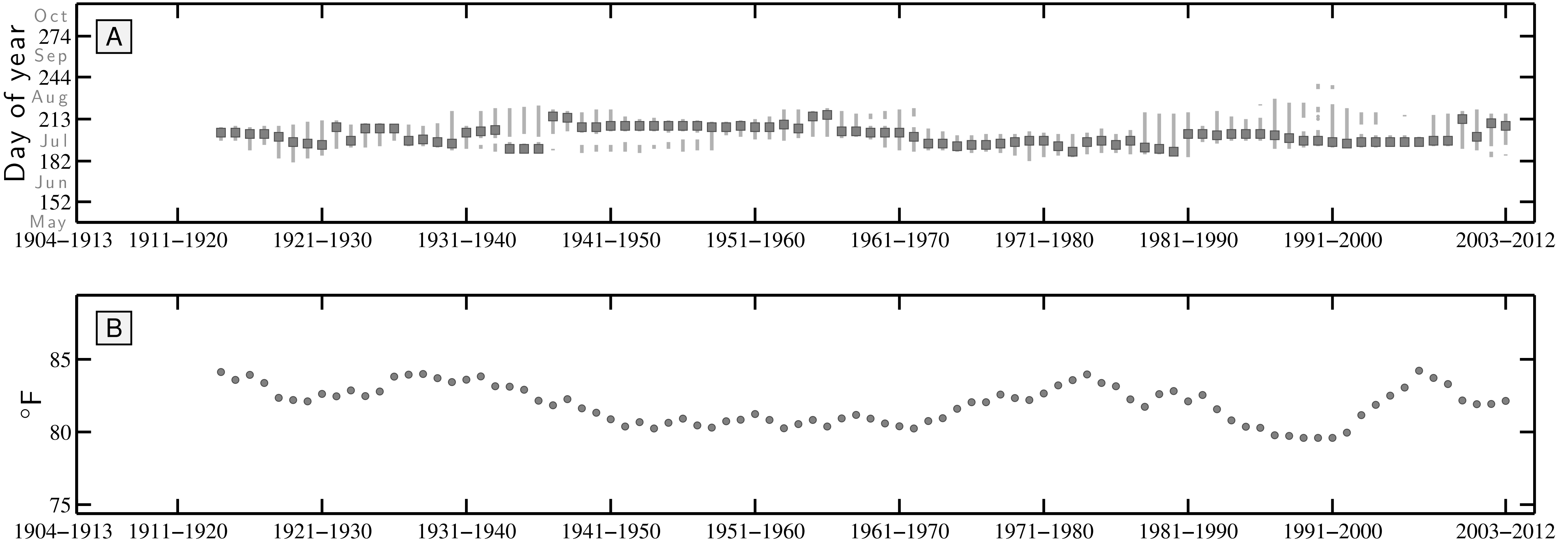

10 year Winter Teletherm dynamics for GRAND RPDS FOREST LAB, MN:

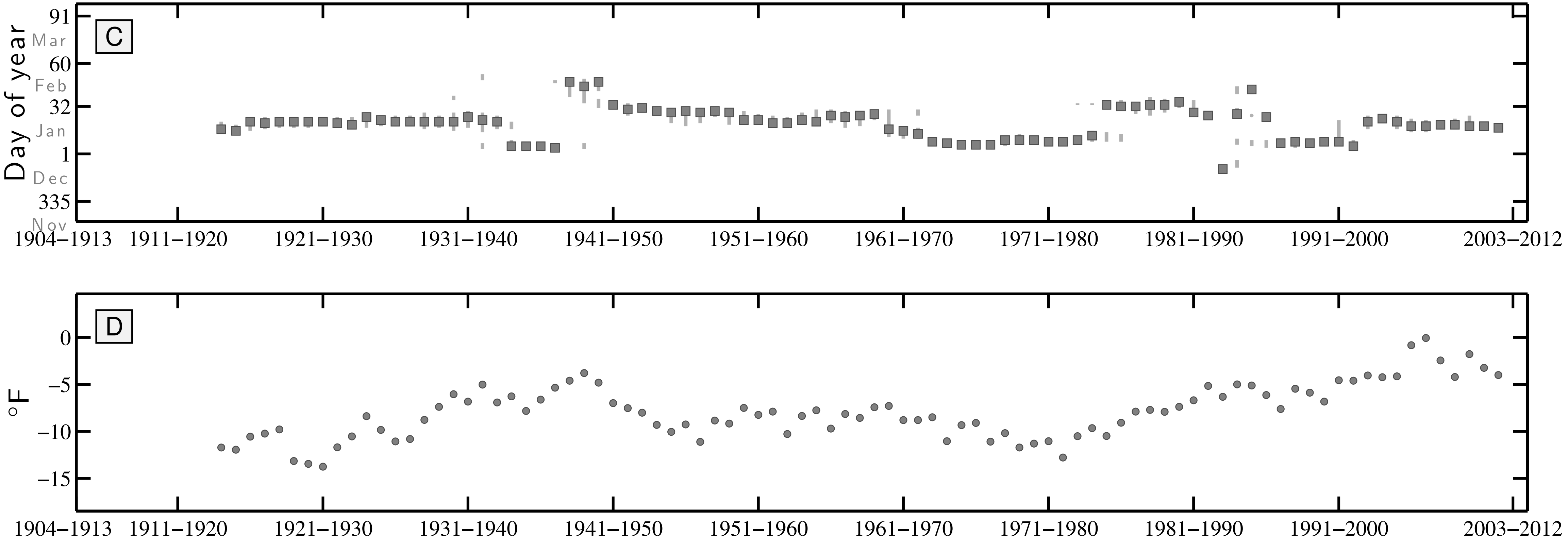

10 year Summer Teletherm dynamics for ITASCA UNIV OF MINN, MN:

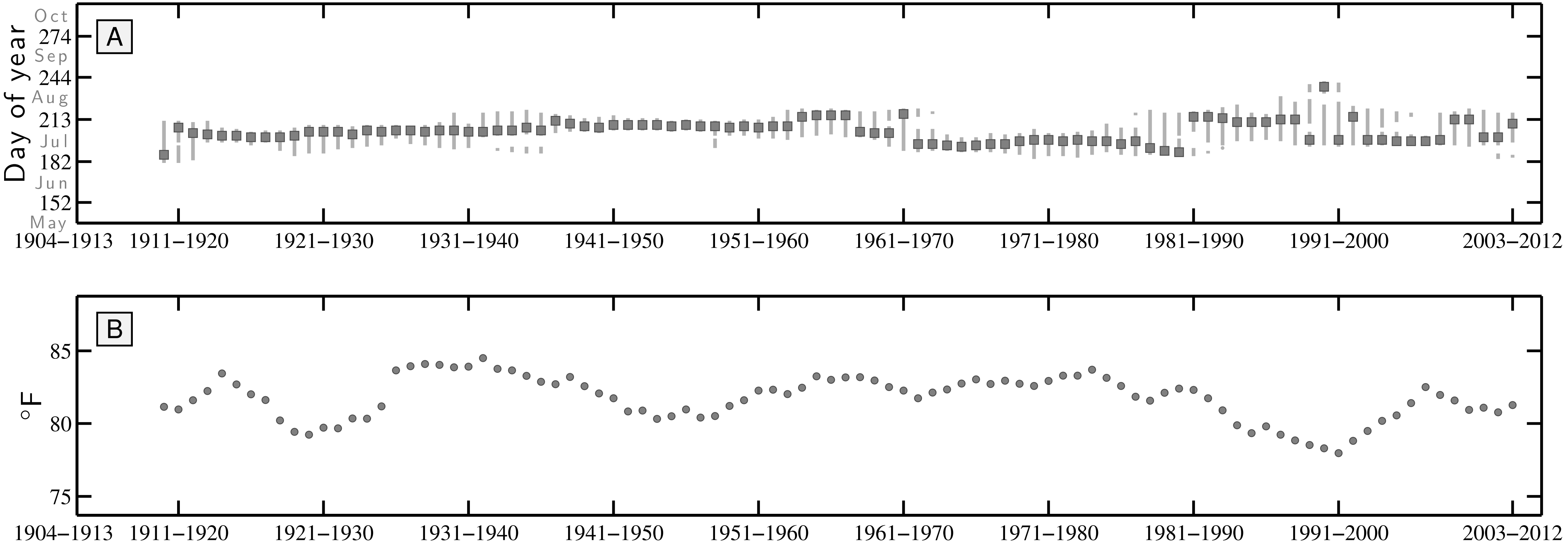

10 year Winter Teletherm dynamics for ITASCA UNIV OF MINN, MN:

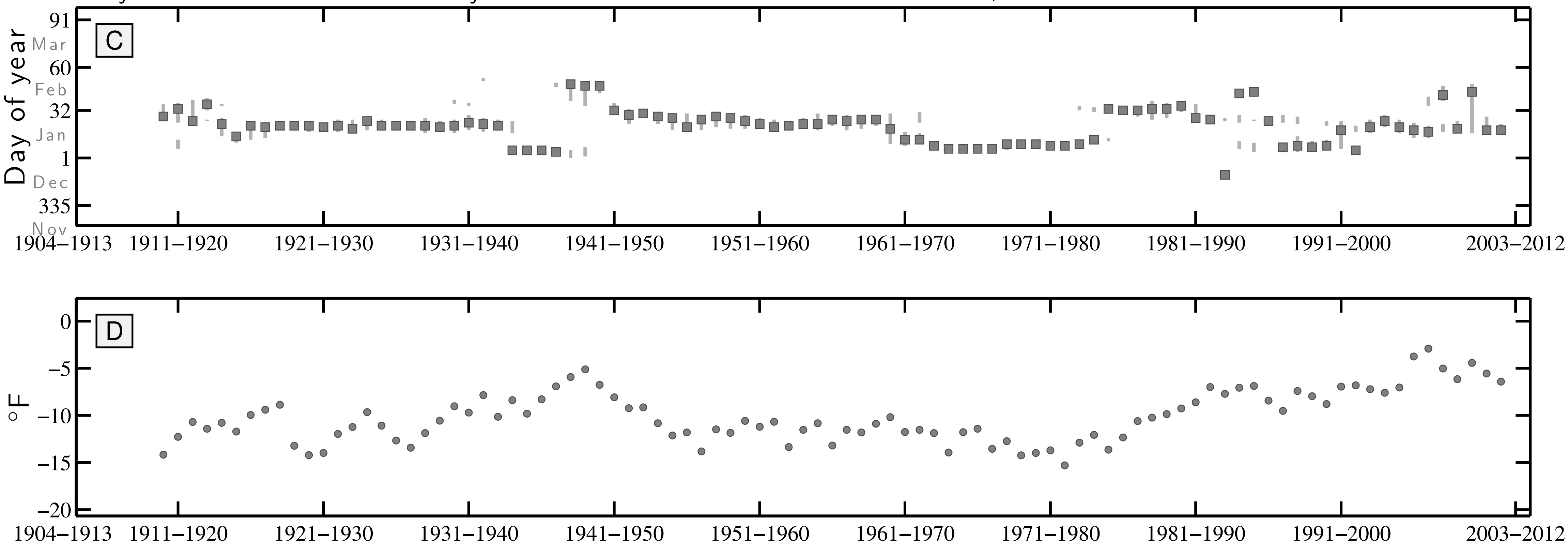

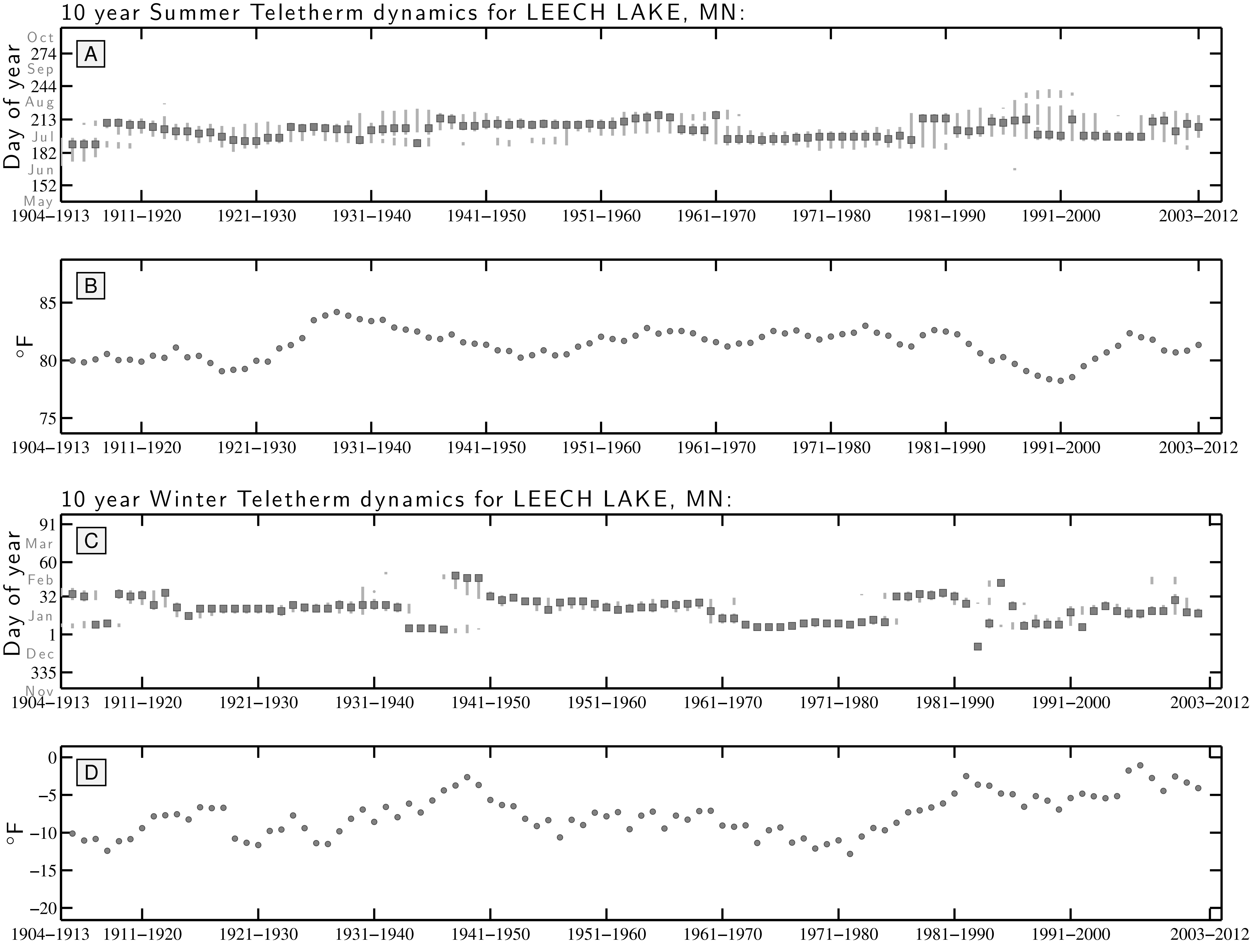
10 year Summer Teletherm dynamics for LEECH LAKE, MN:
A
Day of year
Oct
274
Sep
244
Aug
213
Jul
182
Jun
152
May
1904–1913
1911–1920
1921–1930
1931–1940
1941–1950
1951–1960
1961–1970
1971–1980
1981–1990
1991–2000
2003–2012
B
°F
85
80
75
10 year Winter Teletherm dynamics for LEECH LAKE, MN:
C
91
Mar
60
Feb
32
Jan
1
Dec
335
Nov
D
0
−5
−10
−15
−20

10 year Summer Teletherm dynamics for MARCELL 5NE, MN:

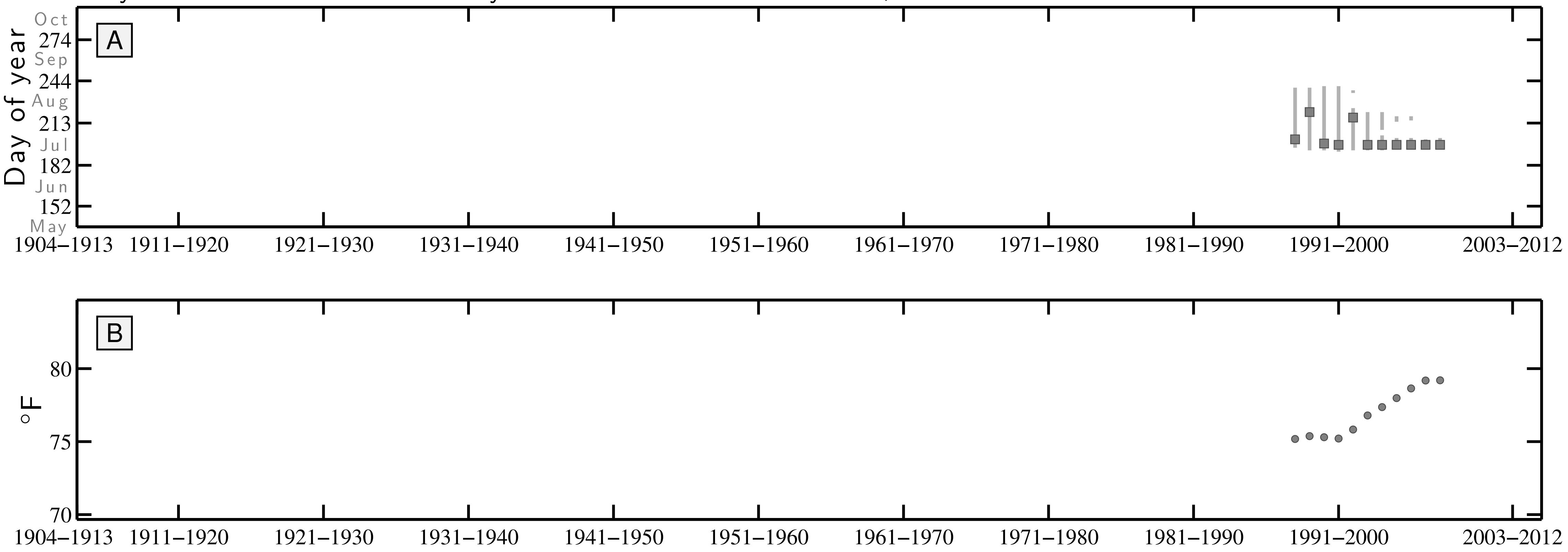

10 year Winter Teletherm dynamics for MARCELL 5NE, MN:

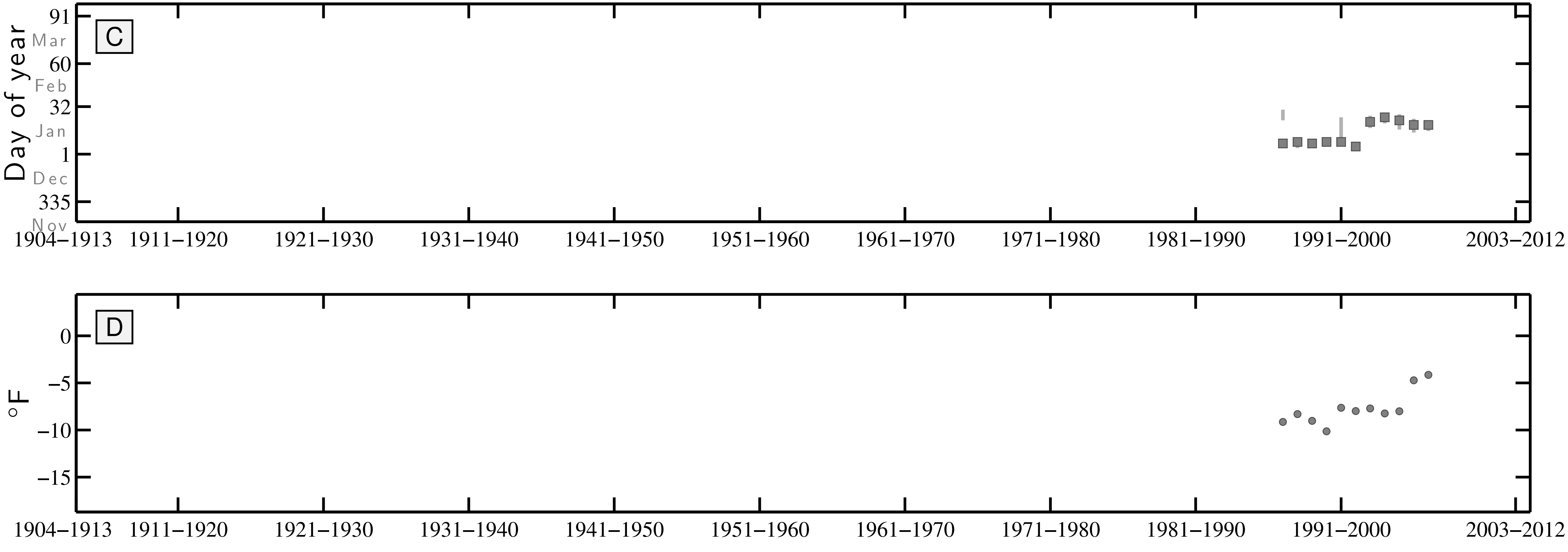

10 year Summer Teletherm dynamics for MILAN 1 NW, MN:

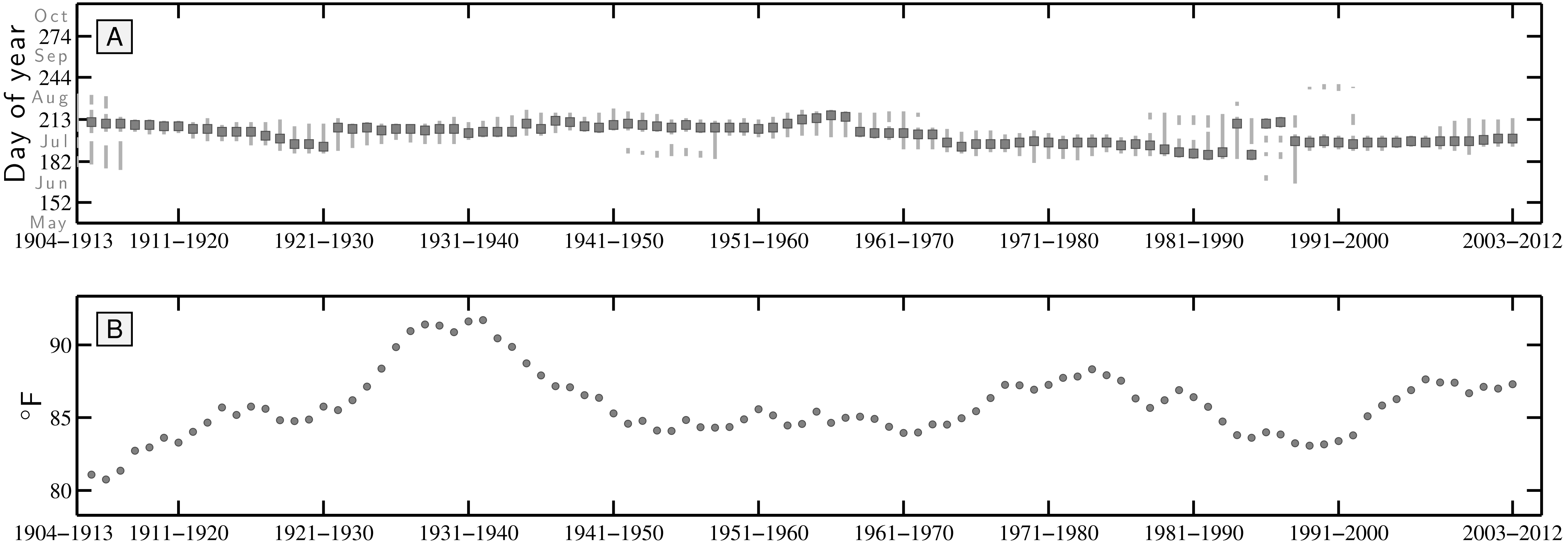

10 year Winter Teletherm dynamics for MILAN 1 NW, MN:

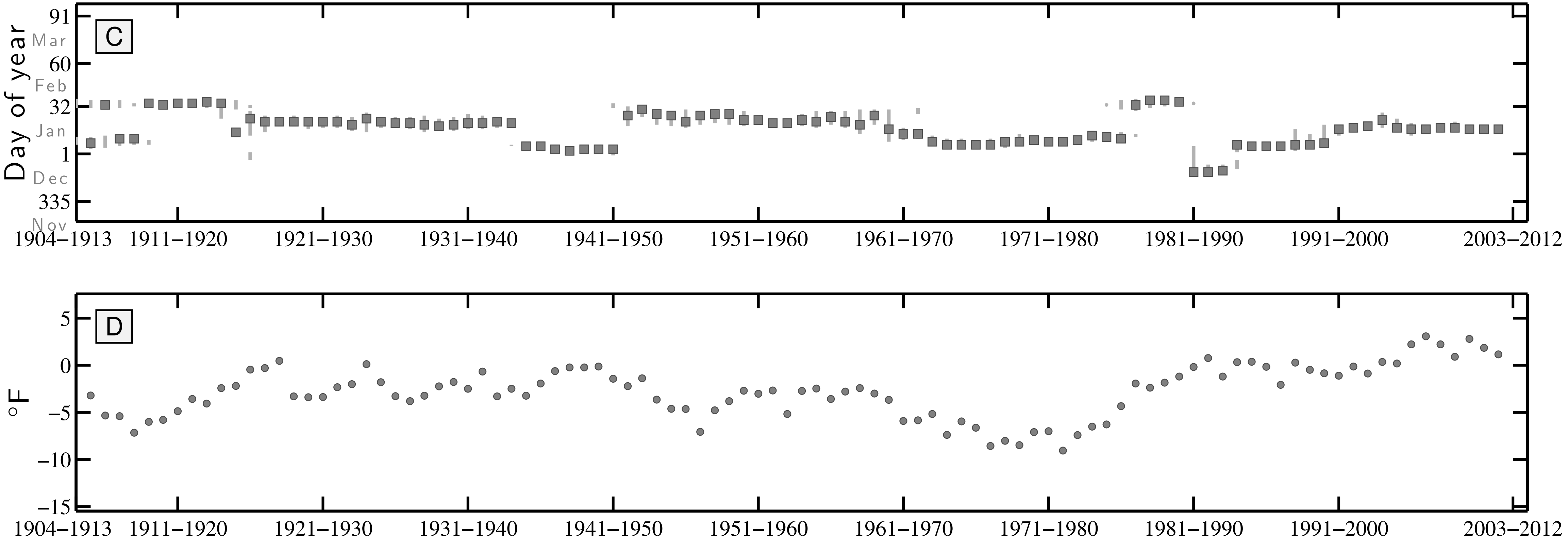

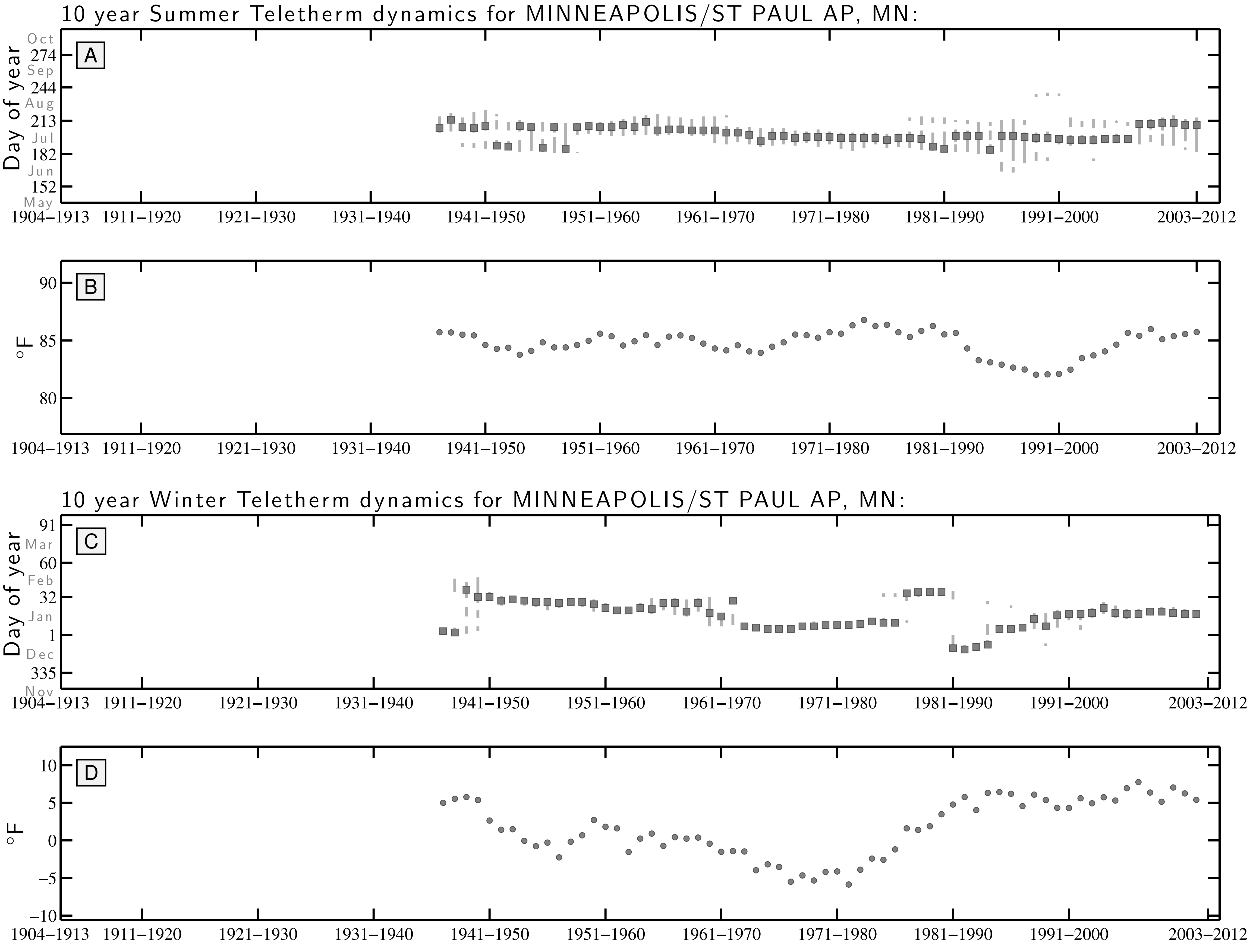

10 year Summer Teletherm dynamics for MINNEAPOLIS/ST PAUL AP, MN:
A
B
Day of year
°F
Oct
274
Sep
244
Aug
213
Jul
182
Jun
152
May
90
85
80
10 year Winter Teletherm dynamics for MINNEAPOLIS/ST PAUL AP, MN:
C
D
91
Mar
60
Feb
32
Jan
1
Dec
335
Nov
10
5
0
−5
−10
1904–1913
1911–1920
1921–1930
1931–1940
1941–1950
1951–1960
1961–1970
1971–1980
1981–1990
1991–2000
2003–2012

10 year Summer Teletherm dynamics for MONTEVIDEO 1 SW, MN:

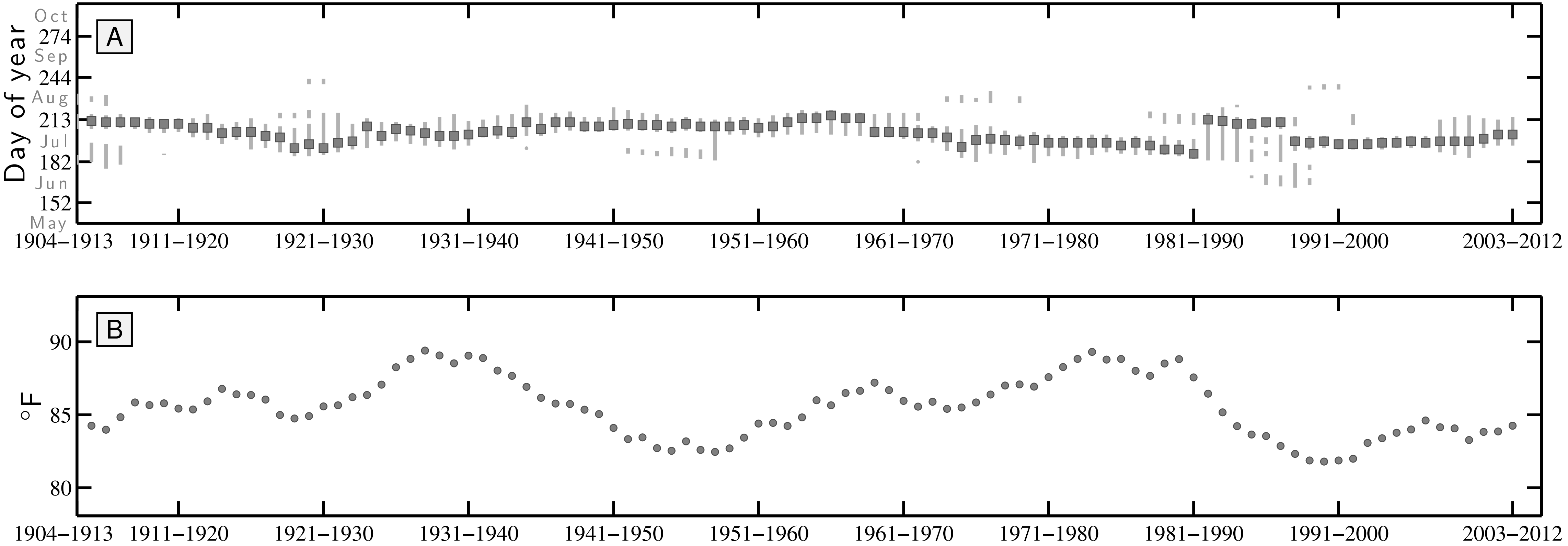

10 year Winter Teletherm dynamics for MONTEVIDEO 1 SW, MN:

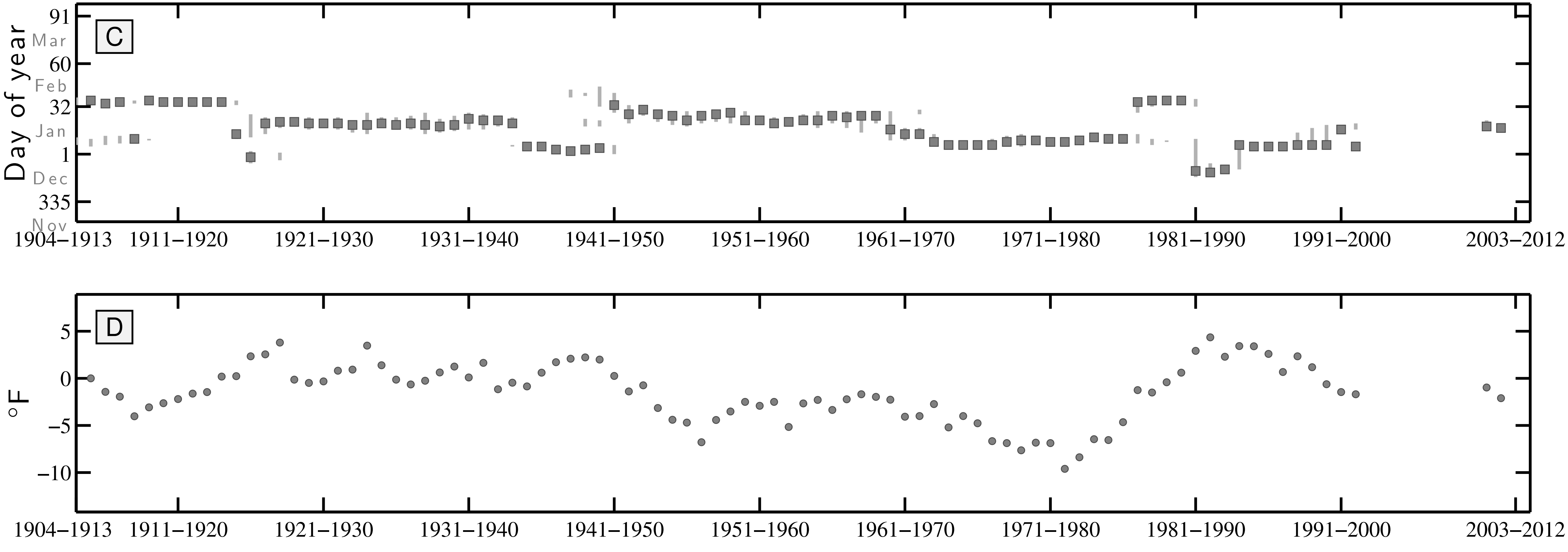

10 year Summer Teletherm dynamics for MORA, MN:

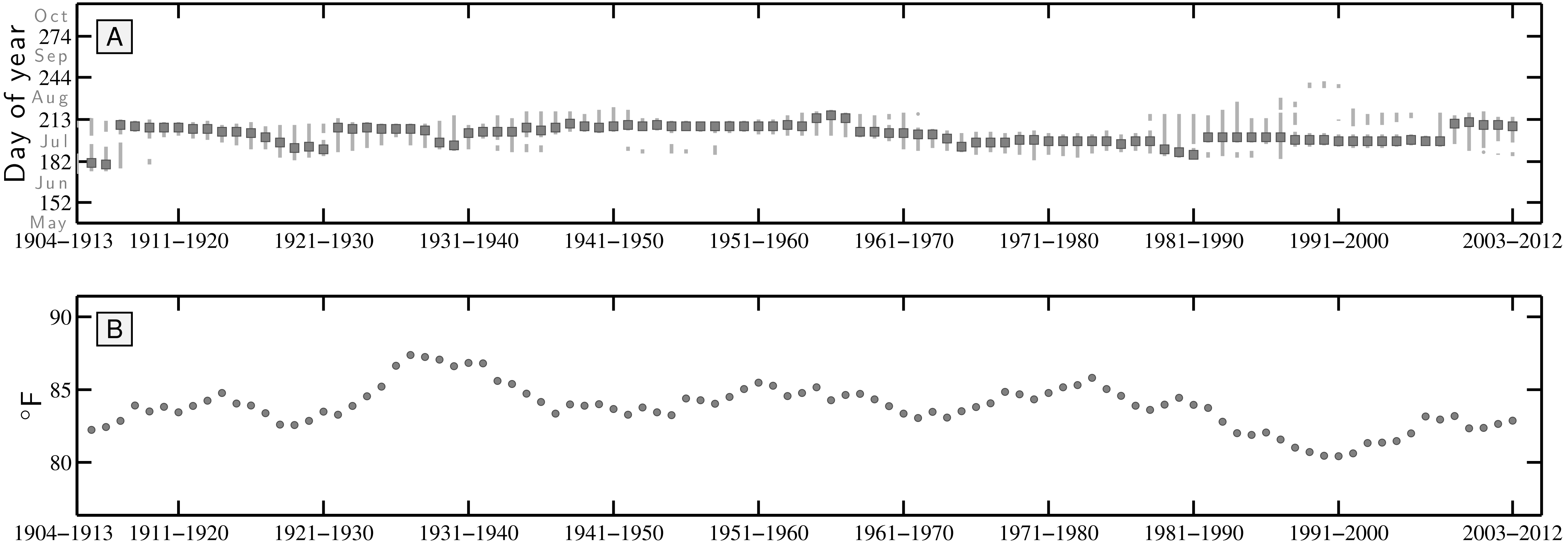

10 year Winter Teletherm dynamics for MORA, MN:

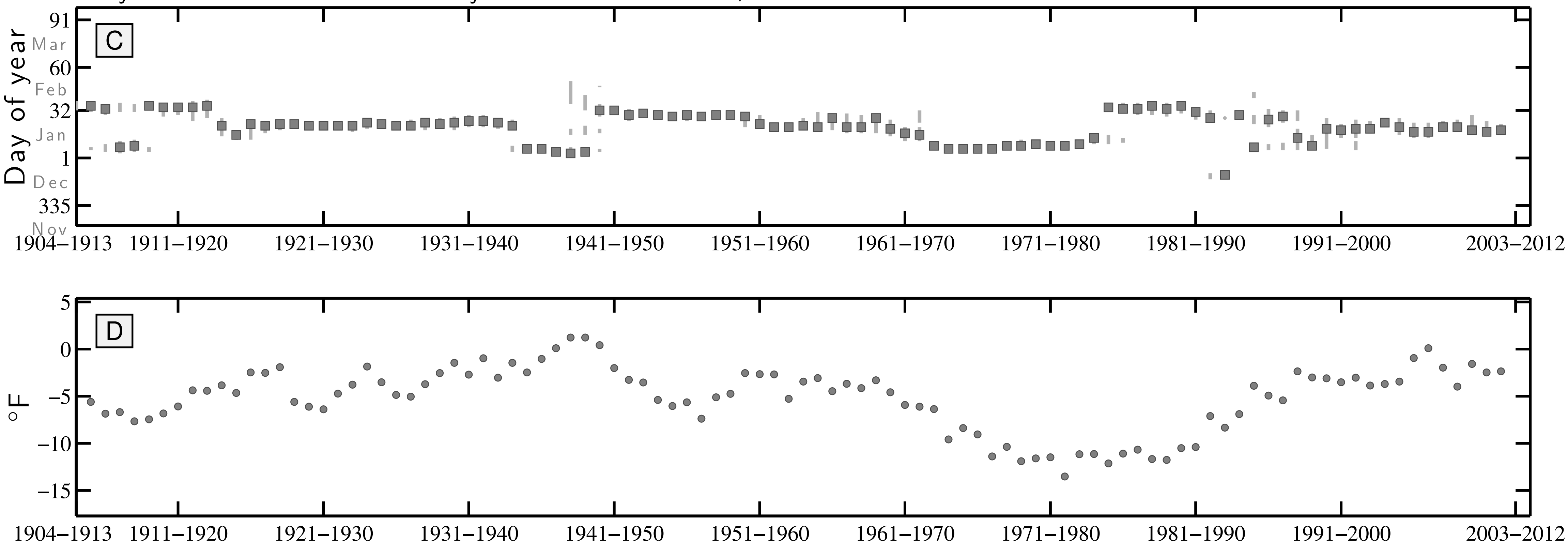

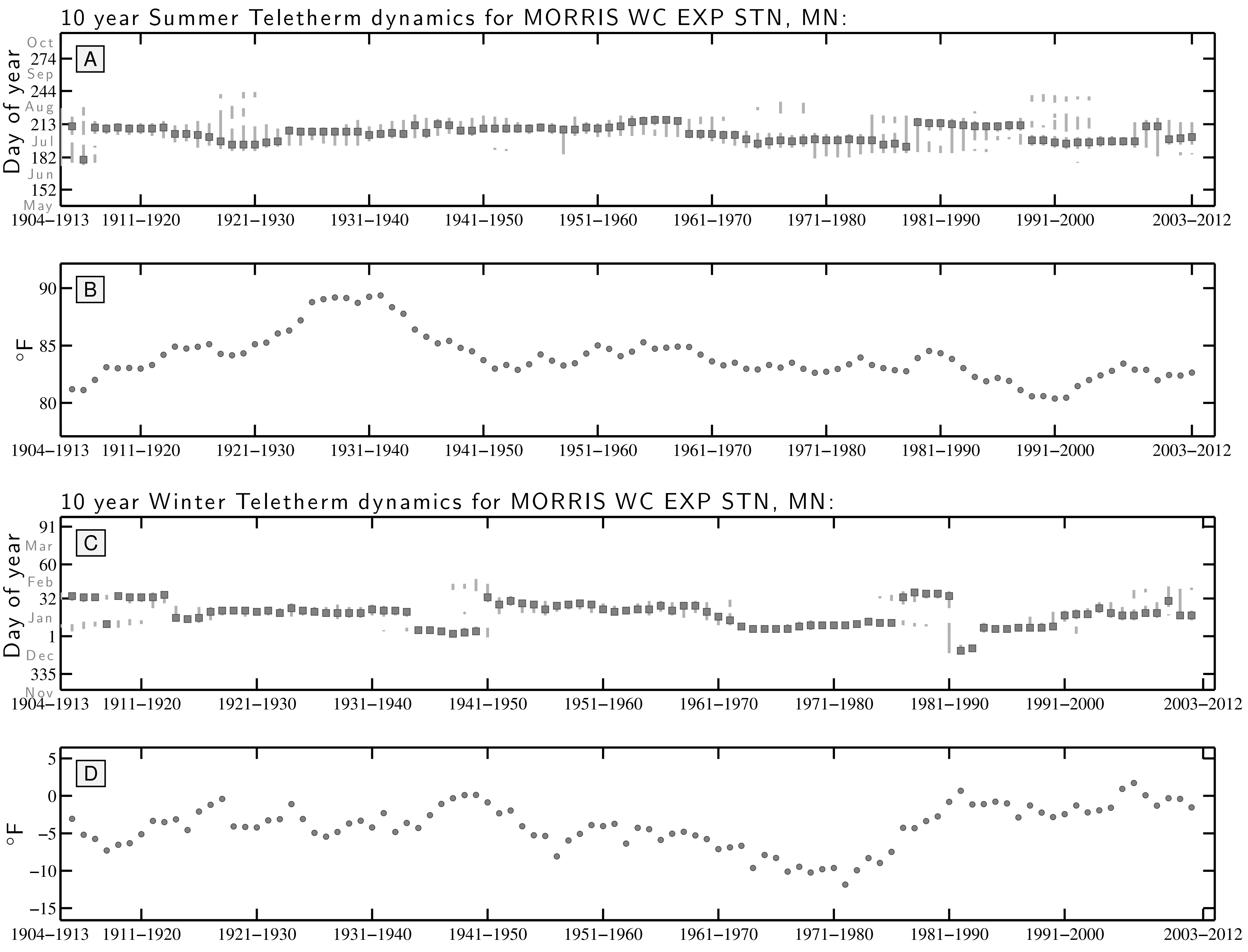
10 year Summer Teletherm dynamics for MORRIS WC EXP STN, MN:
A
Day of year
Oct
274
Sep
244
Aug
213
Jul
182
Jun
152
May
1904–1913
1911–1920
1921–1930
1931–1940
1941–1950
1951–1960
1961–1970
1971–1980
1981–1990
1991–2000
2003–2012
B
°F
90
85
80
10 year Winter Teletherm dynamics for MORRIS WC EXP STN, MN:
C
91
Mar
60
Feb
32
Jan
1
Dec
335
Nov
D
5
0
−5
−10
−15

10 year Summer Teletherm dynamics for NEW ULM 2 SE, MN:

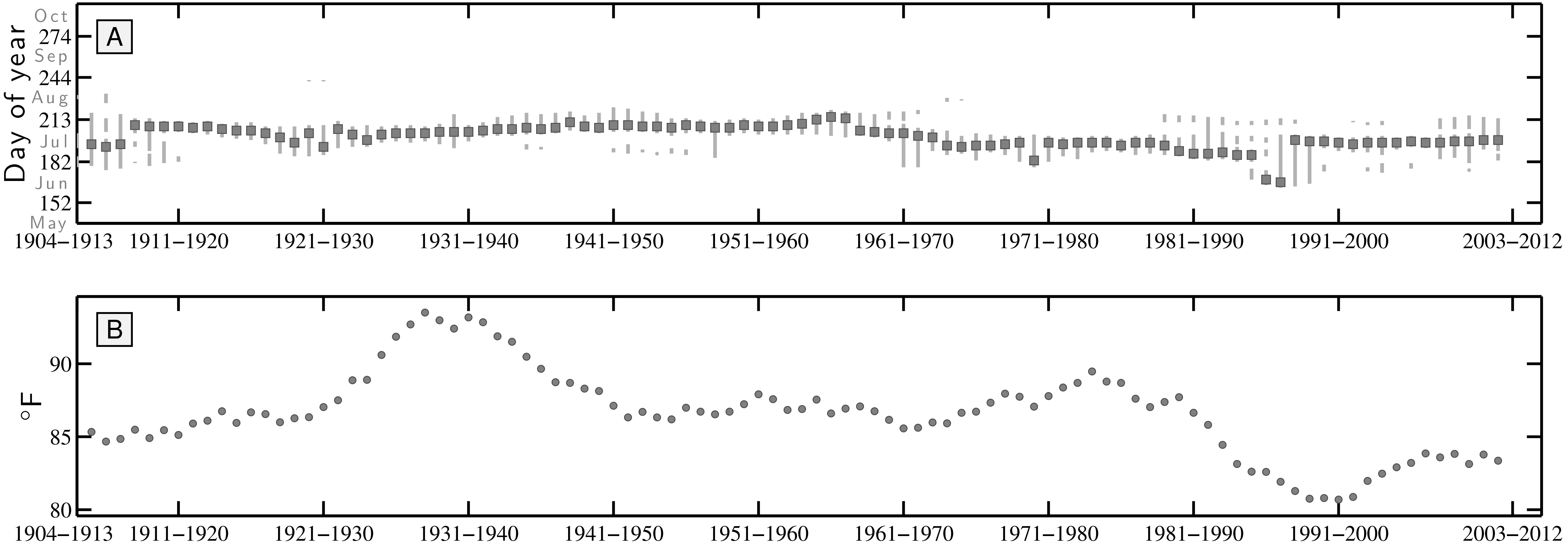

10 year Winter Teletherm dynamics for NEW ULM 2 SE, MN:

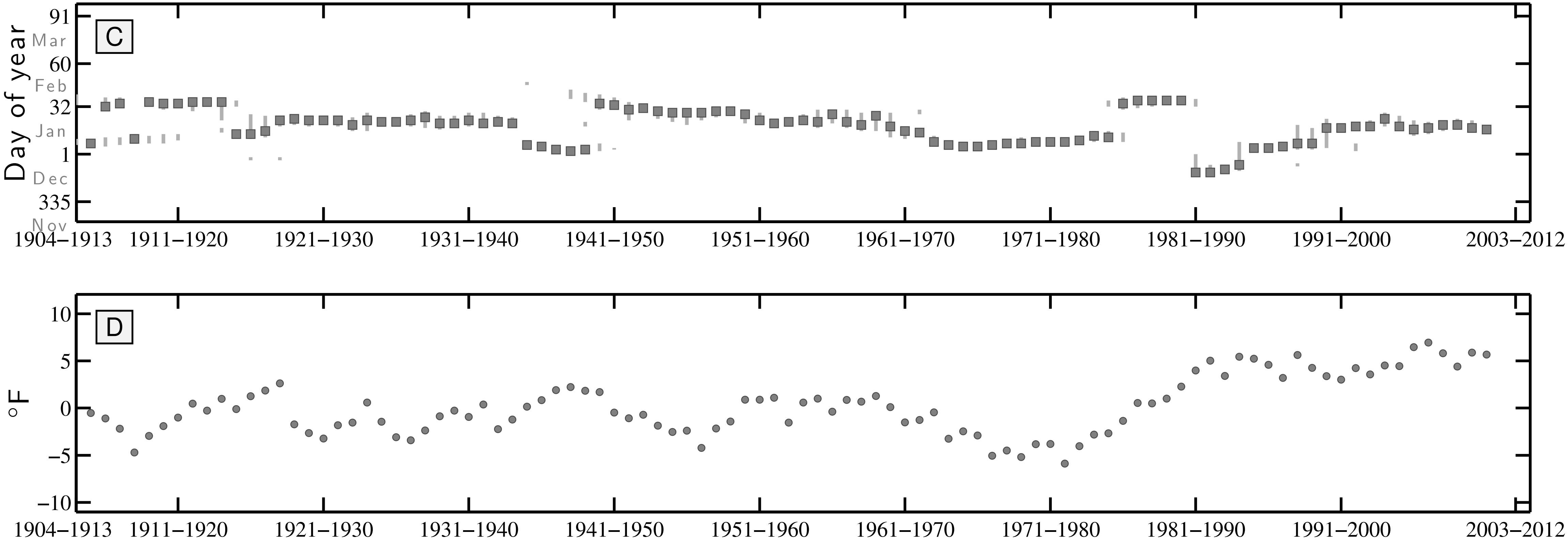

10 year Summer Teletherm dynamics for OLIVIA 3E, MN:

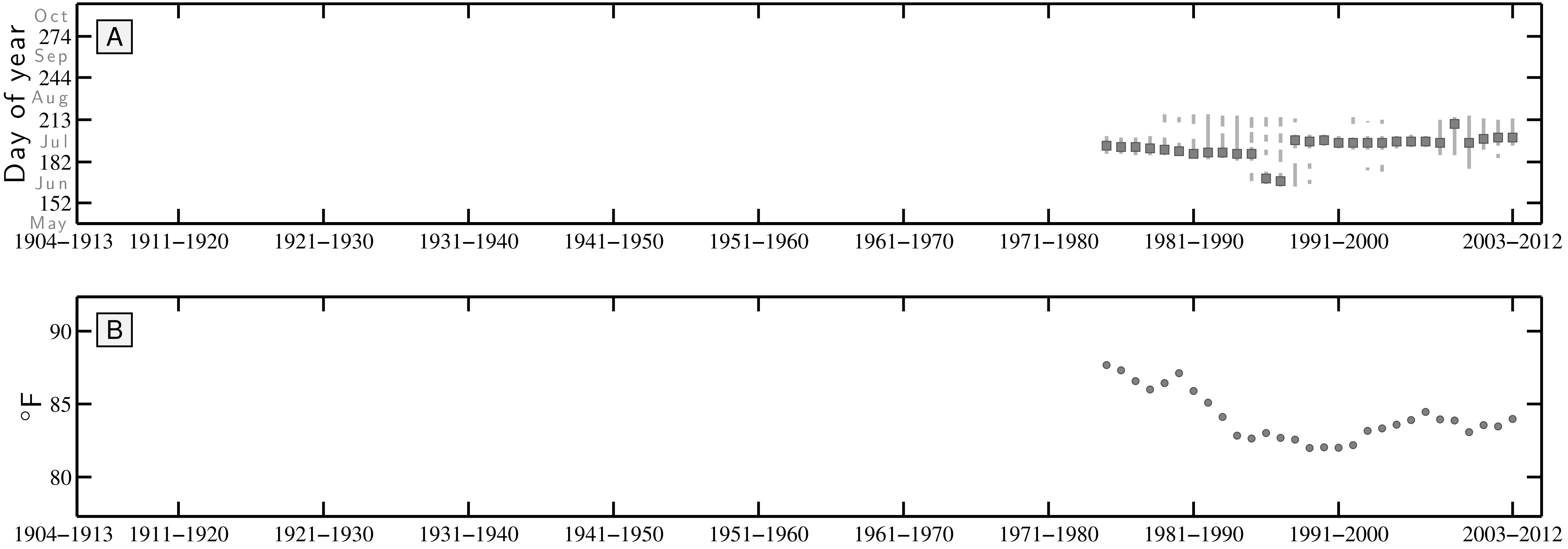

10 year Winter Teletherm dynamics for OLIVIA 3E, MN:

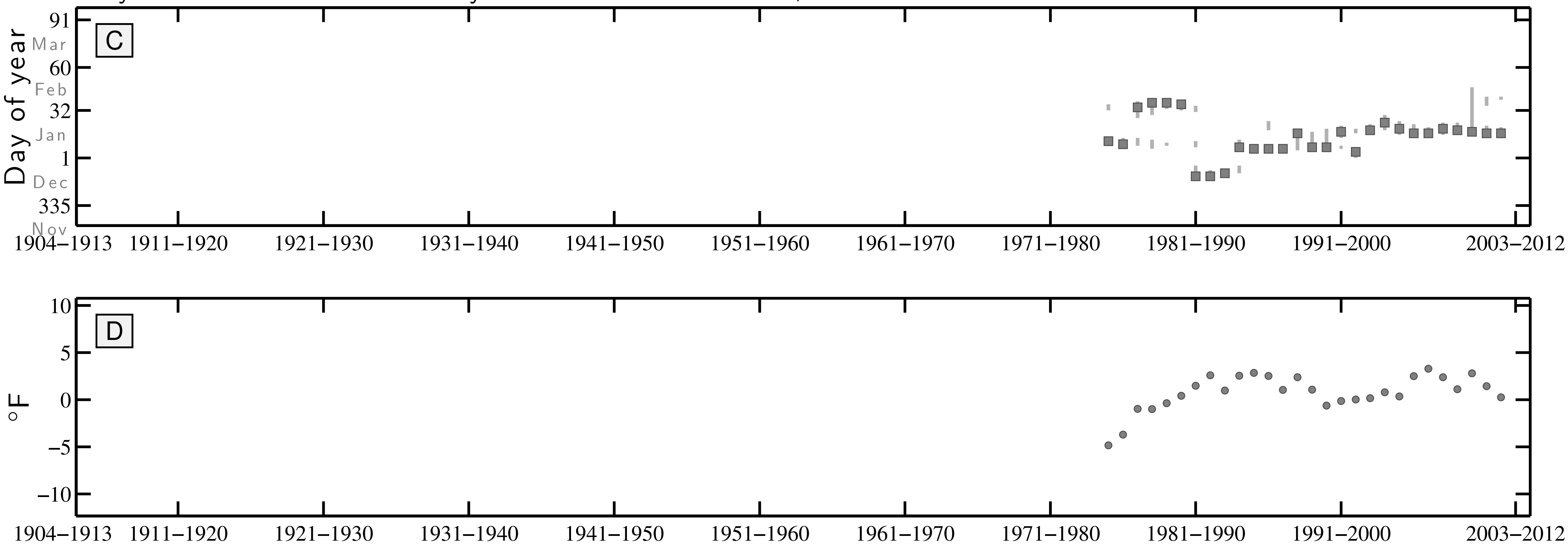

10 year Summer Teletherm dynamics for PARK RAPIDS 2 S, MN:

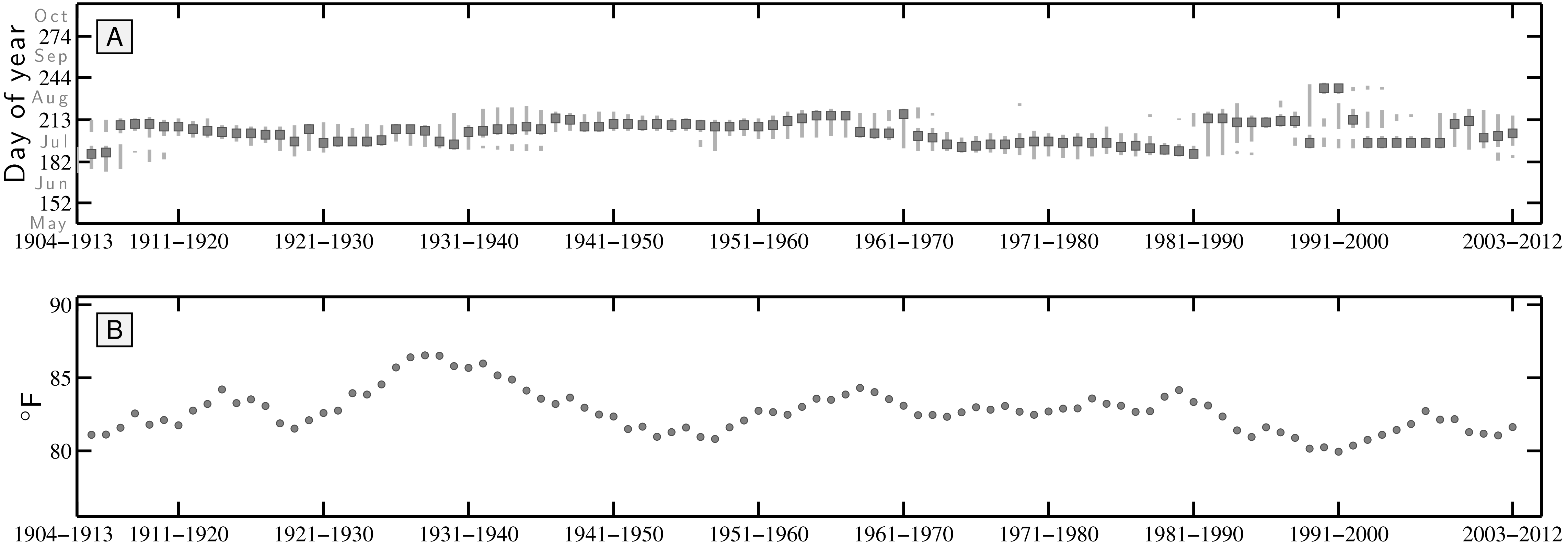

10 year Winter Teletherm dynamics for PARK RAPIDS 2 S, MN:

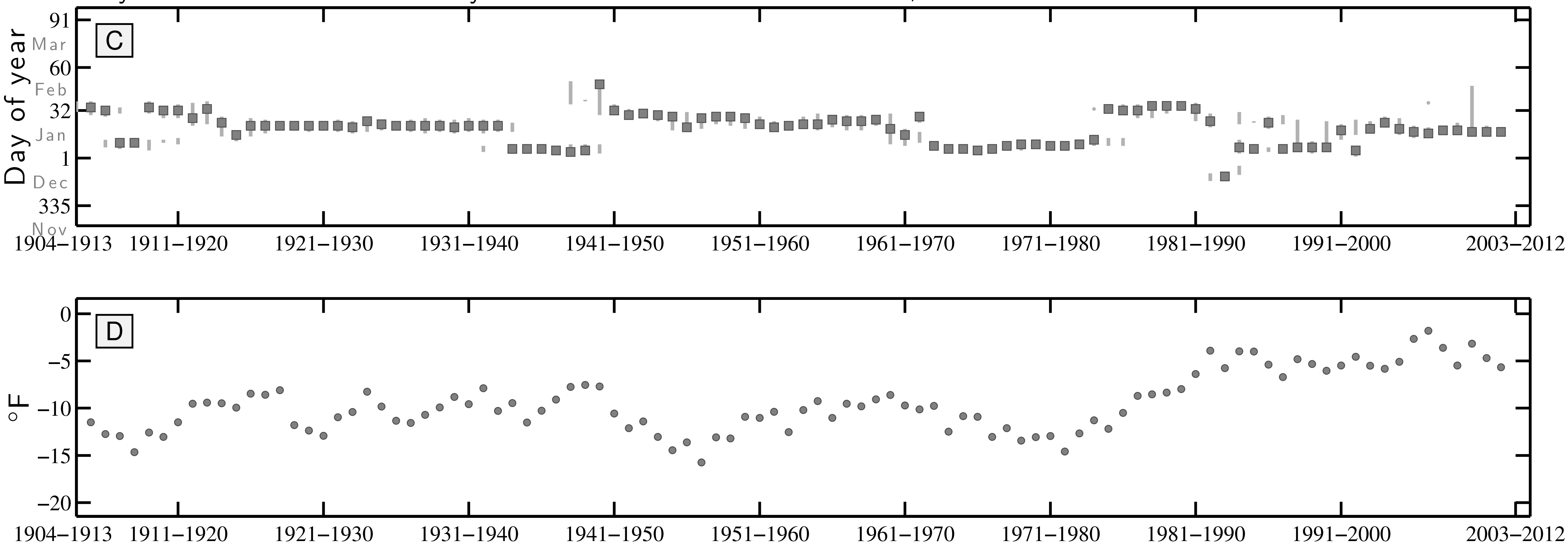

10 year Summer Teletherm dynamics for PINE RIVER DAM, MN:

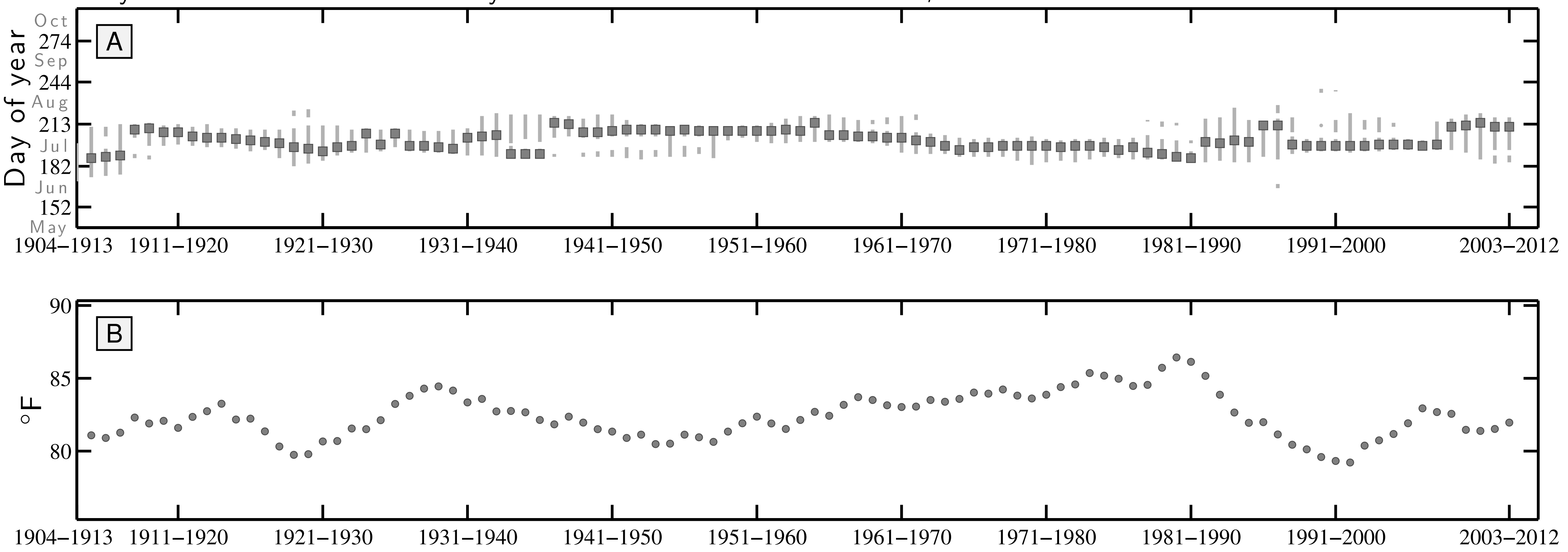

10 year Winter Teletherm dynamics for PINE RIVER DAM, MN:

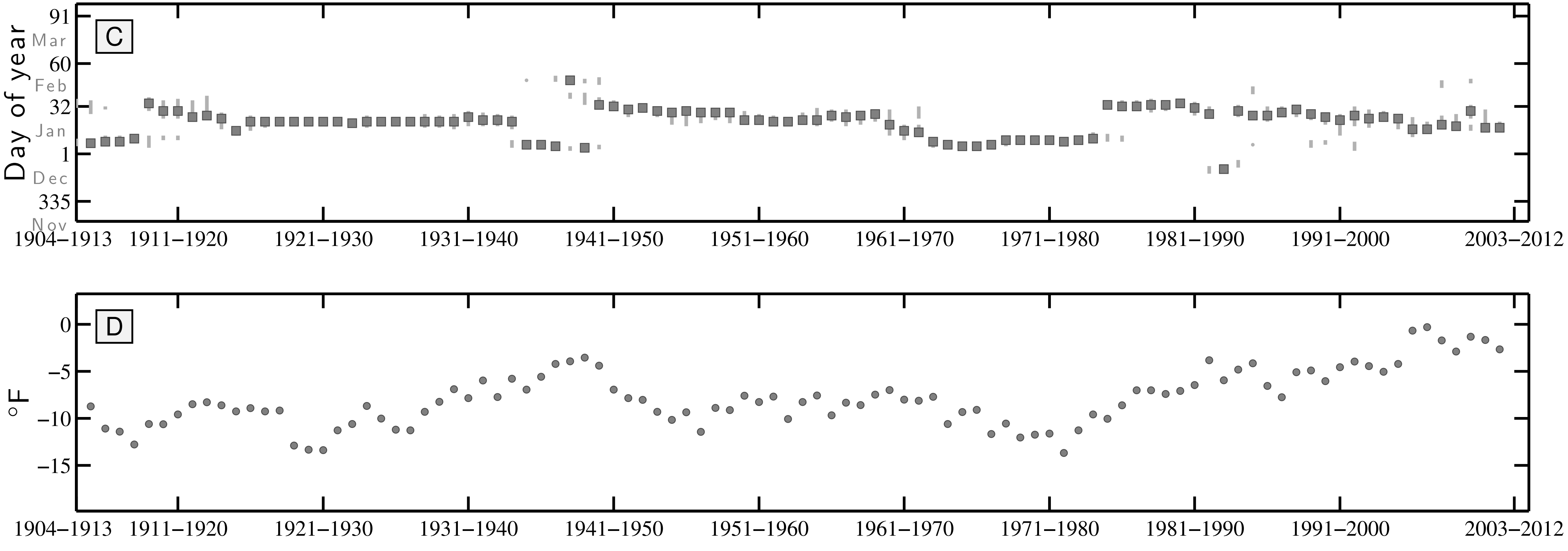

10 year Summer Teletherm dynamics for PIPESTONE, MN:

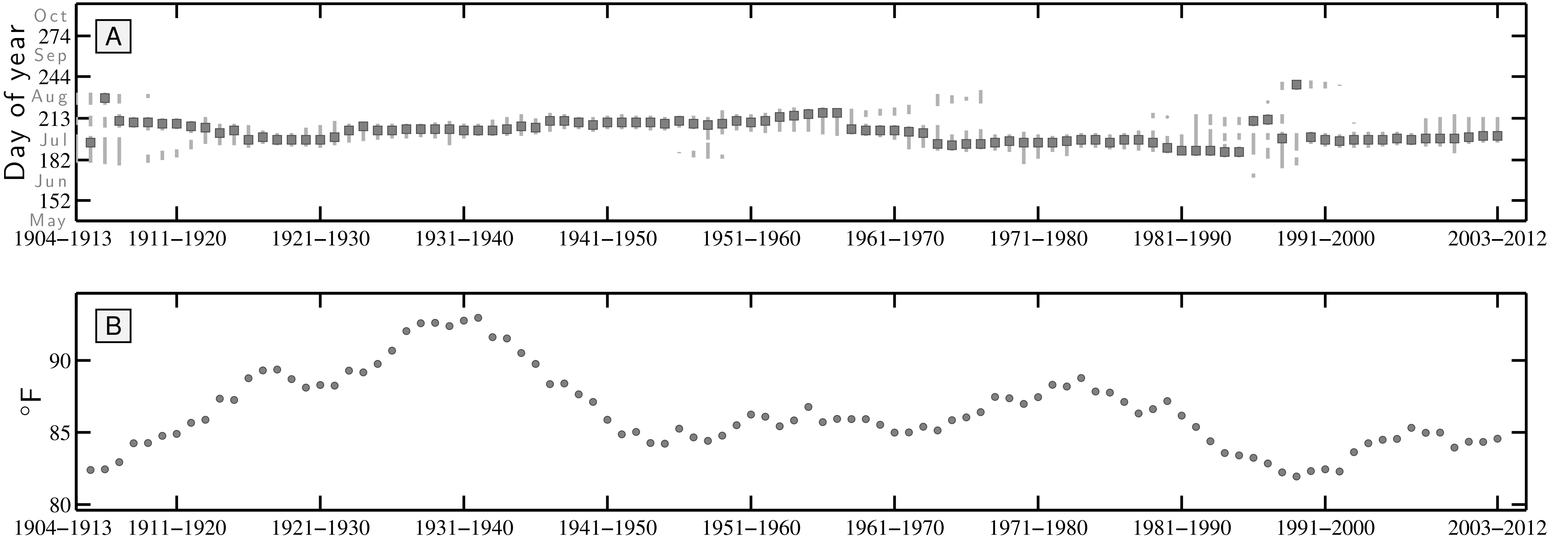

10 year Winter Teletherm dynamics for PIPESTONE, MN:

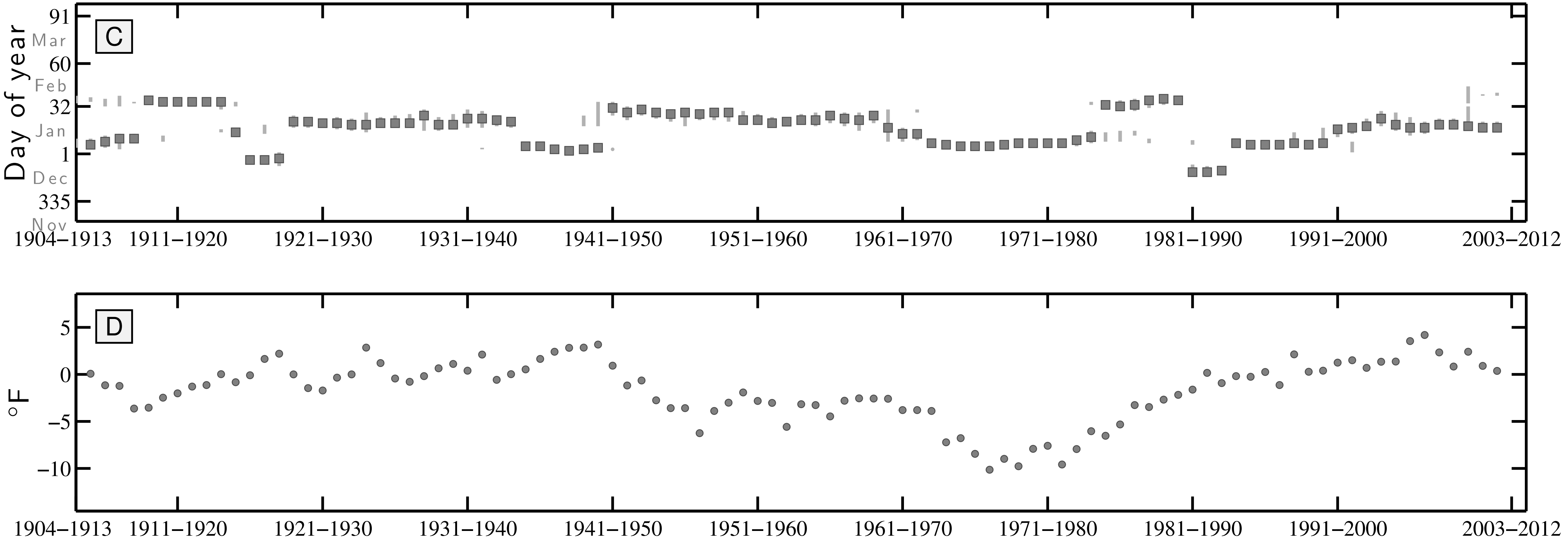

10 year Summer Teletherm dynamics for ROSEAU, MN:

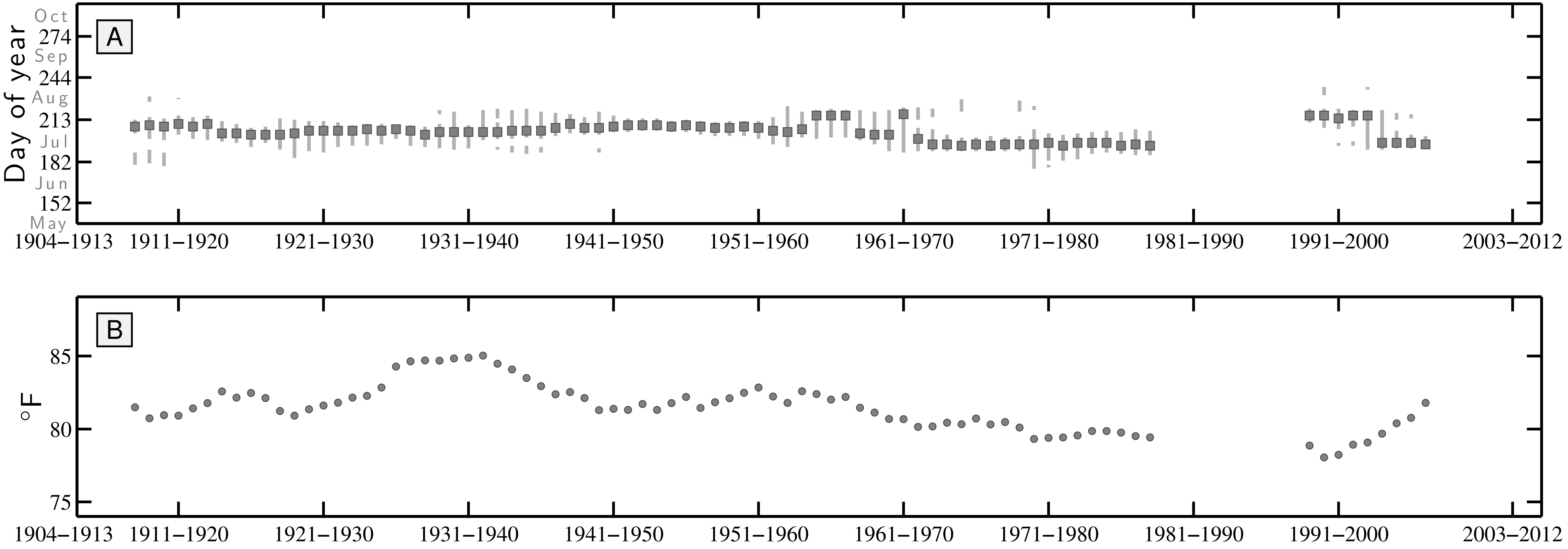

10 year Winter Teletherm dynamics for ROSEAU, MN:

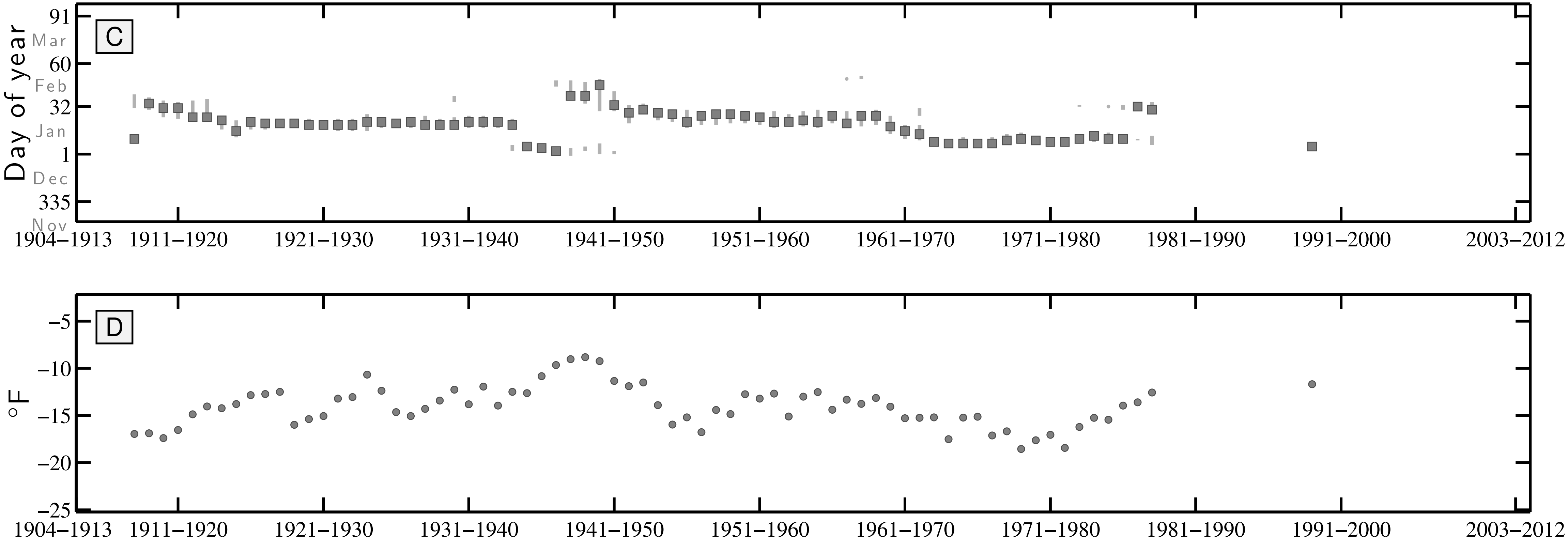

10 year Summer Teletherm dynamics for ST PETER, MN:

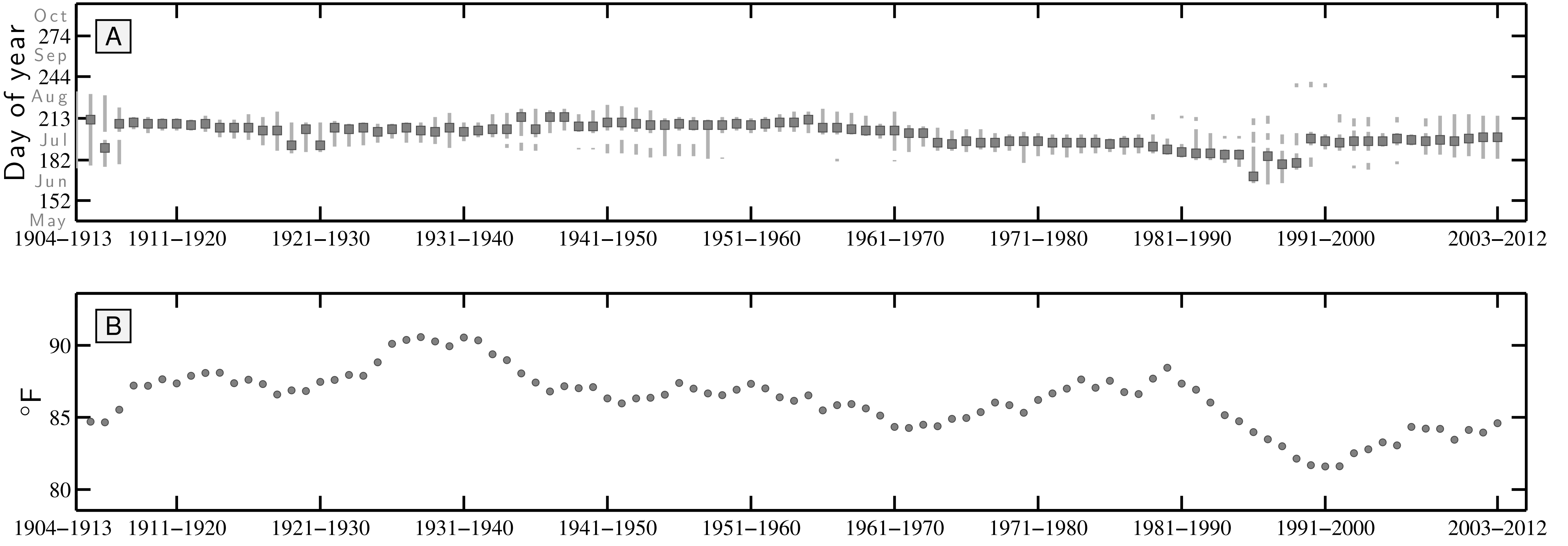

10 year Winter Teletherm dynamics for ST PETER, MN:

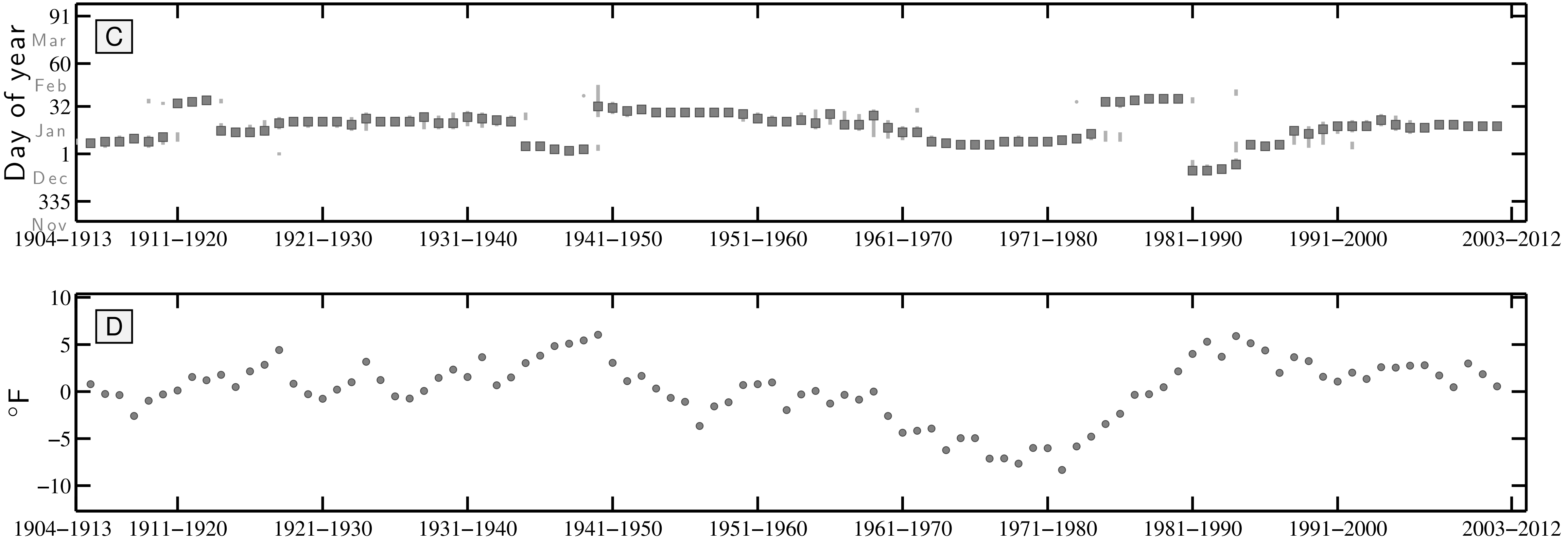

10 year Summer Teletherm dynamics for SANDY LAKE DAM LIBBY, MN:

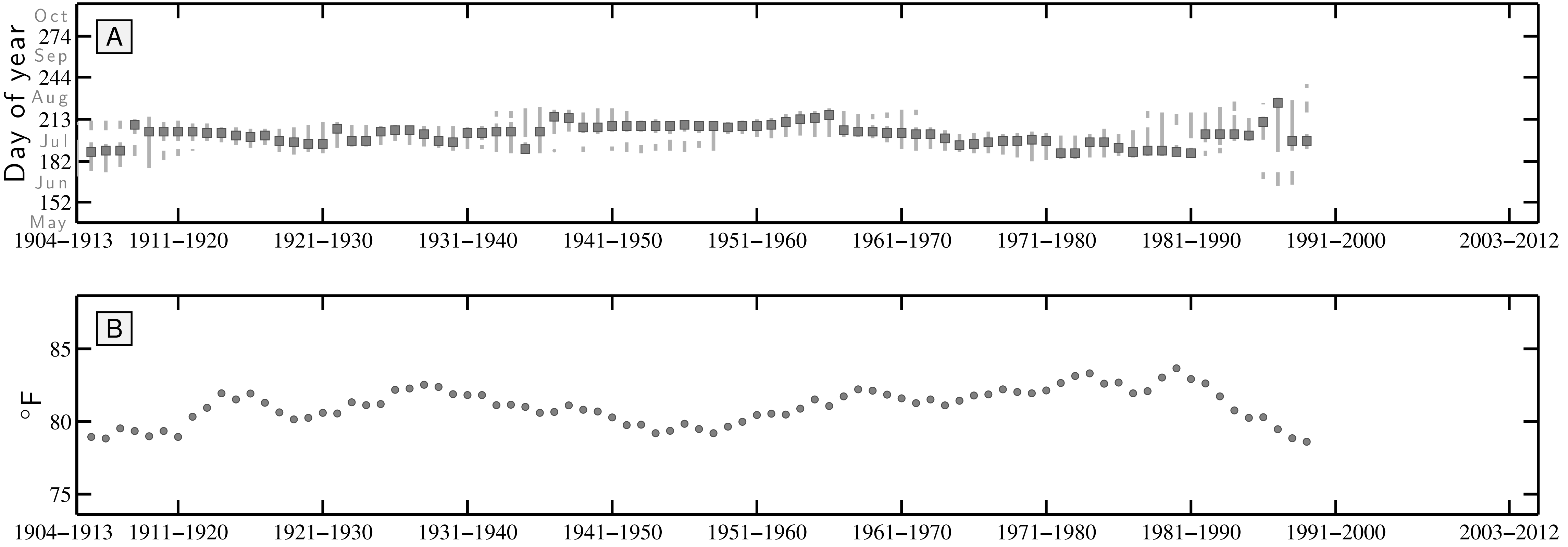

10 year Winter Teletherm dynamics for SANDY LAKE DAM LIBBY, MN:

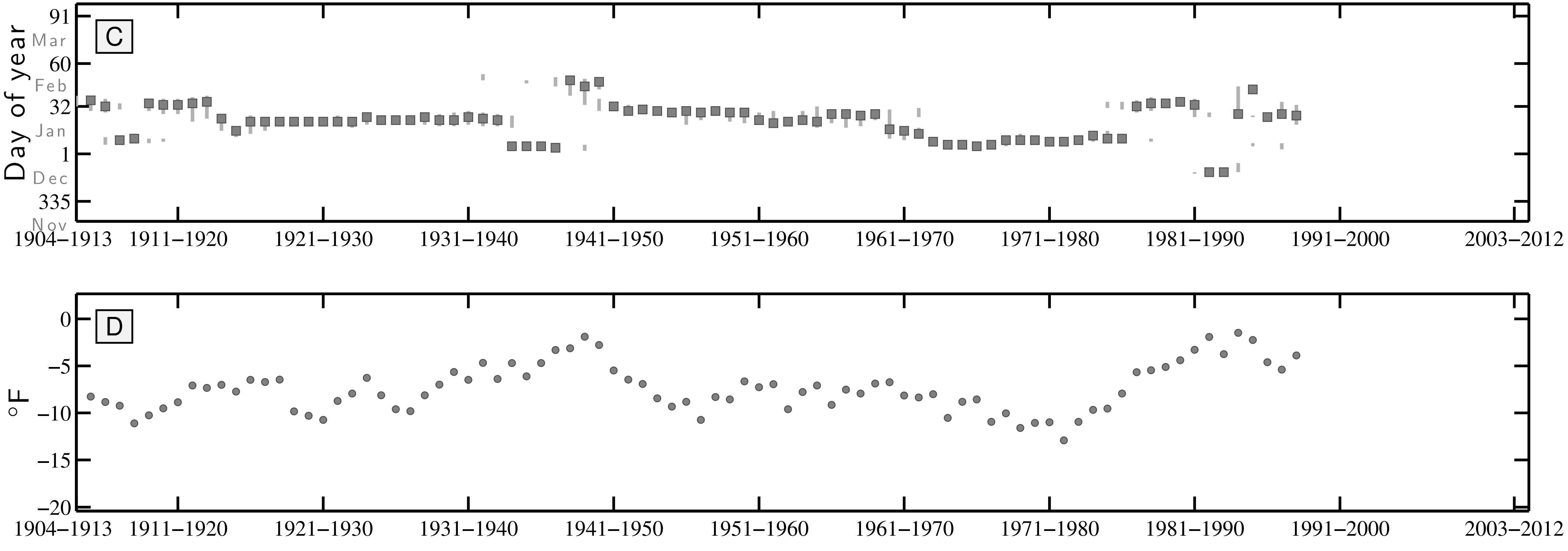

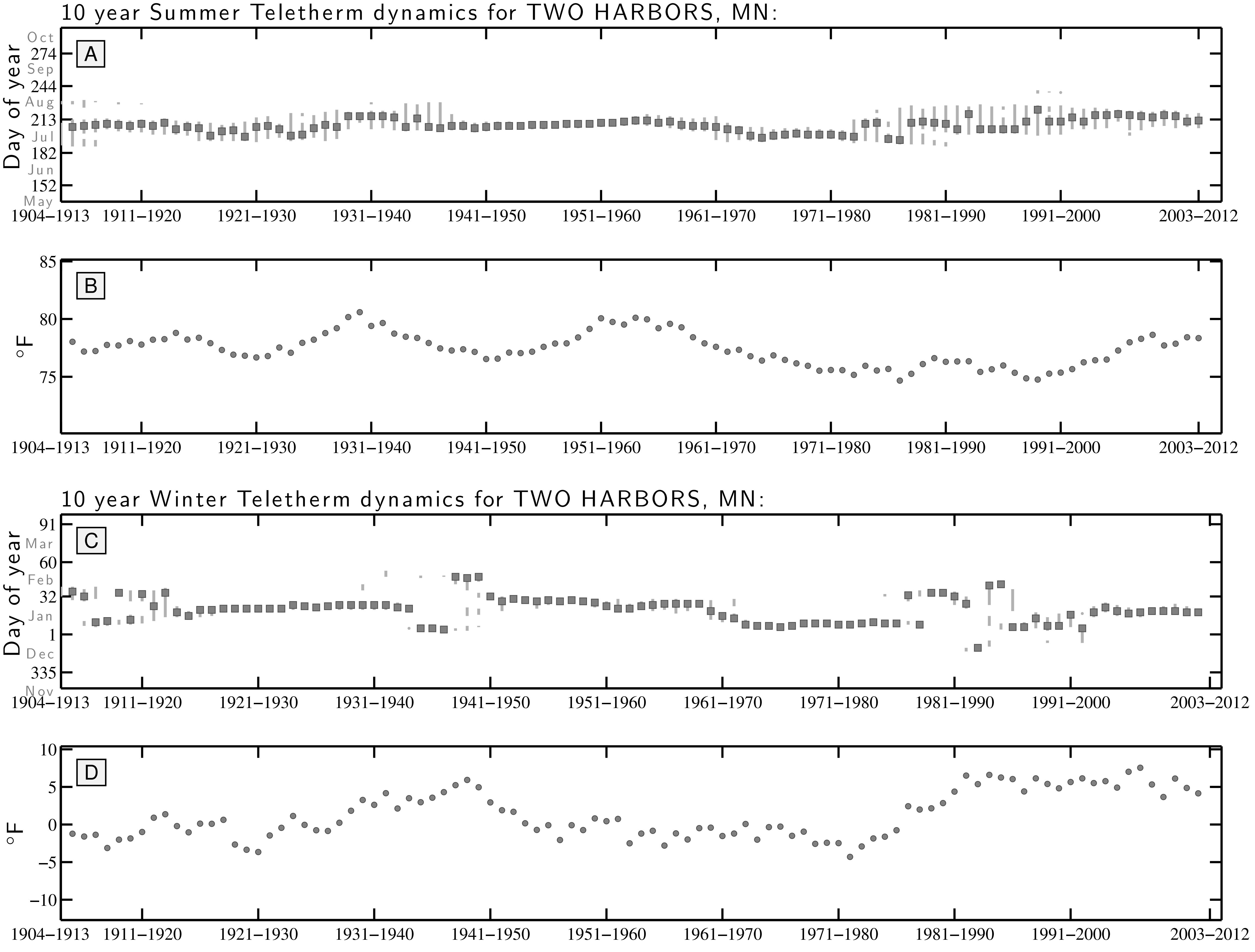
10 year Summer Teletherm dynamics for TWO HARBORS, MN:
A
Day of year
Oct
274
Sep
244
Aug
213
Jul
182
Jun
152
May
1904–1913
1911–1920
1921–1930
1931–1940
1941–1950
1951–1960
1961–1970
1971–1980
1981–1990
1991–2000
2003–2012
B
°F
85
80
75
10 year Winter Teletherm dynamics for TWO HARBORS, MN:
C
91
Mar
60
Feb
32
Jan
1
Dec
335
Nov
D
10
5
0
−5
−10

10 year Summer Teletherm dynamics for WALKER AH GWAH CHING, MN:

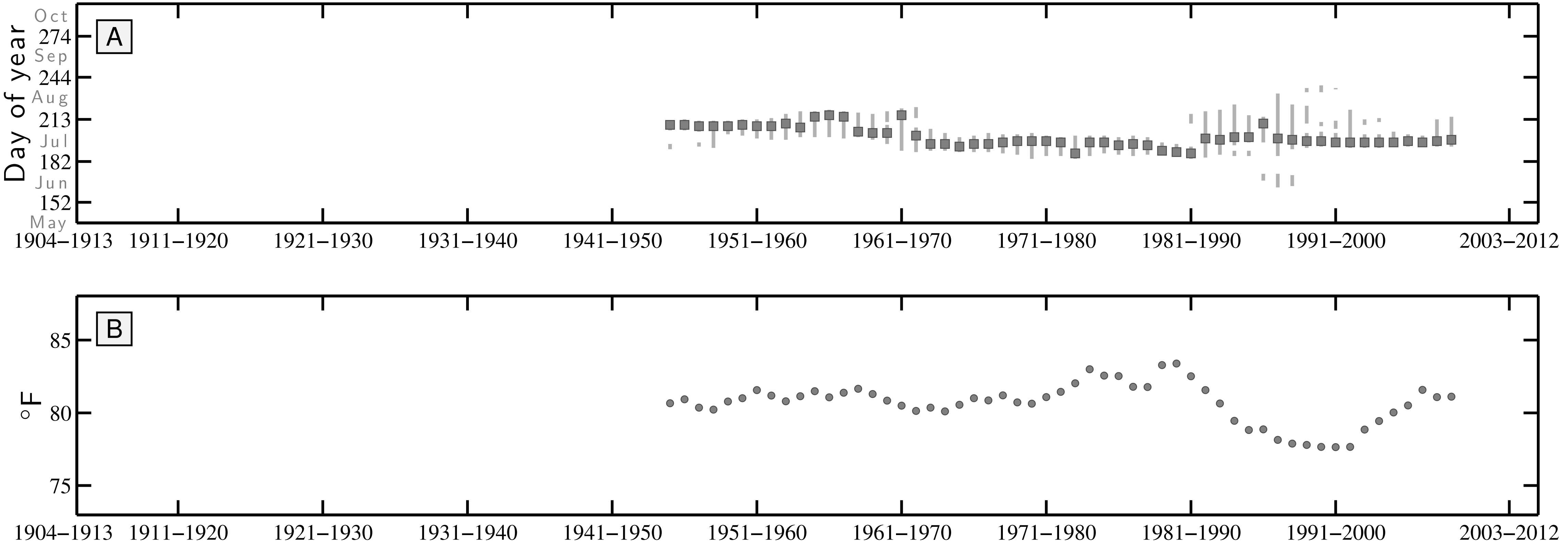

10 year Winter Teletherm dynamics for WALKER AH GWAH CHING, MN:

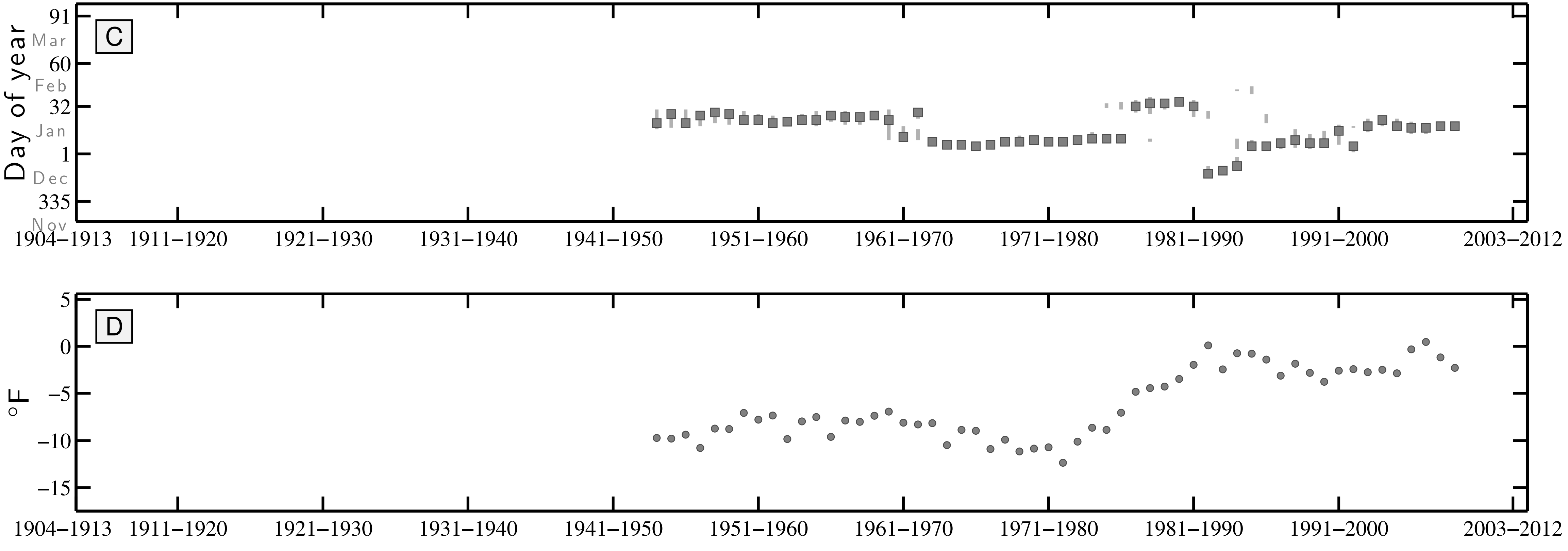

10 year Summer Teletherm dynamics for WINNEBAGO, MN:

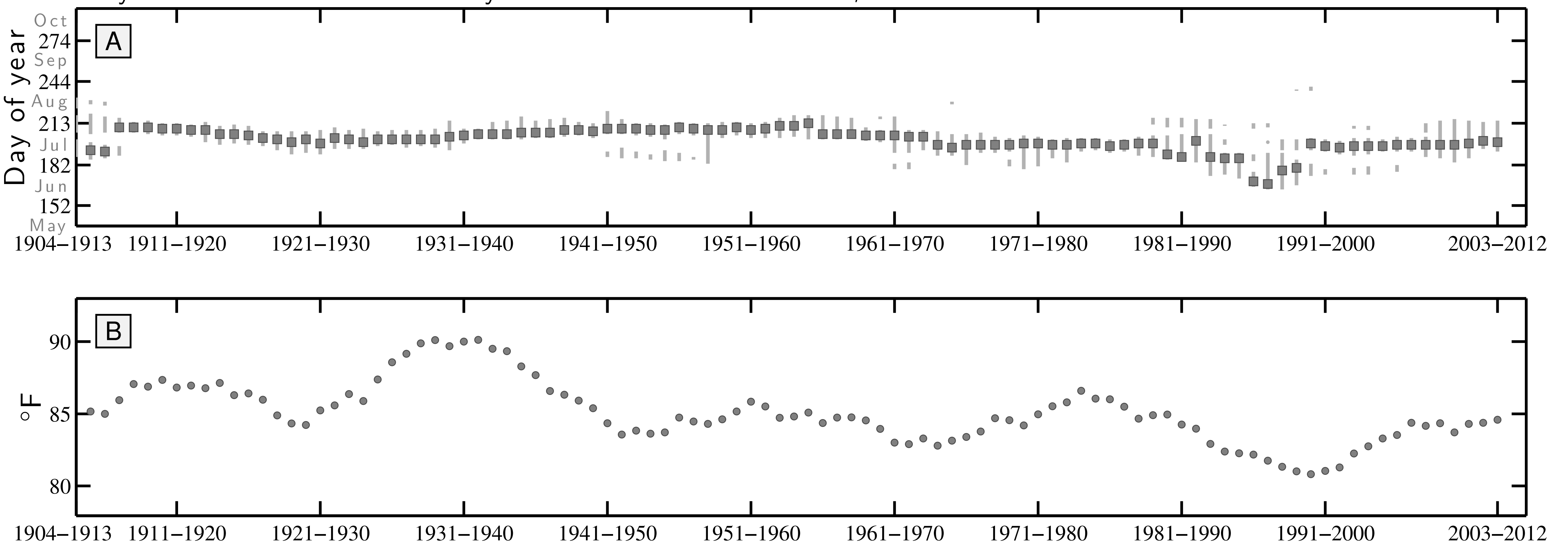

10 year Winter Teletherm dynamics for WINNEBAGO, MN:

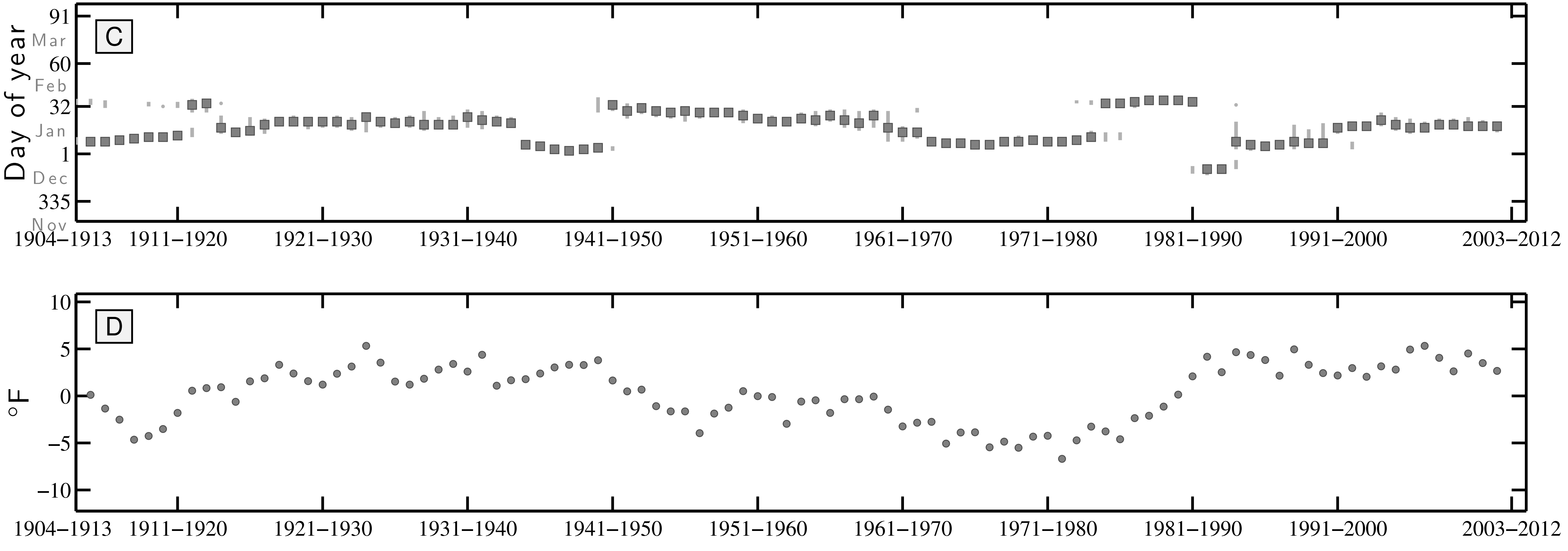

10 year Summer Teletherm dynamics for ZUMBROTA, MN:

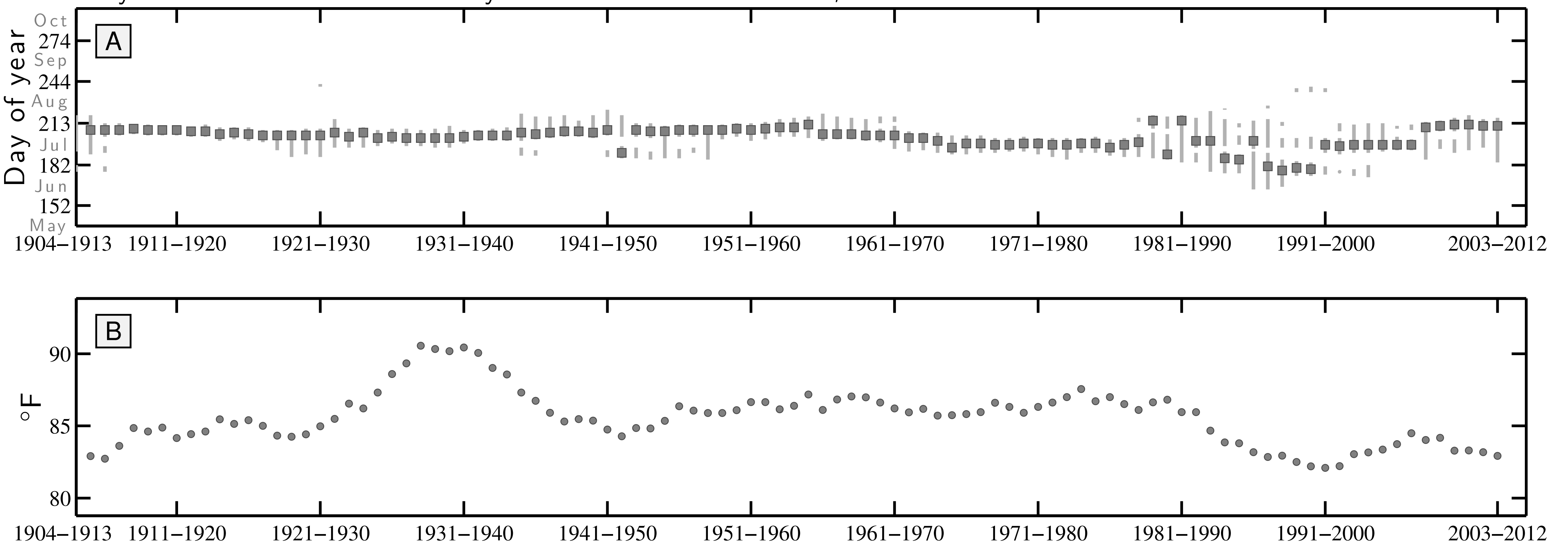

10 year Winter Teletherm dynamics for ZUMBROTA, MN:

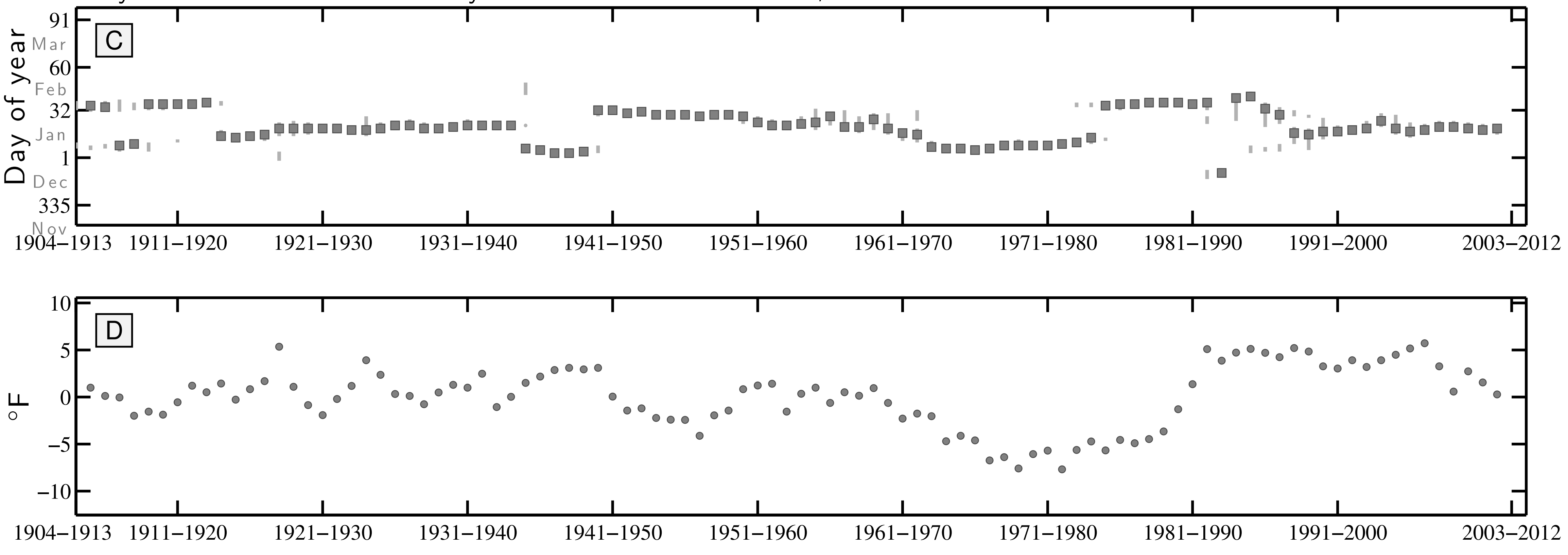

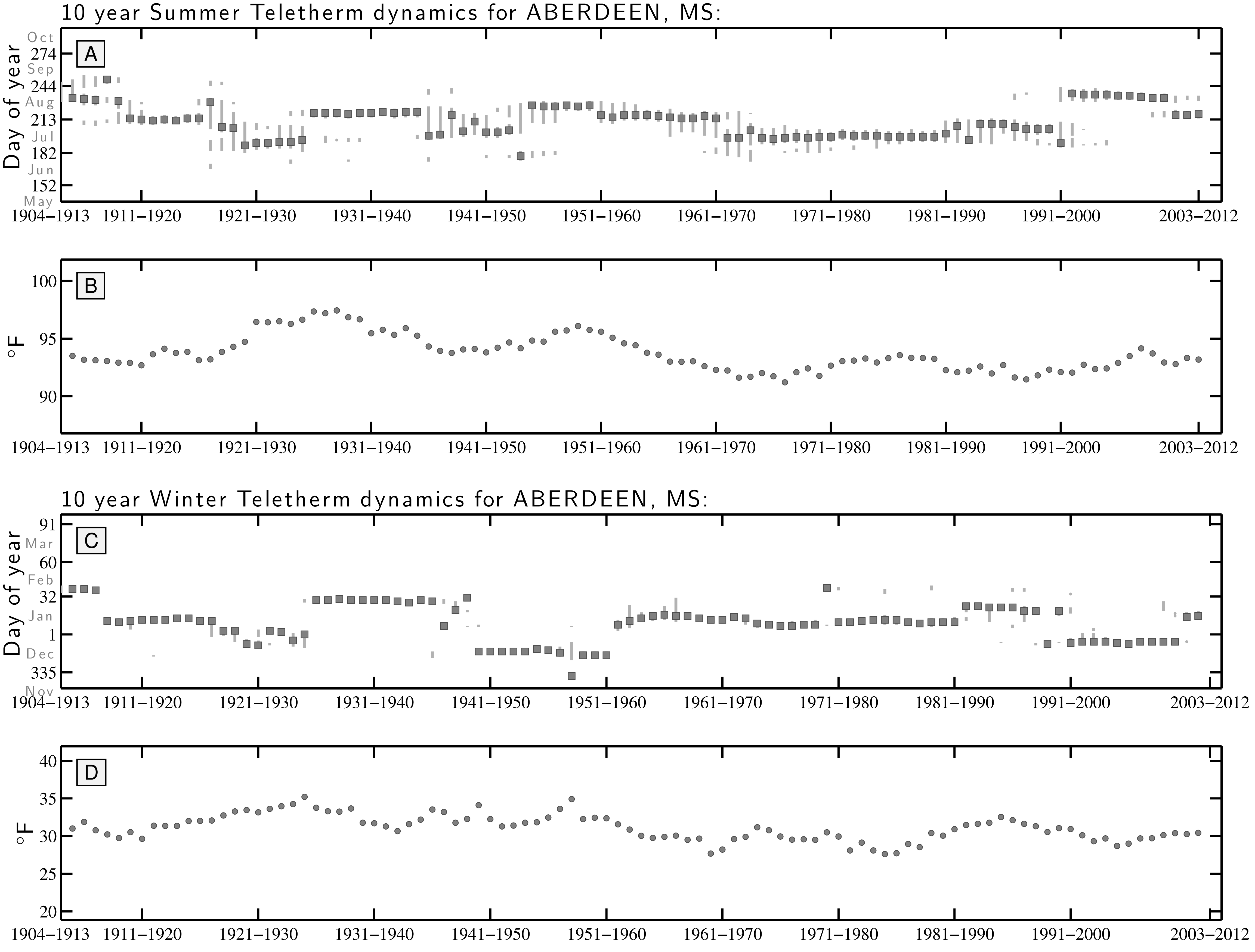

10 year Summer Teletherm dynamics for ABERDEEN, MS:
A
Day of year
Oct
274
Sep
244
Aug
213
Jul
182
Jun
152
May
1904–1913
1911–1920
1921–1930
1931–1940
1941–1950
1951–1960
1961–1970
1971–1980
1981–1990
1991–2000
2003–2012
B
°F
100
95
90
10 year Winter Teletherm dynamics for ABERDEEN, MS:
C
Day of year
91
Mar
60
Feb
32
Jan
1
Dec
335
Nov
D
°F
40
35
30
25
20

10 year Summer Teletherm dynamics for BATESVILLE 2 SW, MS:

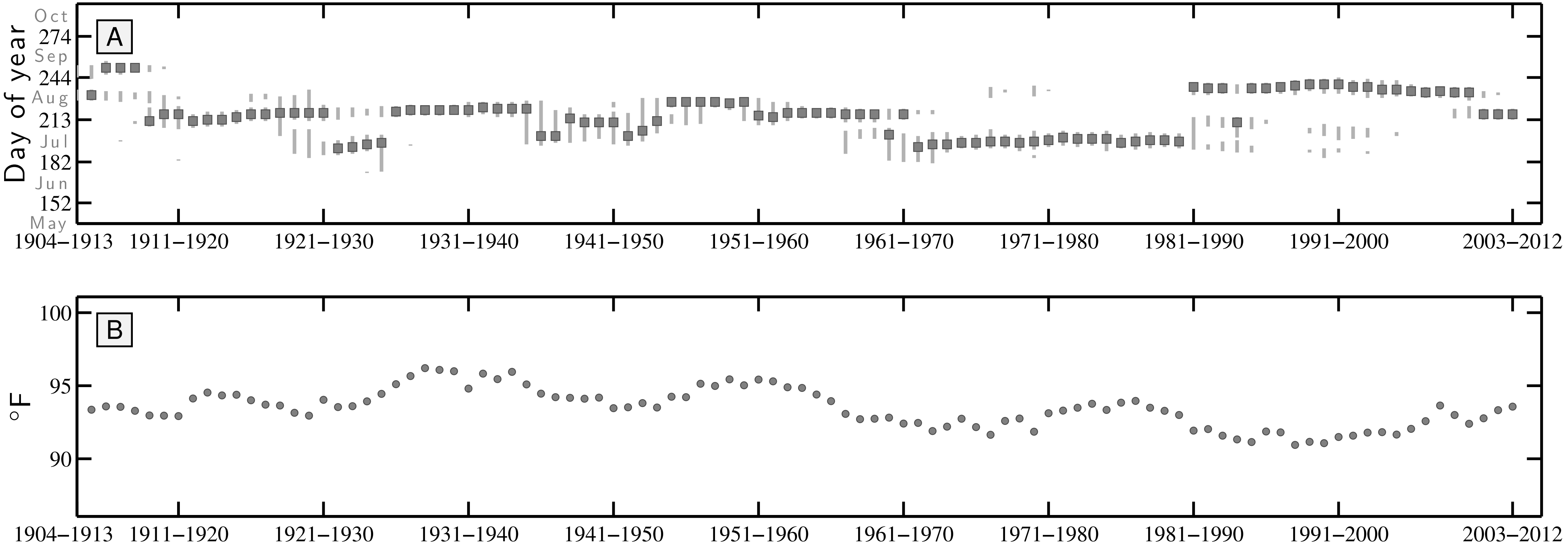

10 year Winter Teletherm dynamics for BATESVILLE 2 SW, MS:

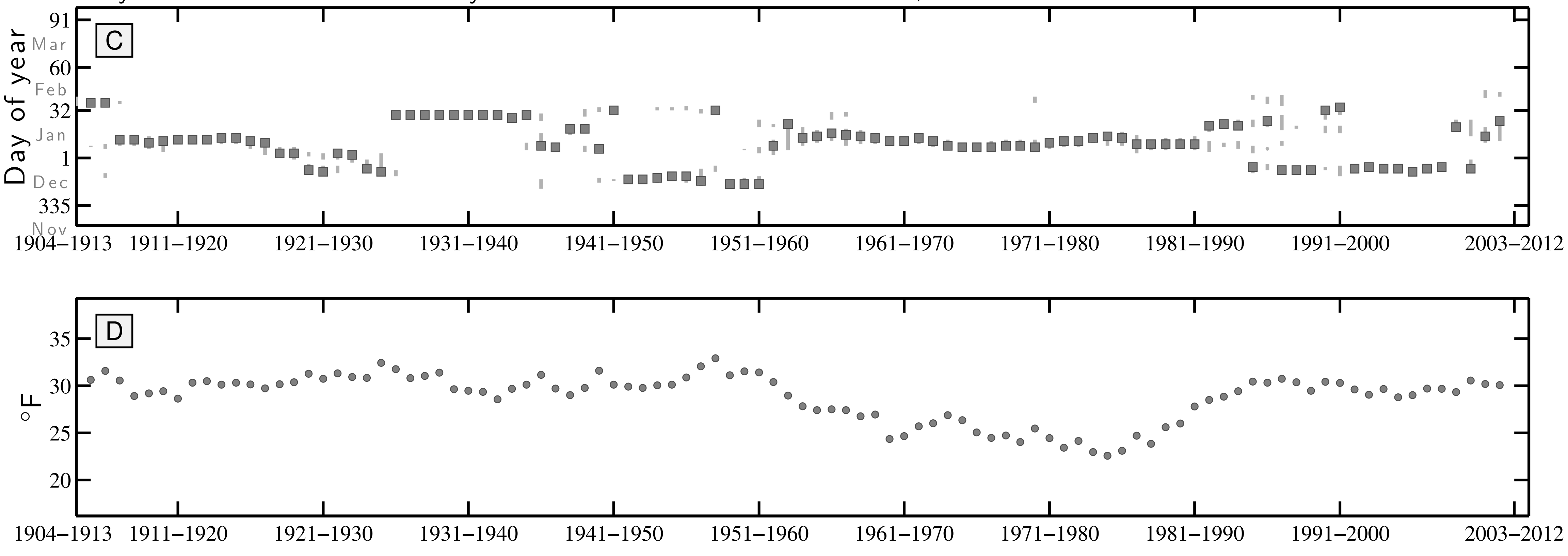

10 year Summer Teletherm dynamics for BOONEVILLE, MS:

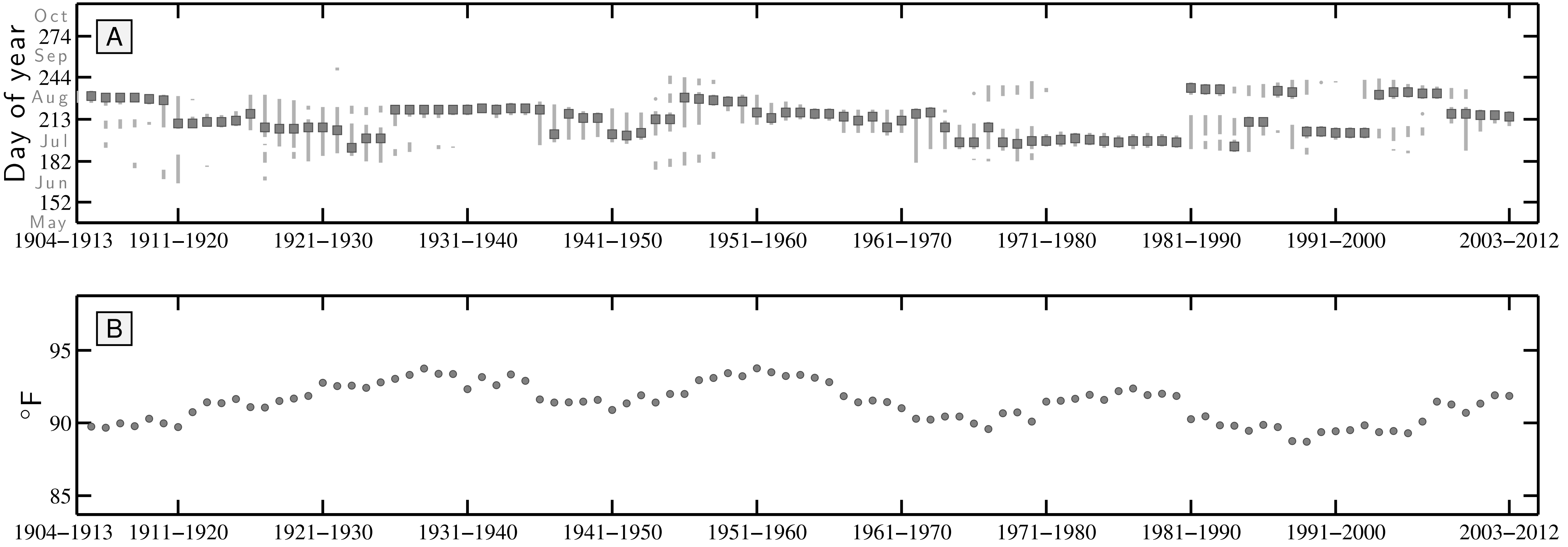

10 year Winter Teletherm dynamics for BOONEVILLE, MS:

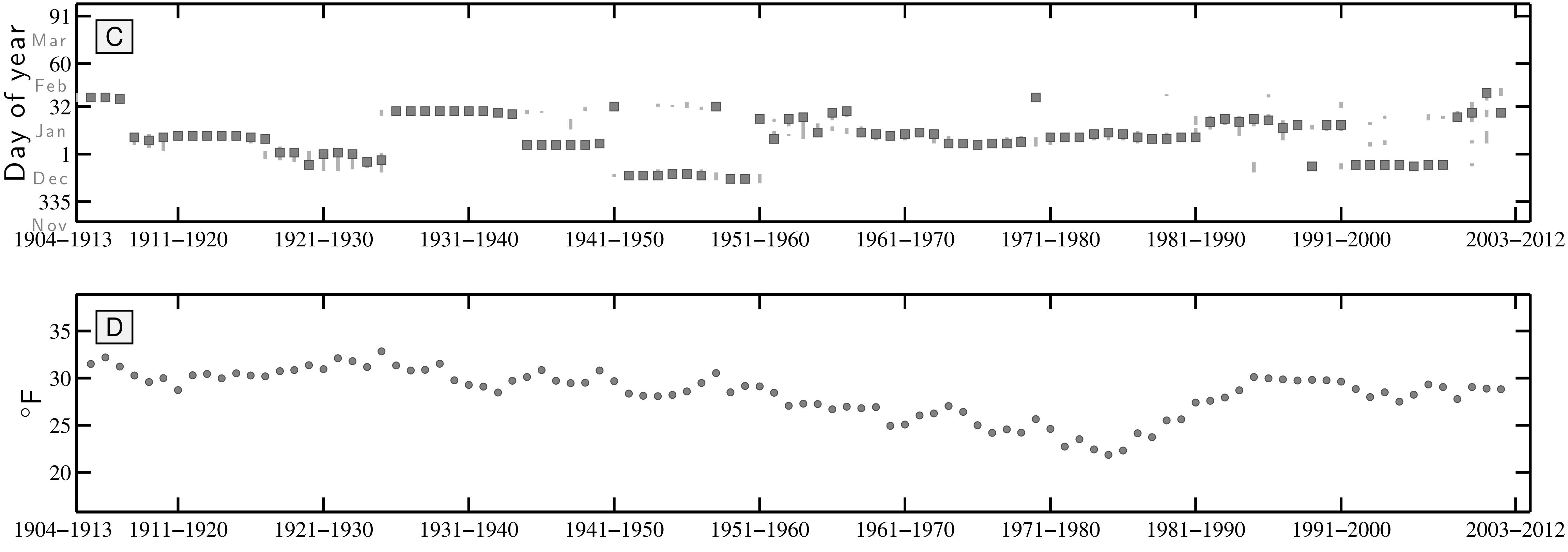

10 year Summer Teletherm dynamics for BROOKHAVEN CITY, MS:

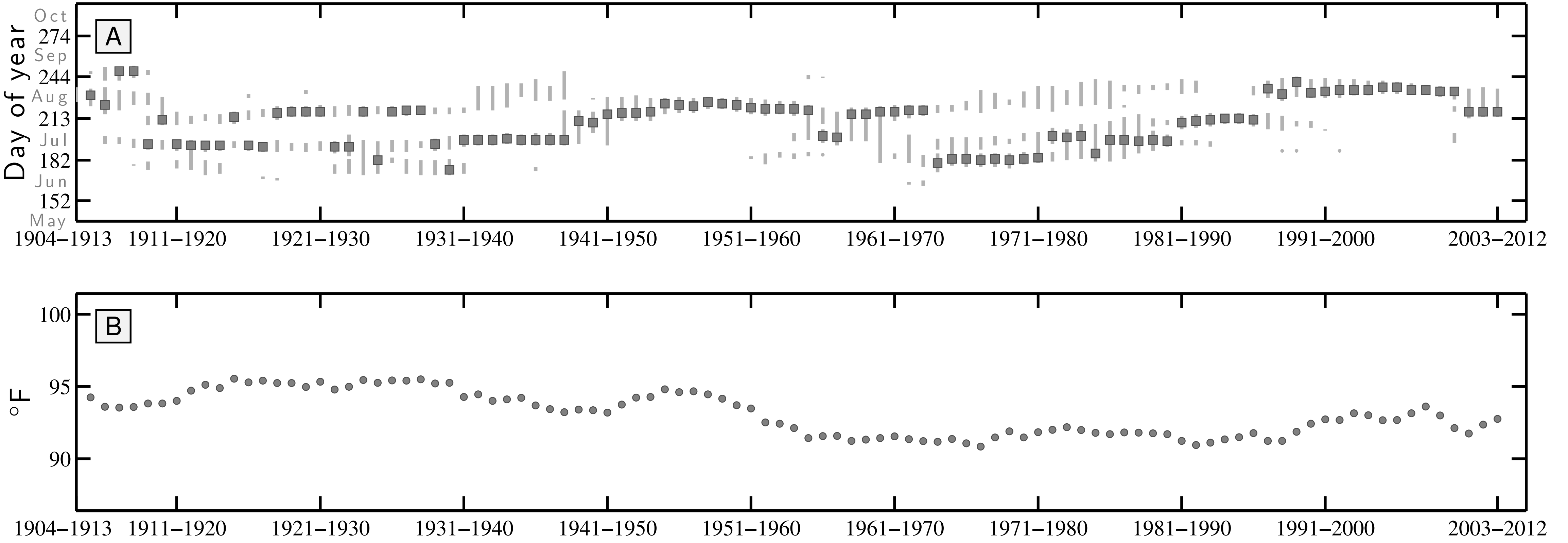

10 year Winter Teletherm dynamics for BROOKHAVEN CITY, MS:

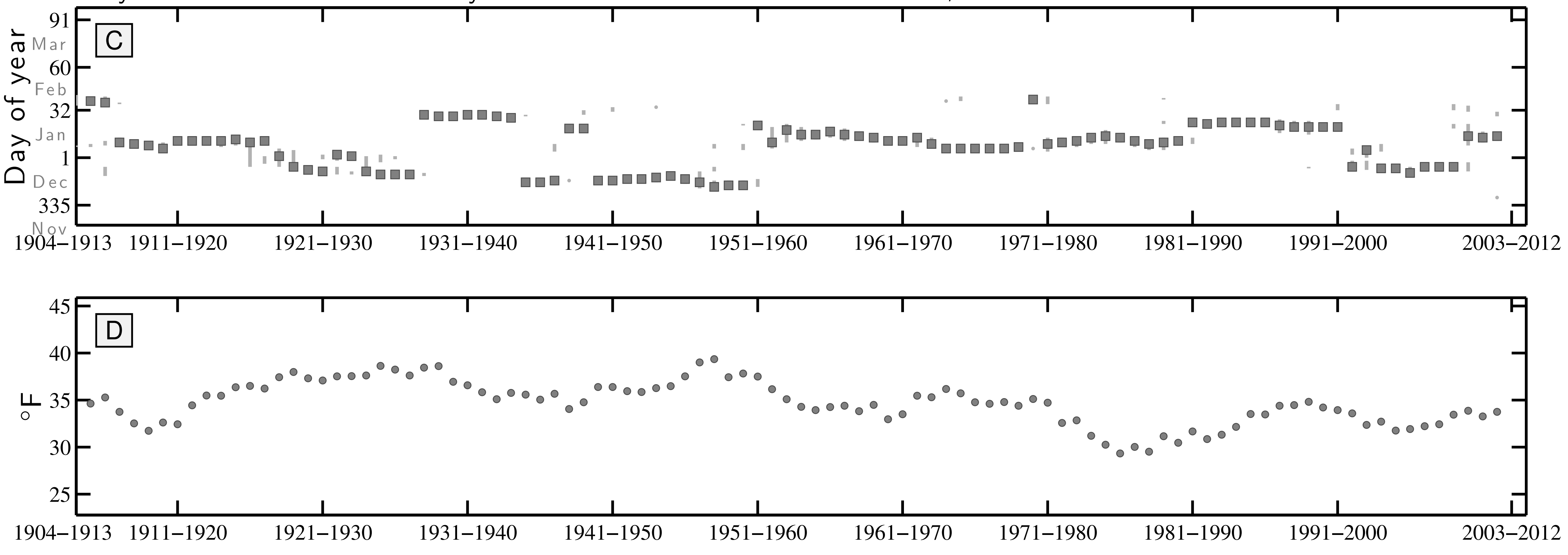

10 year Summer Teletherm dynamics for CANTON 4N, MS:

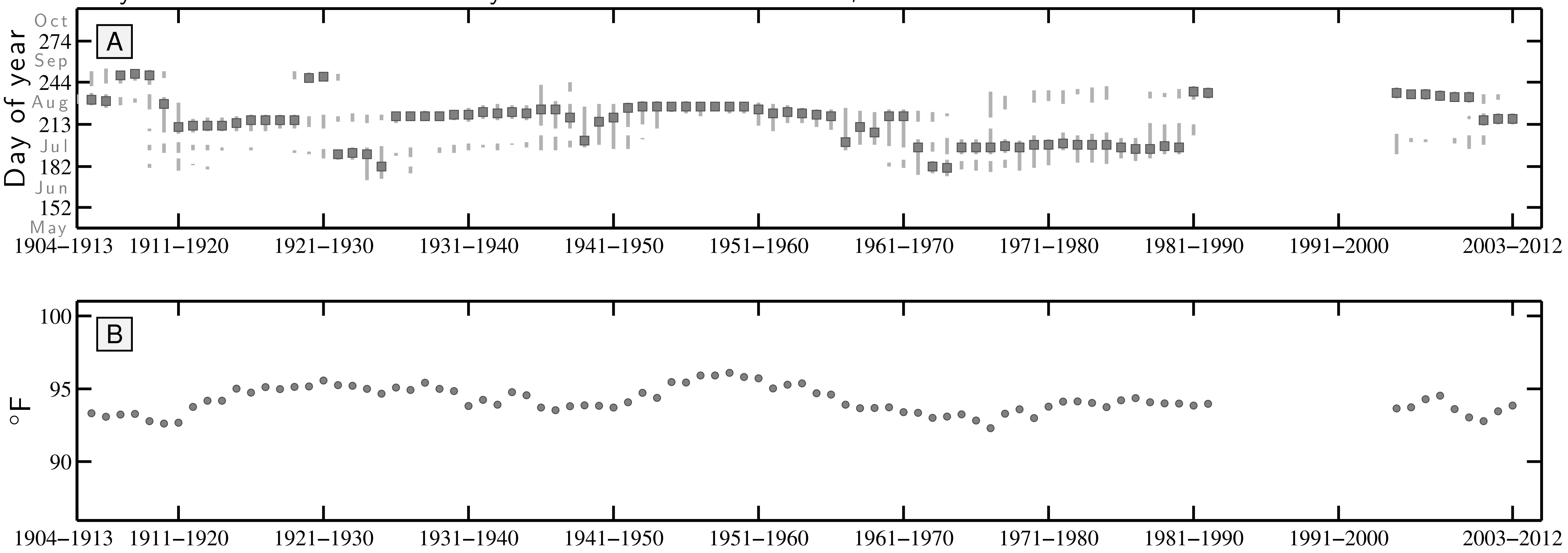

10 year Winter Teletherm dynamics for CANTON 4N, MS:

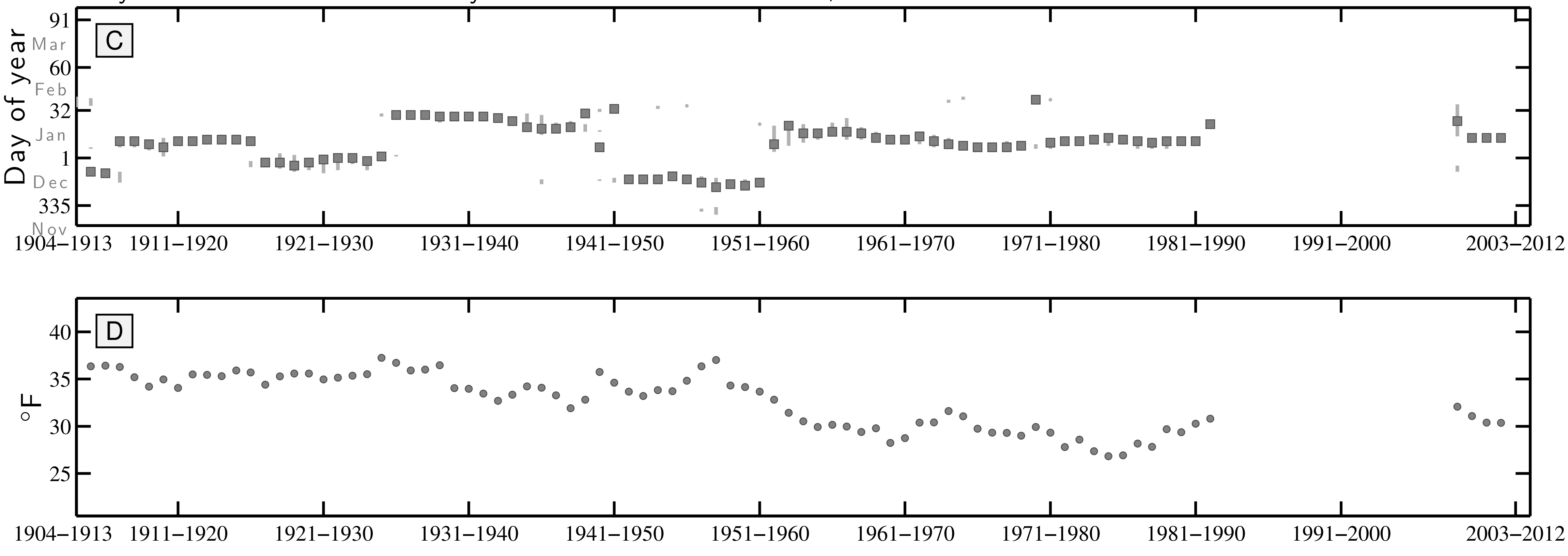

10 year Summer Teletherm dynamics for CLARKSDALE, MS:

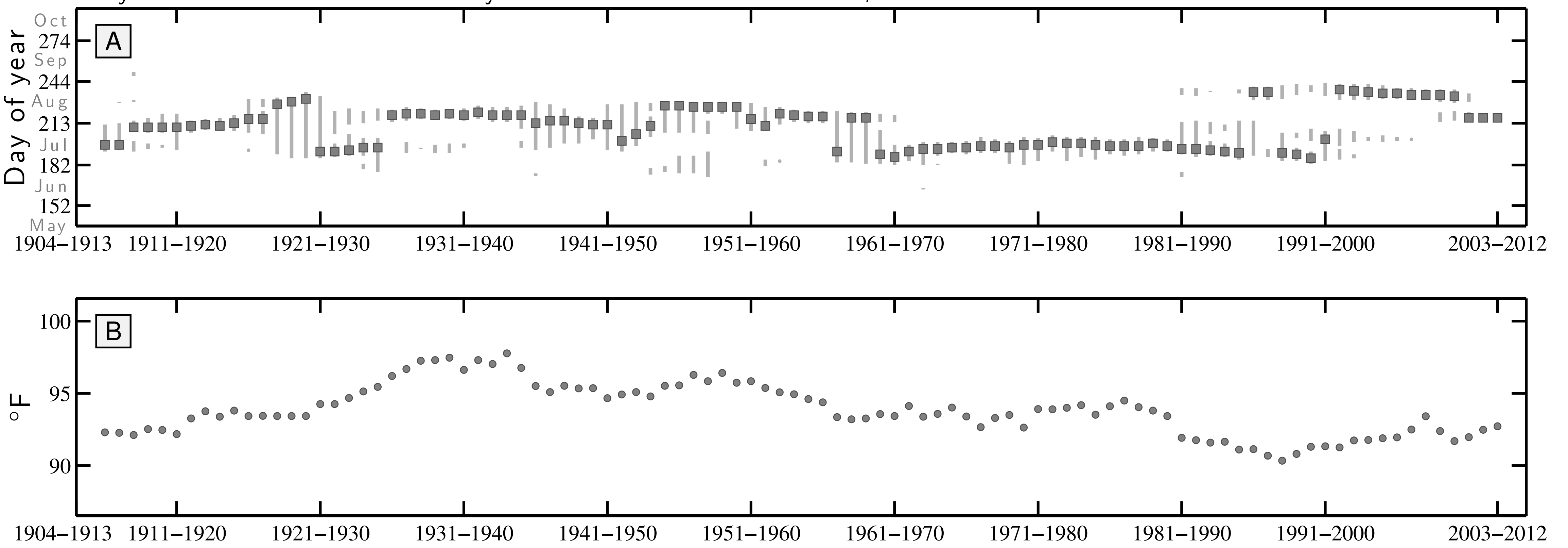

10 year Winter Teletherm dynamics for CLARKSDALE, MS:

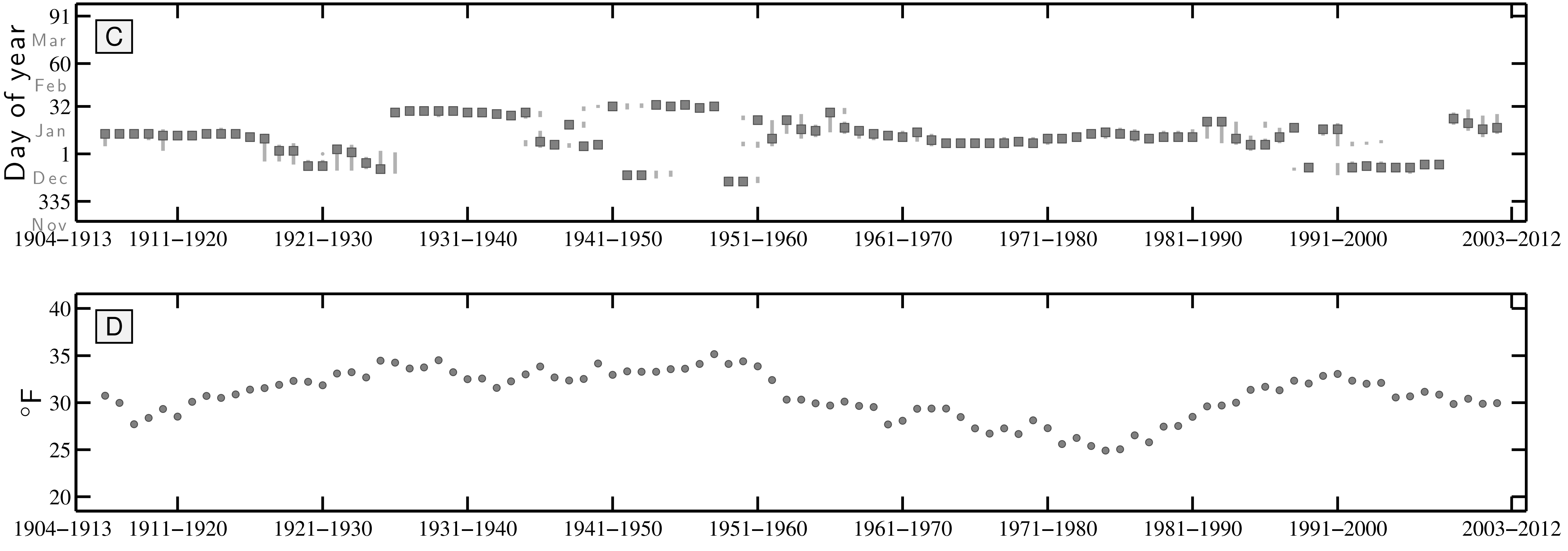

10 year Summer Teletherm dynamics for COLUMBIA, MS:

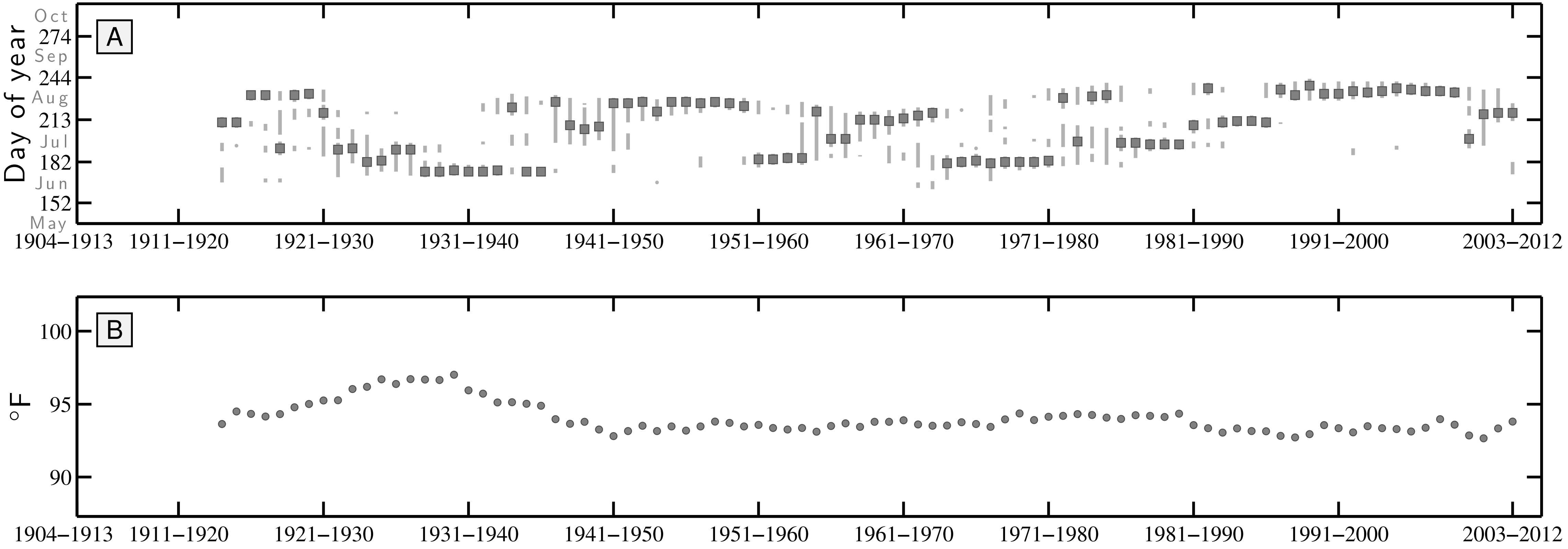

10 year Winter Teletherm dynamics for COLUMBIA, MS:

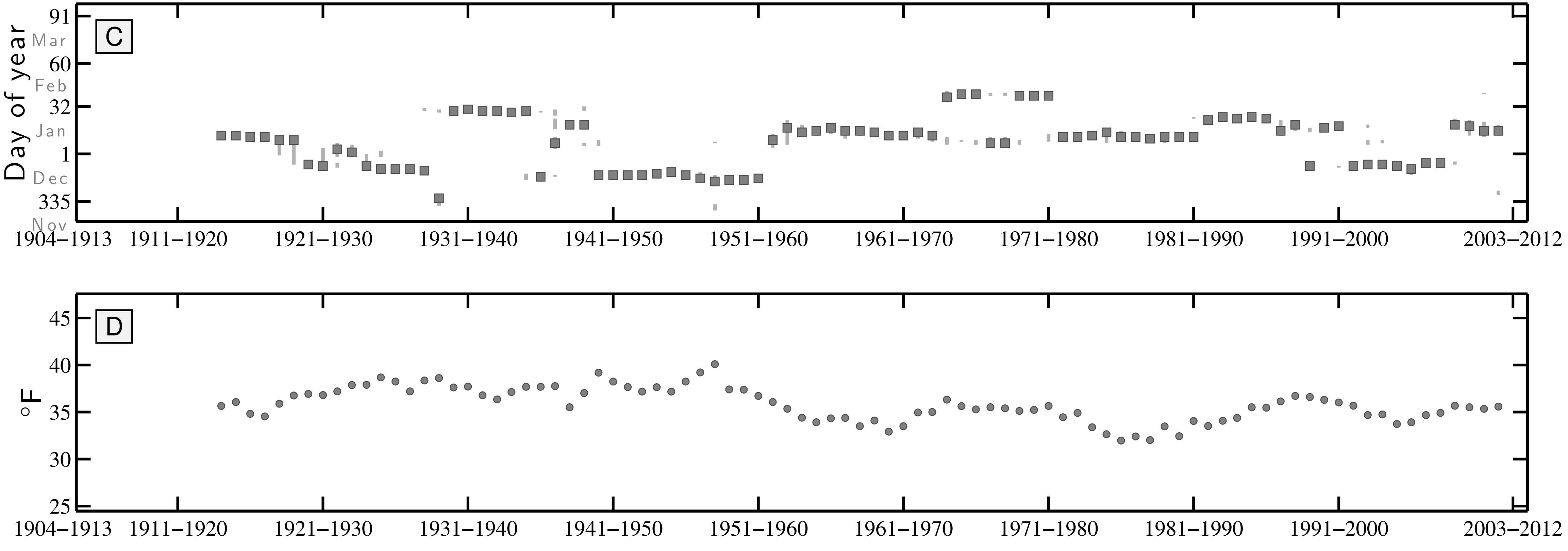

10 year Summer Teletherm dynamics for COLUMBUS, MS:

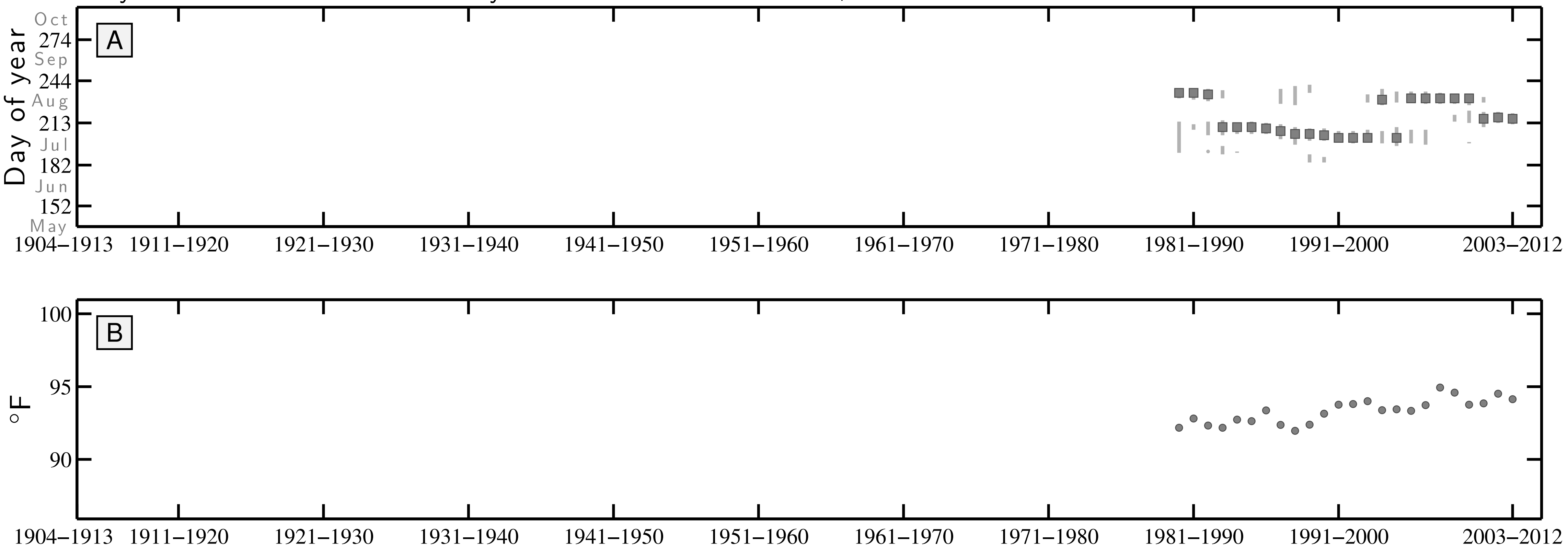

10 year Winter Teletherm dynamics for COLUMBUS, MS:

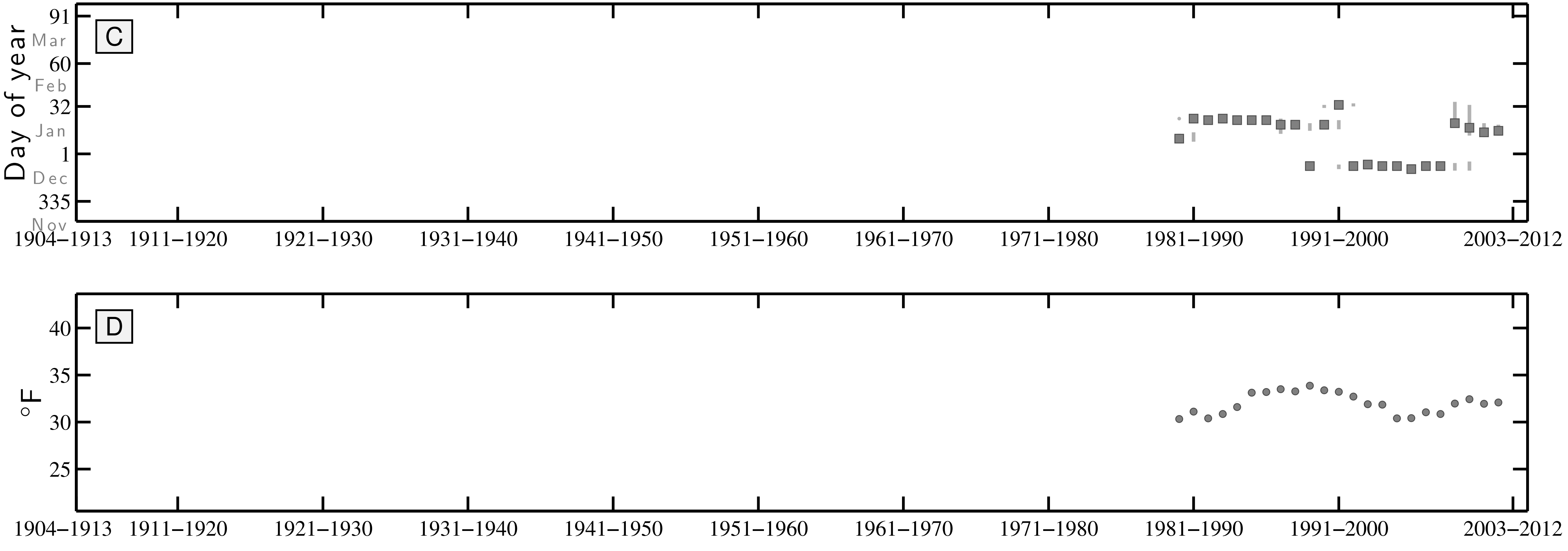

10 year Summer Teletherm dynamics for CORINTH 7 SW, MS:

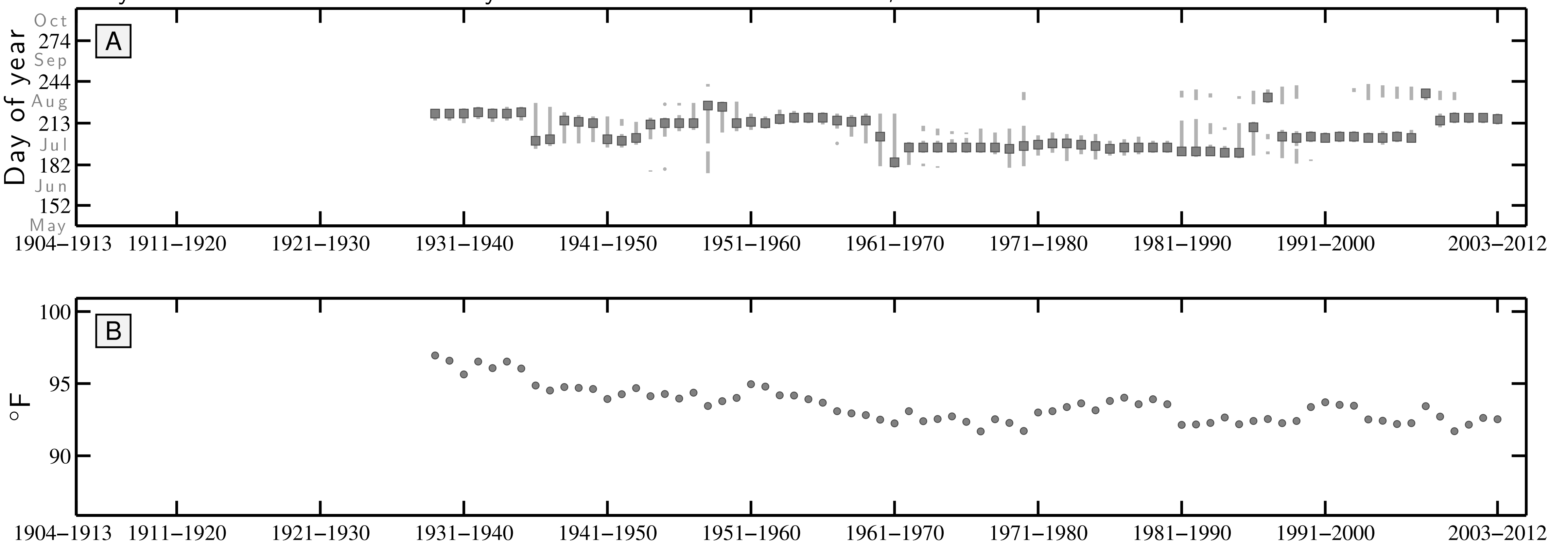

10 year Winter Teletherm dynamics for CORINTH 7 SW, MS:

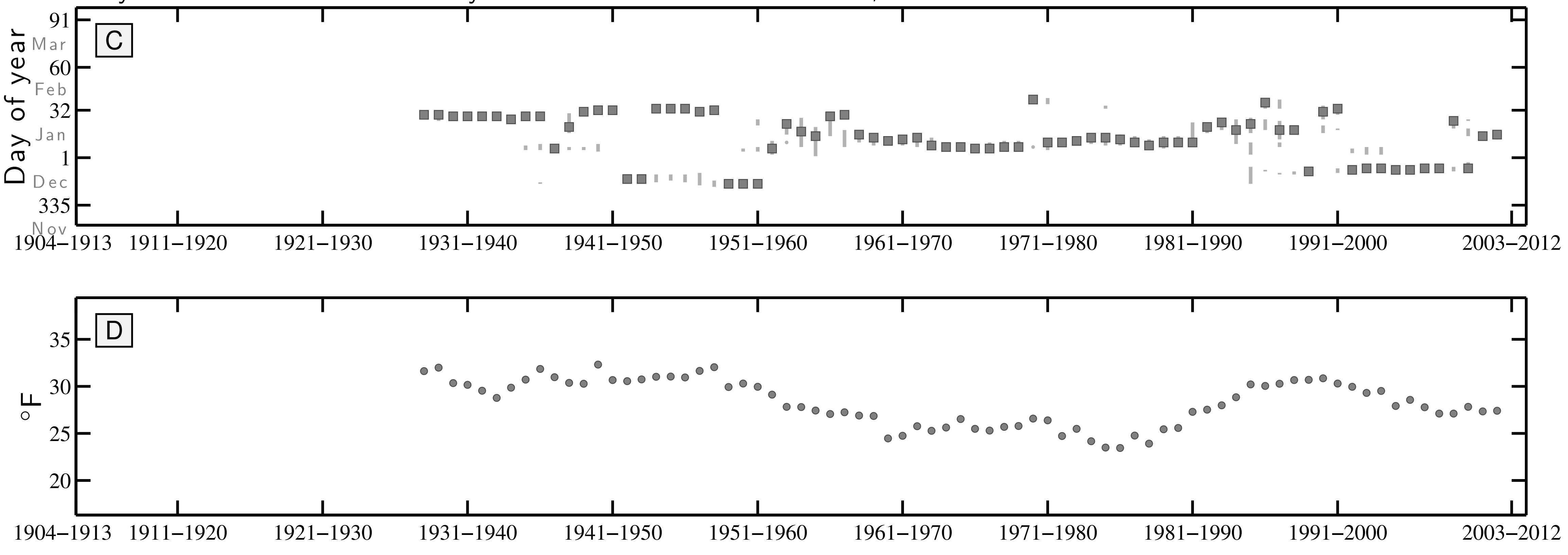

10 year Summer Teletherm dynamics for CRYSTAL SPGS EXP STN, MS:

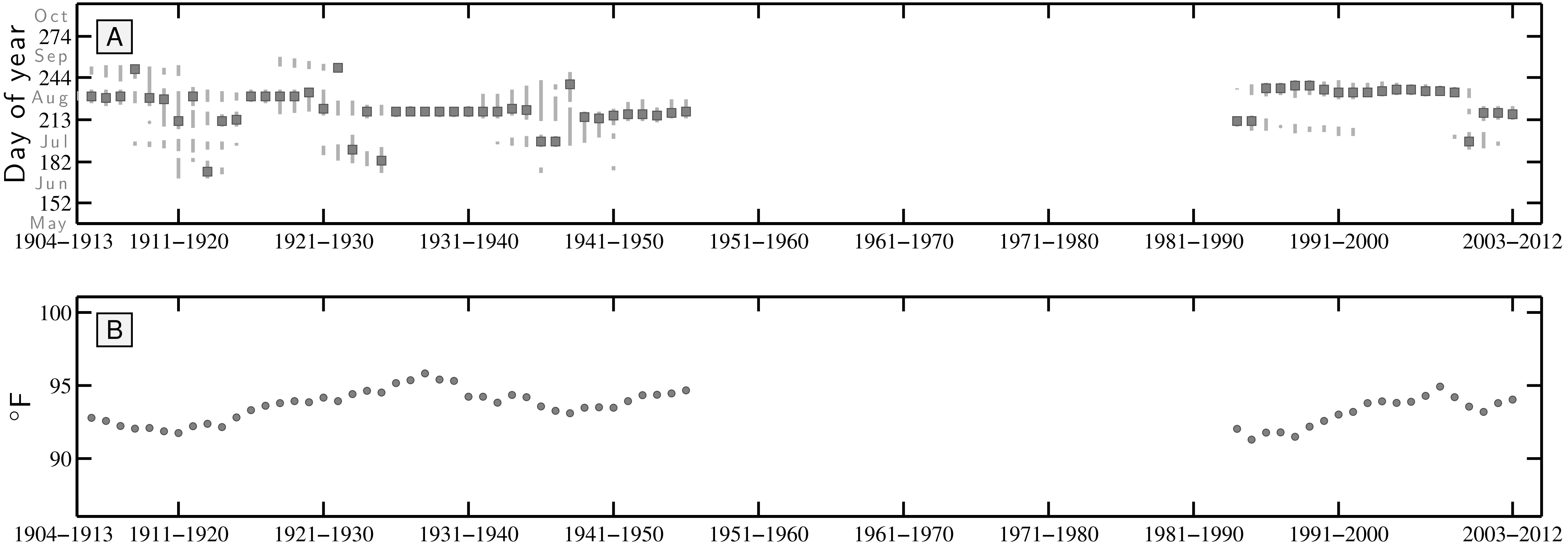

10 year Winter Teletherm dynamics for CRYSTAL SPGS EXP STN, MS:

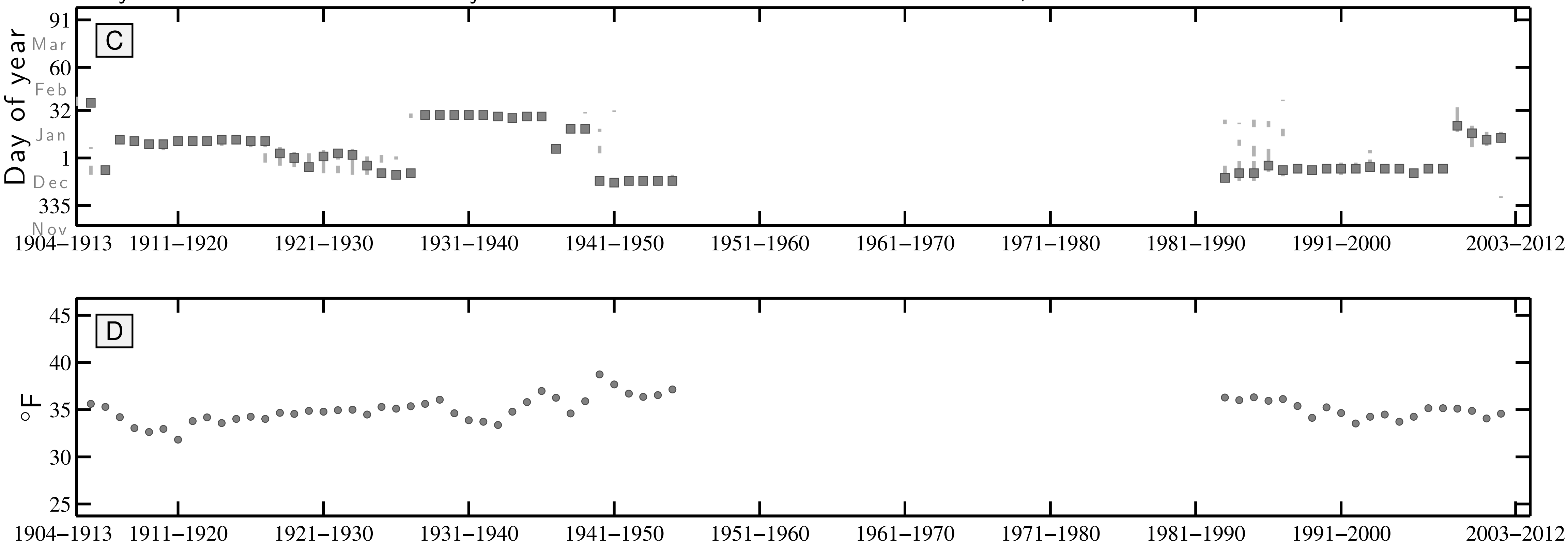

10 year Summer Teletherm dynamics for FOREST, MS:

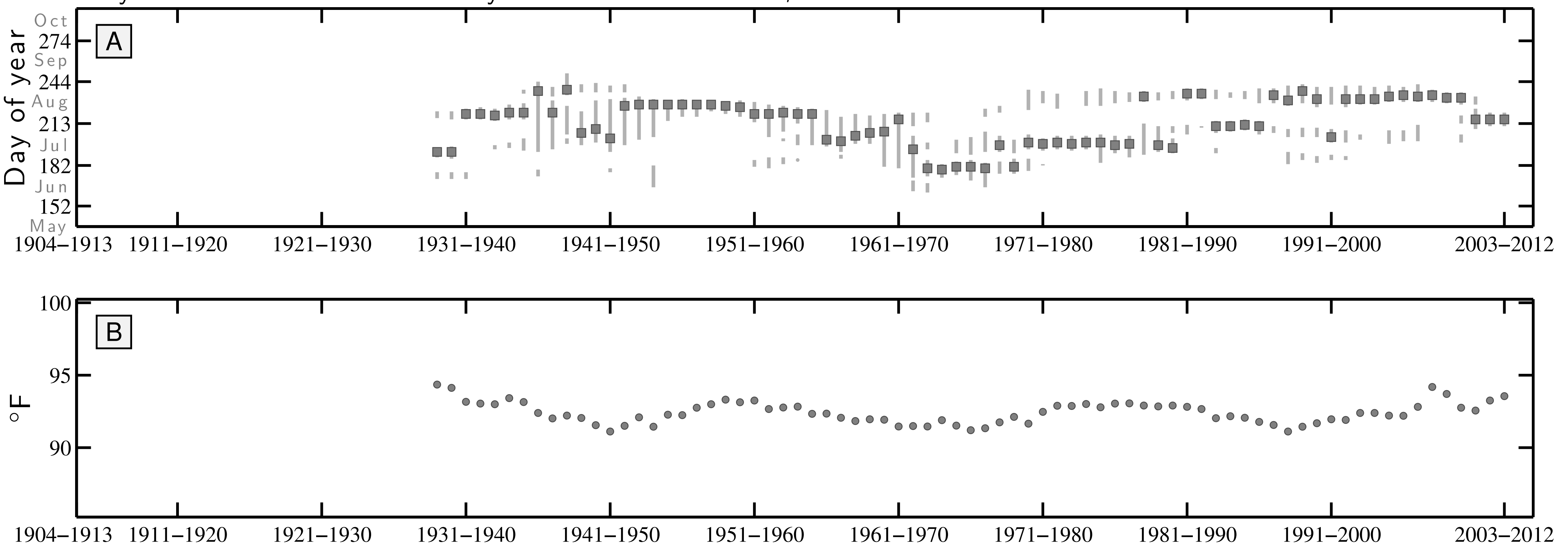

10 year Winter Teletherm dynamics for FOREST, MS:

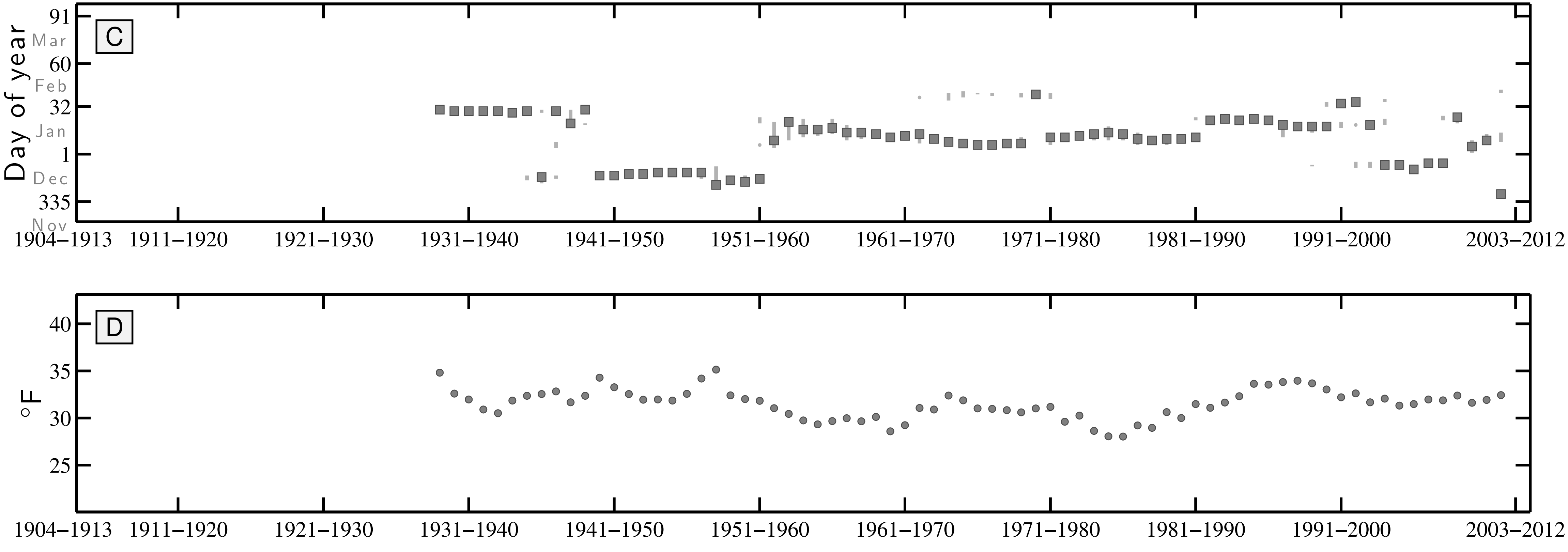

10 year Summer Teletherm dynamics for GREENVILLE, MS:

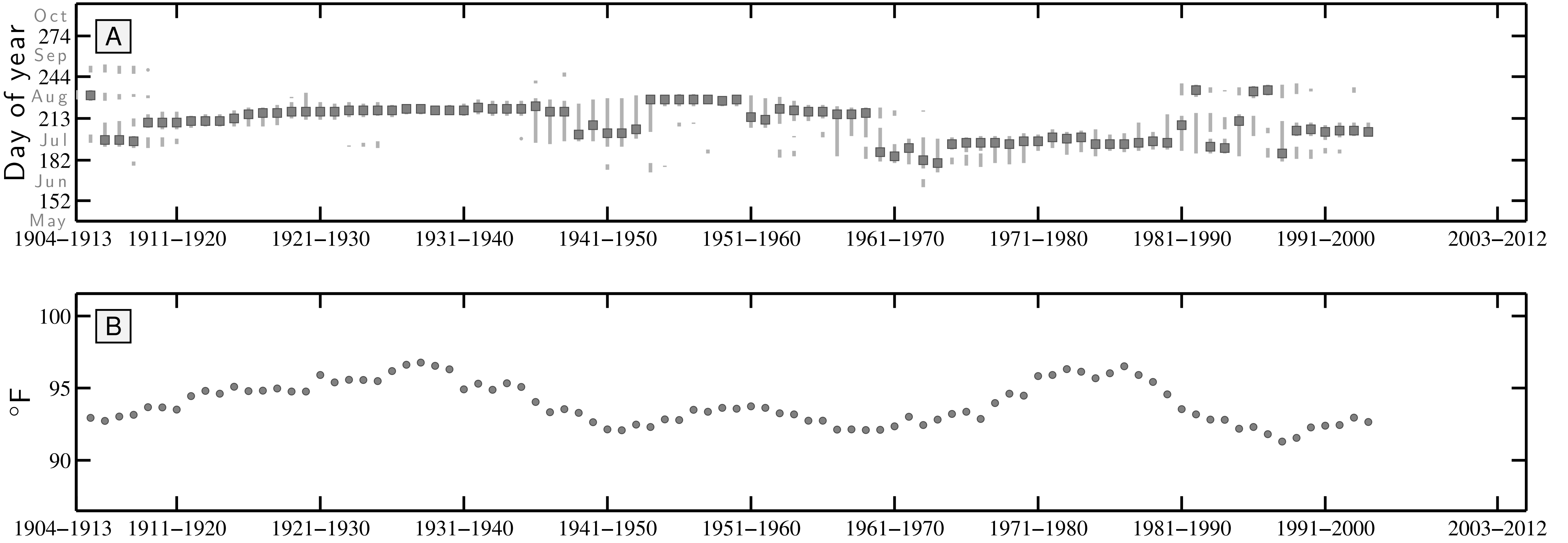

10 year Winter Teletherm dynamics for GREENVILLE, MS:

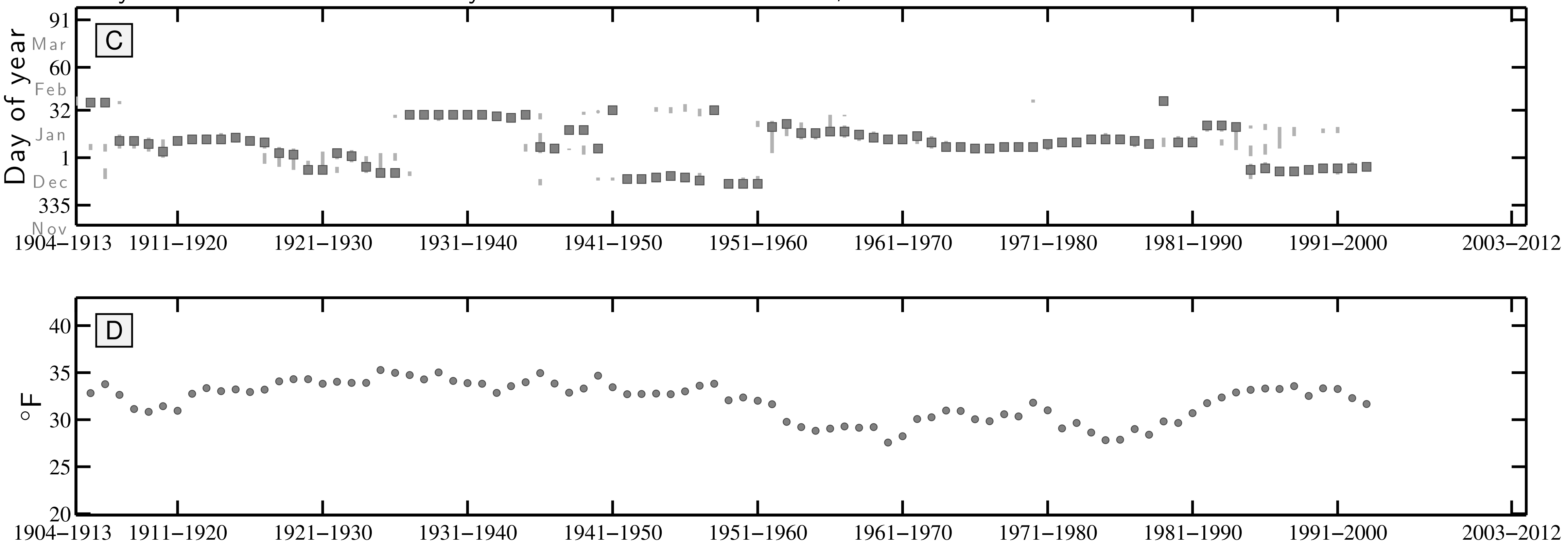

10 year Summer Teletherm dynamics for HATTIESBURG 5SW, MS:

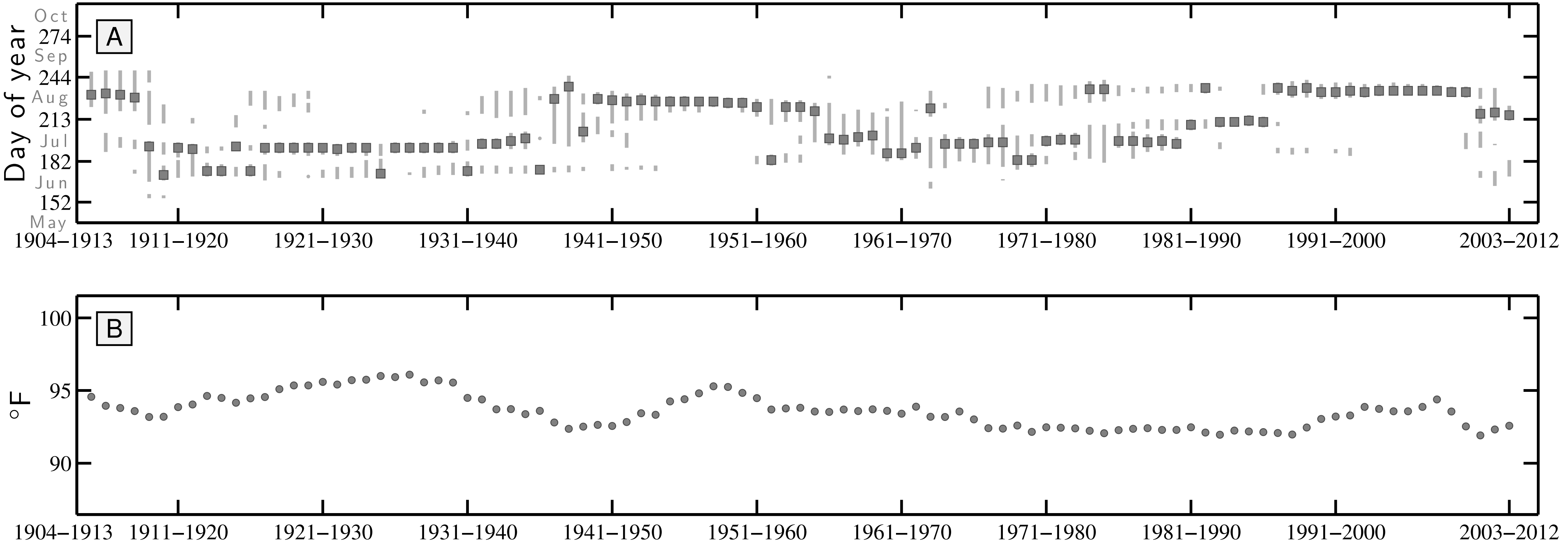

10 year Winter Teletherm dynamics for HATTIESBURG 5SW, MS:

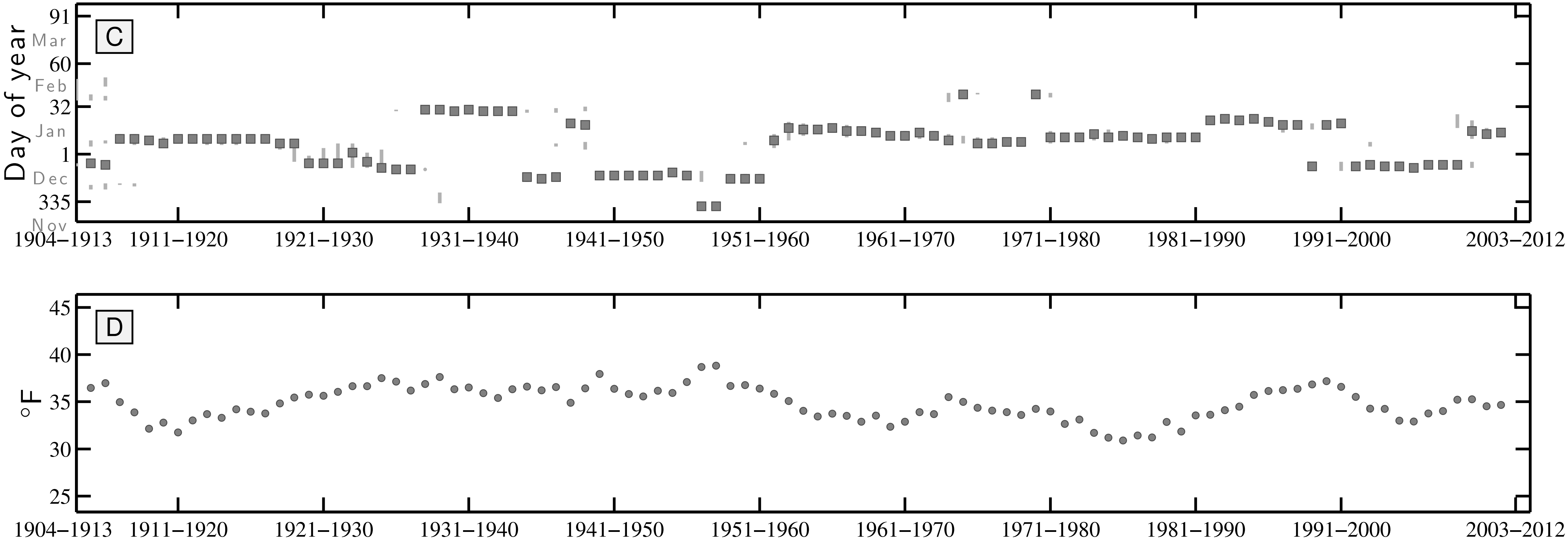

10 year Summer Teletherm dynamics for HERNANDO, MS:

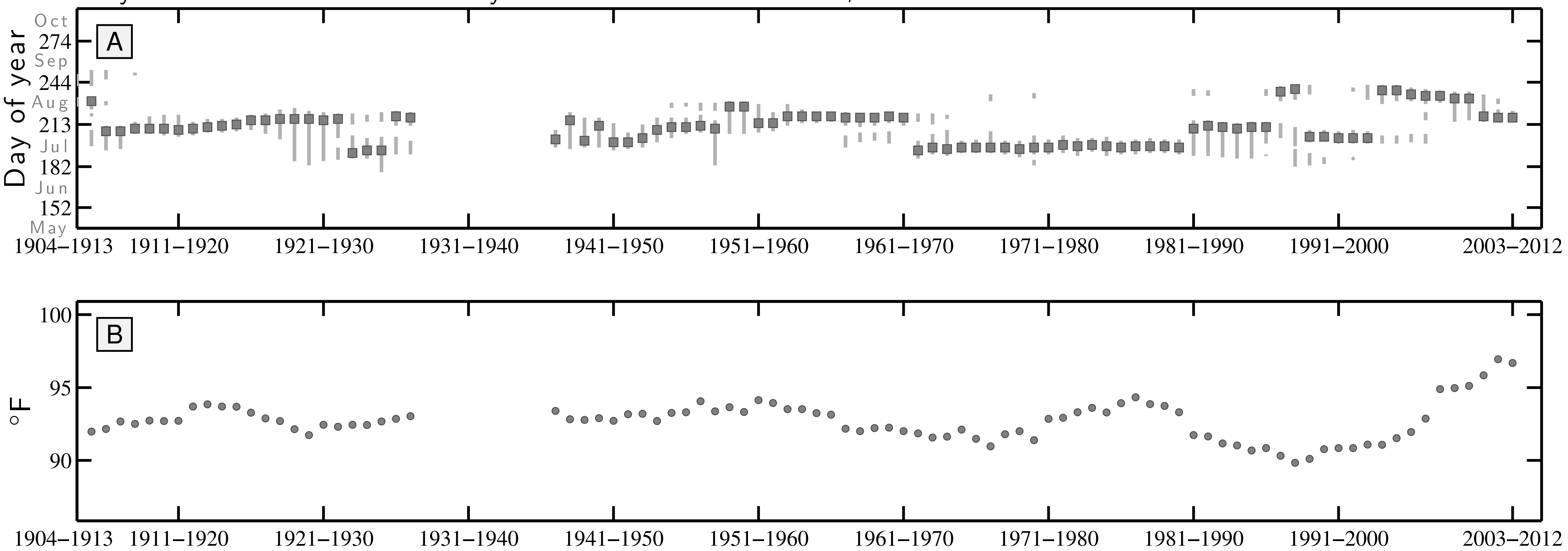

10 year Winter Teletherm dynamics for HERNANDO, MS:

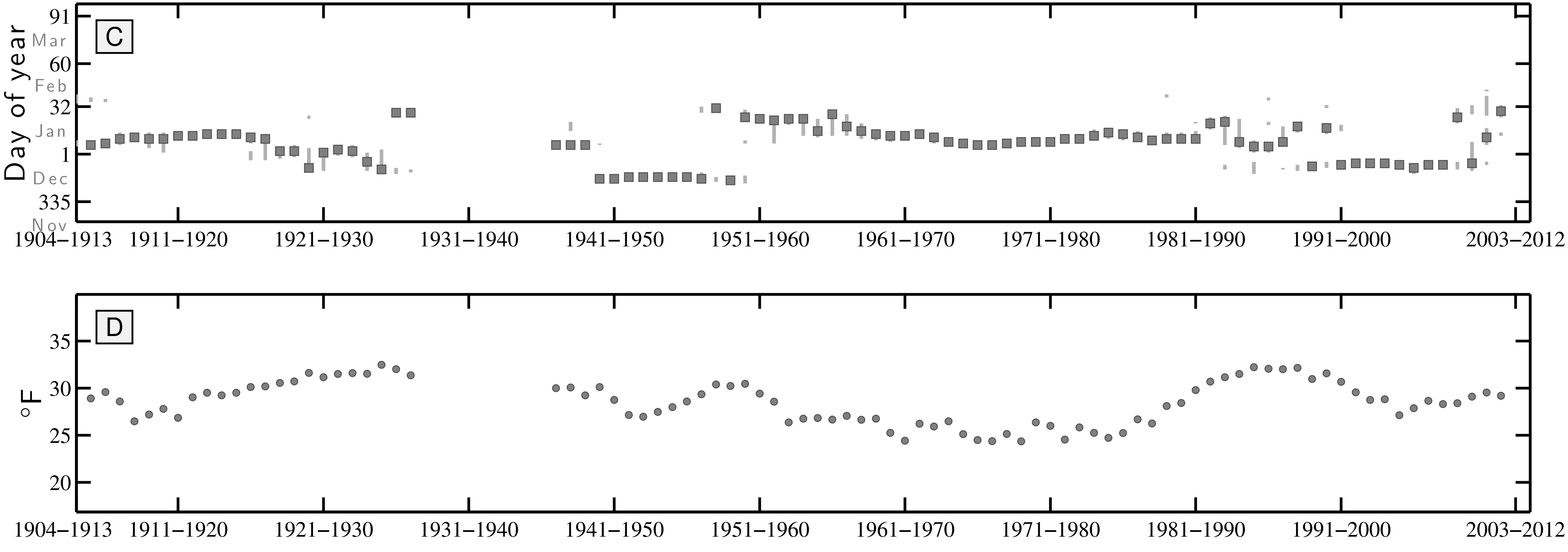

10 year Summer Teletherm dynamics for HOLLY SPRINGS 4 N, MS:

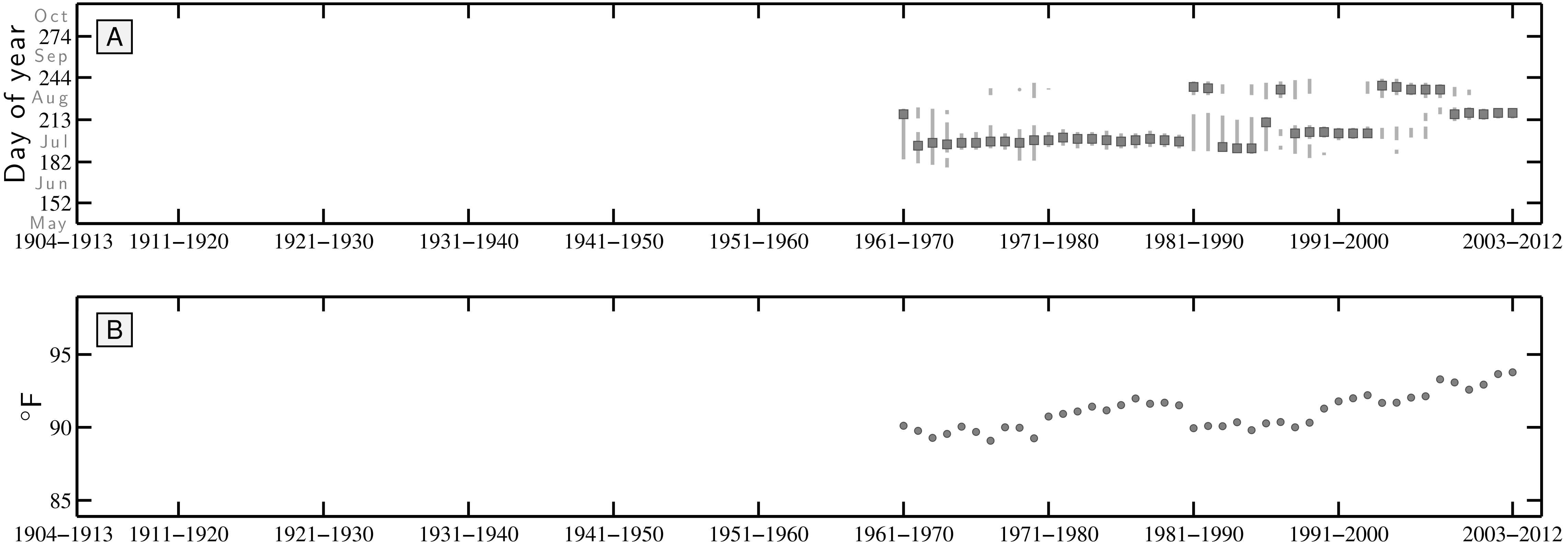

10 year Winter Teletherm dynamics for HOLLY SPRINGS 4 N, MS:

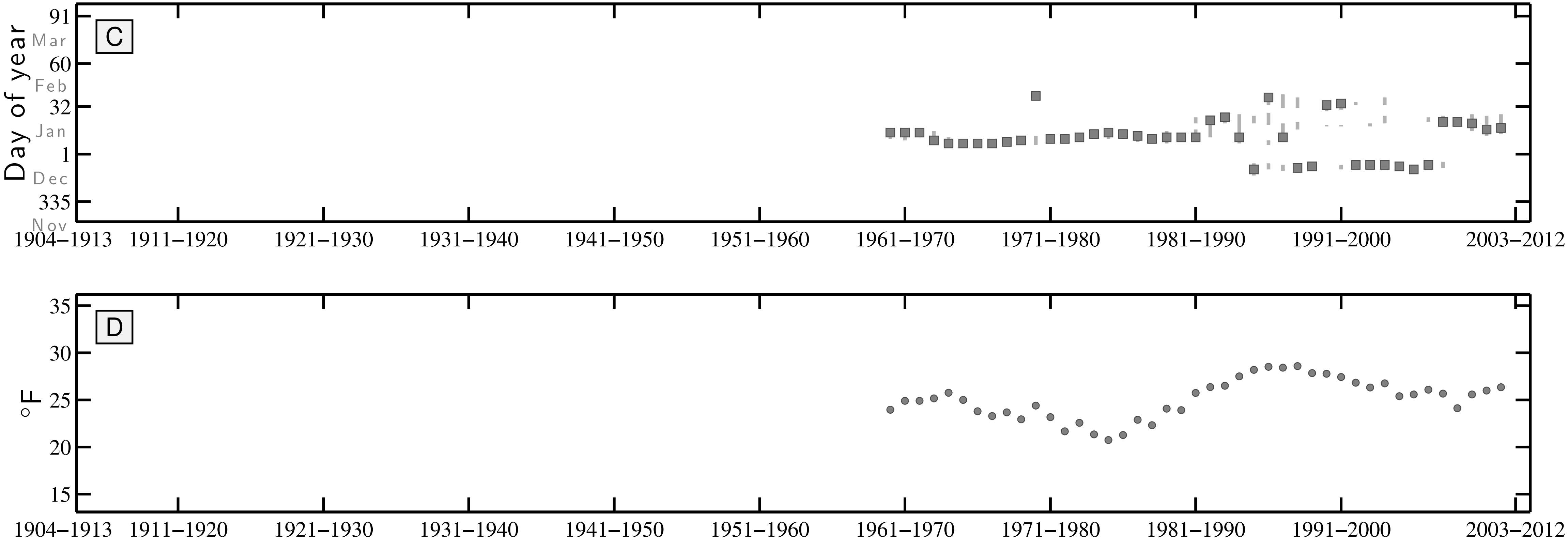

10 year Summer Teletherm dynamics for KOSCIUSKO, MS:

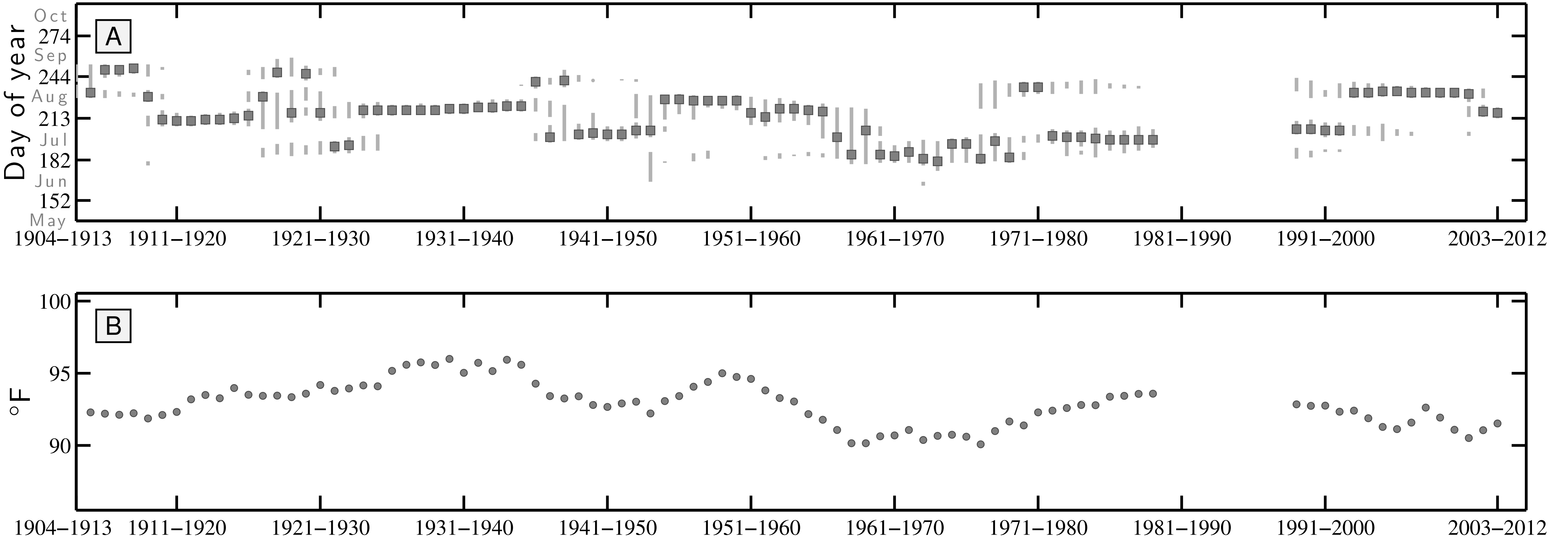

10 year Winter Teletherm dynamics for KOSCIUSKO, MS:

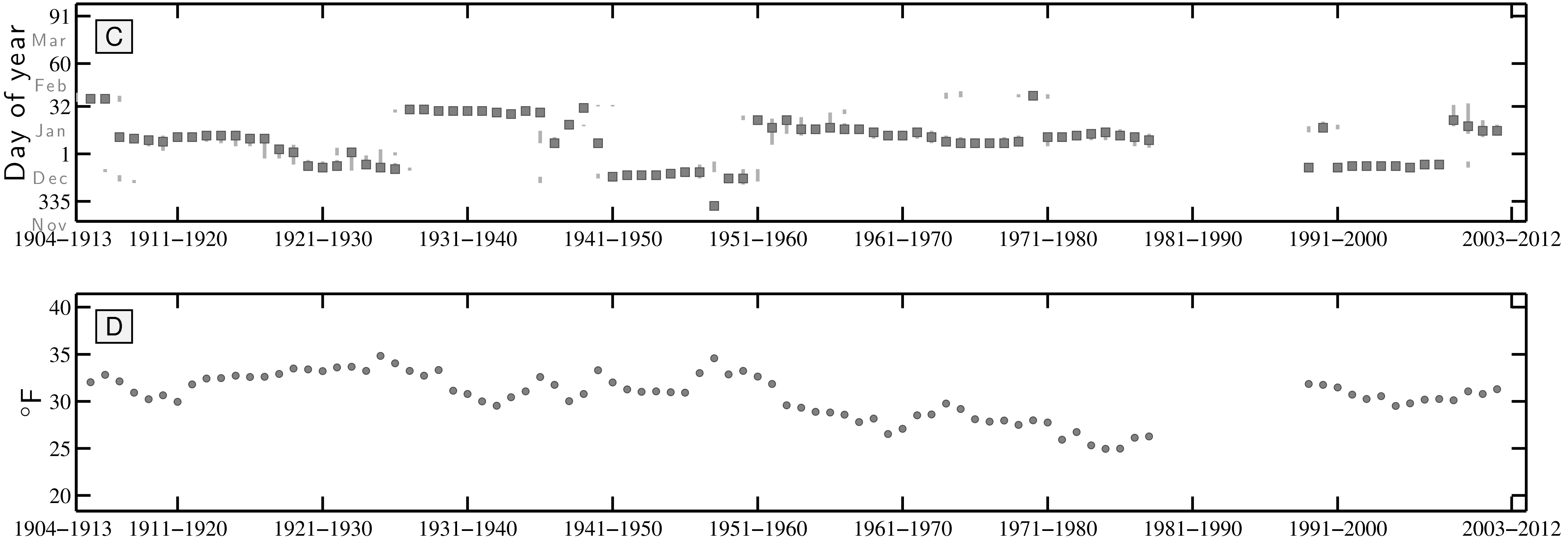

10 year Summer Teletherm dynamics for LAUREL, MS:

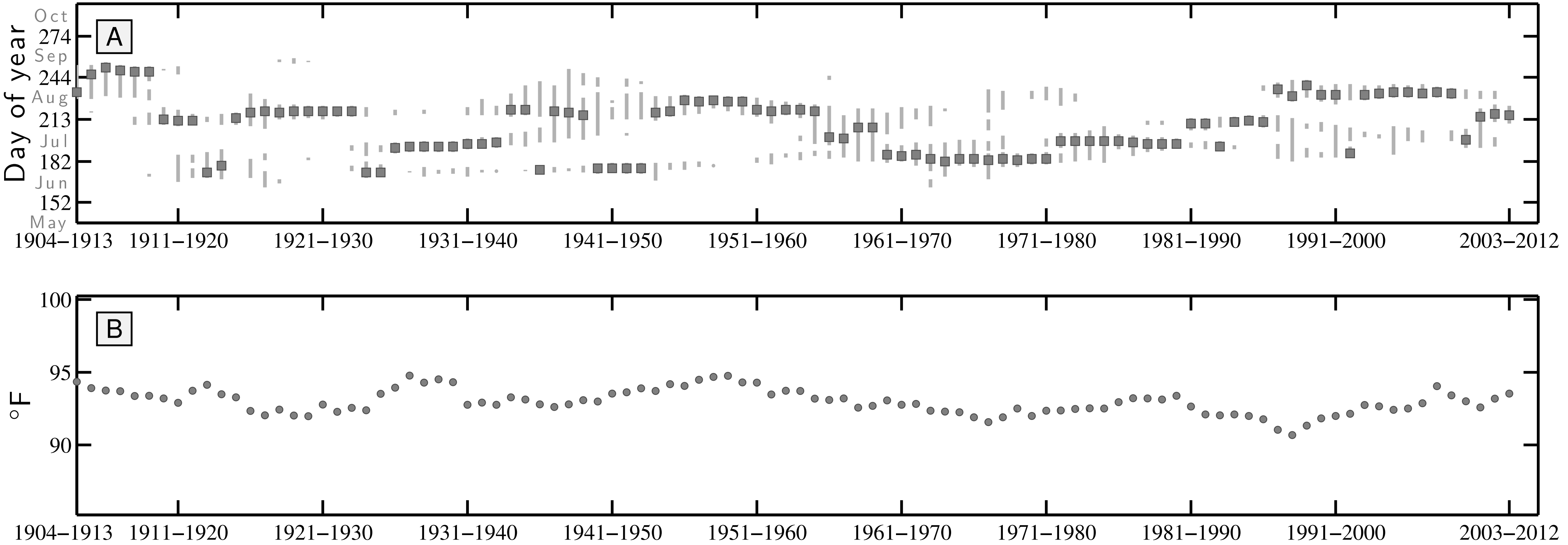

10 year Winter Teletherm dynamics for LAUREL, MS:

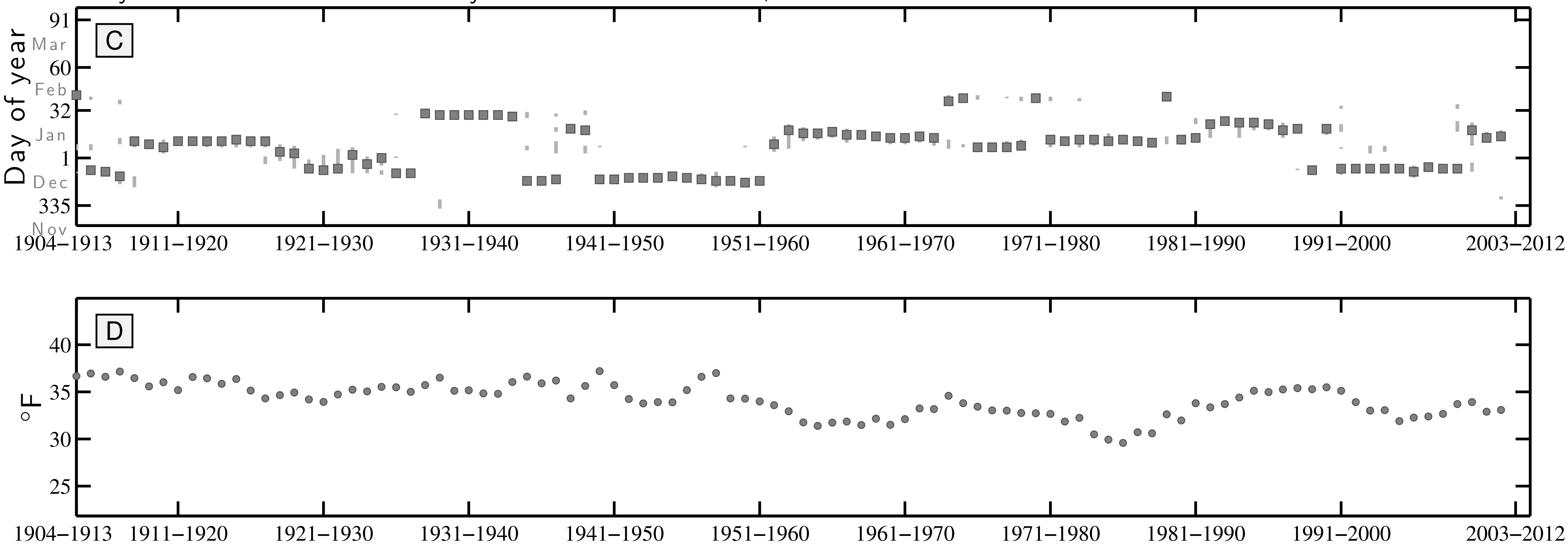

10 year Summer Teletherm dynamics for LOUISVILLE, MS:

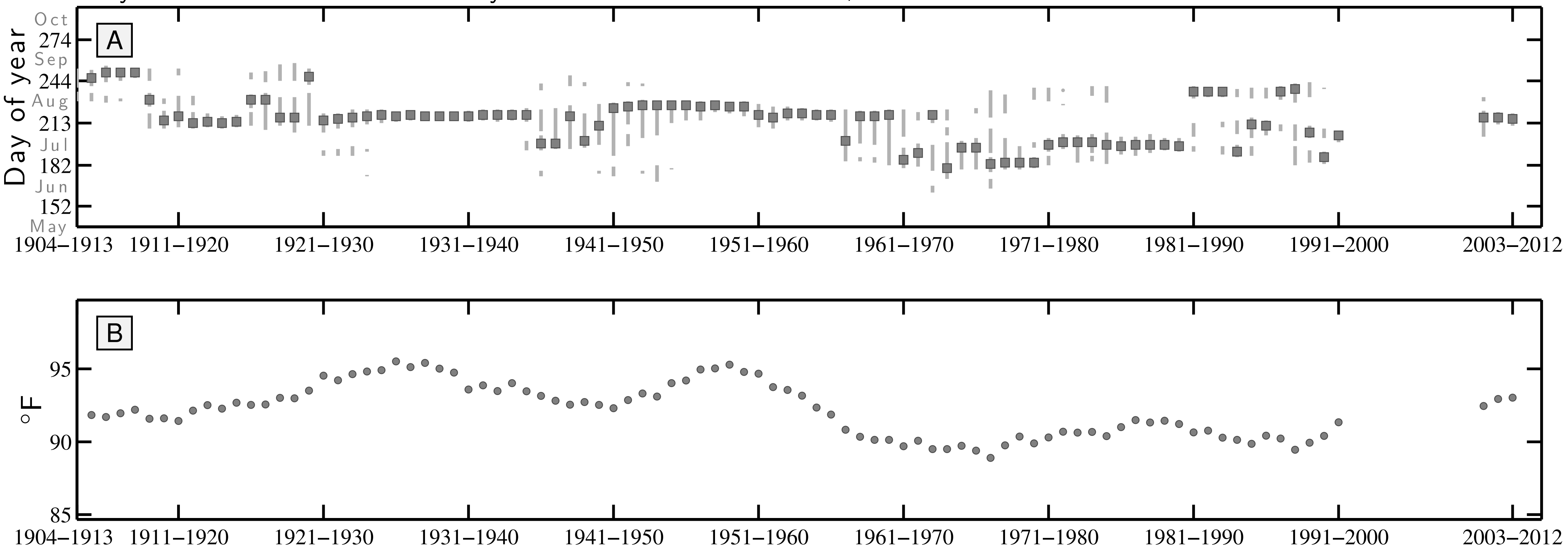

10 year Winter Teletherm dynamics for LOUISVILLE, MS:

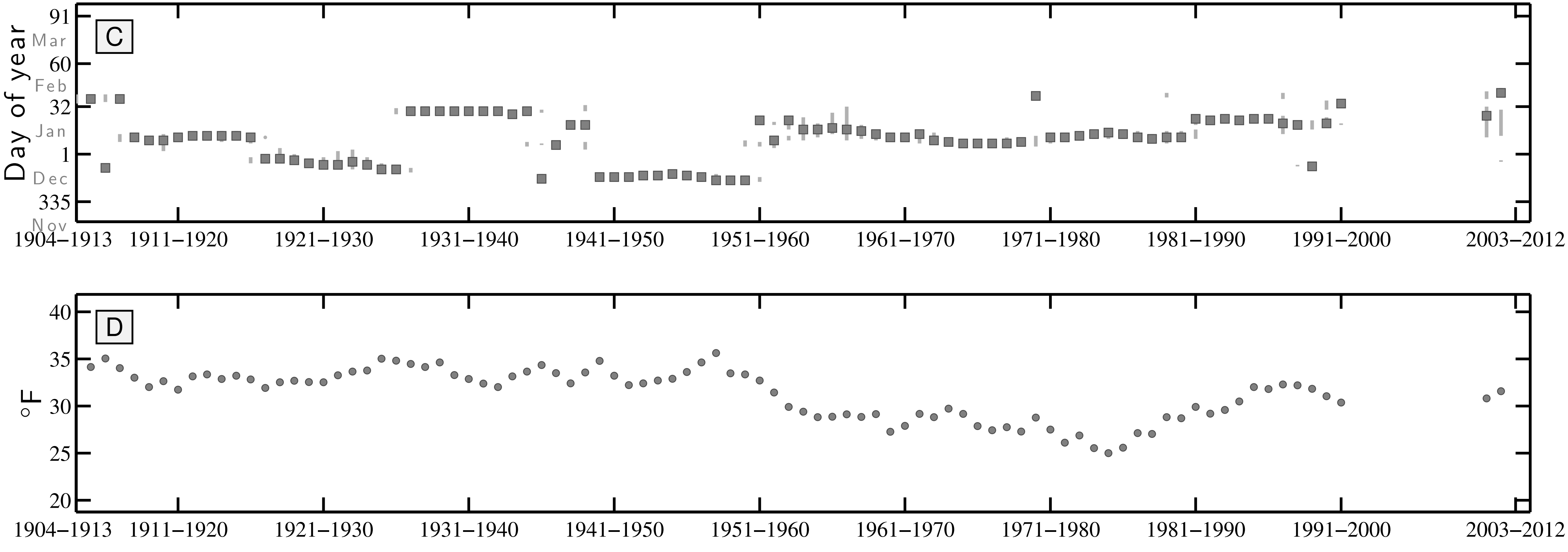

10 year Summer Teletherm dynamics for MONTICELLO, MS:

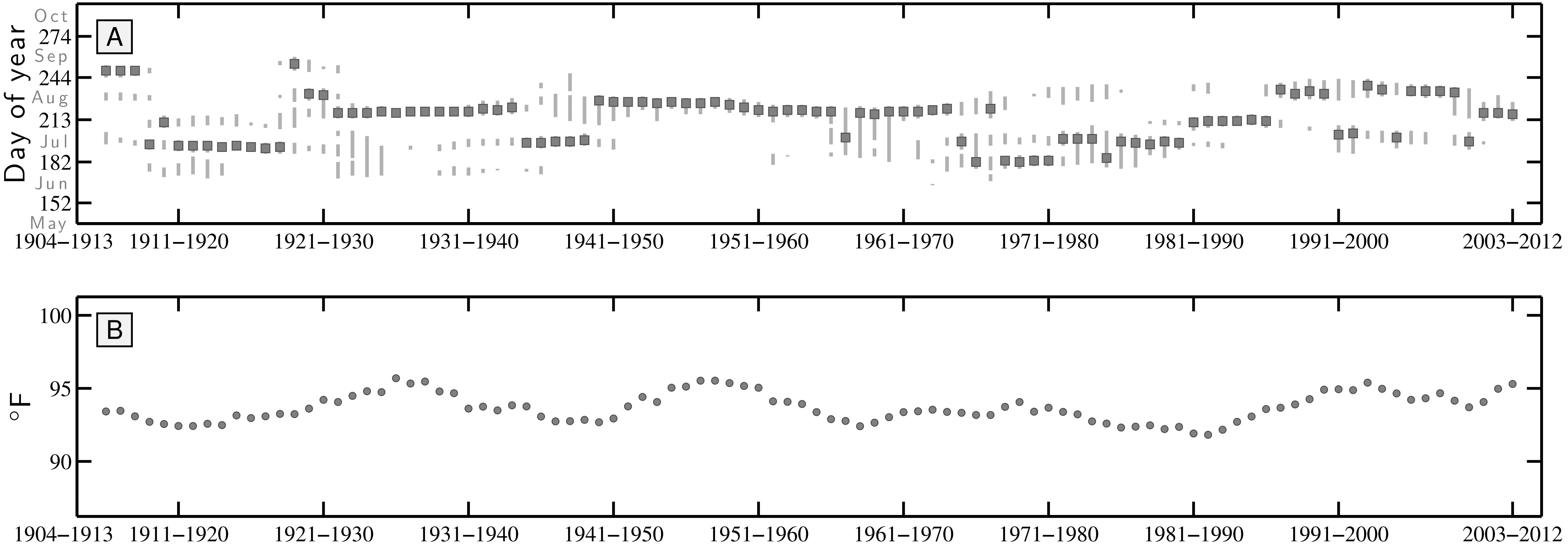

10 year Winter Teletherm dynamics for MONTICELLO, MS:

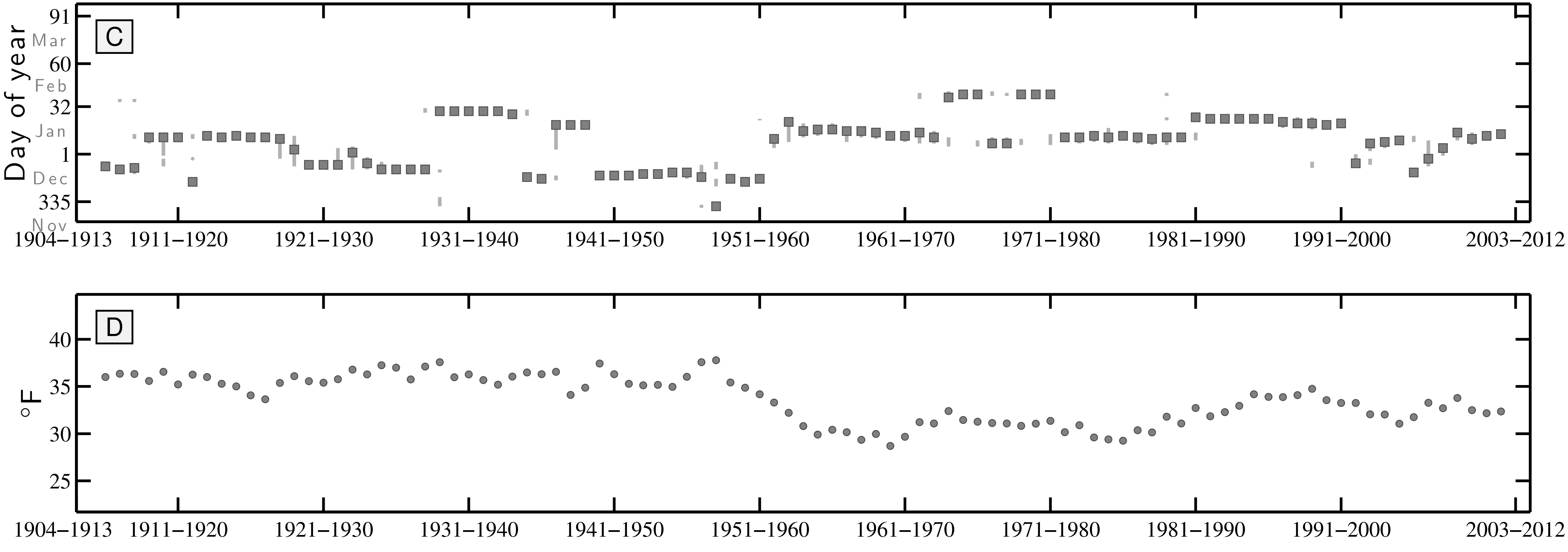

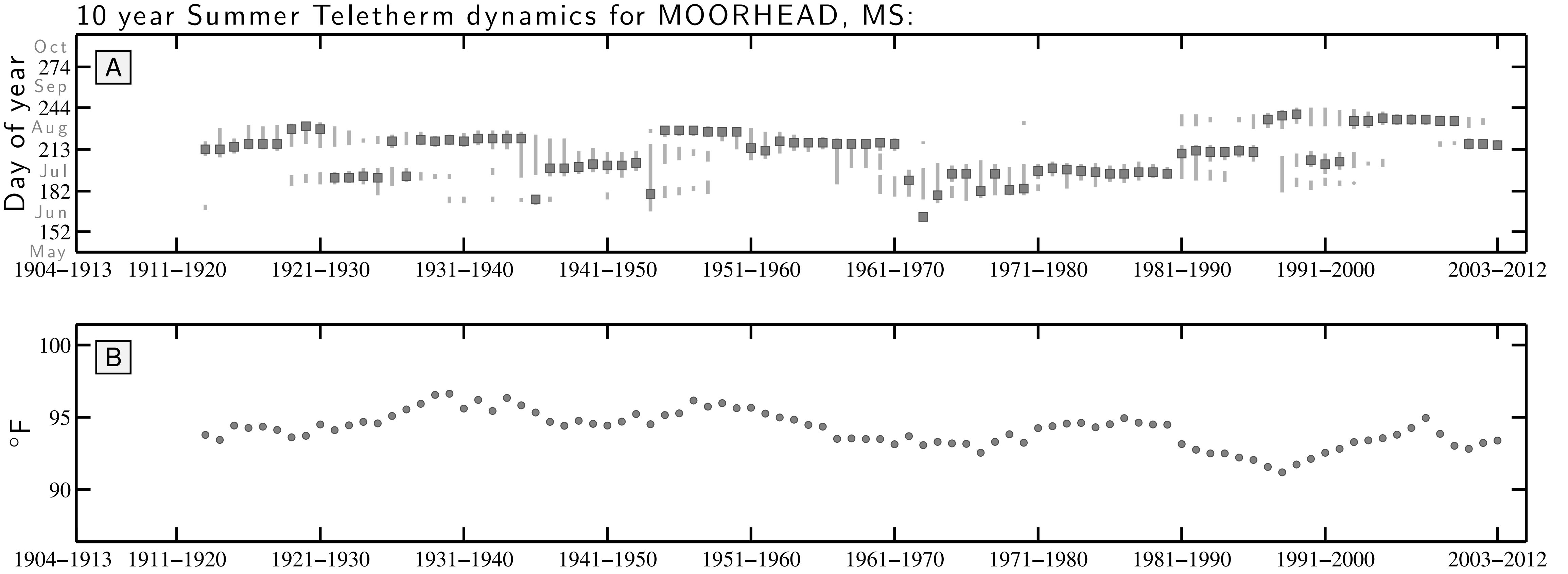

10 year Summer Teletherm dynamics for MOORHEAD, MS:
A
B
Day of year
Oct
274
Sep
244
Aug
213
Jul
182
Jun
152
May
°F
100
95
90
1904–1913
1911–1920
1921–1930
1931–1940
1941–1950
1951–1960
1961–1970
1971–1980
1981–1990
1991–2000
2003–2012

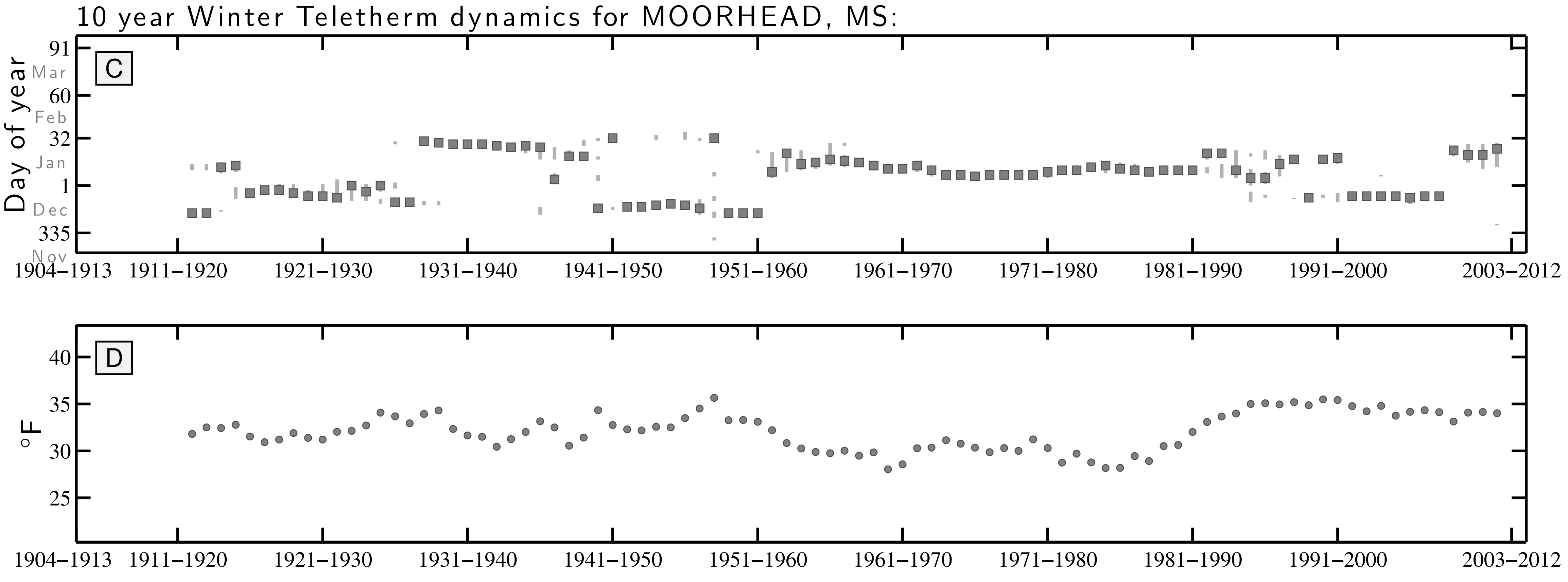

10 year Winter Teletherm dynamics for MOORHEAD, MS:
C
D
Day of year
91
Mar
60
Feb
32
Jan
1
Dec
335
Nov
°F
40
35
30
25
1904–1913
1911–1920
1921–1930
1931–1940
1941–1950
1951–1960
1961–1970
1971–1980
1981–1990
1991–2000
2003–2012

10 year Summer Teletherm dynamics for NATCHEZ, MS:

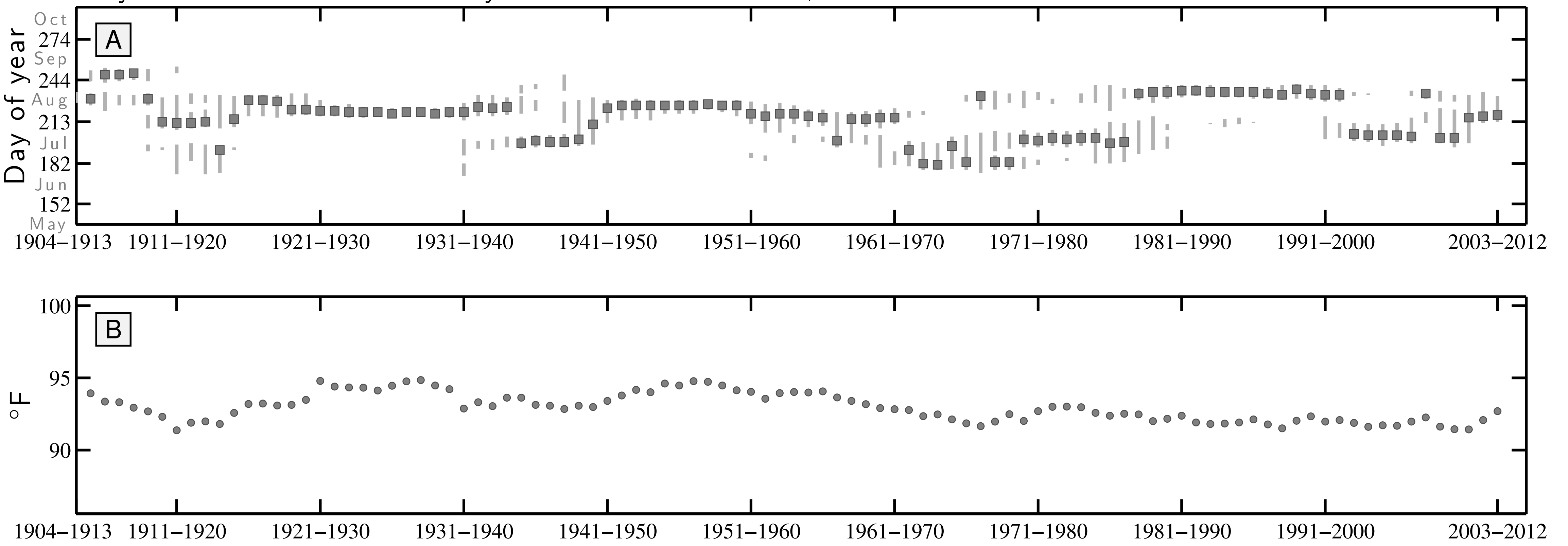

10 year Winter Teletherm dynamics for NATCHEZ, MS:

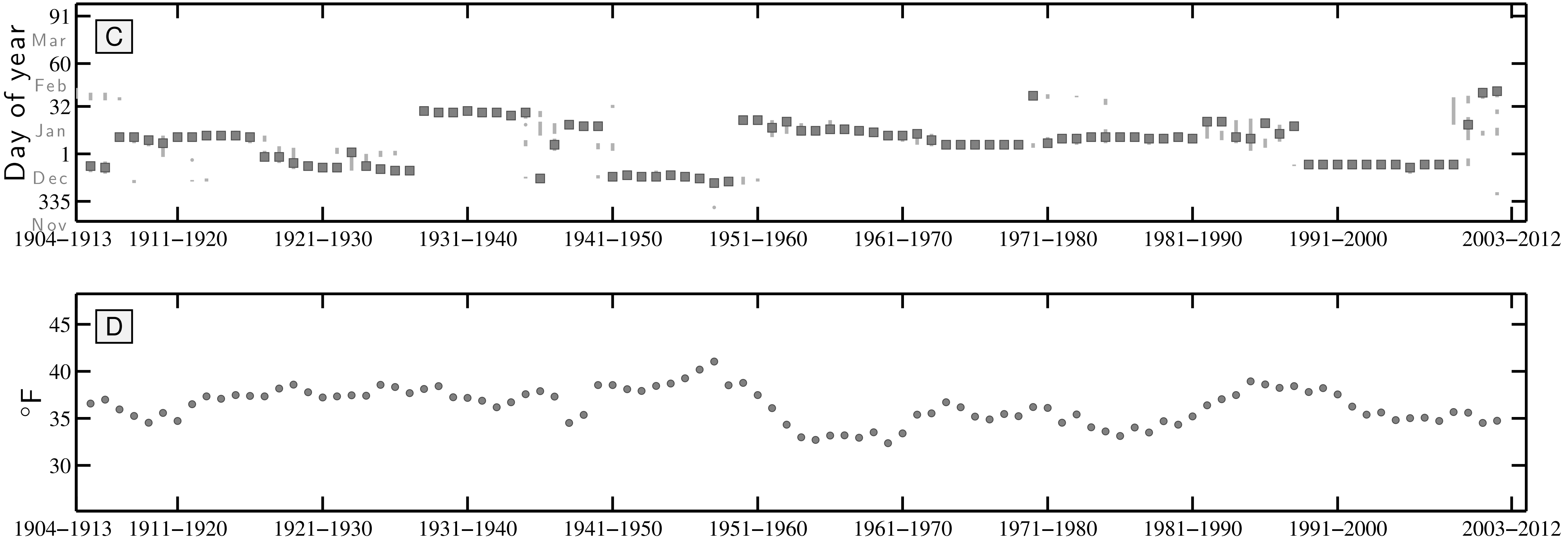

10 year Summer Teletherm dynamics for PASCAGOULA 3 NE, MS:

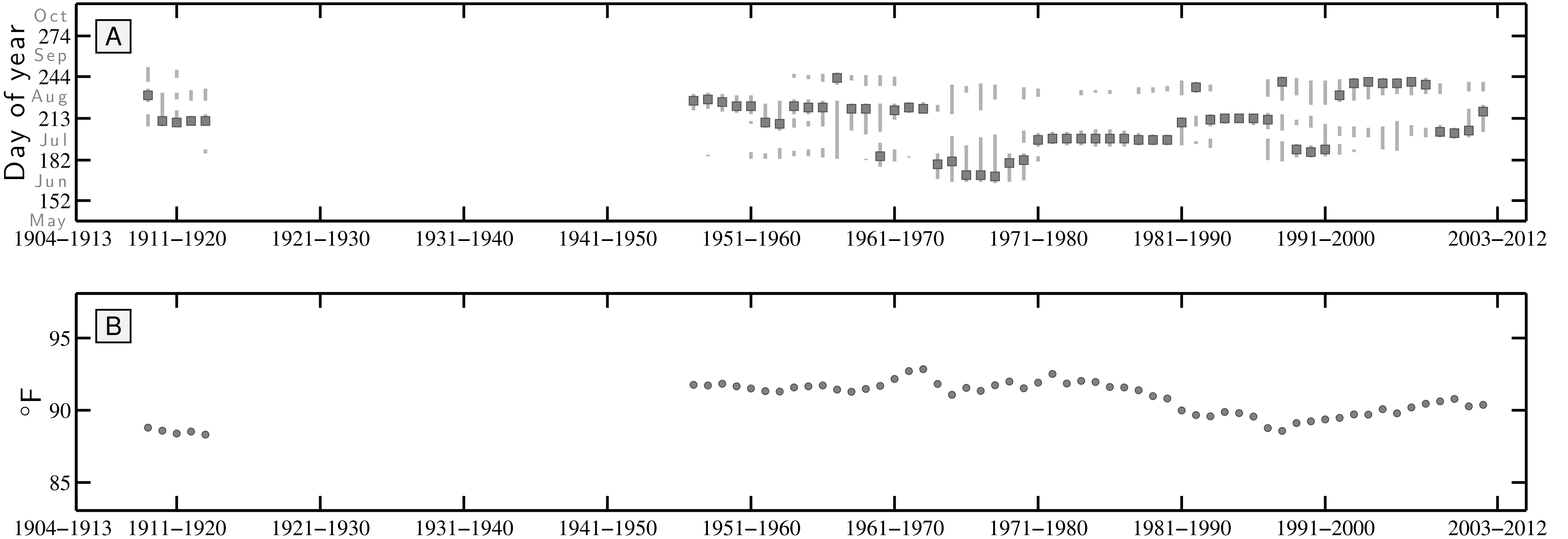

10 year Winter Teletherm dynamics for PASCAGOULA 3 NE, MS:

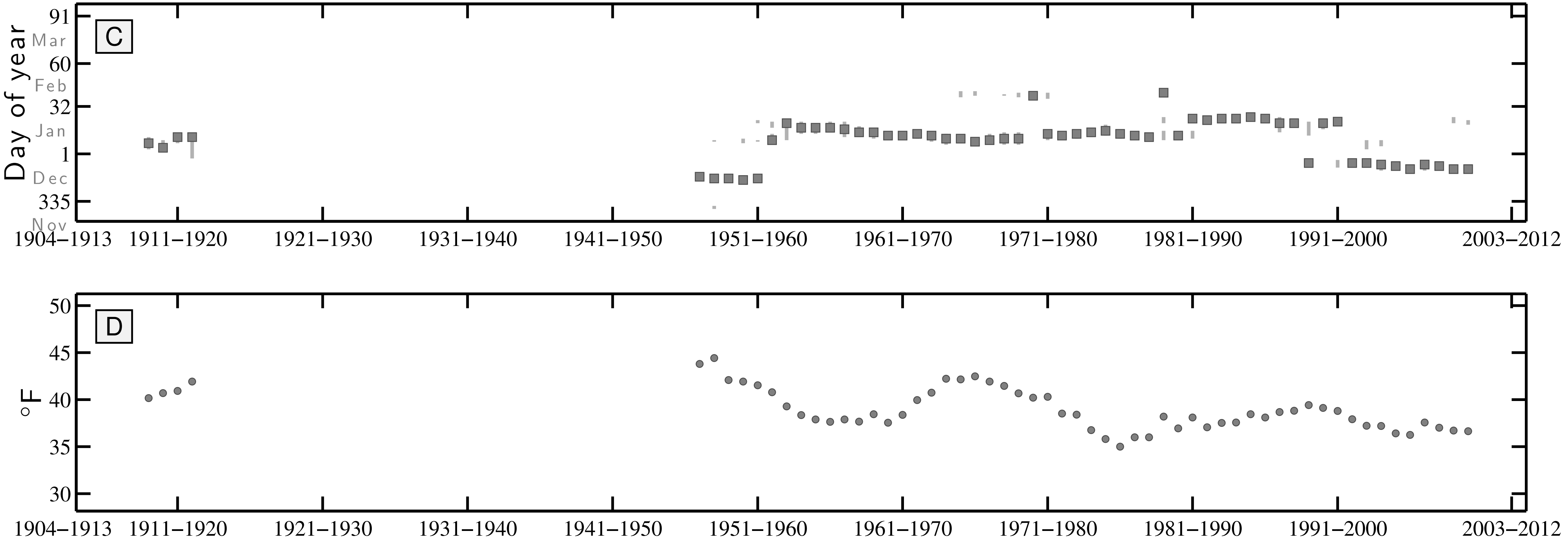

10 year Summer Teletherm dynamics for PONTOTOC EXP STN, MS:

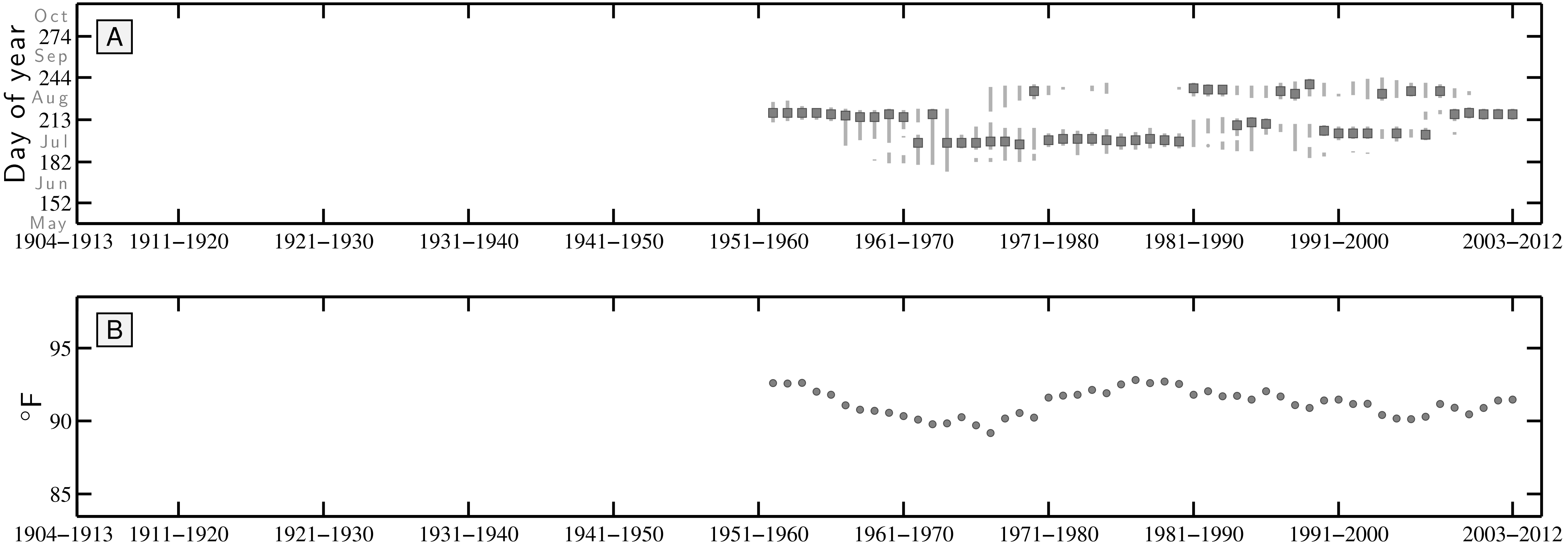

10 year Winter Teletherm dynamics for PONTOTOC EXP STN, MS:

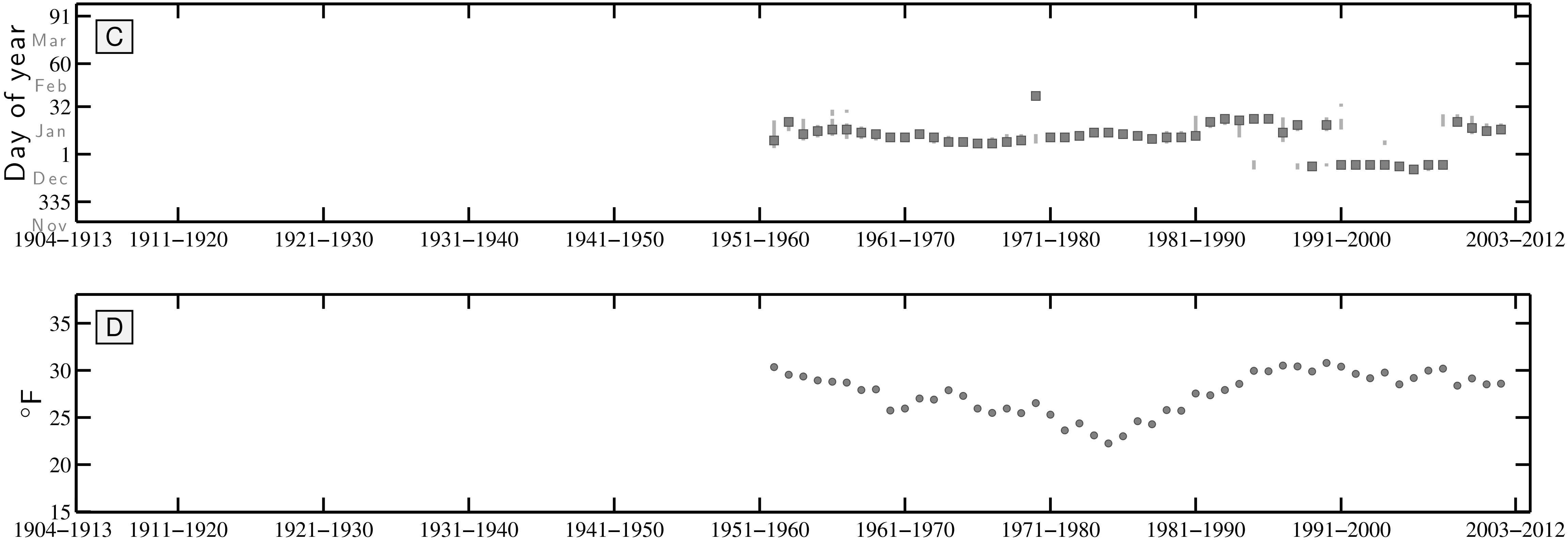

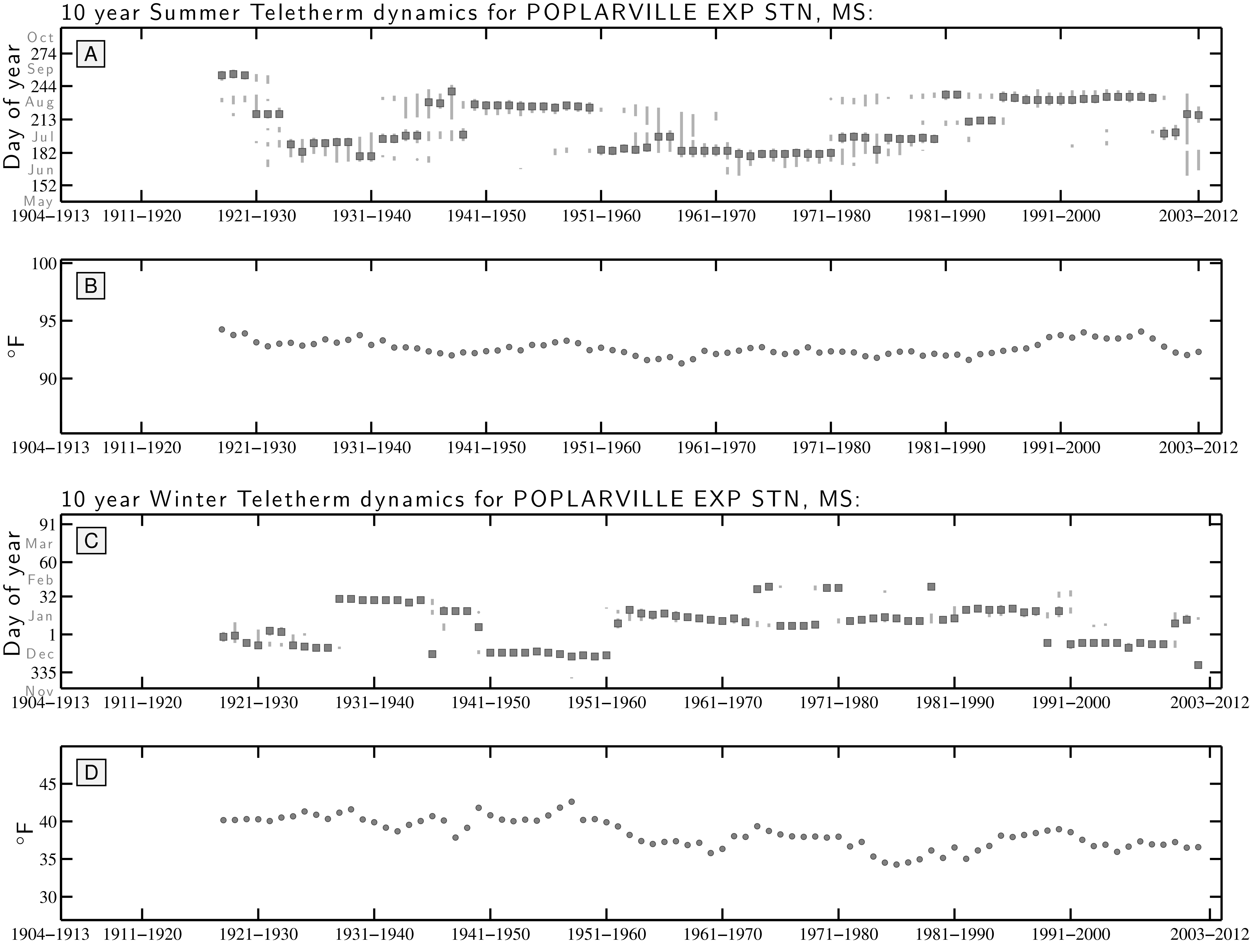
10 year Summer Teletherm dynamics for POPLARVILLE EXP STN, MS:
A
Day of year
Oct
274
Sep
244
Aug
213
Jul
182
Jun
152
May
1904–1913
1911–1920
1921–1930
1931–1940
1941–1950
1951–1960
1961–1970
1971–1980
1981–1990
1991–2000
2003–2012
B
°F
100
95
90
10 year Winter Teletherm dynamics for POPLARVILLE EXP STN, MS:
C
91
Mar
60
Feb
32
Jan
1
Dec
335
Nov
D
45
40
35
30

10 year Summer Teletherm dynamics for PORT GIBSON 1 NE, MS:

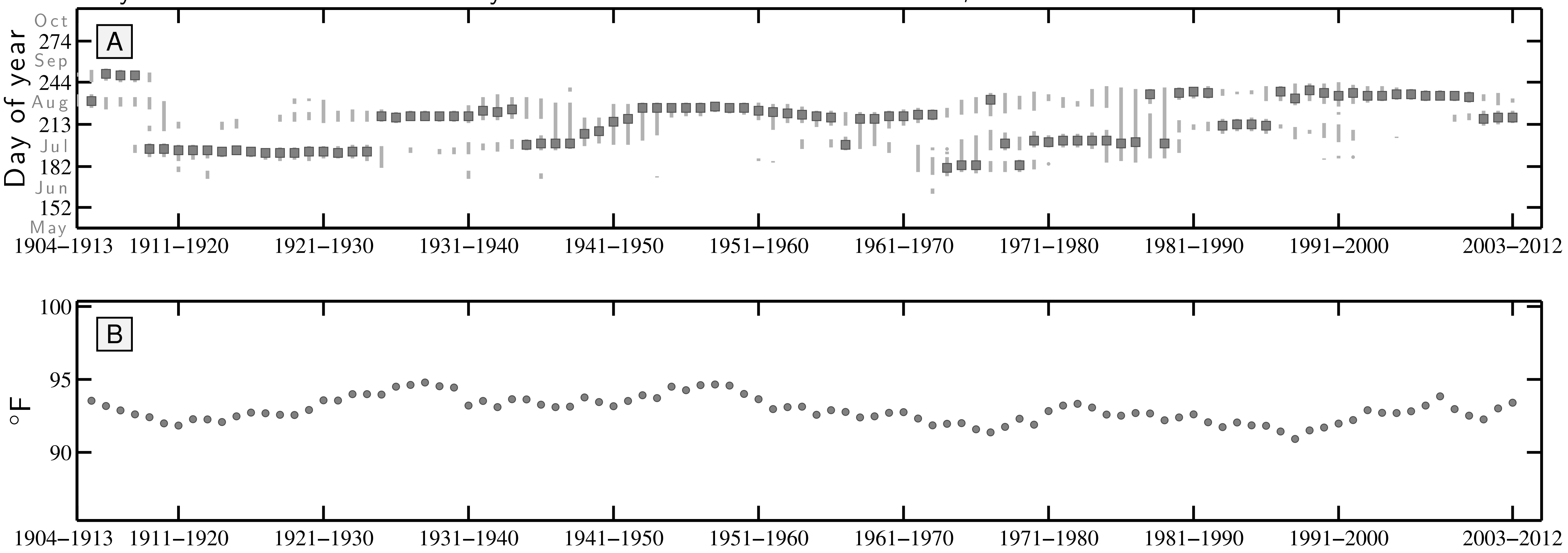

10 year Winter Teletherm dynamics for PORT GIBSON 1 NE, MS:

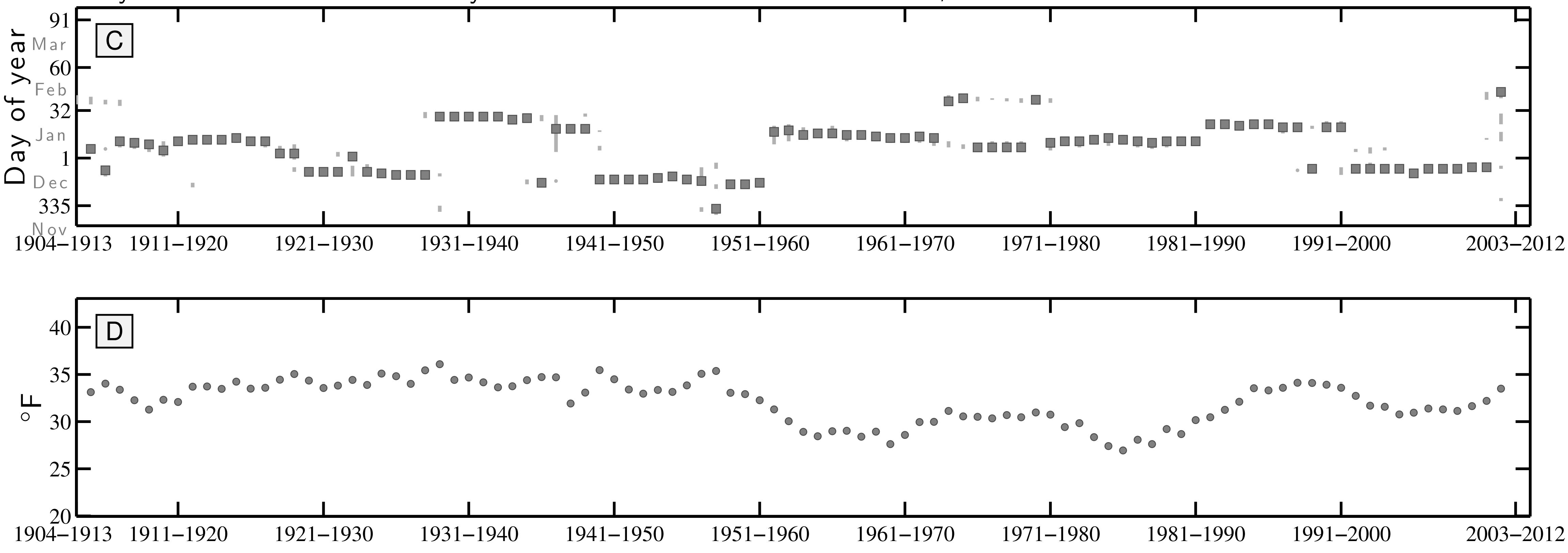

10 year Summer Teletherm dynamics for STATE UNIV, MS:

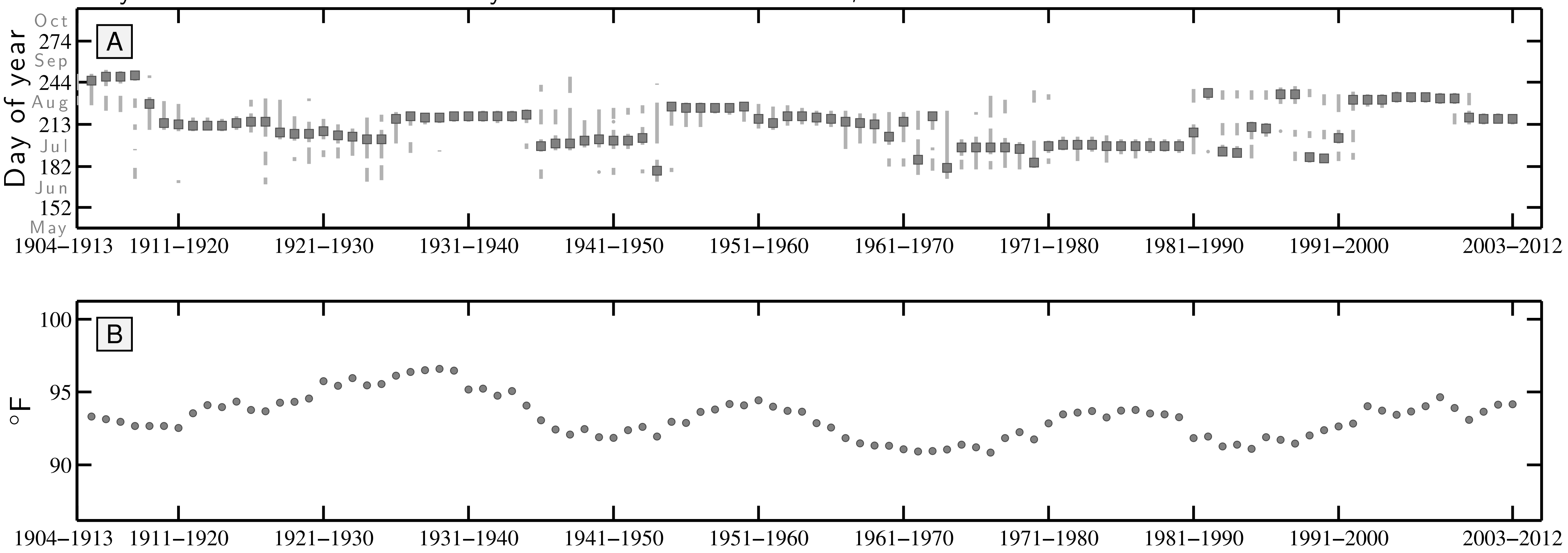

10 year Winter Teletherm dynamics for STATE UNIV, MS:

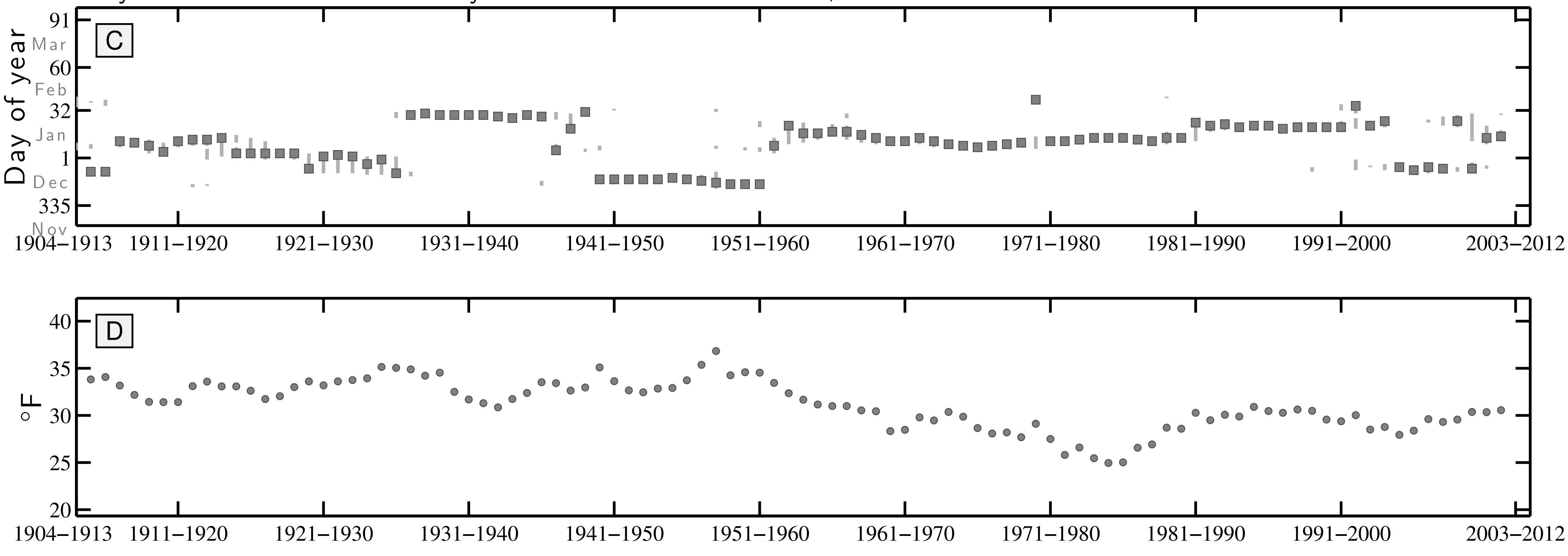

10 year Summer Teletherm dynamics for UNIVERSITY, MS:

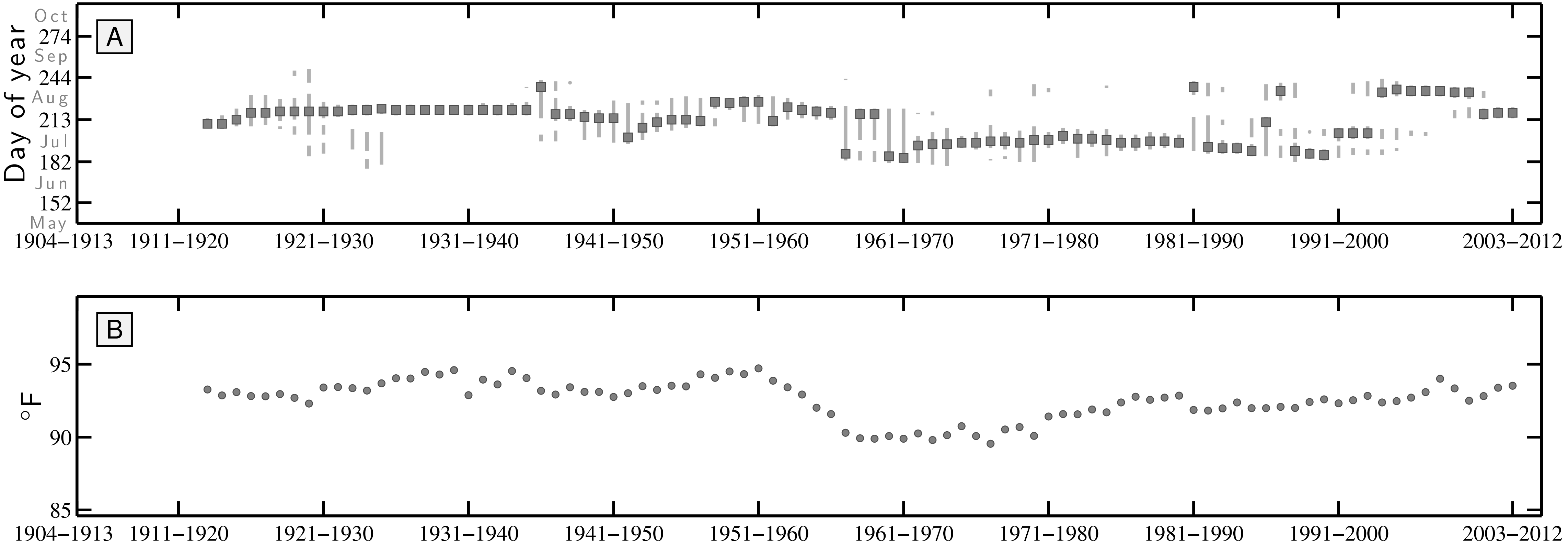

10 year Winter Teletherm dynamics for UNIVERSITY, MS:

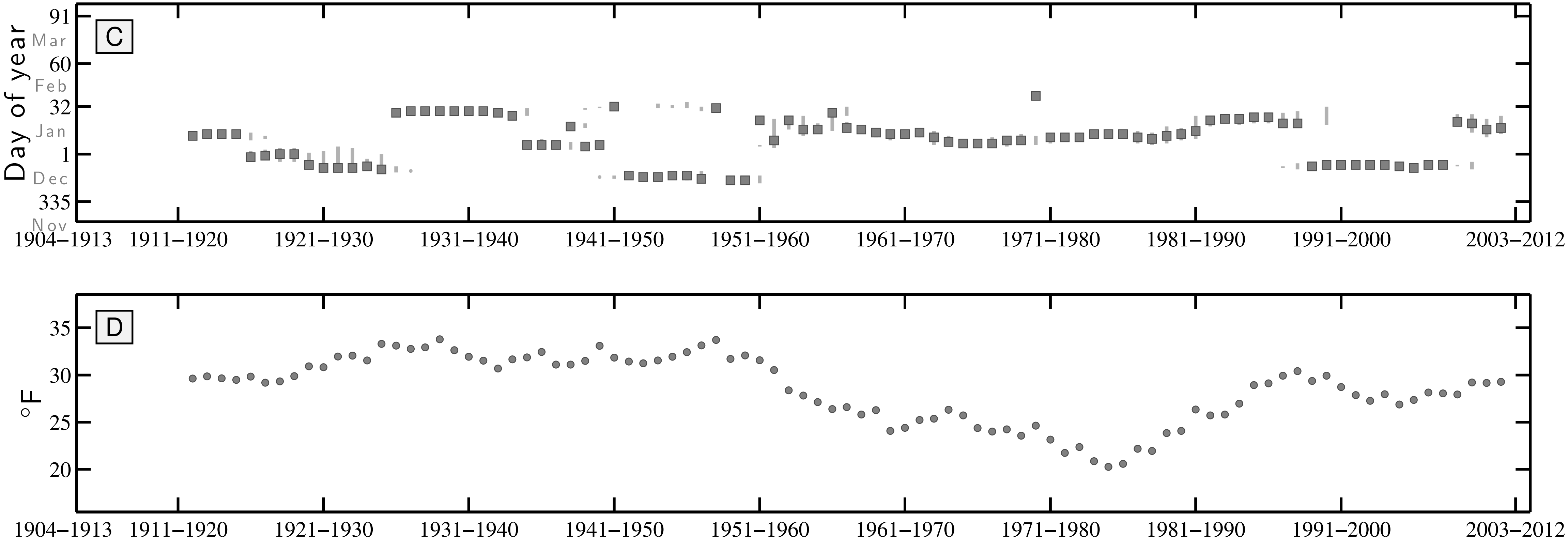

10 year Summer Teletherm dynamics for WATER VALLEY, MS:

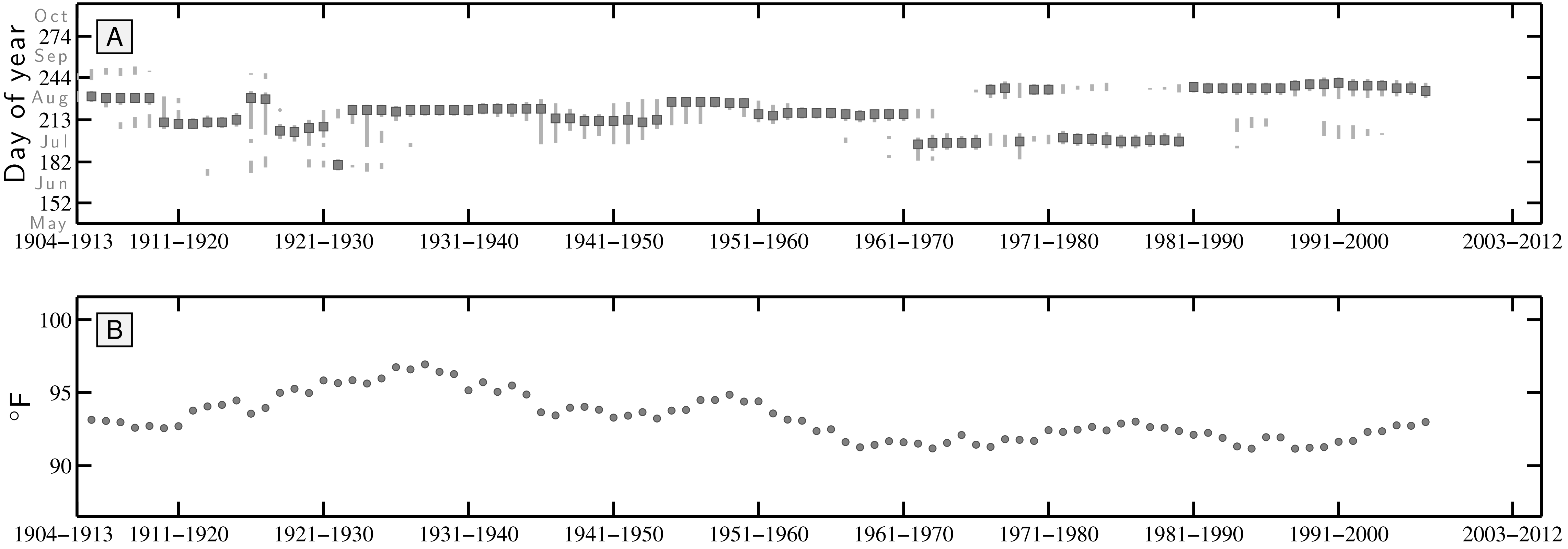

10 year Winter Teletherm dynamics for WATER VALLEY, MS:

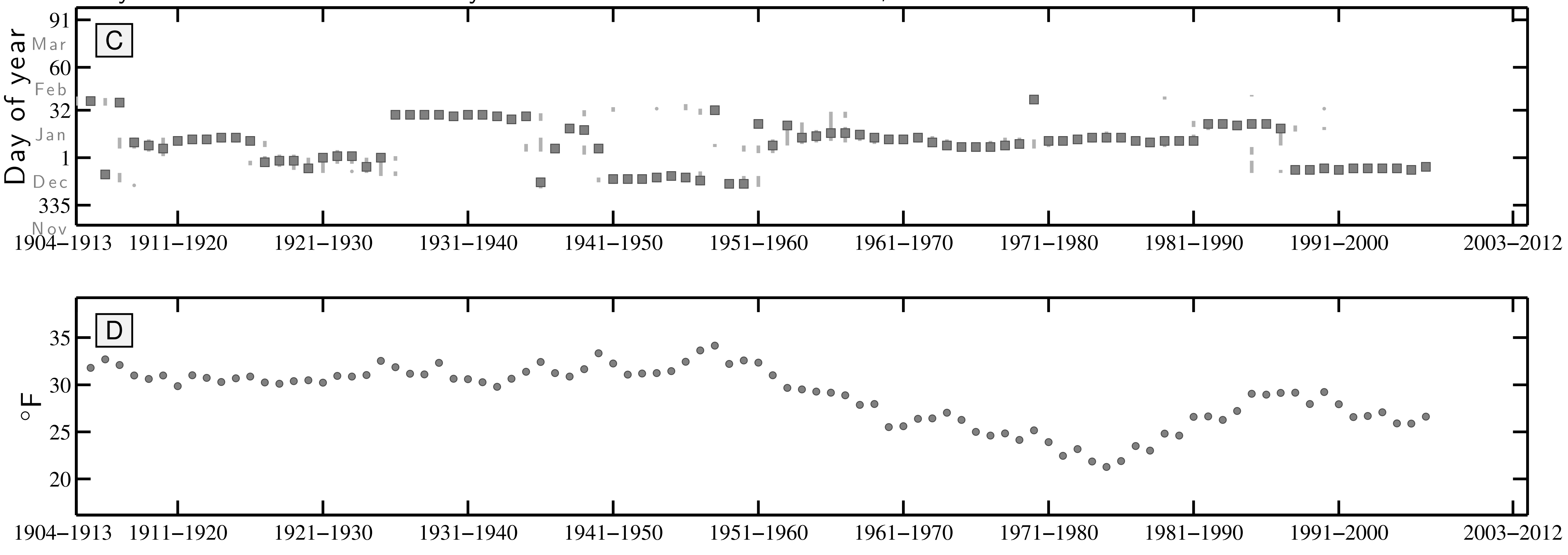

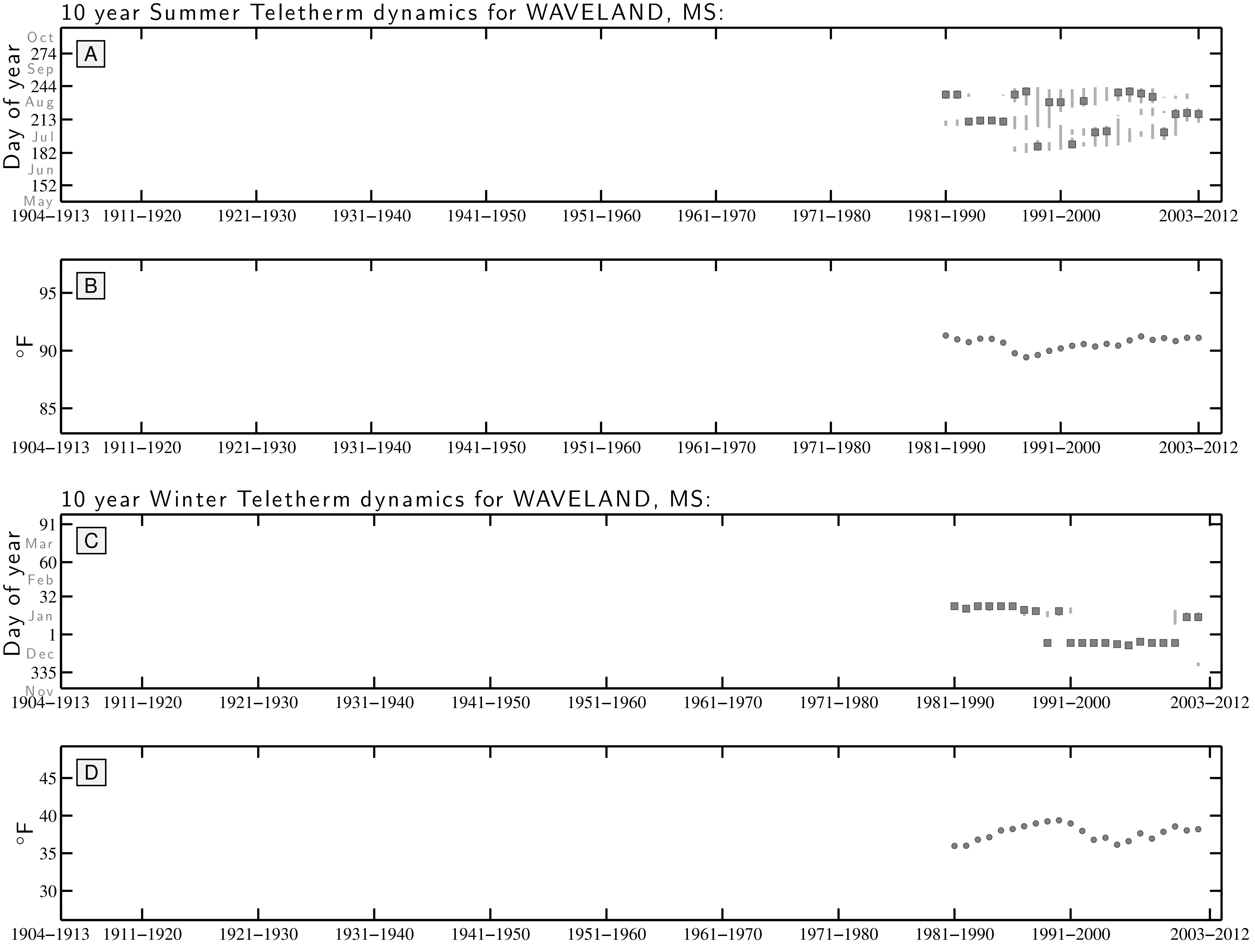

10 year Summer Teletherm dynamics for WAVELAND, MS:
A
Day of year
Oct
274
Sep
244
Aug
213
Jul
182
Jun
152
May
1904–1913
1911–1920
1921–1930
1931–1940
1941–1950
1951–1960
1961–1970
1971–1980
1981–1990
1991–2000
2003–2012
B
°F
95
90
85
10 year Winter Teletherm dynamics for WAVELAND, MS:
C
Day of year
91
Mar
60
Feb
32
Jan
1
Dec
335
Nov
D
°F
45
40
35
30

10 year Summer Teletherm dynamics for WAYNESBORO 2 W, MS:

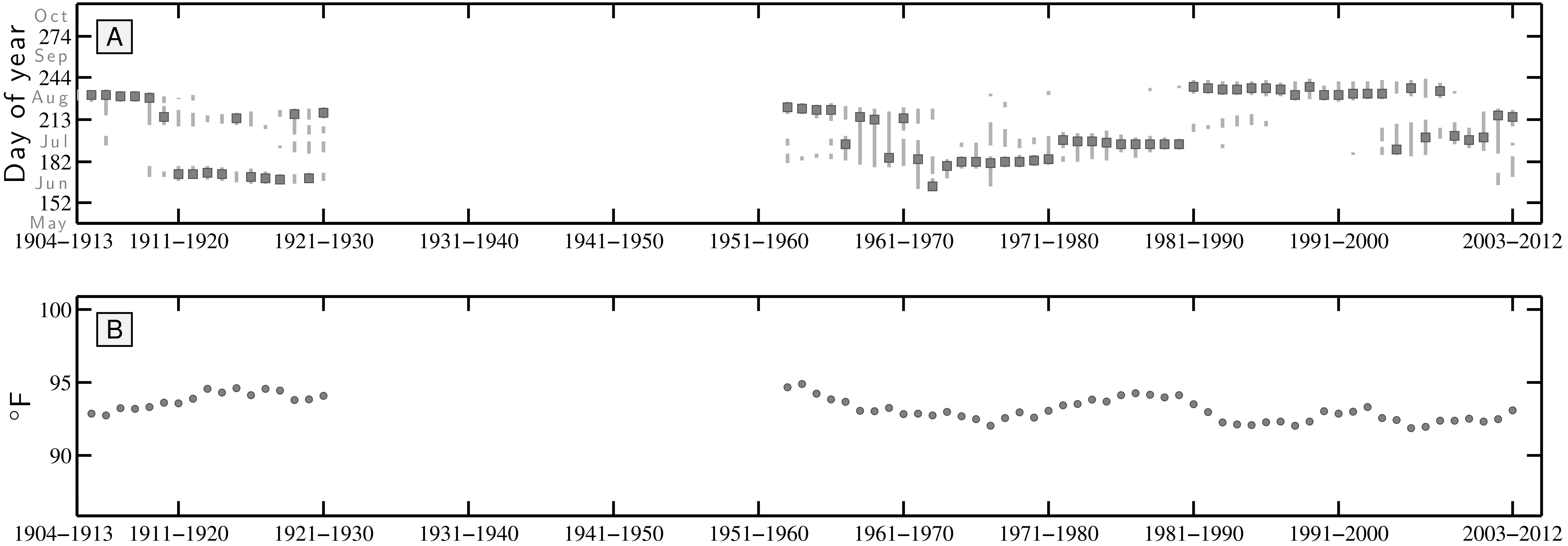

10 year Winter Teletherm dynamics for WAYNESBORO 2 W, MS:

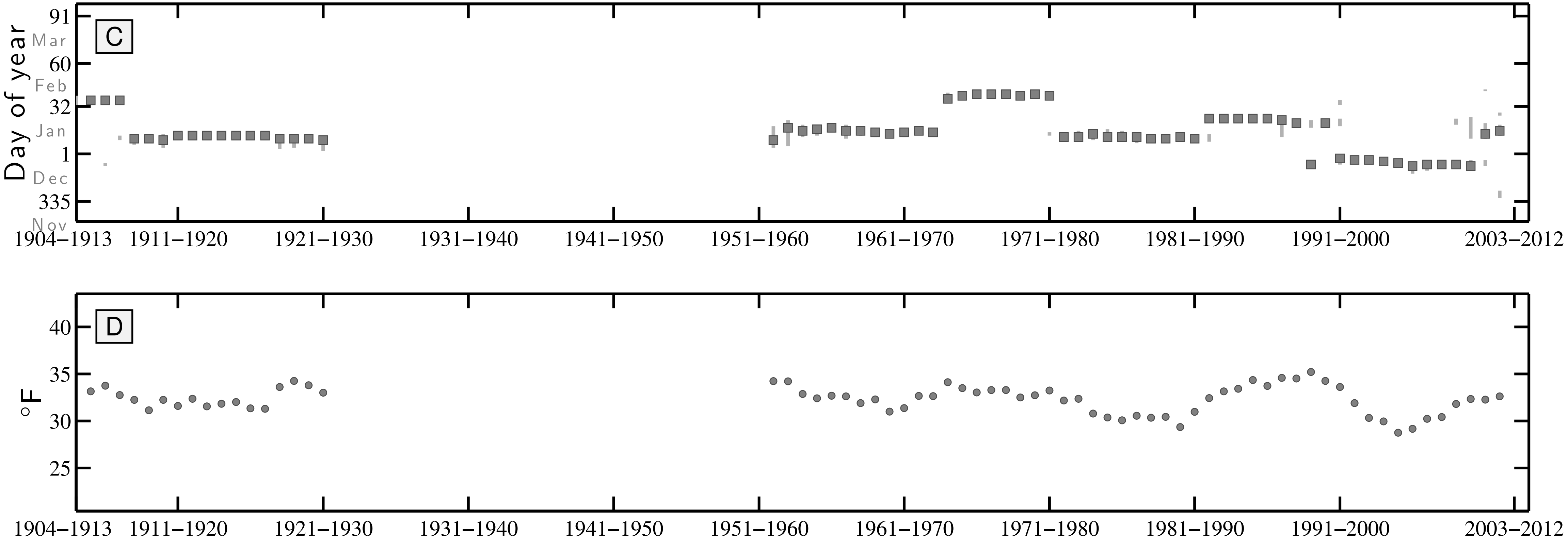

10 year Summer Teletherm dynamics for WOODVILLE 4 ESE, MS:

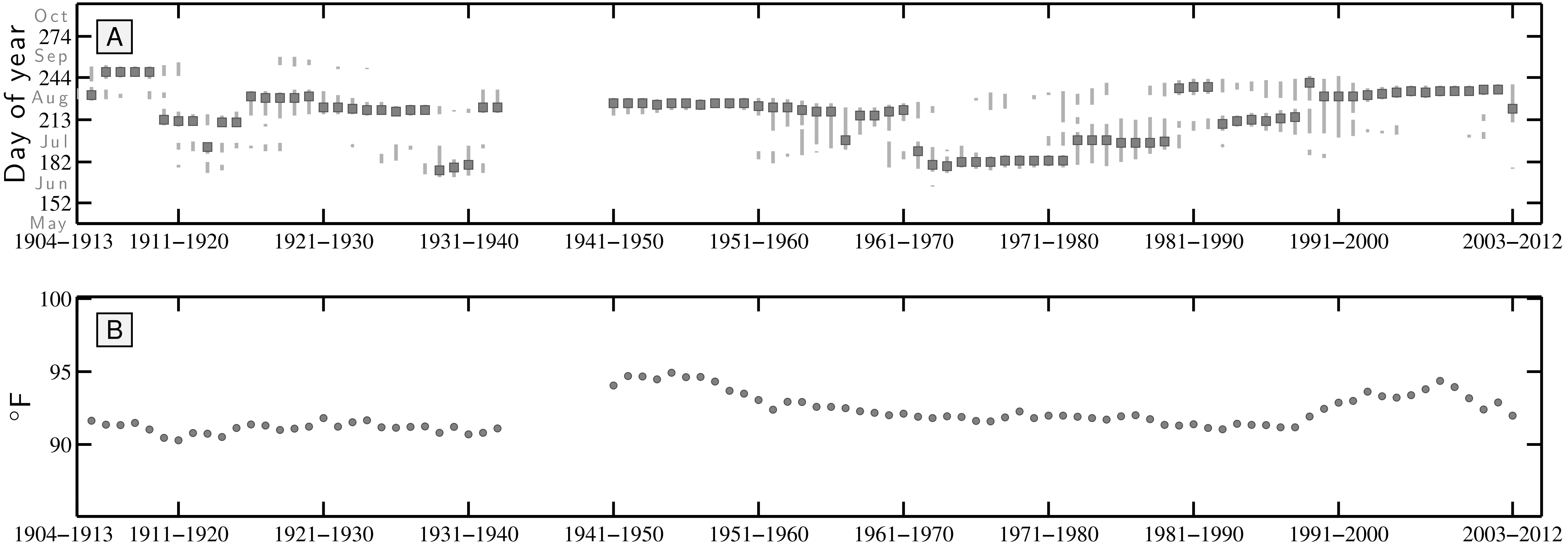

10 year Winter Teletherm dynamics for WOODVILLE 4 ESE, MS:

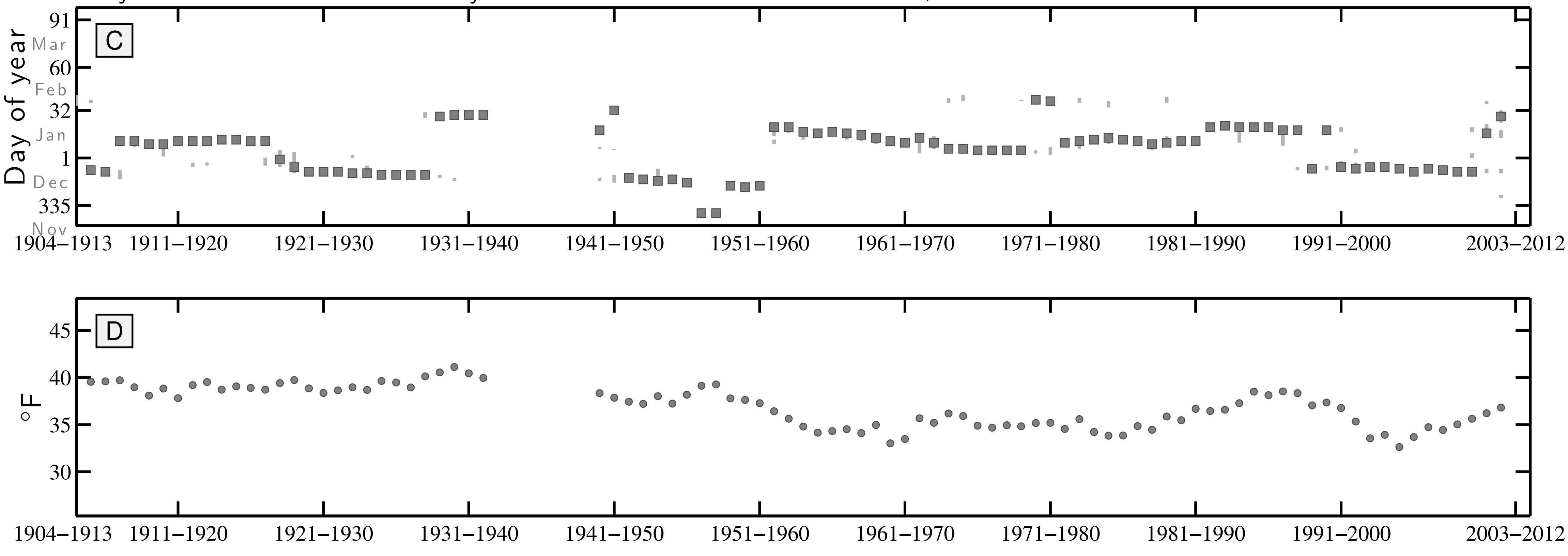

10 year Summer Teletherm dynamics for YAZOO CITY 5 NNE, MS:

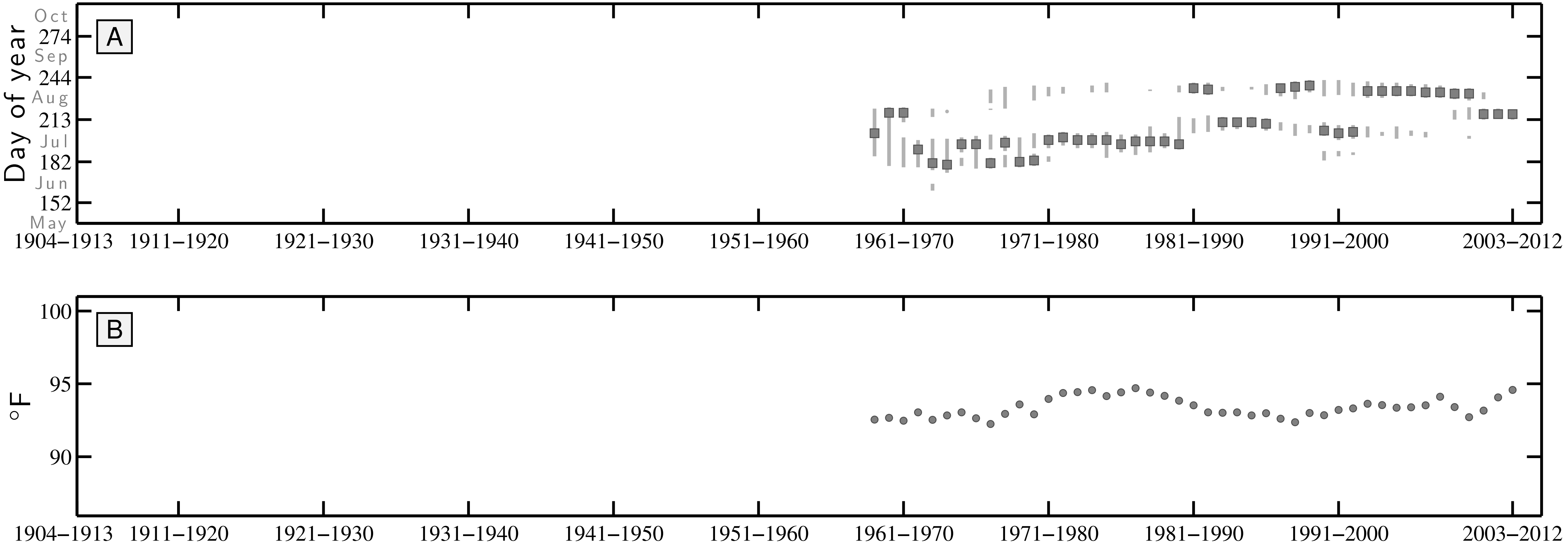

10 year Winter Teletherm dynamics for YAZOO CITY 5 NNE, MS:

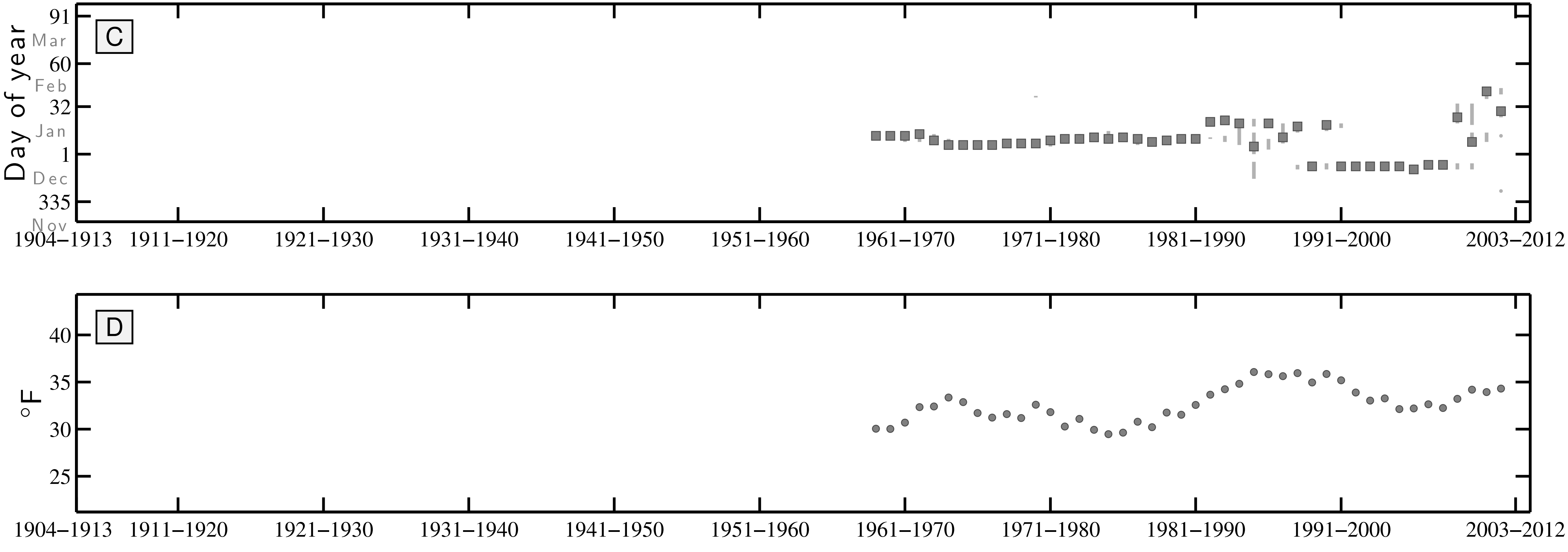

10 year Summer Teletherm dynamics for APPLETON CITY, MO:

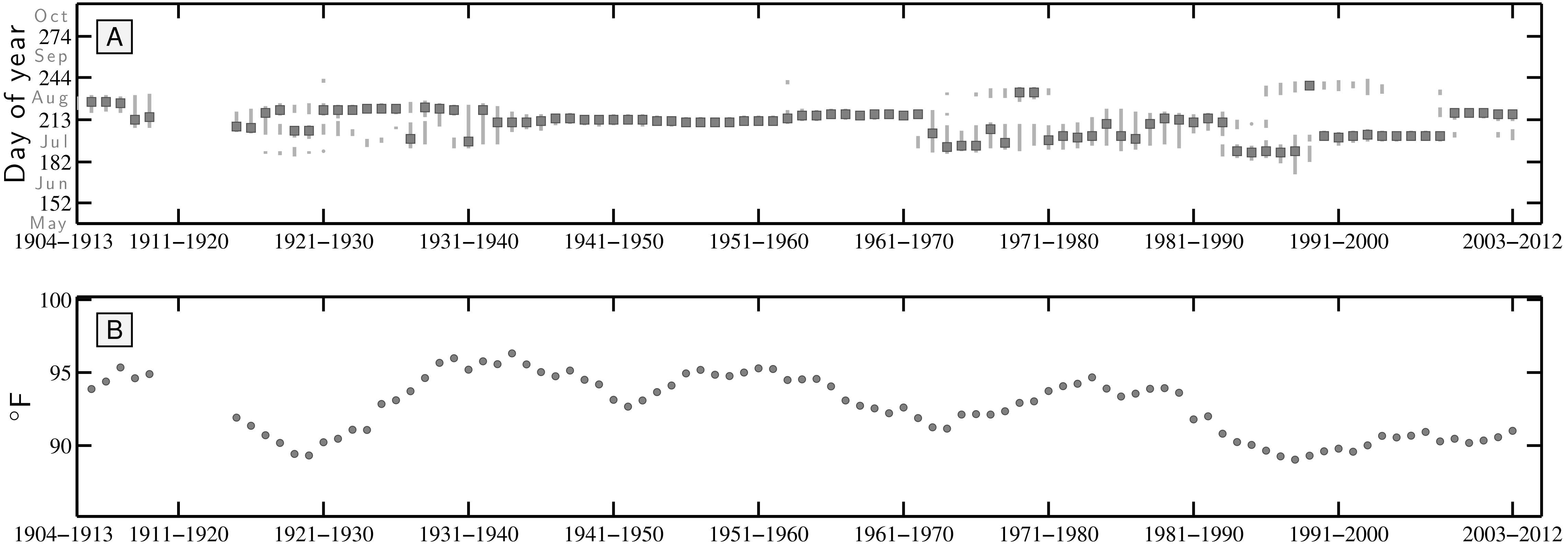

10 year Winter Teletherm dynamics for APPLETON CITY, MO:

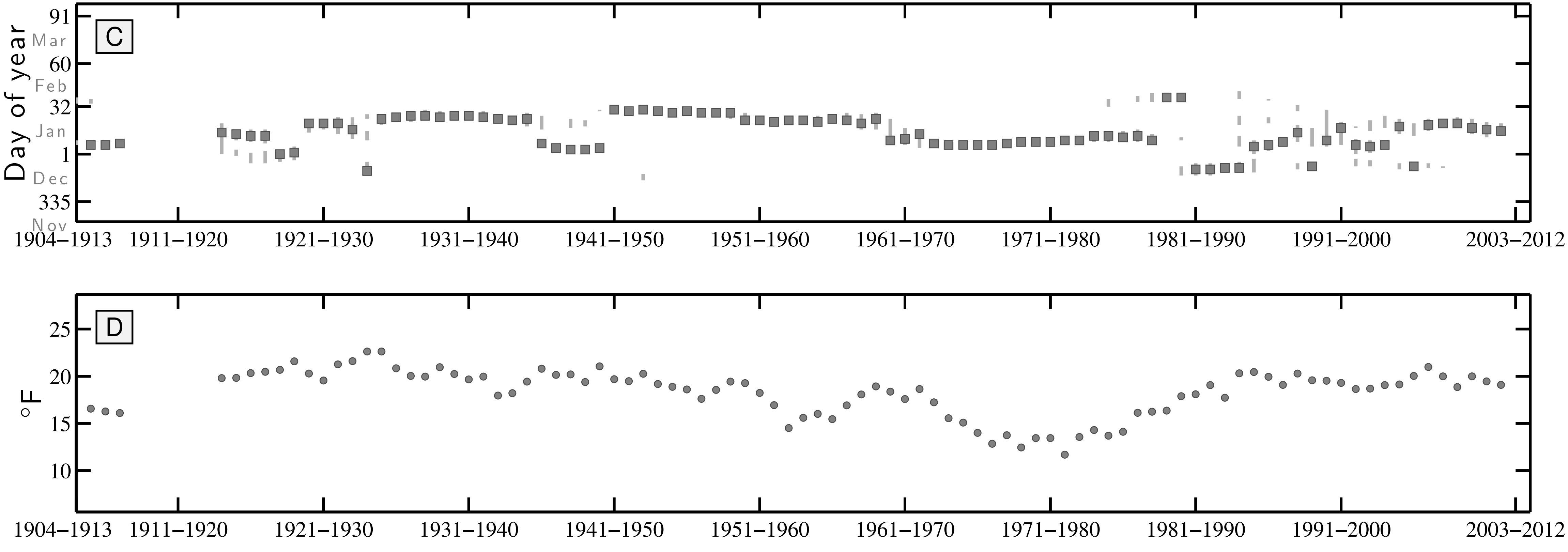

10 year Summer Teletherm dynamics for BOWLING GREEN 1 E, MO:

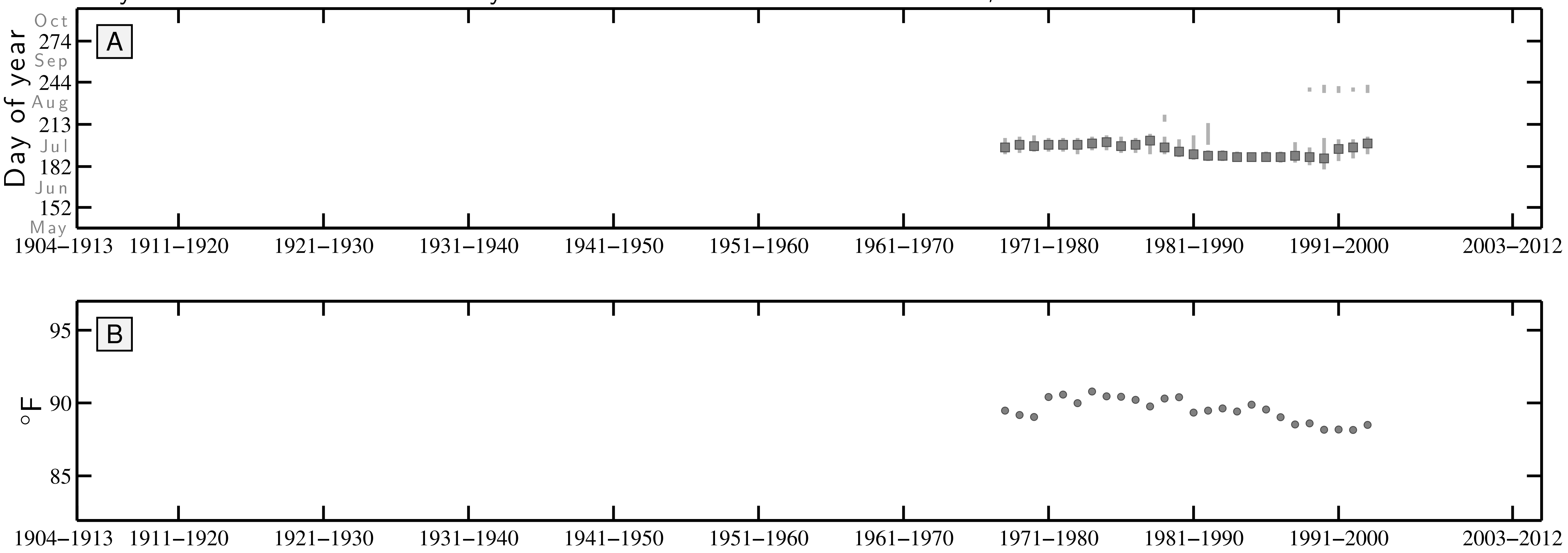

10 year Winter Teletherm dynamics for BOWLING GREEN 1 E, MO:

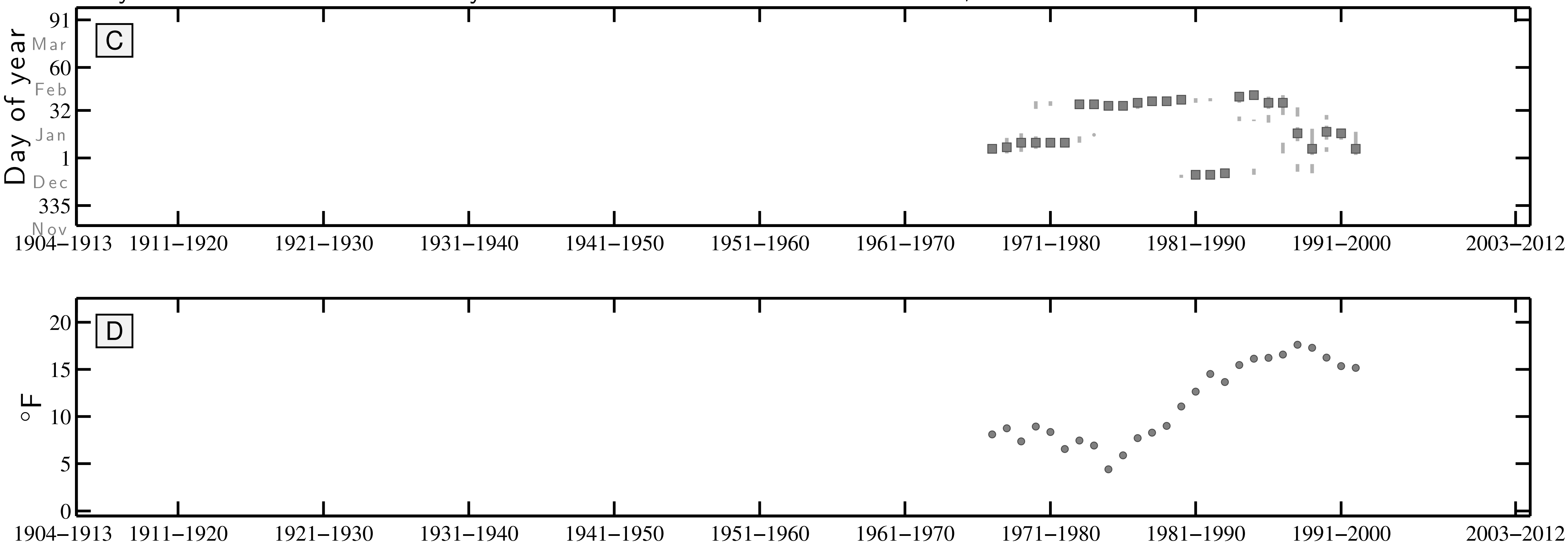

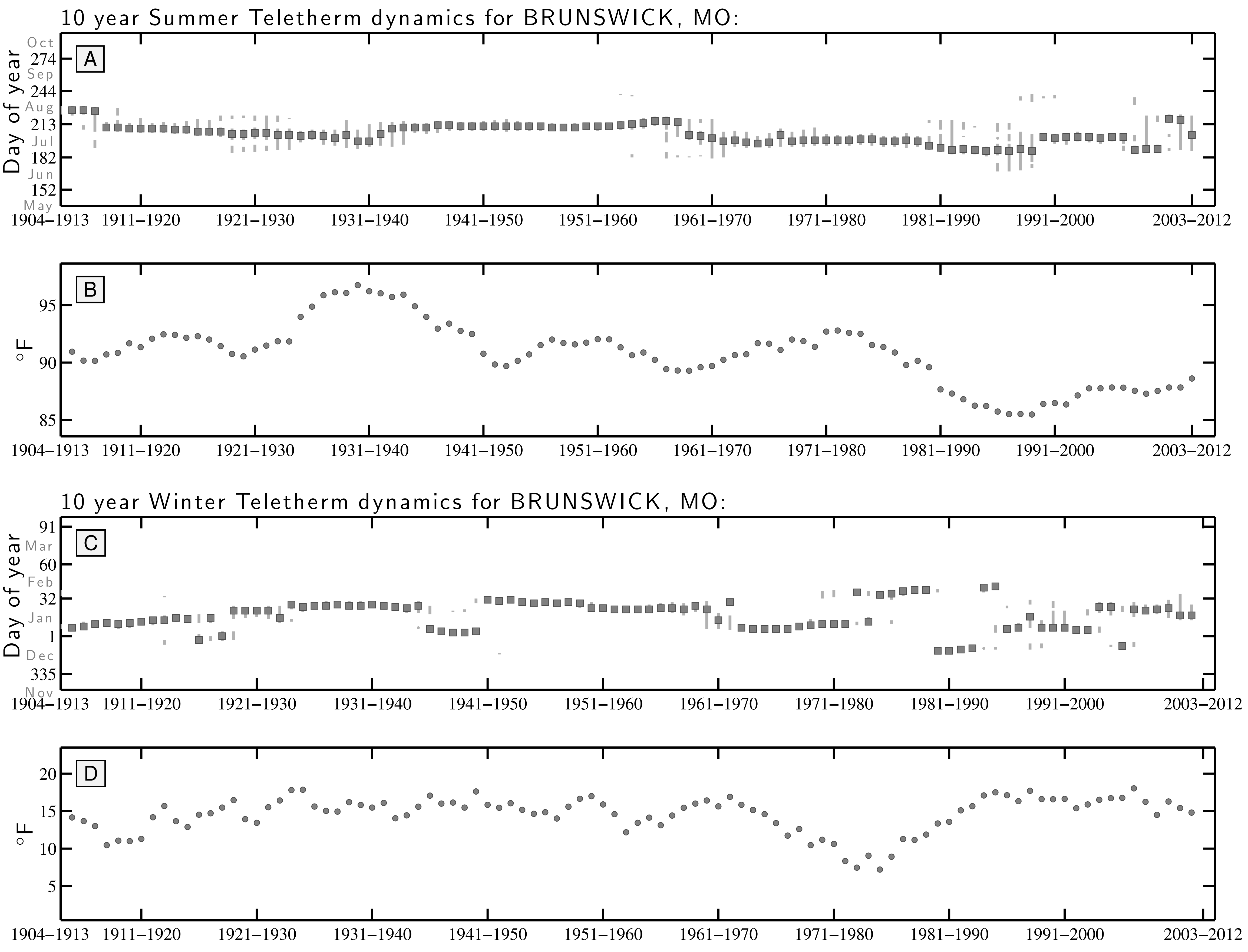
10 year Summer Teletherm dynamics for BRUNSWICK, MO:
A
Day of year
Oct
274
Sep
244
Aug
213
Jul
182
Jun
152
May
B
°F
95
90
85
10 year Winter Teletherm dynamics for BRUNSWICK, MO:
C
Day of year
91
Mar
60
Feb
32
Jan
1
Dec
335
Nov
D
°F
20
15
10
5
1904–1913
1911–1920
1921–1930
1931–1940
1941–1950
1951–1960
1961–1970
1971–1980
1981–1990
1991–2000
2003–2012

10 year Summer Teletherm dynamics for CARUTHERSVILLE, MO:

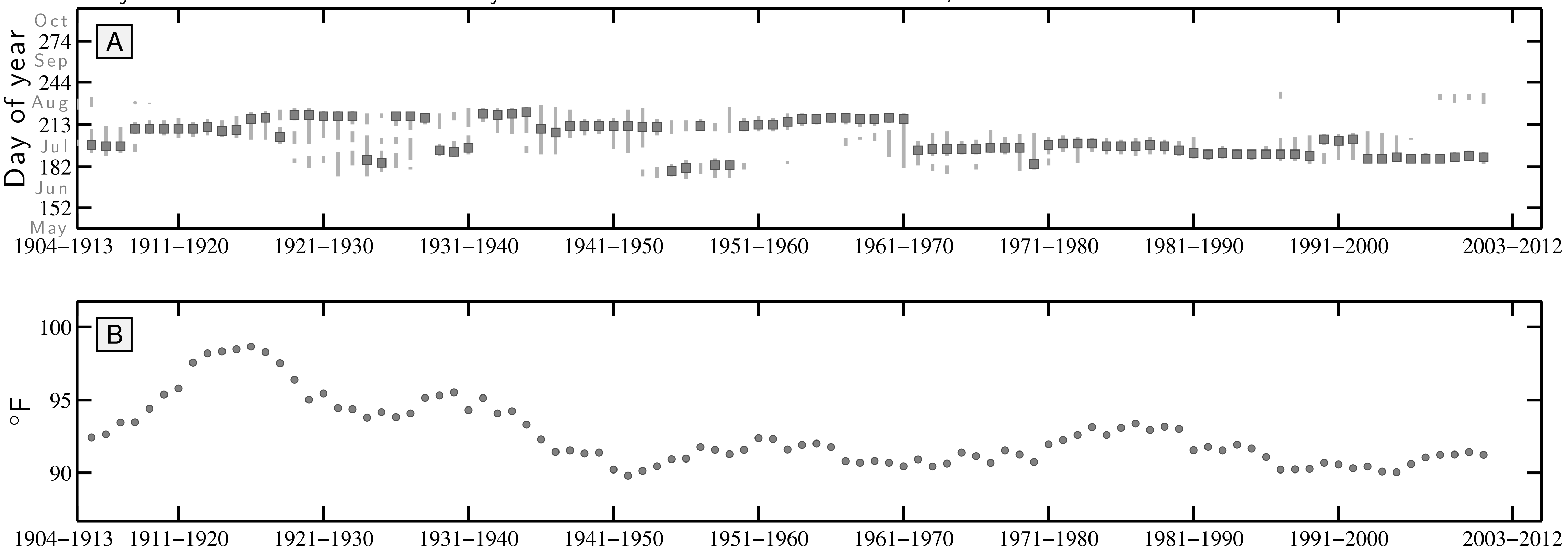

10 year Winter Teletherm dynamics for CARUTHERSVILLE, MO:

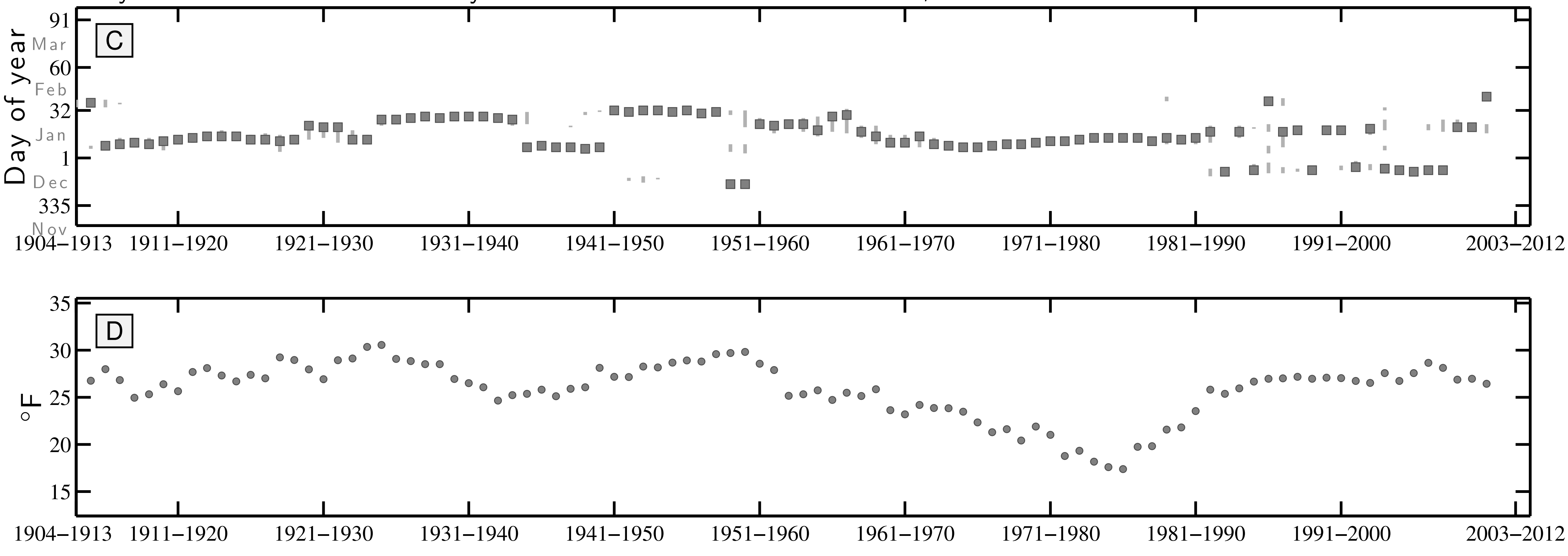

10 year Summer Teletherm dynamics for CLINTON, MO:

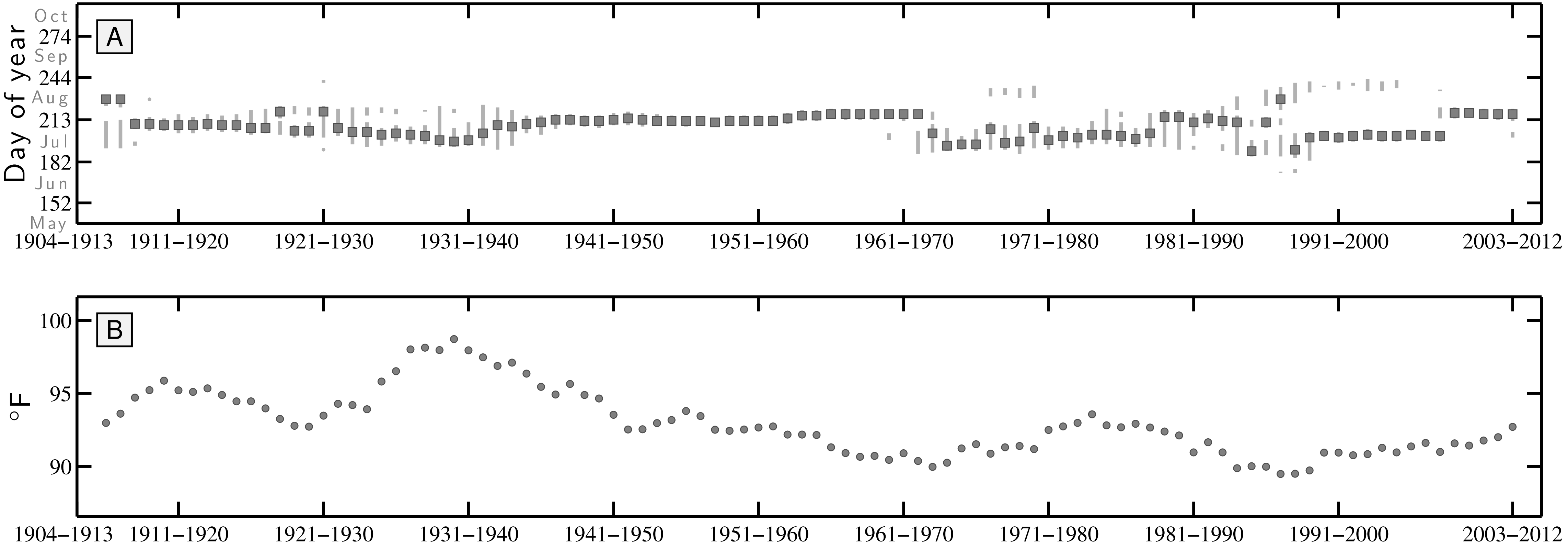

10 year Winter Teletherm dynamics for CLINTON, MO:

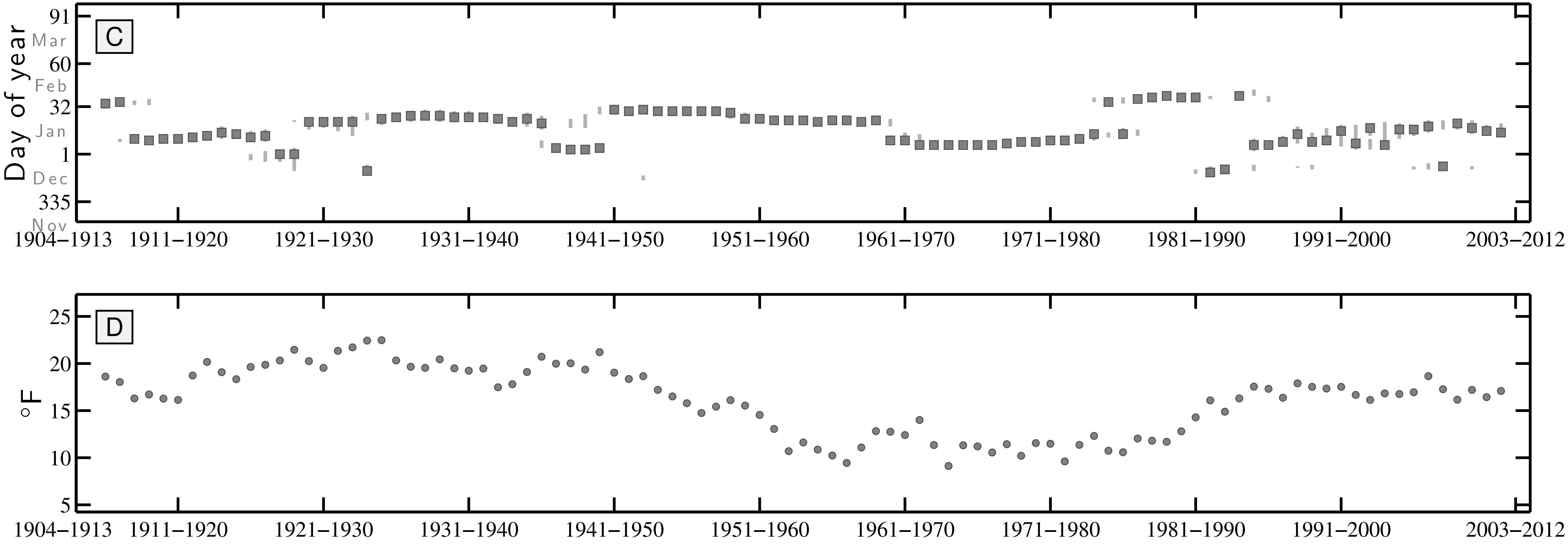

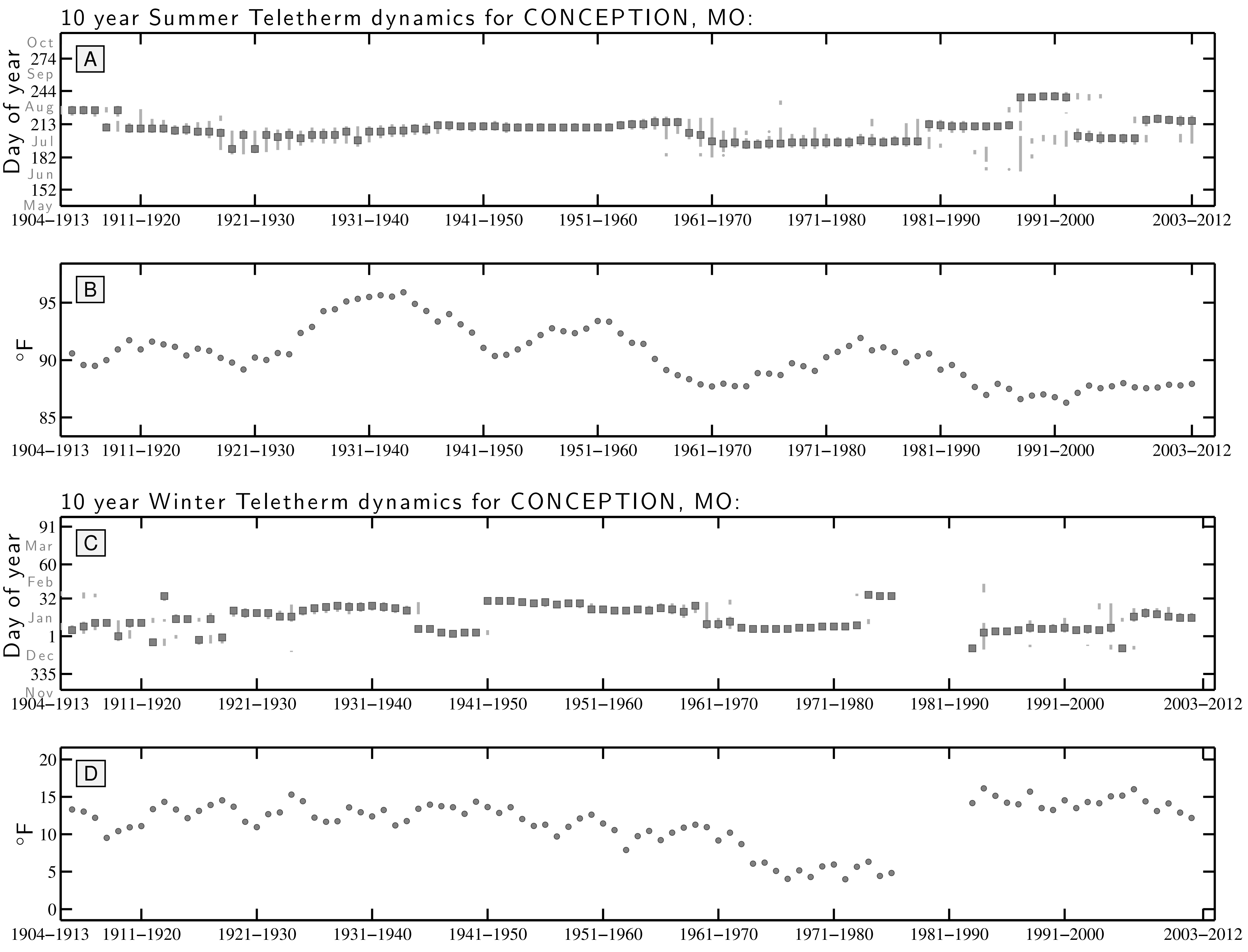
10 year Summer Teletherm dynamics for CONCEPTION, MO:
A
B
Day of year
°F
Oct
274
Sep
244
Aug
213
Jul
182
Jun
152
May
95
90
85
1904–1913
1911–1920
1921–1930
1931–1940
1941–1950
1951–1960
1961–1970
1971–1980
1981–1990
1991–2000
2003–2012
10 year Winter Teletherm dynamics for CONCEPTION, MO:
C
D
91
Mar
60
Feb
32
Jan
1
Dec
335
Nov
20
15
10
5
0

10 year Summer Teletherm dynamics for DONIPHAN, MO:

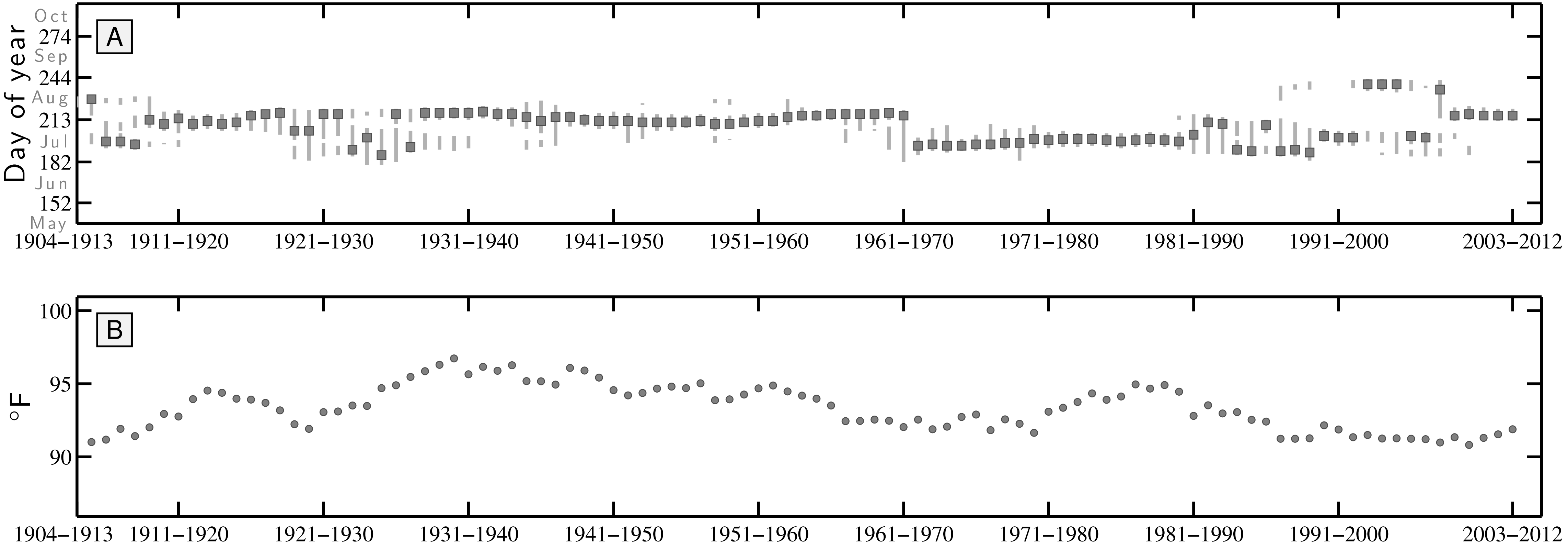

10 year Winter Teletherm dynamics for DONIPHAN, MO:

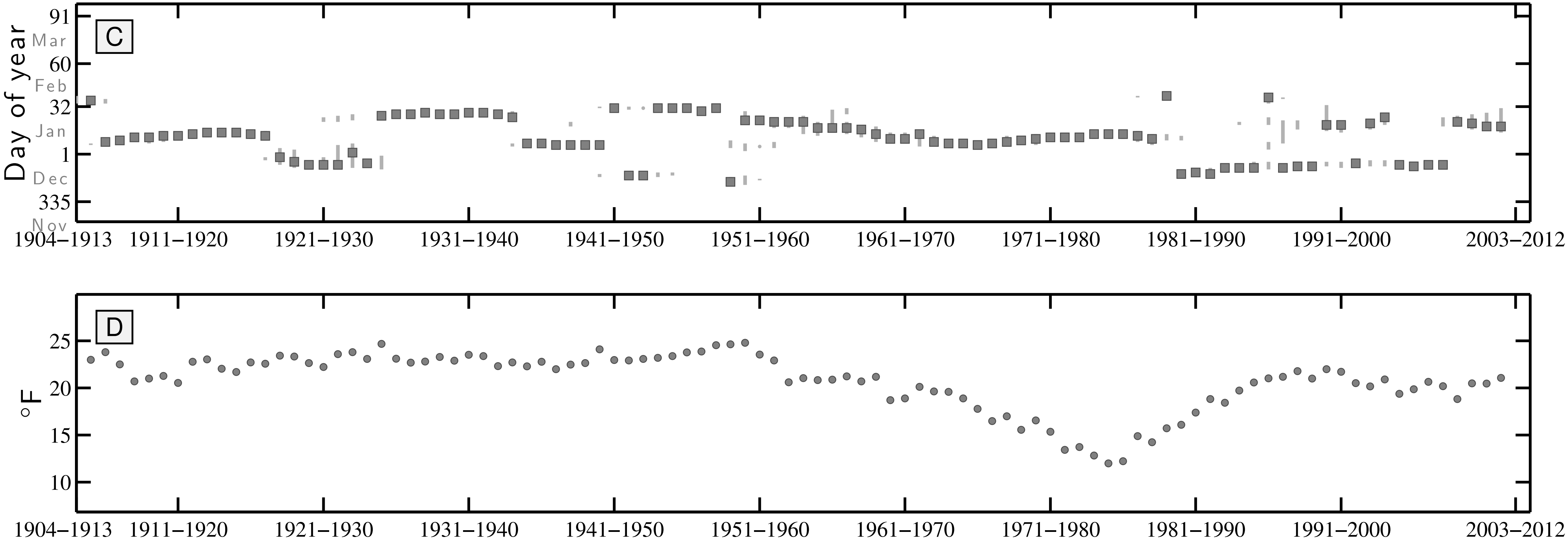

10 year Summer Teletherm dynamics for FARMINGTON, MO:

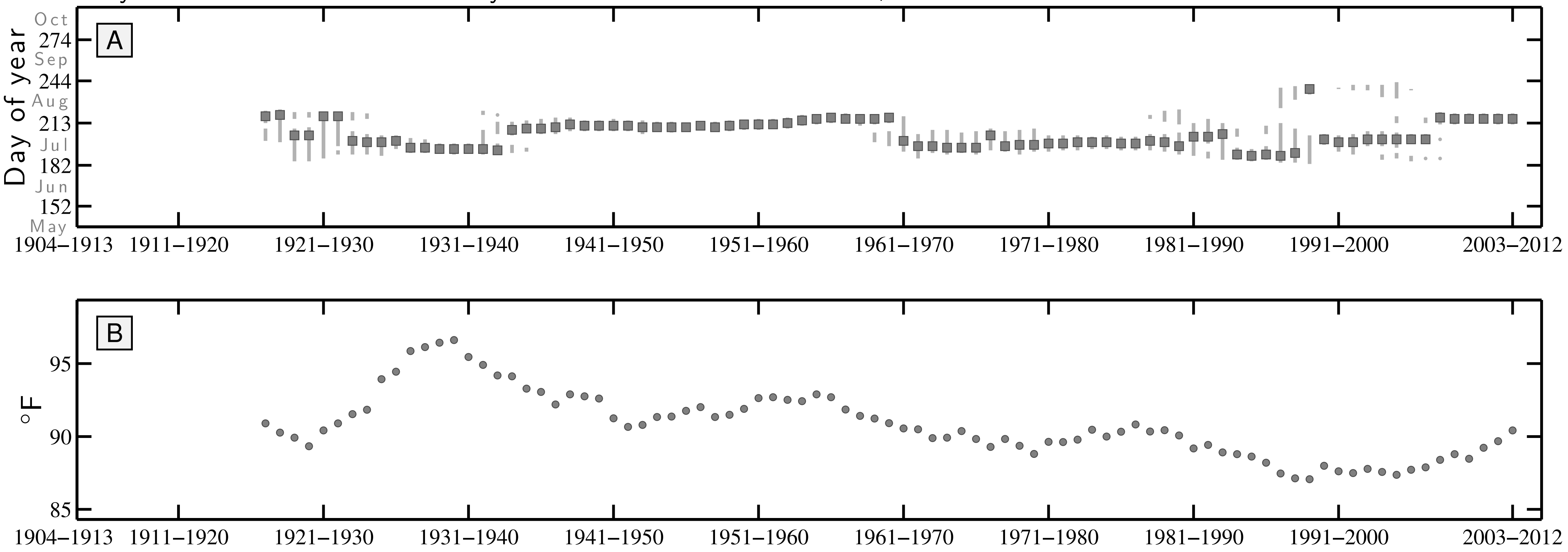

10 year Winter Teletherm dynamics for FARMINGTON, MO:

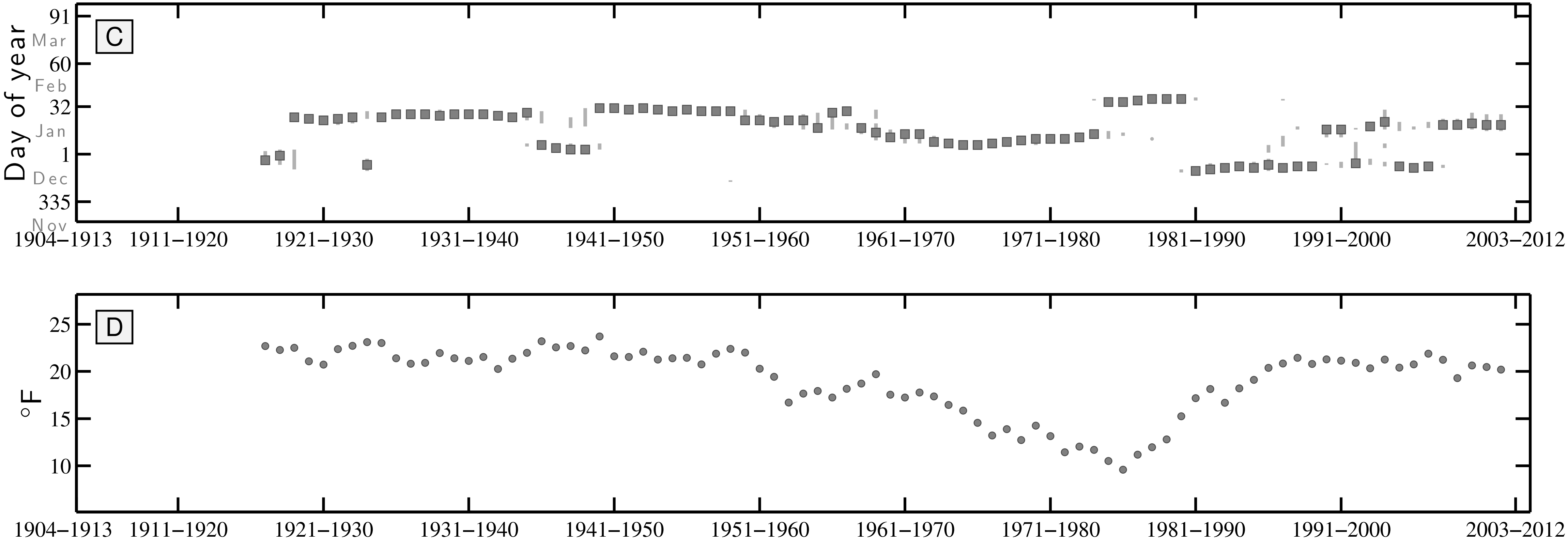

10 year Summer Teletherm dynamics for JEFFERSON CITY WTP, MO:

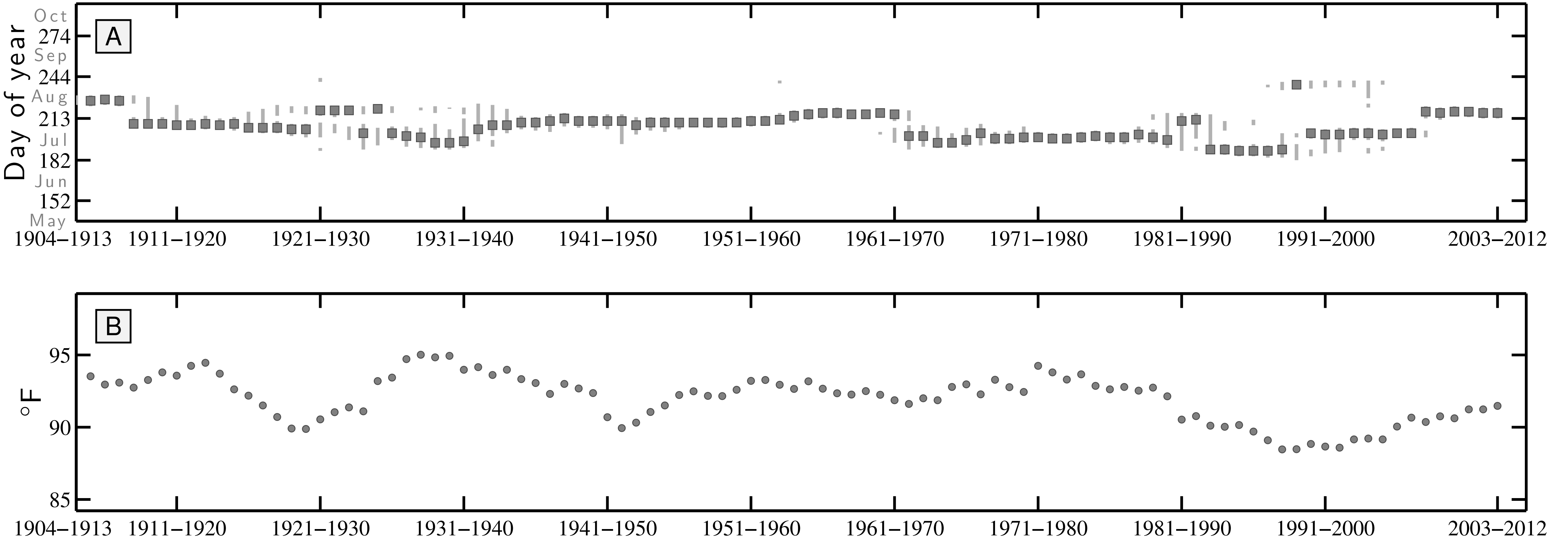

10 year Winter Teletherm dynamics for JEFFERSON CITY WTP, MO:

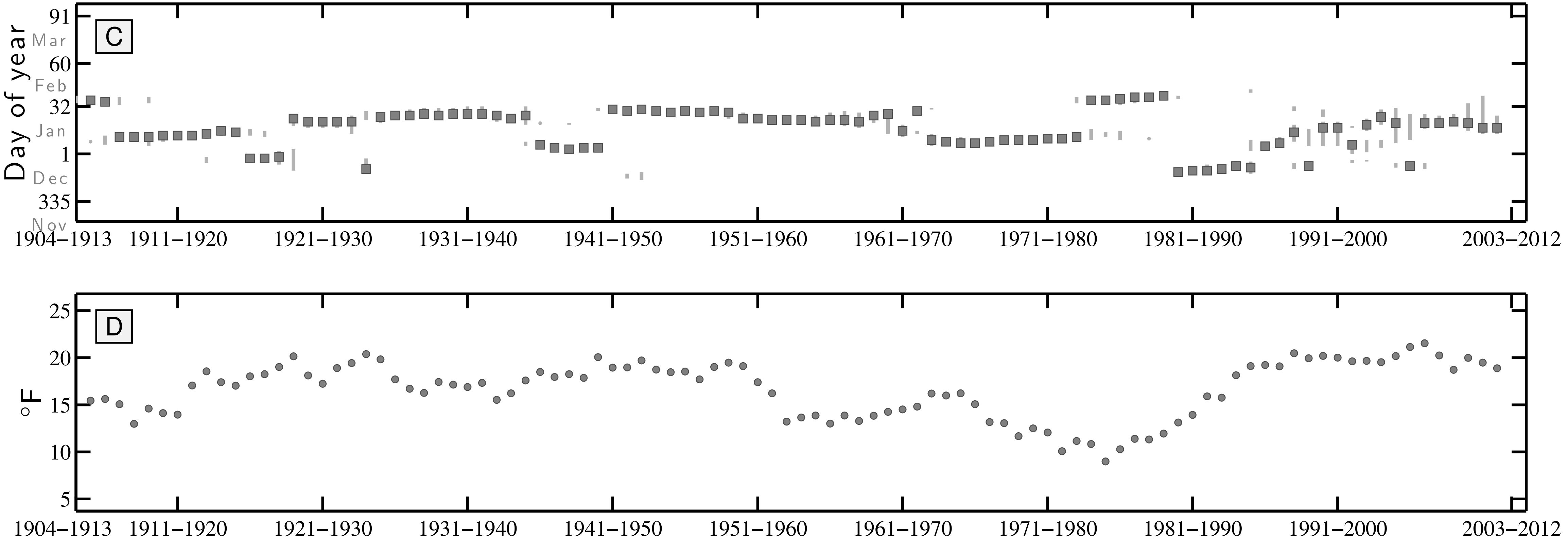

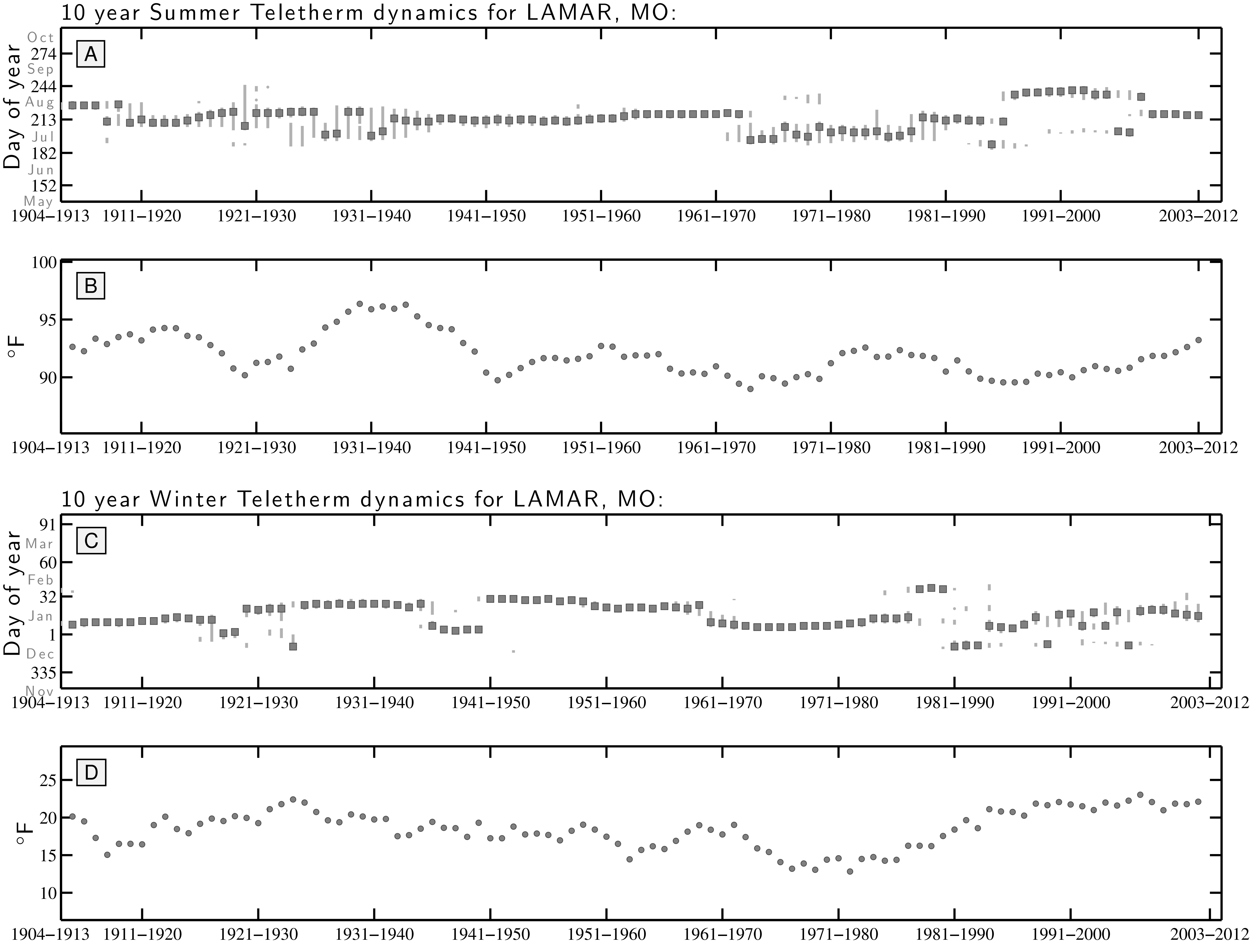
10 year Summer Teletherm dynamics for LAMAR, MO:
A
B
Day of year
°F
Oct
274
Sep
244
Aug
213
Jul
182
Jun
152
May
100
95
90
1904–1913
1911–1920
1921–1930
1931–1940
1941–1950
1951–1960
1961–1970
1971–1980
1981–1990
1991–2000
2003–2012
10 year Winter Teletherm dynamics for LAMAR, MO:
C
D
91
Mar
60
Feb
32
Jan
1
Dec
335
Nov
25
20
15
10

10 year Summer Teletherm dynamics for LEBANON 2W, MO:

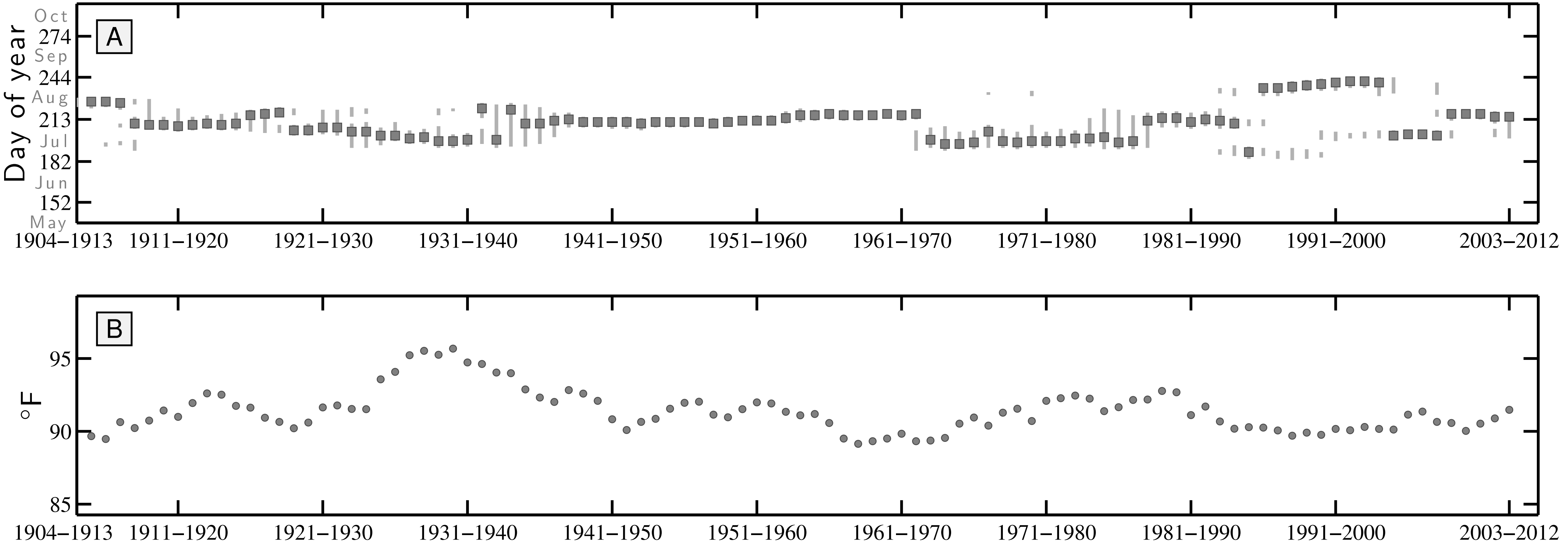

10 year Winter Teletherm dynamics for LEBANON 2W, MO:

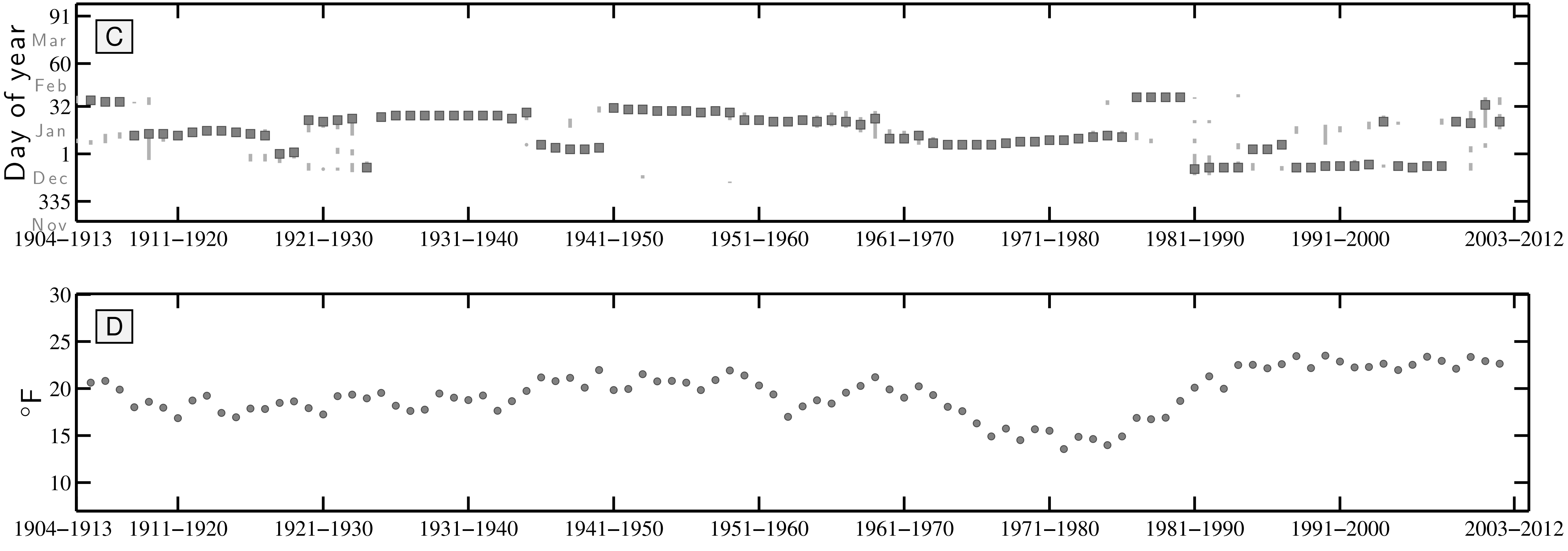

10 year Summer Teletherm dynamics for LEES SUMMIT REED WR, MO:

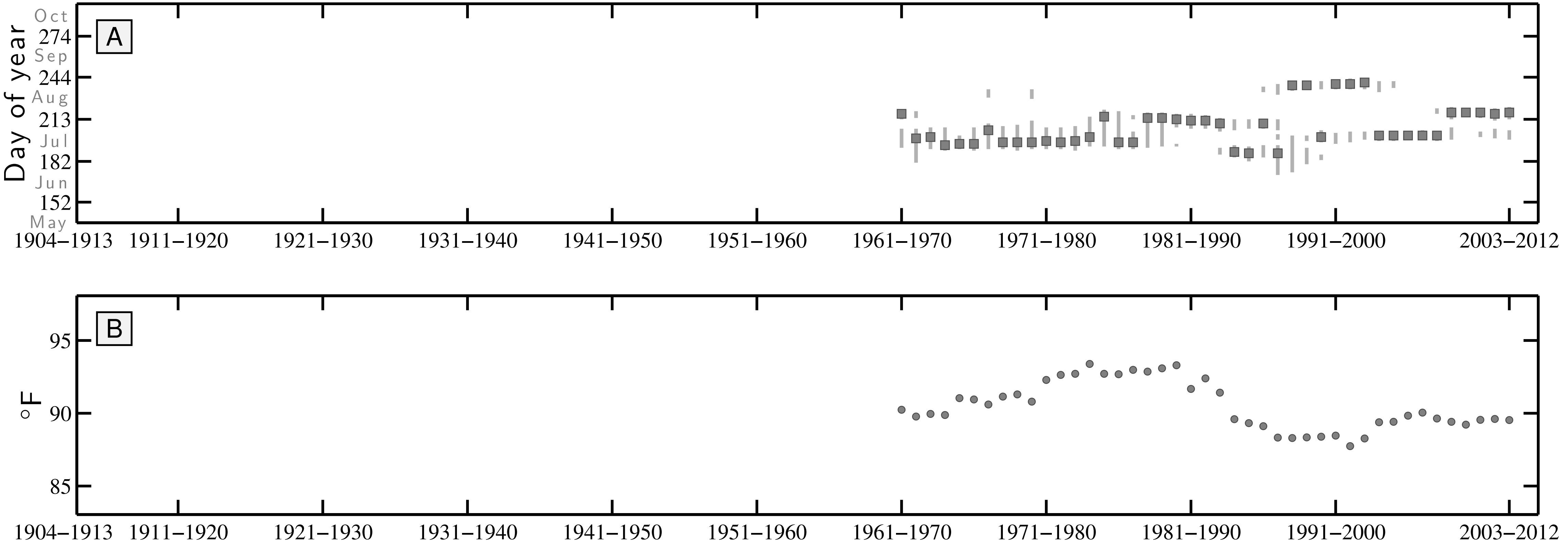

10 year Winter Teletherm dynamics for LEES SUMMIT REED WR, MO:

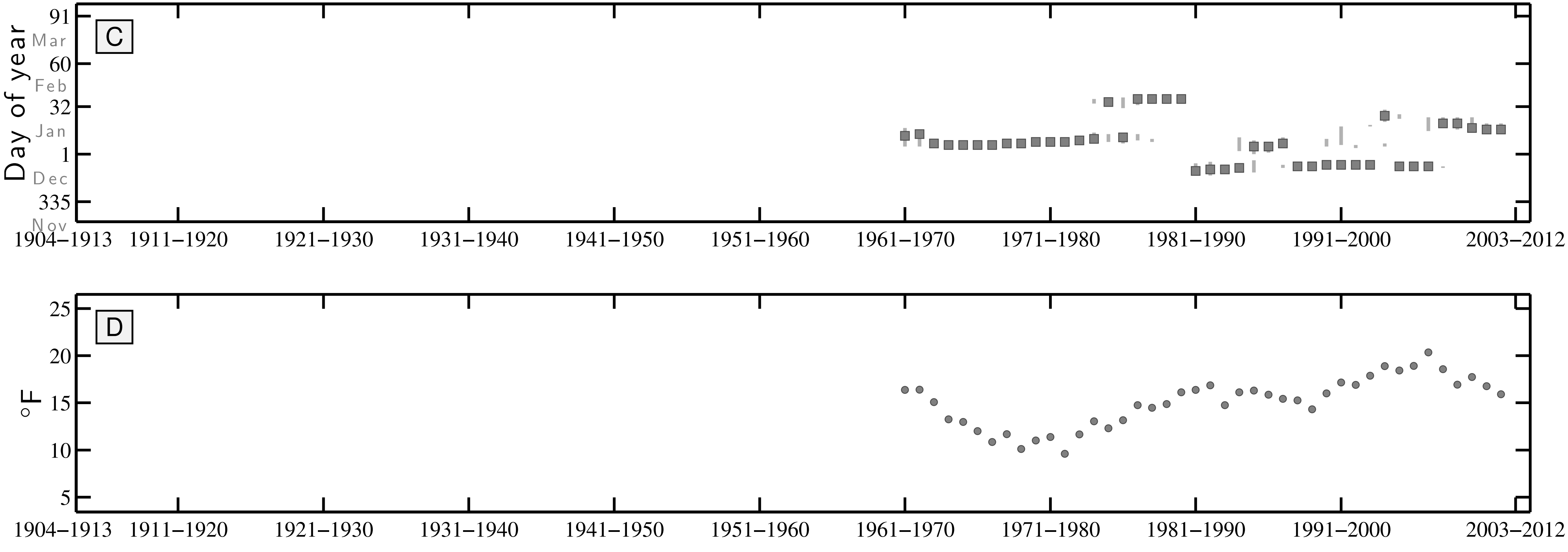

10 year Summer Teletherm dynamics for LEXINGTON 3E, MO:

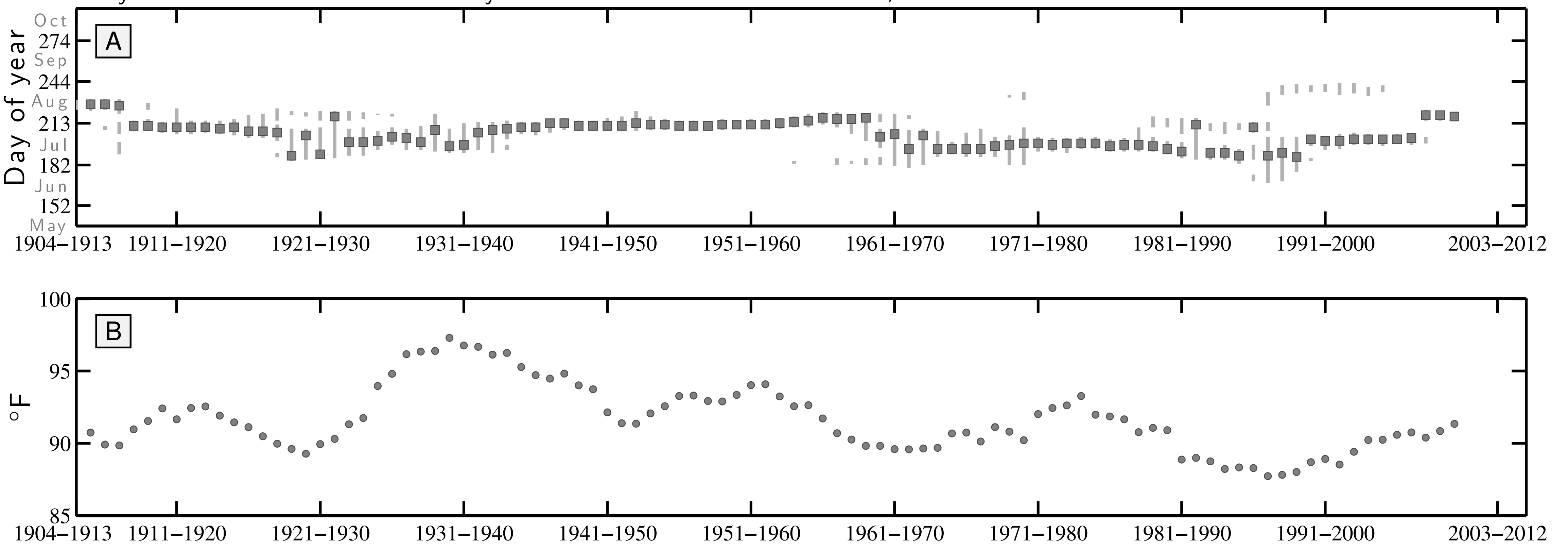

10 year Winter Teletherm dynamics for LEXINGTON 3E, MO:

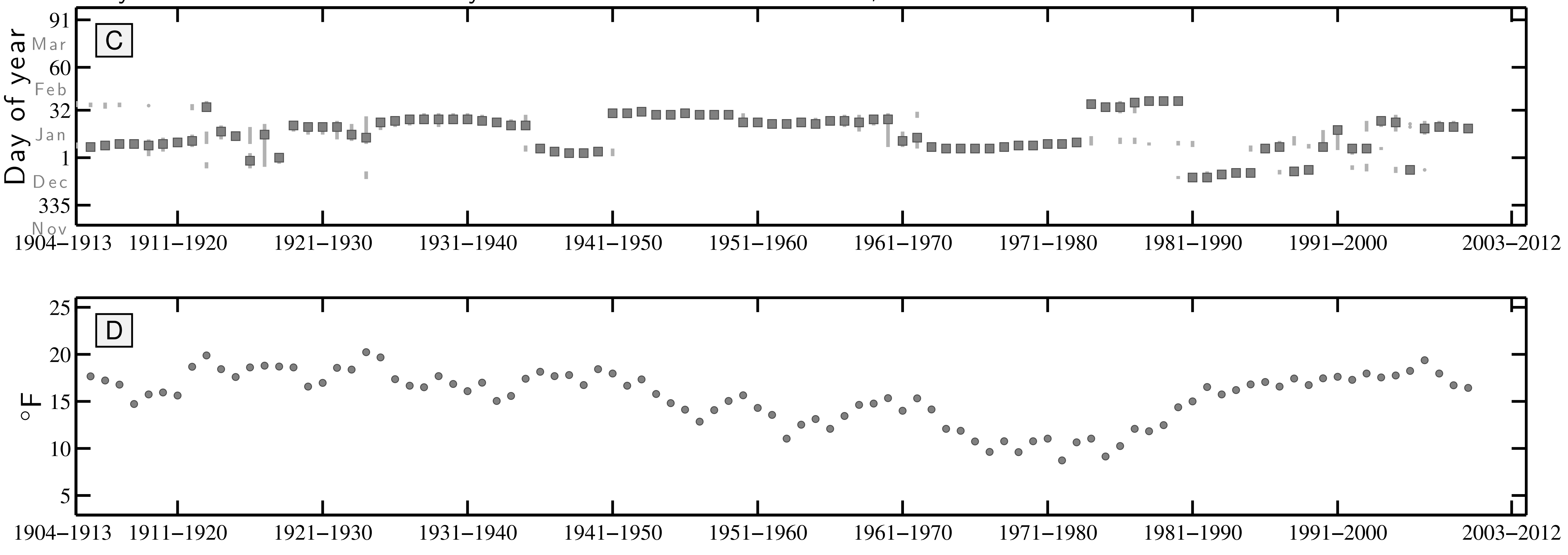

10 year Summer Teletherm dynamics for LOCKWOOD, MO:

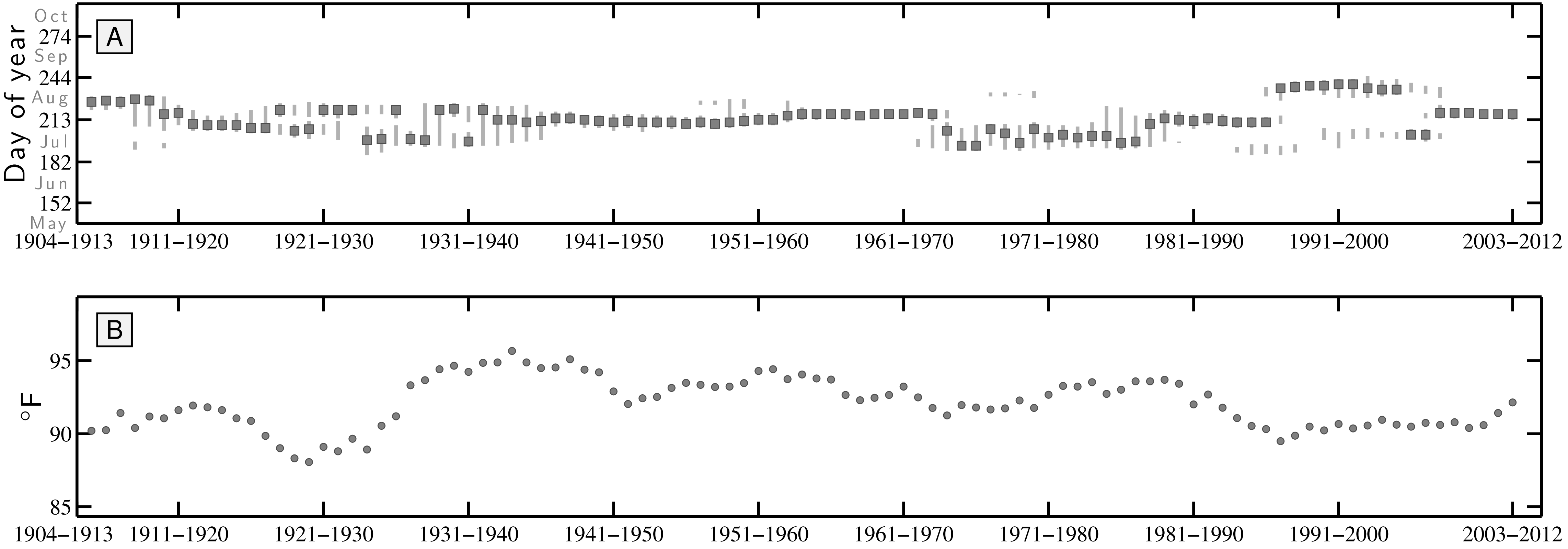

10 year Winter Teletherm dynamics for LOCKWOOD, MO:

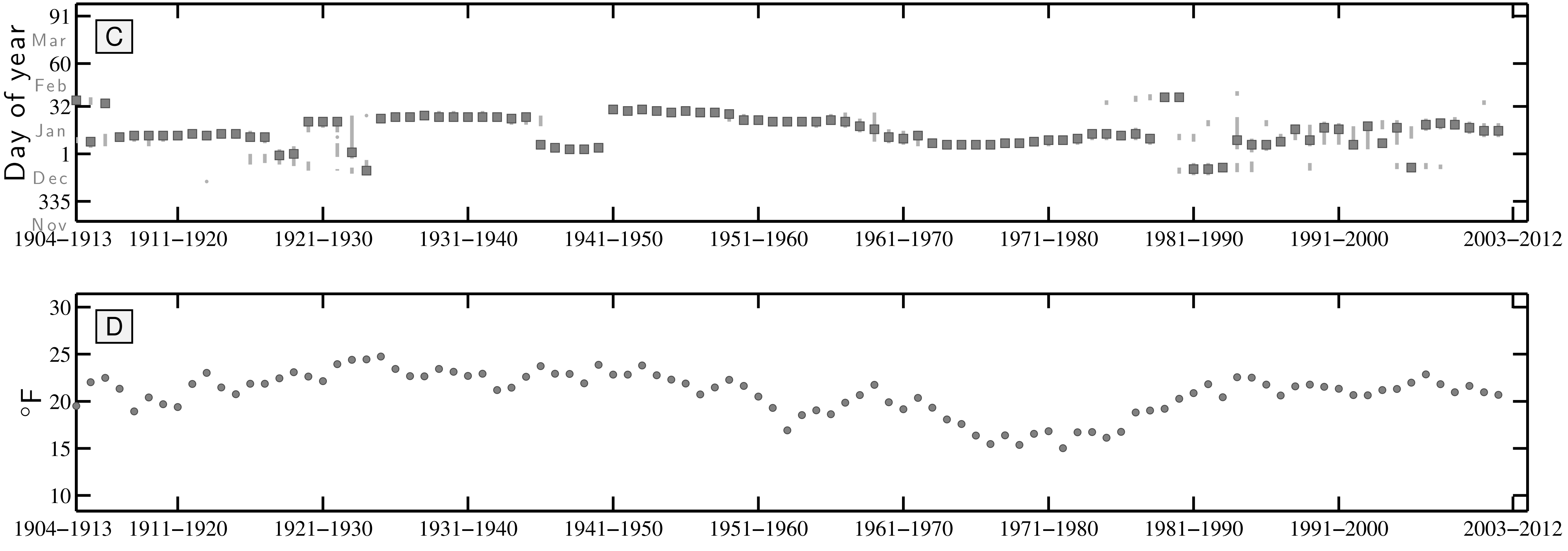

10 year Summer Teletherm dynamics for MARBLE HILL, MO:

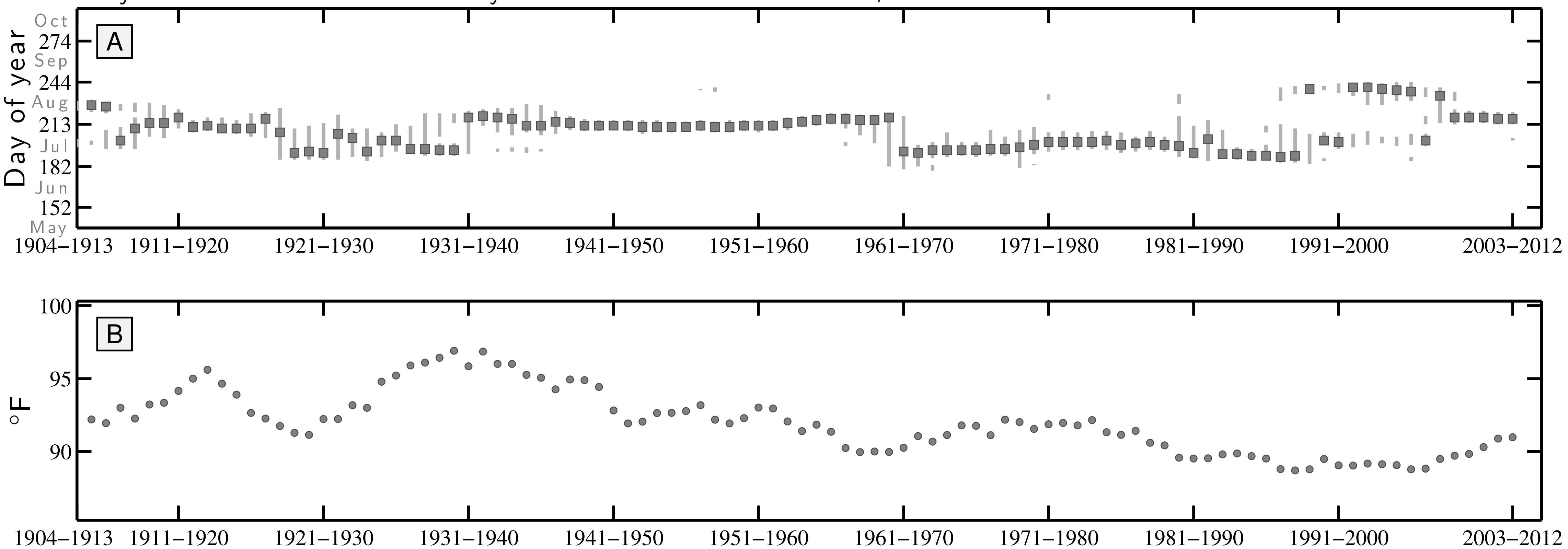

10 year Winter Teletherm dynamics for MARBLE HILL, MO:

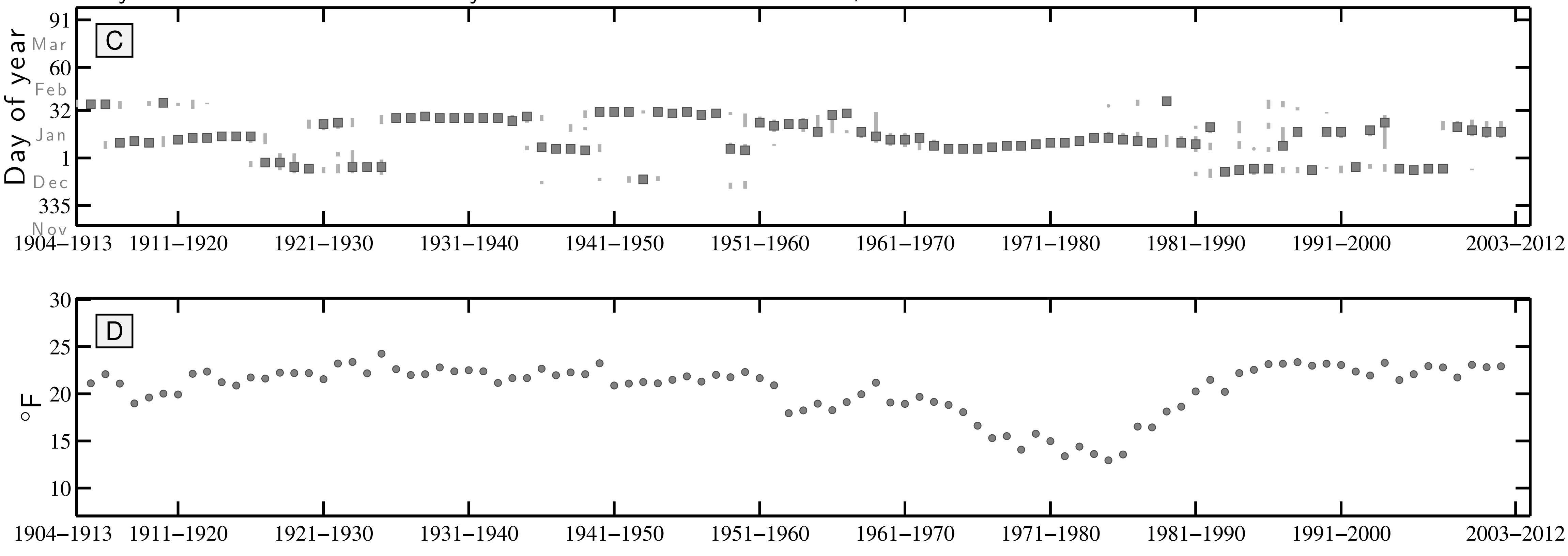

10 year Summer Teletherm dynamics for MEXICO, MO:

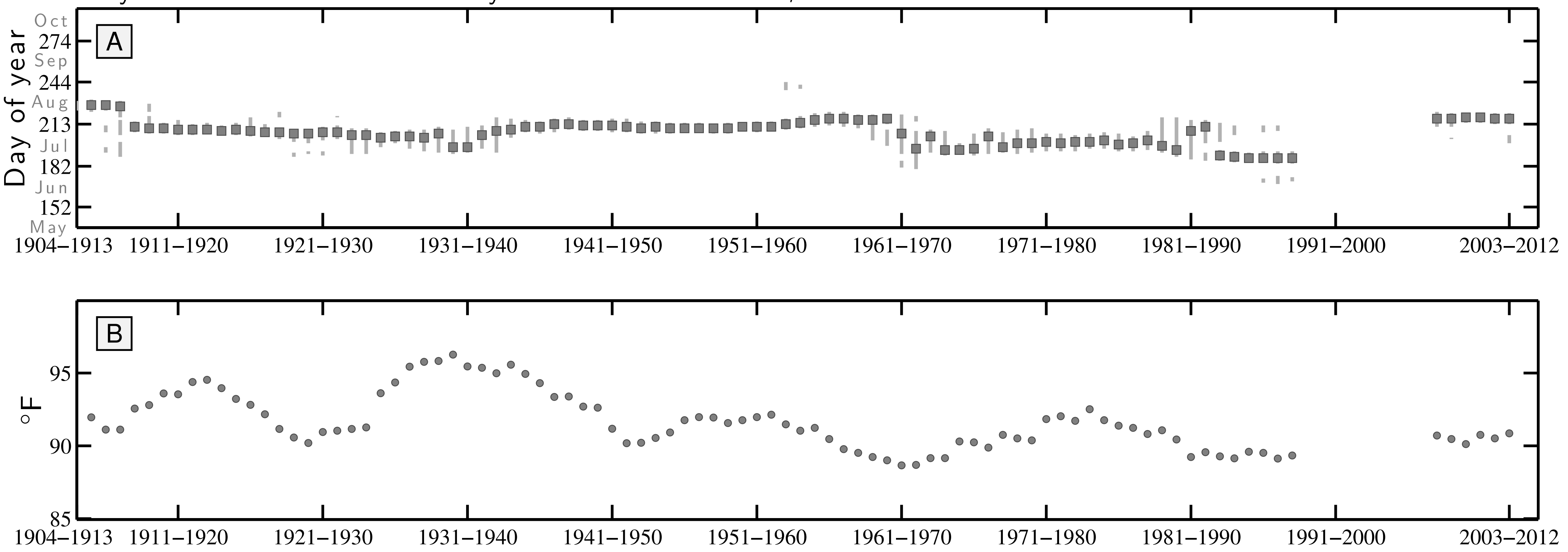

10 year Winter Teletherm dynamics for MEXICO, MO:

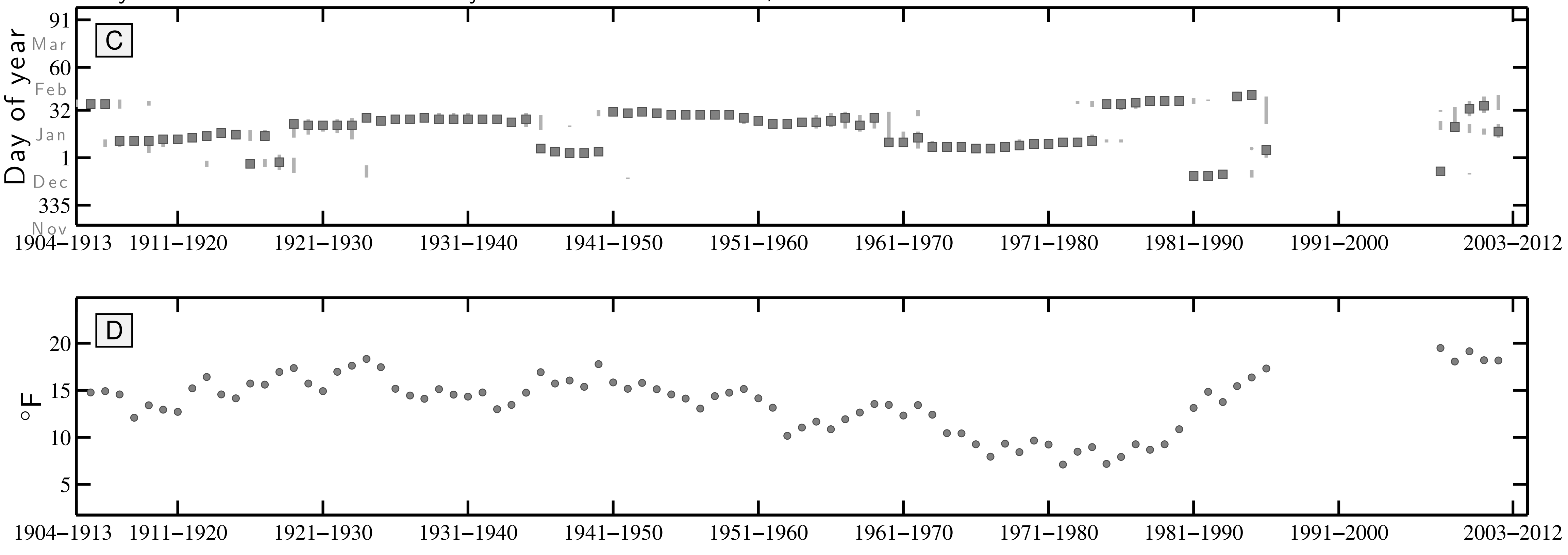

10 year Summer Teletherm dynamics for MOBERLY, MO:

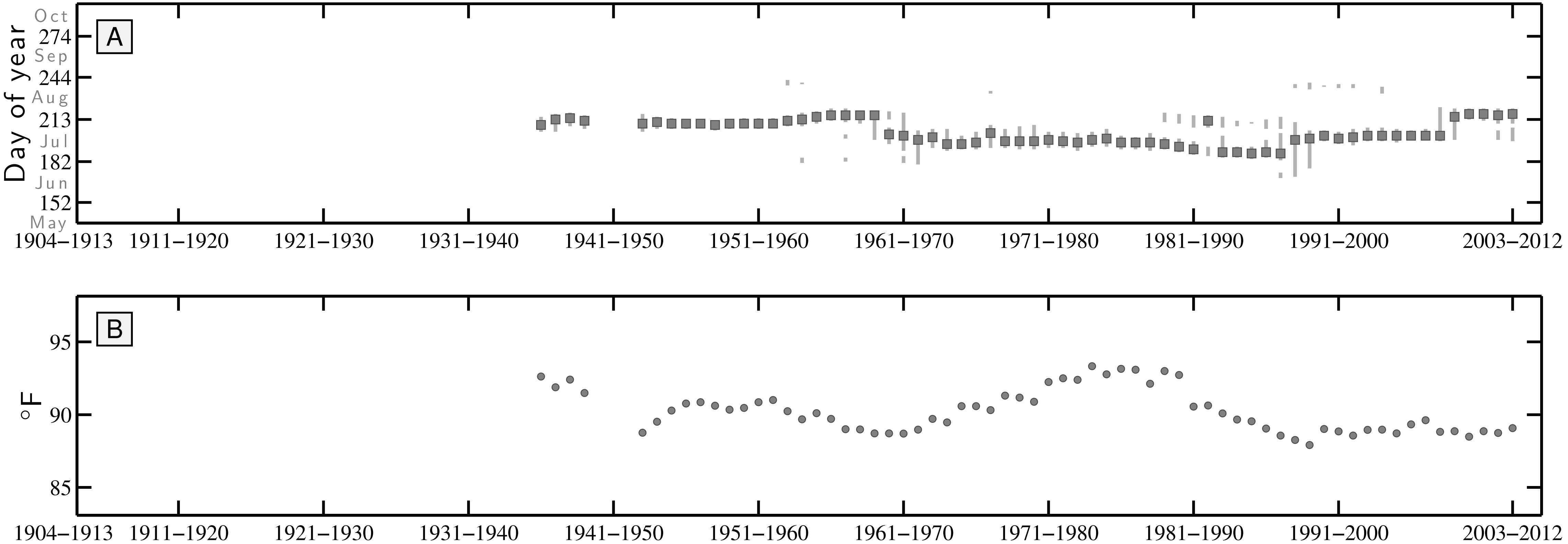

10 year Winter Teletherm dynamics for MOBERLY, MO:

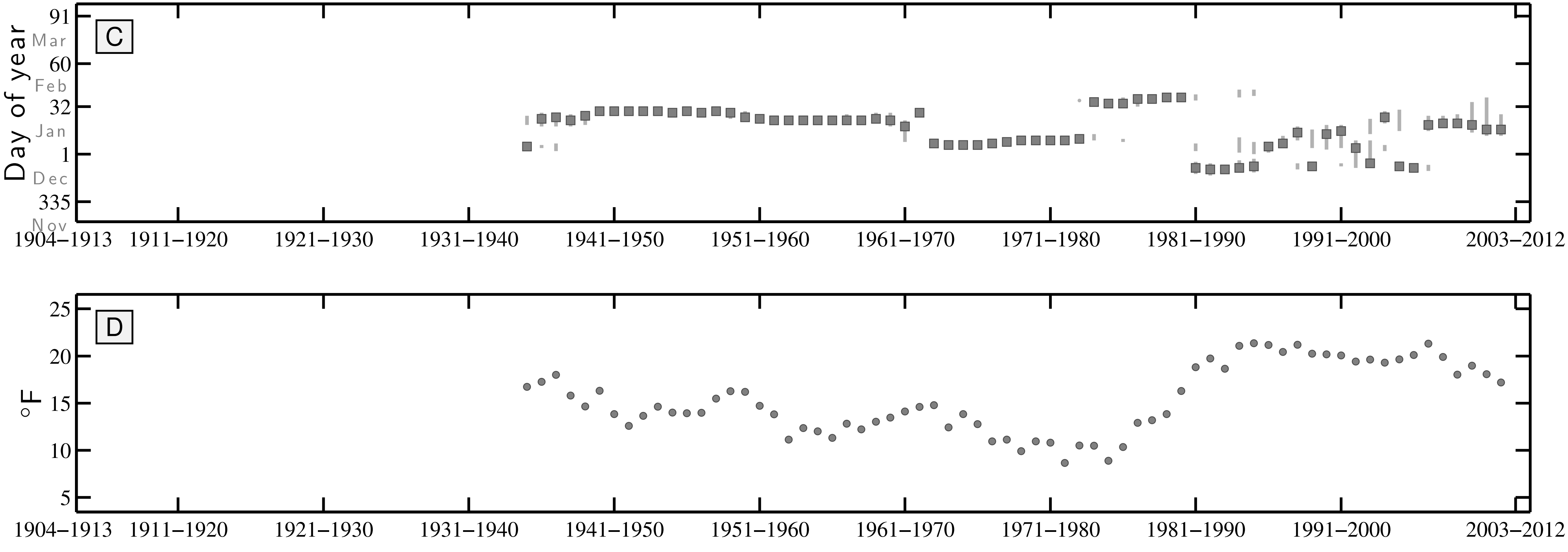

10 year Summer Teletherm dynamics for MTN GROVE 2 N, MO:

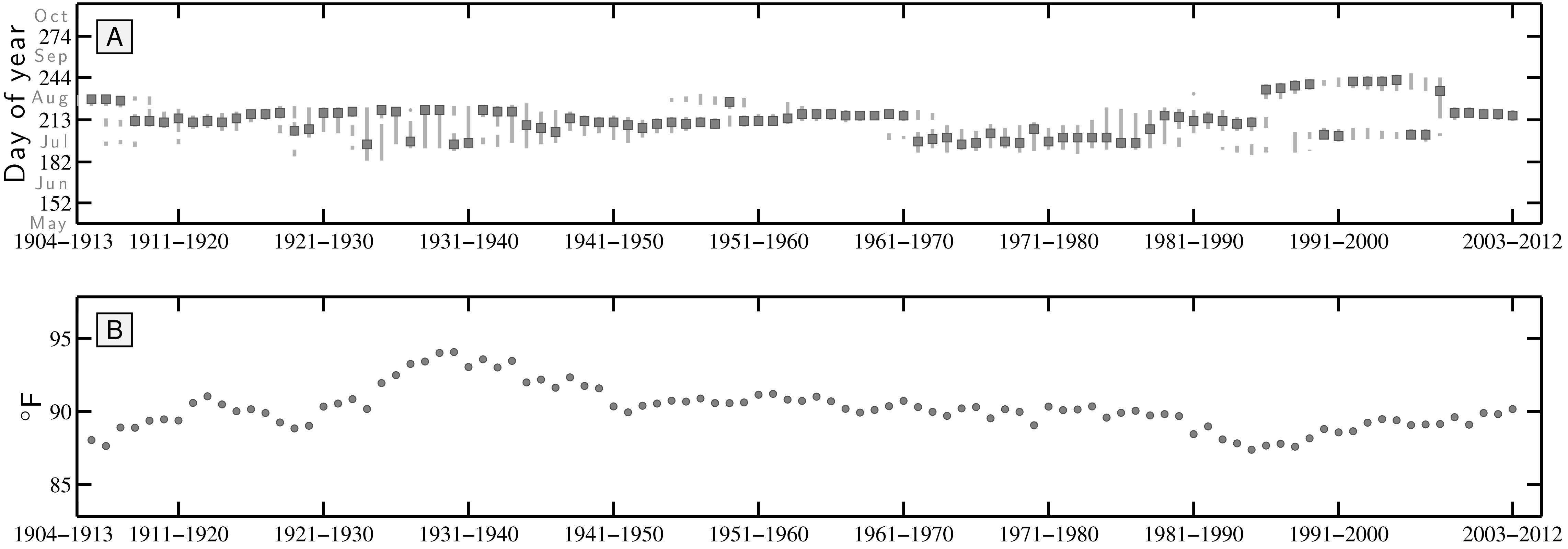

10 year Winter Teletherm dynamics for MTN GROVE 2 N, MO:

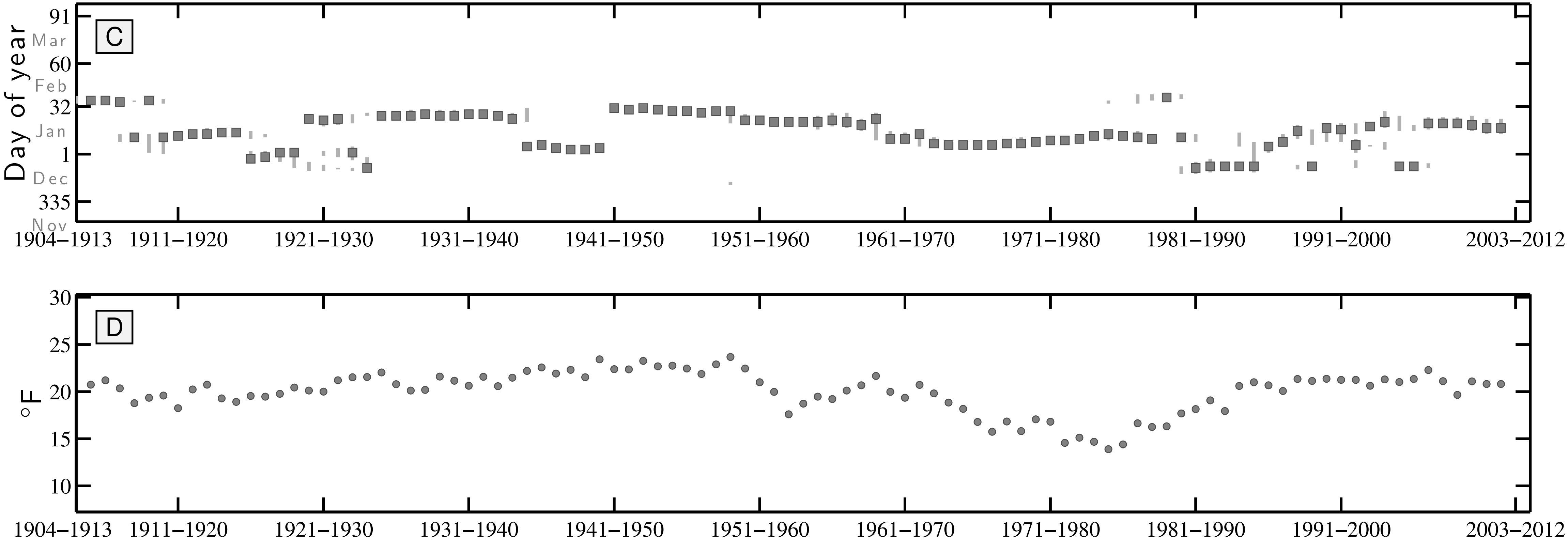

10 year Summer Teletherm dynamics for NEOSHO, MO:

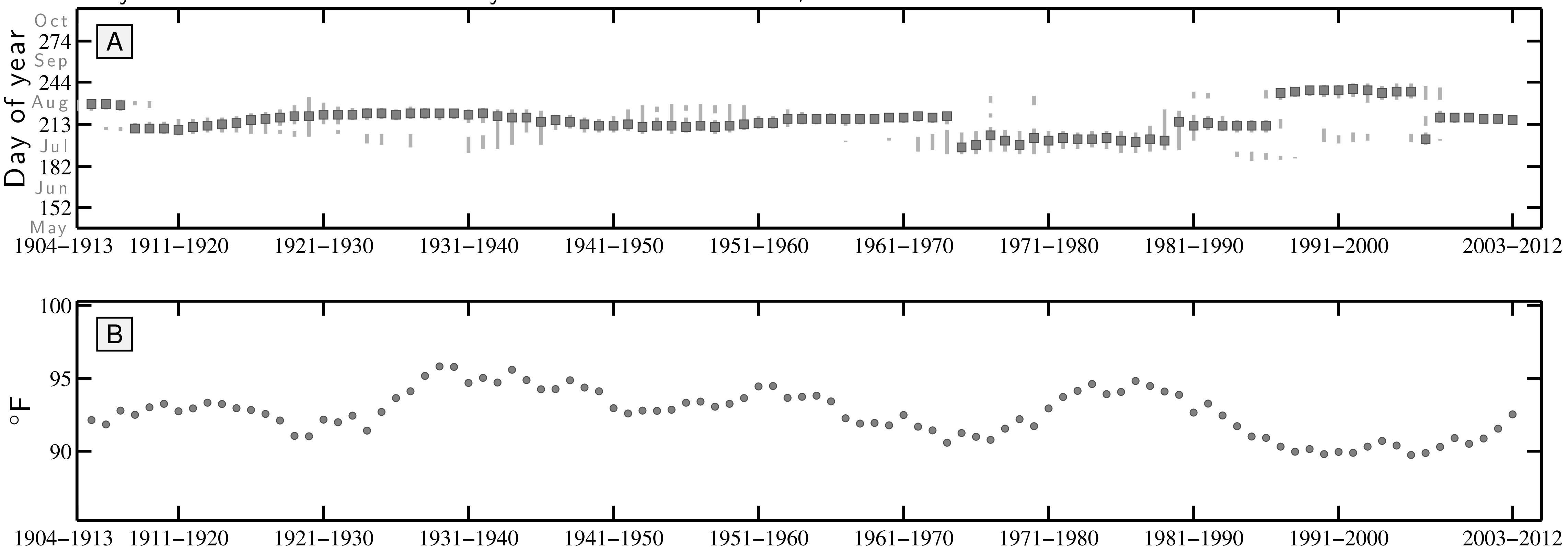

10 year Winter Teletherm dynamics for NEOSHO, MO:

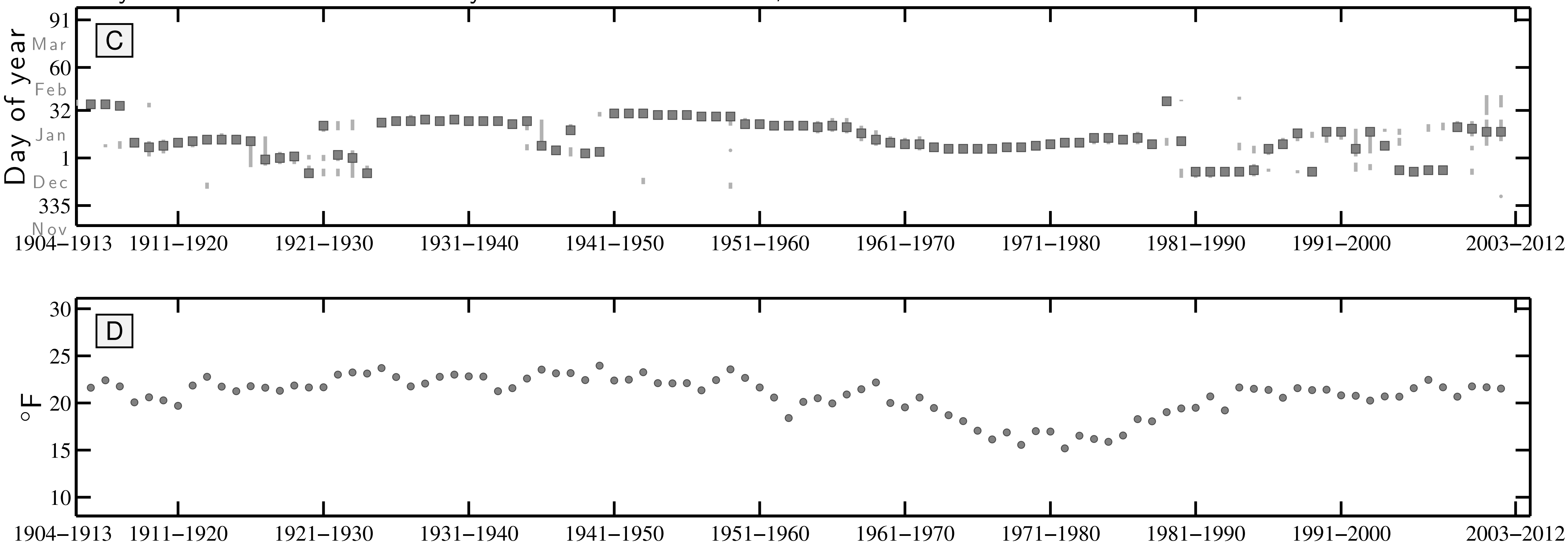

10 year Summer Teletherm dynamics for ROLLA UNI OF MISSOURI, MO:

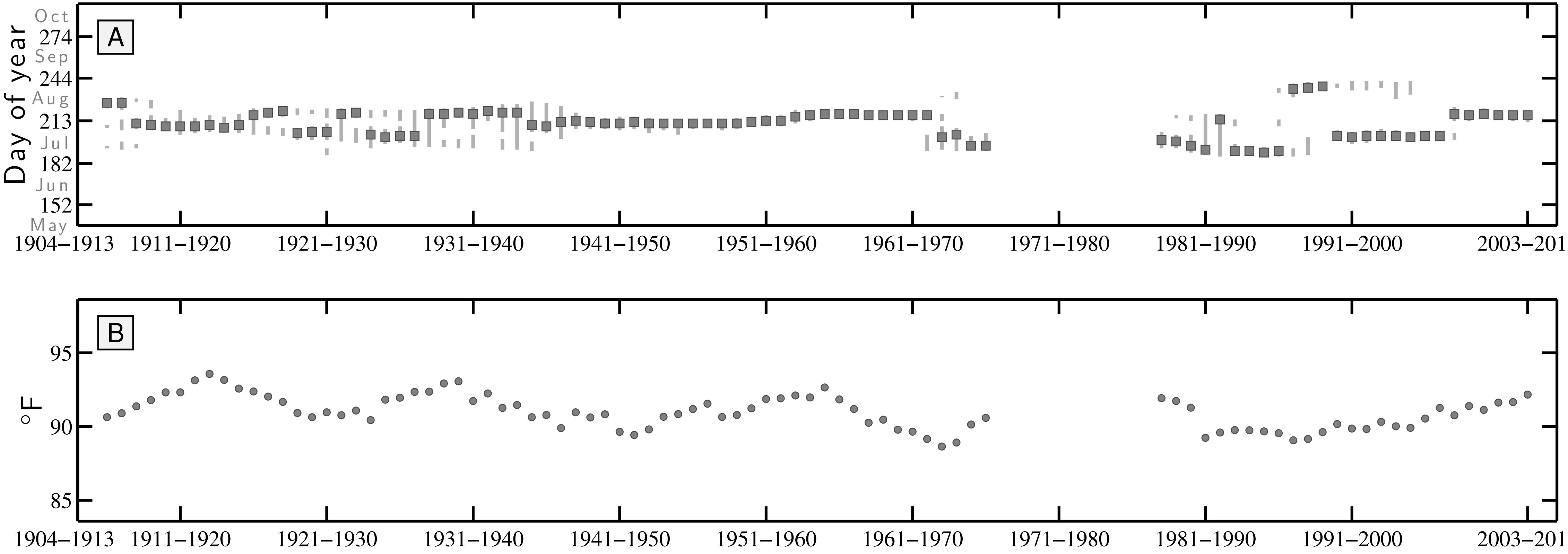

10 year Winter Teletherm dynamics for ROLLA UNI OF MISSOURI, MO:

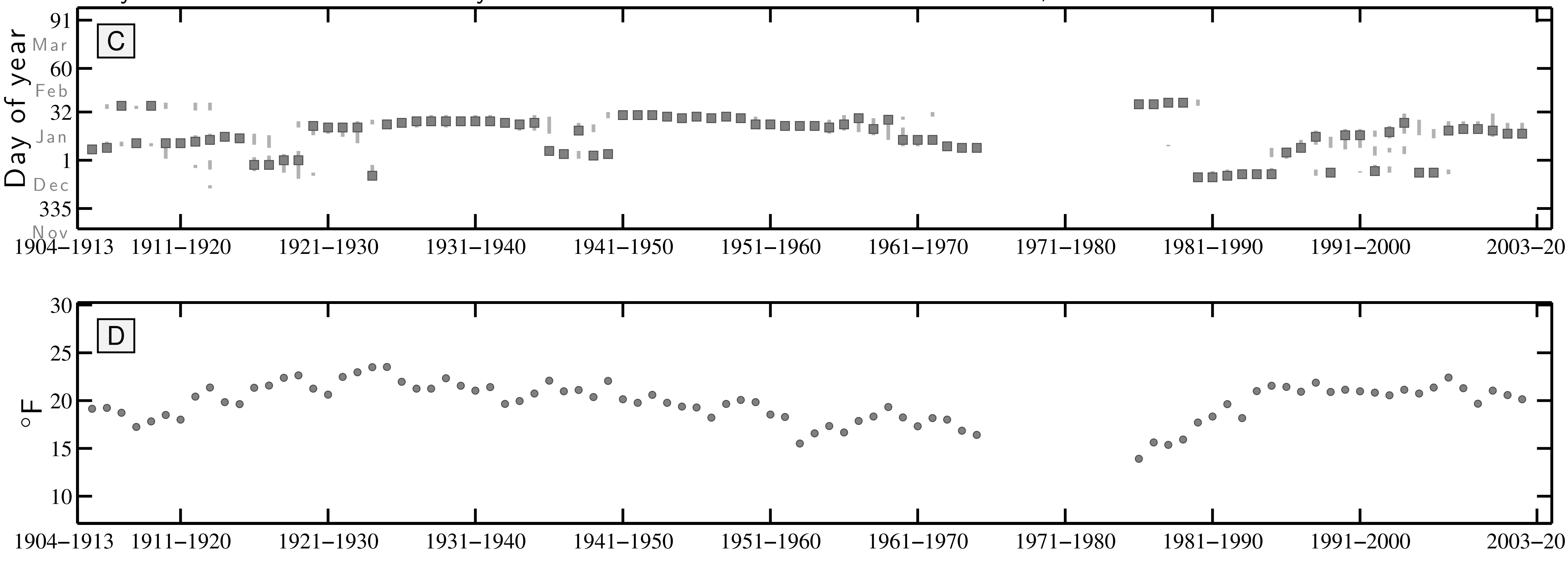

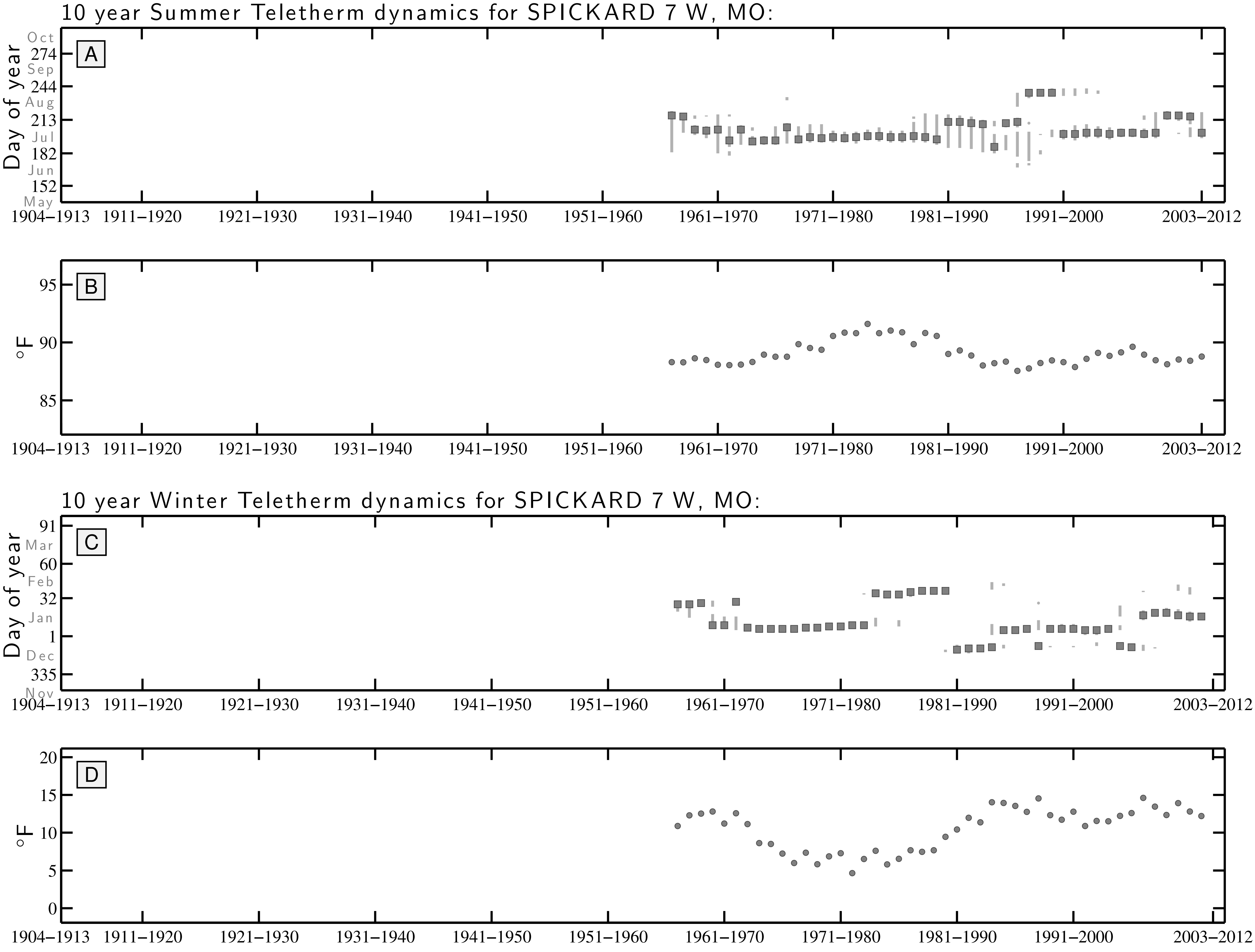
10 year Summer Teletherm dynamics for SPICKARD 7 W, MO:
A
B
Day of year
°F
Oct
274
Sep
244
Aug
213
Jul
182
Jun
152
May
95
90
85
10 year Winter Teletherm dynamics for SPICKARD 7 W, MO:
C
D
91
Mar
60
Feb
32
Jan
1
Dec
335
Nov
20
15
10
5
0
1904–1913
1911–1920
1921–1930
1931–1940
1941–1950
1951–1960
1961–1970
1971–1980
1981–1990
1991–2000
2003–2012

10 year Summer Teletherm dynamics for STEFFENVILLE, MO:

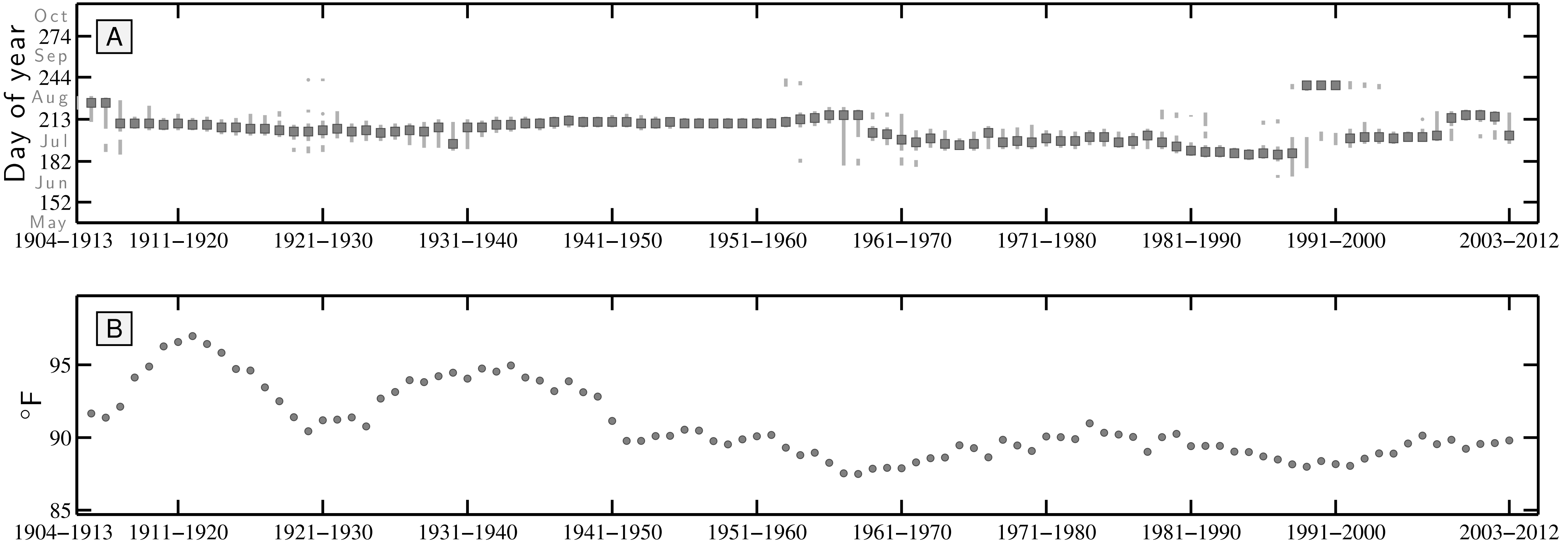

10 year Winter Teletherm dynamics for STEFFENVILLE, MO:

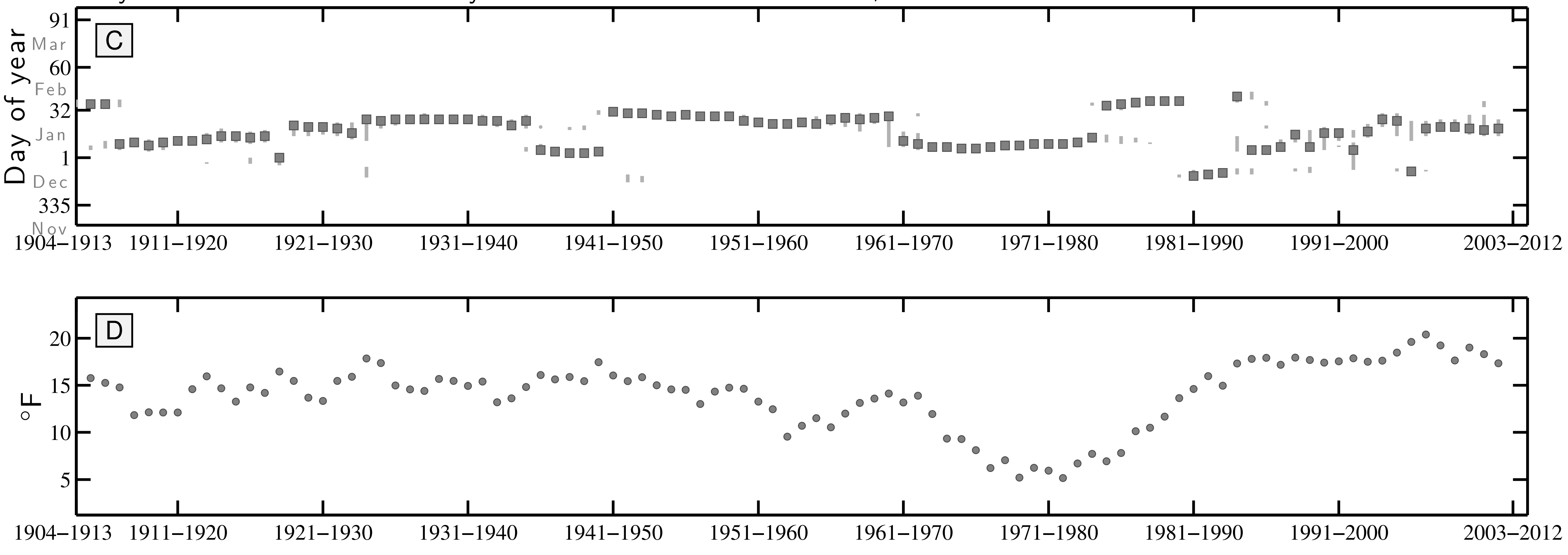

10 year Summer Teletherm dynamics for SWEET SPRINGS, MO:

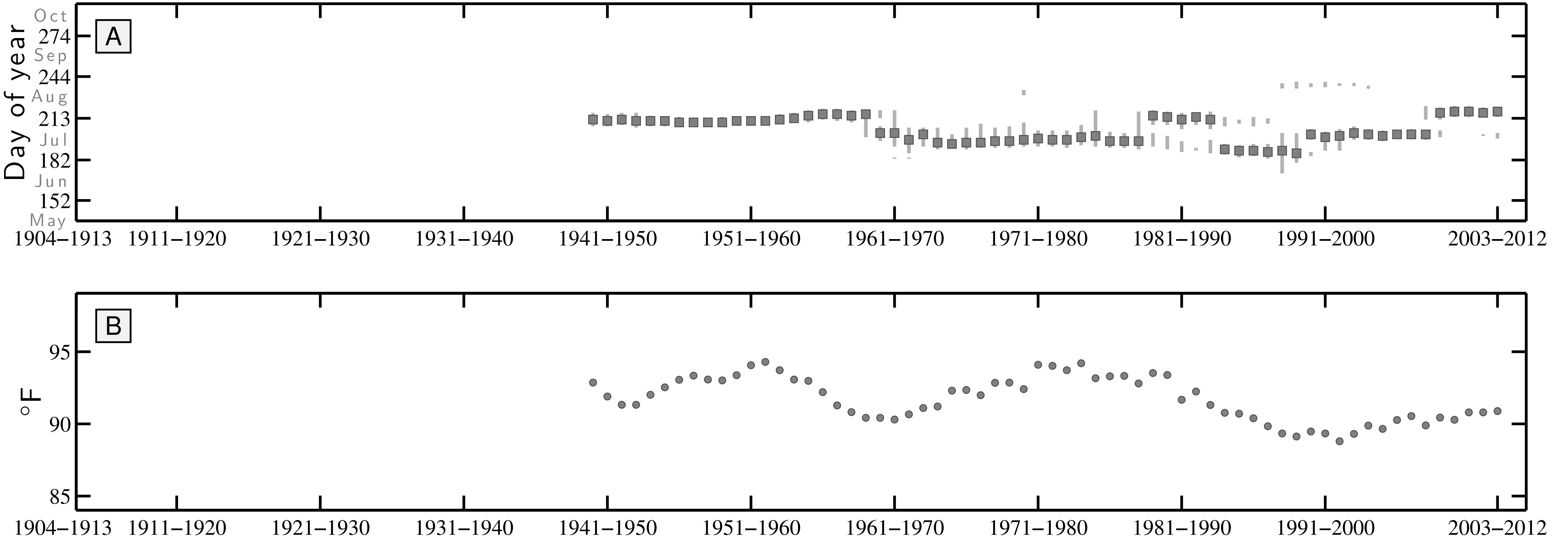

10 year Winter Teletherm dynamics for SWEET SPRINGS, MO:

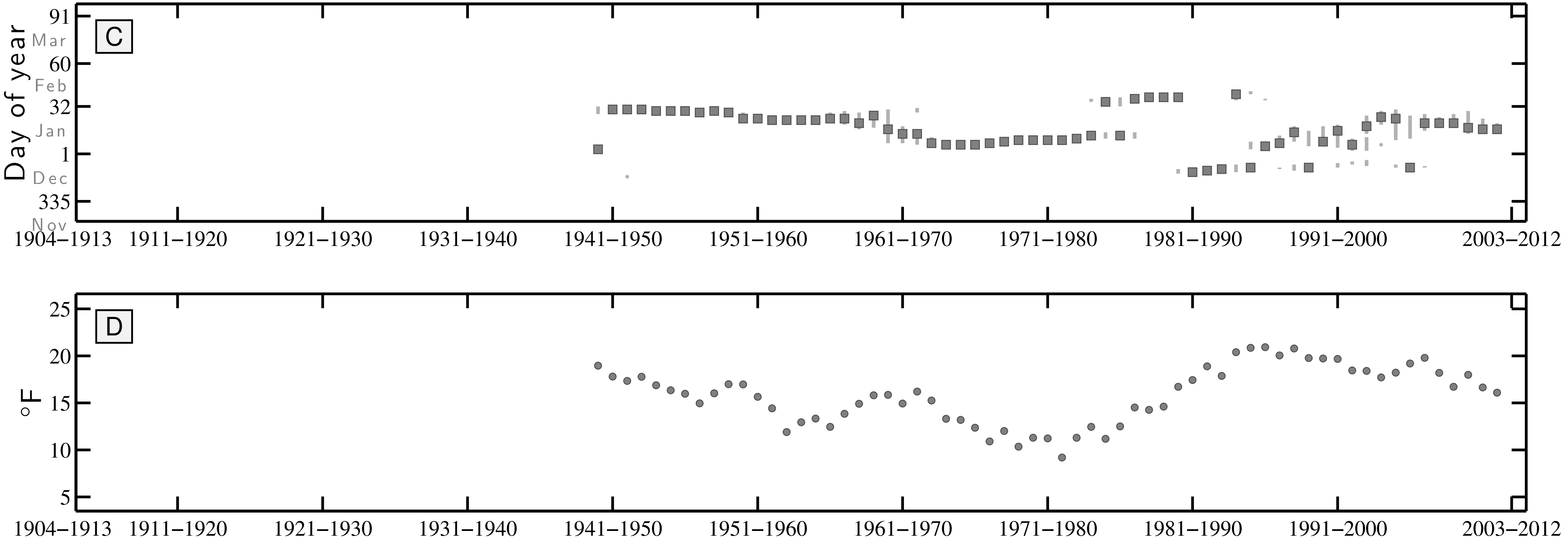

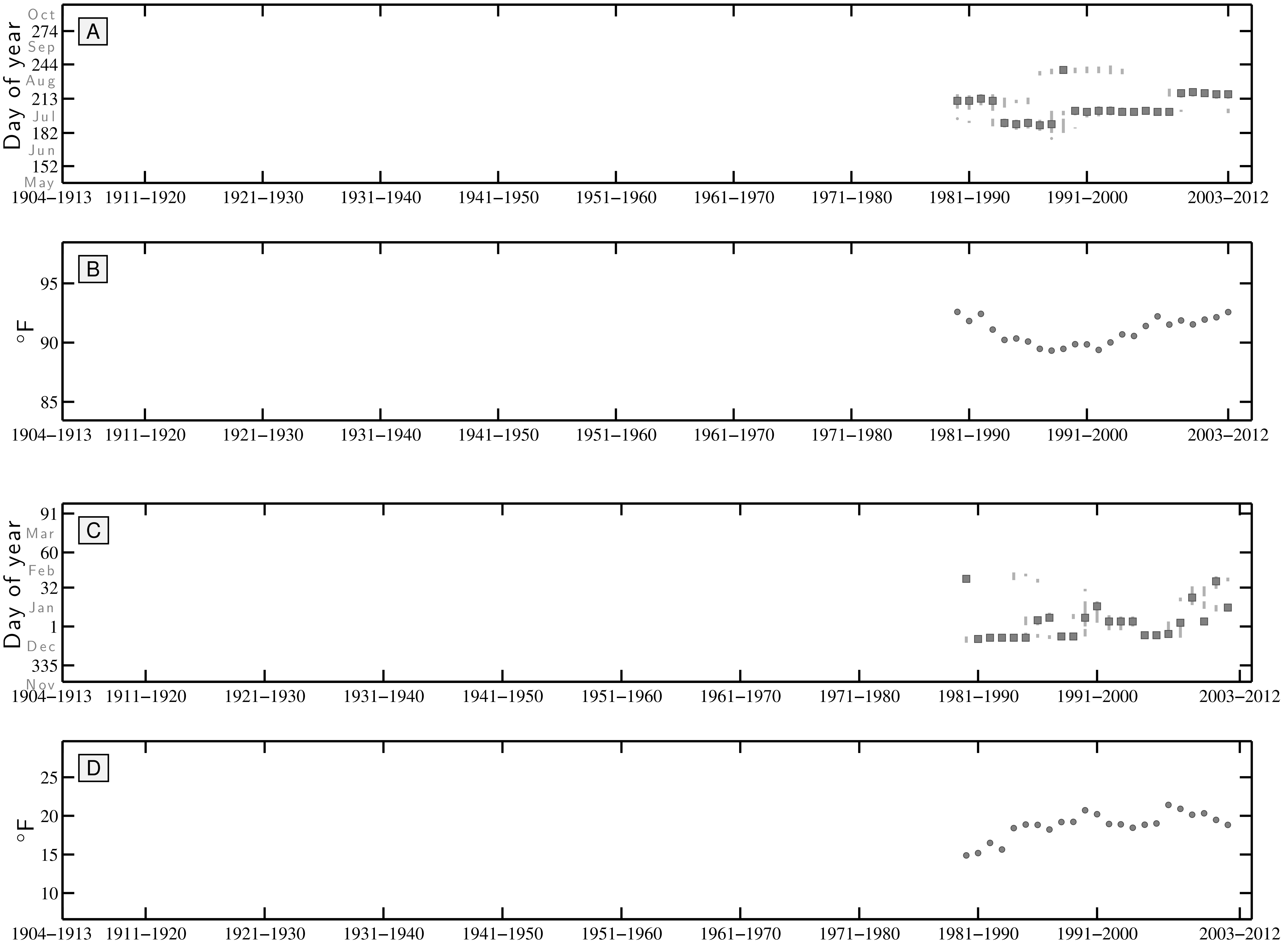
A
Day of year
Oct
274
Sep
244
Aug
213
Jul
182
Jun
152
May
1904–1913
1911–1920
1921–1930
1931–1940
1941–1950
1951–1960
1961–1970
1971–1980
1981–1990
1991–2000
2003–2012
B
°F
95
90
85
C
91
Mar
60
Feb
32
Jan
1
Dec
335
Nov
D
25
20
15
10

10 year Summer Teletherm dynamics for UNIONVILLE, MO:

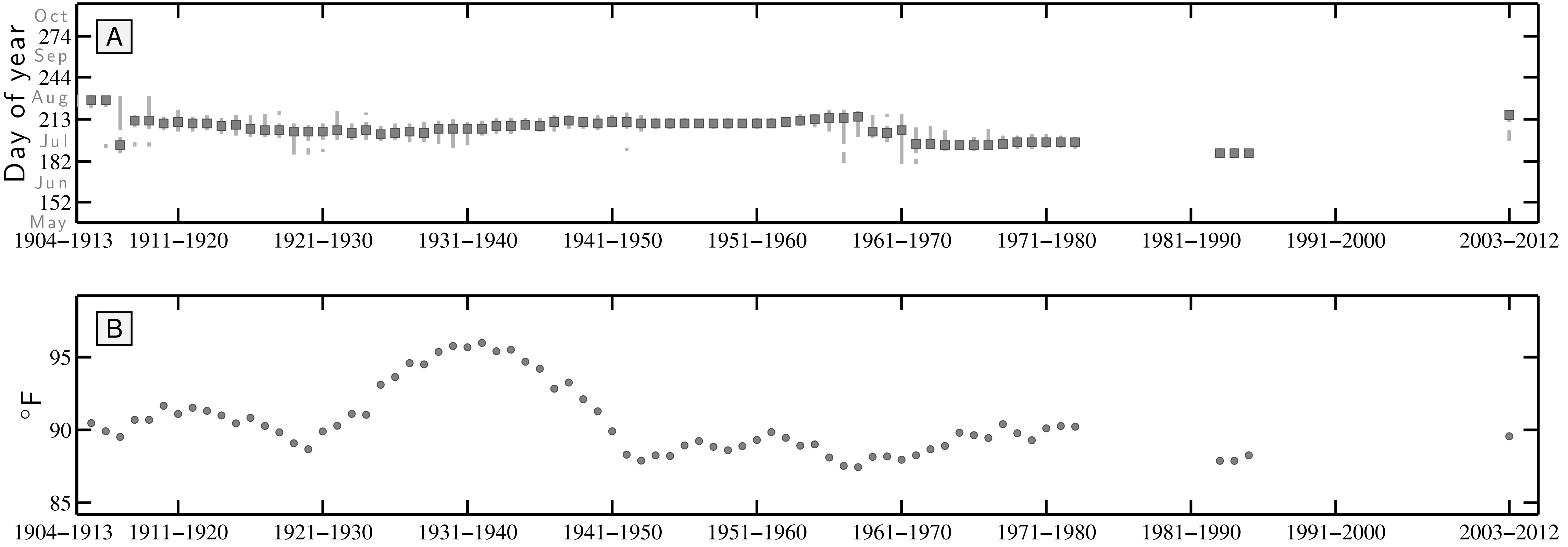

10 year Winter Teletherm dynamics for UNIONVILLE, MO:

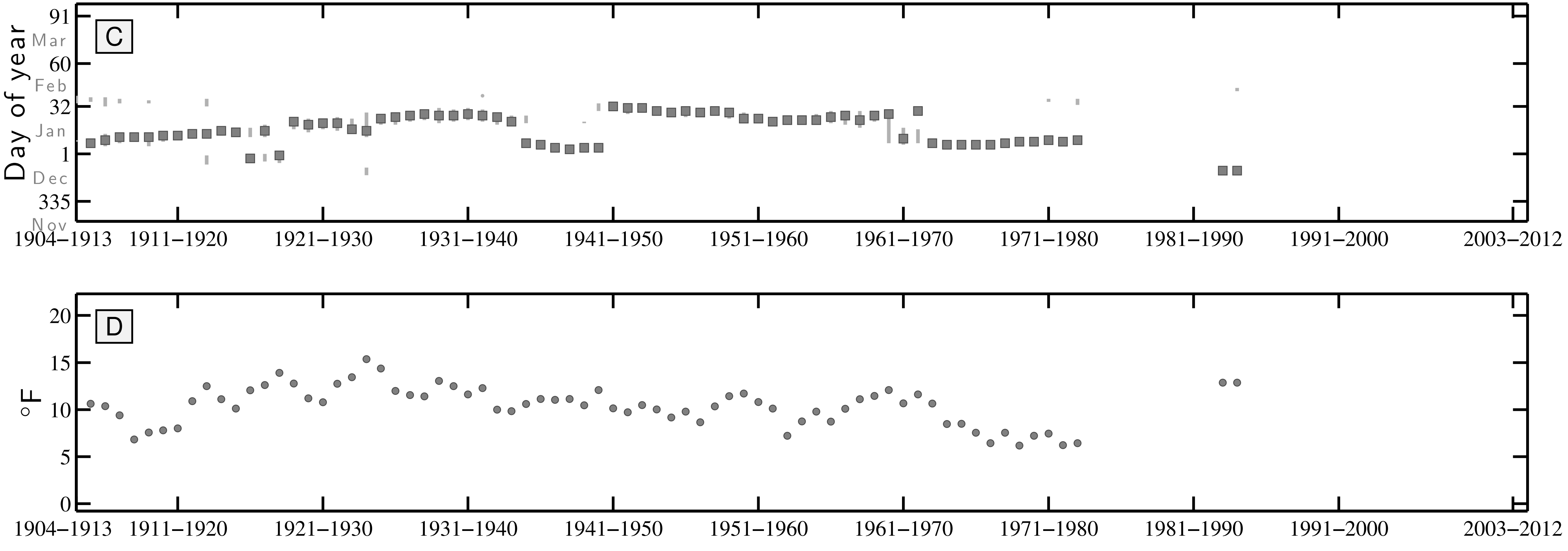

10 year Summer Teletherm dynamics for WARRENTON 1 N, MO:

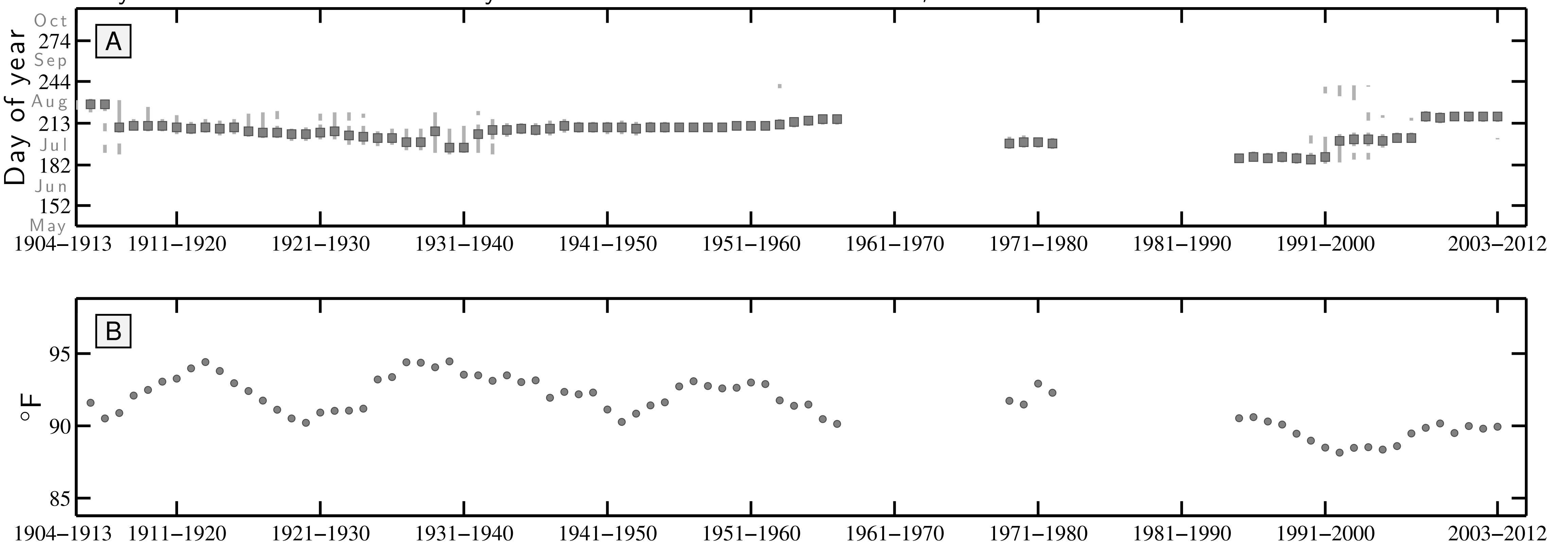

10 year Winter Teletherm dynamics for WARRENTON 1 N, MO:

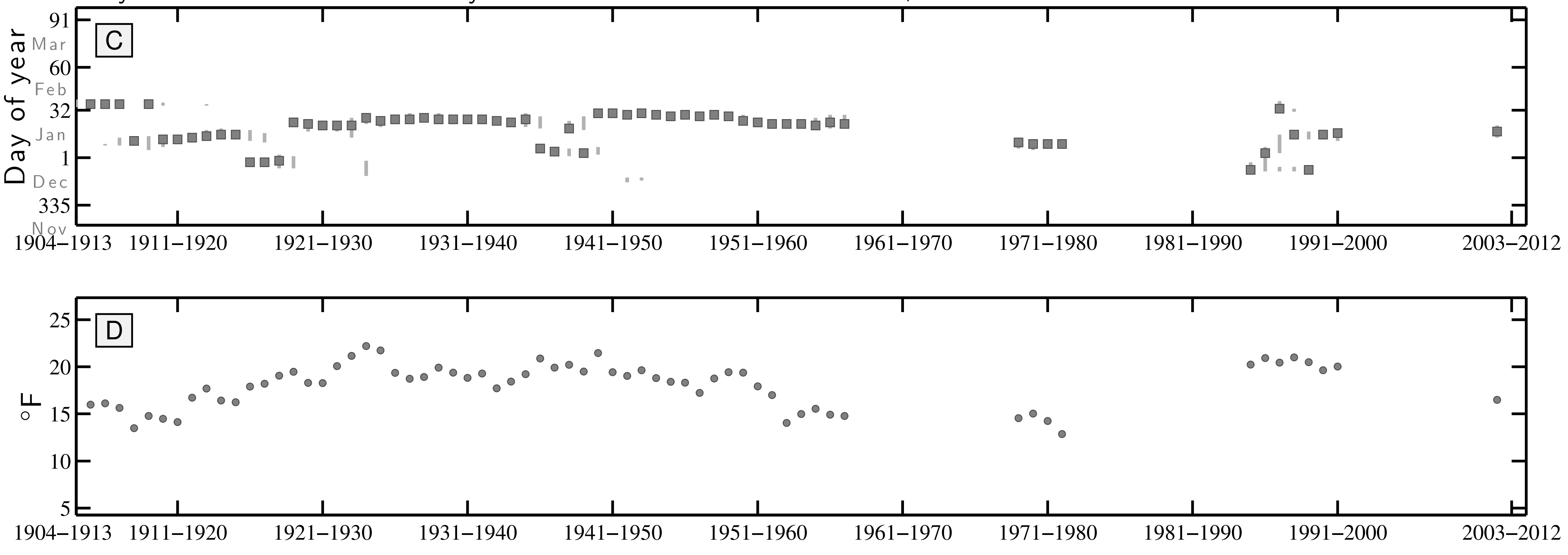

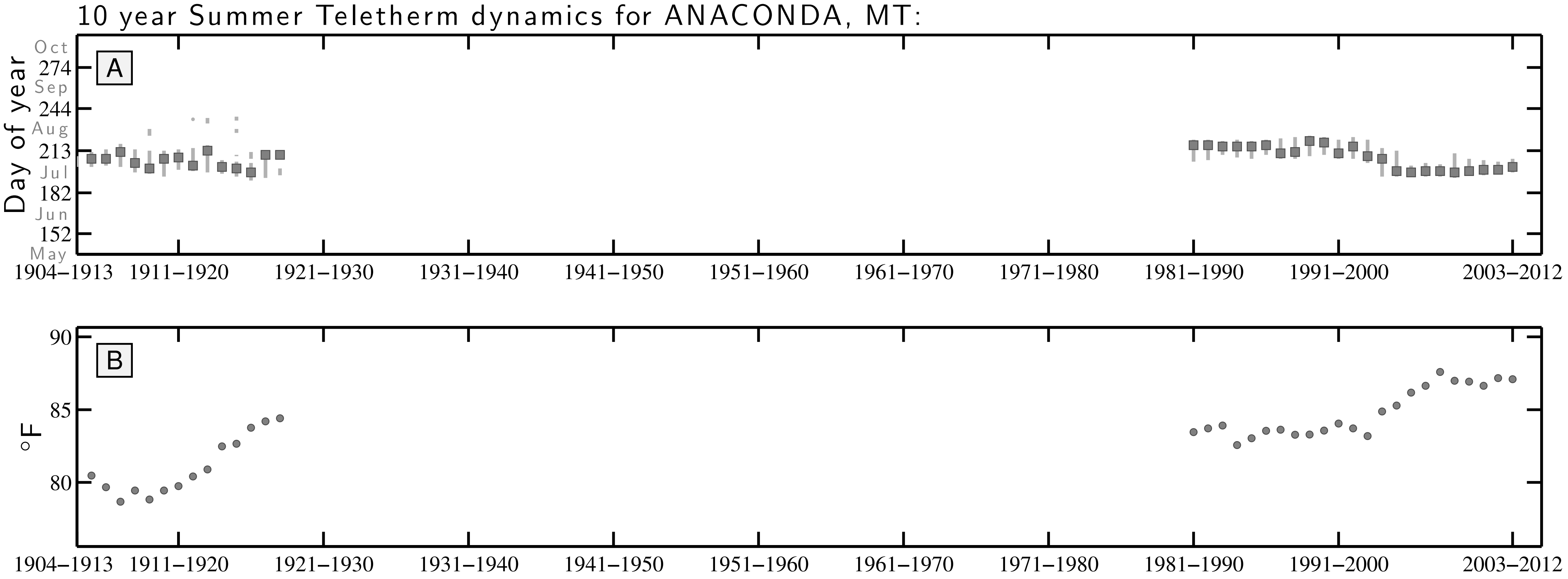
10 year Summer Teletherm dynamics for ANACONDA, MT:
A
B
Day of year
°F
Oct
274
Sep
244
Aug
213
Jul
182
Jun
152
May
90
85
80
1904–1913
1911–1920
1921–1930
1931–1940
1941–1950
1951–1960
1961–1970
1971–1980
1981–1990
1991–2000
2003–2012

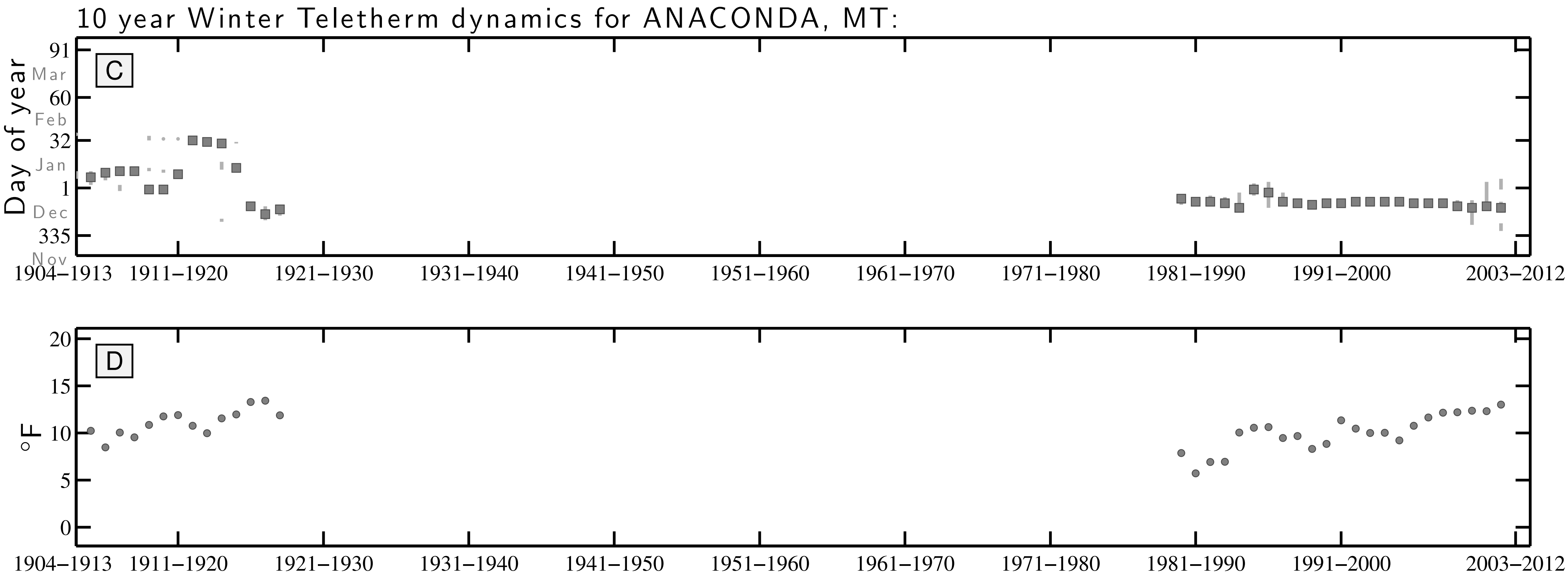
10 year Winter Teletherm dynamics for ANACONDA, MT:
C
D
Day of year
°F
91
Mar
60
Feb
32
Jan
1
Dec
335
Nov
20
15
10
5
0
1904–1913
1911–1920
1921–1930
1931–1940
1941–1950
1951–1960
1961–1970
1971–1980
1981–1990
1991–2000
2003–2012

10 year Summer Teletherm dynamics for AUGUSTA, MT:

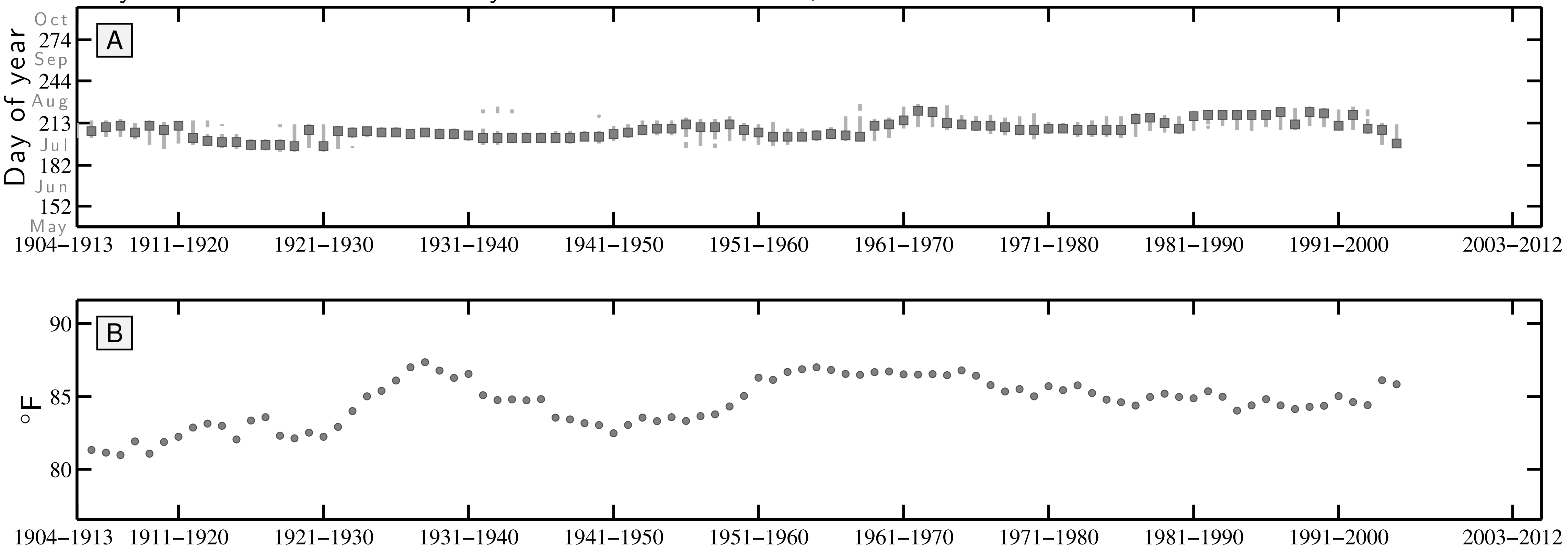

10 year Winter Teletherm dynamics for AUGUSTA, MT:

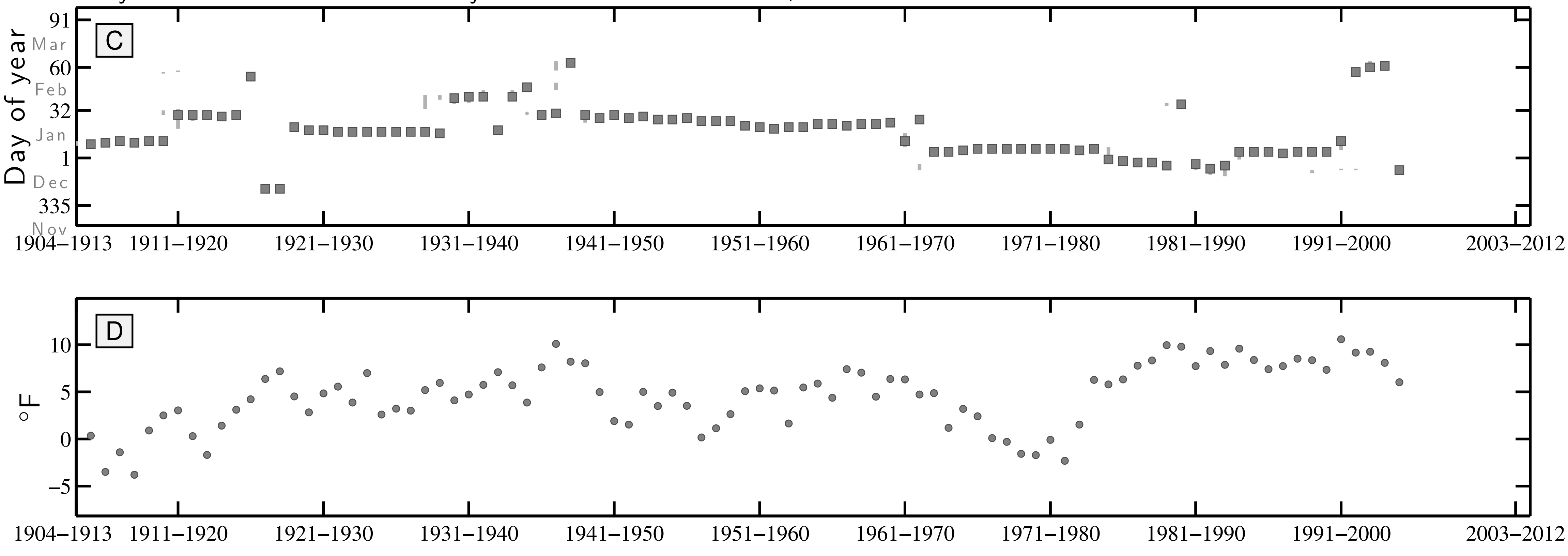

10 year Summer Teletherm dynamics for BIG TIMBER, MT:

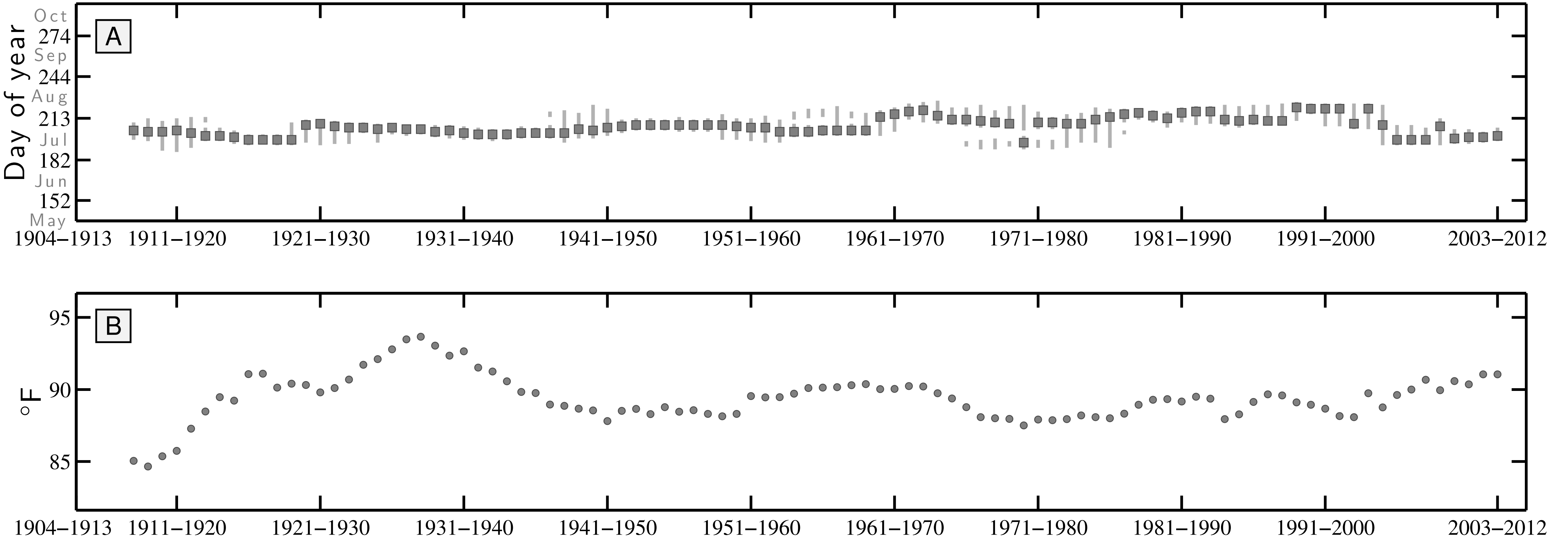

10 year Winter Teletherm dynamics for BIG TIMBER, MT:

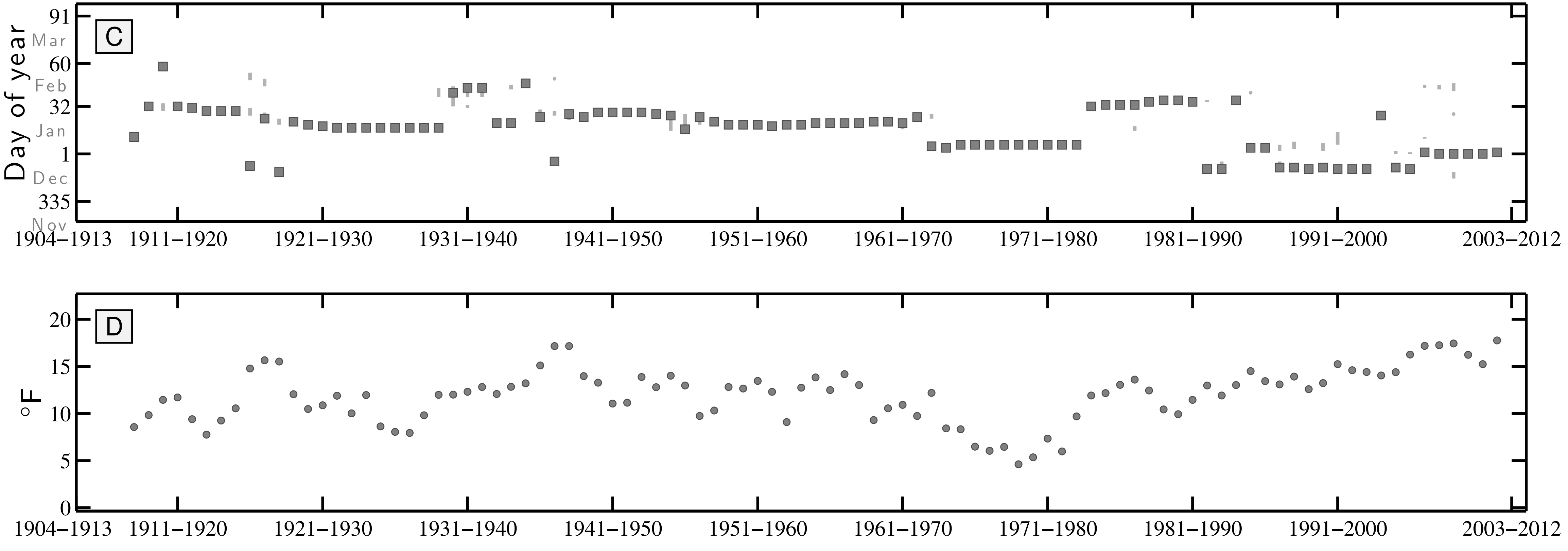

10 year Summer Teletherm dynamics for BOZEMAN MONTANA ST U, MT:

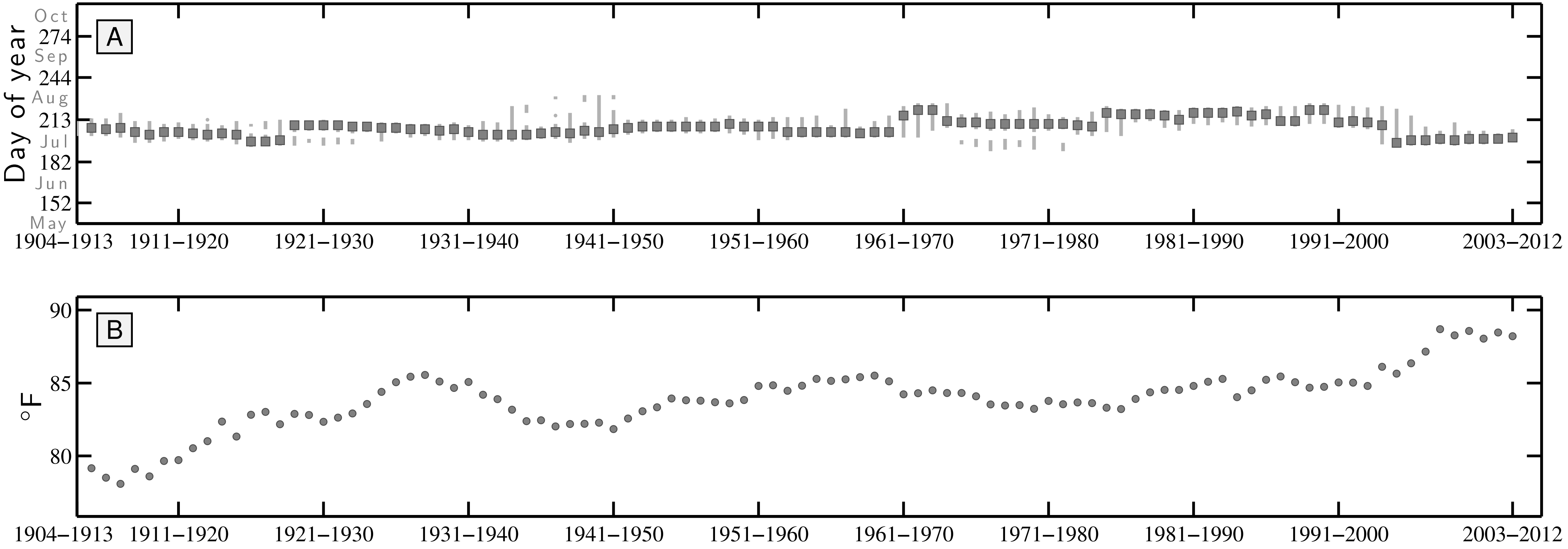

10 year Winter Teletherm dynamics for BOZEMAN MONTANA ST U, MT:

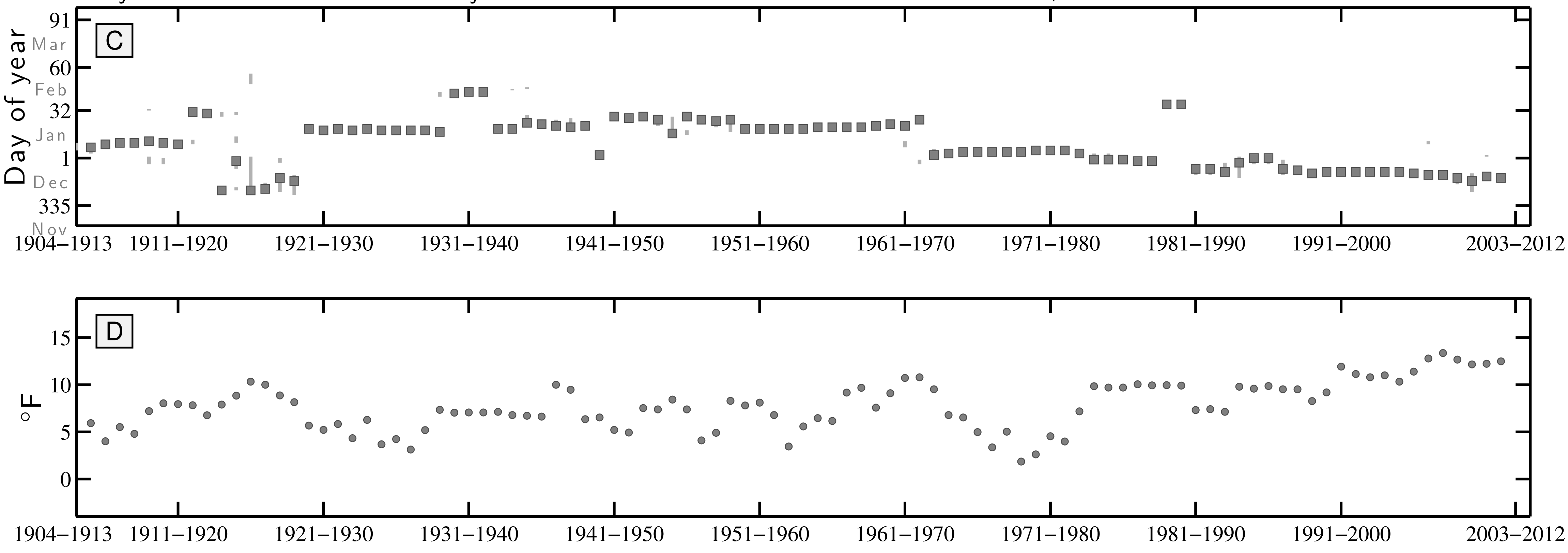

10 year Summer Teletherm dynamics for CASCADE 5 S, MT:

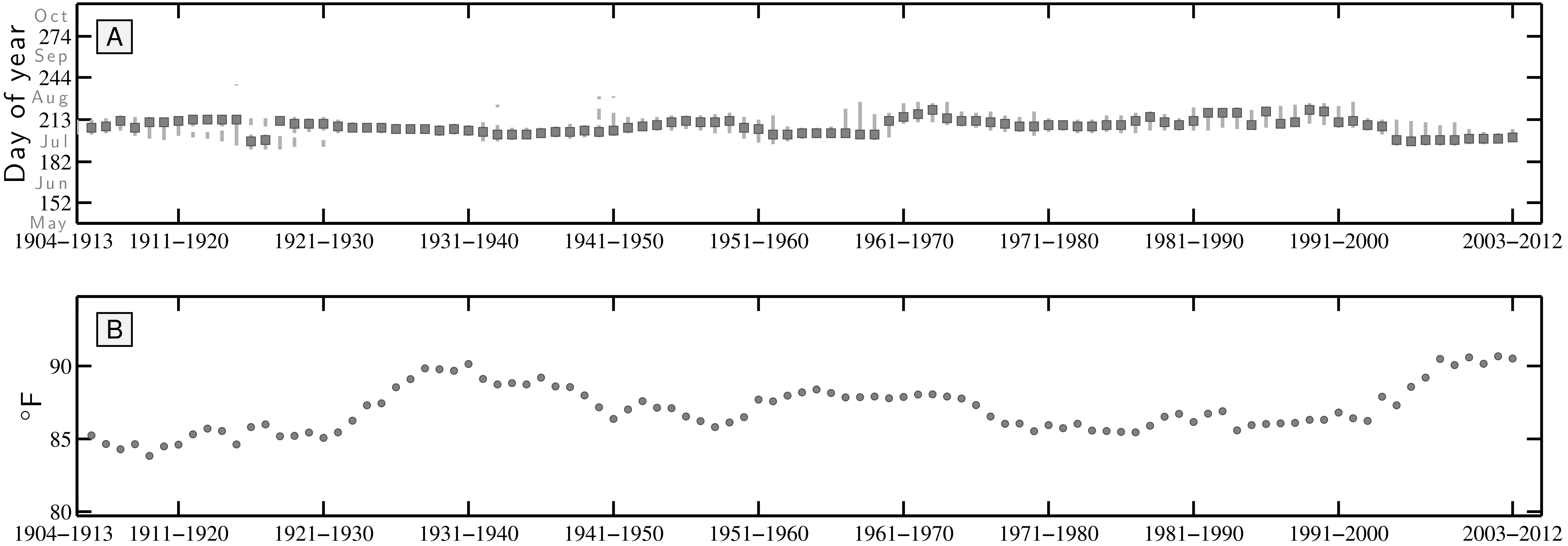

10 year Winter Teletherm dynamics for CASCADE 5 S, MT:

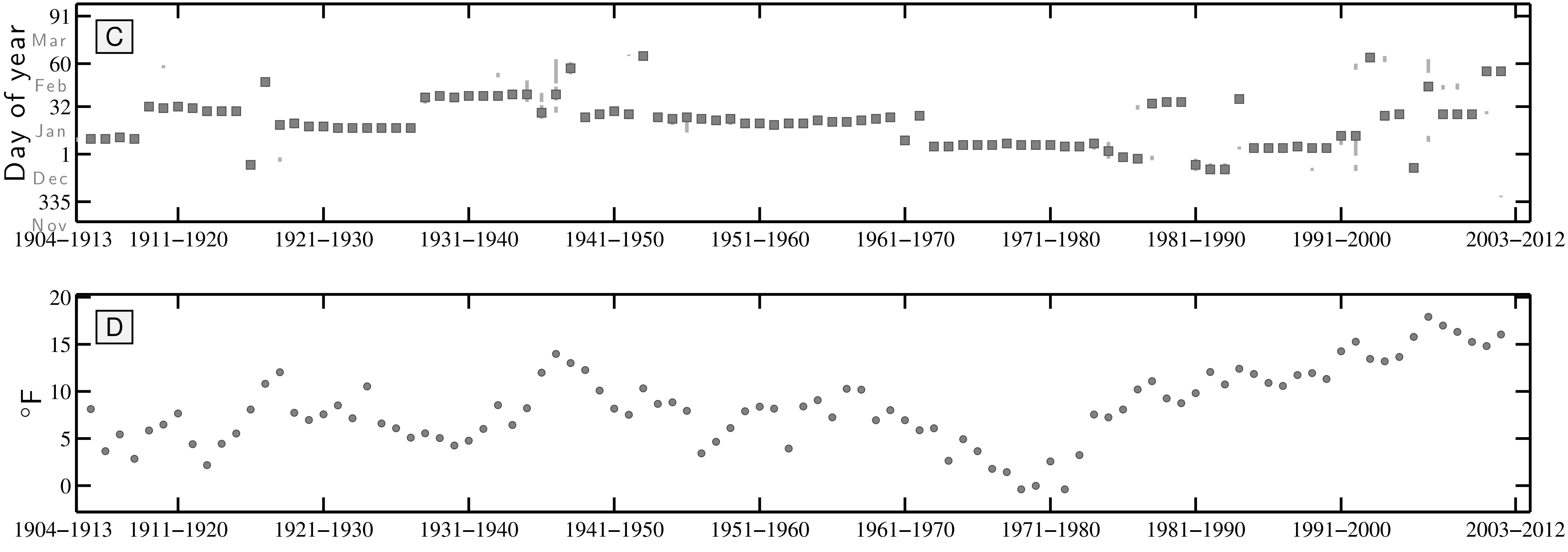

10 year Summer Teletherm dynamics for CHINOOK, MT:

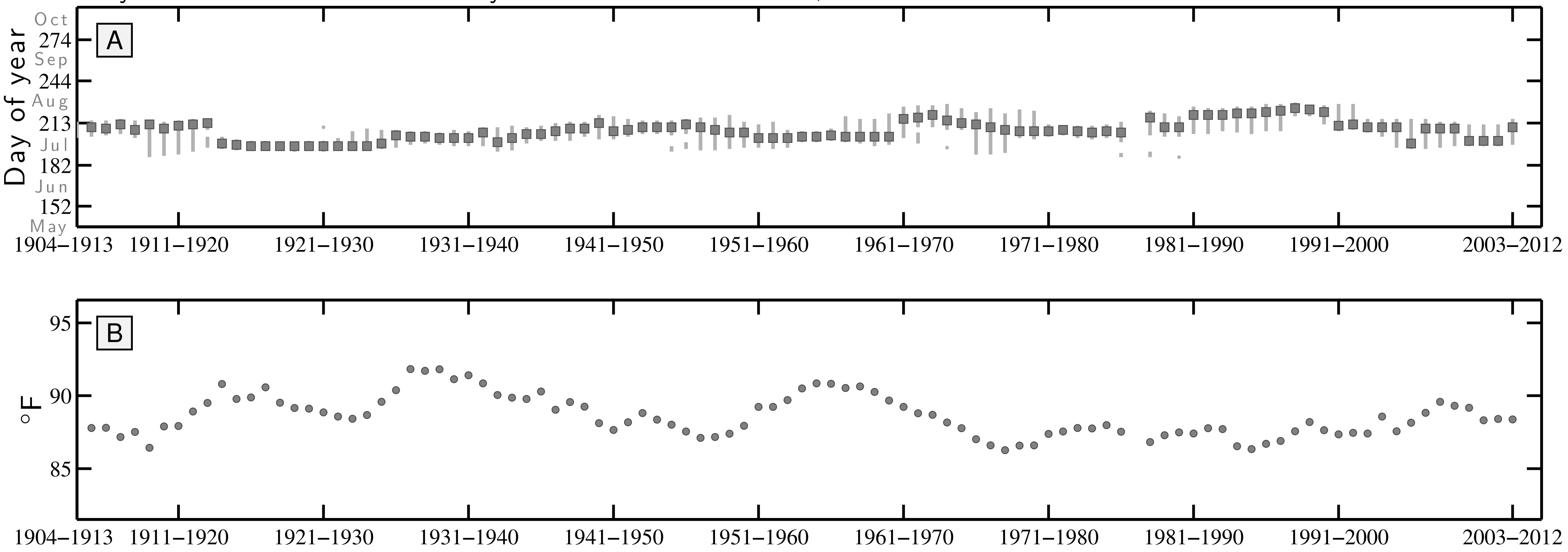

10 year Winter Teletherm dynamics for CHINOOK, MT:

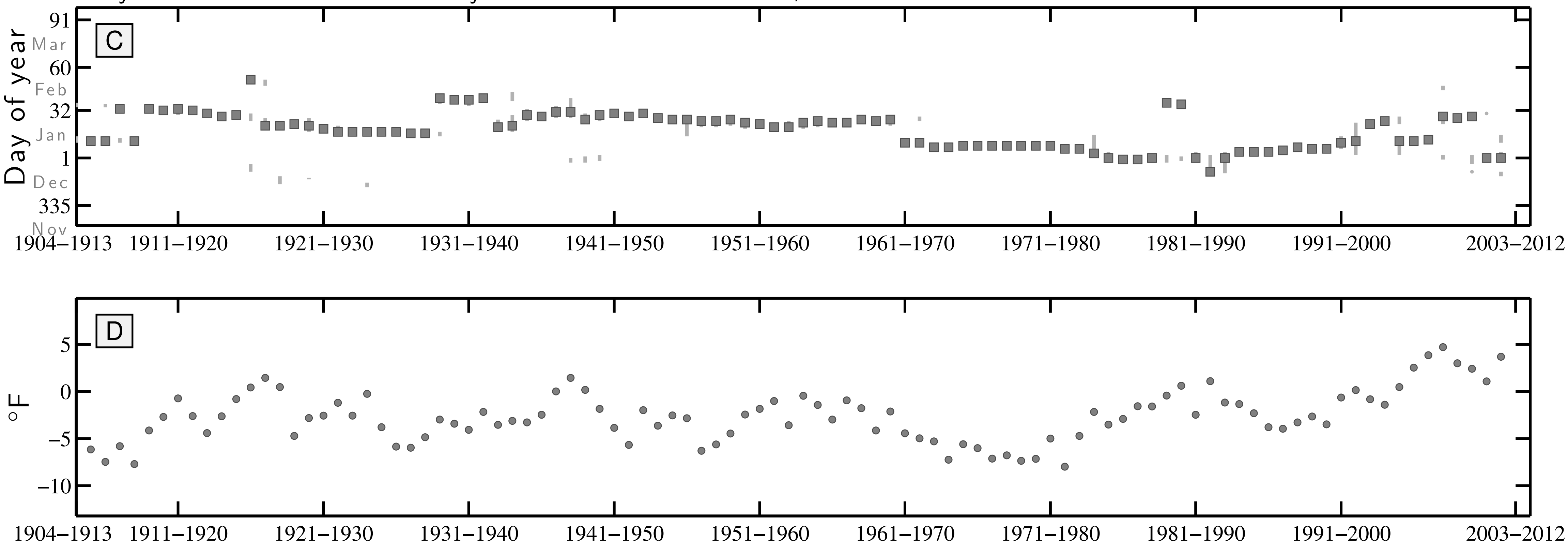

10 year Summer Teletherm dynamics for CHOTEAU, MT:

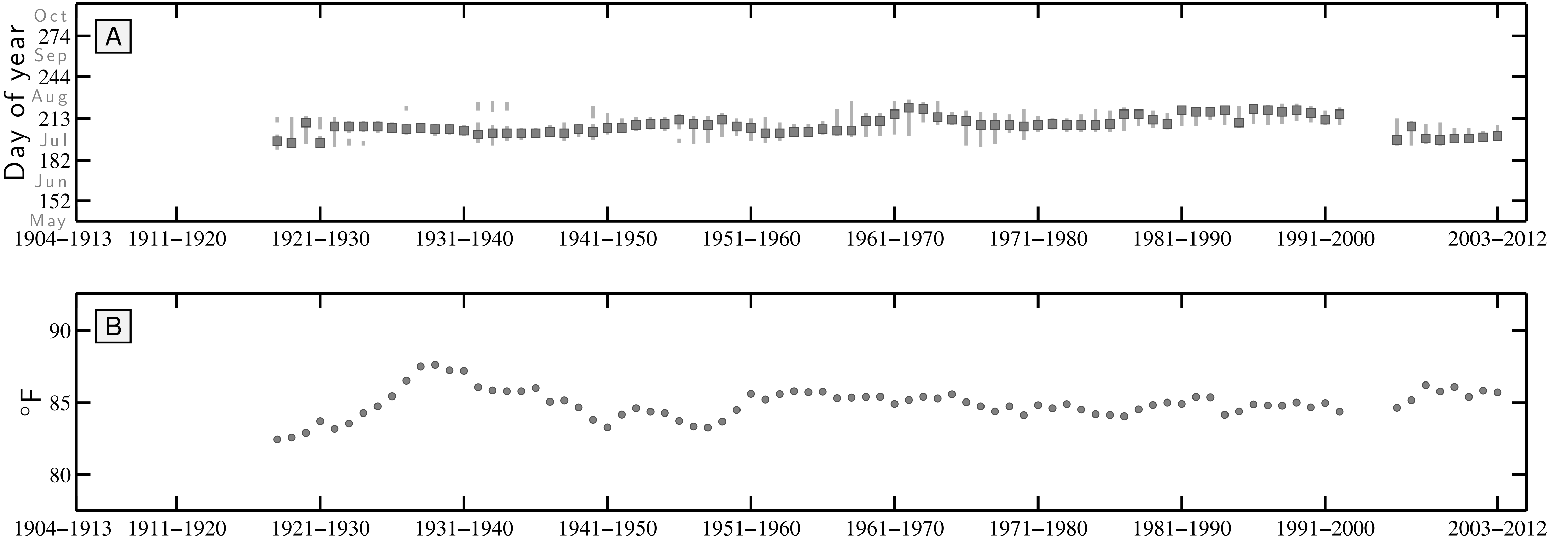

10 year Winter Teletherm dynamics for CHOTEAU, MT:

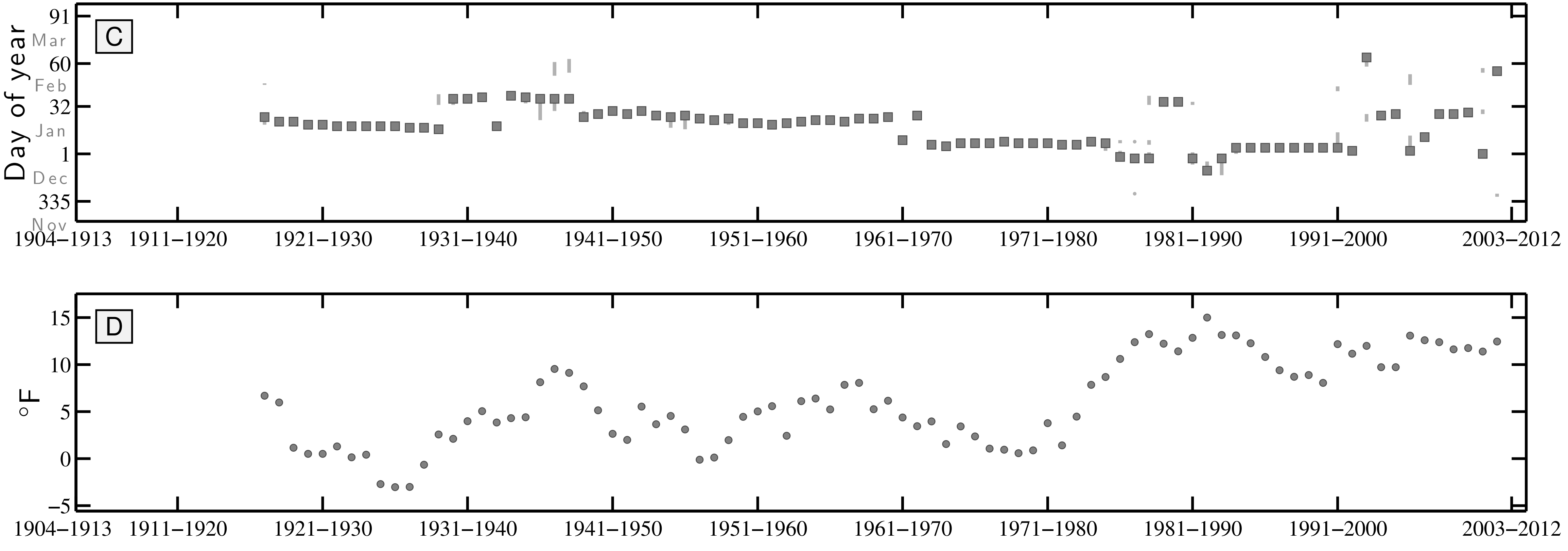

10 year Summer Teletherm dynamics for CUT BANK AP, MT:

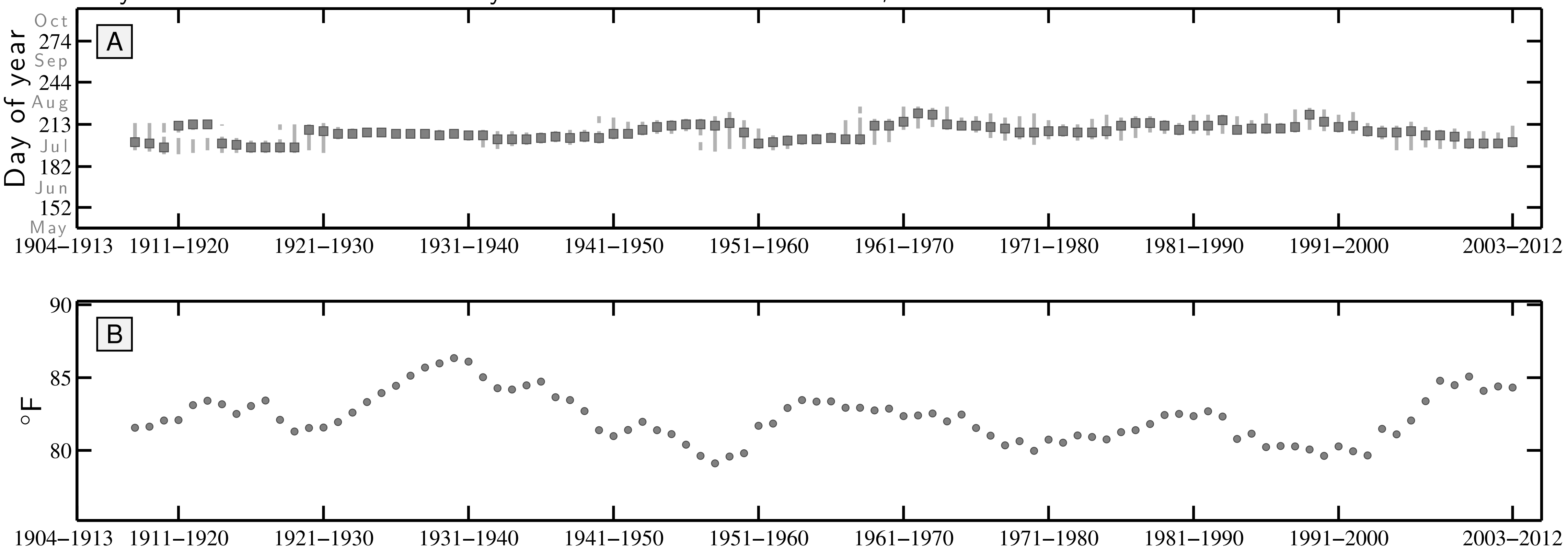

10 year Winter Teletherm dynamics for CUT BANK AP, MT:

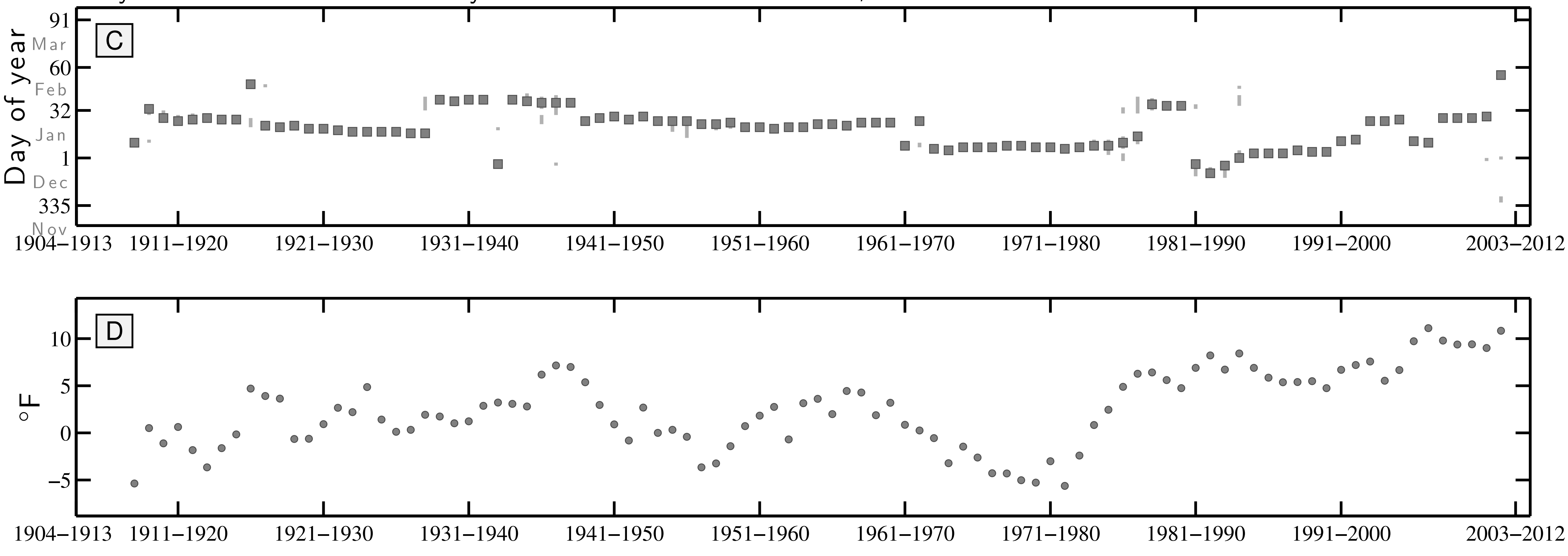

10 year Summer Teletherm dynamics for DILLON WMCE, MT:

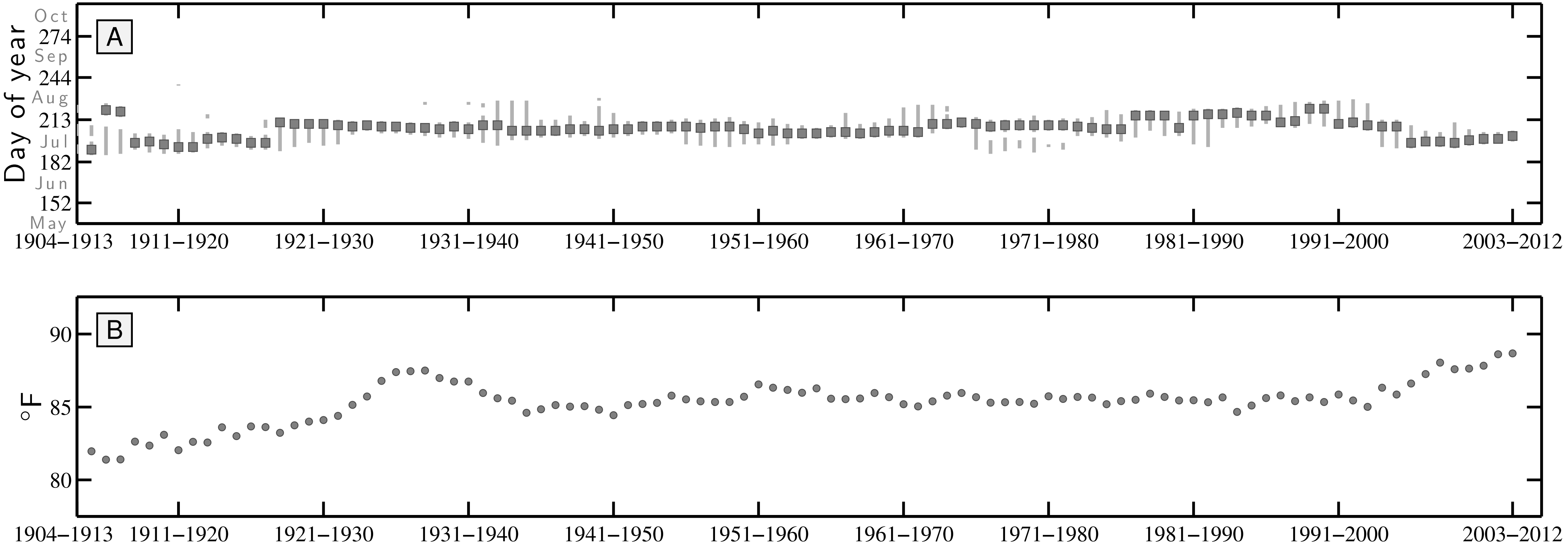

10 year Winter Teletherm dynamics for DILLON WMCE, MT:

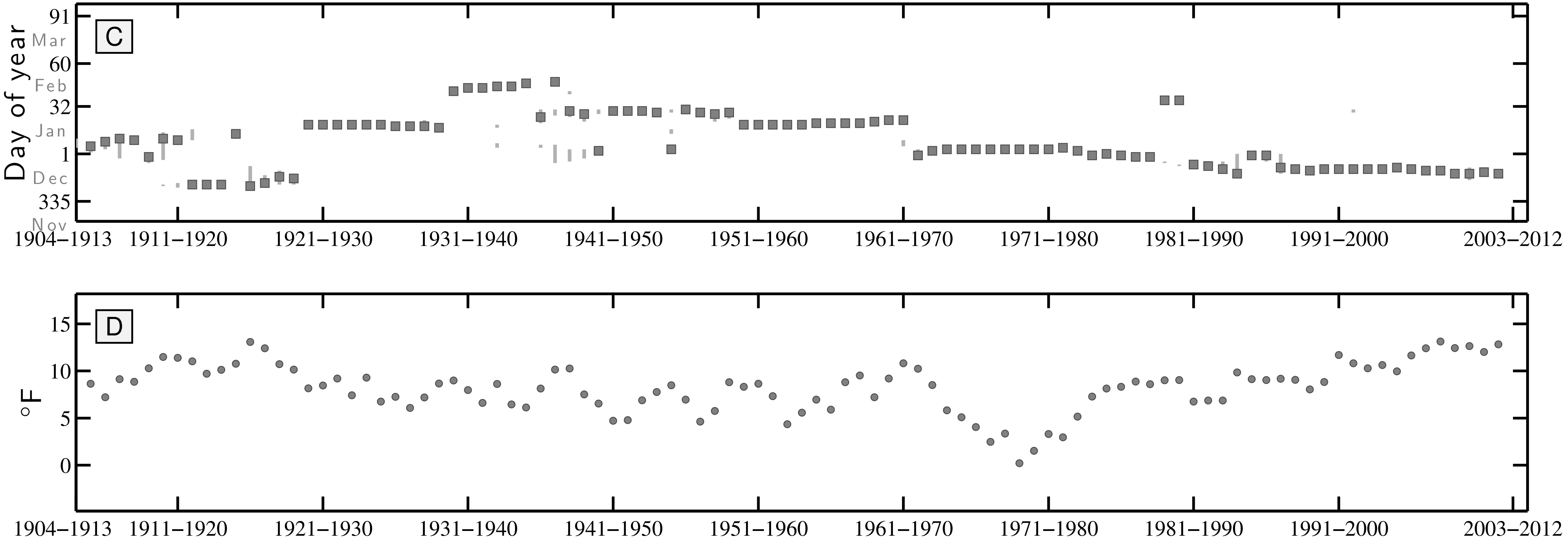

10 year Summer Teletherm dynamics for EKALAKA, MT:

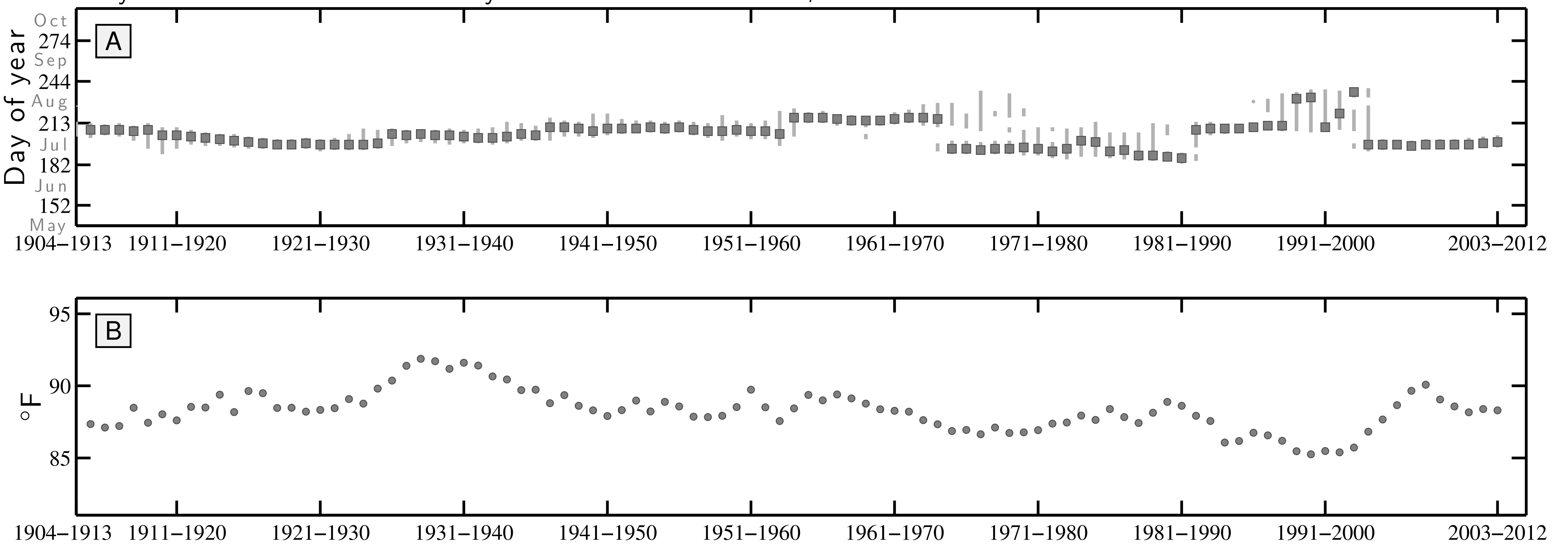

10 year Winter Teletherm dynamics for EKALAKA, MT:

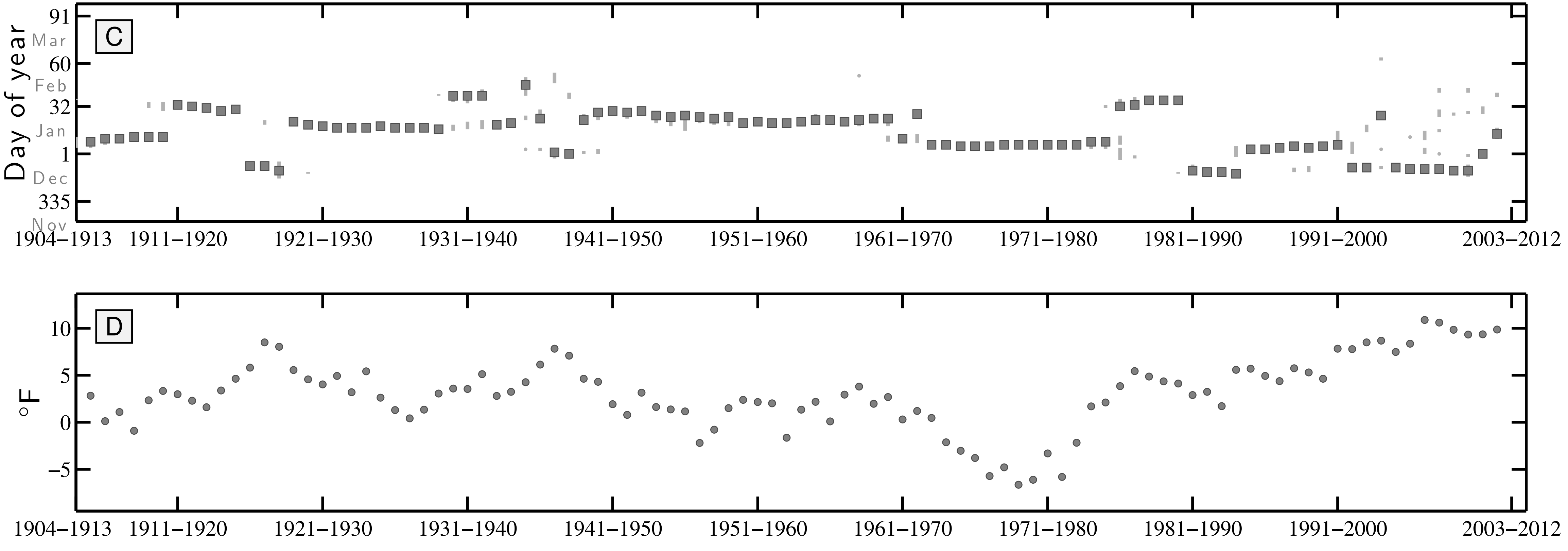

10 year Summer Teletherm dynamics for ENNIS, MT:

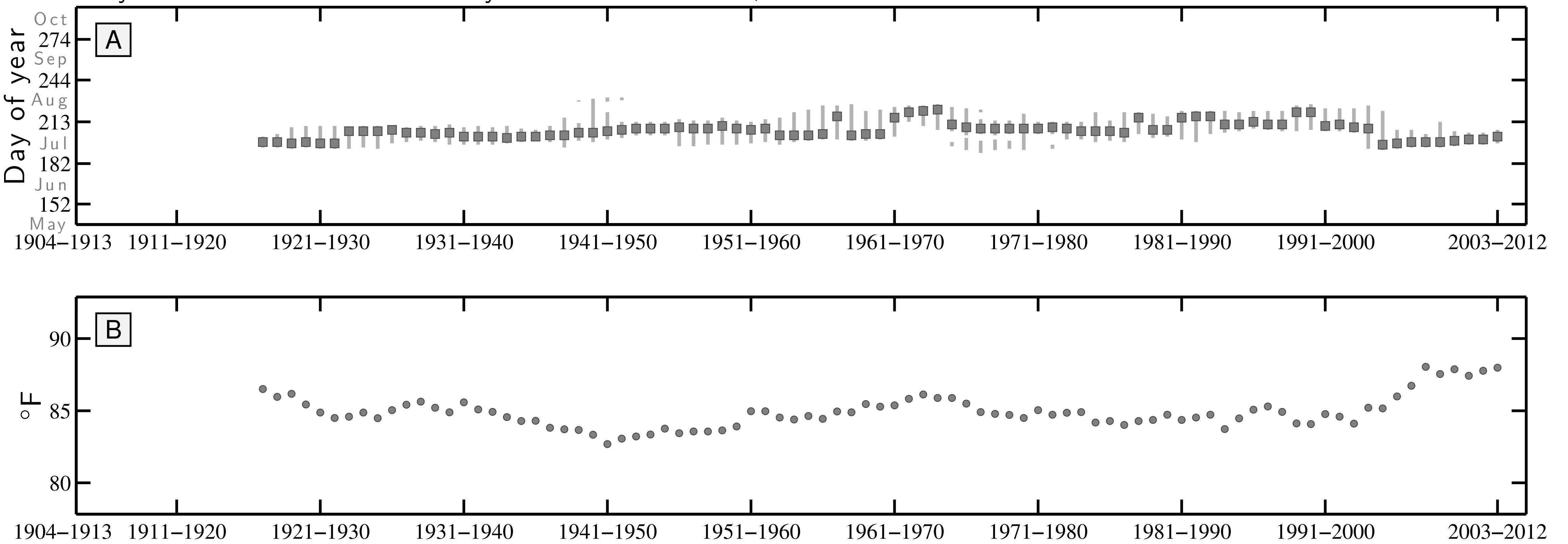

10 year Winter Teletherm dynamics for ENNIS, MT:

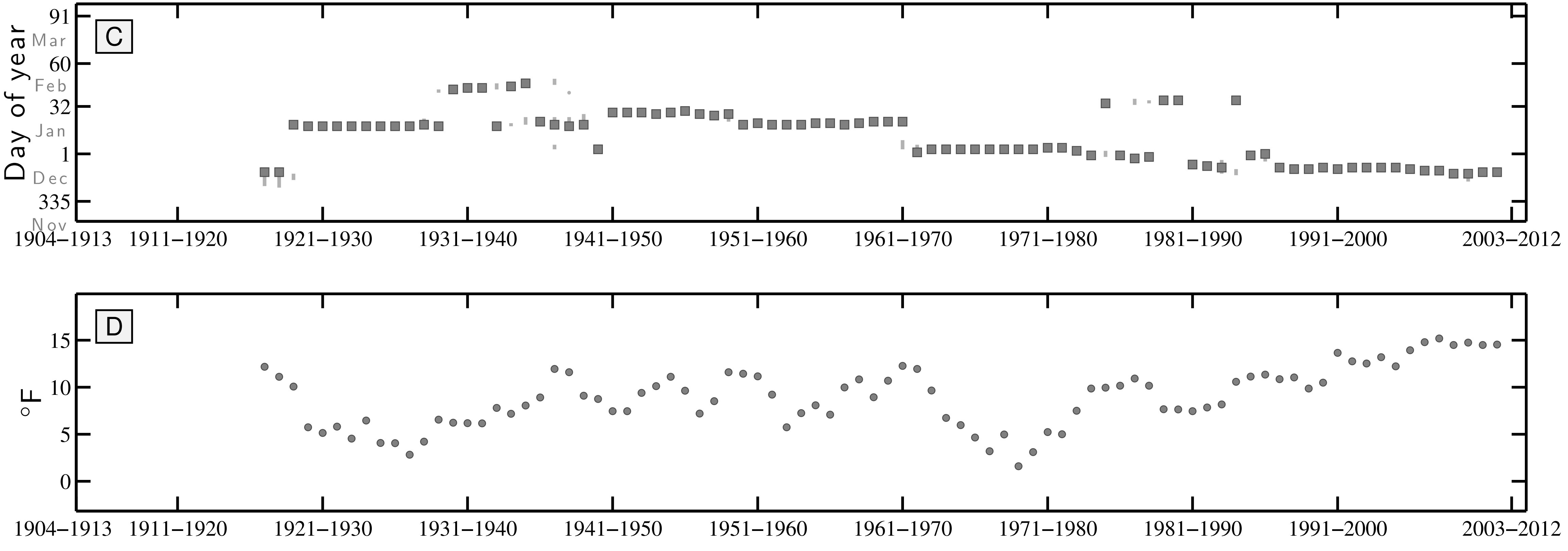

10 year Summer Teletherm dynamics for FLATWILLOW 4 ENE, MT:

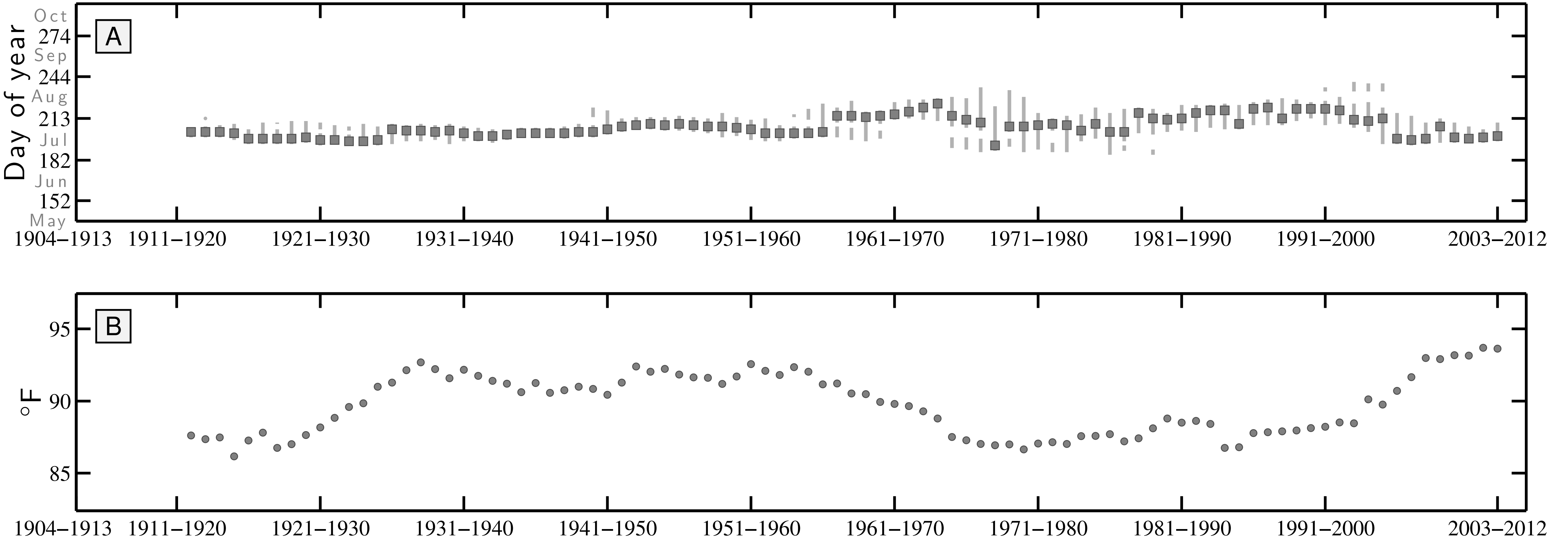

10 year Winter Teletherm dynamics for FLATWILLOW 4 ENE, MT:

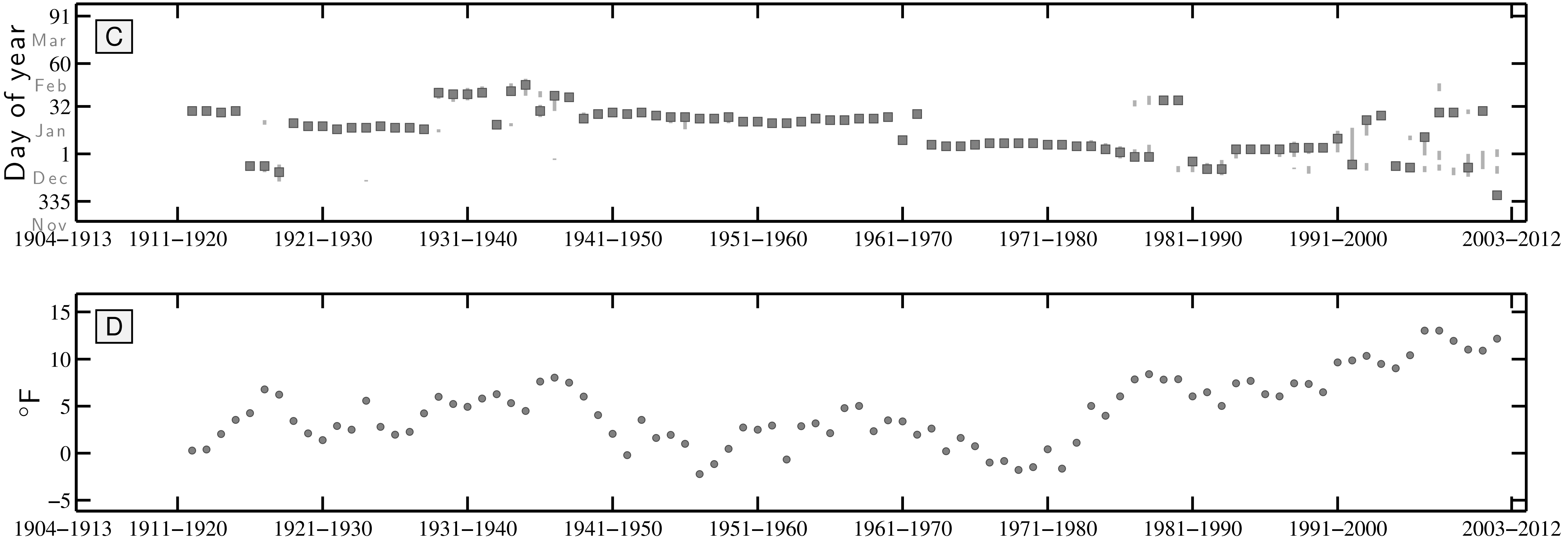

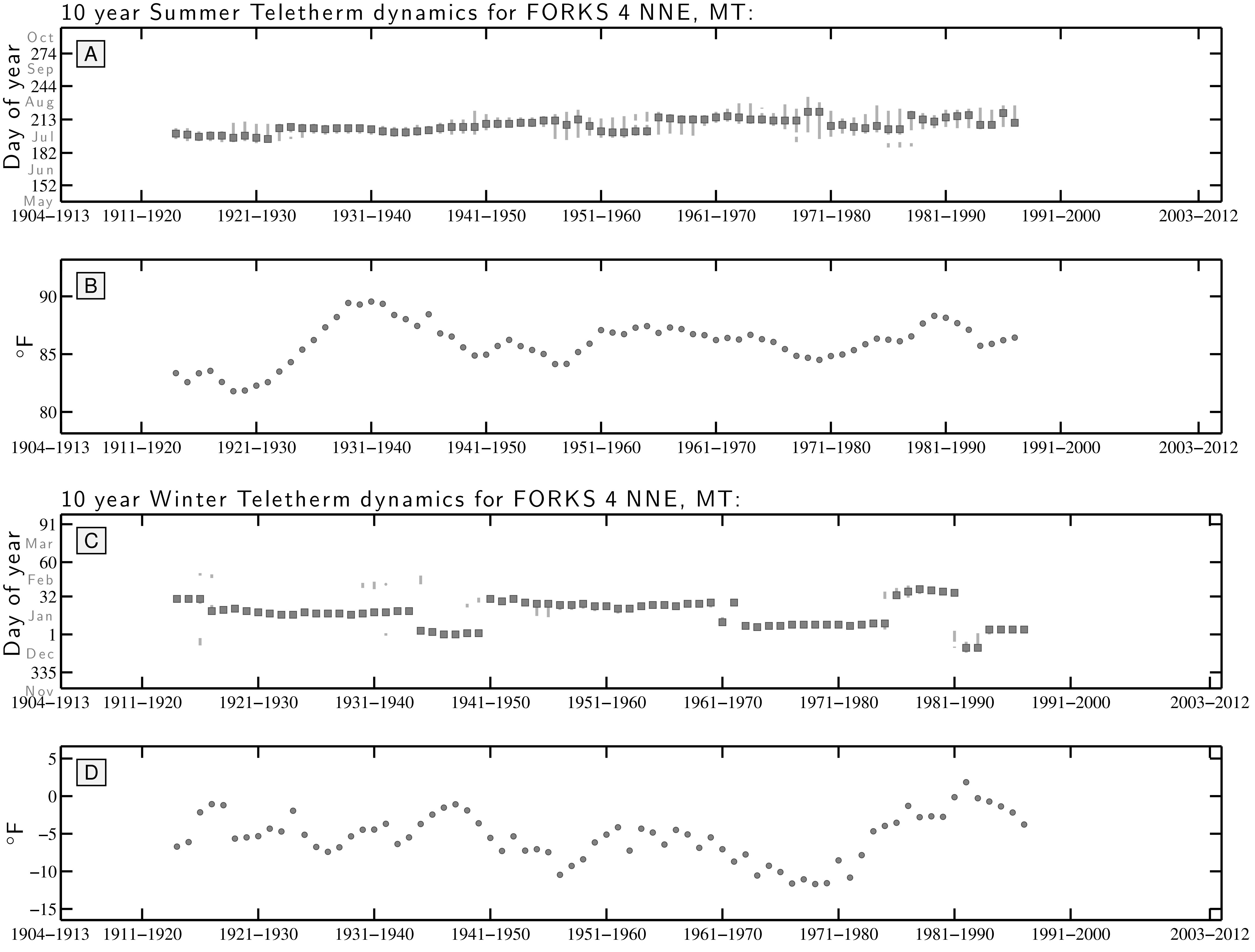

10 year Summer Teletherm dynamics for FORKS 4 NNE, MT:
A
B
Day of year
°F
Oct
274
Sep
244
Aug
213
Jul
182
Jun
152
May
90
85
80
10 year Winter Teletherm dynamics for FORKS 4 NNE, MT:
C
D
91
Mar
60
Feb
32
Jan
1
Dec
335
Nov
5
0
−5
−10
−15
1904–1913
1911–1920
1921–1930
1931–1940
1941–1950
1951–1960
1961–1970
1971–1980
1981–1990
1991–2000
2003–2012

10 year Summer Teletherm dynamics for FT ASSINNIBOINE, MT:

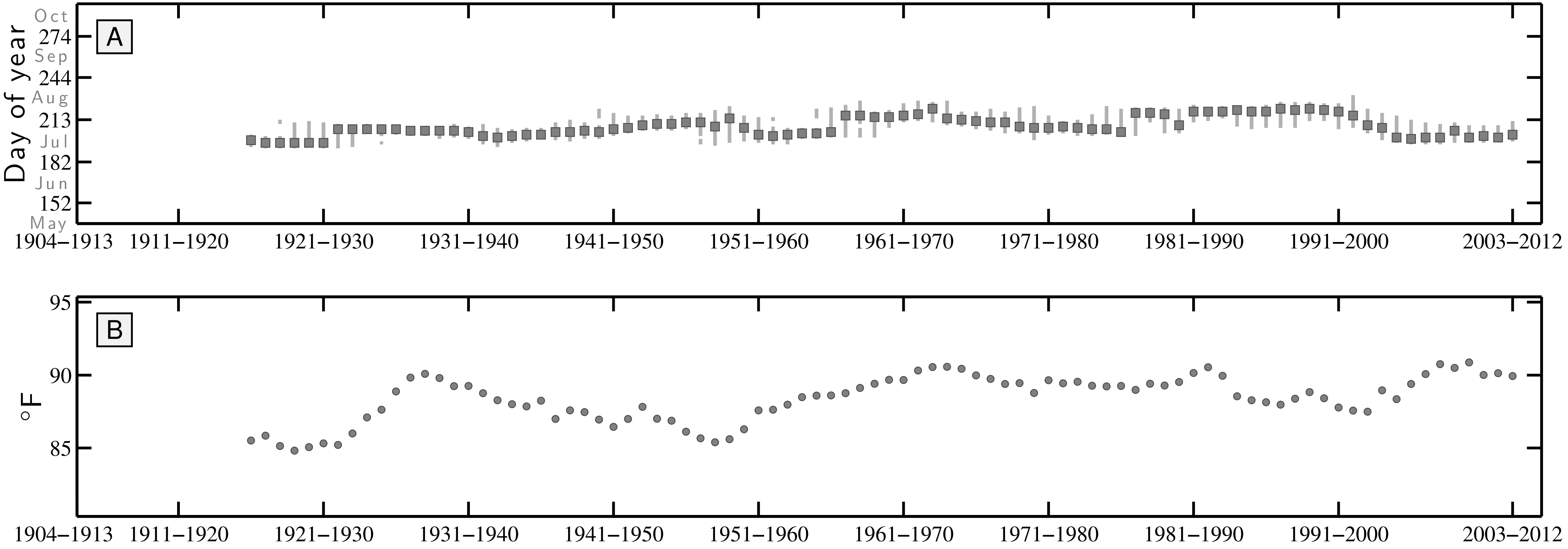

10 year Winter Teletherm dynamics for FT ASSINNIBOINE, MT:

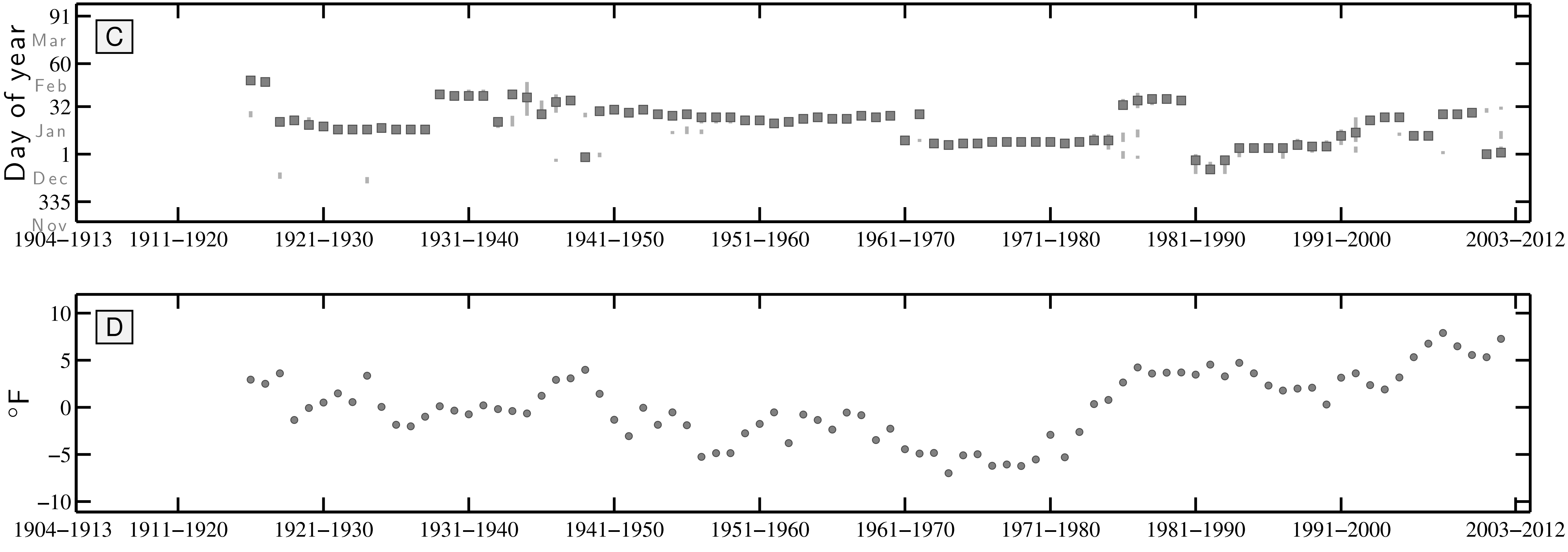

10 year Summer Teletherm dynamics for FORTINE 1 N, MT:

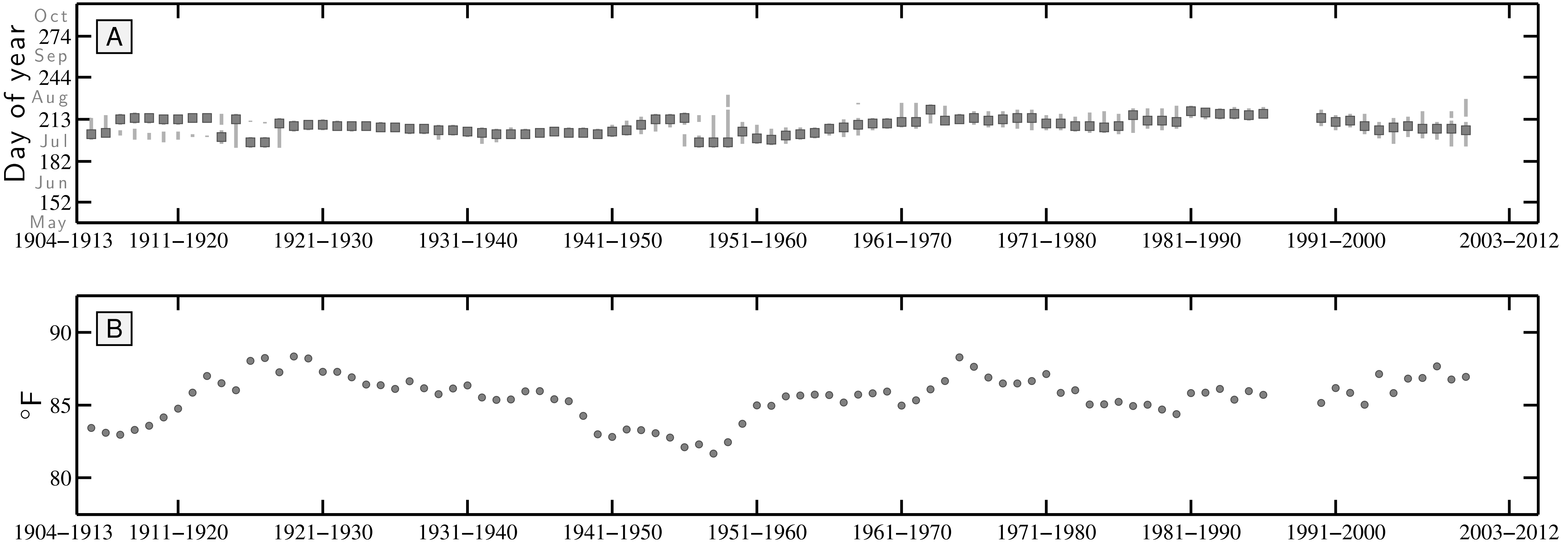

10 year Winter Teletherm dynamics for FORTINE 1 N, MT:

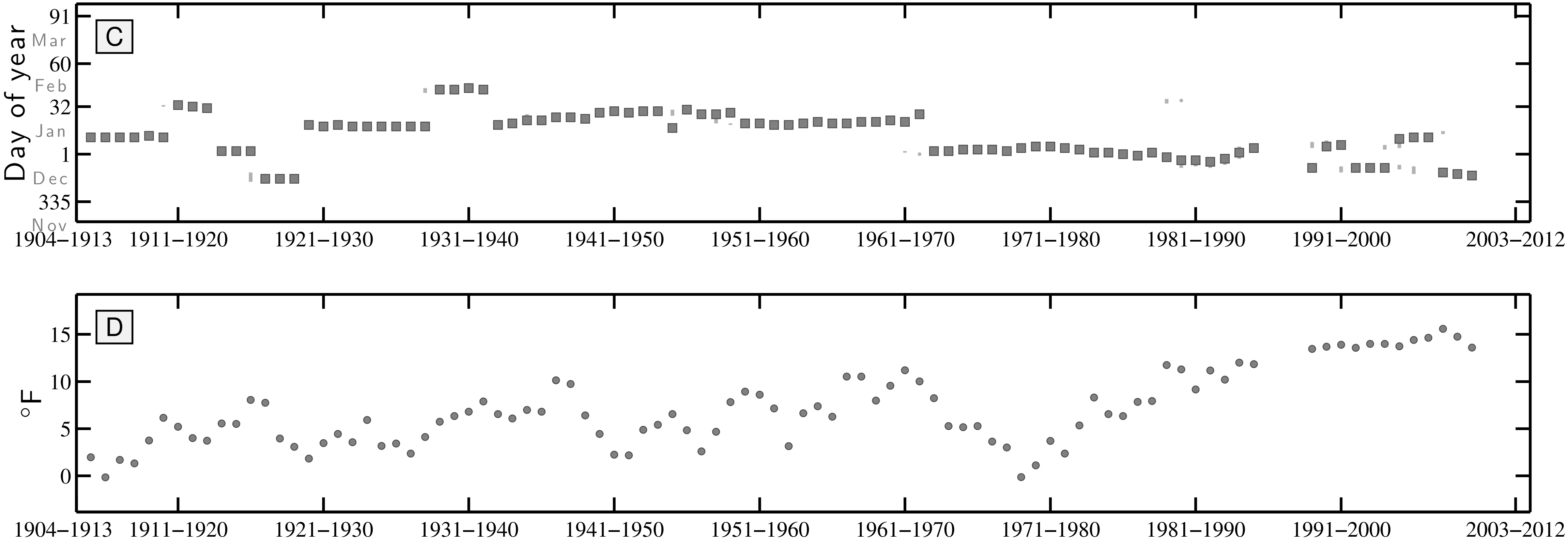

10 year Summer Teletherm dynamics for GLASGOW INTL AP, MT:

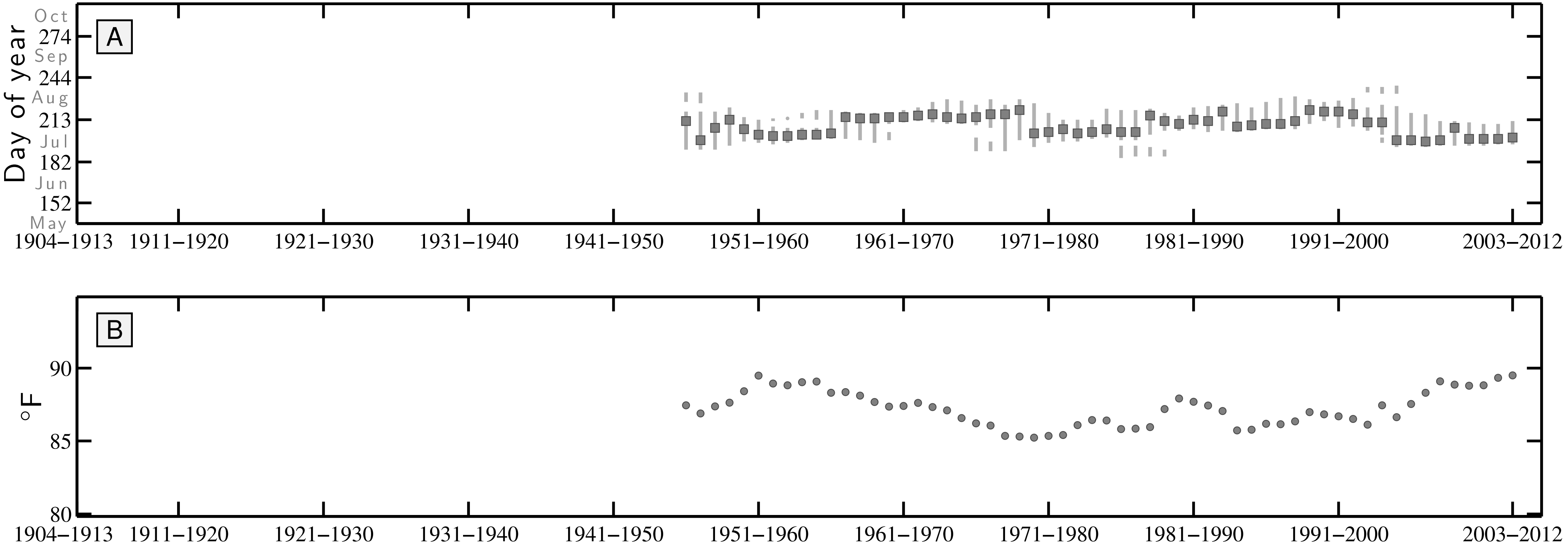

10 year Winter Teletherm dynamics for GLASGOW INTL AP, MT:

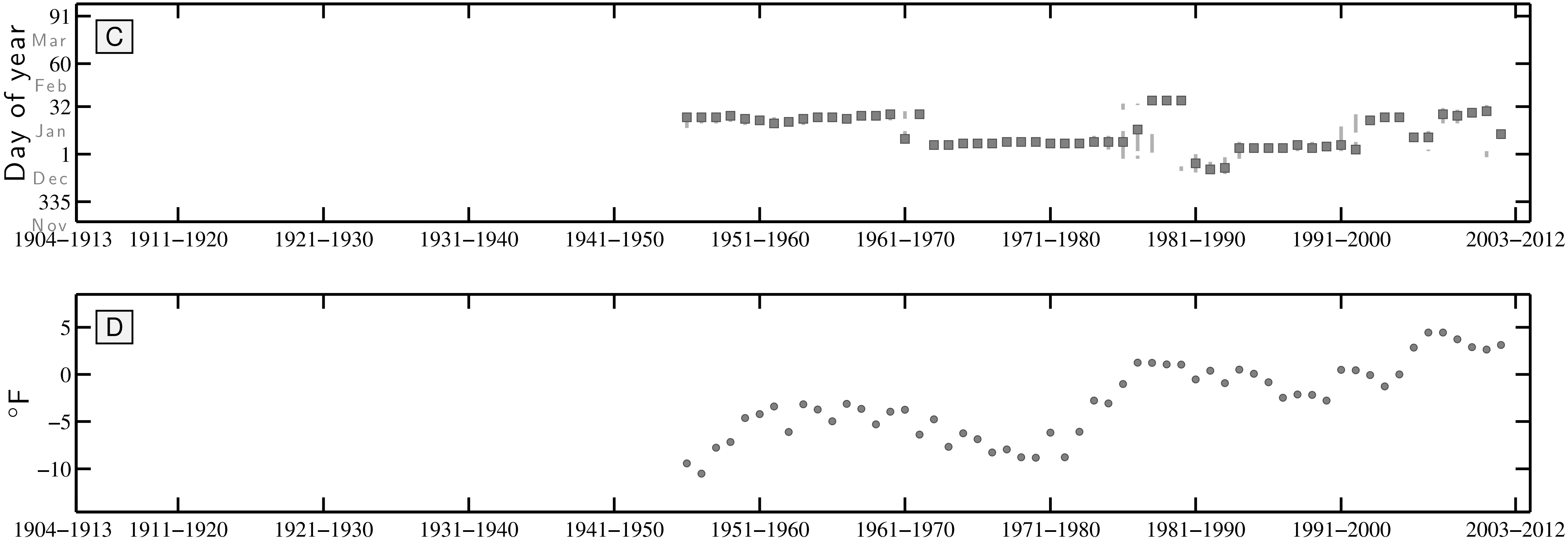

10 year Summer Teletherm dynamics for GLENDIVE, MT:

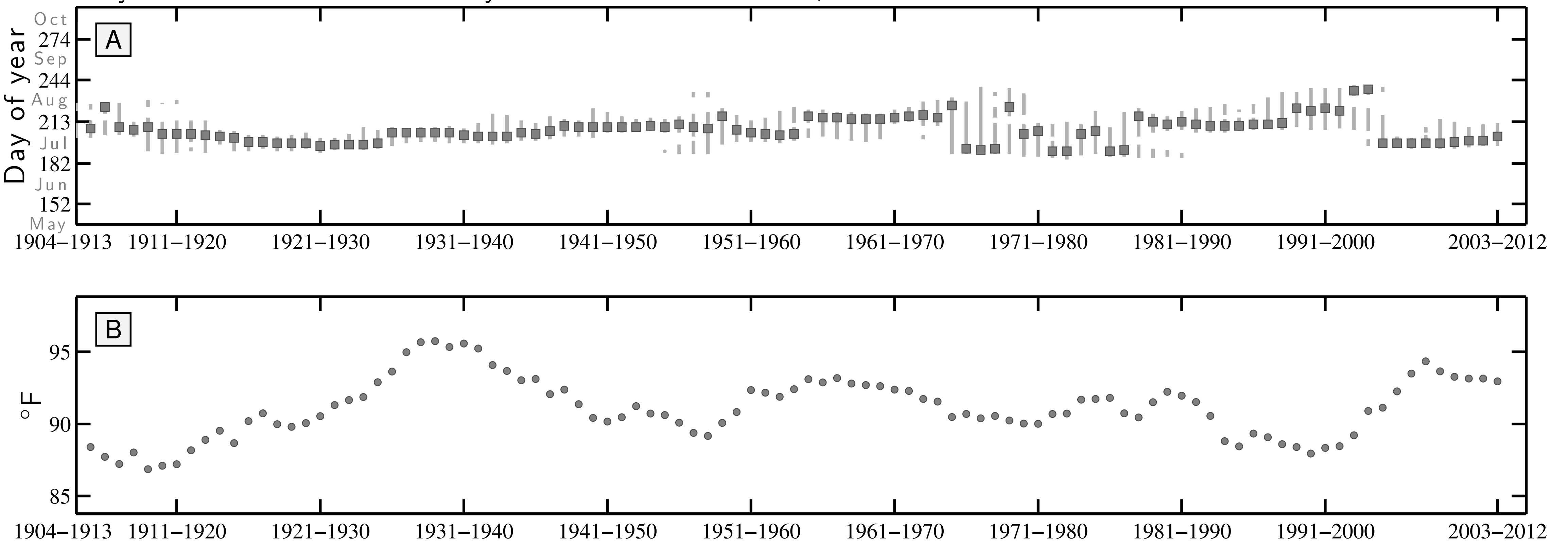

10 year Winter Teletherm dynamics for GLENDIVE, MT:

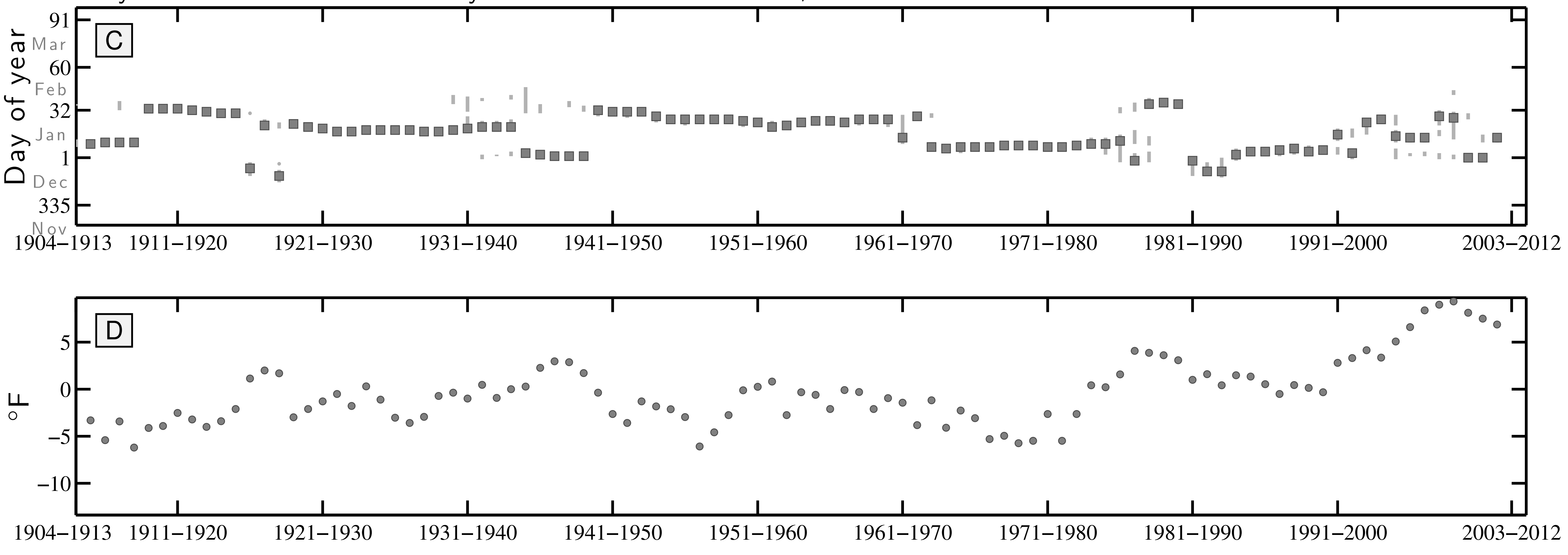

10 year Summer Teletherm dynamics for GREAT FALLS AP, MT:

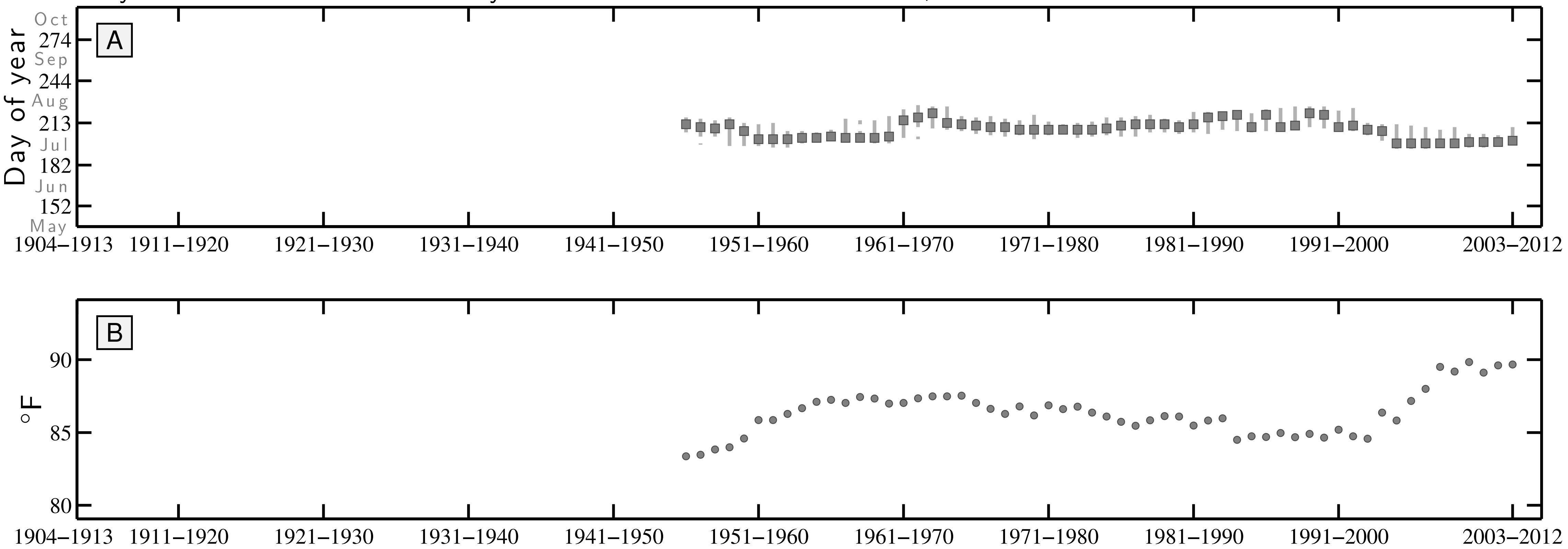

10 year Winter Teletherm dynamics for GREAT FALLS AP, MT:

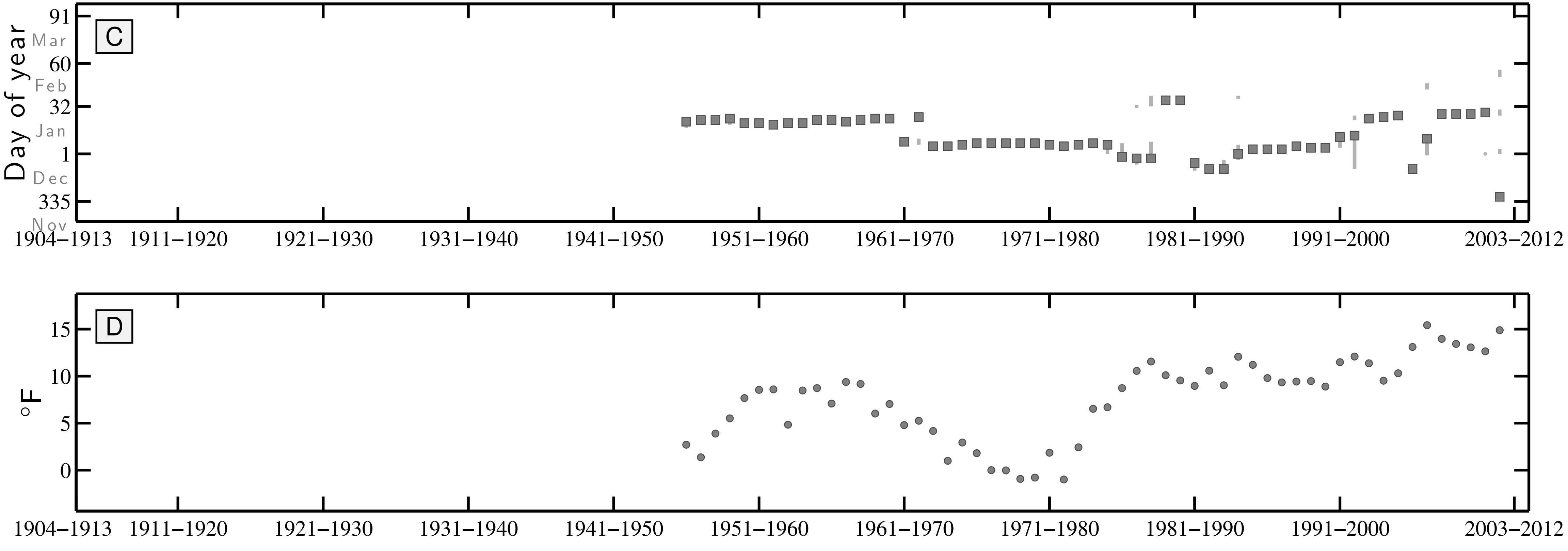

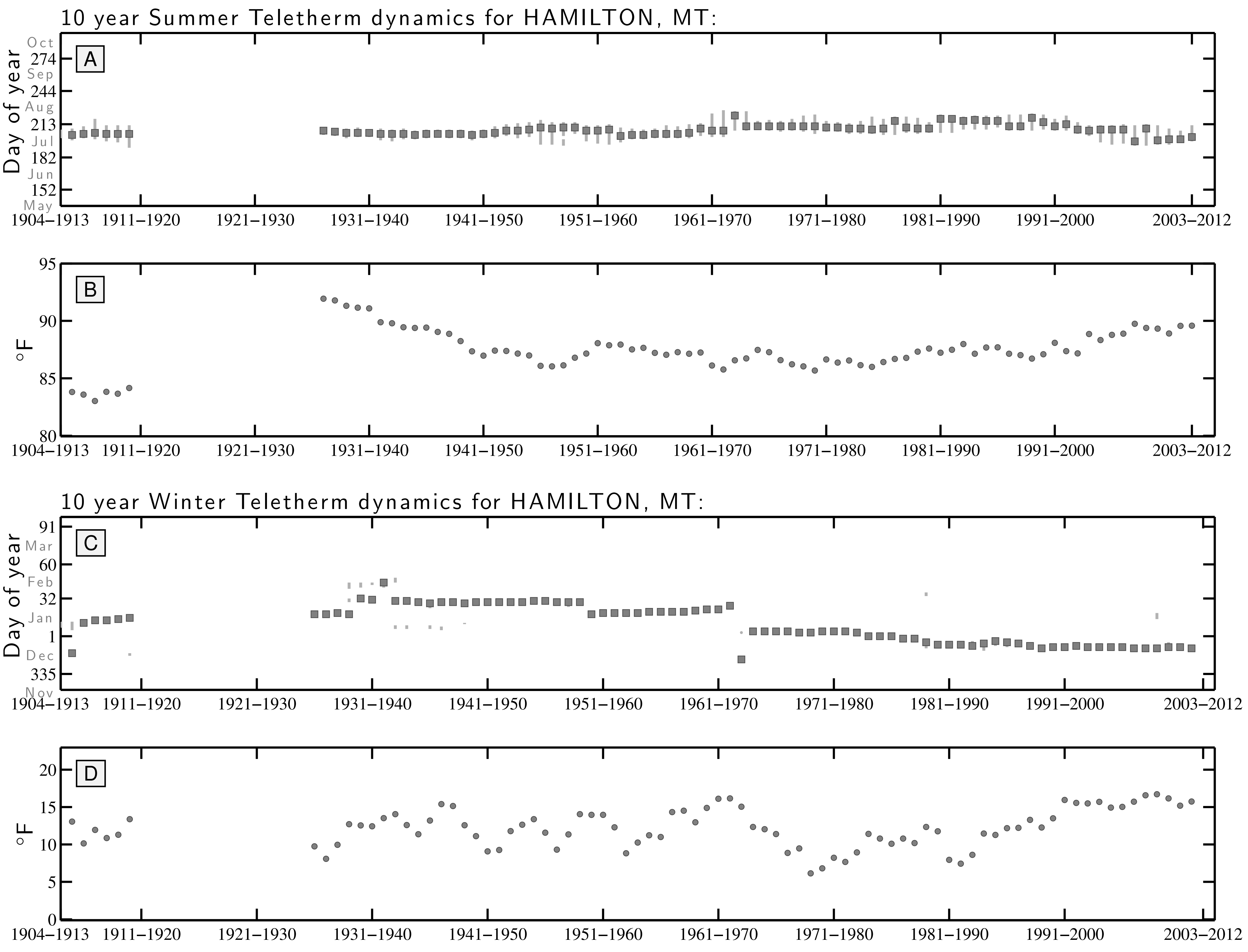
10 year Summer Teletherm dynamics for HAMILTON, MT:
A
Day of year
Oct
274
Sep
244
Aug
213
Jul
182
Jun
152
May
B
°F
95
90
85
80
10 year Winter Teletherm dynamics for HAMILTON, MT:
C
Day of year
91
Mar
60
Feb
32
Jan
1
Dec
335
Nov
D
°F
20
15
10
5
0
1904–1913
1911–1920
1921–1930
1931–1940
1941–1950
1951–1960
1961–1970
1971–1980
1981–1990
1991–2000
2003–2012

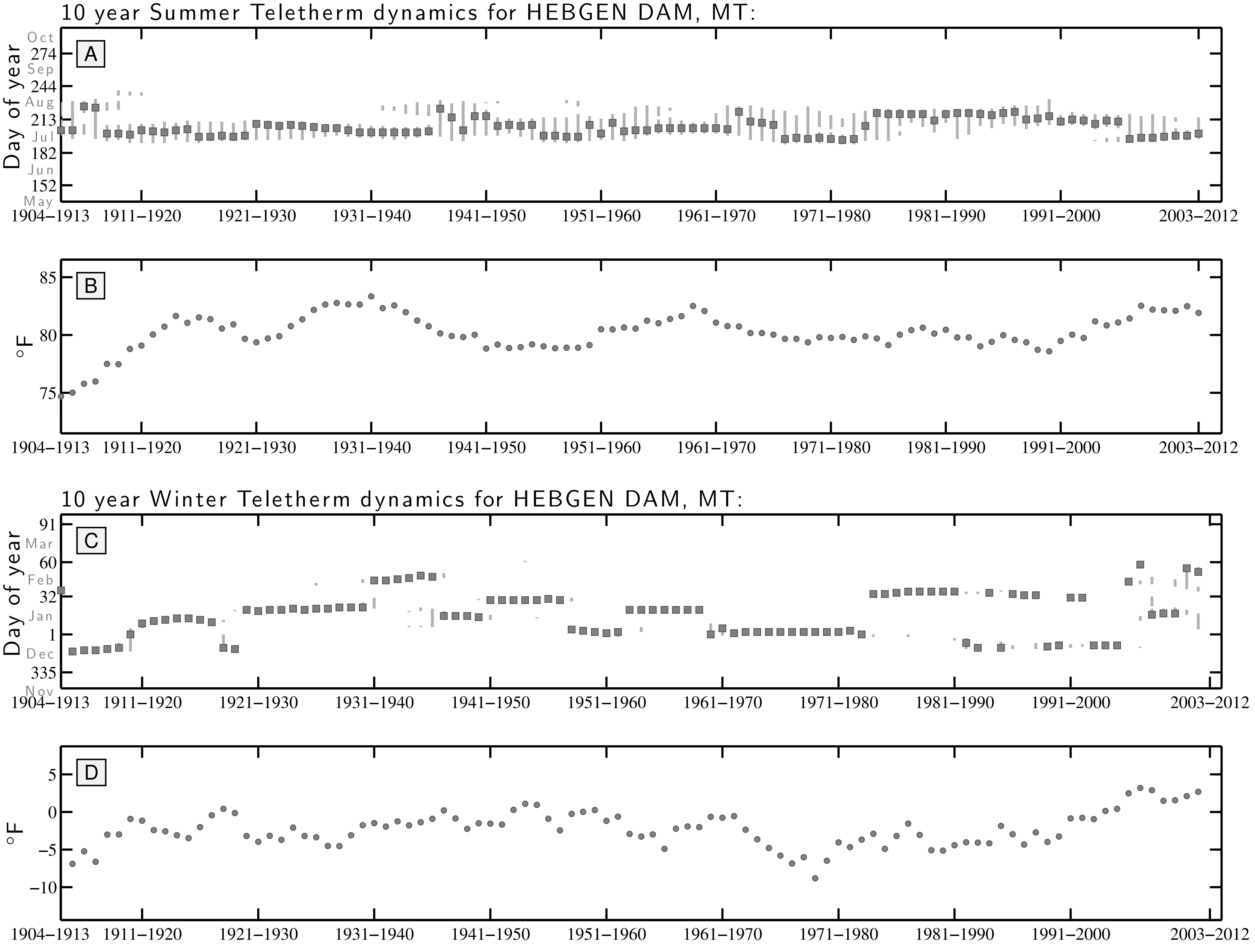

10 year Summer Teletherm dynamics for HEBGEN DAM, MT:
A
Day of year
Oct
274
Sep
244
Aug
213
Jul
182
Jun
152
May
1904–1913
1911–1920
1921–1930
1931–1940
1941–1950
1951–1960
1961–1970
1971–1980
1981–1990
1991–2000
2003–2012
B
°F
85
80
75
10 year Winter Teletherm dynamics for HEBGEN DAM, MT:
C
91
Mar
60
Feb
32
Jan
1
Dec
335
Nov
D
5
0
−5
−10

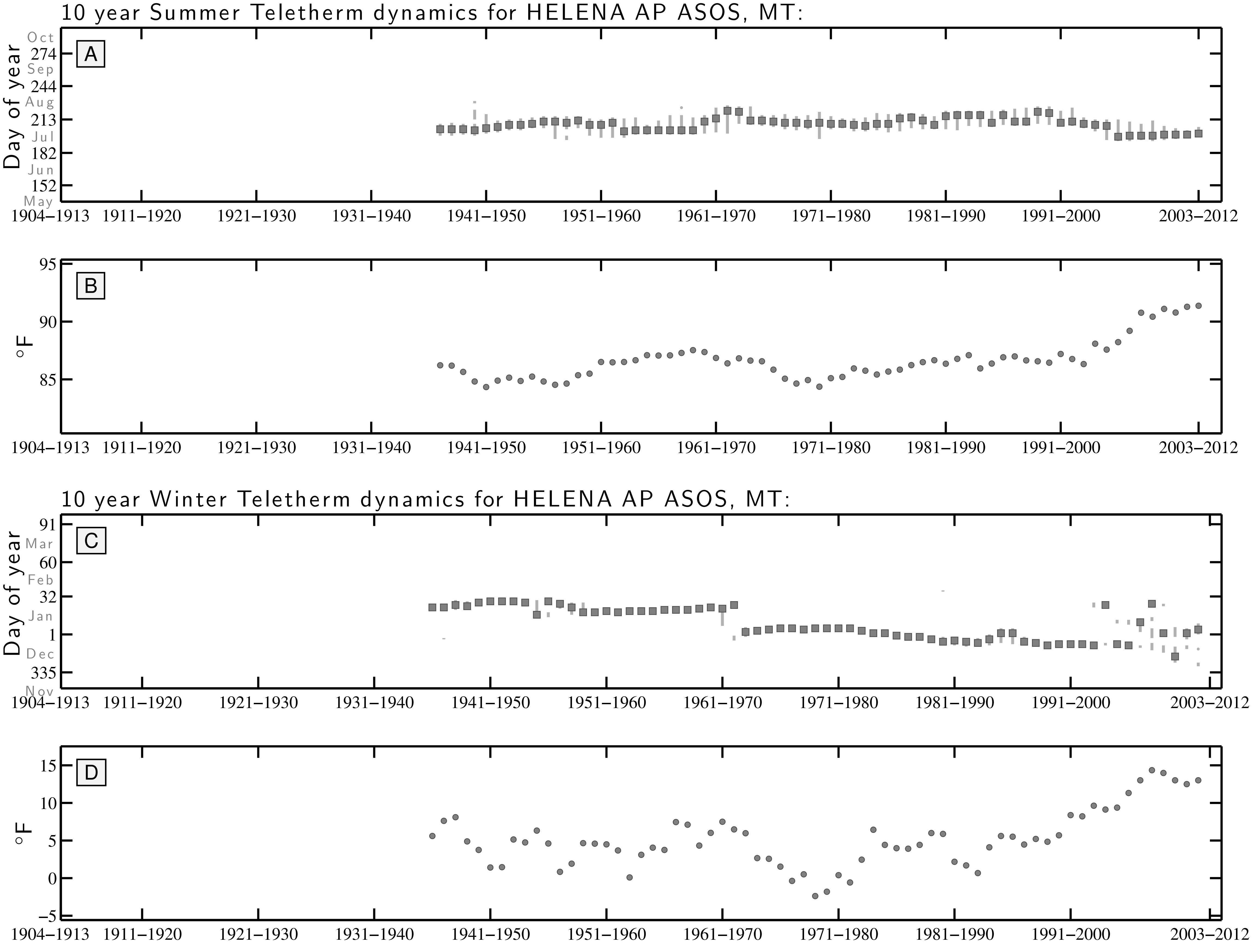
10 year Summer Teletherm dynamics for HELENA AP ASOS, MT:
A
B
Day of year
°F
10 year Winter Teletherm dynamics for HELENA AP ASOS, MT:
C
D
1904–1913
1911–1920
1921–1930
1931–1940
1941–1950
1951–1960
1961–1970
1971–1980
1981–1990
1991–2000
2003–2012

10 year Summer Teletherm dynamics for HUNTLEY EXP STN, MT:

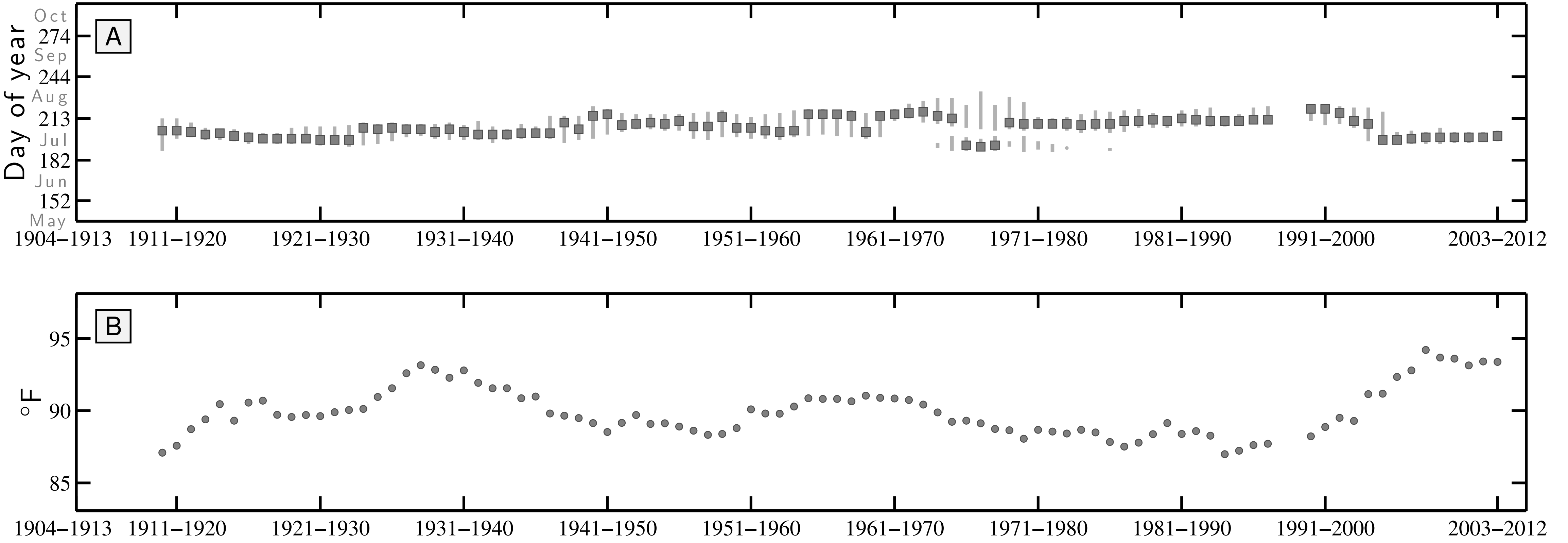

10 year Winter Teletherm dynamics for HUNTLEY EXP STN, MT:

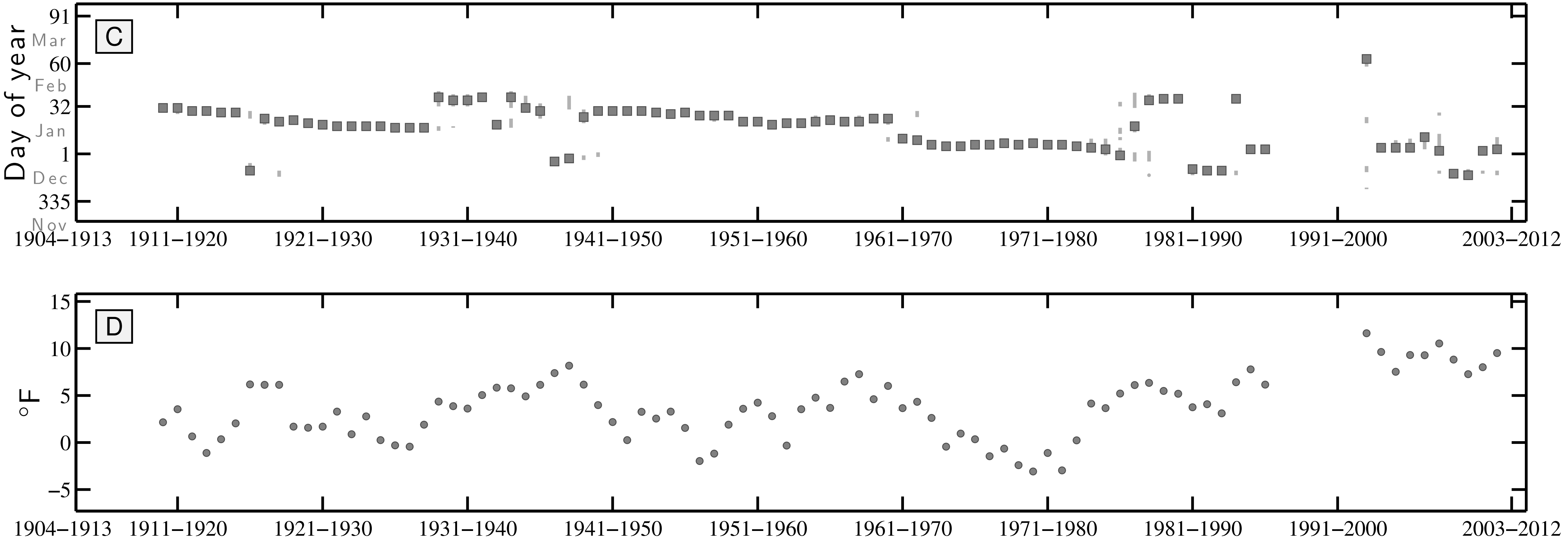

10 year Summer Teletherm dynamics for HYSHAM 25 SSE, MT:

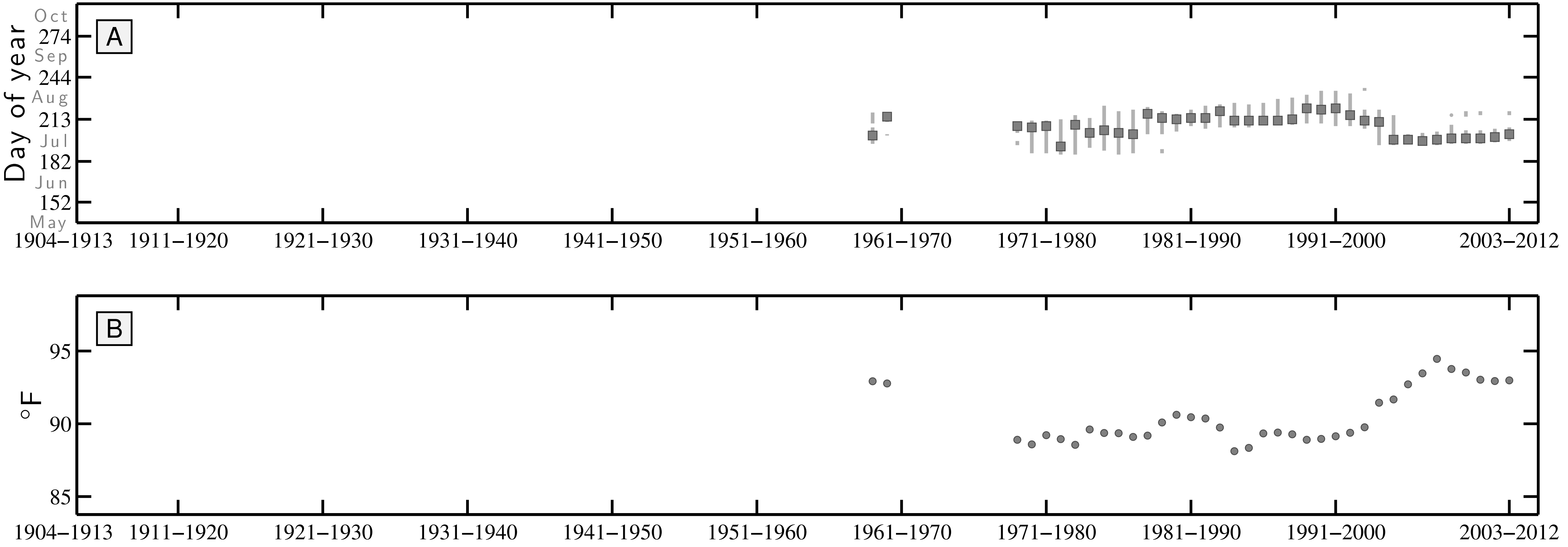

10 year Winter Teletherm dynamics for HYSHAM 25 SSE, MT:

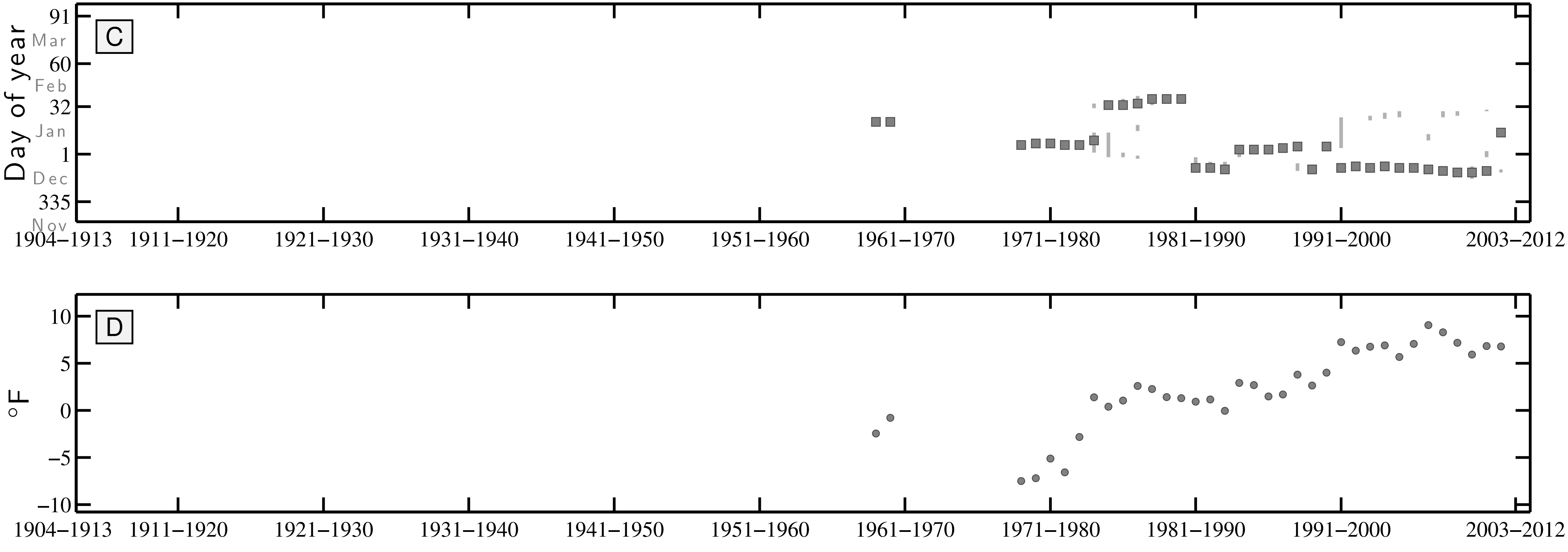

10 year Summer Teletherm dynamics for JORDAN, MT:

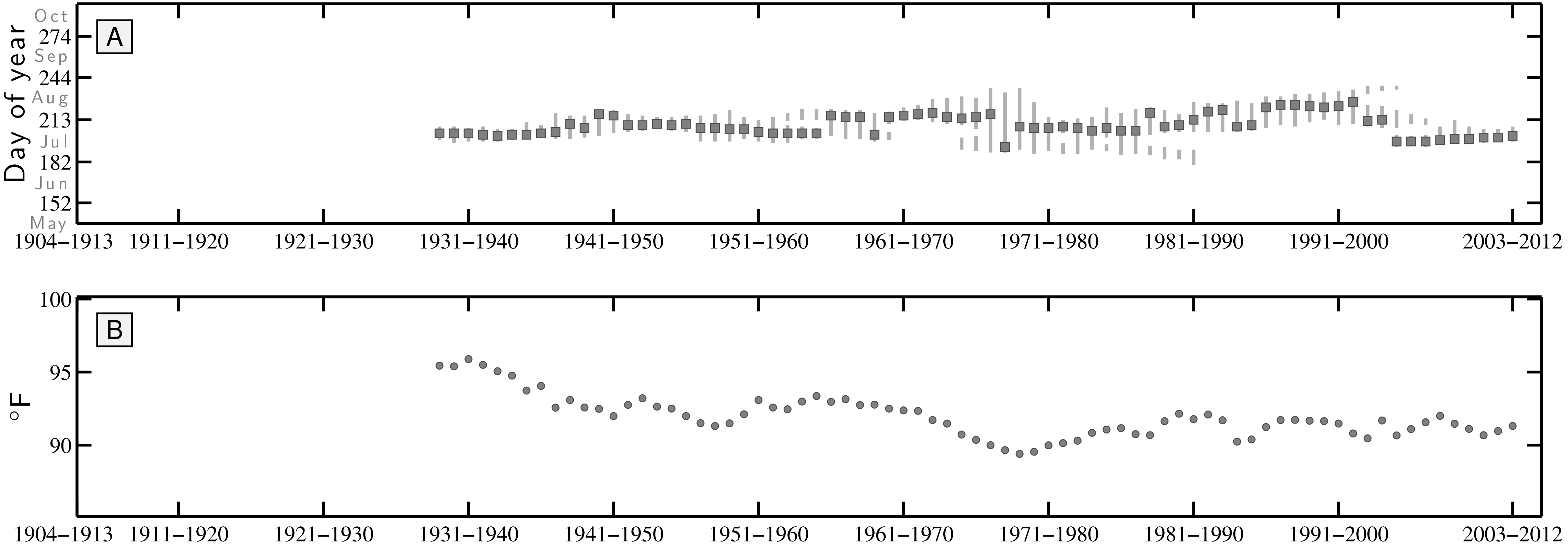

10 year Winter Teletherm dynamics for JORDAN, MT:

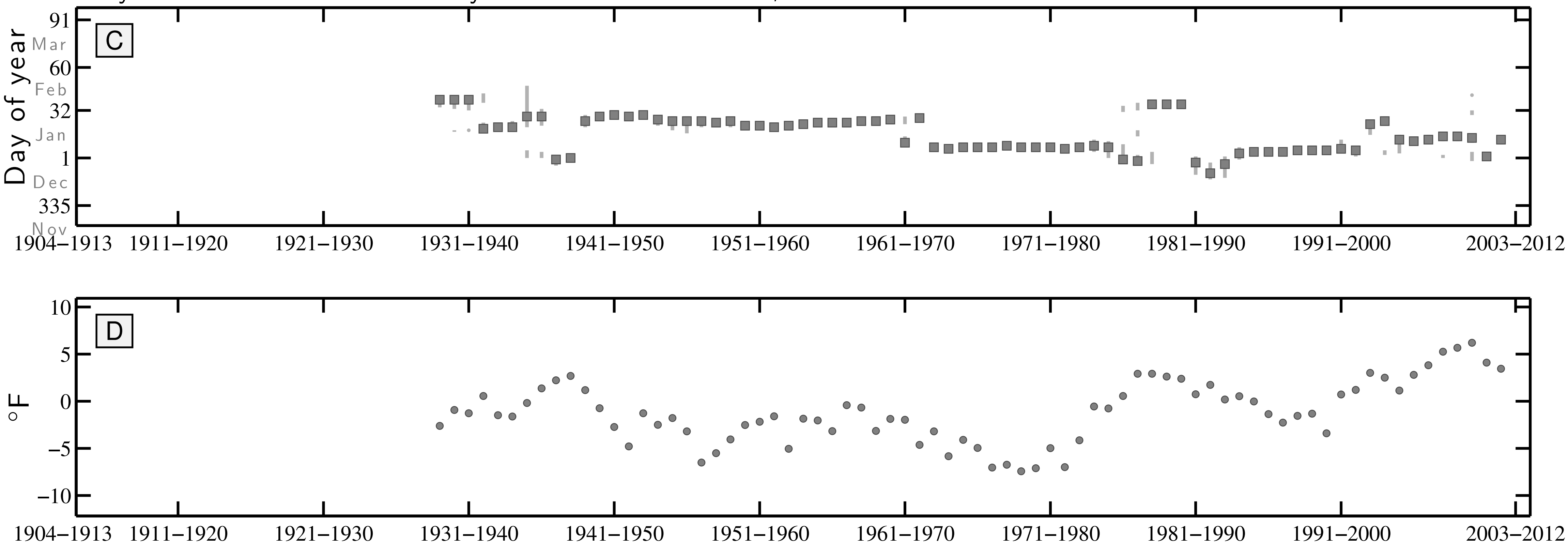

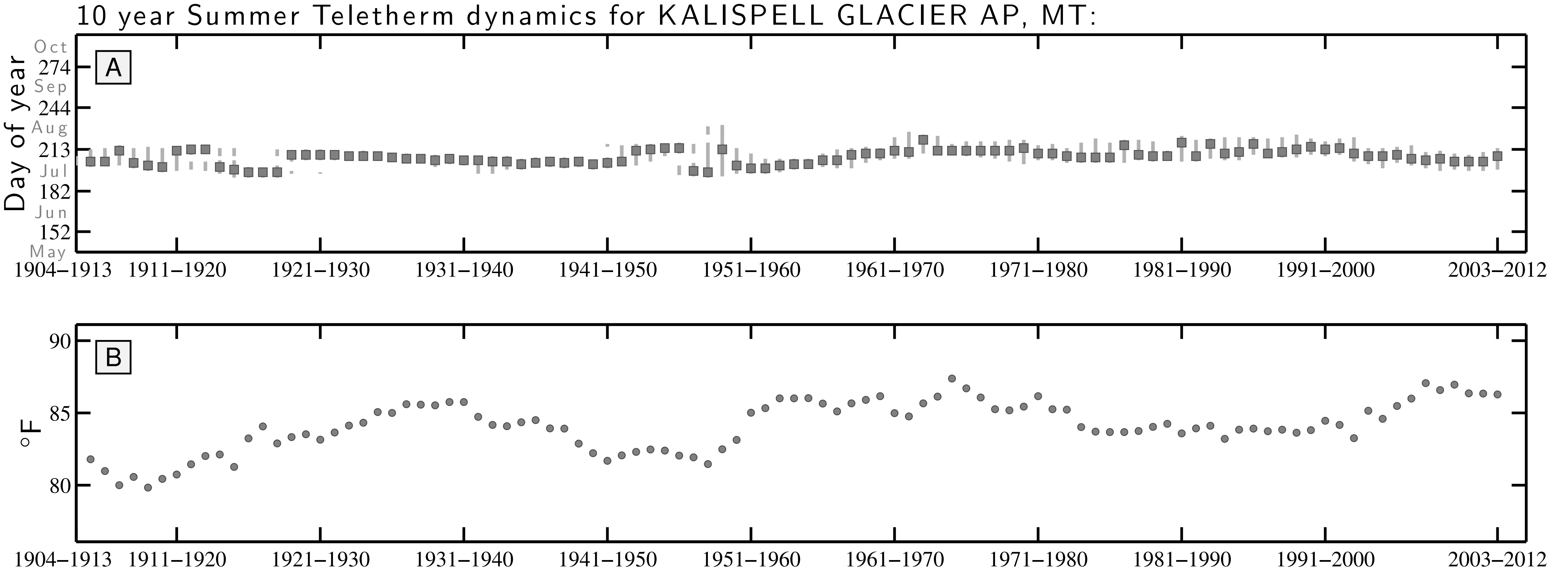
10 year Summer Teletherm dynamics for KALISPELL GLACIER AP, MT:
A
B
Day of year
°F
Oct
274
Sep
244
Aug
213
Jul
182
Jun
152
May
90
85
80
1904–1913
1911–1920
1921–1930
1931–1940
1941–1950
1951–1960
1961–1970
1971–1980
1981–1990
1991–2000
2003–2012

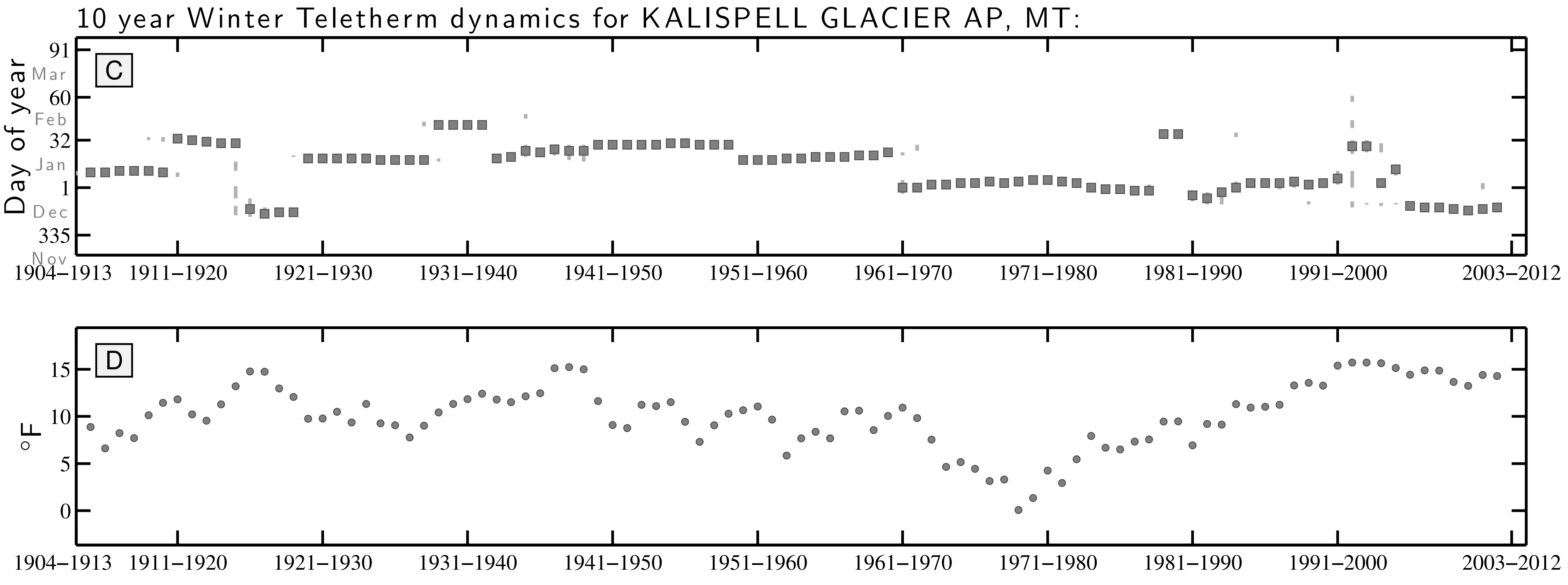
10 year Winter Teletherm dynamics for KALISPELL GLACIER AP, MT:
C
D
Day of year
°F
91
Mar
60
Feb
32
Jan
1
Dec
335
Nov
15
10
5
0
1904–1913
1911–1920
1921–1930
1931–1940
1941–1950
1951–1960
1961–1970
1971–1980
1981–1990
1991–2000
2003–2012

10 year Summer Teletherm dynamics for LIBBY 1 NE RS, MT:

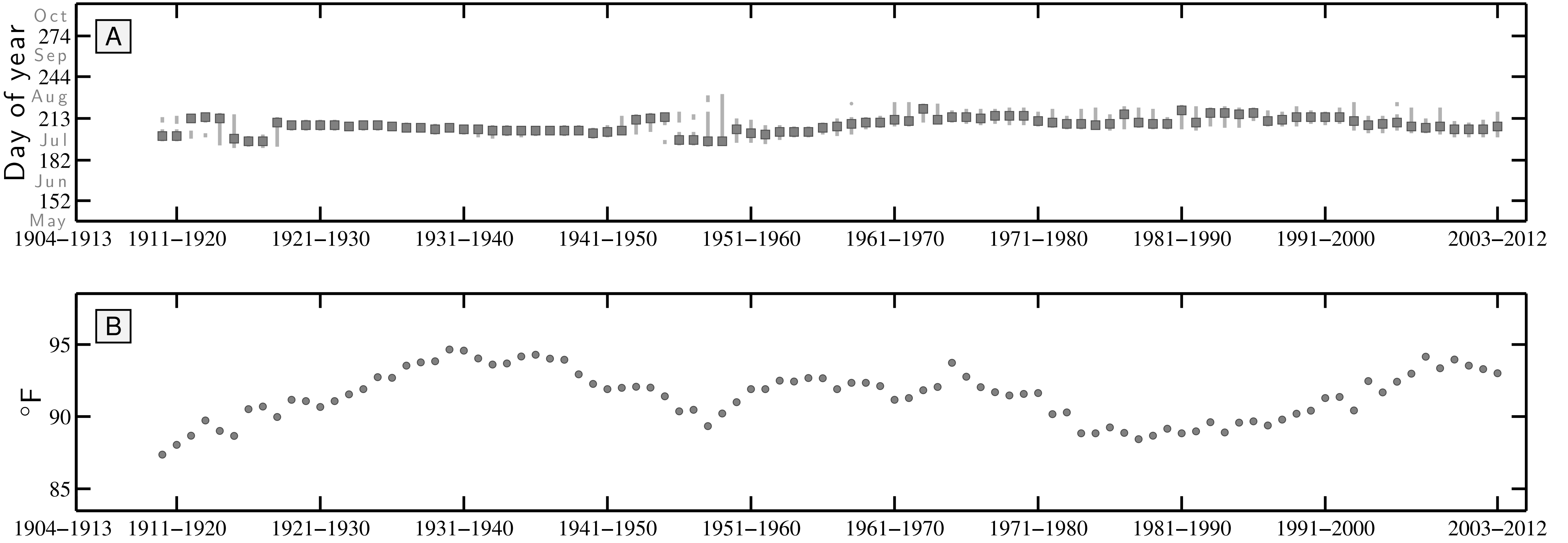

10 year Winter Teletherm dynamics for LIBBY 1 NE RS, MT:

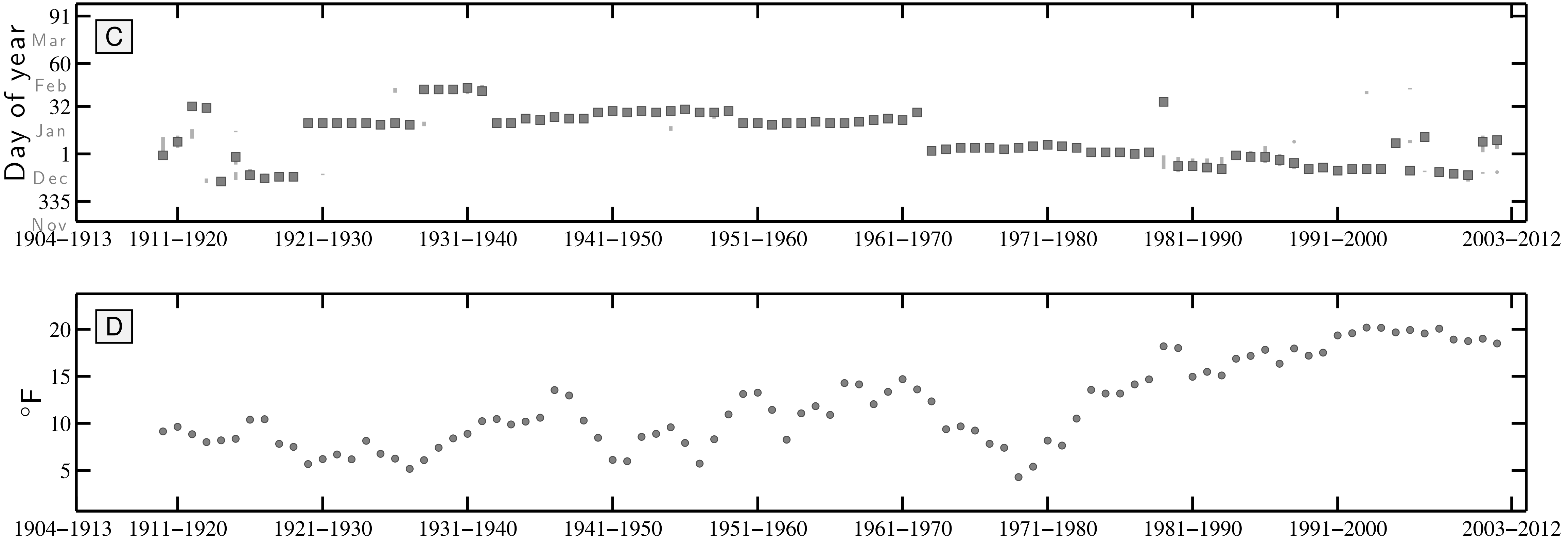

10 year Summer Teletherm dynamics for LIVINGSTON 12 S, MT:

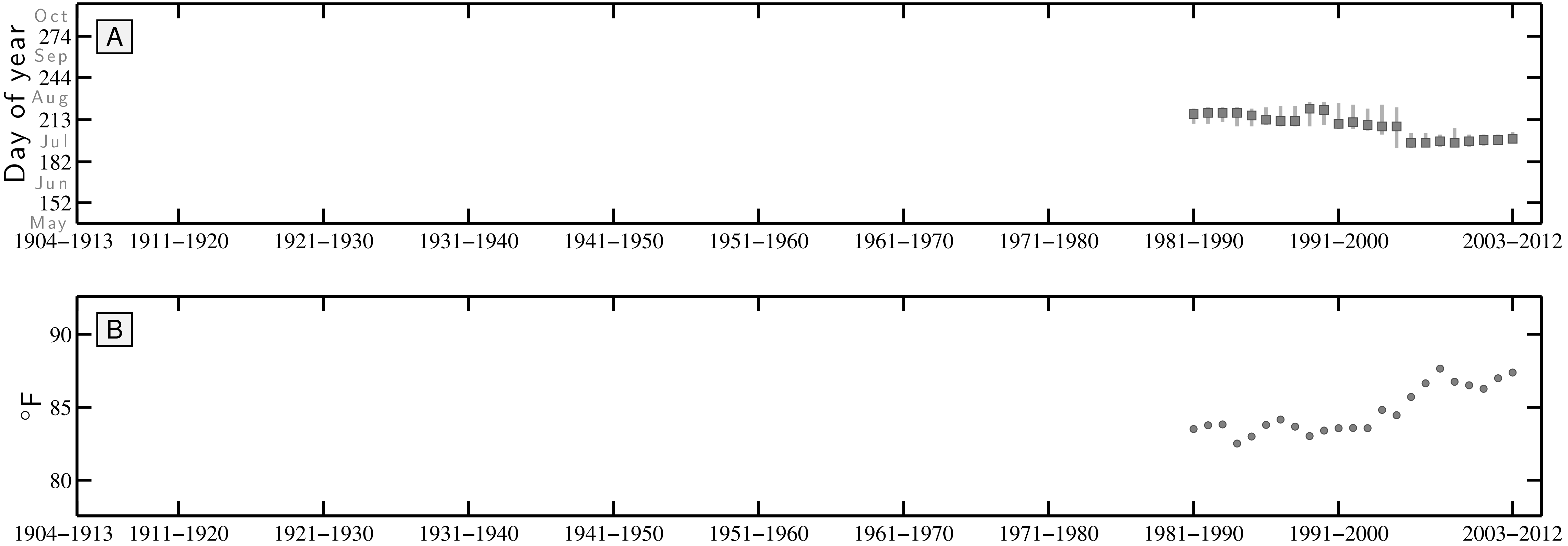

10 year Winter Teletherm dynamics for LIVINGSTON 12 S, MT:

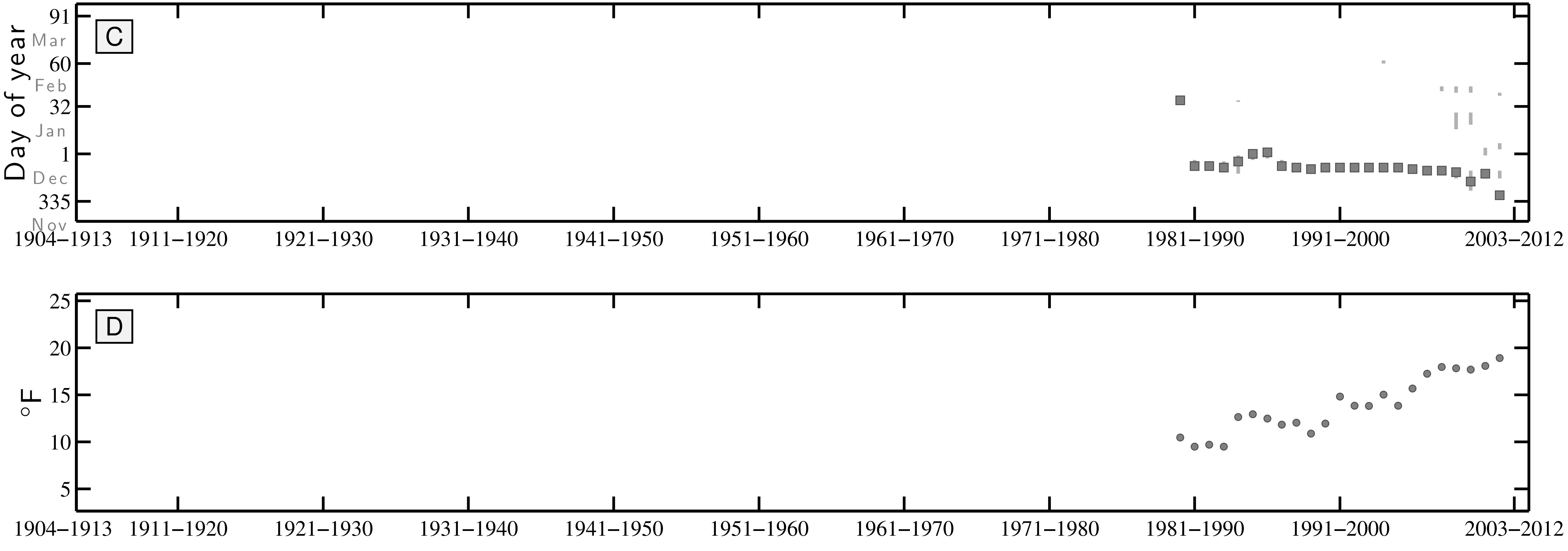

10 year Summer Teletherm dynamics for MALTA 7 E, MT:

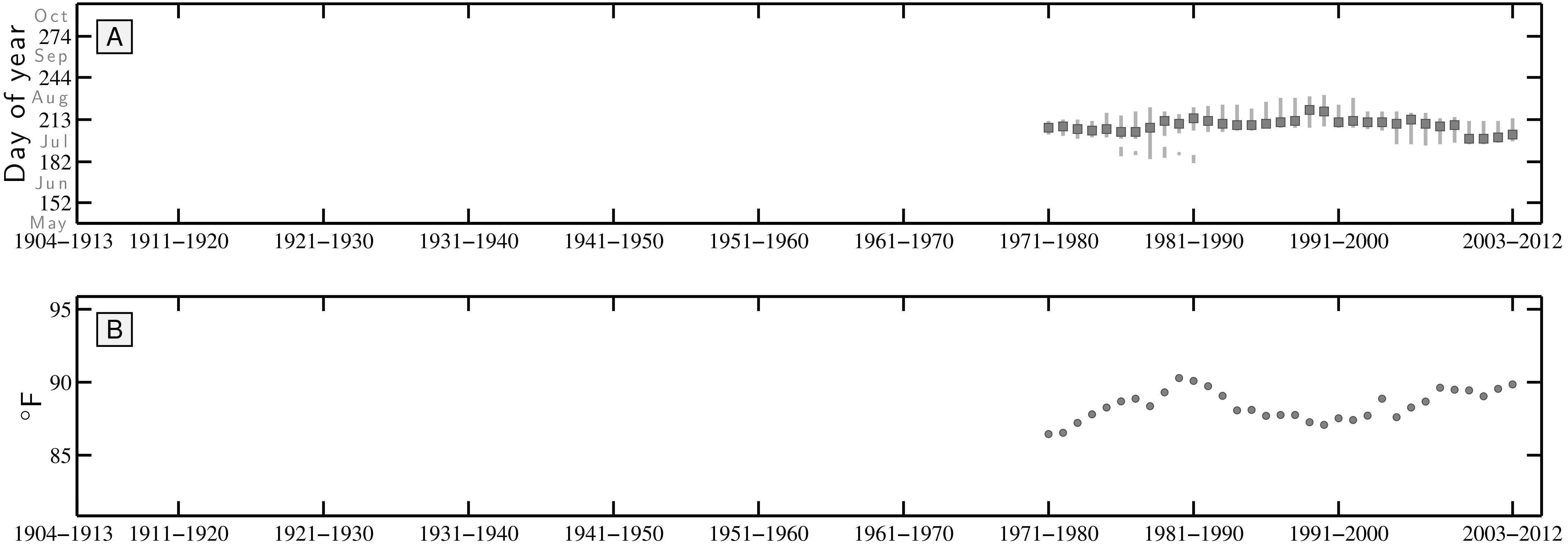

10 year Winter Teletherm dynamics for MALTA 7 E, MT:

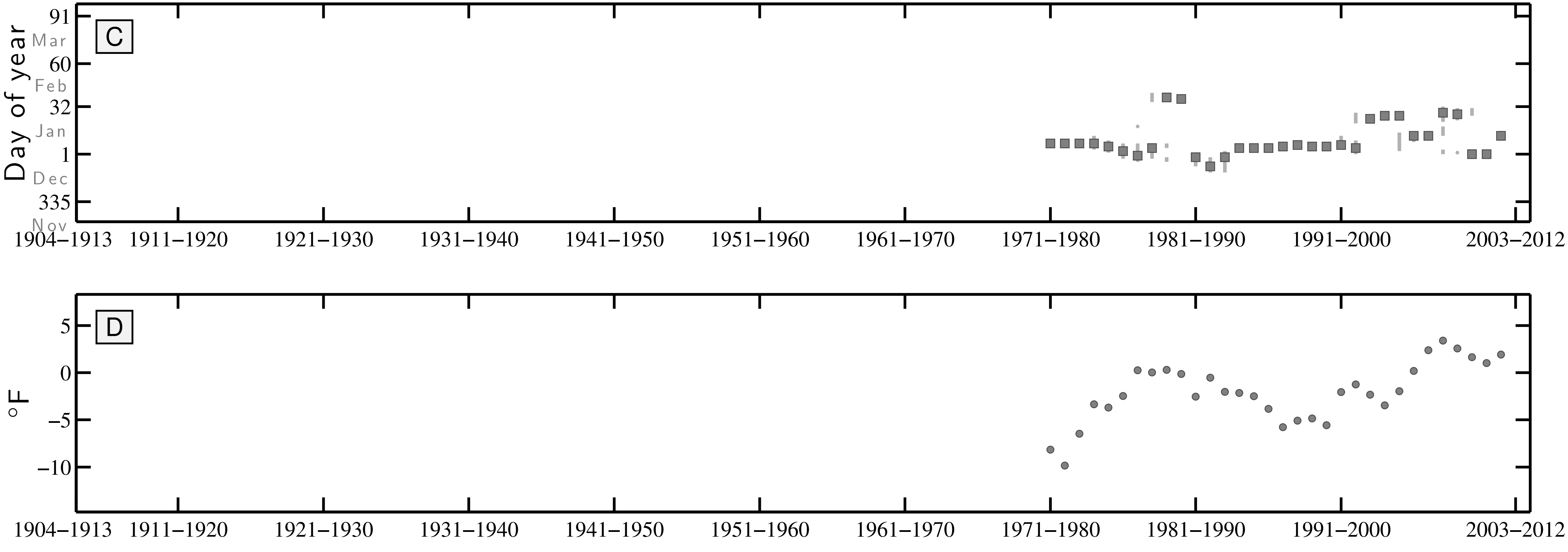

10 year Summer Teletherm dynamics for MEDICINE LAKE 3 SE, MT:

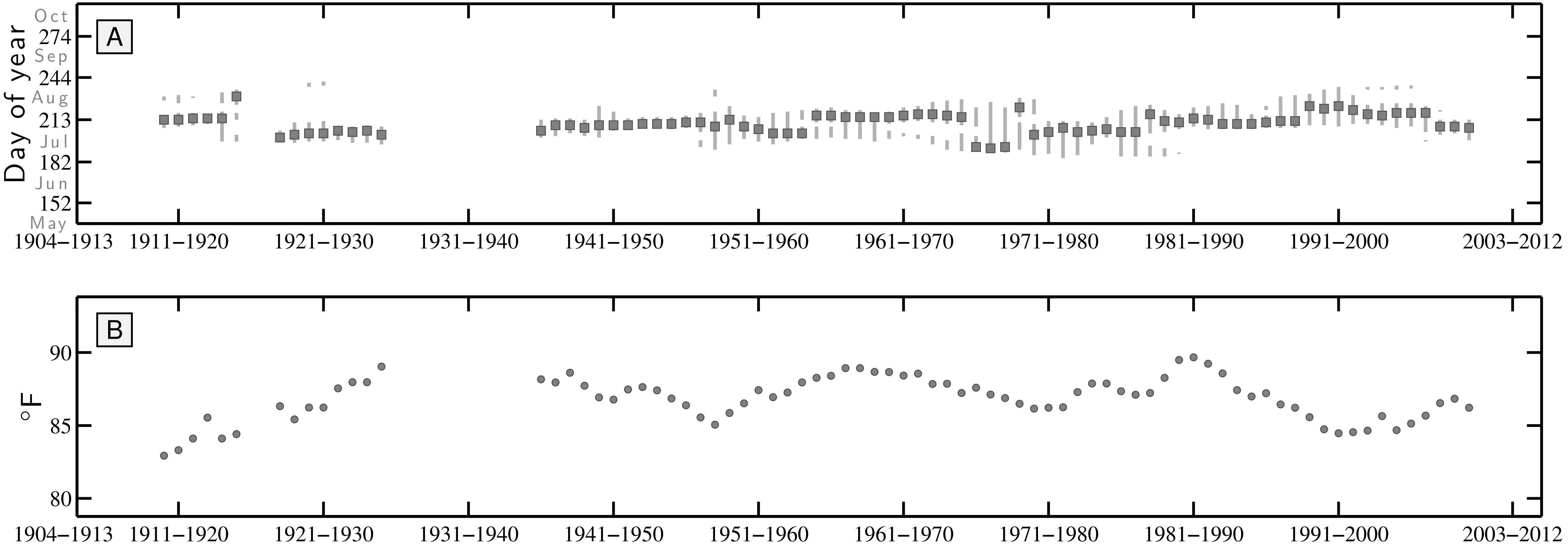

10 year Winter Teletherm dynamics for MEDICINE LAKE 3 SE, MT:

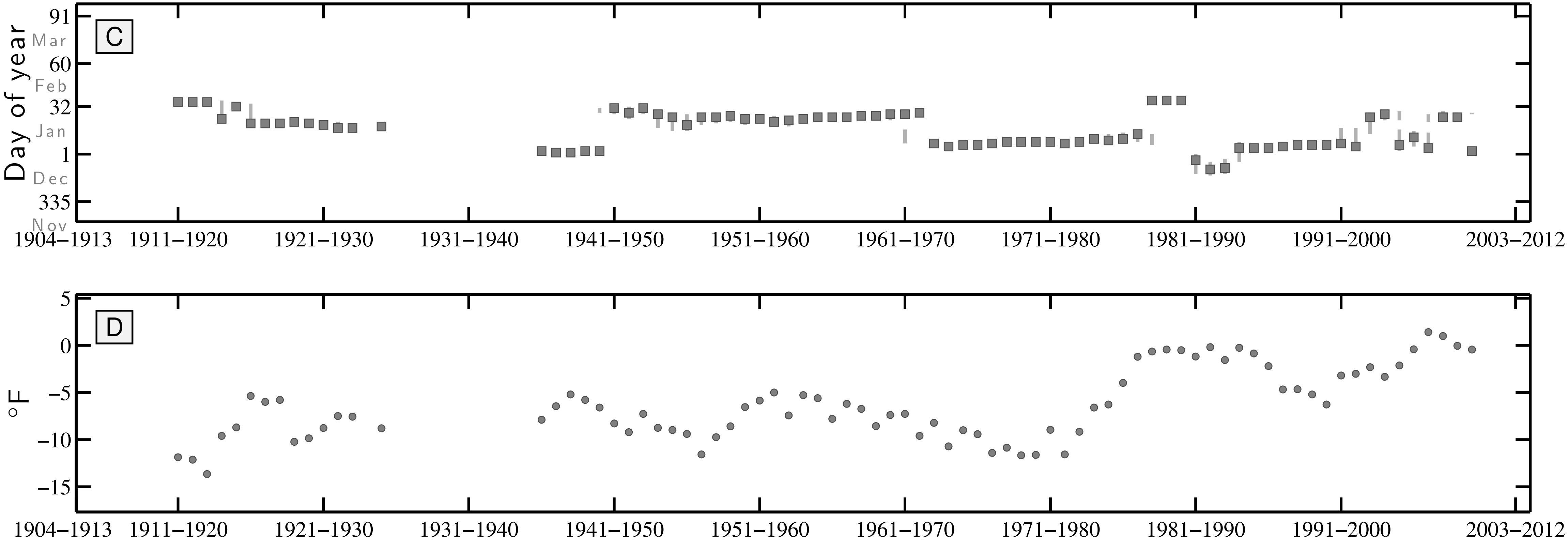

10 year Summer Teletherm dynamics for MILDRED 5 N, MT:

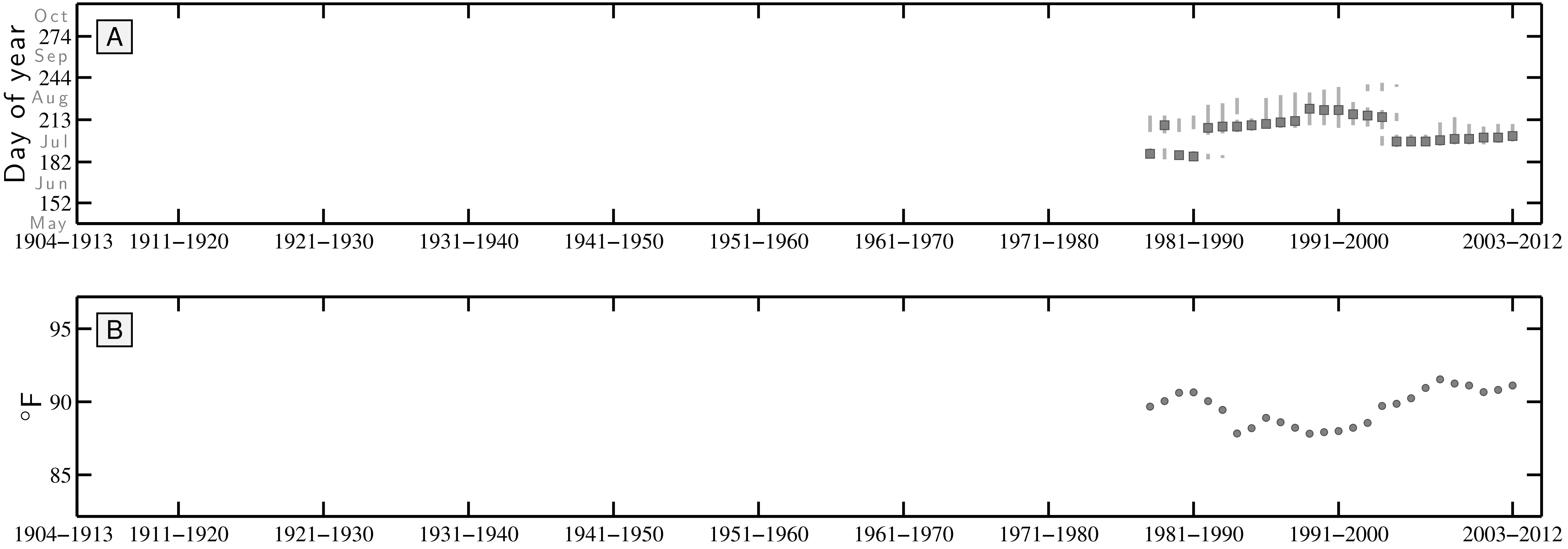

10 year Winter Teletherm dynamics for MILDRED 5 N, MT:

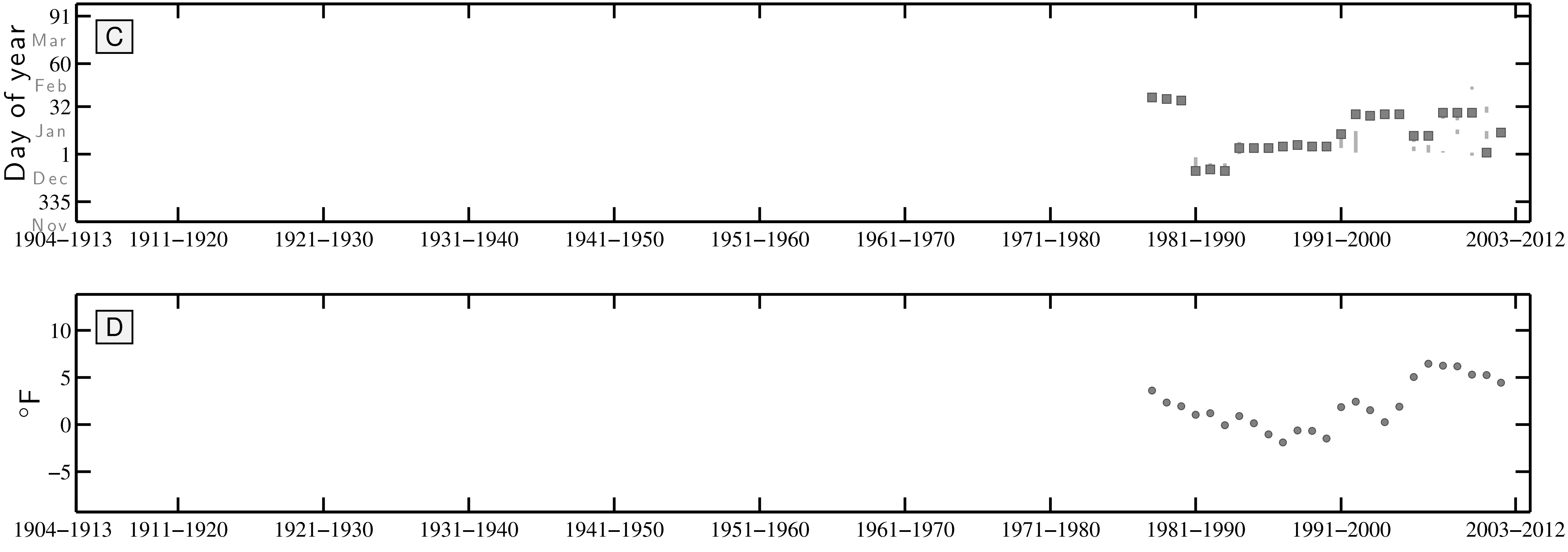

10 year Summer Teletherm dynamics for MILES CITY AP, MT:

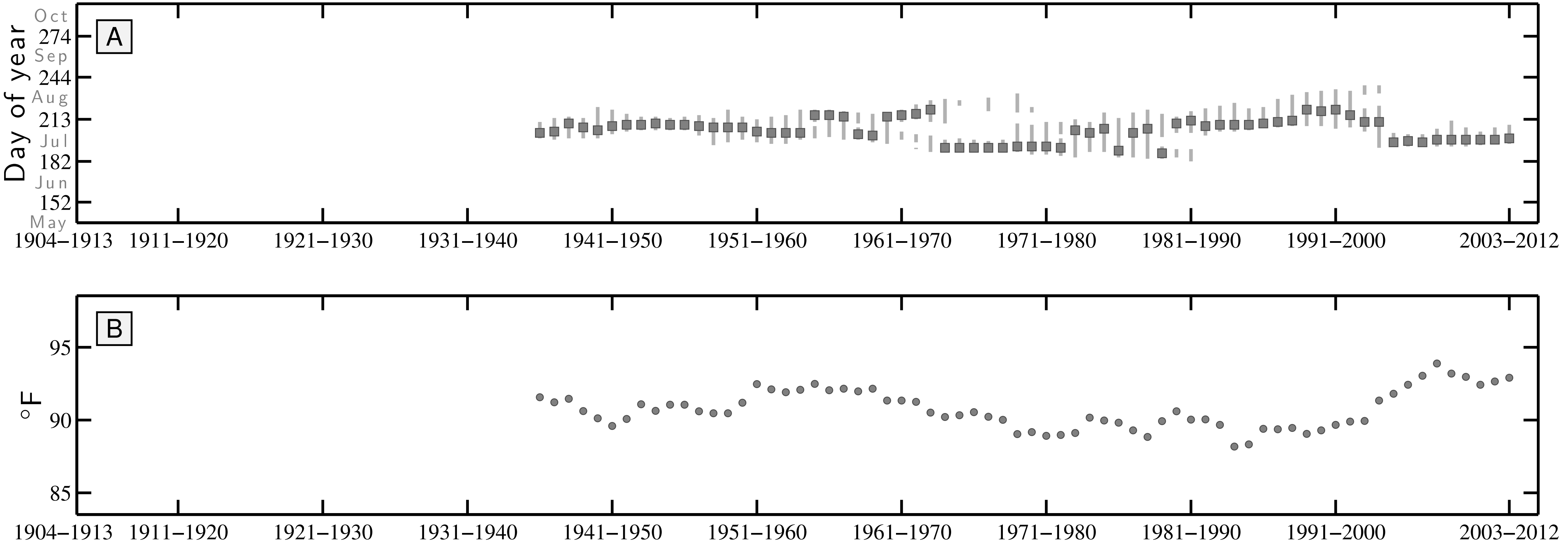

10 year Winter Teletherm dynamics for MILES CITY AP, MT:

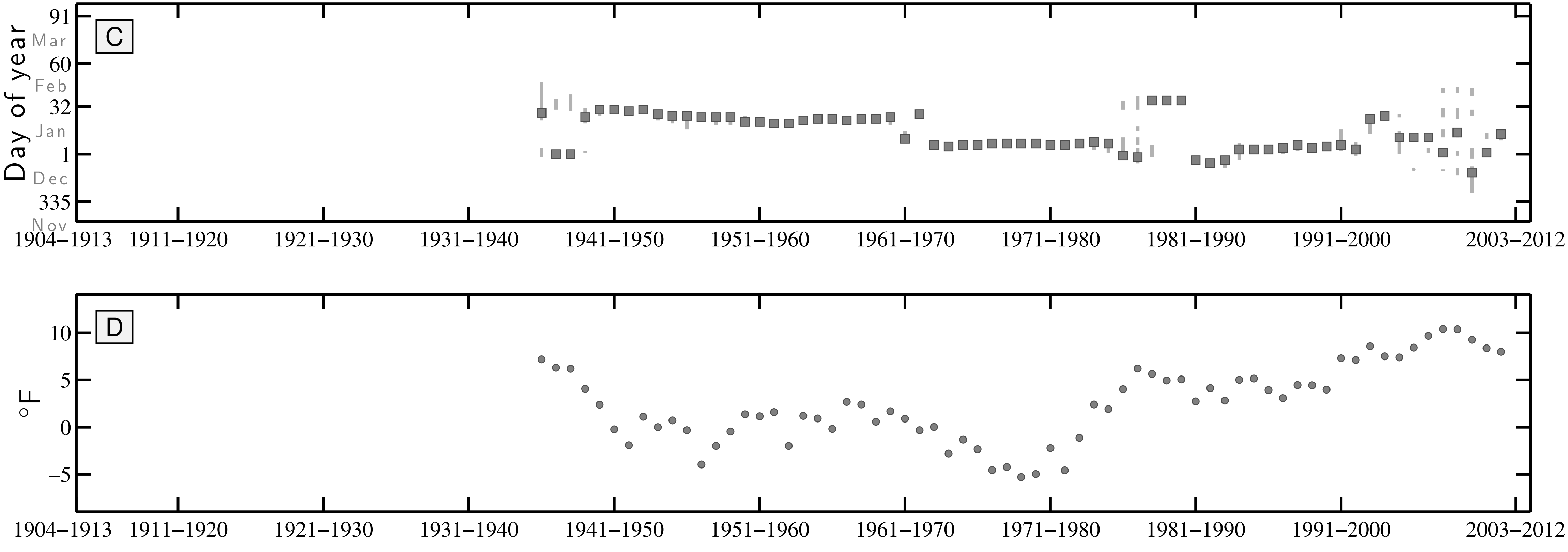

10 year Summer Teletherm dynamics for MOCCASIN EXP STN, MT:

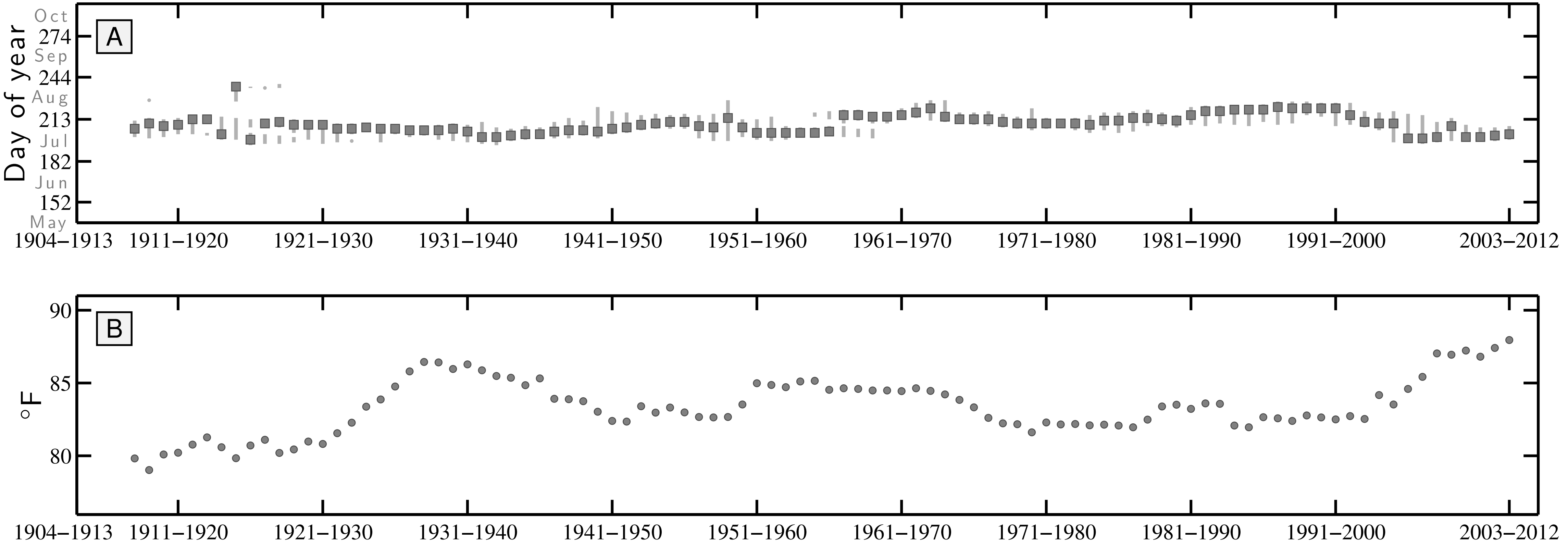

10 year Winter Teletherm dynamics for MOCCASIN EXP STN, MT:

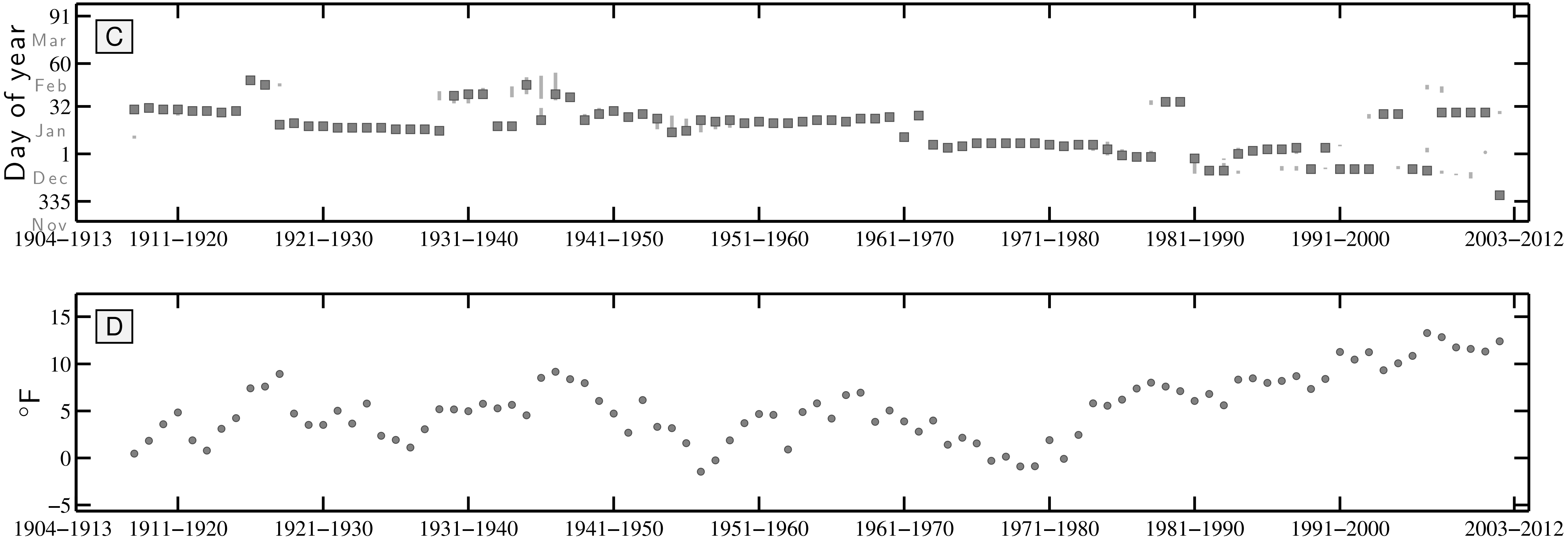

10 year Summer Teletherm dynamics for NORRIS MADISON PH, MT:

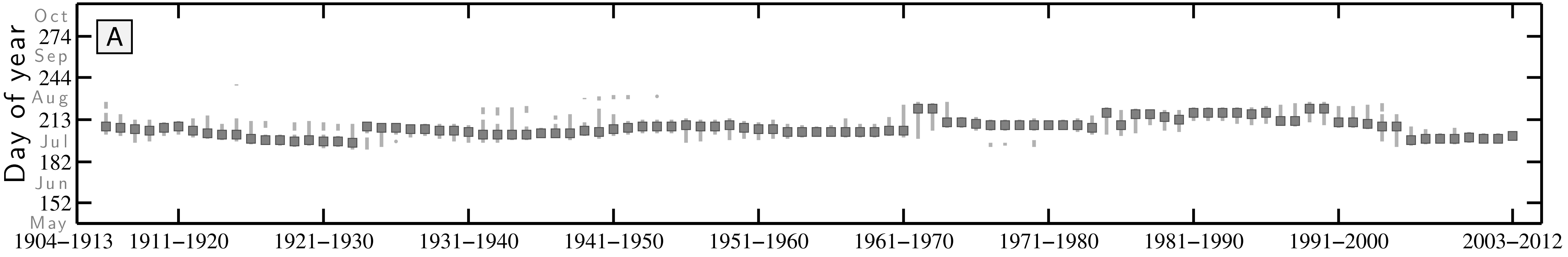

10 year Winter Teletherm dynamics for NORRIS MADISON PH, MT:

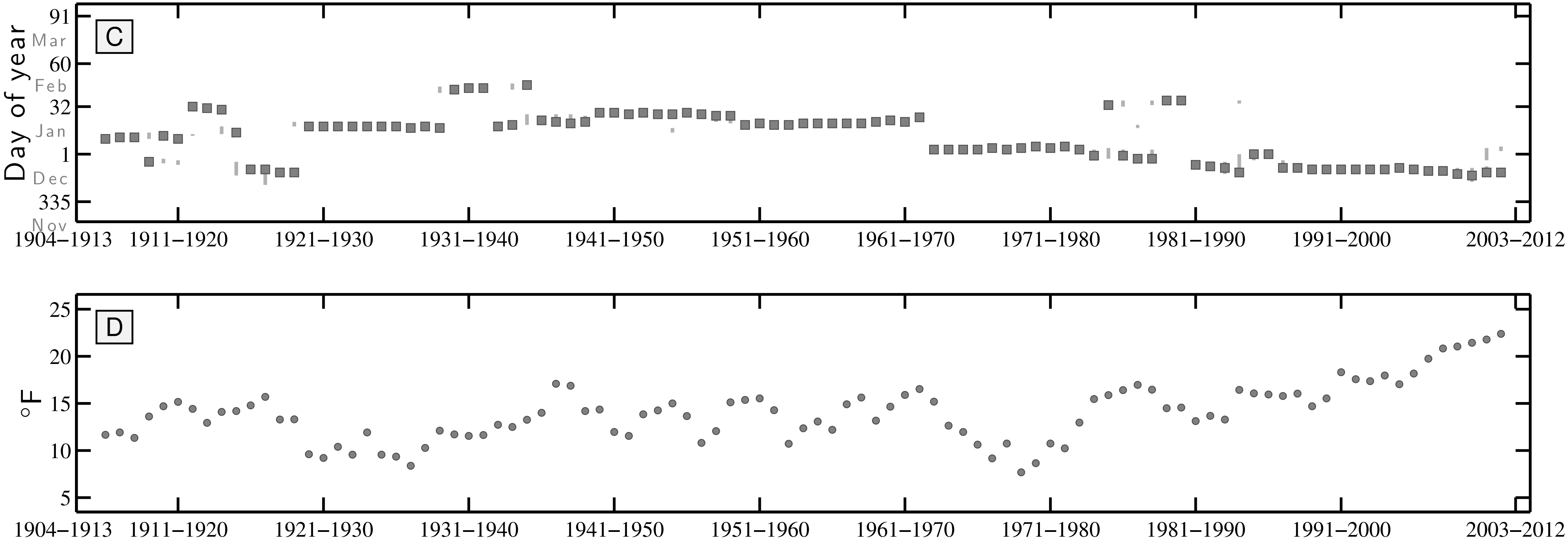

10 year Summer Teletherm dynamics for PHILIPSBURG RS, MT:

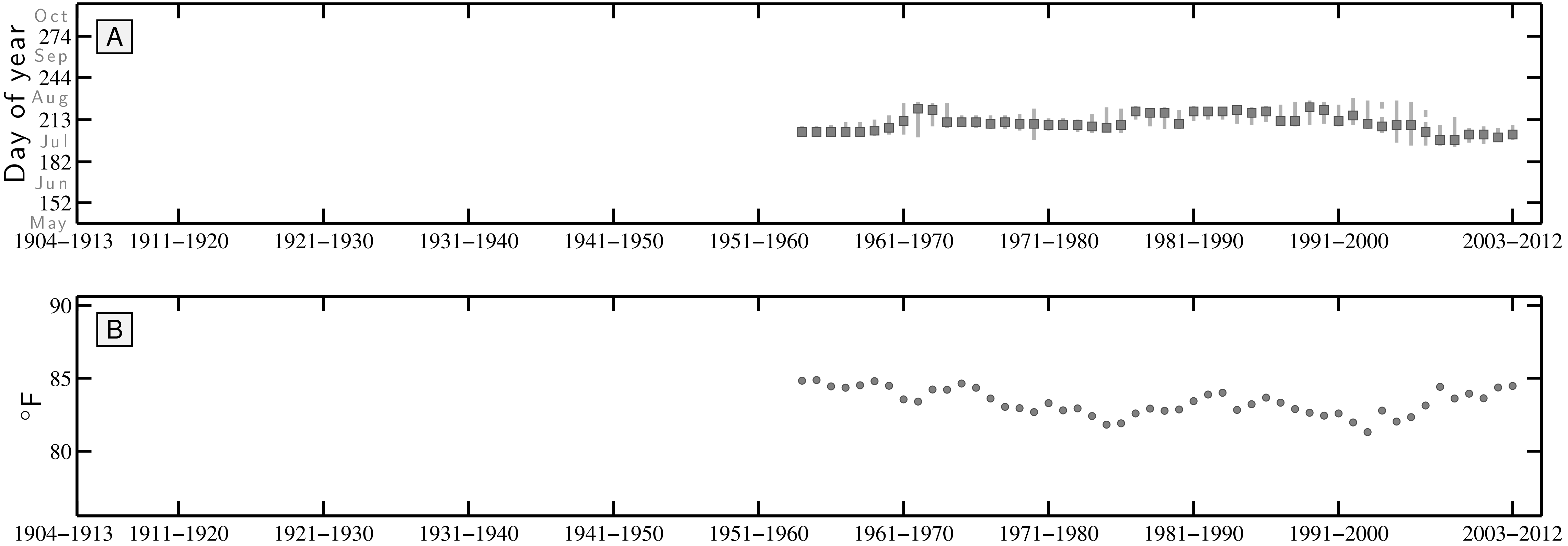

10 year Winter Teletherm dynamics for PHILIPSBURG RS, MT:

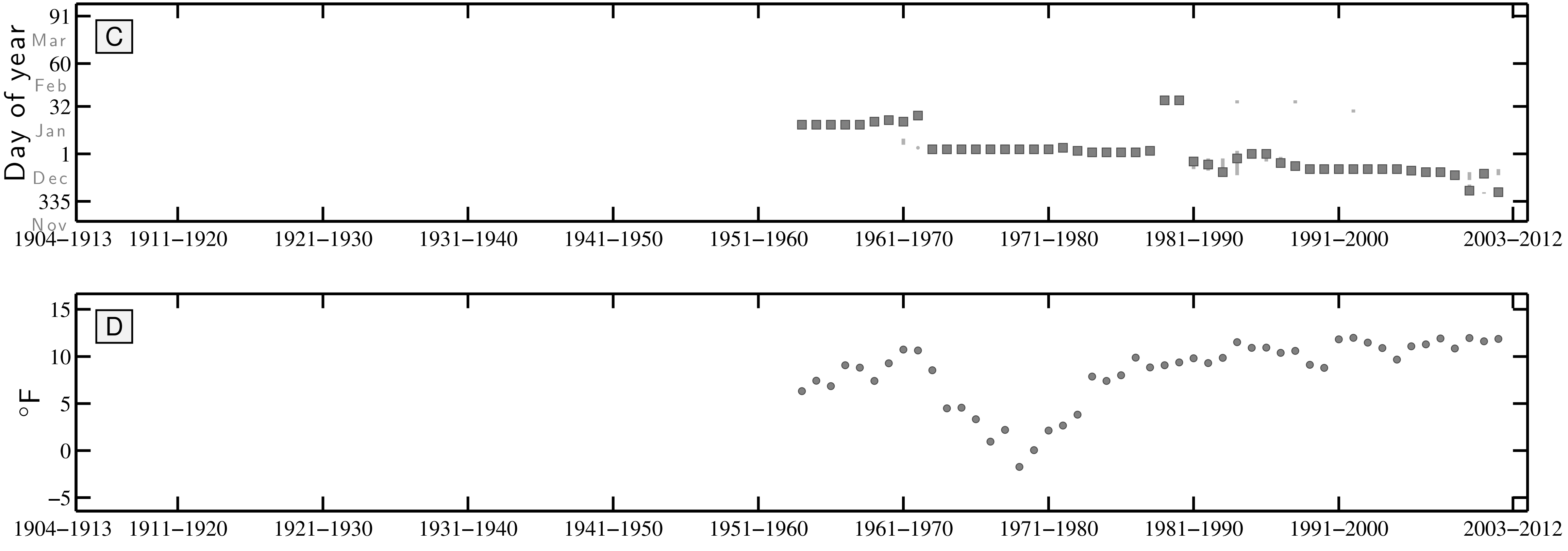

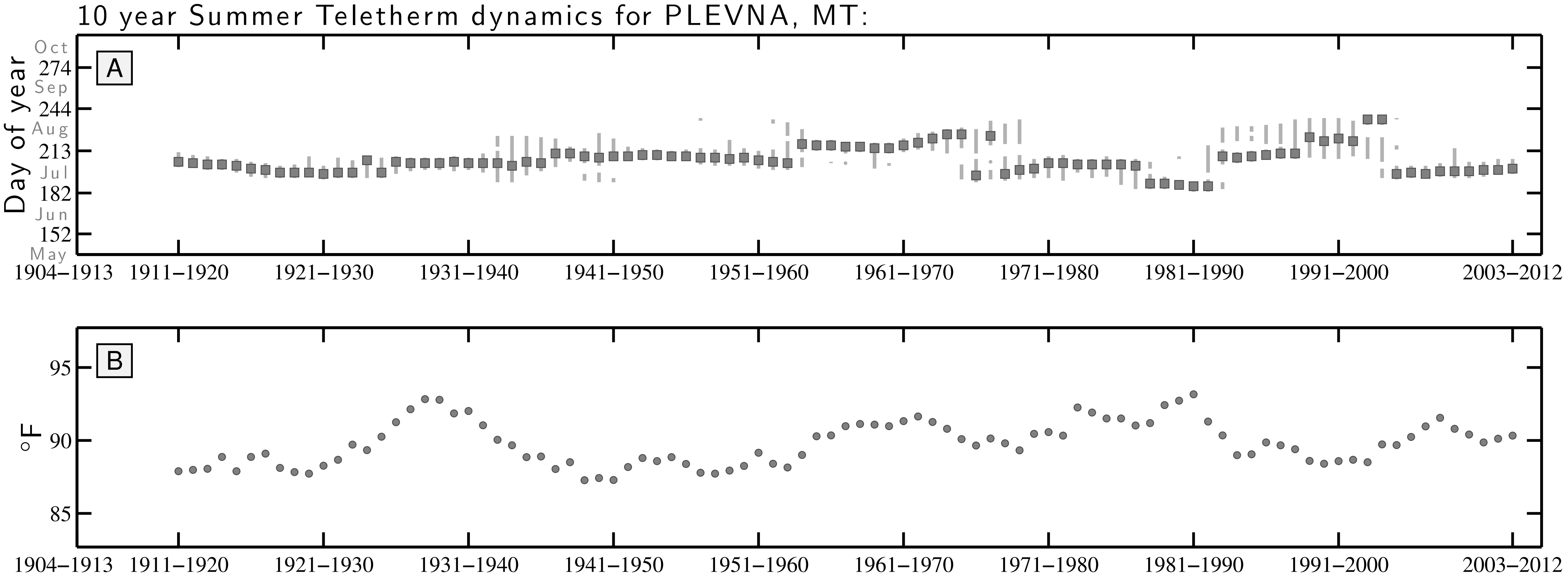

10 year Summer Teletherm dynamics for PLEVNA, MT:
A
B
Day of year
°F
Oct
274
Sep
244
Aug
213
Jul
182
Jun
152
May
95
90
85
1904–1913
1911–1920
1921–1930
1931–1940
1941–1950
1951–1960
1961–1970
1971–1980
1981–1990
1991–2000
2003–2012

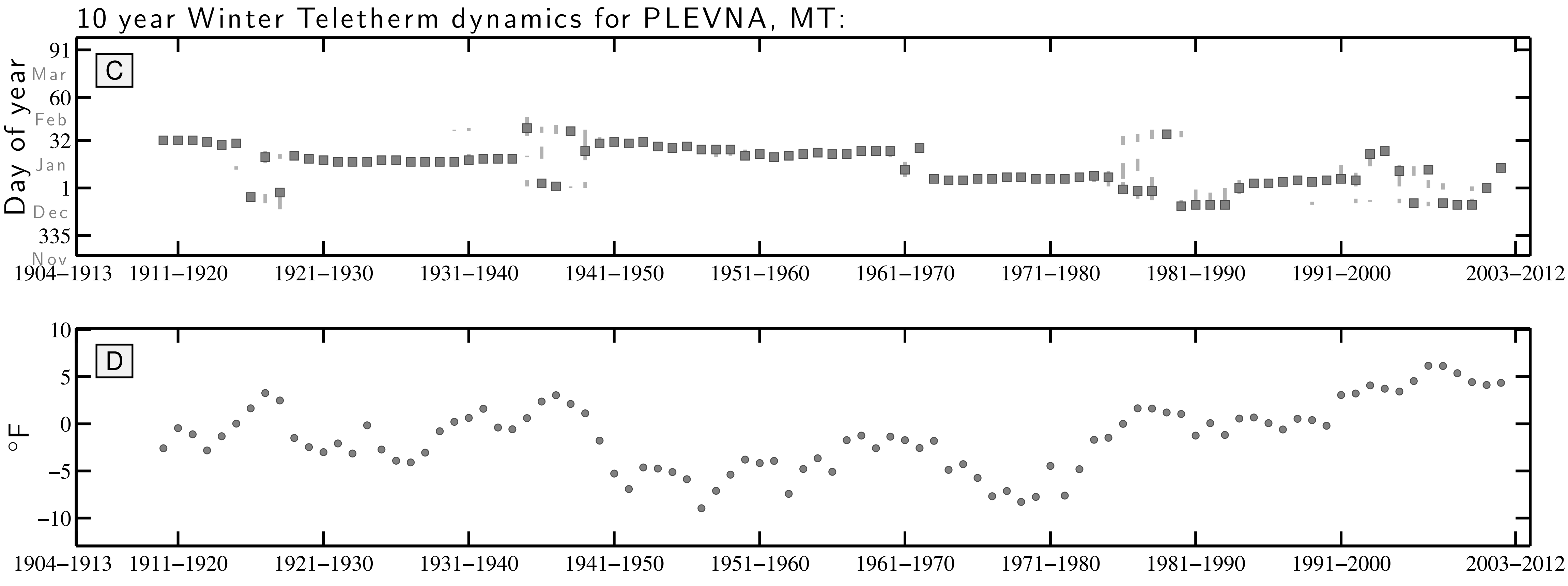

10 year Winter Teletherm dynamics for PLEVNA, MT:
C
D
Day of year
°F
91
Mar
60
Feb
32
Jan
1
Dec
335
Nov
10
5
0
−5
−10
1904–1913
1911–1920
1921–1930
1931–1940
1941–1950
1951–1960
1961–1970
1971–1980
1981–1990
1991–2000
2003–2012

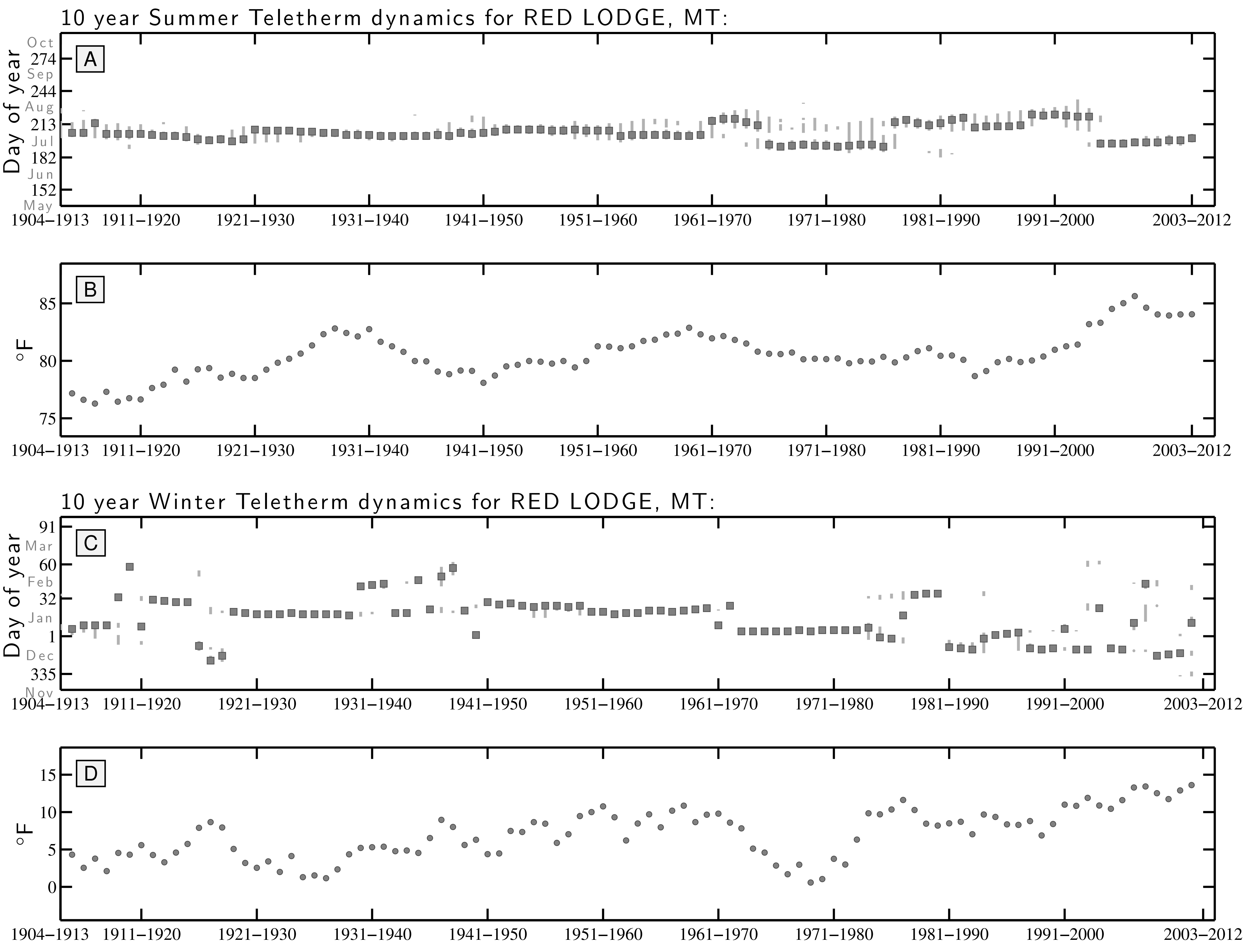
10 year Summer Teletherm dynamics for RED LODGE, MT:
A
Day of year
Oct
274
Sep
244
Aug
213
Jul
182
Jun
152
May
1904–1913
1911–1920
1921–1930
1931–1940
1941–1950
1951–1960
1961–1970
1971–1980
1981–1990
1991–2000
2003–2012
B
°F
85
80
75
10 year Winter Teletherm dynamics for RED LODGE, MT:
C
91
Mar
60
Feb
32
Jan
1
Dec
335
Nov
D
15
10
5
0

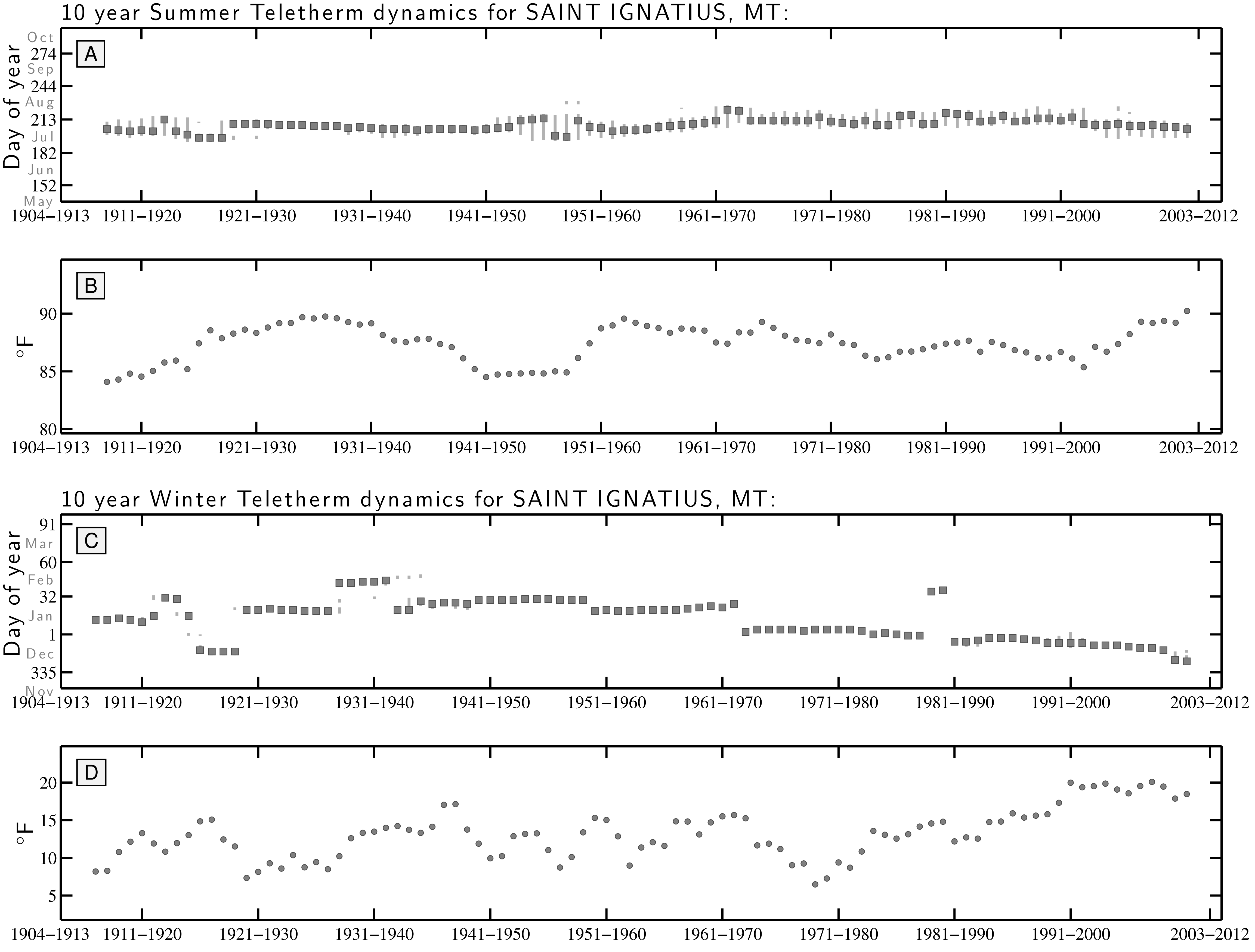
10 year Summer Teletherm dynamics for SAINT IGNATIUS, MT:
A
Day of year
Oct
274
Sep
244
Aug
213
Jul
182
Jun
152
May
B
°F
90
85
80
10 year Winter Teletherm dynamics for SAINT IGNATIUS, MT:
C
Day of year
91
Mar
60
Feb
32
Jan
1
Dec
335
Nov
D
°F
20
15
10
5
1904–1913
1911–1920
1921–1930
1931–1940
1941–1950
1951–1960
1961–1970
1971–1980
1981–1990
1991–2000
2003–2012

10 year Summer Teletherm dynamics for SAINT REGIS 1 NE, MT:

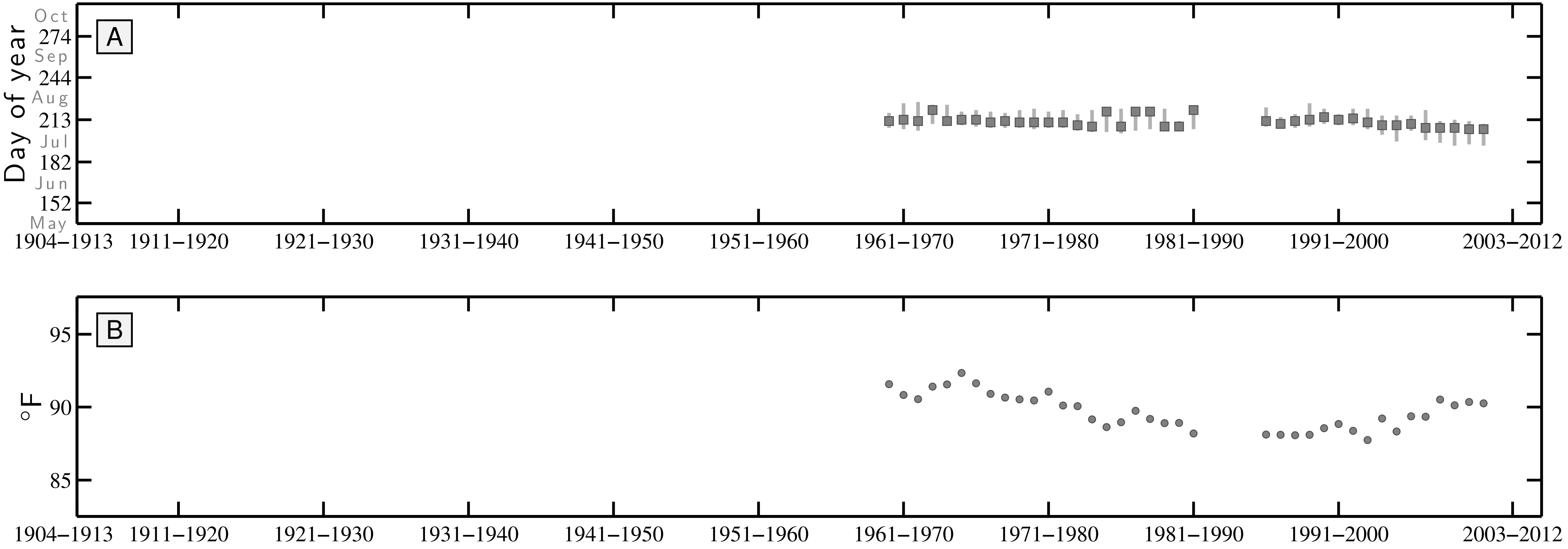

10 year Winter Teletherm dynamics for SAINT REGIS 1 NE, MT:

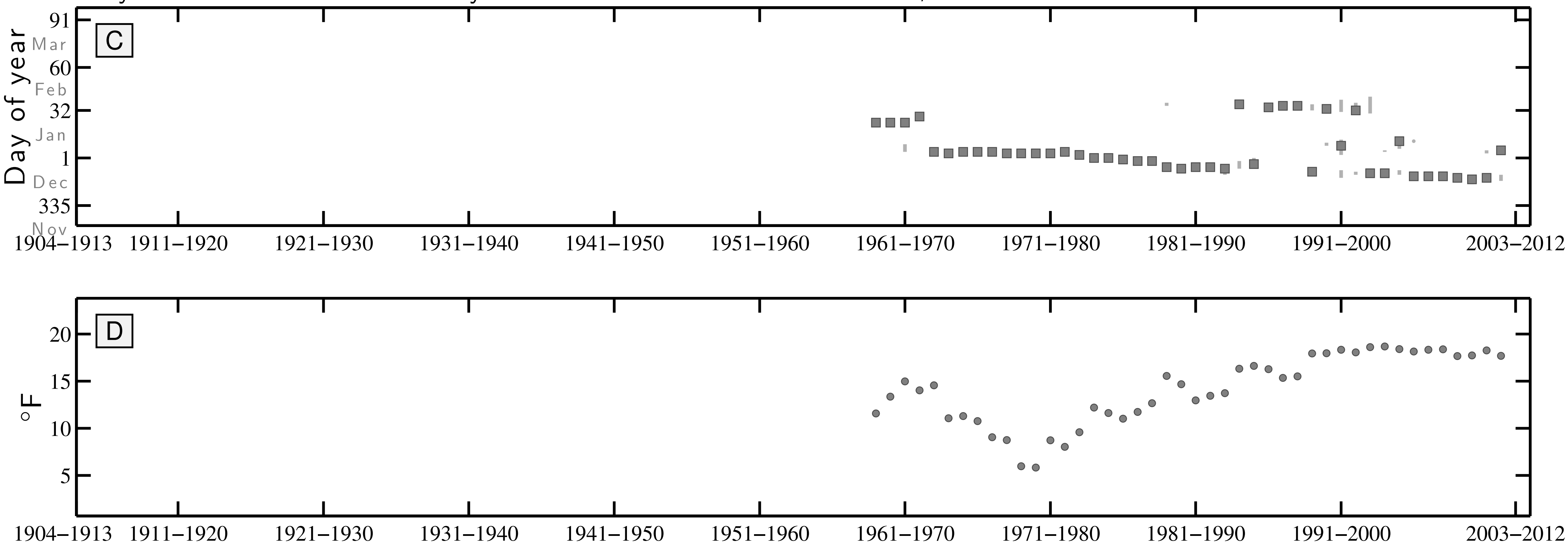

10 year Summer Teletherm dynamics for SAVAGE, MT:

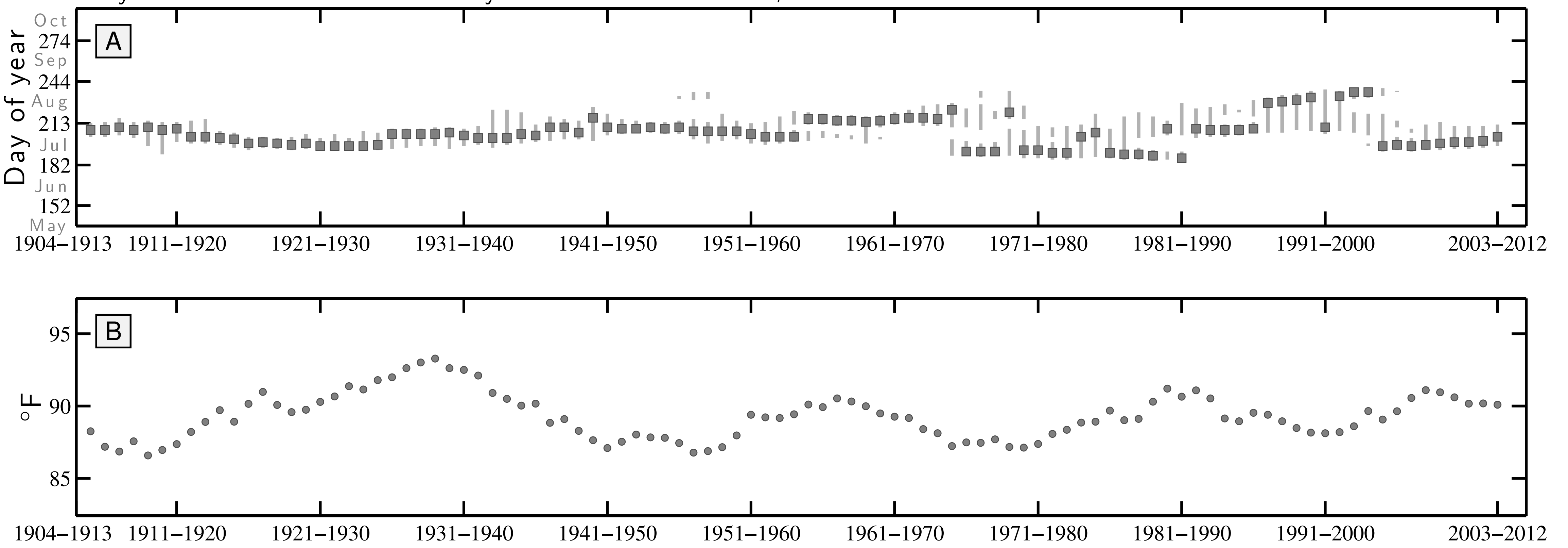

10 year Winter Teletherm dynamics for SAVAGE, MT:

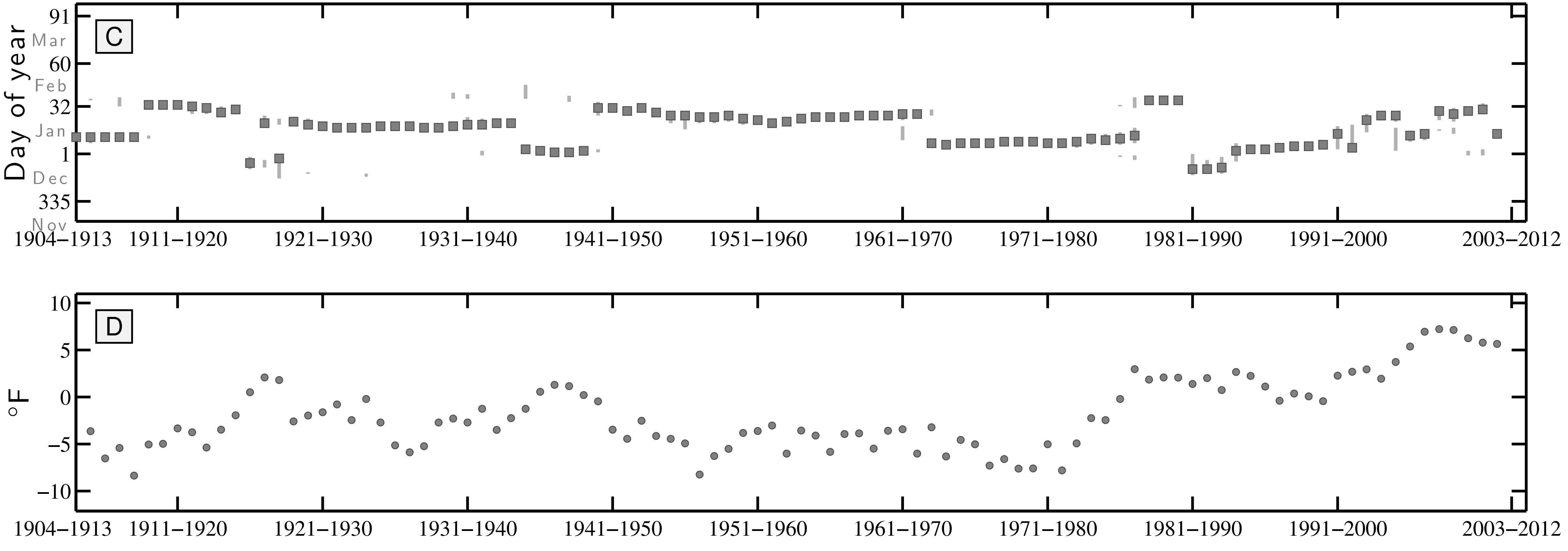

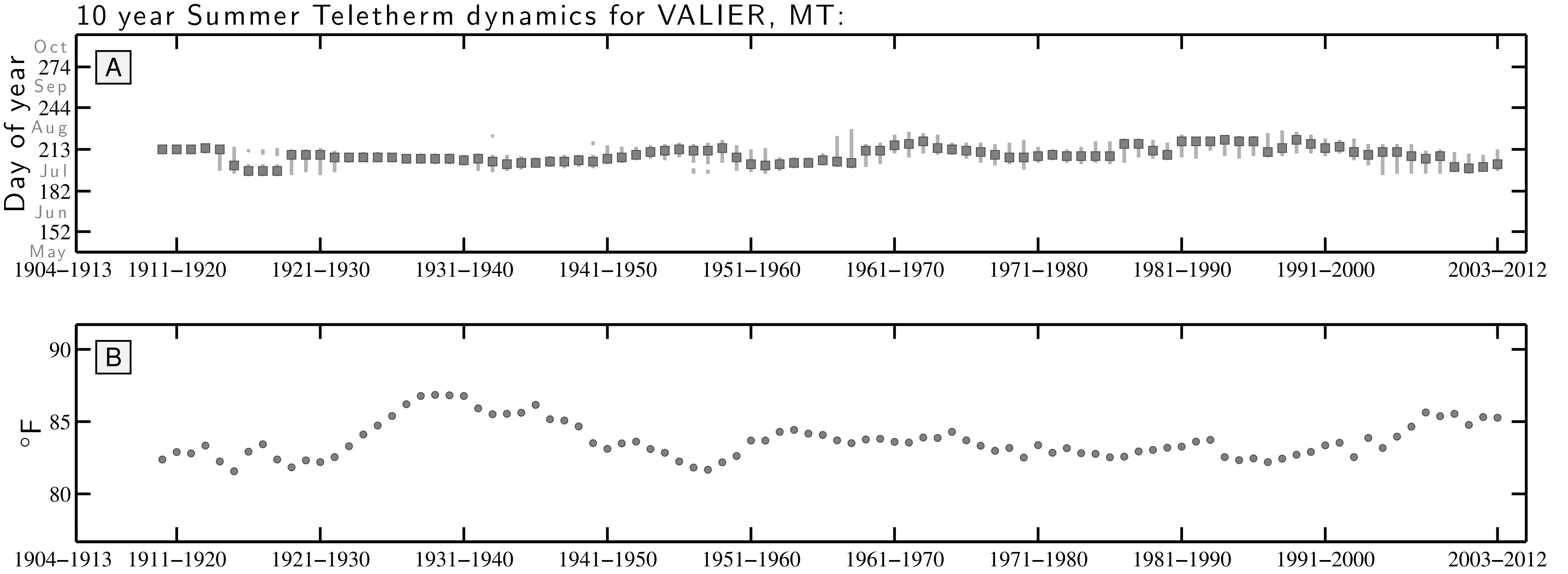
10 year Summer Teletherm dynamics for VALIER, MT:
A
B
Day of year
°F
Oct
274
Sep
244
Aug
213
Jul
182
Jun
152
May
90
85
80
1904–1913
1911–1920
1921–1930
1931–1940
1941–1950
1951–1960
1961–1970
1971–1980
1981–1990
1991–2000
2003–2012

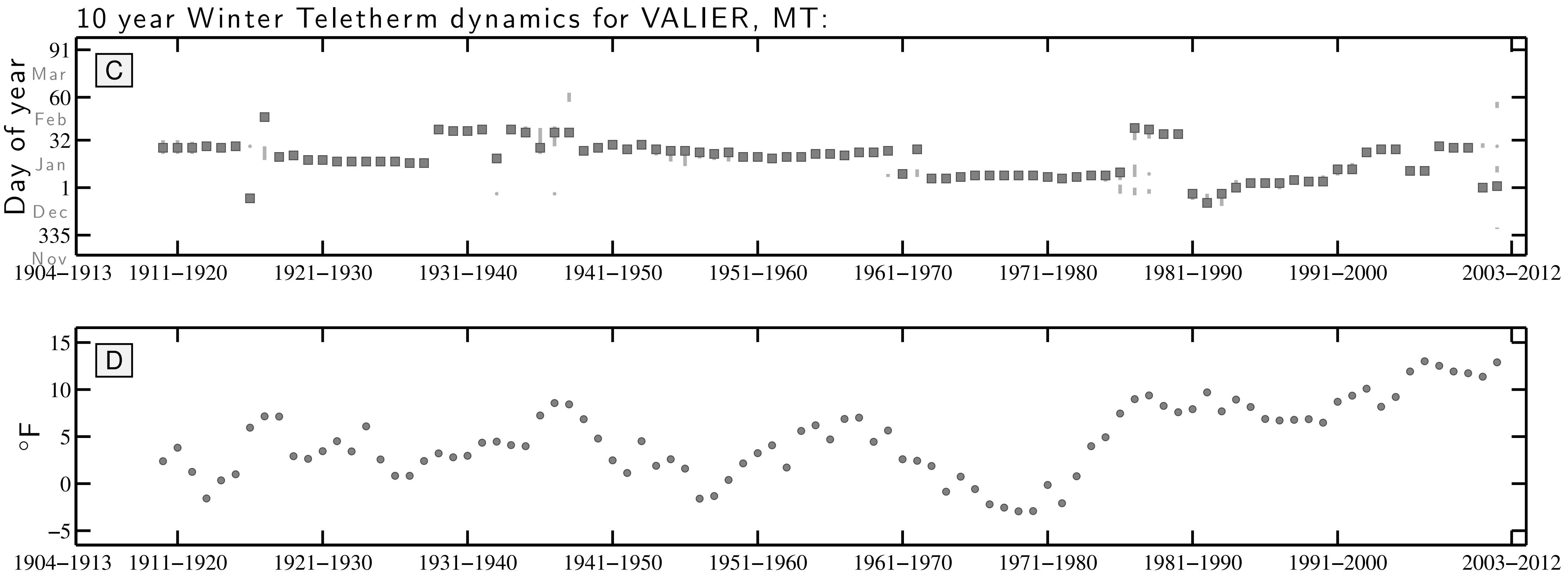
10 year Winter Teletherm dynamics for VALIER, MT:
C
D
Day of year
°F
91
Mar
60
Feb
32
Jan
1
Dec
335
Nov
15
10
5
0
−5
1904–1913
1911–1920
1921–1930
1931–1940
1941–1950
1951–1960
1961–1970
1971–1980
1981–1990
1991–2000
2003–2012

10 year Summer Teletherm dynamics for VIDA 6 NE, MT:

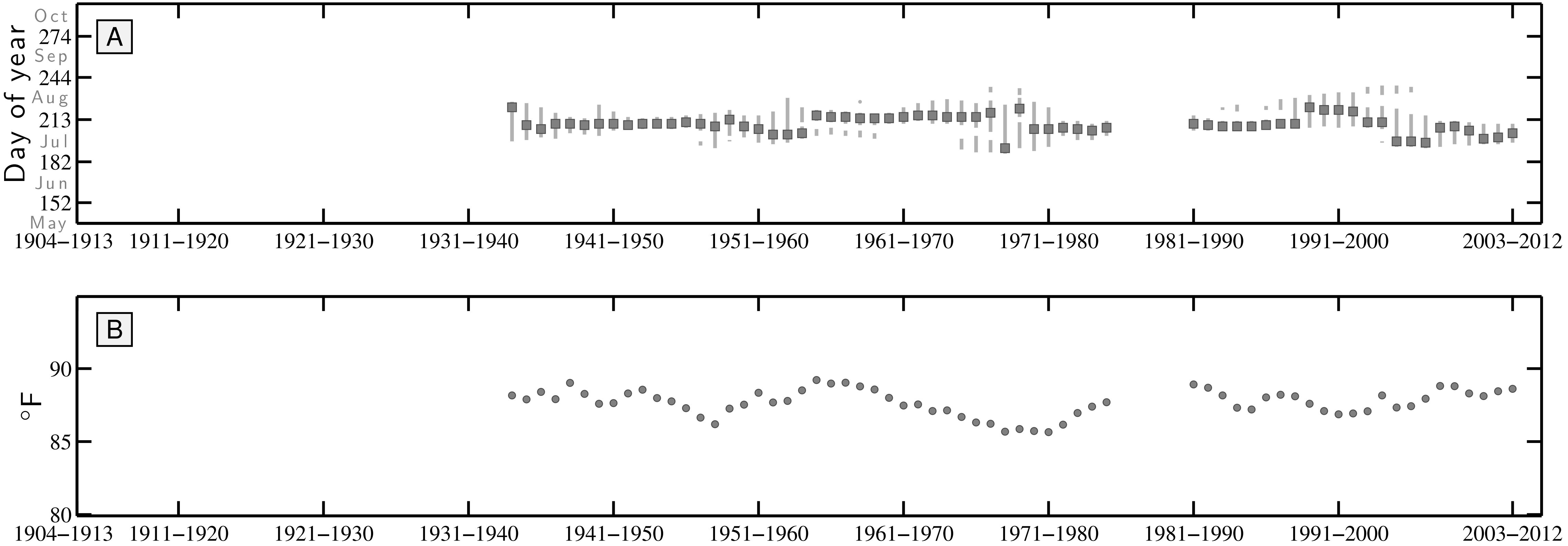

10 year Winter Teletherm dynamics for VIDA 6 NE, MT:

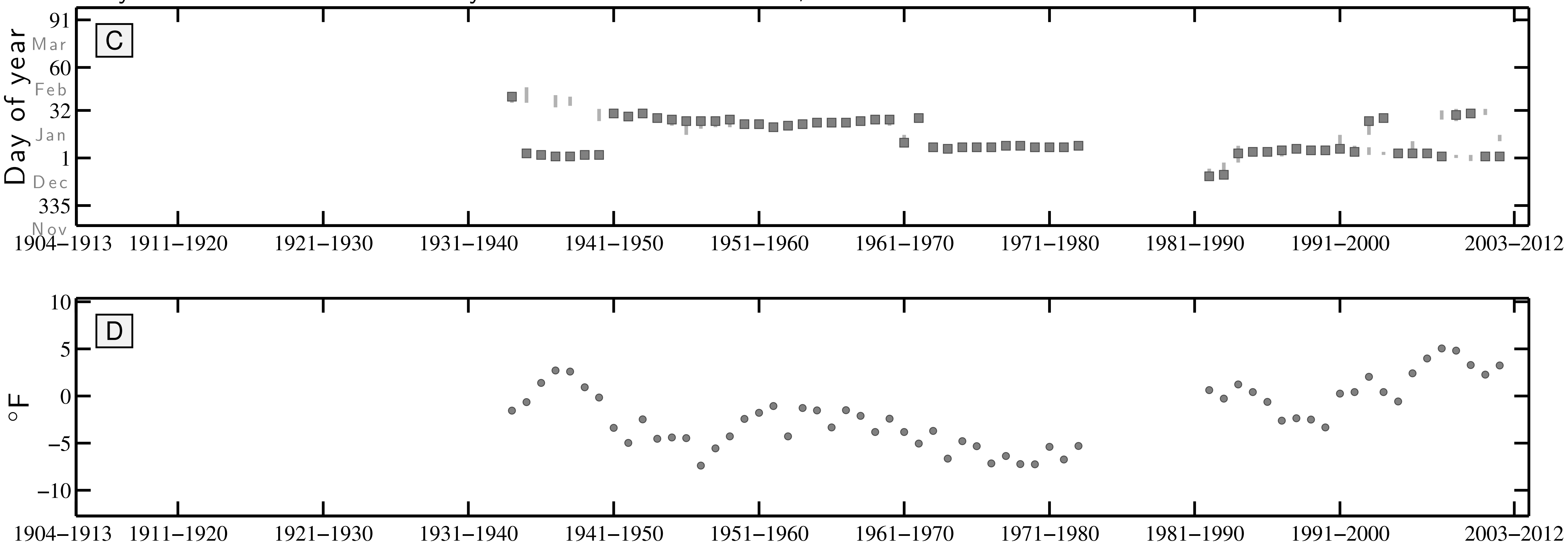

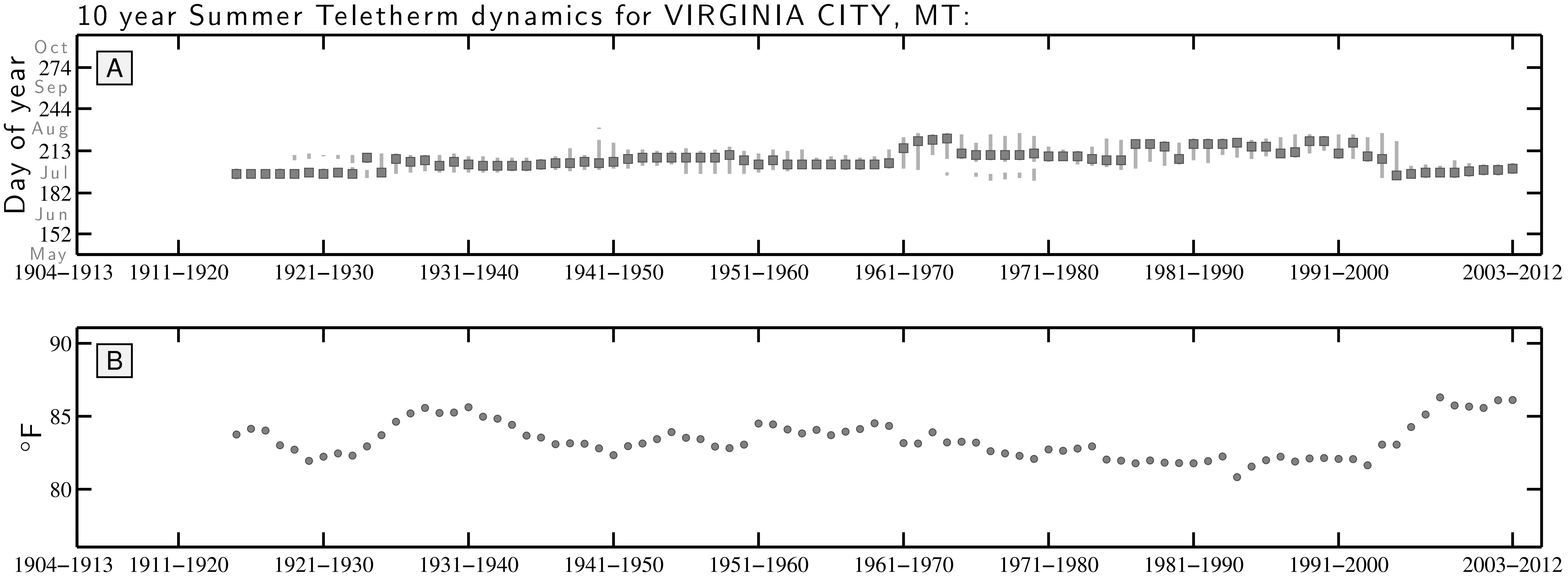

10 year Summer Teletherm dynamics for VIRGINIA CITY, MT:
A
B
Day of year
°F
Oct
274
Sep
244
Aug
213
Jul
182
Jun
152
May
90
85
80
1904–1913
1911–1920
1921–1930
1931–1940
1941–1950
1951–1960
1961–1970
1971–1980
1981–1990
1991–2000
2003–2012

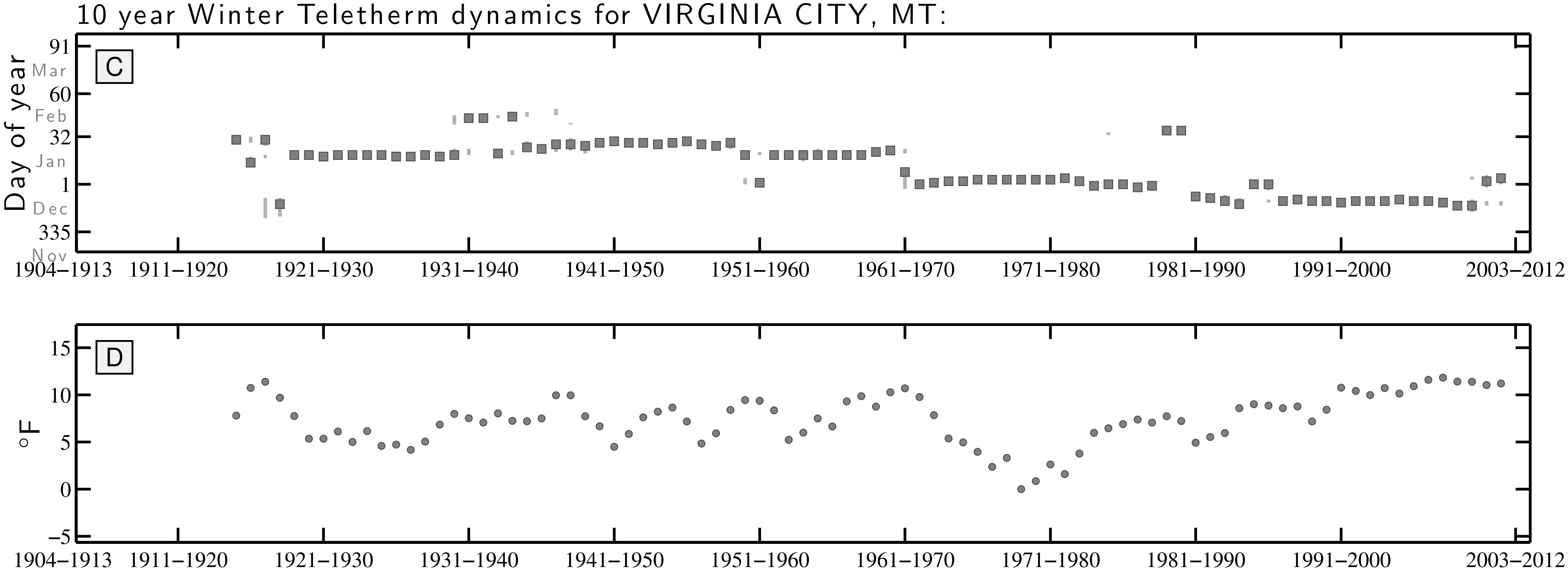

10 year Winter Teletherm dynamics for VIRGINIA CITY, MT:
C
D
Day of year
°F
91
Mar
60
Feb
32
Jan
1
Dec
335
Nov
15
10
5
0
−5
1904–1913
1911–1920
1921–1930
1931–1940
1941–1950
1951–1960
1961–1970
1971–1980
1981–1990
1991–2000
2003–2012

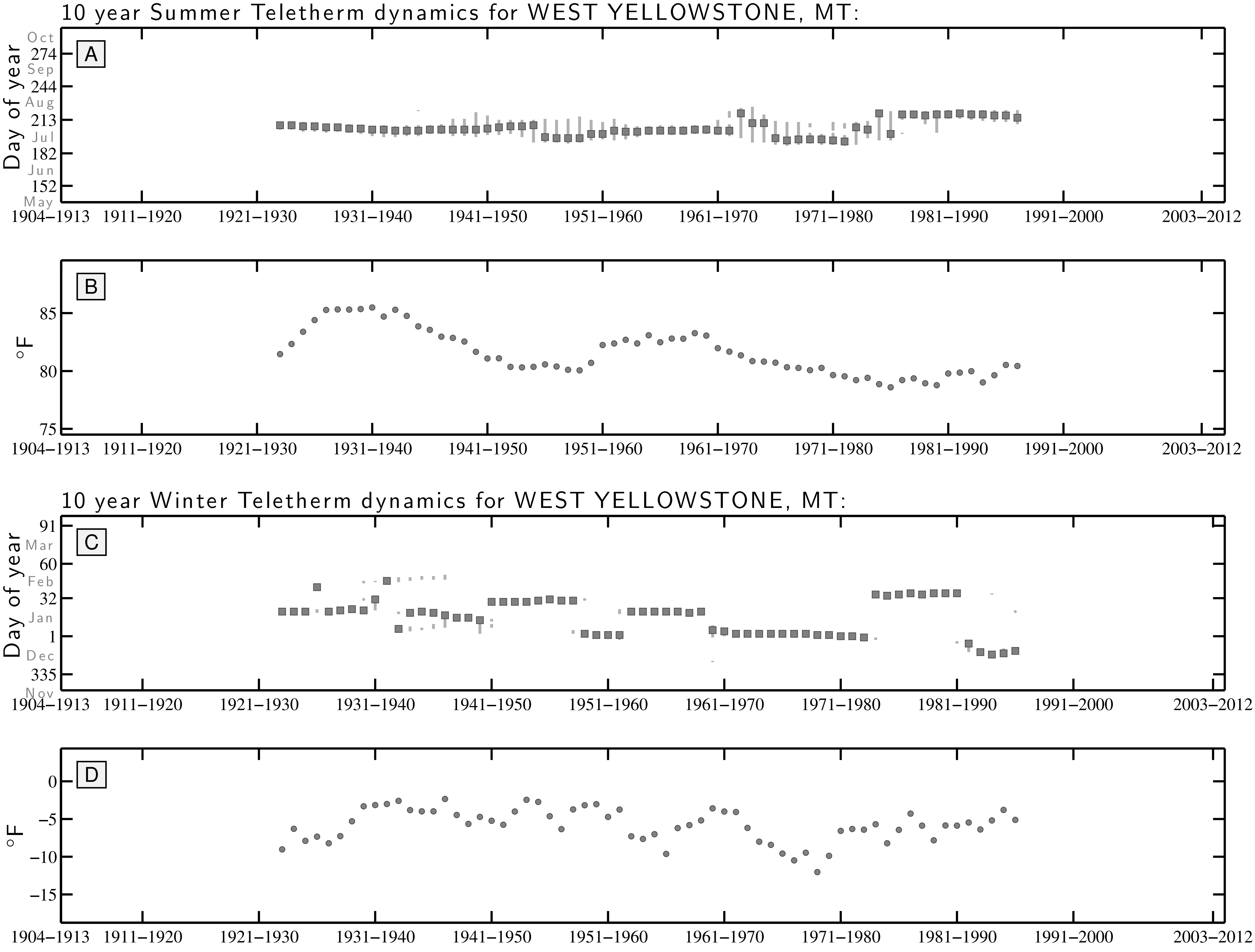

10 year Summer Teletherm dynamics for WEST YELLOWSTONE, MT:
A
B
Day of year
°F
Oct
274
Sep
244
Aug
213
Jul
182
Jun
152
May
85
80
75
1904–1913
1911–1920
1921–1930
1931–1940
1941–1950
1951–1960
1961–1970
1971–1980
1981–1990
1991–2000
2003–2012
10 year Winter Teletherm dynamics for WEST YELLOWSTONE, MT:
C
D
91
Mar
60
Feb
32
Jan
1
Dec
335
Nov
0
−5
−10
−15

10 year Summer Teletherm dynamics for WHITE SULPHUR SPRNGS 2, MT:

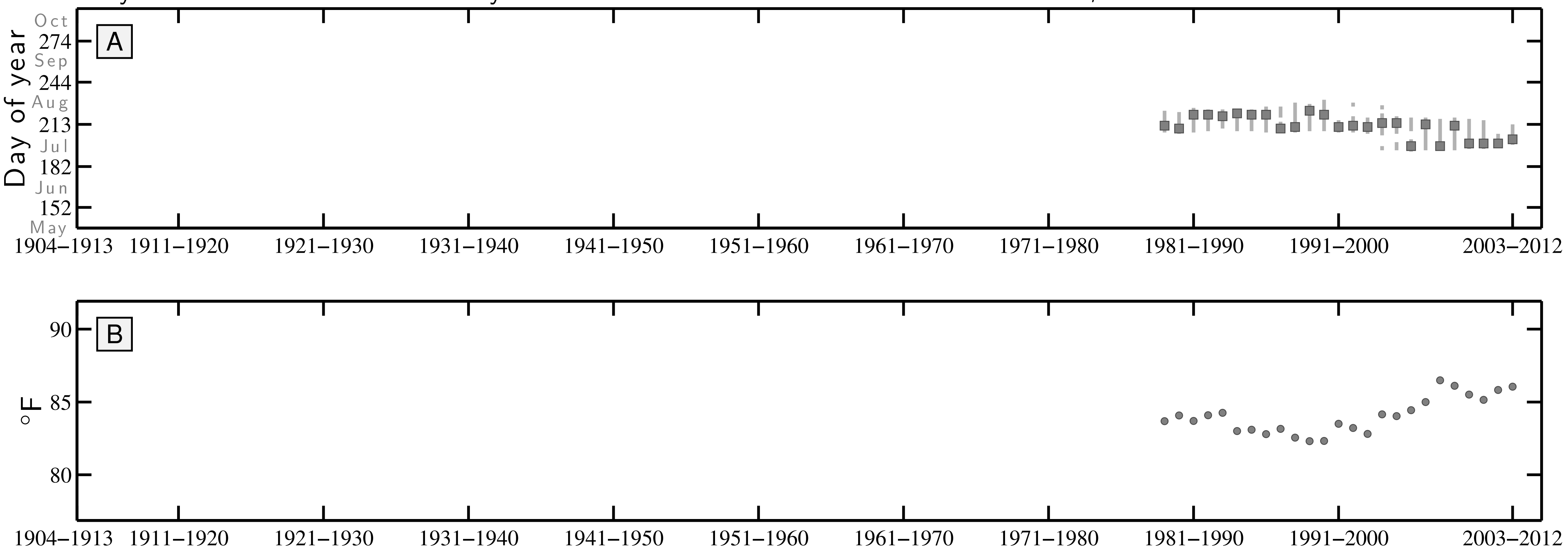

10 year Winter Teletherm dynamics for WHITE SULPHUR SPRNGS 2, MT:

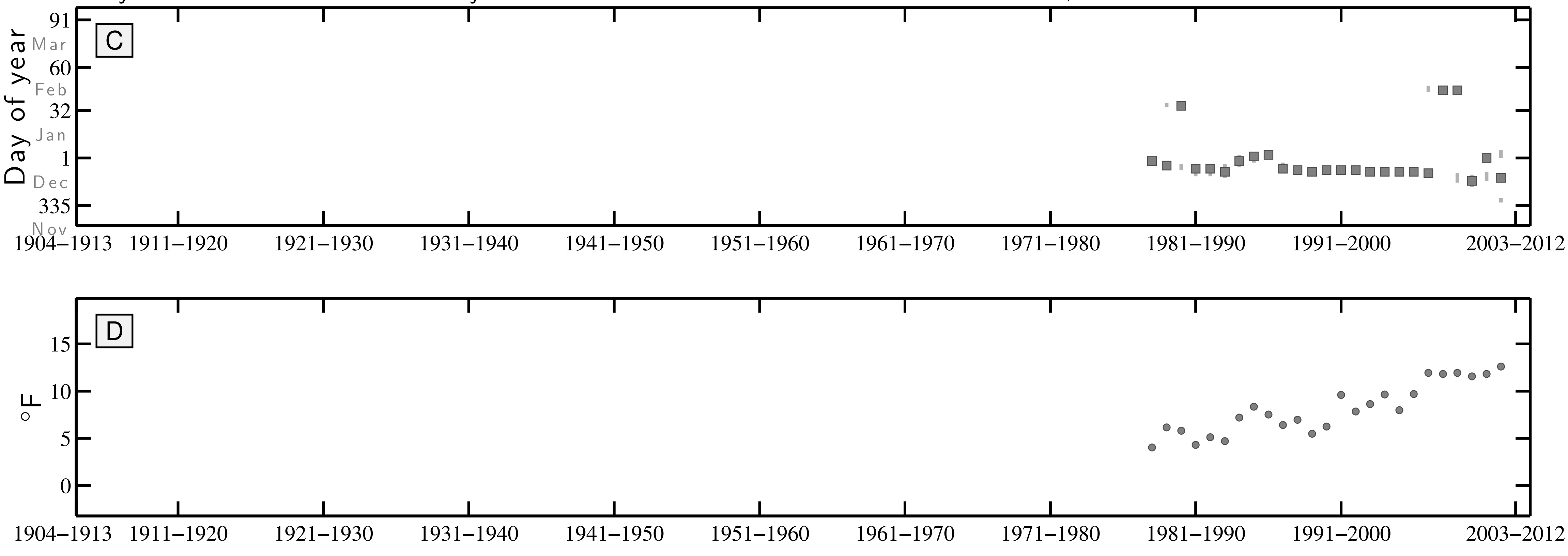

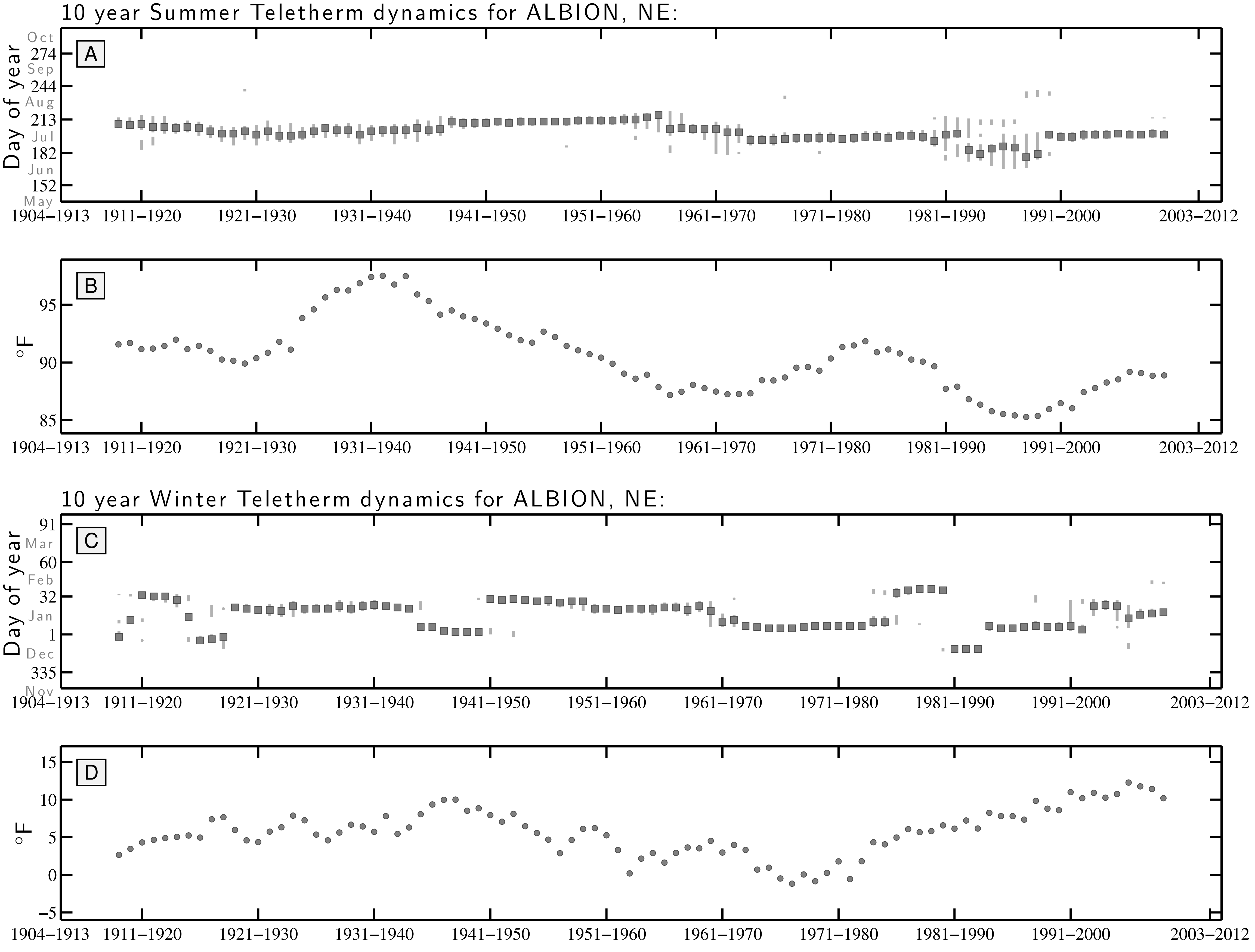
10 year Summer Teletherm dynamics for ALBION, NE:
A
Day of year
Oct
274
Sep
244
Aug
213
Jul
182
Jun
152
May
1904–1913
1911–1920
1921–1930
1931–1940
1941–1950
1951–1960
1961–1970
1971–1980
1981–1990
1991–2000
2003–2012
B
°F
95
90
85
10 year Winter Teletherm dynamics for ALBION, NE:
C
Day of year
91
Mar
60
Feb
32
Jan
1
Dec
335
Nov
D
°F
15
10
5
0
−5

10 year Summer Teletherm dynamics for ALLIANCE 1WNW, NE:

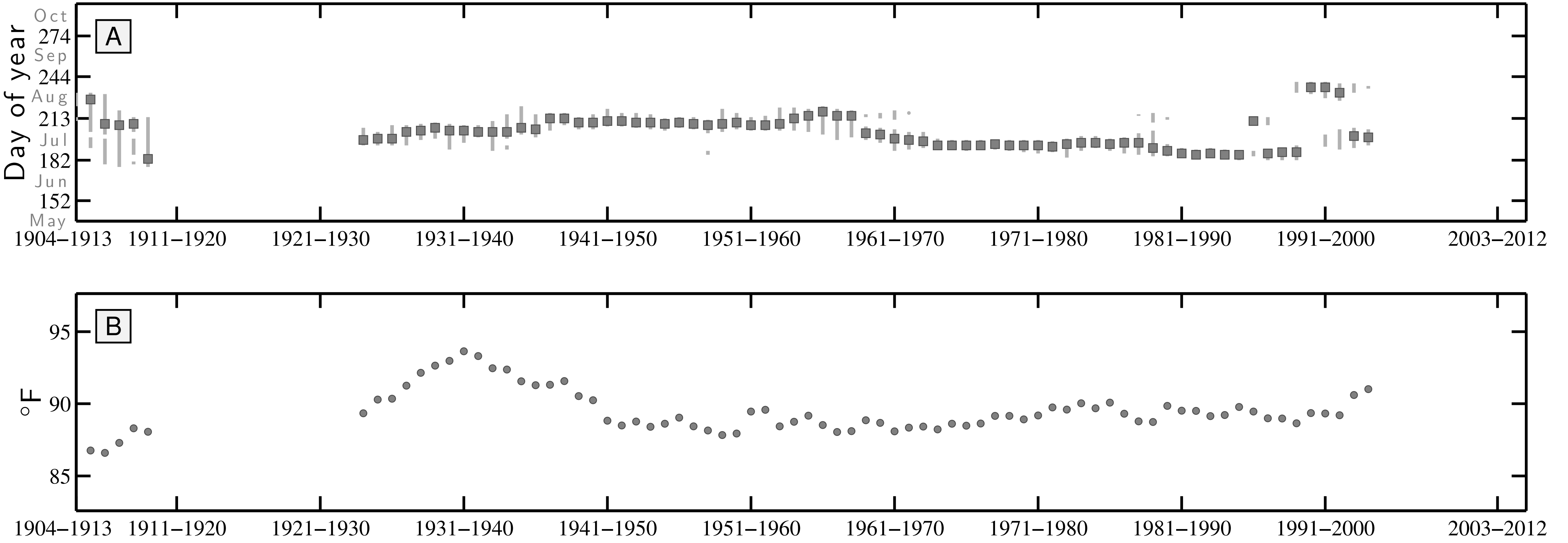

10 year Winter Teletherm dynamics for ALLIANCE 1WNW, NE:

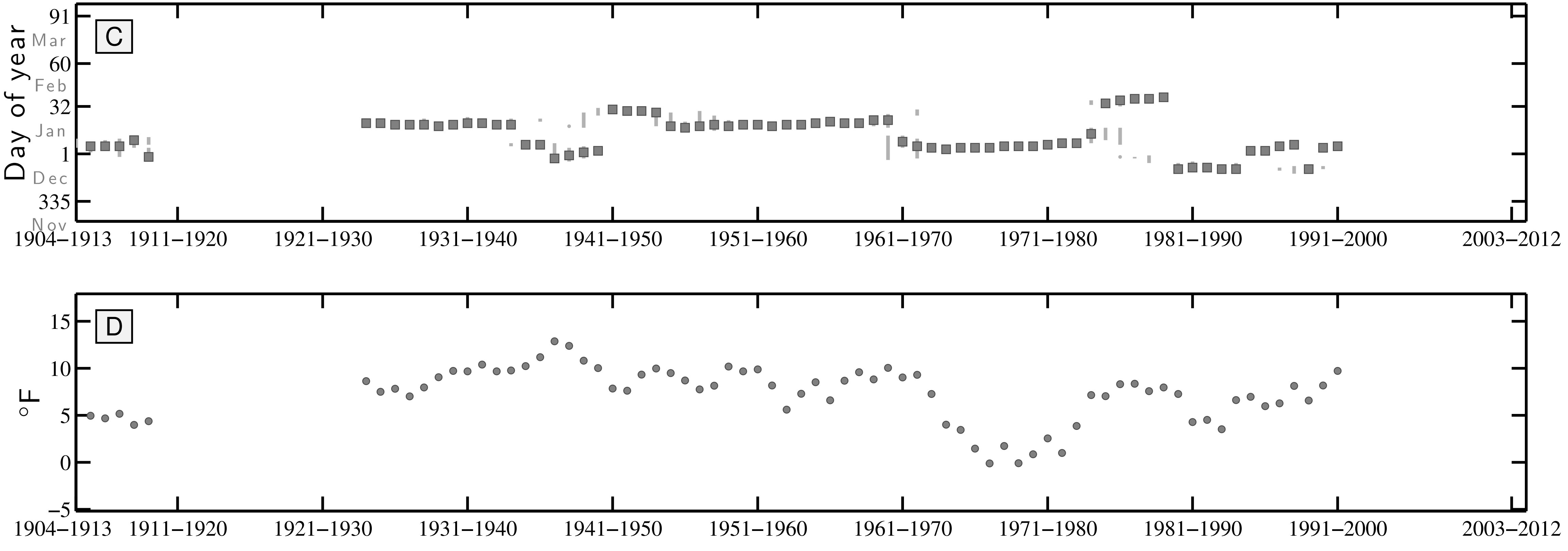

10 year Summer Teletherm dynamics for ASHLAND NO 2, NE:

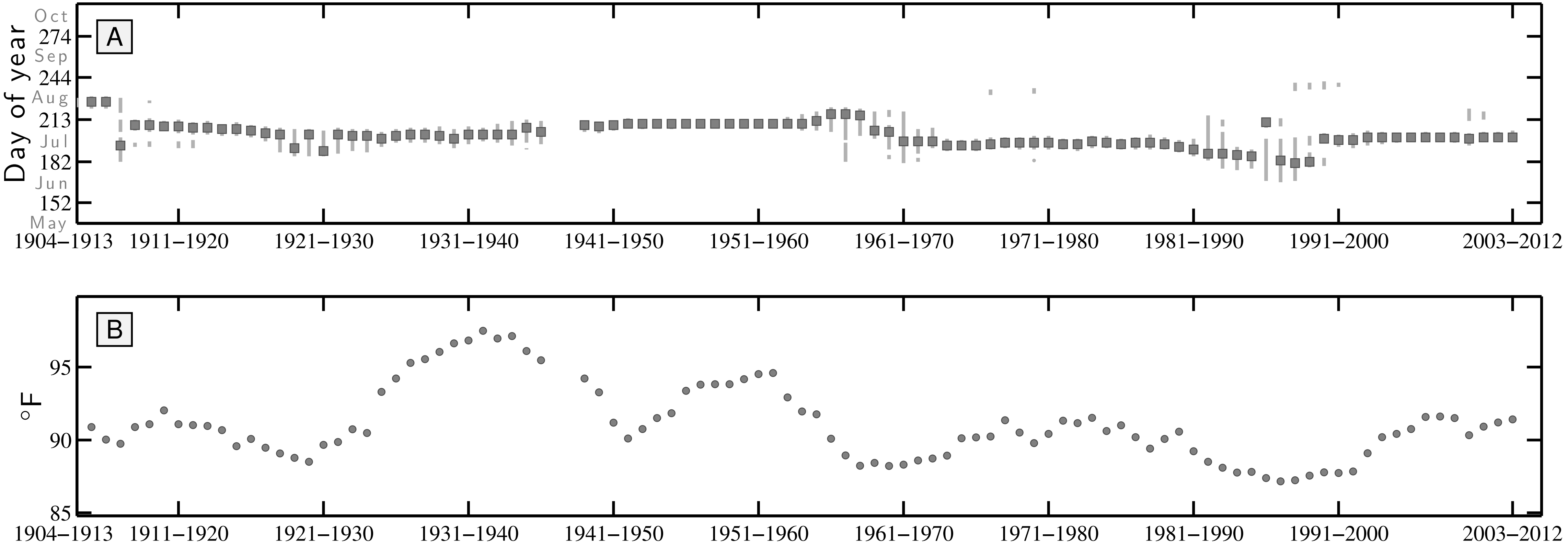

10 year Winter Teletherm dynamics for ASHLAND NO 2, NE:

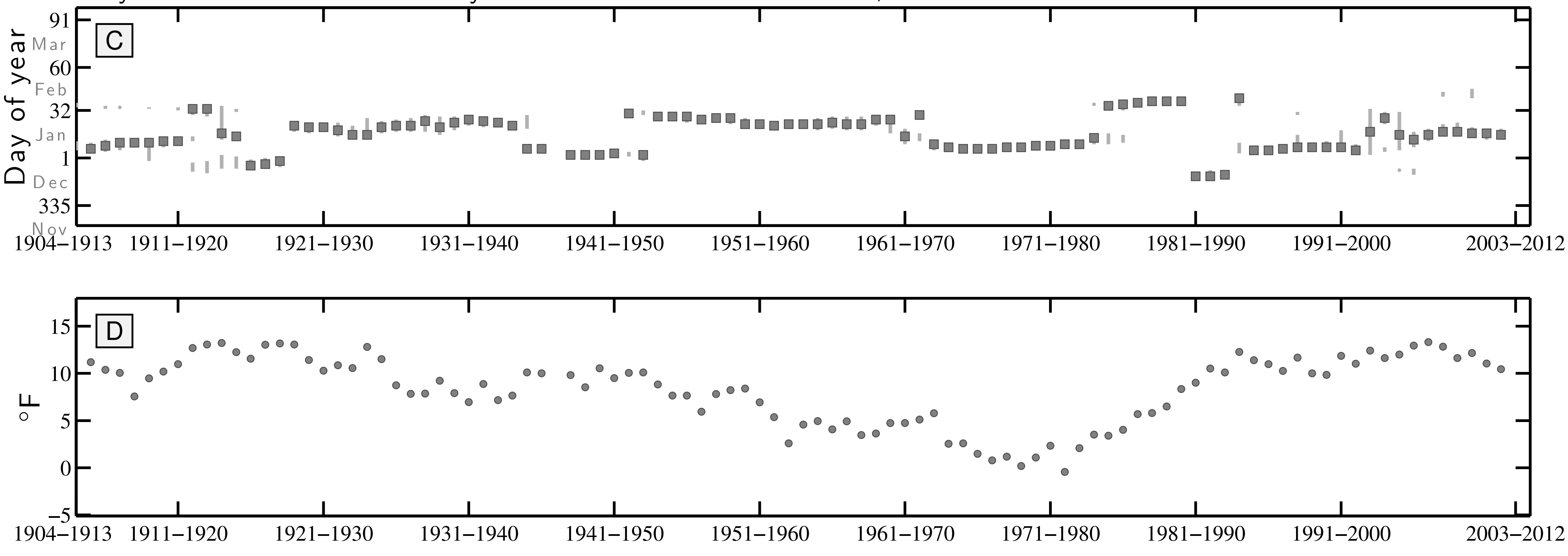

10 year Summer Teletherm dynamics for ATKINSON 3SW, NE:

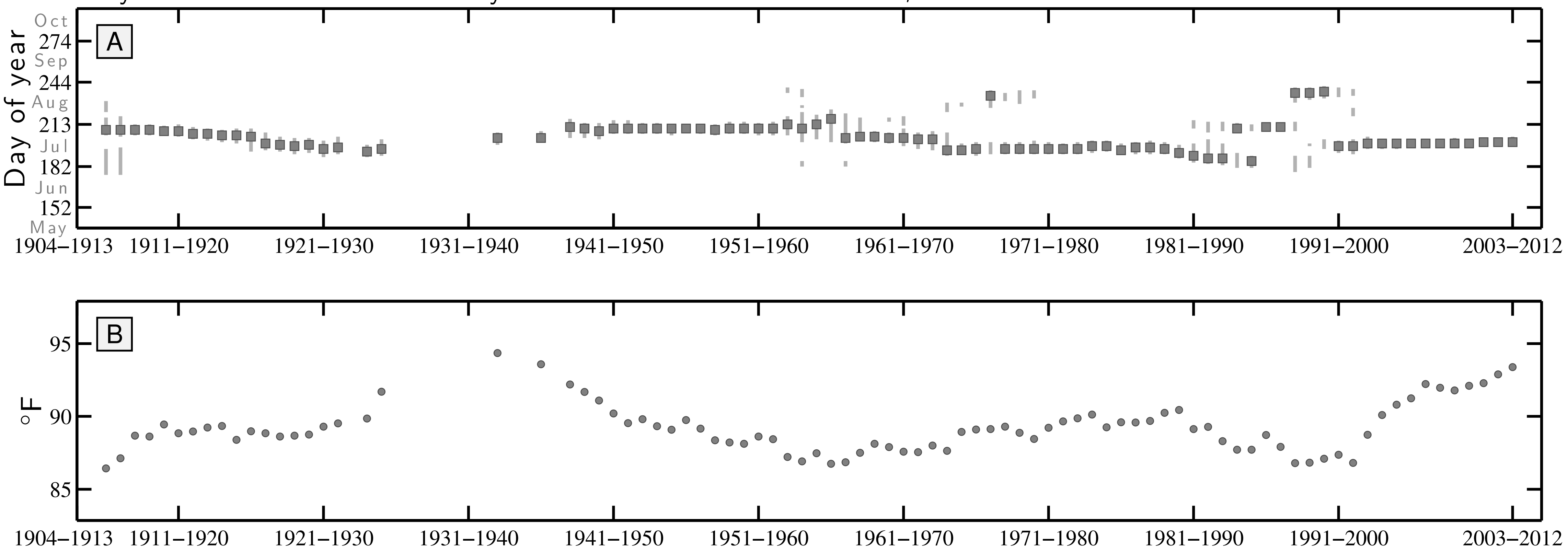

10 year Winter Teletherm dynamics for ATKINSON 3SW, NE:

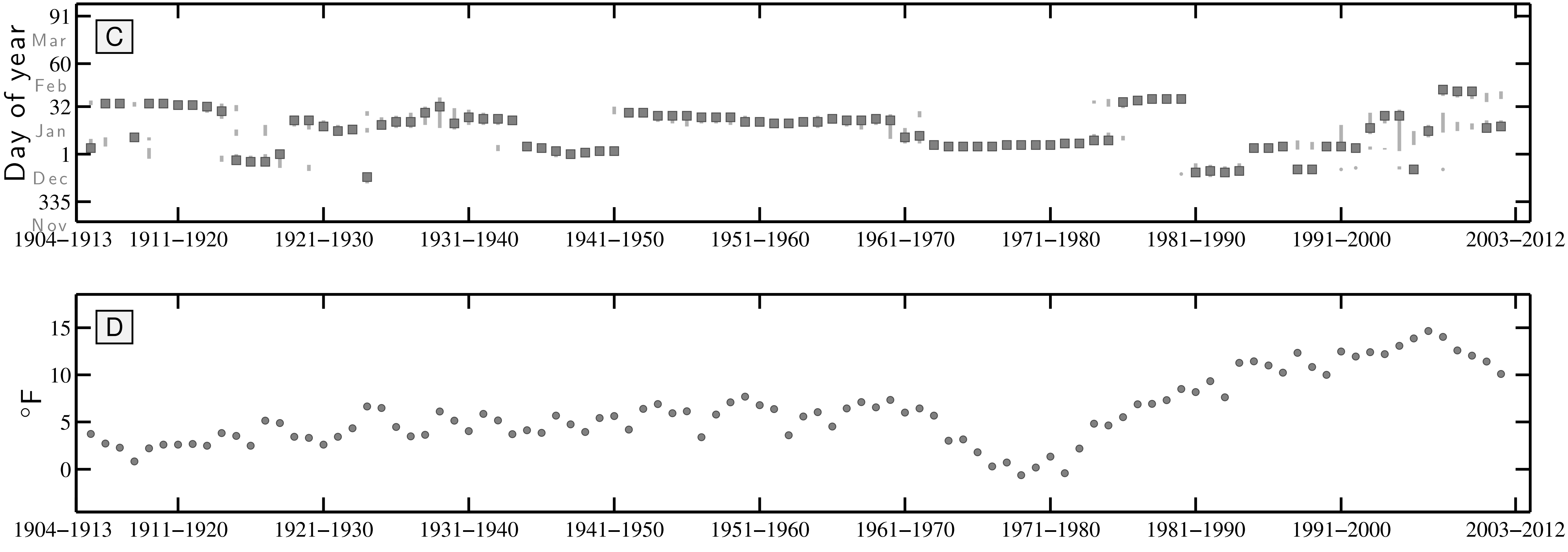

10 year Summer Teletherm dynamics for AUBURN 5 ESE, NE:

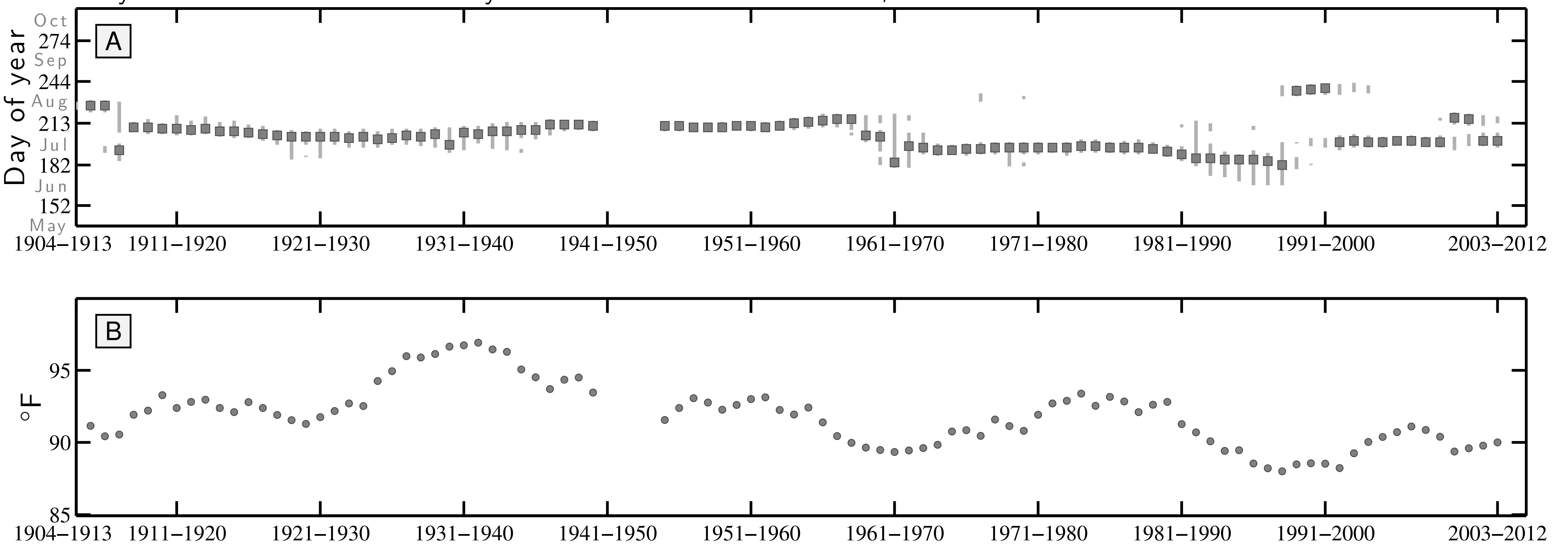

10 year Winter Teletherm dynamics for AUBURN 5 ESE, NE:

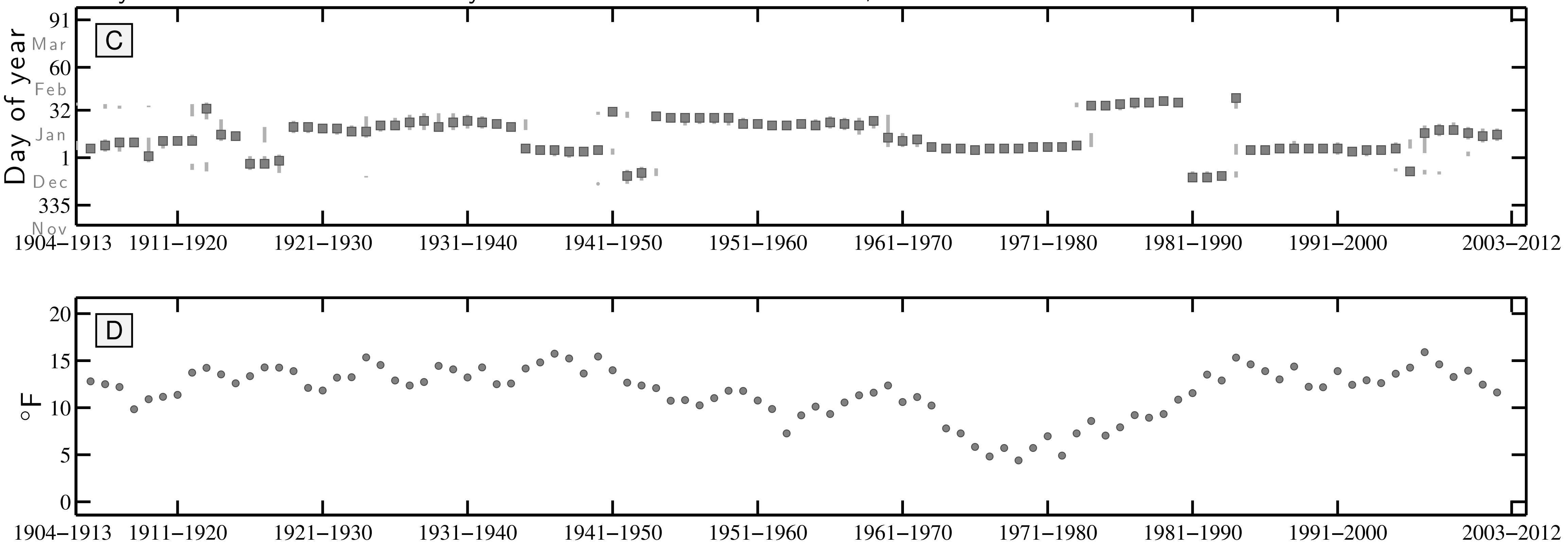

10 year Summer Teletherm dynamics for BEATRICE 1N, NE:

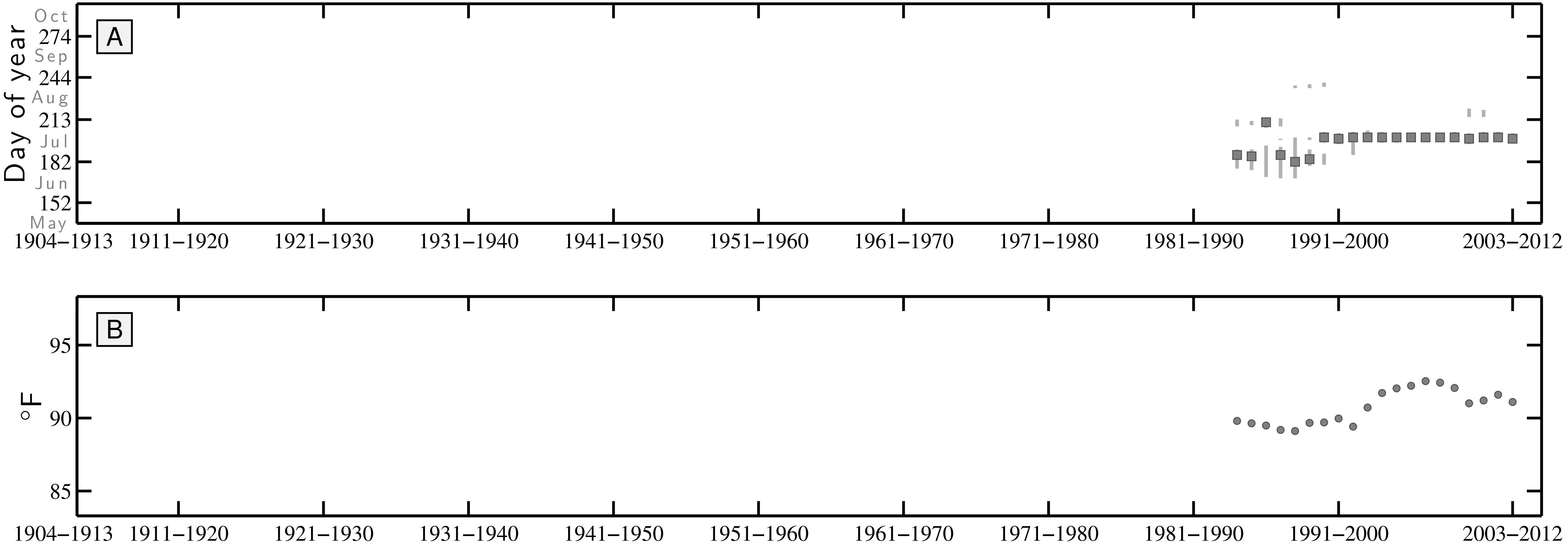

10 year Winter Teletherm dynamics for BEATRICE 1N, NE:

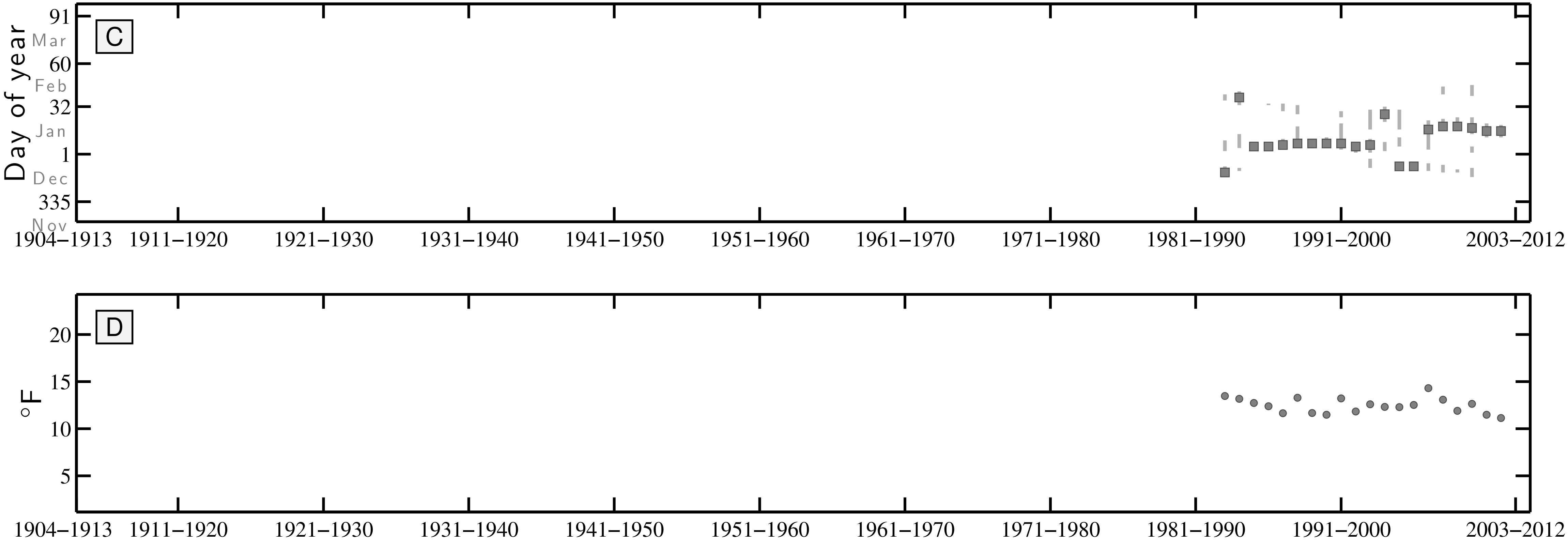

10 year Summer Teletherm dynamics for BEAVER CITY, NE:

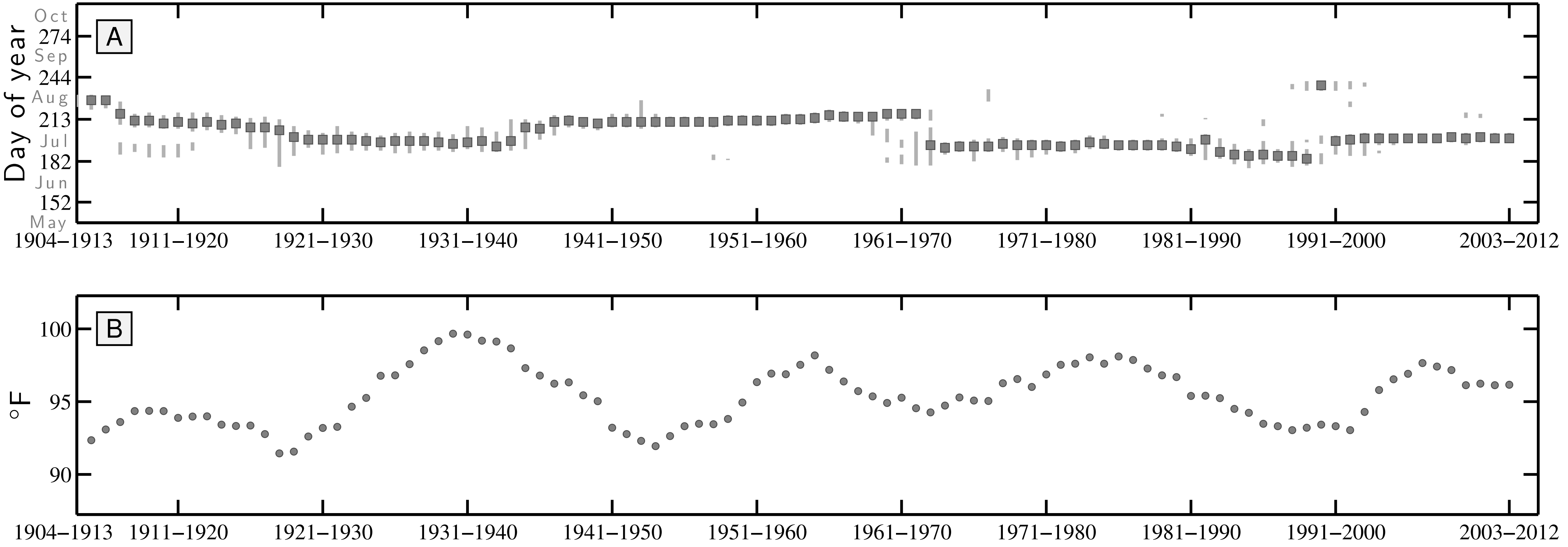

10 year Winter Teletherm dynamics for BEAVER CITY, NE:

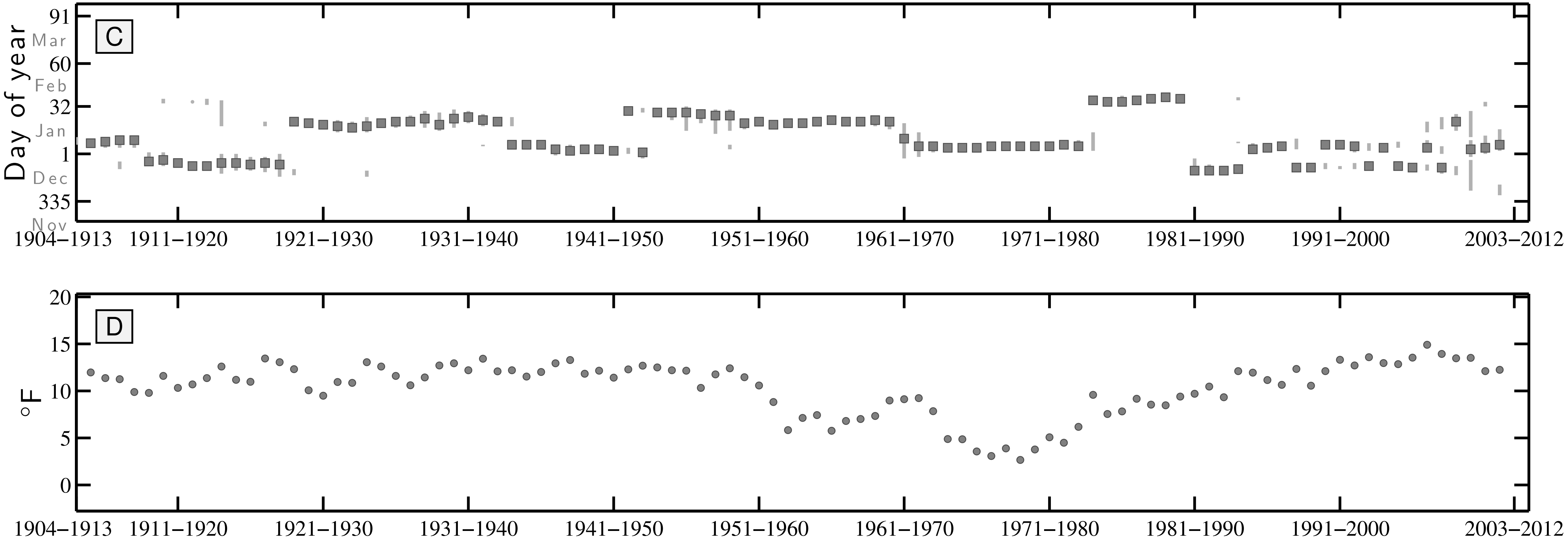

10 year Summer Teletherm dynamics for BRIDGEPORT, NE:

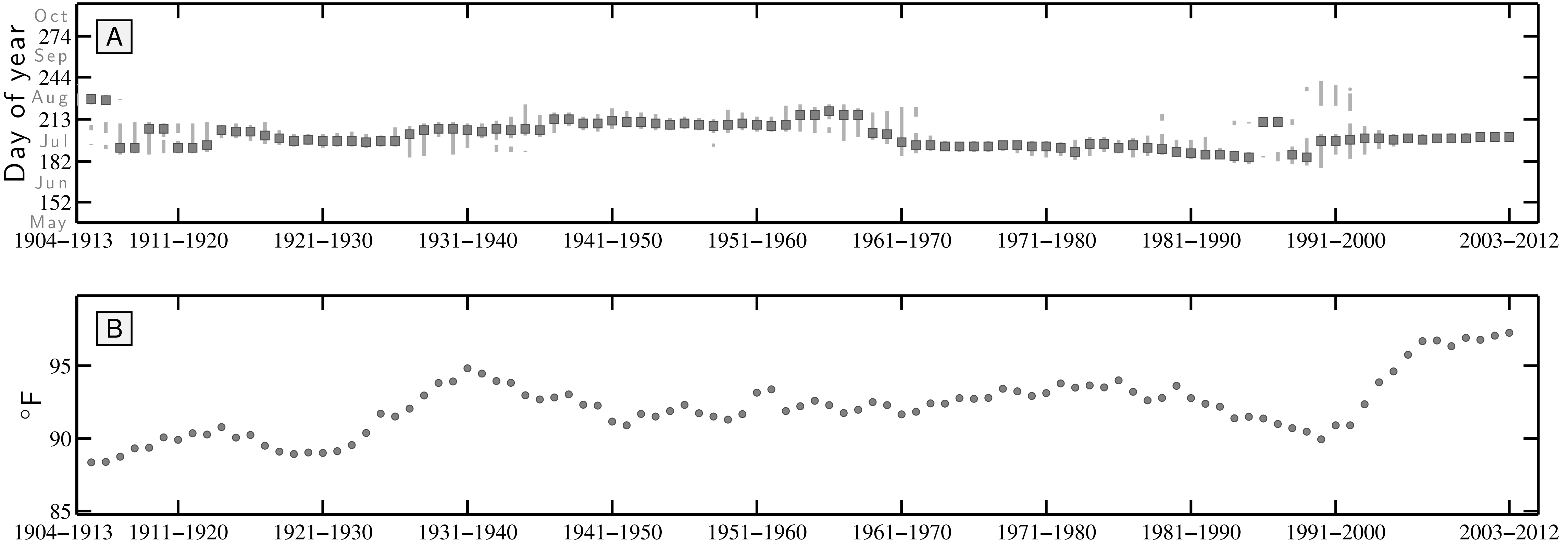

10 year Winter Teletherm dynamics for BRIDGEPORT, NE:

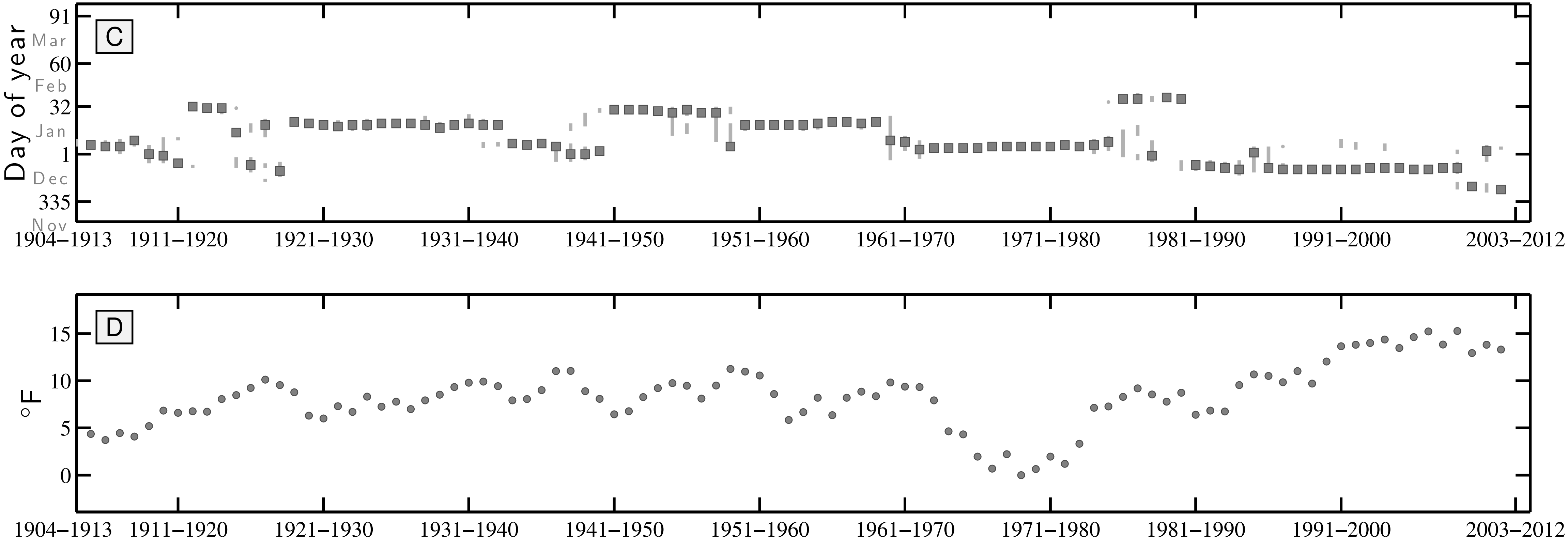

10 year Summer Teletherm dynamics for BROKEN BOW 2 W, NE:

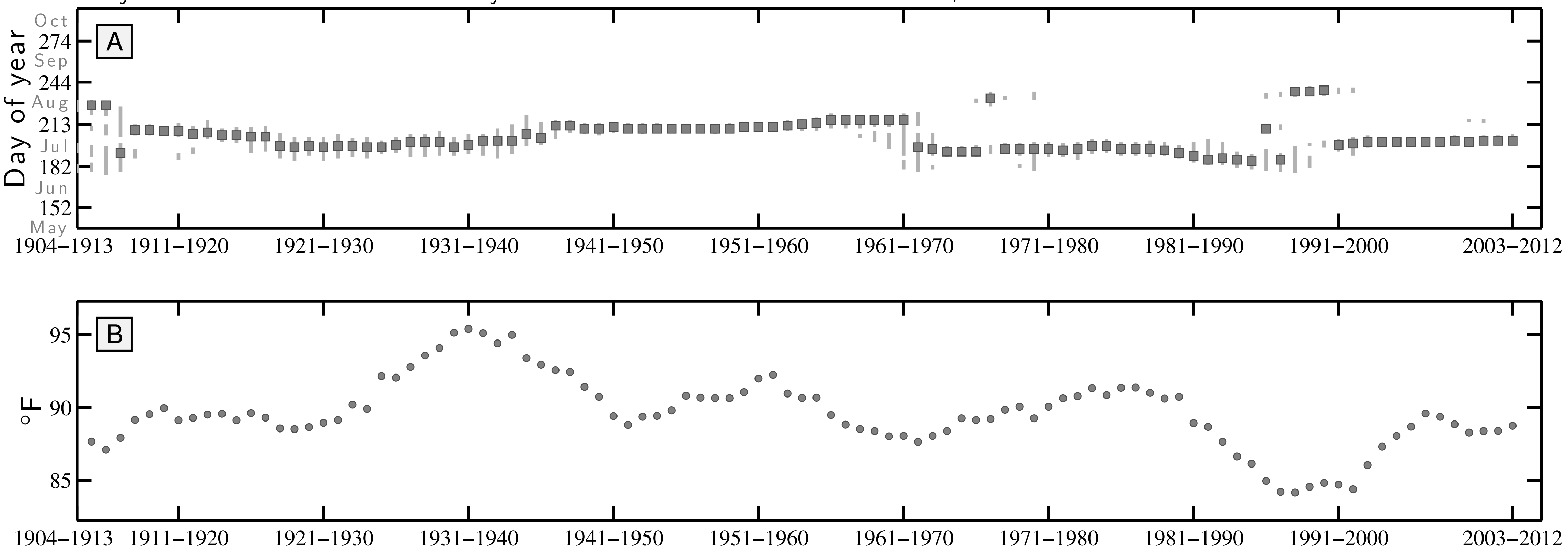

10 year Winter Teletherm dynamics for BROKEN BOW 2 W, NE:

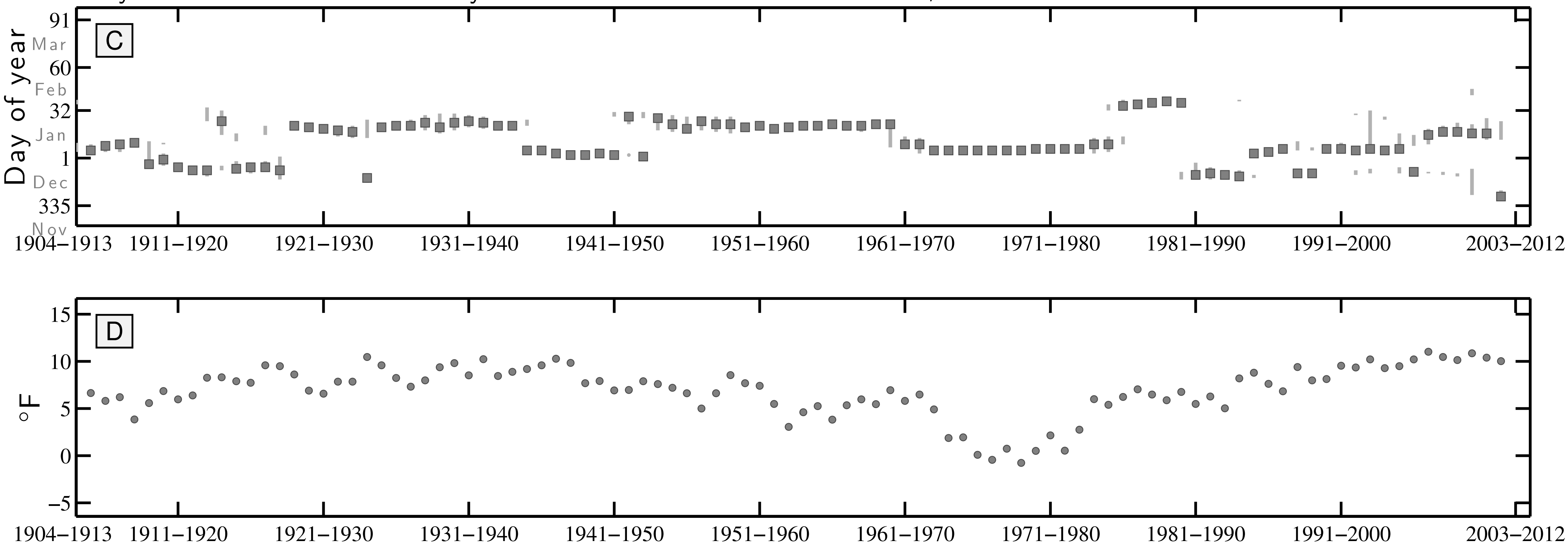

10 year Summer Teletherm dynamics for CRETE, NE:

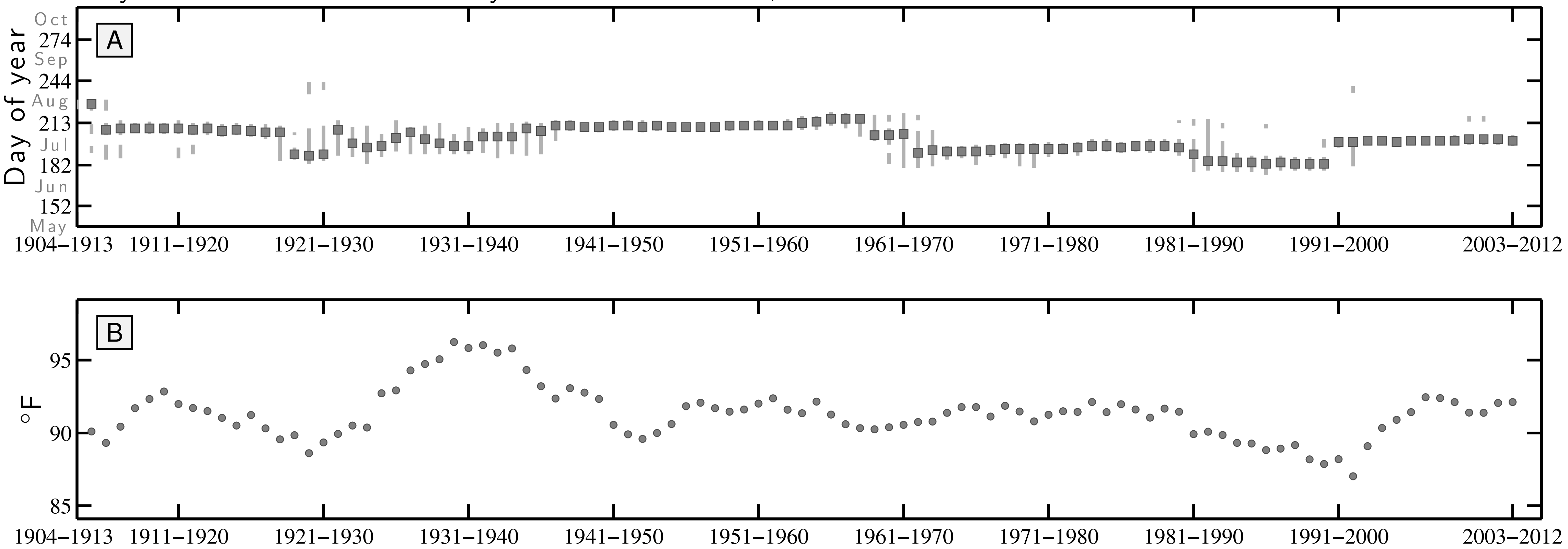

10 year Winter Teletherm dynamics for CRETE, NE:

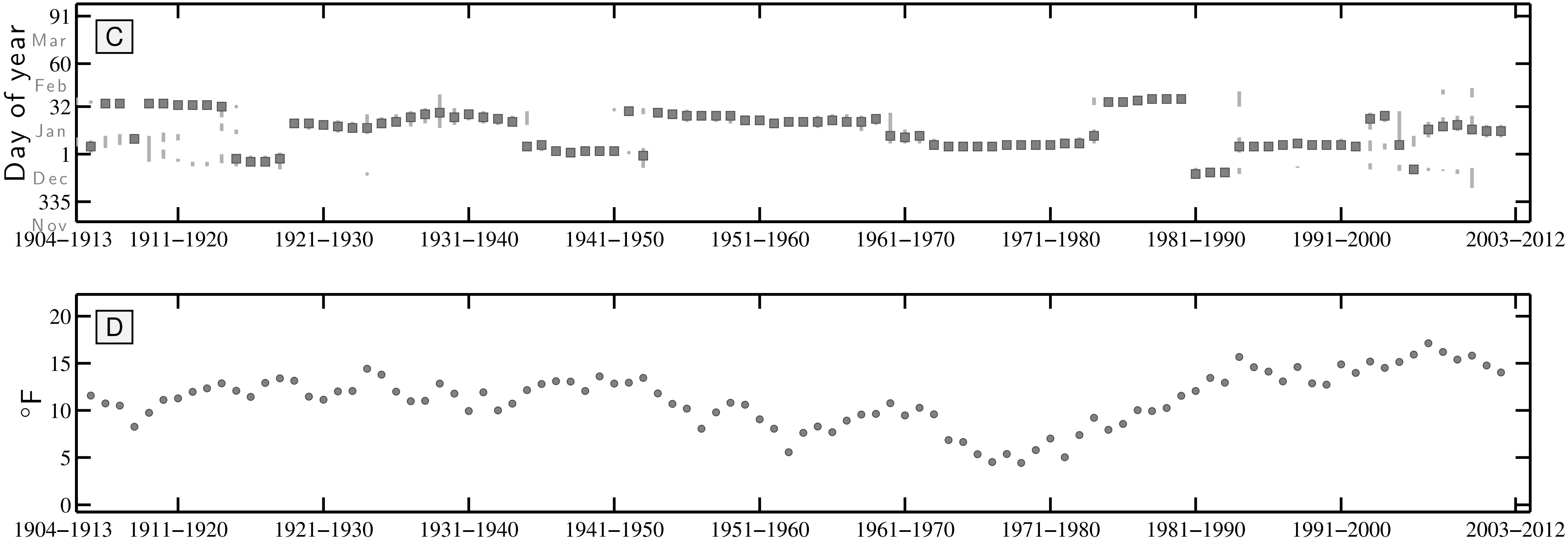

10 year Summer Teletherm dynamics for CURTIS 3NNE, NE:

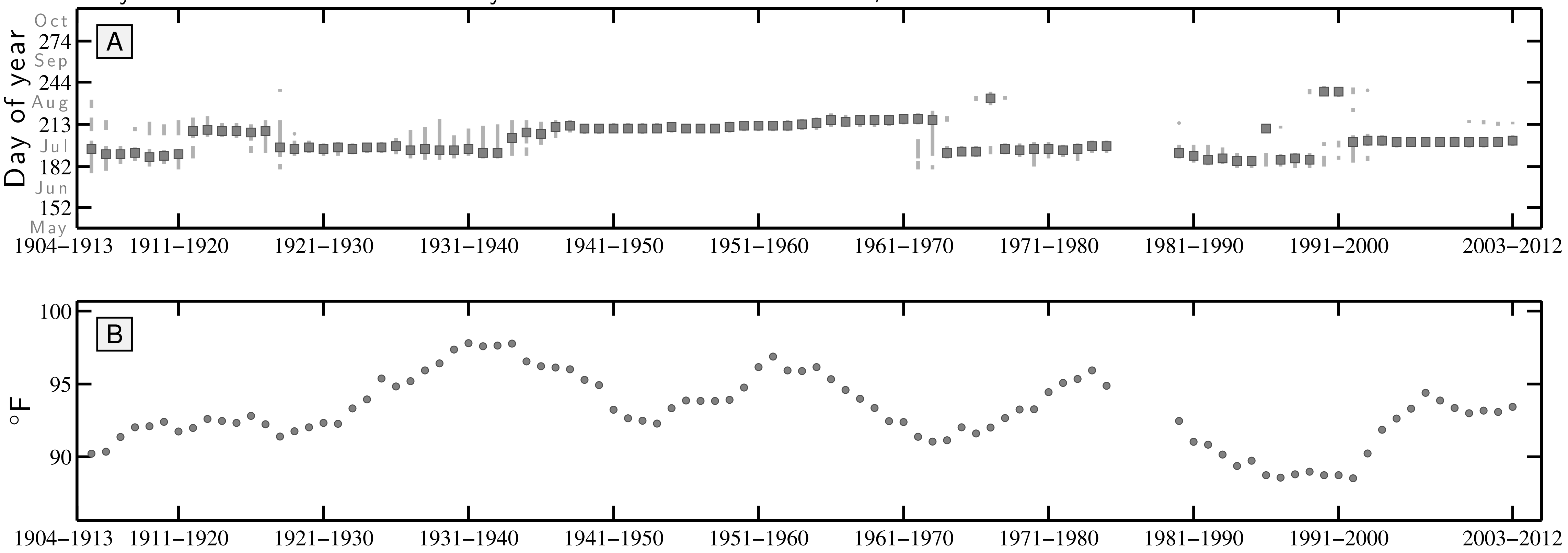

10 year Winter Teletherm dynamics for CURTIS 3NNE, NE:

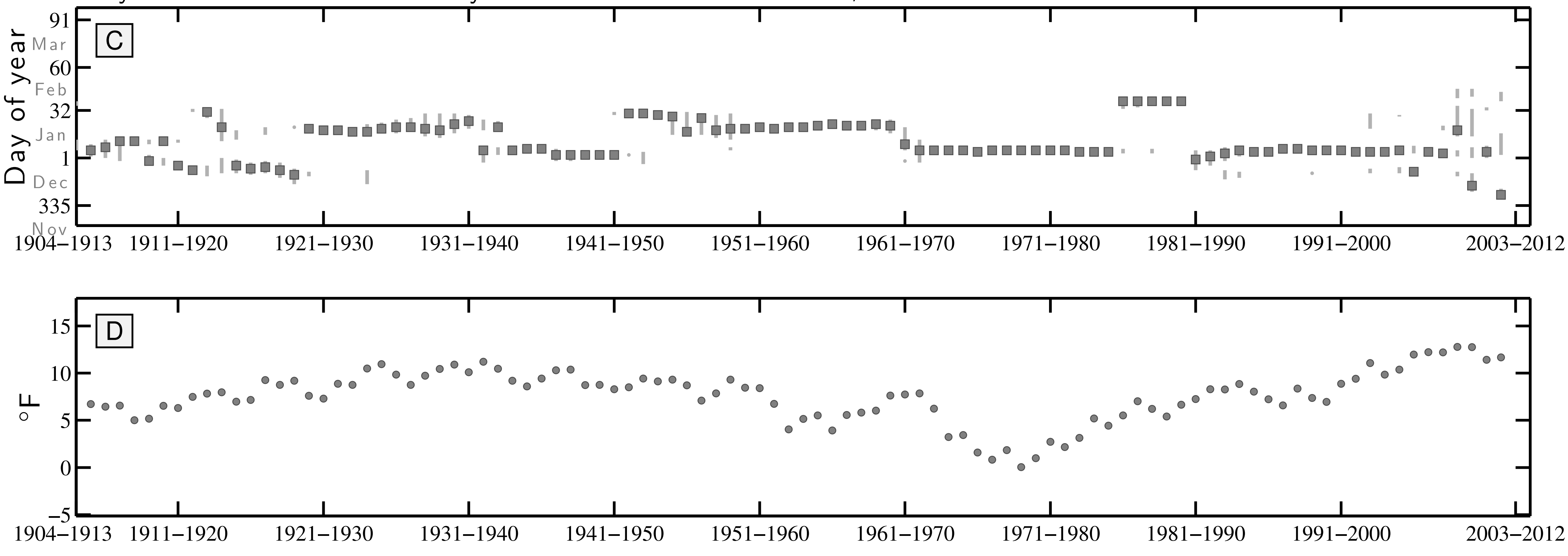

10 year Summer Teletherm dynamics for DAVID CITY, NE:

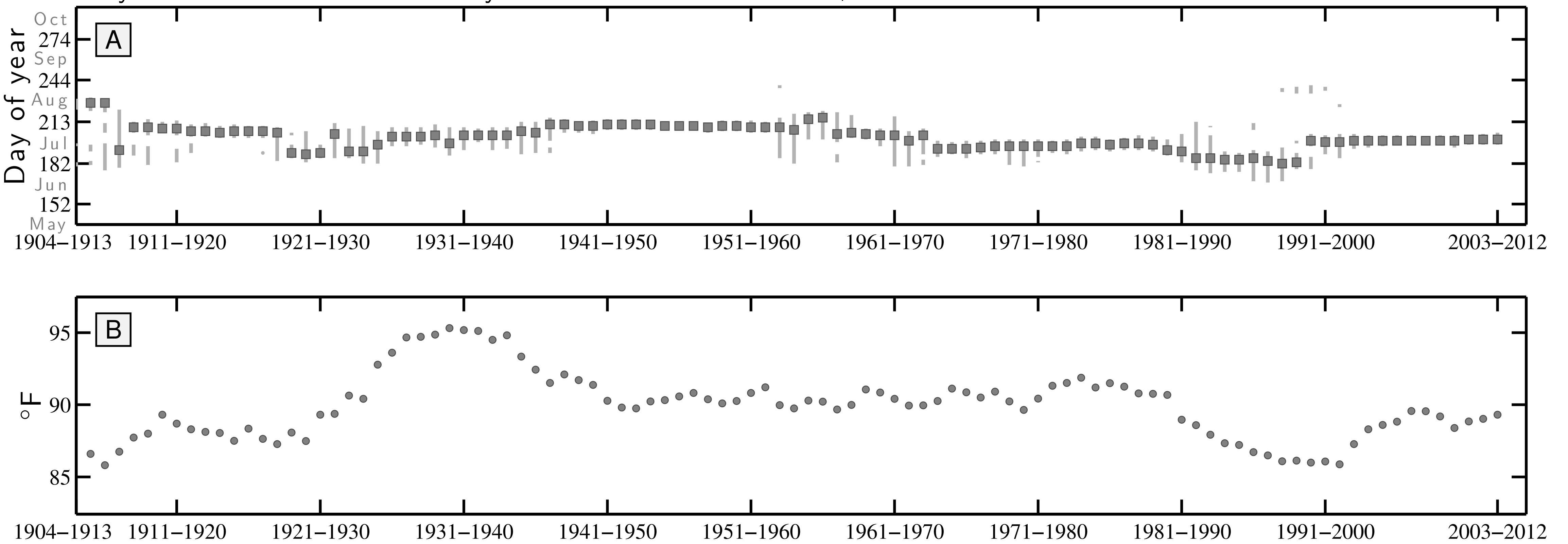

10 year Winter Teletherm dynamics for DAVID CITY, NE:

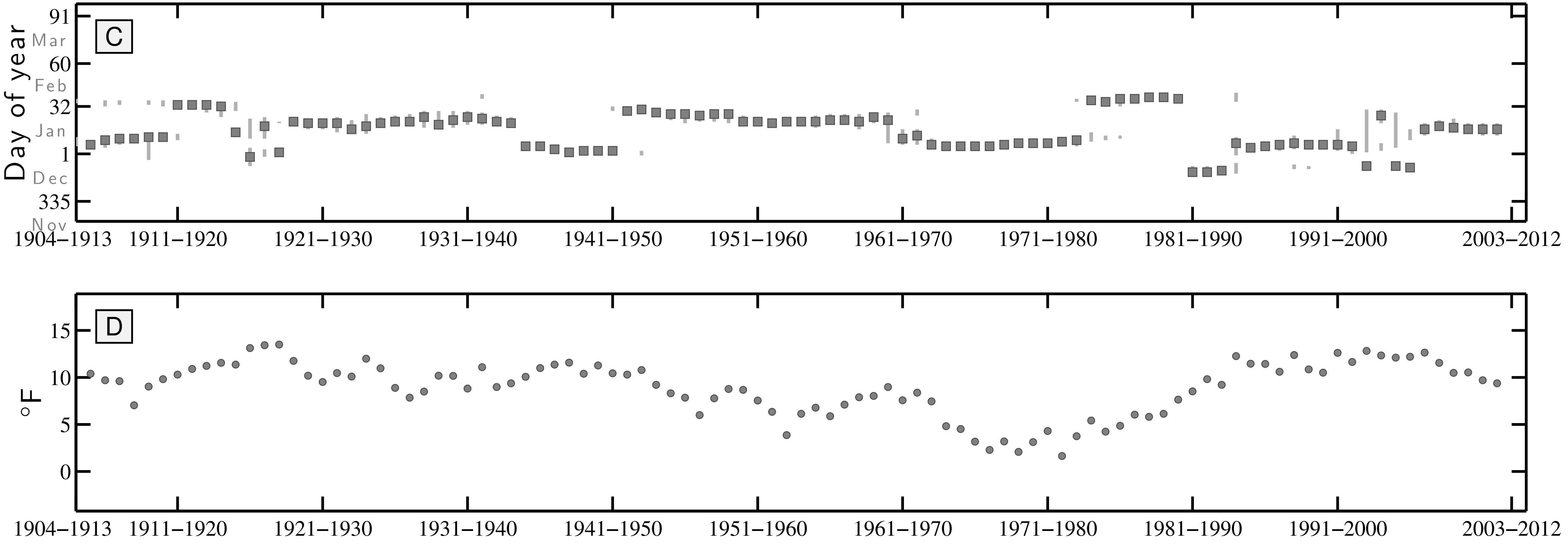

10 year Summer Teletherm dynamics for FAIRBURY 5S, NE:

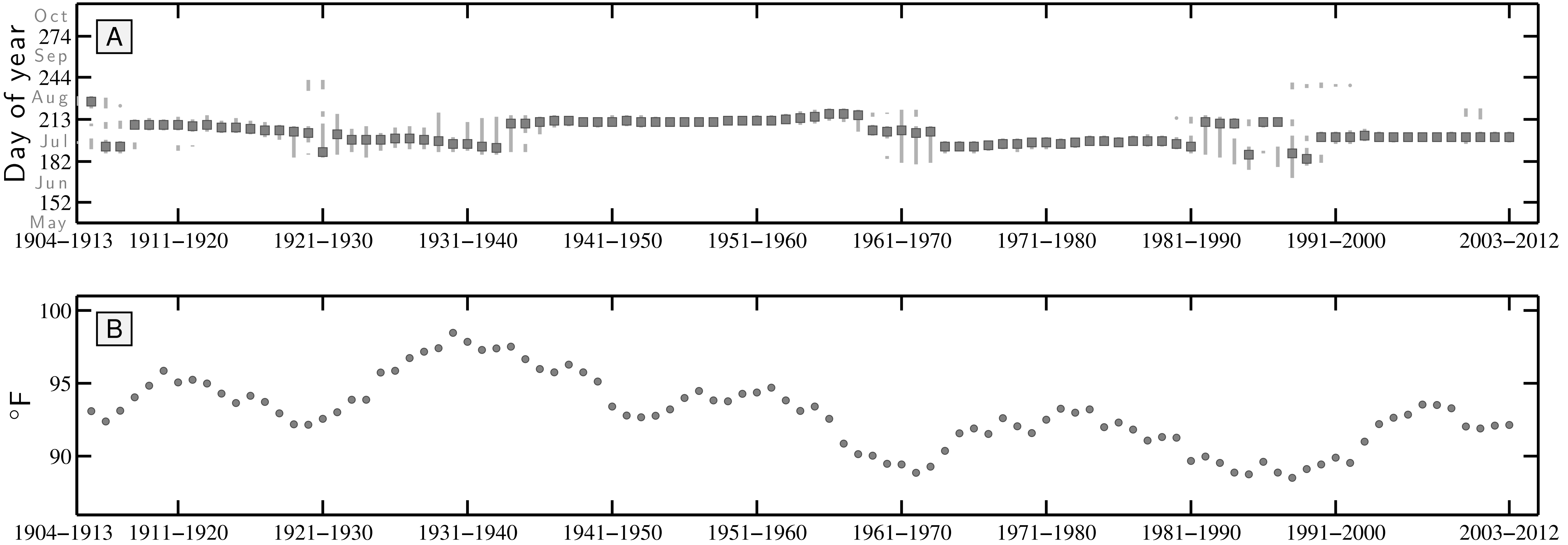

10 year Winter Teletherm dynamics for FAIRBURY 5S, NE:

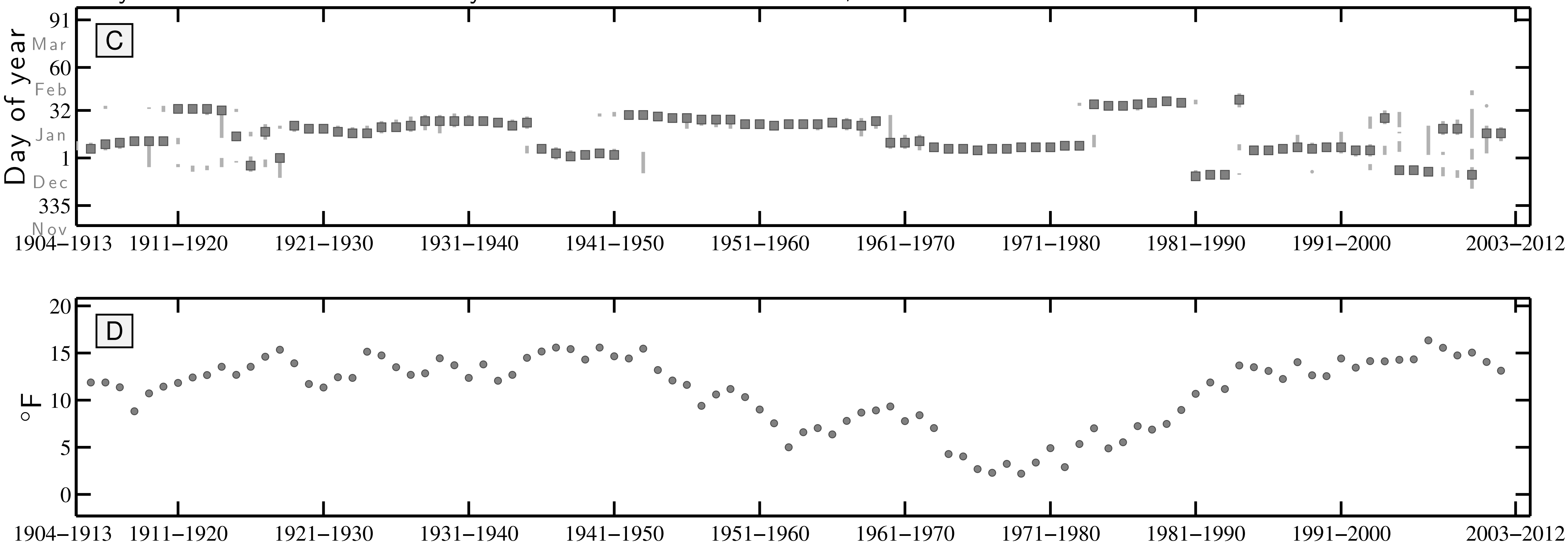

10 year Summer Teletherm dynamics for FAIRMONT, NE:

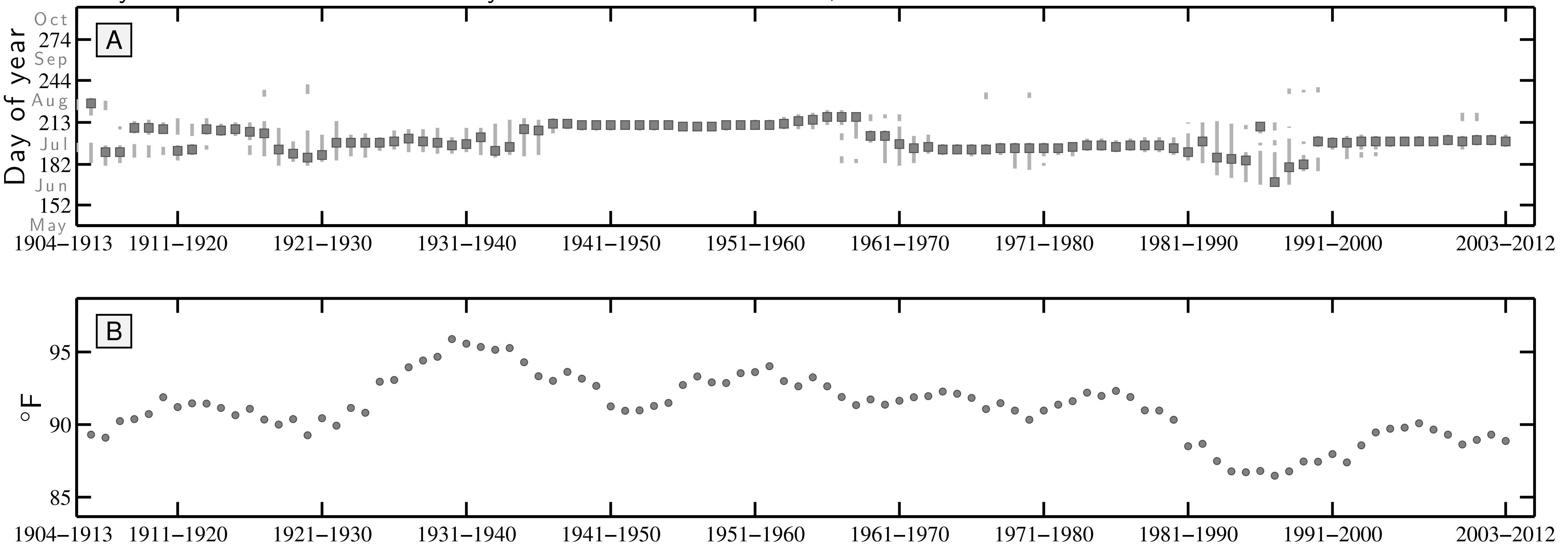

10 year Winter Teletherm dynamics for FAIRMONT, NE:

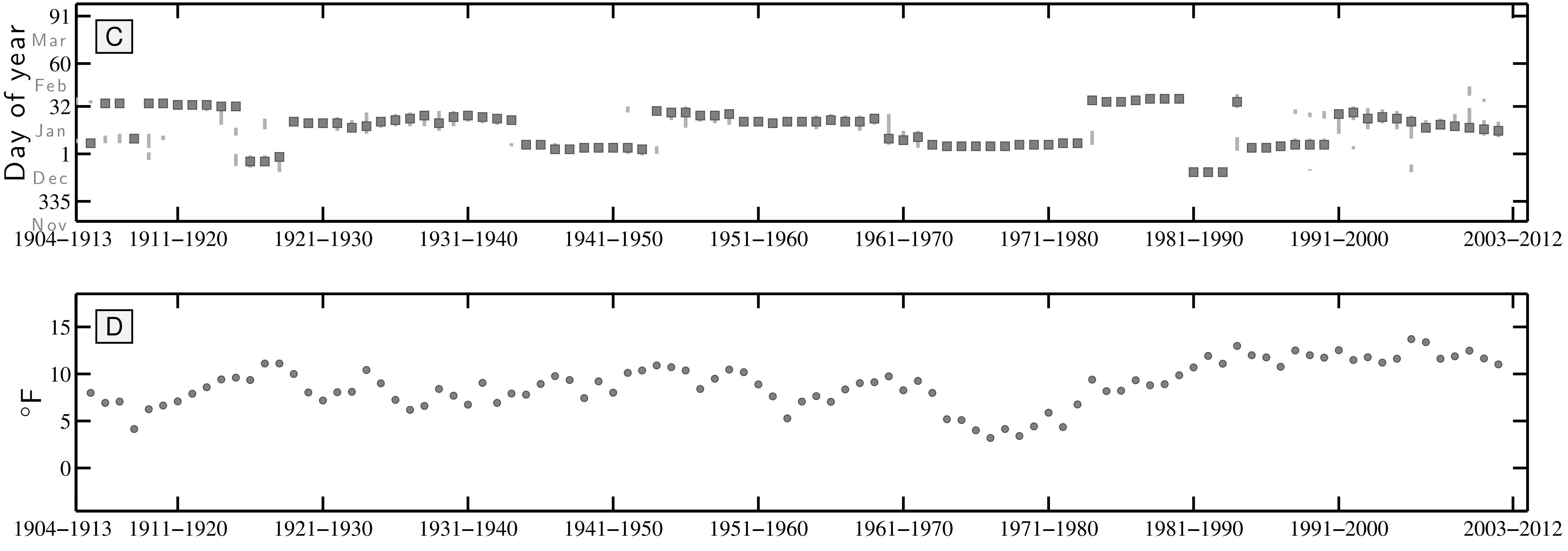

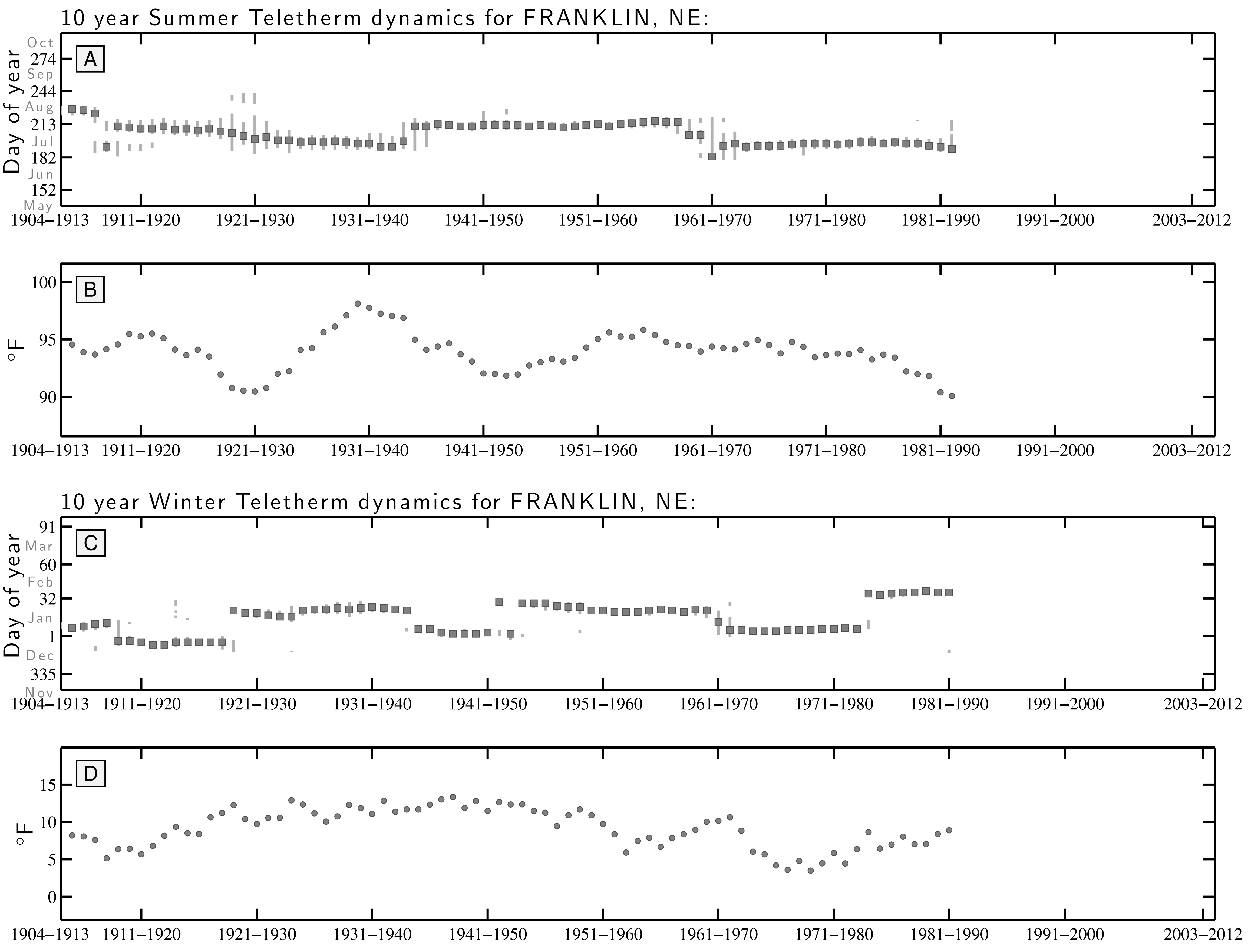

10 year Summer Teletherm dynamics for FRANKLIN, NE:
A
Day of year
Oct
274
Sep
244
Aug
213
Jul
182
Jun
152
May
B
°F
100
95
90
1904–1913
1911–1920
1921–1930
1931–1940
1941–1950
1951–1960
1961–1970
1971–1980
1981–1990
1991–2000
2003–2012
10 year Winter Teletherm dynamics for FRANKLIN, NE:
C
Day of year
91
Mar
60
Feb
32
Jan
1
Dec
335
Nov
D
°F
15
10
5
0

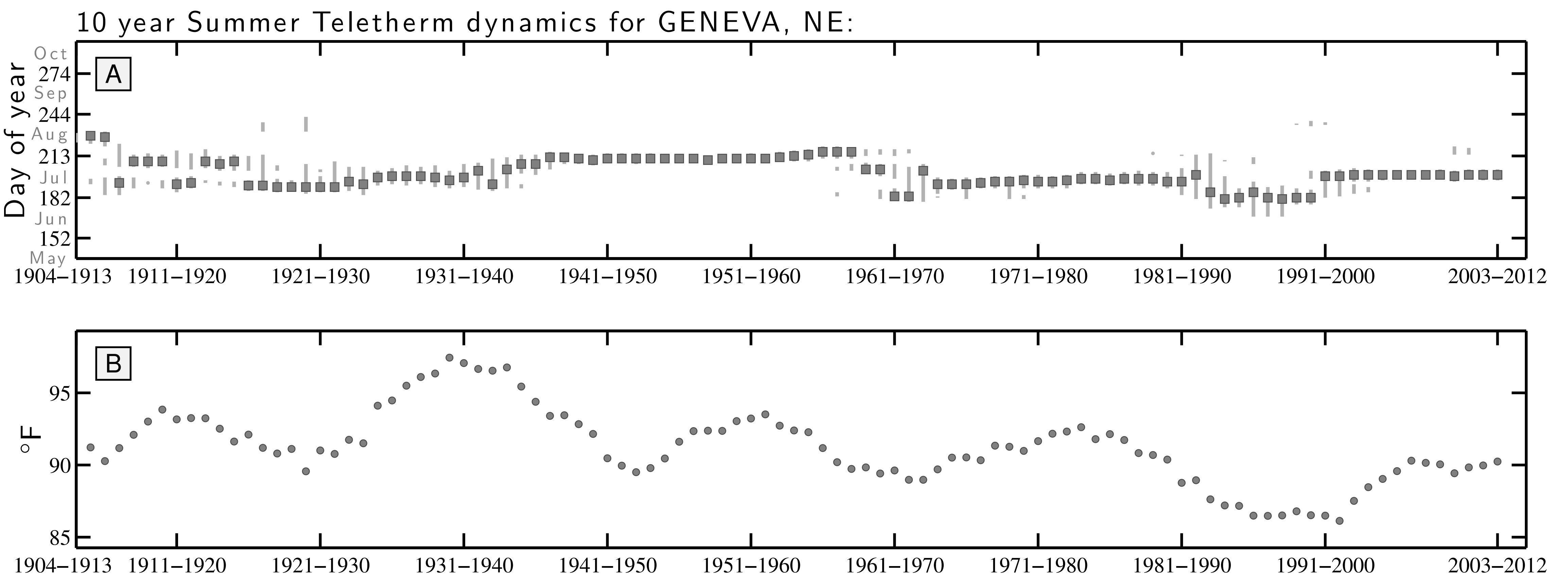
10 year Summer Teletherm dynamics for GENEVA, NE:
A
B
Day of year
°F
Oct
274
Sep
244
Aug
213
Jul
182
Jun
152
May
95
90
85
1904–1913
1911–1920
1921–1930
1931–1940
1941–1950
1951–1960
1961–1970
1971–1980
1981–1990
1991–2000
2003–2012

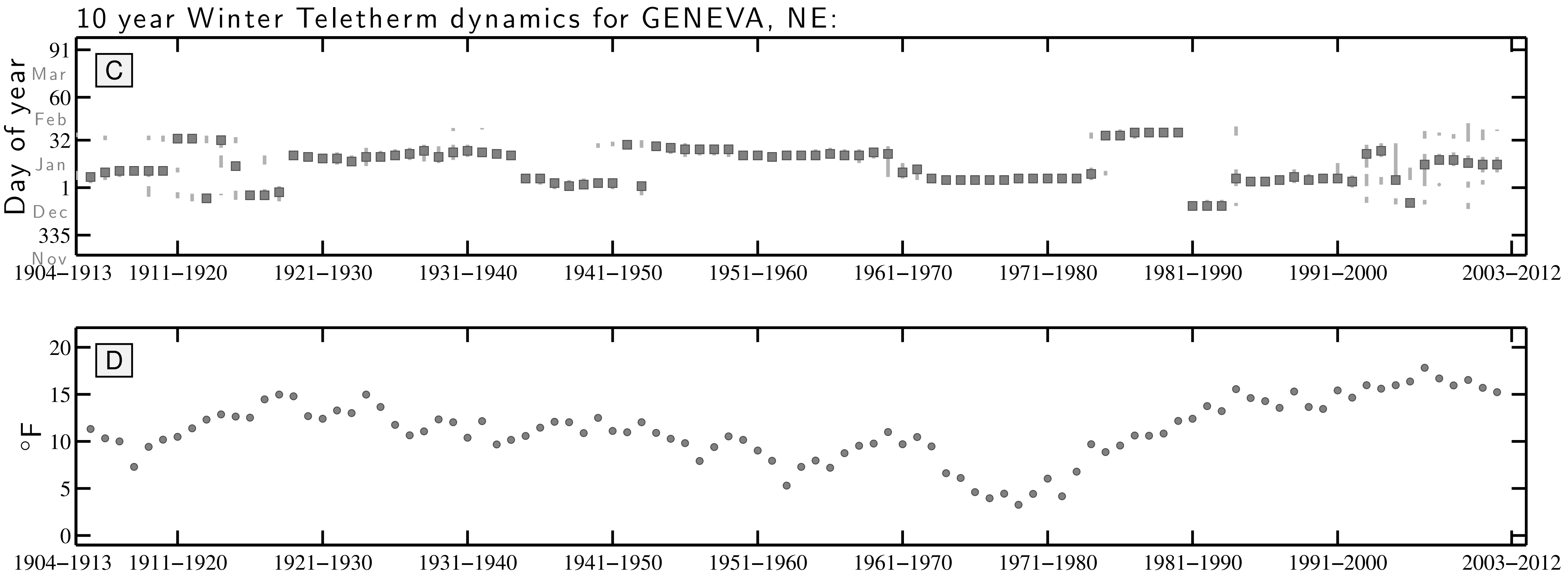
10 year Winter Teletherm dynamics for GENEVA, NE:
C
D
Day of year
°F
91
Mar
60
Feb
32
Jan
1
Dec
335
Nov
20
15
10
5
0
1904–1913
1911–1920
1921–1930
1931–1940
1941–1950
1951–1960
1961–1970
1971–1980
1981–1990
1991–2000
2003–2012

10 year Summer Teletherm dynamics for GENOA 2 W, NE:

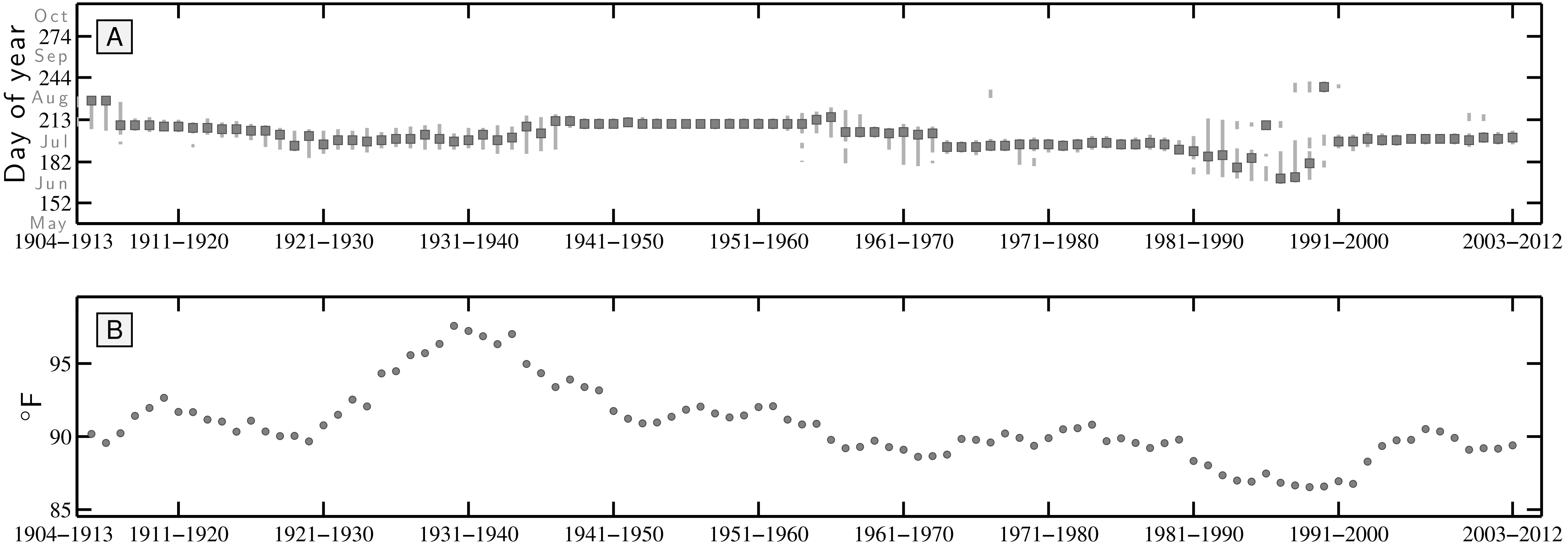

10 year Winter Teletherm dynamics for GENOA 2 W, NE:

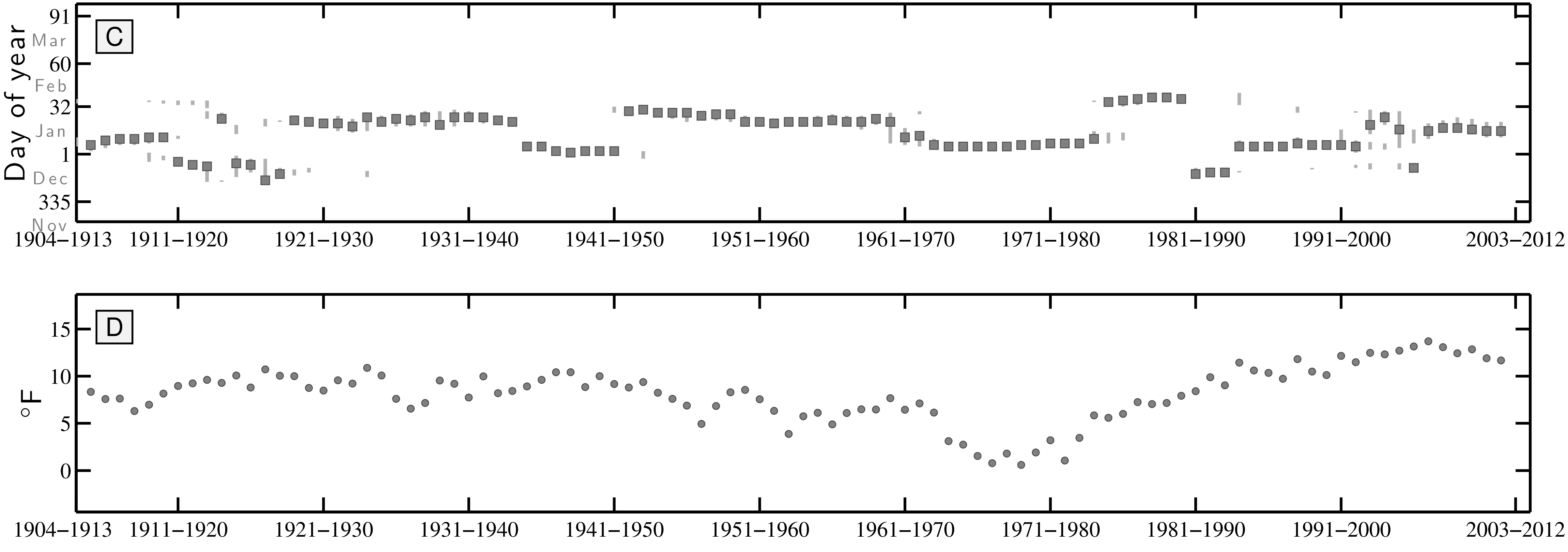

10 year Summer Teletherm dynamics for GOTHENBURG, NE:

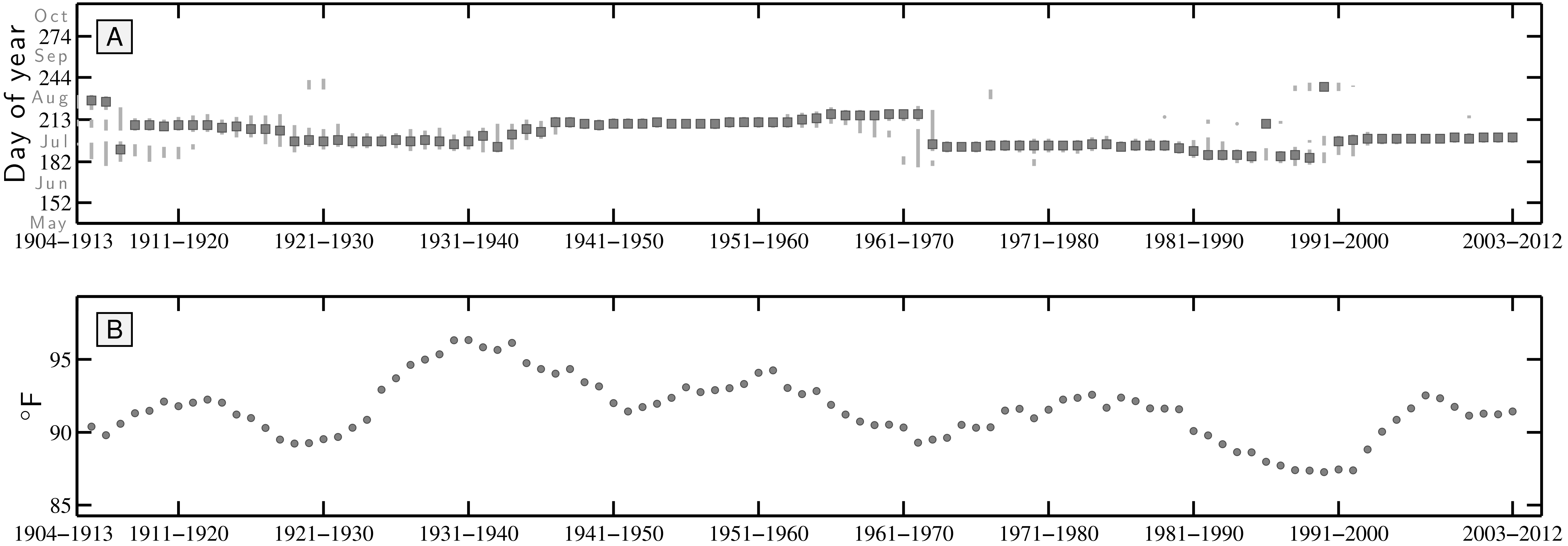

10 year Winter Teletherm dynamics for GOTHENBURG, NE:

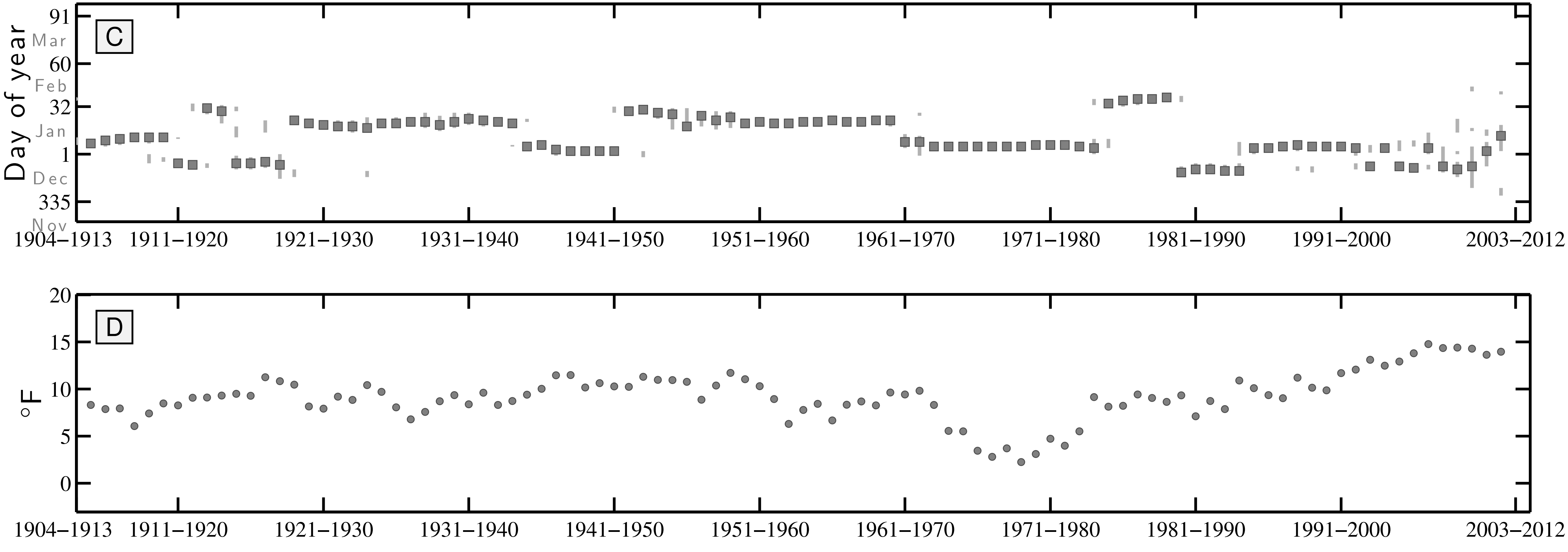

10 year Summer Teletherm dynamics for HARRISON, NE:

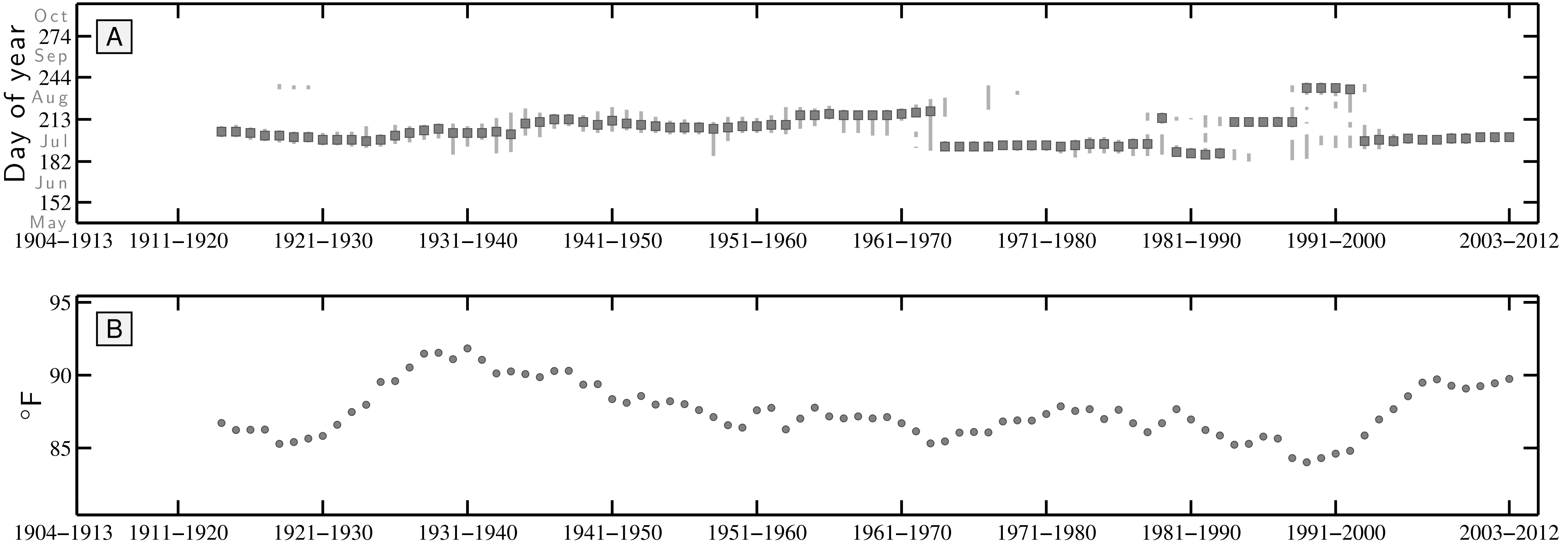

10 year Winter Teletherm dynamics for HARRISON, NE:

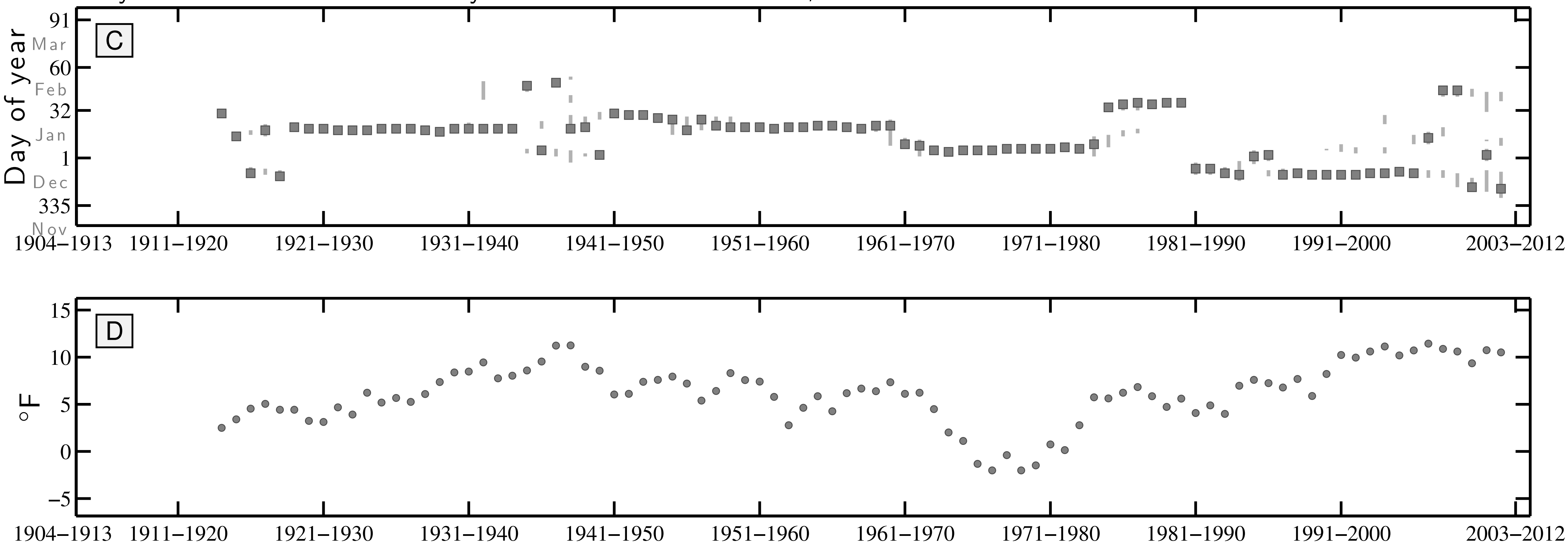

10 year Summer Teletherm dynamics for HARTINGTON, NE:

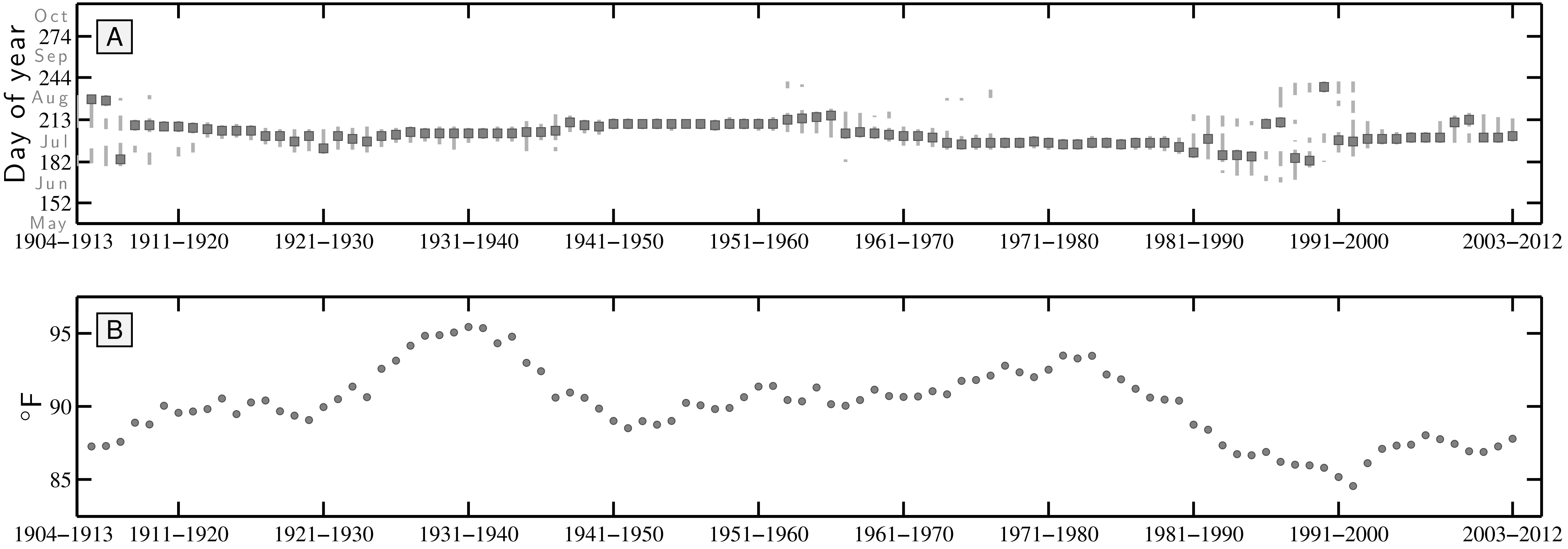

10 year Winter Teletherm dynamics for HARTINGTON, NE:

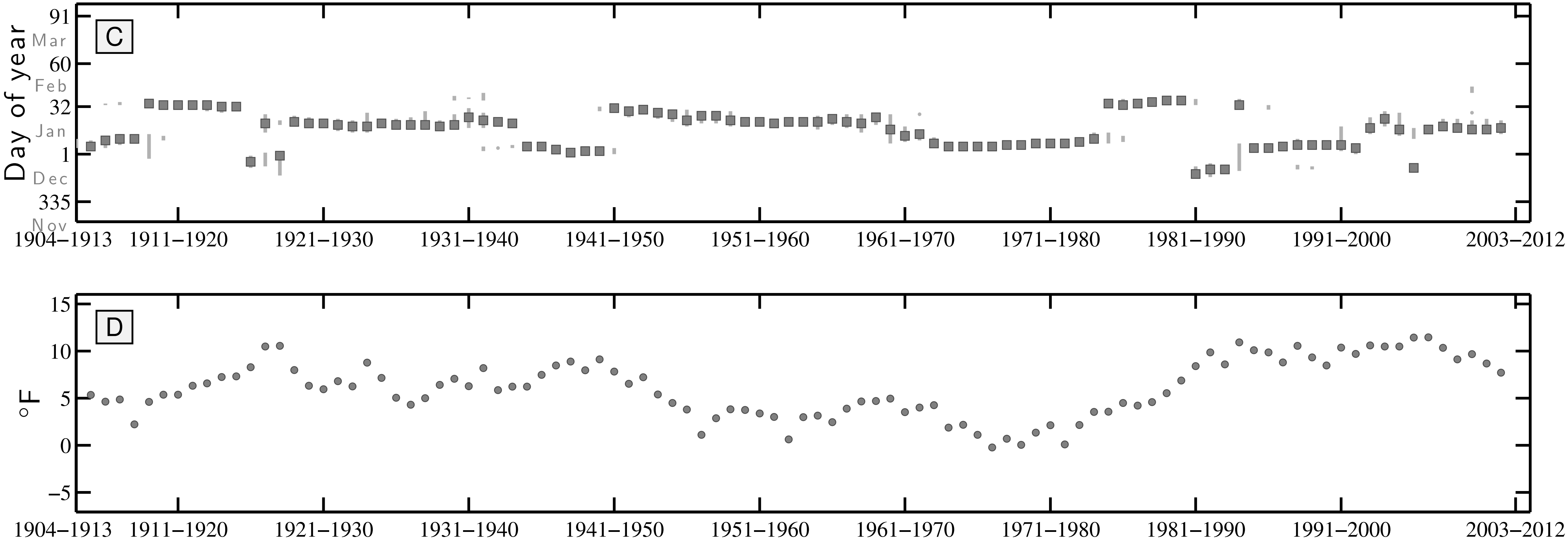

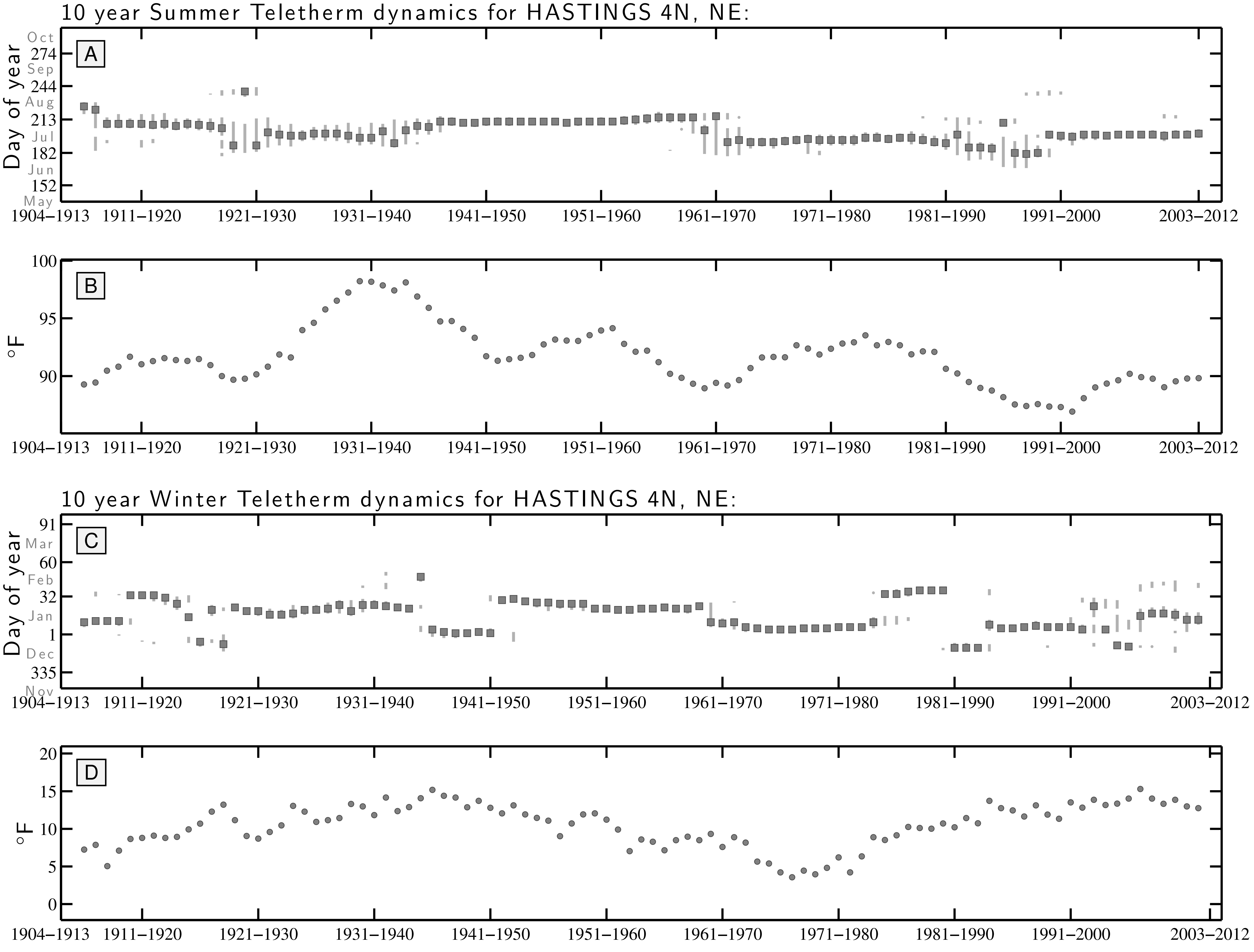

10 year Summer Teletherm dynamics for HASTINGS 4N, NE:
A
Day of year
Oct
274
Sep
244
Aug
213
Jul
182
Jun
152
May
1904–1913
1911–1920
1921–1930
1931–1940
1941–1950
1951–1960
1961–1970
1971–1980
1981–1990
1991–2000
2003–2012
B
°F
100
95
90
10 year Winter Teletherm dynamics for HASTINGS 4N, NE:
C
91
Mar
60
Feb
32
Jan
1
Dec
335
Nov
D
20
15
10
5
0

10 year Summer Teletherm dynamics for HAY SPRINGS 12 S, NE:

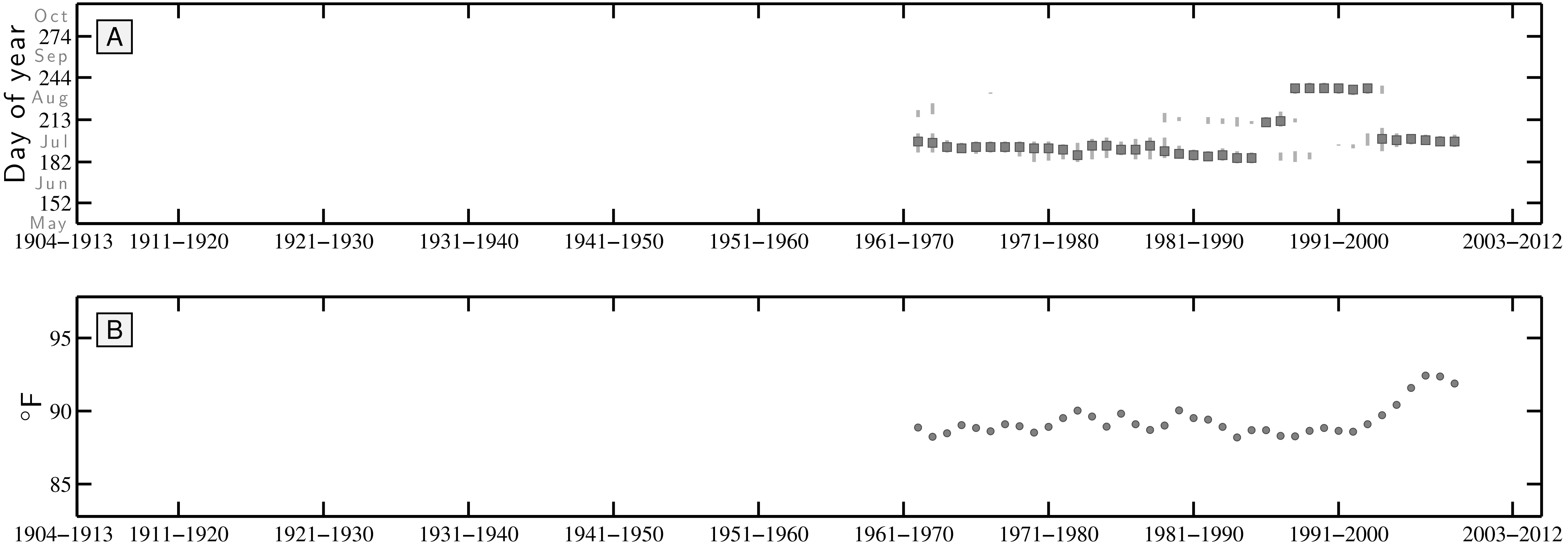

10 year Winter Teletherm dynamics for HAY SPRINGS 12 S, NE:

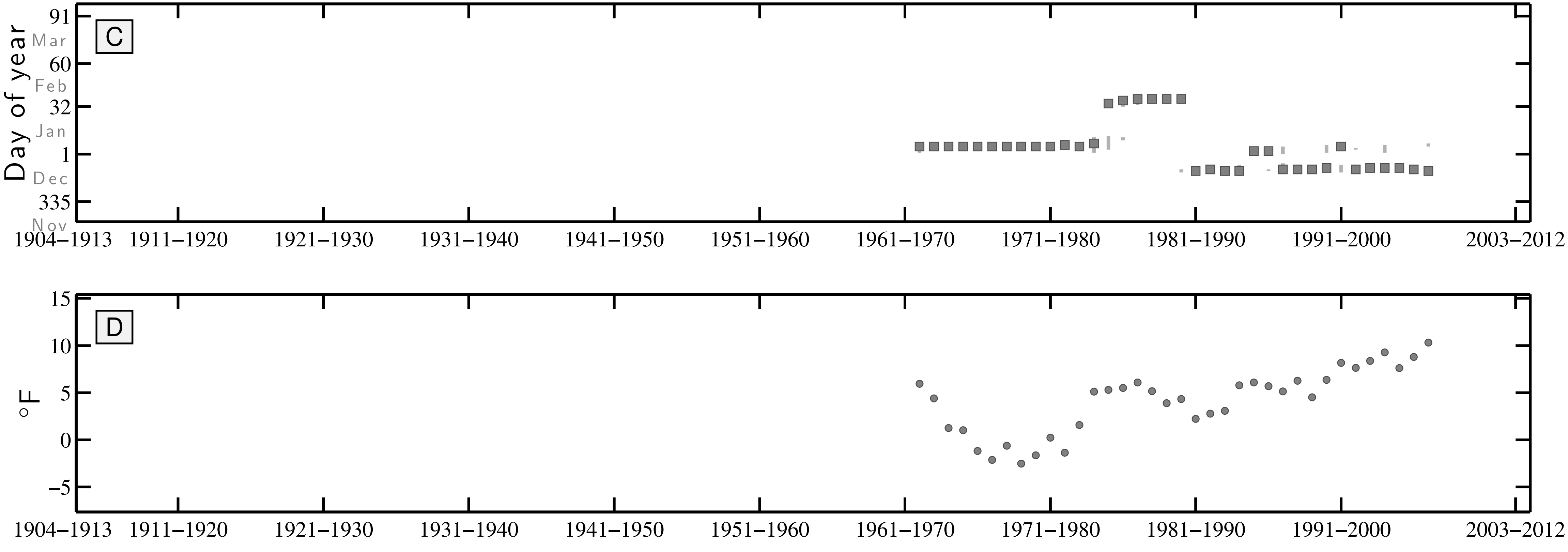

10 year Summer Teletherm dynamics for HEBRON, NE:

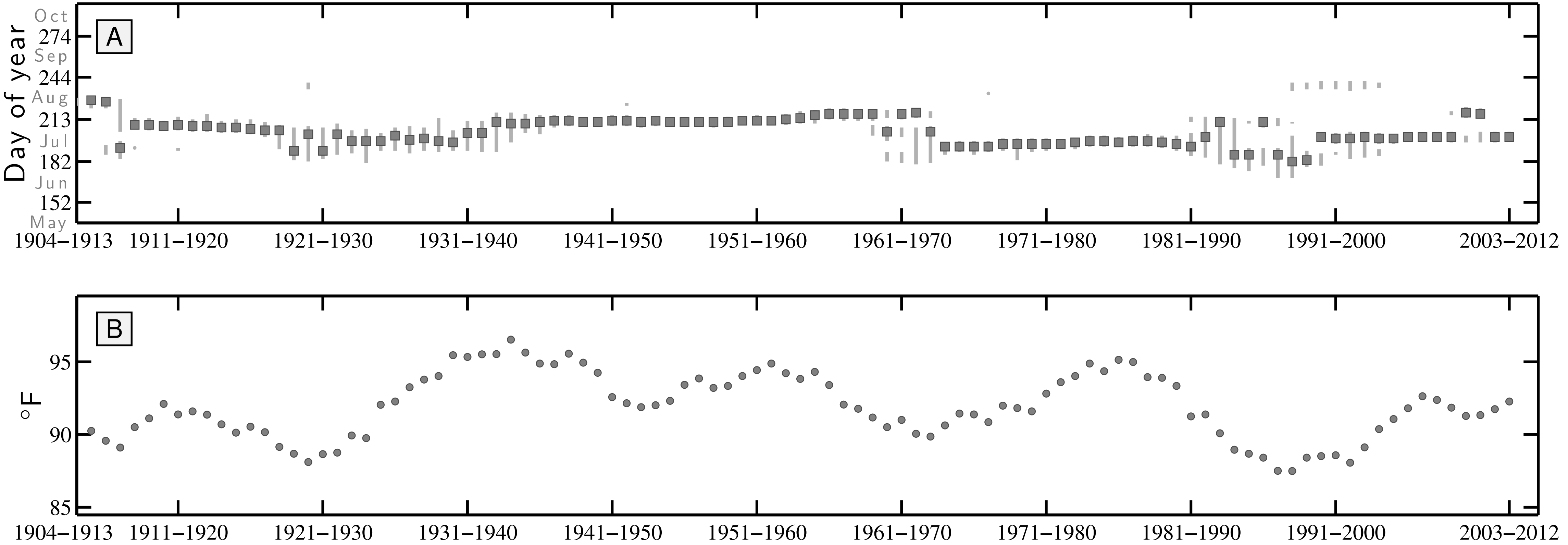

10 year Winter Teletherm dynamics for HEBRON, NE:

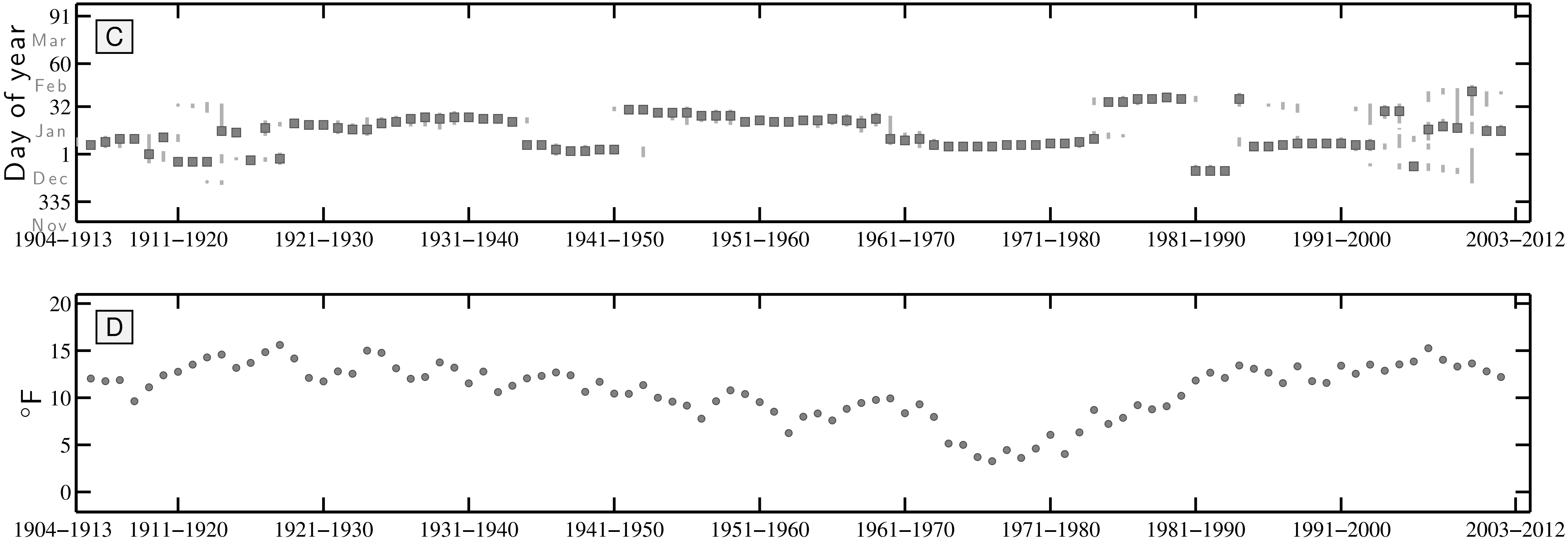

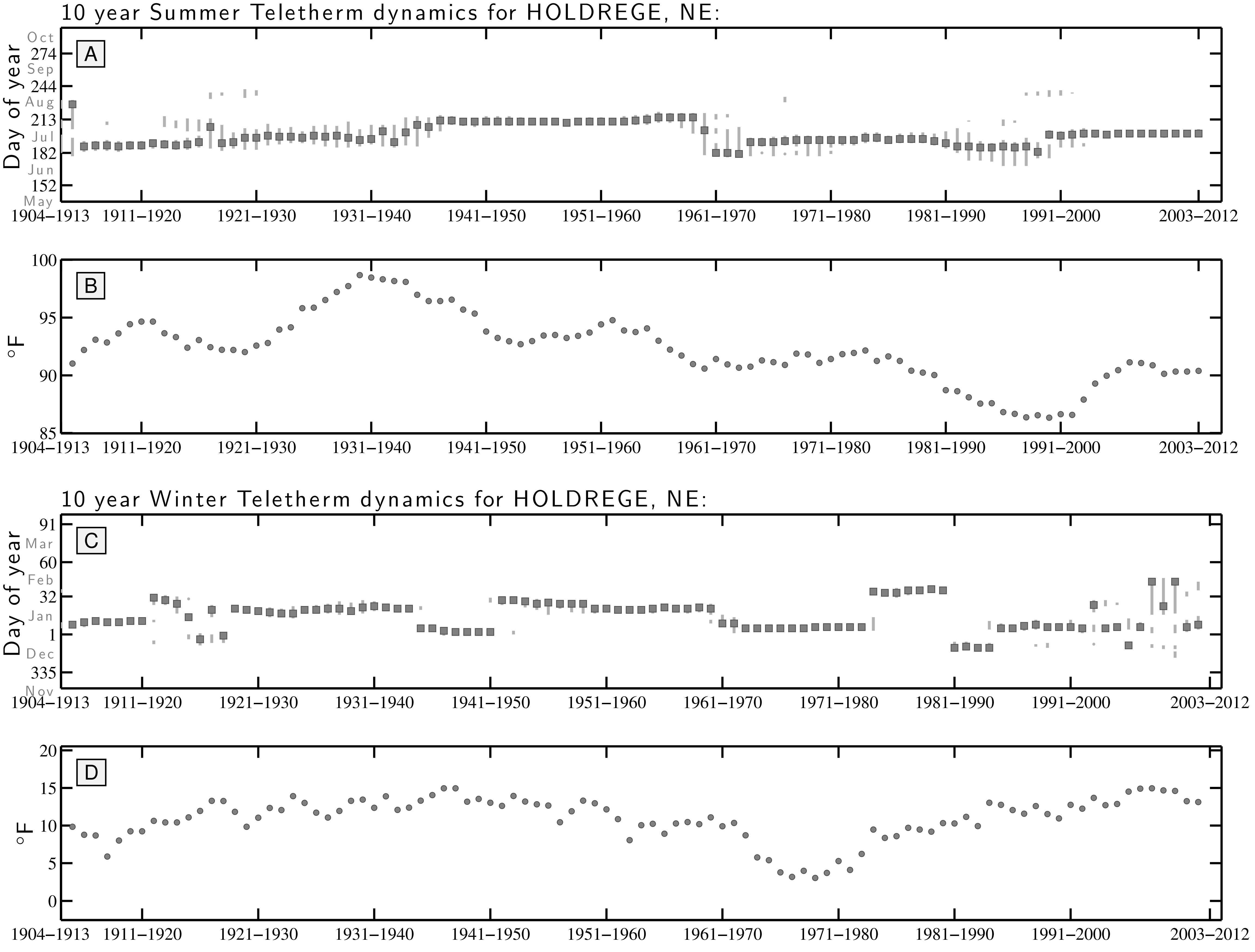
10 year Summer Teletherm dynamics for HOLDREGE, NE:
A
Day of year
May
152
Jun
182
Jul
213
Aug
244
Sep
274
Oct
B
°F
85
90
95
100
10 year Winter Teletherm dynamics for HOLDREGE, NE:
C
Day of year
Nov
335
Dec
1
Jan
32
Feb
60
Mar
91
D
°F
0
5
10
15
20
1904–1913
1911–1920
1921–1930
1931–1940
1941–1950
1951–1960
1961–1970
1971–1980
1981–1990
1991–2000
2003–2012

10 year Summer Teletherm dynamics for IMPERIAL, NE:

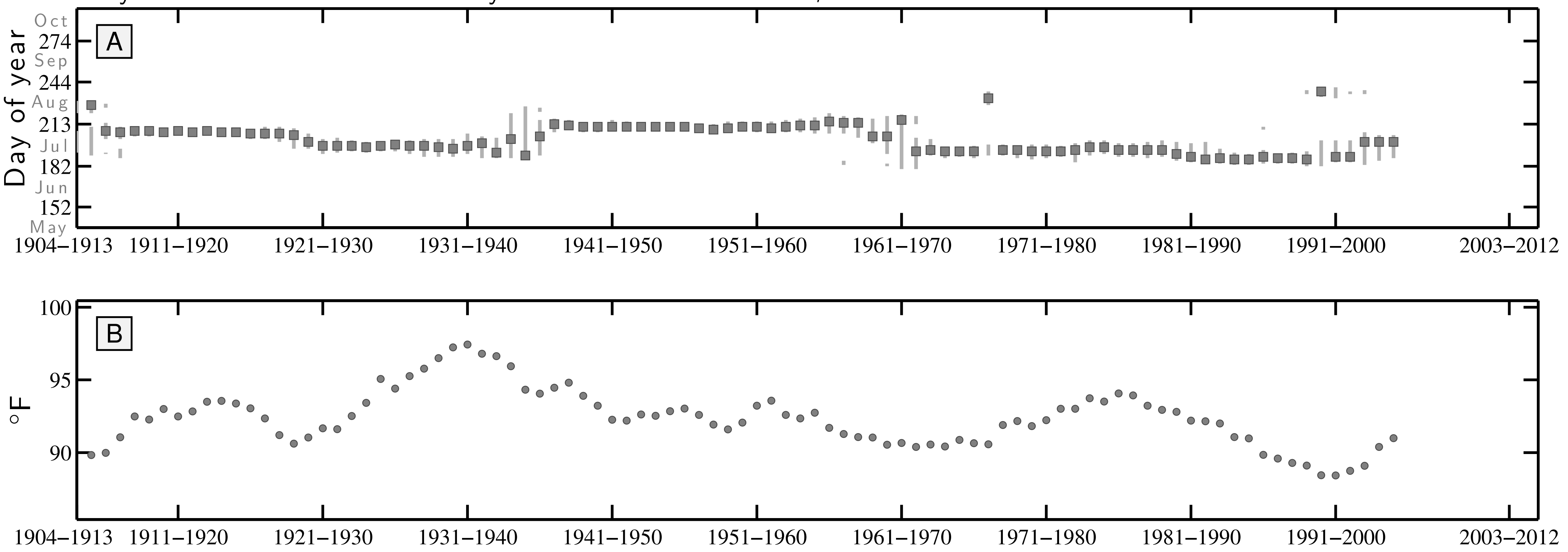

10 year Winter Teletherm dynamics for IMPERIAL, NE:

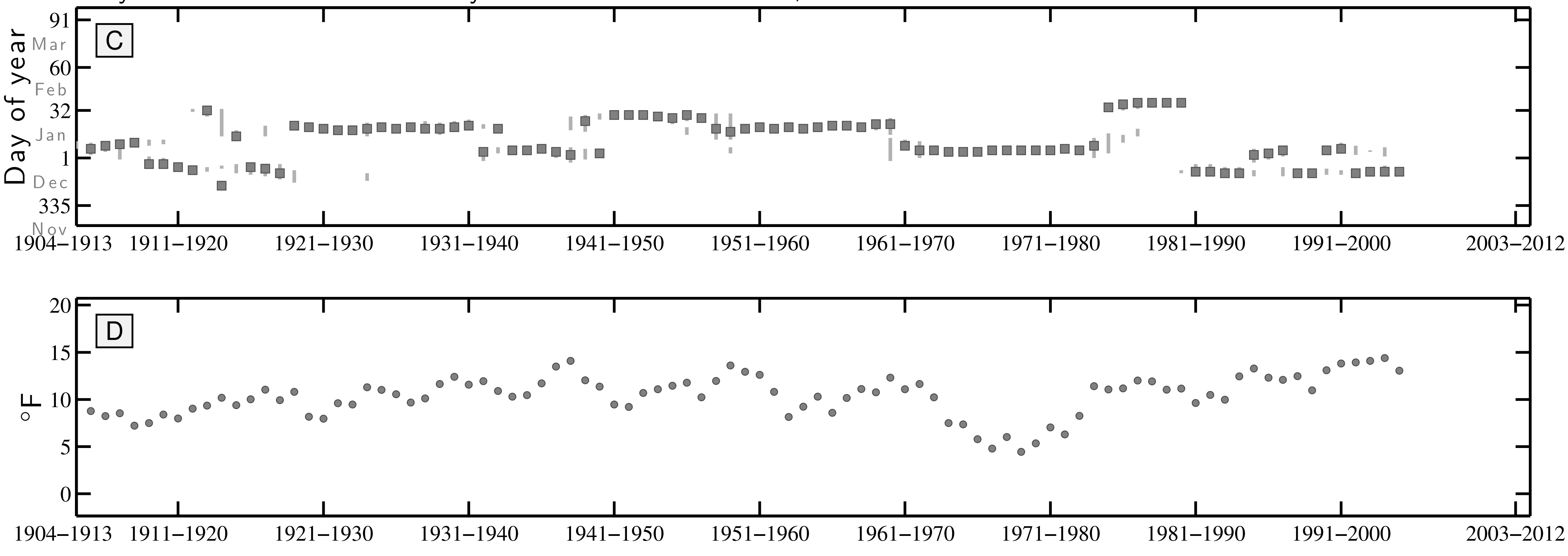

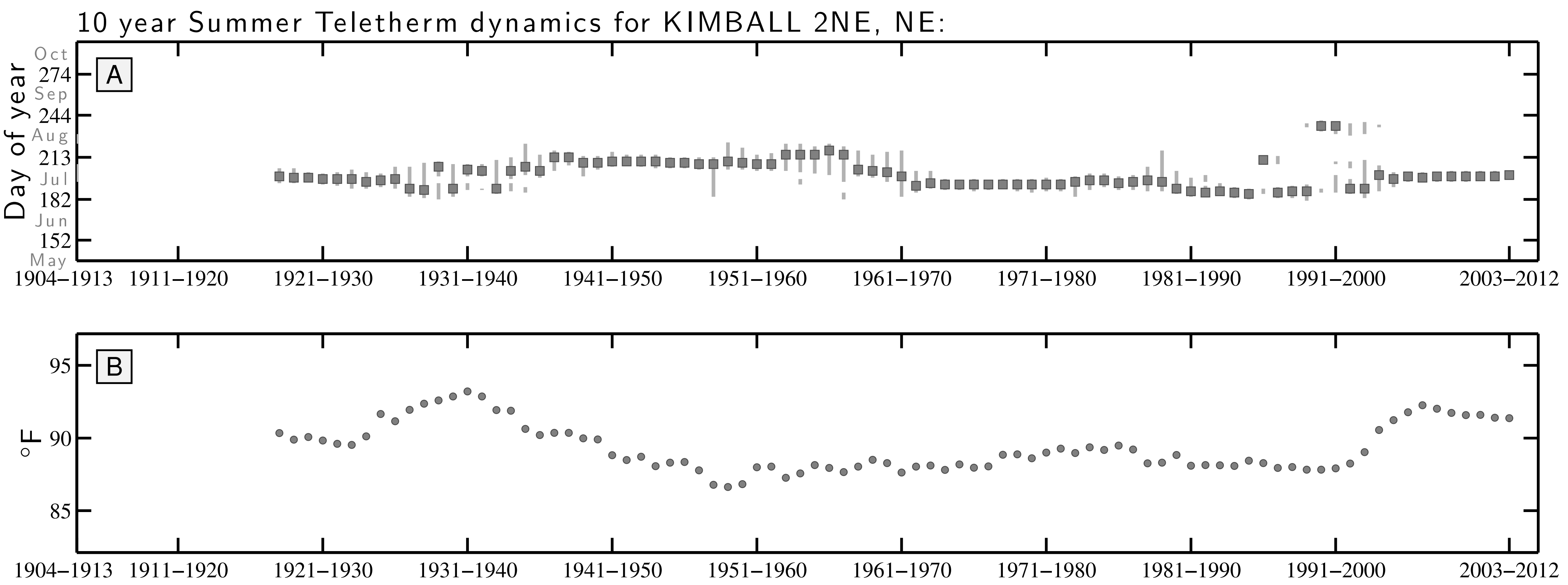
10 year Summer Teletherm dynamics for KIMBALL 2NE, NE:
A
B
Day of year
°F
Oct
274
Sep
244
Aug
213
Jul
182
Jun
152
May
95
90
85
1904–1913
1911–1920
1921–1930
1931–1940
1941–1950
1951–1960
1961–1970
1971–1980
1981–1990
1991–2000
2003–2012

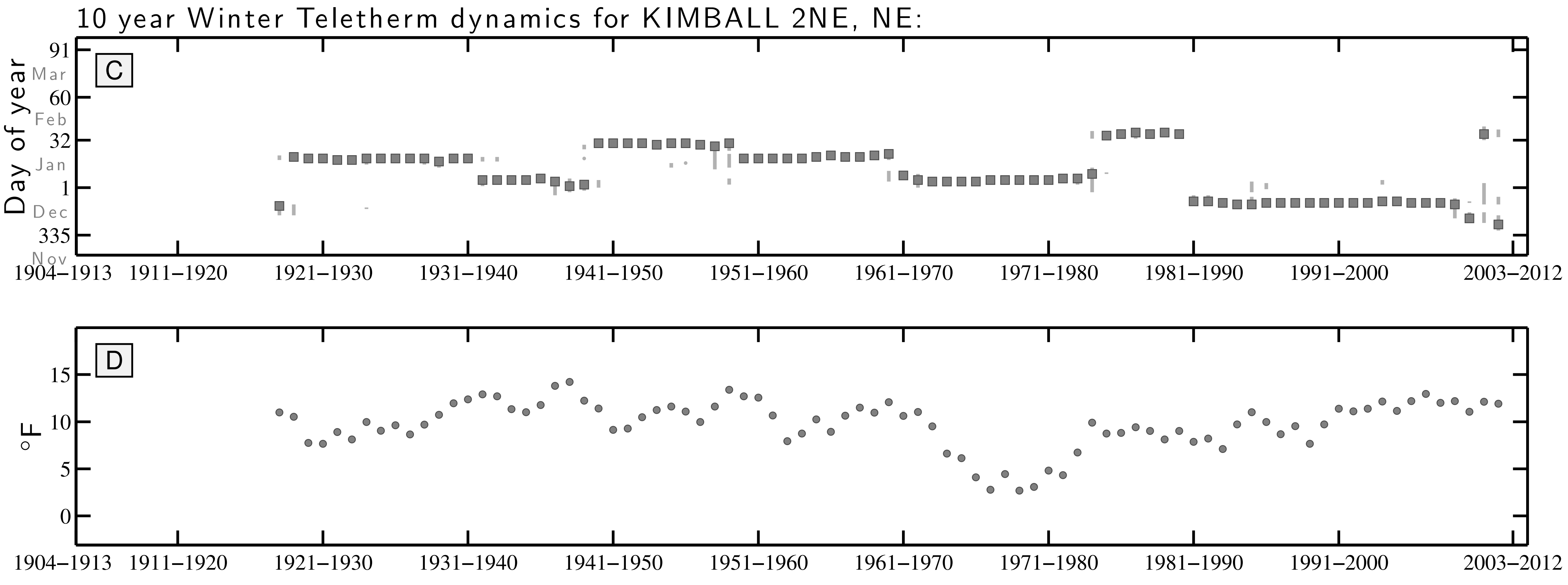
10 year Winter Teletherm dynamics for KIMBALL 2NE, NE:
C
D
Day of year
°F
91
Mar
60
Feb
32
Jan
1
Dec
335
Nov
15
10
5
0
1904–1913
1911–1920
1921–1930
1931–1940
1941–1950
1951–1960
1961–1970
1971–1980
1981–1990
1991–2000
2003–2012

10 year Summer Teletherm dynamics for LODGEPOLE, NE:

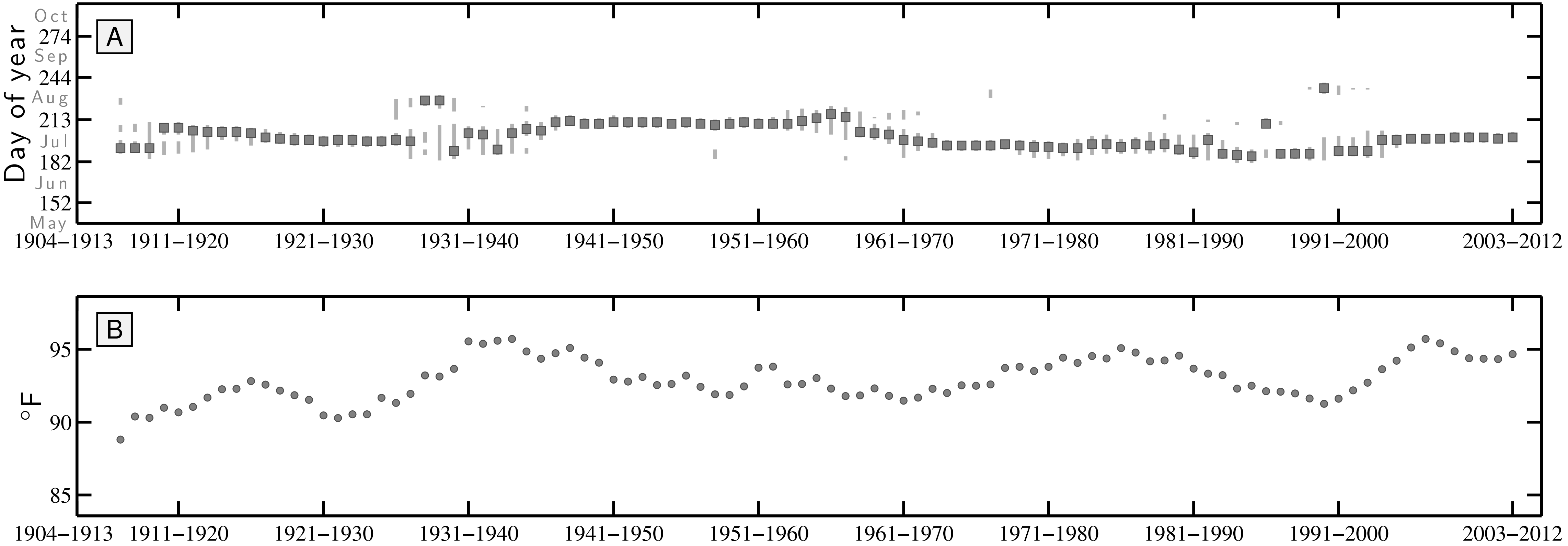

10 year Winter Teletherm dynamics for LODGEPOLE, NE:

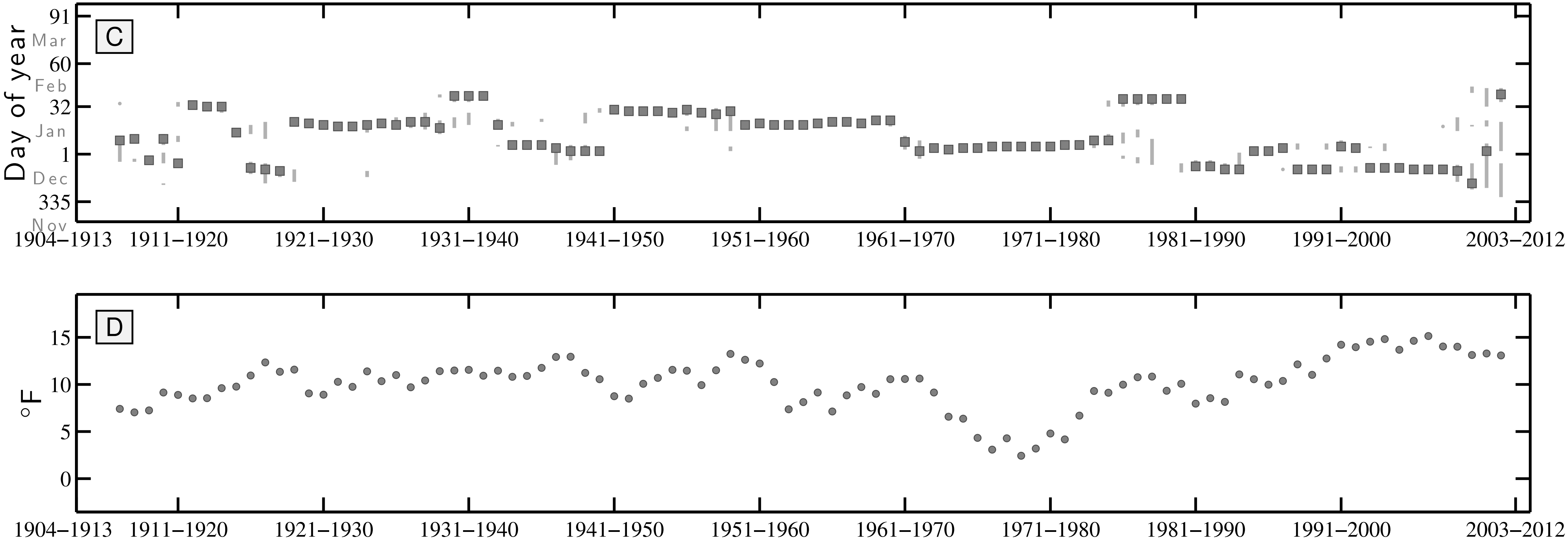

10 year Summer Teletherm dynamics for LOUP CITY, NE:

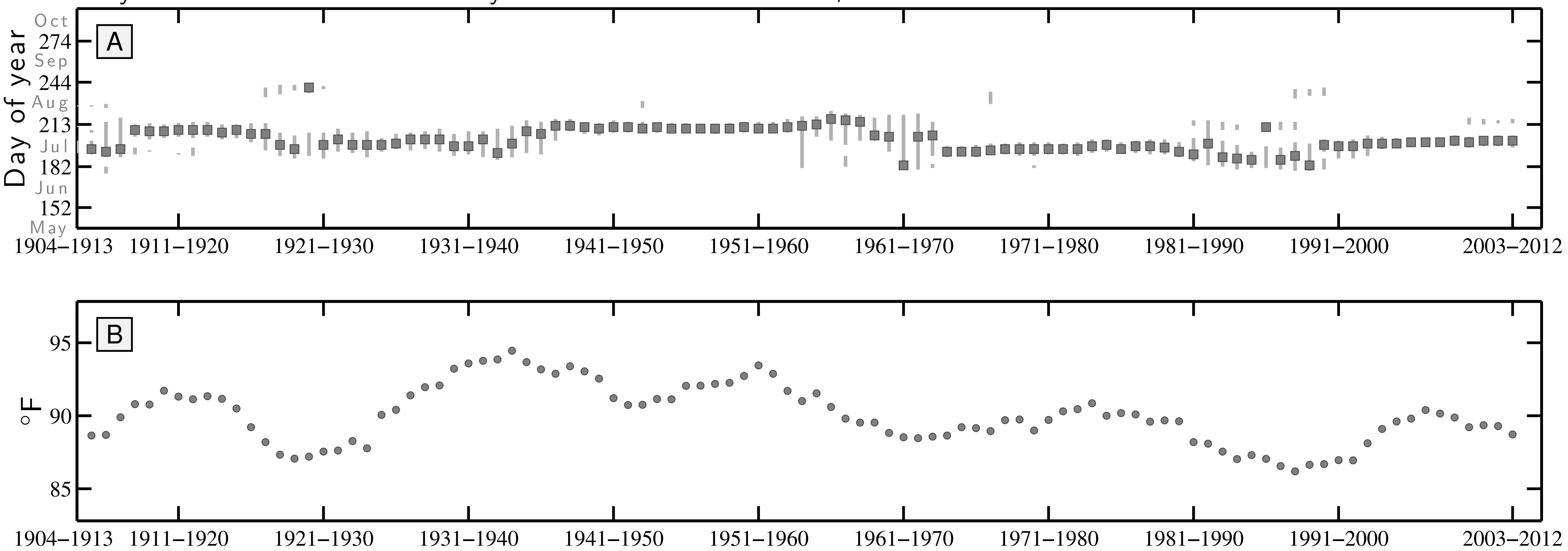

10 year Winter Teletherm dynamics for LOUP CITY, NE:

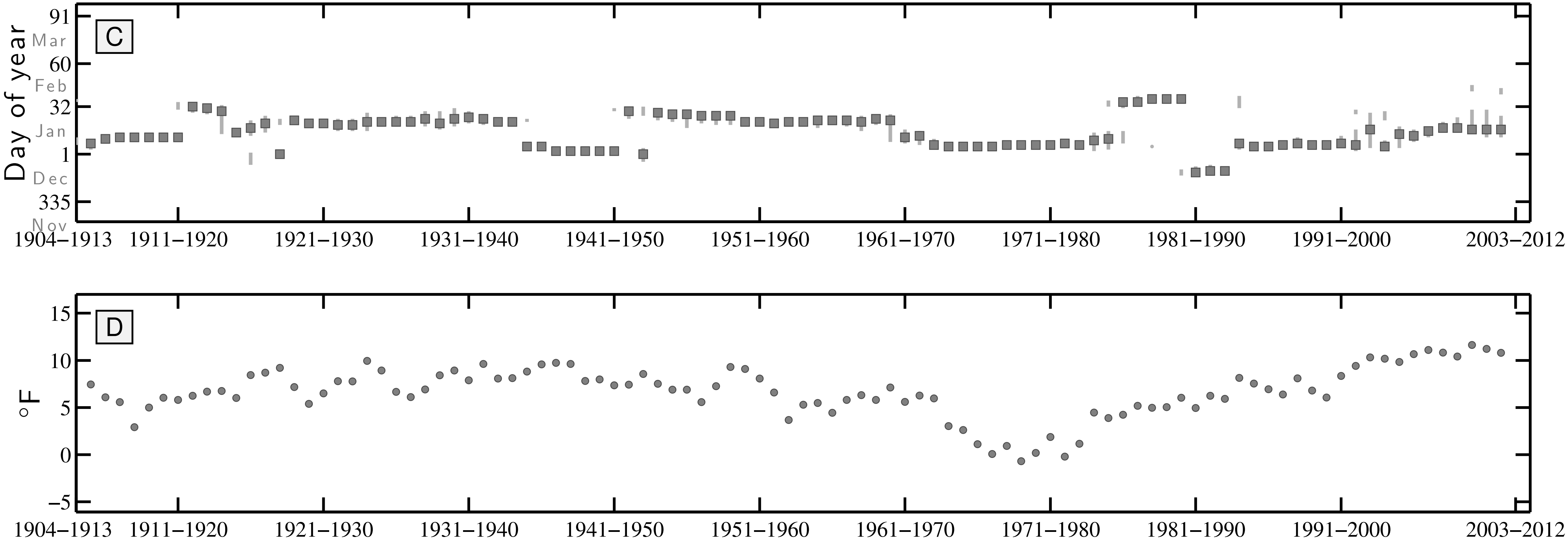

10 year Summer Teletherm dynamics for MADISON, NE:

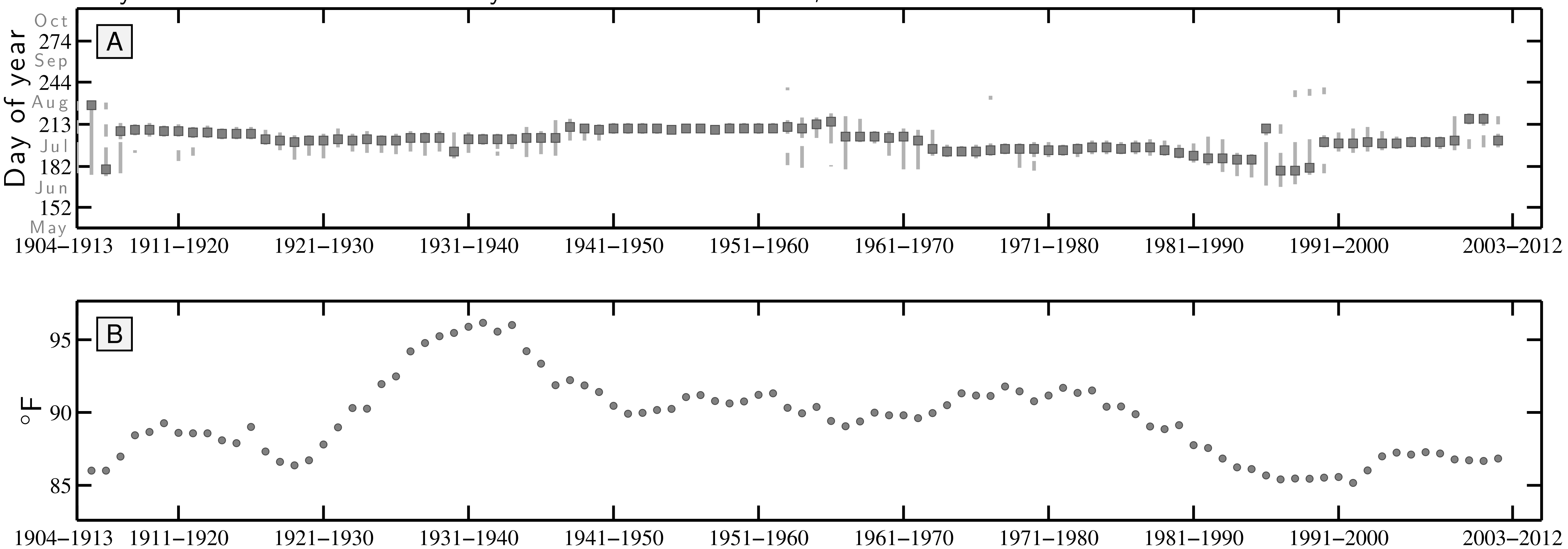

10 year Winter Teletherm dynamics for MADISON, NE:

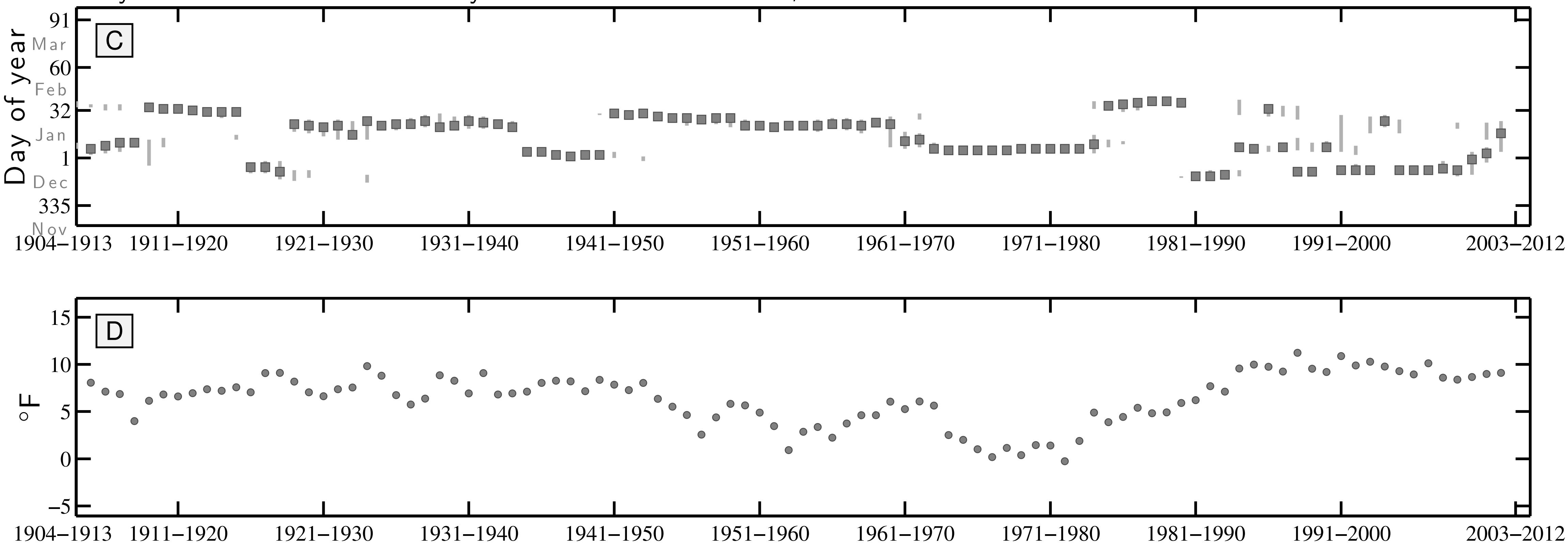

10 year Summer Teletherm dynamics for MC COOK, NE:

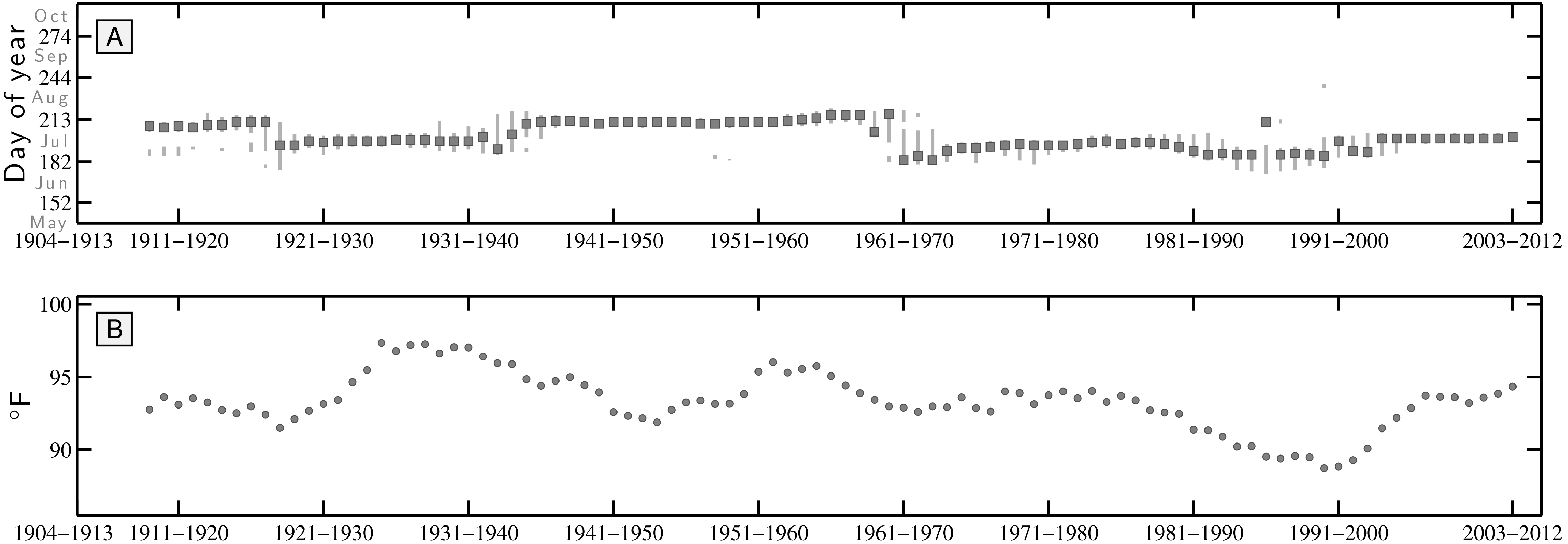

10 year Winter Teletherm dynamics for MC COOK, NE:

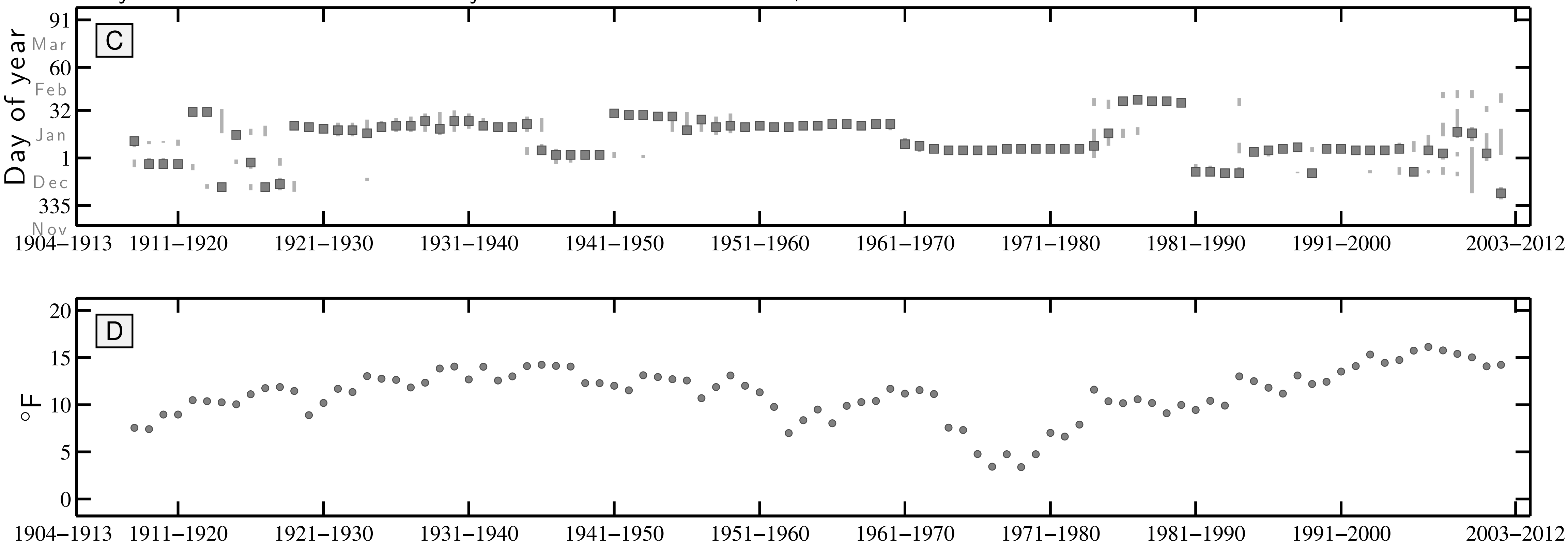

10 year Summer Teletherm dynamics for MERRIMAN, NE:

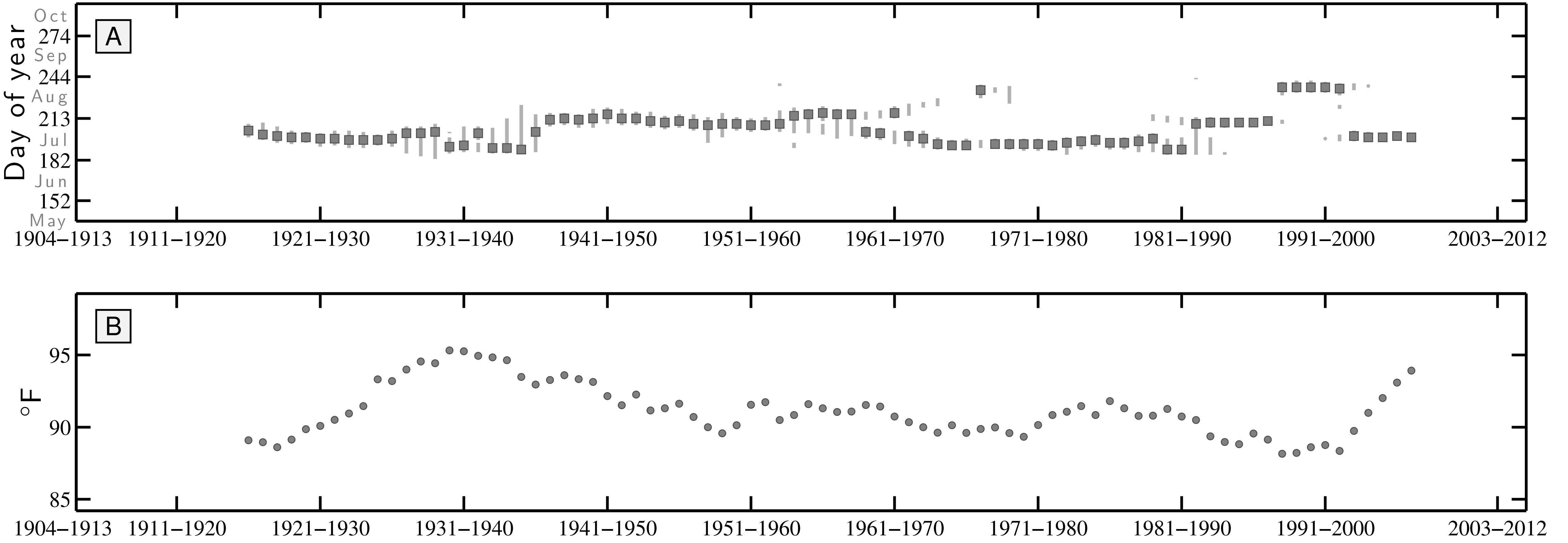

10 year Winter Teletherm dynamics for MERRIMAN, NE:

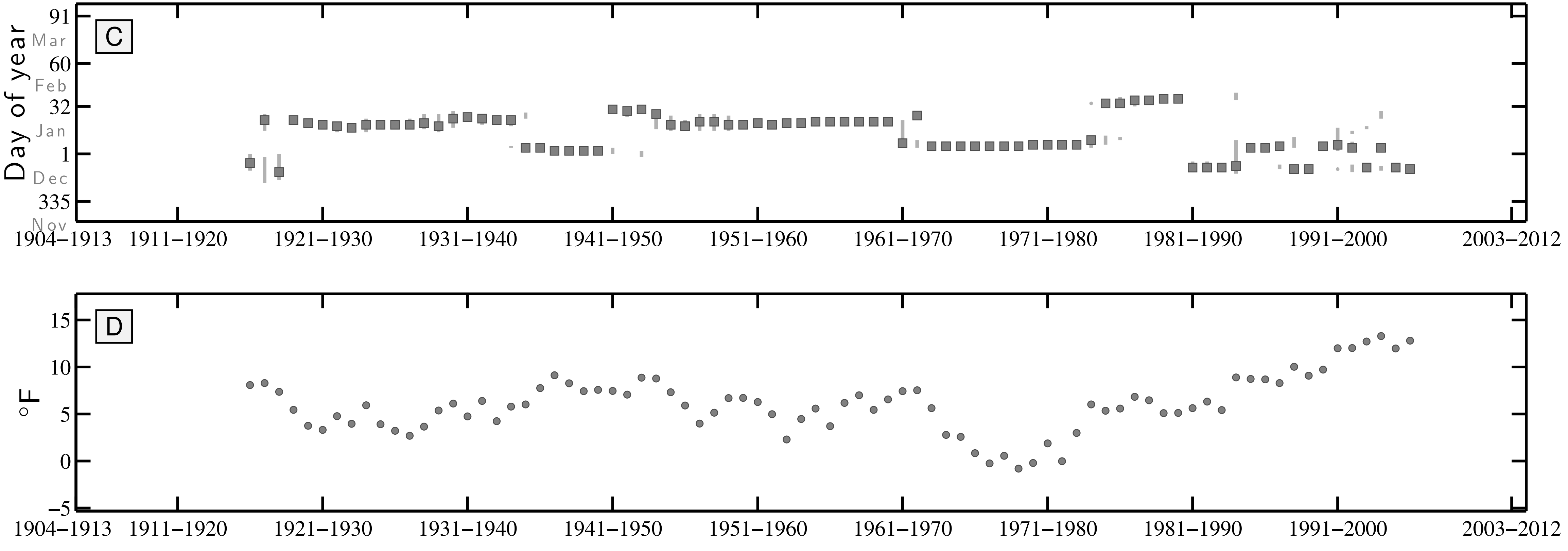

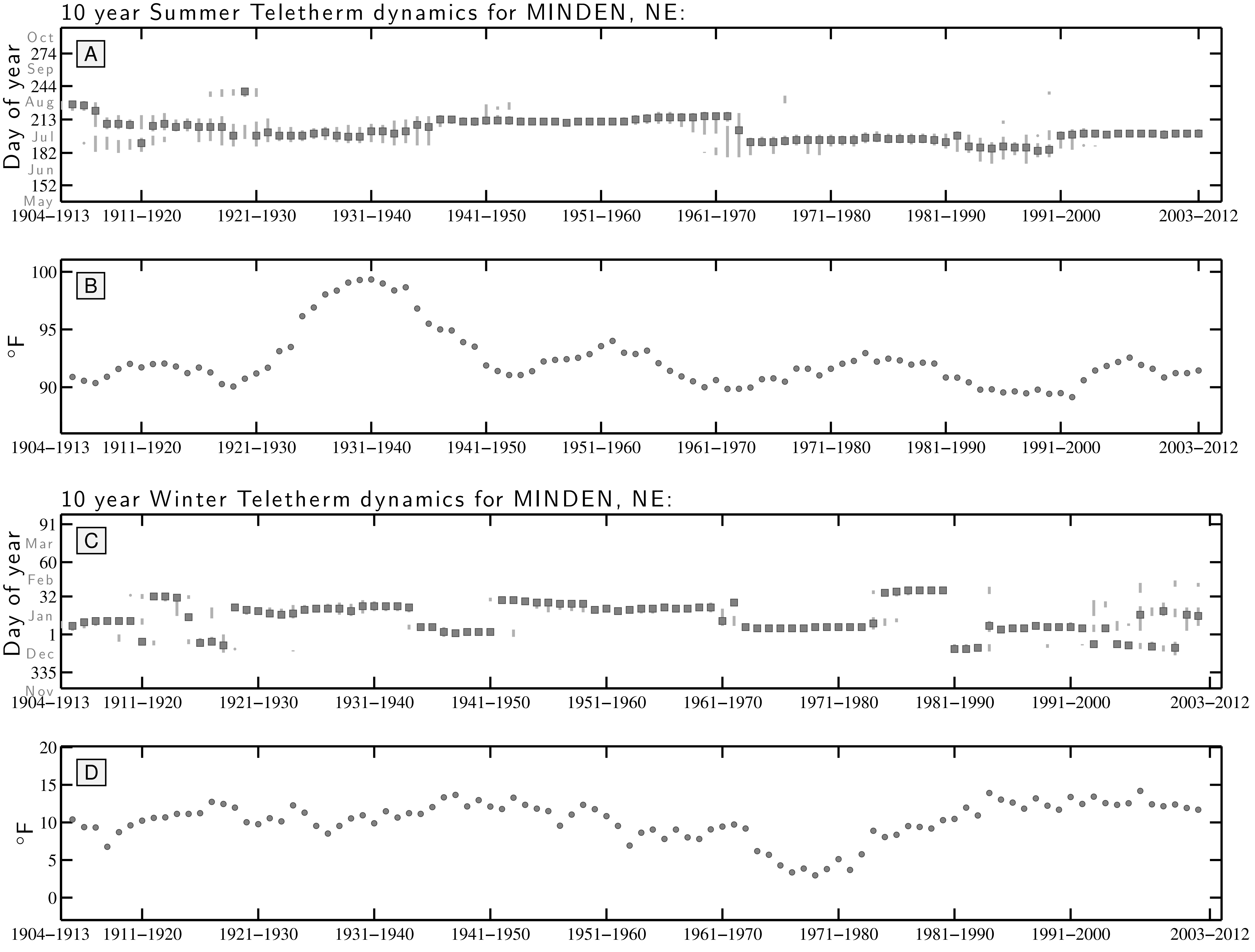
10 year Summer Teletherm dynamics for MINDEN, NE:
A
B
Day of year
°F
Oct
274
Sep
244
Aug
213
Jul
182
Jun
152
May
100
95
90
10 year Winter Teletherm dynamics for MINDEN, NE:
C
D
91
Mar
60
Feb
32
Jan
1
Dec
335
Nov
20
15
10
5
0
1904–1913
1911–1920
1921–1930
1931–1940
1941–1950
1951–1960
1961–1970
1971–1980
1981–1990
1991–2000
2003–2012

10 year Summer Teletherm dynamics for NORTH LOUP, NE:

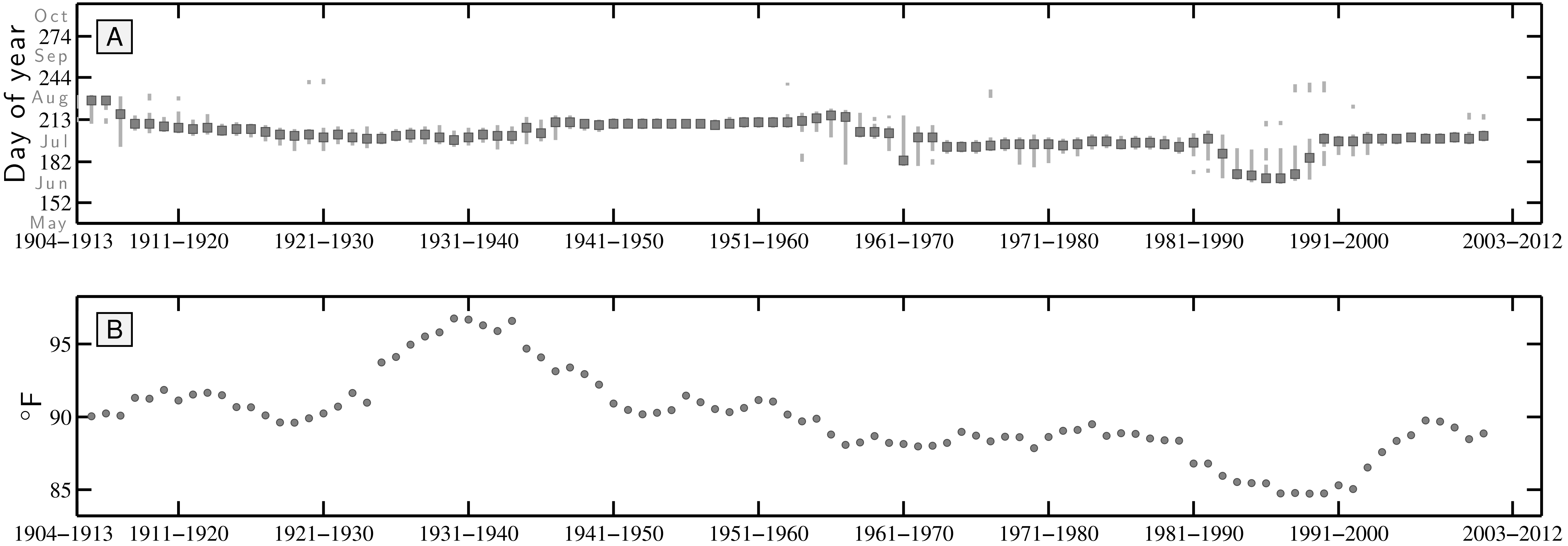

10 year Winter Teletherm dynamics for NORTH LOUP, NE:

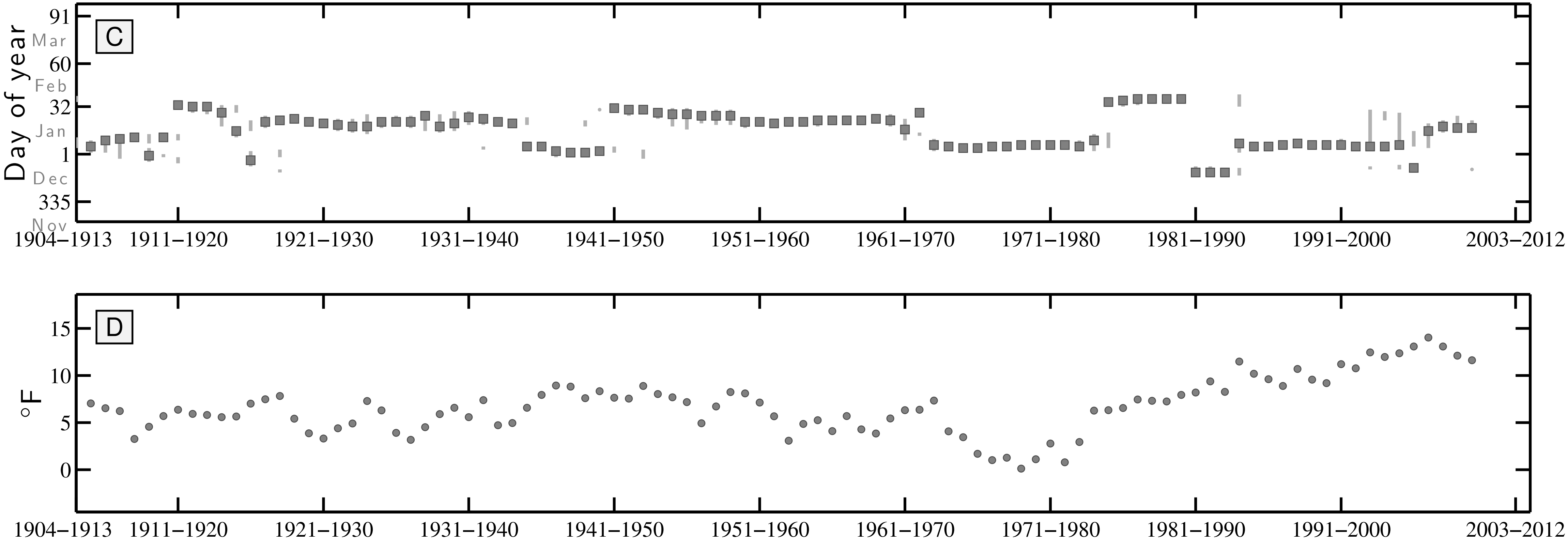

10 year Summer Teletherm dynamics for OAKDALE, NE:

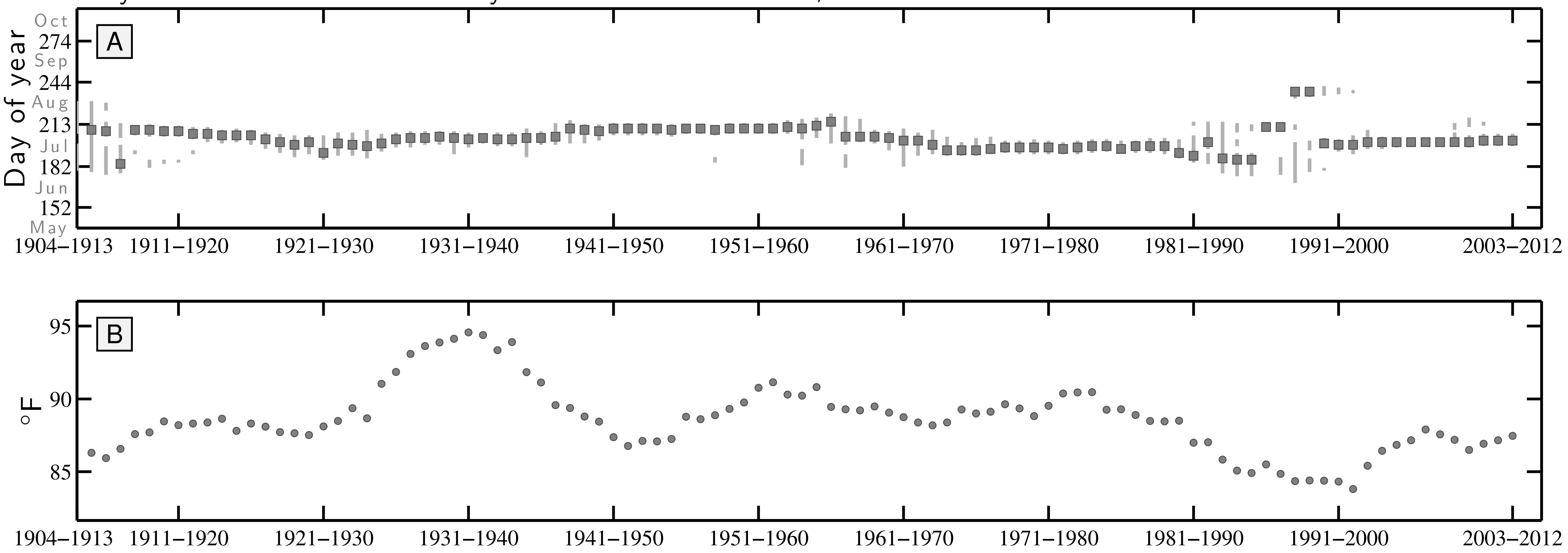

10 year Winter Teletherm dynamics for OAKDALE, NE:

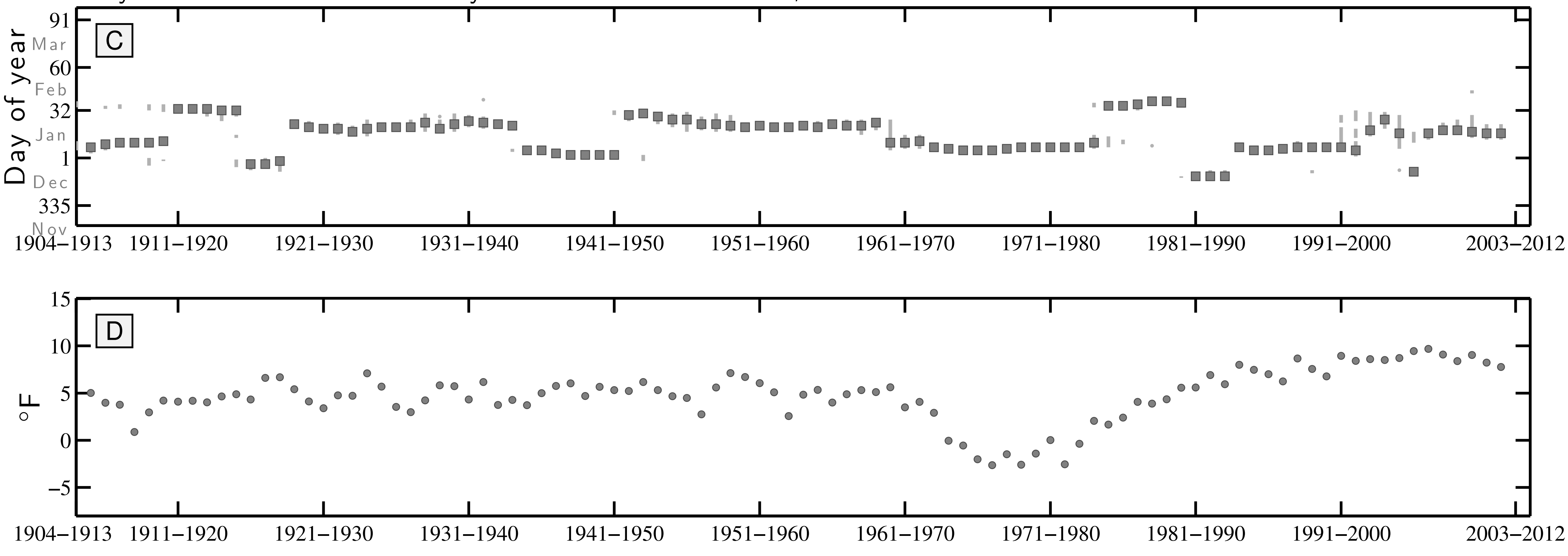

10 year Summer Teletherm dynamics for PAWNEE CITY, NE:

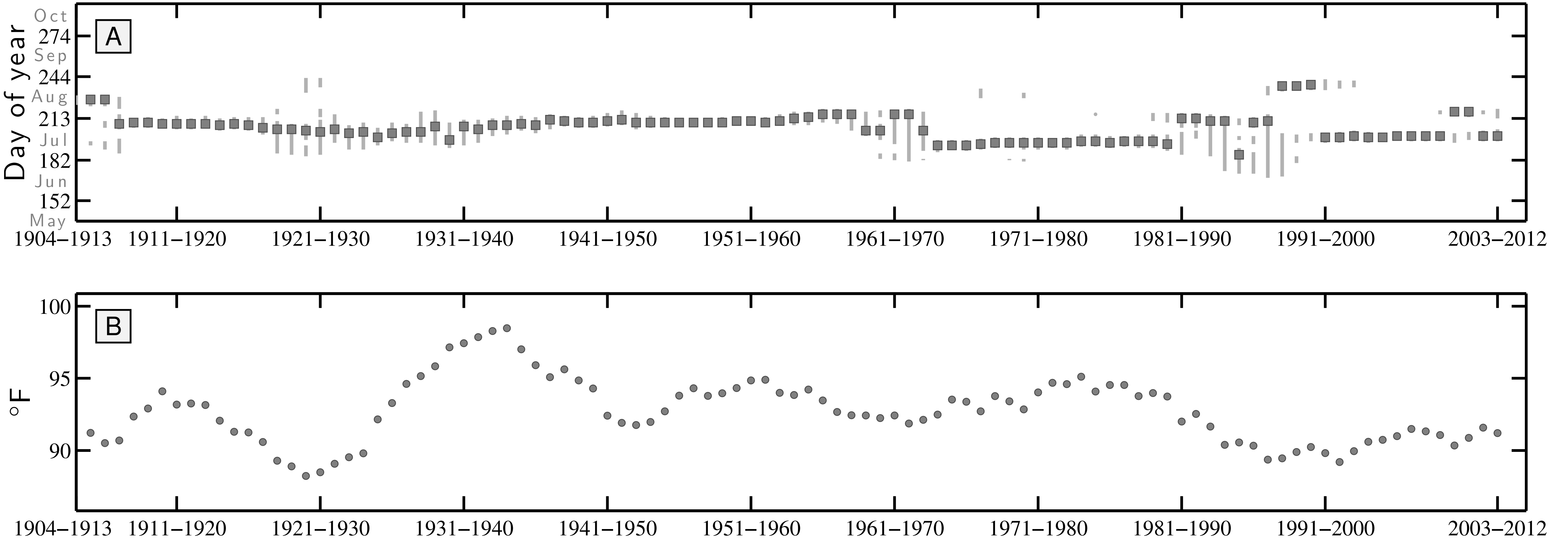

10 year Winter Teletherm dynamics for PAWNEE CITY, NE:

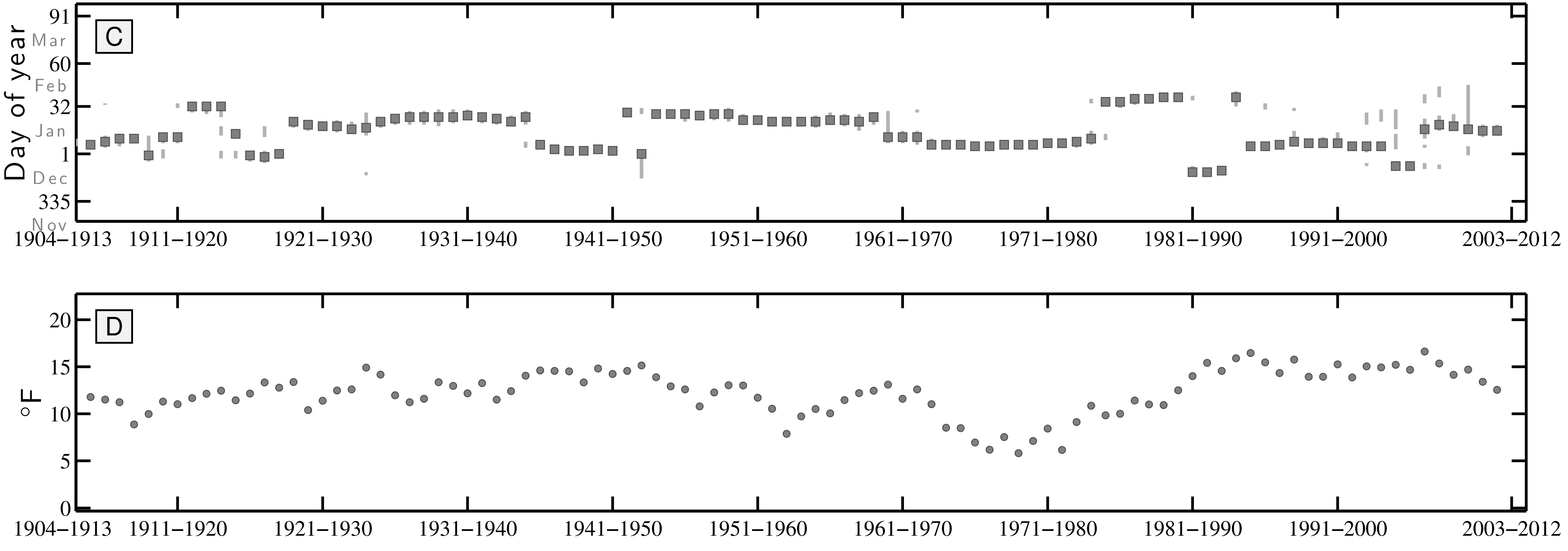

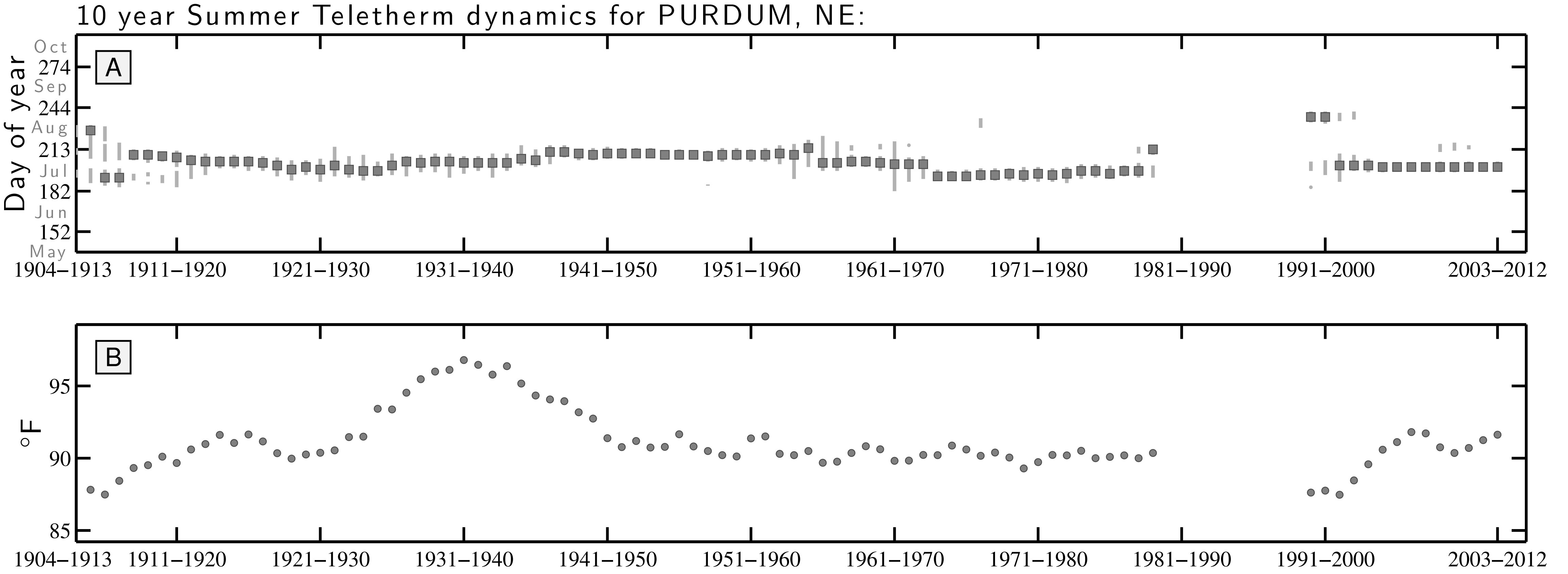

10 year Summer Teletherm dynamics for PURDUM, NE:
A
B
Day of year
°F
Oct
274
Sep
244
Aug
213
Jul
182
Jun
152
May
95
90
85
1904–1913
1911–1920
1921–1930
1931–1940
1941–1950
1951–1960
1961–1970
1971–1980
1981–1990
1991–2000
2003–2012

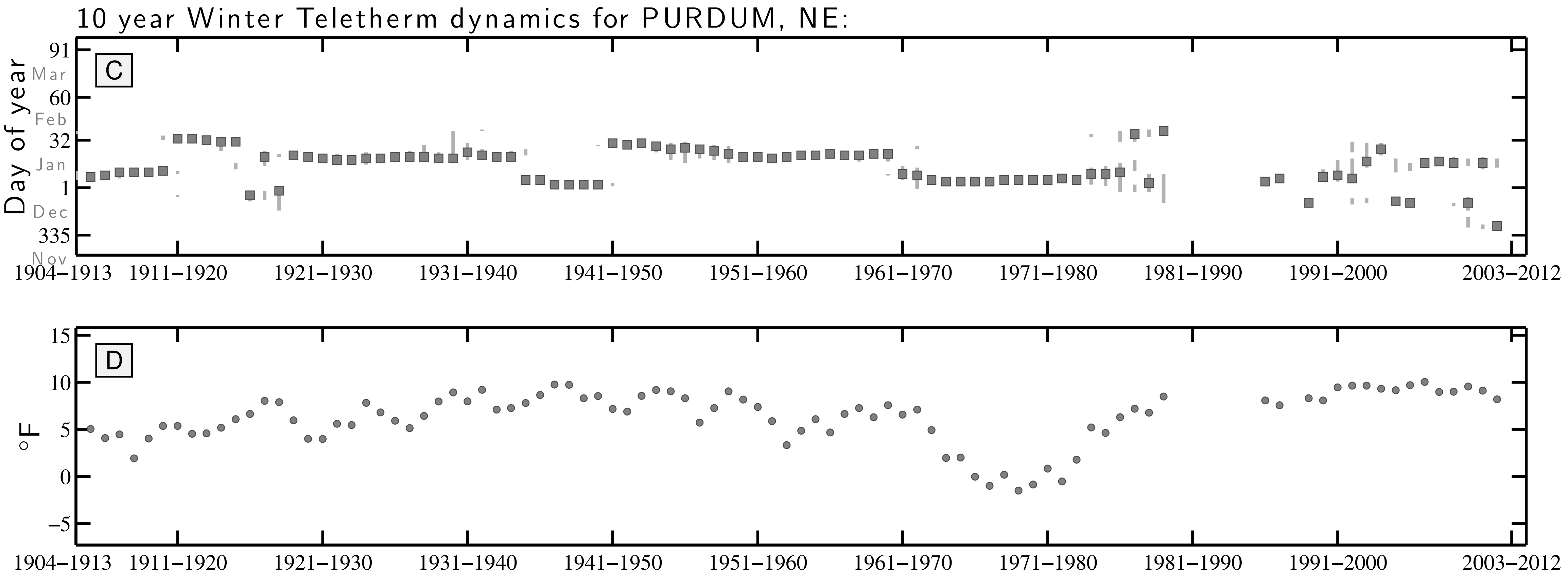

10 year Winter Teletherm dynamics for PURDUM, NE:
C
D
Day of year
°F
91
Mar
60
Feb
32
Jan
1
Dec
335
Nov
15
10
5
0
−5
1904–1913
1911–1920
1921–1930
1931–1940
1941–1950
1951–1960
1961–1970
1971–1980
1981–1990
1991–2000
2003–2012

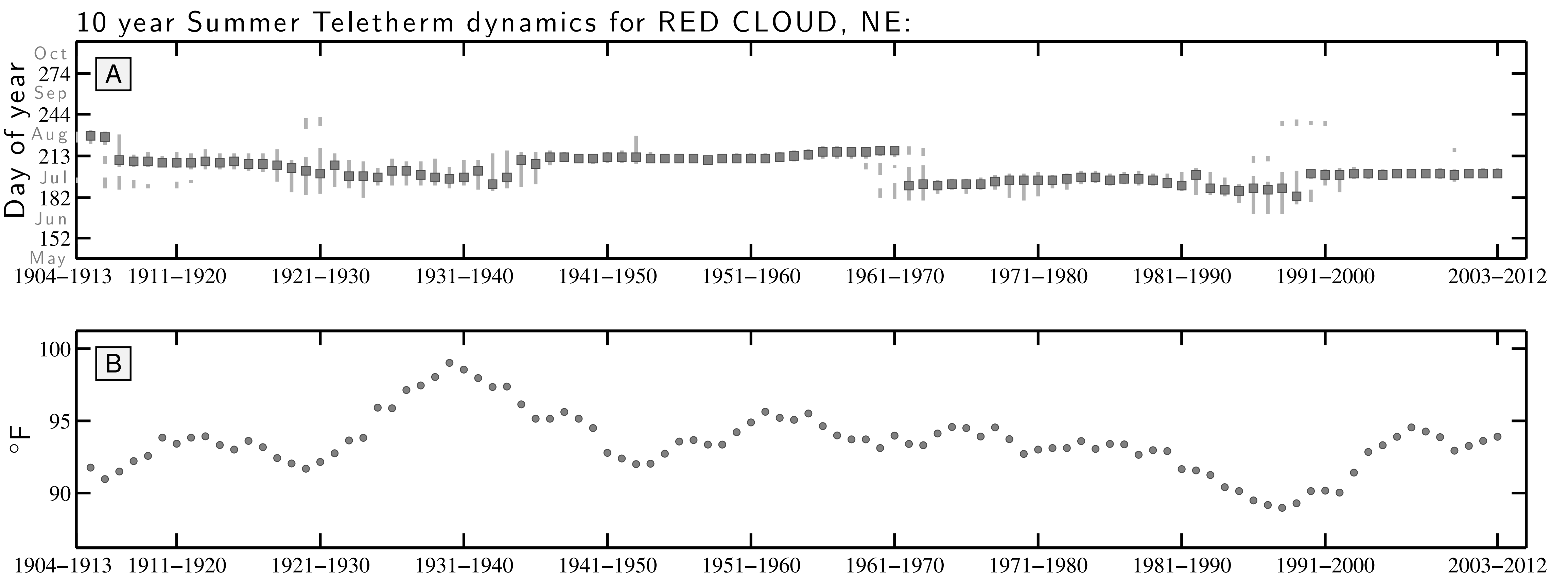
10 year Summer Teletherm dynamics for RED CLOUD, NE:
A
B
Day of year
°F
Oct
274
Sep
244
Aug
213
Jul
182
Jun
152
May
100
95
90
1904–1913
1911–1920
1921–1930
1931–1940
1941–1950
1951–1960
1961–1970
1971–1980
1981–1990
1991–2000
2003–2012

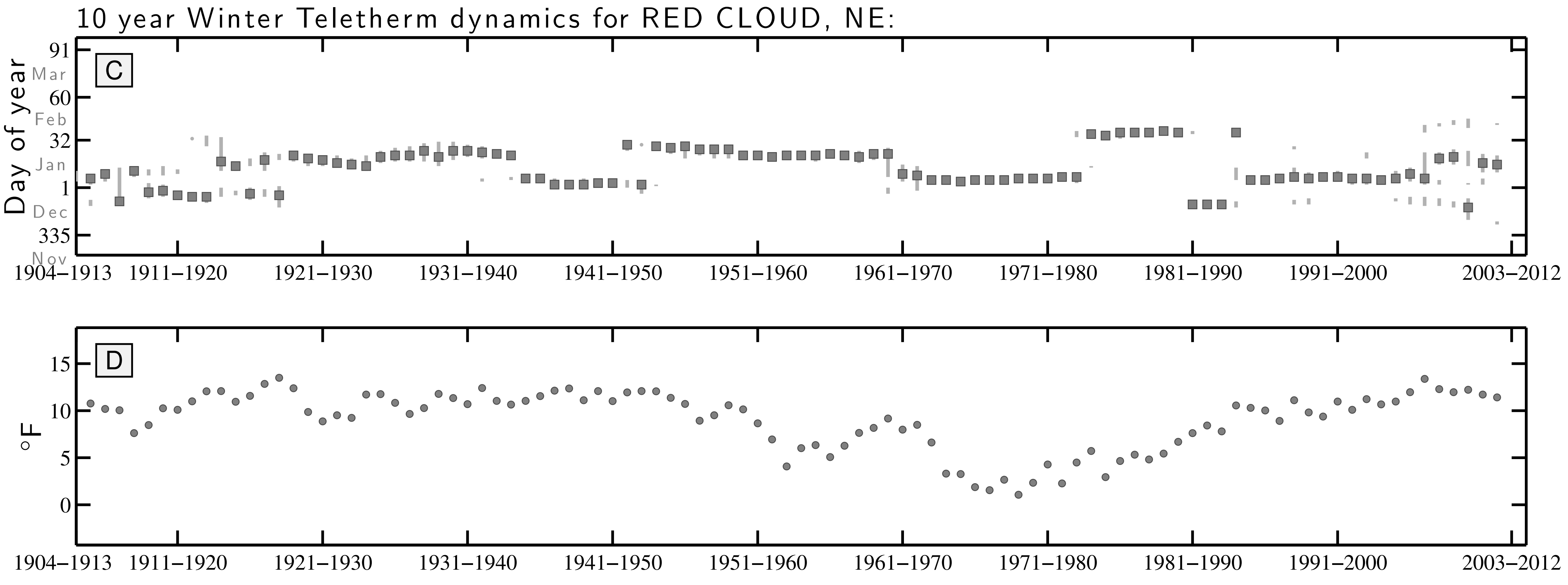
10 year Winter Teletherm dynamics for RED CLOUD, NE:
C
D
Day of year
°F
91
Mar
60
Feb
32
Jan
1
Dec
335
Nov
15
10
5
0
1904–1913
1911–1920
1921–1930
1931–1940
1941–1950
1951–1960
1961–1970
1971–1980
1981–1990
1991–2000
2003–2012

10 year Summer Teletherm dynamics for SAINT PAUL 4N, NE:

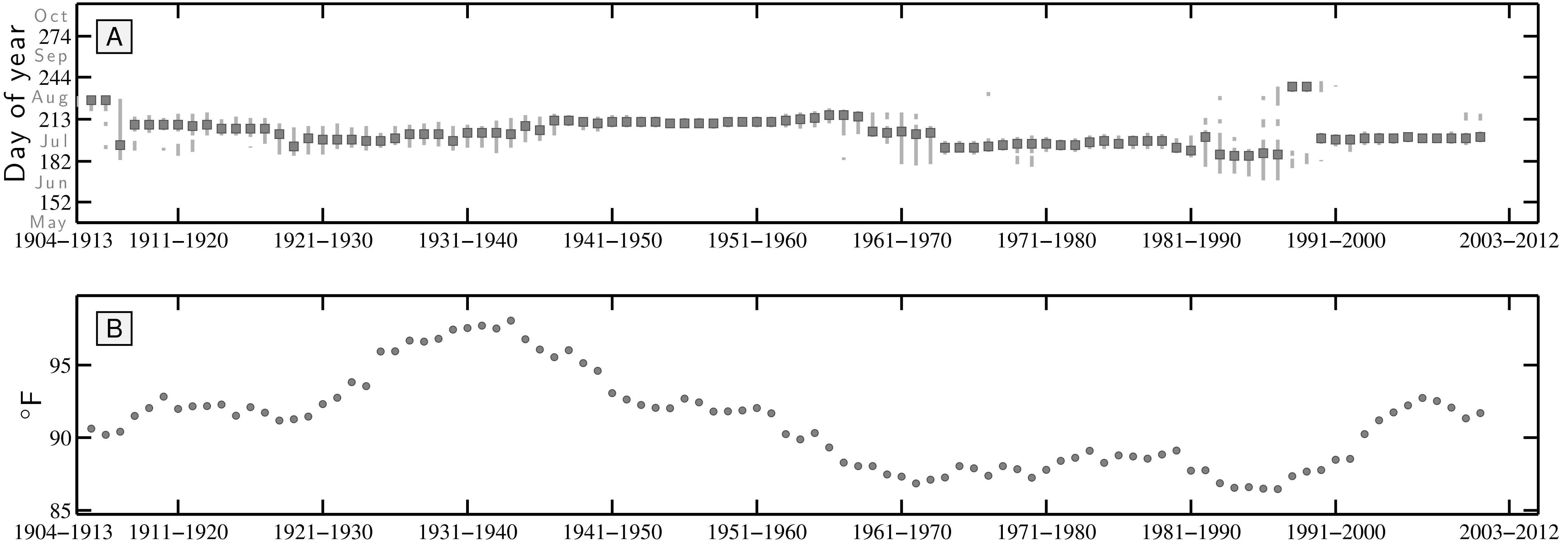

10 year Winter Teletherm dynamics for SAINT PAUL 4N, NE:

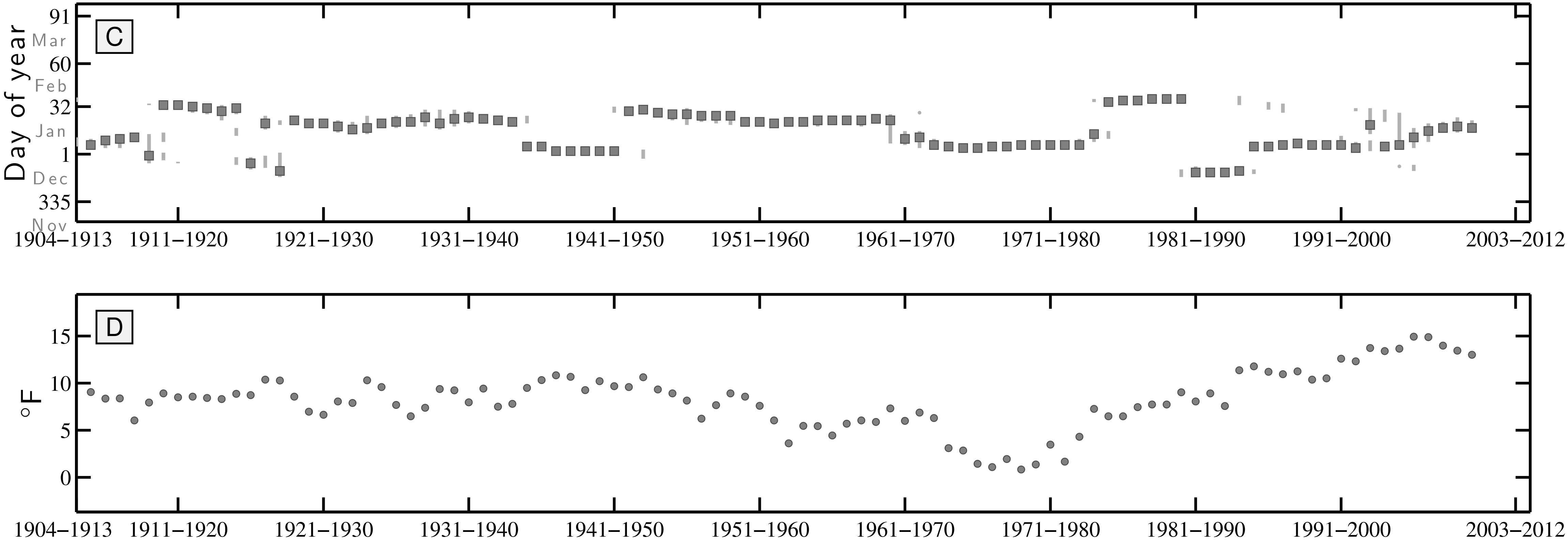

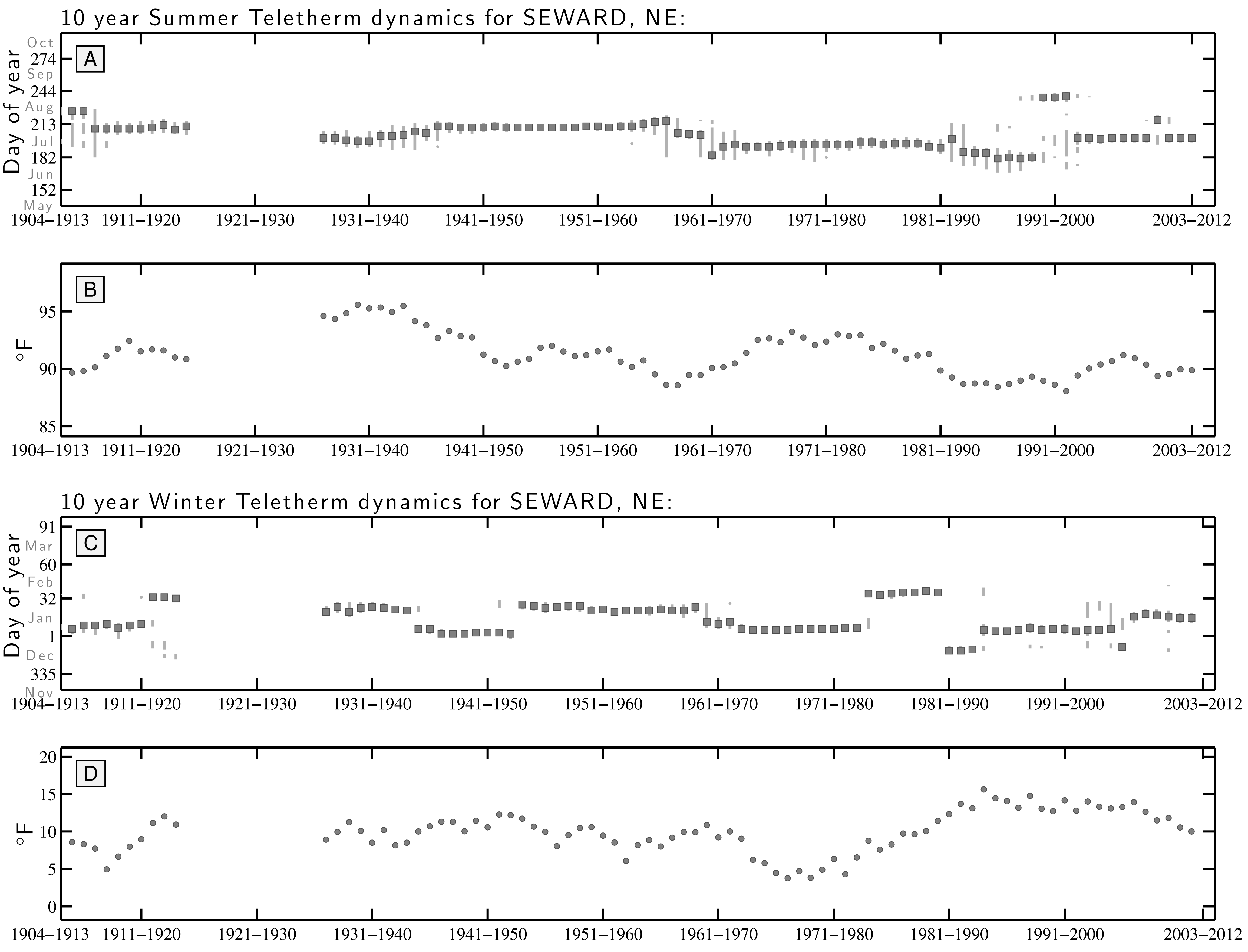
10 year Summer Teletherm dynamics for SEWARD, NE:
A
B
Day of year
°F
10 year Winter Teletherm dynamics for SEWARD, NE:
C
D
1904–1913
1911–1920
1921–1930
1931–1940
1941–1950
1951–1960
1961–1970
1971–1980
1981–1990
1991–2000
2003–2012

10 year Summer Teletherm dynamics for STAPLETON 5W, NE:

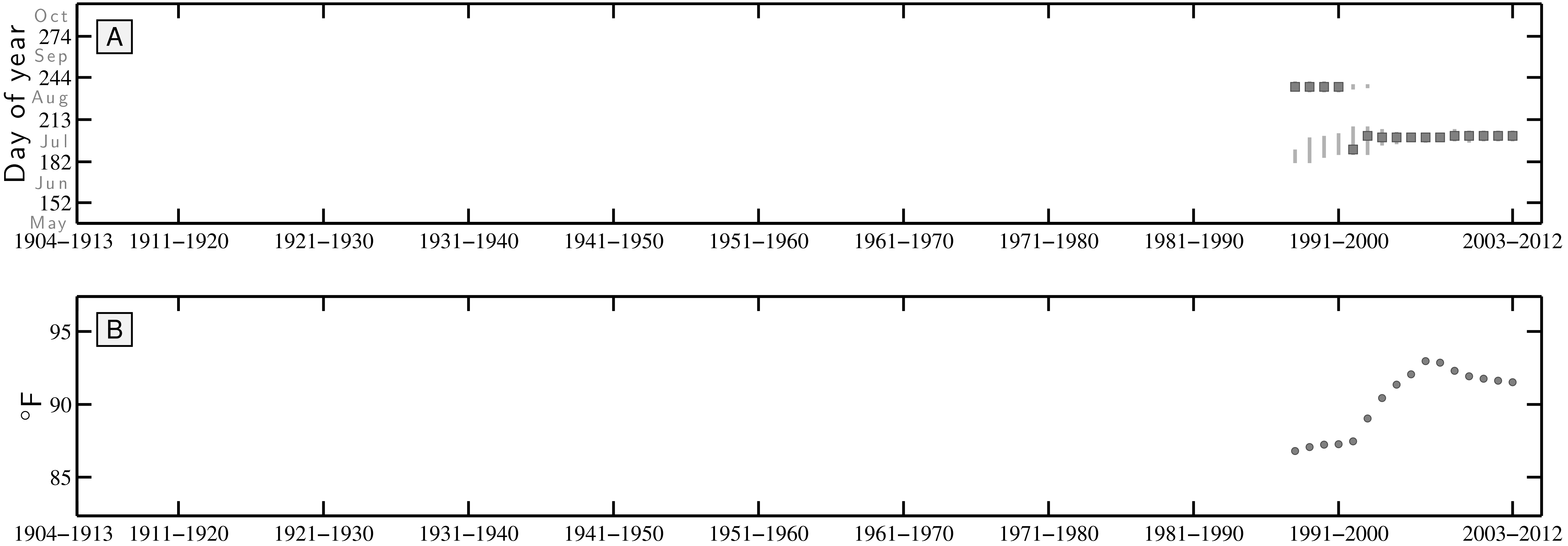

10 year Winter Teletherm dynamics for STAPLETON 5W, NE:

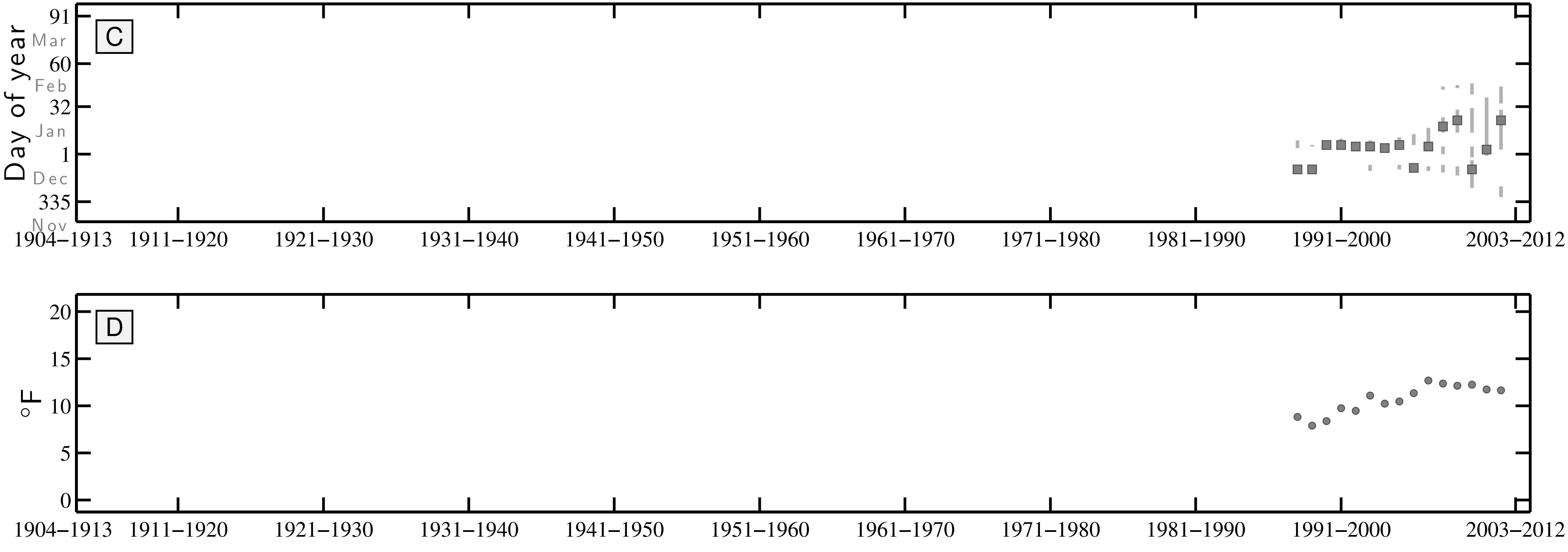

10 year Summer Teletherm dynamics for SYRACUSE, NE:

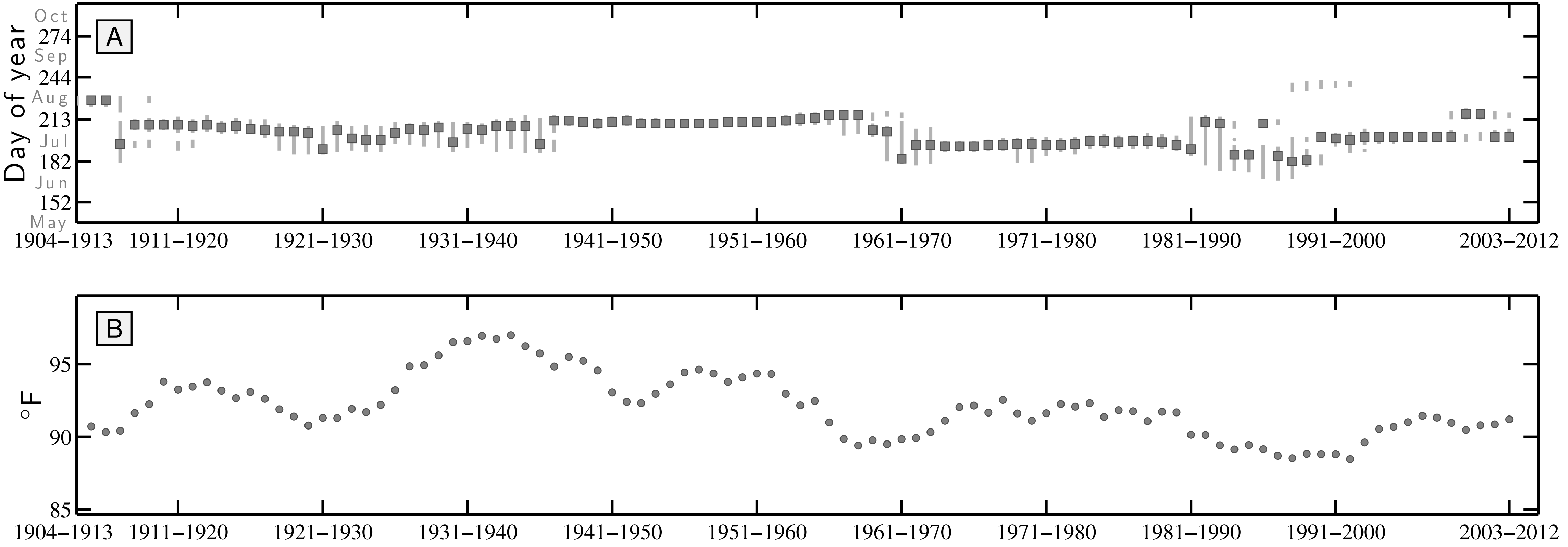

10 year Winter Teletherm dynamics for SYRACUSE, NE:

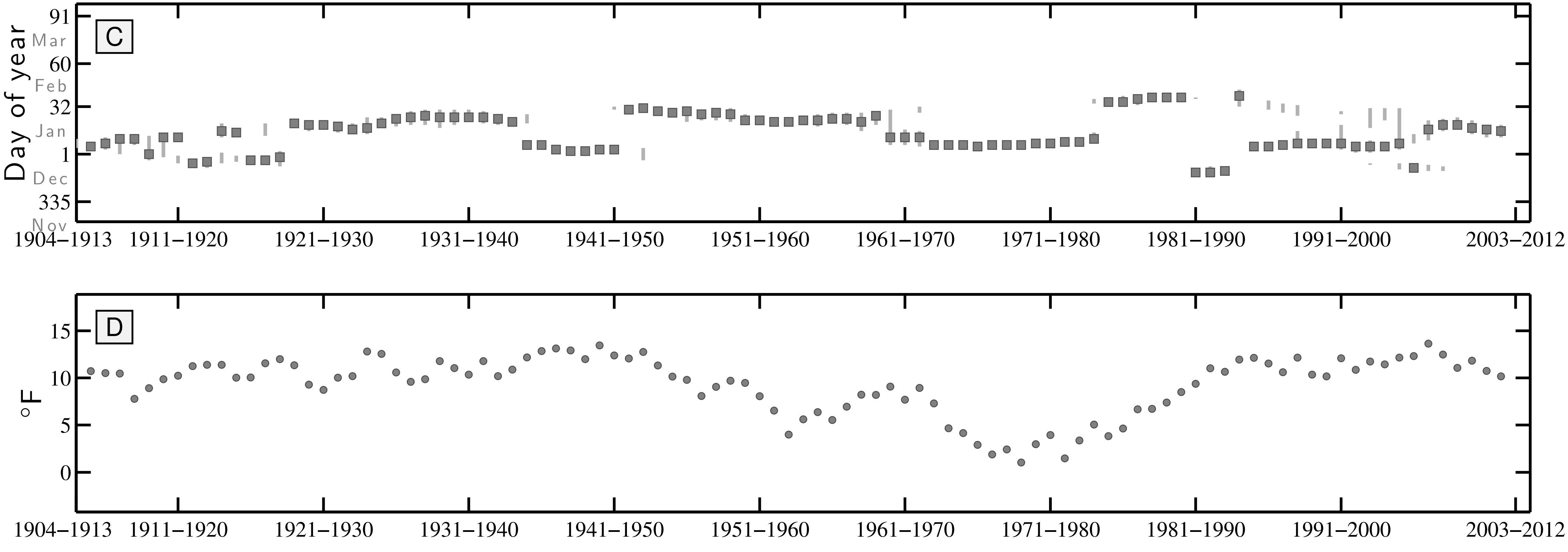

10 year Summer Teletherm dynamics for TECUMSEH 1S, NE:

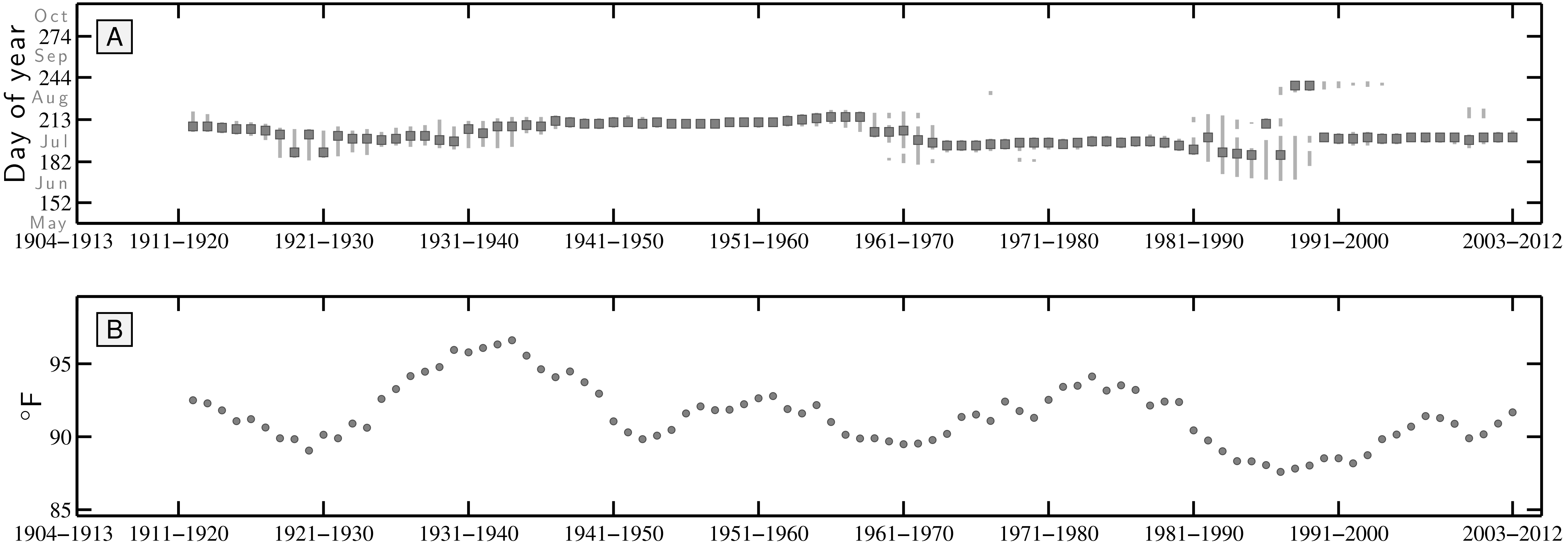

10 year Winter Teletherm dynamics for TECUMSEH 1S, NE:

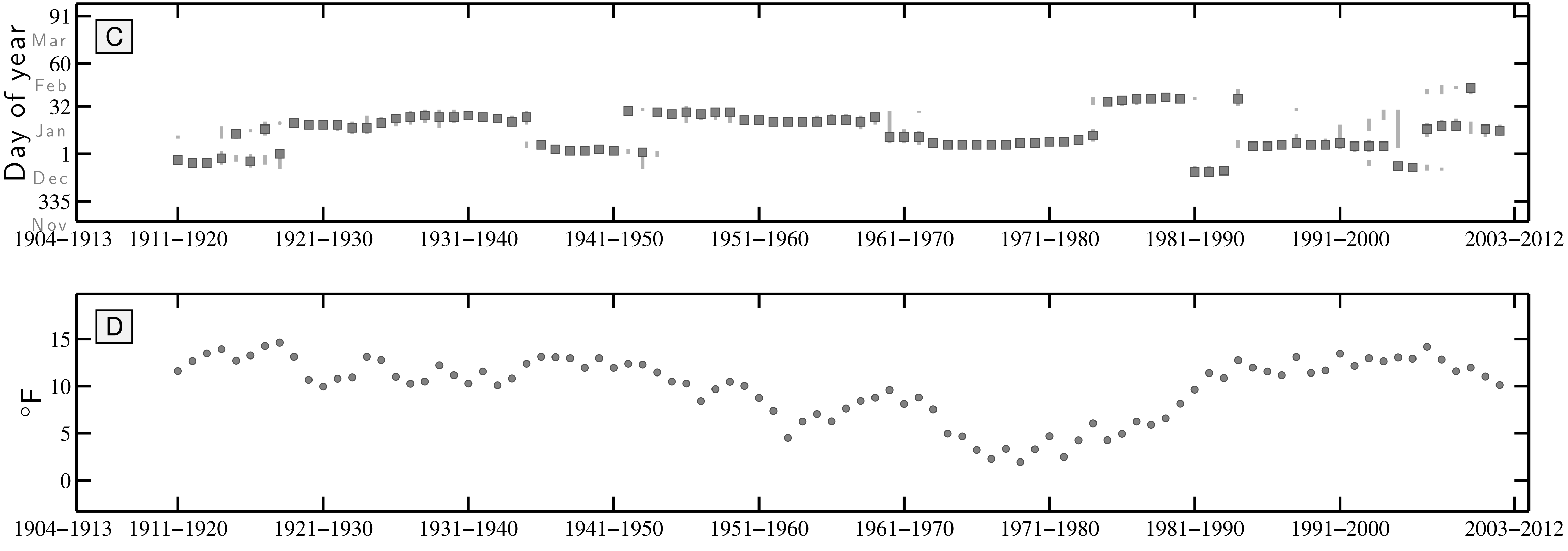

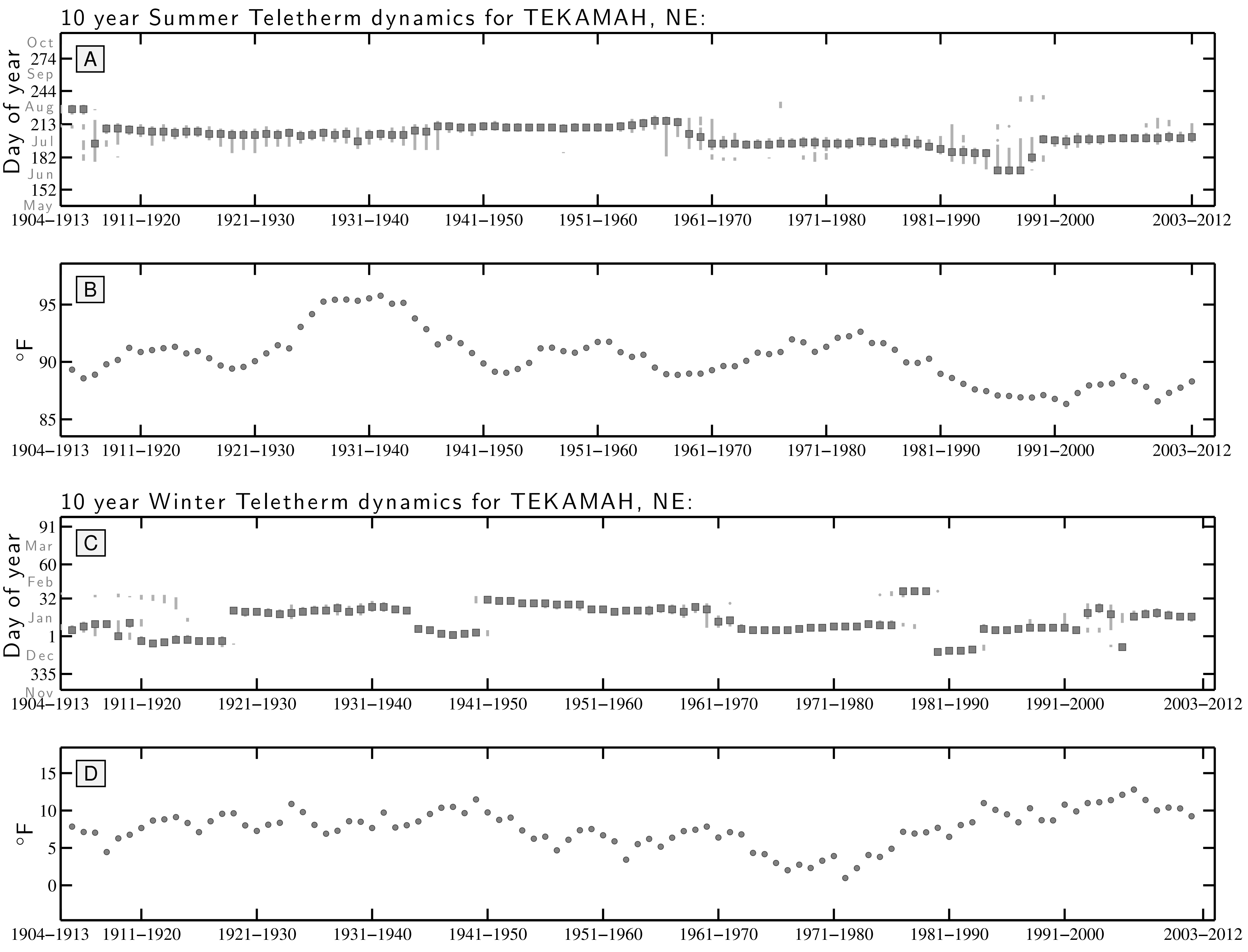
10 year Summer Teletherm dynamics for TEKAMAH, NE:
A
B
Day of year
°F
Oct
274
Sep
244
Aug
213
Jul
182
Jun
152
May
95
90
85
1904–1913
1911–1920
1921–1930
1931–1940
1941–1950
1951–1960
1961–1970
1971–1980
1981–1990
1991–2000
2003–2012
10 year Winter Teletherm dynamics for TEKAMAH, NE:
C
D
91
Mar
60
Feb
32
Jan
1
Dec
335
Nov
15
10
5
0

10 year Summer Teletherm dynamics for WAKEFIELD, NE:

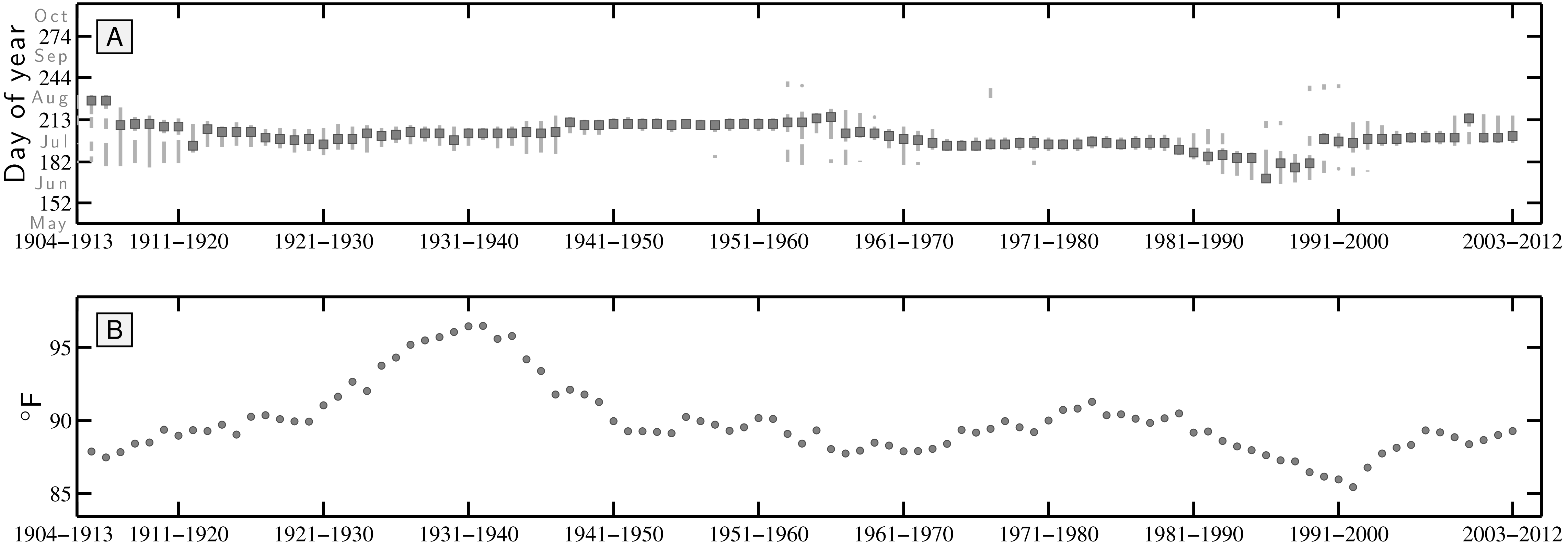

10 year Winter Teletherm dynamics for WAKEFIELD, NE:

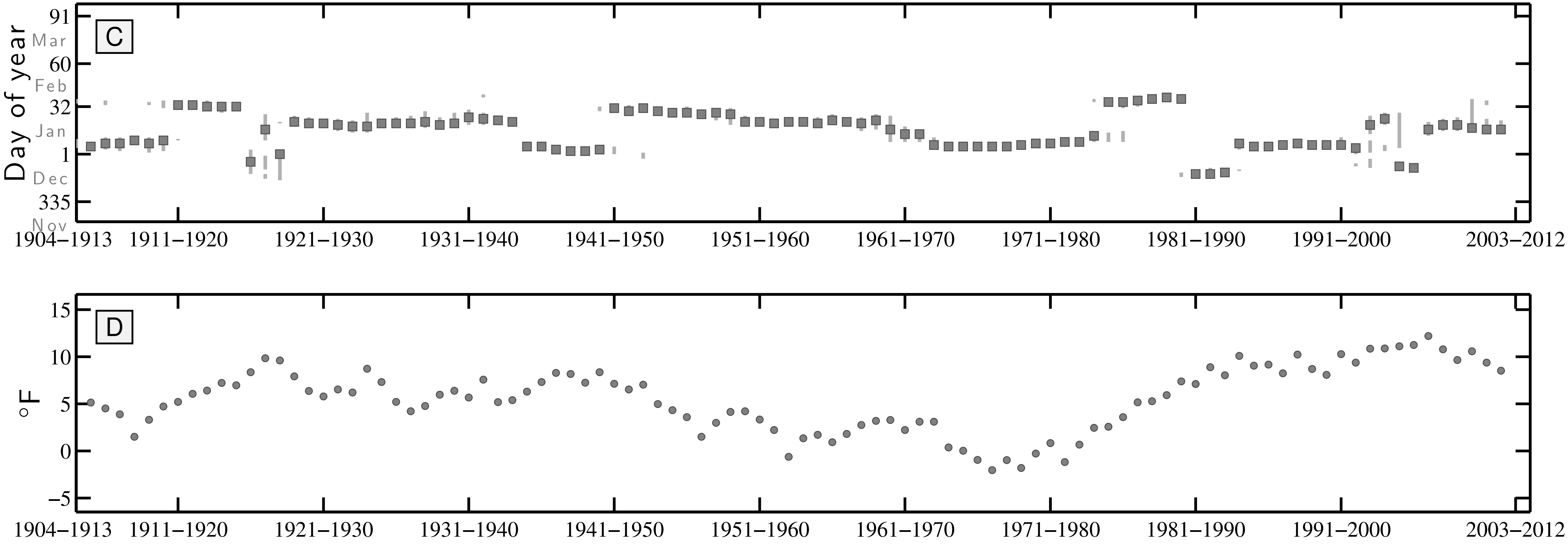

10 year Summer Teletherm dynamics for WEEPING WATER, NE:

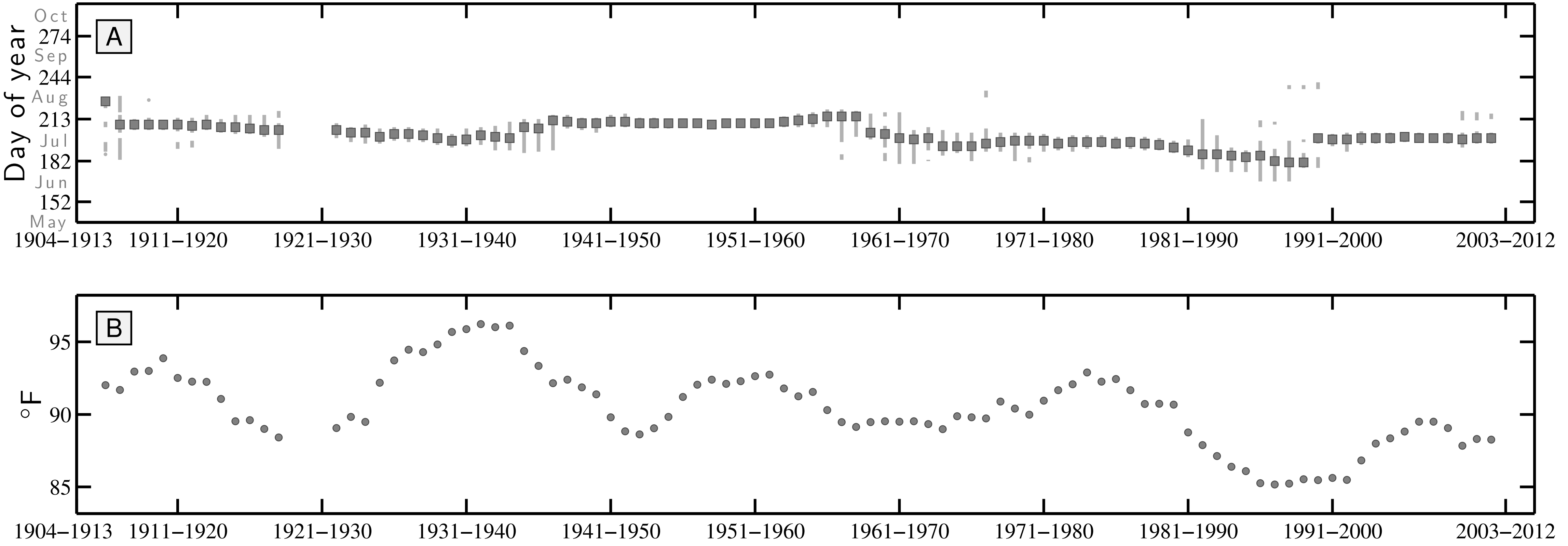

10 year Winter Teletherm dynamics for WEEPING WATER, NE:

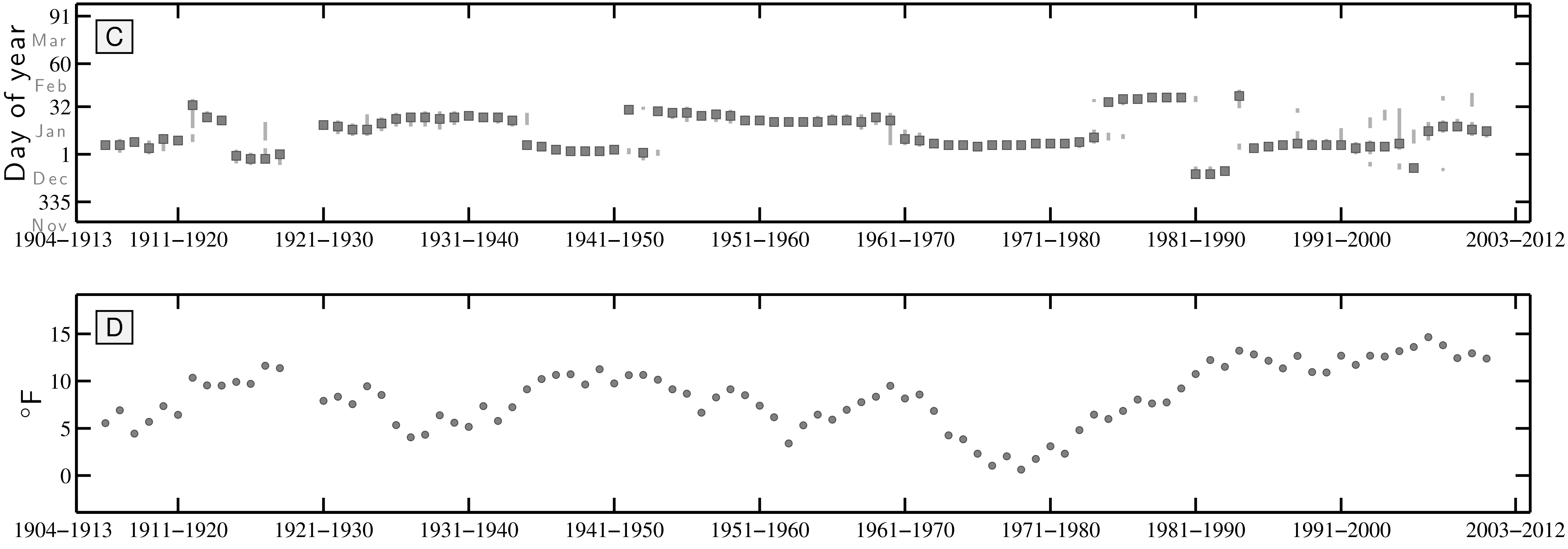

10 year Summer Teletherm dynamics for YORK, NE:

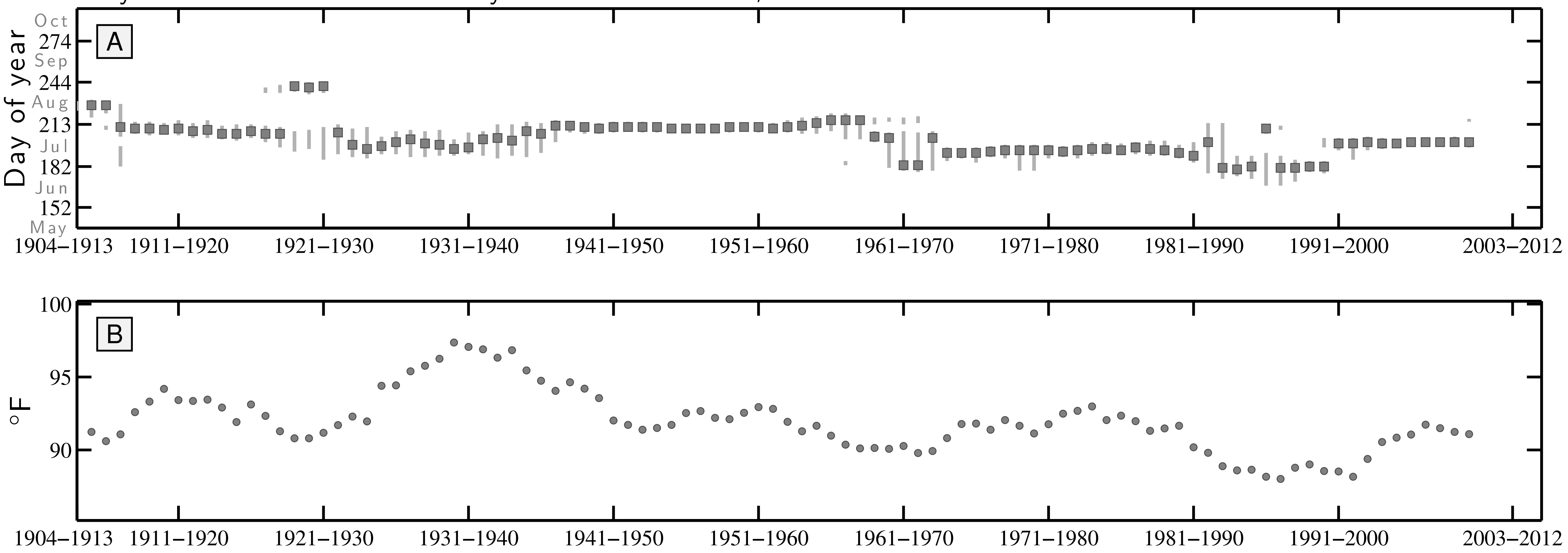

10 year Winter Teletherm dynamics for YORK, NE:

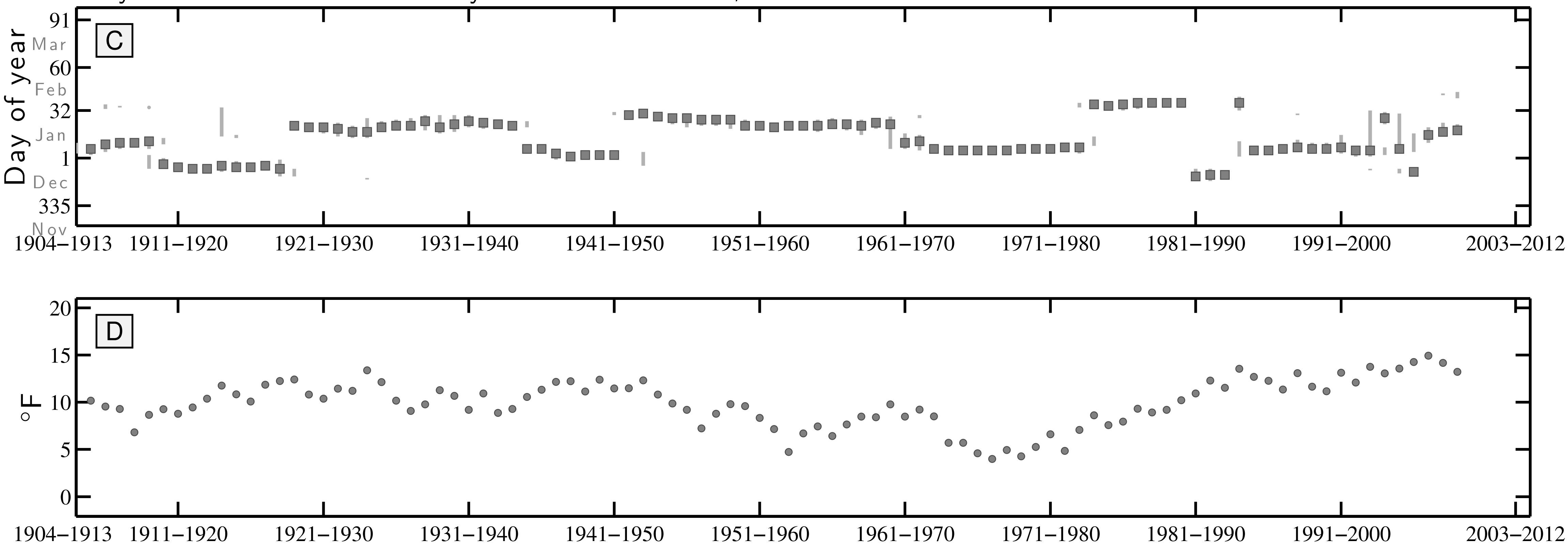

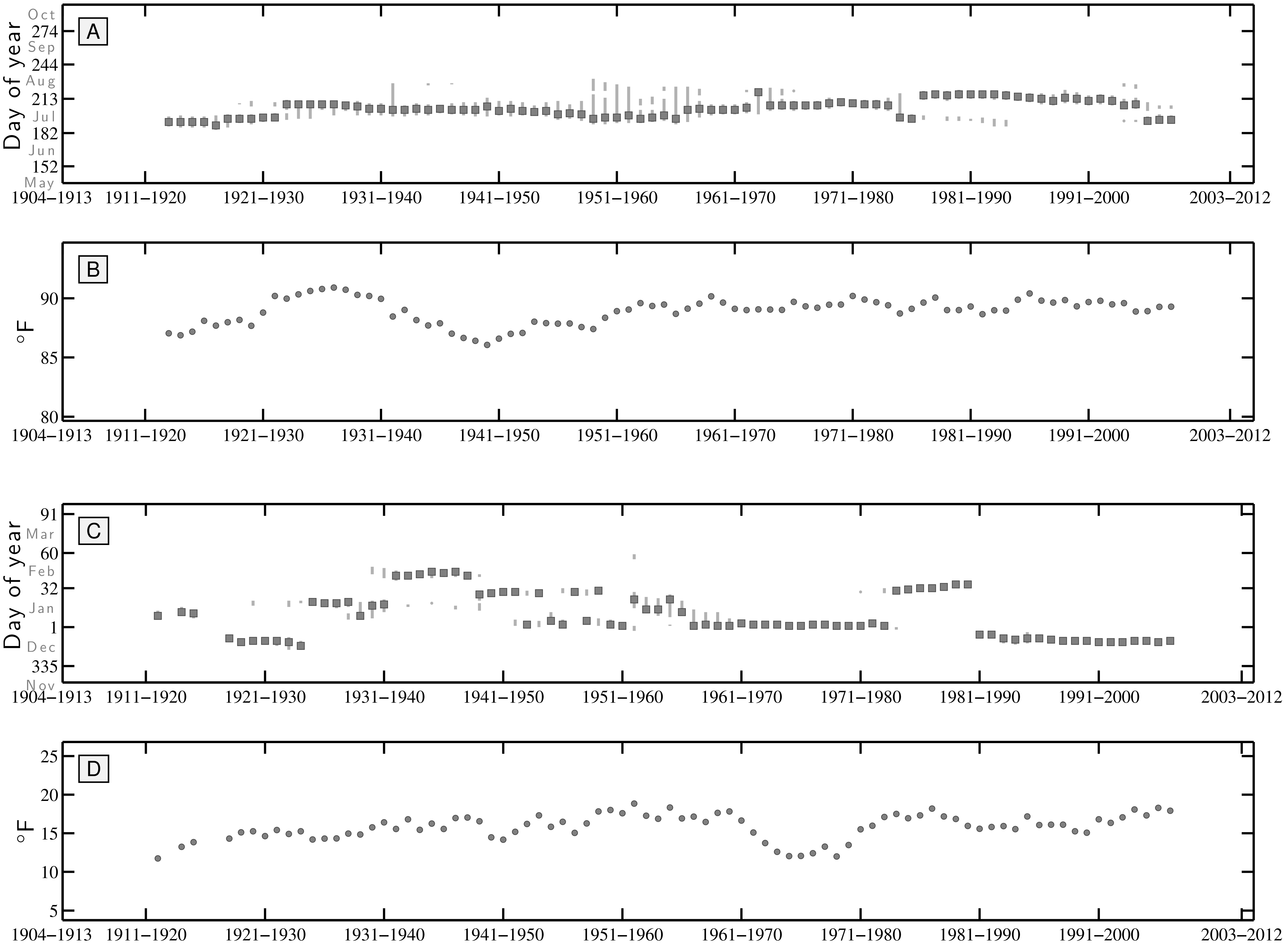

A
Day of year
Oct
274
Sep
244
Aug
213
Jul
182
Jun
152
May
1904–1913
1911–1920
1921–1930
1931–1940
1941–1950
1951–1960
1961–1970
1971–1980
1981–1990
1991–2000
2003–2012
B
°F
90
85
80
C
91
Mar
60
Feb
32
Jan
1
Dec
335
Nov
D
25
20
15
10
5

10 year Summer Teletherm dynamics for BATTLE MOUNTAIN 4SE, NV:

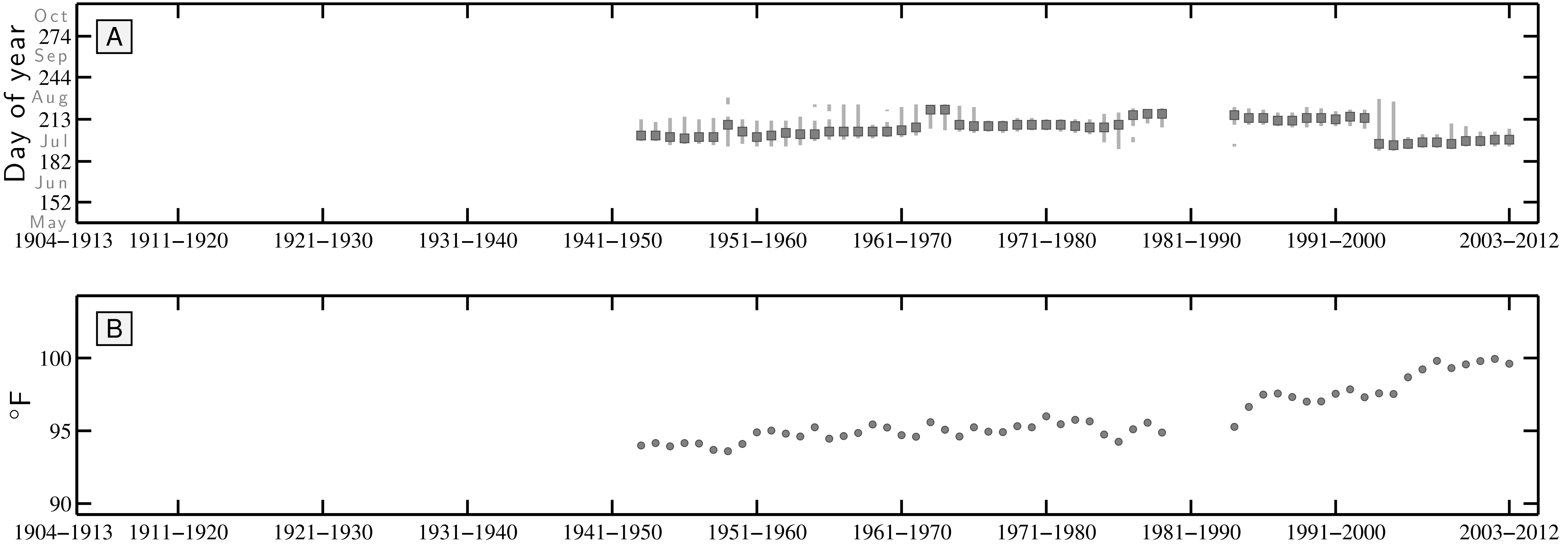

10 year Winter Teletherm dynamics for BATTLE MOUNTAIN 4SE, NV:

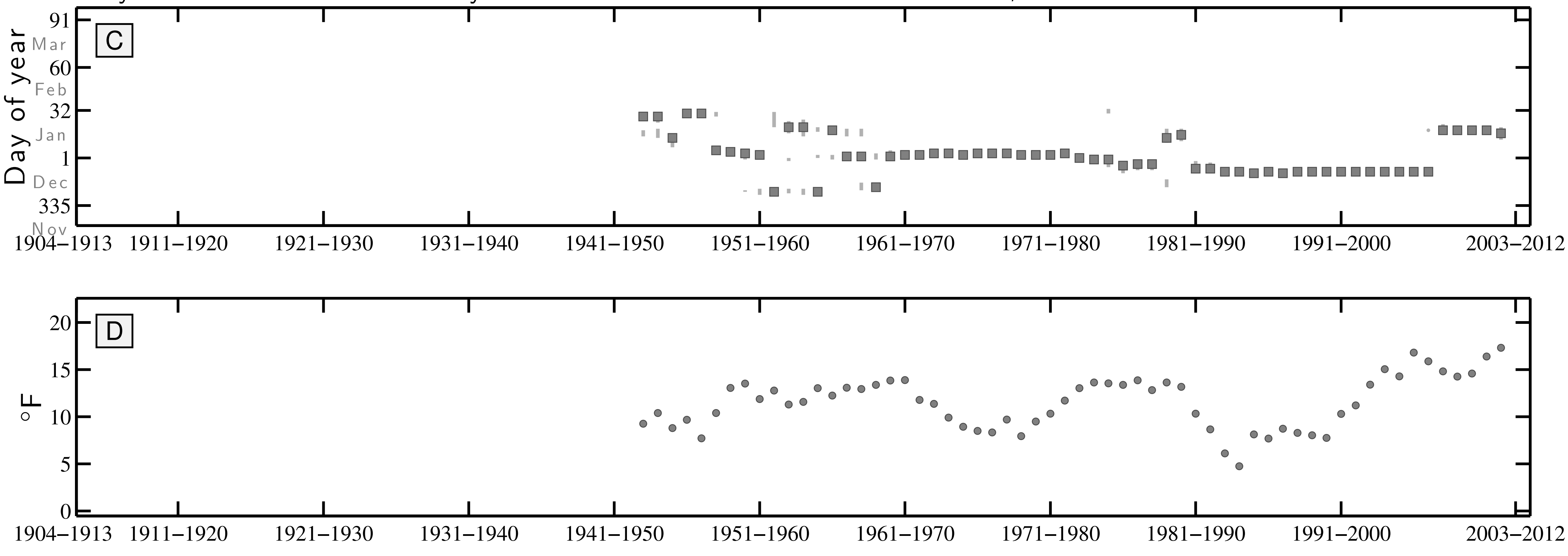

10 year Summer Teletherm dynamics for BOULDER CITY, NV:

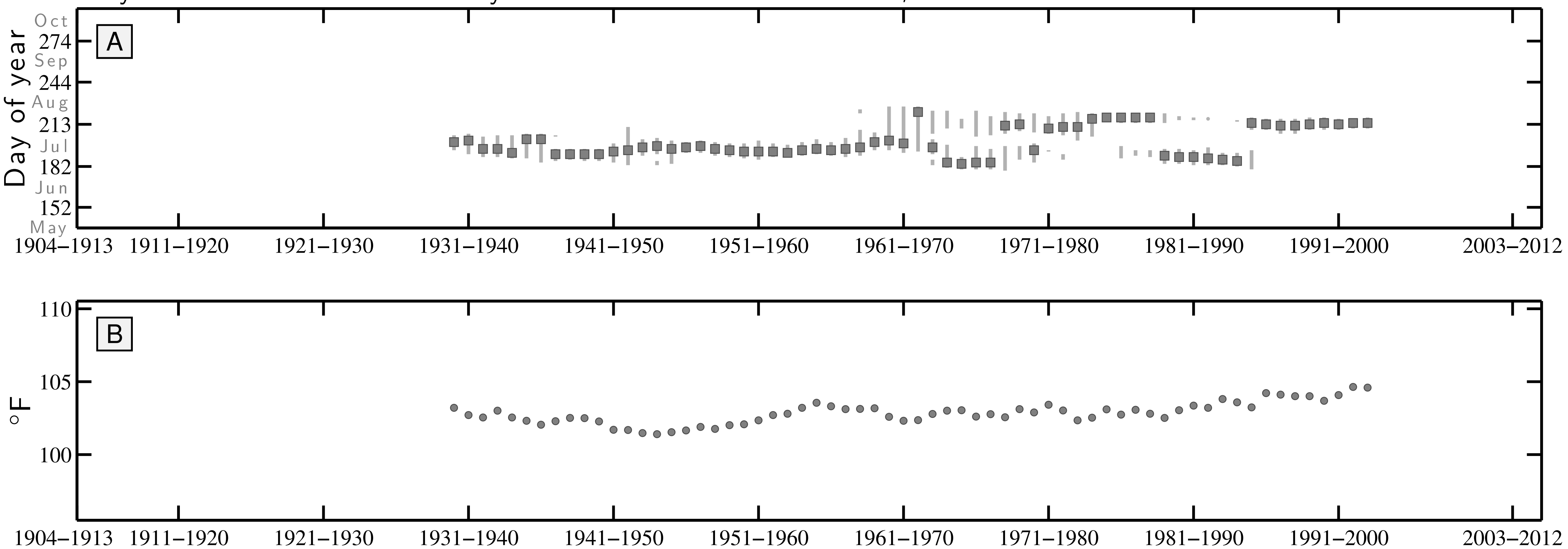

10 year Winter Teletherm dynamics for BOULDER CITY, NV:

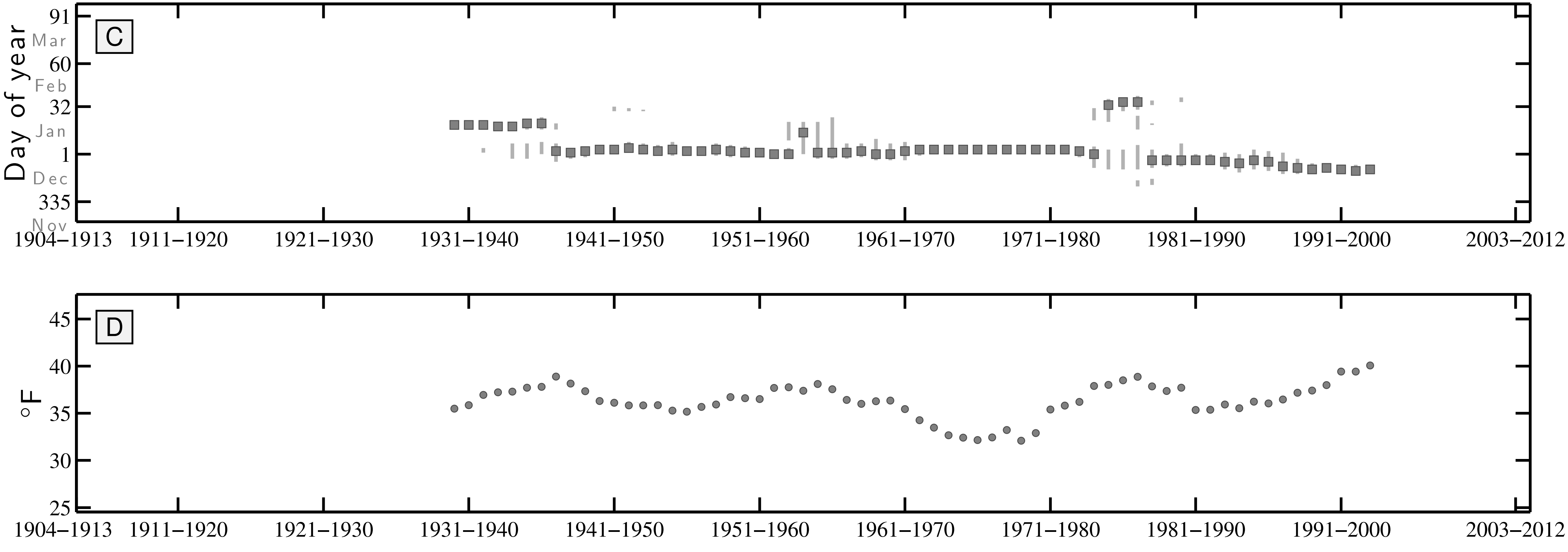

10 year Summer Teletherm dynamics for ELKO RGNL AP, NV:

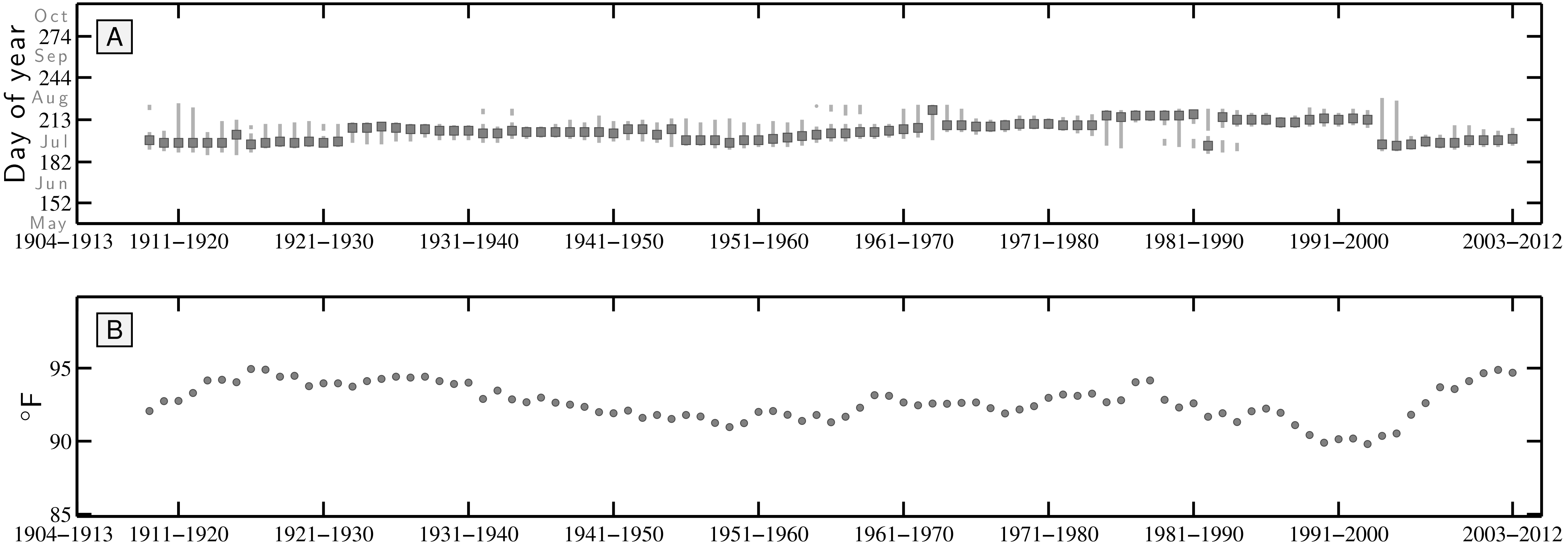

10 year Winter Teletherm dynamics for ELKO RGNL AP, NV:

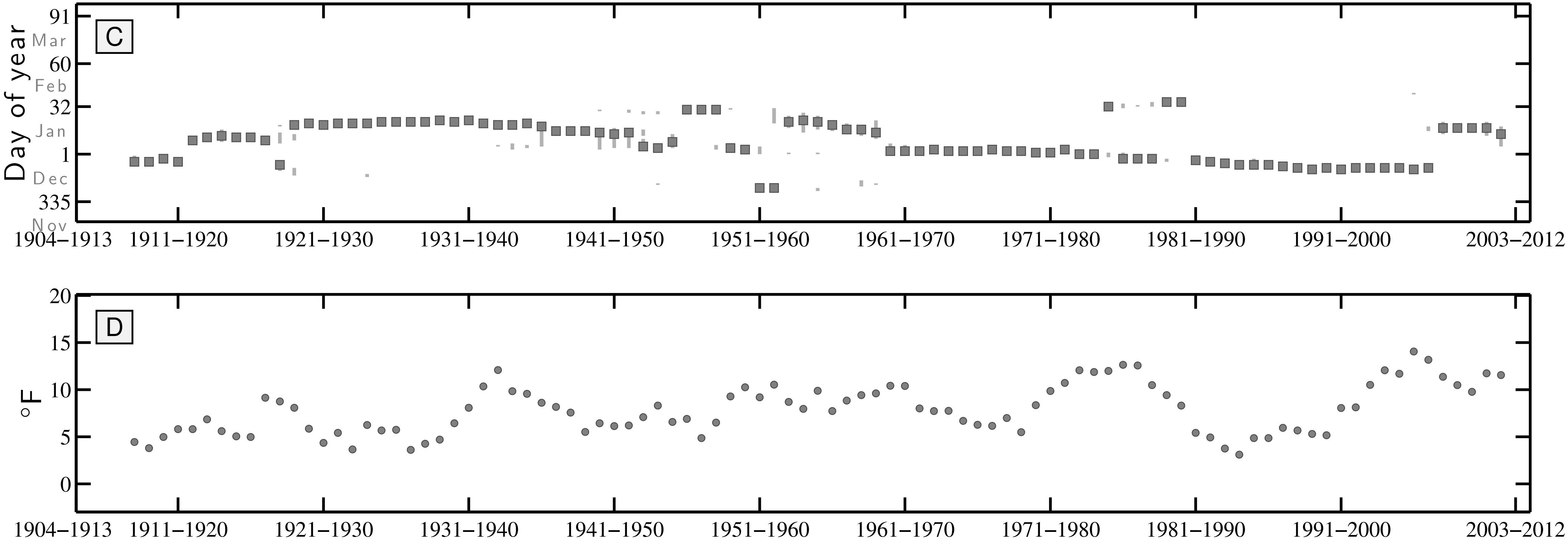

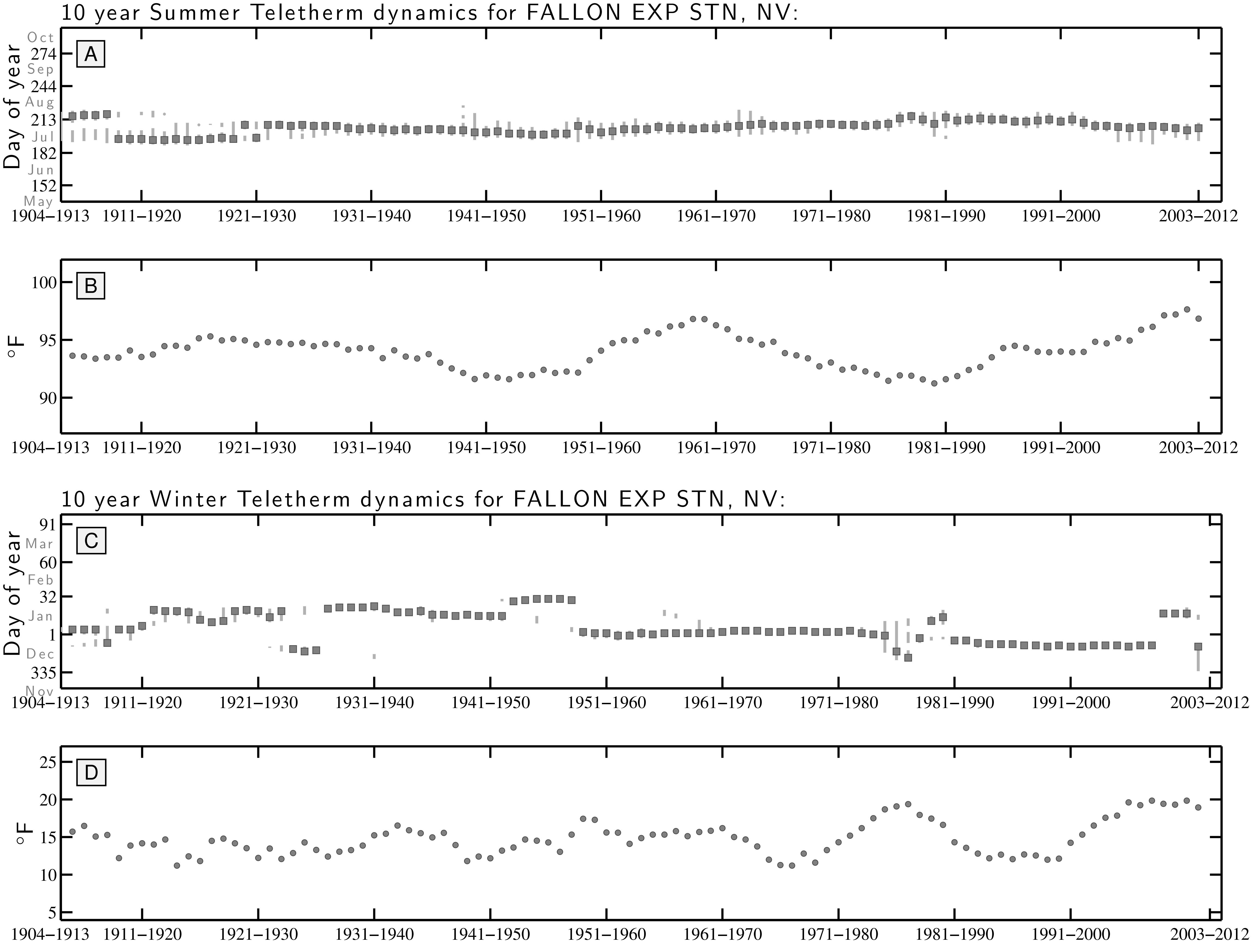
10 year Summer Teletherm dynamics for FALLON EXP STN, NV:
A
Day of year
Oct
274
Sep
244
Aug
213
Jul
182
Jun
152
May
1904–1913
1911–1920
1921–1930
1931–1940
1941–1950
1951–1960
1961–1970
1971–1980
1981–1990
1991–2000
2003–2012
B
°F
100
95
90
10 year Winter Teletherm dynamics for FALLON EXP STN, NV:
C
91
Mar
60
Feb
32
Jan
1
Dec
335
Nov
D
25
20
15
10
5

10 year Summer Teletherm dynamics for GOLCONDA, NV:

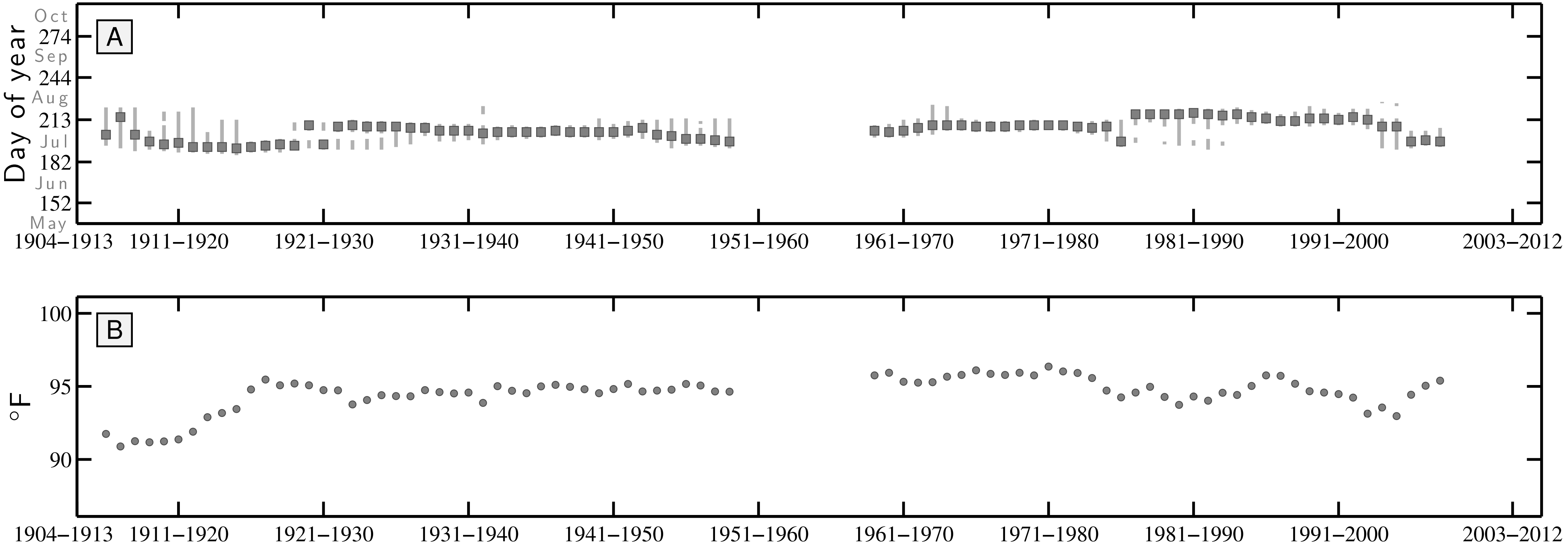

10 year Winter Teletherm dynamics for GOLCONDA, NV:

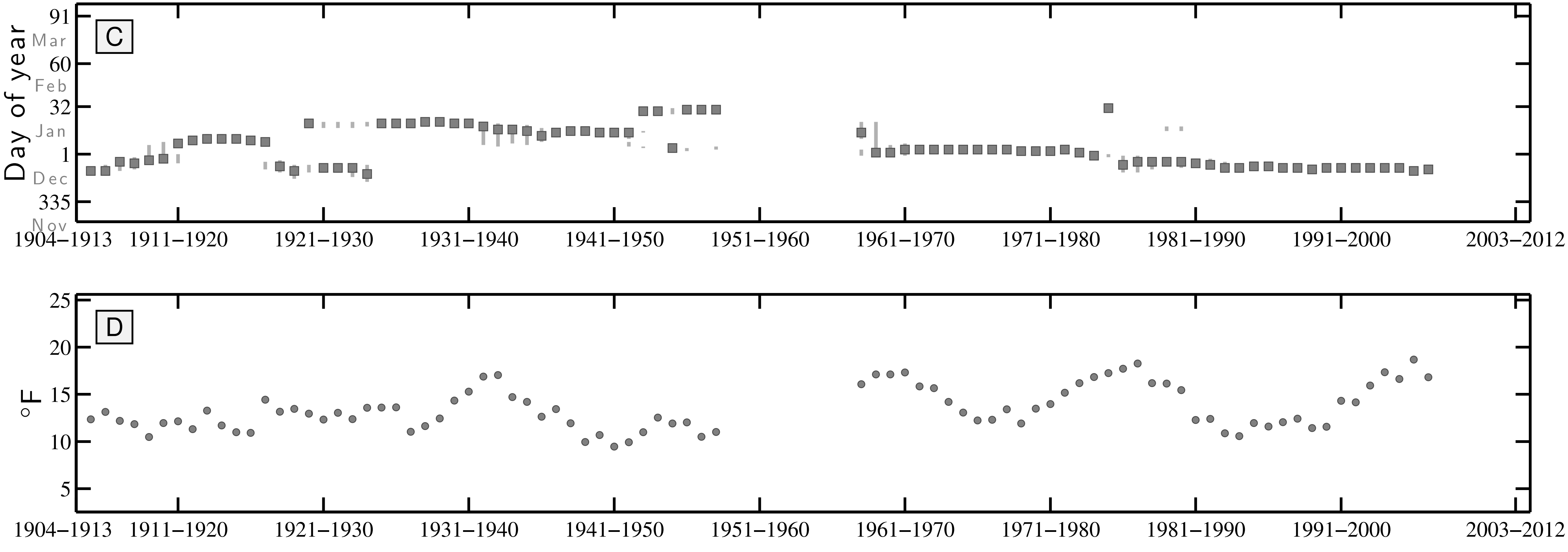

10 year Summer Teletherm dynamics for LOVELOCK, NV:

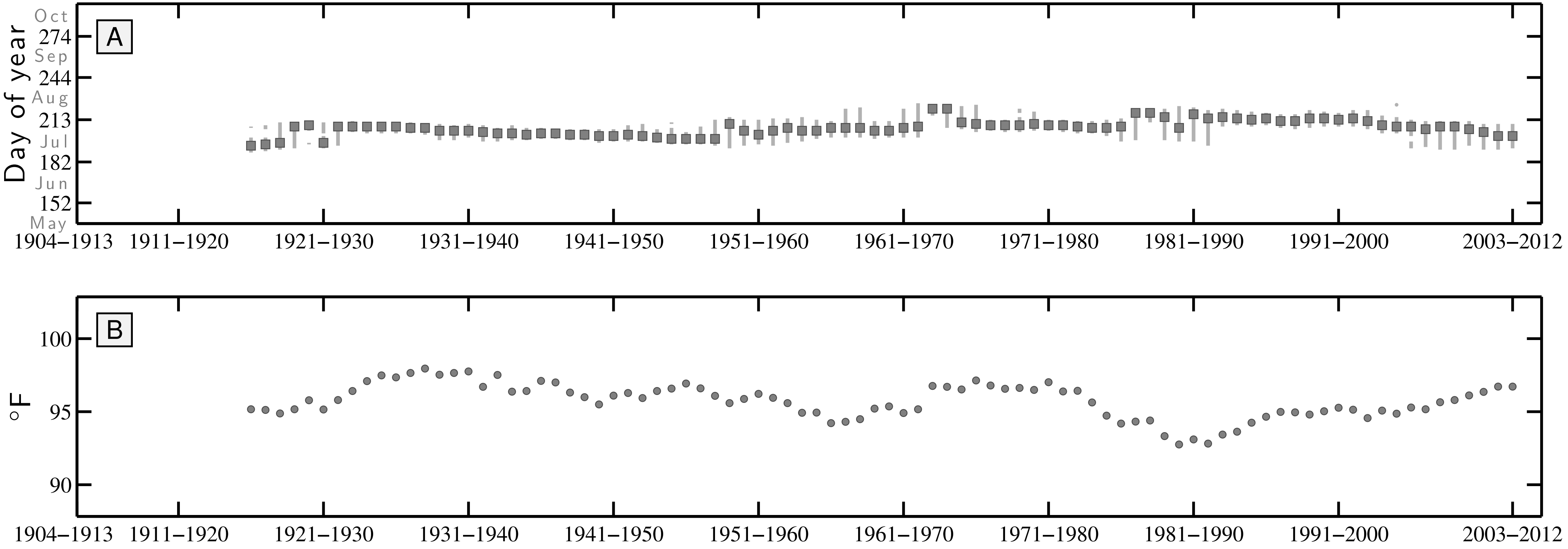

10 year Winter Teletherm dynamics for LOVELOCK, NV:

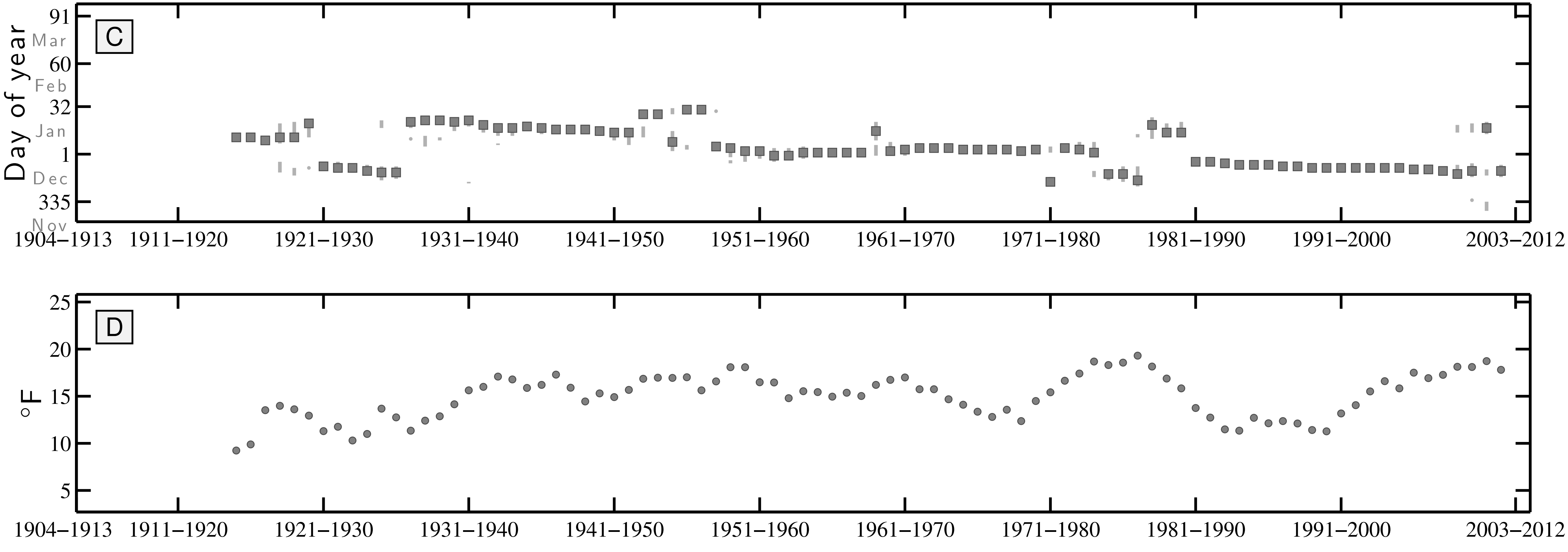

10 year Summer Teletherm dynamics for MCGILL, NV:

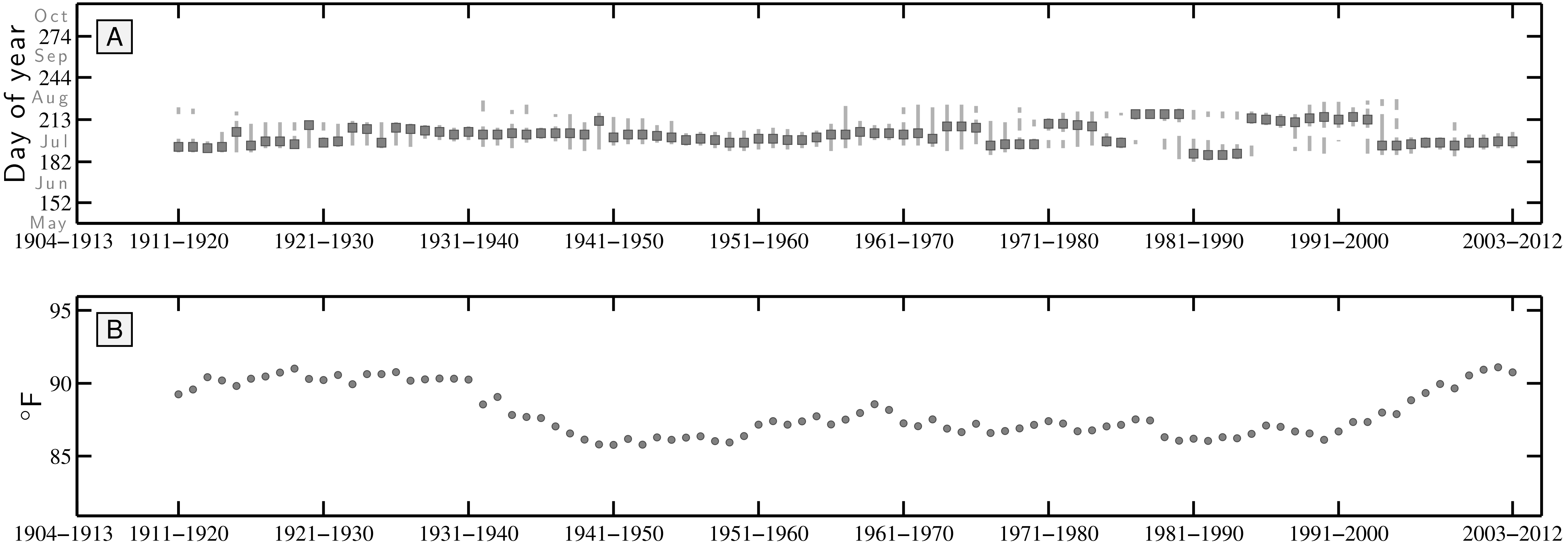

10 year Winter Teletherm dynamics for MCGILL, NV:

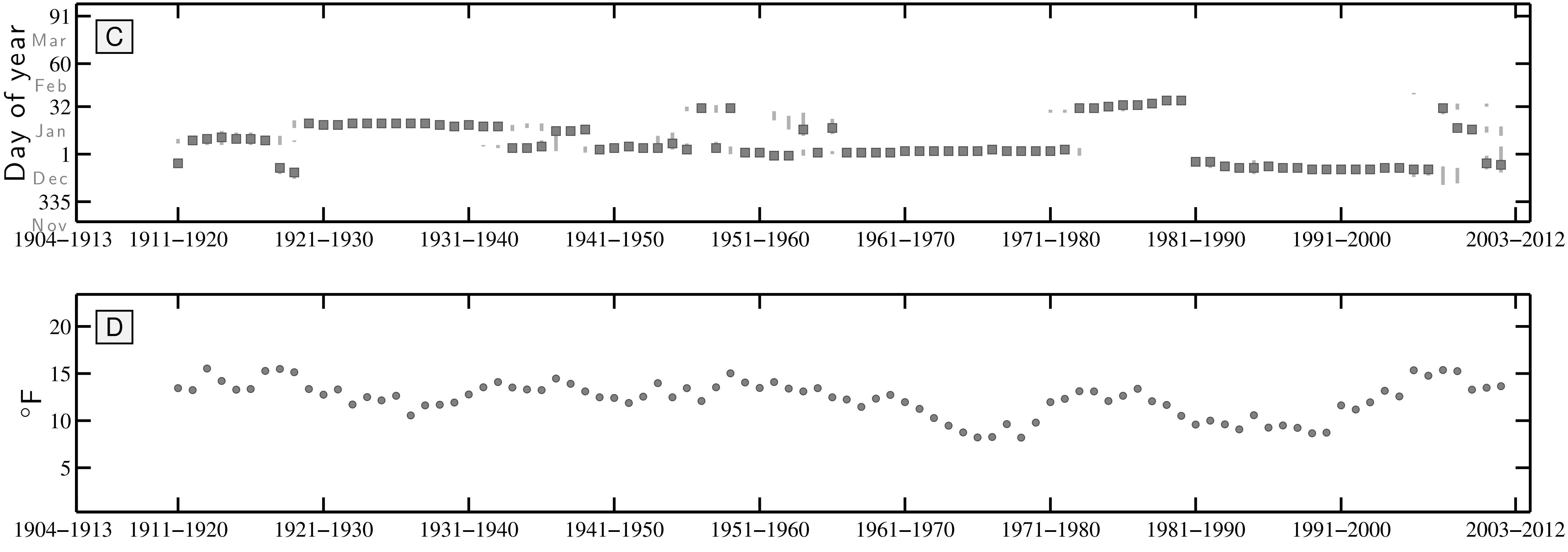

10 year Summer Teletherm dynamics for MINA, NV:

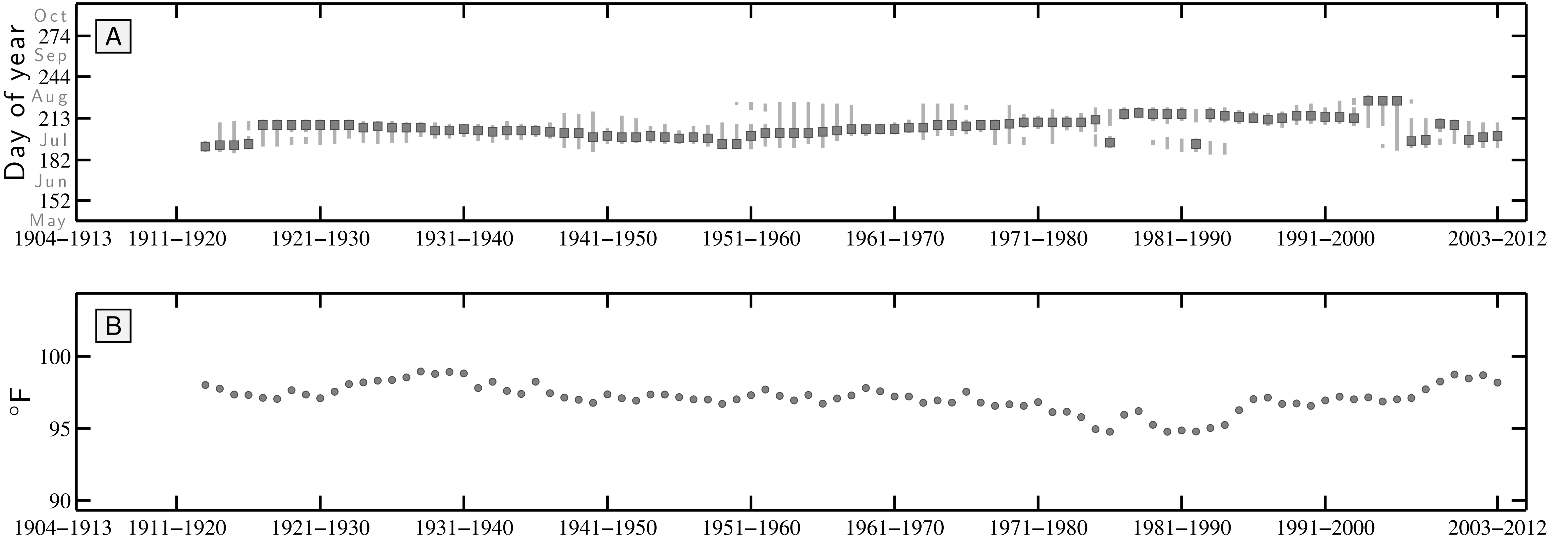

10 year Winter Teletherm dynamics for MINA, NV:

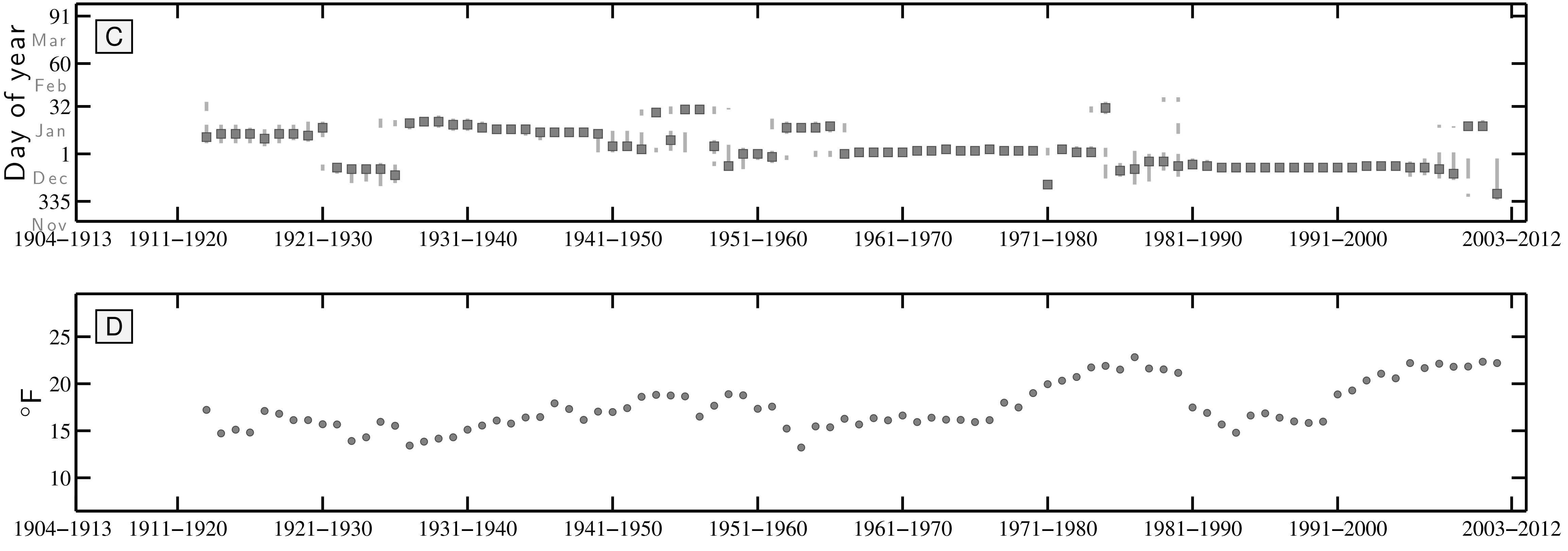

10 year Summer Teletherm dynamics for RENO AP, NV:

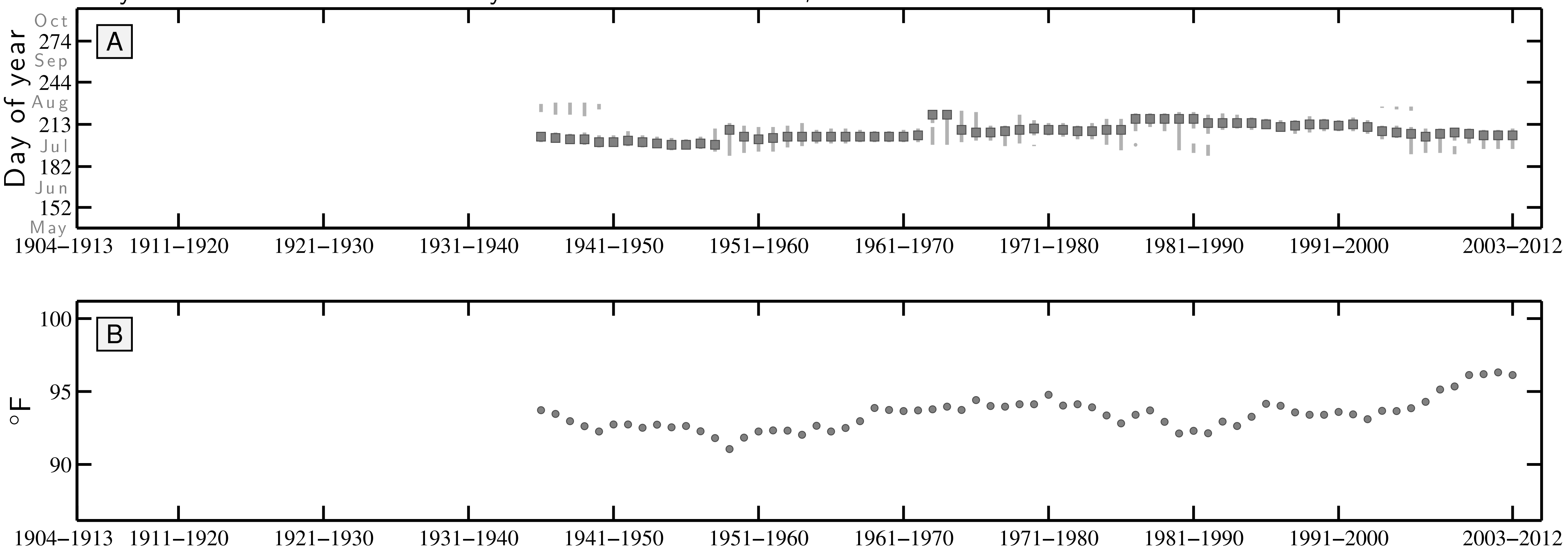

10 year Winter Teletherm dynamics for RENO AP, NV:

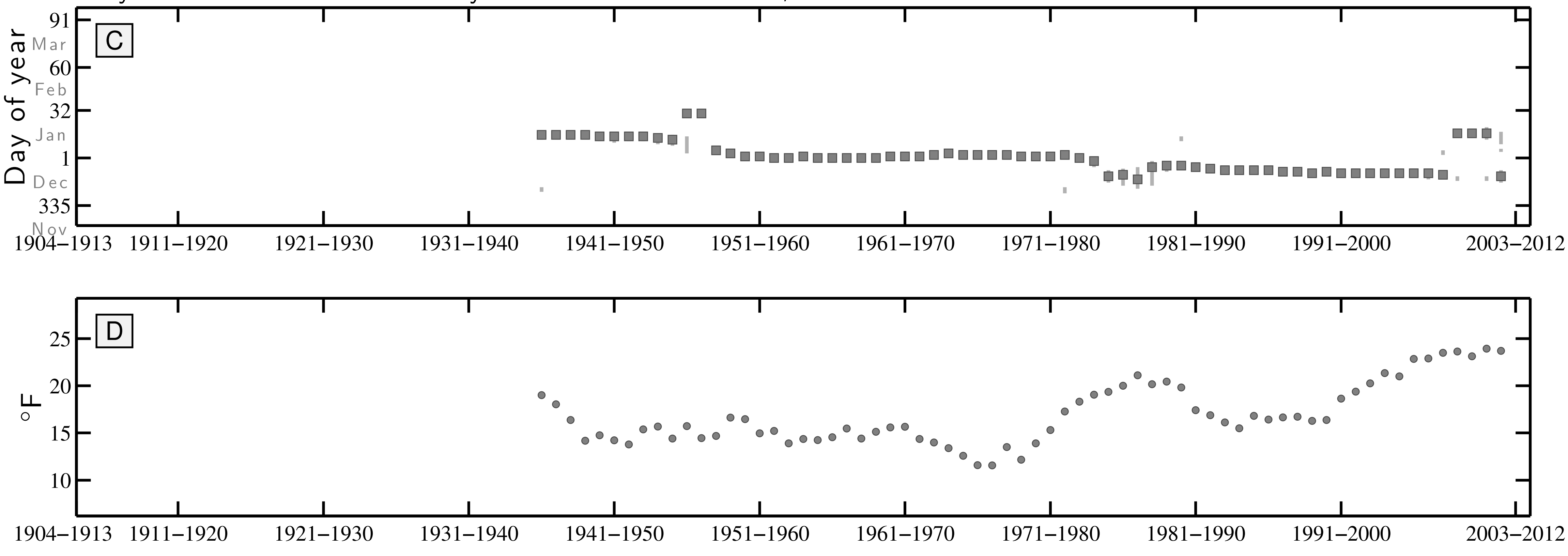

10 year Summer Teletherm dynamics for SEARCHLIGHT, NV:

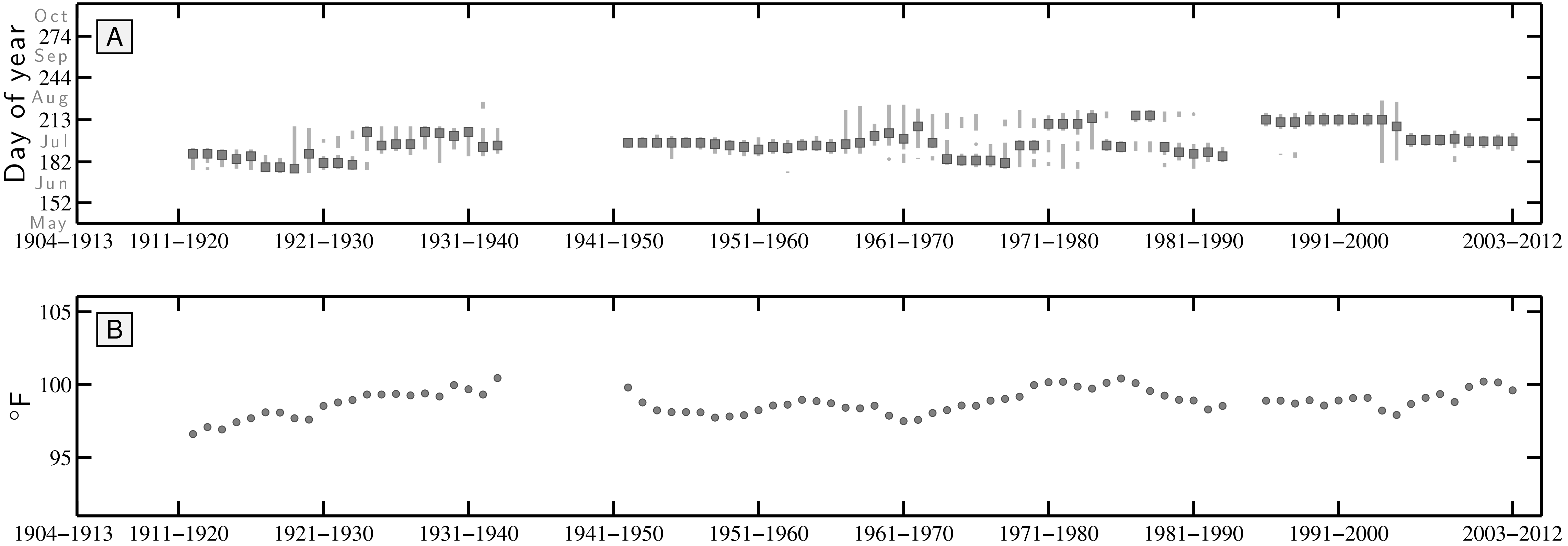

10 year Winter Teletherm dynamics for SEARCHLIGHT, NV:

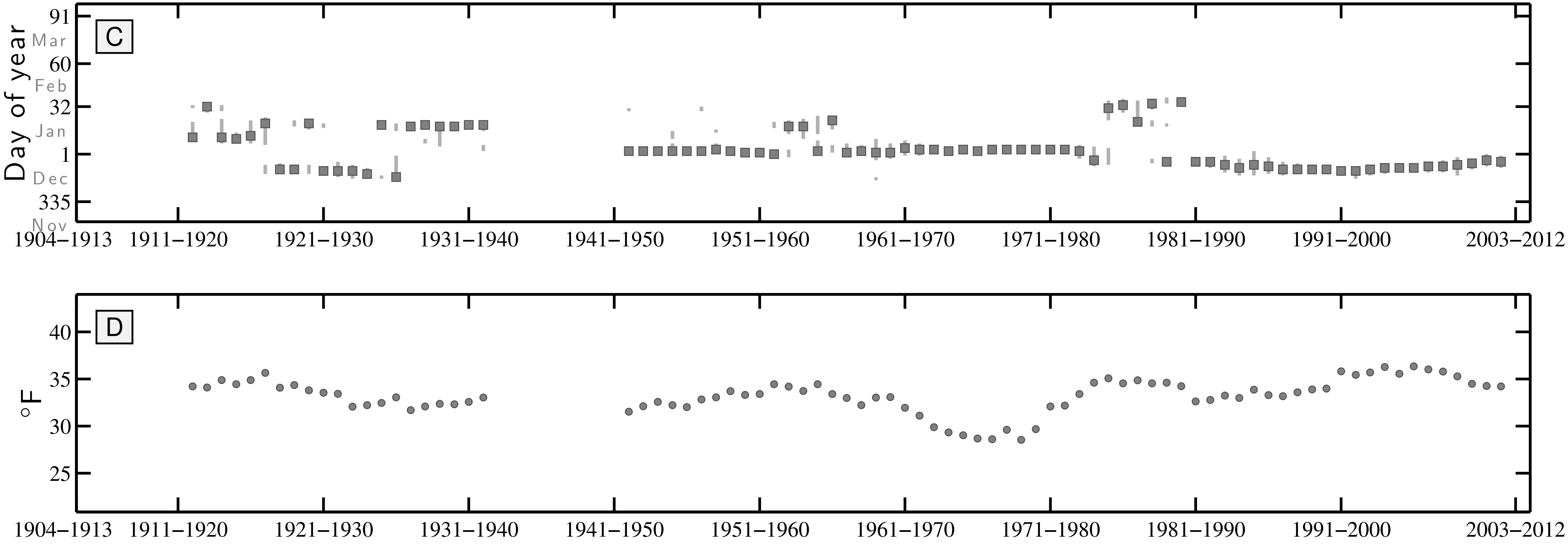

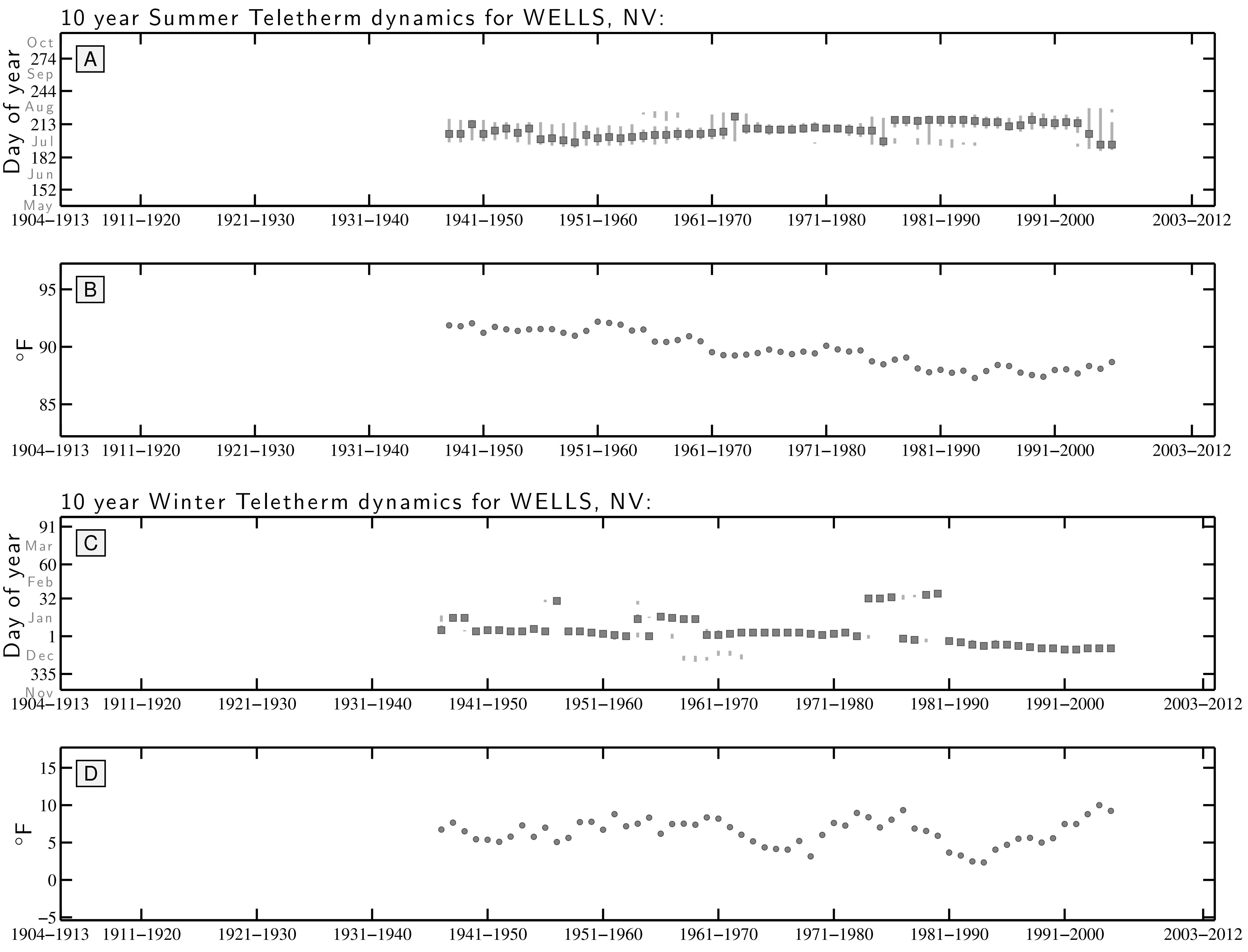
10 year Summer Teletherm dynamics for WELLS, NV:
A
B
Day of year
°F
Oct
274
Sep
244
Aug
213
Jul
182
Jun
152
May
95
90
85
10 year Winter Teletherm dynamics for WELLS, NV:
C
D
91
Mar
60
Feb
32
Jan
1
Dec
335
Nov
15
10
5
0
−5
1904–1913
1911–1920
1921–1930
1931–1940
1941–1950
1951–1960
1961–1970
1971–1980
1981–1990
1991–2000
2003–2012

10 year Summer Teletherm dynamics for WINNEMUCCA AP, NV:

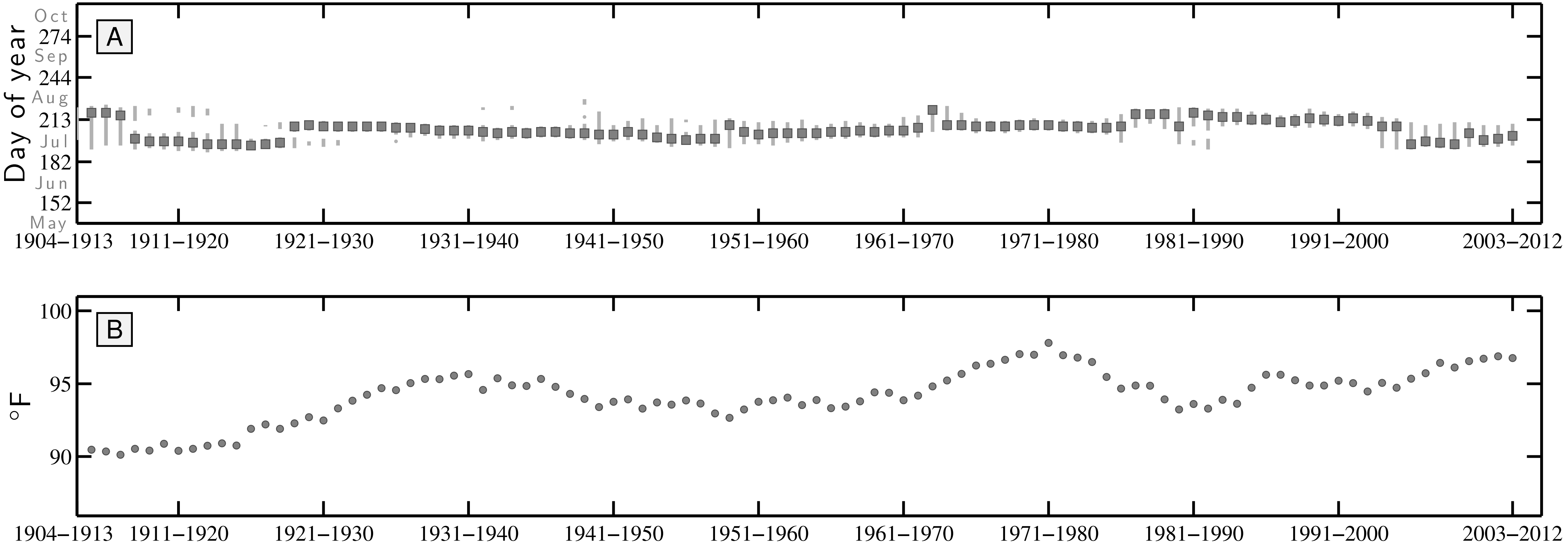

10 year Winter Teletherm dynamics for WINNEMUCCA AP, NV:

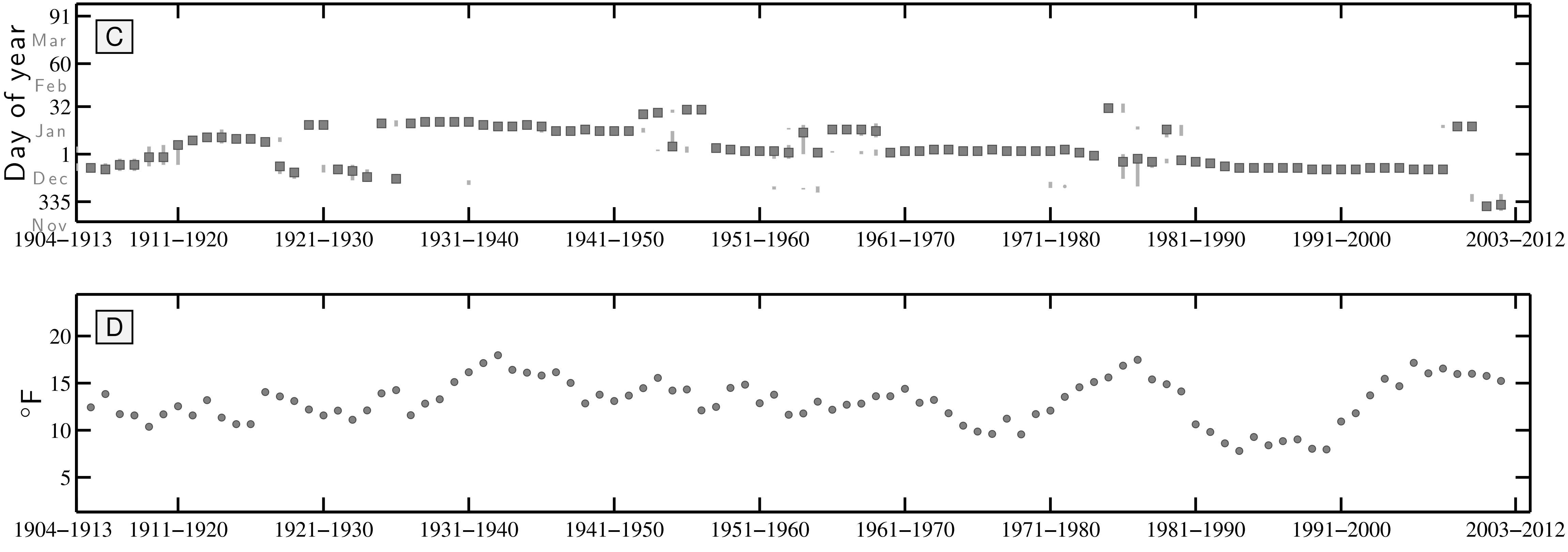

10 year Summer Teletherm dynamics for BETHLEHEM 2, NH:

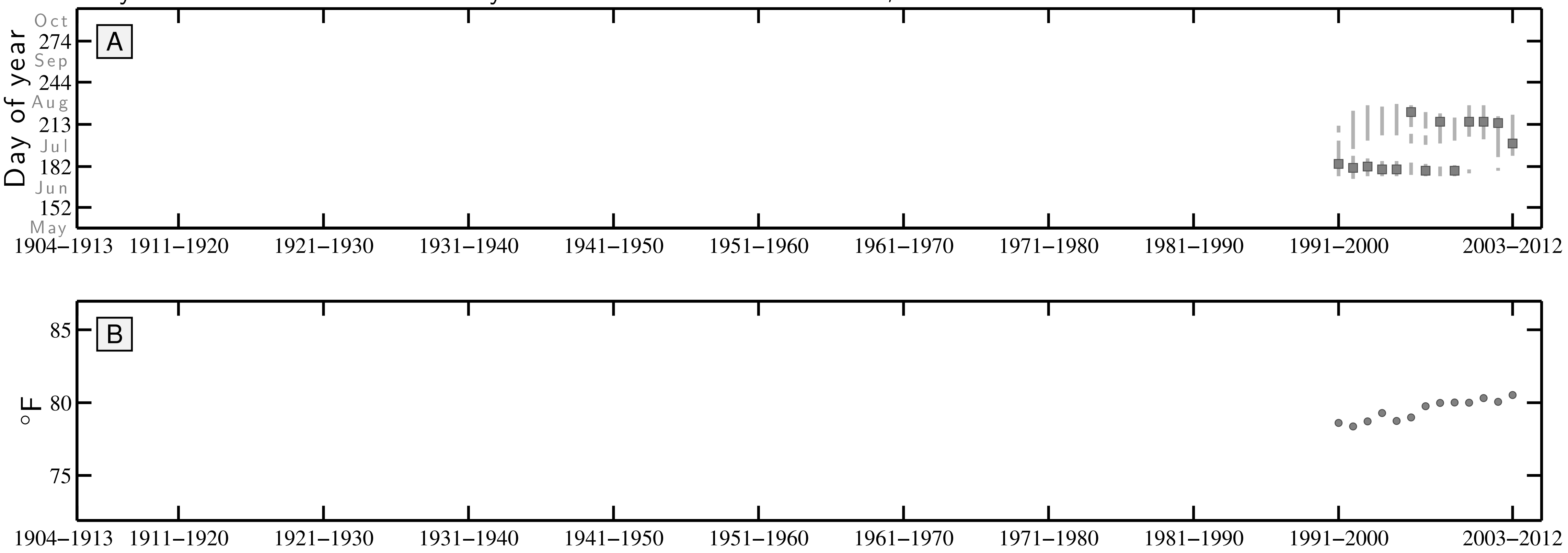

10 year Winter Teletherm dynamics for BETHLEHEM 2, NH:

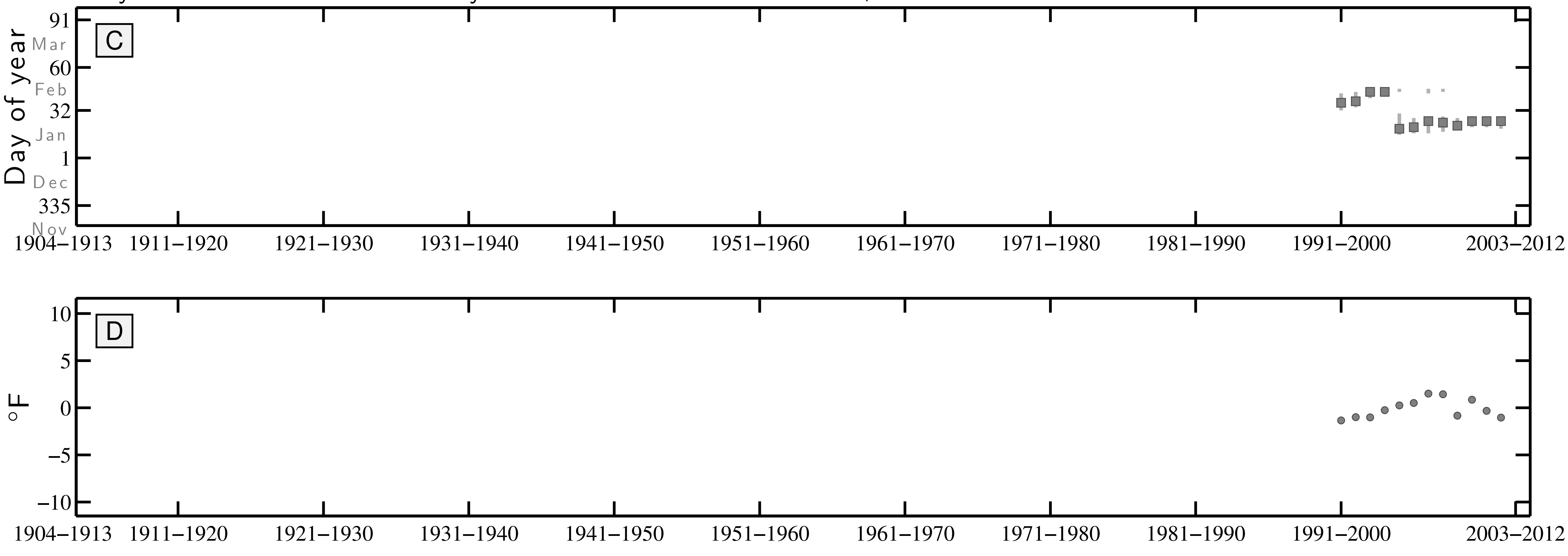

10 year Summer Teletherm dynamics for DURHAM, NH:

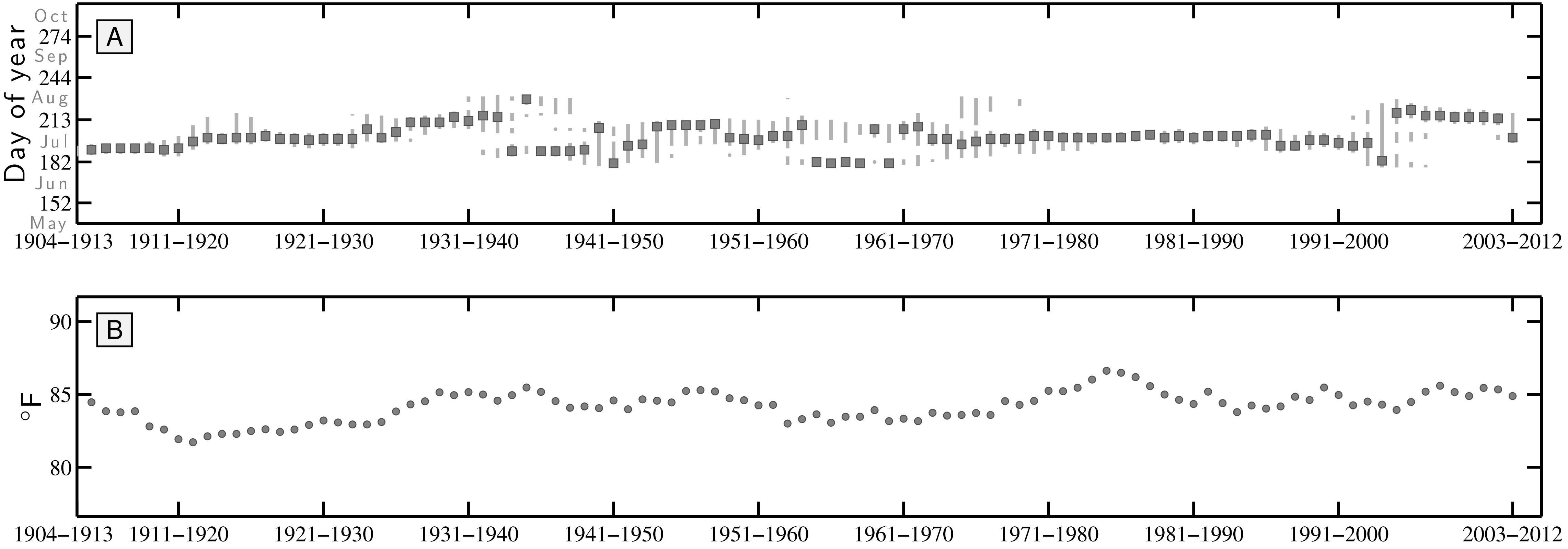

10 year Winter Teletherm dynamics for DURHAM, NH:

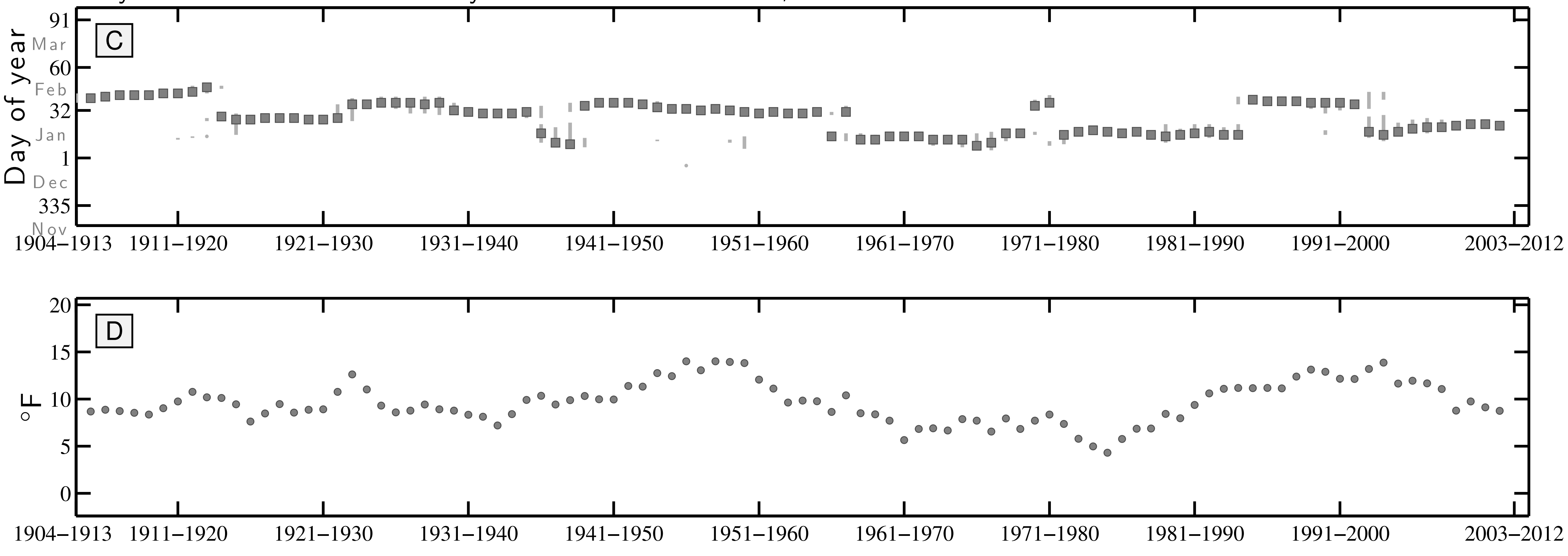

10 year Summer Teletherm dynamics for FIRST CONNECTICUT LAKE, NH:

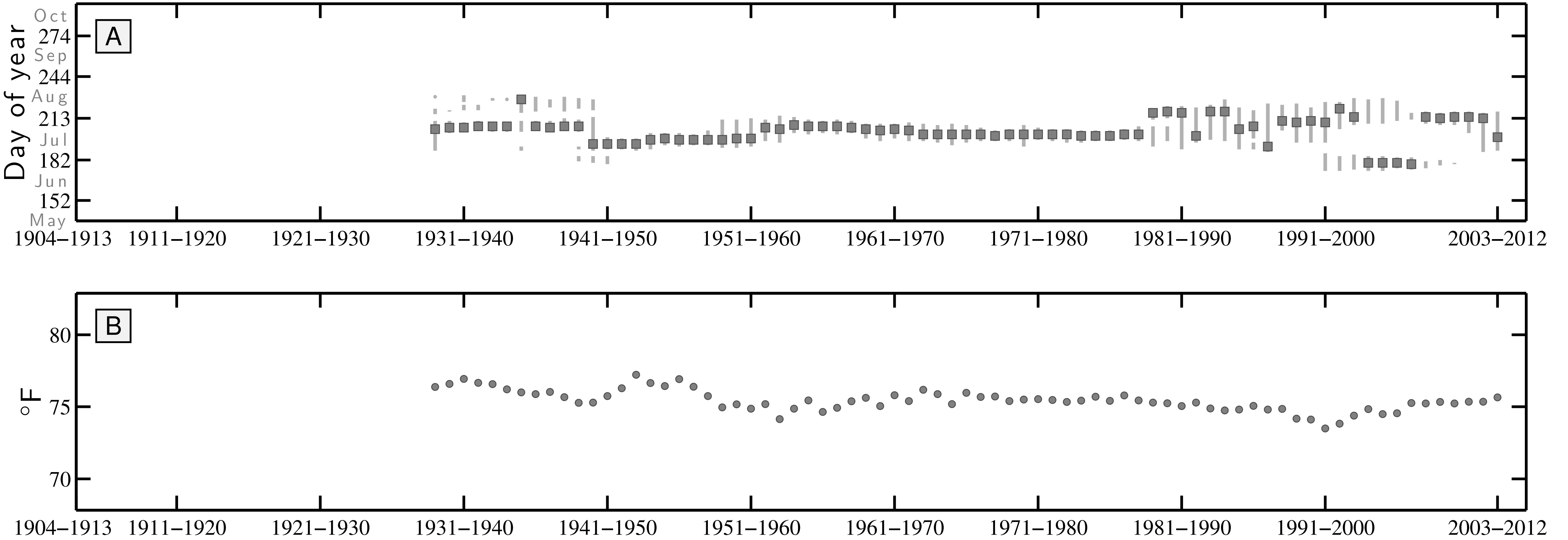

10 year Winter Teletherm dynamics for FIRST CONNECTICUT LAKE, NH:

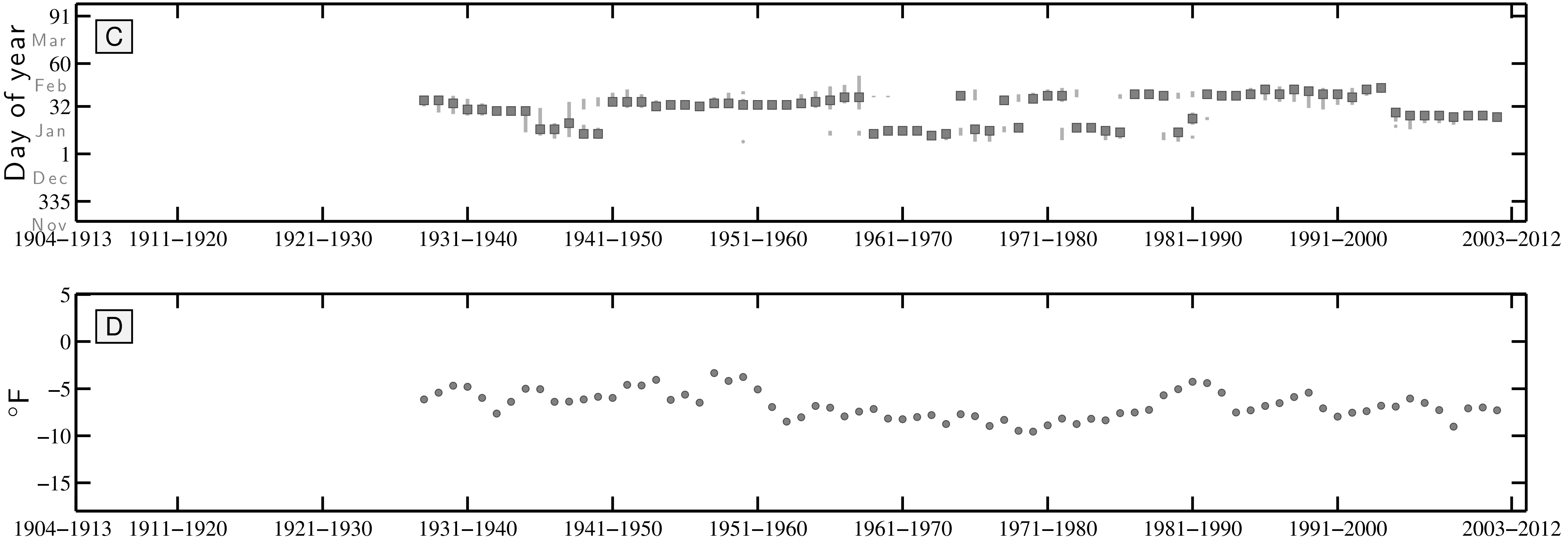

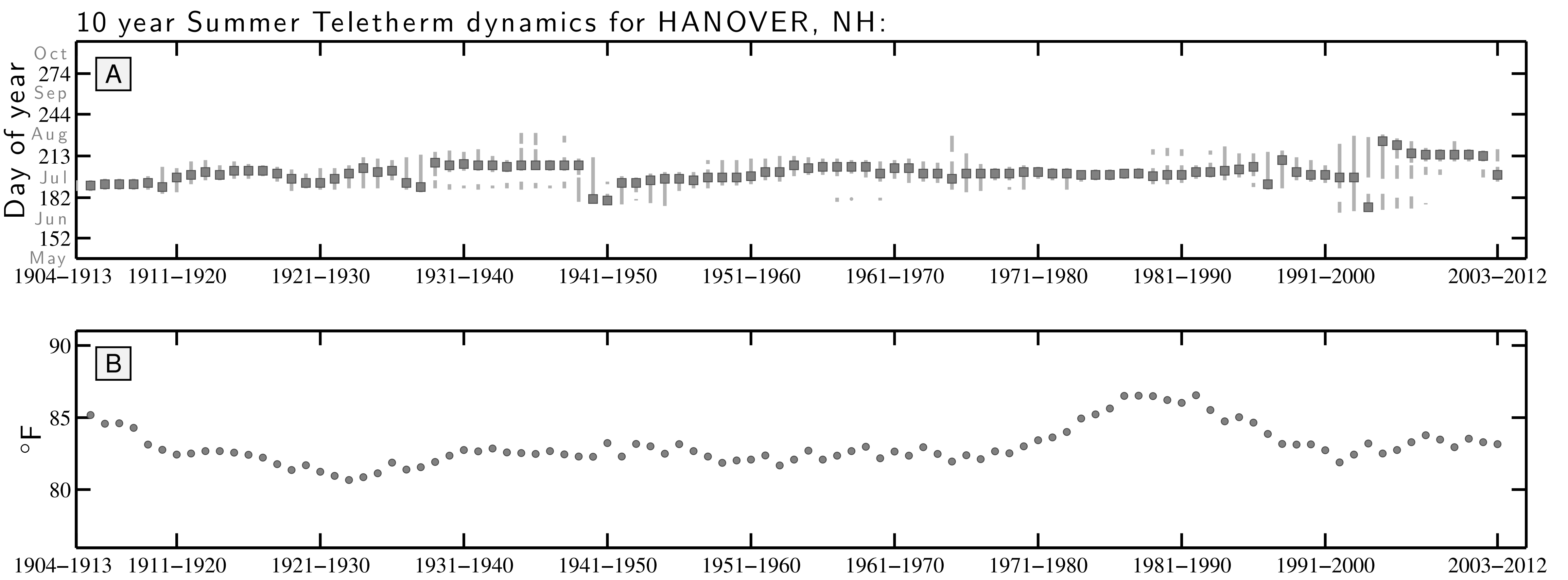
10 year Summer Teletherm dynamics for HANOVER, NH:
A
B
Day of year
°F
Oct
274
Sep
244
Aug
213
Jul
182
Jun
152
May
90
85
80
1904–1913
1911–1920
1921–1930
1931–1940
1941–1950
1951–1960
1961–1970
1971–1980
1981–1990
1991–2000
2003–2012

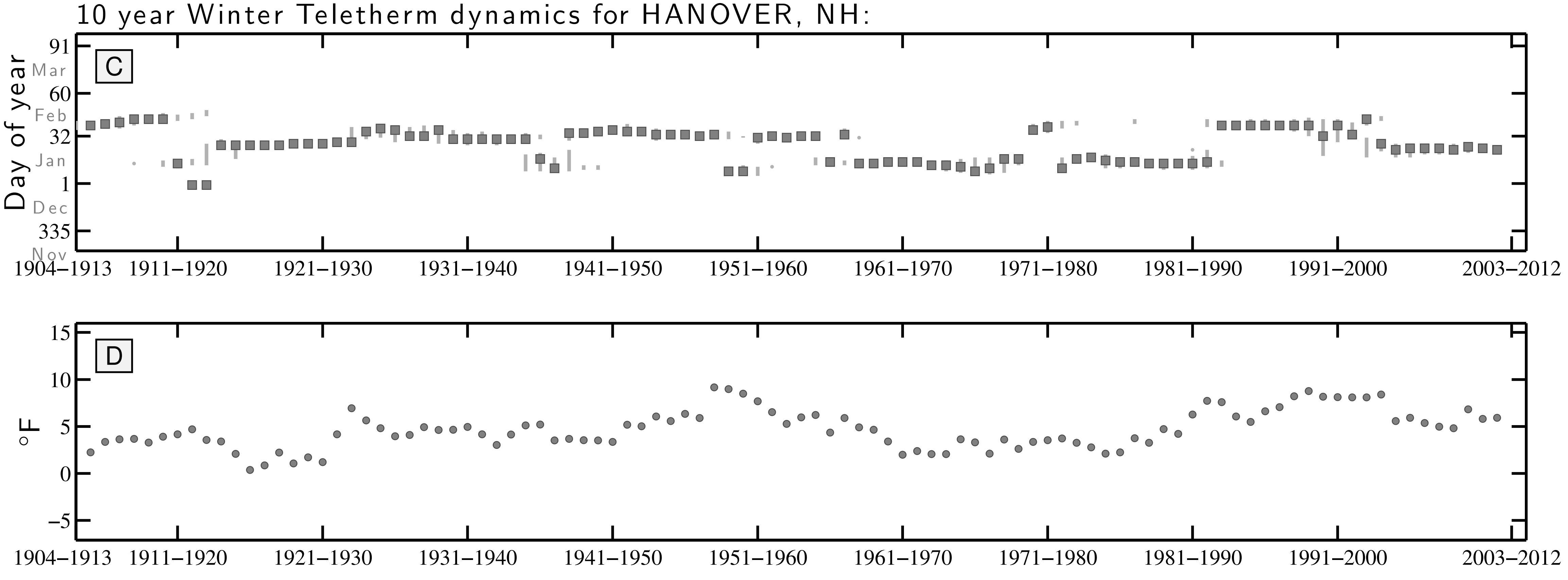
10 year Winter Teletherm dynamics for HANOVER, NH:
C
D
Day of year
°F
91
Mar
60
Feb
32
Jan
1
Dec
335
Nov
15
10
5
0
−5
1904–1913
1911–1920
1921–1930
1931–1940
1941–1950
1951–1960
1961–1970
1971–1980
1981–1990
1991–2000
2003–2012

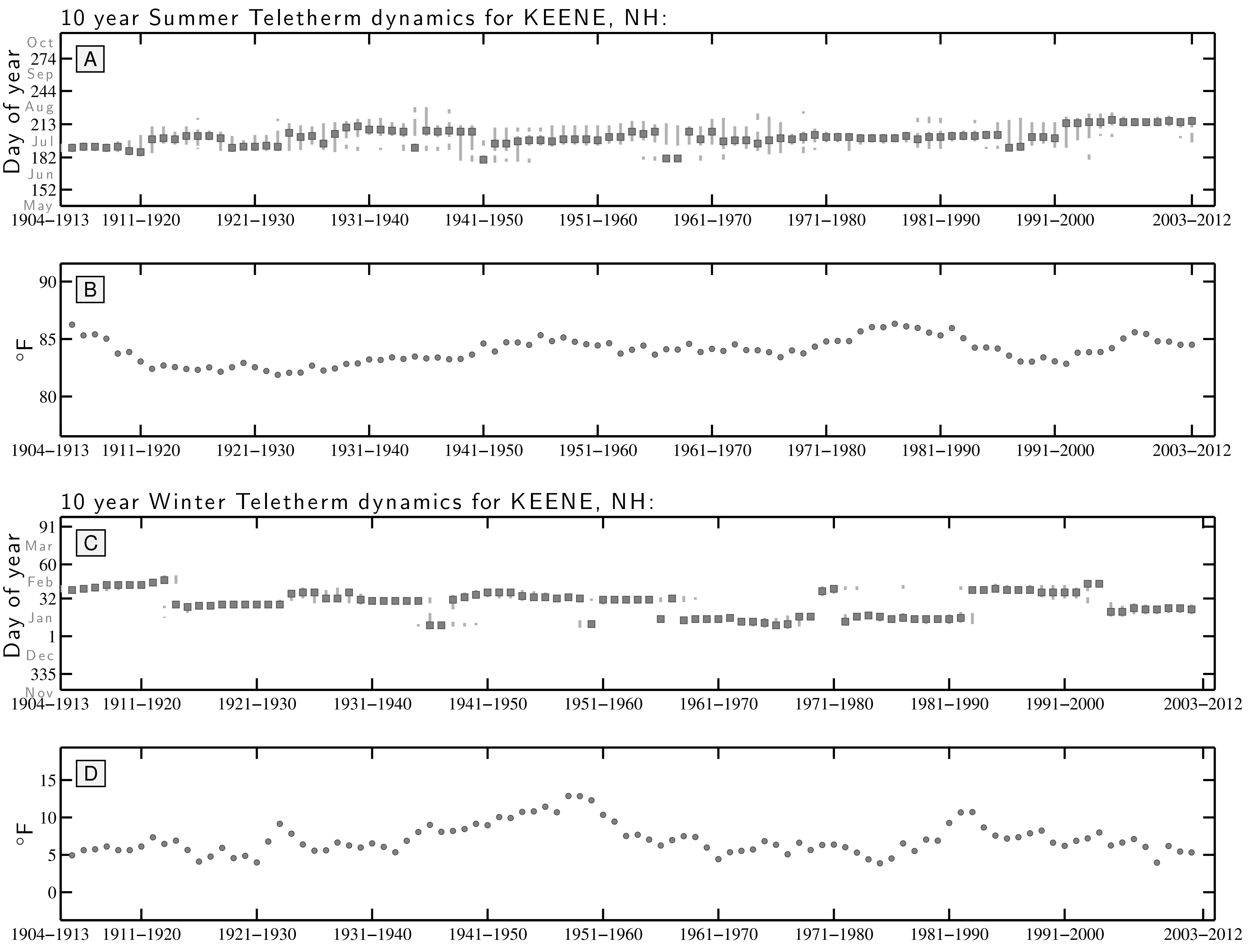

10 year Summer Teletherm dynamics for KEENE, NH:
A
Day of year
Oct
274
Sep
244
Aug
213
Jul
182
Jun
152
May
1904–1913
1911–1920
1921–1930
1931–1940
1941–1950
1951–1960
1961–1970
1971–1980
1981–1990
1991–2000
2003–2012
B
°F
90
85
80
10 year Winter Teletherm dynamics for KEENE, NH:
C
91
Mar
60
Feb
32
Jan
1
Dec
335
Nov
D
15
10
5
0

10 year Summer Teletherm dynamics for ATLANTIC CITY, NJ:

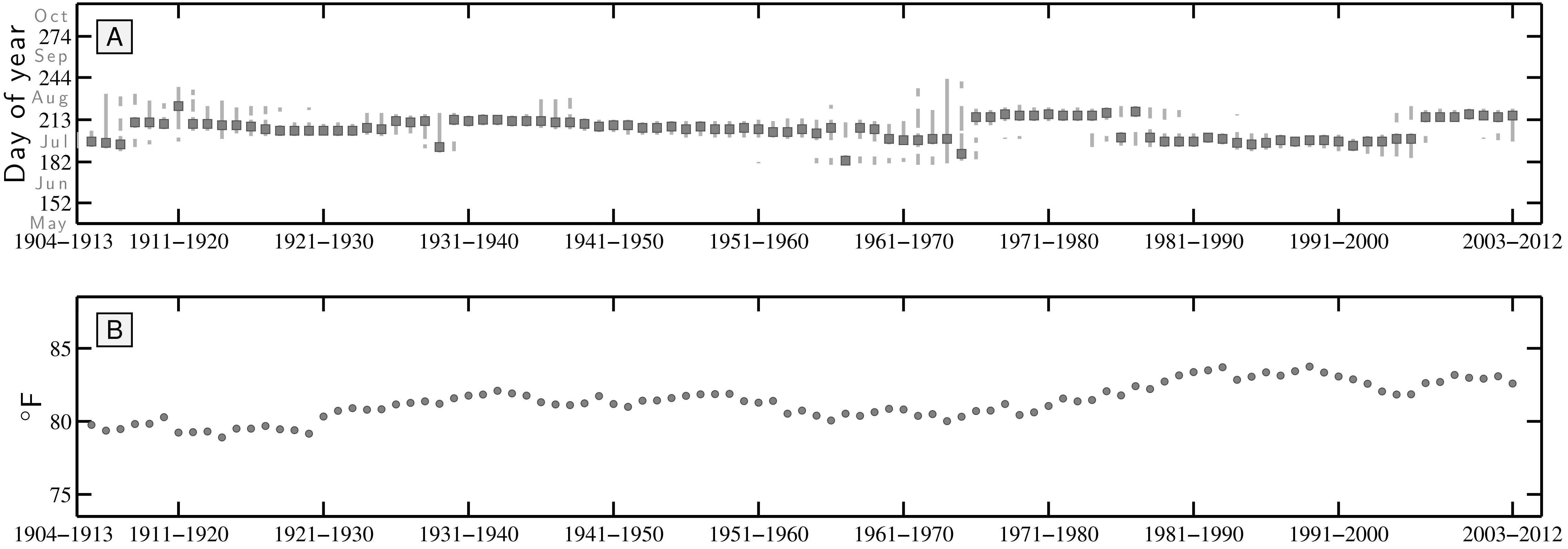

10 year Winter Teletherm dynamics for ATLANTIC CITY, NJ:

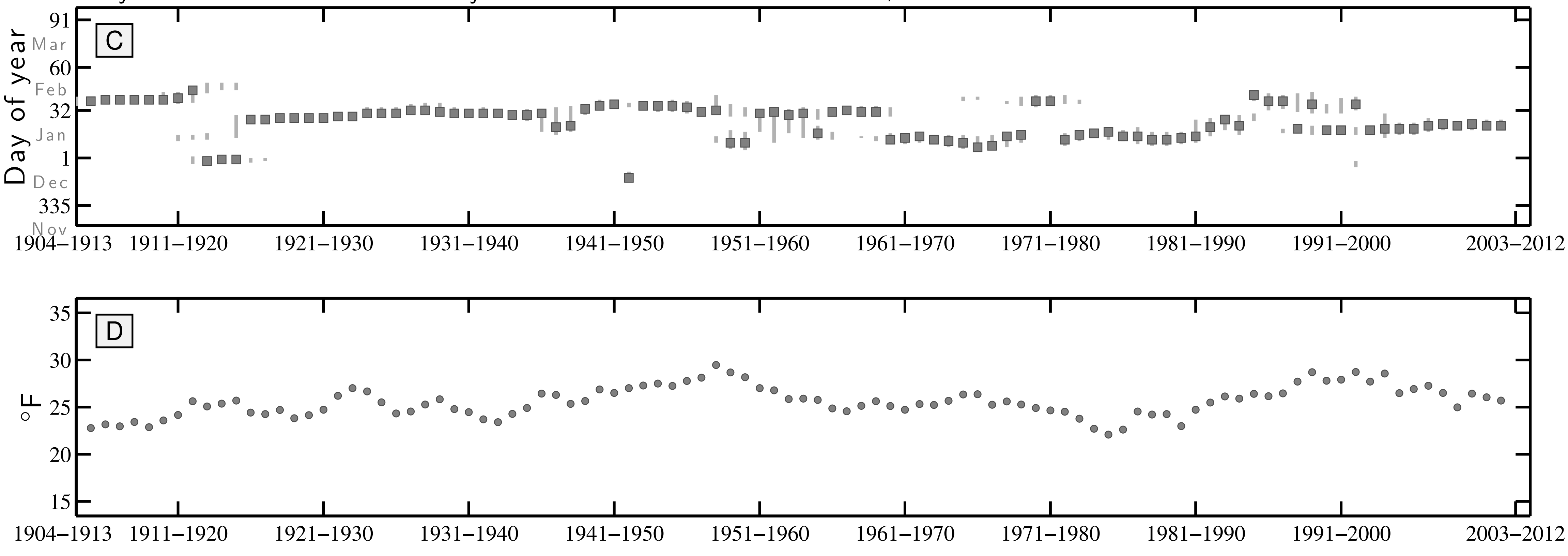

10 year Summer Teletherm dynamics for BELVIDERE BRG, NJ:

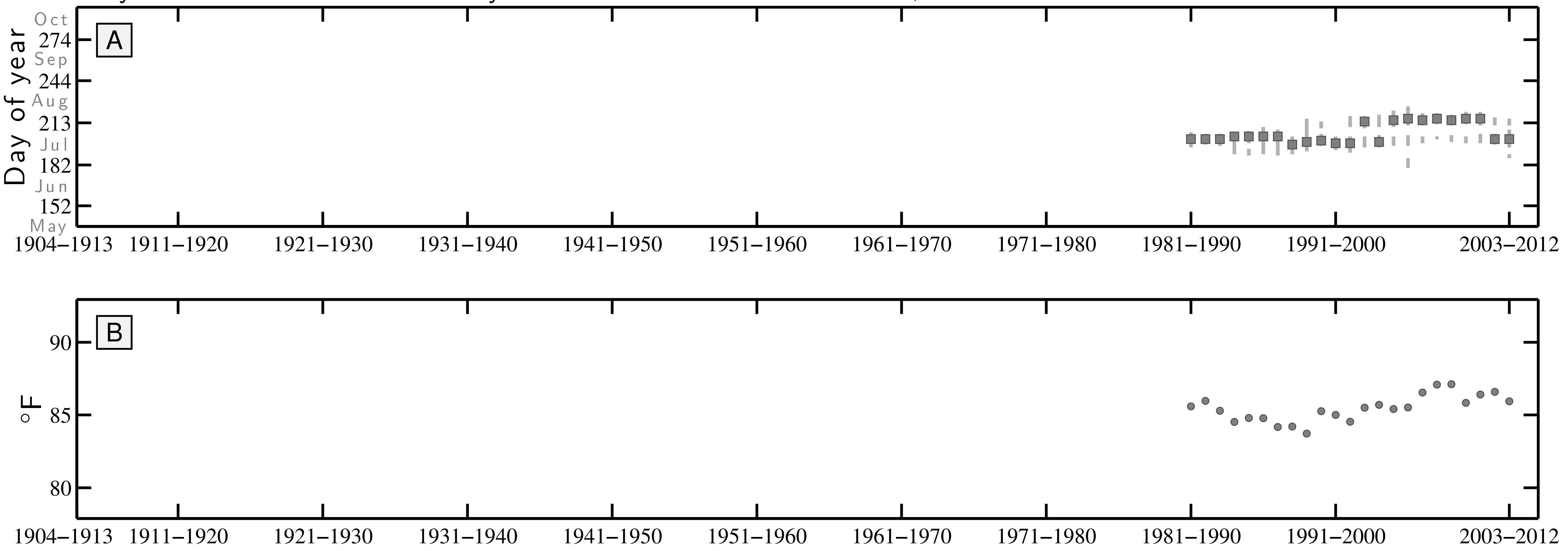

10 year Winter Teletherm dynamics for BELVIDERE BRG, NJ:

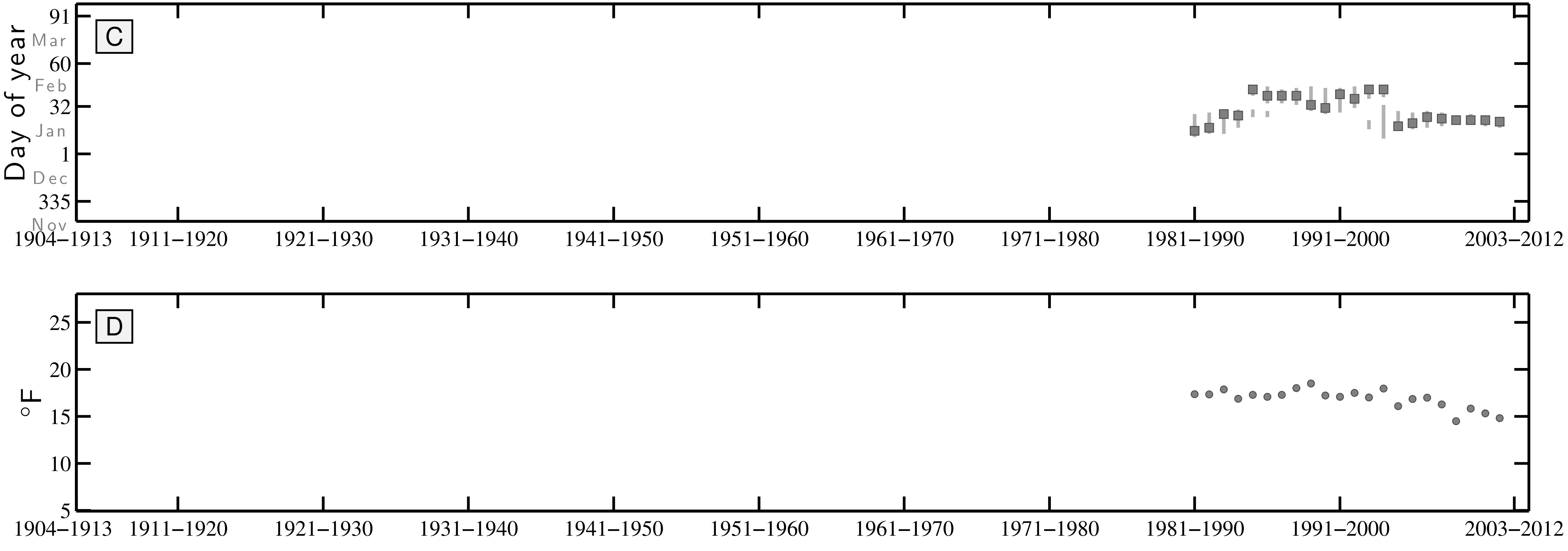

10 year Summer Teletherm dynamics for BOONTON 1 SE, NJ:

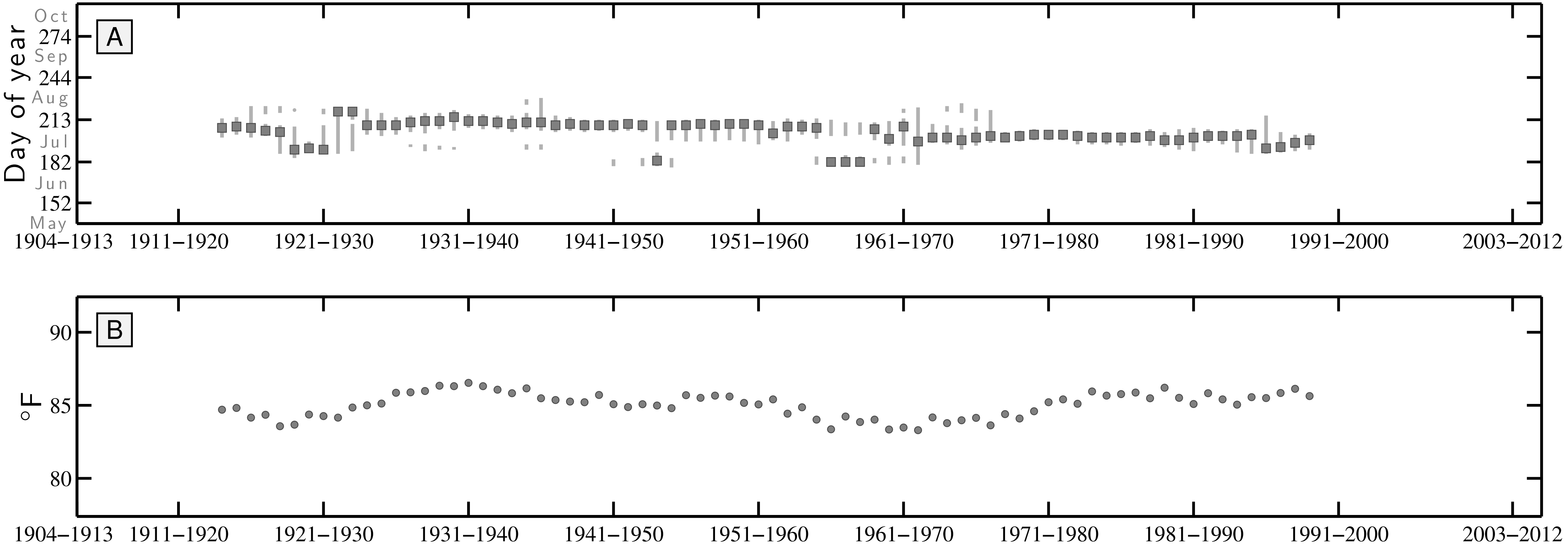

10 year Winter Teletherm dynamics for BOONTON 1 SE, NJ:

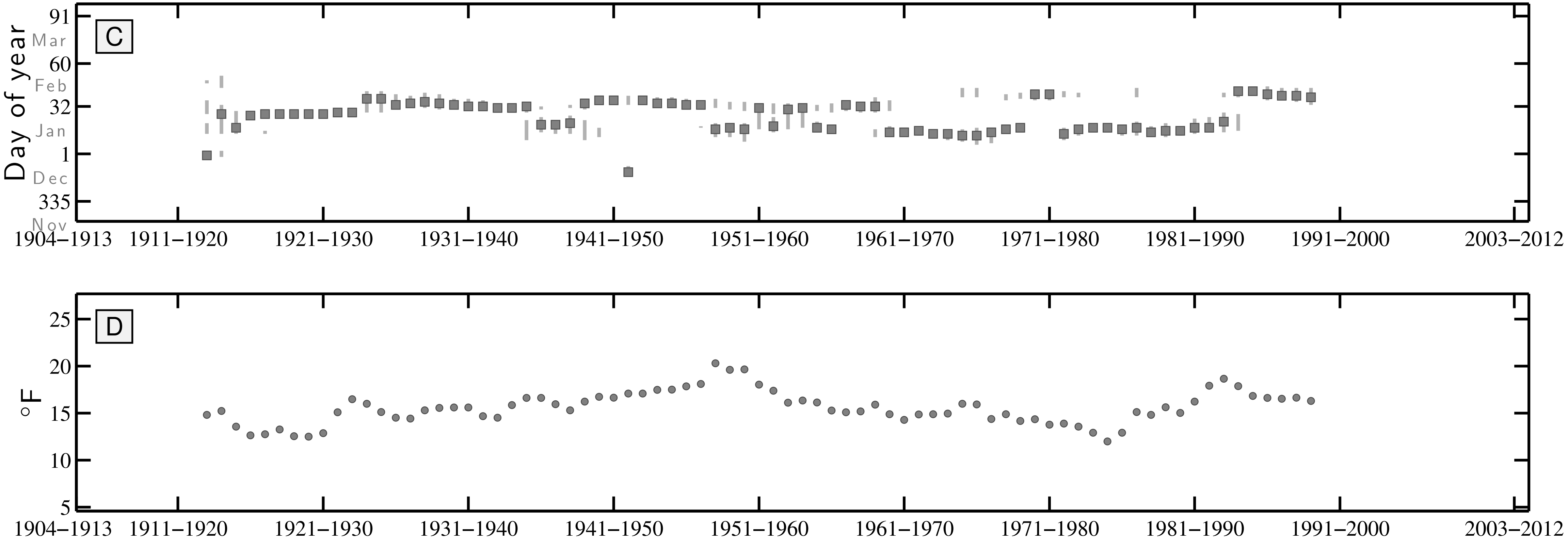

10 year Summer Teletherm dynamics for CHARLOTTEBURG RSVR, NJ:

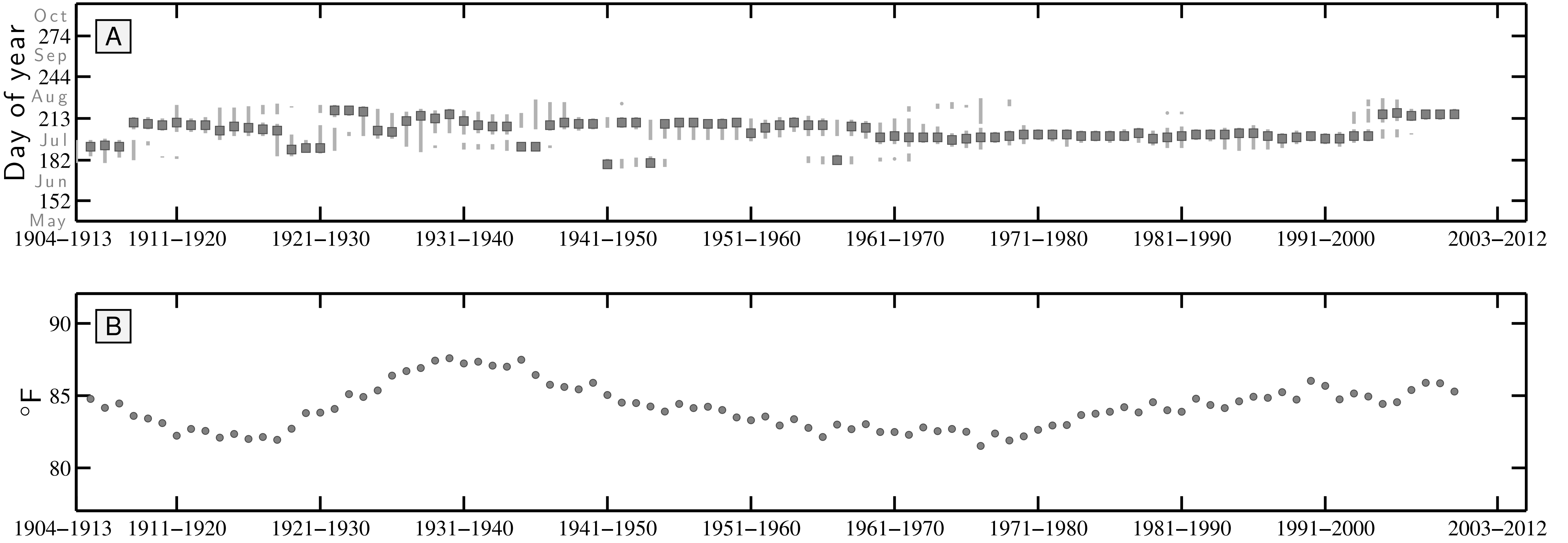

10 year Winter Teletherm dynamics for CHARLOTTEBURG RSVR, NJ:

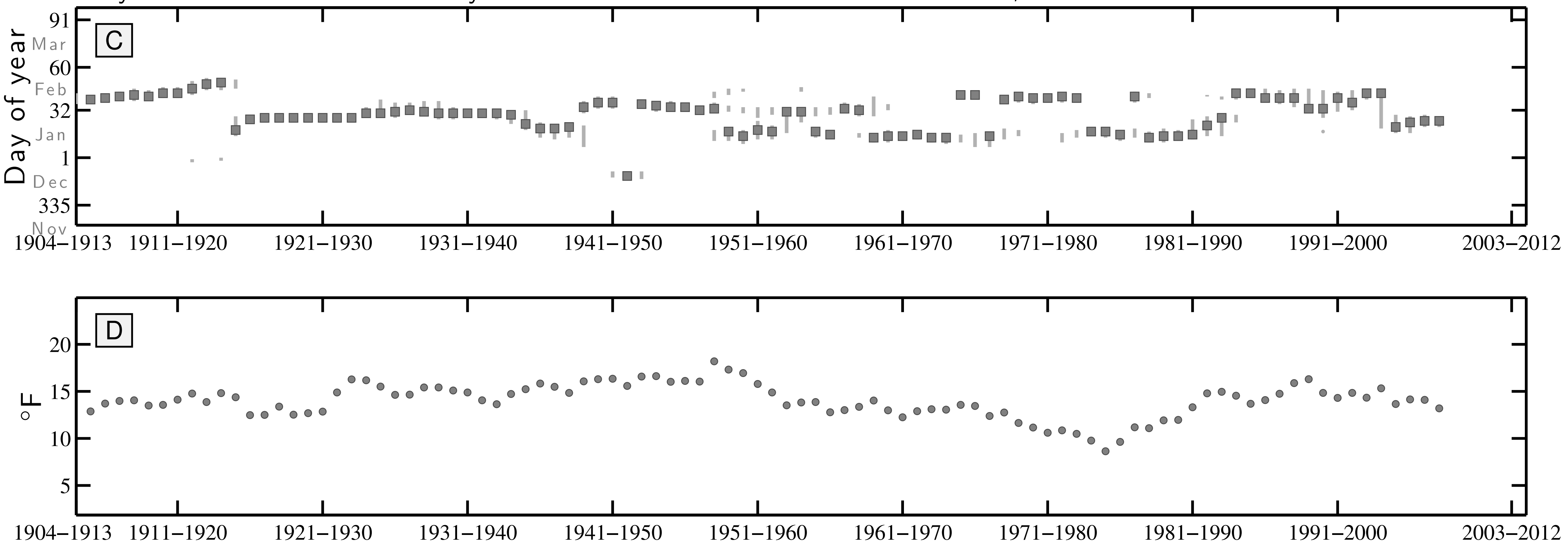

10 year Summer Teletherm dynamics for FLEMINGTON 5 NNW, NJ:

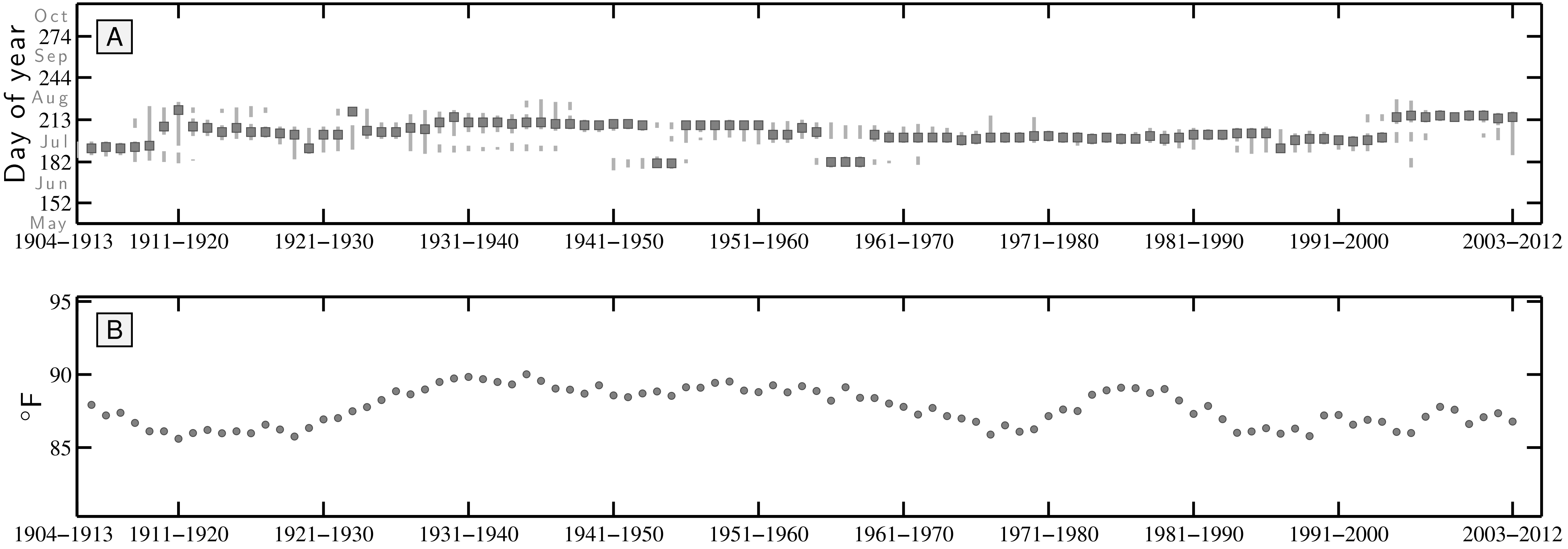

10 year Winter Teletherm dynamics for FLEMINGTON 5 NNW, NJ:

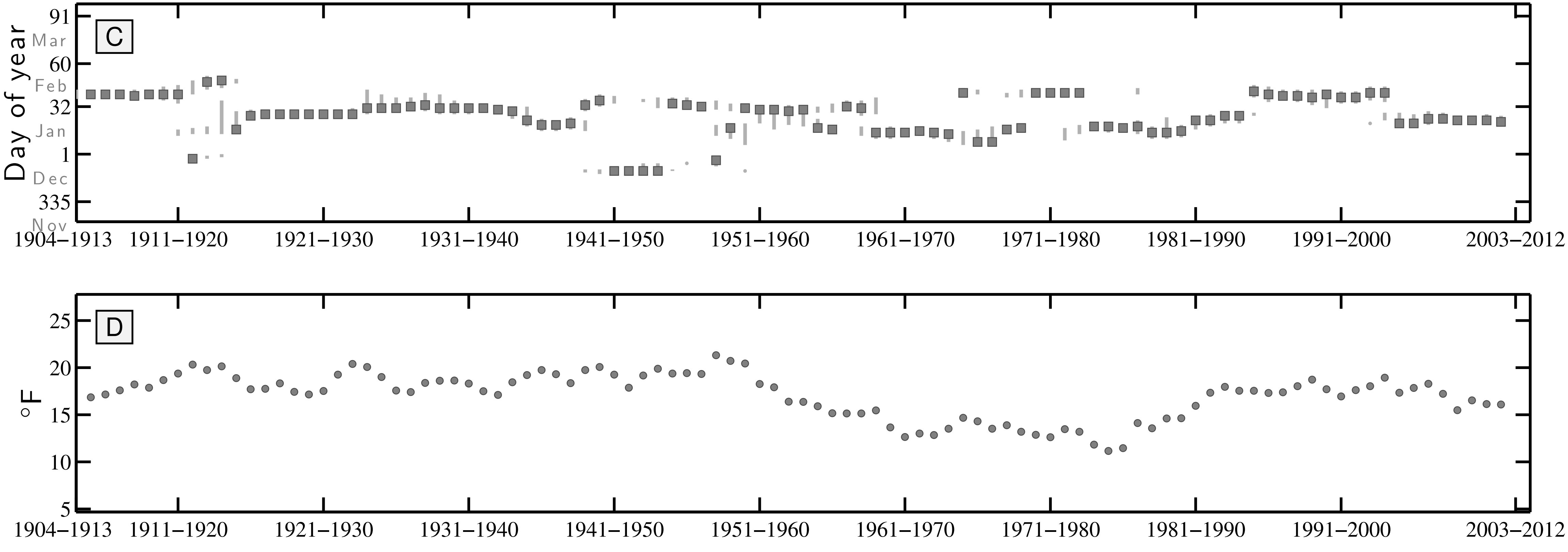

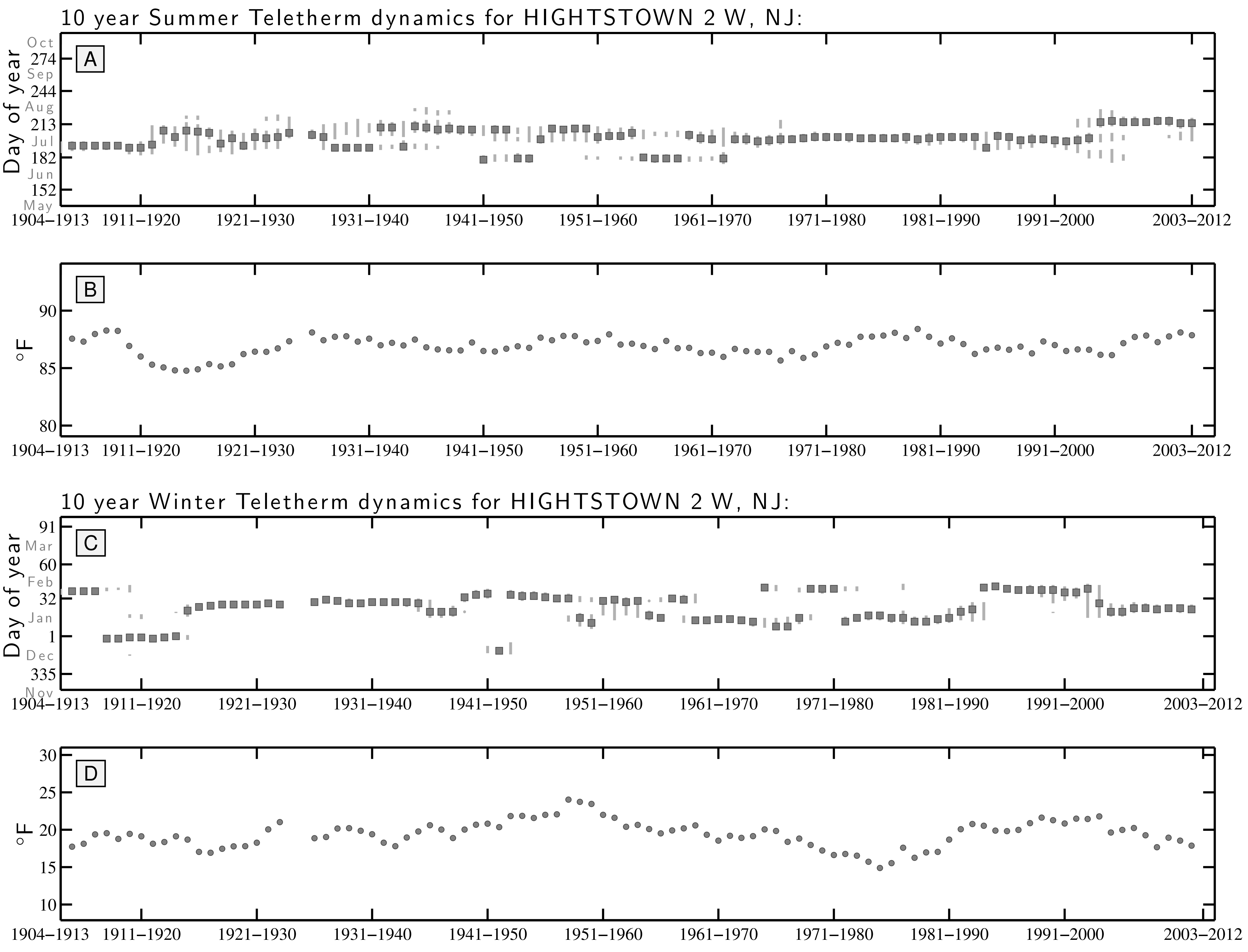
10 year Summer Teletherm dynamics for HIGHTSTOWN 2 W, NJ:
A
Day of year
Oct
274
Sep
244
Aug
213
Jul
182
Jun
152
May
1904–1913
1911–1920
1921–1930
1931–1940
1941–1950
1951–1960
1961–1970
1971–1980
1981–1990
1991–2000
2003–2012
B
°F
90
85
80
10 year Winter Teletherm dynamics for HIGHTSTOWN 2 W, NJ:
C
91
Mar
60
Feb
32
Jan
1
Dec
335
Nov
D
30
25
20
15
10

10 year Summer Teletherm dynamics for INDIAN MILLS 2 W, NJ:

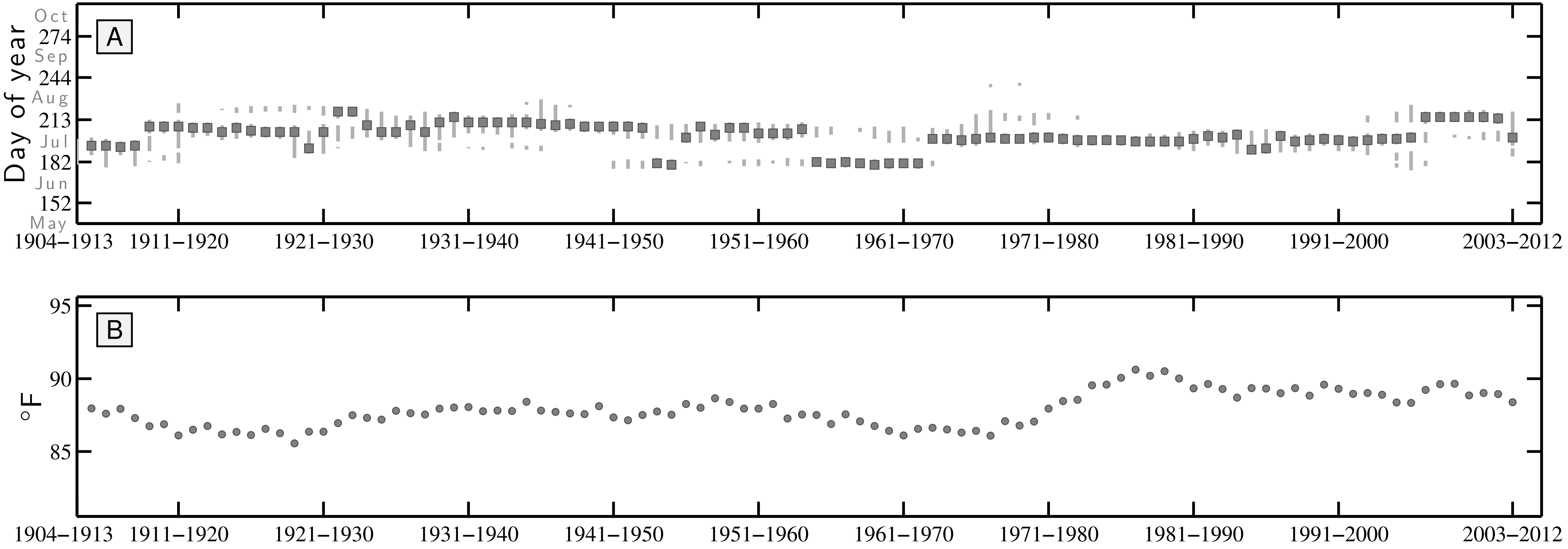

10 year Winter Teletherm dynamics for INDIAN MILLS 2 W, NJ:

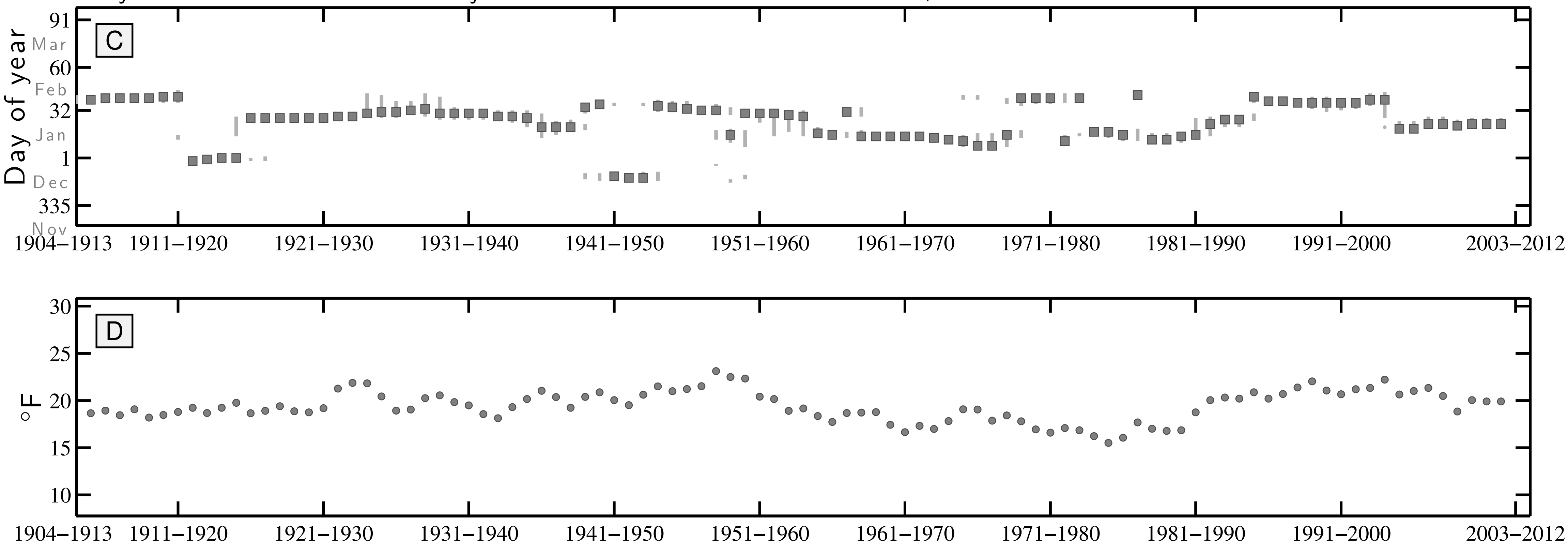

10 year Summer Teletherm dynamics for LONG BRANCH OAKHURST, NJ:

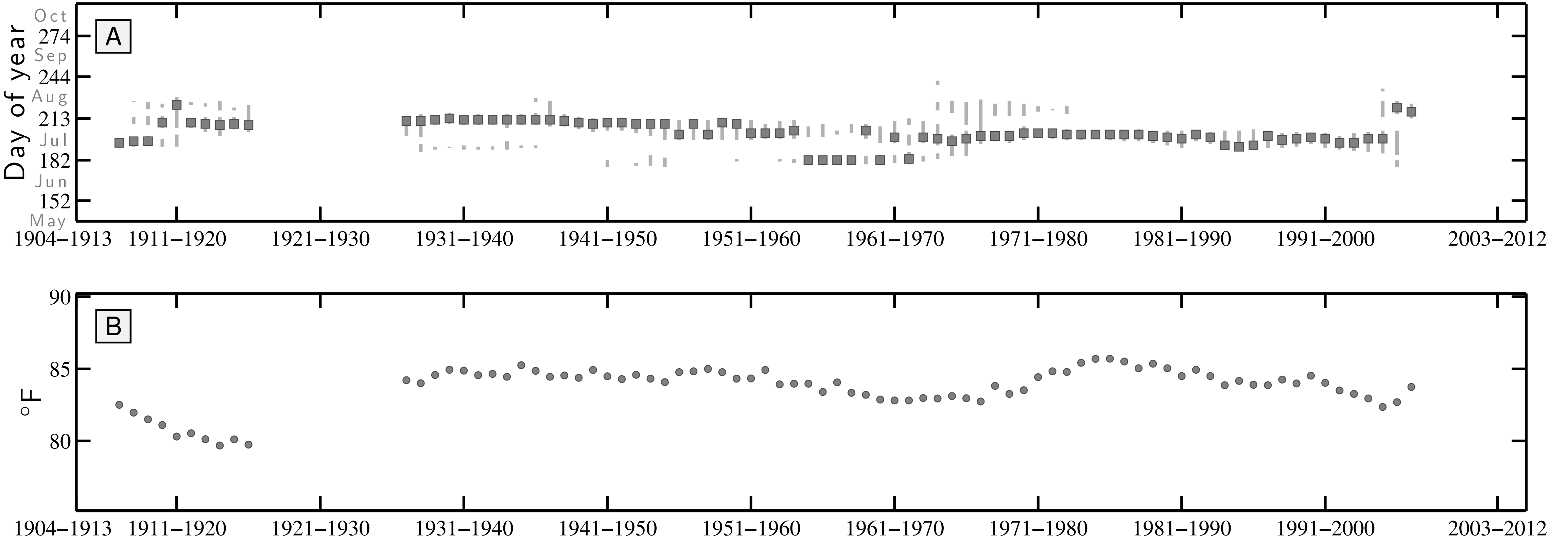

10 year Winter Teletherm dynamics for LONG BRANCH OAKHURST, NJ:

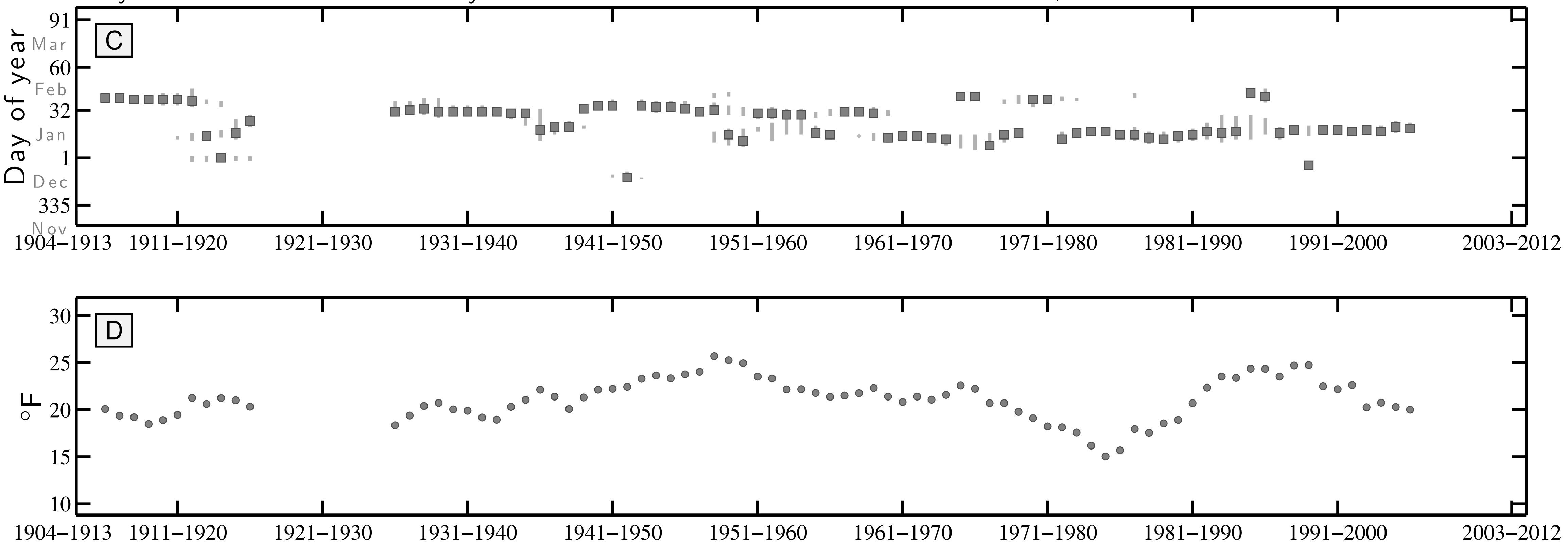

10 year Summer Teletherm dynamics for MOORESTOWN, NJ:

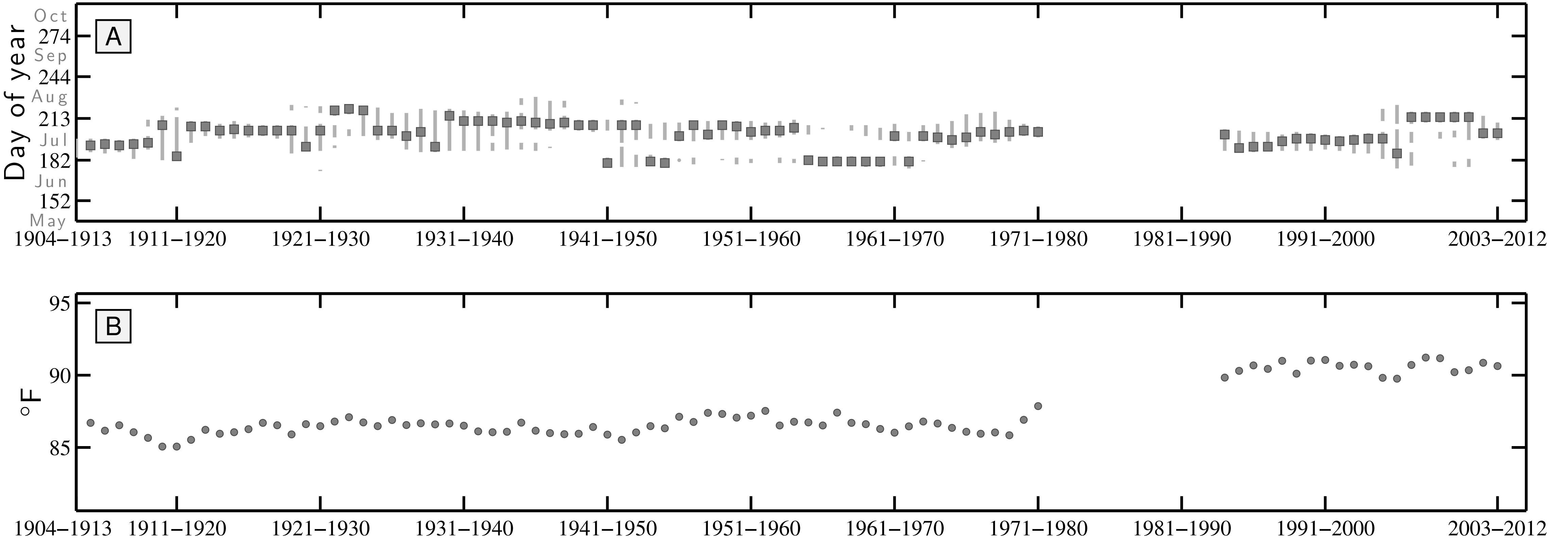

10 year Winter Teletherm dynamics for MOORESTOWN, NJ:

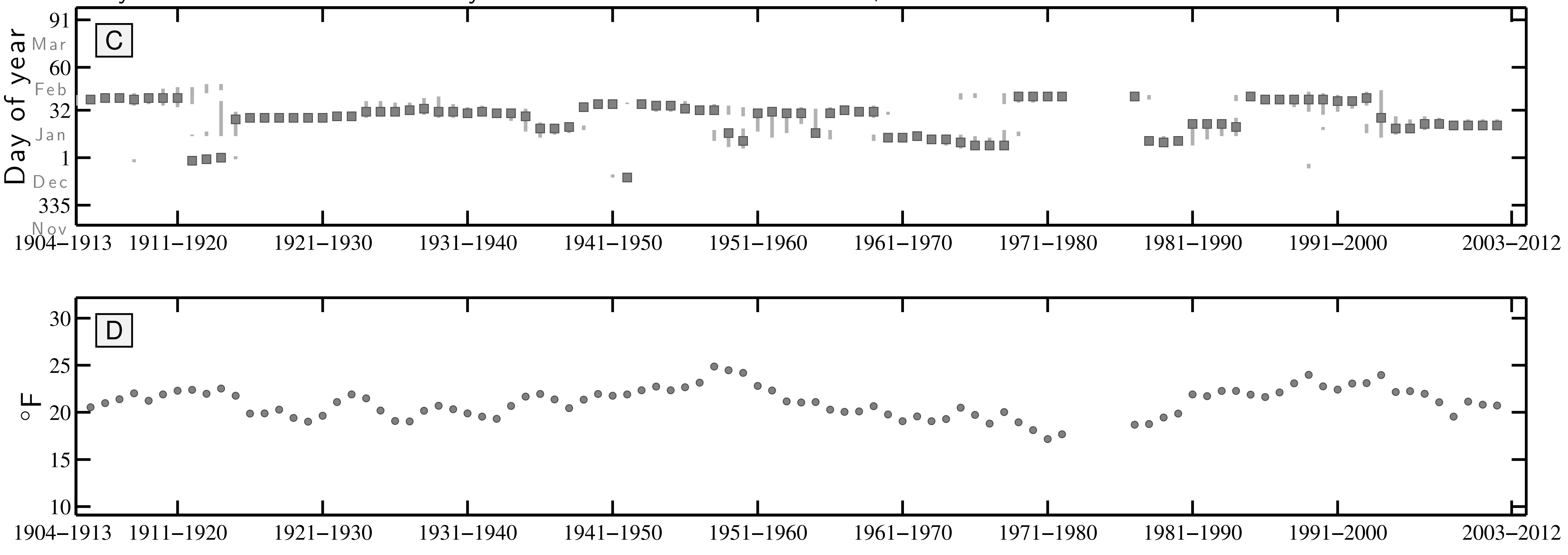

10 year Summer Teletherm dynamics for NEW BRUNSWICK 3 SE, NJ:

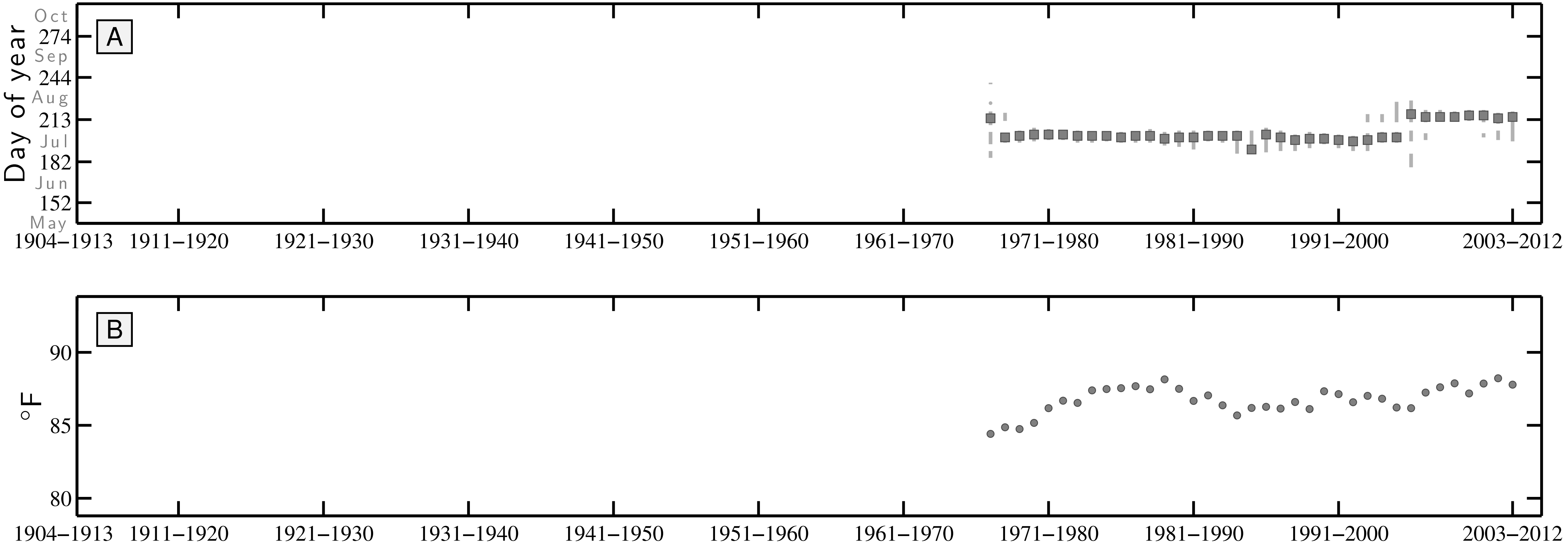

10 year Winter Teletherm dynamics for NEW BRUNSWICK 3 SE, NJ:

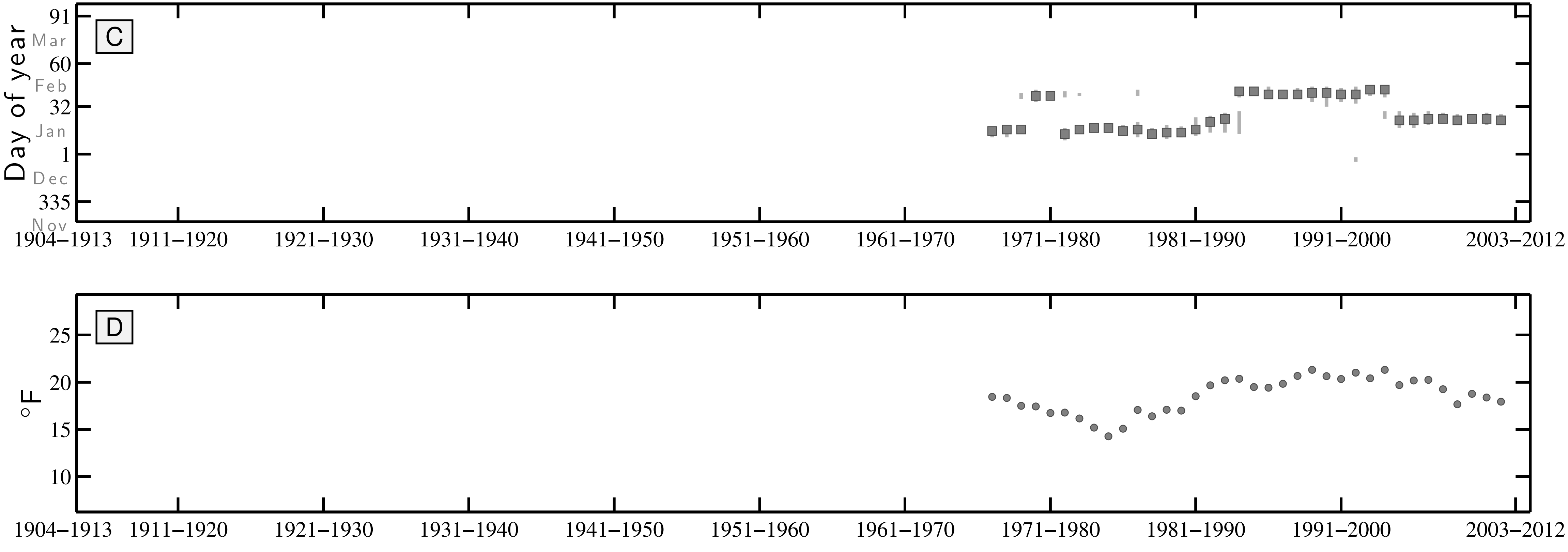

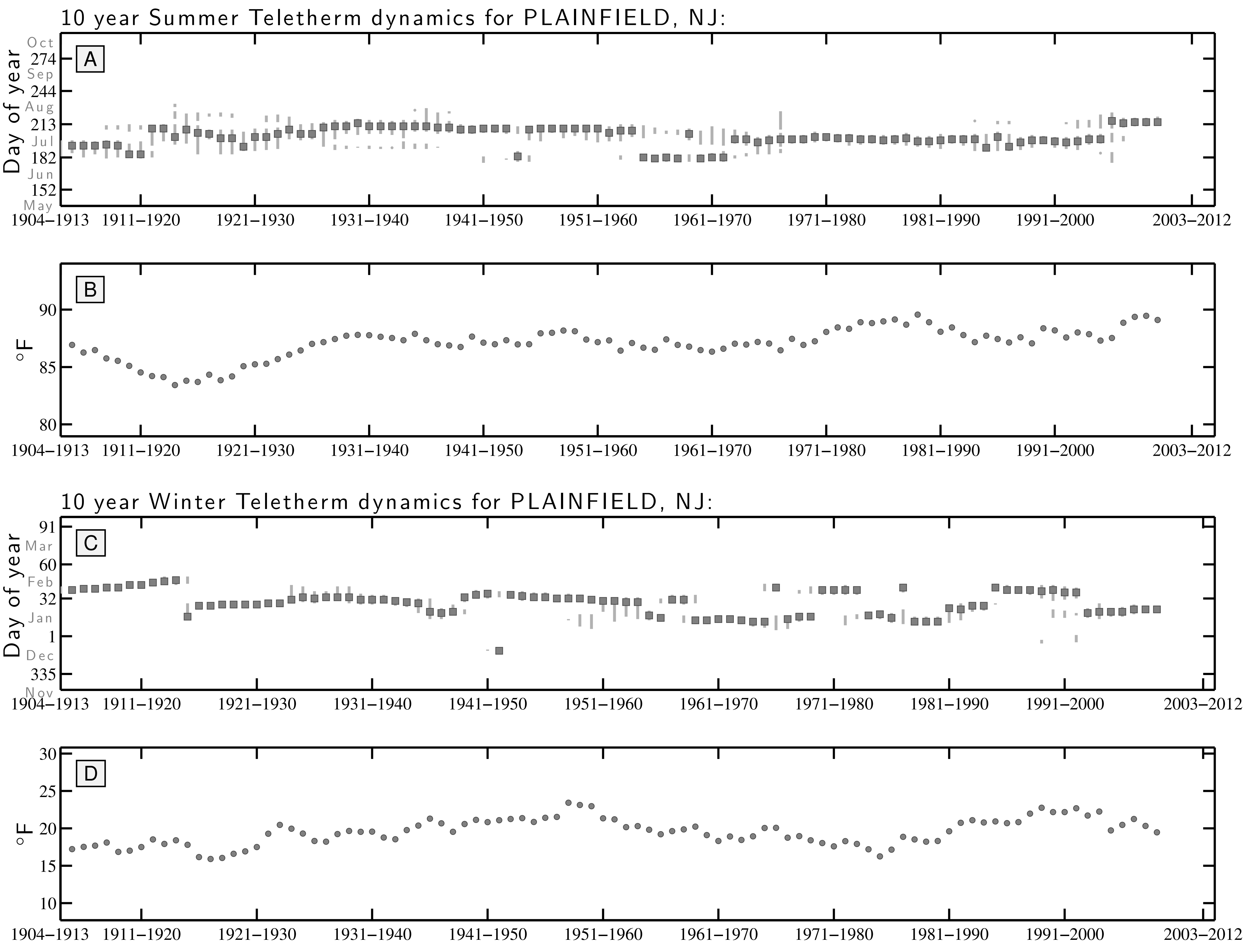
10 year Summer Teletherm dynamics for PLAINFIELD, NJ:
A
Day of year
Oct
274
Sep
244
Aug
213
Jul
182
Jun
152
May
1904–1913
1911–1920
1921–1930
1931–1940
1941–1950
1951–1960
1961–1970
1971–1980
1981–1990
1991–2000
2003–2012
B
°F
90
85
80
10 year Winter Teletherm dynamics for PLAINFIELD, NJ:
C
Day of year
91
Mar
60
Feb
32
Jan
1
Dec
335
Nov
D
°F
30
25
20
15
10

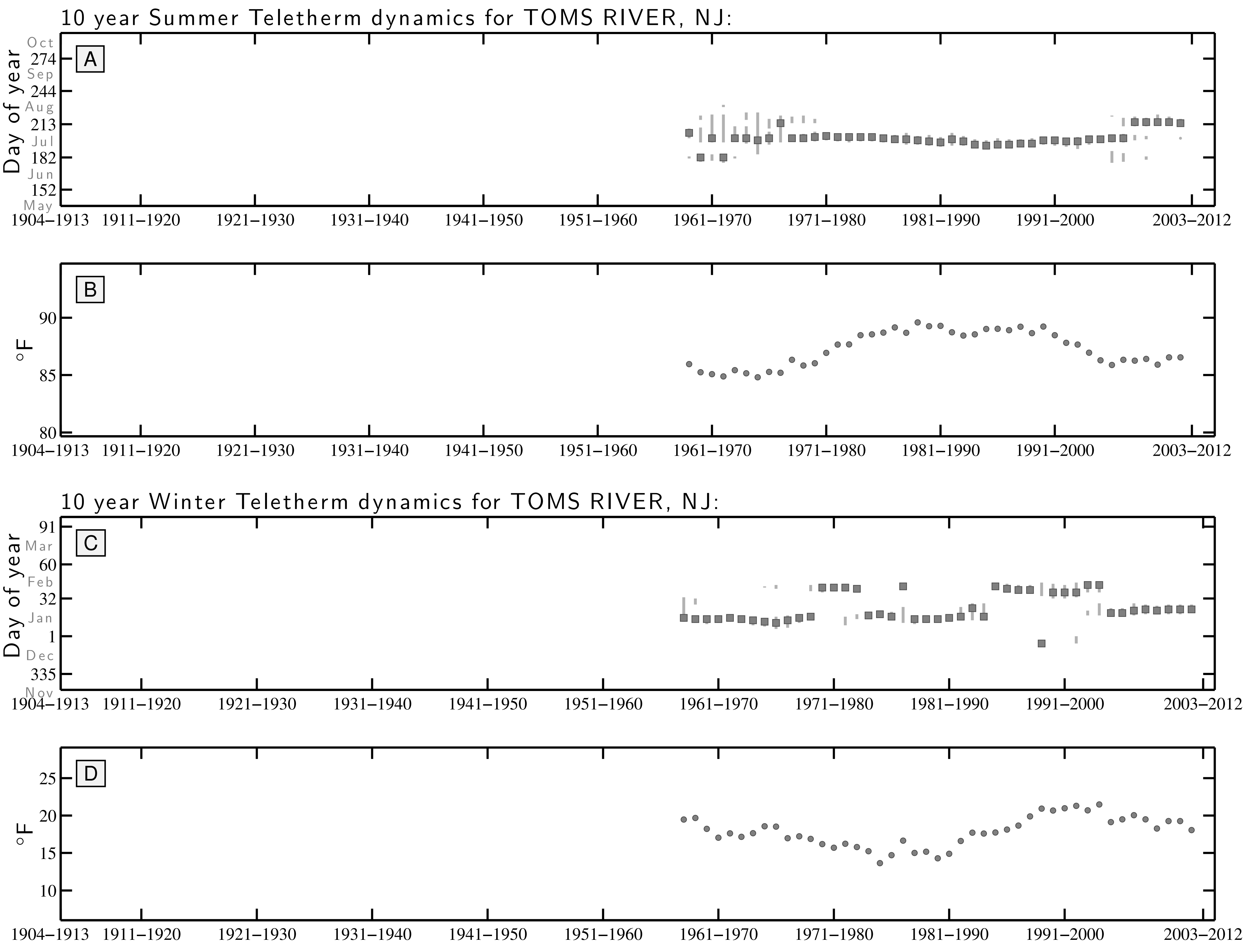
10 year Summer Teletherm dynamics for TOMS RIVER, NJ:
A
B
Day of year
°F
10 year Winter Teletherm dynamics for TOMS RIVER, NJ:
C
D
1904–1913
1911–1920
1921–1930
1931–1940
1941–1950
1951–1960
1961–1970
1971–1980
1981–1990
1991–2000
2003–2012

10 year Summer Teletherm dynamics for AZTEC RUINS NM, NM:

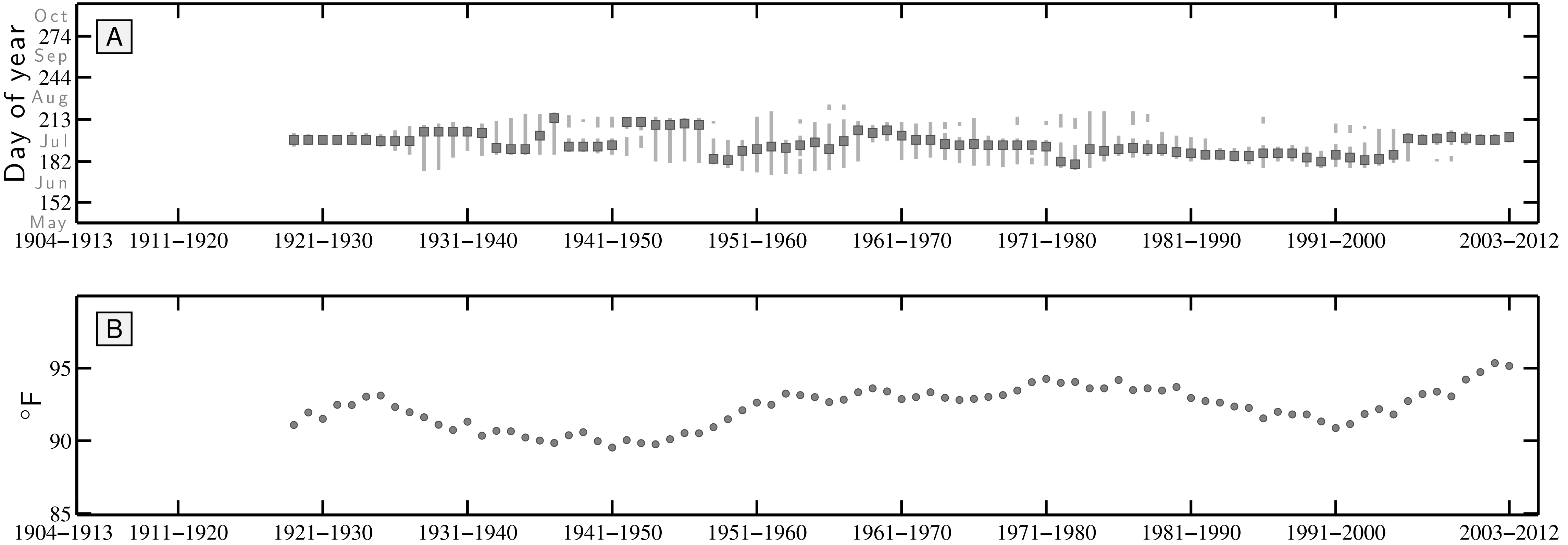

10 year Winter Teletherm dynamics for AZTEC RUINS NM, NM:

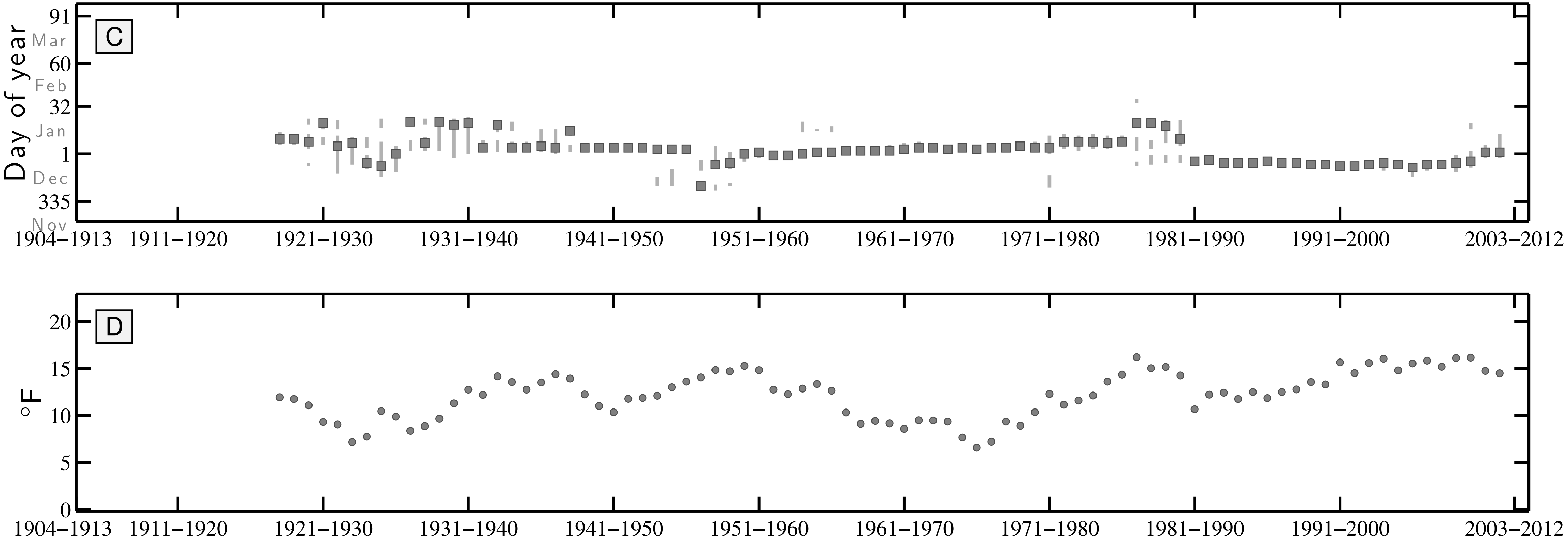

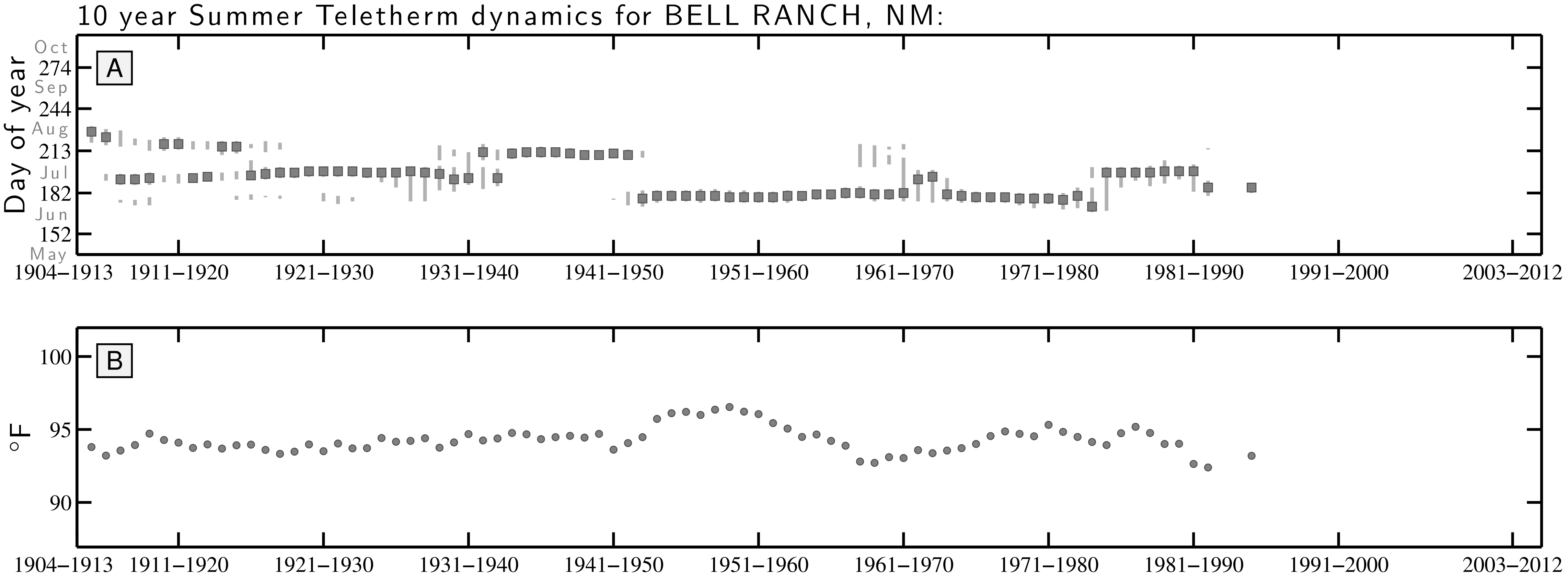
10 year Summer Teletherm dynamics for BELL RANCH, NM:
A
B
Day of year
°F
Oct
274
Sep
244
Aug
213
Jul
182
Jun
152
May
100
95
90
1904–1913
1911–1920
1921–1930
1931–1940
1941–1950
1951–1960
1961–1970
1971–1980
1981–1990
1991–2000
2003–2012

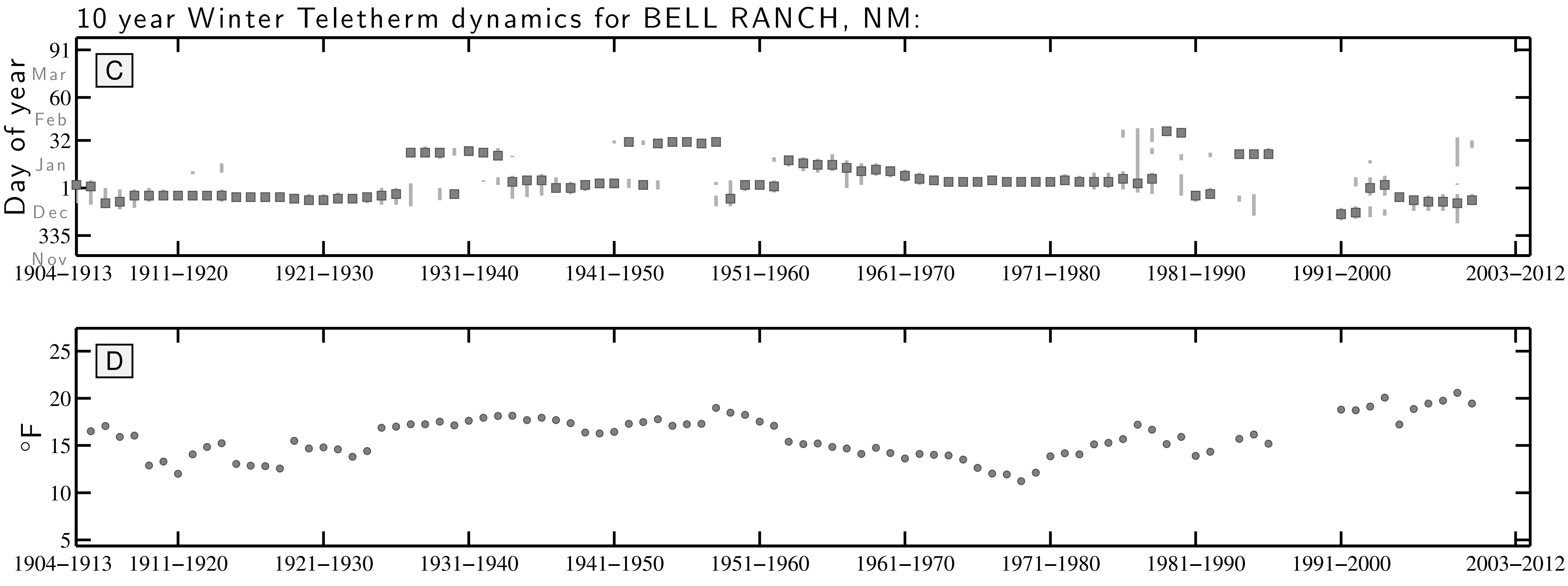
10 year Winter Teletherm dynamics for BELL RANCH, NM:
C
D
Day of year
°F
91
Mar
60
Feb
32
Jan
1
Dec
335
Nov
25
20
15
10
5
1904–1913
1911–1920
1921–1930
1931–1940
1941–1950
1951–1960
1961–1970
1971–1980
1981–1990
1991–2000
2003–2012

10 year Summer Teletherm dynamics for CARLSBAD, NM:

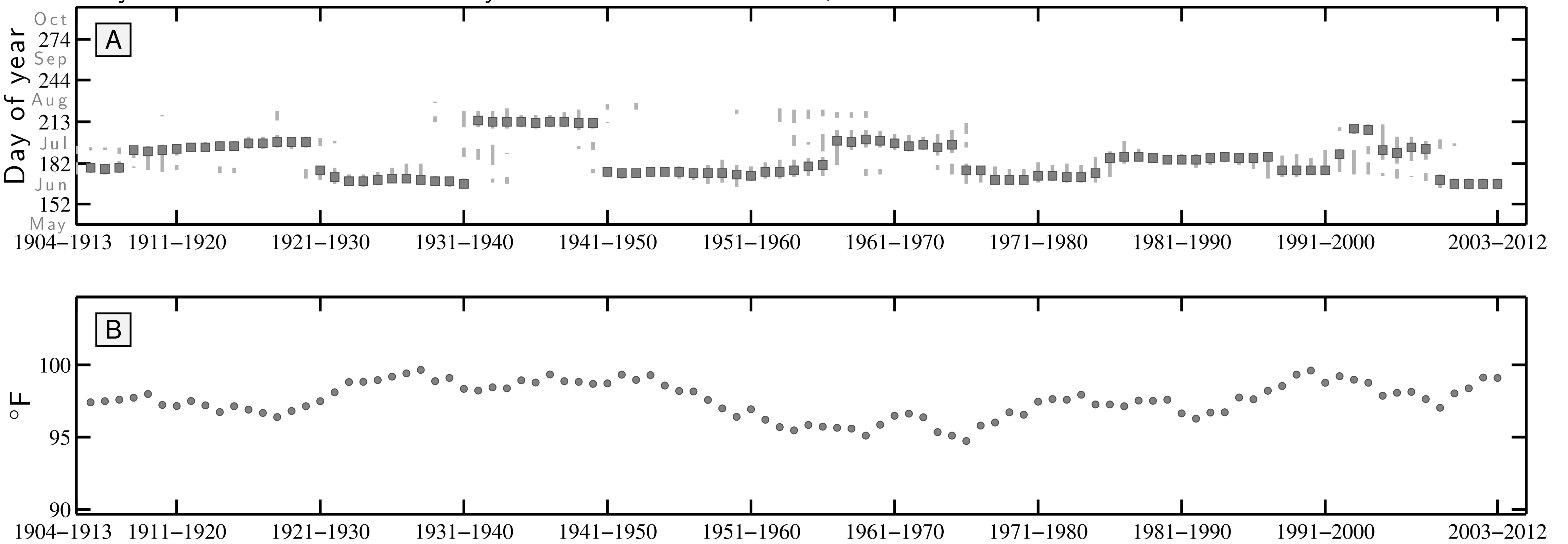

10 year Winter Teletherm dynamics for CARLSBAD, NM:

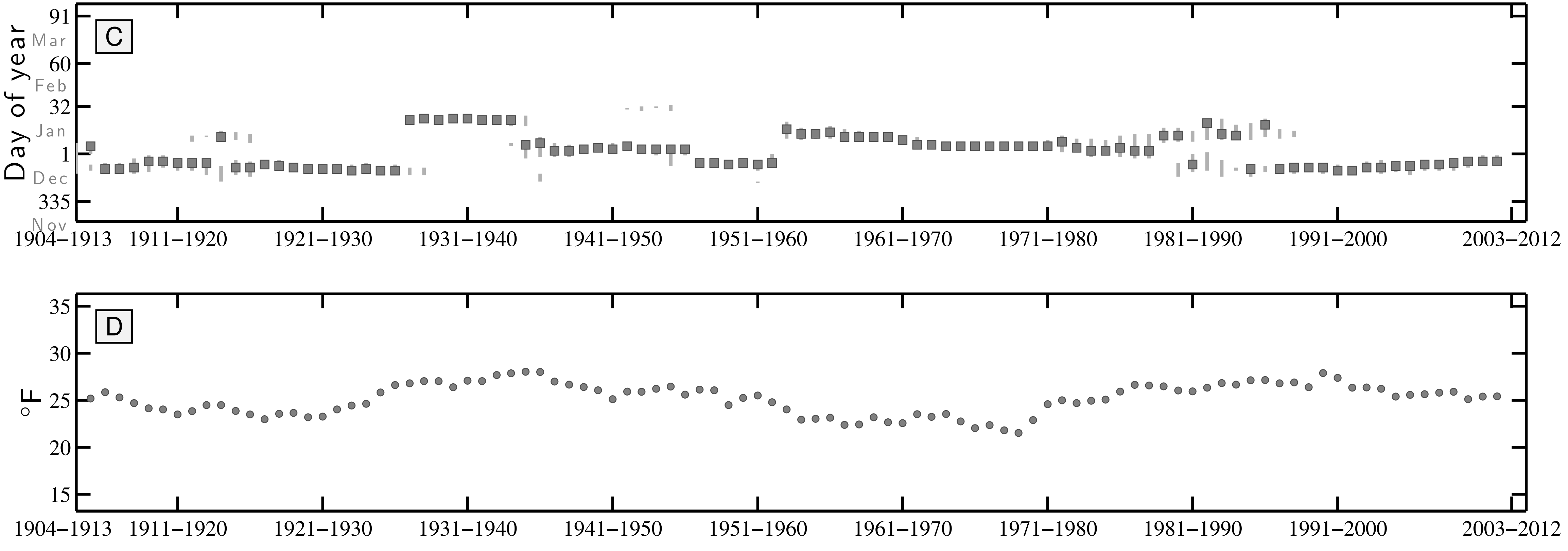

10 year Summer Teletherm dynamics for CARRIZOZO 1SW, NM:

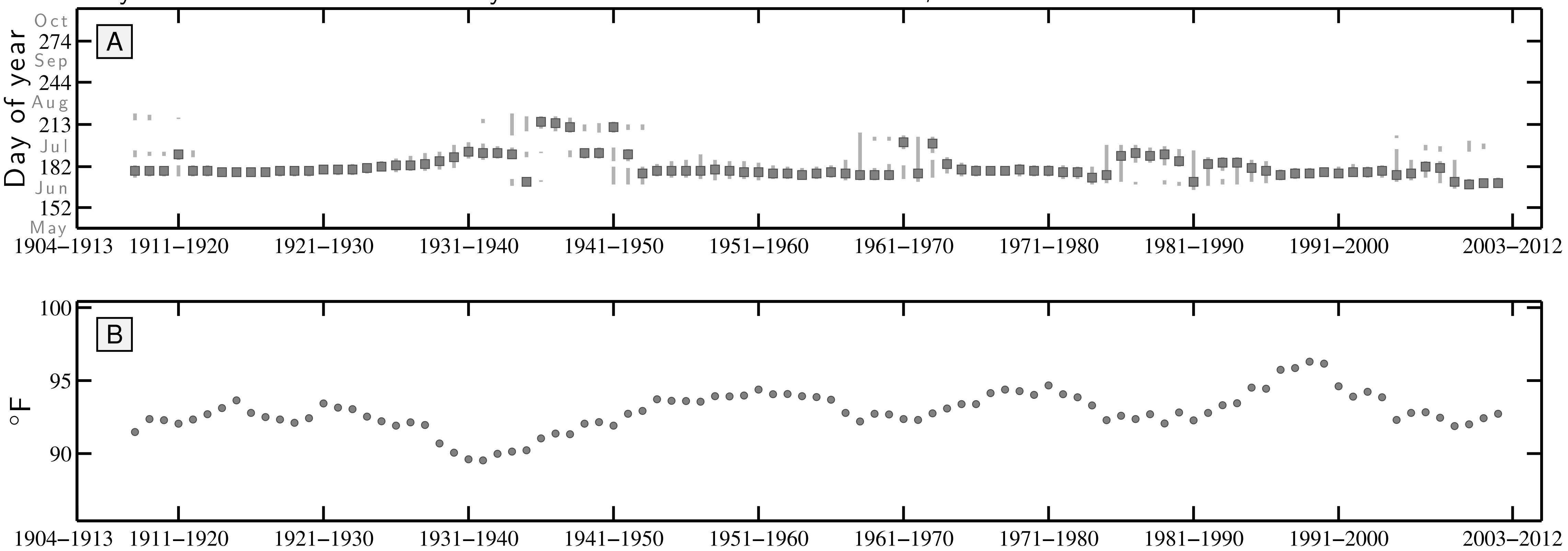

10 year Winter Teletherm dynamics for CARRIZOZO 1SW, NM:

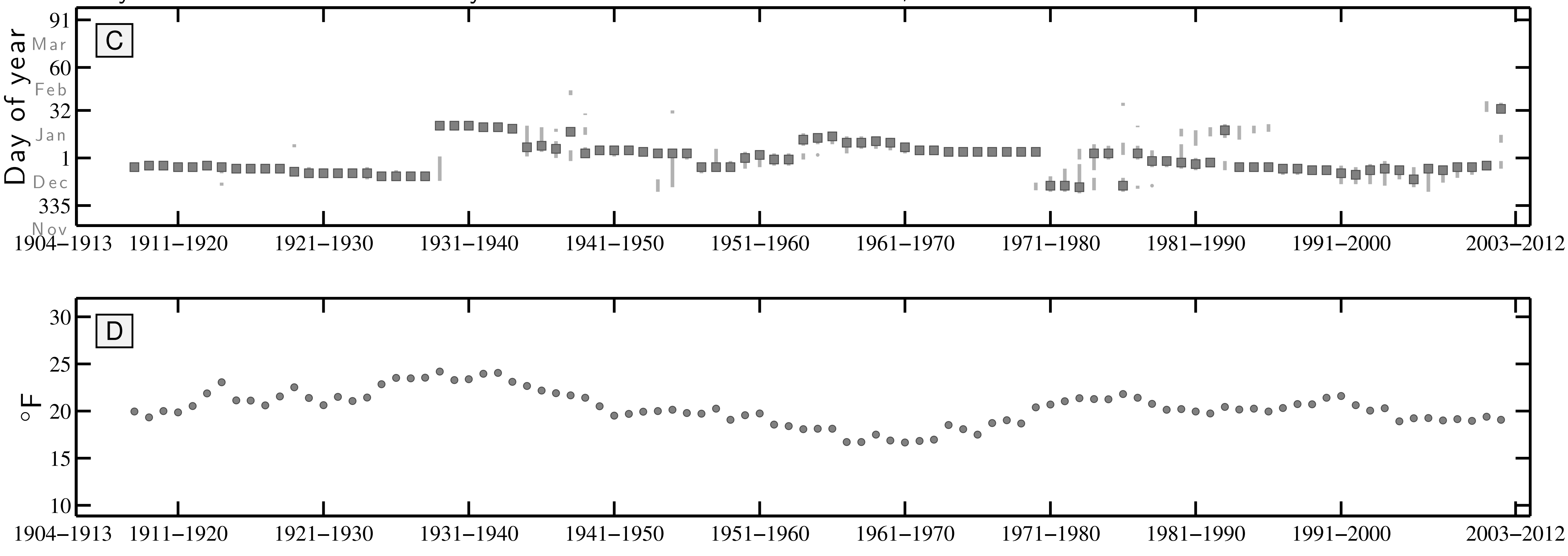

10 year Summer Teletherm dynamics for CHAMA, NM:

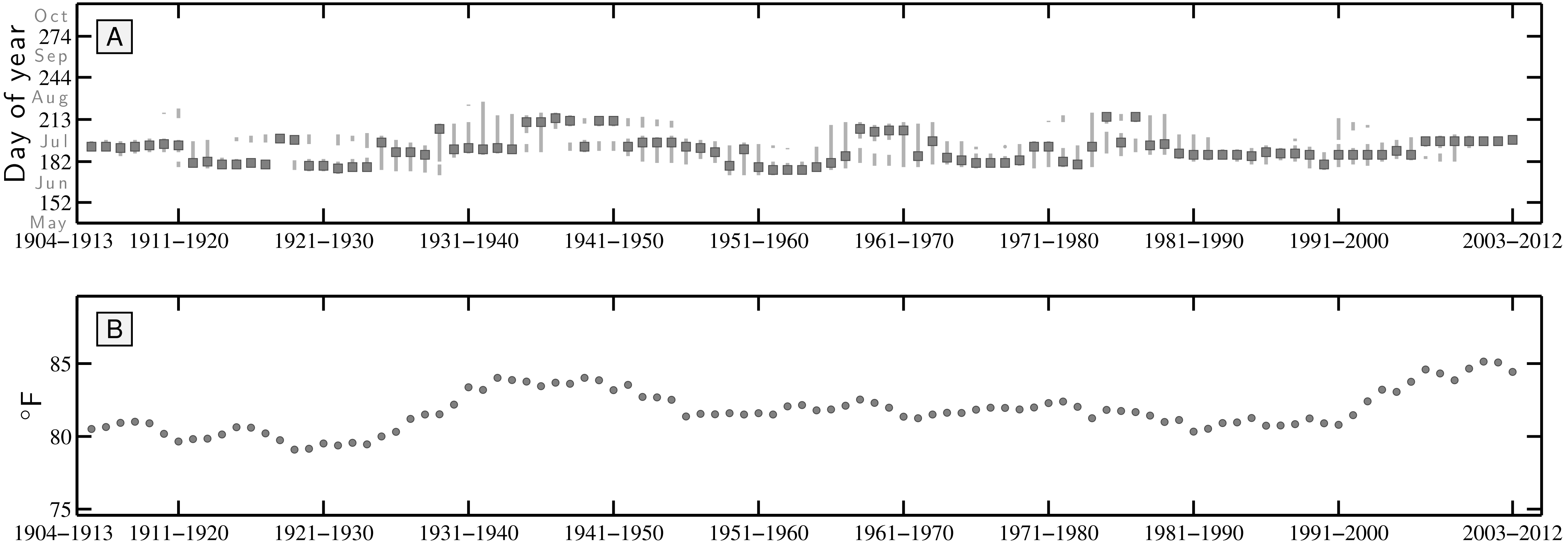

10 year Winter Teletherm dynamics for CHAMA, NM:

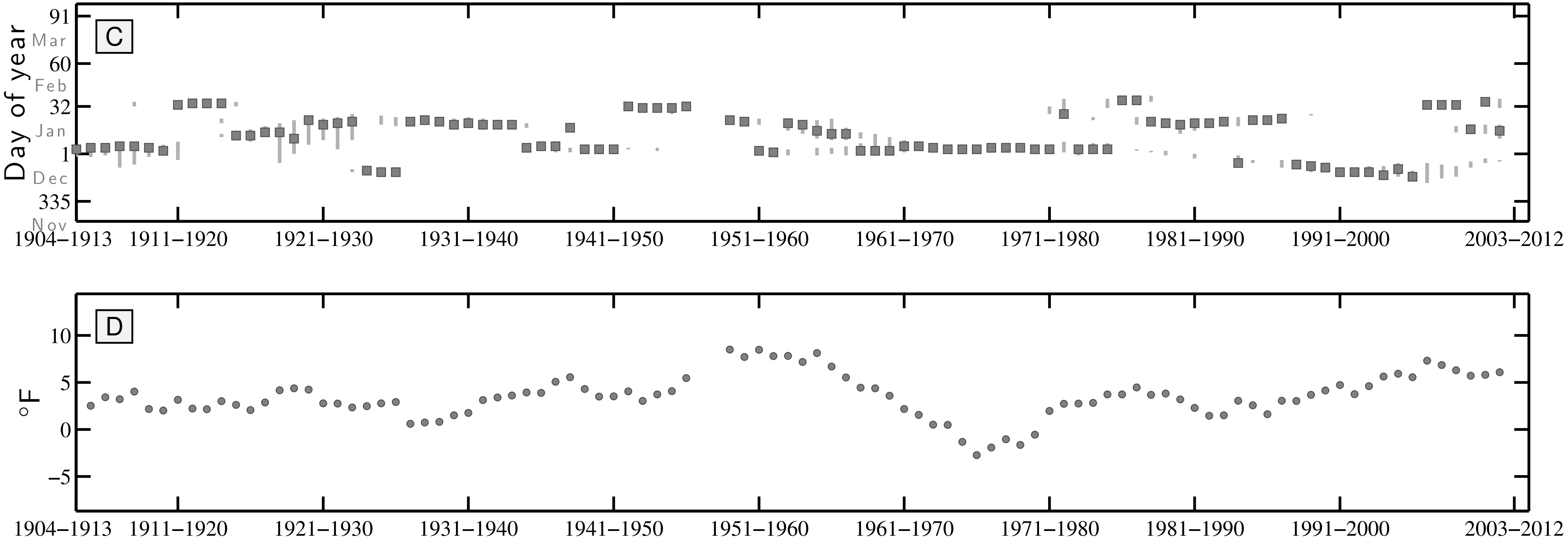

10 year Summer Teletherm dynamics for CIMARRON 4 SW, NM:

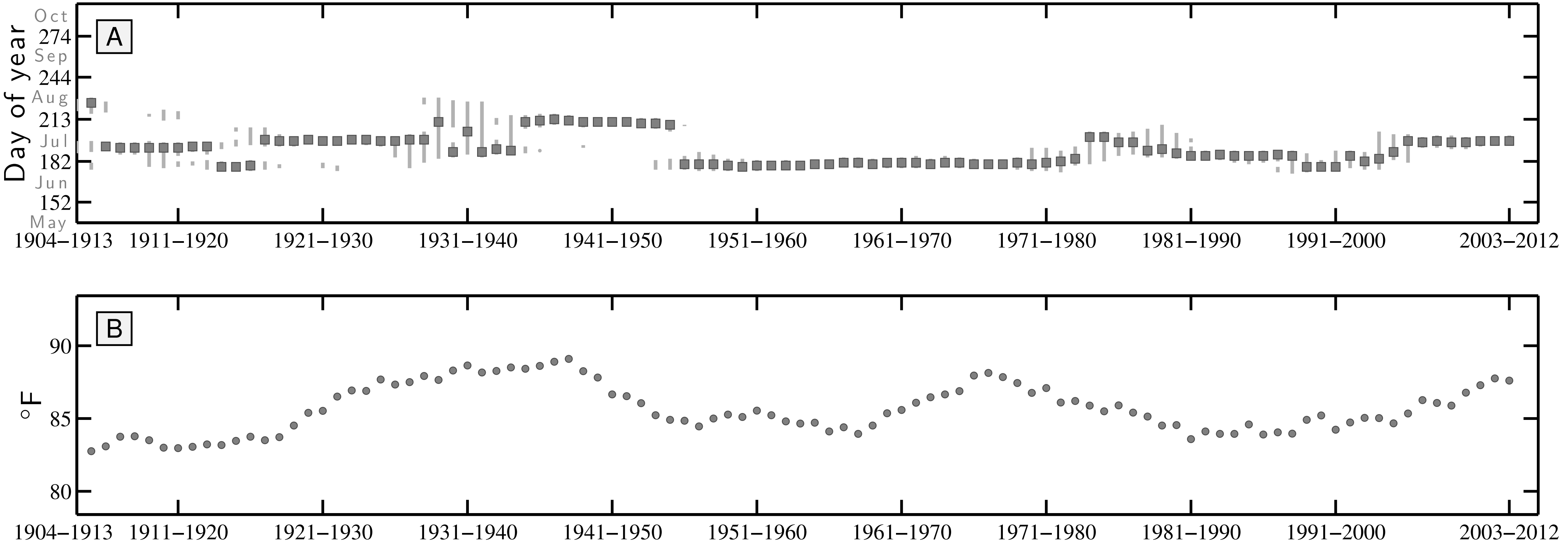

10 year Winter Teletherm dynamics for CIMARRON 4 SW, NM:

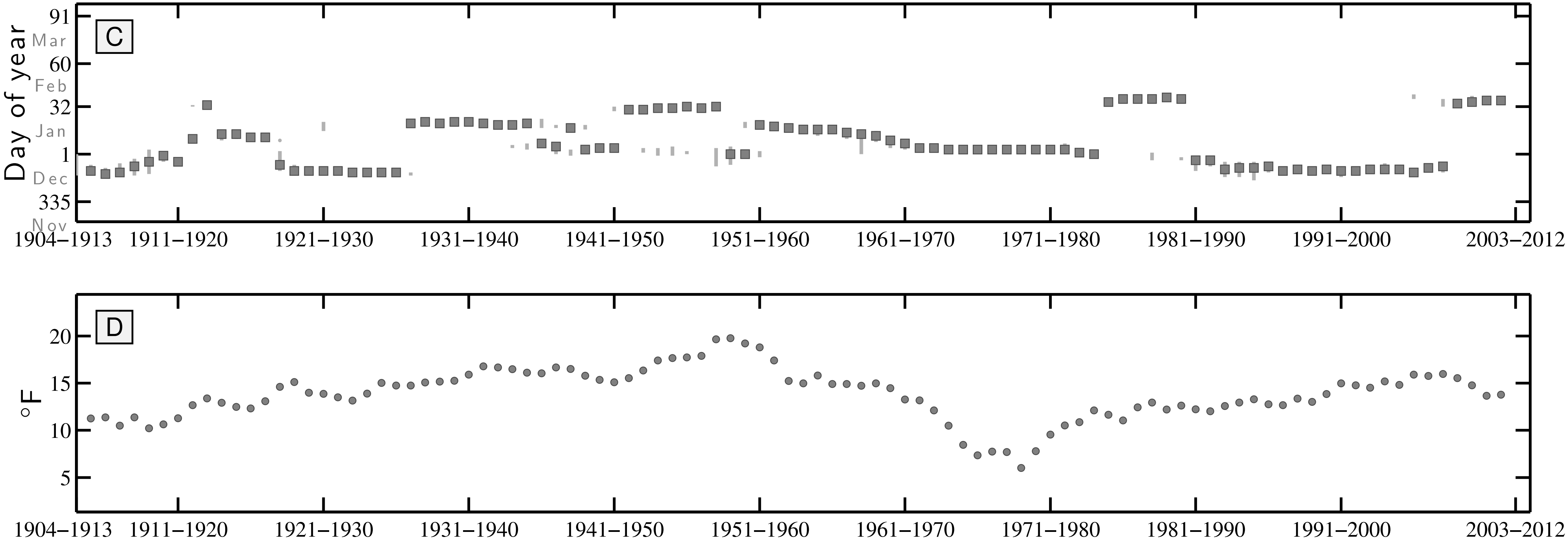

10 year Summer Teletherm dynamics for CLAYTON MUNI ARPK AP, NM:

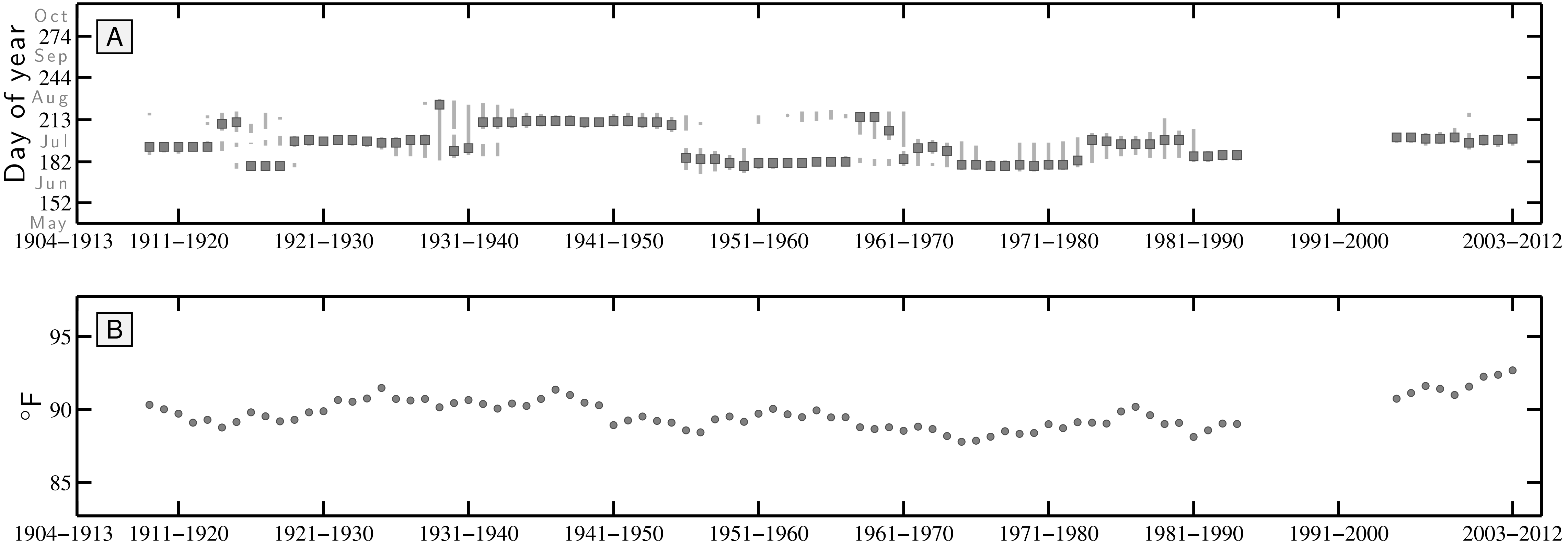

10 year Winter Teletherm dynamics for CLAYTON MUNI ARPK AP, NM:

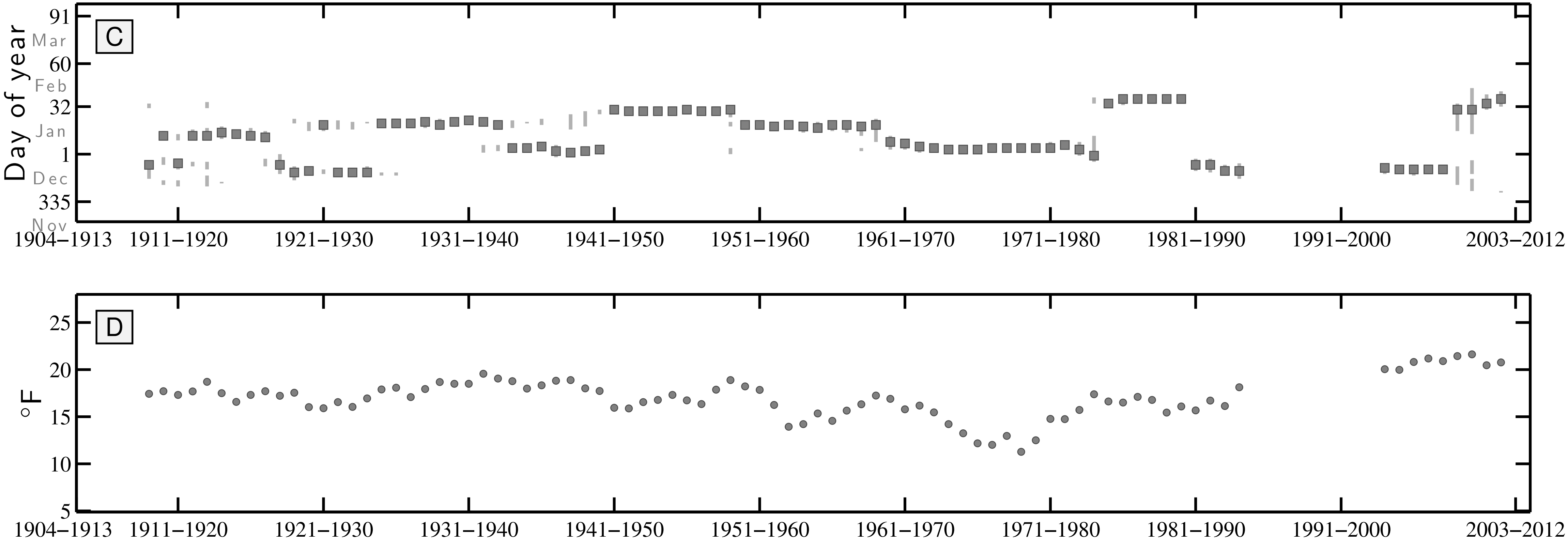

10 year Summer Teletherm dynamics for DULCE, NM:

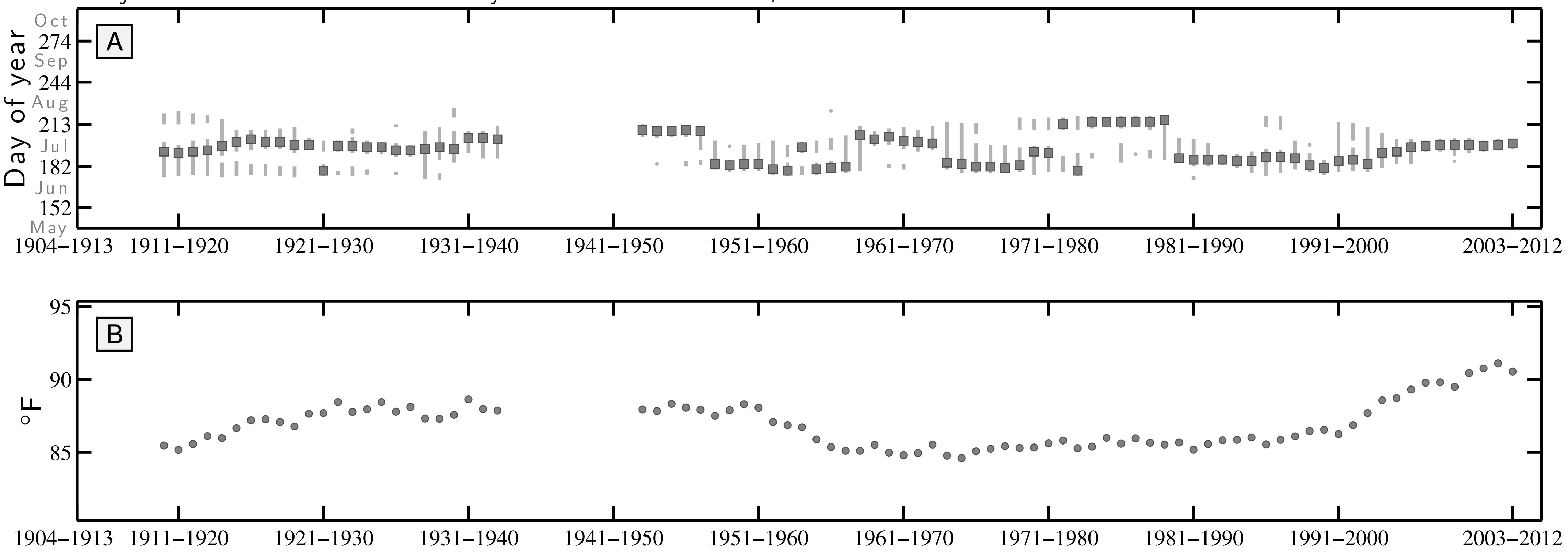

10 year Winter Teletherm dynamics for DULCE, NM:

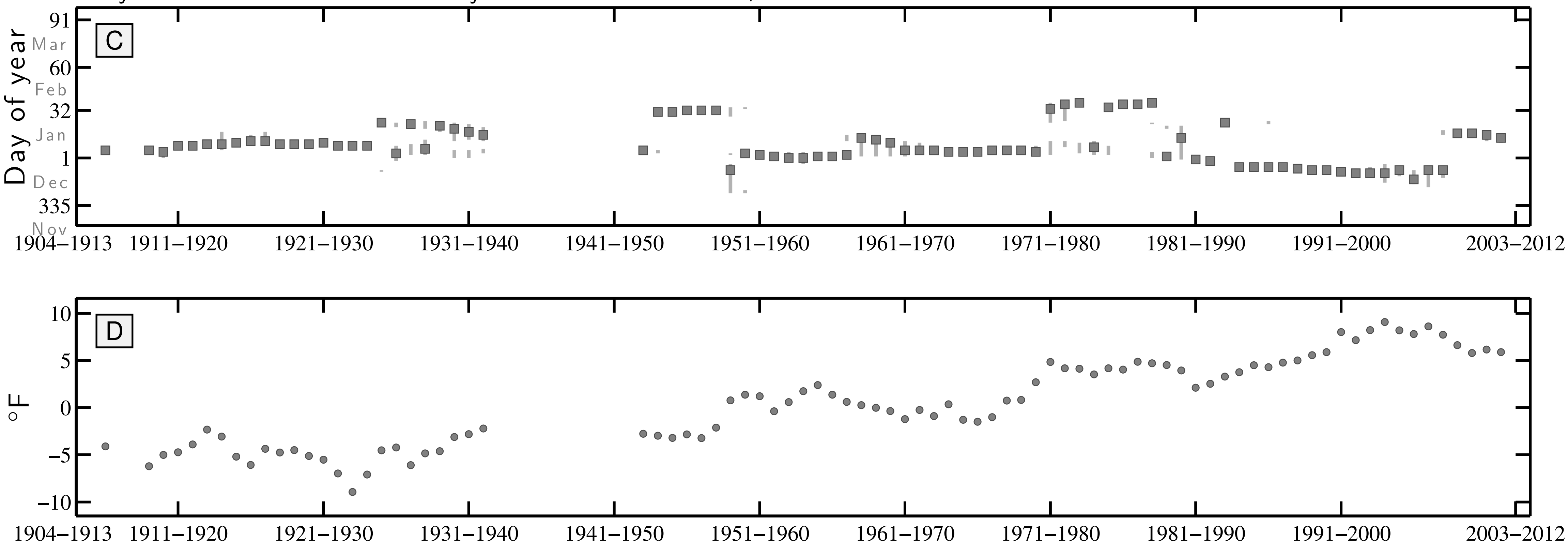

10 year Summer Teletherm dynamics for ELEPHANT BUTTE DAM, NM:

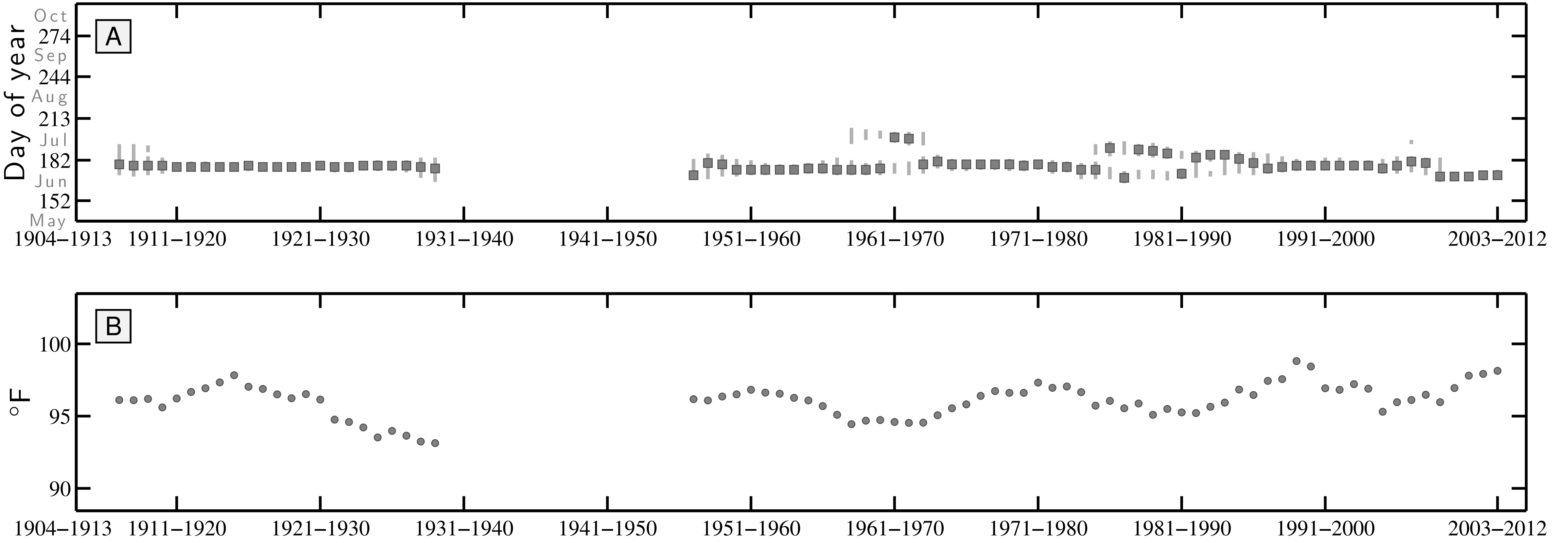

10 year Winter Teletherm dynamics for ELEPHANT BUTTE DAM, NM:

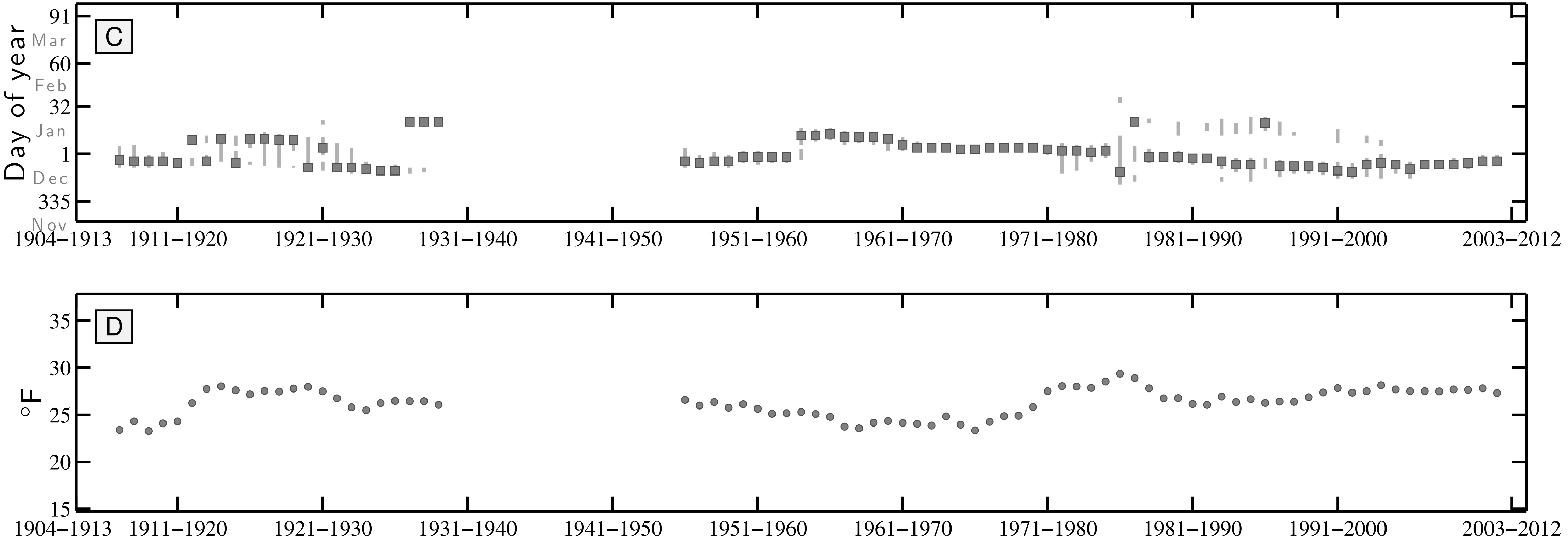

10 year Summer Teletherm dynamics for FT BAYARD, NM:

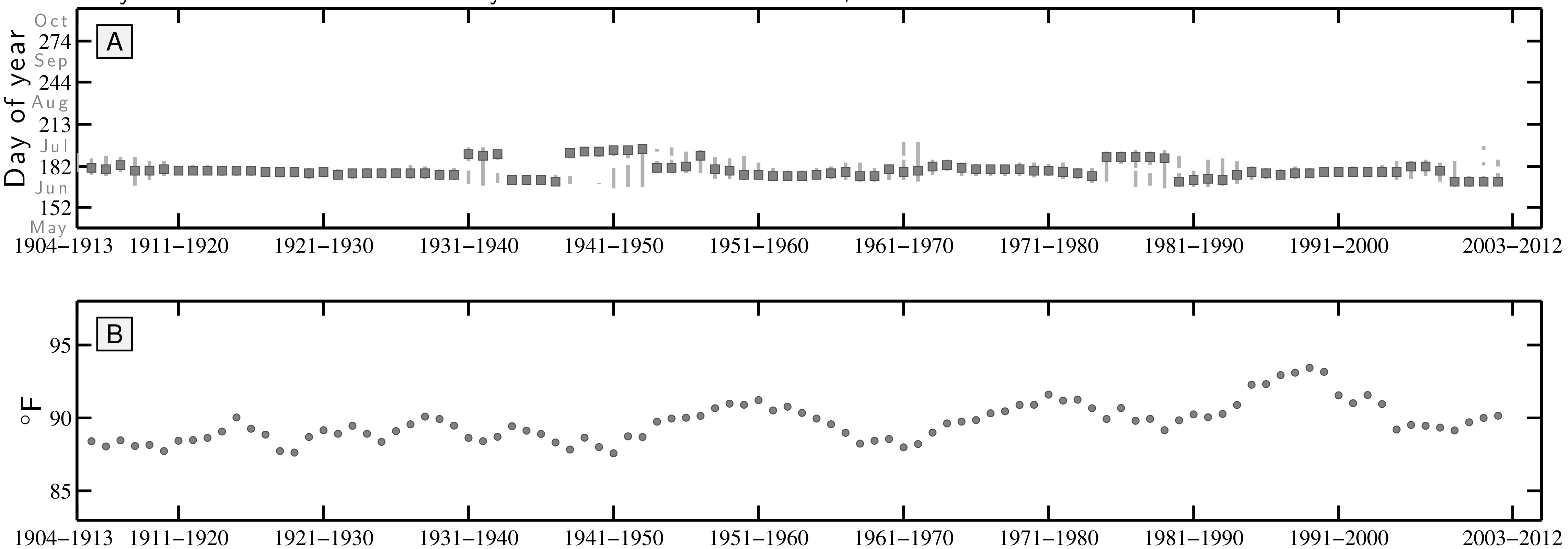

10 year Winter Teletherm dynamics for FT BAYARD, NM:

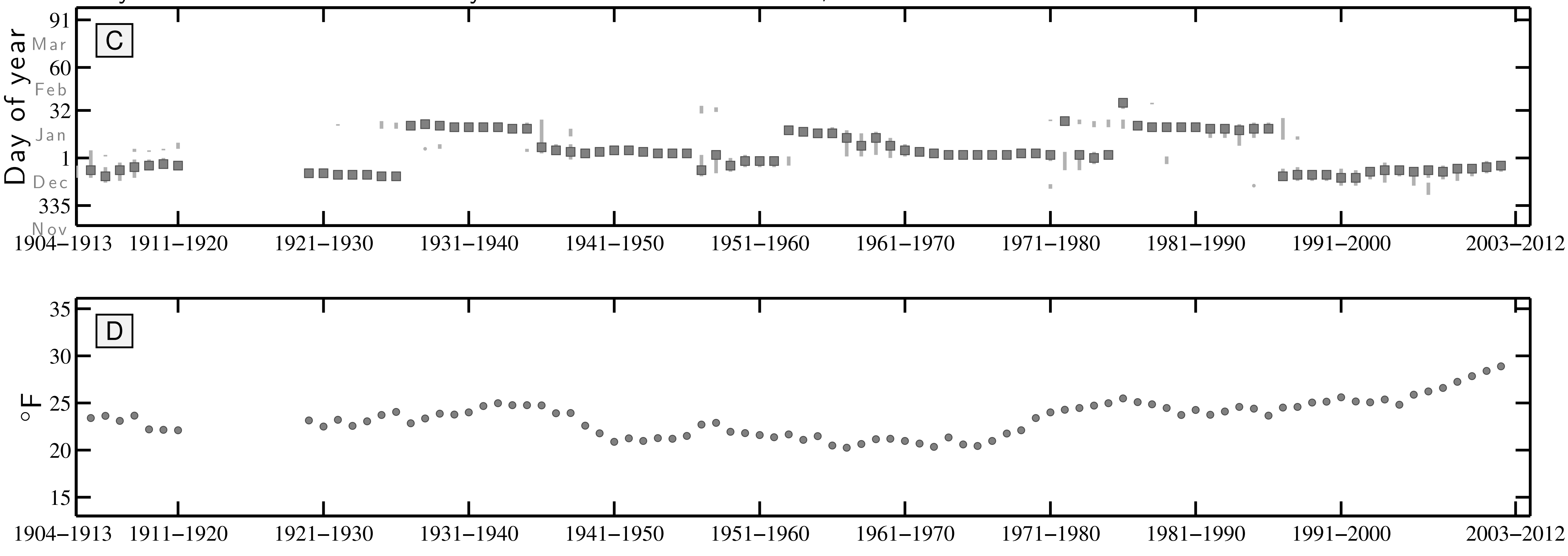

10 year Summer Teletherm dynamics for FT SUMNER, NM:

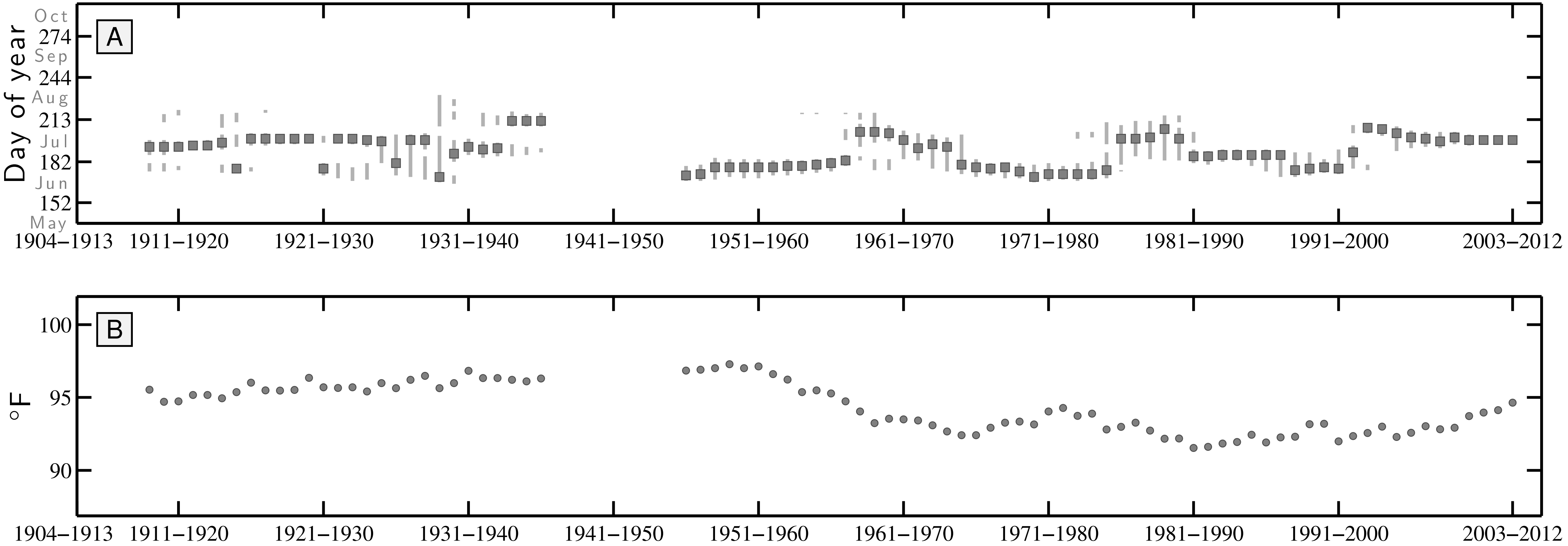

10 year Winter Teletherm dynamics for FT SUMNER, NM:

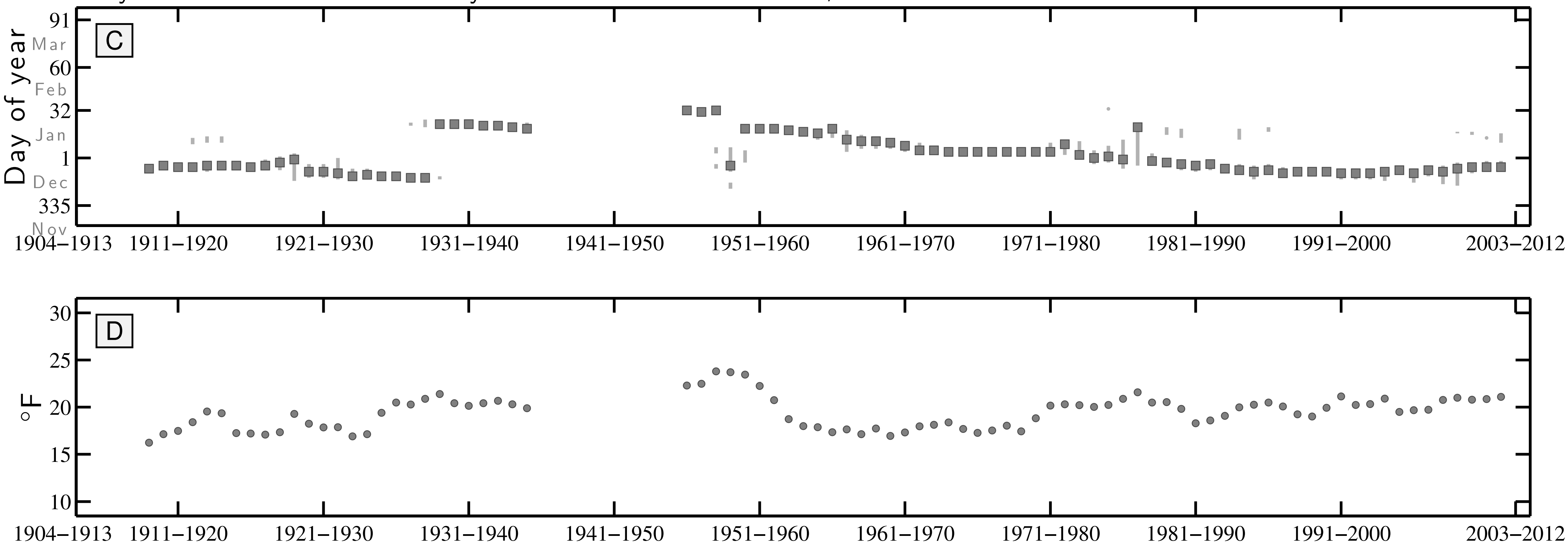

10 year Summer Teletherm dynamics for GAGE, NM:

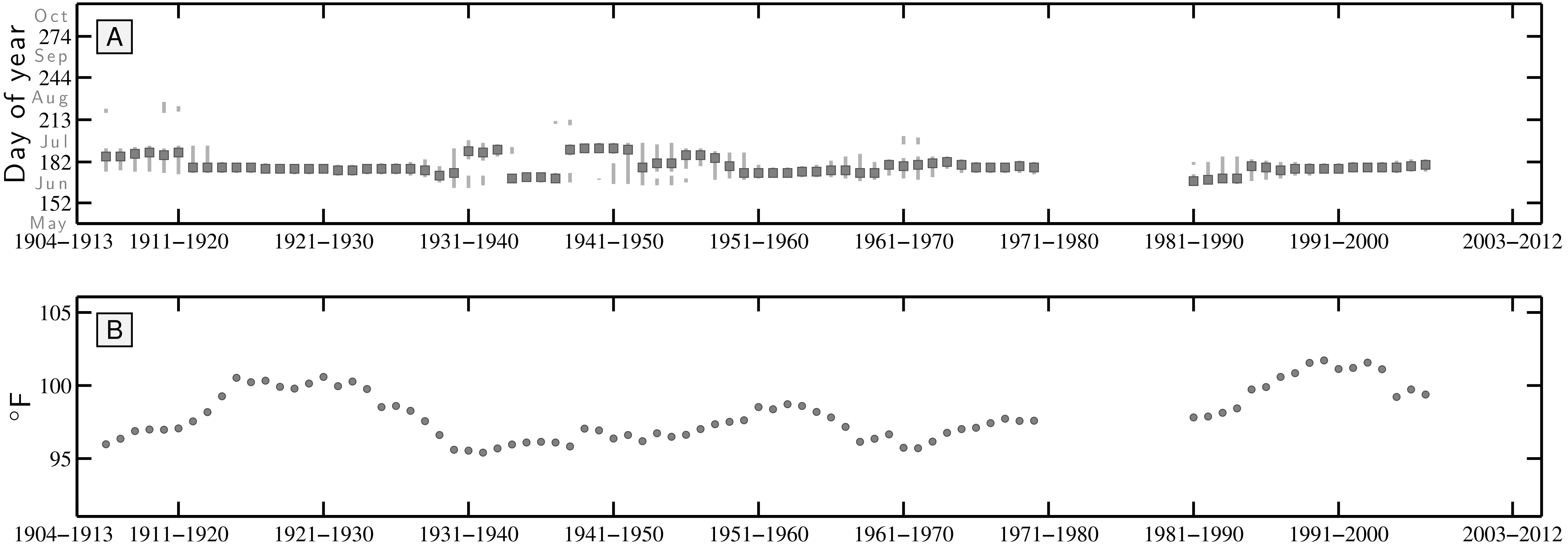

10 year Winter Teletherm dynamics for GAGE, NM:

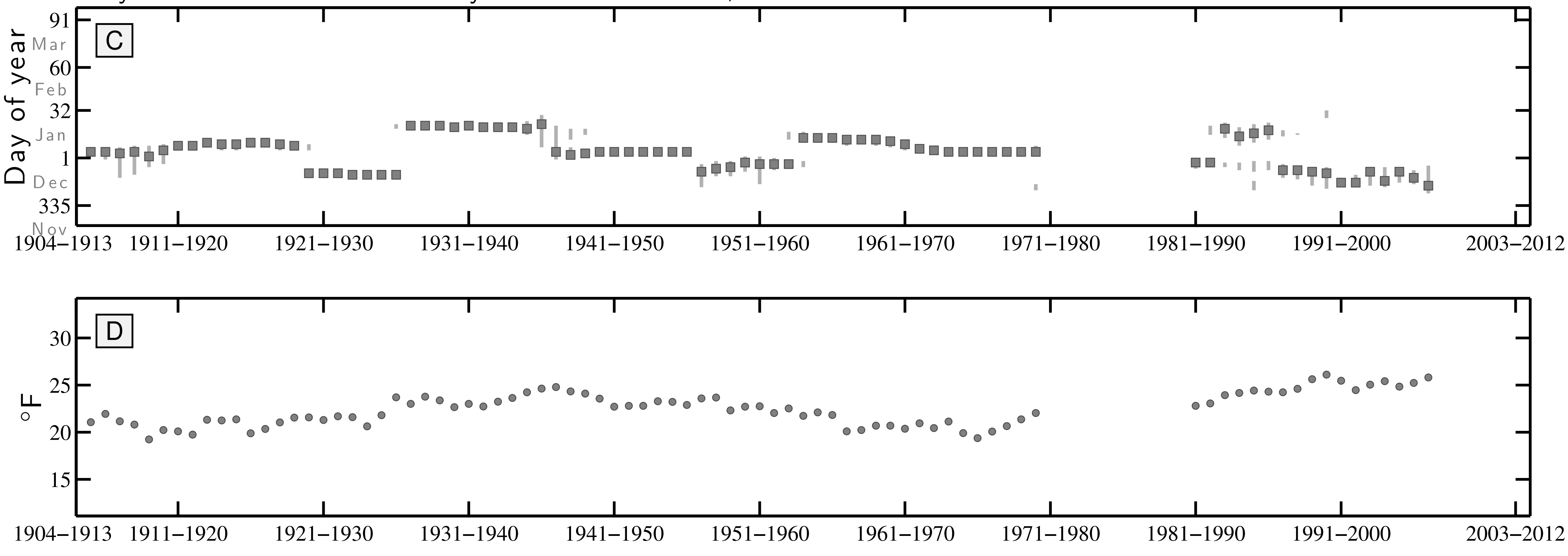

10 year Summer Teletherm dynamics for JEMEZ SPRINGS, NM:

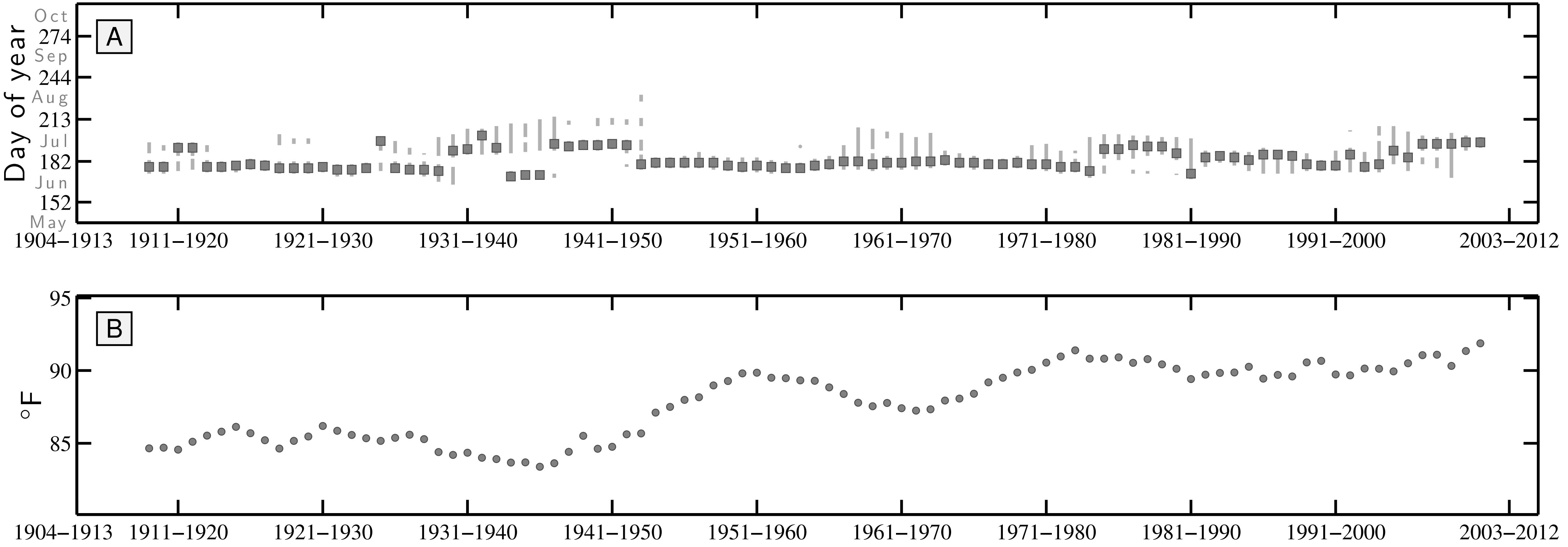

10 year Winter Teletherm dynamics for JEMEZ SPRINGS, NM:

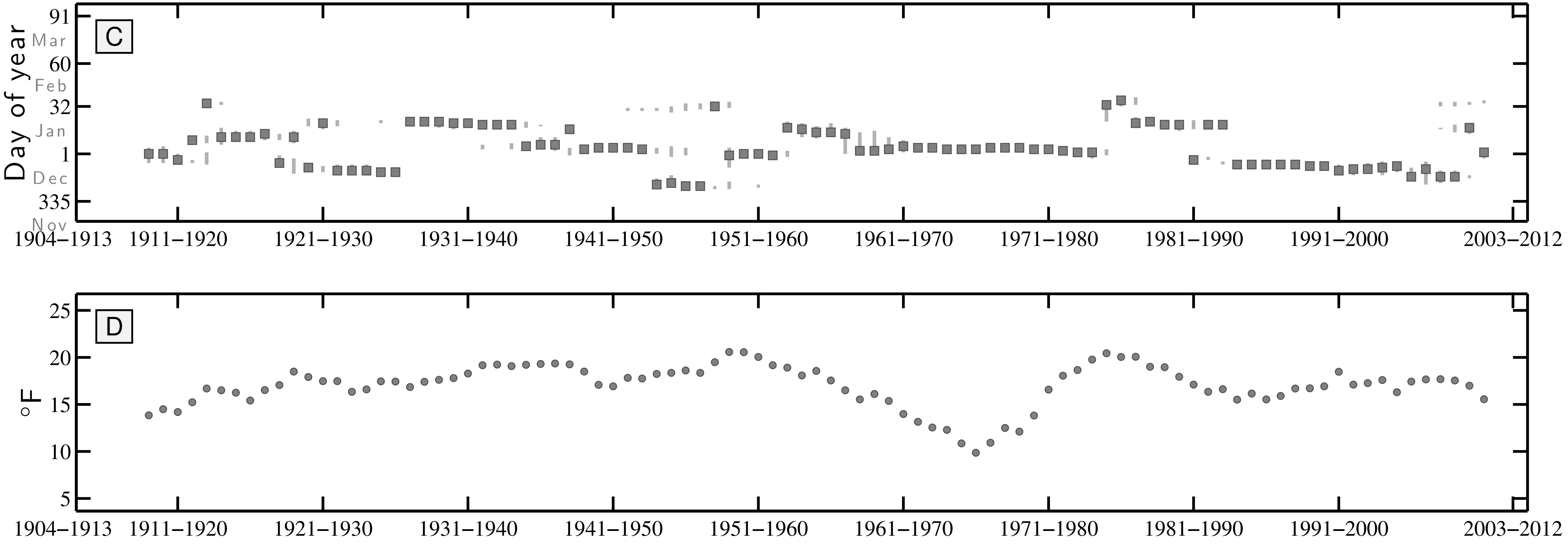

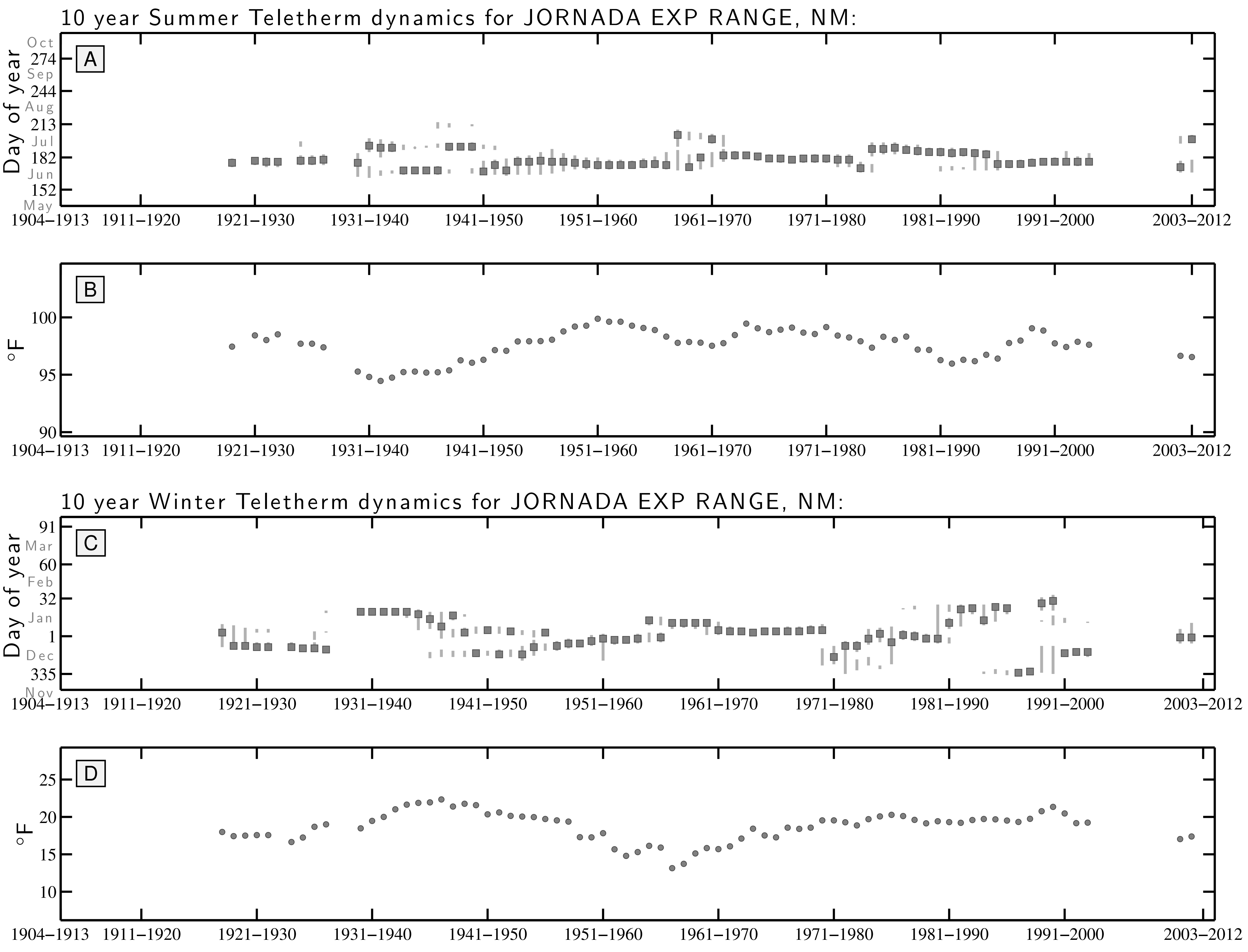
10 year Summer Teletherm dynamics for JORNADA EXP RANGE, NM:
A
B
Day of year
°F
Oct
274
Sep
244
Aug
213
Jul
182
Jun
152
May
100
95
90
1904–1913
1911–1920
1921–1930
1931–1940
1941–1950
1951–1960
1961–1970
1971–1980
1981–1990
1991–2000
2003–2012
10 year Winter Teletherm dynamics for JORNADA EXP RANGE, NM:
C
D
91
Mar
60
Feb
32
Jan
1
Dec
335
Nov
25
20
15
10

10 year Summer Teletherm dynamics for LAS VEGAS WWTP, NM:

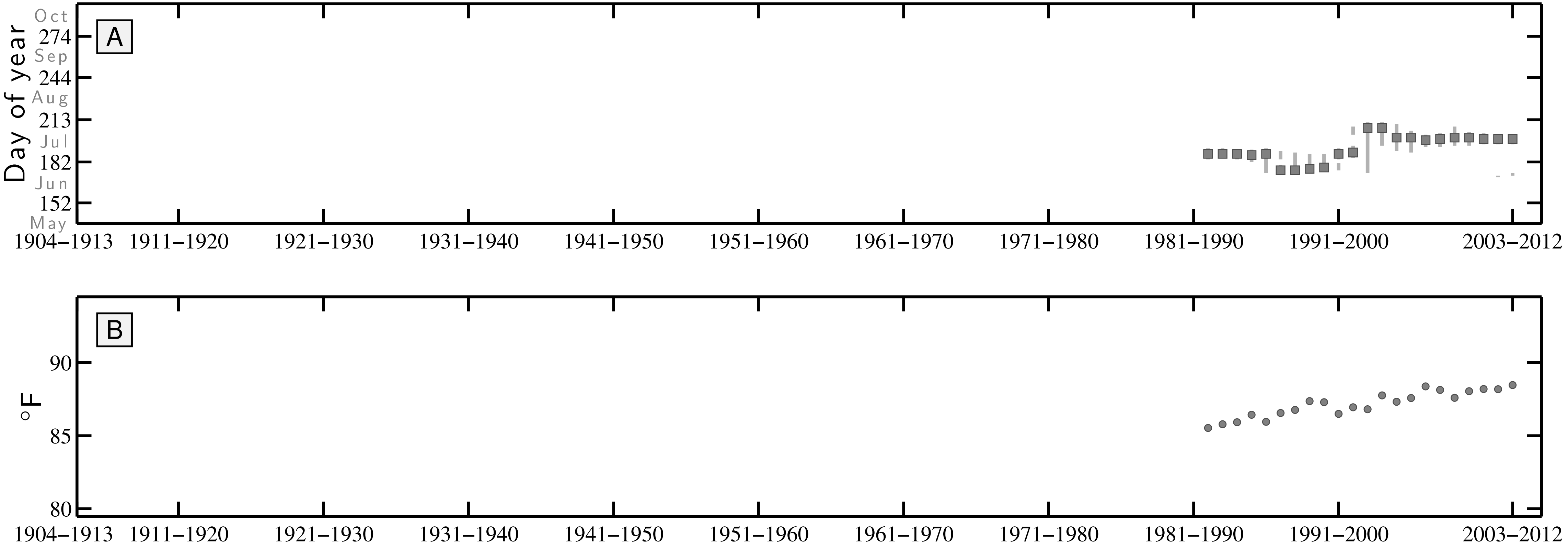

10 year Winter Teletherm dynamics for LAS VEGAS WWTP, NM:

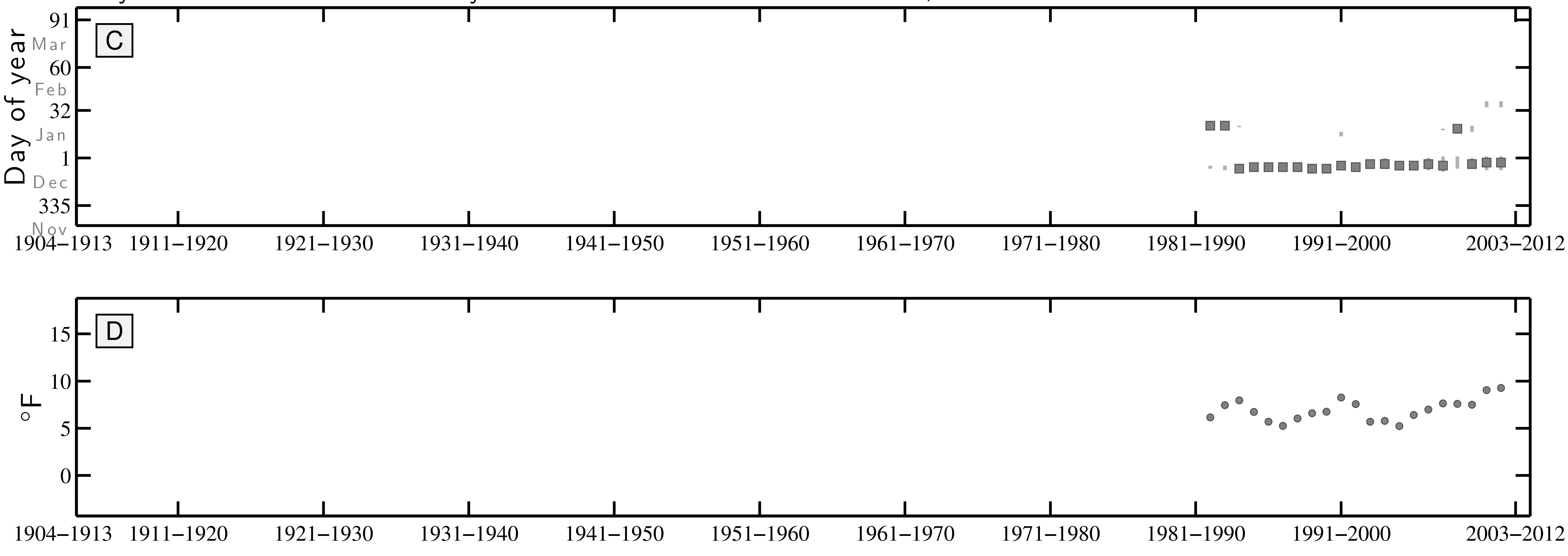

10 year Summer Teletherm dynamics for LOS LUNAS 3 SSW, NM:

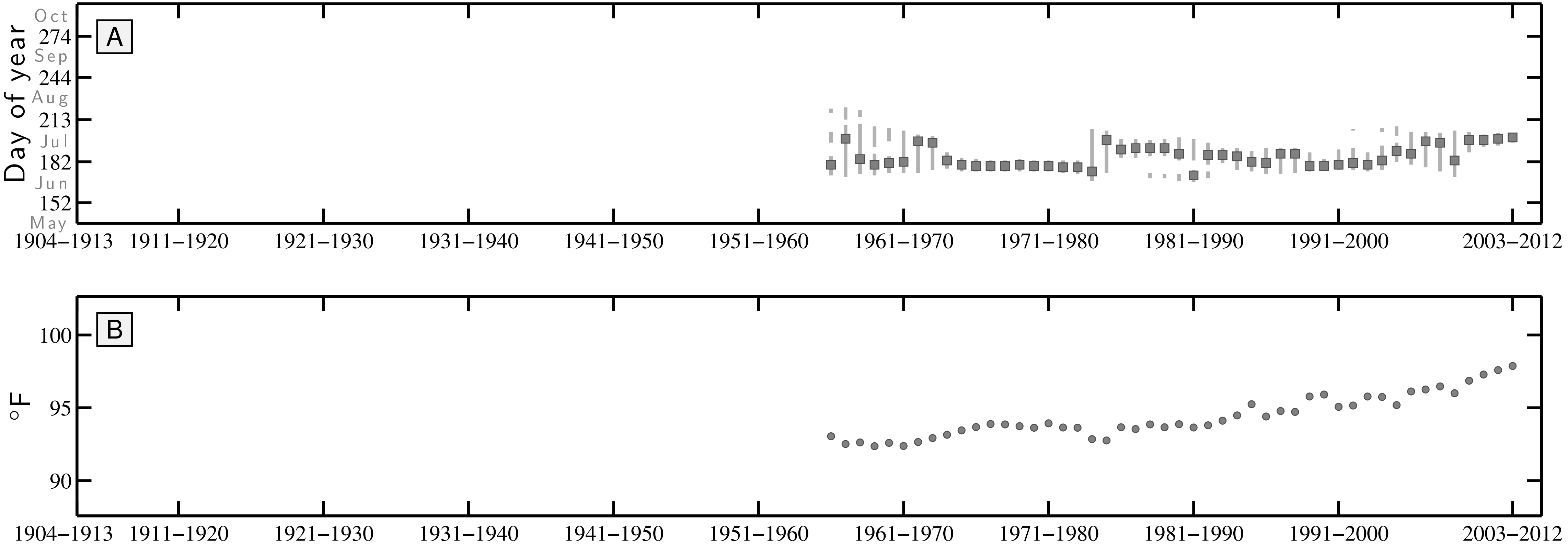

10 year Winter Teletherm dynamics for LOS LUNAS 3 SSW, NM:

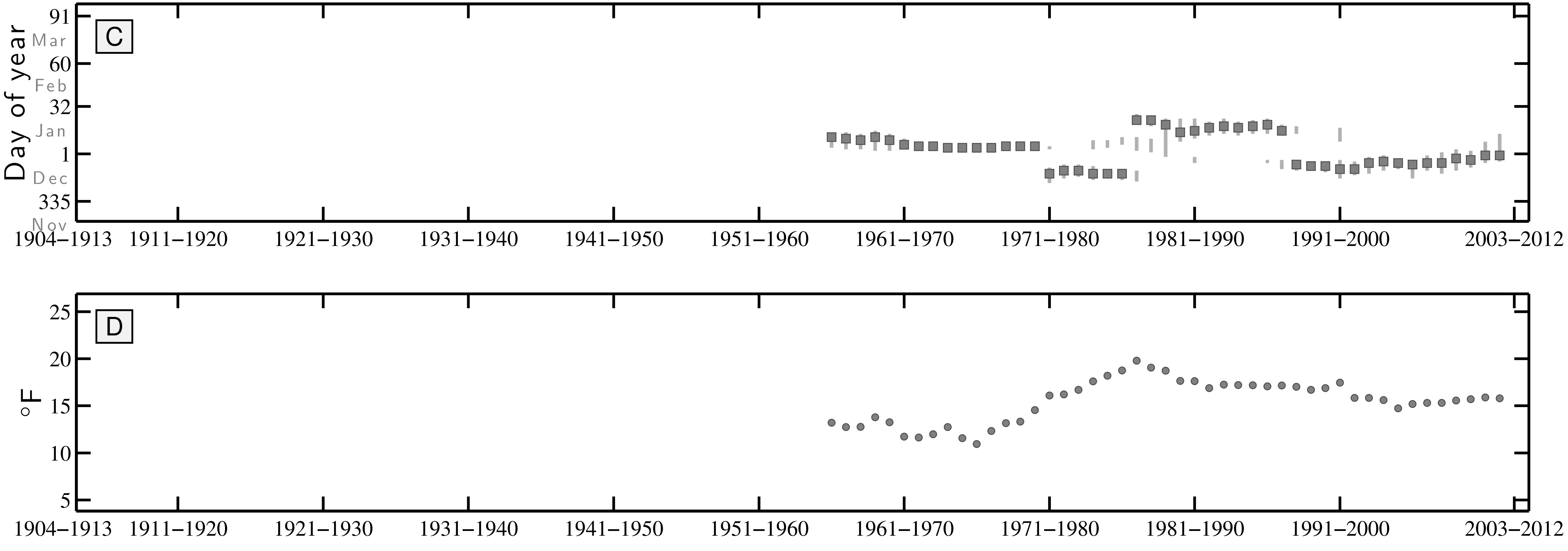

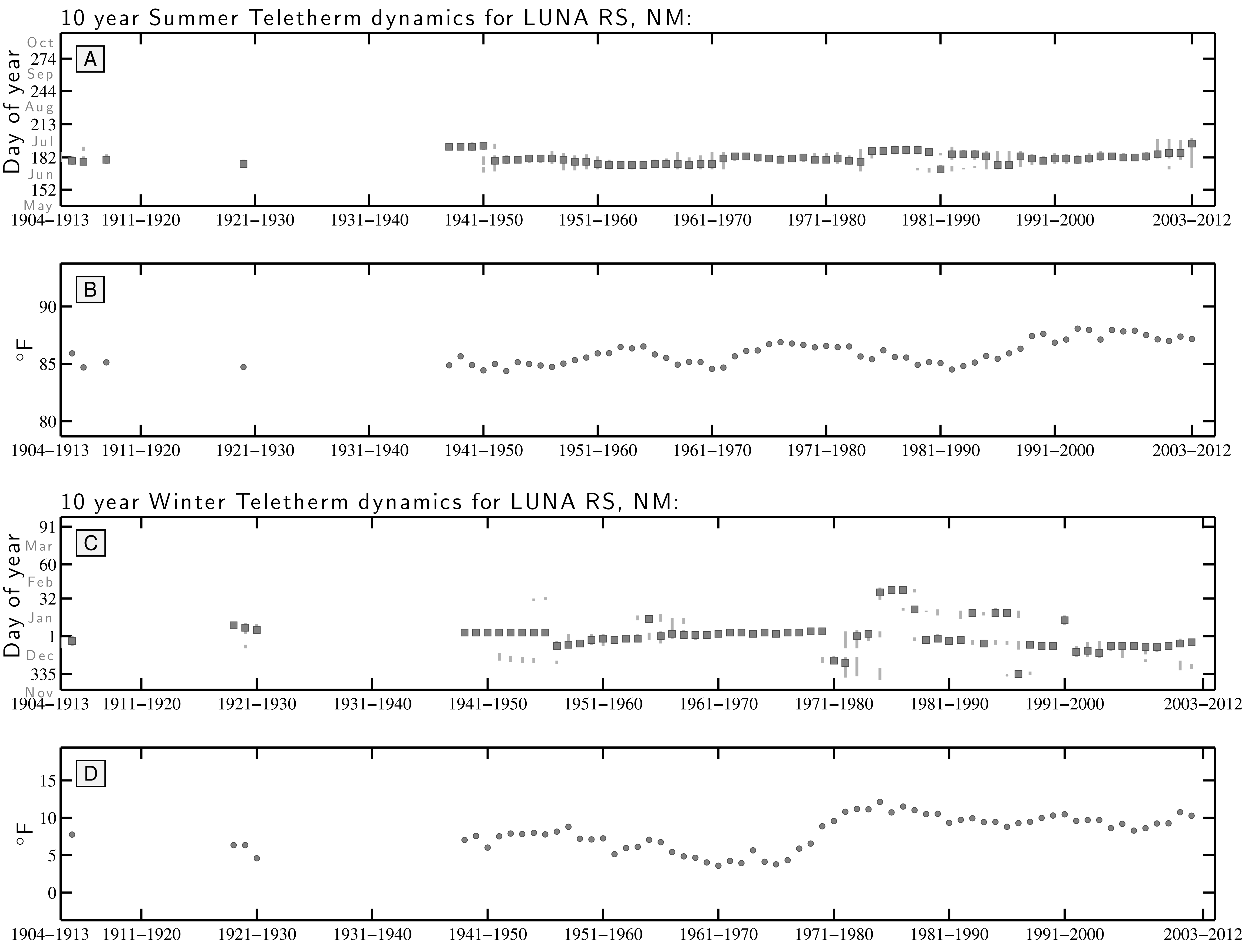
10 year Summer Teletherm dynamics for LUNA RS, NM:
A
B
Day of year
°F
Oct
274
Sep
244
Aug
213
Jul
182
Jun
152
May
90
85
80
1904–1913
1911–1920
1921–1930
1931–1940
1941–1950
1951–1960
1961–1970
1971–1980
1981–1990
1991–2000
2003–2012
10 year Winter Teletherm dynamics for LUNA RS, NM:
C
D
91
Mar
60
Feb
32
Jan
1
Dec
335
Nov
15
10
5
0

10 year Summer Teletherm dynamics for MTN PARK, NM:

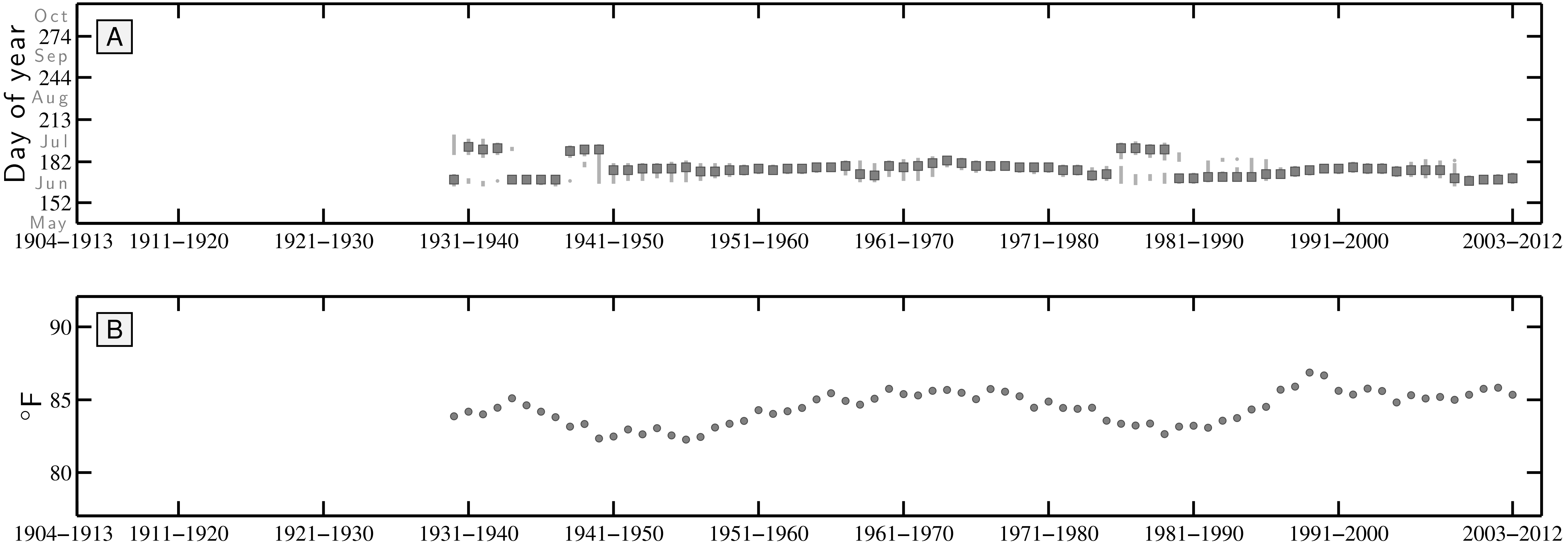

10 year Winter Teletherm dynamics for MTN PARK, NM:

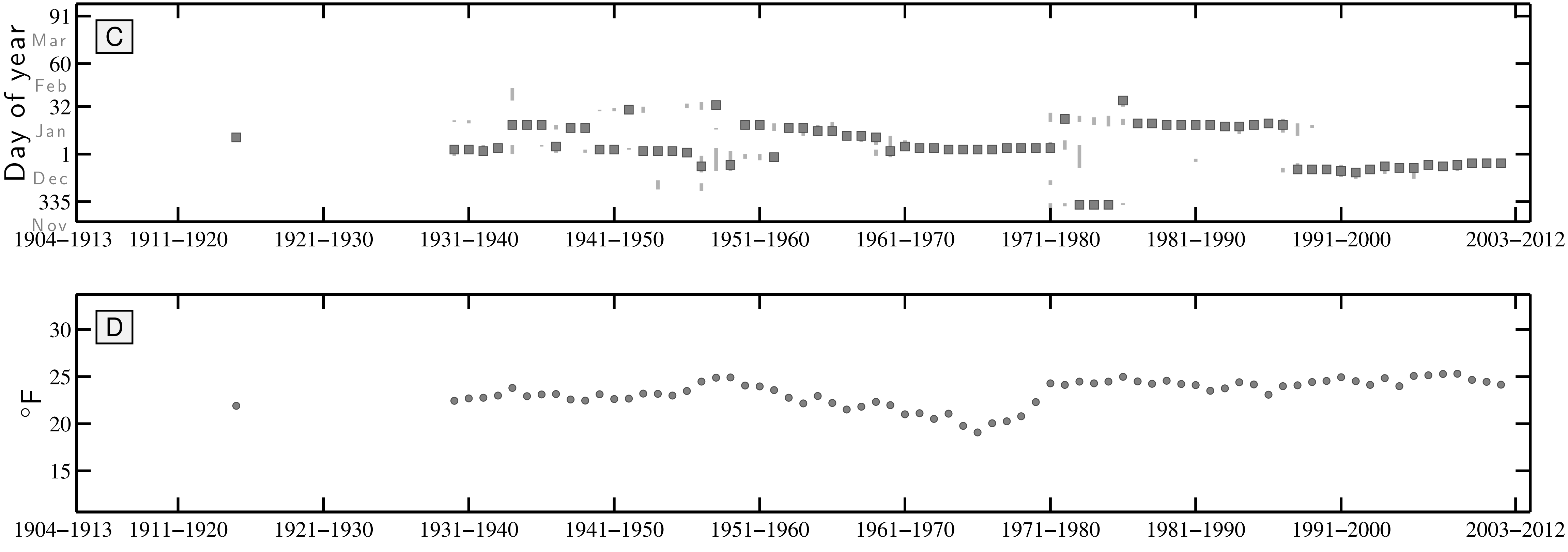

10 year Summer Teletherm dynamics for MOUNTAINAIR, NM:

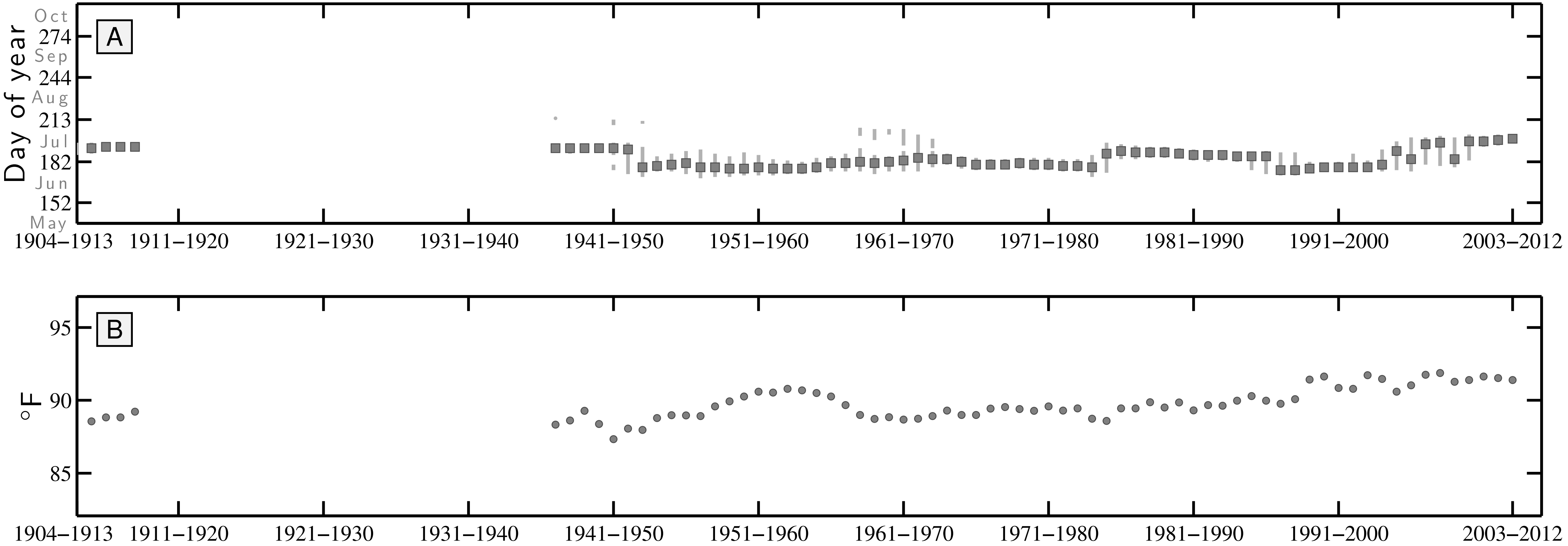

10 year Winter Teletherm dynamics for MOUNTAINAIR, NM:

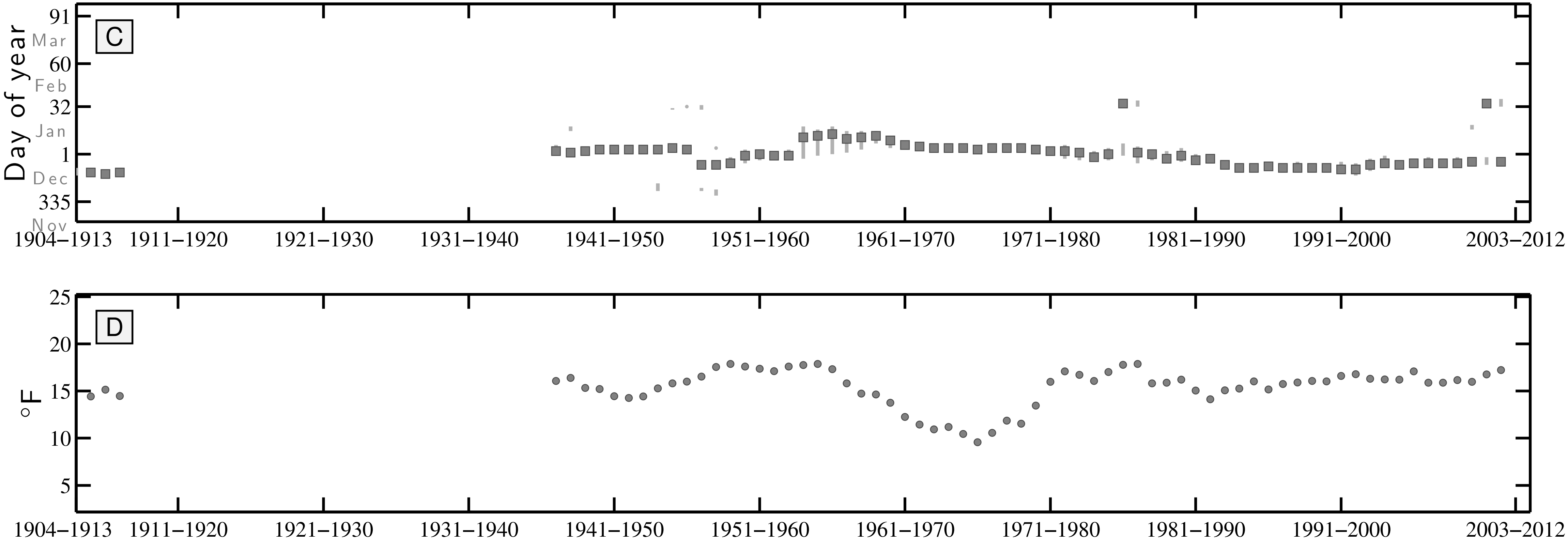

10 year Summer Teletherm dynamics for OROGRANDE, NM:

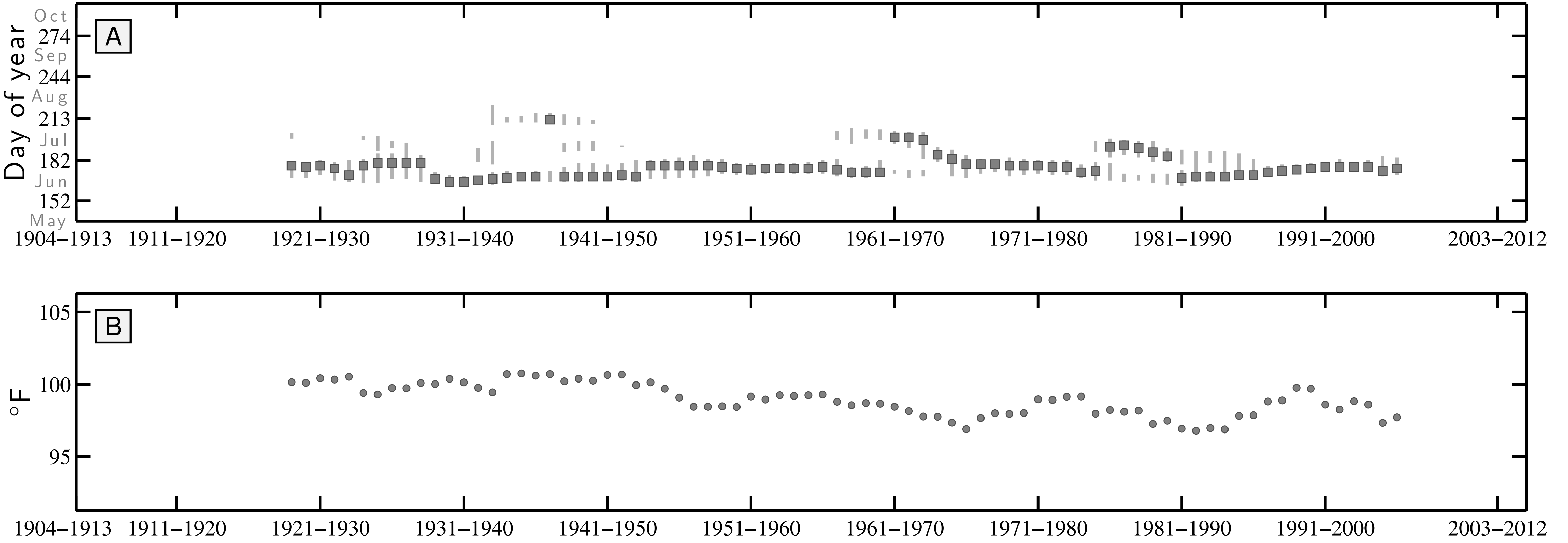

10 year Winter Teletherm dynamics for OROGRANDE, NM:

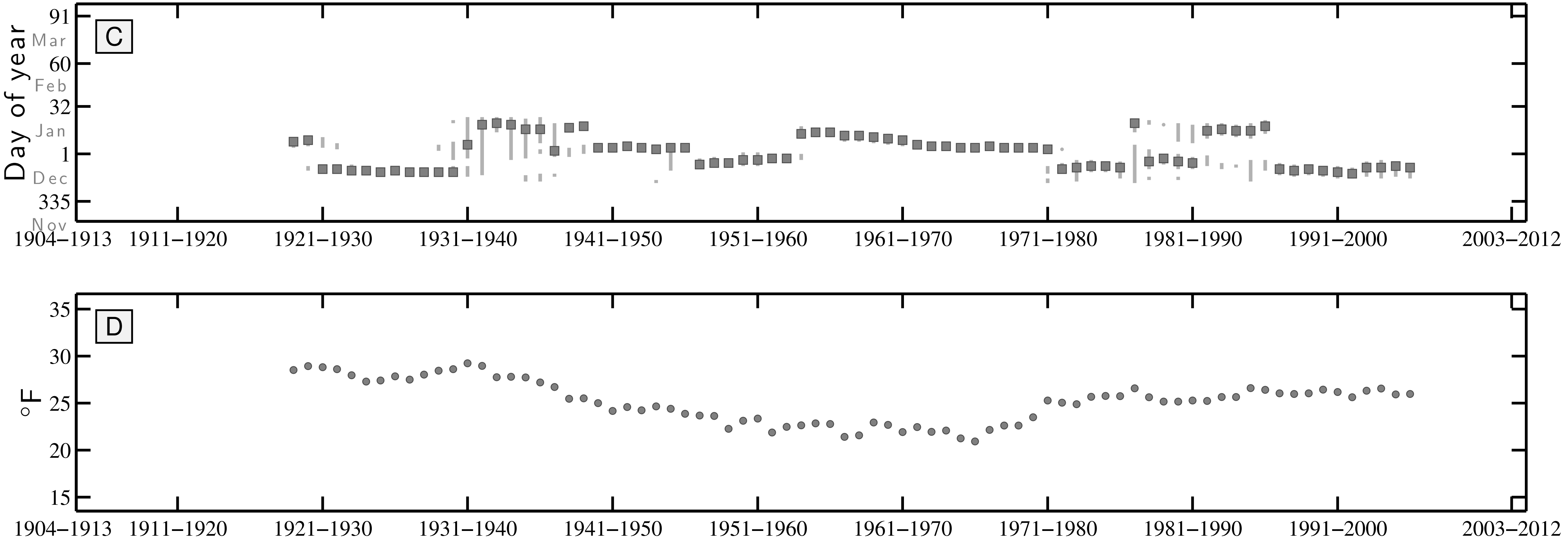

10 year Summer Teletherm dynamics for RED RIVER, NM:

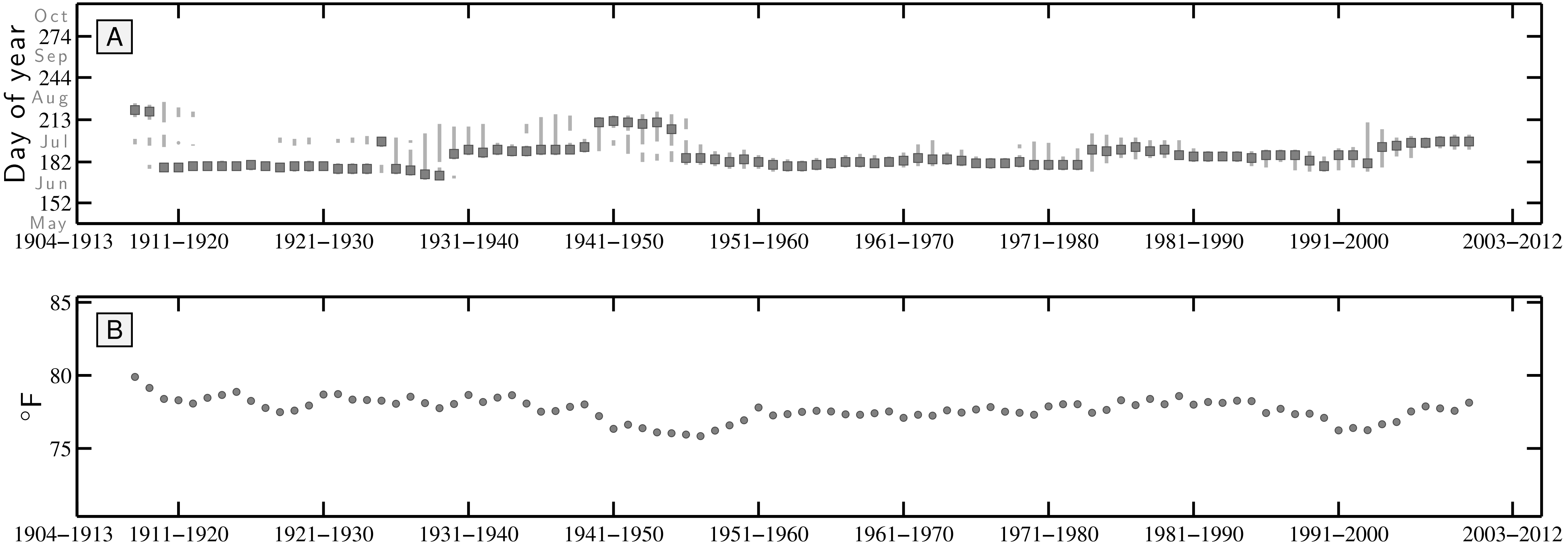

10 year Winter Teletherm dynamics for RED RIVER, NM:

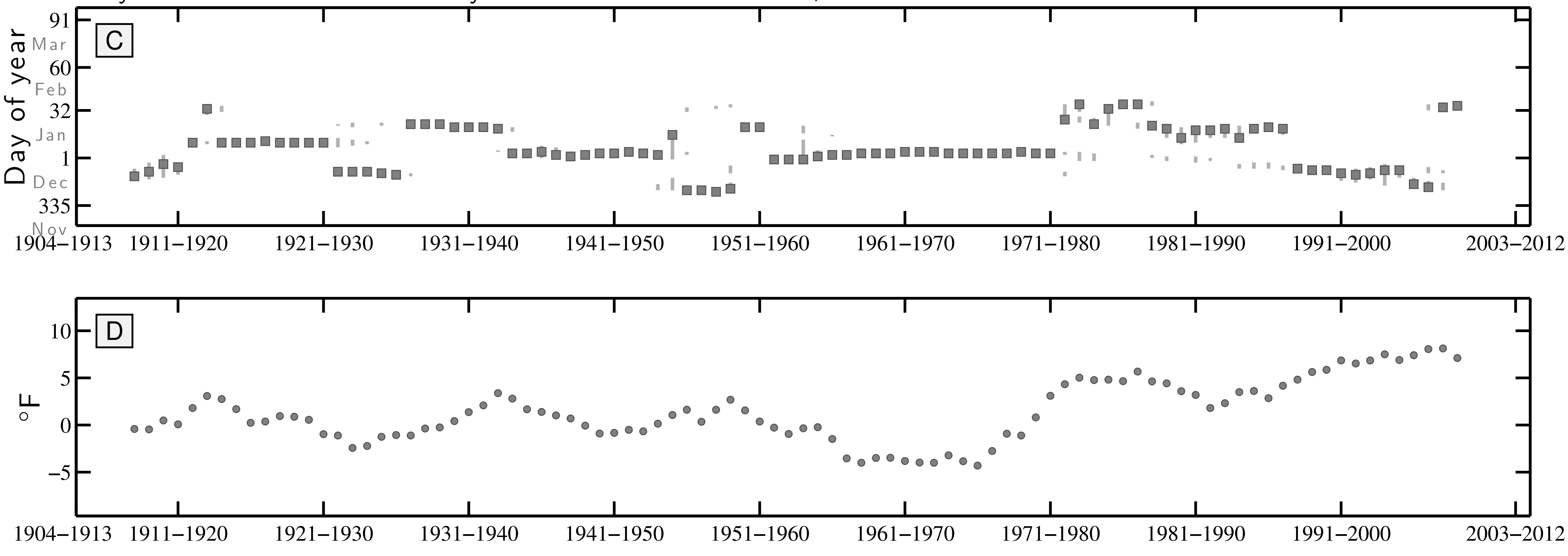

10 year Summer Teletherm dynamics for ROSWELL IND AP, NM:

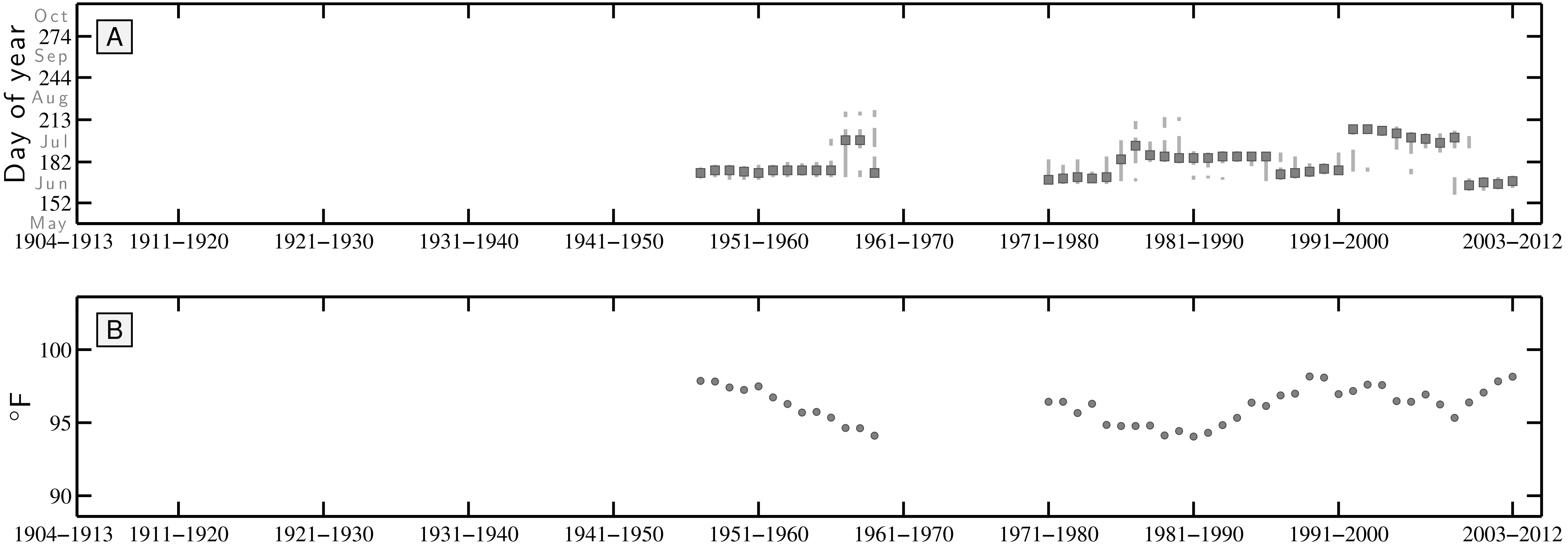

10 year Winter Teletherm dynamics for ROSWELL IND AP, NM:

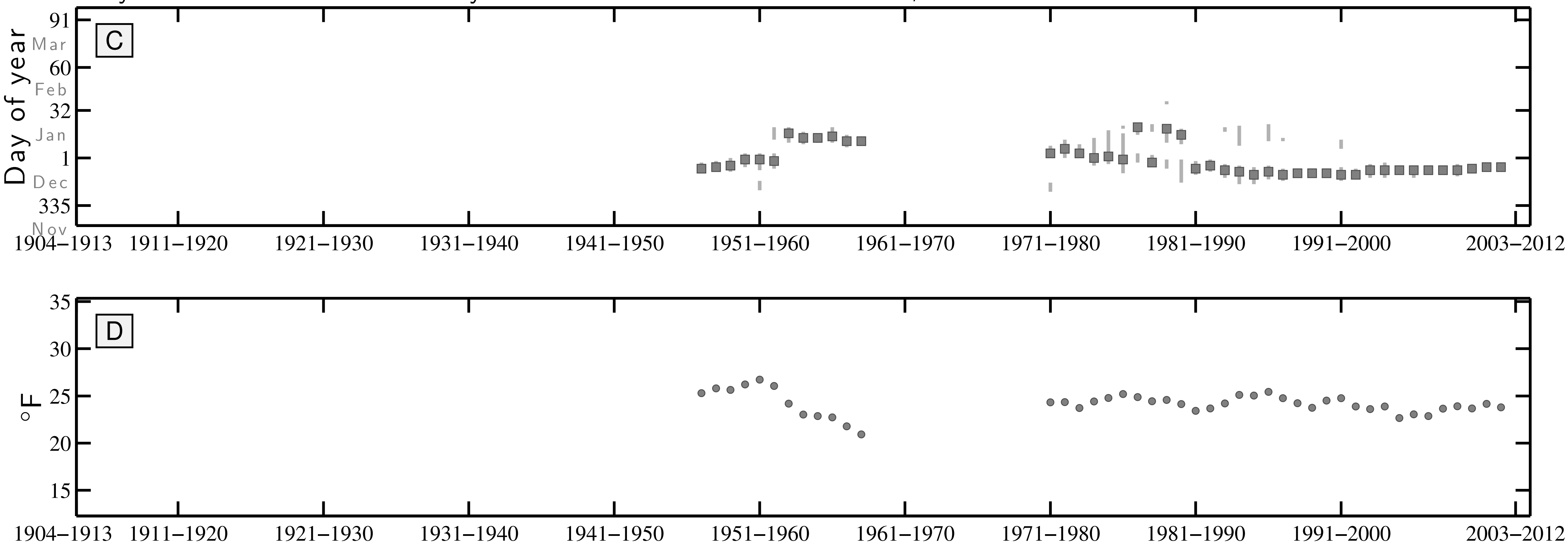

10 year Summer Teletherm dynamics for SAN JON, NM:

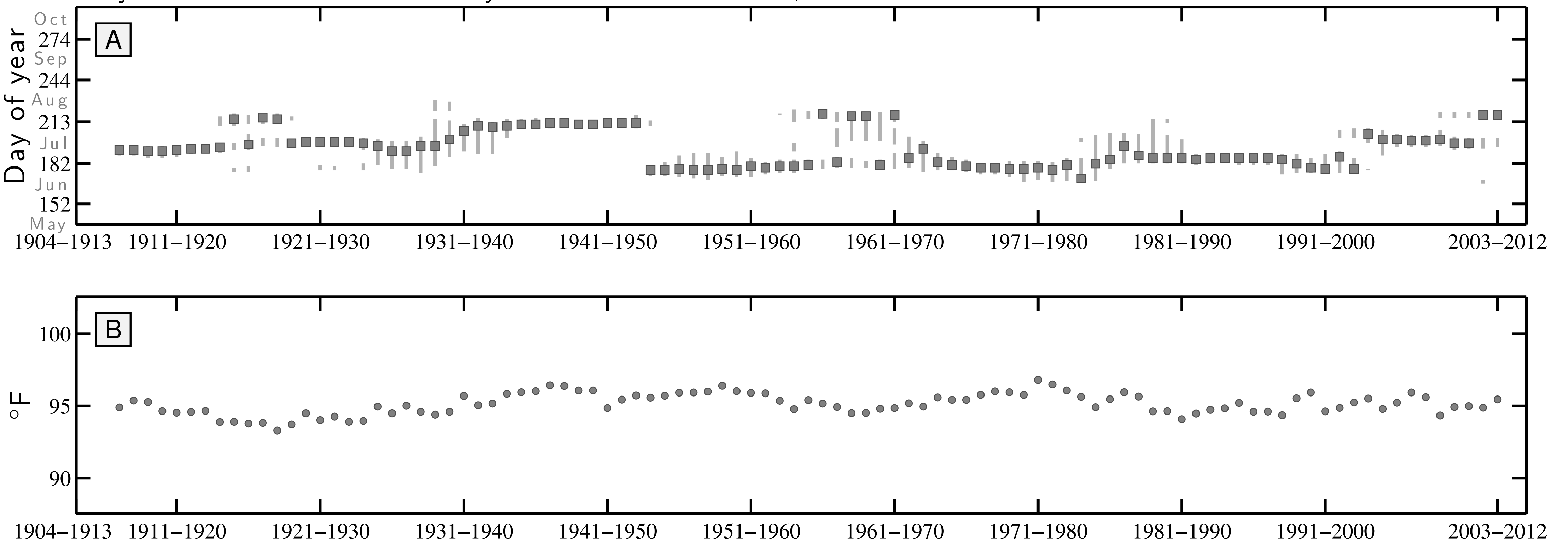

10 year Winter Teletherm dynamics for SAN JON, NM:

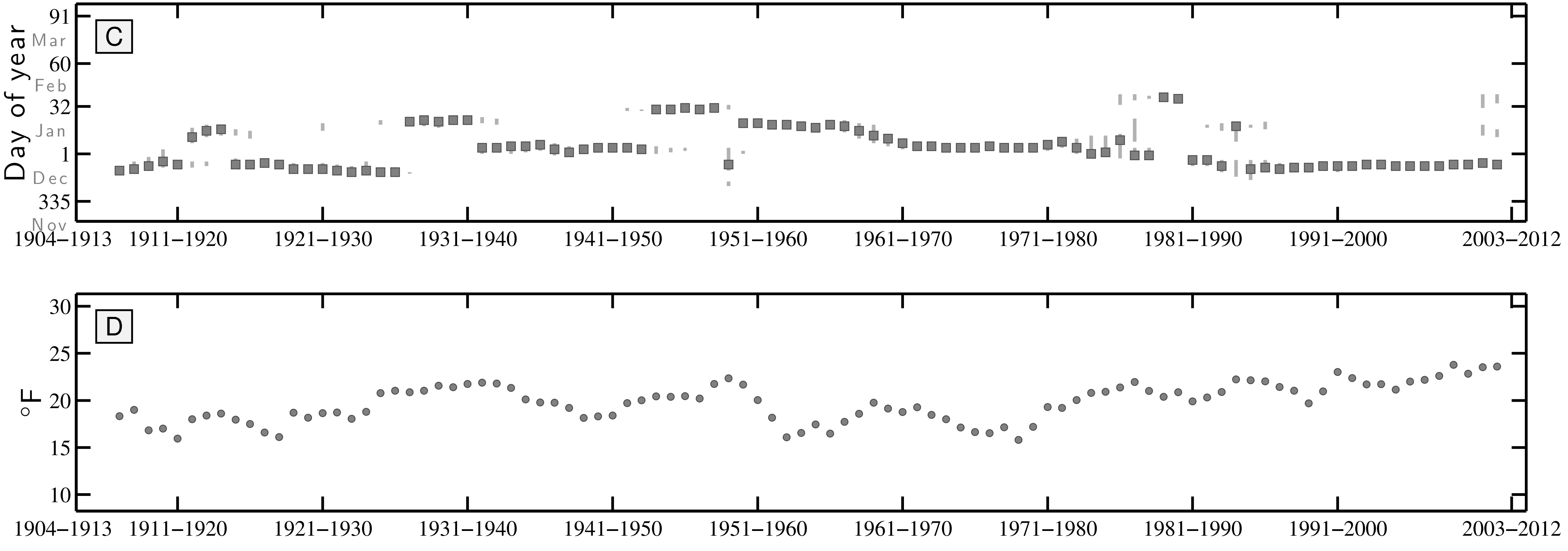

10 year Summer Teletherm dynamics for SANTA ROSA, NM:

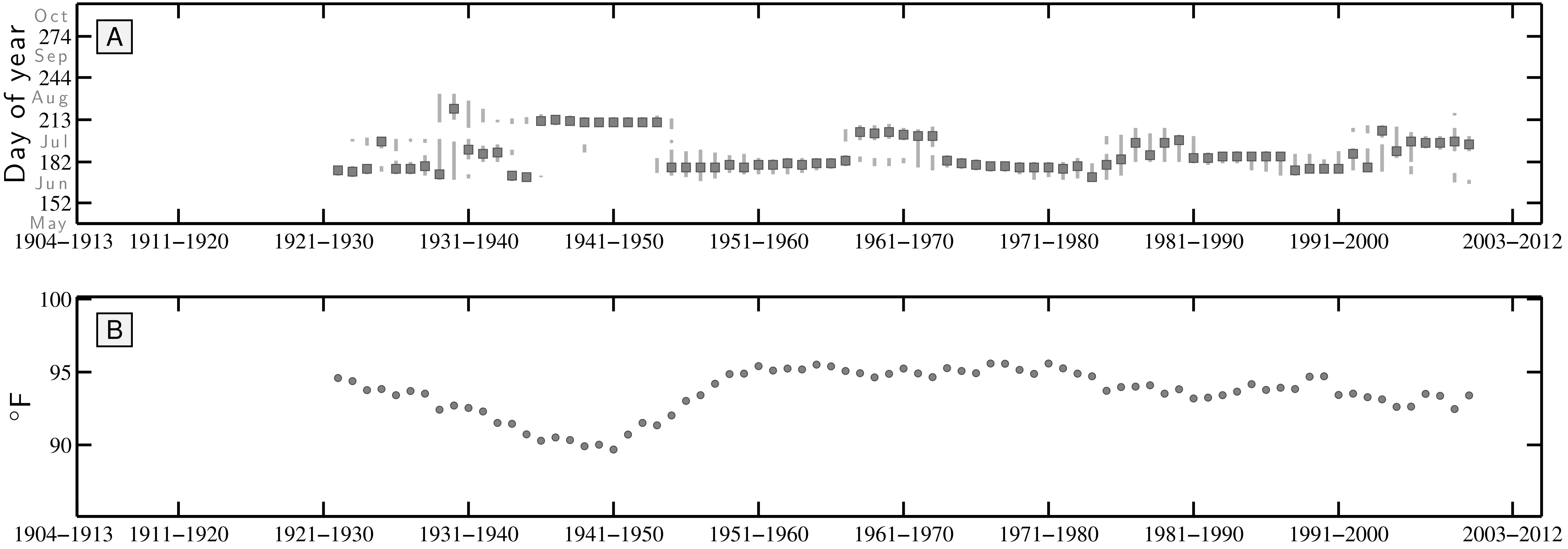

10 year Winter Teletherm dynamics for SANTA ROSA, NM:

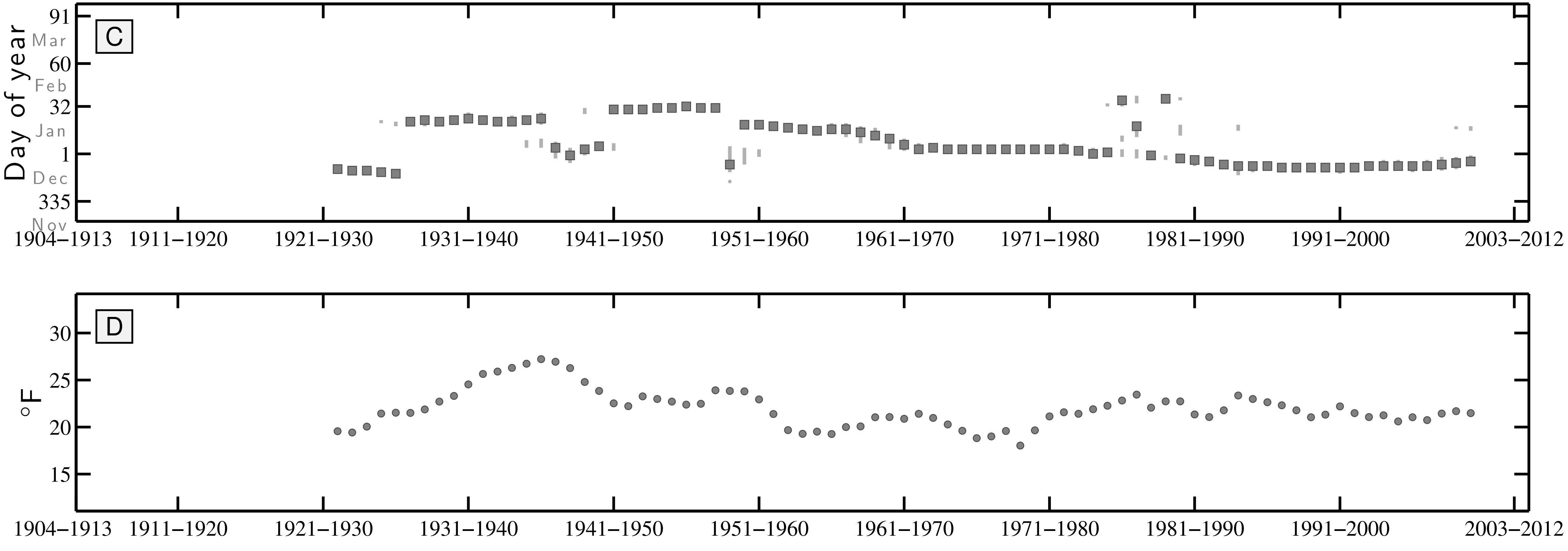

10 year Summer Teletherm dynamics for SOCORRO, NM:

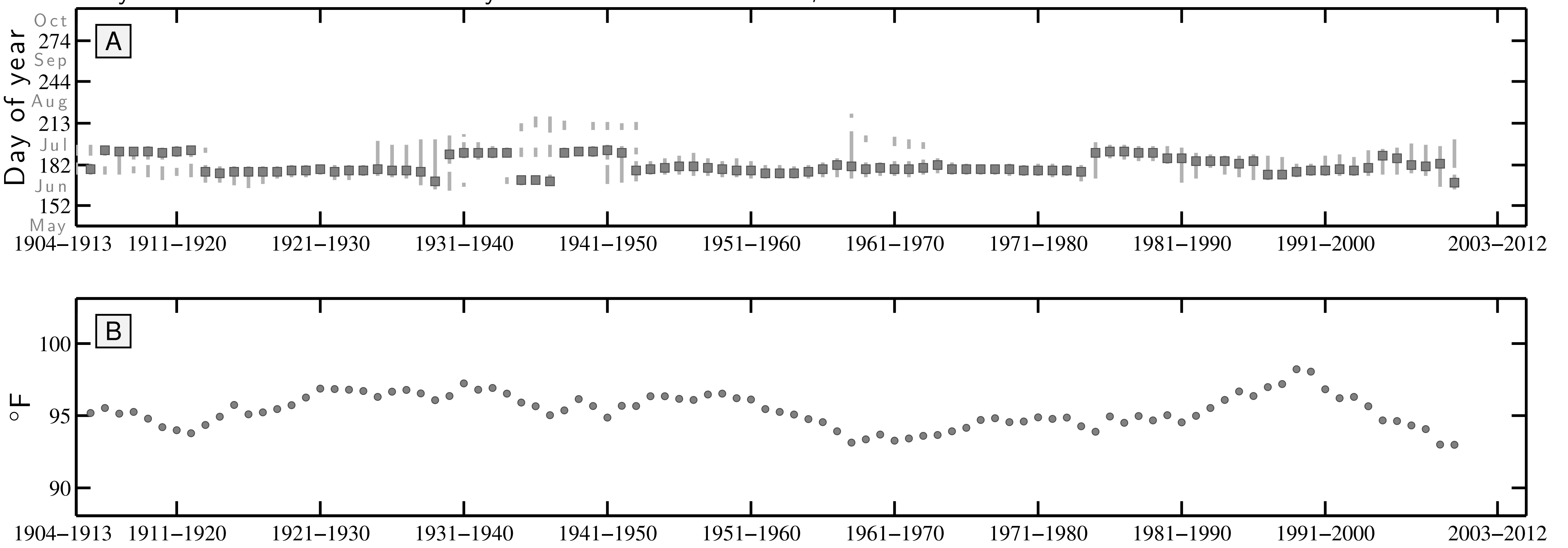

10 year Winter Teletherm dynamics for SOCORRO, NM:

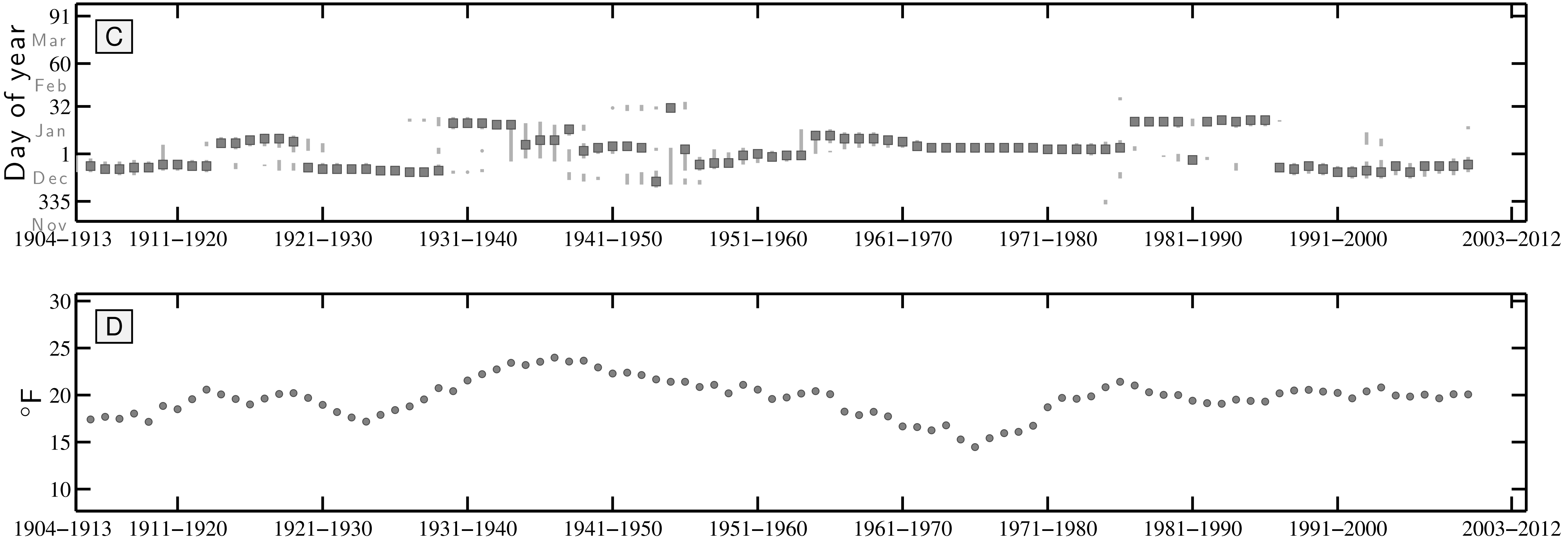

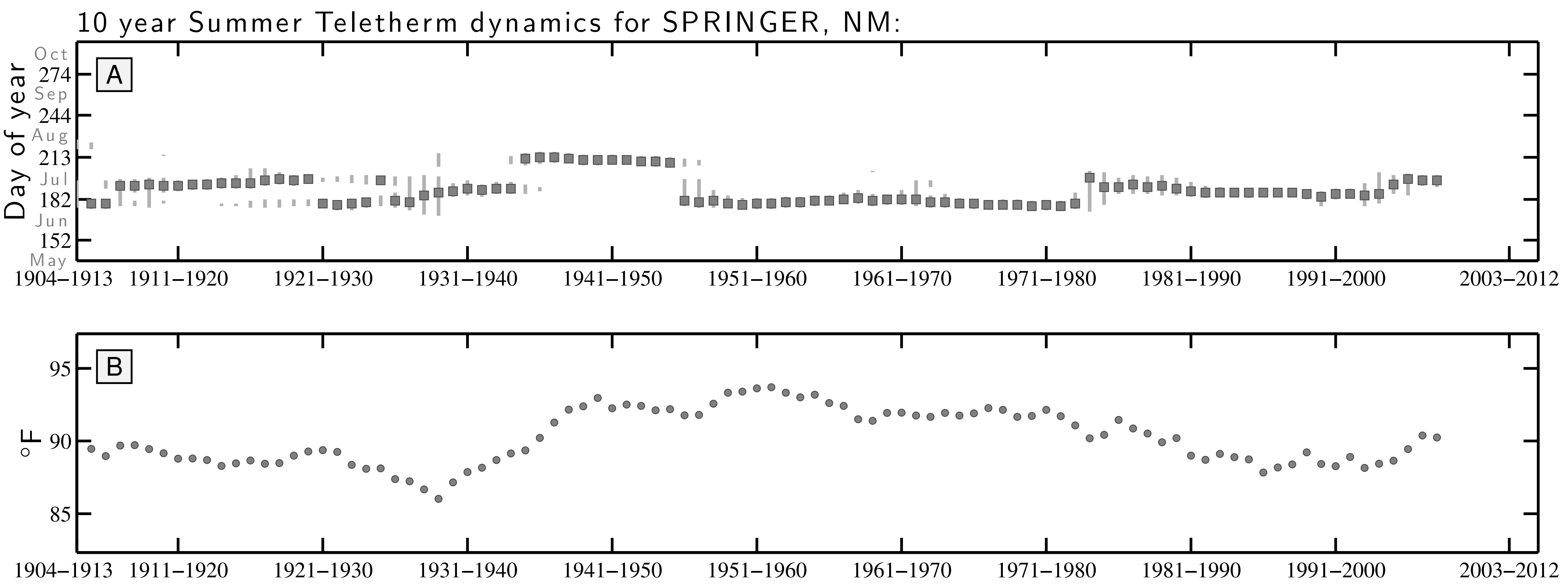
10 year Summer Teletherm dynamics for SPRINGER, NM:
A
B
Day of year
Oct
274
Sep
244
Aug
213
Jul
182
Jun
152
May
°F
95
90
85
1904–1913
1911–1920
1921–1930
1931–1940
1941–1950
1951–1960
1961–1970
1971–1980
1981–1990
1991–2000
2003–2012

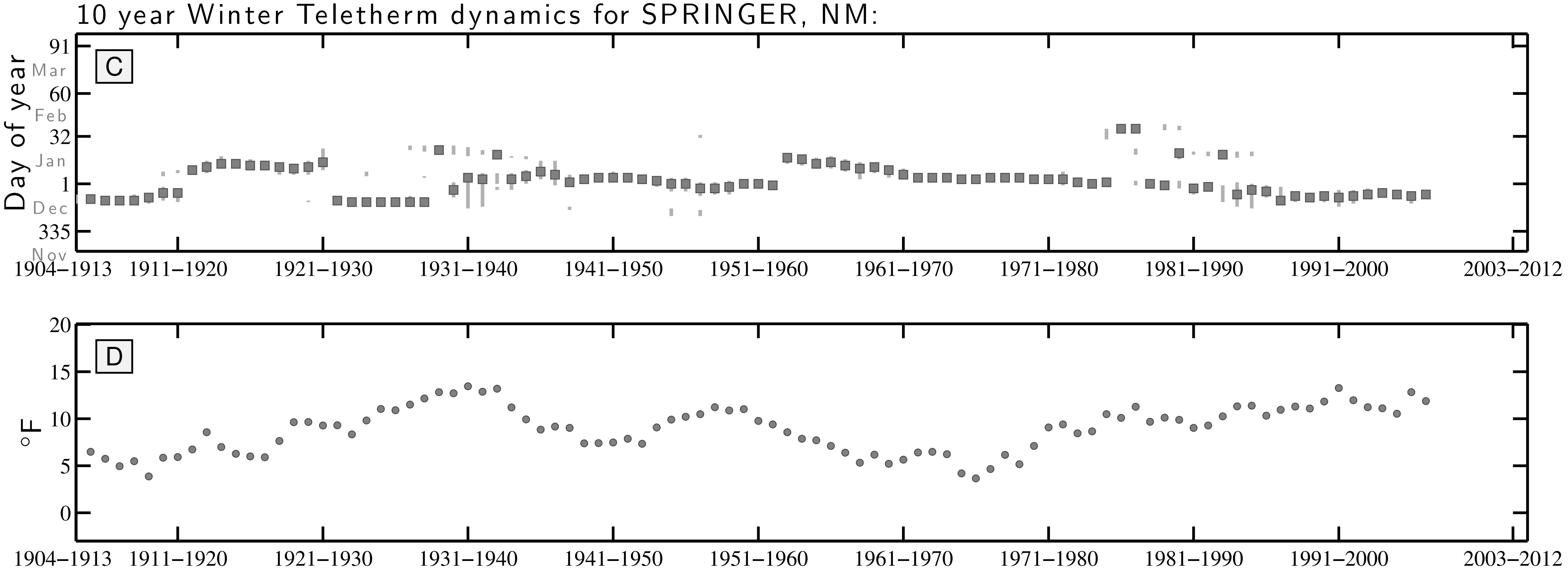
10 year Winter Teletherm dynamics for SPRINGER, NM:
C
D
Day of year
91
Mar
60
Feb
32
Jan
1
Dec
335
Nov
°F
20
15
10
5
0
1904–1913
1911–1920
1921–1930
1931–1940
1941–1950
1951–1960
1961–1970
1971–1980
1981–1990
1991–2000
2003–2012

10 year Summer Teletherm dynamics for STATE UNIV, NM:

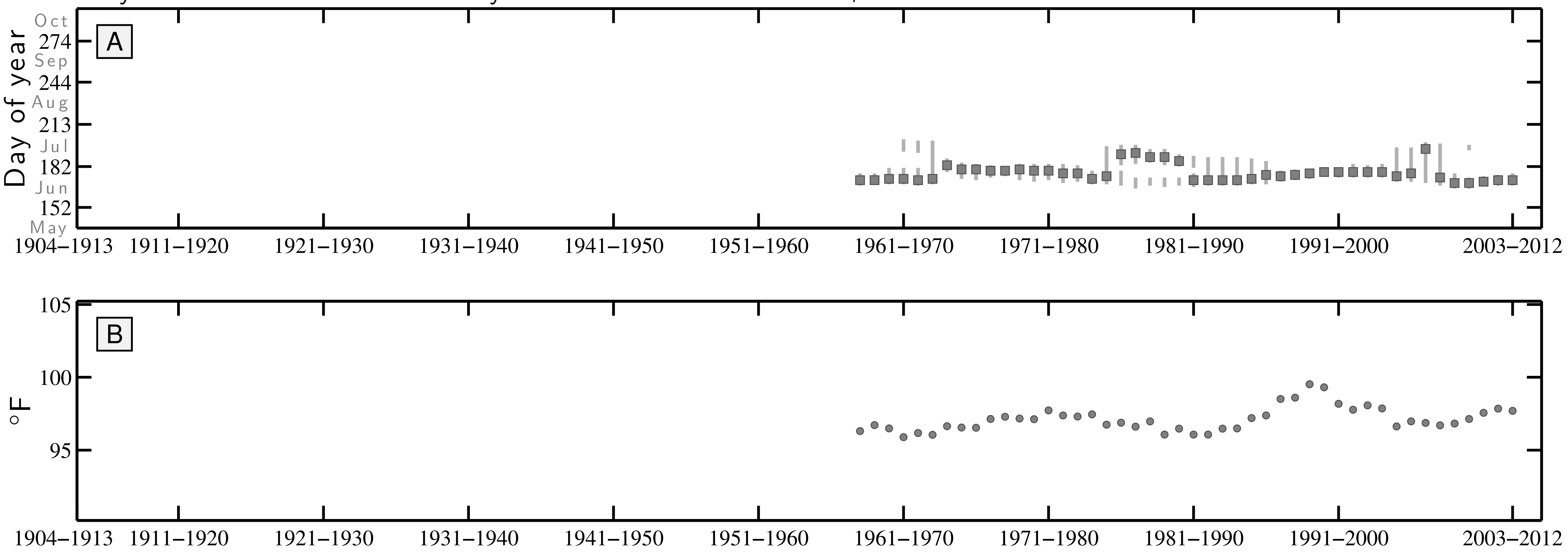

10 year Winter Teletherm dynamics for STATE UNIV, NM:

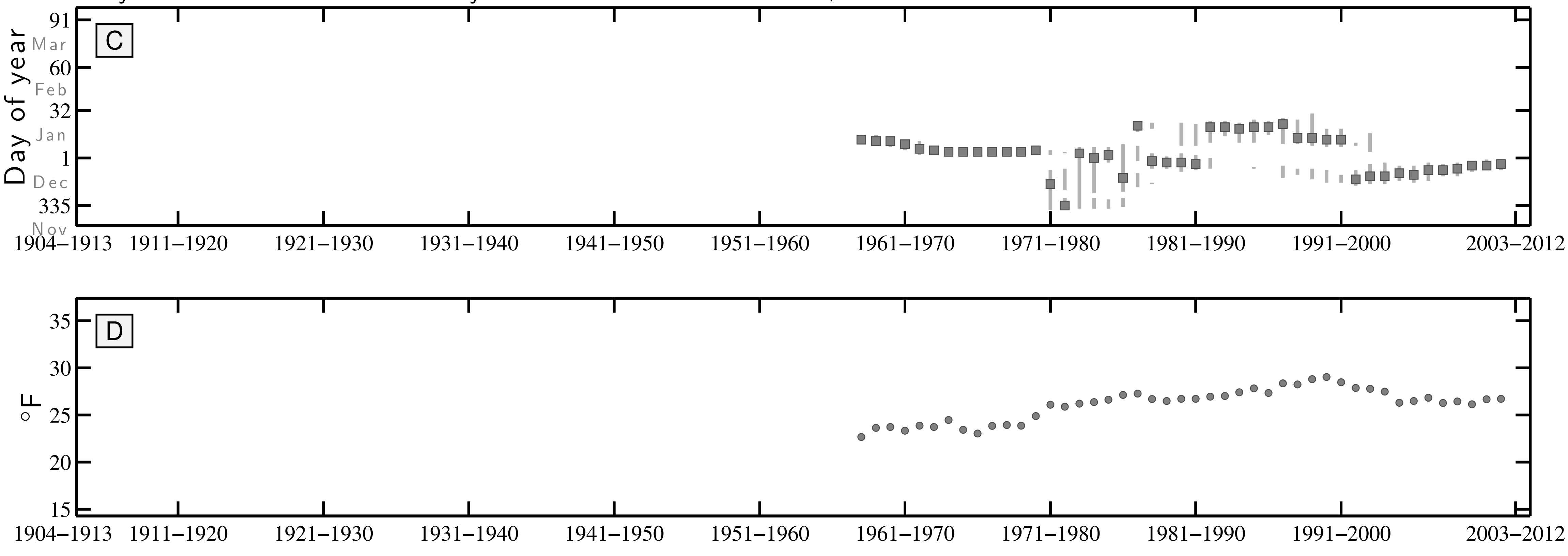

10 year Summer Teletherm dynamics for TUCUMCARI 4 NE, NM:

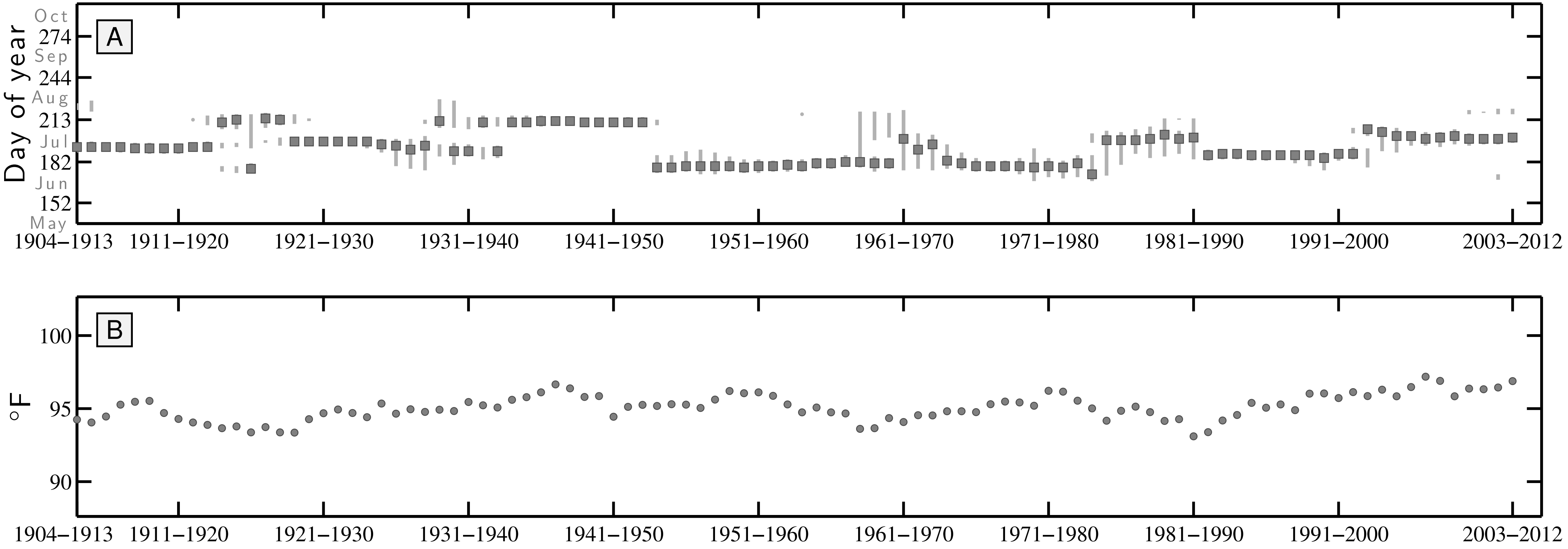

10 year Winter Teletherm dynamics for TUCUMCARI 4 NE, NM:

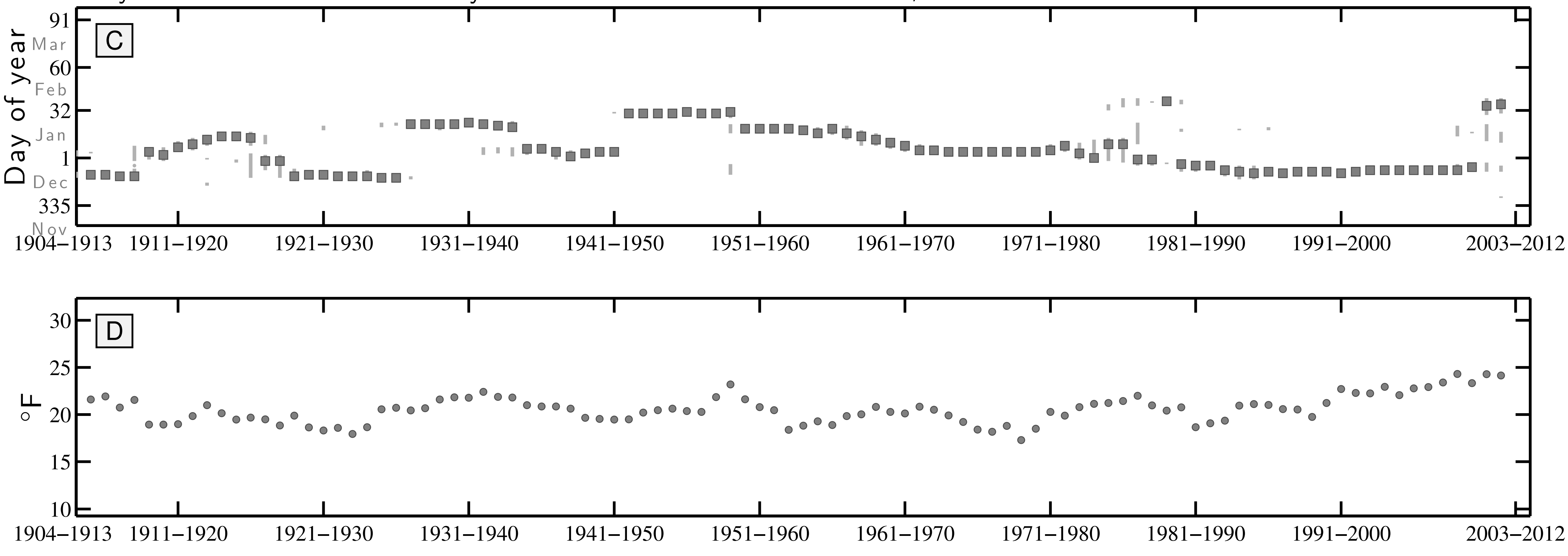

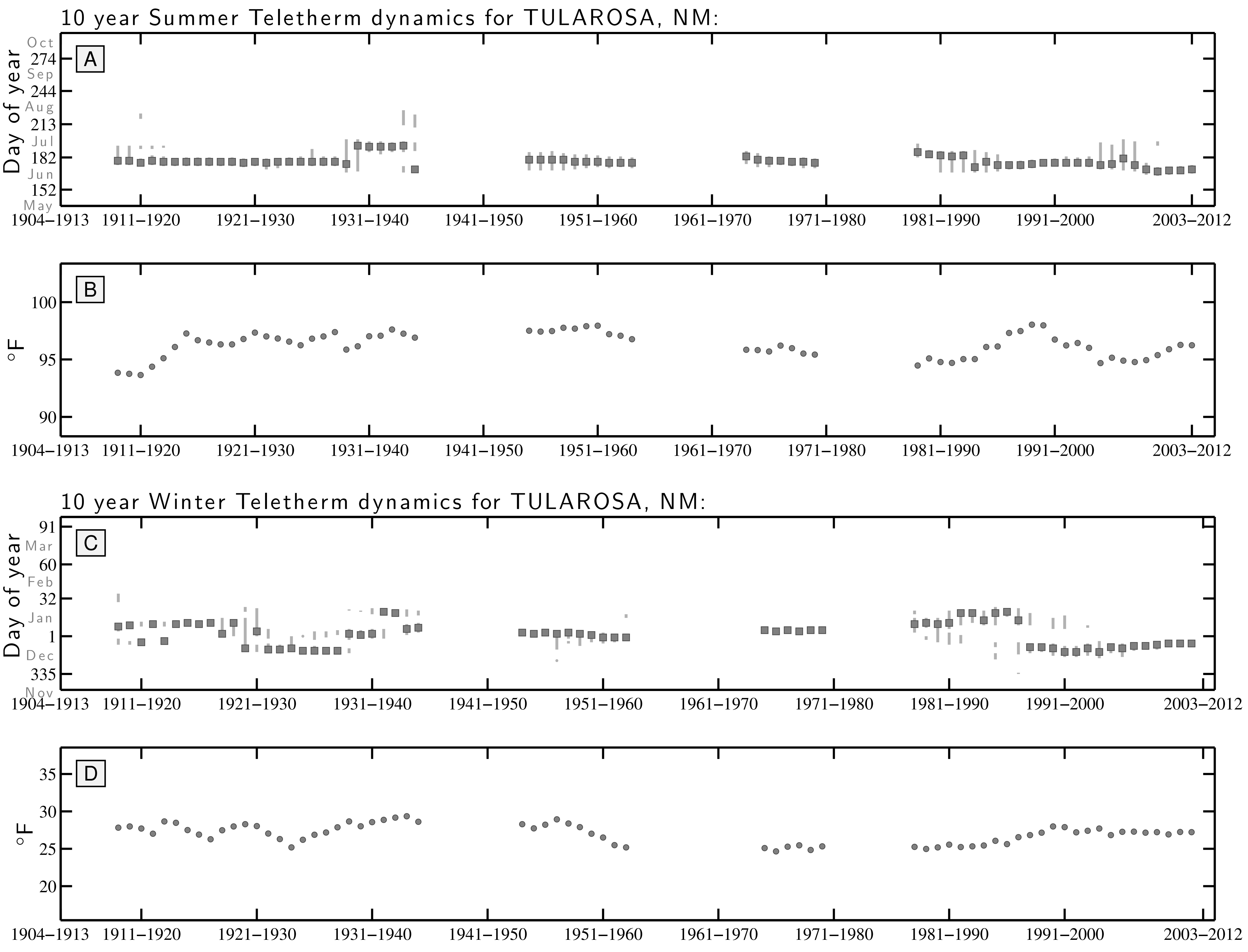

10 year Summer Teletherm dynamics for TULAROSA, NM:
A
Day of year
Oct
274
Sep
244
Aug
213
Jul
182
Jun
152
May
1904–1913
1911–1920
1921–1930
1931–1940
1941–1950
1951–1960
1961–1970
1971–1980
1981–1990
1991–2000
2003–2012
B
°F
100
95
90
10 year Winter Teletherm dynamics for TULAROSA, NM:
C
91
Mar
60
Feb
32
Jan
1
Dec
335
Nov
D
35
30
25
20

10 year Summer Teletherm dynamics for ADDISON, NY:

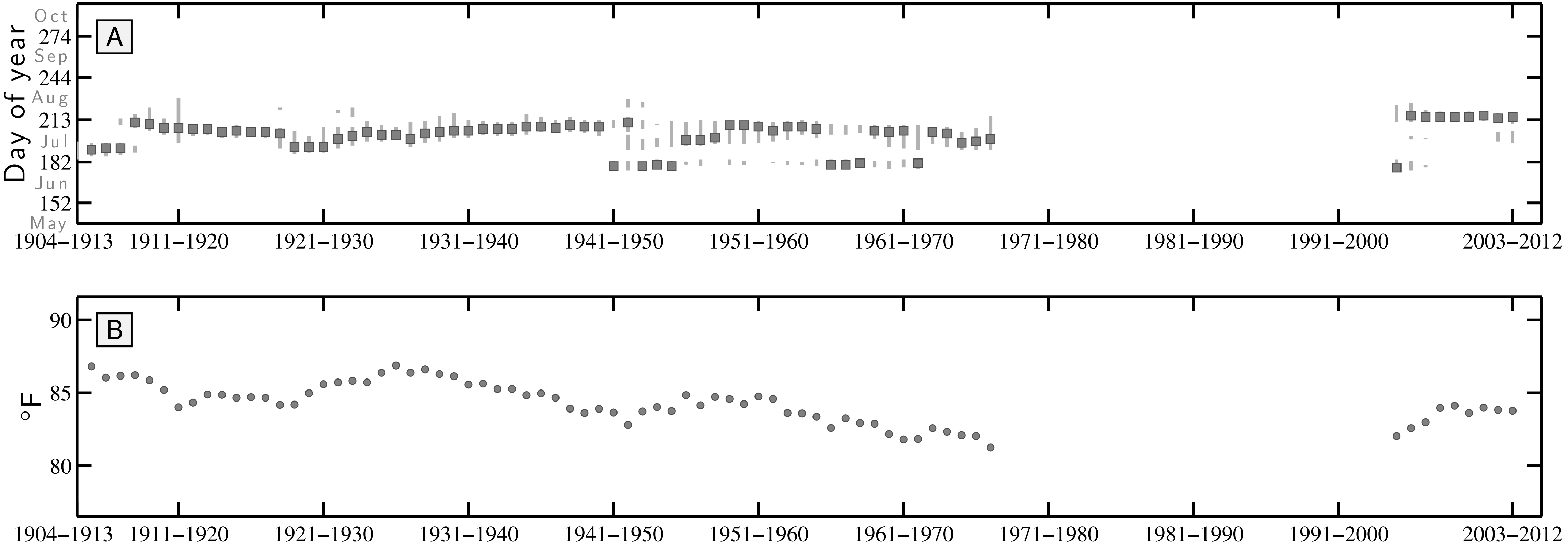

10 year Winter Teletherm dynamics for ADDISON, NY:

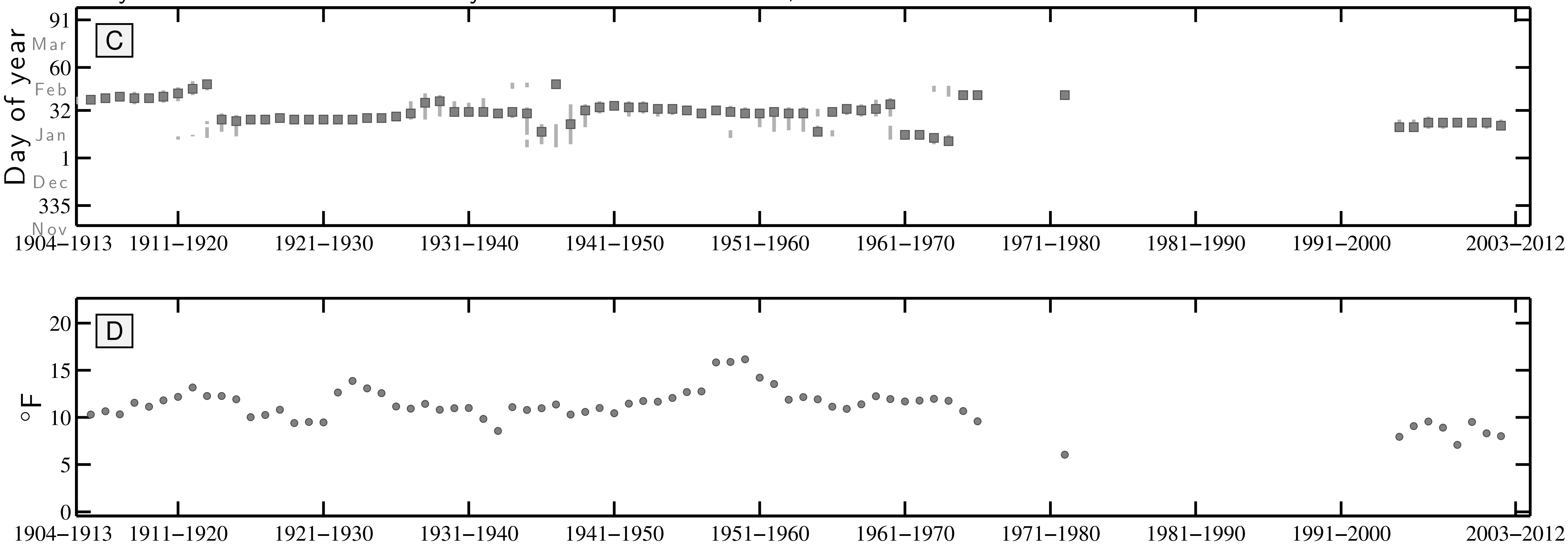

10 year Summer Teletherm dynamics for ALBANY INTL AP, NY:

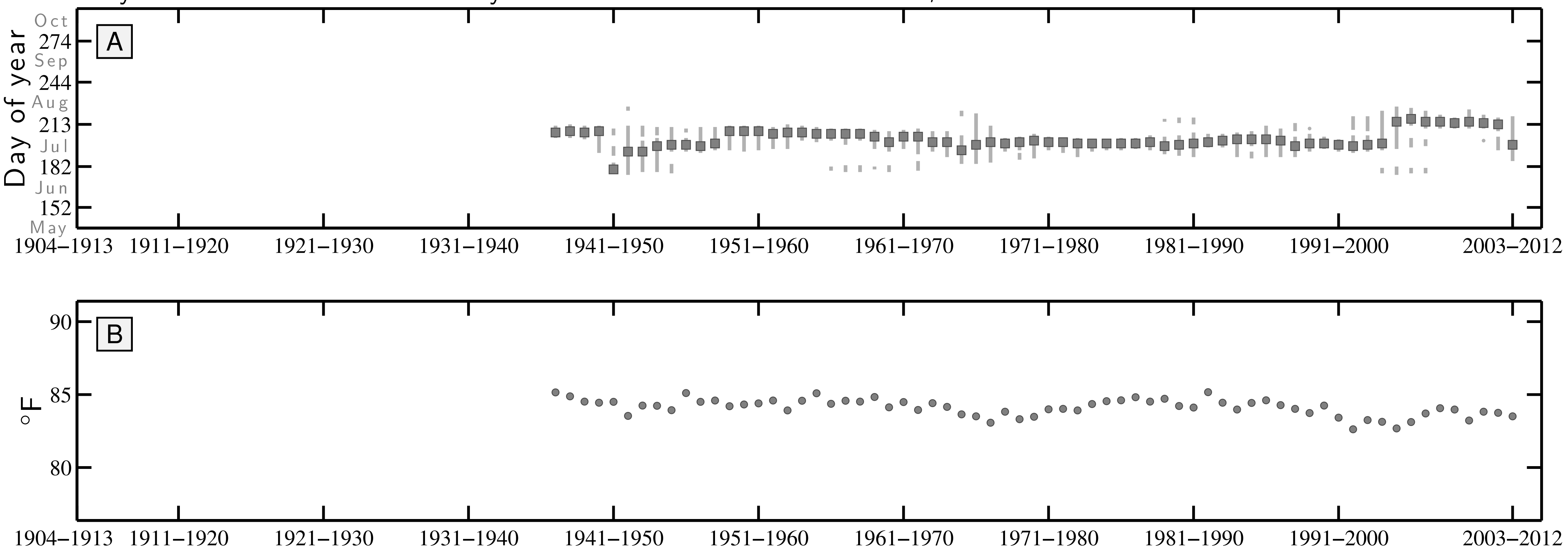

10 year Winter Teletherm dynamics for ALBANY INTL AP, NY:

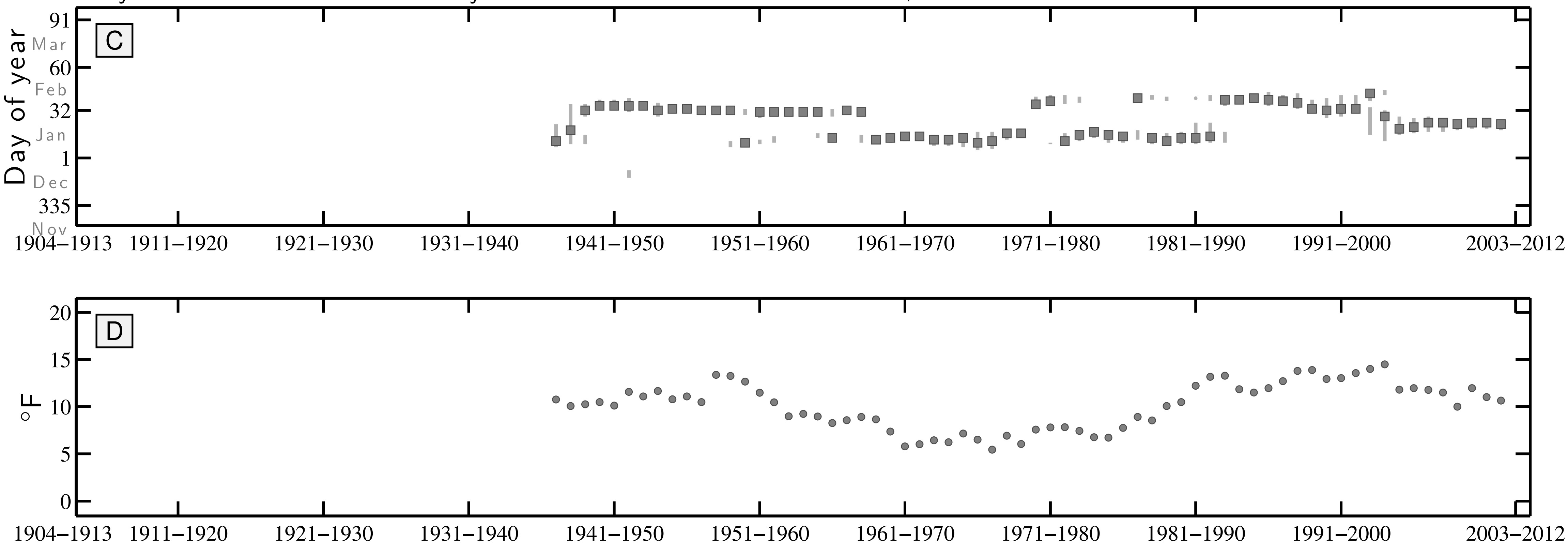

10 year Summer Teletherm dynamics for ALFRED, NY:

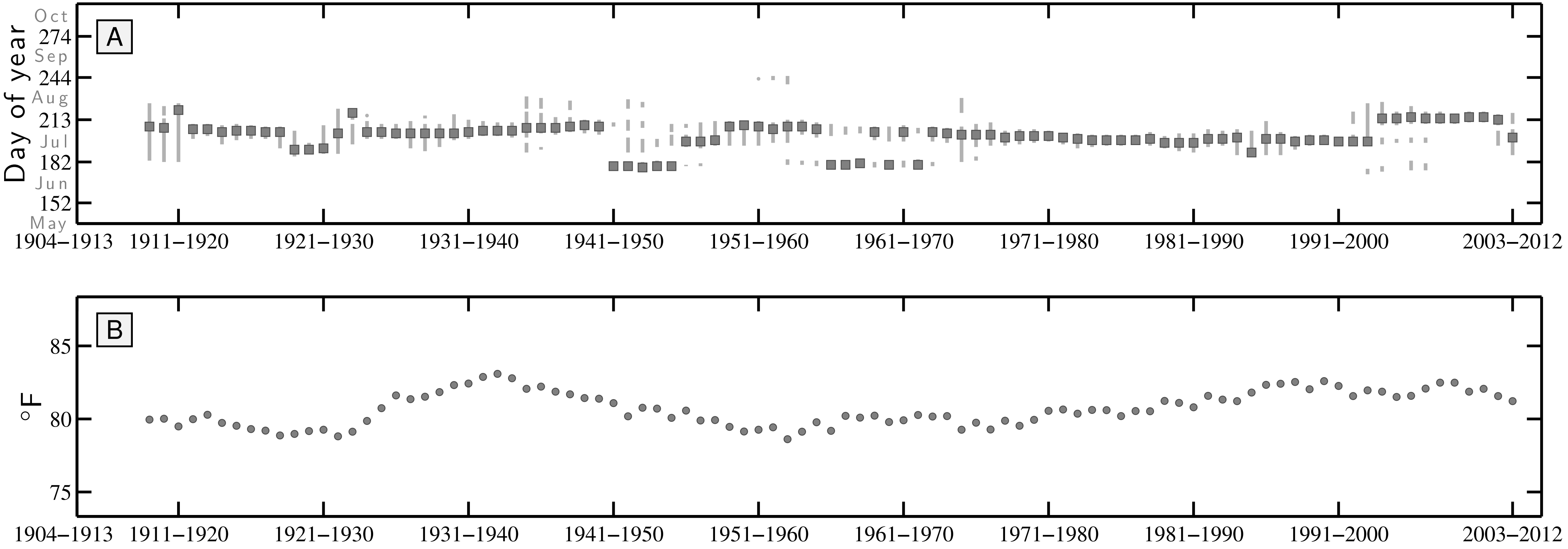

10 year Winter Teletherm dynamics for ALFRED, NY:

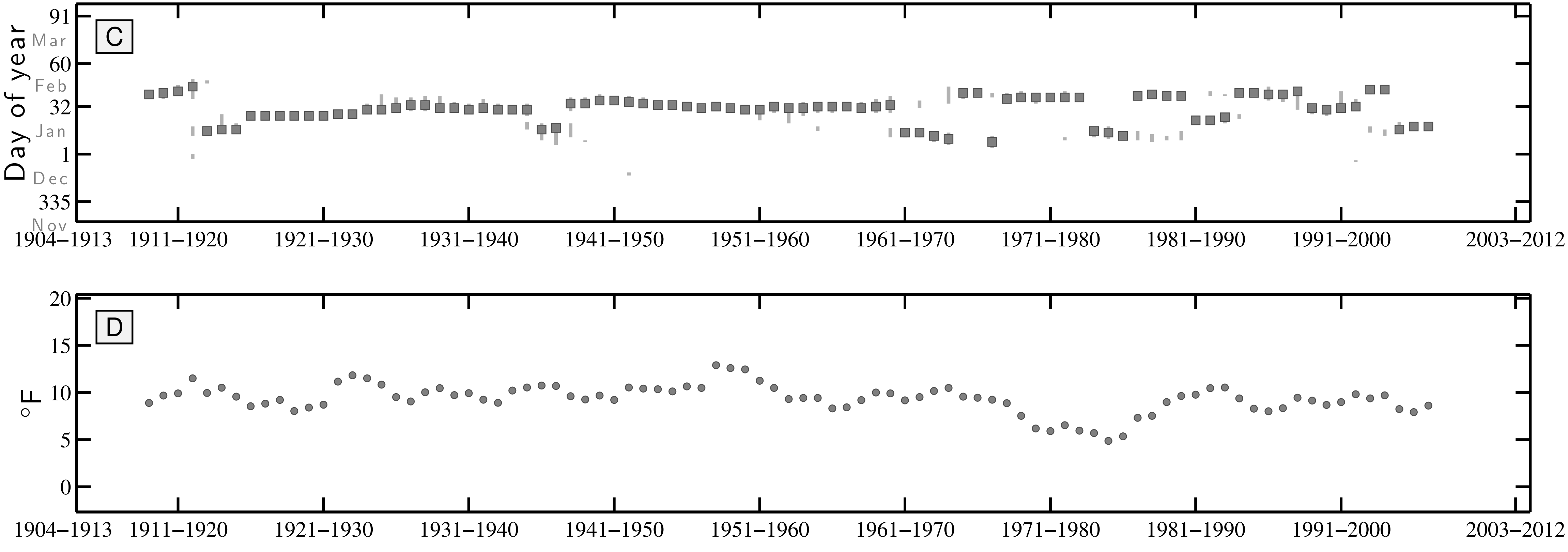

10 year Summer Teletherm dynamics for ALLEGANY SP, NY:

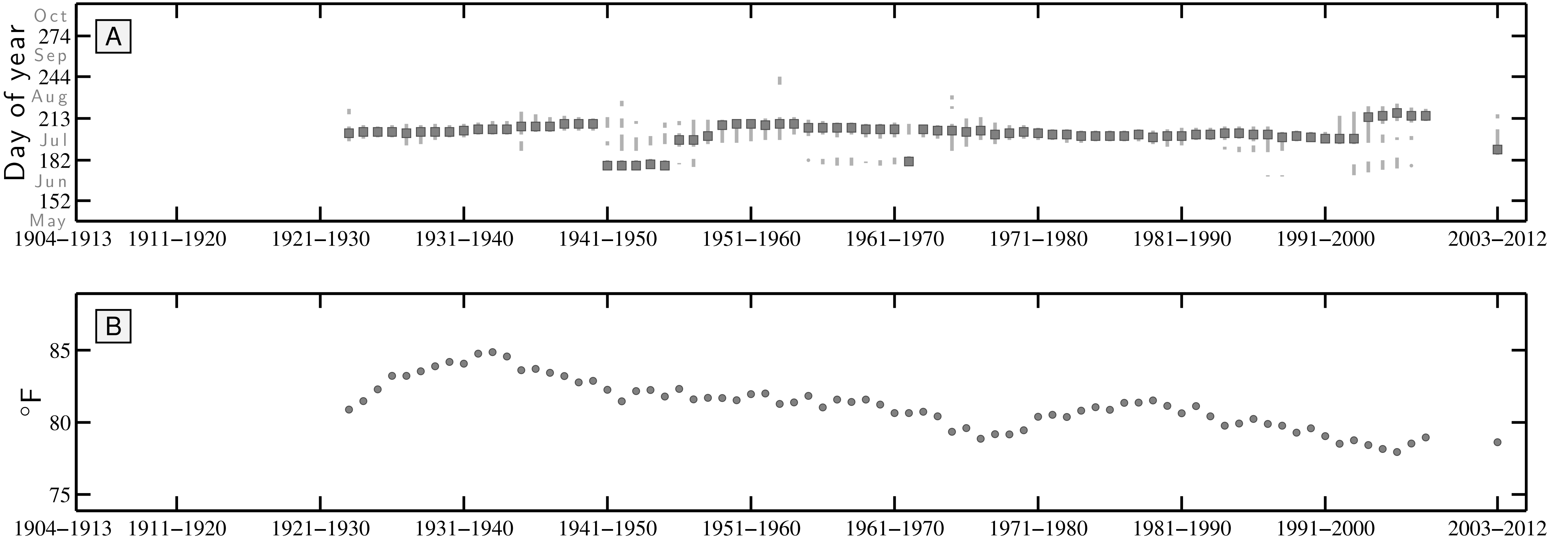

10 year Winter Teletherm dynamics for ALLEGANY SP, NY:

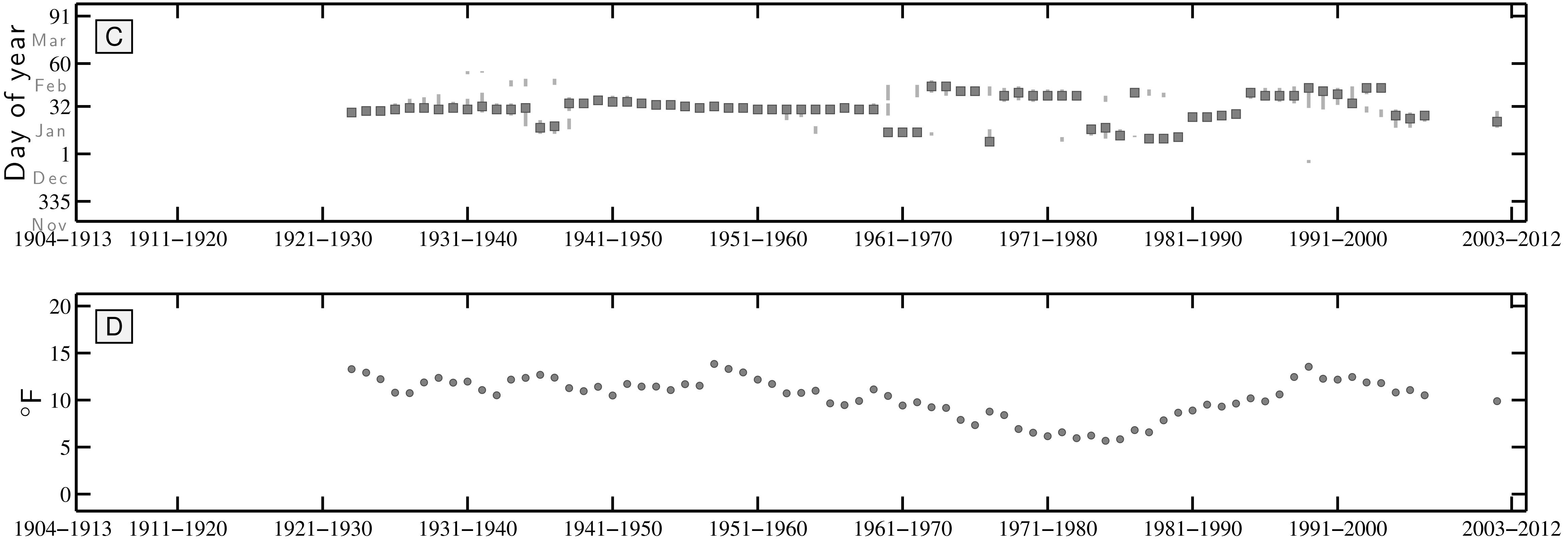

10 year Summer Teletherm dynamics for ANGELICA, NY:

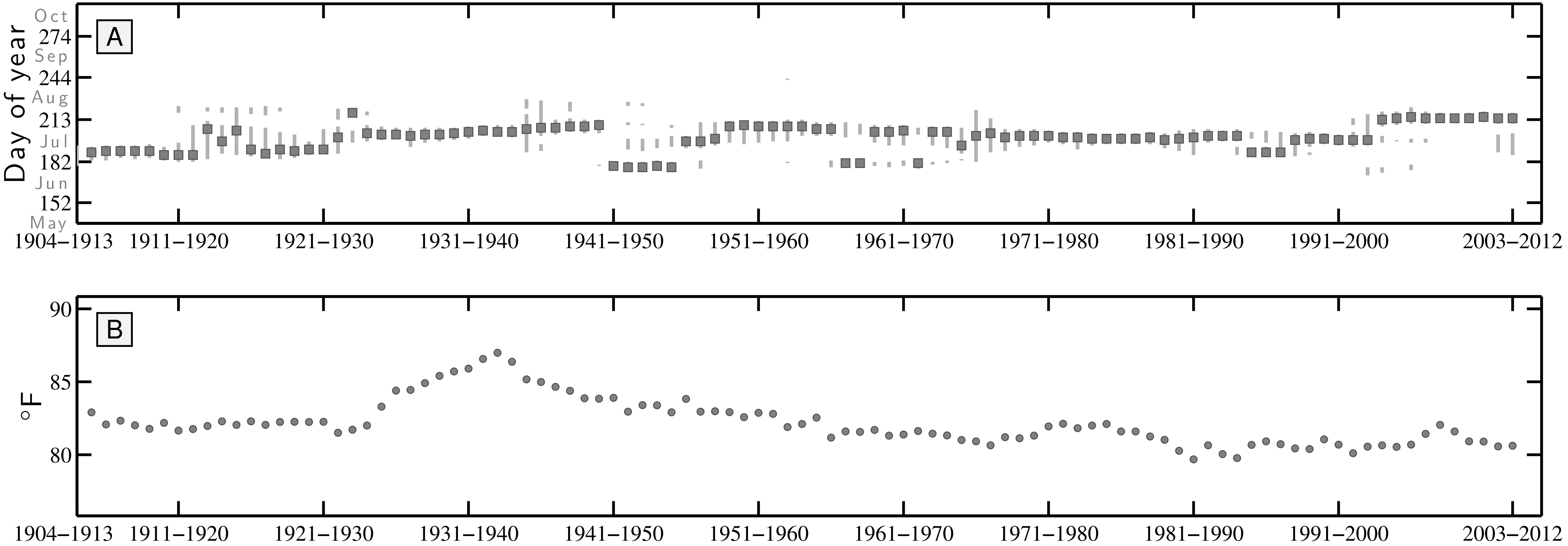

10 year Winter Teletherm dynamics for ANGELICA, NY:

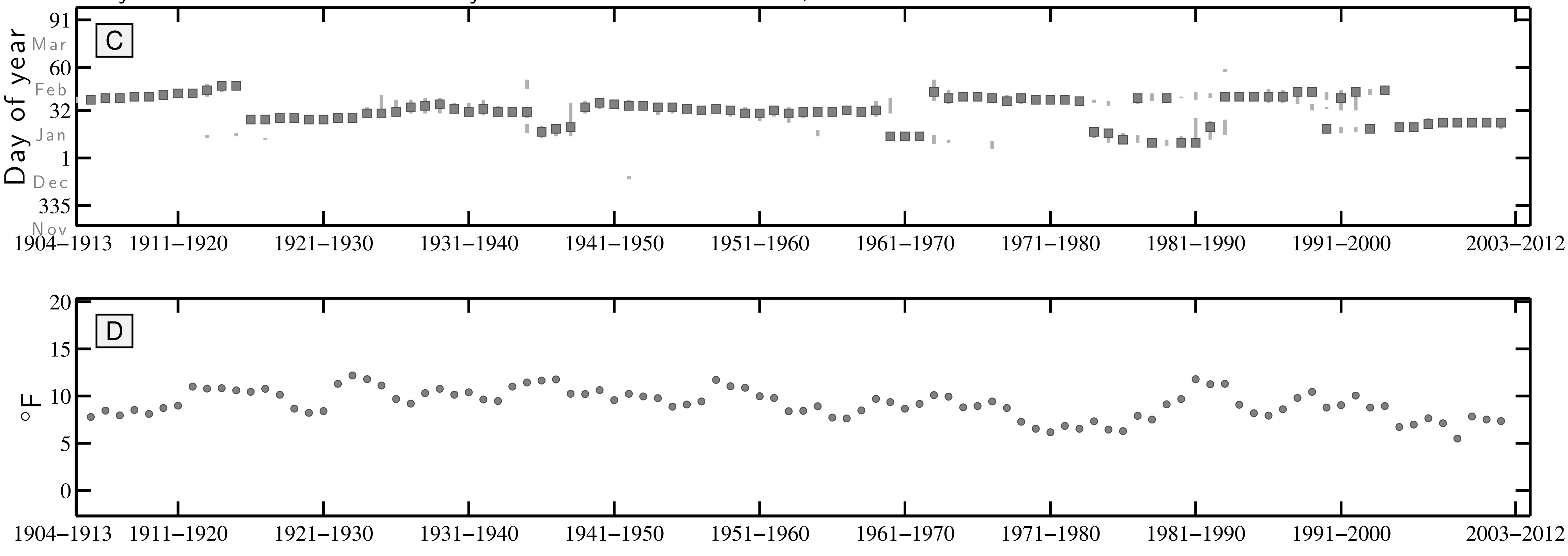

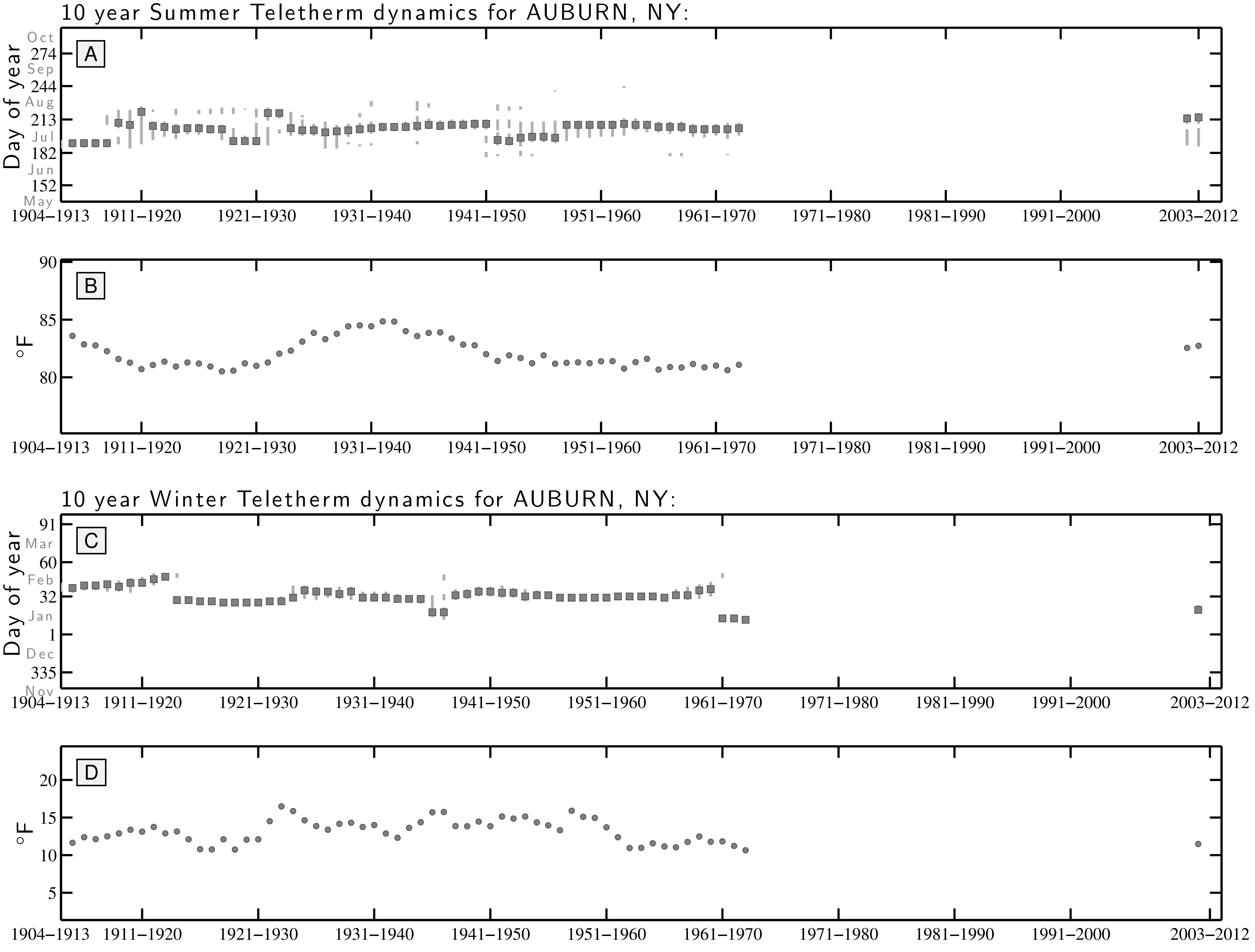
10 year Summer Teletherm dynamics for AUBURN, NY:
A
Day of year
Oct
274
Sep
244
Aug
213
Jul
182
Jun
152
May
B
°F
90
85
80
10 year Winter Teletherm dynamics for AUBURN, NY:
C
Day of year
91
Mar
60
Feb
32
Jan
1
Dec
335
Nov
D
°F
20
15
10
5
1904–1913
1911–1920
1921–1930
1931–1940
1941–1950
1951–1960
1961–1970
1971–1980
1981–1990
1991–2000
2003–2012

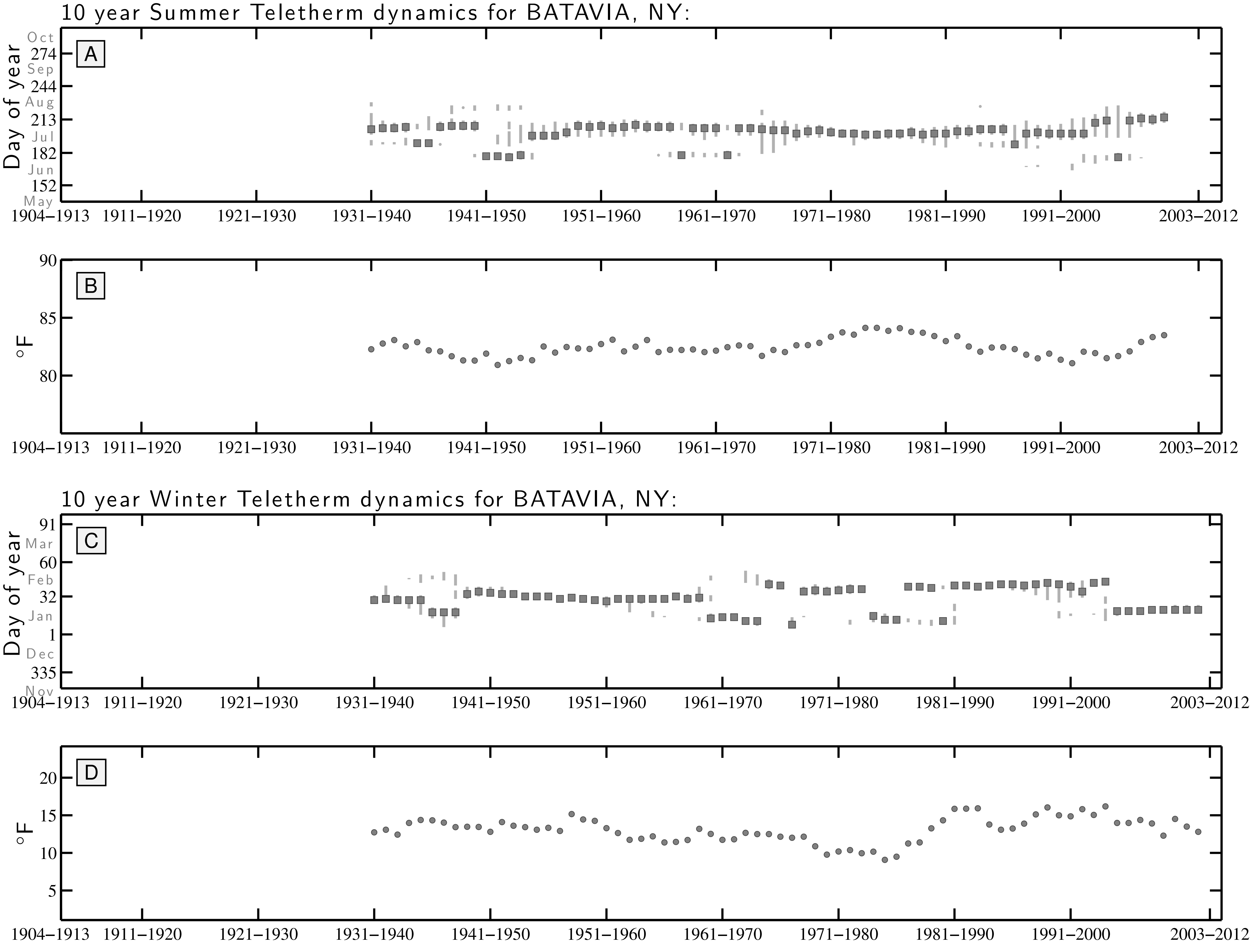
10 year Summer Teletherm dynamics for BATAVIA, NY:
A
Day of year
Oct
274
Sep
244
Aug
213
Jul
182
Jun
152
May
B
°F
90
85
80
10 year Winter Teletherm dynamics for BATAVIA, NY:
C
Day of year
91
Mar
60
Feb
32
Jan
1
Dec
335
Nov
D
°F
20
15
10
5
1904–1913
1911–1920
1921–1930
1931–1940
1941–1950
1951–1960
1961–1970
1971–1980
1981–1990
1991–2000
2003–2012

10 year Summer Teletherm dynamics for BINGHAMTON GREATER AP, NY:

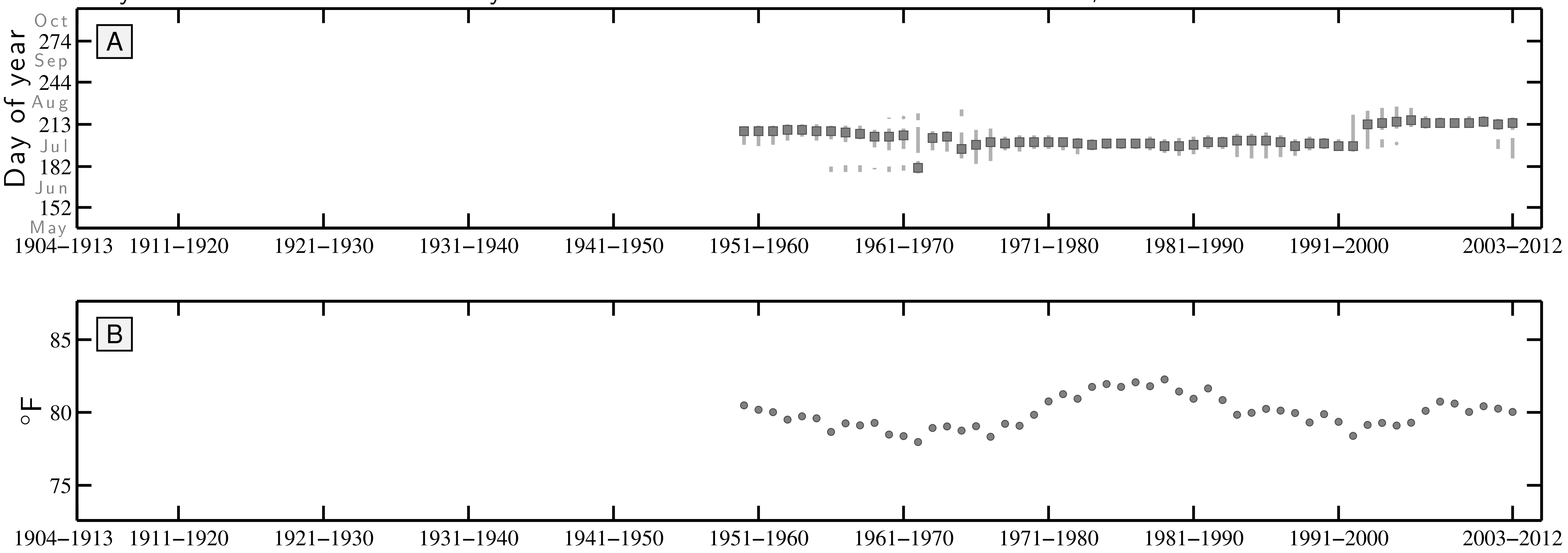

10 year Winter Teletherm dynamics for BINGHAMTON GREATER AP, NY:

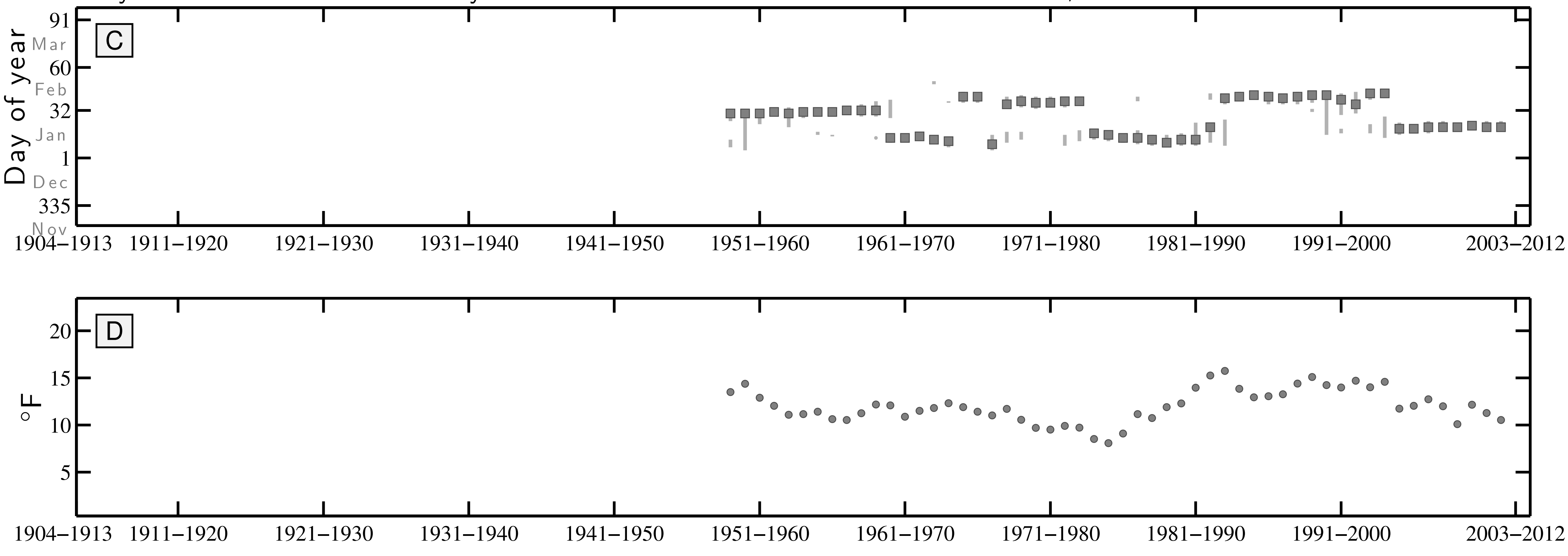

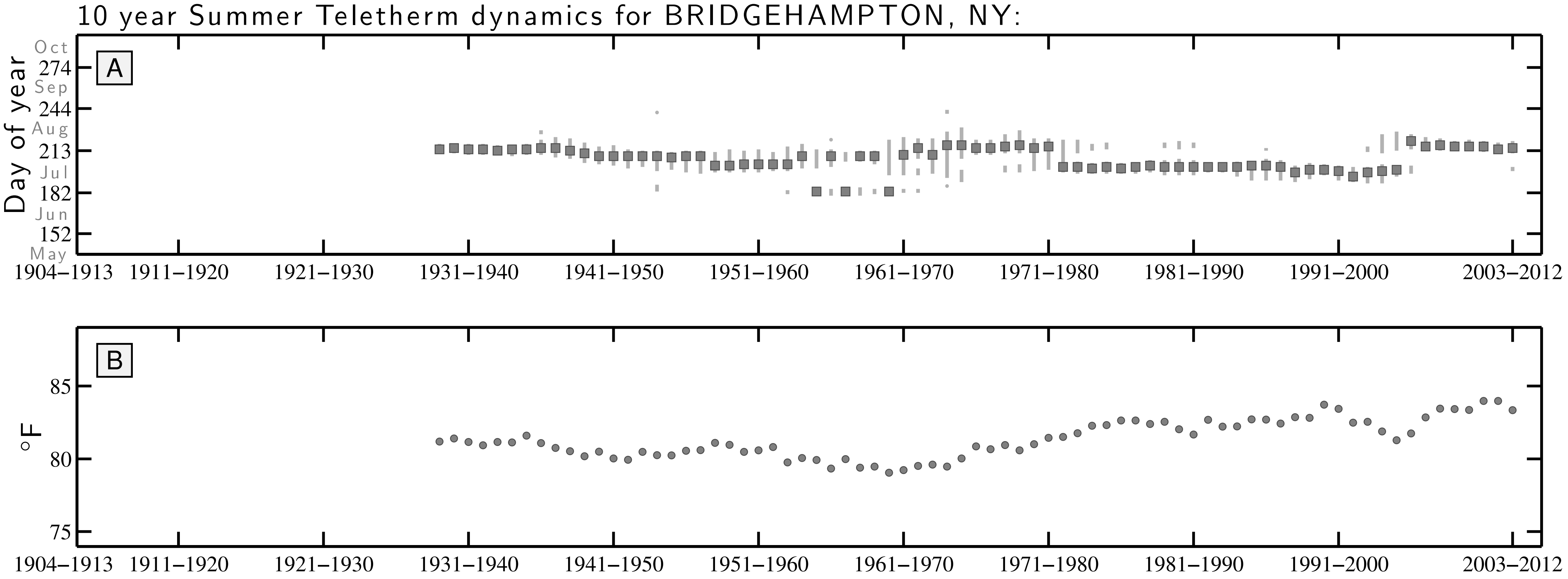
10 year Summer Teletherm dynamics for BRIDGEHAMPTON, NY:
A
B
Day of year
May
152
Jun
182
Jul
213
Aug
244
Sep
274
Oct
°F
75
80
85
1904–1913
1911–1920
1921–1930
1931–1940
1941–1950
1951–1960
1961–1970
1971–1980
1981–1990
1991–2000
2003–2012

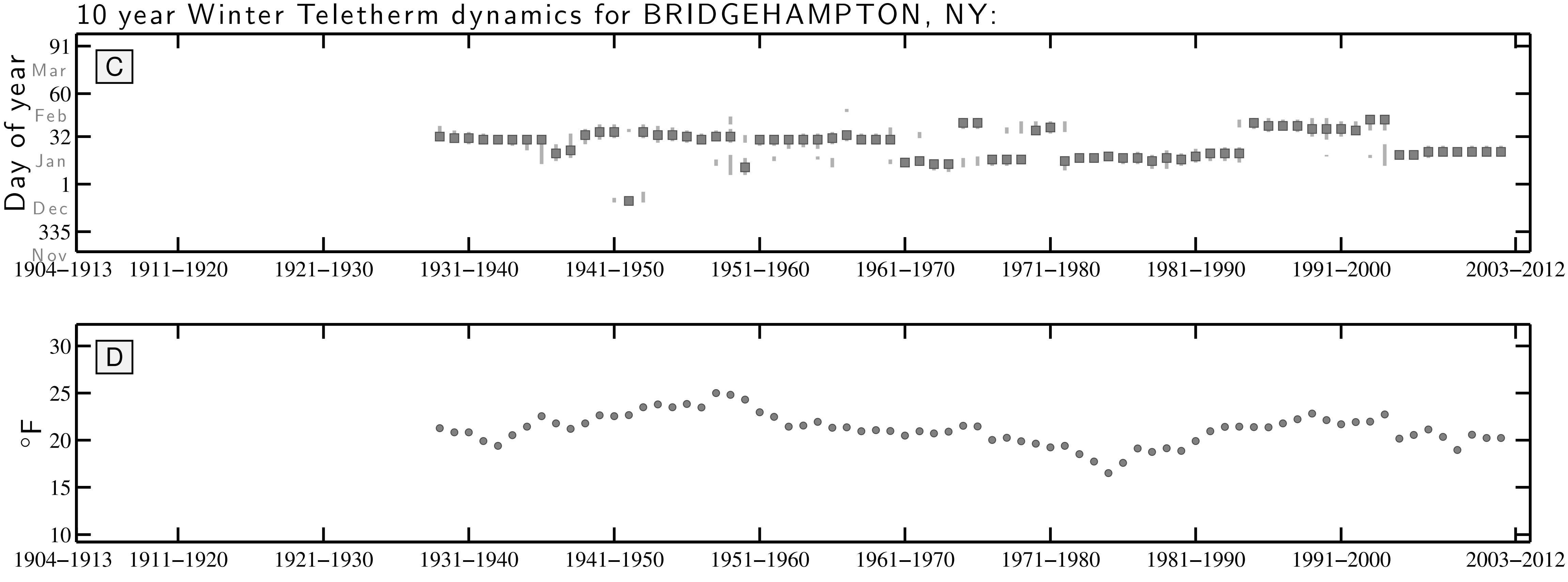
10 year Winter Teletherm dynamics for BRIDGEHAMPTON, NY:
C
D
Day of year
Nov
335
Dec
1
Jan
32
Feb
60
Mar
91
°F
10
15
20
25
30
1904–1913
1911–1920
1921–1930
1931–1940
1941–1950
1951–1960
1961–1970
1971–1980
1981–1990
1991–2000
2003–2012

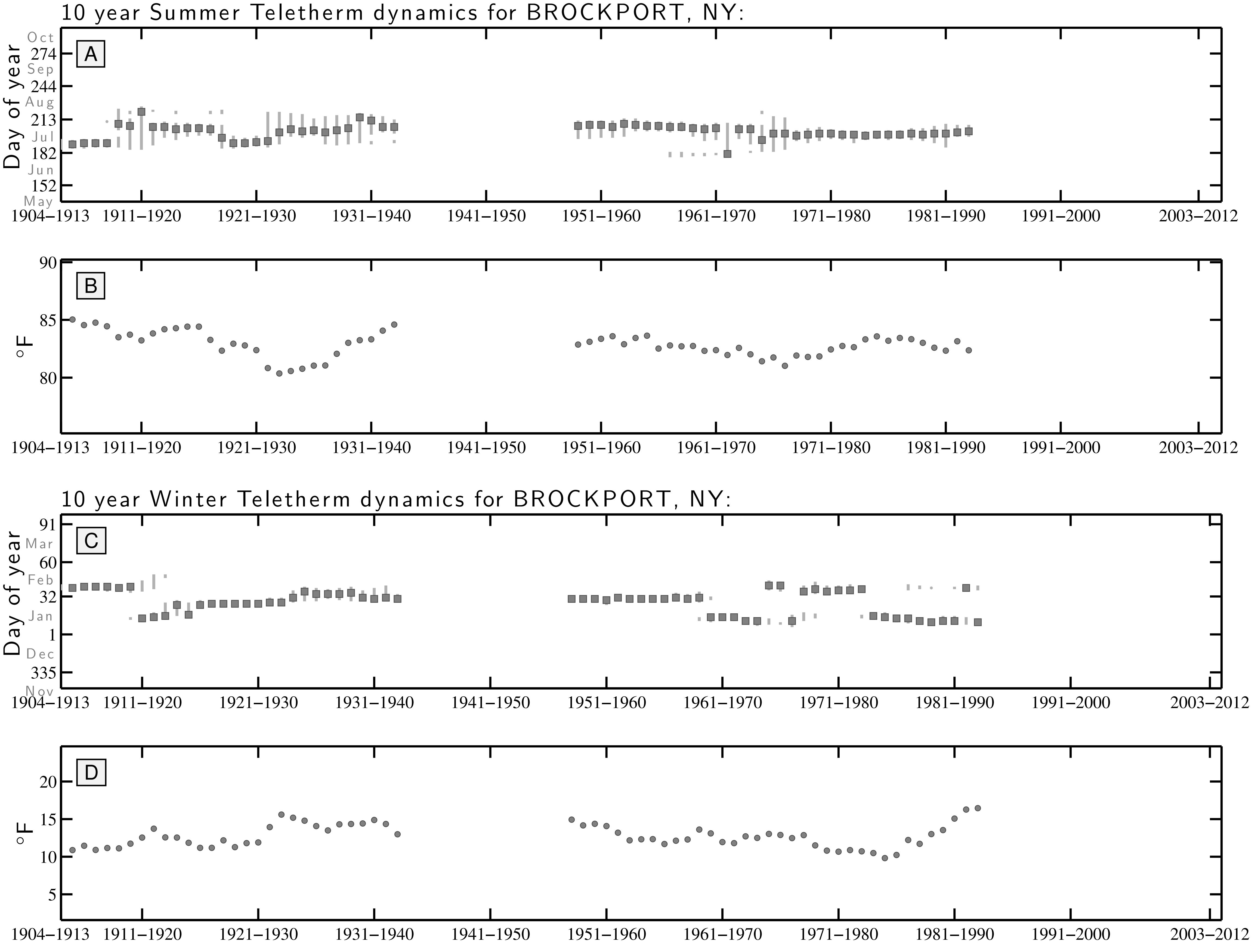
10 year Summer Teletherm dynamics for BROCKPORT, NY:
A
B
Day of year
Oct
274
Sep
244
Aug
213
Jul
182
Jun
152
May
°F
90
85
80
1904–1913
1911–1920
1921–1930
1931–1940
1941–1950
1951–1960
1961–1970
1971–1980
1981–1990
1991–2000
2003–2012
10 year Winter Teletherm dynamics for BROCKPORT, NY:
C
D
Day of year
91
Mar
60
Feb
32
Jan
1
Dec
335
Nov
°F
20
15
10
5

10 year Summer Teletherm dynamics for BUFFALO NIAGARA INTL, NY:

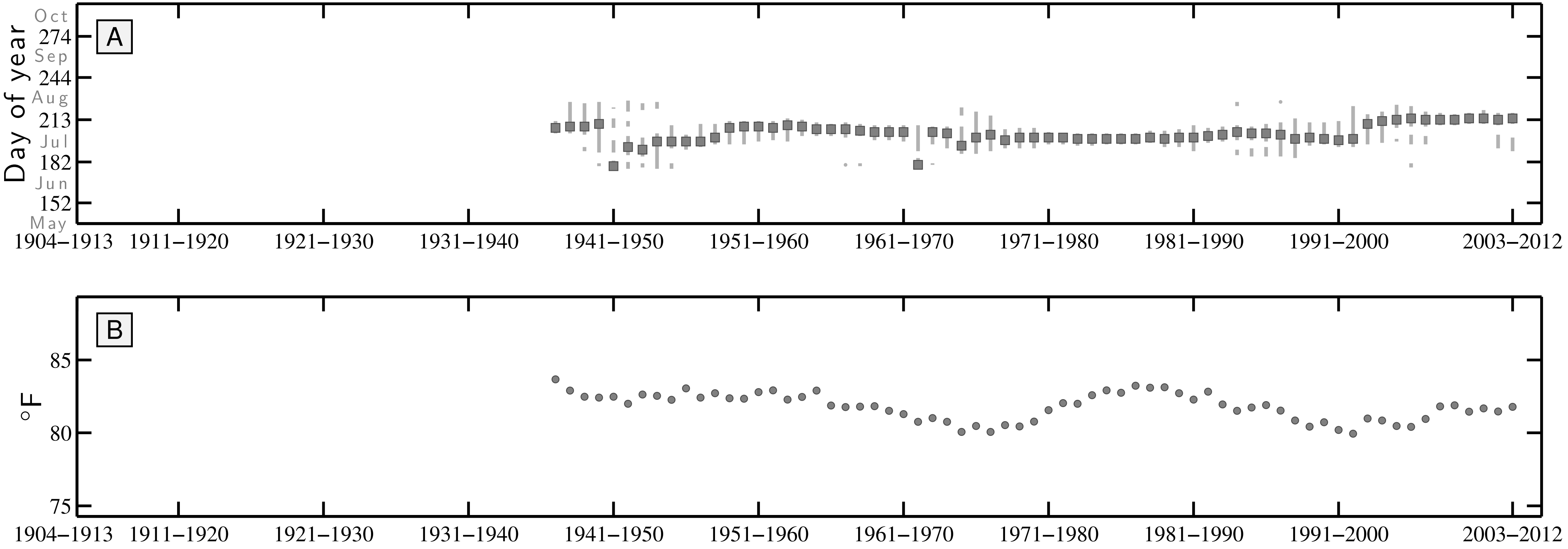

10 year Winter Teletherm dynamics for BUFFALO NIAGARA INTL, NY:

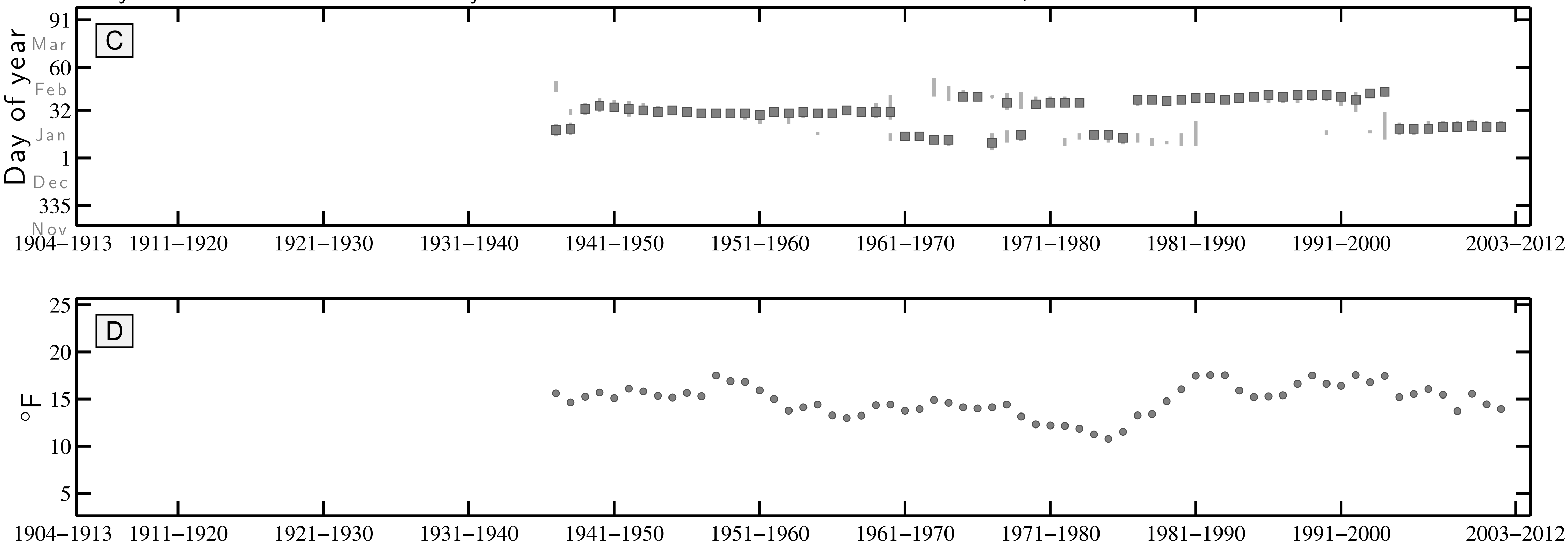

10 year Summer Teletherm dynamics for CANTON 4 SE, NY:

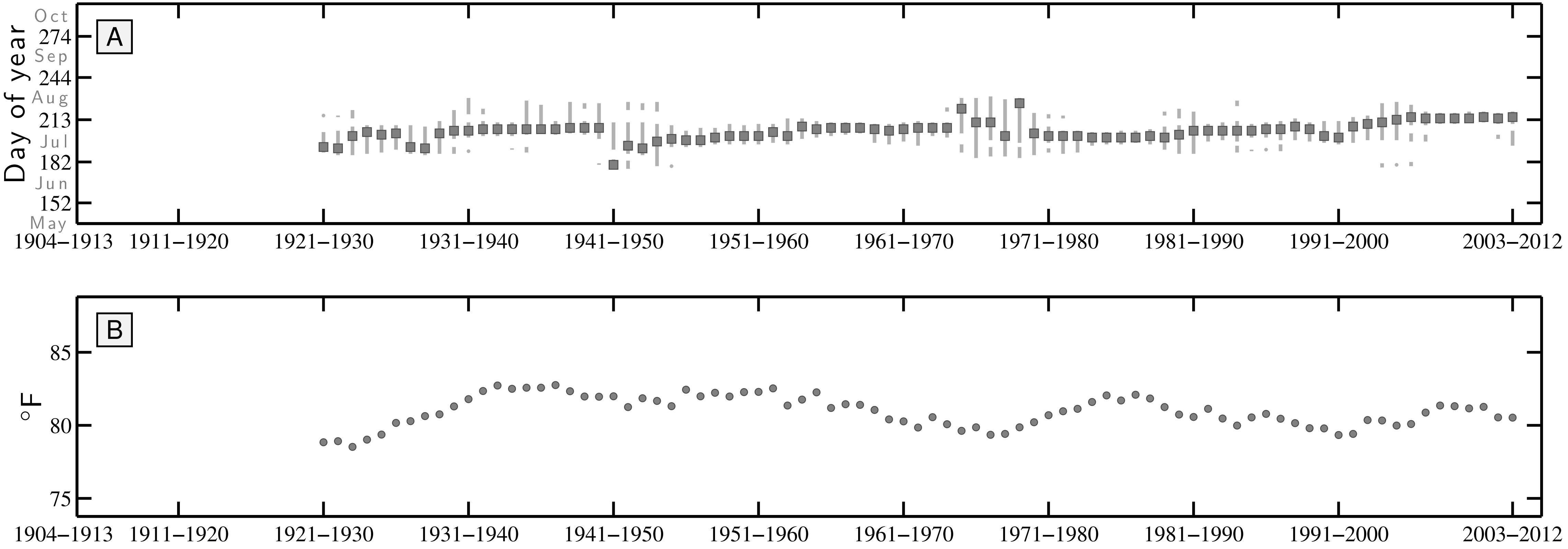

10 year Winter Teletherm dynamics for CANTON 4 SE, NY:

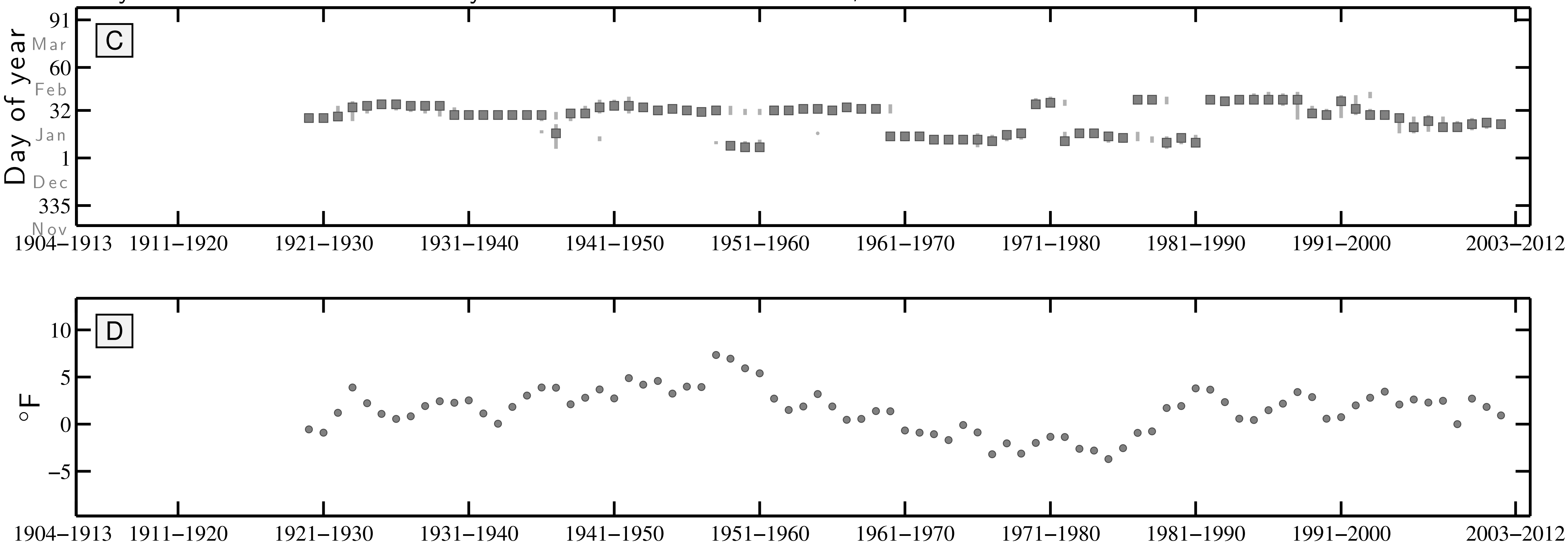

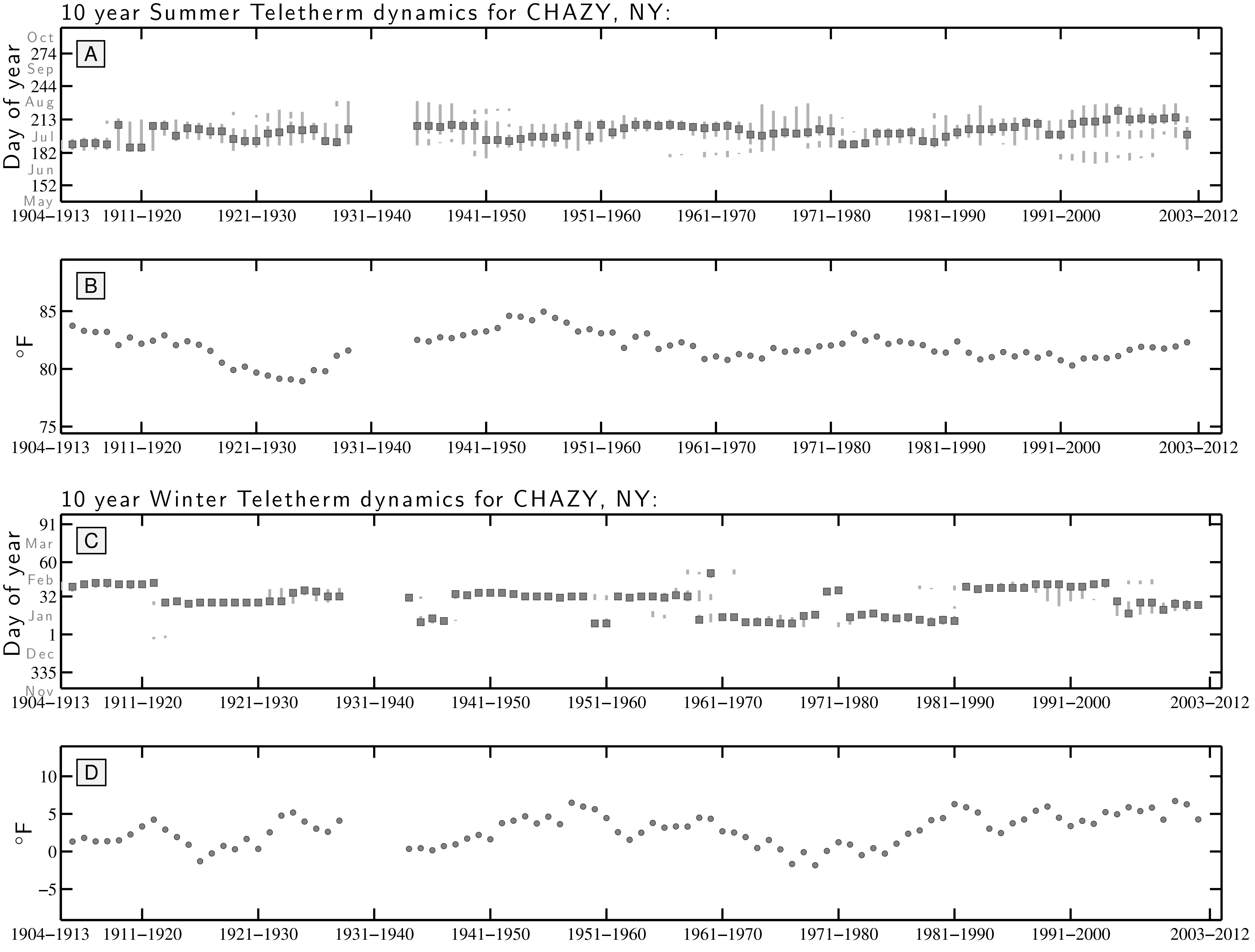
10 year Summer Teletherm dynamics for CHAZY, NY:
A
B
Day of year
°F
Oct
274
Sep
244
Aug
213
Jul
182
Jun
152
May
85
80
75
1904–1913
1911–1920
1921–1930
1931–1940
1941–1950
1951–1960
1961–1970
1971–1980
1981–1990
1991–2000
2003–2012
10 year Winter Teletherm dynamics for CHAZY, NY:
C
D
91
Mar
60
Feb
32
Jan
1
Dec
335
Nov
10
5
0
−5

10 year Summer Teletherm dynamics for COOPERSTOWN, NY:

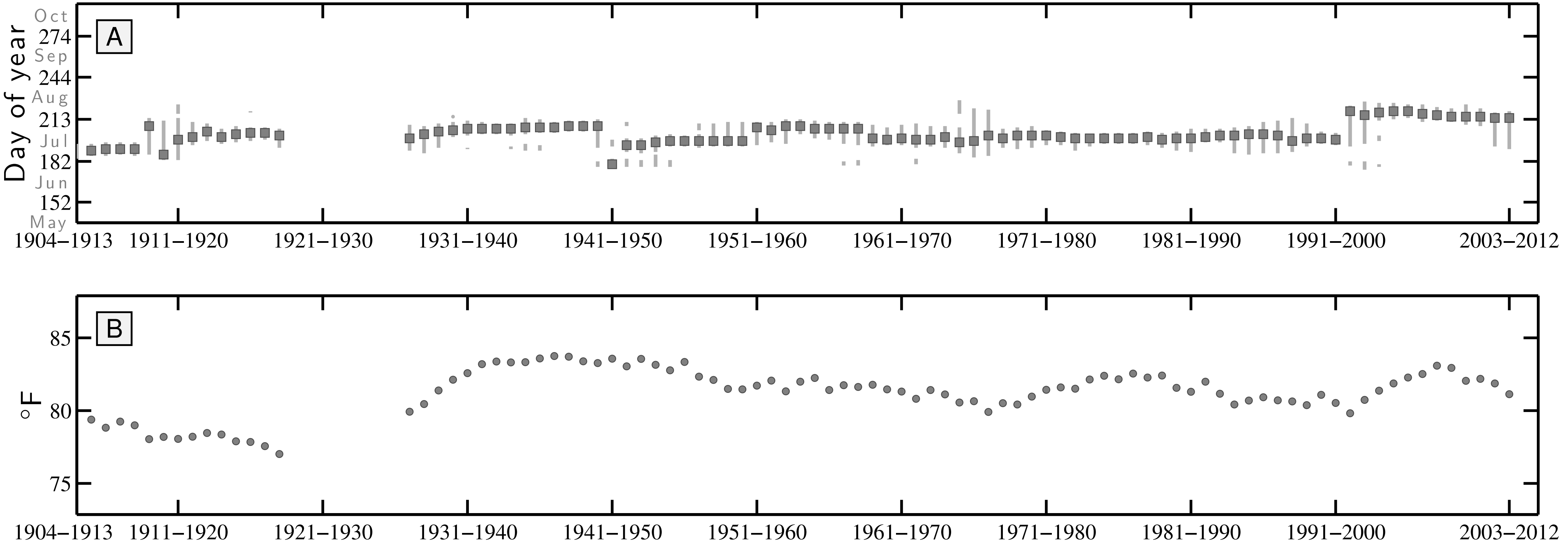

10 year Winter Teletherm dynamics for COOPERSTOWN, NY:

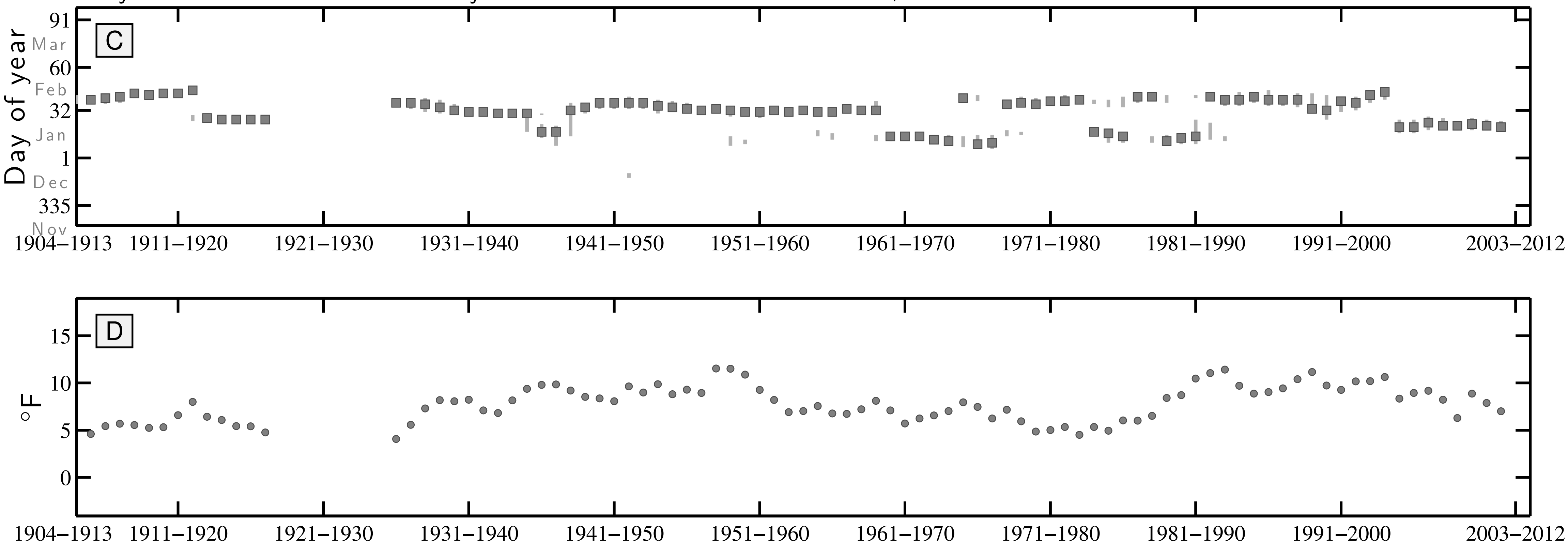

10 year Summer Teletherm dynamics for CORTLAND, NY:

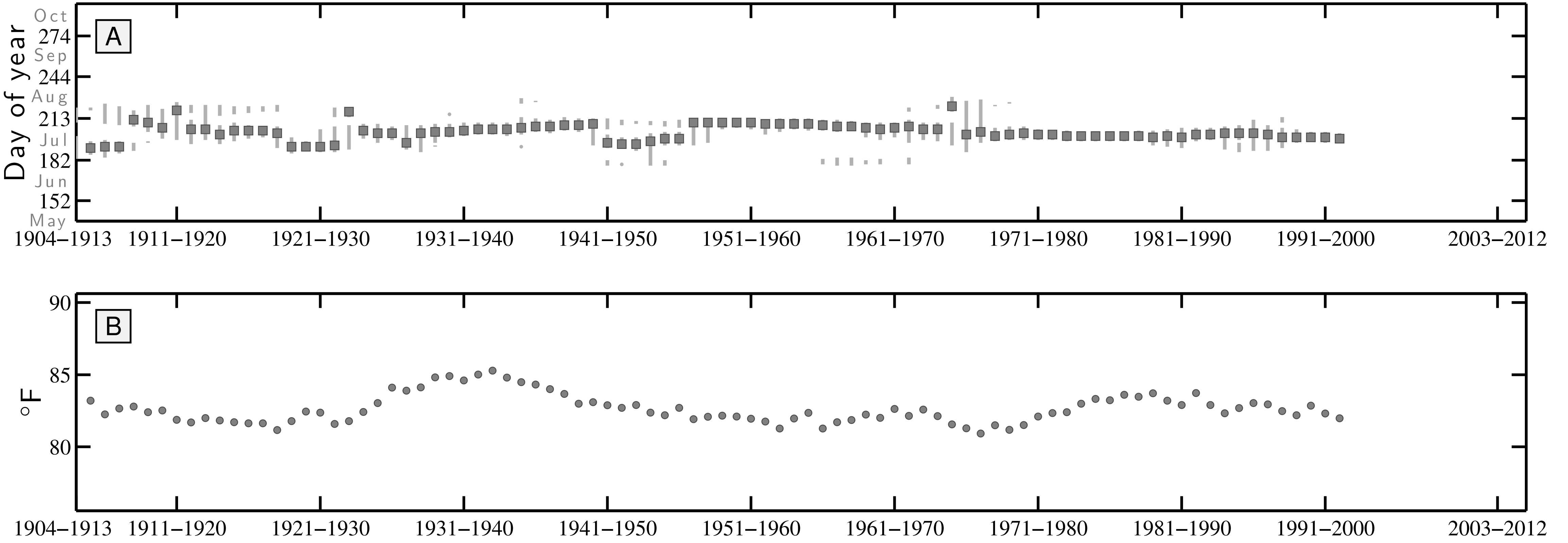

10 year Winter Teletherm dynamics for CORTLAND, NY:

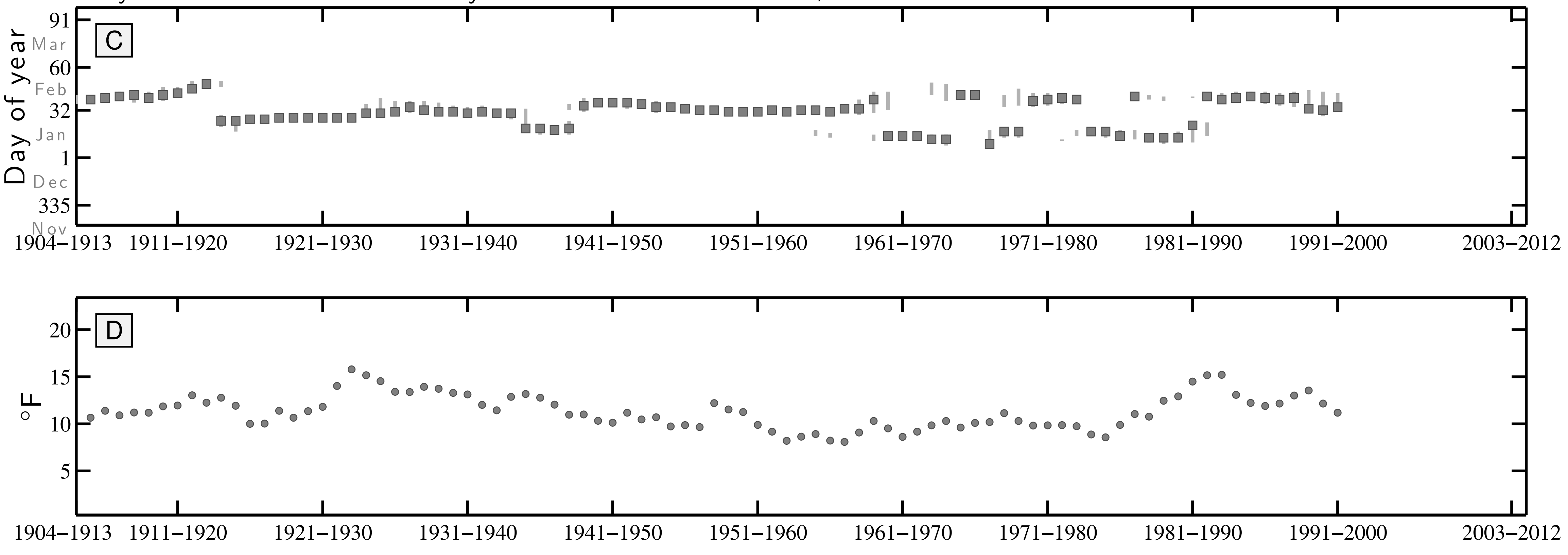

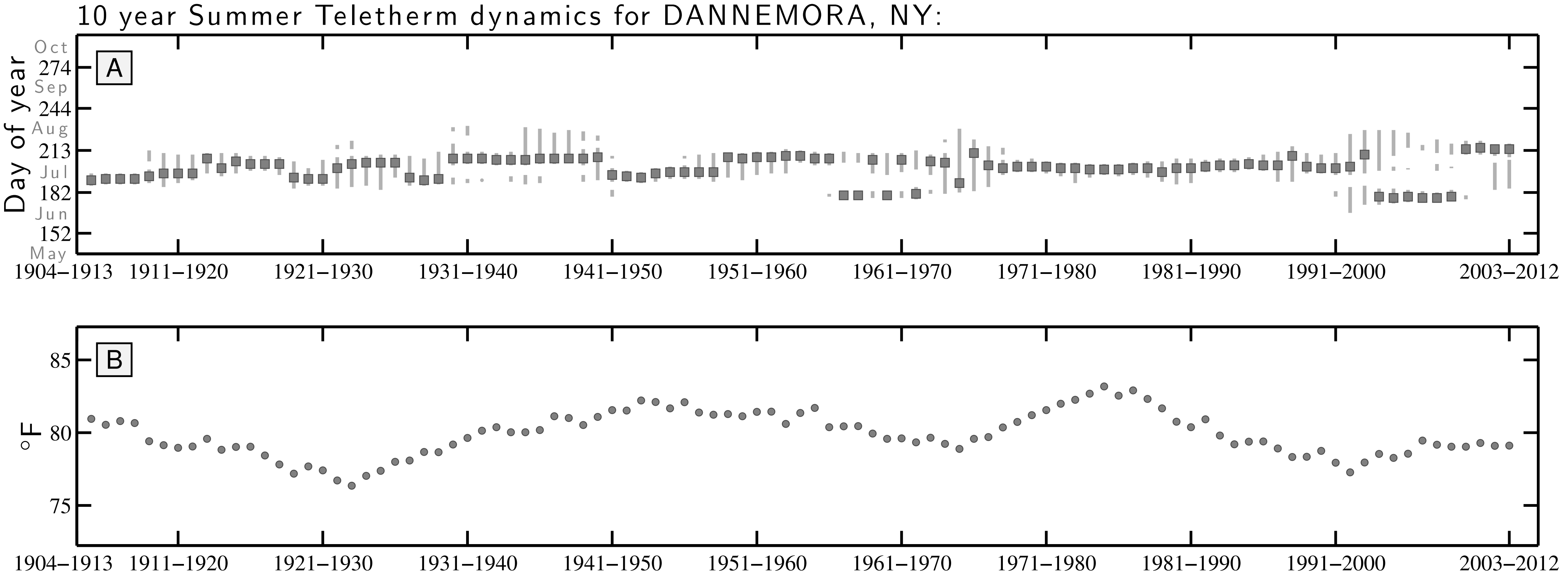
10 year Summer Teletherm dynamics for DANNEMORA, NY:
A
B
Day of year
°F
Oct
274
Sep
244
Aug
213
Jul
182
Jun
152
May
85
80
75
1904–1913
1911–1920
1921–1930
1931–1940
1941–1950
1951–1960
1961–1970
1971–1980
1981–1990
1991–2000
2003–2012
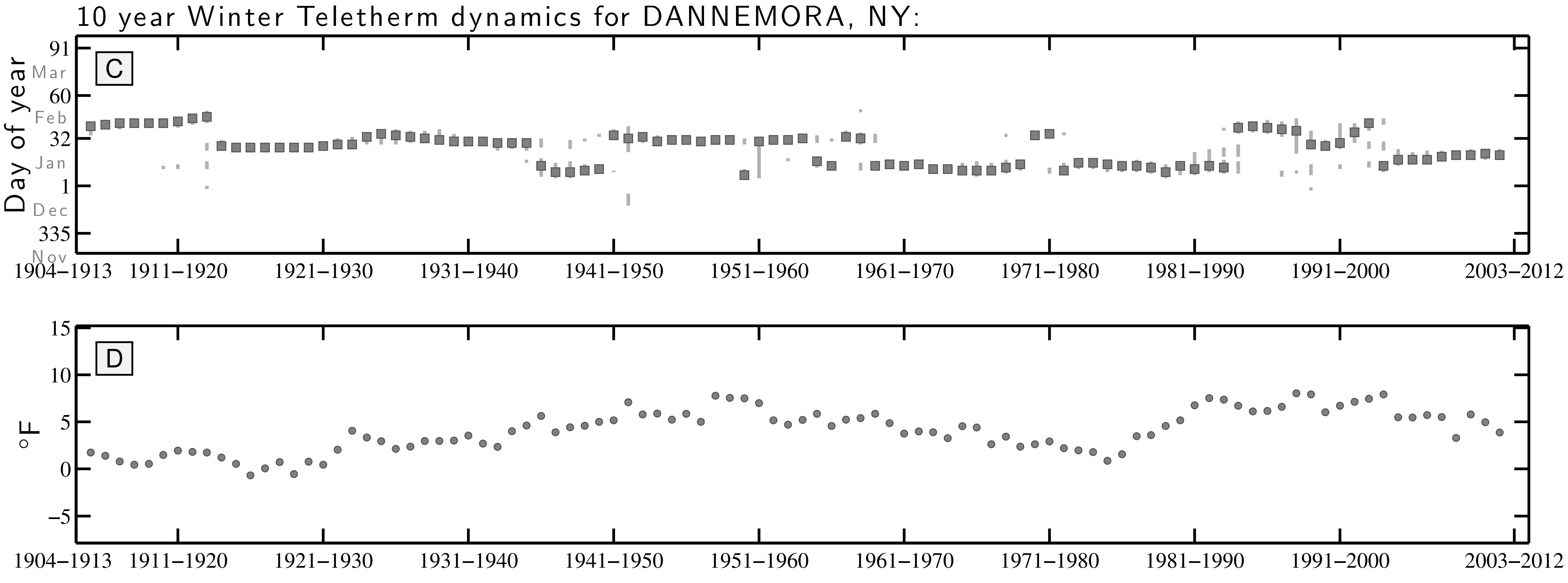
10 year Winter Teletherm dynamics for DANNEMORA, NY:
C
D
Day of year
°F
91
Mar
60
Feb
32
Jan
1
Dec
335
Nov
15
10
5
0
−5
1904–1913
1911–1920
1921–1930
1931–1940
1941–1950
1951–1960
1961–1970
1971–1980
1981–1990
1991–2000
2003–2012

10 year Summer Teletherm dynamics for DANSVILLE, NY:

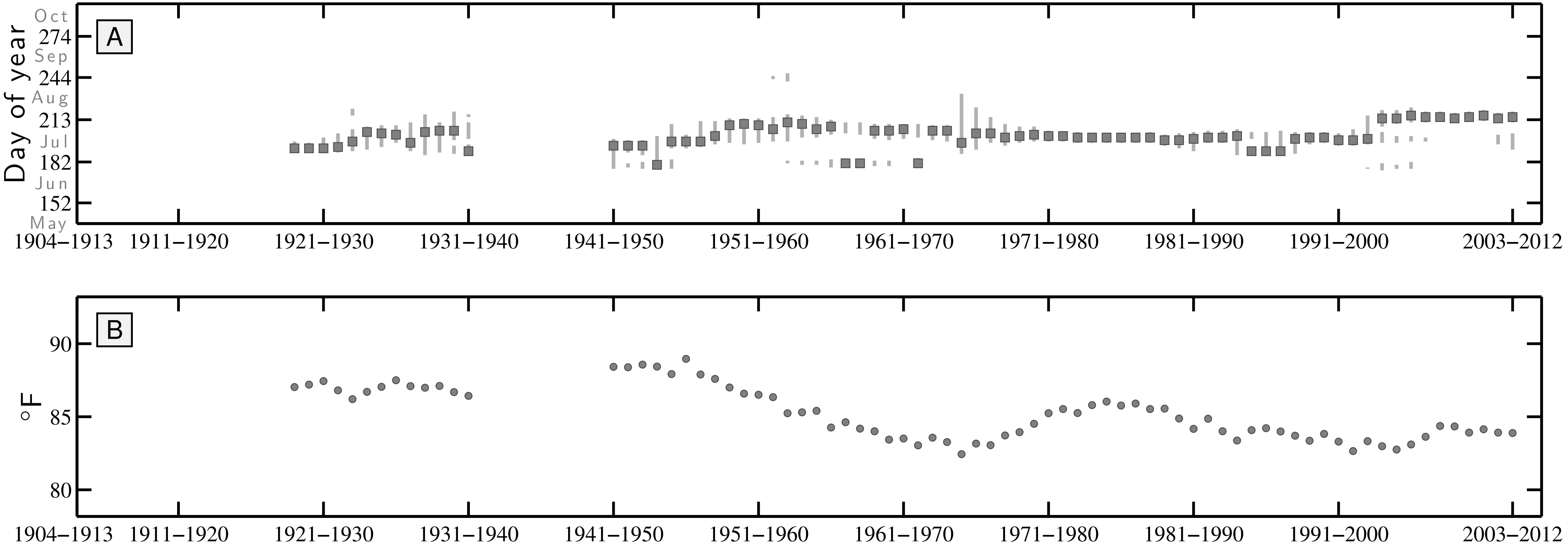

10 year Winter Teletherm dynamics for DANSVILLE, NY:

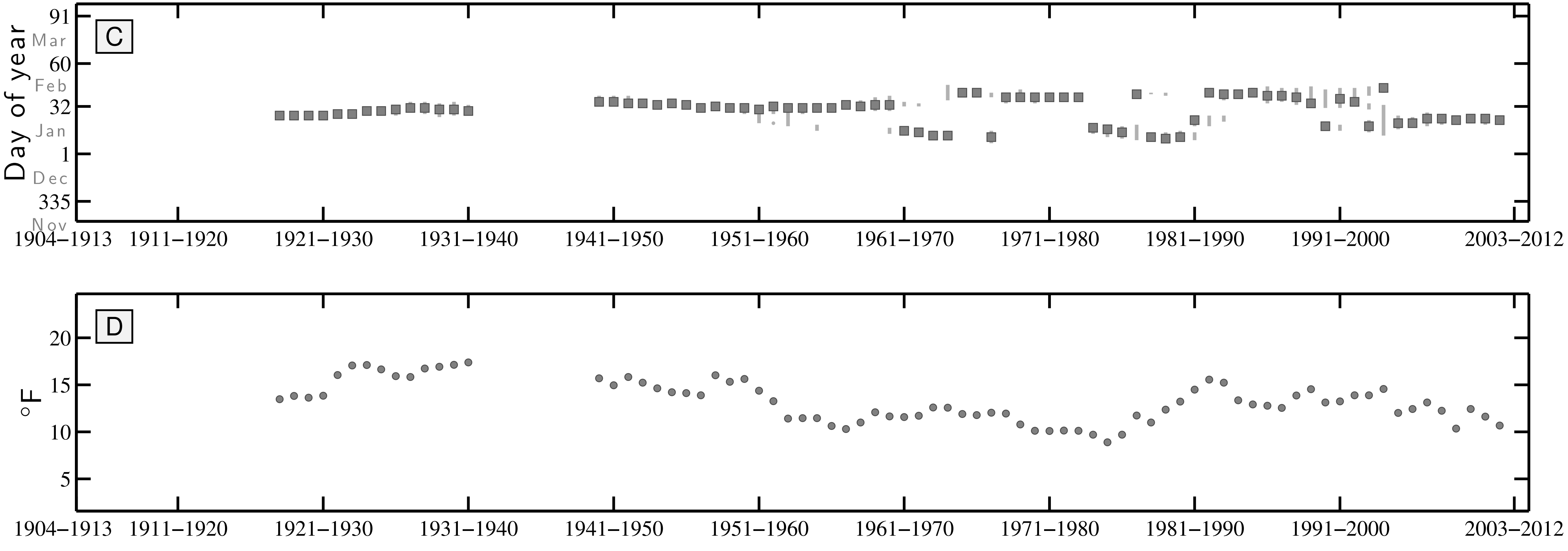

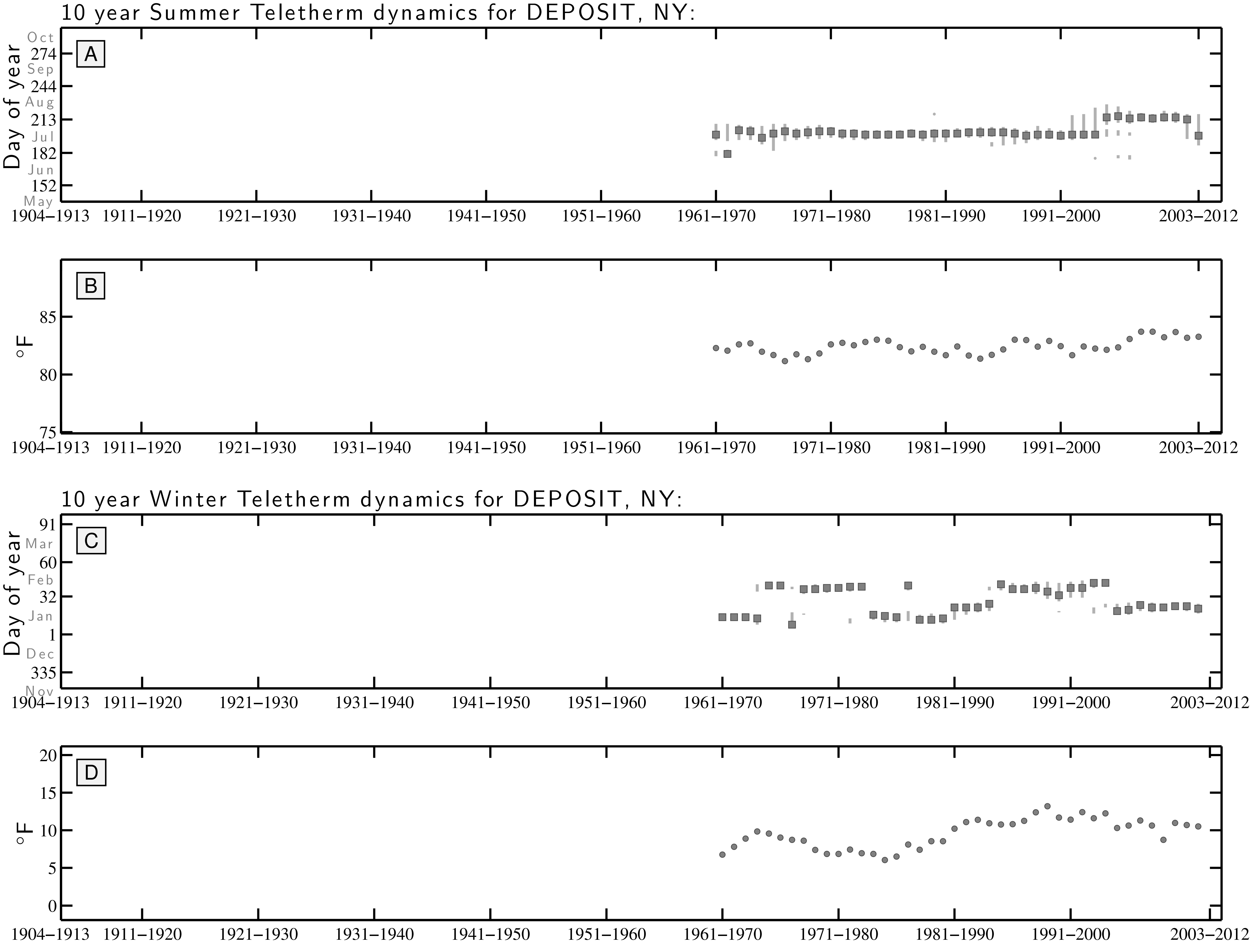
10 year Summer Teletherm dynamics for DEPOSIT, NY:
A
B
Day of year
°F
Oct
274
Sep
244
Aug
213
Jul
182
Jun
152
May
85
80
75
1904–1913
1911–1920
1921–1930
1931–1940
1941–1950
1951–1960
1961–1970
1971–1980
1981–1990
1991–2000
2003–2012
10 year Winter Teletherm dynamics for DEPOSIT, NY:
C
D
91
Mar
60
Feb
32
Jan
1
Dec
335
Nov
20
15
10
5
0

10 year Summer Teletherm dynamics for DOBBS FERRY ARDSLEY, NY:

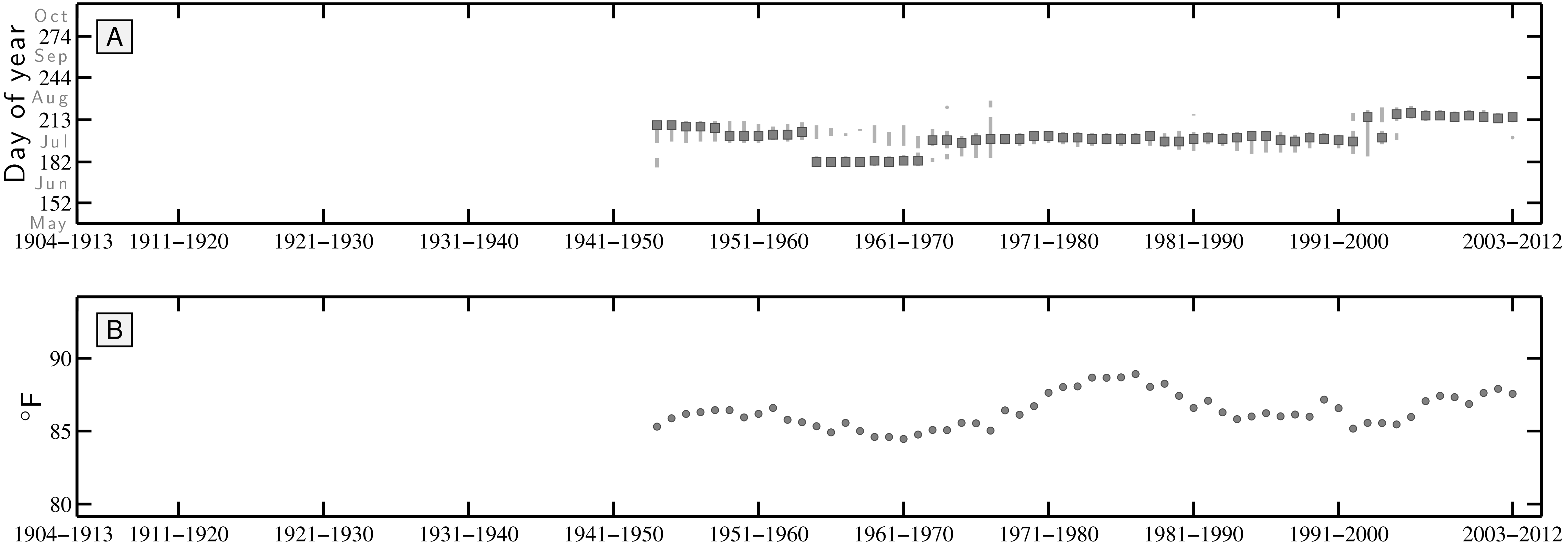

10 year Winter Teletherm dynamics for DOBBS FERRY ARDSLEY, NY:

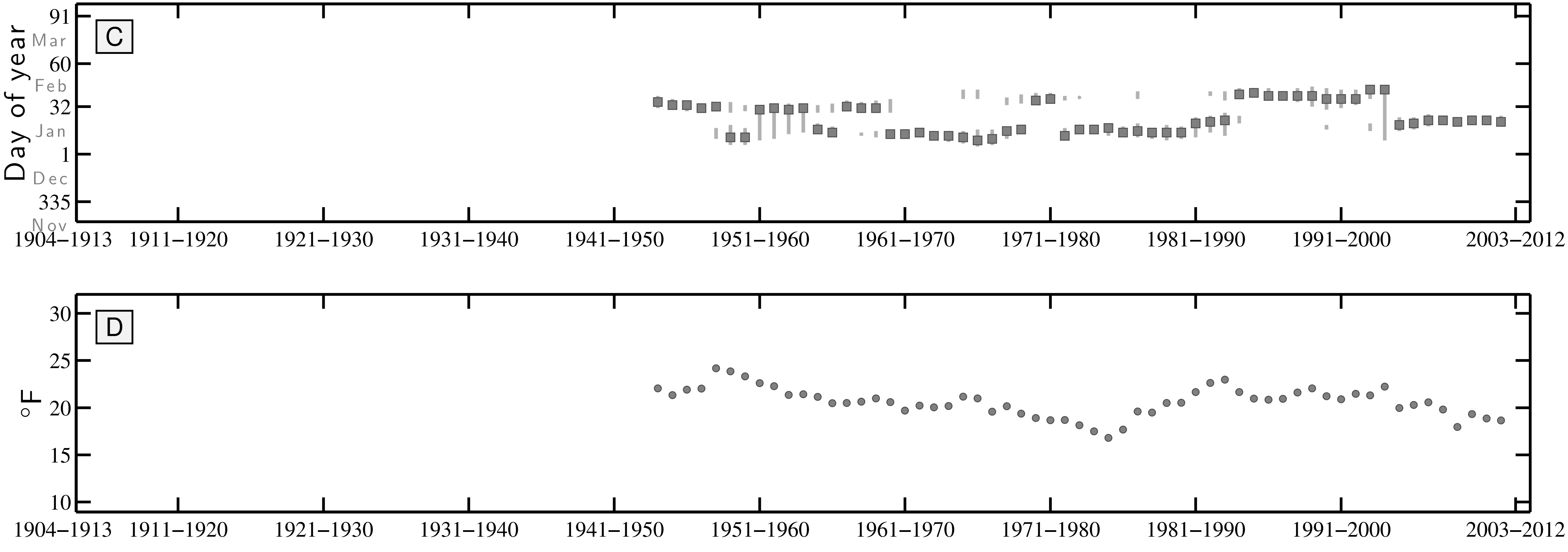

10 year Summer Teletherm dynamics for ELMIRA, NY:

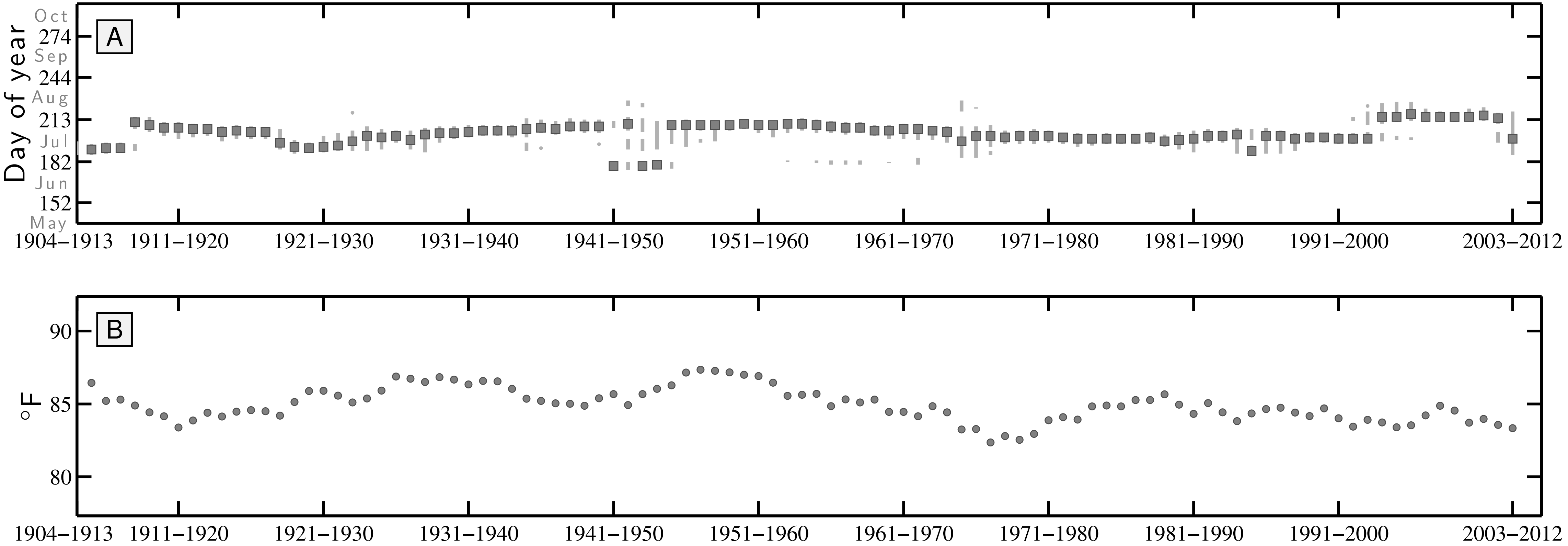

10 year Winter Teletherm dynamics for ELMIRA, NY:

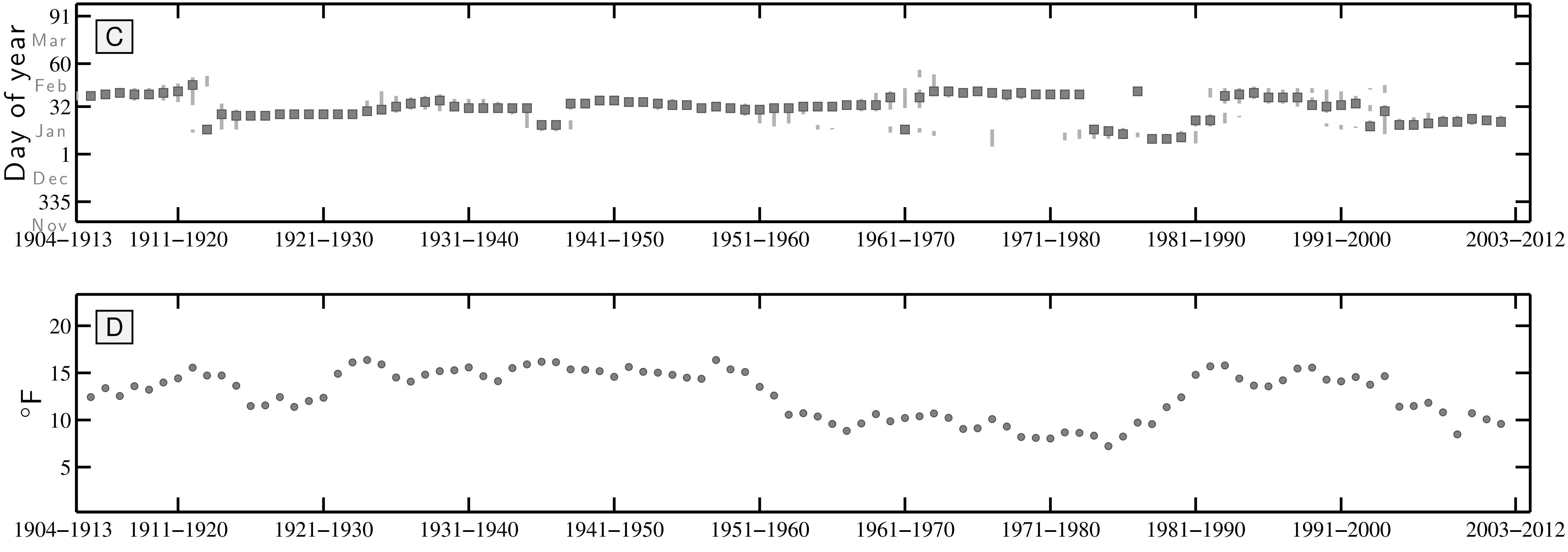

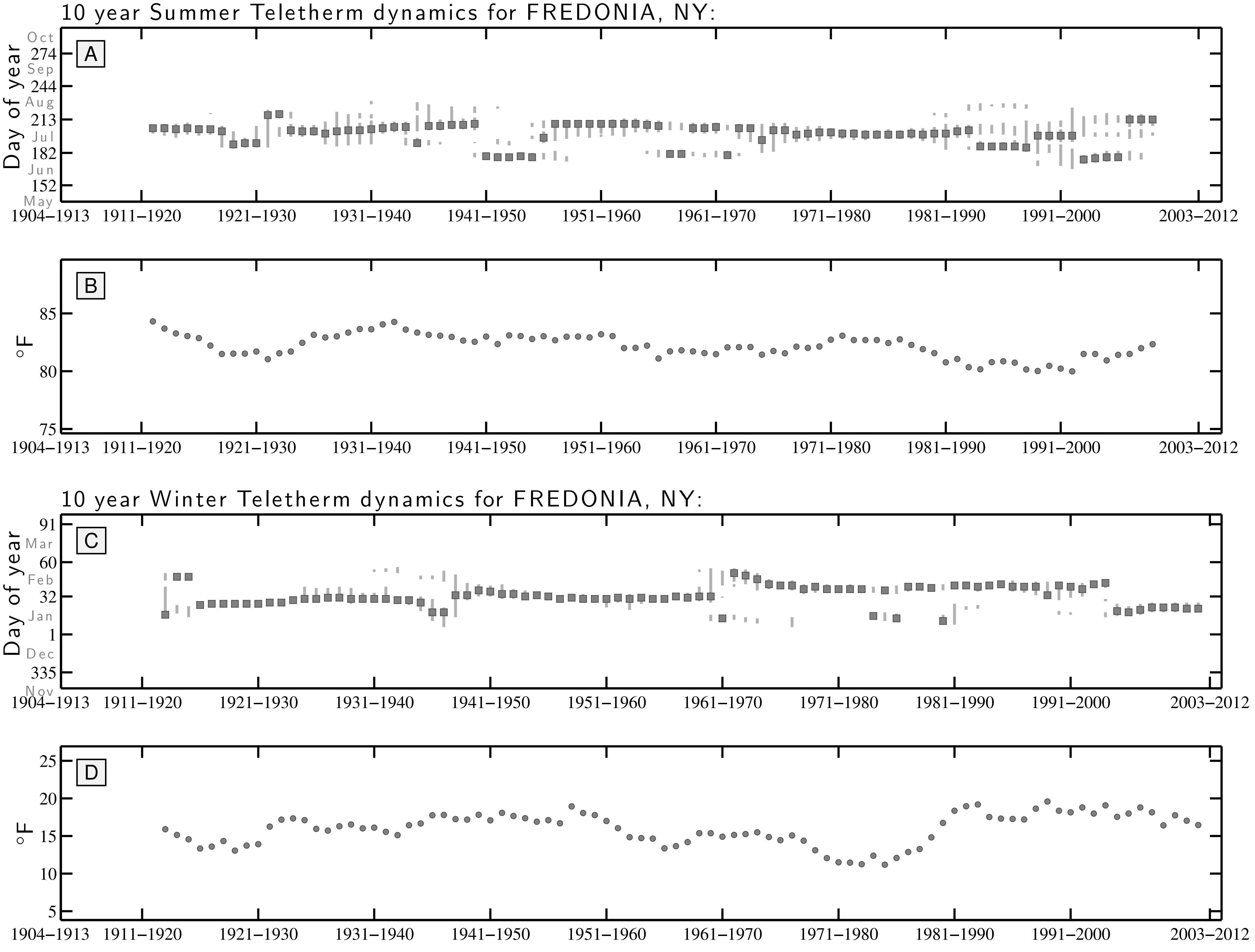

10 year Summer Teletherm dynamics for FREDONIA, NY:
A
Day of year
Oct
274
Sep
244
Aug
213
Jul
182
Jun
152
May
1904–1913
1911–1920
1921–1930
1931–1940
1941–1950
1951–1960
1961–1970
1971–1980
1981–1990
1991–2000
2003–2012
B
°F
85
80
75
10 year Winter Teletherm dynamics for FREDONIA, NY:
C
91
Mar
60
Feb
32
Jan
1
Dec
335
Nov
D
25
20
15
10
5

10 year Summer Teletherm dynamics for GENEVA RSCH FARM, NY:

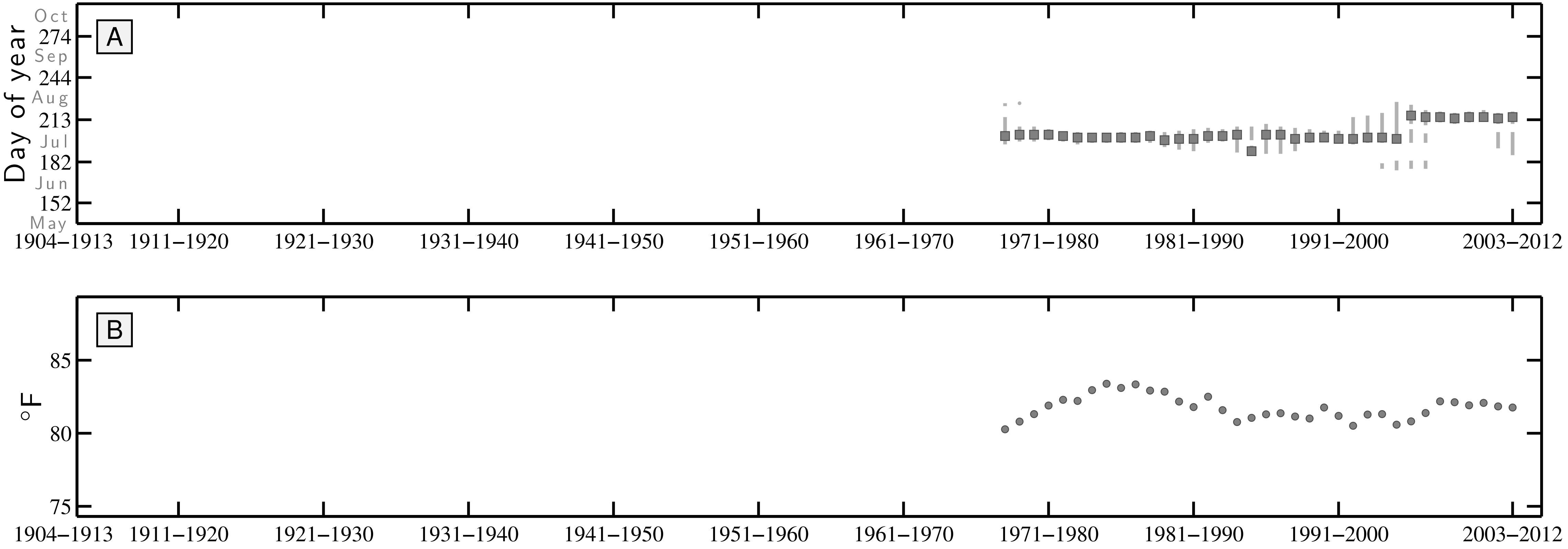

10 year Winter Teletherm dynamics for GENEVA RSCH FARM, NY:

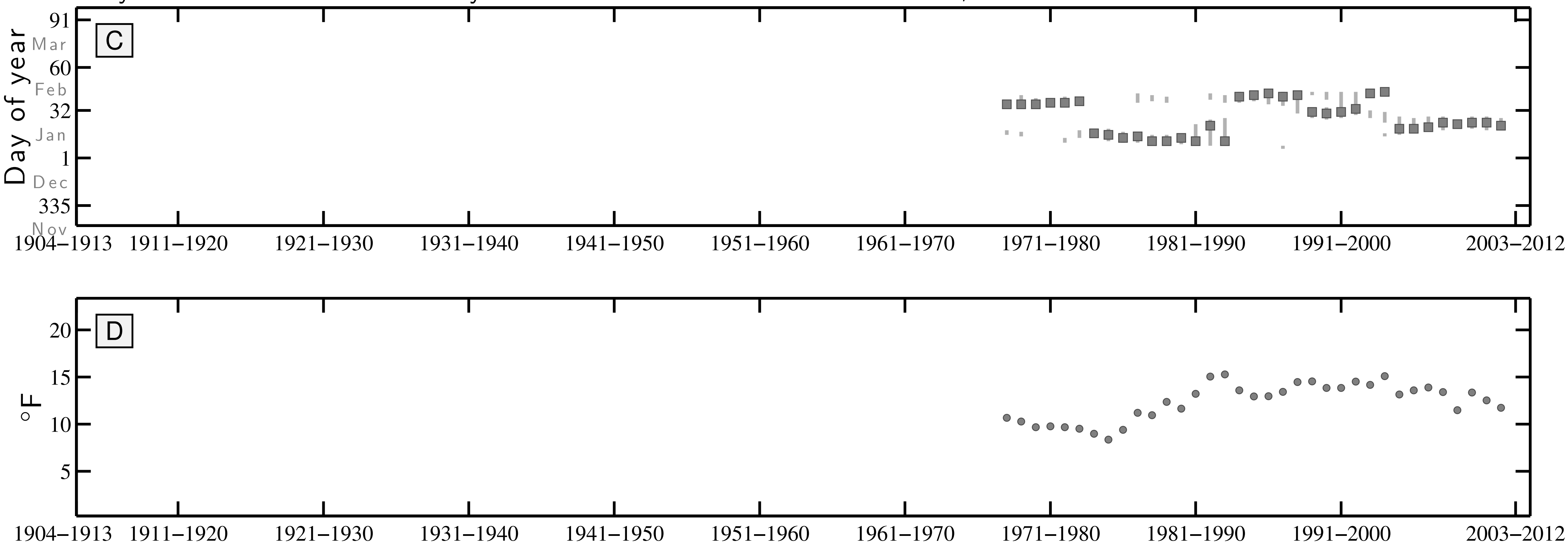

10 year Summer Teletherm dynamics for GLENHAM, NY:

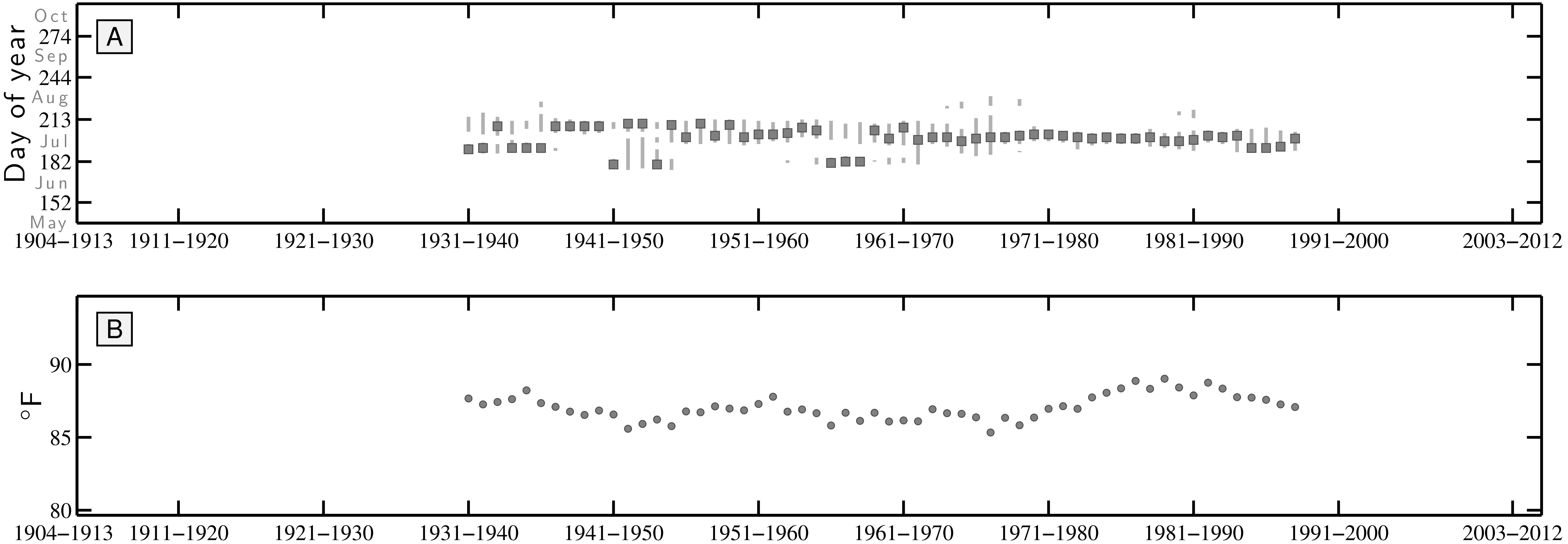

10 year Winter Teletherm dynamics for GLENHAM, NY:

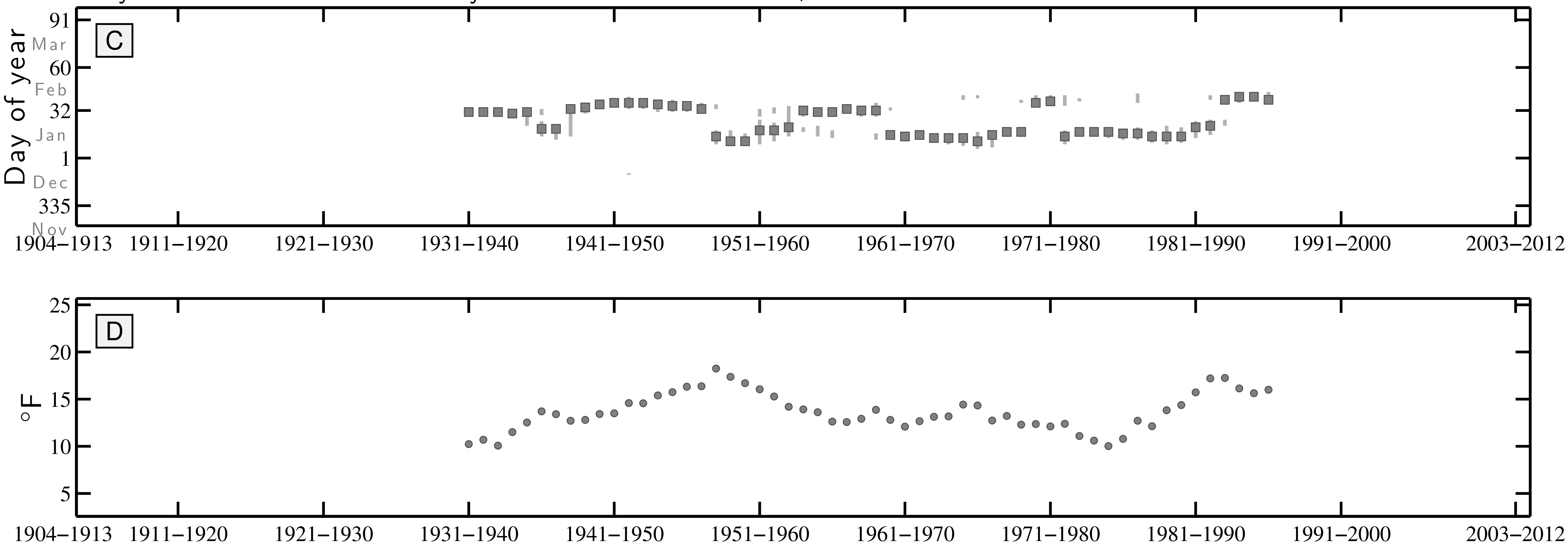

10 year Summer Teletherm dynamics for GLOVERSVILLE, NY:

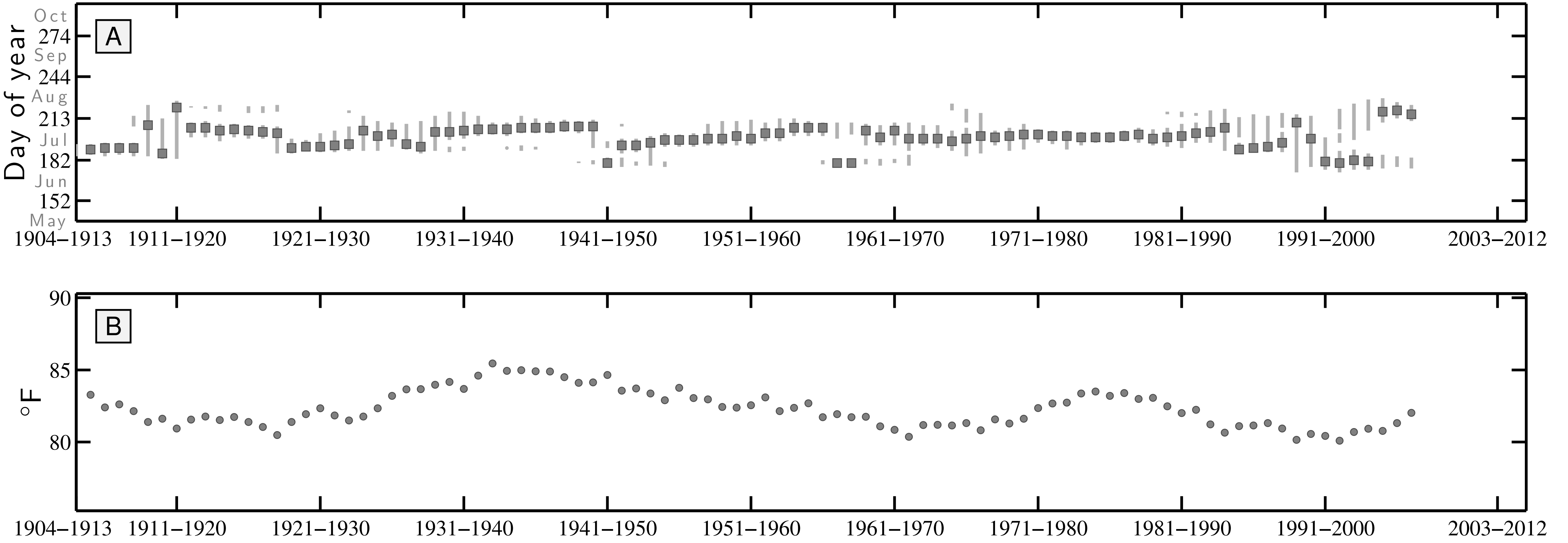

10 year Winter Teletherm dynamics for GLOVERSVILLE, NY:

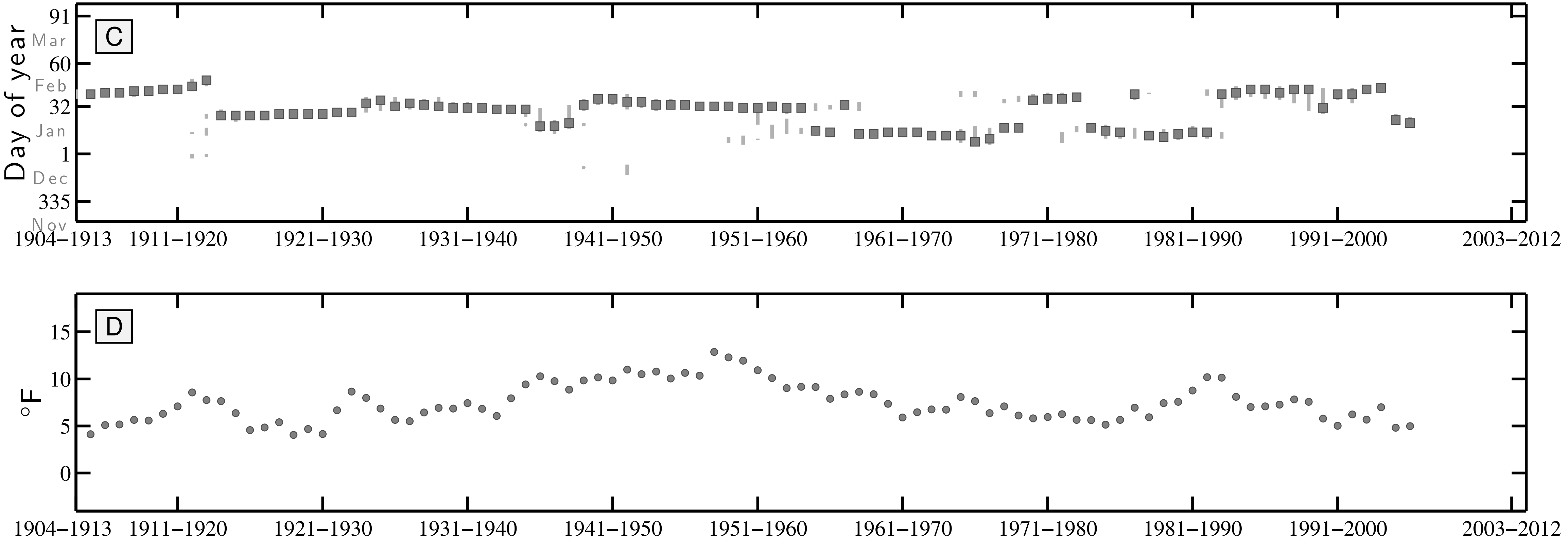

10 year Summer Teletherm dynamics for HEMLOCK, NY:

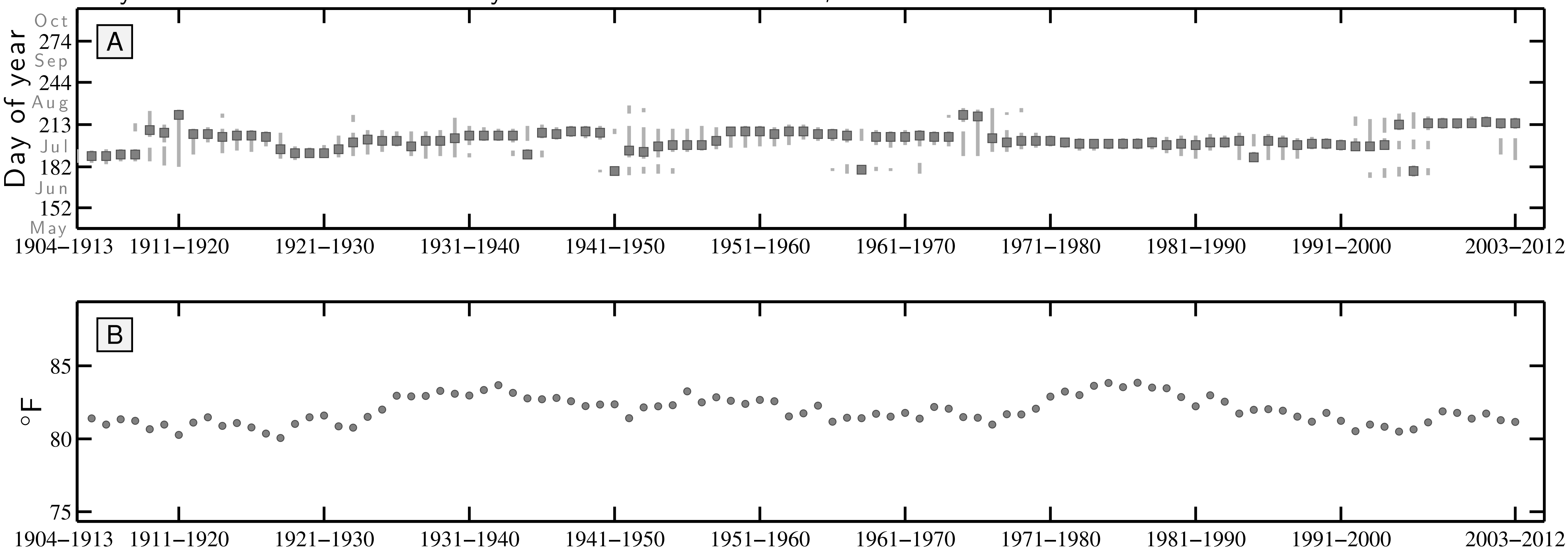

10 year Winter Teletherm dynamics for HEMLOCK, NY:

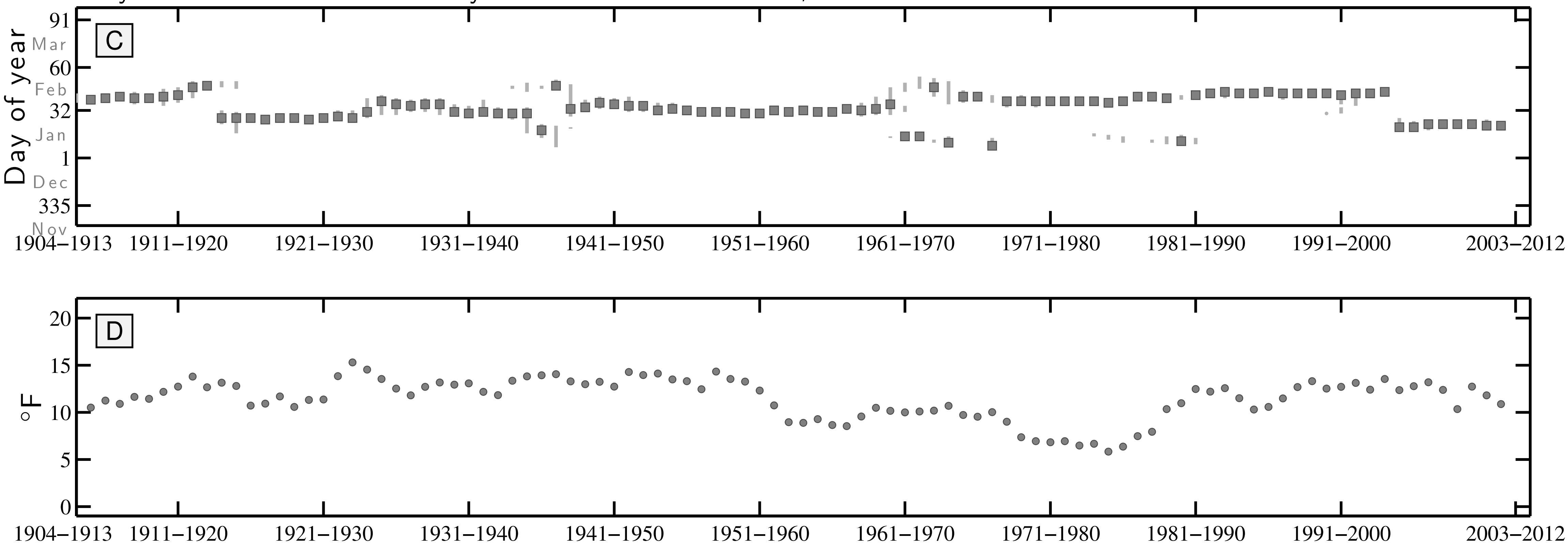

10 year Summer Teletherm dynamics for INDIAN LAKE 2SW, NY:

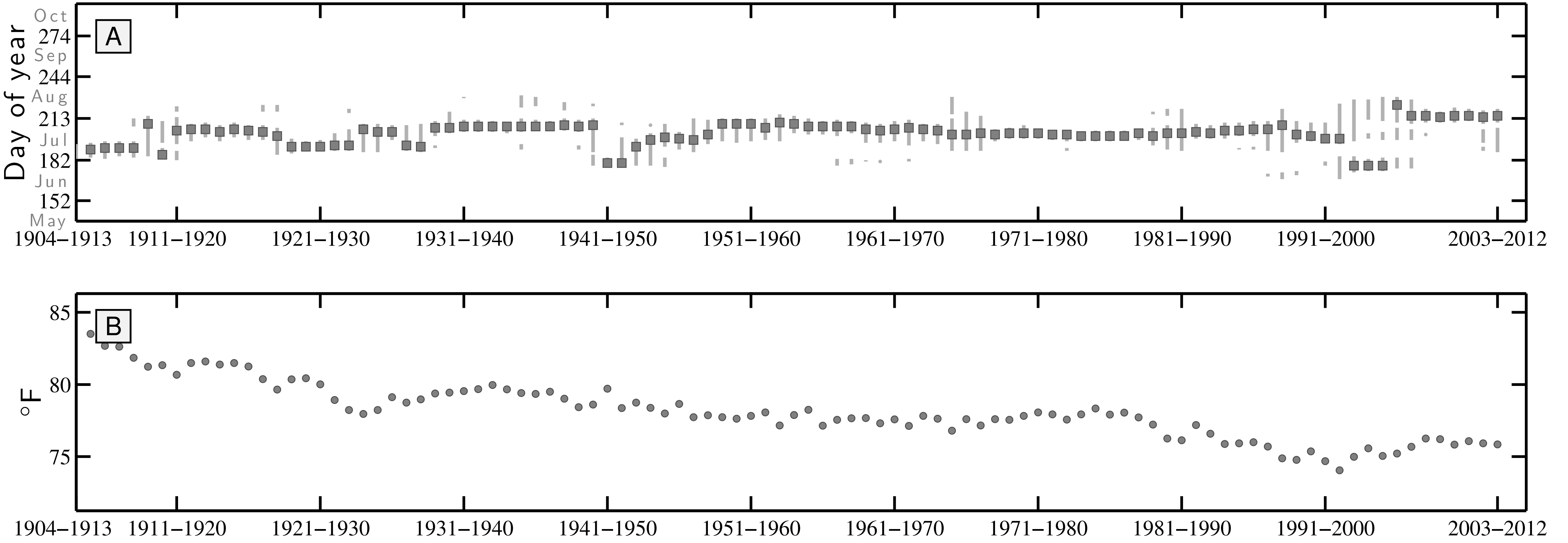

10 year Winter Teletherm dynamics for INDIAN LAKE 2SW, NY:

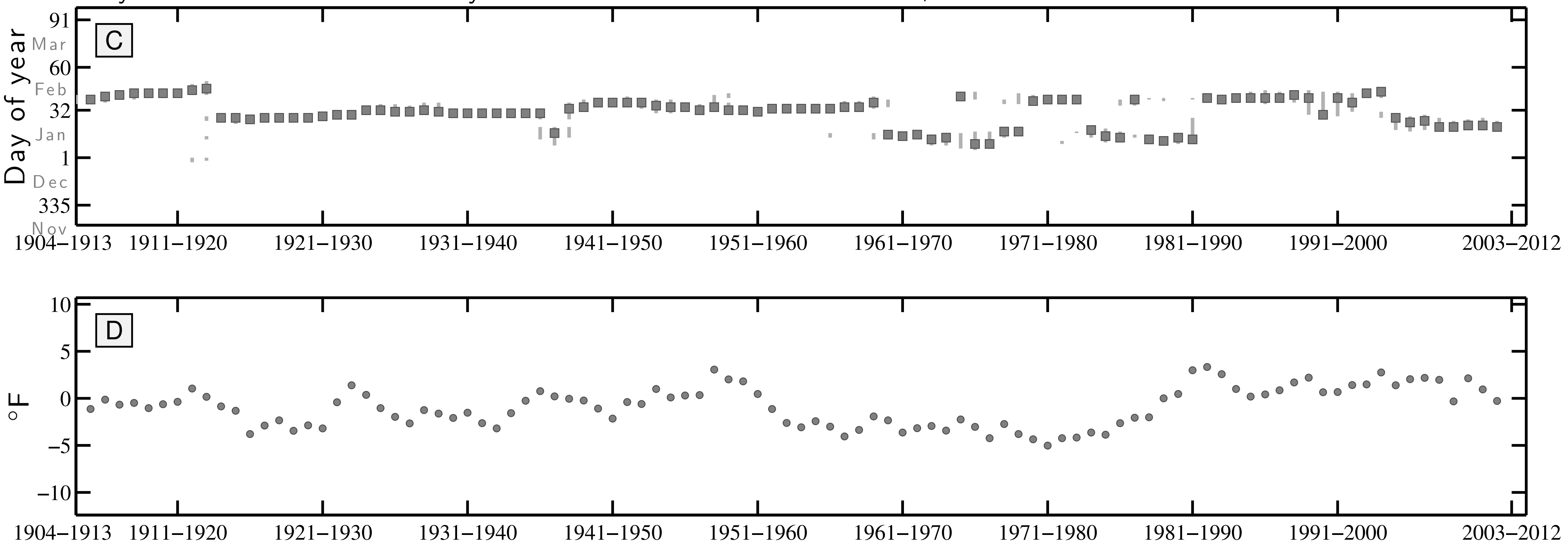

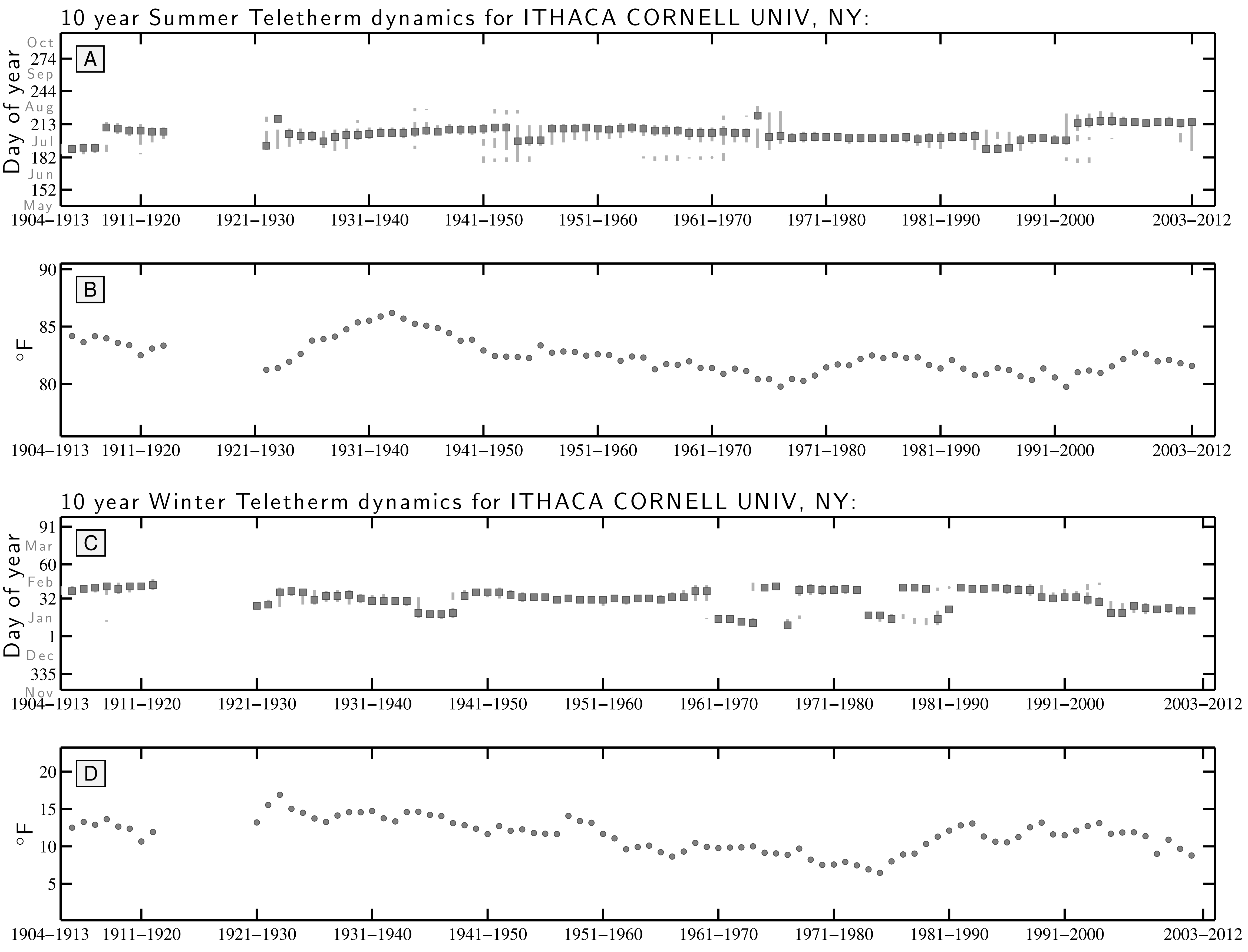

10 year Summer Teletherm dynamics for ITHACA CORNELL UNIV, NY:
A
Day of year
Oct
274
Sep
244
Aug
213
Jul
182
Jun
152
May
1904–1913
1911–1920
1921–1930
1931–1940
1941–1950
1951–1960
1961–1970
1971–1980
1981–1990
1991–2000
2003–2012
B
°F
90
85
80
10 year Winter Teletherm dynamics for ITHACA CORNELL UNIV, NY:
C
Day of year
91
Mar
60
Feb
32
Jan
1
Dec
335
Nov
D
°F
20
15
10
5

10 year Summer Teletherm dynamics for LAKE PLACID 2 S, NY:

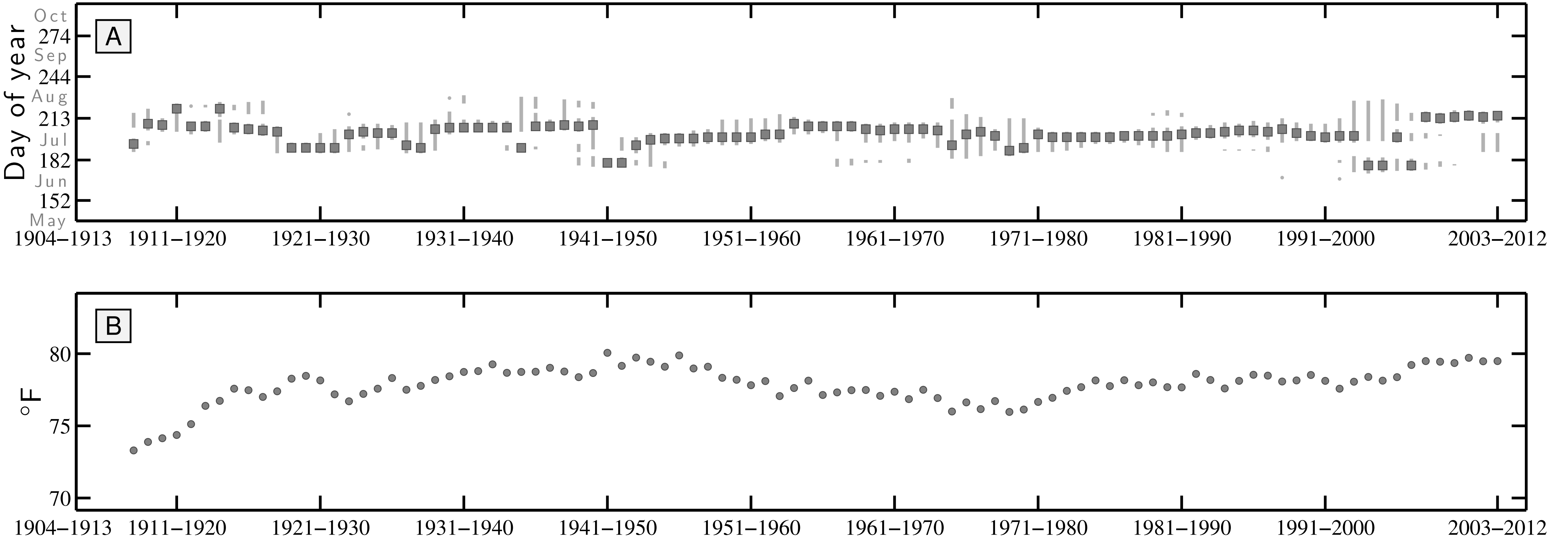

10 year Winter Teletherm dynamics for LAKE PLACID 2 S, NY:

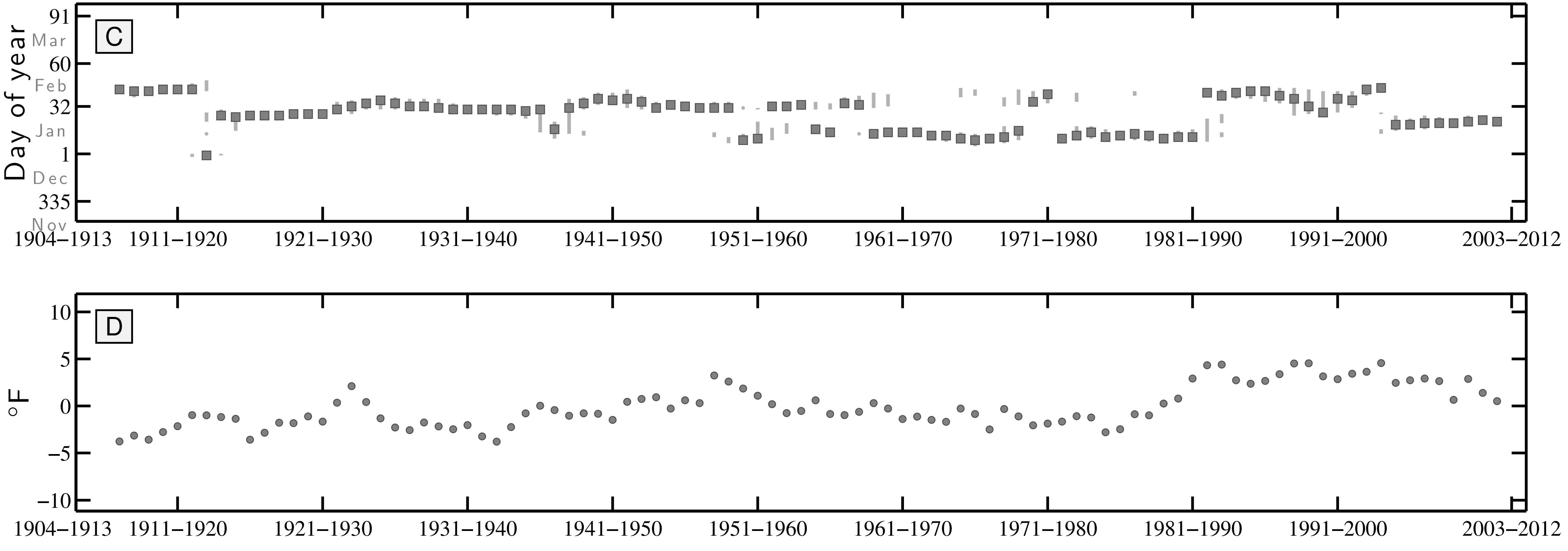

10 year Summer Teletherm dynamics for LAWRENCEVILLE 3 SW, NY:

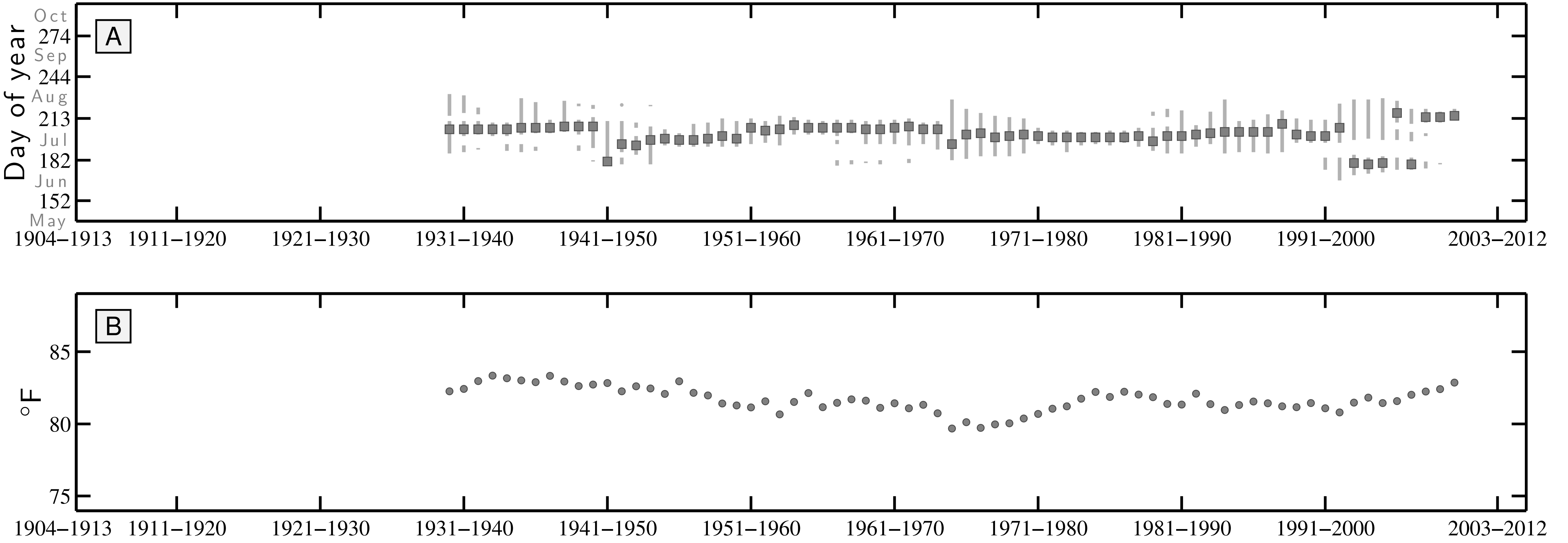

10 year Winter Teletherm dynamics for LAWRENCEVILLE 3 SW, NY:

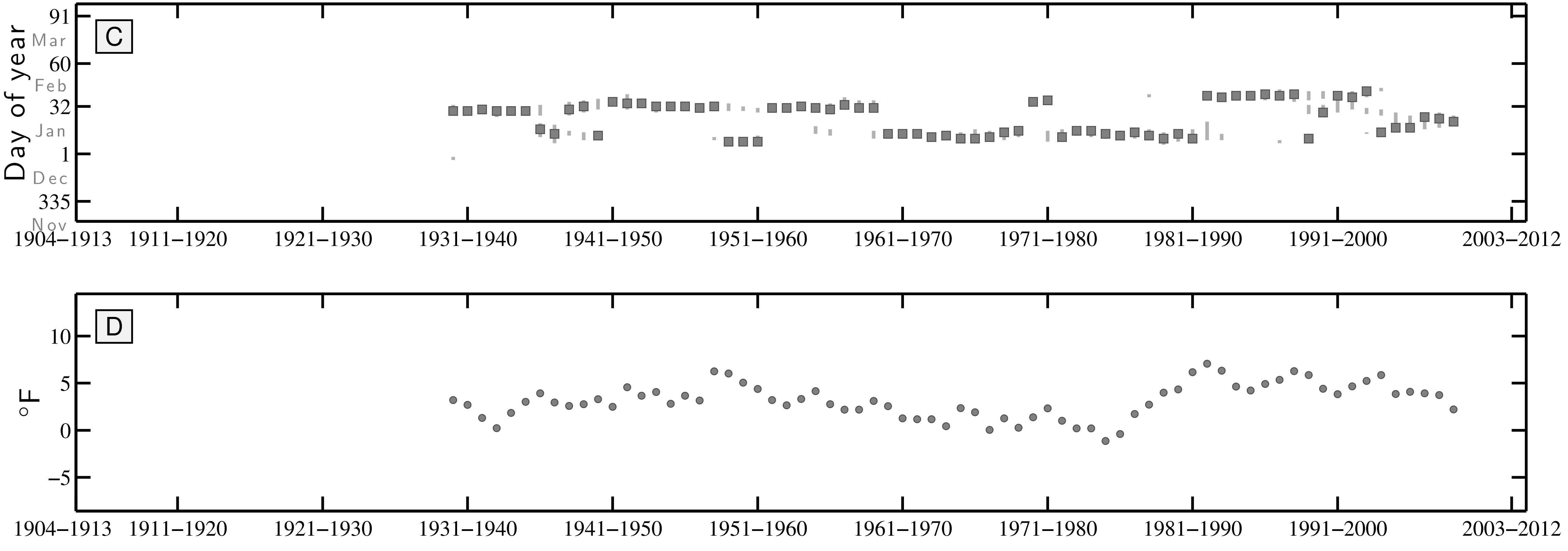

10 year Summer Teletherm dynamics for LITTLE FALLS CITY RSVR, NY:

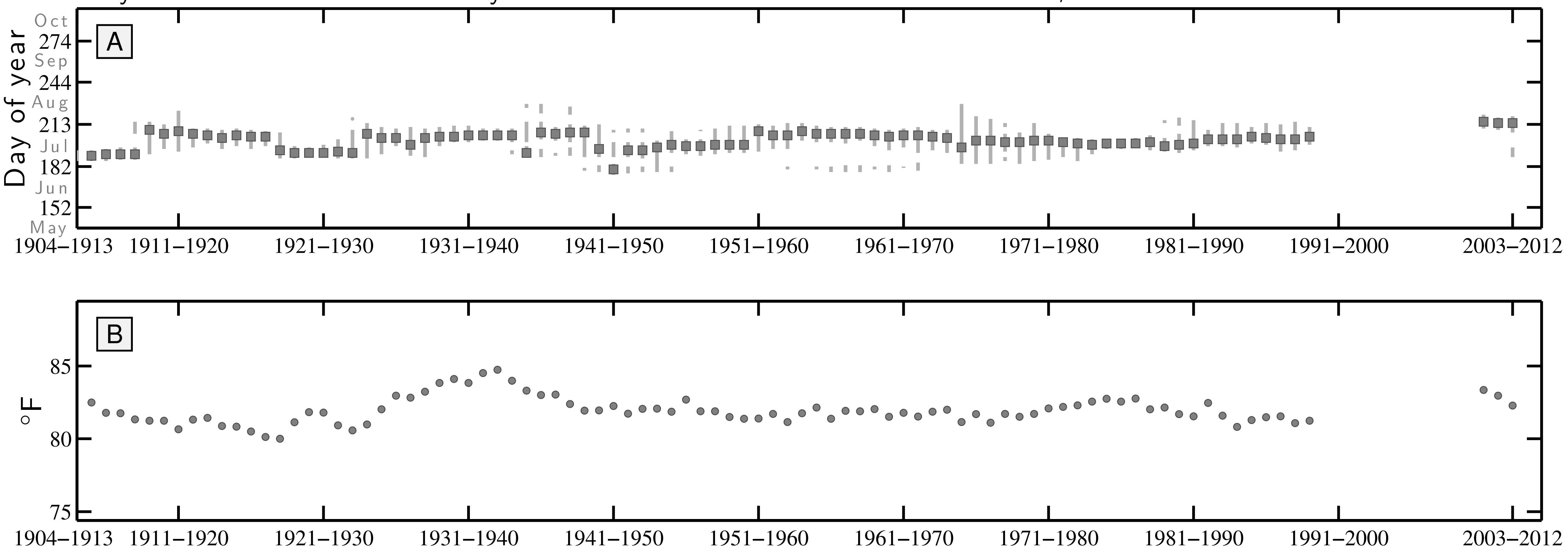

10 year Winter Teletherm dynamics for LITTLE FALLS CITY RSVR, NY:

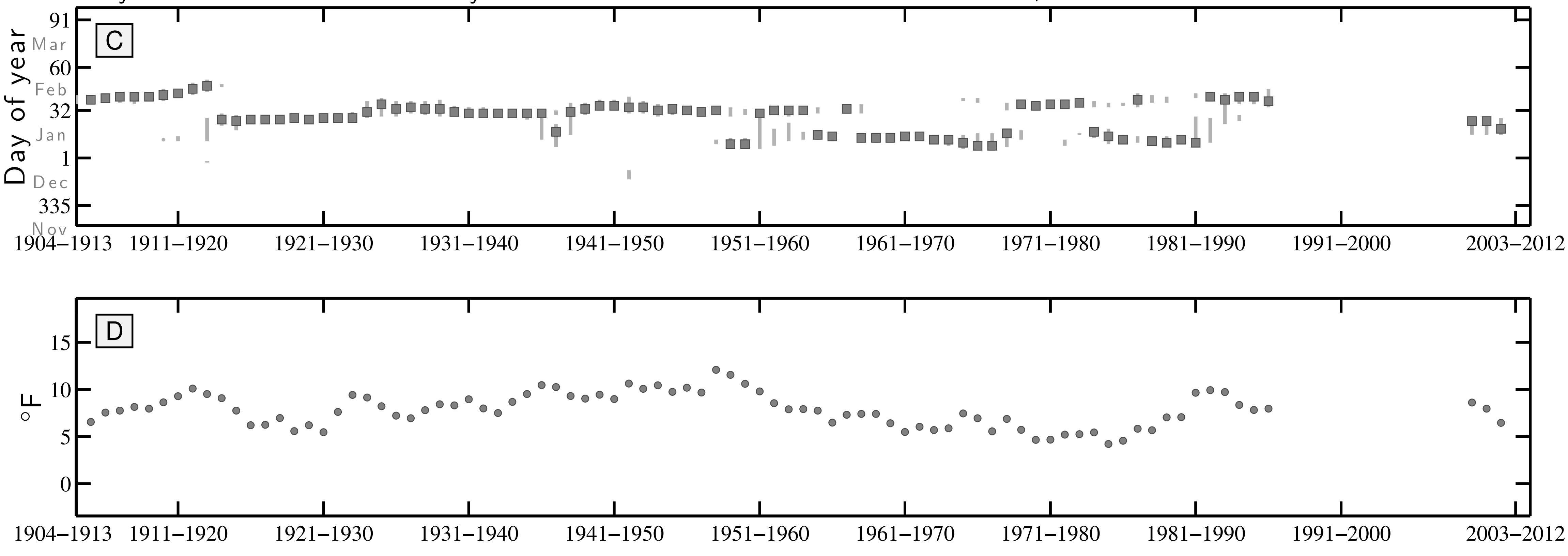

10 year Summer Teletherm dynamics for LITTLE FALLS MILL ST, NY:

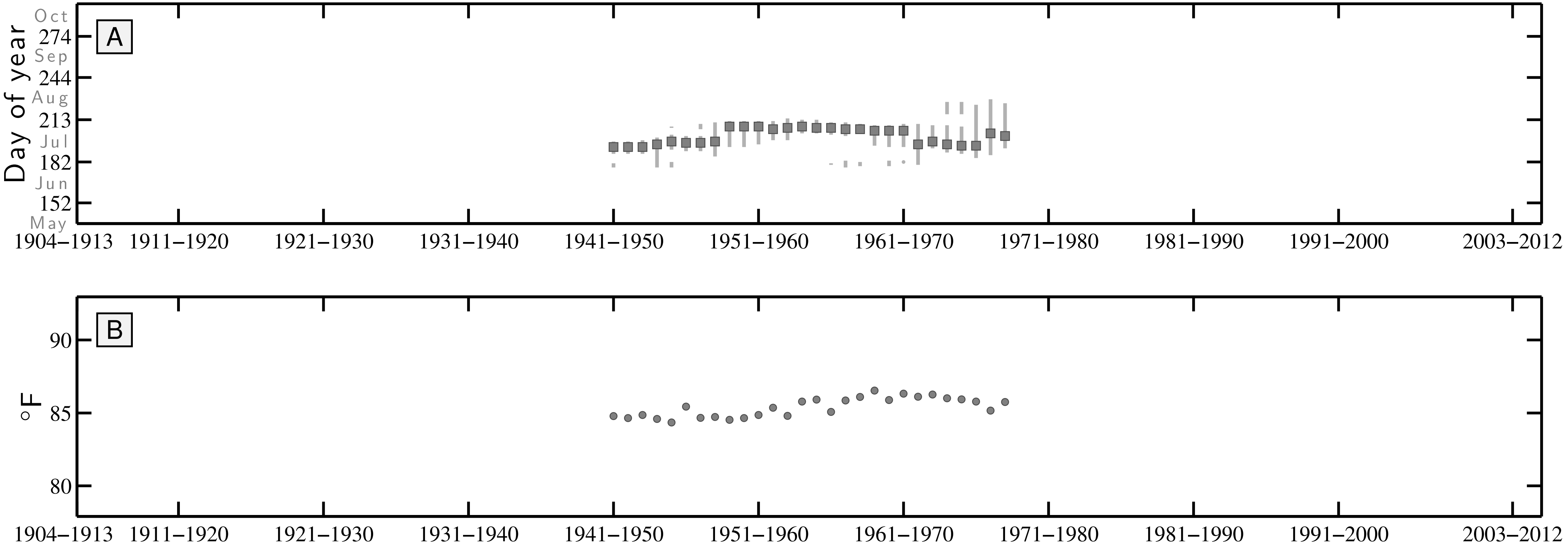

10 year Winter Teletherm dynamics for LITTLE FALLS MILL ST, NY:

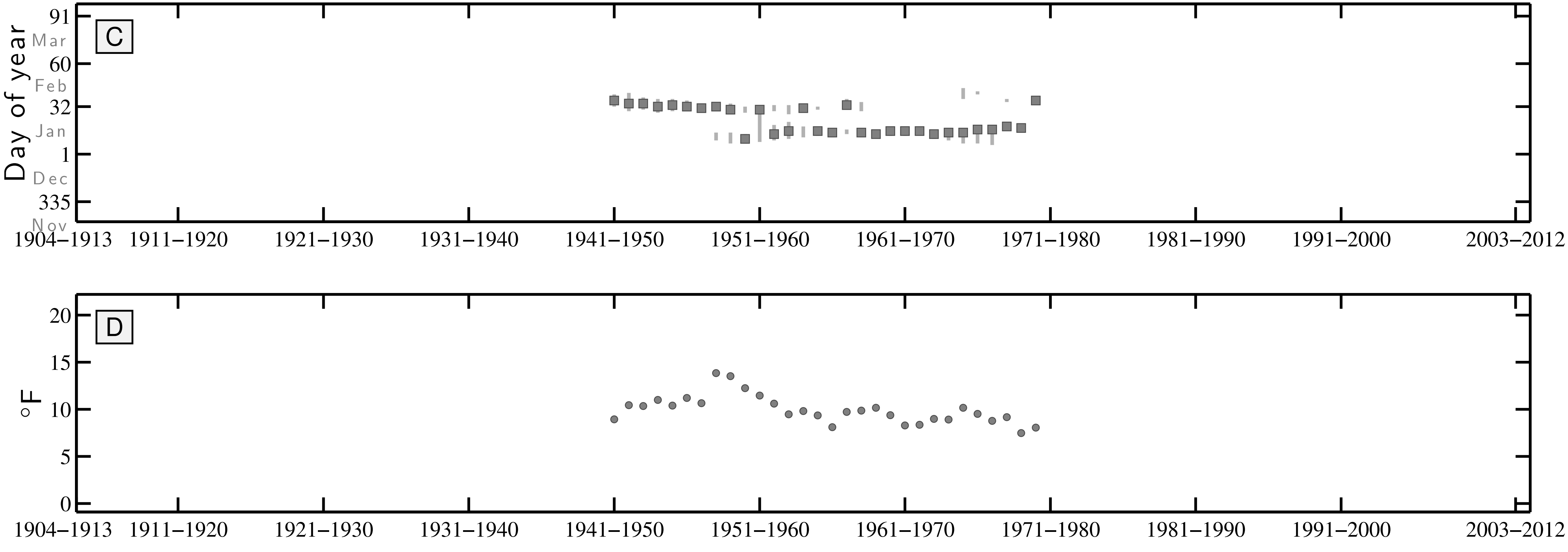

10 year Summer Teletherm dynamics for LOCKPORT 3 S, NY:

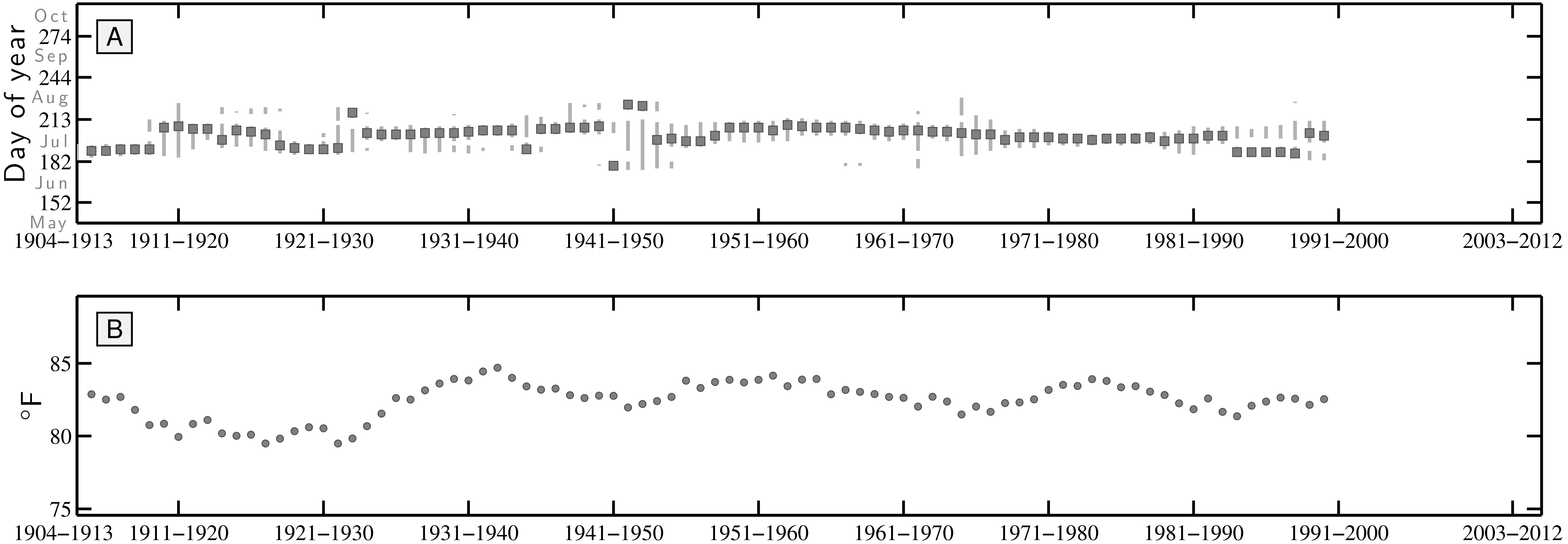

10 year Winter Teletherm dynamics for LOCKPORT 3 S, NY:

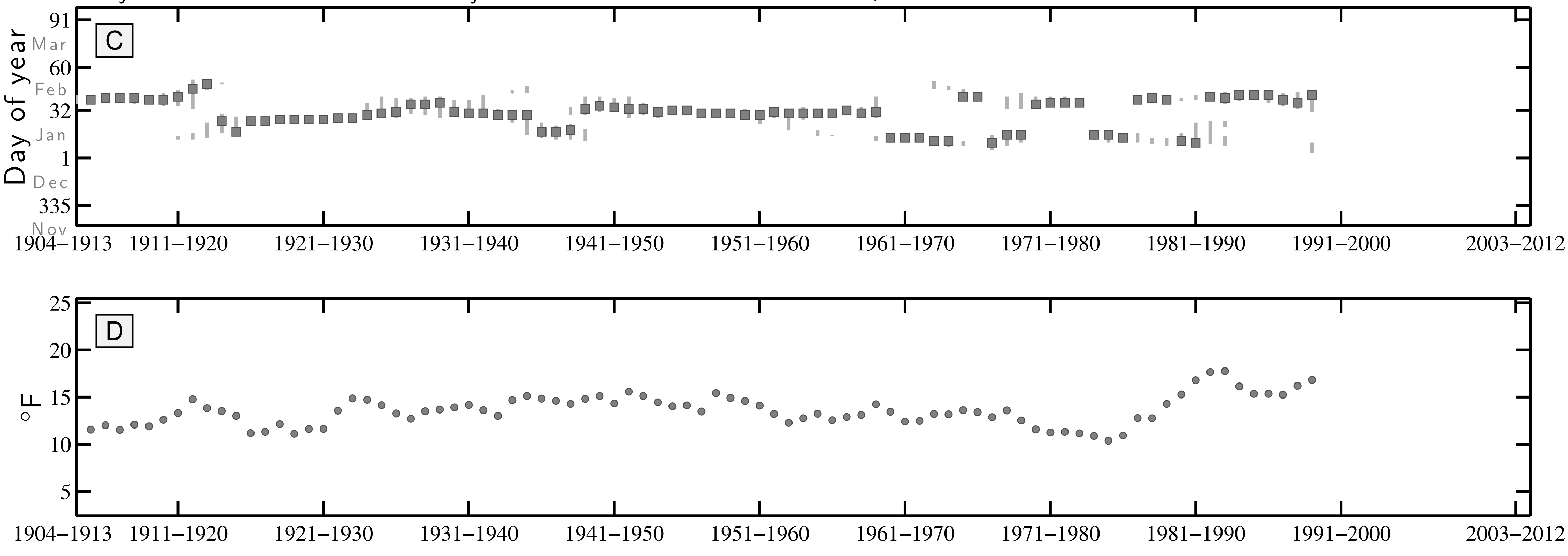

10 year Summer Teletherm dynamics for LOWVILLE, NY:

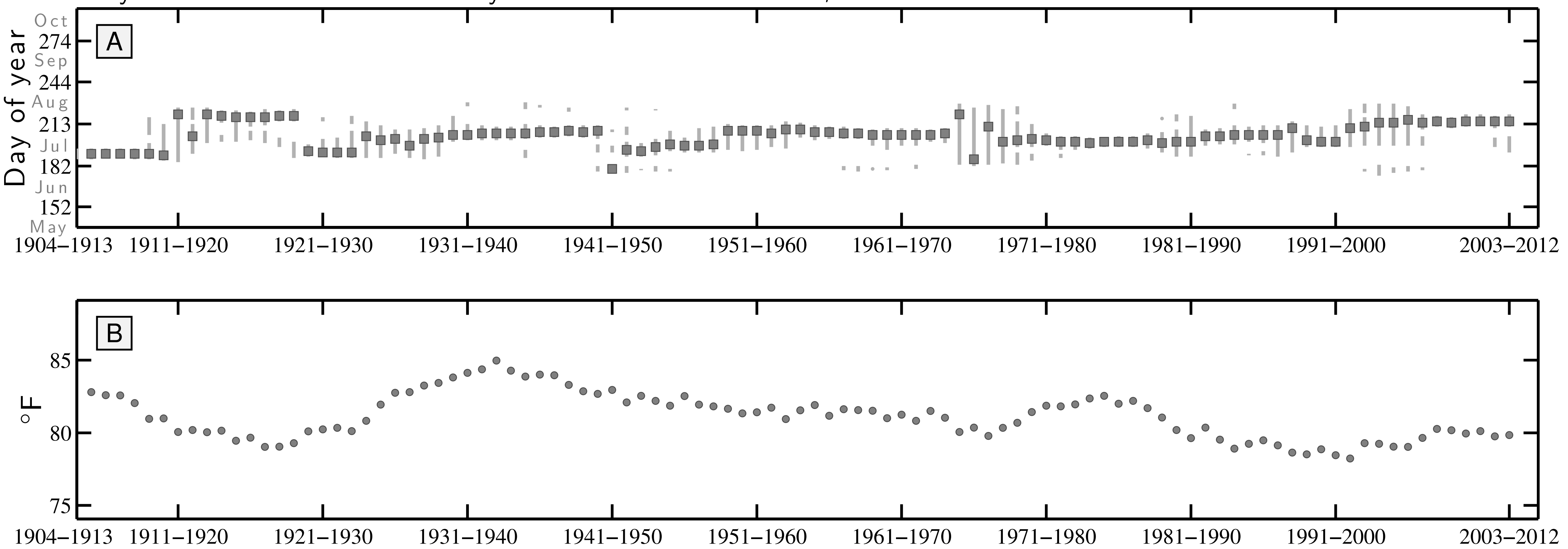

10 year Winter Teletherm dynamics for LOWVILLE, NY:

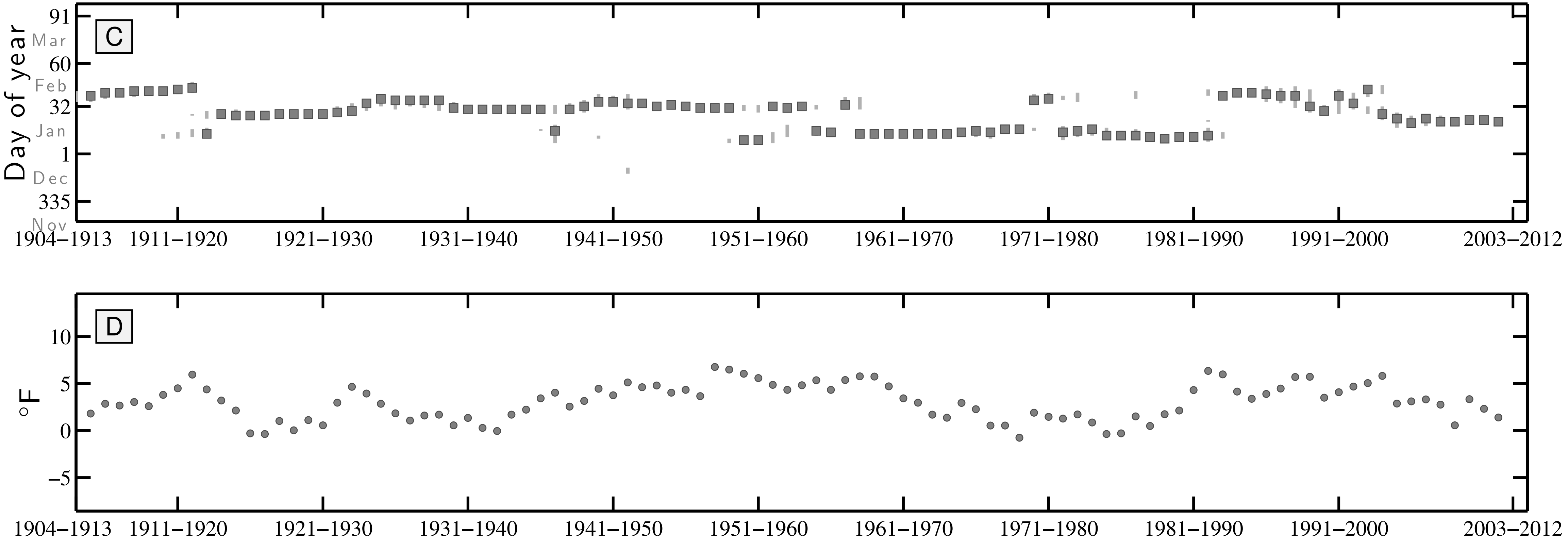

10 year Summer Teletherm dynamics for MALONE, NY:

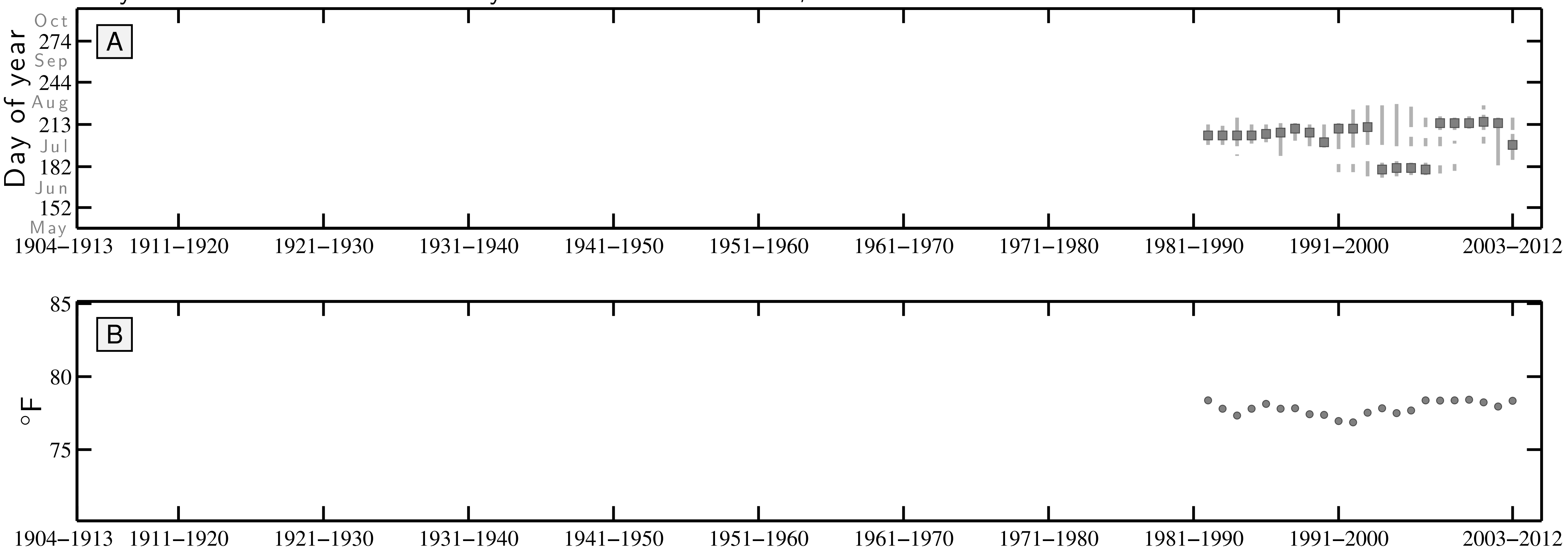

10 year Winter Teletherm dynamics for MALONE, NY:

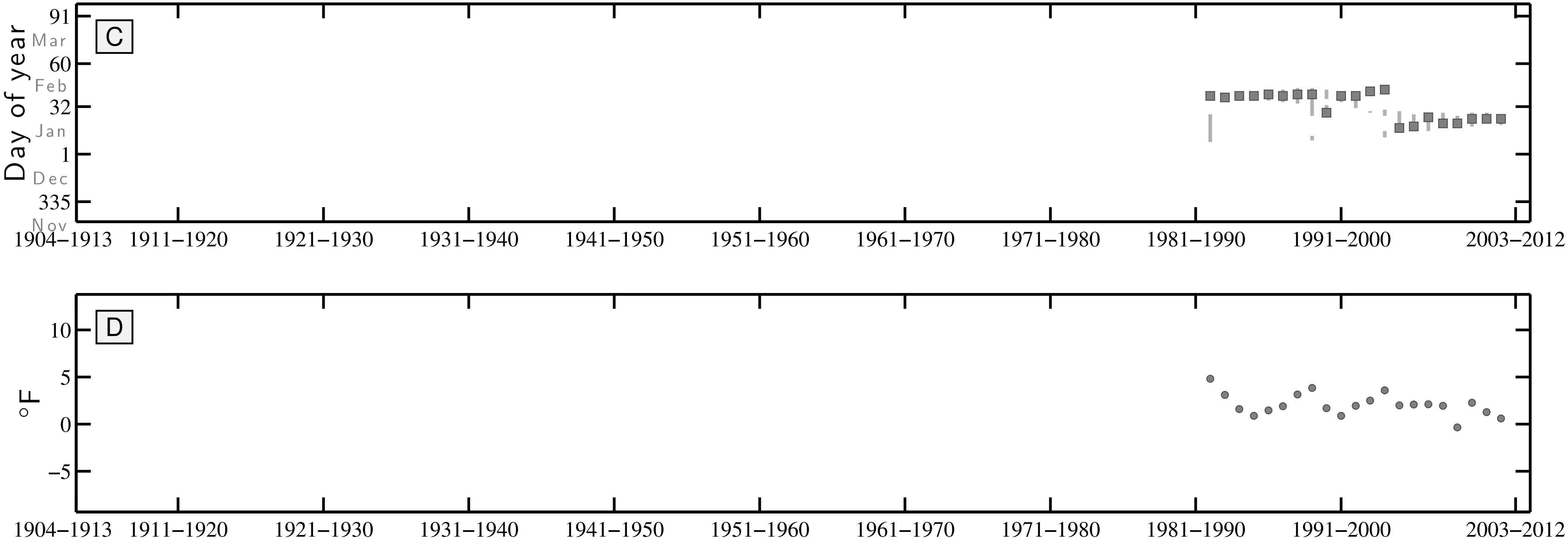

10 year Summer Teletherm dynamics for MARYLAND 9 SW, NY:

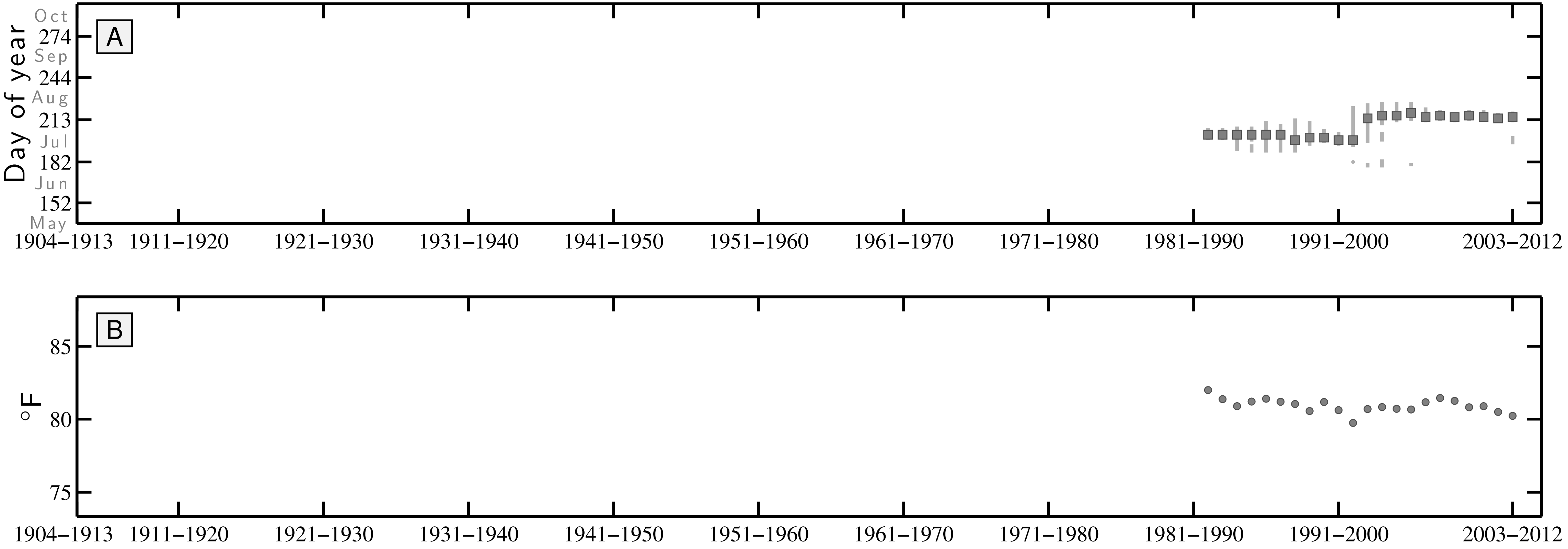

10 year Winter Teletherm dynamics for MARYLAND 9 SW, NY:

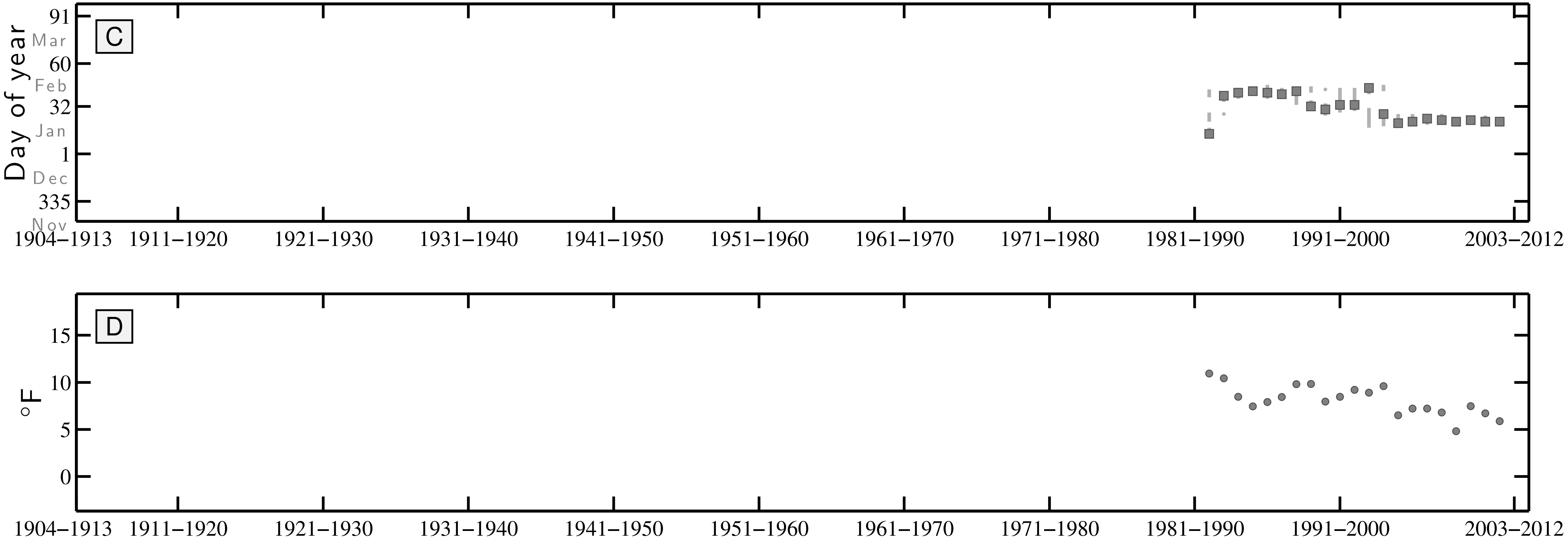

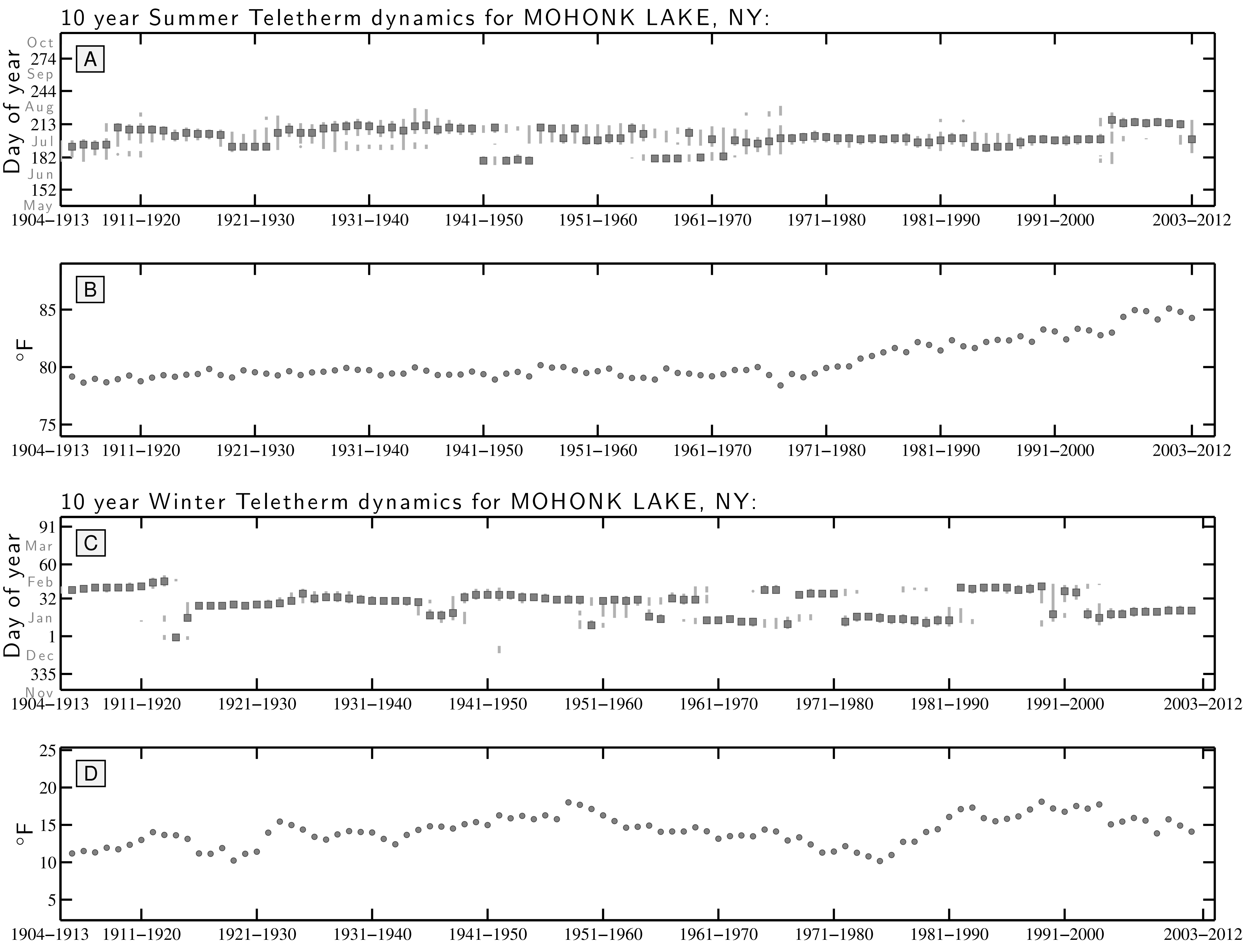
10 year Summer Teletherm dynamics for MOHONK LAKE, NY:
A
Day of year
Oct
274
Sep
244
Aug
213
Jul
182
Jun
152
May
B
°F
85
80
75
10 year Winter Teletherm dynamics for MOHONK LAKE, NY:
C
Day of year
91
Mar
60
Feb
32
Jan
1
Dec
335
Nov
D
°F
25
20
15
10
5
1904–1913
1911–1920
1921–1930
1931–1940
1941–1950
1951–1960
1961–1970
1971–1980
1981–1990
1991–2000
2003–2012

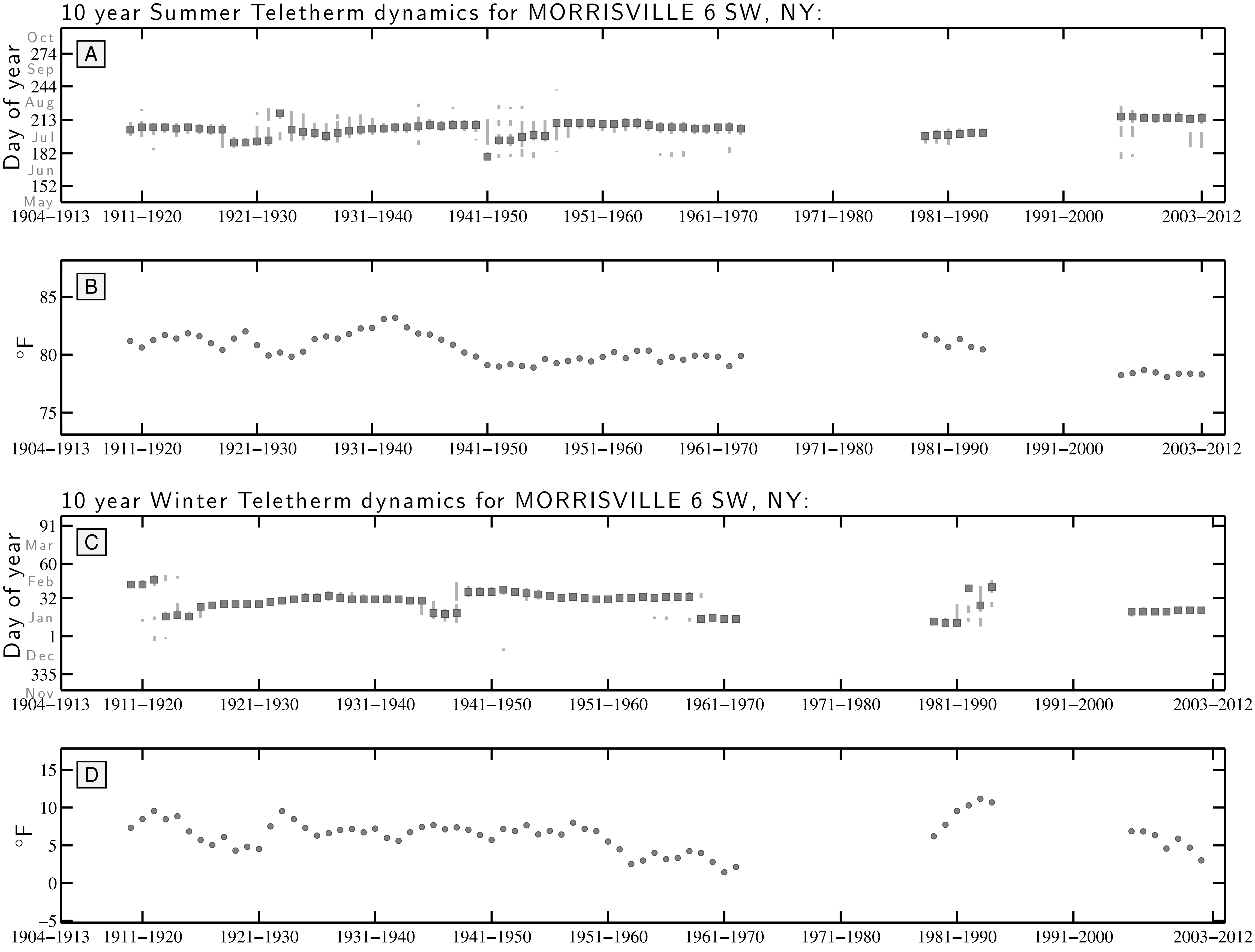

10 year Summer Teletherm dynamics for MORRISVILLE 6 SW, NY:
A
Day of year
Oct
274
Sep
244
Aug
213
Jul
182
Jun
152
May
B
°F
85
80
75
10 year Winter Teletherm dynamics for MORRISVILLE 6 SW, NY:
C
Day of year
91
Mar
60
Feb
32
Jan
1
Dec
335
Nov
D
°F
15
10
5
0
−5
1904–1913
1911–1920
1921–1930
1931–1940
1941–1950
1951–1960
1961–1970
1971–1980
1981–1990
1991–2000
2003–2012

10 year Summer Teletherm dynamics for NY CITY CNTRL PARK, NY:

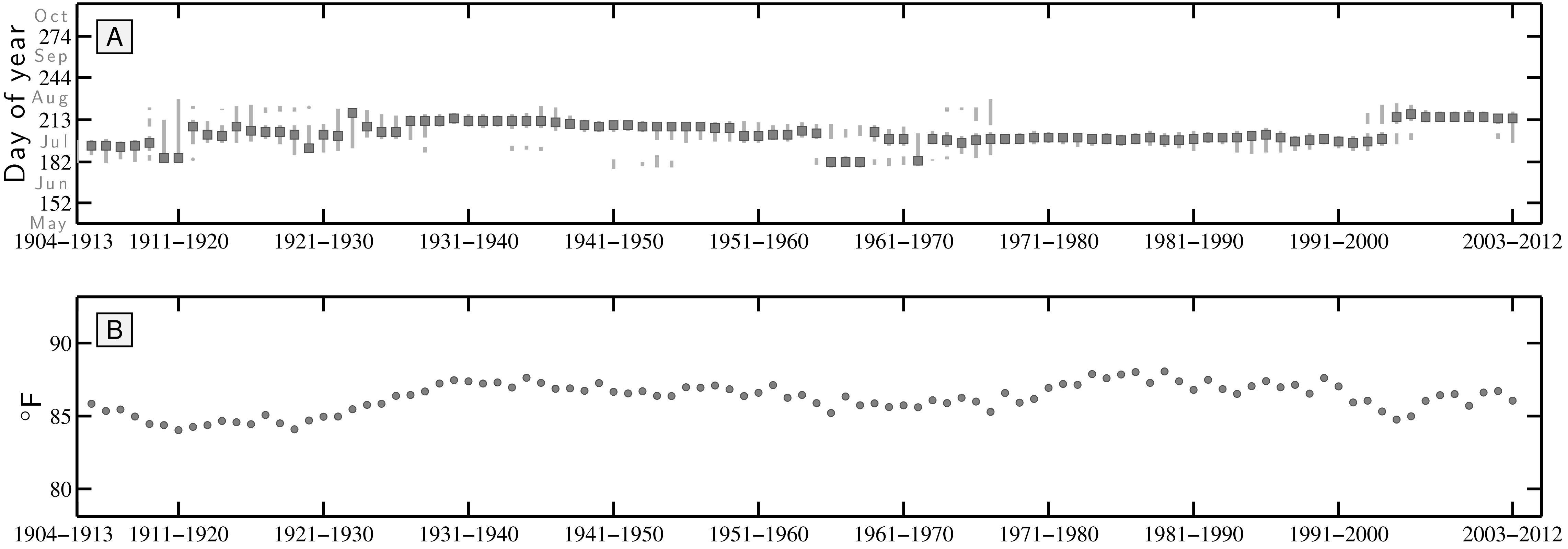

10 year Winter Teletherm dynamics for NY CITY CNTRL PARK, NY:

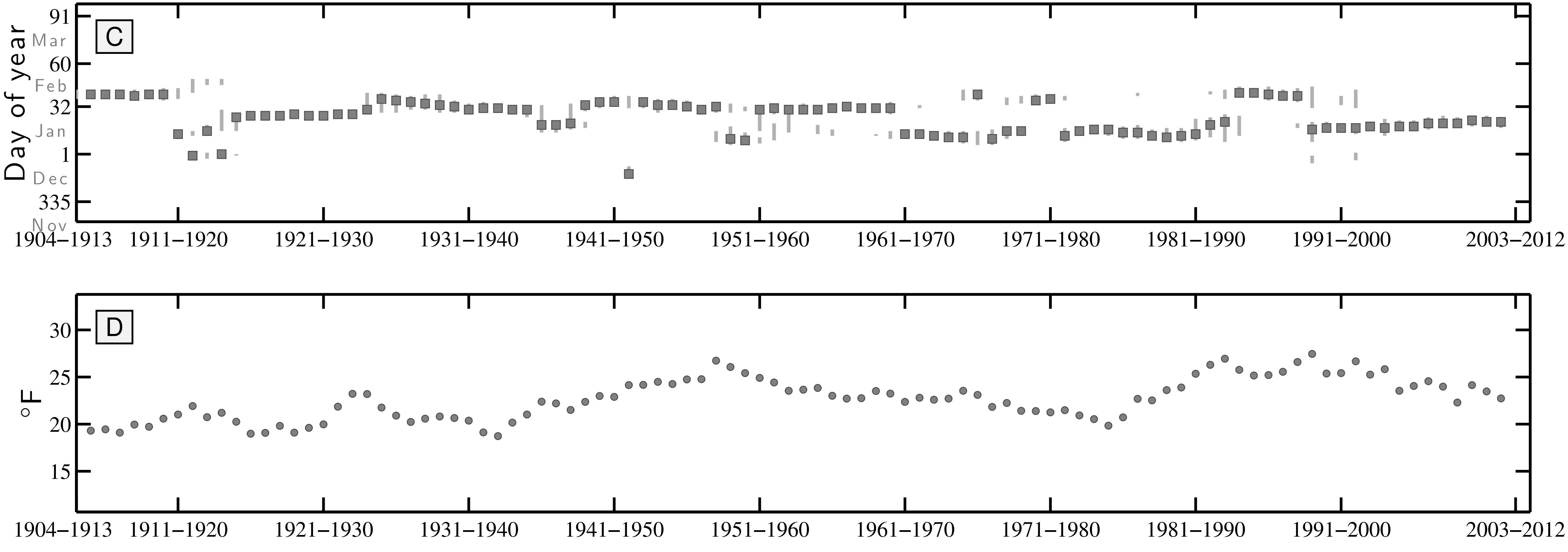

10 year Summer Teletherm dynamics for NORWICH, NY:

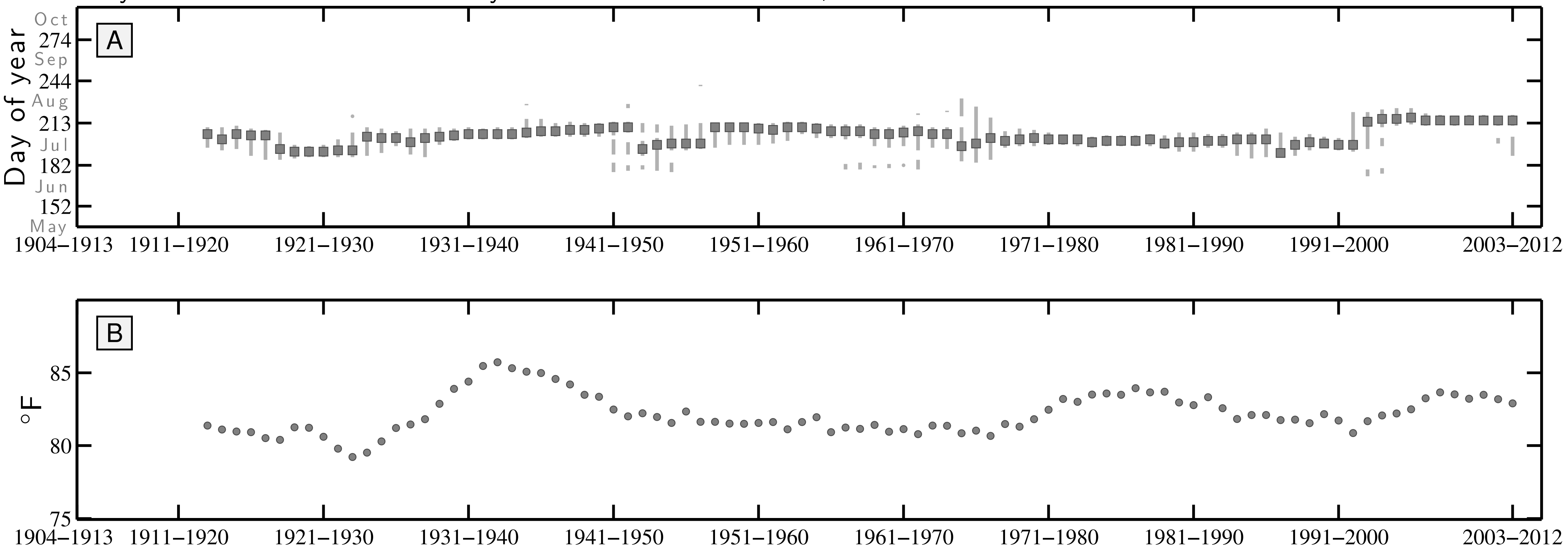

10 year Winter Teletherm dynamics for NORWICH, NY:

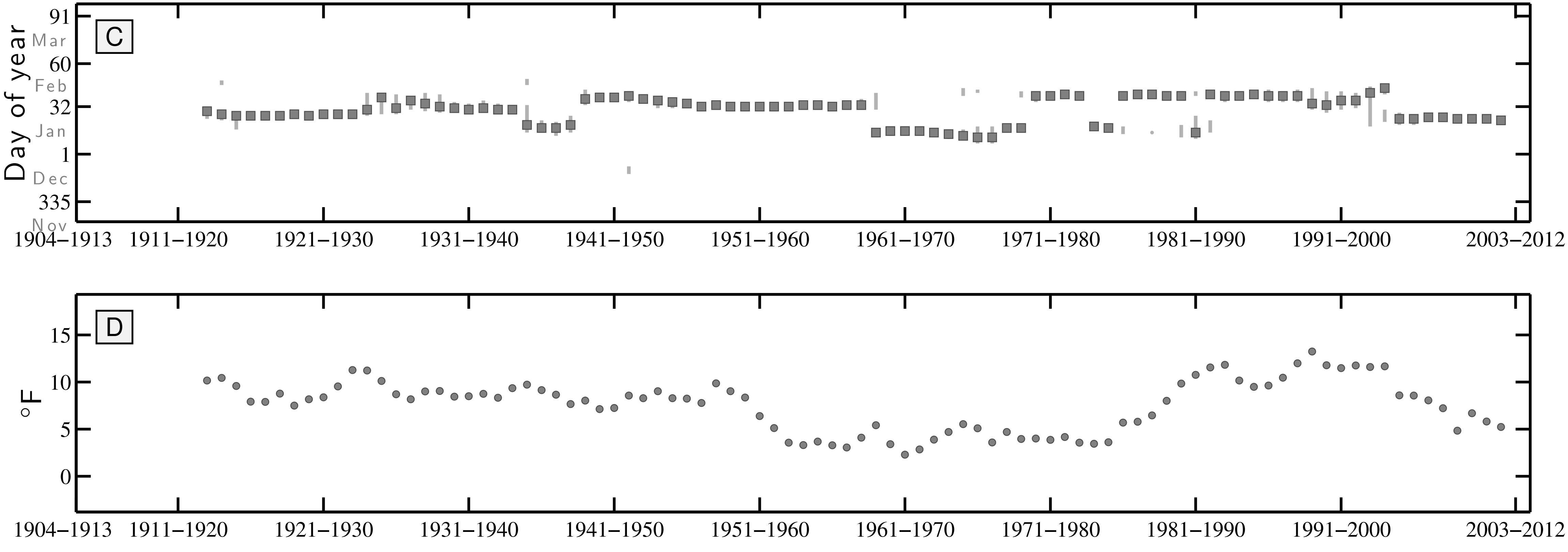

10 year Summer Teletherm dynamics for OGDENSBURG 4 NE, NY:

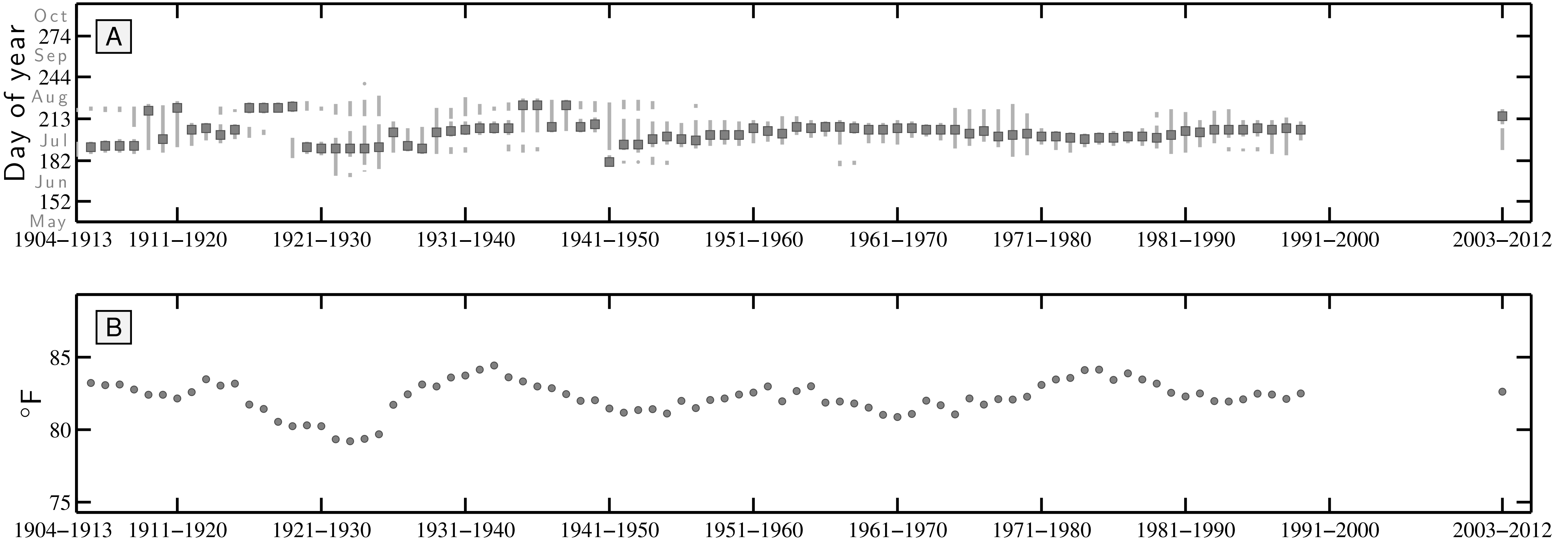

10 year Winter Teletherm dynamics for OGDENSBURG 4 NE, NY:

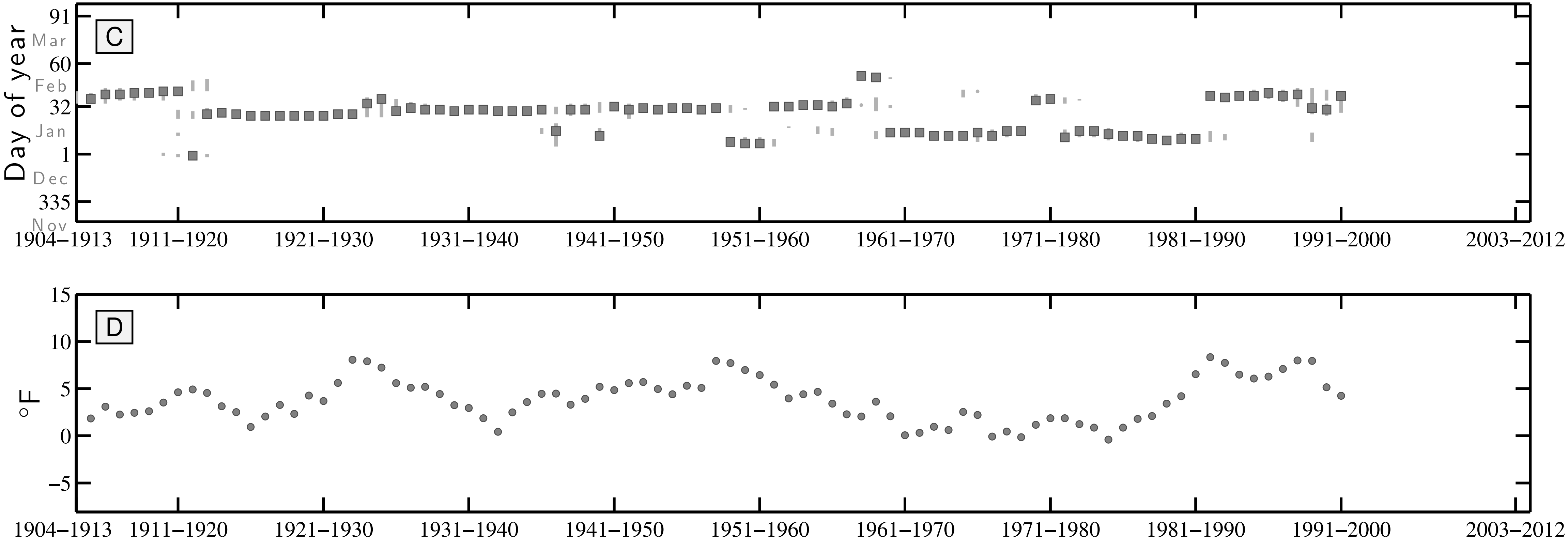

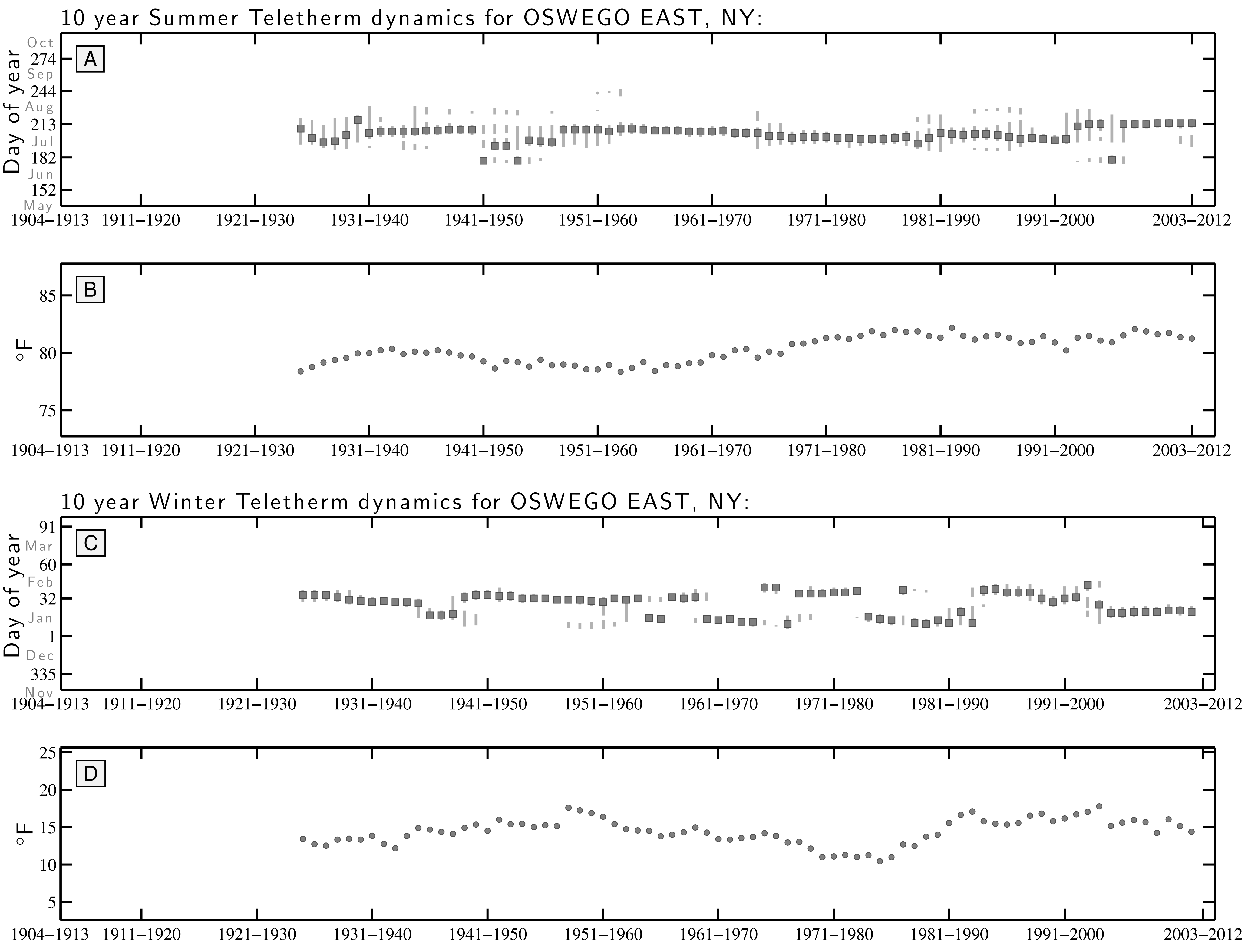
10 year Summer Teletherm dynamics for OSWEGO EAST, NY:
A
B
Day of year
°F
Oct
274
Sep
244
Aug
213
Jul
182
Jun
152
May
85
80
75
10 year Winter Teletherm dynamics for OSWEGO EAST, NY:
C
D
91
Mar
60
Feb
32
Jan
1
Dec
335
Nov
25
20
15
10
5
1904–1913
1911–1920
1921–1930
1931–1940
1941–1950
1951–1960
1961–1970
1971–1980
1981–1990
1991–2000
2003–2012

10 year Summer Teletherm dynamics for PORT JERVIS, NY:

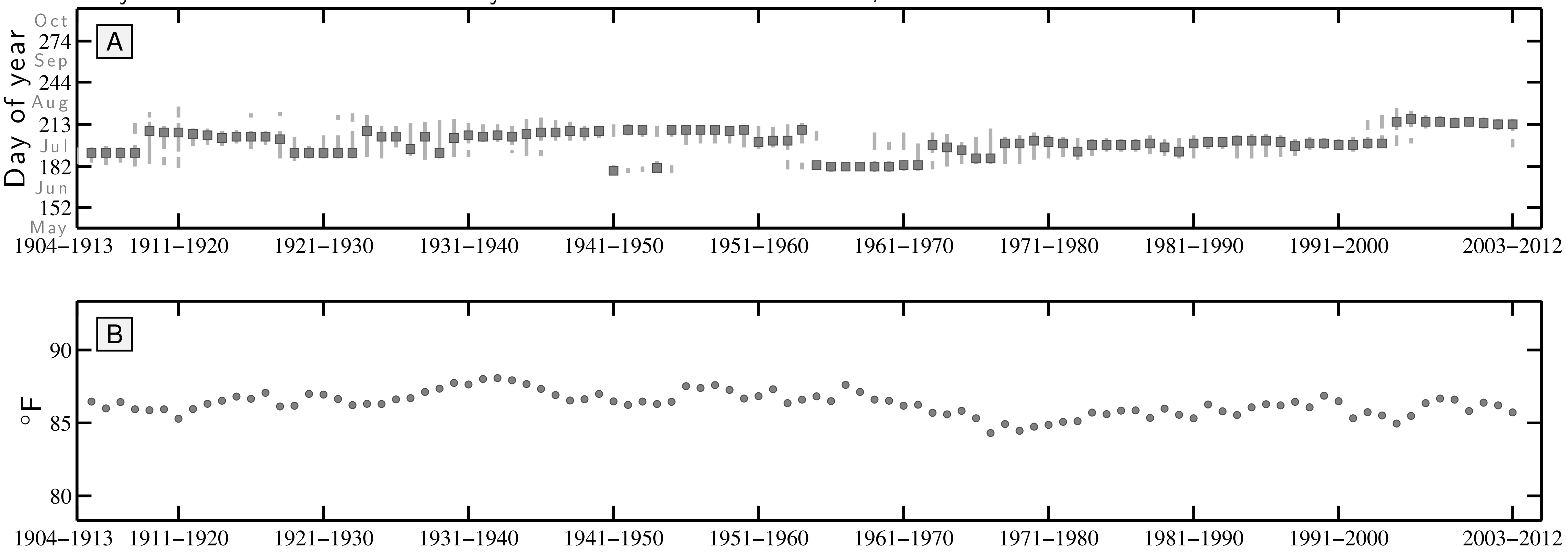

10 year Winter Teletherm dynamics for PORT JERVIS, NY:

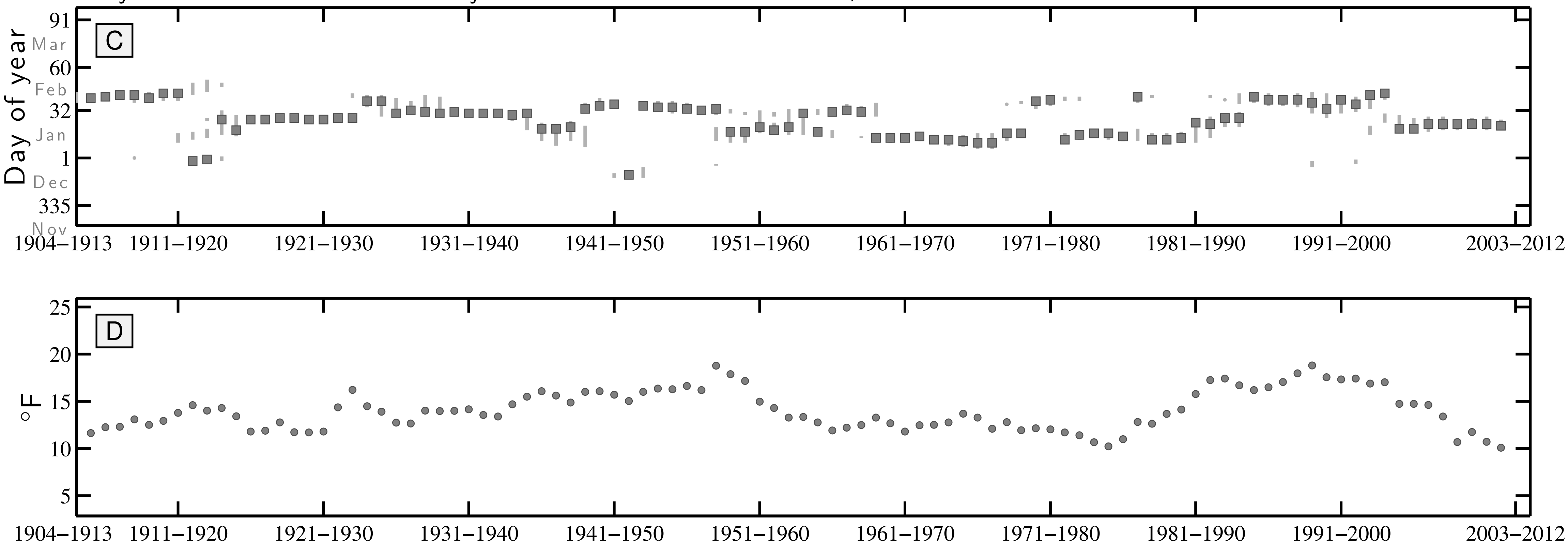

10 year Summer Teletherm dynamics for POUGHKEEPSIE, NY:

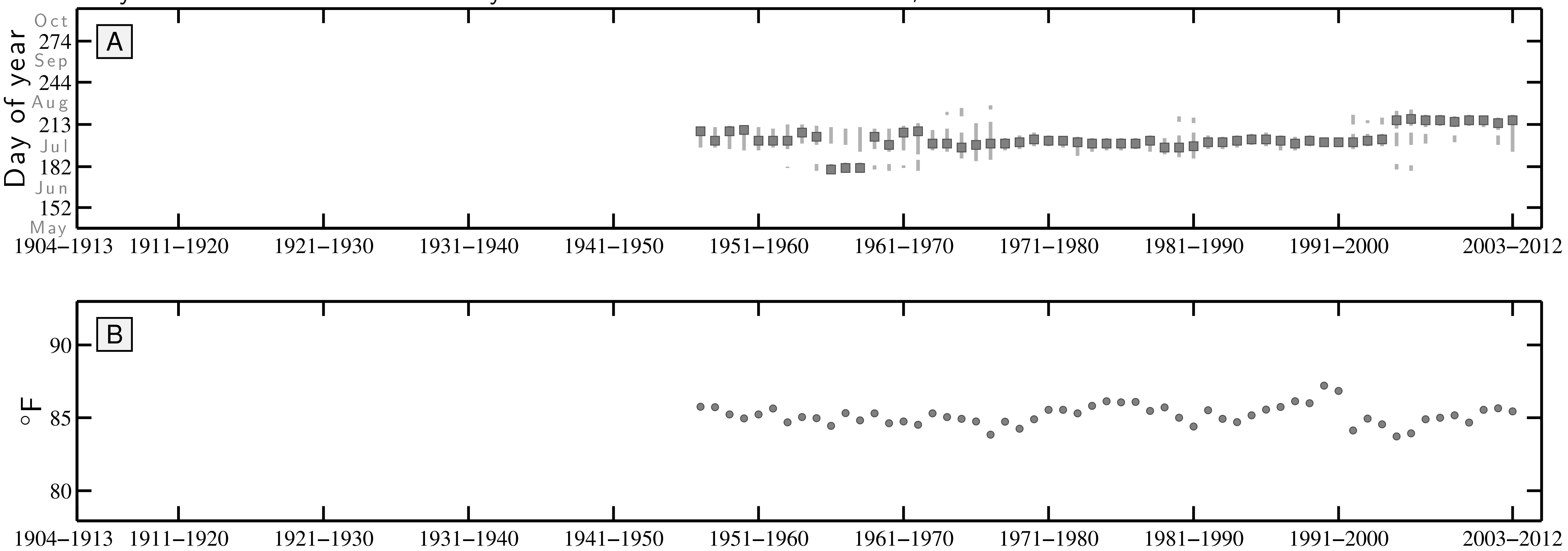

10 year Winter Teletherm dynamics for POUGHKEEPSIE, NY:

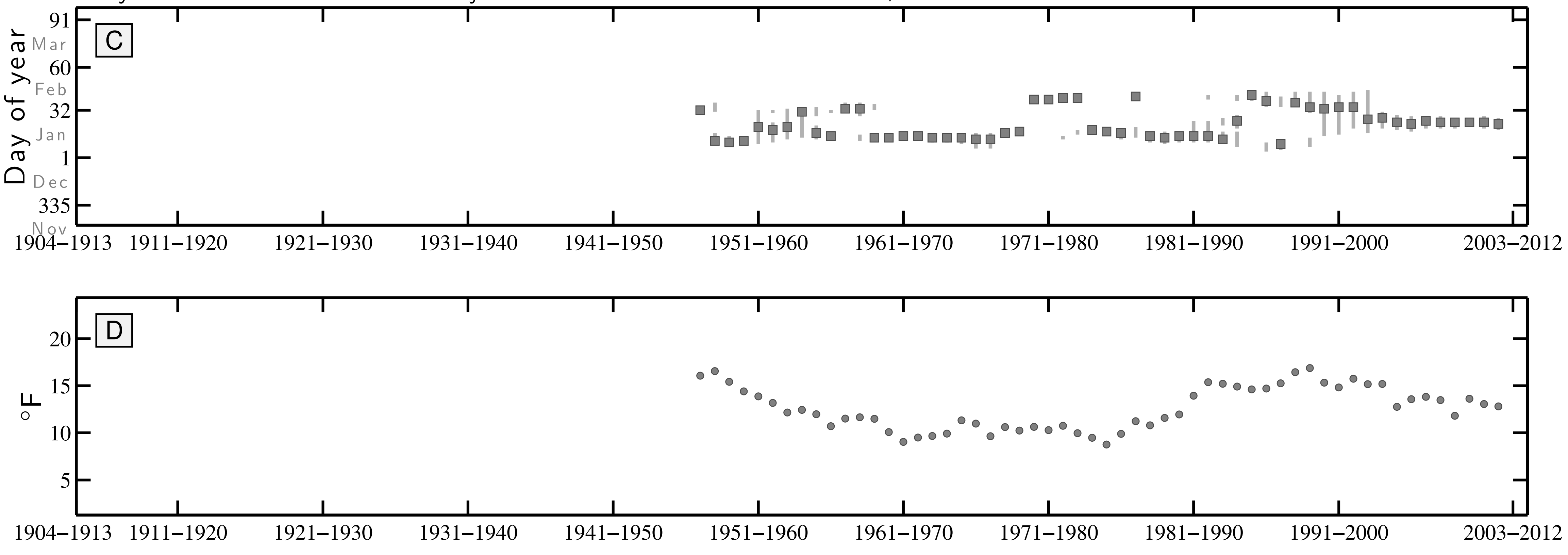

10 year Summer Teletherm dynamics for ROCHESTER INTL AP, NY:

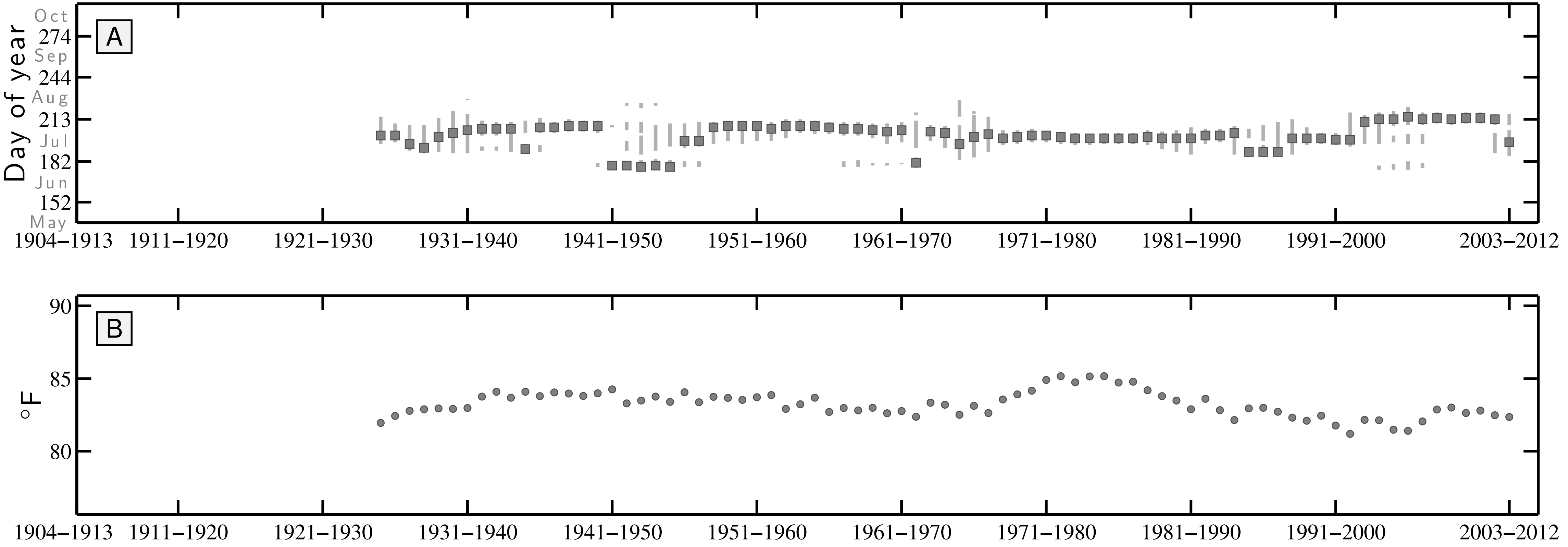

10 year Winter Teletherm dynamics for ROCHESTER INTL AP, NY:

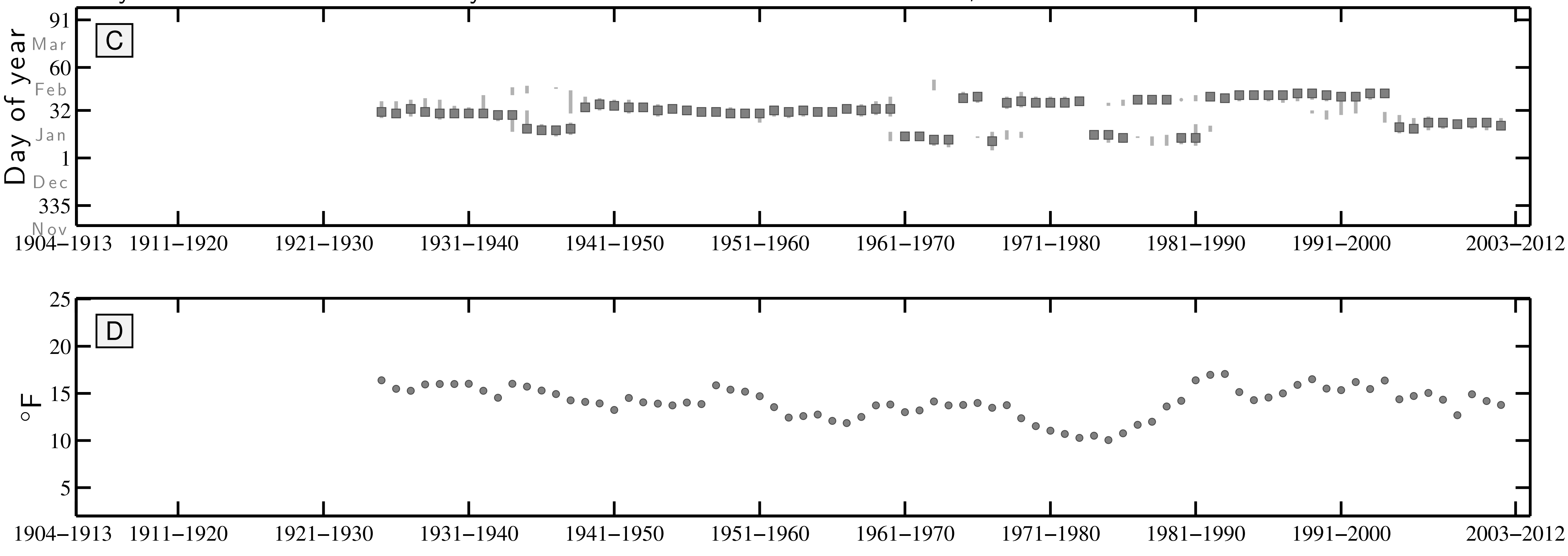

10 year Summer Teletherm dynamics for SARATOGA SPRINGS 4 SW, NY:

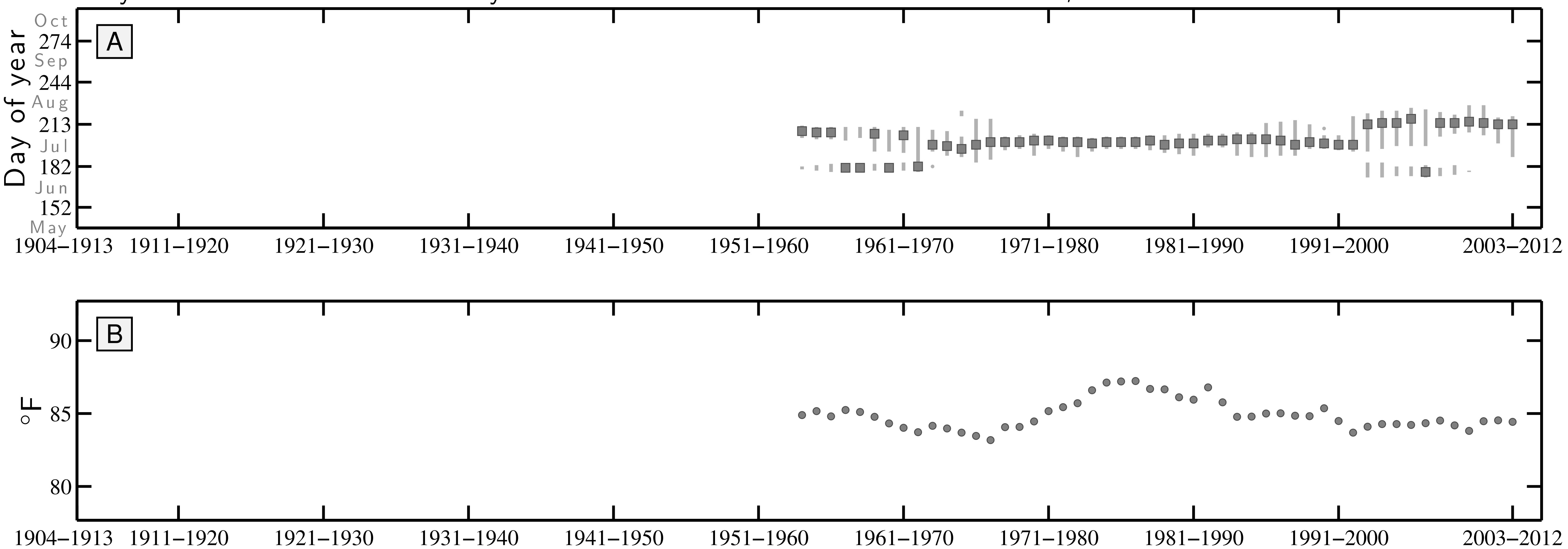

10 year Winter Teletherm dynamics for SARATOGA SPRINGS 4 SW, NY:

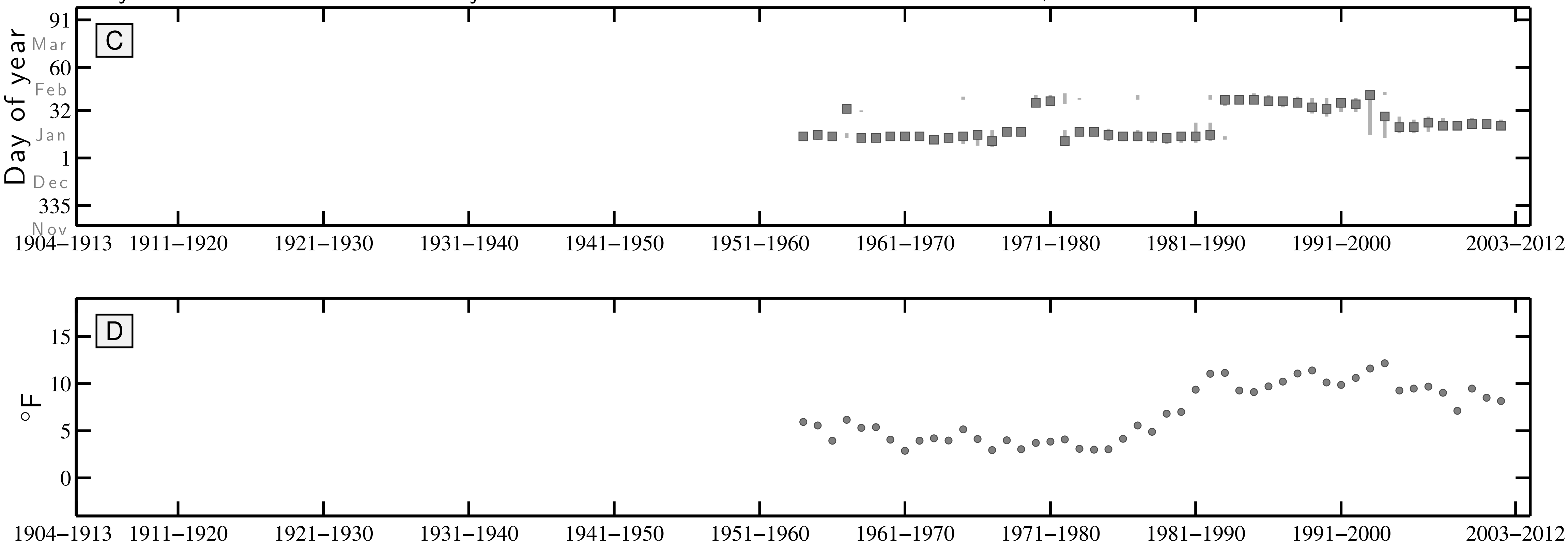

10 year Summer Teletherm dynamics for SETAUKET STRONG, NY:

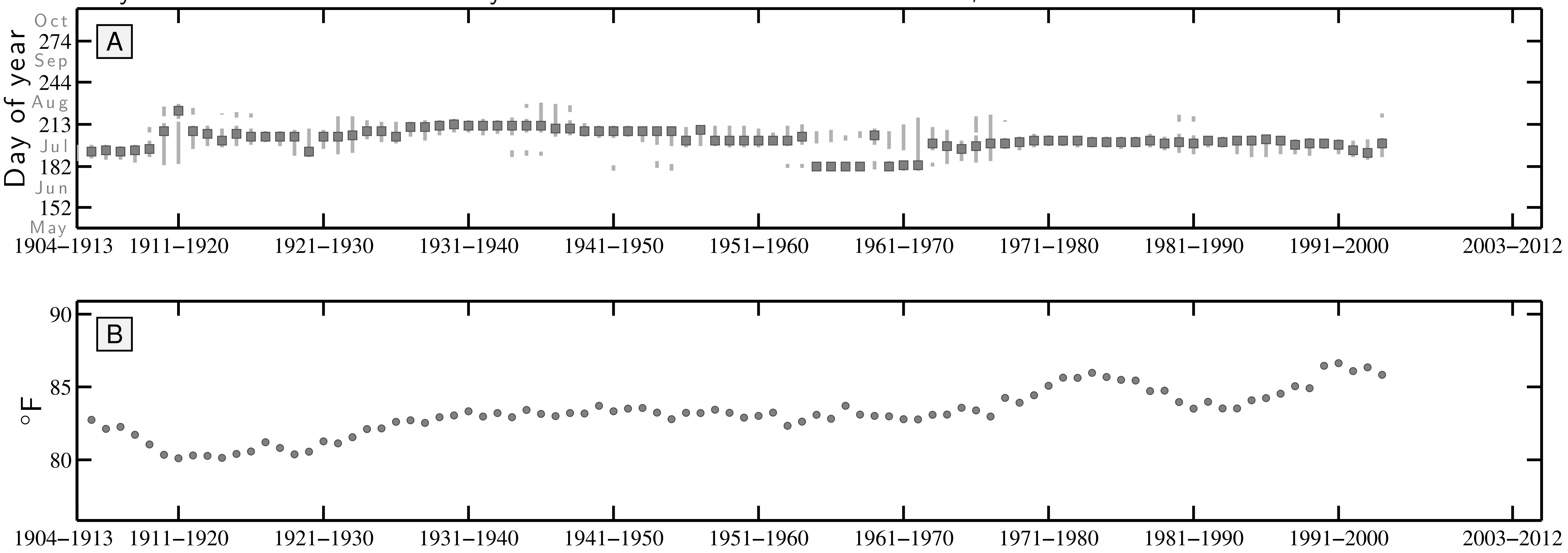

10 year Winter Teletherm dynamics for SETAUKET STRONG, NY:

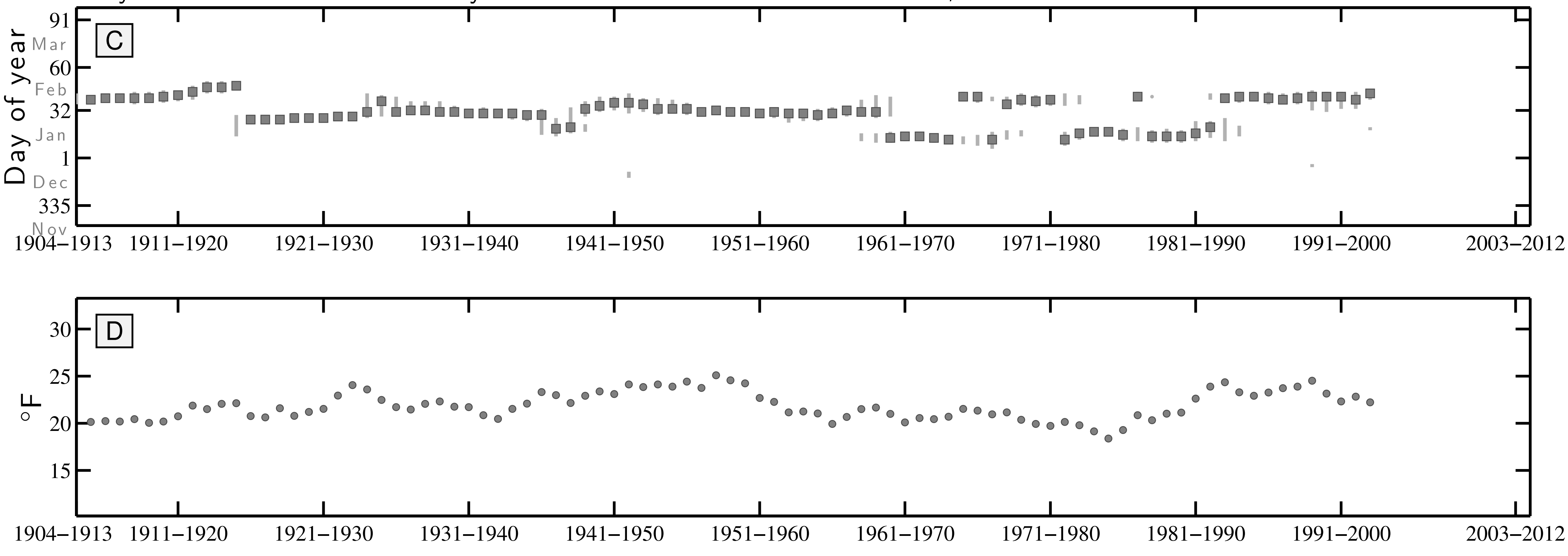

10 year Summer Teletherm dynamics for STILLWATER RSVR, NY:

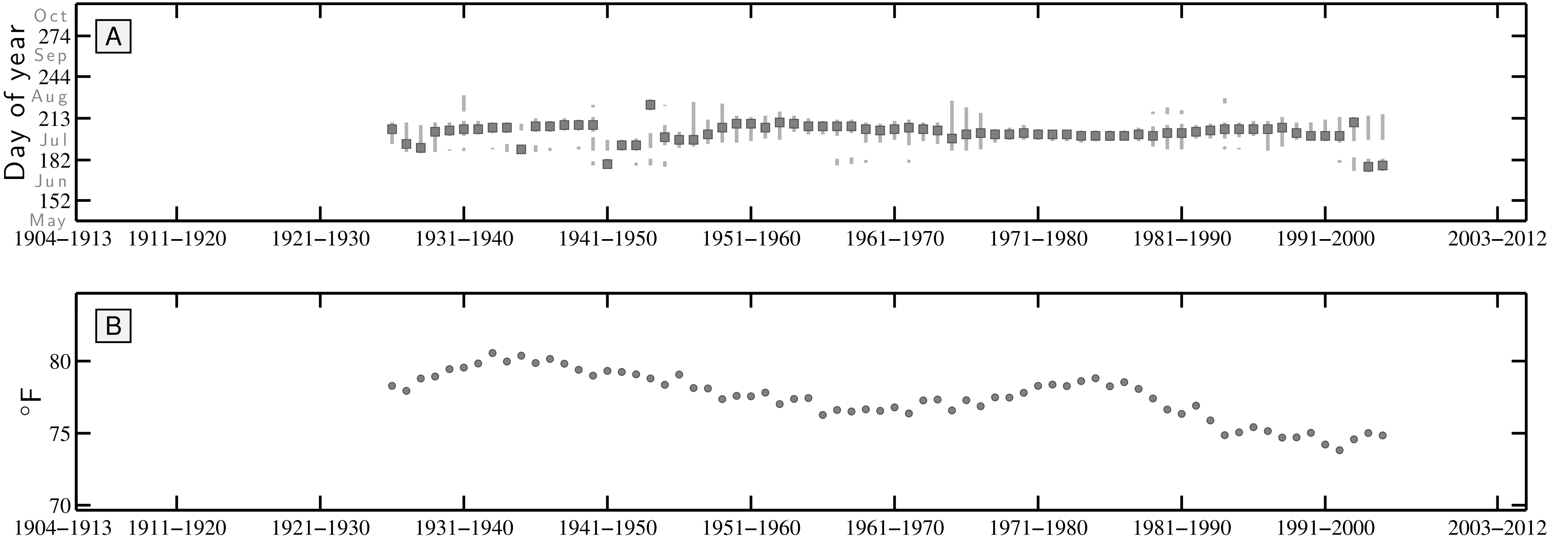

10 year Winter Teletherm dynamics for STILLWATER RSVR, NY:

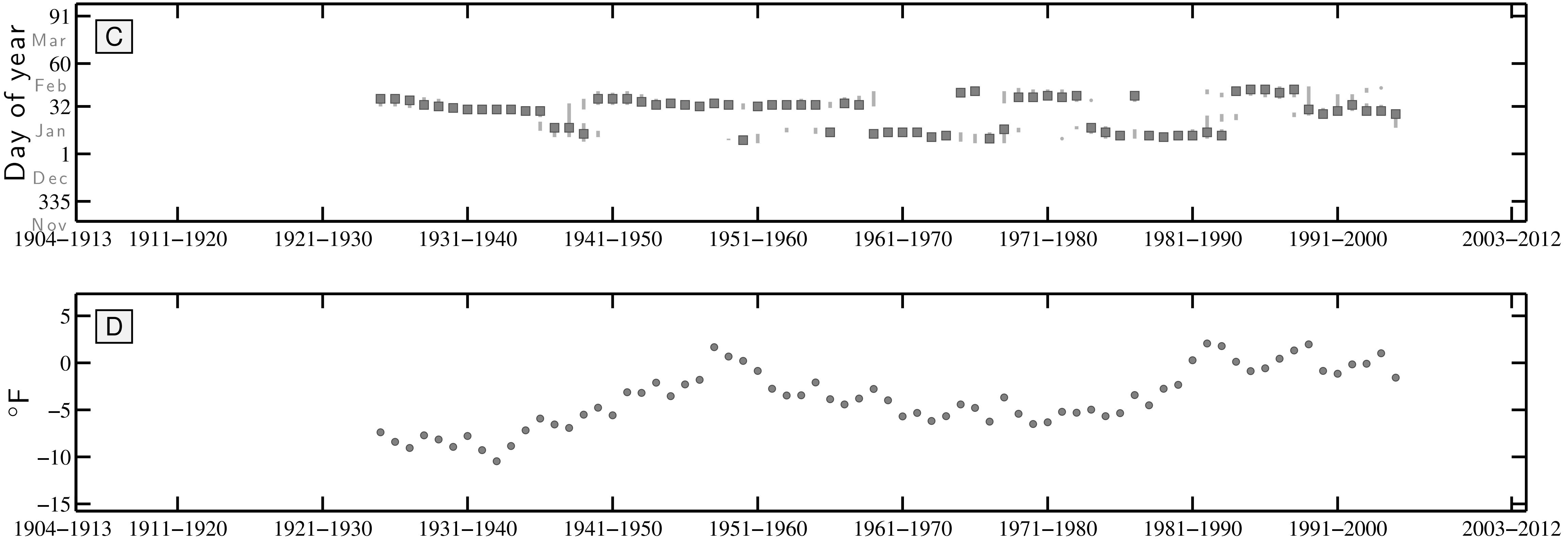

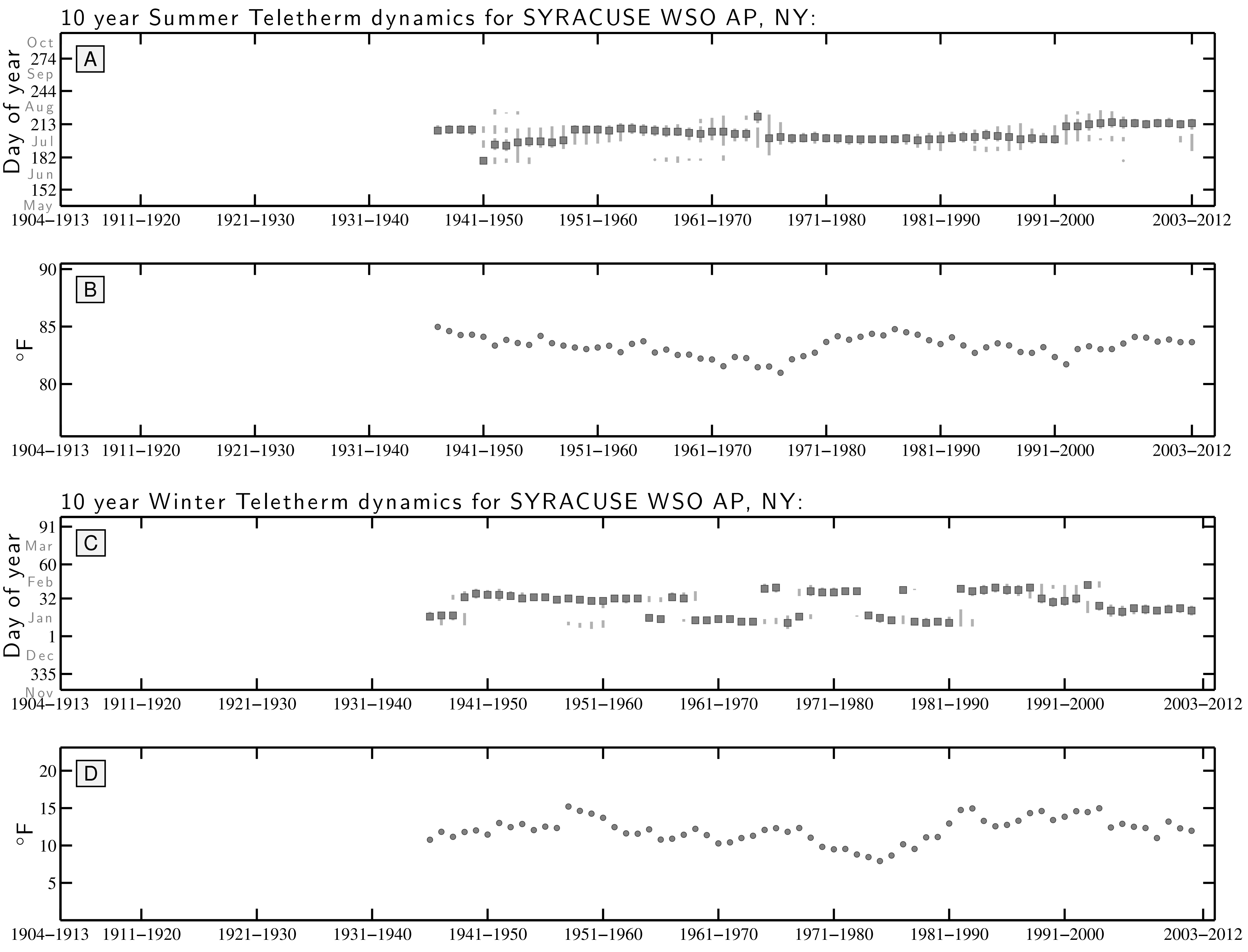
10 year Summer Teletherm dynamics for SYRACUSE WSO AP, NY:
A
B
Day of year
°F
Oct
274
Sep
244
Aug
213
Jul
182
Jun
152
May
90
85
80
1904–1913
1911–1920
1921–1930
1931–1940
1941–1950
1951–1960
1961–1970
1971–1980
1981–1990
1991–2000
2003–2012
10 year Winter Teletherm dynamics for SYRACUSE WSO AP, NY:
C
D
91
Mar
60
Feb
32
Jan
1
Dec
335
Nov
20
15
10
5

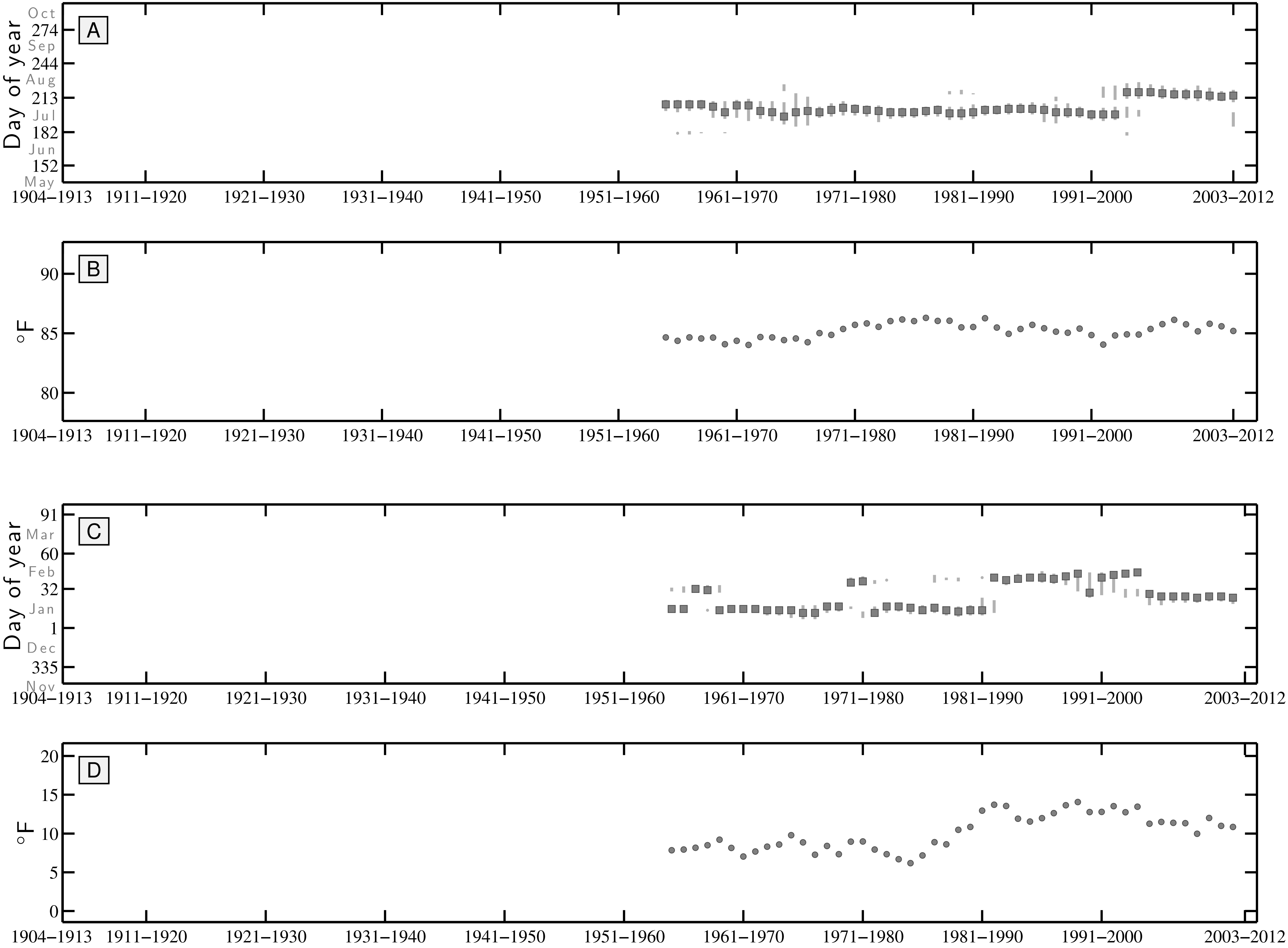
A
Day of year
Oct
274
Sep
244
Aug
213
Jul
182
Jun
152
May
1904–1913
1911–1920
1921–1930
1931–1940
1941–1950
1951–1960
1961–1970
1971–1980
1981–1990
1991–2000
2003–2012
B
°F
90
85
80
C
91
Mar
60
Feb
32
Jan
1
Dec
335
Nov
D
20
15
10
5
0

10 year Summer Teletherm dynamics for TUPPER LAKE SUNMOUNT, NY:

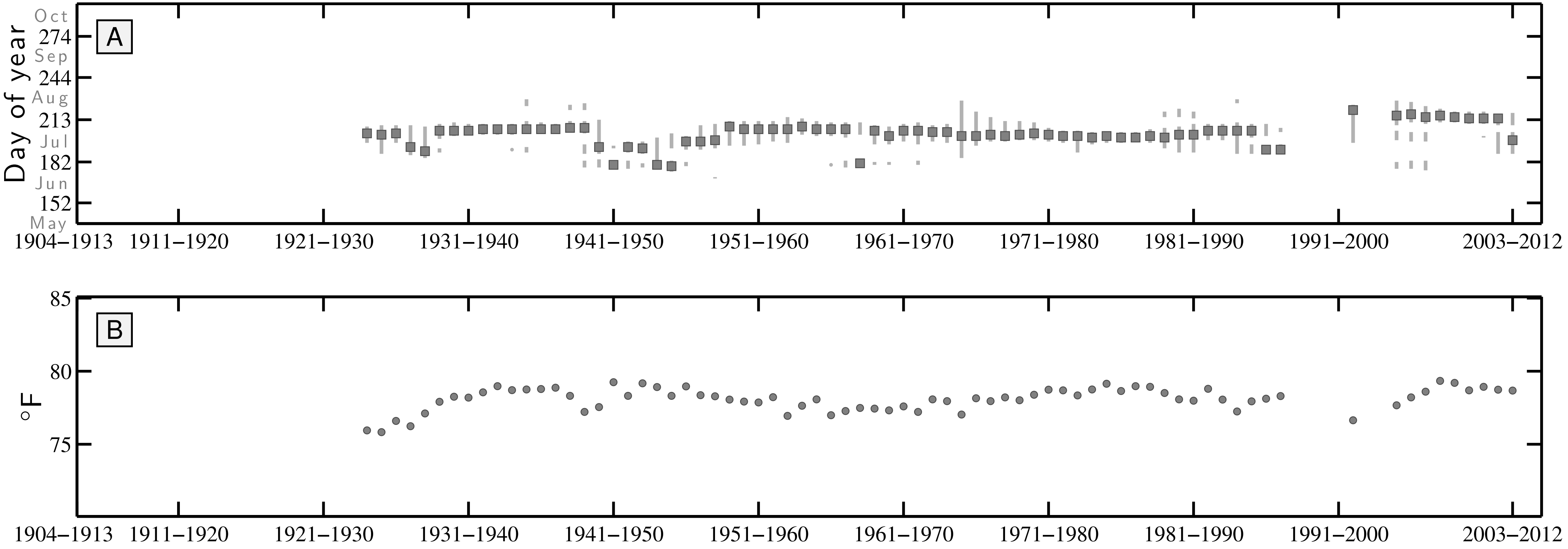

10 year Winter Teletherm dynamics for TUPPER LAKE SUNMOUNT, NY:

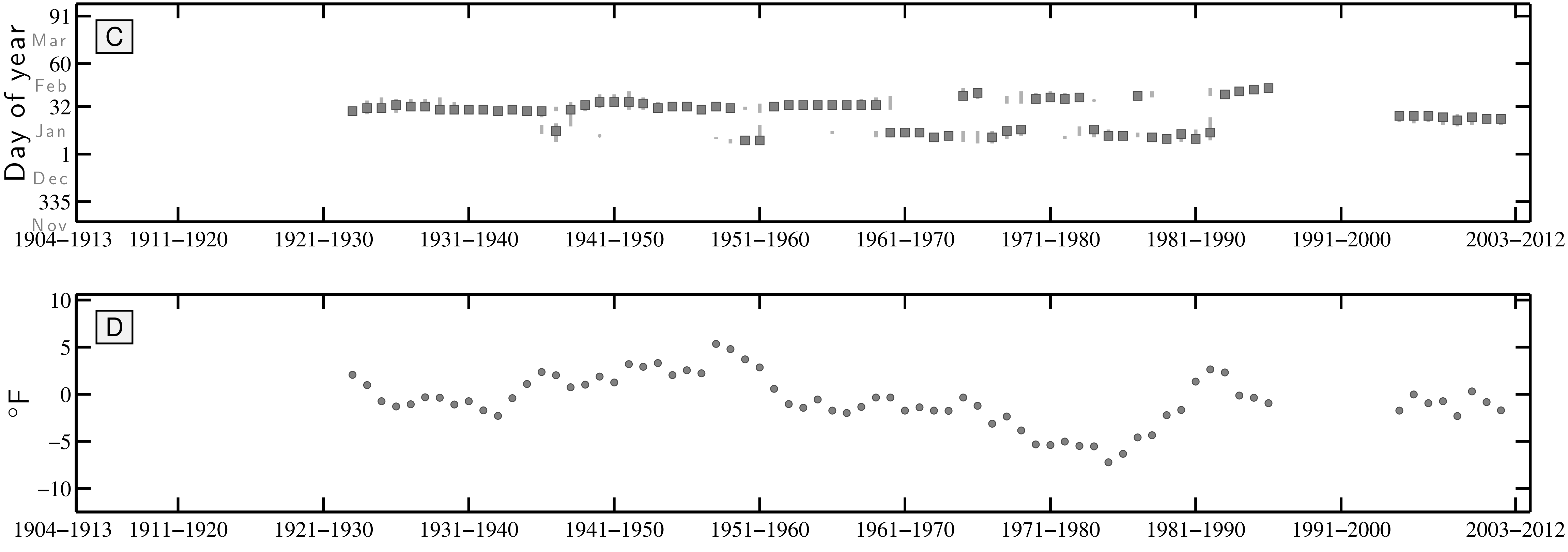

10 year Summer Teletherm dynamics for UTICA FAA AP, NY:

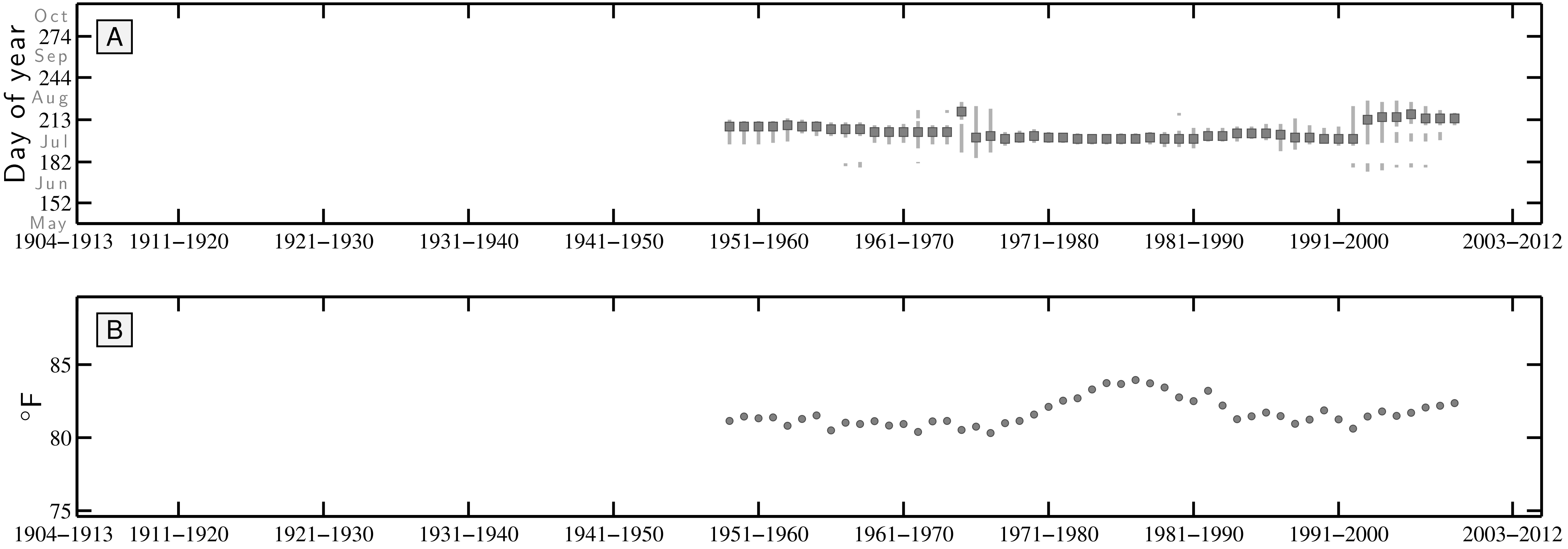

10 year Winter Teletherm dynamics for UTICA FAA AP, NY:

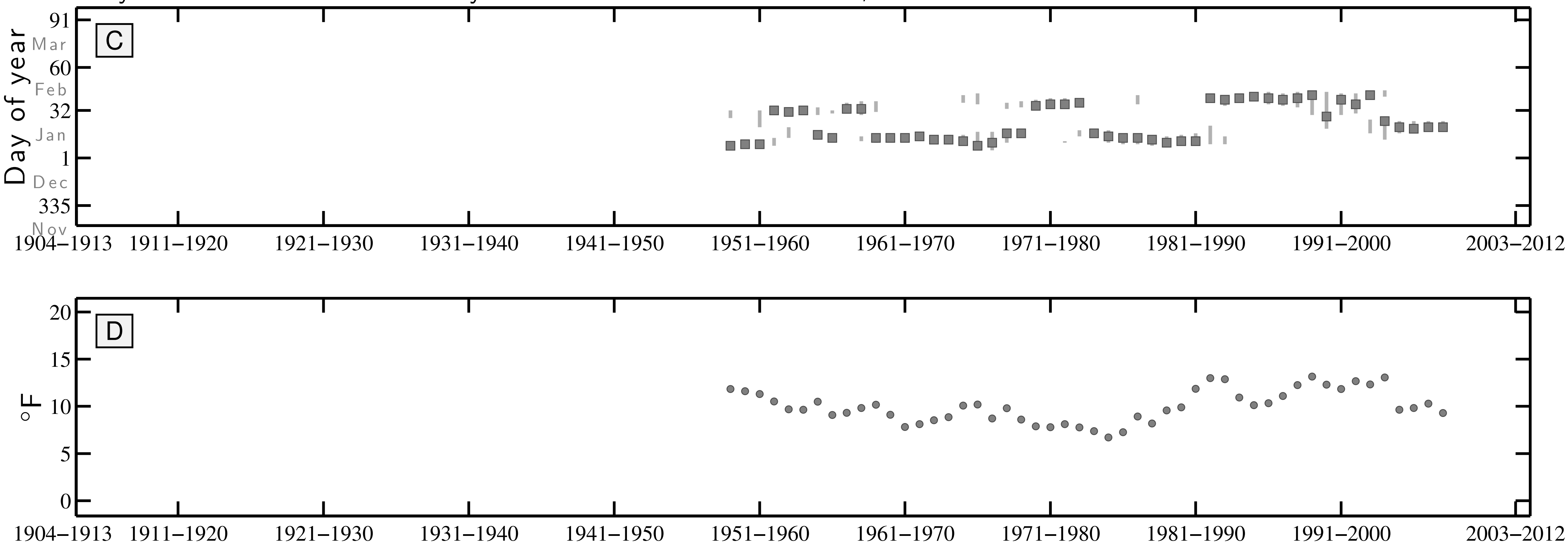

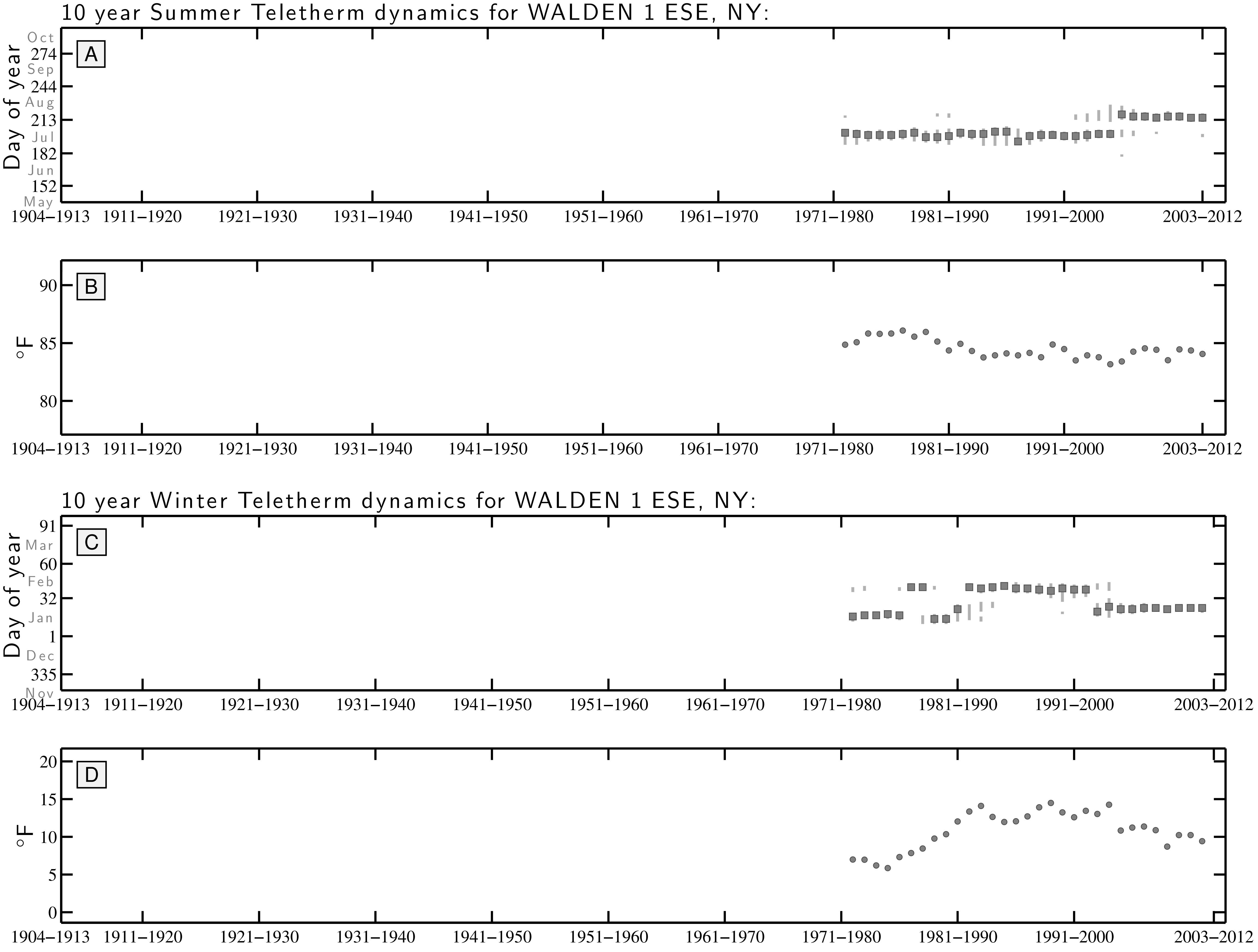
10 year Summer Teletherm dynamics for WALDEN 1 ESE, NY:
A
B
Day of year
°F
Oct
274
Sep
244
Aug
213
Jul
182
Jun
152
May
90
85
80
1904–1913
1911–1920
1921–1930
1931–1940
1941–1950
1951–1960
1961–1970
1971–1980
1981–1990
1991–2000
2003–2012
10 year Winter Teletherm dynamics for WALDEN 1 ESE, NY:
C
D
91
Mar
60
Feb
32
Jan
1
Dec
335
Nov
20
15
10
5
0

10 year Summer Teletherm dynamics for WALES, NY:

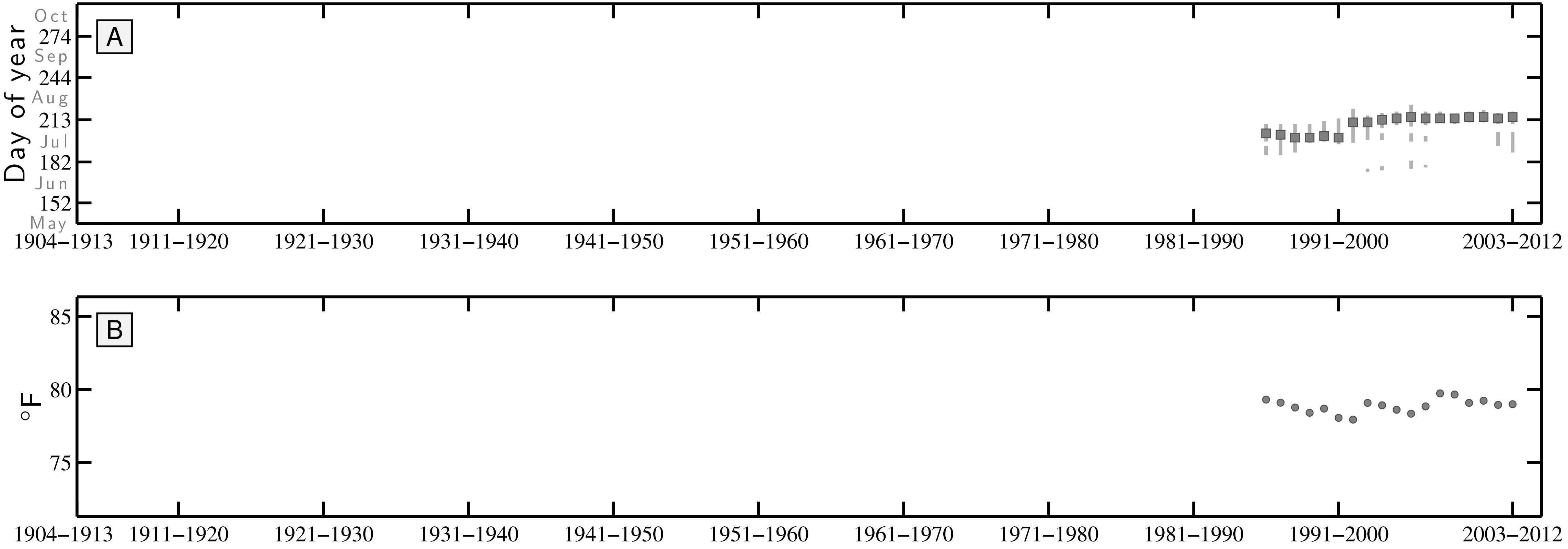

10 year Winter Teletherm dynamics for WALES, NY:

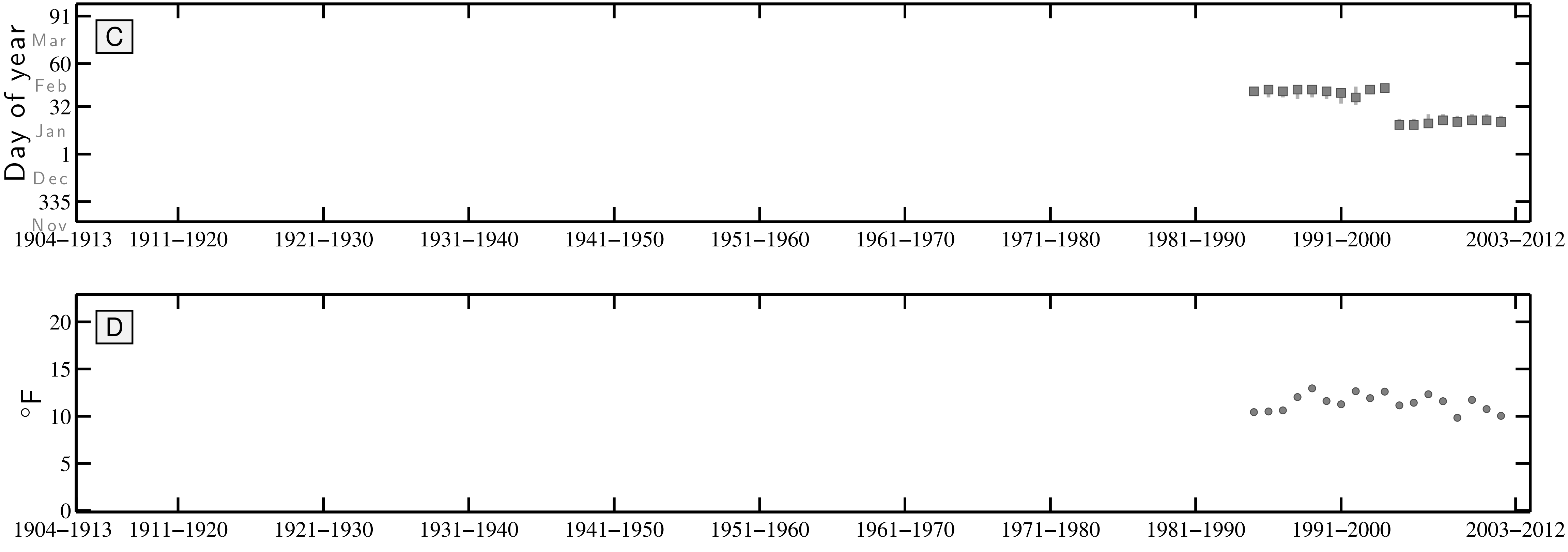

10 year Summer Teletherm dynamics for WANAKENA RNGR SCHOOL, NY:

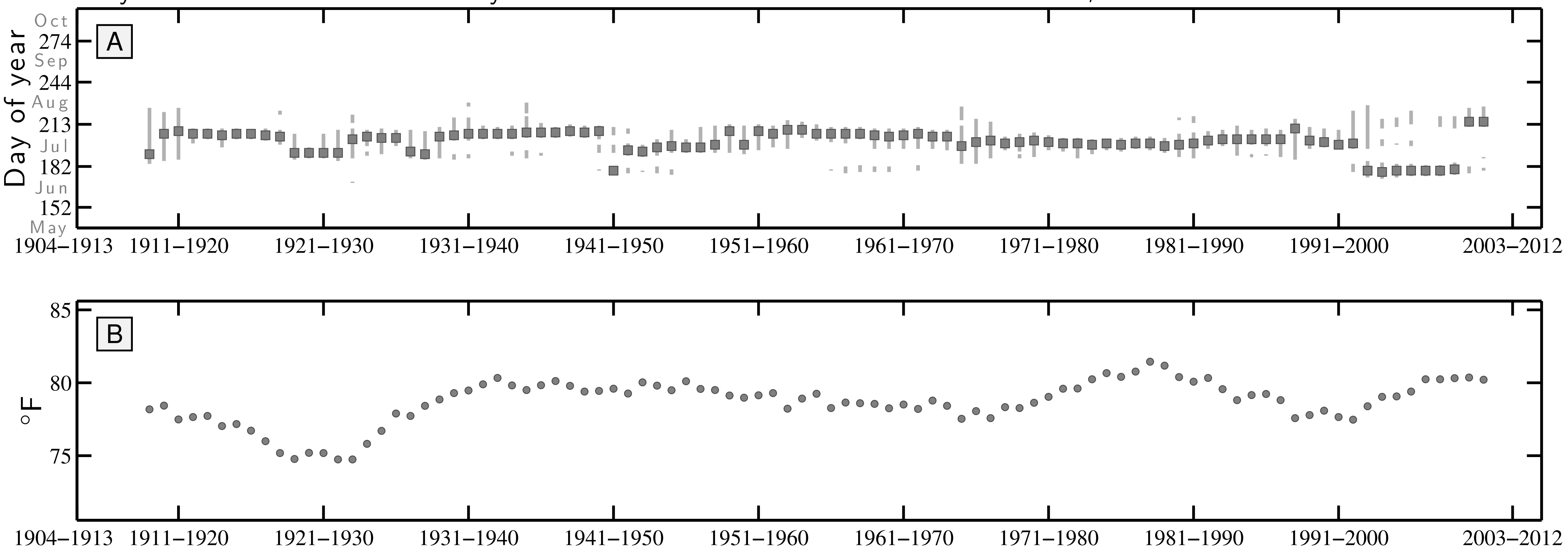

10 year Winter Teletherm dynamics for WANAKENA RNGR SCHOOL, NY:

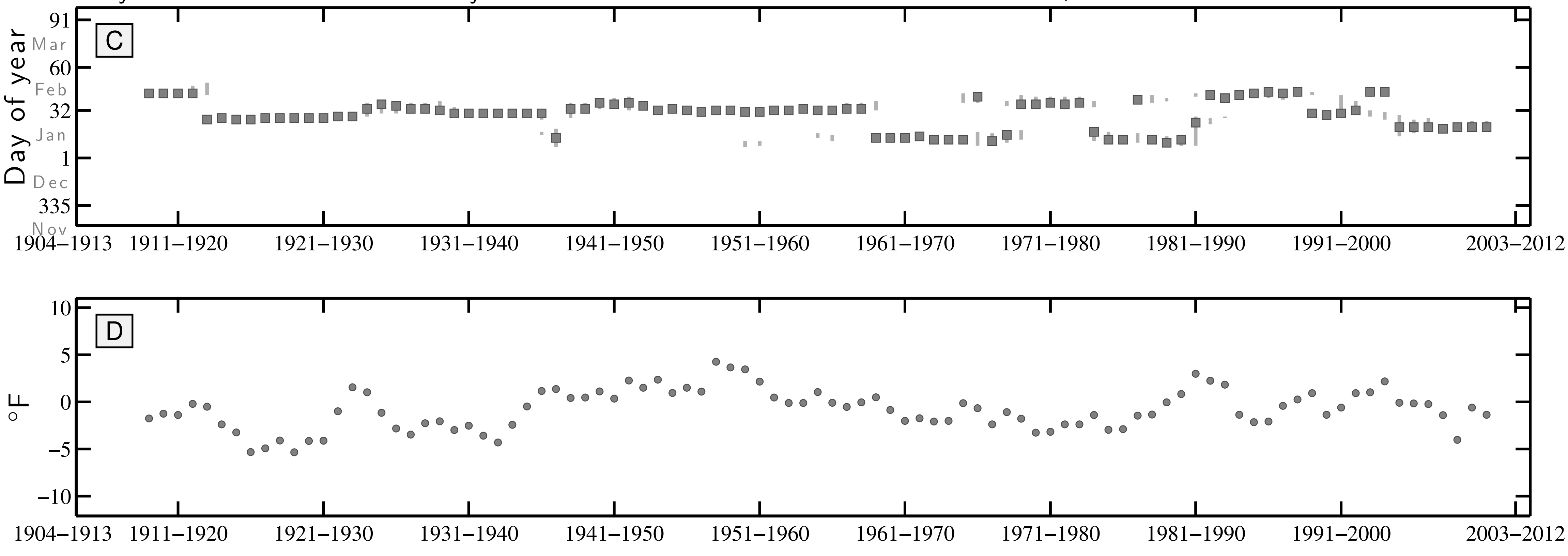

10 year Summer Teletherm dynamics for WATERTOWN, NY:

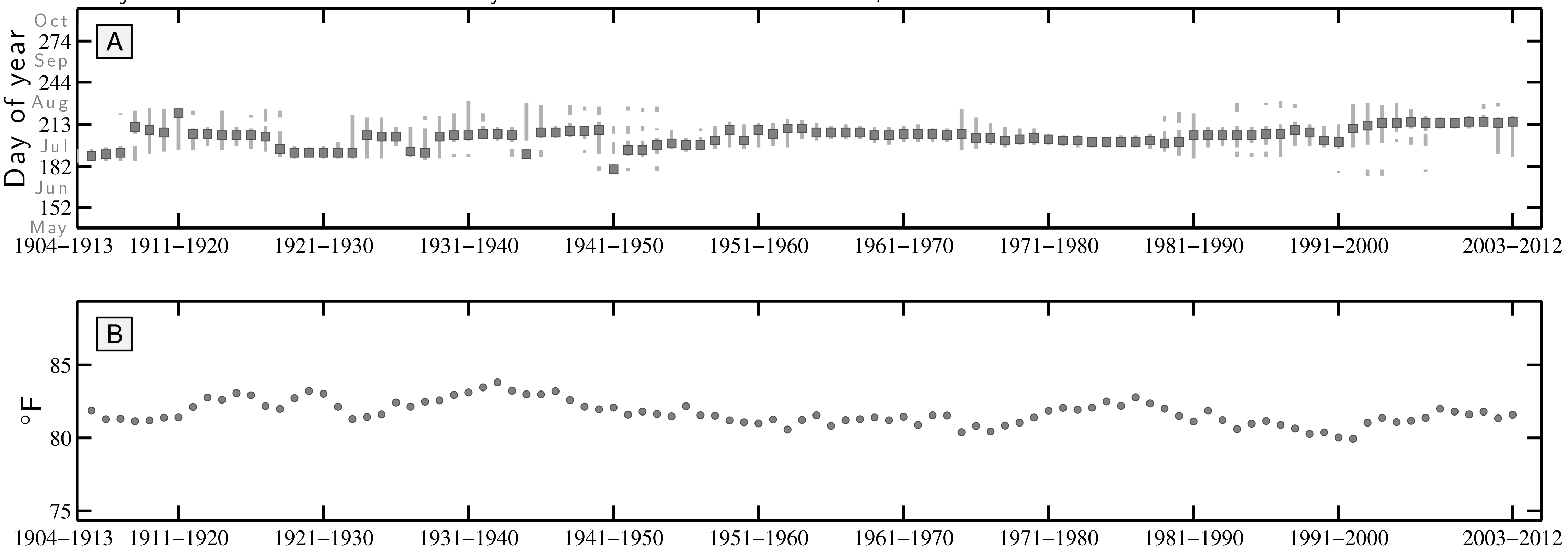

10 year Winter Teletherm dynamics for WATERTOWN, NY:

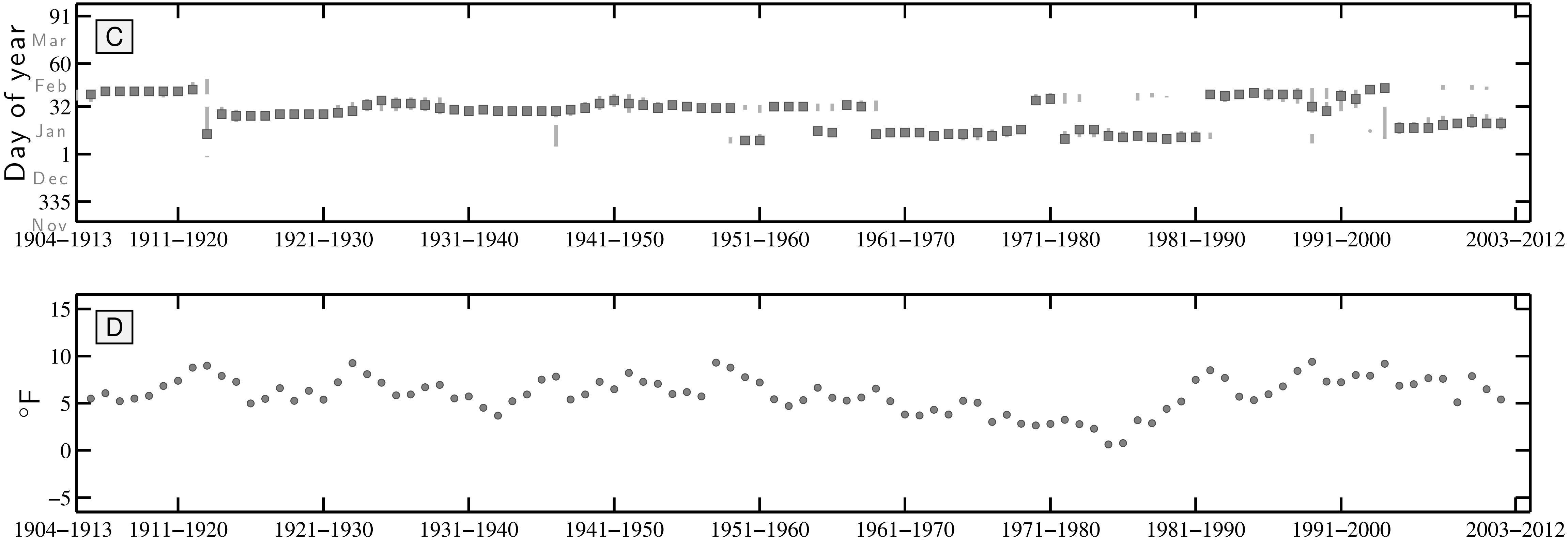

10 year Summer Teletherm dynamics for WEST POINT, NY:

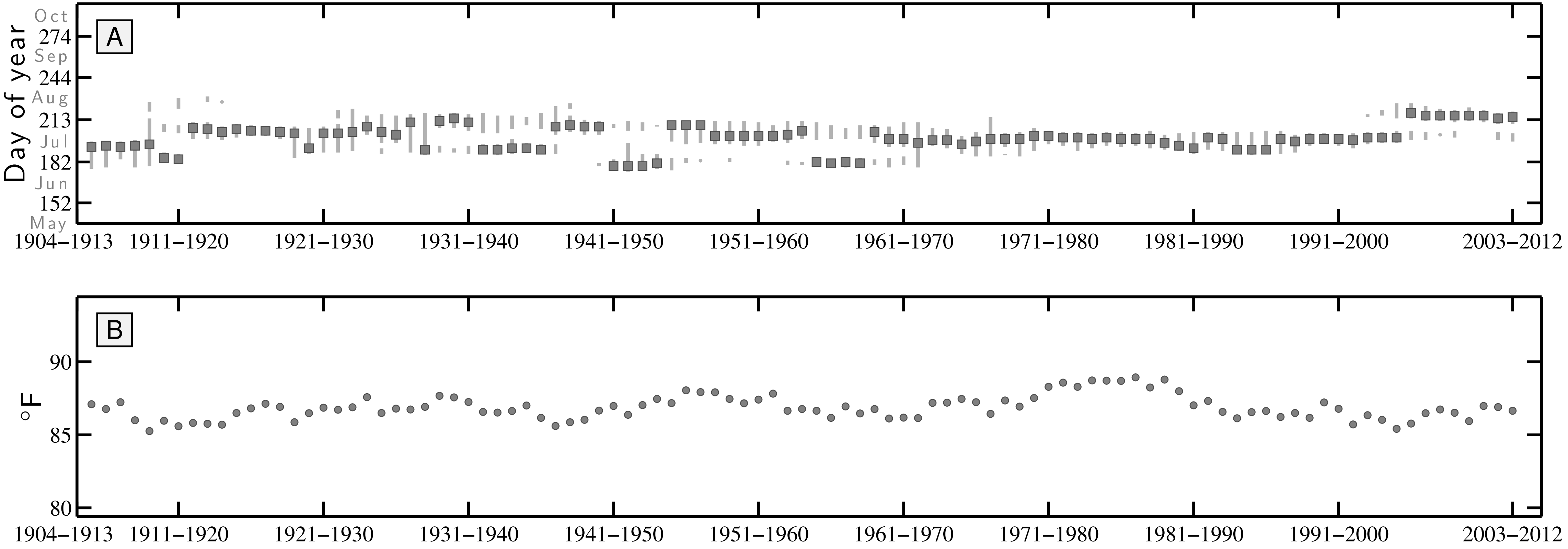

10 year Winter Teletherm dynamics for WEST POINT, NY:

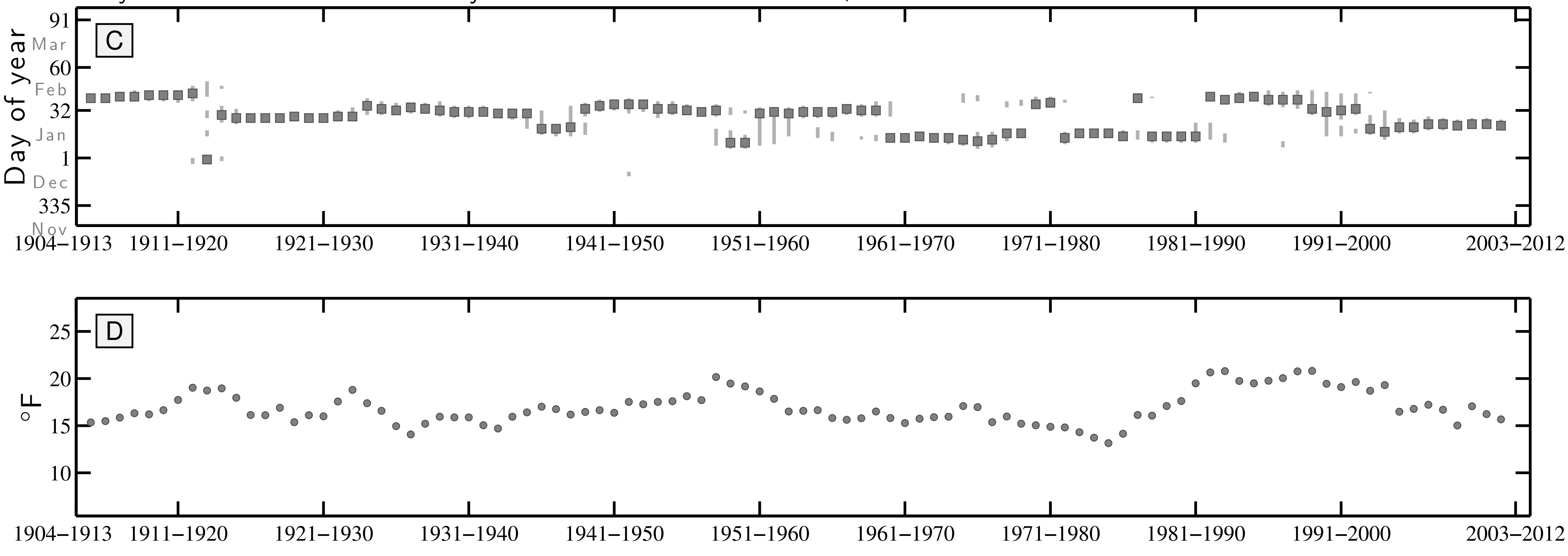

10 year Summer Teletherm dynamics for YORKTOWN HEIGHTS 1 W, NY:

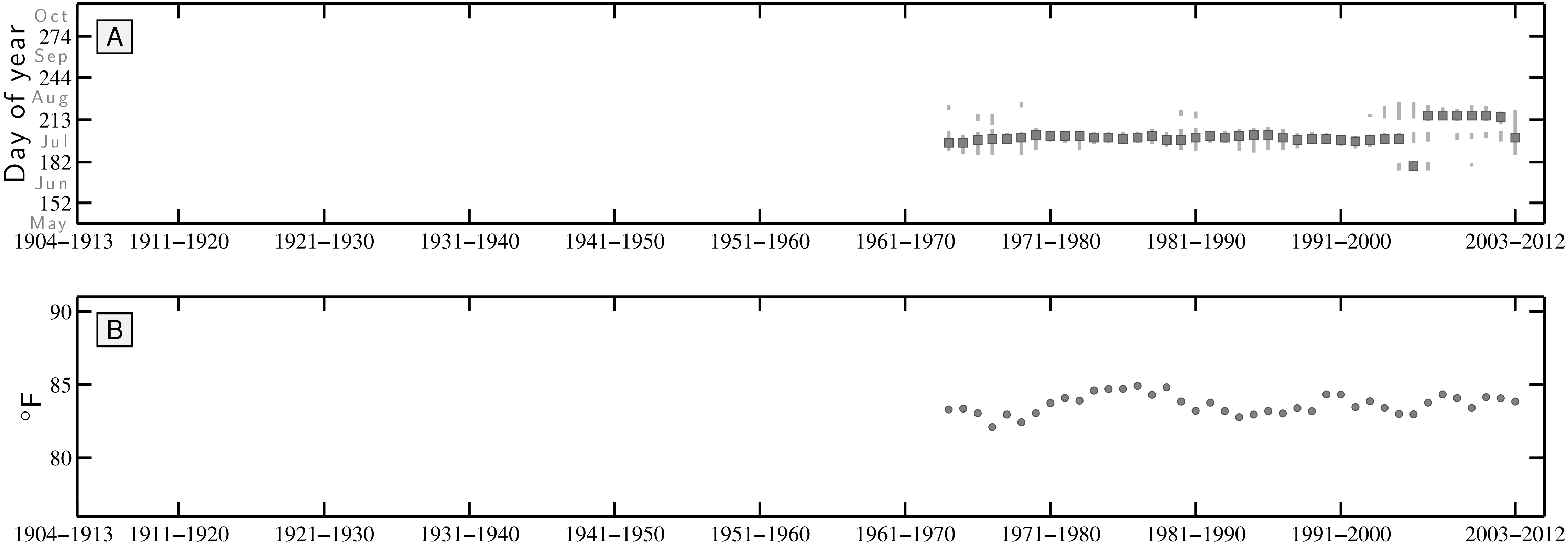

10 year Winter Teletherm dynamics for YORKTOWN HEIGHTS 1 W, NY:

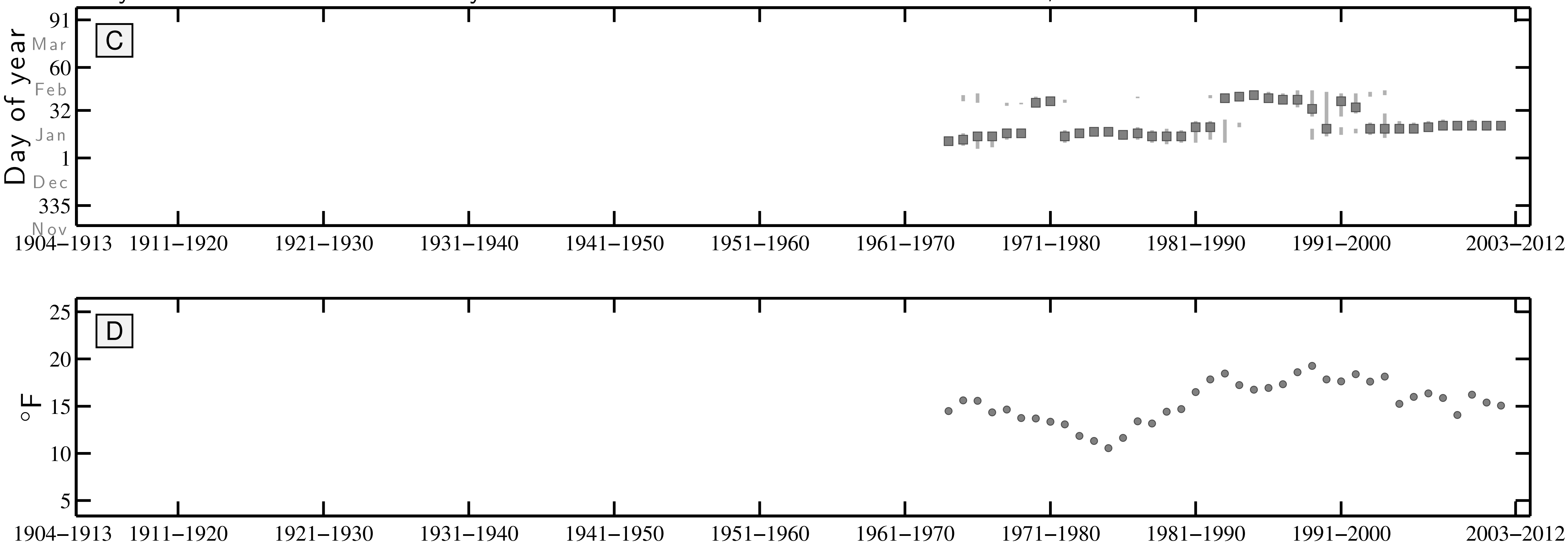

10 year Summer Teletherm dynamics for ALBEMARLE, NC:

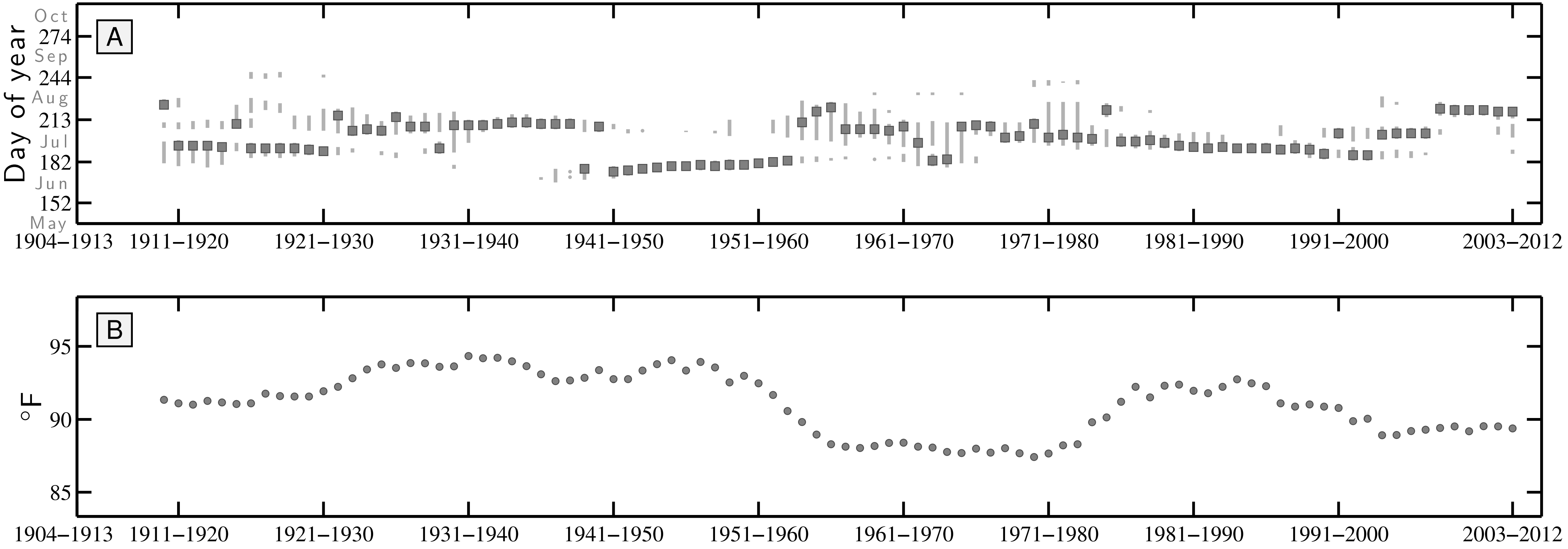

10 year Winter Teletherm dynamics for ALBEMARLE, NC:

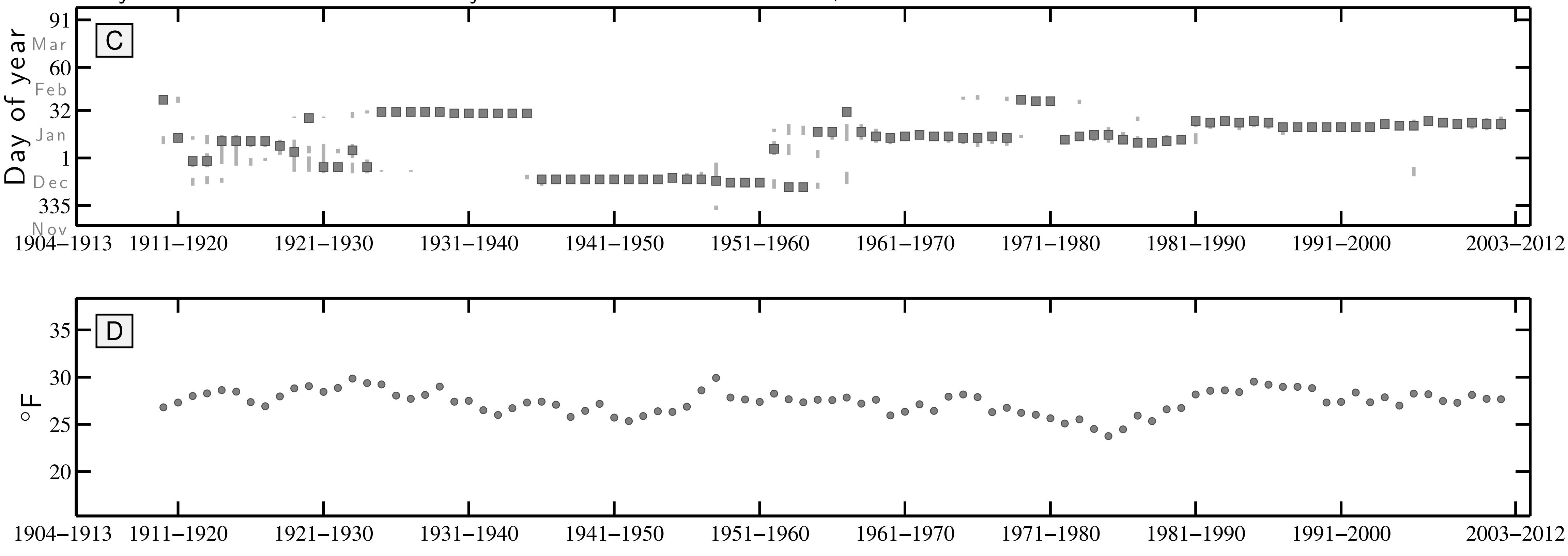

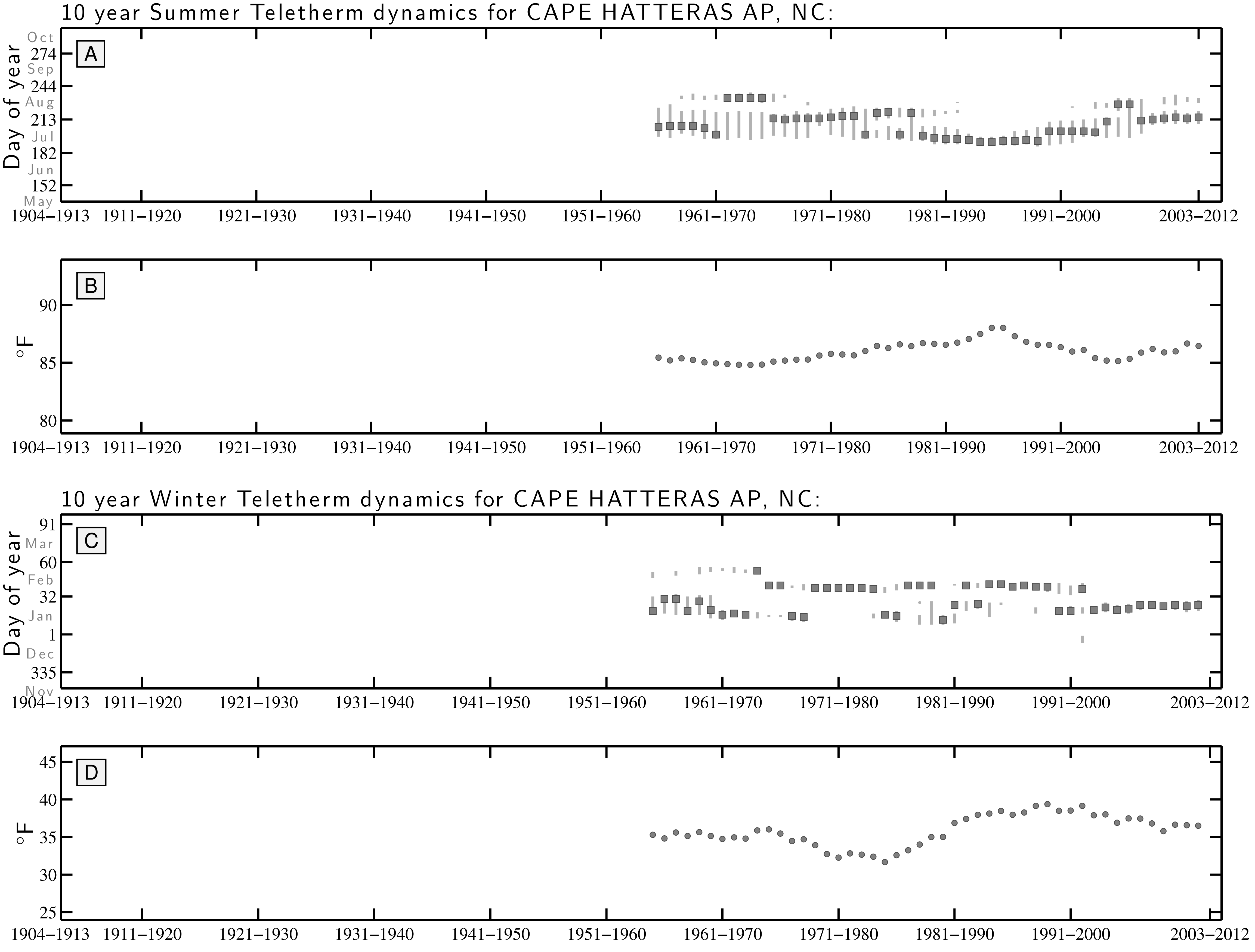

10 year Summer Teletherm dynamics for CAPE HATTERAS AP, NC:
A
Day of year
Oct
274
Sep
244
Aug
213
Jul
182
Jun
152
May
1904–1913
1911–1920
1921–1930
1931–1940
1941–1950
1951–1960
1961–1970
1971–1980
1981–1990
1991–2000
2003–2012
B
°F
90
85
80
10 year Winter Teletherm dynamics for CAPE HATTERAS AP, NC:
C
91
Mar
60
Feb
32
Jan
1
Dec
335
Nov
D
45
40
35
30
25

10 year Summer Teletherm dynamics for CHAPEL HILL 2 W, NC:

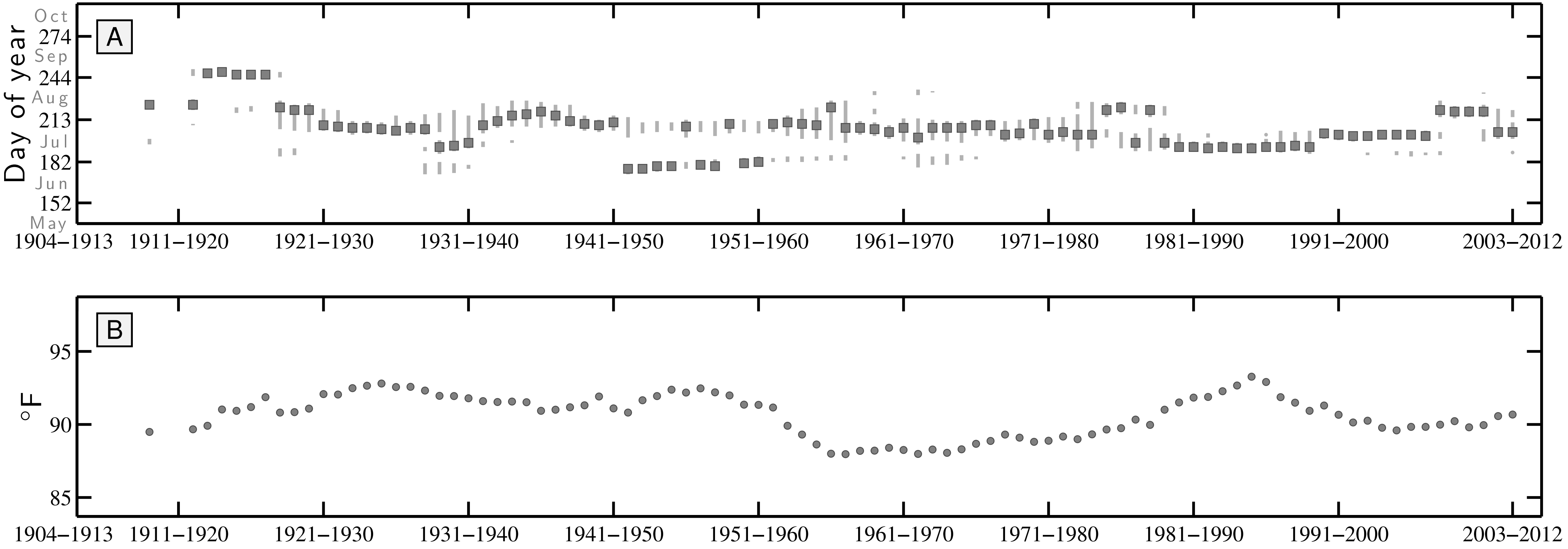

10 year Winter Teletherm dynamics for CHAPEL HILL 2 W, NC:

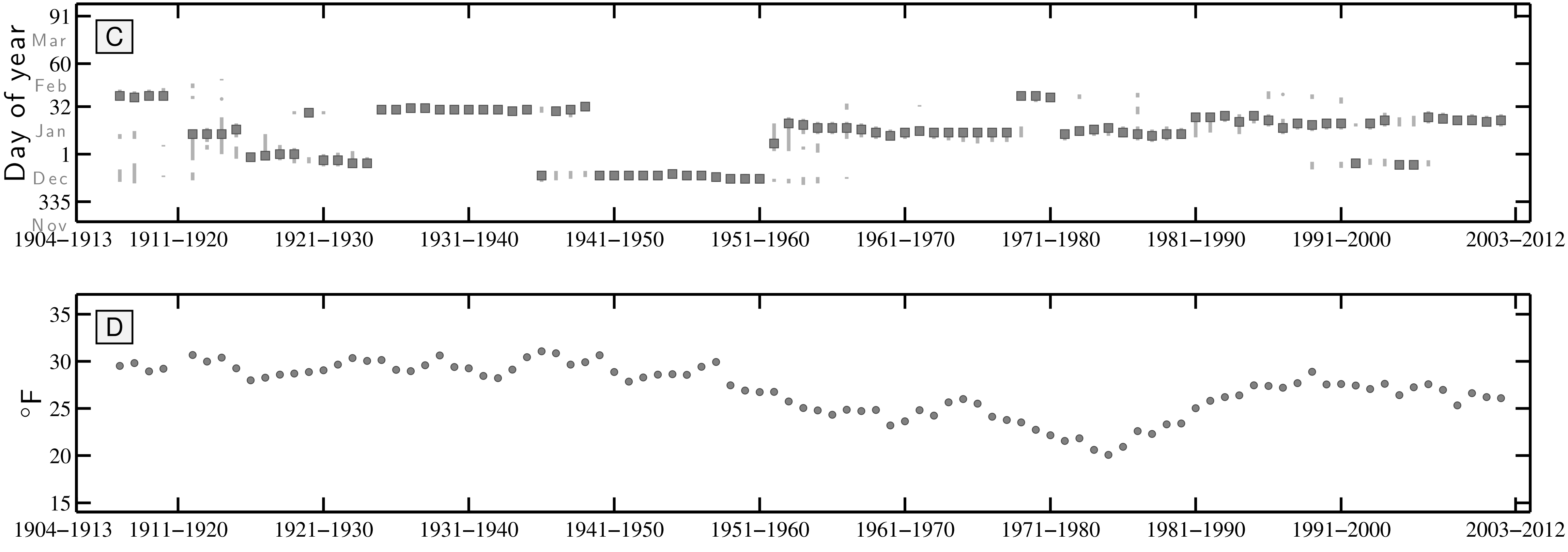

10 year Summer Teletherm dynamics for EDENTON, NC:

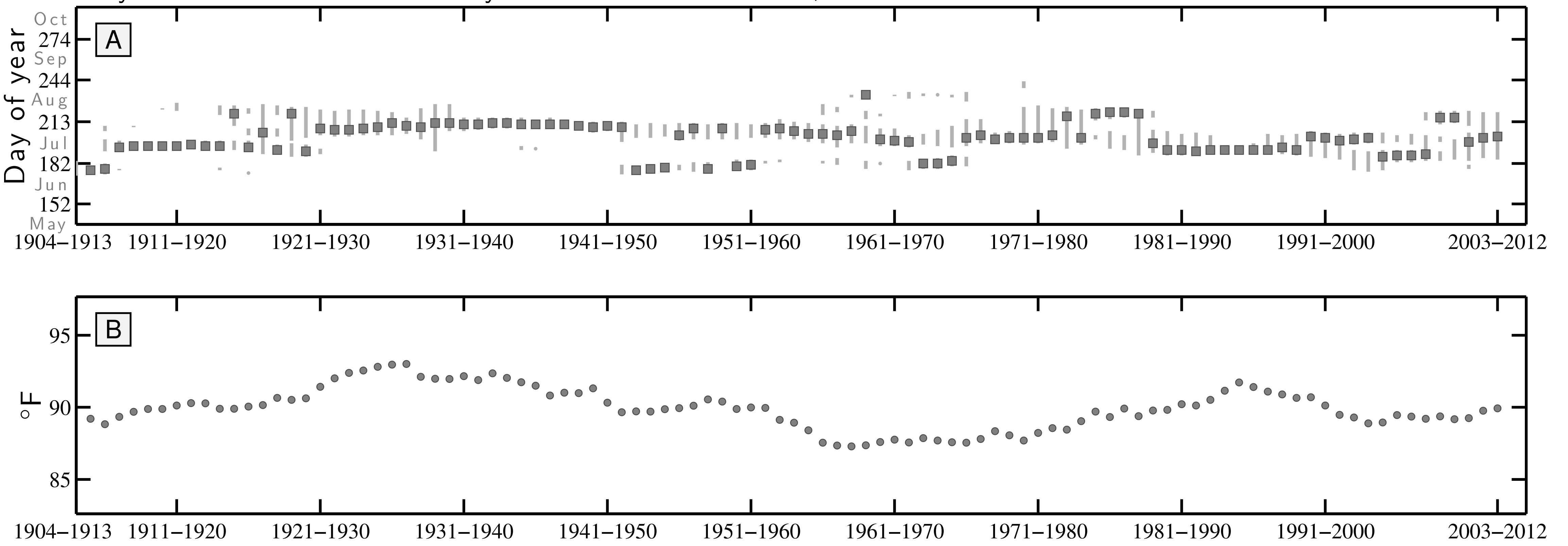

10 year Winter Teletherm dynamics for EDENTON, NC:

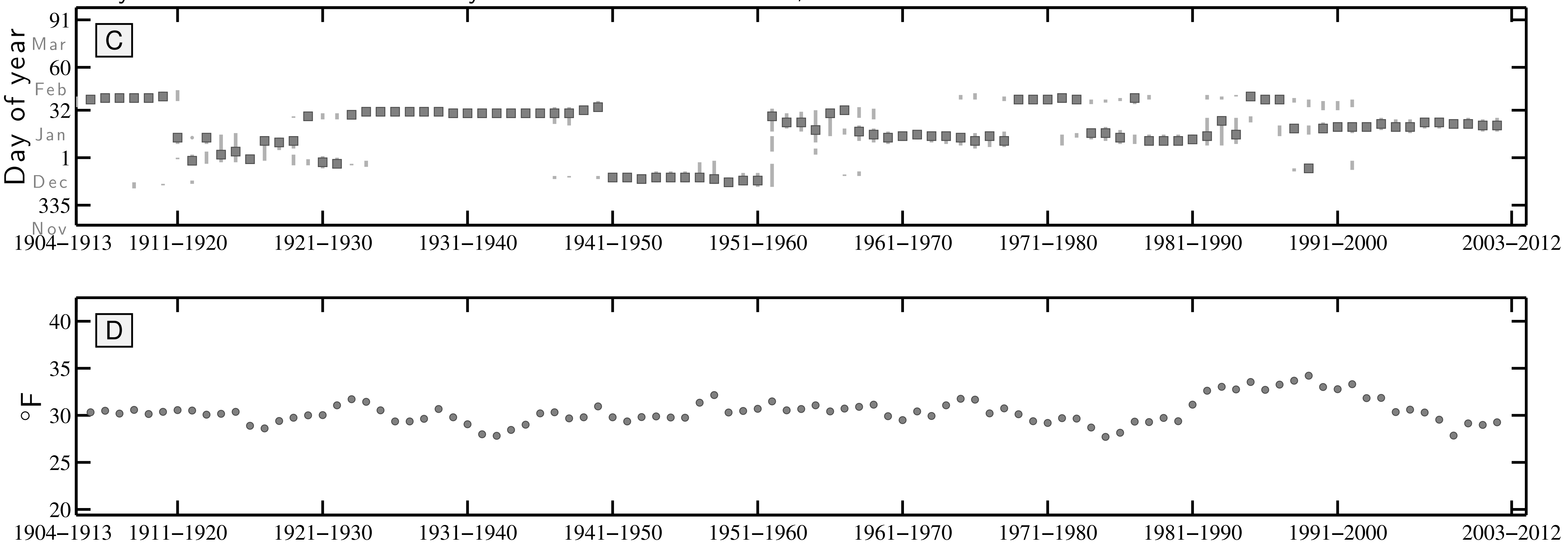

10 year Summer Teletherm dynamics for ELIZABETH CITY, NC:

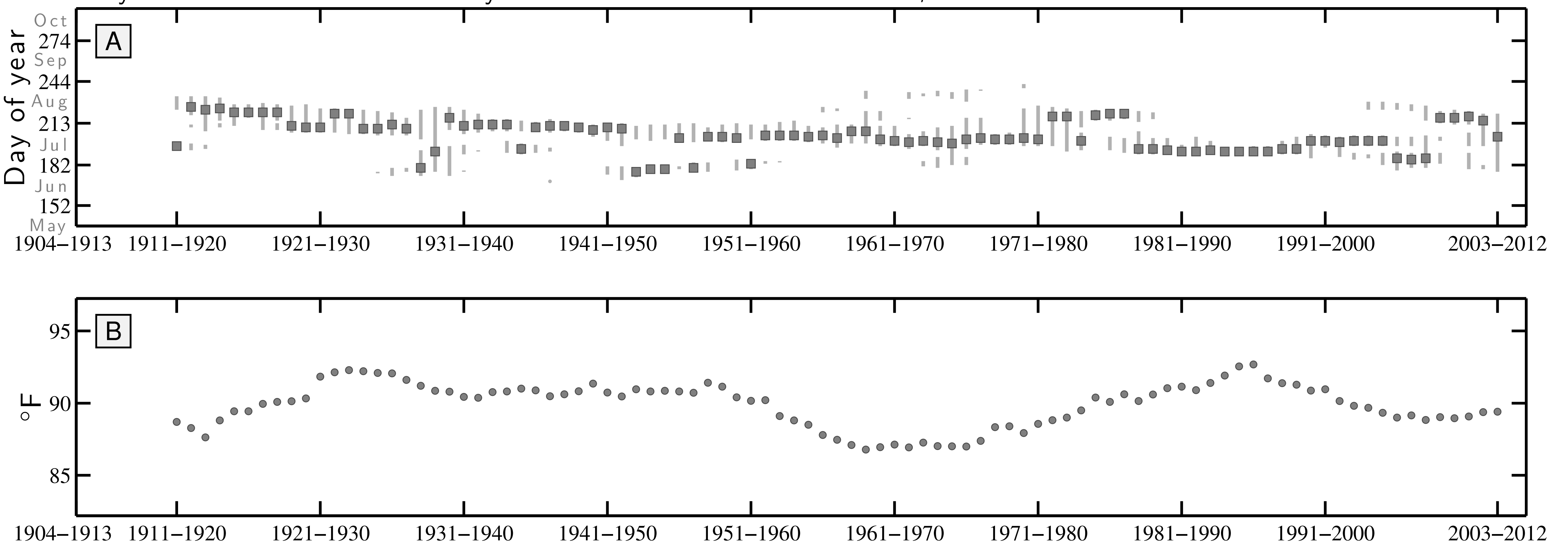

10 year Winter Teletherm dynamics for ELIZABETH CITY, NC:

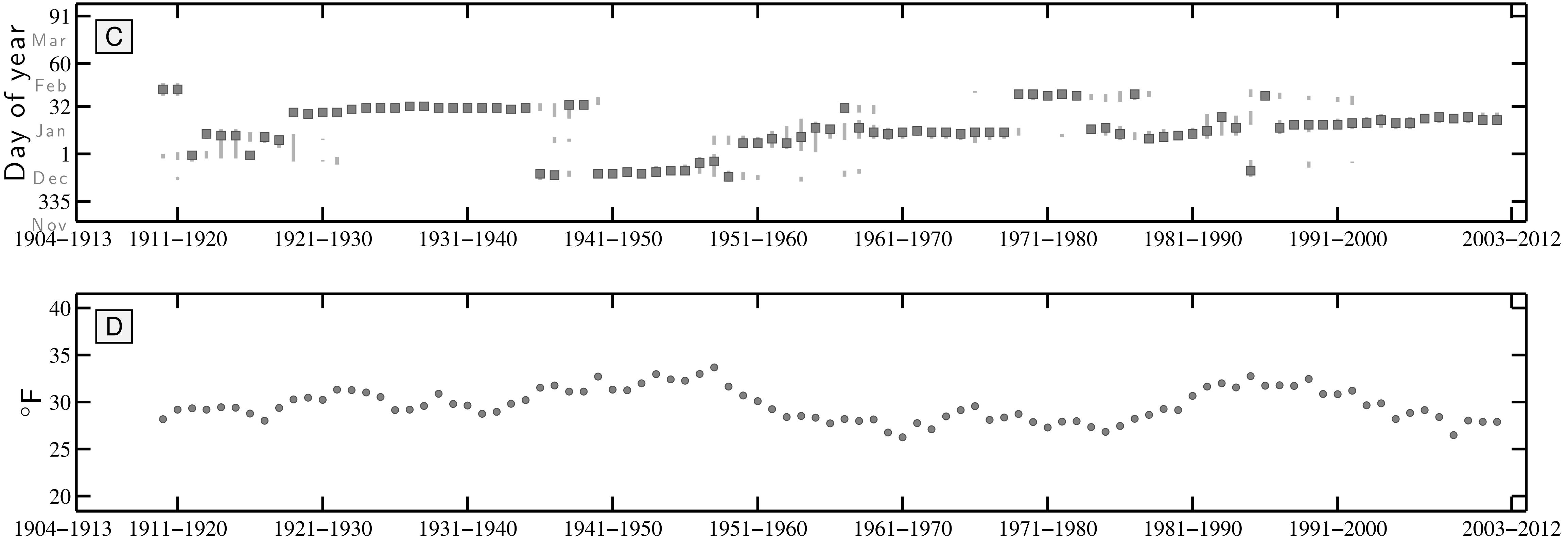

10 year Summer Teletherm dynamics for FAYETTEVILLE PWC, NC:

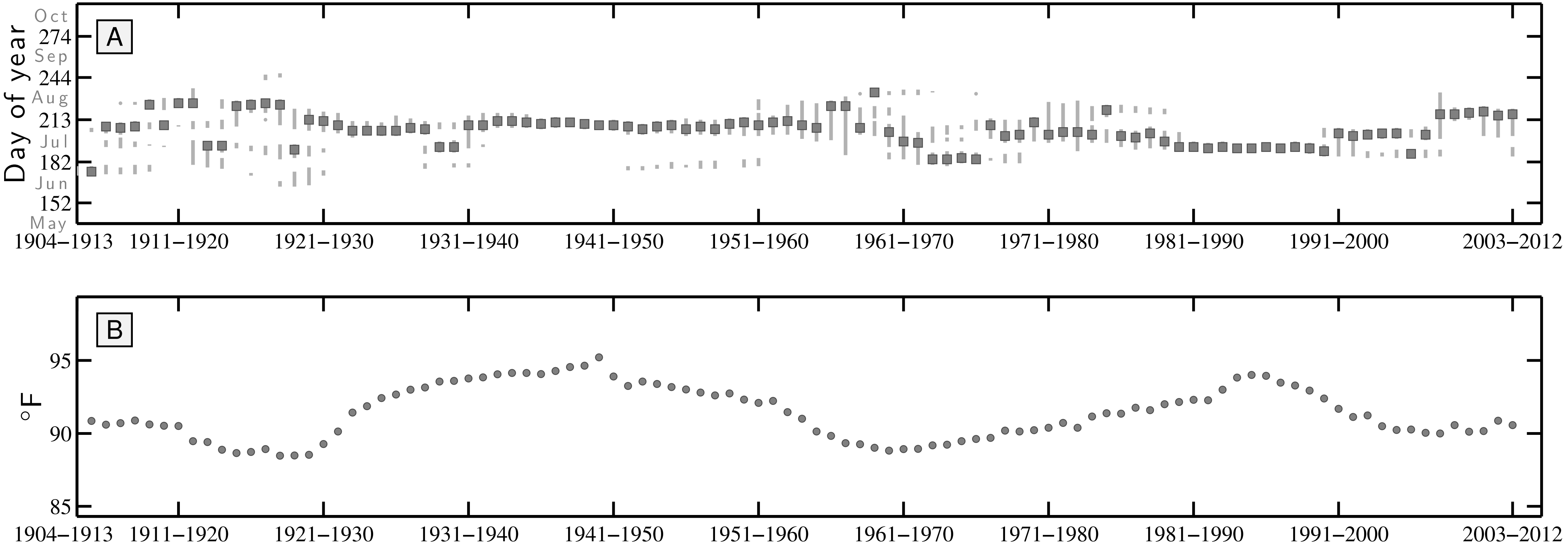

10 year Winter Teletherm dynamics for FAYETTEVILLE PWC, NC:

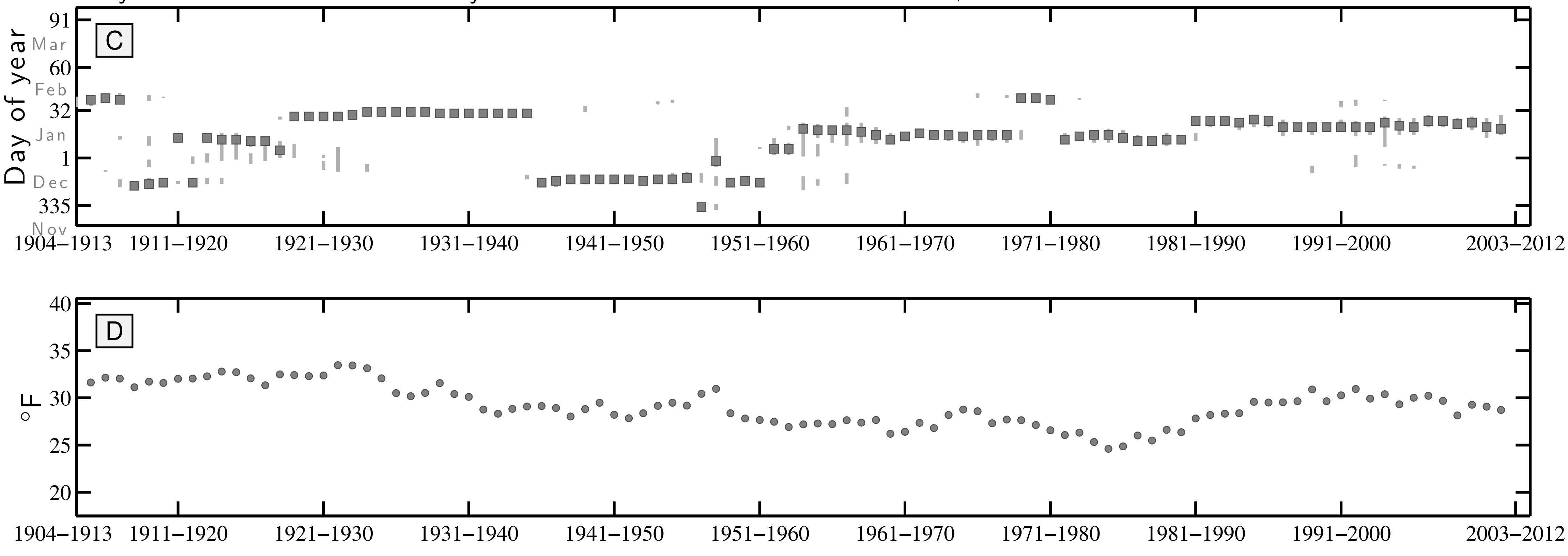

10 year Summer Teletherm dynamics for GOLDSBORO 4 SE, NC:

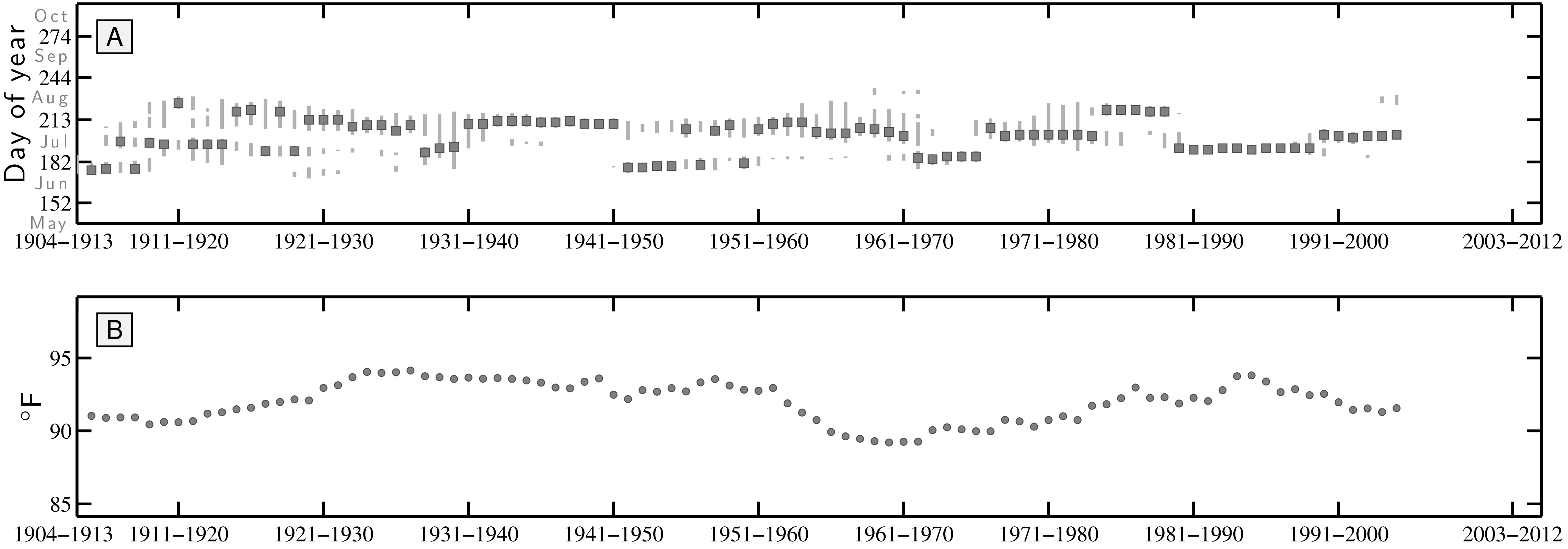

10 year Winter Teletherm dynamics for GOLDSBORO 4 SE, NC:

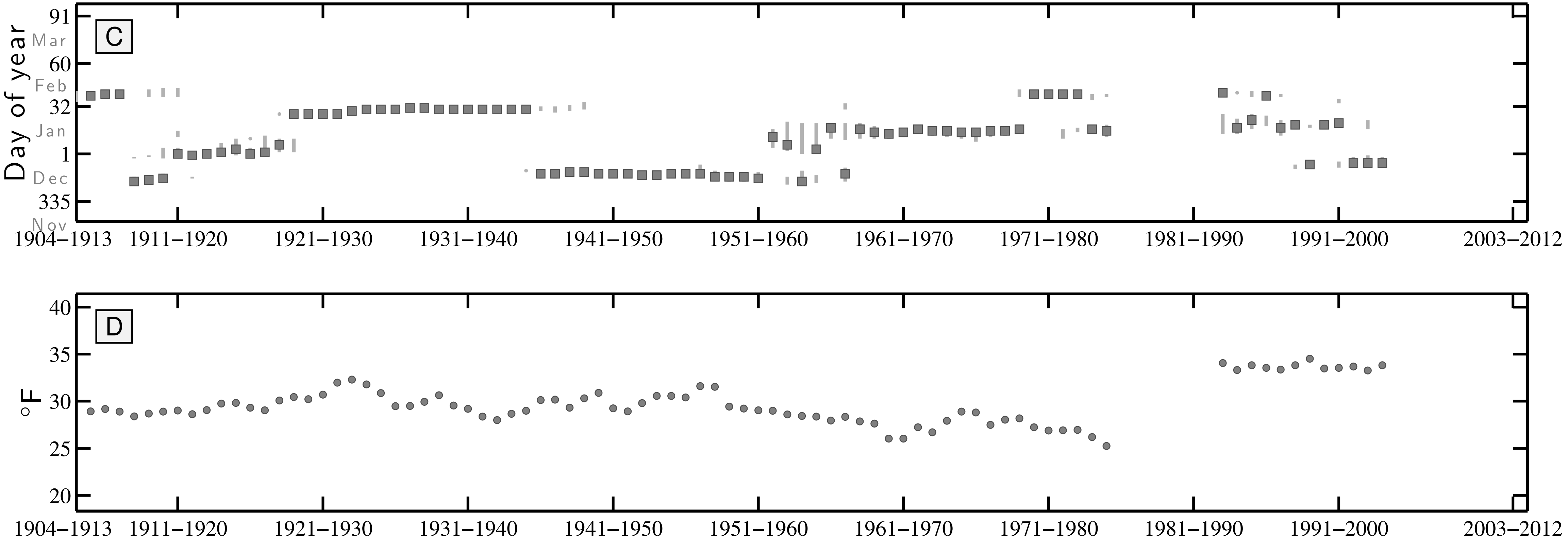

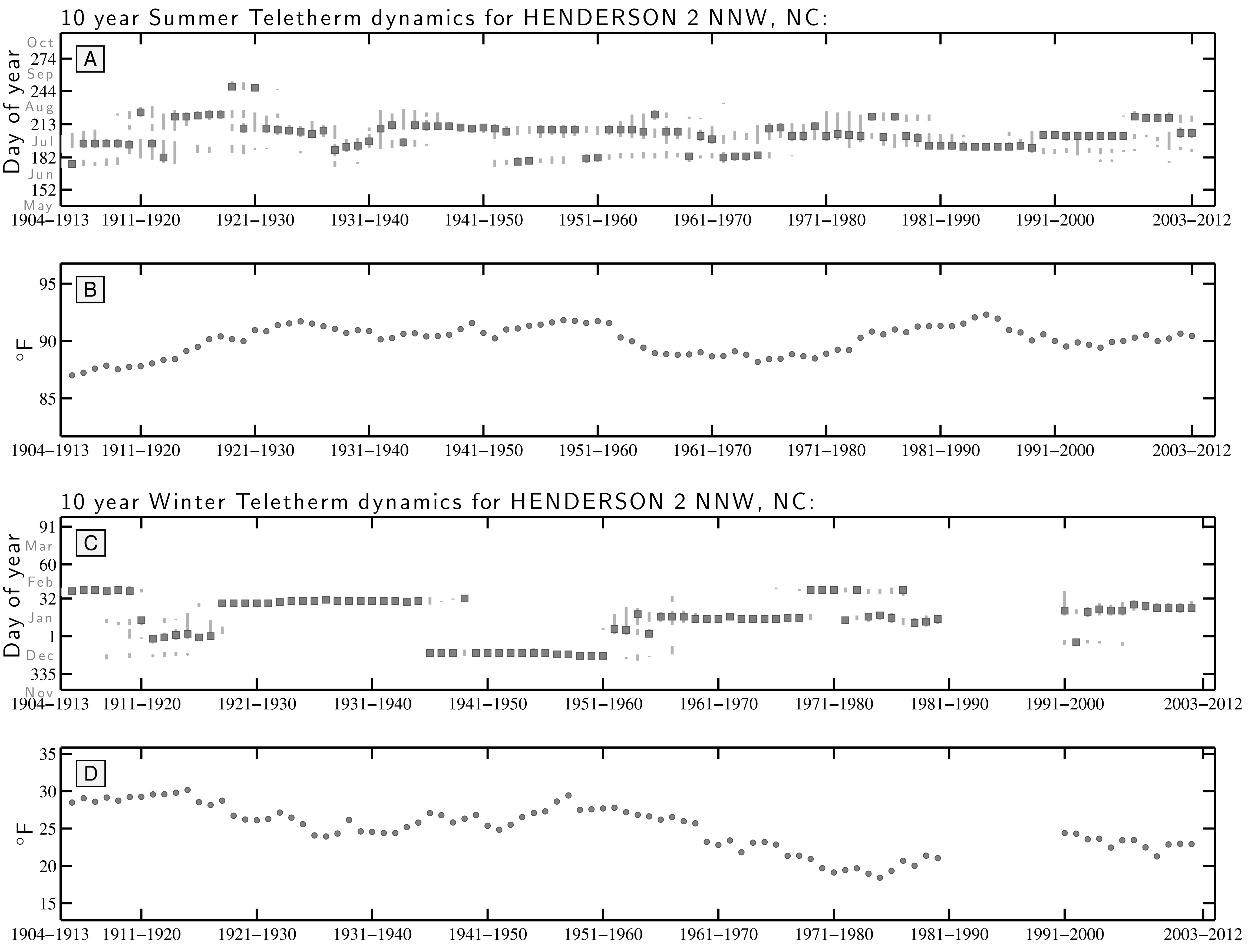
10 year Summer Teletherm dynamics for HENDERSON 2 NNW, NC:
A
B
10 year Winter Teletherm dynamics for HENDERSON 2 NNW, NC:
C
D
Day of year
°F
Oct
274
Sep
244
Aug
213
Jul
182
Jun
152
May
95
90
85
91
Mar
60
Feb
32
Jan
1
Dec
335
Nov
35
30
25
20
15
1904–1913
1911–1920
1921–1930
1931–1940
1941–1950
1951–1960
1961–1970
1971–1980
1981–1990
1991–2000
2003–2012

10 year Summer Teletherm dynamics for HENDERSONVILLE 1 NE, NC:

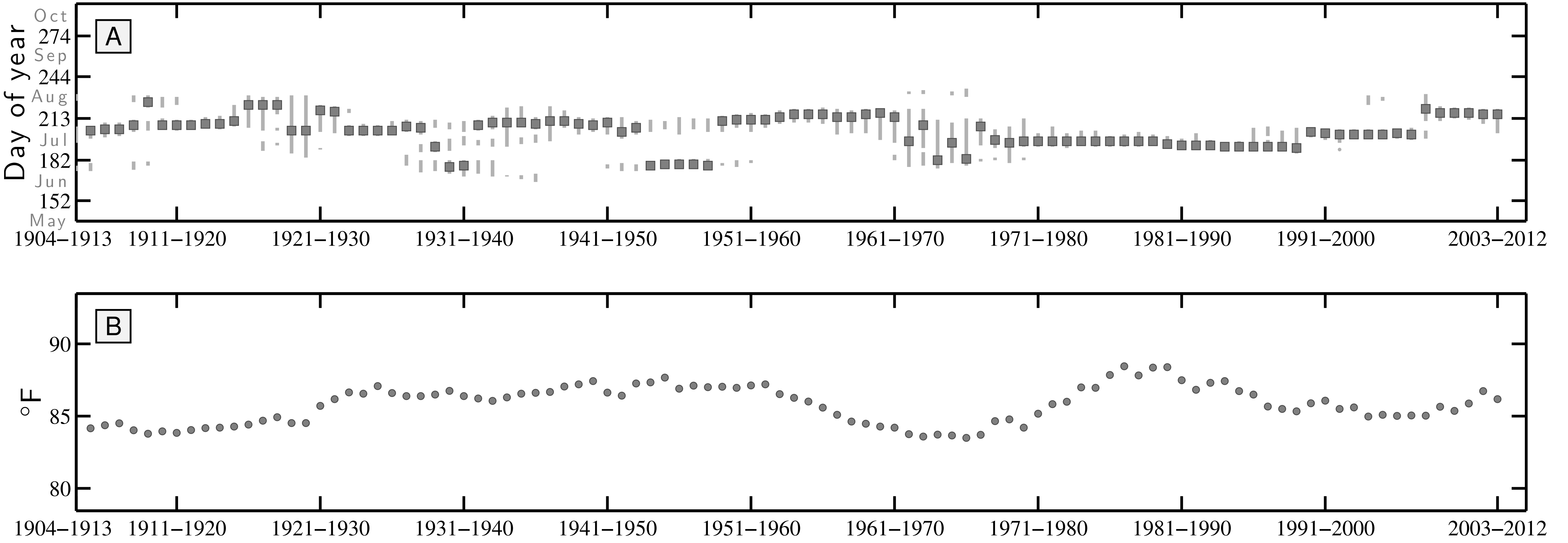

10 year Winter Teletherm dynamics for HENDERSONVILLE 1 NE, NC:

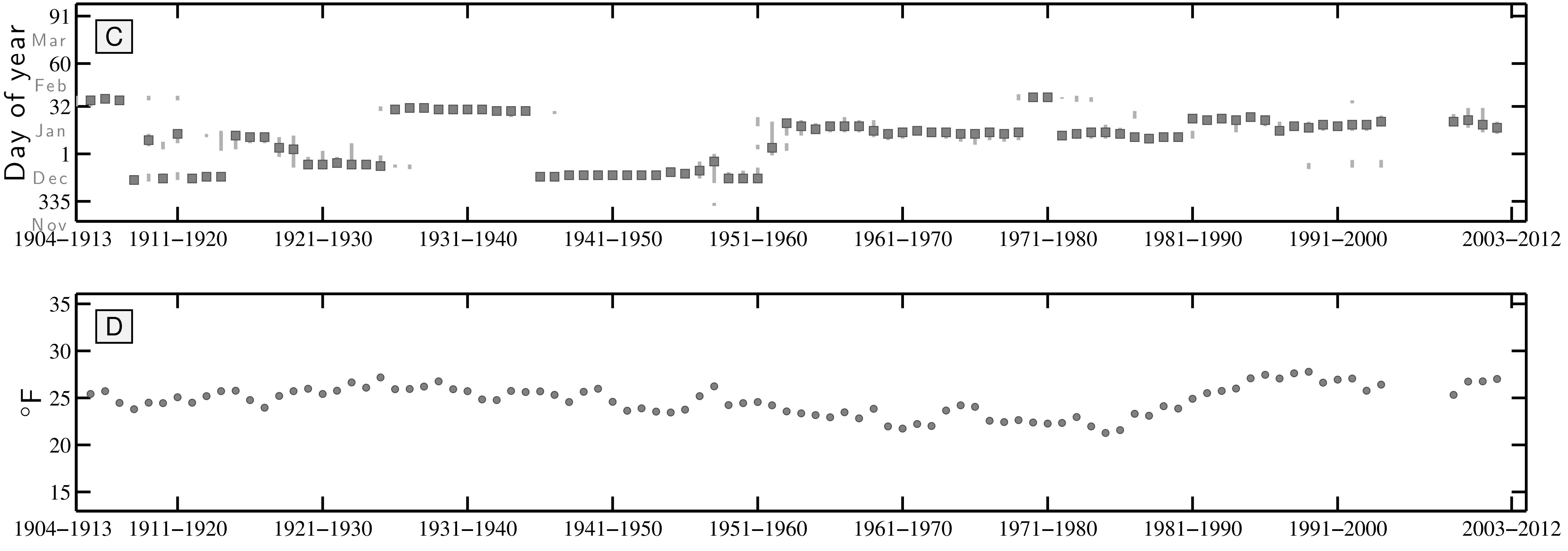

10 year Summer Teletherm dynamics for HIGHLANDS, NC:

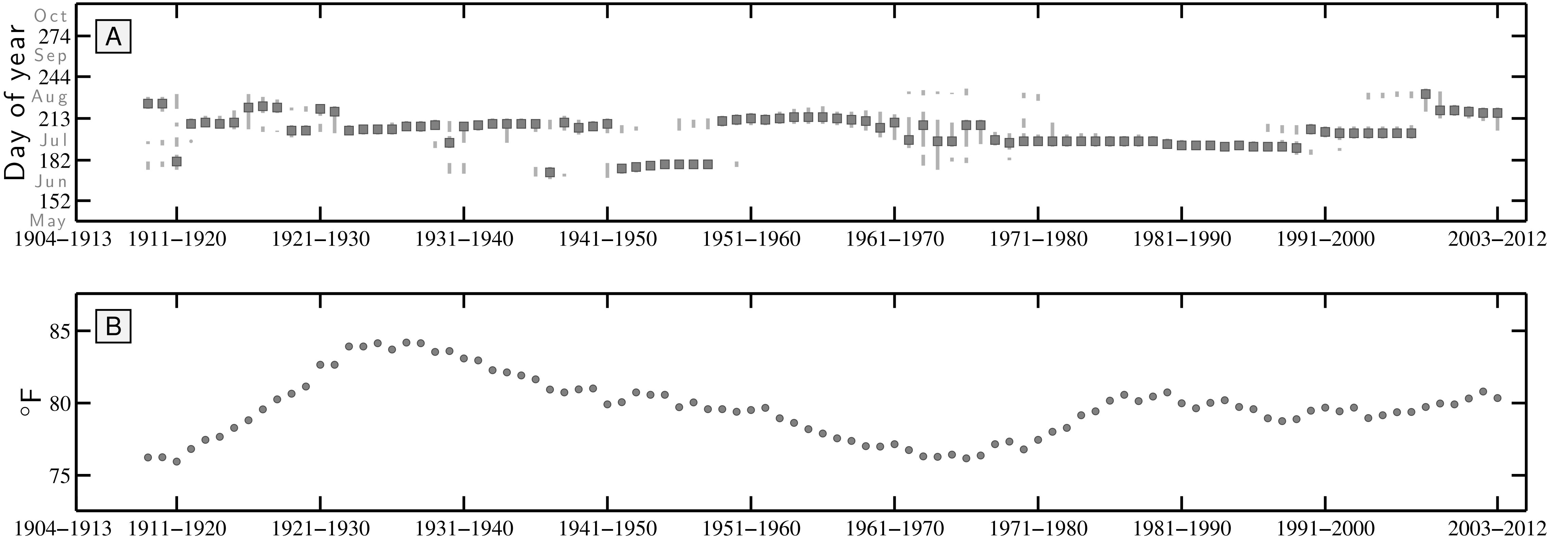

10 year Winter Teletherm dynamics for HIGHLANDS, NC:

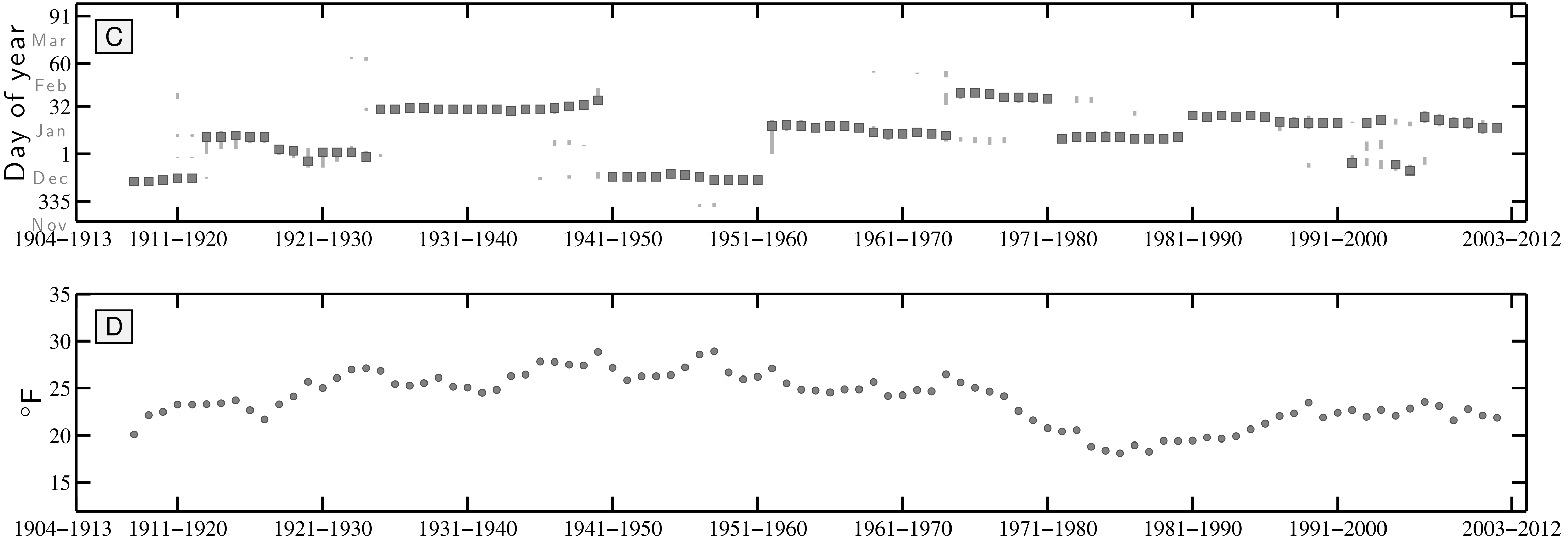

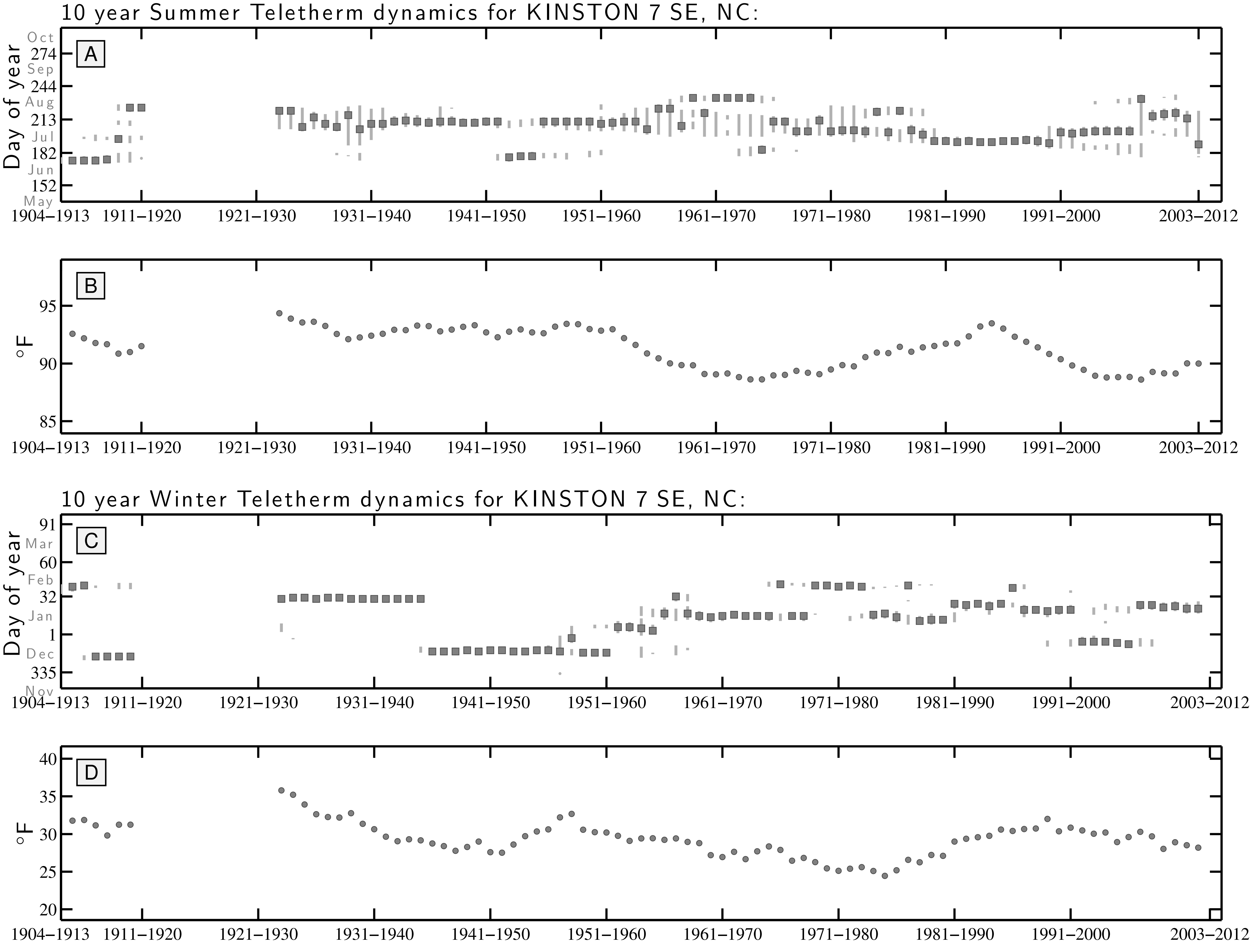
10 year Summer Teletherm dynamics for KINSTON 7 SE, NC:
A
B
Day of year
°F
Oct
274
Sep
244
Aug
213
Jul
182
Jun
152
May
95
90
85
10 year Winter Teletherm dynamics for KINSTON 7 SE, NC:
C
D
91
Mar
60
Feb
32
Jan
1
Dec
335
Nov
40
35
30
25
20
1904–1913
1911–1920
1921–1930
1931–1940
1941–1950
1951–1960
1961–1970
1971–1980
1981–1990
1991–2000
2003–2012

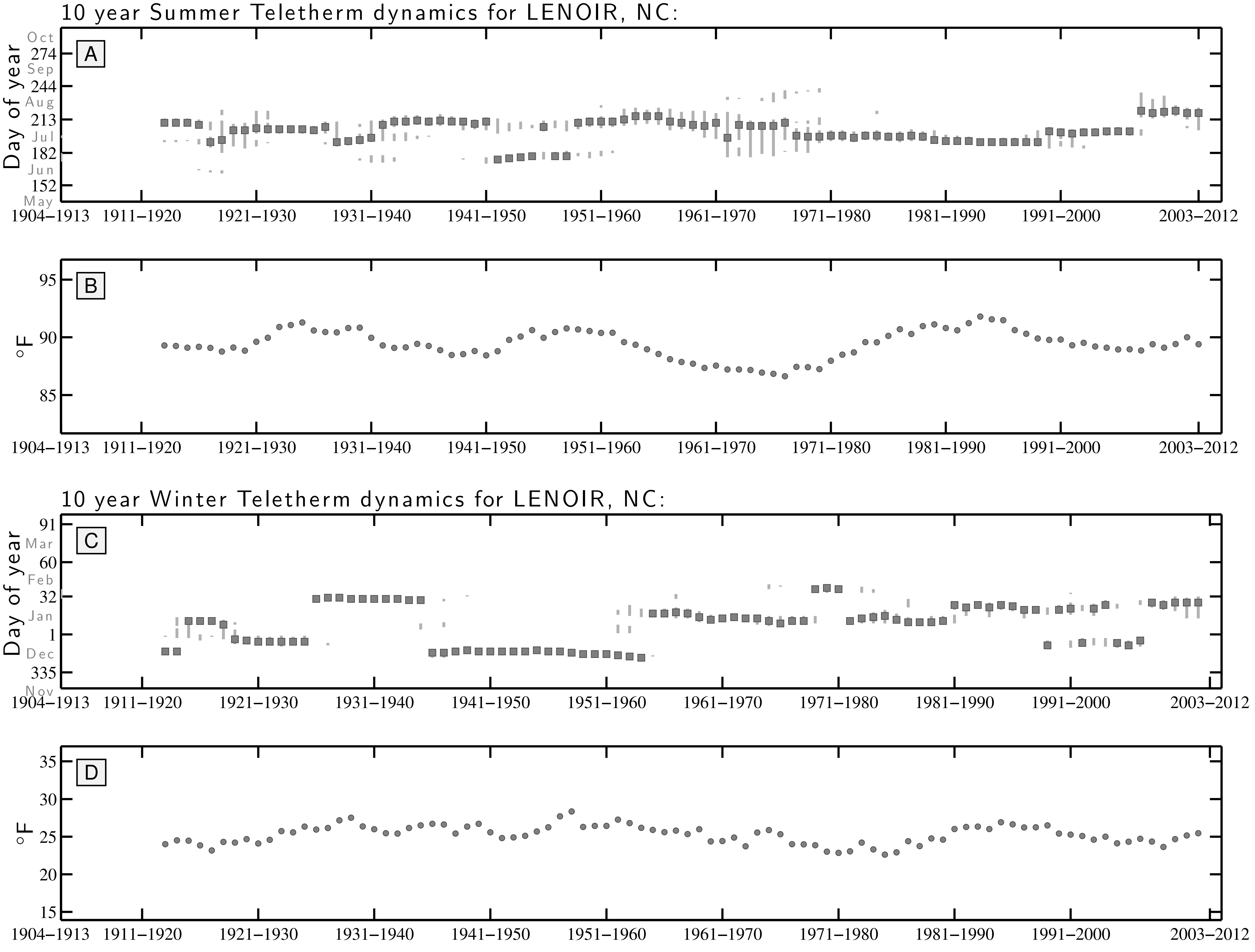
10 year Summer Teletherm dynamics for LENOIR, NC:
A
Day of year
Oct
274
Sep
244
Aug
213
Jul
182
Jun
152
May
B
°F
95
90
85
1904–1913
1911–1920
1921–1930
1931–1940
1941–1950
1951–1960
1961–1970
1971–1980
1981–1990
1991–2000
2003–2012
10 year Winter Teletherm dynamics for LENOIR, NC:
C
Day of year
91
Mar
60
Feb
32
Jan
1
Dec
335
Nov
D
°F
35
30
25
20
15

10 year Summer Teletherm dynamics for LOUISBURG, NC:

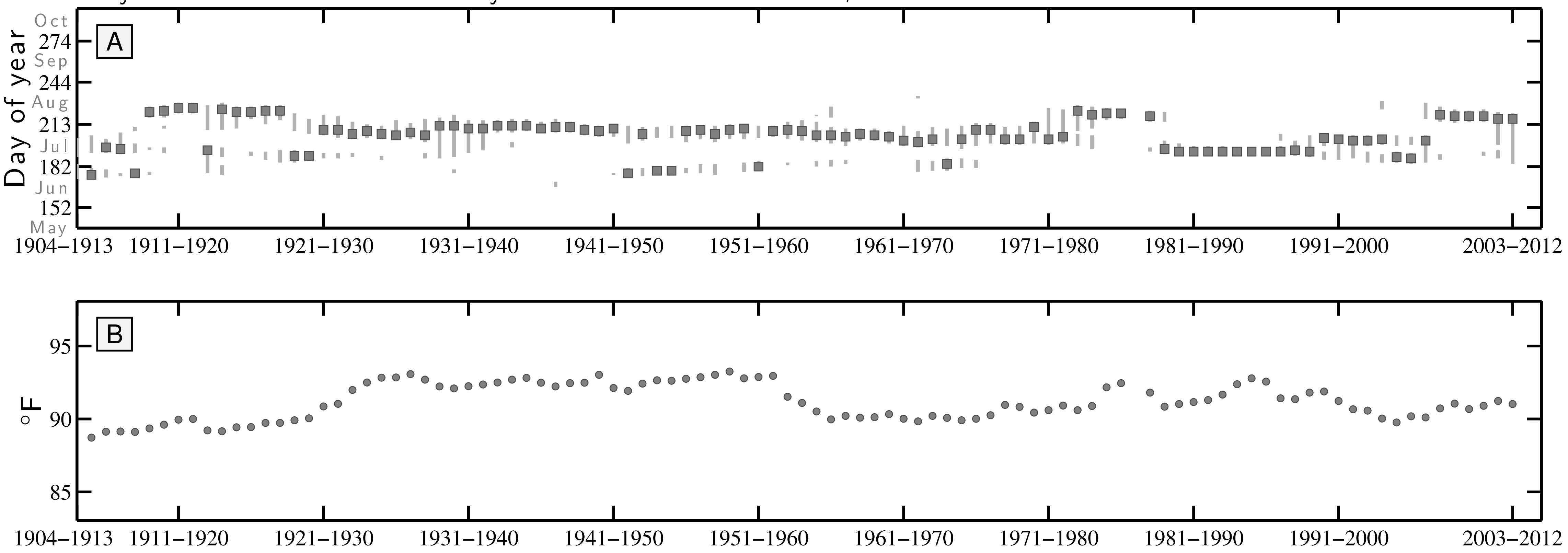

10 year Winter Teletherm dynamics for LOUISBURG, NC:

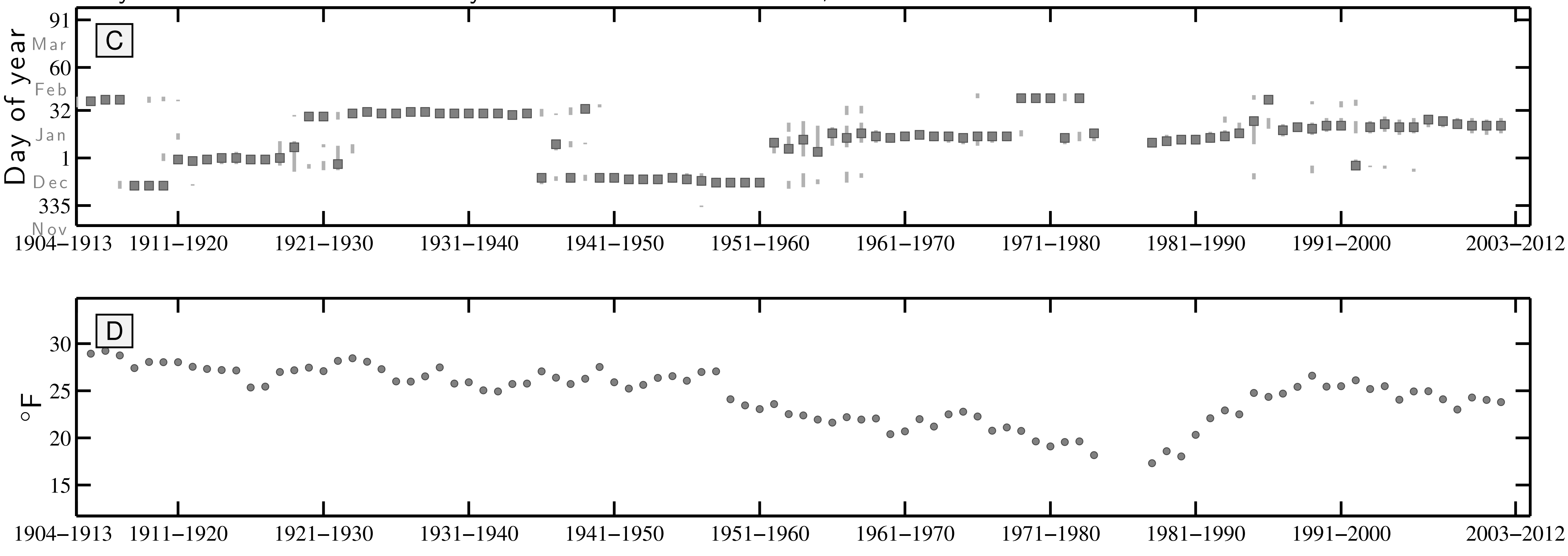

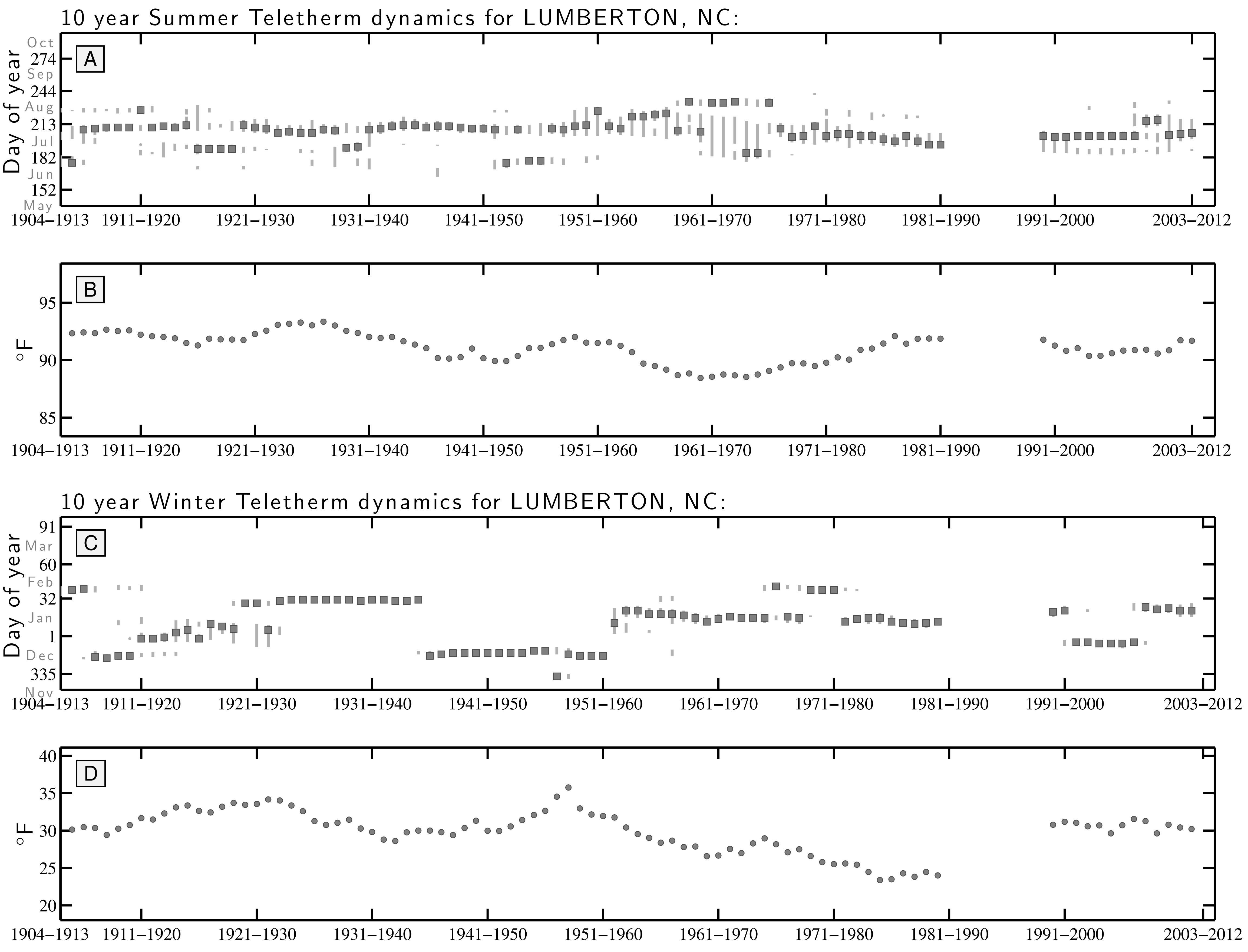

10 year Summer Teletherm dynamics for LUMBERTON, NC:
A
Day of year
Oct
274
Sep
244
Aug
213
Jul
182
Jun
152
May
1904–1913
1911–1920
1921–1930
1931–1940
1941–1950
1951–1960
1961–1970
1971–1980
1981–1990
1991–2000
2003–2012
B
°F
95
90
85
10 year Winter Teletherm dynamics for LUMBERTON, NC:
C
Day of year
91
Mar
60
Feb
32
Jan
1
Dec
335
Nov
D
°F
40
35
30
25
20

10 year Summer Teletherm dynamics for MARION 2 NW, NC:

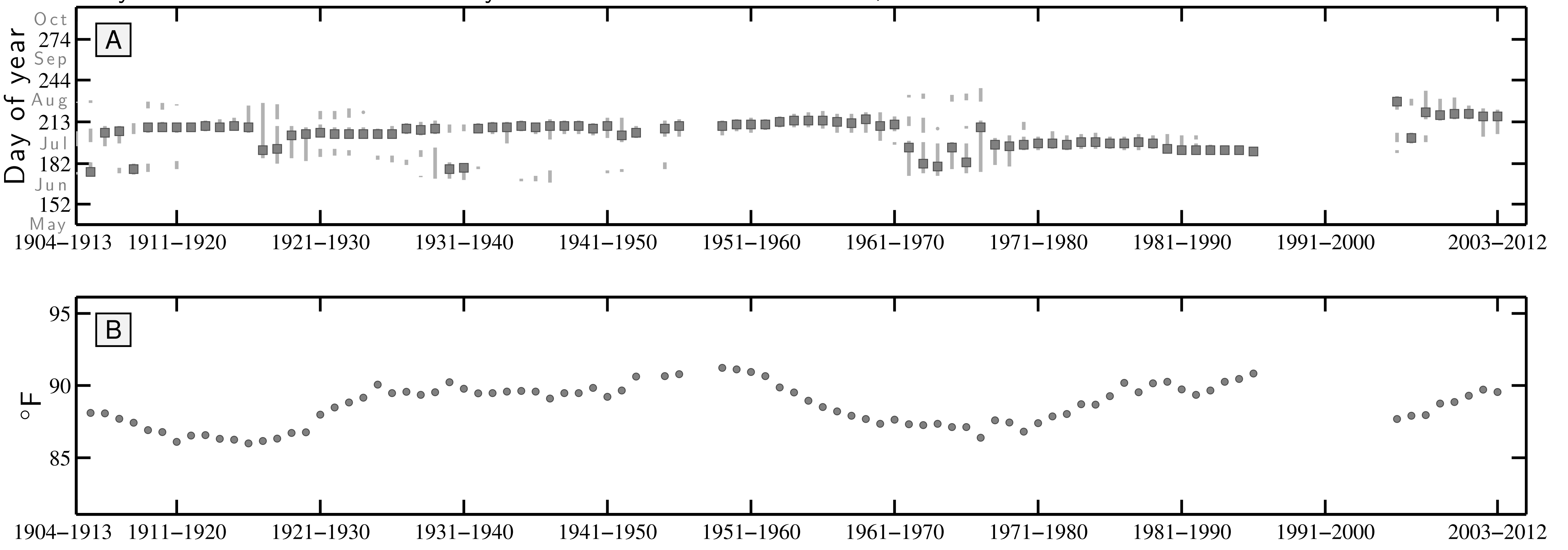

10 year Winter Teletherm dynamics for MARION 2 NW, NC:

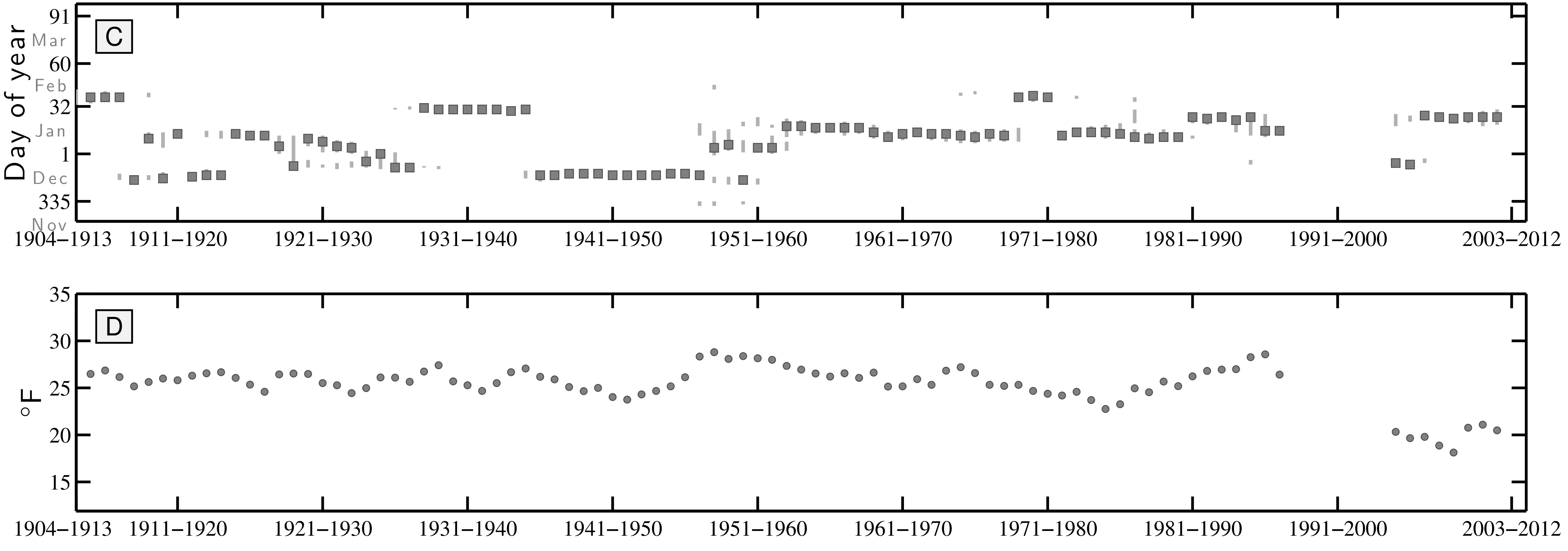

10 year Summer Teletherm dynamics for MARSHALL, NC:

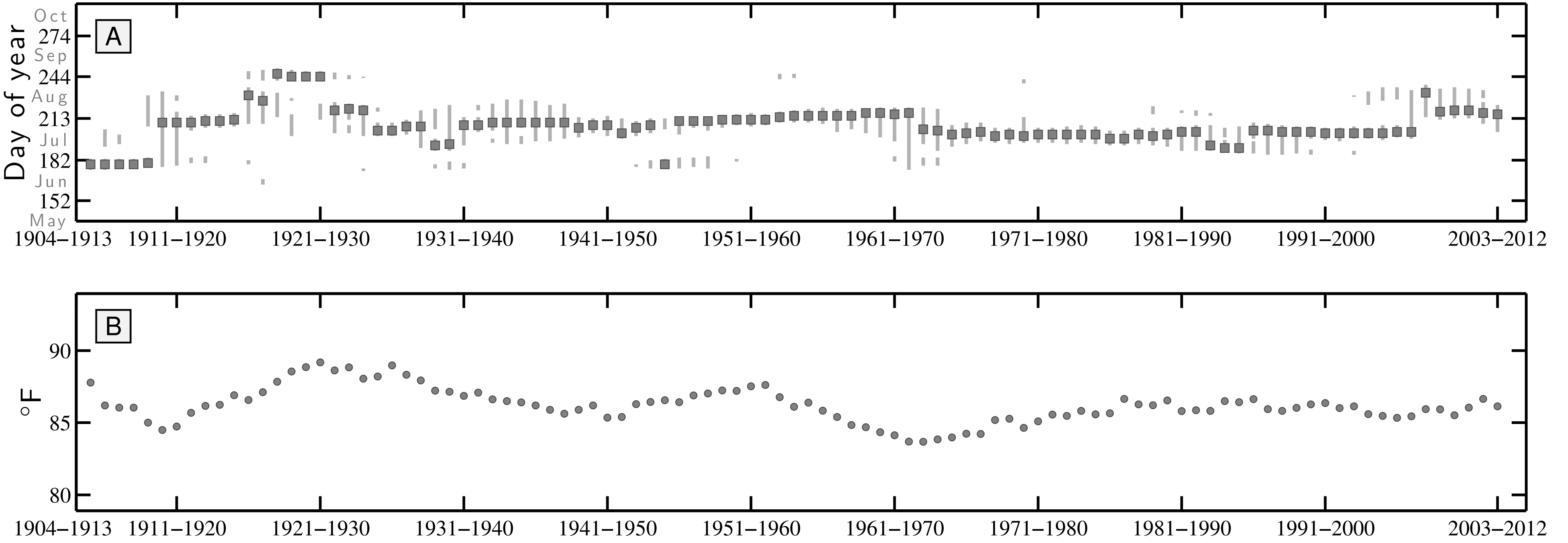

10 year Winter Teletherm dynamics for MARSHALL, NC:

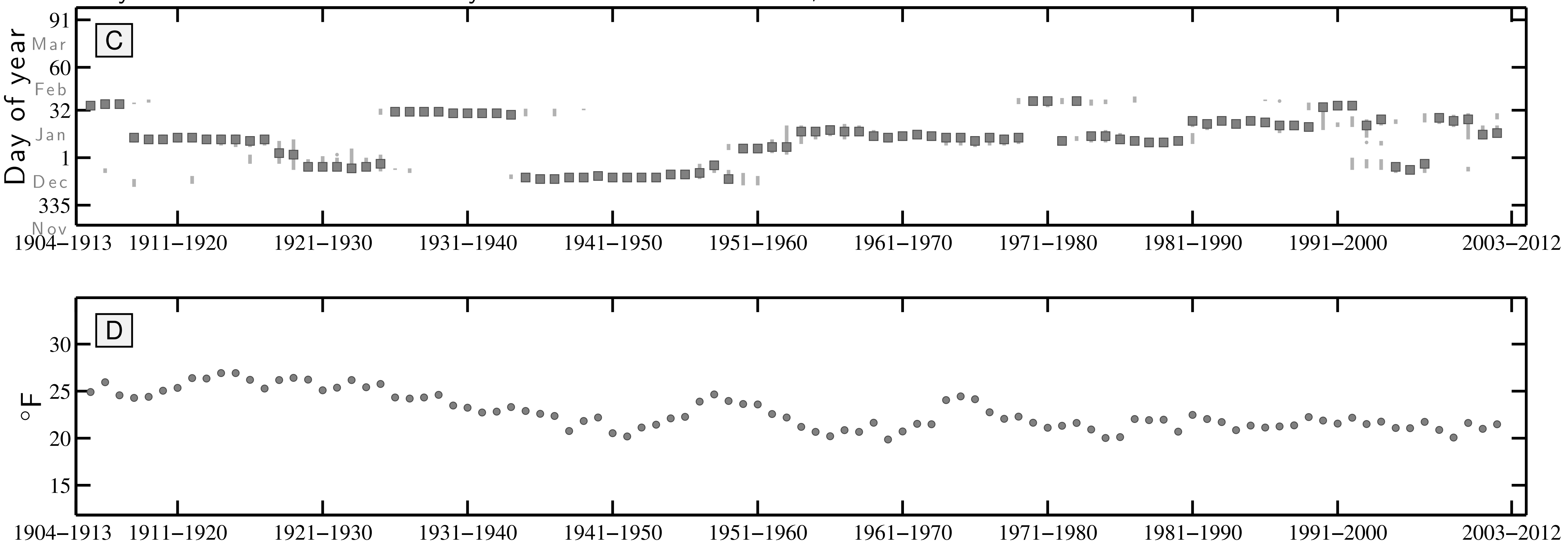

10 year Summer Teletherm dynamics for MONROE 2 SE, NC:

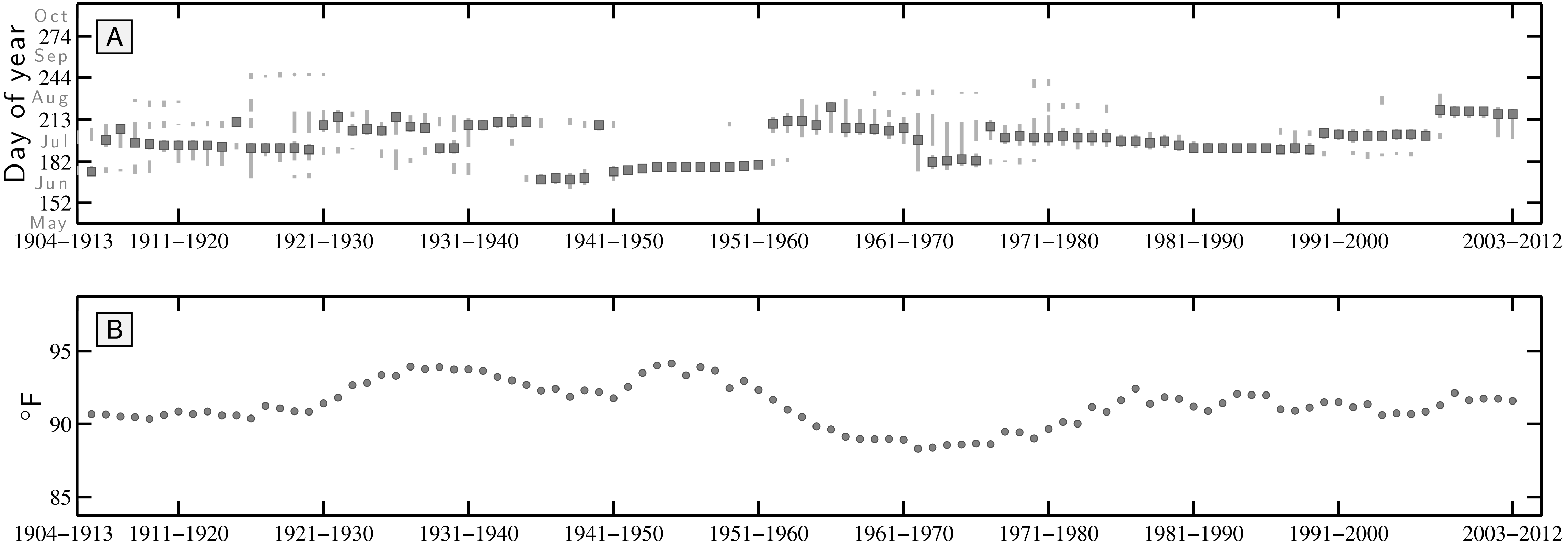

10 year Winter Teletherm dynamics for MONROE 2 SE, NC:

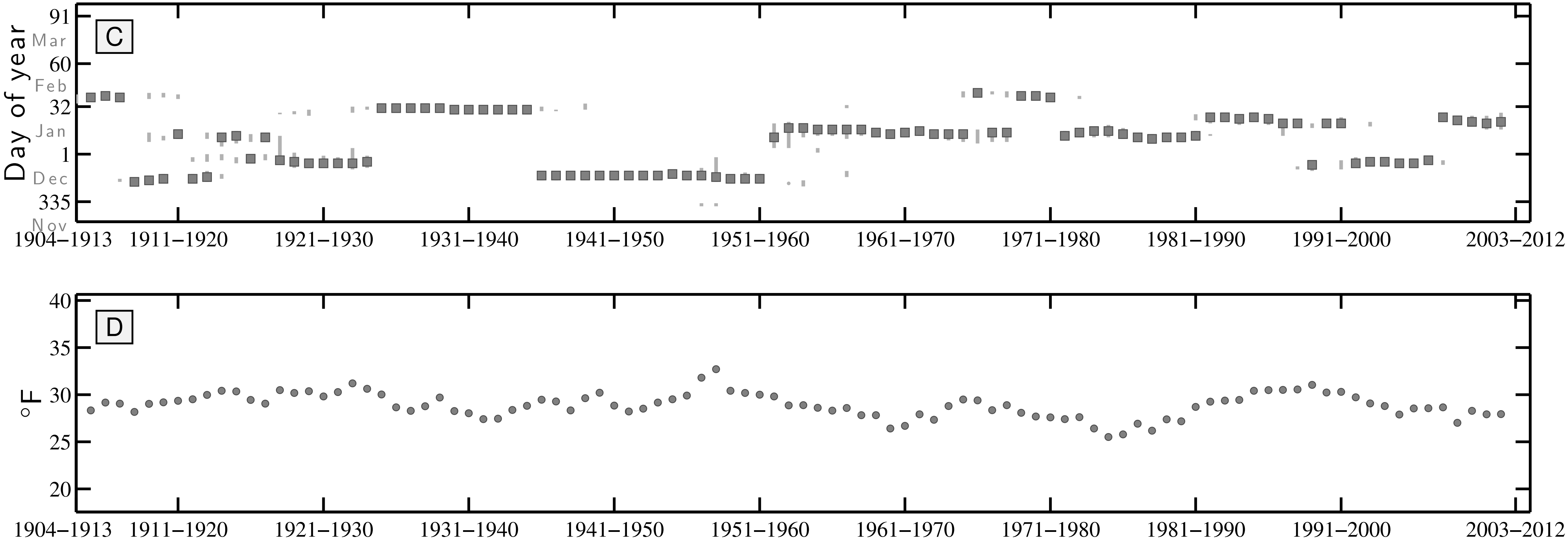

10 year Summer Teletherm dynamics for MOREHEAD CITY 2 WNW, NC:

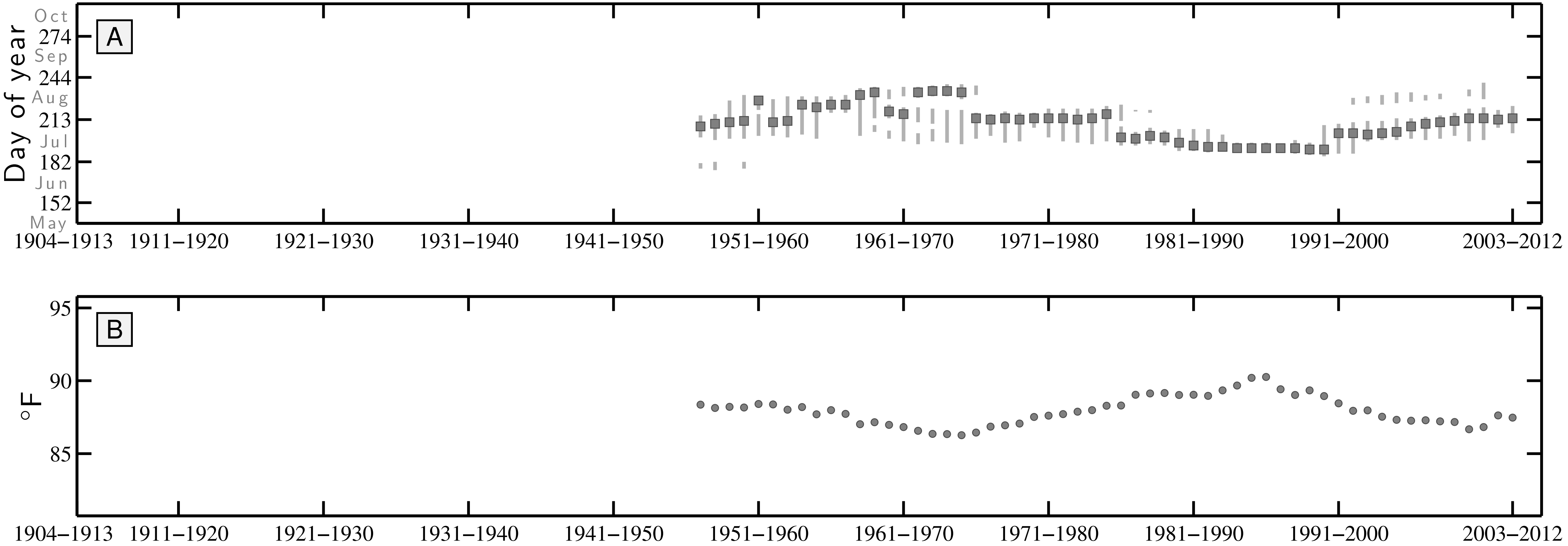

10 year Winter Teletherm dynamics for MOREHEAD CITY 2 WNW, NC:

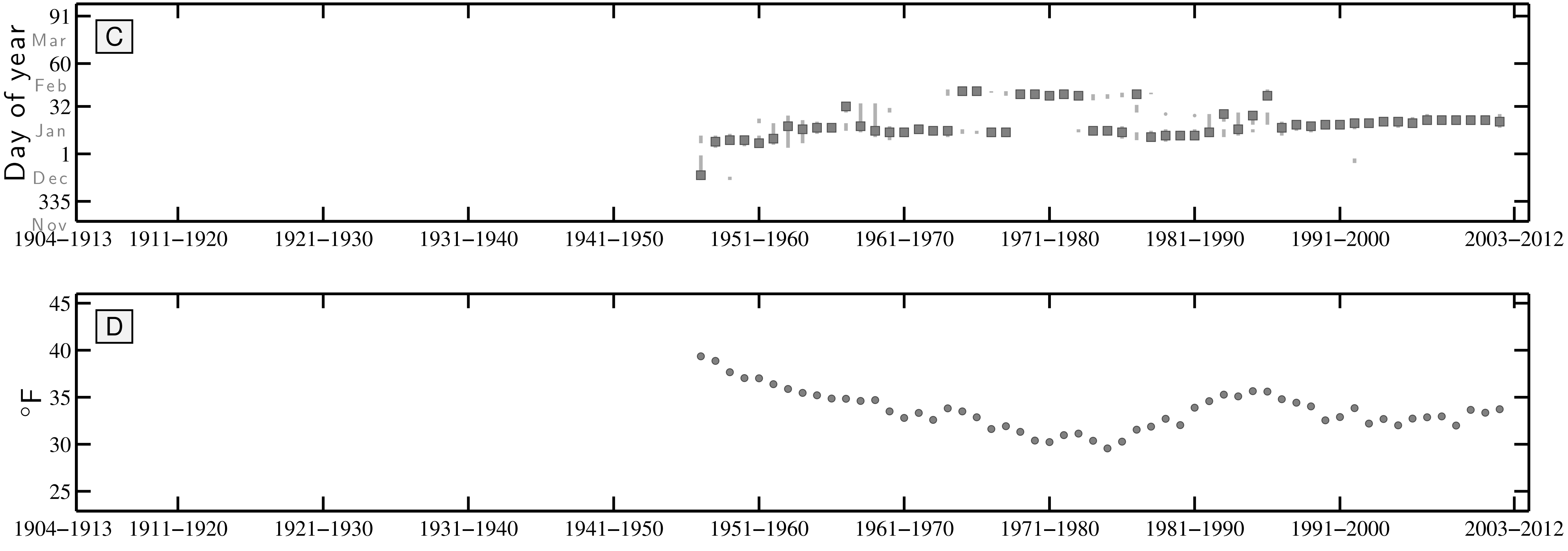

10 year Summer Teletherm dynamics for MORGANTON, NC:

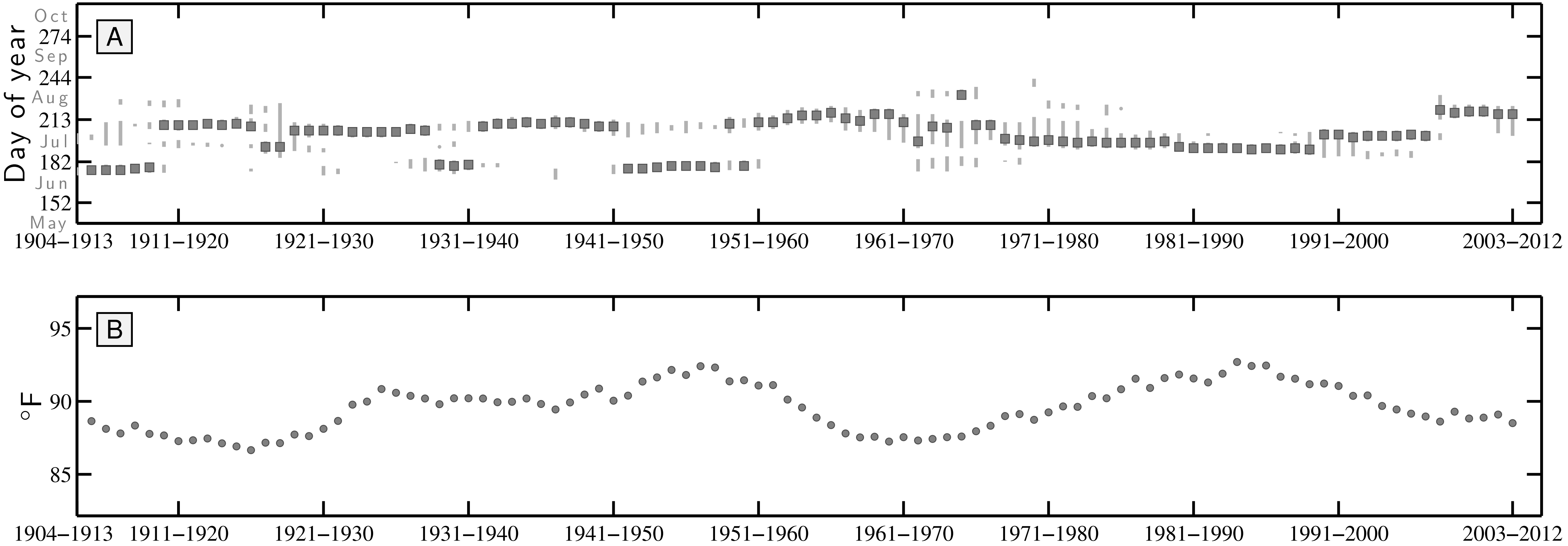

10 year Winter Teletherm dynamics for MORGANTON, NC:

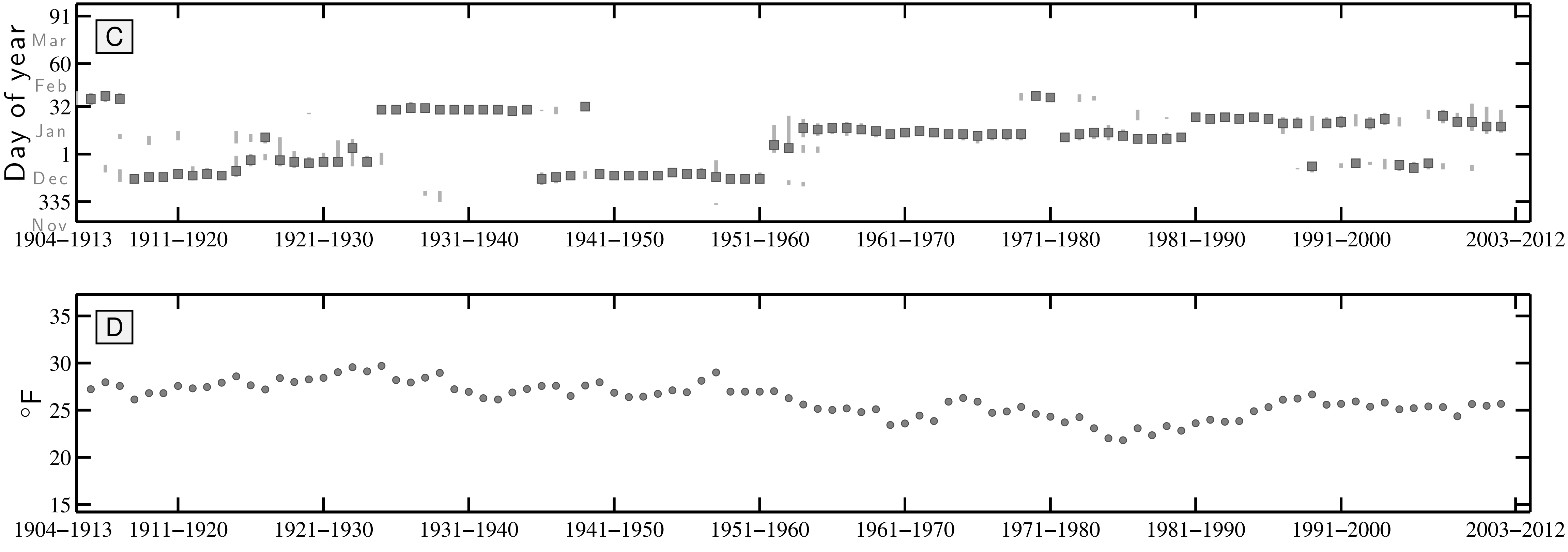

10 year Summer Teletherm dynamics for MT AIRY 2 W, NC:

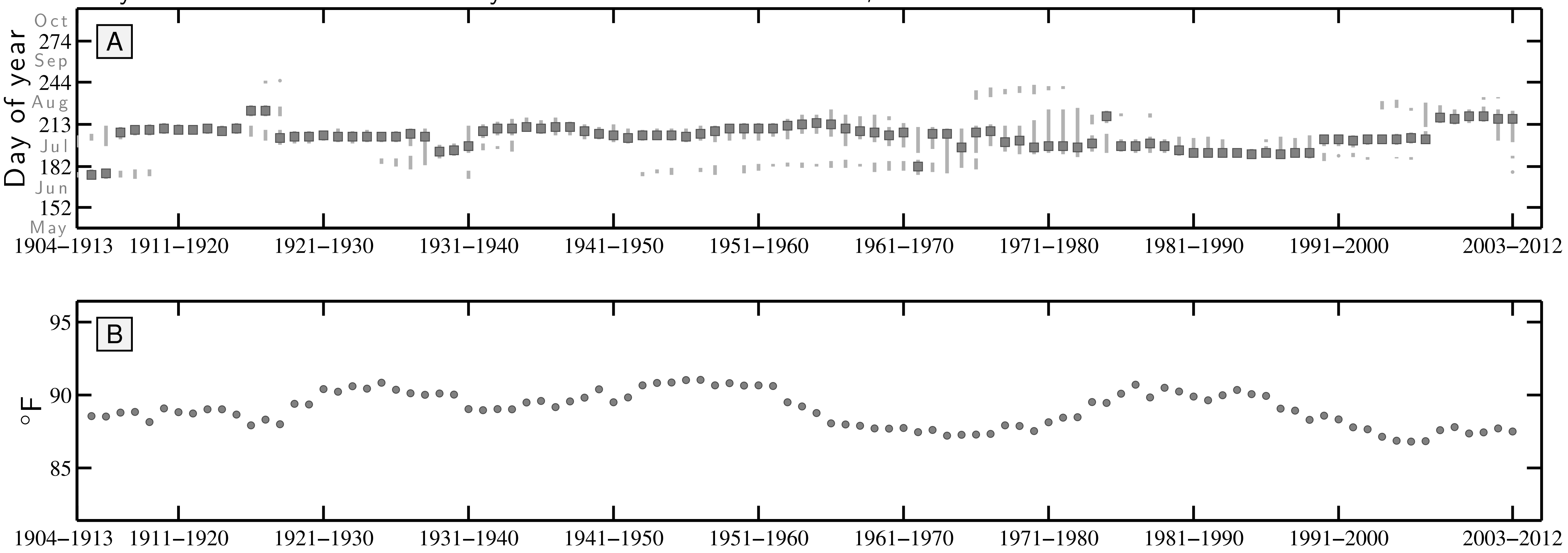

10 year Winter Teletherm dynamics for MT AIRY 2 W, NC:

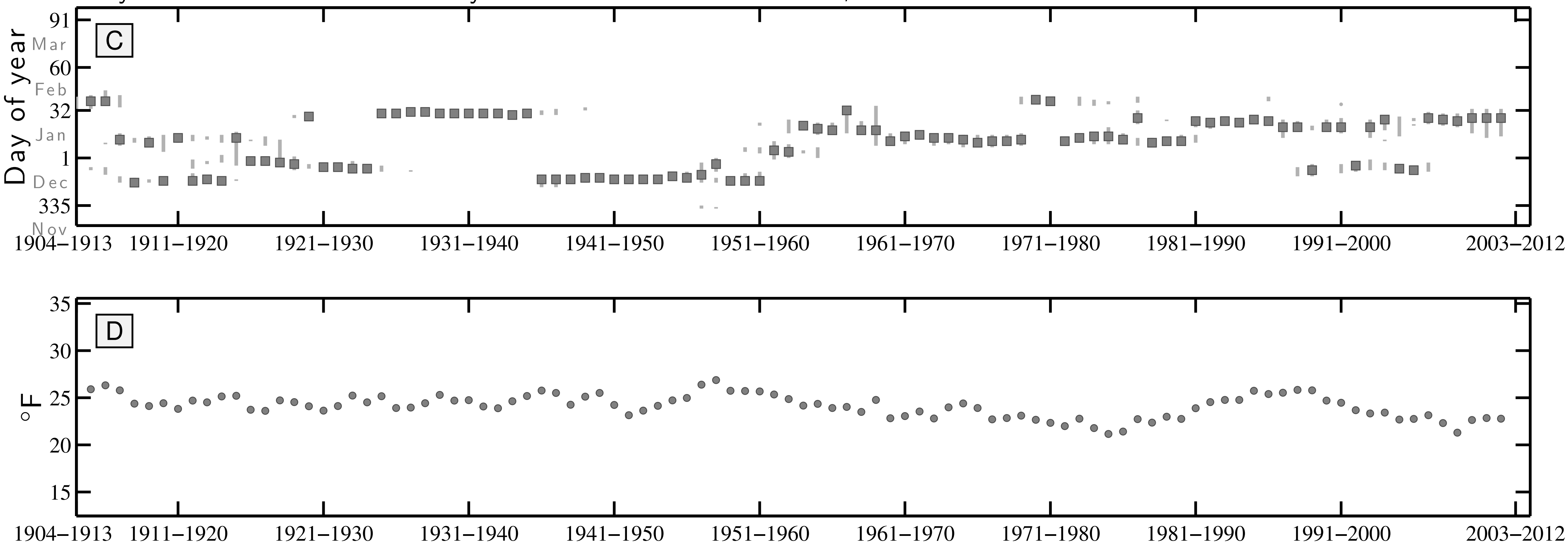

10 year Summer Teletherm dynamics for REIDSVILLE 2 NW, NC:

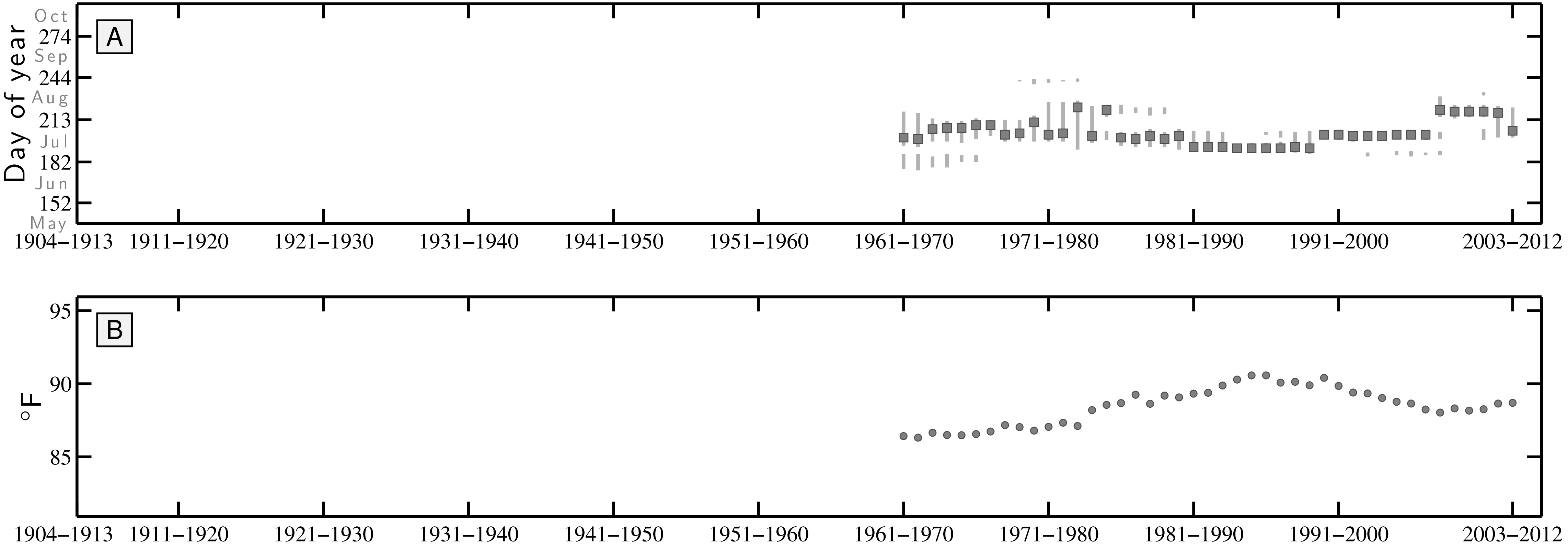

10 year Winter Teletherm dynamics for REIDSVILLE 2 NW, NC:

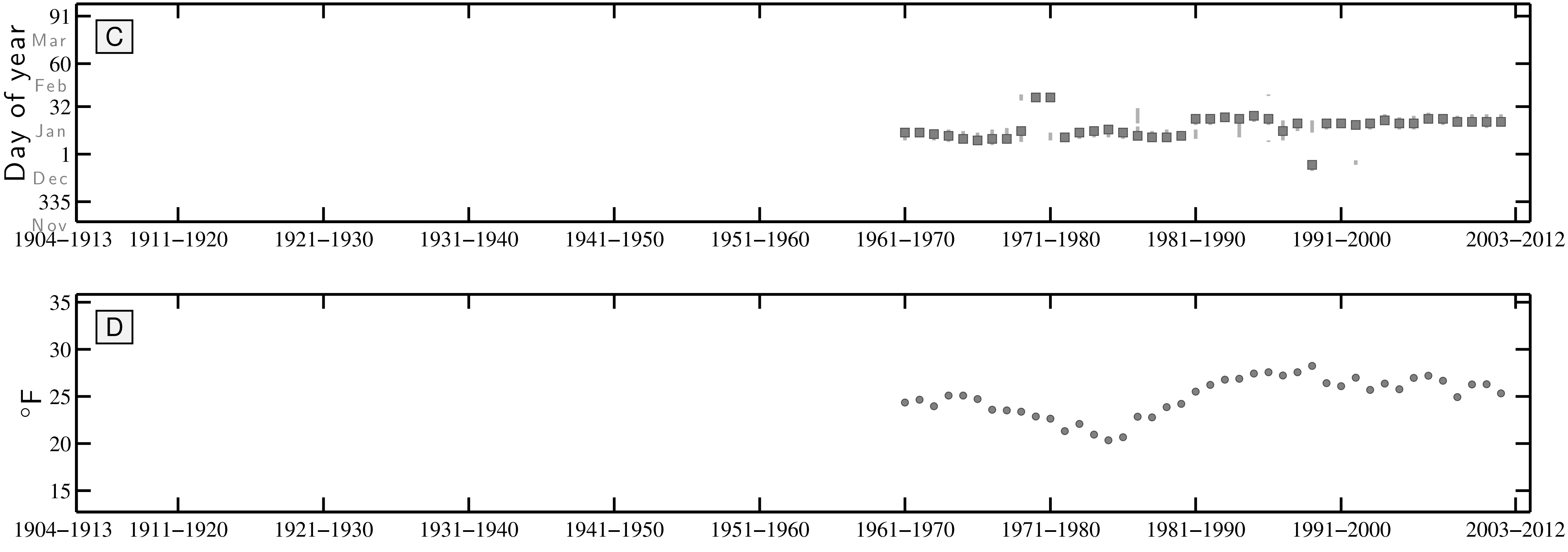

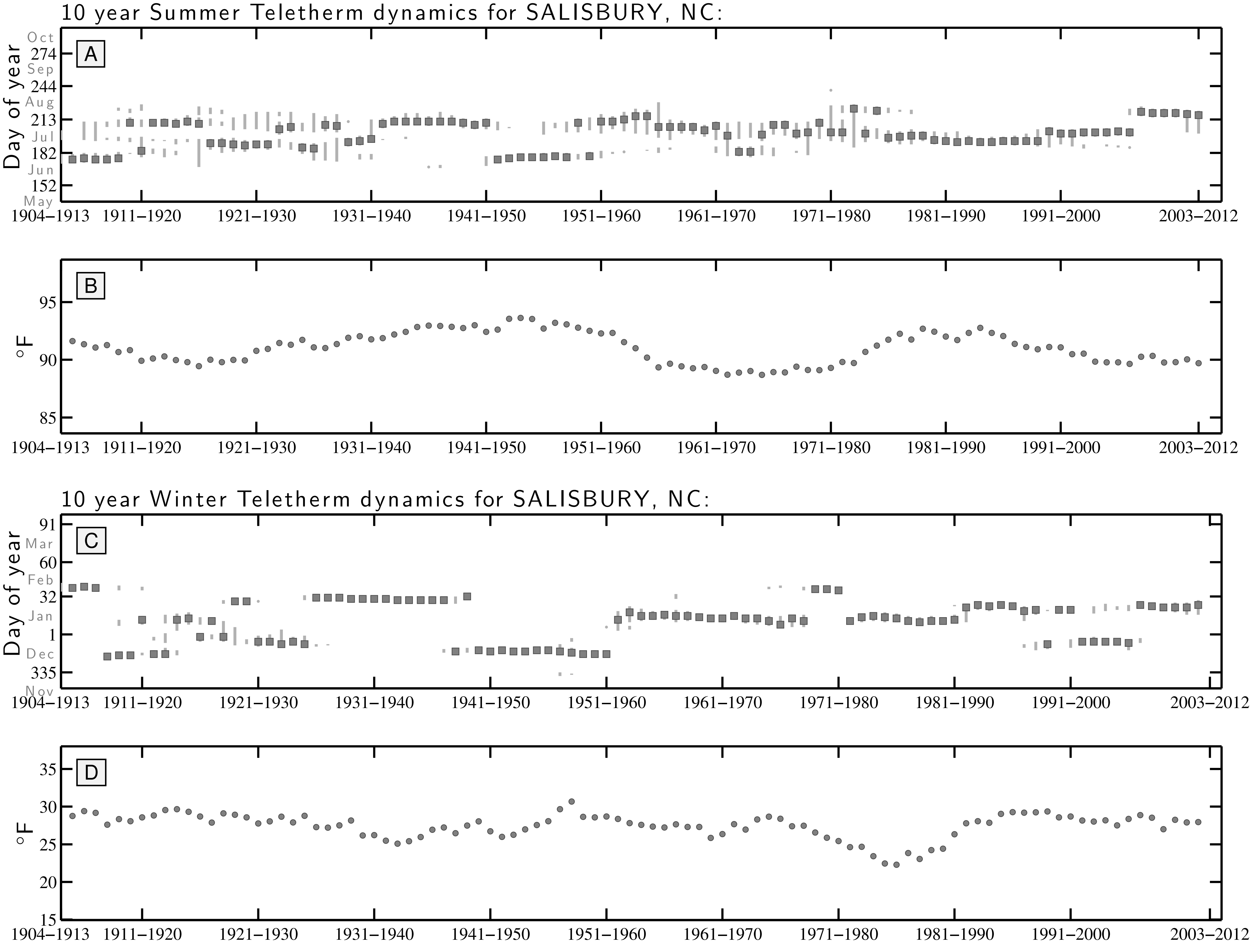

10 year Summer Teletherm dynamics for SALISBURY, NC:
A
Day of year
Oct
274
Sep
244
Aug
213
Jul
182
Jun
152
May
1904–1913
1911–1920
1921–1930
1931–1940
1941–1950
1951–1960
1961–1970
1971–1980
1981–1990
1991–2000
2003–2012
B
°F
95
90
85
10 year Winter Teletherm dynamics for SALISBURY, NC:
C
91
Mar
60
Feb
32
Jan
1
Dec
335
Nov
D
35
30
25
20
15

10 year Summer Teletherm dynamics for SMITHFIELD, NC:

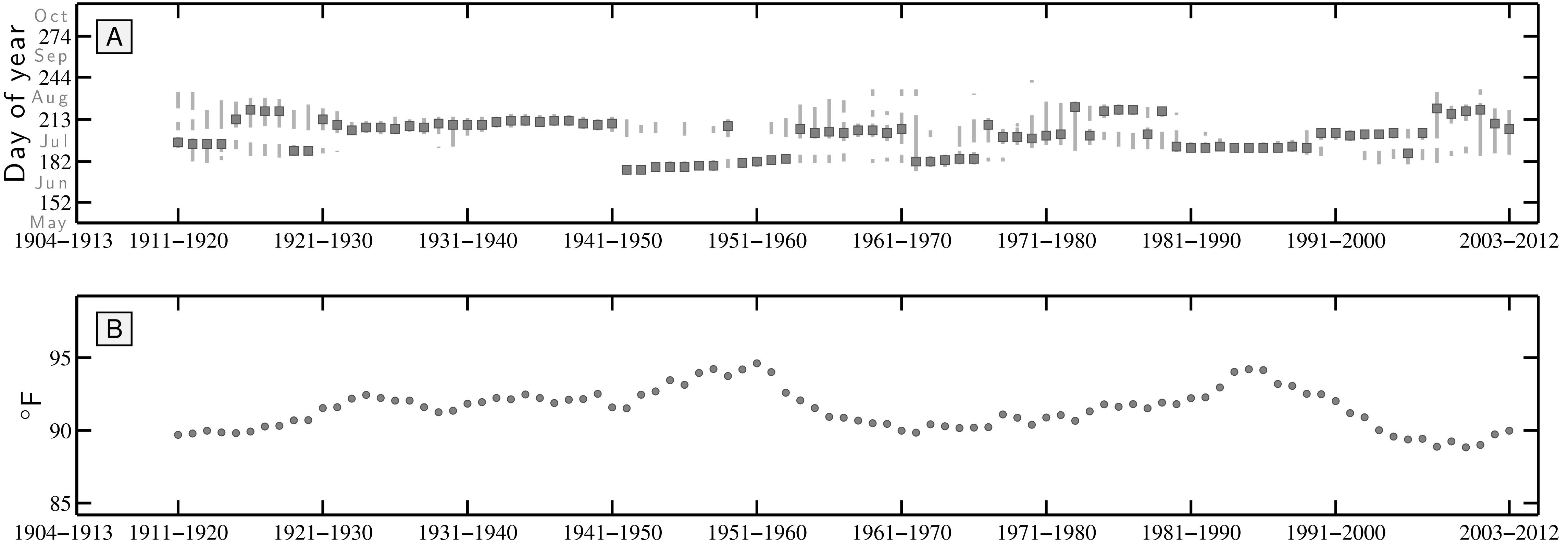

10 year Winter Teletherm dynamics for SMITHFIELD, NC:

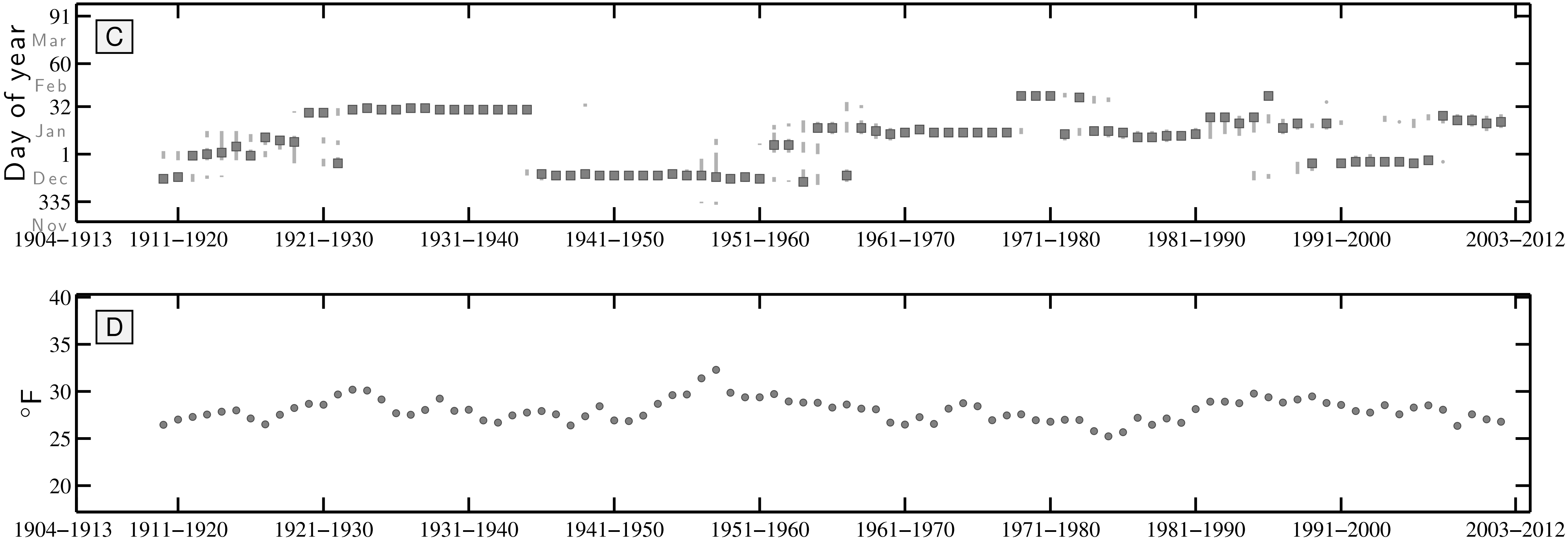

10 year Summer Teletherm dynamics for SOUTHPORT 5 N, NC:

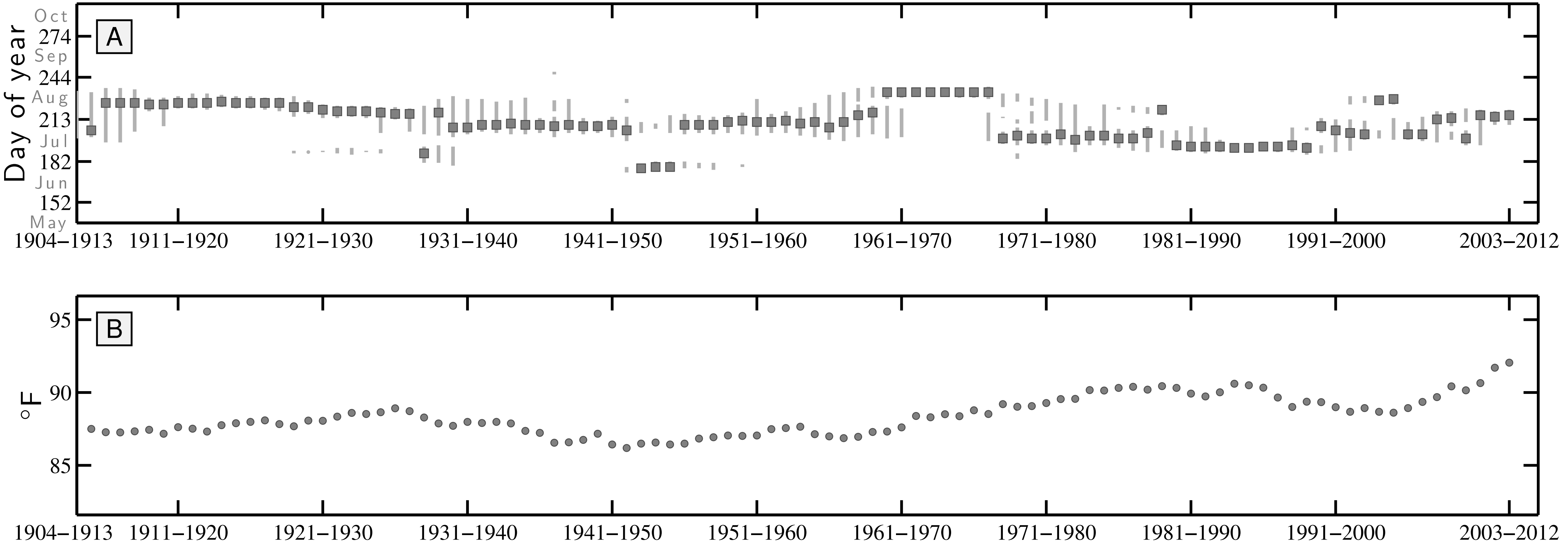

10 year Winter Teletherm dynamics for SOUTHPORT 5 N, NC:

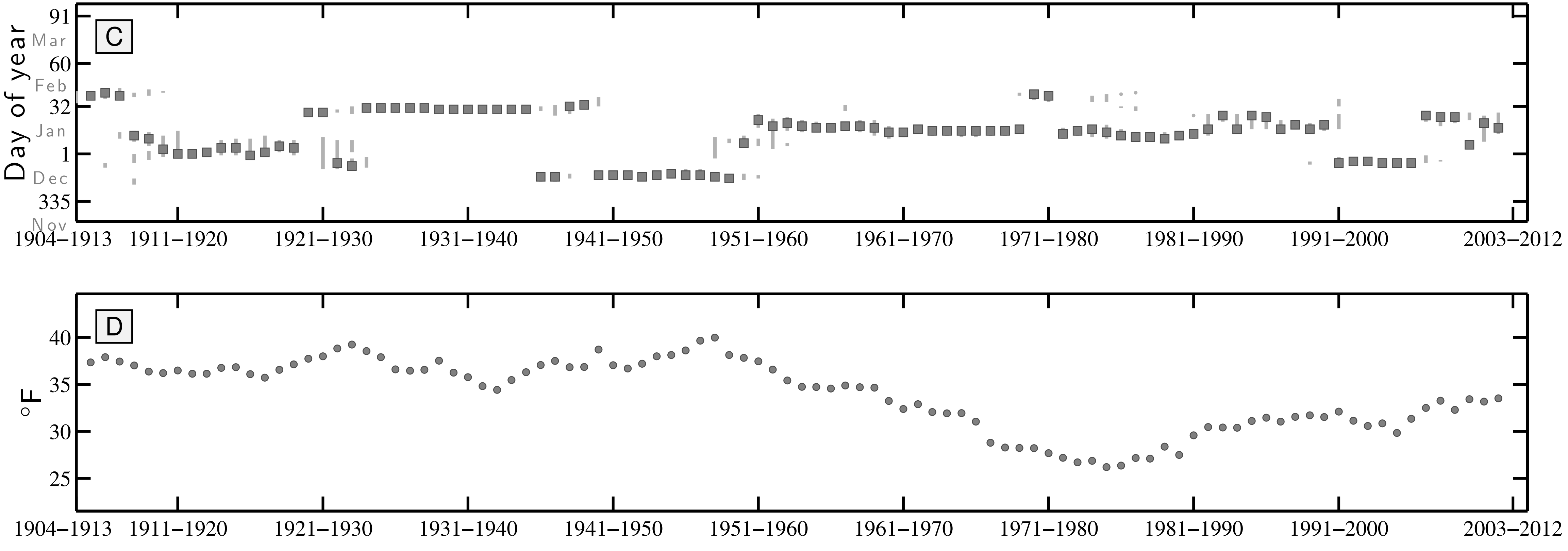

10 year Summer Teletherm dynamics for STATESVILLE 2 NNE, NC:

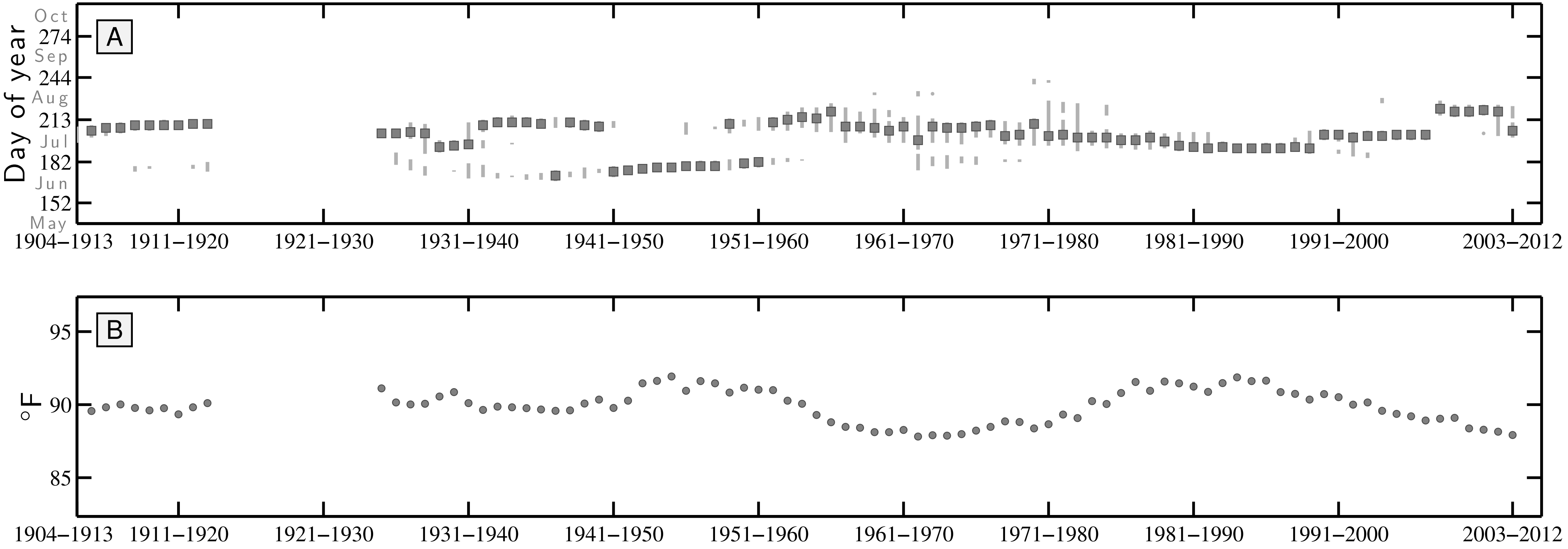

10 year Winter Teletherm dynamics for STATESVILLE 2 NNE, NC:

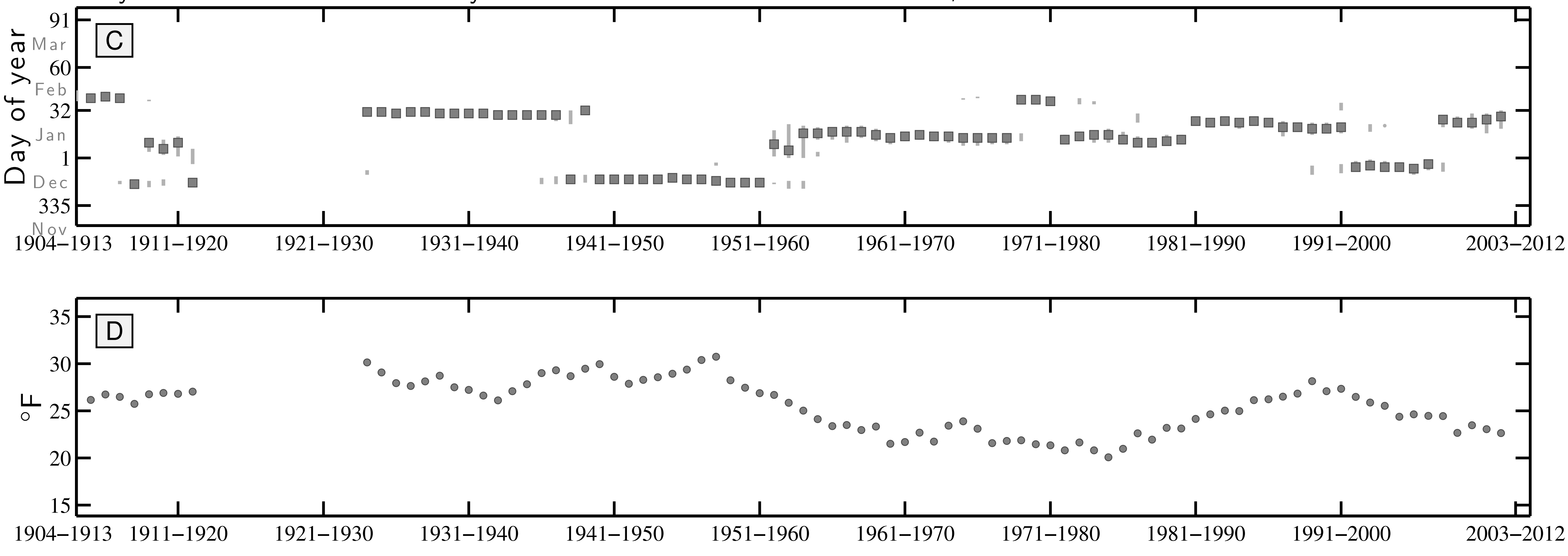

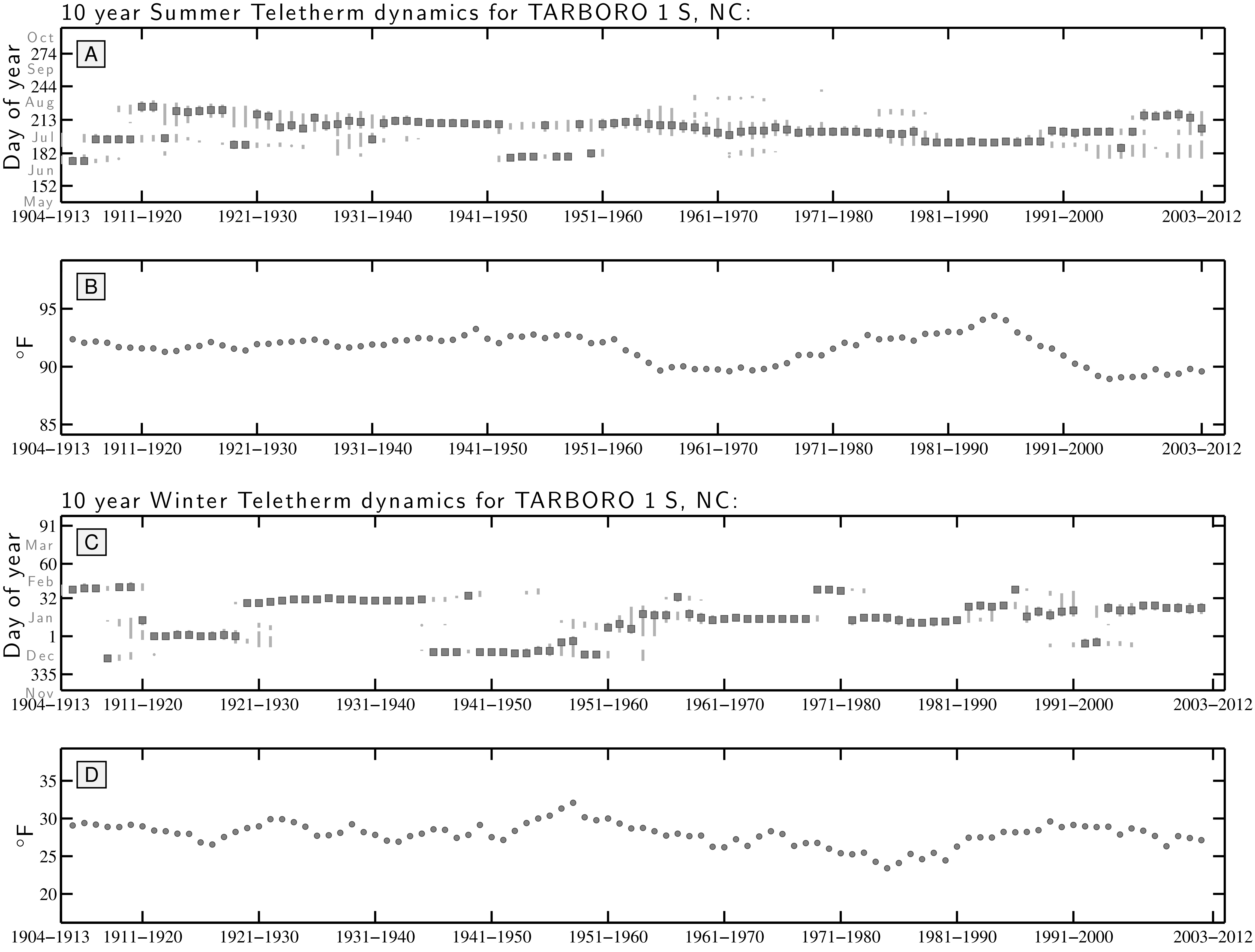
10 year Summer Teletherm dynamics for TARBORO 1 S, NC:
A
B
Day of year
°F
Oct
274
Sep
244
Aug
213
Jul
182
Jun
152
May
95
90
85
10 year Winter Teletherm dynamics for TARBORO 1 S, NC:
C
D
91
Mar
60
Feb
32
Jan
1
Dec
335
Nov
35
30
25
20
1904–1913
1911–1920
1921–1930
1931–1940
1941–1950
1951–1960
1961–1970
1971–1980
1981–1990
1991–2000
2003–2012

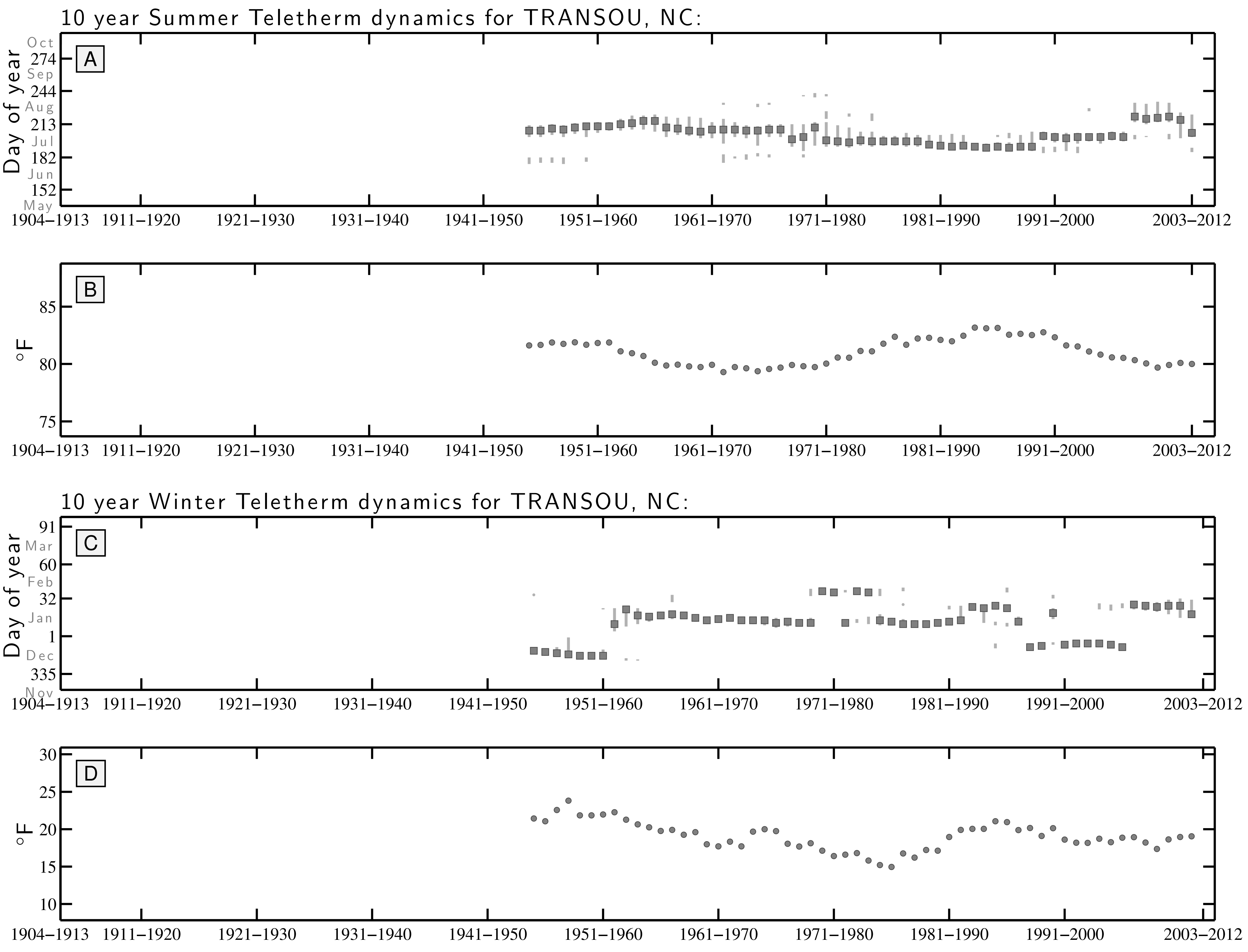

10 year Summer Teletherm dynamics for TRANSOU, NC:
A
Day of year
Oct
274
Sep
244
Aug
213
Jul
182
Jun
152
May
1904–1913
1911–1920
1921–1930
1931–1940
1941–1950
1951–1960
1961–1970
1971–1980
1981–1990
1991–2000
2003–2012
B
°F
85
80
75
10 year Winter Teletherm dynamics for TRANSOU, NC:
C
91
Mar
60
Feb
32
Jan
1
Dec
335
Nov
D
30
25
20
15
10

10 year Summer Teletherm dynamics for WAYNESVILLE 1 E, NC:

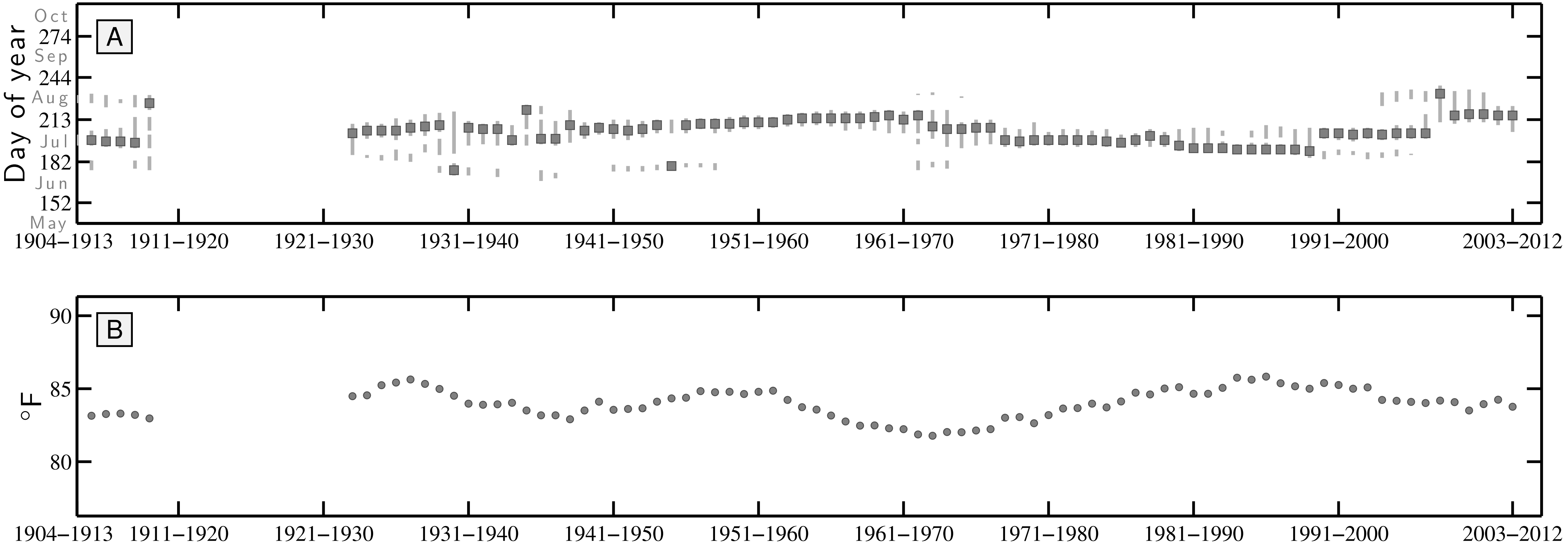

10 year Winter Teletherm dynamics for WAYNESVILLE 1 E, NC:

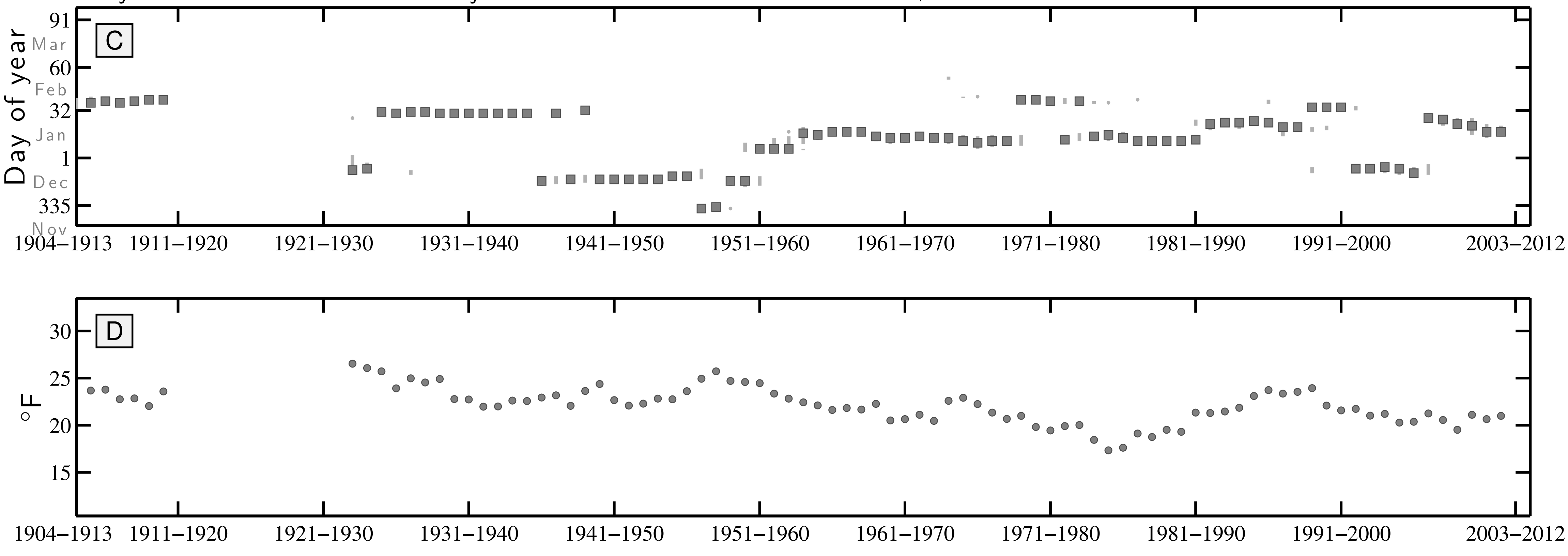

10 year Summer Teletherm dynamics for WILSON 3 SW, NC:

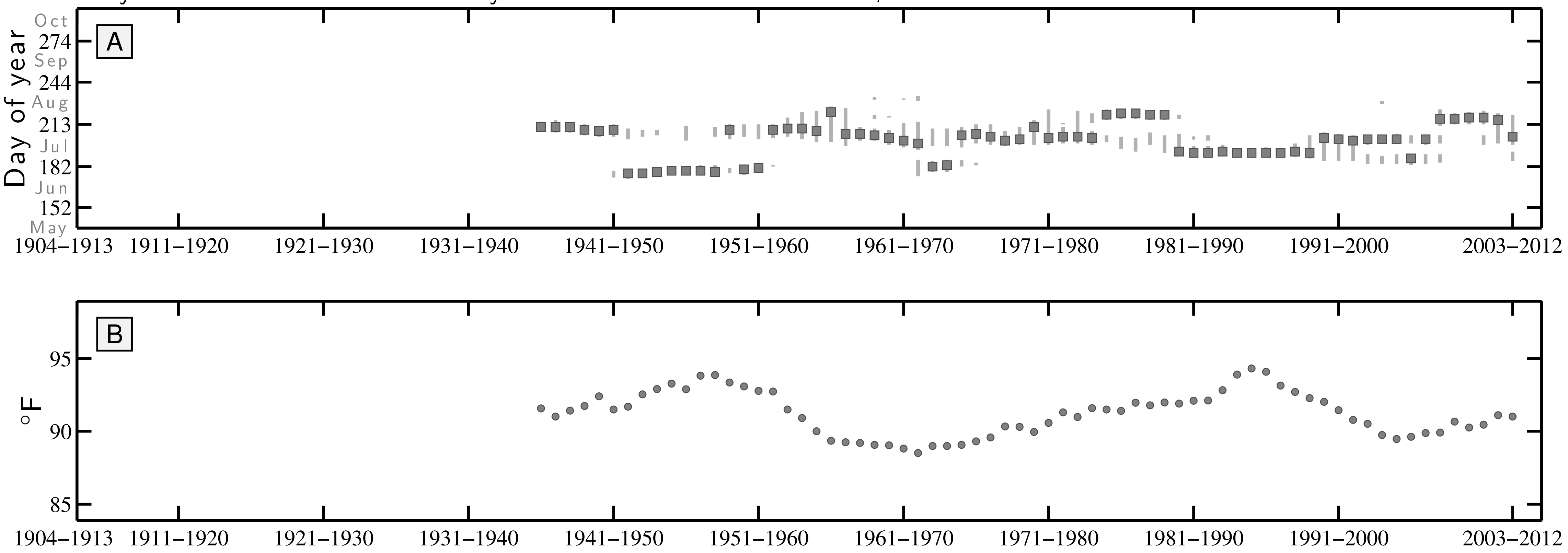

10 year Winter Teletherm dynamics for WILSON 3 SW, NC:

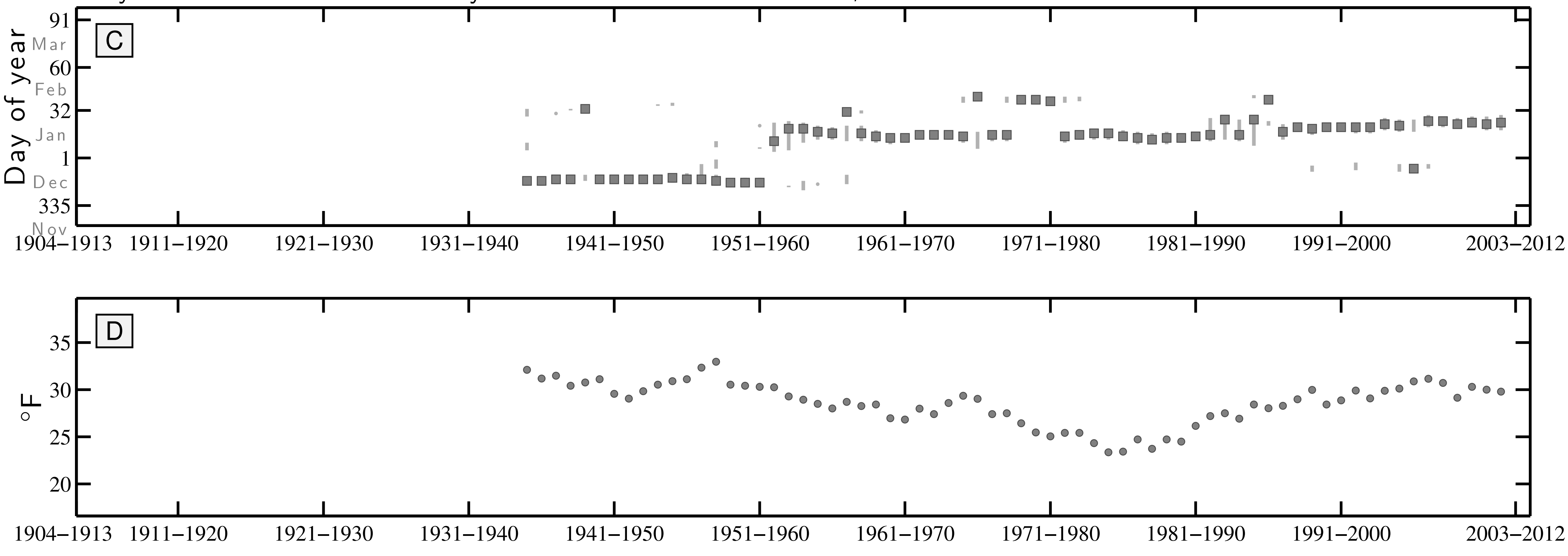

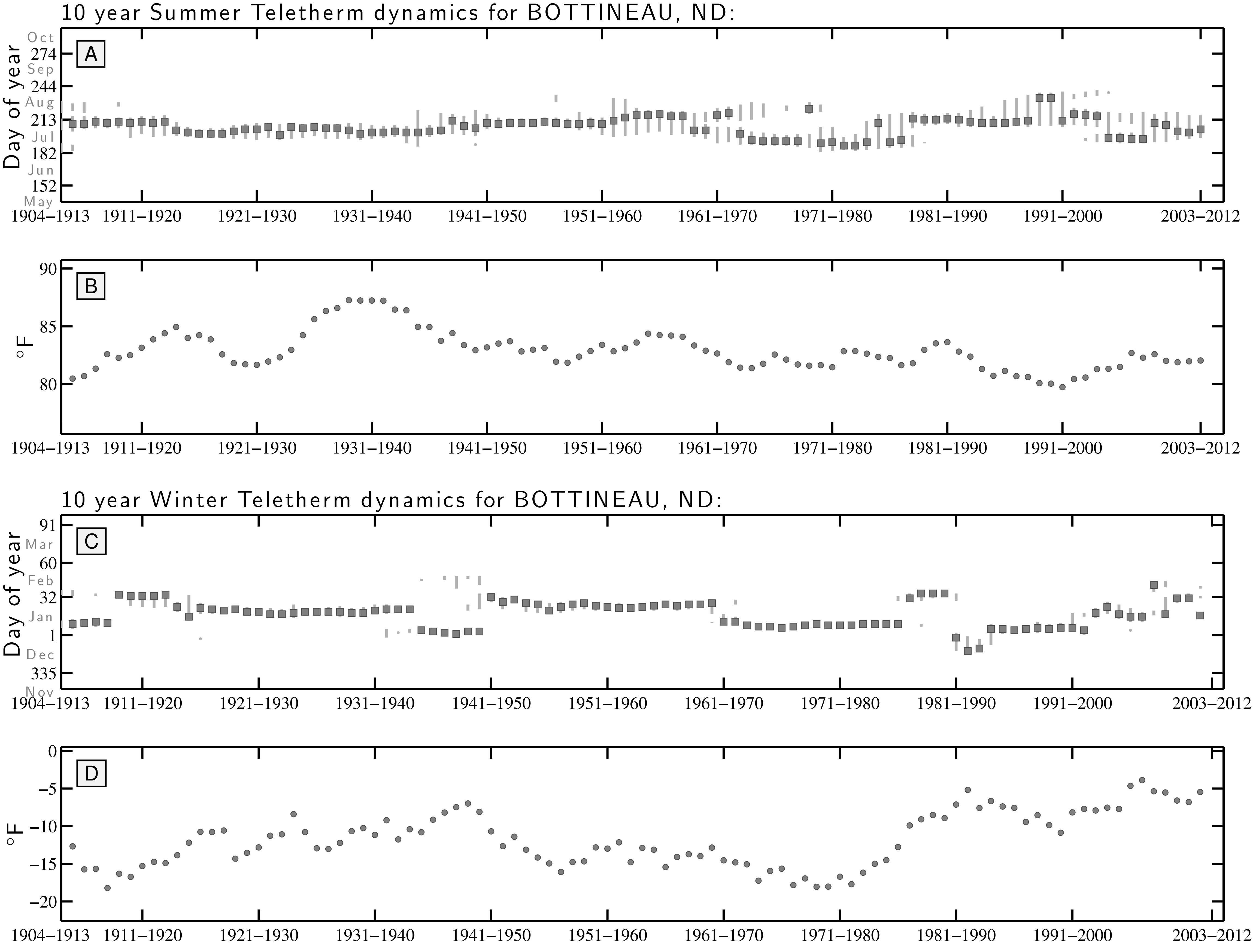
10 year Summer Teletherm dynamics for BOTTINEAU, ND:
A
Day of year
Oct
274
Sep
244
Aug
213
Jul
182
Jun
152
May
1904–1913
1911–1920
1921–1930
1931–1940
1941–1950
1951–1960
1961–1970
1971–1980
1981–1990
1991–2000
2003–2012
B
°F
90
85
80
10 year Winter Teletherm dynamics for BOTTINEAU, ND:
C
91
Mar
60
Feb
32
Jan
1
Dec
335
Nov
D
0
−5
−10
−15
−20

10 year Summer Teletherm dynamics for CASSELTON AGRONOMY FM, ND:

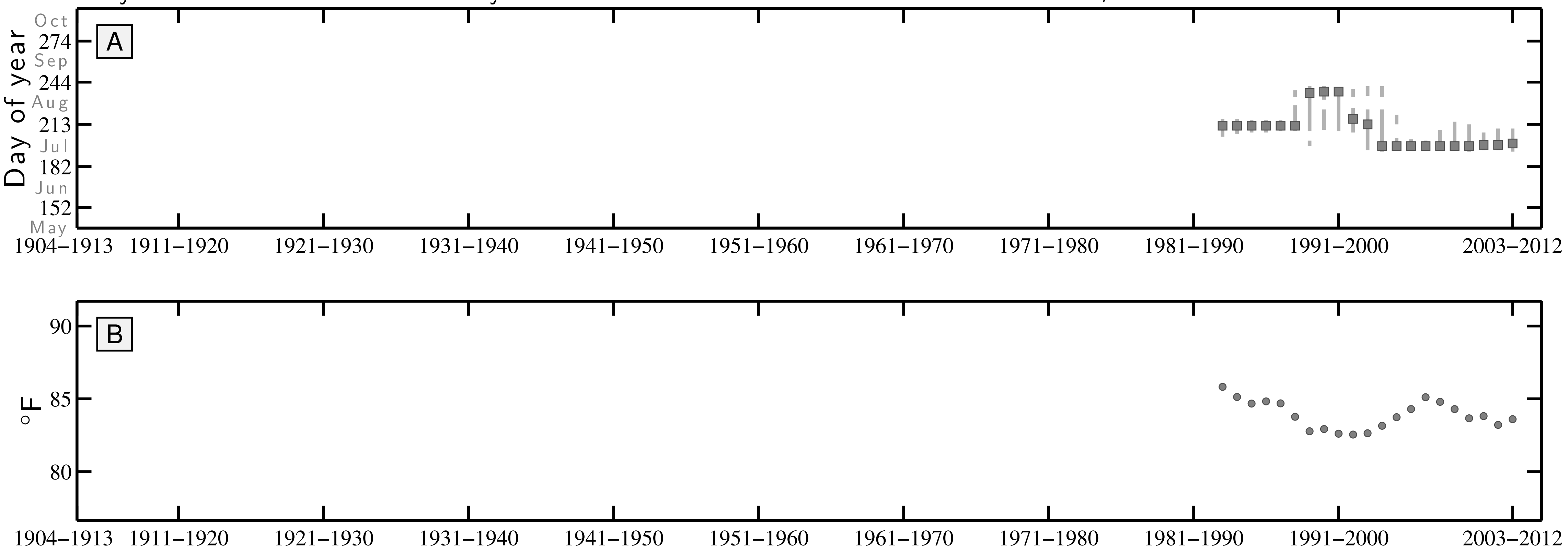

10 year Winter Teletherm dynamics for CASSELTON AGRONOMY FM, ND:

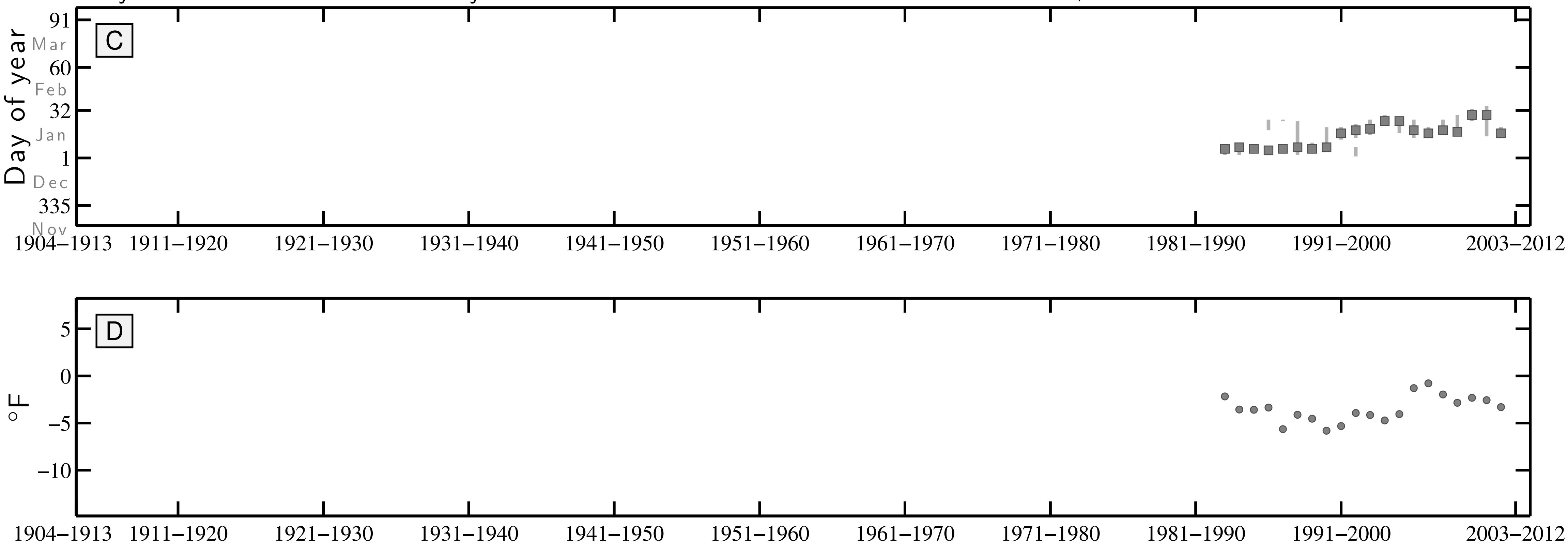

10 year Summer Teletherm dynamics for CROSBY, ND:

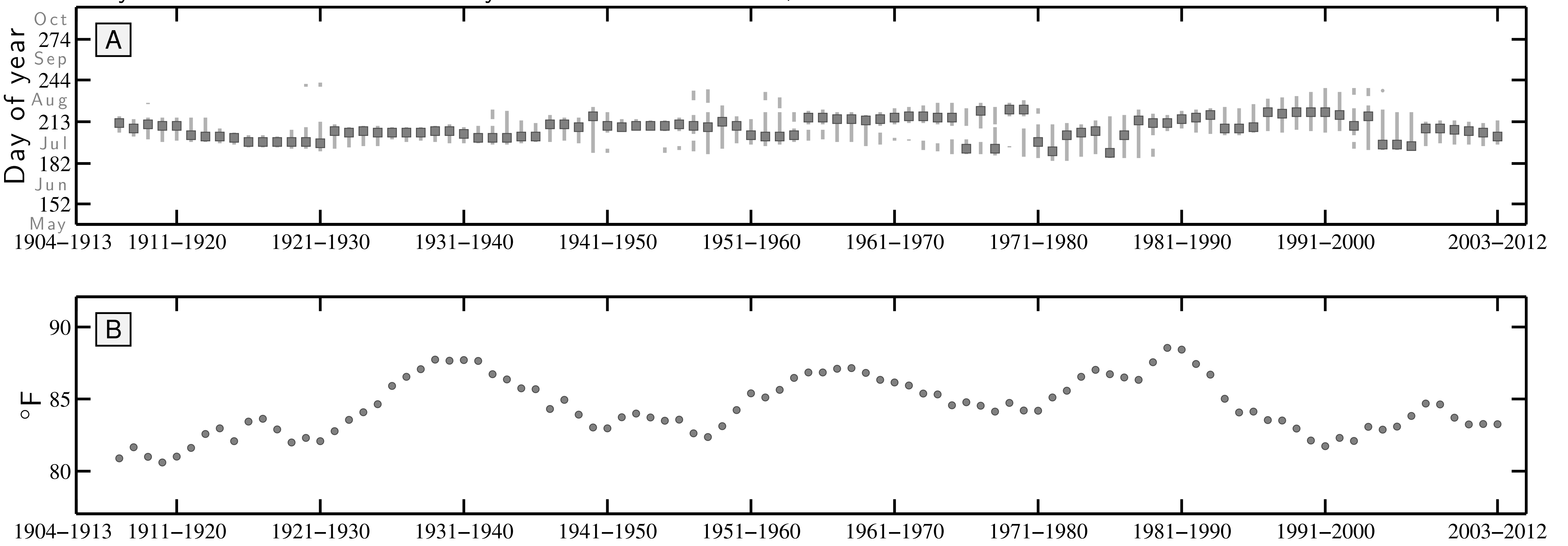

10 year Winter Teletherm dynamics for CROSBY, ND:

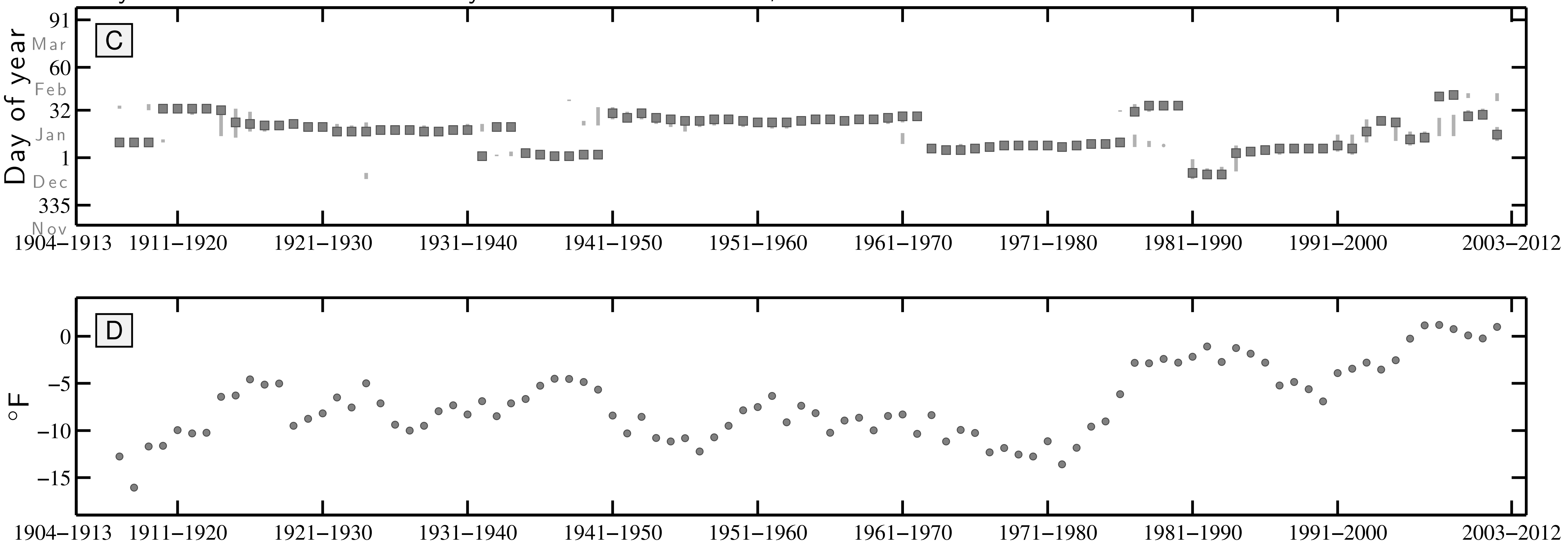

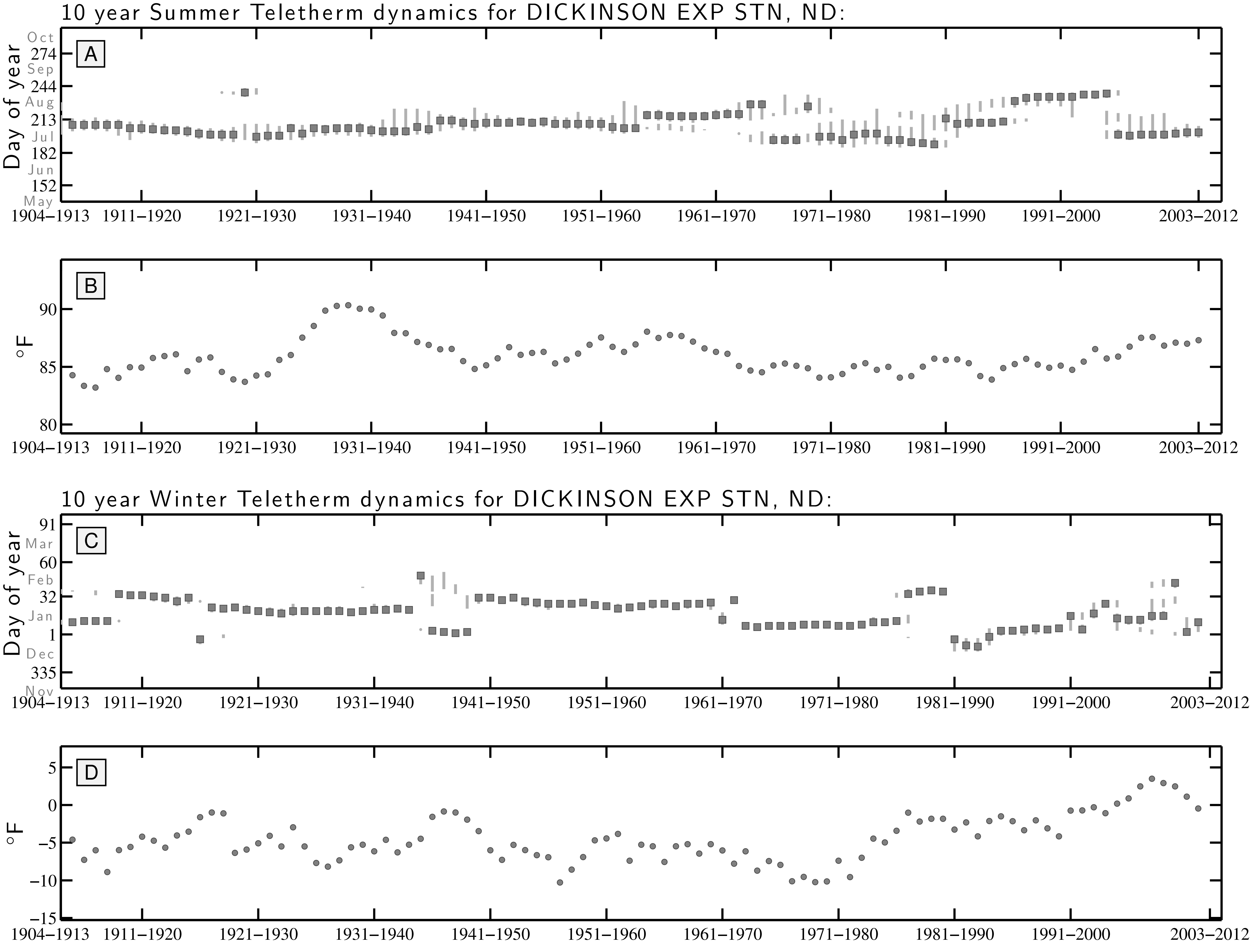
10 year Summer Teletherm dynamics for DICKINSON EXP STN, ND:
A
Day of year
Oct
274
Sep
244
Aug
213
Jul
182
Jun
152
May
1904–1913
1911–1920
1921–1930
1931–1940
1941–1950
1951–1960
1961–1970
1971–1980
1981–1990
1991–2000
2003–2012
B
°F
90
85
80
10 year Winter Teletherm dynamics for DICKINSON EXP STN, ND:
C
91
Mar
60
Feb
32
Jan
1
Dec
335
Nov
D
5
0
−5
−10
−15

10 year Summer Teletherm dynamics for DUNN CENTER 1E, ND:

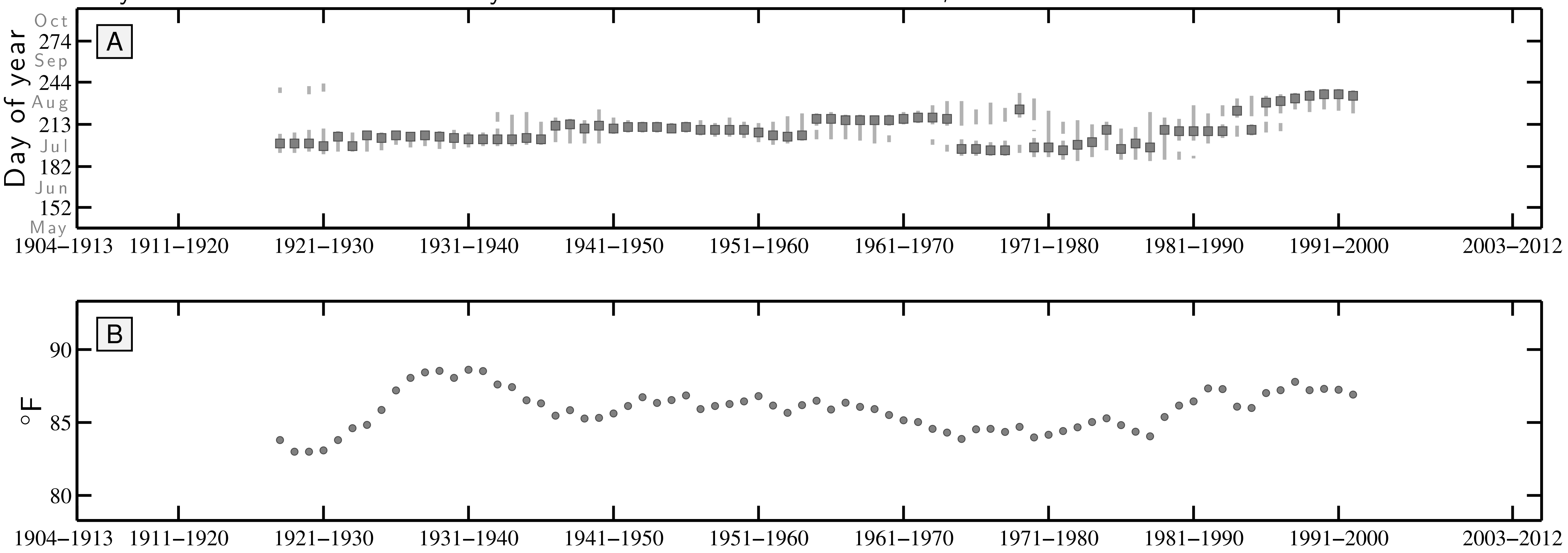

10 year Winter Teletherm dynamics for DUNN CENTER 1E, ND:

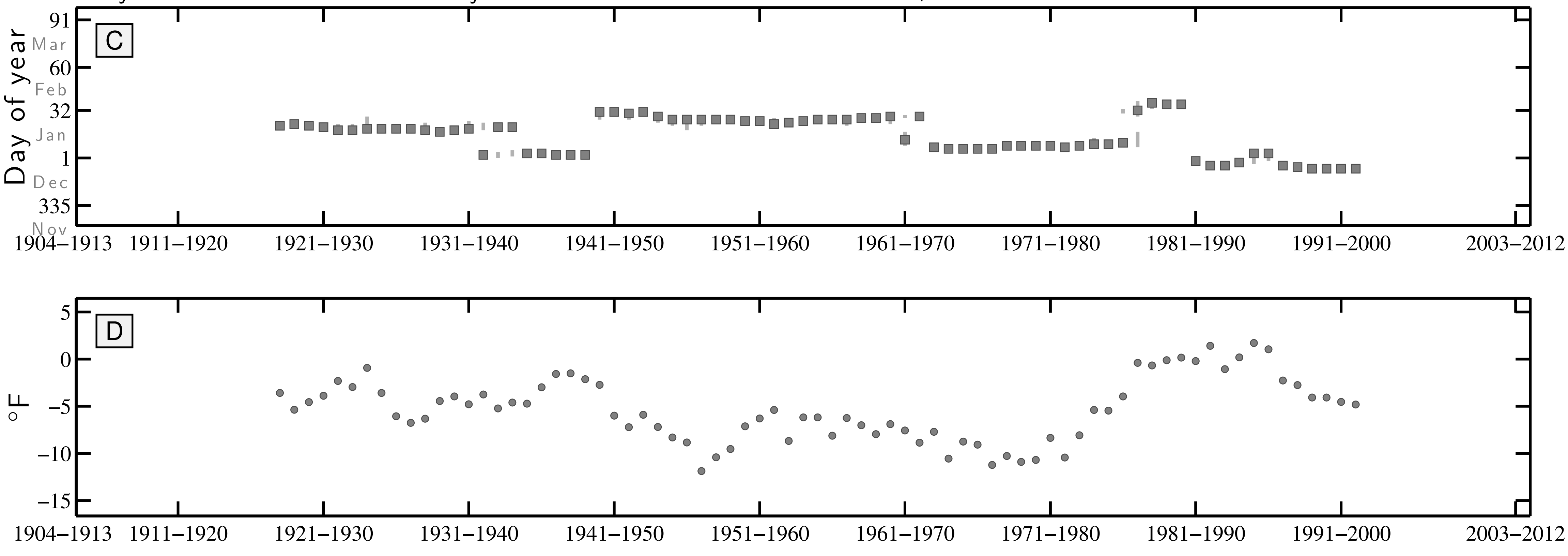

10 year Summer Teletherm dynamics for FT YATES 4 SW, ND:

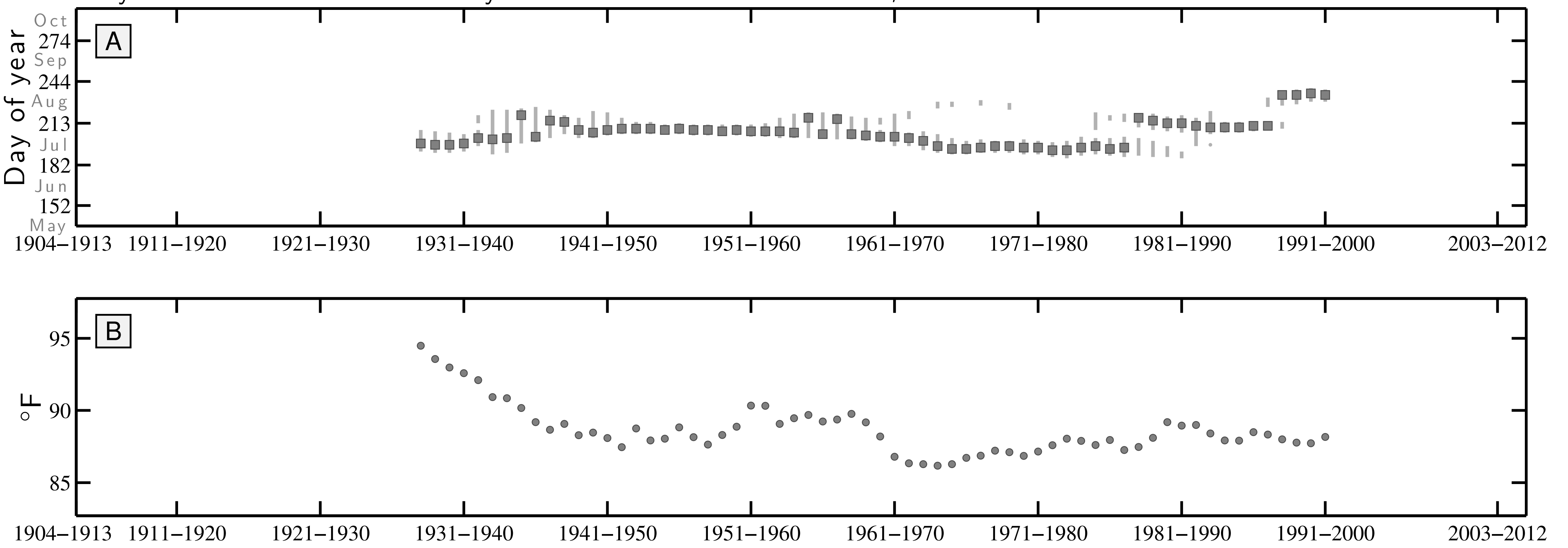

10 year Winter Teletherm dynamics for FT YATES 4 SW, ND:

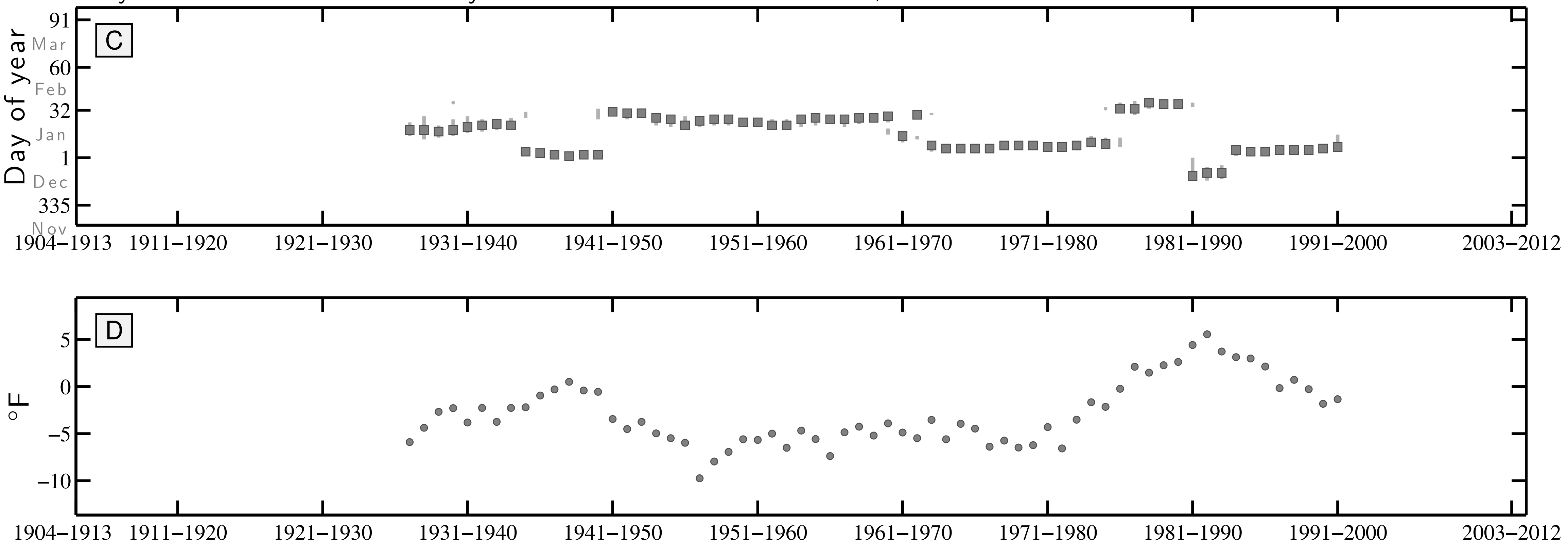

10 year Summer Teletherm dynamics for FULLERTON 1 ESE, ND:

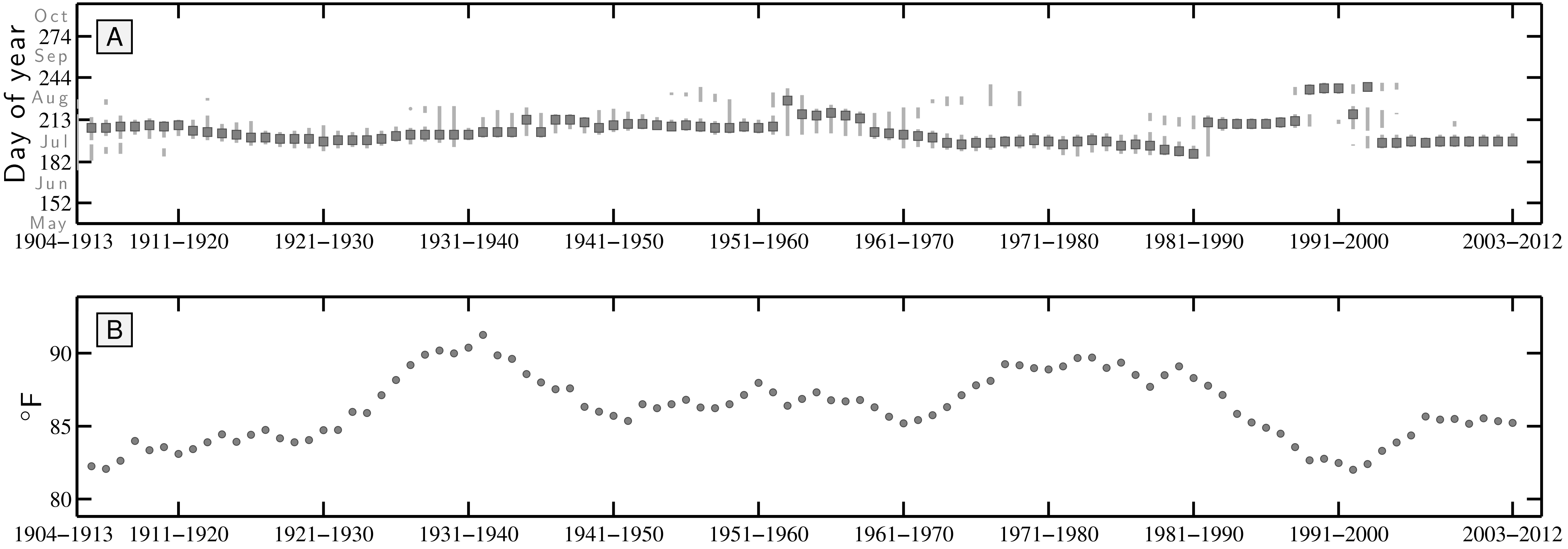

10 year Winter Teletherm dynamics for FULLERTON 1 ESE, ND:

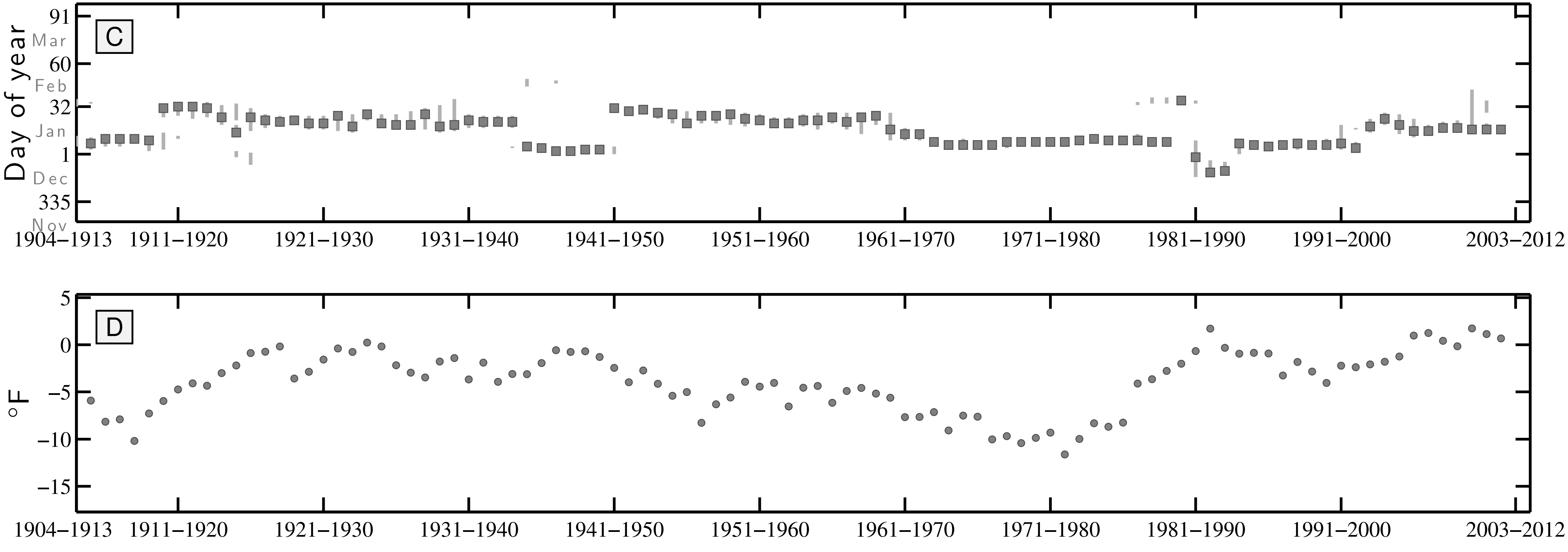

10 year Summer Teletherm dynamics for GRAFTON, ND:

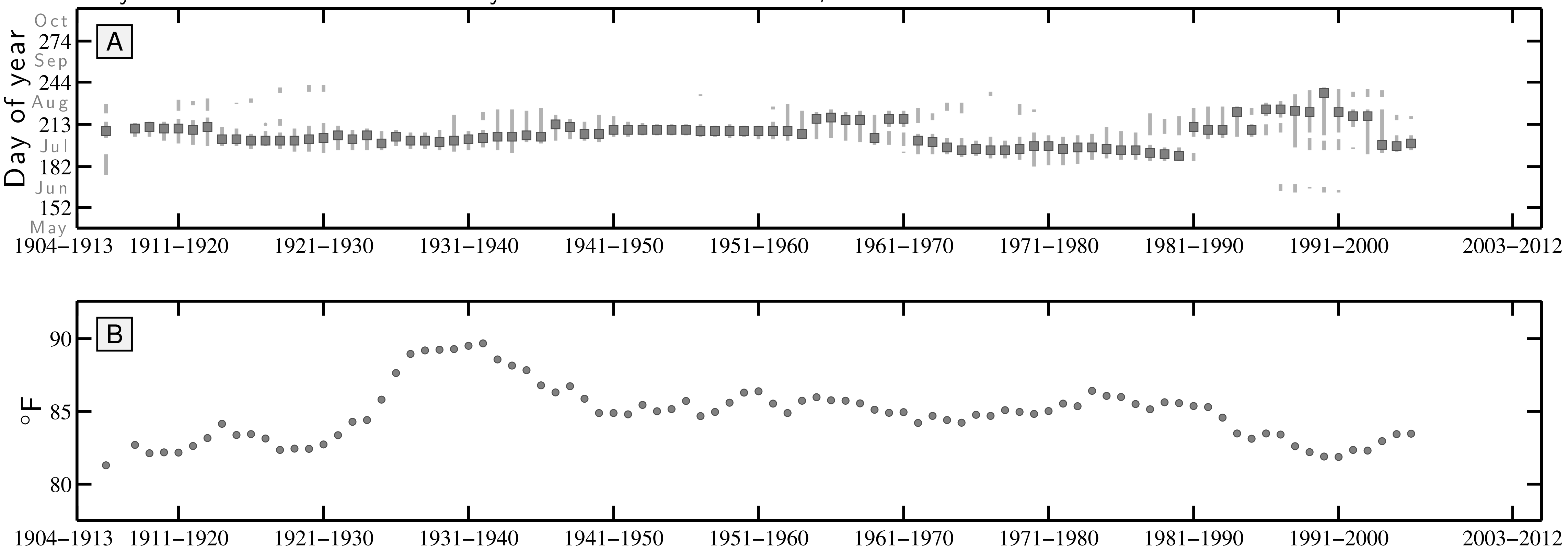

10 year Winter Teletherm dynamics for GRAFTON, ND:

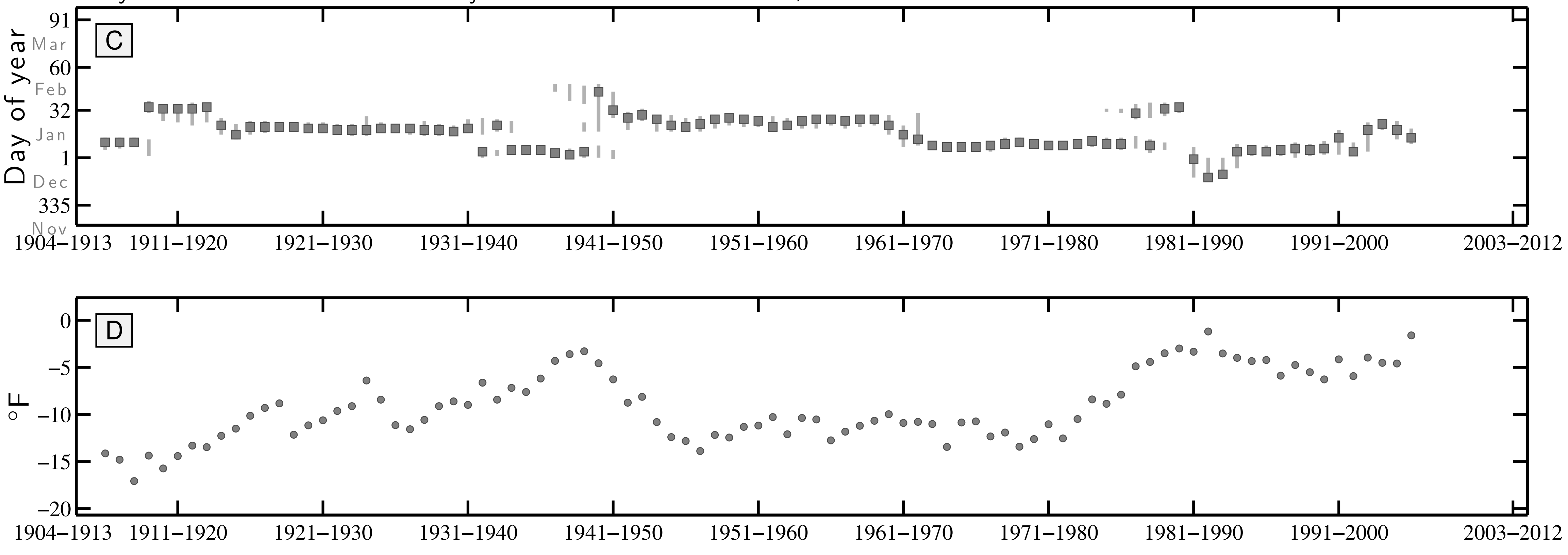

10 year Summer Teletherm dynamics for GRAND FORKS UNIV NWS, ND:

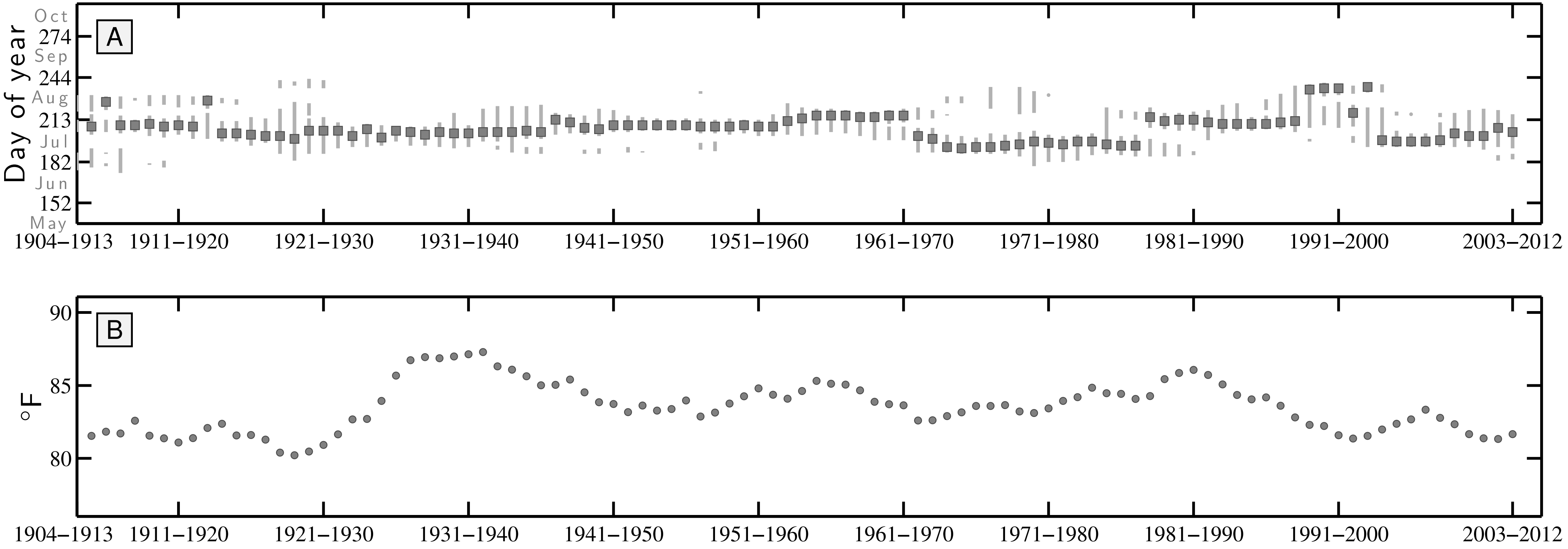

10 year Winter Teletherm dynamics for GRAND FORKS UNIV NWS, ND:

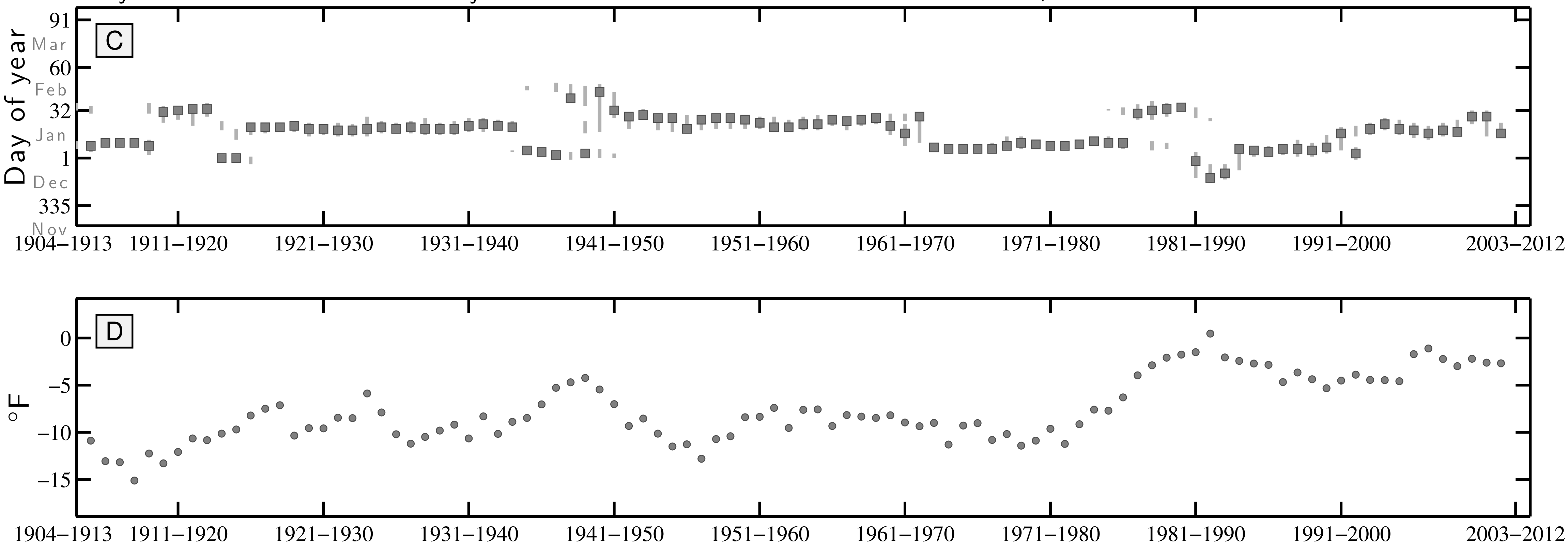

10 year Summer Teletherm dynamics for HETTINGER, ND:

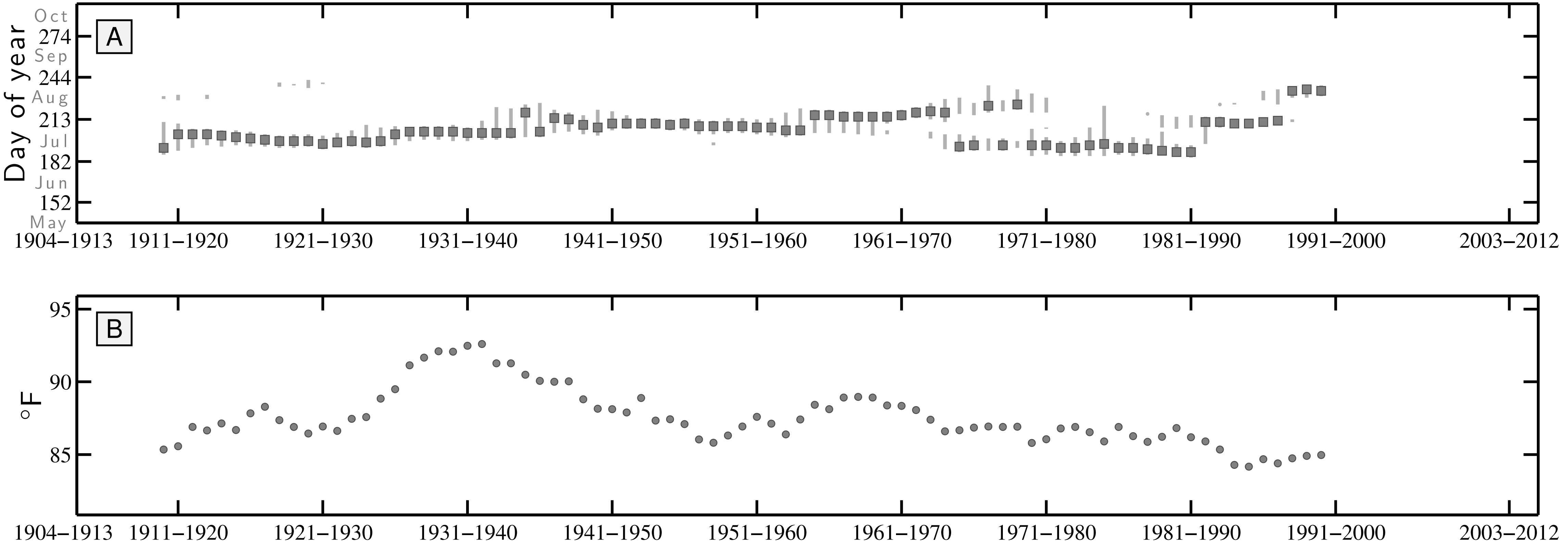

10 year Winter Teletherm dynamics for HETTINGER, ND:

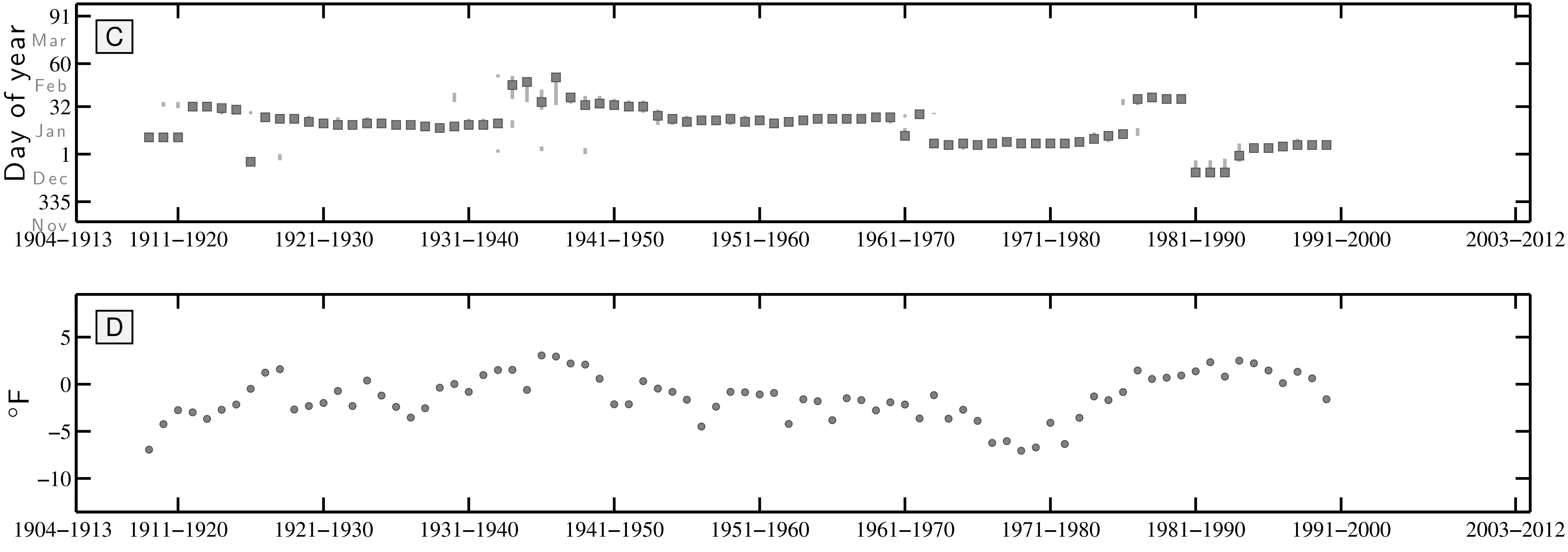

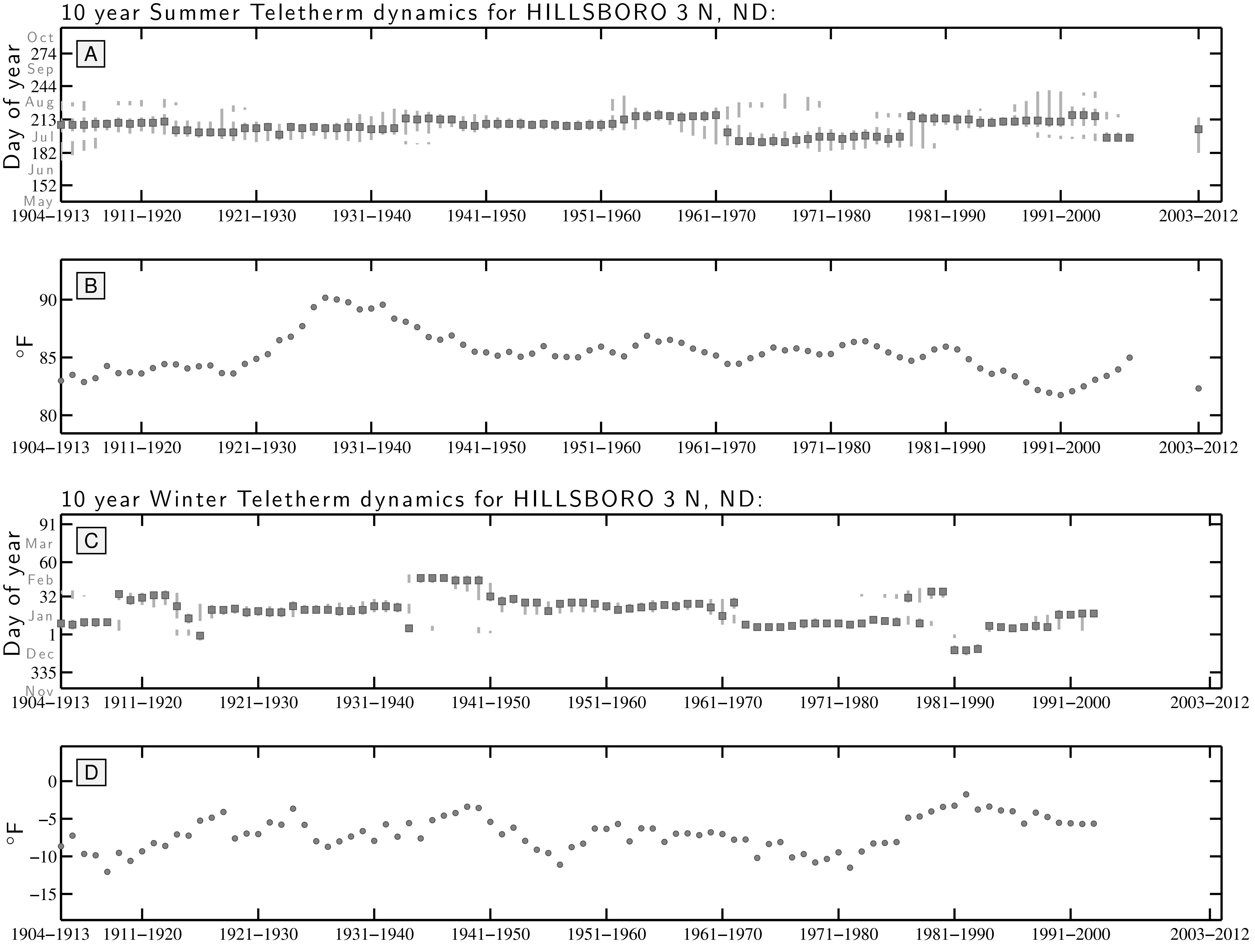
10 year Summer Teletherm dynamics for HILLSBORO 3 N, ND:
A
Day of year
Oct
274
Sep
244
Aug
213
Jul
182
Jun
152
May
1904–1913
1911–1920
1921–1930
1931–1940
1941–1950
1951–1960
1961–1970
1971–1980
1981–1990
1991–2000
2003–2012
B
°F
90
85
80
10 year Winter Teletherm dynamics for HILLSBORO 3 N, ND:
C
91
Mar
60
Feb
32
Jan
1
Dec
335
Nov
D
0
−5
−10
−15

10 year Summer Teletherm dynamics for JAMESTOWN STATE HOSP, ND:

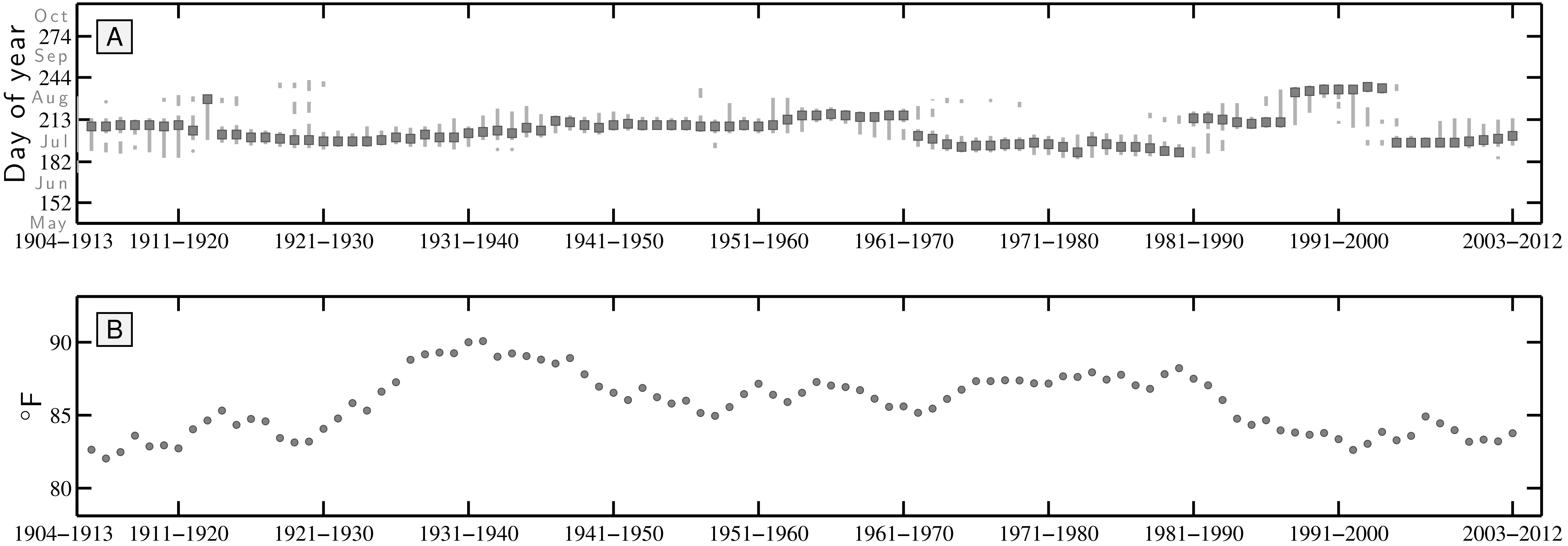

10 year Winter Teletherm dynamics for JAMESTOWN STATE HOSP, ND:

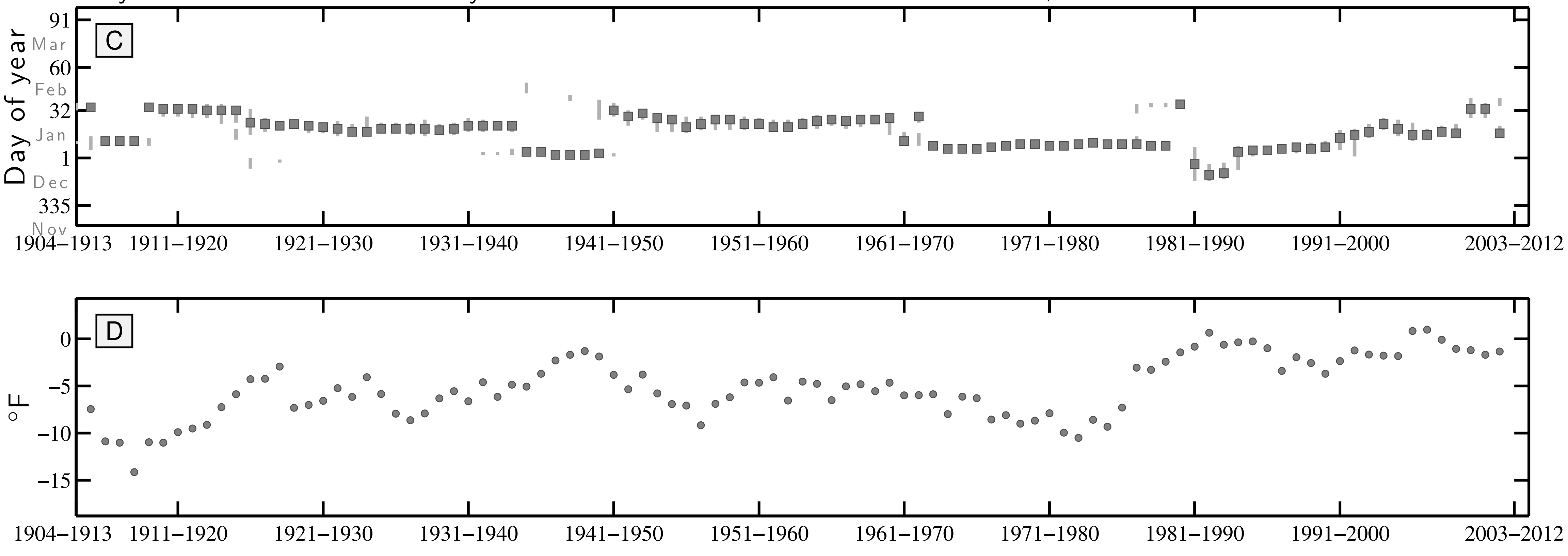

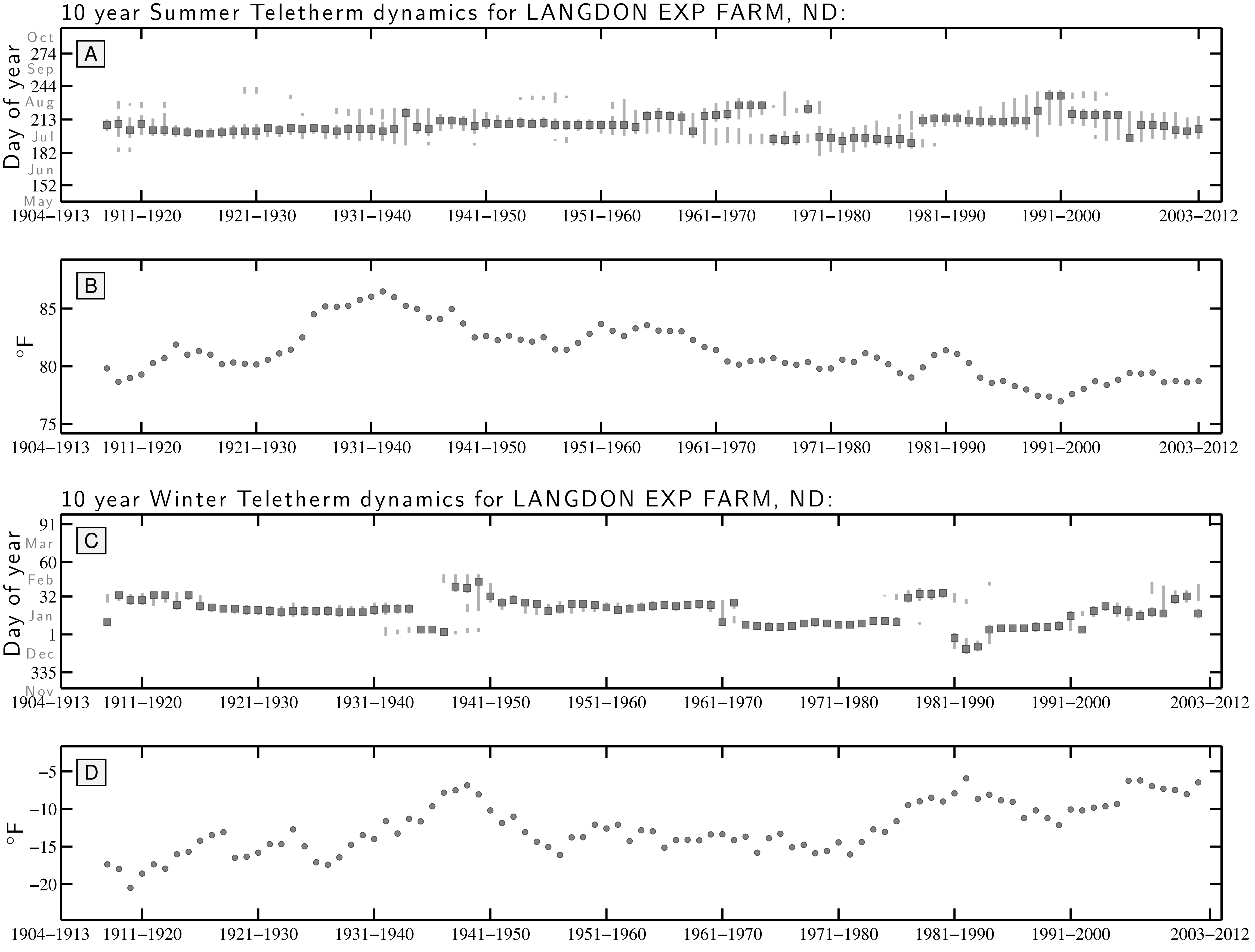
10 year Summer Teletherm dynamics for LANGDON EXP FARM, ND:
A
Day of year
Oct
274
Sep
244
Aug
213
Jul
182
Jun
152
May
1904–1913
1911–1920
1921–1930
1931–1940
1941–1950
1951–1960
1961–1970
1971–1980
1981–1990
1991–2000
2003–2012
B
°F
85
80
75
10 year Winter Teletherm dynamics for LANGDON EXP FARM, ND:
C
Day of year
91
Mar
60
Feb
32
Jan
1
Dec
335
Nov
D
°F
−5
−10
−15
−20

10 year Summer Teletherm dynamics for LISBON, ND:

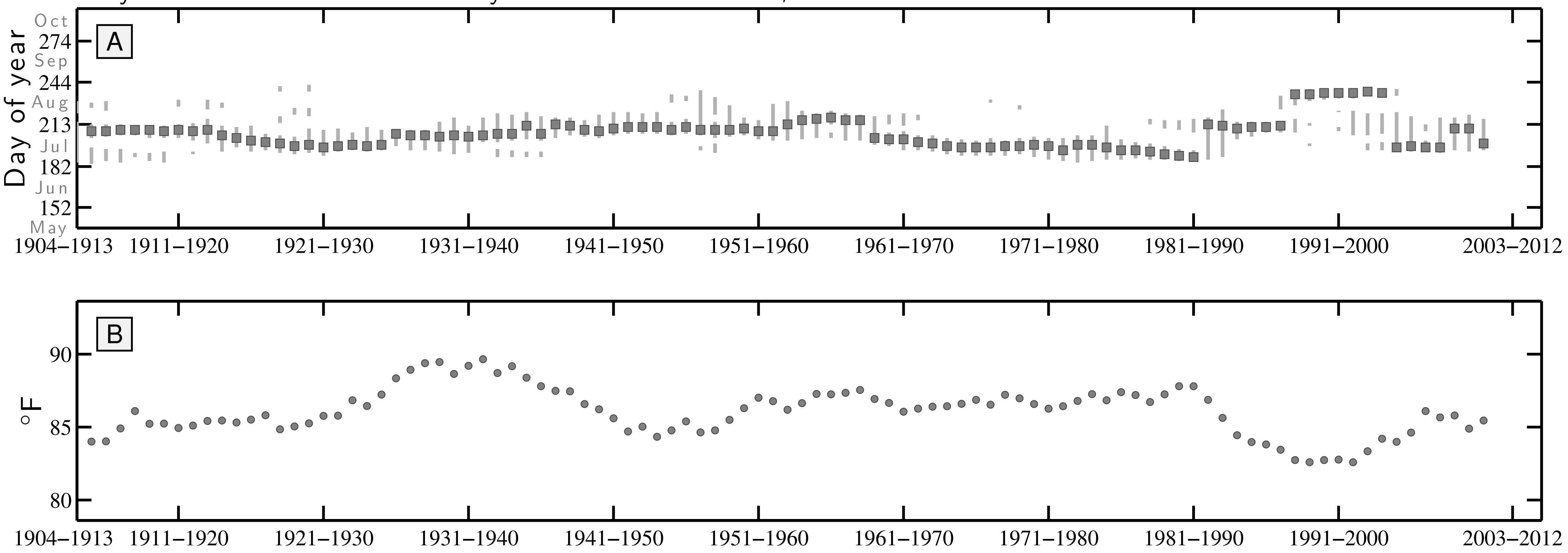

10 year Winter Teletherm dynamics for LISBON, ND:

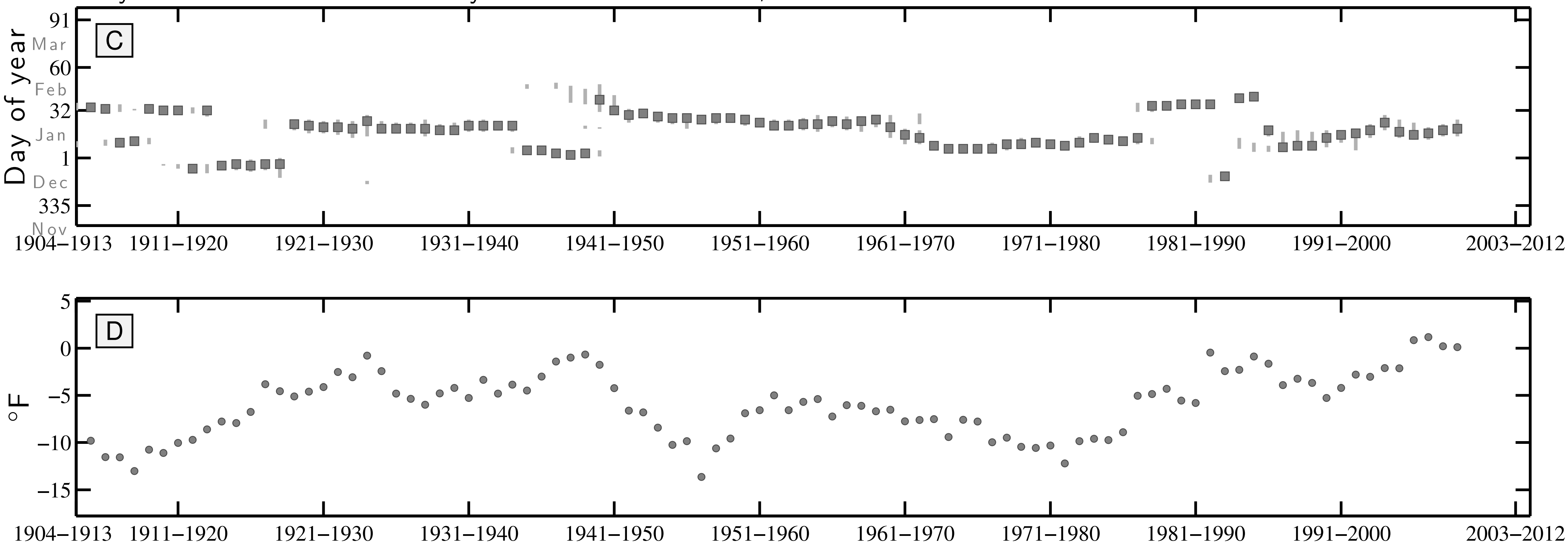

10 year Summer Teletherm dynamics for MANDAN EXP STN, ND:

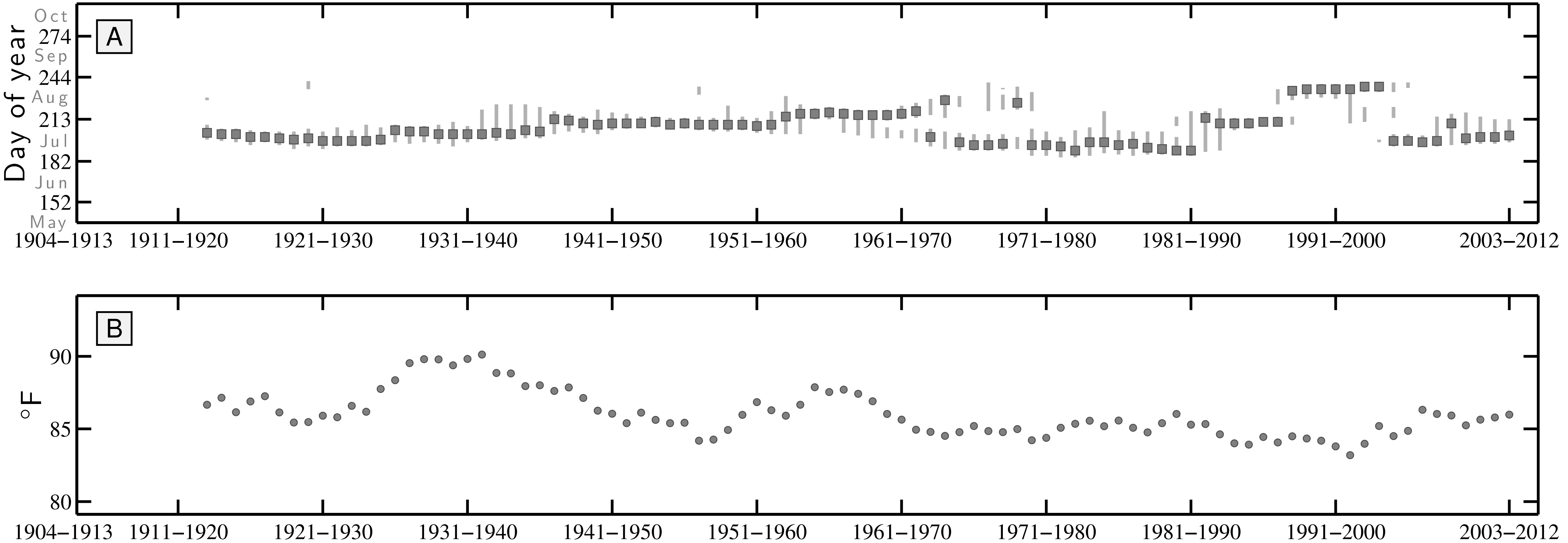

10 year Winter Teletherm dynamics for MANDAN EXP STN, ND:

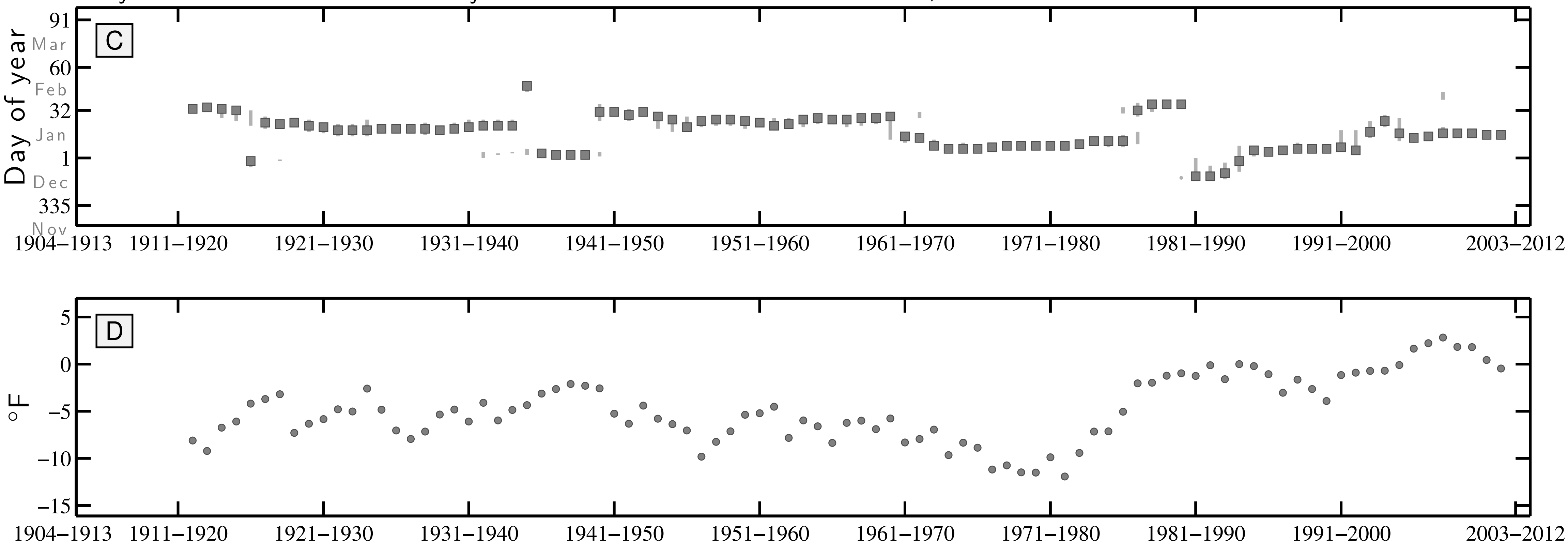

10 year Summer Teletherm dynamics for MOFFIT 3 SE, ND:

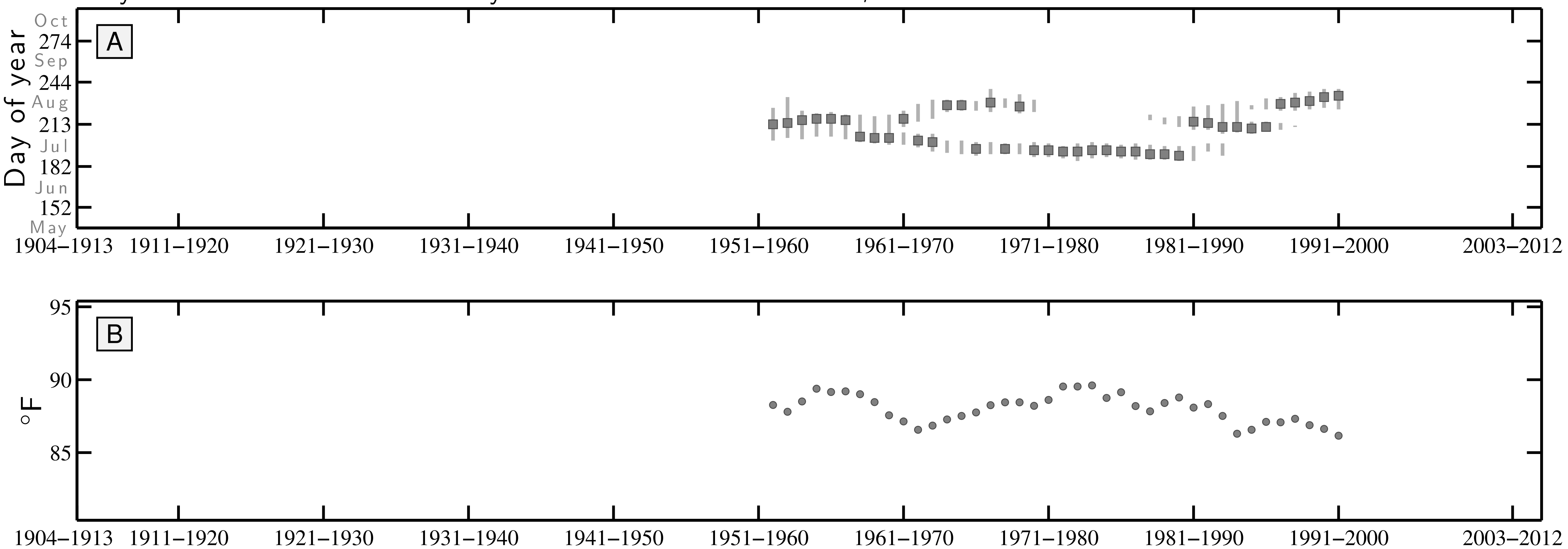

10 year Winter Teletherm dynamics for MOFFIT 3 SE, ND:

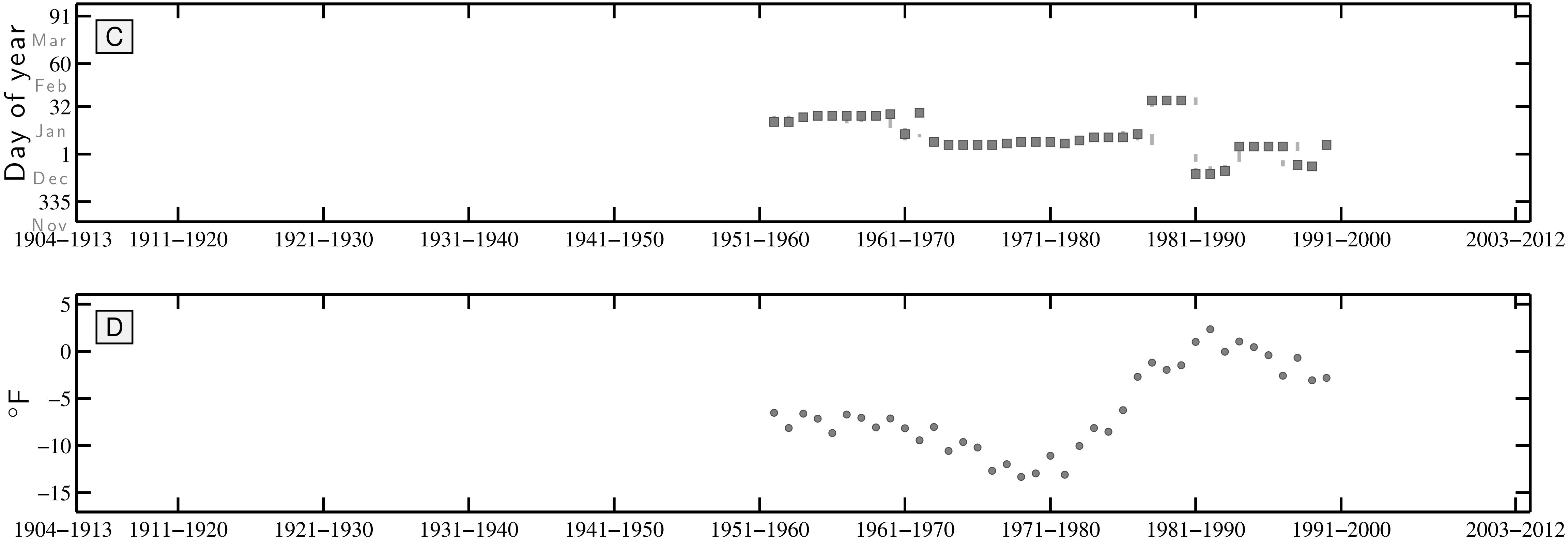

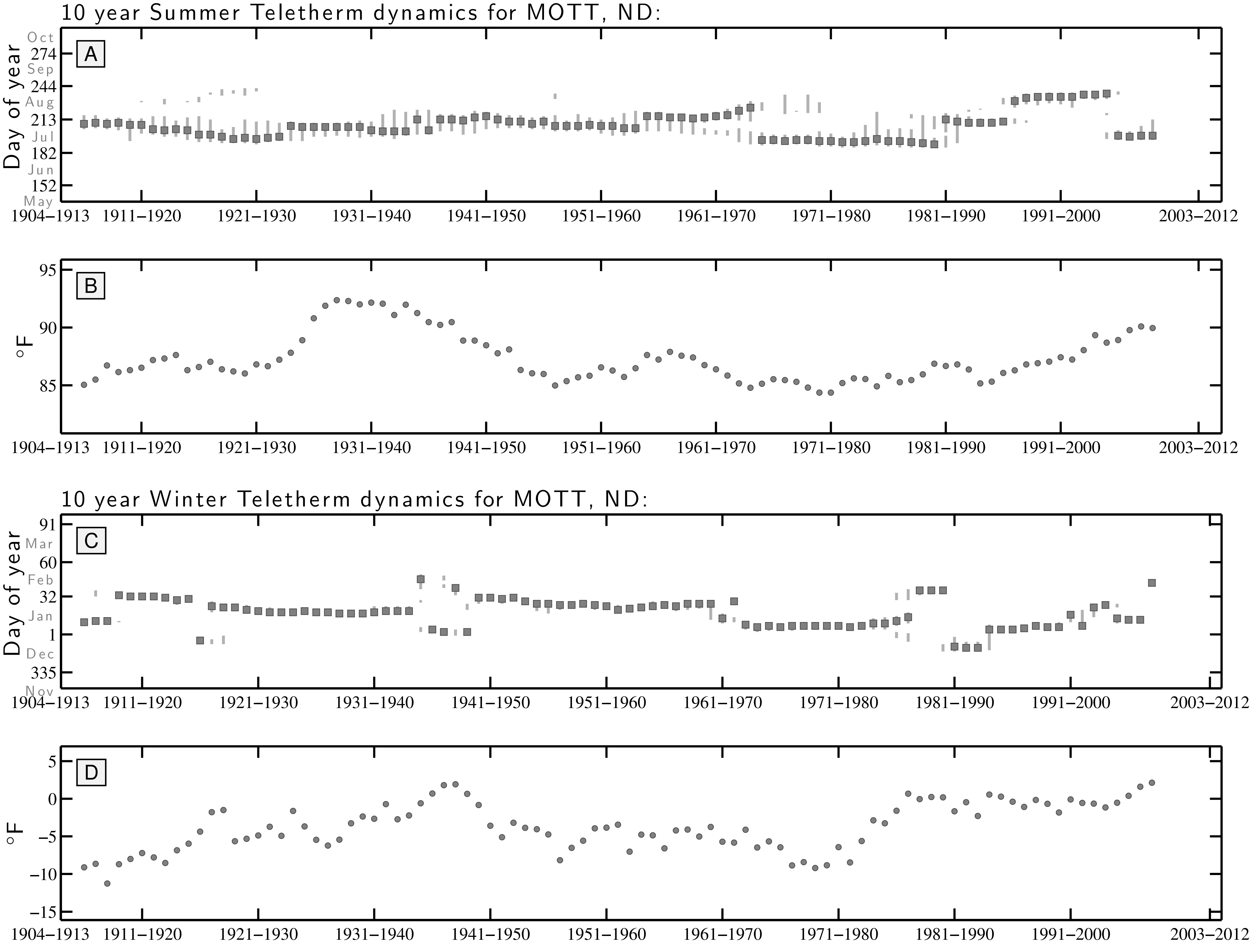
10 year Summer Teletherm dynamics for MOTT, ND:
A
Day of year
Oct
274
Sep
244
Aug
213
Jul
182
Jun
152
May
B
°F
95
90
85
10 year Winter Teletherm dynamics for MOTT, ND:
C
Day of year
91
Mar
60
Feb
32
Jan
1
Dec
335
Nov
D
°F
5
0
−5
−10
−15
1904–1913
1911–1920
1921–1930
1931–1940
1941–1950
1951–1960
1961–1970
1971–1980
1981–1990
1991–2000
2003–2012

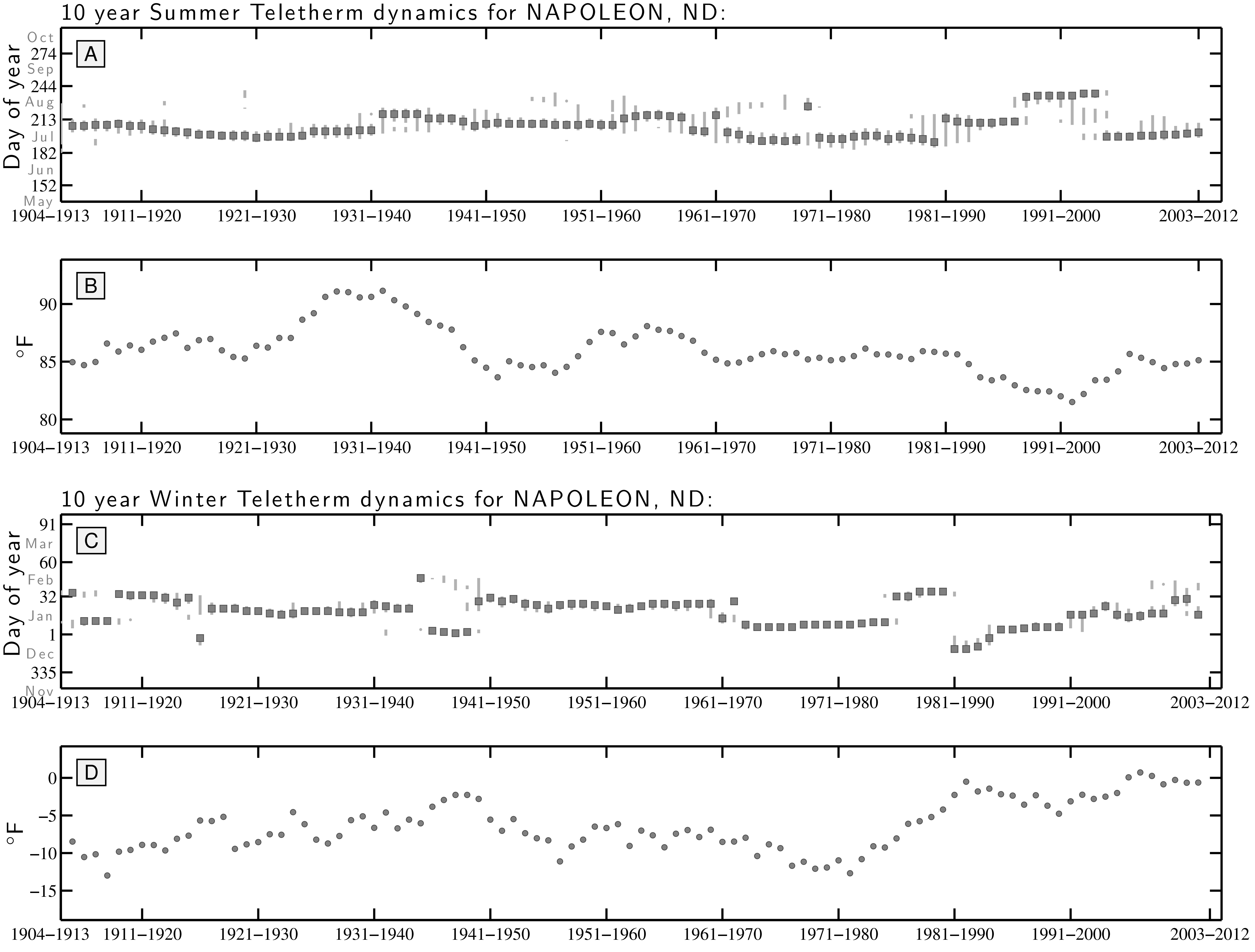
10 year Summer Teletherm dynamics for NAPOLEON, ND:
A
Day of year
Oct
274
Sep
244
Aug
213
Jul
182
Jun
152
May
1904–1913
1911–1920
1921–1930
1931–1940
1941–1950
1951–1960
1961–1970
1971–1980
1981–1990
1991–2000
2003–2012
B
°F
90
85
80
10 year Winter Teletherm dynamics for NAPOLEON, ND:
C
91
Mar
60
Feb
32
Jan
1
Dec
335
Nov
D
0
−5
−10
−15

10 year Summer Teletherm dynamics for NEW ENGLAND, ND:

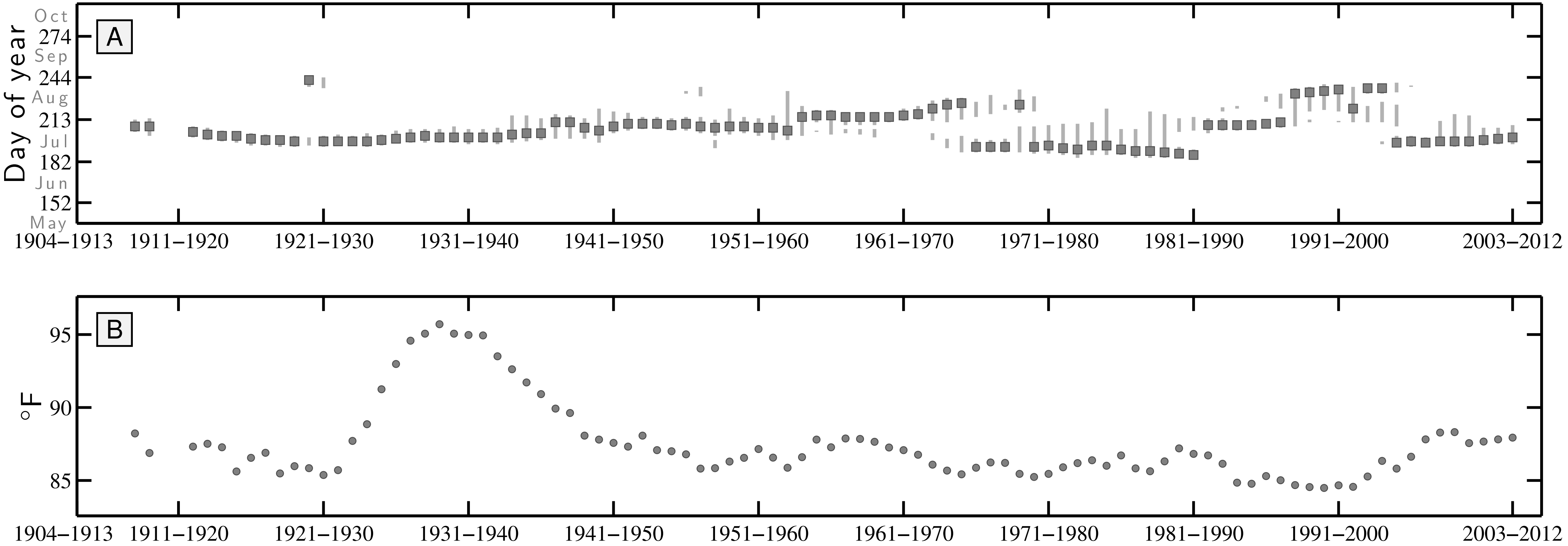

10 year Winter Teletherm dynamics for NEW ENGLAND, ND:

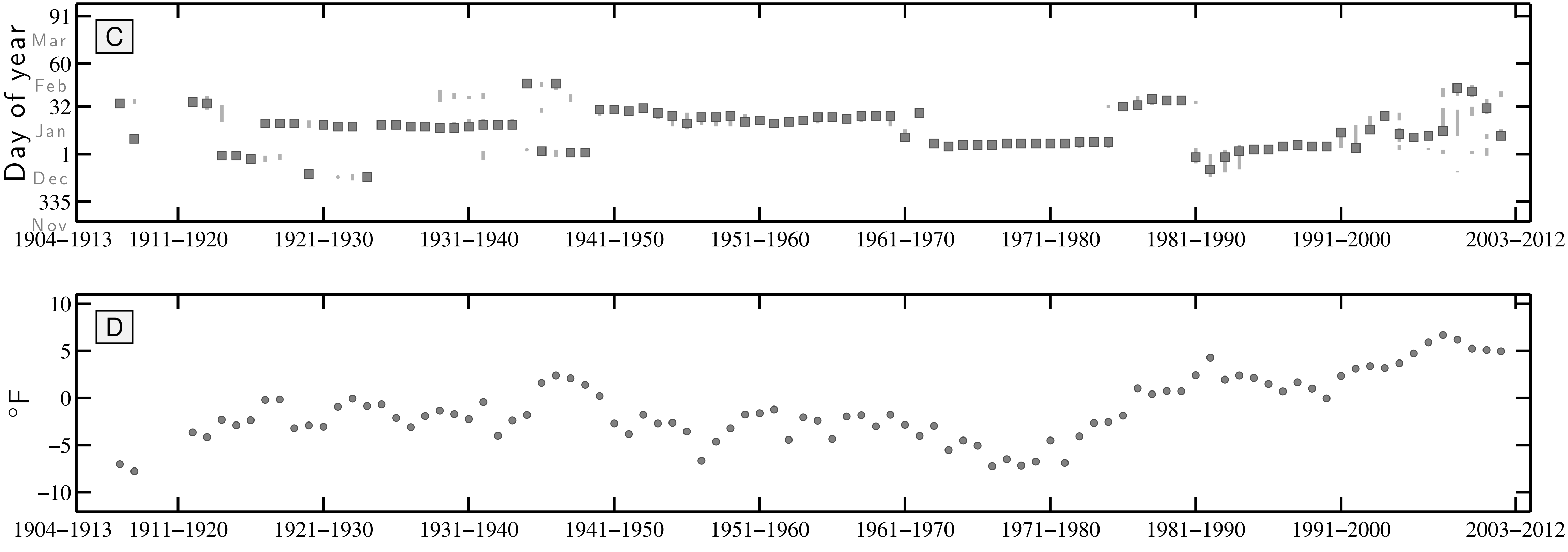

10 year Summer Teletherm dynamics for PEMBINA, ND:

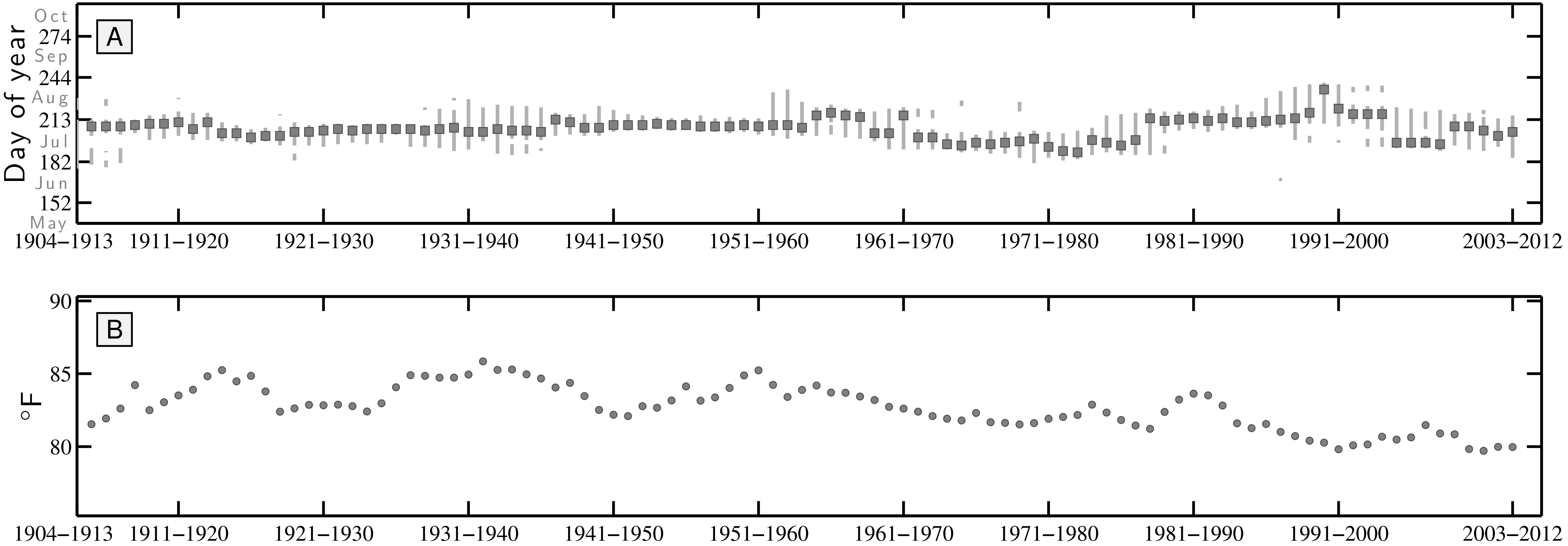

10 year Winter Teletherm dynamics for PEMBINA, ND:

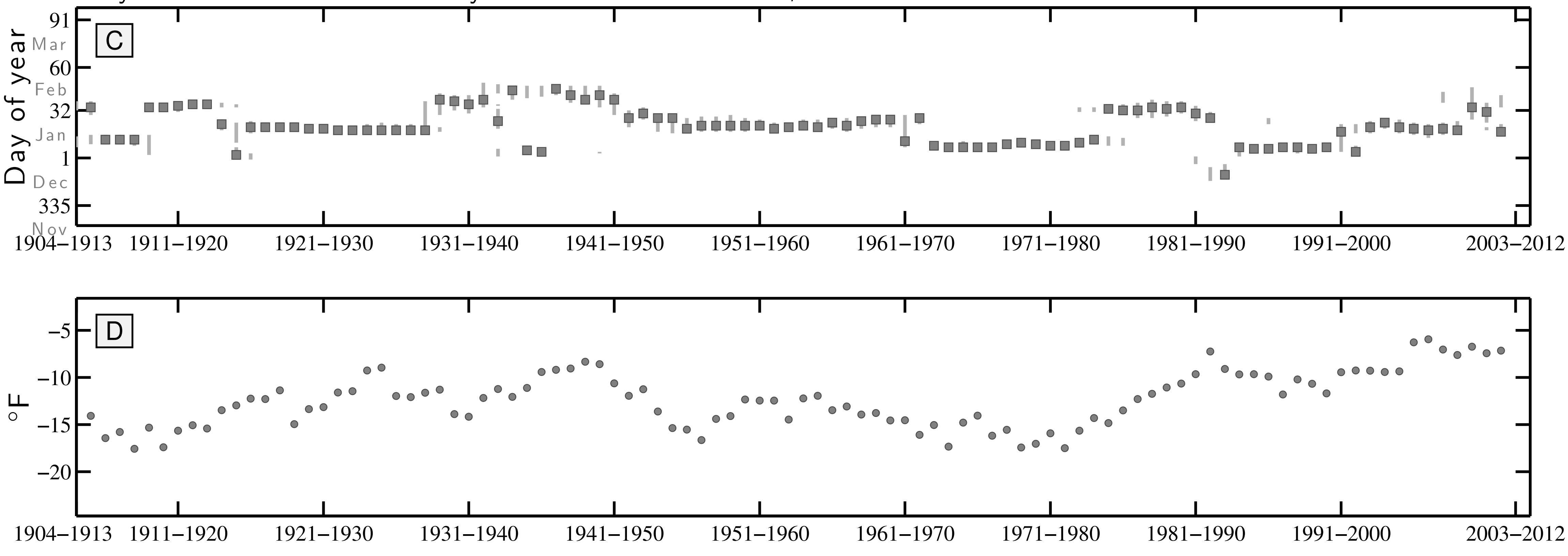

10 year Summer Teletherm dynamics for RICHARDTON ABBEY, ND:

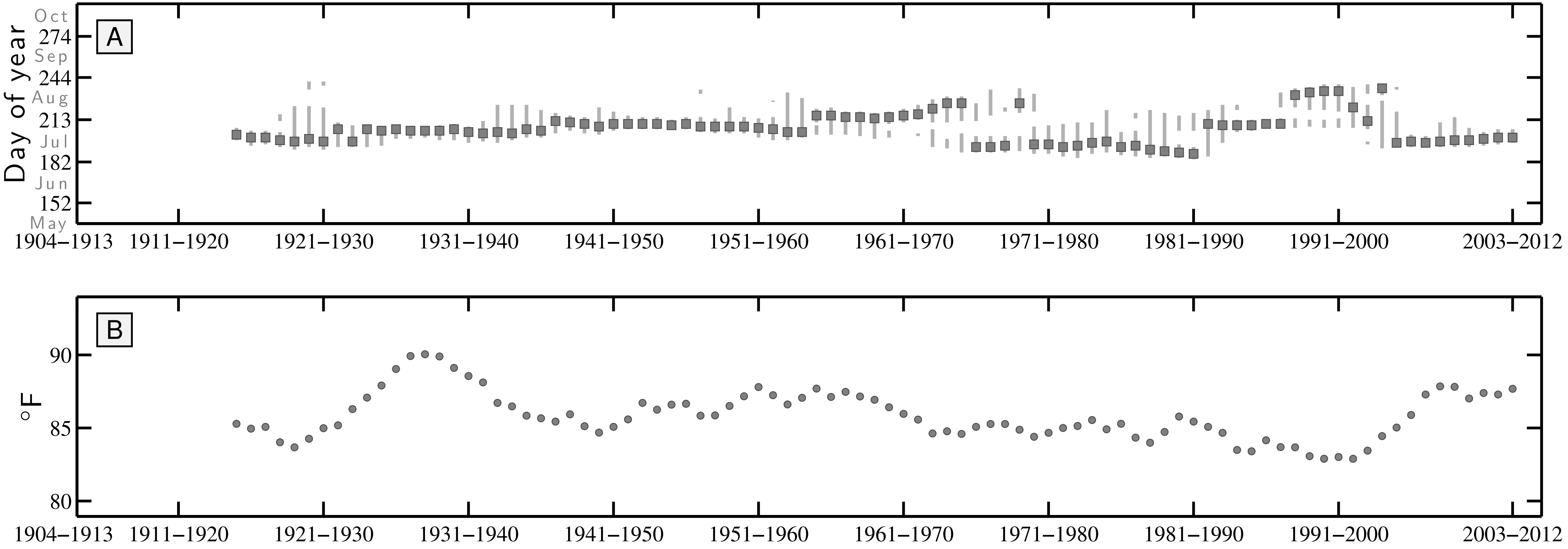

10 year Winter Teletherm dynamics for RICHARDTON ABBEY, ND:

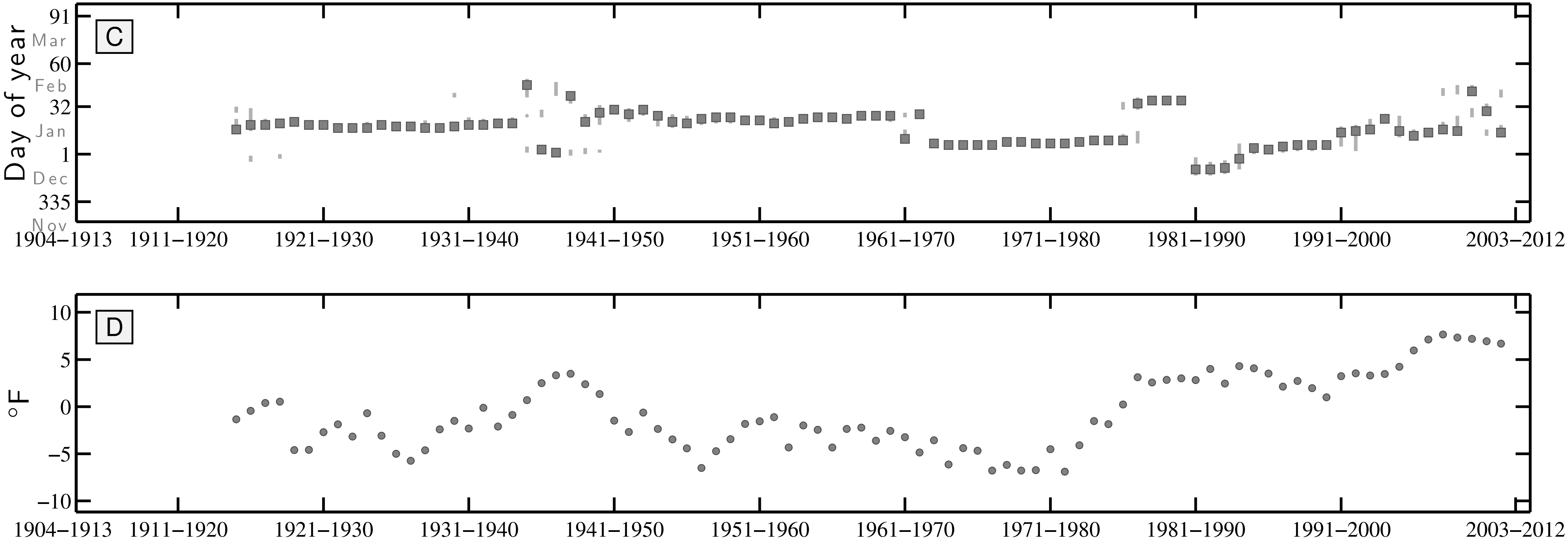

10 year Summer Teletherm dynamics for TOWNER 2 NE, ND:

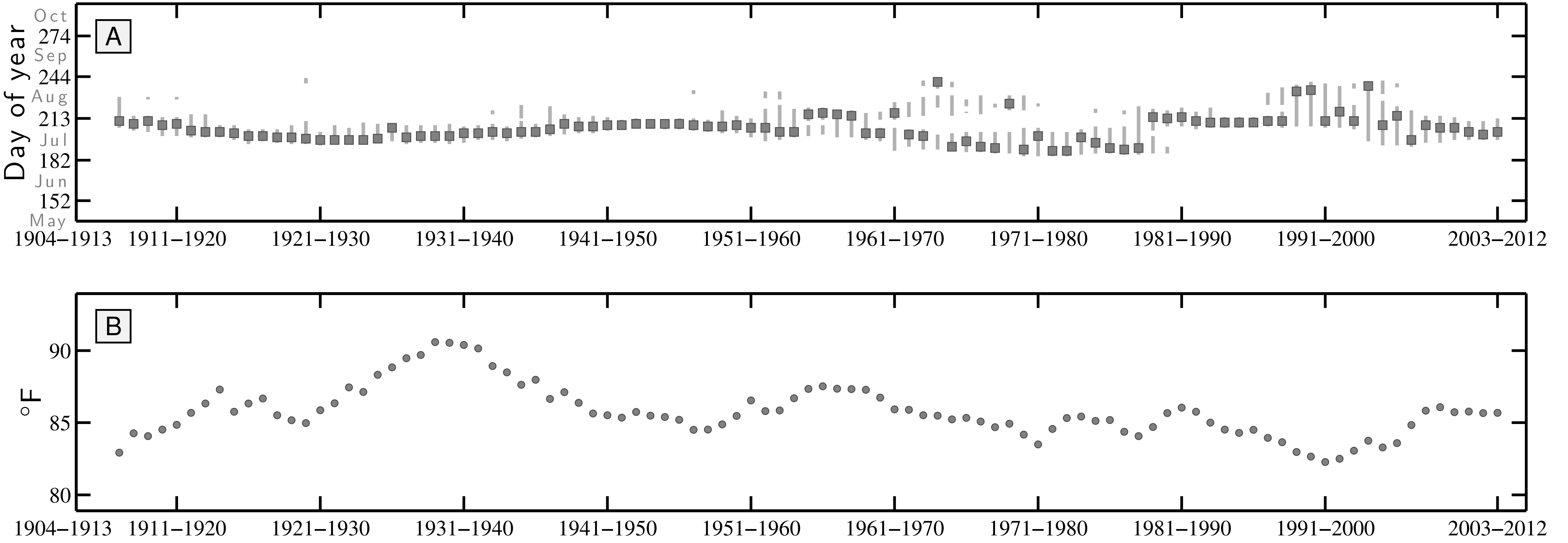

10 year Winter Teletherm dynamics for TOWNER 2 NE, ND:

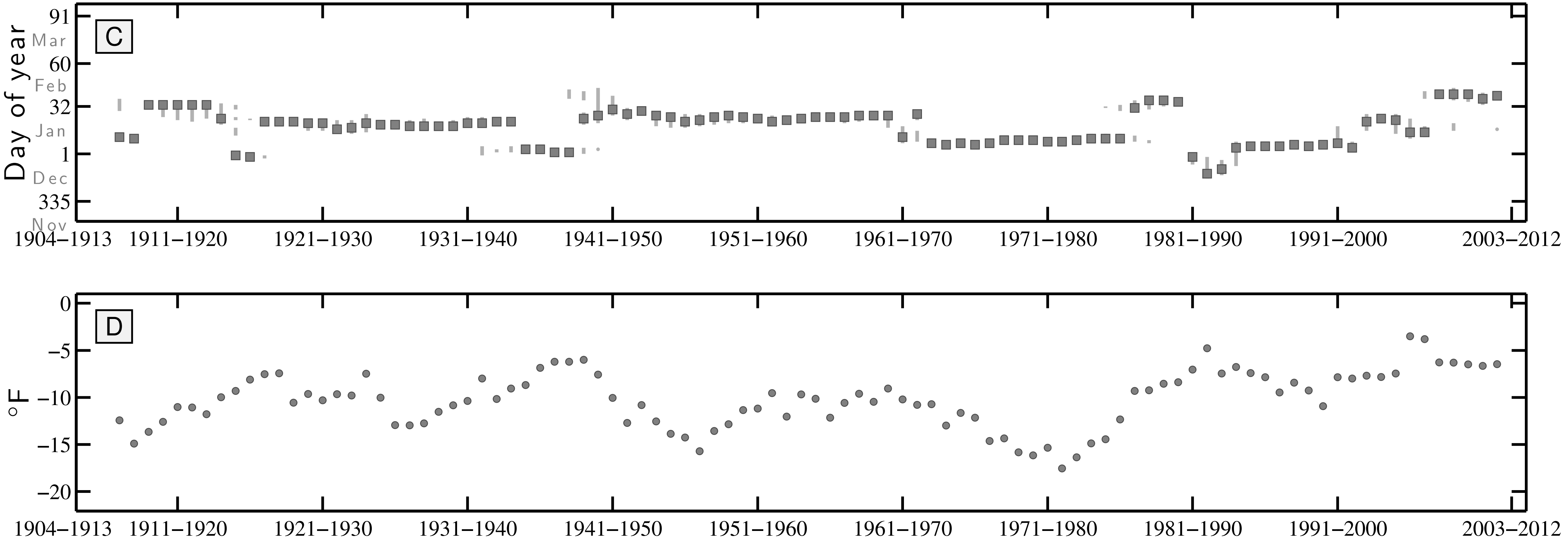

10 year Summer Teletherm dynamics for WAHPETON 3 N, ND:

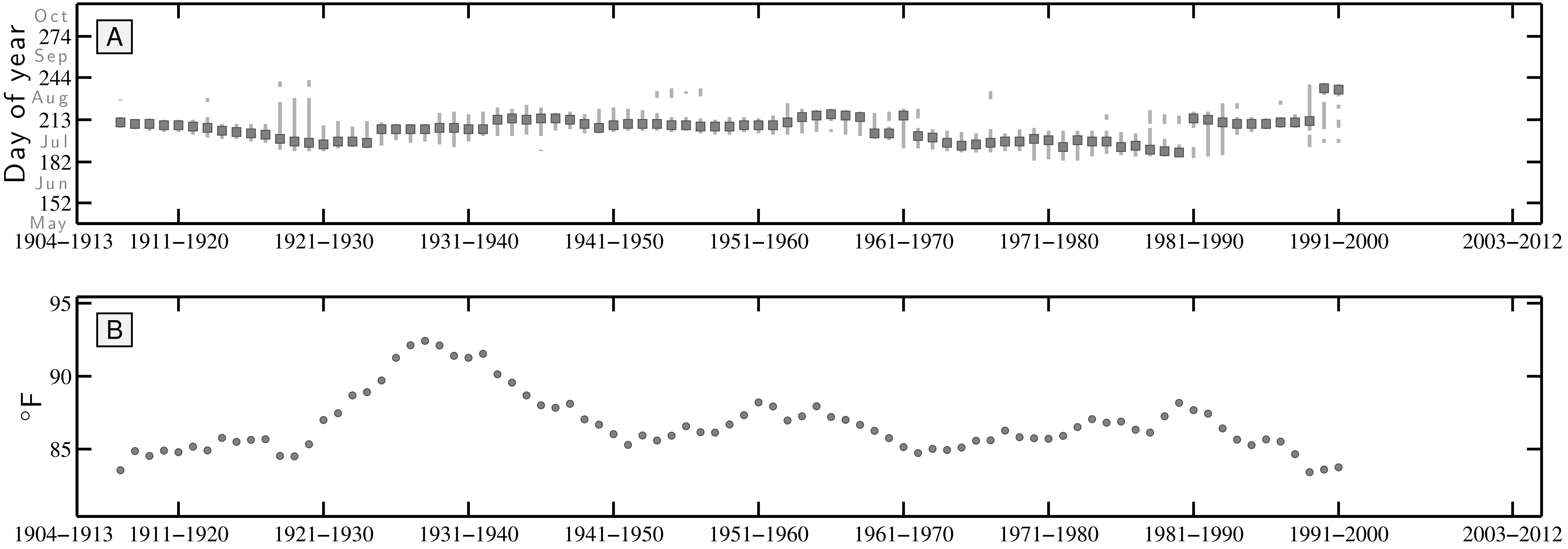

10 year Winter Teletherm dynamics for WAHPETON 3 N, ND:

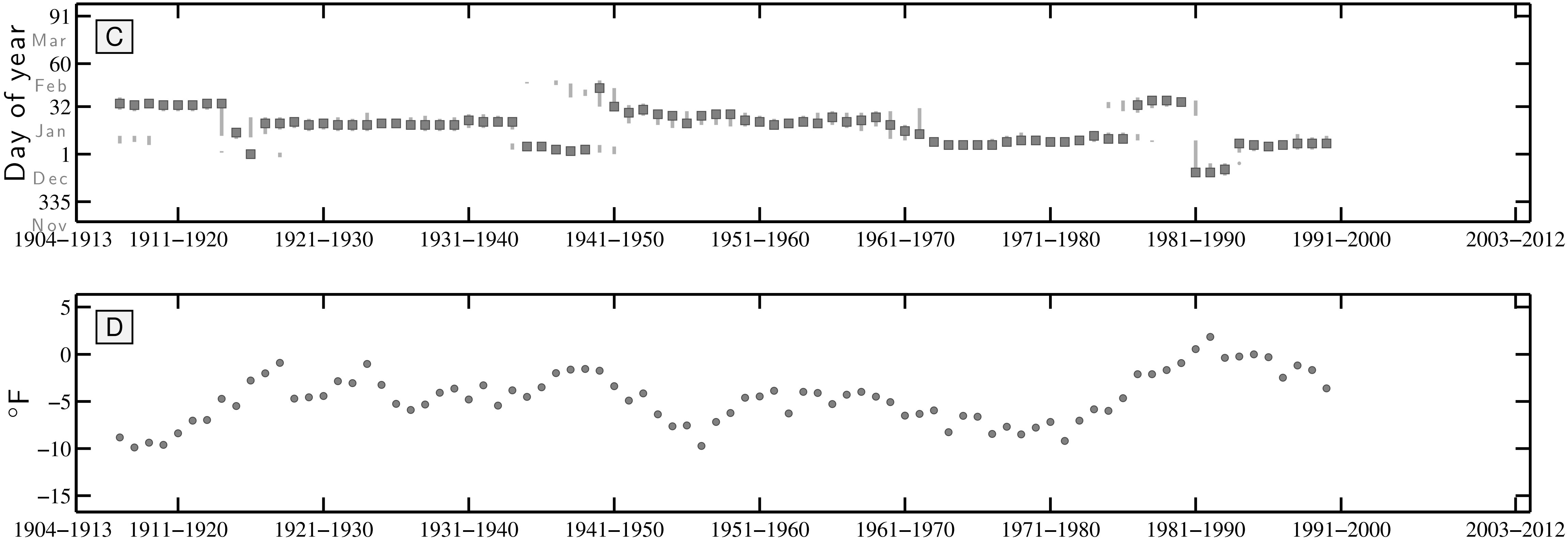

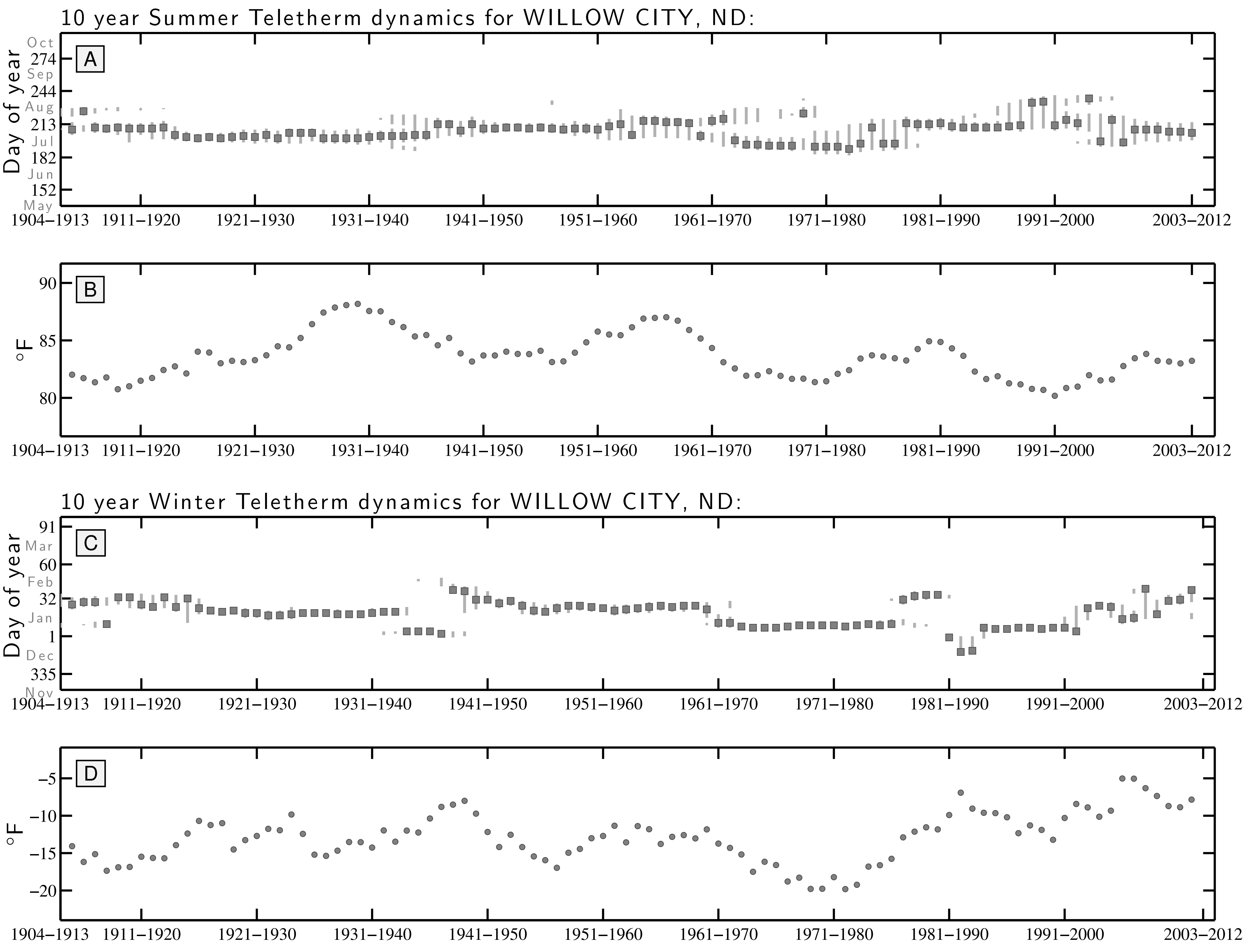
10 year Summer Teletherm dynamics for WILLOW CITY, ND:
A
B
Day of year
°F
Oct
274
Sep
244
Aug
213
Jul
182
Jun
152
May
90
85
80
1904–1913
1911–1920
1921–1930
1931–1940
1941–1950
1951–1960
1961–1970
1971–1980
1981–1990
1991–2000
2003–2012
10 year Winter Teletherm dynamics for WILLOW CITY, ND:
C
D
91
Mar
60
Feb
32
Jan
1
Dec
335
Nov
−5
−10
−15
−20

10 year Summer Teletherm dynamics for BUCYRUS, OH:

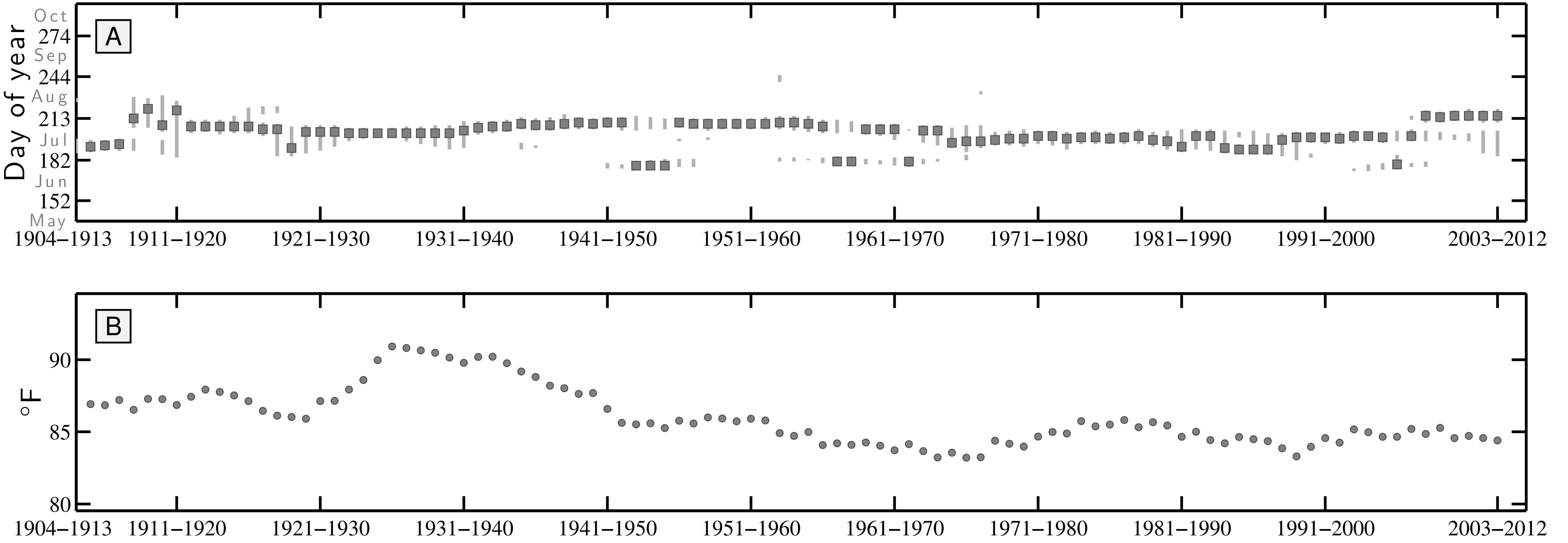

10 year Winter Teletherm dynamics for BUCYRUS, OH:

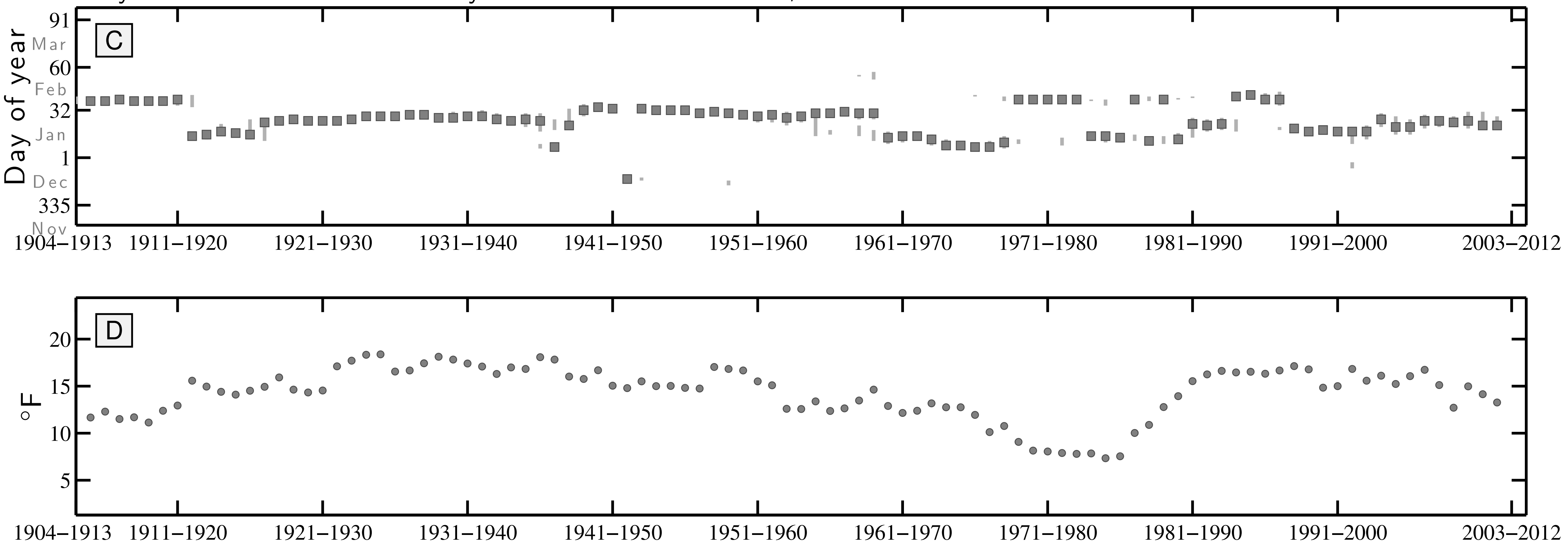

10 year Summer Teletherm dynamics for CADIZ, OH:

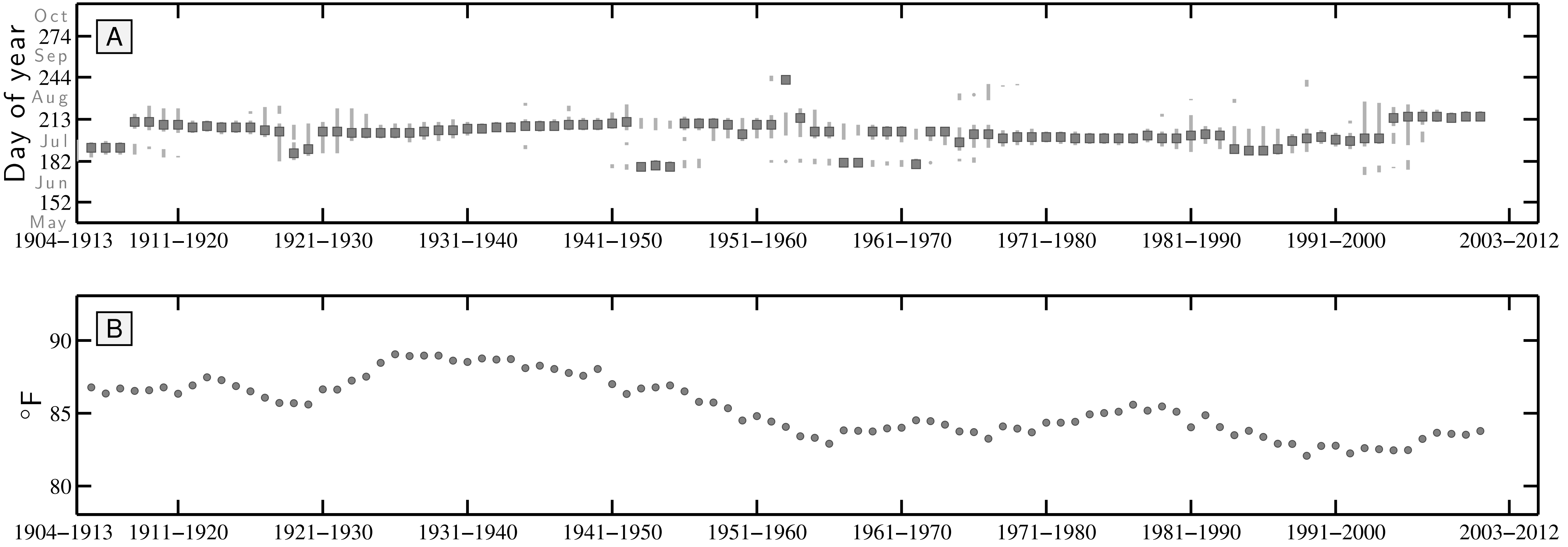

10 year Winter Teletherm dynamics for CADIZ, OH:

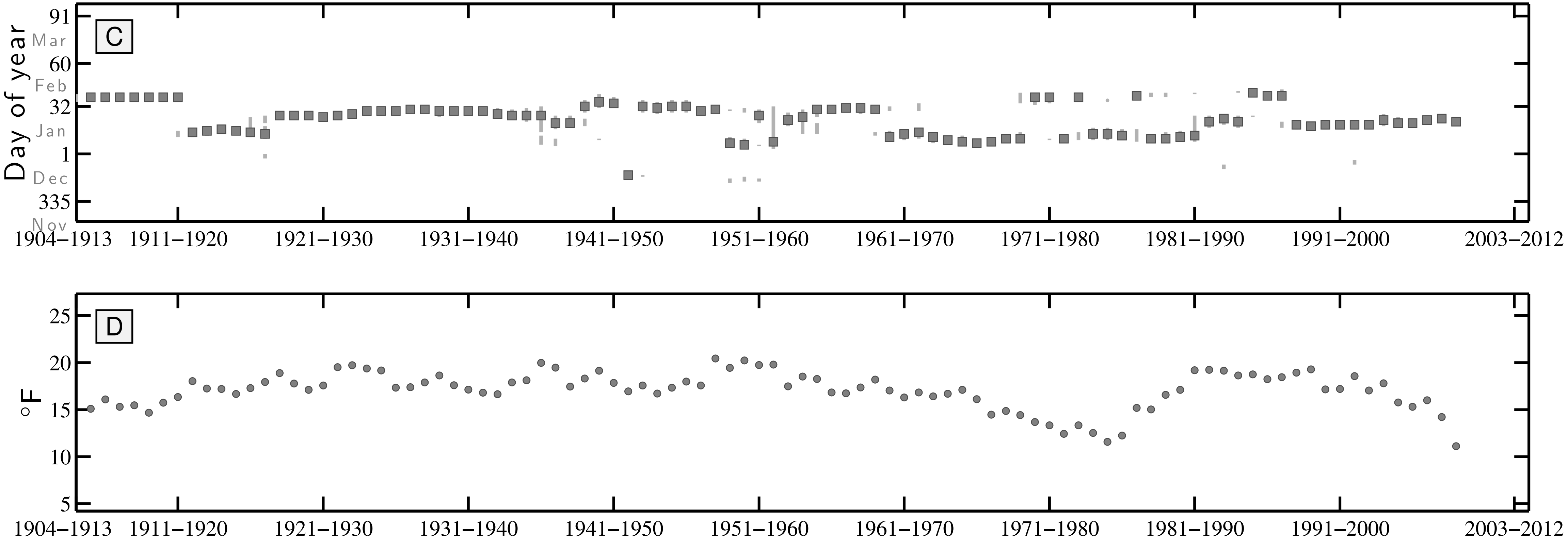

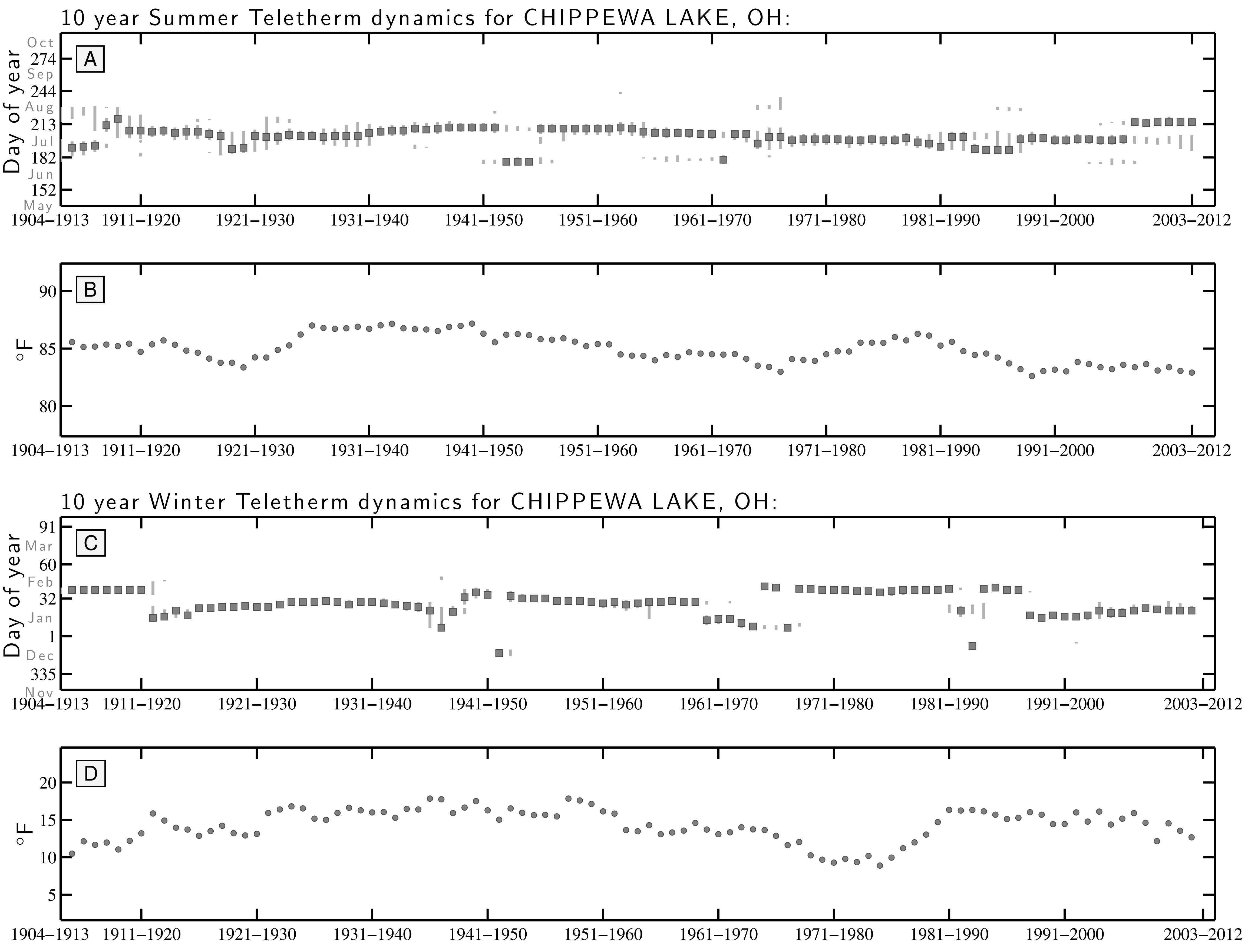

10 year Summer Teletherm dynamics for CHIPPEWA LAKE, OH:
A
Day of year
Oct
274
Sep
244
Aug
213
Jul
182
Jun
152
May
1904–1913
1911–1920
1921–1930
1931–1940
1941–1950
1951–1960
1961–1970
1971–1980
1981–1990
1991–2000
2003–2012
B
°F
90
85
80
10 year Winter Teletherm dynamics for CHIPPEWA LAKE, OH:
C
91
Mar
60
Feb
32
Jan
1
Dec
335
Nov
D
20
15
10
5

10 year Summer Teletherm dynamics for CIRCLEVILLE, OH:

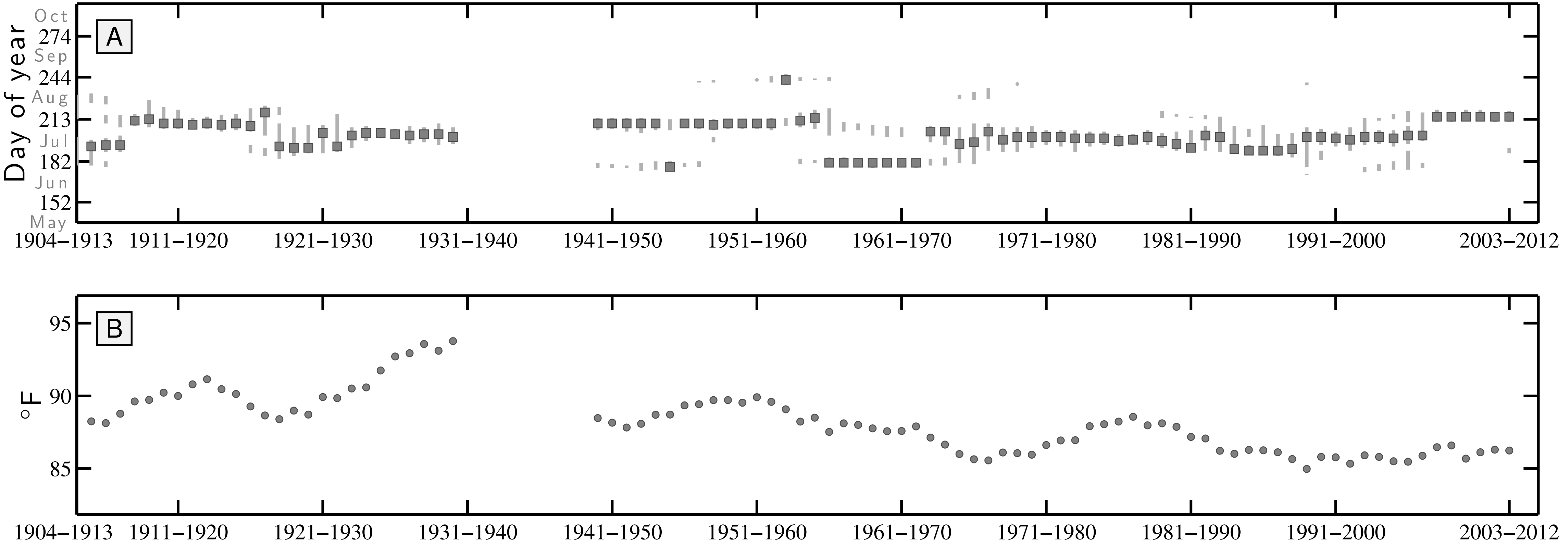

10 year Winter Teletherm dynamics for CIRCLEVILLE, OH:

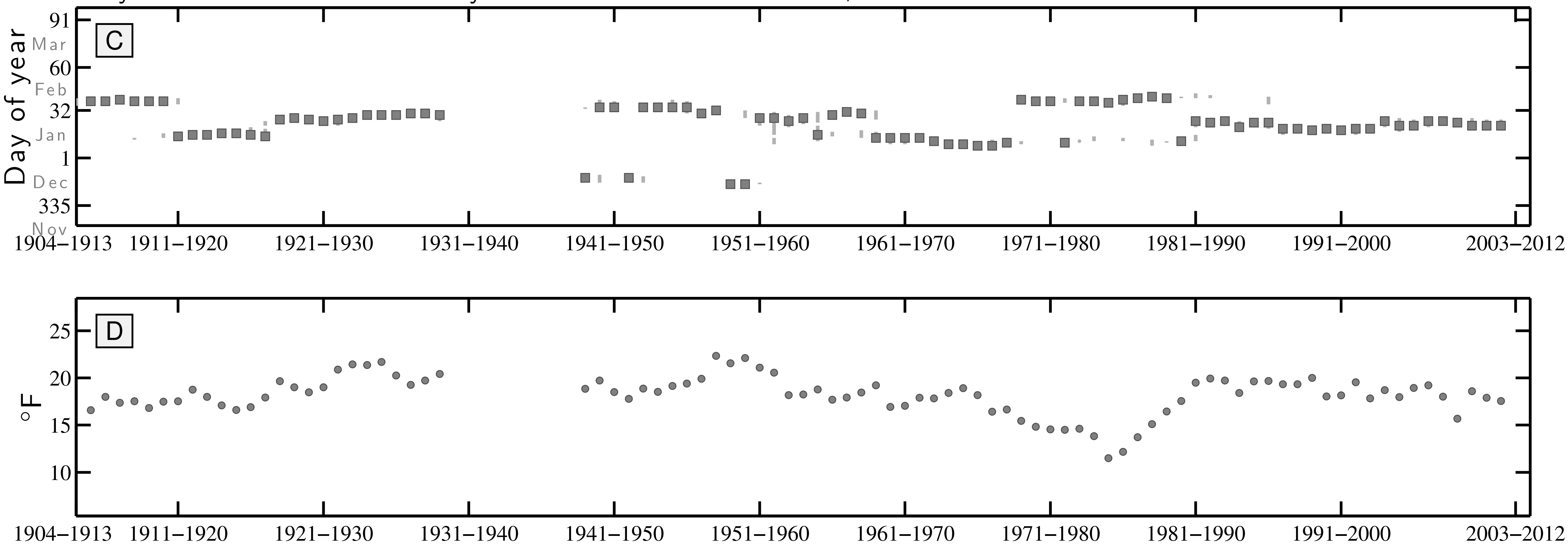

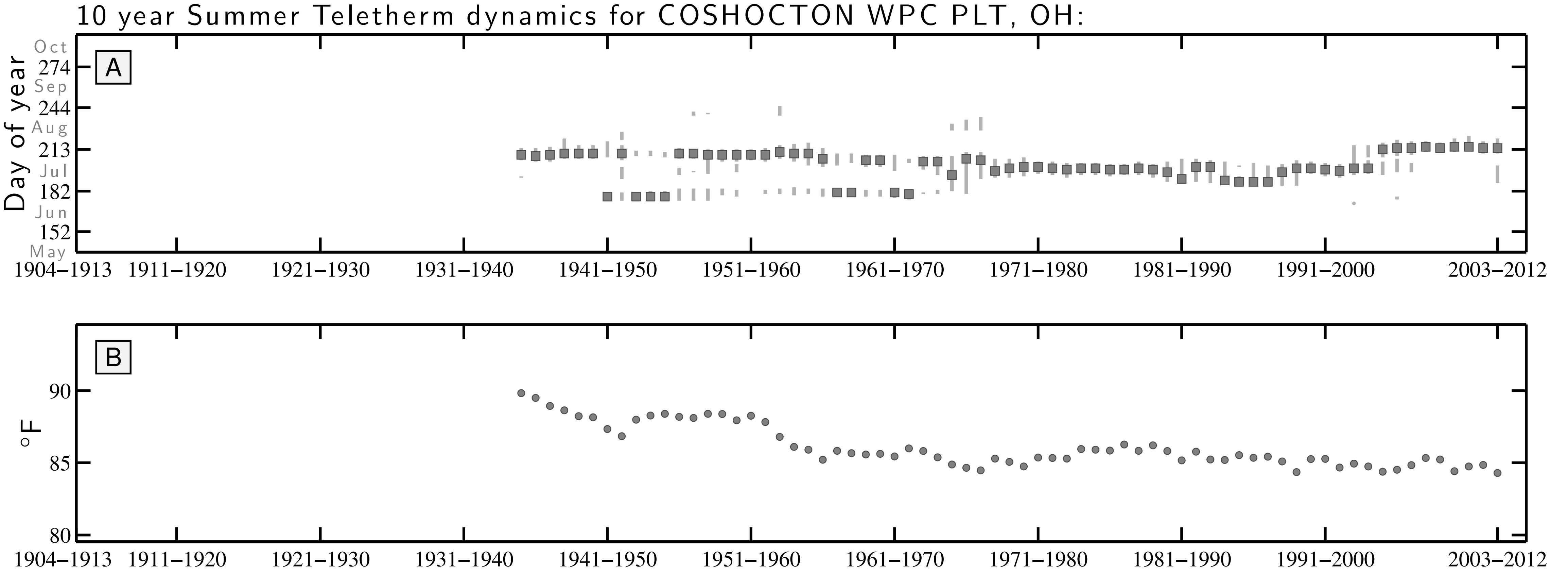
10 year Summer Teletherm dynamics for COSHOCTON WPC PLT, OH:
A
B
Day of year
°F
Oct
274
Sep
244
Aug
213
Jul
182
Jun
152
May
90
85
80
1904–1913
1911–1920
1921–1930
1931–1940
1941–1950
1951–1960
1961–1970
1971–1980
1981–1990
1991–2000
2003–2012

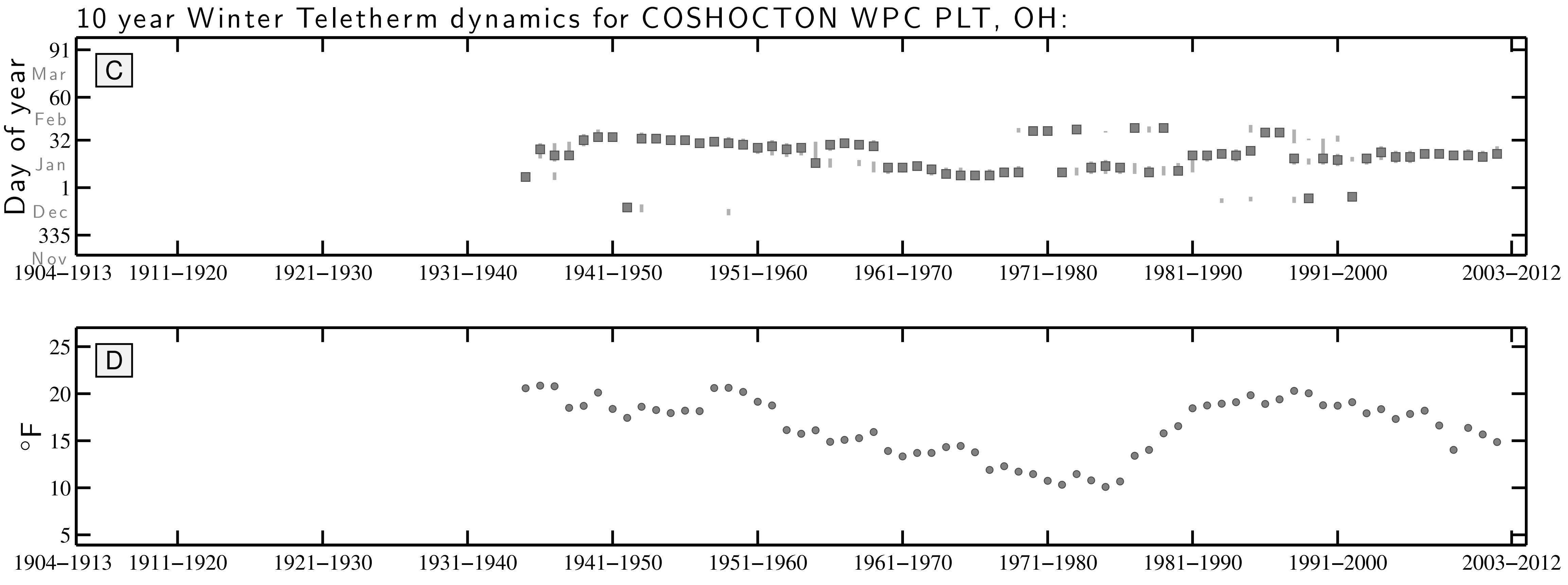
10 year Winter Teletherm dynamics for COSHOCTON WPC PLT, OH:
C
D
Day of year
°F
91
Mar
60
Feb
32
Jan
1
Dec
335
Nov
25
20
15
10
5
1904–1913
1911–1920
1921–1930
1931–1940
1941–1950
1951–1960
1961–1970
1971–1980
1981–1990
1991–2000
2003–2012

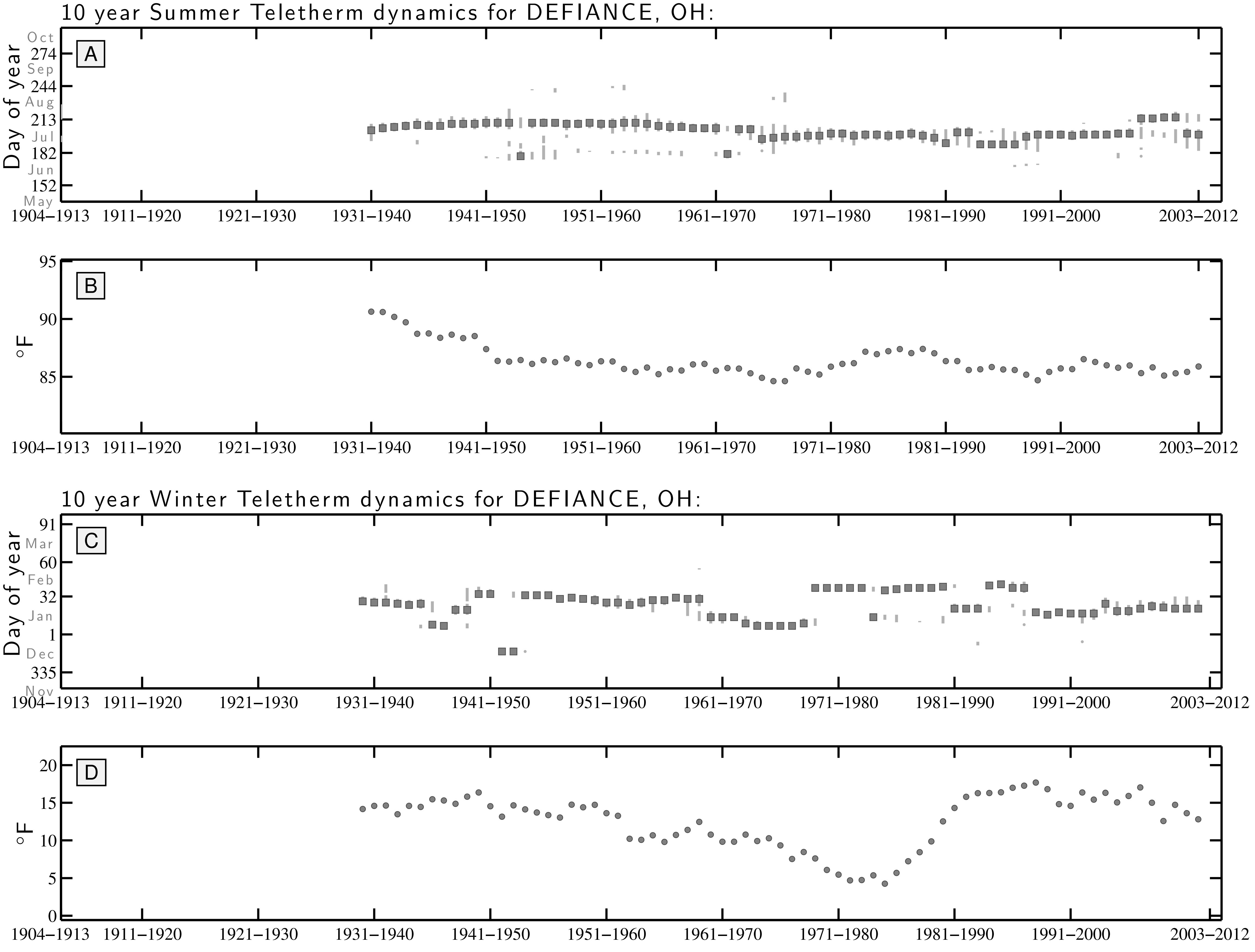
10 year Summer Teletherm dynamics for DEFIANCE, OH:
A
B
Day of year
°F
Oct
274
Sep
244
Aug
213
Jul
182
Jun
152
May
95
90
85
1904–1913
1911–1920
1921–1930
1931–1940
1941–1950
1951–1960
1961–1970
1971–1980
1981–1990
1991–2000
2003–2012
10 year Winter Teletherm dynamics for DEFIANCE, OH:
C
D
91
Mar
60
Feb
32
Jan
1
Dec
335
Nov
20
15
10
5
0

10 year Summer Teletherm dynamics for DELAWARE, OH:

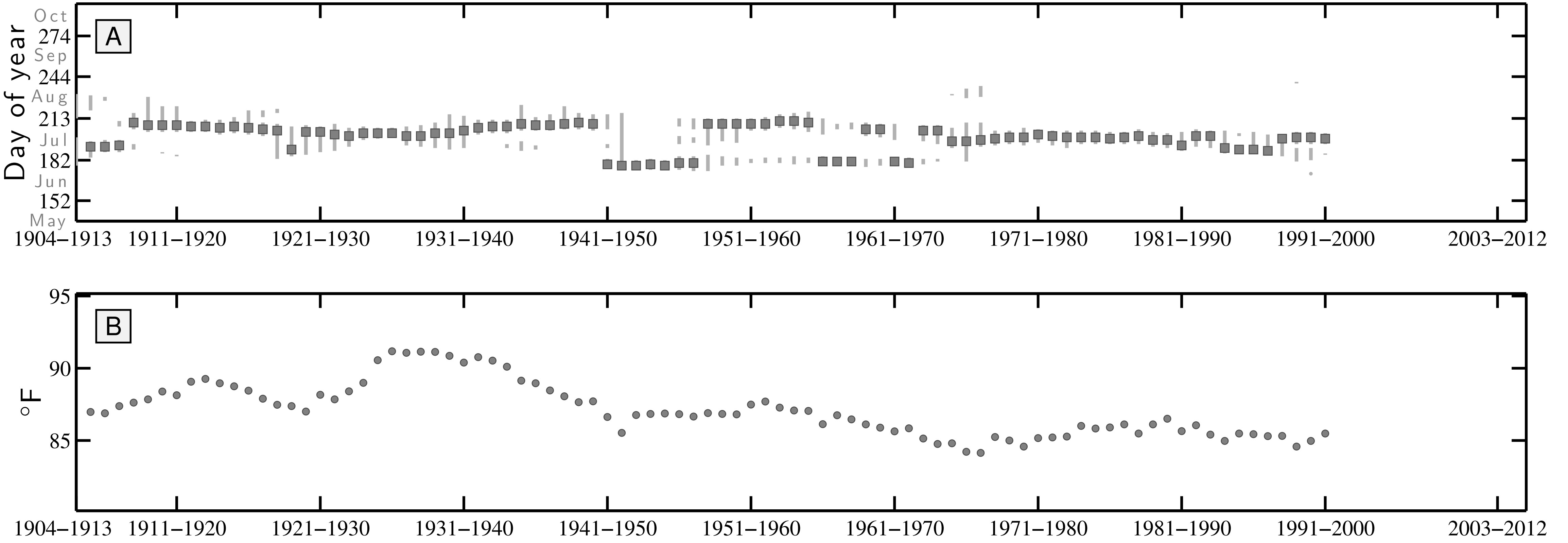

10 year Winter Teletherm dynamics for DELAWARE, OH:

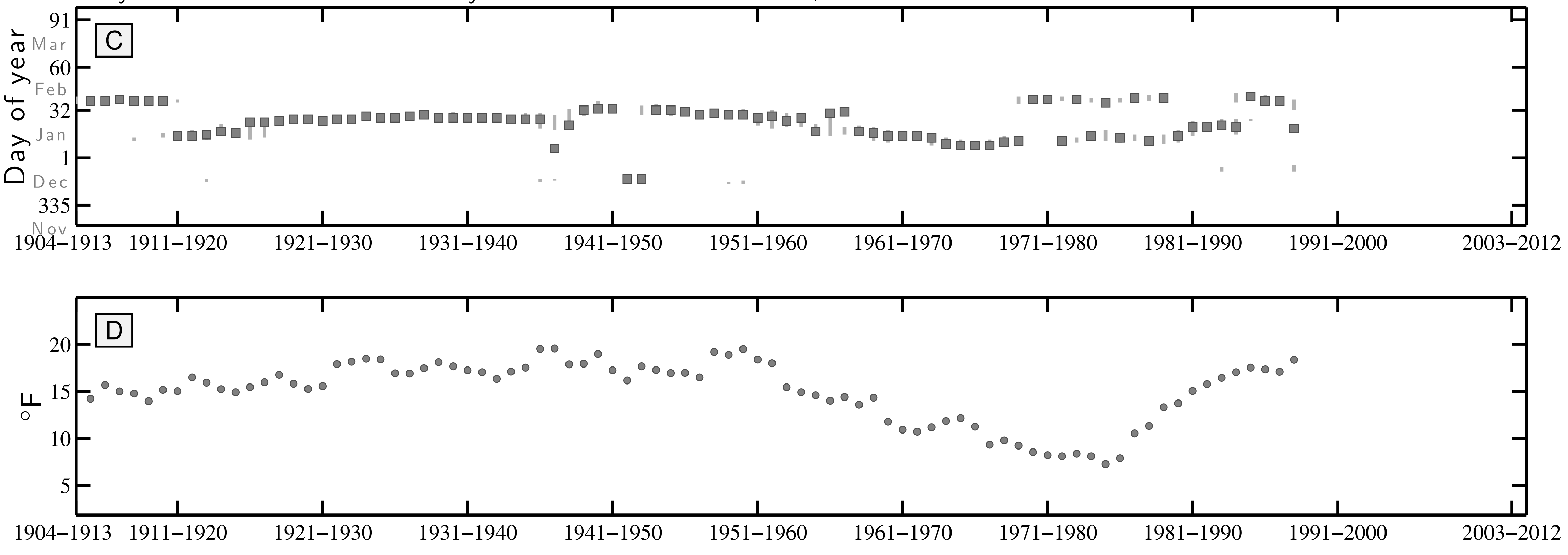

10 year Summer Teletherm dynamics for FINDLAY WPCC, OH:

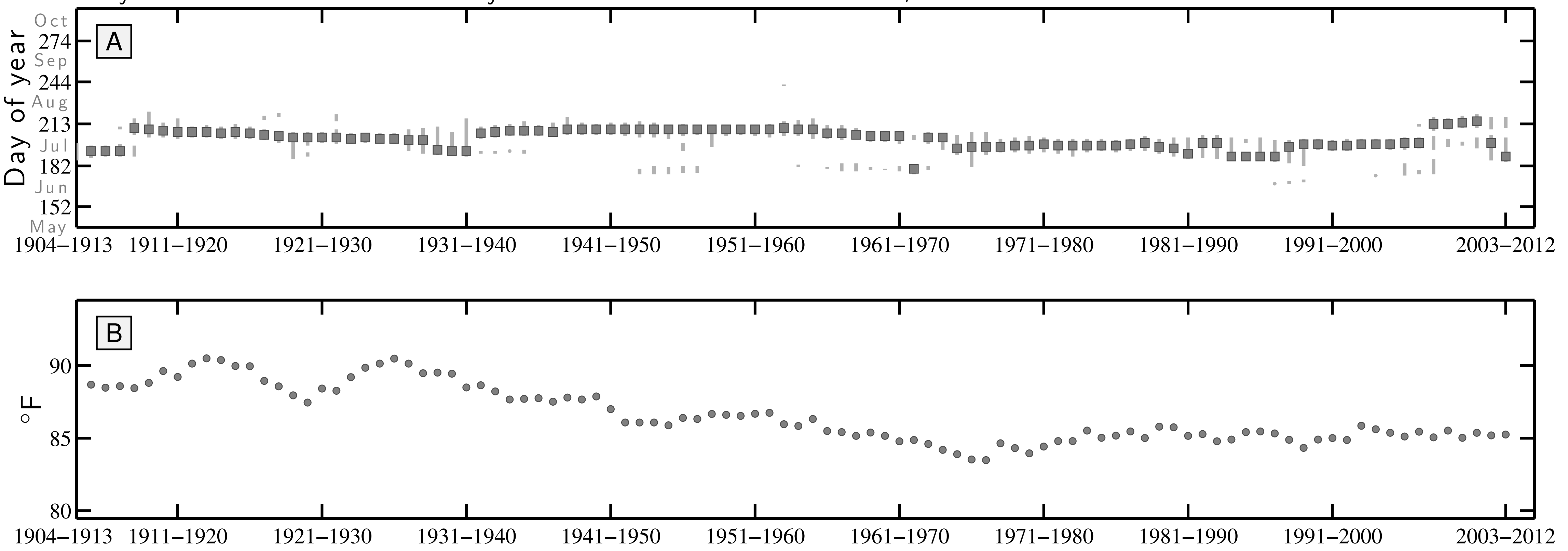

10 year Winter Teletherm dynamics for FINDLAY WPCC, OH:

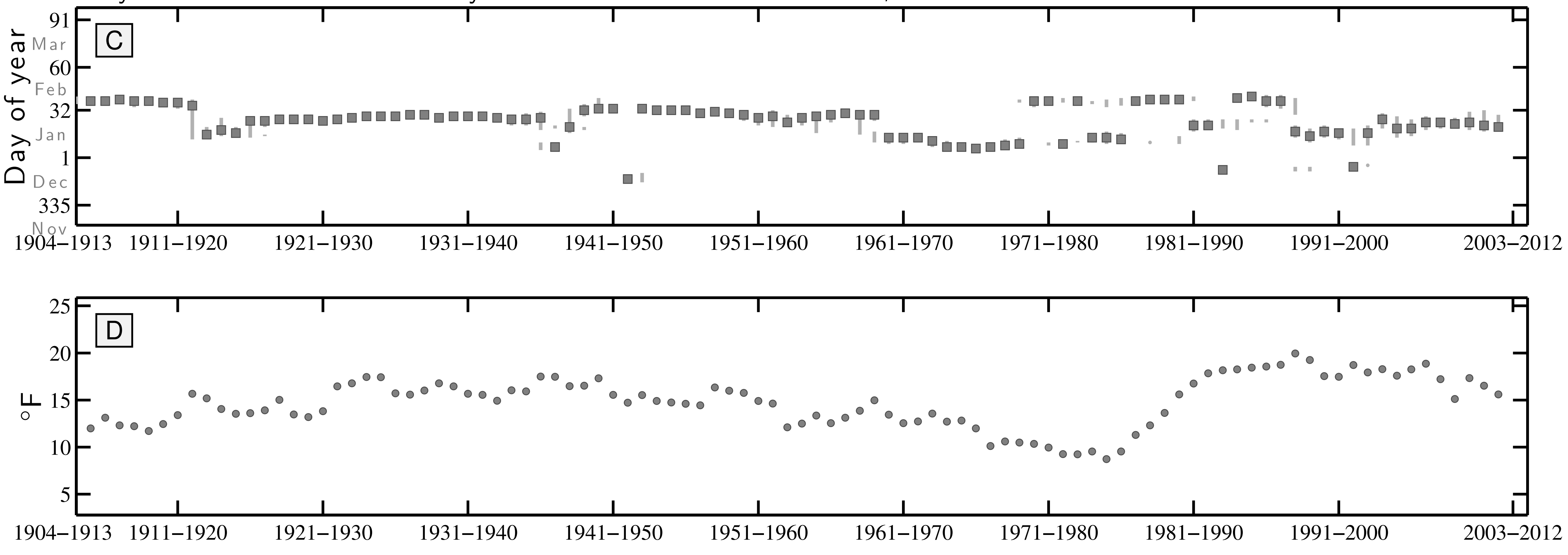

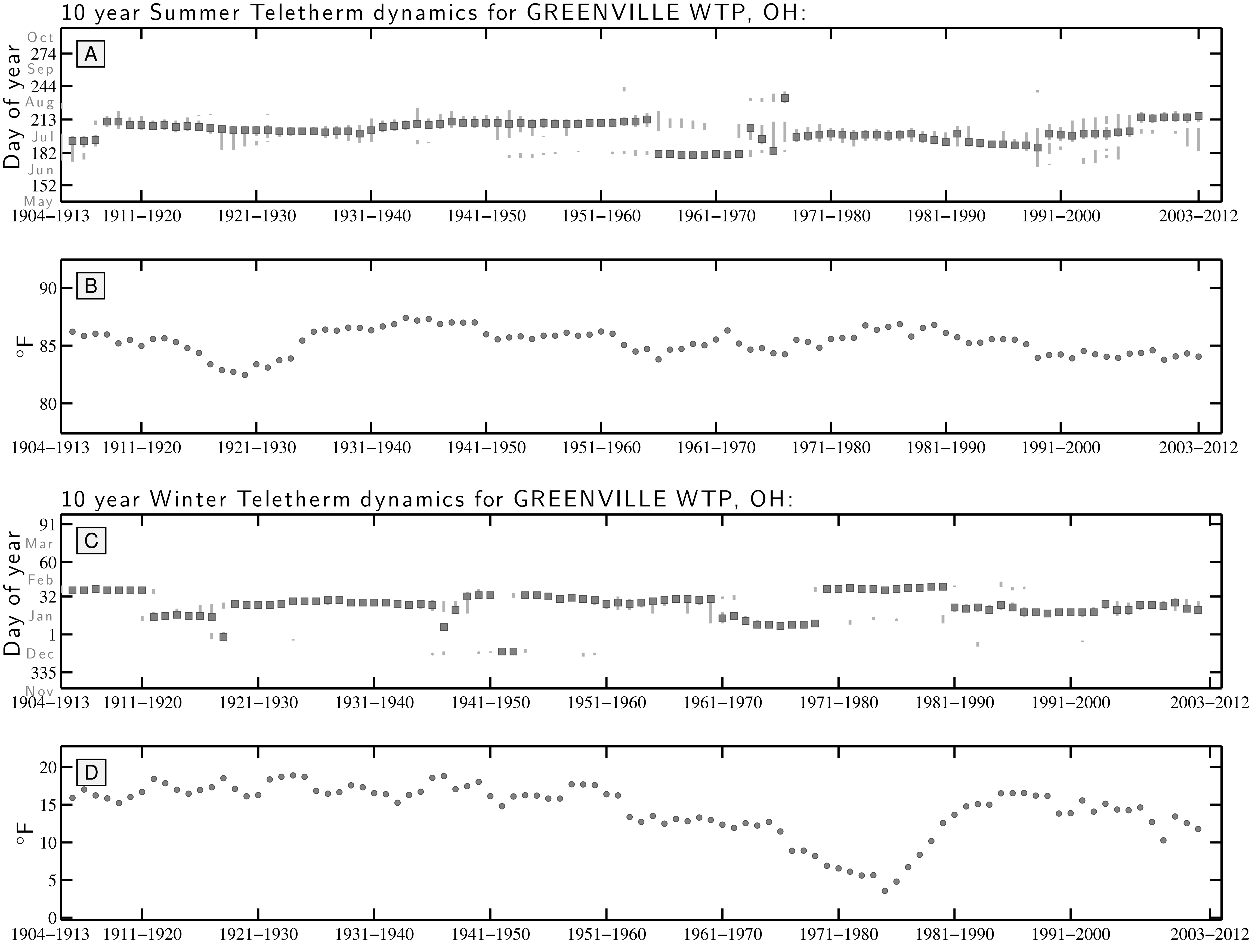

10 year Summer Teletherm dynamics for GREENVILLE WTP, OH:
A
Day of year
Oct
274
Sep
244
Aug
213
Jul
182
Jun
152
May
1904–1913
1911–1920
1921–1930
1931–1940
1941–1950
1951–1960
1961–1970
1971–1980
1981–1990
1991–2000
2003–2012
B
°F
90
85
80
10 year Winter Teletherm dynamics for GREENVILLE WTP, OH:
C
91
Mar
60
Feb
32
Jan
1
Dec
335
Nov
D
20
15
10
5
0

10 year Summer Teletherm dynamics for HILLSBORO, OH:

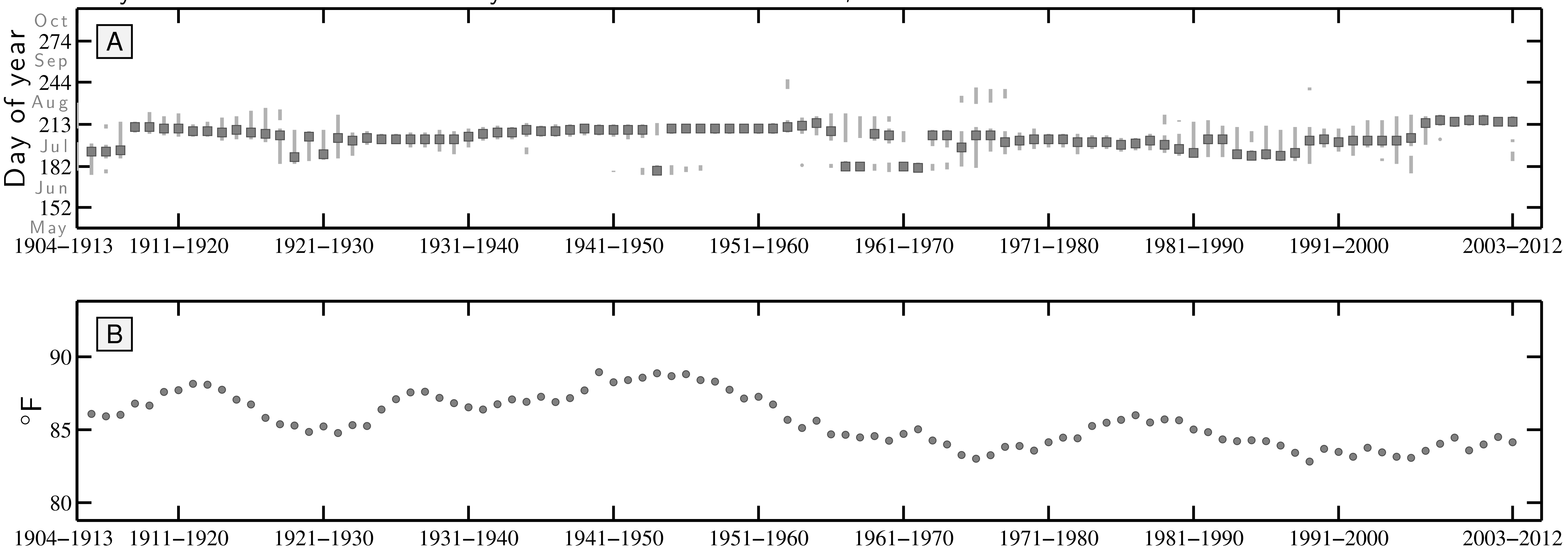

10 year Winter Teletherm dynamics for HILLSBORO, OH:

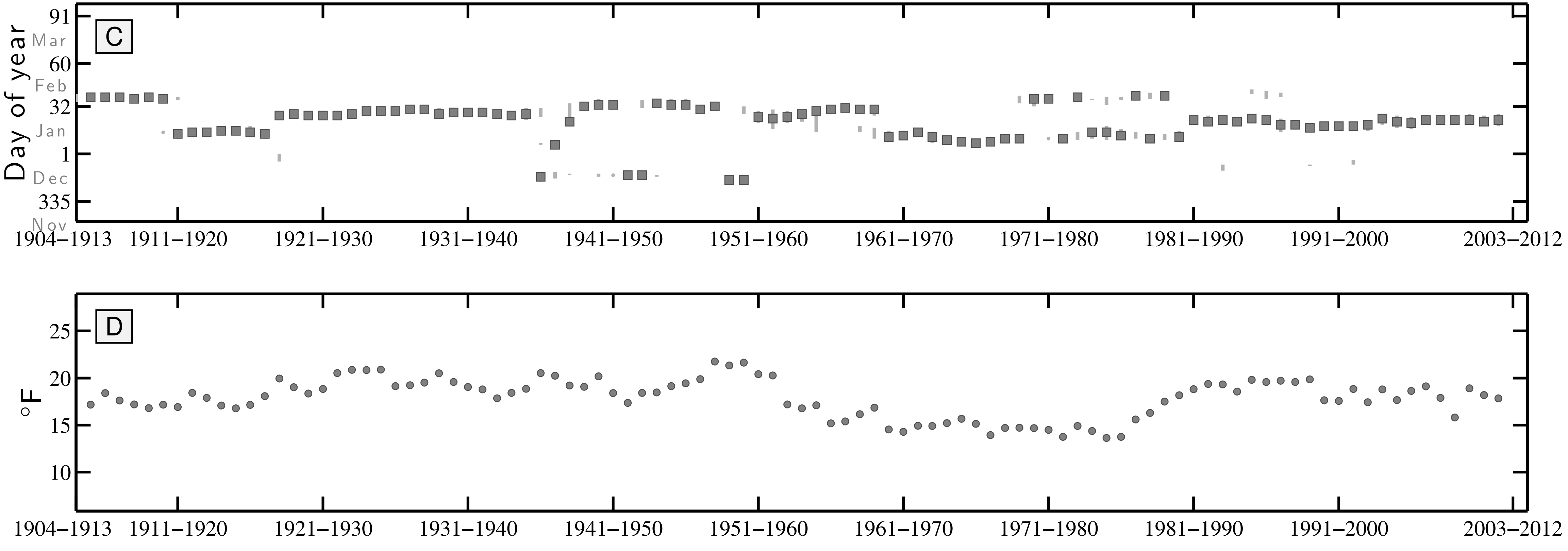

10 year Summer Teletherm dynamics for HIRAM, OH:

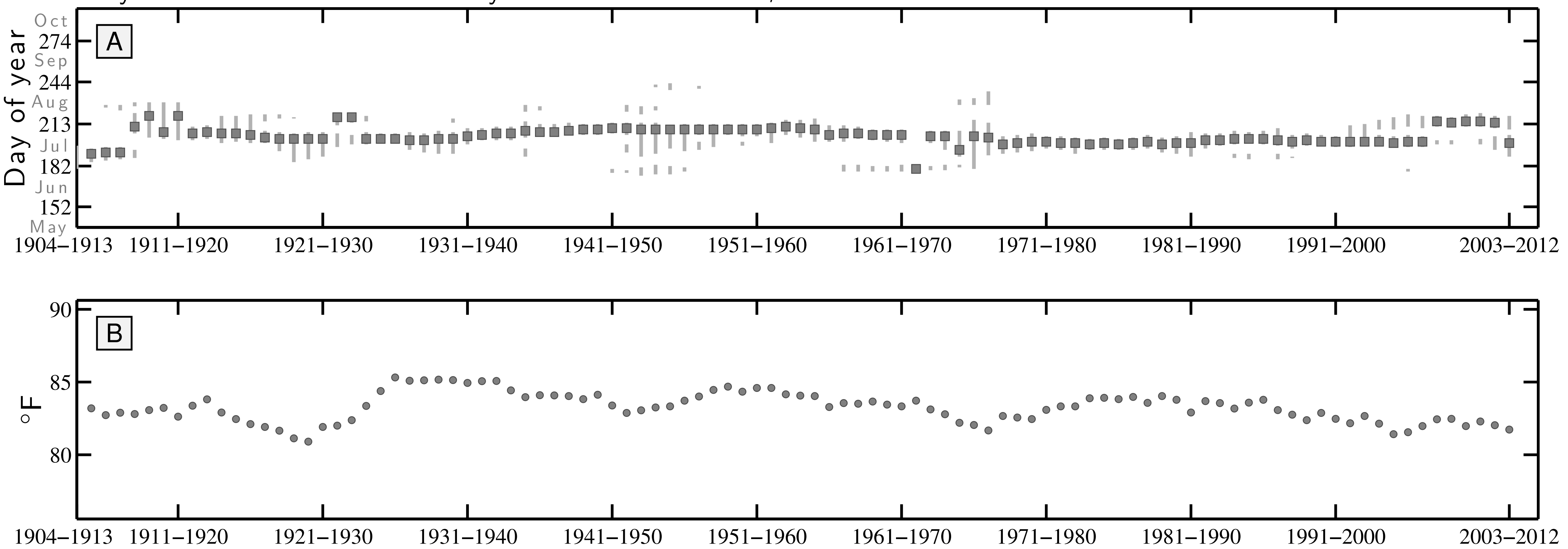

10 year Winter Teletherm dynamics for HIRAM, OH:

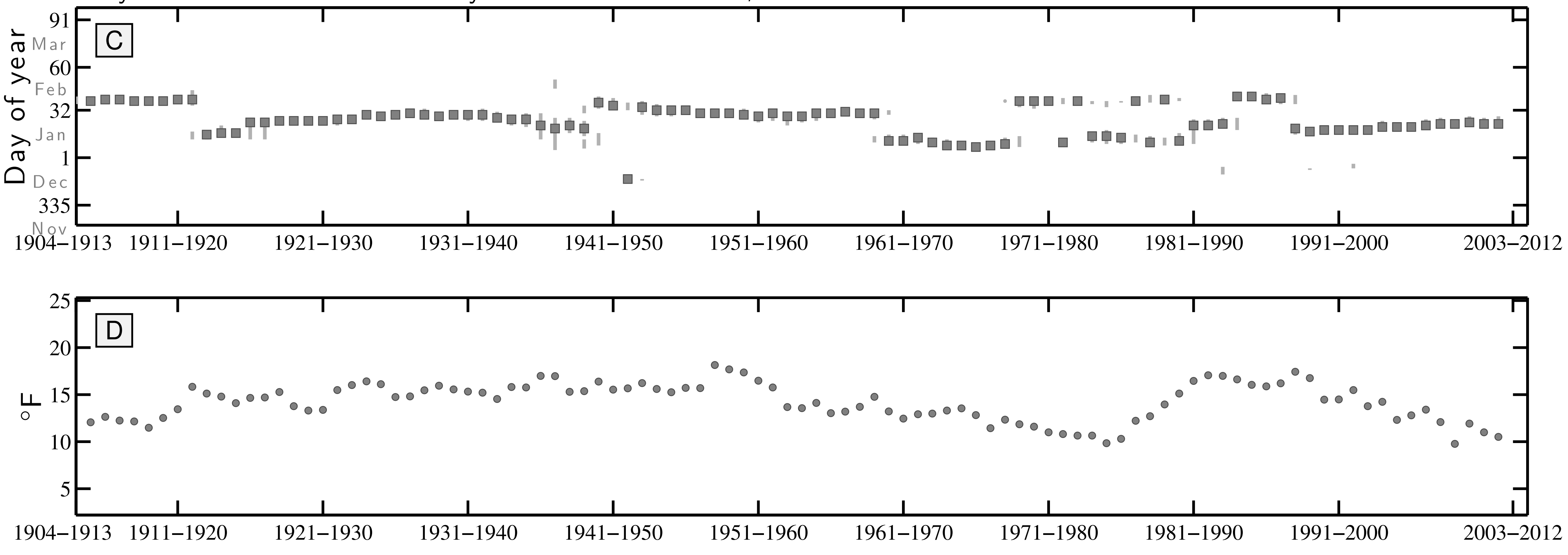

10 year Summer Teletherm dynamics for KENTON, OH:

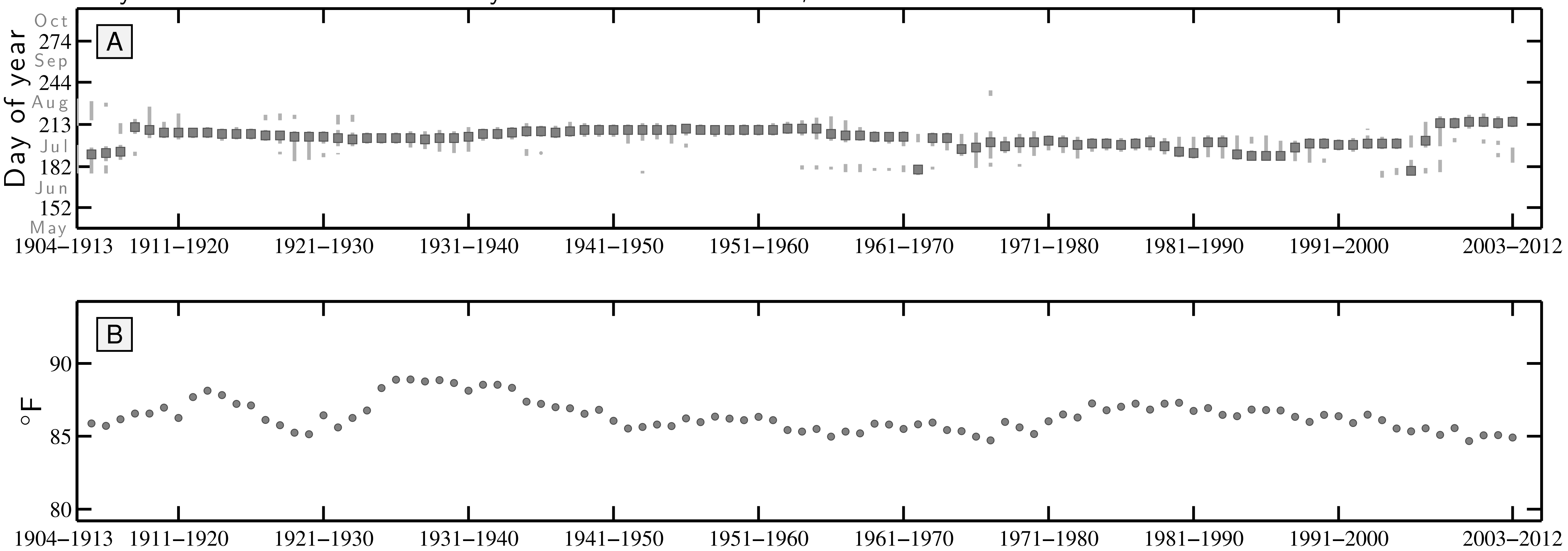

10 year Winter Teletherm dynamics for KENTON, OH:

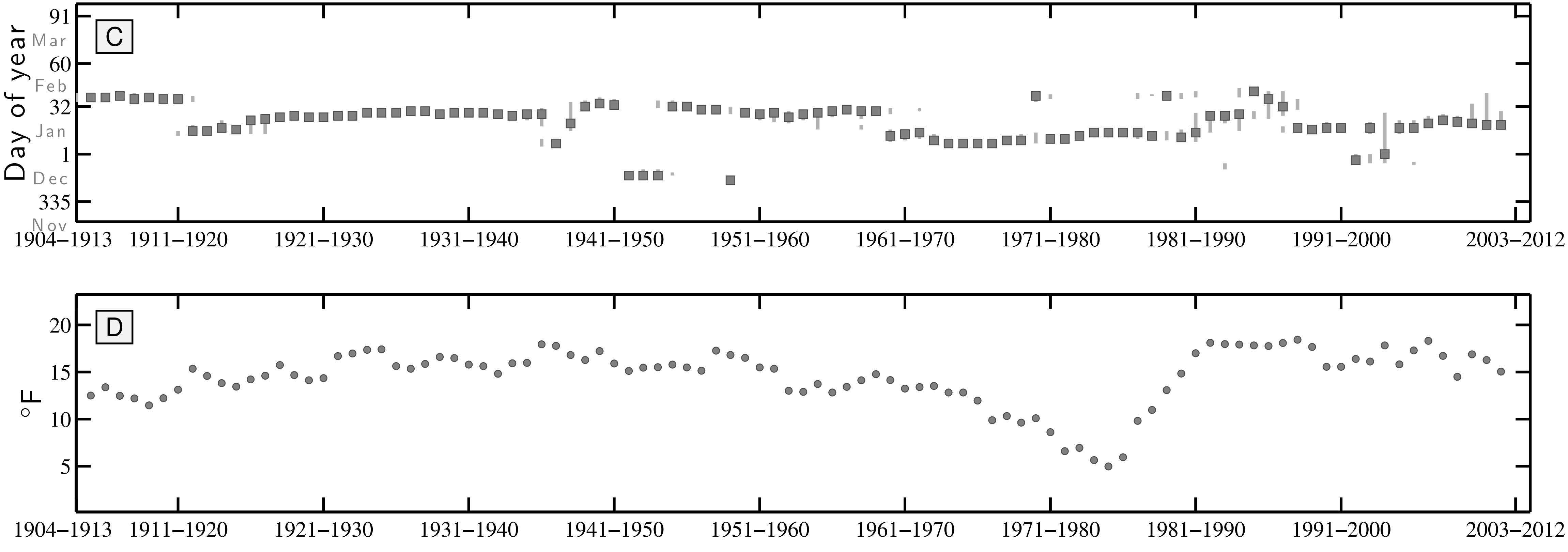

10 year Summer Teletherm dynamics for MC CONNELLSVILLE LK 7, OH:

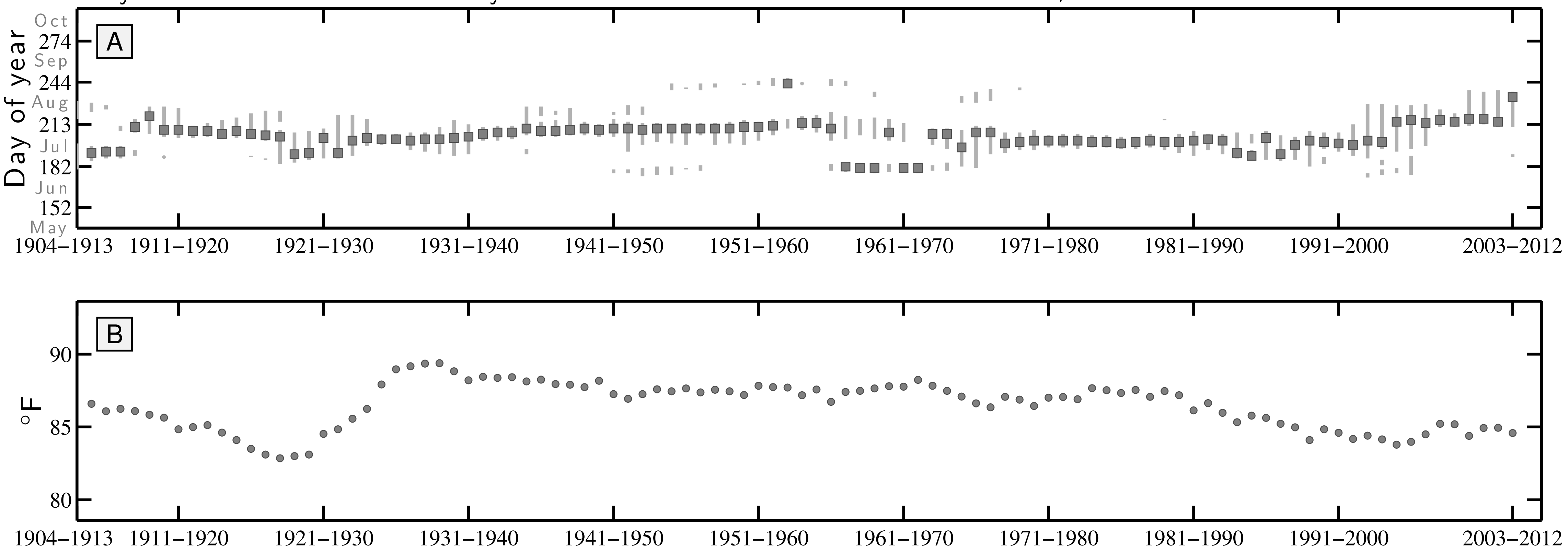

10 year Winter Teletherm dynamics for MC CONNELLSVILLE LK 7, OH:

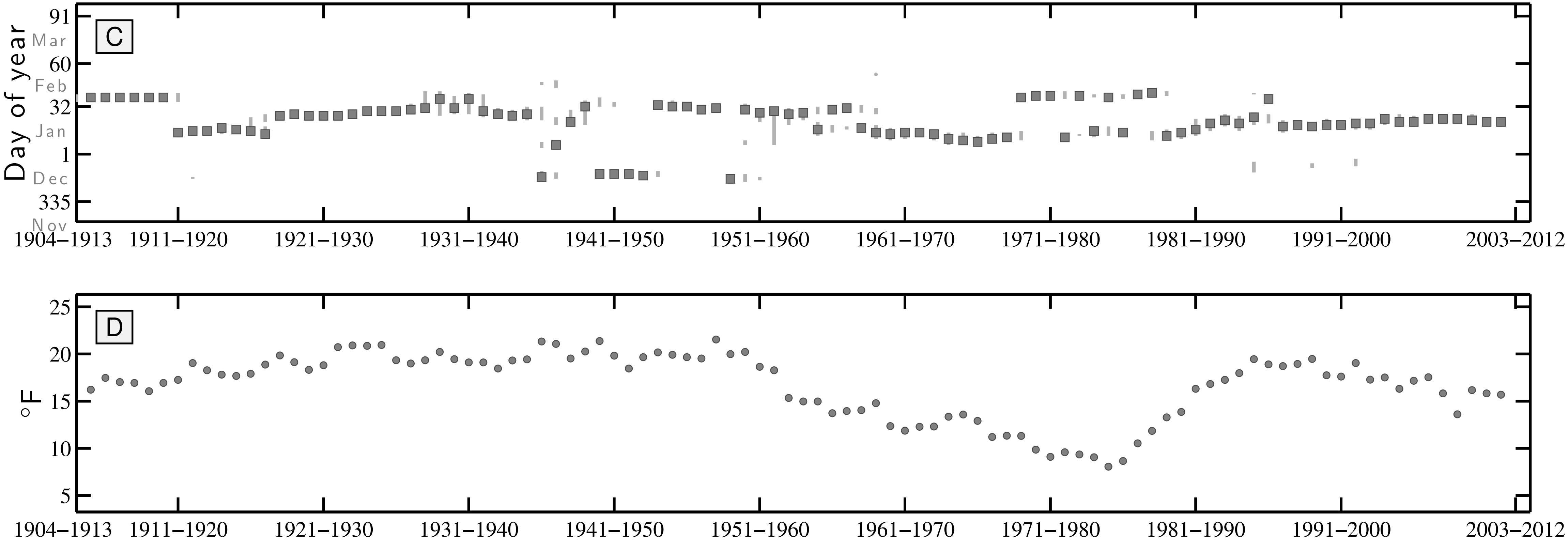

10 year Summer Teletherm dynamics for MILLERSBURG, OH:

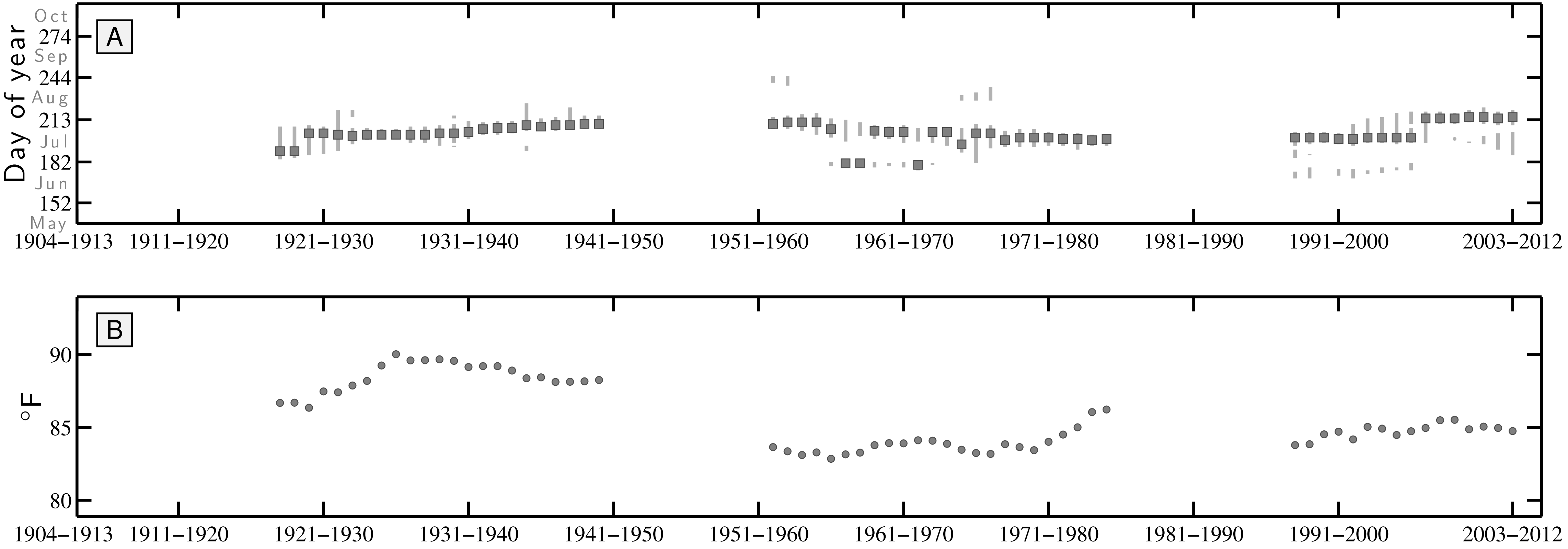

10 year Winter Teletherm dynamics for MILLERSBURG, OH:

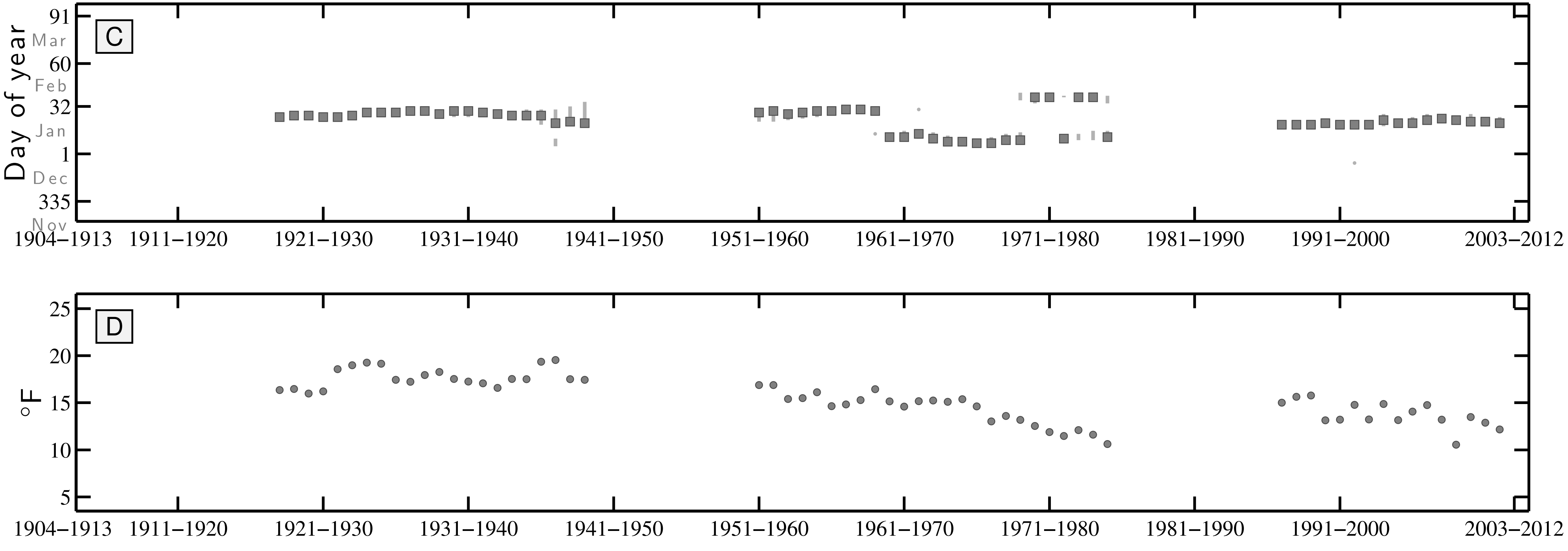

10 year Summer Teletherm dynamics for MILLPORT 4 NE, OH:

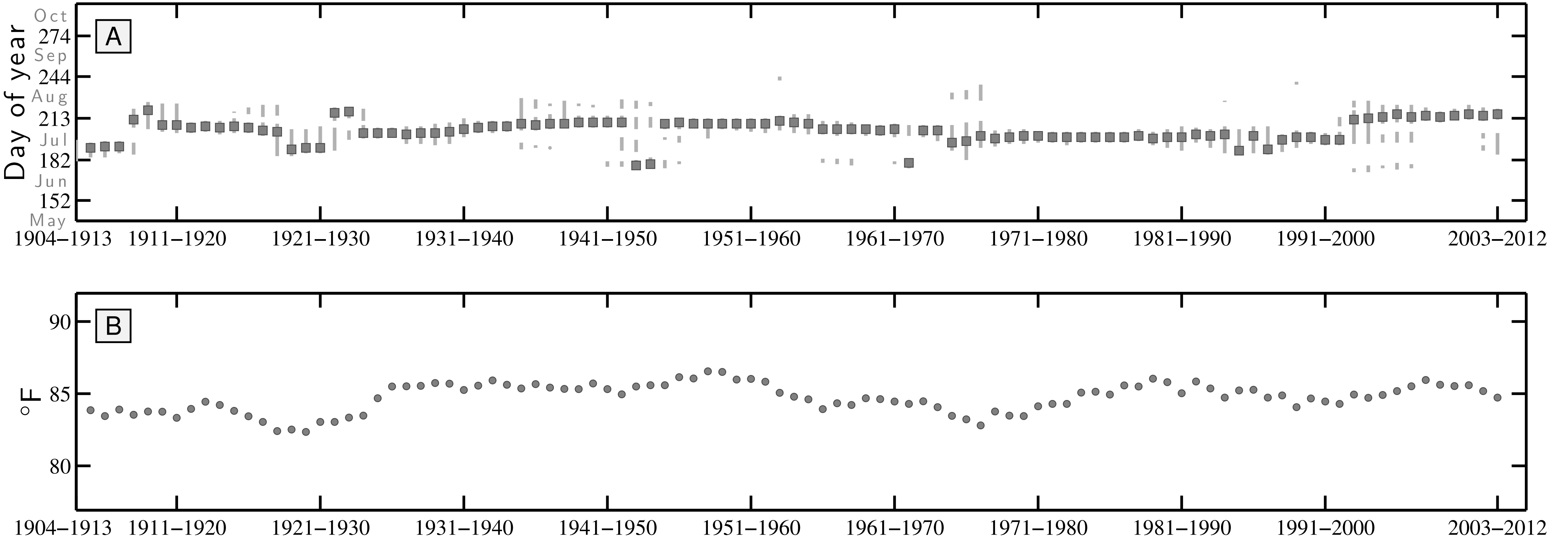

10 year Winter Teletherm dynamics for MILLPORT 4 NE, OH:

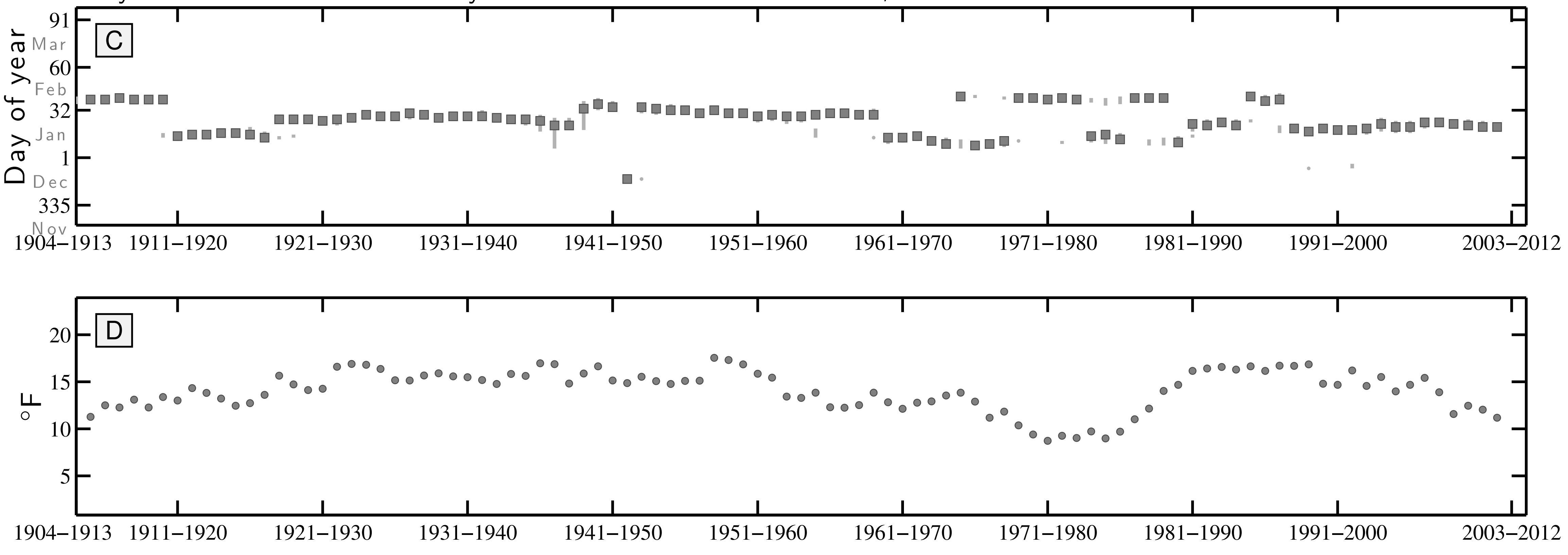

10 year Summer Teletherm dynamics for NORWALK WWTP, OH:

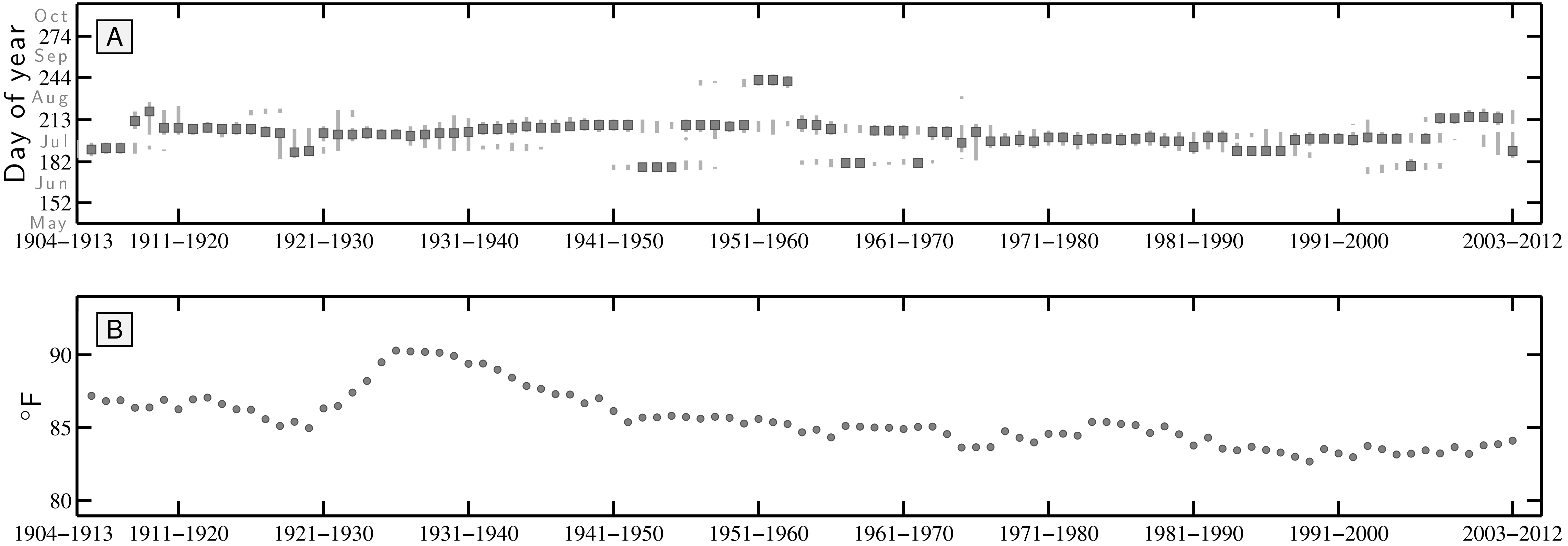

10 year Winter Teletherm dynamics for NORWALK WWTP, OH:

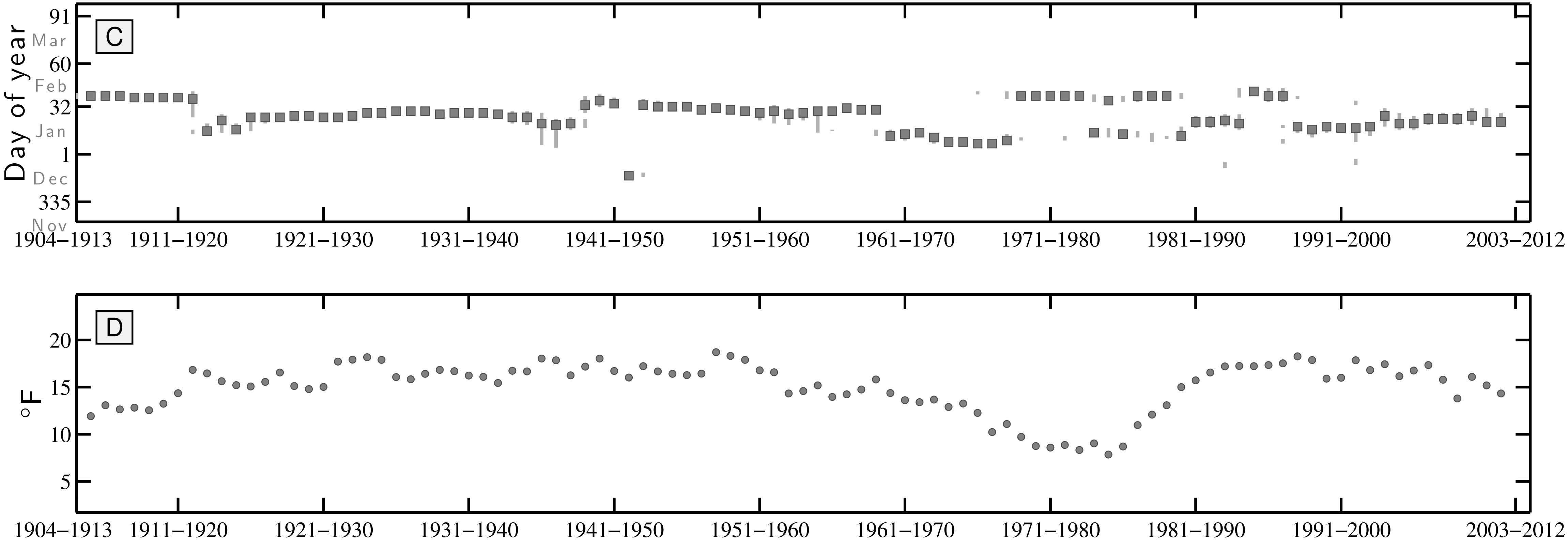

10 year Summer Teletherm dynamics for OBERLIN, OH:

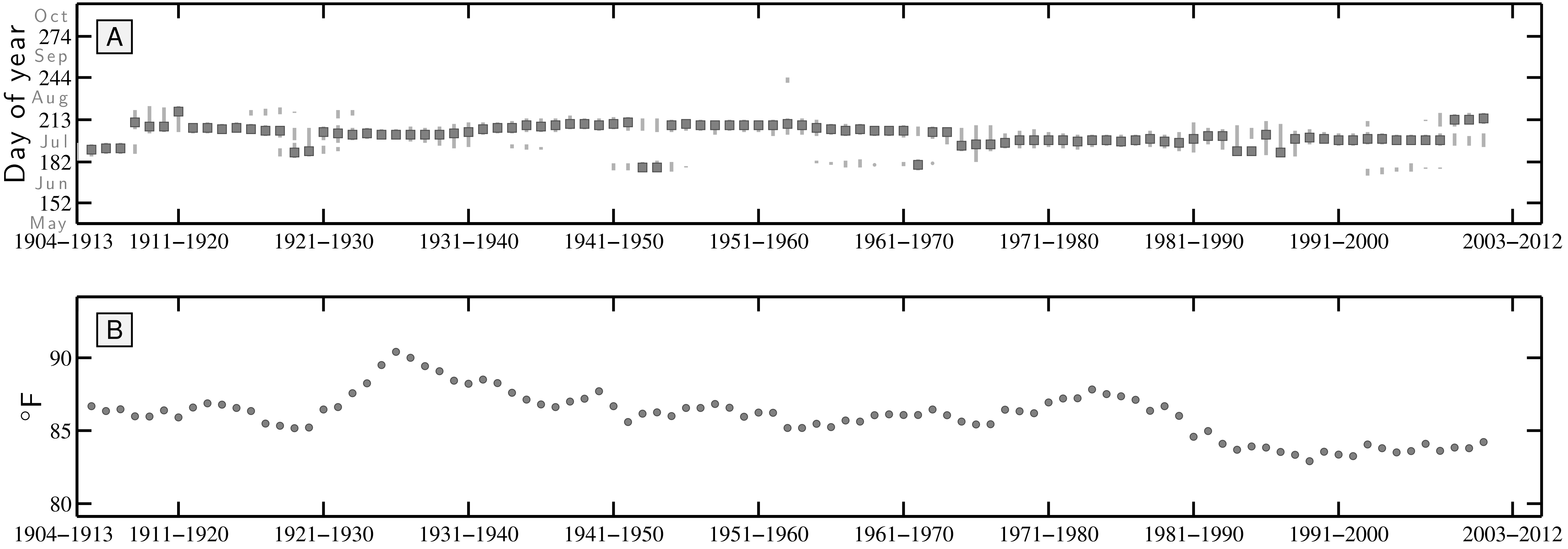

10 year Winter Teletherm dynamics for OBERLIN, OH:

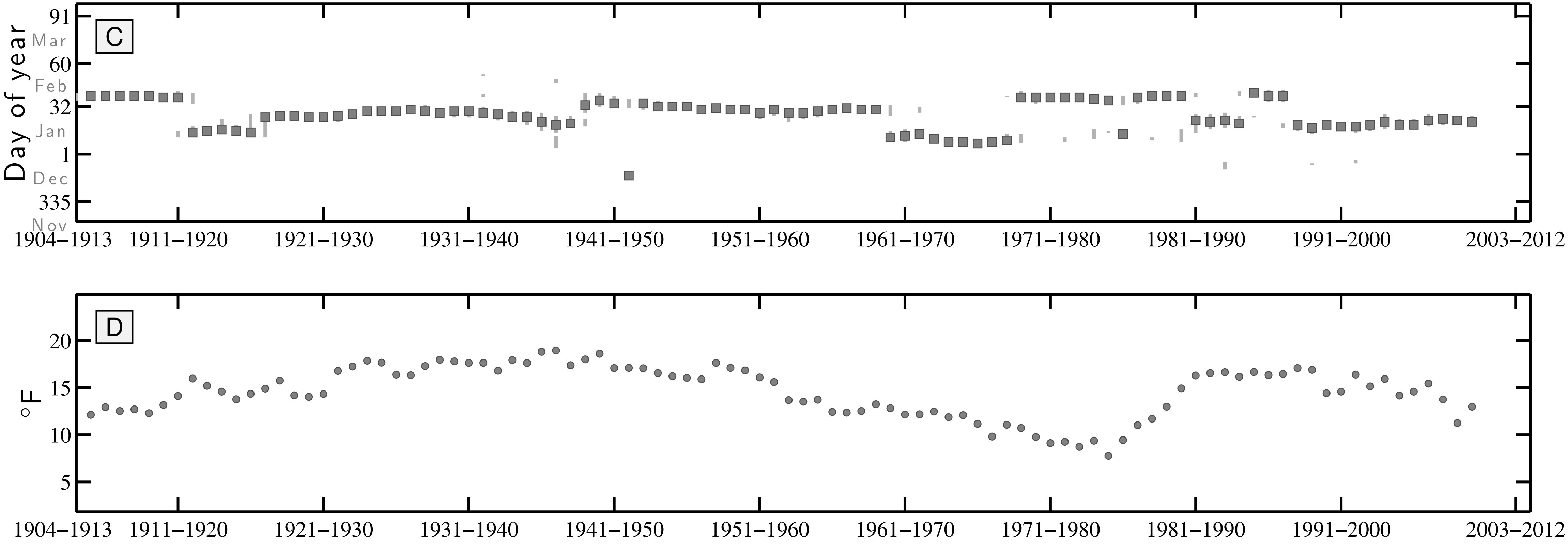

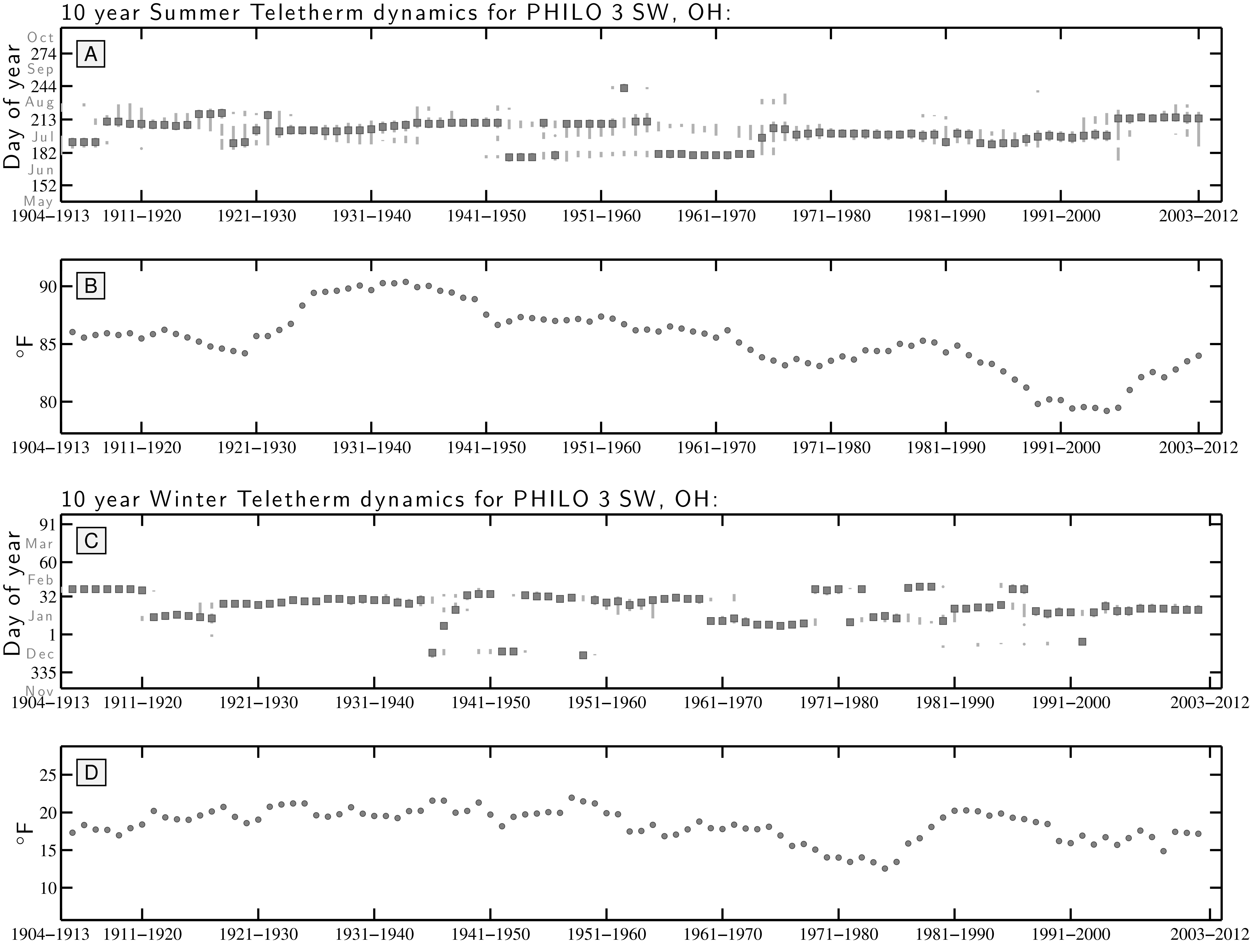
10 year Summer Teletherm dynamics for PHILO 3 SW, OH:
A
Day of year
Oct
274
Sep
244
Aug
213
Jul
182
Jun
152
May
1904–1913
1911–1920
1921–1930
1931–1940
1941–1950
1951–1960
1961–1970
1971–1980
1981–1990
1991–2000
2003–2012
B
°F
90
85
80
10 year Winter Teletherm dynamics for PHILO 3 SW, OH:
C
91
Mar
60
Feb
32
Jan
1
Dec
335
Nov
D
25
20
15
10

10 year Summer Teletherm dynamics for PORTSMOUTH-SCIOTOVILLE, OH:

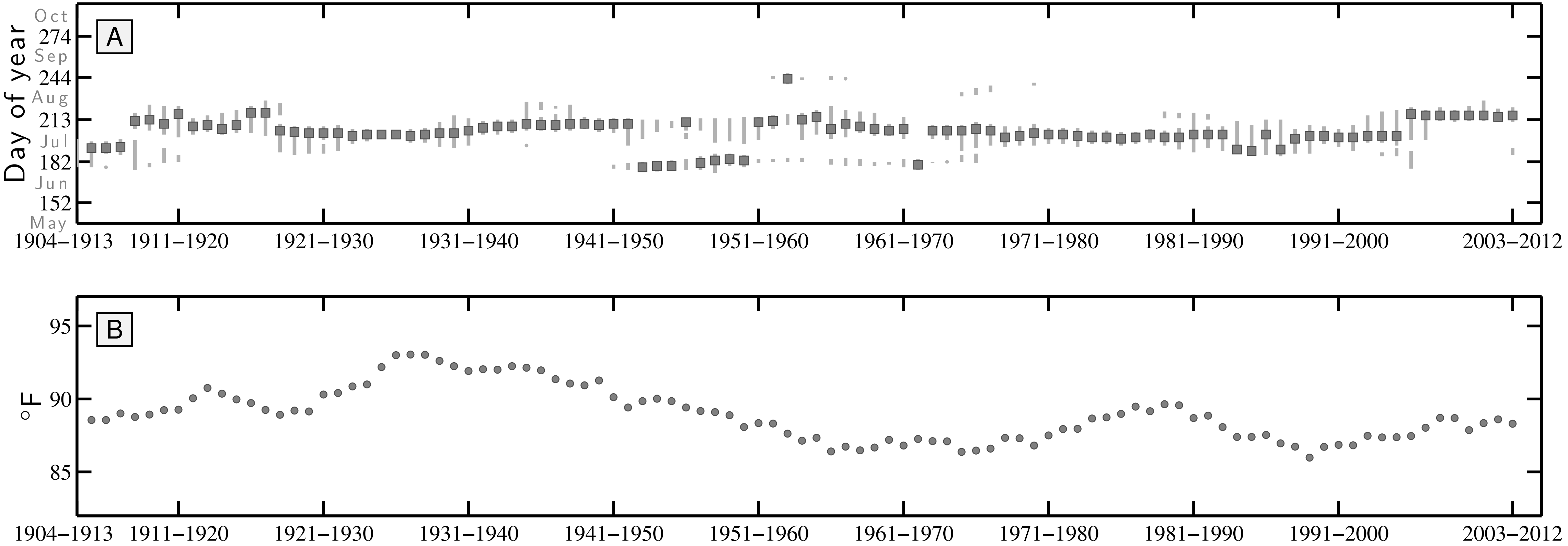

10 year Winter Teletherm dynamics for PORTSMOUTH-SCIOTOVILLE, OH:

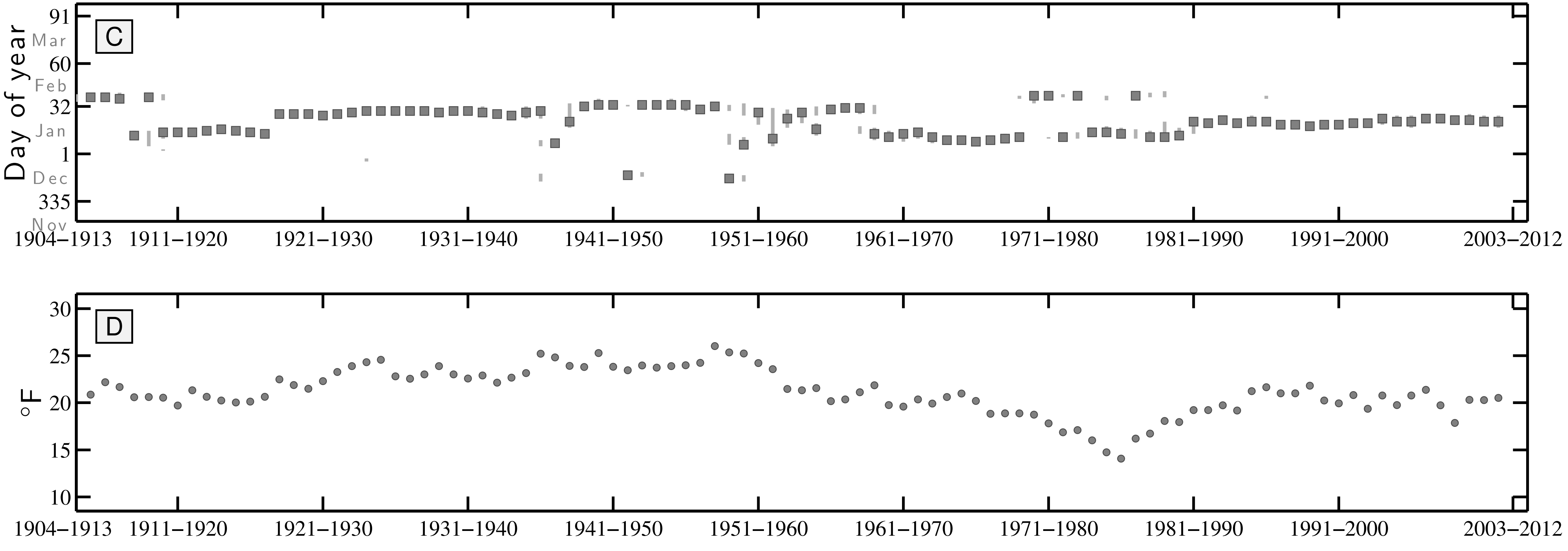

10 year Summer Teletherm dynamics for TIFFIN, OH:

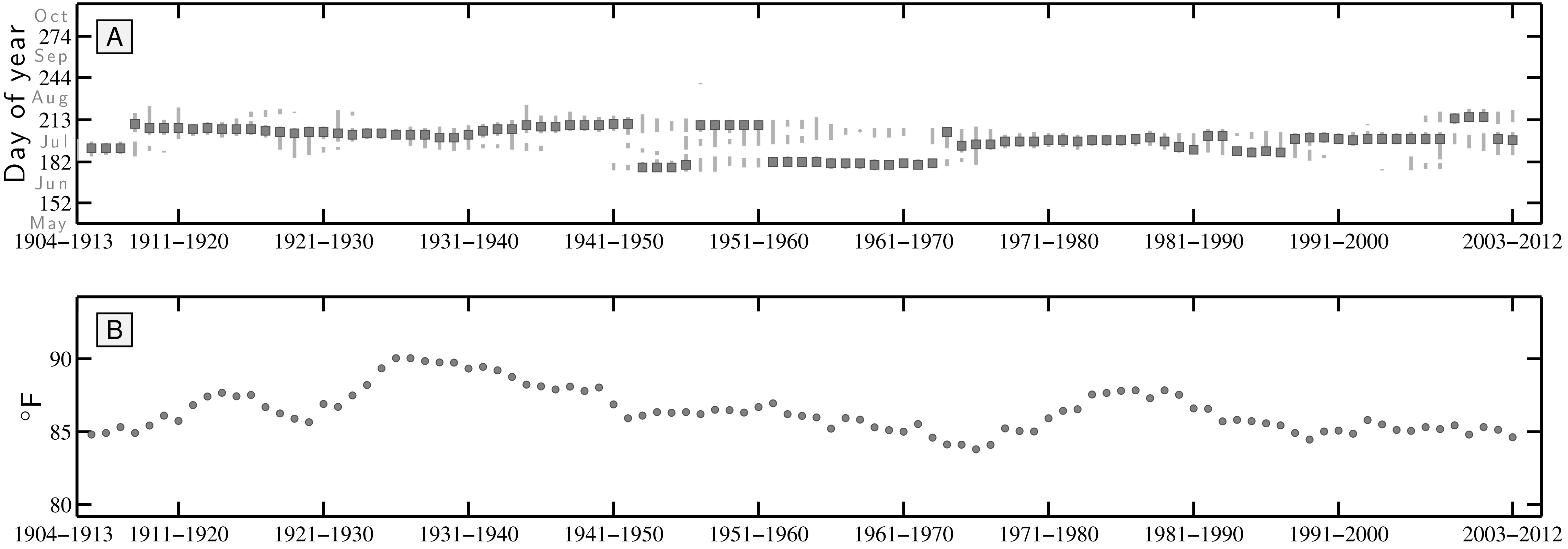

10 year Winter Teletherm dynamics for TIFFIN, OH:

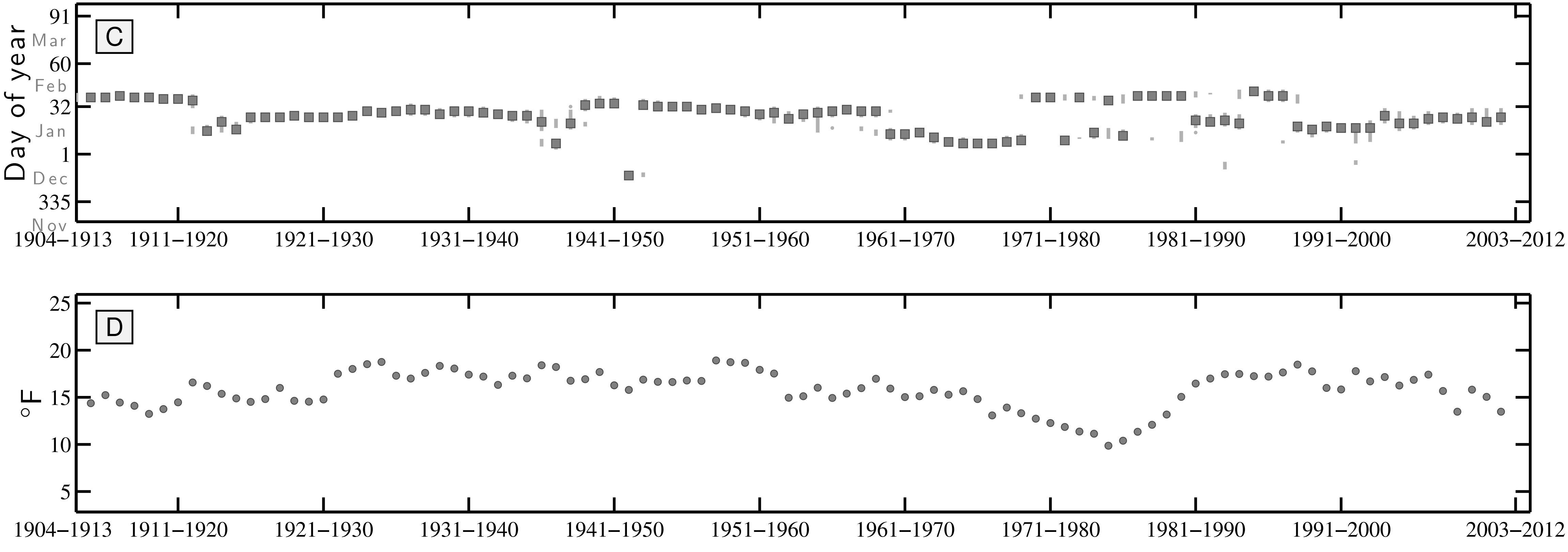

10 year Summer Teletherm dynamics for UPPER SANDUSKY, OH:

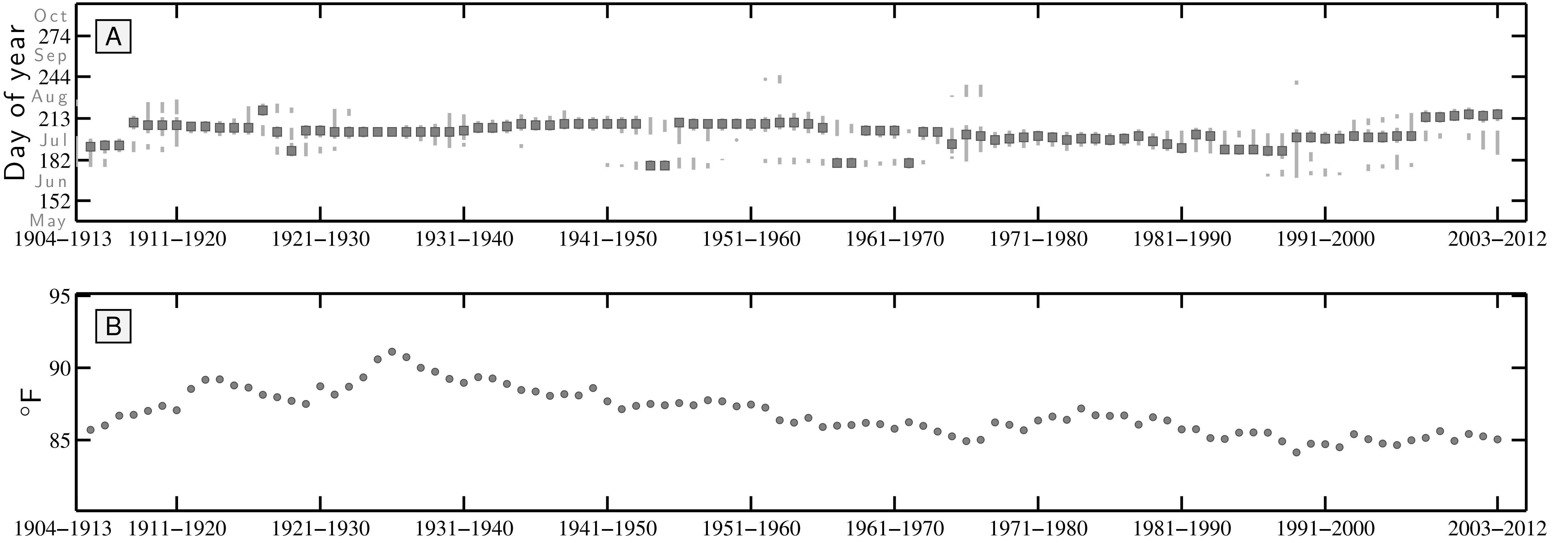

10 year Winter Teletherm dynamics for UPPER SANDUSKY, OH:

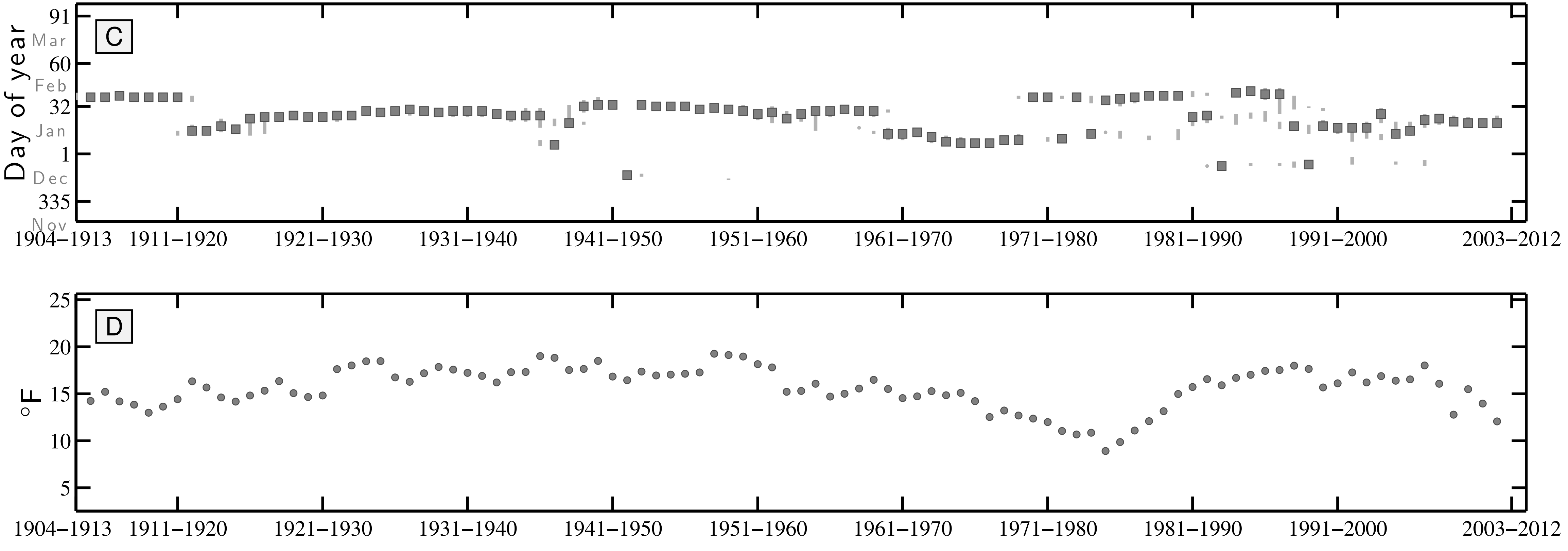

10 year Summer Teletherm dynamics for URBANA WWTP, OH:

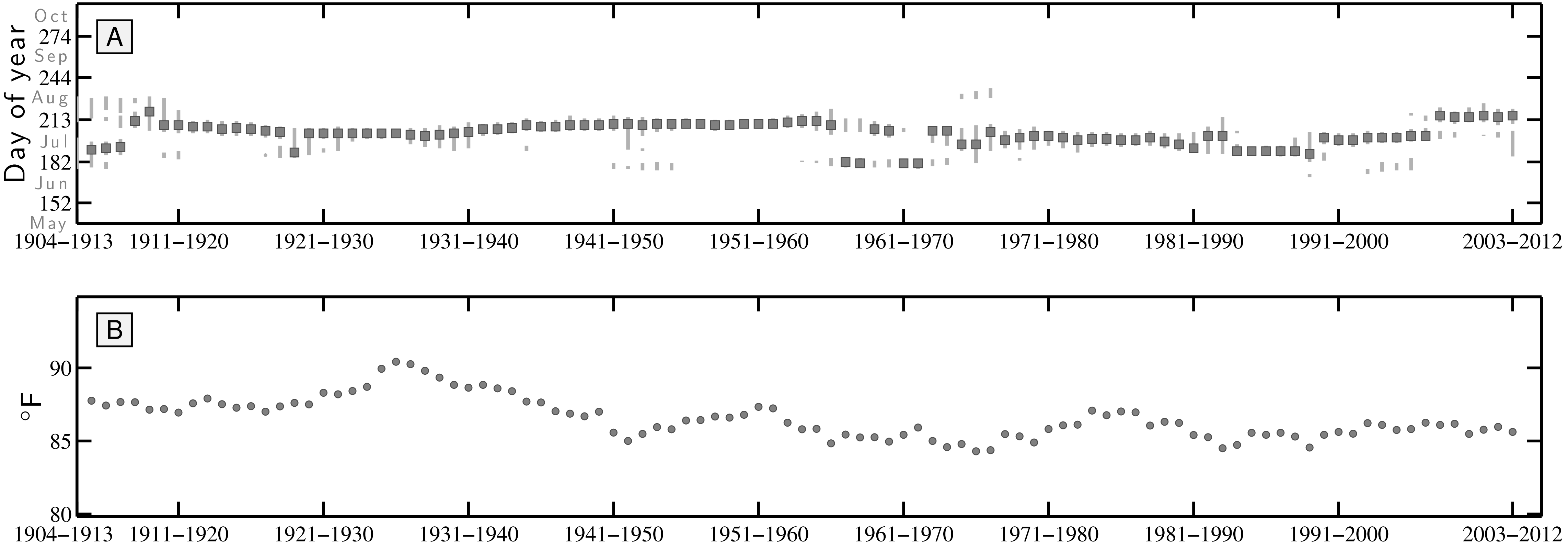

10 year Winter Teletherm dynamics for URBANA WWTP, OH:

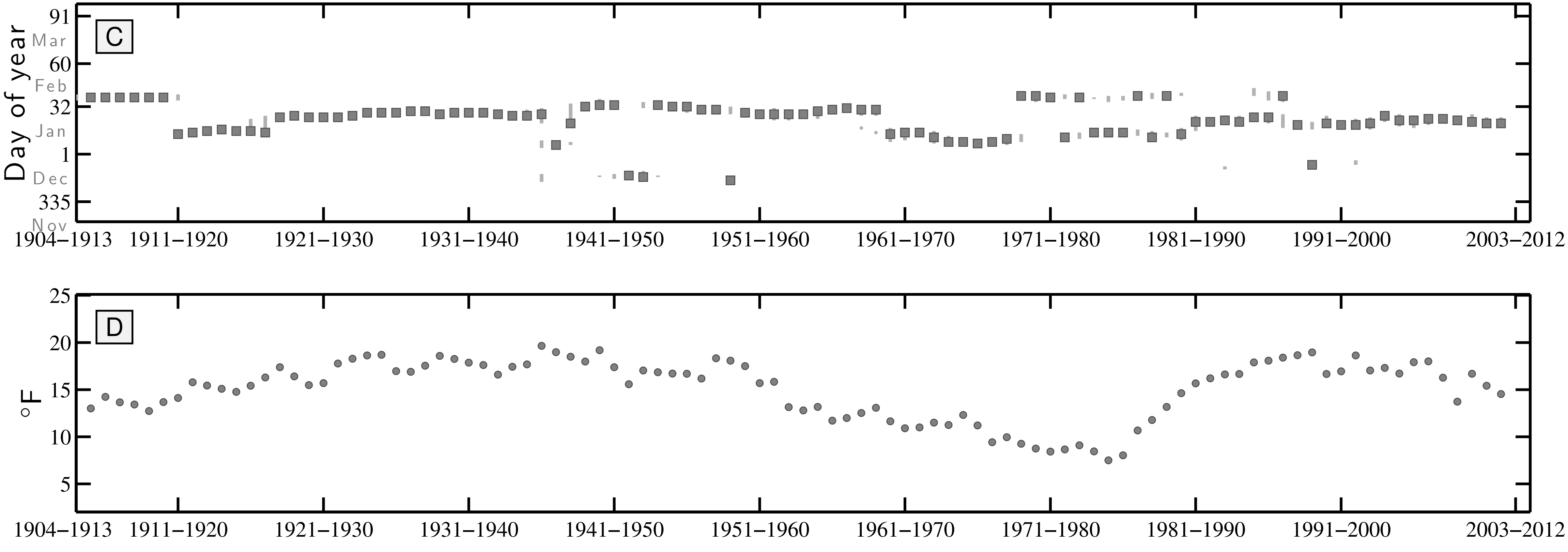

10 year Summer Teletherm dynamics for WARREN 3 S, OH:

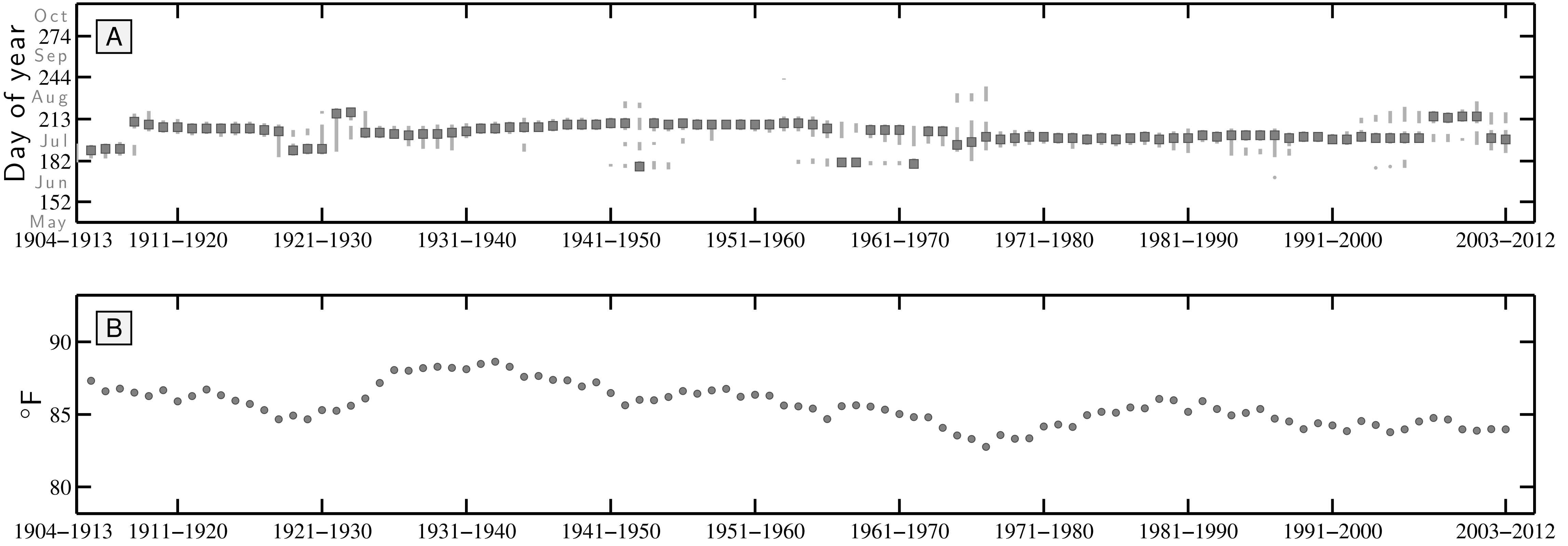

10 year Winter Teletherm dynamics for WARREN 3 S, OH:

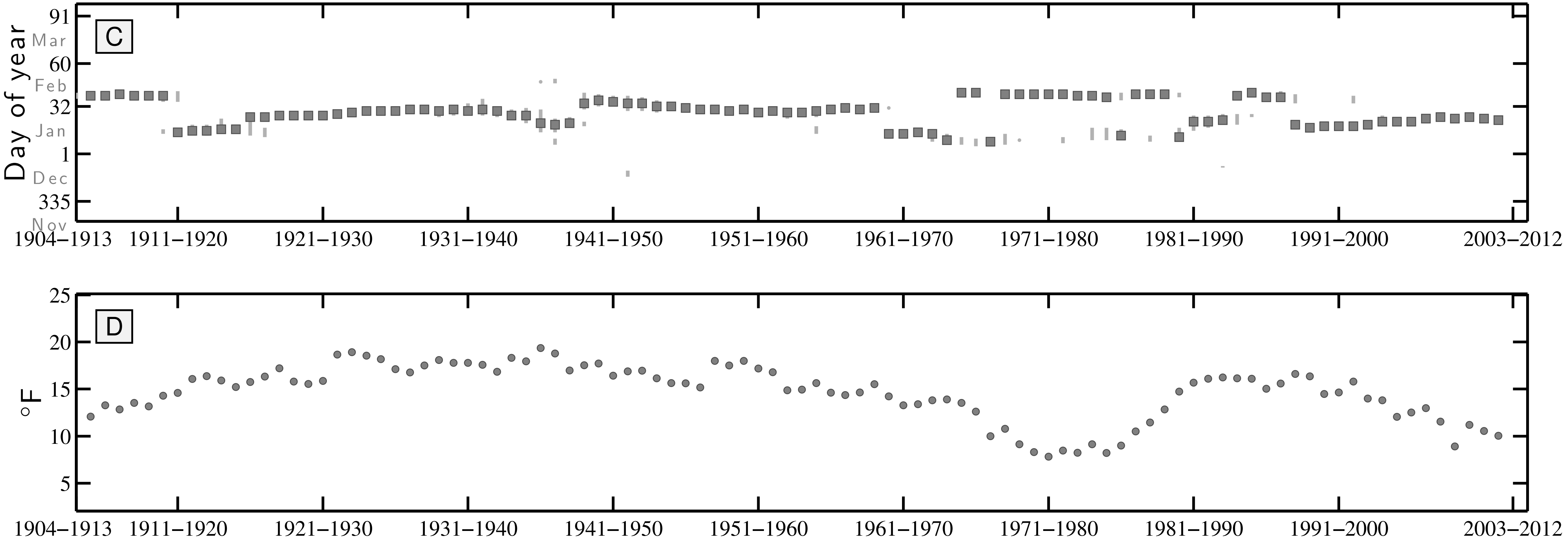

10 year Summer Teletherm dynamics for WAUSEON WTP, OH:

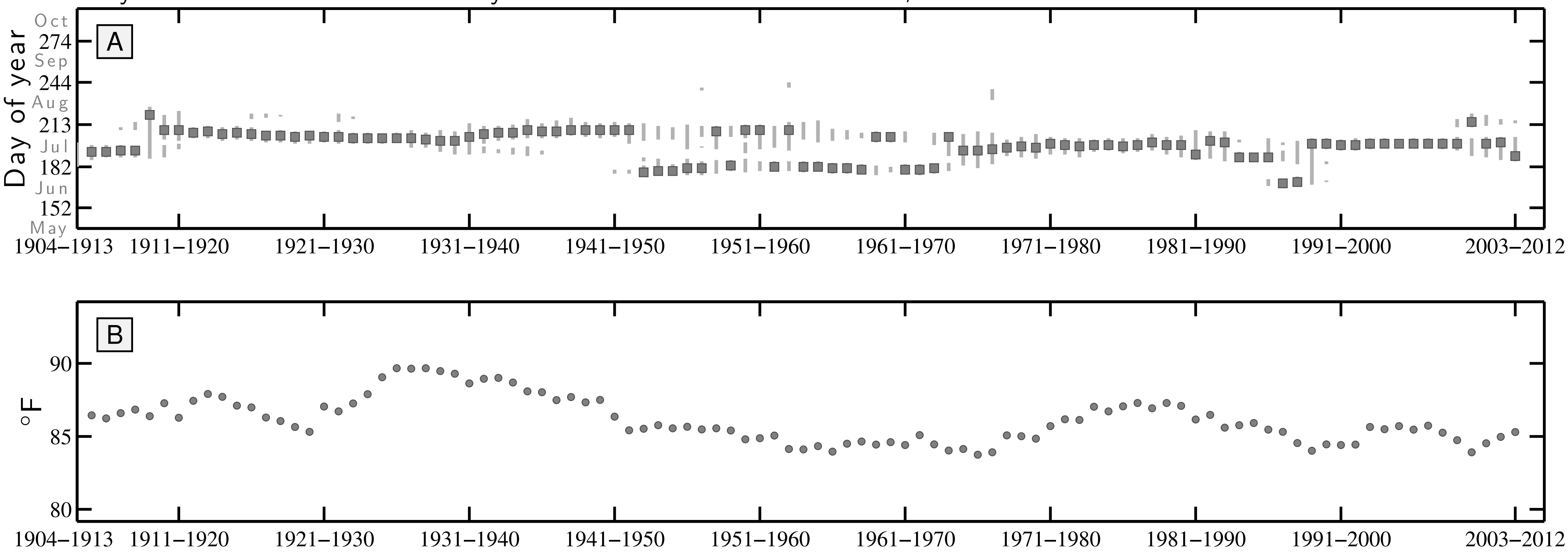

10 year Winter Teletherm dynamics for WAUSEON WTP, OH:

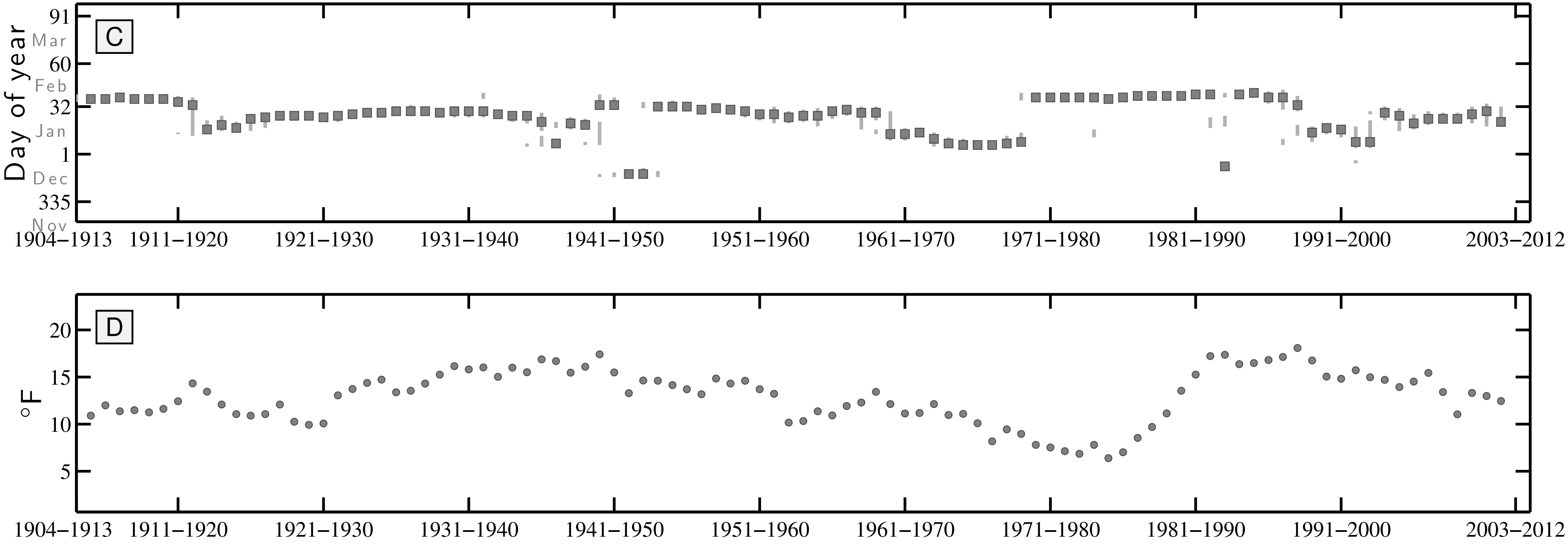

10 year Summer Teletherm dynamics for WAVERLY, OH:

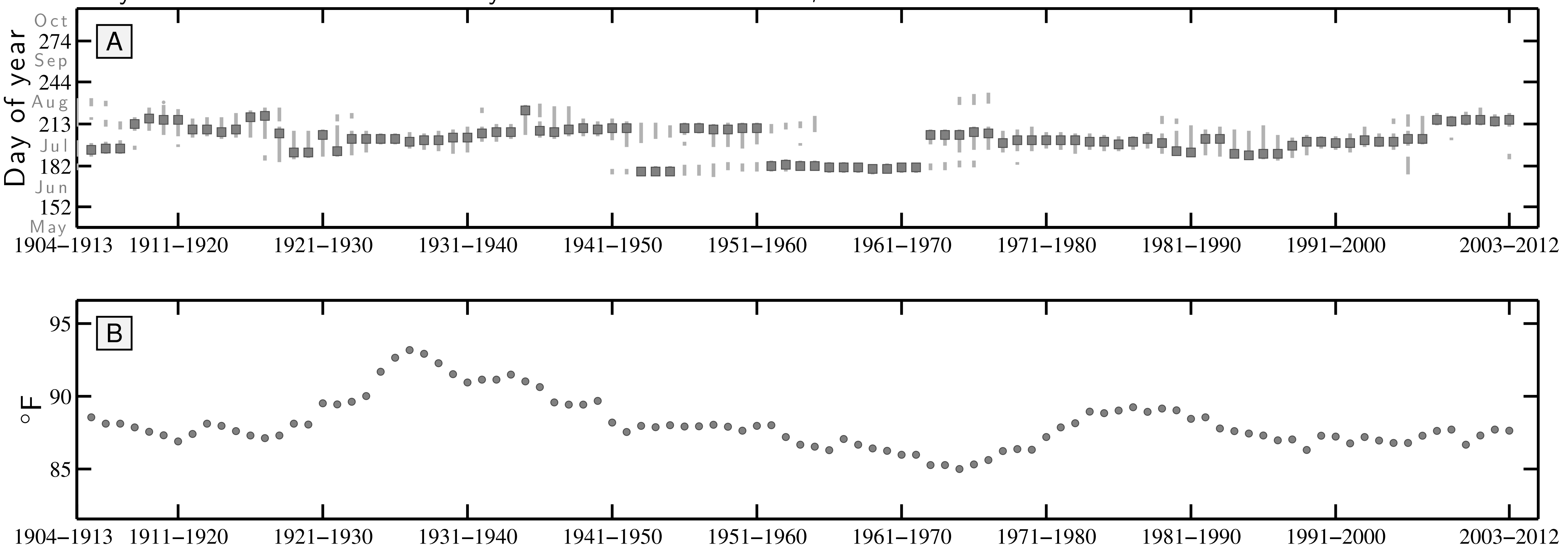

10 year Winter Teletherm dynamics for WAVERLY, OH:

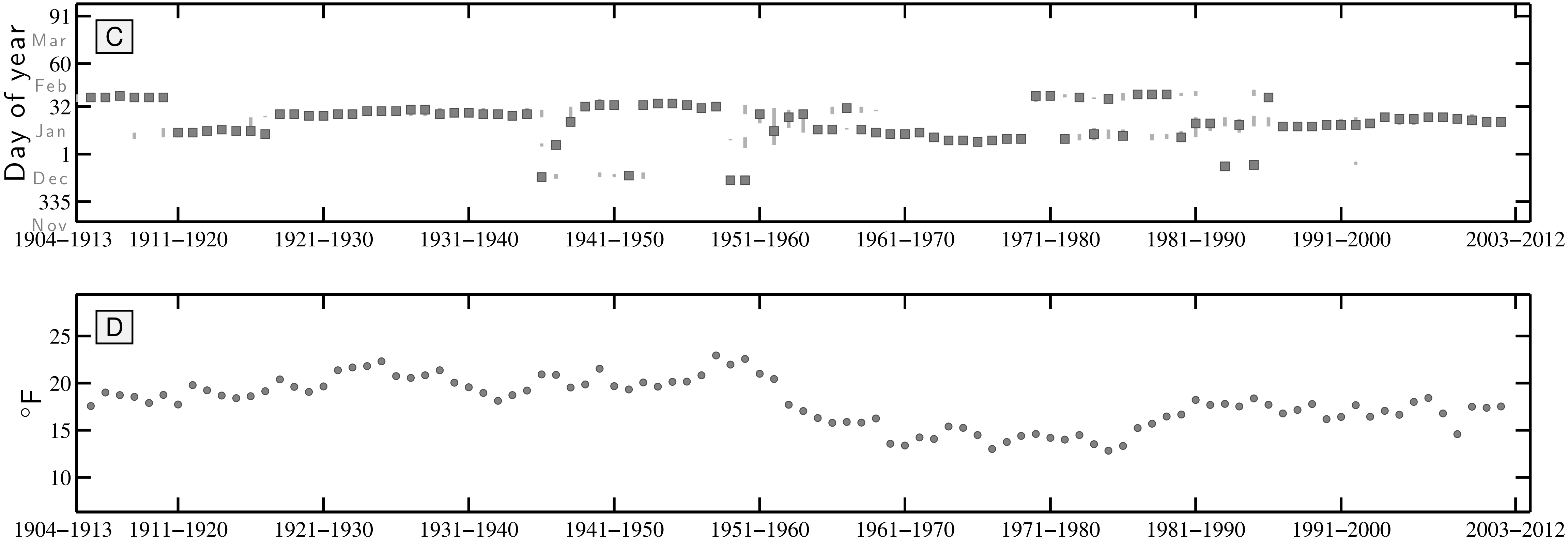

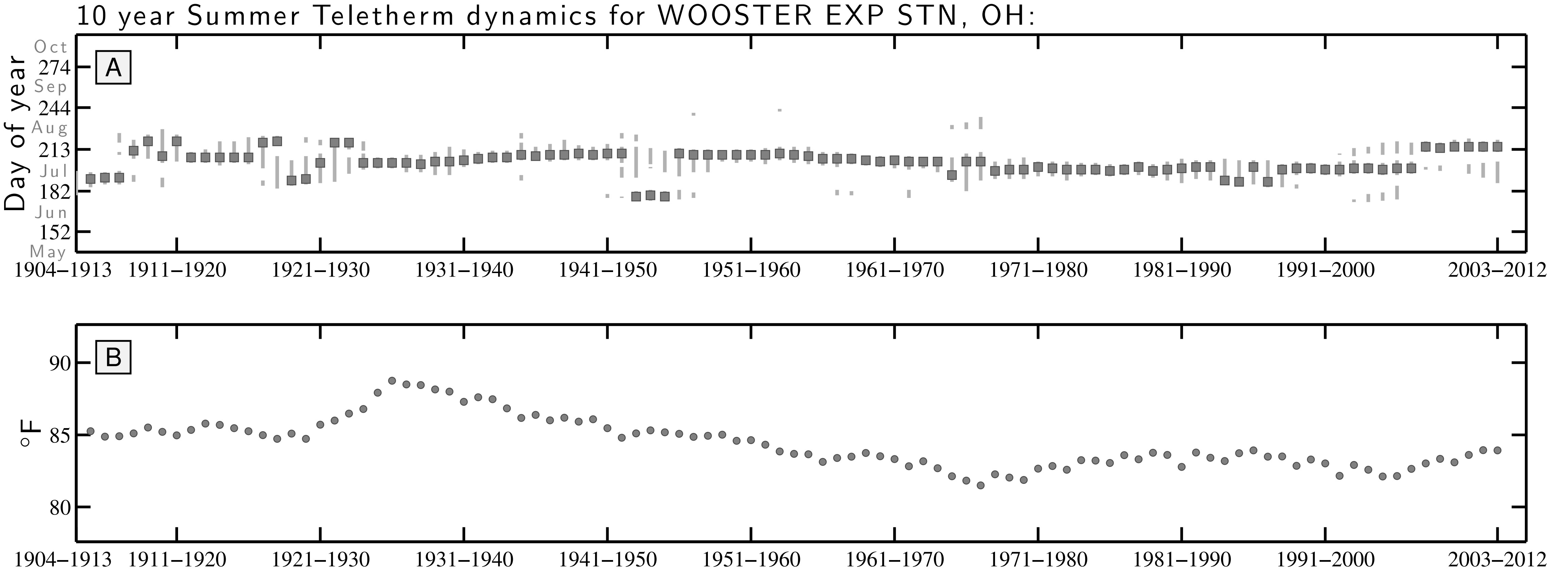
10 year Summer Teletherm dynamics for WOOSTER EXP STN, OH:
A
B
Day of year
°F
Oct
274
Sep
244
Aug
213
Jul
182
Jun
152
May
90
85
80
1904–1913
1911–1920
1921–1930
1931–1940
1941–1950
1951–1960
1961–1970
1971–1980
1981–1990
1991–2000
2003–2012

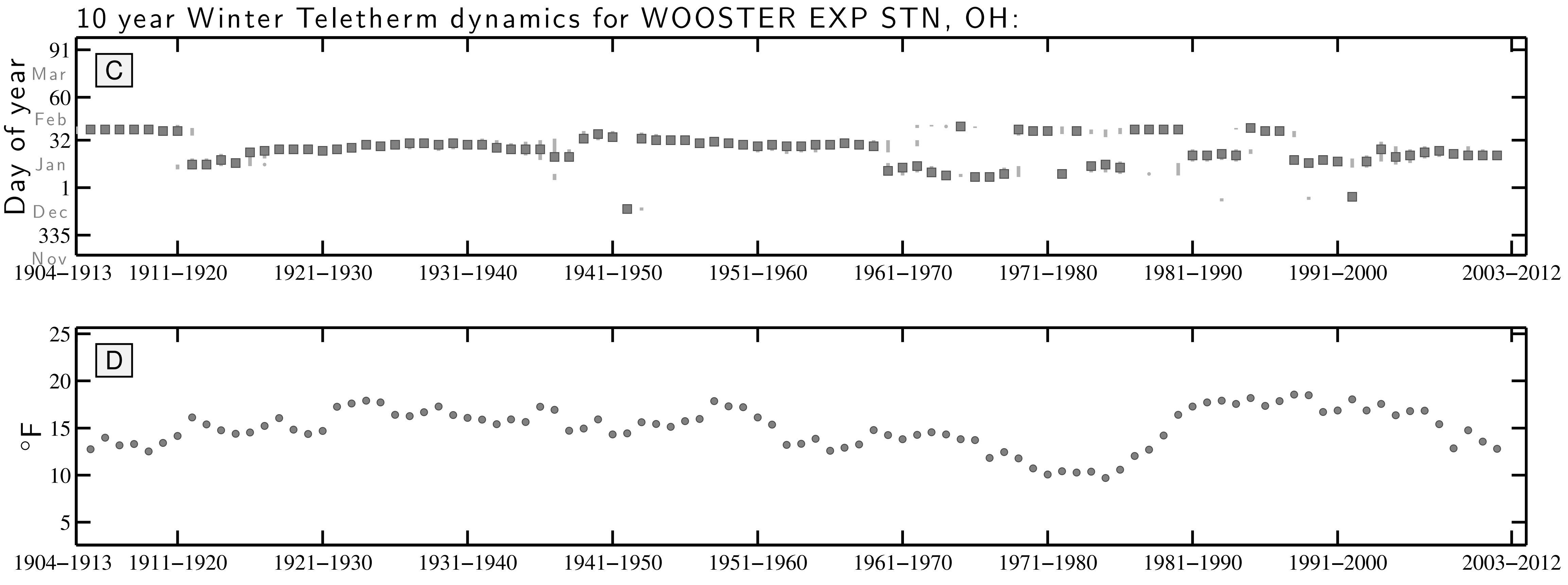
10 year Winter Teletherm dynamics for WOOSTER EXP STN, OH:
C
D
Day of year
°F
91
Mar
60
Feb
32
Jan
1
Dec
335
Nov
25
20
15
10
5
1904–1913
1911–1920
1921–1930
1931–1940
1941–1950
1951–1960
1961–1970
1971–1980
1981–1990
1991–2000
2003–2012

10 year Summer Teletherm dynamics for ADA, OK:

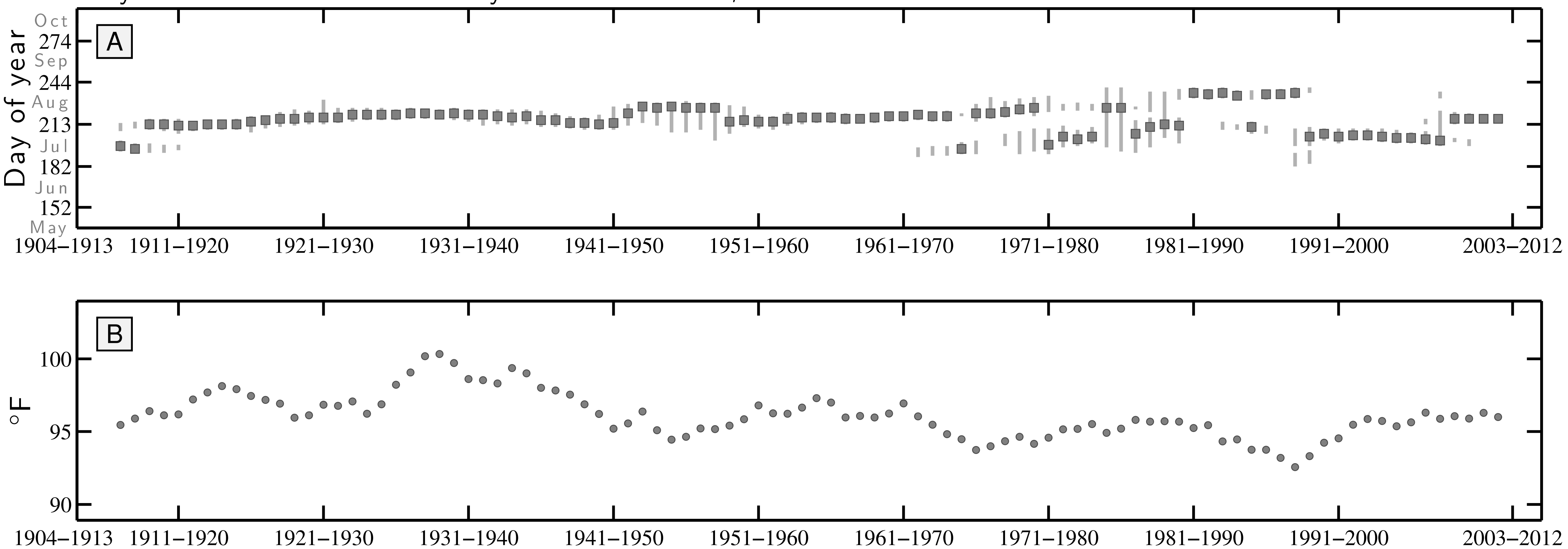

10 year Winter Teletherm dynamics for ADA, OK:

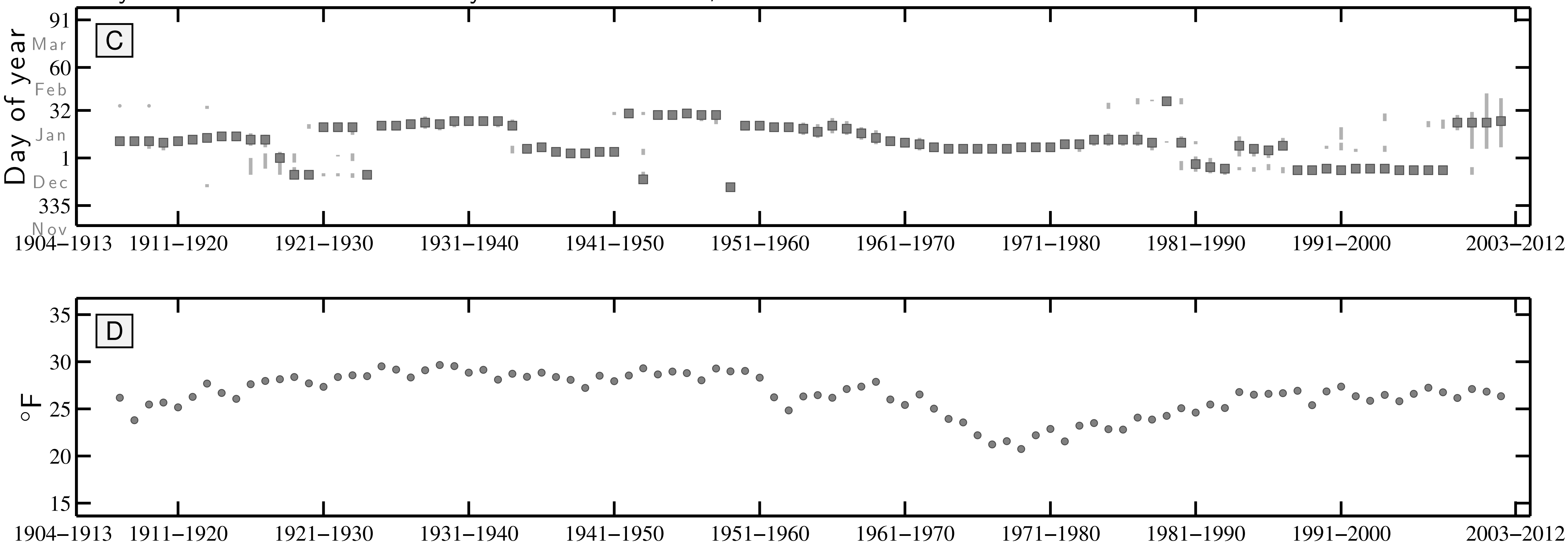

10 year Summer Teletherm dynamics for ALTUS IRIG RSCH STN, OK:

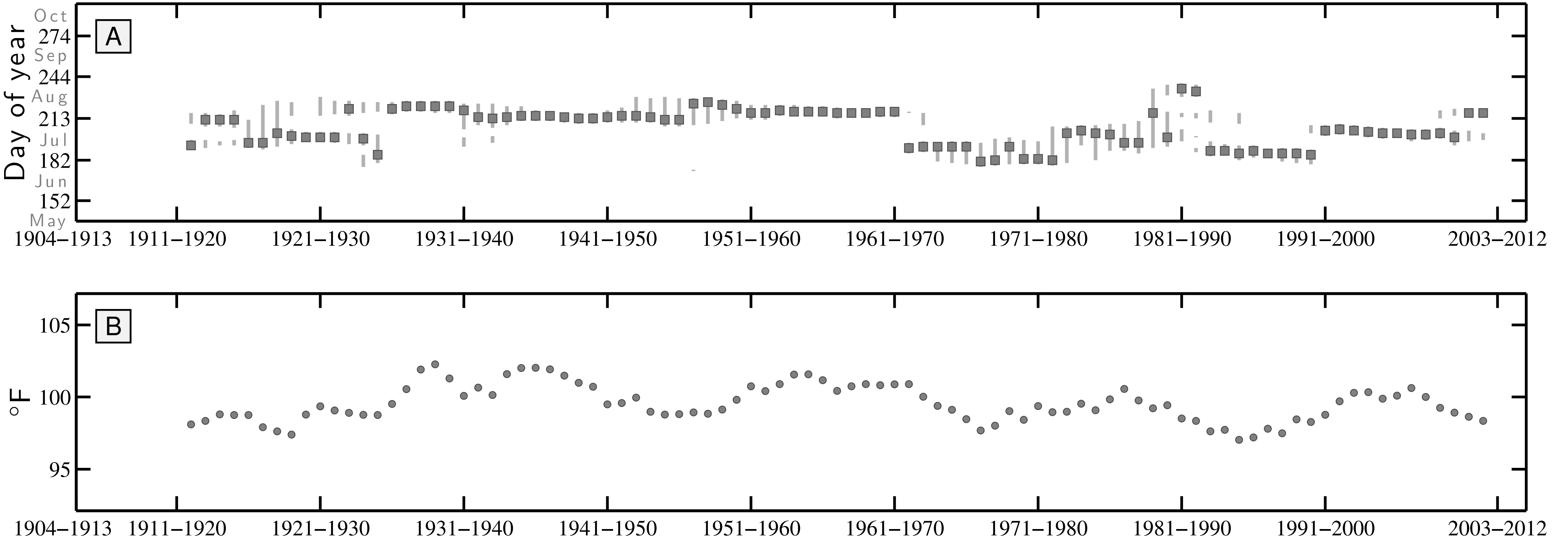

10 year Winter Teletherm dynamics for ALTUS IRIG RSCH STN, OK:

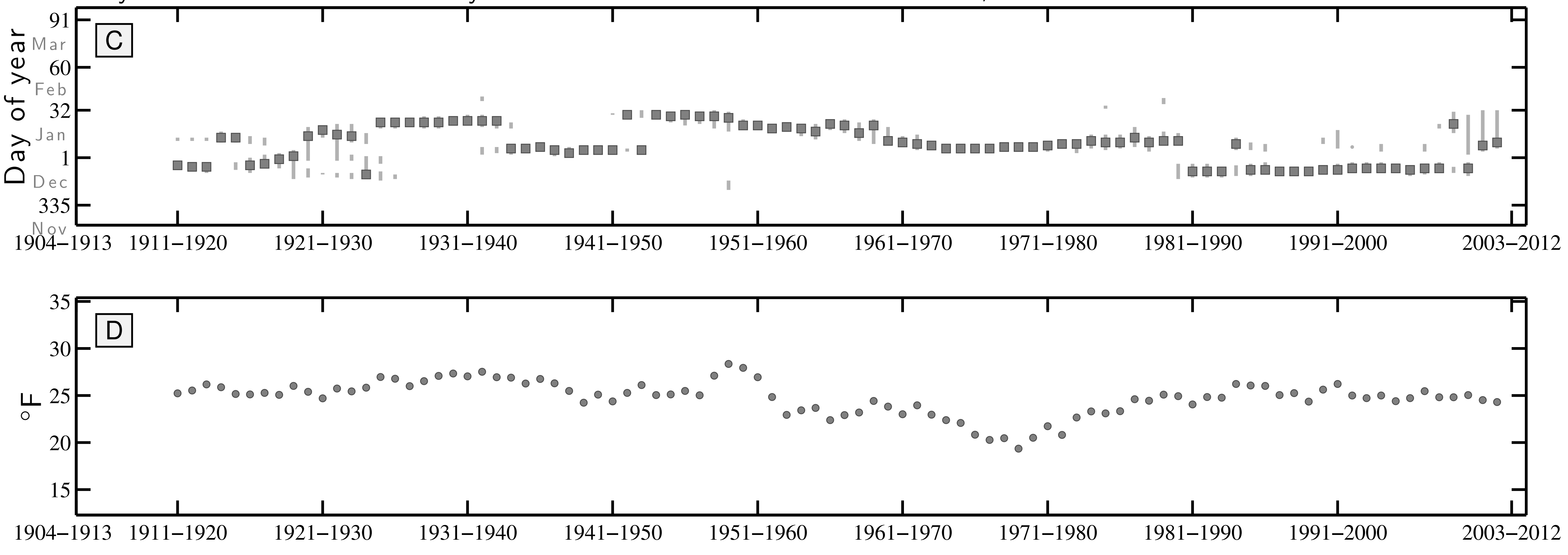

10 year Summer Teletherm dynamics for ANTLERS, OK:

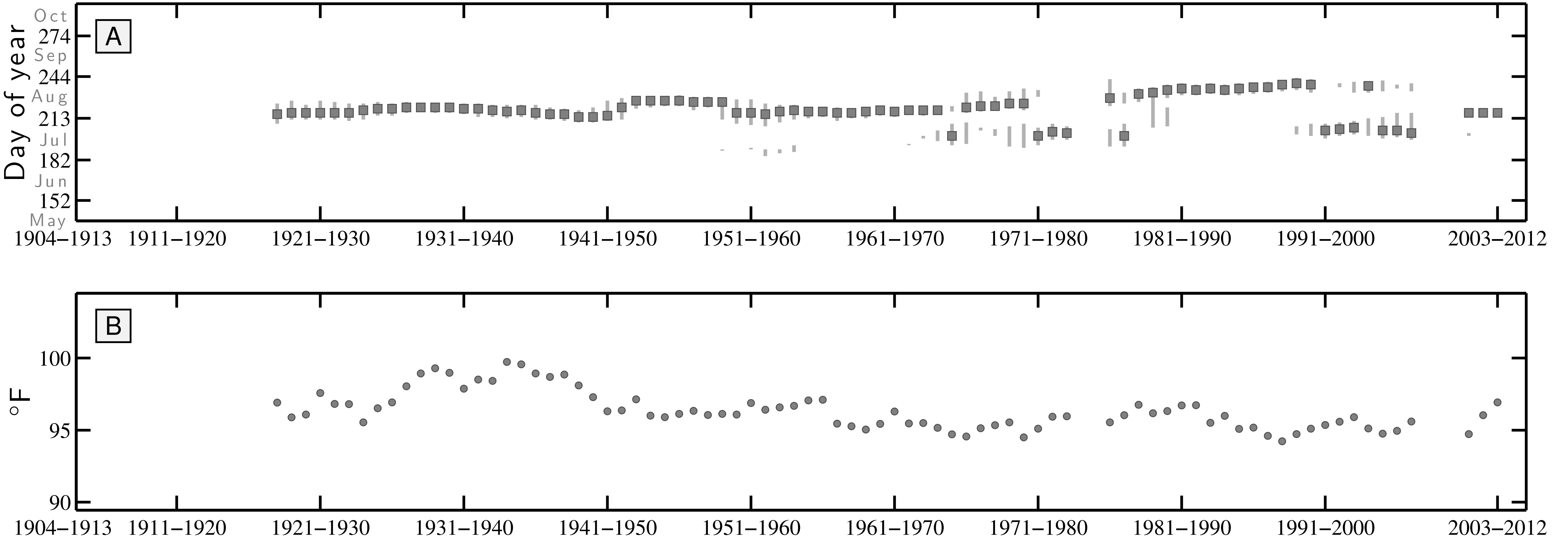

10 year Winter Teletherm dynamics for ANTLERS, OK:

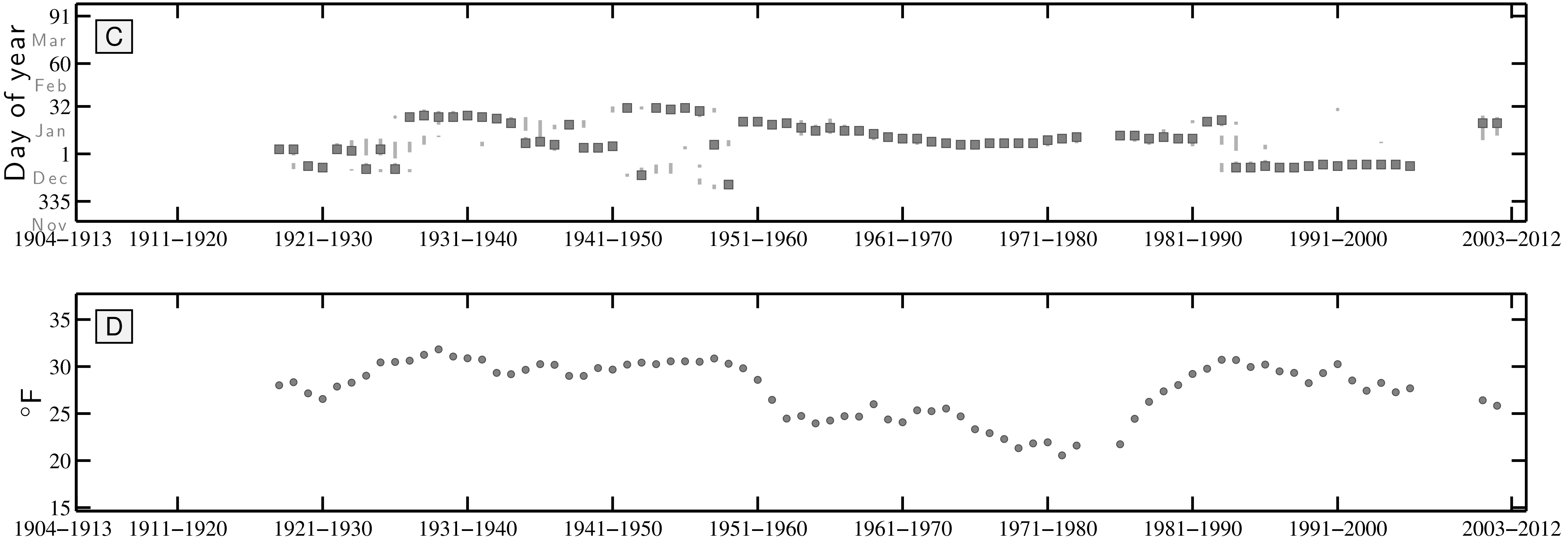

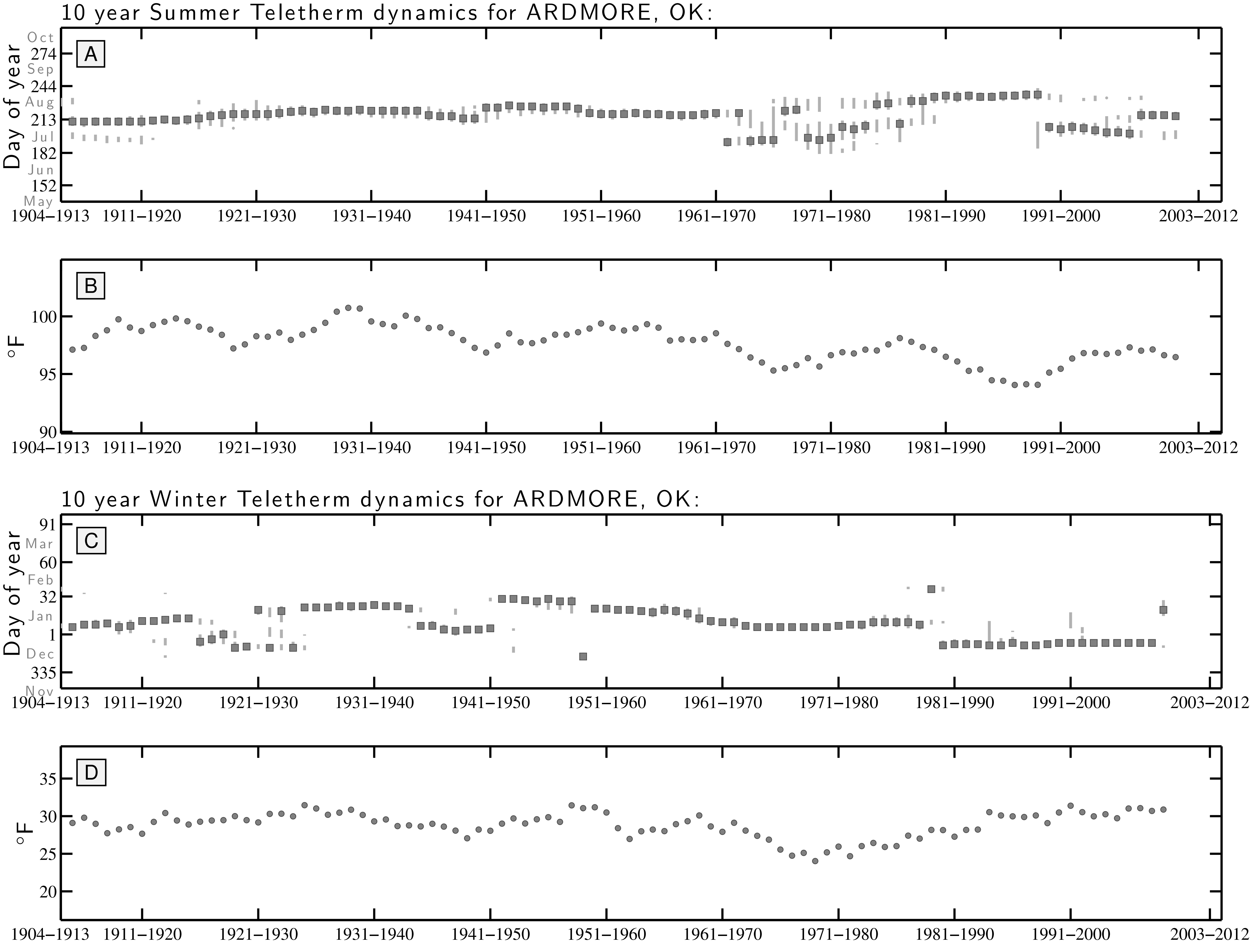

10 year Summer Teletherm dynamics for ARDMORE, OK:
A
Day of year
Oct
274
Sep
244
Aug
213
Jul
182
Jun
152
May
1904–1913
1911–1920
1921–1930
1931–1940
1941–1950
1951–1960
1961–1970
1971–1980
1981–1990
1991–2000
2003–2012
B
°F
100
95
90
10 year Winter Teletherm dynamics for ARDMORE, OK:
C
91
Mar
60
Feb
32
Jan
1
Dec
335
Nov
D
35
30
25
20

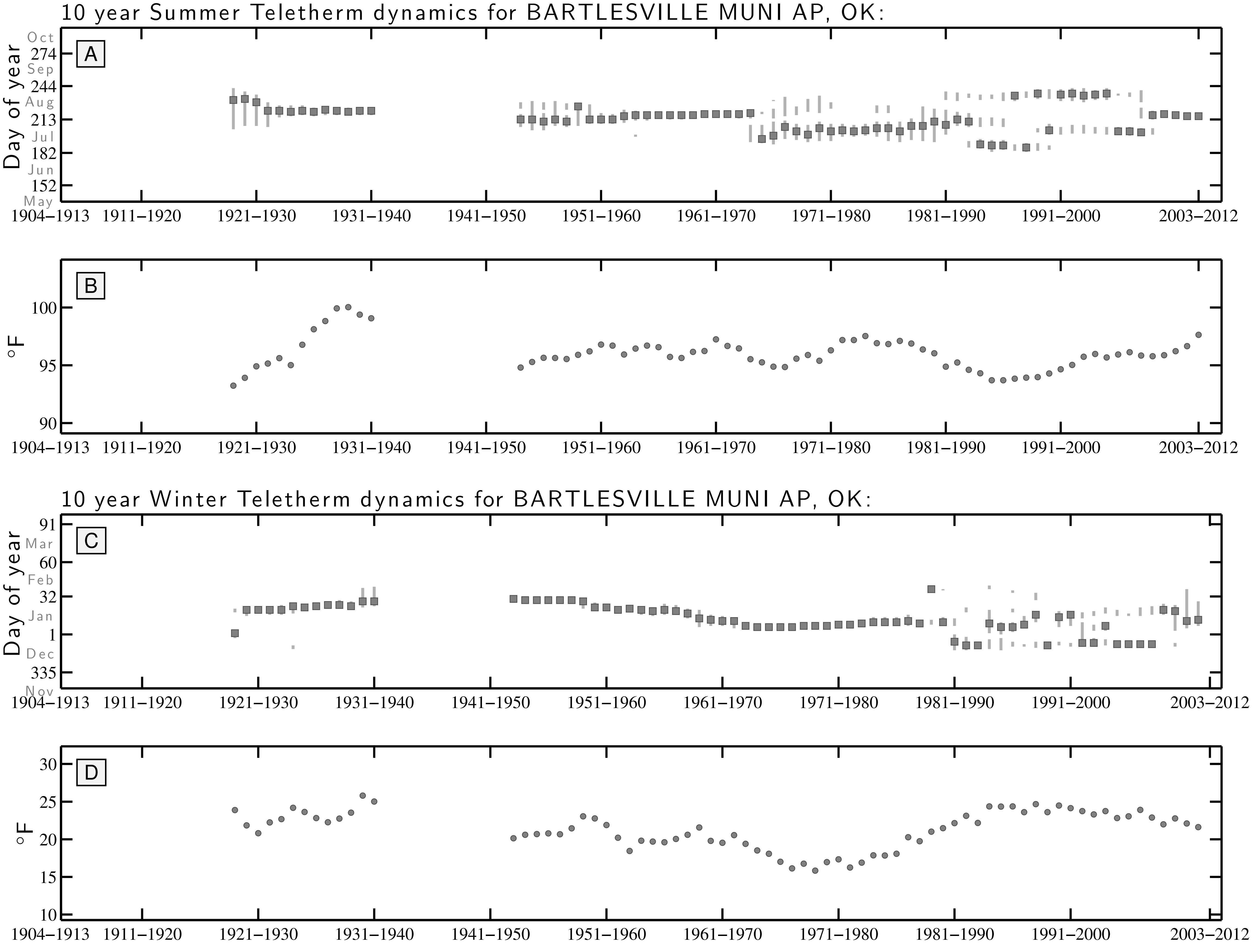
10 year Summer Teletherm dynamics for BARTLESVILLE MUNI AP, OK:
A
B
Day of year
Oct
274
Sep
244
Aug
213
Jul
182
Jun
152
May
°F
100
95
90
10 year Winter Teletherm dynamics for BARTLESVILLE MUNI AP, OK:
C
D
91
Mar
60
Feb
32
Jan
1
Dec
335
Nov
30
25
20
15
10
1904–1913
1911–1920
1921–1930
1931–1940
1941–1950
1951–1960
1961–1970
1971–1980
1981–1990
1991–2000
2003–2012

10 year Summer Teletherm dynamics for BEAVER, OK:

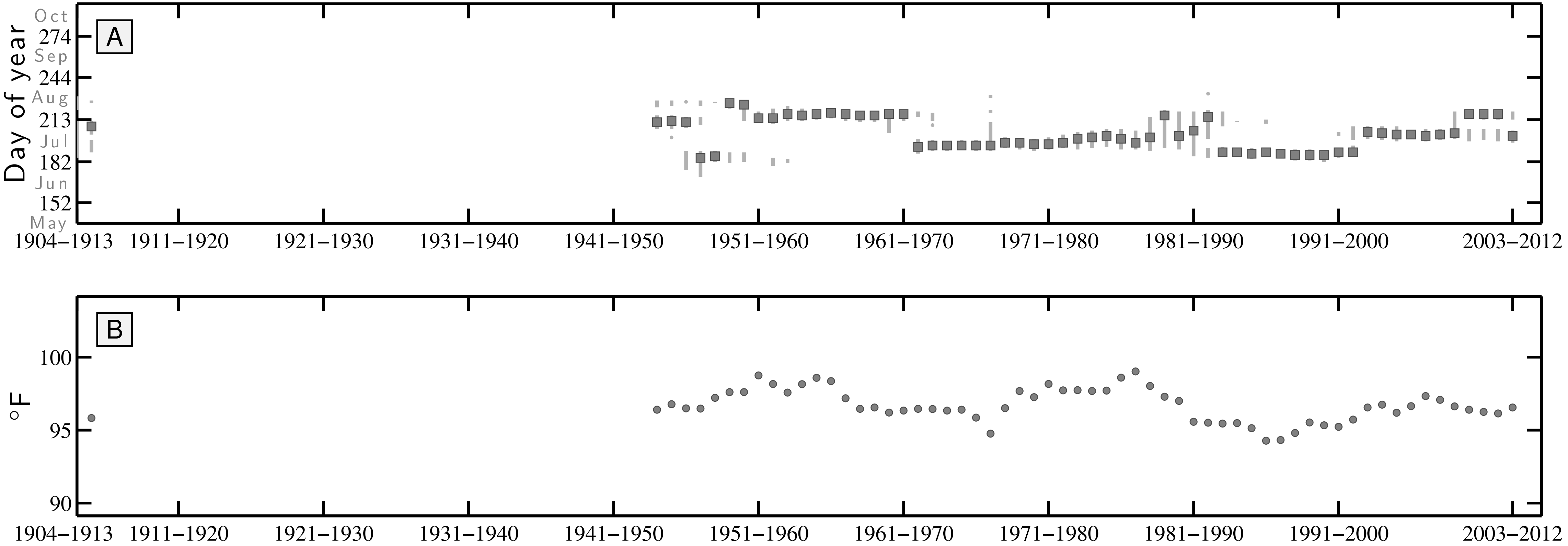

10 year Winter Teletherm dynamics for BEAVER, OK:

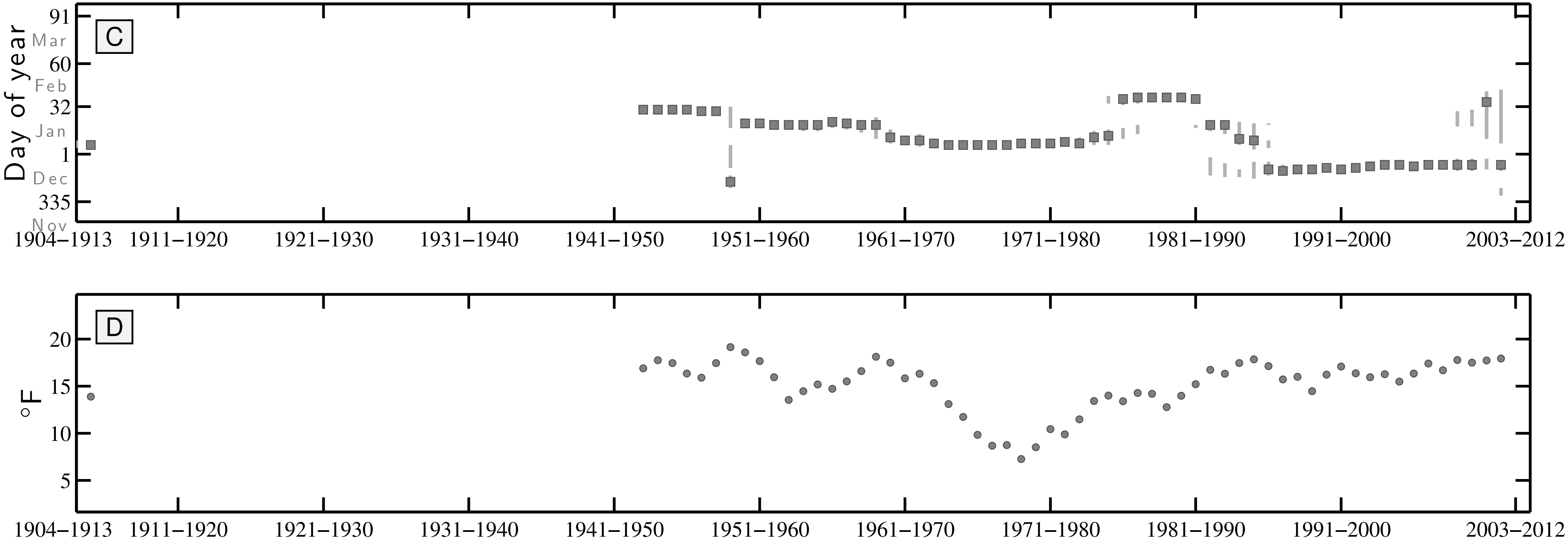

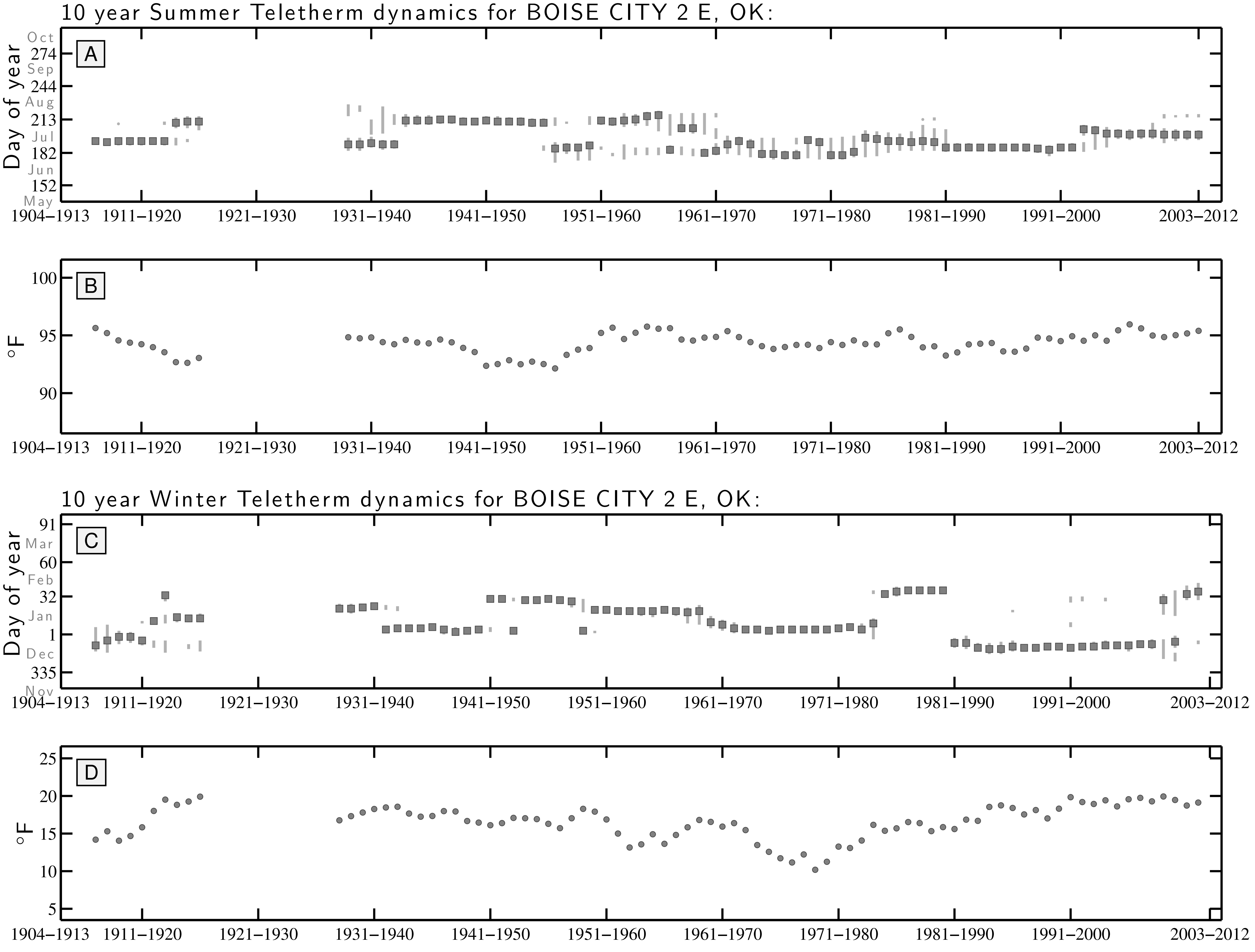

10 year Summer Teletherm dynamics for BOISE CITY 2 E, OK:
A
Day of year
Oct
274
Sep
244
Aug
213
Jul
182
Jun
152
May
1904–1913
1911–1920
1921–1930
1931–1940
1941–1950
1951–1960
1961–1970
1971–1980
1981–1990
1991–2000
2003–2012
B
°F
100
95
90
10 year Winter Teletherm dynamics for BOISE CITY 2 E, OK:
C
Day of year
91
Mar
60
Feb
32
Jan
1
Dec
335
Nov
D
°F
25
20
15
10
5

10 year Summer Teletherm dynamics for BUFFALO 2 SSW, OK:

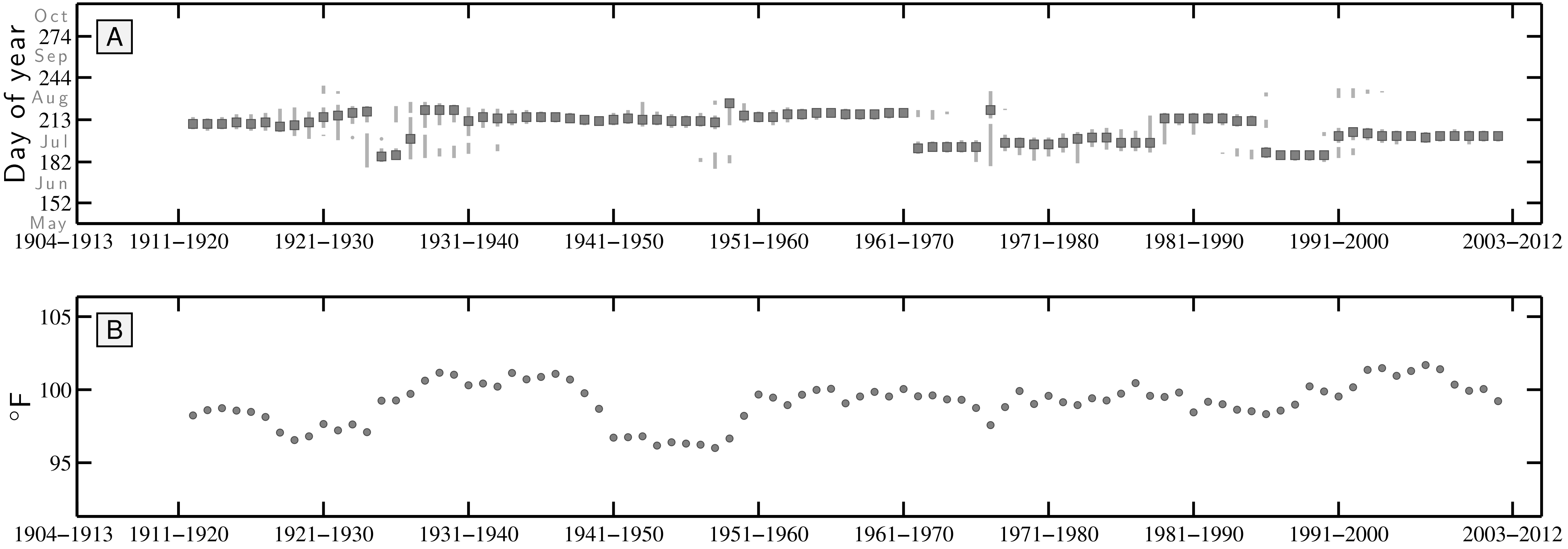

10 year Winter Teletherm dynamics for BUFFALO 2 SSW, OK:

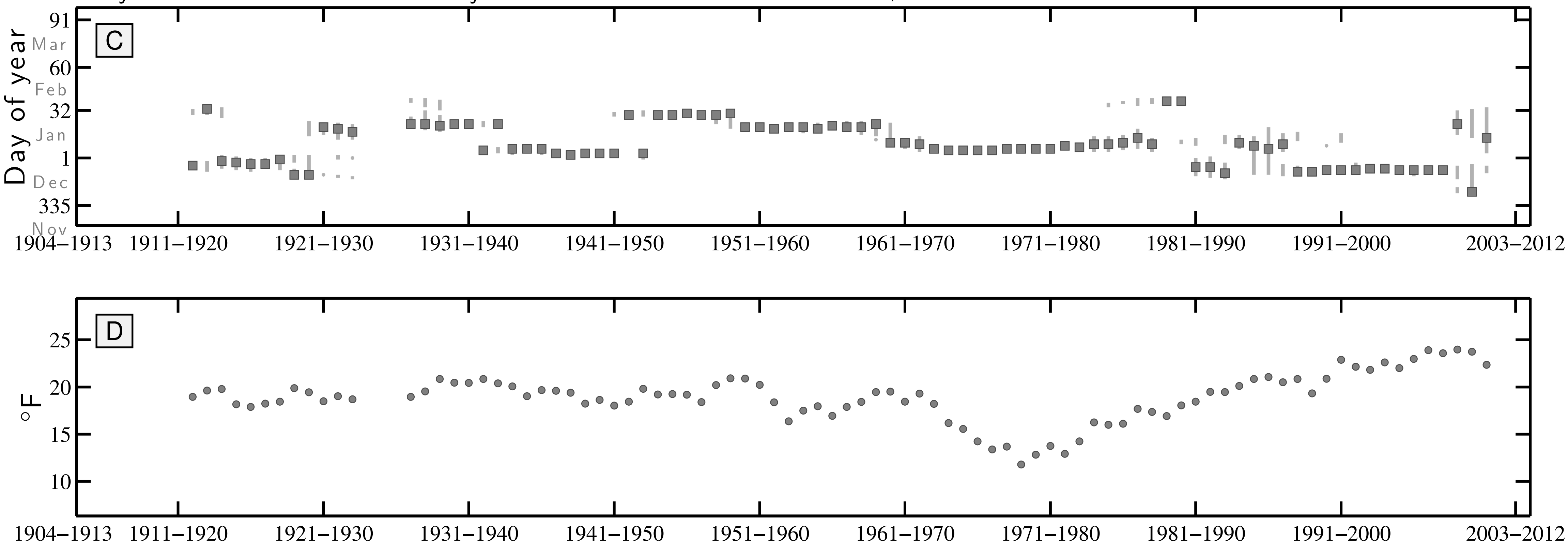

10 year Summer Teletherm dynamics for CARNEGIE 5 NE, OK:

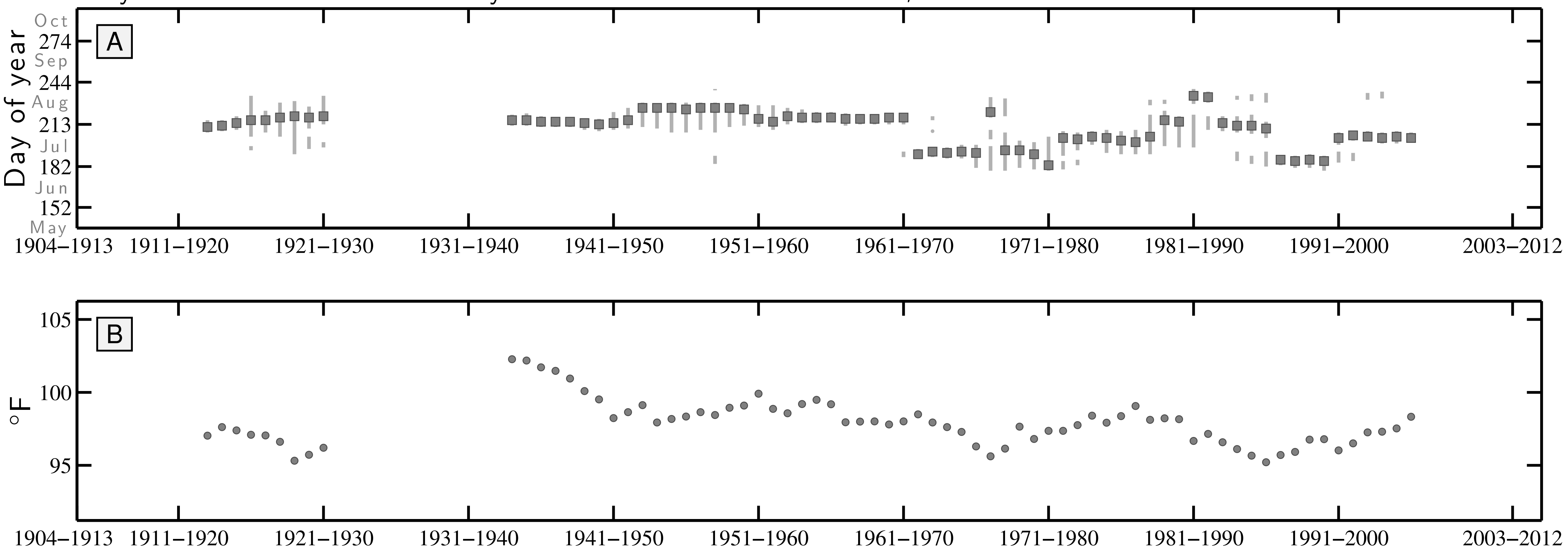

10 year Winter Teletherm dynamics for CARNEGIE 5 NE, OK:

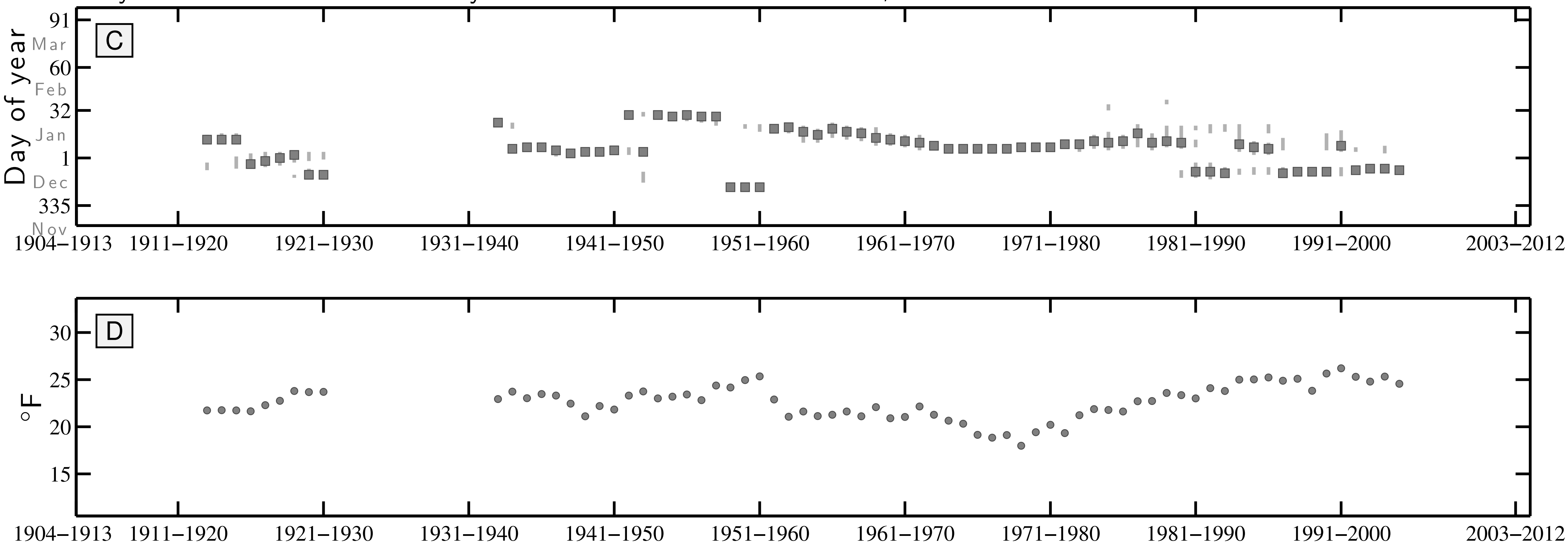

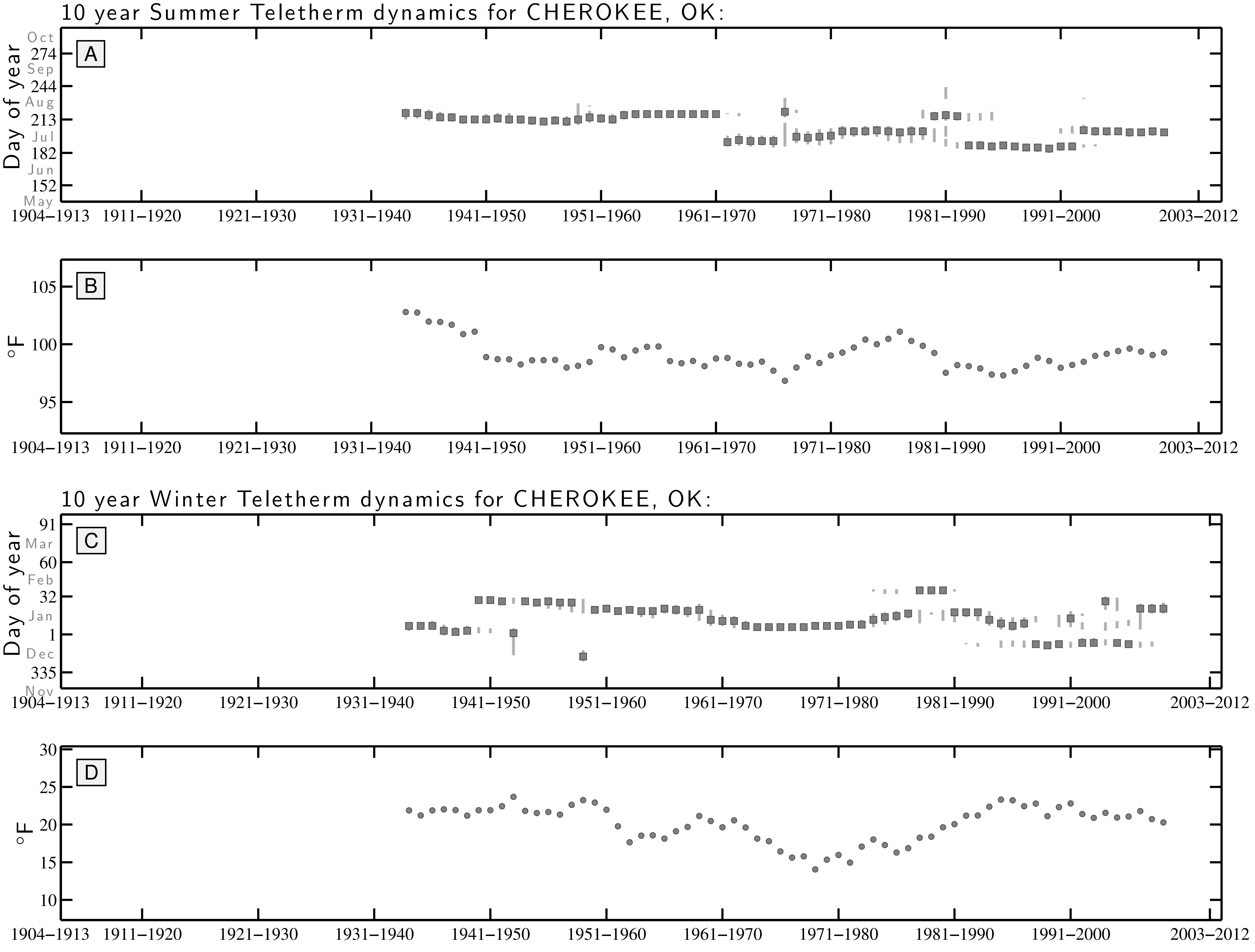
10 year Summer Teletherm dynamics for CHEROKEE, OK:
A
B
Day of year
°F
Oct
274
Sep
244
Aug
213
Jul
182
Jun
152
May
105
100
95
1904–1913
1911–1920
1921–1930
1931–1940
1941–1950
1951–1960
1961–1970
1971–1980
1981–1990
1991–2000
2003–2012
10 year Winter Teletherm dynamics for CHEROKEE, OK:
C
D
91
Mar
60
Feb
32
Jan
1
Dec
335
Nov
30
25
20
15
10

10 year Summer Teletherm dynamics for CLAREMORE 2 ENE, OK:

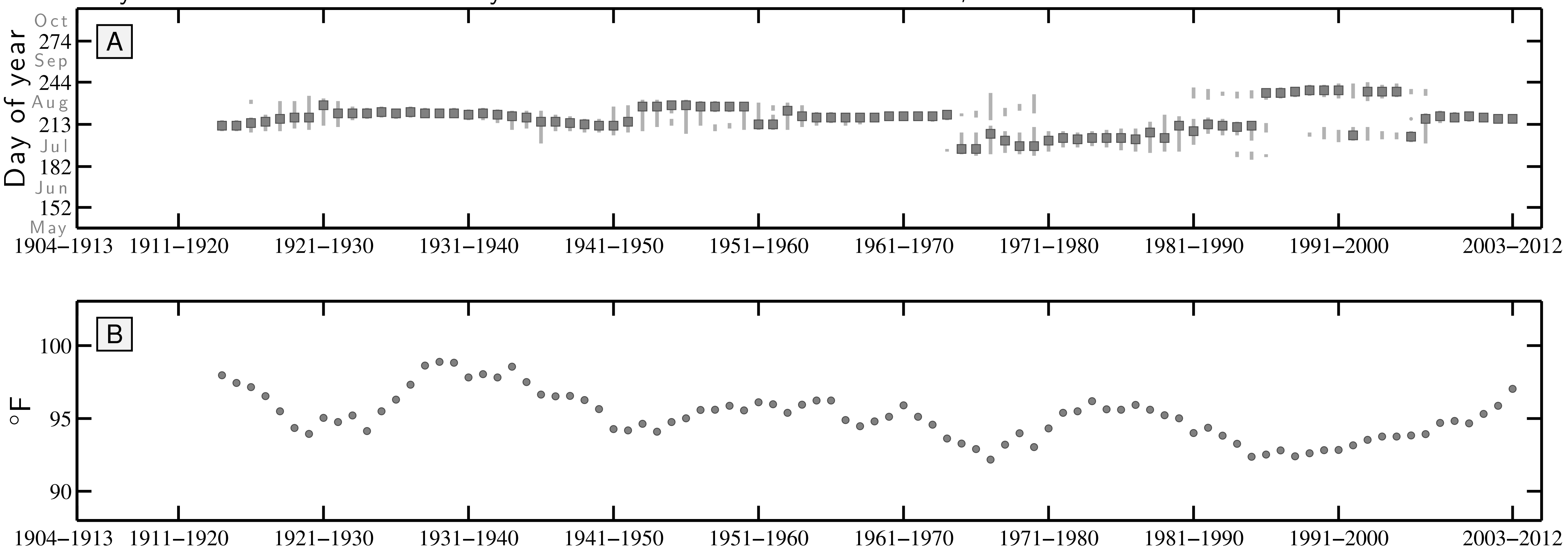

10 year Winter Teletherm dynamics for CLAREMORE 2 ENE, OK:

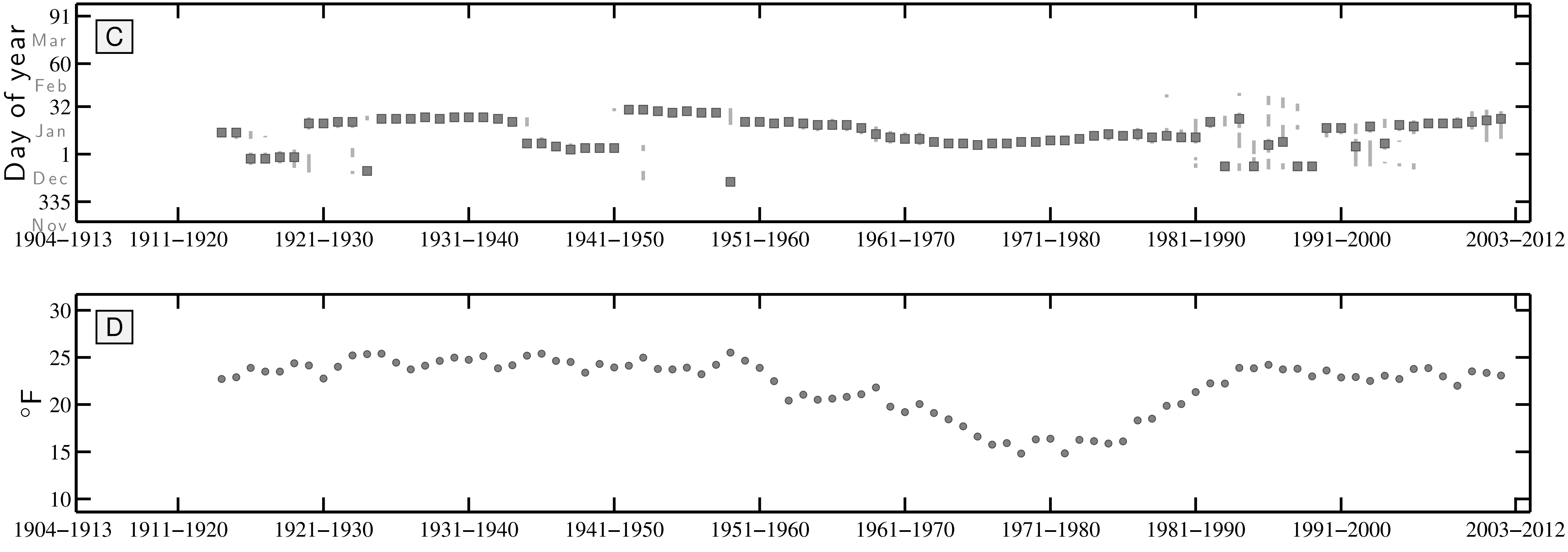

10 year Summer Teletherm dynamics for DURANT, OK:

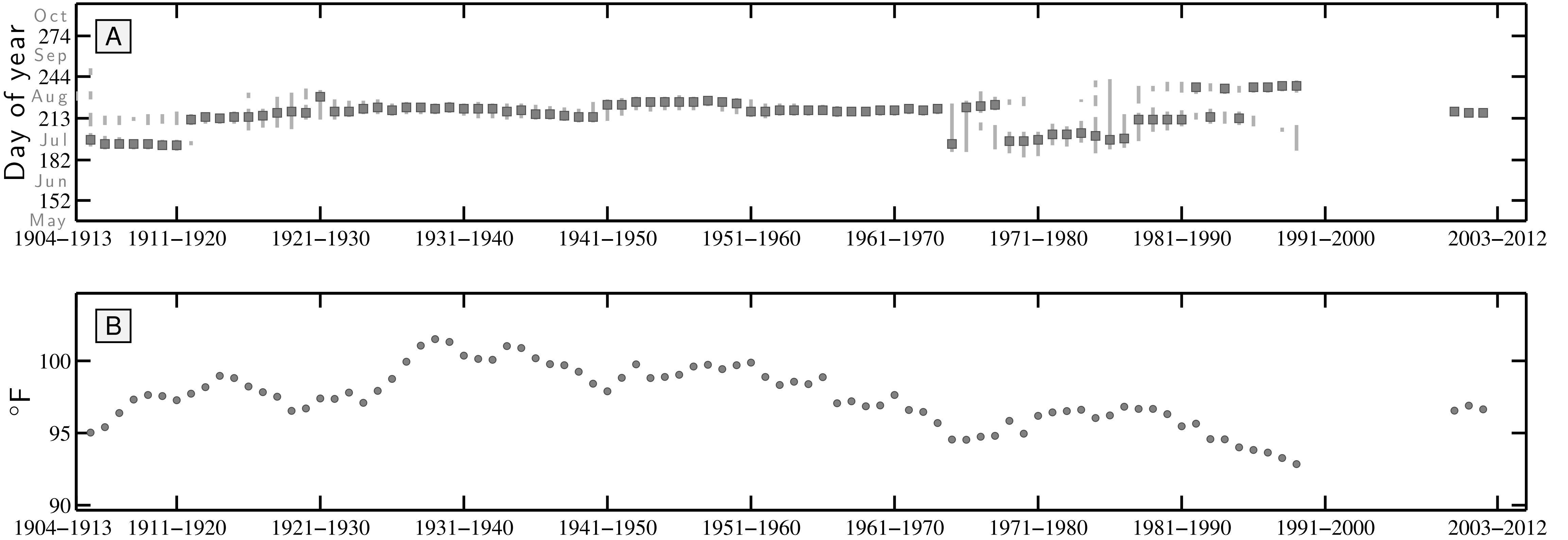

10 year Winter Teletherm dynamics for DURANT, OK:

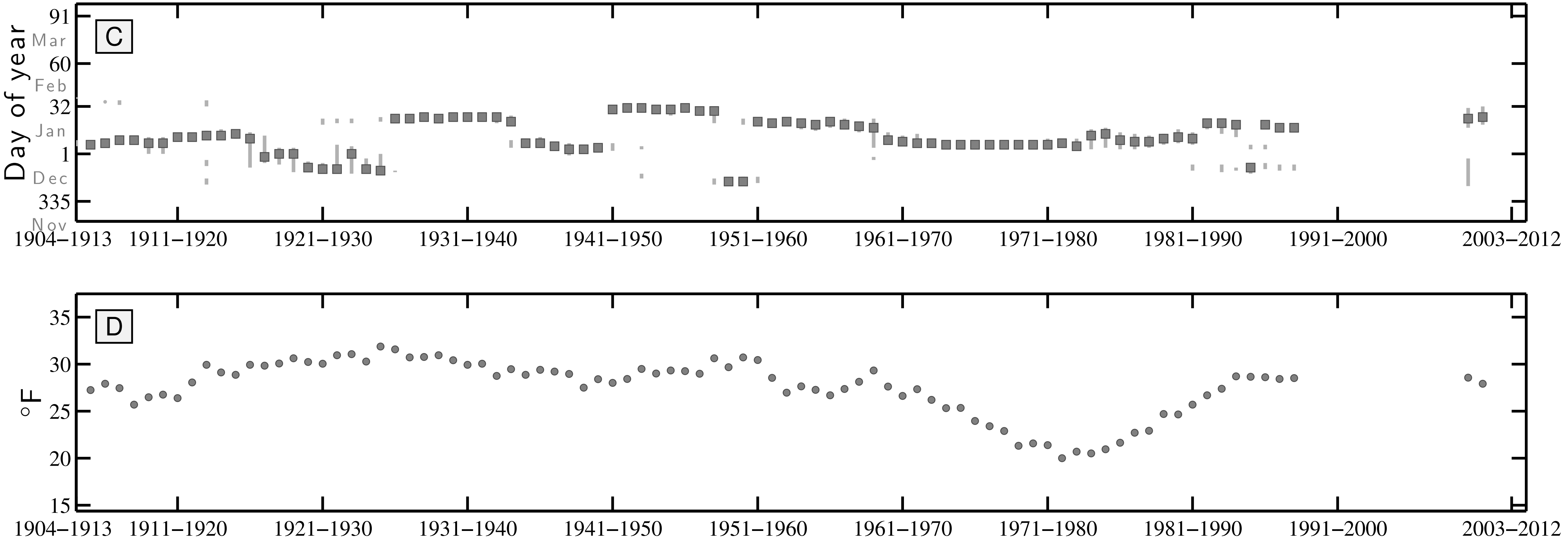

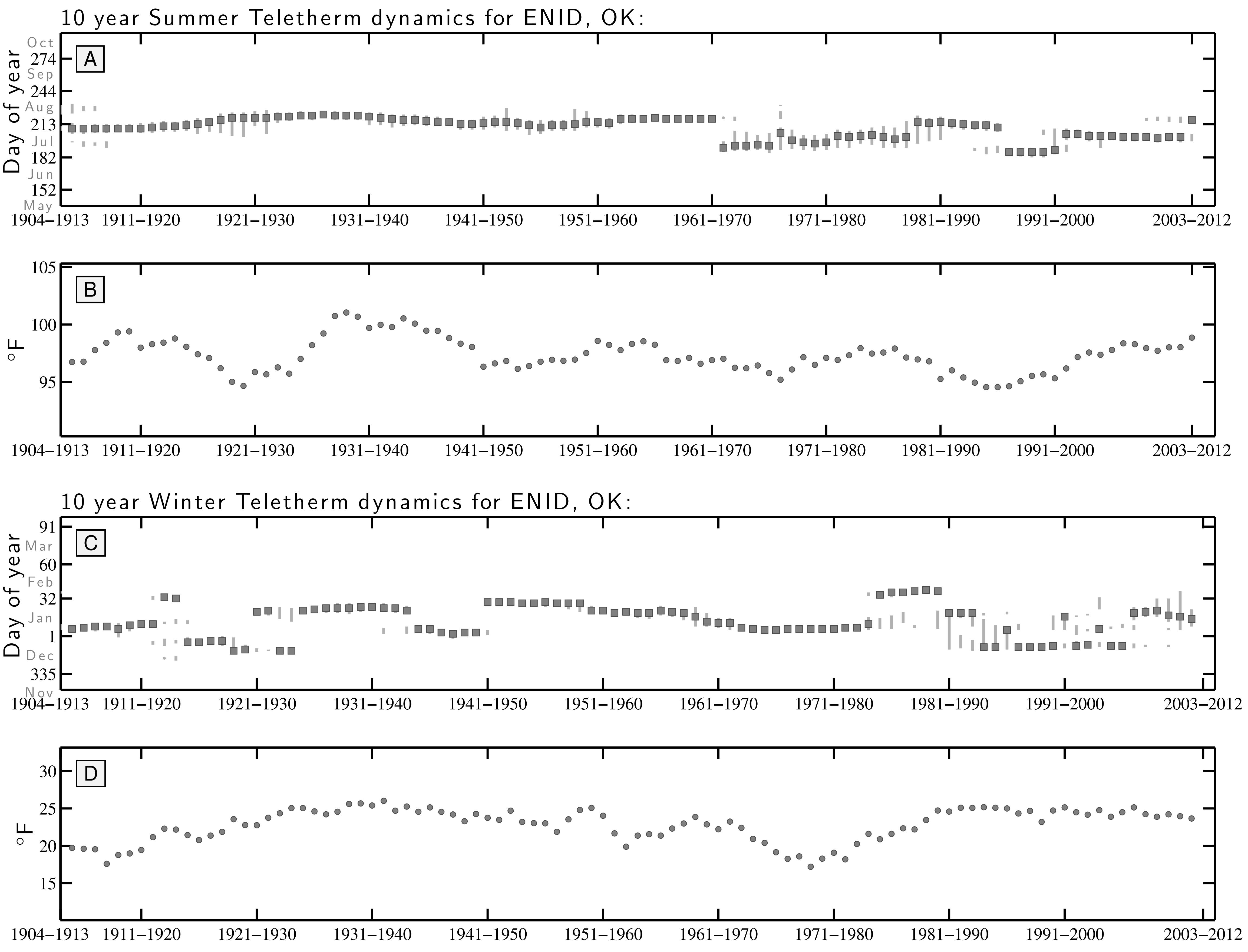
10 year Summer Teletherm dynamics for ENID, OK:
A
B
Day of year
°F
Oct
274
Sep
244
Aug
213
Jul
182
Jun
152
May
105
100
95
10 year Winter Teletherm dynamics for ENID, OK:
C
D
91
Mar
60
Feb
32
Jan
1
Dec
335
Nov
30
25
20
15
1904–1913
1911–1920
1921–1930
1931–1940
1941–1950
1951–1960
1961–1970
1971–1980
1981–1990
1991–2000
2003–2012

10 year Summer Teletherm dynamics for ERICK, OK:

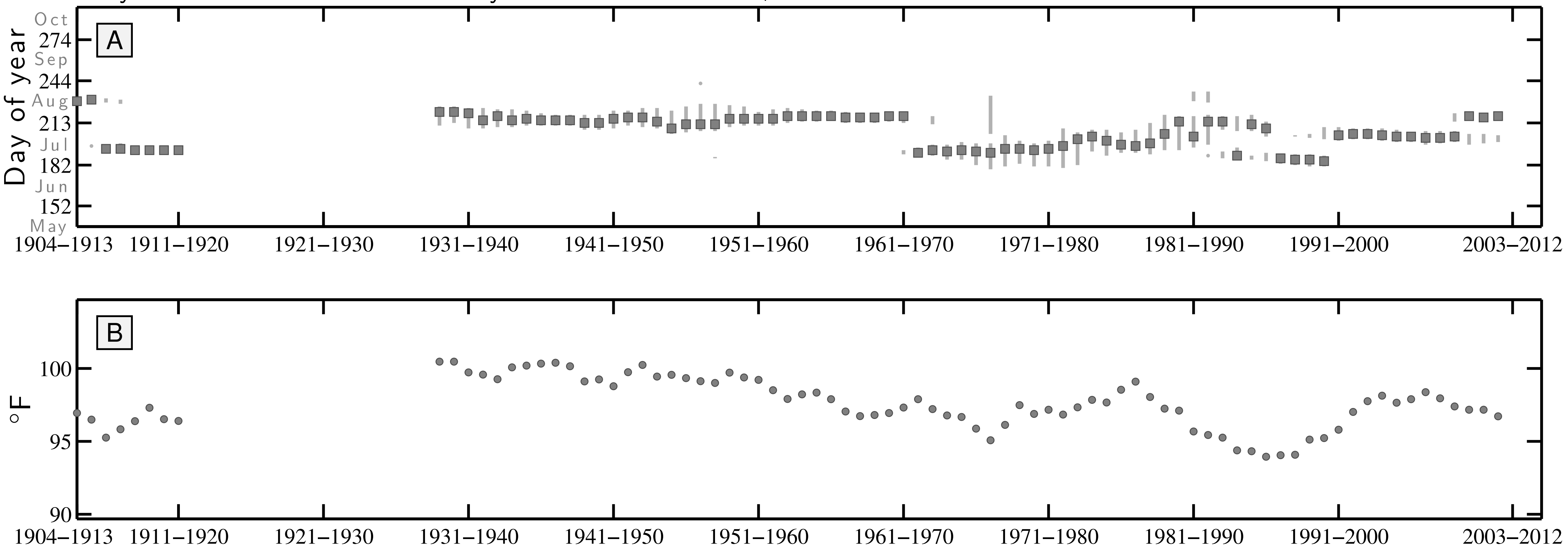

10 year Winter Teletherm dynamics for ERICK, OK:

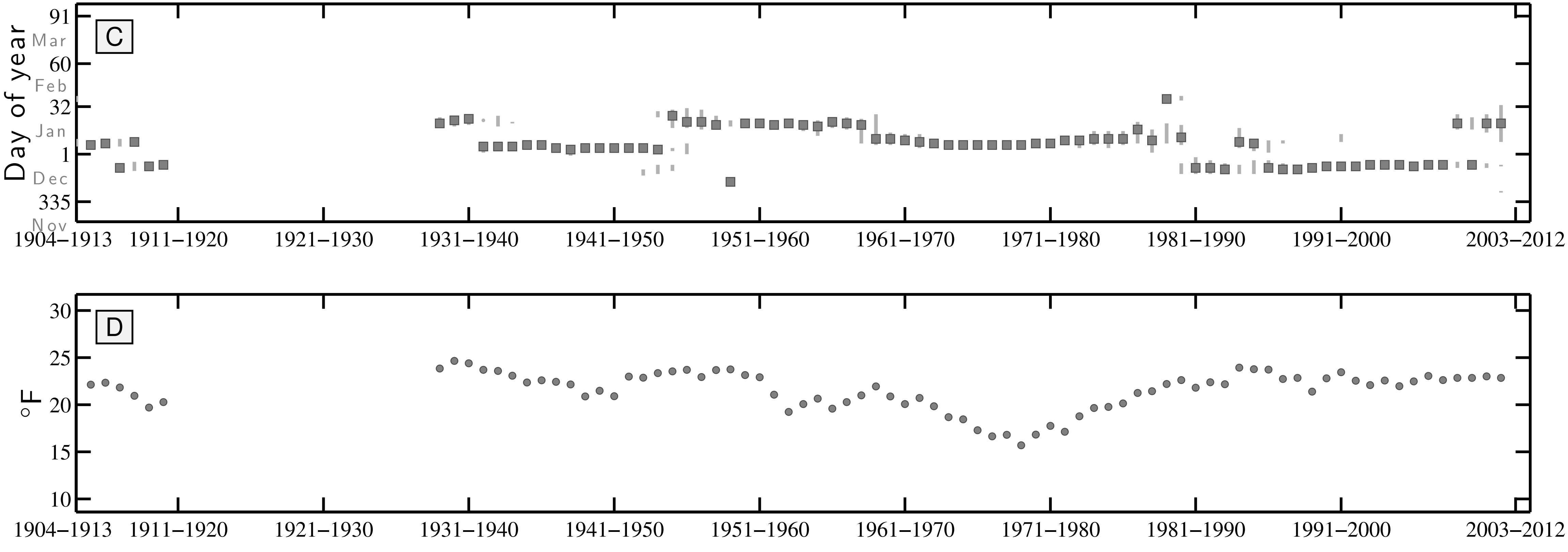

10 year Summer Teletherm dynamics for GEARY, OK:

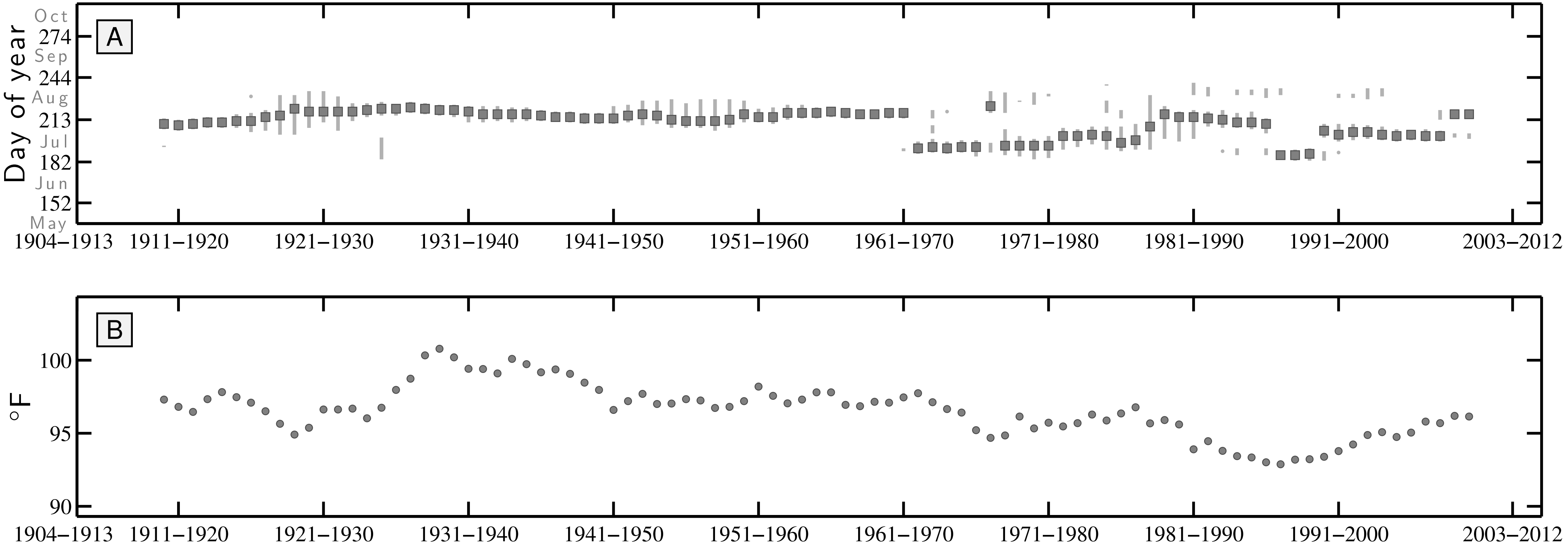

10 year Winter Teletherm dynamics for GEARY, OK:

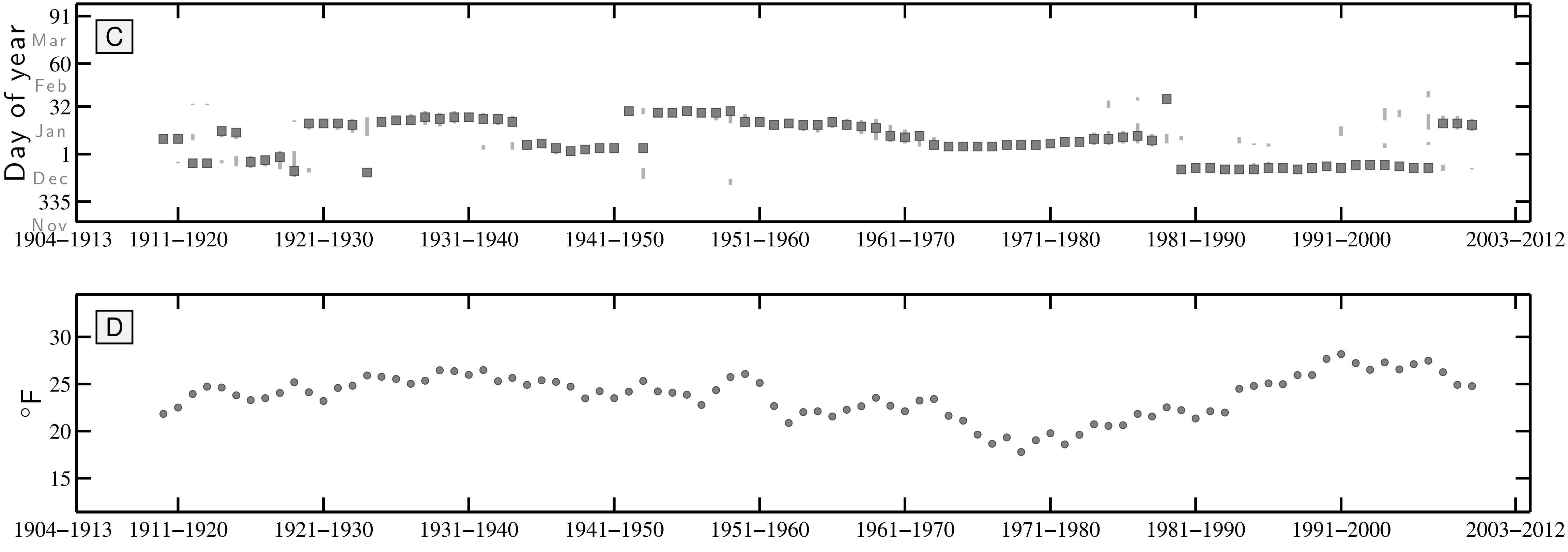

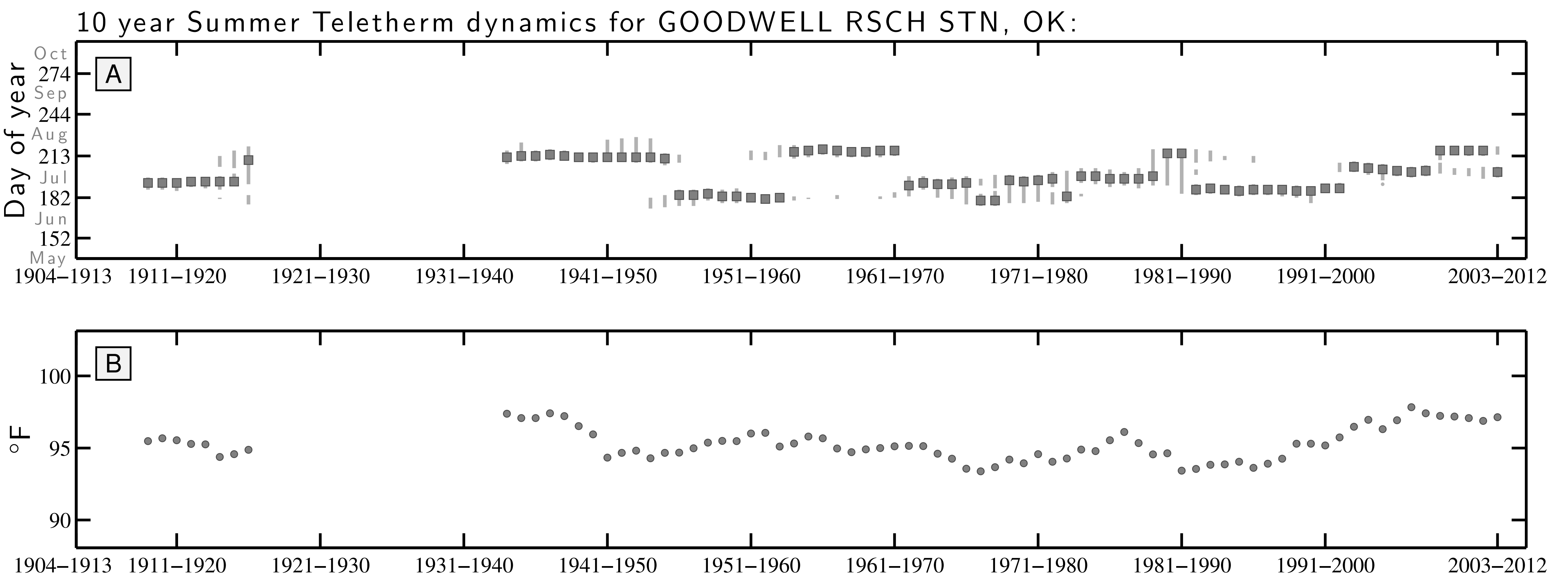
10 year Summer Teletherm dynamics for GOODWELL RSCH STN, OK:
A
B
Day of year
°F
May
Jun
Jul
Aug
Sep
Oct
152
182
213
244
274
90
95
100
1904–1913
1911–1920
1921–1930
1931–1940
1941–1950
1951–1960
1961–1970
1971–1980
1981–1990
1991–2000
2003–2012

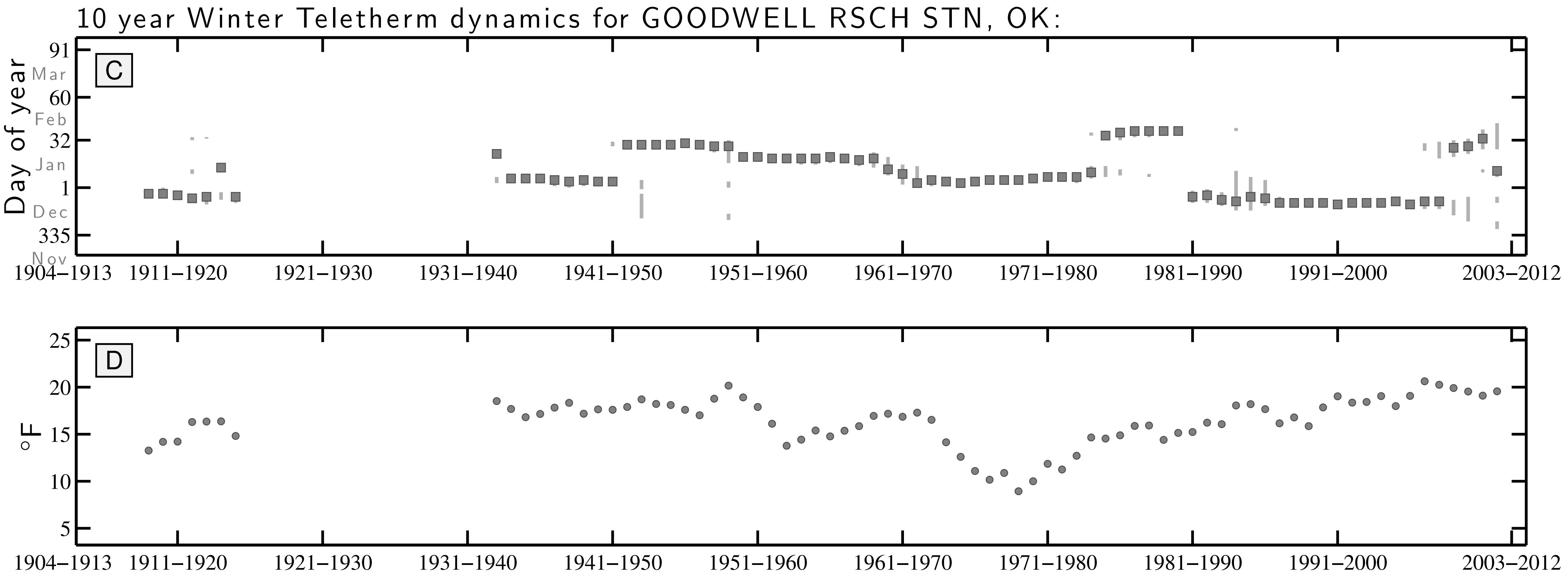
10 year Winter Teletherm dynamics for GOODWELL RSCH STN, OK:
C
D
Day of year
°F
Nov
Dec
Jan
Feb
Mar
335
1
32
60
91
5
10
15
20
25
1904–1913
1911–1920
1921–1930
1931–1940
1941–1950
1951–1960
1961–1970
1971–1980
1981–1990
1991–2000
2003–2012

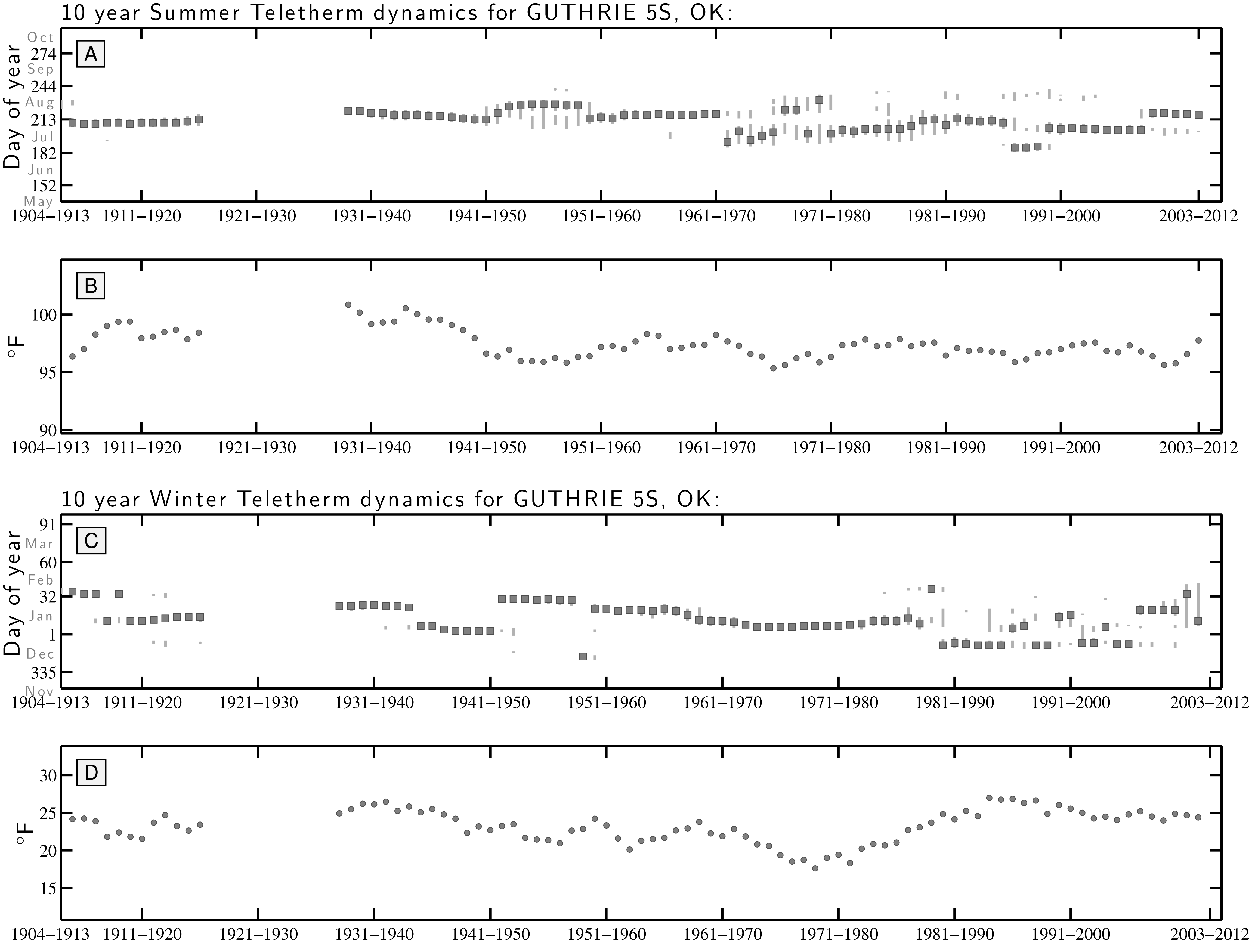
10 year Summer Teletherm dynamics for GUTHRIE 5S, OK:
A
B
Day of year
°F
May
152
Jun
182
Jul
213
Aug
244
Sep
274
Oct
90
95
100
1904–1913
1911–1920
1921–1930
1931–1940
1941–1950
1951–1960
1961–1970
1971–1980
1981–1990
1991–2000
2003–2012
10 year Winter Teletherm dynamics for GUTHRIE 5S, OK:
C
D
Nov
335
Dec
1
Jan
32
Feb
60
Mar
91
15
20
25
30

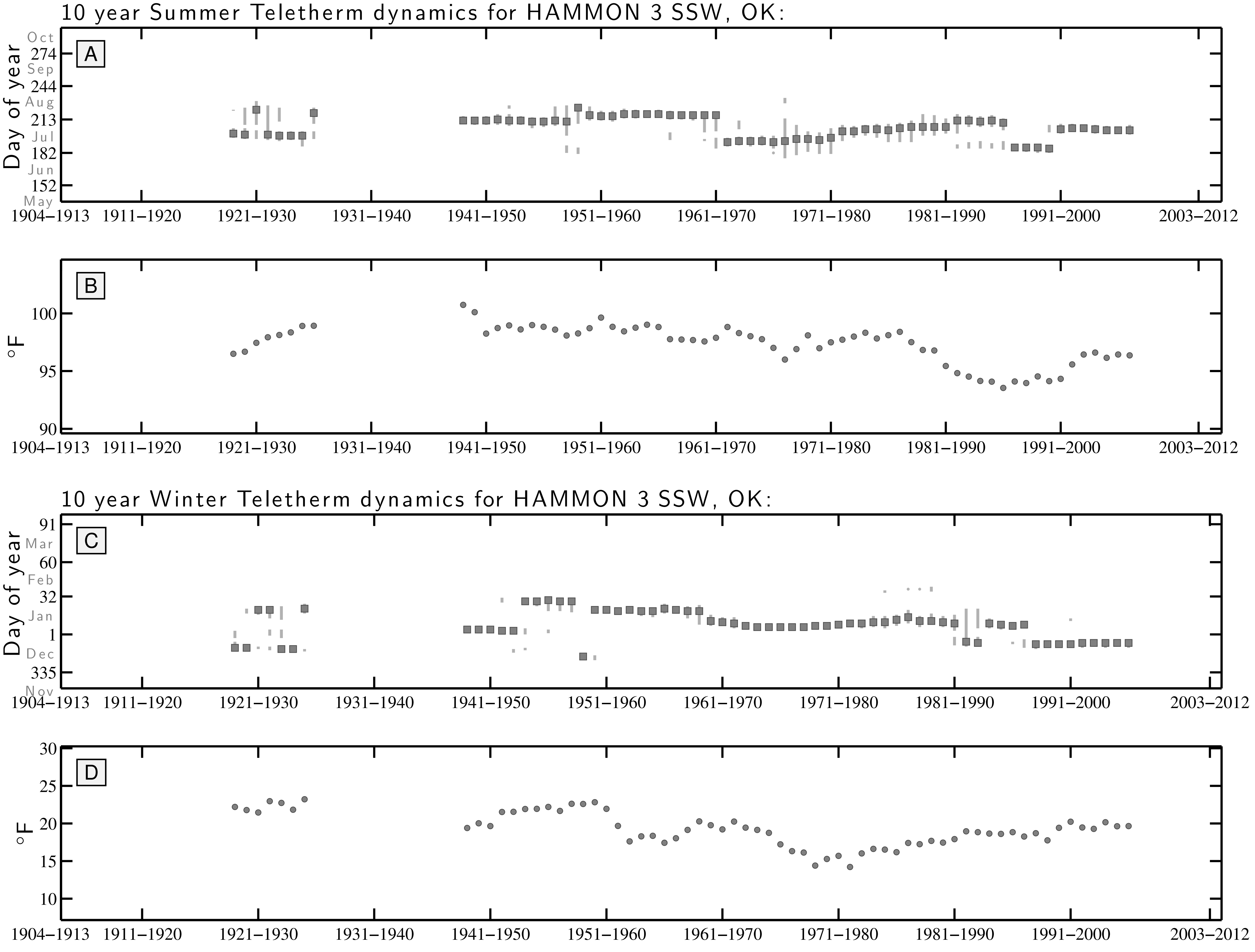

10 year Summer Teletherm dynamics for HAMMON 3 SSW, OK:
A
Day of year
Oct
274
Sep
244
Aug
213
Jul
182
Jun
152
May
1904–1913
1911–1920
1921–1930
1931–1940
1941–1950
1951–1960
1961–1970
1971–1980
1981–1990
1991–2000
2003–2012
B
°F
100
95
90
10 year Winter Teletherm dynamics for HAMMON 3 SSW, OK:
C
91
Mar
60
Feb
32
Jan
1
Dec
335
Nov
D
30
25
20
15
10

10 year Summer Teletherm dynamics for HENNESSEY 4 ESE, OK:

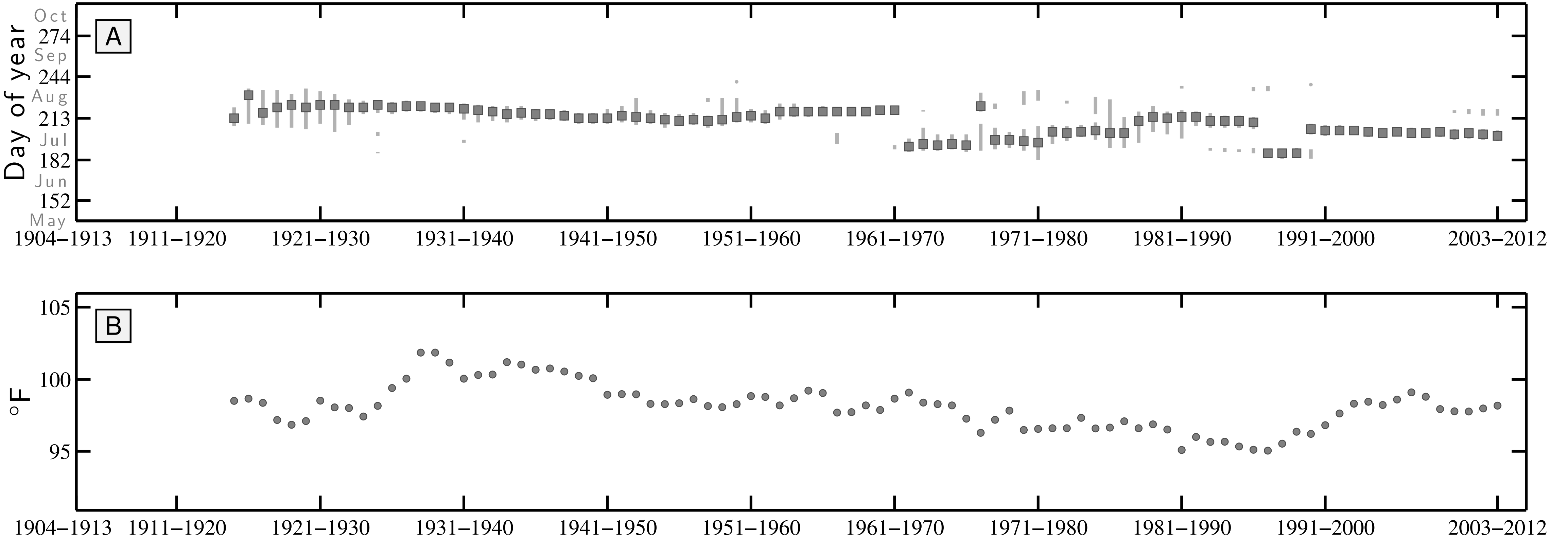

10 year Winter Teletherm dynamics for HENNESSEY 4 ESE, OK:

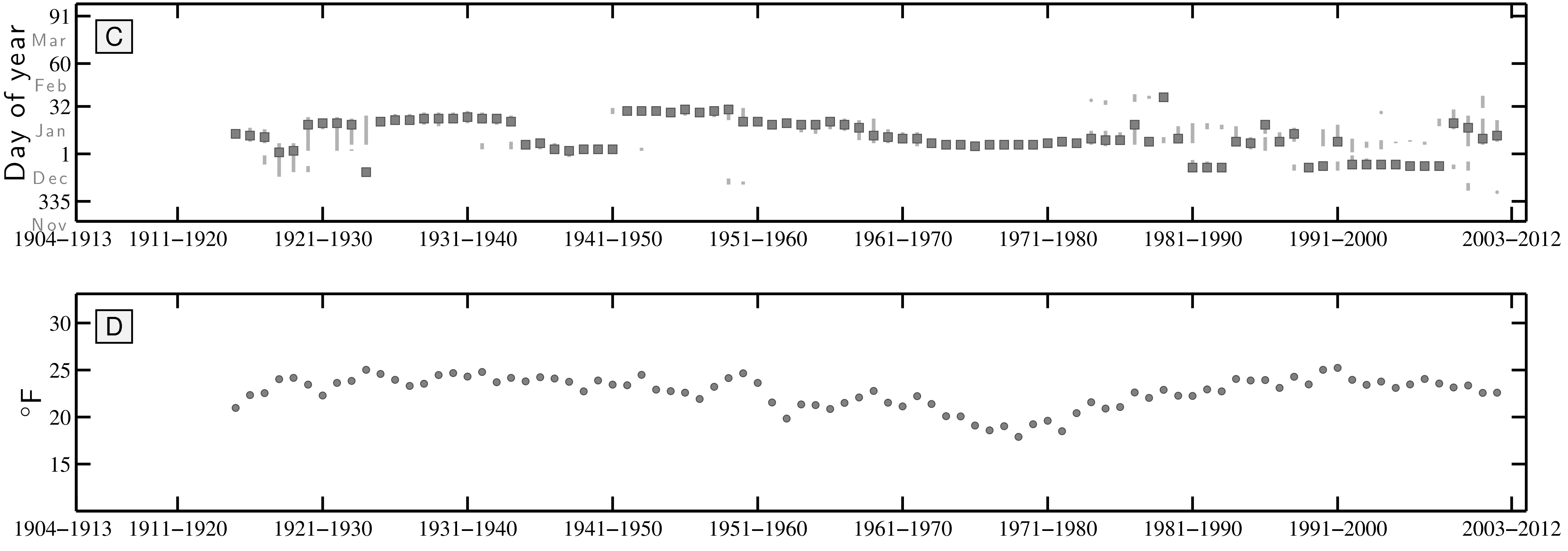

10 year Summer Teletherm dynamics for HOBART MUNI AP, OK:

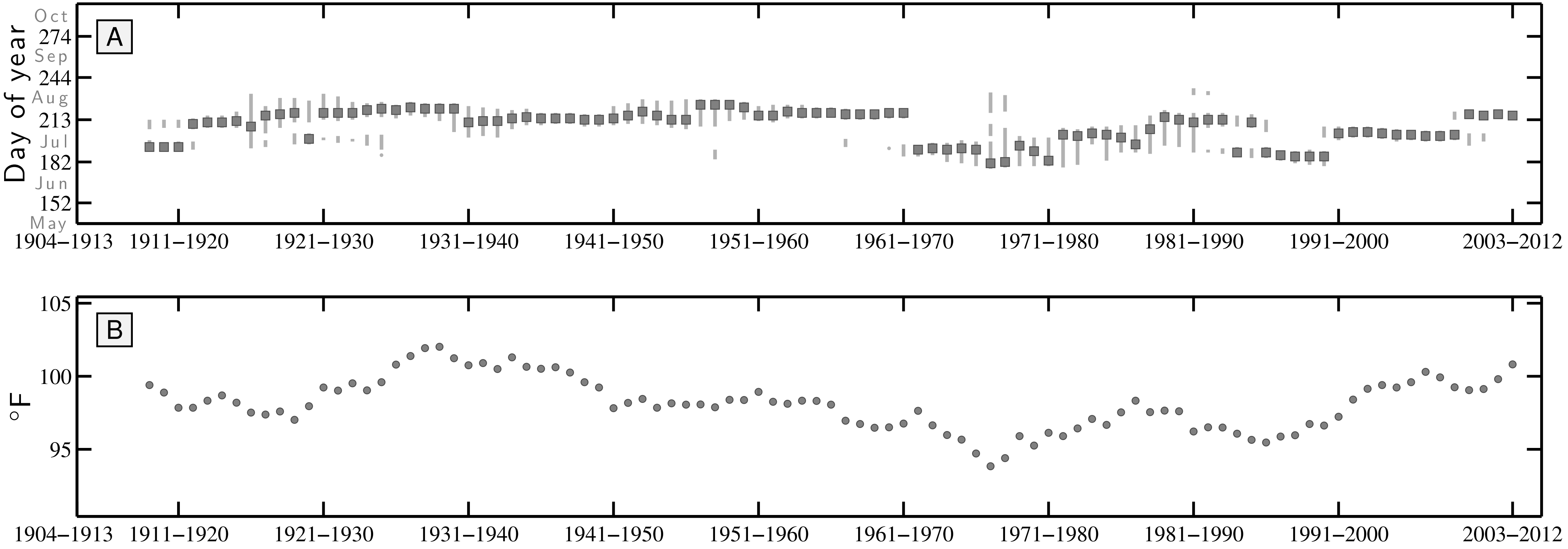

10 year Winter Teletherm dynamics for HOBART MUNI AP, OK:

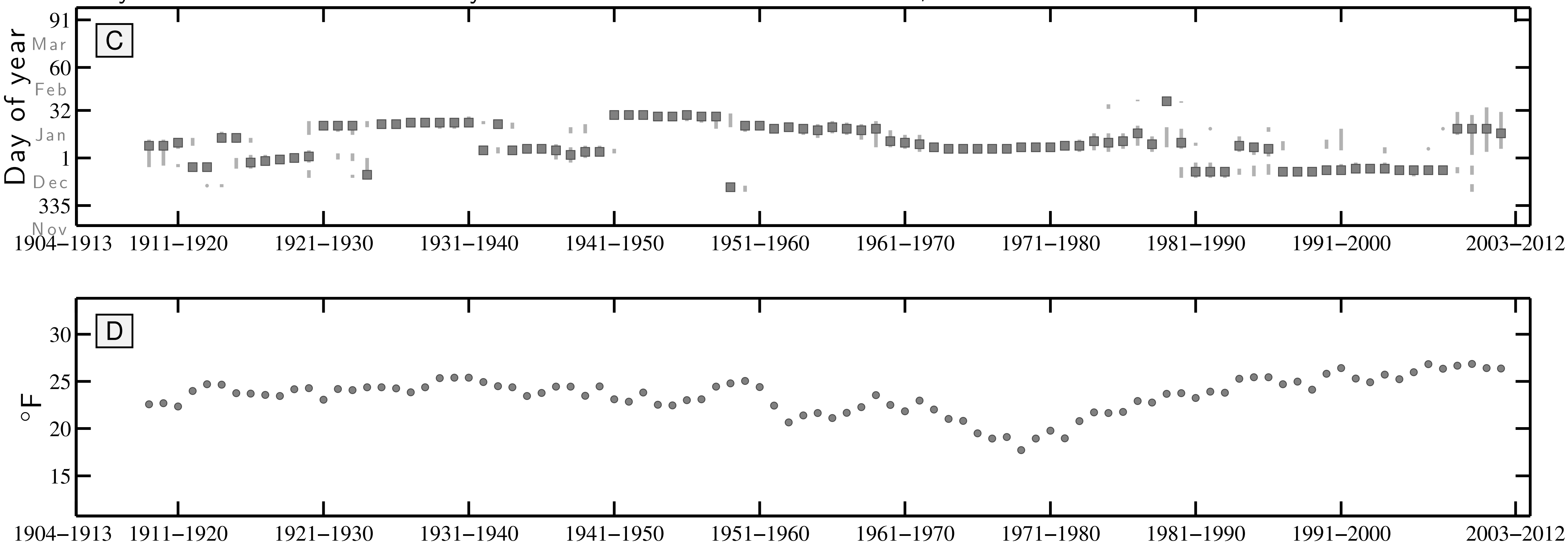

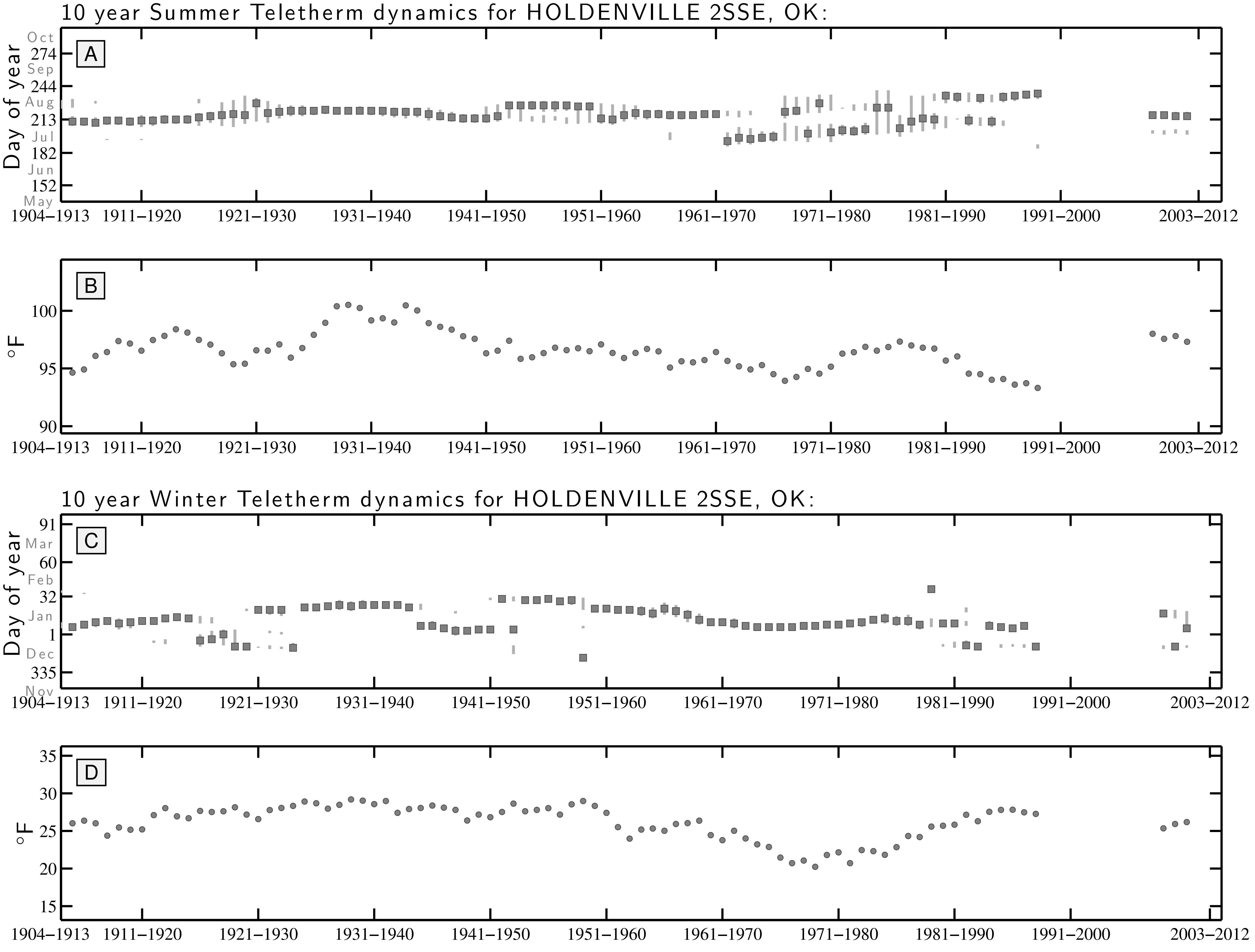
10 year Summer Teletherm dynamics for HOLDENVILLE 2SSE, OK:
A
B
Day of year
°F
Oct
274
Sep
244
Aug
213
Jul
182
Jun
152
May
100
95
90
1904–1913
1911–1920
1921–1930
1931–1940
1941–1950
1951–1960
1961–1970
1971–1980
1981–1990
1991–2000
2003–2012
10 year Winter Teletherm dynamics for HOLDENVILLE 2SSE, OK:
C
D
91
Mar
60
Feb
32
Jan
1
Dec
335
Nov
35
30
25
20
15

10 year Summer Teletherm dynamics for HOOKER, OK:

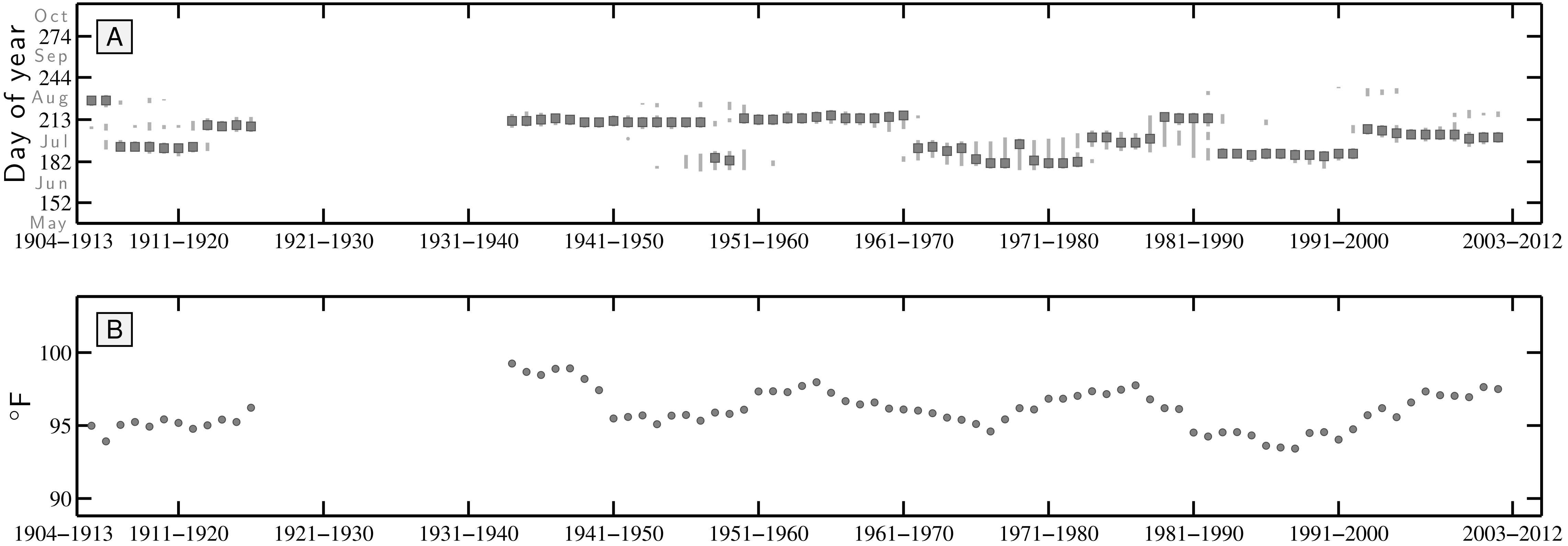

10 year Winter Teletherm dynamics for HOOKER, OK:

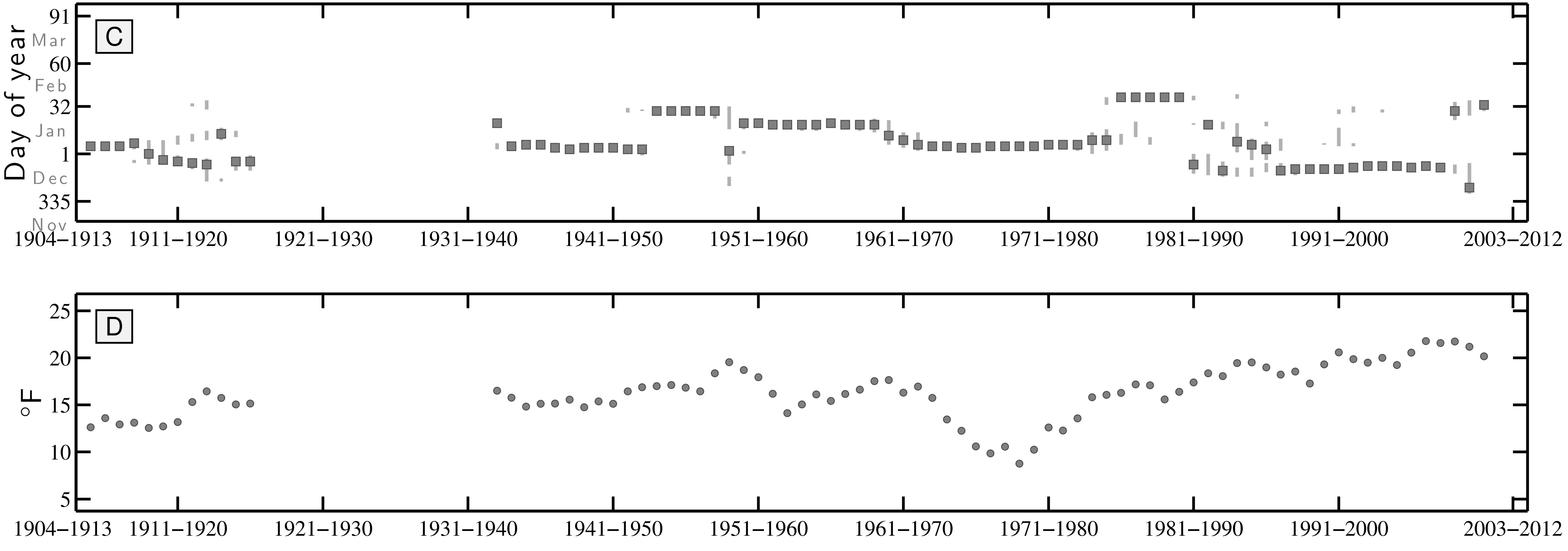

10 year Summer Teletherm dynamics for JEFFERSON, OK:

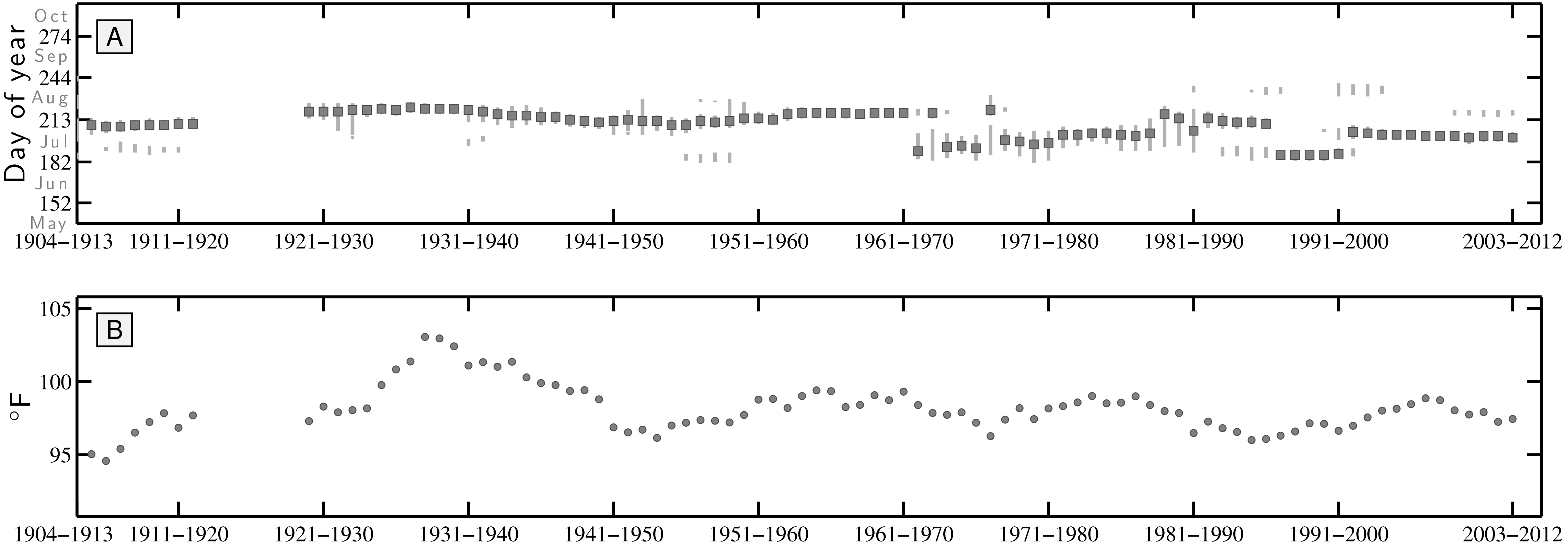

10 year Winter Teletherm dynamics for JEFFERSON, OK:

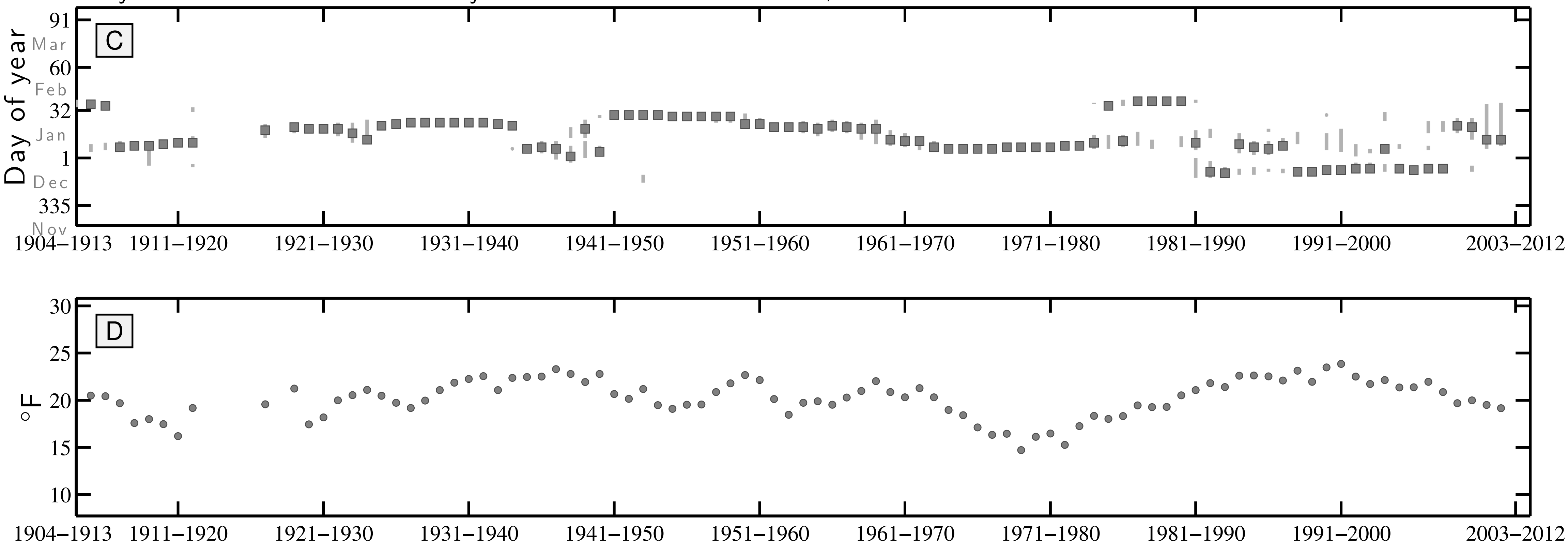

10 year Summer Teletherm dynamics for KENTON, OK:

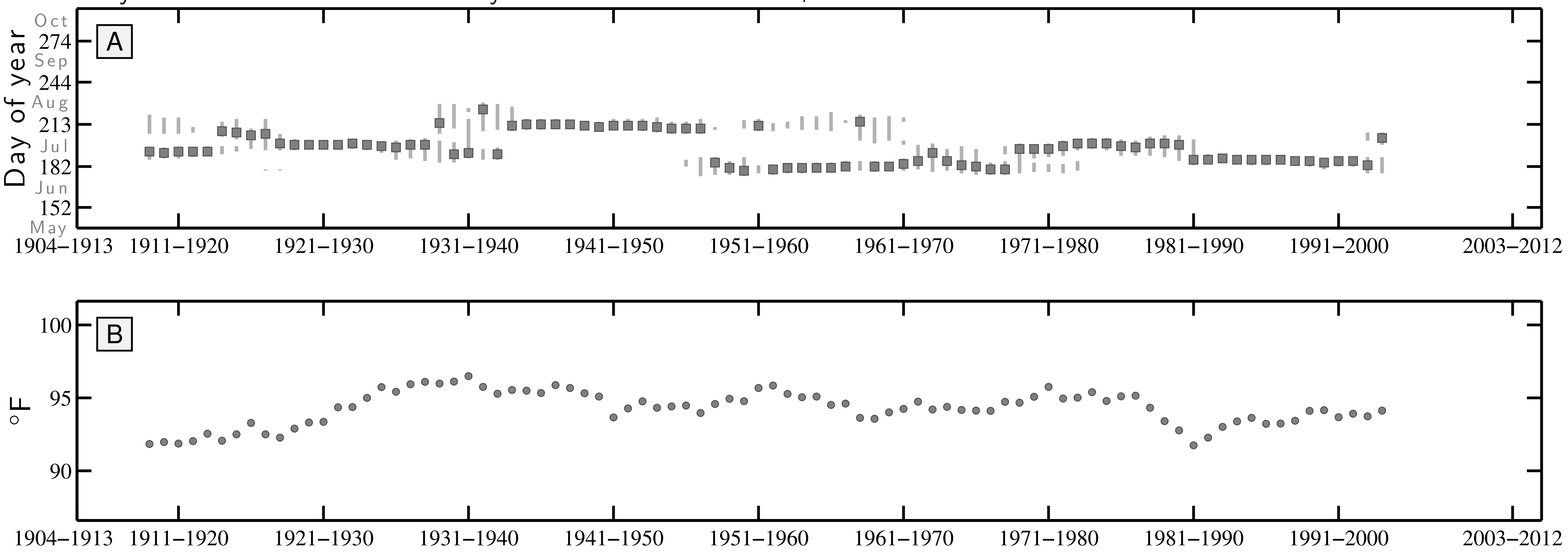

10 year Winter Teletherm dynamics for KENTON, OK:

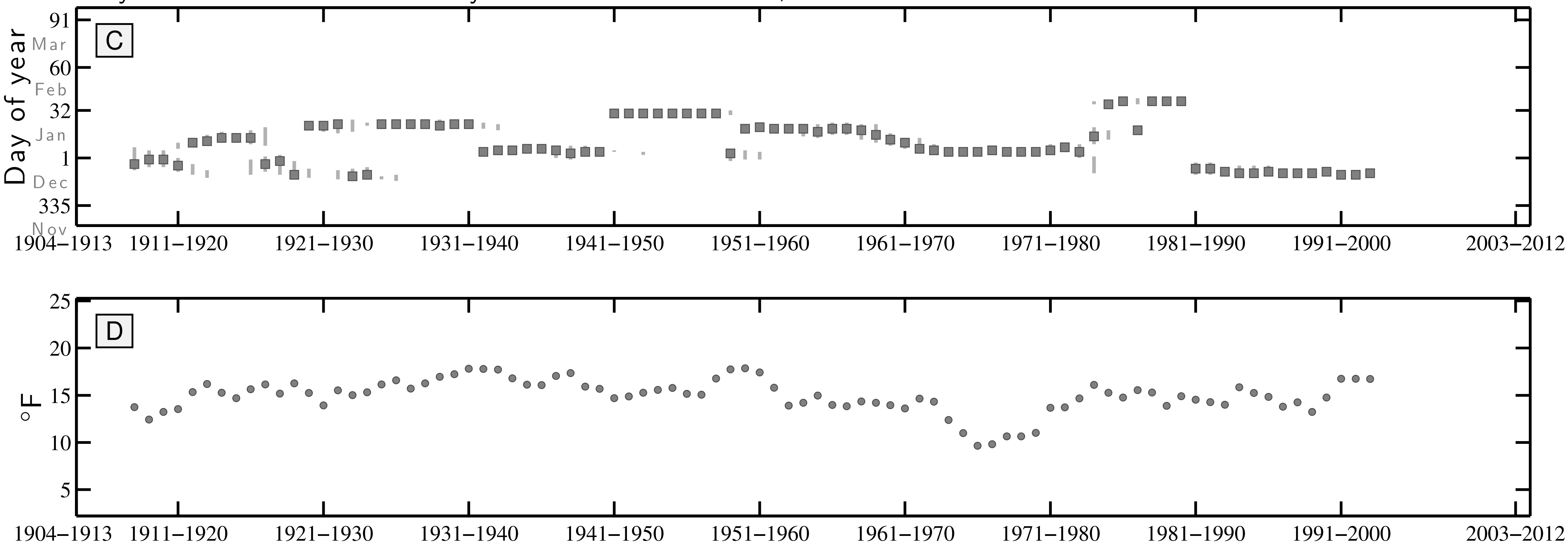

10 year Summer Teletherm dynamics for KINGFISHER, OK:

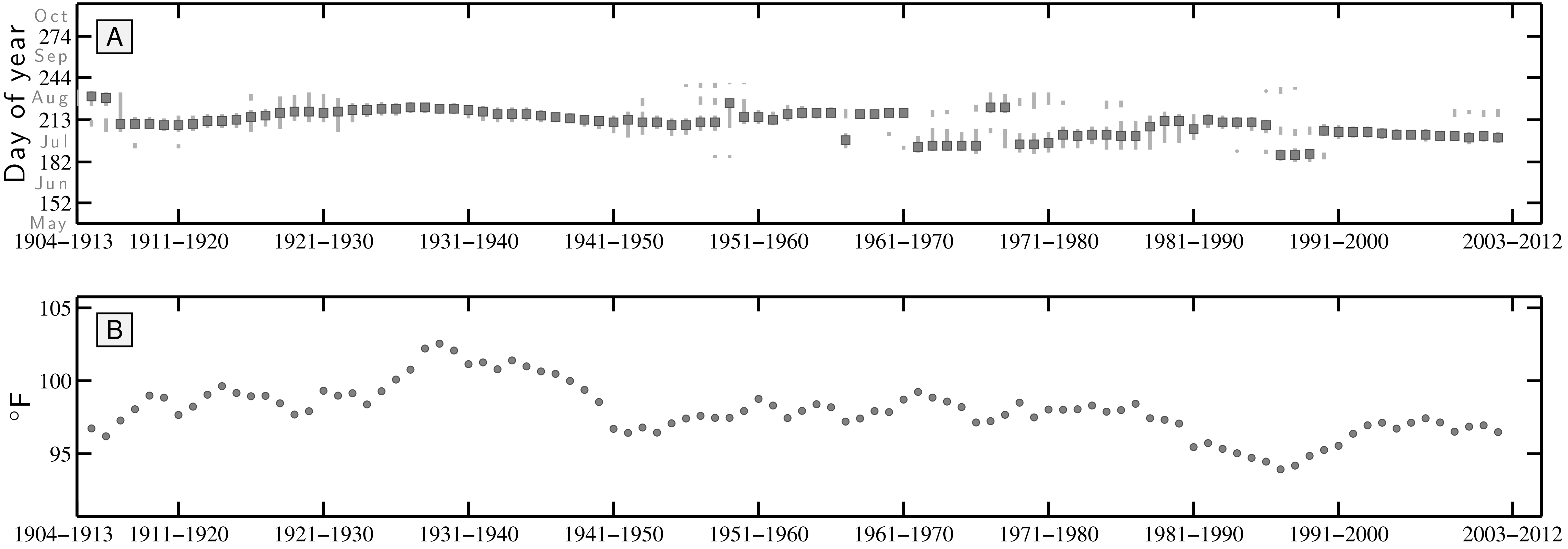

10 year Winter Teletherm dynamics for KINGFISHER, OK:

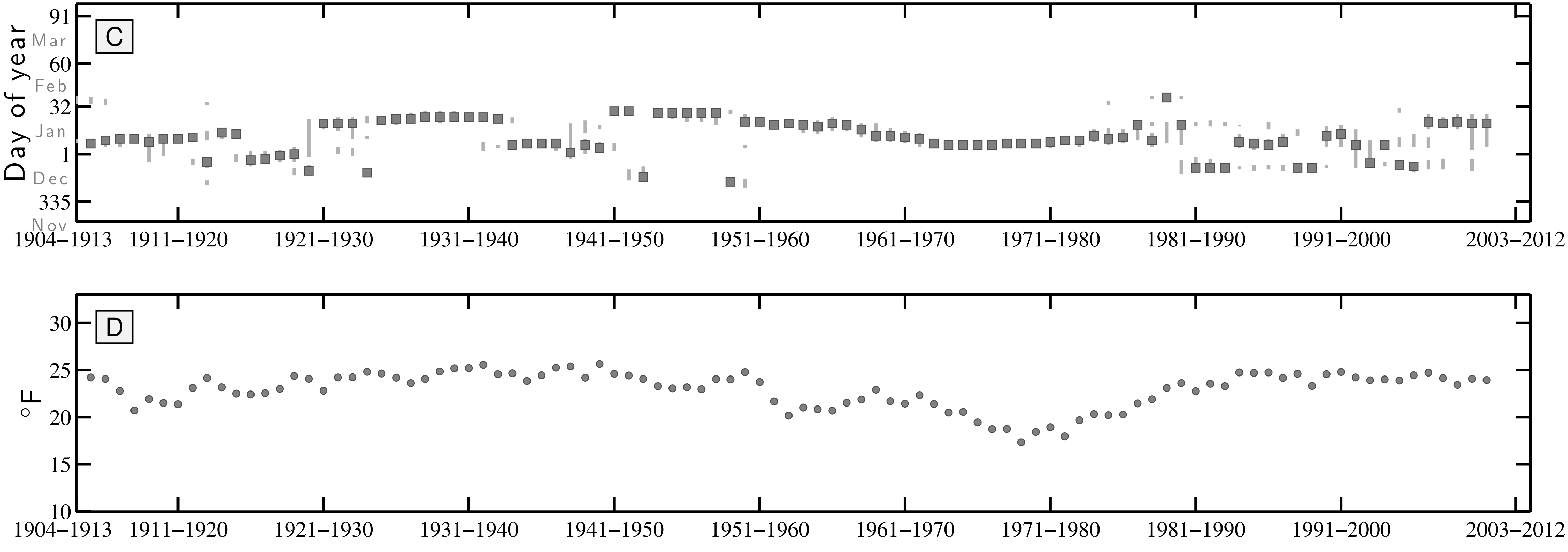

10 year Summer Teletherm dynamics for LAWTON, OK:

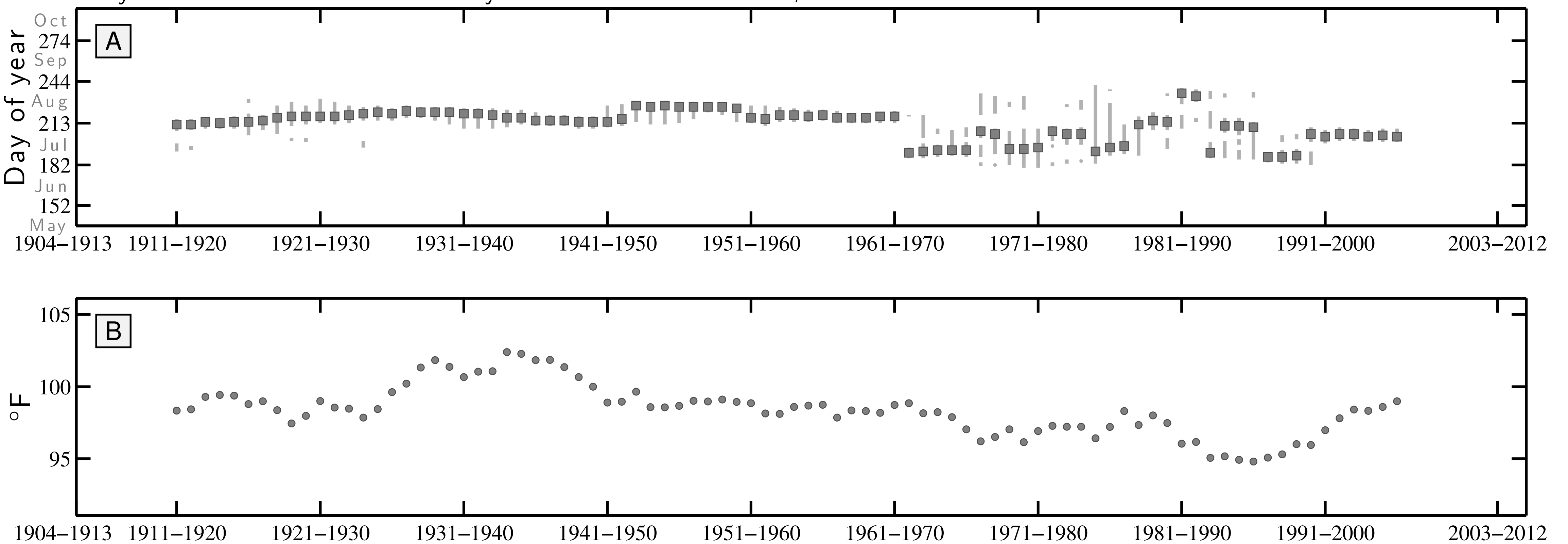

10 year Winter Teletherm dynamics for LAWTON, OK:

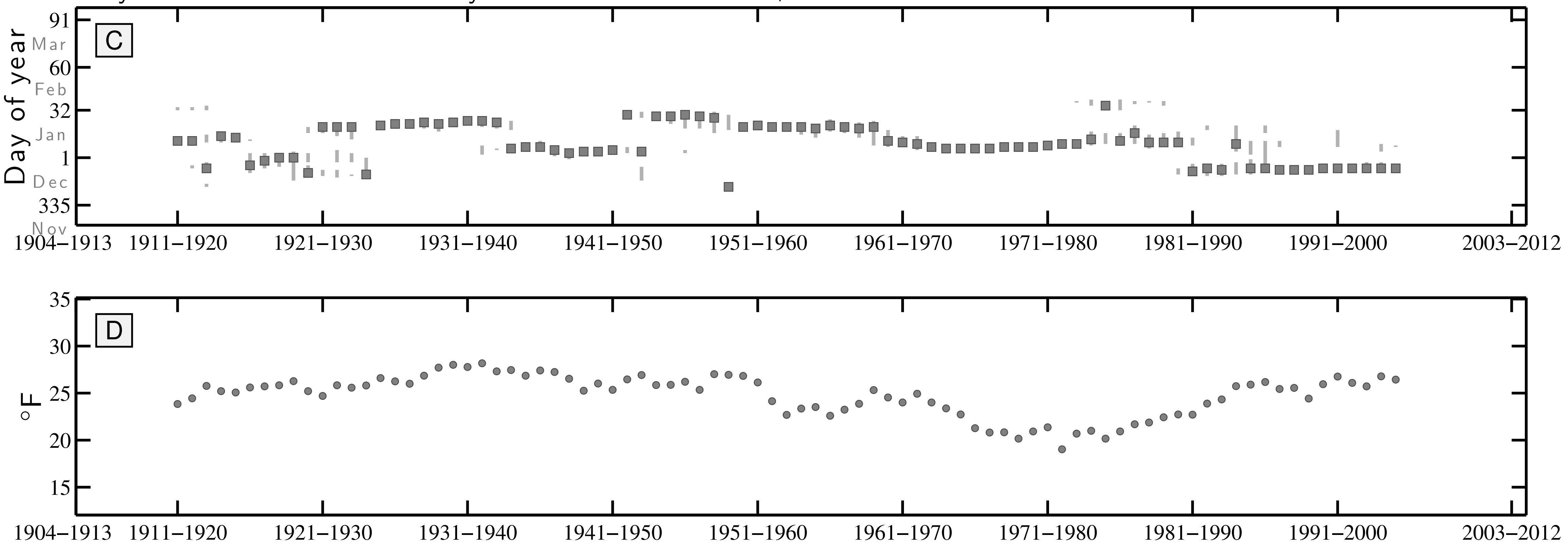

10 year Summer Teletherm dynamics for MANGUM, OK:

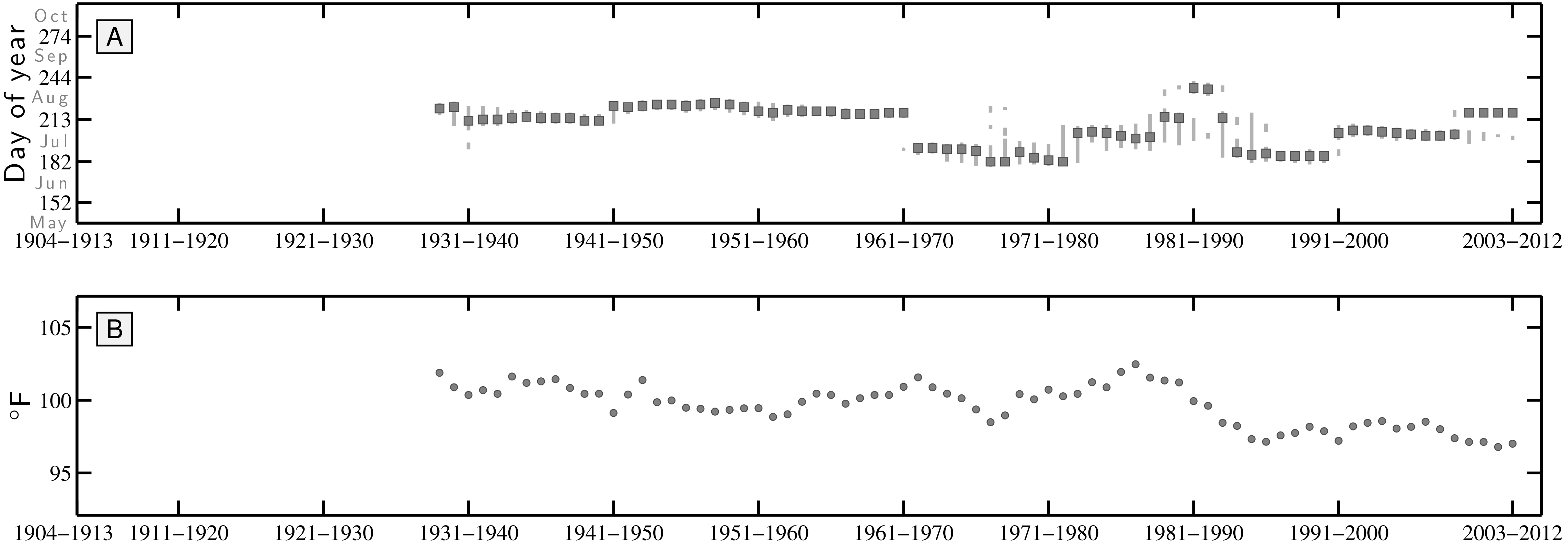

10 year Winter Teletherm dynamics for MANGUM, OK:

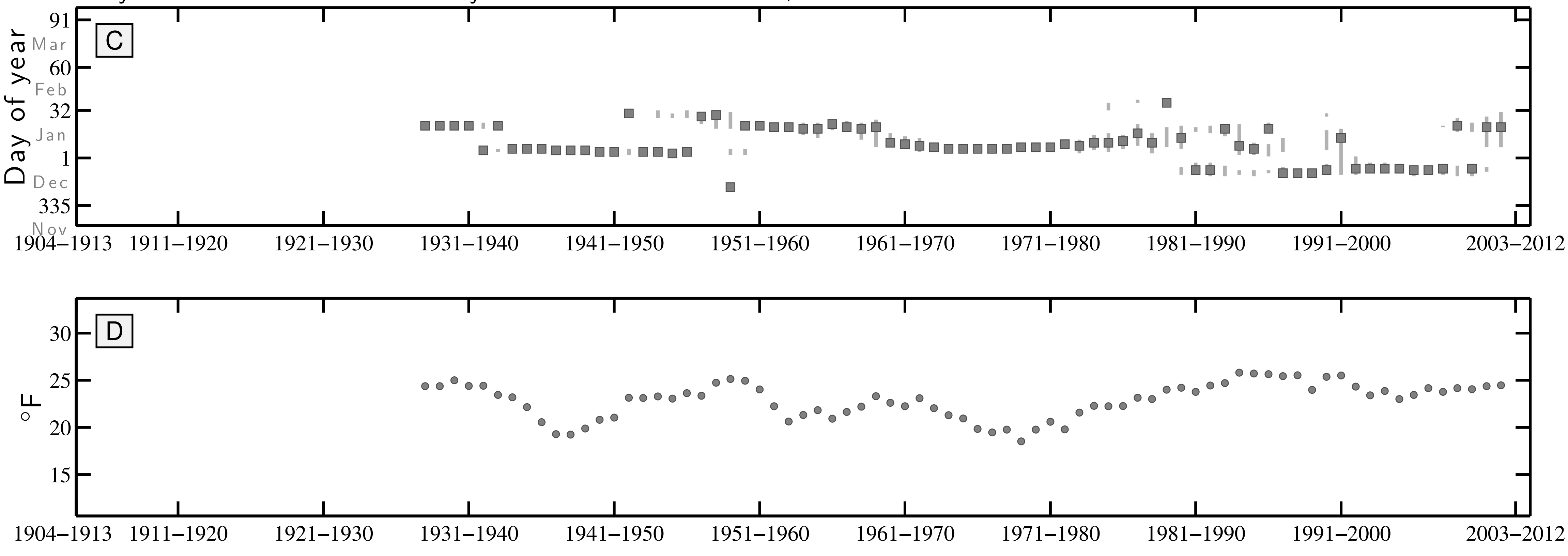

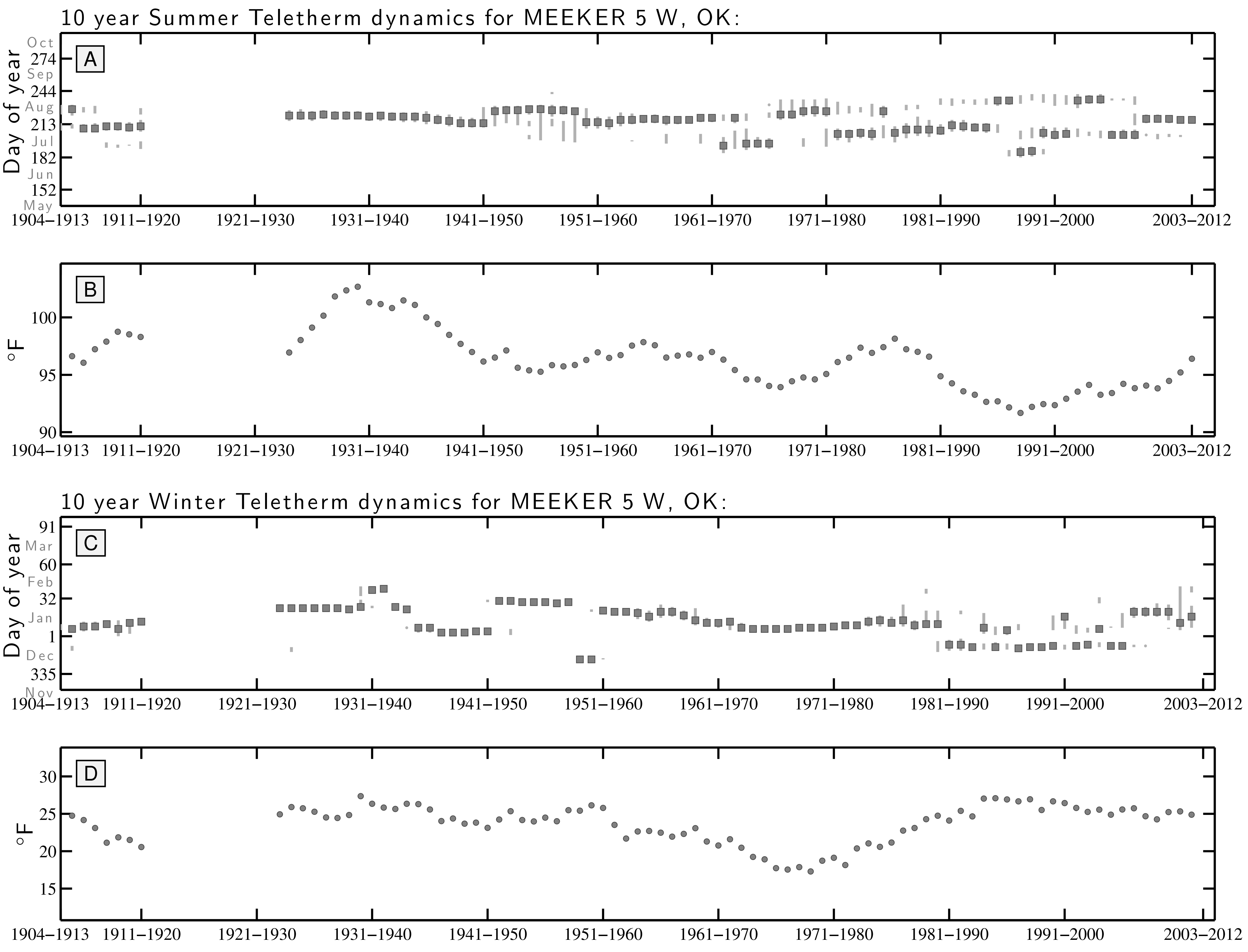
10 year Summer Teletherm dynamics for MEEKER 5 W, OK:
A
B
10 year Winter Teletherm dynamics for MEEKER 5 W, OK:
C
D
Day of year
°F
Oct
274
Sep
244
Aug
213
Jul
182
Jun
152
May
100
95
90
91
Mar
60
Feb
32
Jan
1
Dec
335
Nov
30
25
20
15
1904–1913
1911–1920
1921–1930
1931–1940
1941–1950
1951–1960
1961–1970
1971–1980
1981–1990
1991–2000
2003–2012

10 year Summer Teletherm dynamics for MIAMI, OK:

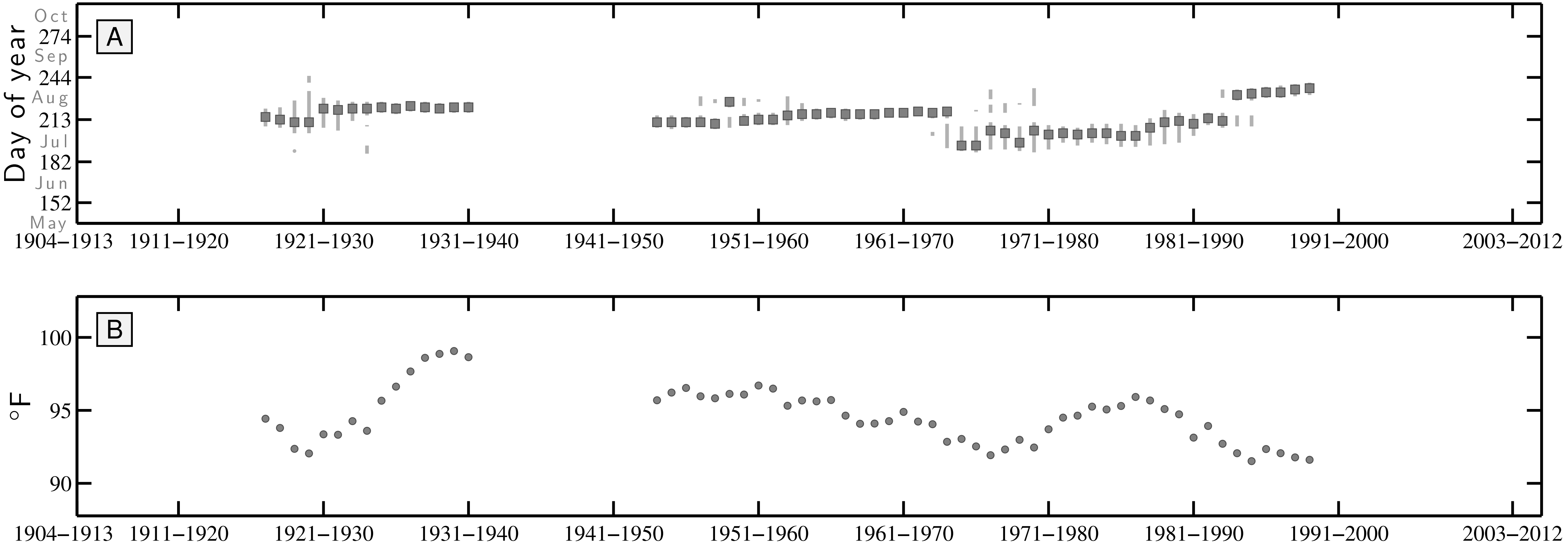

10 year Winter Teletherm dynamics for MIAMI, OK:

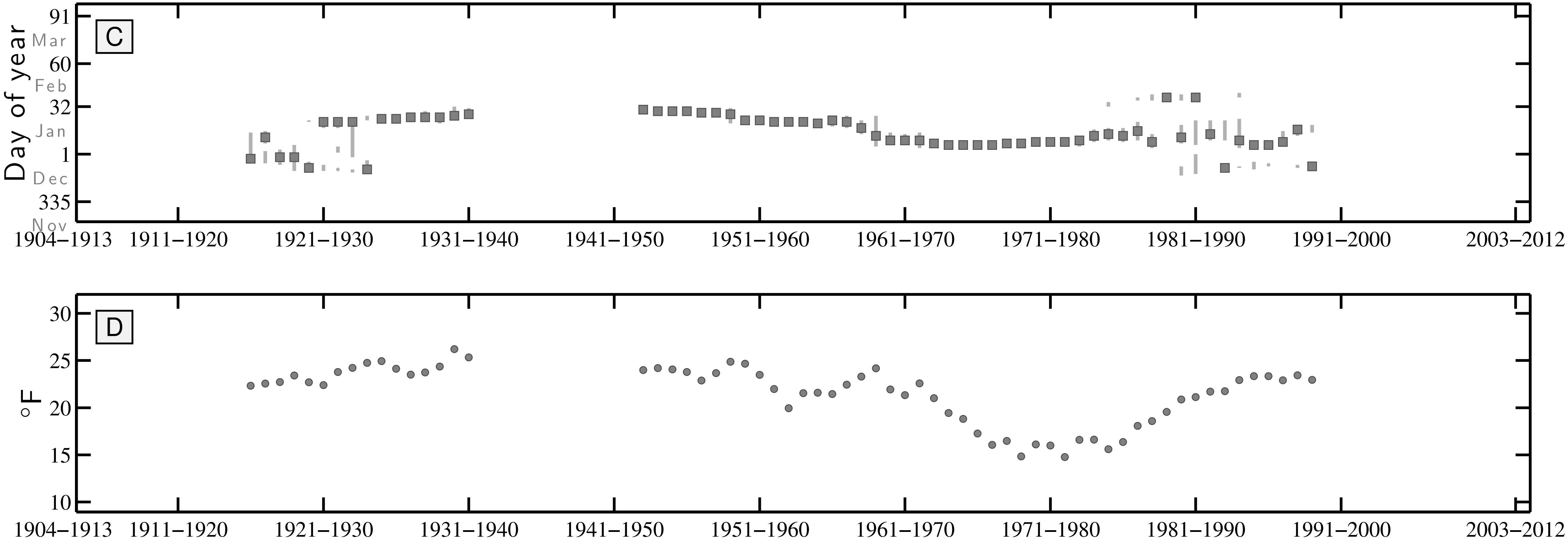

10 year Summer Teletherm dynamics for MUSKOGEE, OK:

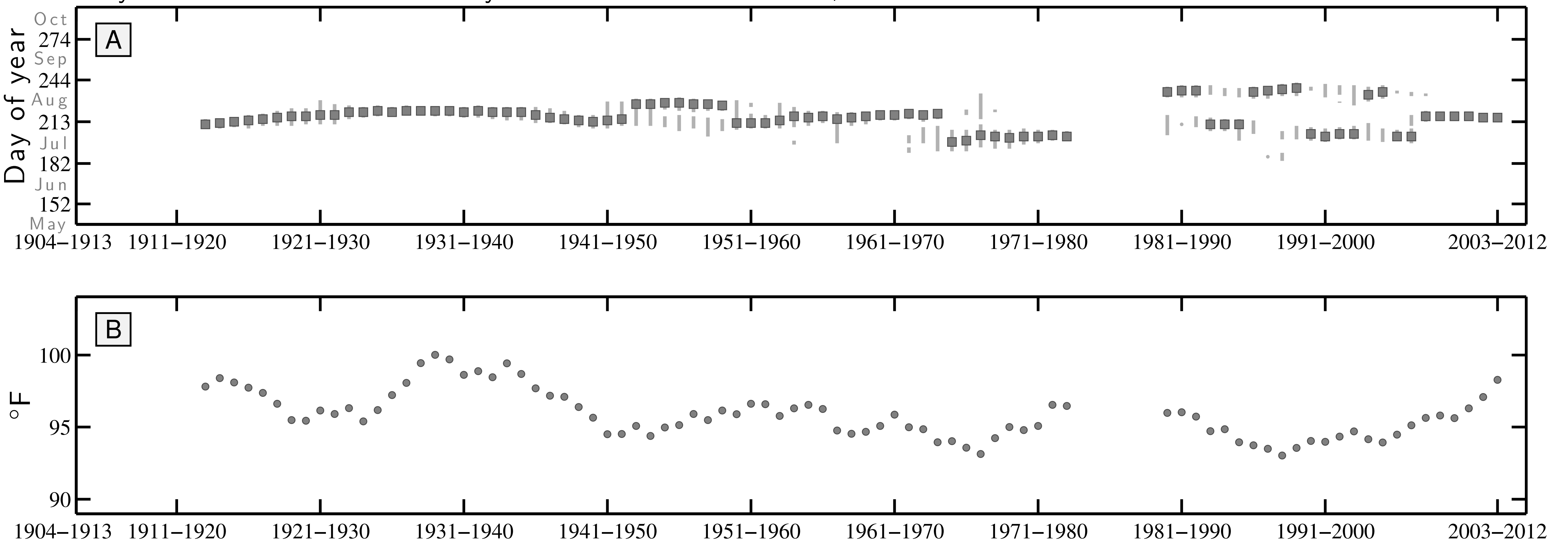

10 year Winter Teletherm dynamics for MUSKOGEE, OK:

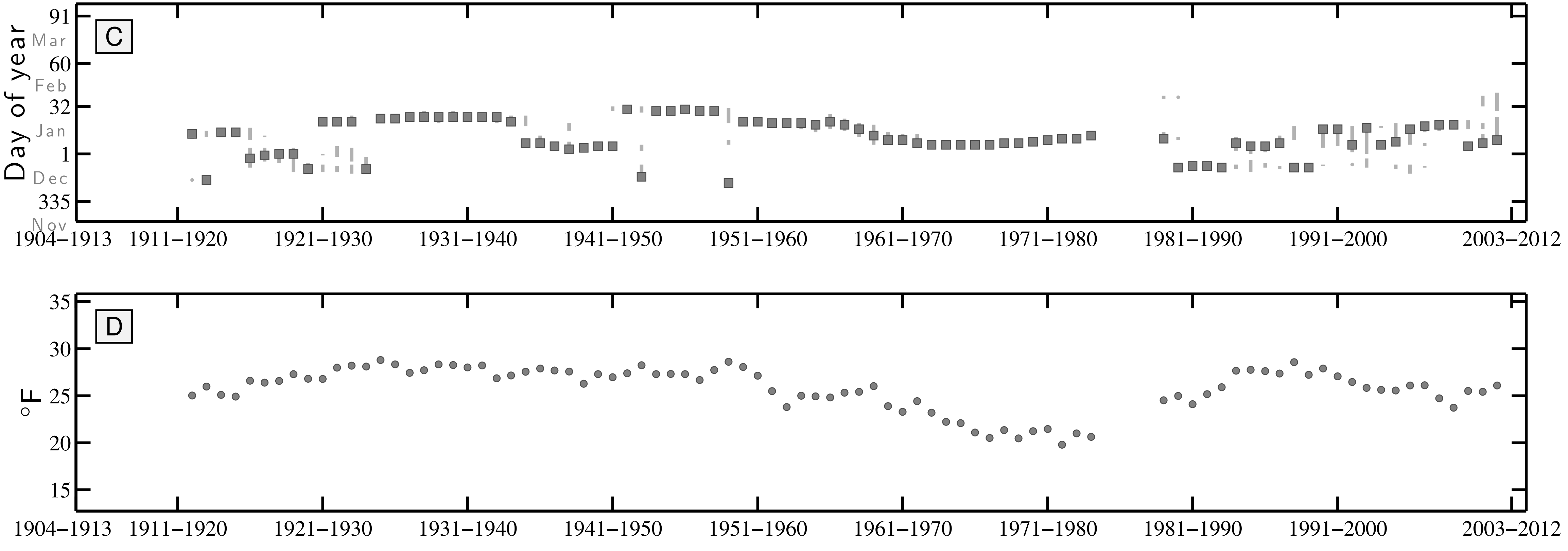

10 year Summer Teletherm dynamics for MUTUAL, OK:

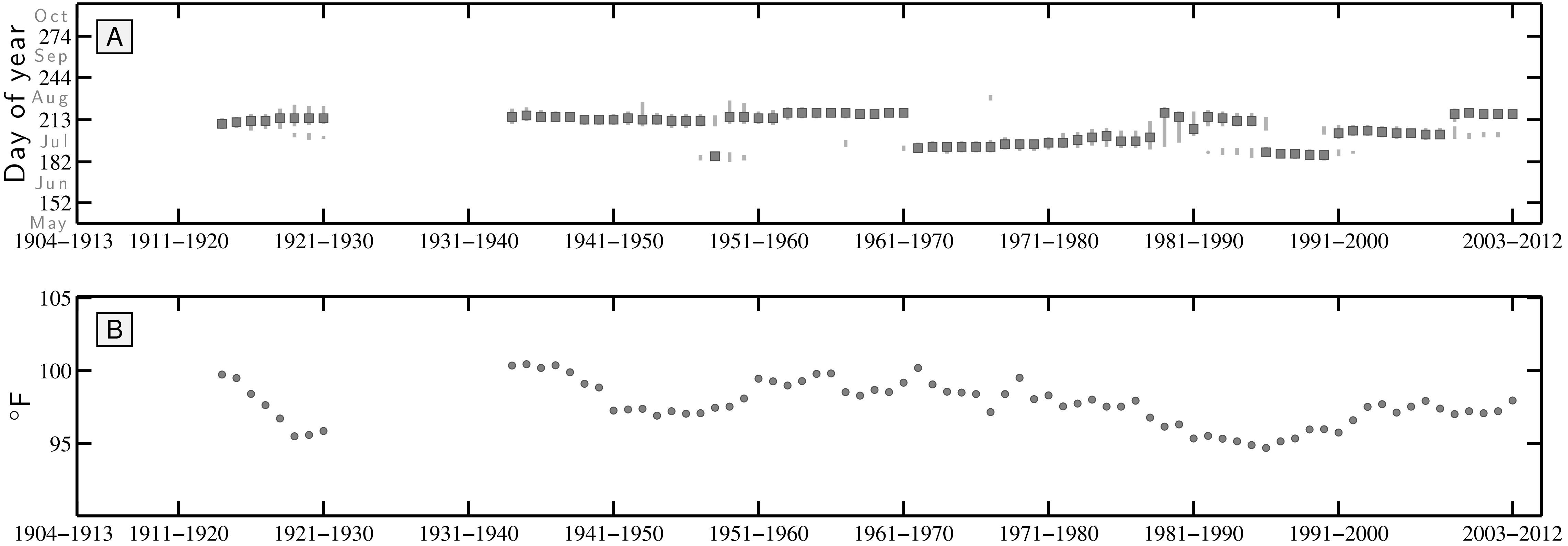

10 year Winter Teletherm dynamics for MUTUAL, OK:

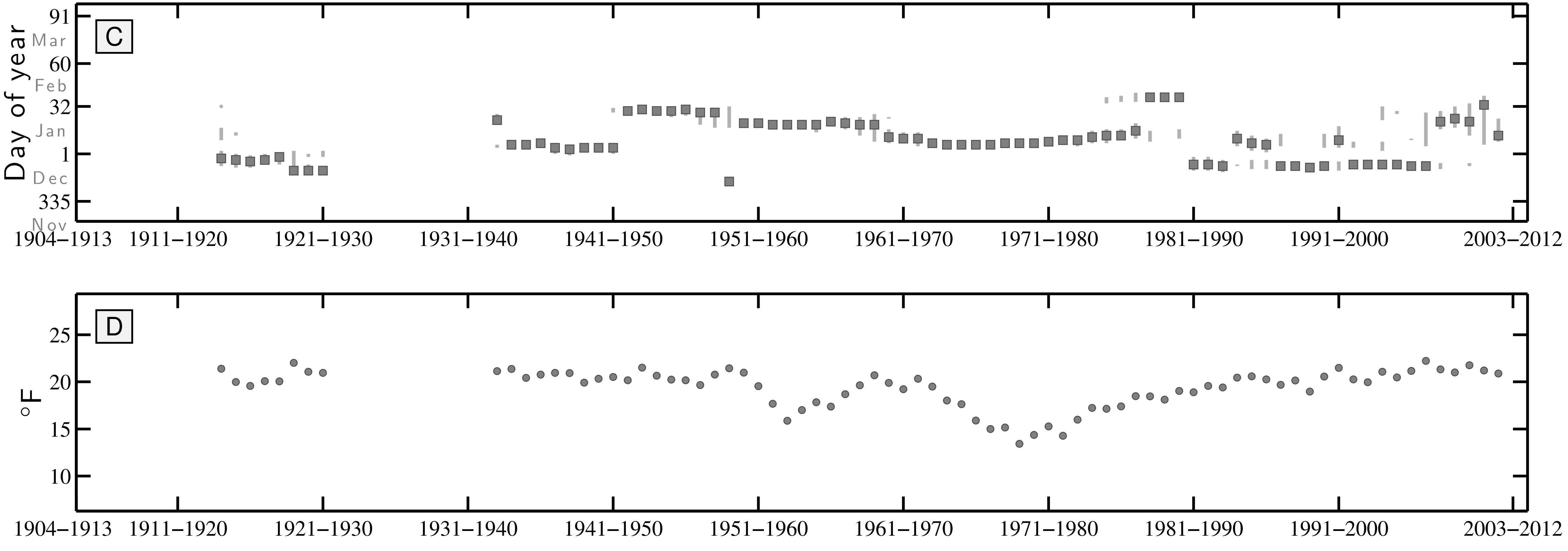

10 year Summer Teletherm dynamics for NEWKIRK 1NW, OK:

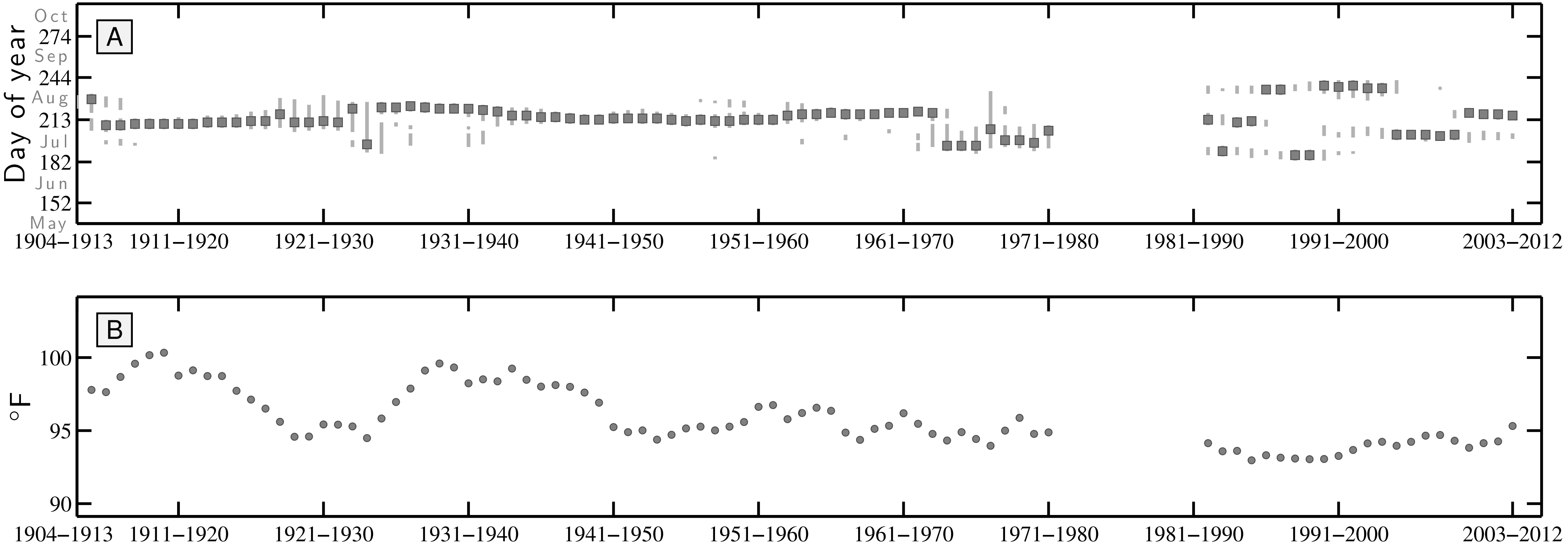

10 year Winter Teletherm dynamics for NEWKIRK 1NW, OK:

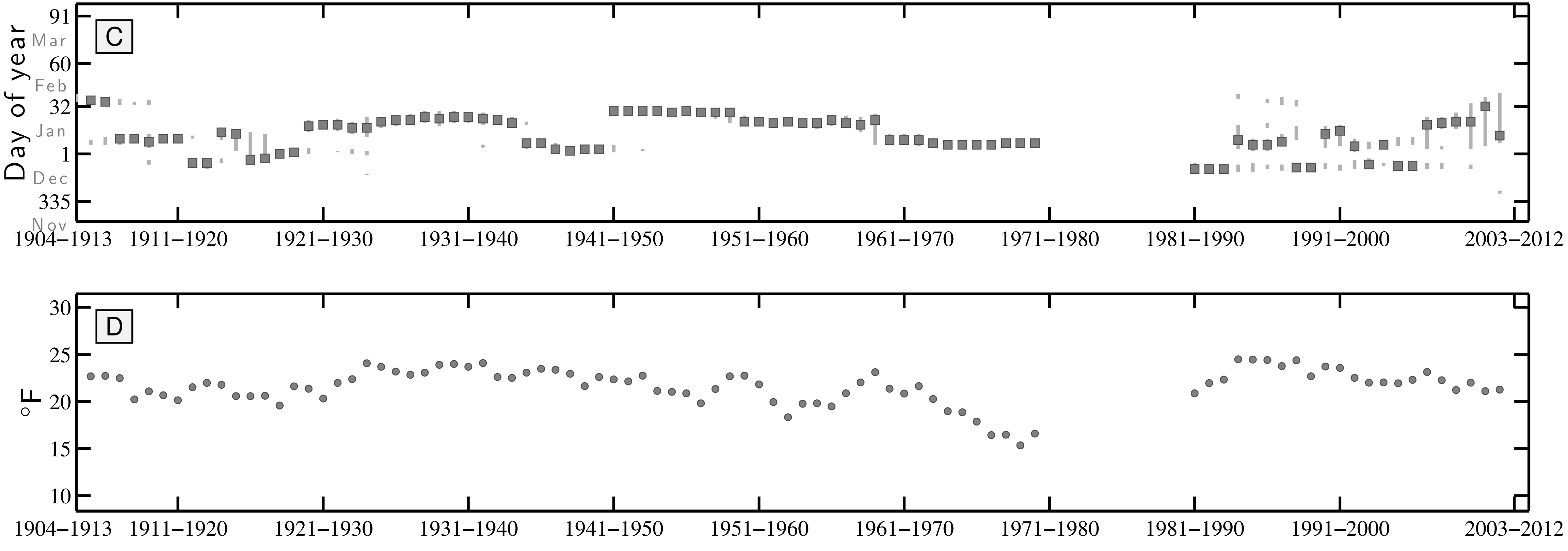

10 year Summer Teletherm dynamics for OKEENE, OK:

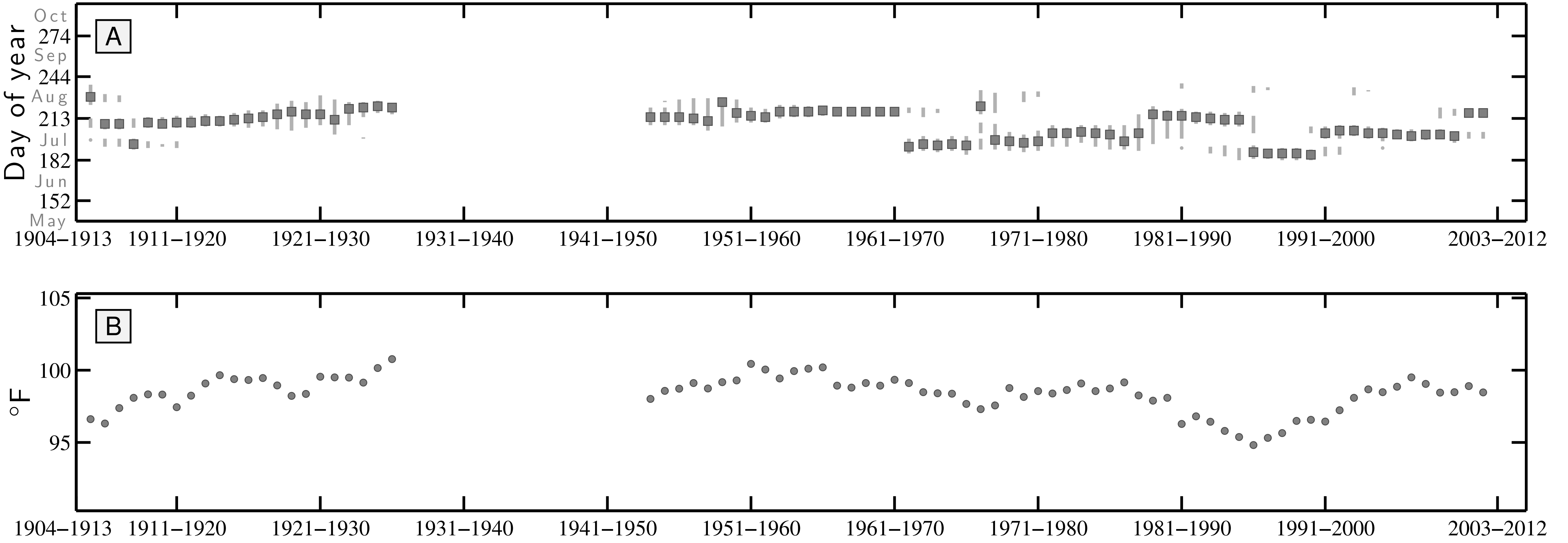

10 year Winter Teletherm dynamics for OKEENE, OK:

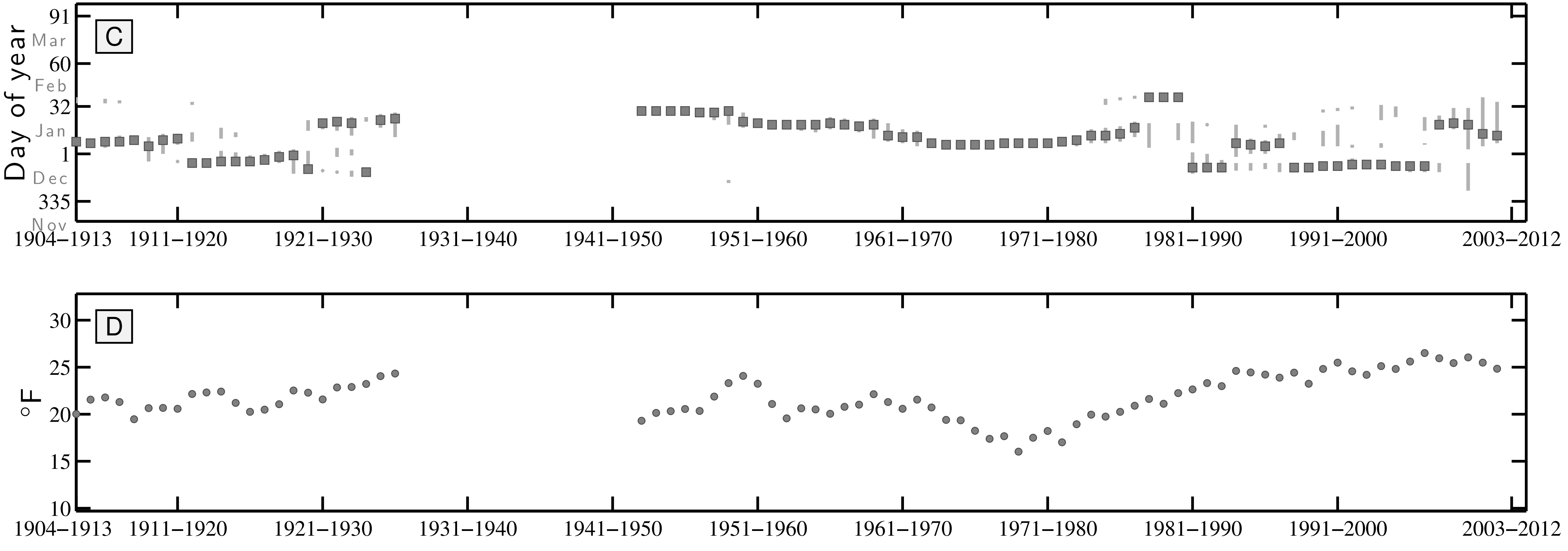

10 year Summer Teletherm dynamics for OKEMAH, OK:

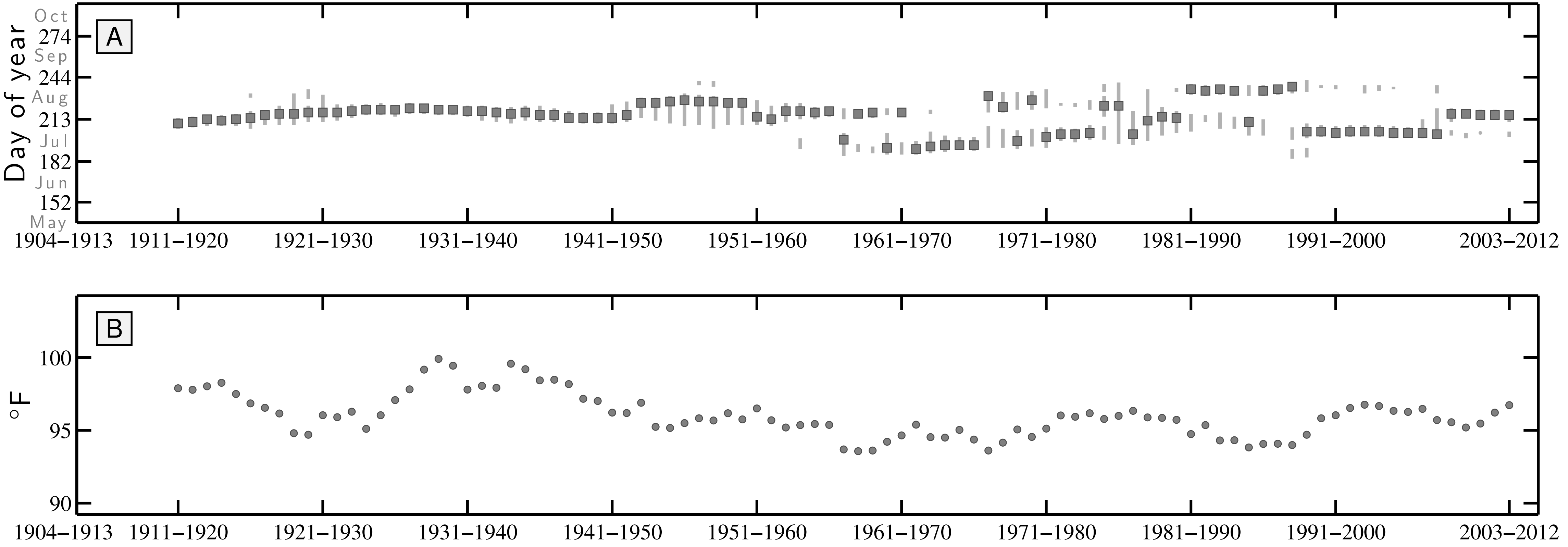

10 year Winter Teletherm dynamics for OKEMAH, OK:

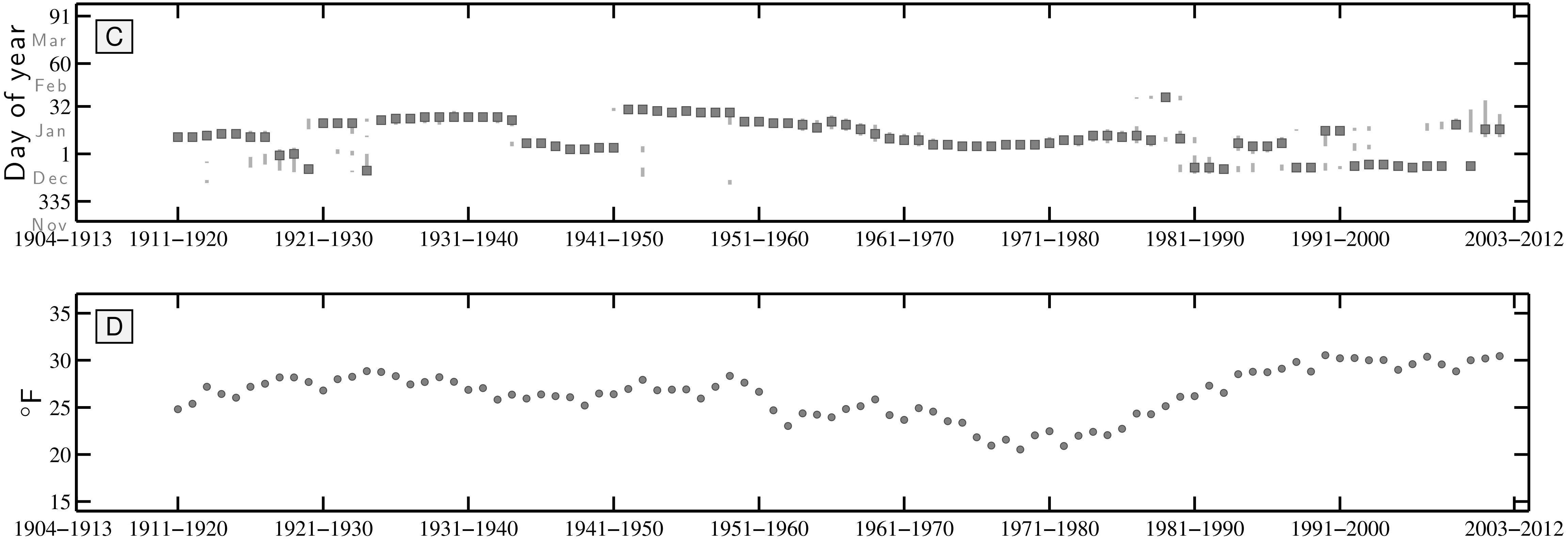

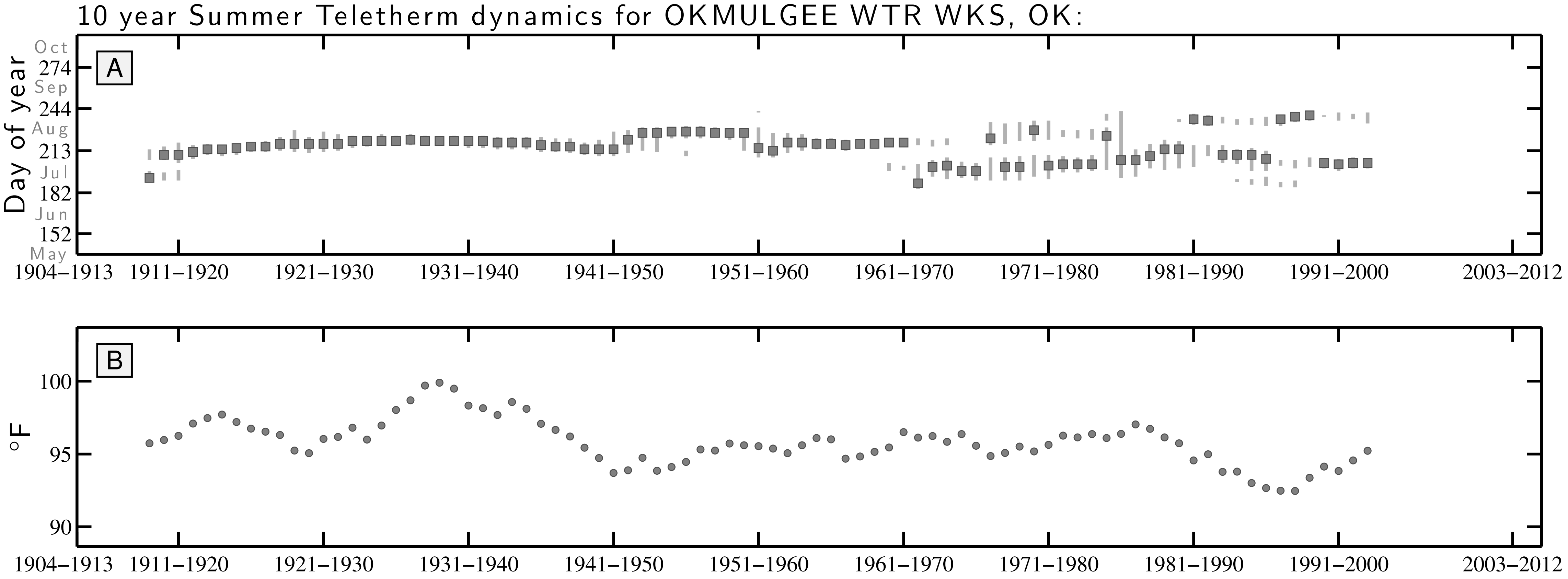
10 year Summer Teletherm dynamics for OKMULGEE WTR WKS, OK:
A
B
Day of year
°F
Oct
274
Sep
244
Aug
213
Jul
182
Jun
152
May
100
95
90
1904–1913
1911–1920
1921–1930
1931–1940
1941–1950
1951–1960
1961–1970
1971–1980
1981–1990
1991–2000
2003–2012

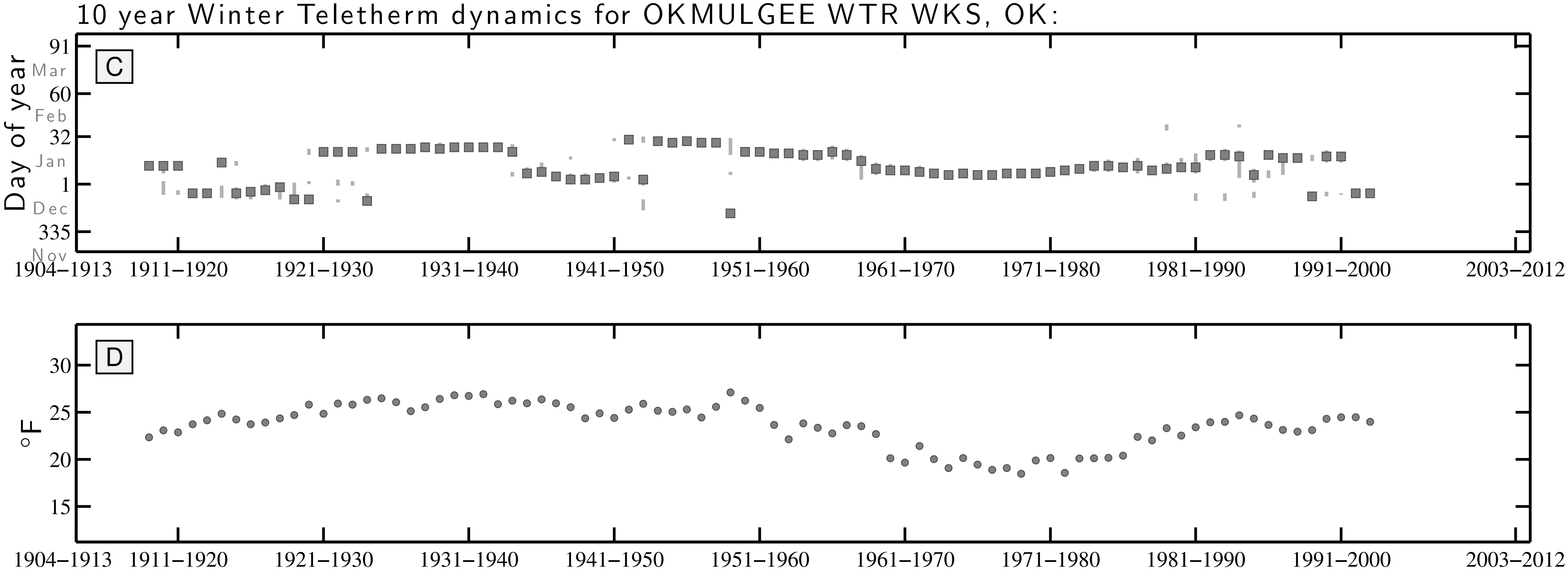
10 year Winter Teletherm dynamics for OKMULGEE WTR WKS, OK:
C
D
Day of year
°F
91
Mar
60
Feb
32
Jan
1
Dec
335
Nov
30
25
20
15
1904–1913
1911–1920
1921–1930
1931–1940
1941–1950
1951–1960
1961–1970
1971–1980
1981–1990
1991–2000
2003–2012

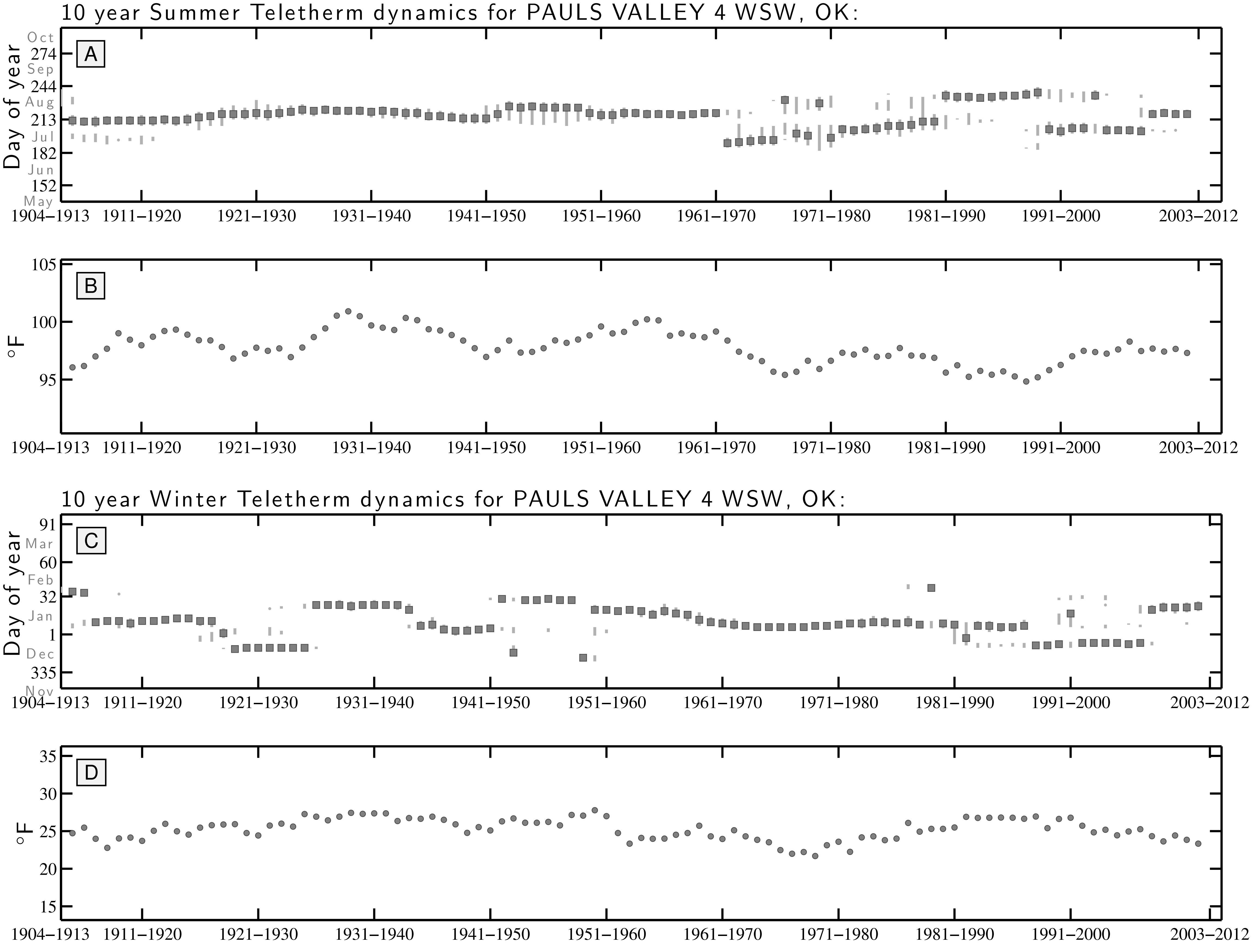
10 year Summer Teletherm dynamics for PAULS VALLEY 4 WSW, OK:
A
B
Day of year
°F
10 year Winter Teletherm dynamics for PAULS VALLEY 4 WSW, OK:
C
D
Day of year
°F
1904–1913
1911–1920
1921–1930
1931–1940
1941–1950
1951–1960
1961–1970
1971–1980
1981–1990
1991–2000
2003–2012

10 year Summer Teletherm dynamics for PAWHUSKA, OK:

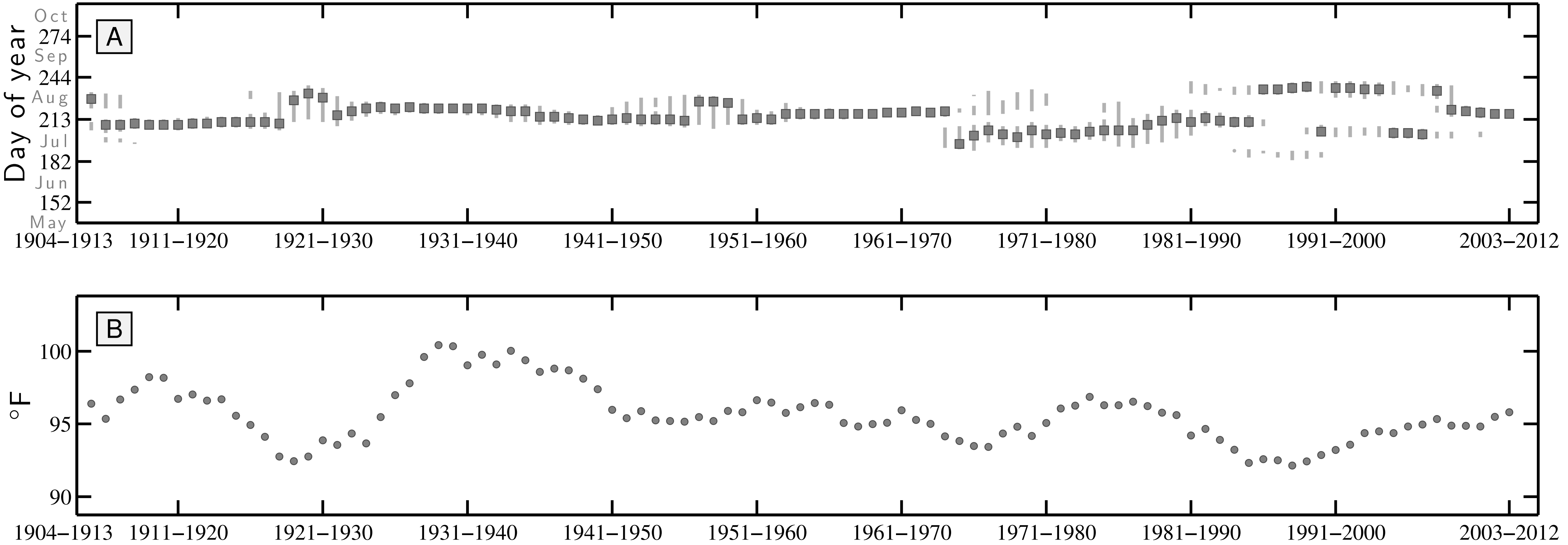

10 year Winter Teletherm dynamics for PAWHUSKA, OK:

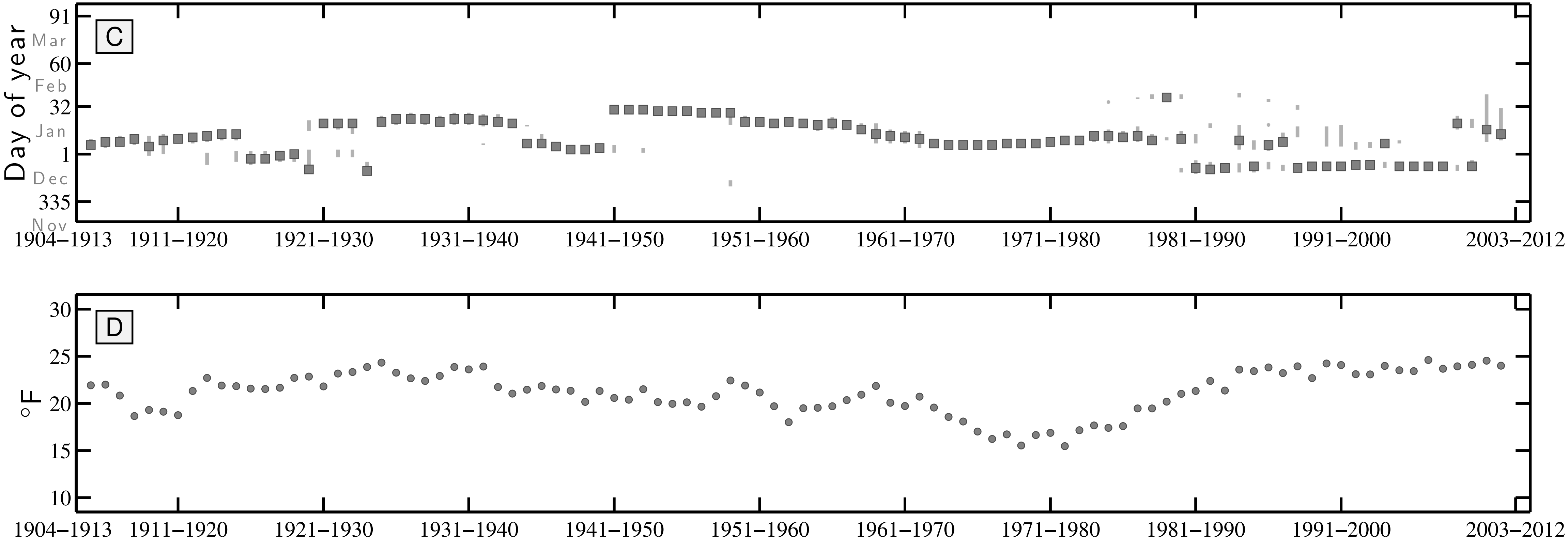

10 year Summer Teletherm dynamics for PERRY, OK:

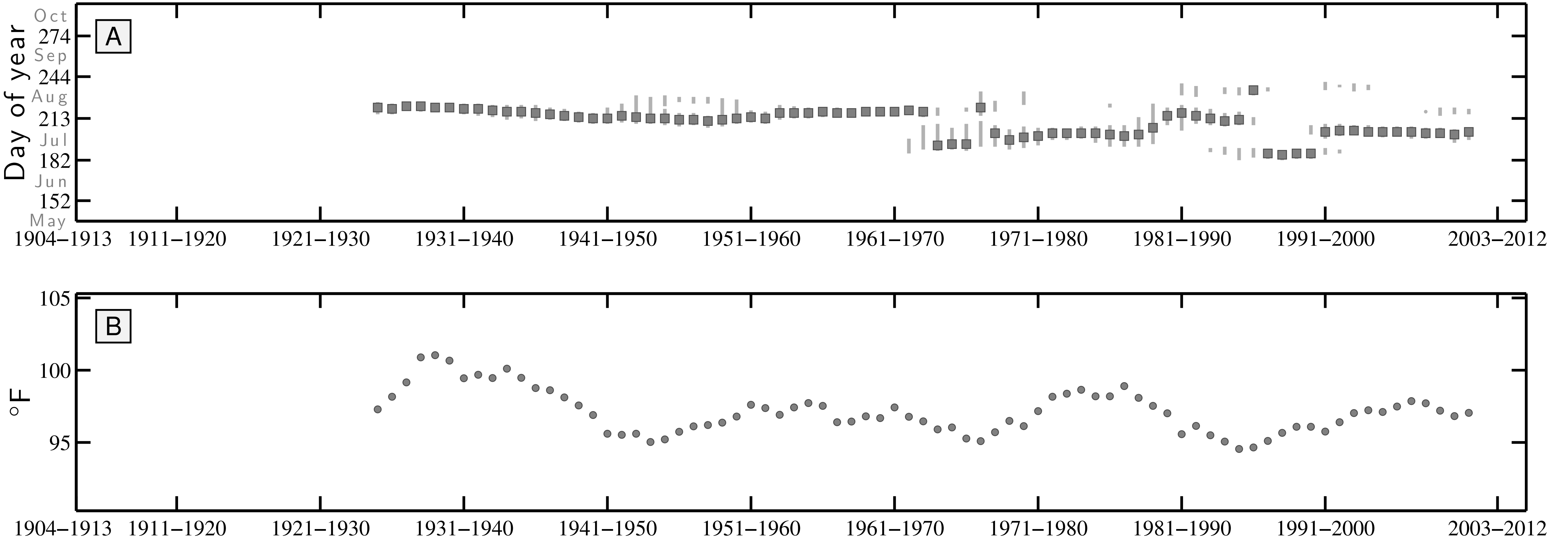

10 year Winter Teletherm dynamics for PERRY, OK:

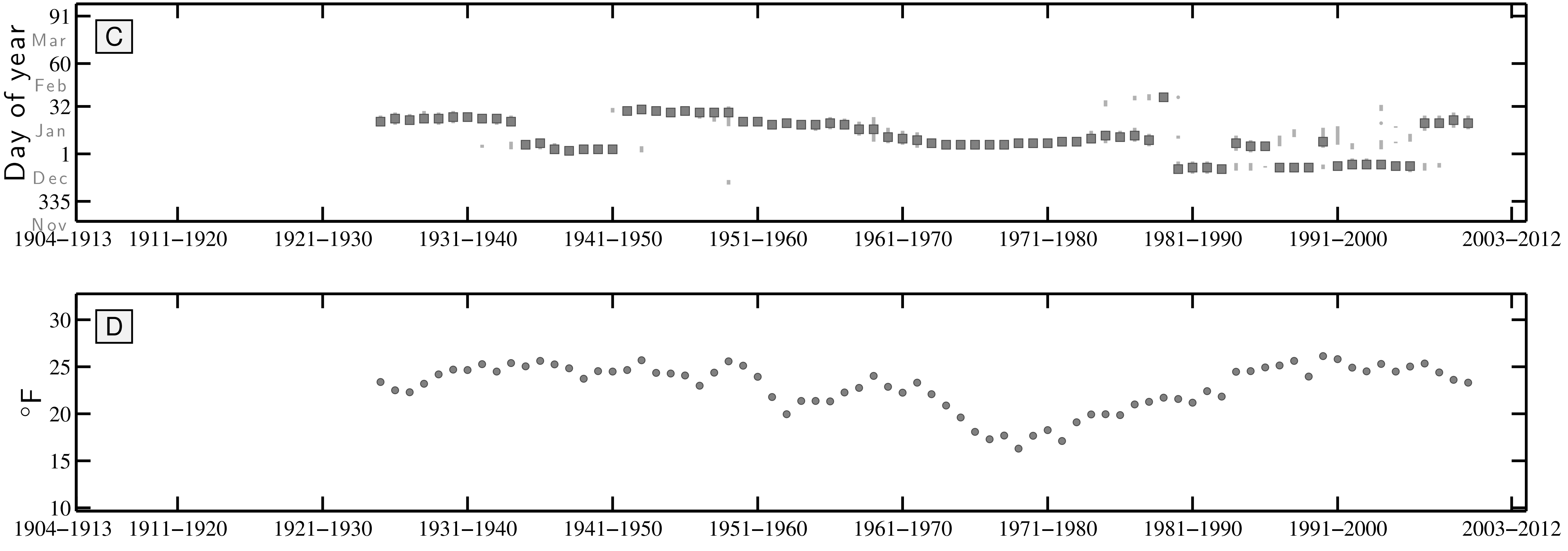

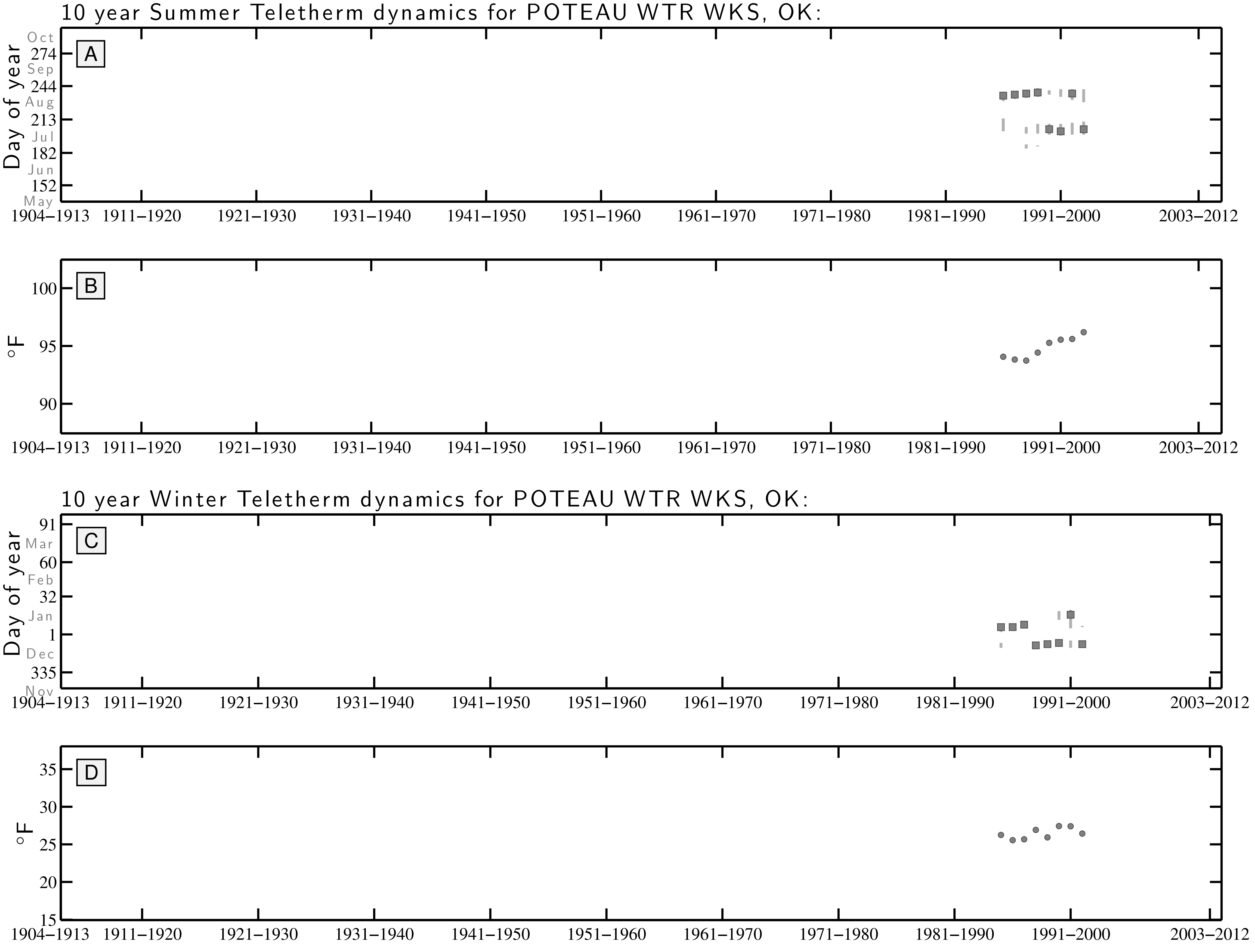
10 year Summer Teletherm dynamics for POTEAU WTR WKS, OK:
A
Day of year
Oct
274
Sep
244
Aug
213
Jul
182
Jun
152
May
B
°F
100
95
90
1904–1913
1911–1920
1921–1930
1931–1940
1941–1950
1951–1960
1961–1970
1971–1980
1981–1990
1991–2000
2003–2012
10 year Winter Teletherm dynamics for POTEAU WTR WKS, OK:
C
Day of year
91
Mar
60
Feb
32
Jan
1
Dec
335
Nov
D
°F
35
30
25
20
15

10 year Summer Teletherm dynamics for STILLWATER 2 W, OK:

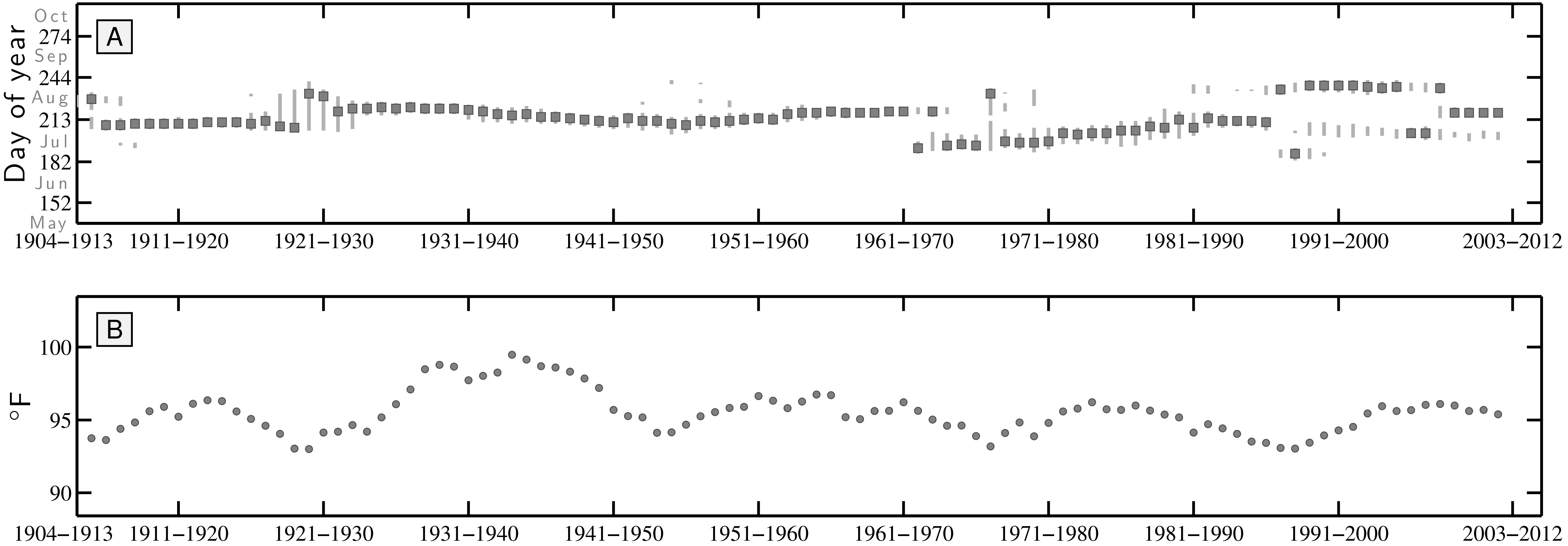

10 year Winter Teletherm dynamics for STILLWATER 2 W, OK:

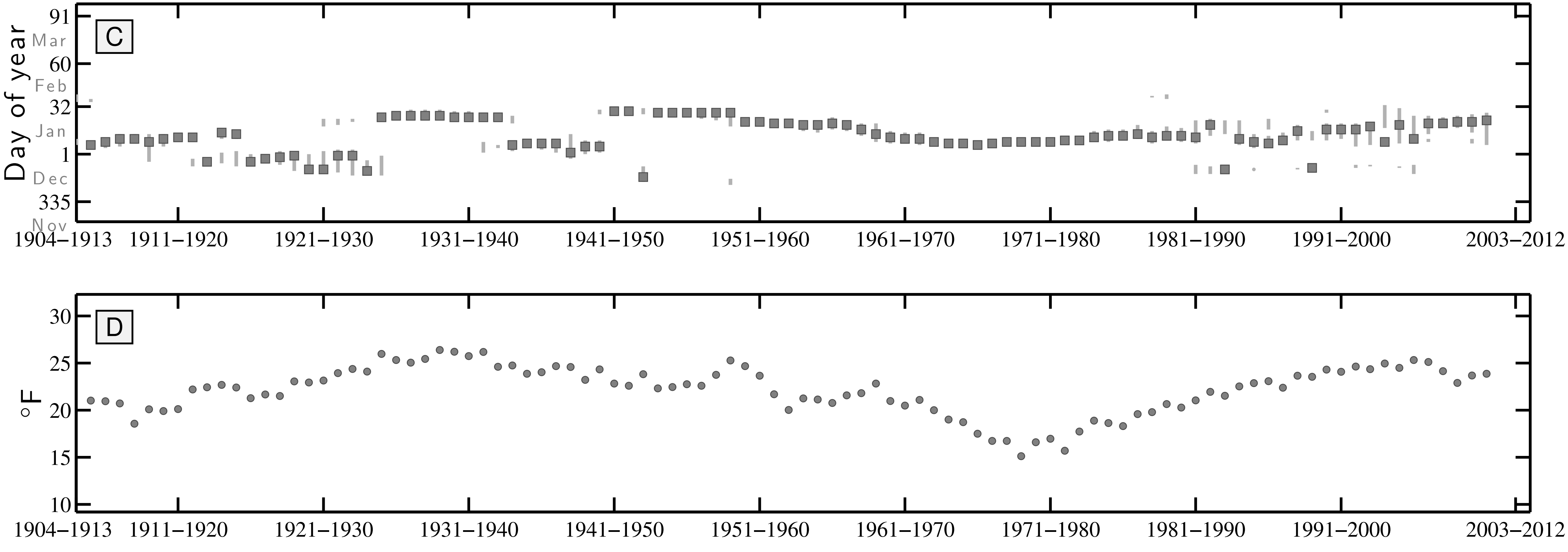

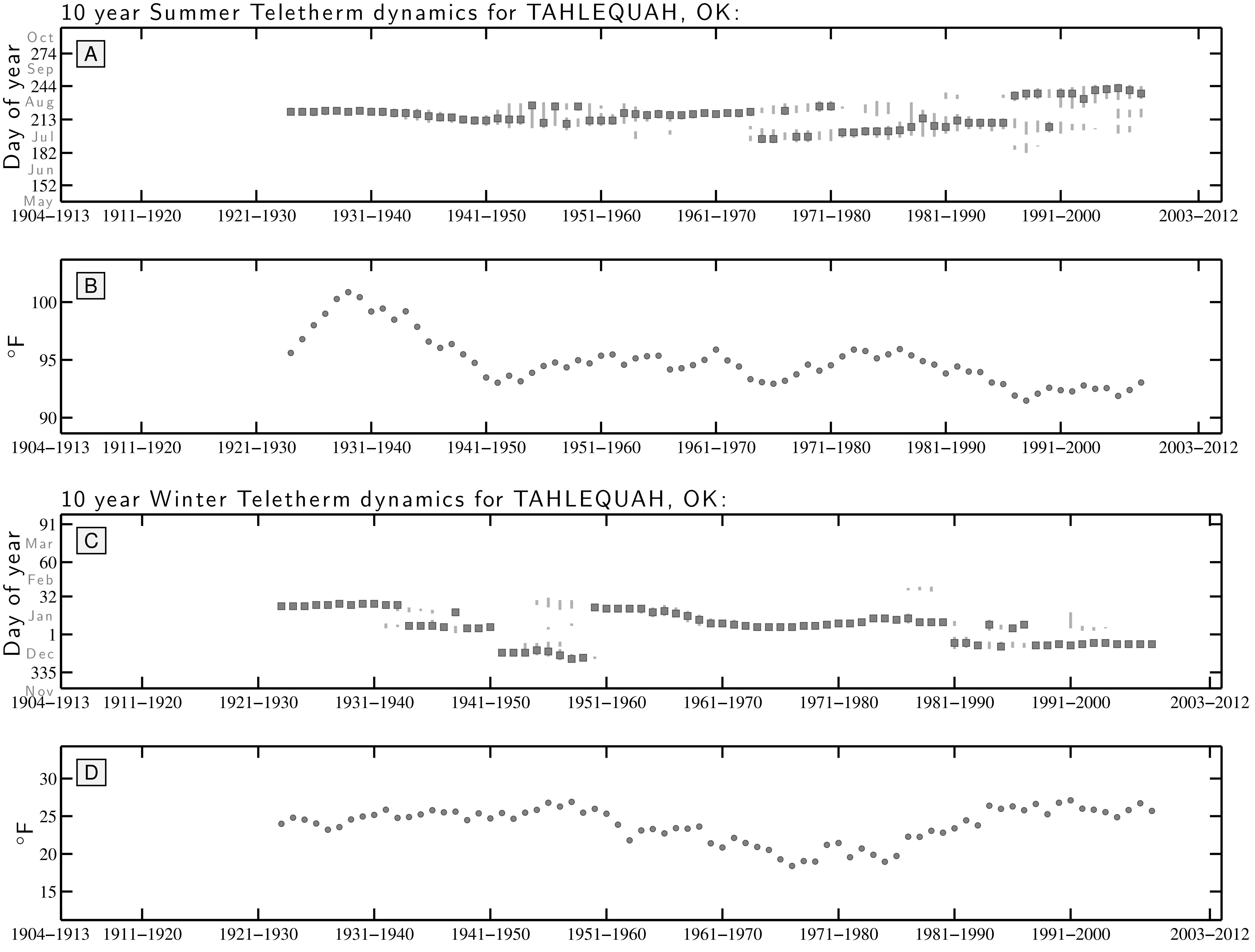
10 year Summer Teletherm dynamics for TAHLEQUAH, OK:
A
Day of year
Oct
274
Sep
244
Aug
213
Jul
182
Jun
152
May
1904–1913
1911–1920
1921–1930
1931–1940
1941–1950
1951–1960
1961–1970
1971–1980
1981–1990
1991–2000
2003–2012
B
°F
100
95
90
10 year Winter Teletherm dynamics for TAHLEQUAH, OK:
C
91
Mar
60
Feb
32
Jan
1
Dec
335
Nov
D
30
25
20
15

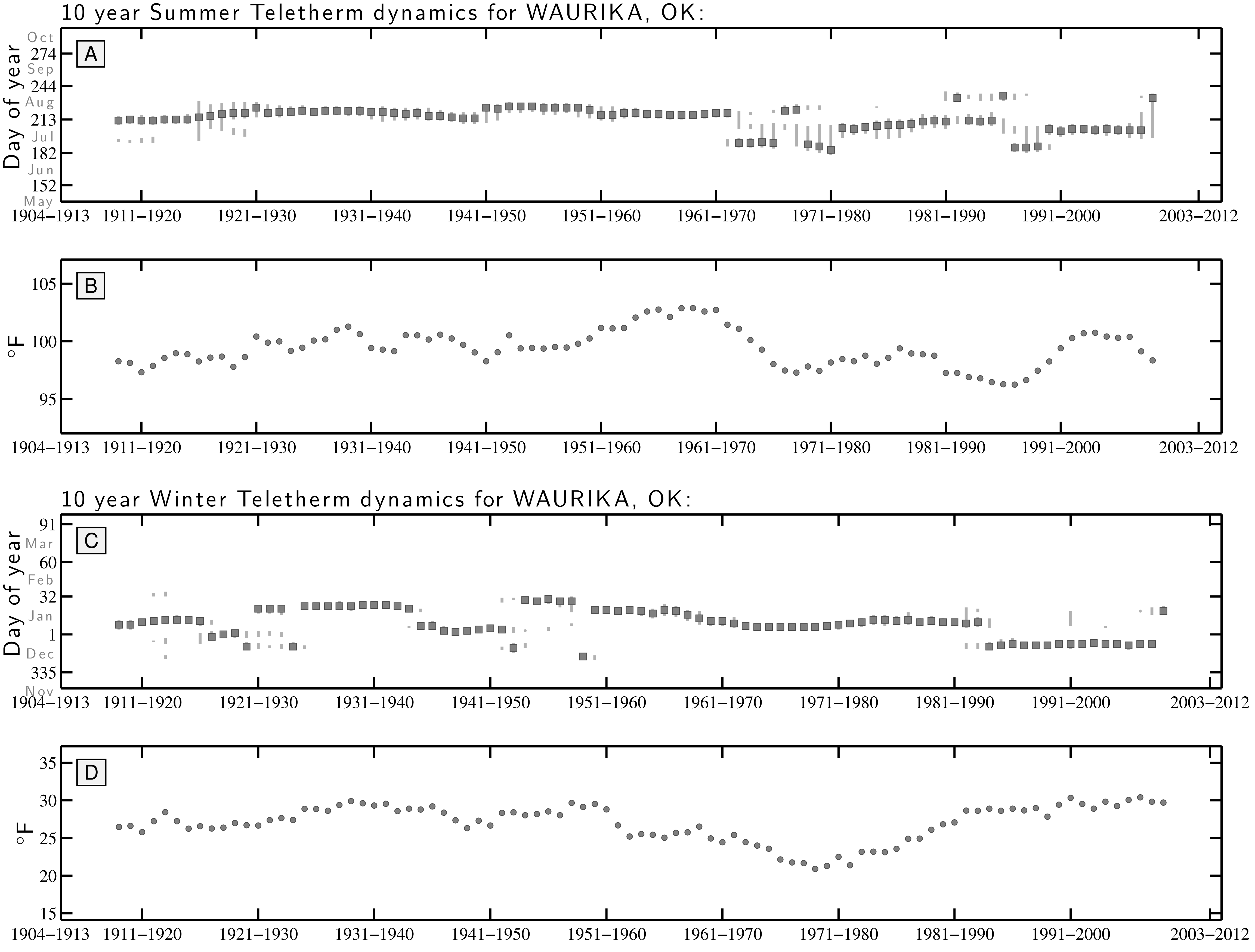
10 year Summer Teletherm dynamics for WAURIKA, OK:
A
Day of year
152
182
213
244
274
May
Jun
Jul
Aug
Sep
Oct
B
°F
95
100
105
1904–1913
1911–1920
1921–1930
1931–1940
1941–1950
1951–1960
1961–1970
1971–1980
1981–1990
1991–2000
2003–2012
10 year Winter Teletherm dynamics for WAURIKA, OK:
C
Day of year
335
1
32
60
91
Nov
Dec
Jan
Feb
Mar
D
°F
15
20
25
30
35

10 year Summer Teletherm dynamics for WEATHERFORD, OK:

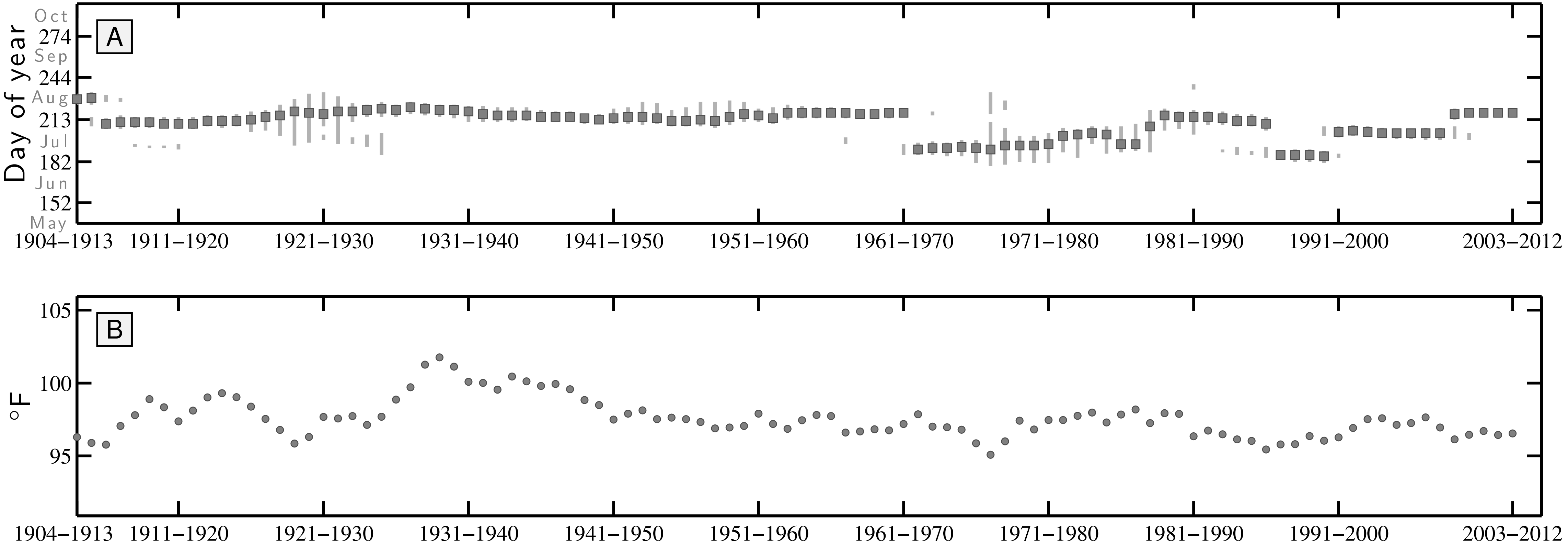

10 year Winter Teletherm dynamics for WEATHERFORD, OK:

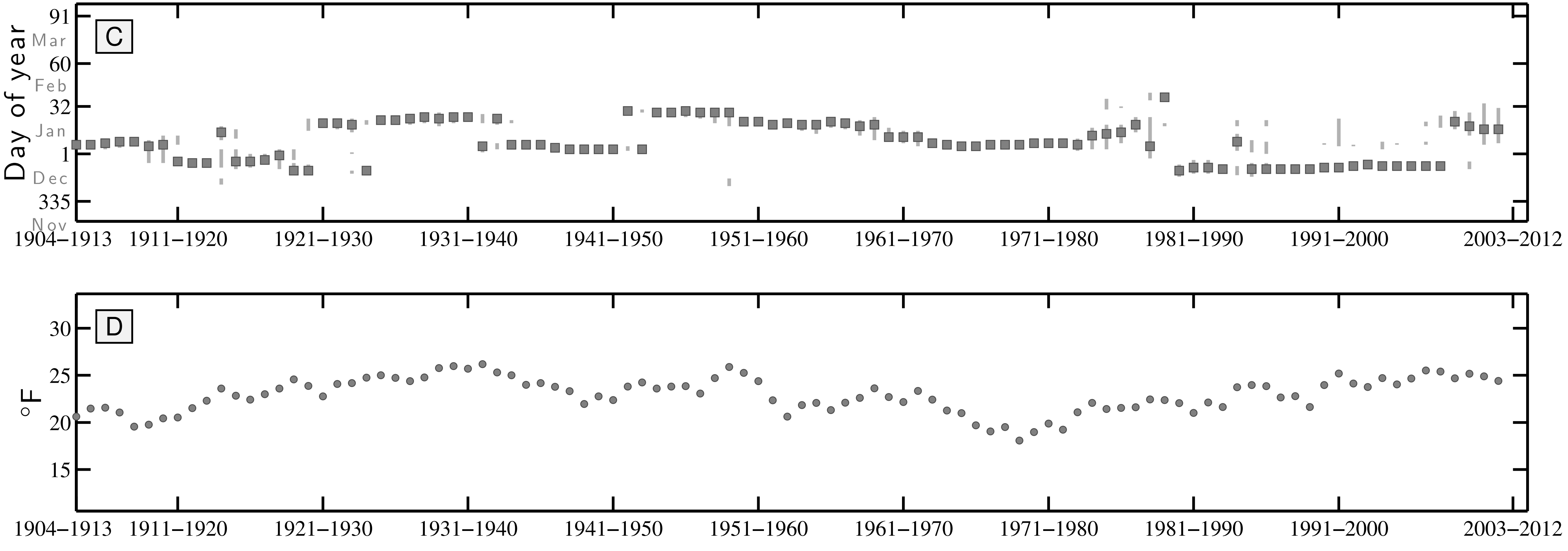

10 year Summer Teletherm dynamics for WEBBERS FALLS 5 WSW, OK:

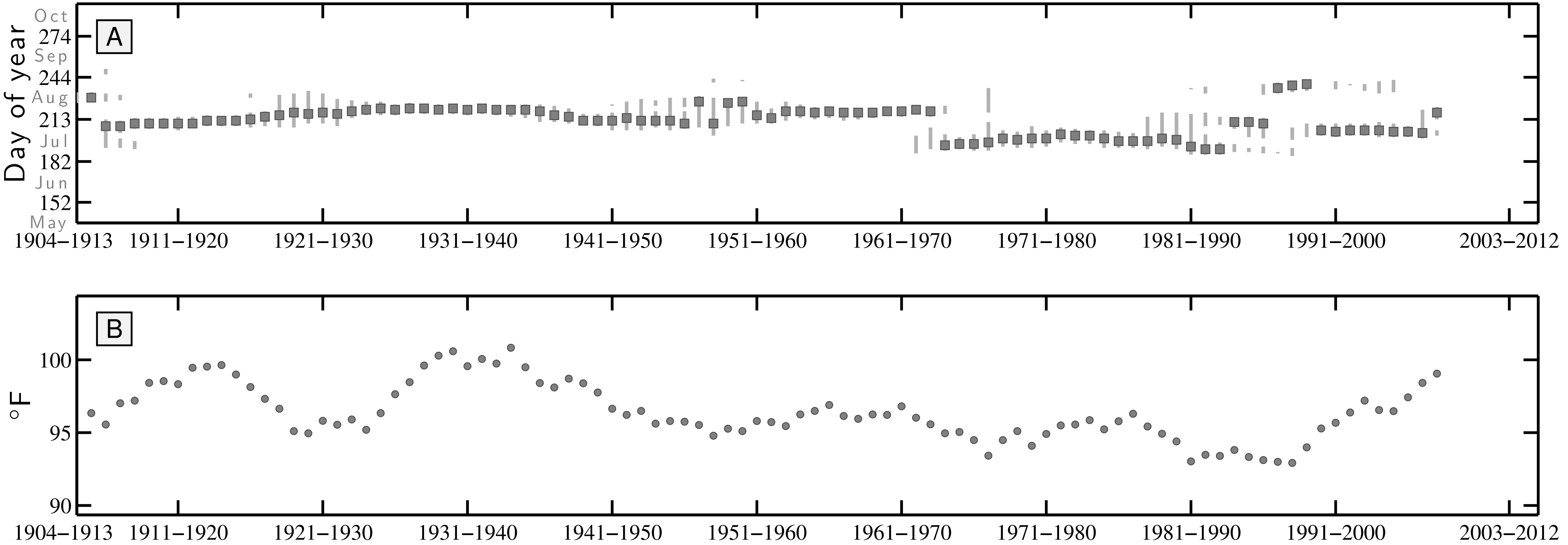

10 year Winter Teletherm dynamics for WEBBERS FALLS 5 WSW, OK:

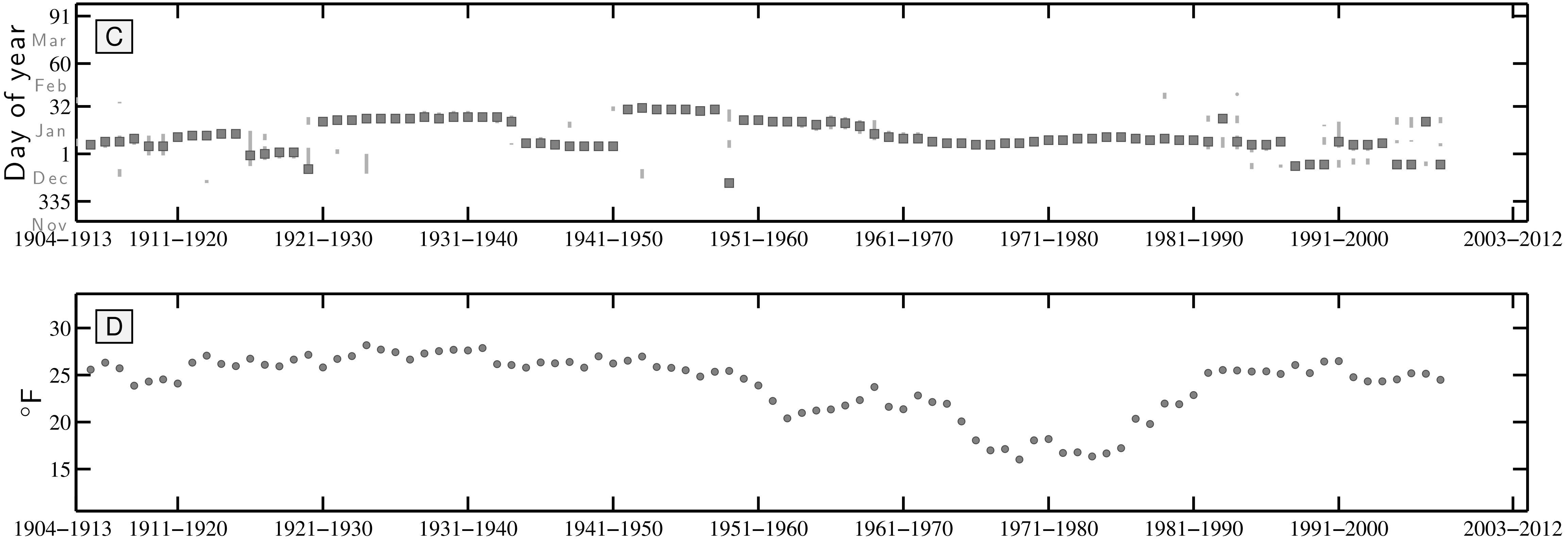

10 year Summer Teletherm dynamics for ASHLAND, OR:

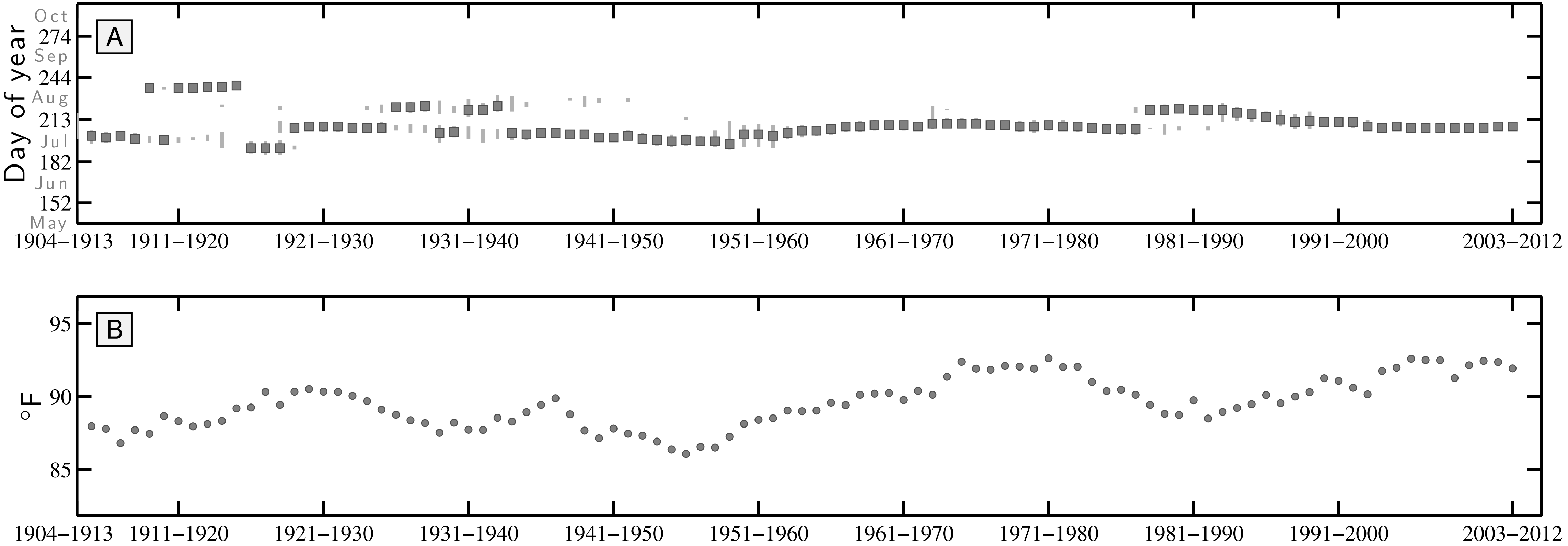

10 year Winter Teletherm dynamics for ASHLAND, OR:

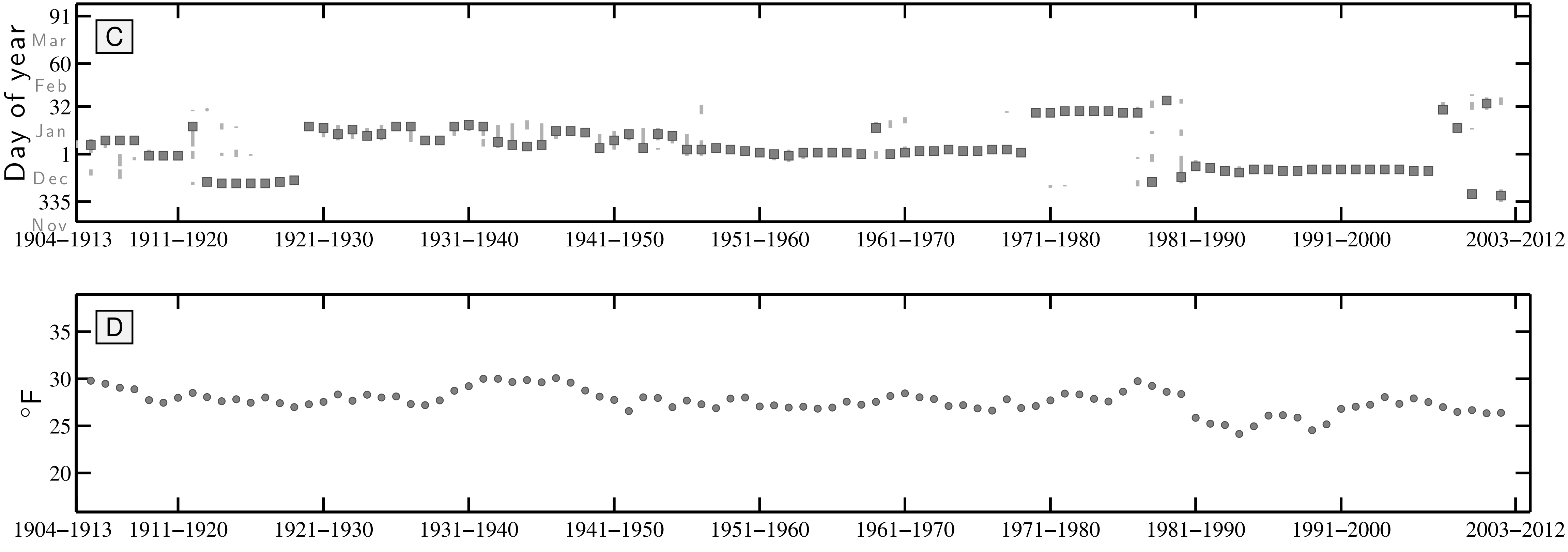

10 year Summer Teletherm dynamics for ASTORIA AP PORT OF, OR:

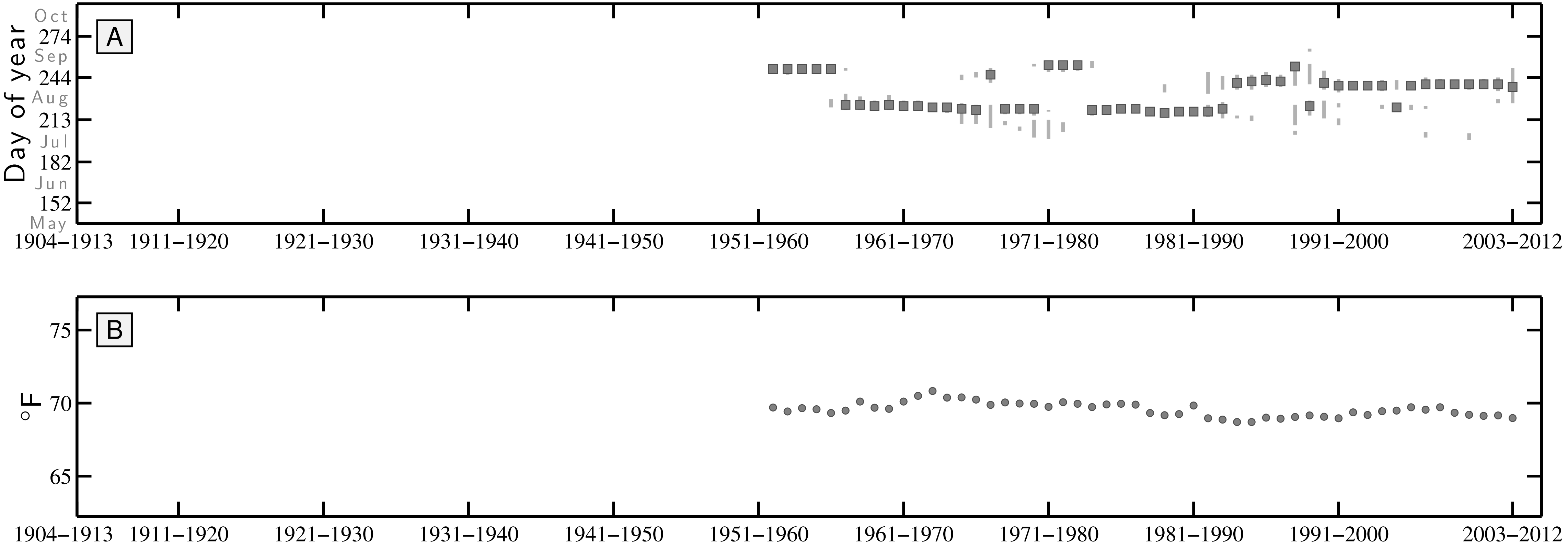

10 year Winter Teletherm dynamics for ASTORIA AP PORT OF, OR:

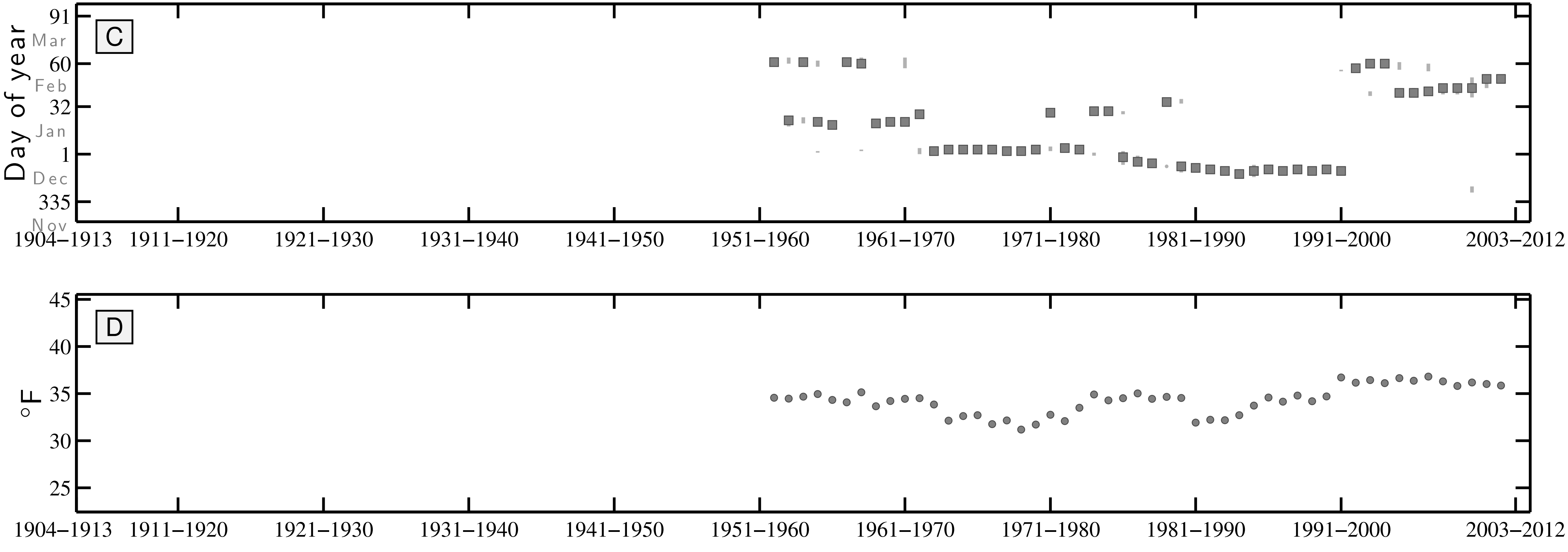

10 year Summer Teletherm dynamics for BAKER CITY AP, OR:

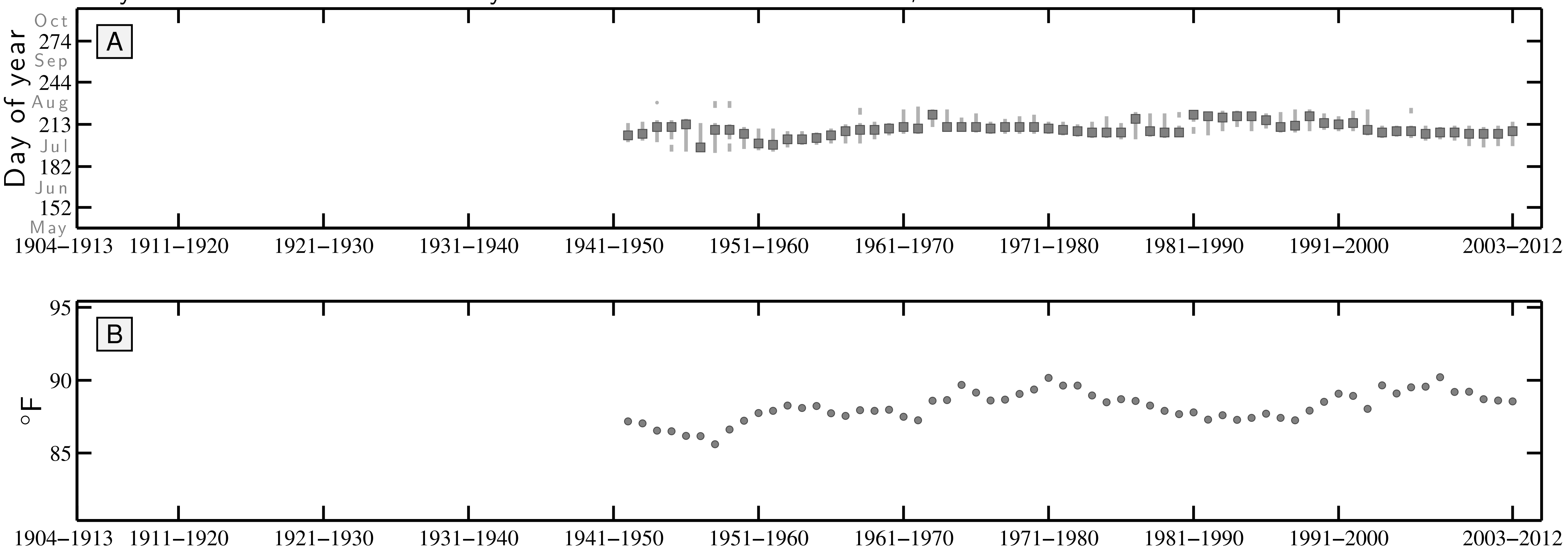

10 year Winter Teletherm dynamics for BAKER CITY AP, OR:

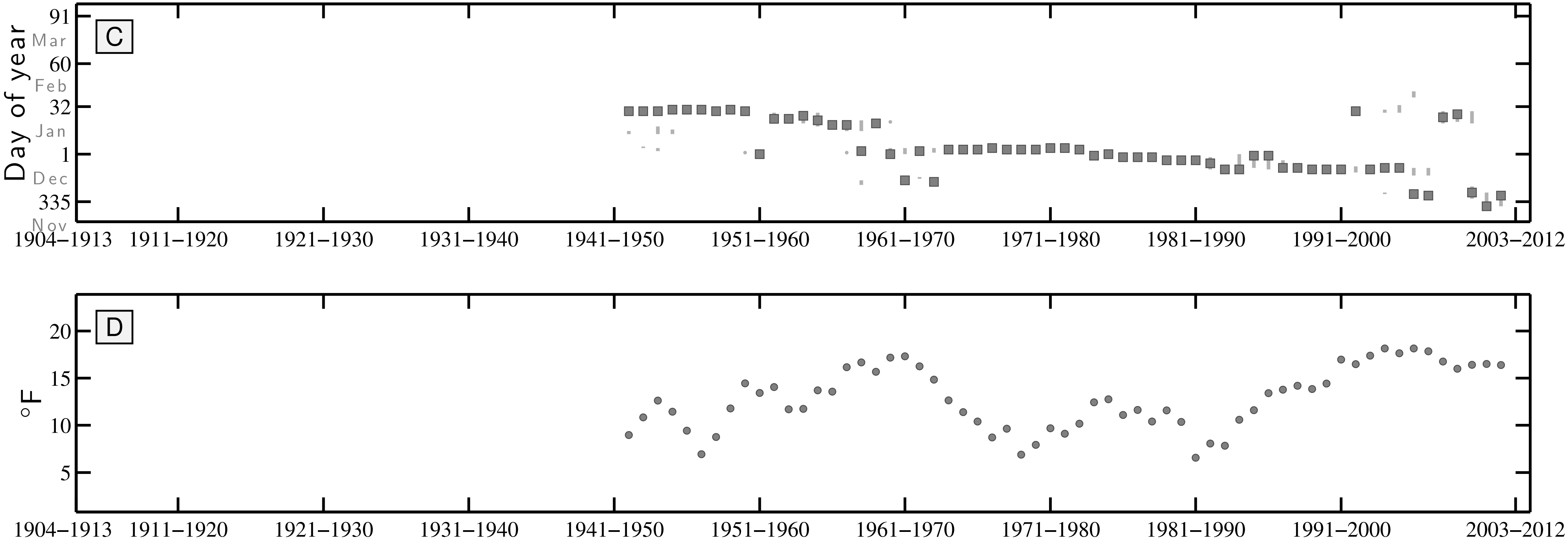

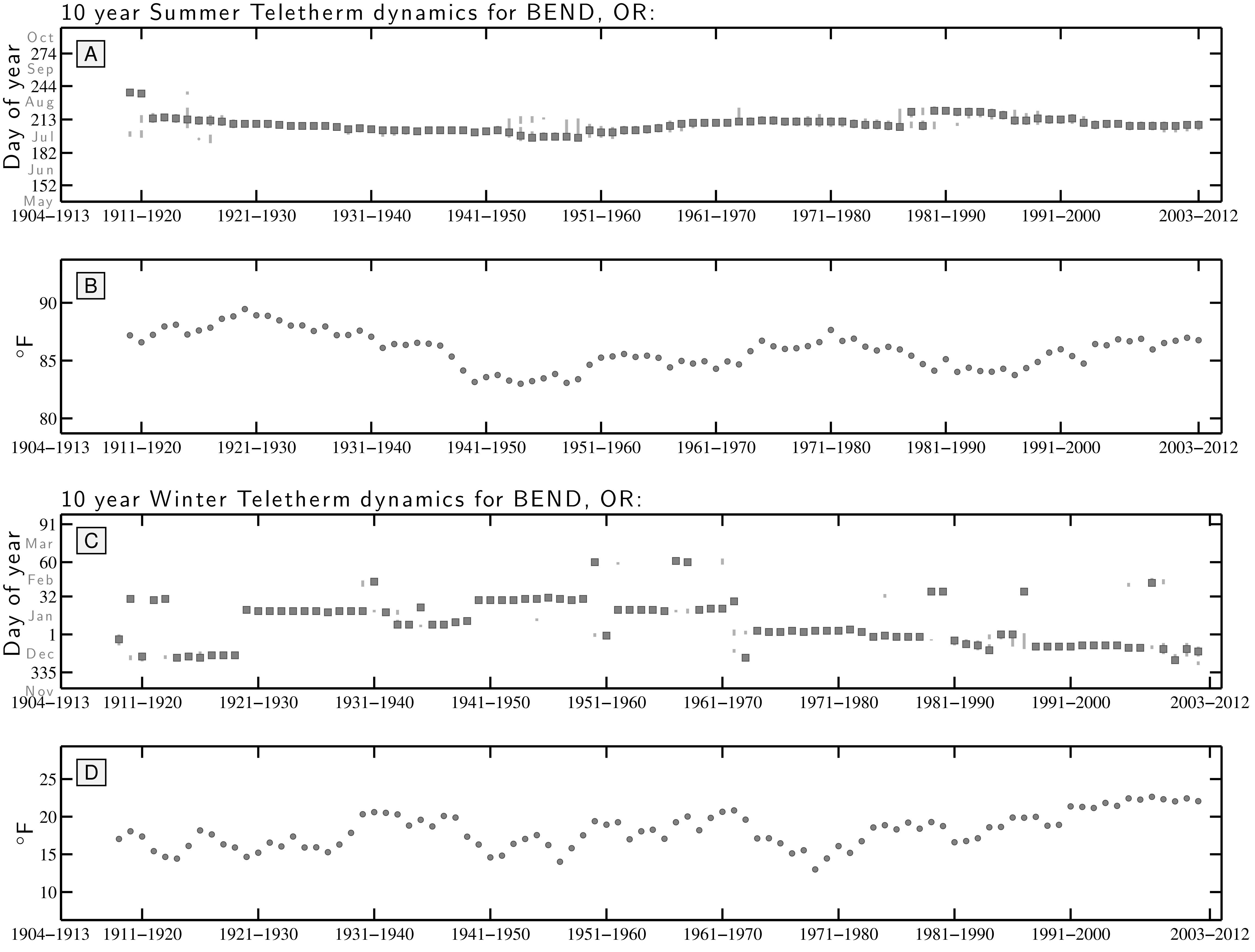

10 year Summer Teletherm dynamics for BEND, OR:
A
B
10 year Winter Teletherm dynamics for BEND, OR:
C
D
Day of year
°F
Oct
274
Sep
244
Aug
213
Jul
182
Jun
152
May
90
85
80
91
Mar
60
Feb
32
Jan
1
Dec
335
Nov
25
20
15
10
1904–1913
1911–1920
1921–1930
1931–1940
1941–1950
1951–1960
1961–1970
1971–1980
1981–1990
1991–2000
2003–2012

10 year Summer Teletherm dynamics for BROOKINGS 2 SE, OR:

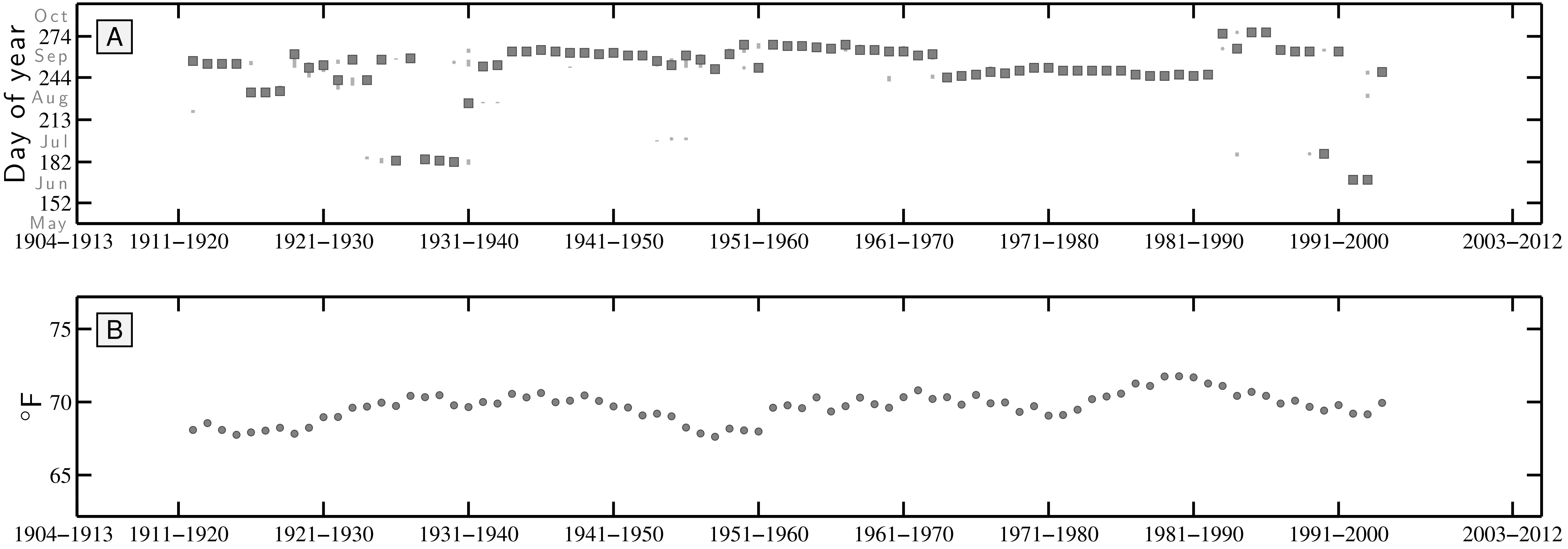

10 year Winter Teletherm dynamics for BROOKINGS 2 SE, OR:

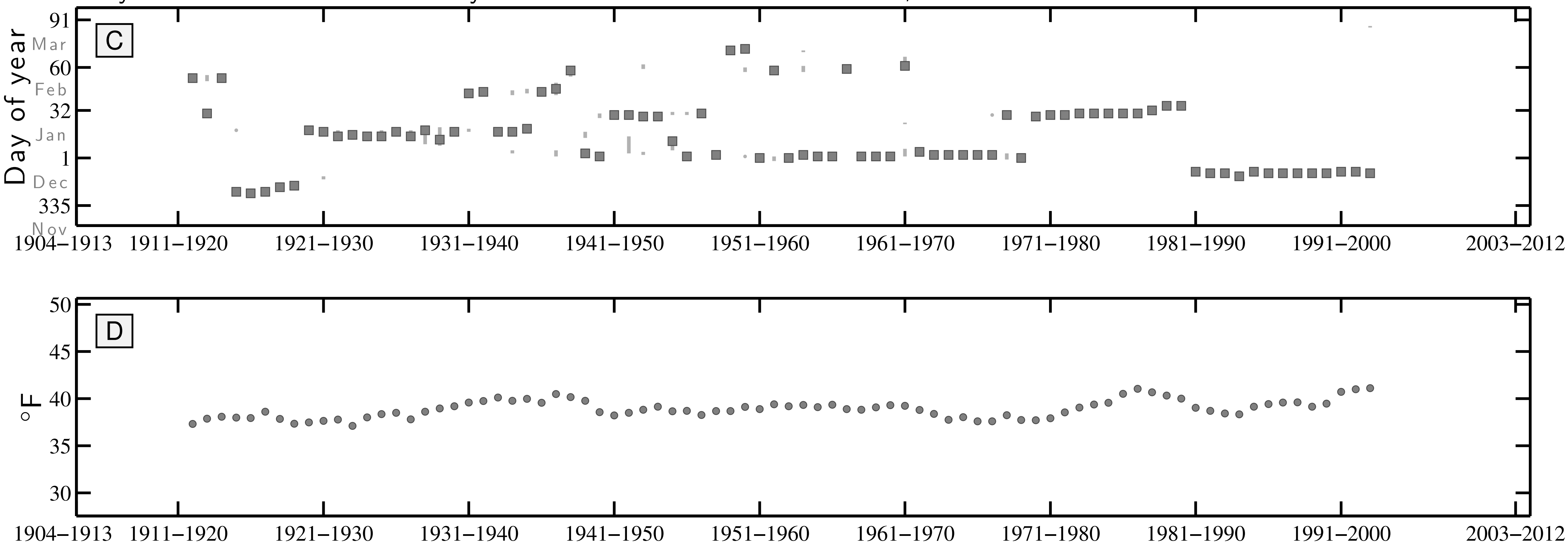

10 year Summer Teletherm dynamics for CASCADIA, OR:

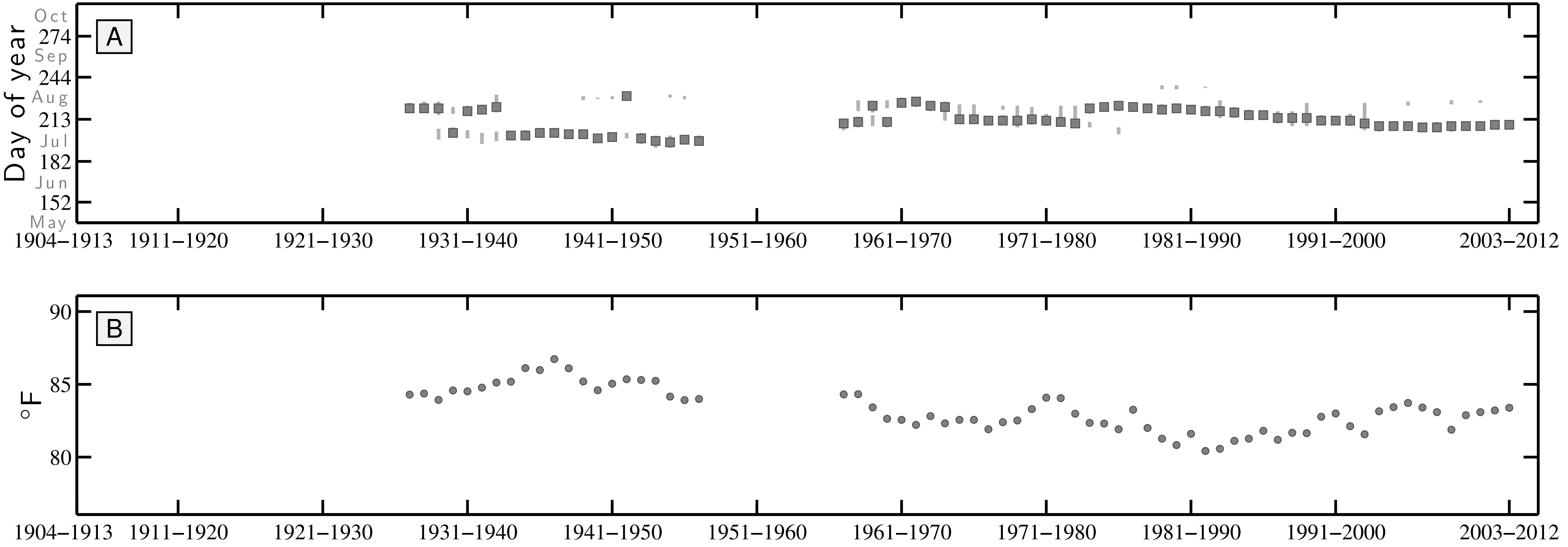

10 year Winter Teletherm dynamics for CASCADIA, OR:

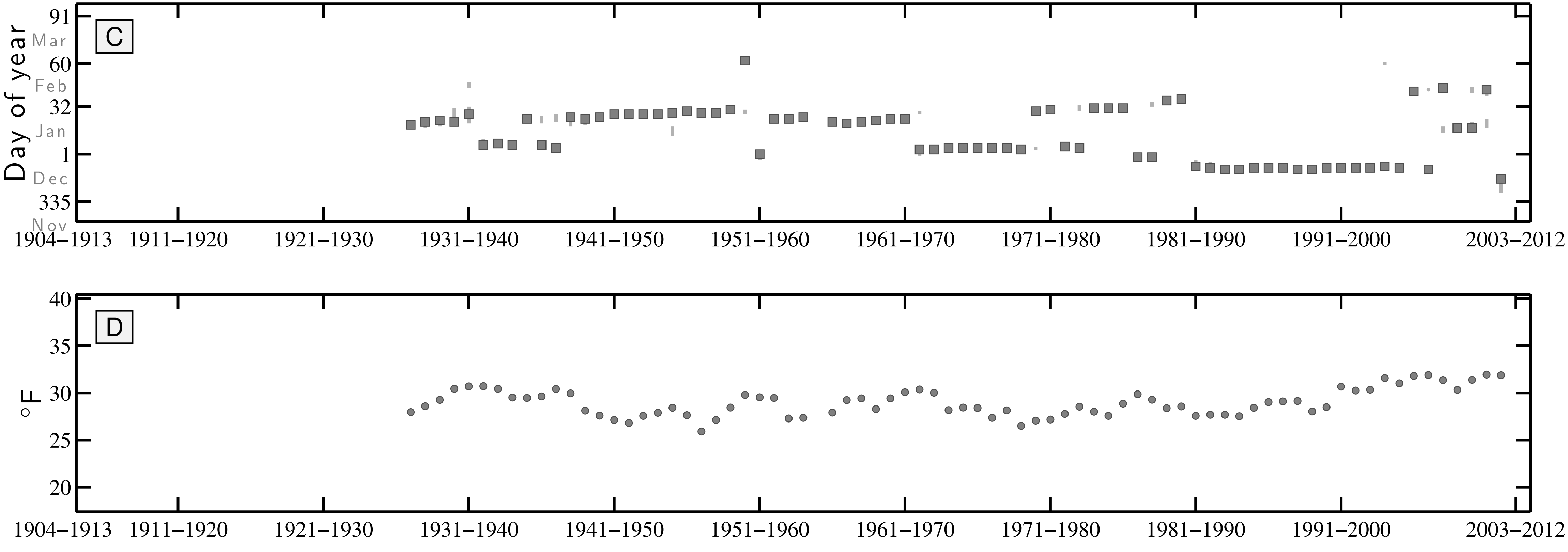

10 year Summer Teletherm dynamics for CONDON, OR:

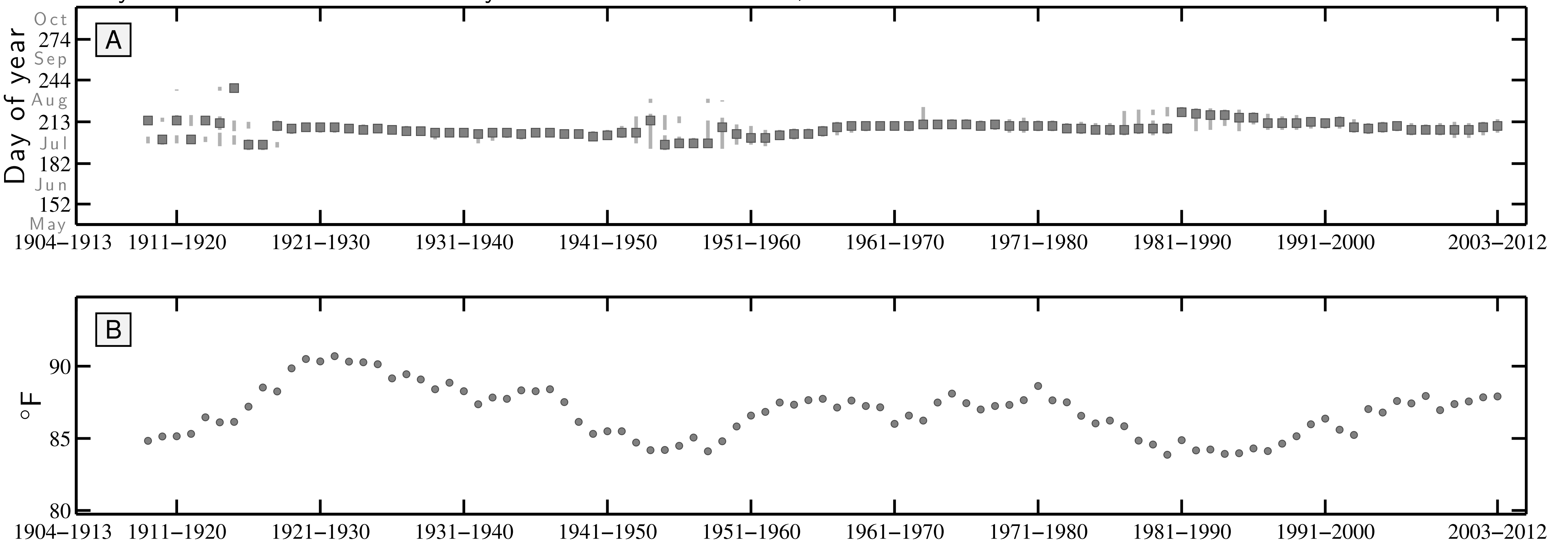

10 year Winter Teletherm dynamics for CONDON, OR:

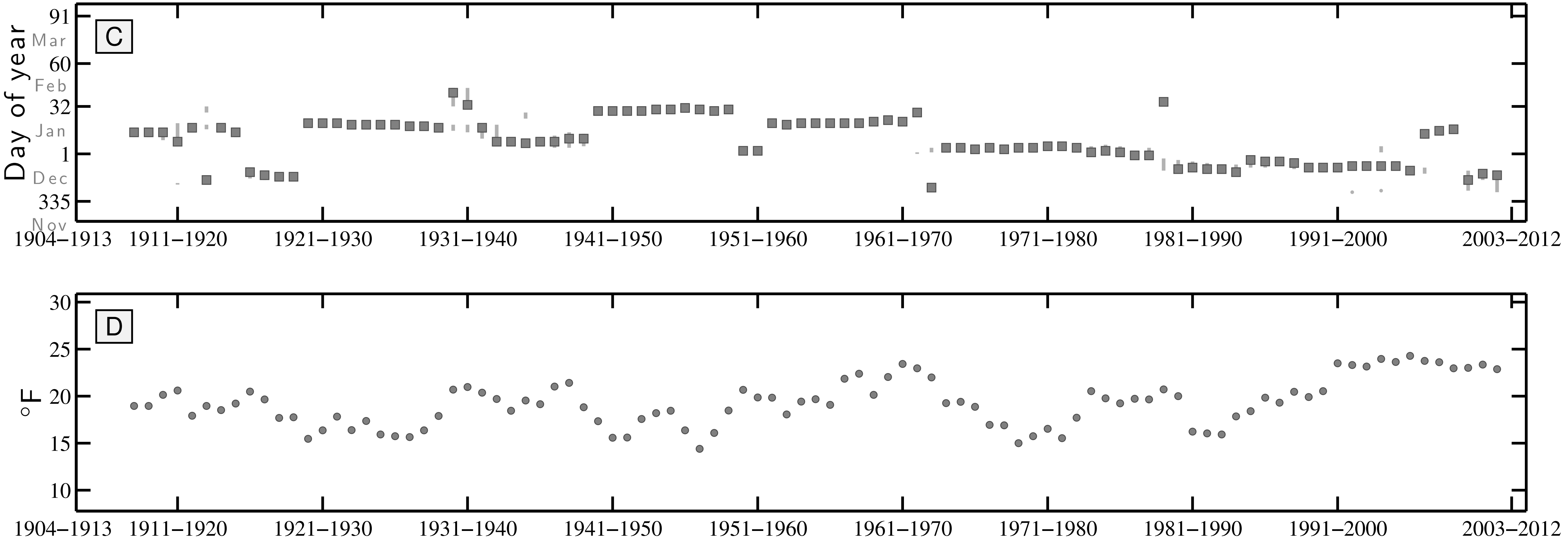

10 year Summer Teletherm dynamics for CORVALLIS STATE UNIV, OR:

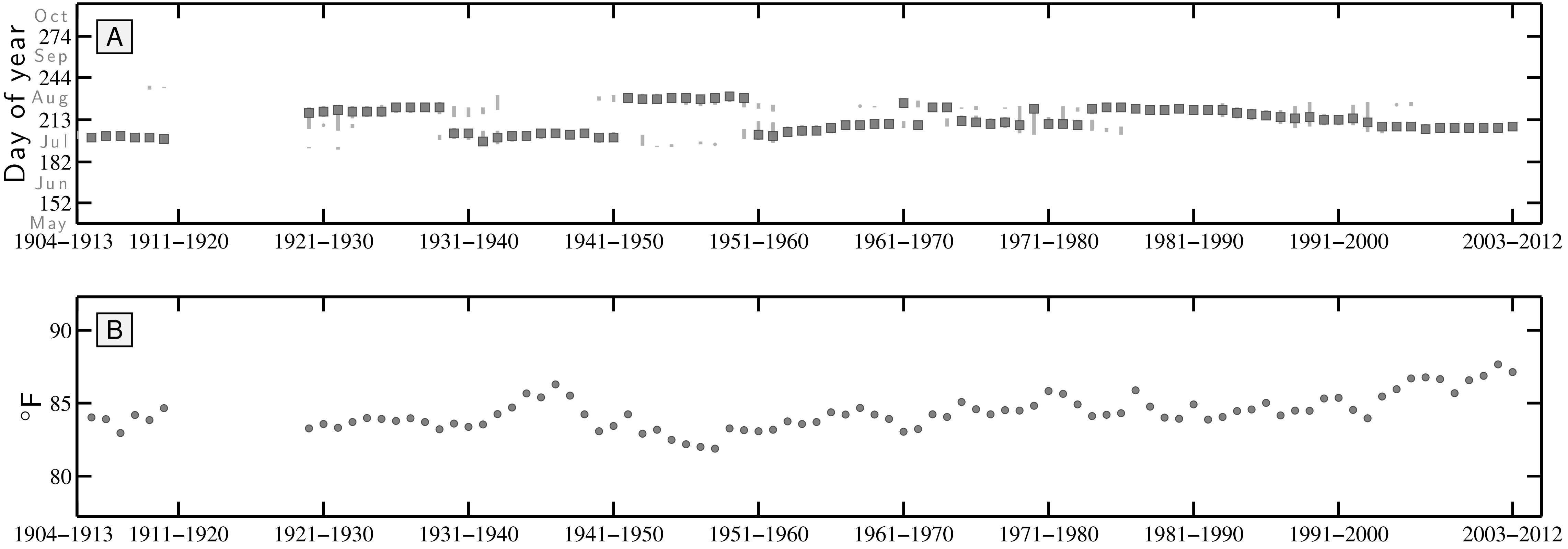

10 year Winter Teletherm dynamics for CORVALLIS STATE UNIV, OR:

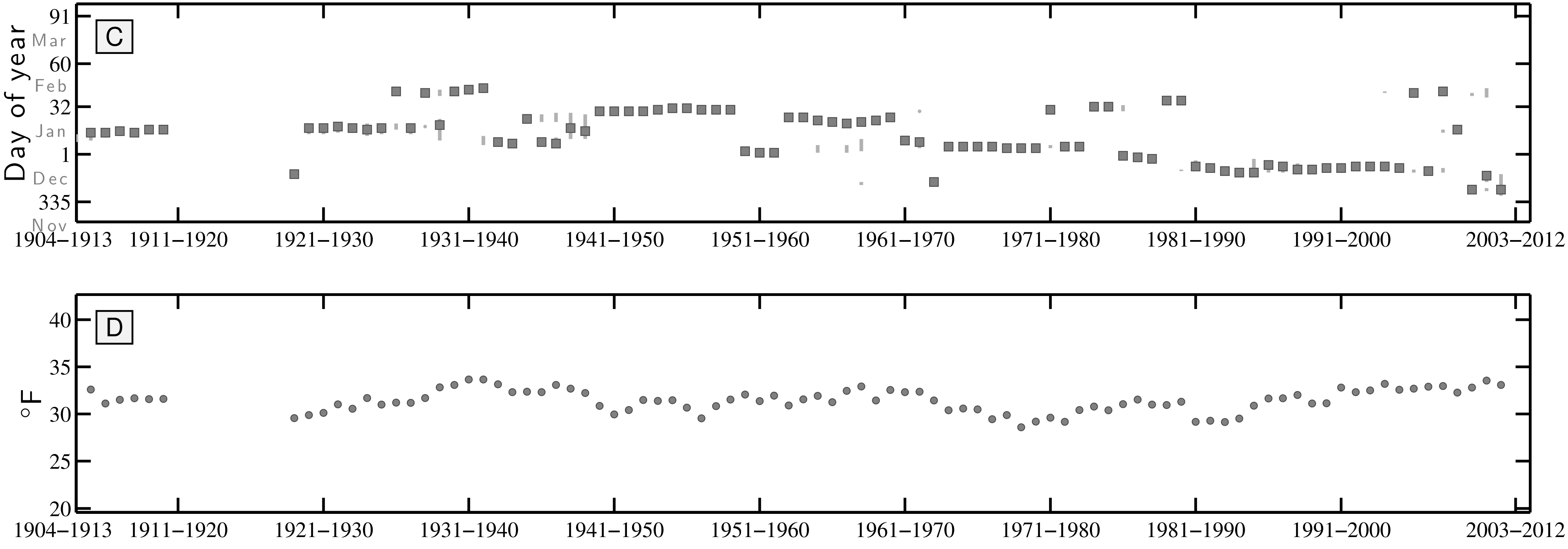

10 year Summer Teletherm dynamics for COTTAGE GROVE 1 NNE, OR:

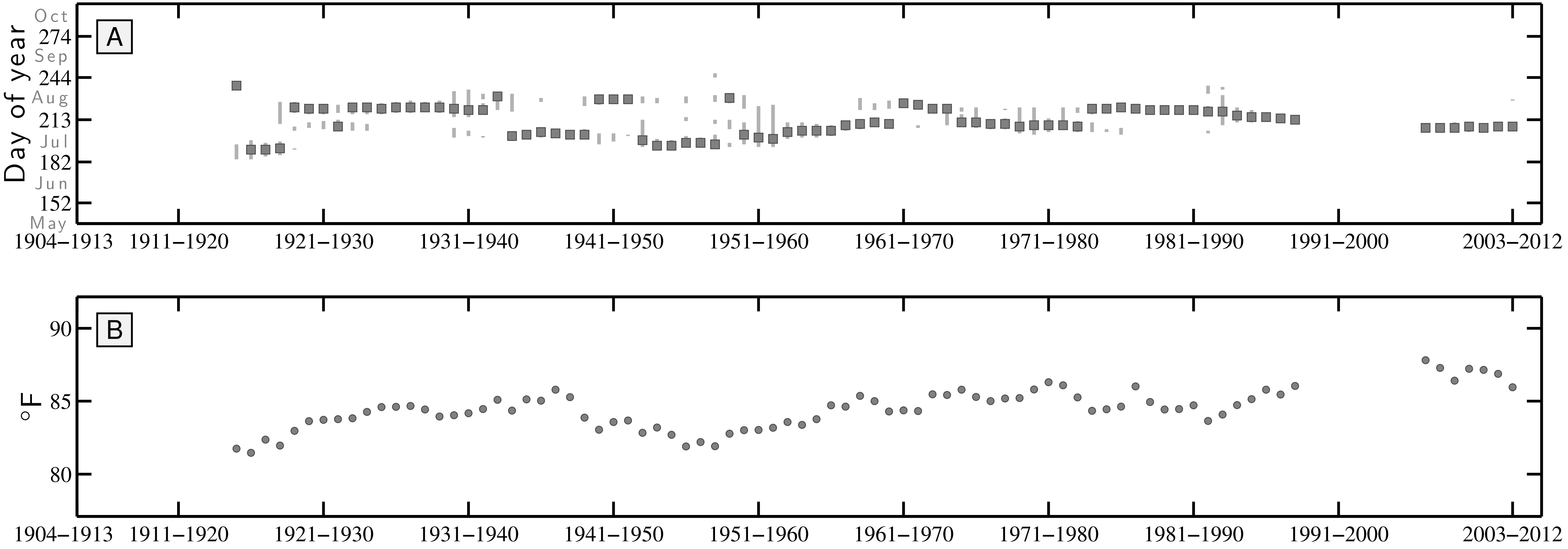

10 year Winter Teletherm dynamics for COTTAGE GROVE 1 NNE, OR:

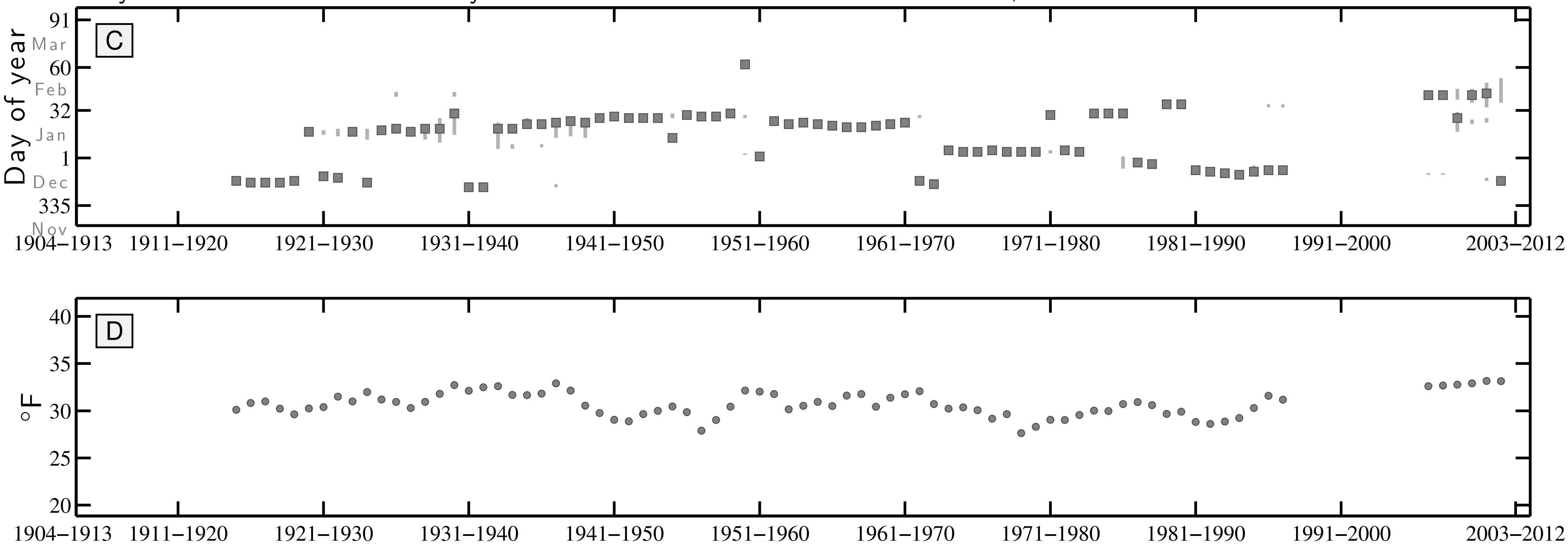

10 year Summer Teletherm dynamics for CRATER LAKE NPS HQ, OR:

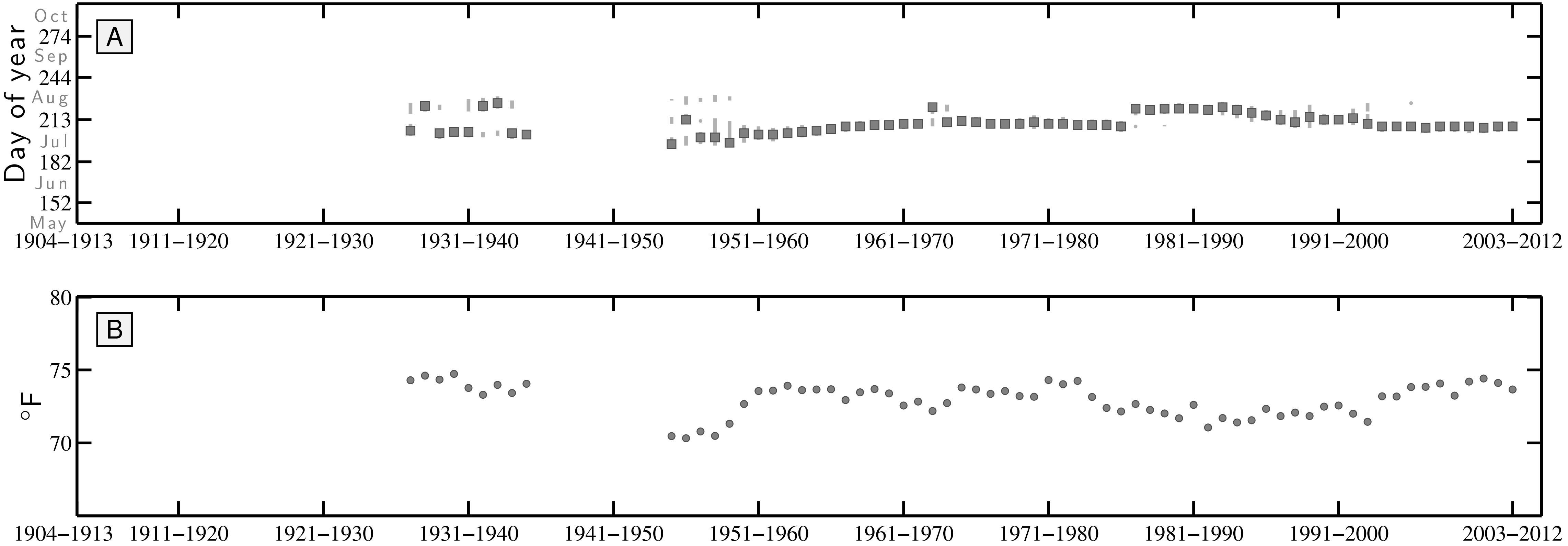

10 year Winter Teletherm dynamics for CRATER LAKE NPS HQ, OR:

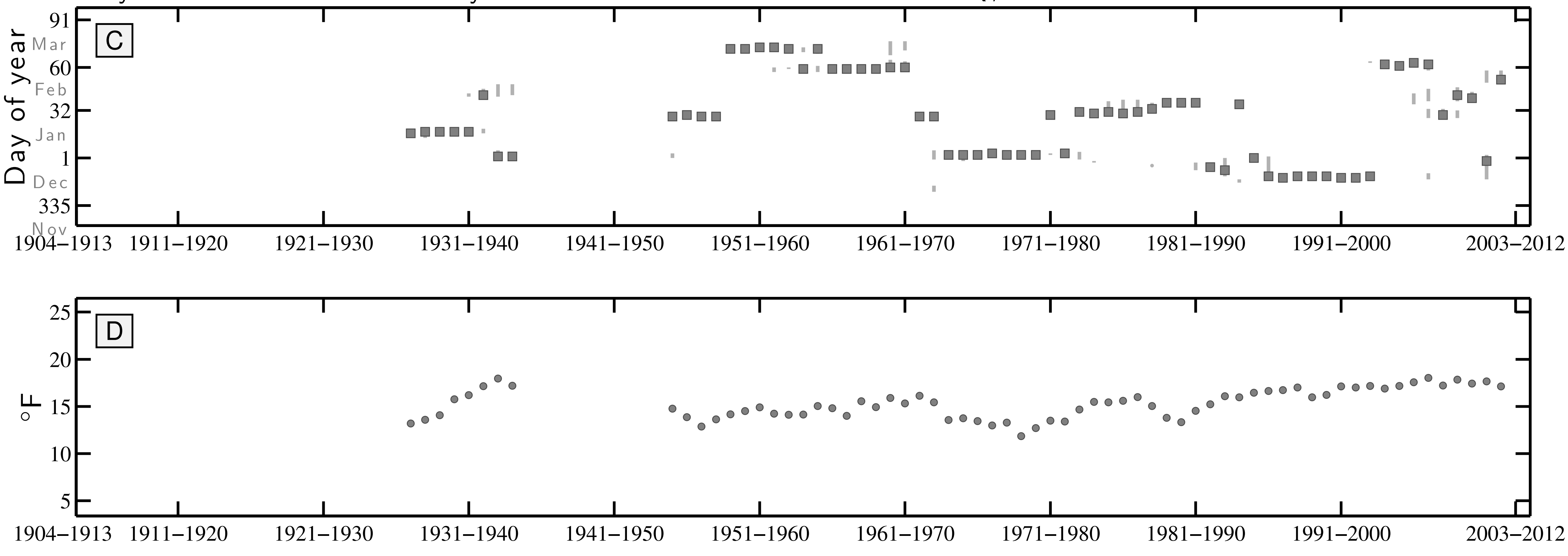

10 year Summer Teletherm dynamics for DANNER, OR:

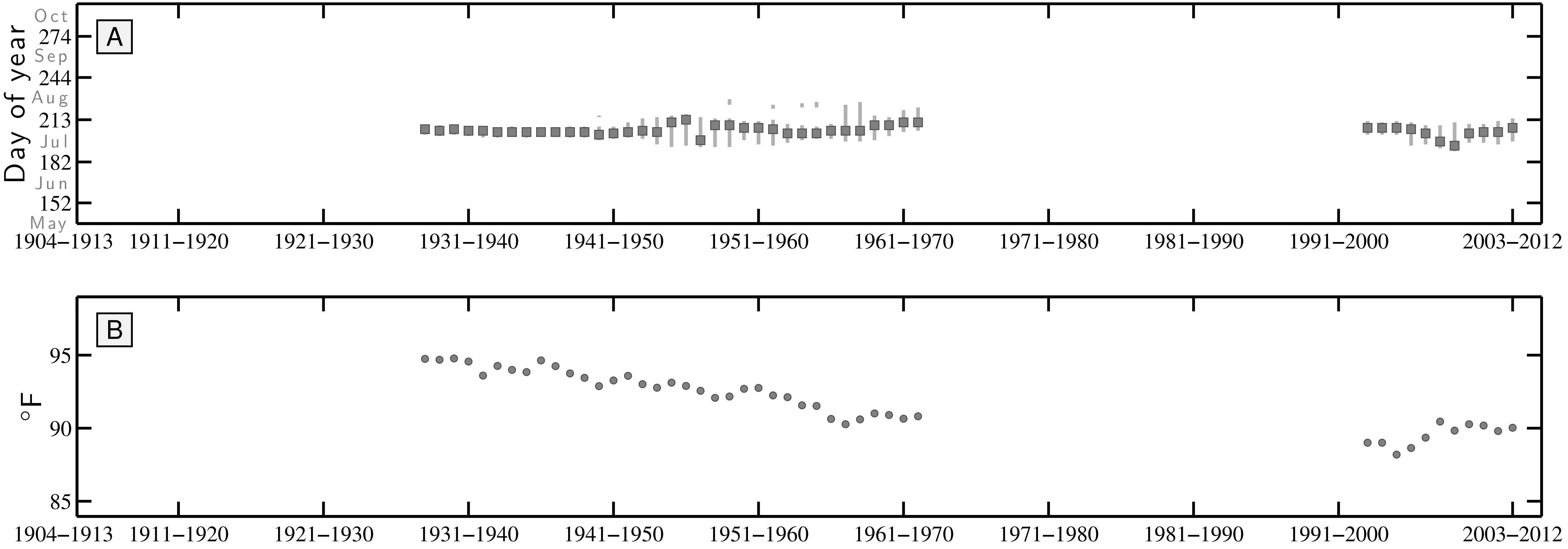

10 year Winter Teletherm dynamics for DANNER, OR:

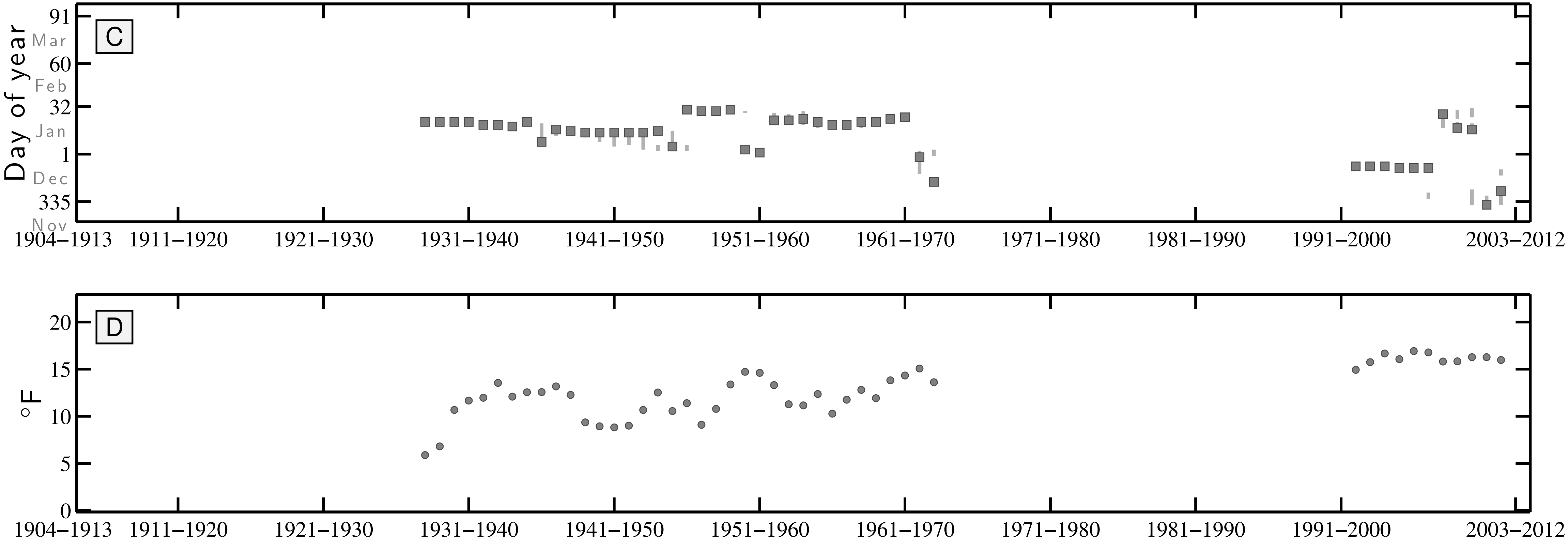

10 year Summer Teletherm dynamics for DRAIN, OR:

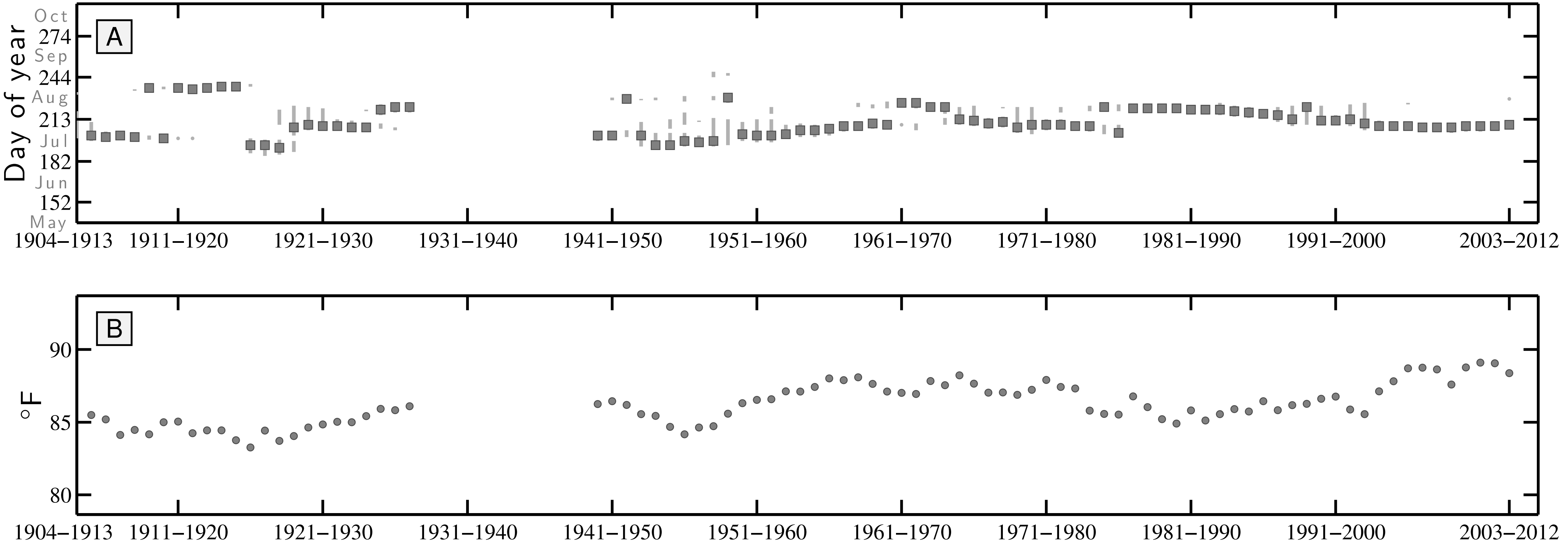

10 year Winter Teletherm dynamics for DRAIN, OR:

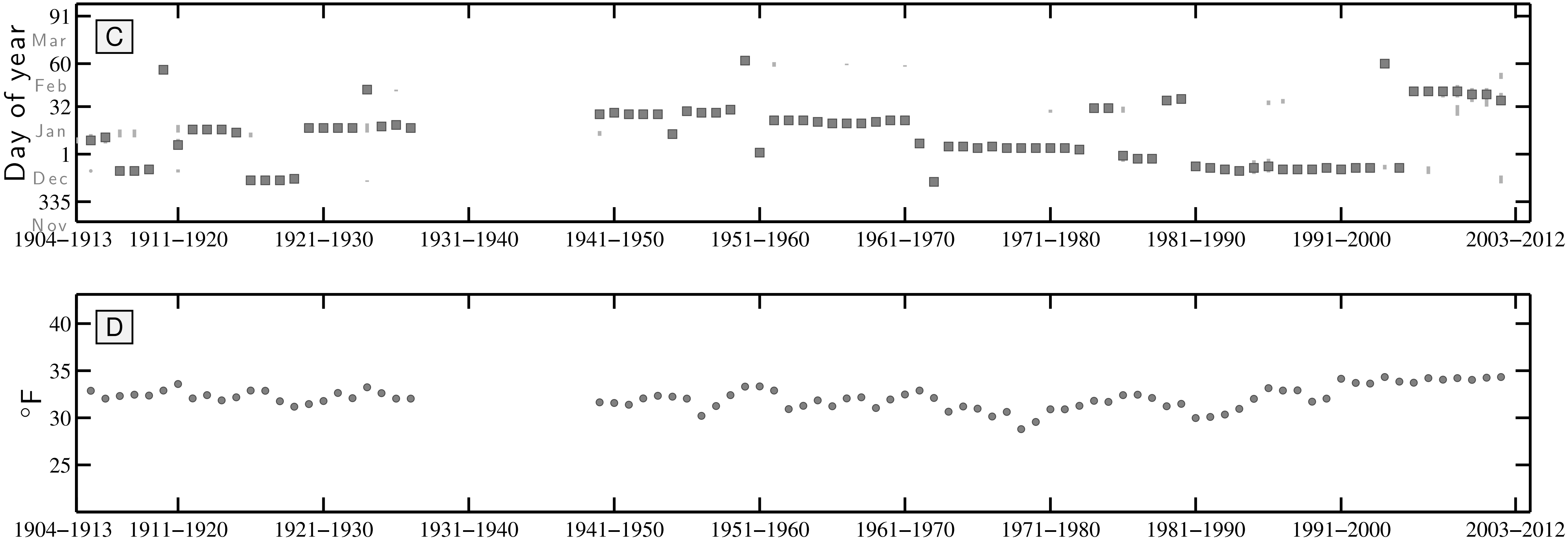

10 year Summer Teletherm dynamics for DUFUR, OR:

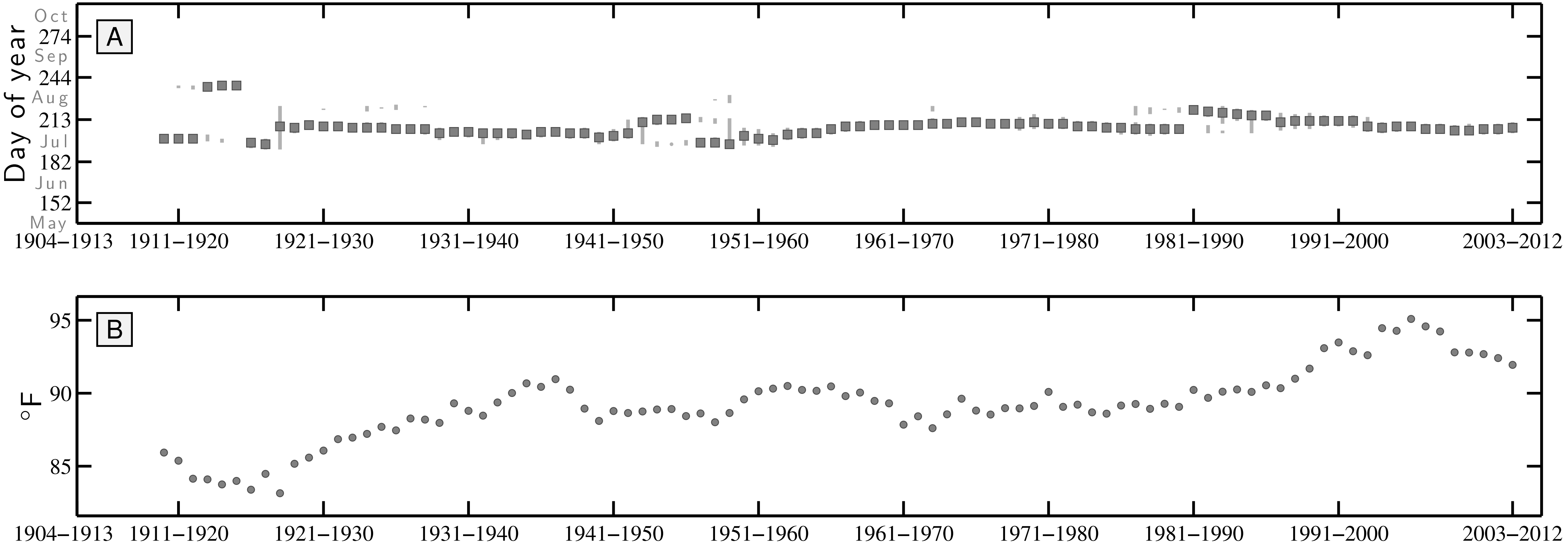

10 year Winter Teletherm dynamics for DUFUR, OR:

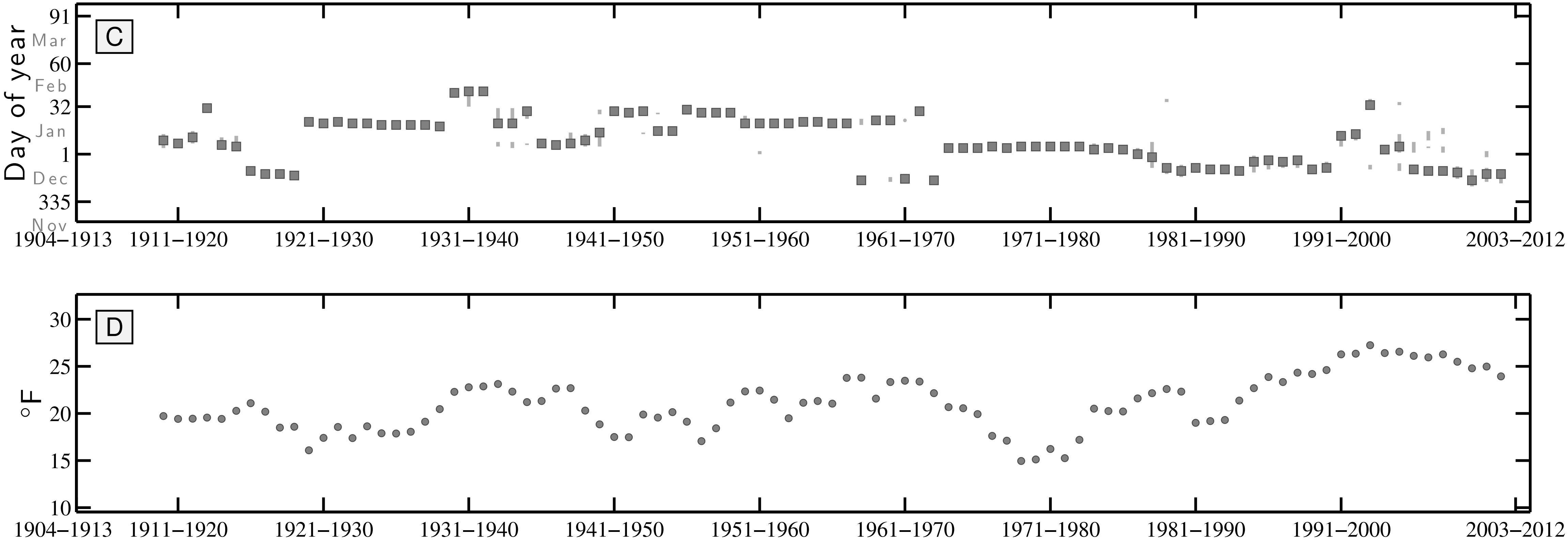

10 year Summer Teletherm dynamics for FOREST GROVE, OR:

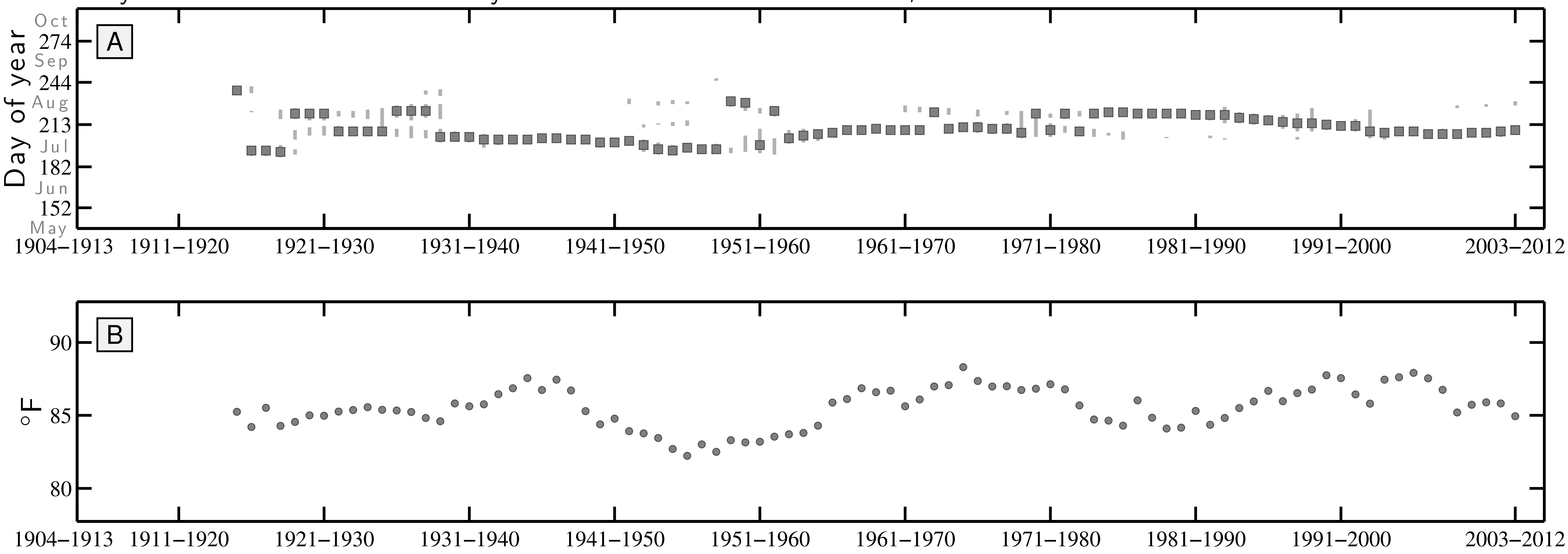

10 year Winter Teletherm dynamics for FOREST GROVE, OR:

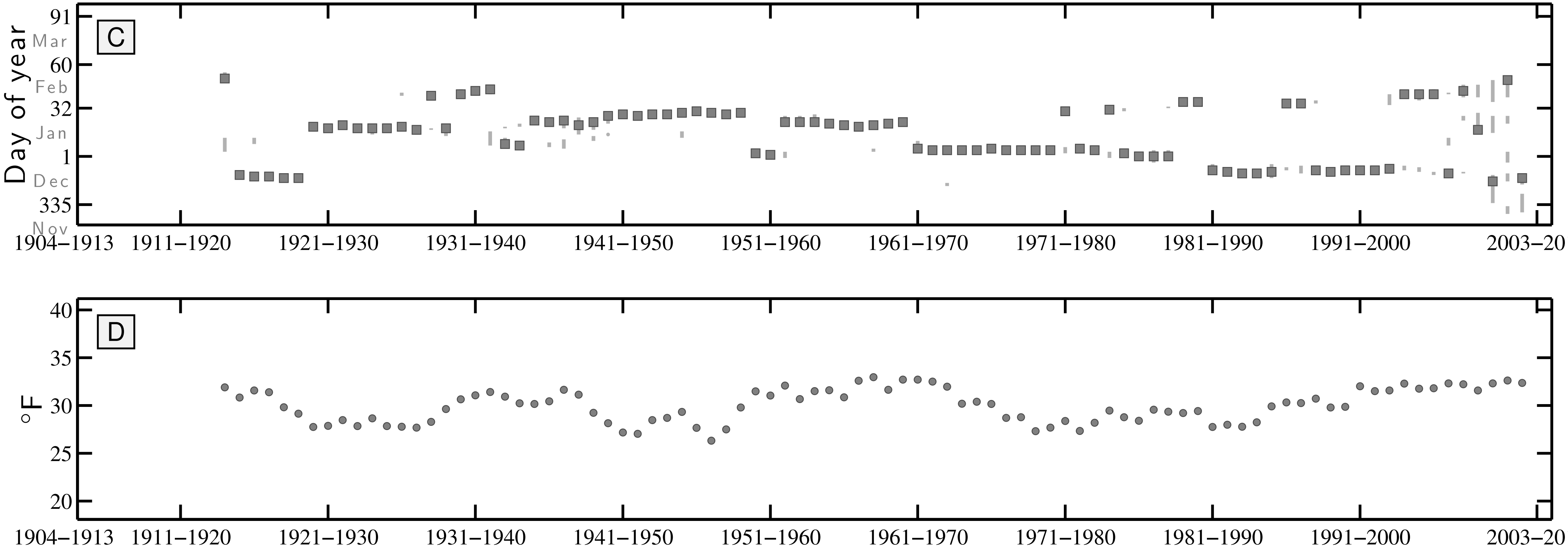

10 year Summer Teletherm dynamics for FREMONT 5 NW, OR:

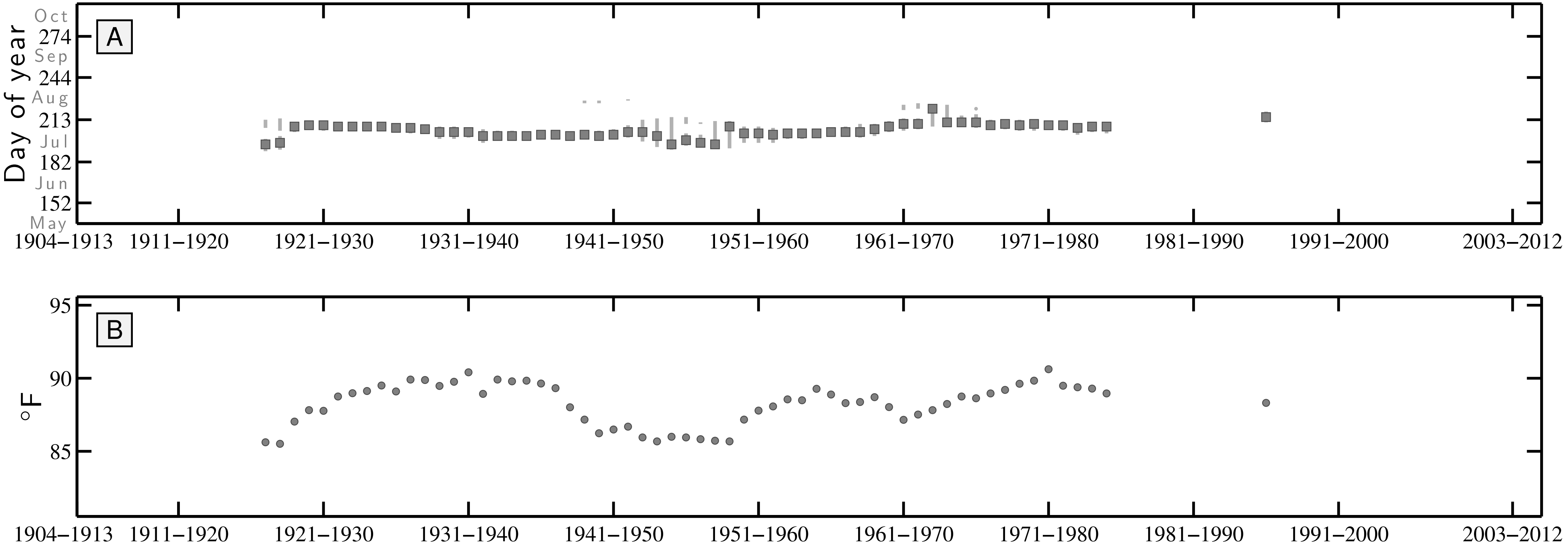

10 year Winter Teletherm dynamics for FREMONT 5 NW, OR:

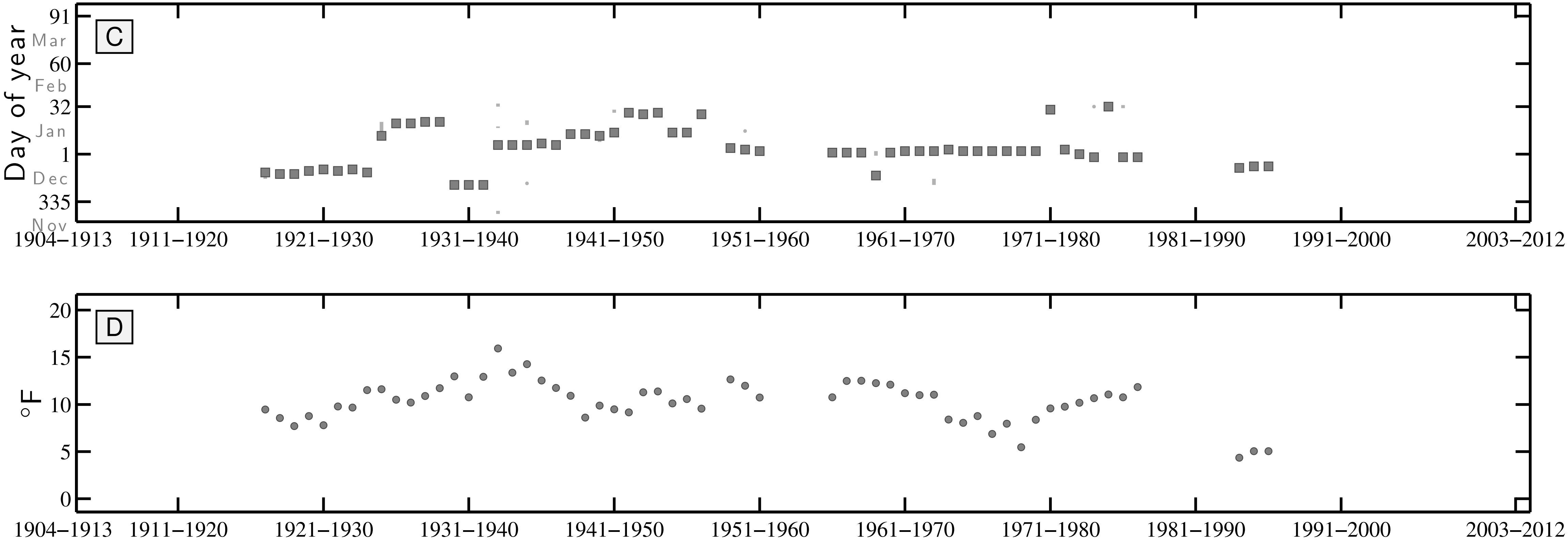

10 year Summer Teletherm dynamics for GRANTS PASS, OR:

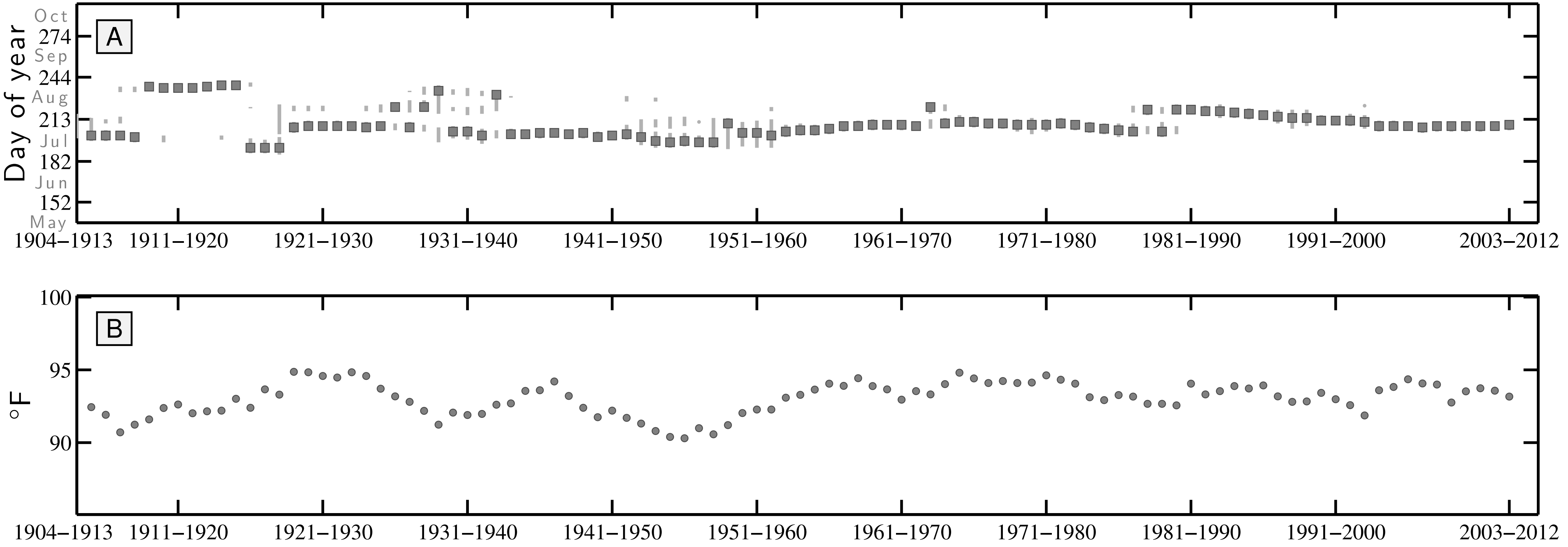

10 year Winter Teletherm dynamics for GRANTS PASS, OR:

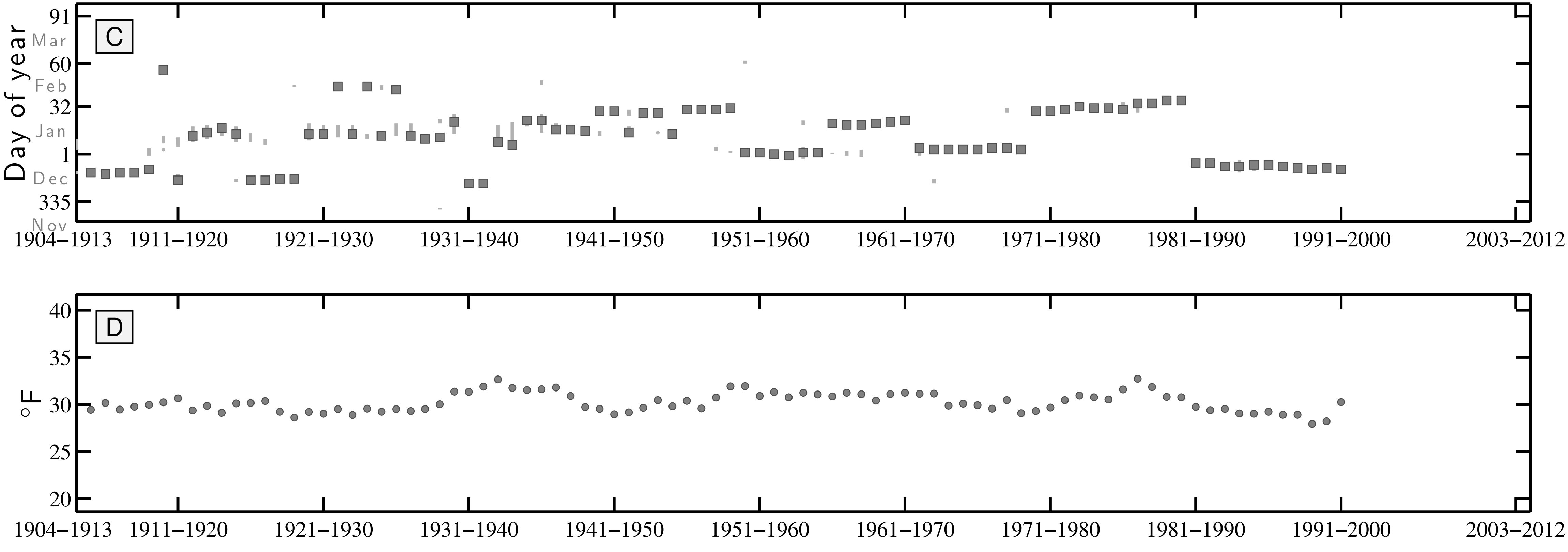

10 year Summer Teletherm dynamics for HEADWORKS PORTLAND WTR, OR:

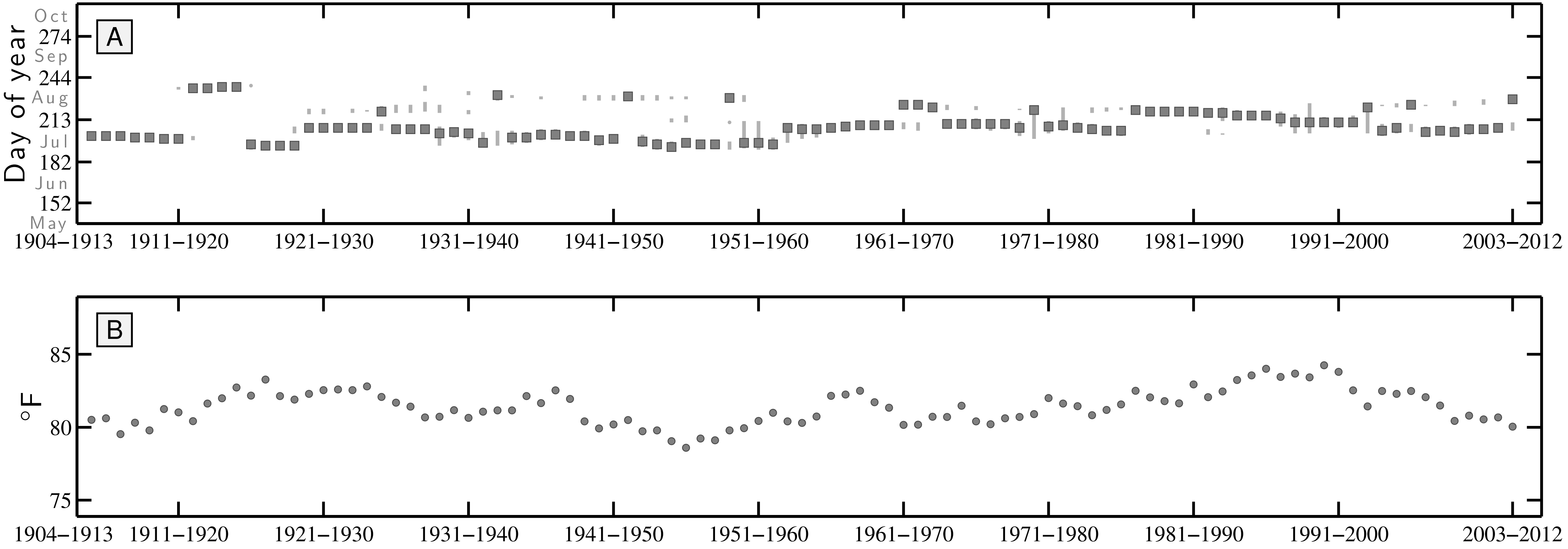

10 year Winter Teletherm dynamics for HEADWORKS PORTLAND WTR, OR:

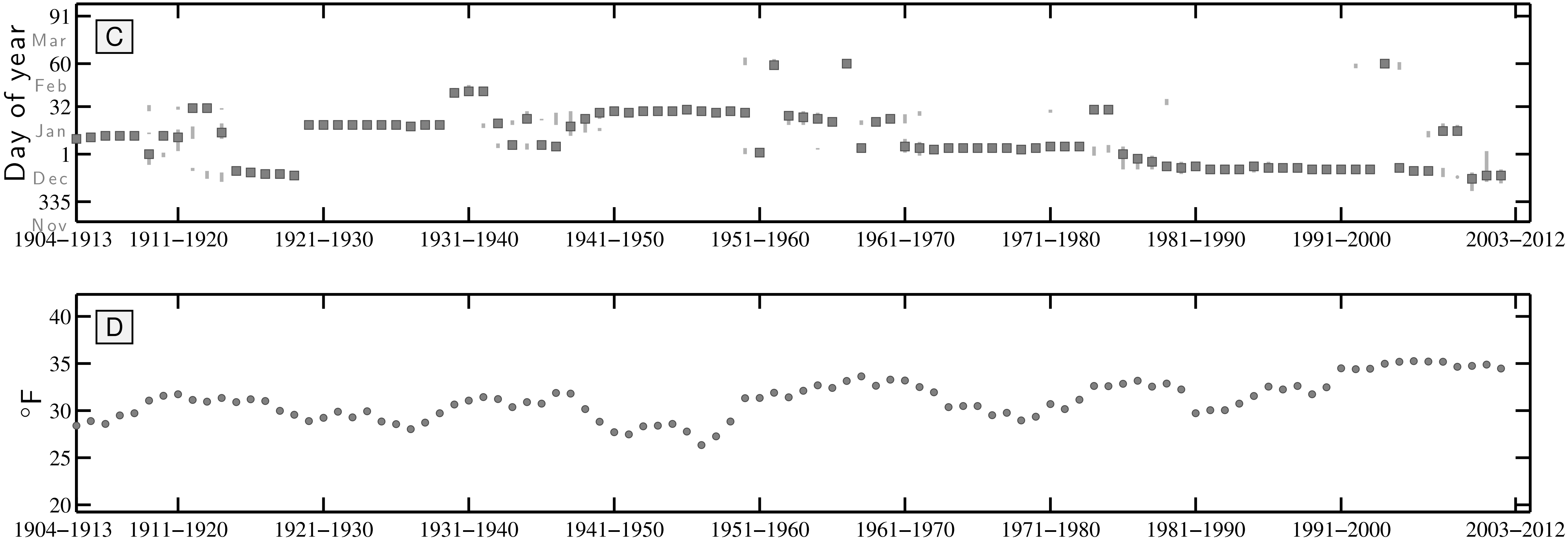

10 year Summer Teletherm dynamics for HEPPNER, OR:

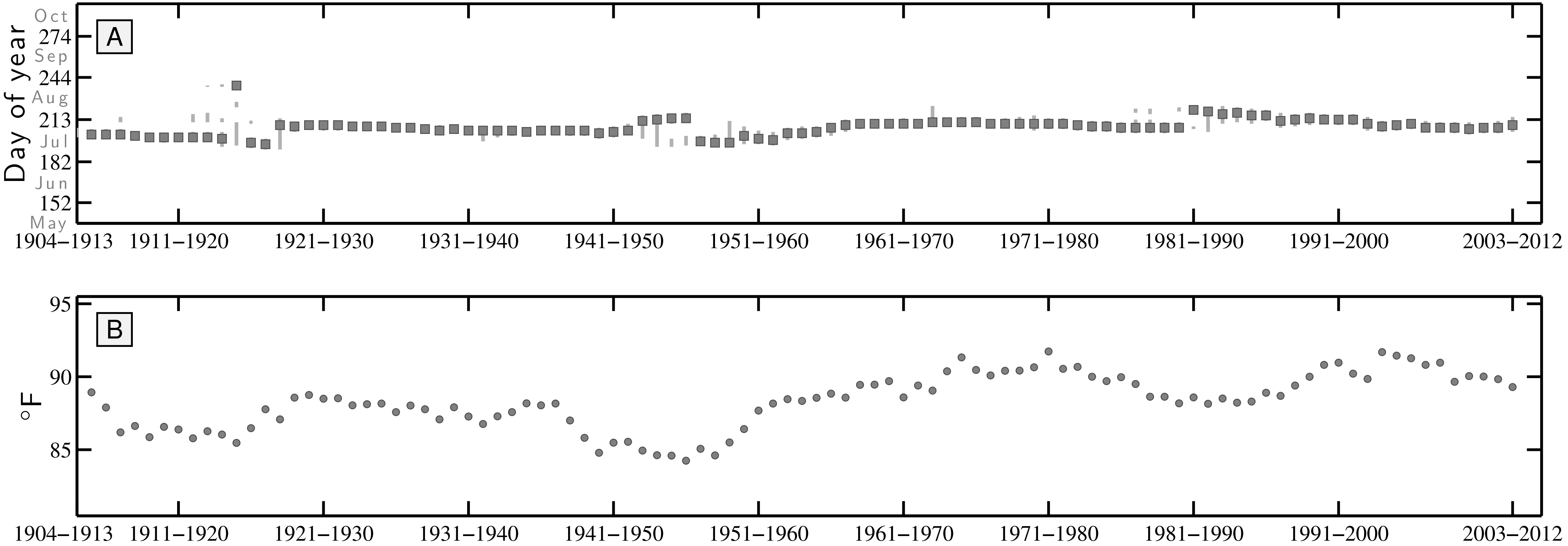

10 year Winter Teletherm dynamics for HEPPNER, OR:

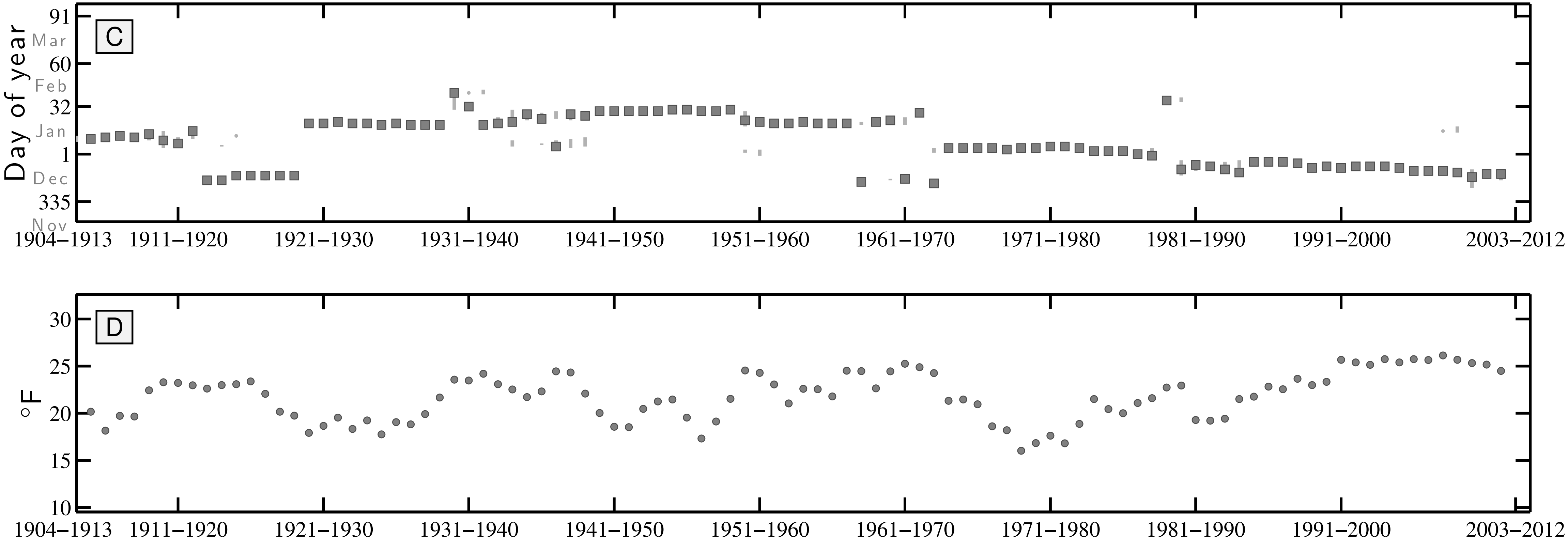

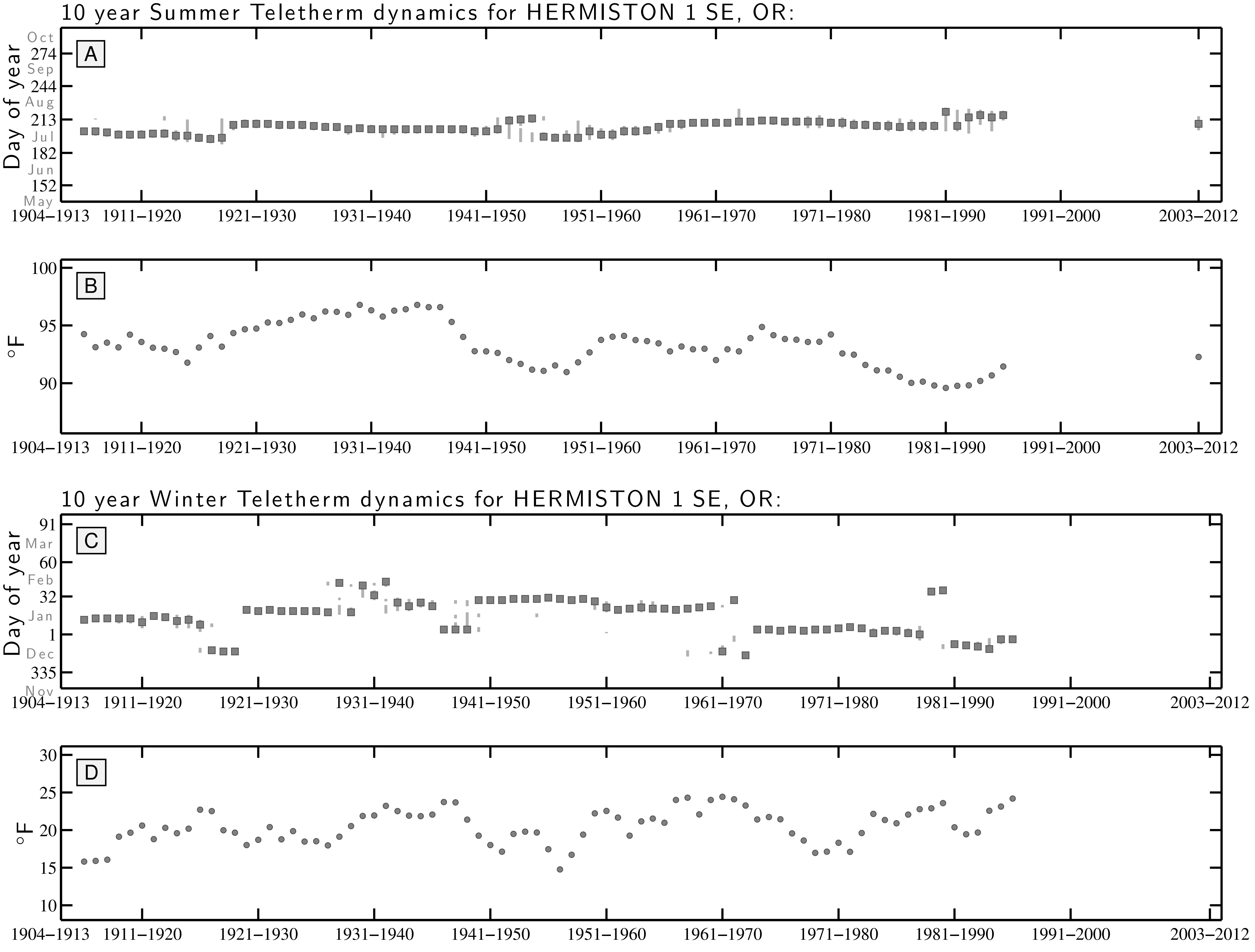
10 year Summer Teletherm dynamics for HERMISTON 1 SE, OR:
A
B
Day of year
°F
Oct
274
Sep
244
Aug
213
Jul
182
Jun
152
May
100
95
90
1904–1913
1911–1920
1921–1930
1931–1940
1941–1950
1951–1960
1961–1970
1971–1980
1981–1990
1991–2000
2003–2012
10 year Winter Teletherm dynamics for HERMISTON 1 SE, OR:
C
D
91
Mar
60
Feb
32
Jan
1
Dec
335
Nov
30
25
20
15
10

10 year Summer Teletherm dynamics for HOOD RIVER EXP STN, OR:

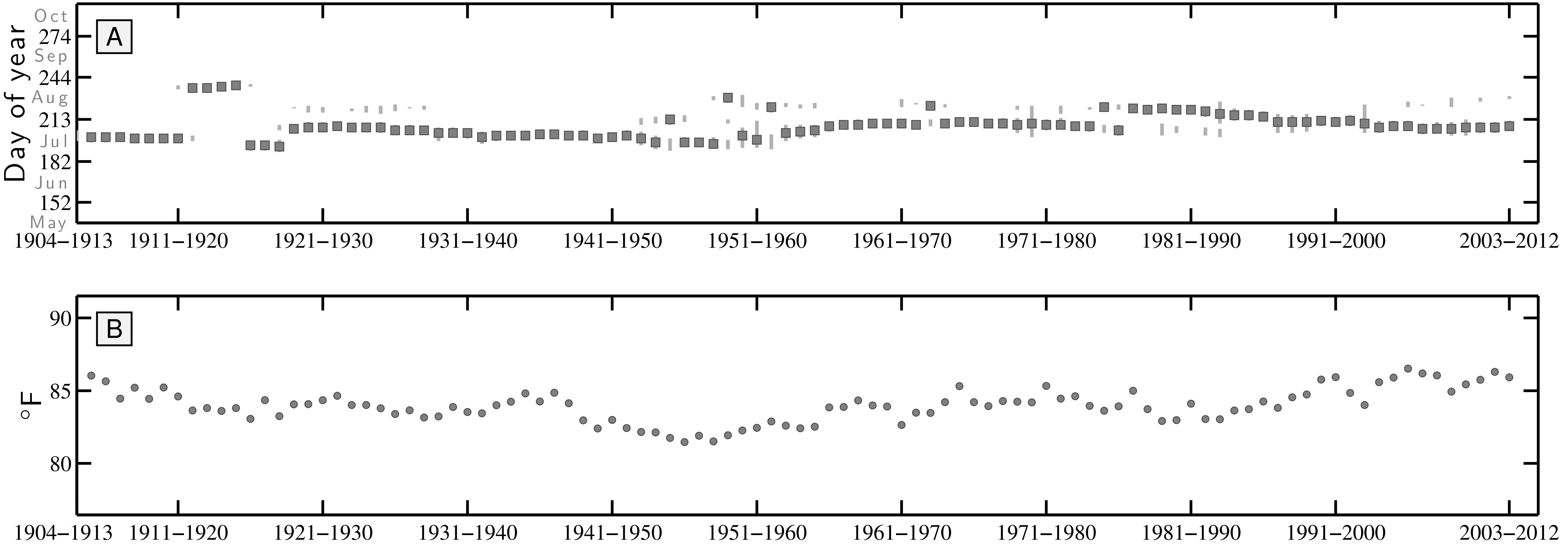

10 year Winter Teletherm dynamics for HOOD RIVER EXP STN, OR:

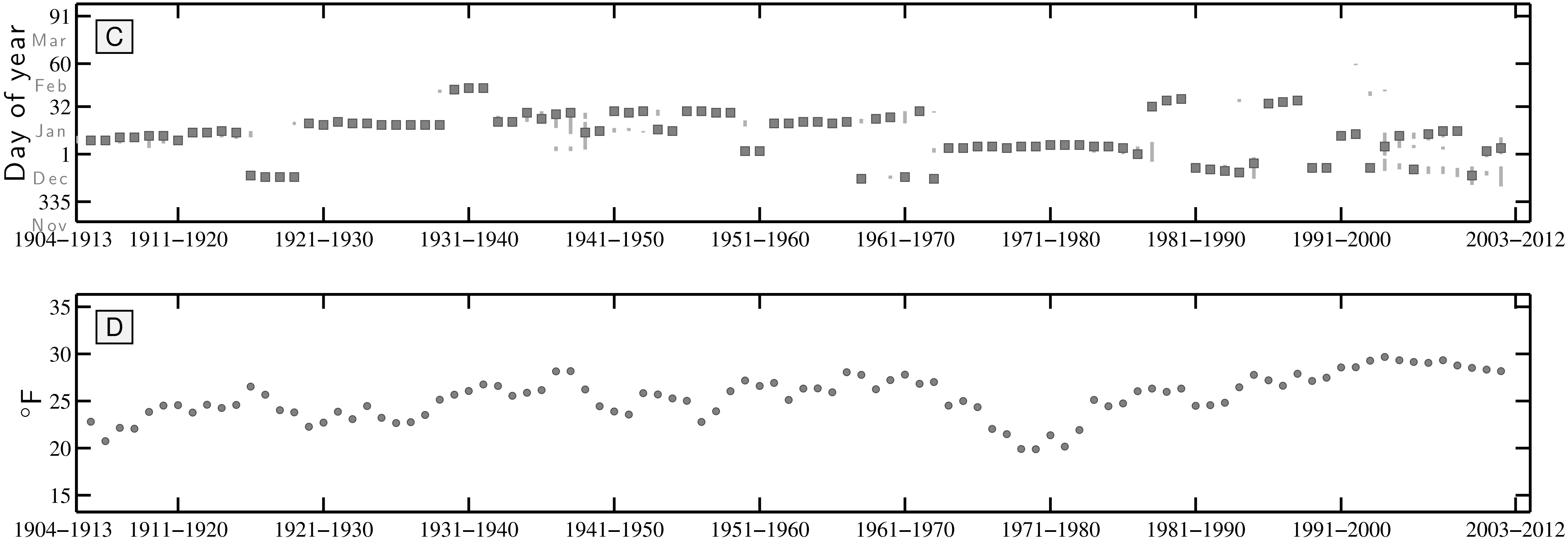

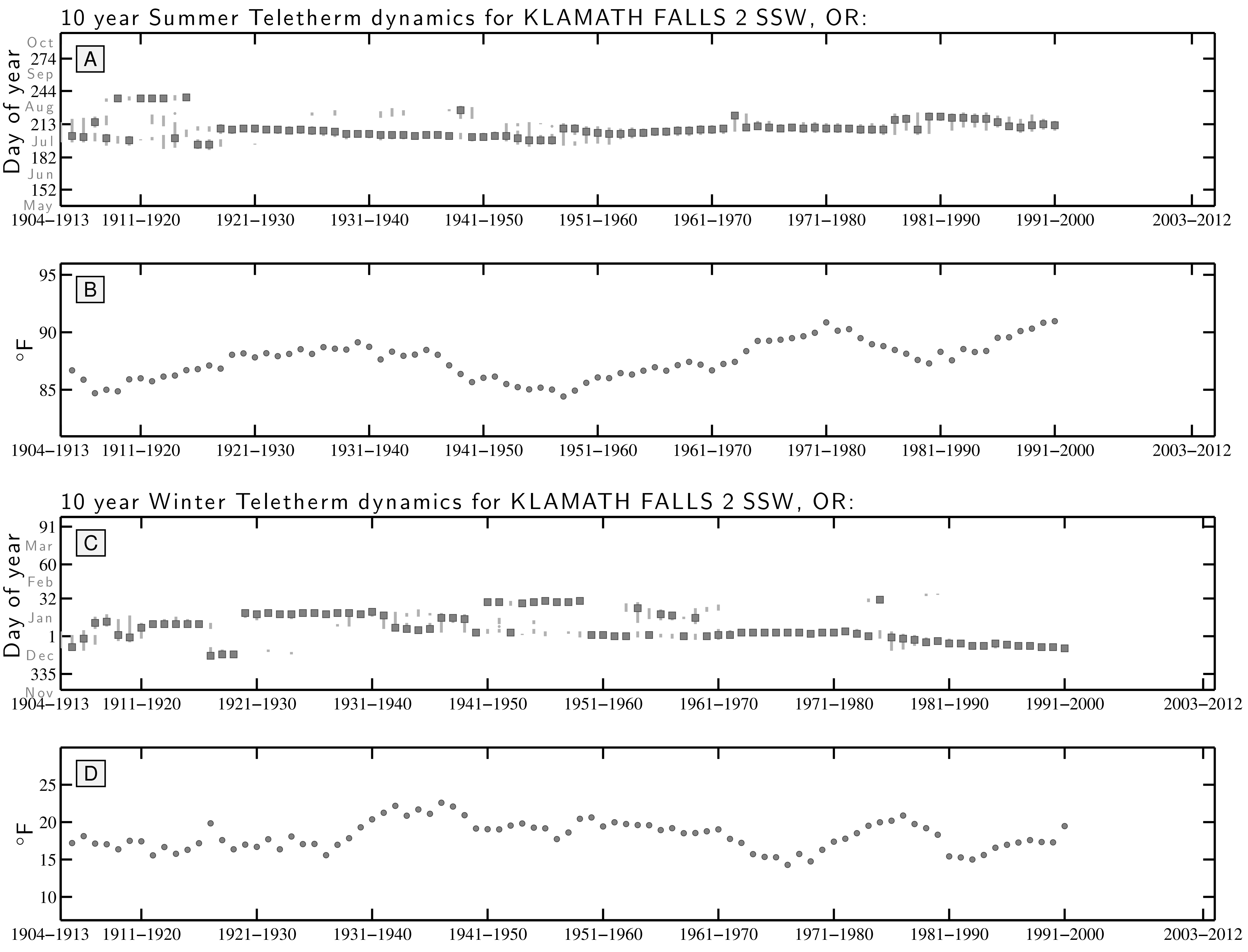

10 year Summer Teletherm dynamics for KLAMATH FALLS 2 SSW, OR:
A
B
Day of year
°F
10 year Winter Teletherm dynamics for KLAMATH FALLS 2 SSW, OR:
C
D
1904–1913
1911–1920
1921–1930
1931–1940
1941–1950
1951–1960
1961–1970
1971–1980
1981–1990
1991–2000
2003–2012

10 year Summer Teletherm dynamics for LAKEVIEW 2 NNW, OR:

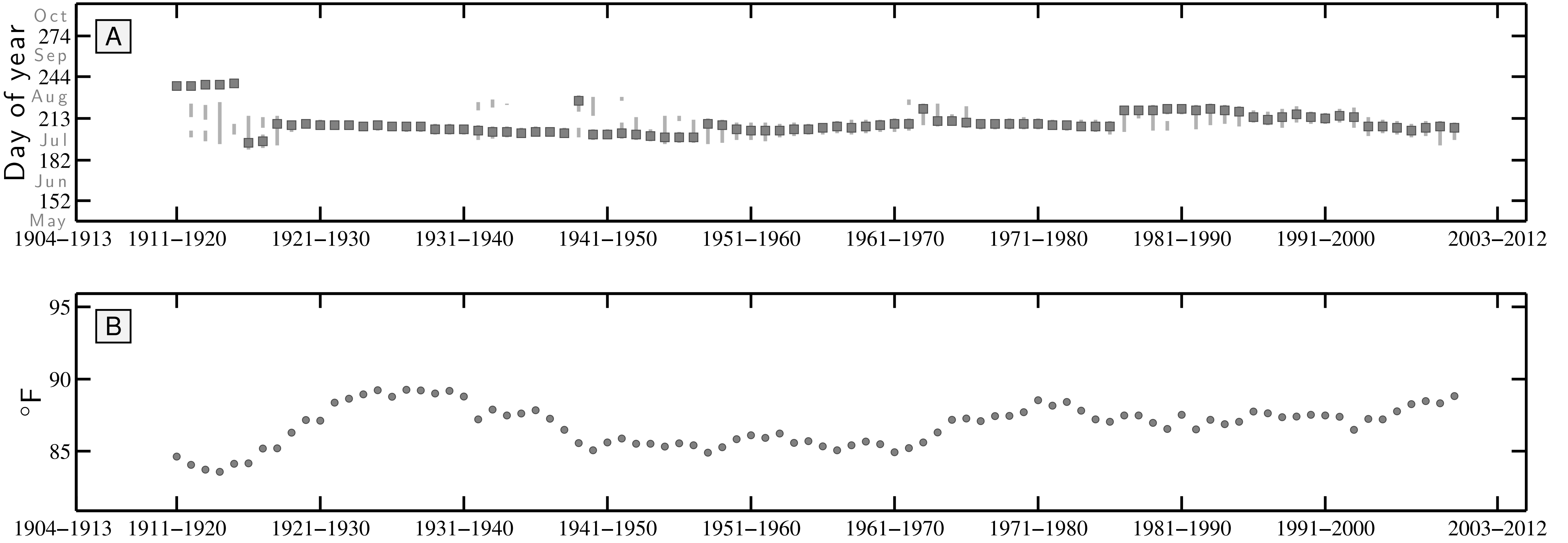

10 year Winter Teletherm dynamics for LAKEVIEW 2 NNW, OR:

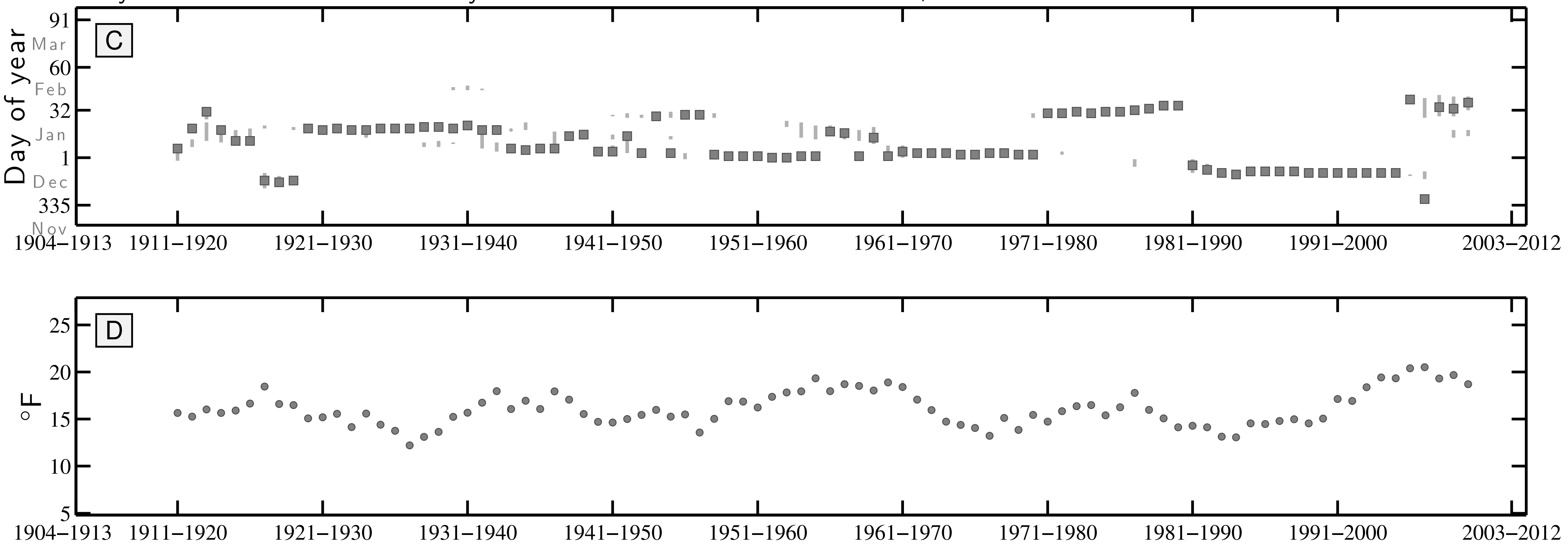

10 year Summer Teletherm dynamics for MALHEUR REFUGE HQ, OR:

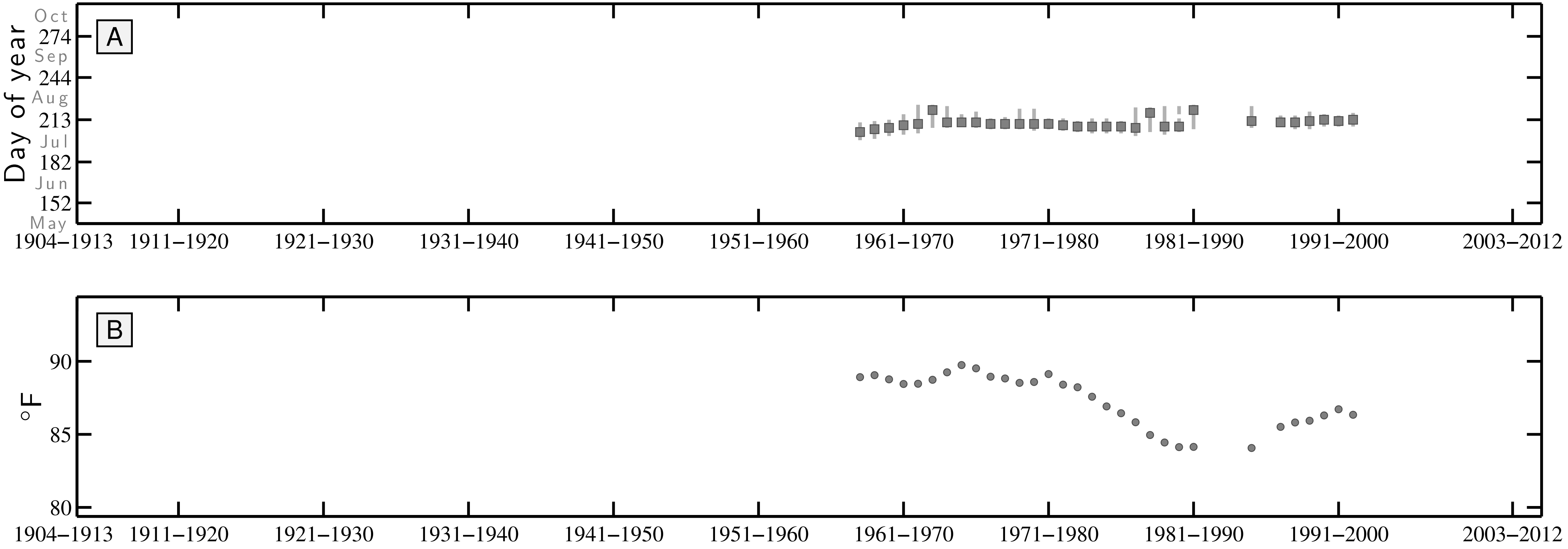

10 year Winter Teletherm dynamics for MALHEUR REFUGE HQ, OR:

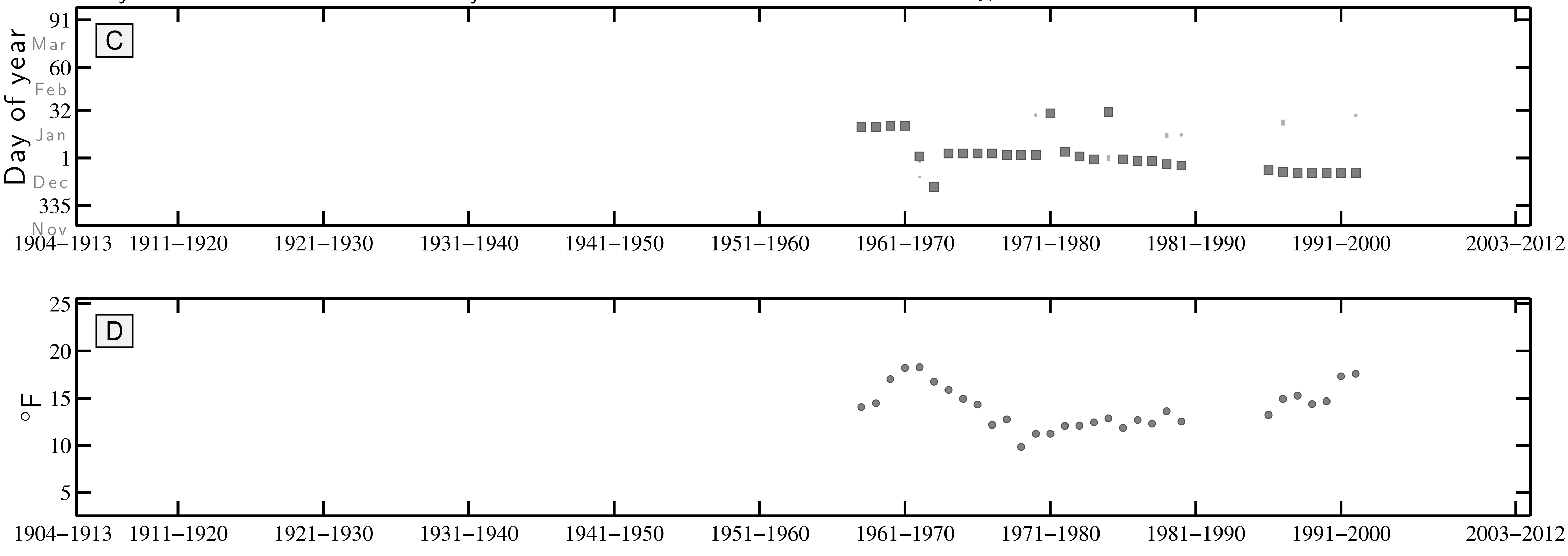

10 year Summer Teletherm dynamics for MCKENZIE BRG RS, OR:

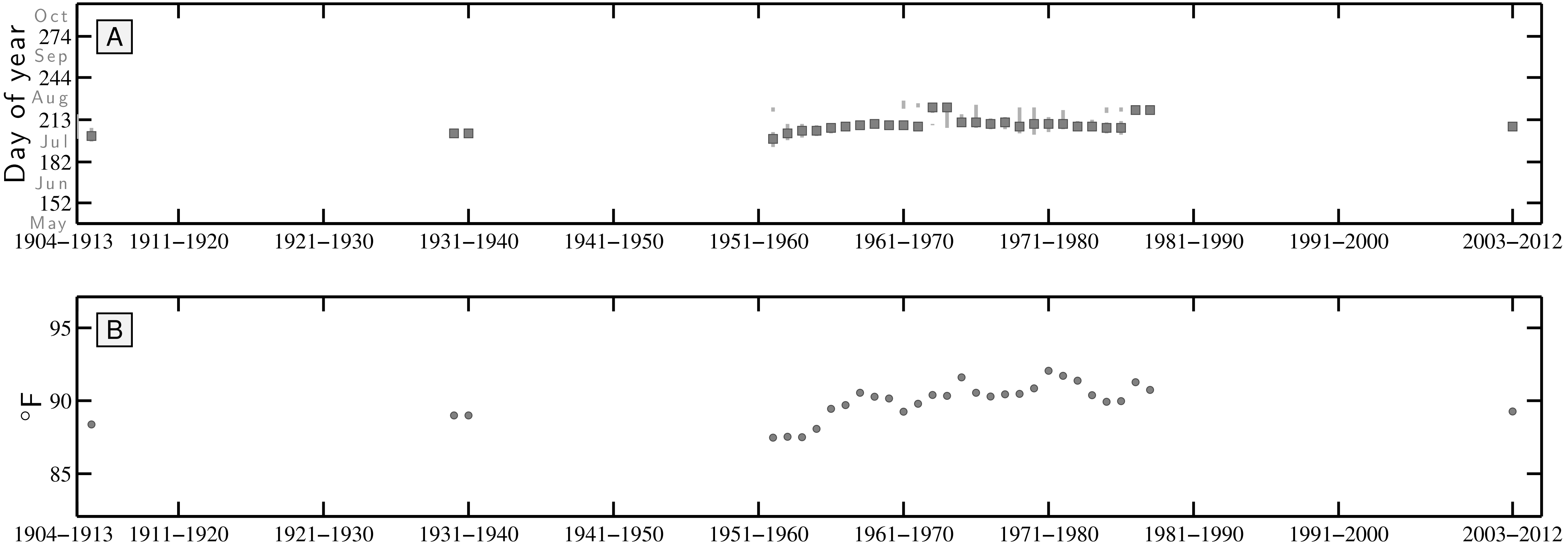

10 year Winter Teletherm dynamics for MCKENZIE BRG RS, OR:

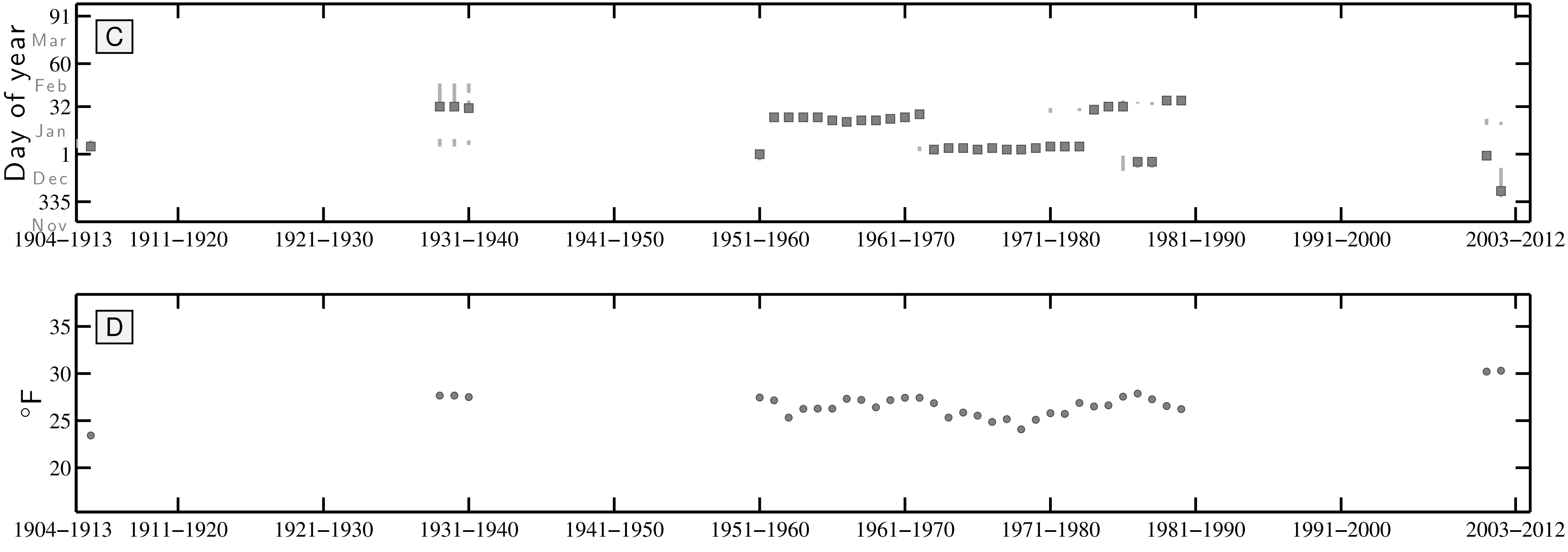

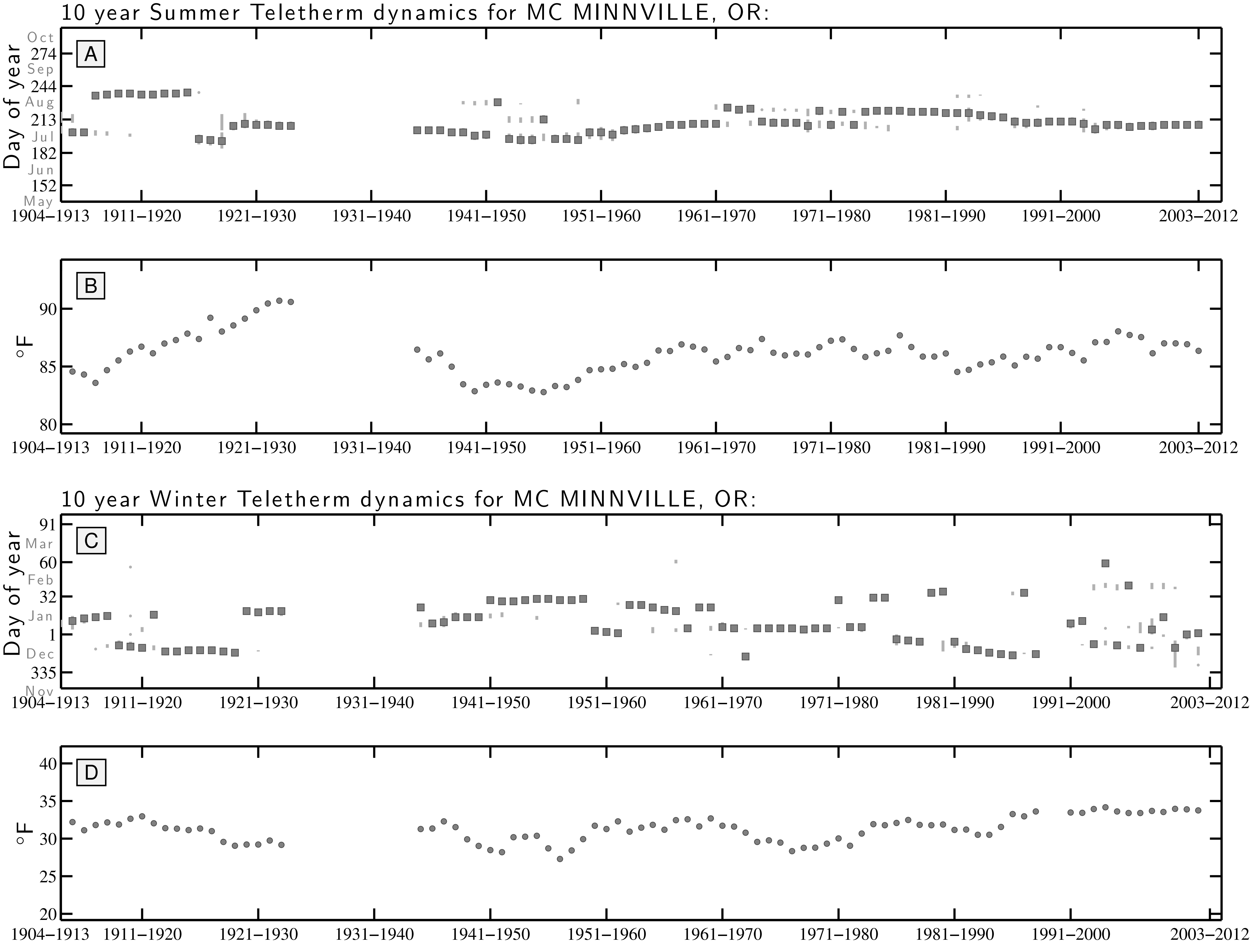
10 year Summer Teletherm dynamics for MC MINNVILLE, OR:
A
Day of year
Oct
274
Sep
244
Aug
213
Jul
182
Jun
152
May
B
°F
90
85
80
10 year Winter Teletherm dynamics for MC MINNVILLE, OR:
C
Day of year
91
Mar
60
Feb
32
Jan
1
Dec
335
Nov
D
°F
40
35
30
25
20
1904–1913
1911–1920
1921–1930
1931–1940
1941–1950
1951–1960
1961–1970
1971–1980
1981–1990
1991–2000
2003–2012

10 year Summer Teletherm dynamics for MILTON FREEWATER, OR:

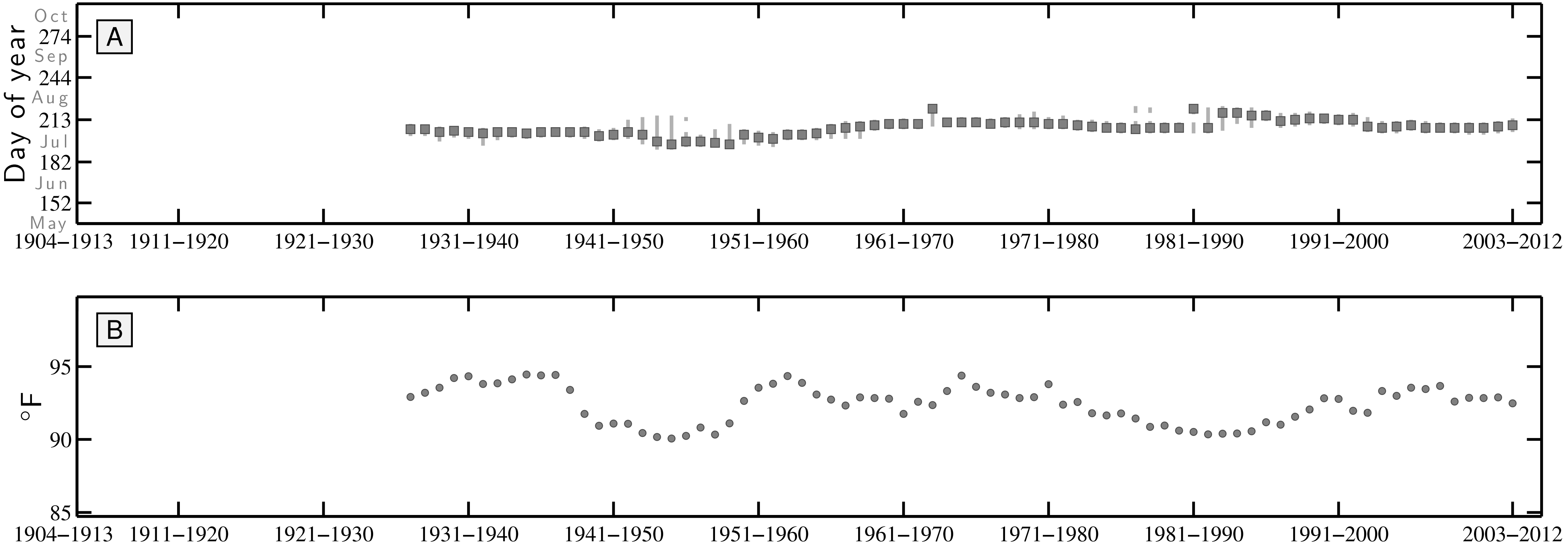

10 year Winter Teletherm dynamics for MILTON FREEWATER, OR:

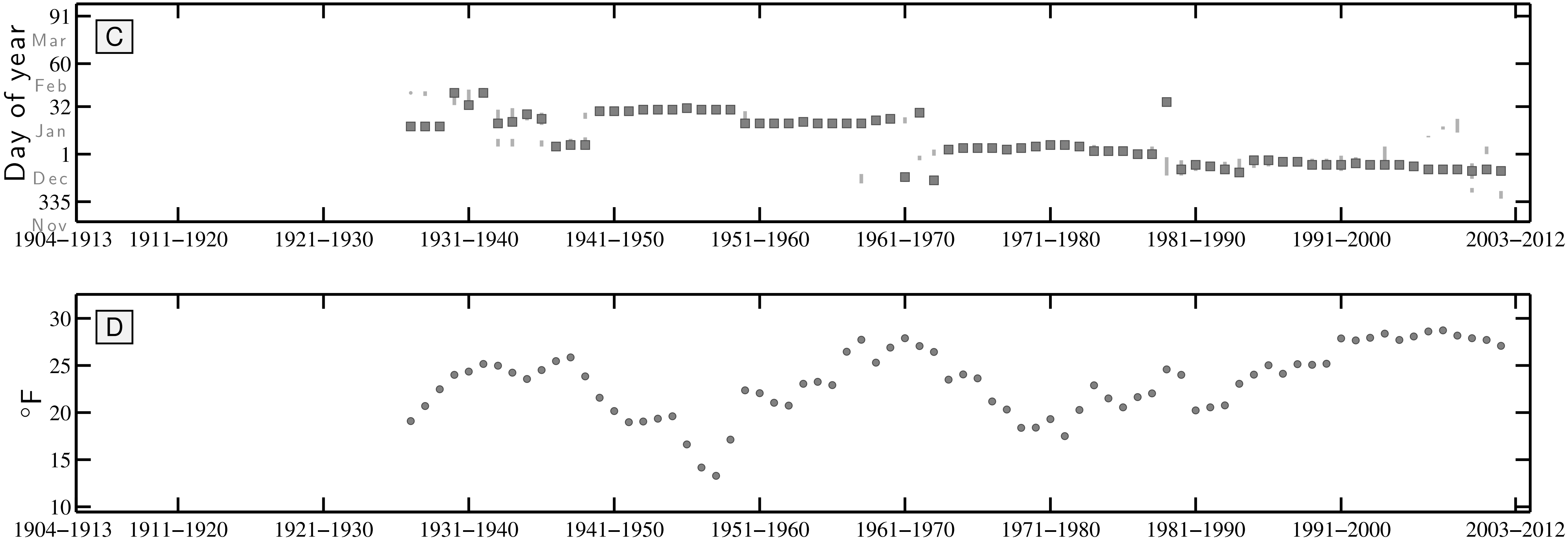

10 year Summer Teletherm dynamics for MORO, OR:

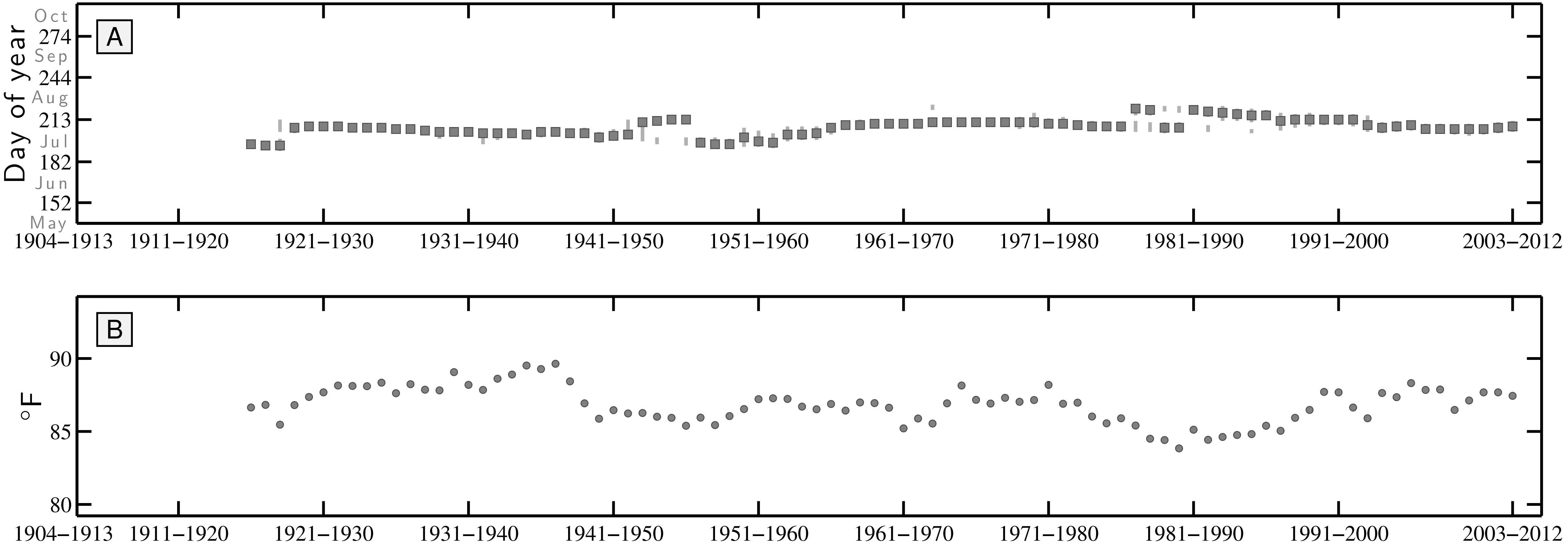

10 year Winter Teletherm dynamics for MORO, OR:

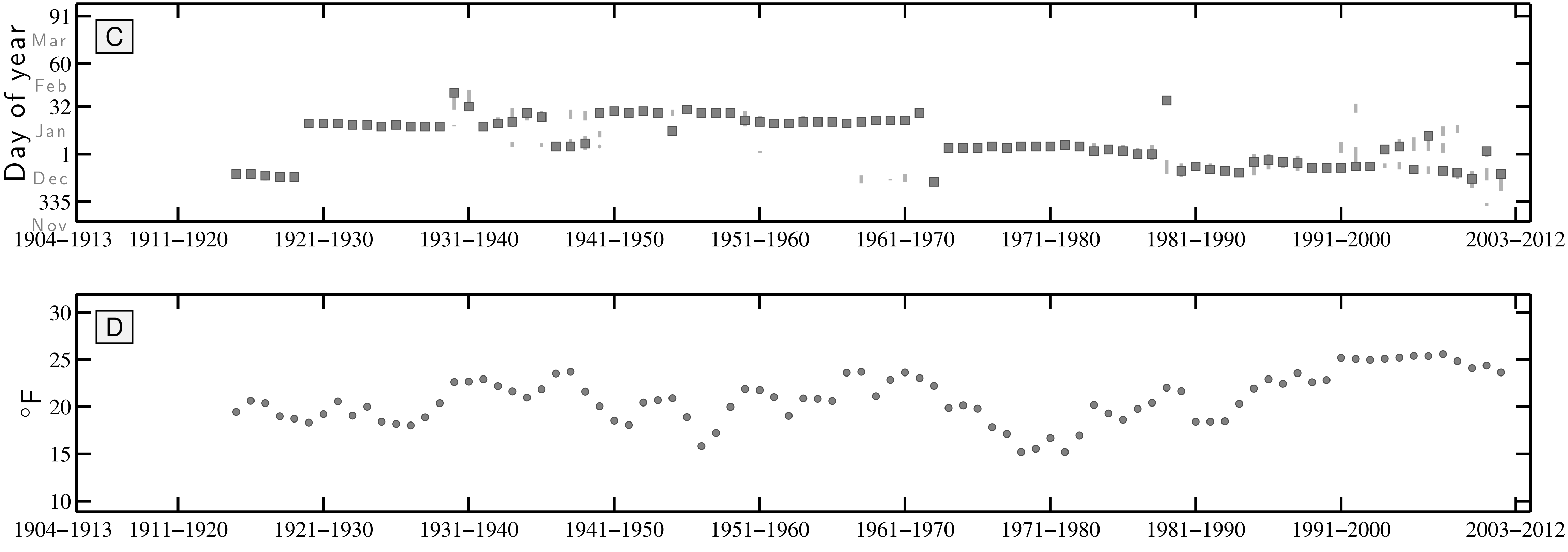

10 year Summer Teletherm dynamics for NEWPORT, OR:

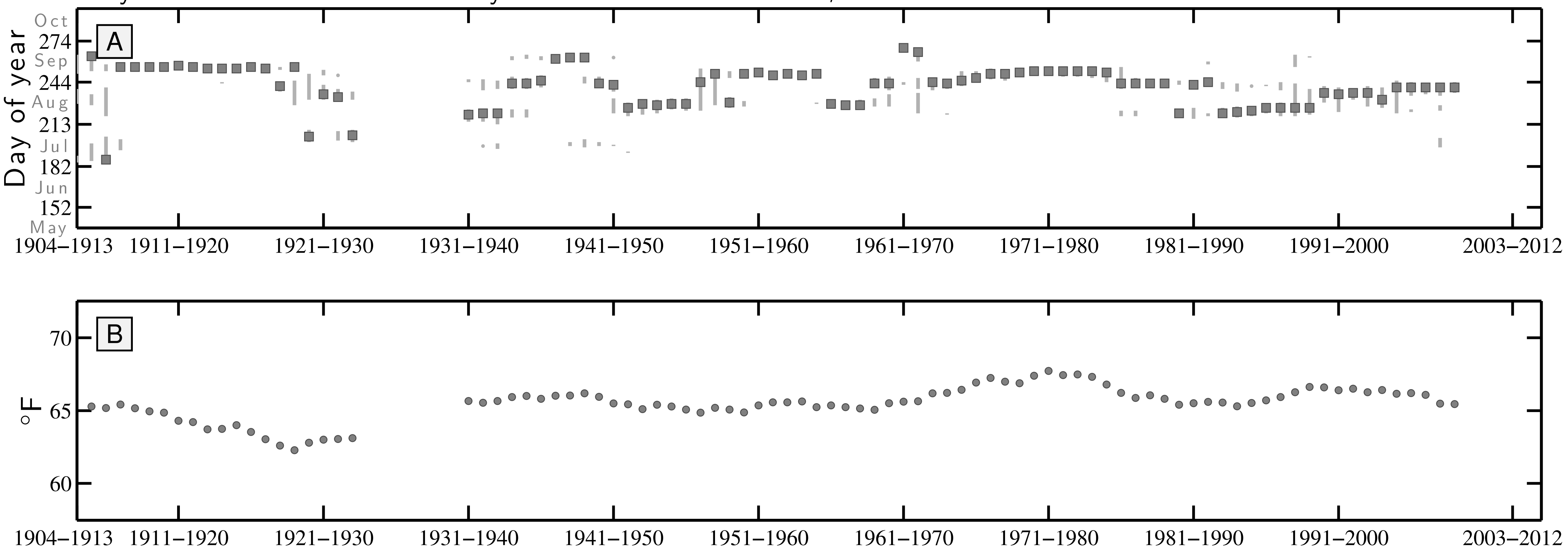

10 year Winter Teletherm dynamics for NEWPORT, OR:

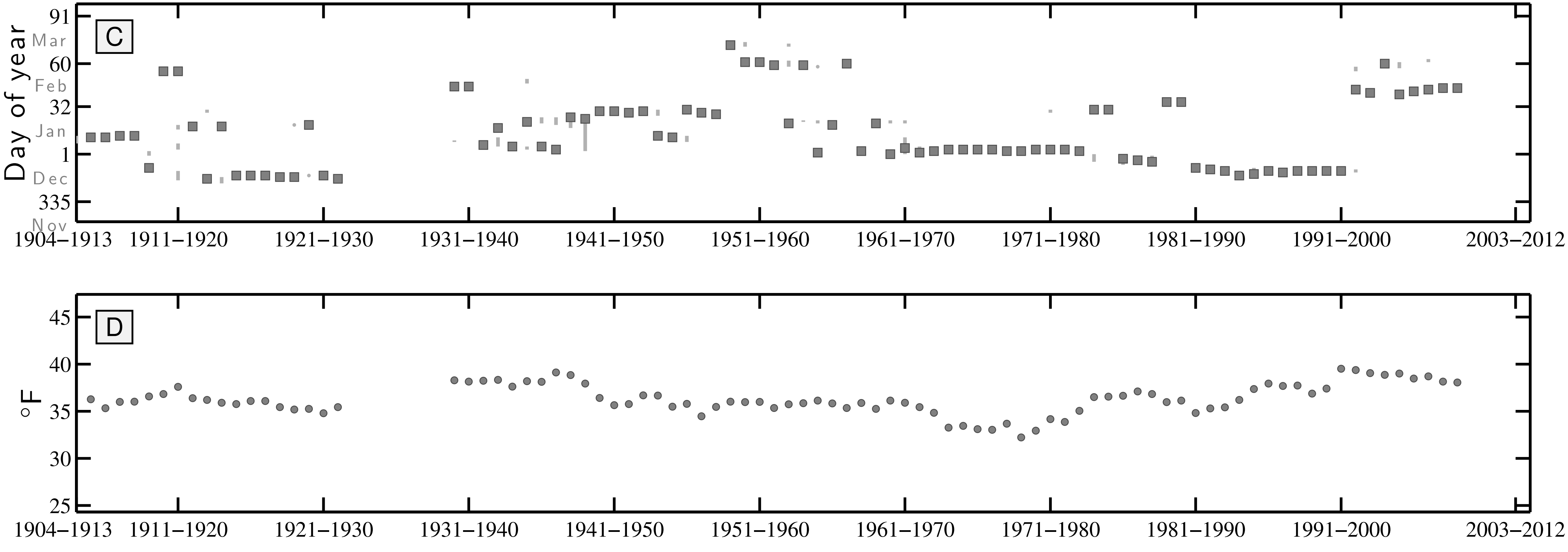

10 year Summer Teletherm dynamics for NORTH BEND FCWOS, OR:

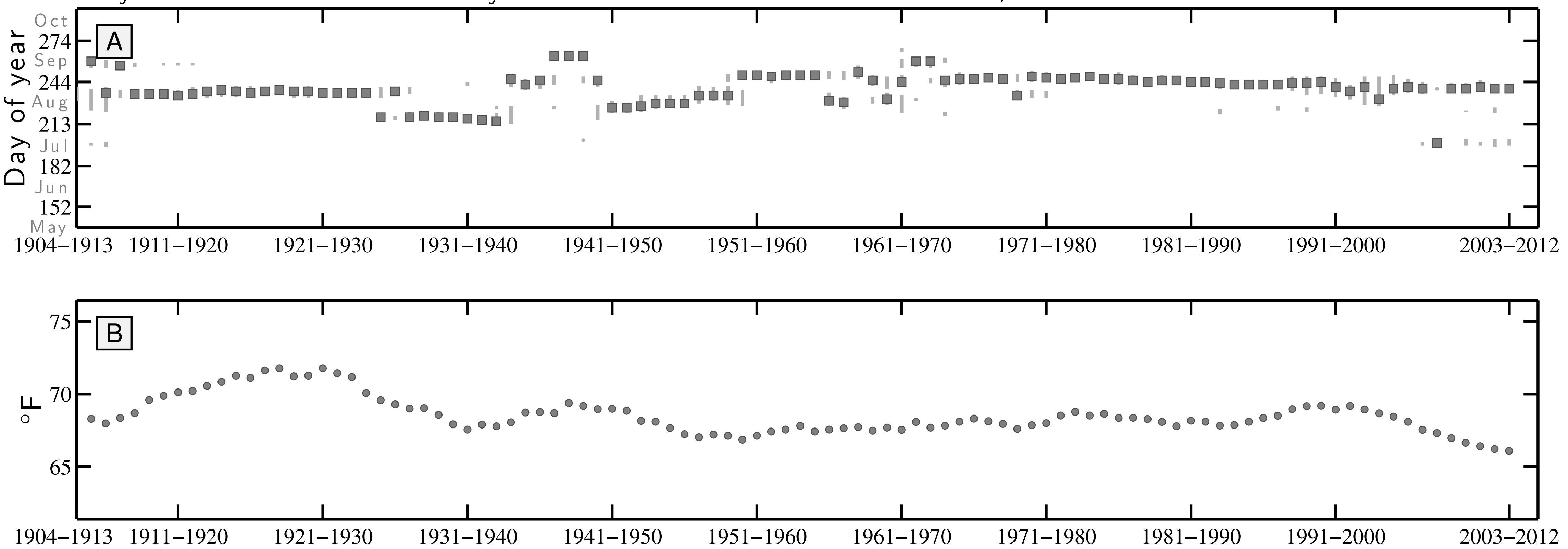

10 year Winter Teletherm dynamics for NORTH BEND FCWOS, OR:

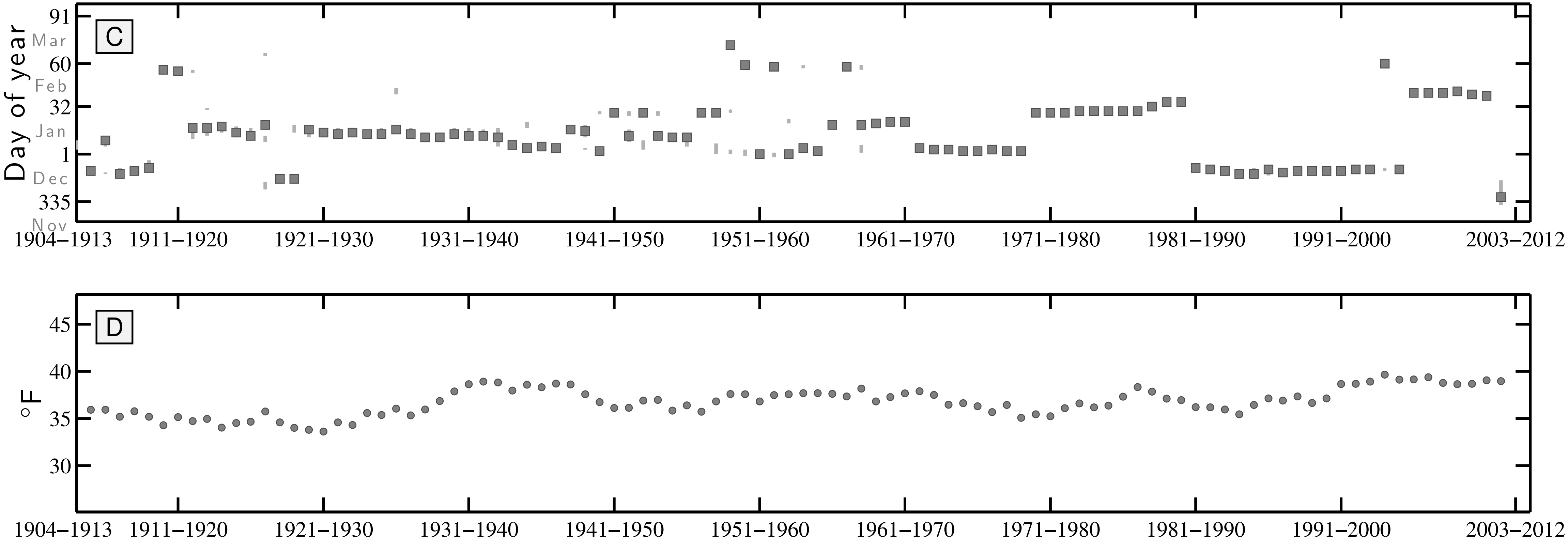

10 year Summer Teletherm dynamics for PAISLEY, OR:

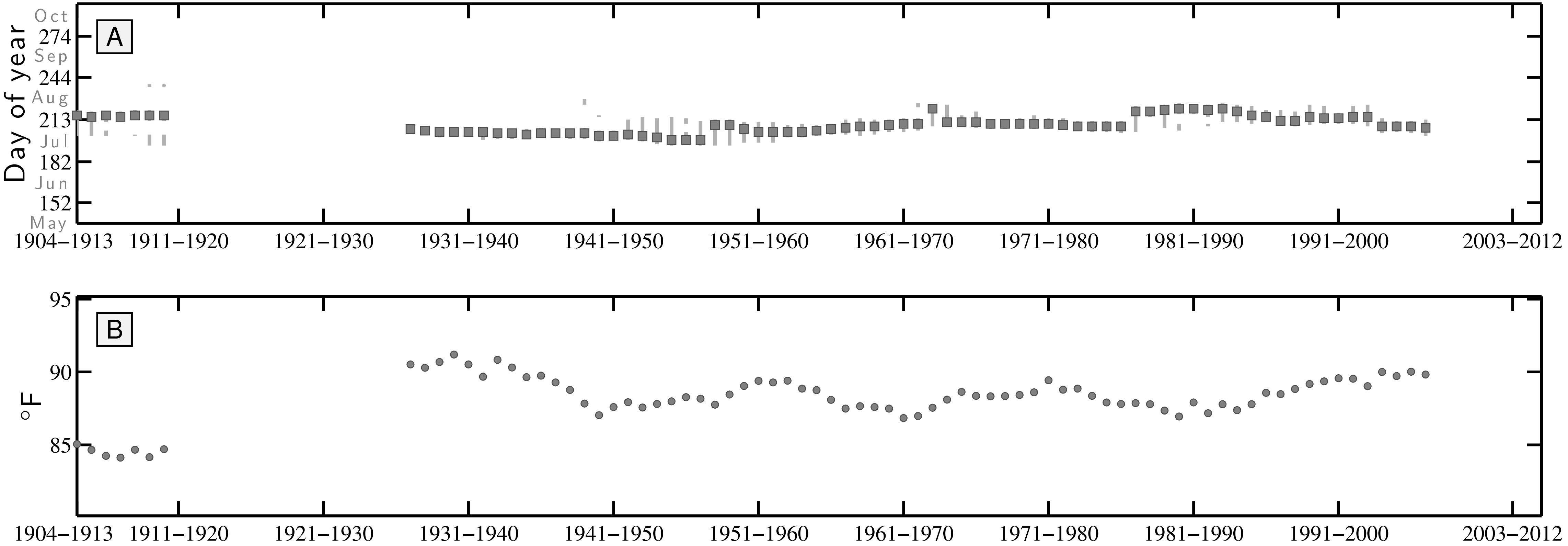

10 year Winter Teletherm dynamics for PAISLEY, OR:

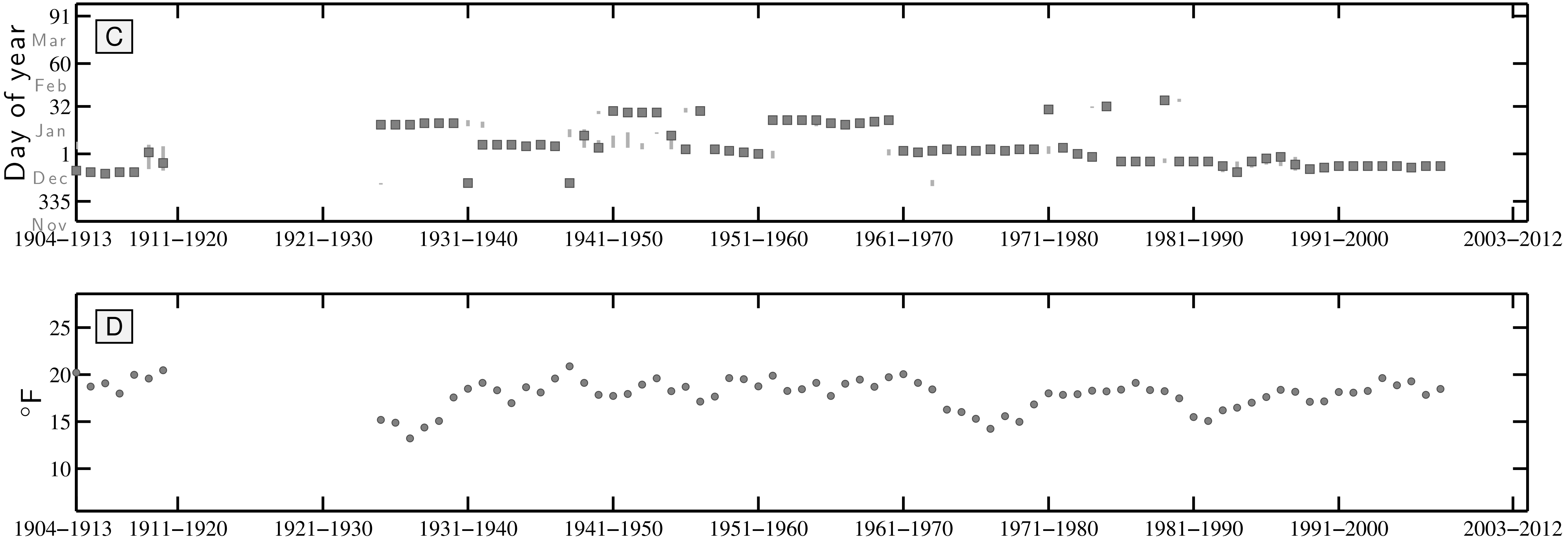

10 year Summer Teletherm dynamics for PILOT ROCK 1 SE, OR:

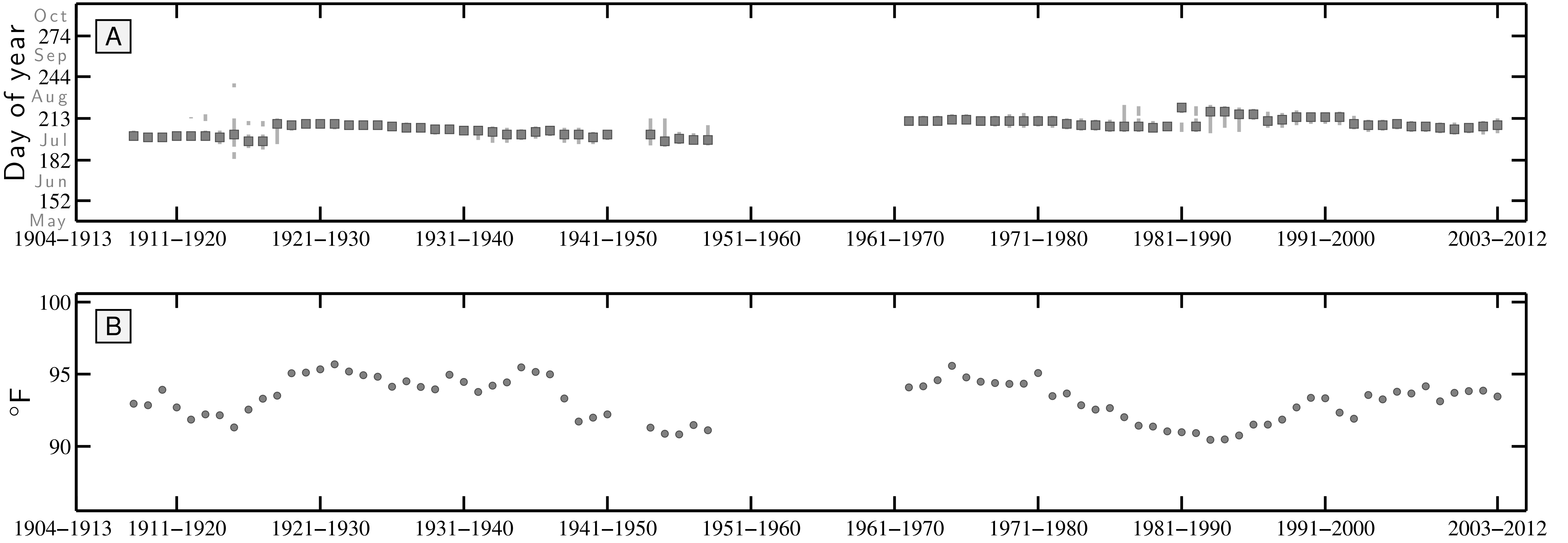

10 year Winter Teletherm dynamics for PILOT ROCK 1 SE, OR:

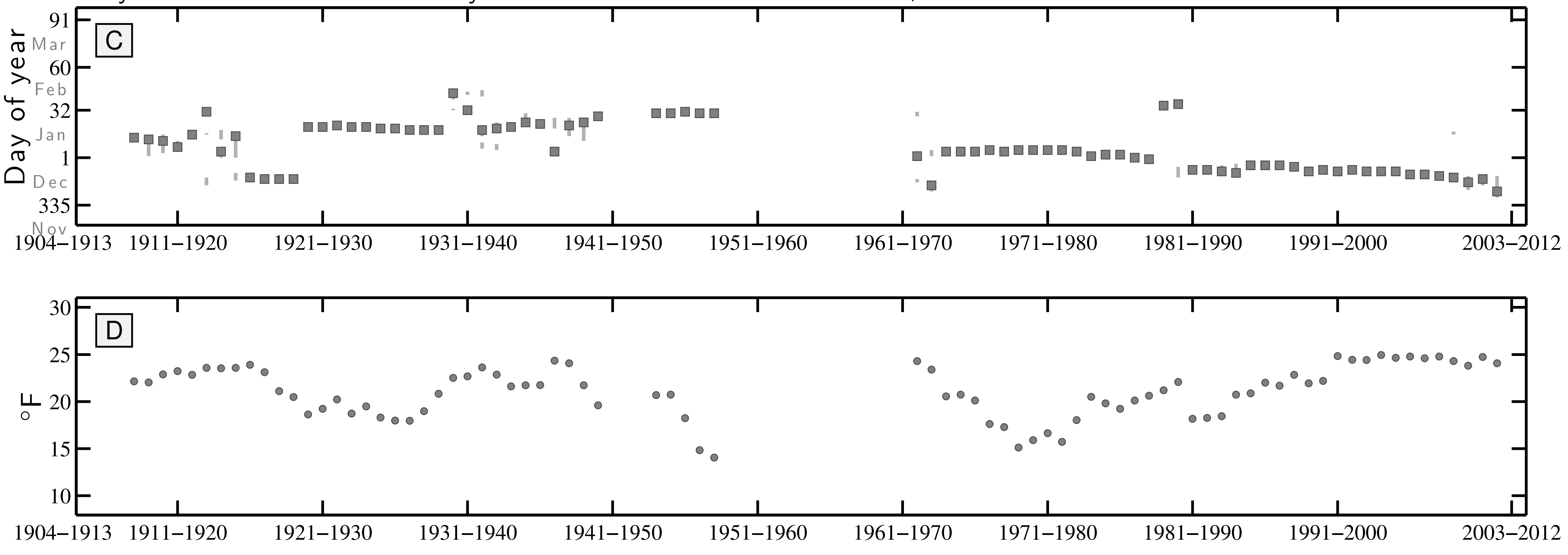

10 year Summer Teletherm dynamics for PRINEVILLE, OR:

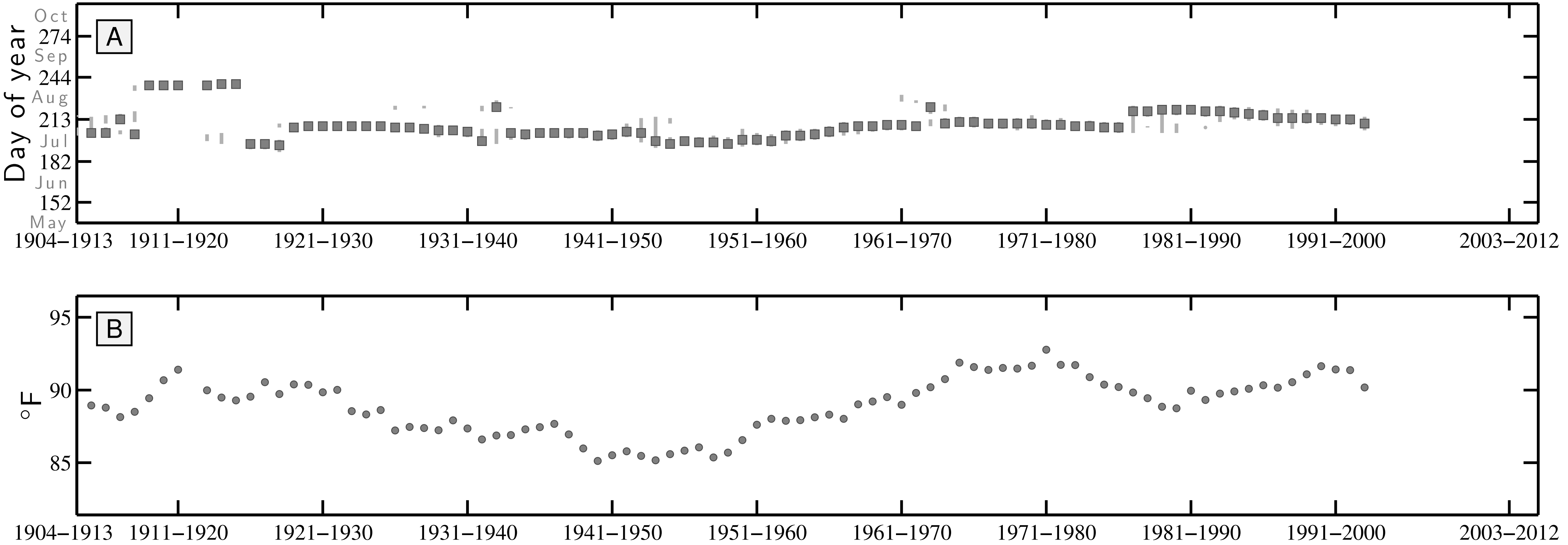

10 year Winter Teletherm dynamics for PRINEVILLE, OR:

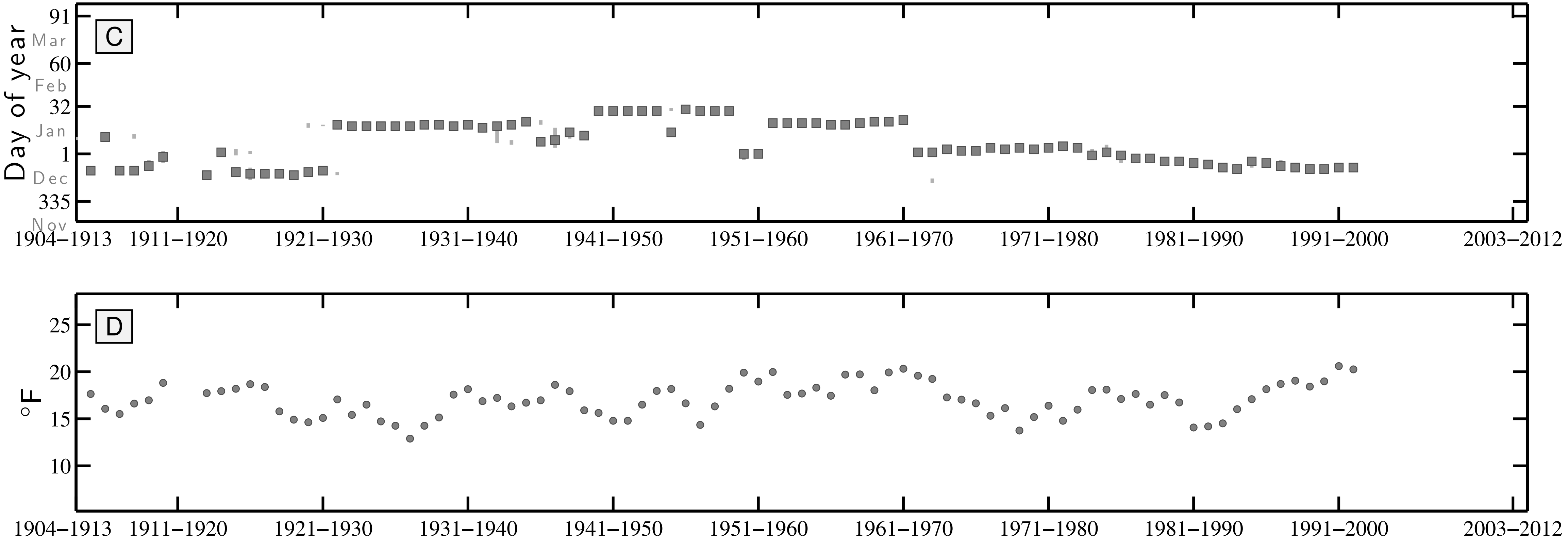

10 year Summer Teletherm dynamics for PROSPECT 2 SW, OR:

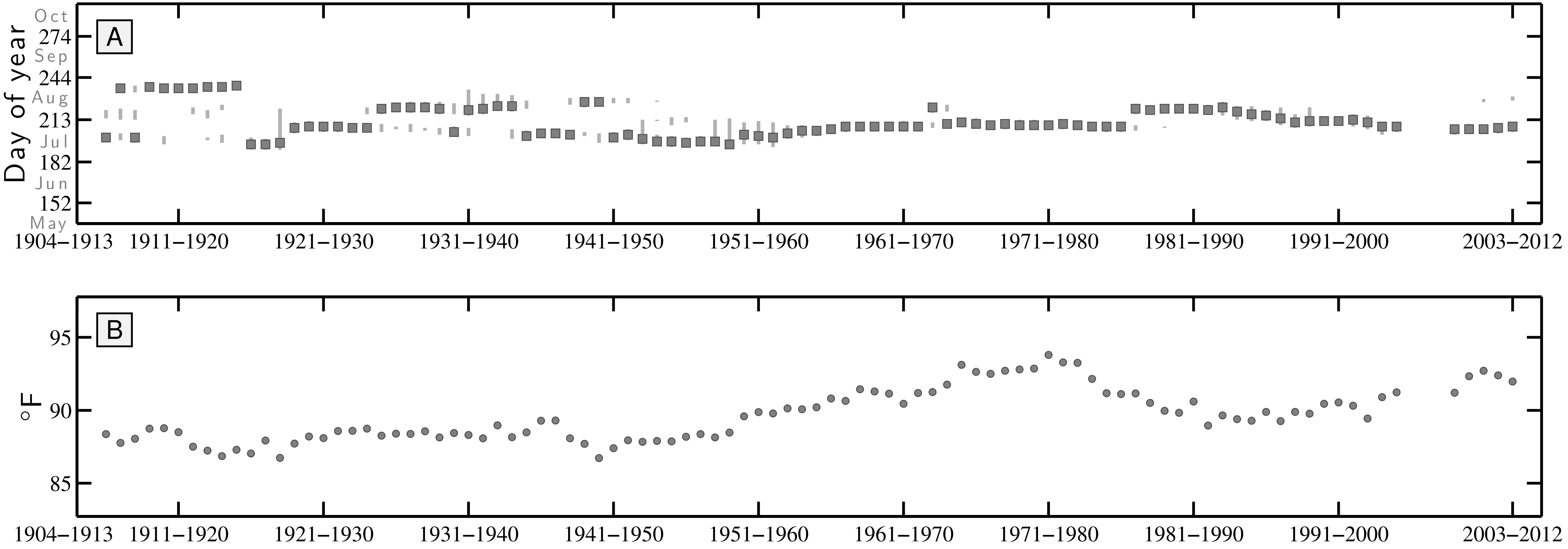

10 year Winter Teletherm dynamics for PROSPECT 2 SW, OR:

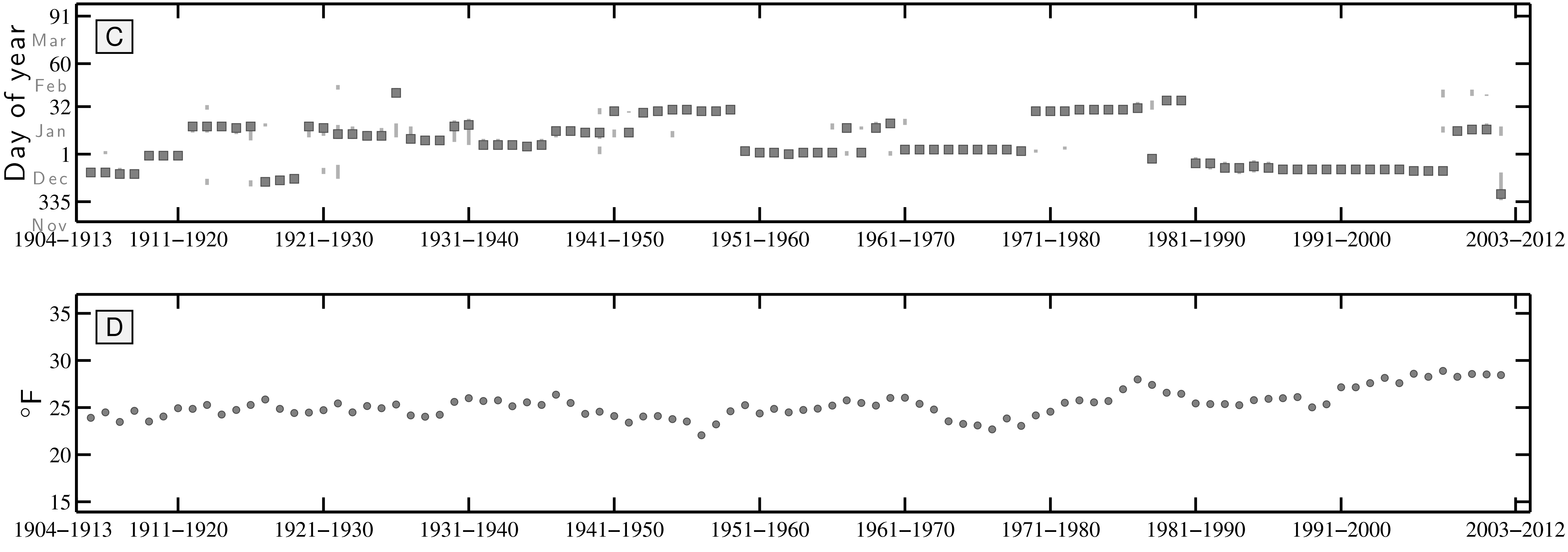

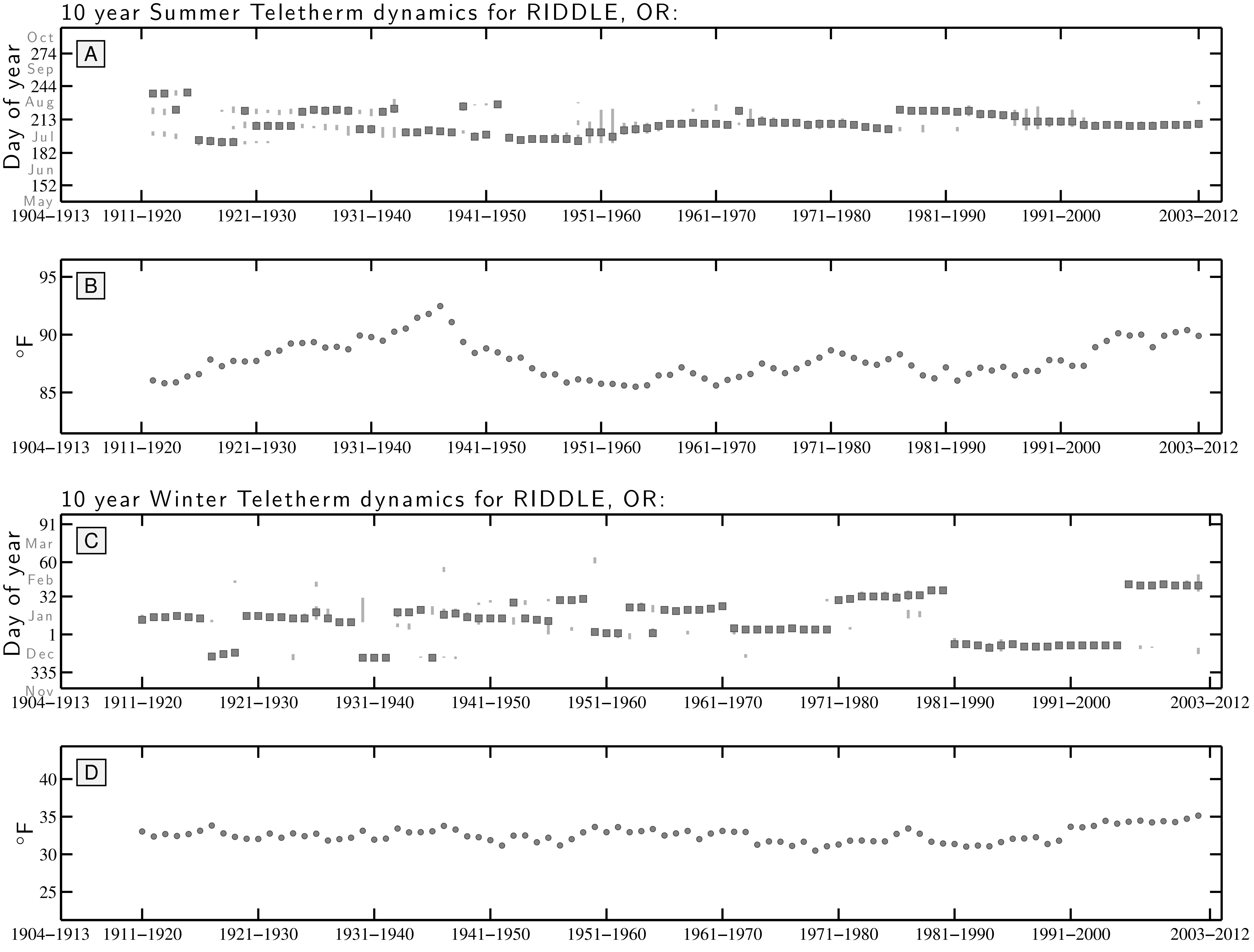

10 year Summer Teletherm dynamics for RIDDLE, OR:
A
Day of year
Oct
274
Sep
244
Aug
213
Jul
182
Jun
152
May
1904–1913
1911–1920
1921–1930
1931–1940
1941–1950
1951–1960
1961–1970
1971–1980
1981–1990
1991–2000
2003–2012
B
°F
95
90
85
10 year Winter Teletherm dynamics for RIDDLE, OR:
C
91
Mar
60
Feb
32
Jan
1
Dec
335
Nov
D
40
35
30
25

10 year Summer Teletherm dynamics for ROSEBURG KQEN, OR:

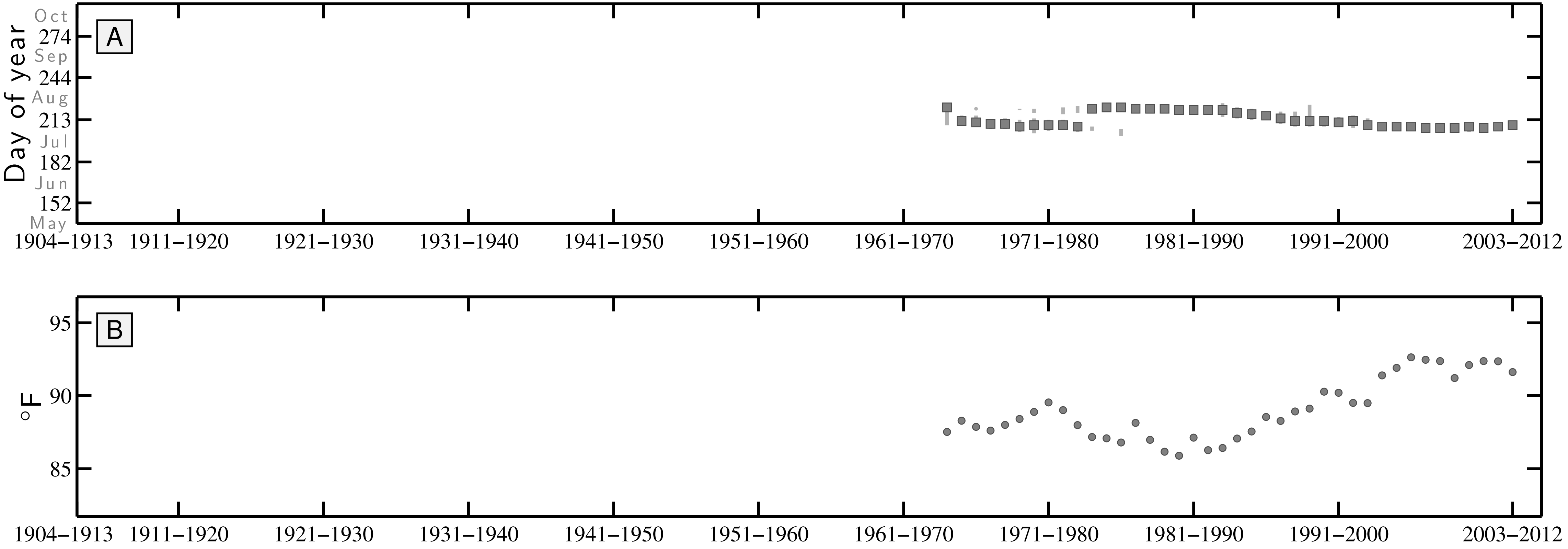

10 year Winter Teletherm dynamics for ROSEBURG KQEN, OR:

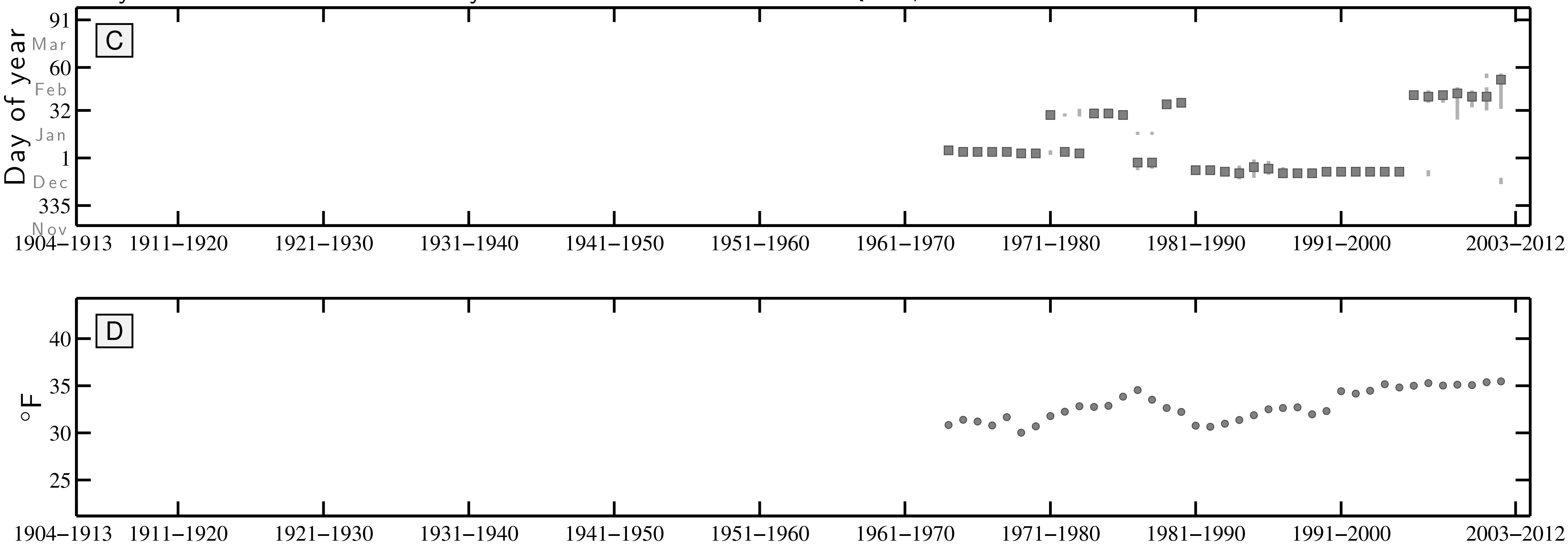

10 year Summer Teletherm dynamics for THREE LYNX, OR:

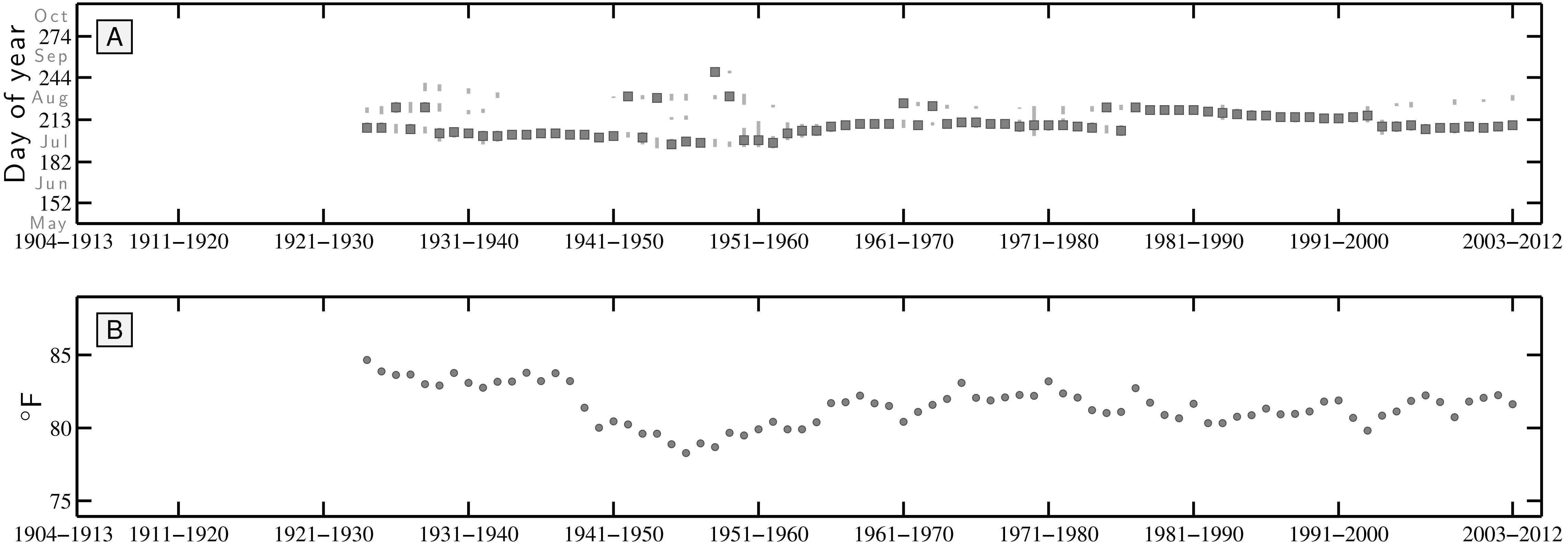

10 year Winter Teletherm dynamics for THREE LYNX, OR:

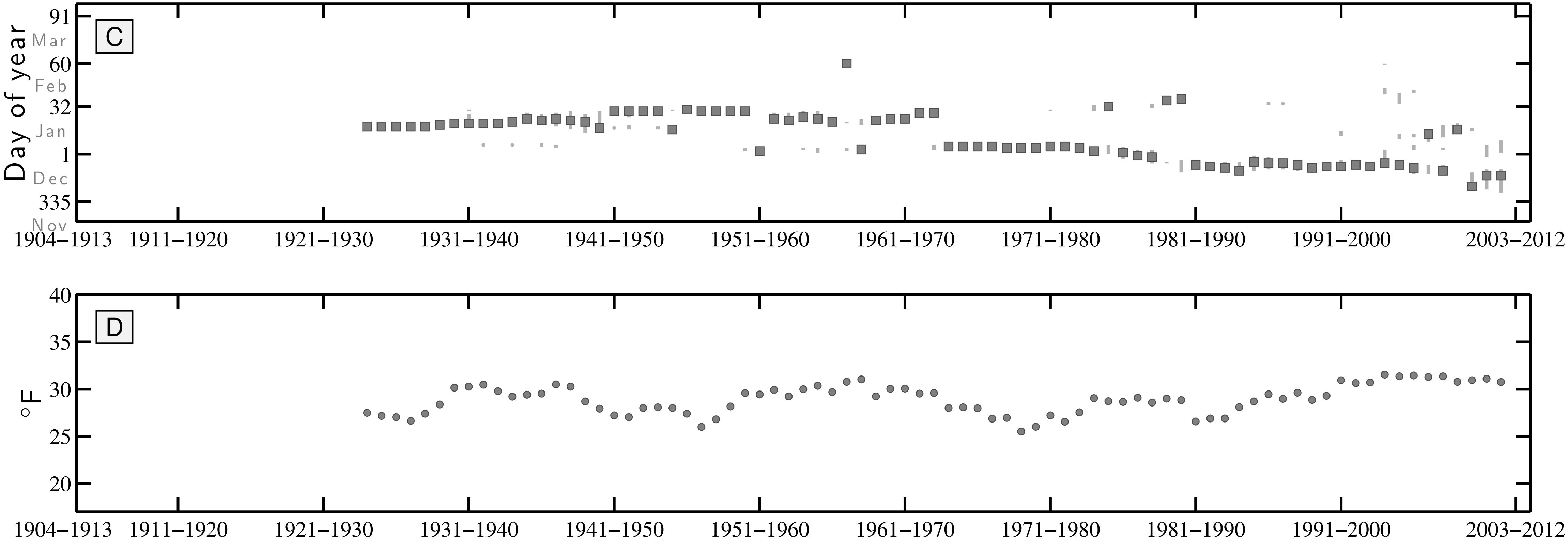

10 year Summer Teletherm dynamics for TILLAMOOK 1 W, OR:

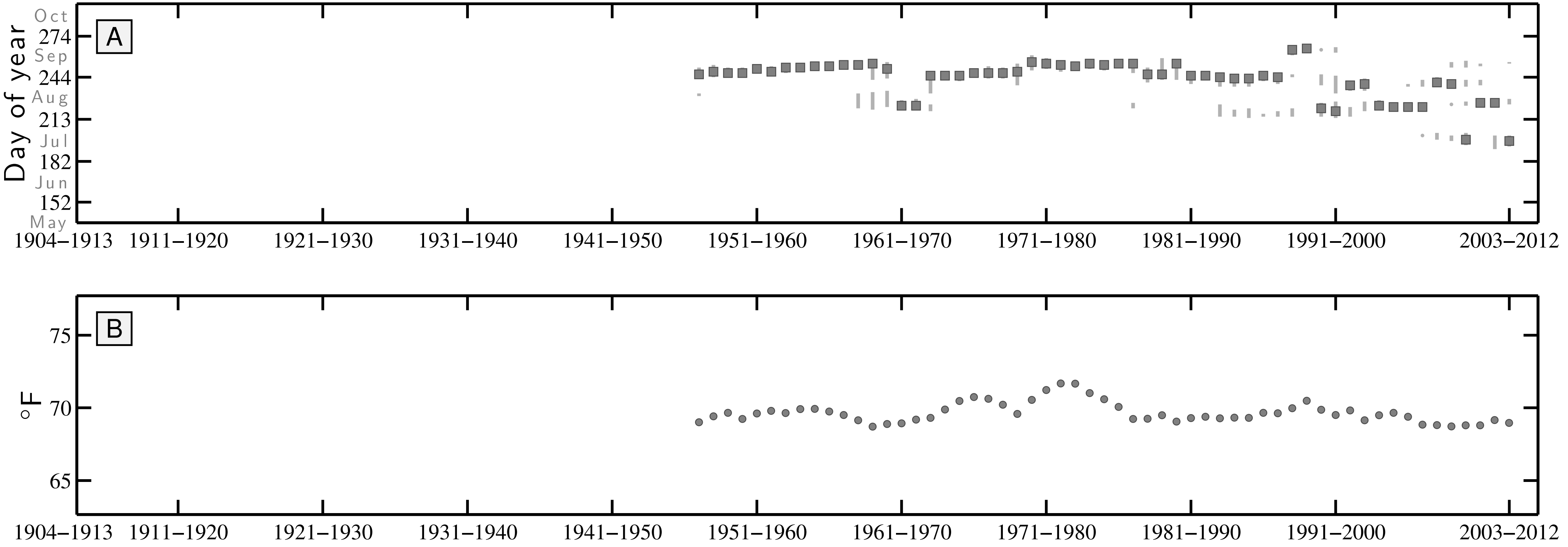

10 year Winter Teletherm dynamics for TILLAMOOK 1 W, OR:

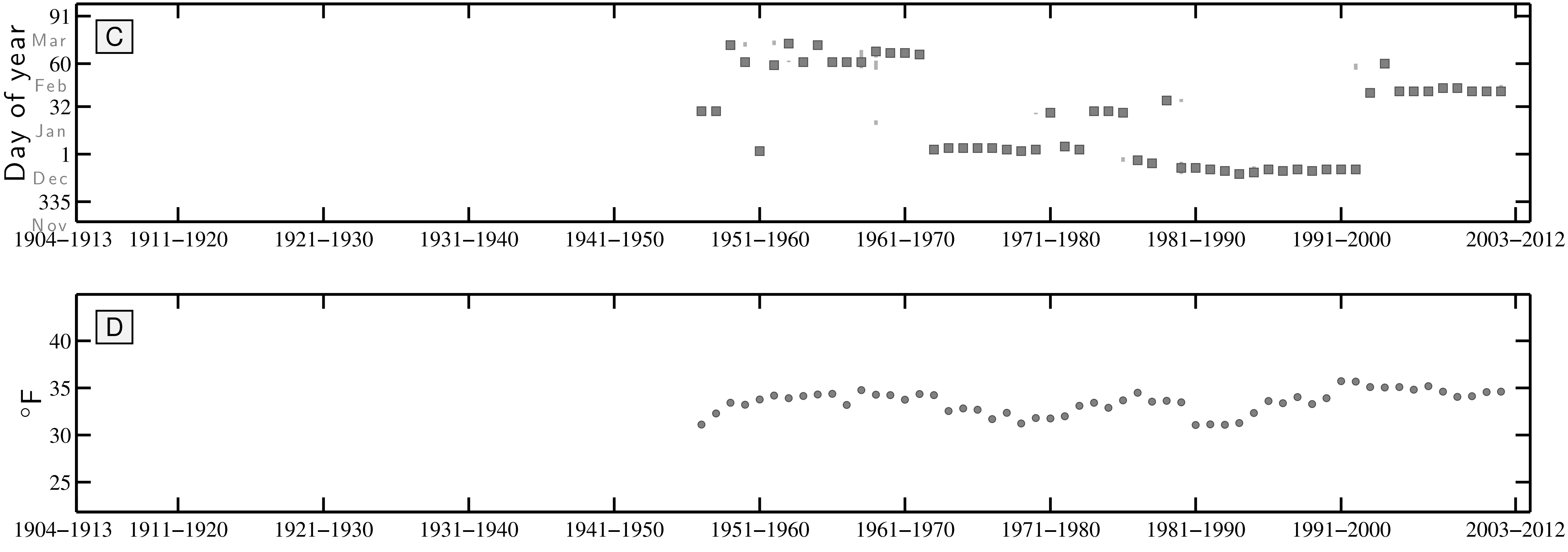

10 year Summer Teletherm dynamics for UNION EXP STN, OR:

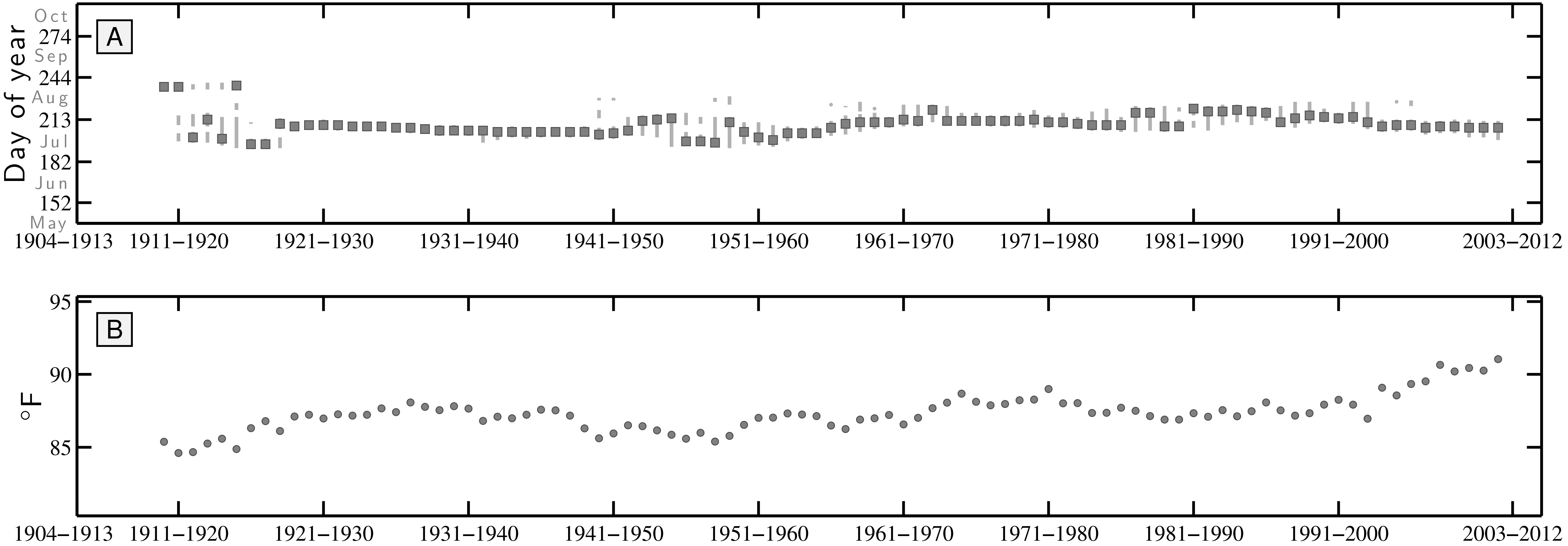

10 year Winter Teletherm dynamics for UNION EXP STN, OR:

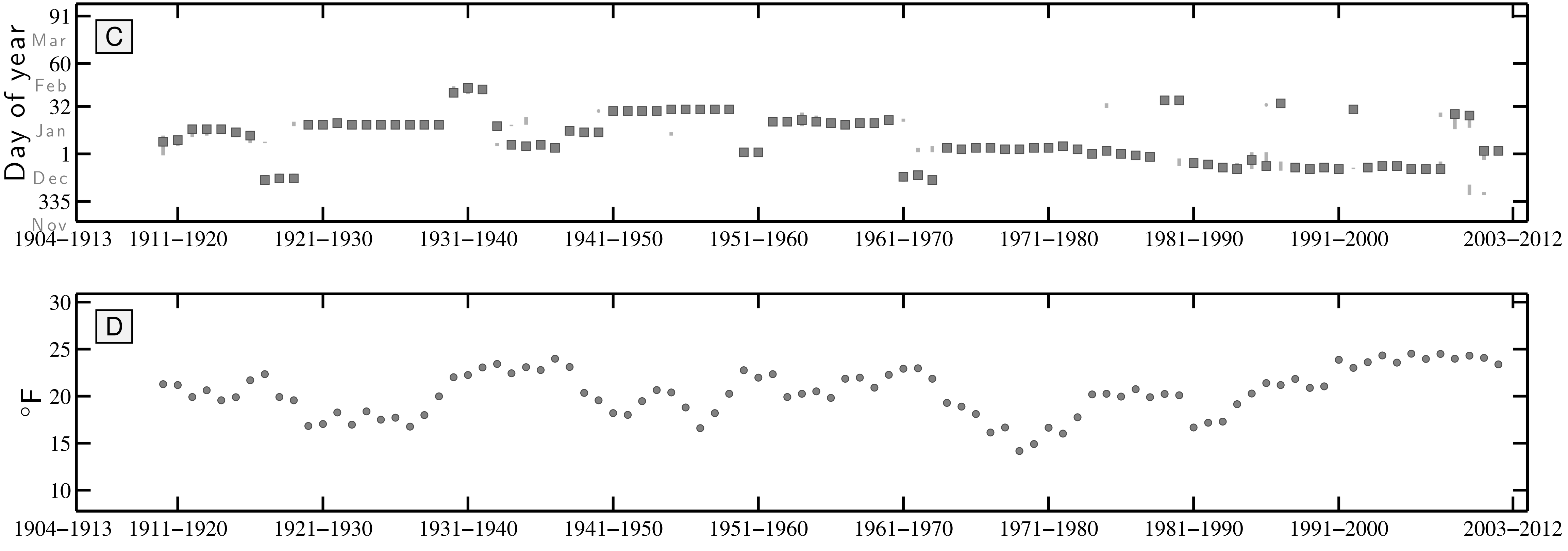

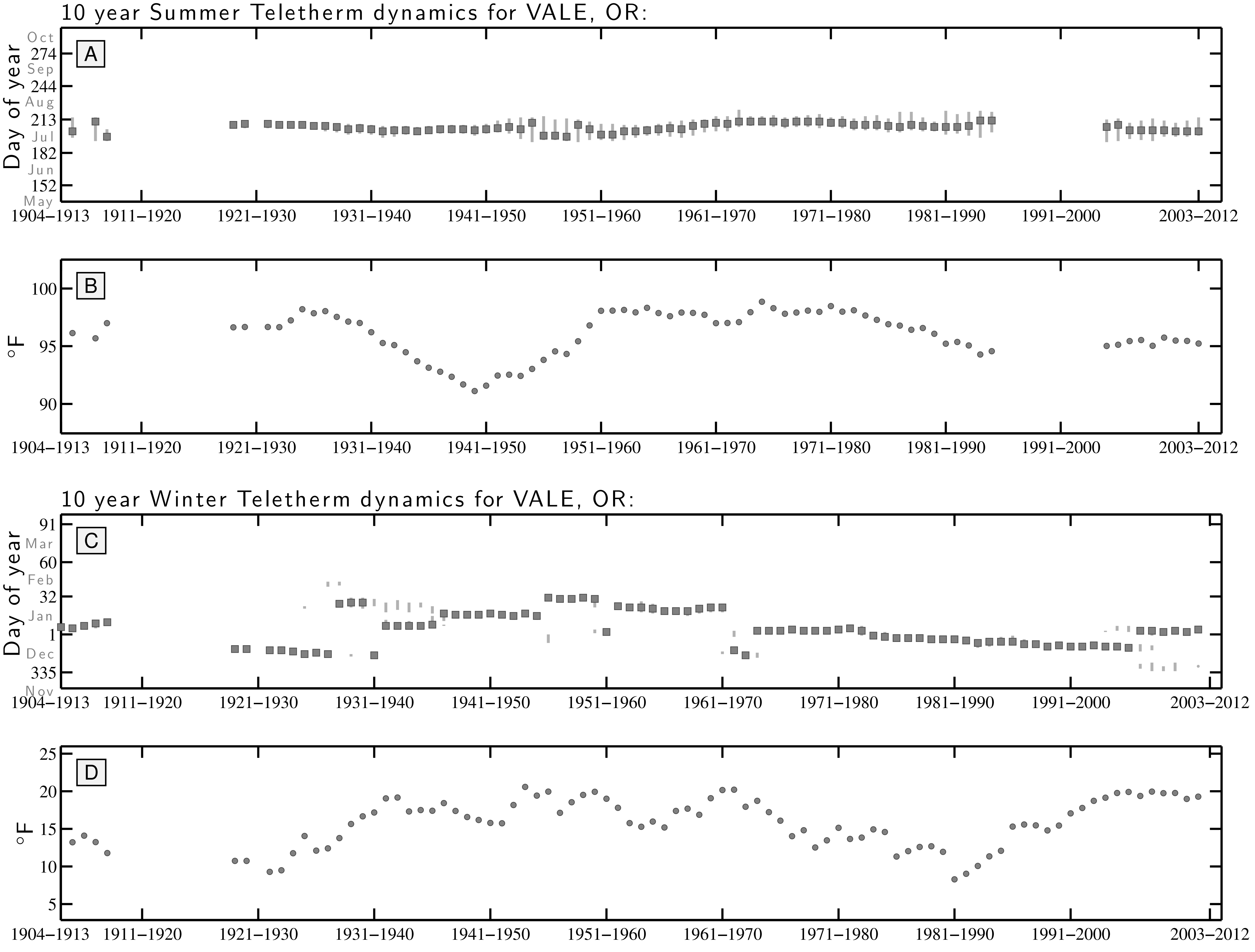

10 year Summer Teletherm dynamics for VALE, OR:
A
Day of year
Oct
274
Sep
244
Aug
213
Jul
182
Jun
152
May
B
°F
100
95
90
10 year Winter Teletherm dynamics for VALE, OR:
C
91
Mar
60
Feb
32
Jan
1
Dec
335
Nov
D
25
20
15
10
5
1904–1913
1911–1920
1921–1930
1931–1940
1941–1950
1951–1960
1961–1970
1971–1980
1981–1990
1991–2000
2003–2012

10 year Summer Teletherm dynamics for WALLOWA, OR:

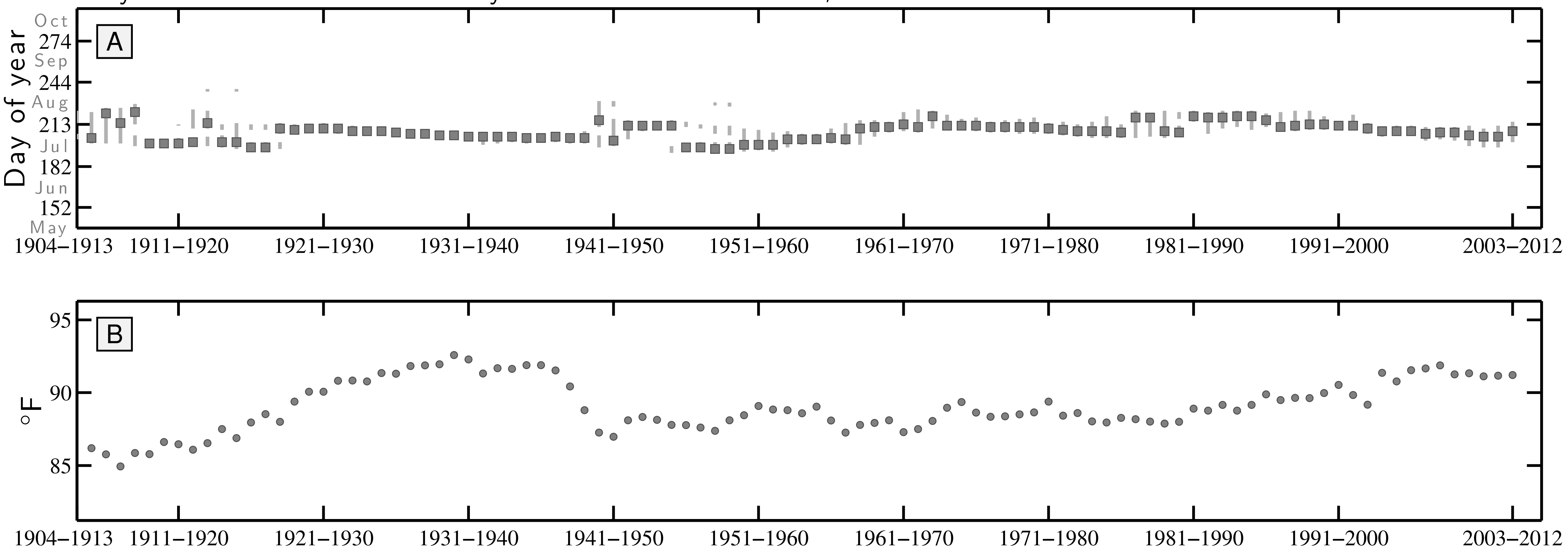

10 year Winter Teletherm dynamics for WALLOWA, OR:

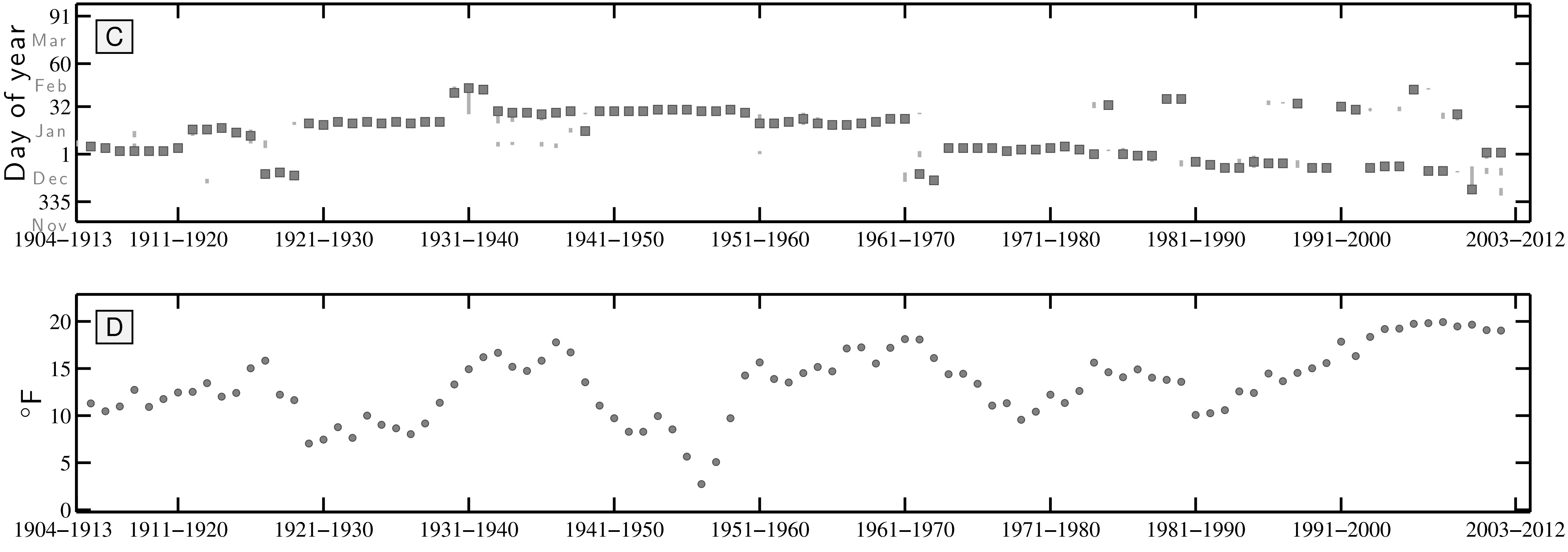

10 year Summer Teletherm dynamics for ALLENTOWN AP, PA:

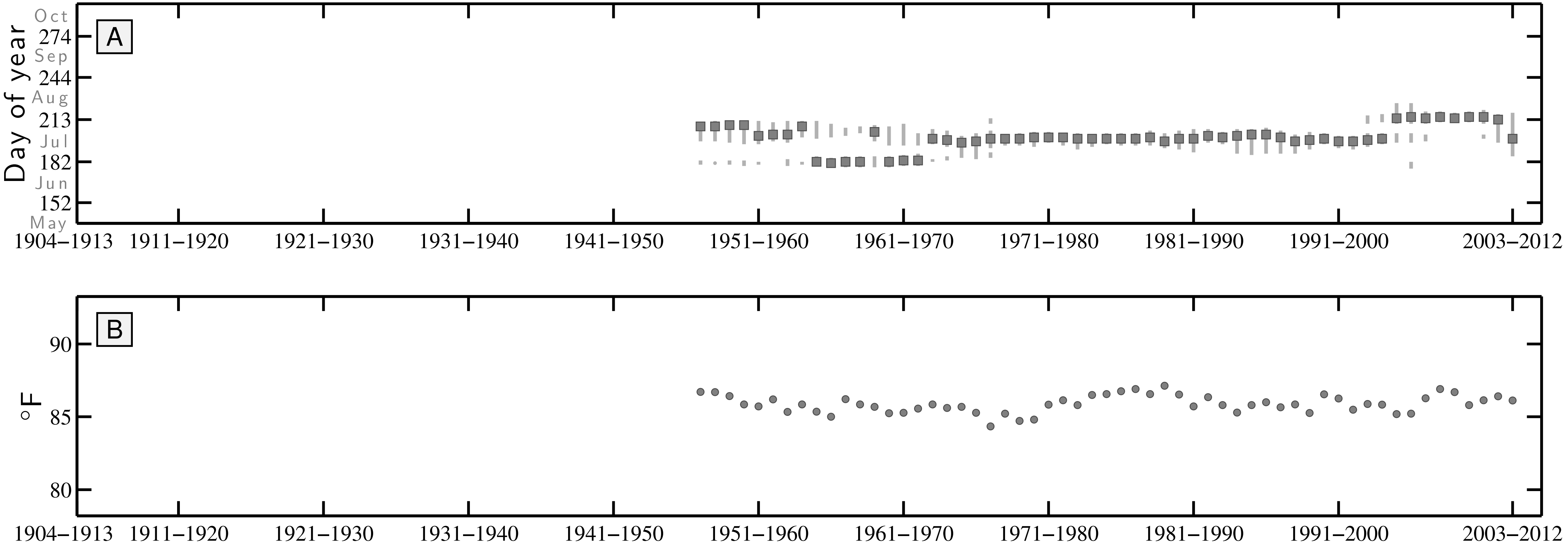

10 year Winter Teletherm dynamics for ALLENTOWN AP, PA:

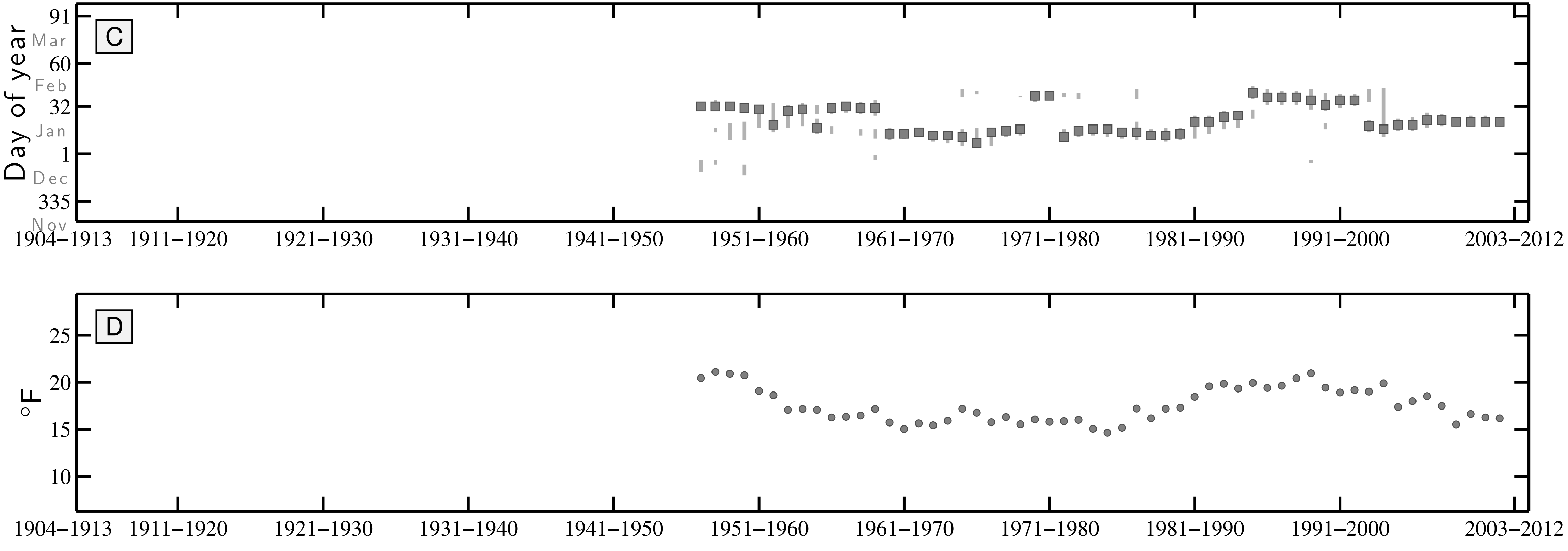

10 year Summer Teletherm dynamics for CHAMBERSBURG 1 ESE, PA:

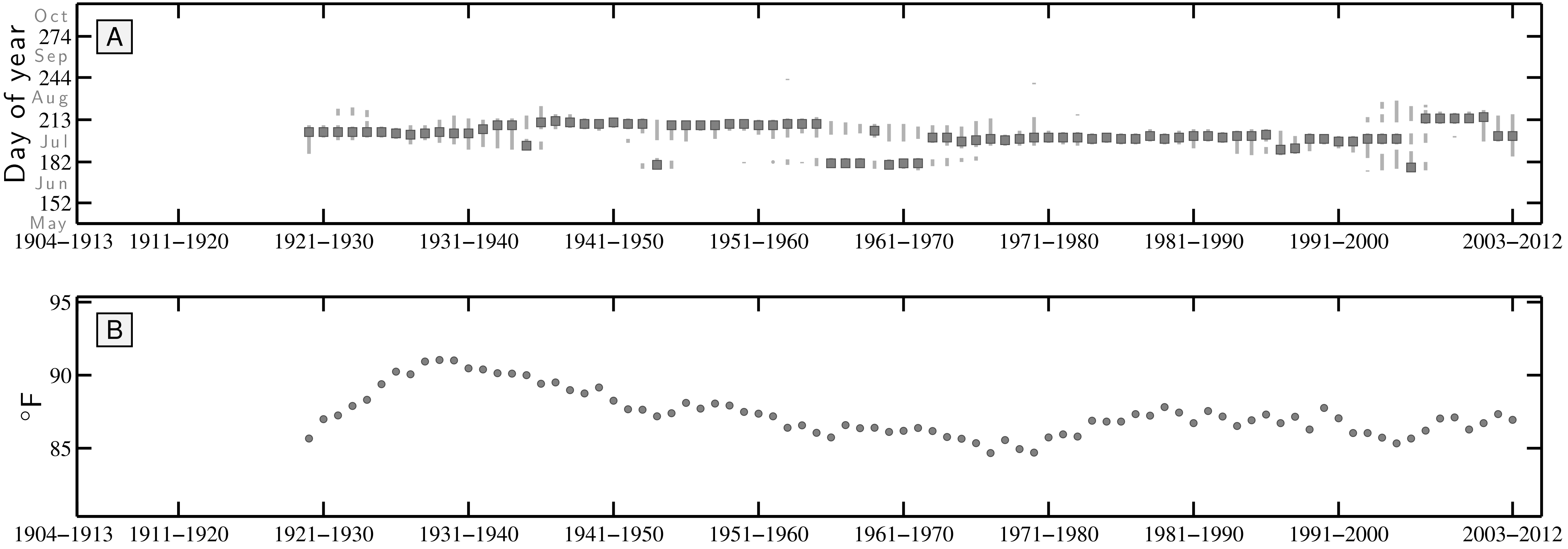

10 year Winter Teletherm dynamics for CHAMBERSBURG 1 ESE, PA:

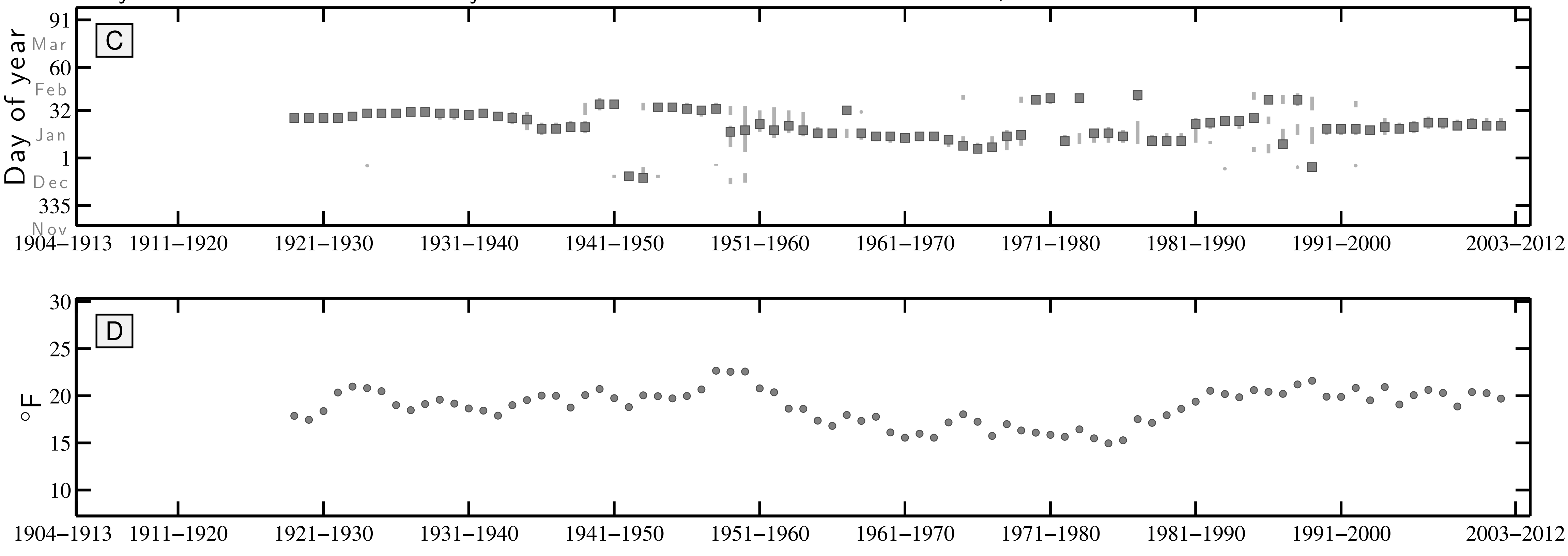

10 year Summer Teletherm dynamics for EISENHOWER NHS, PA:

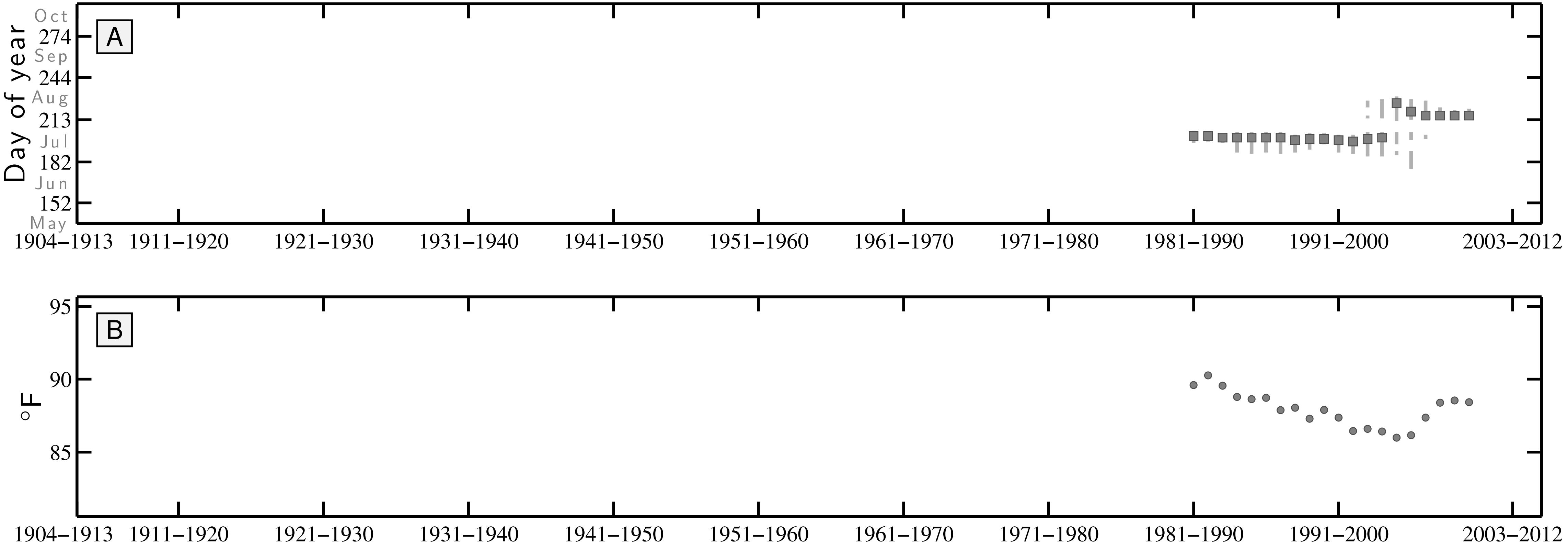

10 year Winter Teletherm dynamics for EISENHOWER NHS, PA:

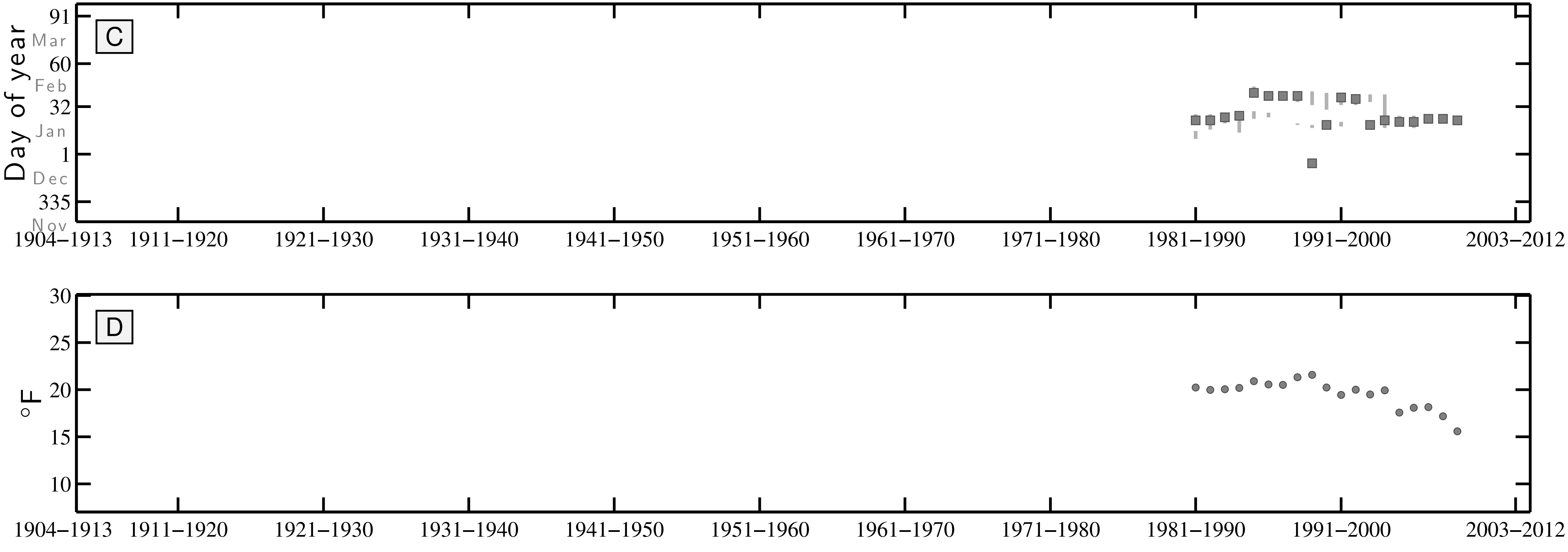

10 year Summer Teletherm dynamics for ERIE WSO AP, PA:

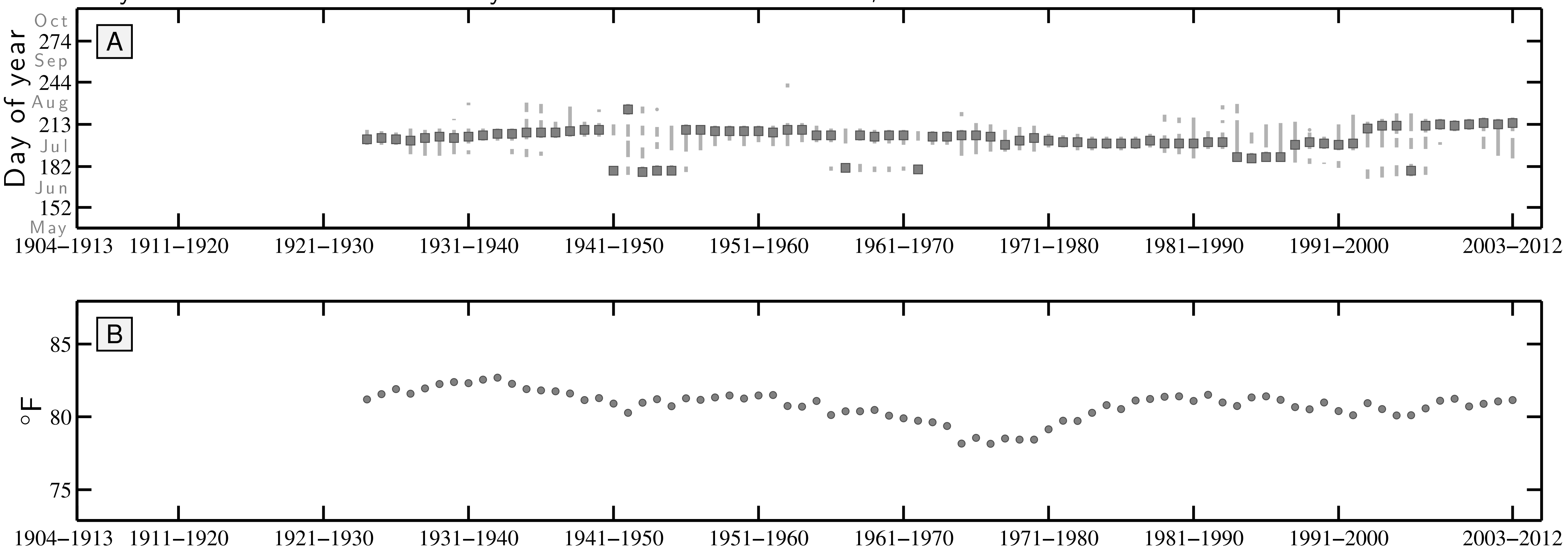

10 year Winter Teletherm dynamics for ERIE WSO AP, PA:

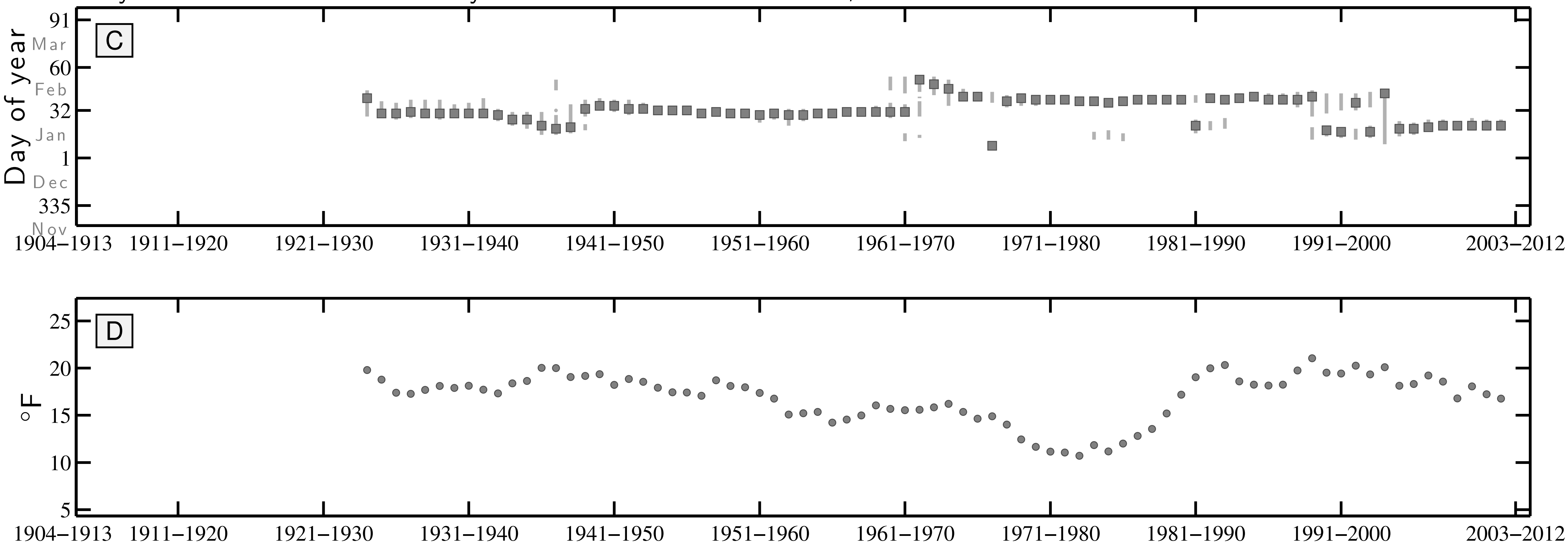

10 year Summer Teletherm dynamics for FRANKLIN, PA:

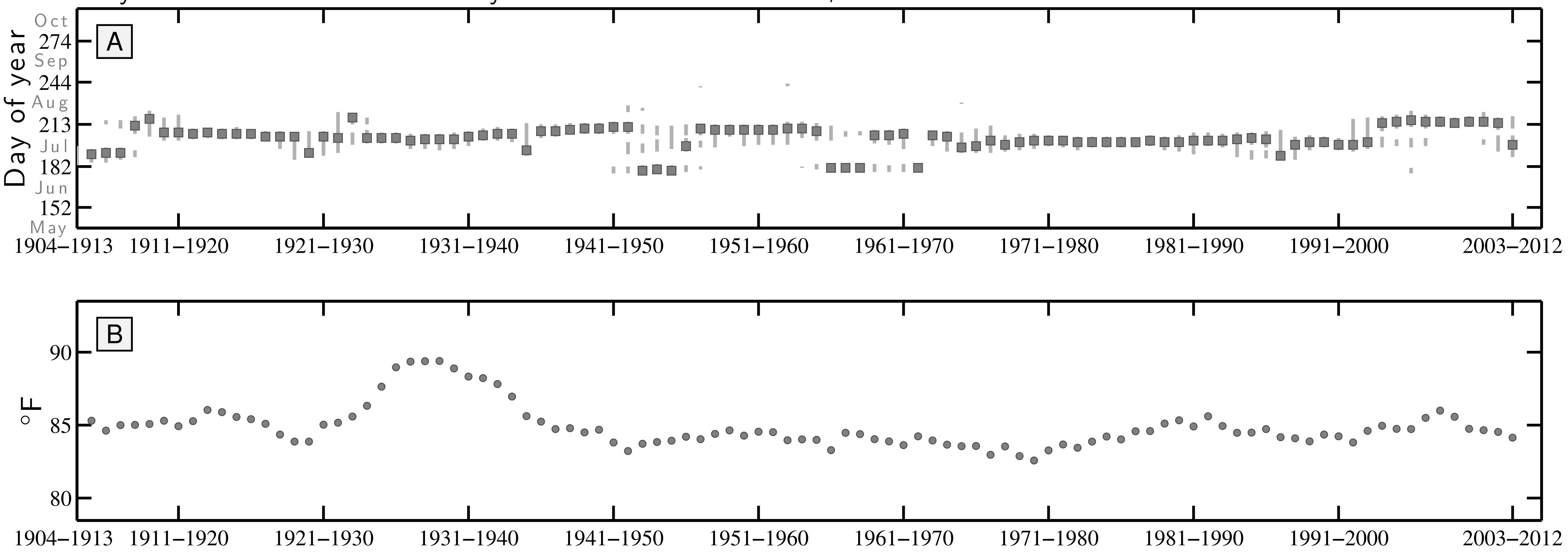

10 year Winter Teletherm dynamics for FRANKLIN, PA:

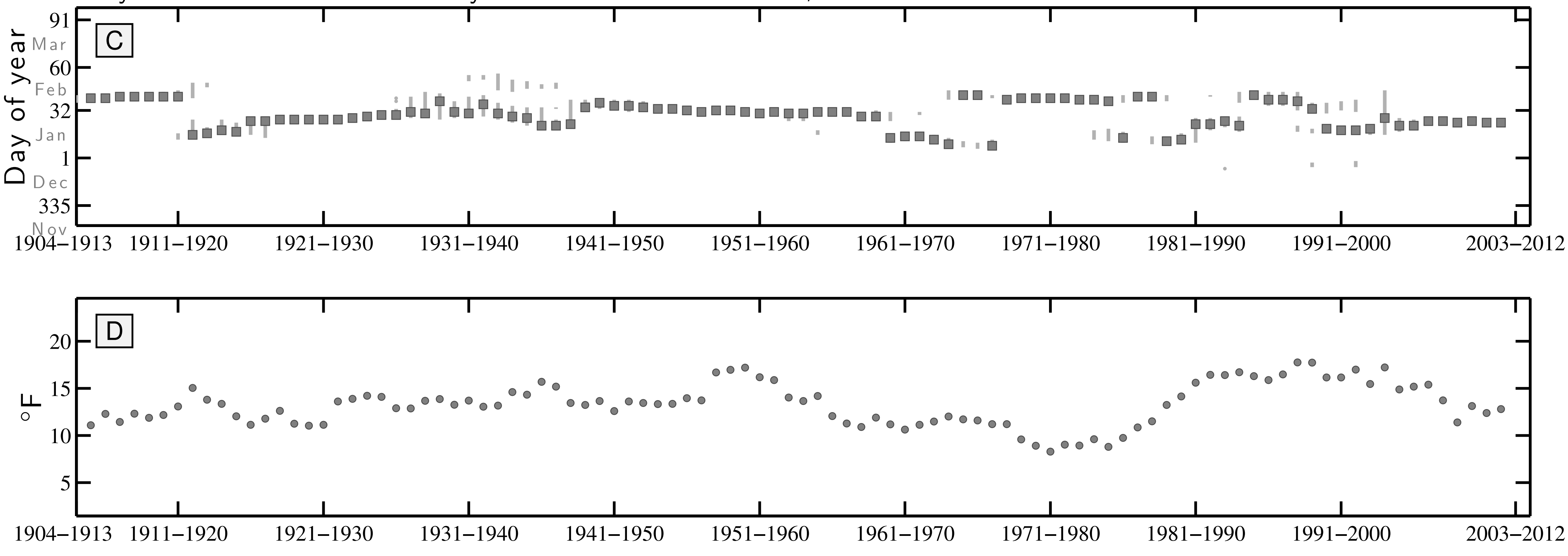

10 year Summer Teletherm dynamics for GREENVILLE 2 NE, PA:

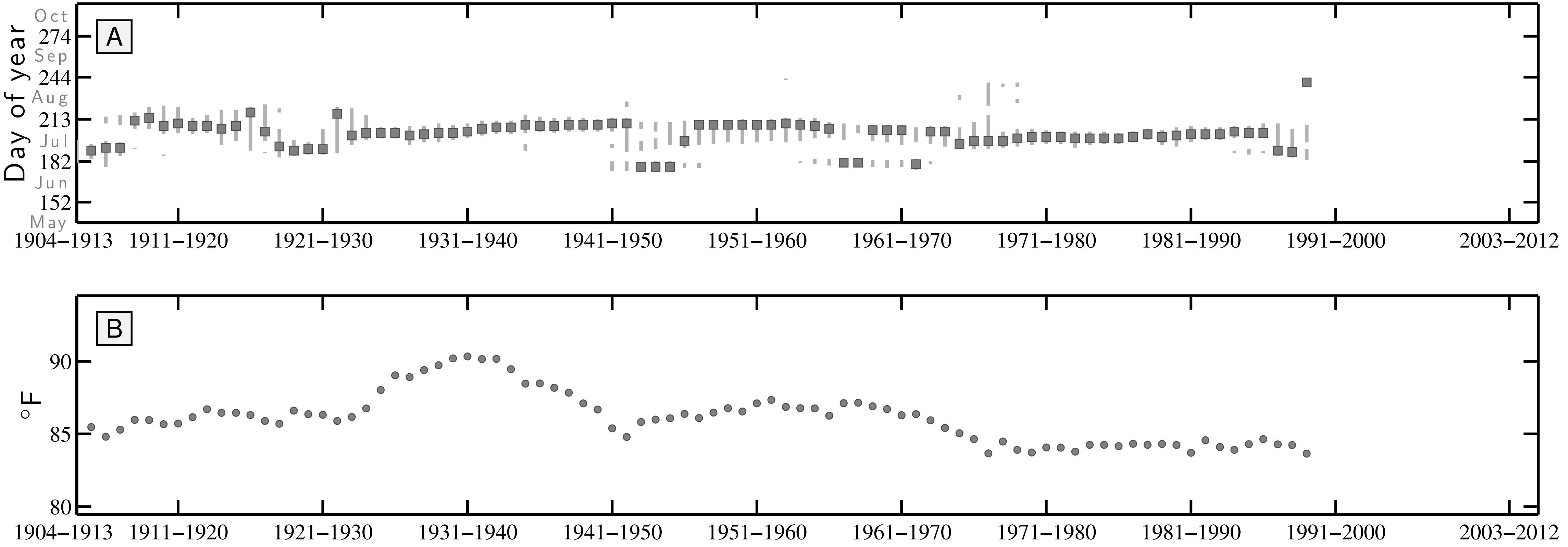

10 year Winter Teletherm dynamics for GREENVILLE 2 NE, PA:

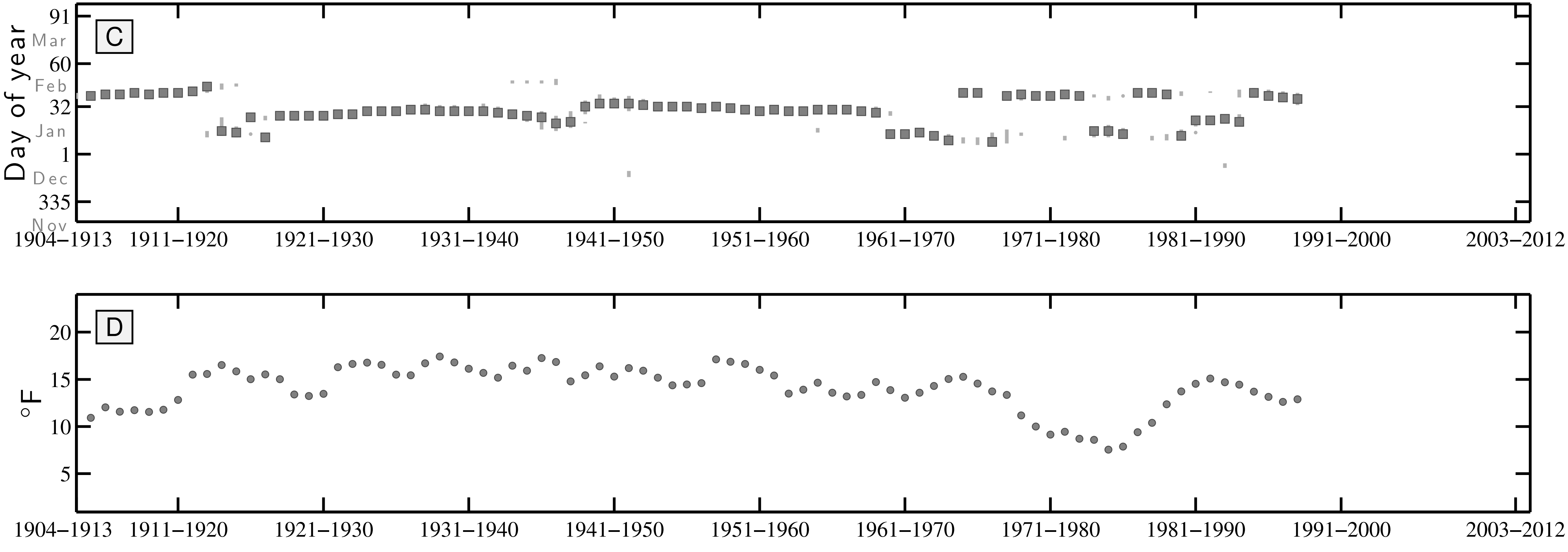

10 year Summer Teletherm dynamics for JOHNSTOWN, PA:

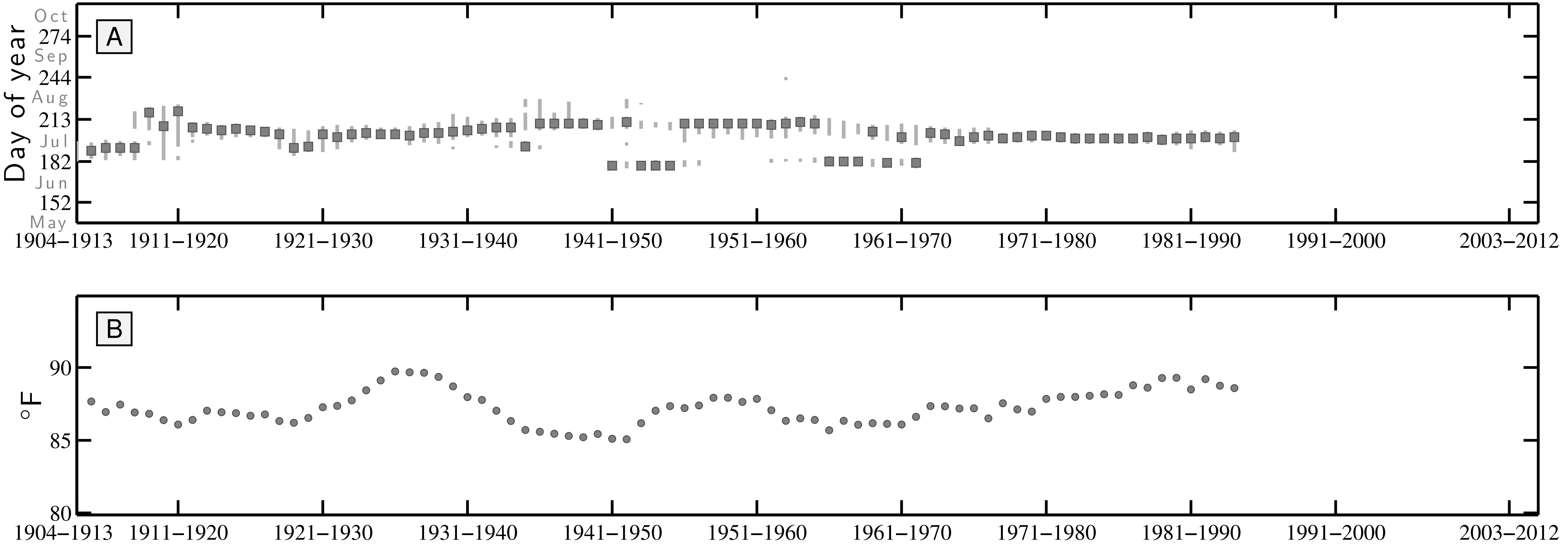

10 year Winter Teletherm dynamics for JOHNSTOWN, PA:

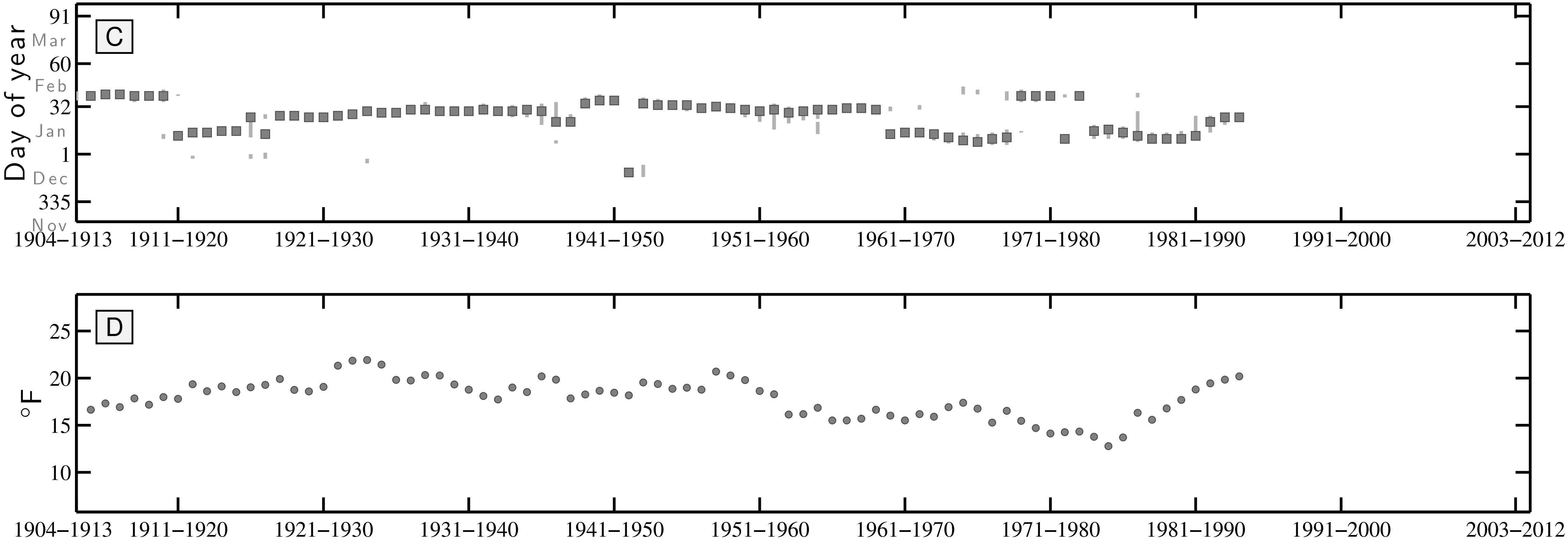

10 year Summer Teletherm dynamics for LEBANON 2 W, PA:

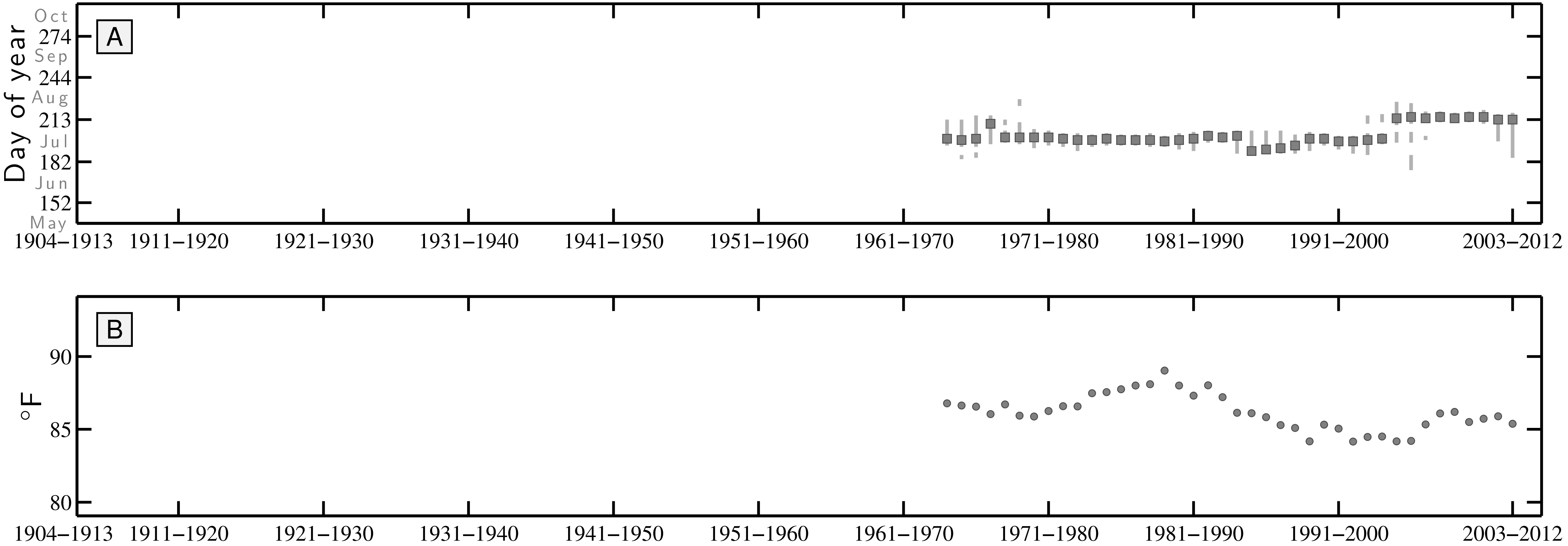

10 year Winter Teletherm dynamics for LEBANON 2 W, PA:

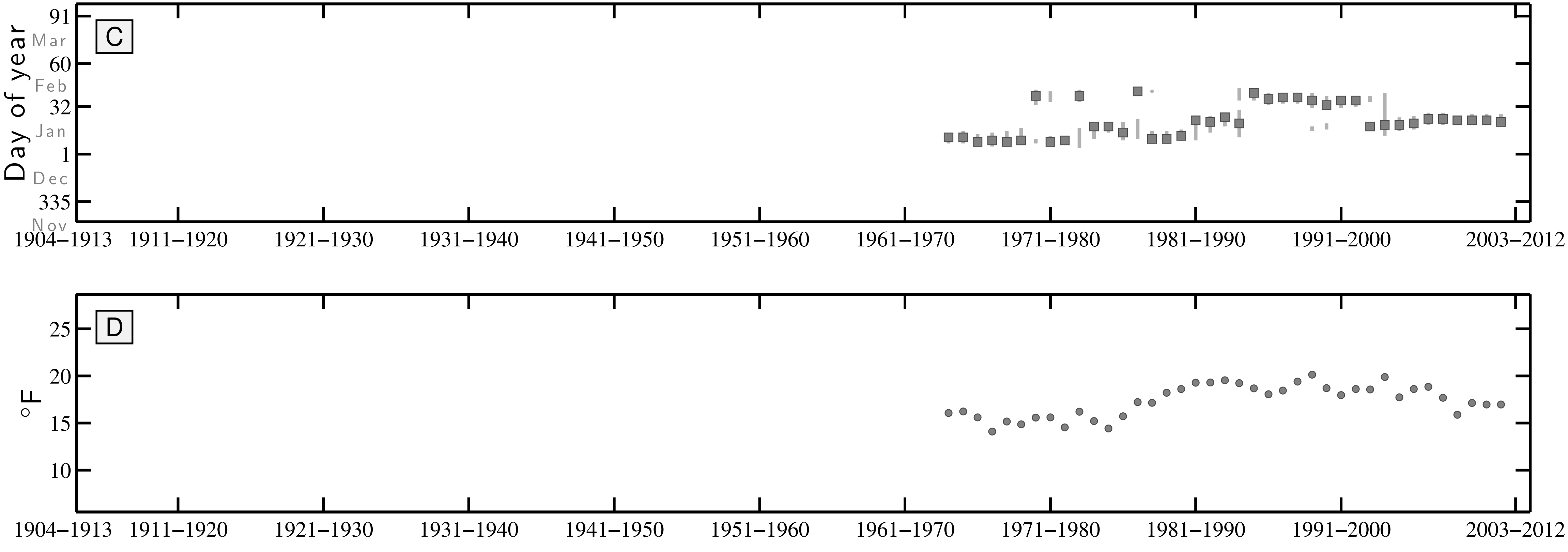

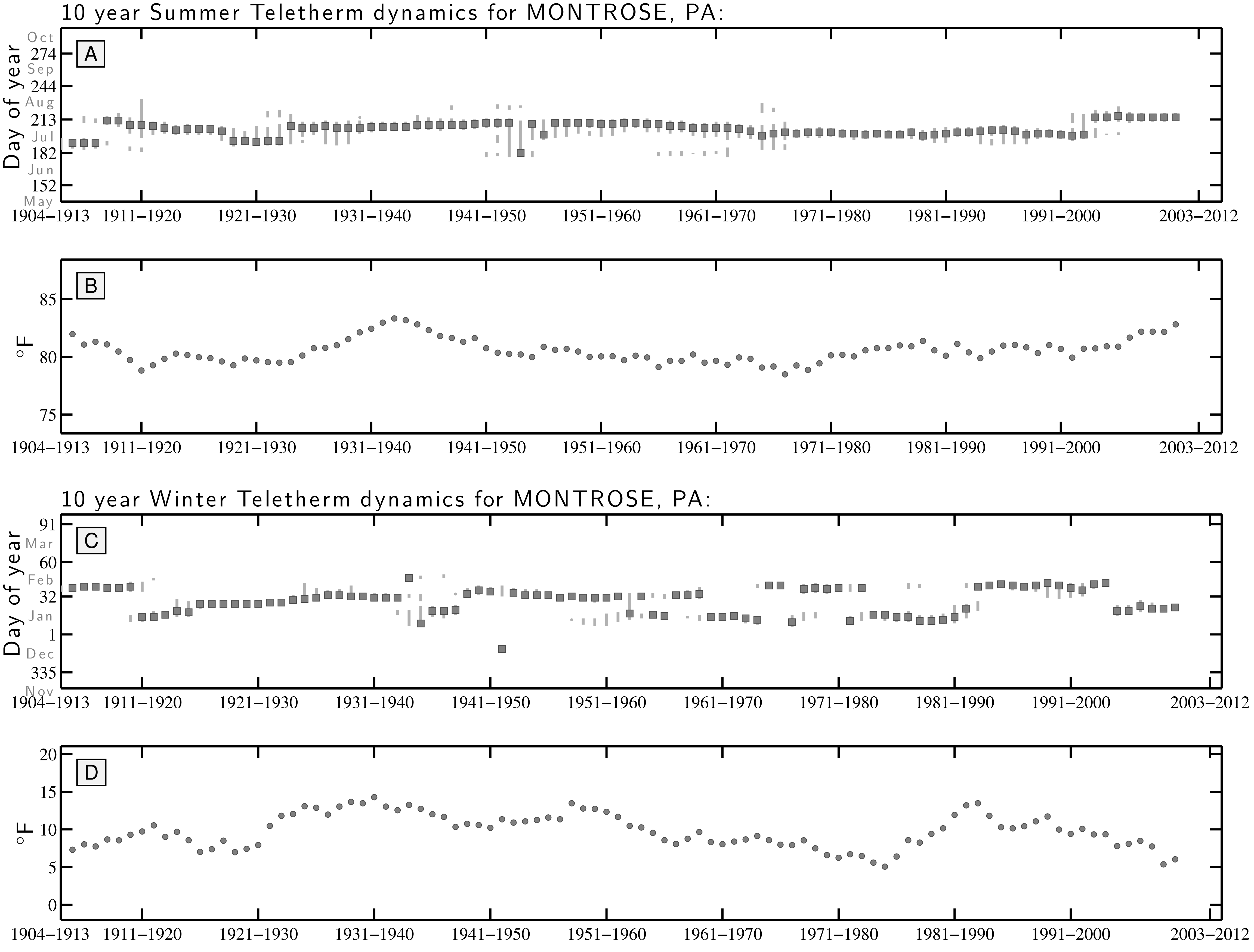
10 year Summer Teletherm dynamics for MONTROSE, PA:
A
B
Day of year
°F
Oct
274
Sep
244
Aug
213
Jul
182
Jun
152
May
85
80
75
10 year Winter Teletherm dynamics for MONTROSE, PA:
C
D
91
Mar
60
Feb
32
Jan
1
Dec
335
Nov
20
15
10
5
0
1904–1913
1911–1920
1921–1930
1931–1940
1941–1950
1951–1960
1961–1970
1971–1980
1981–1990
1991–2000
2003–2012

10 year Summer Teletherm dynamics for NEW CASTLE 1 N, PA:

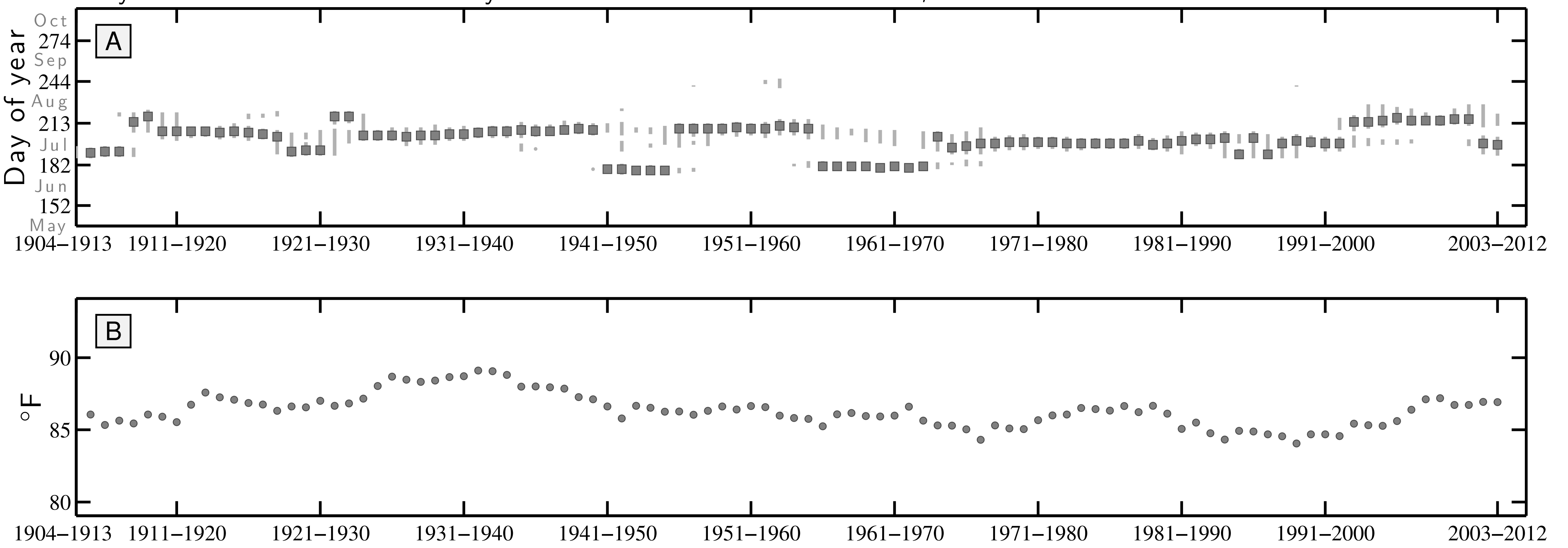

10 year Winter Teletherm dynamics for NEW CASTLE 1 N, PA:

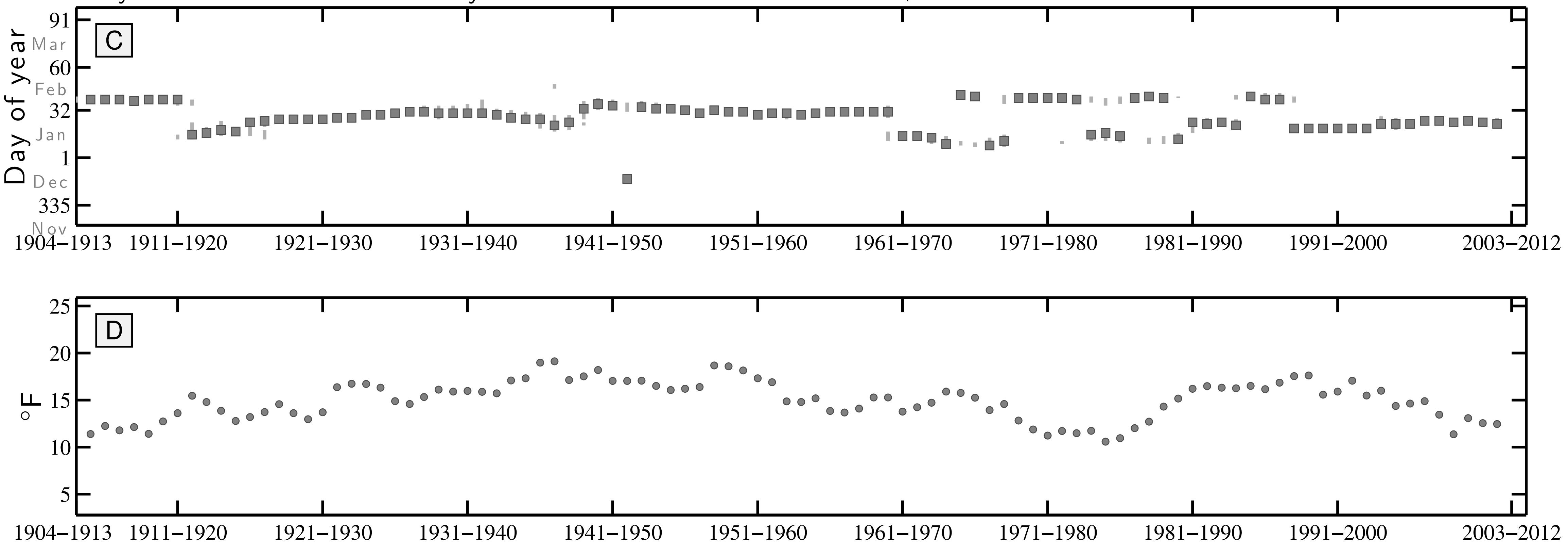

10 year Summer Teletherm dynamics for PALMERTON, PA:

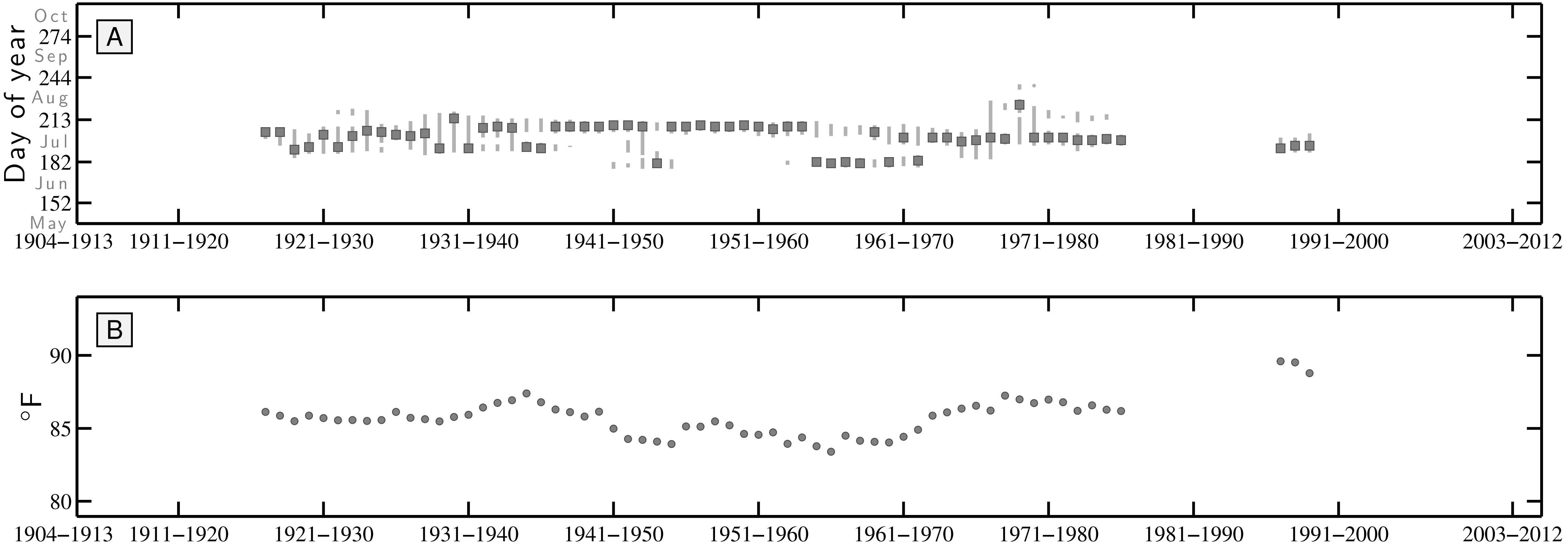

10 year Winter Teletherm dynamics for PALMERTON, PA:

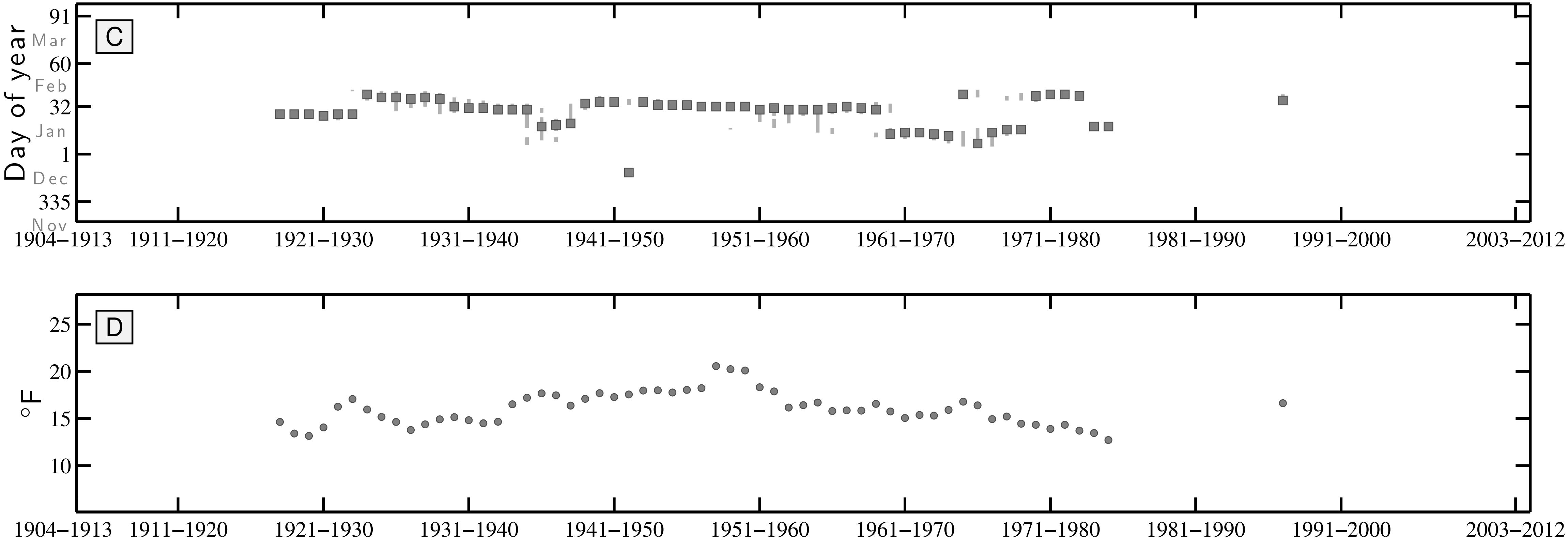

10 year Summer Teletherm dynamics for PLEASANT MT 1 W, PA:

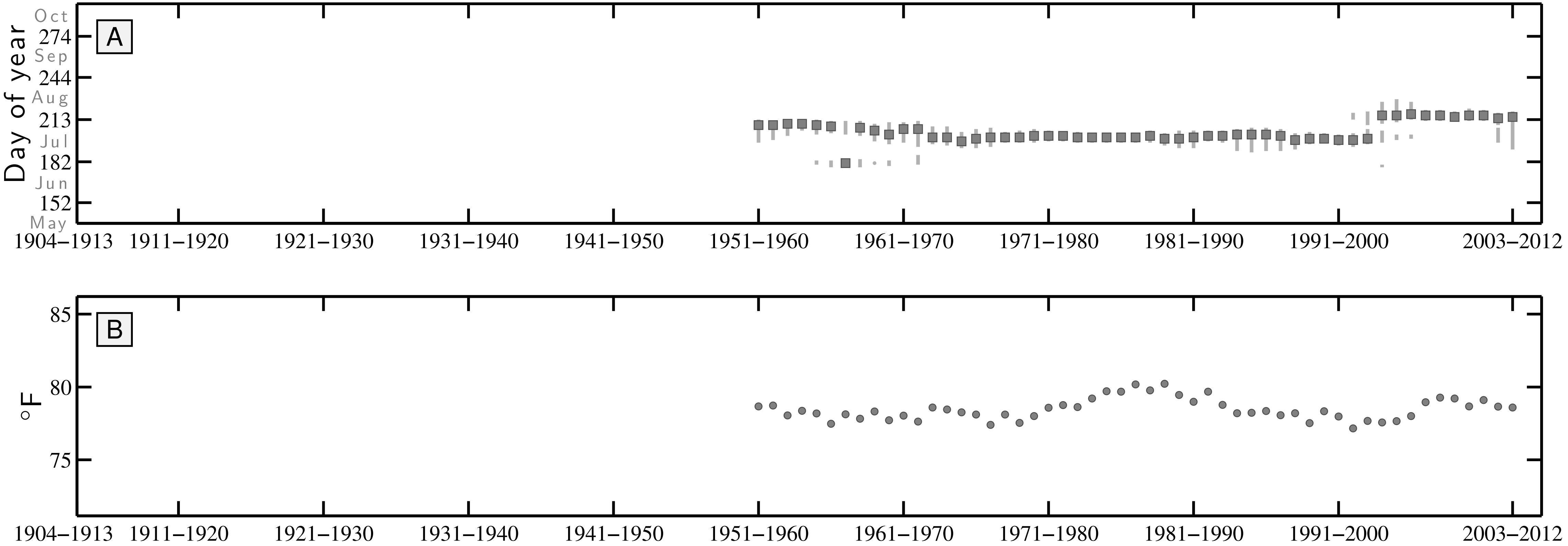

10 year Winter Teletherm dynamics for PLEASANT MT 1 W, PA:

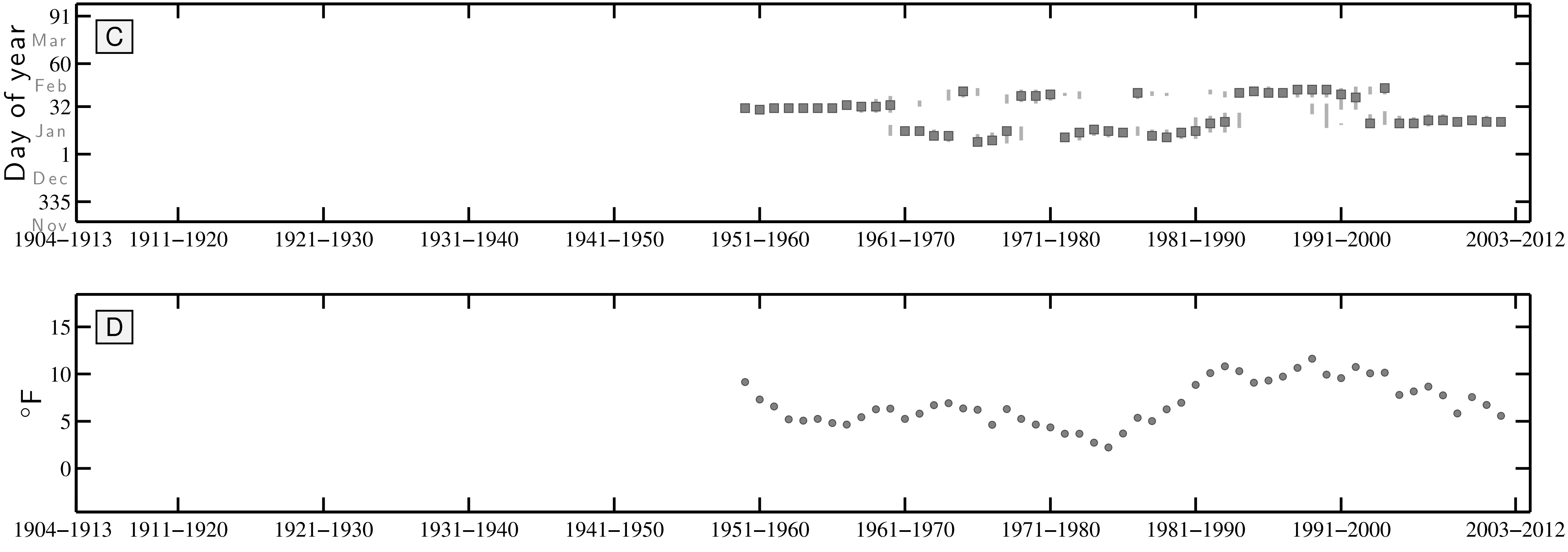

10 year Summer Teletherm dynamics for READING 4 NNW, PA:

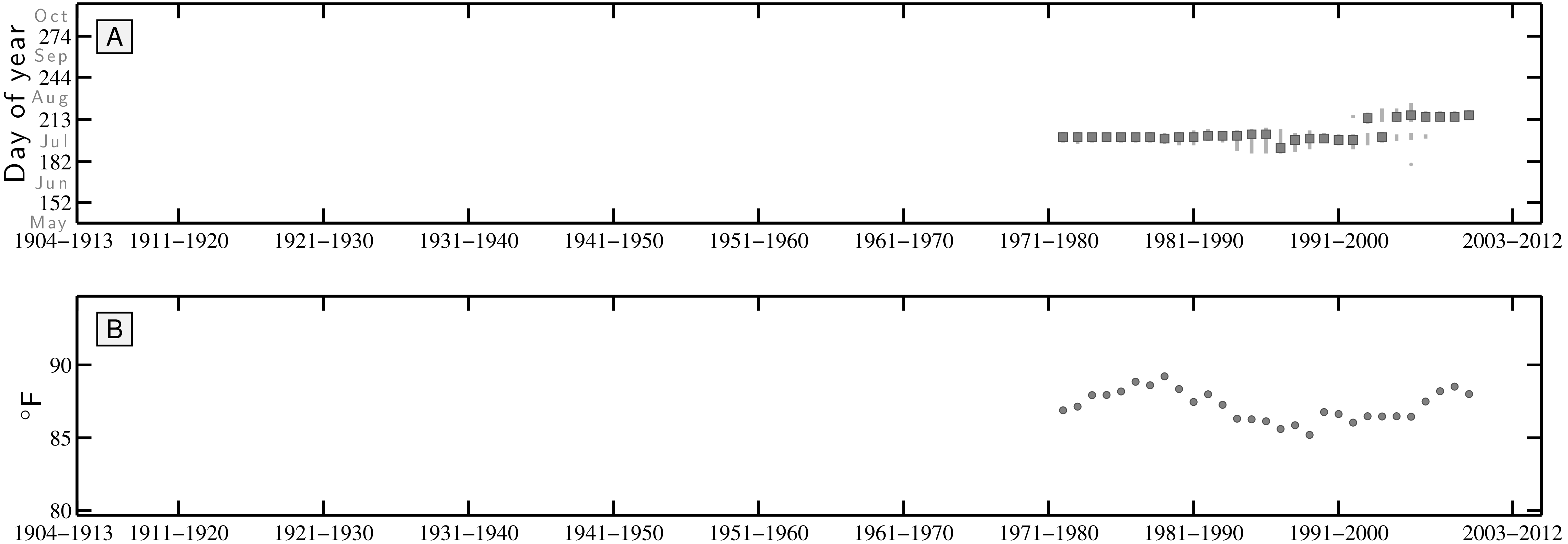

10 year Winter Teletherm dynamics for READING 4 NNW, PA:

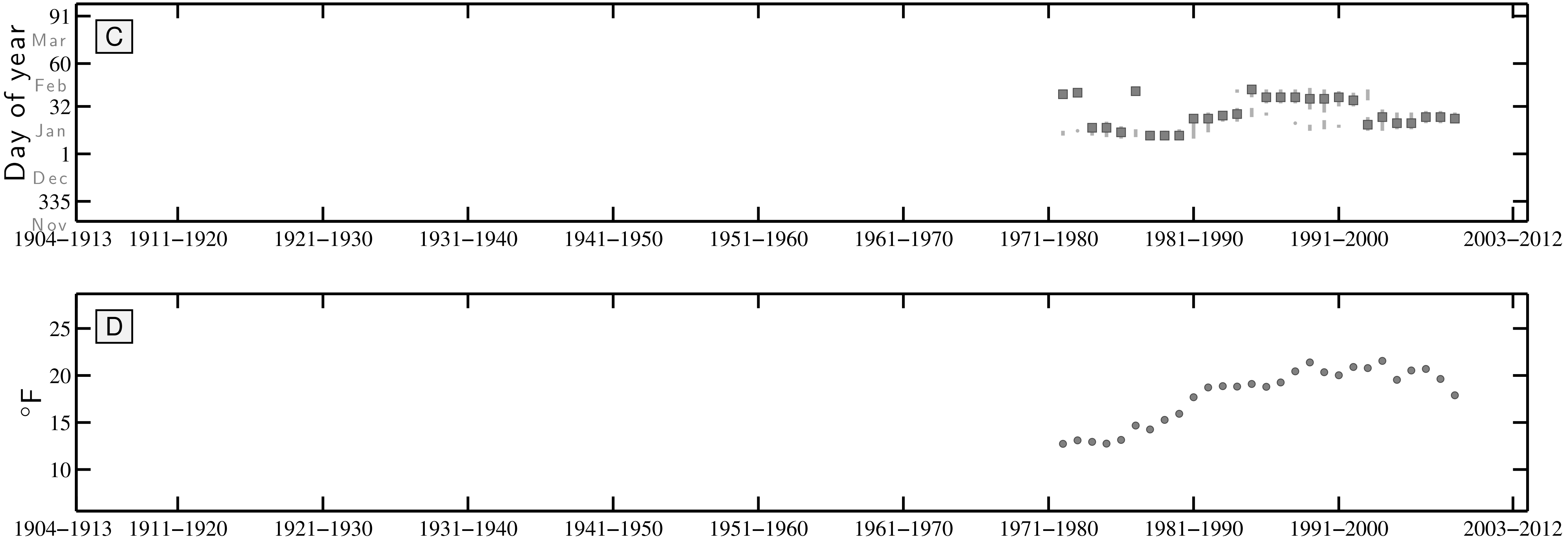

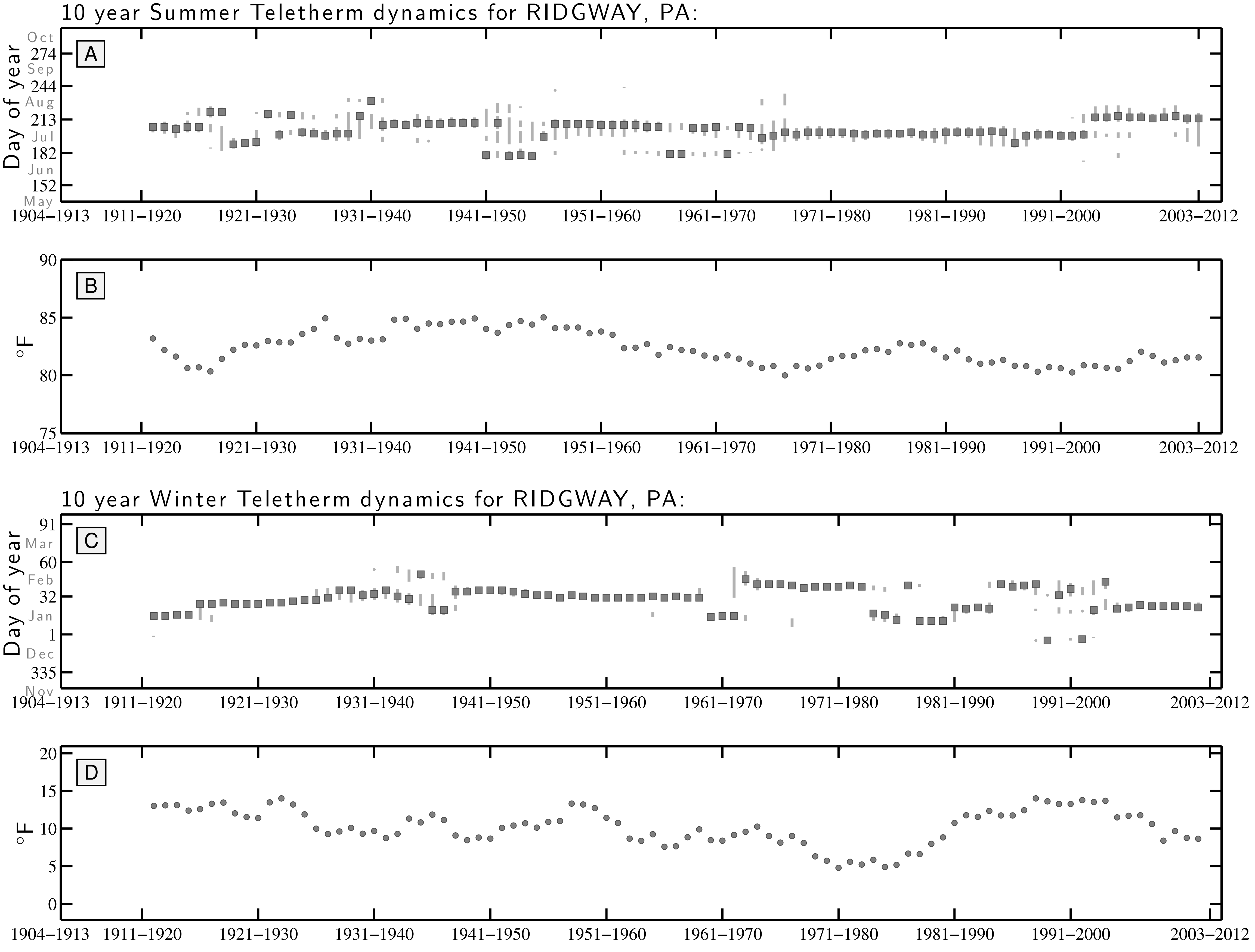

10 year Summer Teletherm dynamics for RIDGWAY, PA:
A
Day of year
Oct
274
Sep
244
Aug
213
Jul
182
Jun
152
May
B
°F
90
85
80
75
10 year Winter Teletherm dynamics for RIDGWAY, PA:
C
Day of year
91
Mar
60
Feb
32
Jan
1
Dec
335
Nov
D
°F
20
15
10
5
0
1904–1913
1911–1920
1921–1930
1931–1940
1941–1950
1951–1960
1961–1970
1971–1980
1981–1990
1991–2000
2003–2012

10 year Summer Teletherm dynamics for SELINSGROVE 2 S, PA:

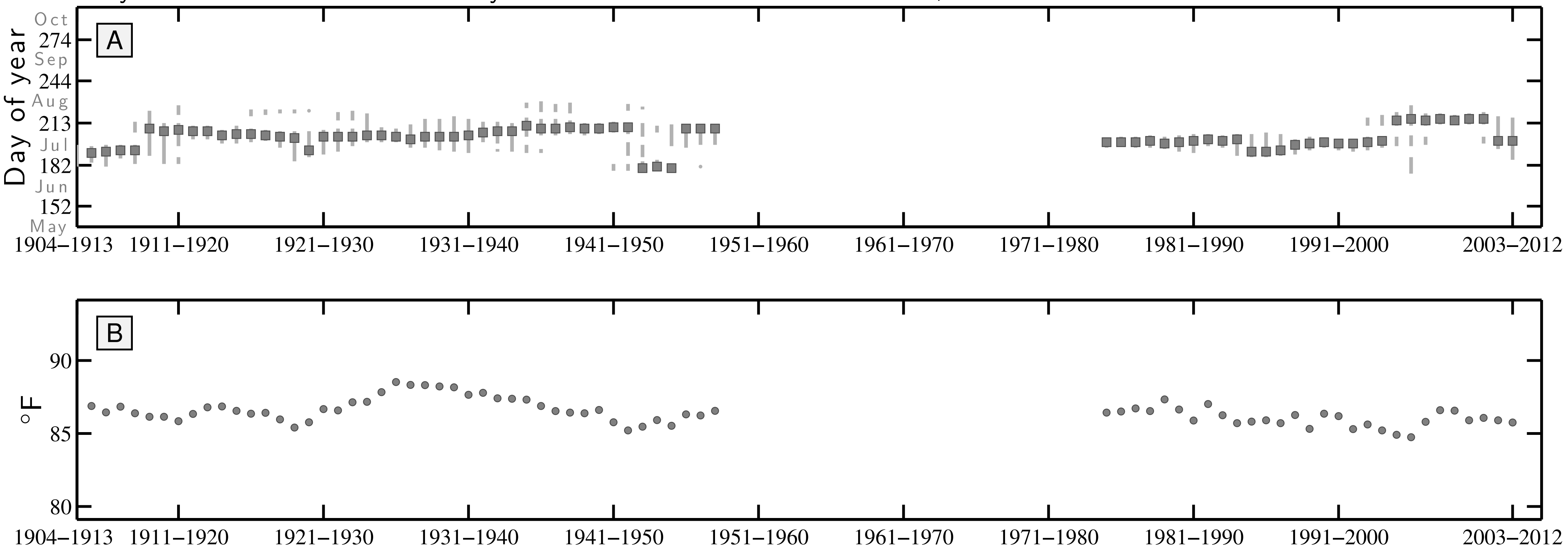

10 year Winter Teletherm dynamics for SELINSGROVE 2 S, PA:

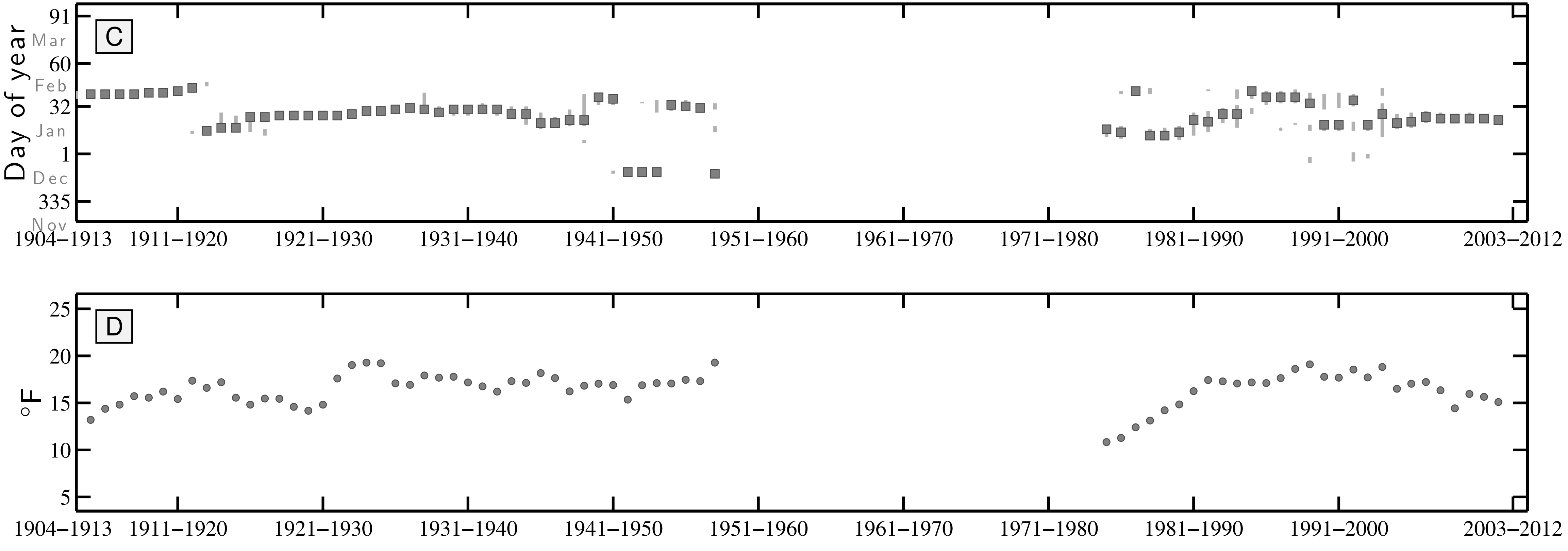

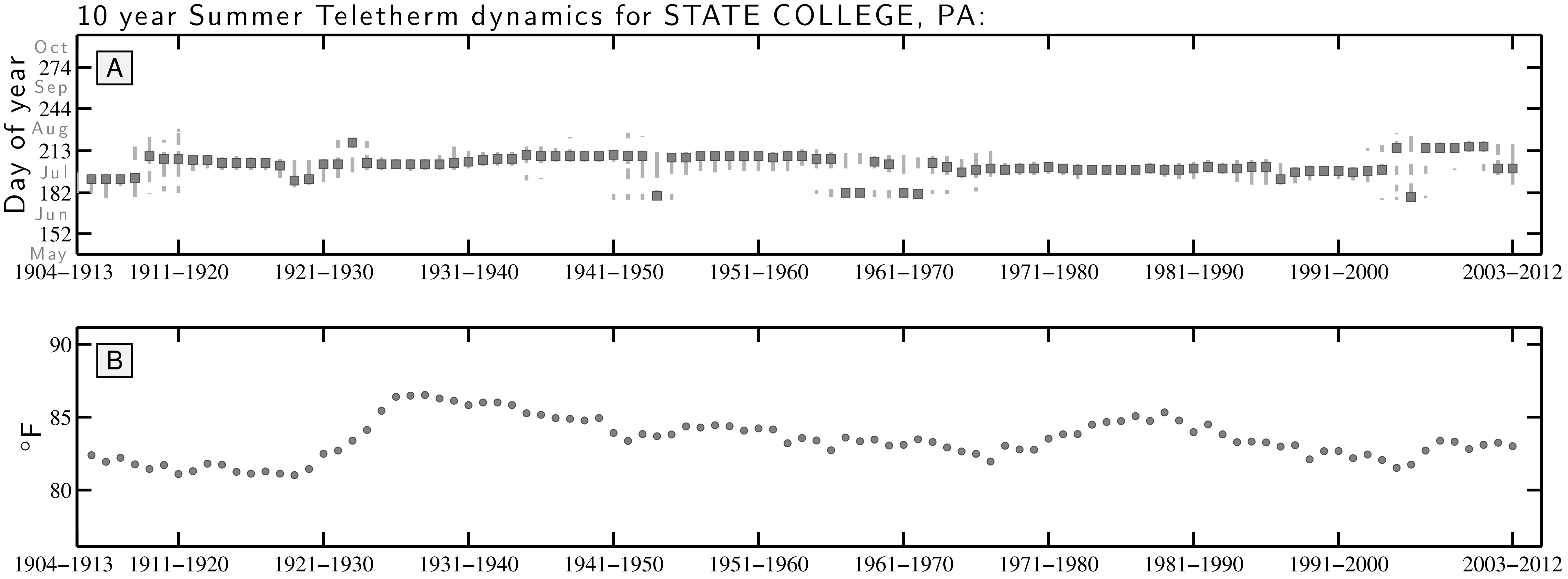

10 year Summer Teletherm dynamics for STATE COLLEGE, PA:
A
Day of year
Oct
274
Sep
244
Aug
213
Jul
182
Jun
152
May
1904–1913
1911–1920
1921–1930
1931–1940
1941–1950
1951–1960
1961–1970
1971–1980
1981–1990
1991–2000
2003–2012
B
°F
90
85
80

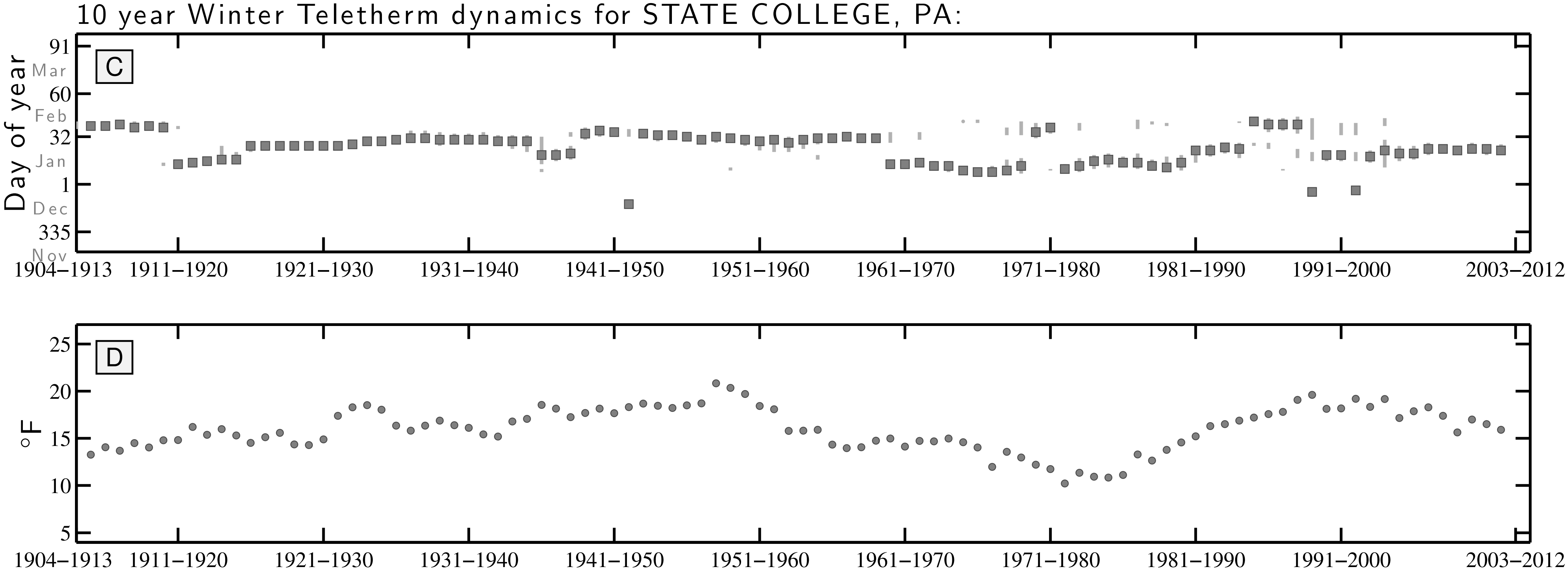

10 year Winter Teletherm dynamics for STATE COLLEGE, PA:
C
Day of year
91
Mar
60
Feb
32
Jan
1
Dec
335
Nov
1904–1913
1911–1920
1921–1930
1931–1940
1941–1950
1951–1960
1961–1970
1971–1980
1981–1990
1991–2000
2003–2012
D
°F
25
20
15
10
5

10 year Summer Teletherm dynamics for STROUDSBURG, PA:

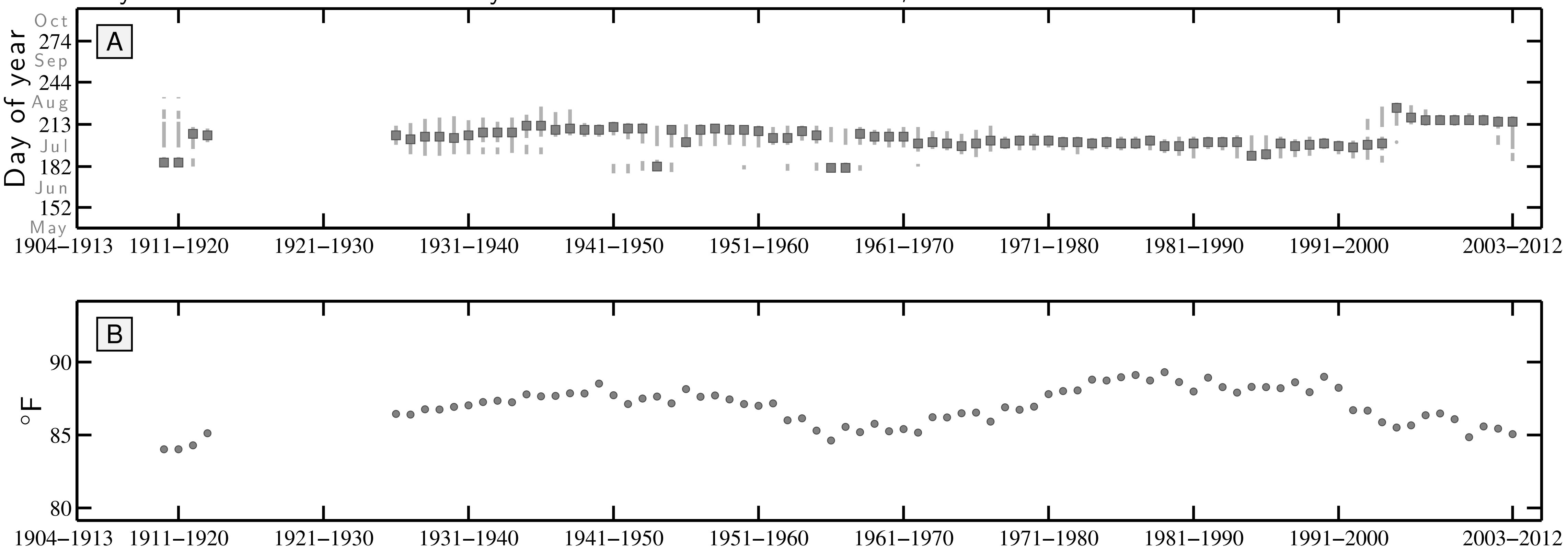

10 year Winter Teletherm dynamics for STROUDSBURG, PA:

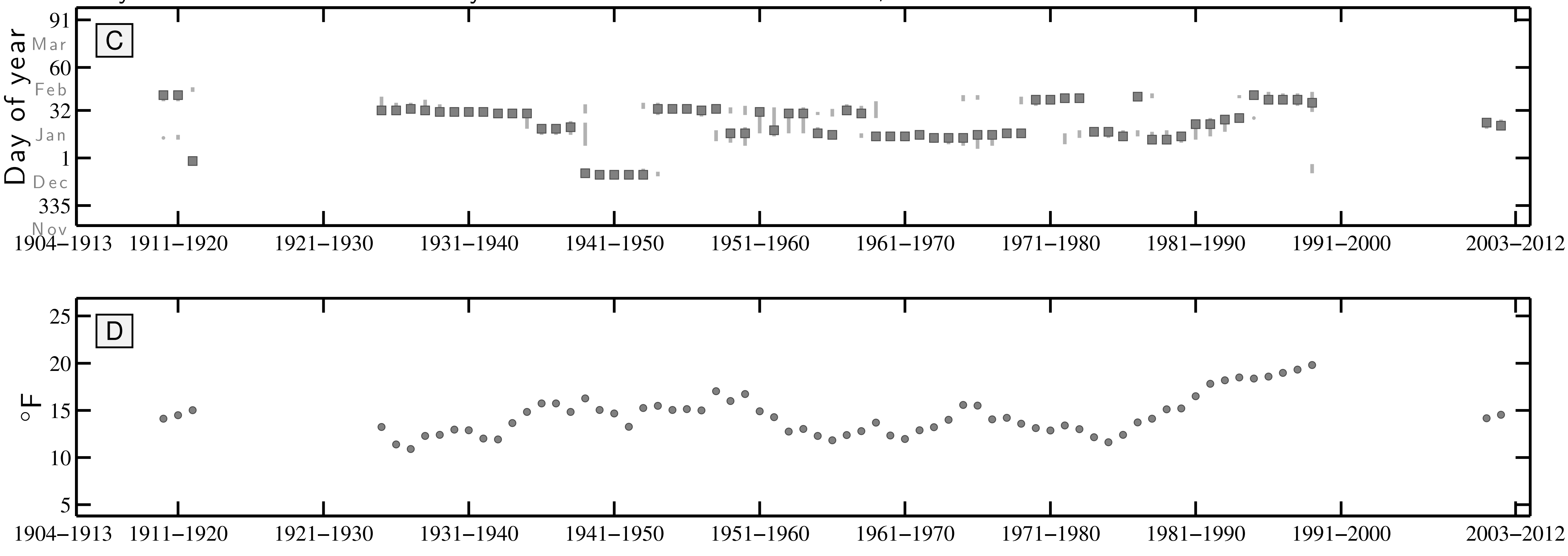

10 year Summer Teletherm dynamics for TOWANDA 1 S, PA:

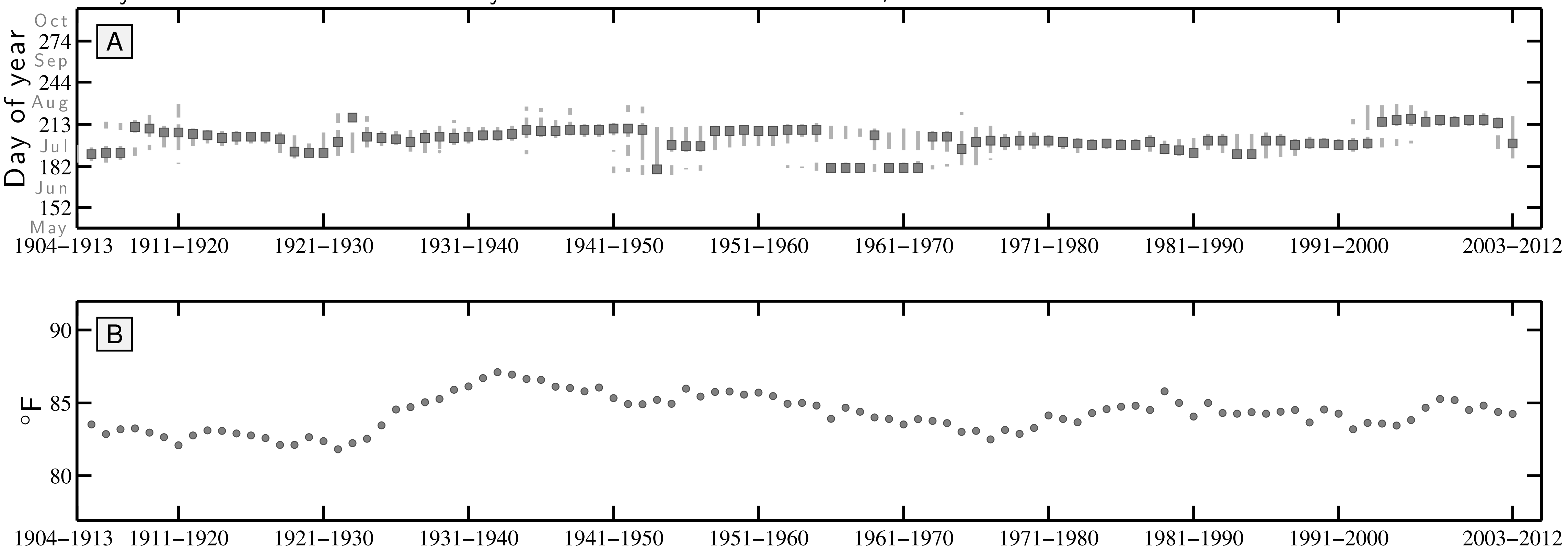

10 year Winter Teletherm dynamics for TOWANDA 1 S, PA:

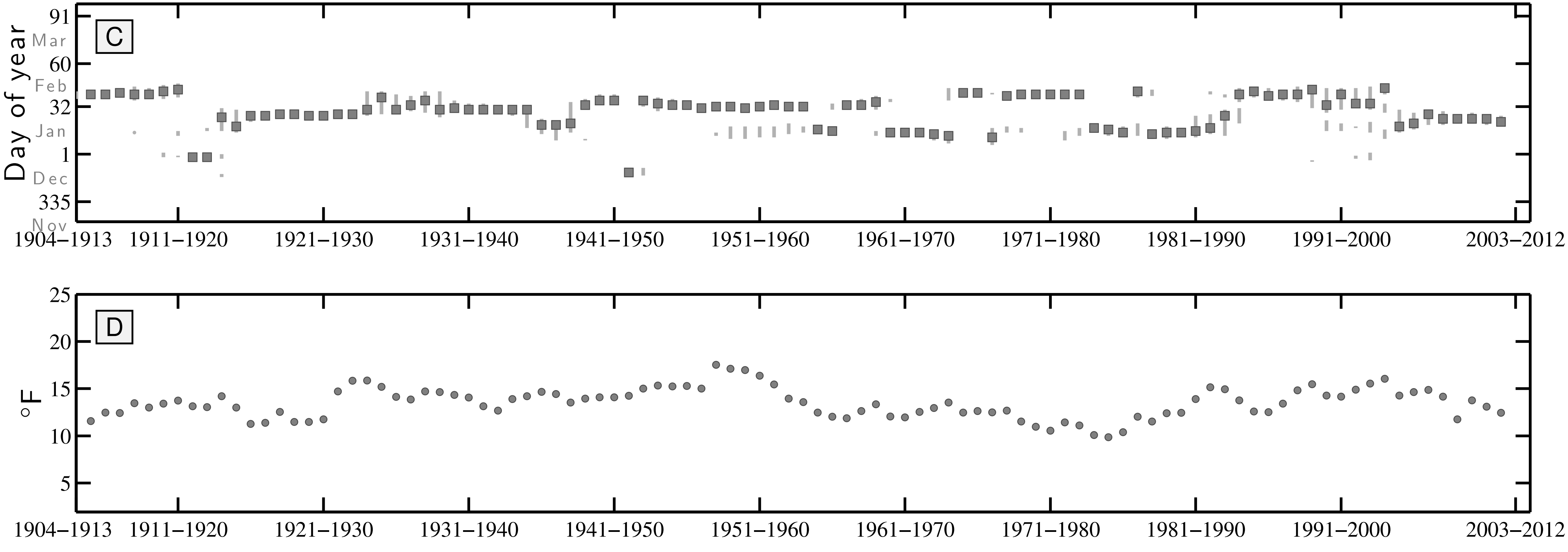

10 year Summer Teletherm dynamics for UNIONTOWN 1 NE, PA:

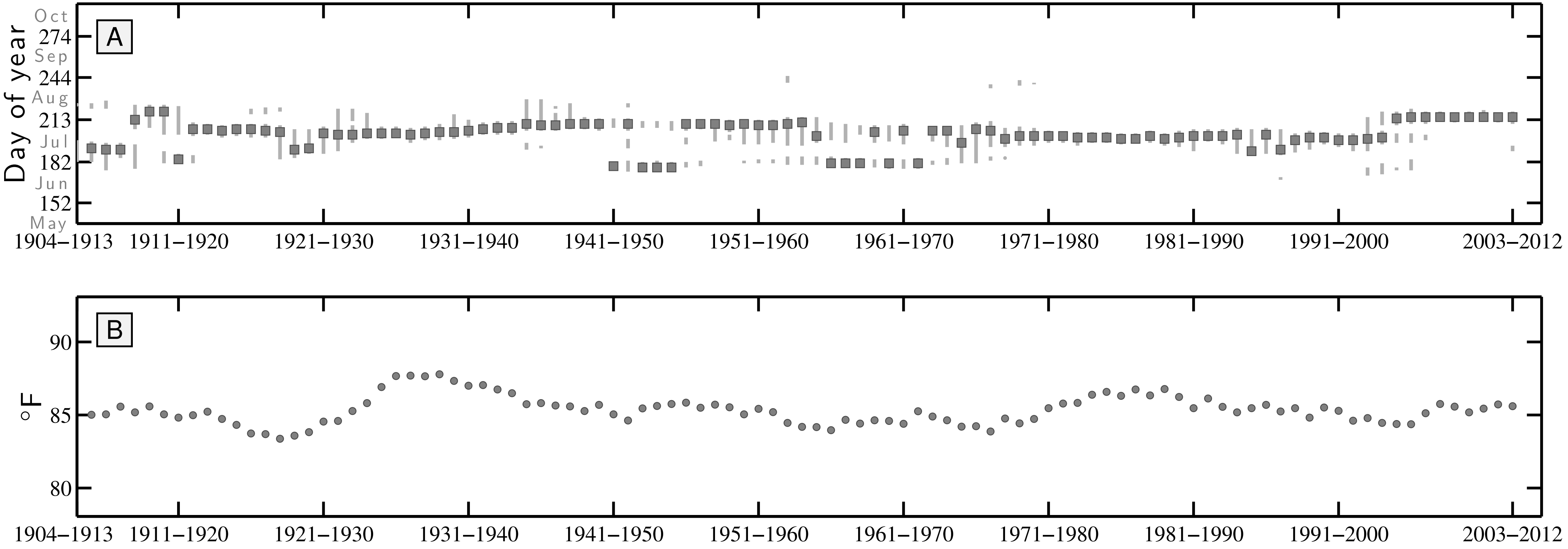

10 year Winter Teletherm dynamics for UNIONTOWN 1 NE, PA:

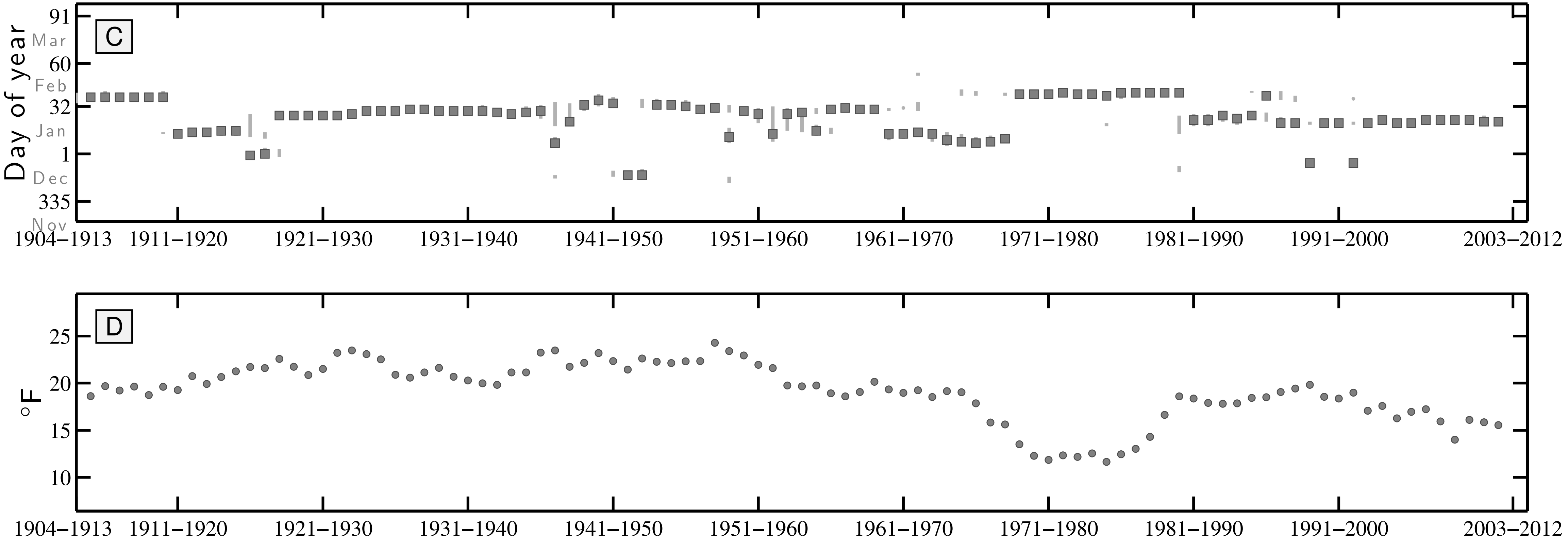

10 year Summer Teletherm dynamics for WARREN, PA:

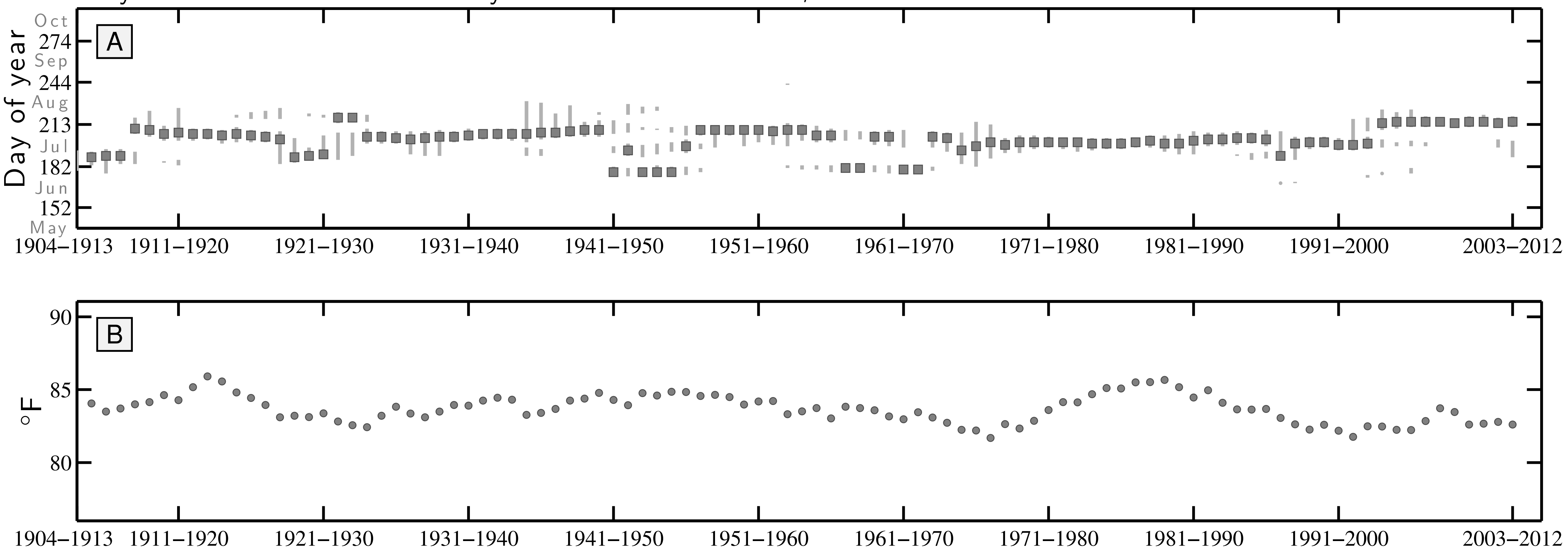

10 year Winter Teletherm dynamics for WARREN, PA:

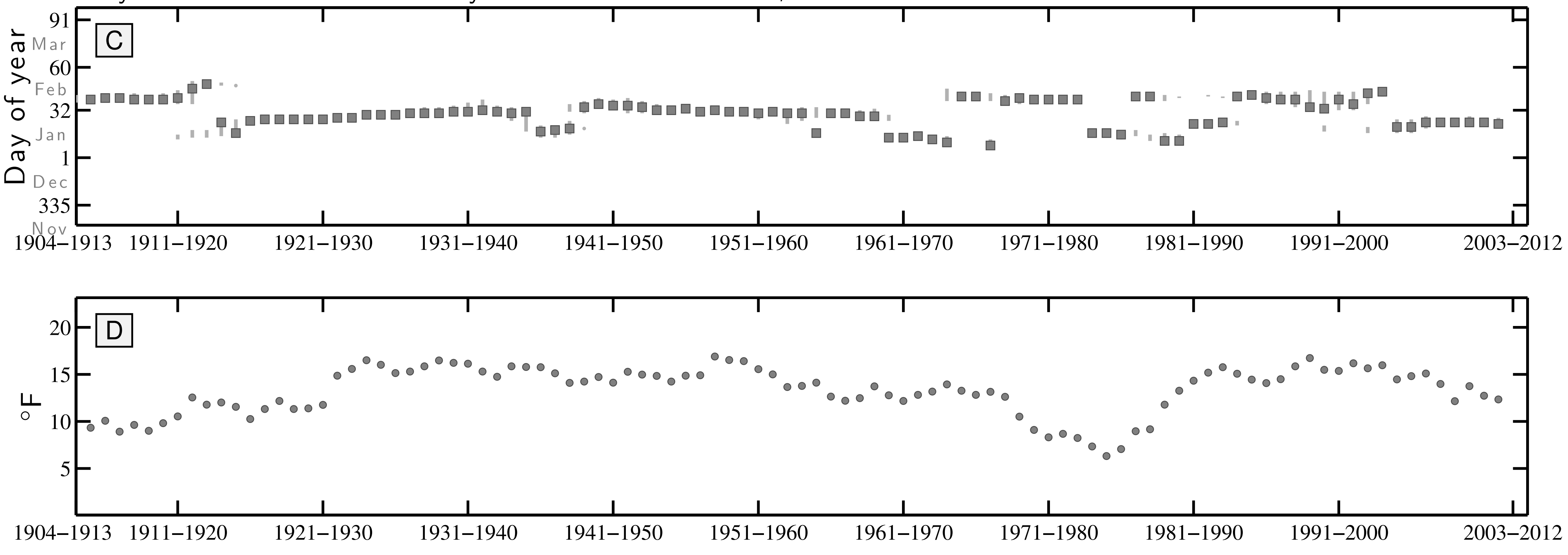

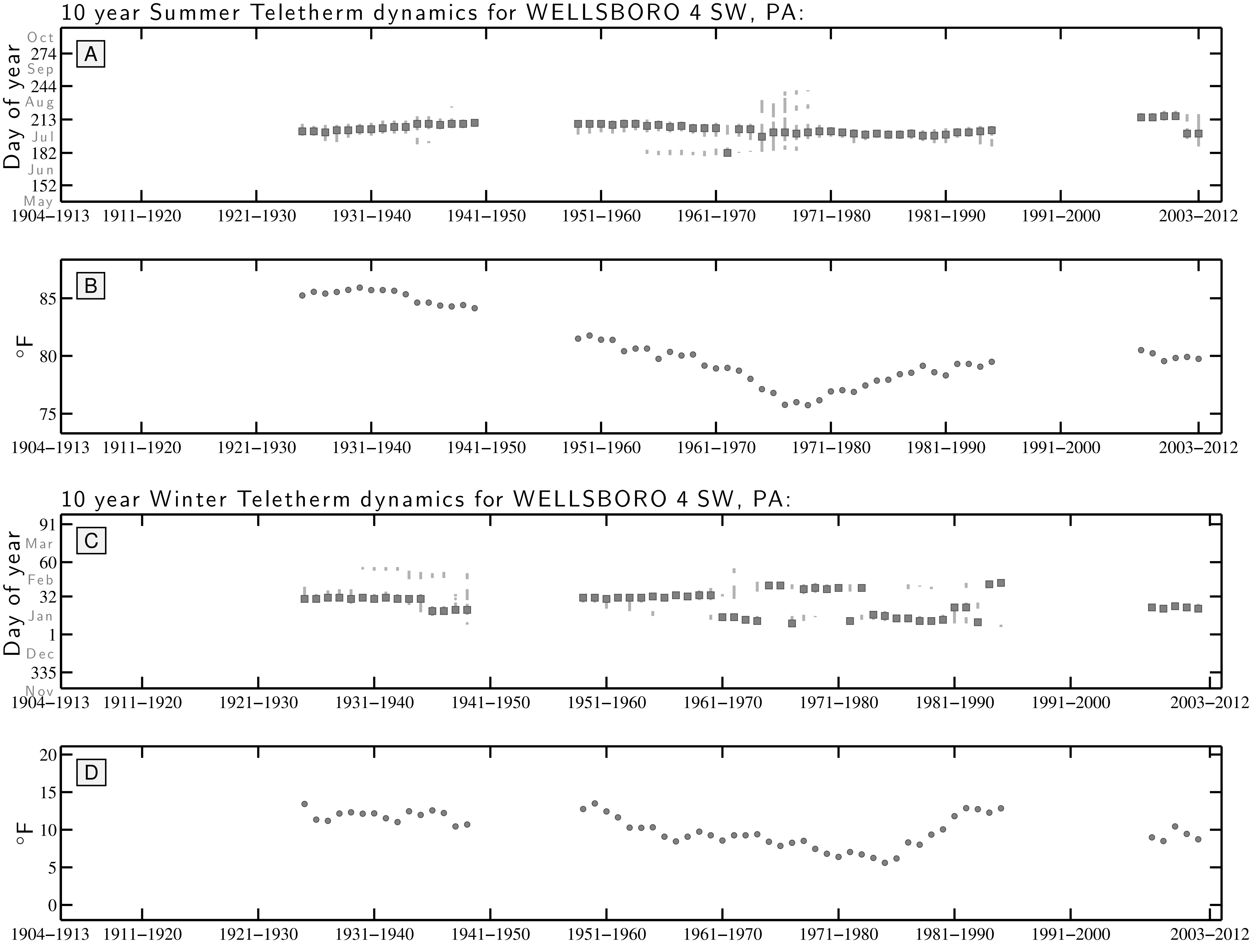

10 year Summer Teletherm dynamics for WELLSBORO 4 SW, PA:
A
B
Day of year
°F
Oct
274
Sep
244
Aug
213
Jul
182
Jun
152
May
85
80
75
1904–1913
1911–1920
1921–1930
1931–1940
1941–1950
1951–1960
1961–1970
1971–1980
1981–1990
1991–2000
2003–2012
10 year Winter Teletherm dynamics for WELLSBORO 4 SW, PA:
C
D
91
Mar
60
Feb
32
Jan
1
Dec
335
Nov
20
15
10
5
0

10 year Summer Teletherm dynamics for WEST CHESTER 2 NW, PA:

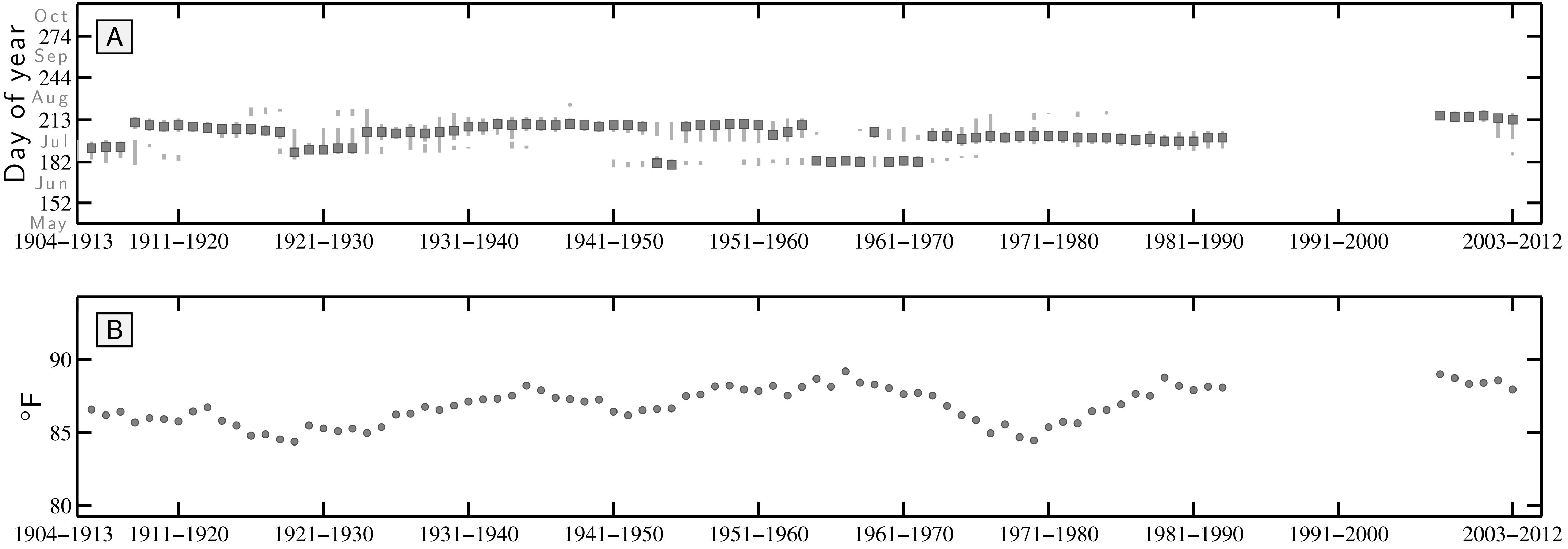

10 year Winter Teletherm dynamics for WEST CHESTER 2 NW, PA:

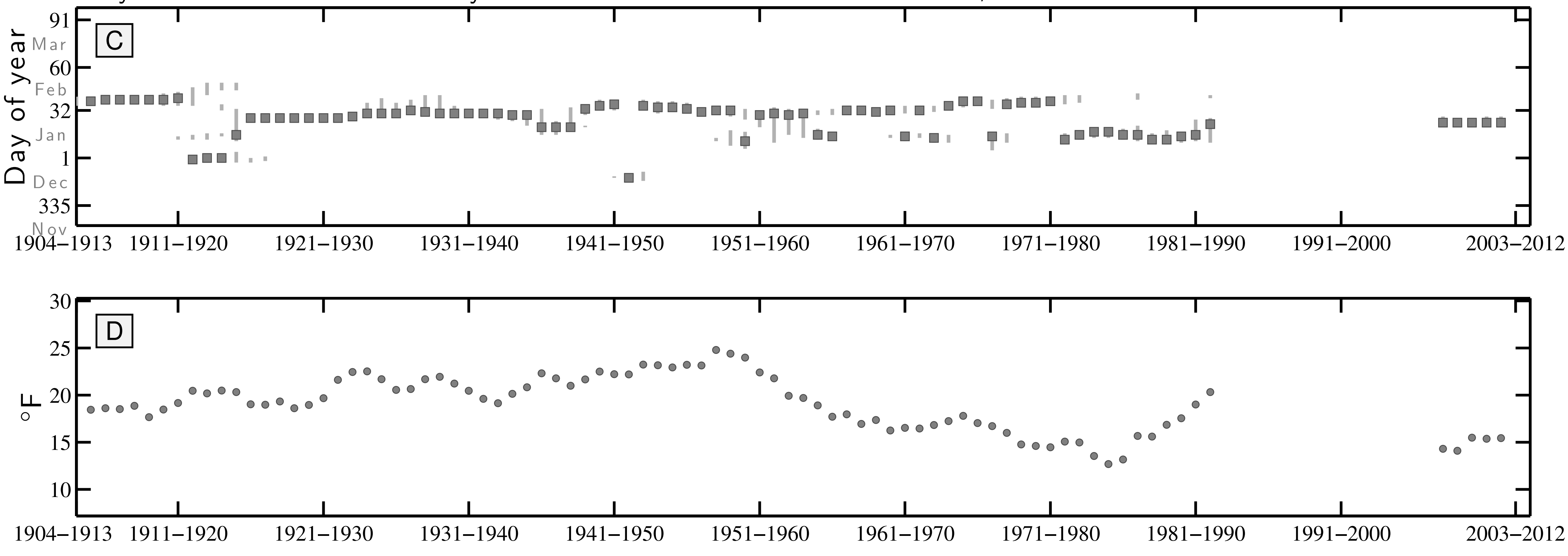

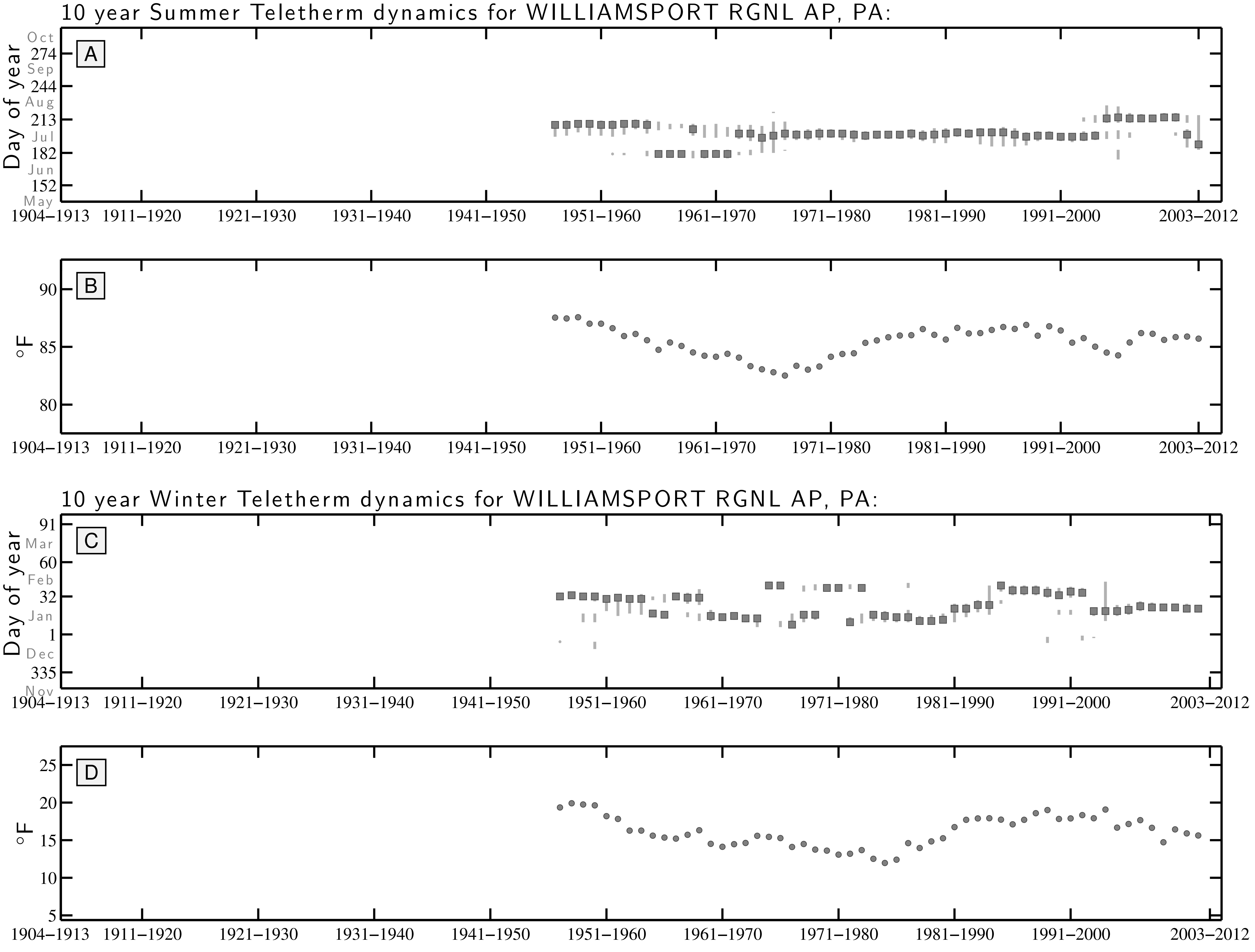

10 year Summer Teletherm dynamics for WILLIAMSPORT RGNL AP, PA:
A
Day of year
Oct
274
Sep
244
Aug
213
Jul
182
Jun
152
May
1904–1913
1911–1920
1921–1930
1931–1940
1941–1950
1951–1960
1961–1970
1971–1980
1981–1990
1991–2000
2003–2012
B
°F
90
85
80
10 year Winter Teletherm dynamics for WILLIAMSPORT RGNL AP, PA:
C
91
Mar
60
Feb
32
Jan
1
Dec
335
Nov
D
25
20
15
10
5

10 year Summer Teletherm dynamics for YORK 3 SSW PUMP, PA:

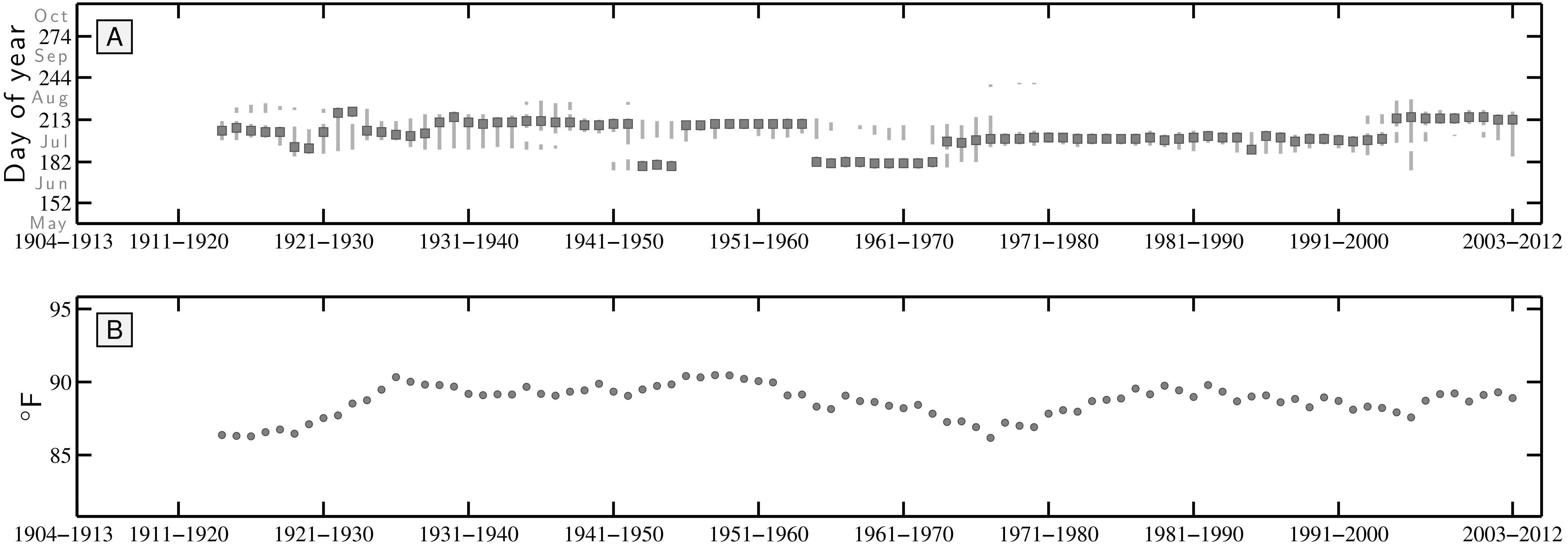

10 year Winter Teletherm dynamics for YORK 3 SSW PUMP, PA:

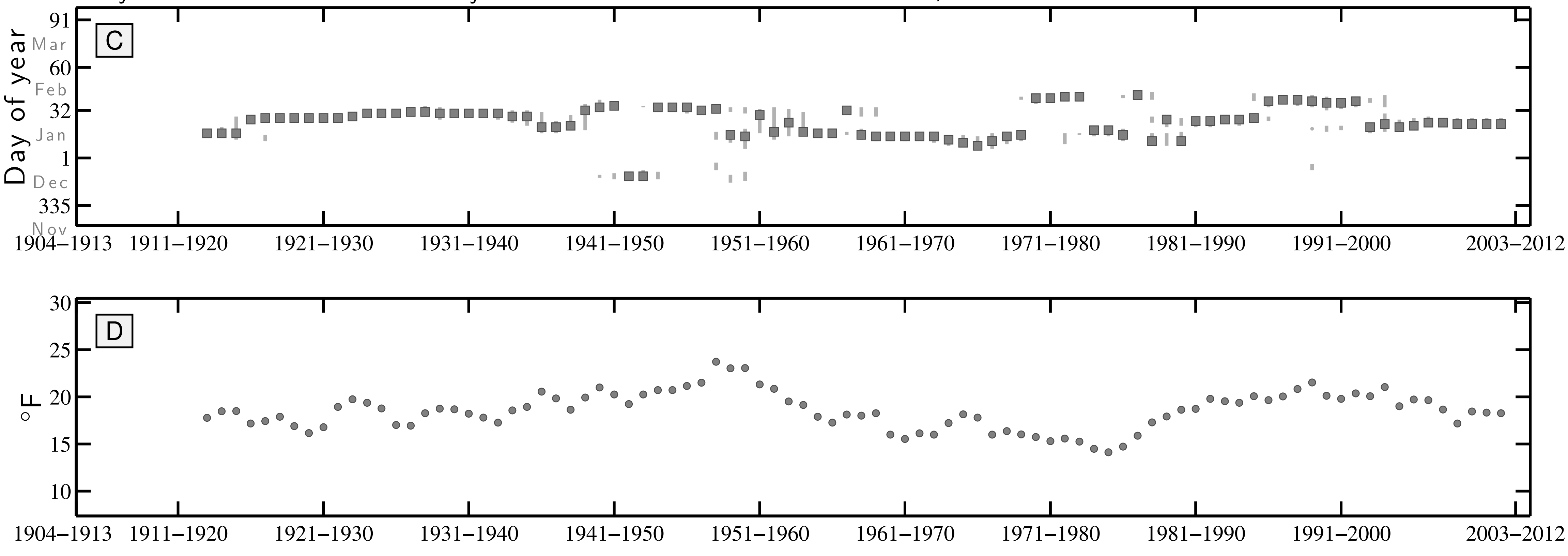

10 year Summer Teletherm dynamics for BLOCK ISLAND STATE AP, RI:

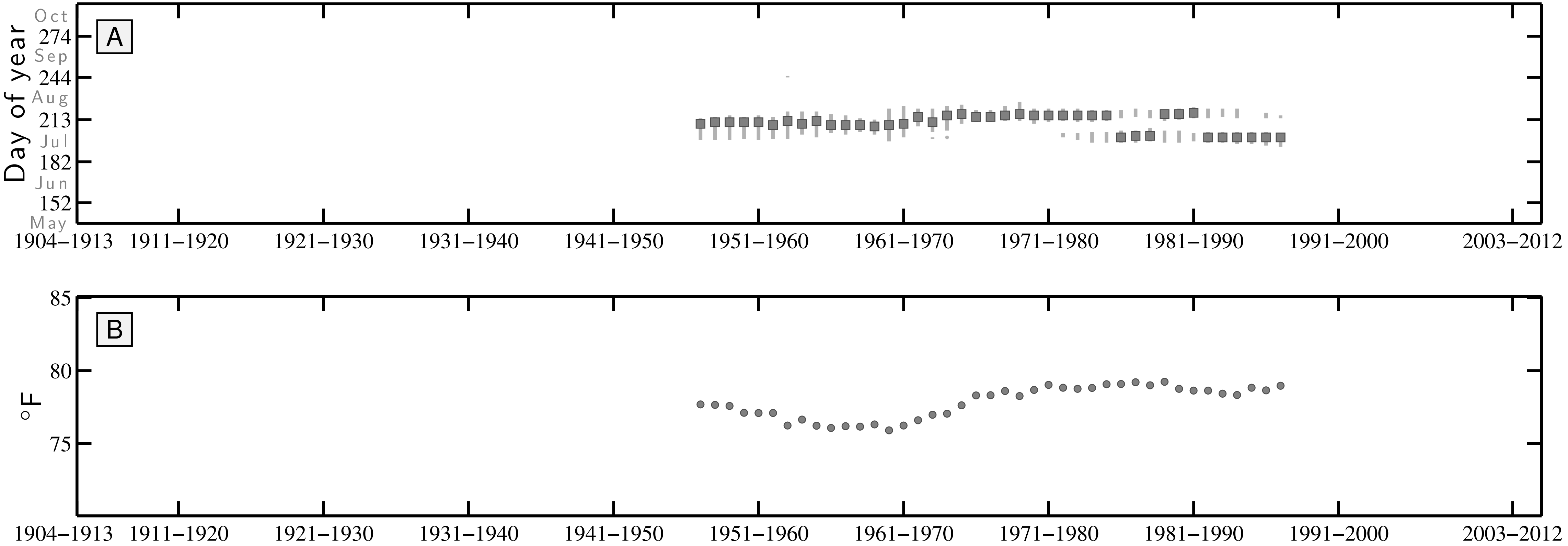

10 year Winter Teletherm dynamics for BLOCK ISLAND STATE AP, RI:

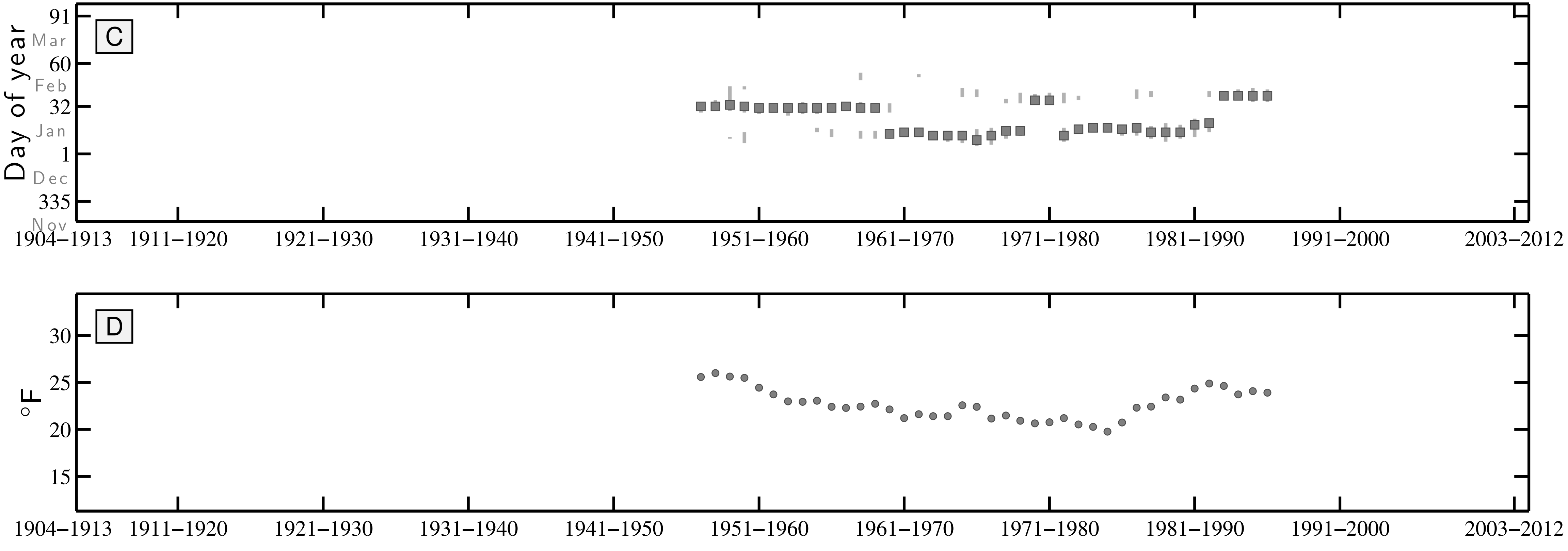

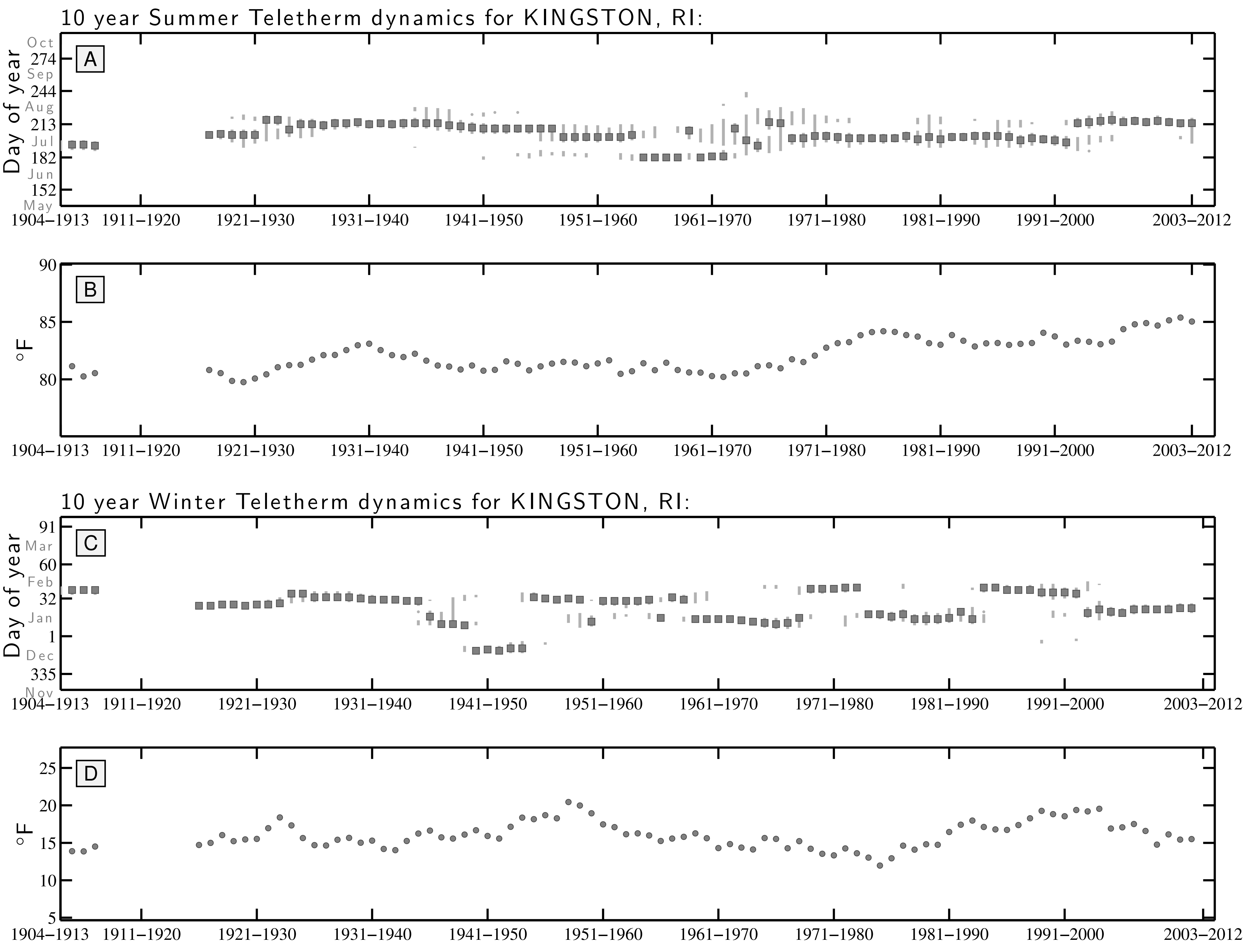
10 year Summer Teletherm dynamics for KINGSTON, RI:
A
Day of year
Oct
274
Sep
244
Aug
213
Jul
182
Jun
152
May
1904–1913
1911–1920
1921–1930
1931–1940
1941–1950
1951–1960
1961–1970
1971–1980
1981–1990
1991–2000
2003–2012
B
°F
90
85
80
10 year Winter Teletherm dynamics for KINGSTON, RI:
C
Day of year
91
Mar
60
Feb
32
Jan
1
Dec
335
Nov
D
°F
25
20
15
10
5

10 year Summer Teletherm dynamics for PROVIDENCE WSO AP, RI:

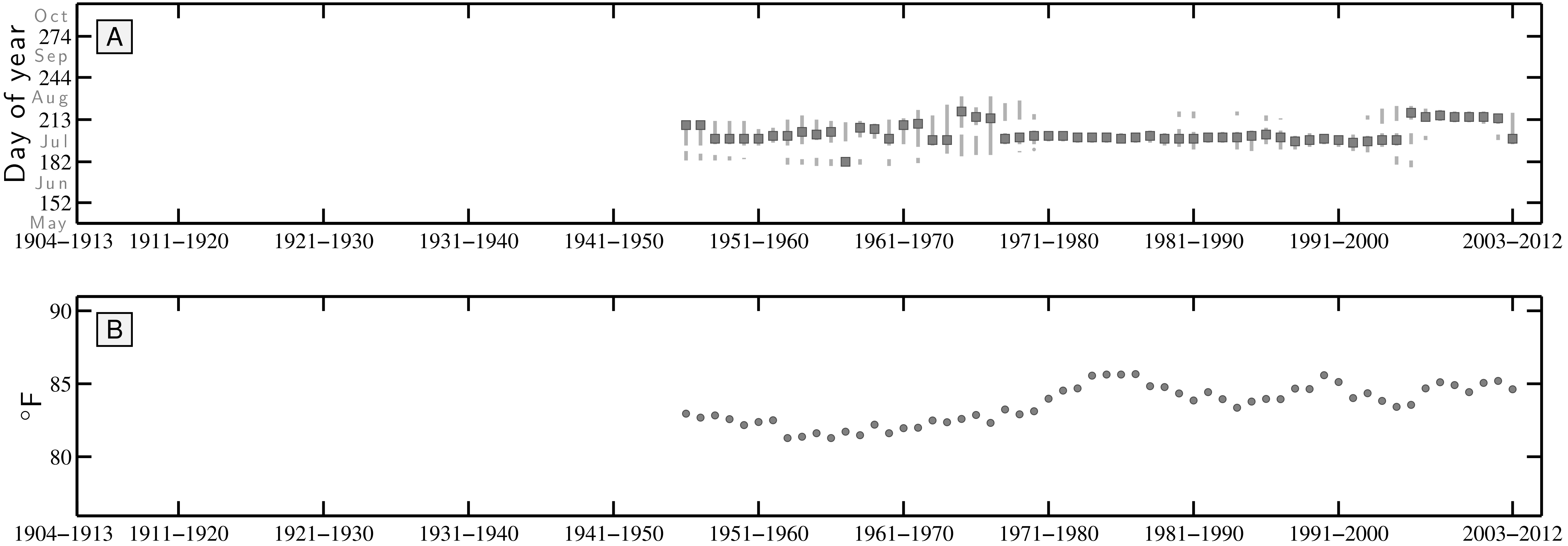

10 year Winter Teletherm dynamics for PROVIDENCE WSO AP, RI:

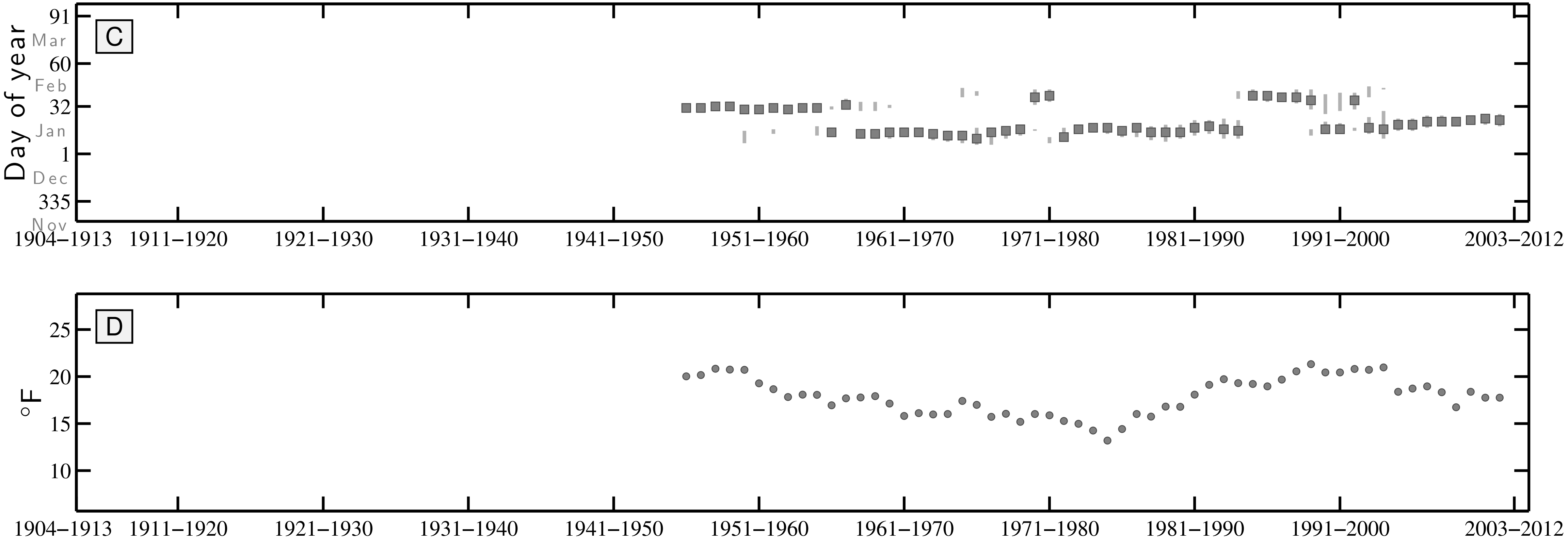

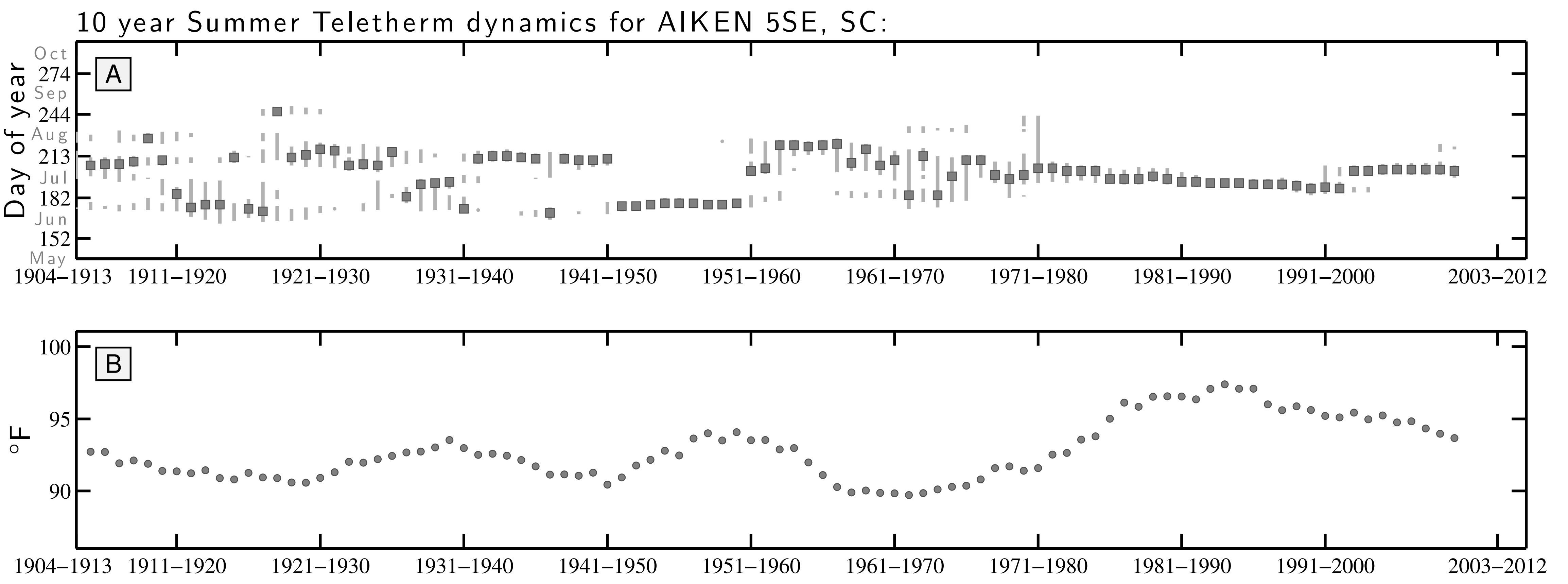

10 year Summer Teletherm dynamics for AIKEN 5SE, SC:
A
B
Day of year
°F
Oct
Sep
Aug
Jul
Jun
May
274
244
213
182
152
100
95
90
1904–1913
1911–1920
1921–1930
1931–1940
1941–1950
1951–1960
1961–1970
1971–1980
1981–1990
1991–2000
2003–2012

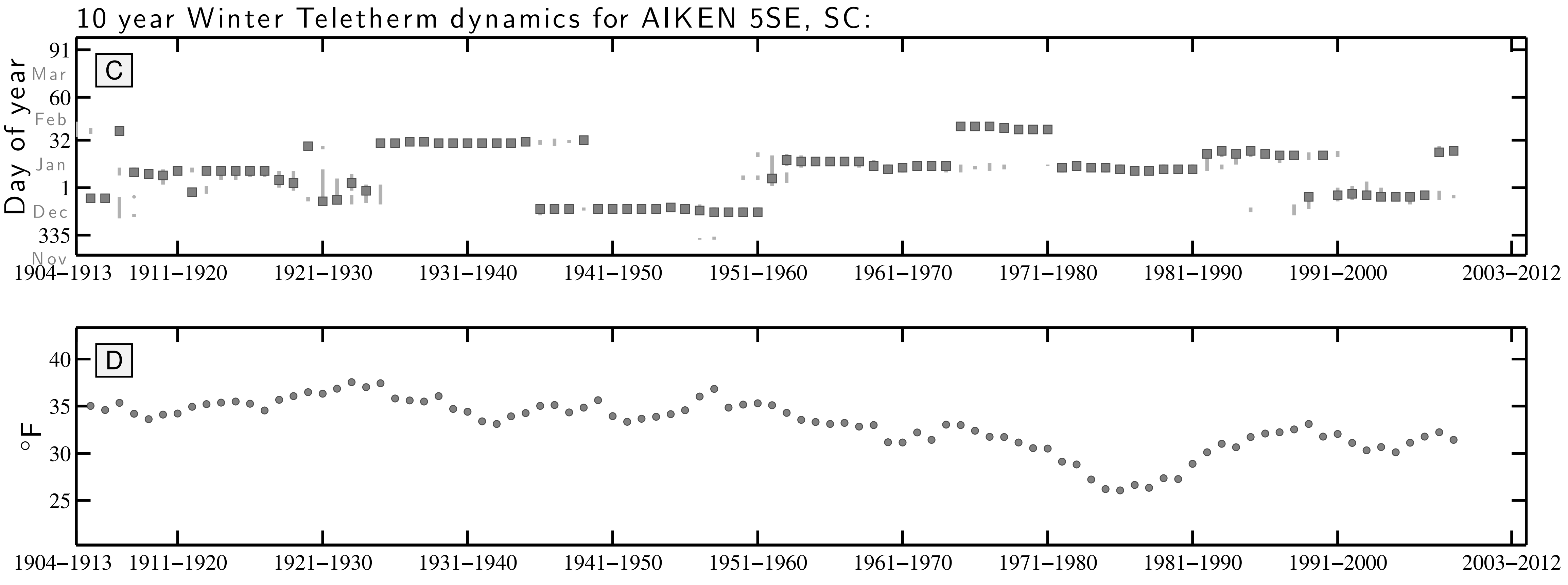

10 year Winter Teletherm dynamics for AIKEN 5SE, SC:
C
D
Day of year
°F
Mar
Feb
Jan
Dec
Nov
91
60
32
1
335
40
35
30
25
1904–1913
1911–1920
1921–1930
1931–1940
1941–1950
1951–1960
1961–1970
1971–1980
1981–1990
1991–2000
2003–2012

10 year Summer Teletherm dynamics for ANDERSON, SC:

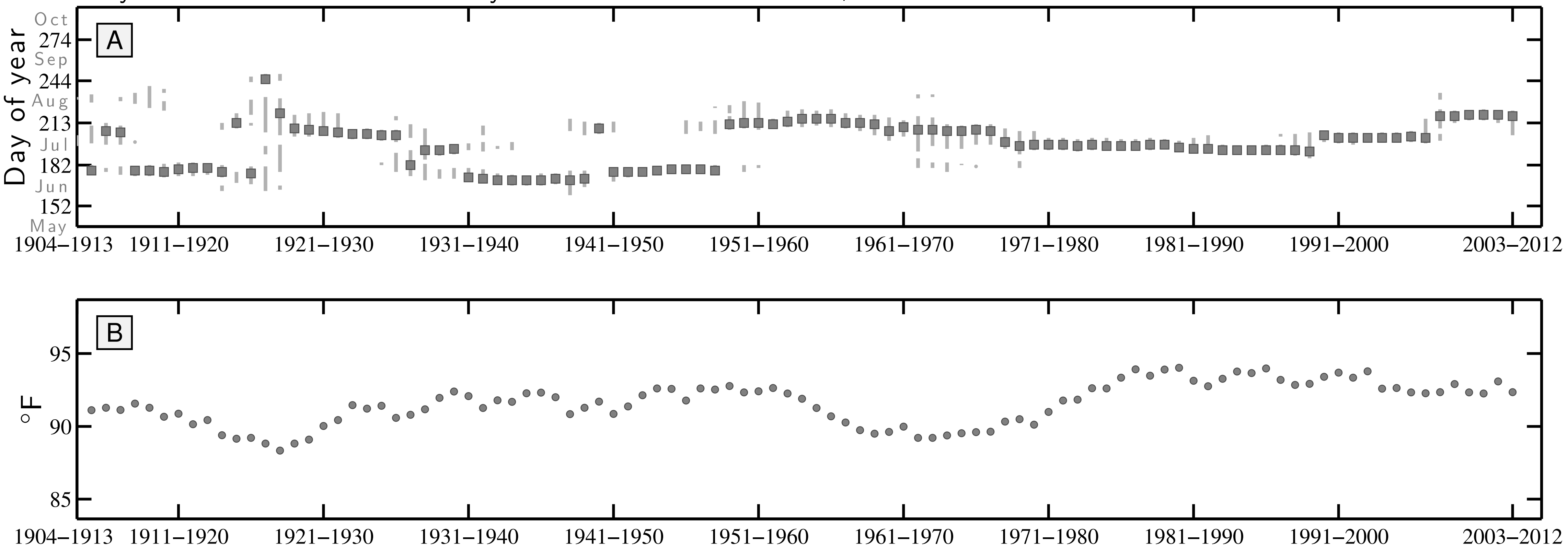

10 year Winter Teletherm dynamics for ANDERSON, SC:

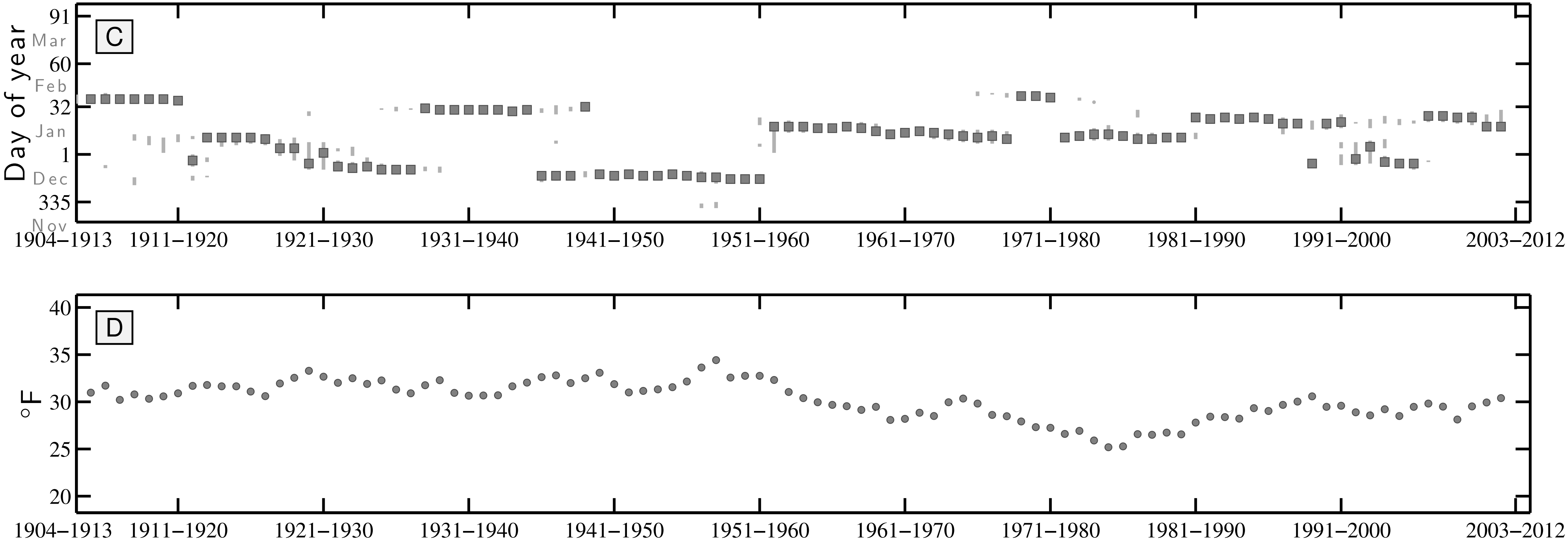

10 year Summer Teletherm dynamics for BEAUFORT WWTP, SC:

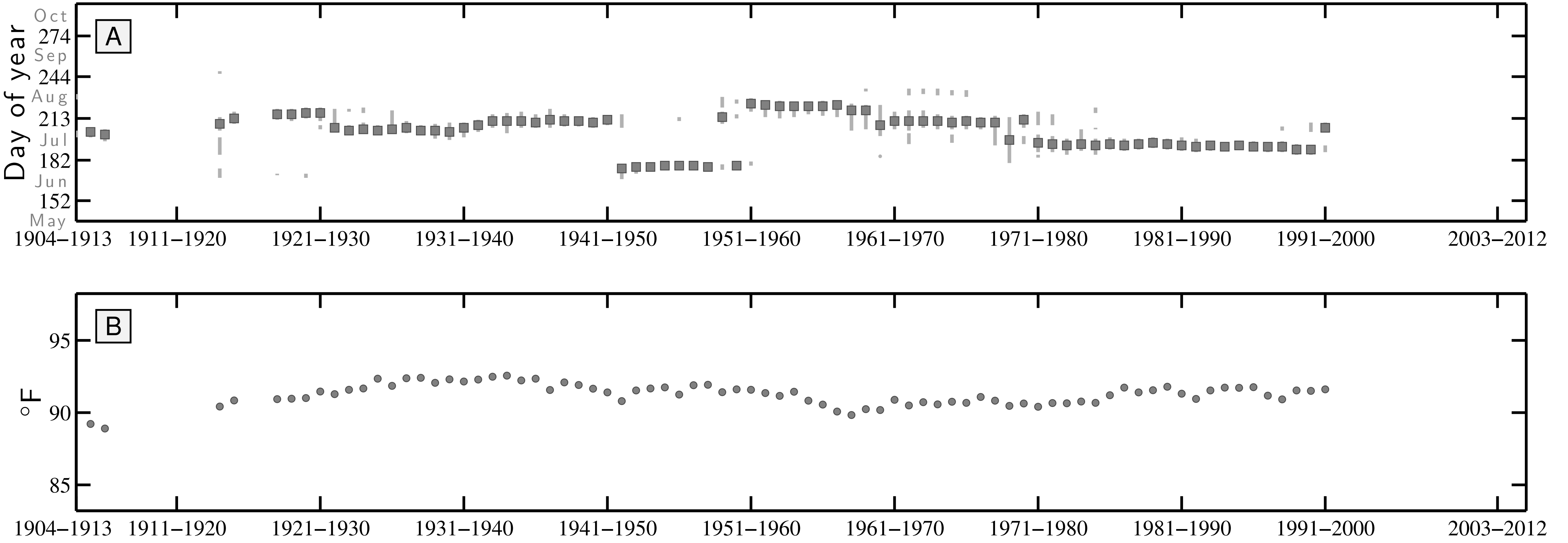

10 year Winter Teletherm dynamics for BEAUFORT WWTP, SC:

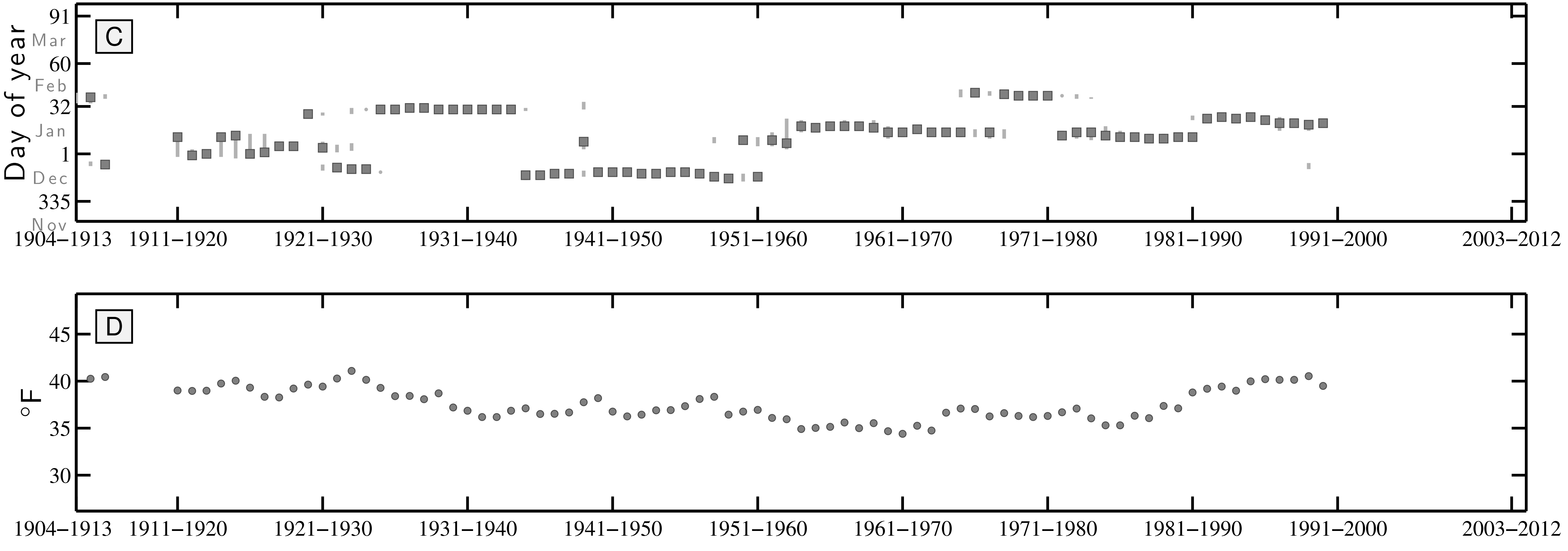

10 year Summer Teletherm dynamics for BLACKVILLE 3 W, SC:

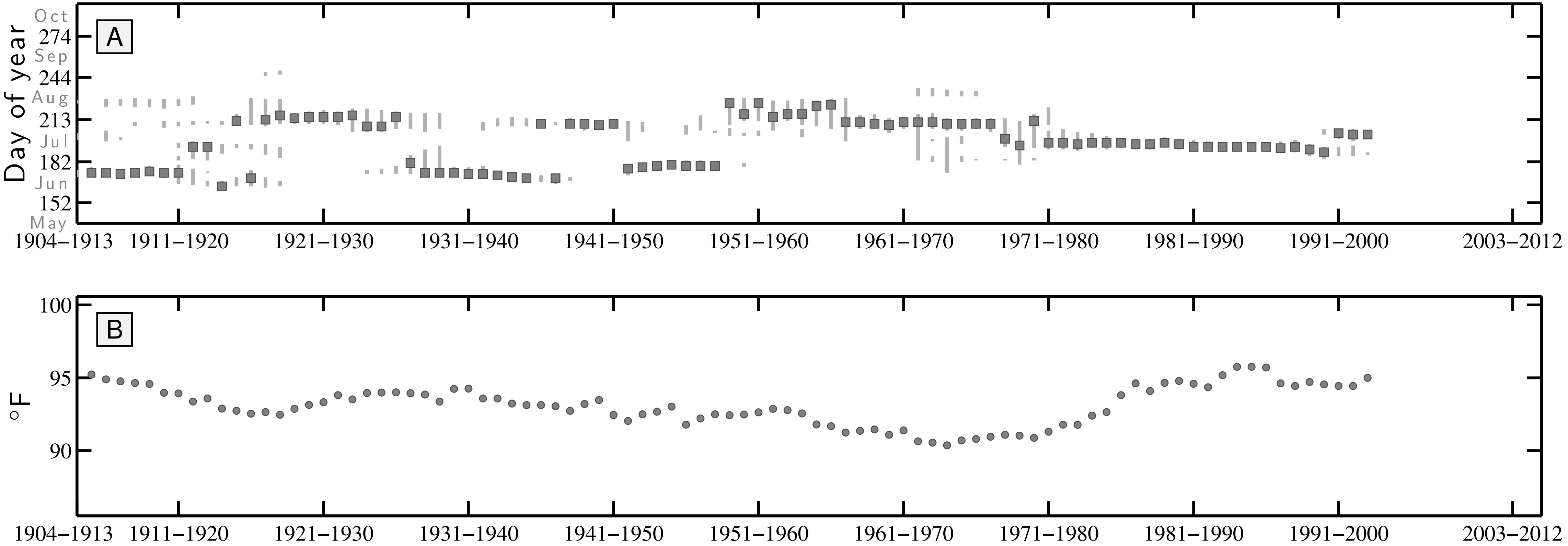

10 year Winter Teletherm dynamics for BLACKVILLE 3 W, SC:

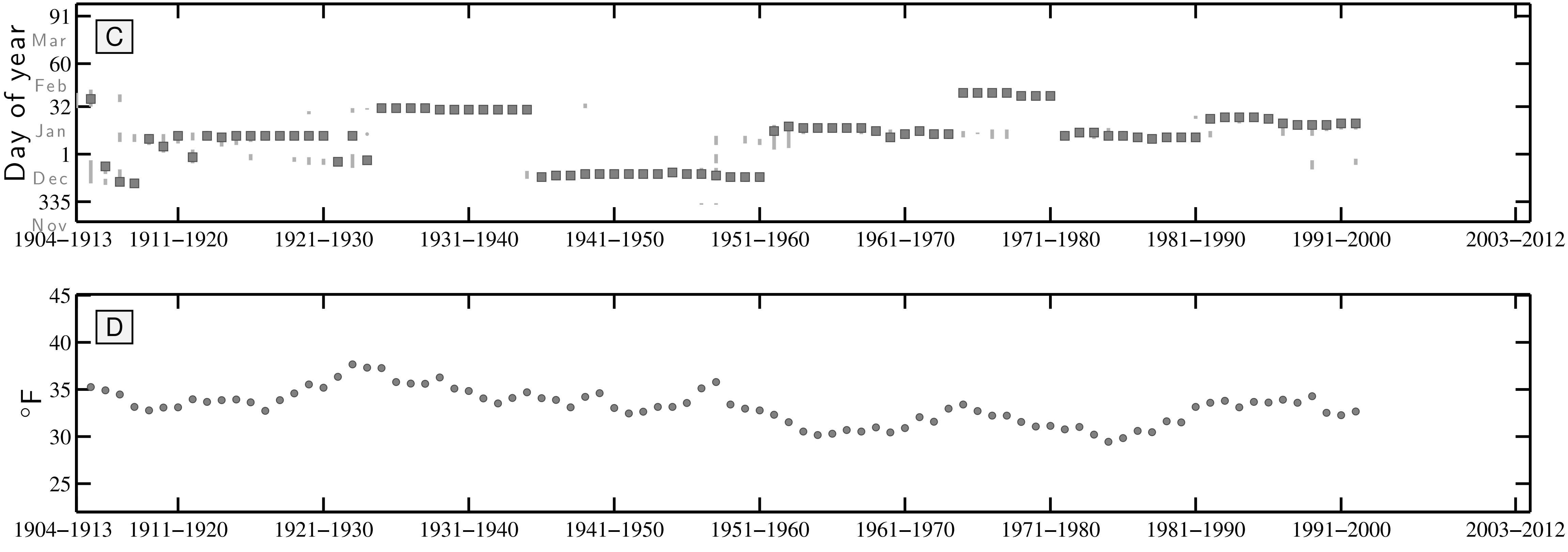

10 year Summer Teletherm dynamics for CALHOUN FALLS, SC:

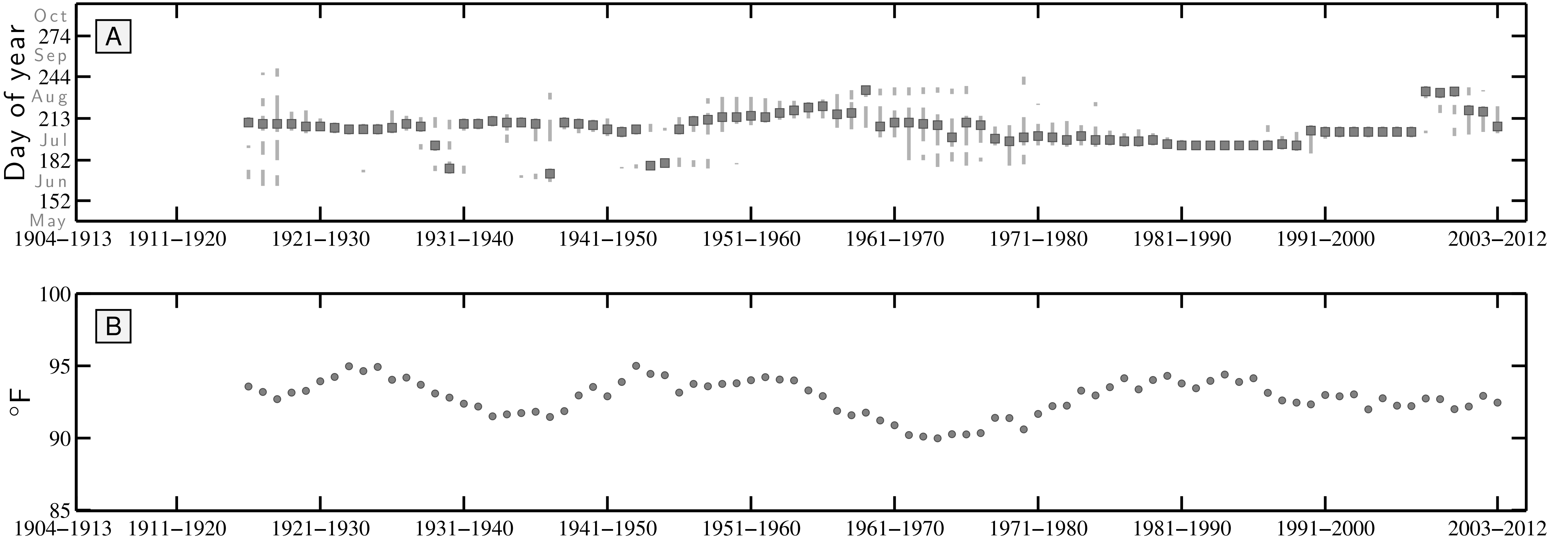

10 year Winter Teletherm dynamics for CALHOUN FALLS, SC:

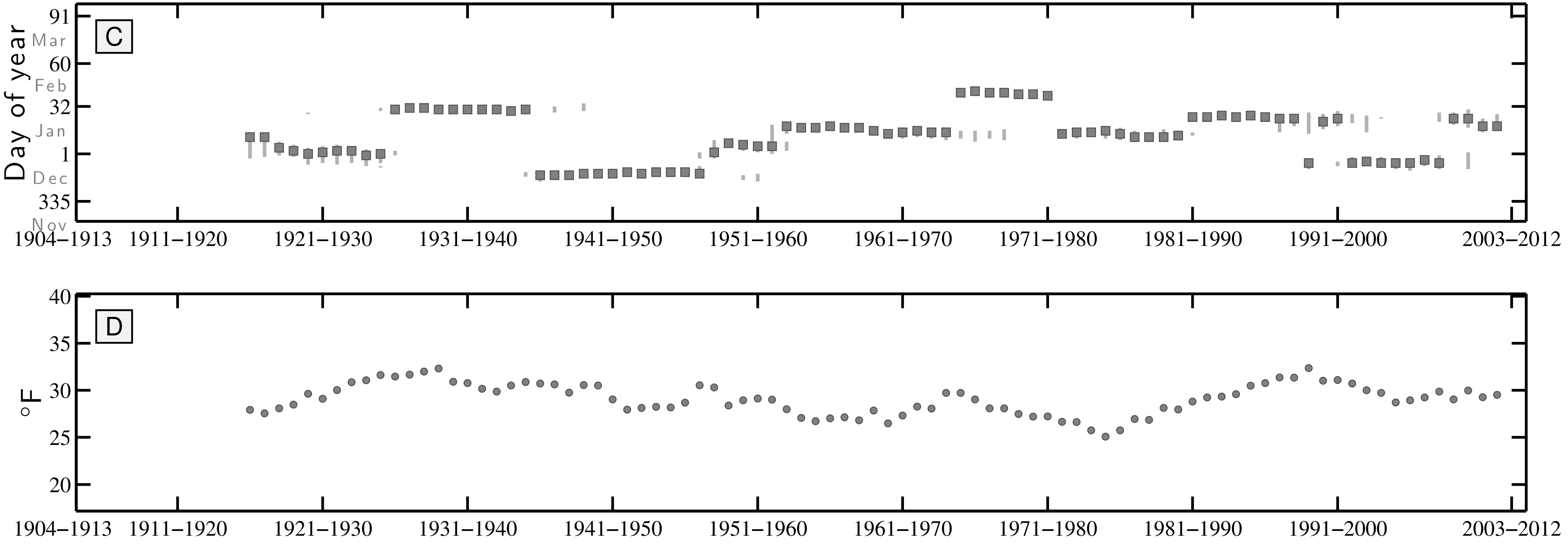

10 year Summer Teletherm dynamics for CAMDEN 3 W, SC:

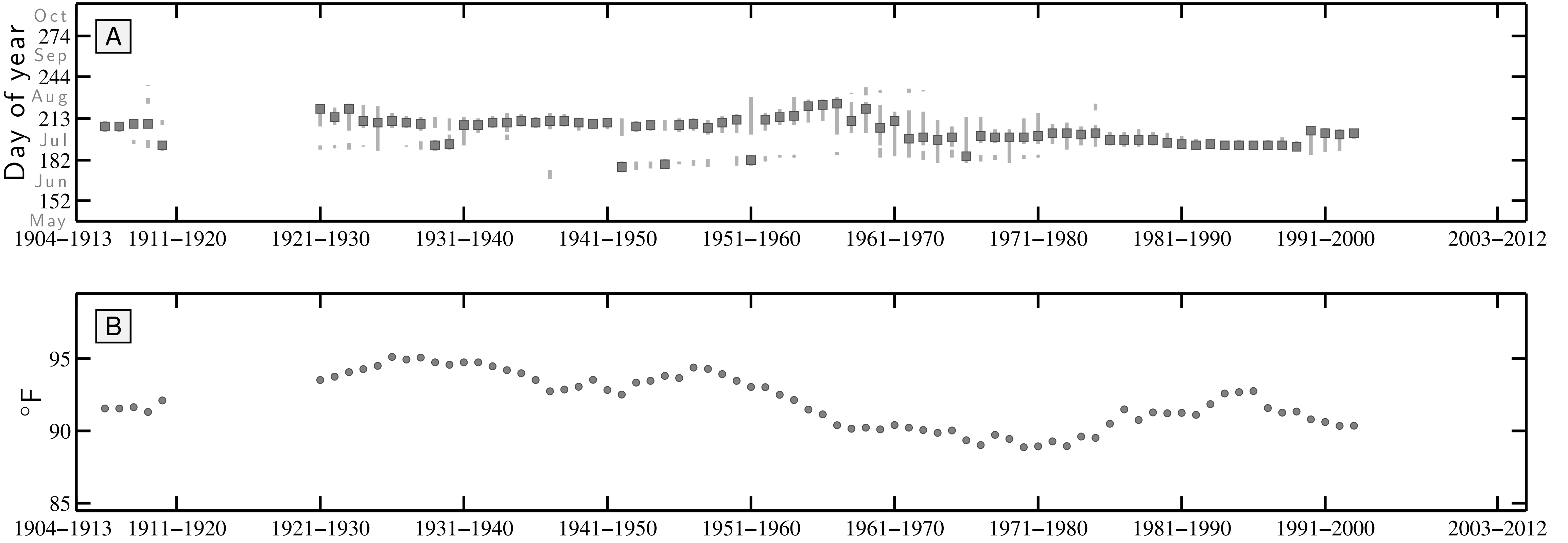

10 year Winter Teletherm dynamics for CAMDEN 3 W, SC:

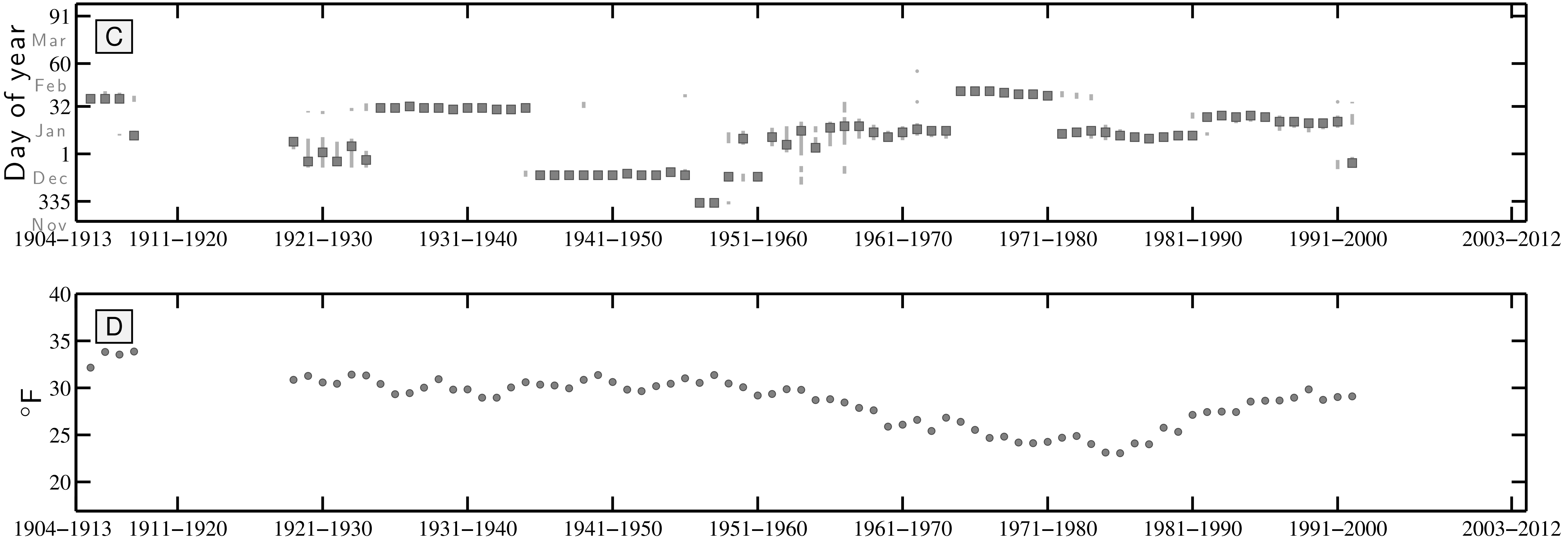

10 year Summer Teletherm dynamics for CHARLESTON CITY, SC:

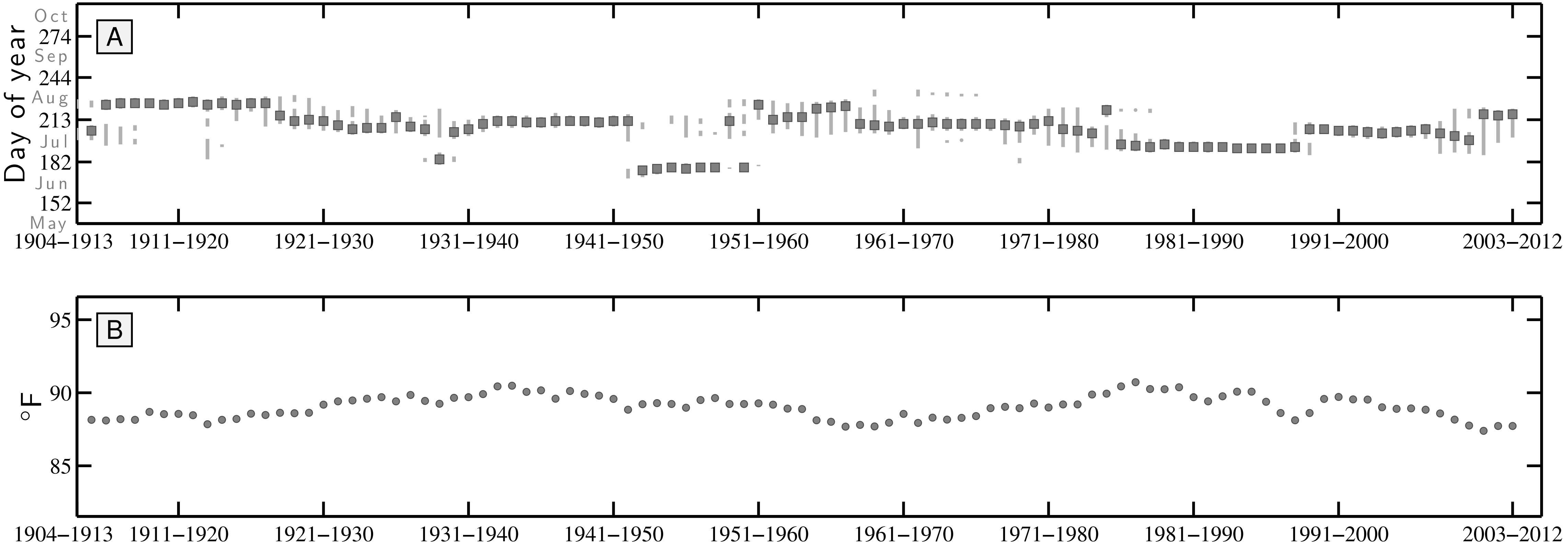

10 year Winter Teletherm dynamics for CHARLESTON CITY, SC:

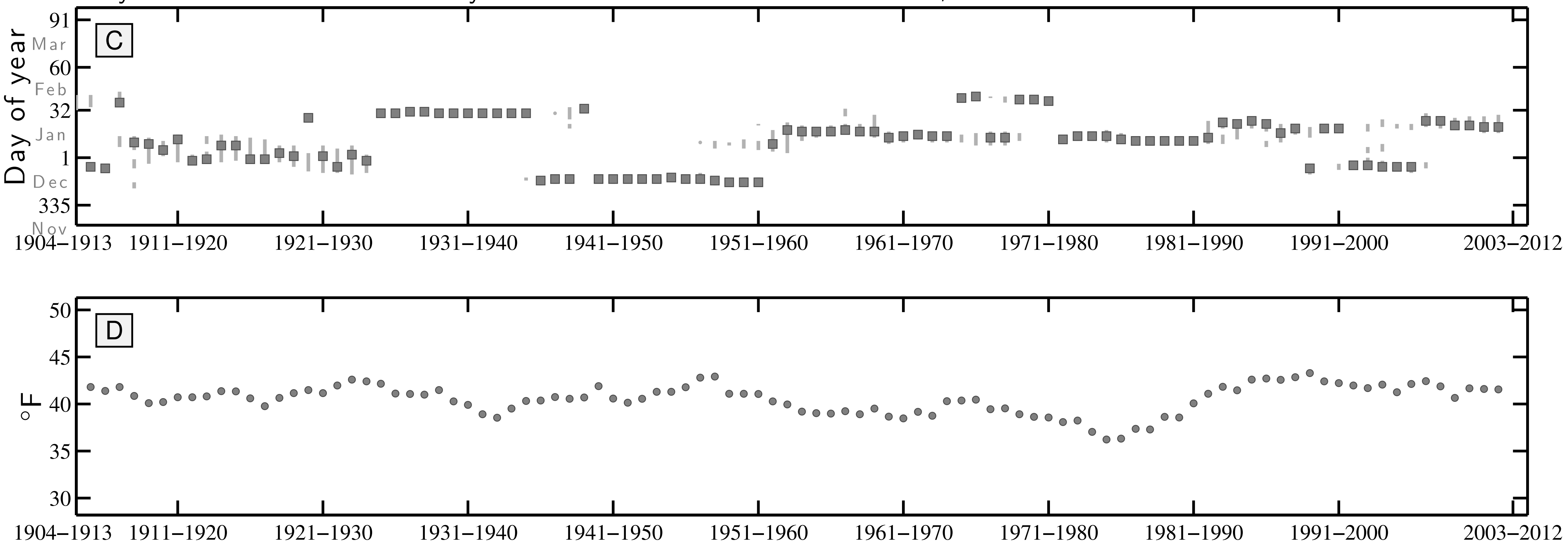

10 year Summer Teletherm dynamics for CHERAW, SC:

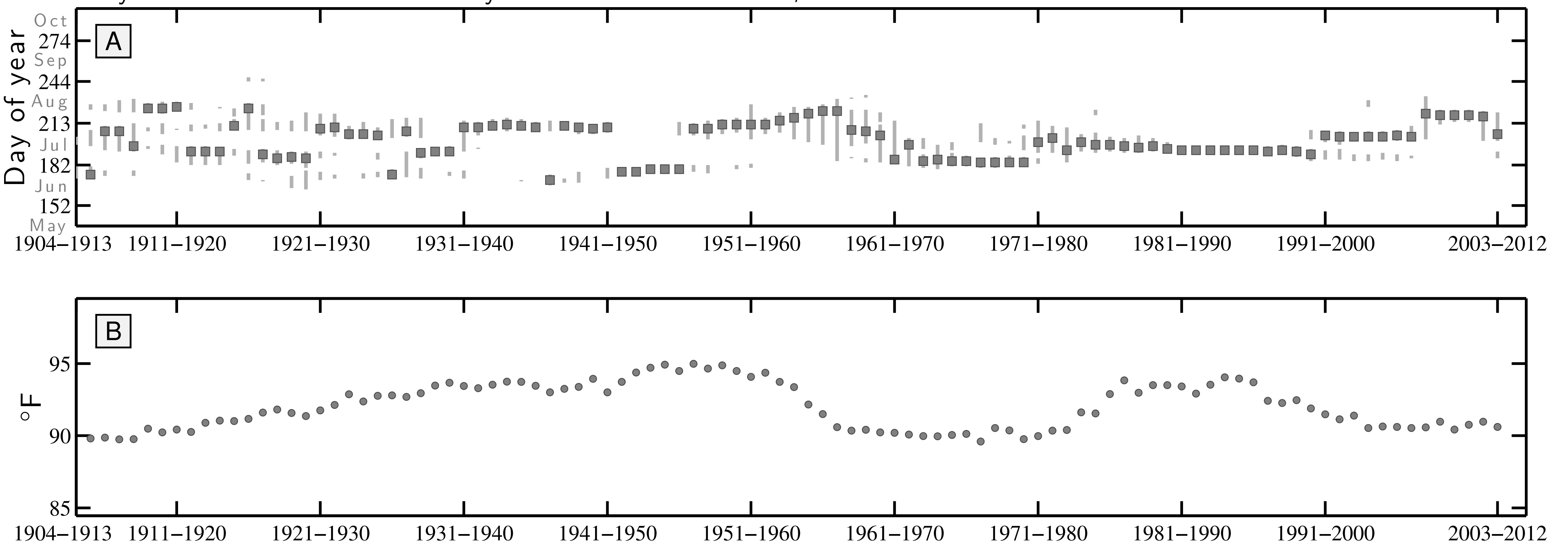

10 year Winter Teletherm dynamics for CHERAW, SC:

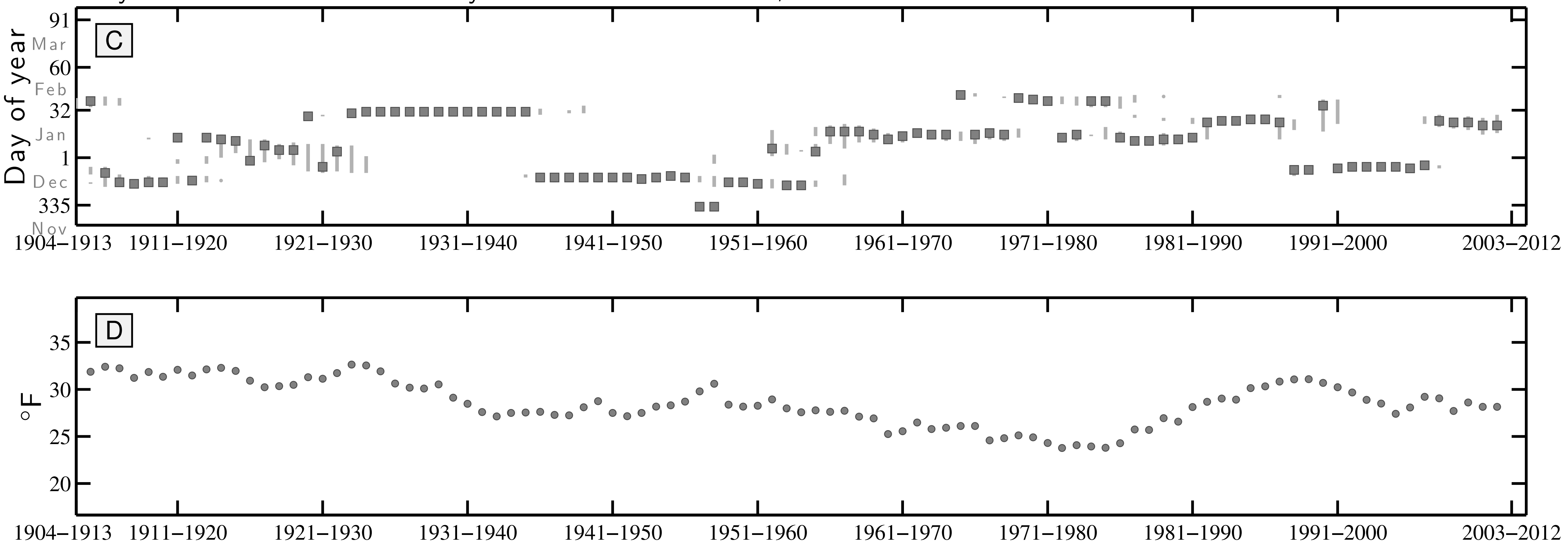

10 year Summer Teletherm dynamics for CLEMSON UNIV, SC:

10 year Winter Teletherm dynamics for CLEMSON UNIV, SC:

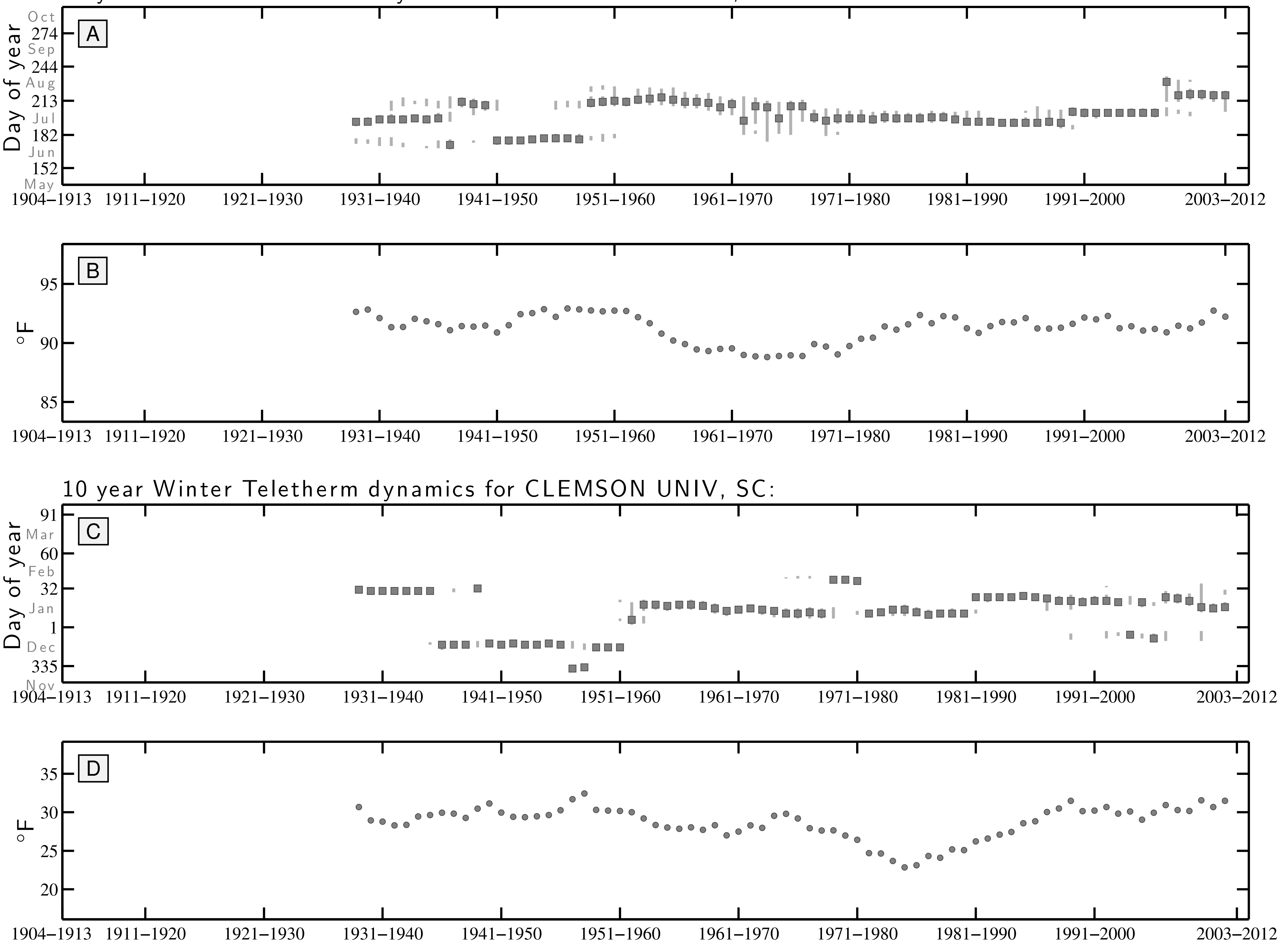

10 year Summer Teletherm dynamics for COLUMBIA UNIV OF SC, SC:

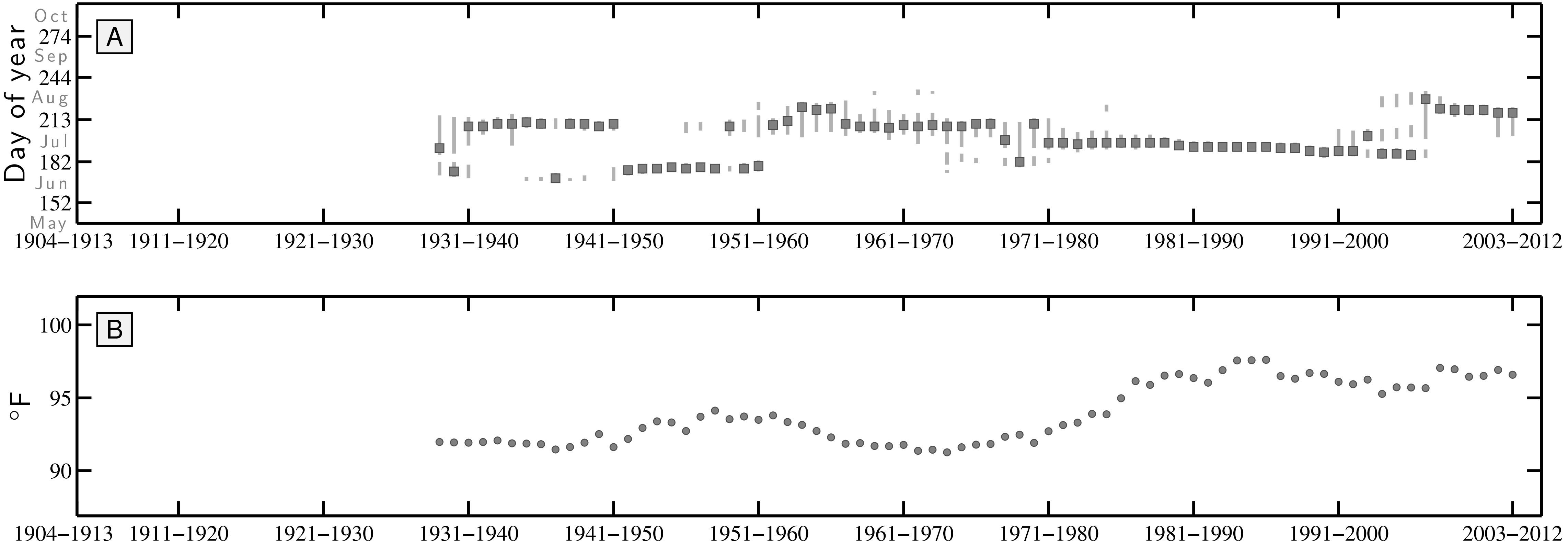

10 year Winter Teletherm dynamics for COLUMBIA UNIV OF SC, SC:

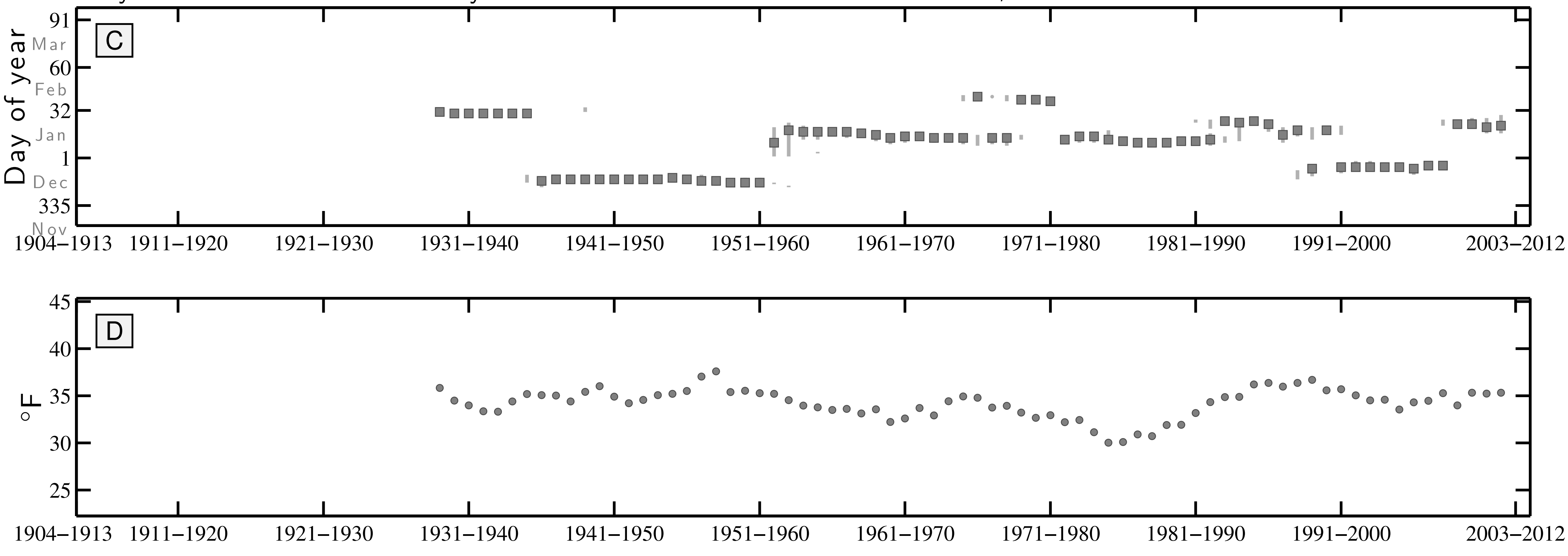

10 year Summer Teletherm dynamics for CONWAY, SC:

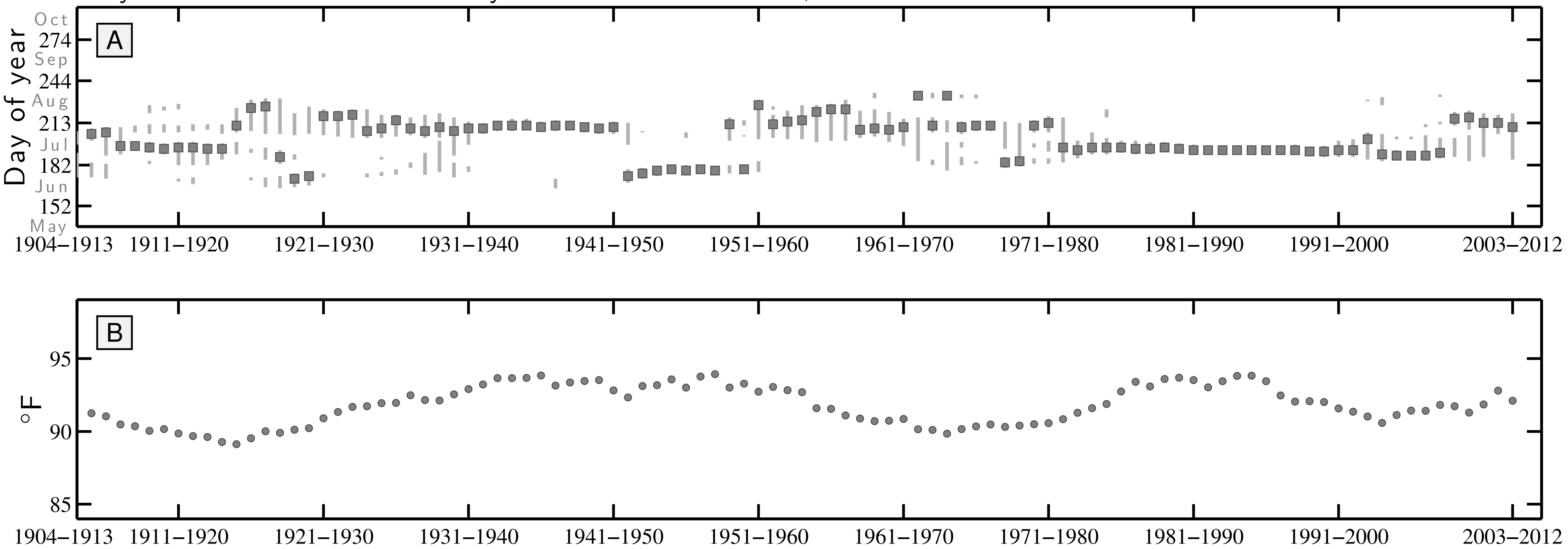

10 year Winter Teletherm dynamics for CONWAY, SC:

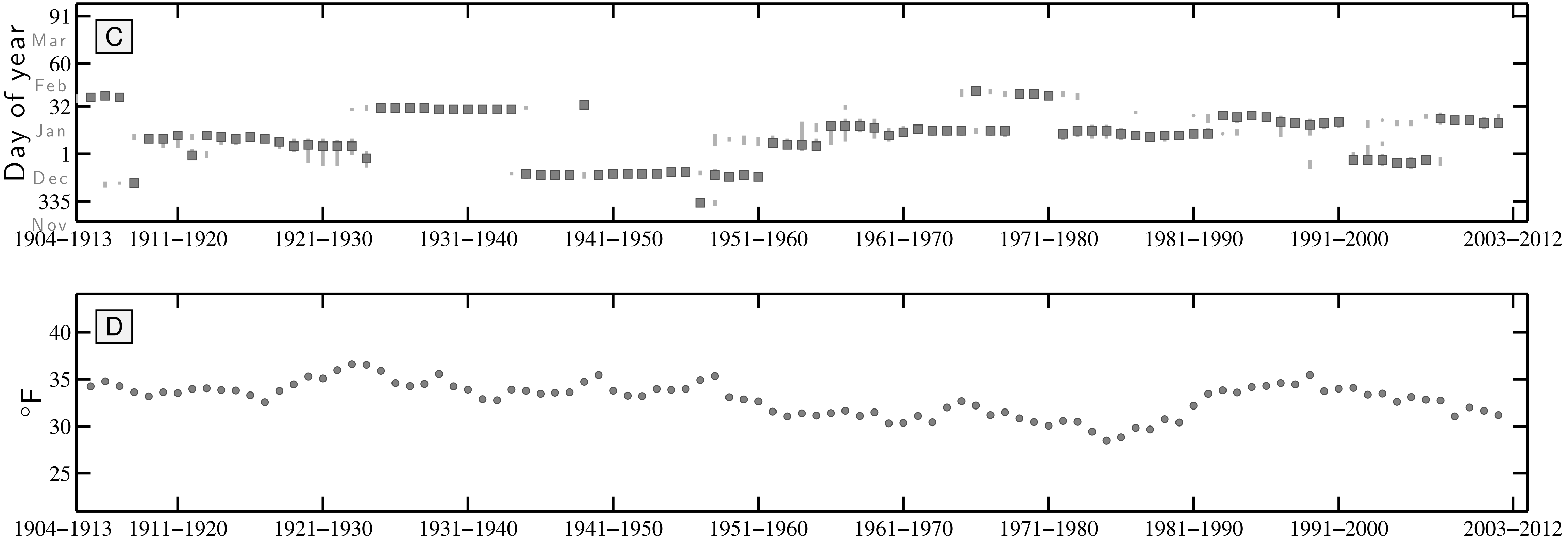

10 year Summer Teletherm dynamics for DARLINGTON, SC:

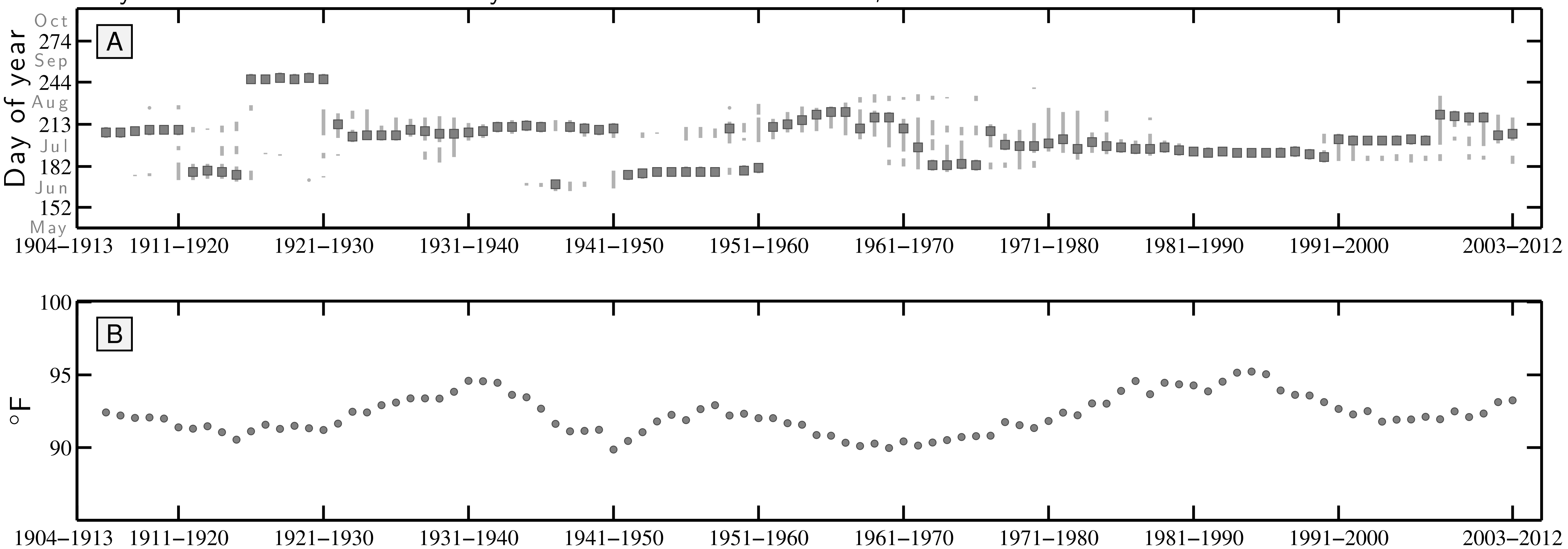

10 year Winter Teletherm dynamics for DARLINGTON, SC:

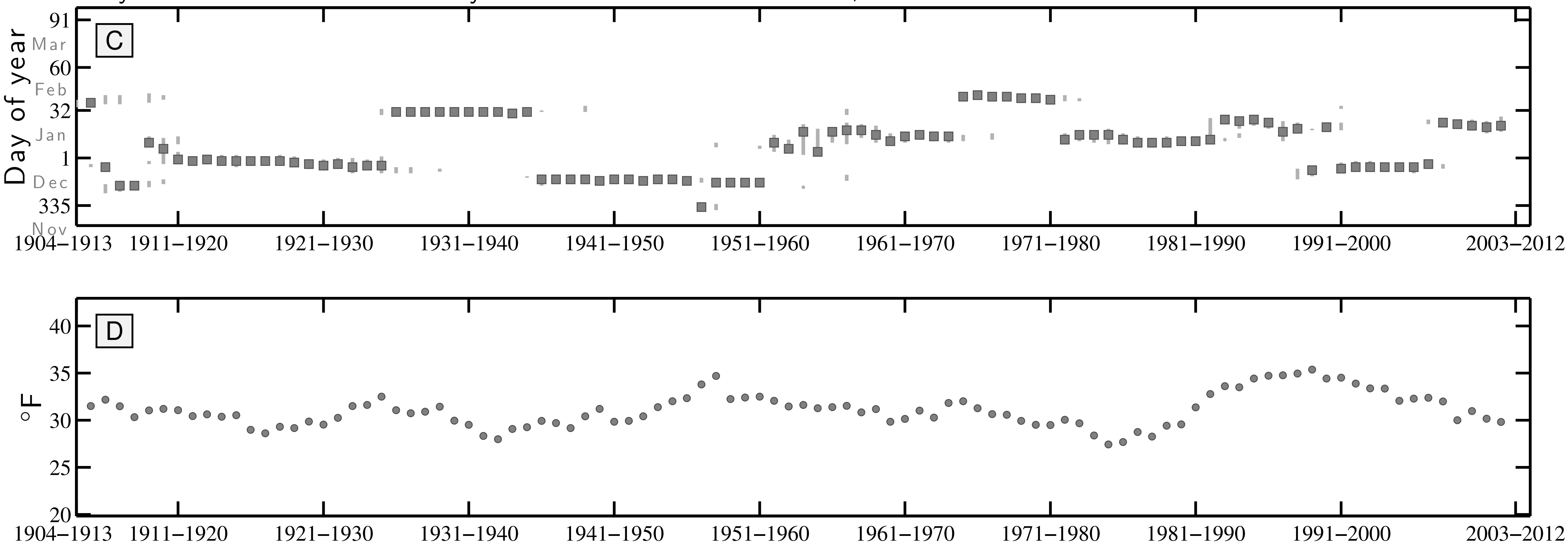

10 year Summer Teletherm dynamics for GEORGETOWN 2 E, SC:

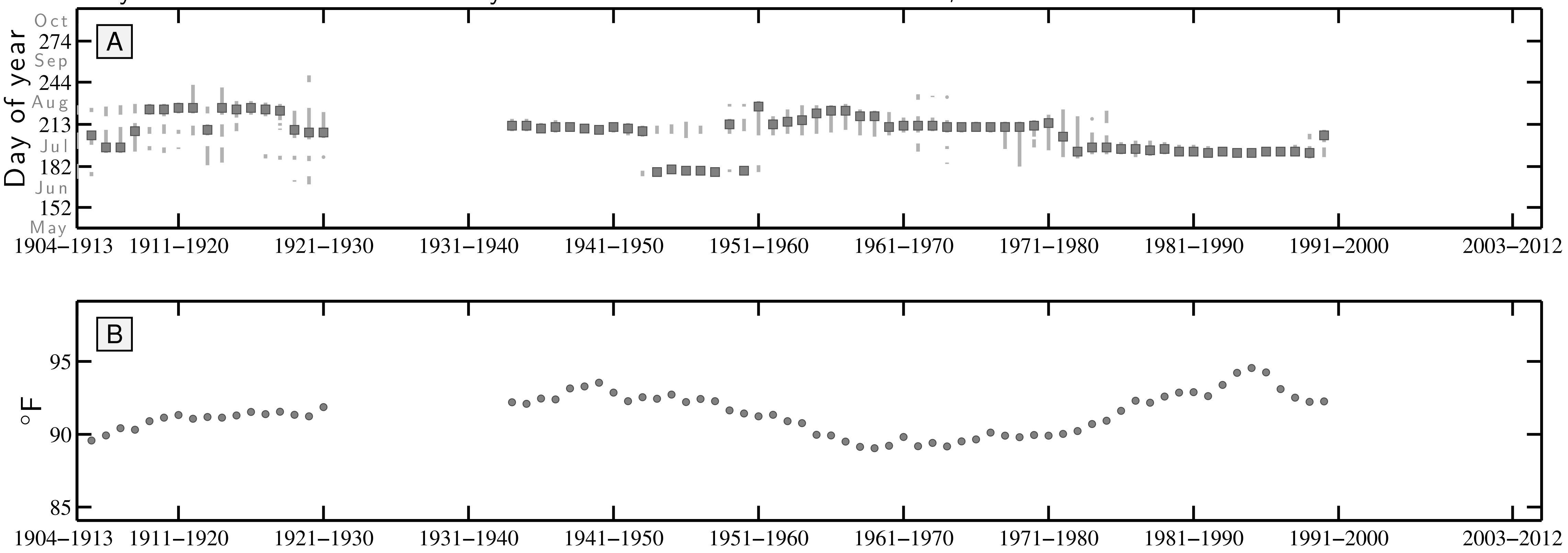

10 year Winter Teletherm dynamics for GEORGETOWN 2 E, SC:

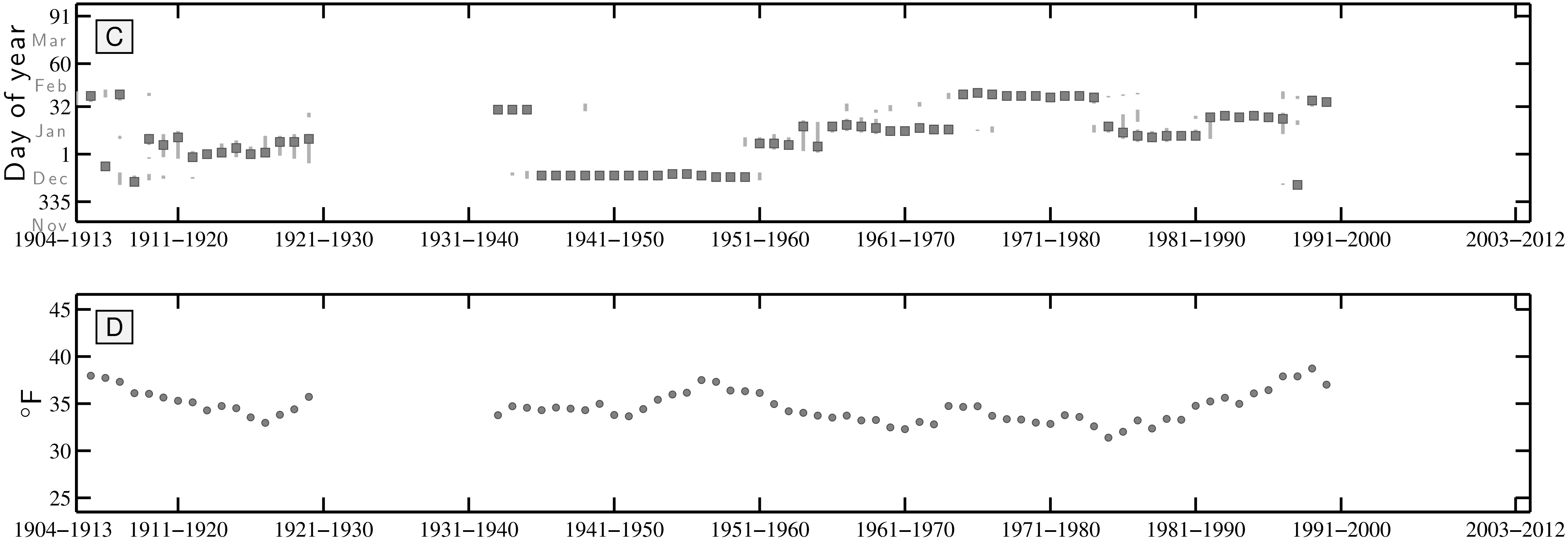

10 year Summer Teletherm dynamics for GRNVL SPART INTL AP, SC:

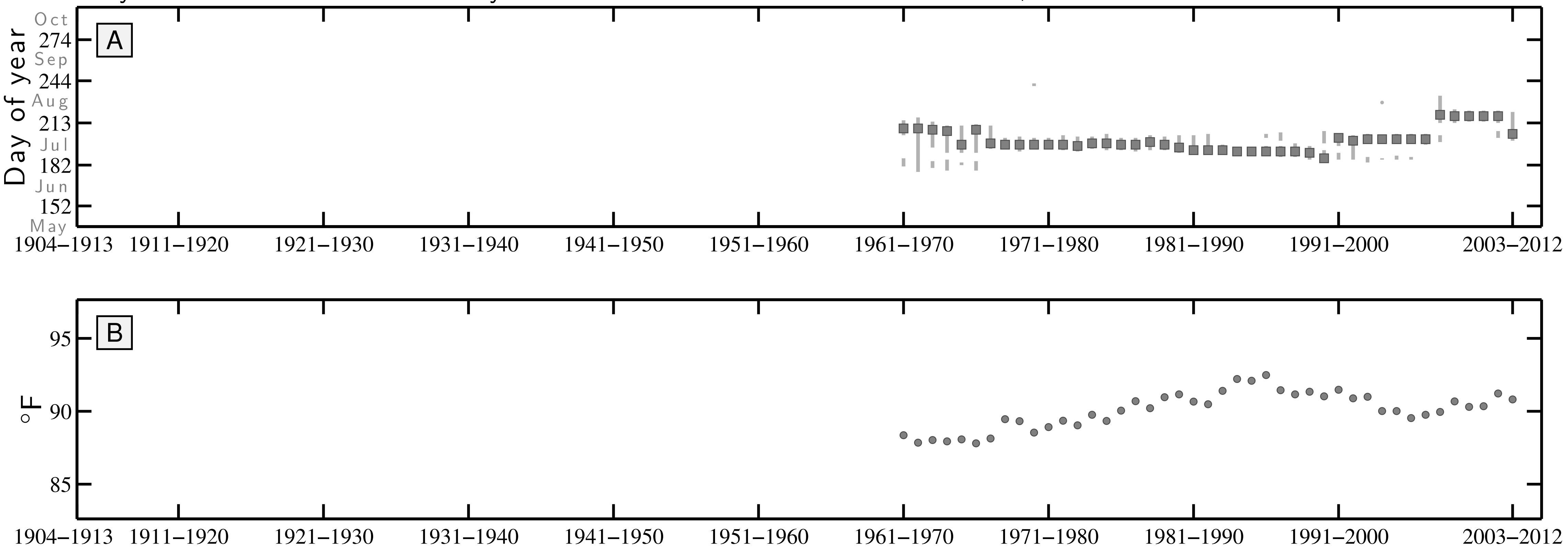

10 year Winter Teletherm dynamics for GRNVL SPART INTL AP, SC:

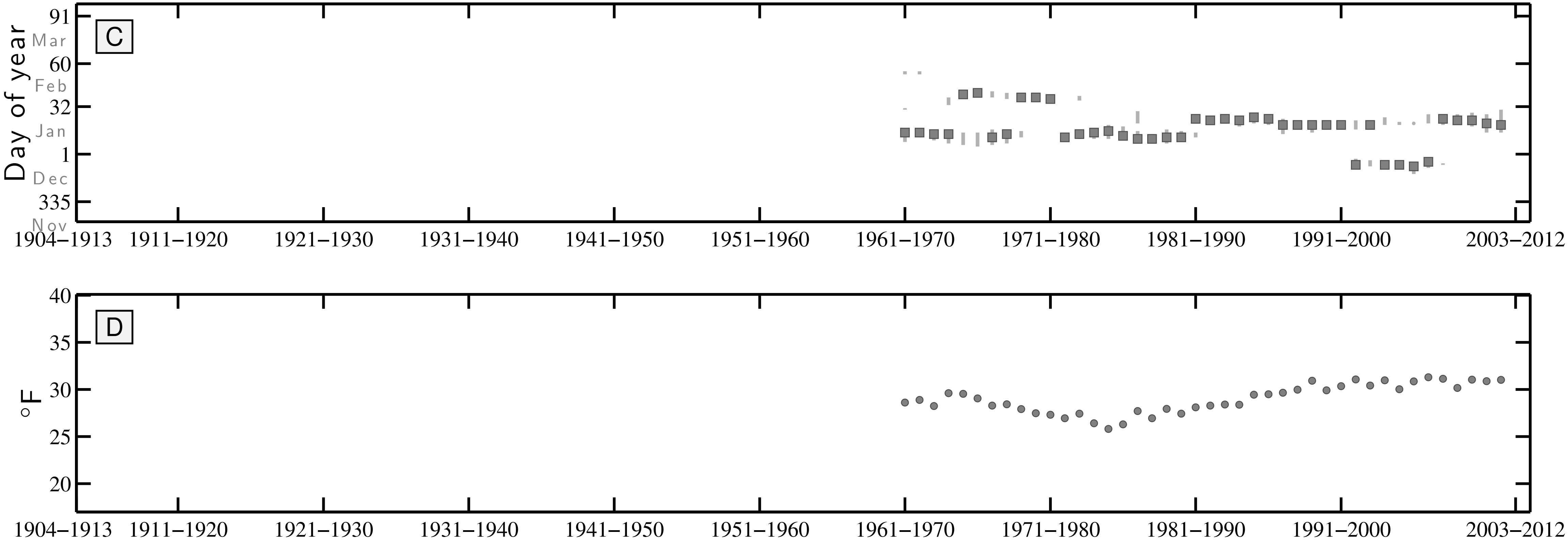

10 year Summer Teletherm dynamics for GREENWOOD, SC:

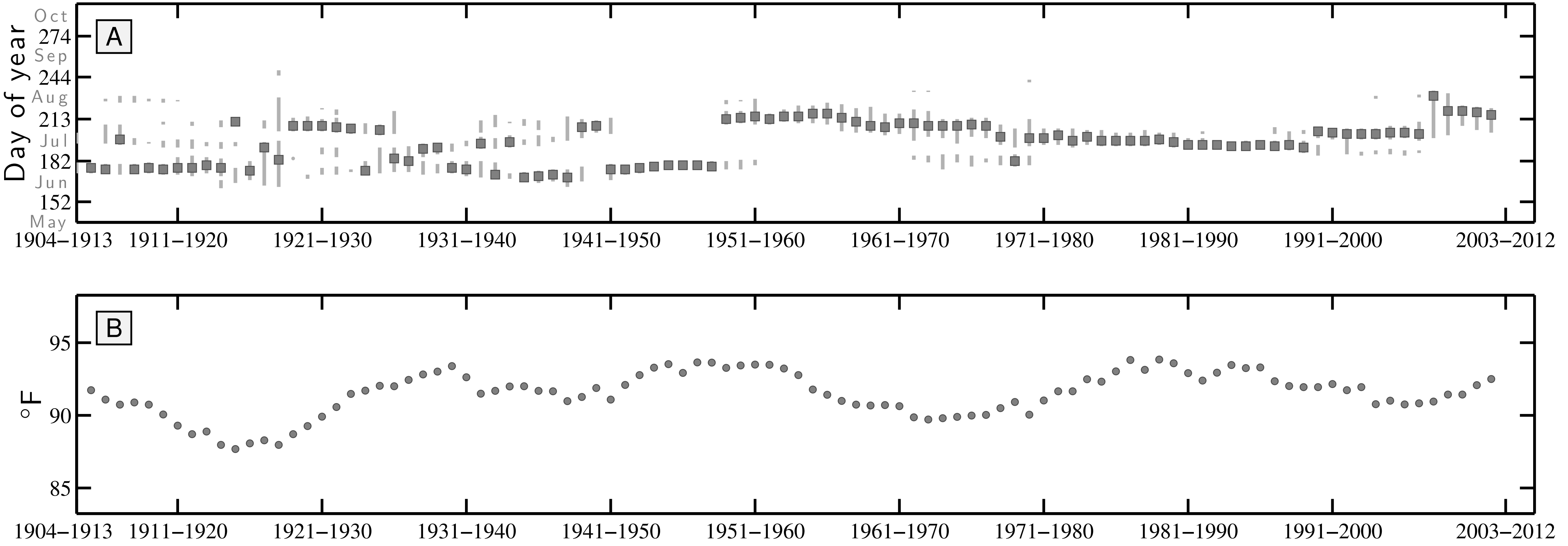

10 year Winter Teletherm dynamics for GREENWOOD, SC:

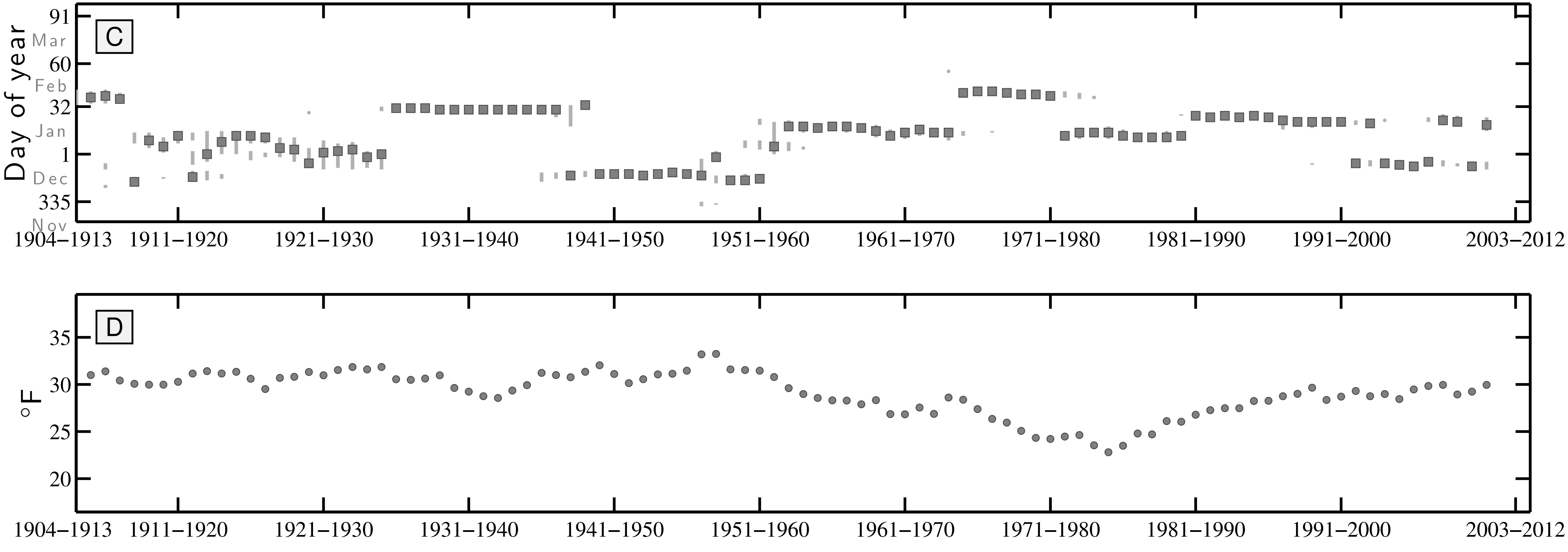

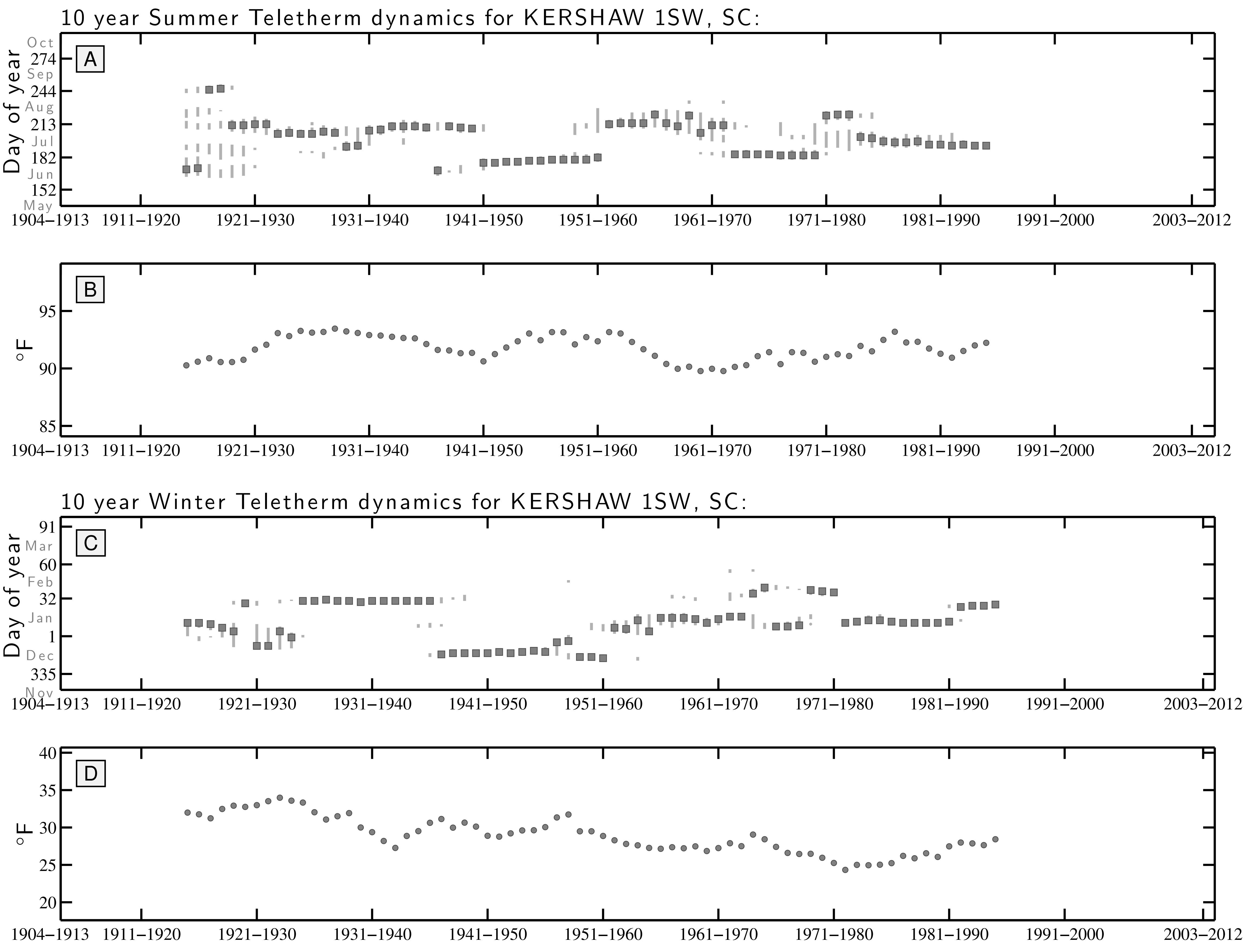

10 year Summer Teletherm dynamics for KERSHAW 1SW, SC:
A
Day of year
Oct
274
Sep
244
Aug
213
Jul
182
Jun
152
May
1904–1913
1911–1920
1921–1930
1931–1940
1941–1950
1951–1960
1961–1970
1971–1980
1981–1990
1991–2000
2003–2012
B
°F
95
90
85
10 year Winter Teletherm dynamics for KERSHAW 1SW, SC:
C
91
Mar
60
Feb
32
Jan
1
Dec
335
Nov
D
40
35
30
25
20

10 year Summer Teletherm dynamics for KINGSTREE, SC:

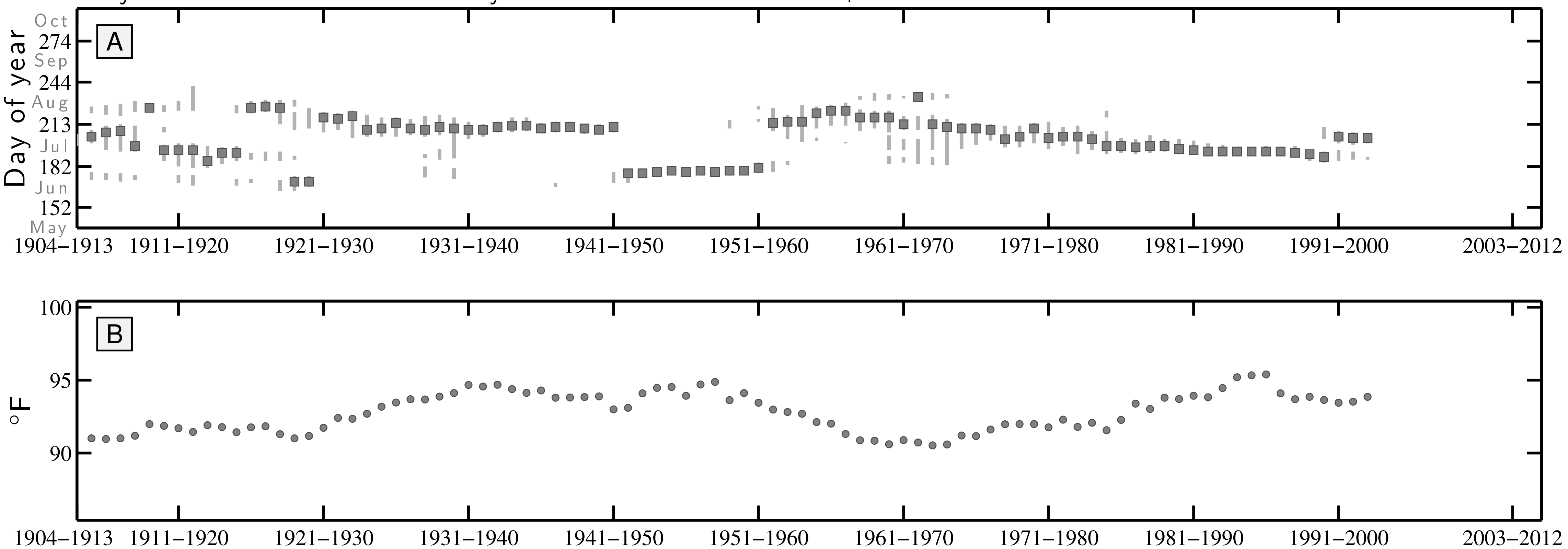

10 year Winter Teletherm dynamics for KINGSTREE, SC:

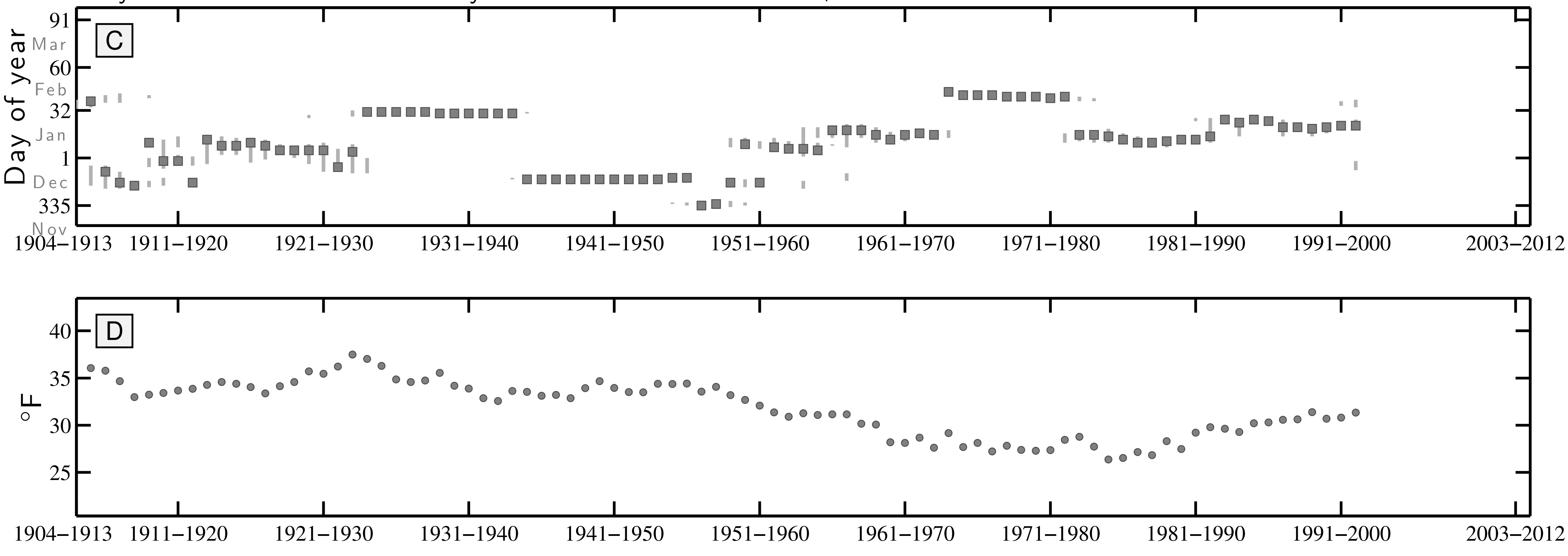

10 year Summer Teletherm dynamics for LAURENS, SC:

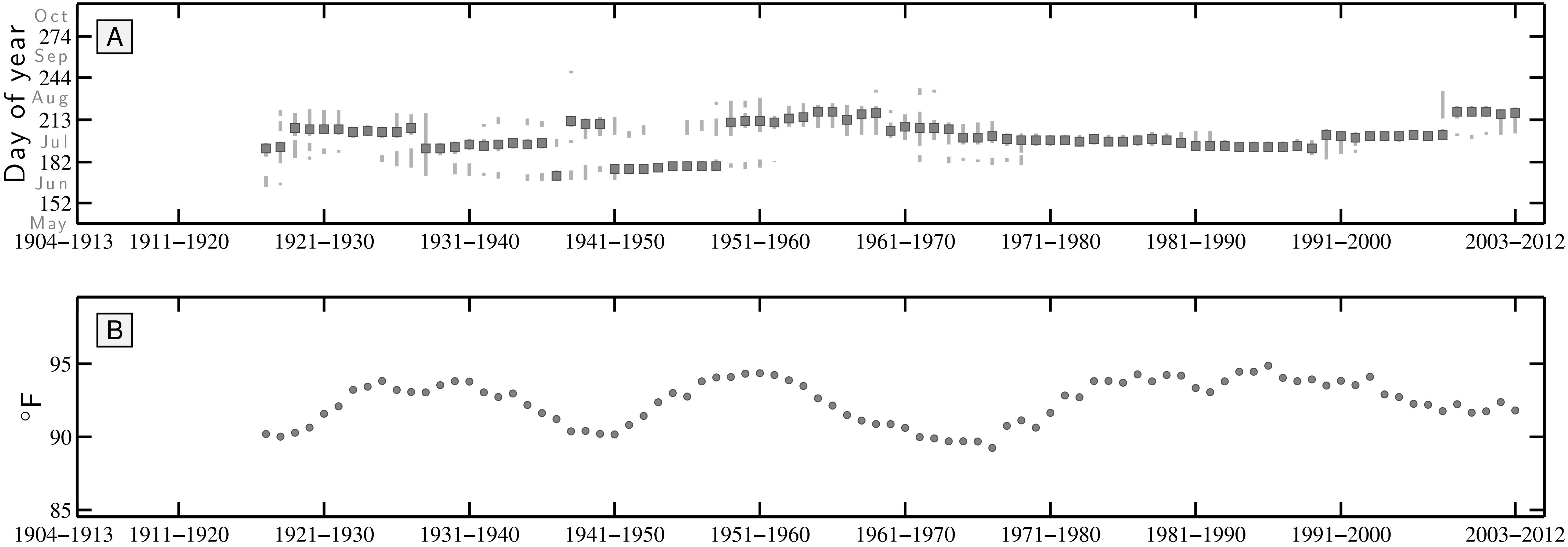

10 year Winter Teletherm dynamics for LAURENS, SC:

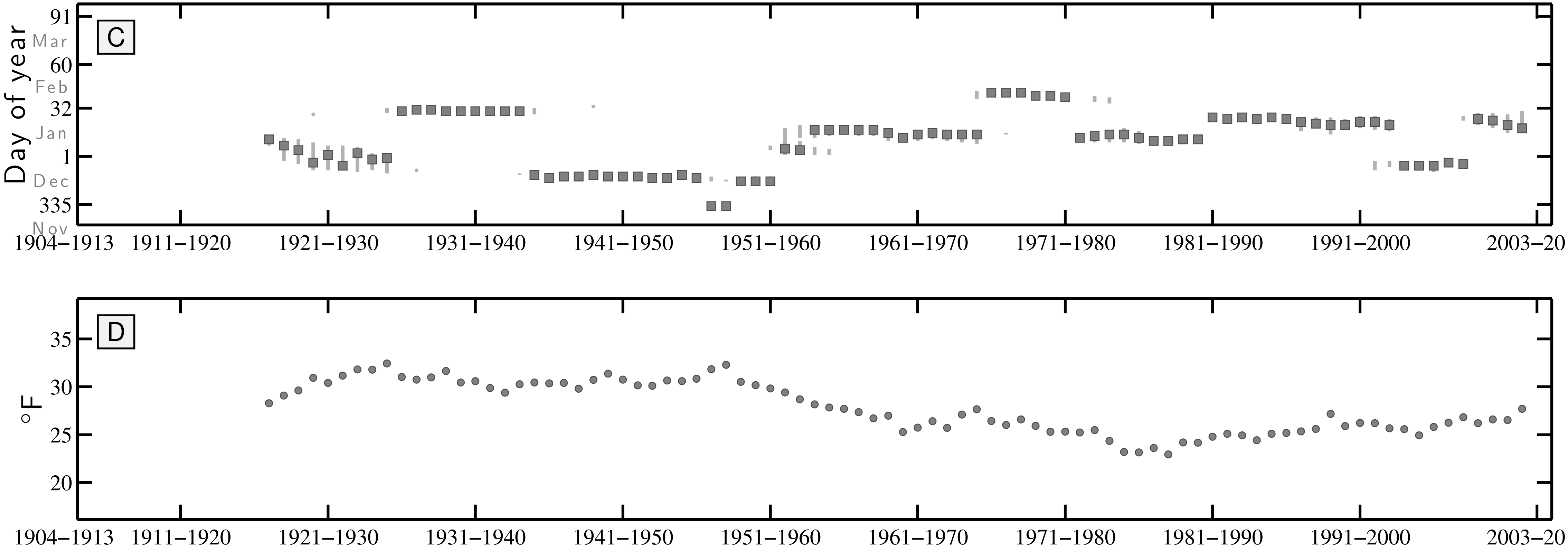

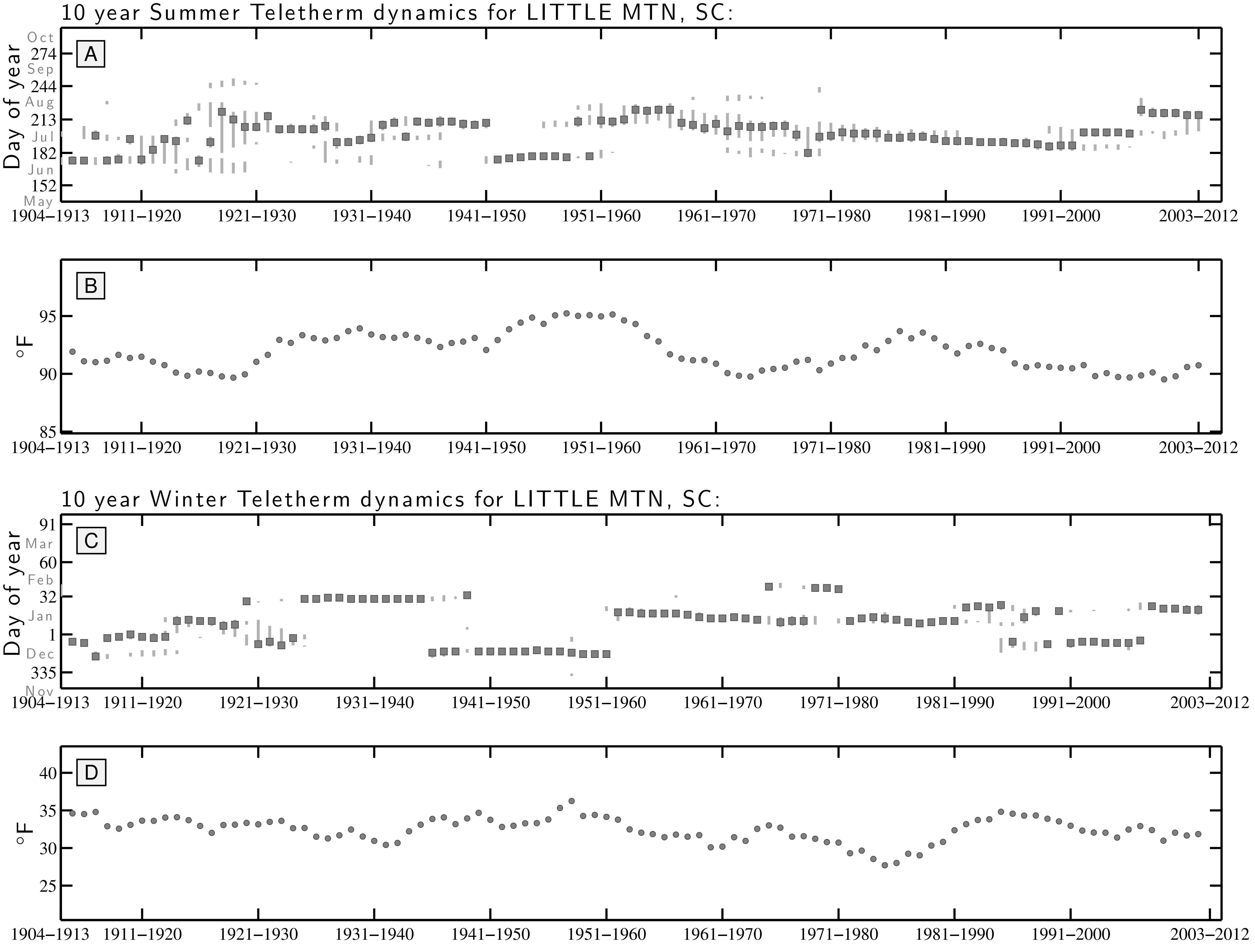

10 year Summer Teletherm dynamics for LITTLE MTN, SC:
A
Day of year
Oct
274
Sep
244
Aug
213
Jul
182
Jun
152
May
B
°F
95
90
85
10 year Winter Teletherm dynamics for LITTLE MTN, SC:
C
91
Mar
60
Feb
32
Jan
1
Dec
335
Nov
D
40
35
30
25
1904–1913
1911–1920
1921–1930
1931–1940
1941–1950
1951–1960
1961–1970
1971–1980
1981–1990
1991–2000
2003–2012

10 year Summer Teletherm dynamics for NEWBERRY, SC:

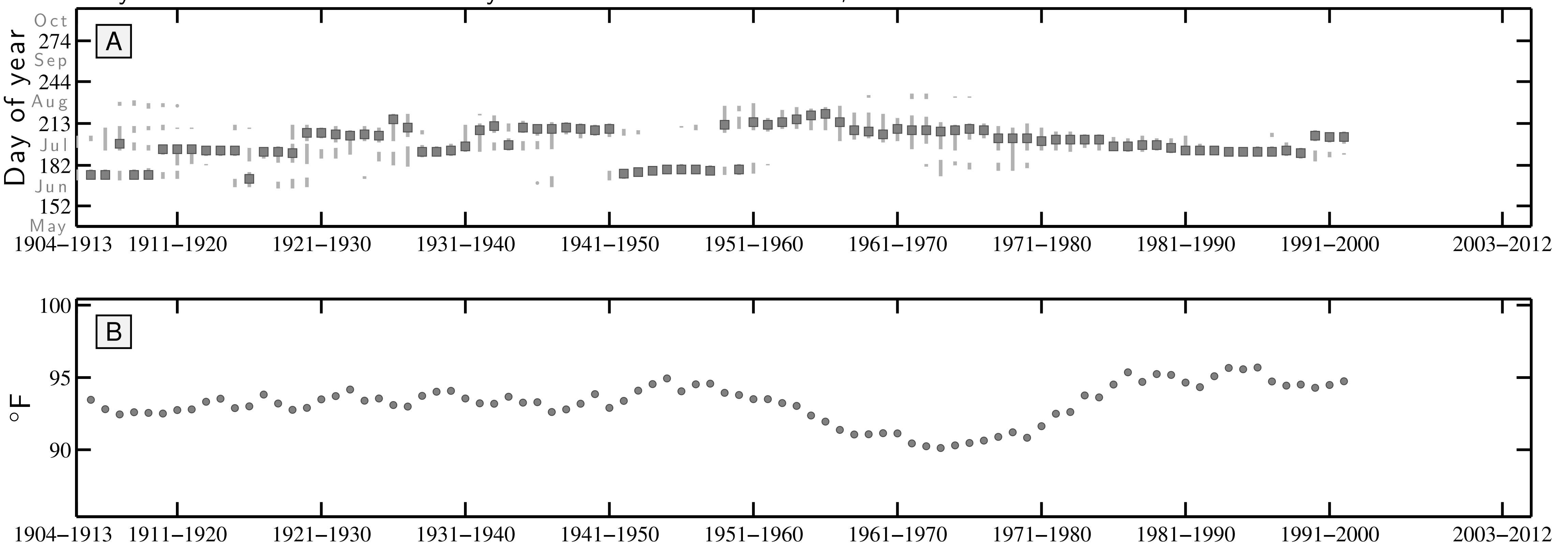

10 year Winter Teletherm dynamics for NEWBERRY, SC:

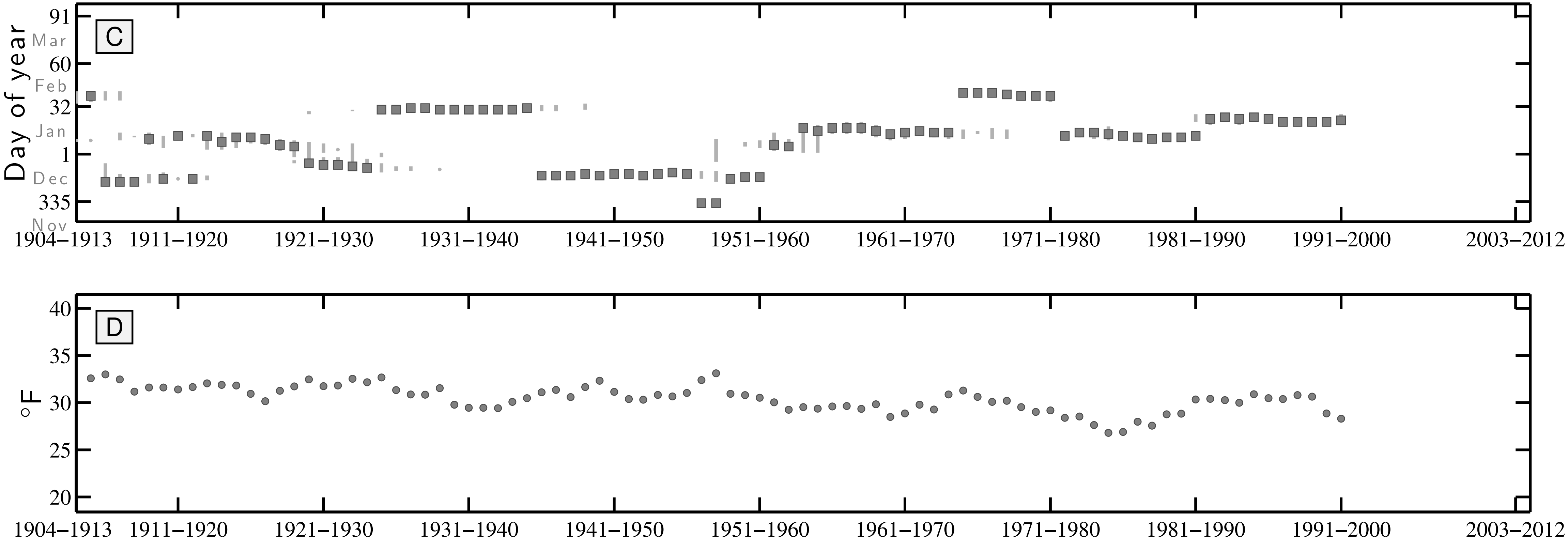

10 year Summer Teletherm dynamics for ORANGEBURG 2, SC:

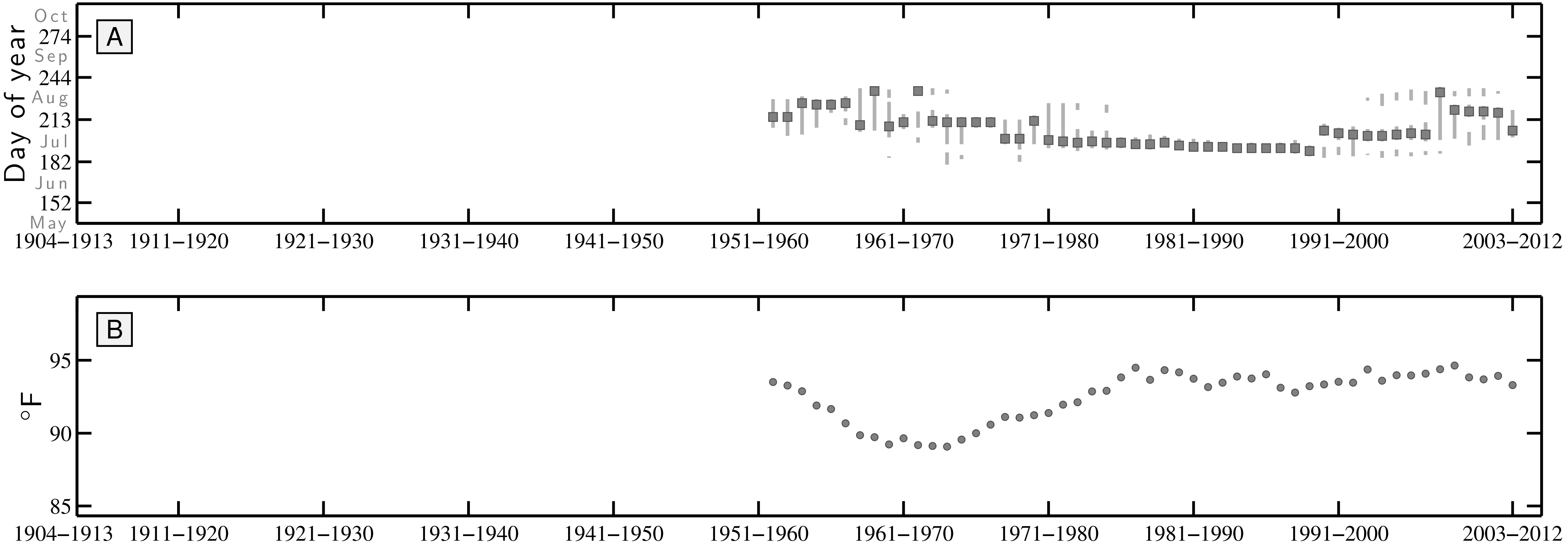

10 year Winter Teletherm dynamics for ORANGEBURG 2, SC:

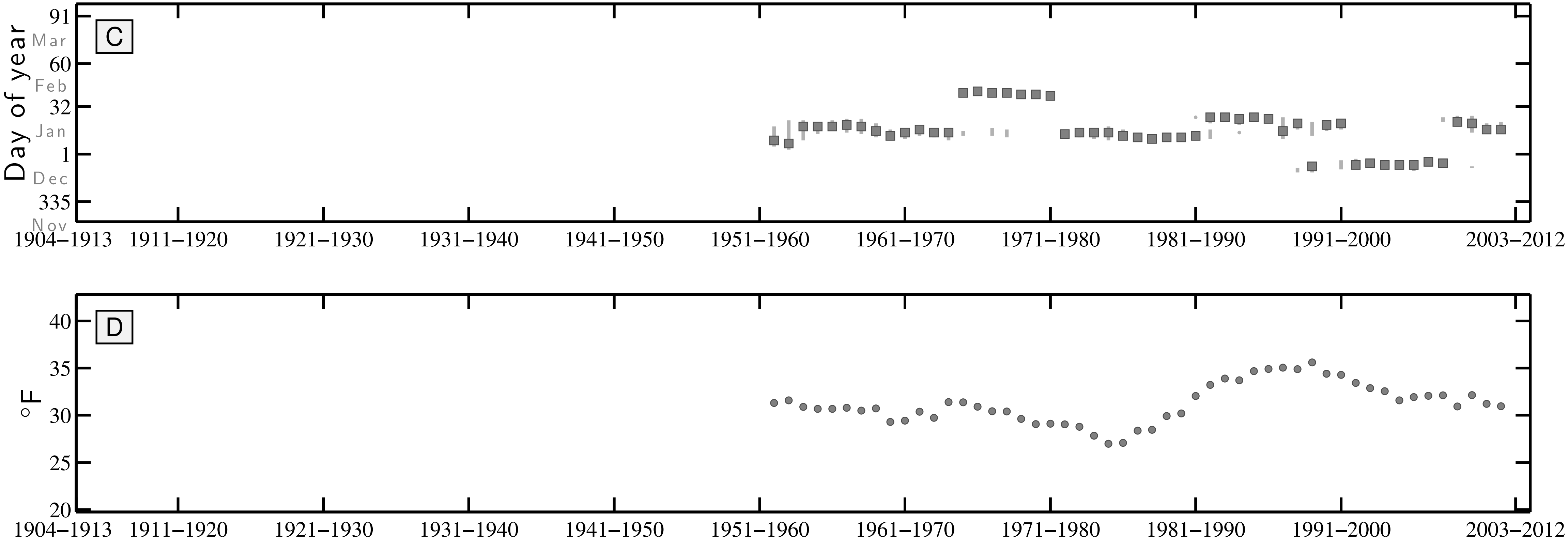

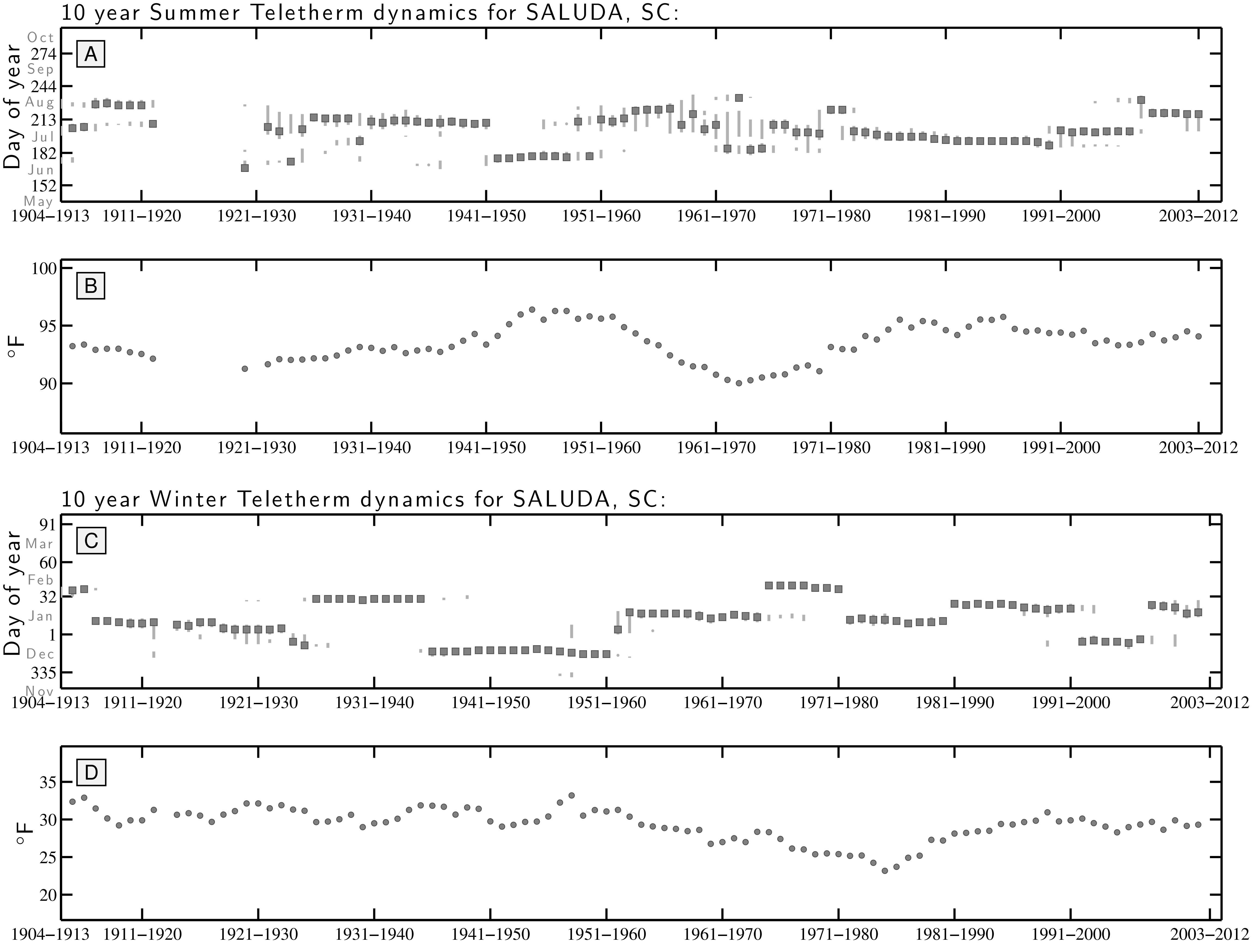
10 year Summer Teletherm dynamics for SALUDA, SC:
A
B
Day of year
°F
Oct
274
Sep
244
Aug
213
Jul
182
Jun
152
May
100
95
90
1904–1913
1911–1920
1921–1930
1931–1940
1941–1950
1951–1960
1961–1970
1971–1980
1981–1990
1991–2000
2003–2012
10 year Winter Teletherm dynamics for SALUDA, SC:
C
D
91
Mar
60
Feb
32
Jan
1
Dec
335
Nov
35
30
25
20

10 year Summer Teletherm dynamics for SANTUCK, SC:

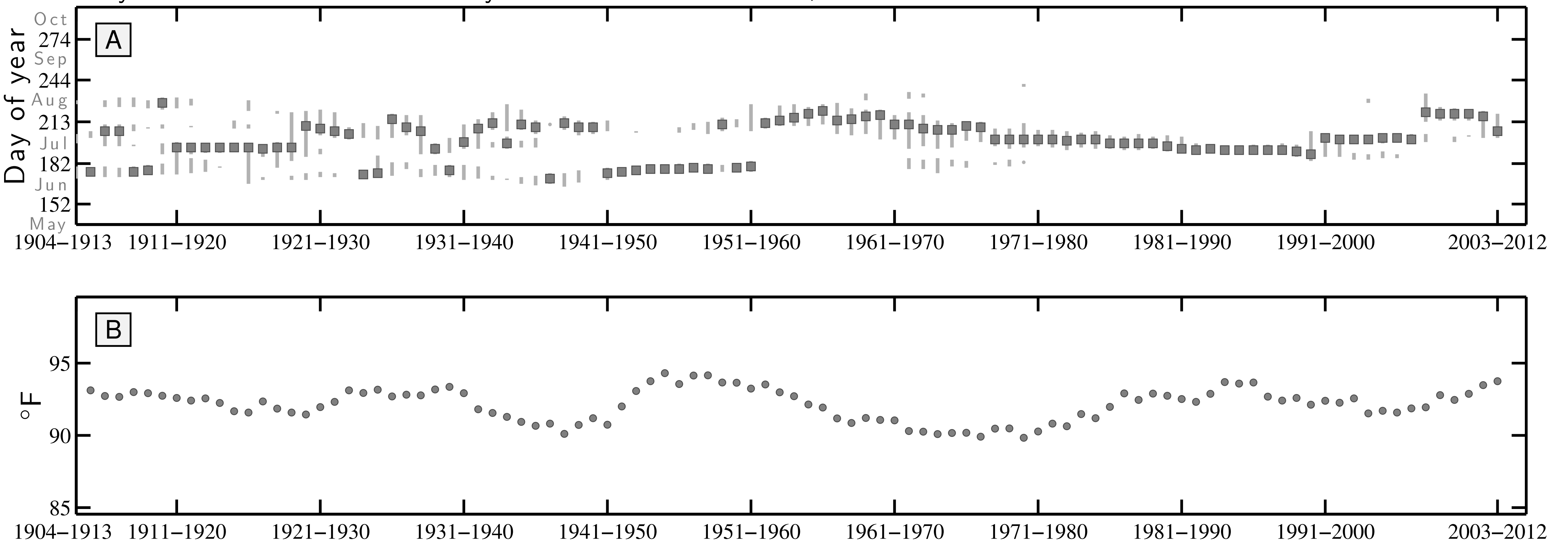

10 year Winter Teletherm dynamics for SANTUCK, SC:

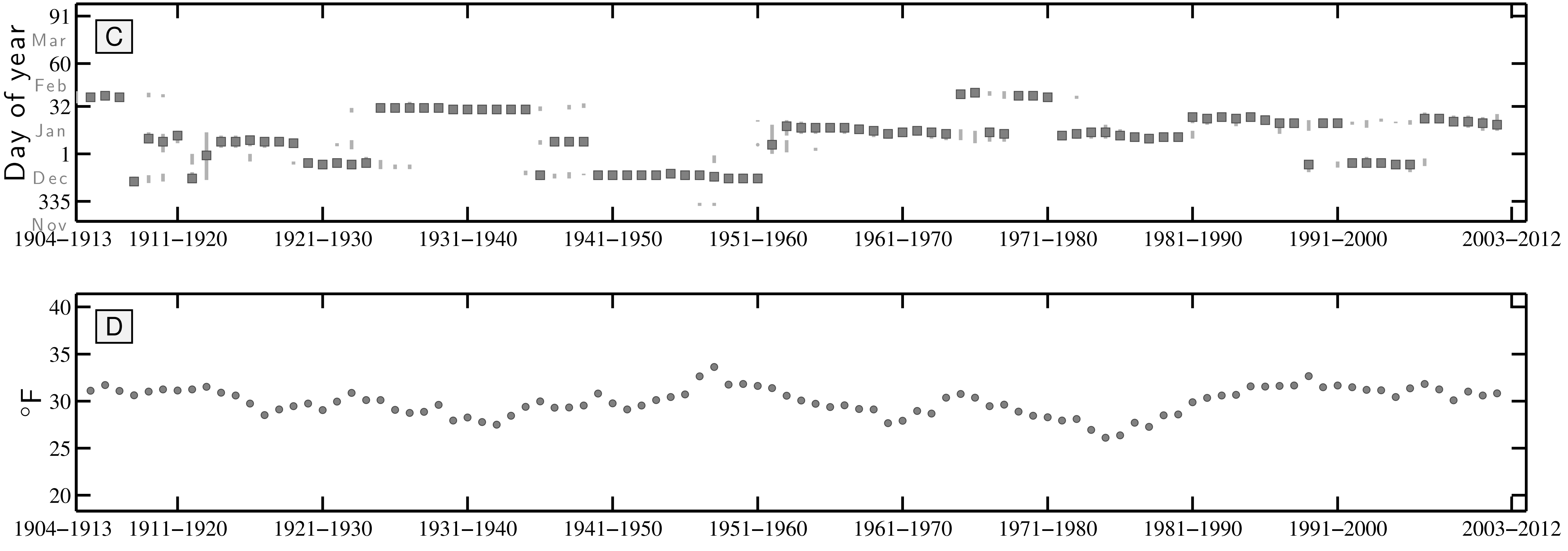

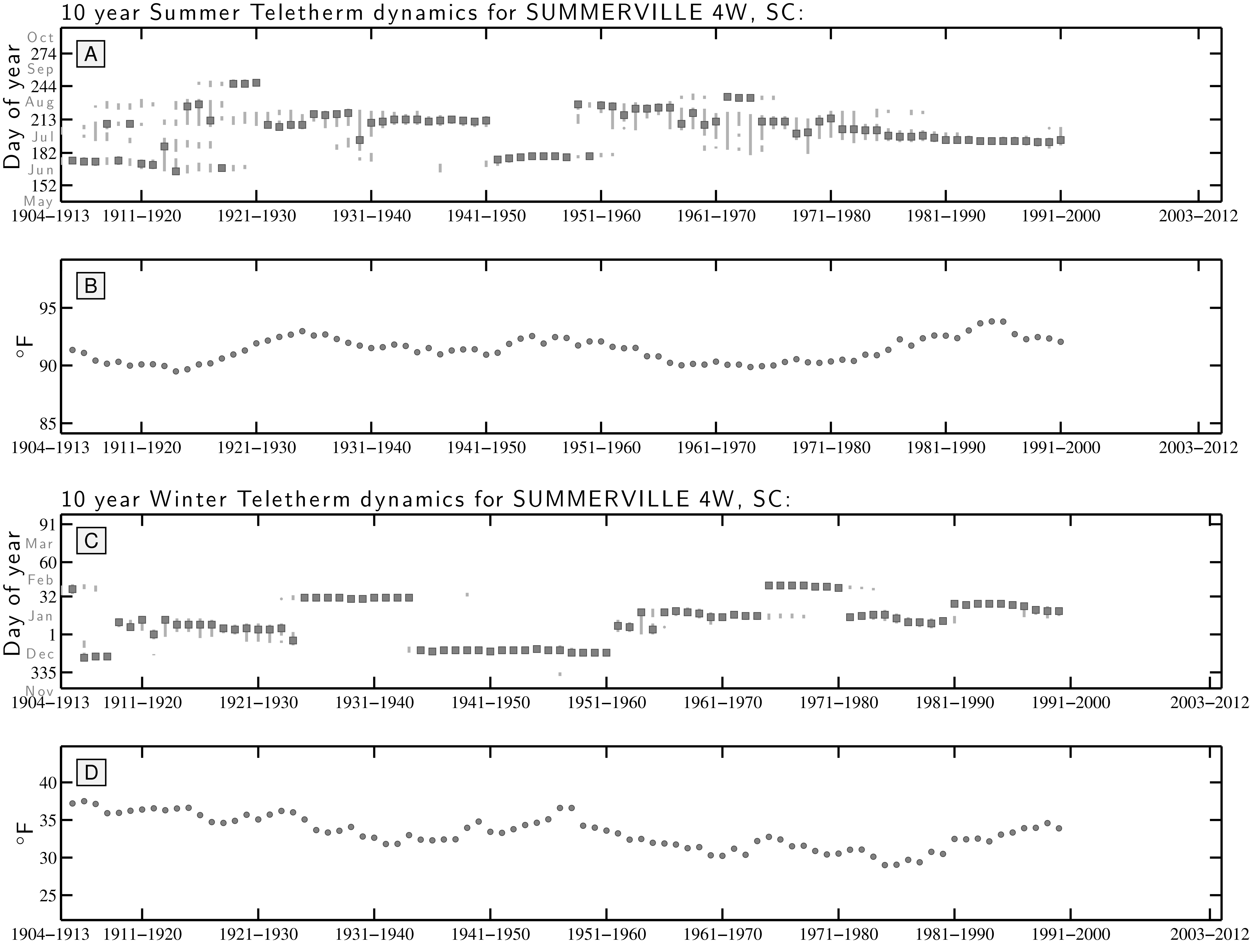
10 year Summer Teletherm dynamics for SUMMERVILLE 4W, SC:
A
Day of year
Oct
274
Sep
244
Aug
213
Jul
182
Jun
152
May
1904–1913
1911–1920
1921–1930
1931–1940
1941–1950
1951–1960
1961–1970
1971–1980
1981–1990
1991–2000
2003–2012
B
°F
95
90
85
10 year Winter Teletherm dynamics for SUMMERVILLE 4W, SC:
C
Day of year
91
Mar
60
Feb
32
Jan
1
Dec
335
Nov
D
°F
40
35
30
25

10 year Summer Teletherm dynamics for SUMTER, SC:

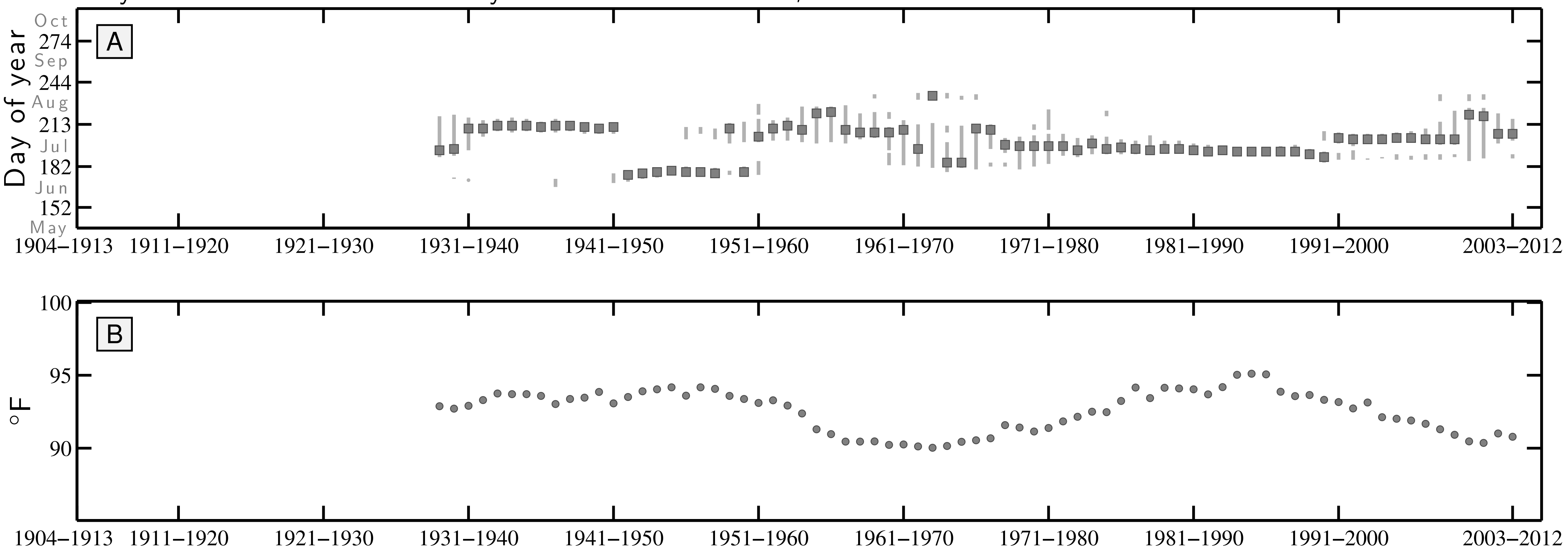

10 year Winter Teletherm dynamics for SUMTER, SC:

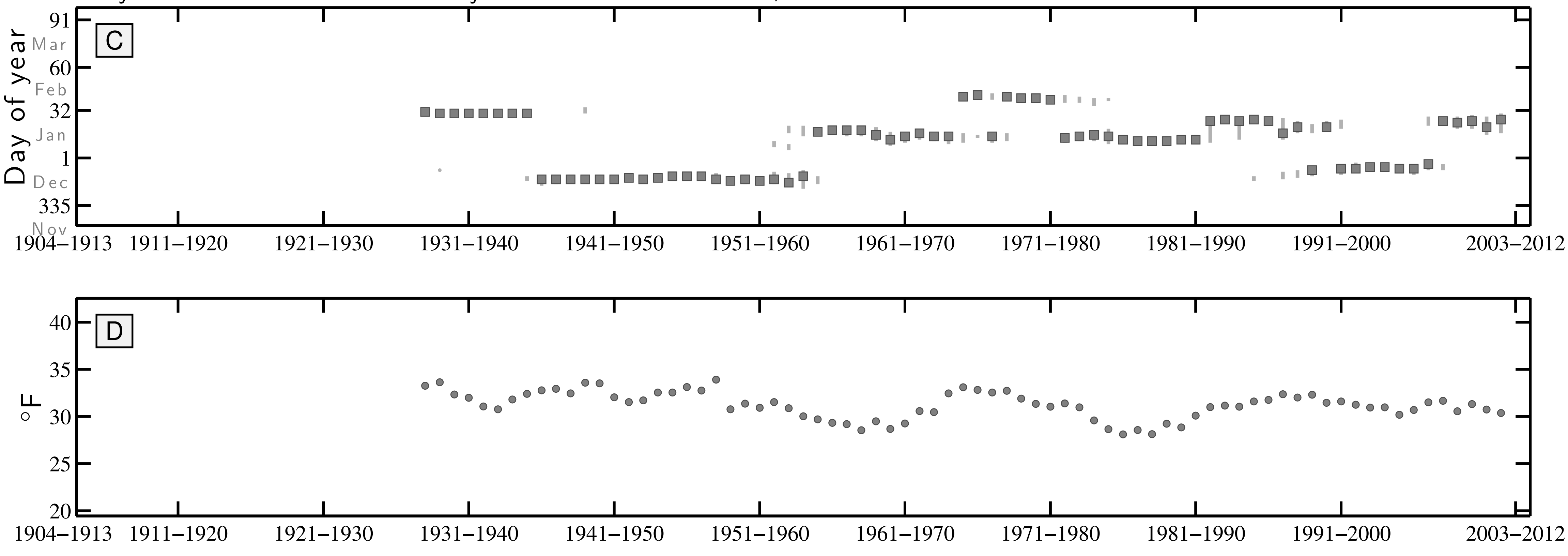

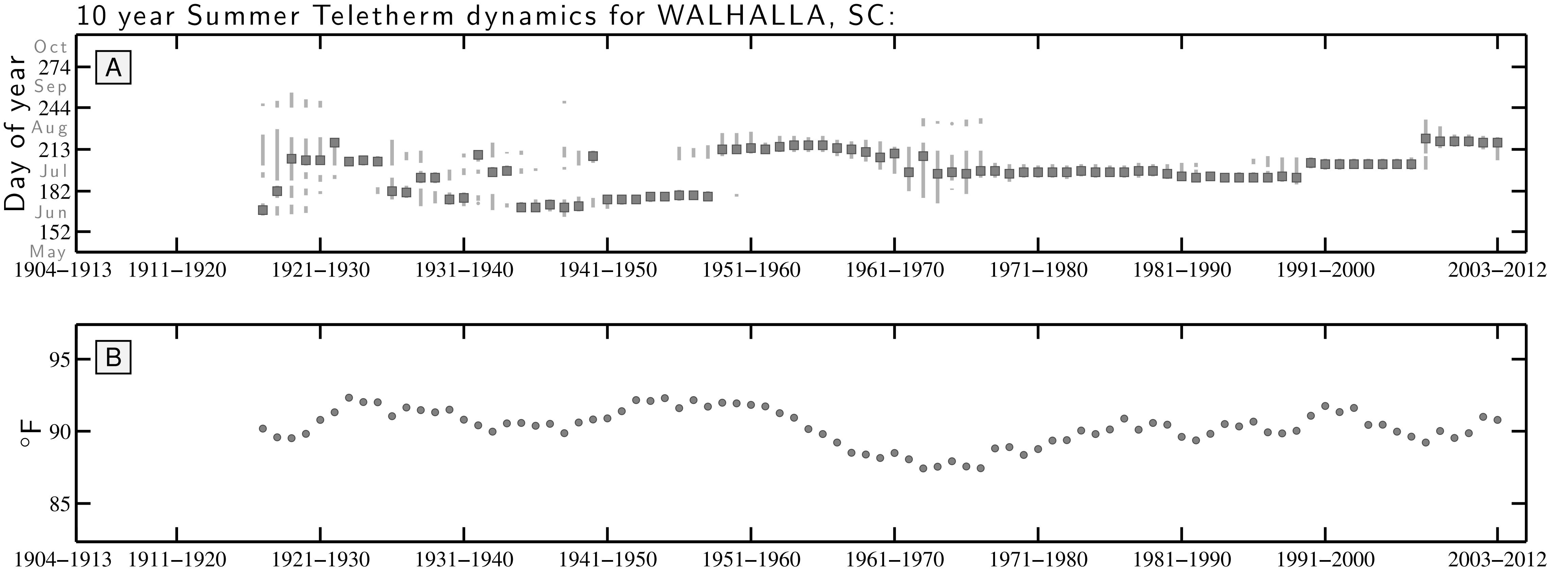

10 year Summer Teletherm dynamics for WALHALLA, SC:
A
B
Day of year
°F
Oct
274
Sep
244
Aug
213
Jul
182
Jun
152
May
95
90
85
1904–1913
1911–1920
1921–1930
1931–1940
1941–1950
1951–1960
1961–1970
1971–1980
1981–1990
1991–2000
2003–2012

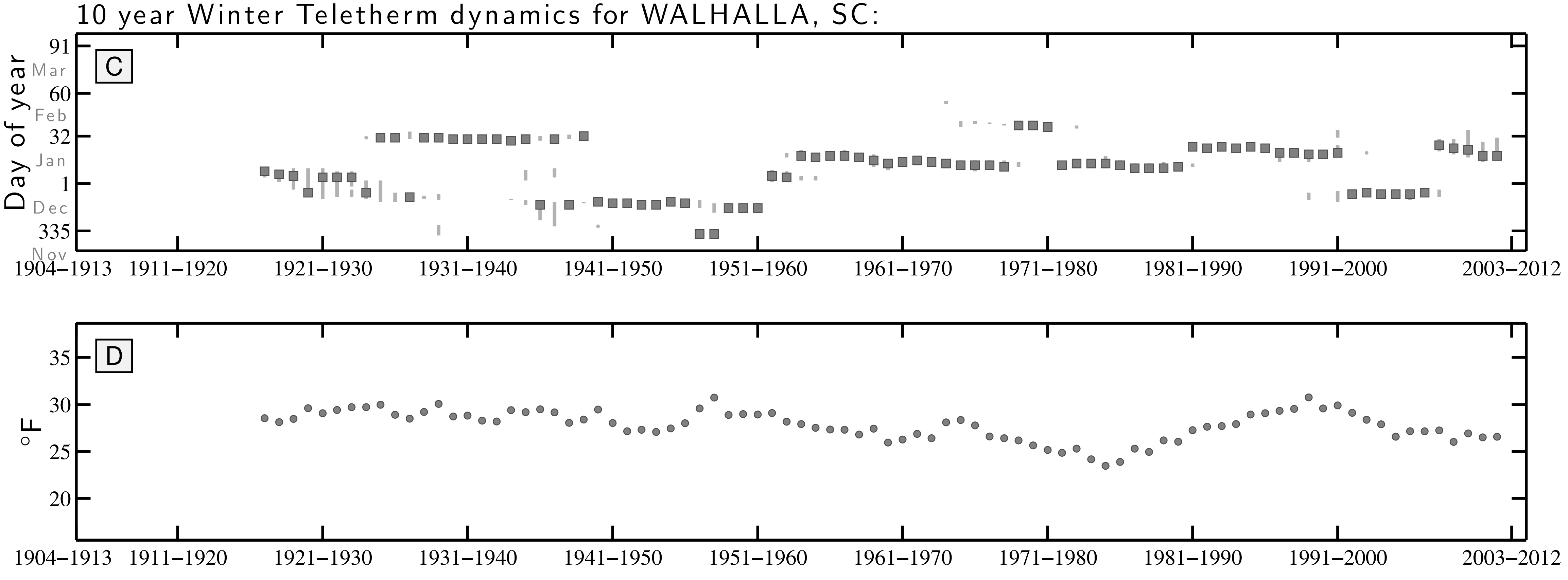

10 year Winter Teletherm dynamics for WALHALLA, SC:
C
D
Day of year
°F
91
Mar
60
Feb
32
Jan
1
Dec
335
Nov
35
30
25
20
1904–1913
1911–1920
1921–1930
1931–1940
1941–1950
1951–1960
1961–1970
1971–1980
1981–1990
1991–2000
2003–2012

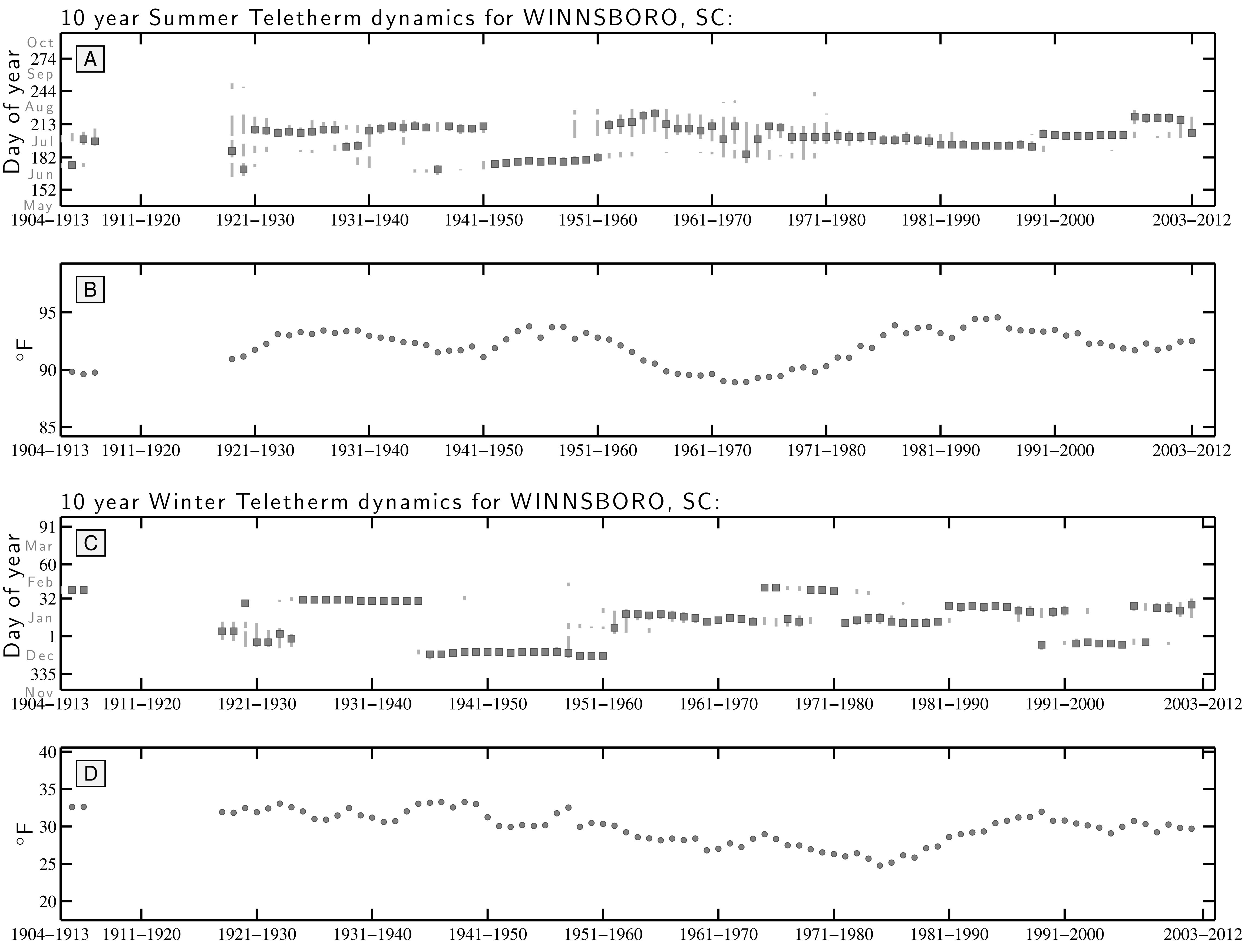
10 year Summer Teletherm dynamics for WINNSBORO, SC:
A
Day of year
Oct
274
Sep
244
Aug
213
Jul
182
Jun
152
May
B
°F
95
90
85
10 year Winter Teletherm dynamics for WINNSBORO, SC:
C
Day of year
91
Mar
60
Feb
32
Jan
1
Dec
335
Nov
D
°F
40
35
30
25
20
1904–1913
1911–1920
1921–1930
1931–1940
1941–1950
1951–1960
1961–1970
1971–1980
1981–1990
1991–2000
2003–2012

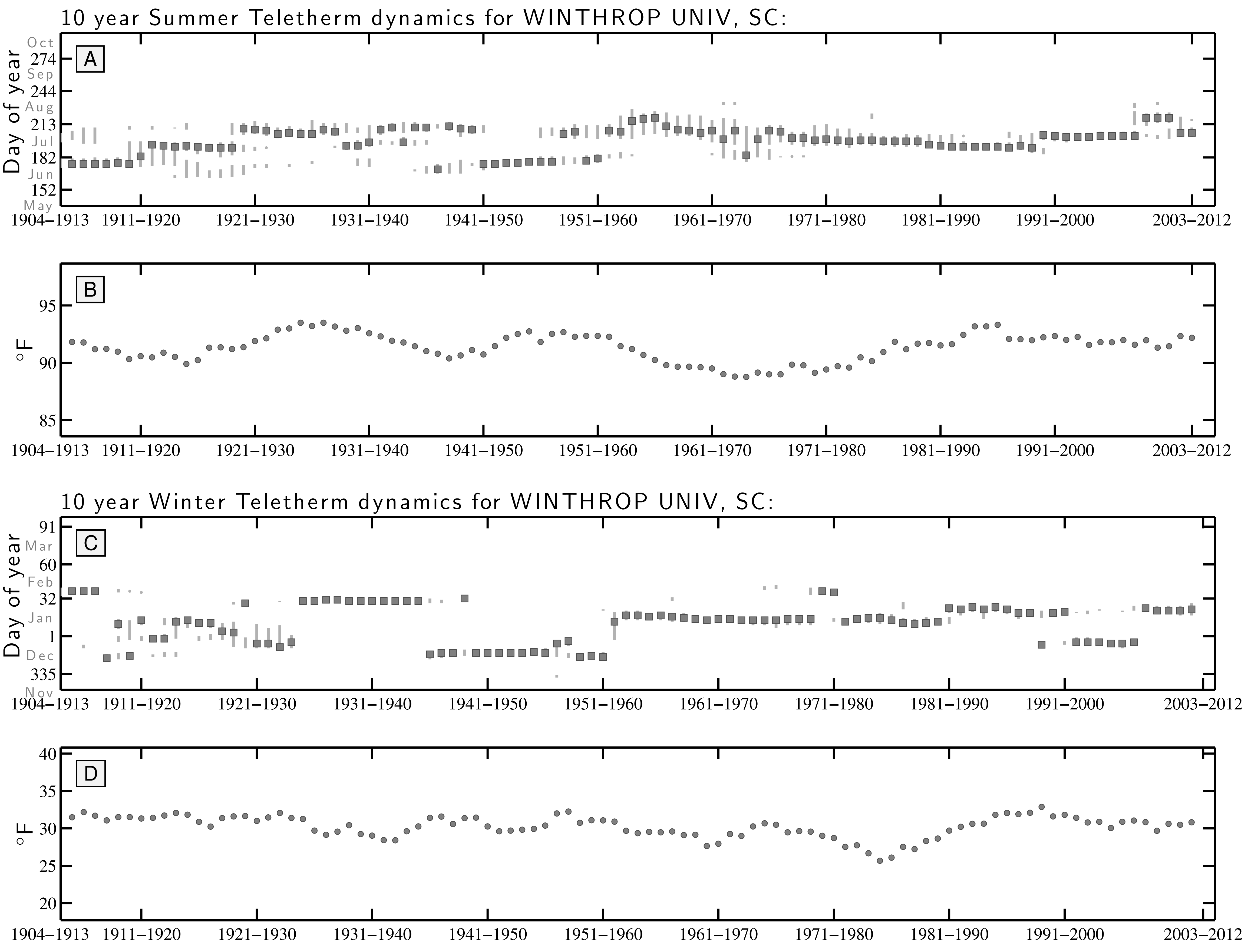

10 year Summer Teletherm dynamics for WINTHROP UNIV, SC:
A
B
10 year Winter Teletherm dynamics for WINTHROP UNIV, SC:
C
D
Day of year
°F
Oct
274
Sep
244
Aug
213
Jul
182
Jun
152
May
95
90
85
91
Mar
60
Feb
32
Jan
1
Dec
335
Nov
40
35
30
25
20
1904–1913
1911–1920
1921–1930
1931–1940
1941–1950
1951–1960
1961–1970
1971–1980
1981–1990
1991–2000
2003–2012

10 year Summer Teletherm dynamics for YEMASSEE, SC:

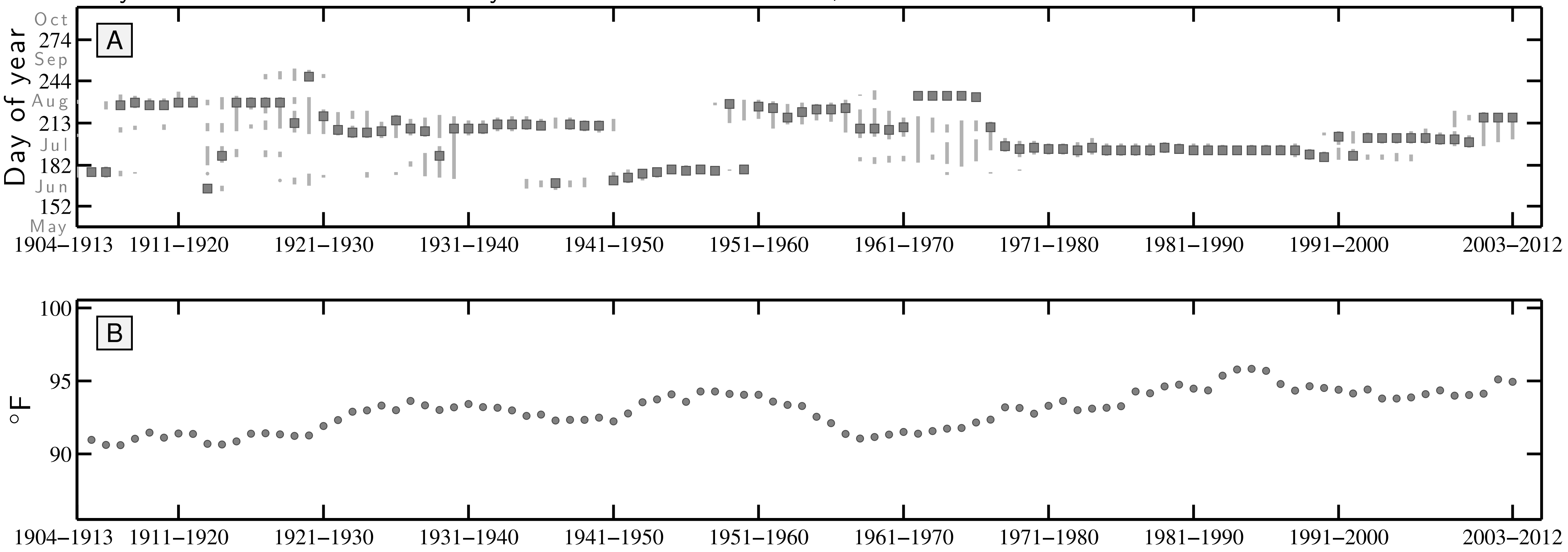

10 year Winter Teletherm dynamics for YEMASSEE, SC:

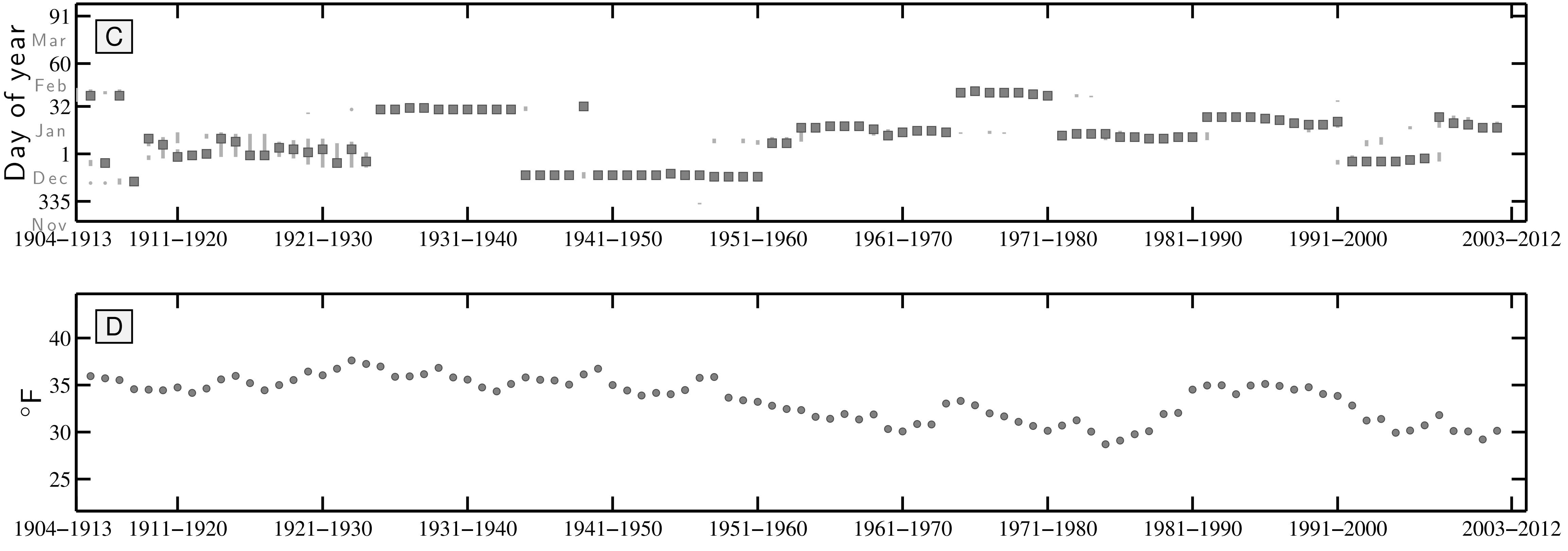

10 year Summer Teletherm dynamics for ABERDEEN RGNL AP, SD:

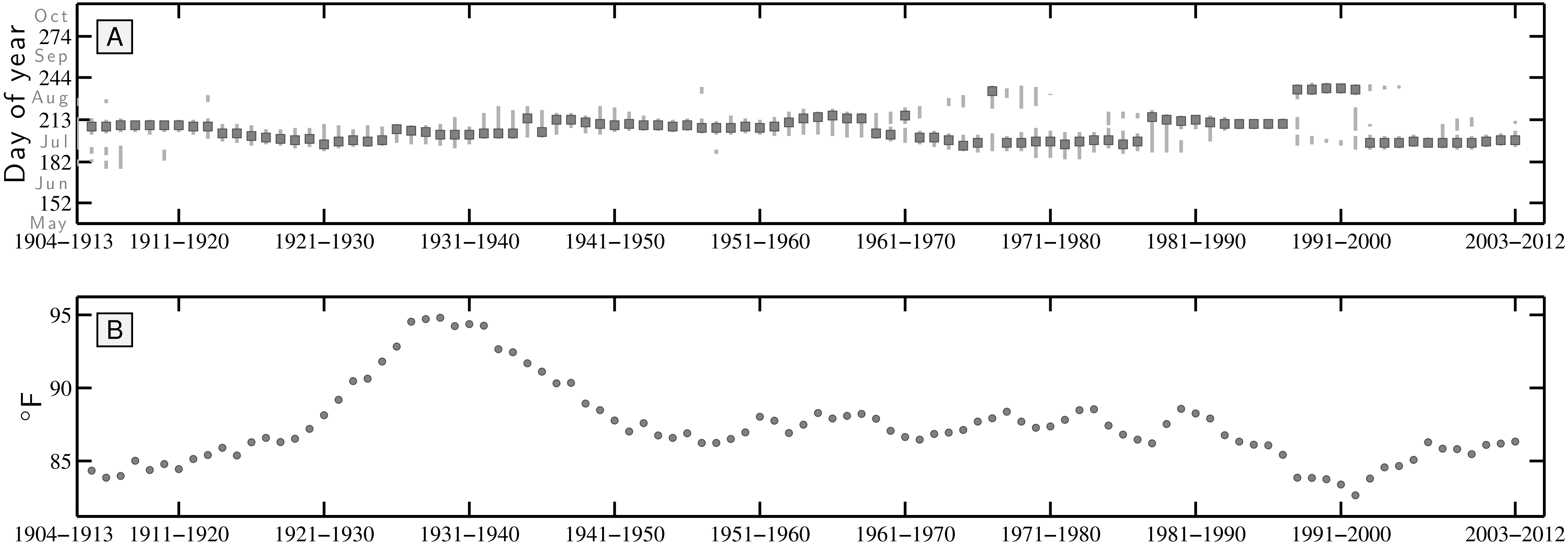

10 year Winter Teletherm dynamics for ABERDEEN RGNL AP, SD:

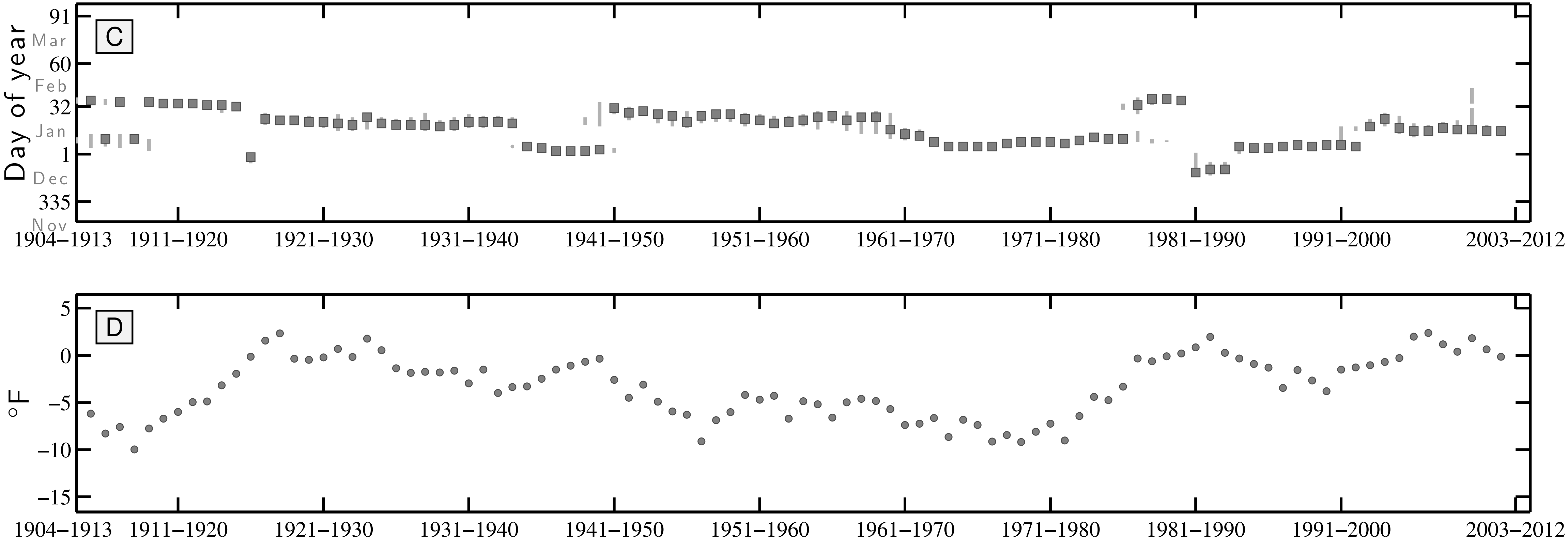

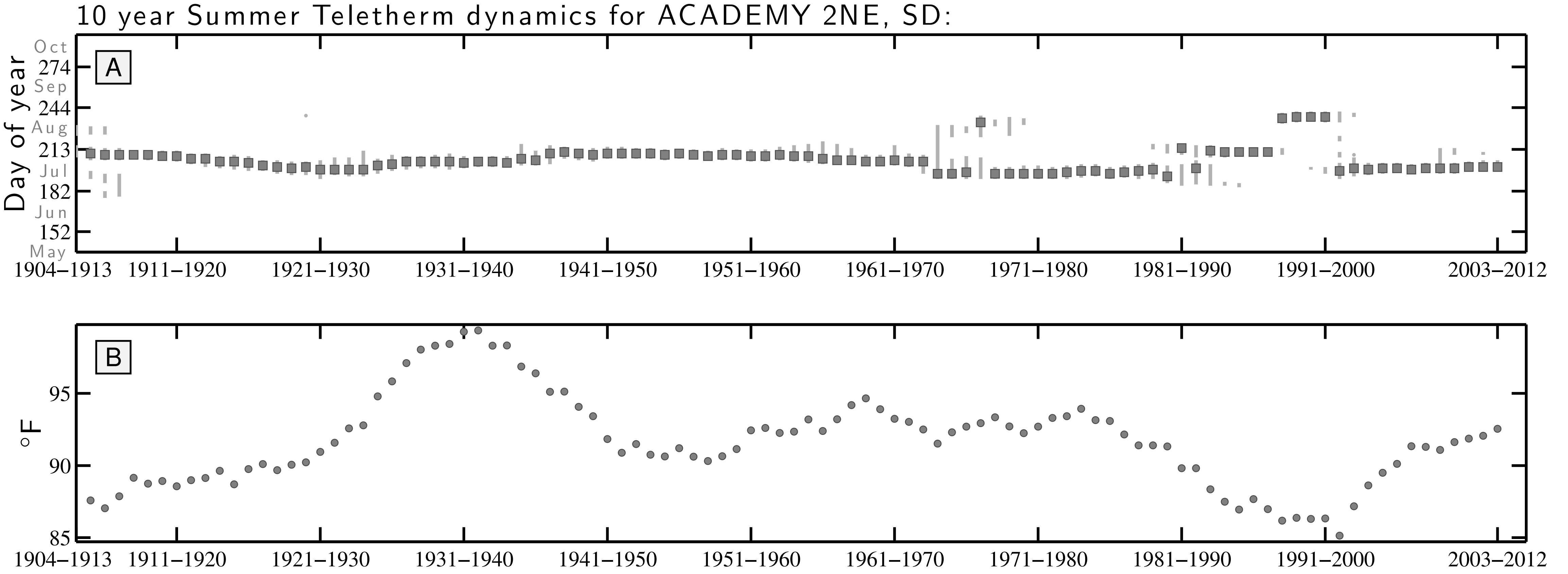

10 year Summer Teletherm dynamics for ACADEMY 2NE, SD:
A
Day of year
Oct
274
Sep
244
Aug
213
Jul
182
Jun
152
May
B
°F
95
90
85
1904–1913
1911–1920
1921–1930
1931–1940
1941–1950
1951–1960
1961–1970
1971–1980
1981–1990
1991–2000
2003–2012

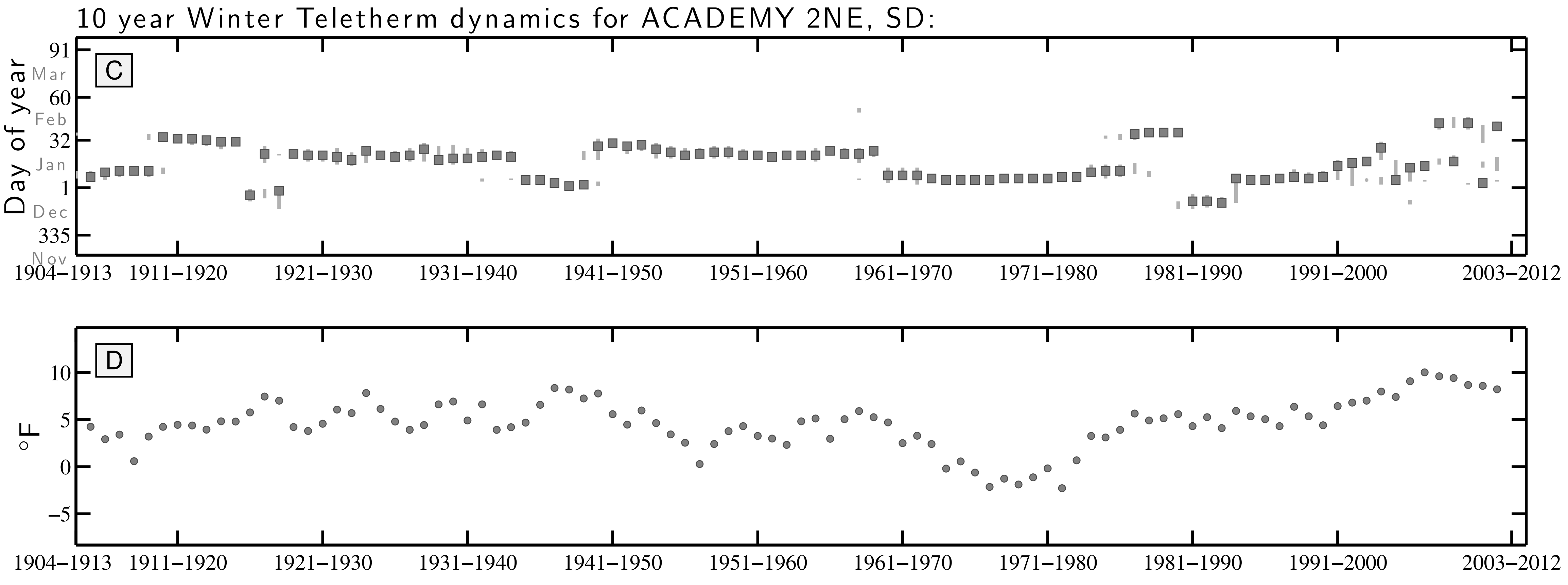

10 year Winter Teletherm dynamics for ACADEMY 2NE, SD:
C
Day of year
91
Mar
60
Feb
32
Jan
1
Dec
335
Nov
D
°F
10
5
0
−5
1904–1913
1911–1920
1921–1930
1931–1940
1941–1950
1951–1960
1961–1970
1971–1980
1981–1990
1991–2000
2003–2012

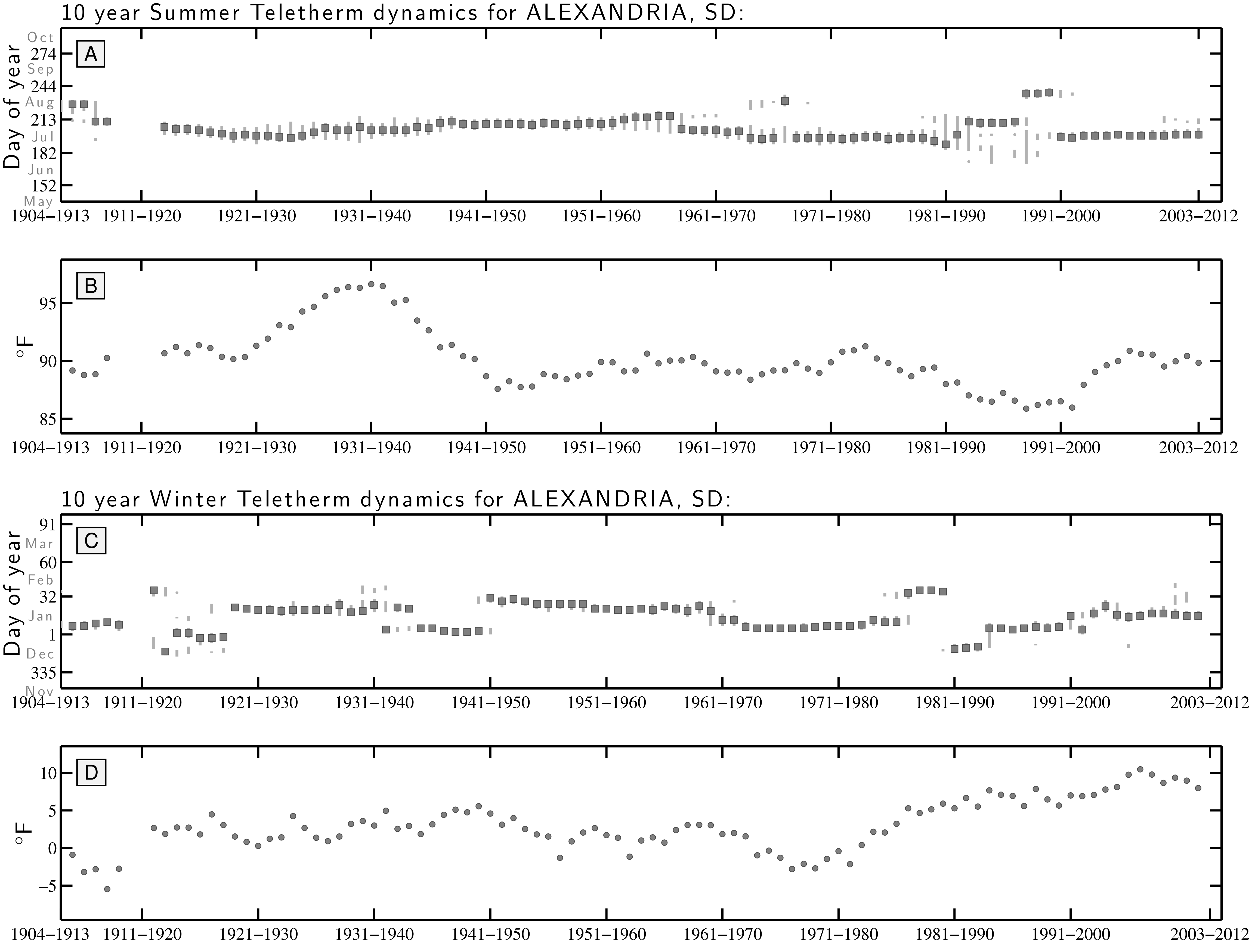
10 year Summer Teletherm dynamics for ALEXANDRIA, SD:
A
B
Day of year
°F
Oct
274
Sep
244
Aug
213
Jul
182
Jun
152
May
95
90
85
1904–1913
1911–1920
1921–1930
1931–1940
1941–1950
1951–1960
1961–1970
1971–1980
1981–1990
1991–2000
2003–2012
10 year Winter Teletherm dynamics for ALEXANDRIA, SD:
C
D
91
Mar
60
Feb
32
Jan
1
Dec
335
Nov
10
5
0
−5

10 year Summer Teletherm dynamics for CANTON, SD:

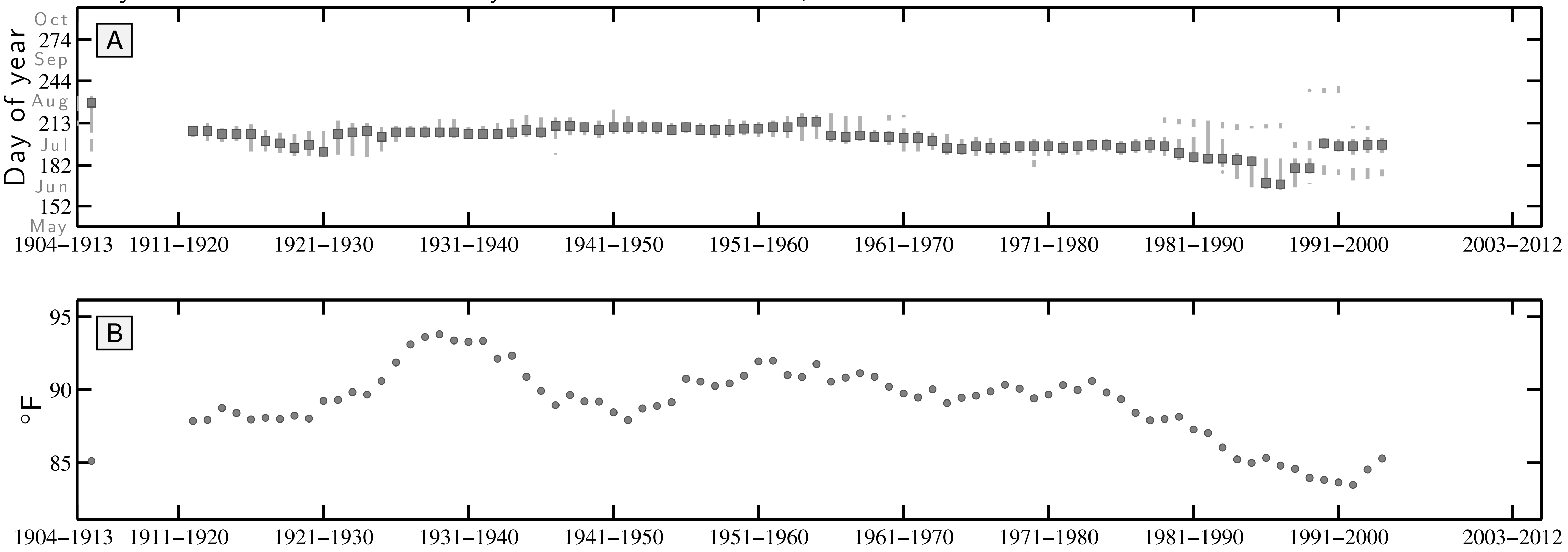

10 year Winter Teletherm dynamics for CANTON, SD:

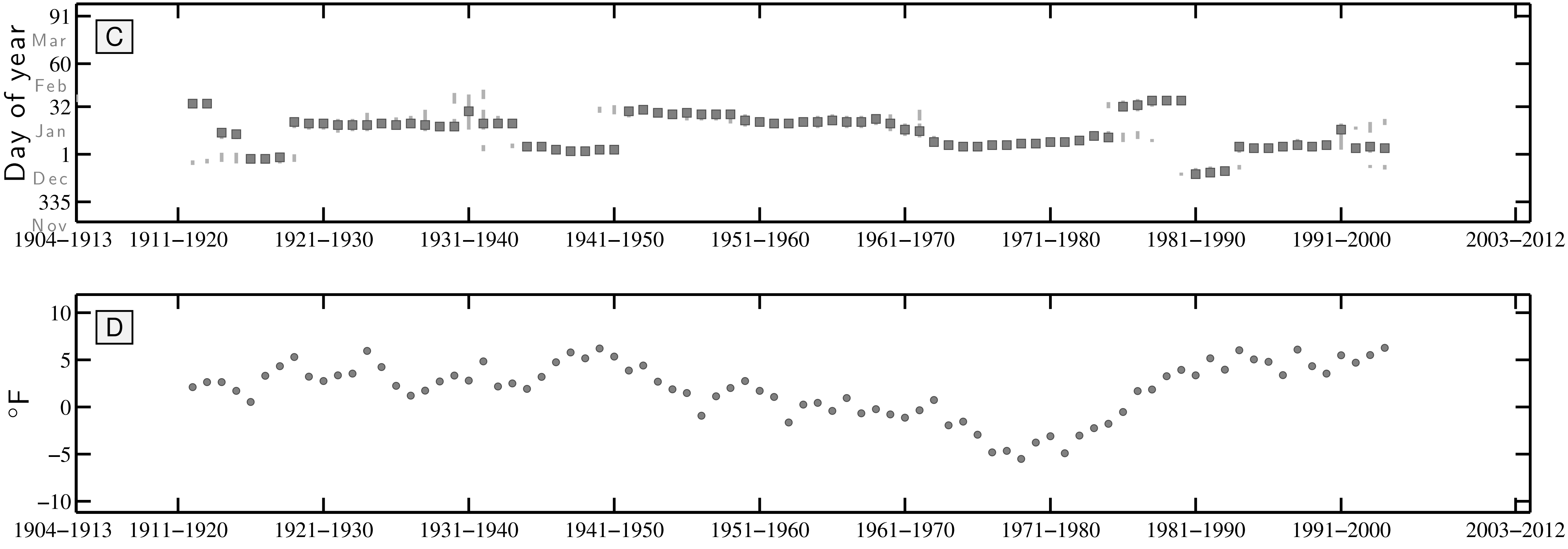

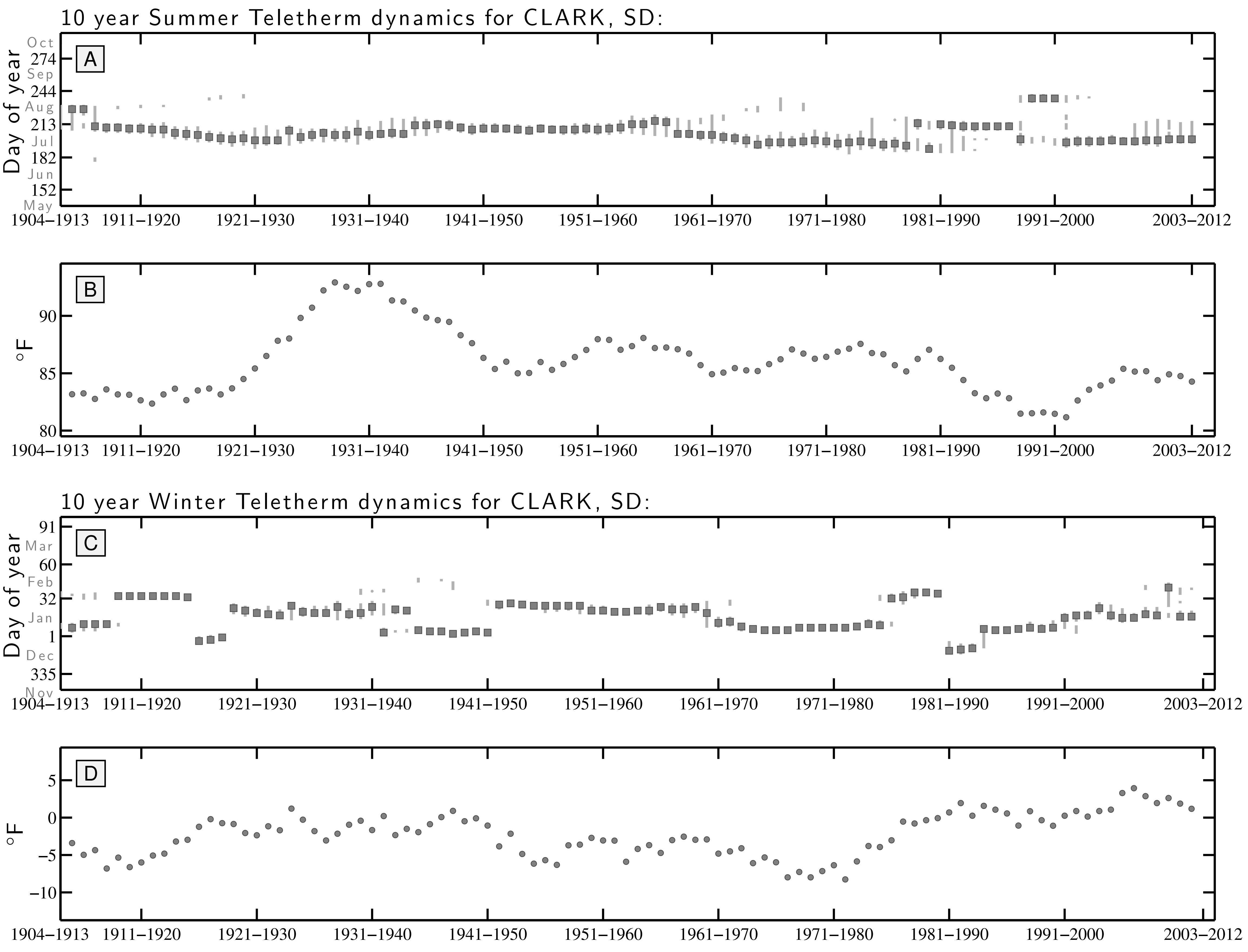
10 year Summer Teletherm dynamics for CLARK, SD:
A
B
Day of year
°F
Oct
274
Sep
244
Aug
213
Jul
182
Jun
152
May
90
85
80
1904–1913
1911–1920
1921–1930
1931–1940
1941–1950
1951–1960
1961–1970
1971–1980
1981–1990
1991–2000
2003–2012
10 year Winter Teletherm dynamics for CLARK, SD:
C
D
91
Mar
60
Feb
32
Jan
1
Dec
335
Nov
5
0
−5
−10

10 year Summer Teletherm dynamics for COTTONWOOD 2 E, SD:

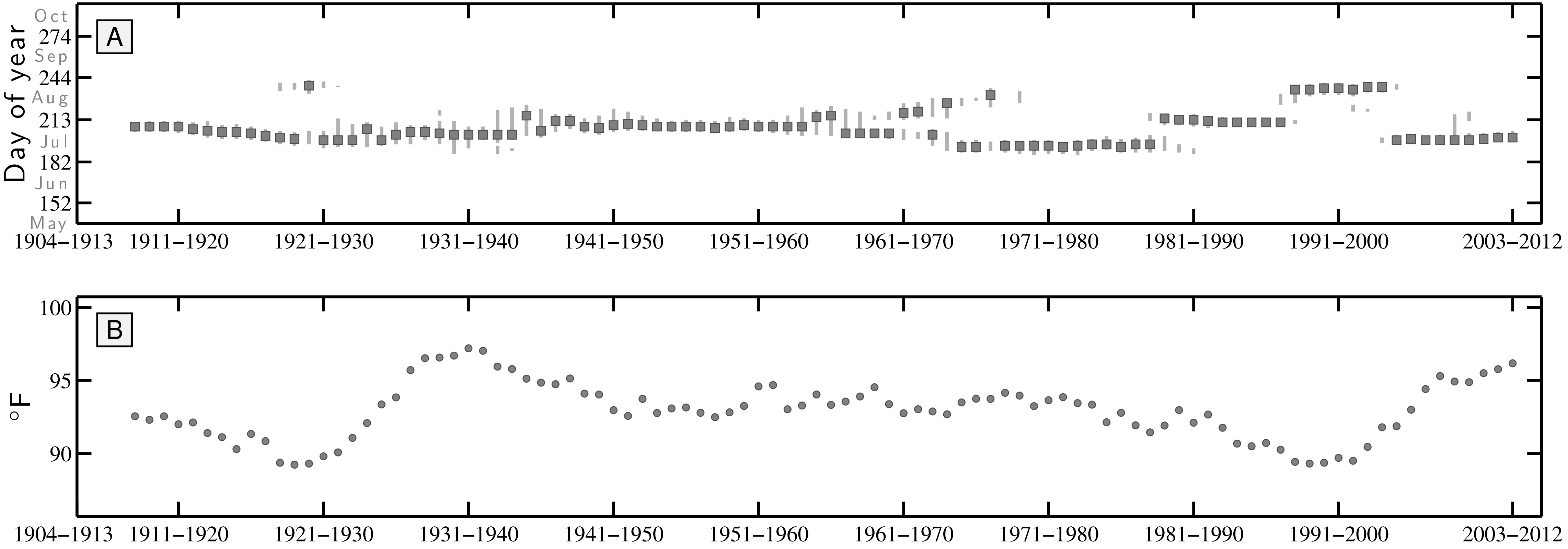

10 year Winter Teletherm dynamics for COTTONWOOD 2 E, SD:

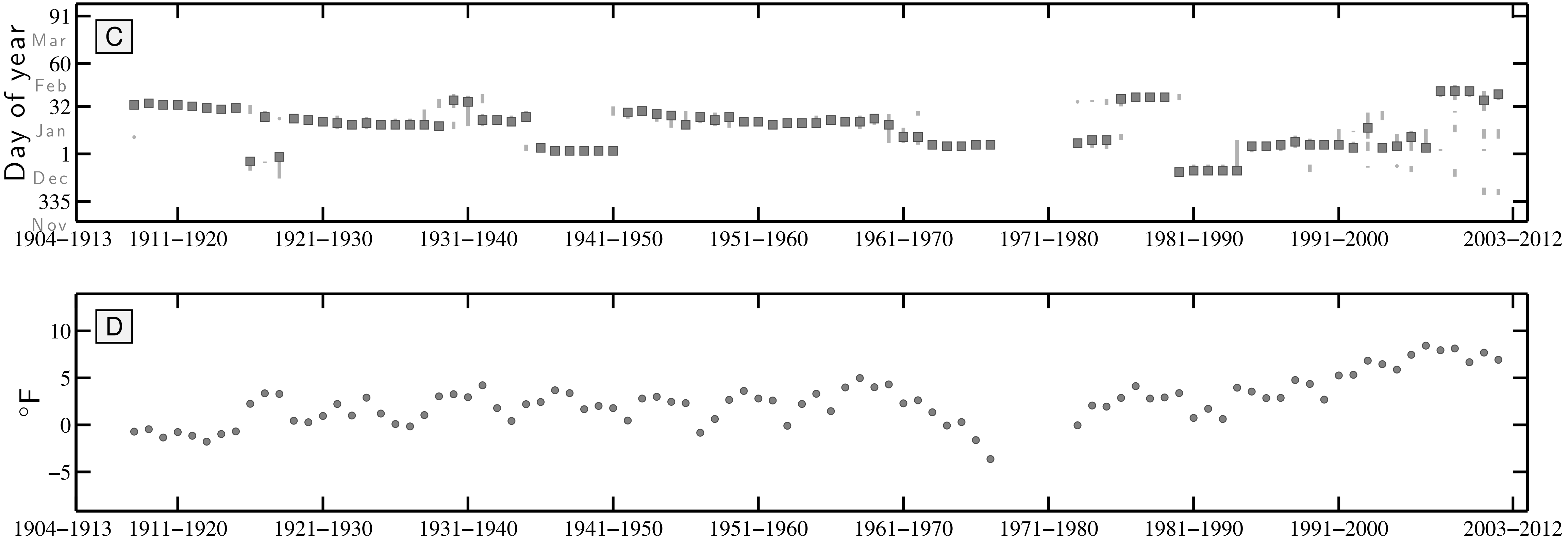

10 year Summer Teletherm dynamics for DUPREE, SD:

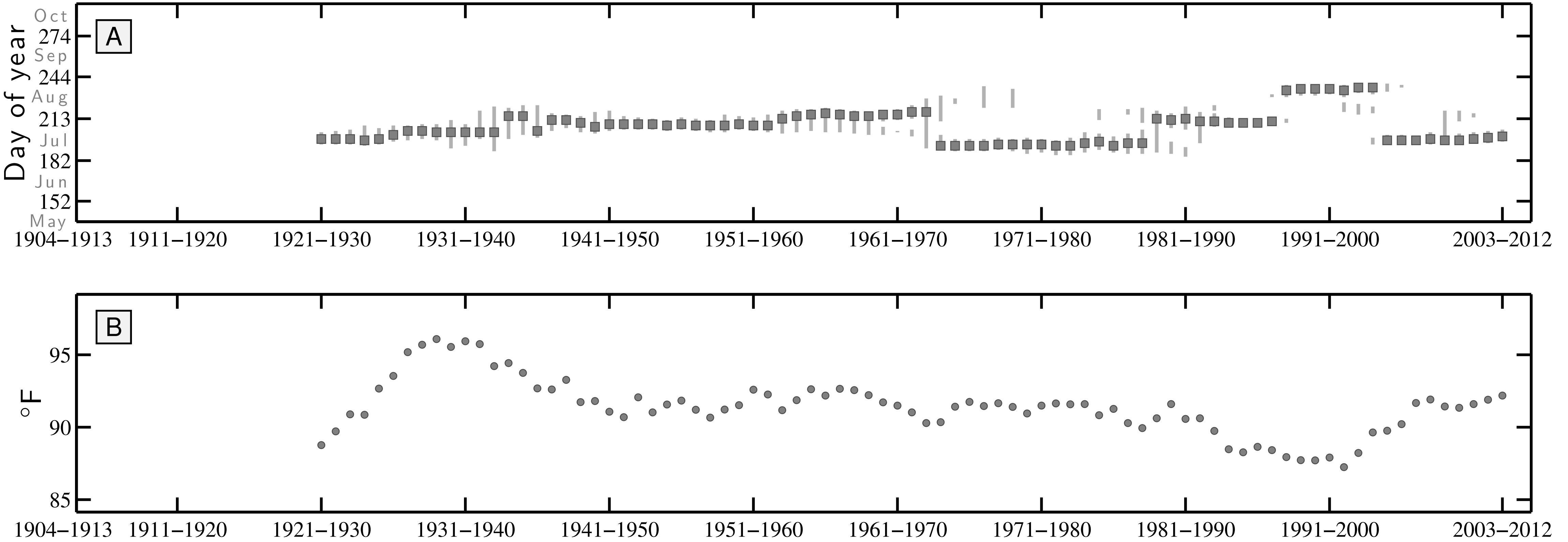

10 year Winter Teletherm dynamics for DUPREE, SD:

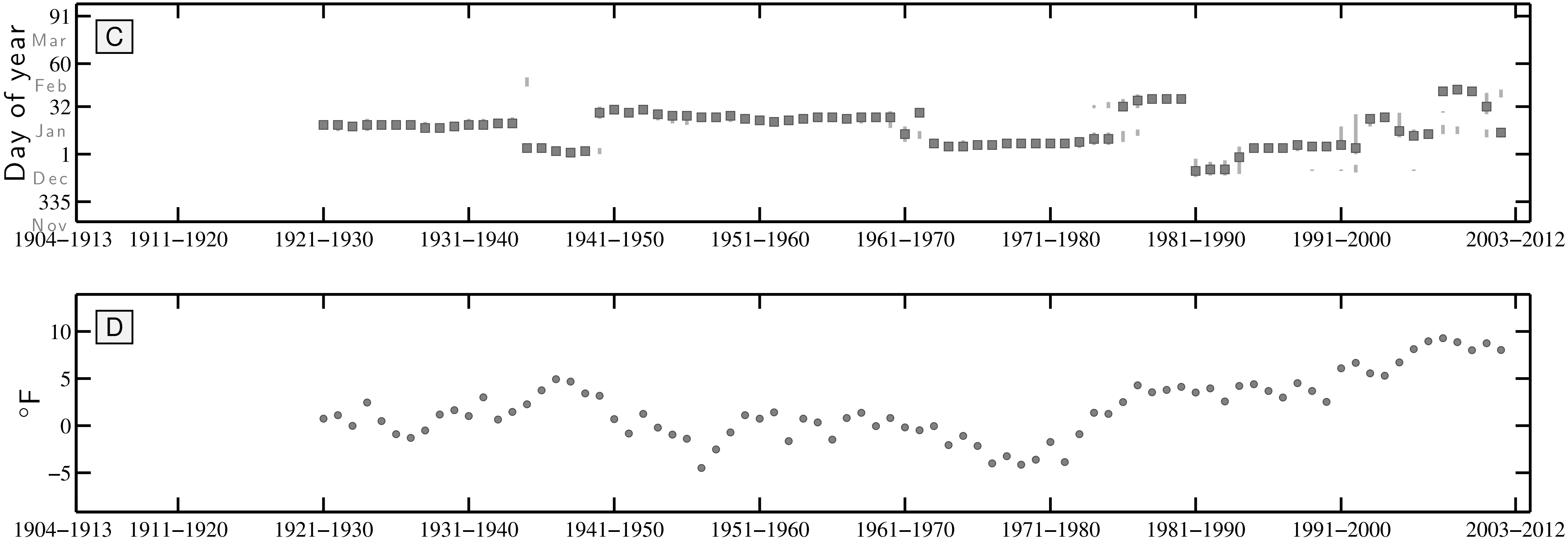

10 year Summer Teletherm dynamics for EUREKA, SD:

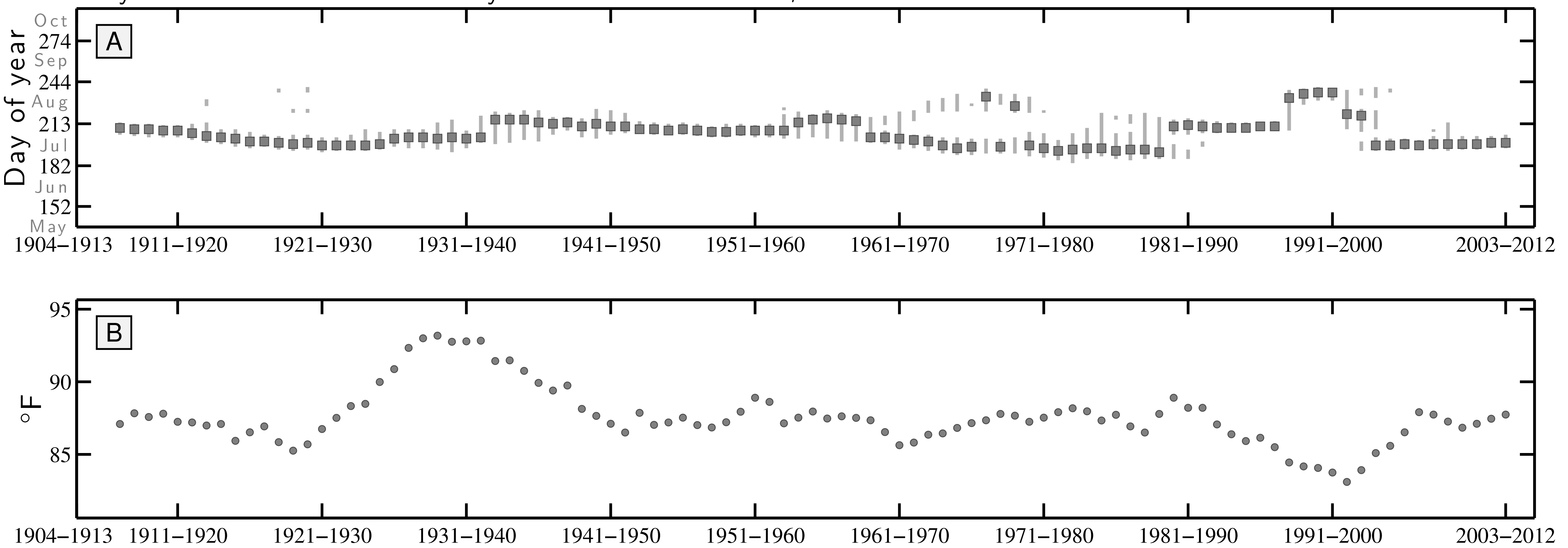

10 year Winter Teletherm dynamics for EUREKA, SD:

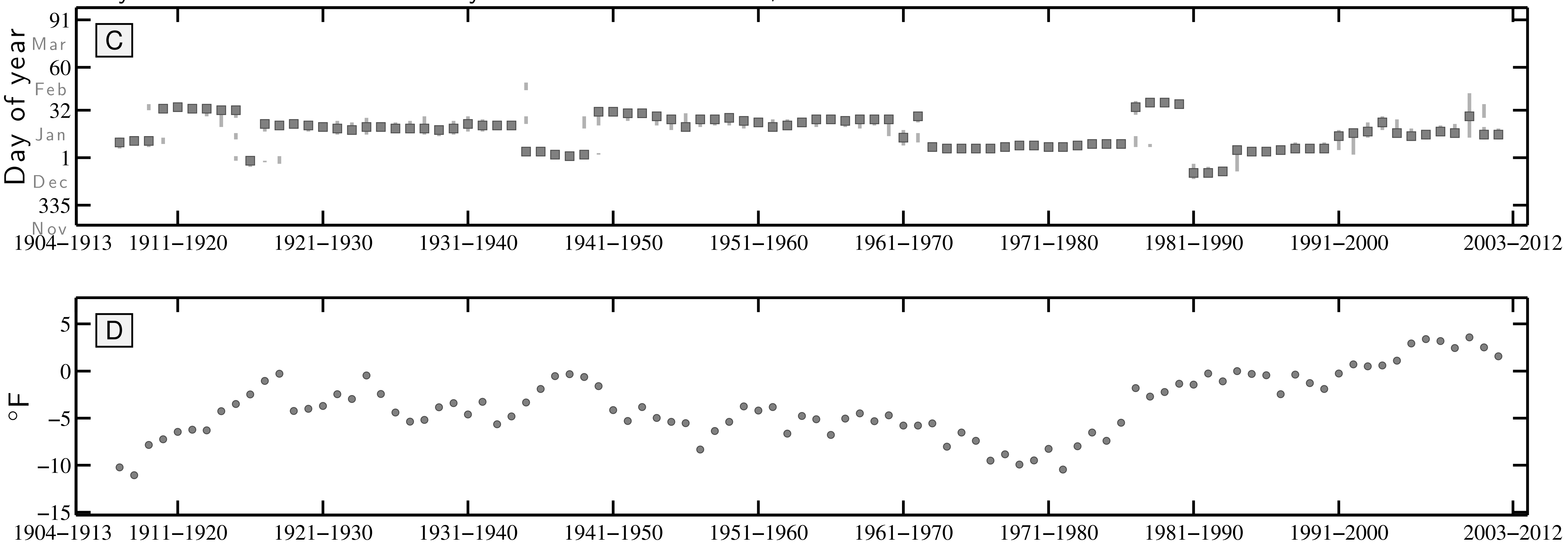

10 year Summer Teletherm dynamics for FAULKTON 1 NW, SD:

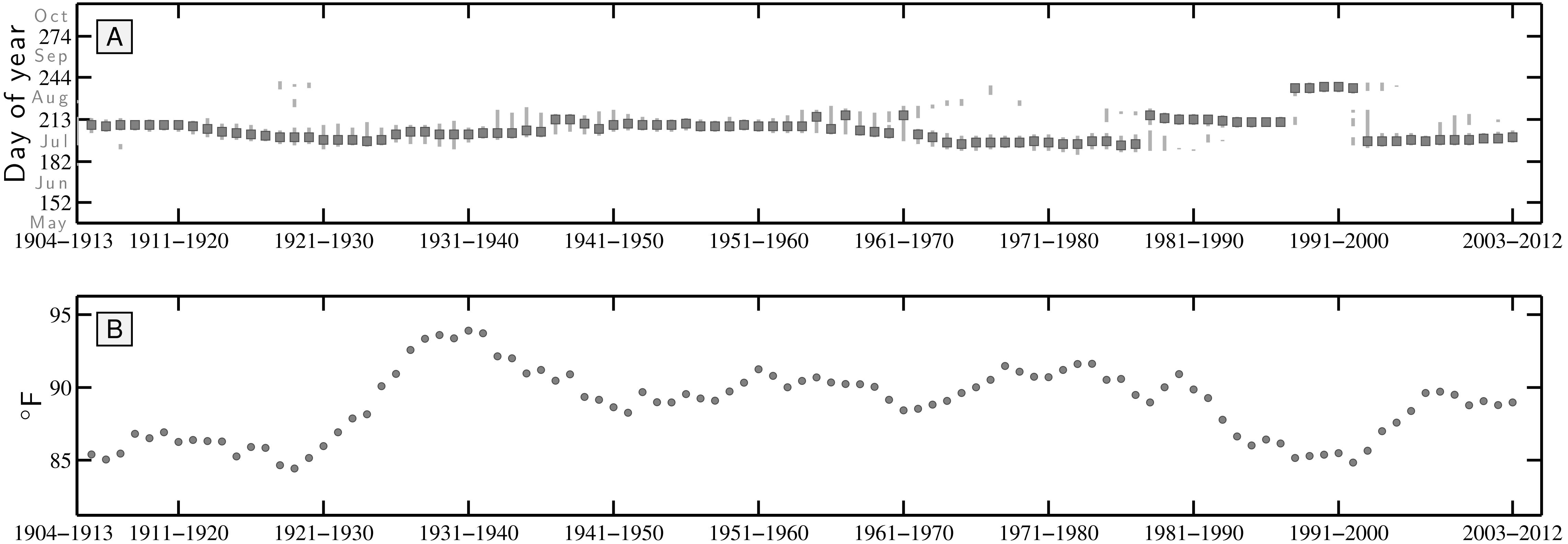

10 year Winter Teletherm dynamics for FAULKTON 1 NW, SD:

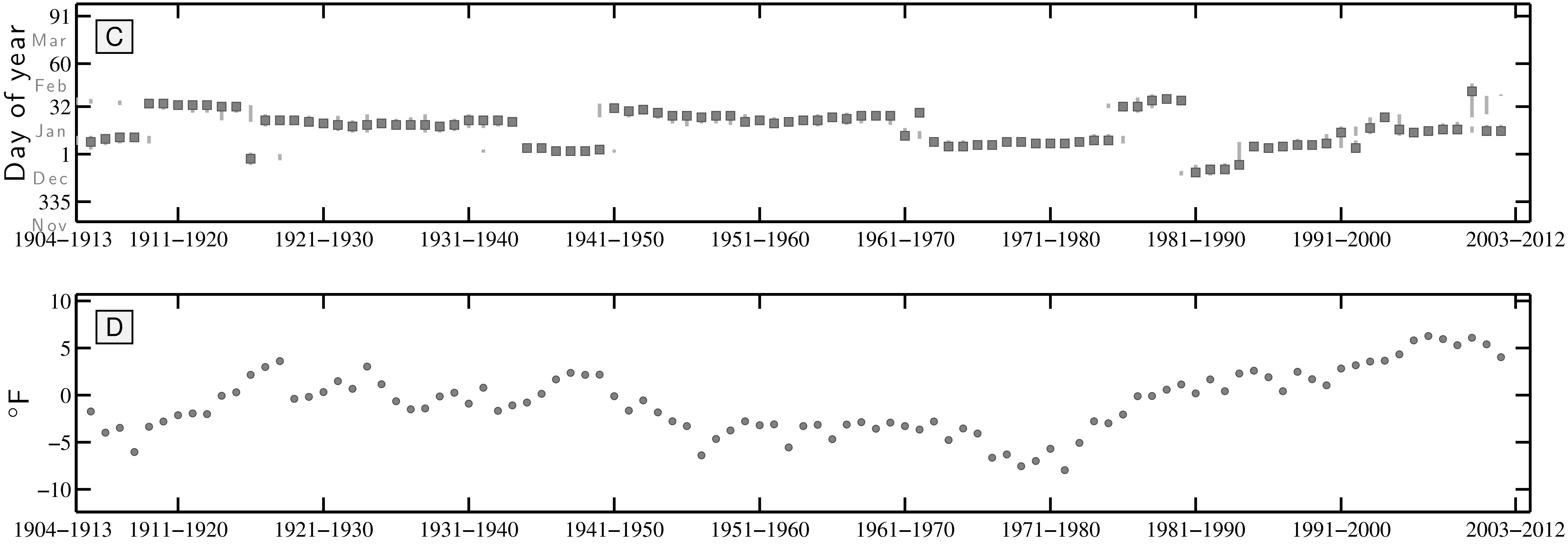

10 year Summer Teletherm dynamics for FORESTBURG 3 NE, SD:

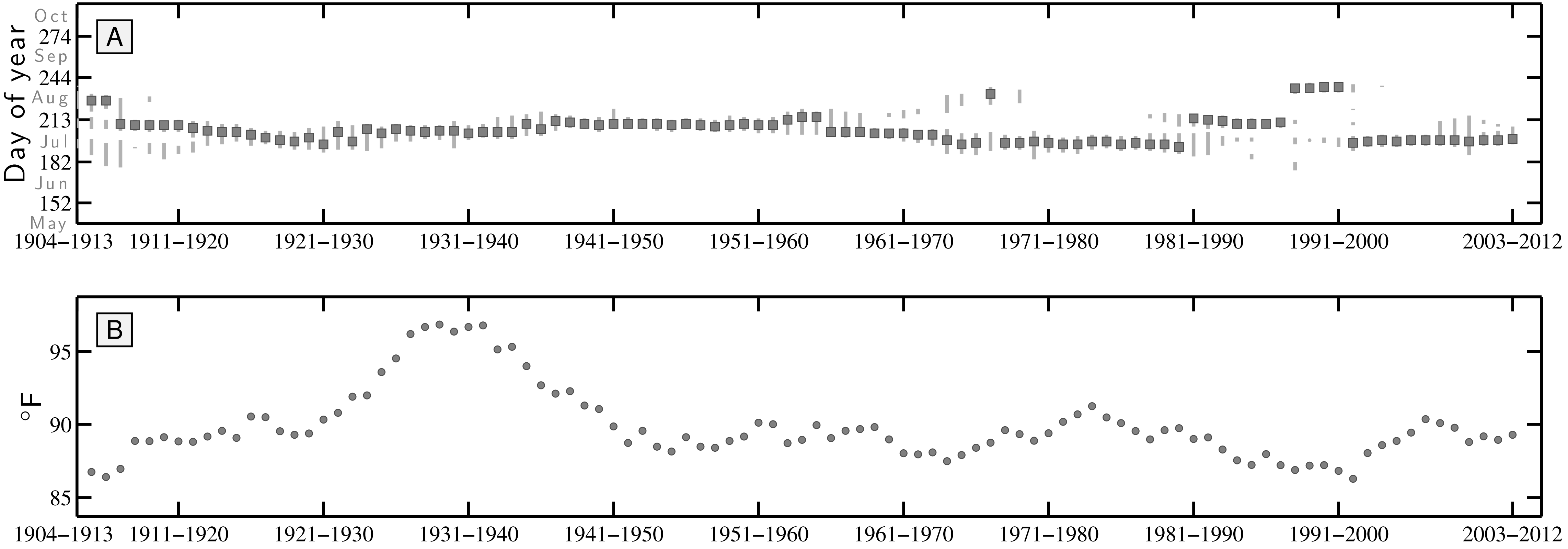

10 year Winter Teletherm dynamics for FORESTBURG 3 NE, SD:

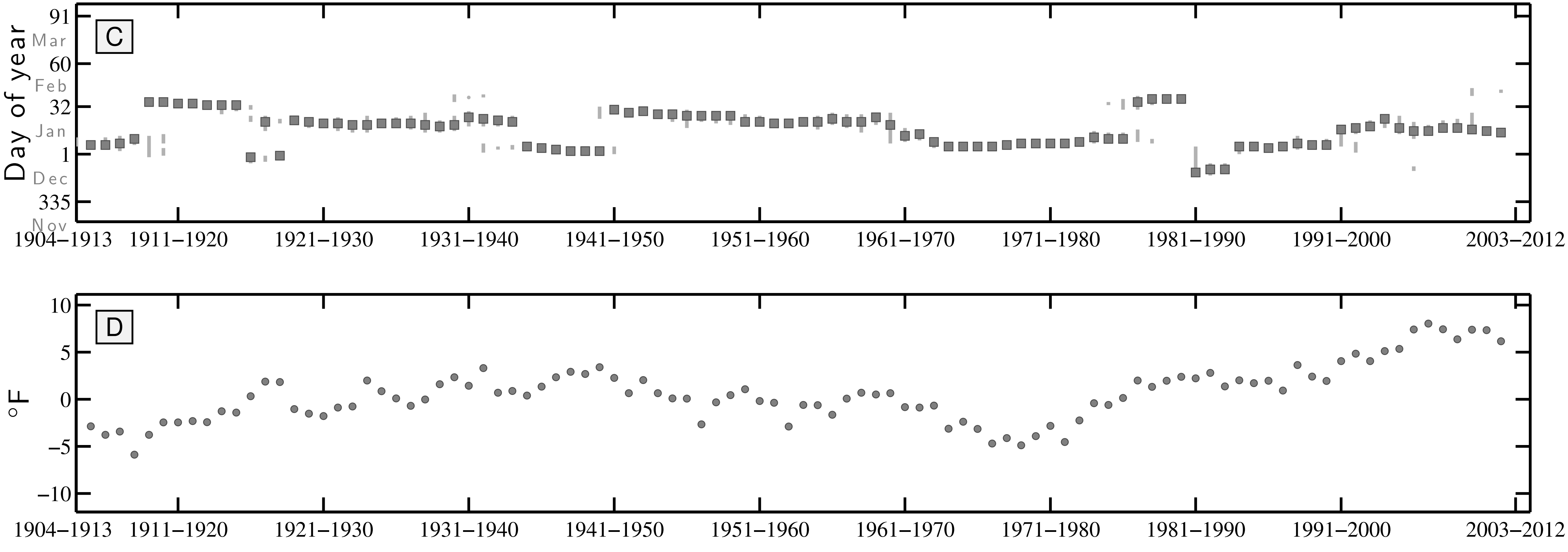

10 year Summer Teletherm dynamics for GANN VALLEY 4NW, SD:

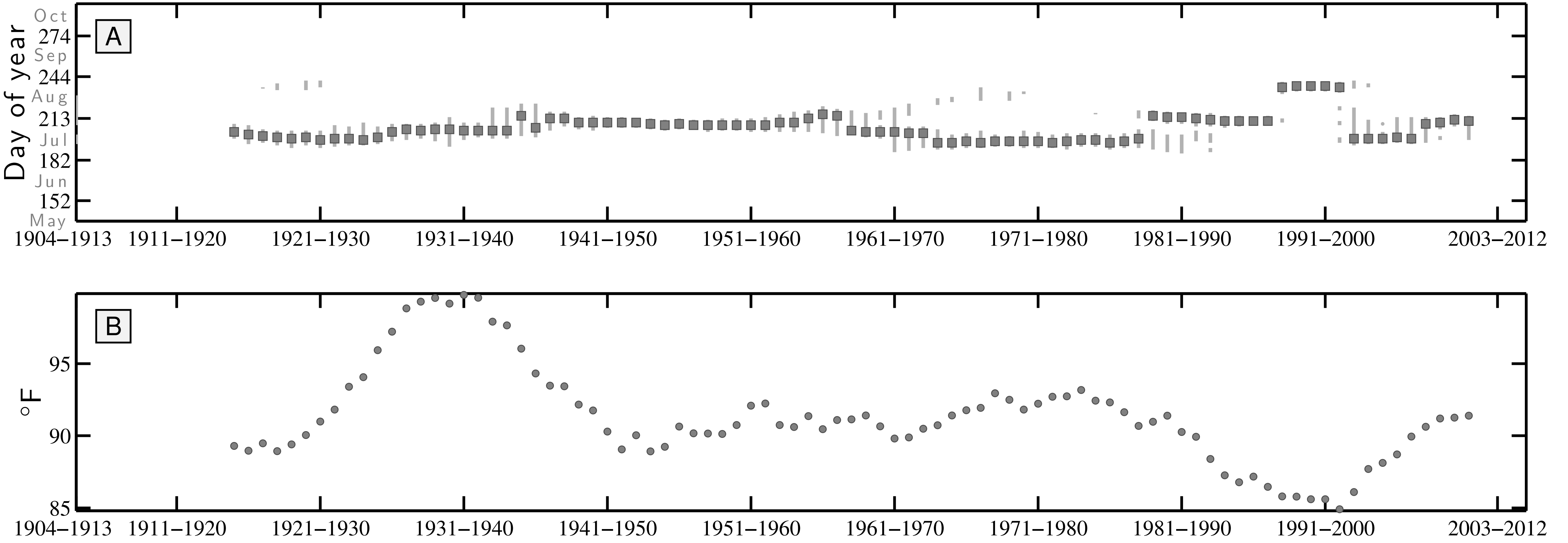

10 year Winter Teletherm dynamics for GANN VALLEY 4NW, SD:

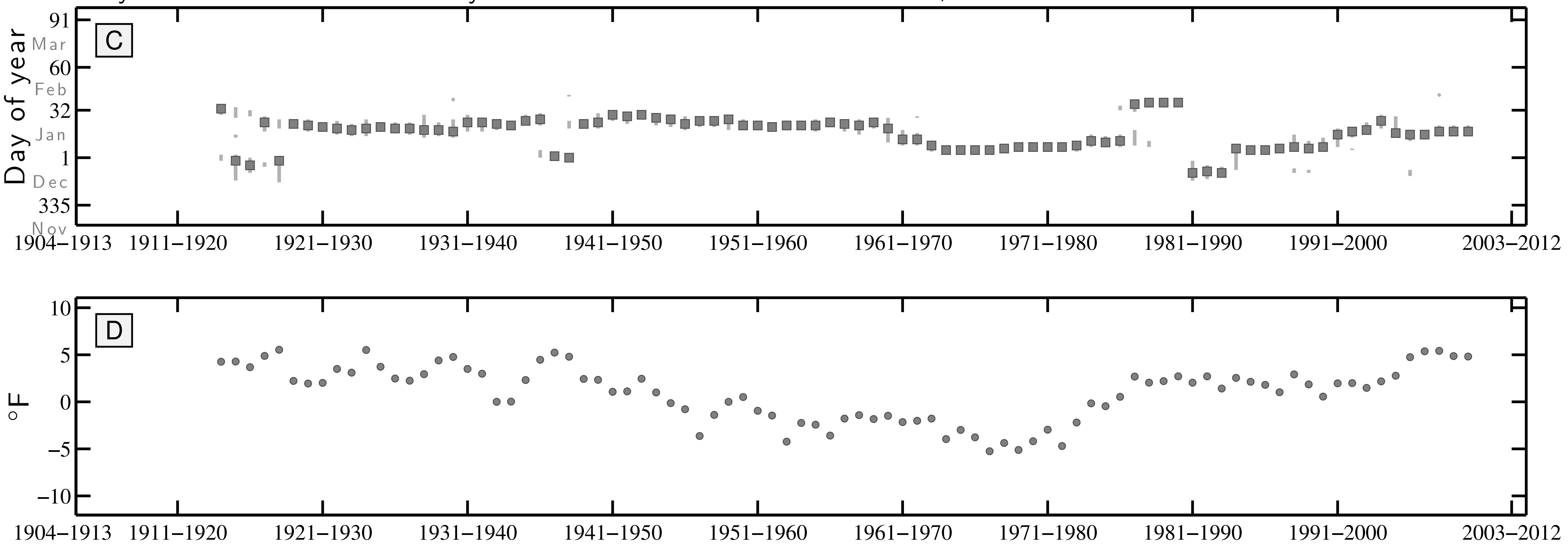

10 year Summer Teletherm dynamics for HIGHMORE 1 W, SD:

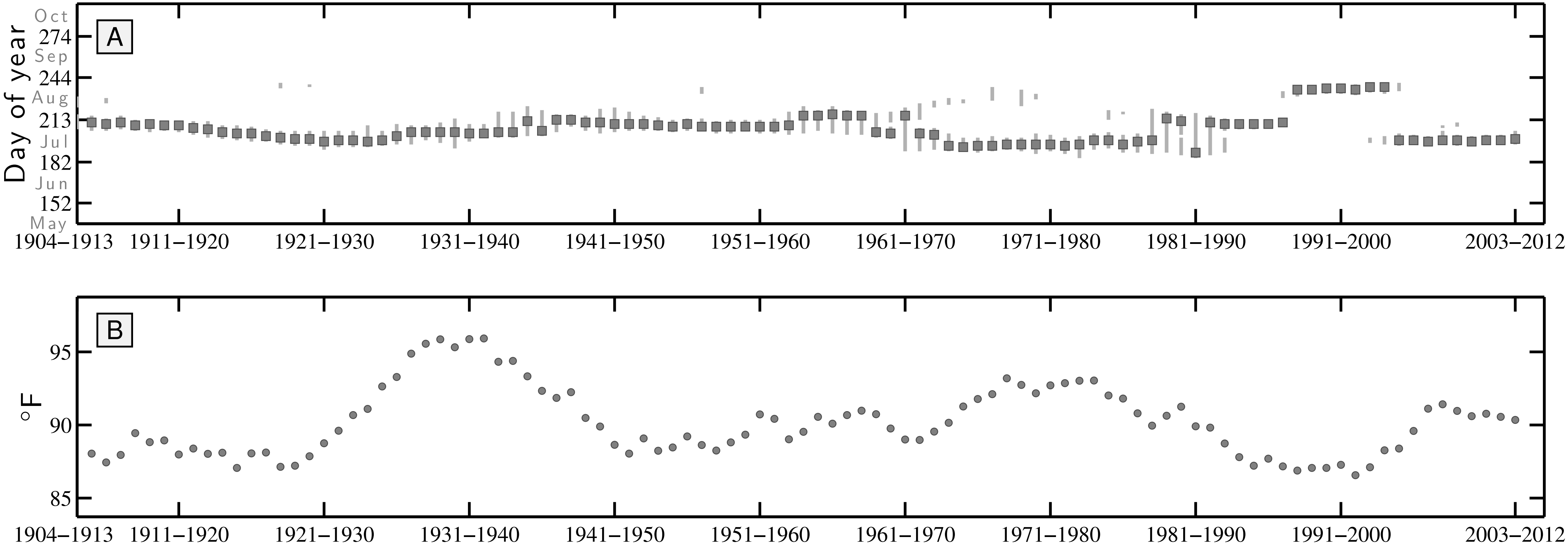

10 year Winter Teletherm dynamics for HIGHMORE 1 W, SD:

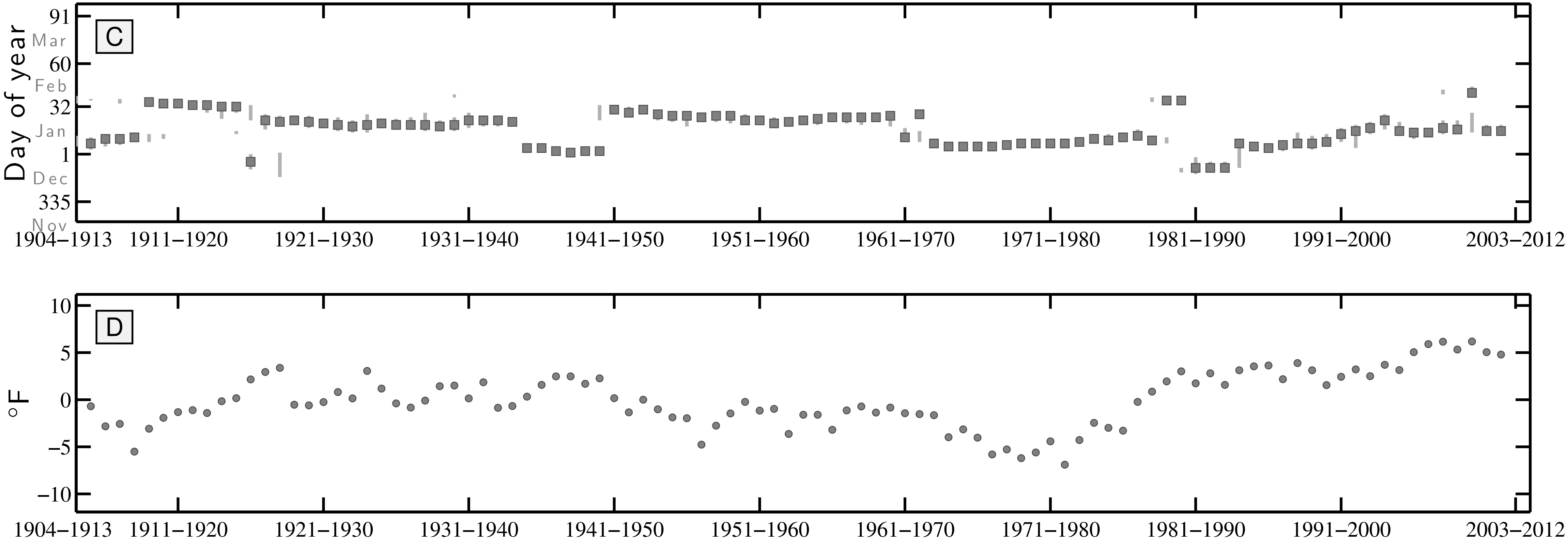

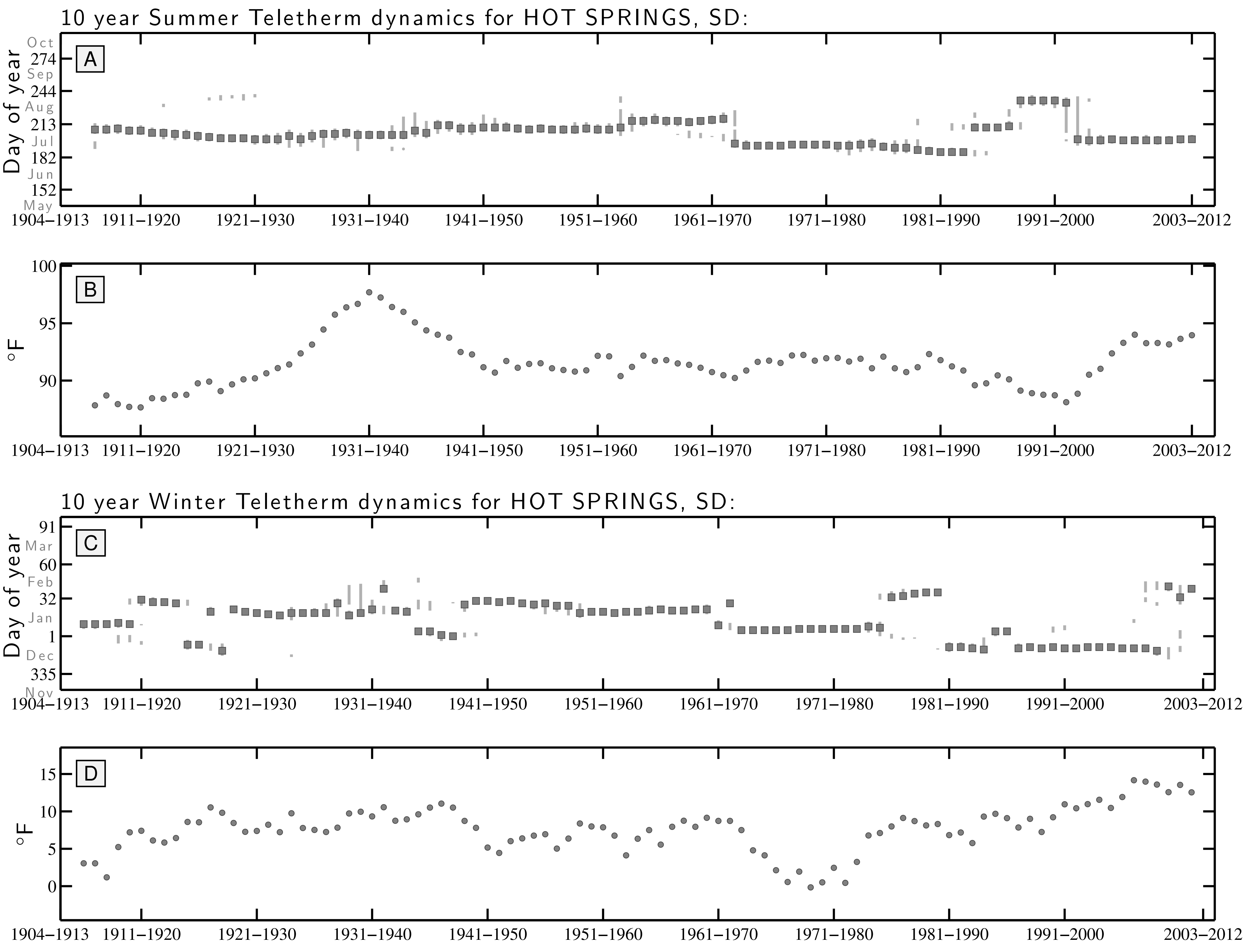

10 year Summer Teletherm dynamics for HOT SPRINGS, SD:
A
Day of year
Oct
274
Sep
244
Aug
213
Jul
182
Jun
152
May
1904–1913
1911–1920
1921–1930
1931–1940
1941–1950
1951–1960
1961–1970
1971–1980
1981–1990
1991–2000
2003–2012
B
°F
100
95
90
10 year Winter Teletherm dynamics for HOT SPRINGS, SD:
C
91
Mar
60
Feb
32
Jan
1
Dec
335
Nov
D
15
10
5
0

10 year Summer Teletherm dynamics for HOWARD, SD:

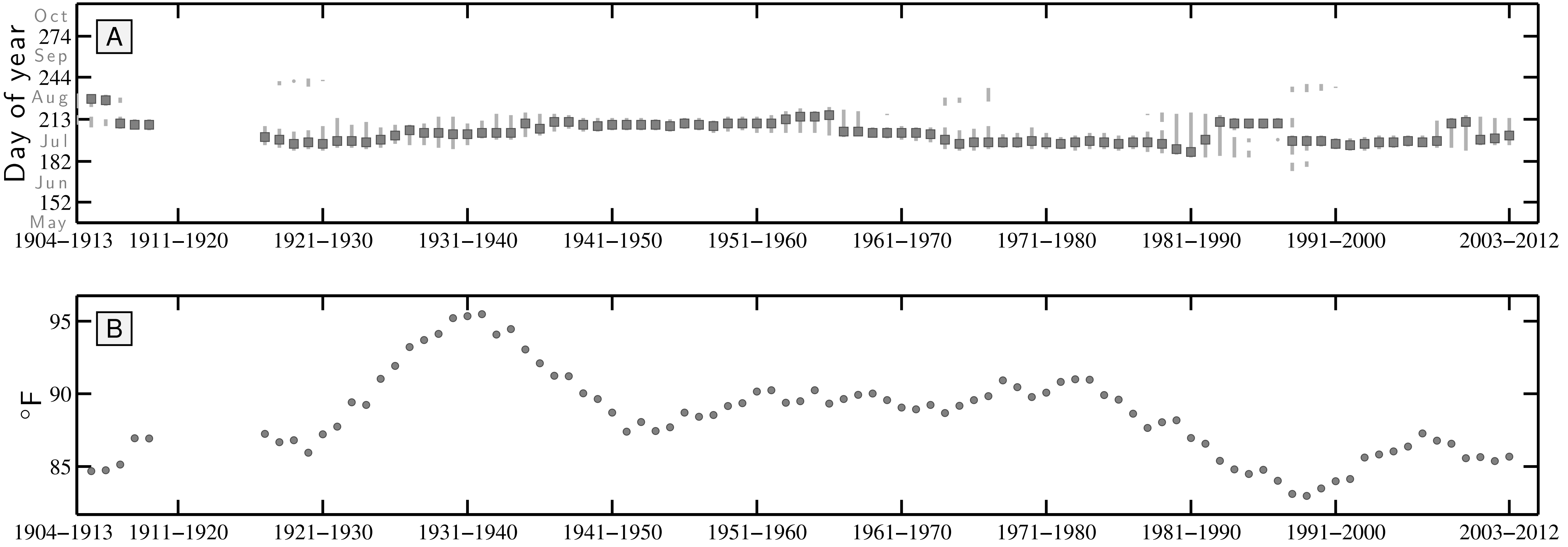

10 year Winter Teletherm dynamics for HOWARD, SD:

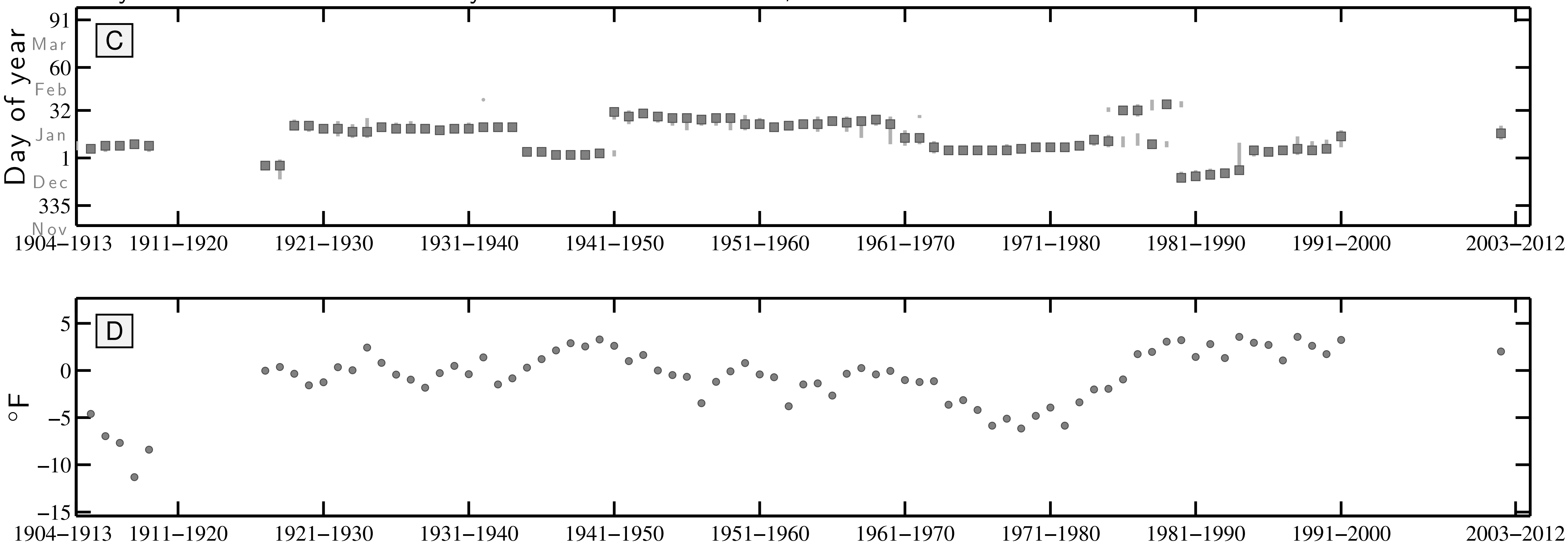

10 year Summer Teletherm dynamics for KENNEBEC, SD:

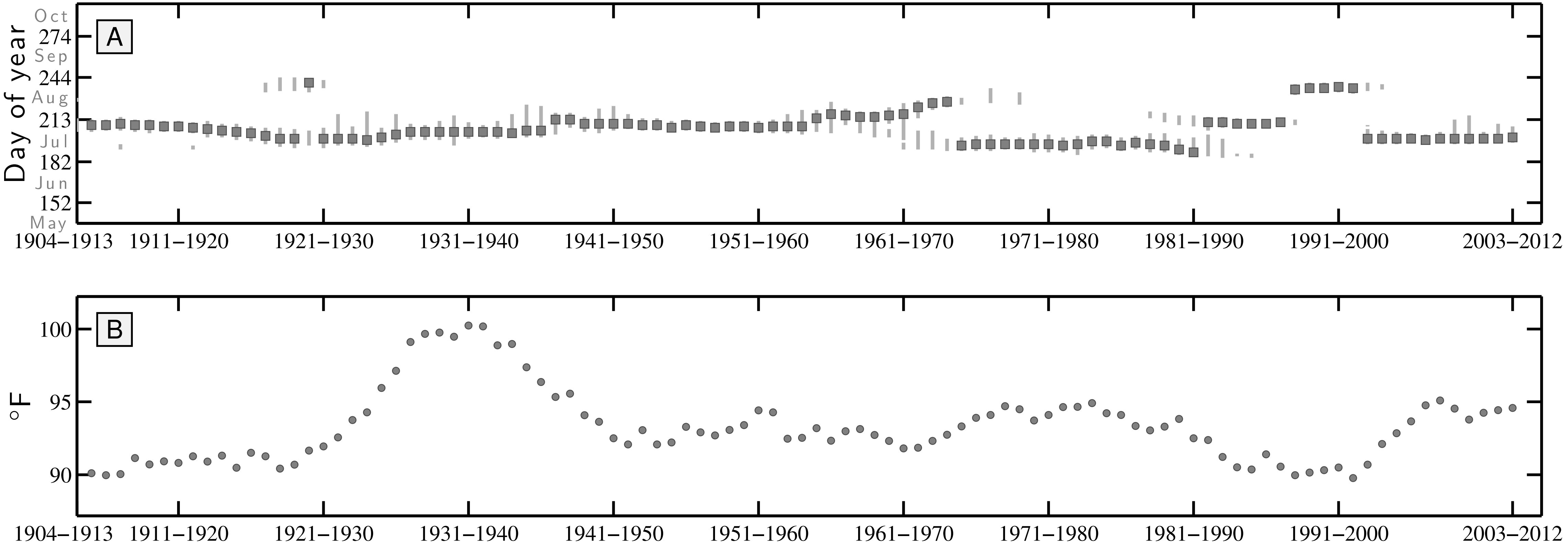

10 year Winter Teletherm dynamics for KENNEBEC, SD:

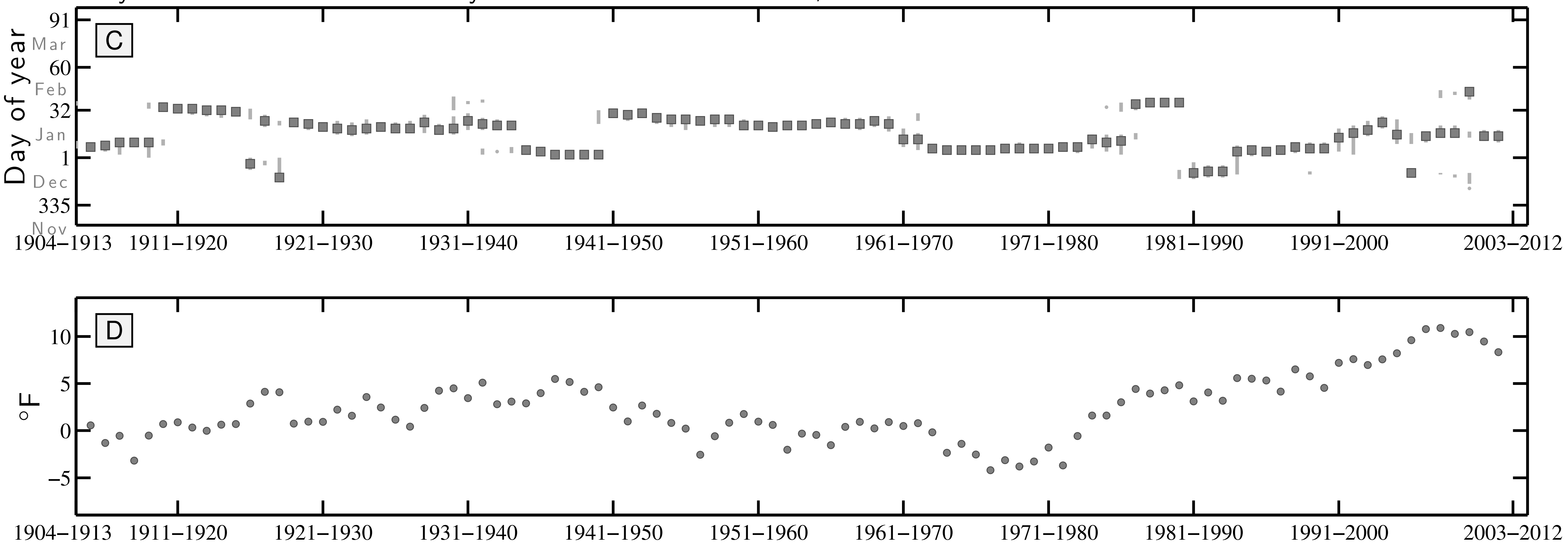

10 year Summer Teletherm dynamics for MELLETTE 4 W, SD:

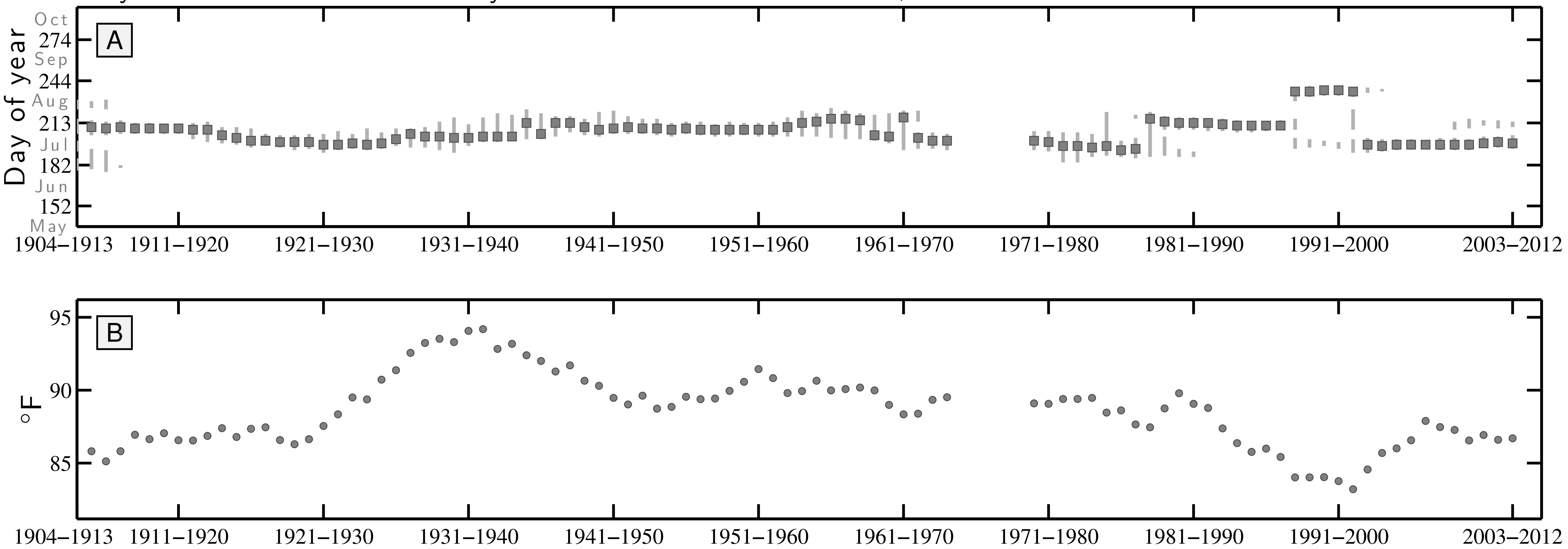

10 year Winter Teletherm dynamics for MELLETTE 4 W, SD:

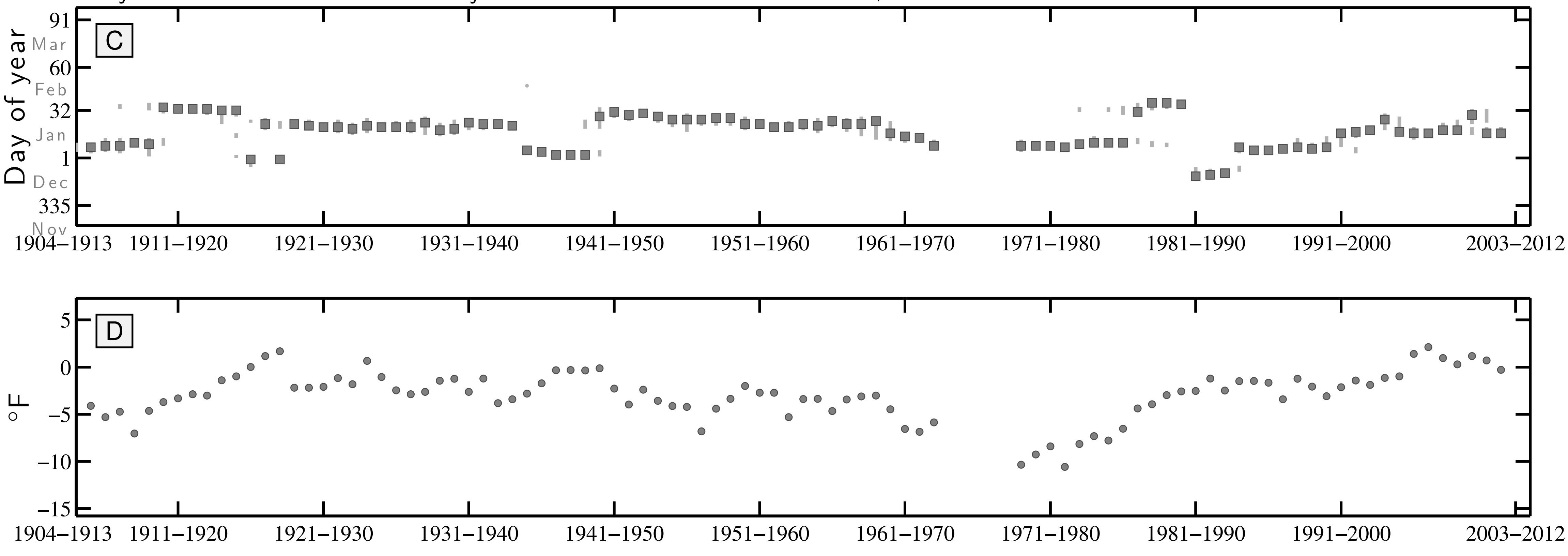

10 year Summer Teletherm dynamics for MENNO, SD:

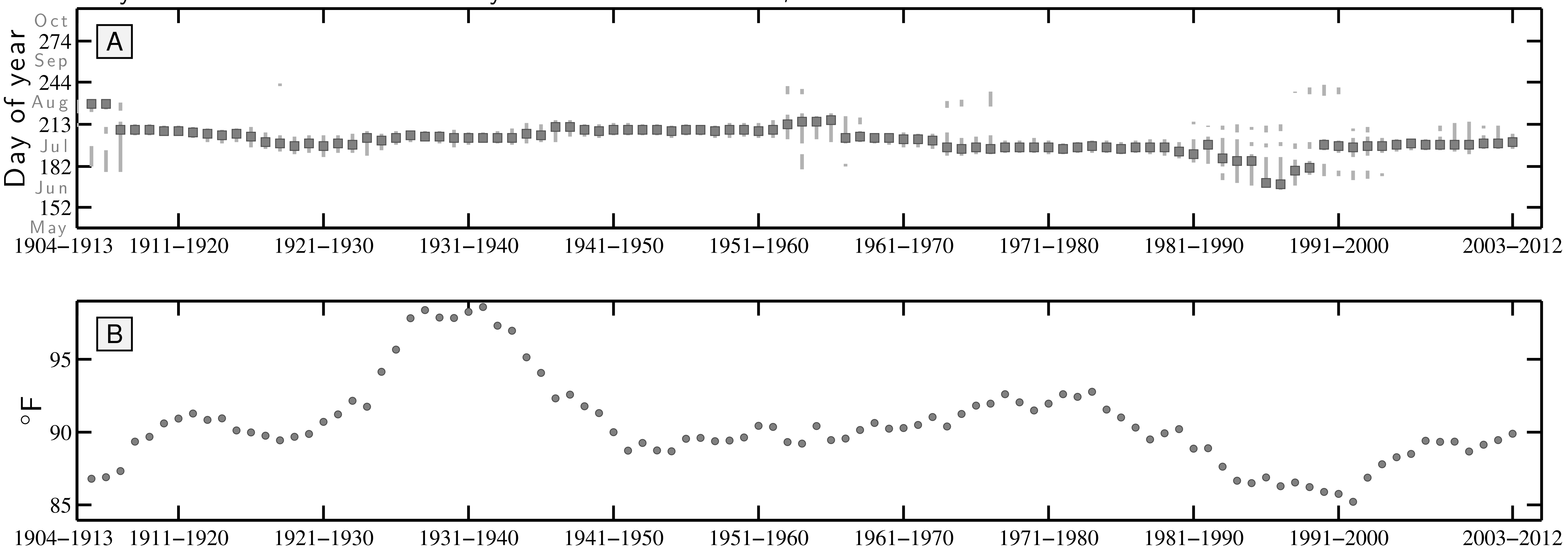

10 year Winter Teletherm dynamics for MENNO, SD:

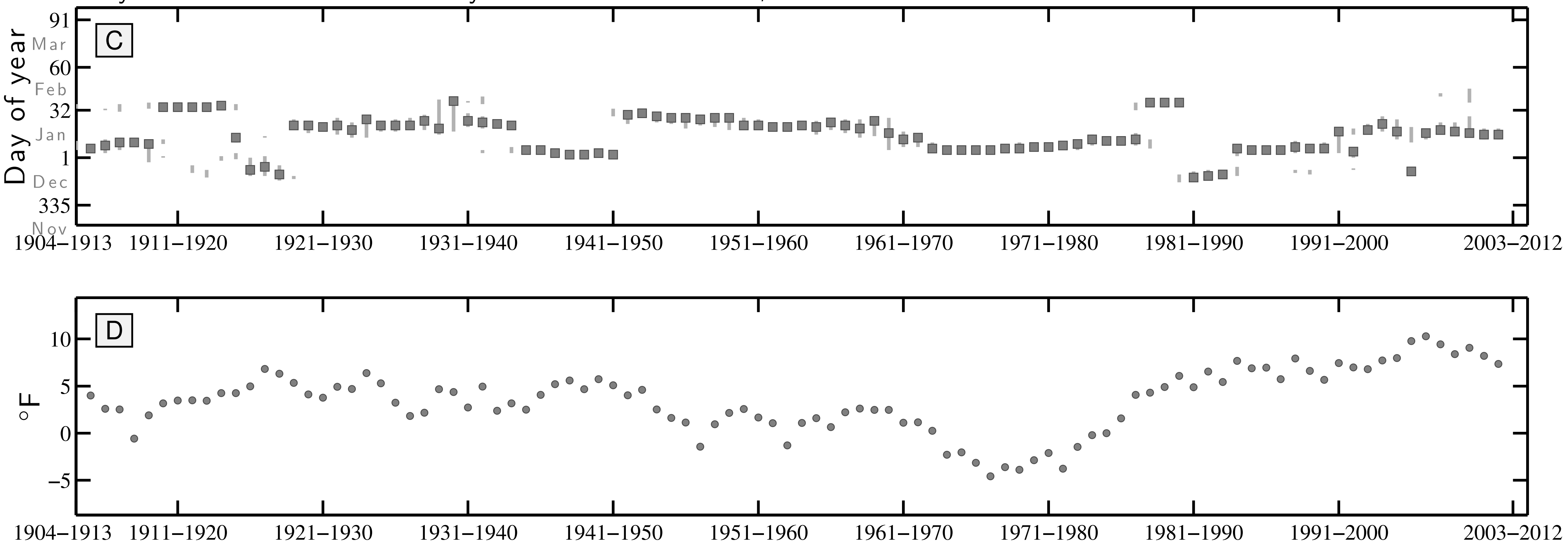

10 year Summer Teletherm dynamics for MILBANK 4 NW, SD:

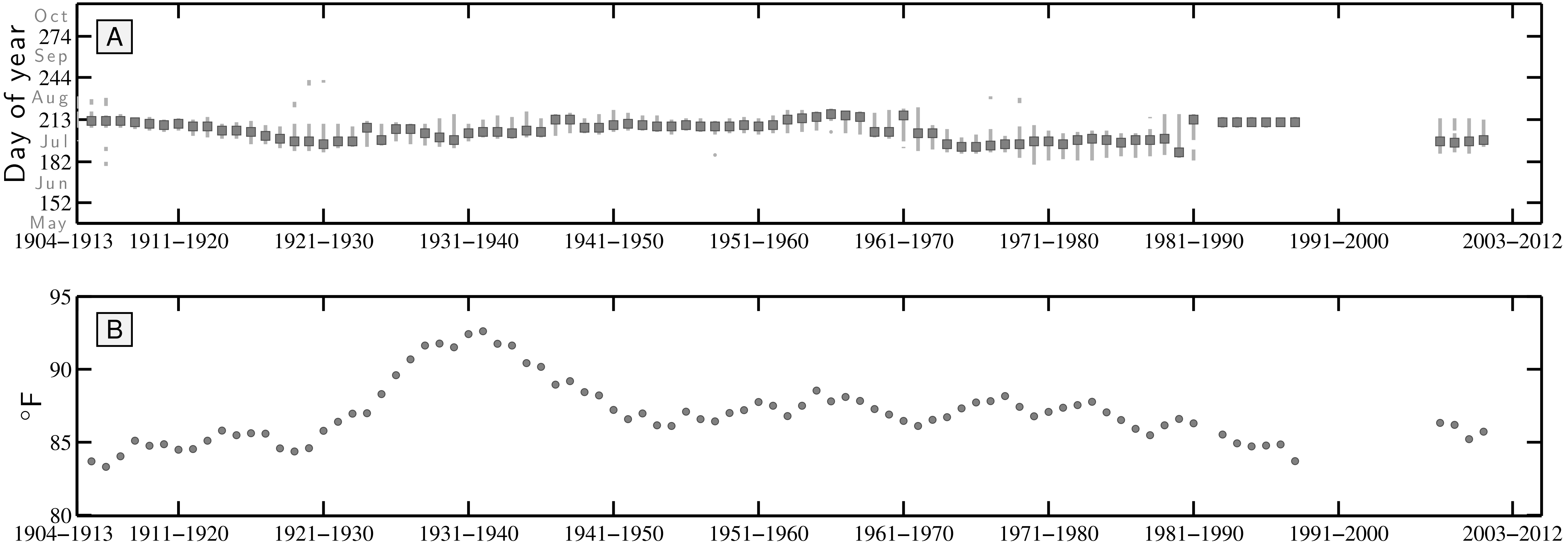

10 year Winter Teletherm dynamics for MILBANK 4 NW, SD:

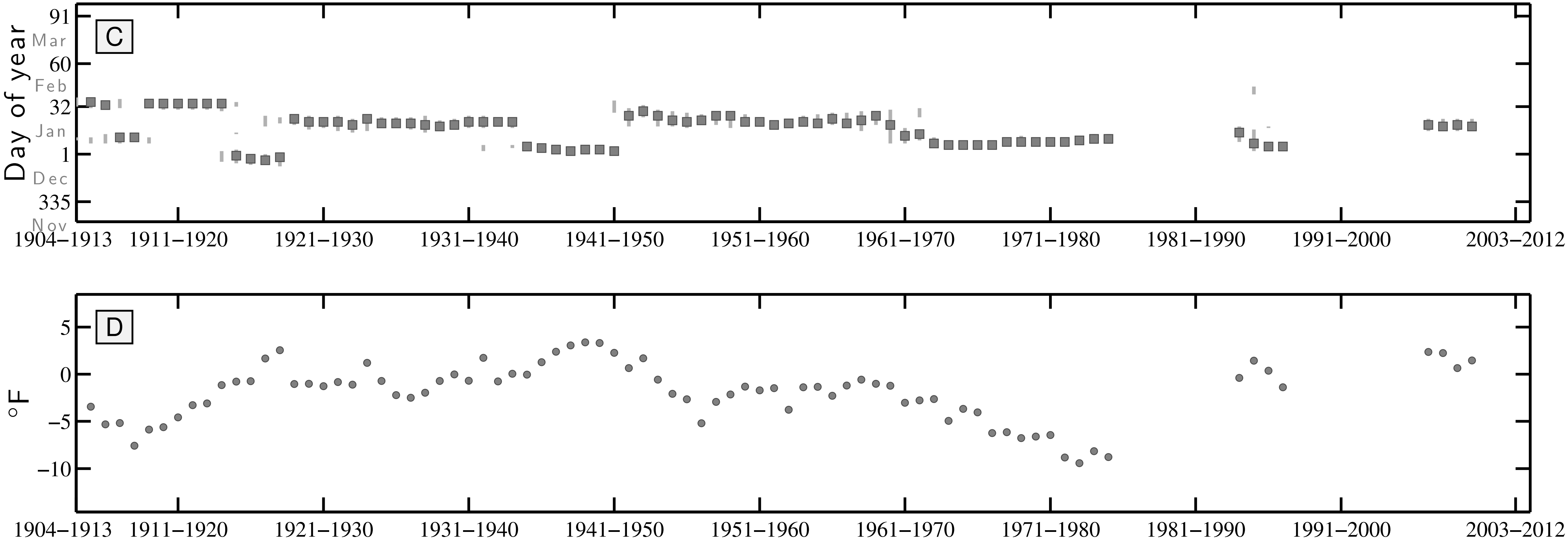

10 year Summer Teletherm dynamics for MURDO, SD:

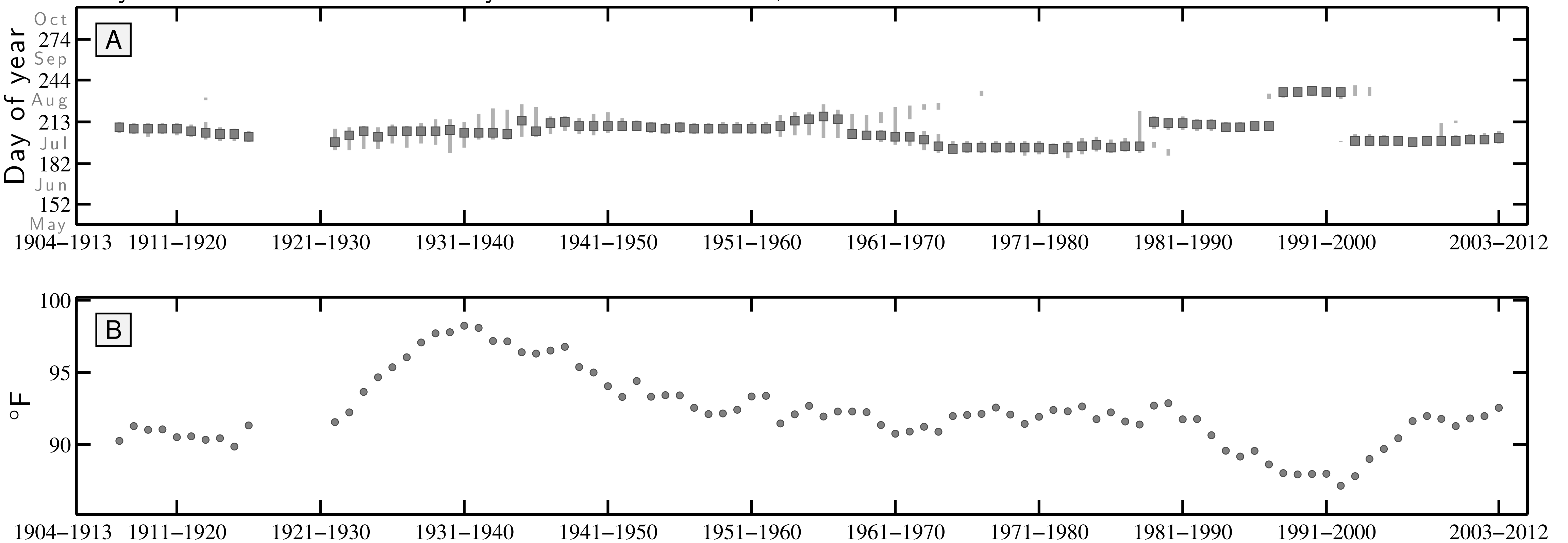

10 year Winter Teletherm dynamics for MURDO, SD:

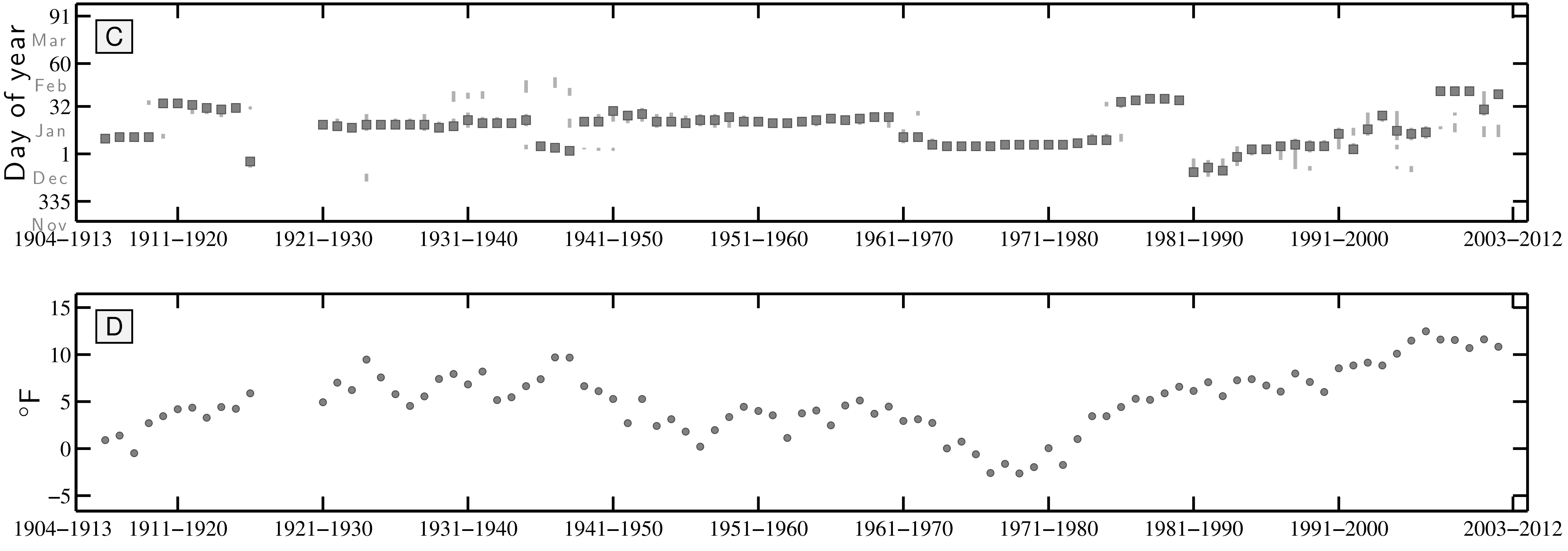

10 year Summer Teletherm dynamics for OAHE DAM, SD:

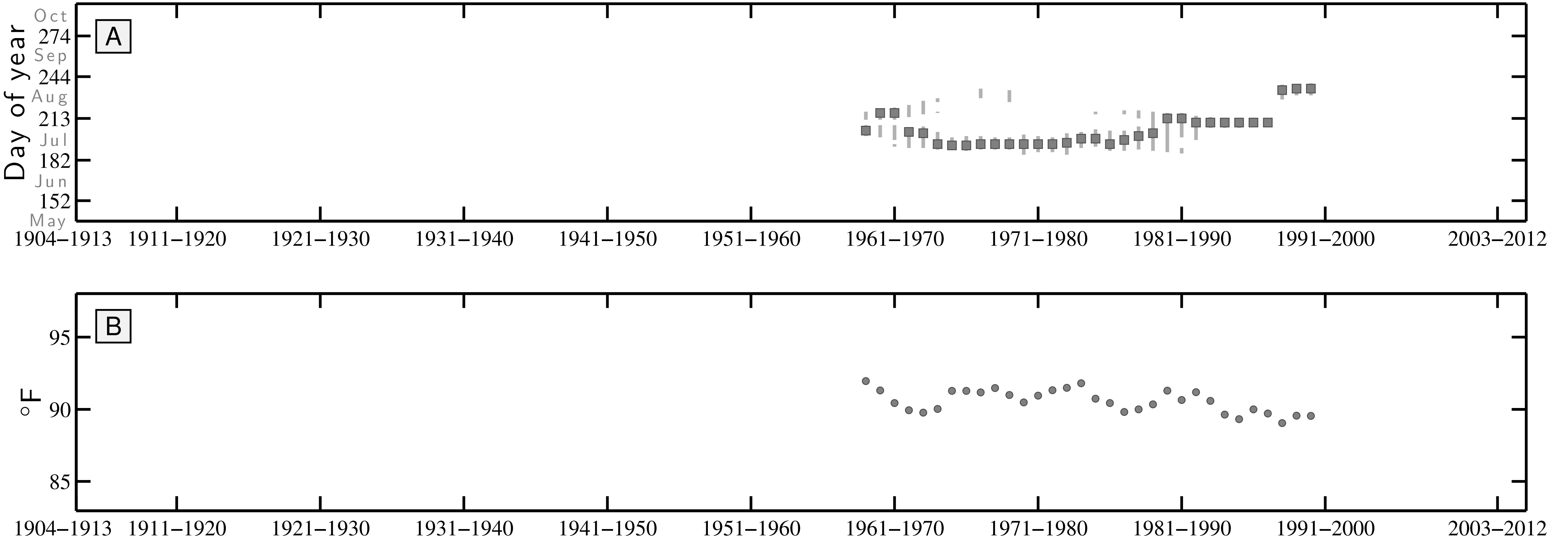

10 year Winter Teletherm dynamics for OAHE DAM, SD:

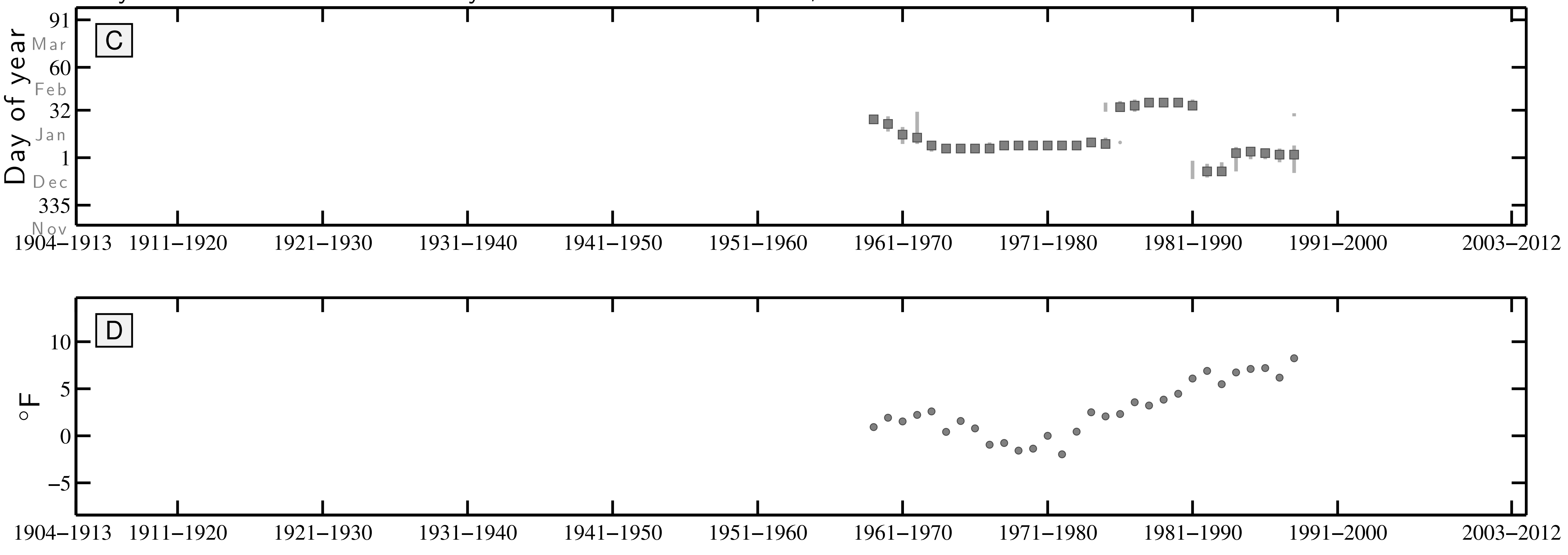

10 year Summer Teletherm dynamics for PIERRE RGNL AP, SD:

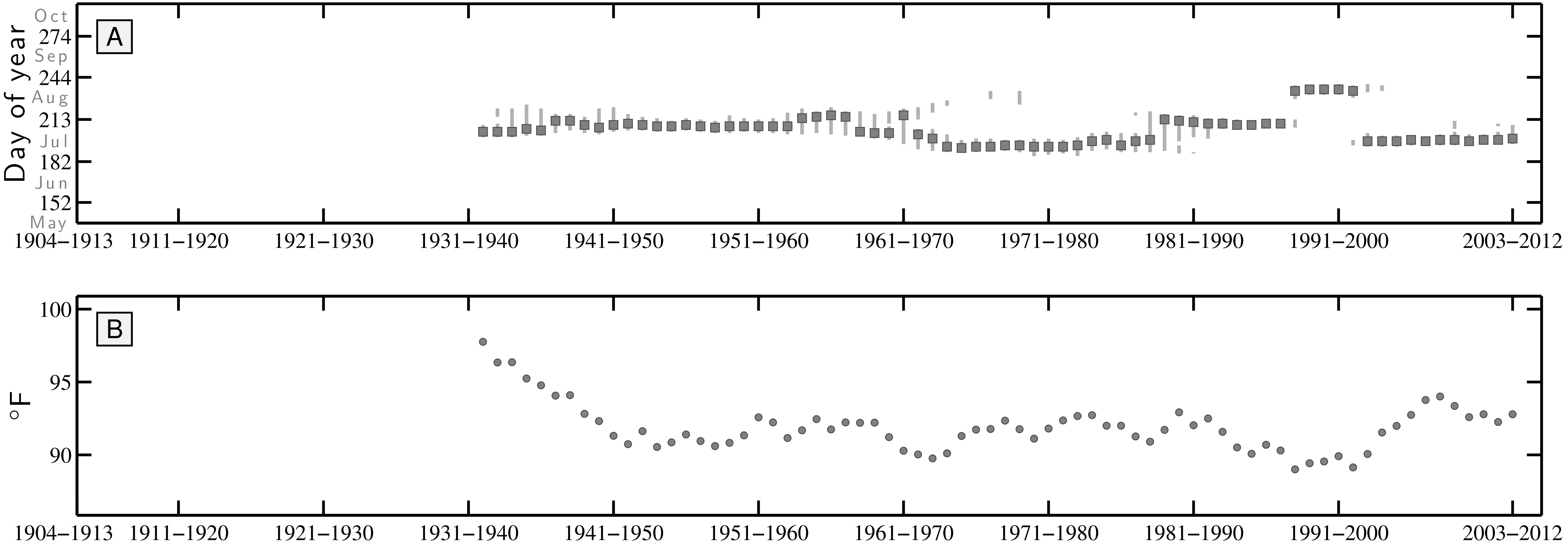

10 year Winter Teletherm dynamics for PIERRE RGNL AP, SD:

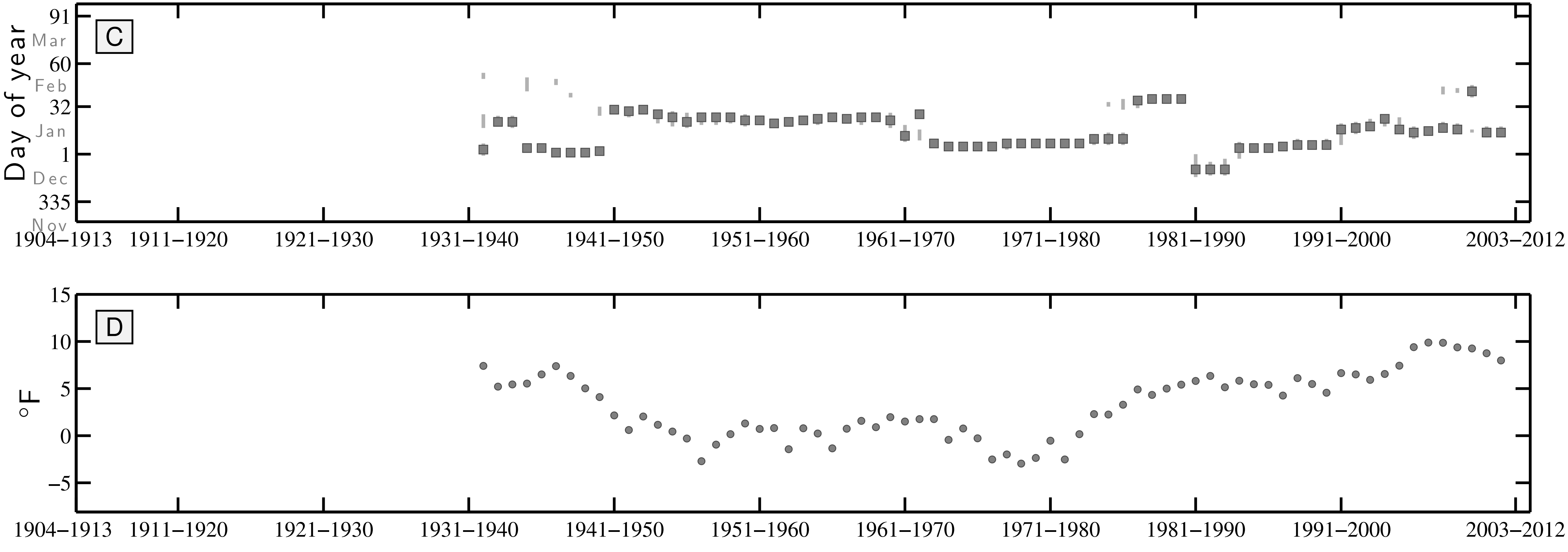

10 year Summer Teletherm dynamics for RAPID CITY 4NW, SD:

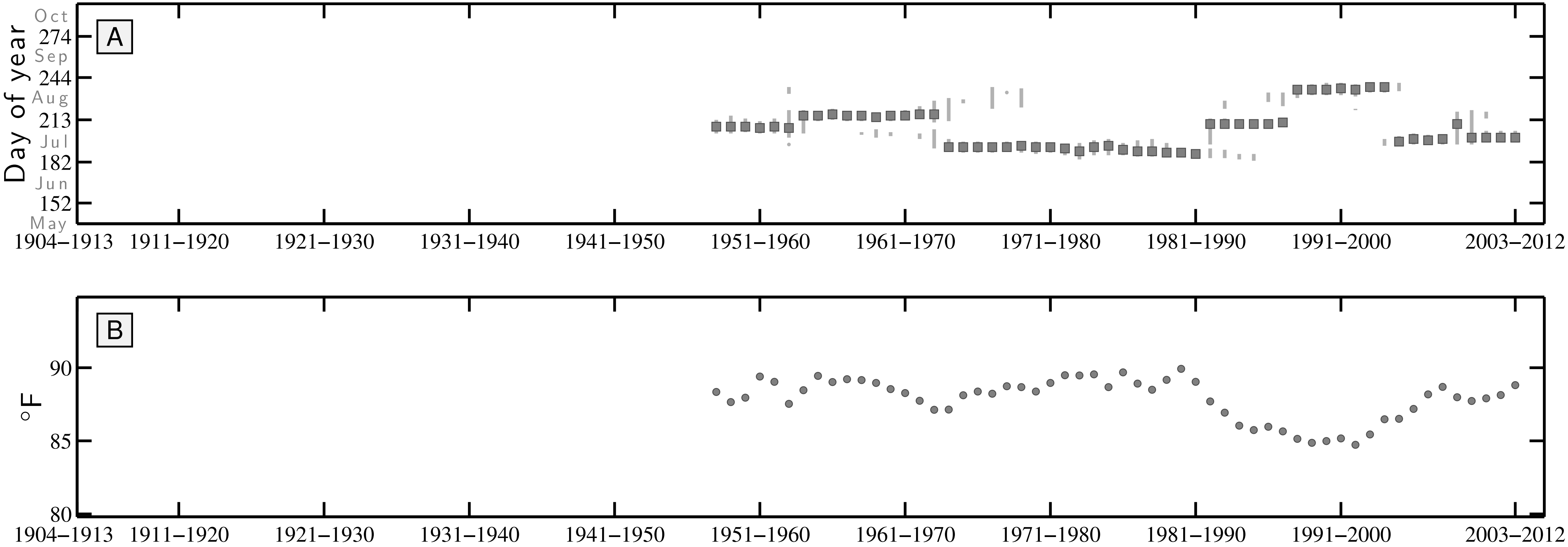

10 year Winter Teletherm dynamics for RAPID CITY 4NW, SD:

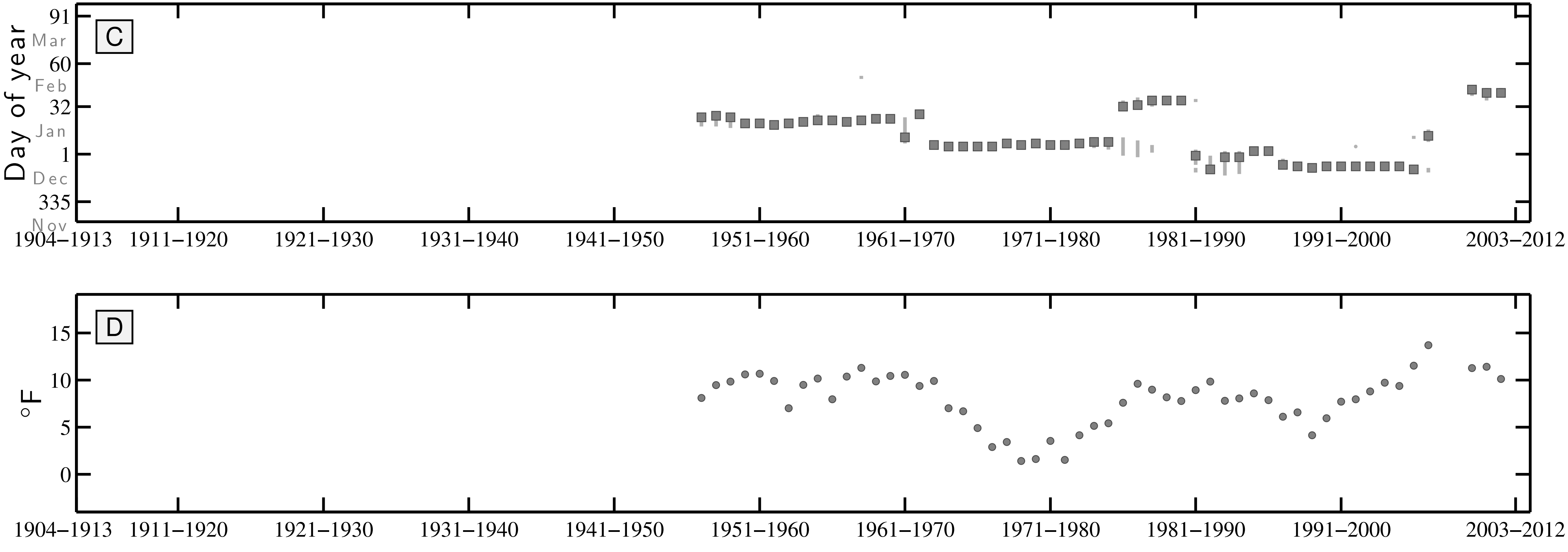

10 year Summer Teletherm dynamics for VERMILLION 2 SE, SD:

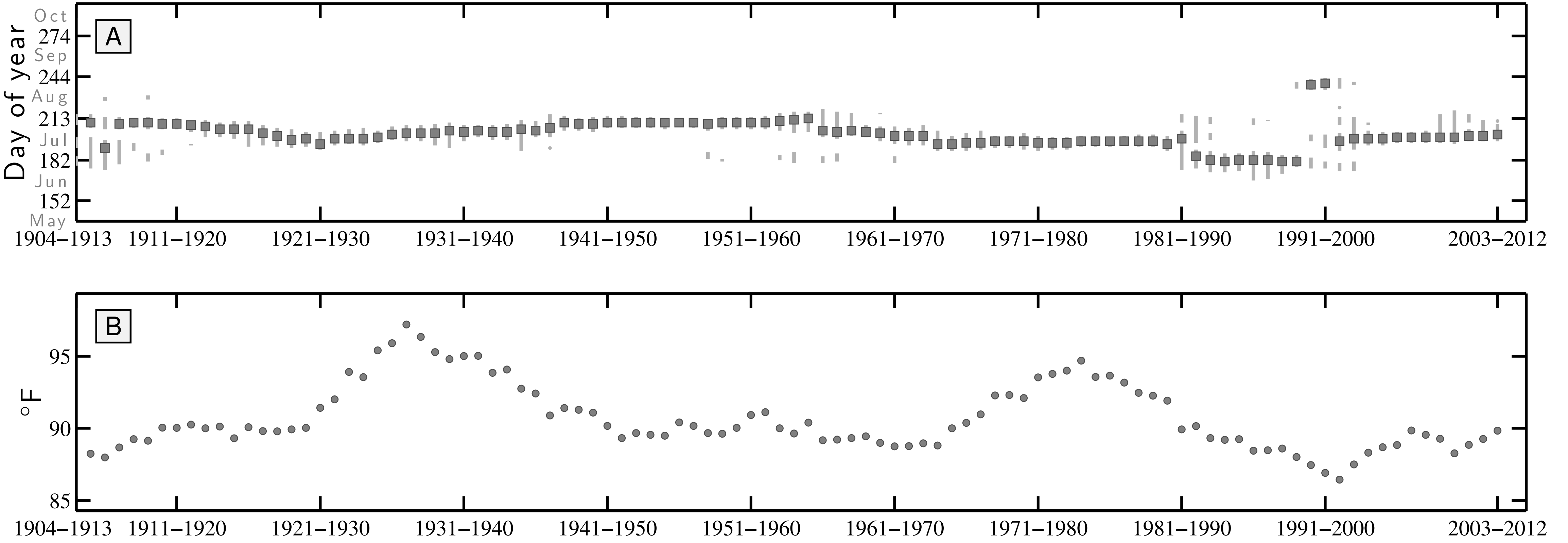

10 year Winter Teletherm dynamics for VERMILLION 2 SE, SD:

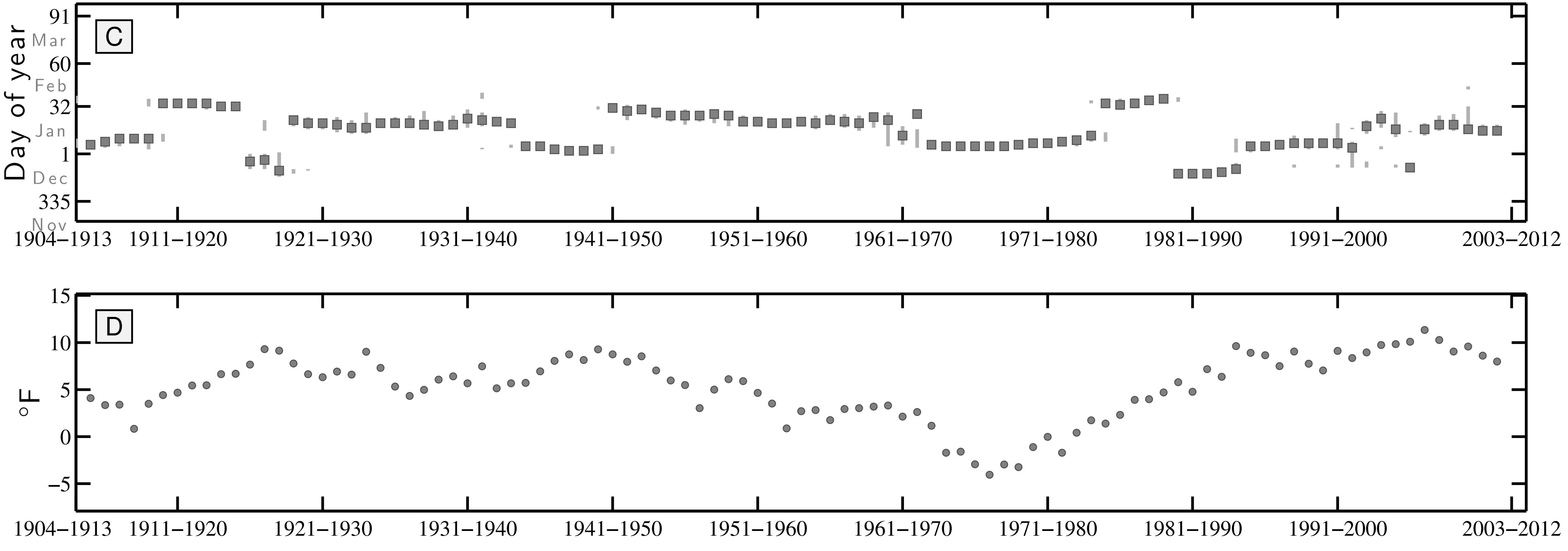

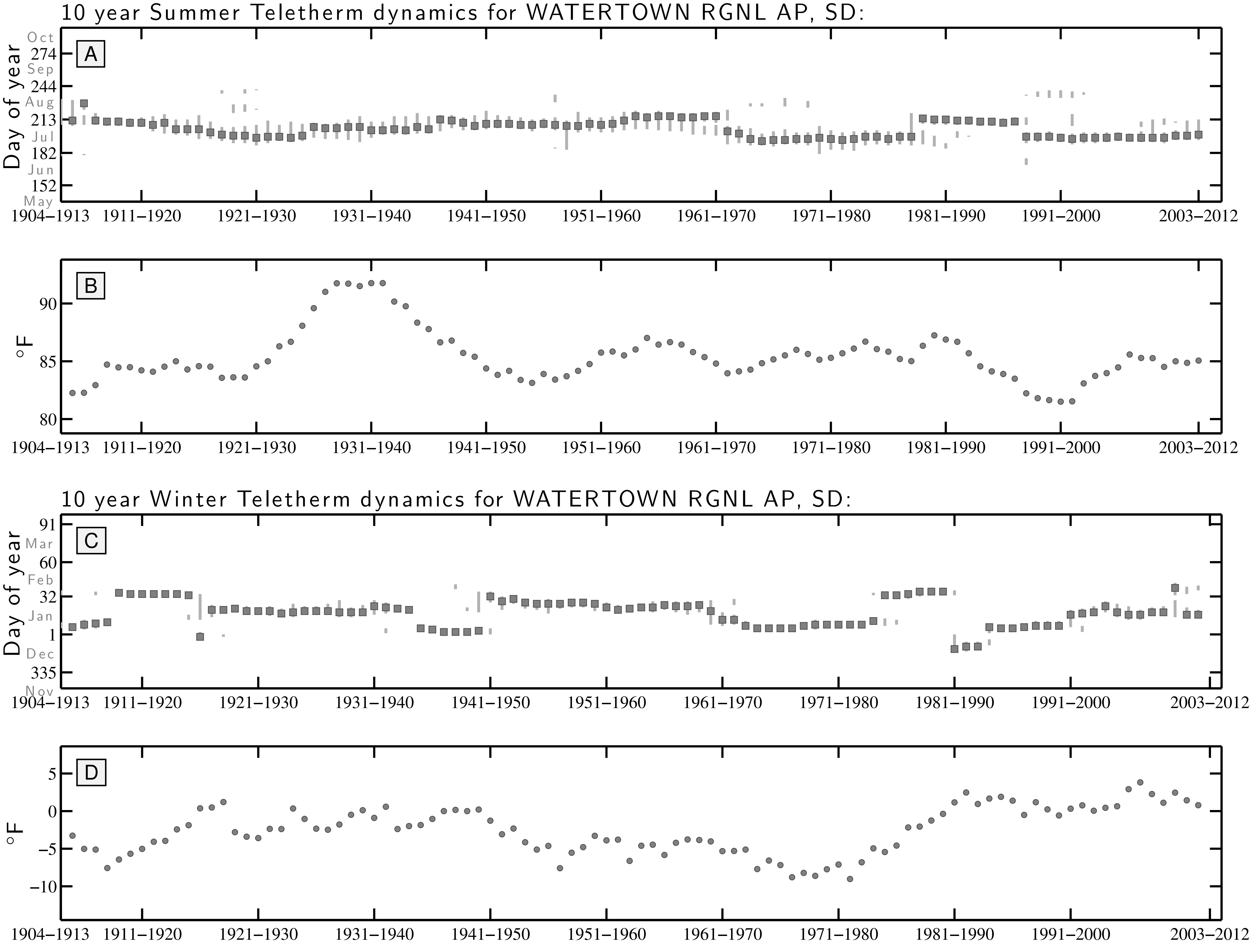
10 year Summer Teletherm dynamics for WATERTOWN RGNL AP, SD:
A
B
Day of year
°F
Oct
Sep
Aug
Jul
Jun
May
274
244
213
182
152
90
85
80
1904–1913
1911–1920
1921–1930
1931–1940
1941–1950
1951–1960
1961–1970
1971–1980
1981–1990
1991–2000
2003–2012
10 year Winter Teletherm dynamics for WATERTOWN RGNL AP, SD:
C
D
Mar
Feb
Jan
Dec
Nov
91
60
32
1
335
5
0
−5
−10

10 year Summer Teletherm dynamics for WOOD, SD:

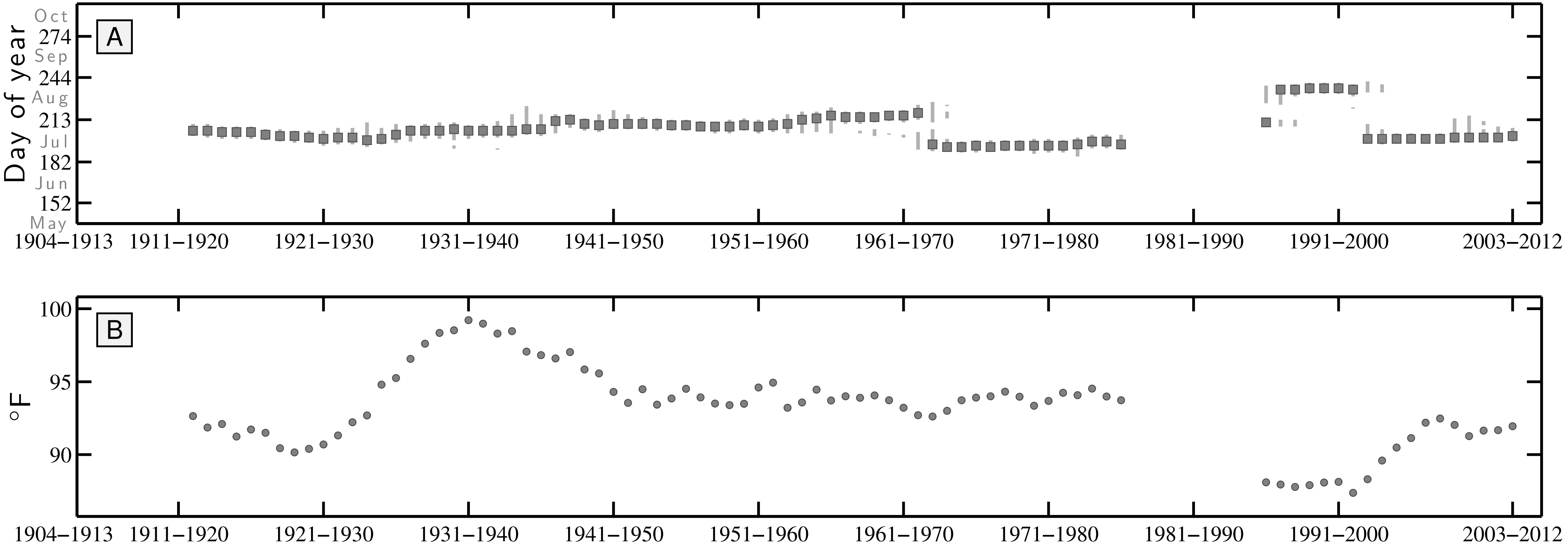

10 year Winter Teletherm dynamics for WOOD, SD:

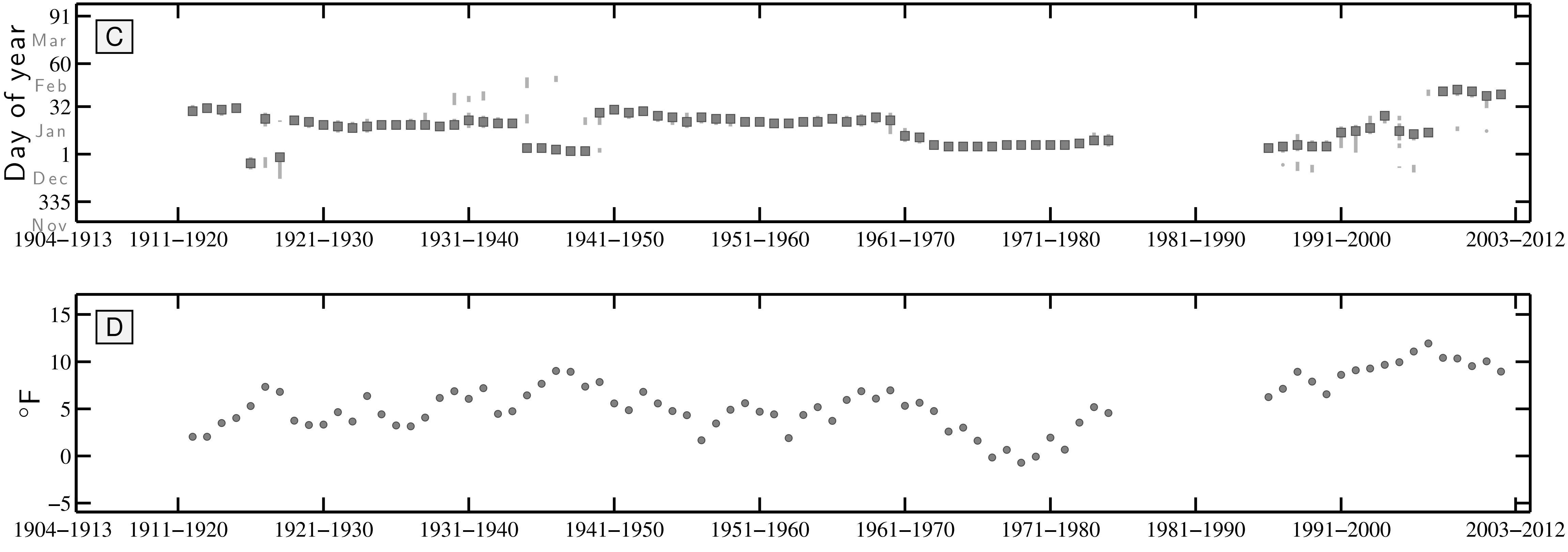

10 year Summer Teletherm dynamics for CLARKSVILLE WWTP, TN:

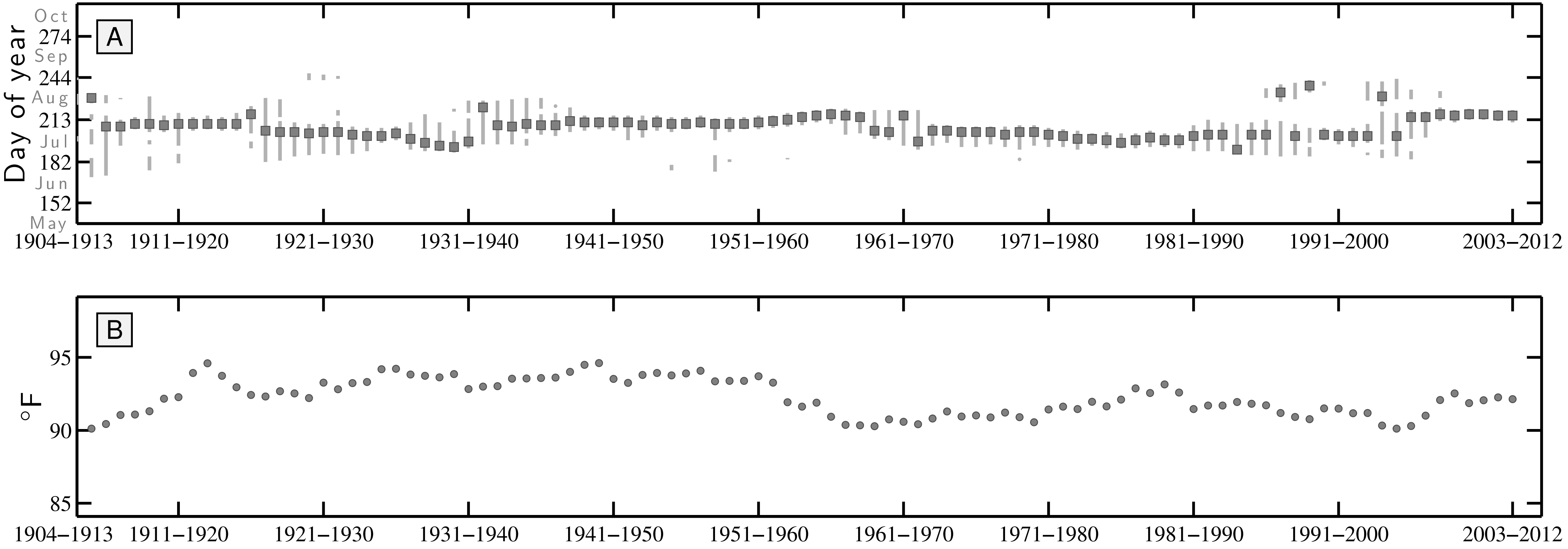

10 year Winter Teletherm dynamics for CLARKSVILLE WWTP, TN:

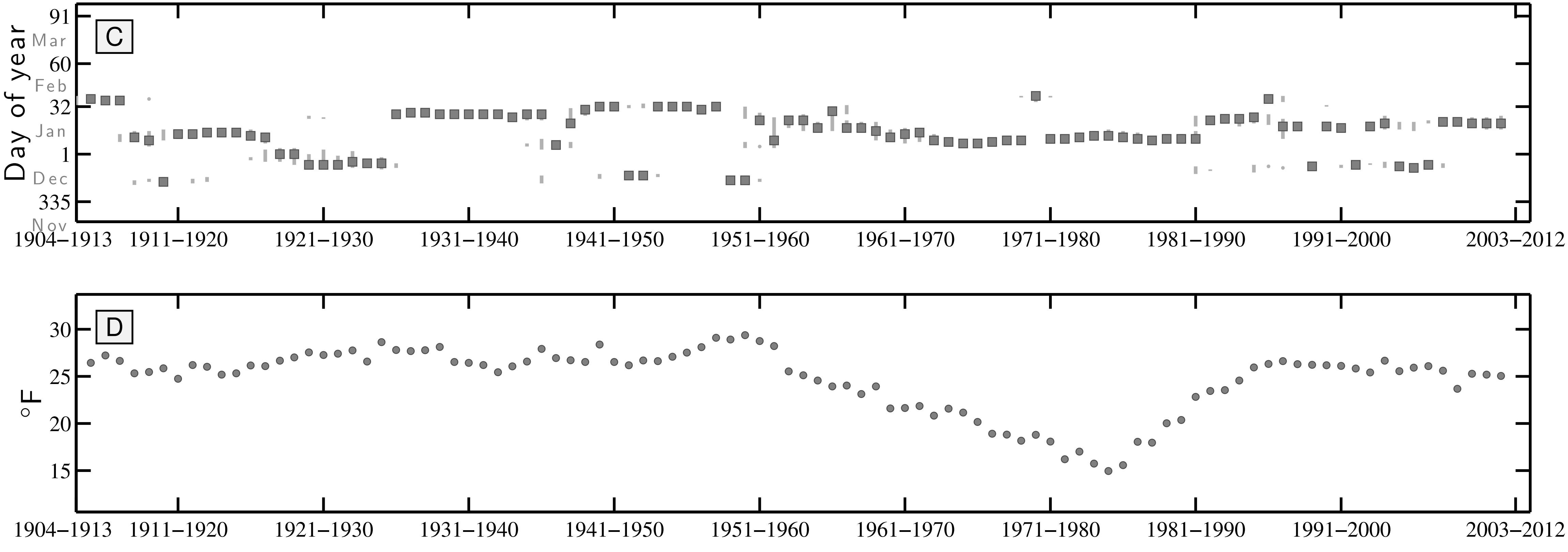

10 year Summer Teletherm dynamics for COPPERHILL, TN:

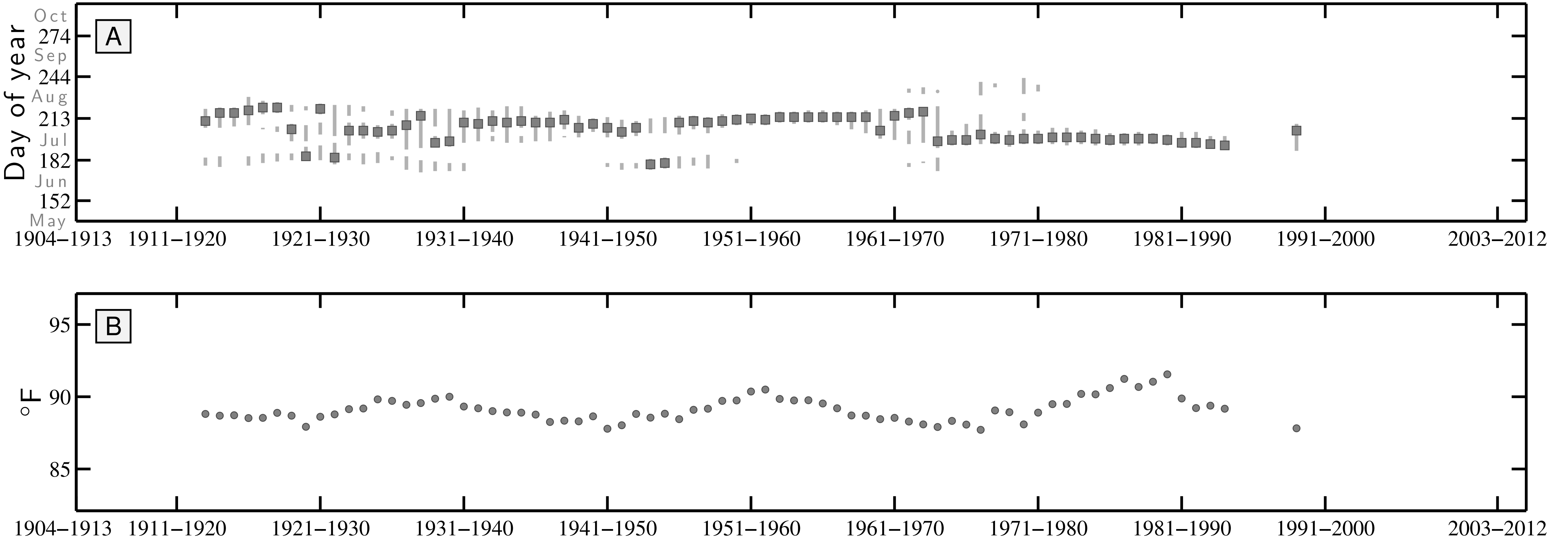

10 year Winter Teletherm dynamics for COPPERHILL, TN:

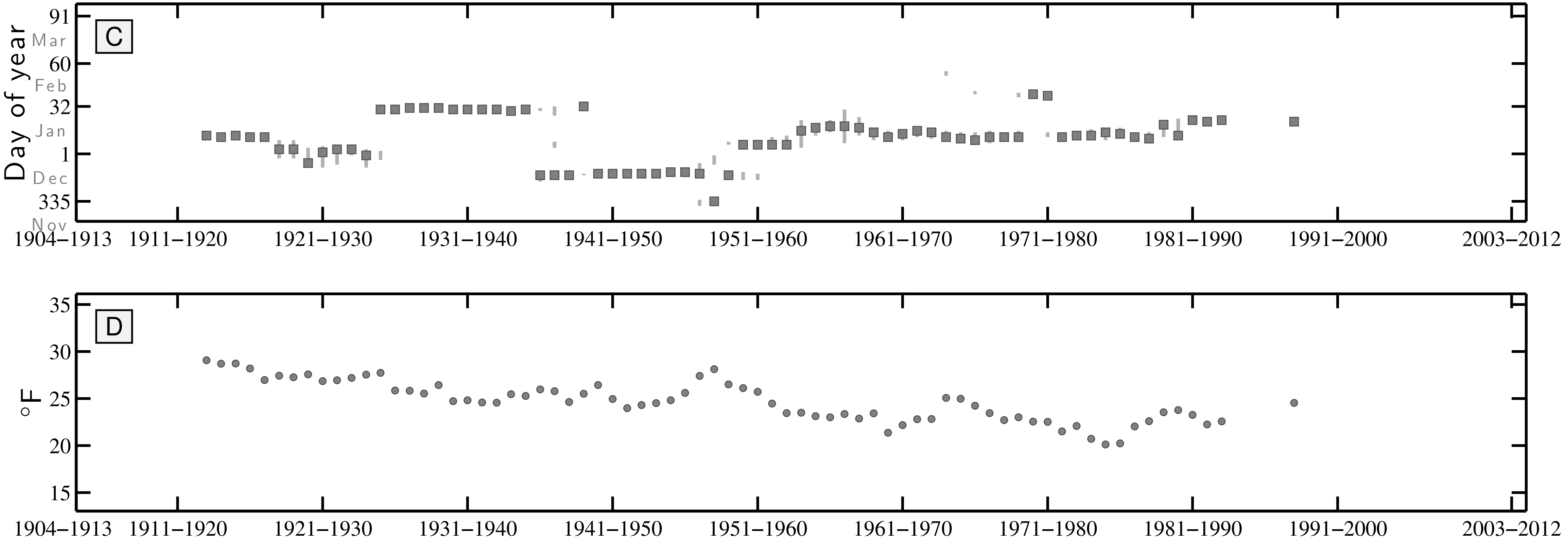

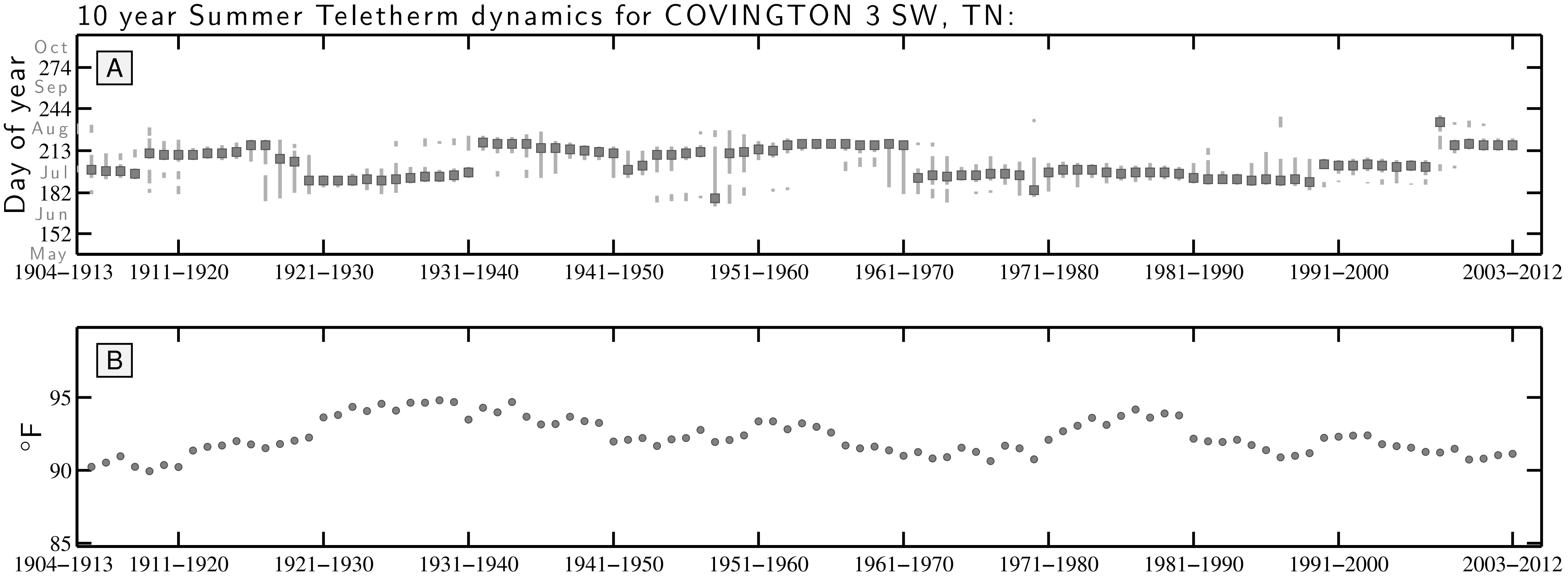
10 year Summer Teletherm dynamics for COVINGTON 3 SW, TN:
A
B
Day of year
°F
Oct
274
Sep
244
Aug
213
Jul
182
Jun
152
May
95
90
85
1904–1913
1911–1920
1921–1930
1931–1940
1941–1950
1951–1960
1961–1970
1971–1980
1981–1990
1991–2000
2003–2012

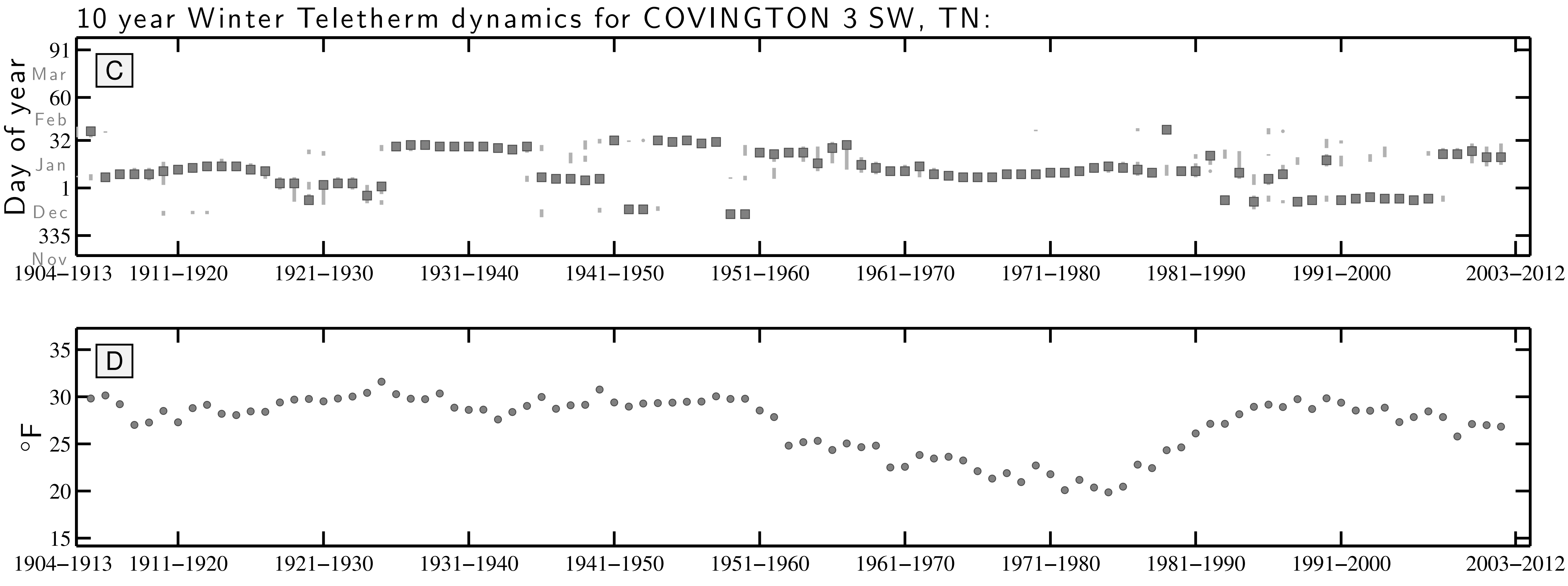
10 year Winter Teletherm dynamics for COVINGTON 3 SW, TN:
C
D
Day of year
°F
91
Mar
60
Feb
32
Jan
1
Dec
335
Nov
35
30
25
20
15
1904–1913
1911–1920
1921–1930
1931–1940
1941–1950
1951–1960
1961–1970
1971–1980
1981–1990
1991–2000
2003–2012

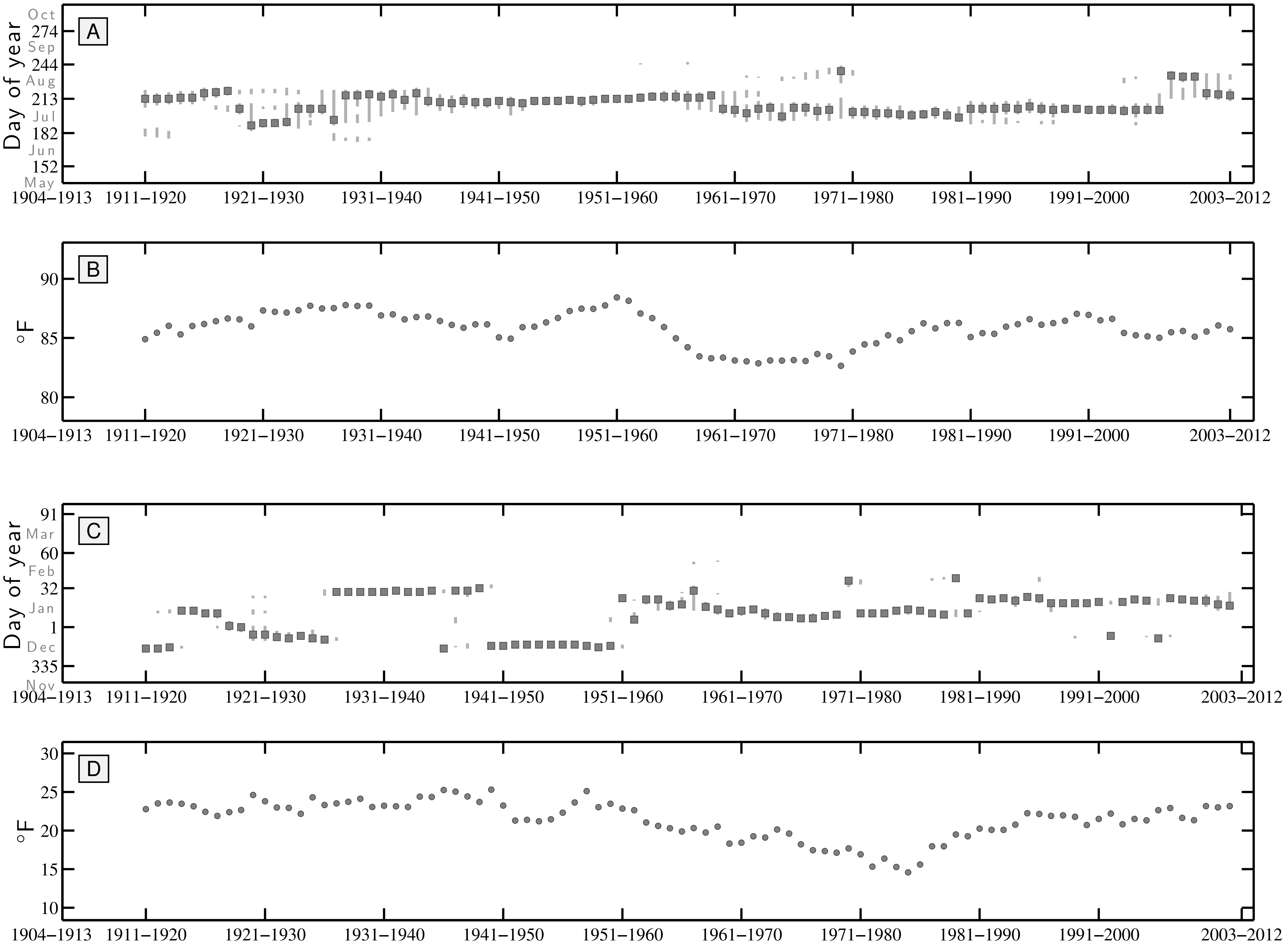
A
Day of year
Oct
274
Sep
244
Aug
213
Jul
182
Jun
152
May
B
°F
90
85
80
C
Day of year
91
Mar
60
Feb
32
Jan
1
Dec
335
Nov
D
°F
30
25
20
15
10
1904–1913
1911–1920
1921–1930
1931–1940
1941–1950
1951–1960
1961–1970
1971–1980
1981–1990
1991–2000
2003–2012

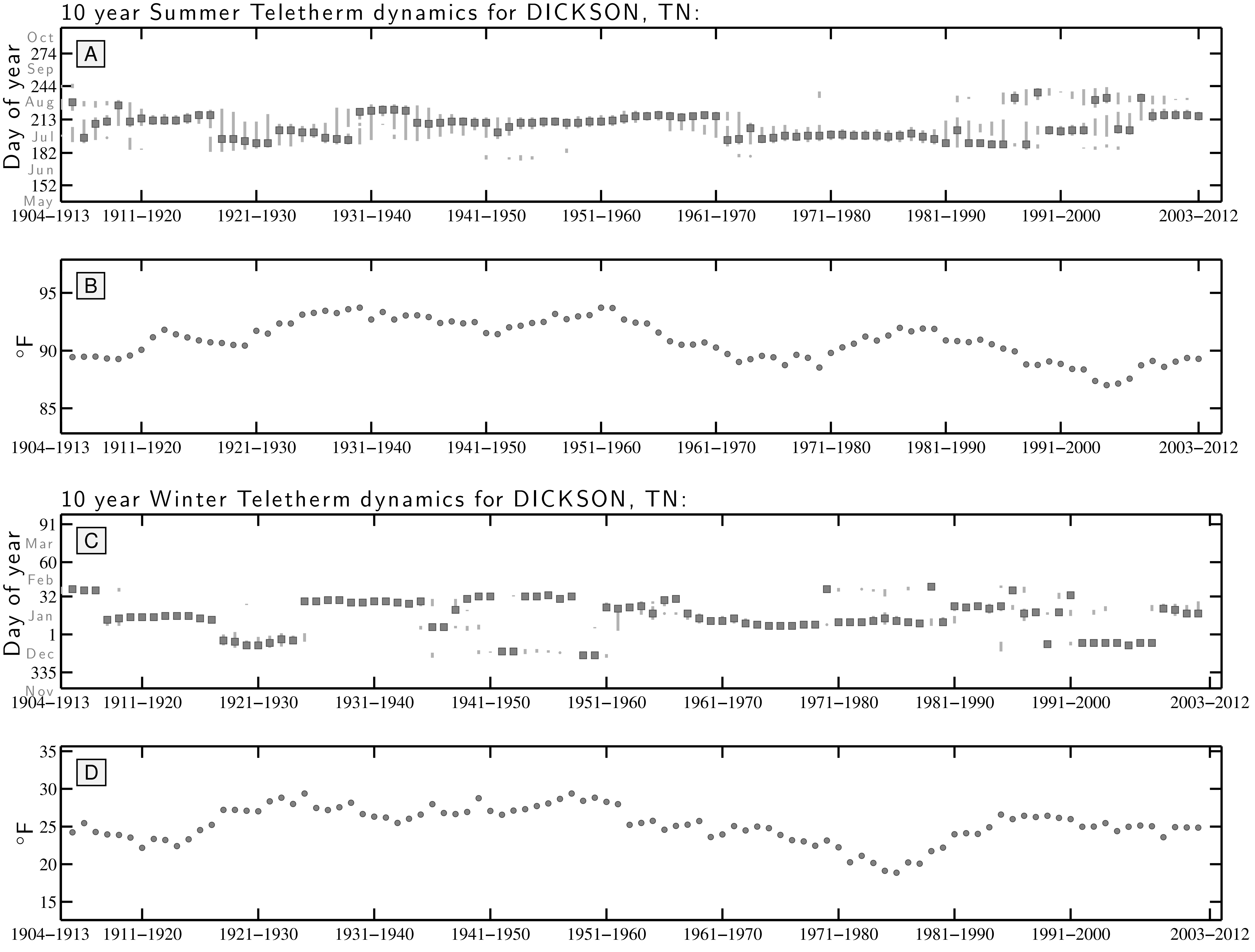
10 year Summer Teletherm dynamics for DICKSON, TN:
A
Day of year
Oct
274
Sep
244
Aug
213
Jul
182
Jun
152
May
B
°F
95
90
85
10 year Winter Teletherm dynamics for DICKSON, TN:
C
Day of year
91
Mar
60
Feb
32
Jan
1
Dec
335
Nov
D
°F
35
30
25
20
15
1904–1913
1911–1920
1921–1930
1931–1940
1941–1950
1951–1960
1961–1970
1971–1980
1981–1990
1991–2000
2003–2012

10 year Summer Teletherm dynamics for DOVER 1 W, TN:

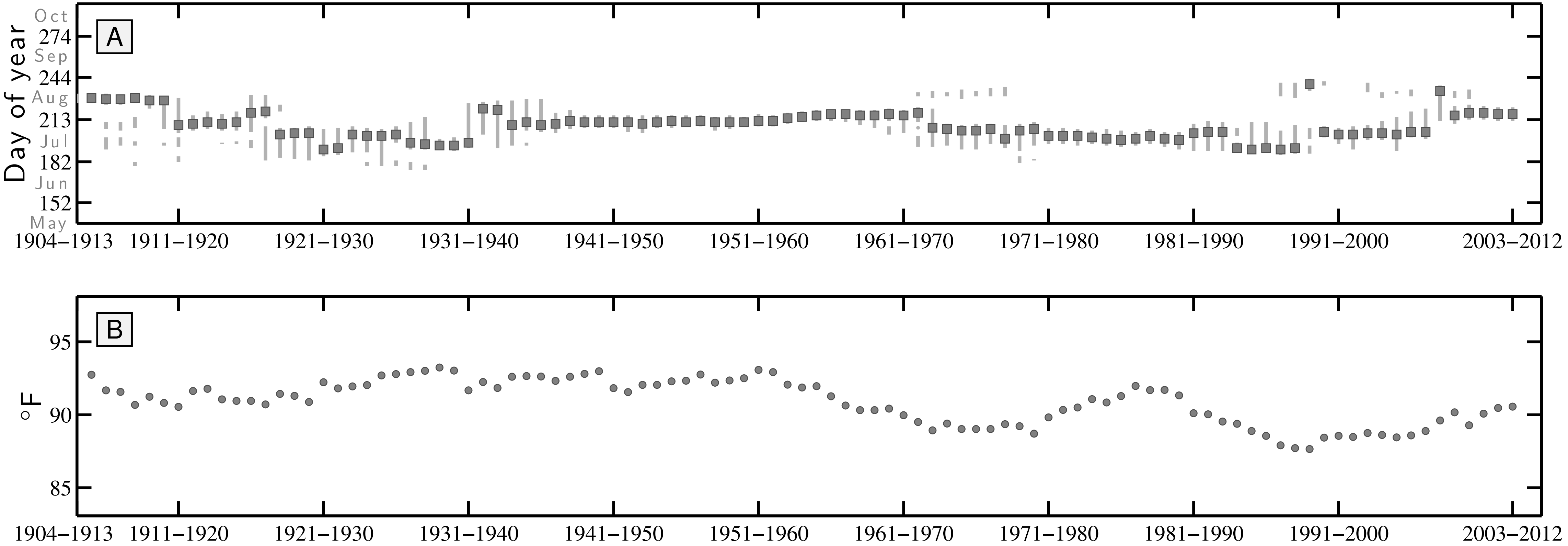

10 year Winter Teletherm dynamics for DOVER 1 W, TN:

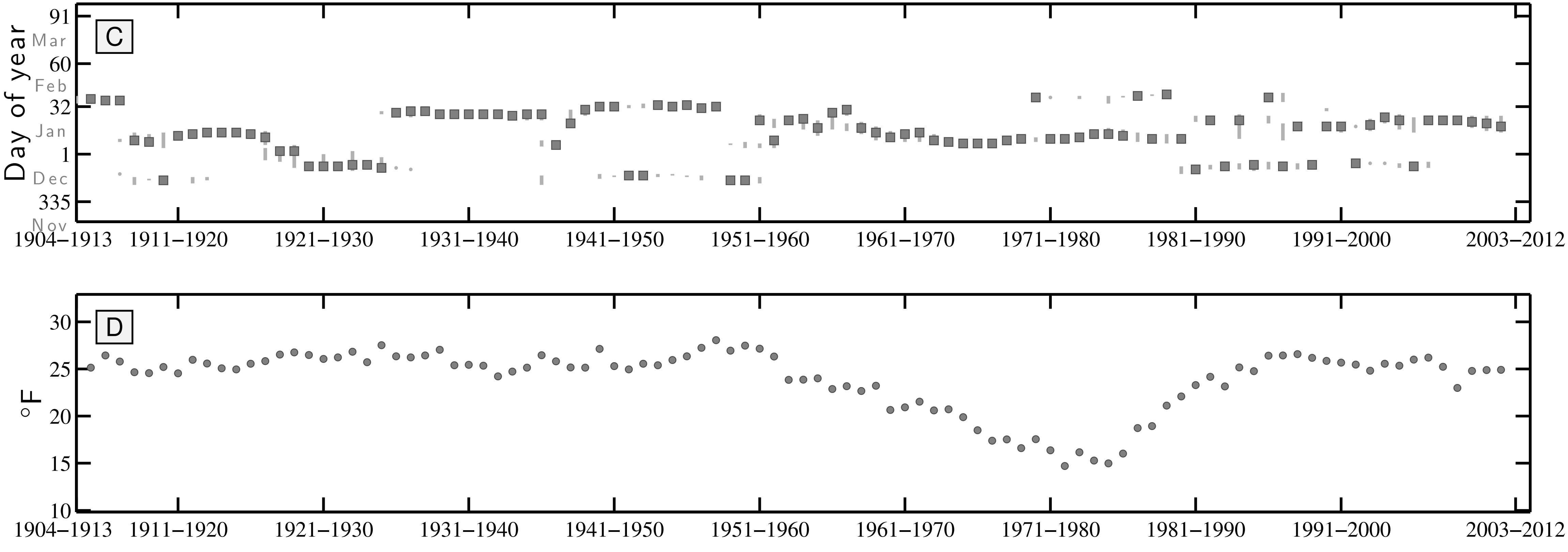

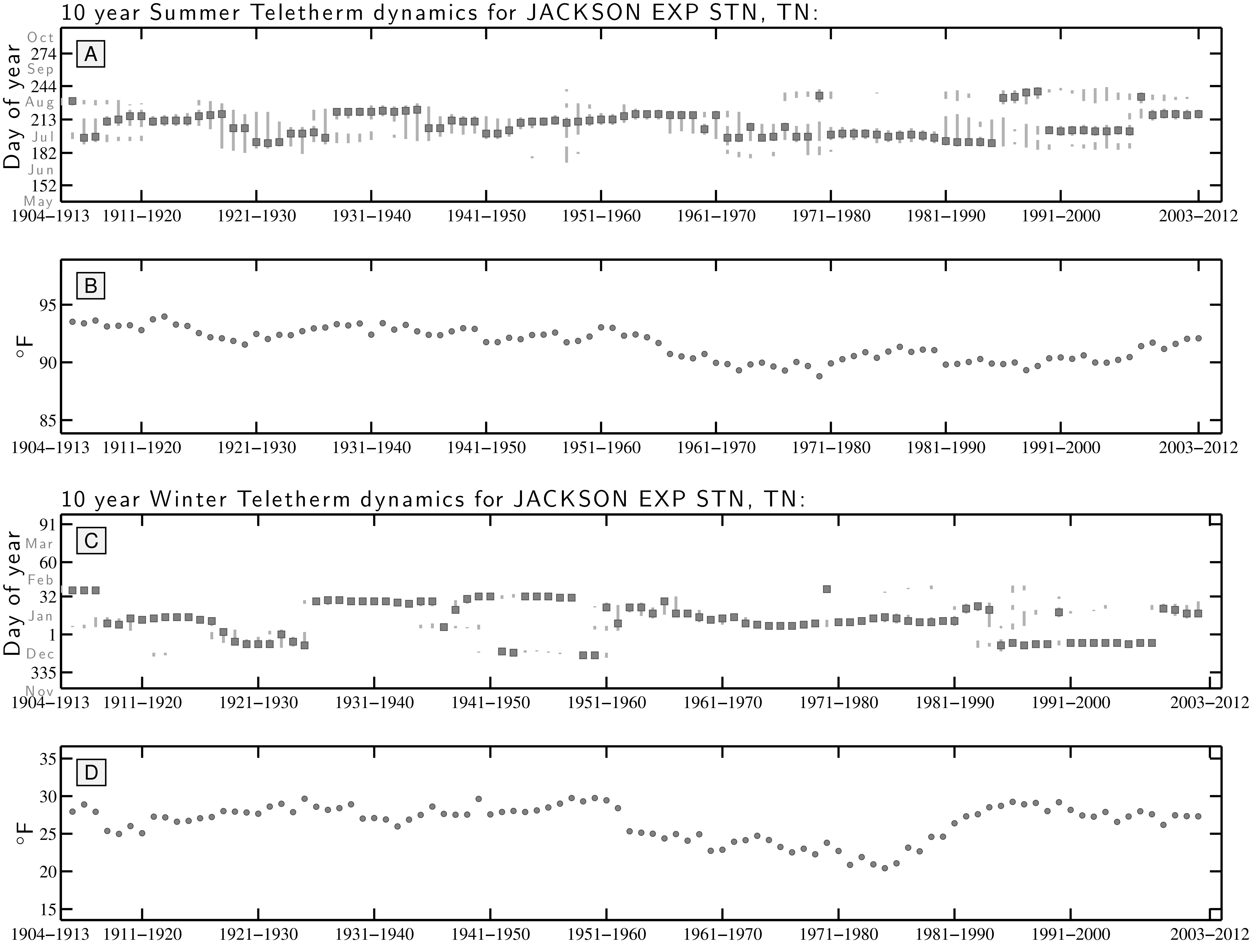
10 year Summer Teletherm dynamics for JACKSON EXP STN, TN:
A
B
Day of year
°F
Oct
274
Sep
244
Aug
213
Jul
182
Jun
152
May
95
90
85
1904–1913
1911–1920
1921–1930
1931–1940
1941–1950
1951–1960
1961–1970
1971–1980
1981–1990
1991–2000
2003–2012
10 year Winter Teletherm dynamics for JACKSON EXP STN, TN:
C
D
91
Mar
60
Feb
32
Jan
1
Dec
335
Nov
35
30
25
20
15

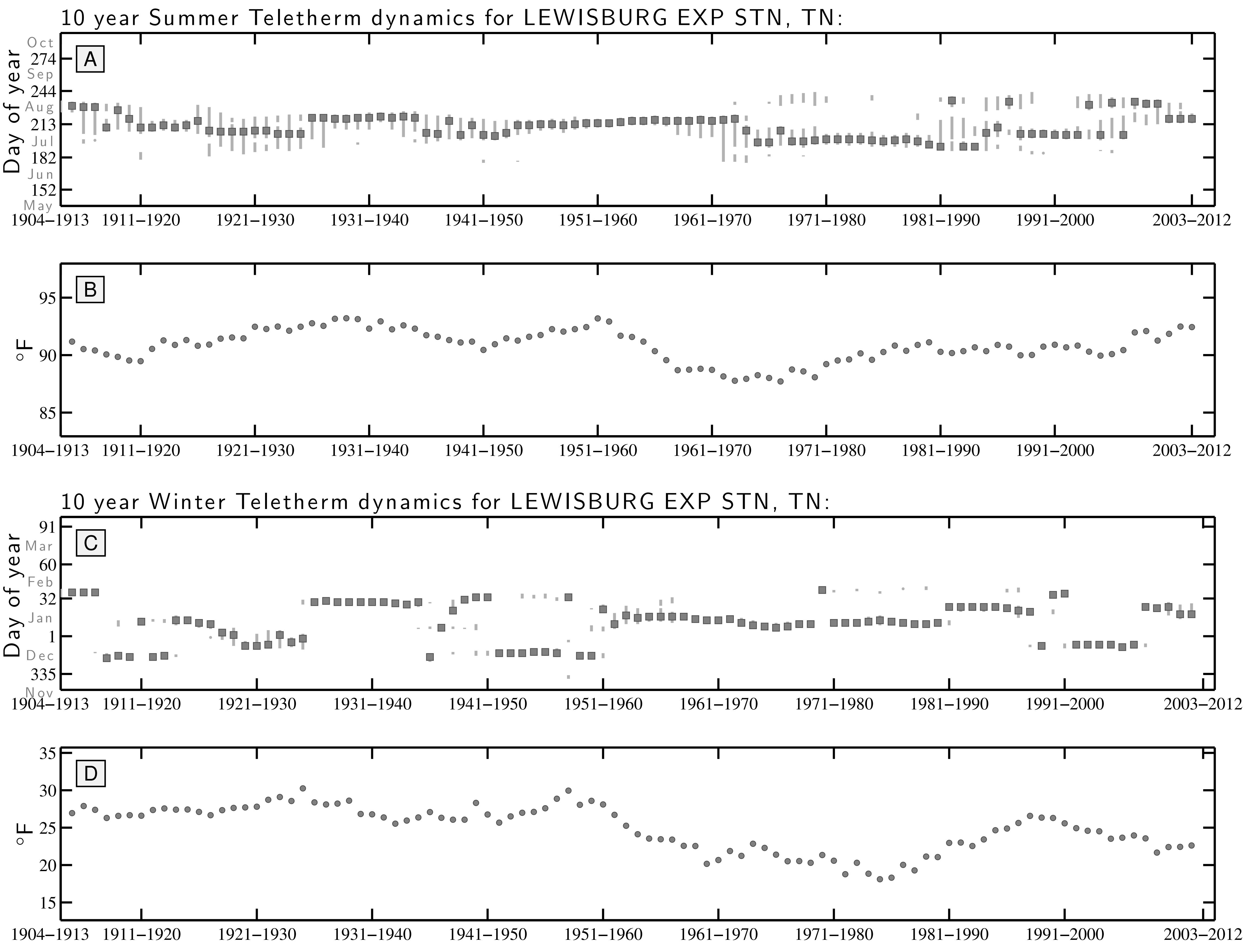
10 year Summer Teletherm dynamics for LEWISBURG EXP STN, TN:
A
Day of year
Oct
274
Sep
244
Aug
213
Jul
182
Jun
152
May
1904–1913
1911–1920
1921–1930
1931–1940
1941–1950
1951–1960
1961–1970
1971–1980
1981–1990
1991–2000
2003–2012
B
°F
95
90
85
10 year Winter Teletherm dynamics for LEWISBURG EXP STN, TN:
C
91
Mar
60
Feb
32
Jan
1
Dec
335
Nov
D
35
30
25
20
15

10 year Summer Teletherm dynamics for MC MINNVILLE, TN:

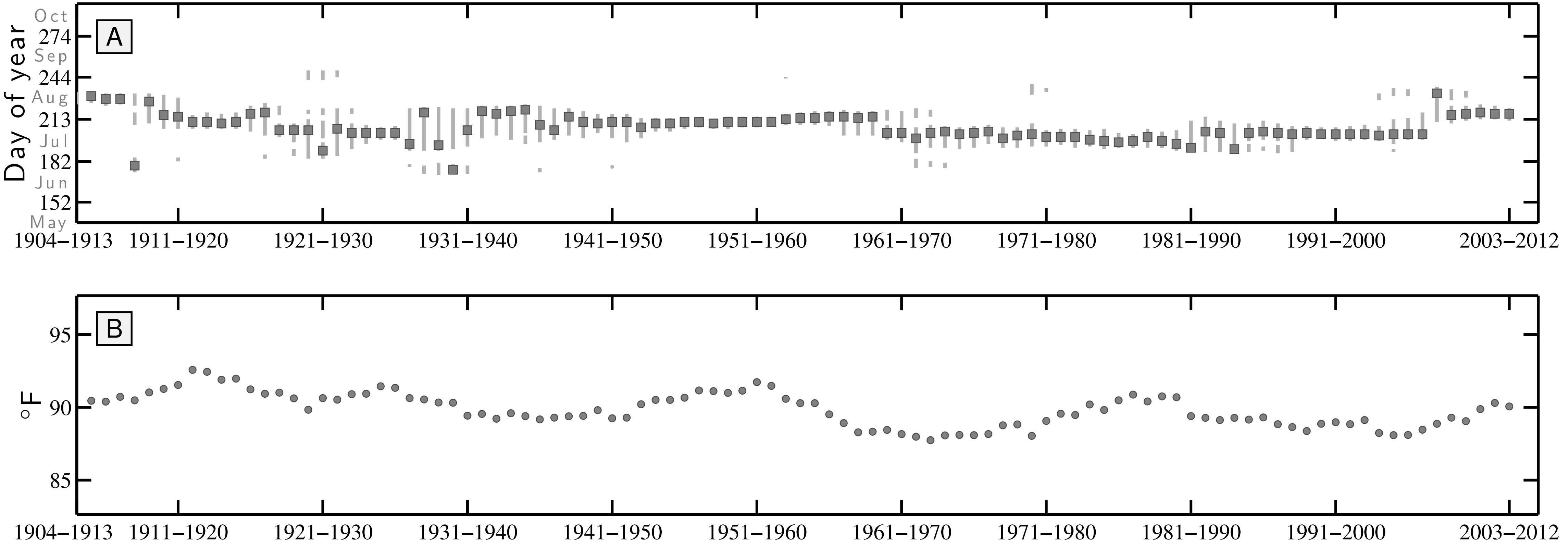

10 year Winter Teletherm dynamics for MC MINNVILLE, TN:

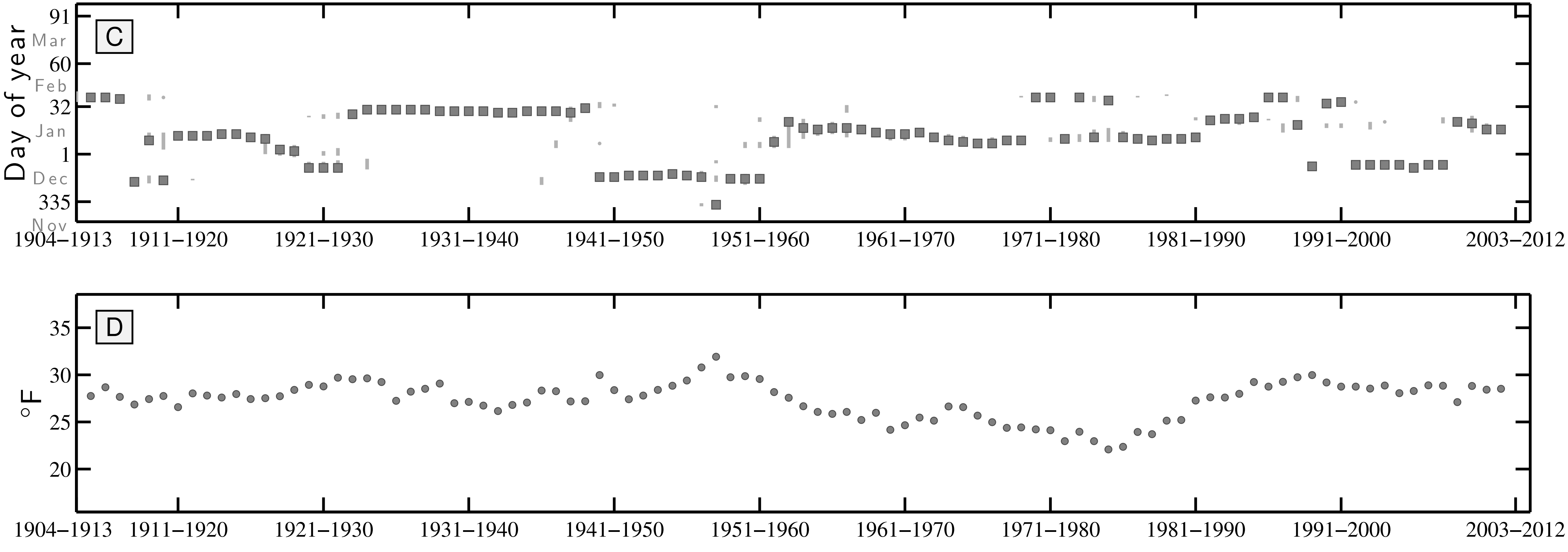

10 year Summer Teletherm dynamics for MURFREESBORO 5 N, TN:

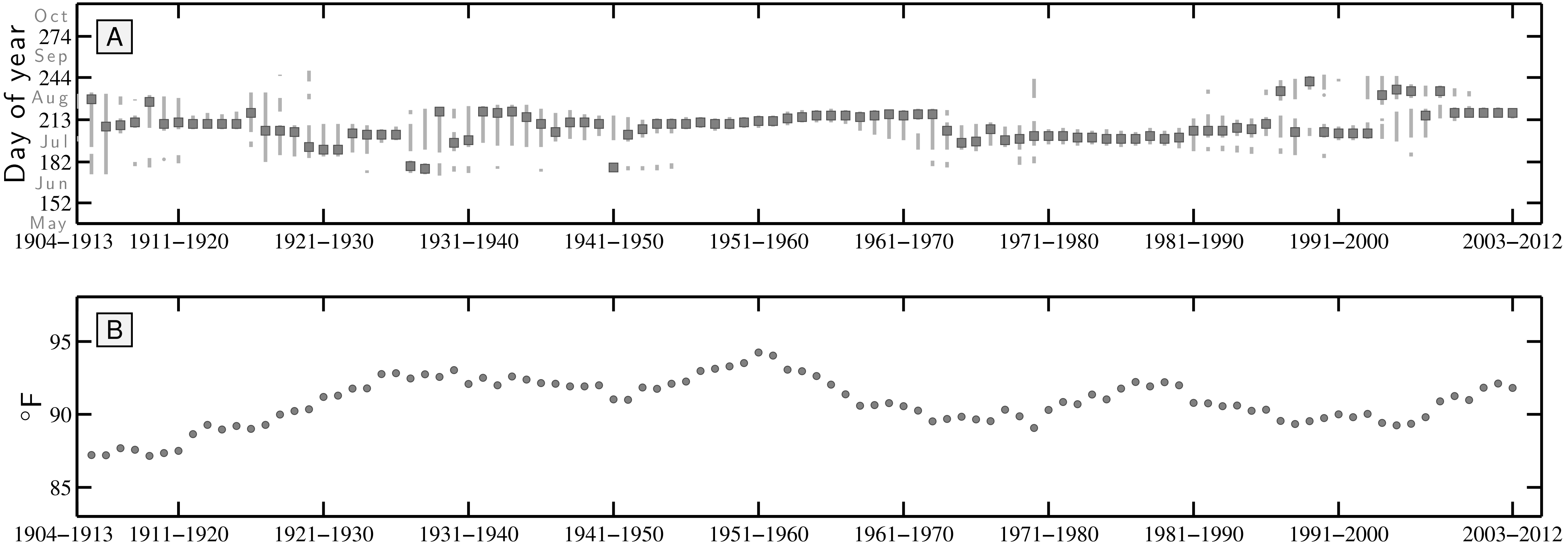

10 year Winter Teletherm dynamics for MURFREESBORO 5 N, TN:

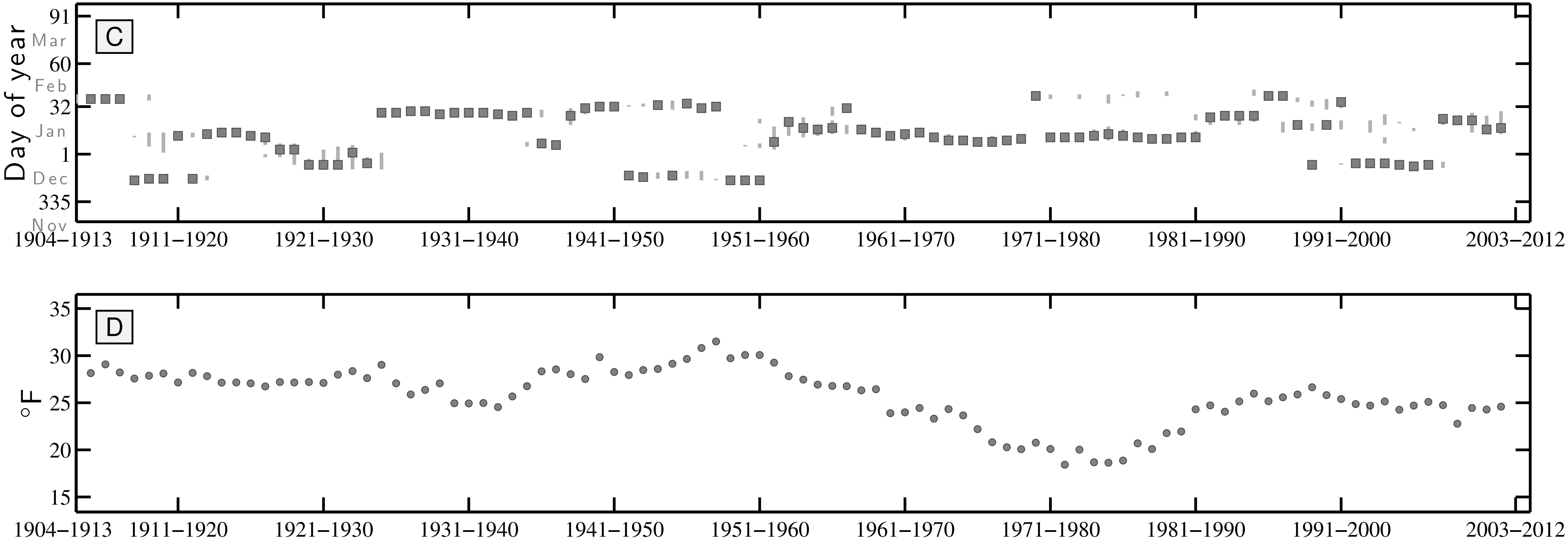

10 year Summer Teletherm dynamics for NEWPORT 1 NW, TN:

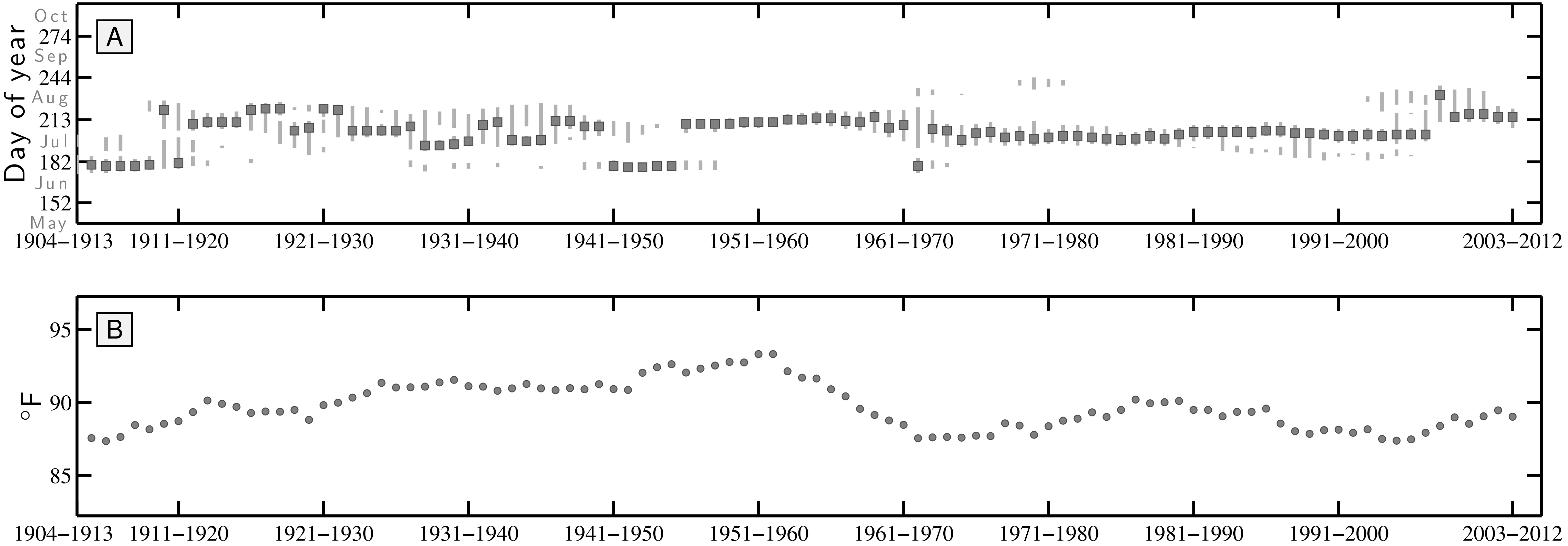

10 year Winter Teletherm dynamics for NEWPORT 1 NW, TN:

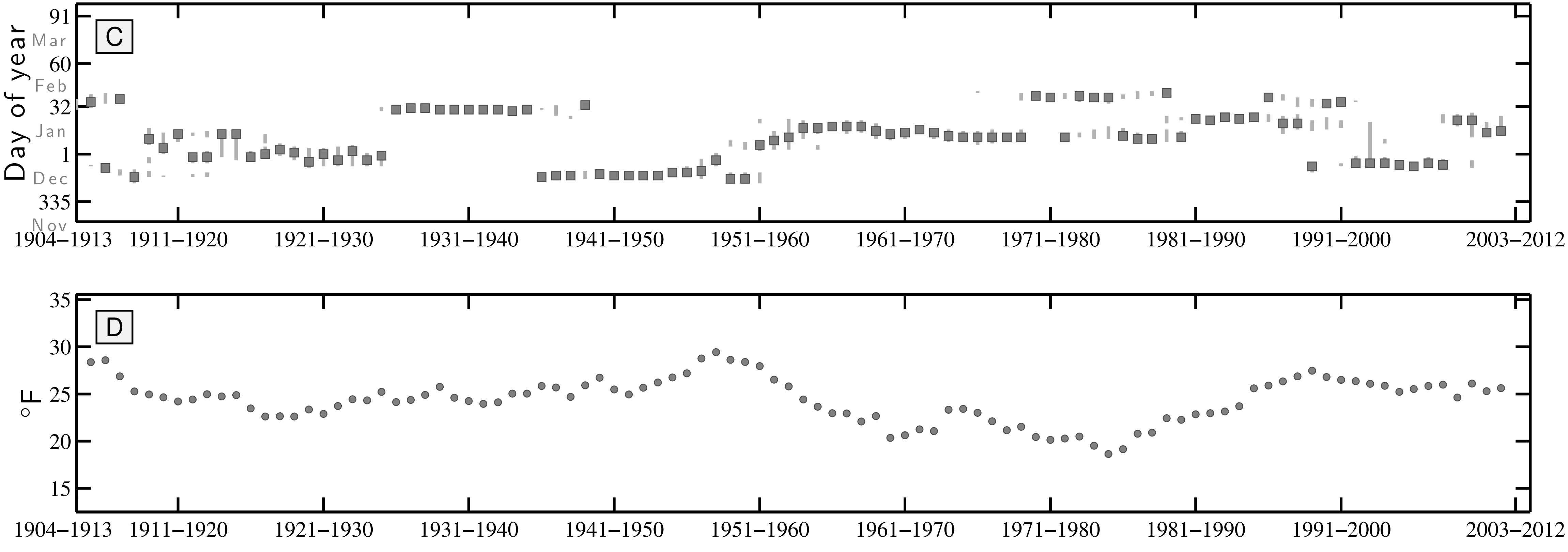

10 year Summer Teletherm dynamics for ROGERSVILLE 1 NE, TN:

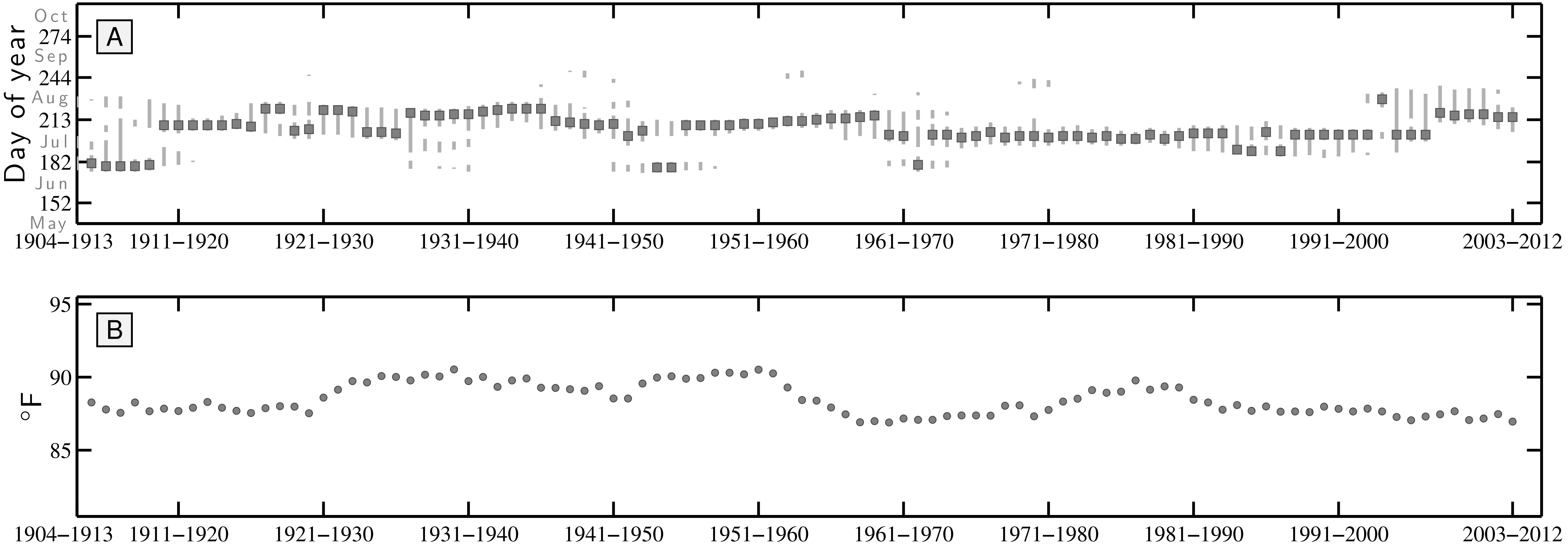

10 year Winter Teletherm dynamics for ROGERSVILLE 1 NE, TN:

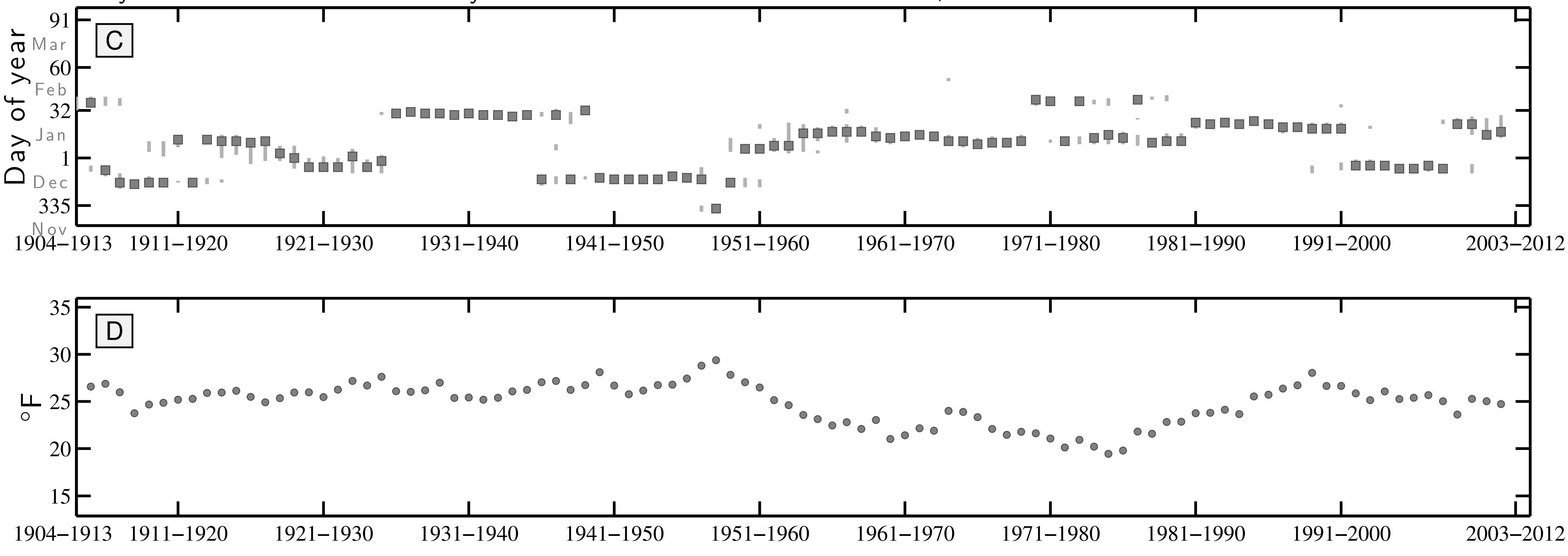

10 year Summer Teletherm dynamics for TULLAHOMA, TN:

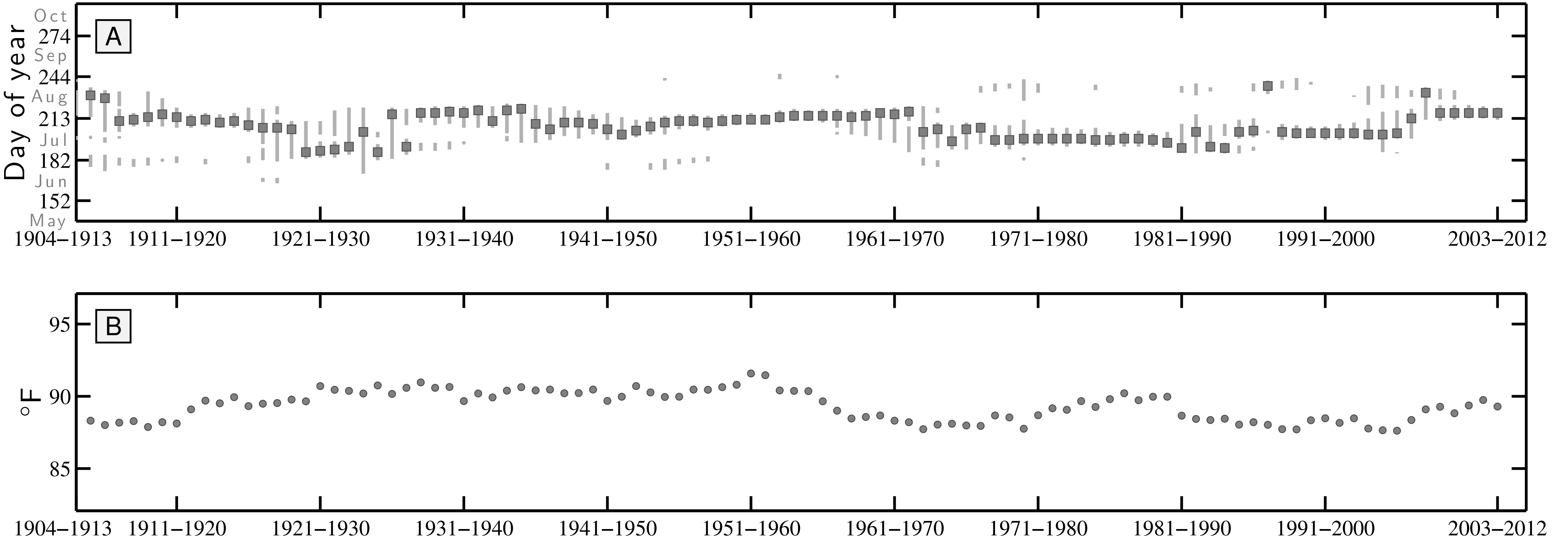

10 year Winter Teletherm dynamics for TULLAHOMA, TN:

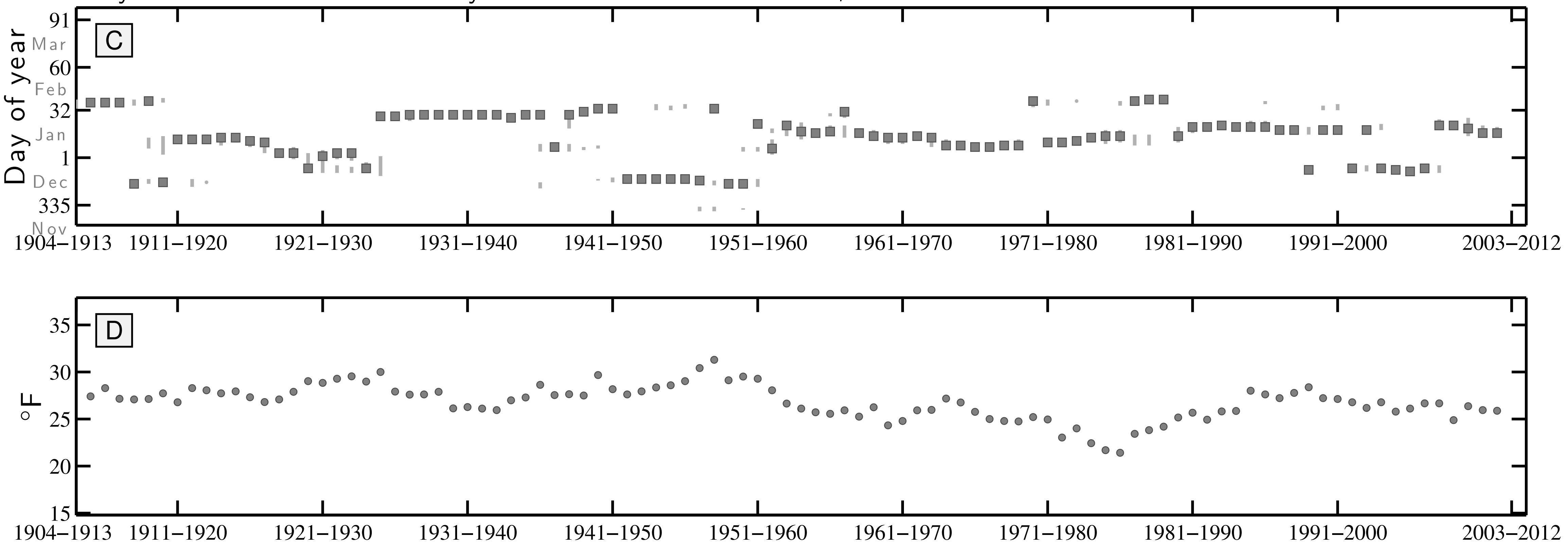

10 year Summer Teletherm dynamics for UNION CITY, TN:

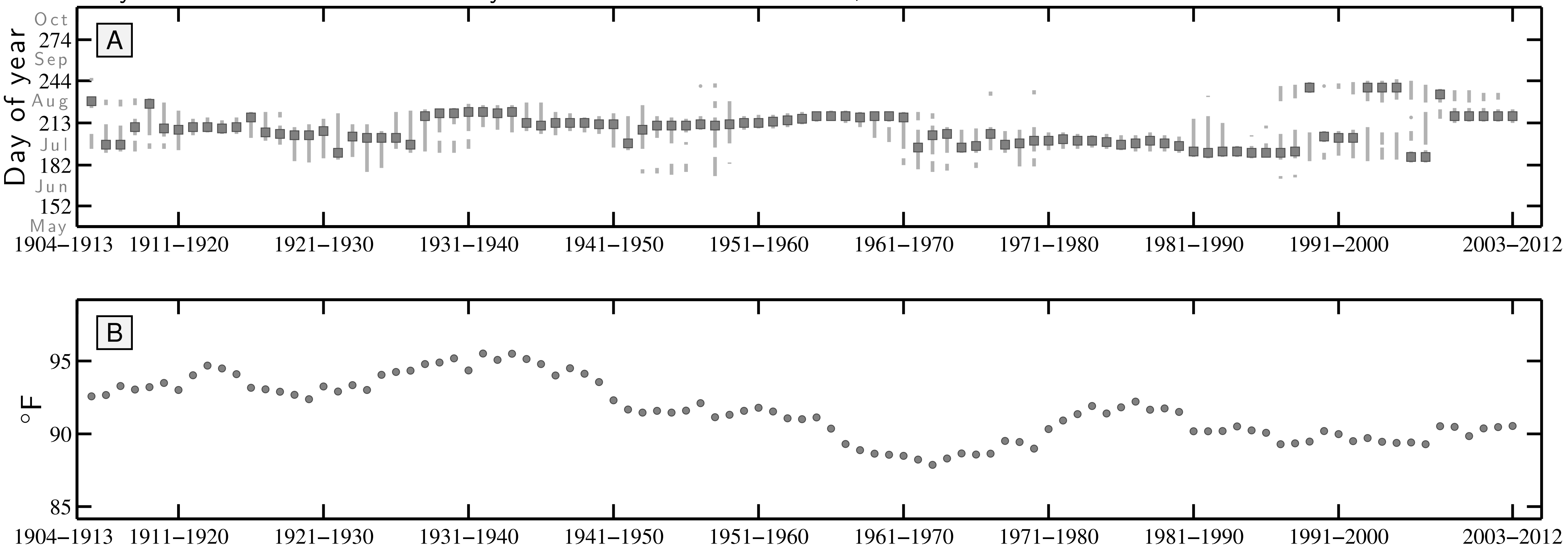

10 year Winter Teletherm dynamics for UNION CITY, TN:

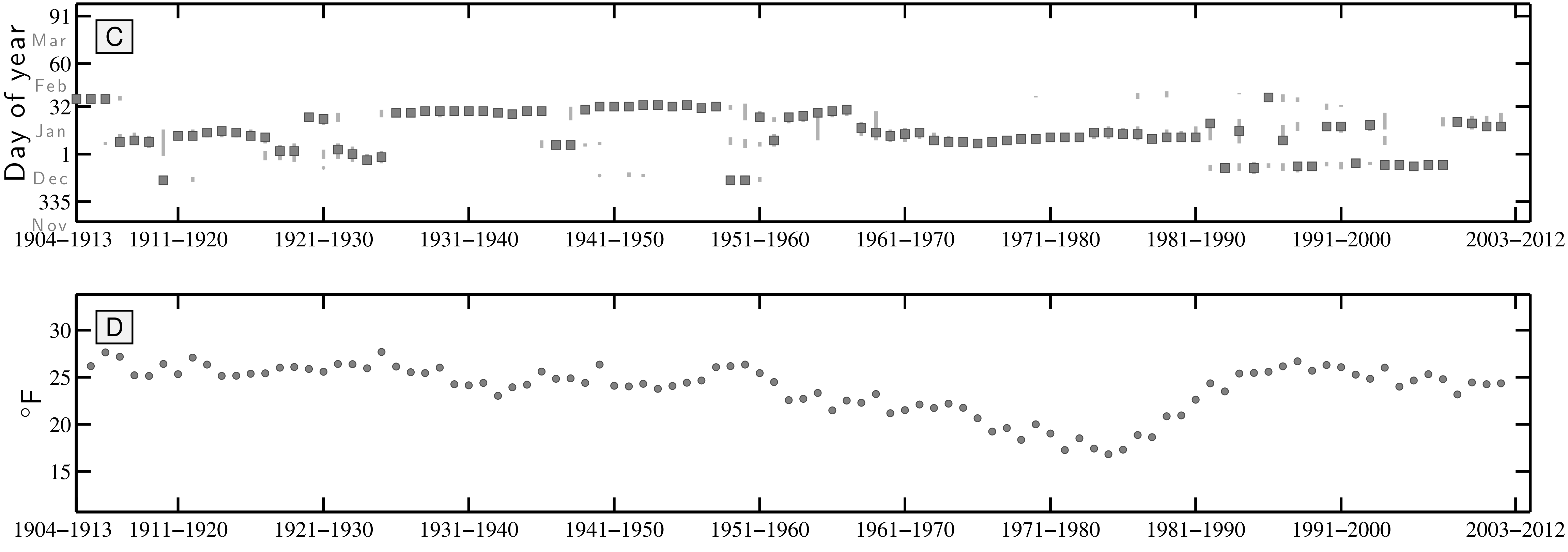

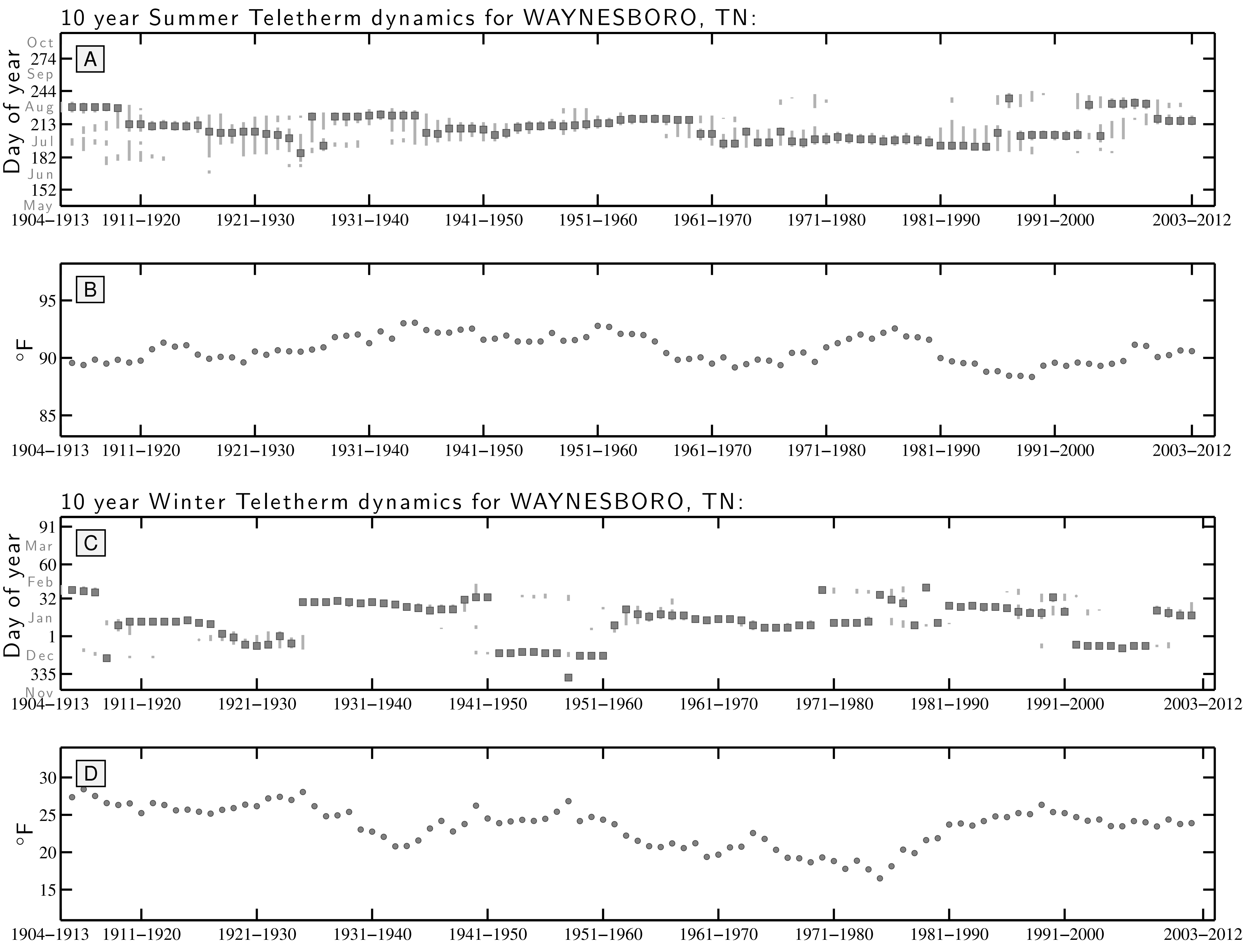
10 year Summer Teletherm dynamics for WAYNESBORO, TN:
A
Day of year
Oct
274
Sep
244
Aug
213
Jul
182
Jun
152
May
1904–1913
1911–1920
1921–1930
1931–1940
1941–1950
1951–1960
1961–1970
1971–1980
1981–1990
1991–2000
2003–2012
B
°F
95
90
85
10 year Winter Teletherm dynamics for WAYNESBORO, TN:
C
91
Mar
60
Feb
32
Jan
1
Dec
335
Nov
D
30
25
20
15

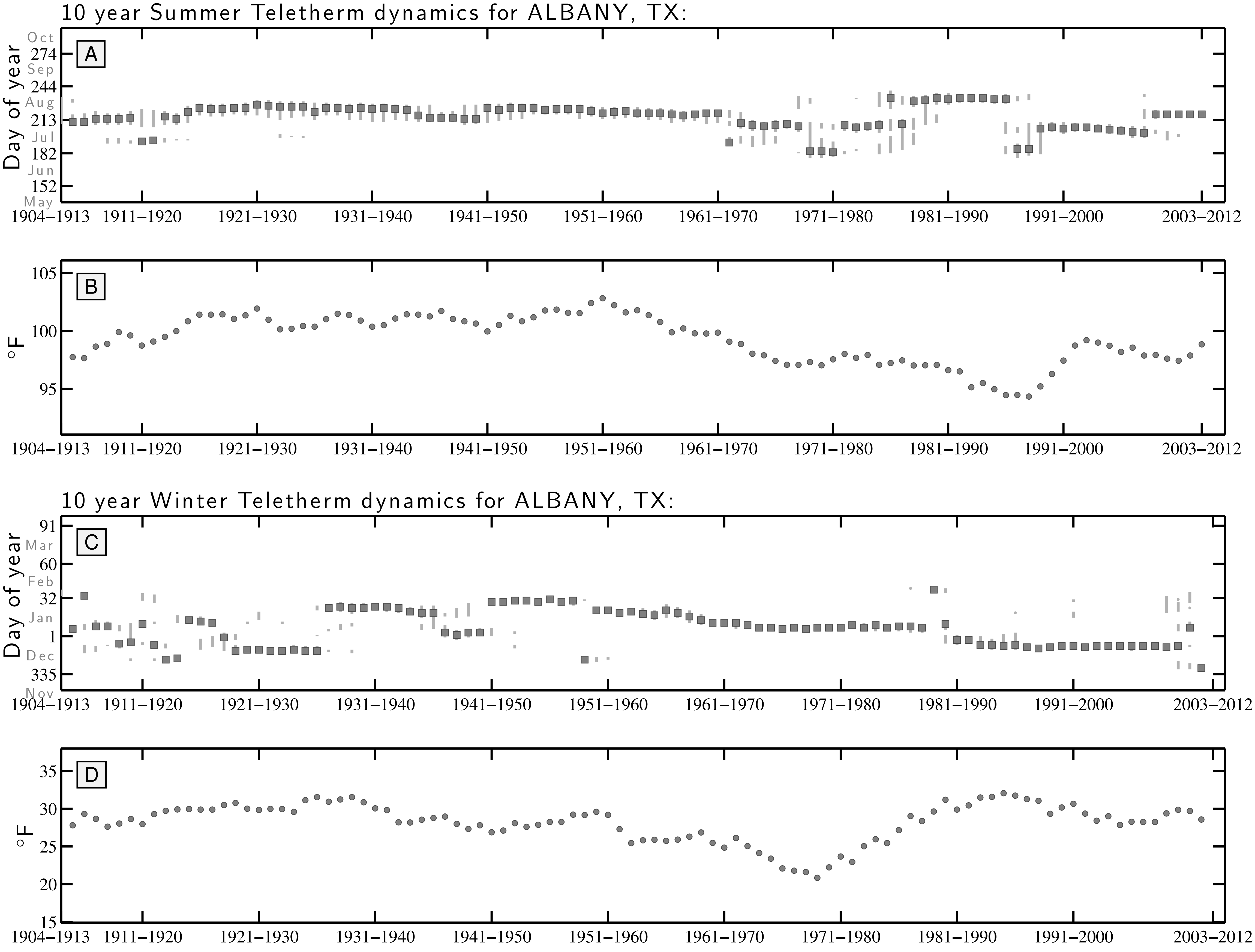
10 year Summer Teletherm dynamics for ALBANY, TX:
A
Day of year
Oct
274
Sep
244
Aug
213
Jul
182
Jun
152
May
B
°F
105
100
95
10 year Winter Teletherm dynamics for ALBANY, TX:
C
Day of year
91
Mar
60
Feb
32
Jan
1
Dec
335
Nov
D
°F
35
30
25
20
15
1904–1913
1911–1920
1921–1930
1931–1940
1941–1950
1951–1960
1961–1970
1971–1980
1981–1990
1991–2000
2003–2012

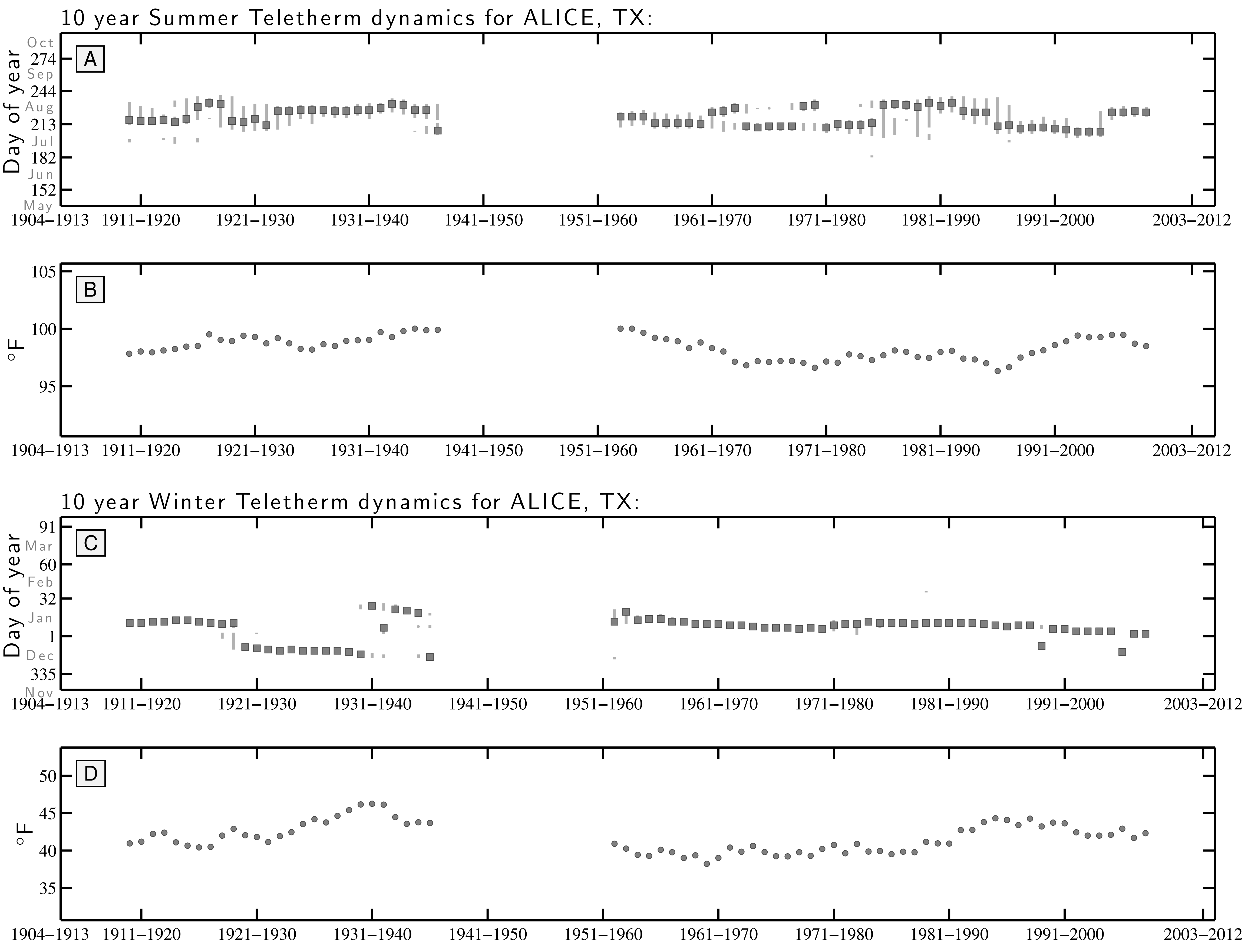
10 year Summer Teletherm dynamics for ALICE, TX:
A
Day of year
Oct
274
Sep
244
Aug
213
Jul
182
Jun
152
May
B
°F
105
100
95
10 year Winter Teletherm dynamics for ALICE, TX:
C
Day of year
91
Mar
60
Feb
32
Jan
1
Dec
335
Nov
D
°F
50
45
40
35
1904–1913
1911–1920
1921–1930
1931–1940
1941–1950
1951–1960
1961–1970
1971–1980
1981–1990
1991–2000
2003–2012

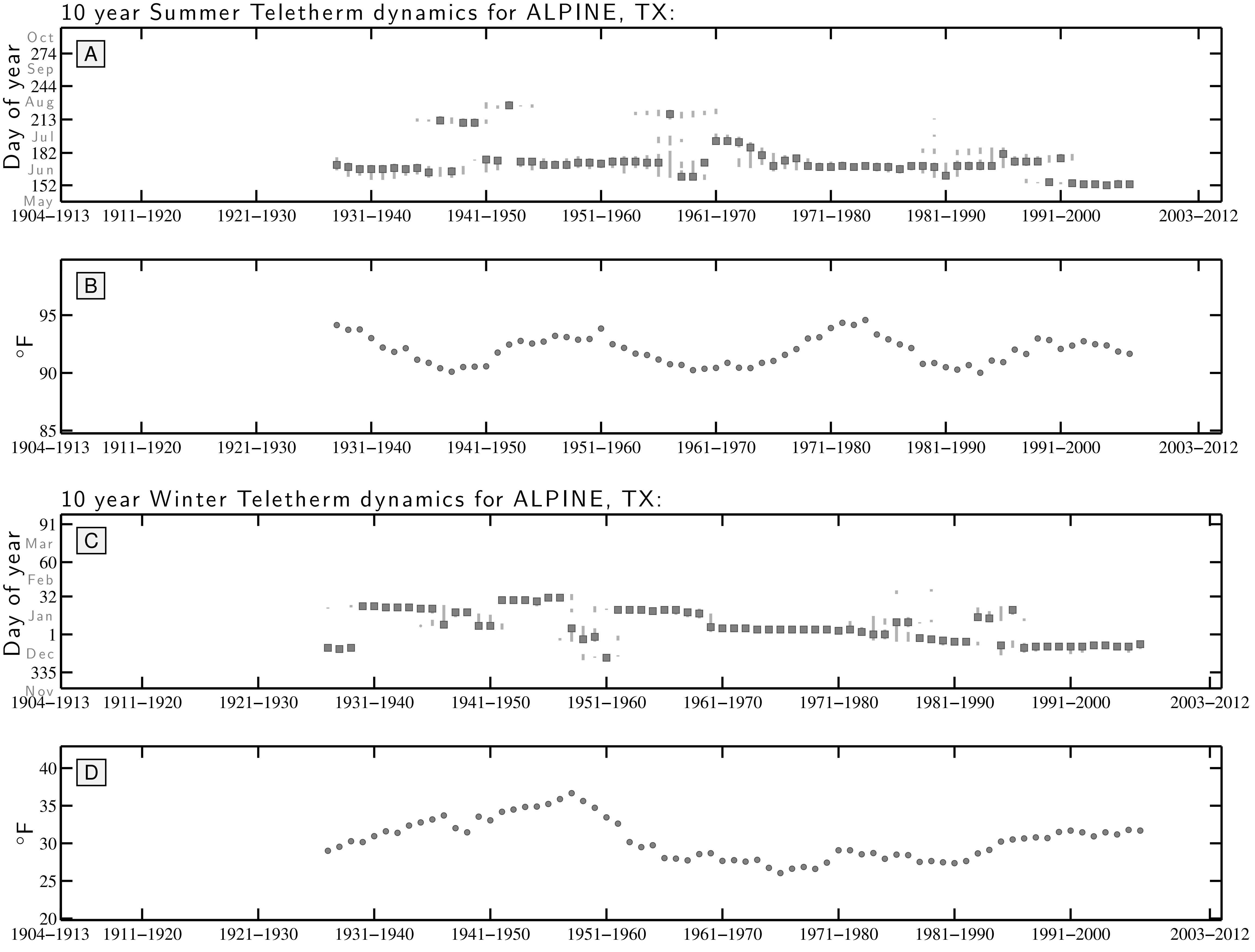
10 year Summer Teletherm dynamics for ALPINE, TX:
A
B
Day of year
°F
Oct
274
Sep
244
Aug
213
Jul
182
Jun
152
May
95
90
85
10 year Winter Teletherm dynamics for ALPINE, TX:
C
D
91
Mar
60
Feb
32
Jan
1
Dec
335
Nov
40
35
30
25
20
1904–1913
1911–1920
1921–1930
1931–1940
1941–1950
1951–1960
1961–1970
1971–1980
1981–1990
1991–2000
2003–2012

10 year Summer Teletherm dynamics for BALLINGER 2 NW, TX:

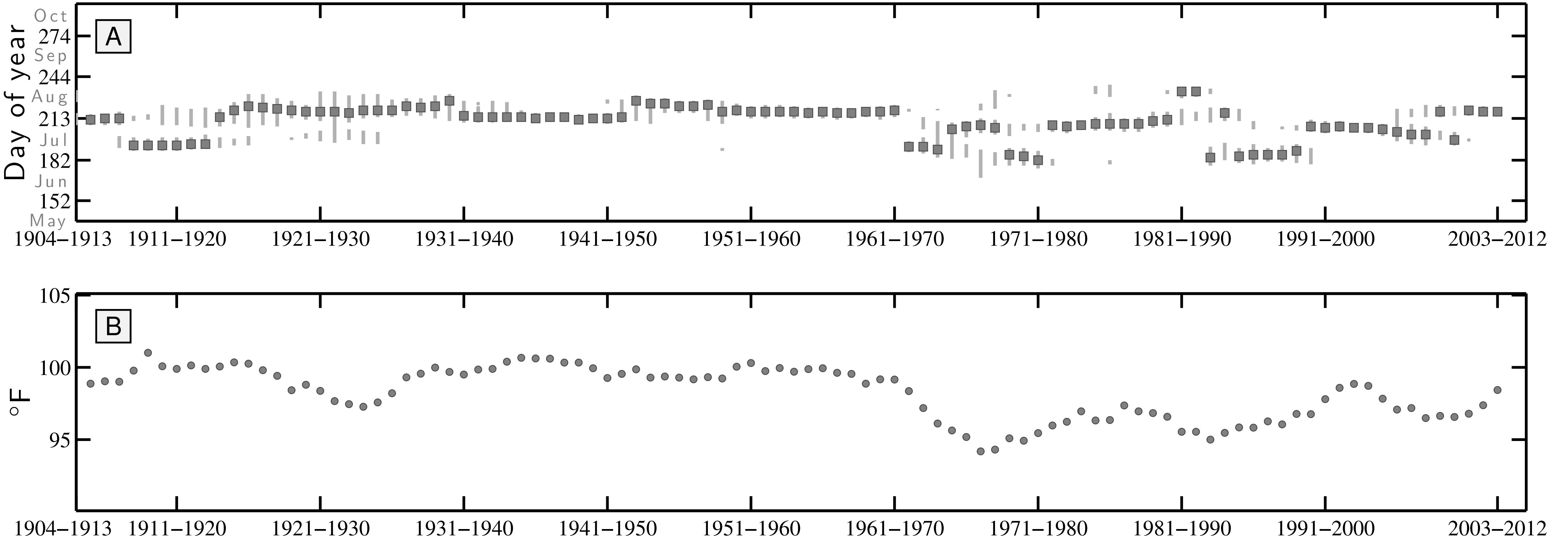

10 year Winter Teletherm dynamics for BALLINGER 2 NW, TX:

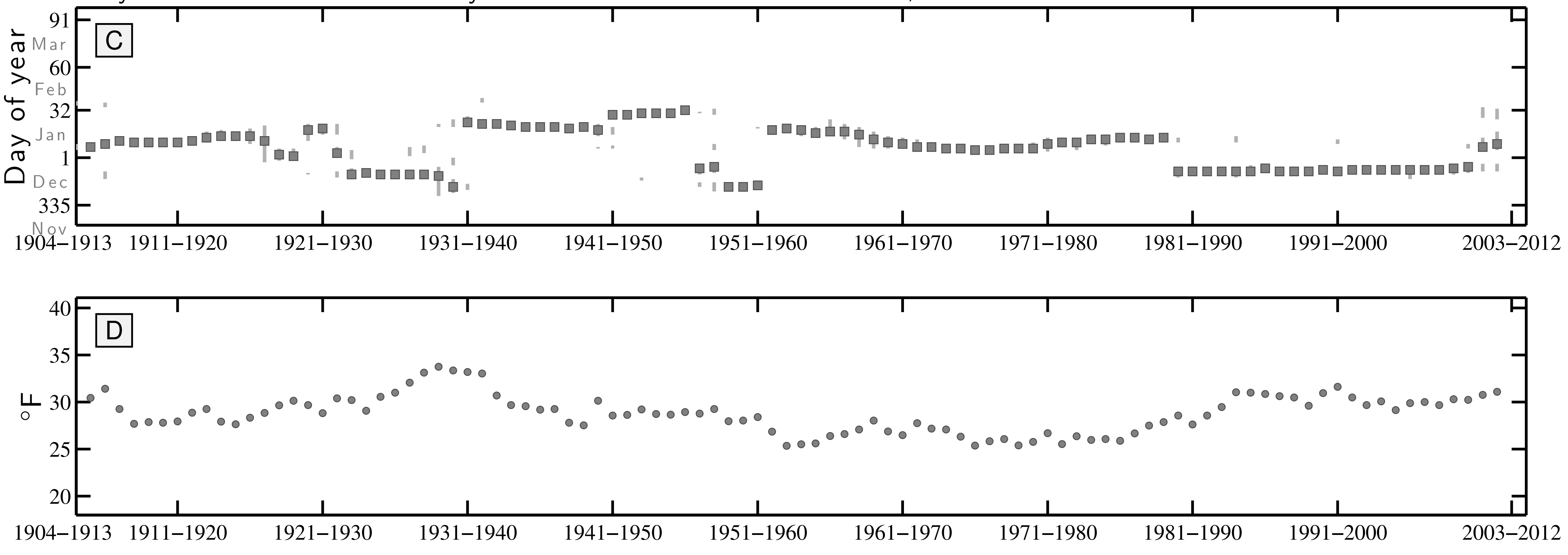

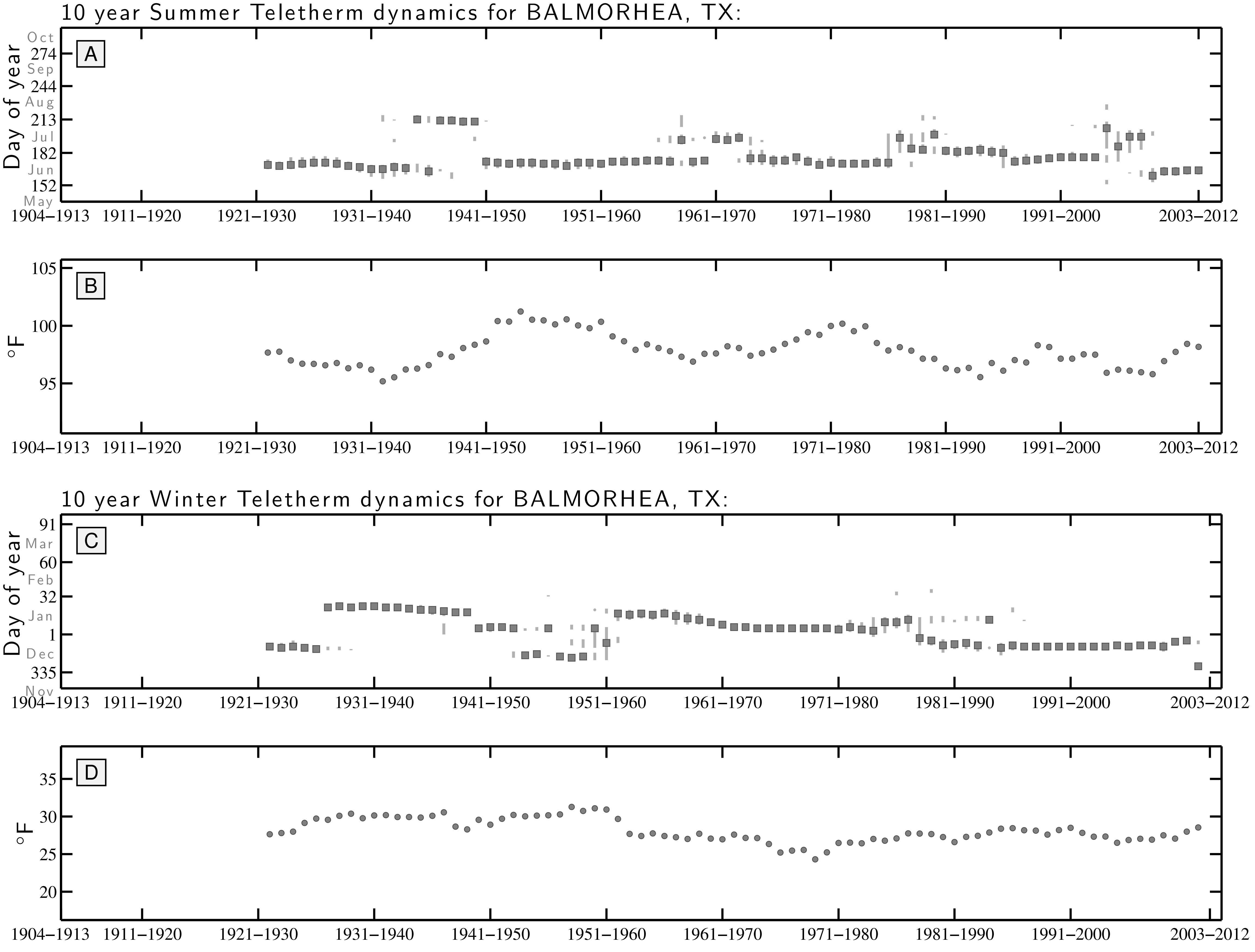

10 year Summer Teletherm dynamics for BALMORHEA, TX:
A
Day of year
Oct
274
Sep
244
Aug
213
Jul
182
Jun
152
May
B
°F
105
100
95
10 year Winter Teletherm dynamics for BALMORHEA, TX:
C
Day of year
91
Mar
60
Feb
32
Jan
1
Dec
335
Nov
D
°F
35
30
25
20
1904–1913
1911–1920
1921–1930
1931–1940
1941–1950
1951–1960
1961–1970
1971–1980
1981–1990
1991–2000
2003–2012

10 year Summer Teletherm dynamics for BEEVILLE 5 NE, TX:

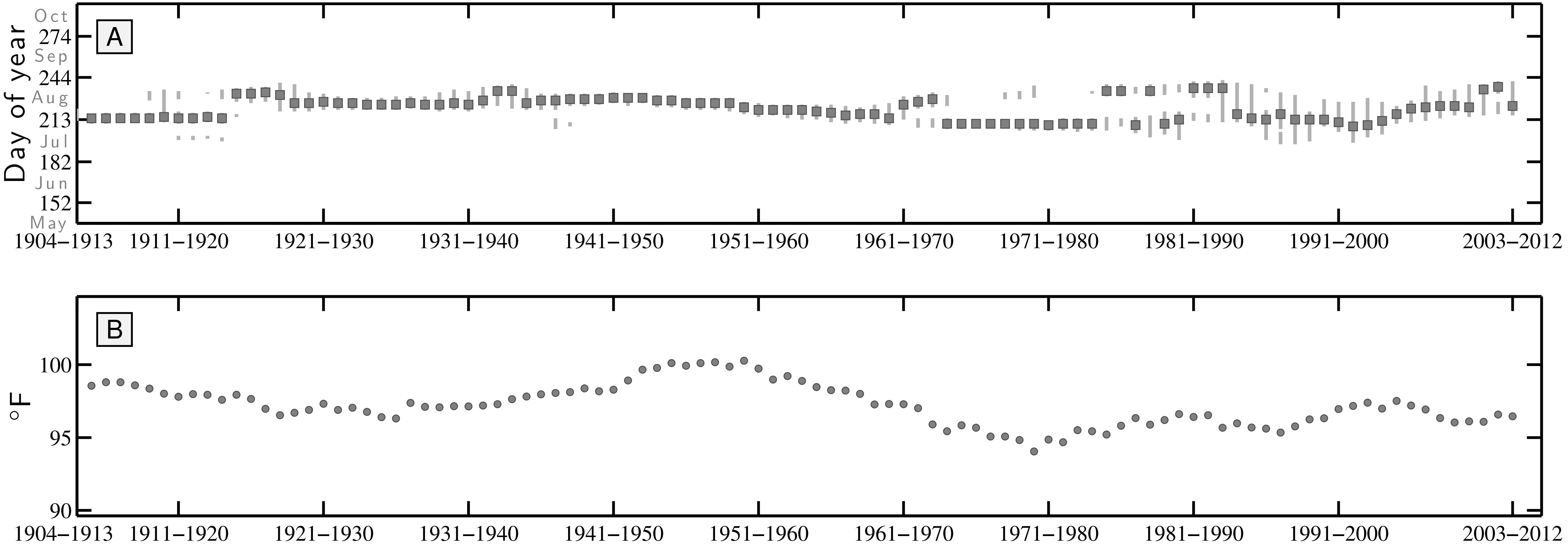

10 year Winter Teletherm dynamics for BEEVILLE 5 NE, TX:

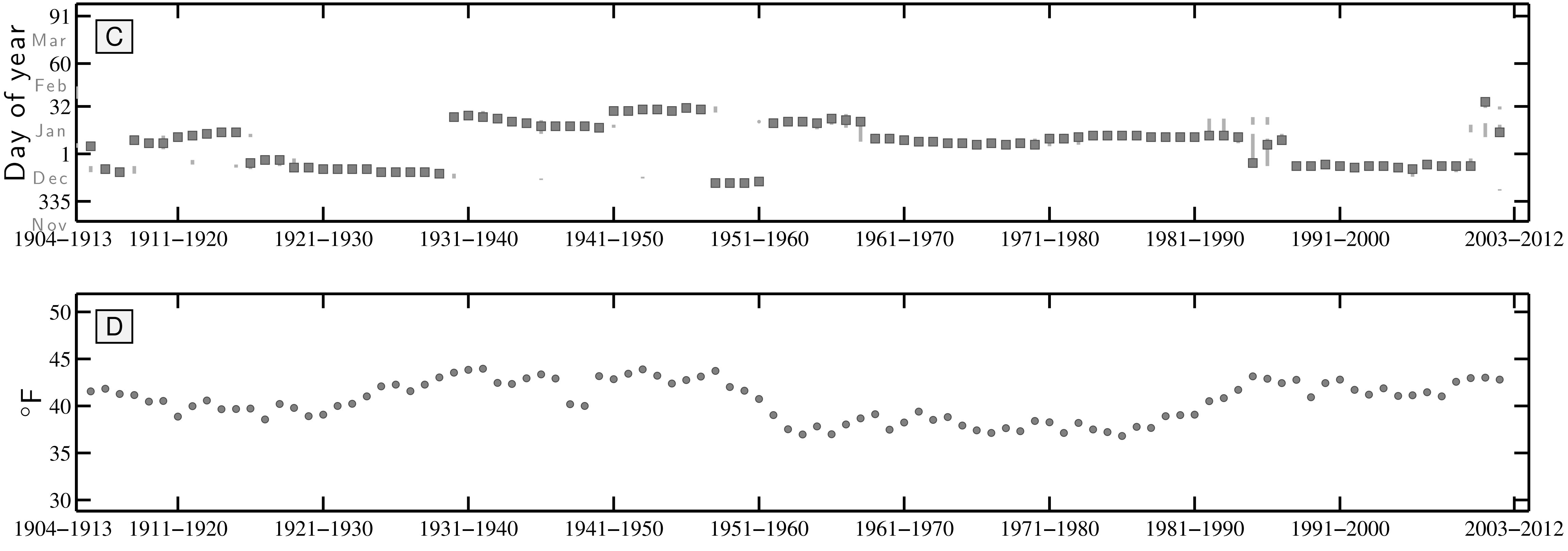

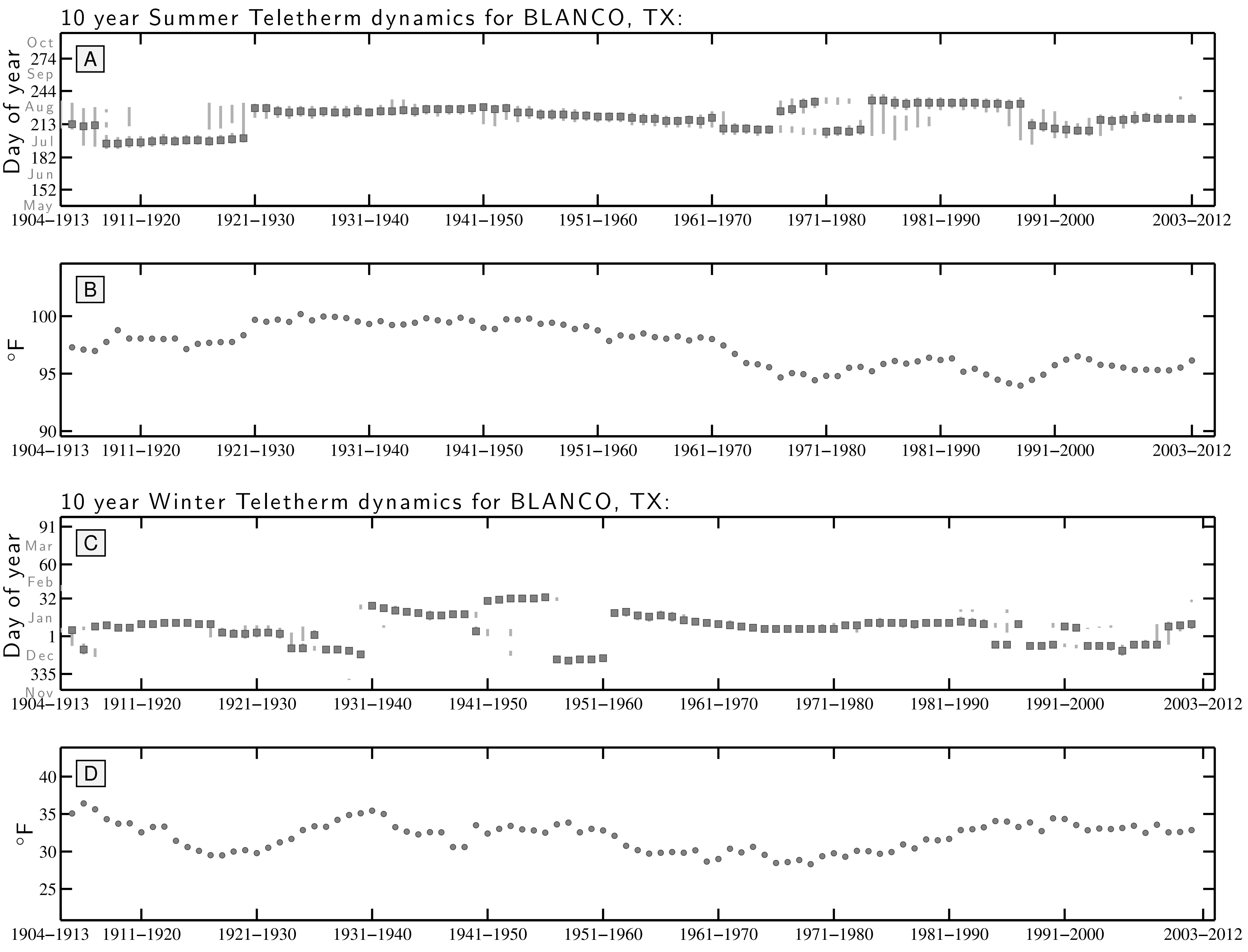
10 year Summer Teletherm dynamics for BLANCO, TX:
A
B
10 year Winter Teletherm dynamics for BLANCO, TX:
C
D
Day of year
°F
Oct
274
Sep
244
Aug
213
Jul
182
Jun
152
May
100
95
90
91
Mar
60
Feb
32
Jan
1
Dec
335
Nov
40
35
30
25
1904–1913
1911–1920
1921–1930
1931–1940
1941–1950
1951–1960
1961–1970
1971–1980
1981–1990
1991–2000
2003–2012

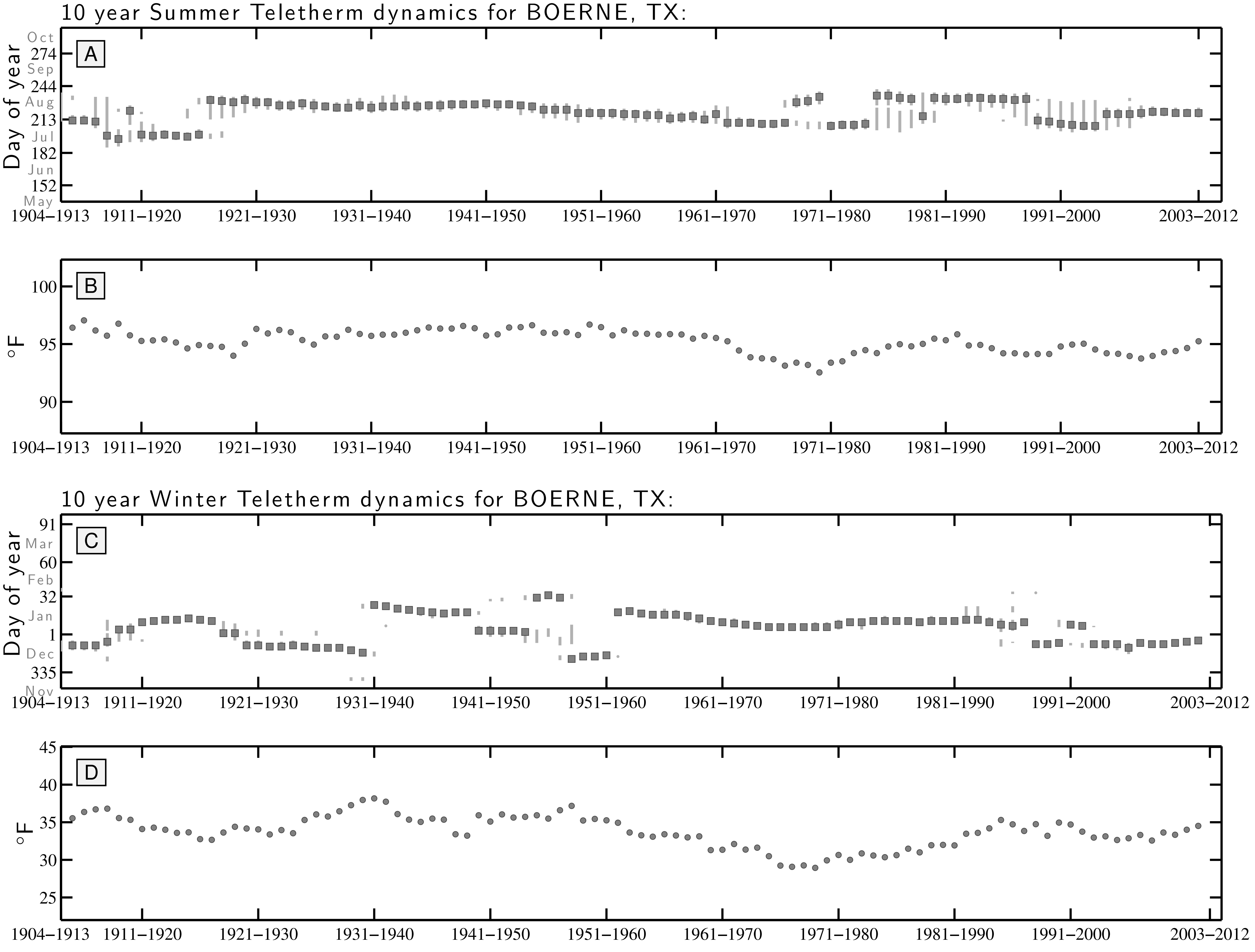

10 year Summer Teletherm dynamics for BOERNE, TX:
A
Day of year
Oct
274
Sep
244
Aug
213
Jul
182
Jun
152
May
1904–1913
1911–1920
1921–1930
1931–1940
1941–1950
1951–1960
1961–1970
1971–1980
1981–1990
1991–2000
2003–2012
B
°F
100
95
90
10 year Winter Teletherm dynamics for BOERNE, TX:
C
91
Mar
60
Feb
32
Jan
1
Dec
335
Nov
D
45
40
35
30
25

10 year Summer Teletherm dynamics for BOYS RANCH, TX:

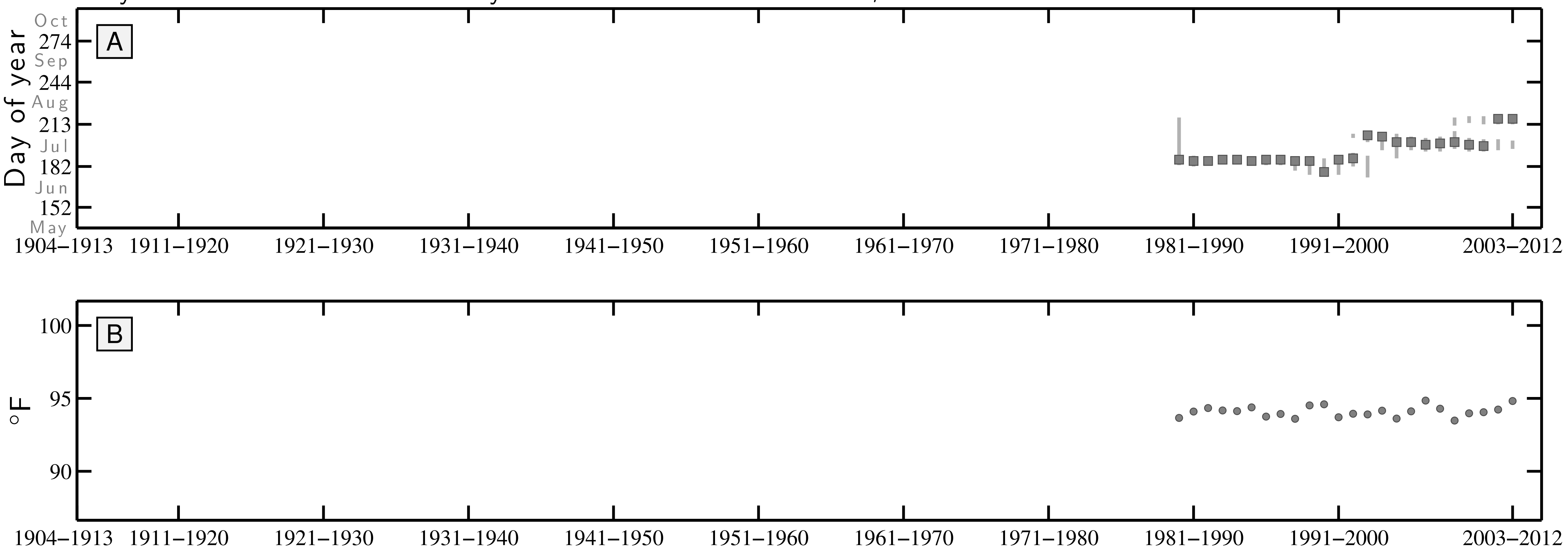

10 year Winter Teletherm dynamics for BOYS RANCH, TX:

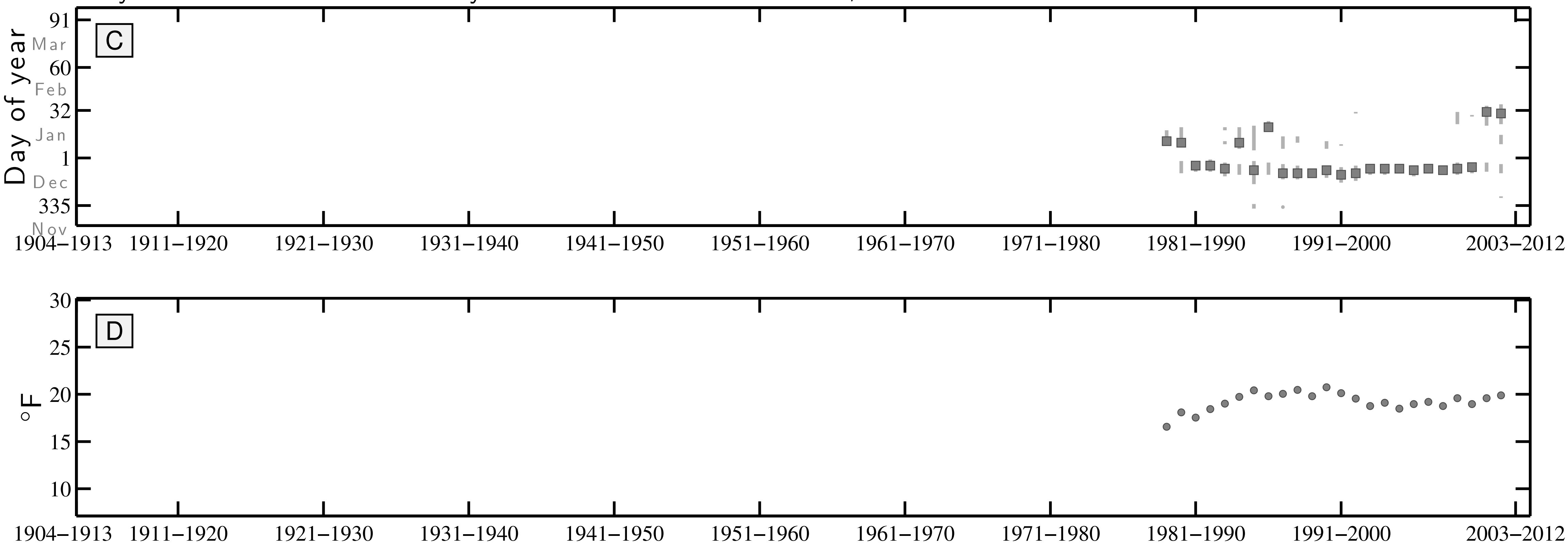

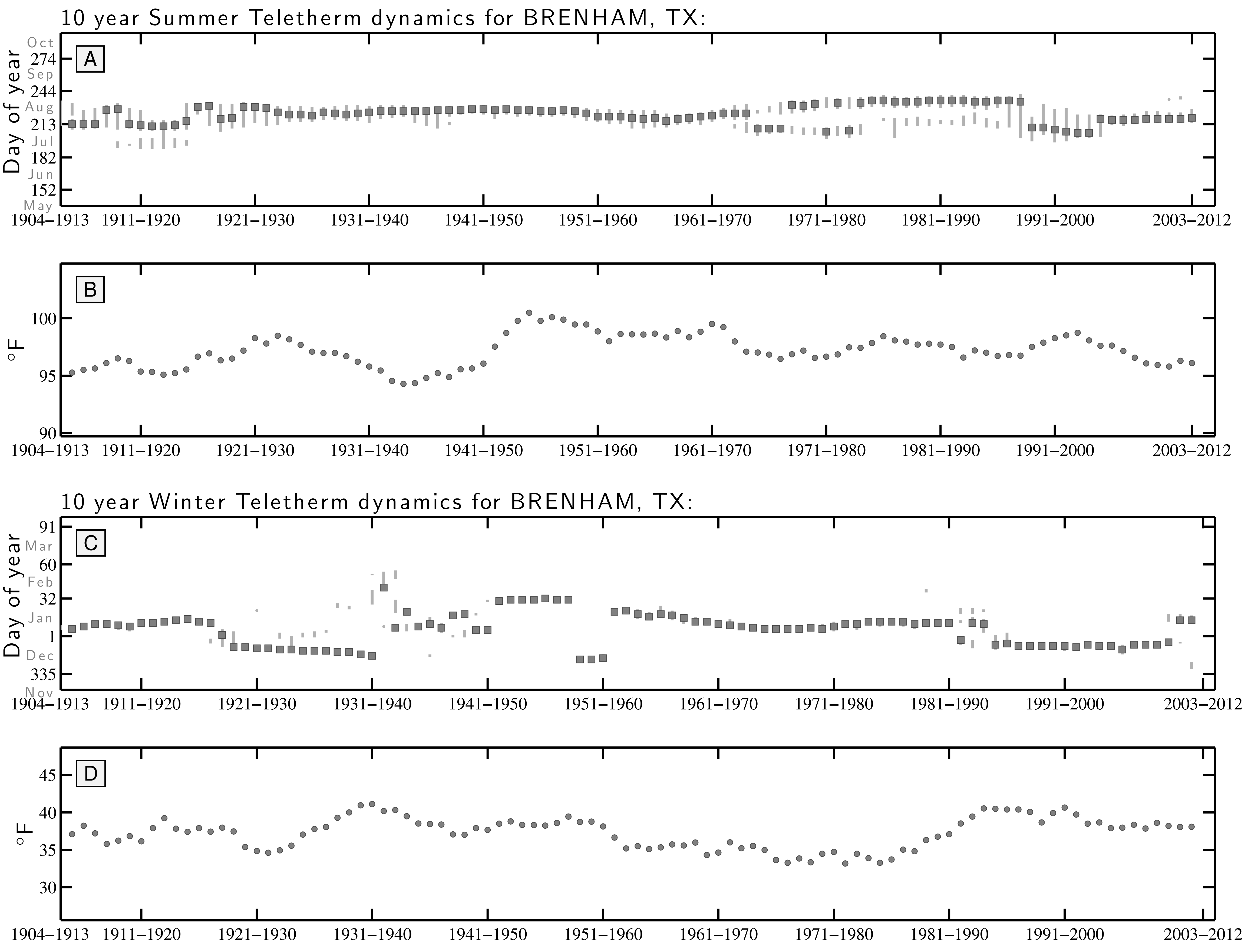
10 year Summer Teletherm dynamics for BRENHAM, TX:
A
Day of year
Oct
274
Sep
244
Aug
213
Jul
182
Jun
152
May
1904–1913
1911–1920
1921–1930
1931–1940
1941–1950
1951–1960
1961–1970
1971–1980
1981–1990
1991–2000
2003–2012
B
°F
100
95
90
10 year Winter Teletherm dynamics for BRENHAM, TX:
C
91
Mar
60
Feb
32
Jan
1
Dec
335
Nov
D
45
40
35
30

10 year Summer Teletherm dynamics for BROWNWOOD 2ENE, TX:

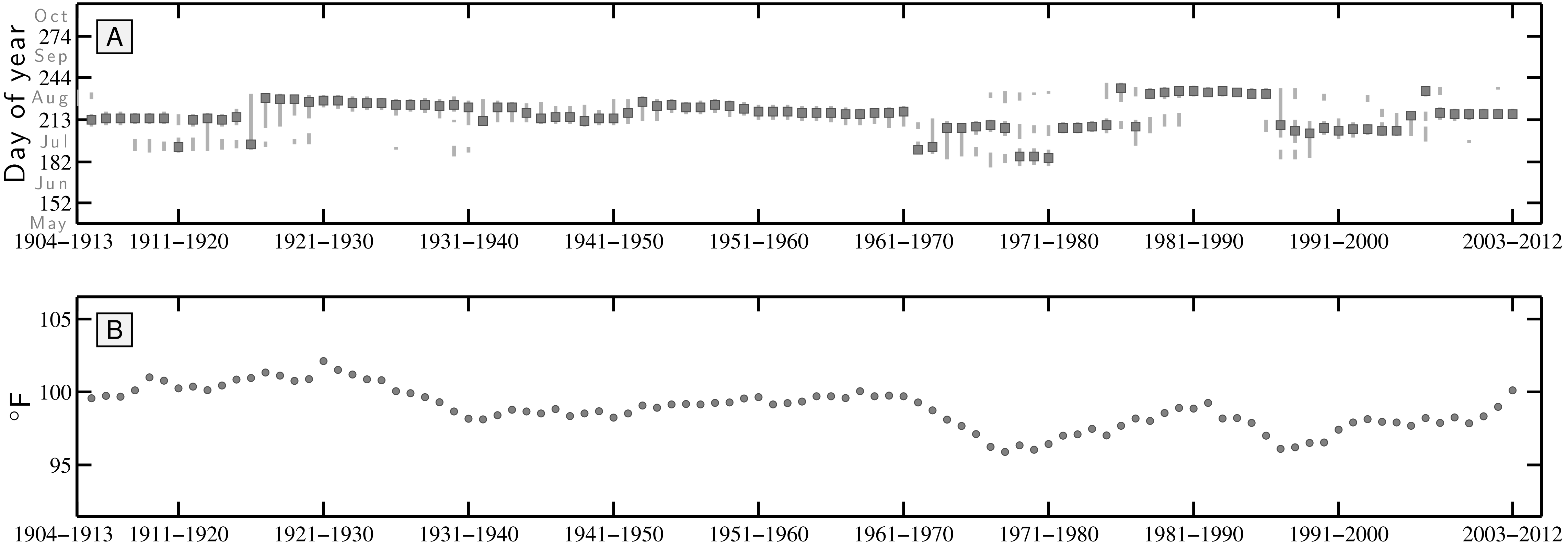

10 year Winter Teletherm dynamics for BROWNWOOD 2ENE, TX:

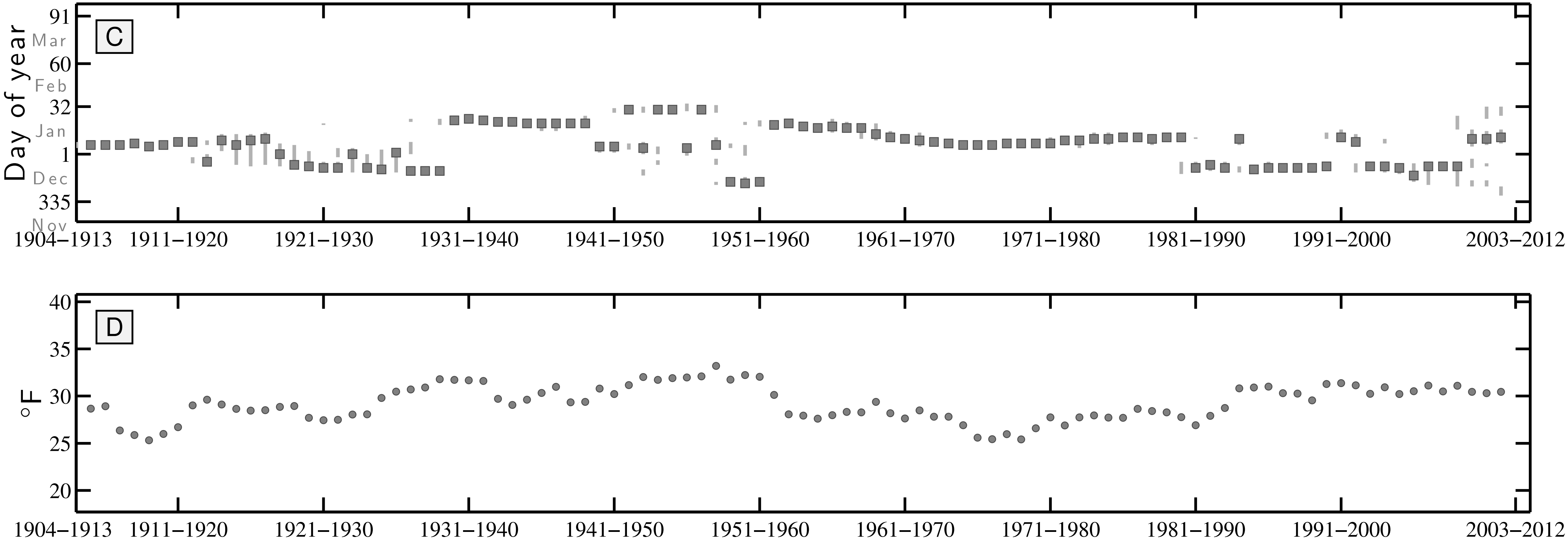

10 year Summer Teletherm dynamics for CATARINA, TX:

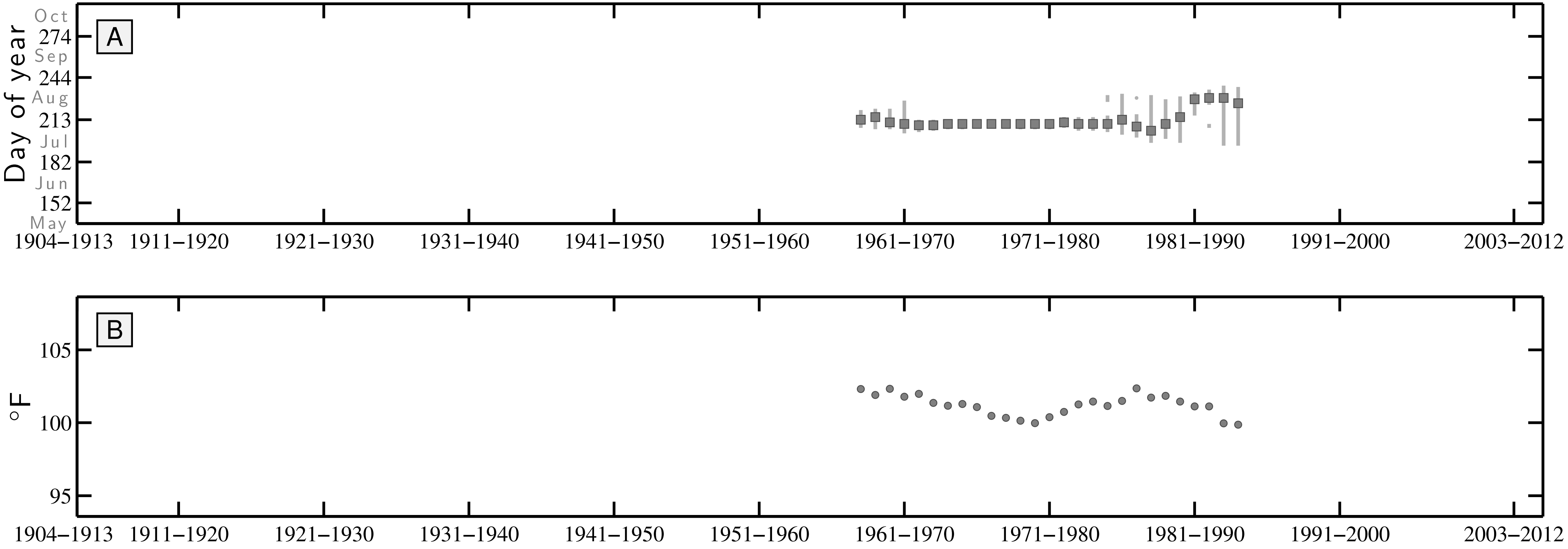

10 year Winter Teletherm dynamics for CATARINA, TX:

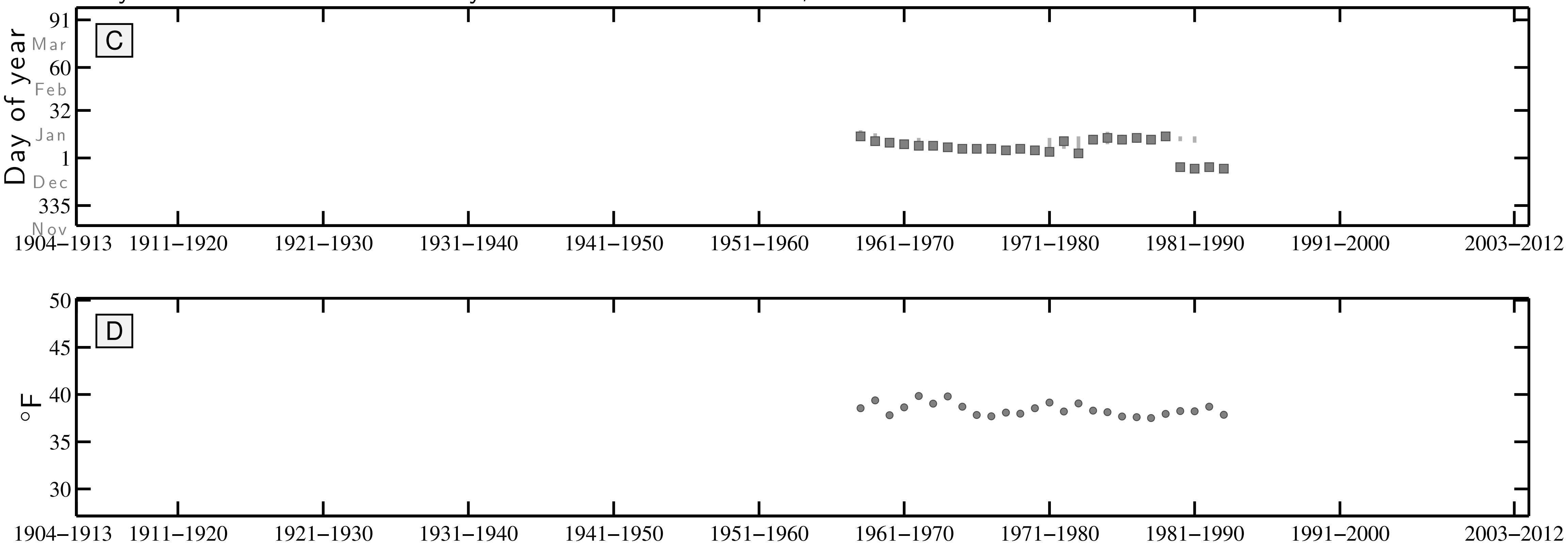

10 year Summer Teletherm dynamics for CLARKSVILLE 2NE, TX:

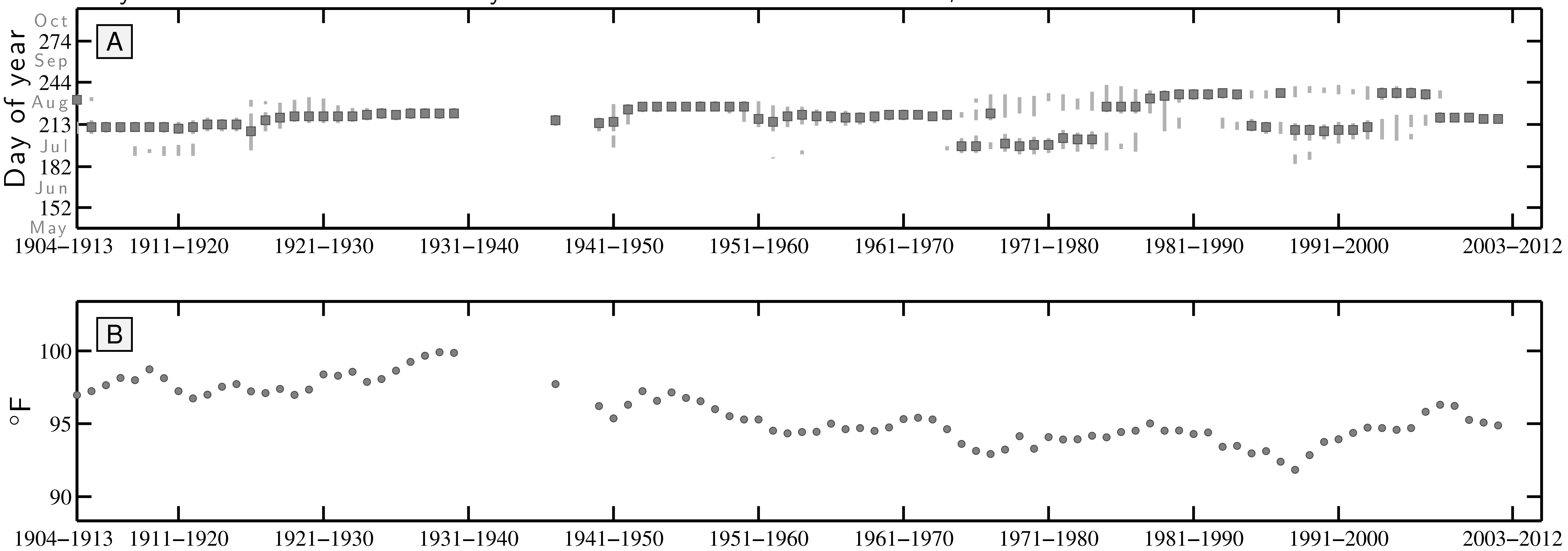

10 year Winter Teletherm dynamics for CLARKSVILLE 2NE, TX:

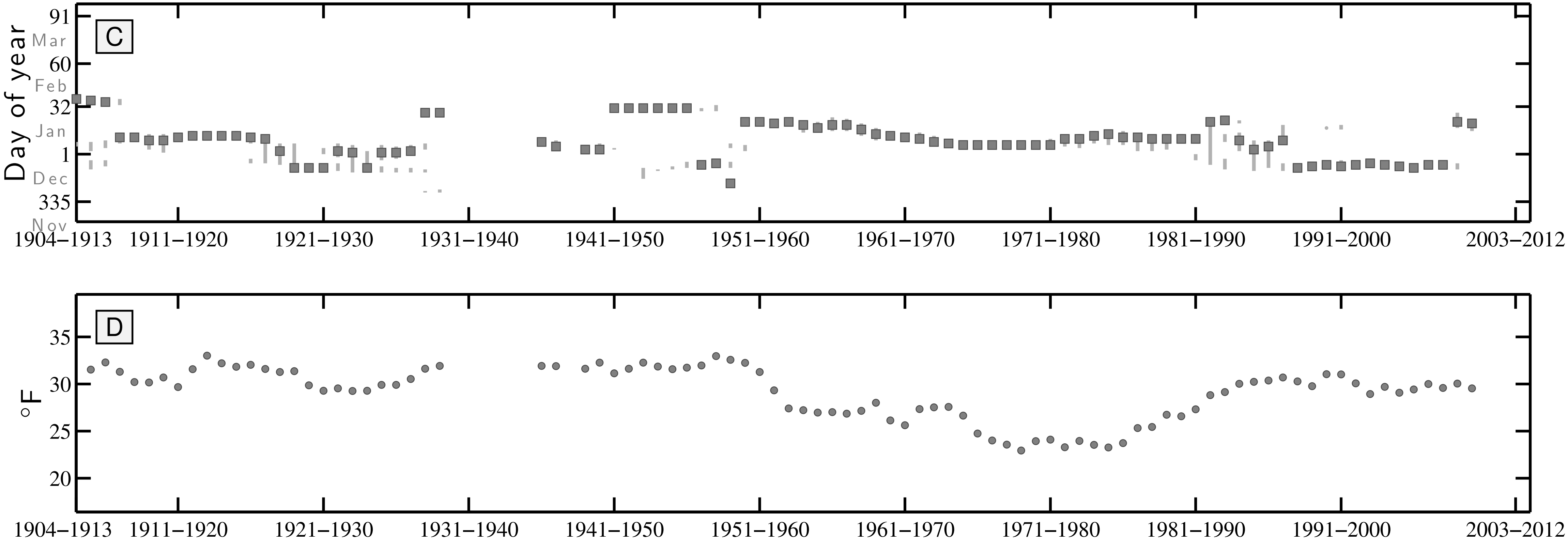

10 year Summer Teletherm dynamics for CORPUS CHRISTI AP, TX:

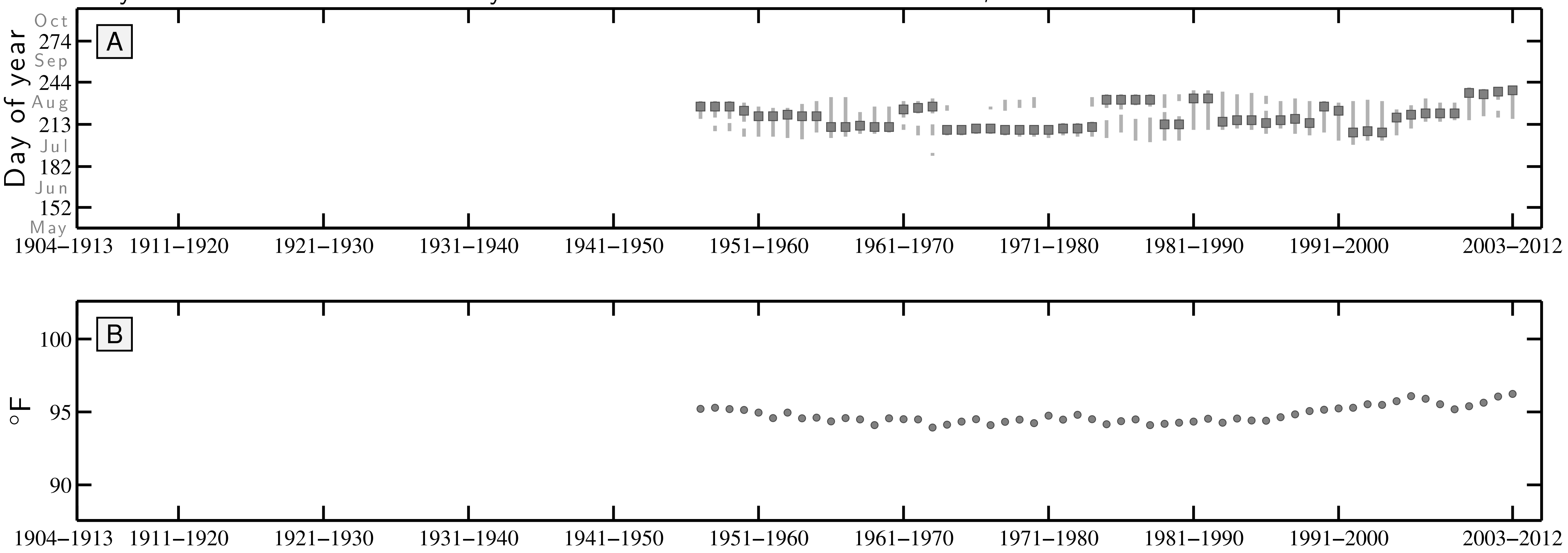

10 year Winter Teletherm dynamics for CORPUS CHRISTI AP, TX:

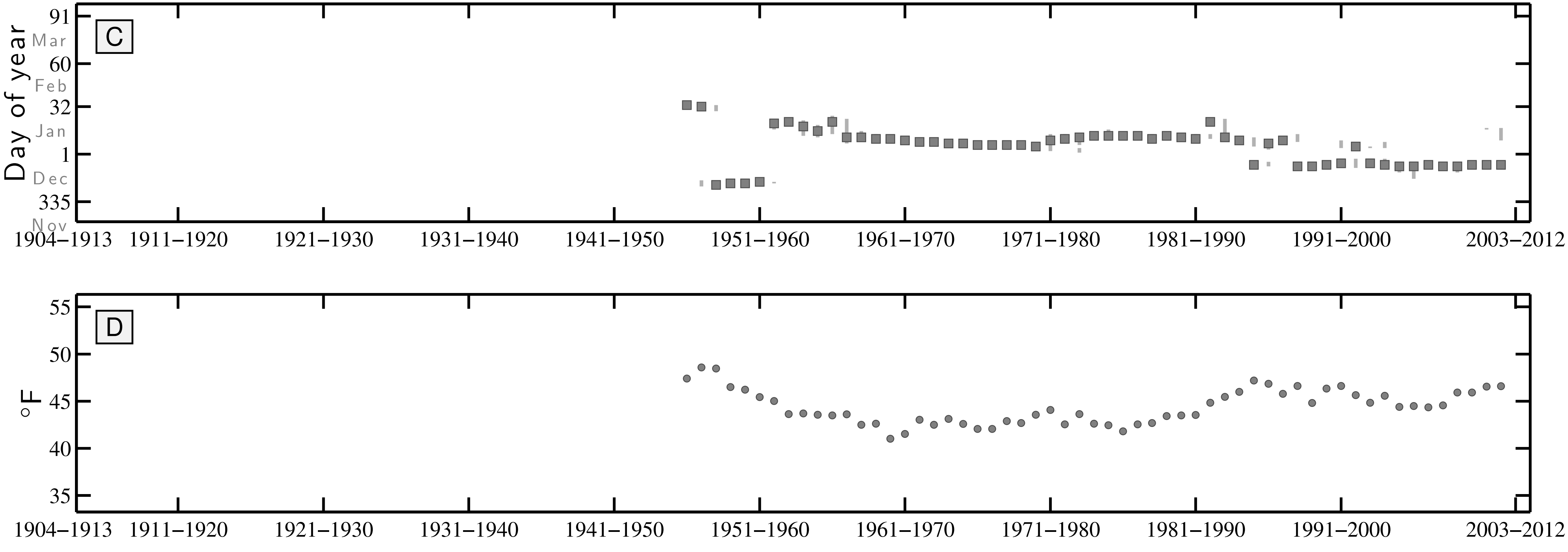

10 year Summer Teletherm dynamics for CORSICANA, TX:

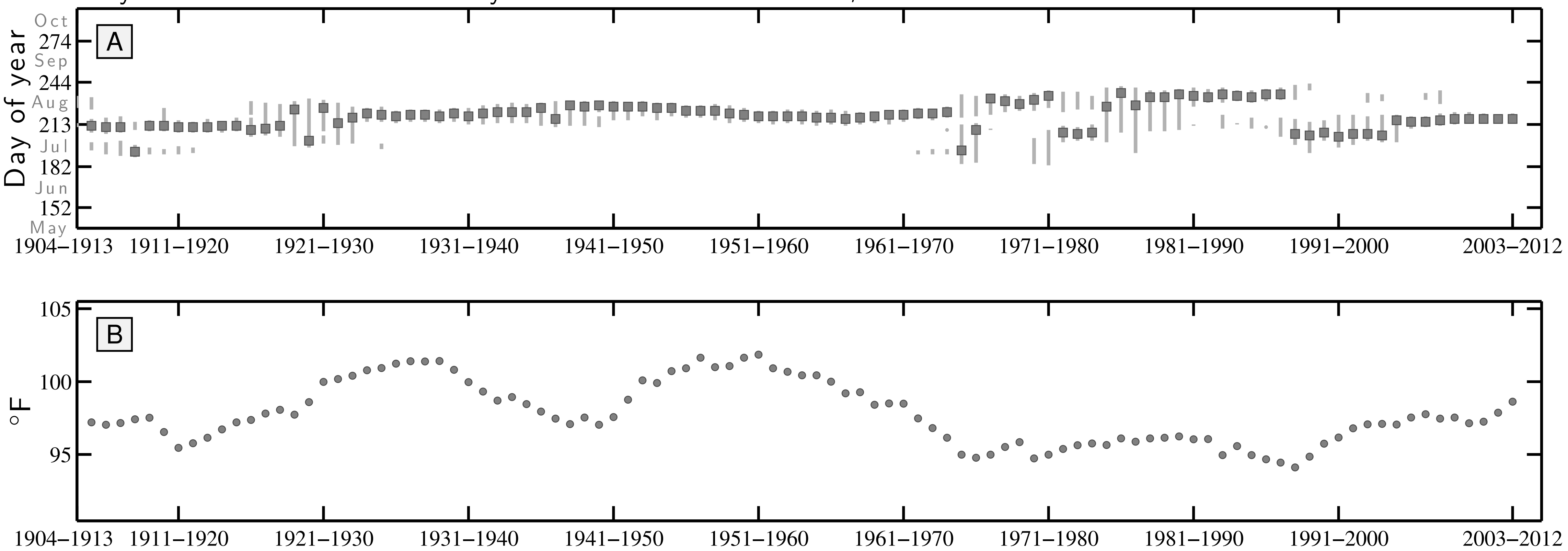

10 year Winter Teletherm dynamics for CORSICANA, TX:

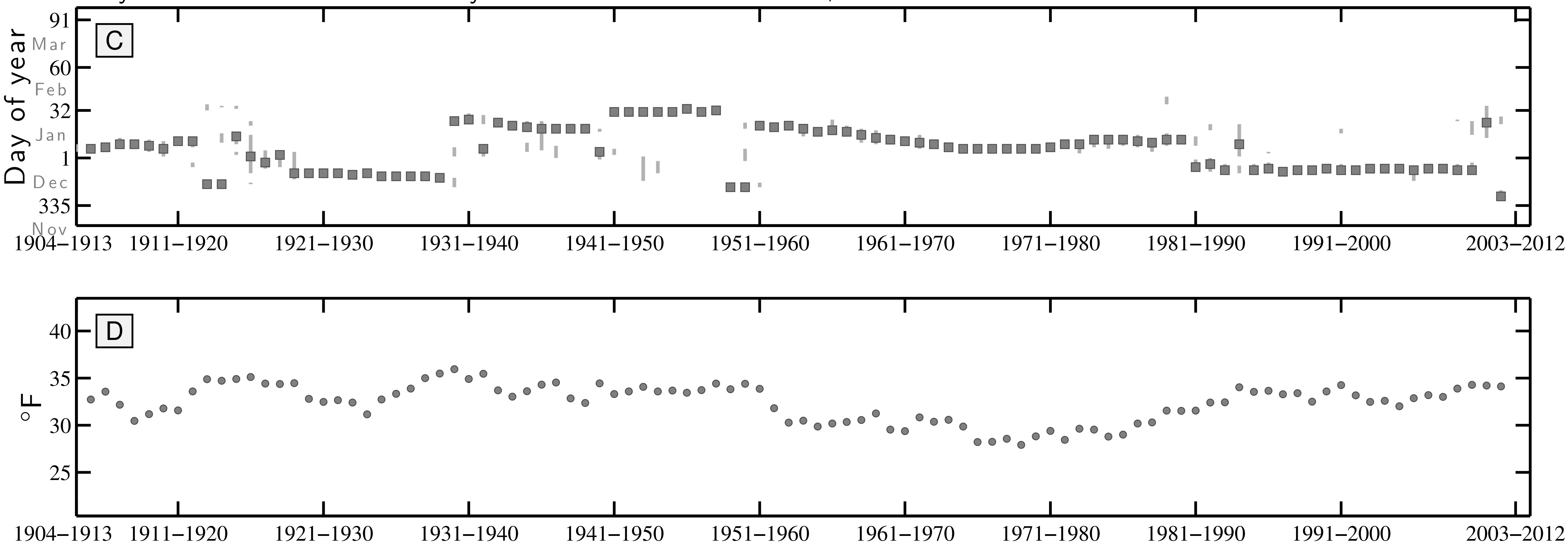

10 year Summer Teletherm dynamics for CROSBYTON, TX:

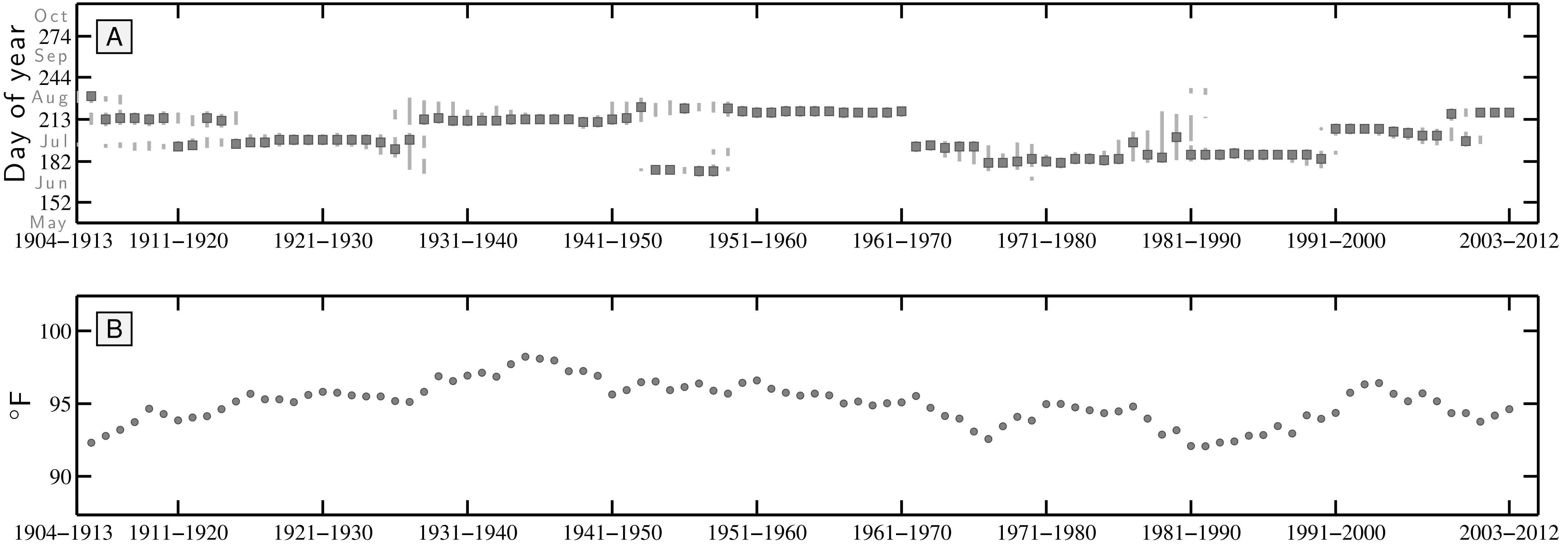

10 year Winter Teletherm dynamics for CROSBYTON, TX:

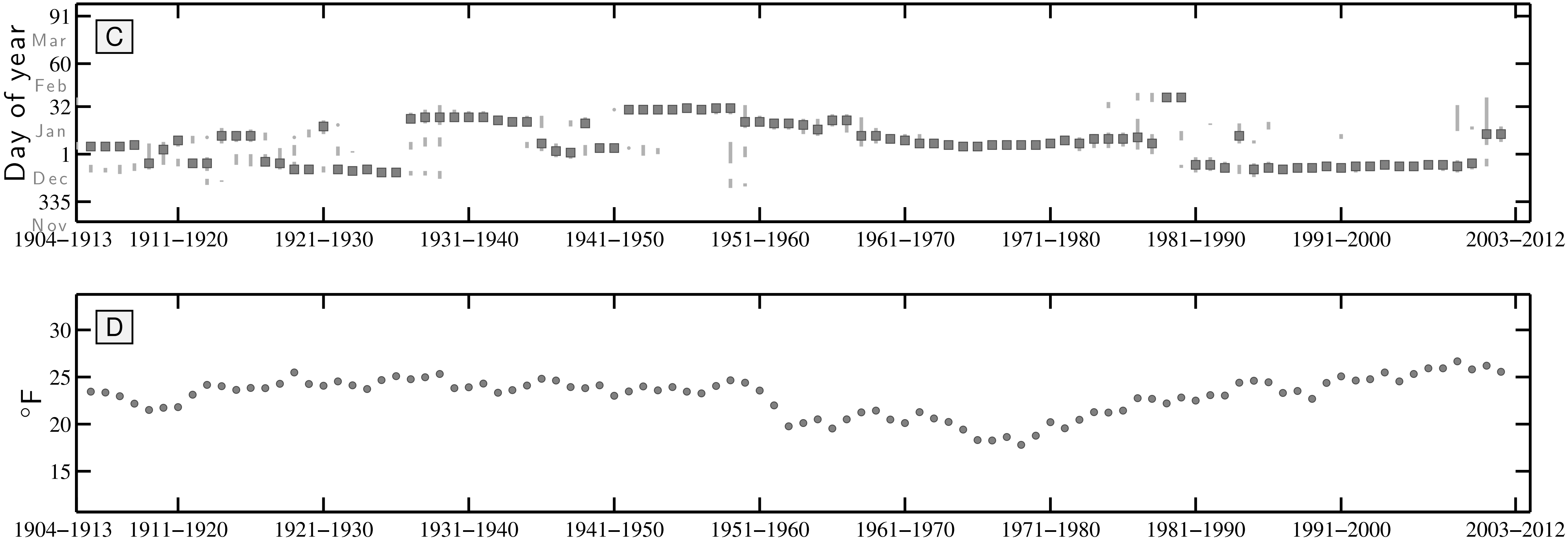

10 year Summer Teletherm dynamics for DANEVANG 1 W, TX:

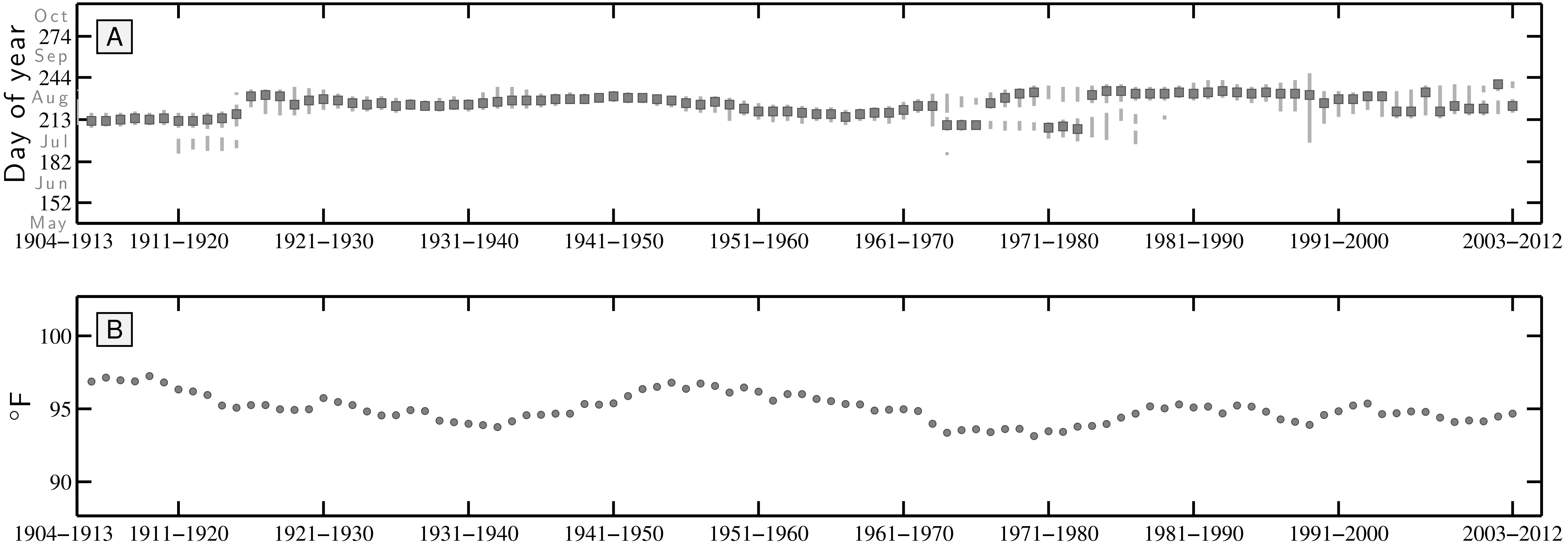

10 year Winter Teletherm dynamics for DANEVANG 1 W, TX:

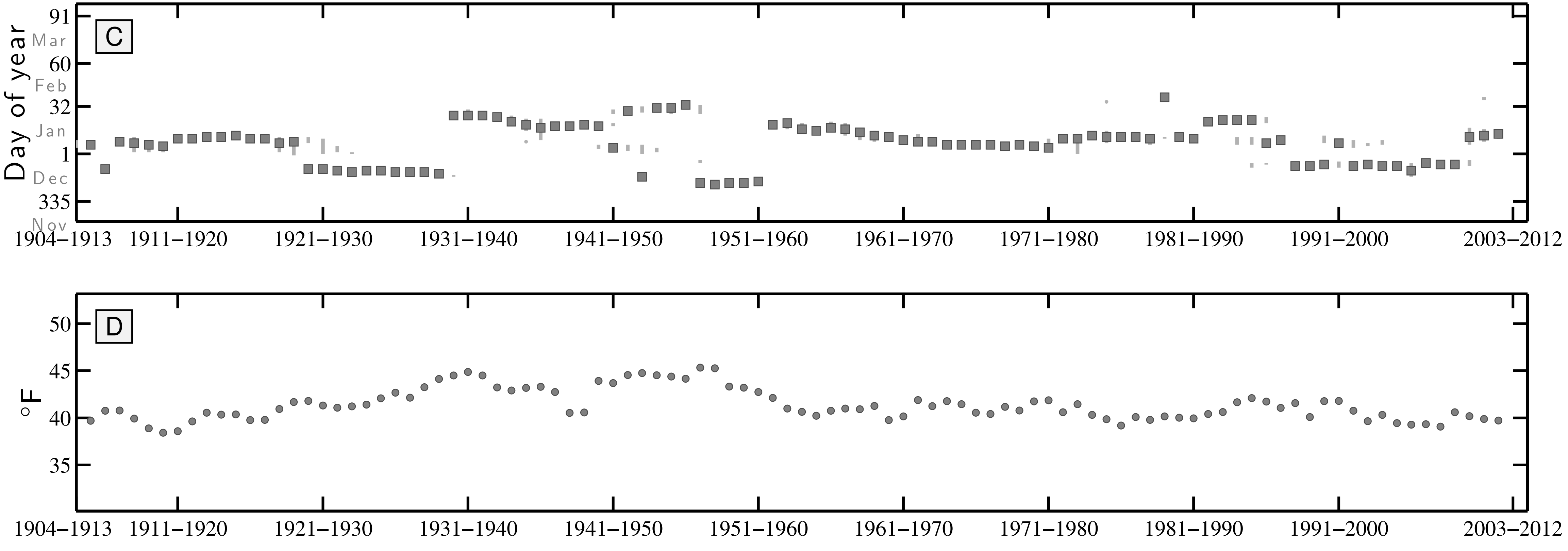

10 year Summer Teletherm dynamics for DUBLIN 2SE, TX:

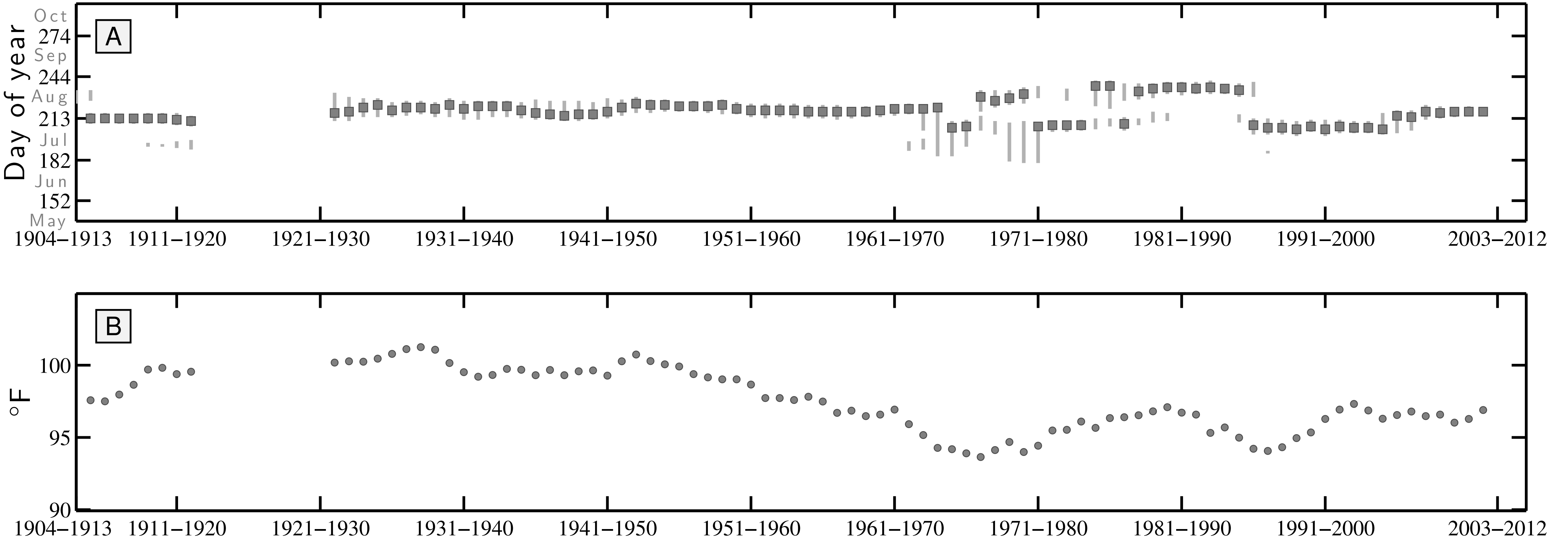

10 year Winter Teletherm dynamics for DUBLIN 2SE, TX:

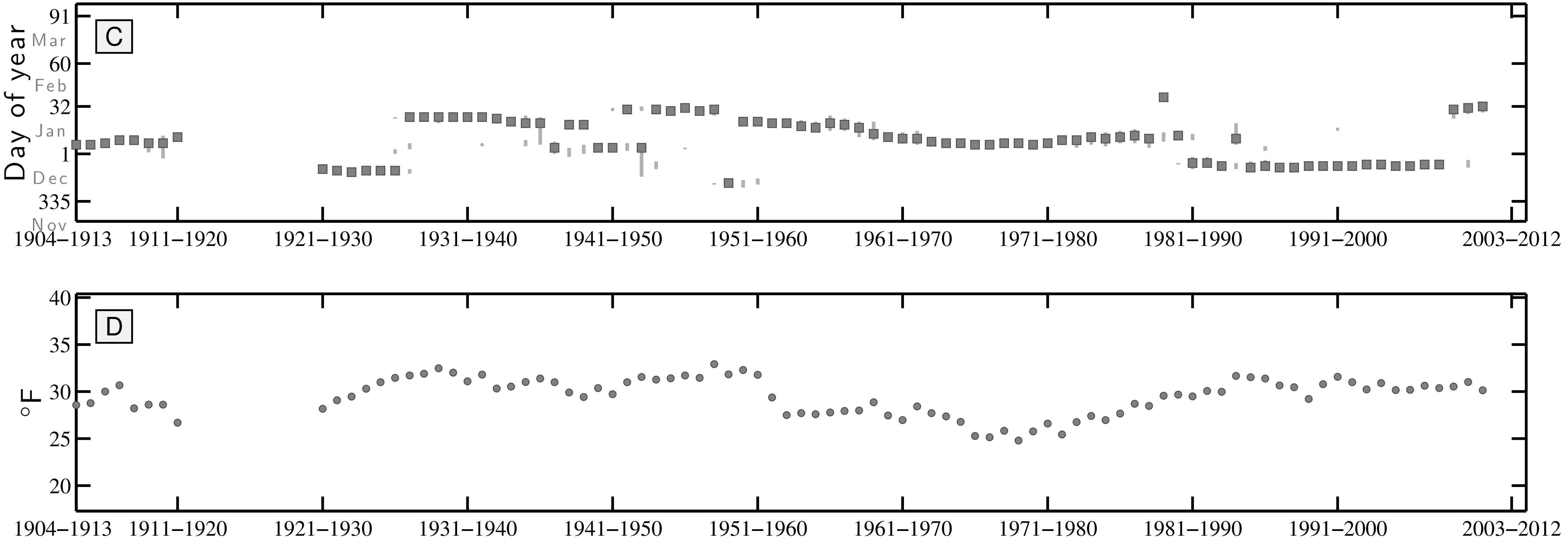

10 year Summer Teletherm dynamics for EAGLE PASS 3N, TX:

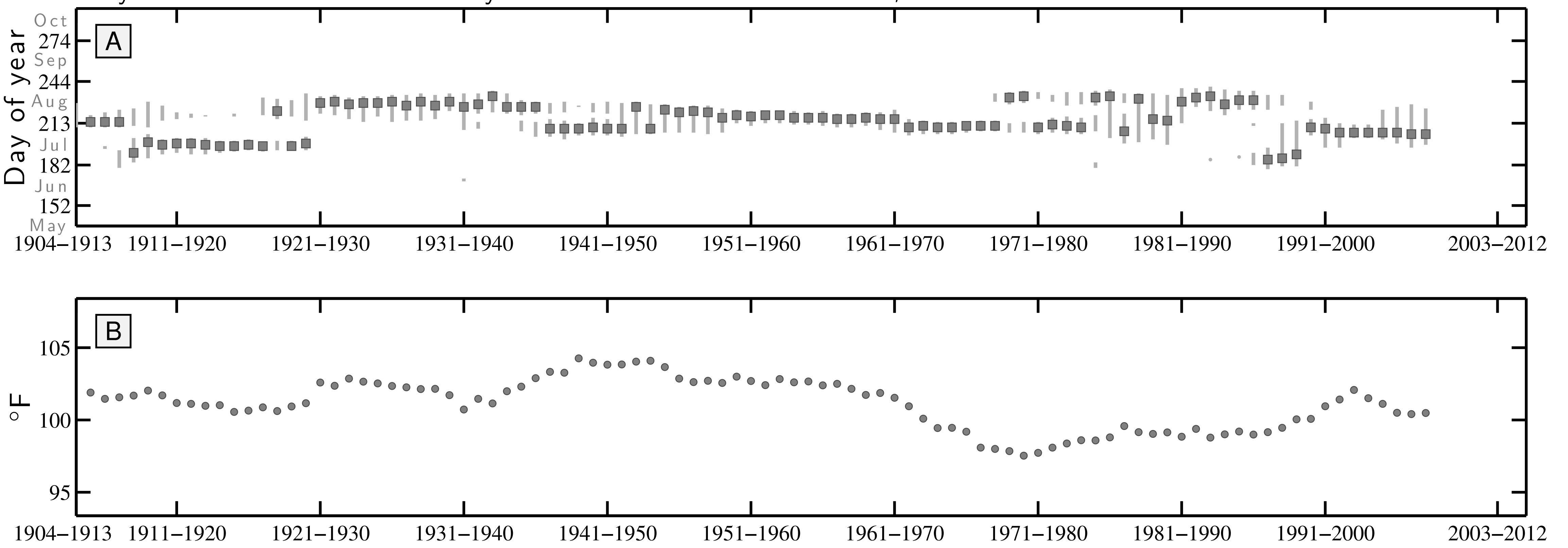

10 year Winter Teletherm dynamics for EAGLE PASS 3N, TX:

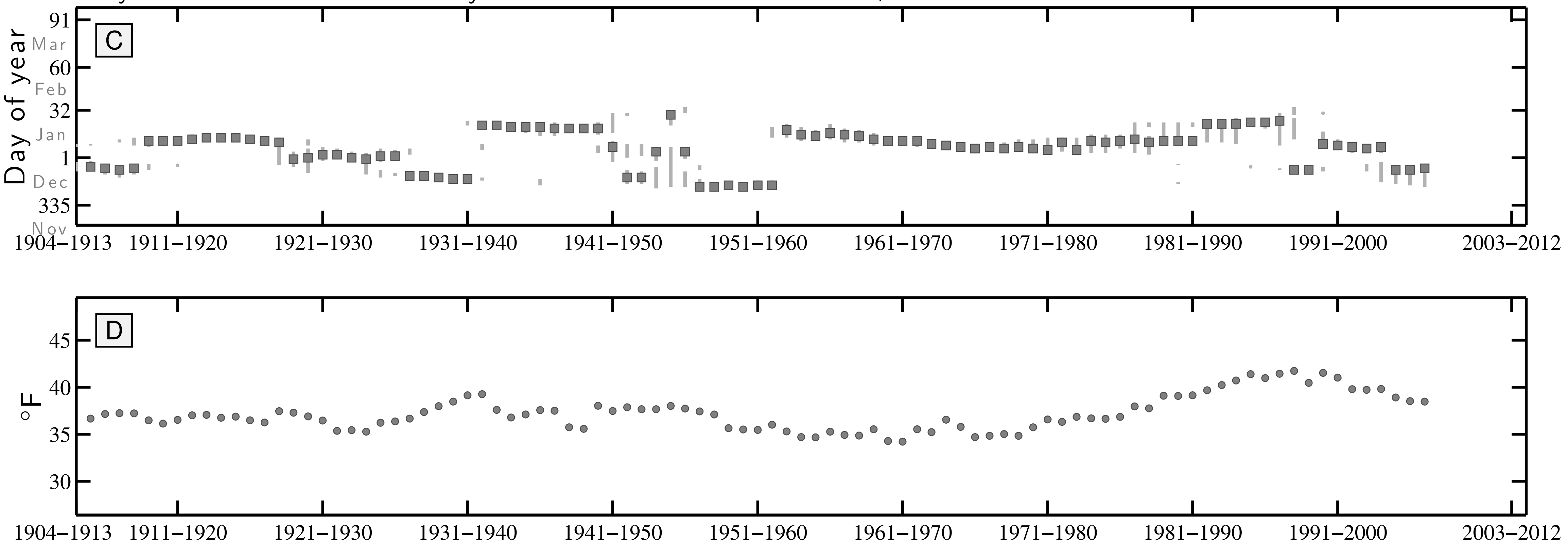

10 year Summer Teletherm dynamics for EL PASO AP, TX:

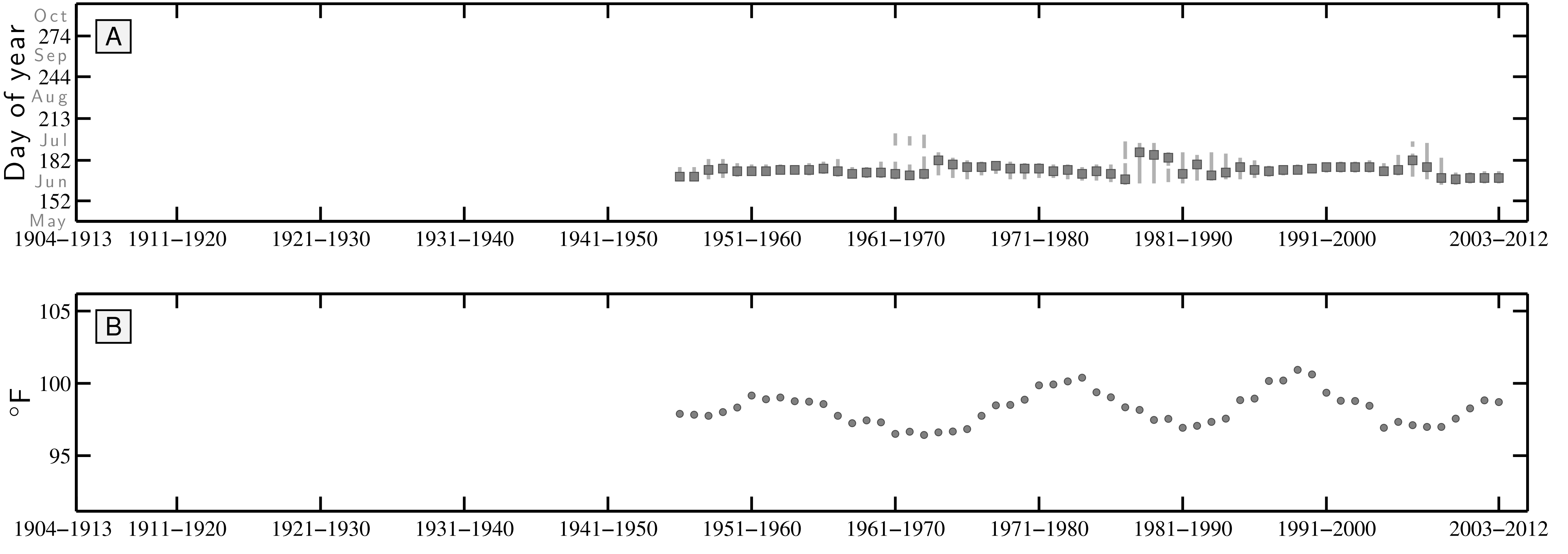

10 year Winter Teletherm dynamics for EL PASO AP, TX:

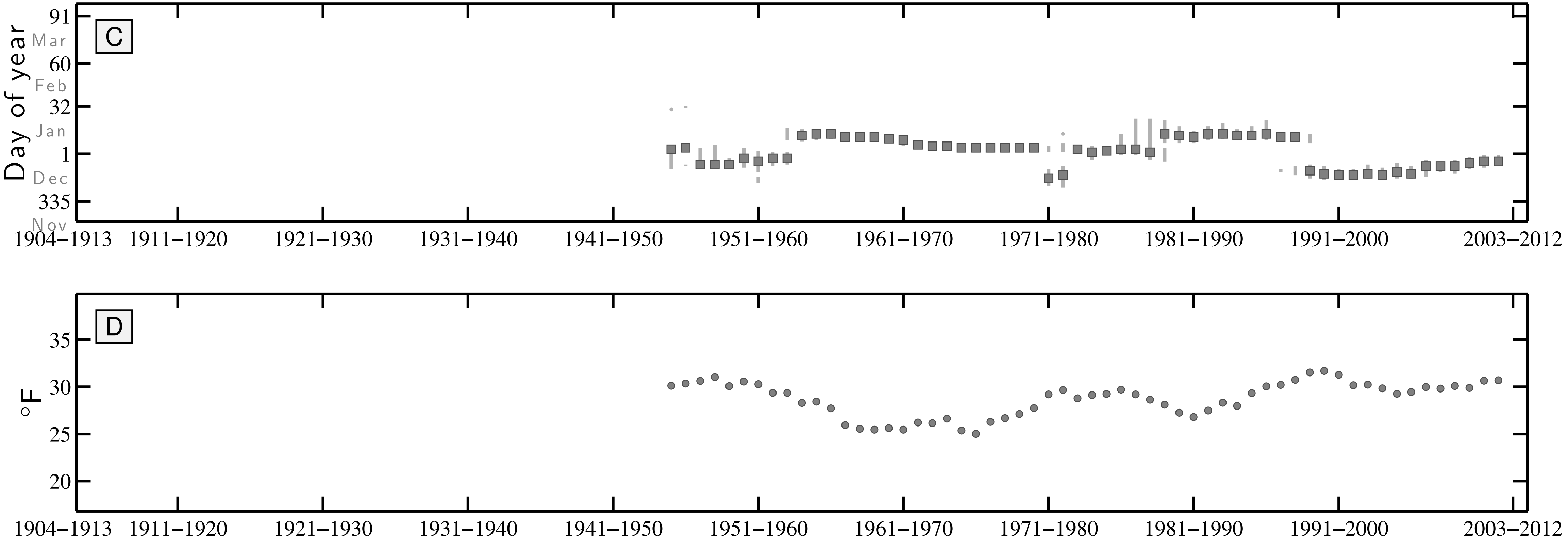

10 year Summer Teletherm dynamics for ENCINAL, TX:

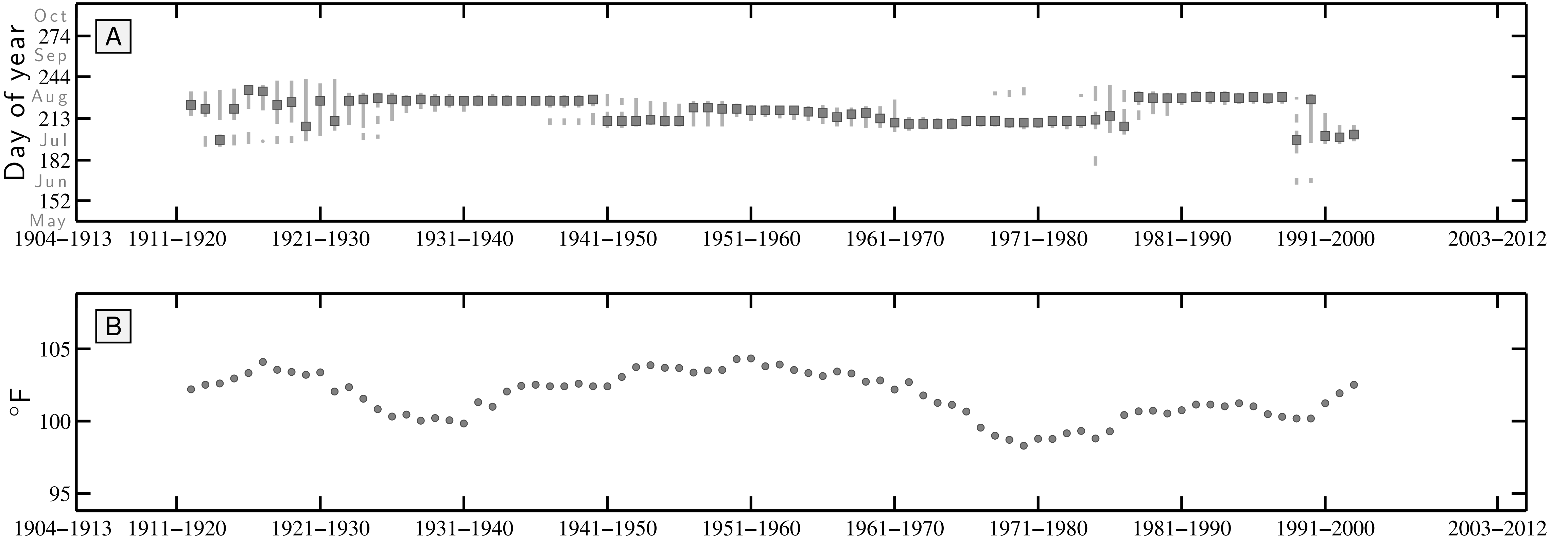

10 year Winter Teletherm dynamics for ENCINAL, TX:

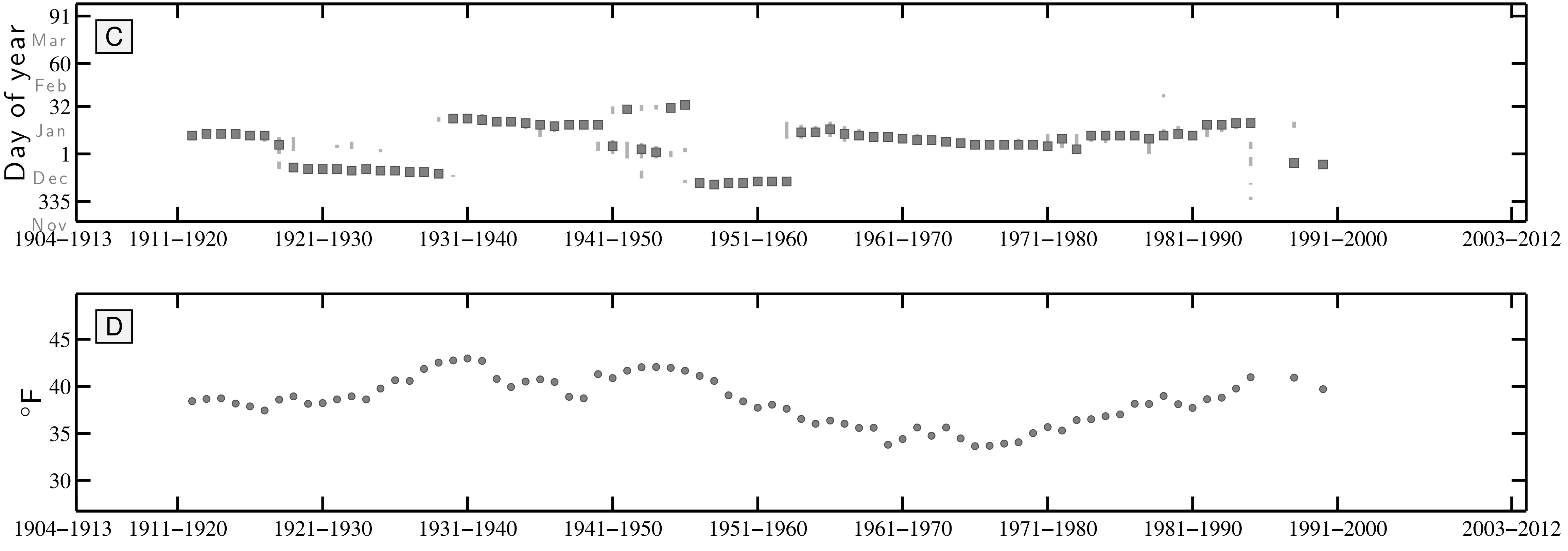

10 year Summer Teletherm dynamics for FALFURRIAS, TX:

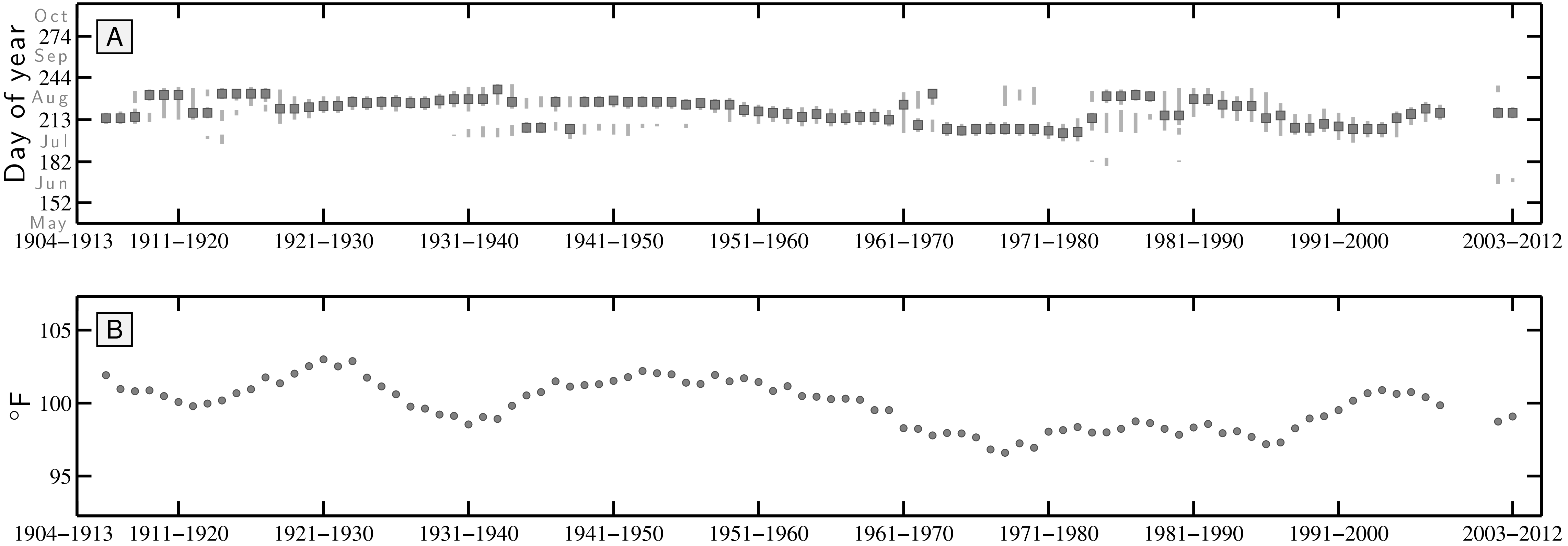

10 year Winter Teletherm dynamics for FALFURRIAS, TX:

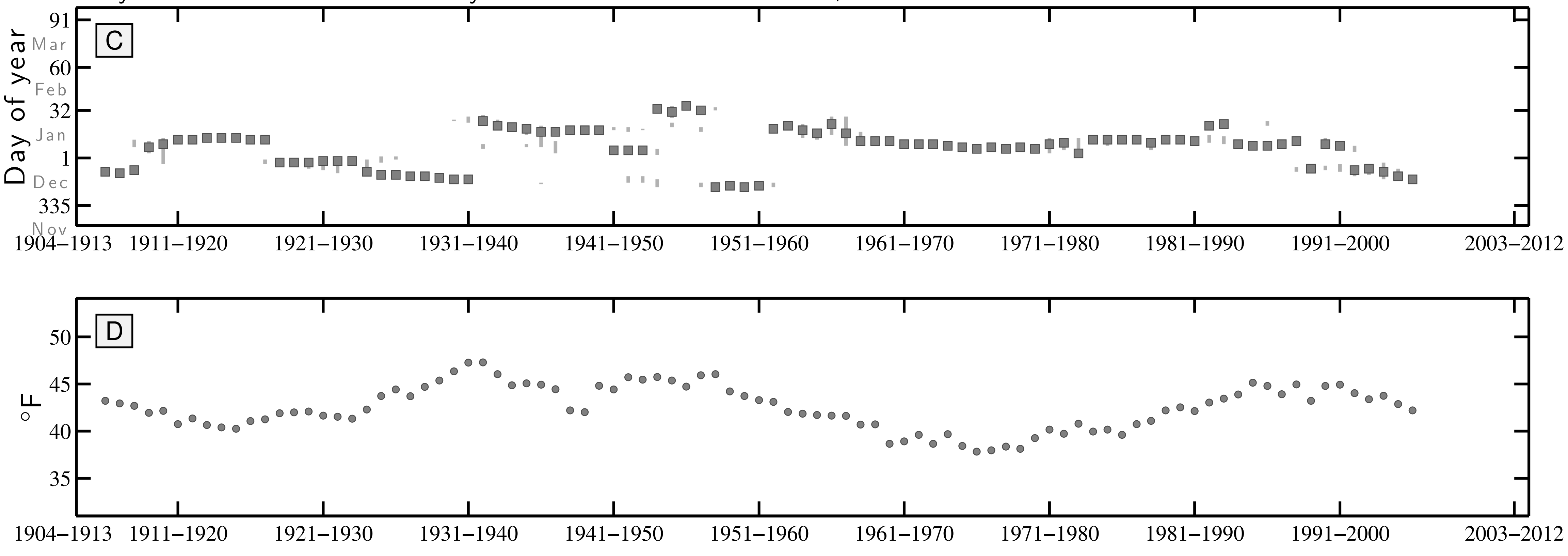

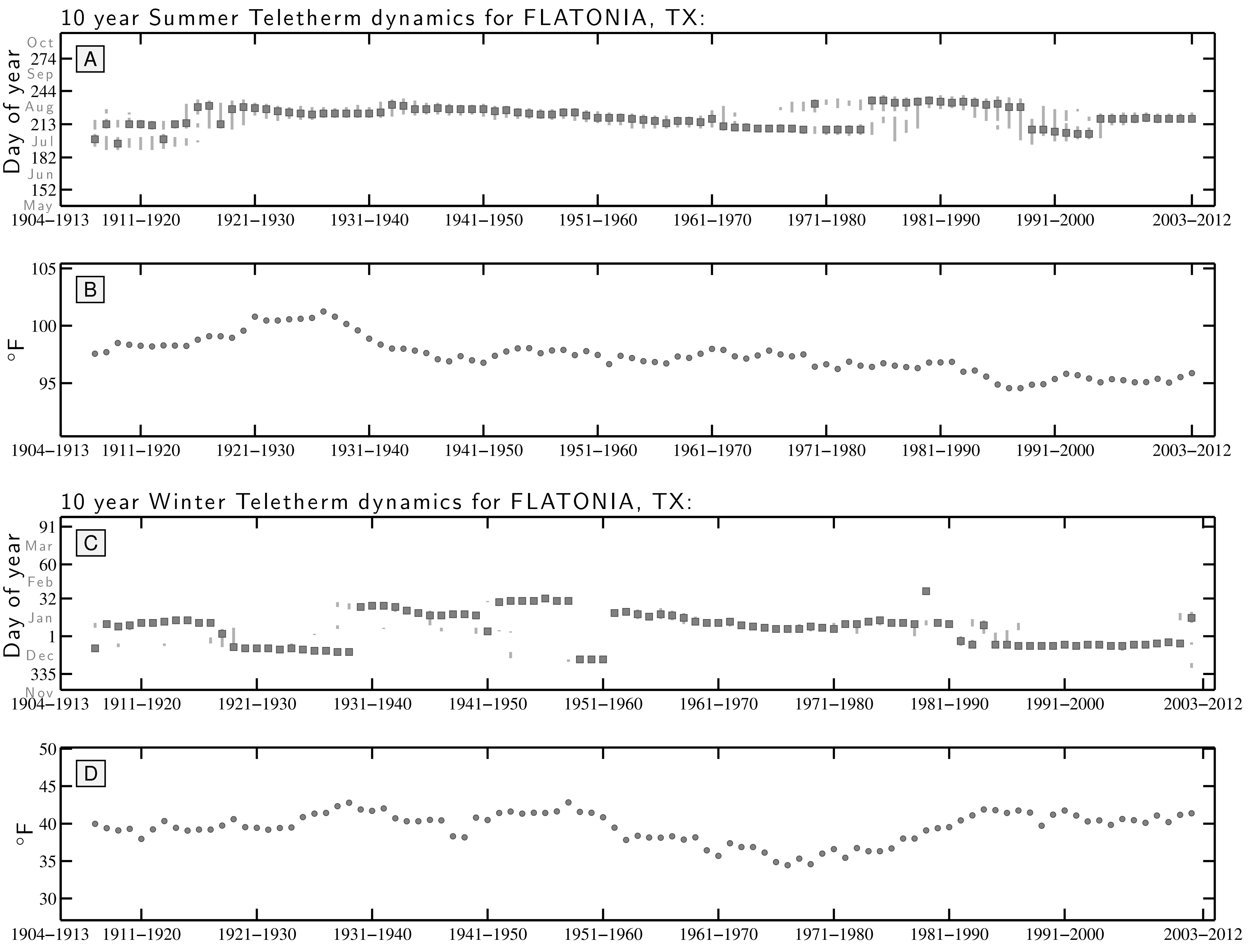
10 year Summer Teletherm dynamics for FLATONIA, TX:
A
Day of year
Oct
274
Sep
244
Aug
213
Jul
182
Jun
152
May
B
°F
105
100
95
1904–1913
1911–1920
1921–1930
1931–1940
1941–1950
1951–1960
1961–1970
1971–1980
1981–1990
1991–2000
2003–2012
10 year Winter Teletherm dynamics for FLATONIA, TX:
C
Day of year
91
Mar
60
Feb
32
Jan
1
Dec
335
Nov
D
°F
50
45
40
35
30

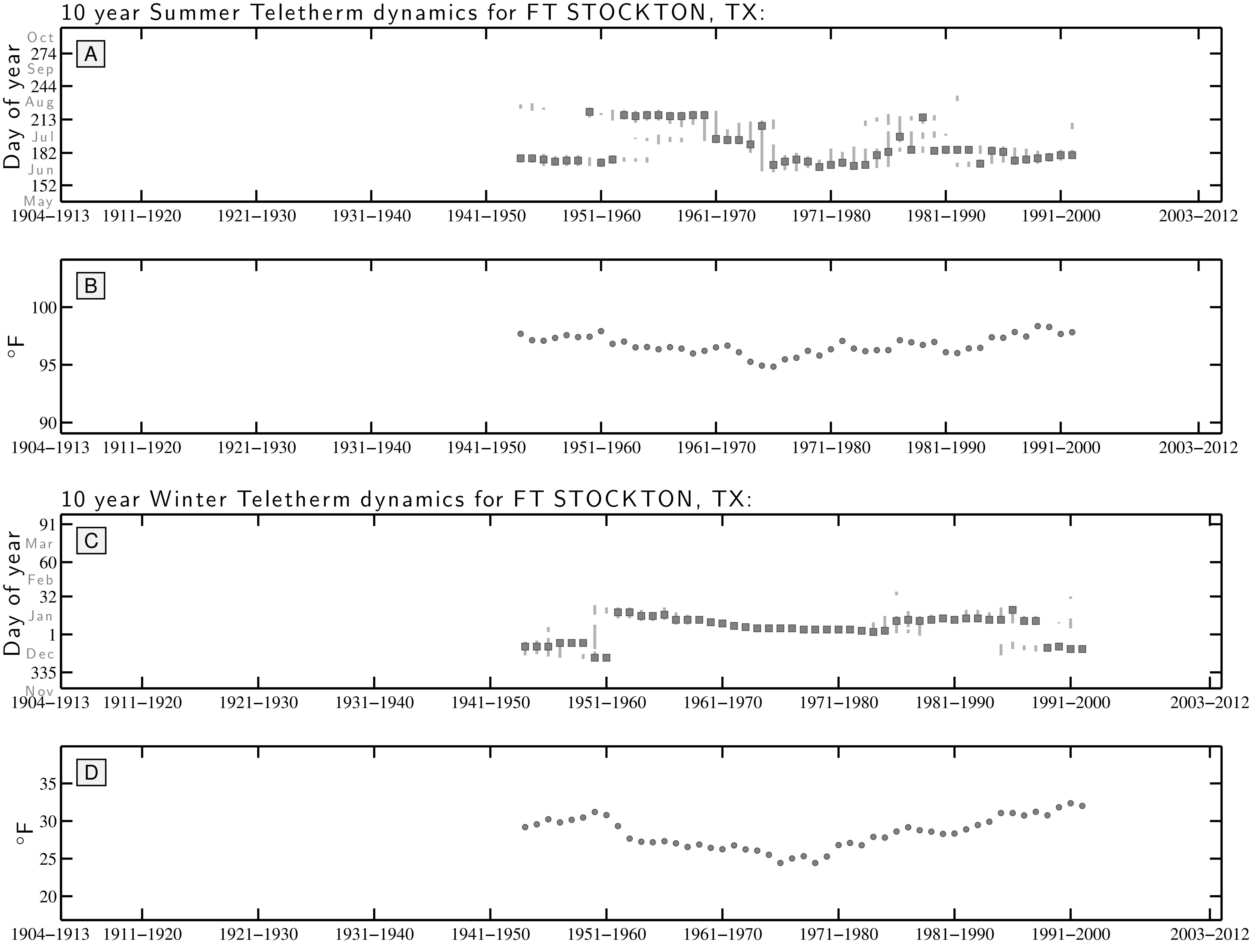
10 year Summer Teletherm dynamics for FT STOCKTON, TX:
A
Day of year
Oct
274
Sep
244
Aug
213
Jul
182
Jun
152
May
1904–1913
1911–1920
1921–1930
1931–1940
1941–1950
1951–1960
1961–1970
1971–1980
1981–1990
1991–2000
2003–2012
B
°F
100
95
90
10 year Winter Teletherm dynamics for FT STOCKTON, TX:
C
91
Mar
60
Feb
32
Jan
1
Dec
335
Nov
D
35
30
25
20

10 year Summer Teletherm dynamics for GAINESVILLE 5 ENE, TX:

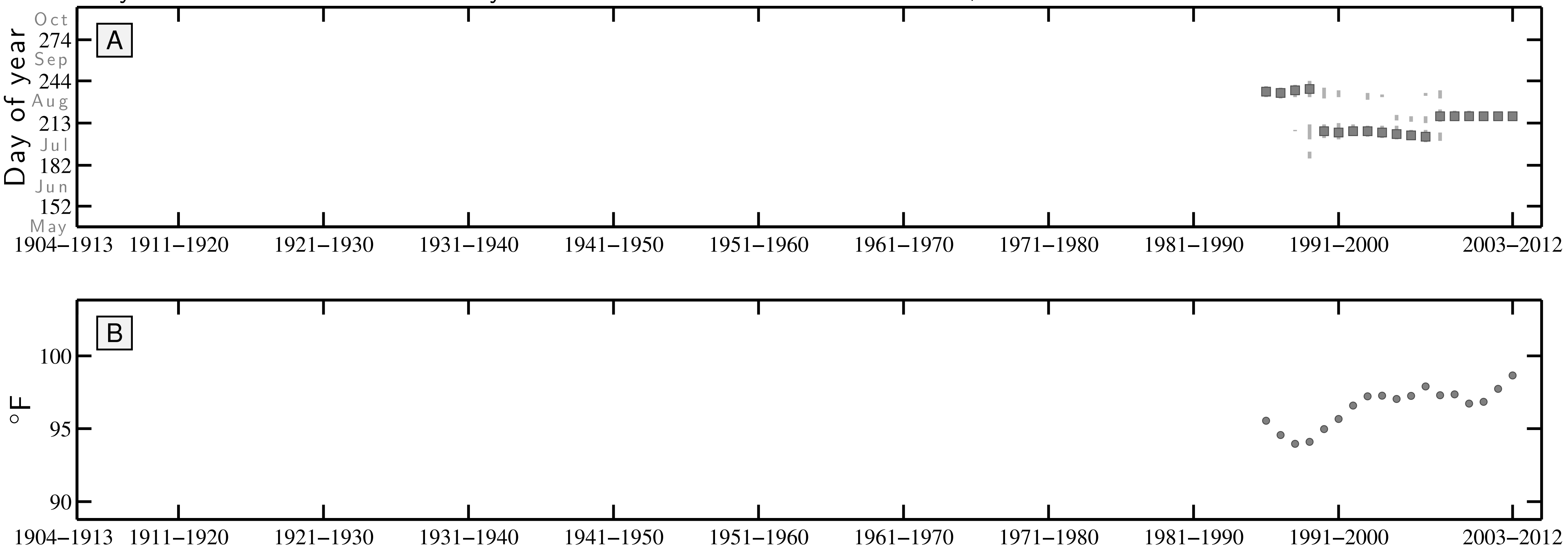

10 year Winter Teletherm dynamics for GAINESVILLE 5 ENE, TX:

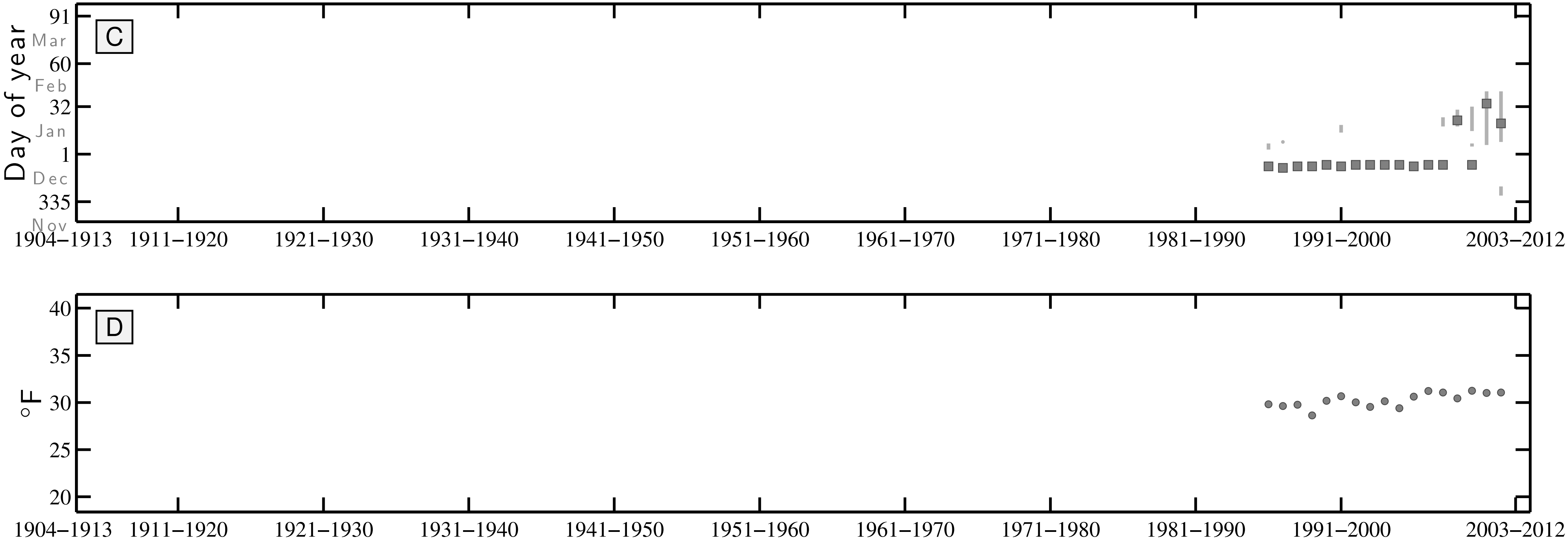

10 year Summer Teletherm dynamics for GREENVILLE KGVL RADIO, TX:

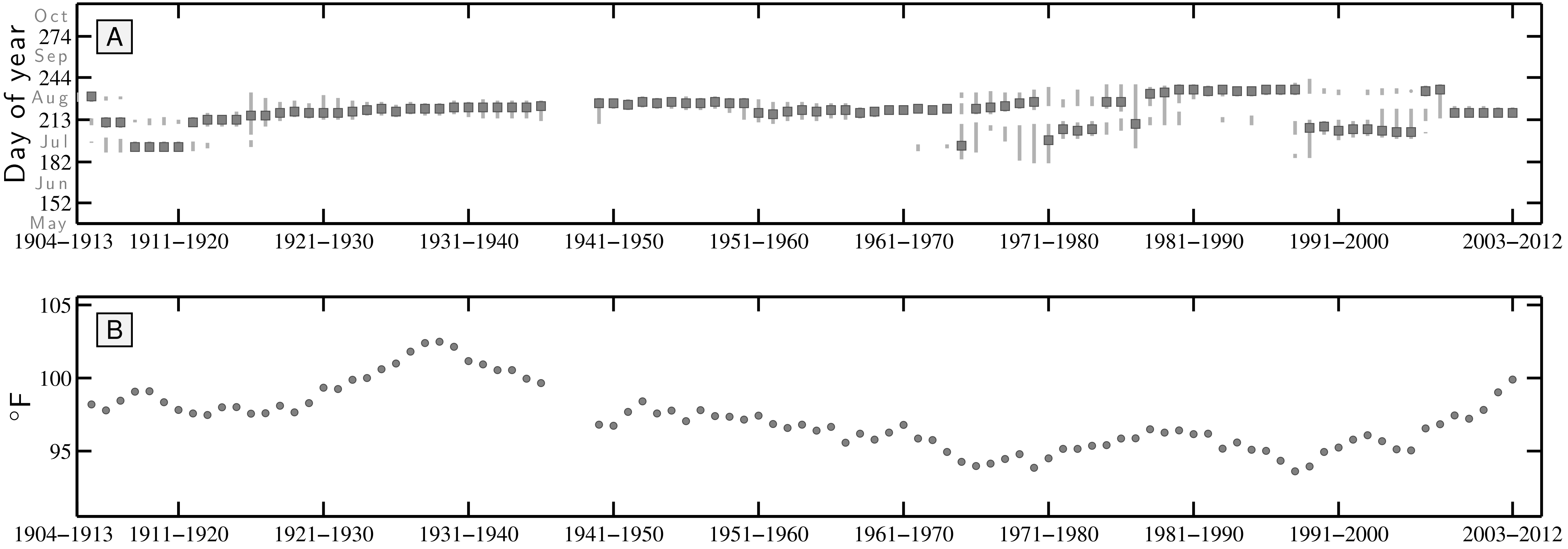

10 year Winter Teletherm dynamics for GREENVILLE KGVL RADIO, TX:

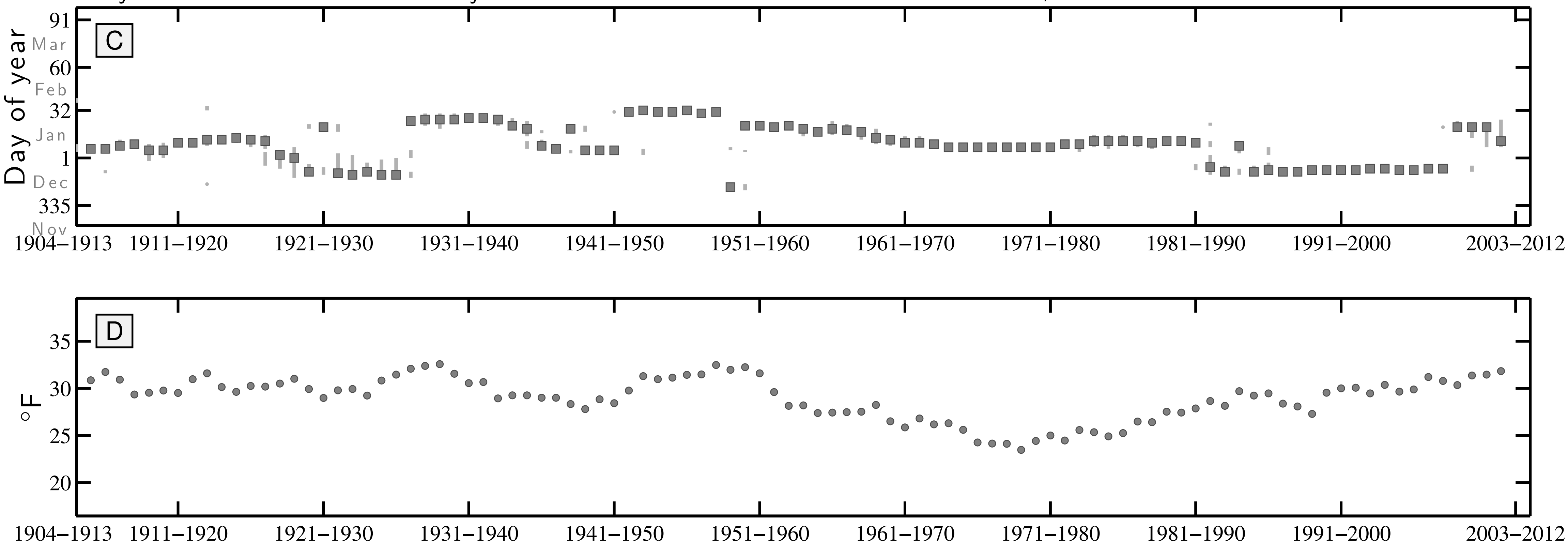

10 year Summer Teletherm dynamics for HALLETTSVILLE 2 N, TX:

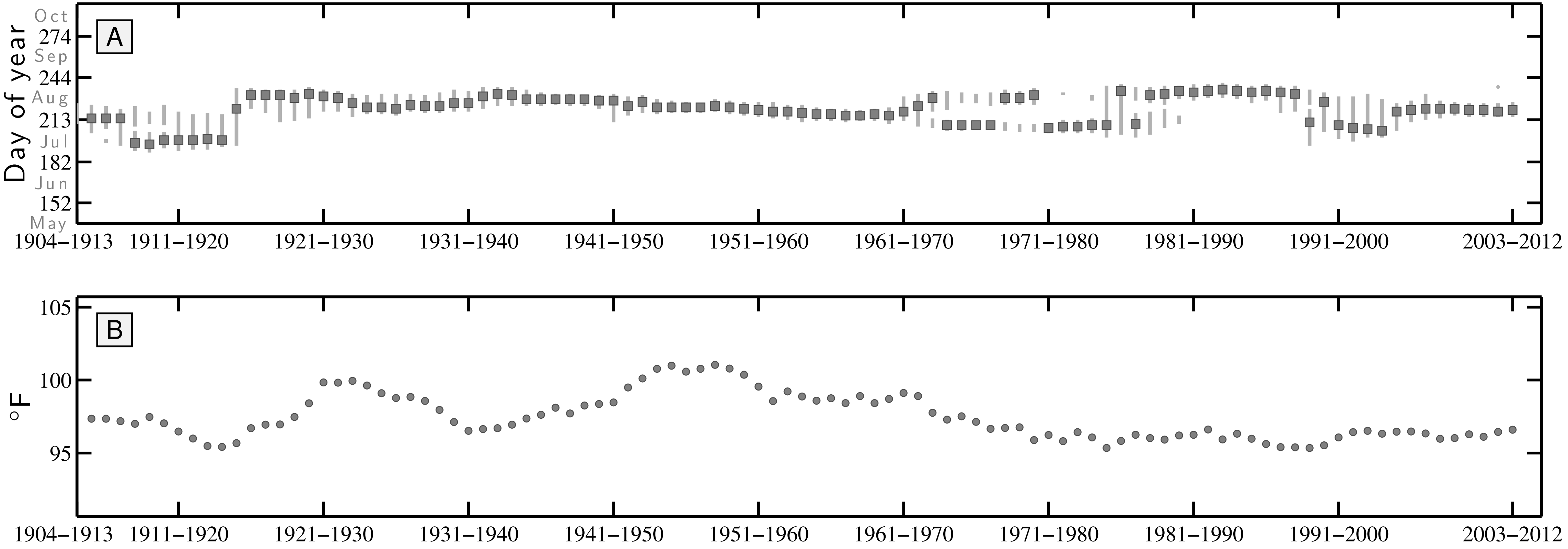

10 year Winter Teletherm dynamics for HALLETTSVILLE 2 N, TX:

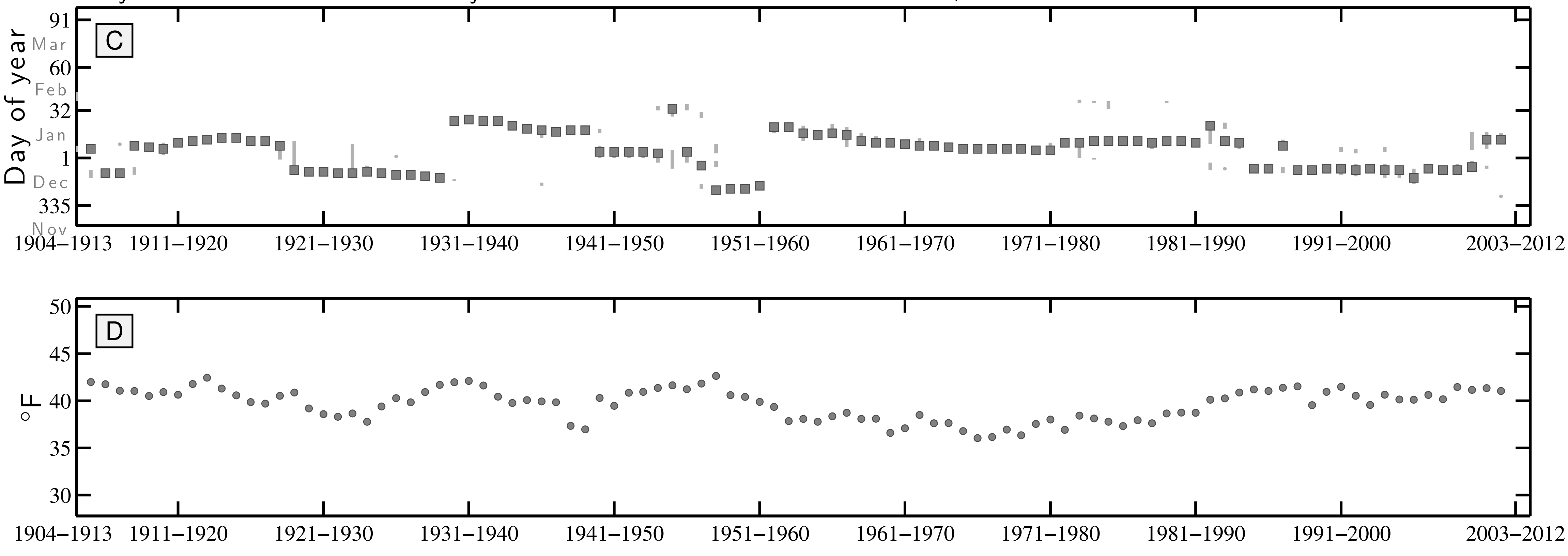

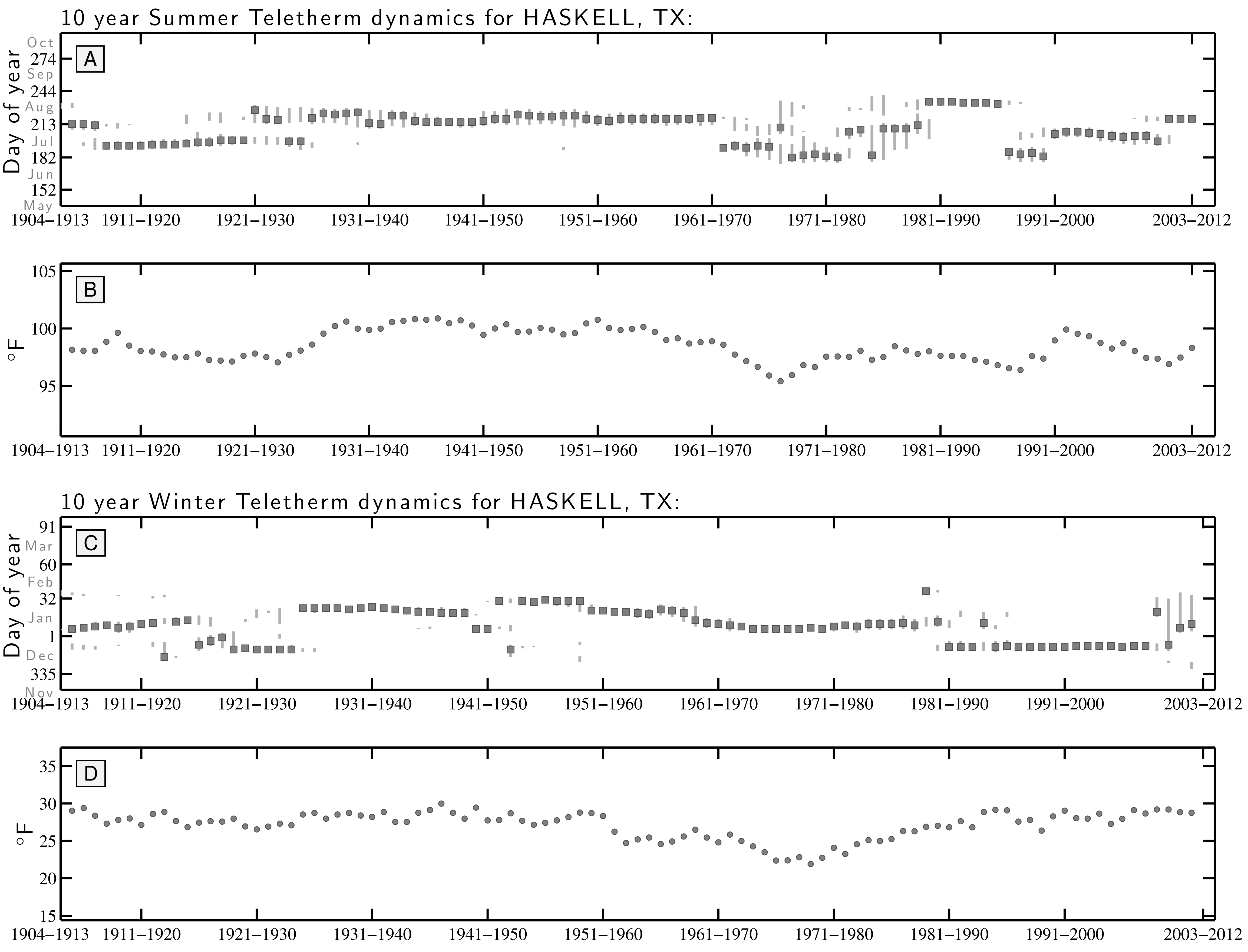
10 year Summer Teletherm dynamics for HASKELL, TX:
A
Day of year
Oct
274
Sep
244
Aug
213
Jul
182
Jun
152
May
1904–1913
1911–1920
1921–1930
1931–1940
1941–1950
1951–1960
1961–1970
1971–1980
1981–1990
1991–2000
2003–2012
B
°F
105
100
95
10 year Winter Teletherm dynamics for HASKELL, TX:
C
Day of year
91
Mar
60
Feb
32
Jan
1
Dec
335
Nov
D
°F
35
30
25
20
15

10 year Summer Teletherm dynamics for LAMPASAS, TX:

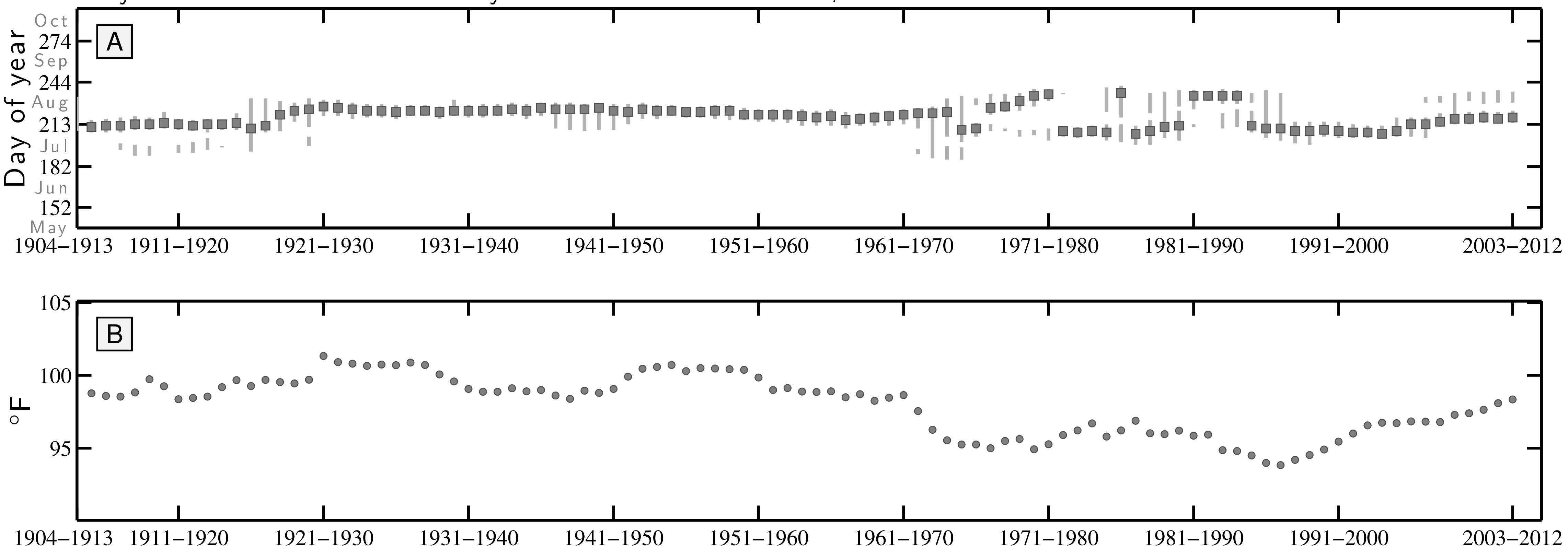

10 year Winter Teletherm dynamics for LAMPASAS, TX:

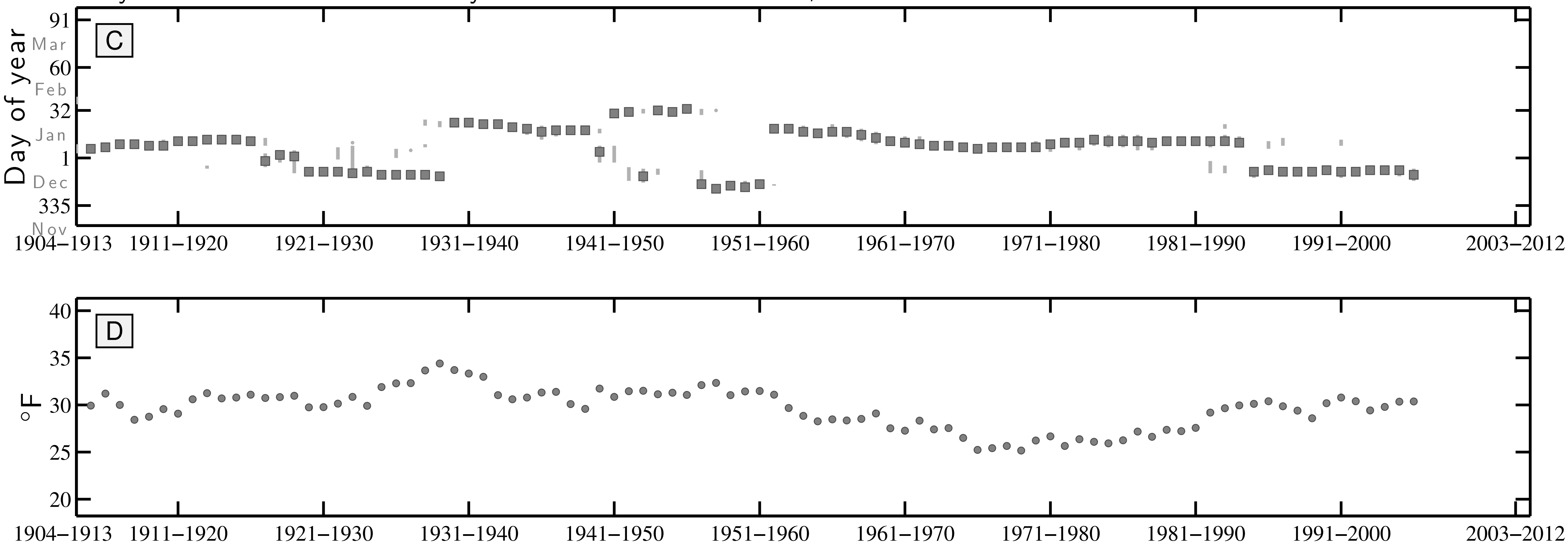

10 year Summer Teletherm dynamics for LIBERTY, TX:

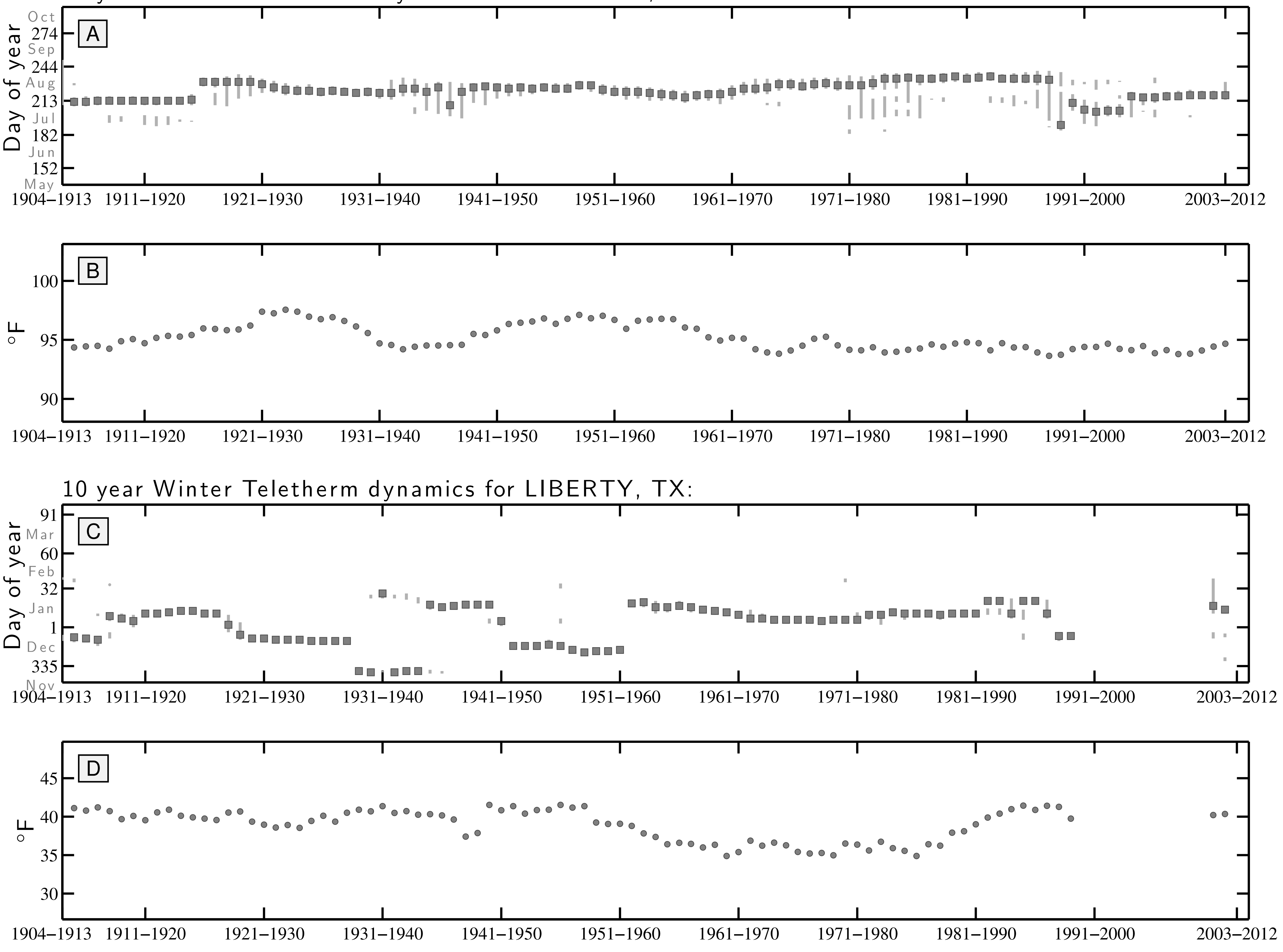

10 year Winter Teletherm dynamics for LIBERTY, TX:

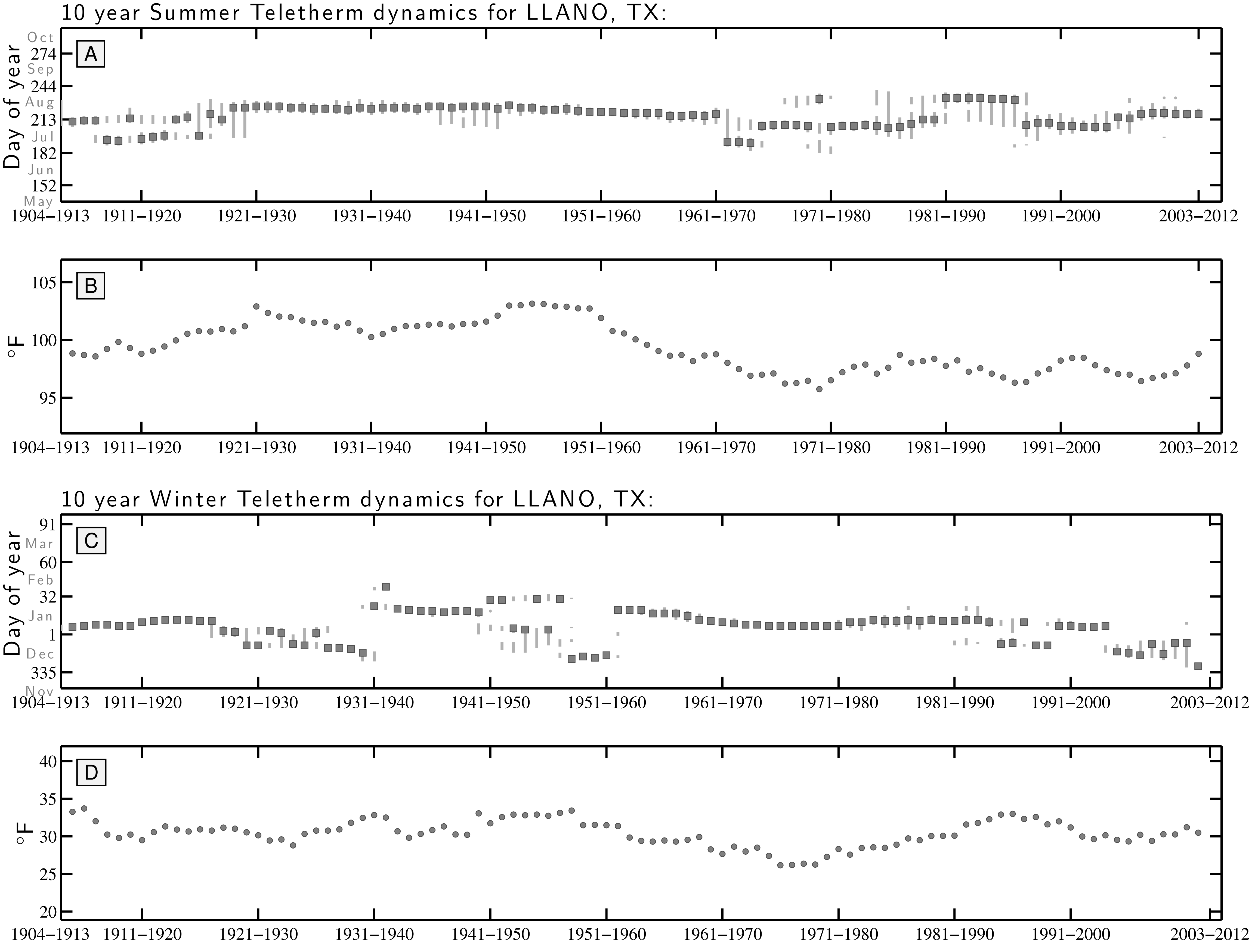
10 year Summer Teletherm dynamics for LLANO, TX:
A
Day of year
Oct
274
Sep
244
Aug
213
Jul
182
Jun
152
May
1904–1913
1911–1920
1921–1930
1931–1940
1941–1950
1951–1960
1961–1970
1971–1980
1981–1990
1991–2000
2003–2012
B
°F
105
100
95
10 year Winter Teletherm dynamics for LLANO, TX:
C
Day of year
91
Mar
60
Feb
32
Jan
1
Dec
335
Nov
D
°F
40
35
30
25
20

10 year Summer Teletherm dynamics for LULING, TX:

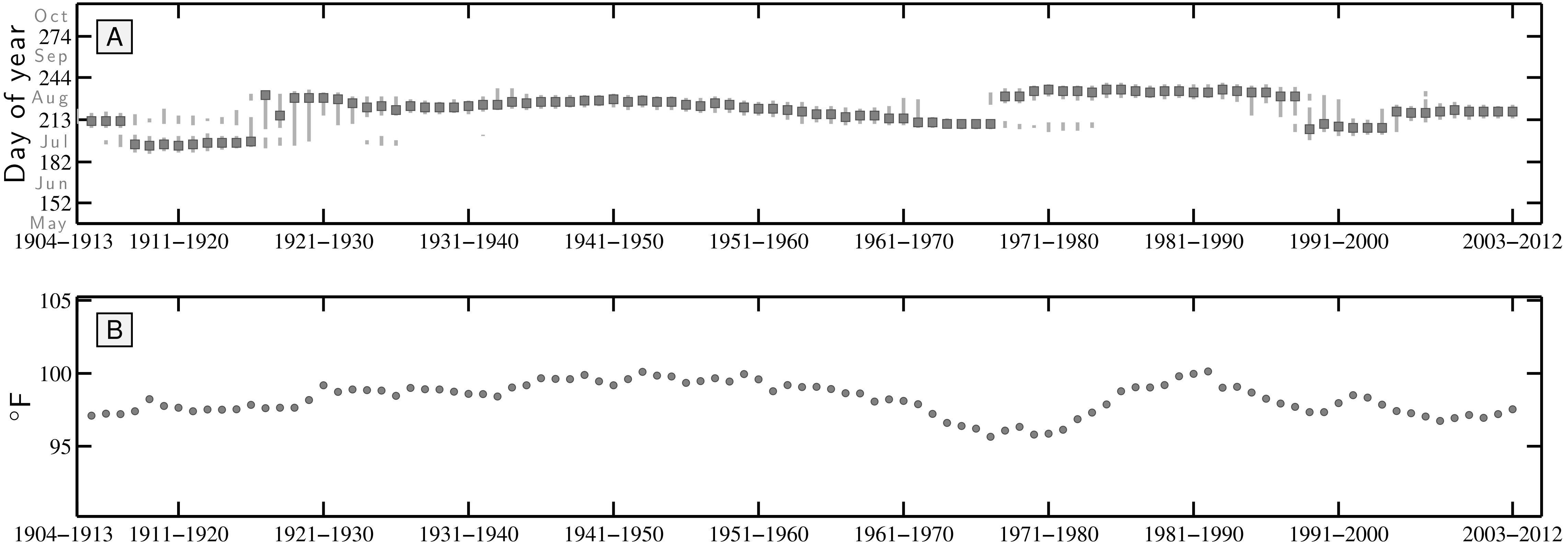

10 year Winter Teletherm dynamics for LULING, TX:

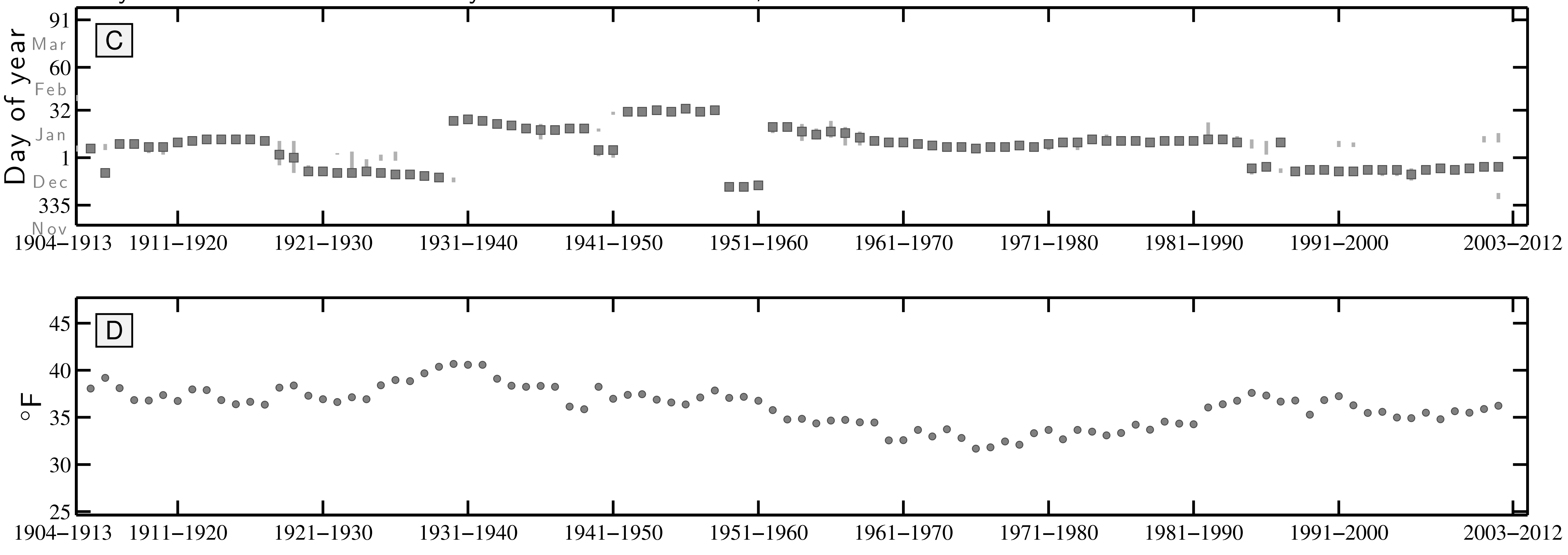

10 year Summer Teletherm dynamics for MARSHALL, TX:

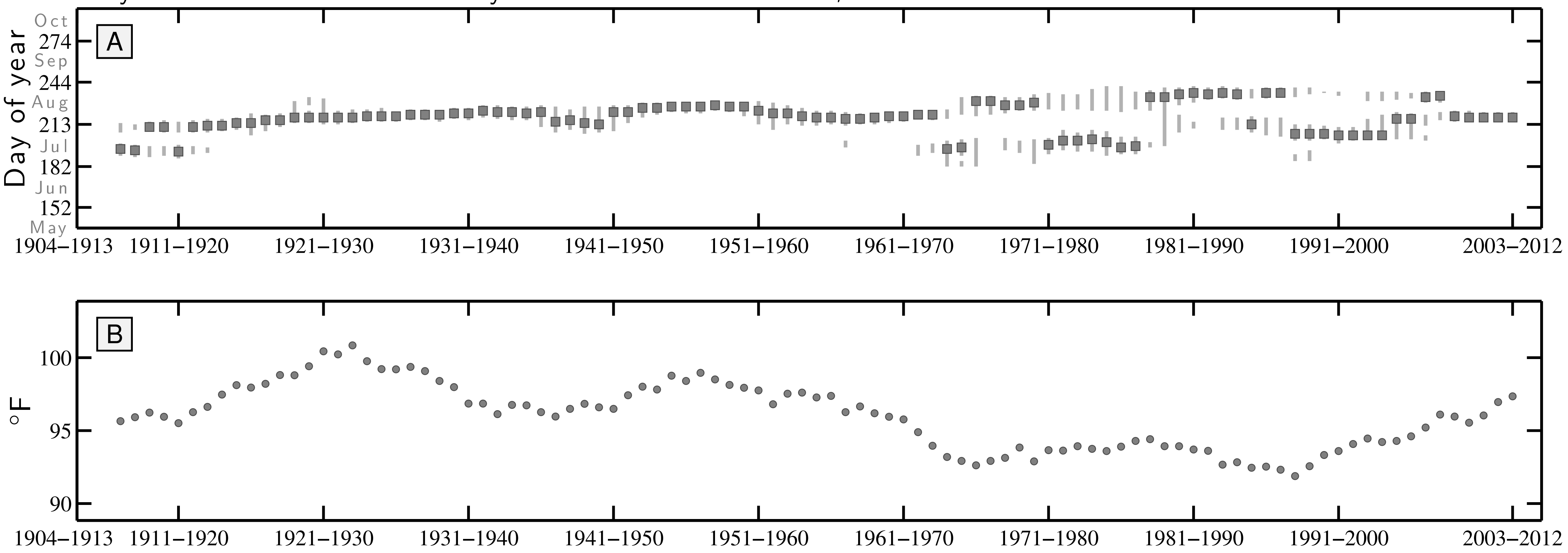

10 year Winter Teletherm dynamics for MARSHALL, TX:

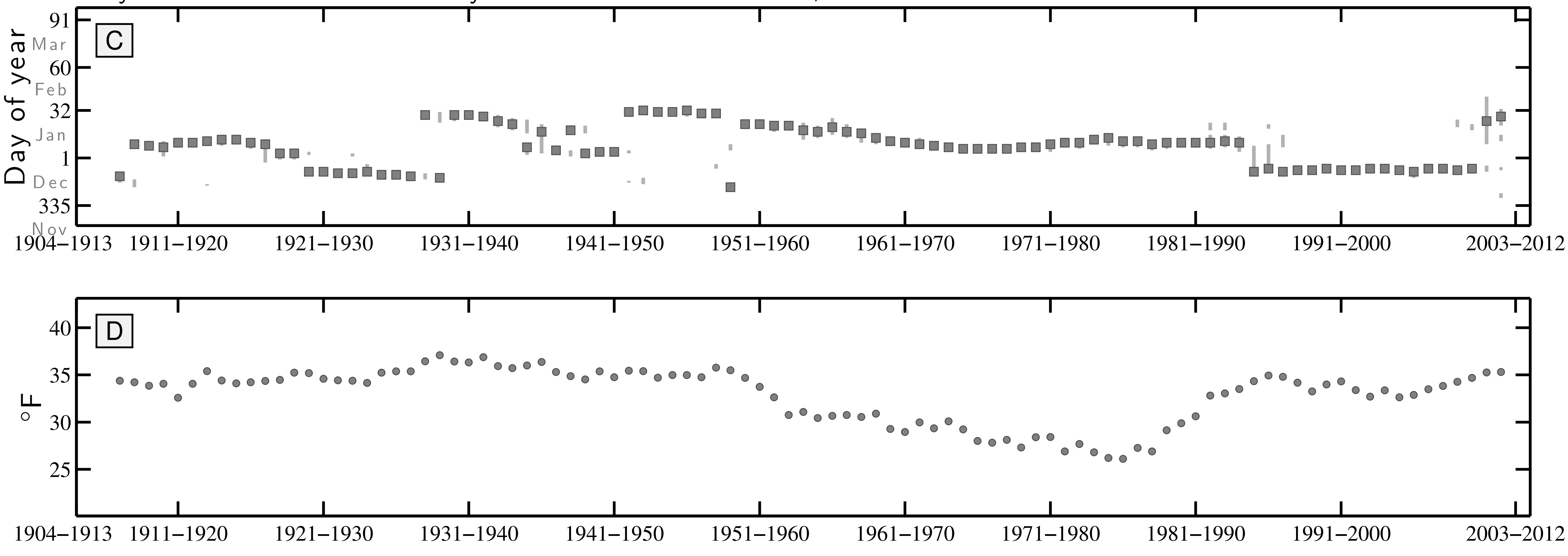

10 year Summer Teletherm dynamics for MCCAMEY, TX:

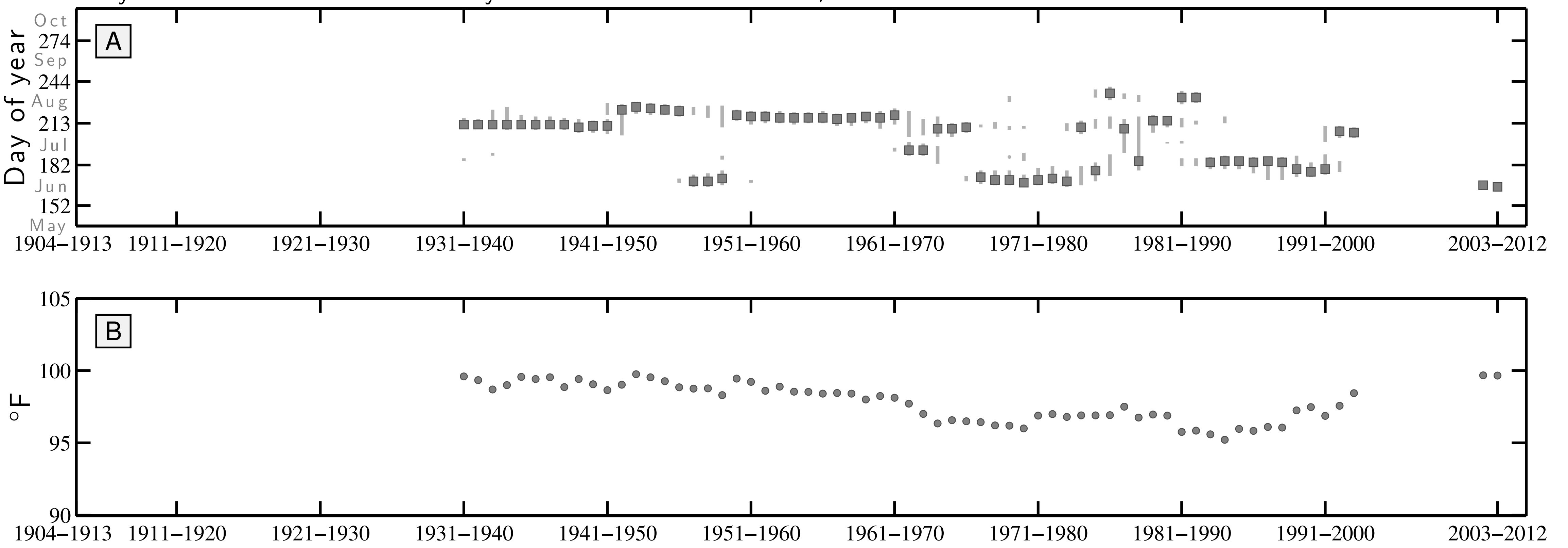

10 year Winter Teletherm dynamics for MCCAMEY, TX:

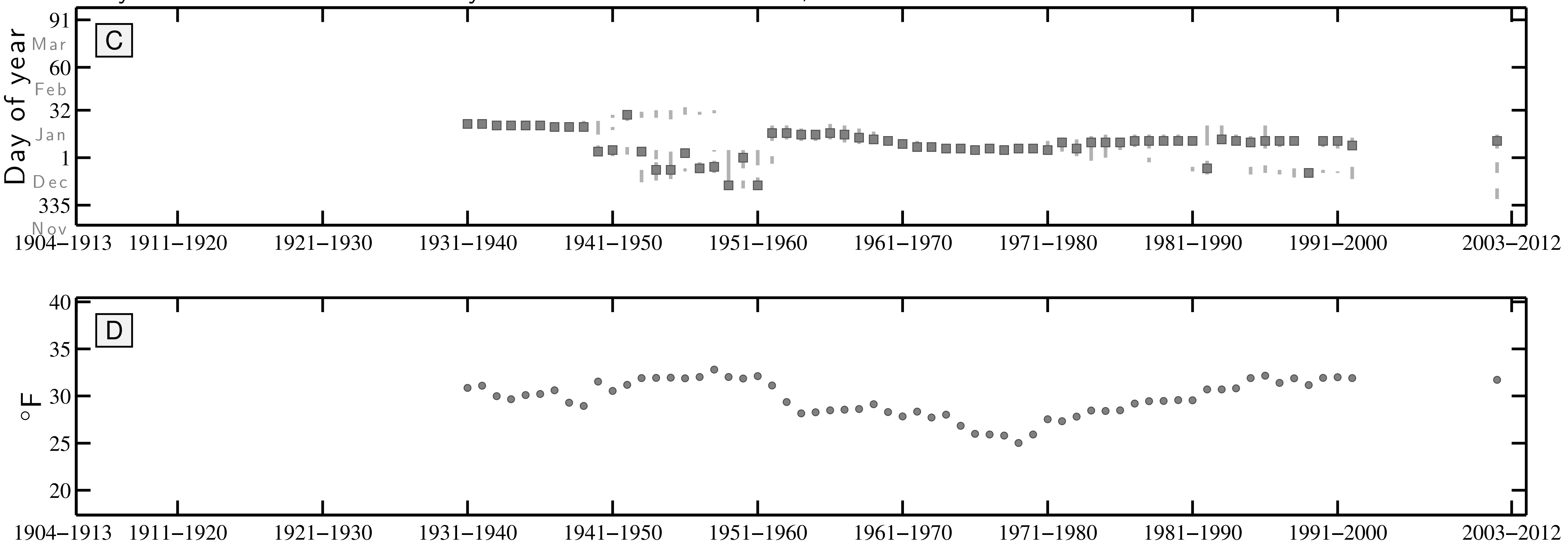

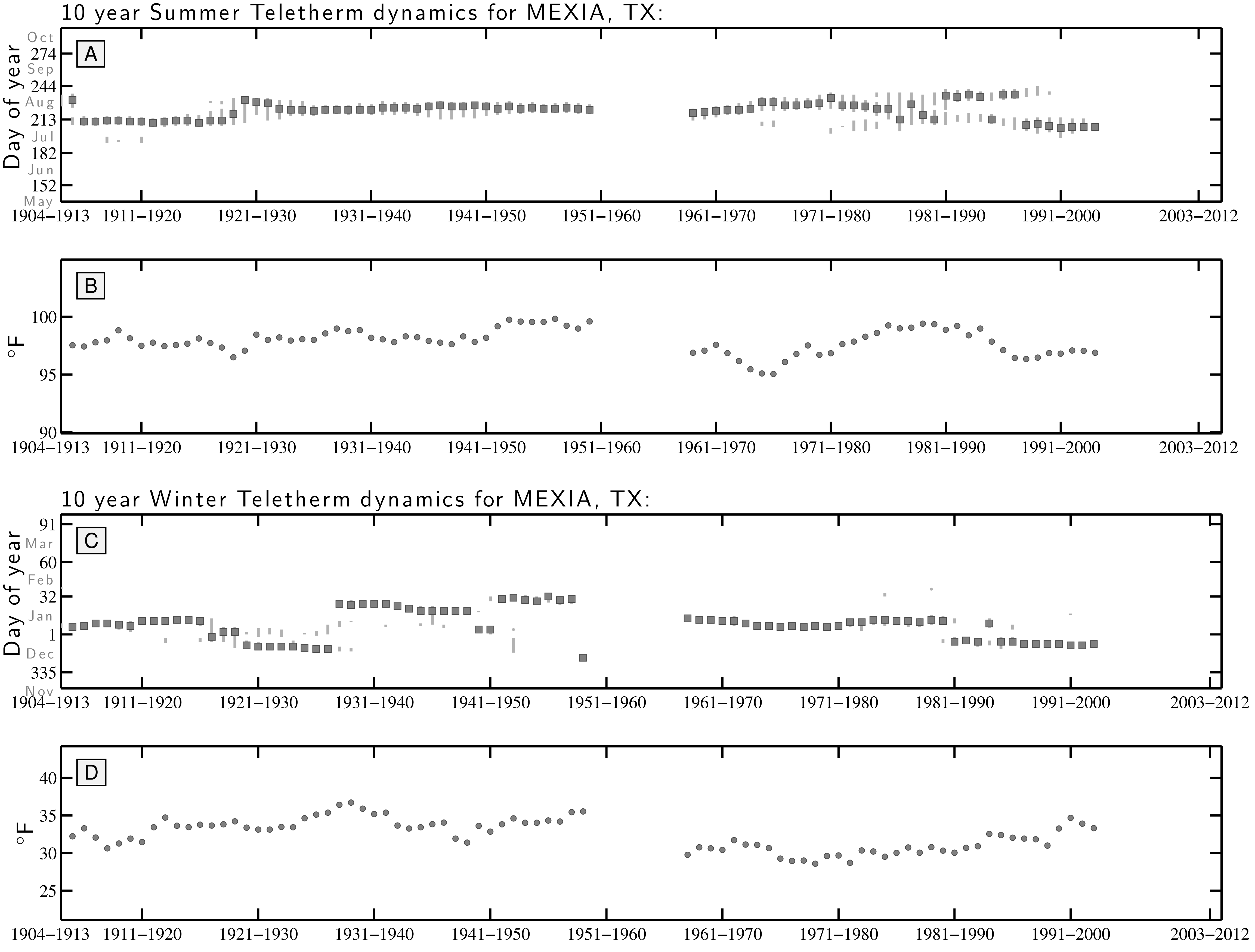
10 year Summer Teletherm dynamics for MEXIA, TX:
A
Day of year
Oct
274
Sep
244
Aug
213
Jul
182
Jun
152
May
1904–1913
1911–1920
1921–1930
1931–1940
1941–1950
1951–1960
1961–1970
1971–1980
1981–1990
1991–2000
2003–2012
B
°F
100
95
90
10 year Winter Teletherm dynamics for MEXIA, TX:
C
91
Mar
60
Feb
32
Jan
1
Dec
335
Nov
D
40
35
30
25

10 year Summer Teletherm dynamics for MIAMI, TX:

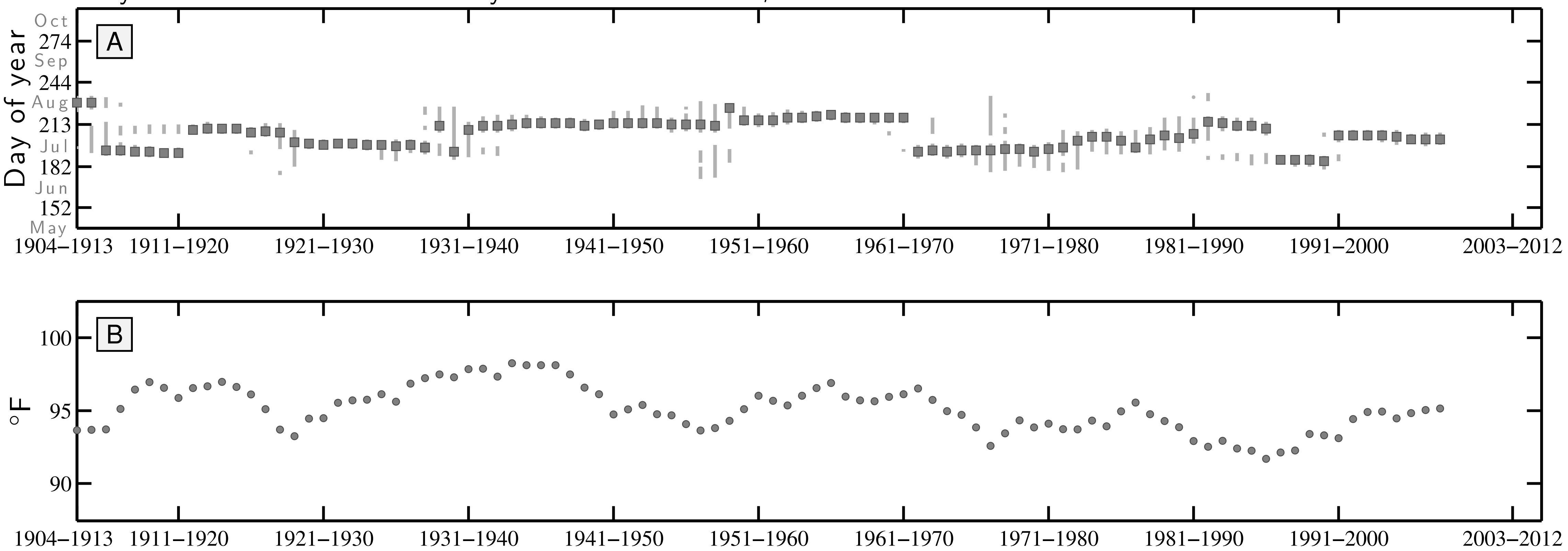

10 year Winter Teletherm dynamics for MIAMI, TX:

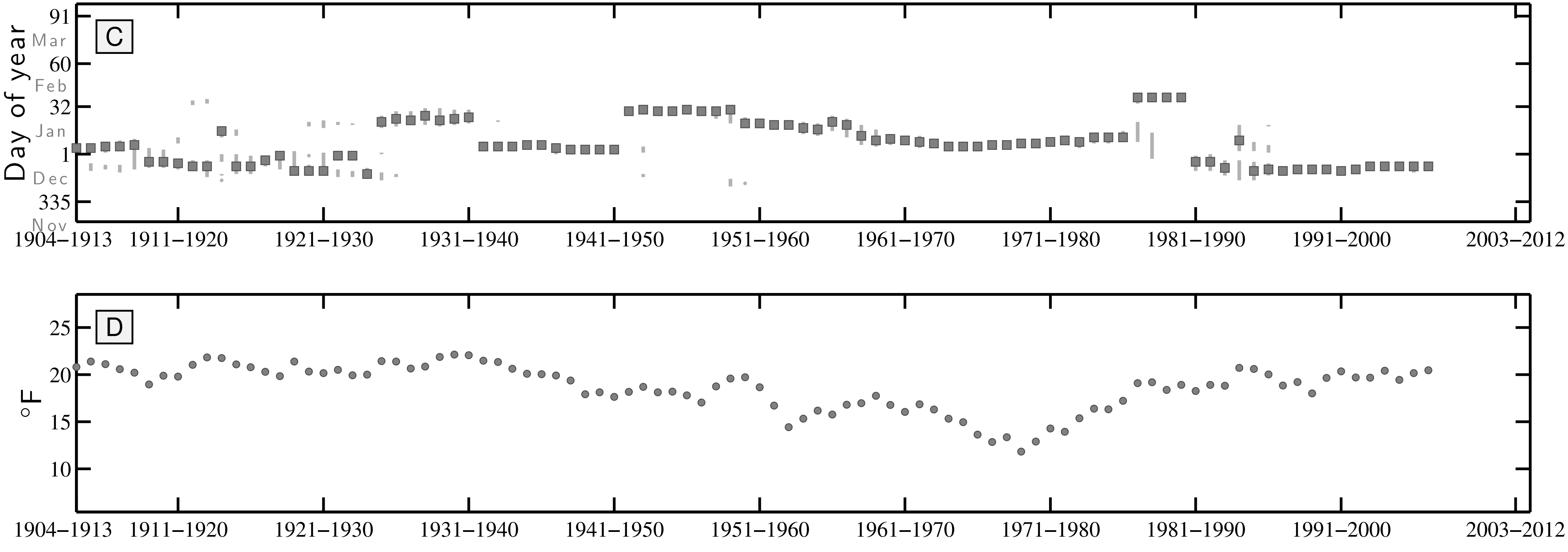

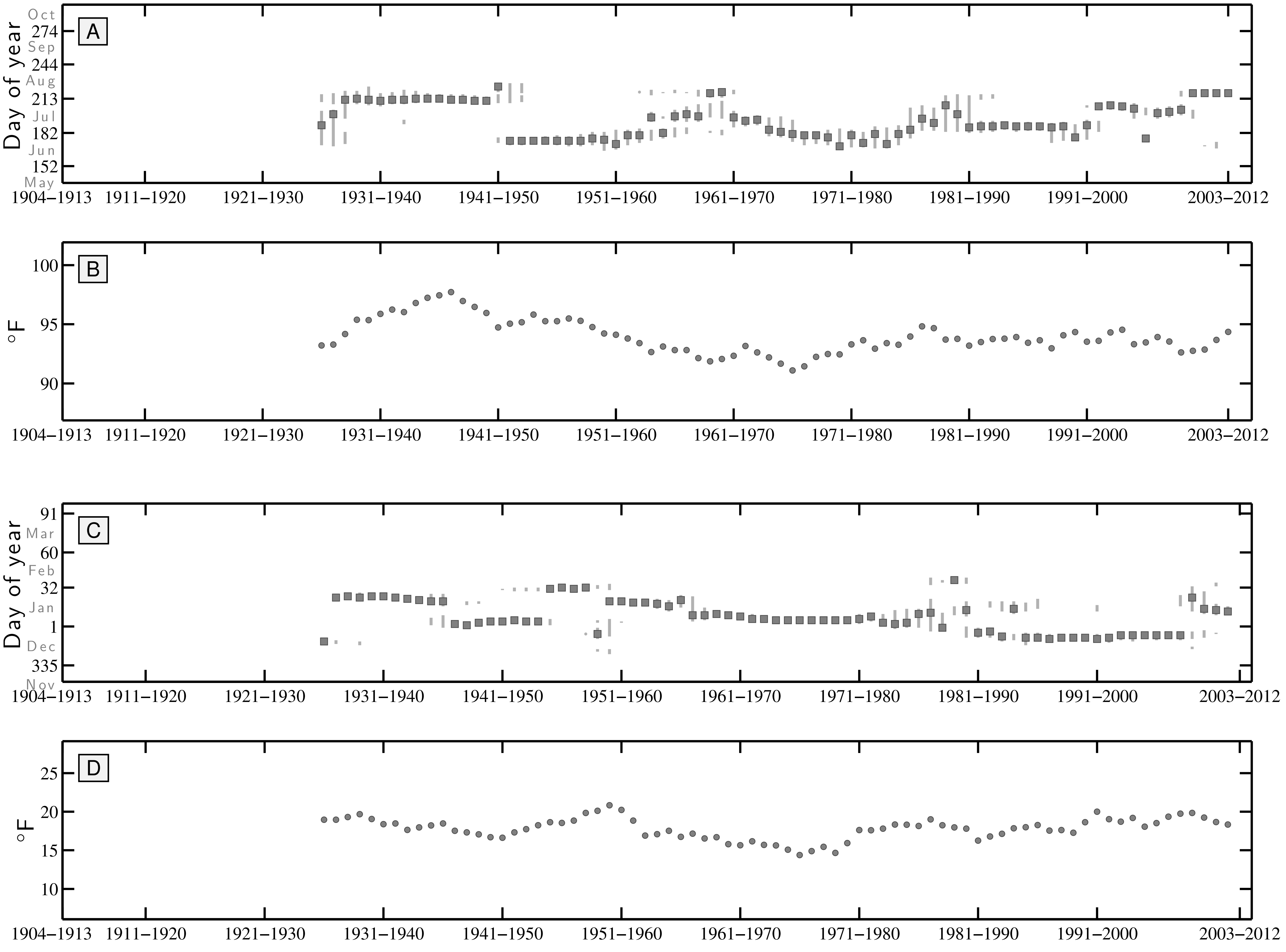
A
B
C
D
Day of year
°F
Oct
274
Sep
244
Aug
213
Jul
182
Jun
152
May
100
95
90
91
Mar
60
Feb
32
Jan
1
Dec
335
Nov
25
20
15
10
1904–1913
1911–1920
1921–1930
1931–1940
1941–1950
1951–1960
1961–1970
1971–1980
1981–1990
1991–2000
2003–2012

10 year Summer Teletherm dynamics for NEW BRAUNFELS, TX:

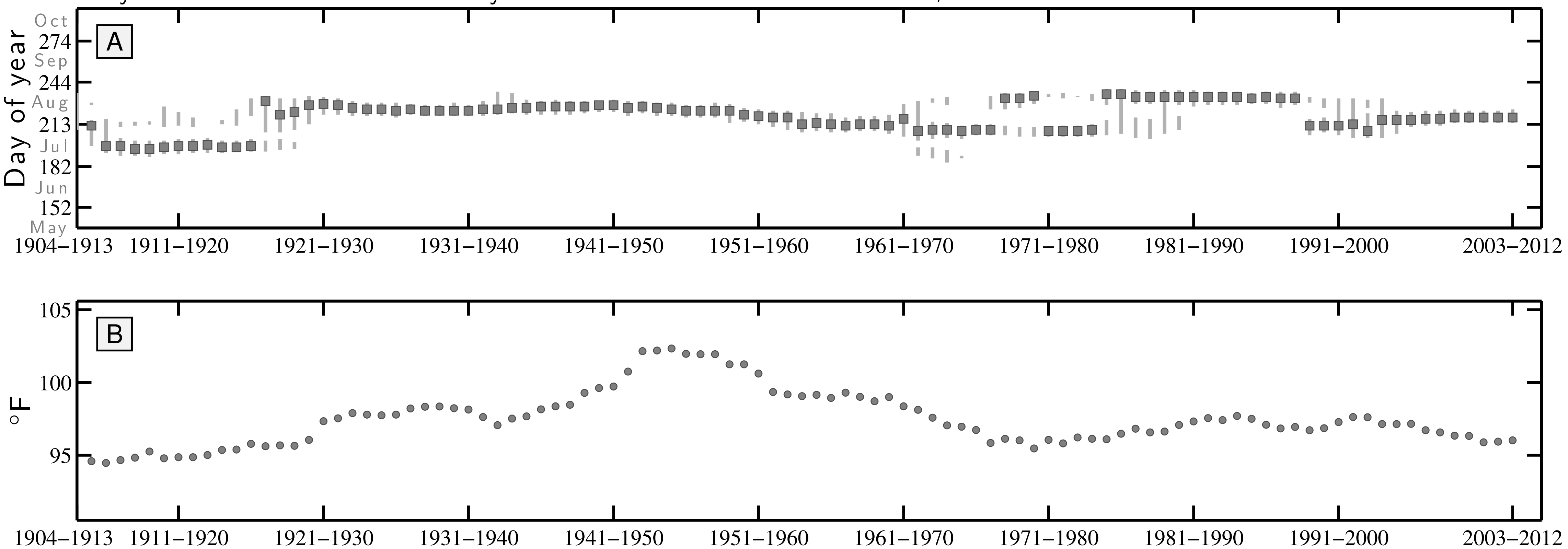

10 year Winter Teletherm dynamics for NEW BRAUNFELS, TX:

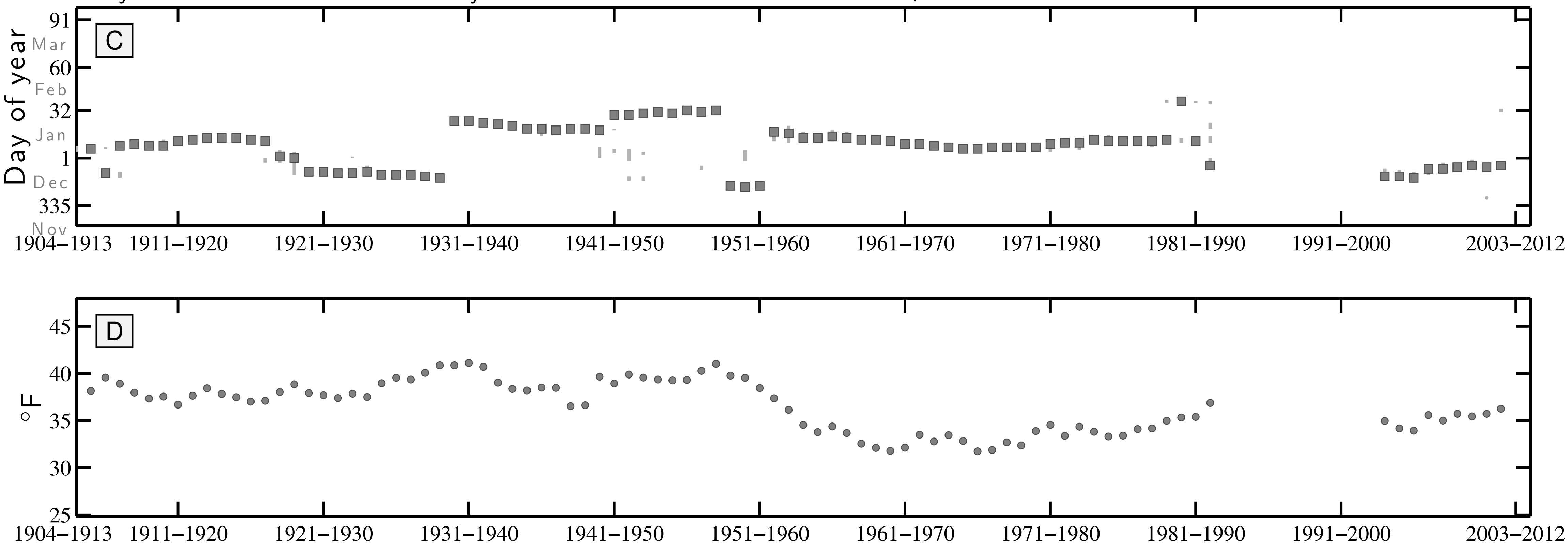

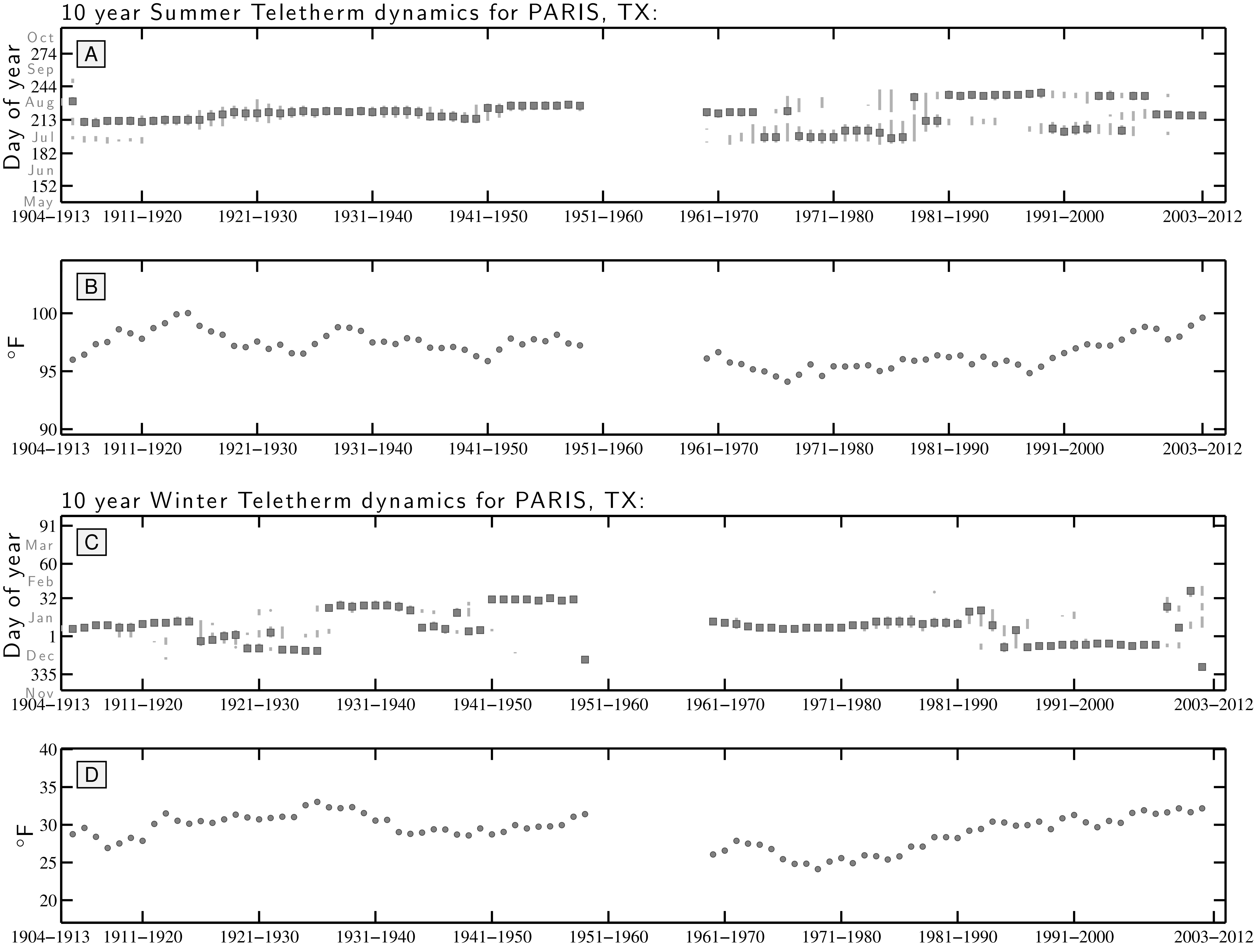
10 year Summer Teletherm dynamics for PARIS, TX:
A
B
Day of year
°F
Oct
274
Sep
244
Aug
213
Jul
182
Jun
152
May
100
95
90
1904–1913
1911–1920
1921–1930
1931–1940
1941–1950
1951–1960
1961–1970
1971–1980
1981–1990
1991–2000
2003–2012
10 year Winter Teletherm dynamics for PARIS, TX:
C
D
91
Mar
60
Feb
32
Jan
1
Dec
335
Nov
40
35
30
25
20

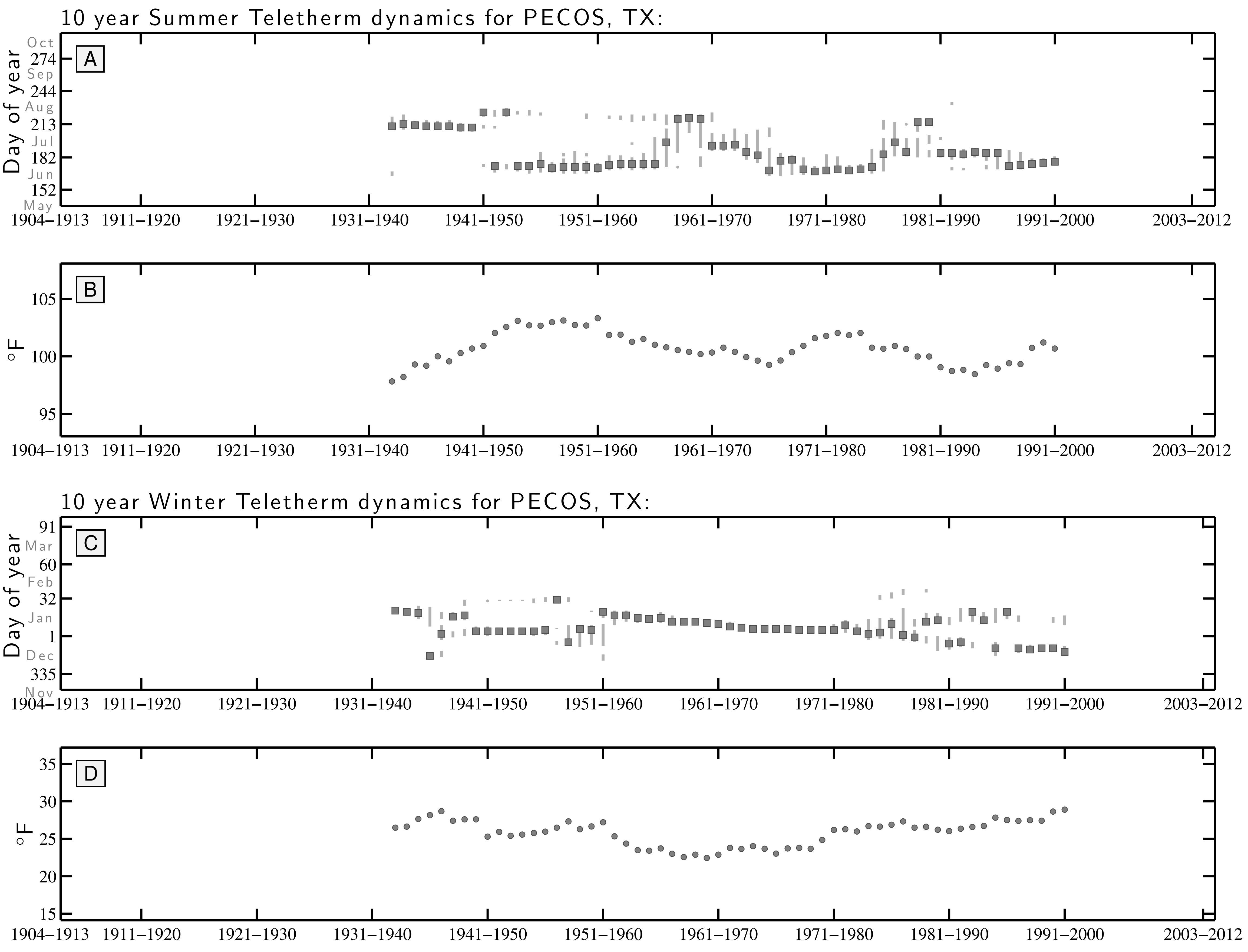
10 year Summer Teletherm dynamics for PECOS, TX:
A
Day of year
Oct
274
Sep
244
Aug
213
Jul
182
Jun
152
May
1904–1913
1911–1920
1921–1930
1931–1940
1941–1950
1951–1960
1961–1970
1971–1980
1981–1990
1991–2000
2003–2012
B
°F
105
100
95
10 year Winter Teletherm dynamics for PECOS, TX:
C
91
Mar
60
Feb
32
Jan
1
Dec
335
Nov
D
35
30
25
20
15

10 year Summer Teletherm dynamics for PLAINVIEW, TX:

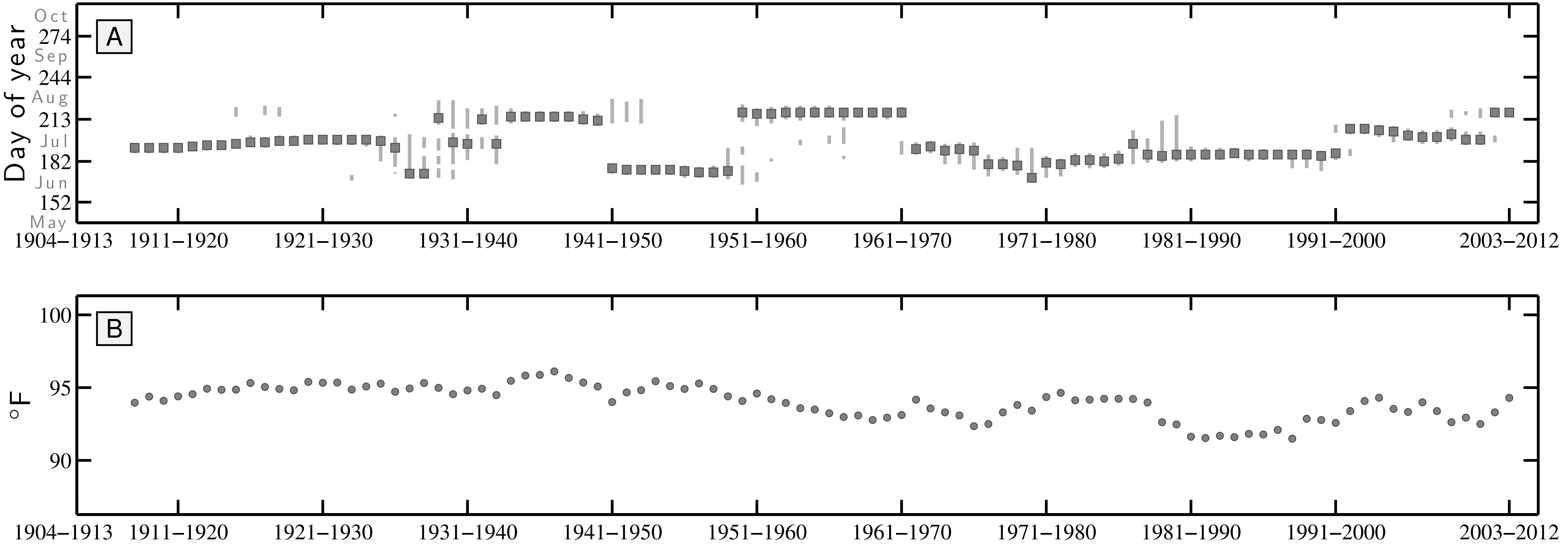

10 year Winter Teletherm dynamics for PLAINVIEW, TX:

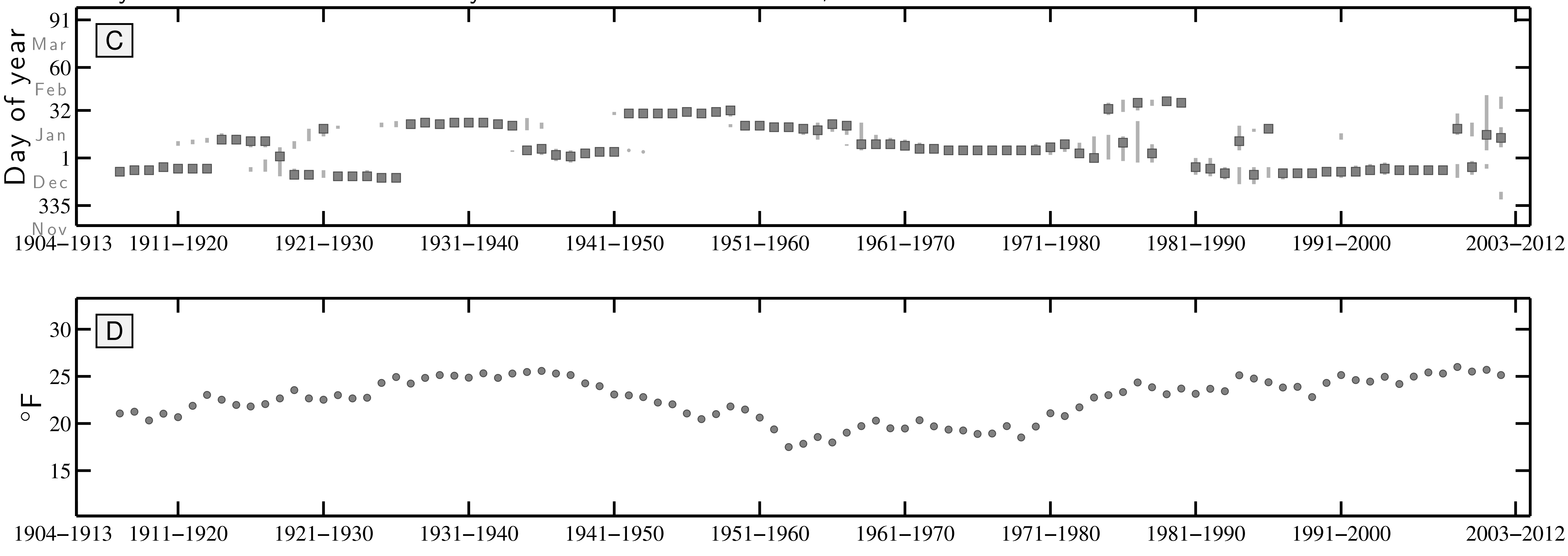

10 year Summer Teletherm dynamics for QUANAH 2 SW, TX:

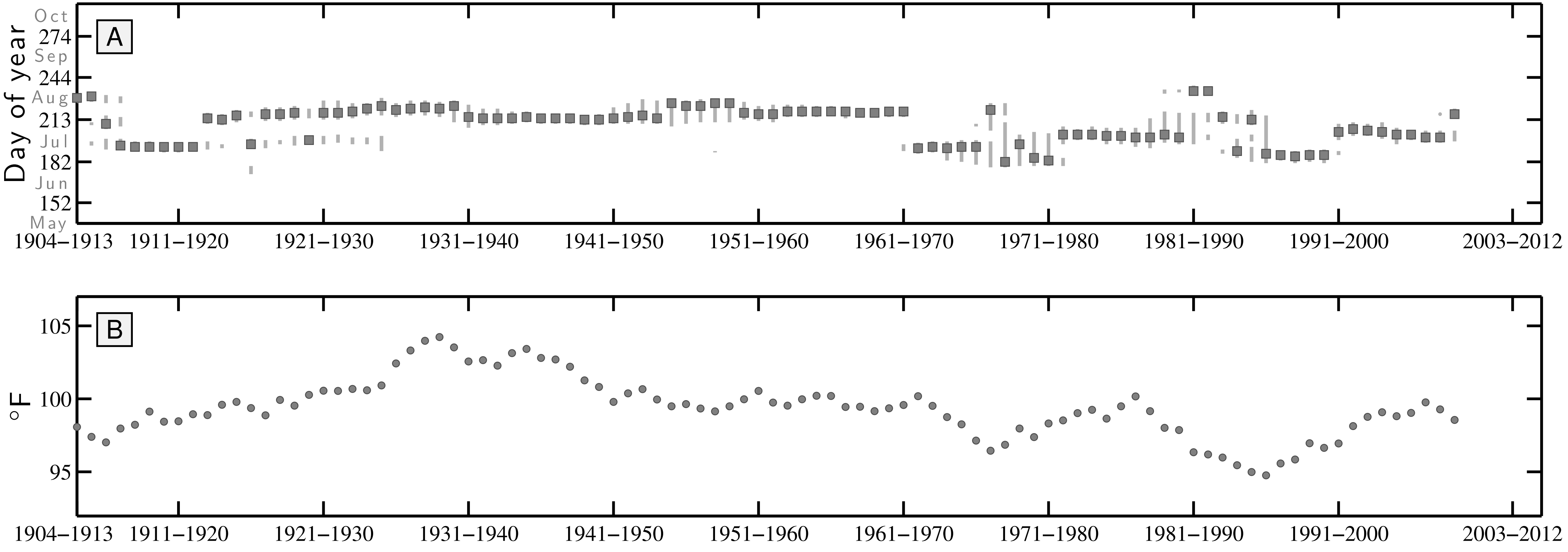

10 year Winter Teletherm dynamics for QUANAH 2 SW, TX:

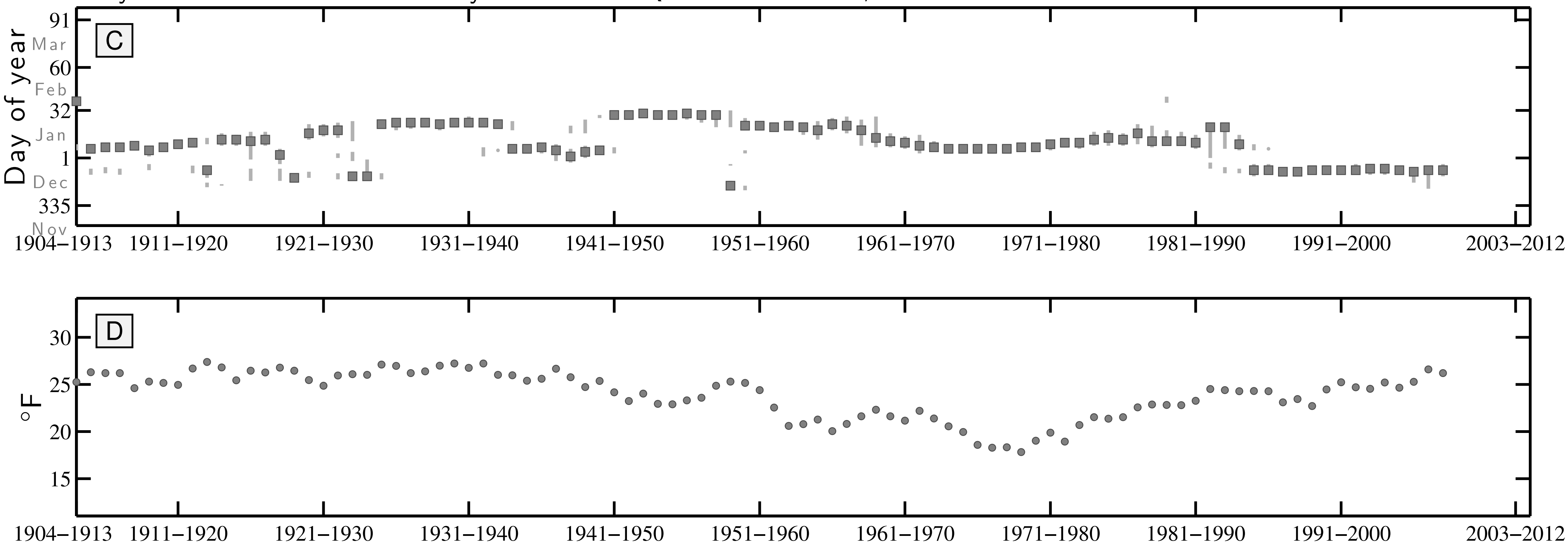

10 year Summer Teletherm dynamics for RIO GRANDE CITY, TX:

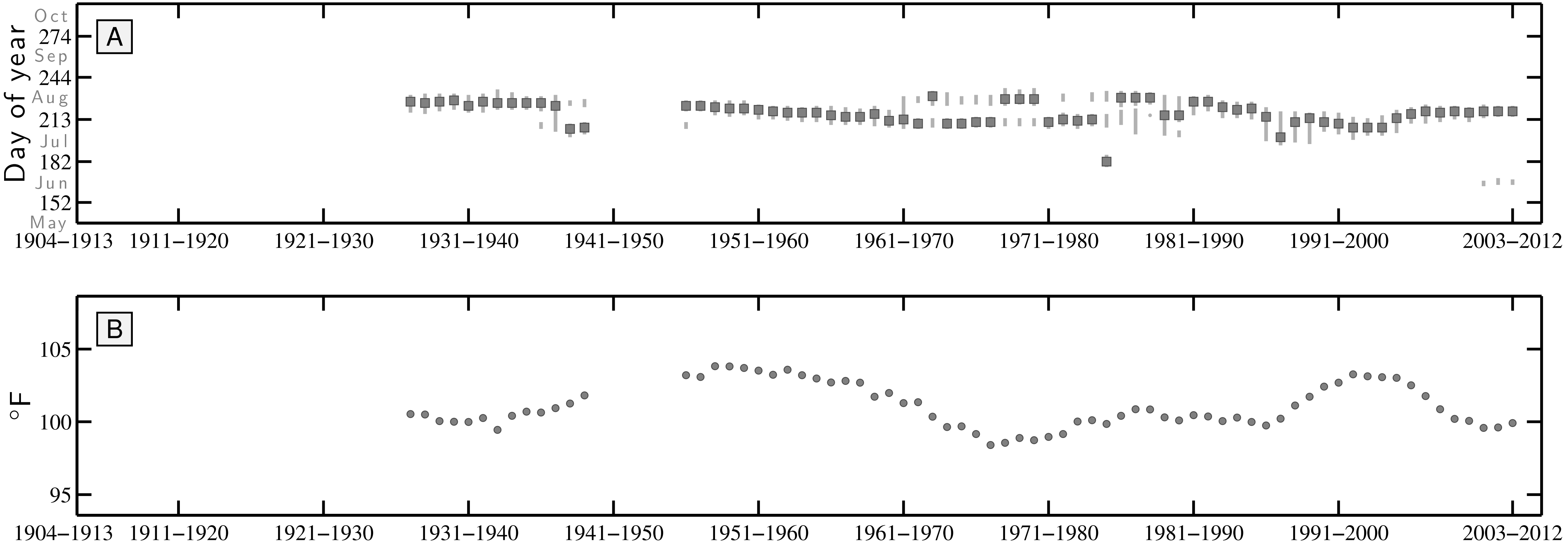

10 year Winter Teletherm dynamics for RIO GRANDE CITY, TX:

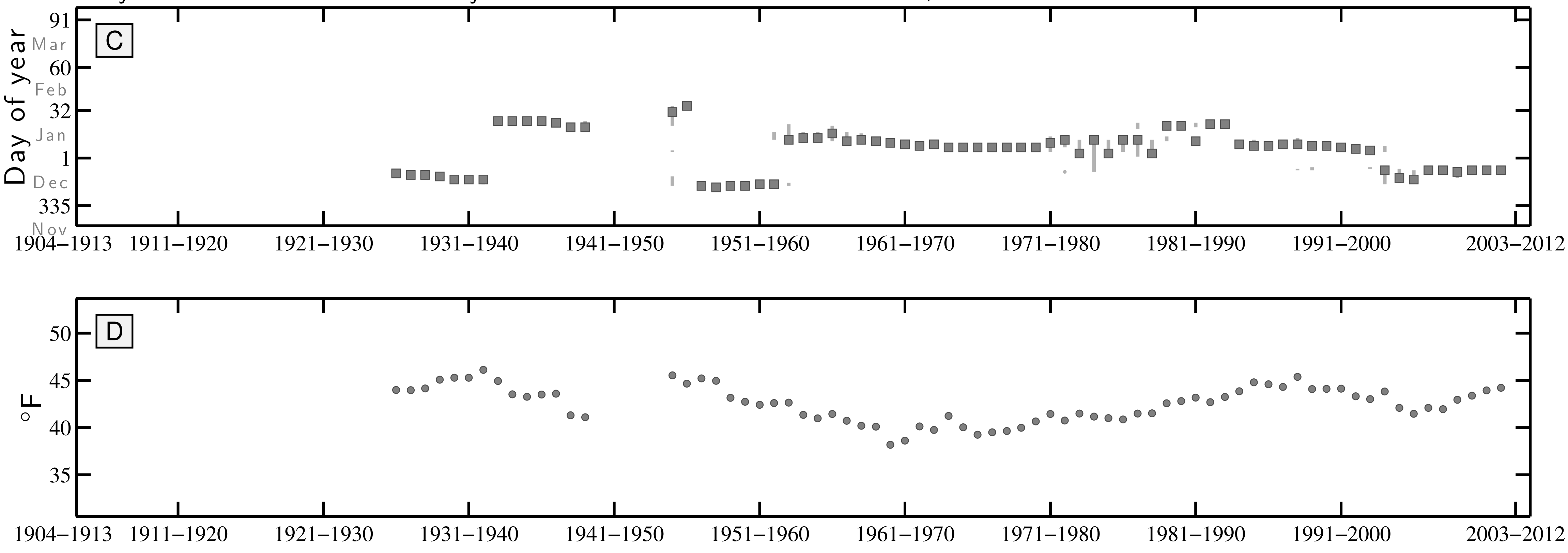

10 year Summer Teletherm dynamics for SAN ANTONIO INTL AP, TX:

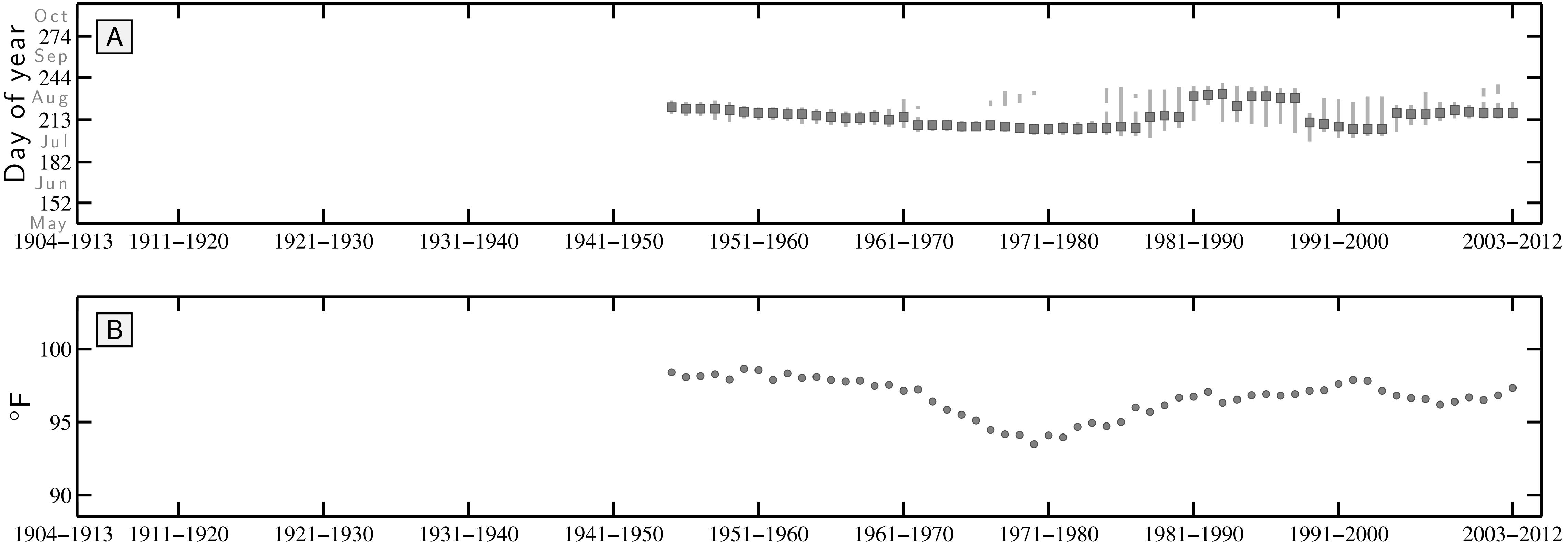

10 year Winter Teletherm dynamics for SAN ANTONIO INTL AP, TX:

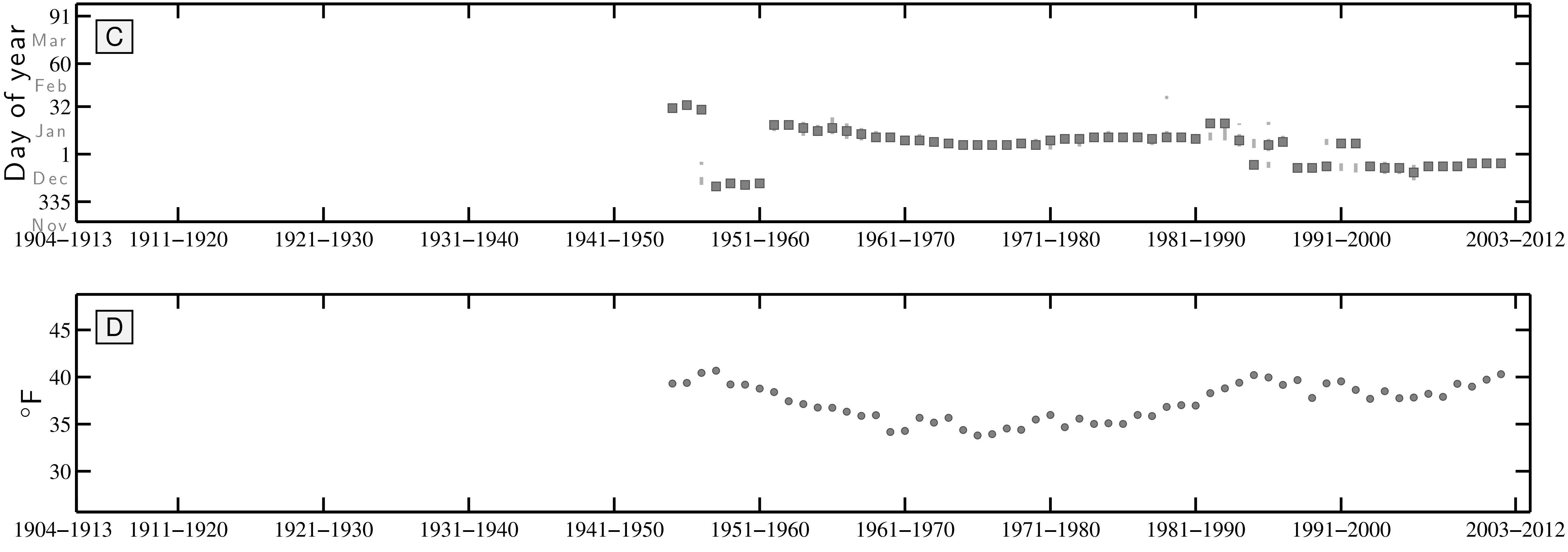

10 year Summer Teletherm dynamics for SEMINOLE, TX:

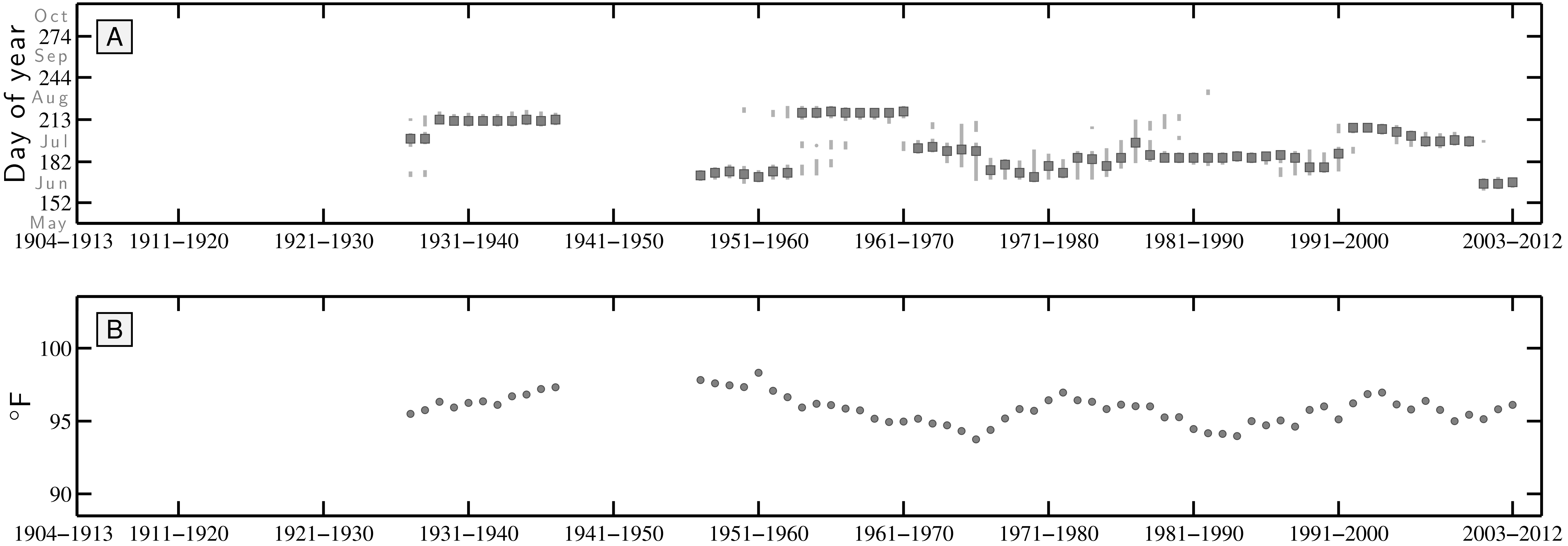

10 year Winter Teletherm dynamics for SEMINOLE, TX:

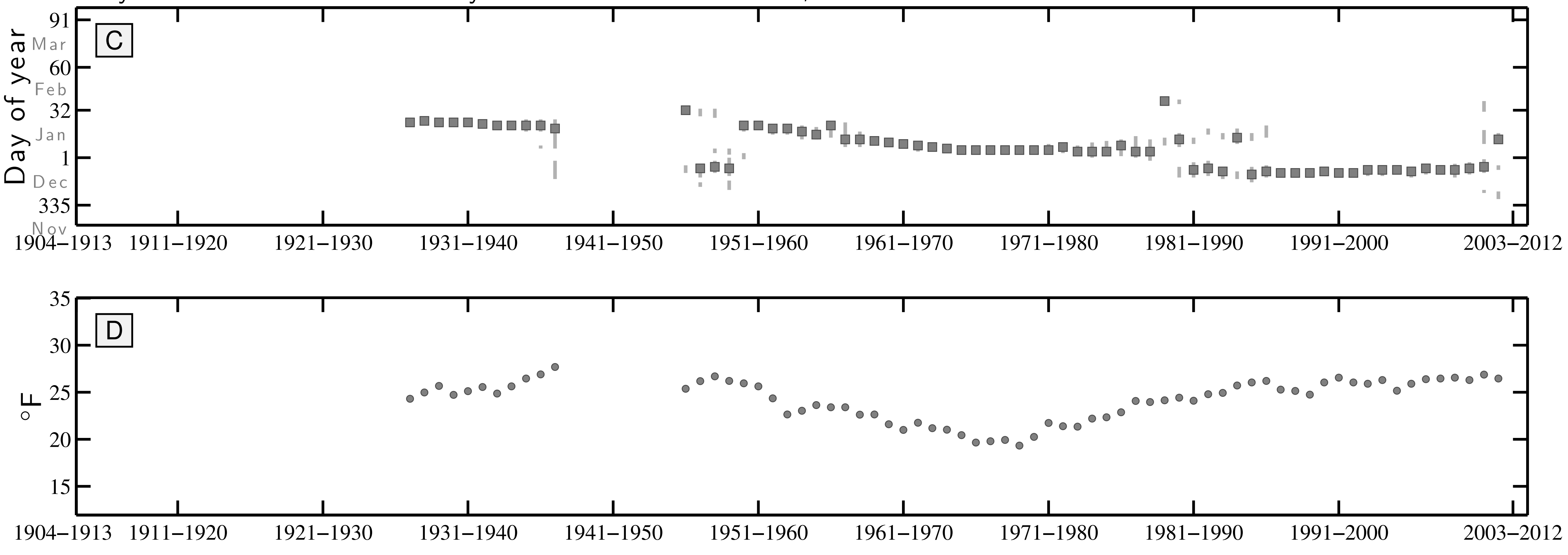

10 year Summer Teletherm dynamics for SNYDER, TX:

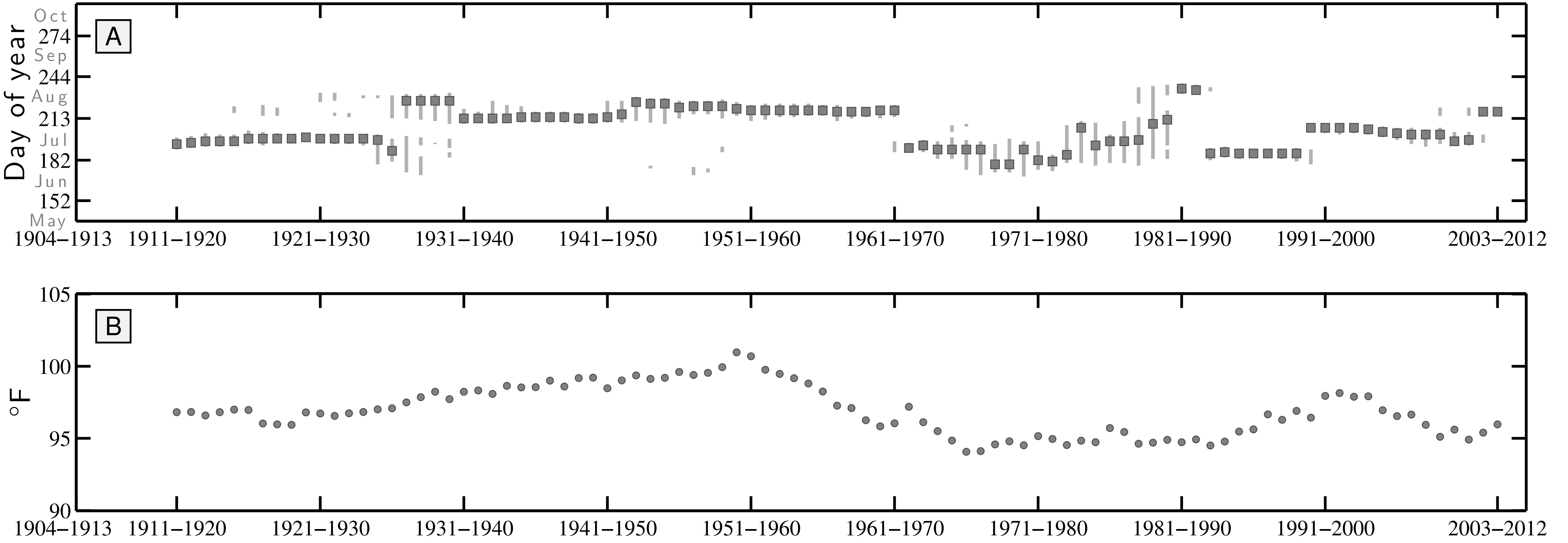

10 year Winter Teletherm dynamics for SNYDER, TX:

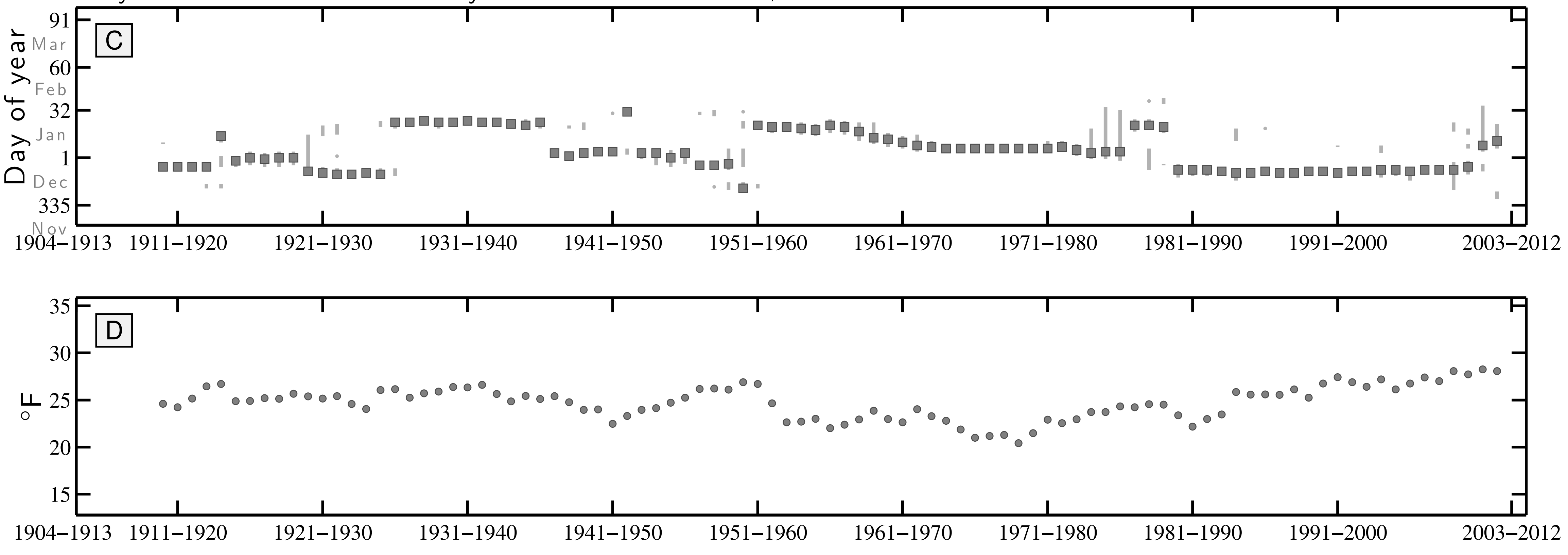

10 year Summer Teletherm dynamics for STRATFORD, TX:

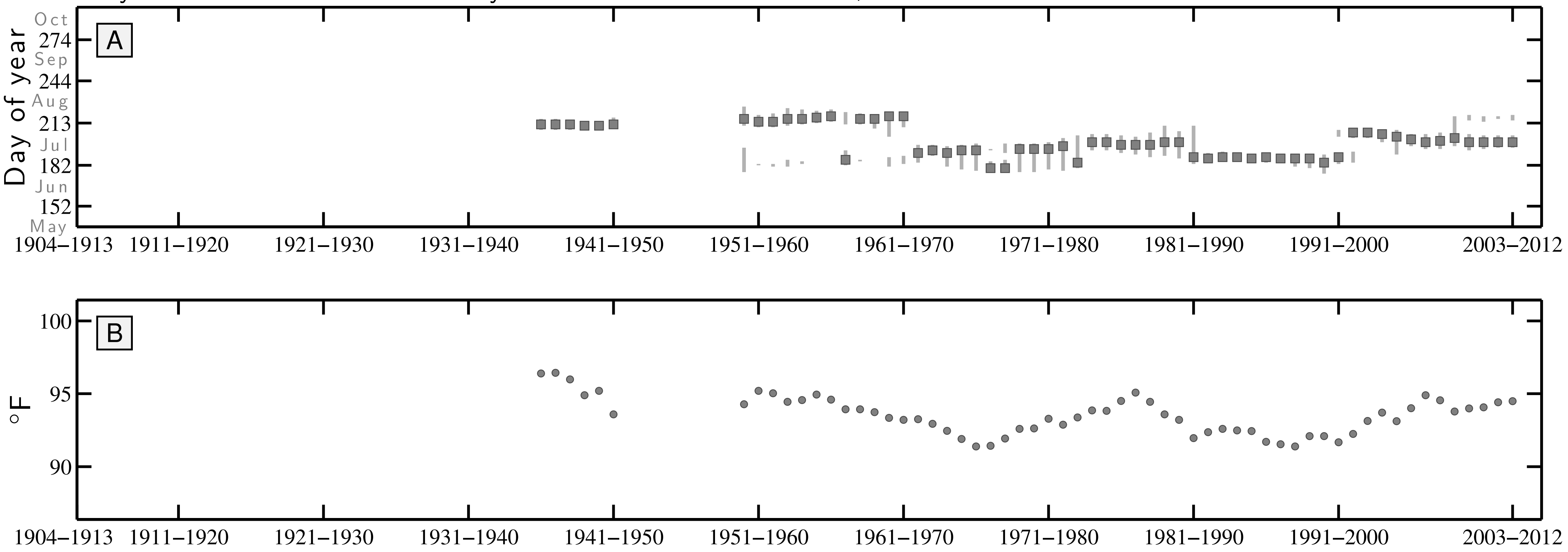

10 year Winter Teletherm dynamics for STRATFORD, TX:

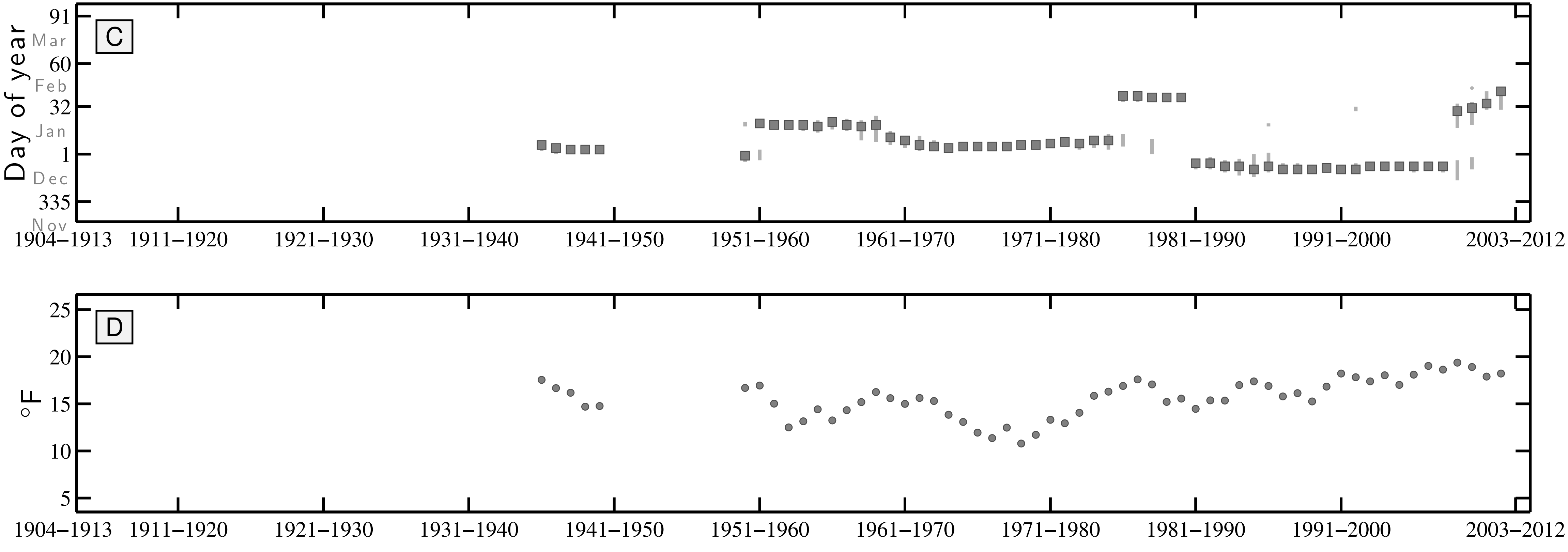

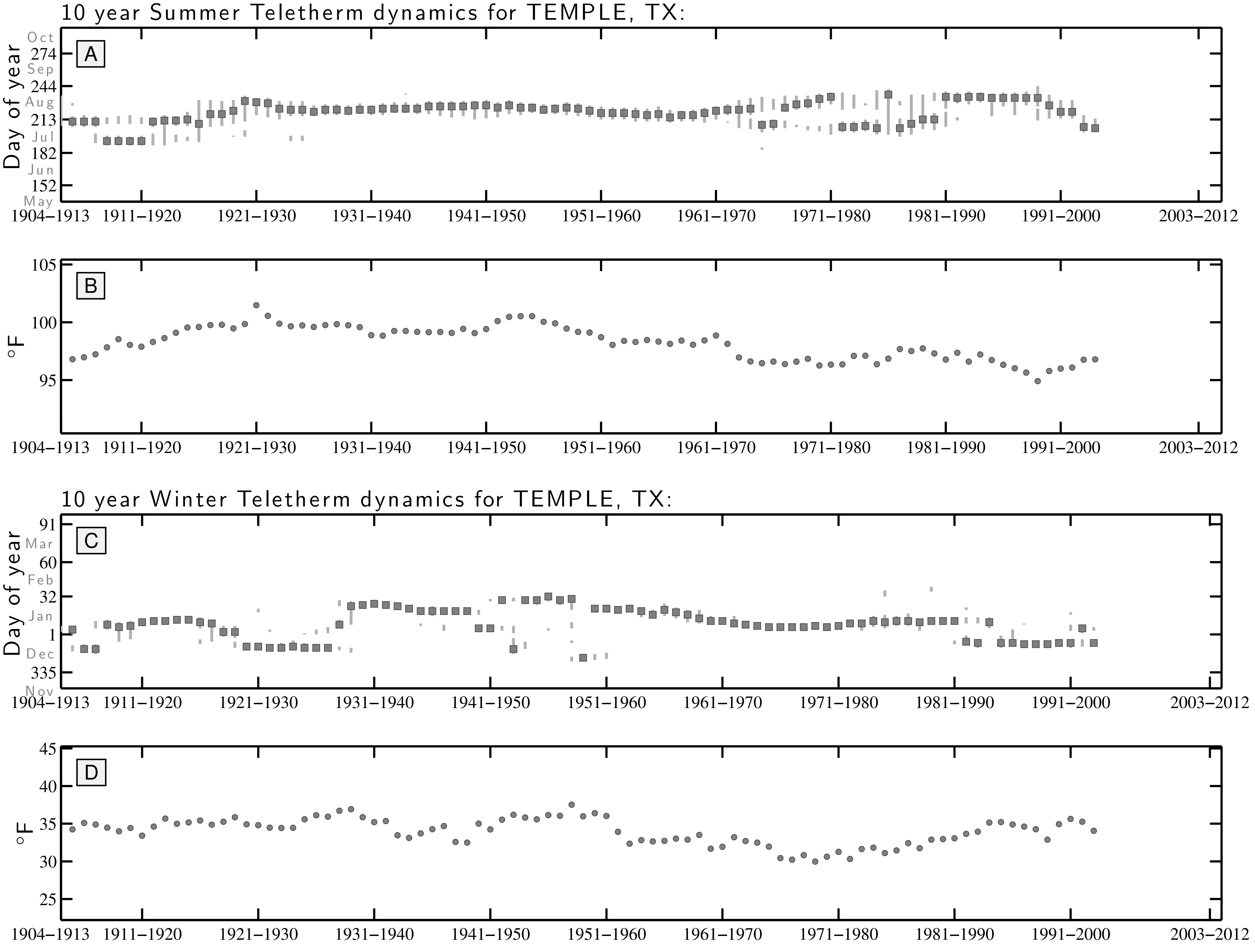
10 year Summer Teletherm dynamics for TEMPLE, TX:
A
Day of year
Oct
274
Sep
244
Aug
213
Jul
182
Jun
152
May
B
°F
105
100
95
10 year Winter Teletherm dynamics for TEMPLE, TX:
C
91
Mar
60
Feb
32
Jan
1
Dec
335
Nov
D
45
40
35
30
25
1904–1913
1911–1920
1921–1930
1931–1940
1941–1950
1951–1960
1961–1970
1971–1980
1981–1990
1991–2000
2003–2012

10 year Summer Teletherm dynamics for WEATHERFORD, TX:

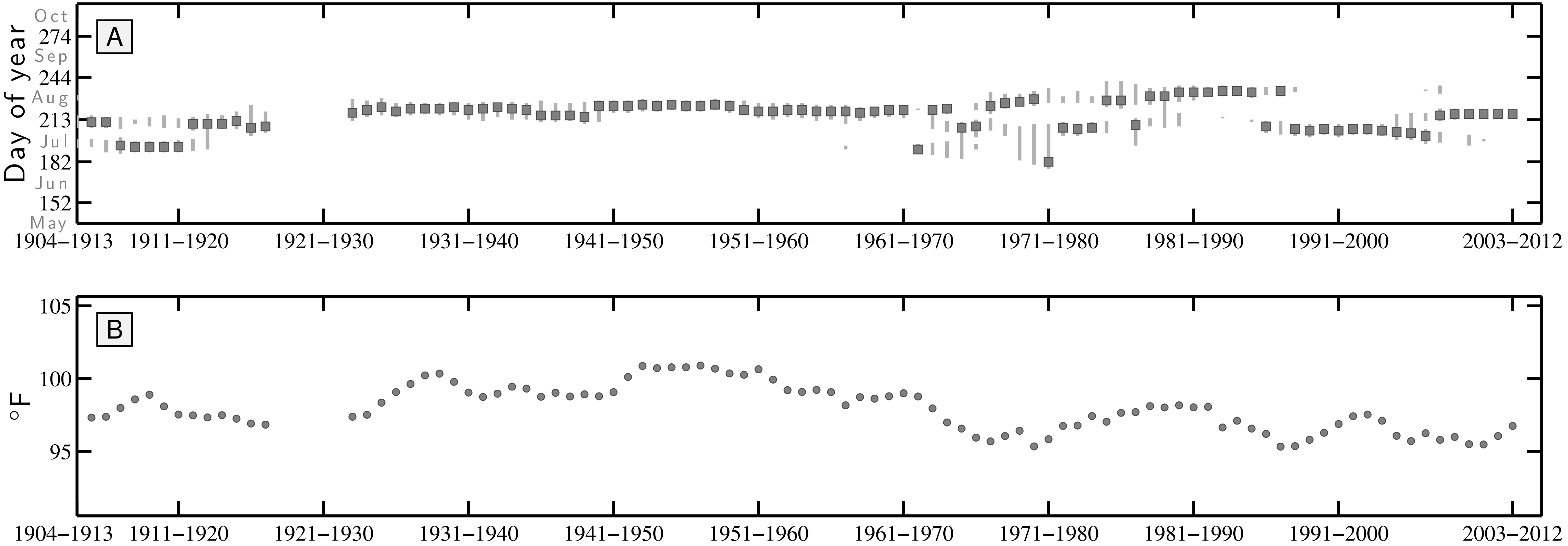

10 year Winter Teletherm dynamics for WEATHERFORD, TX:

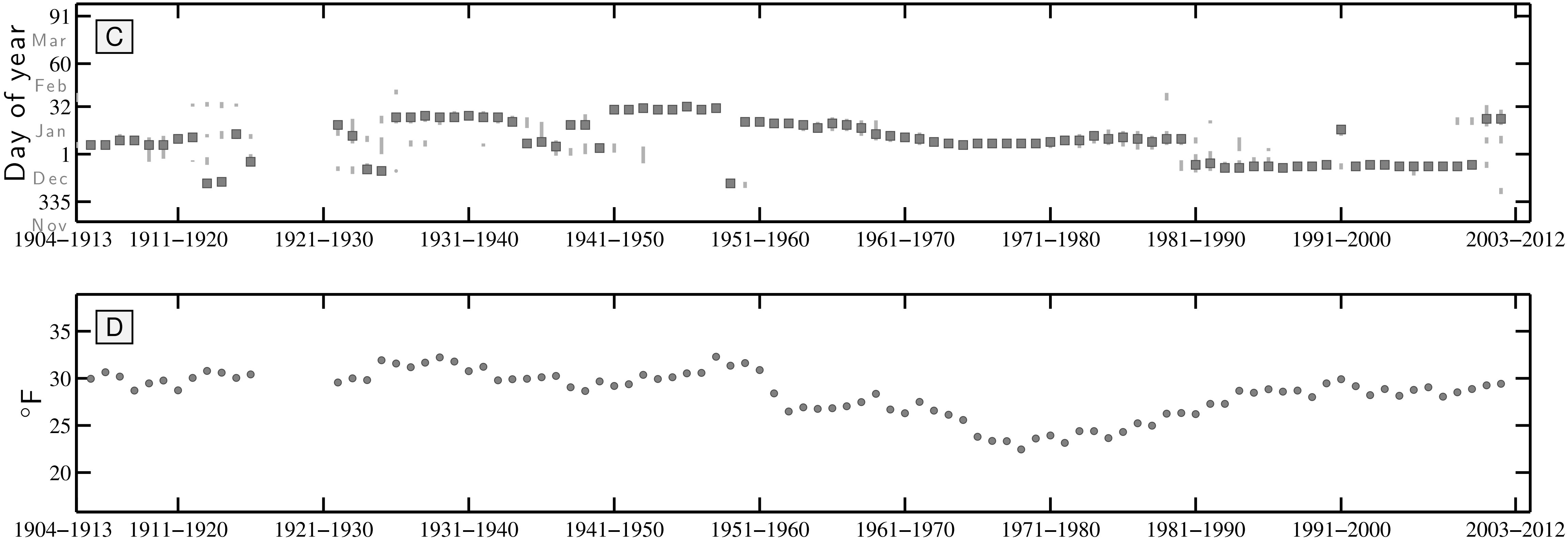

10 year Summer Teletherm dynamics for ALTON, UT:

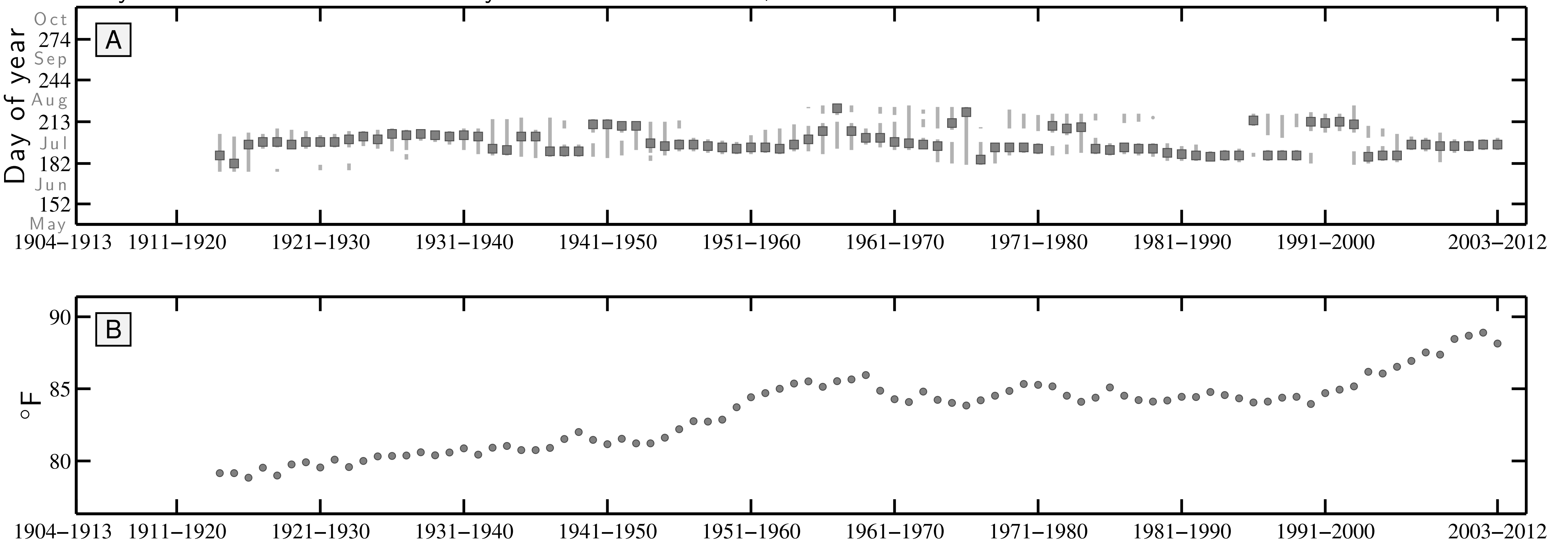

10 year Winter Teletherm dynamics for ALTON, UT:

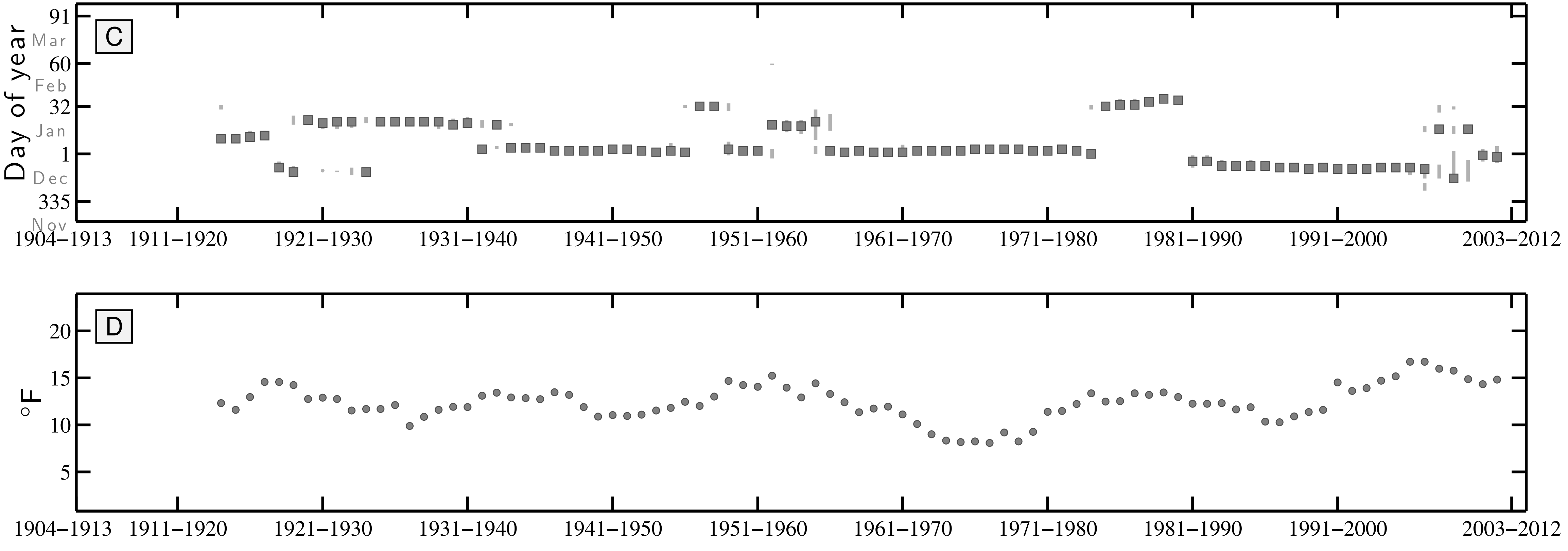

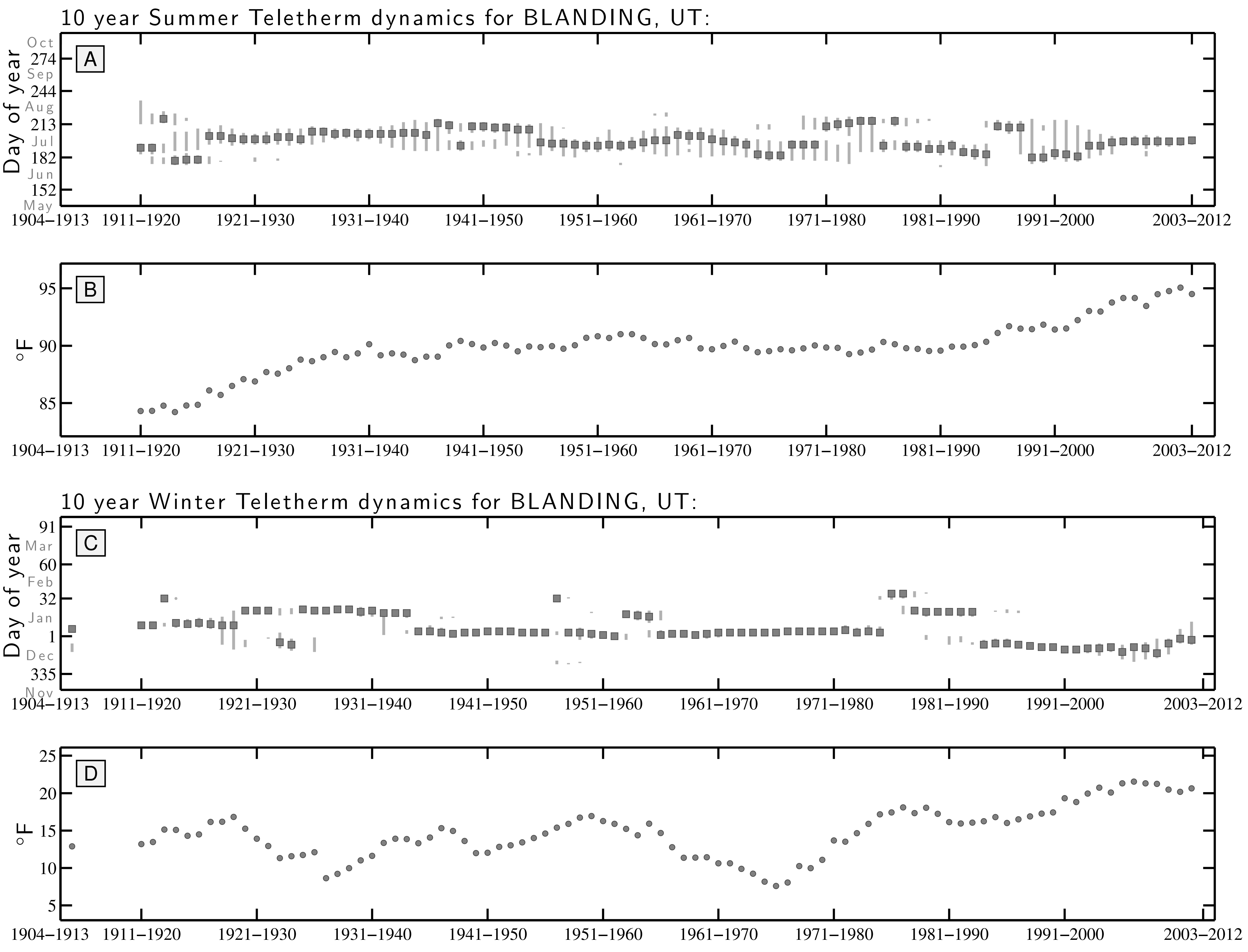

10 year Summer Teletherm dynamics for BLANDING, UT:
A
Day of year
Oct
274
Sep
244
Aug
213
Jul
182
Jun
152
May
1904–1913
1911–1920
1921–1930
1931–1940
1941–1950
1951–1960
1961–1970
1971–1980
1981–1990
1991–2000
2003–2012
B
°F
95
90
85
10 year Winter Teletherm dynamics for BLANDING, UT:
C
Day of year
91
Mar
60
Feb
32
Jan
1
Dec
335
Nov
D
°F
25
20
15
10
5

10 year Summer Teletherm dynamics for BLUFF, UT:

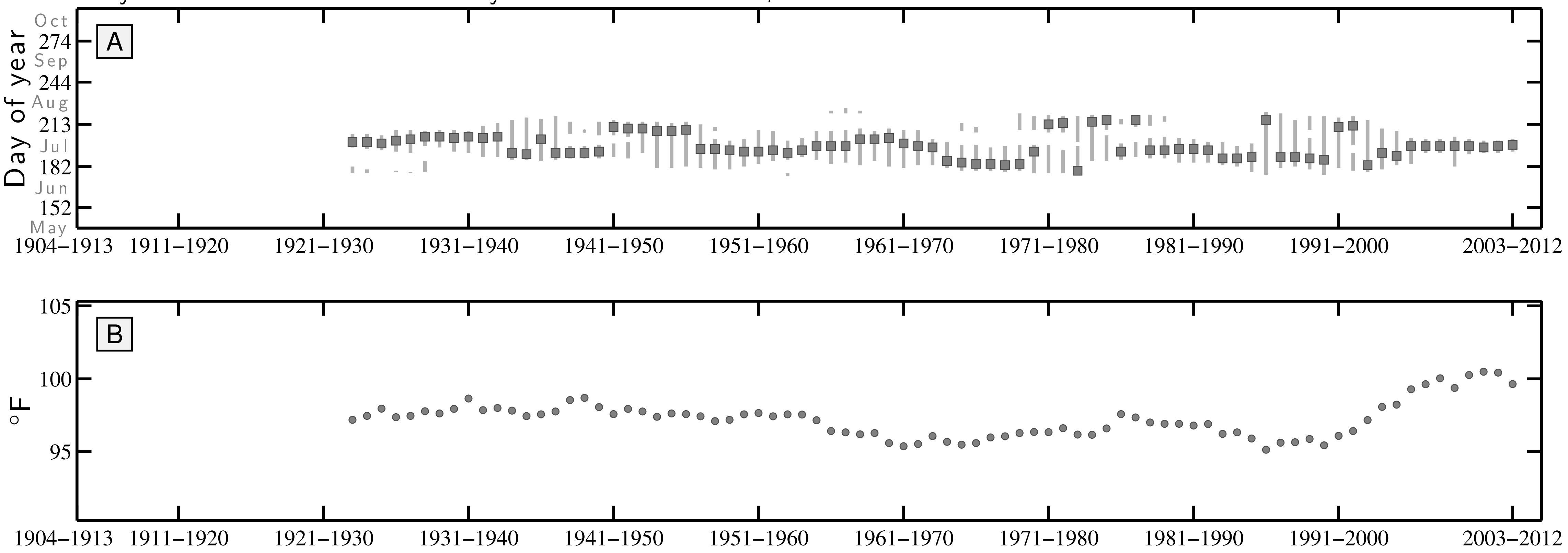

10 year Winter Teletherm dynamics for BLUFF, UT:

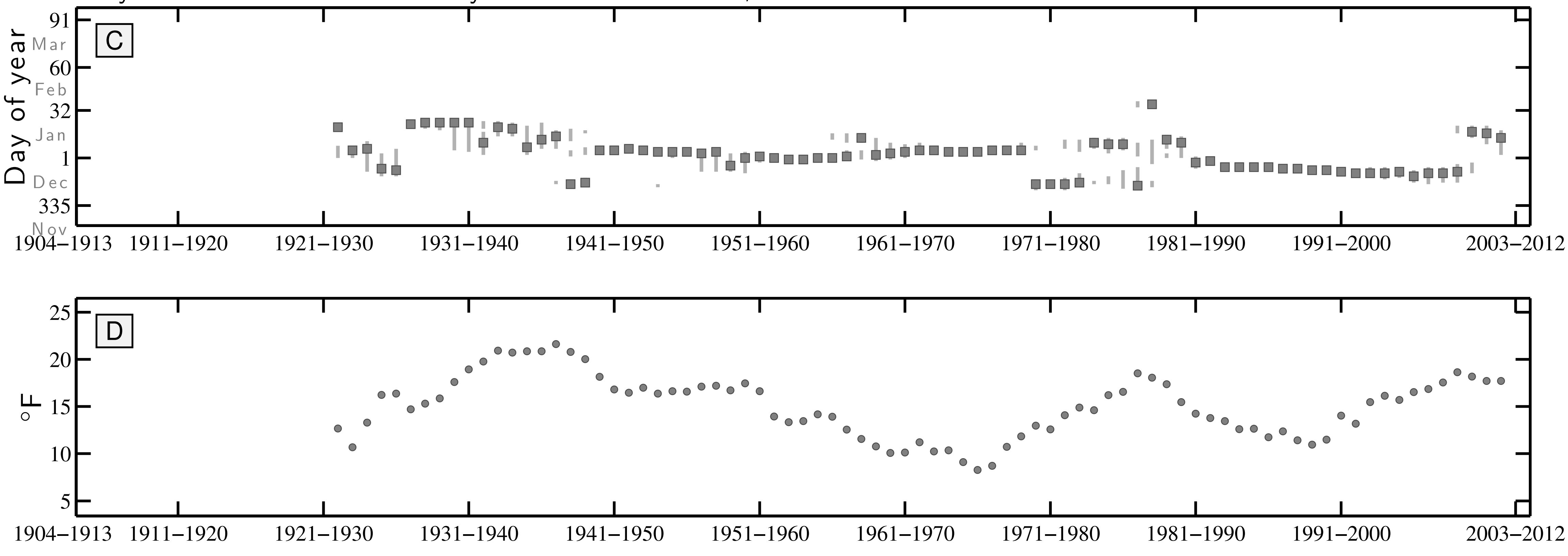

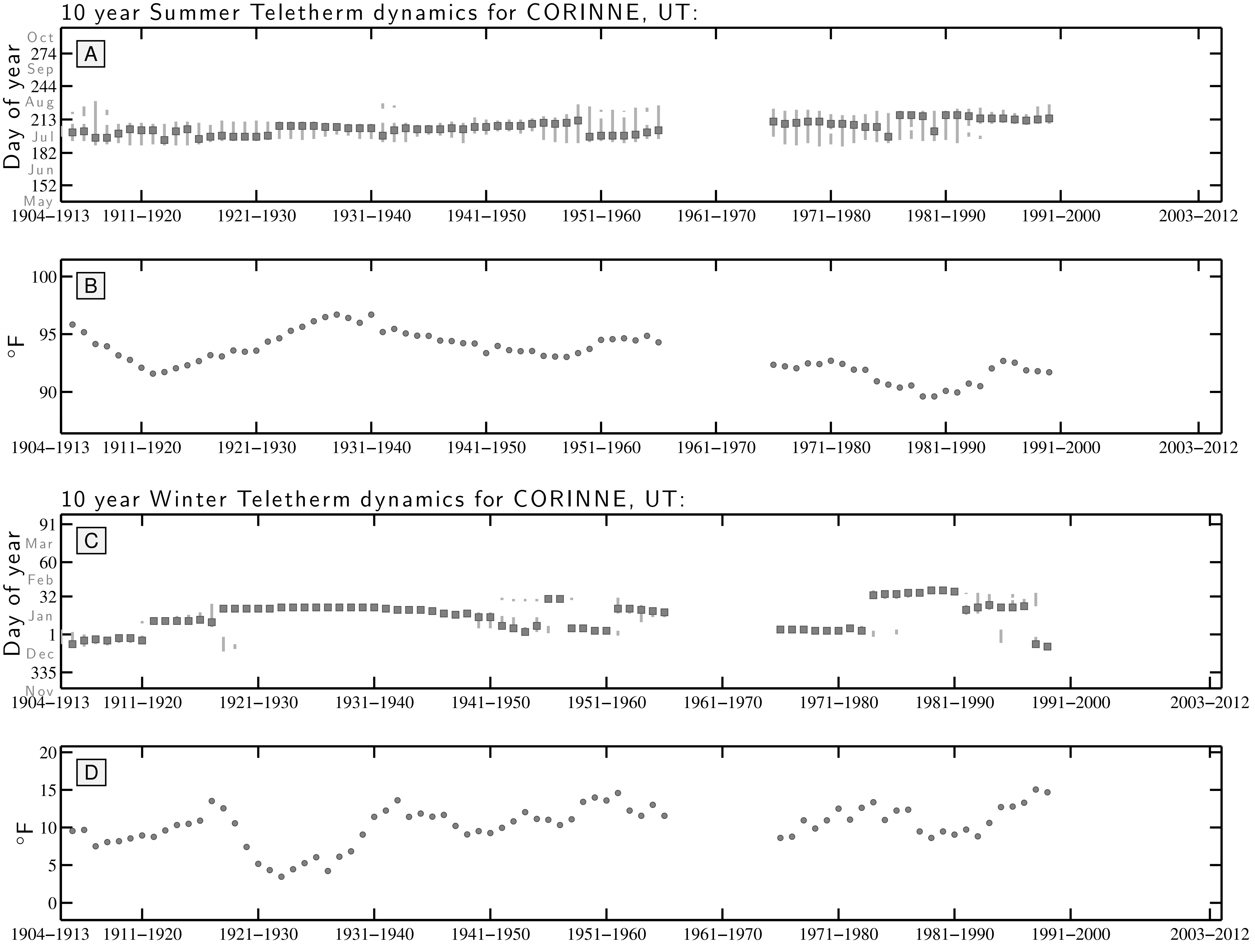
10 year Summer Teletherm dynamics for CORINNE, UT:
A
B
Day of year
°F
10 year Winter Teletherm dynamics for CORINNE, UT:
C
D
1904–1913
1911–1920
1921–1930
1931–1940
1941–1950
1951–1960
1961–1970
1971–1980
1981–1990
1991–2000
2003–2012

10 year Summer Teletherm dynamics for DESERET, UT:

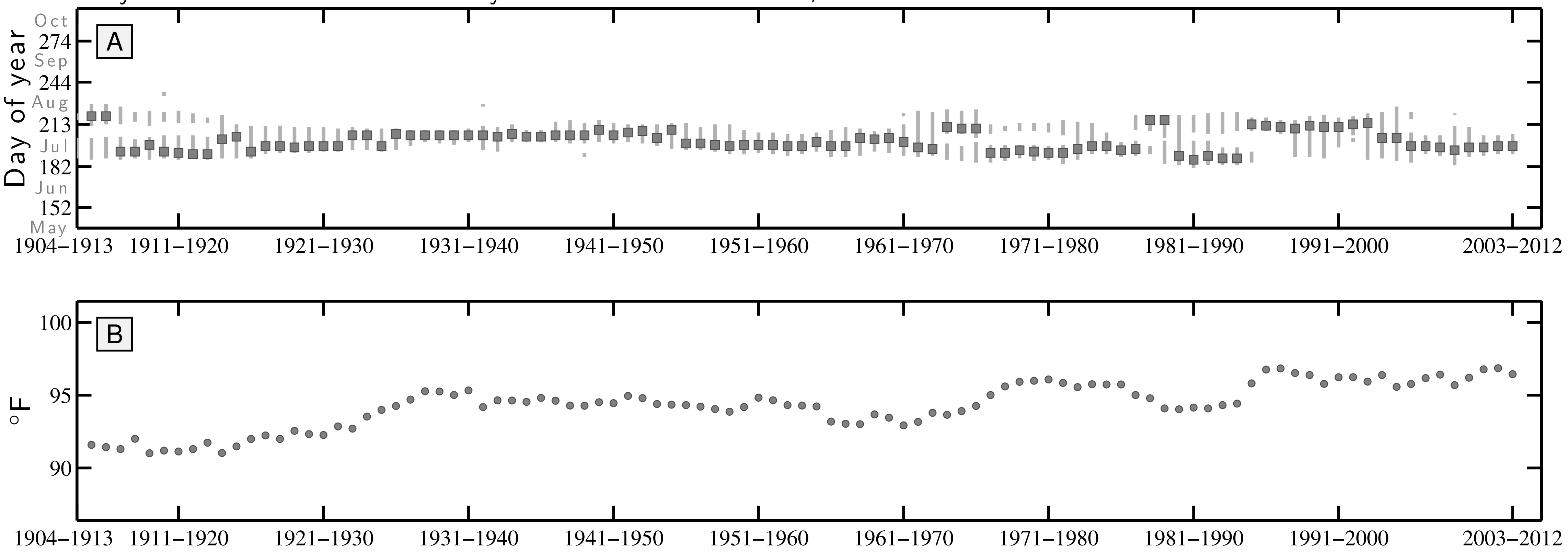

10 year Winter Teletherm dynamics for DESERET, UT:

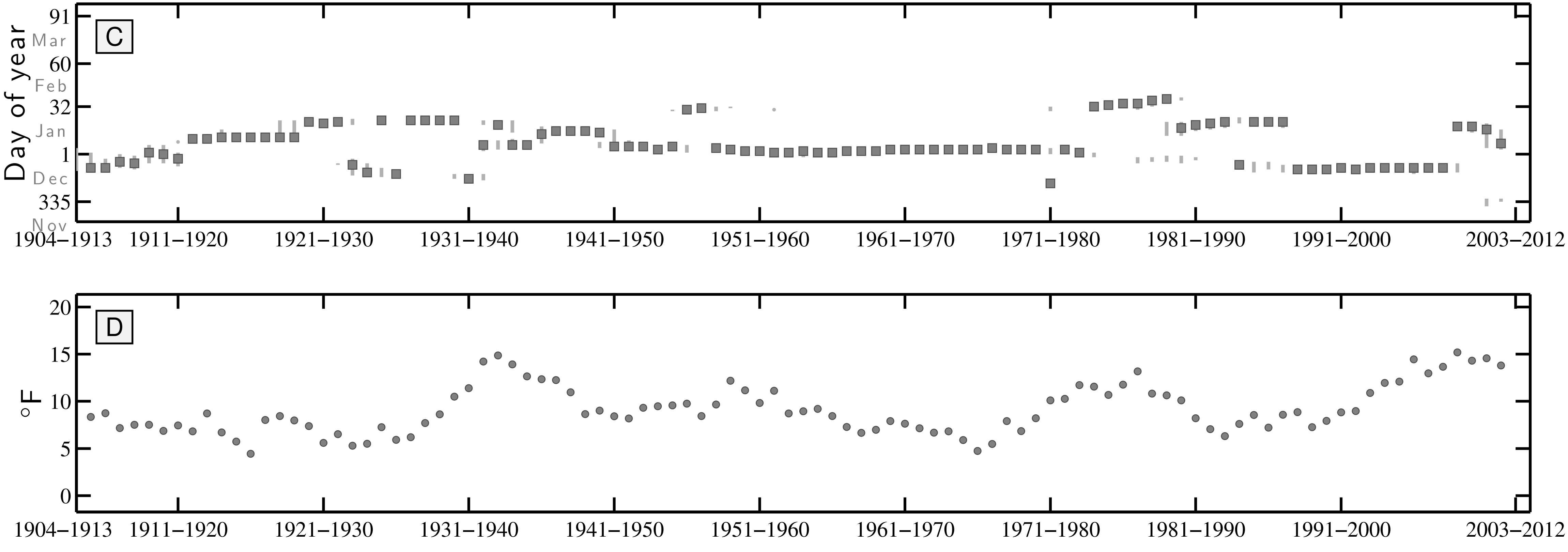

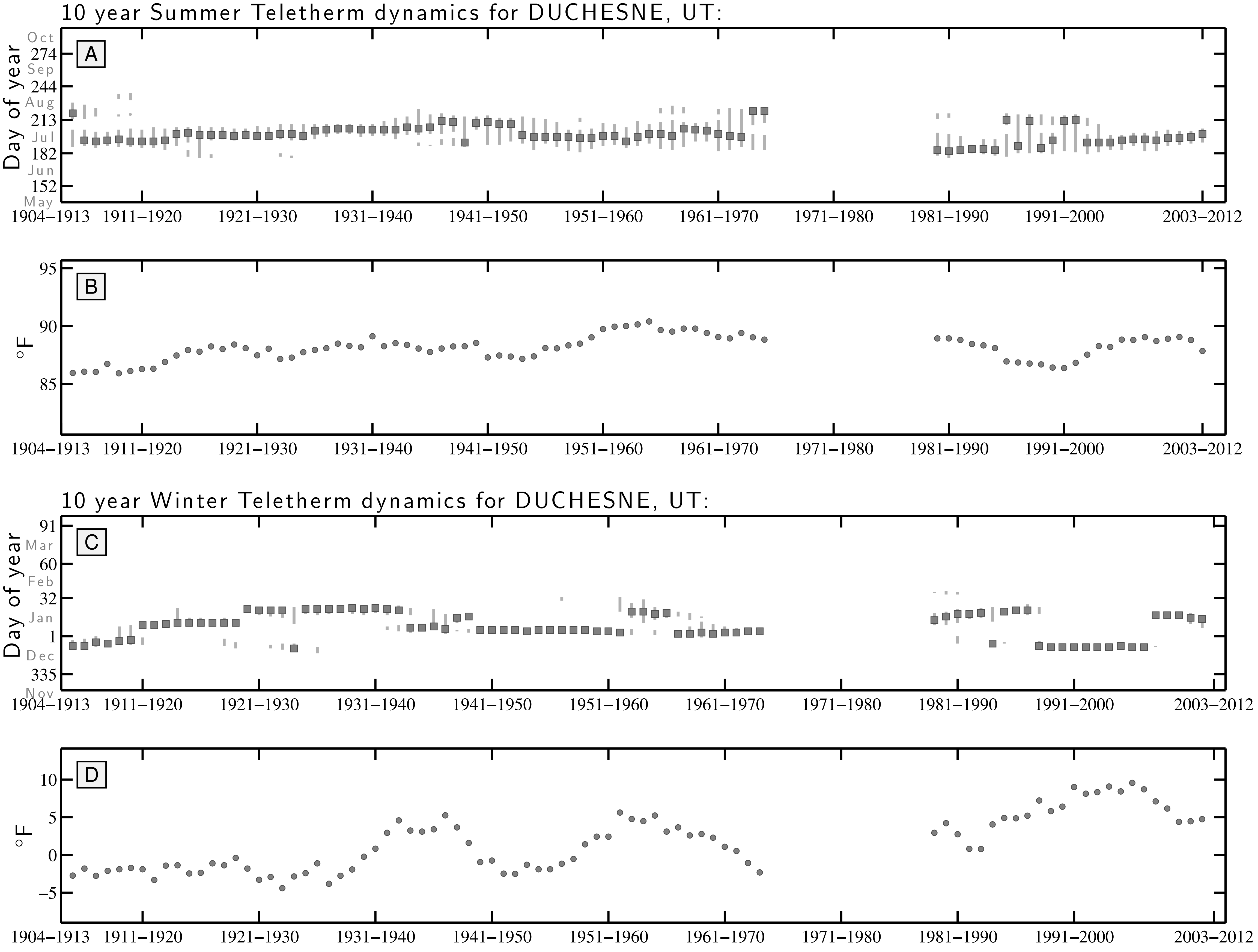

10 year Summer Teletherm dynamics for DUCHESNE, UT:
A
Day of year
Oct
274
Sep
244
Aug
213
Jul
182
Jun
152
May
1904–1913
1911–1920
1921–1930
1931–1940
1941–1950
1951–1960
1961–1970
1971–1980
1981–1990
1991–2000
2003–2012
B
°F
95
90
85
10 year Winter Teletherm dynamics for DUCHESNE, UT:
C
91
Mar
60
Feb
32
Jan
1
Dec
335
Nov
D
10
5
0
−5

10 year Summer Teletherm dynamics for ESCALANTE, UT:

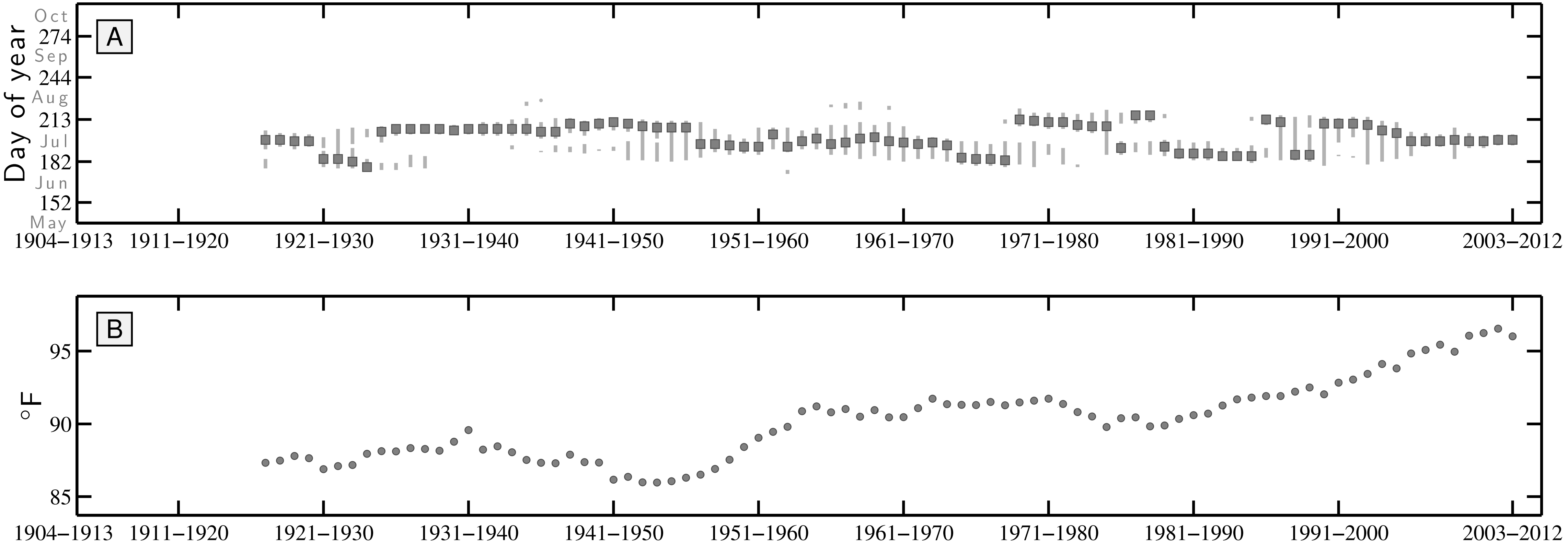

10 year Winter Teletherm dynamics for ESCALANTE, UT:

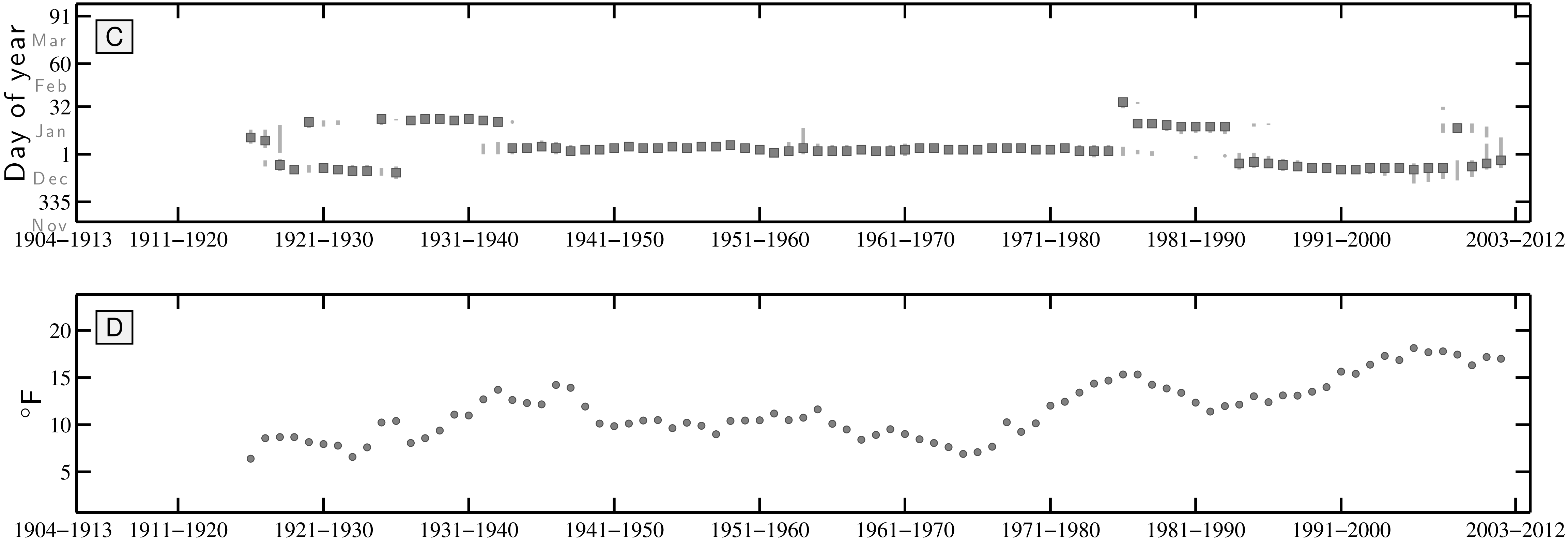

10 year Summer Teletherm dynamics for FARMINGTON 3 NW, UT:

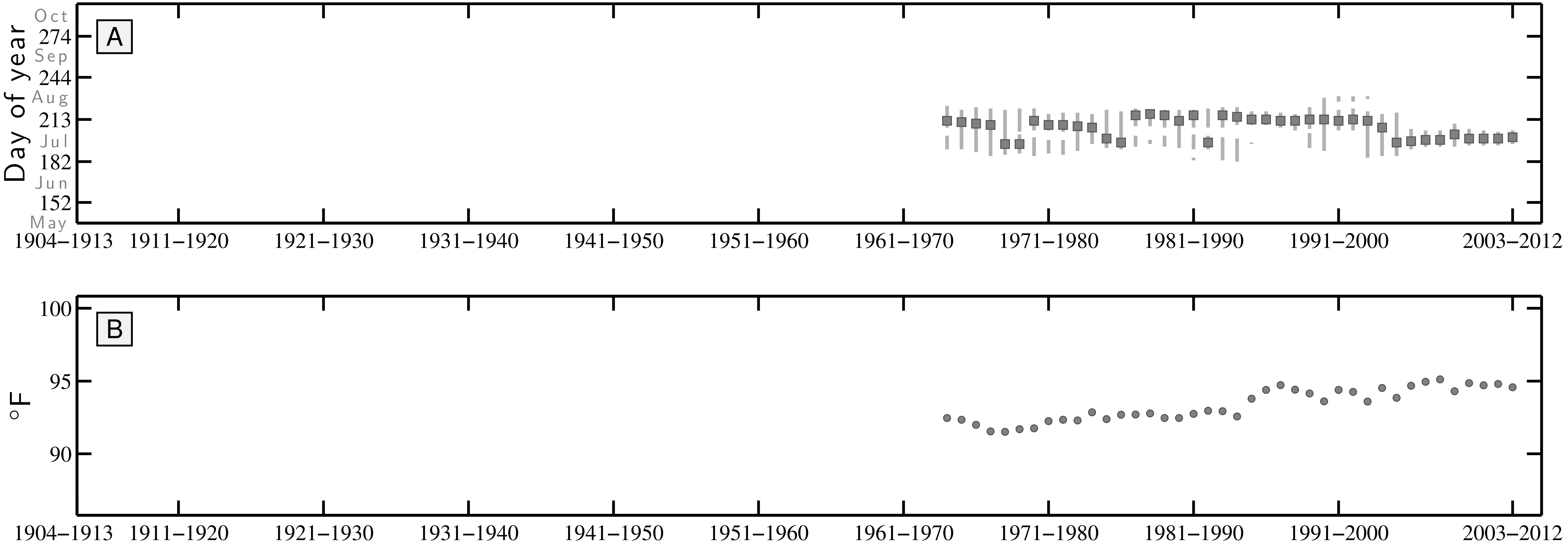

10 year Winter Teletherm dynamics for FARMINGTON 3 NW, UT:

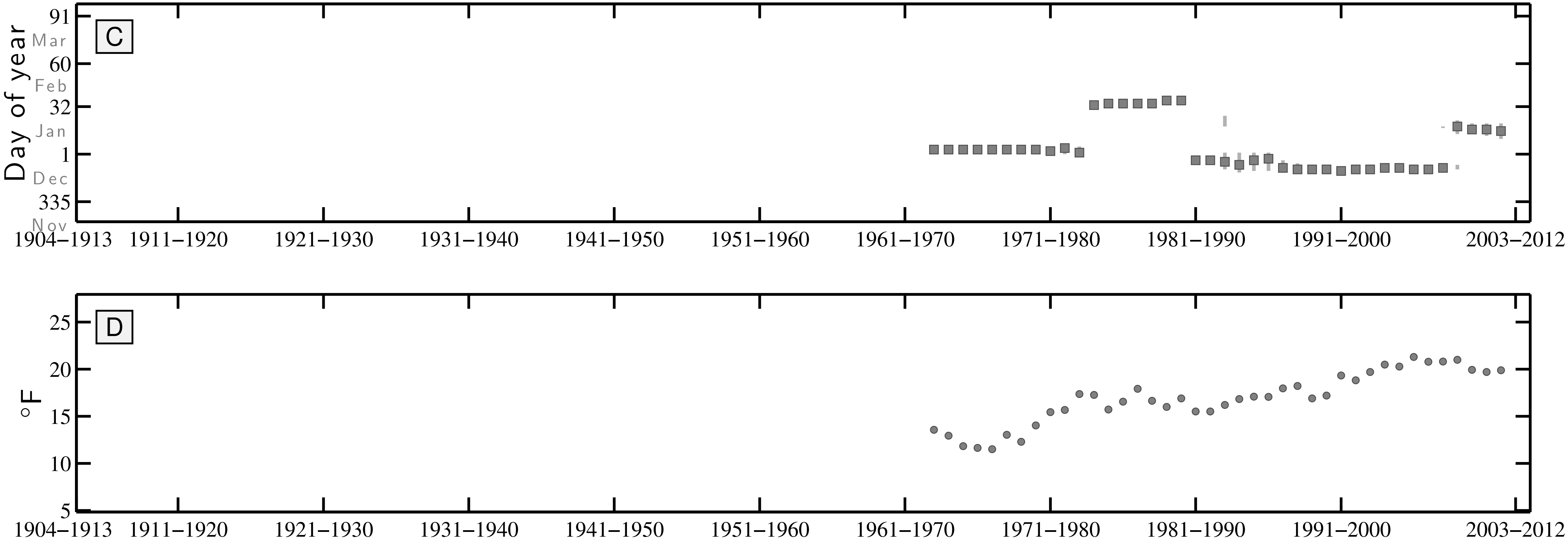

10 year Summer Teletherm dynamics for FILLMORE, UT:

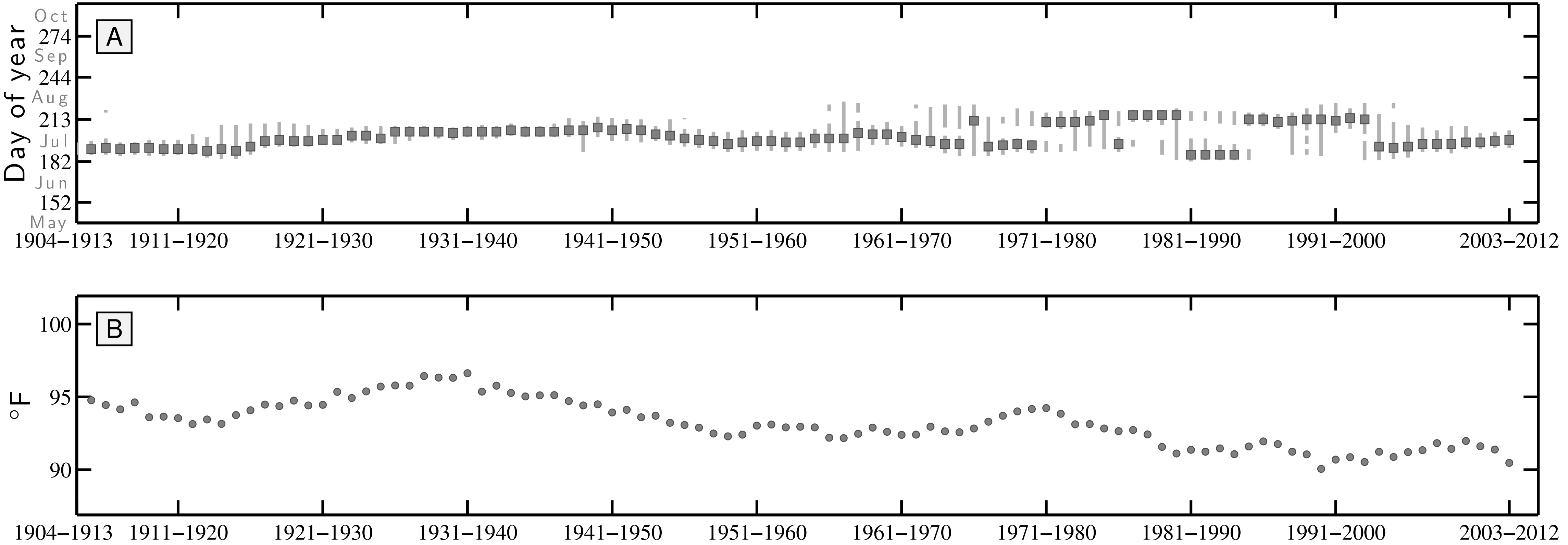

10 year Winter Teletherm dynamics for FILLMORE, UT:

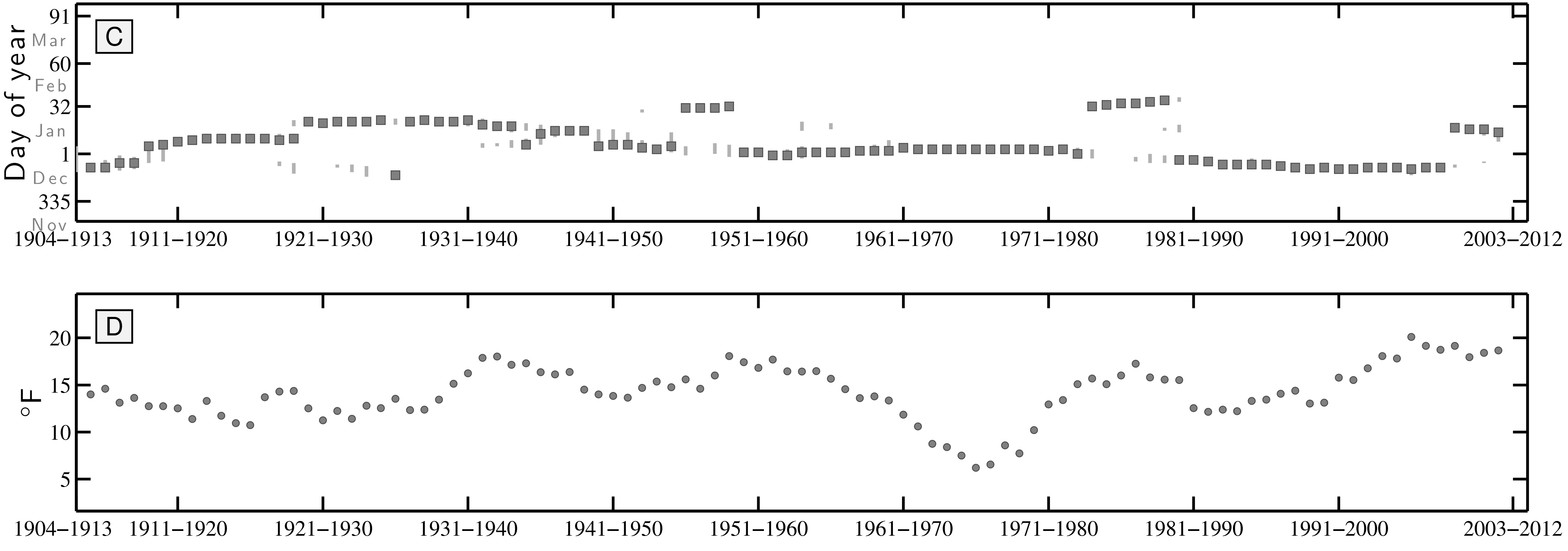

10 year Summer Teletherm dynamics for FT DUCHESNE, UT:

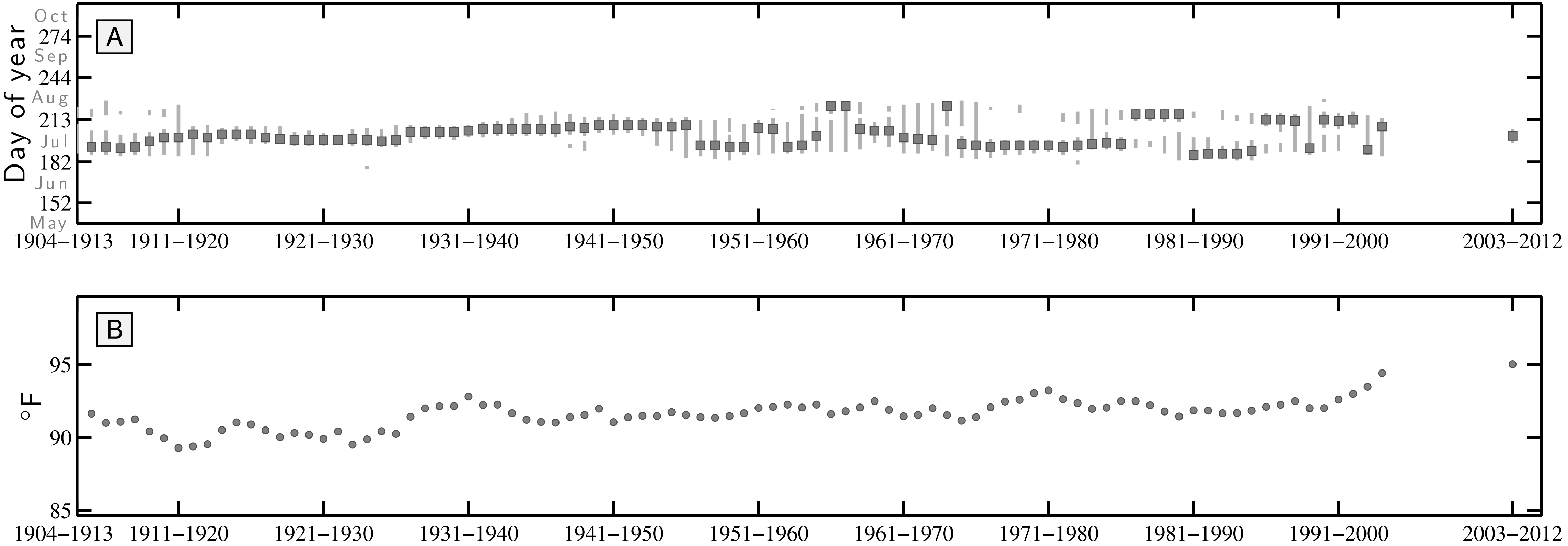

10 year Winter Teletherm dynamics for FT DUCHESNE, UT:

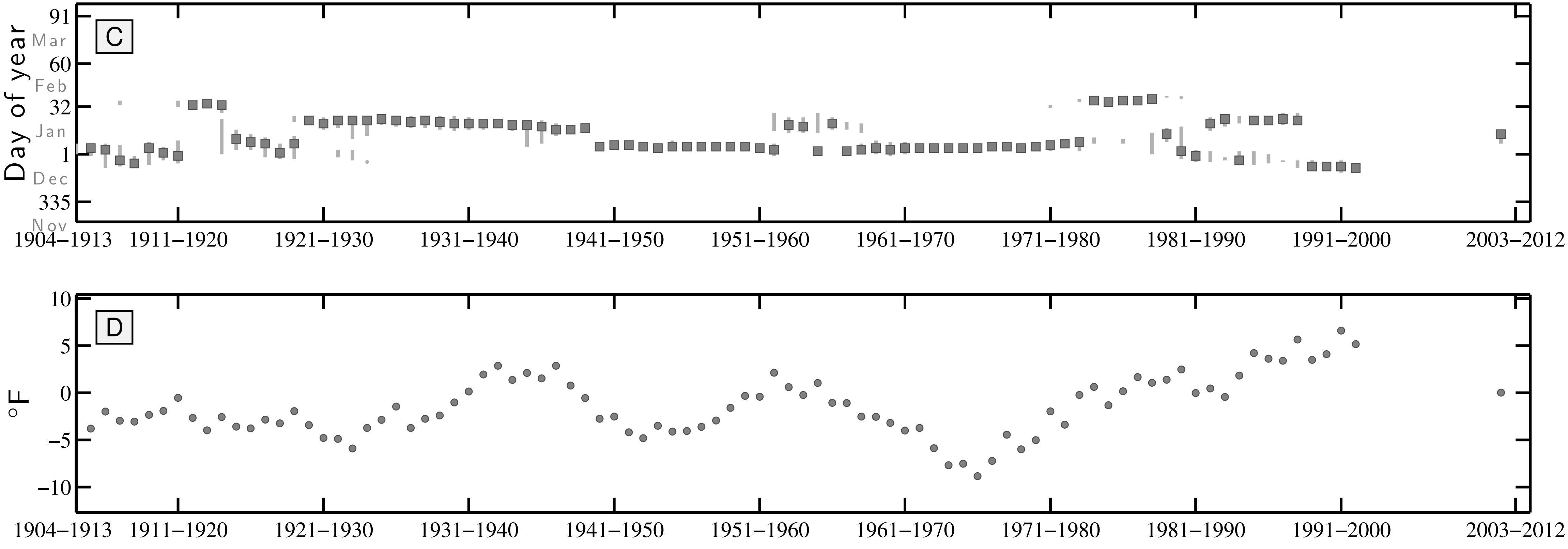

10 year Summer Teletherm dynamics for GREEN RIVER AVIATION, UT:

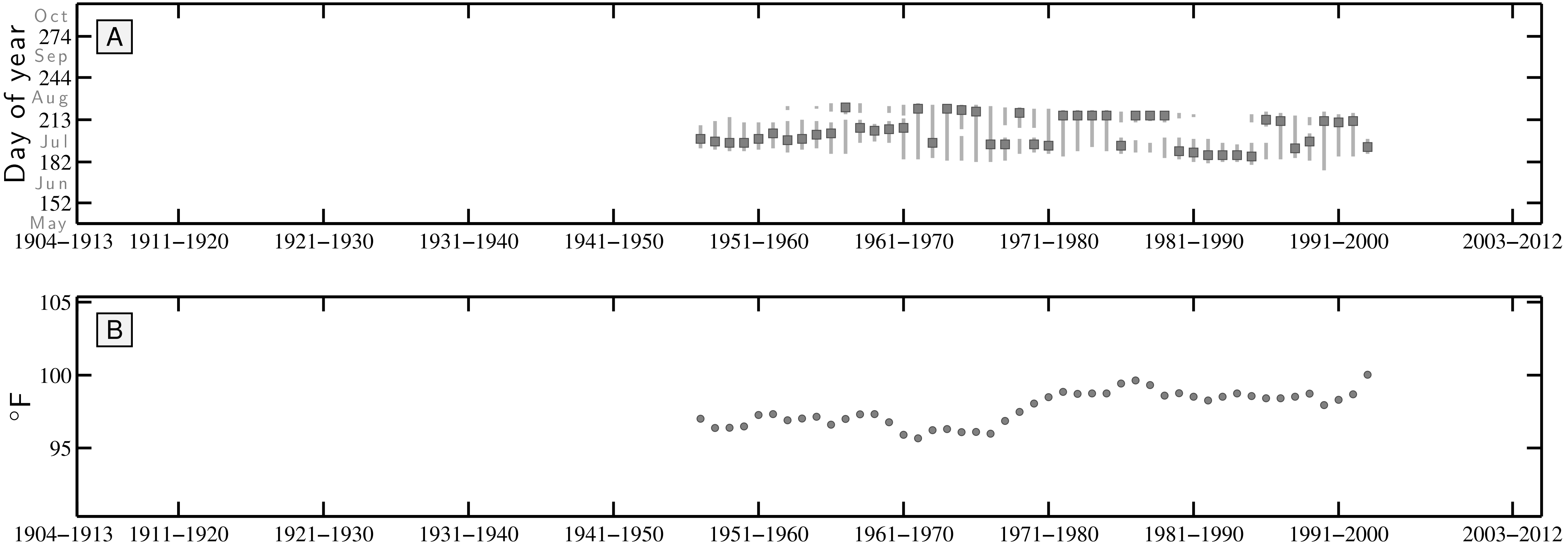

10 year Winter Teletherm dynamics for GREEN RIVER AVIATION, UT:

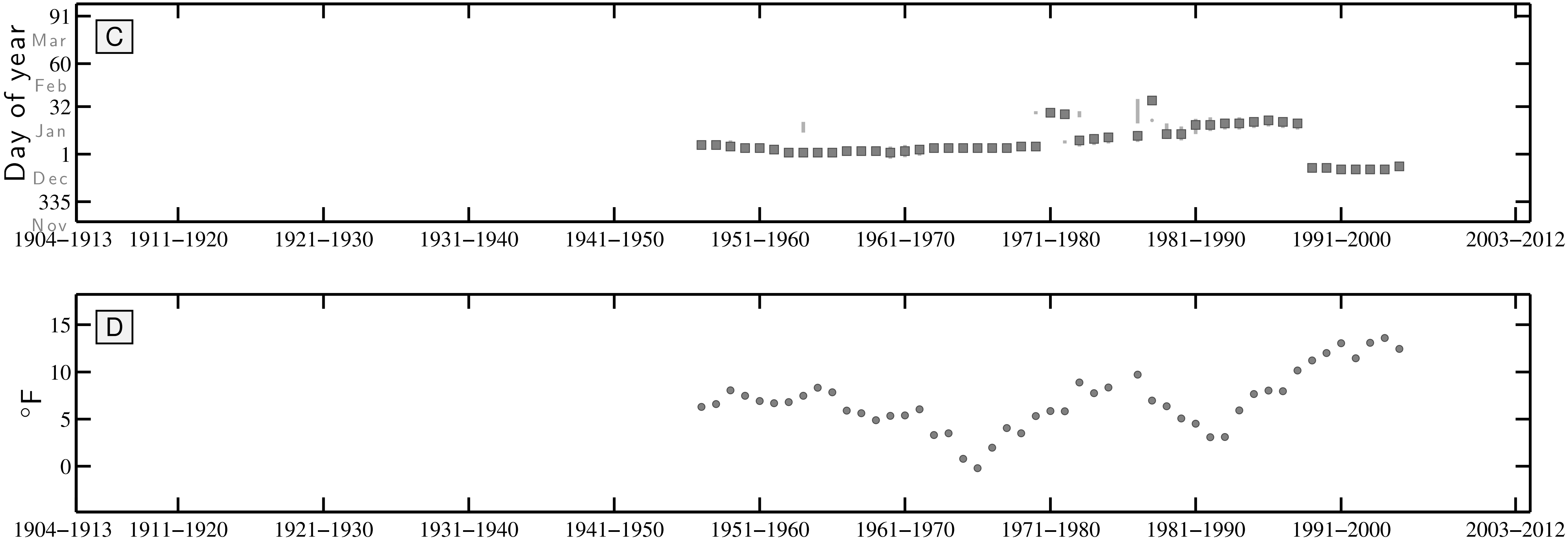

10 year Summer Teletherm dynamics for HANKSVILLE, UT:

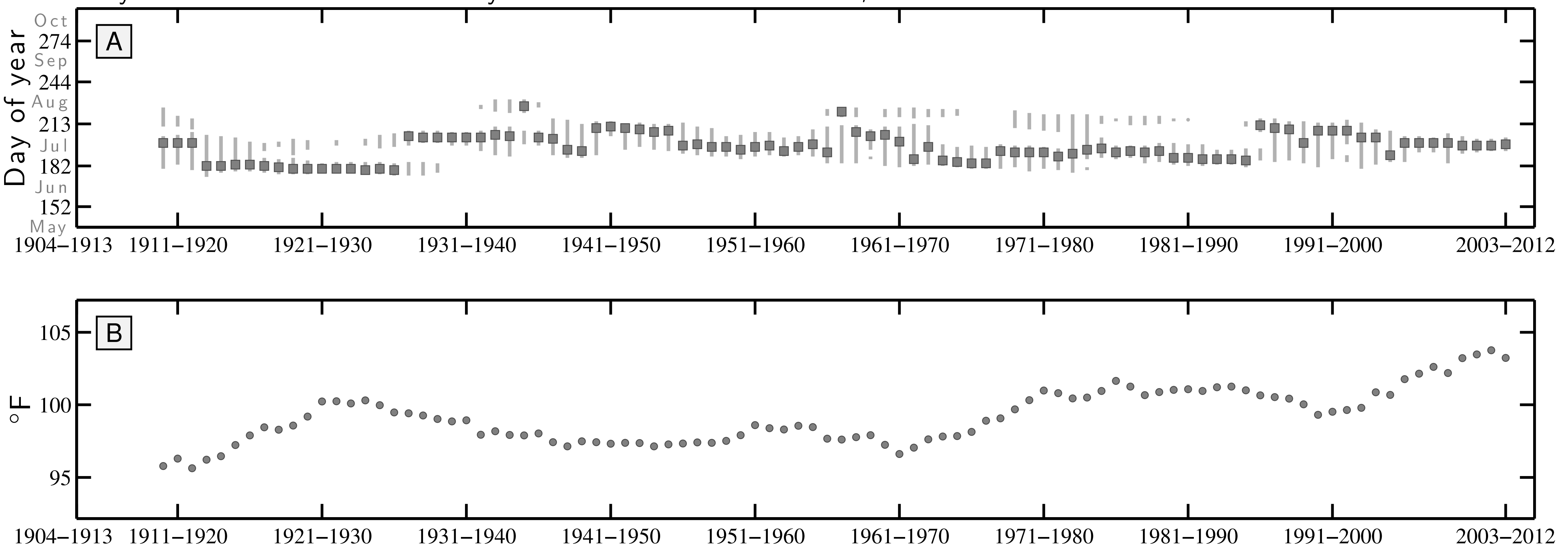

10 year Winter Teletherm dynamics for HANKSVILLE, UT:

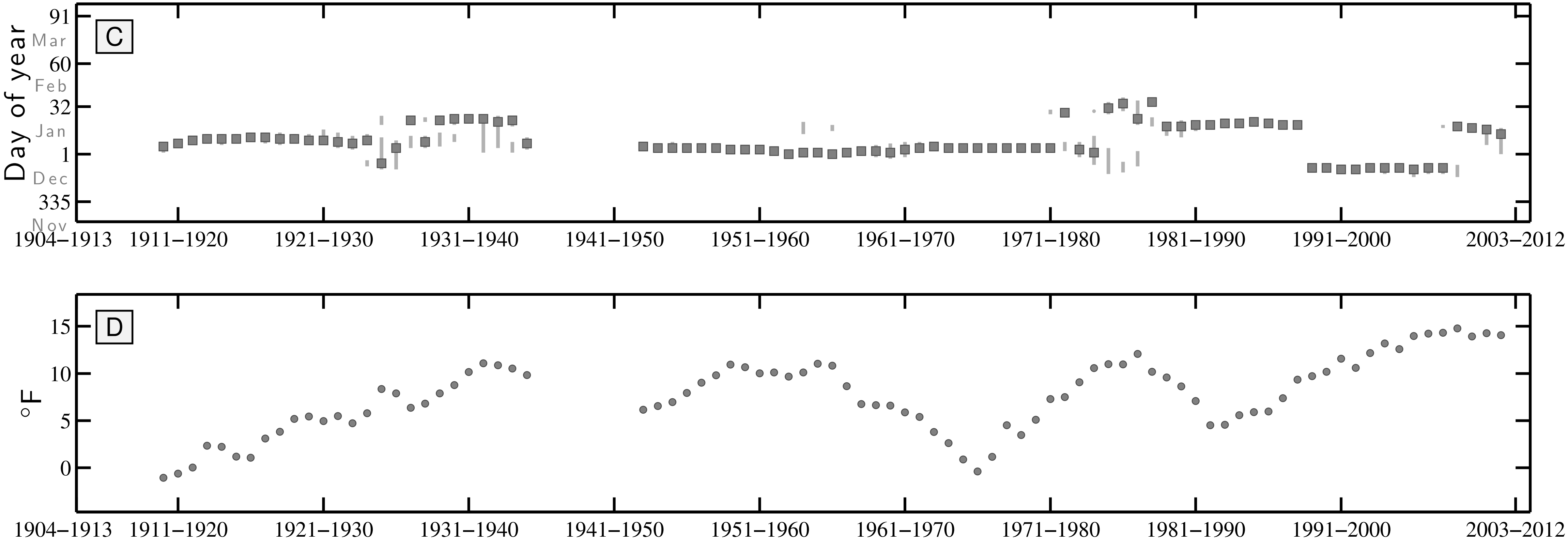

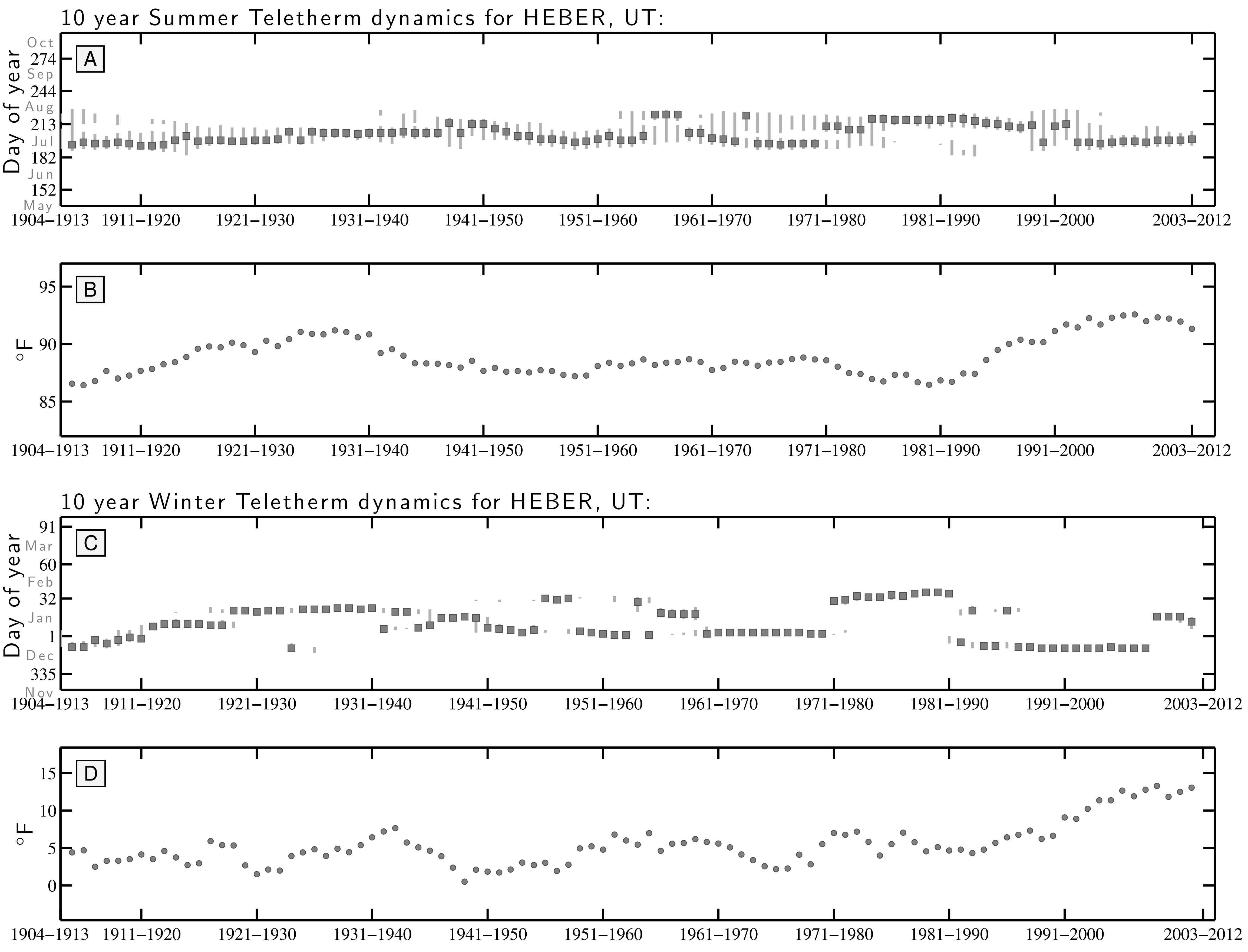
10 year Summer Teletherm dynamics for HEBER, UT:
A
Day of year
Oct
274
Sep
244
Aug
213
Jul
182
Jun
152
May
1904–1913
1911–1920
1921–1930
1931–1940
1941–1950
1951–1960
1961–1970
1971–1980
1981–1990
1991–2000
2003–2012
B
°F
95
90
85
10 year Winter Teletherm dynamics for HEBER, UT:
C
91
Mar
60
Feb
32
Jan
1
Dec
335
Nov
D
15
10
5
0

10 year Summer Teletherm dynamics for KANAB, UT:

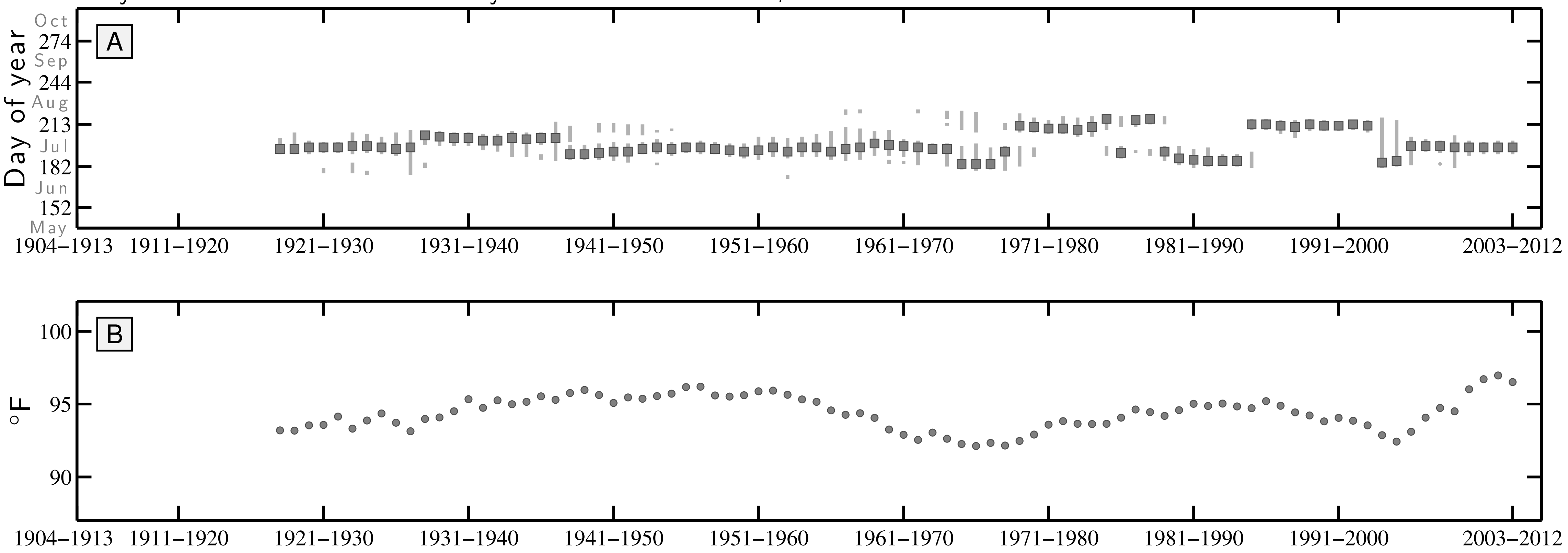

10 year Winter Teletherm dynamics for KANAB, UT:

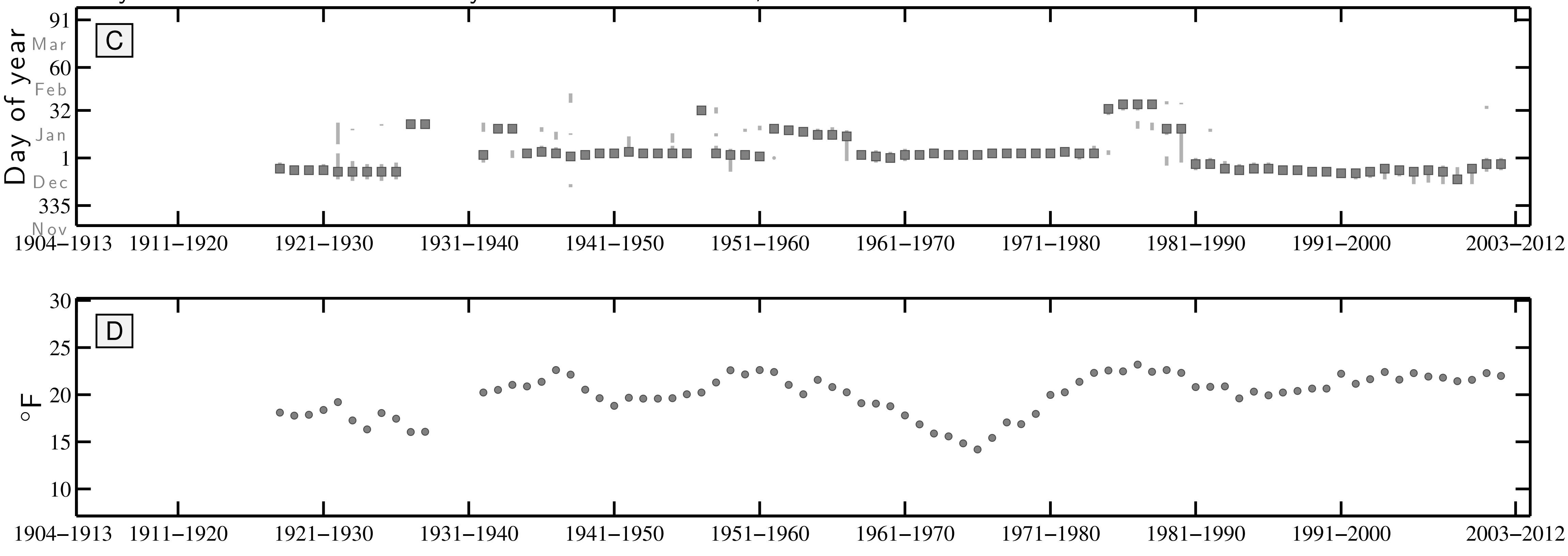

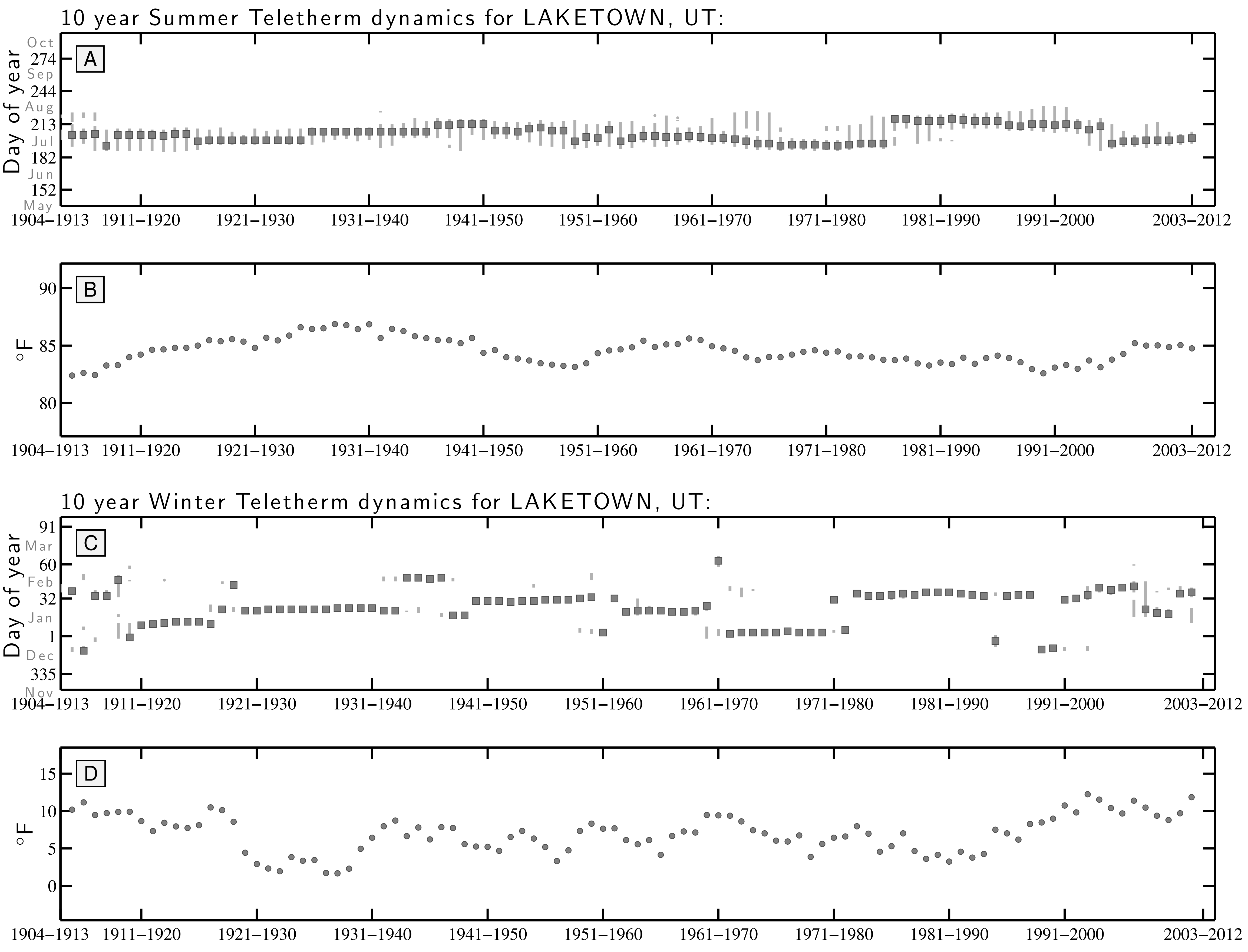

10 year Summer Teletherm dynamics for LAKETOWN, UT:
A
B
Day of year
°F
Oct
274
Sep
244
Aug
213
Jul
182
Jun
152
May
90
85
80
10 year Winter Teletherm dynamics for LAKETOWN, UT:
C
D
91
Mar
60
Feb
32
Jan
1
Dec
335
Nov
15
10
5
0
1904–1913
1911–1920
1921–1930
1931–1940
1941–1950
1951–1960
1961–1970
1971–1980
1981–1990
1991–2000
2003–2012

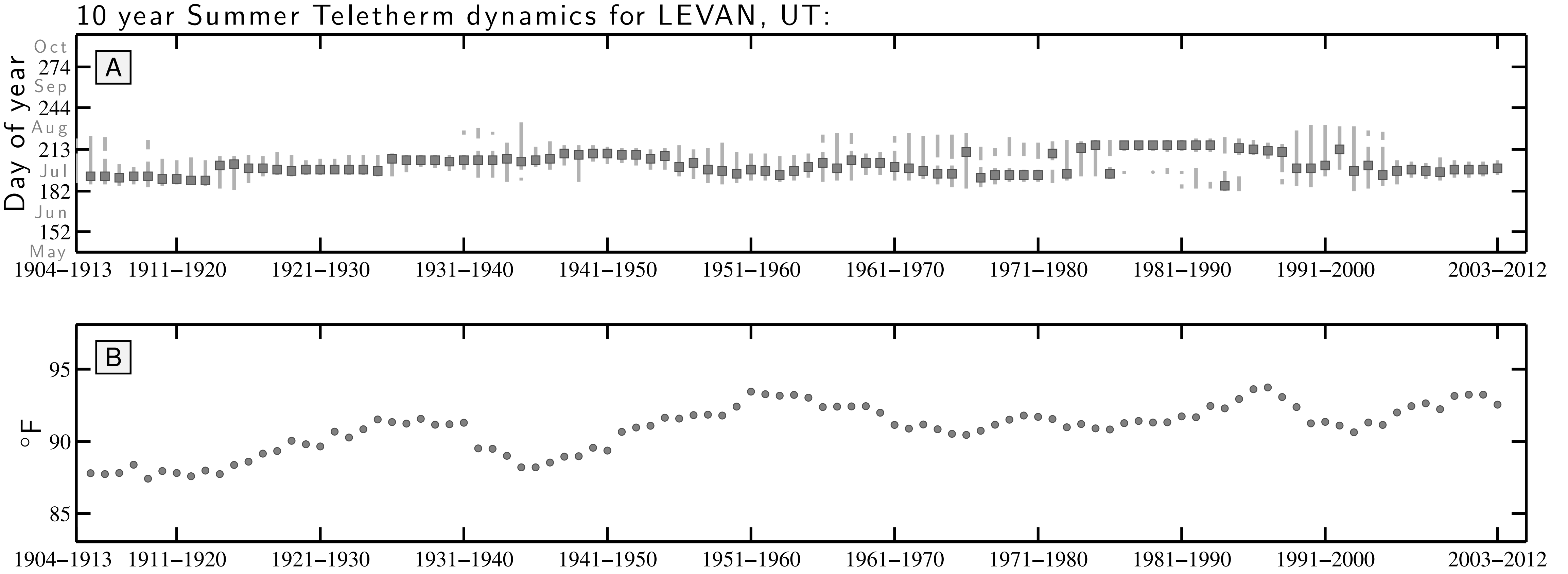
10 year Summer Teletherm dynamics for LEVAN, UT:
A
Day of year
Oct
274
Sep
244
Aug
213
Jul
182
Jun
152
May
B
°F
95
90
85
1904–1913
1911–1920
1921–1930
1931–1940
1941–1950
1951–1960
1961–1970
1971–1980
1981–1990
1991–2000
2003–2012

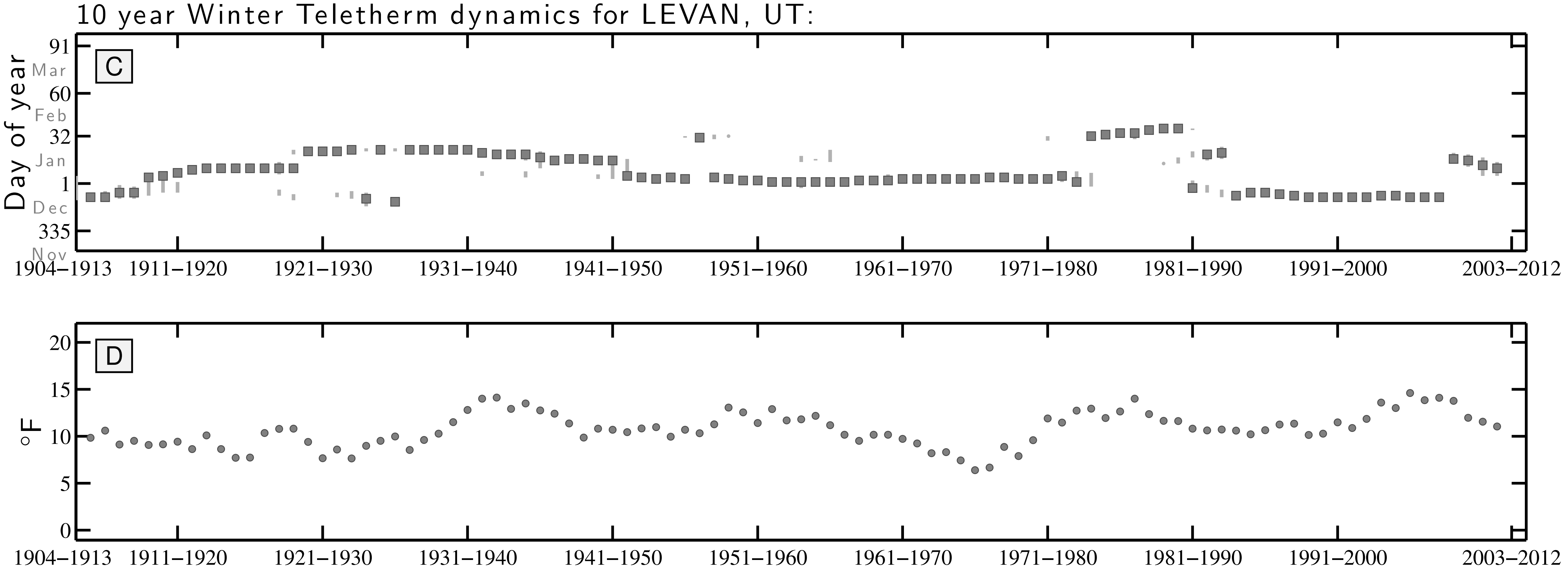
10 year Winter Teletherm dynamics for LEVAN, UT:
C
Day of year
91
Mar
60
Feb
32
Jan
1
Dec
335
Nov
D
°F
20
15
10
5
0
1904–1913
1911–1920
1921–1930
1931–1940
1941–1950
1951–1960
1961–1970
1971–1980
1981–1990
1991–2000
2003–2012

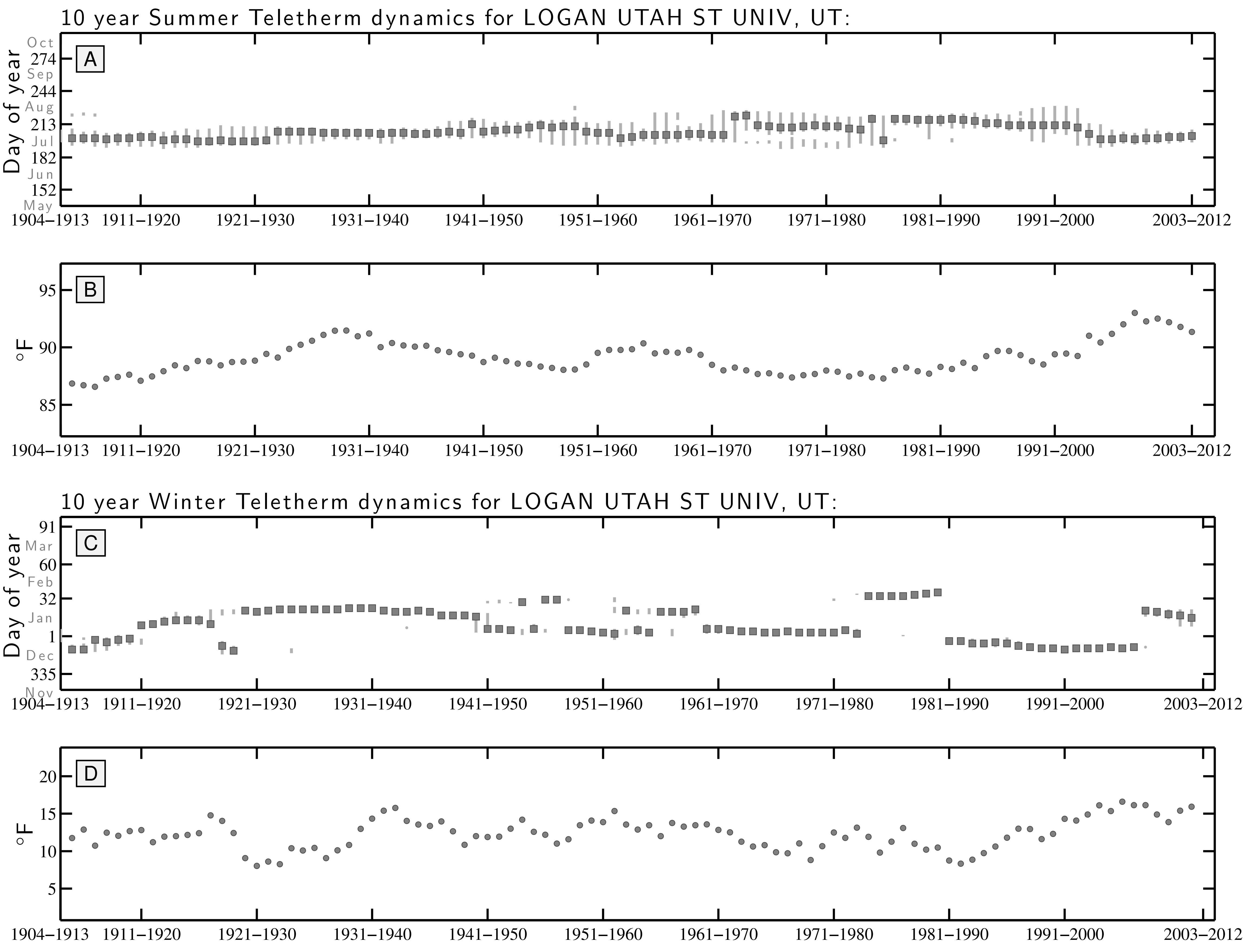
10 year Summer Teletherm dynamics for LOGAN UTAH ST UNIV, UT:
A
Day of year
Oct
274
Sep
244
Aug
213
Jul
182
Jun
152
May
B
°F
95
90
85
1904–1913
1911–1920
1921–1930
1931–1940
1941–1950
1951–1960
1961–1970
1971–1980
1981–1990
1991–2000
2003–2012
10 year Winter Teletherm dynamics for LOGAN UTAH ST UNIV, UT:
C
Day of year
91
Mar
60
Feb
32
Jan
1
Dec
335
Nov
D
°F
20
15
10
5

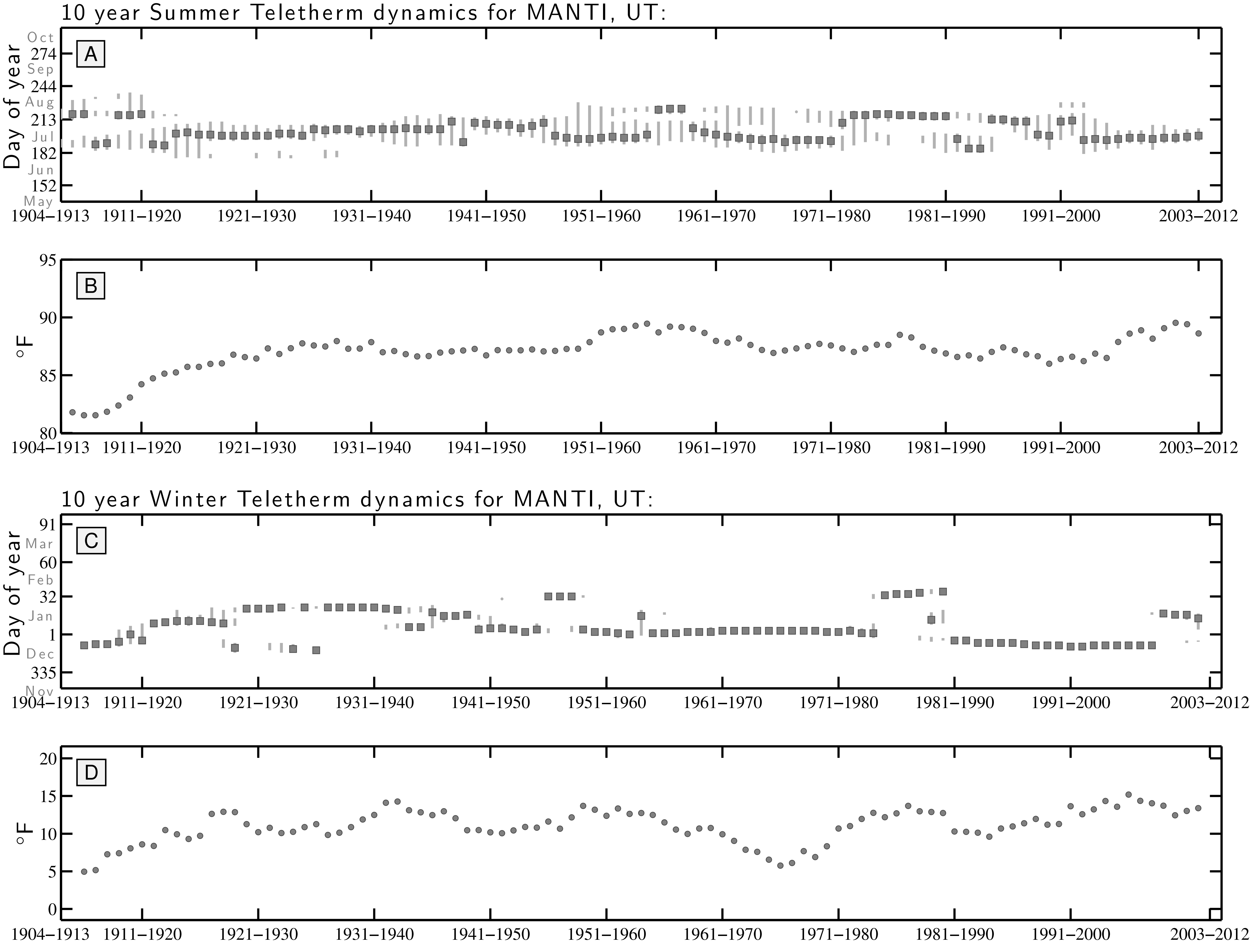

10 year Summer Teletherm dynamics for MANTI, UT:
A
Day of year
Oct
274
Sep
244
Aug
213
Jul
182
Jun
152
May
1904–1913
1911–1920
1921–1930
1931–1940
1941–1950
1951–1960
1961–1970
1971–1980
1981–1990
1991–2000
2003–2012
B
°F
95
90
85
80
10 year Winter Teletherm dynamics for MANTI, UT:
C
91
Mar
60
Feb
32
Jan
1
Dec
335
Nov
D
20
15
10
5
0

10 year Summer Teletherm dynamics for MARYSVALE, UT:

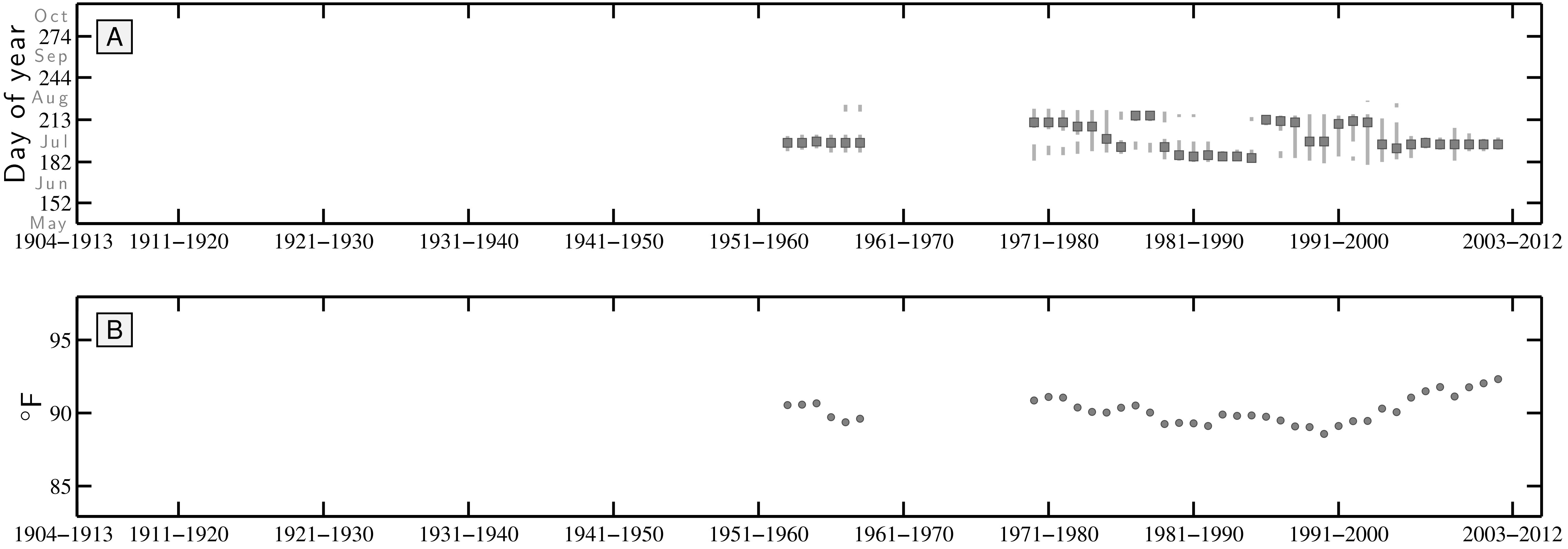

10 year Winter Teletherm dynamics for MARYSVALE, UT:

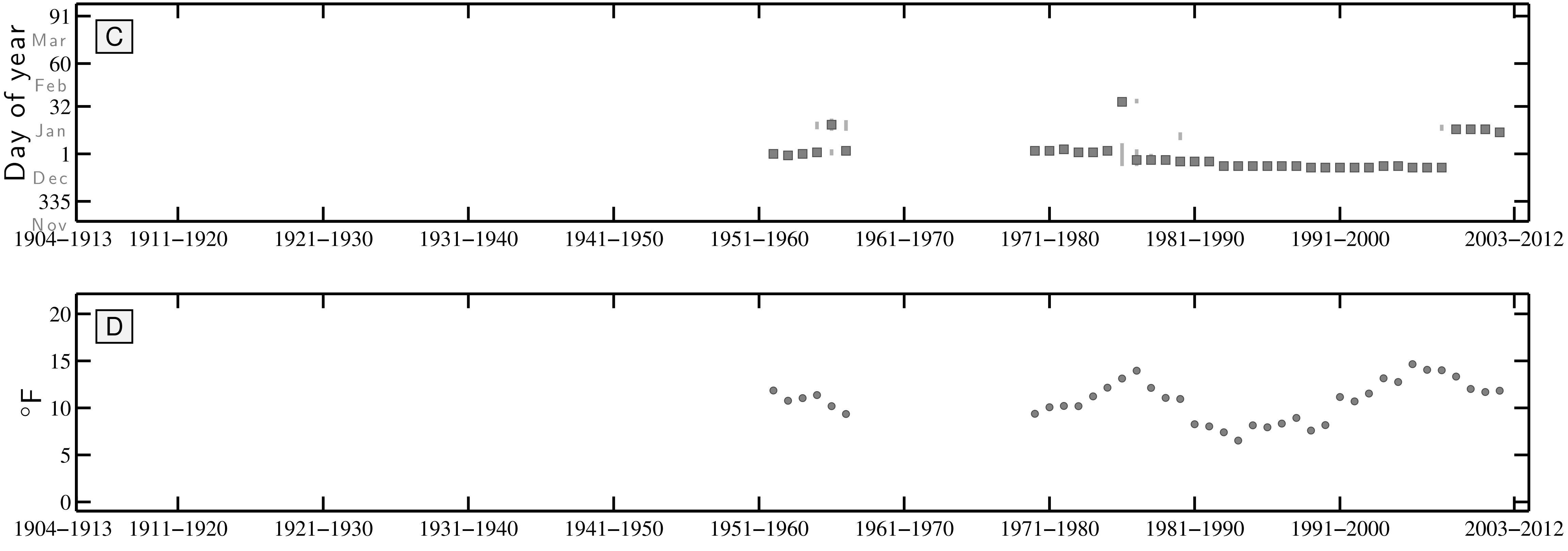

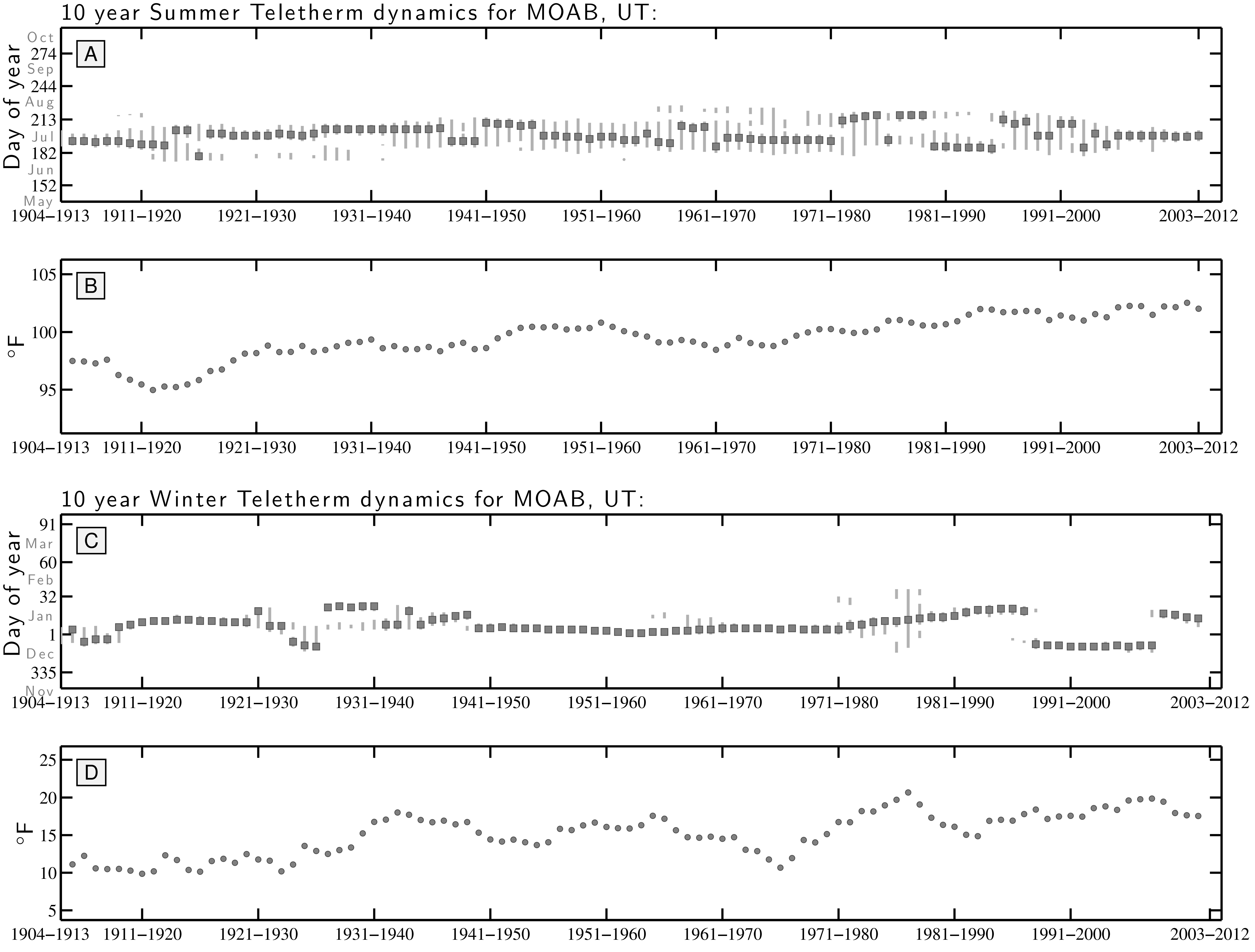
10 year Summer Teletherm dynamics for MOAB, UT:
A
Day of year
Oct
274
Sep
244
Aug
213
Jul
182
Jun
152
May
B
°F
105
100
95
10 year Winter Teletherm dynamics for MOAB, UT:
C
Day of year
91
Mar
60
Feb
32
Jan
1
Dec
335
Nov
D
°F
25
20
15
10
5
1904–1913
1911–1920
1921–1930
1931–1940
1941–1950
1951–1960
1961–1970
1971–1980
1981–1990
1991–2000
2003–2012

10 year Summer Teletherm dynamics for MODENA, UT:

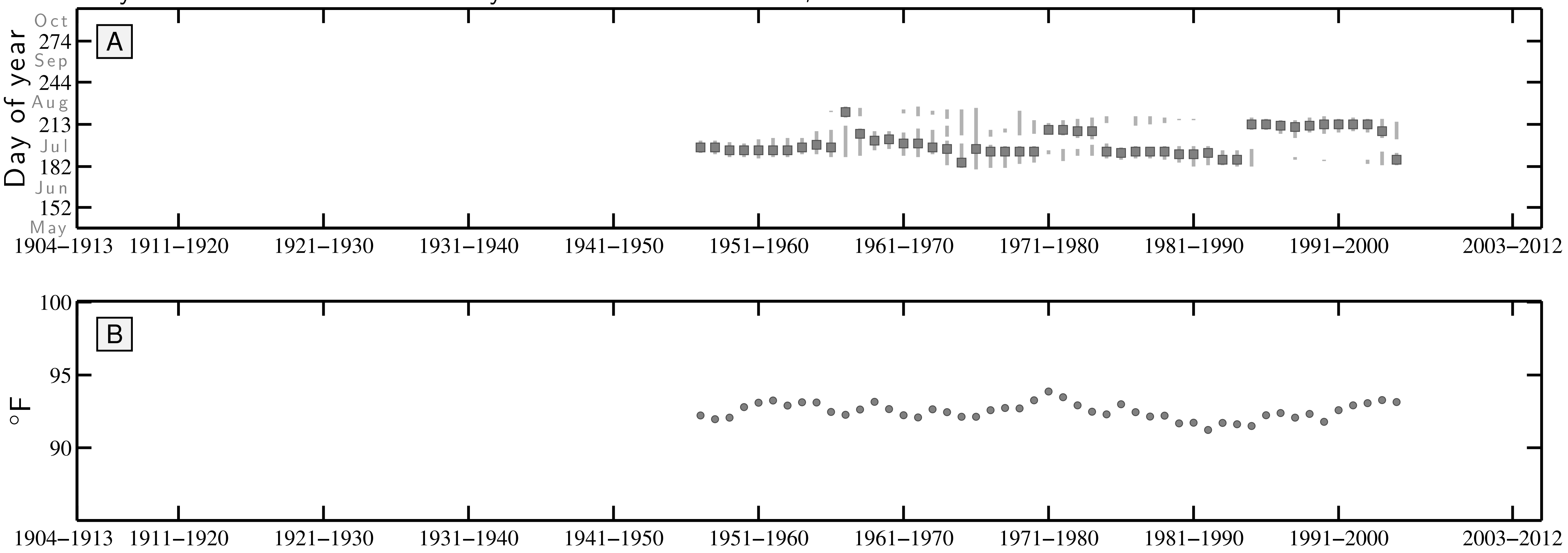

10 year Winter Teletherm dynamics for MODENA, UT:

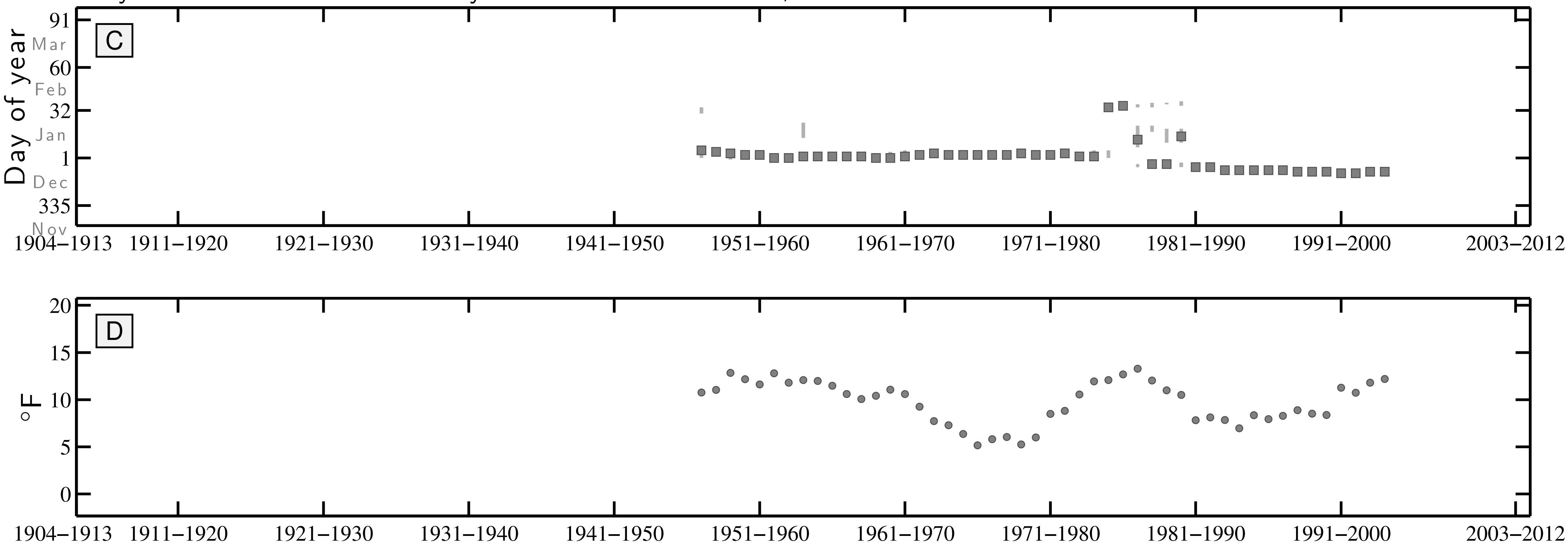

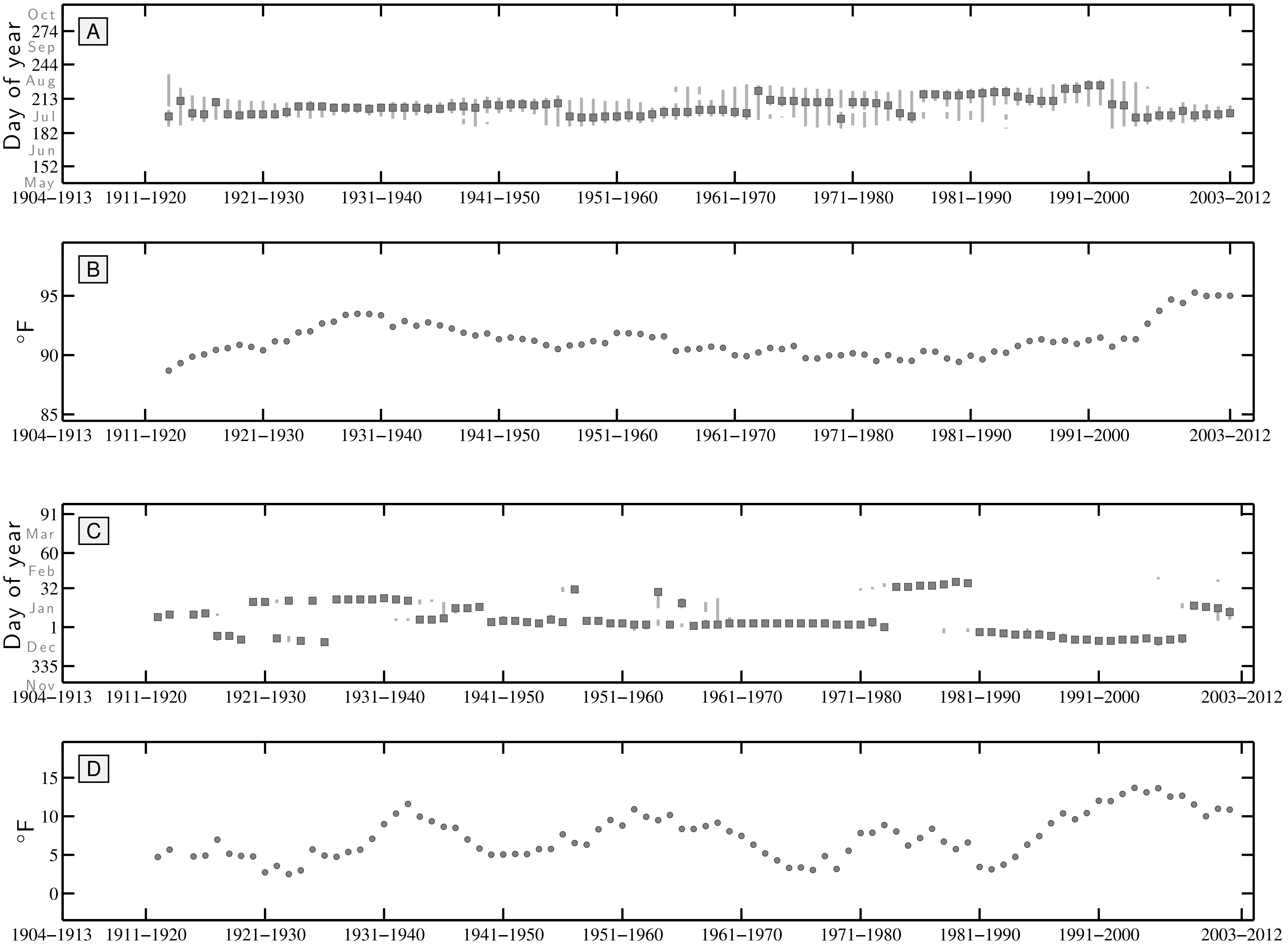
A
B
C
D
Day of year
°F
Oct
274
Sep
244
Aug
213
Jul
182
Jun
152
May
95
90
85
91
Mar
60
Feb
32
Jan
1
Dec
335
Nov
15
10
5
0
1904–1913
1911–1920
1921–1930
1931–1940
1941–1950
1951–1960
1961–1970
1971–1980
1981–1990
1991–2000
2003–2012

10 year Summer Teletherm dynamics for NEPHI, UT:

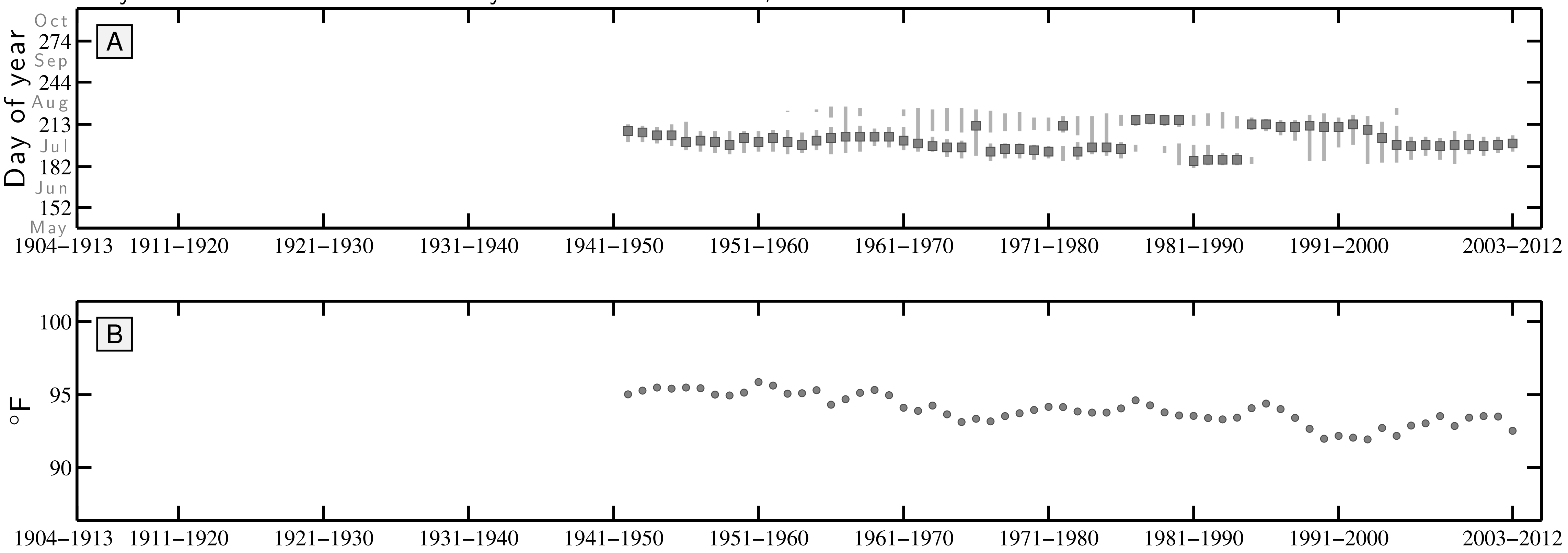

10 year Winter Teletherm dynamics for NEPHI, UT:

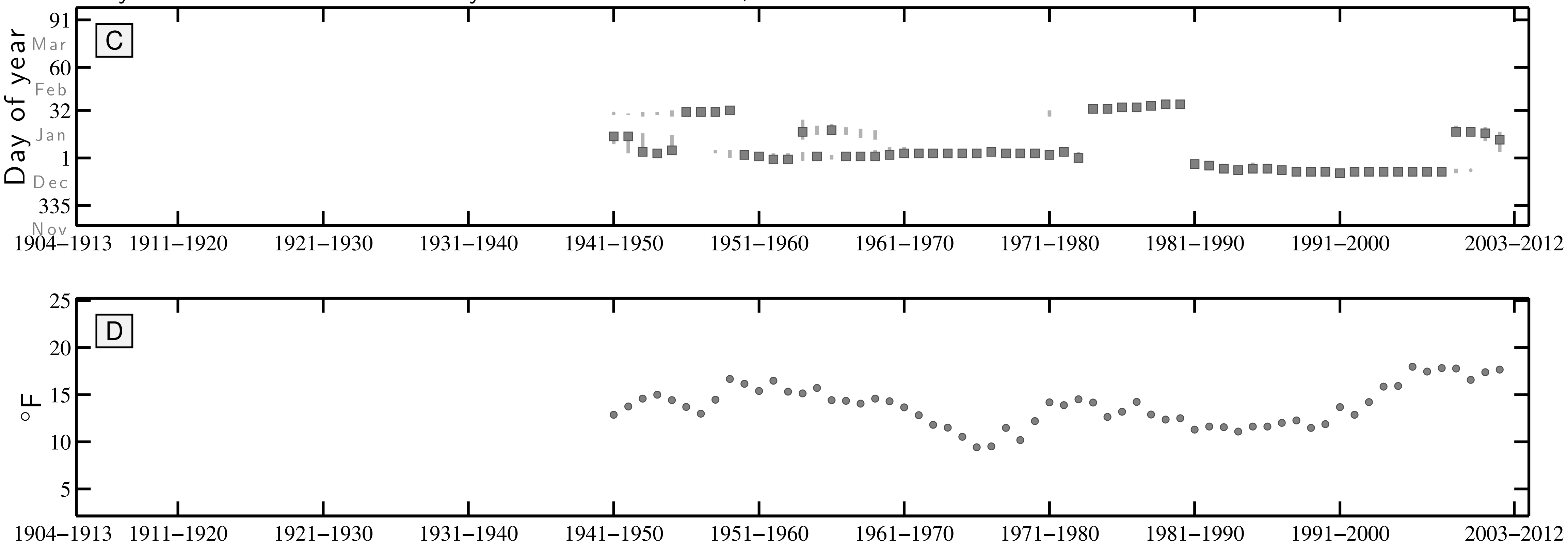

10 year Summer Teletherm dynamics for OGDEN PIONEER P H, UT:

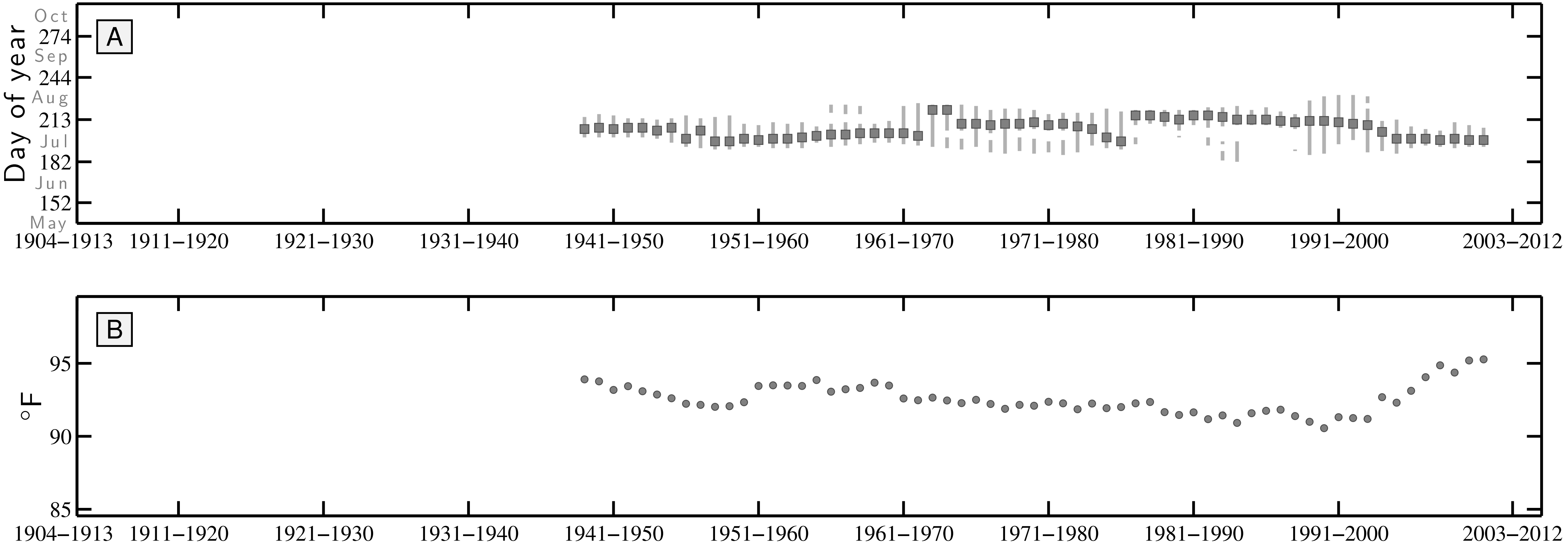

10 year Winter Teletherm dynamics for OGDEN PIONEER P H, UT:

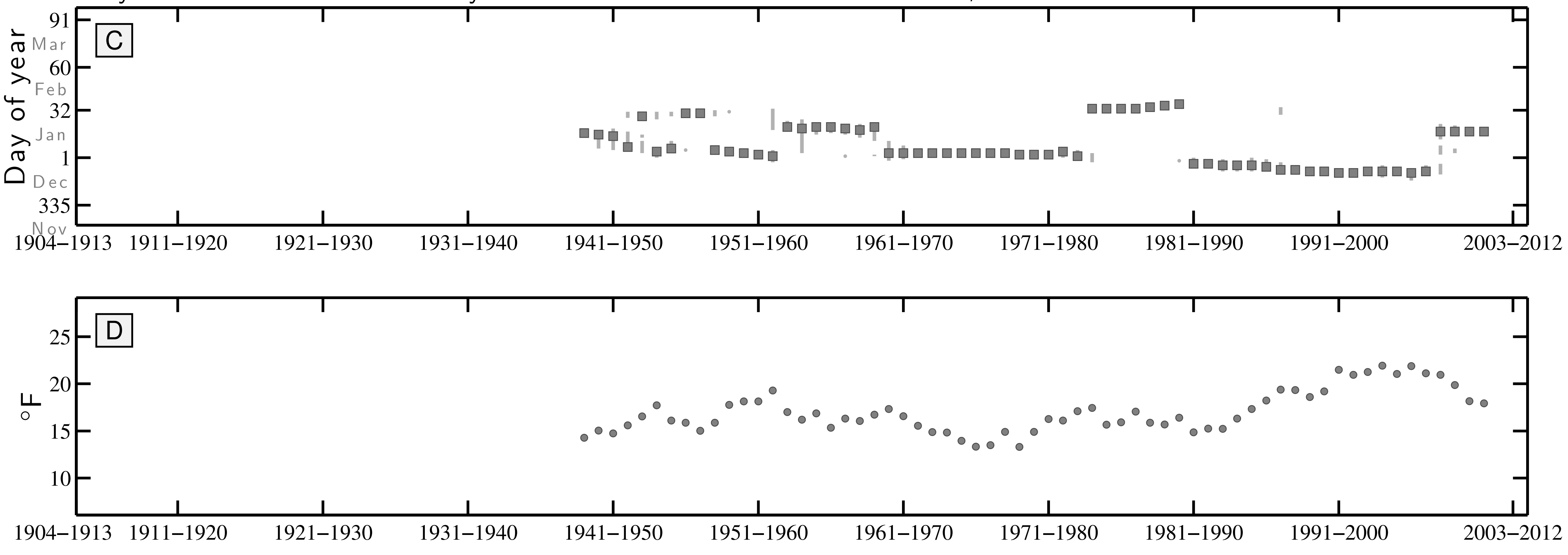

10 year Summer Teletherm dynamics for PANGUITCH, UT:

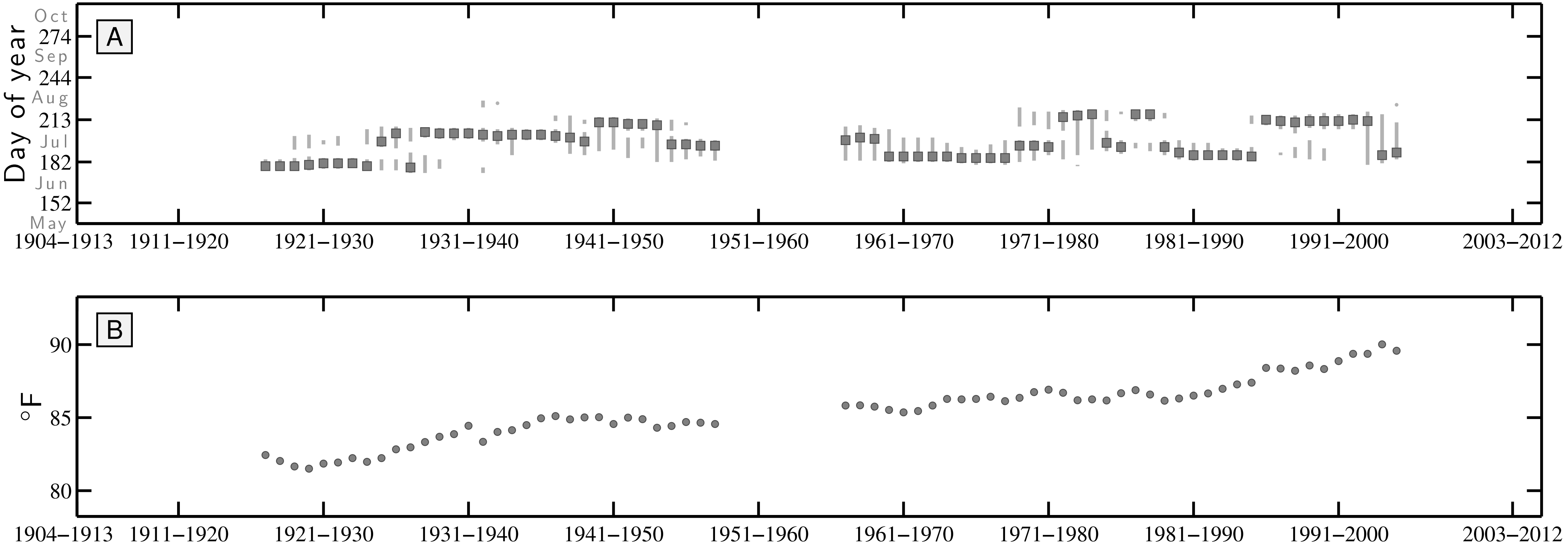

10 year Winter Teletherm dynamics for PANGUITCH, UT:

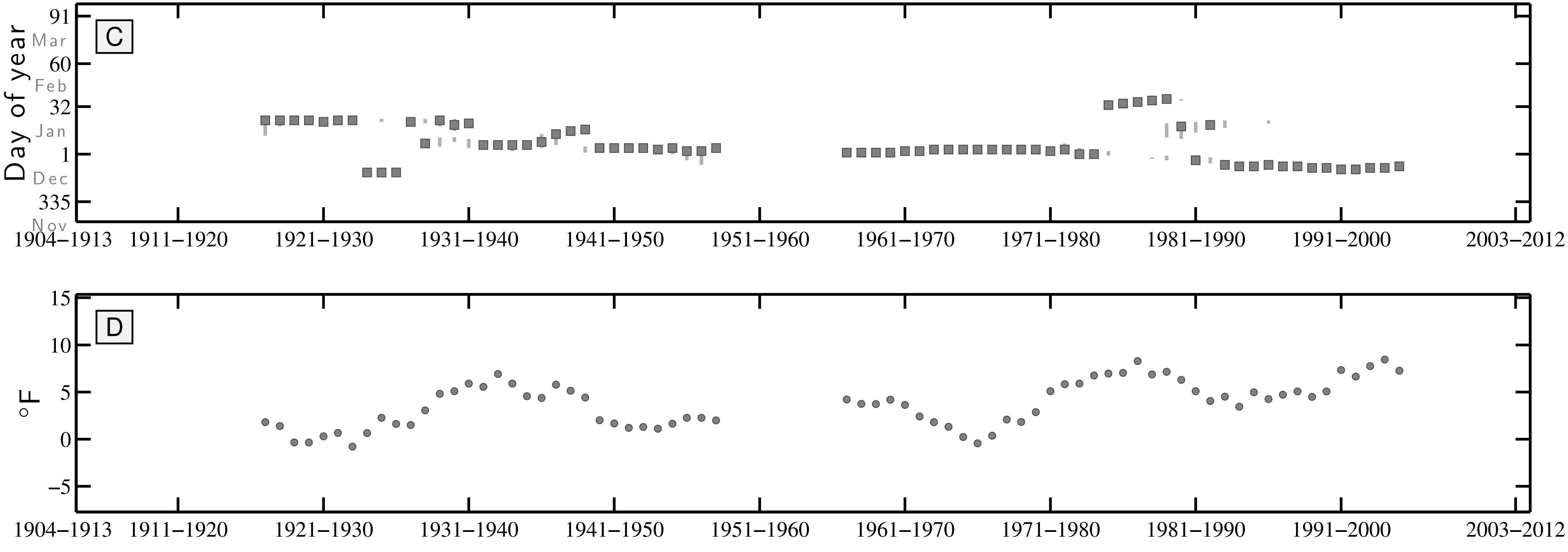

10 year Summer Teletherm dynamics for PAROWAN PWR, UT:

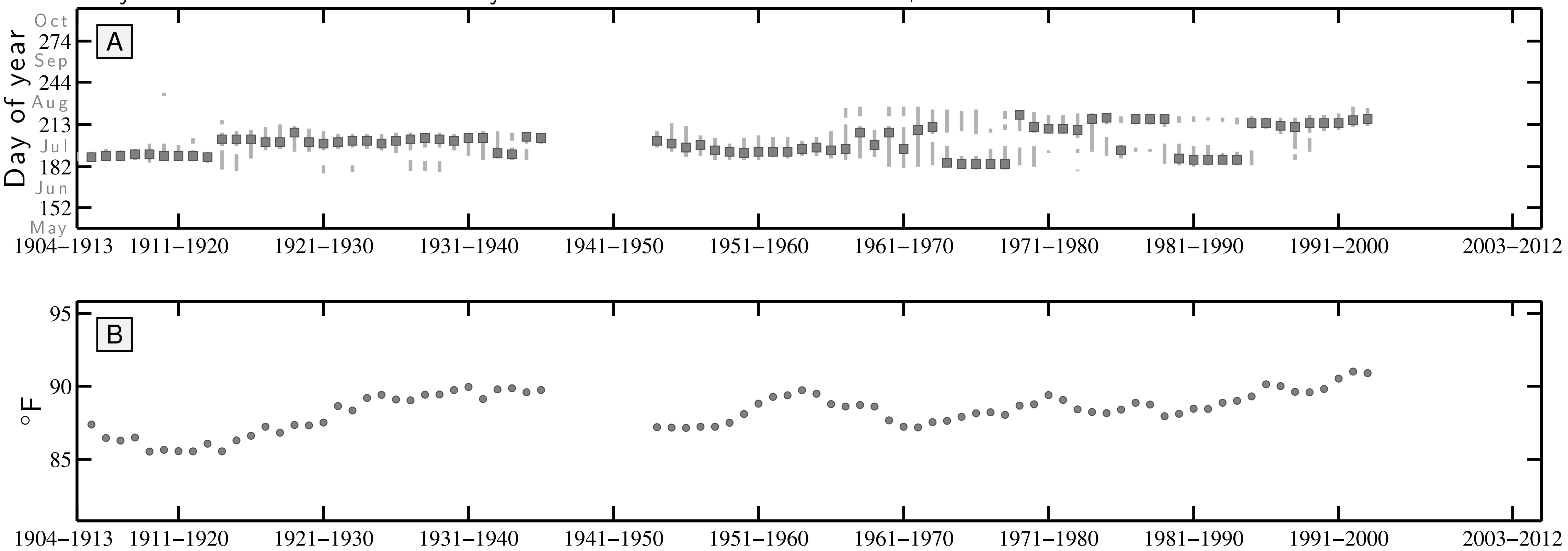

10 year Winter Teletherm dynamics for PAROWAN PWR, UT:

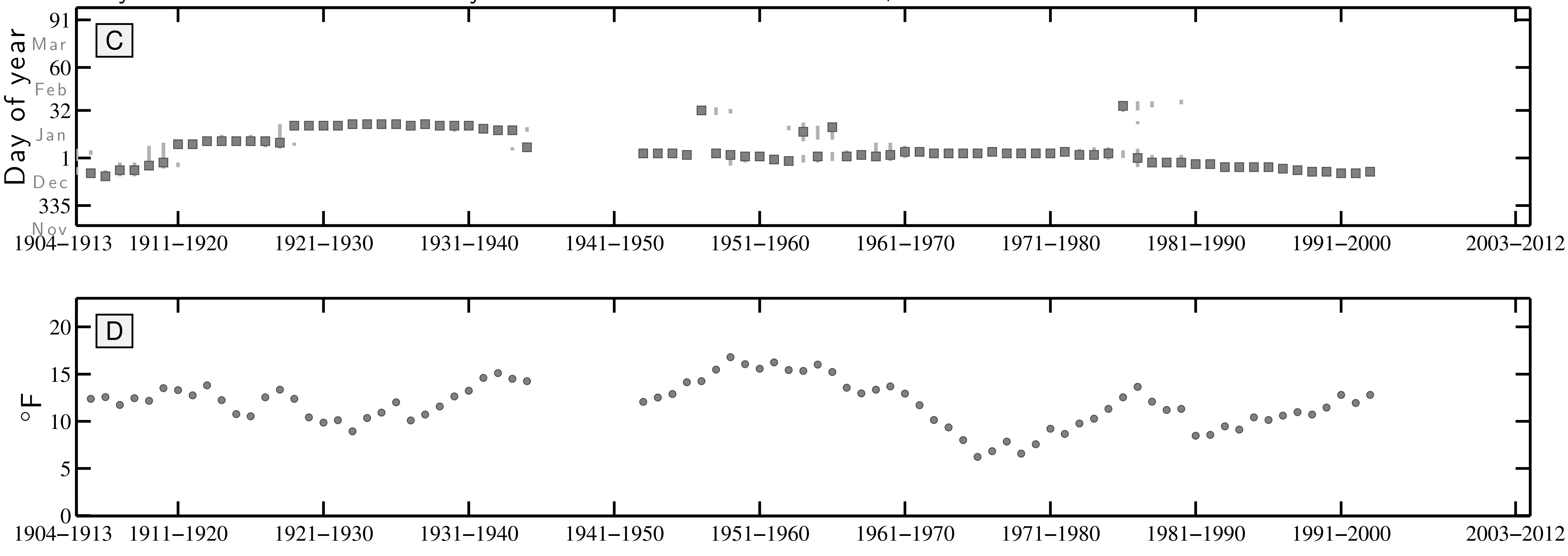

10 year Summer Teletherm dynamics for RICHFIELD RADIO KSVC, UT:

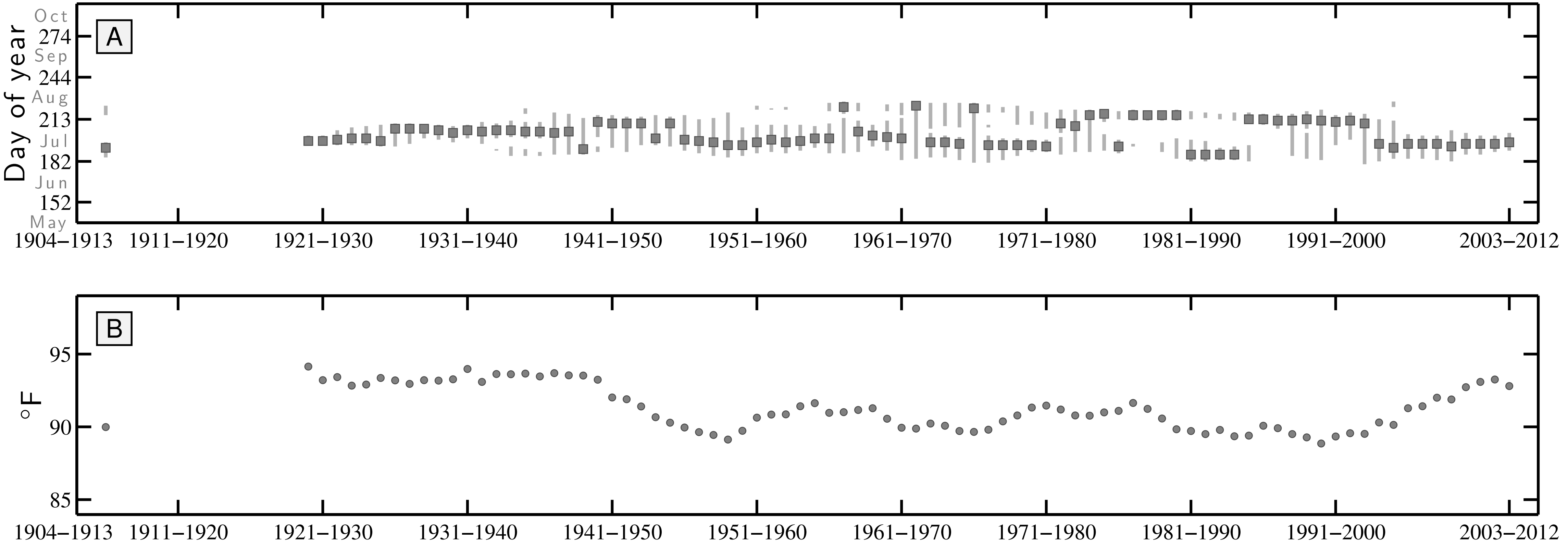

10 year Winter Teletherm dynamics for RICHFIELD RADIO KSVC, UT:

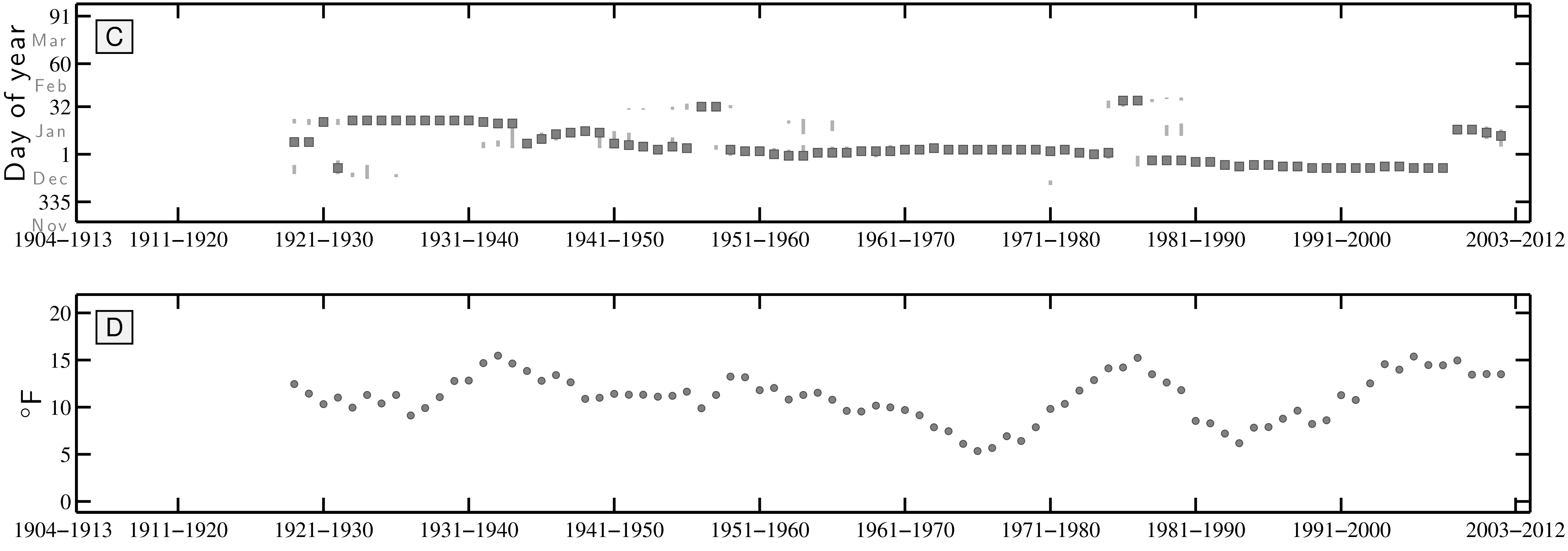

10 year Summer Teletherm dynamics for ST GEORGE, UT:

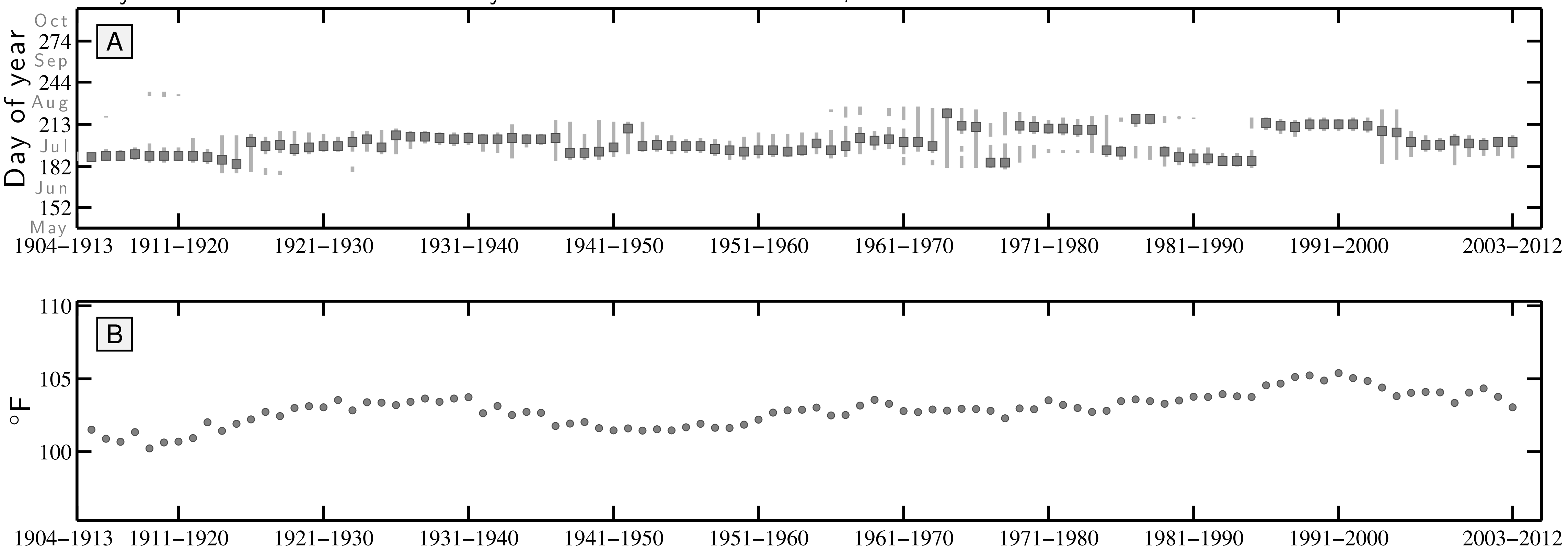

10 year Winter Teletherm dynamics for ST GEORGE, UT:

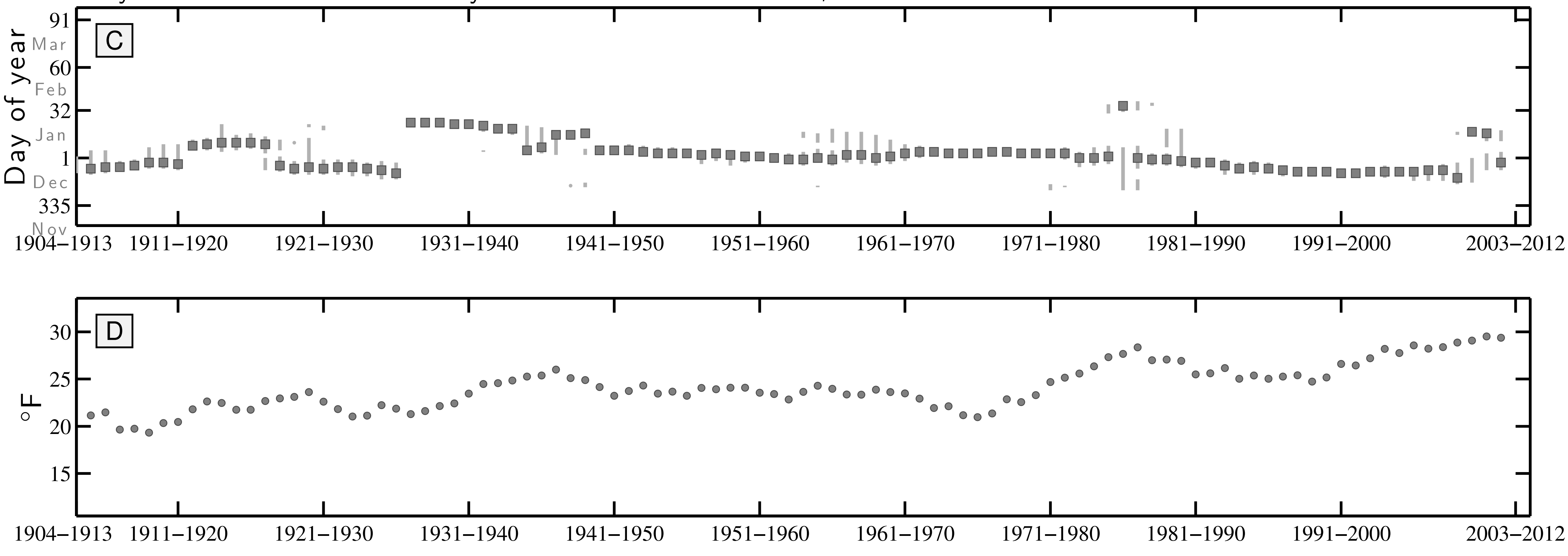

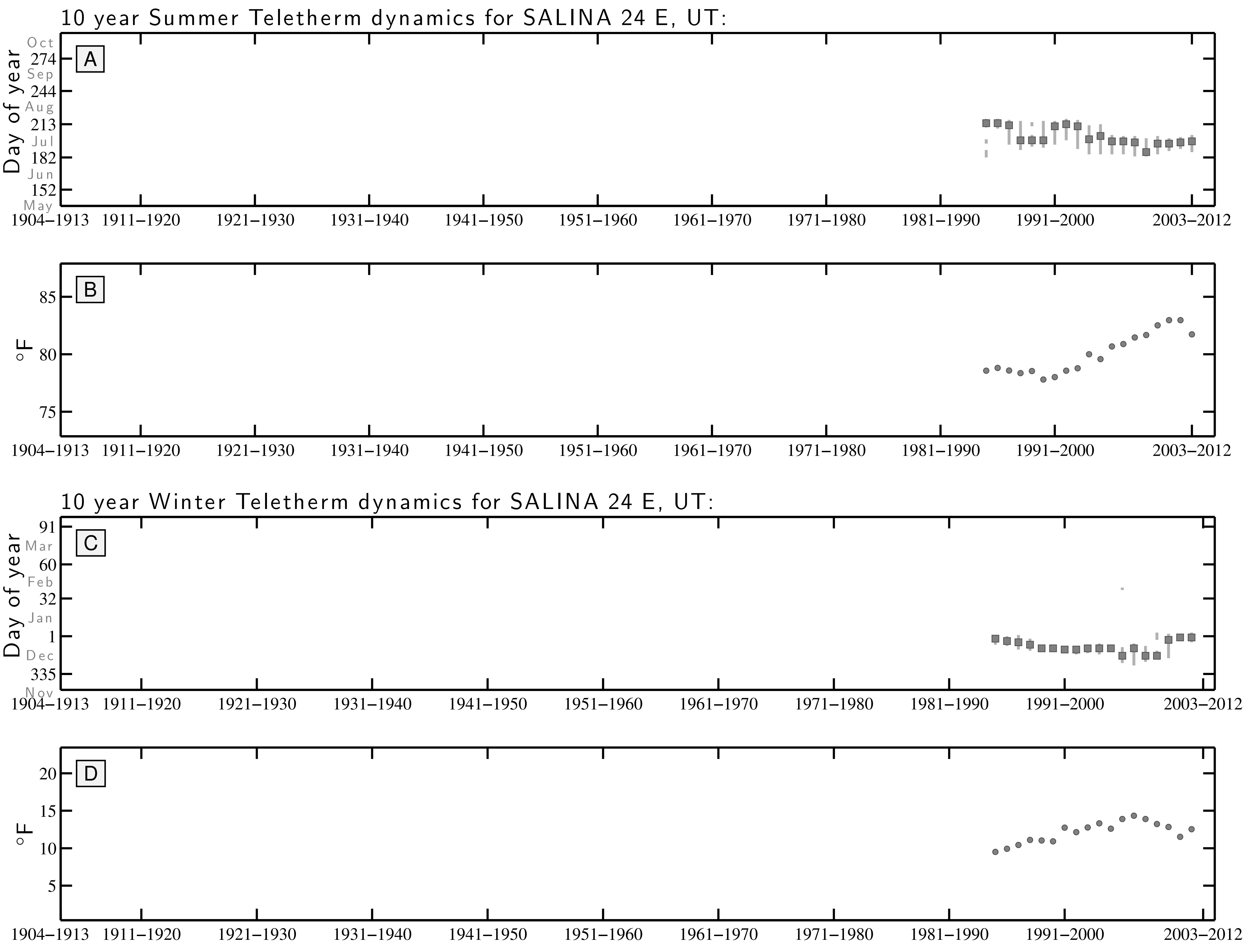

10 year Summer Teletherm dynamics for SALINA 24 E, UT:
A
B
Day of year
°F
Oct
274
Sep
244
Aug
213
Jul
182
Jun
152
May
85
80
75
1904–1913
1911–1920
1921–1930
1931–1940
1941–1950
1951–1960
1961–1970
1971–1980
1981–1990
1991–2000
2003–2012
10 year Winter Teletherm dynamics for SALINA 24 E, UT:
C
D
91
Mar
60
Feb
32
Jan
1
Dec
335
Nov
20
15
10
5

10 year Summer Teletherm dynamics for SCIPIO, UT:

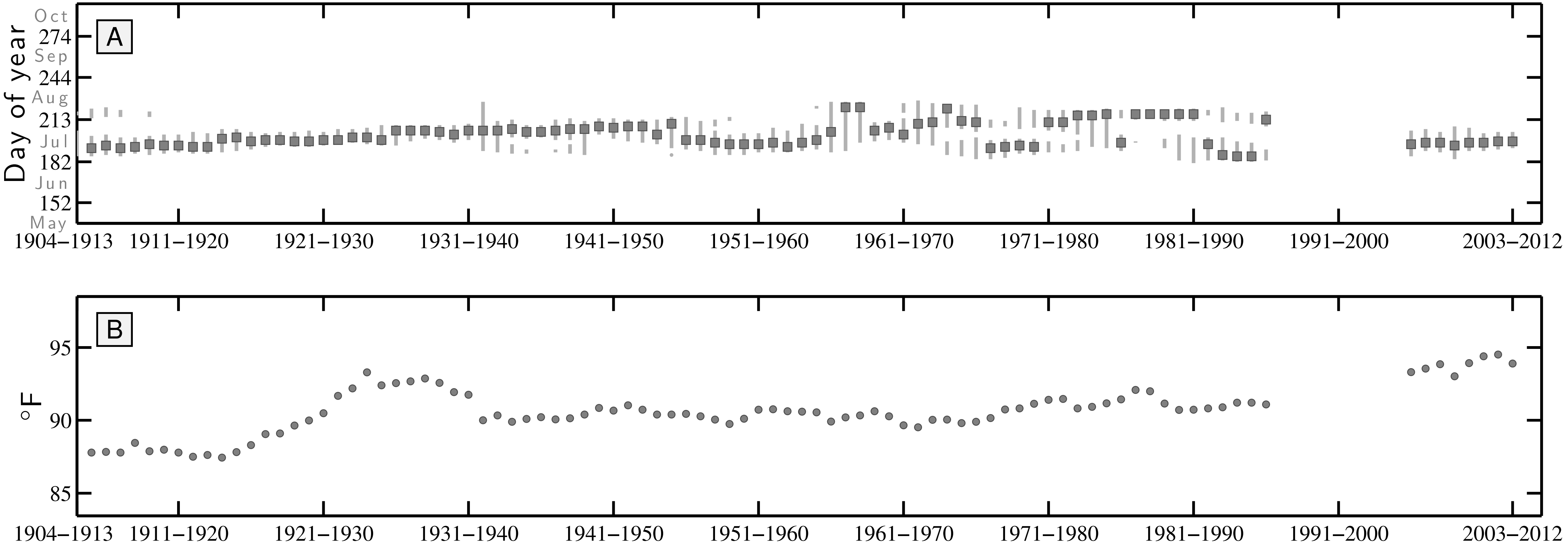

10 year Winter Teletherm dynamics for SCIPIO, UT:

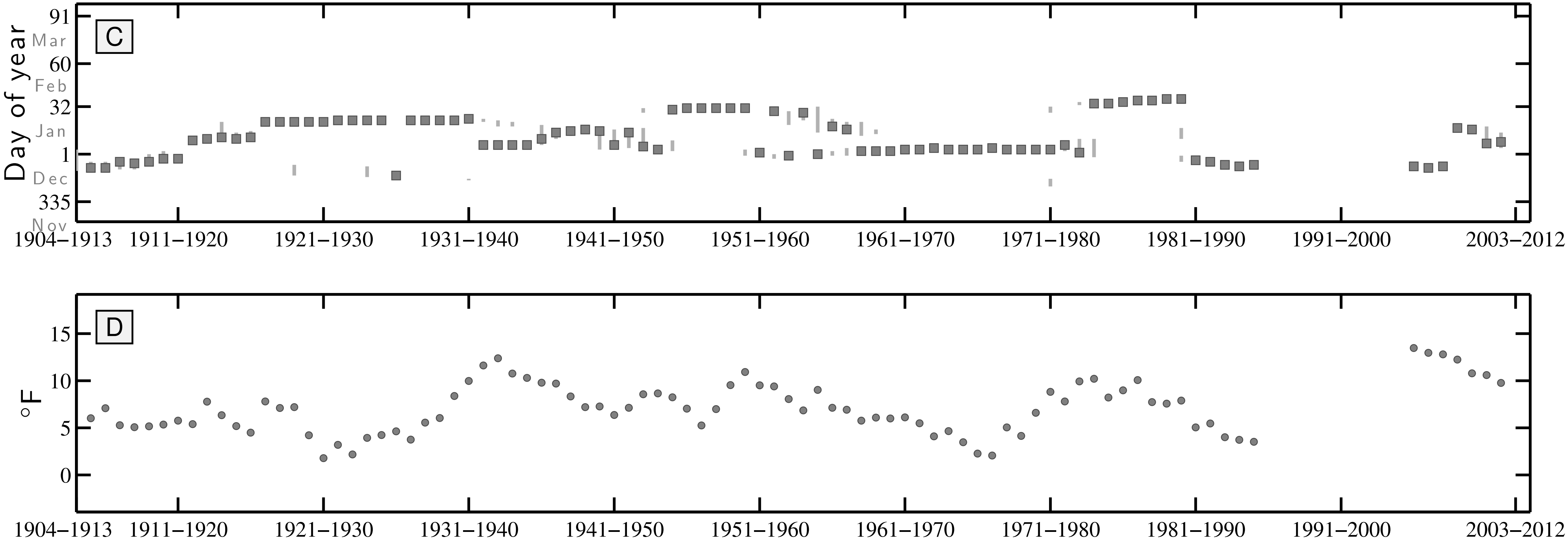

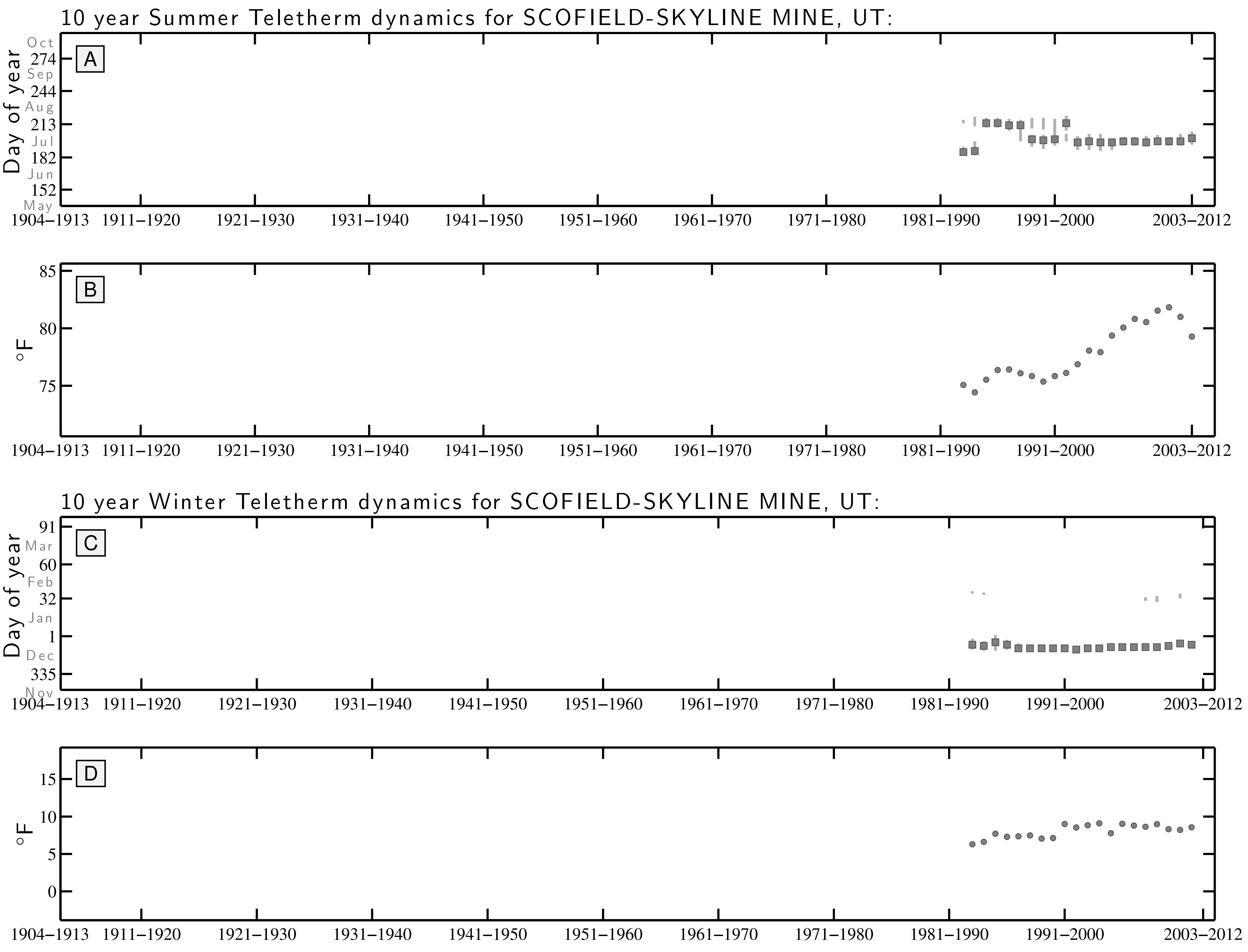
10 year Summer Teletherm dynamics for SCOFIELD-SKYLINE MINE, UT:
A
B
Day of year
°F
10 year Winter Teletherm dynamics for SCOFIELD-SKYLINE MINE, UT:
C
D
1904–1913
1911–1920
1921–1930
1931–1940
1941–1950
1951–1960
1961–1970
1971–1980
1981–1990
1991–2000
2003–2012

10 year Summer Teletherm dynamics for SNAKE CREEK POWERHOUSE, UT:

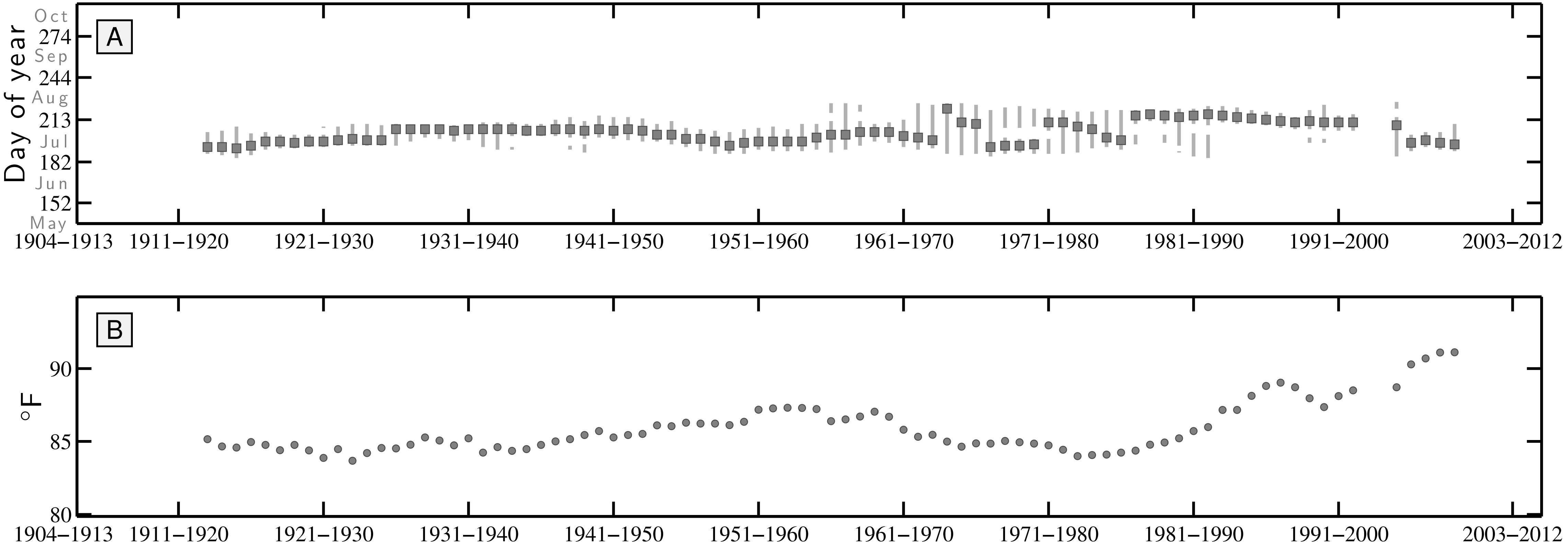

10 year Winter Teletherm dynamics for SNAKE CREEK POWERHOUSE, UT:

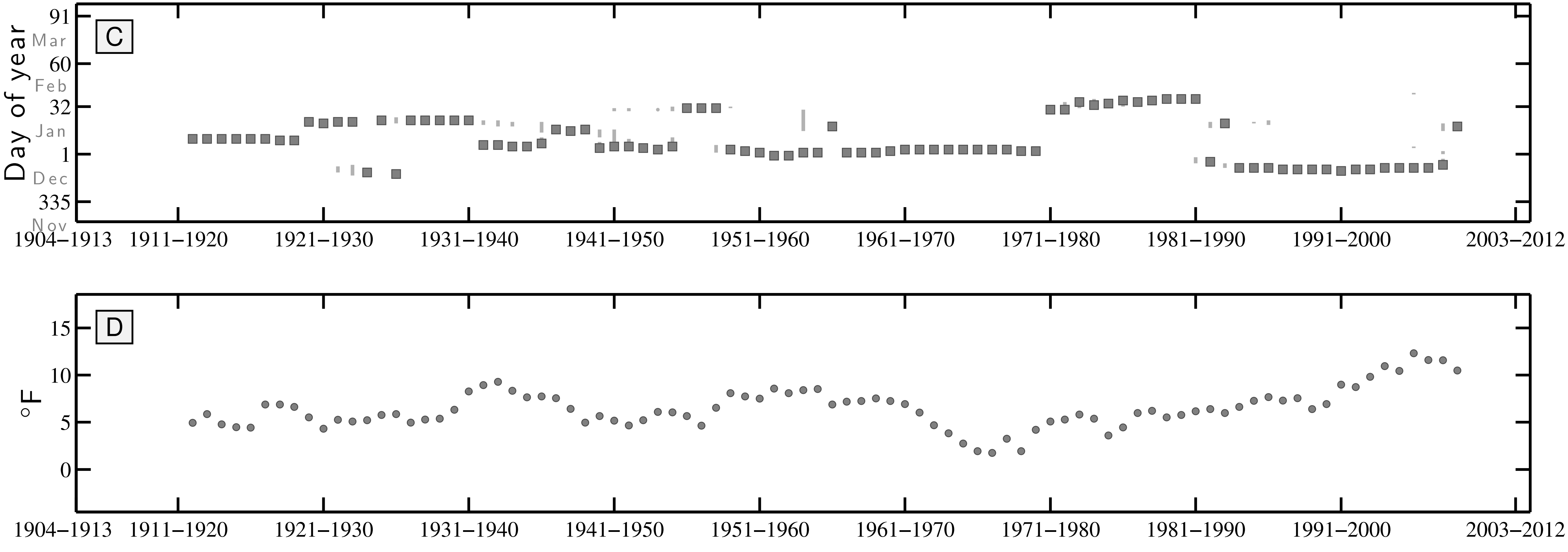

10 year Summer Teletherm dynamics for SPANISH FORK PWR HOUSE, UT:

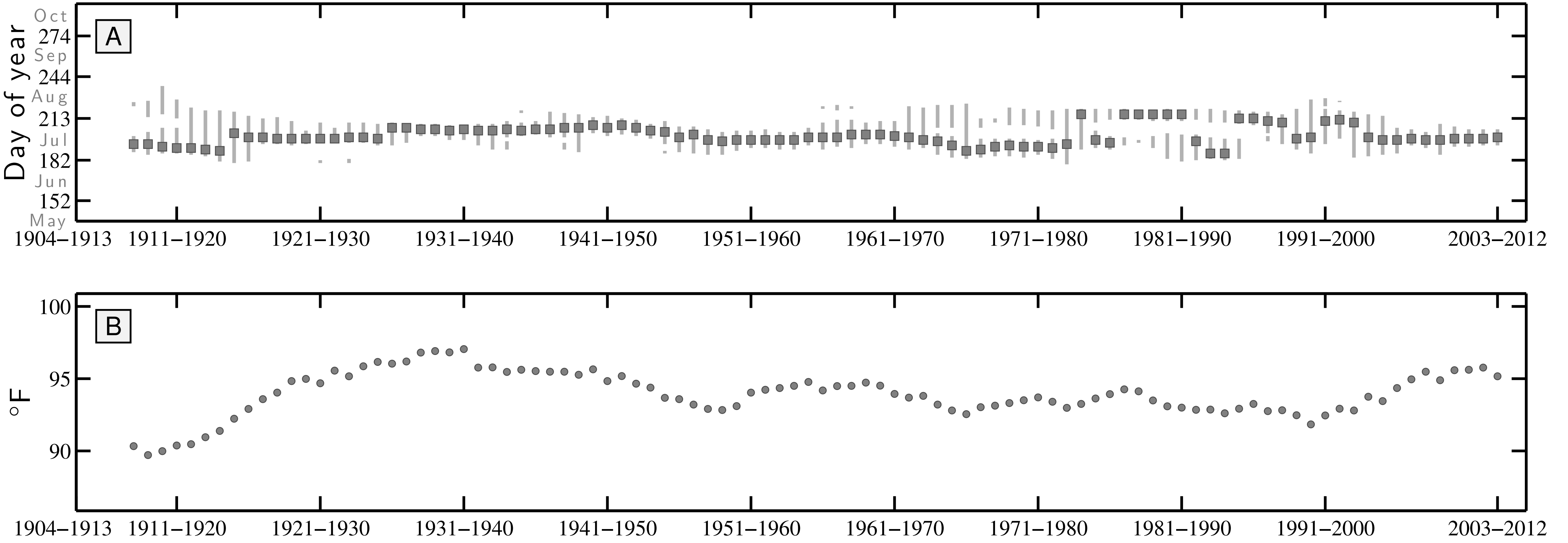

10 year Winter Teletherm dynamics for SPANISH FORK PWR HOUSE, UT:

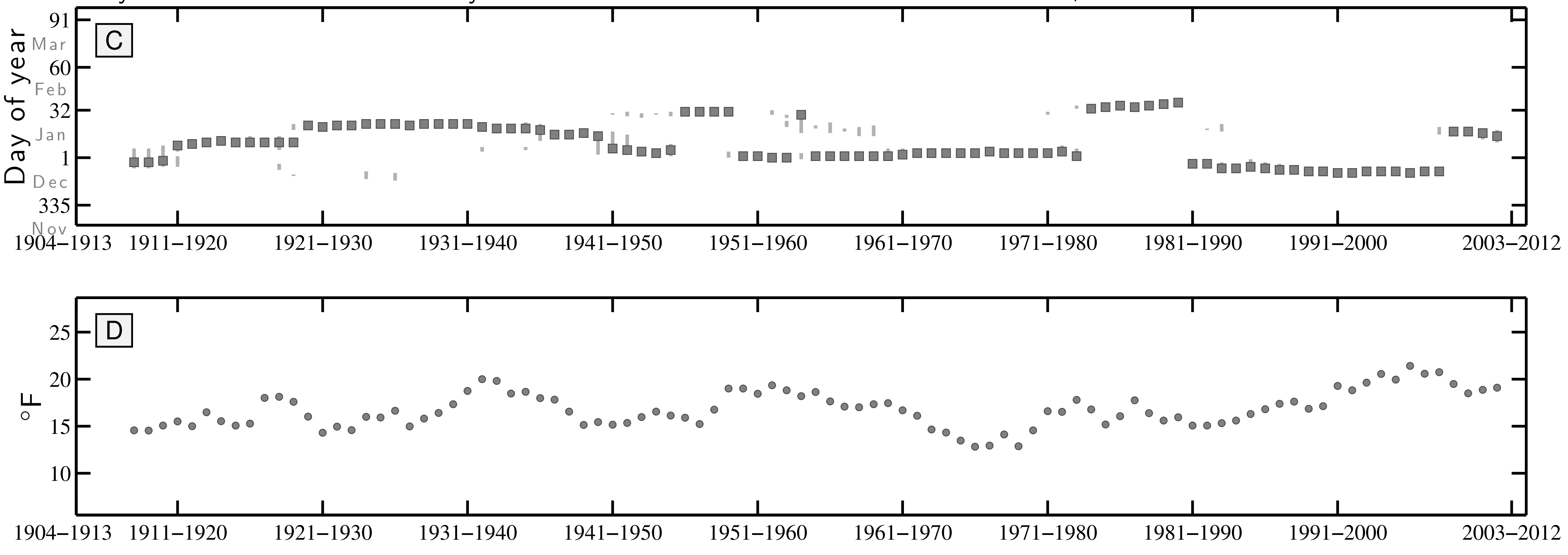

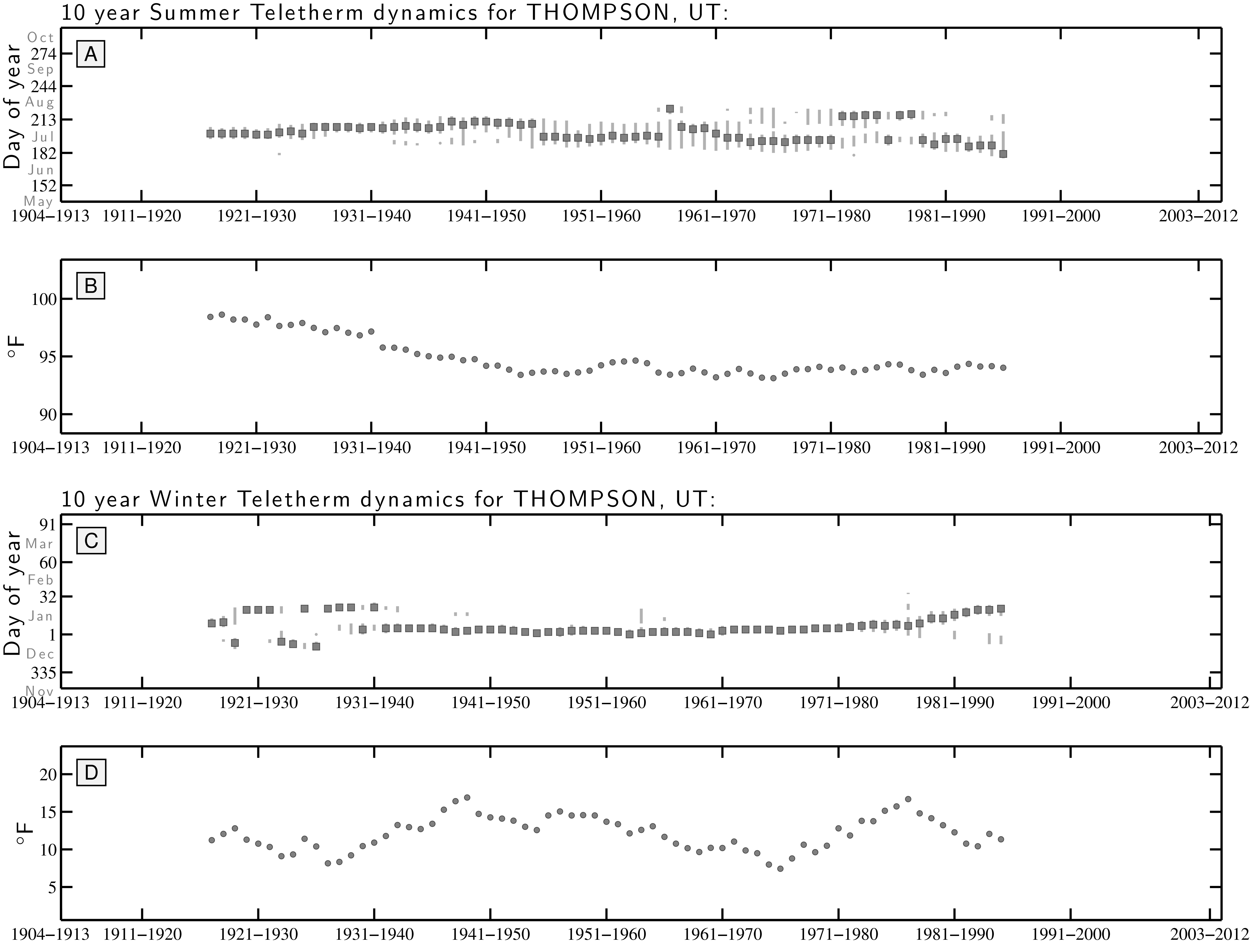
10 year Summer Teletherm dynamics for THOMPSON, UT:
A
B
Day of year
°F
Oct
274
Sep
244
Aug
213
Jul
182
Jun
152
May
100
95
90
1904–1913
1911–1920
1921–1930
1931–1940
1941–1950
1951–1960
1961–1970
1971–1980
1981–1990
1991–2000
2003–2012
10 year Winter Teletherm dynamics for THOMPSON, UT:
C
D
91
Mar
60
Feb
32
Jan
1
Dec
335
Nov
20
15
10
5

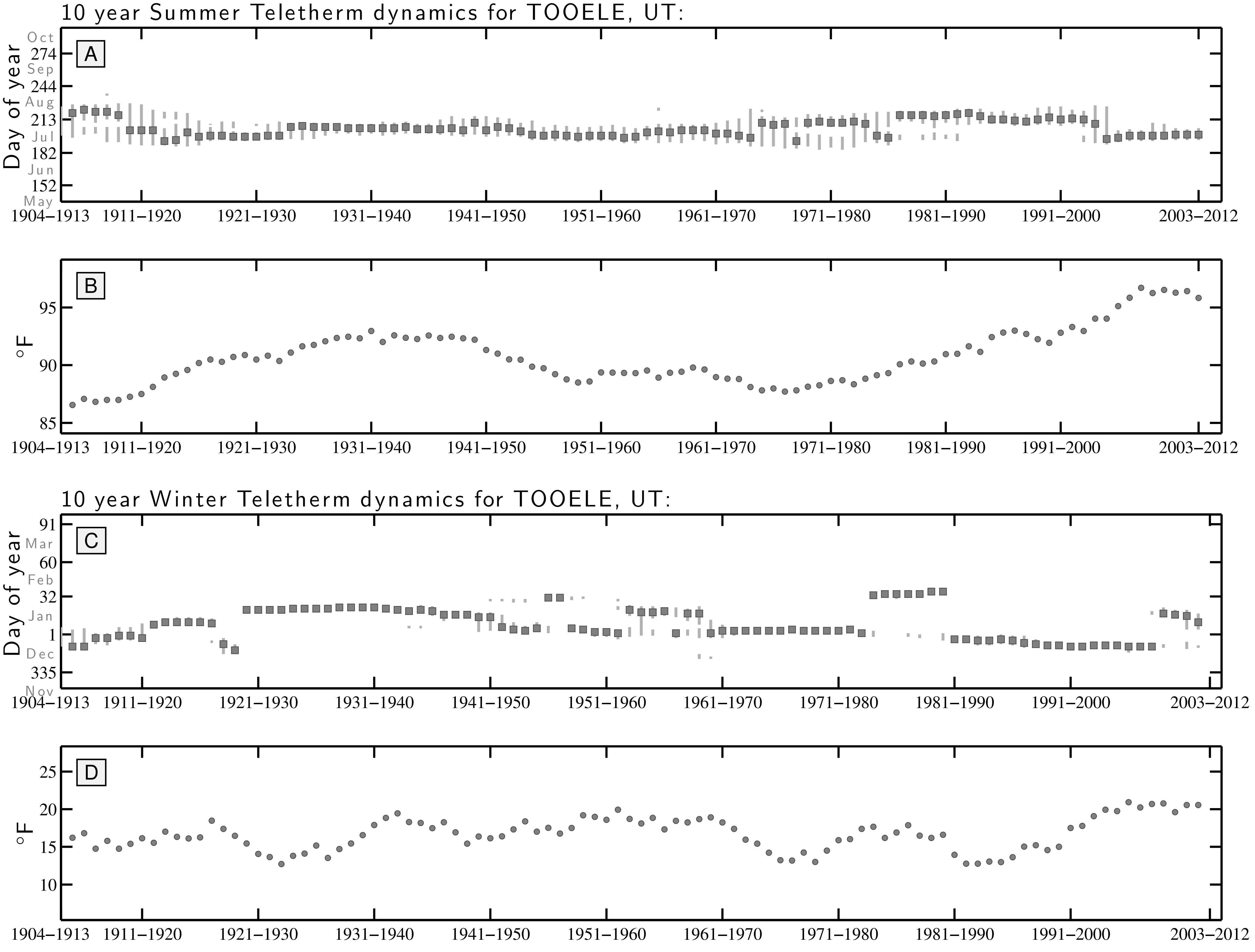

10 year Summer Teletherm dynamics for TOOELE, UT:
A
Day of year
Oct
274
Sep
244
Aug
213
Jul
182
Jun
152
May
1904–1913
1911–1920
1921–1930
1931–1940
1941–1950
1951–1960
1961–1970
1971–1980
1981–1990
1991–2000
2003–2012
B
°F
95
90
85
10 year Winter Teletherm dynamics for TOOELE, UT:
C
91
Mar
60
Feb
32
Jan
1
Dec
335
Nov
D
25
20
15
10

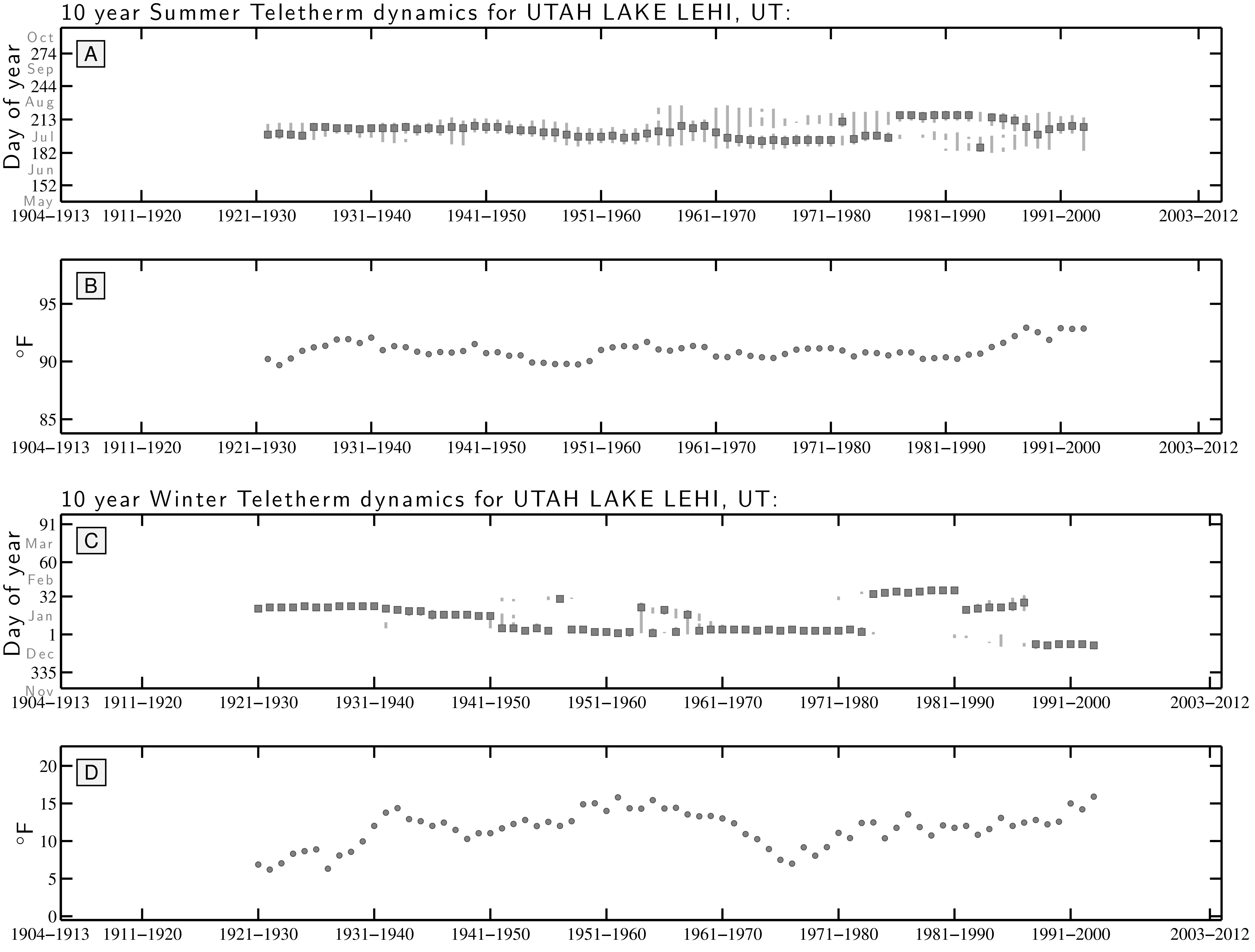
10 year Summer Teletherm dynamics for UTAH LAKE LEHI, UT:
A
B
Day of year
°F
Oct
274
Sep
244
Aug
213
Jul
182
Jun
152
May
95
90
85
1904–1913
1911–1920
1921–1930
1931–1940
1941–1950
1951–1960
1961–1970
1971–1980
1981–1990
1991–2000
2003–2012
10 year Winter Teletherm dynamics for UTAH LAKE LEHI, UT:
C
D
91
Mar
60
Feb
32
Jan
1
Dec
335
Nov
20
15
10
5
0

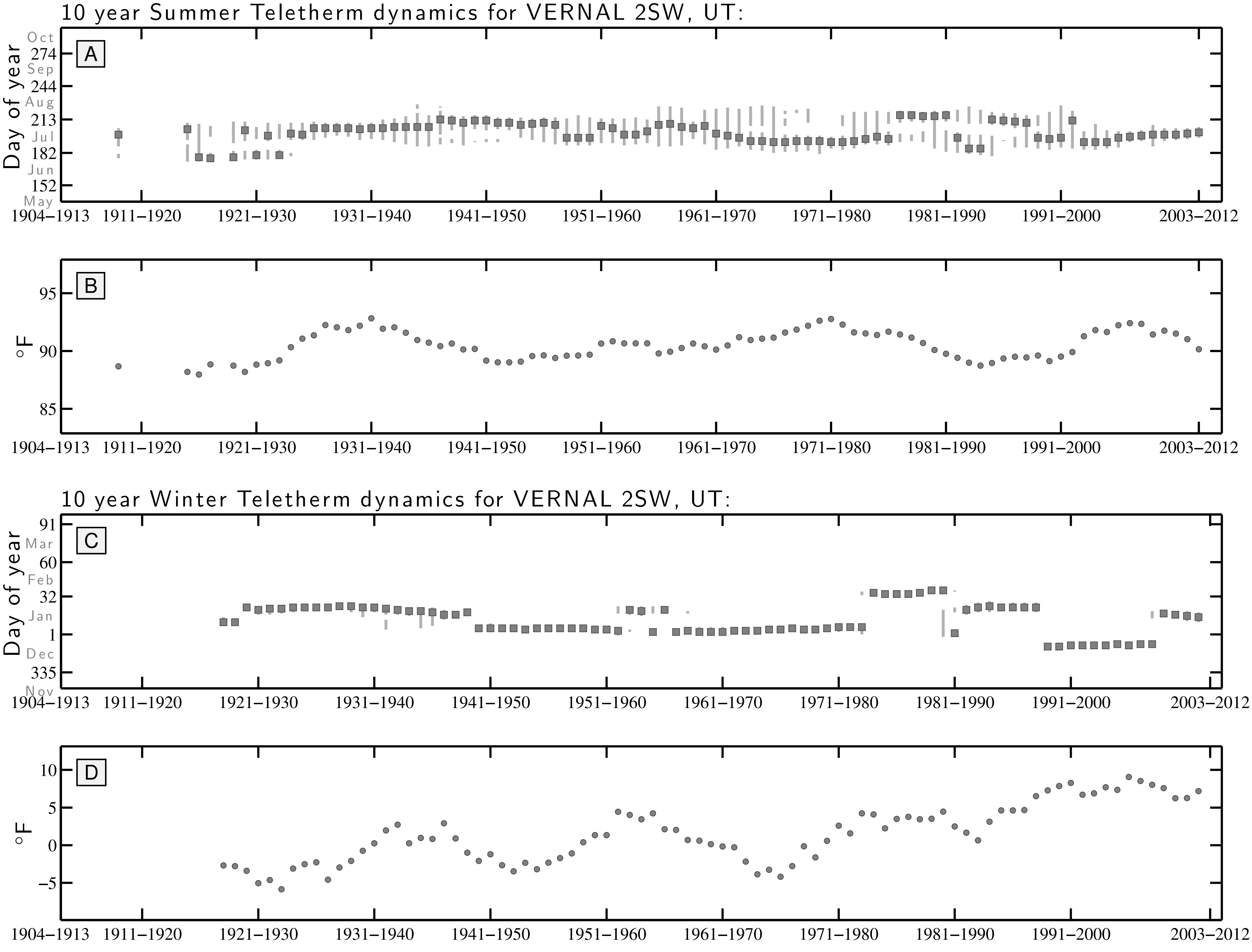
10 year Summer Teletherm dynamics for VERNAL 2SW, UT:
A
B
Day of year
°F
Oct
274
Sep
244
Aug
213
Jul
182
Jun
152
May
95
90
85
10 year Winter Teletherm dynamics for VERNAL 2SW, UT:
C
D
91
Mar
60
Feb
32
Jan
1
Dec
335
Nov
10
5
0
-5
1904–1913
1911–1920
1921–1930
1931–1940
1941–1950
1951–1960
1961–1970
1971–1980
1981–1990
1991–2000
2003–2012

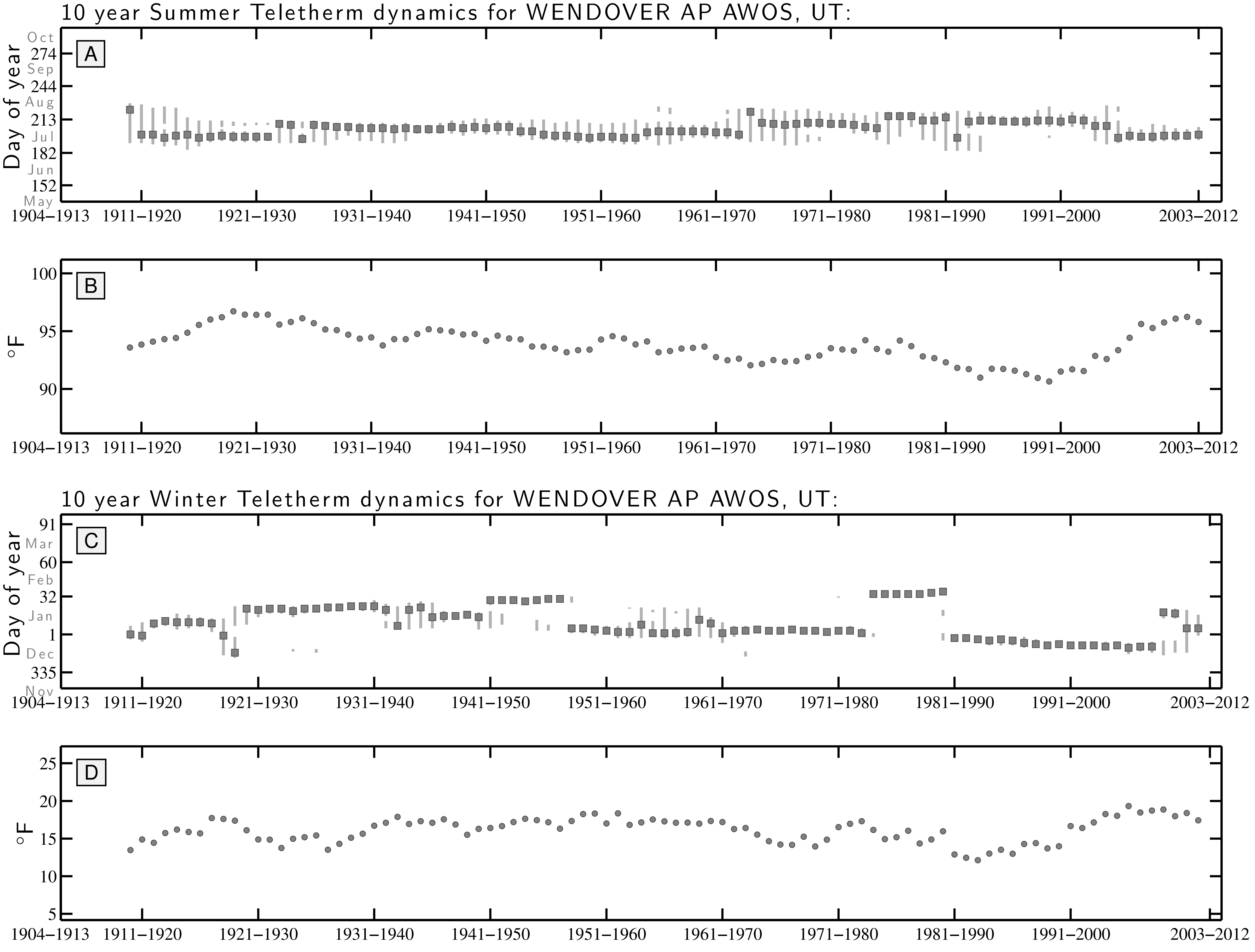

10 year Summer Teletherm dynamics for WENDOVER AP AWOS, UT:
A
B
Day of year
Oct
274
Sep
244
Aug
213
Jul
182
Jun
152
May
°F
100
95
90
1904–1913
1911–1920
1921–1930
1931–1940
1941–1950
1951–1960
1961–1970
1971–1980
1981–1990
1991–2000
2003–2012
10 year Winter Teletherm dynamics for WENDOVER AP AWOS, UT:
C
D
91
Mar
60
Feb
32
Jan
1
Dec
335
Nov
25
20
15
10
5

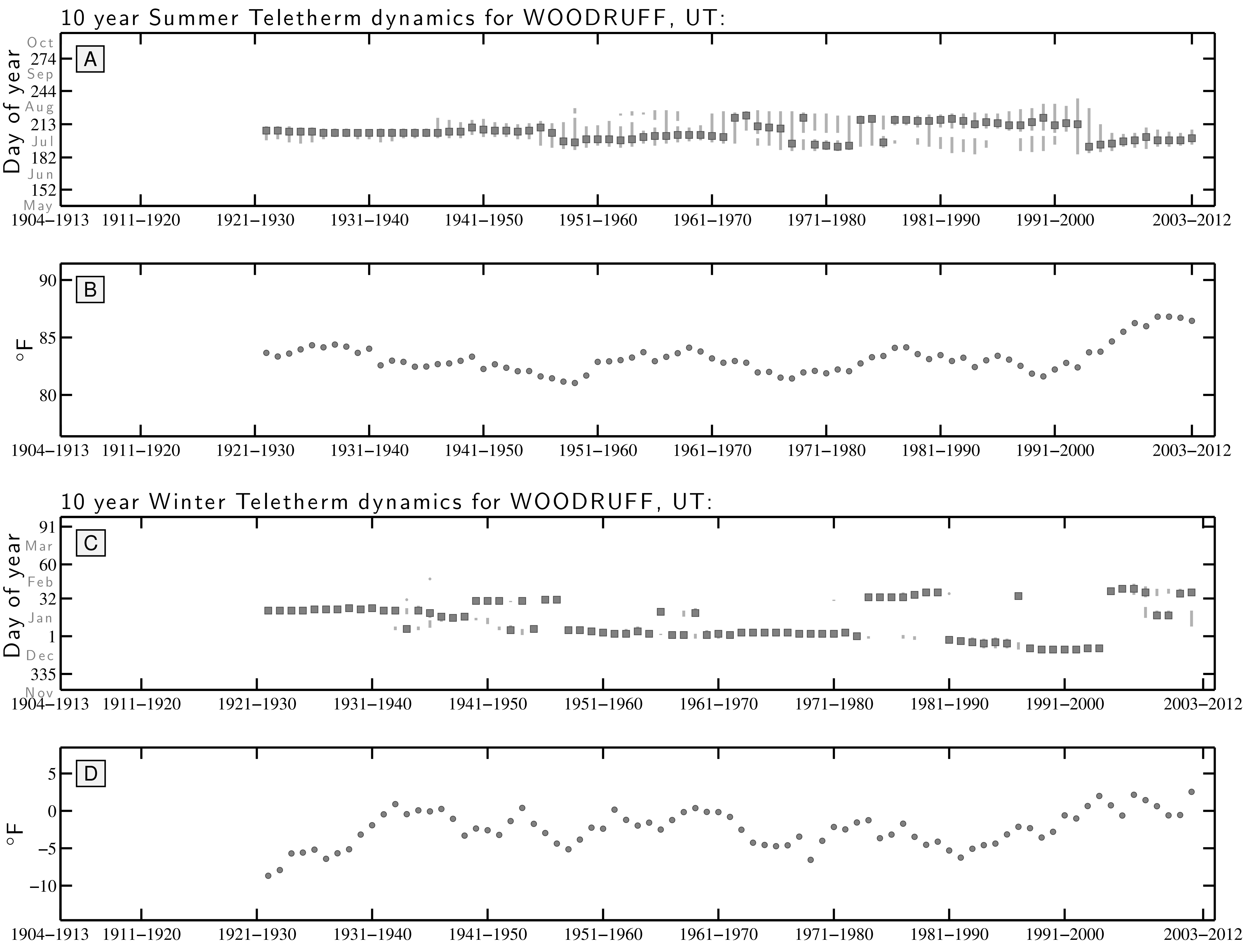

10 year Summer Teletherm dynamics for WOODRUFF, UT:
A
Day of year
Oct
274
Sep
244
Aug
213
Jul
182
Jun
152
May
B
°F
90
85
80
10 year Winter Teletherm dynamics for WOODRUFF, UT:
C
Day of year
91
Mar
60
Feb
32
Jan
1
Dec
335
Nov
D
°F
5
0
−5
−10
1904–1913
1911–1920
1921–1930
1931–1940
1941–1950
1951–1960
1961–1970
1971–1980
1981–1990
1991–2000
2003–2012

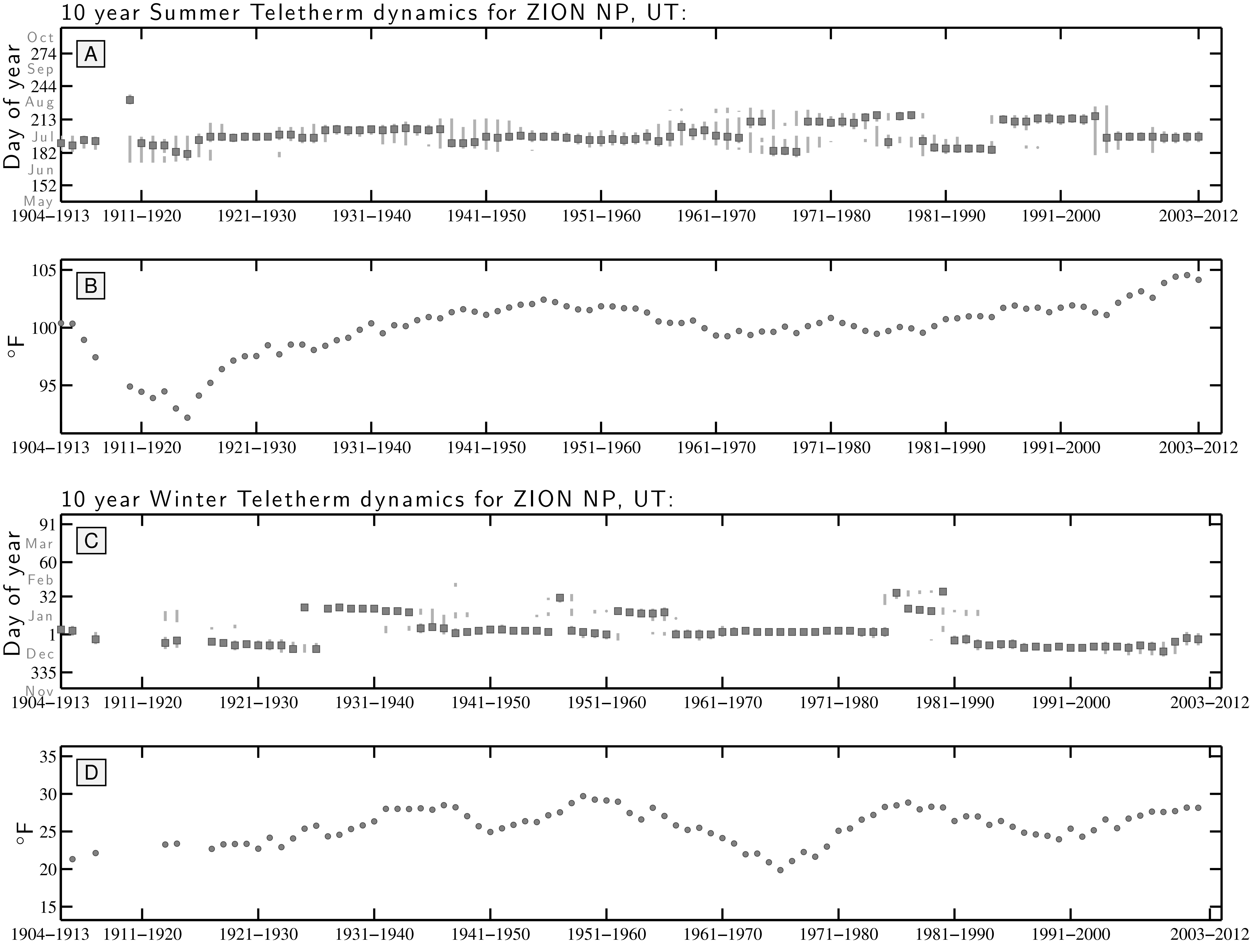
10 year Summer Teletherm dynamics for ZION NP, UT:
A
B
Day of year
°F
Oct
Sep
Aug
Jul
Jun
May
274
244
213
182
152
105
100
95
1904–1913
1911–1920
1921–1930
1931–1940
1941–1950
1951–1960
1961–1970
1971–1980
1981–1990
1991–2000
2003–2012
10 year Winter Teletherm dynamics for ZION NP, UT:
C
D
Mar
Feb
Jan
Dec
Nov
91
60
32
1
335
35
30
25
20
15

10 year Summer Teletherm dynamics for BURLINGTON WSO AP, VT:

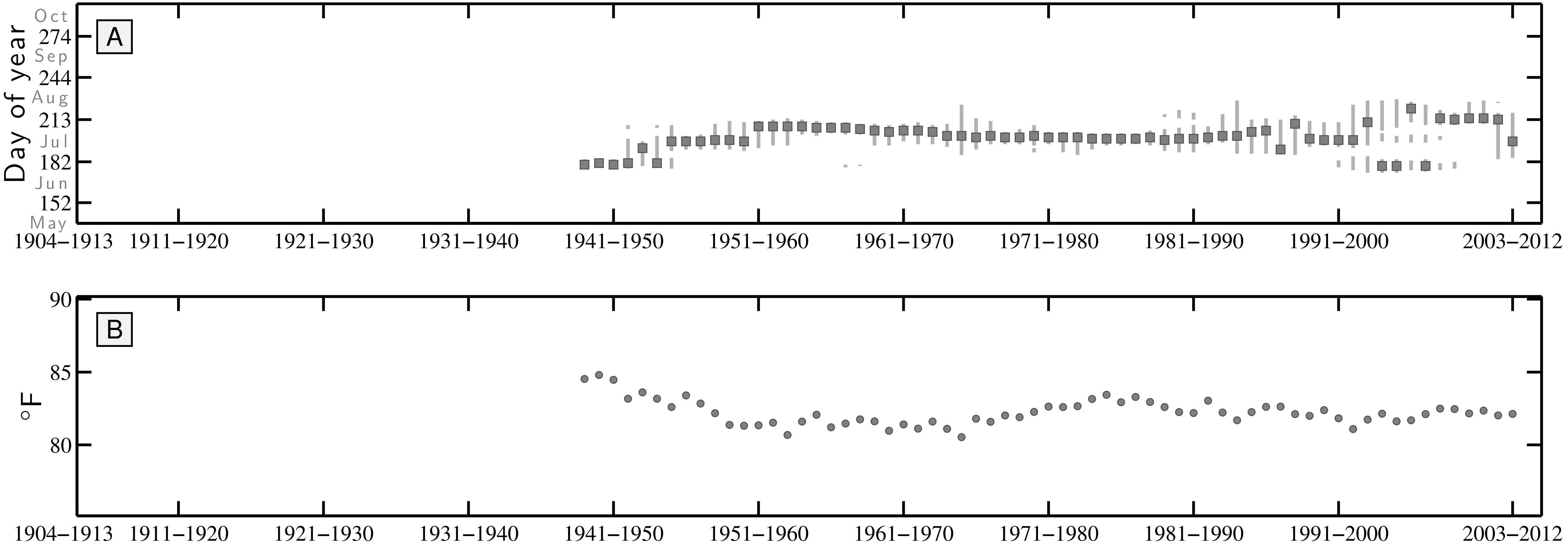

10 year Winter Teletherm dynamics for BURLINGTON WSO AP, VT:

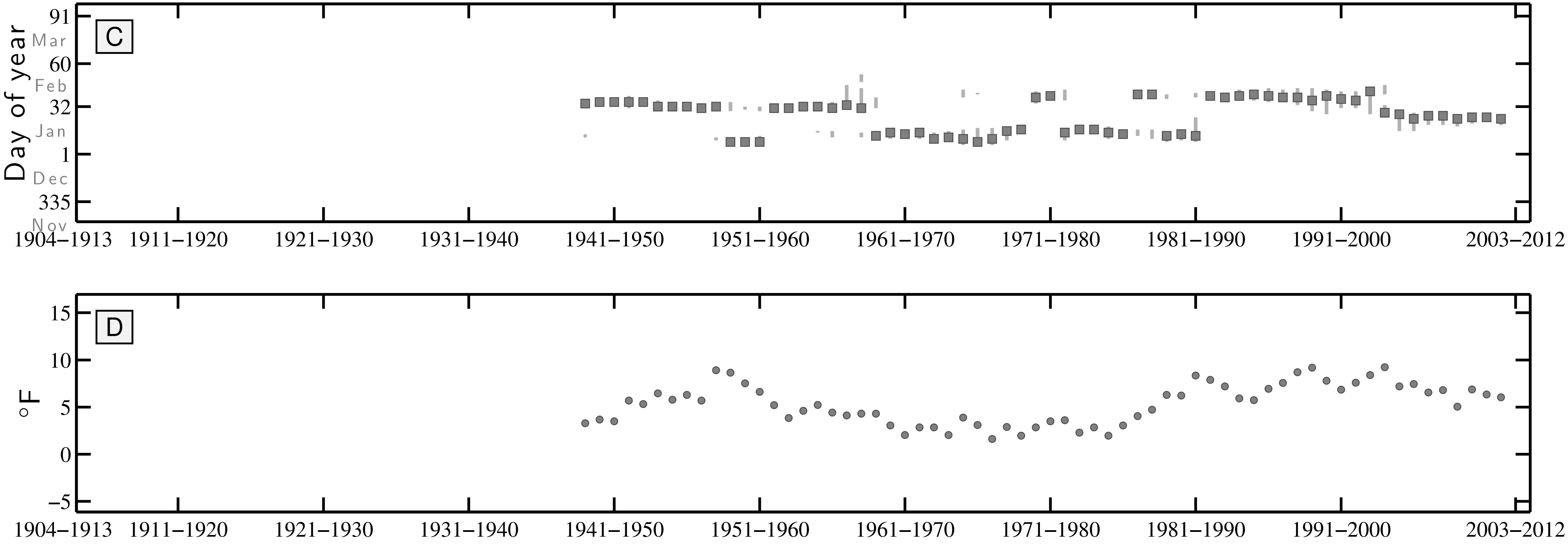

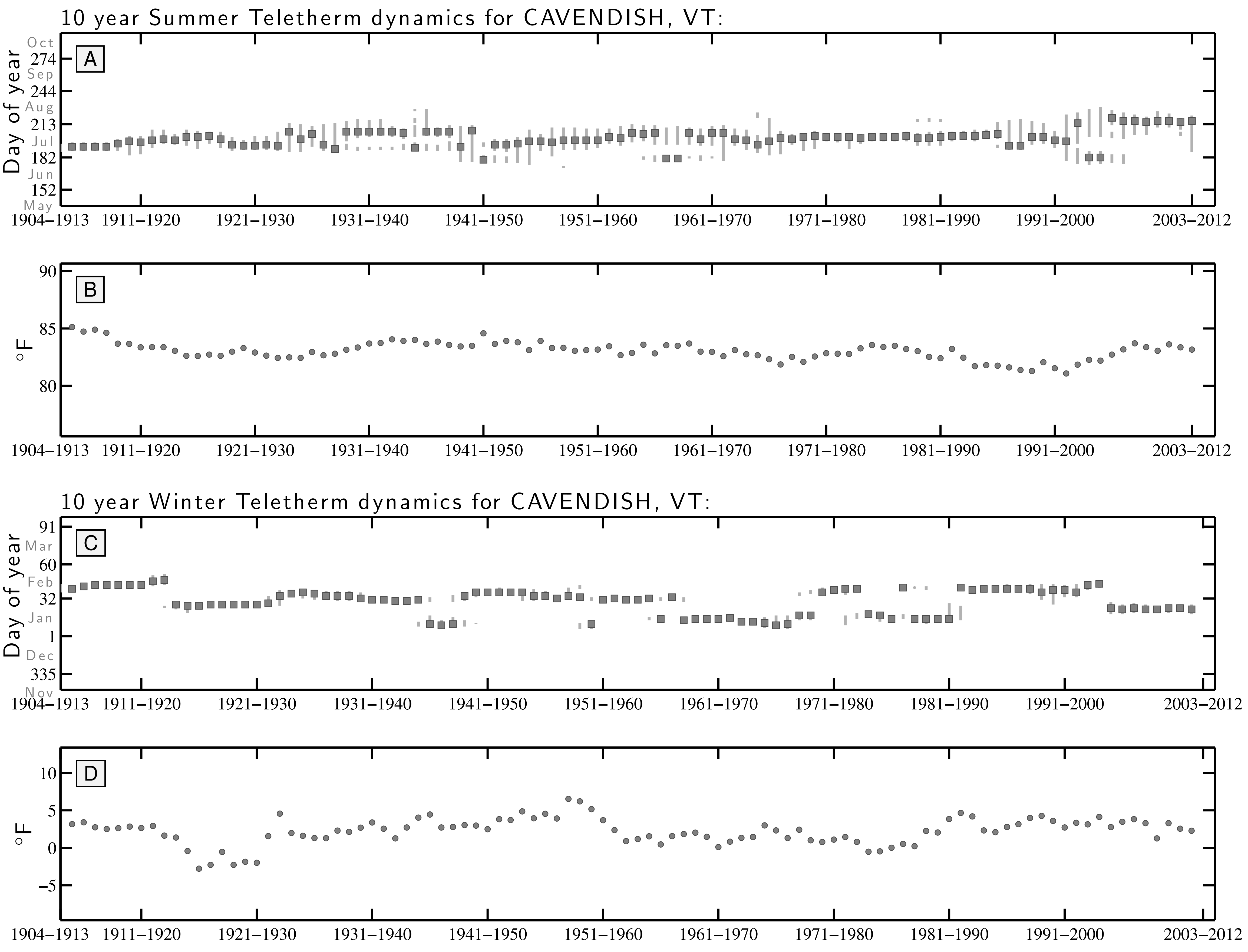
10 year Summer Teletherm dynamics for CAVENDISH, VT:
10 year Winter Teletherm dynamics for CAVENDISH, VT:
A
B
C
D
Day of year
°F
Oct
274
Sep
244
Aug
213
Jul
182
Jun
152
May
91
Mar
60
Feb
32
Jan
1
Dec
335
Nov
90
85
80
10
5
0
−5
1904–1913
1911–1920
1921–1930
1931–1940
1941–1950
1951–1960
1961–1970
1971–1980
1981–1990
1991–2000
2003–2012

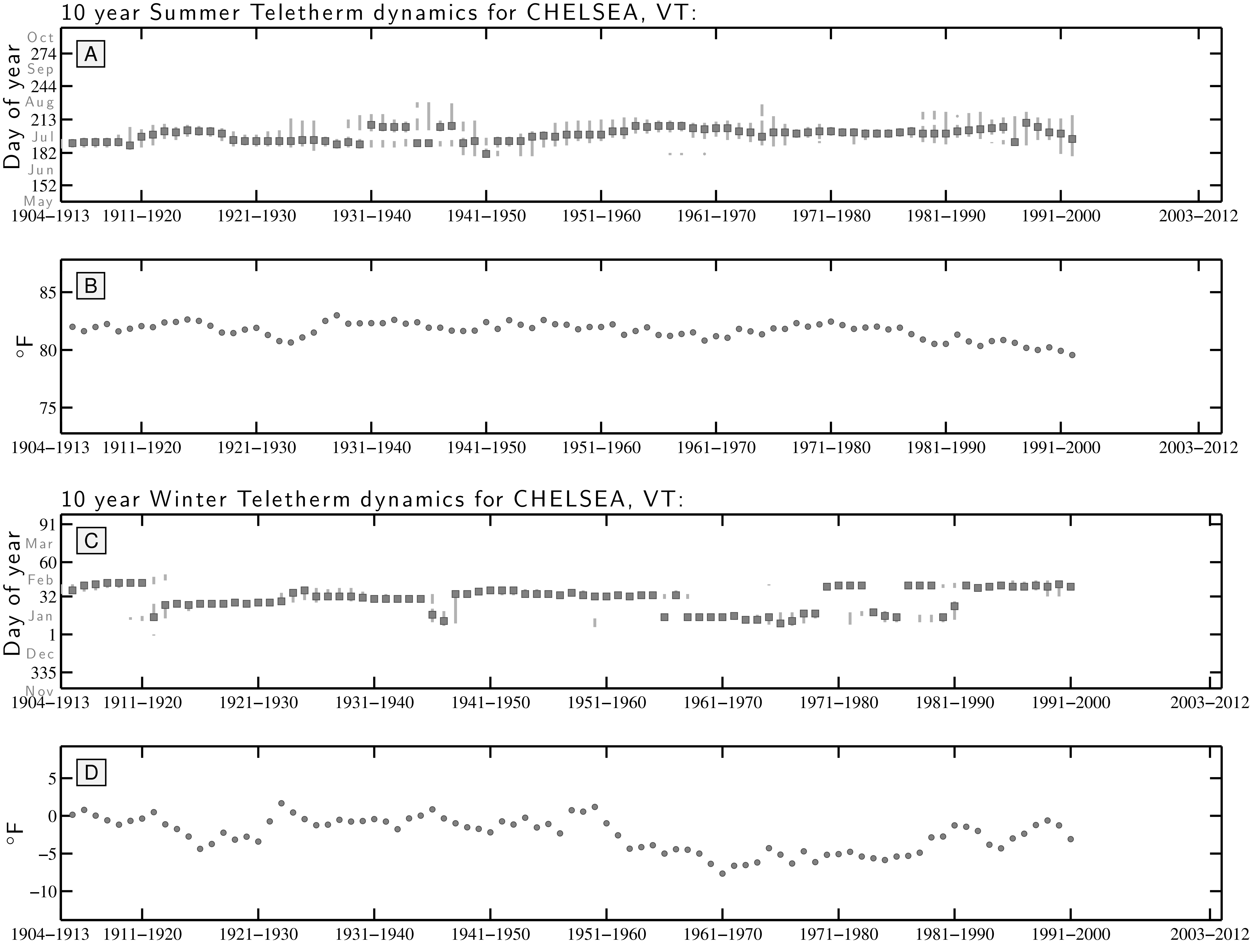
10 year Summer Teletherm dynamics for CHELSEA, VT:
A
B
Day of year
°F
Oct
Sep
Aug
Jul
Jun
May
274
244
213
182
152
85
80
75
1904–1913
1911–1920
1921–1930
1931–1940
1941–1950
1951–1960
1961–1970
1971–1980
1981–1990
1991–2000
2003–2012
10 year Winter Teletherm dynamics for CHELSEA, VT:
C
D
Mar
Feb
Jan
Dec
Nov
91
60
32
1
335
5
0
−5
−10

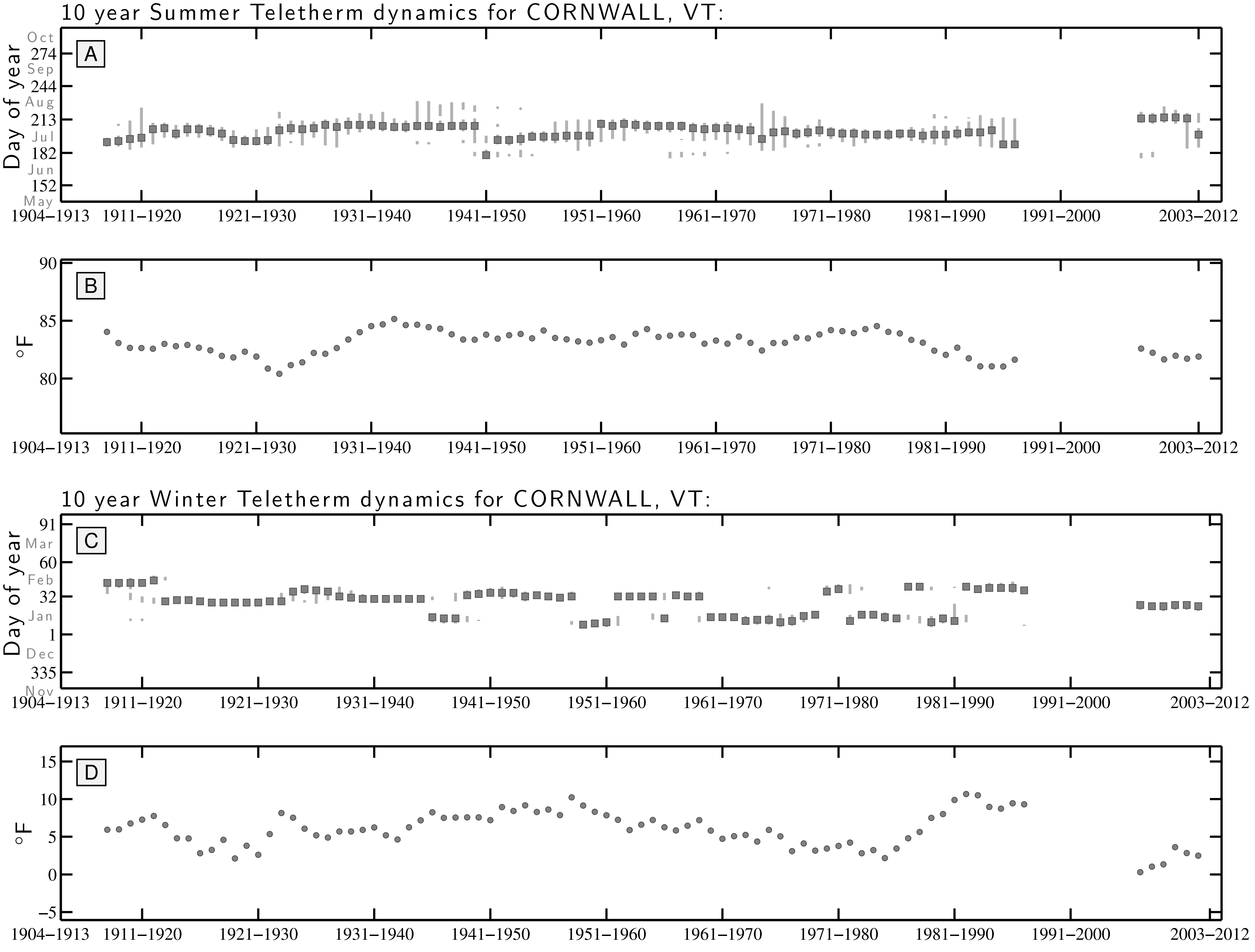
10 year Summer Teletherm dynamics for CORNWALL, VT:
A
Day of year
Oct
274
Sep
244
Aug
213
Jul
182
Jun
152
May
1904–1913
1911–1920
1921–1930
1931–1940
1941–1950
1951–1960
1961–1970
1971–1980
1981–1990
1991–2000
2003–2012
B
°F
90
85
80
10 year Winter Teletherm dynamics for CORNWALL, VT:
C
91
Mar
60
Feb
32
Jan
1
Dec
335
Nov
D
15
10
5
0
−5

10 year Summer Teletherm dynamics for ENOSBURG FALLS, VT:

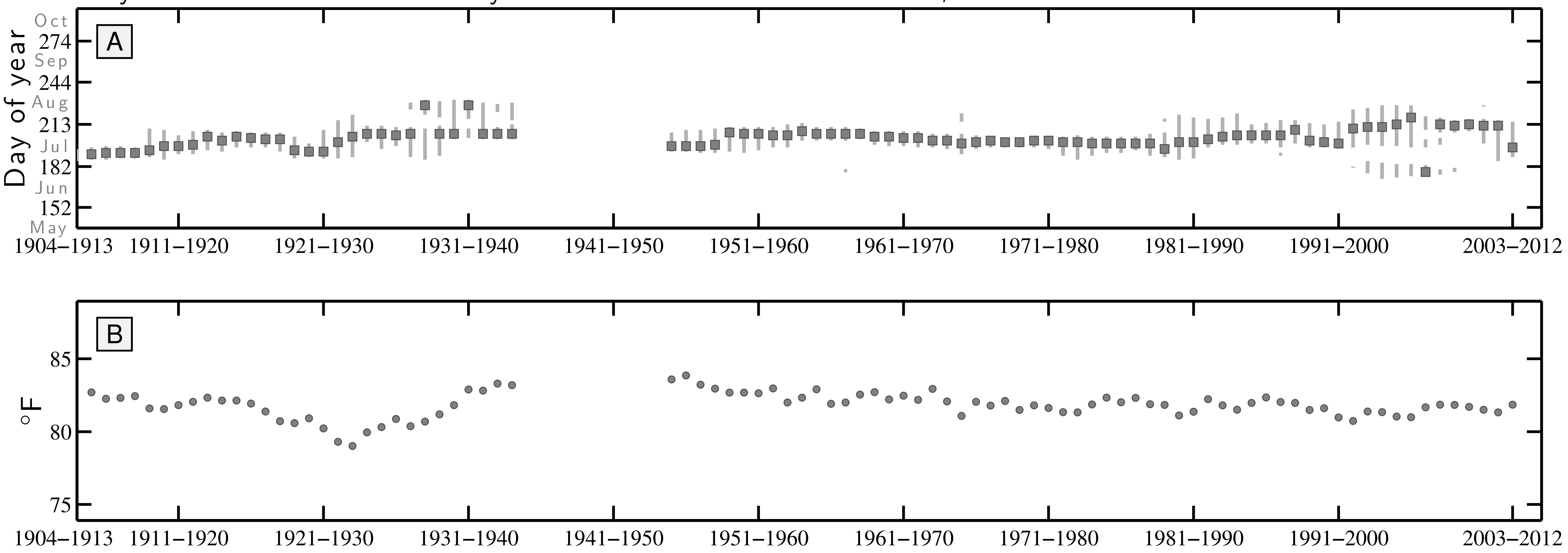

10 year Winter Teletherm dynamics for ENOSBURG FALLS, VT:

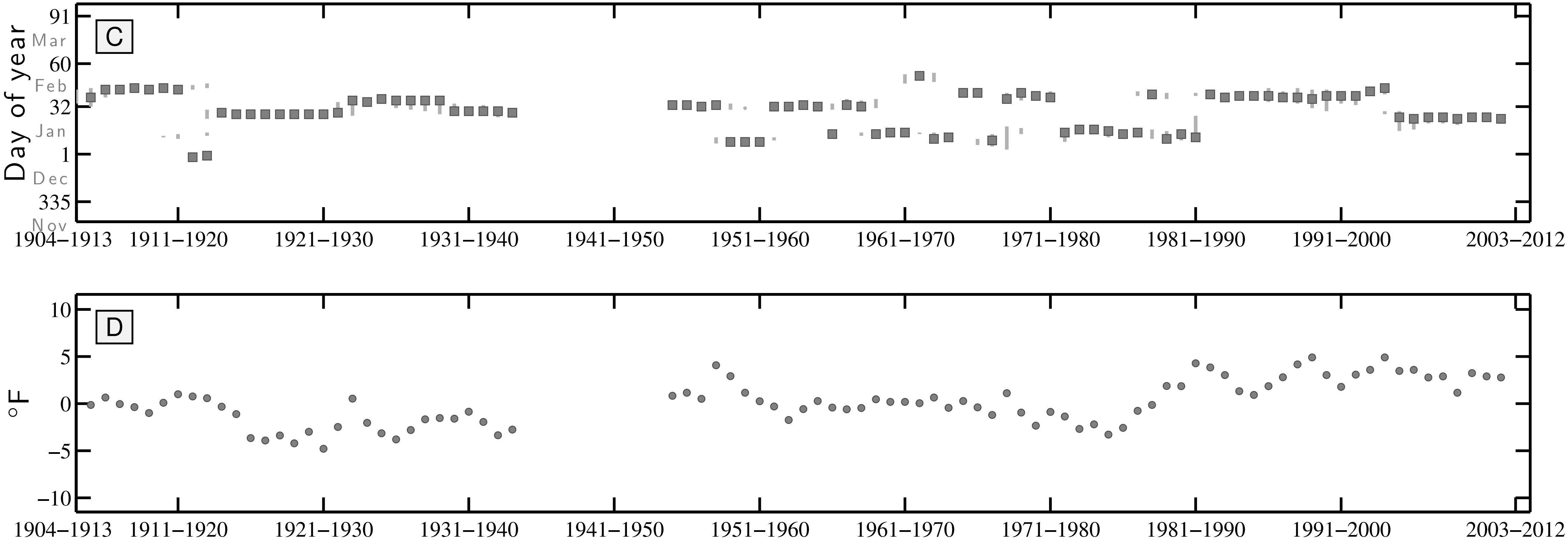

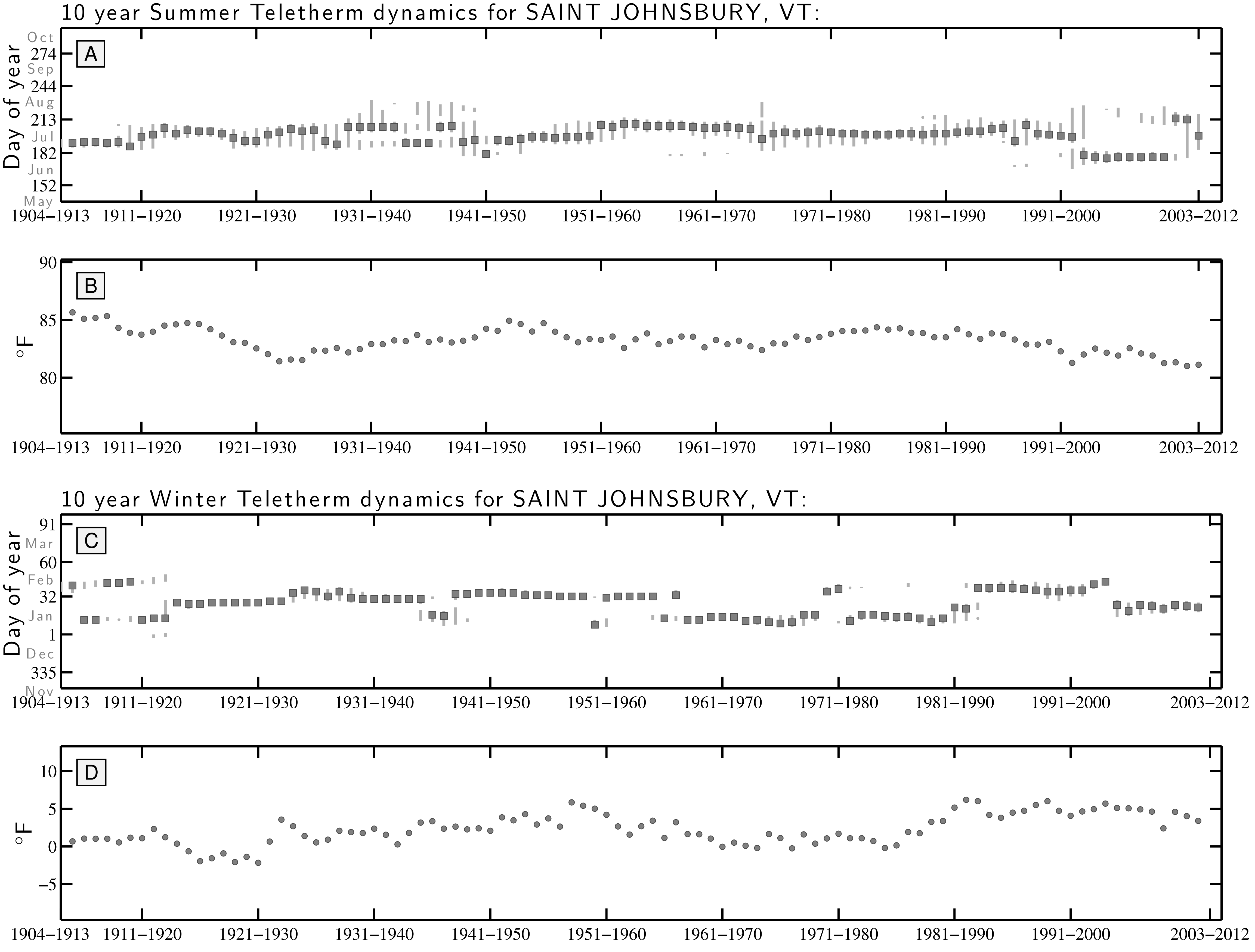

10 year Summer Teletherm dynamics for SAINT JOHNSBURY, VT:
A
B
Day of year
°F
Oct
274
Sep
244
Aug
213
Jul
182
Jun
152
May
90
85
80
1904–1913
1911–1920
1921–1930
1931–1940
1941–1950
1951–1960
1961–1970
1971–1980
1981–1990
1991–2000
2003–2012
10 year Winter Teletherm dynamics for SAINT JOHNSBURY, VT:
C
D
91
Mar
60
Feb
32
Jan
1
Dec
335
Nov
10
5
0
−5

10 year Summer Teletherm dynamics for SOUTH HERO, VT:

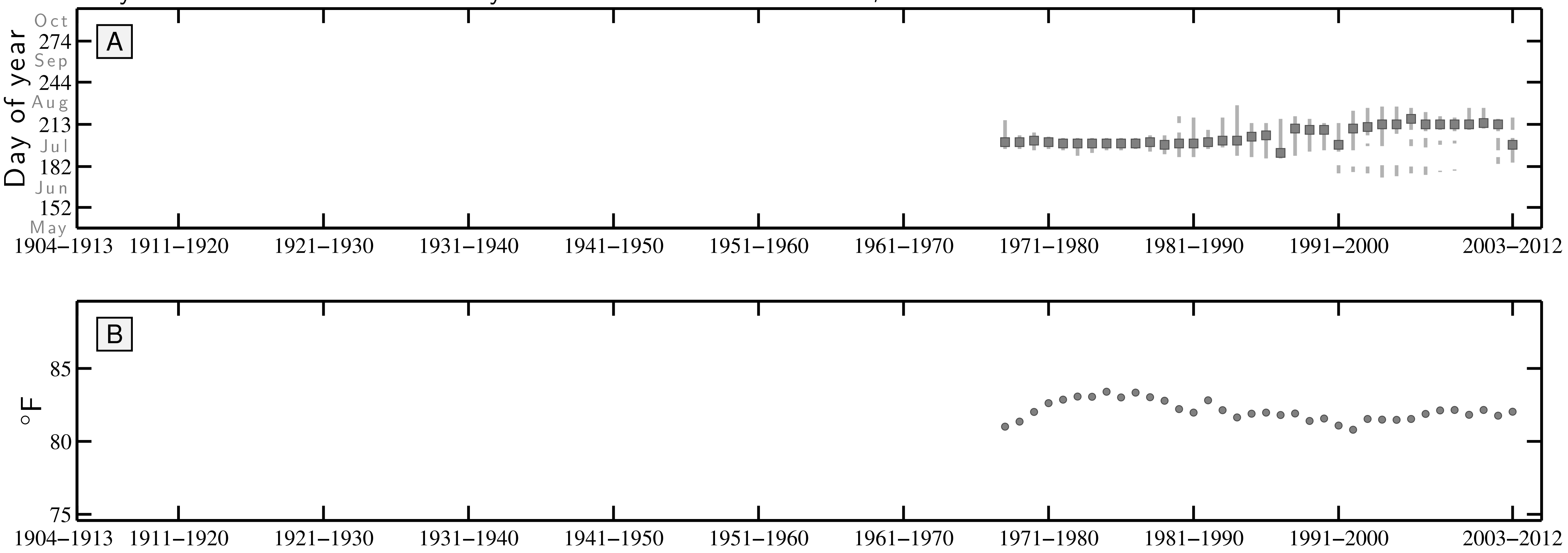

10 year Winter Teletherm dynamics for SOUTH HERO, VT:

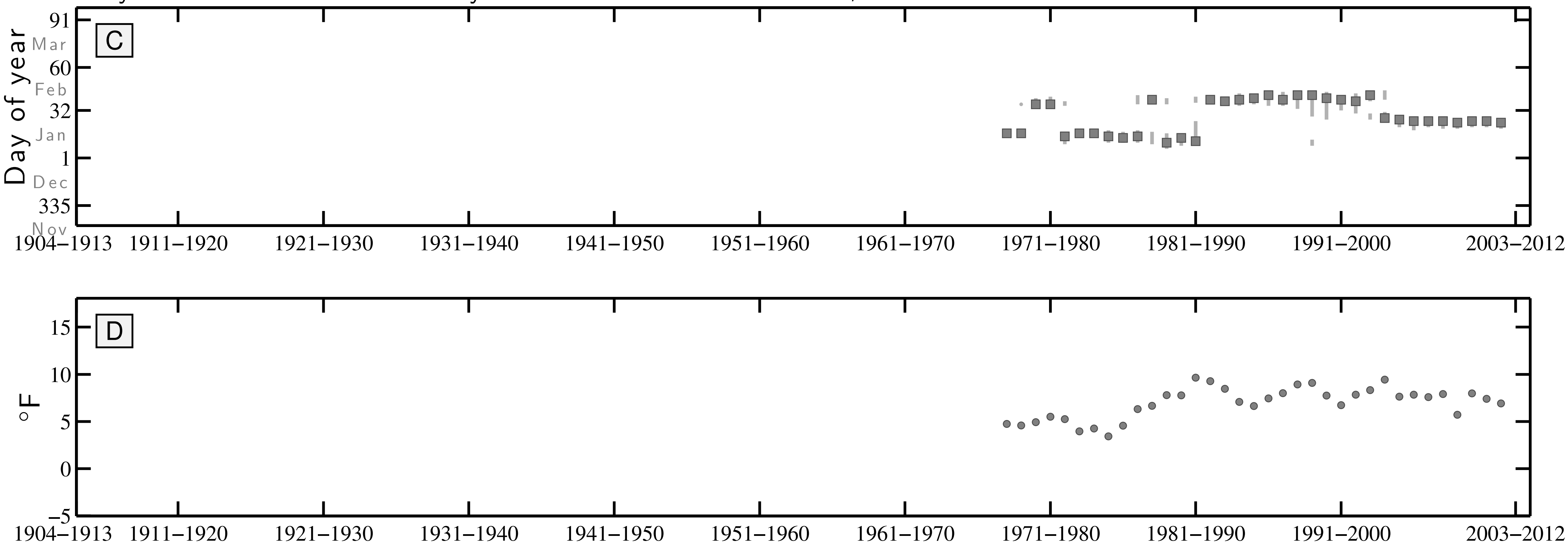

10 year Summer Teletherm dynamics for SOUTH LINCOLN, VT:

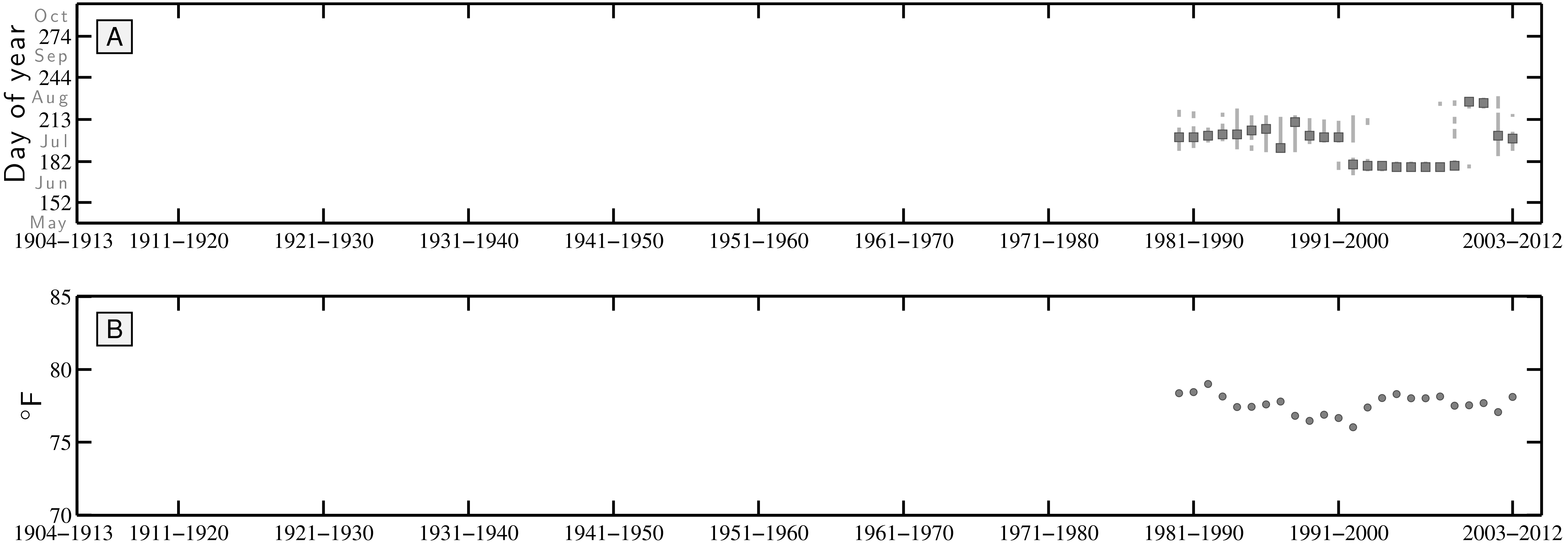

10 year Winter Teletherm dynamics for SOUTH LINCOLN, VT:

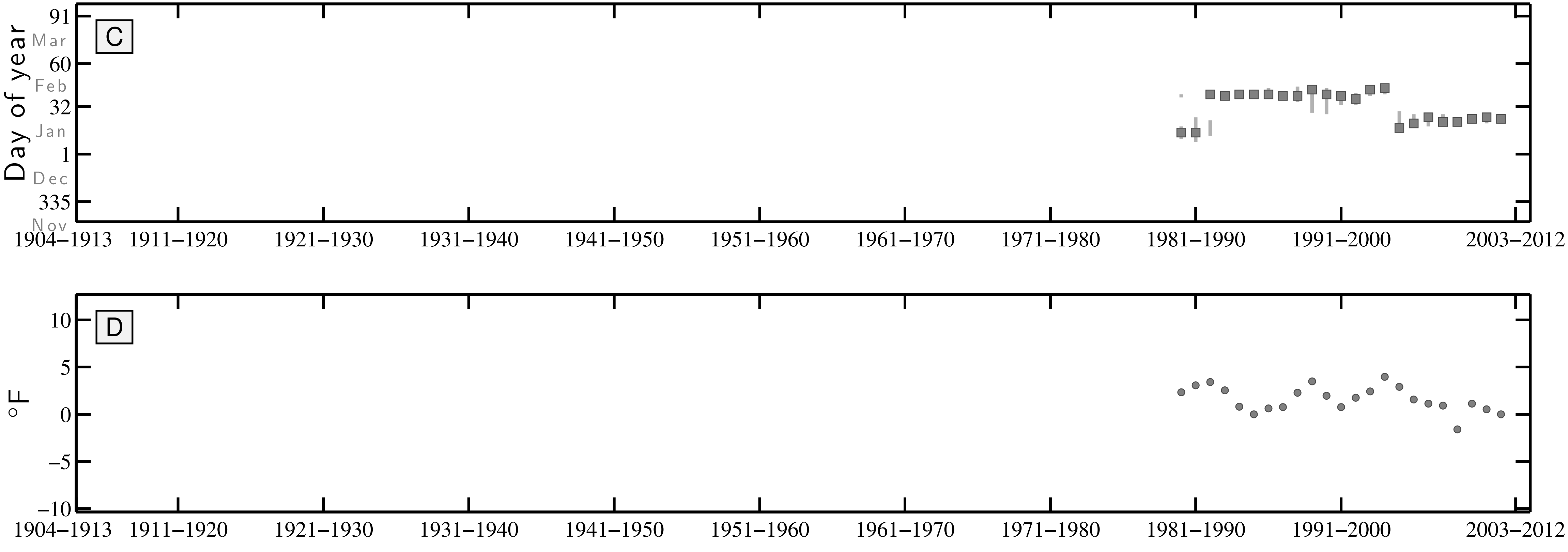

10 year Summer Teletherm dynamics for BLACKSBURG NWSO, VA:

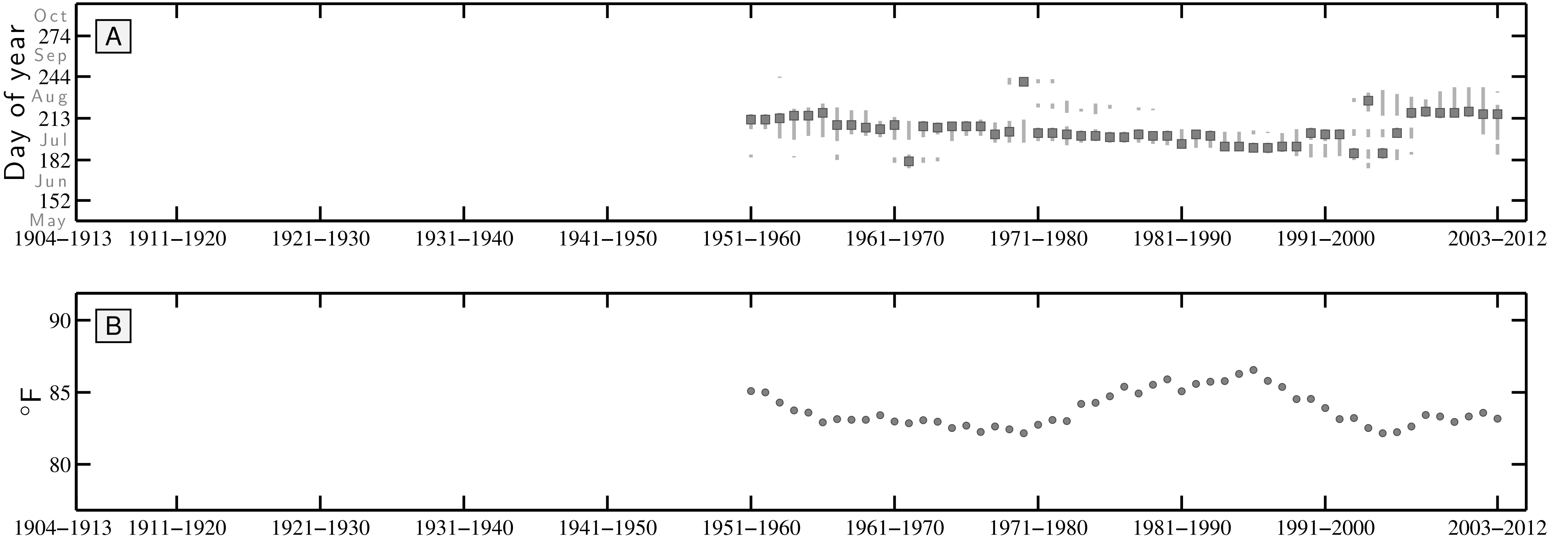

10 year Winter Teletherm dynamics for BLACKSBURG NWSO, VA:

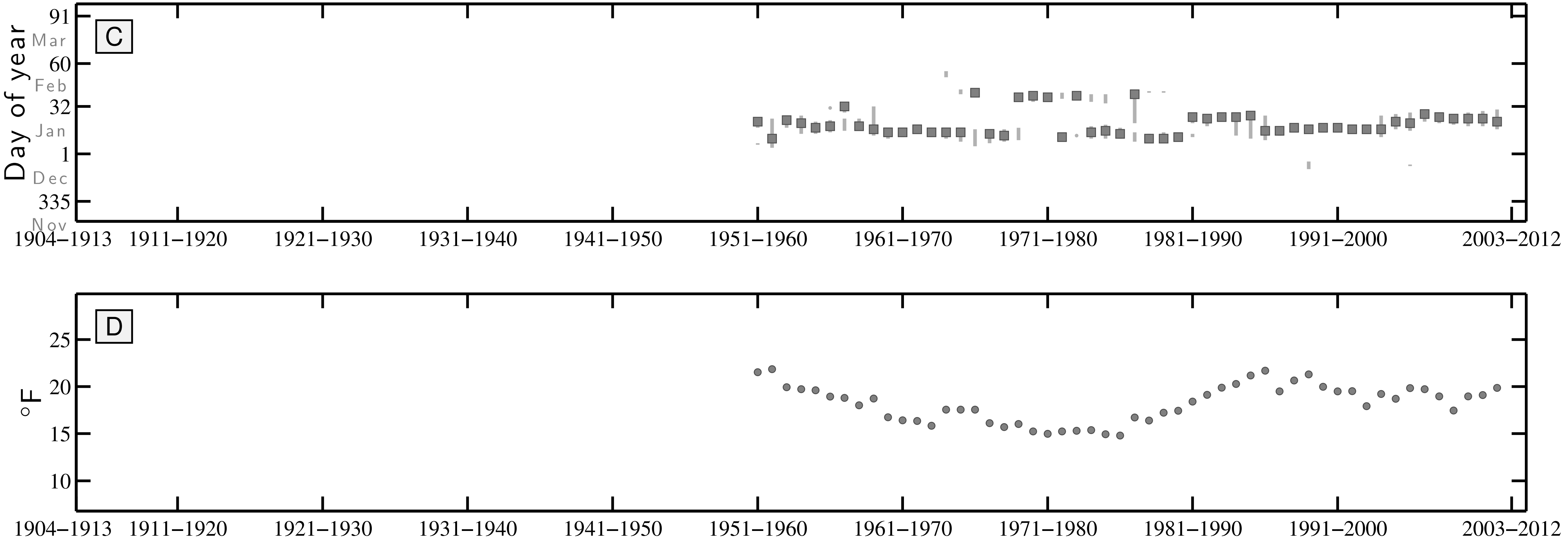

10 year Summer Teletherm dynamics for BREMO BLUFF, VA:

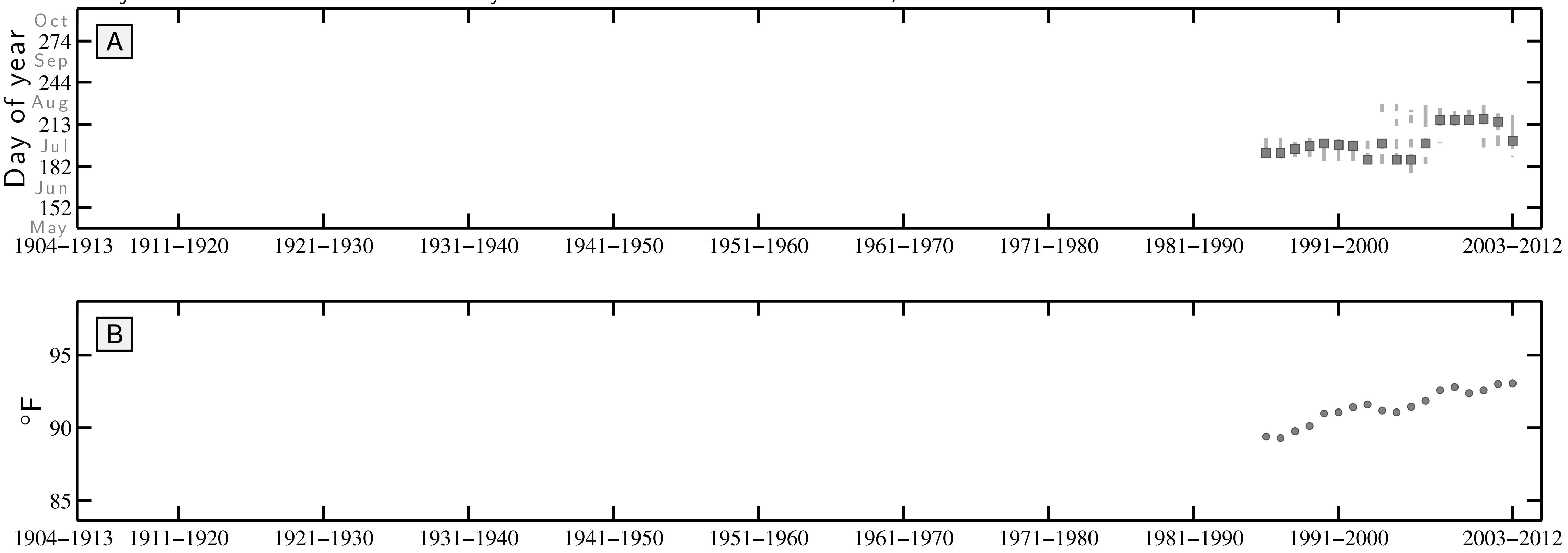

10 year Winter Teletherm dynamics for BREMO BLUFF, VA:

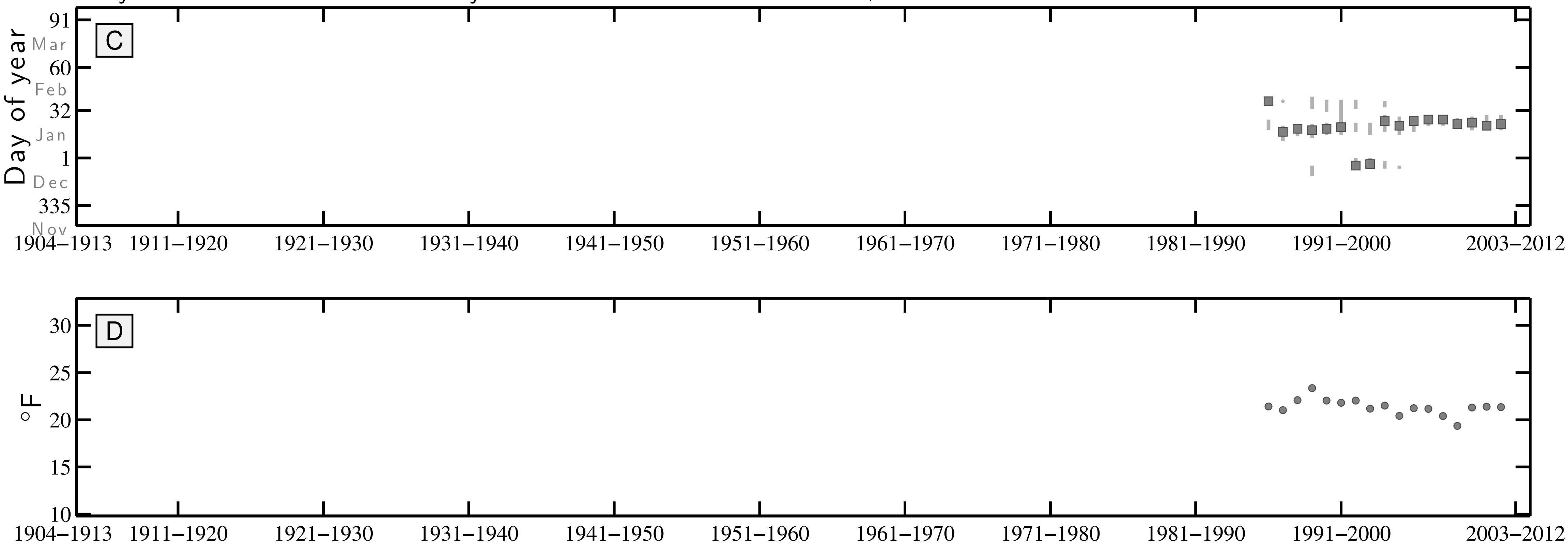

10 year Summer Teletherm dynamics for BURKES GARDEN, VA:

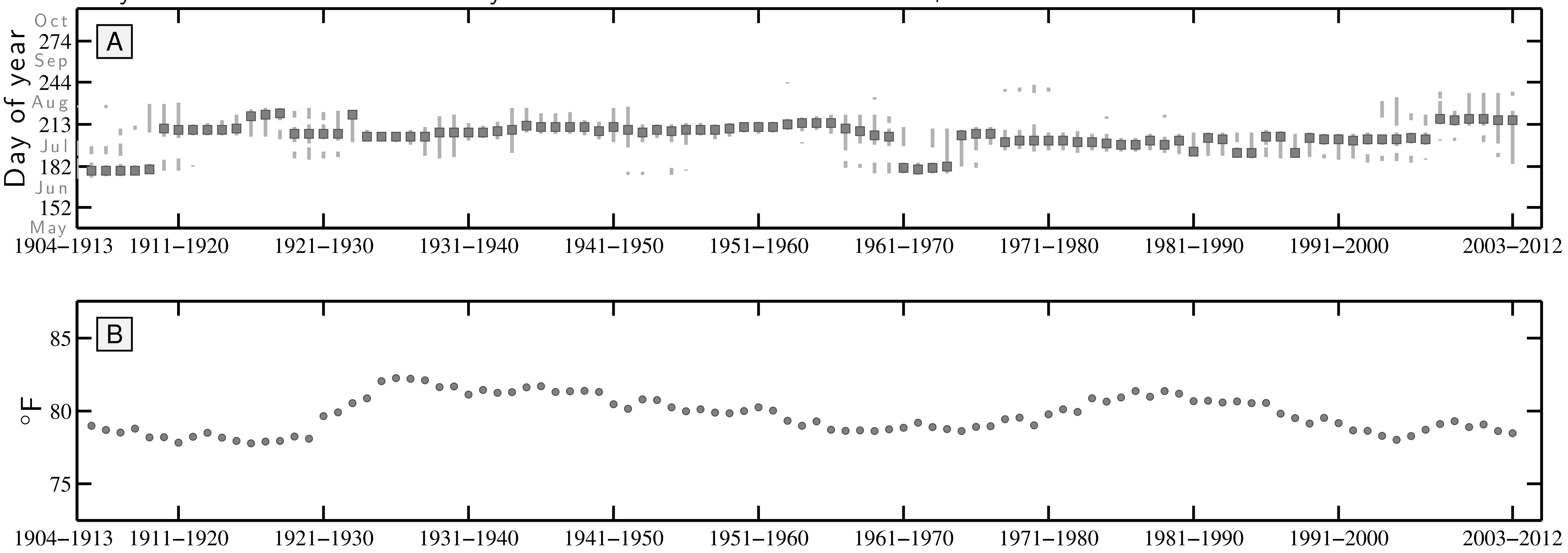

10 year Winter Teletherm dynamics for BURKES GARDEN, VA:

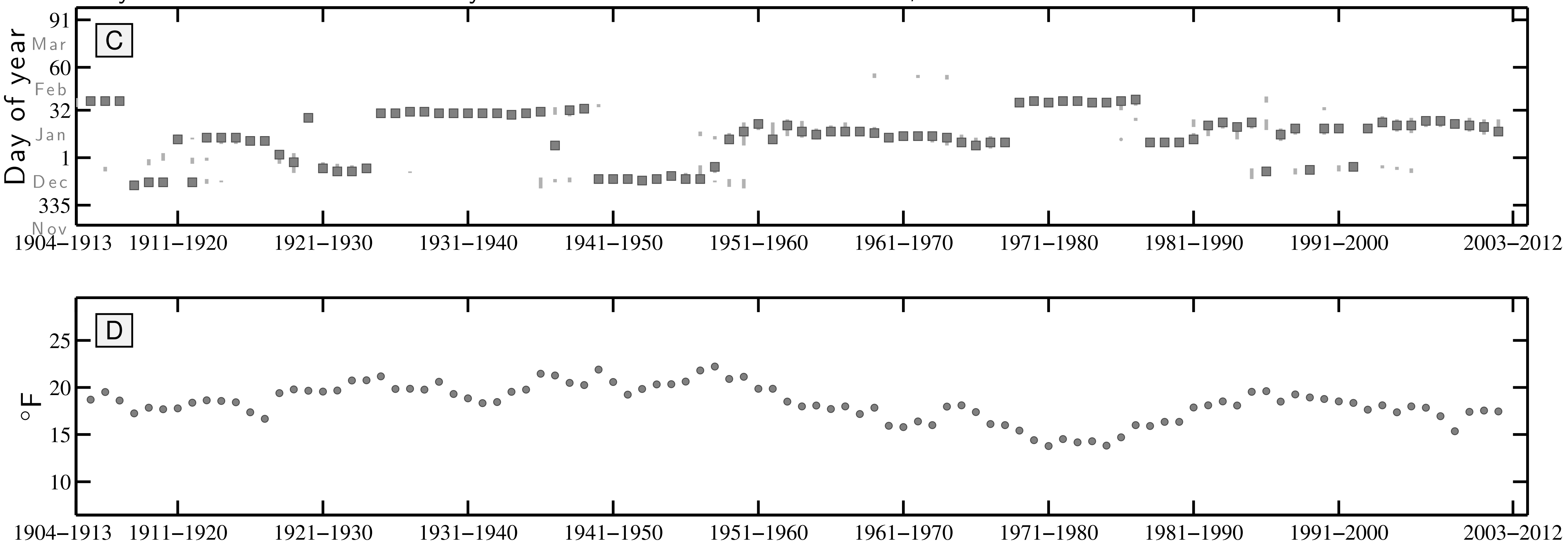

10 year Summer Teletherm dynamics for CHARLOTTESVILLE 2W, VA:

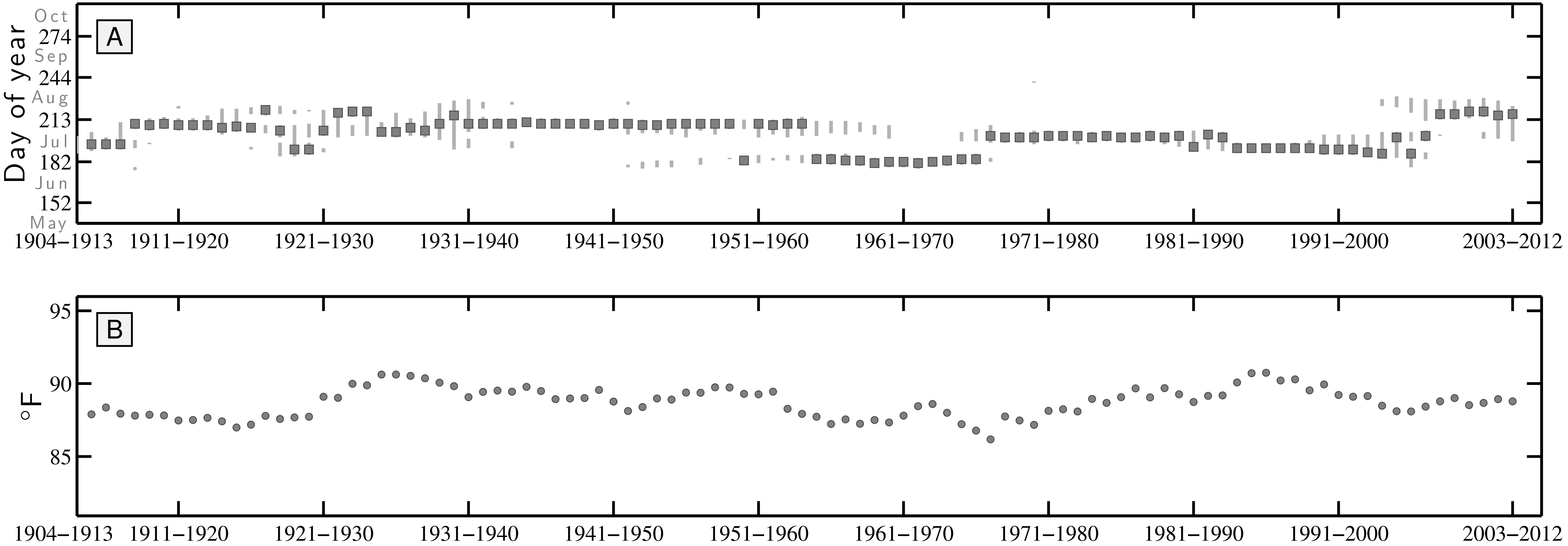

10 year Winter Teletherm dynamics for CHARLOTTESVILLE 2W, VA:

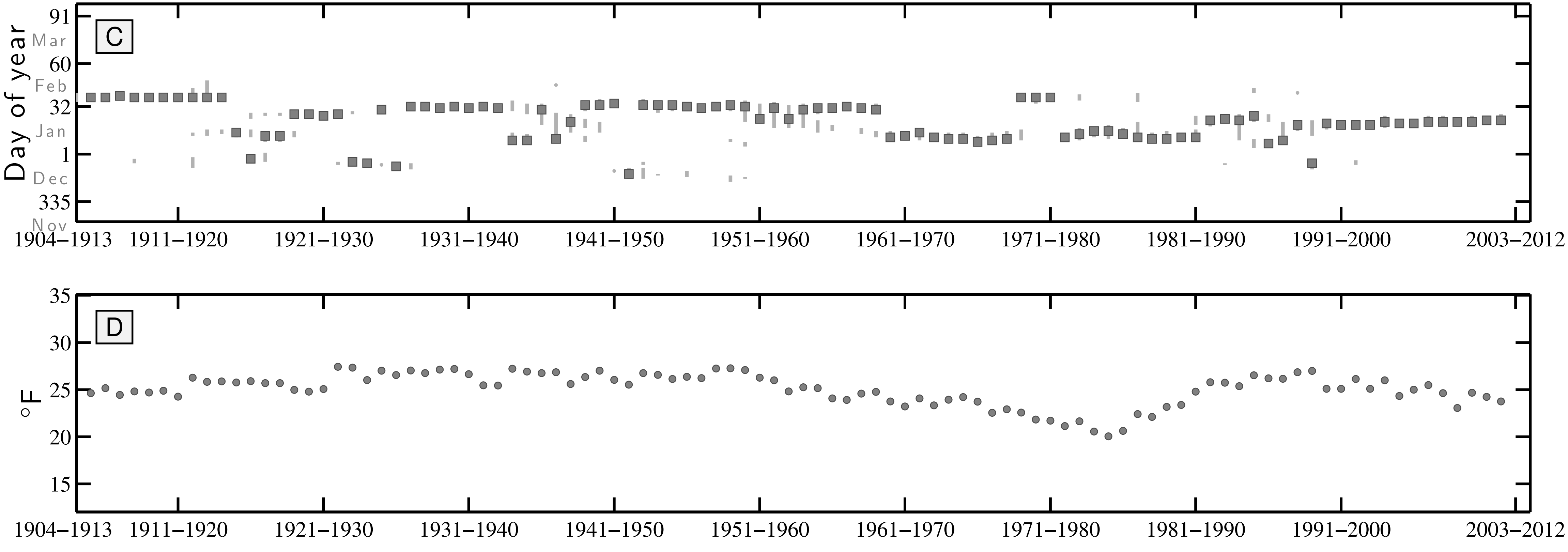

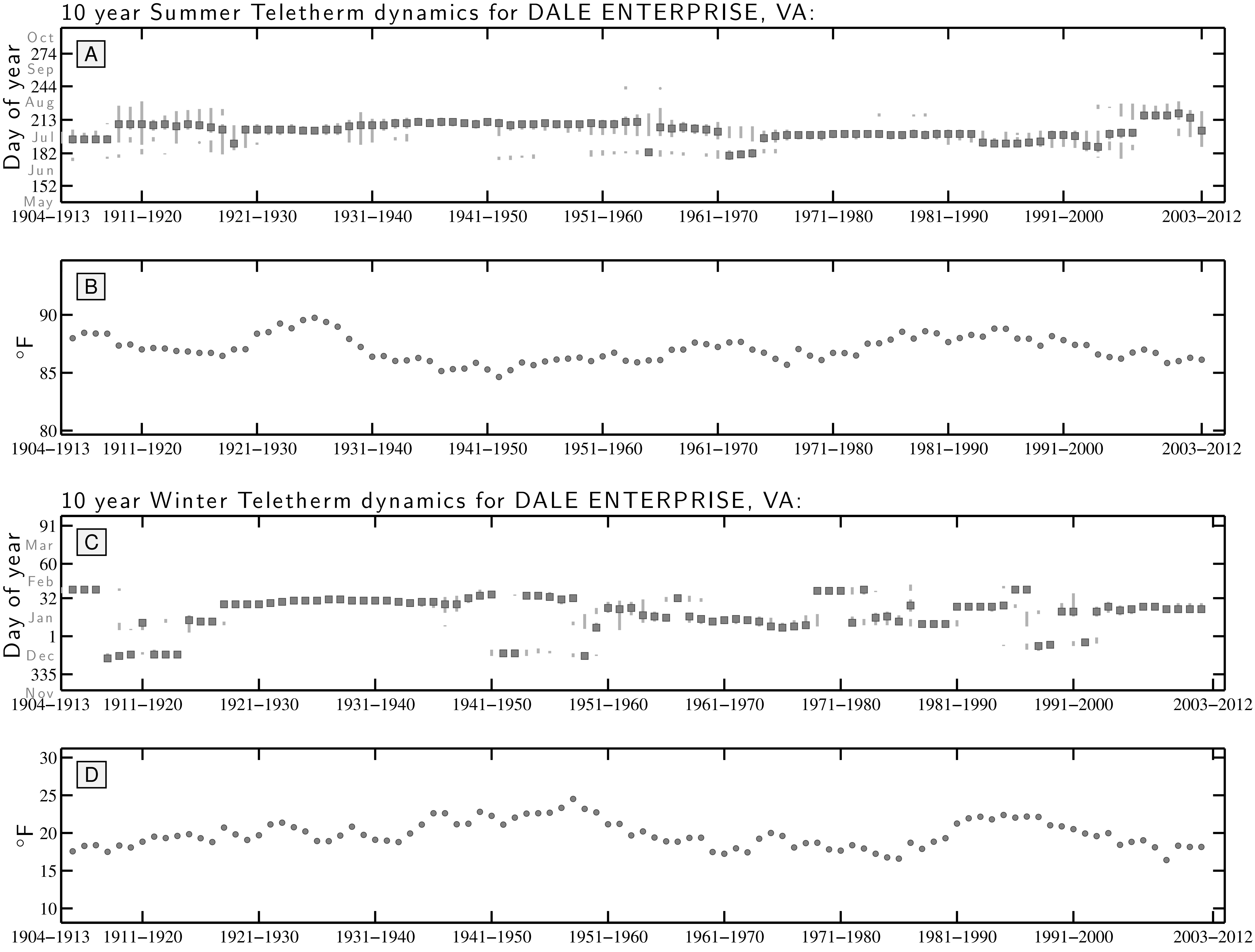
10 year Summer Teletherm dynamics for DALE ENTERPRISE, VA:
A
B
10 year Winter Teletherm dynamics for DALE ENTERPRISE, VA:
C
D
Day of year
°F
Oct
Sep
Aug
Jul
Jun
May
274
244
213
182
152
90
85
80
91
60
32
1
335
Mar
Feb
Jan
Dec
Nov
30
25
20
15
10
1904–1913
1911–1920
1921–1930
1931–1940
1941–1950
1951–1960
1961–1970
1971–1980
1981–1990
1991–2000
2003–2012

10 year Summer Teletherm dynamics for DANVILLE, VA:

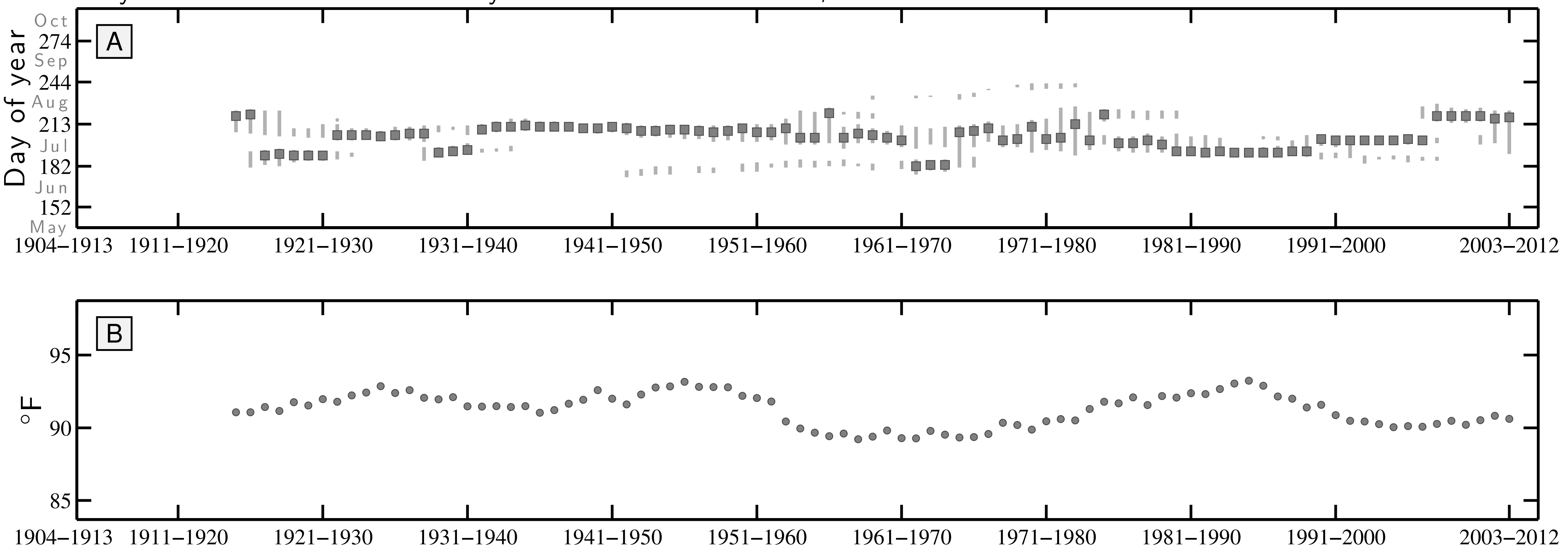

10 year Winter Teletherm dynamics for DANVILLE, VA:

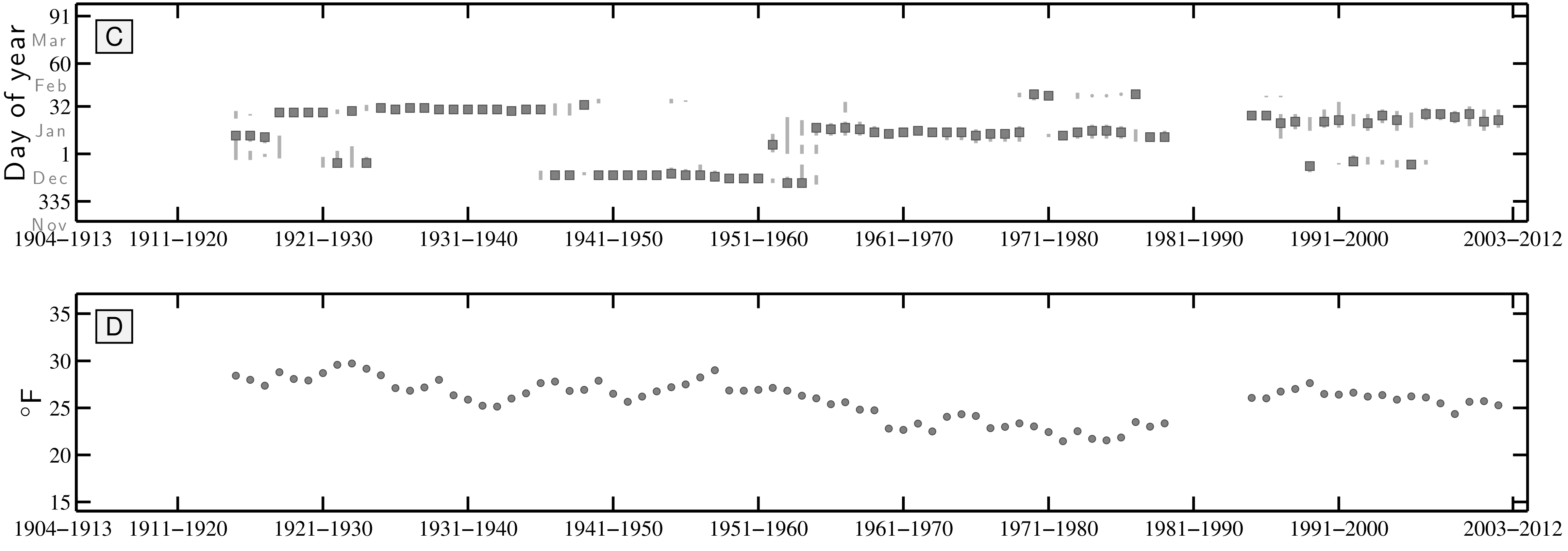

10 year Summer Teletherm dynamics for FARMVILLE 2 N, VA:

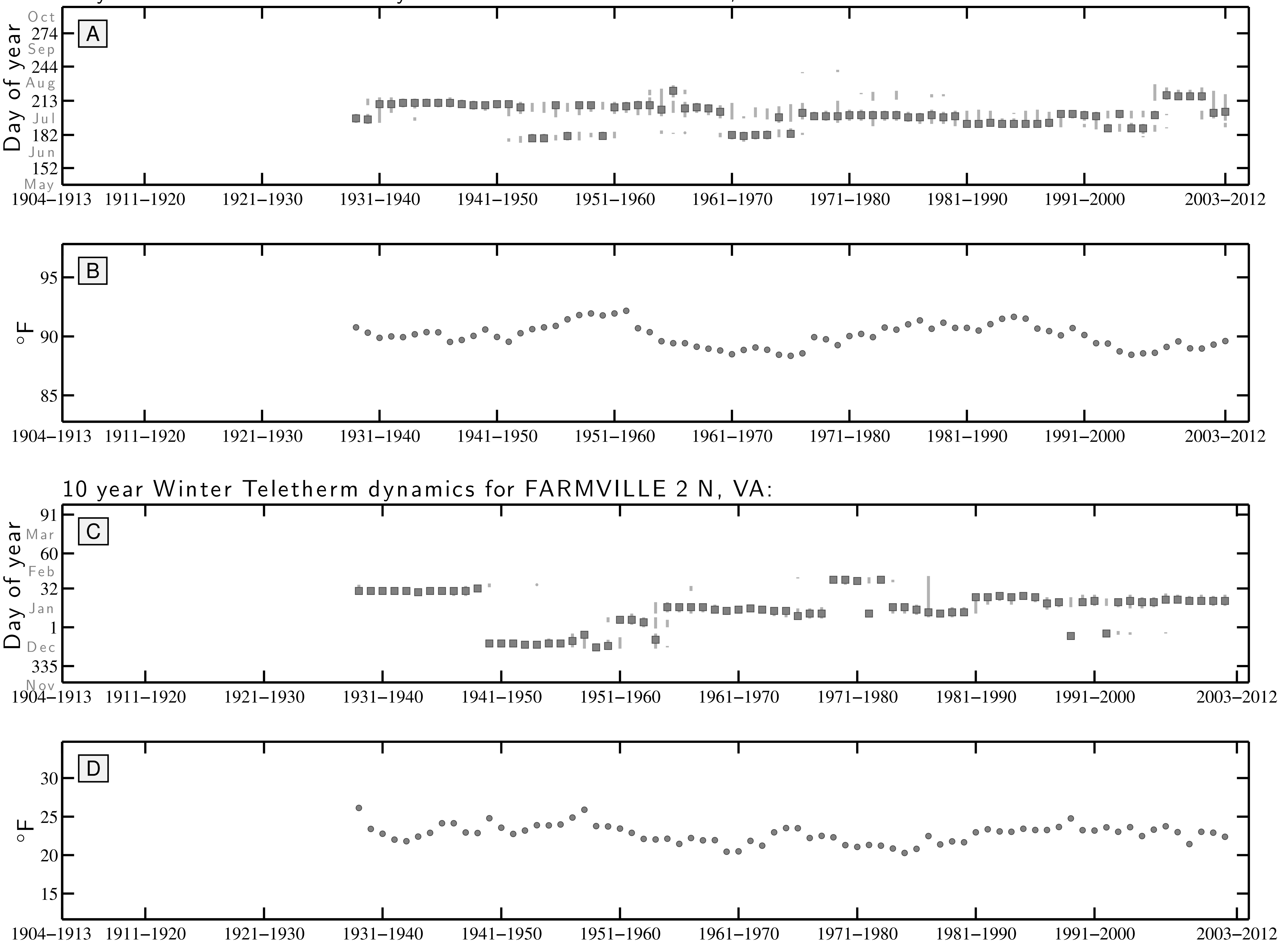

10 year Summer Teletherm dynamics for FREDERICKSBURG NP, VA:

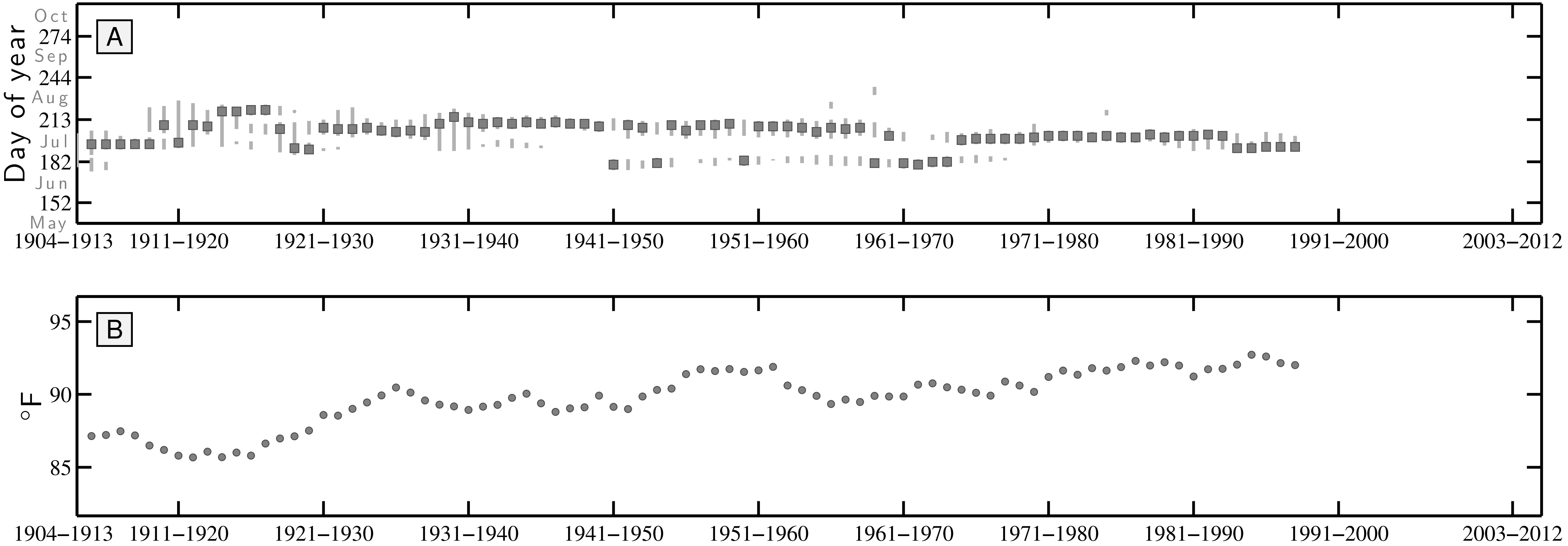

10 year Winter Teletherm dynamics for FREDERICKSBURG NP, VA:

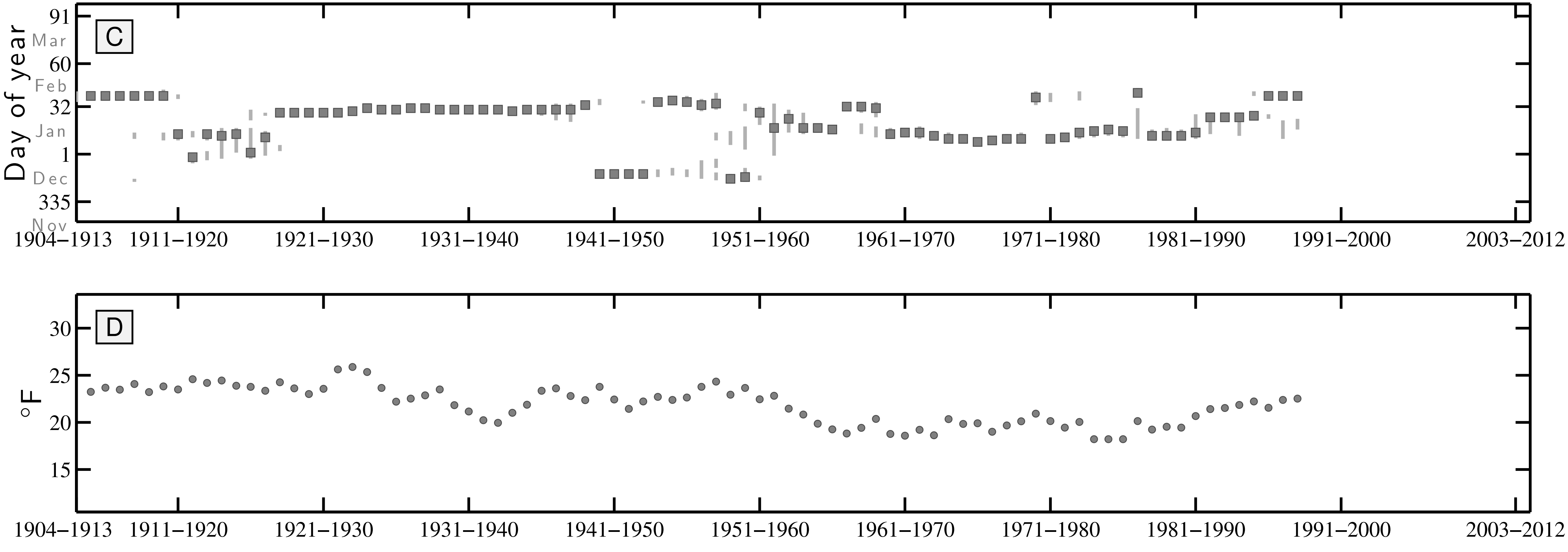

10 year Summer Teletherm dynamics for HOPEWELL, VA:

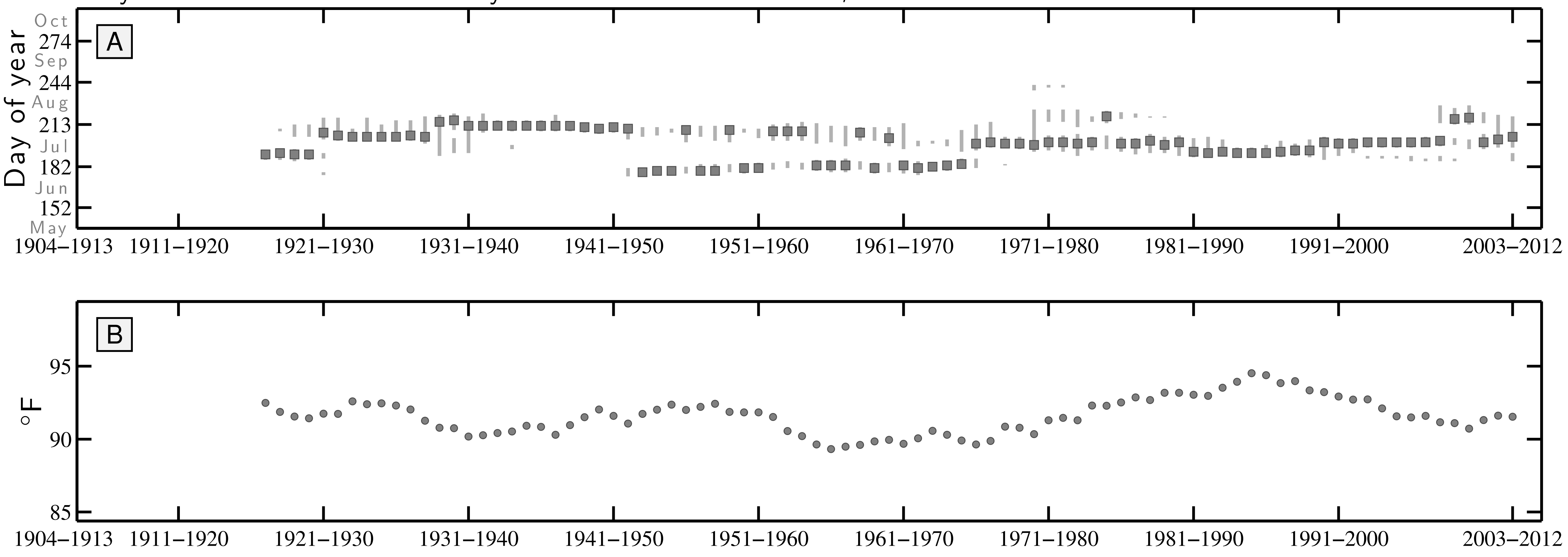

10 year Winter Teletherm dynamics for HOPEWELL, VA:

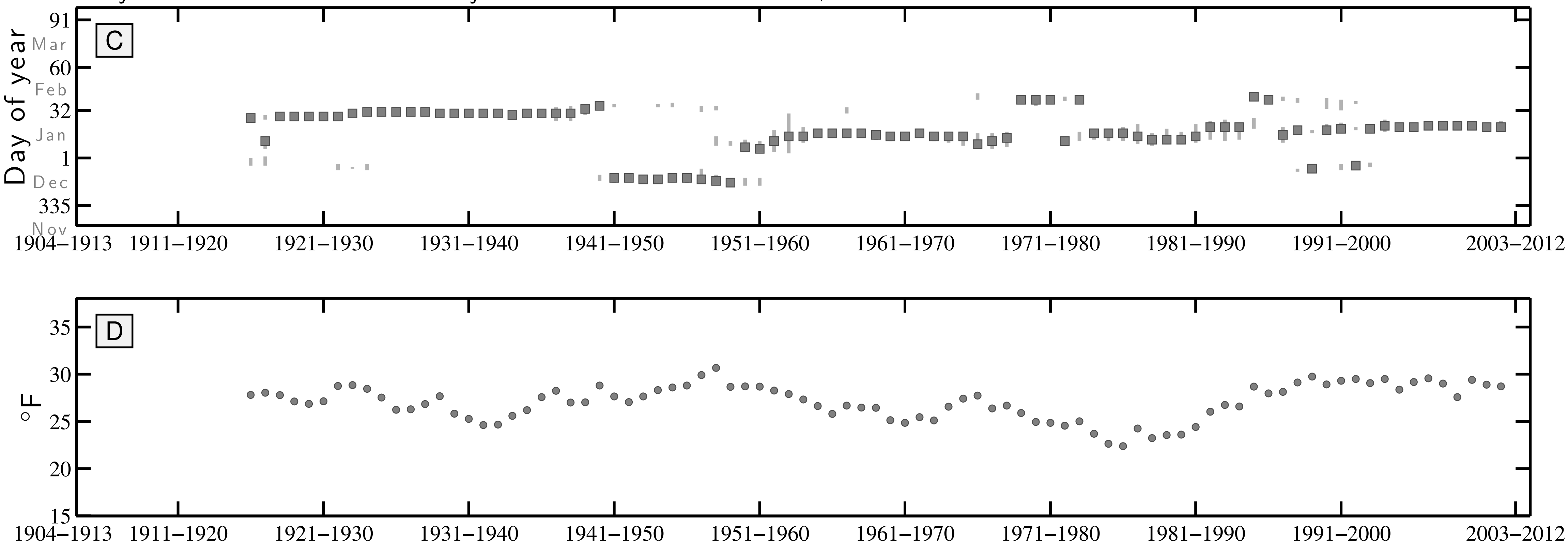

10 year Summer Teletherm dynamics for HOT SPRINGS, VA:

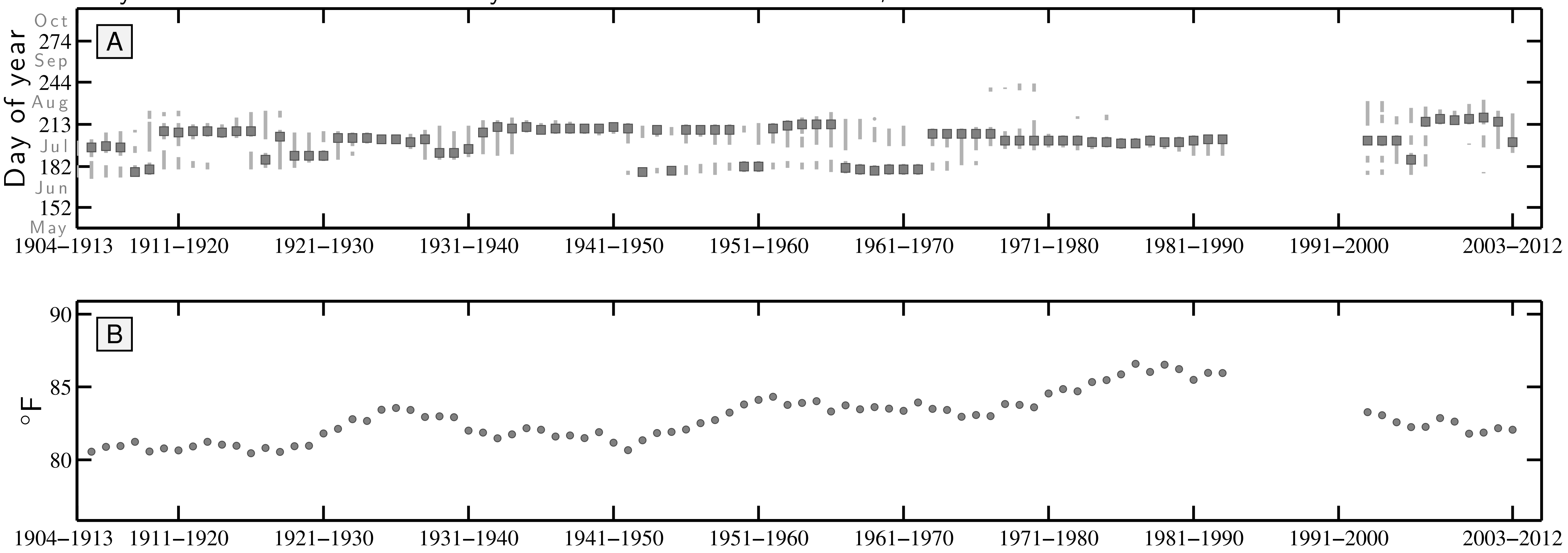

10 year Winter Teletherm dynamics for HOT SPRINGS, VA:

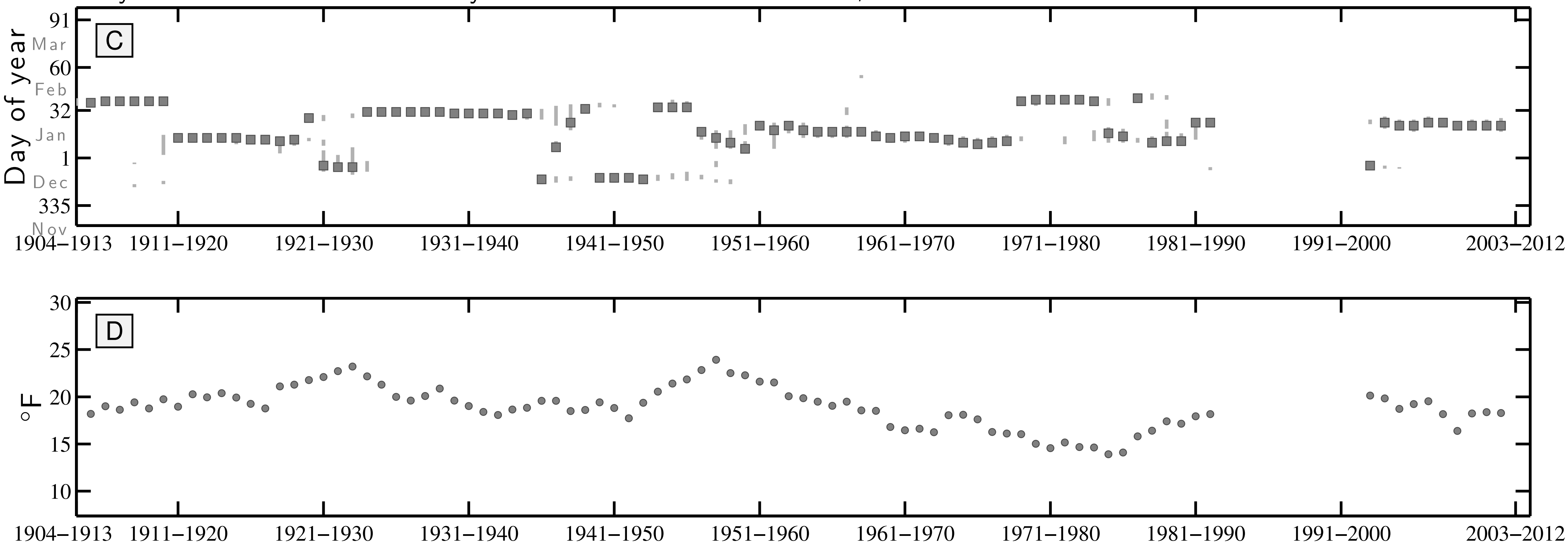

10 year Summer Teletherm dynamics for LEXINGTON, VA:

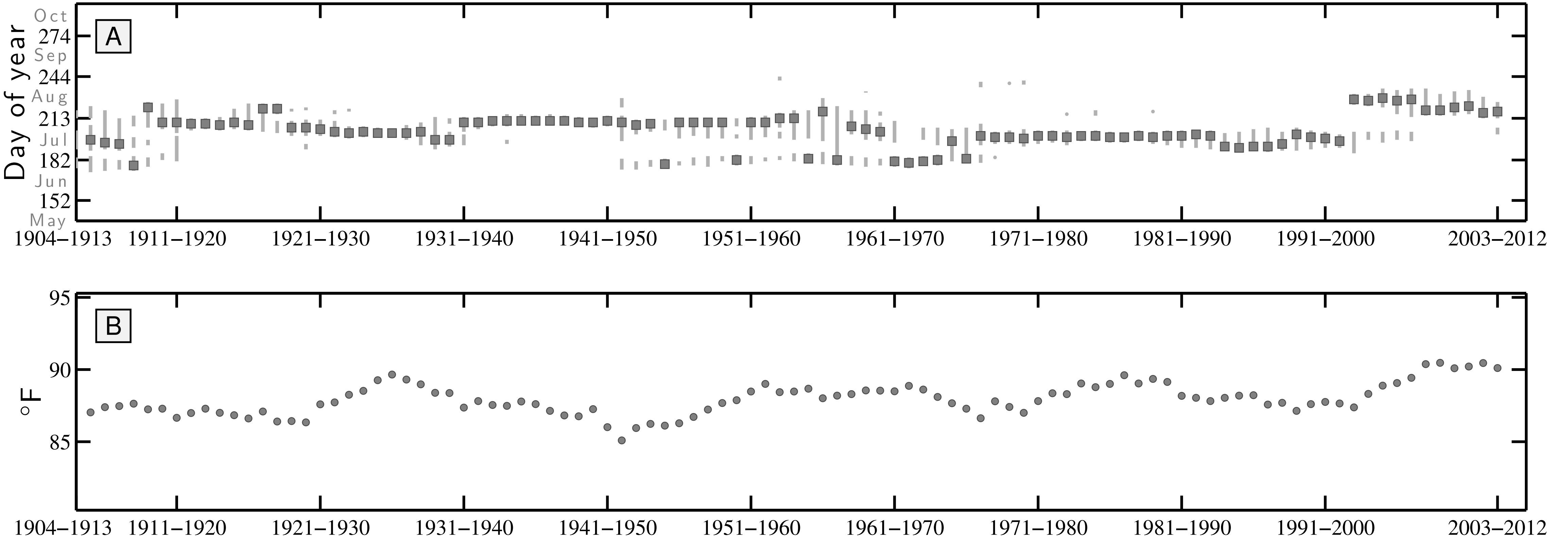

10 year Winter Teletherm dynamics for LEXINGTON, VA:

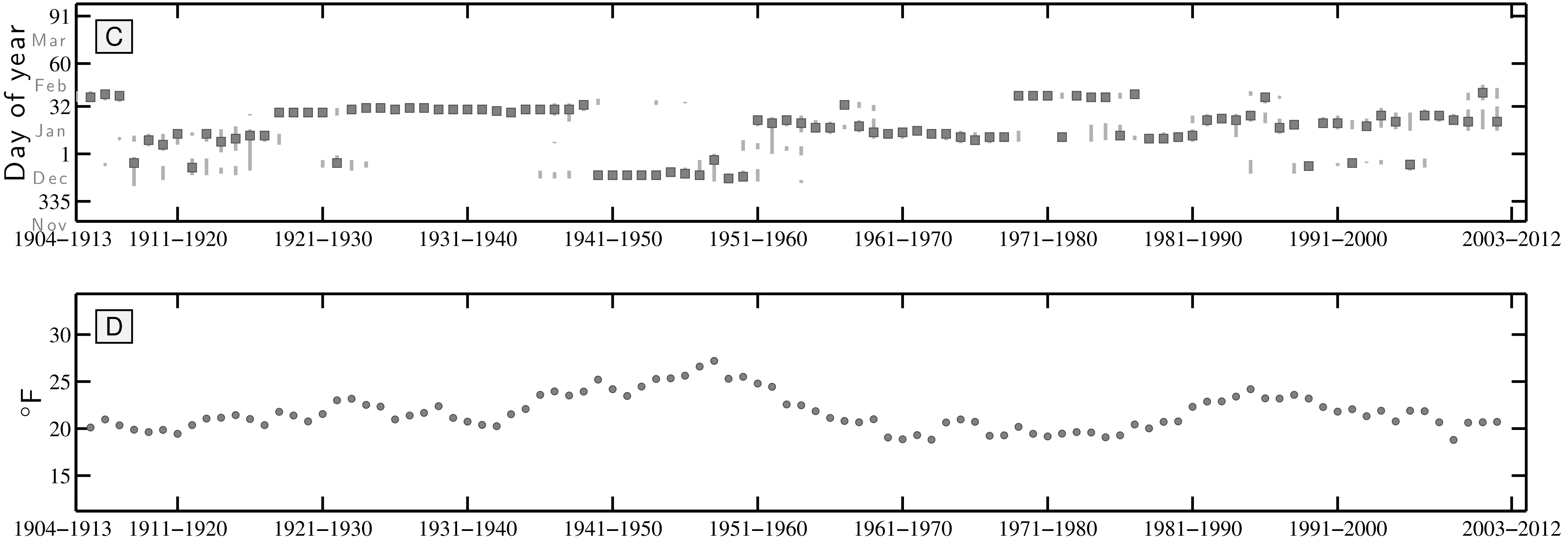

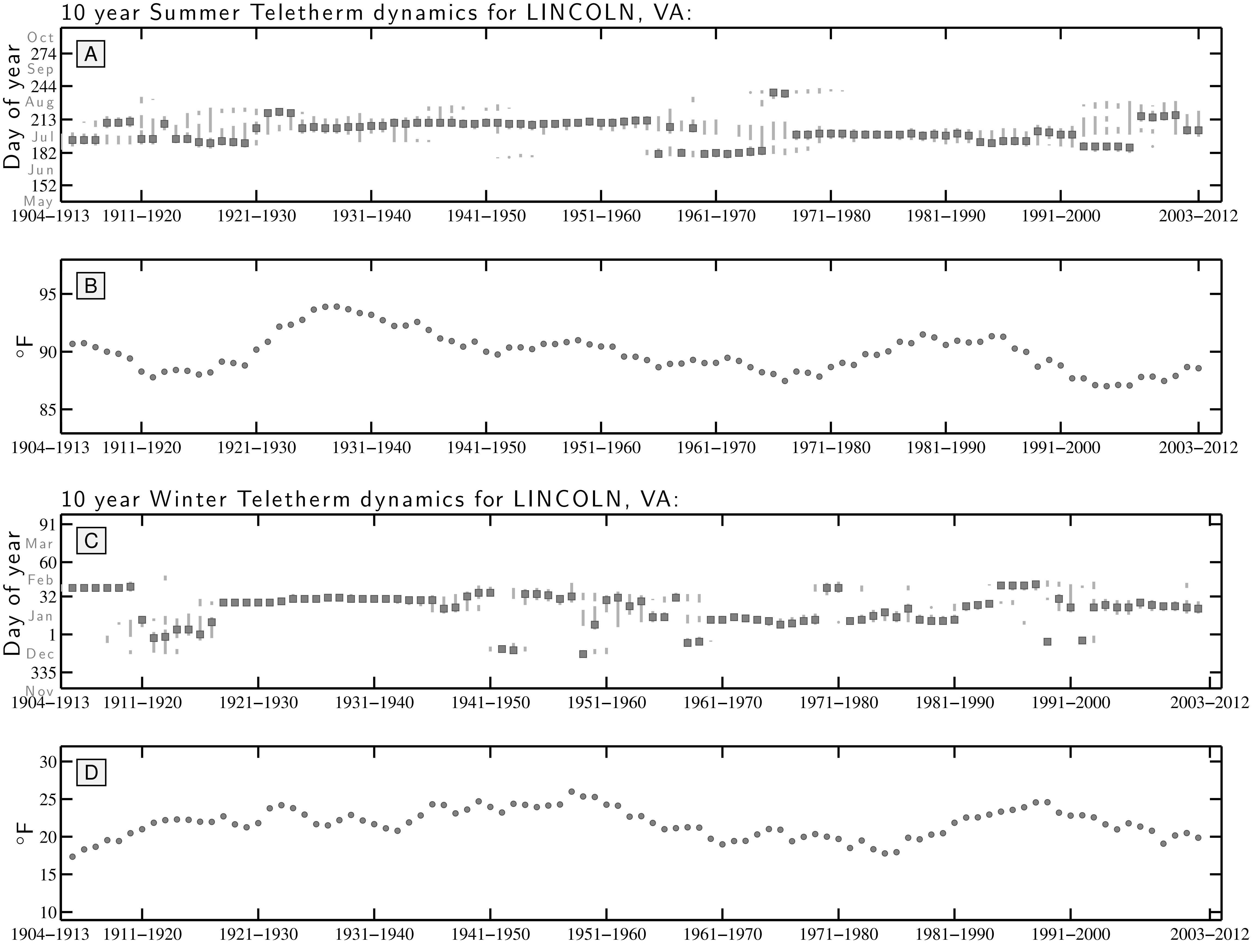
10 year Summer Teletherm dynamics for LINCOLN, VA:
A
B
Day of year
°F
Oct
Sep
Aug
Jul
Jun
May
274
244
213
182
152
95
90
85
10 year Winter Teletherm dynamics for LINCOLN, VA:
C
D
Mar
Feb
Jan
Dec
Nov
91
60
32
1
335
30
25
20
15
10
1904–1913
1911–1920
1921–1930
1931–1940
1941–1950
1951–1960
1961–1970
1971–1980
1981–1990
1991–2000
2003–2012

10 year Summer Teletherm dynamics for NORFOLK INTL AP, VA:

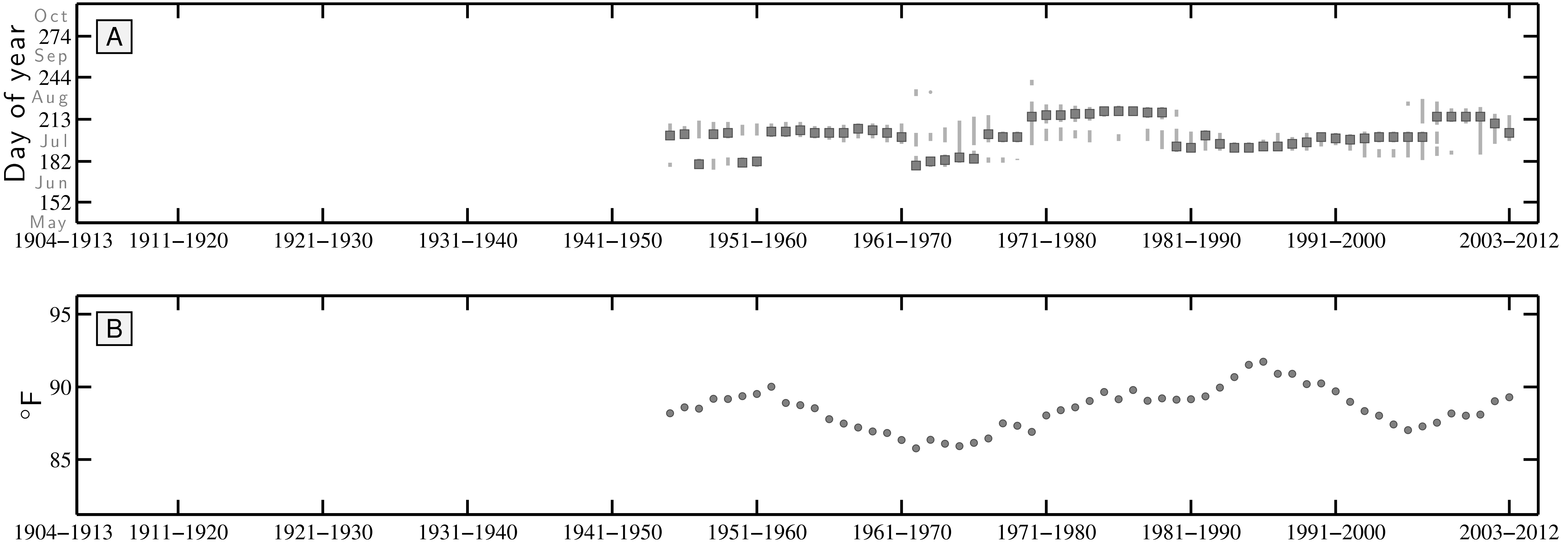

10 year Winter Teletherm dynamics for NORFOLK INTL AP, VA:

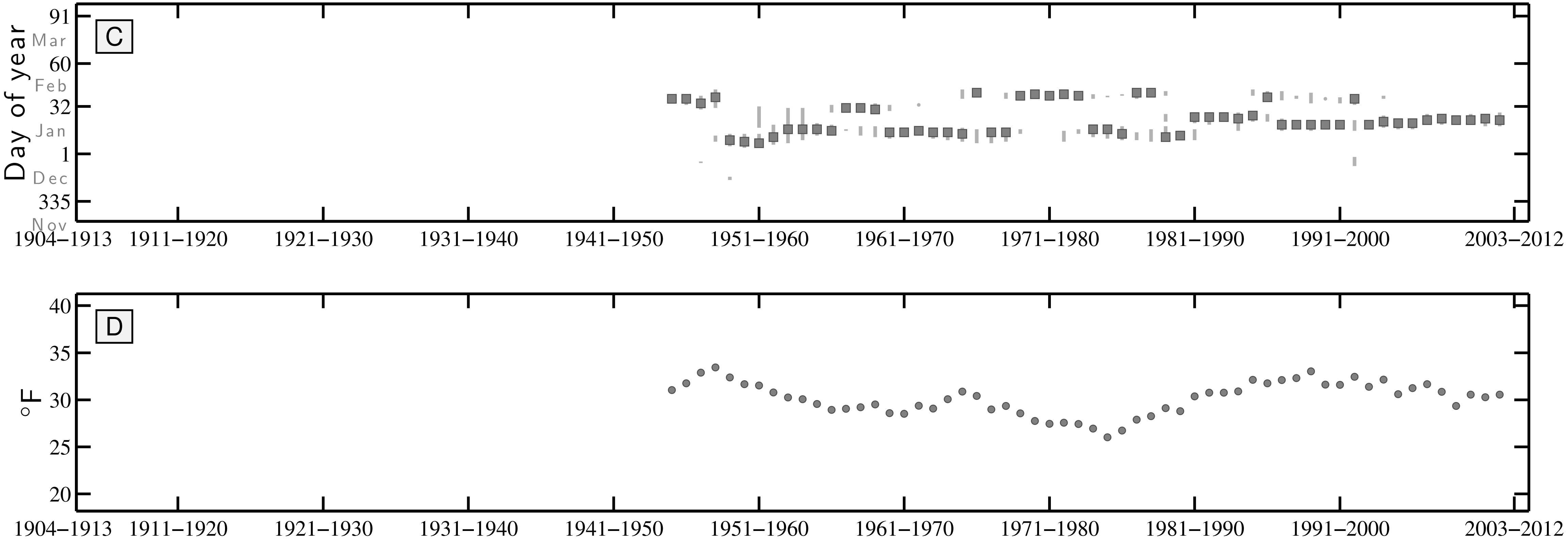

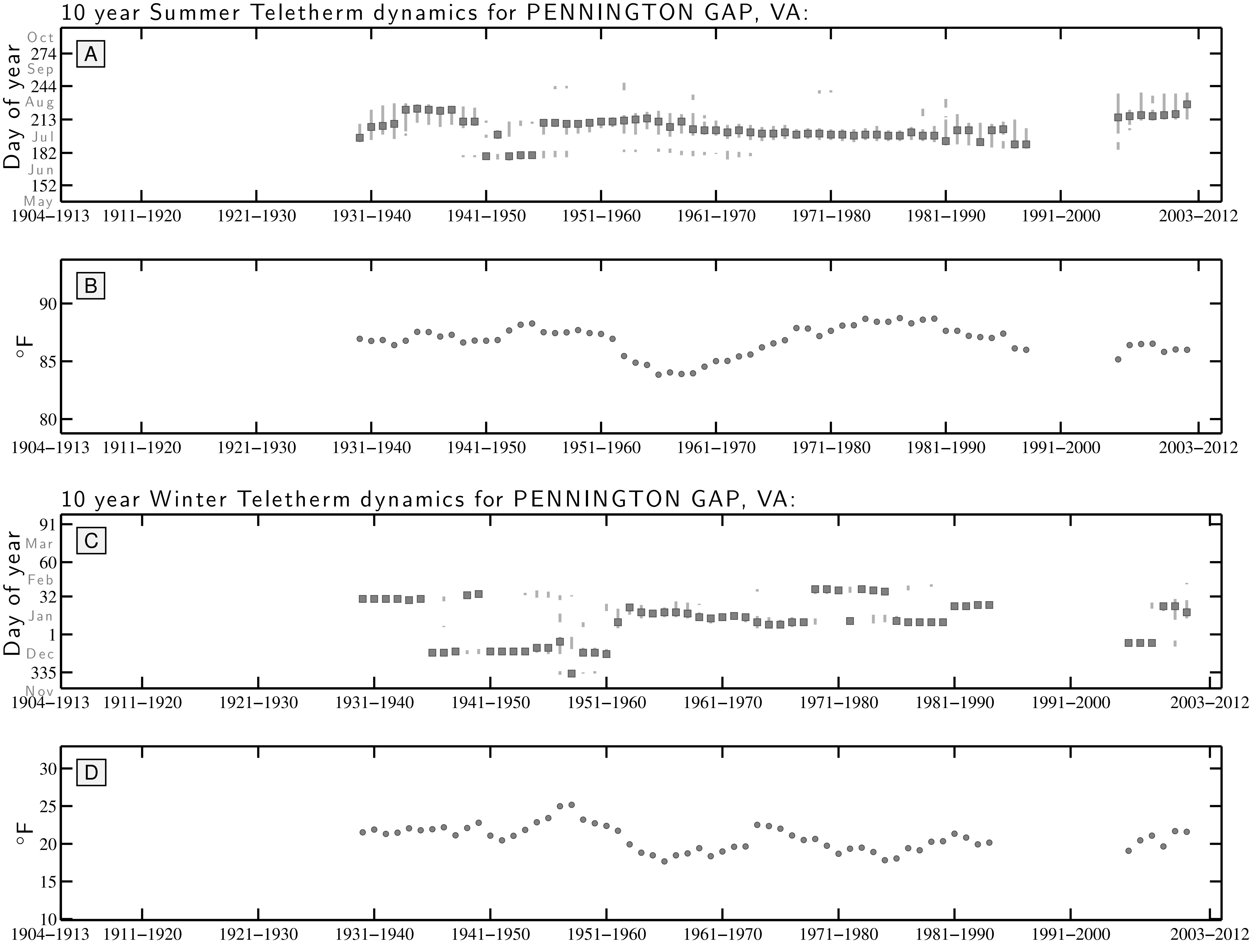
10 year Summer Teletherm dynamics for PENNINGTON GAP, VA:
A
Day of year
Oct
274
Sep
244
Aug
213
Jul
182
Jun
152
May
B
°F
90
85
80
10 year Winter Teletherm dynamics for PENNINGTON GAP, VA:
C
Day of year
91
Mar
60
Feb
32
Jan
1
Dec
335
Nov
D
°F
30
25
20
15
10
1904–1913
1911–1920
1921–1930
1931–1940
1941–1950
1951–1960
1961–1970
1971–1980
1981–1990
1991–2000
2003–2012

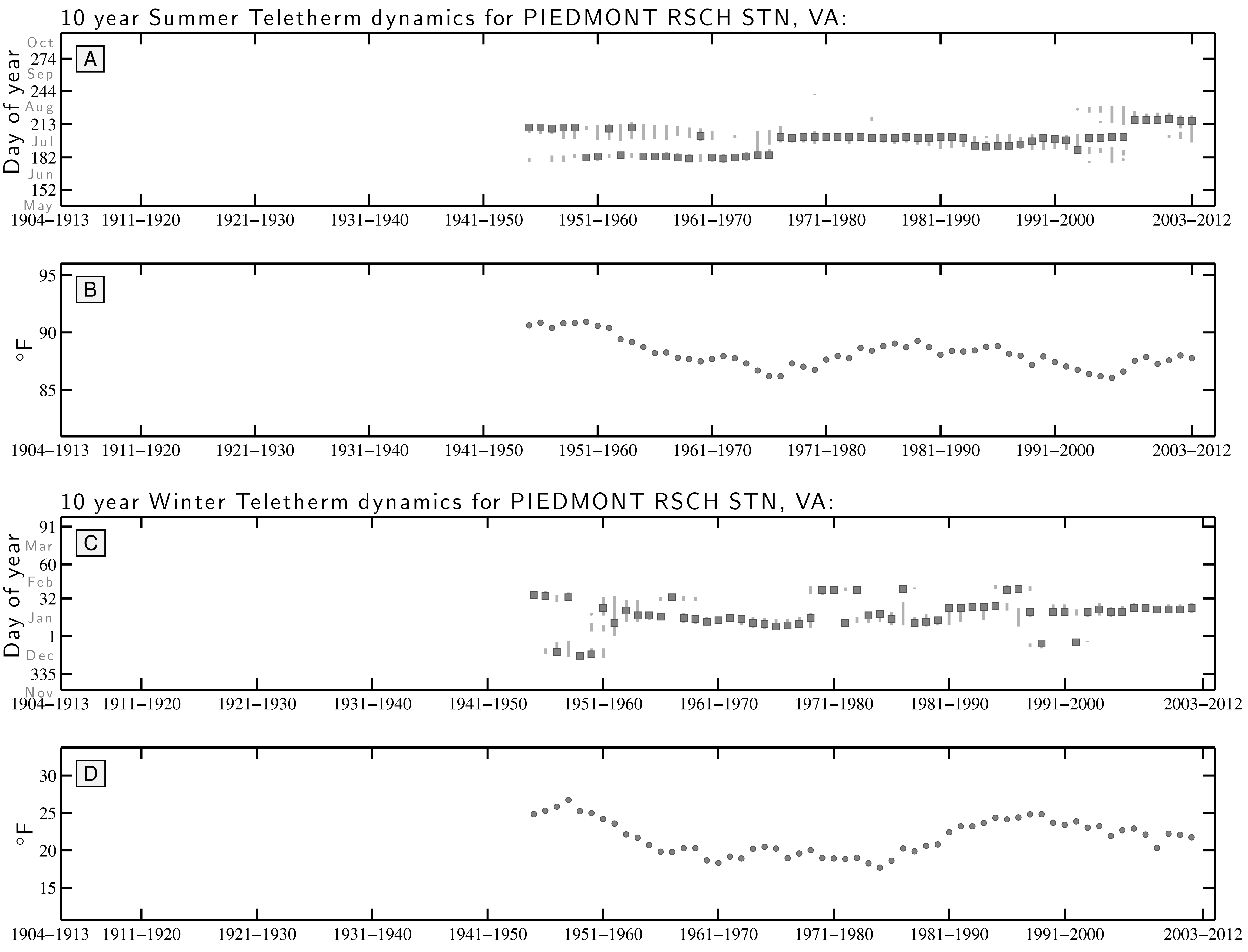

10 year Summer Teletherm dynamics for PIEDMONT RSCH STN, VA:
A
B
Day of year
°F
Oct
274
Sep
244
Aug
213
Jul
182
Jun
152
May
95
90
85
10 year Winter Teletherm dynamics for PIEDMONT RSCH STN, VA:
C
D
91
Mar
60
Feb
32
Jan
1
Dec
335
Nov
30
25
20
15
1904–1913
1911–1920
1921–1930
1931–1940
1941–1950
1951–1960
1961–1970
1971–1980
1981–1990
1991–2000
2003–2012

10 year Summer Teletherm dynamics for ROCKY MT, VA:

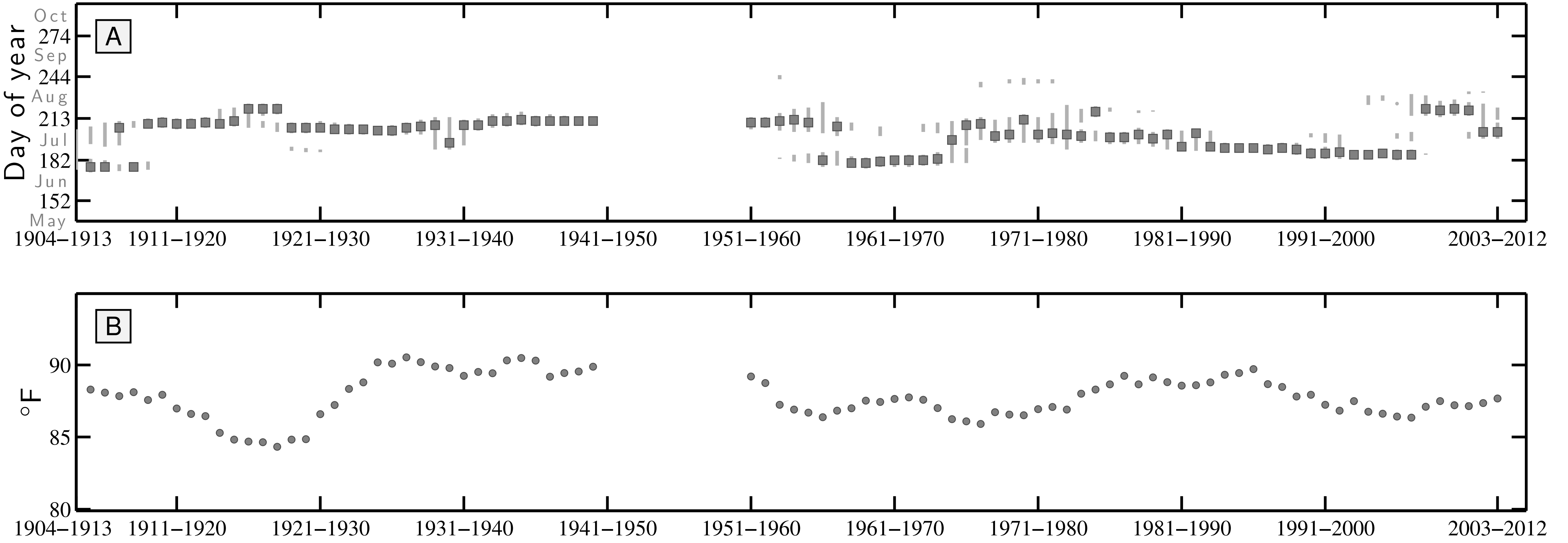

10 year Winter Teletherm dynamics for ROCKY MT, VA:

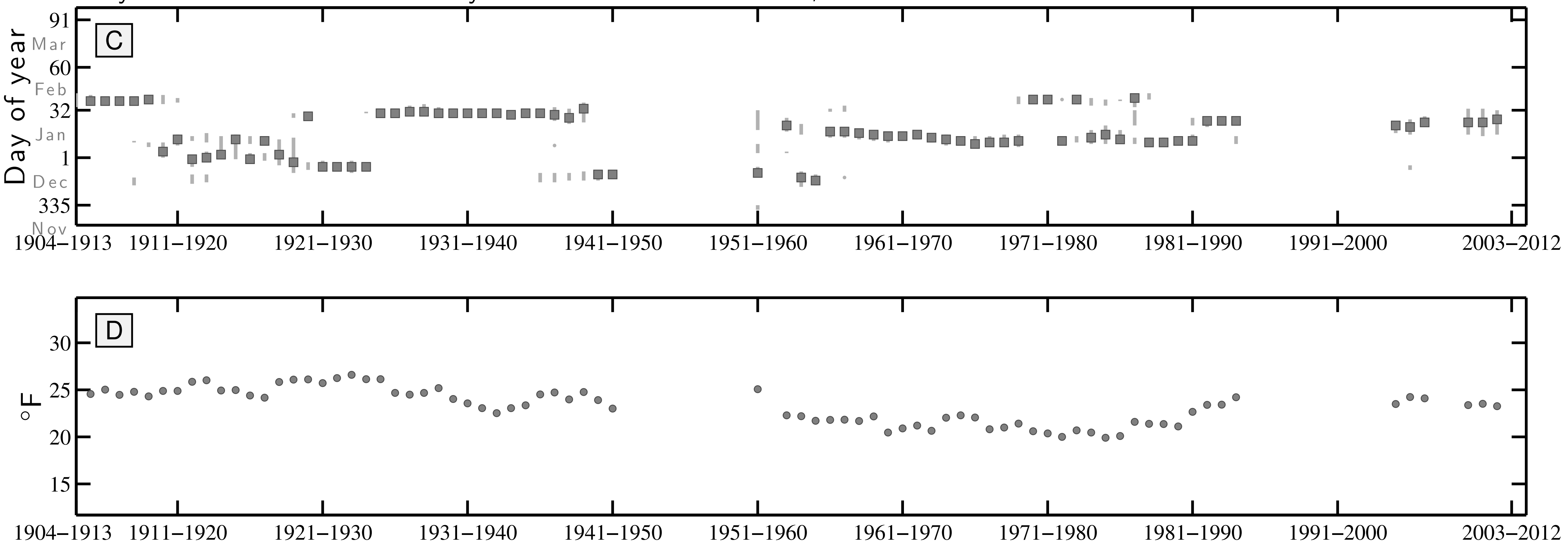

10 year Summer Teletherm dynamics for STAUNTON WATER TRMTMT PLT, VA:

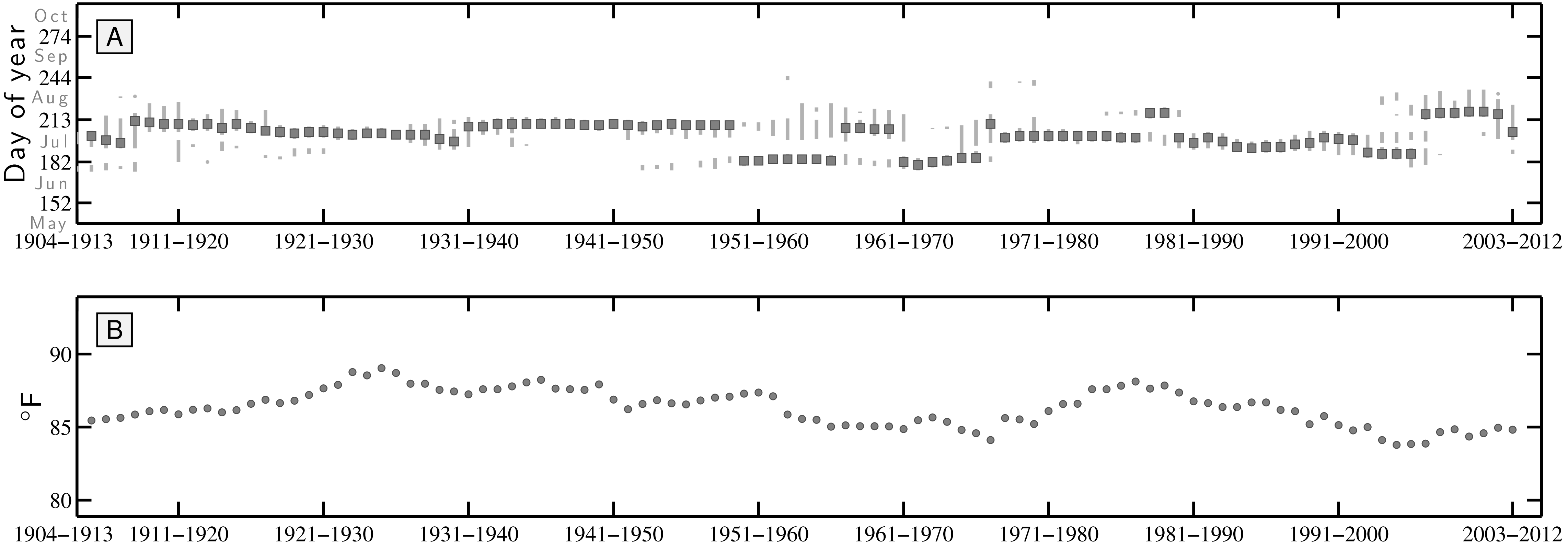

10 year Winter Teletherm dynamics for STAUNTON WATER TRMTMT PLT, VA:

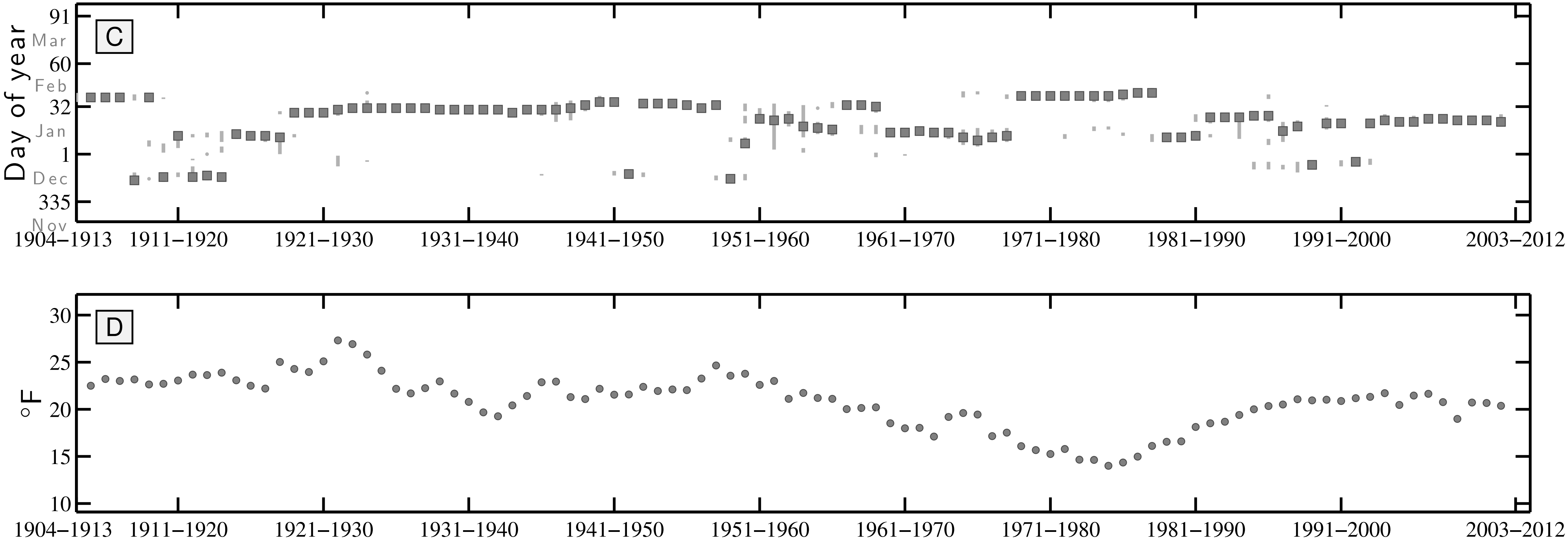

10 year Summer Teletherm dynamics for WILLIAMSBURG 2 N, VA:

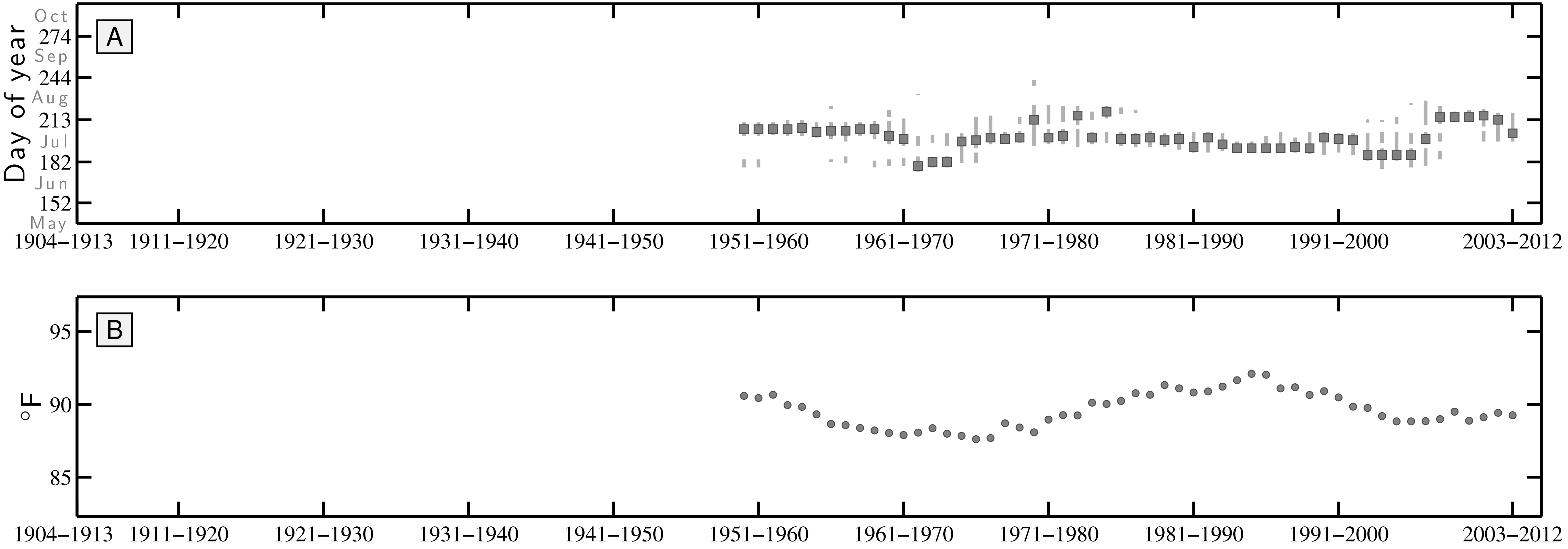

10 year Winter Teletherm dynamics for WILLIAMSBURG 2 N, VA:

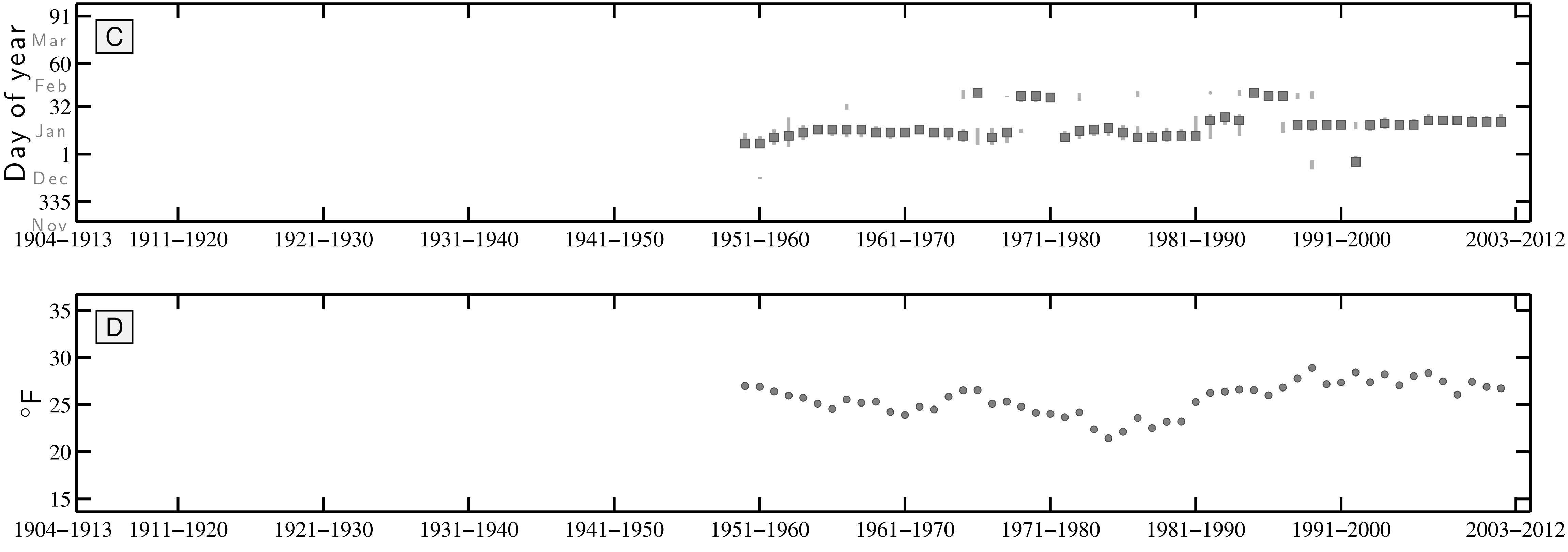

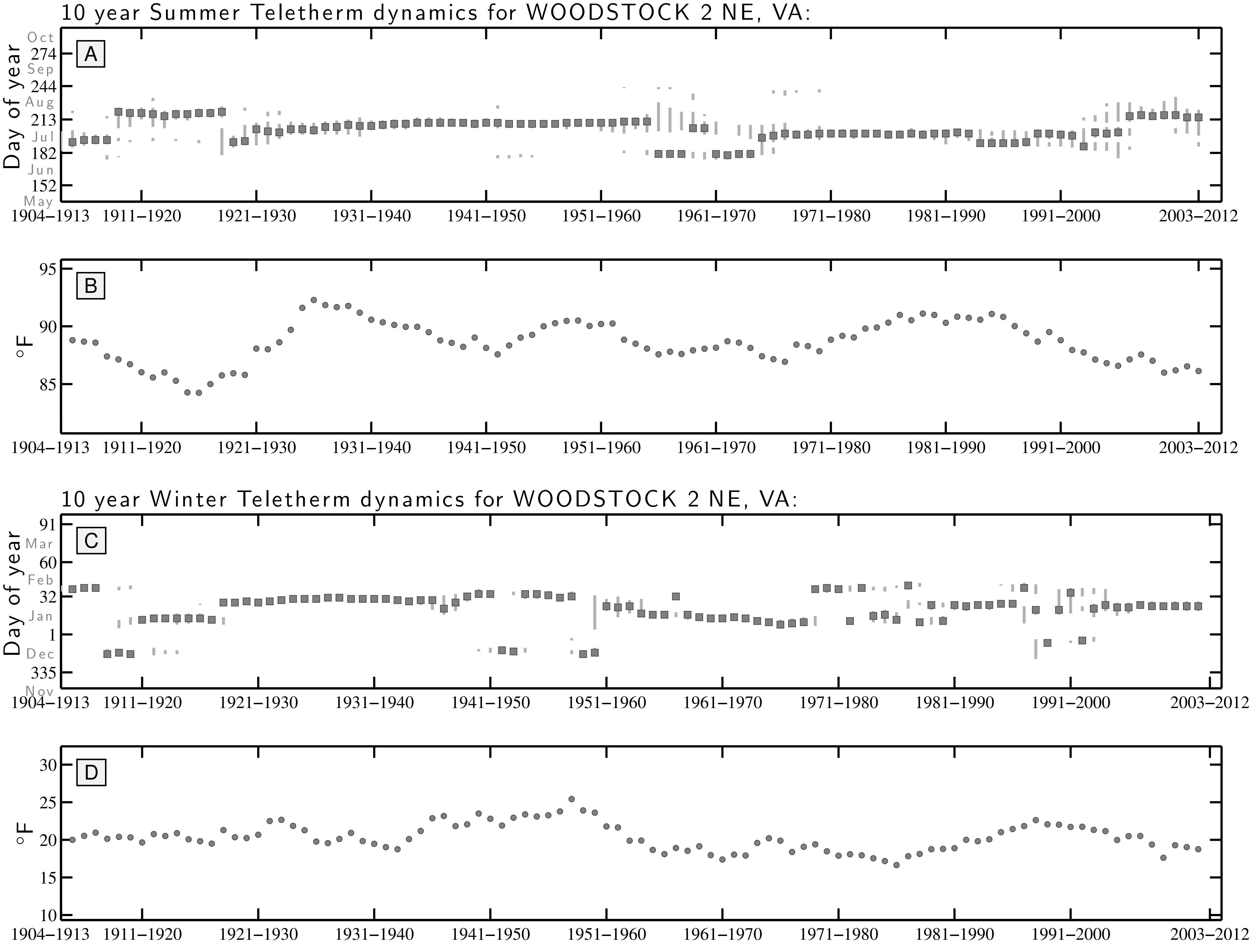
10 year Summer Teletherm dynamics for WOODSTOCK 2 NE, VA:
A
Day of year
Oct
274
Sep
244
Aug
213
Jul
182
Jun
152
May
1904–1913
1911–1920
1921–1930
1931–1940
1941–1950
1951–1960
1961–1970
1971–1980
1981–1990
1991–2000
2003–2012
B
°F
95
90
85
10 year Winter Teletherm dynamics for WOODSTOCK 2 NE, VA:
C
91
Mar
60
Feb
32
Jan
1
Dec
335
Nov
D
30
25
20
15
10

10 year Summer Teletherm dynamics for ABERDEEN, WA:

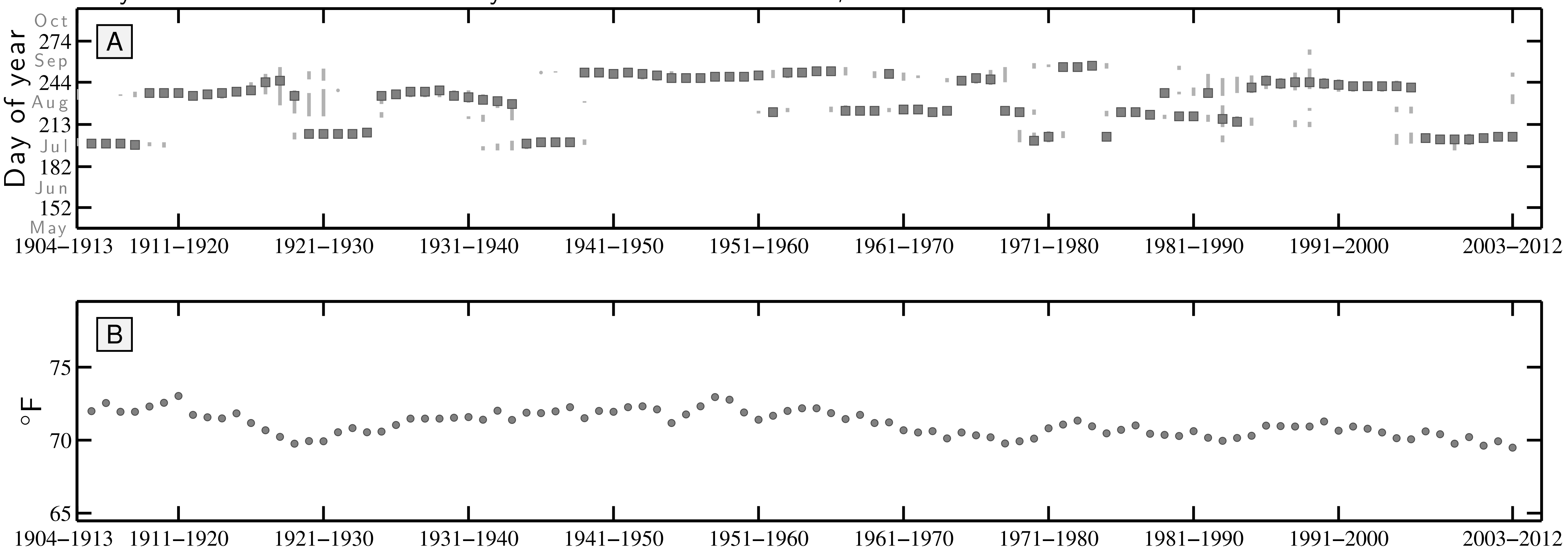

10 year Winter Teletherm dynamics for ABERDEEN, WA:

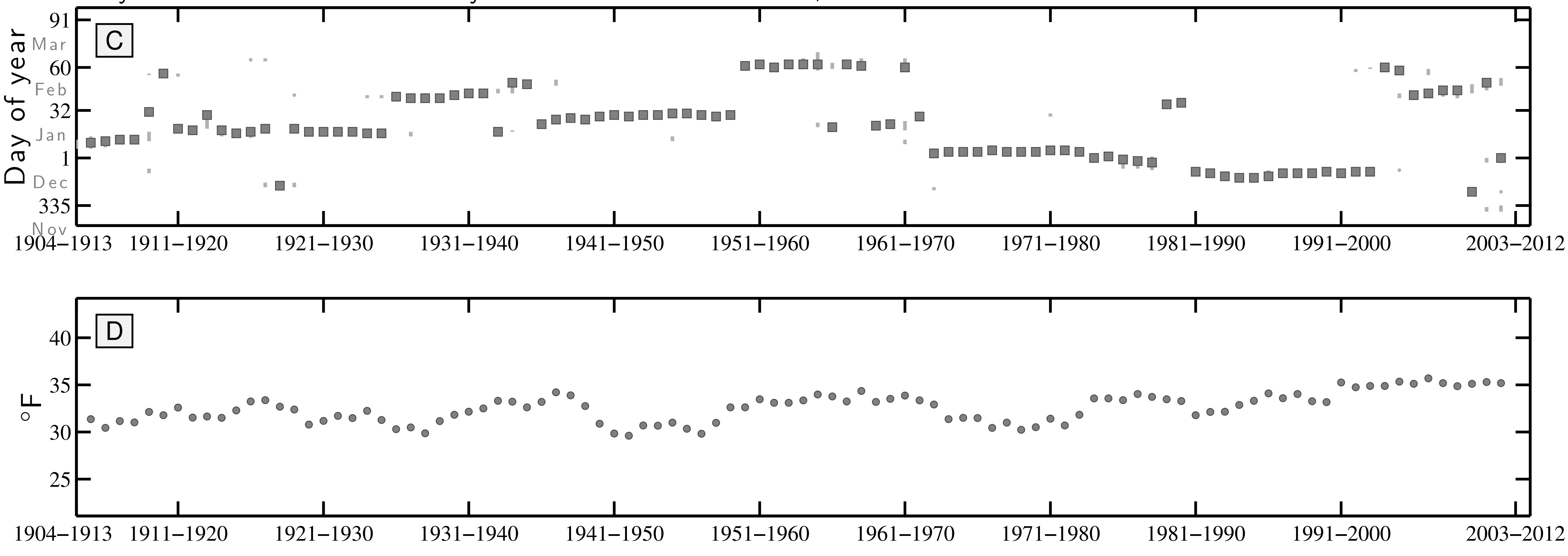

10 year Summer Teletherm dynamics for BELLINGHAM 3 SSW, WA:

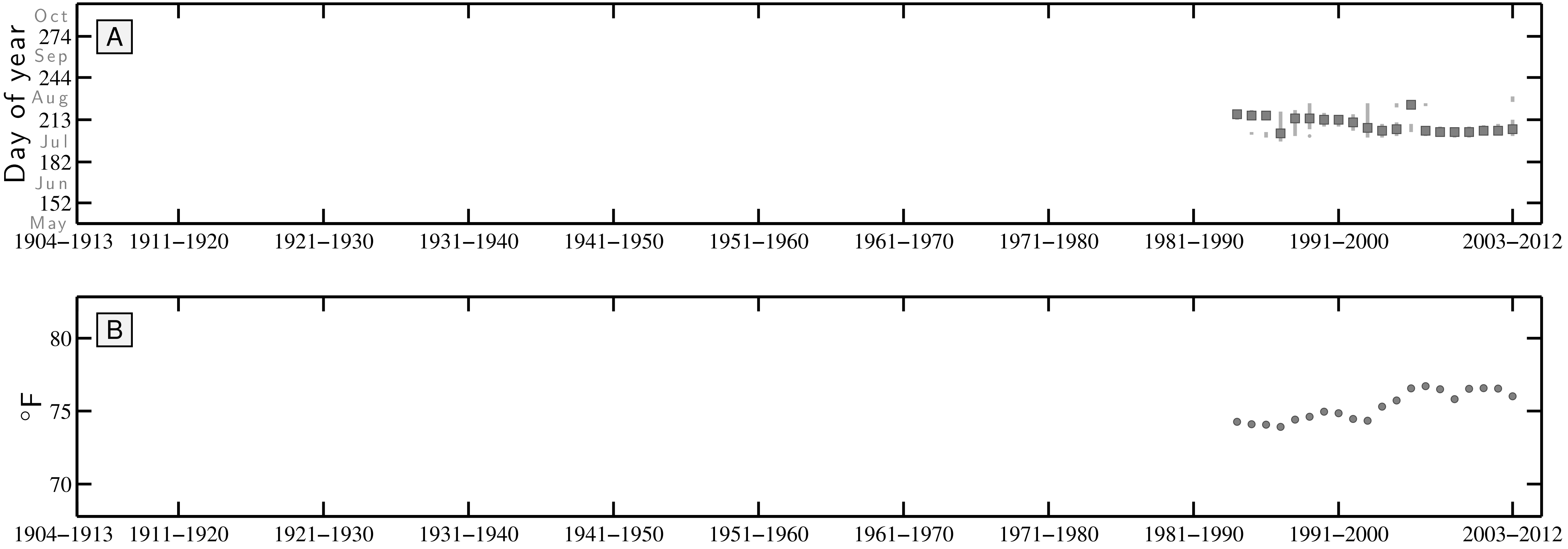

10 year Winter Teletherm dynamics for BELLINGHAM 3 SSW, WA:

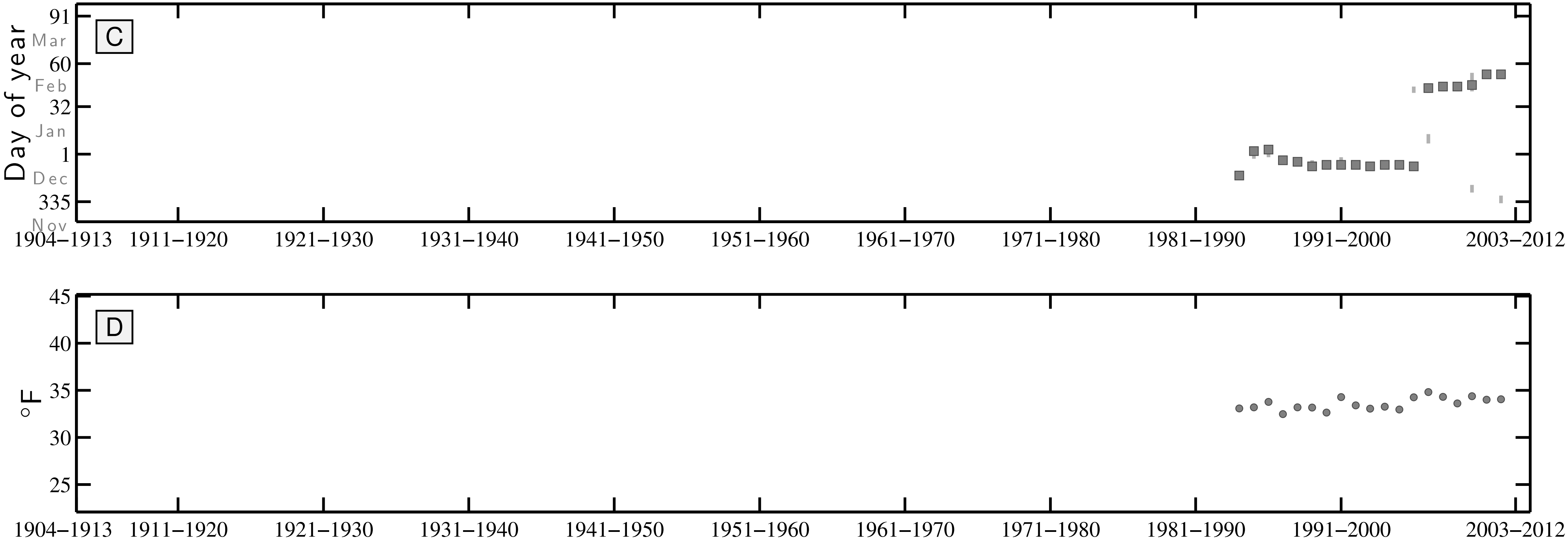

10 year Summer Teletherm dynamics for BLAINE, WA:

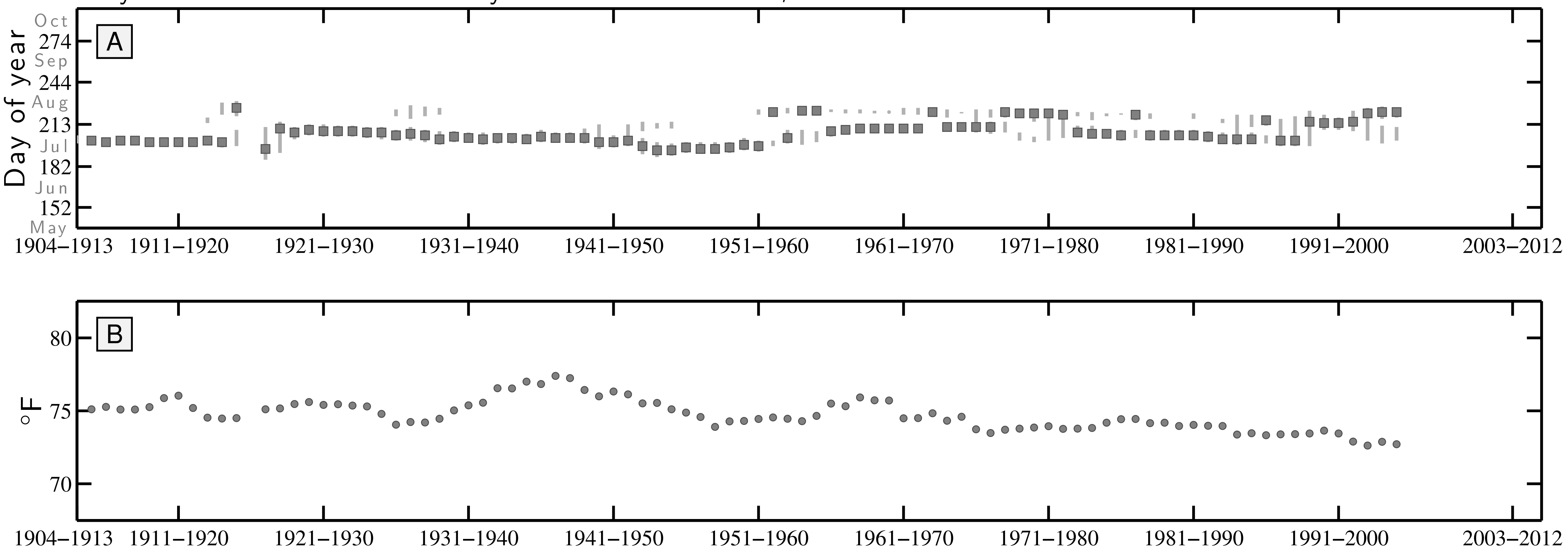

10 year Winter Teletherm dynamics for BLAINE, WA:

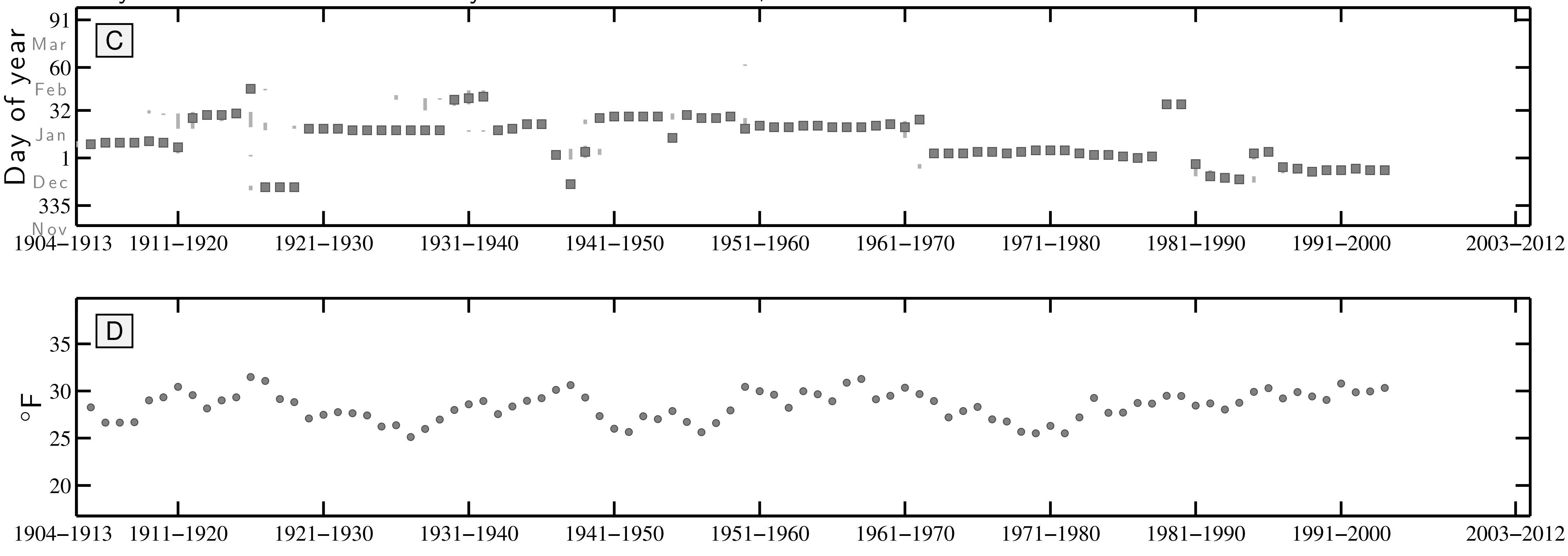

10 year Summer Teletherm dynamics for BUCKLEY 1 NE, WA:

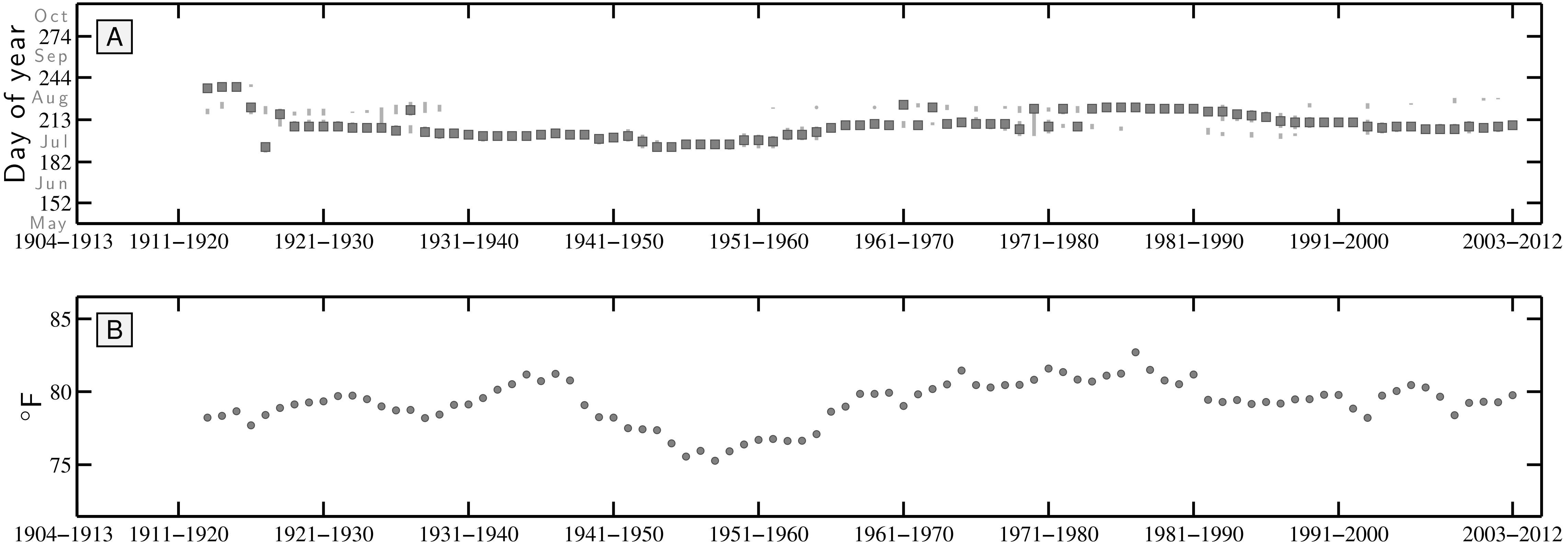

10 year Winter Teletherm dynamics for BUCKLEY 1 NE, WA:

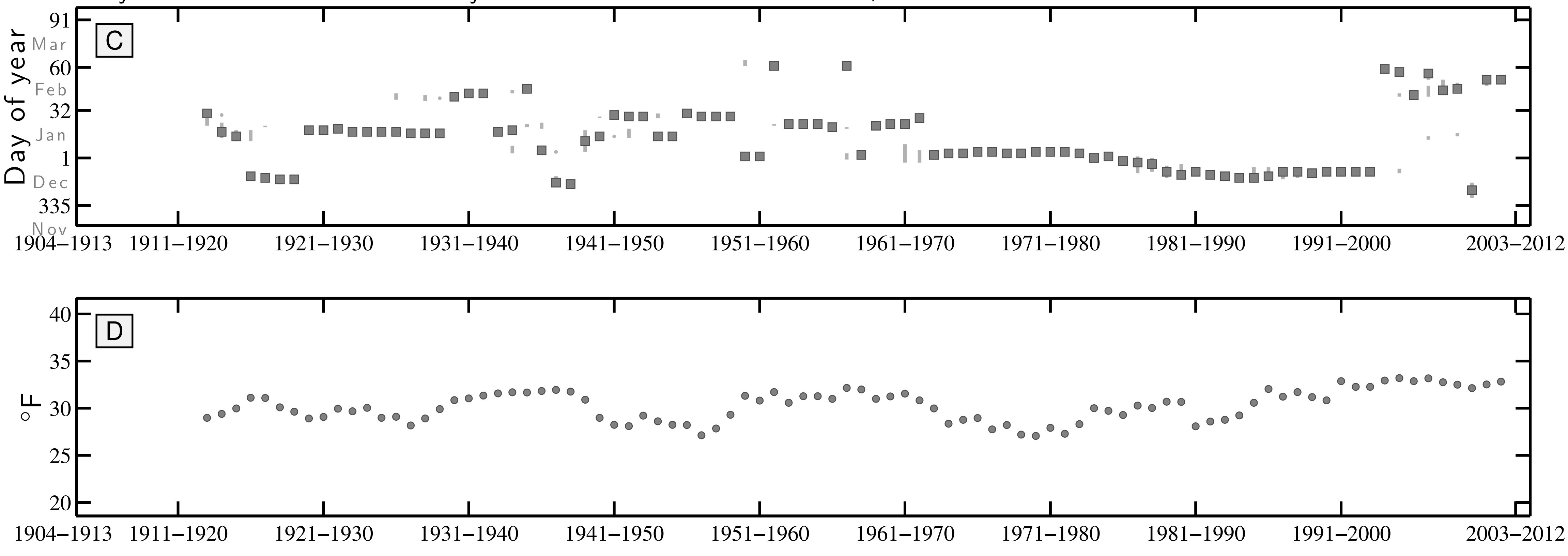

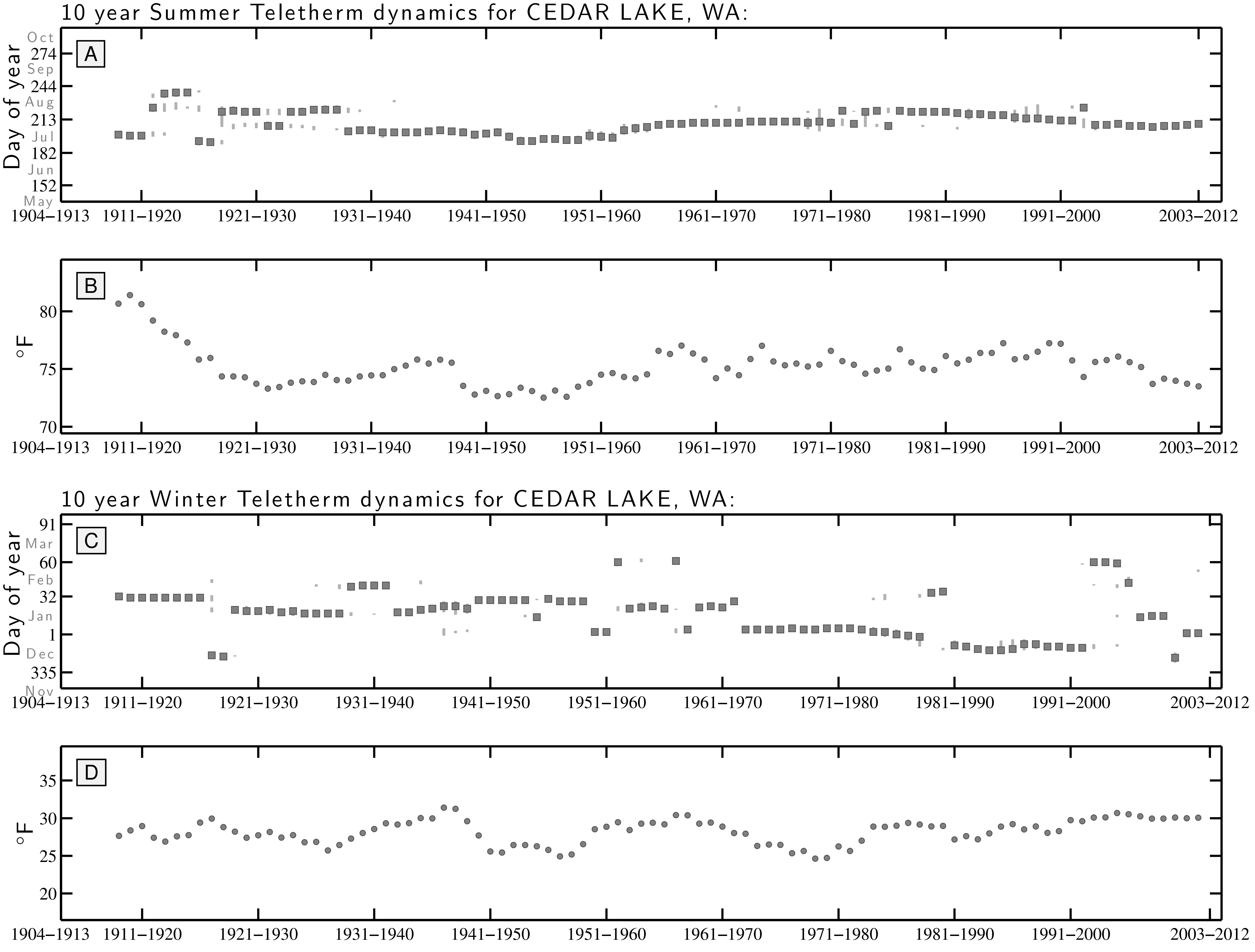
10 year Summer Teletherm dynamics for CEDAR LAKE, WA:
A
Day of year
Oct
274
Sep
244
Aug
213
Jul
182
Jun
152
May
B
°F
80
75
70
10 year Winter Teletherm dynamics for CEDAR LAKE, WA:
C
91
Mar
60
Feb
32
Jan
1
Dec
335
Nov
D
35
30
25
20
1904–1913
1911–1920
1921–1930
1931–1940
1941–1950
1951–1960
1961–1970
1971–1980
1981–1990
1991–2000
2003–2012

10 year Summer Teletherm dynamics for CENTRALIA, WA:

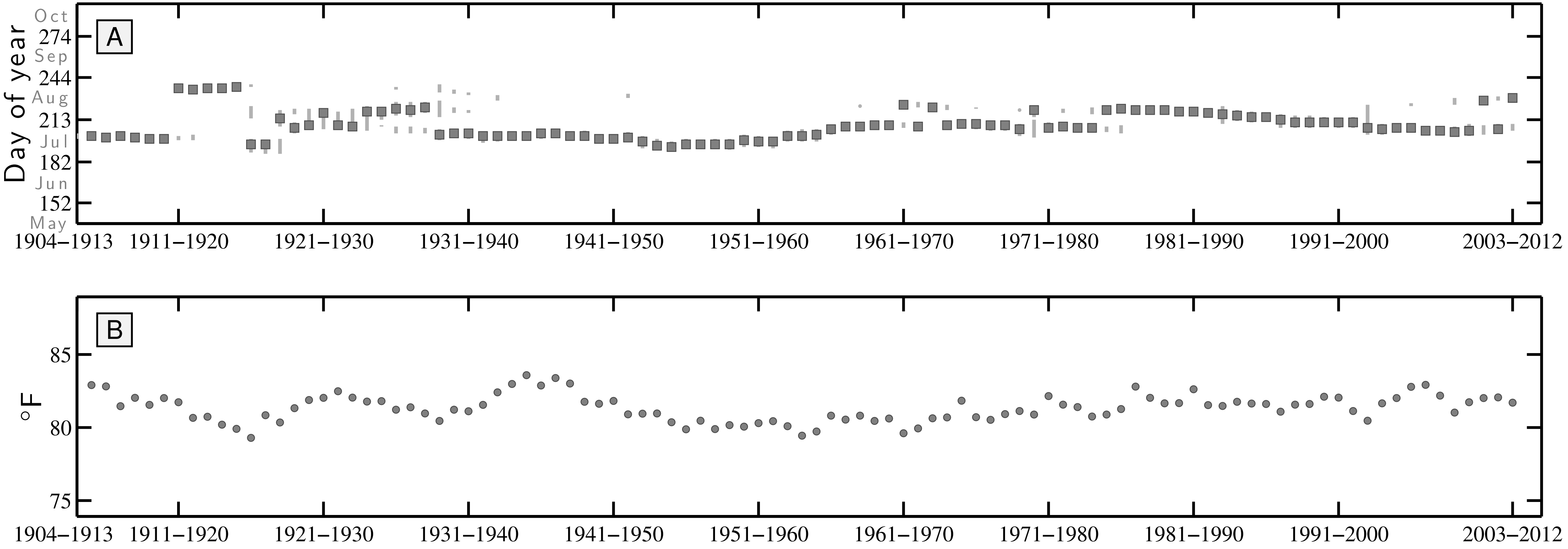

10 year Winter Teletherm dynamics for CENTRALIA, WA:

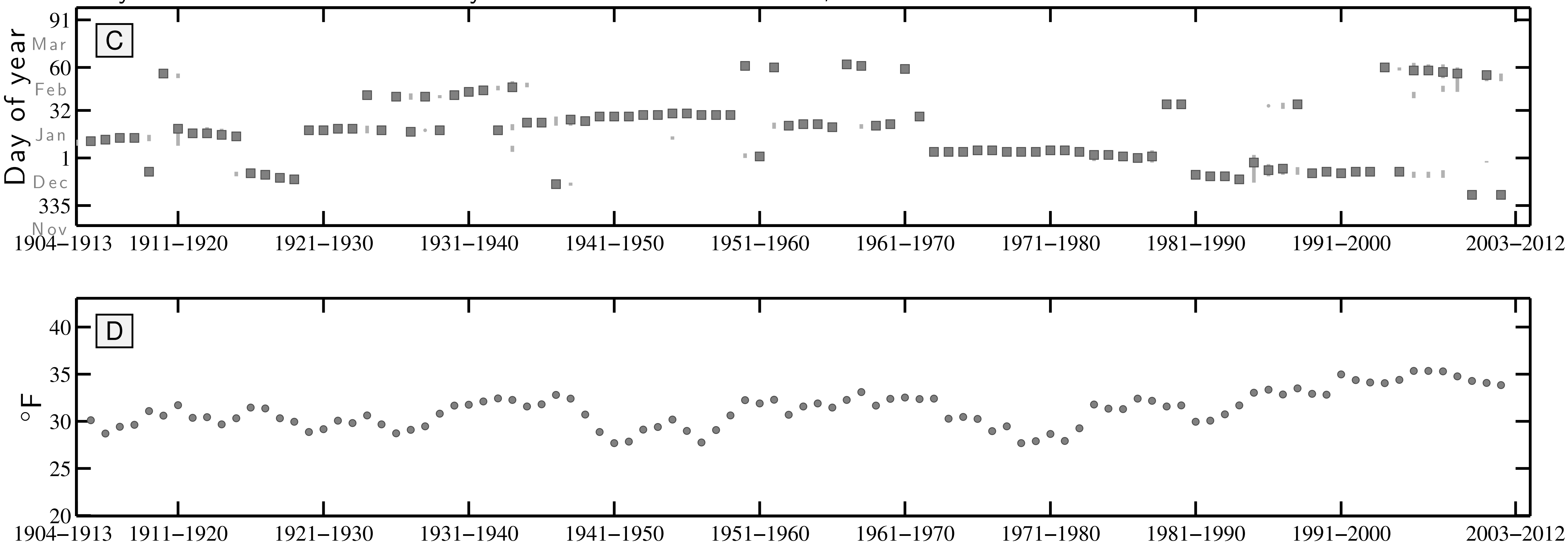

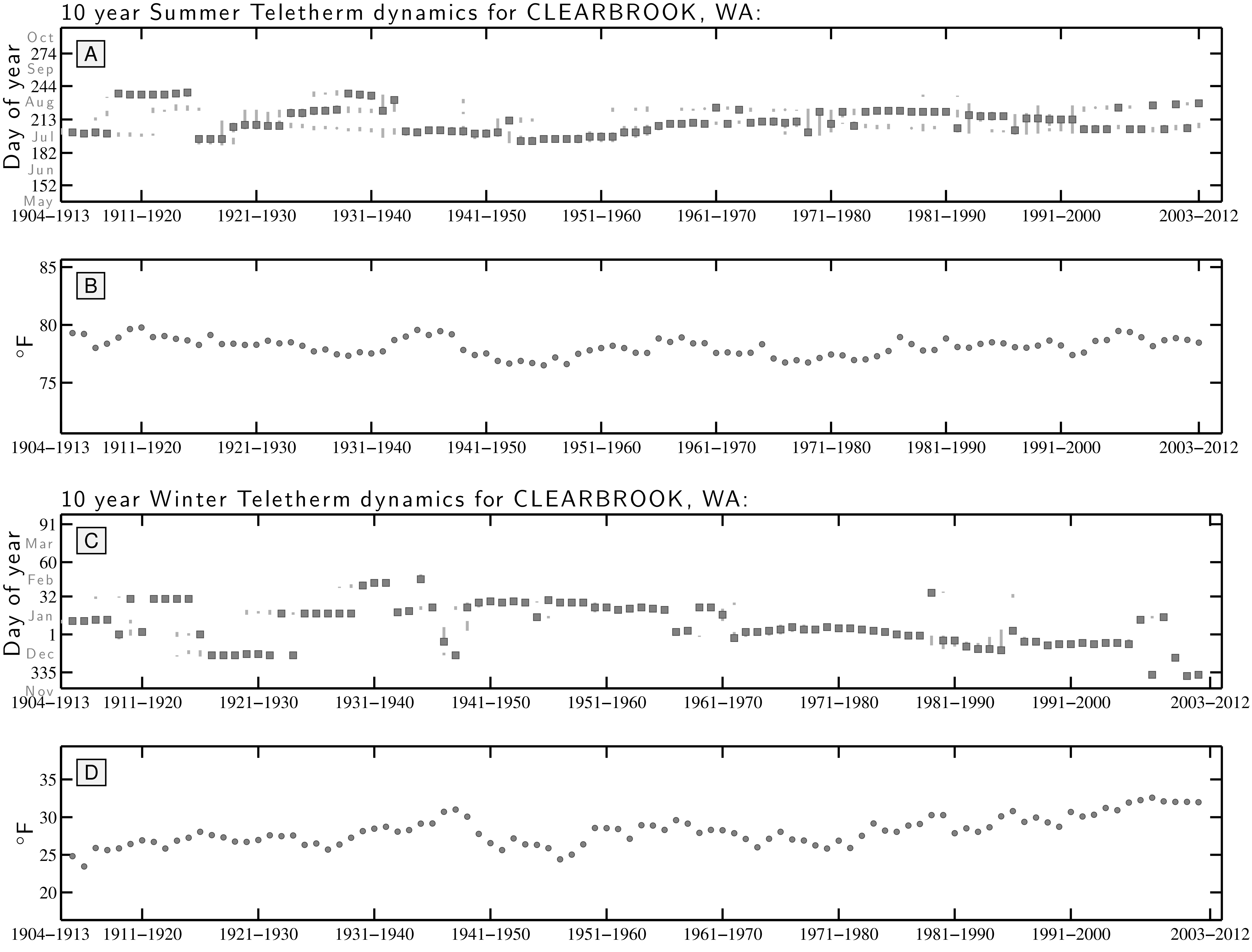
10 year Summer Teletherm dynamics for CLEARBROOK, WA:
A
B
Day of year
°F
Oct
274
Sep
244
Aug
213
Jul
182
Jun
152
May
85
80
75
1904–1913
1911–1920
1921–1930
1931–1940
1941–1950
1951–1960
1961–1970
1971–1980
1981–1990
1991–2000
2003–2012
10 year Winter Teletherm dynamics for CLEARBROOK, WA:
C
D
91
Mar
60
Feb
32
Jan
1
Dec
335
Nov
35
30
25
20

10 year Summer Teletherm dynamics for CLE ELUM, WA:

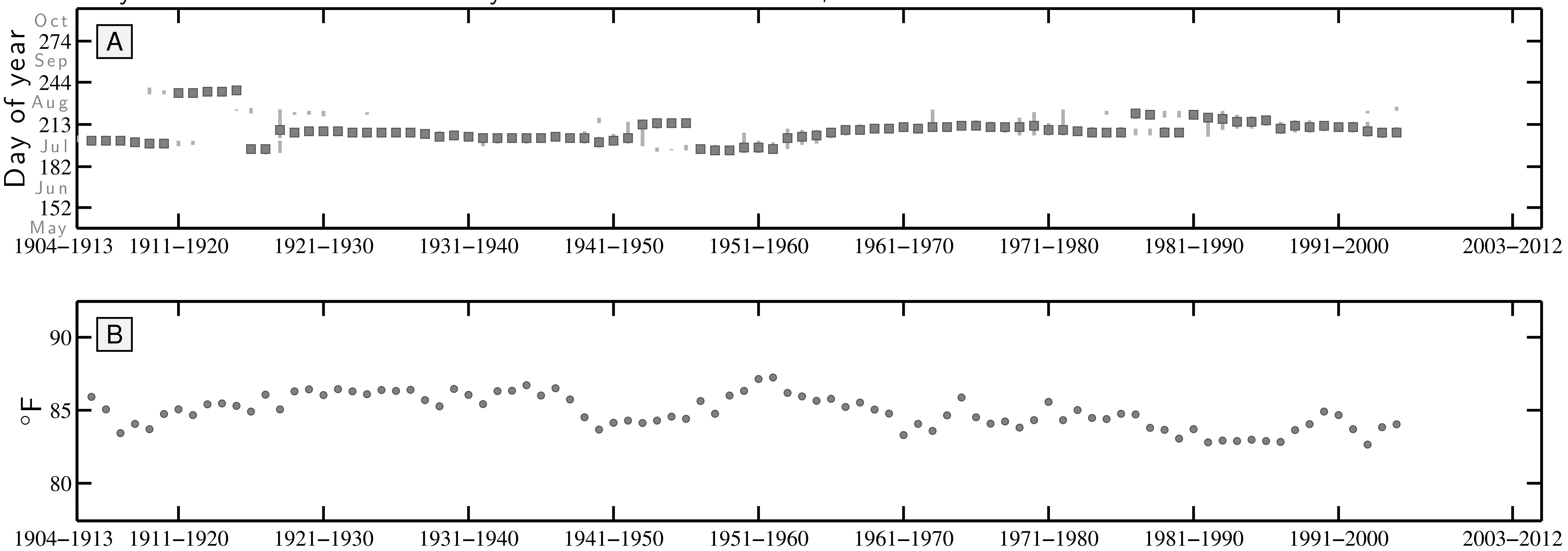

10 year Winter Teletherm dynamics for CLE ELUM, WA:

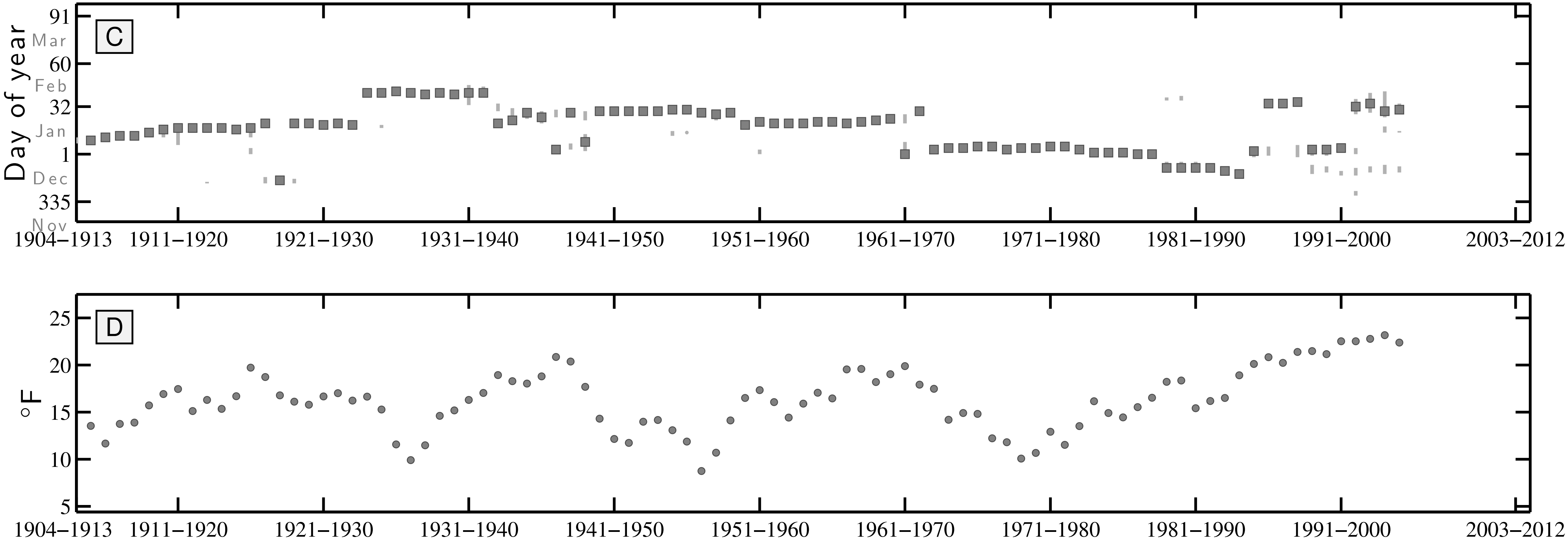

10 year Summer Teletherm dynamics for COLVILLE, WA:

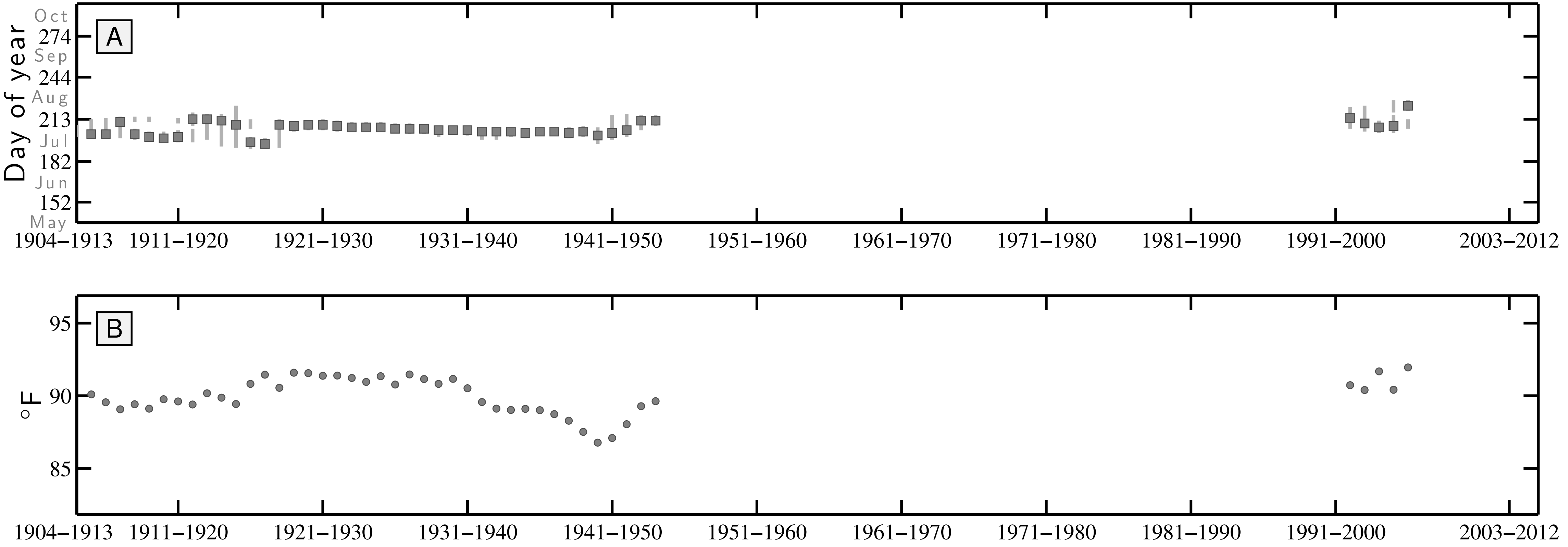

10 year Winter Teletherm dynamics for COLVILLE, WA:

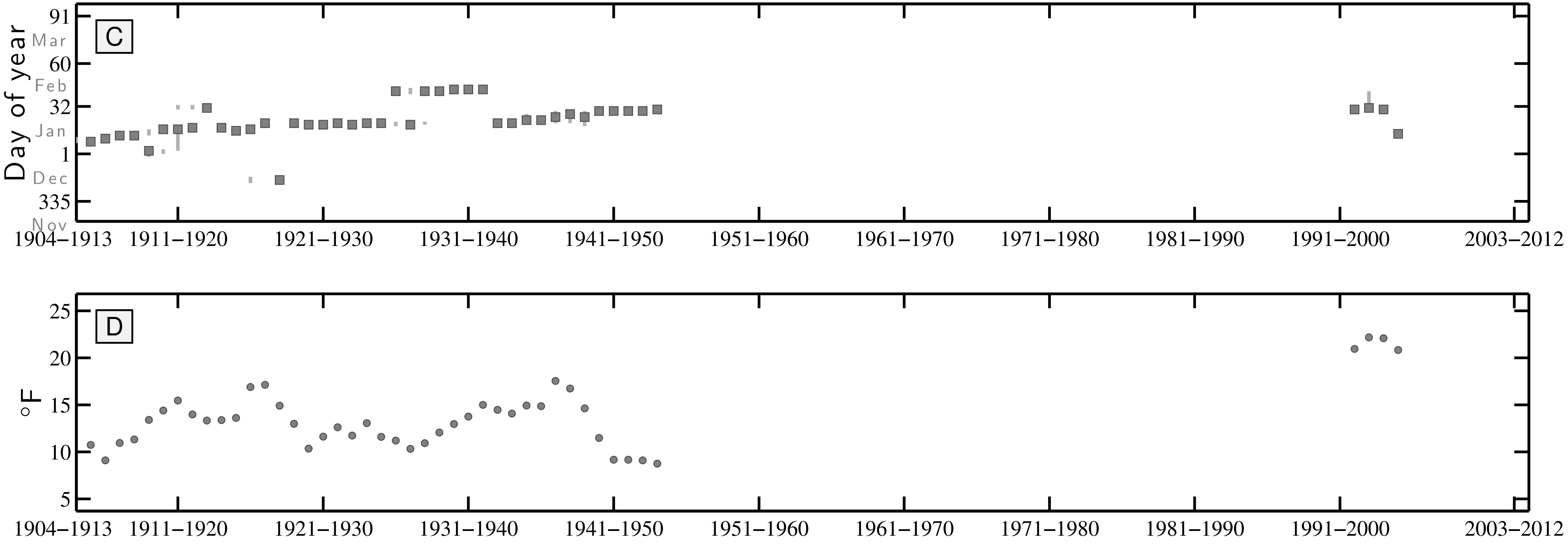

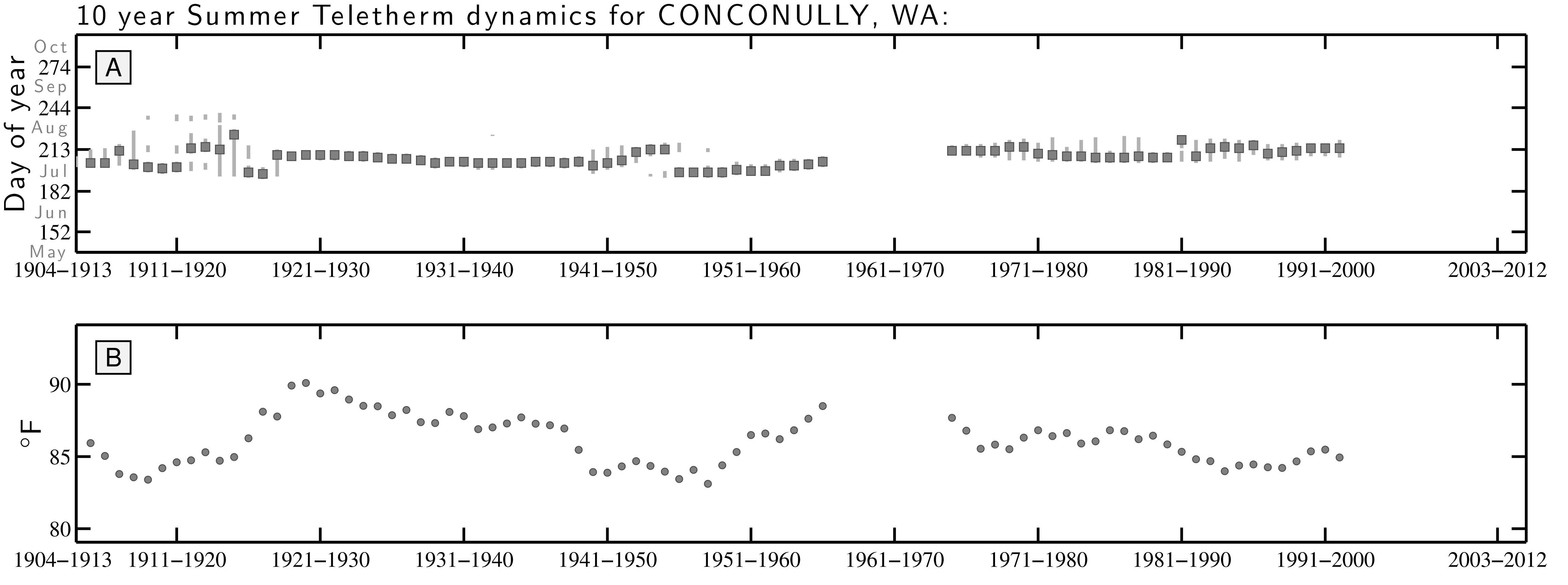
10 year Summer Teletherm dynamics for CONCONULLY, WA:
A
B
Day of year
°F
Oct
274
Sep
244
Aug
213
Jul
182
Jun
152
May
90
85
80
1904–1913
1911–1920
1921–1930
1931–1940
1941–1950
1951–1960
1961–1970
1971–1980
1981–1990
1991–2000
2003–2012

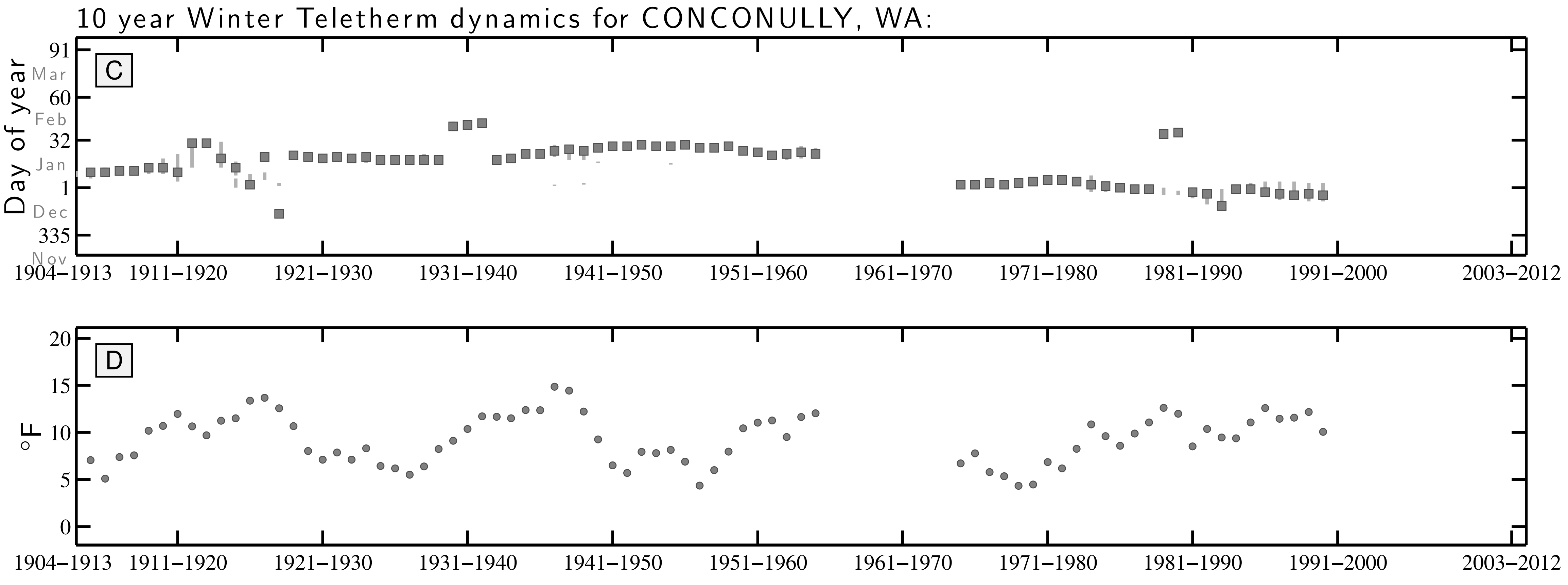
10 year Winter Teletherm dynamics for CONCONULLY, WA:
C
D
Day of year
°F
91
Mar
60
Feb
32
Jan
1
Dec
335
Nov
20
15
10
5
0
1904–1913
1911–1920
1921–1930
1931–1940
1941–1950
1951–1960
1961–1970
1971–1980
1981–1990
1991–2000
2003–2012

10 year Summer Teletherm dynamics for CUSHMAN POWERHOUSE 2, WA:

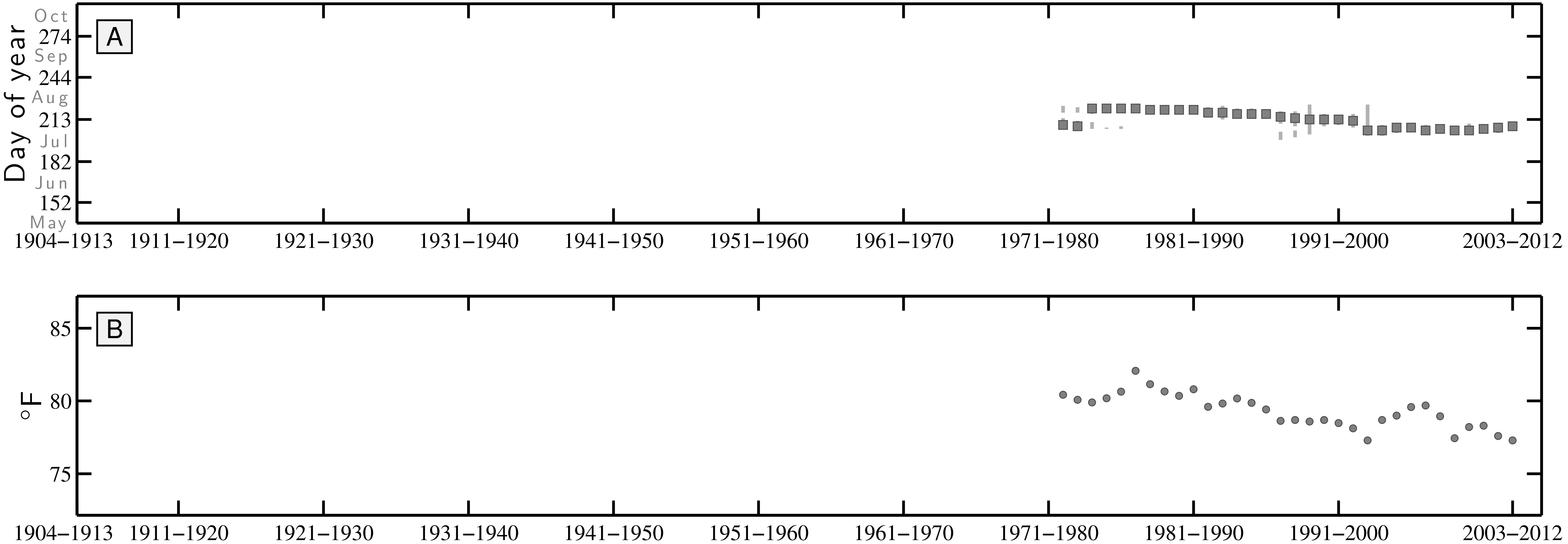

10 year Winter Teletherm dynamics for CUSHMAN POWERHOUSE 2, WA:

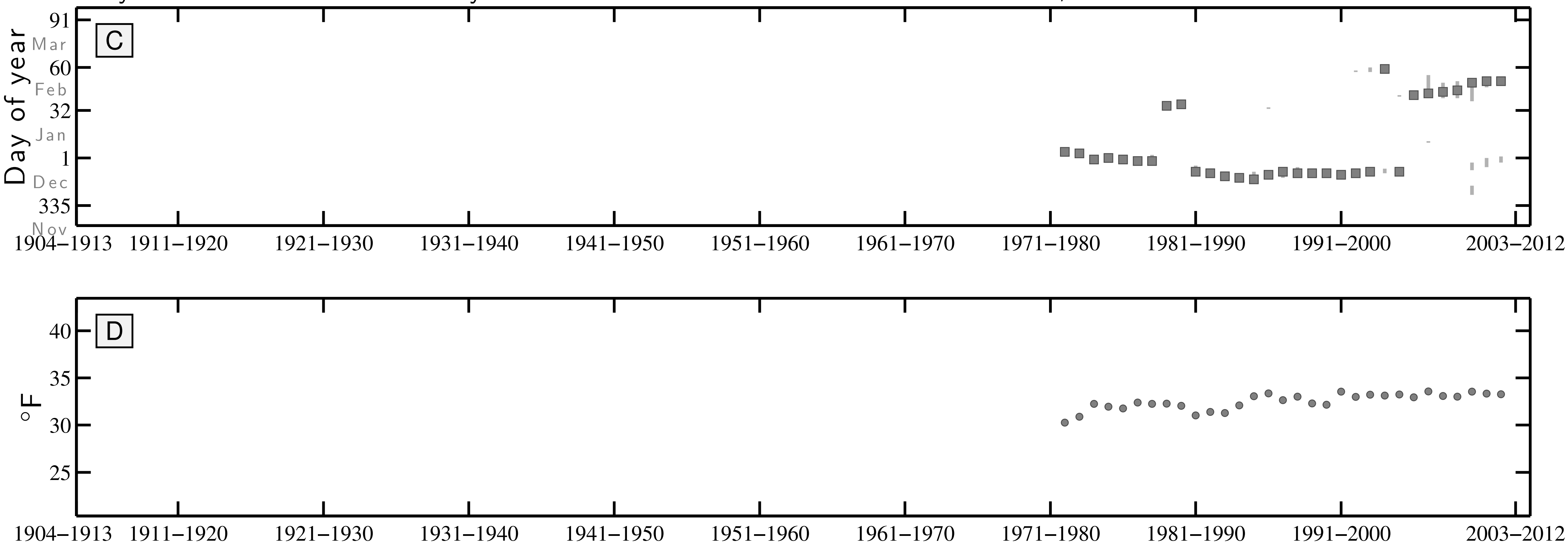

10 year Summer Teletherm dynamics for DAVENPORT, WA:

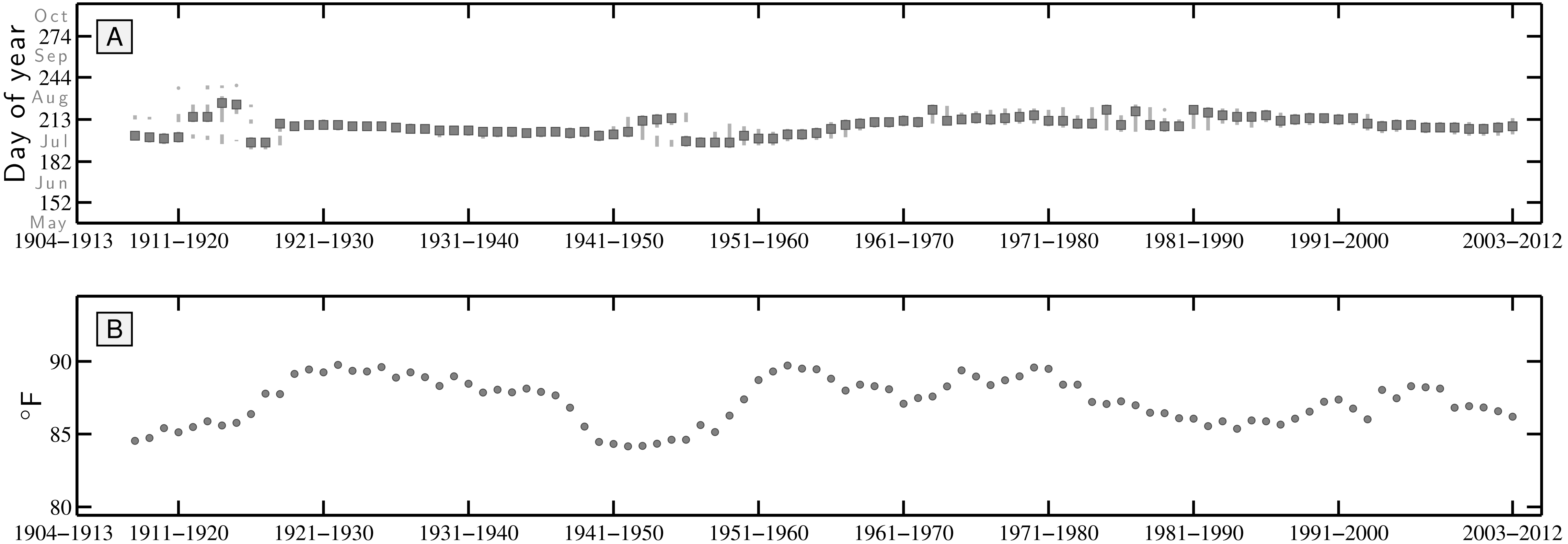

10 year Winter Teletherm dynamics for DAVENPORT, WA:

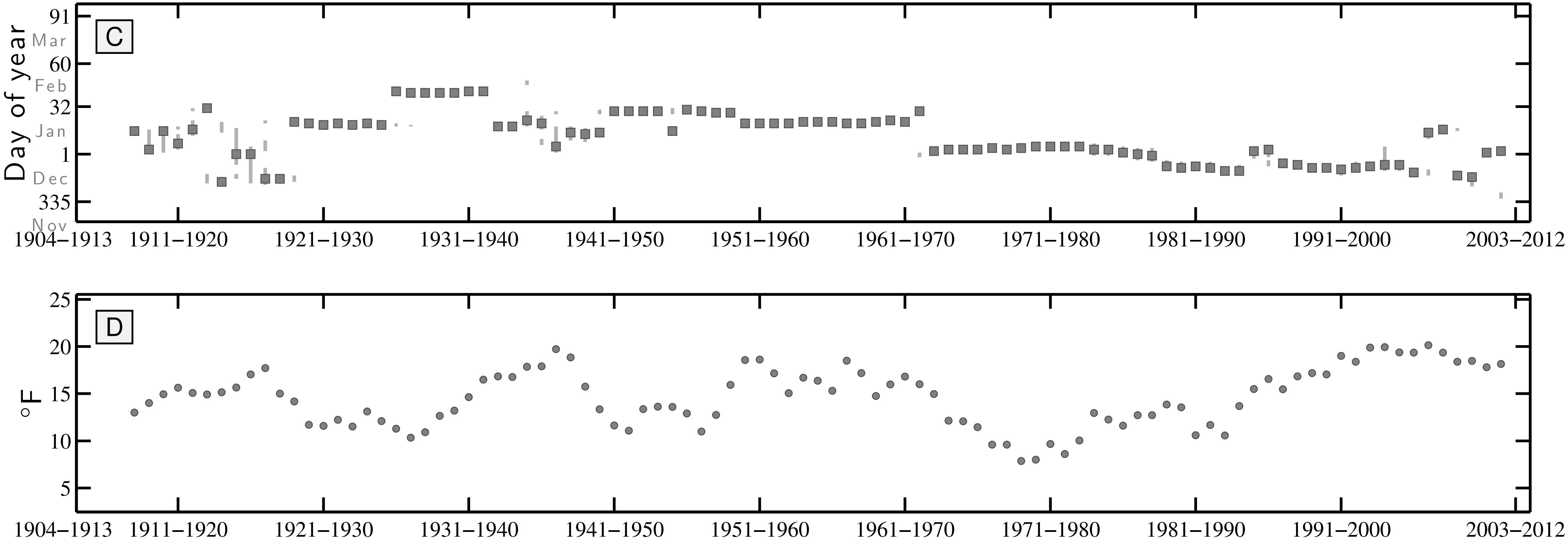

10 year Summer Teletherm dynamics for DAYTON 1 WSW, WA:

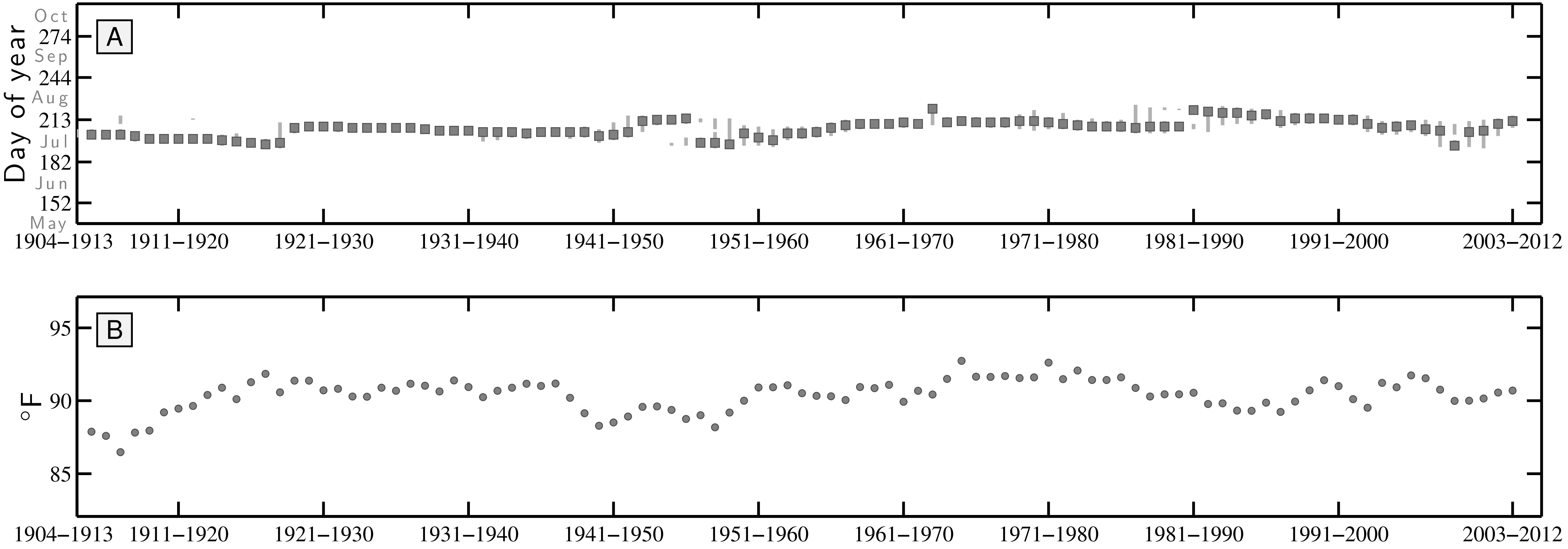

10 year Winter Teletherm dynamics for DAYTON 1 WSW, WA:

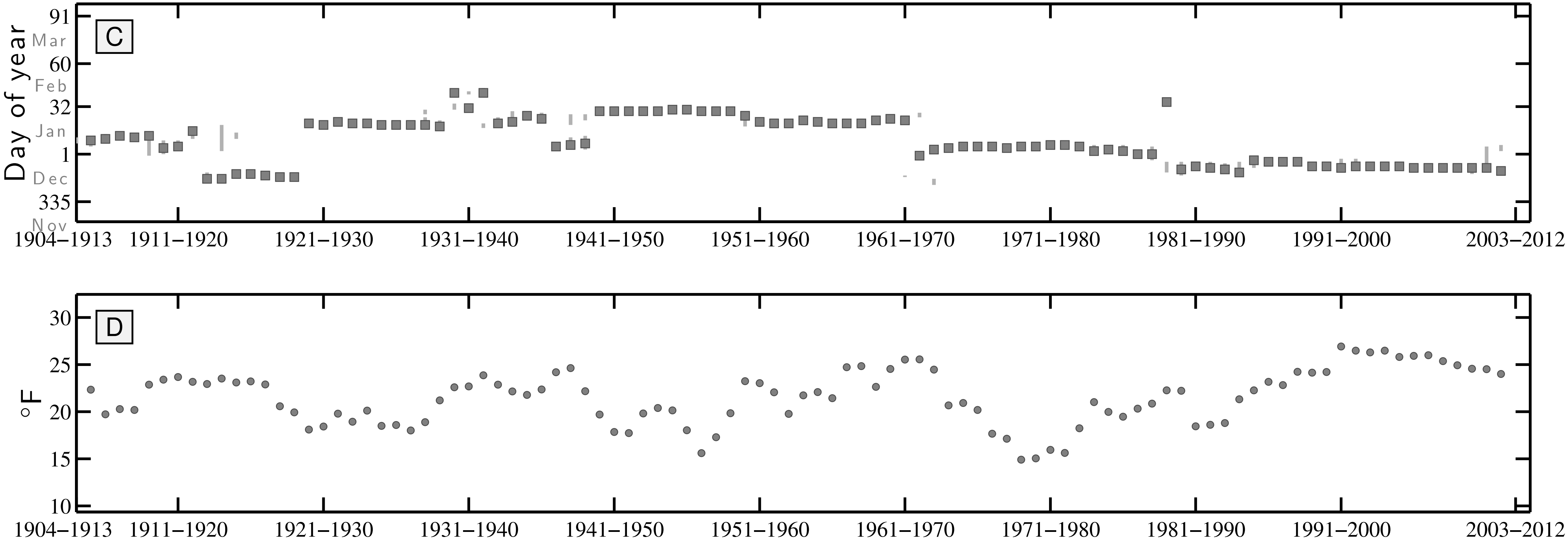

10 year Summer Teletherm dynamics for ELLENSBURG, WA:

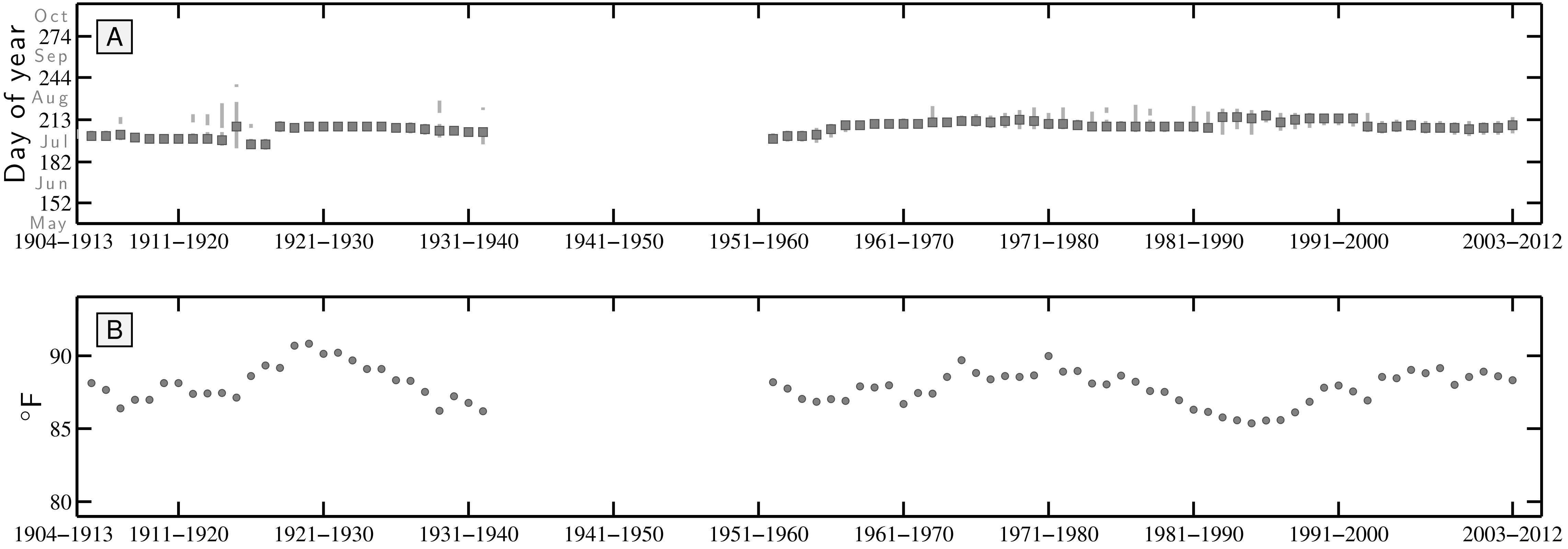

10 year Winter Teletherm dynamics for ELLENSBURG, WA:

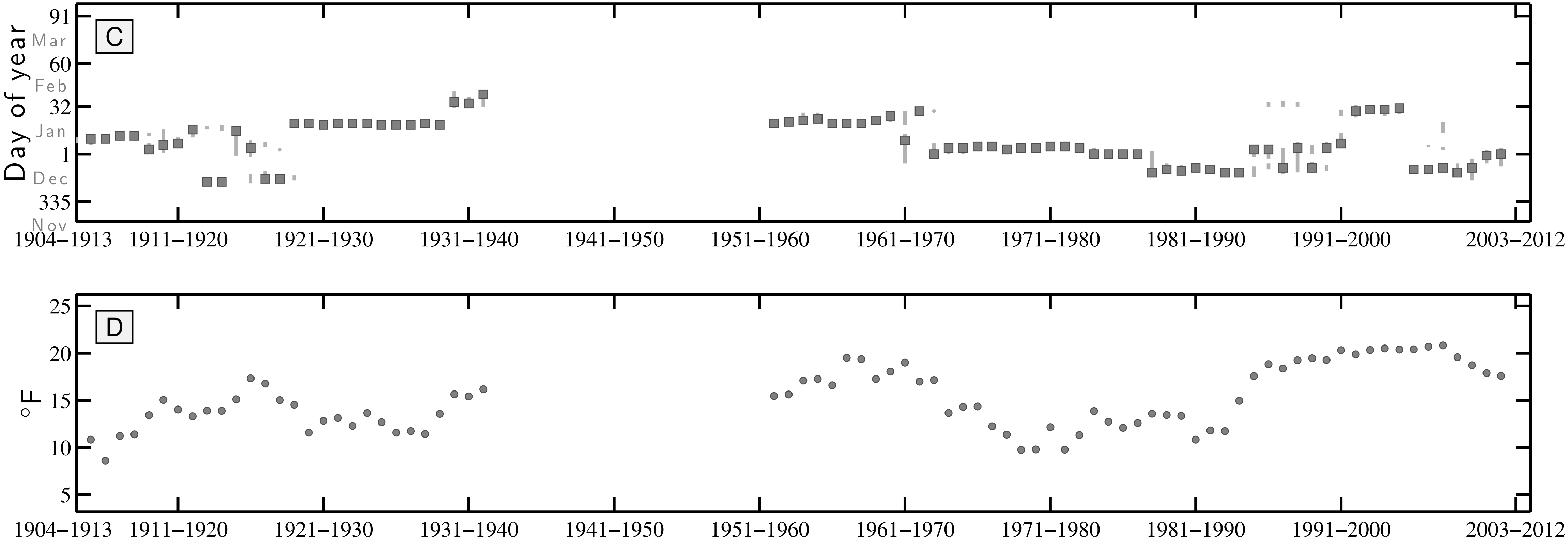

10 year Summer Teletherm dynamics for EVERETT, WA:

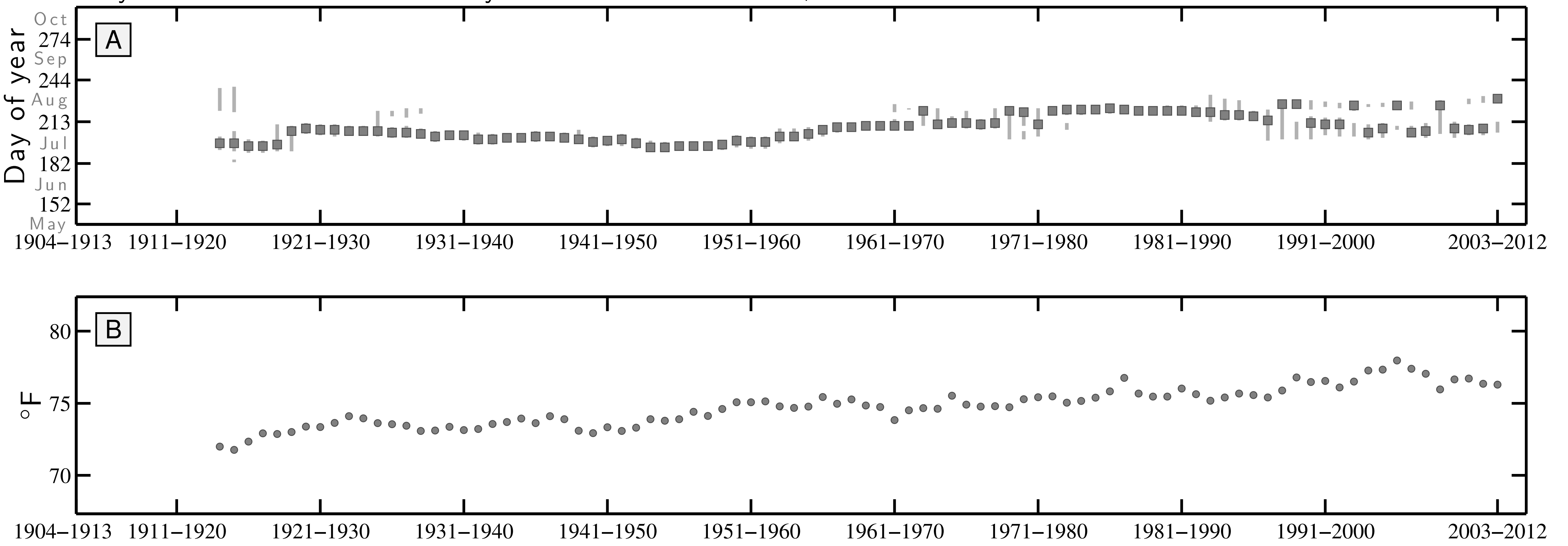

10 year Winter Teletherm dynamics for EVERETT, WA:

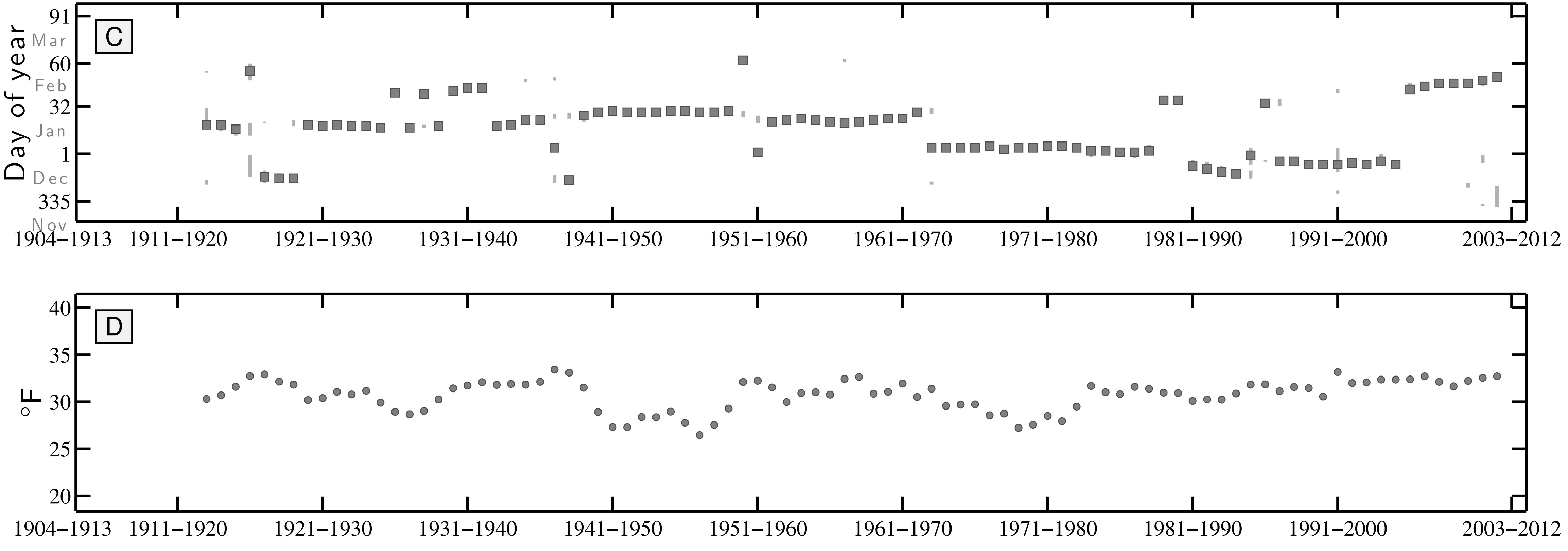

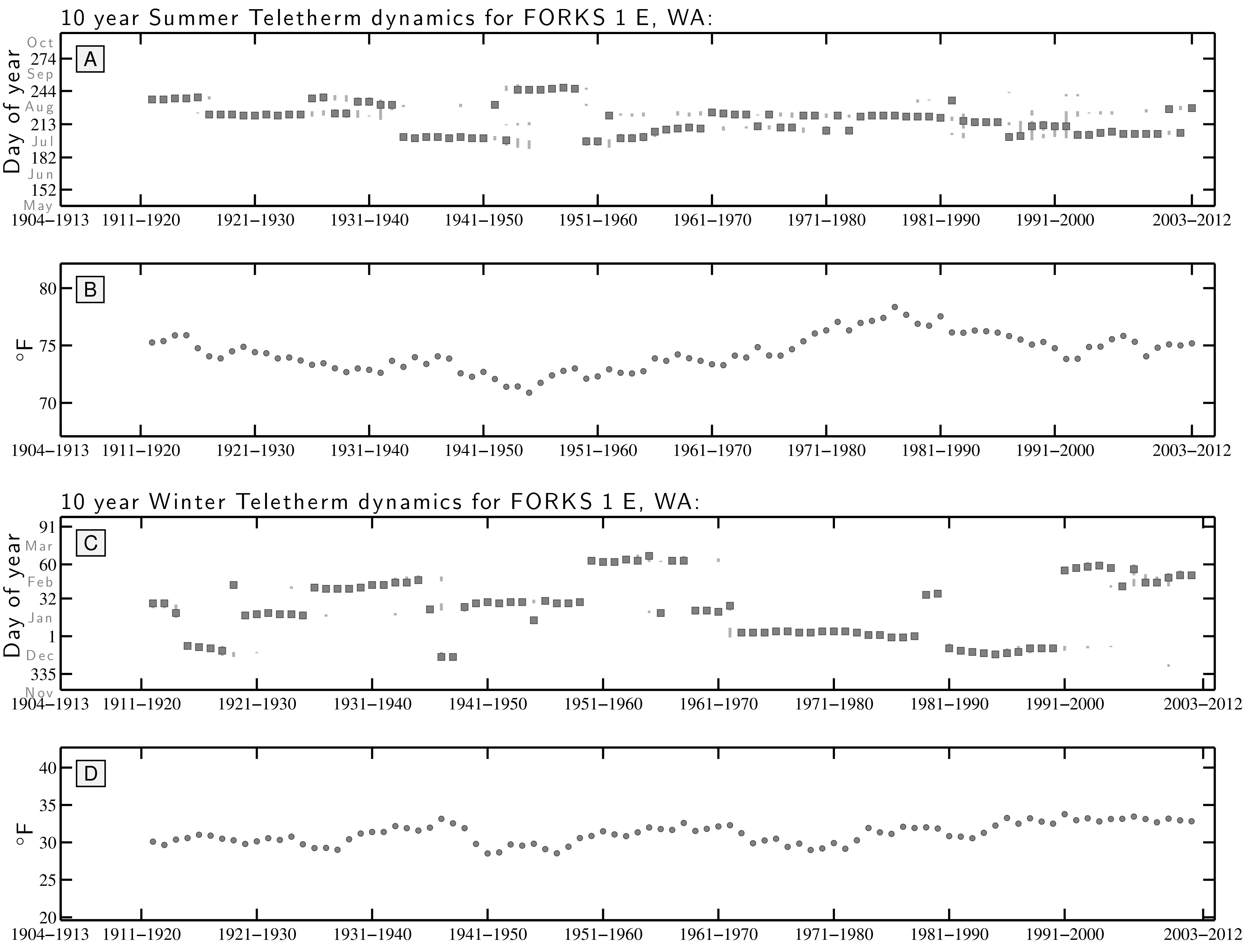
10 year Summer Teletherm dynamics for FORKS 1 E, WA:
A
B
Day of year
°F
Oct
274
Sep
244
Aug
213
Jul
182
Jun
152
May
80
75
70
1904–1913
1911–1920
1921–1930
1931–1940
1941–1950
1951–1960
1961–1970
1971–1980
1981–1990
1991–2000
2003–2012
10 year Winter Teletherm dynamics for FORKS 1 E, WA:
C
D
91
Mar
60
Feb
32
Jan
1
Dec
335
Nov
40
35
30
25
20

10 year Summer Teletherm dynamics for GOLDENDALE, WA:

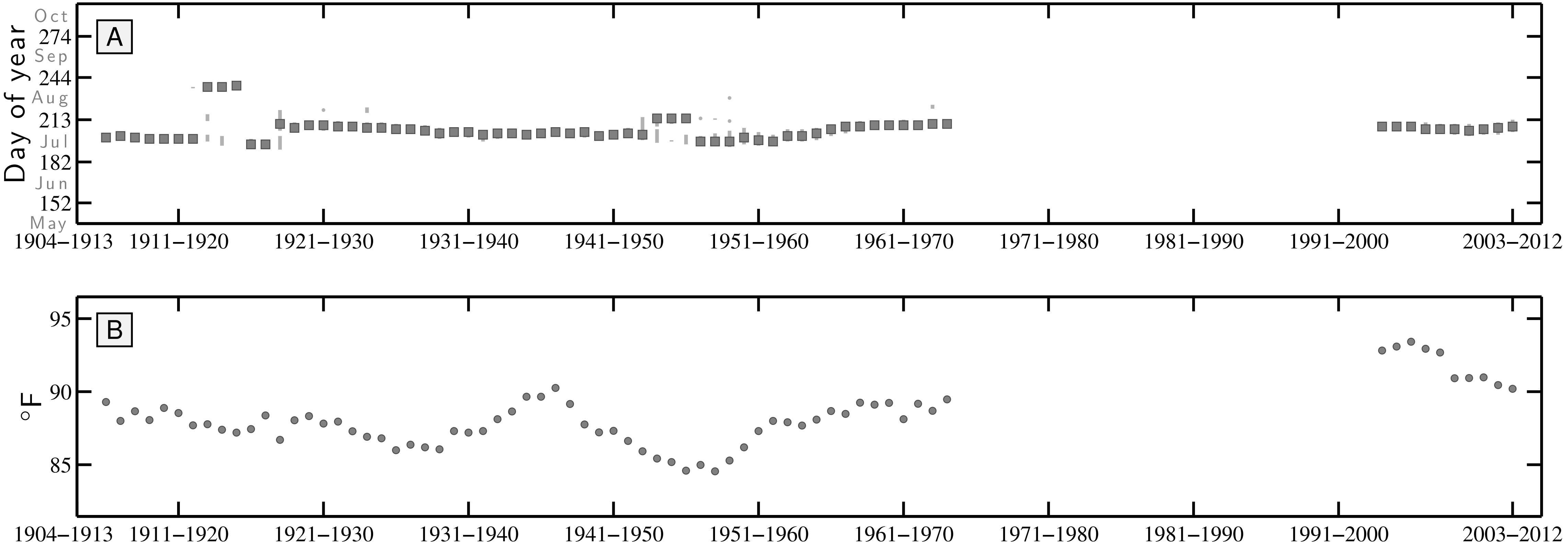

10 year Winter Teletherm dynamics for GOLDENDALE, WA:

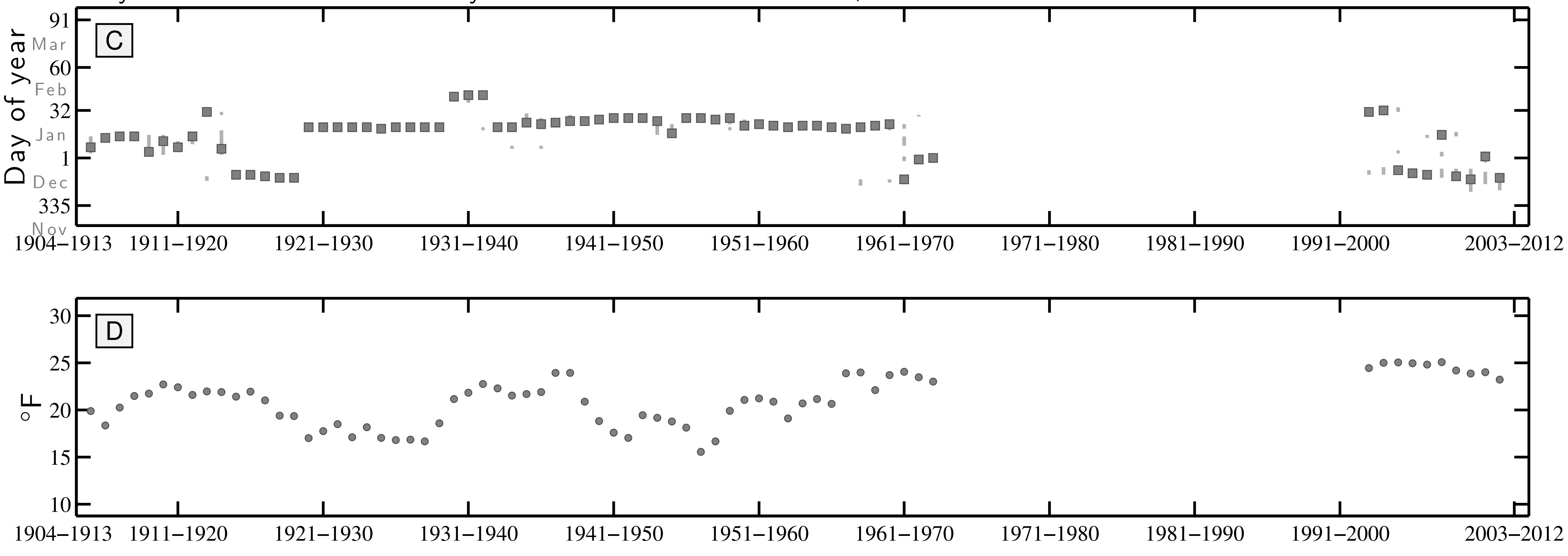

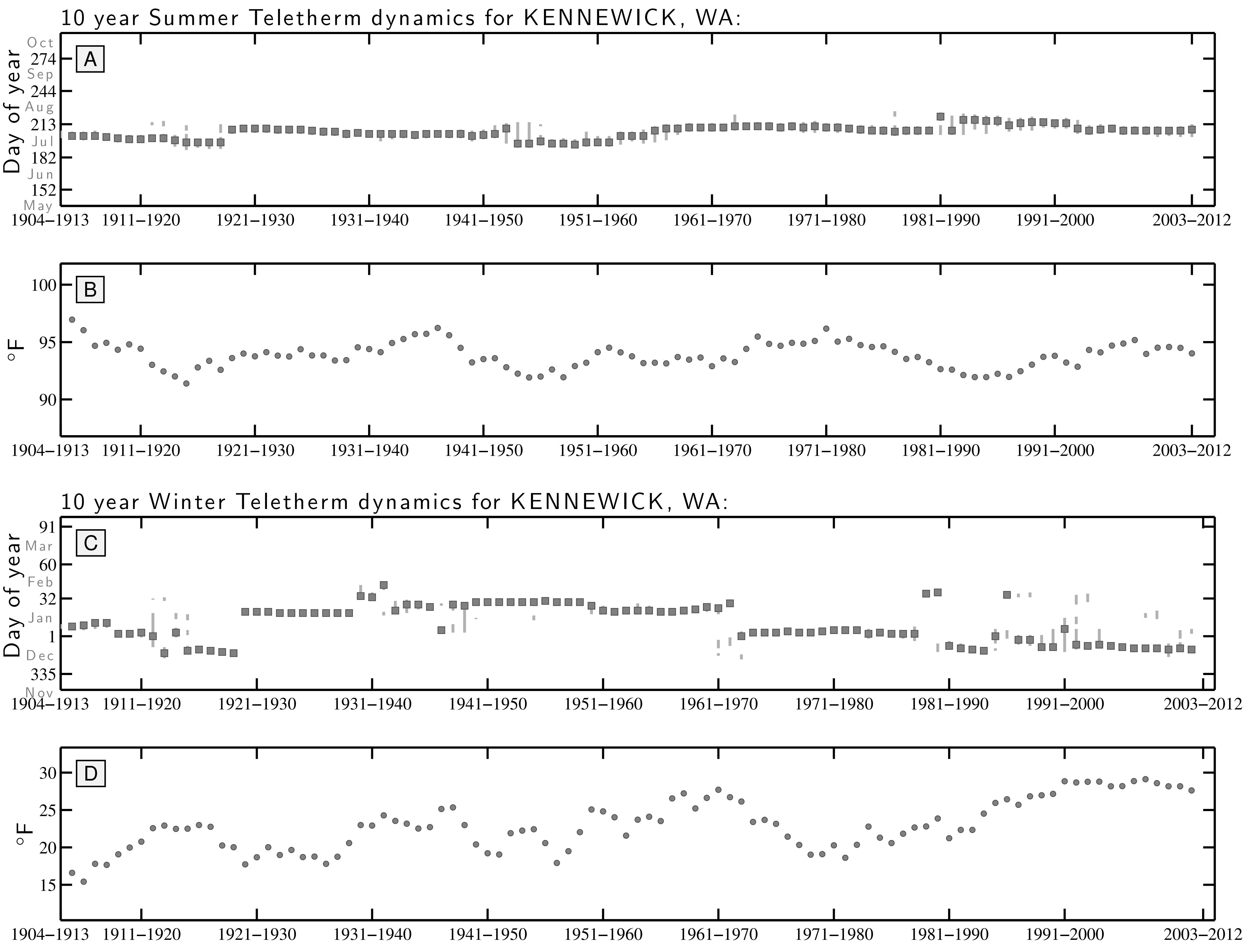
10 year Summer Teletherm dynamics for KENNEWICK, WA:
A
Day of year
Oct
274
Sep
244
Aug
213
Jul
182
Jun
152
May
B
°F
100
95
90
10 year Winter Teletherm dynamics for KENNEWICK, WA:
C
91
Mar
60
Feb
32
Jan
1
Dec
335
Nov
D
30
25
20
15
1904–1913
1911–1920
1921–1930
1931–1940
1941–1950
1951–1960
1961–1970
1971–1980
1981–1990
1991–2000
2003–2012

10 year Summer Teletherm dynamics for LONG BEACH EXP STN, WA:

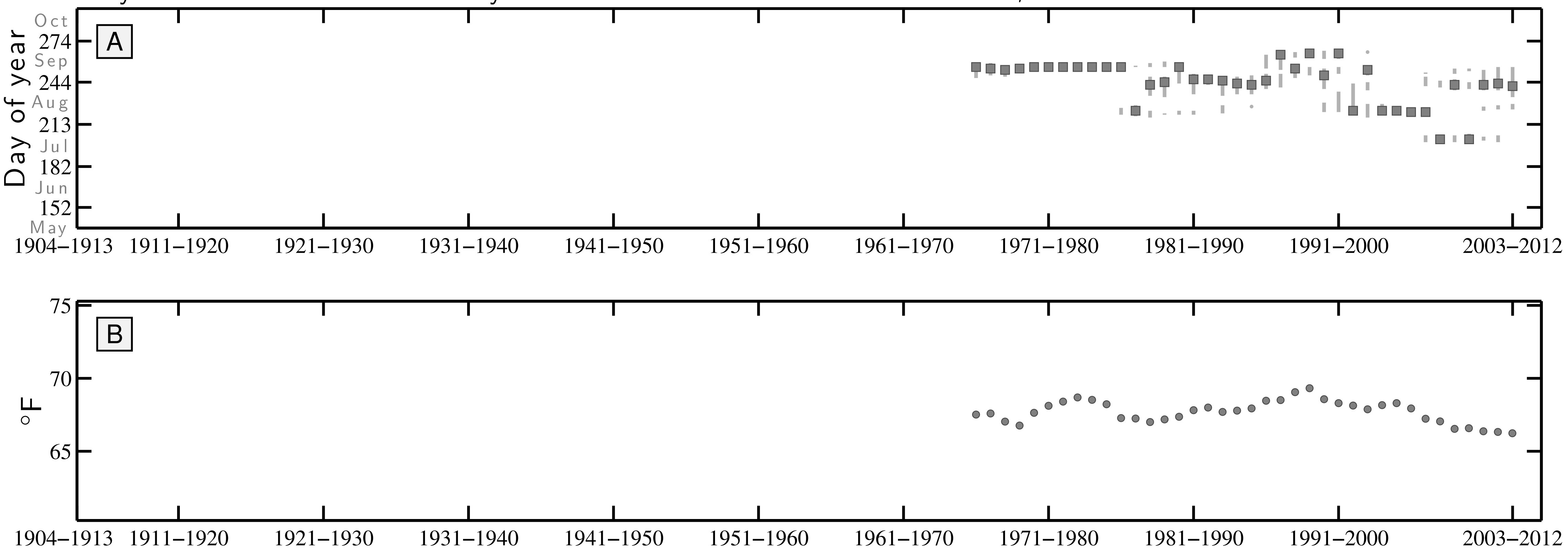

10 year Winter Teletherm dynamics for LONG BEACH EXP STN, WA:

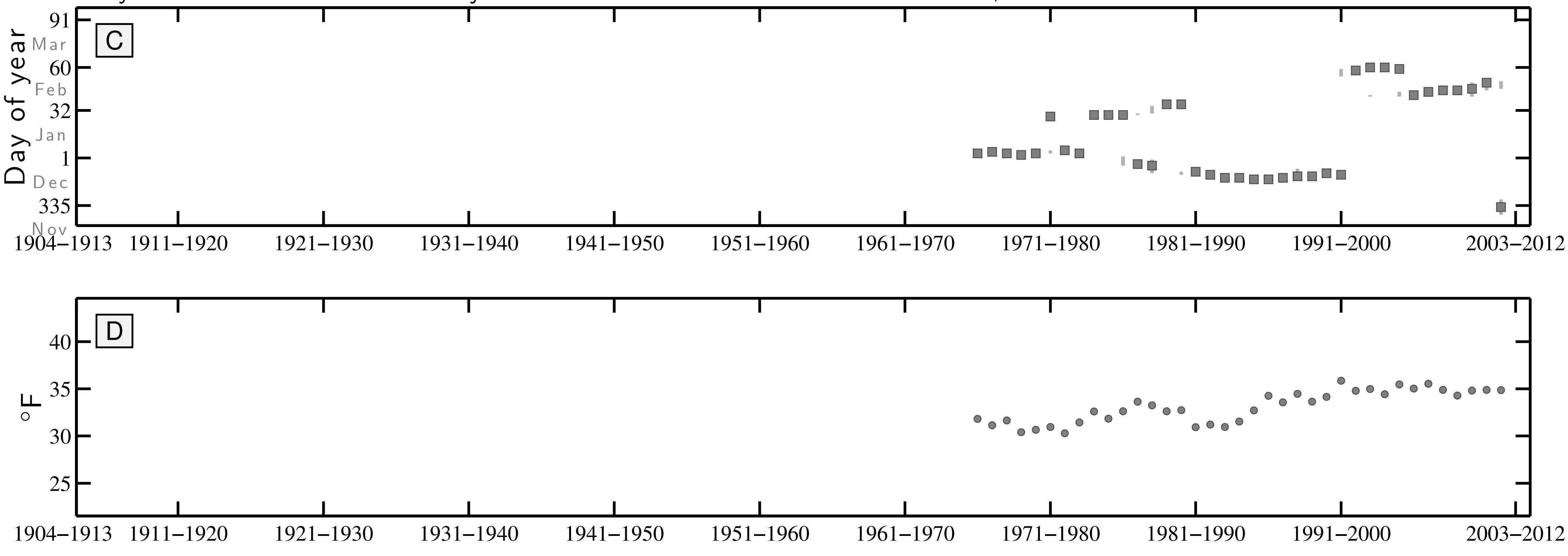

10 year Summer Teletherm dynamics for LONGMIRE RAINIER NPS, WA:

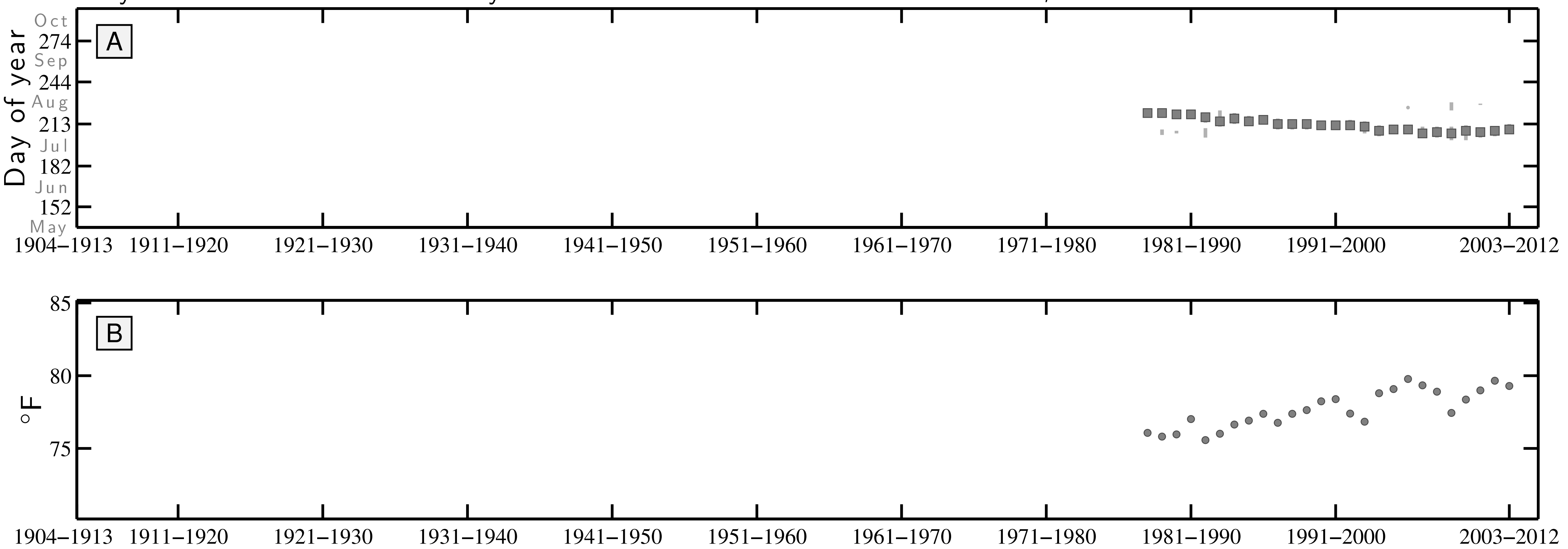

10 year Winter Teletherm dynamics for LONGMIRE RAINIER NPS, WA:

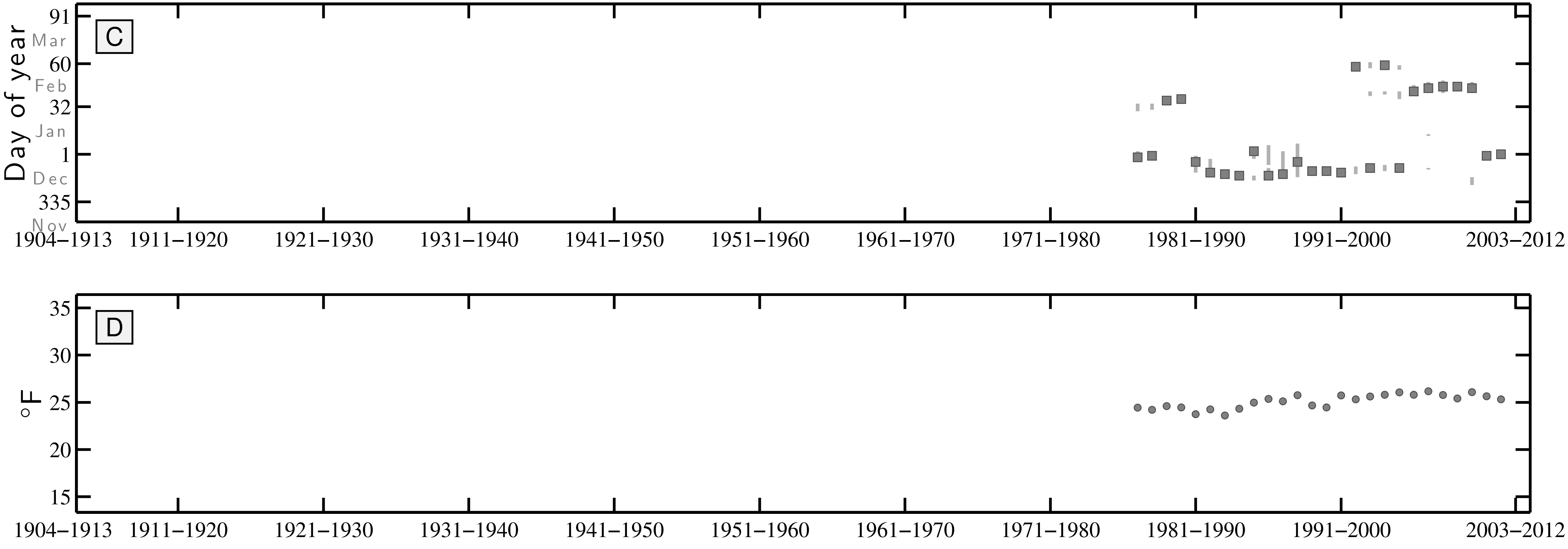

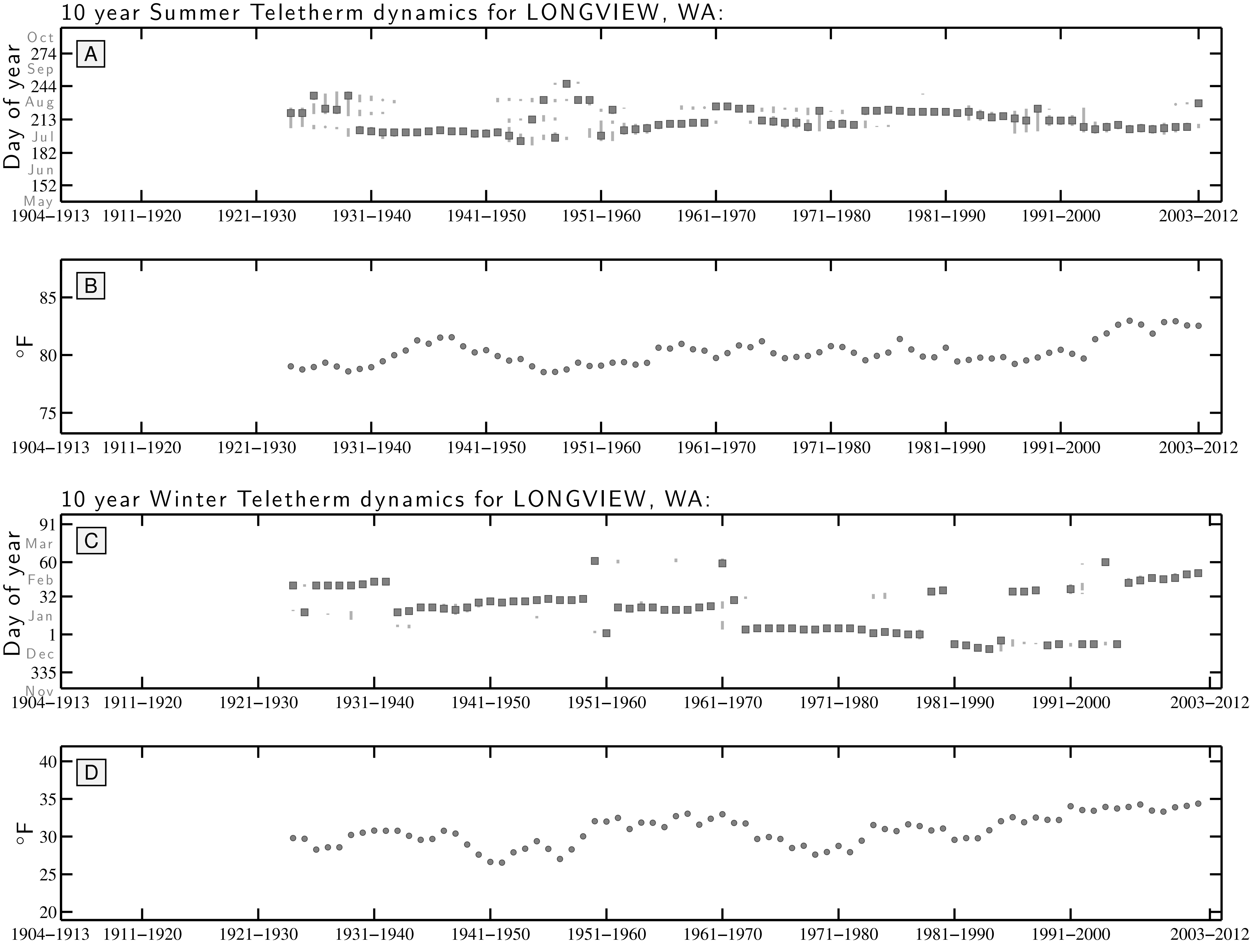
10 year Summer Teletherm dynamics for LONGVIEW, WA:
A
Day of year
Oct
274
Sep
244
Aug
213
Jul
182
Jun
152
May
B
°F
85
80
75
10 year Winter Teletherm dynamics for LONGVIEW, WA:
C
Day of year
91
Mar
60
Feb
32
Jan
1
Dec
335
Nov
D
°F
40
35
30
25
20
1904–1913
1911–1920
1921–1930
1931–1940
1941–1950
1951–1960
1961–1970
1971–1980
1981–1990
1991–2000
2003–2012

10 year Summer Teletherm dynamics for MC MILLIN RSVR, WA:

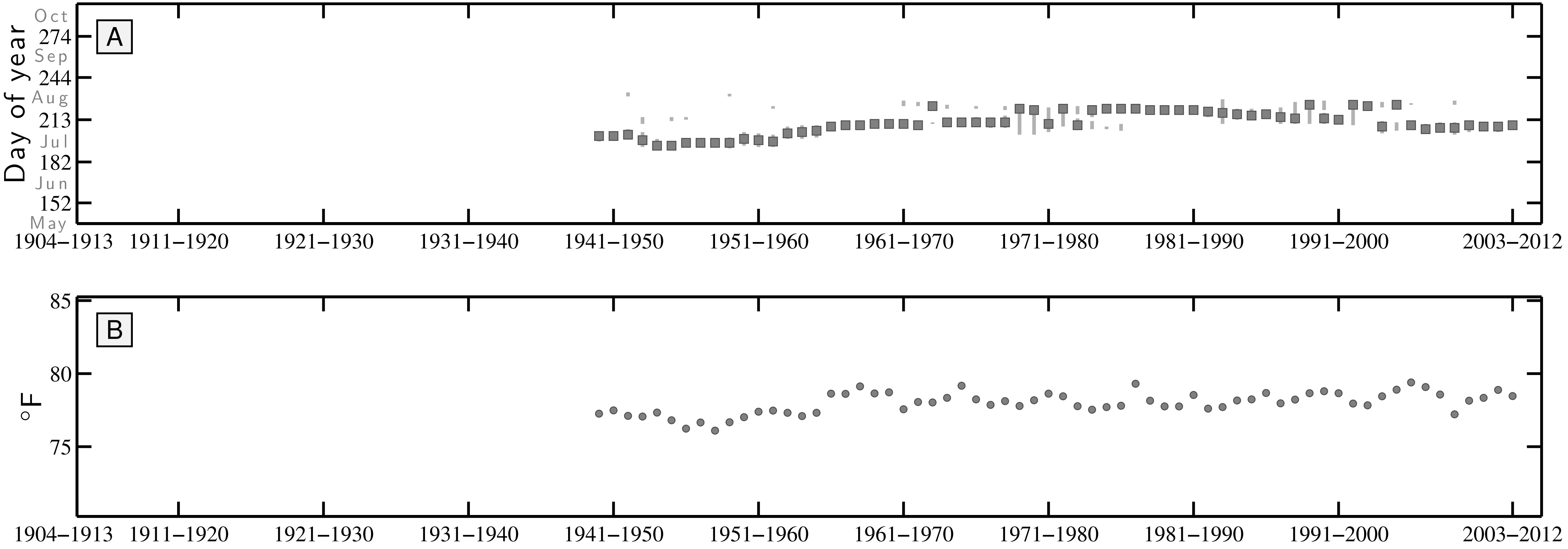

10 year Winter Teletherm dynamics for MC MILLIN RSVR, WA:

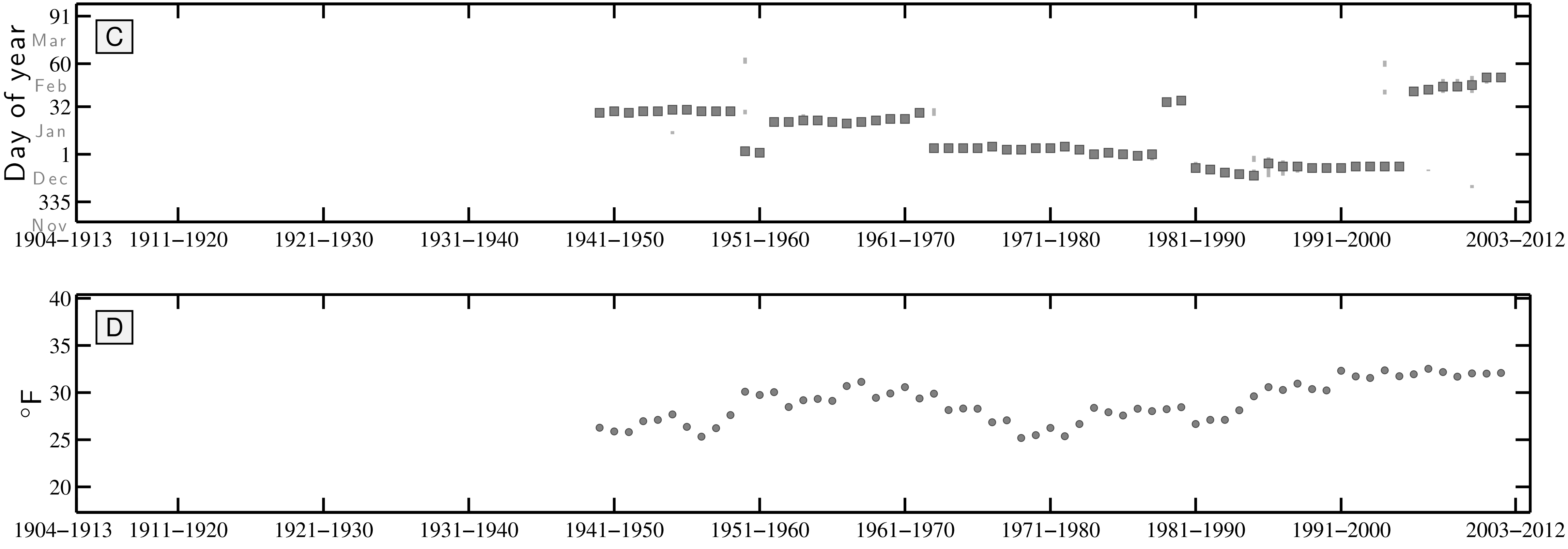

10 year Summer Teletherm dynamics for NORTHPORT, WA:

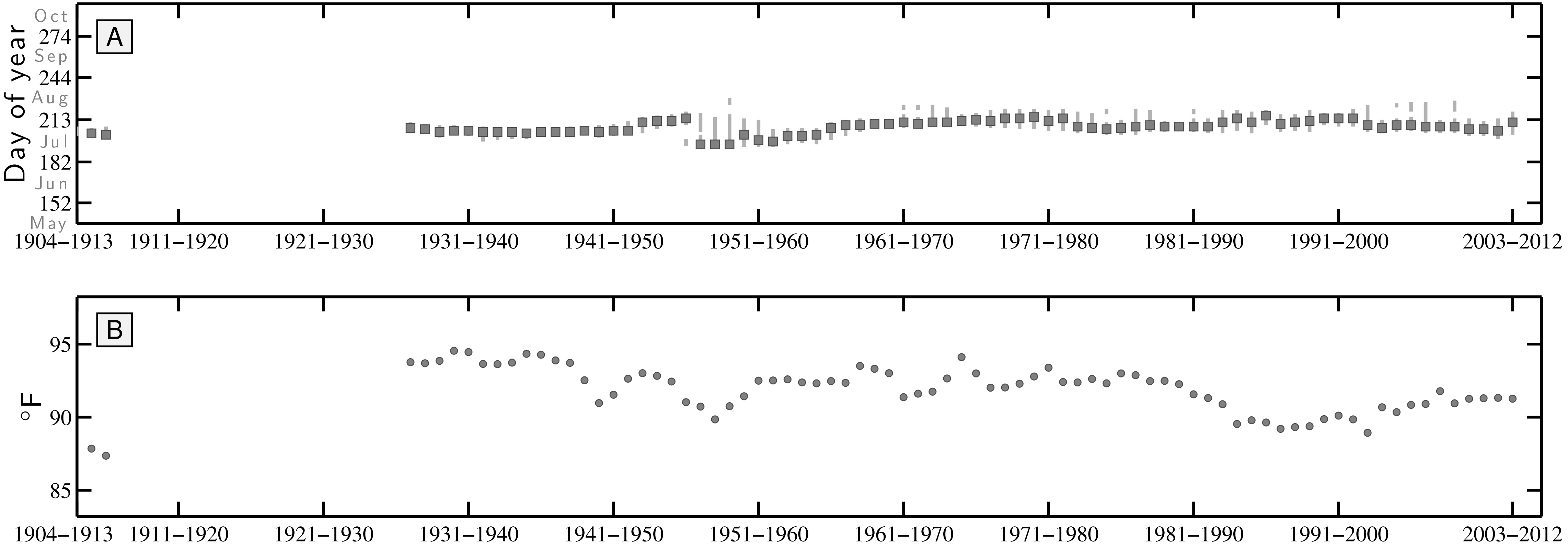

10 year Winter Teletherm dynamics for NORTHPORT, WA:

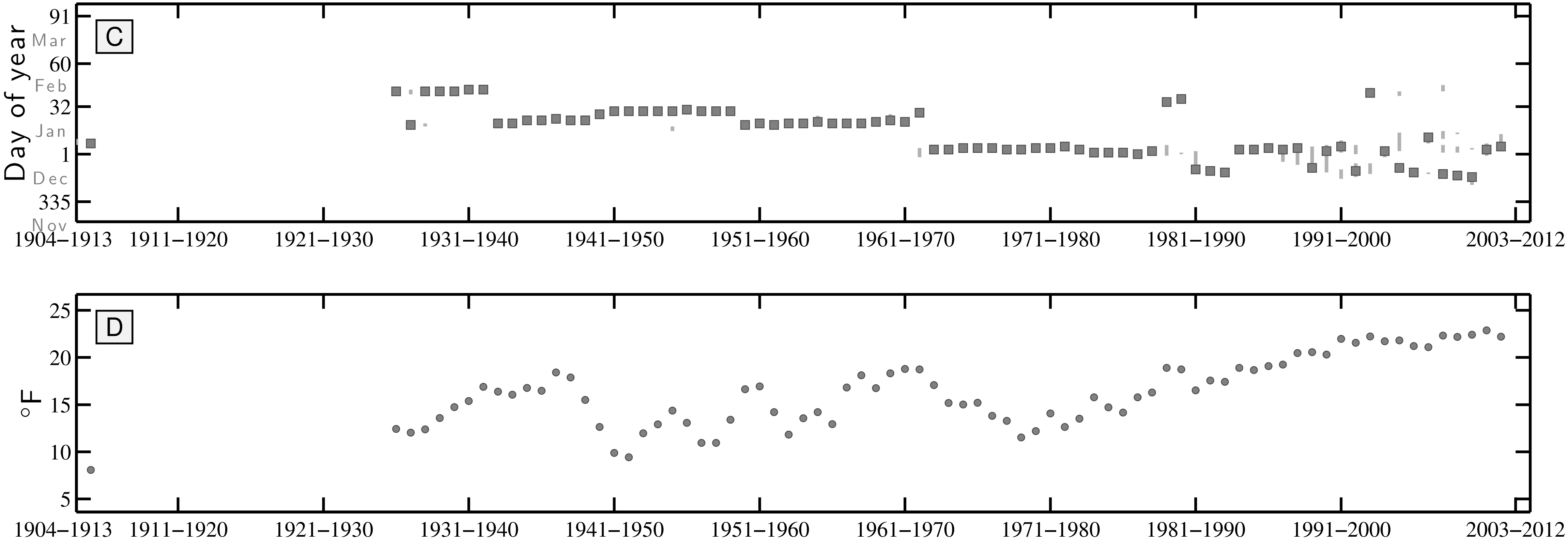

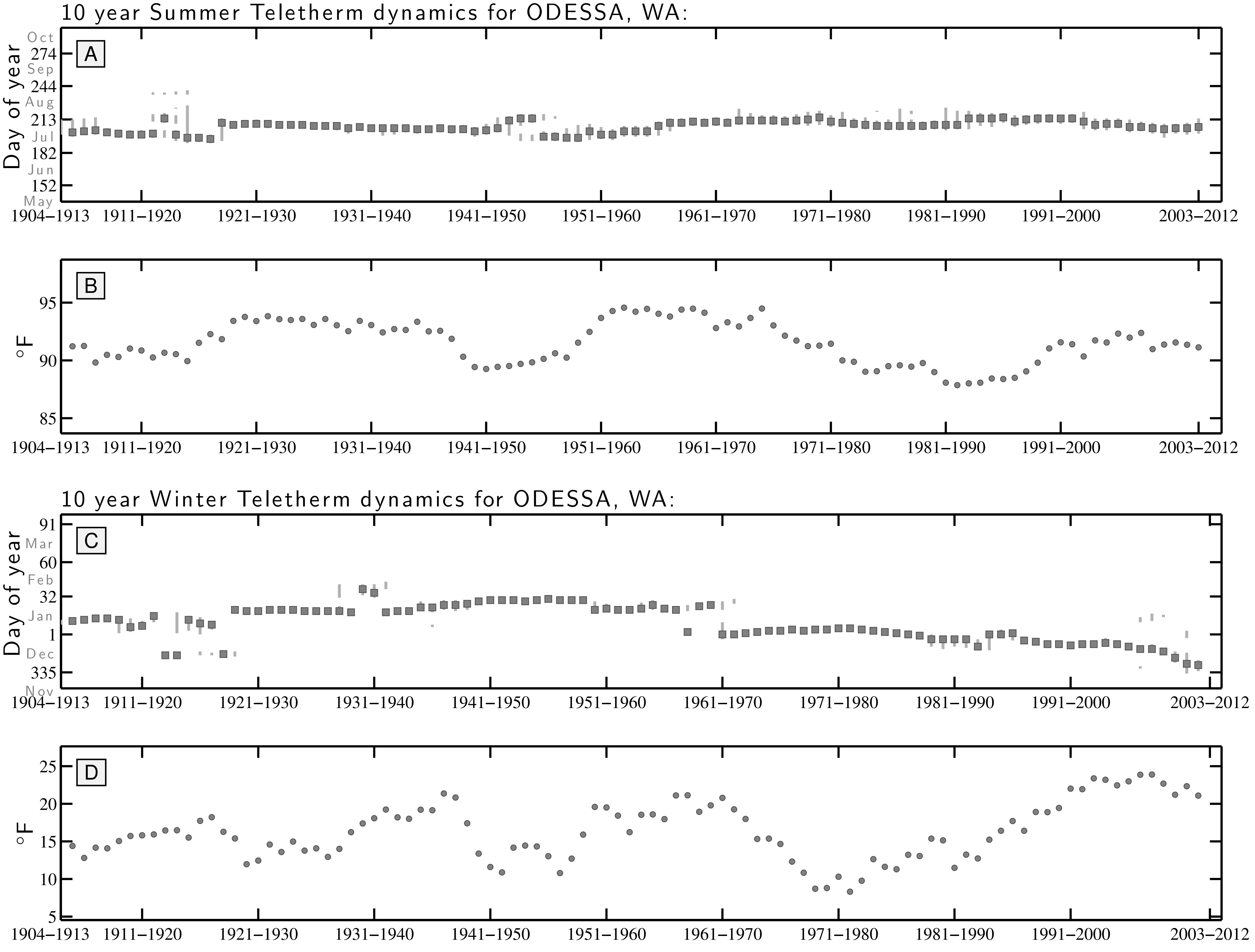
10 year Summer Teletherm dynamics for ODESSA, WA:
A
Day of year
Oct
274
Sep
244
Aug
213
Jul
182
Jun
152
May
1904–1913
1911–1920
1921–1930
1931–1940
1941–1950
1951–1960
1961–1970
1971–1980
1981–1990
1991–2000
2003–2012
B
°F
95
90
85
10 year Winter Teletherm dynamics for ODESSA, WA:
C
Day of year
91
Mar
60
Feb
32
Jan
1
Dec
335
Nov
D
°F
25
20
15
10
5

10 year Summer Teletherm dynamics for OLGA 2 SE, WA:

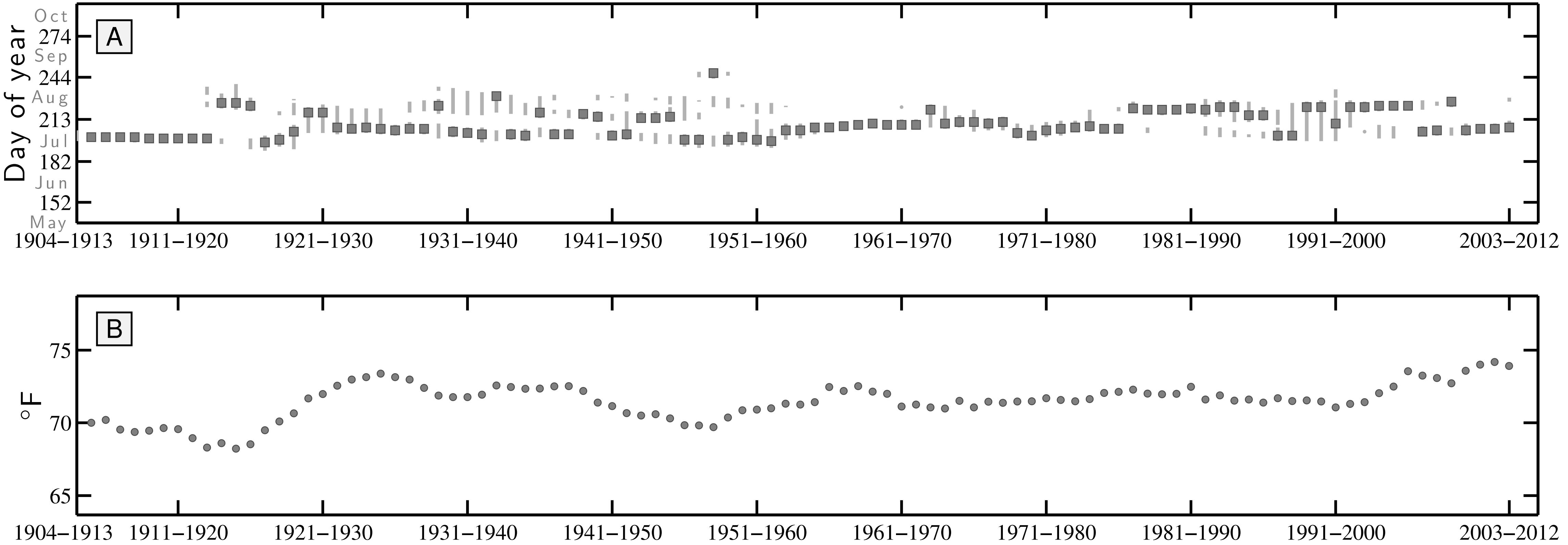

10 year Winter Teletherm dynamics for OLGA 2 SE, WA:

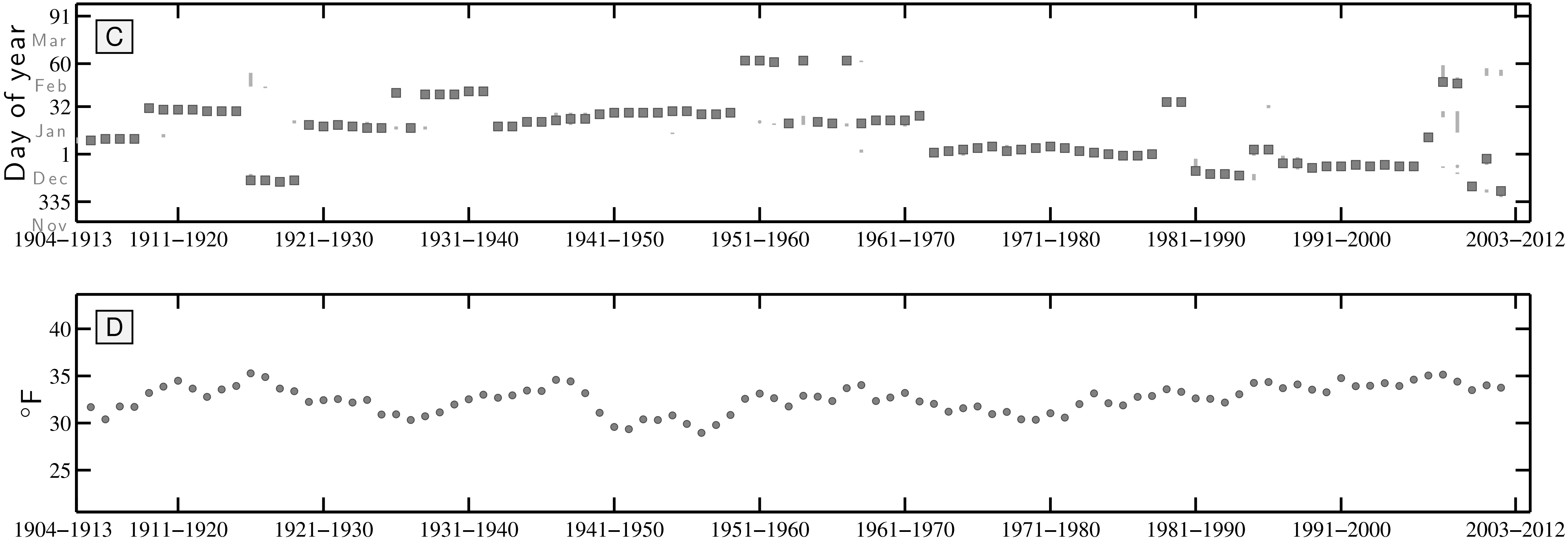

10 year Summer Teletherm dynamics for POMEROY, WA:

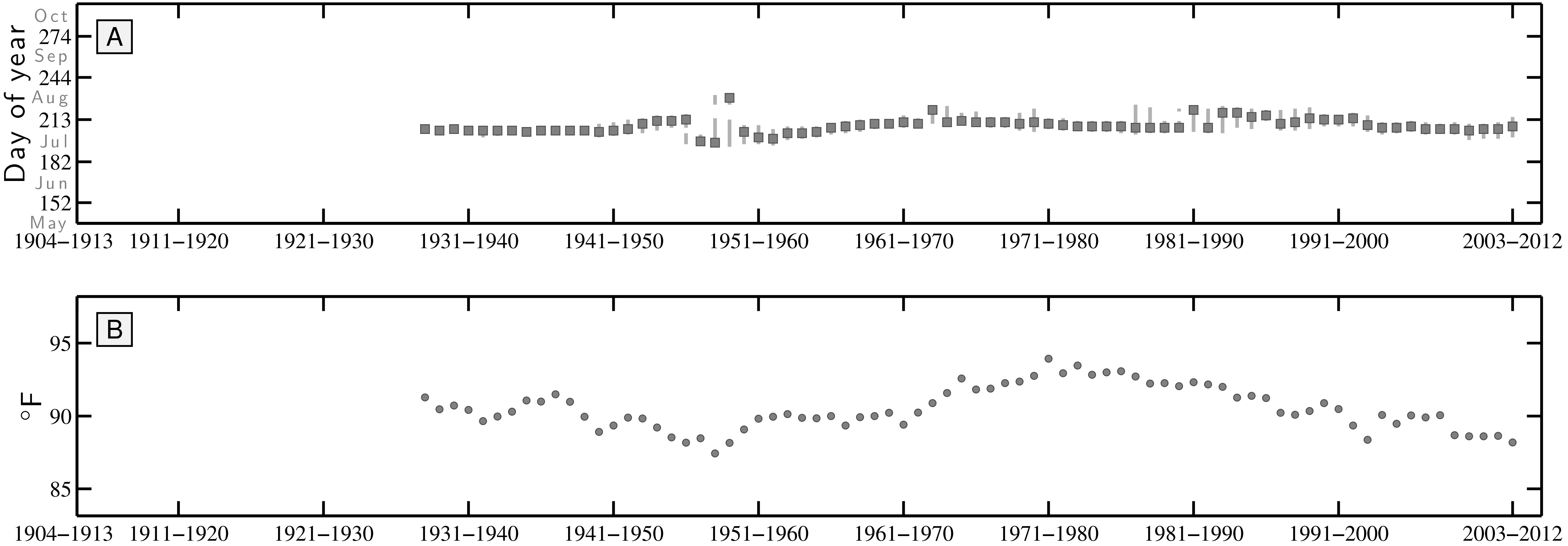

10 year Winter Teletherm dynamics for POMEROY, WA:

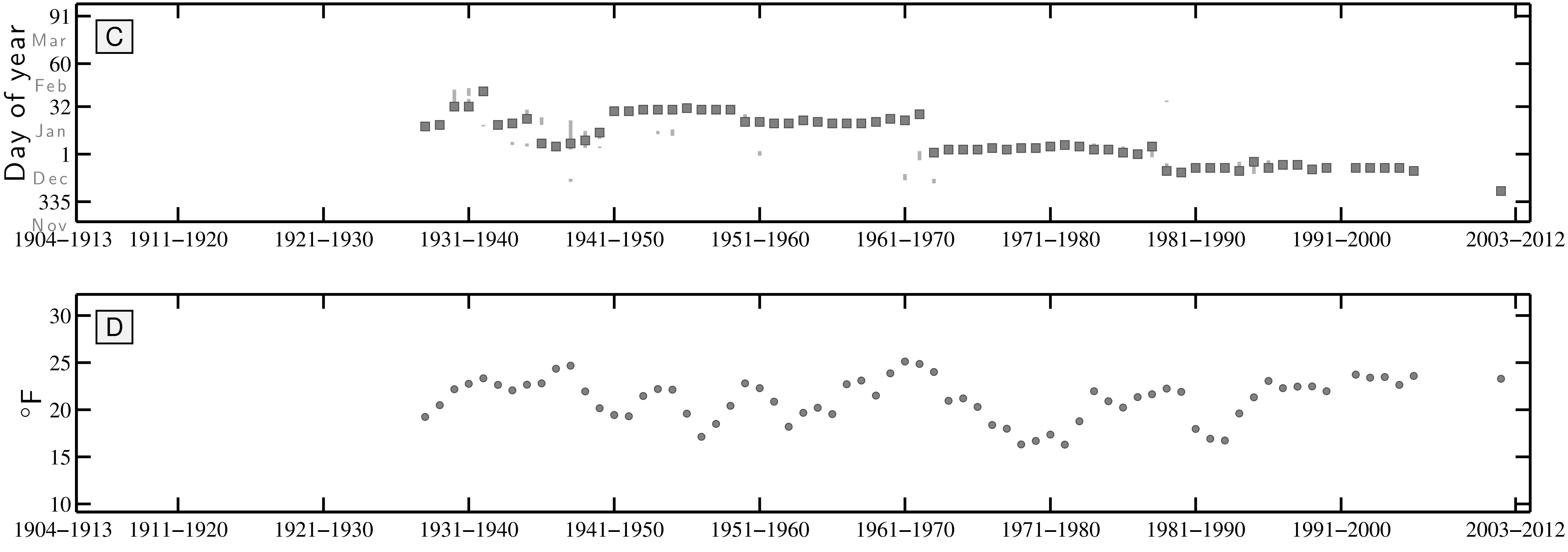

10 year Summer Teletherm dynamics for PORT ANGELES, WA:

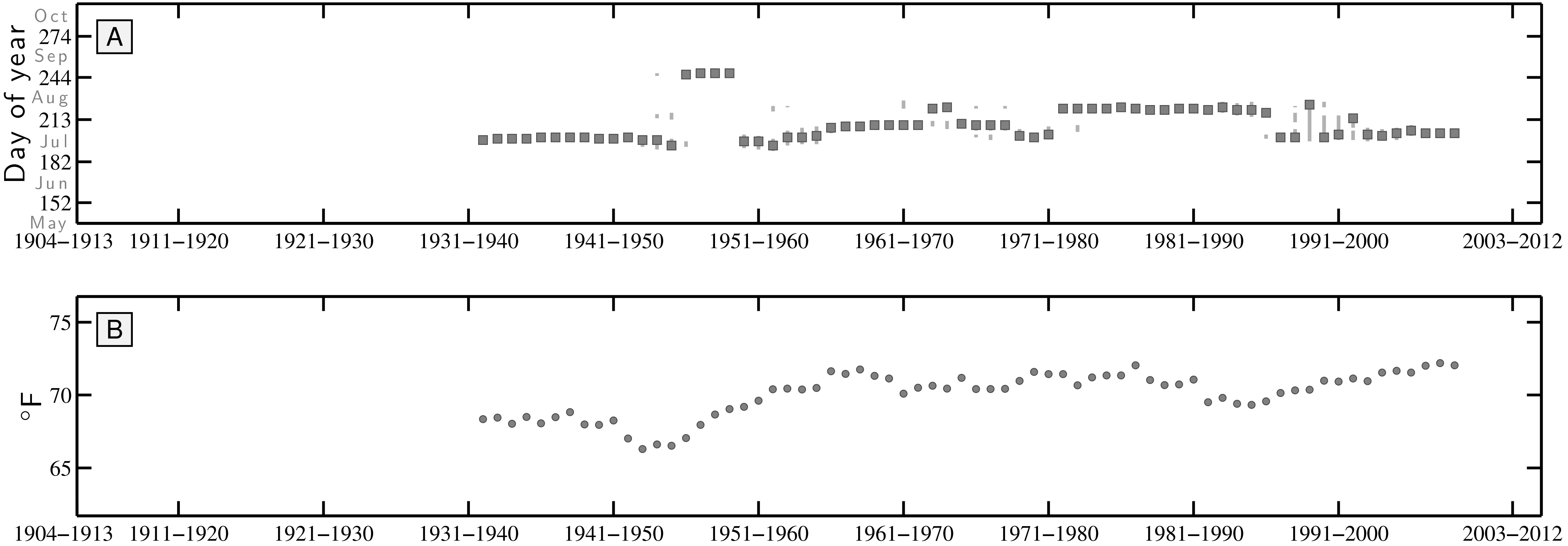

10 year Winter Teletherm dynamics for PORT ANGELES, WA:

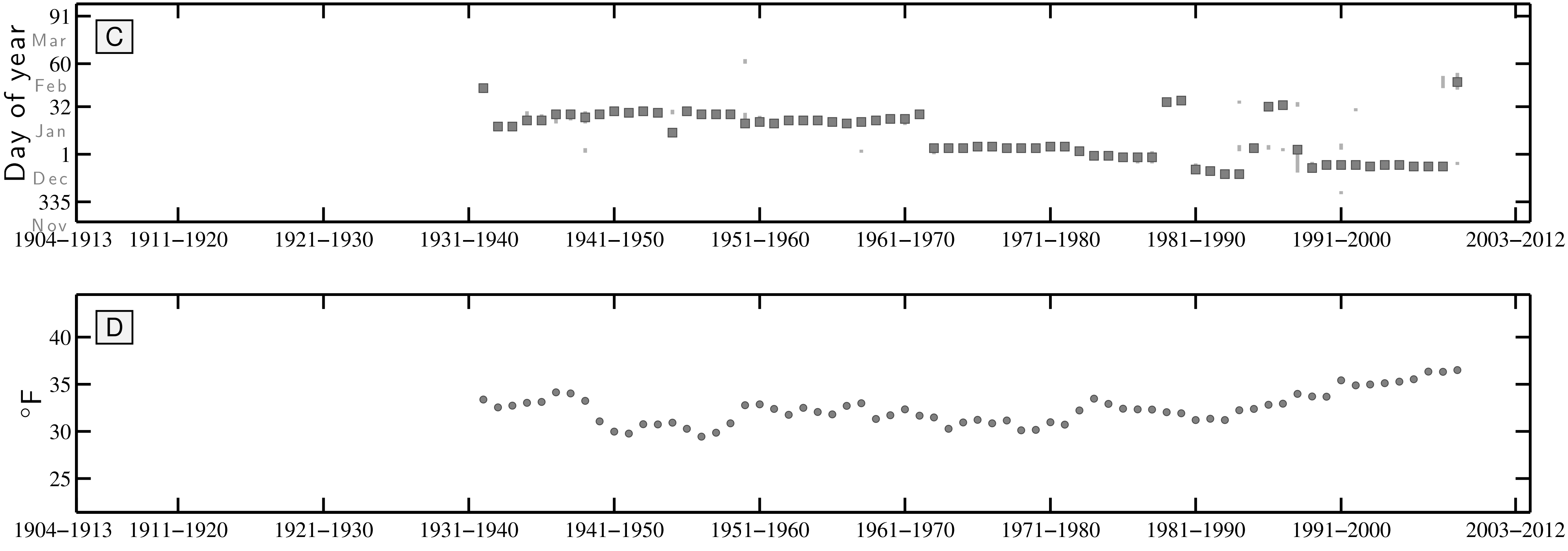

10 year Summer Teletherm dynamics for PORT TOWNSEND, WA:

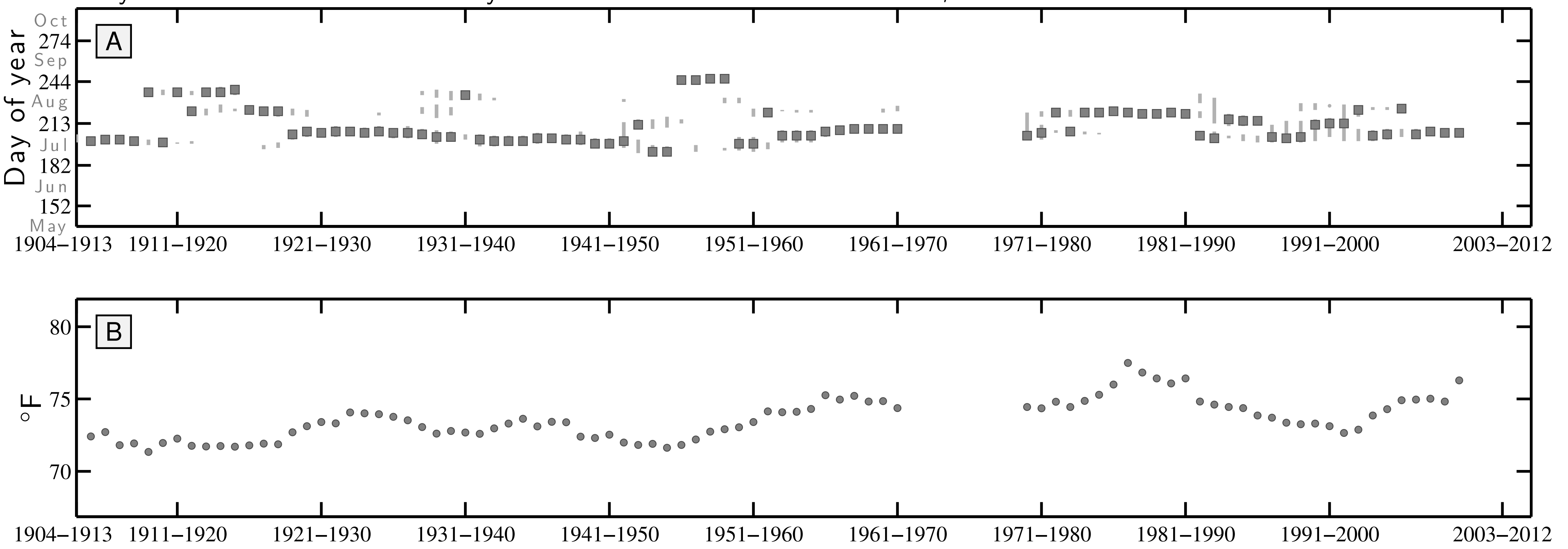

10 year Winter Teletherm dynamics for PORT TOWNSEND, WA:

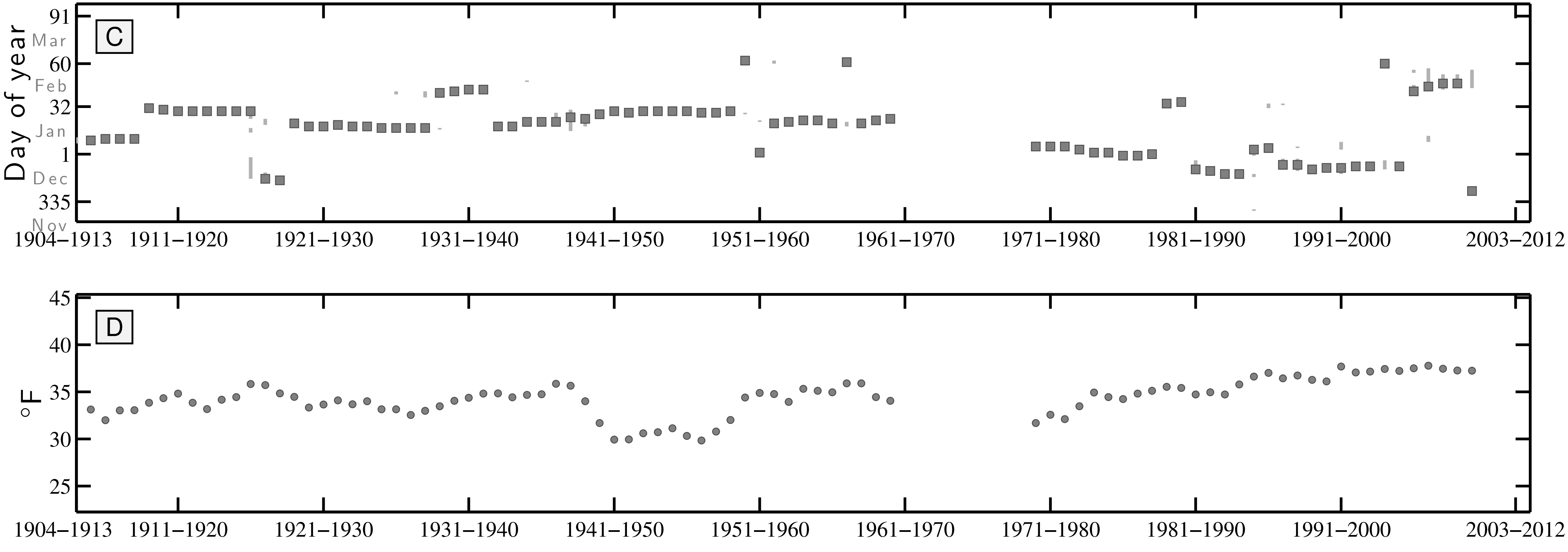

10 year Summer Teletherm dynamics for PULLMAN 2 NW, WA:

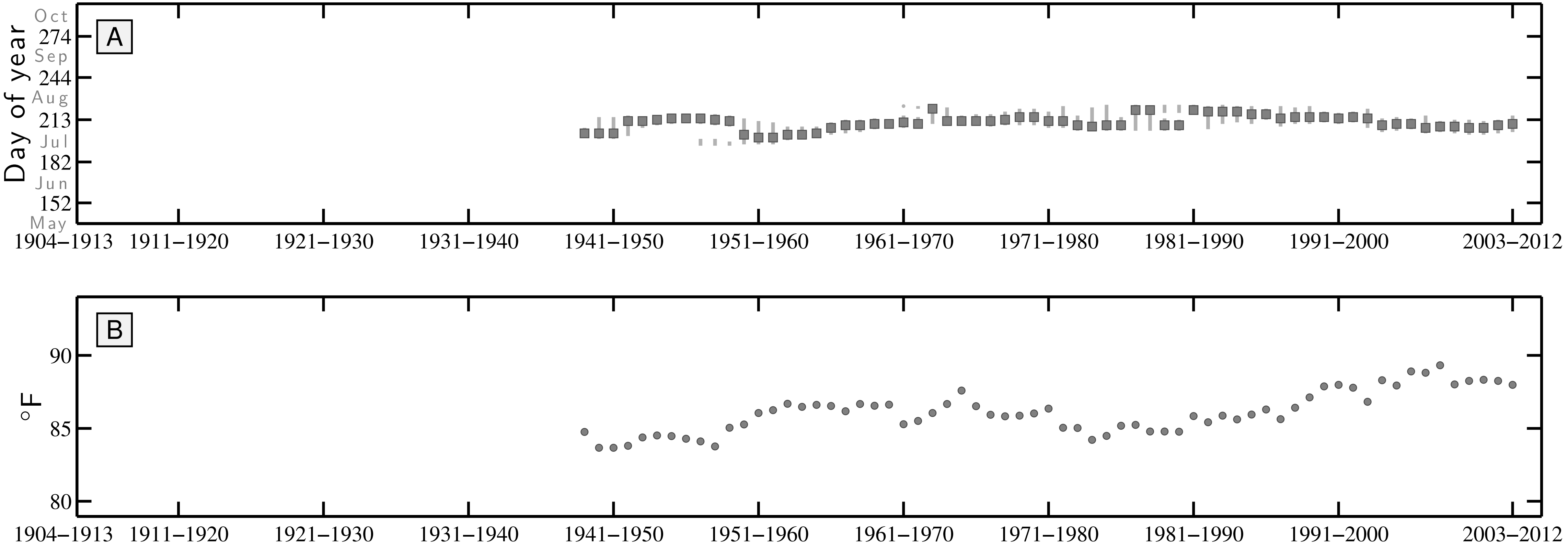

10 year Winter Teletherm dynamics for PULLMAN 2 NW, WA:

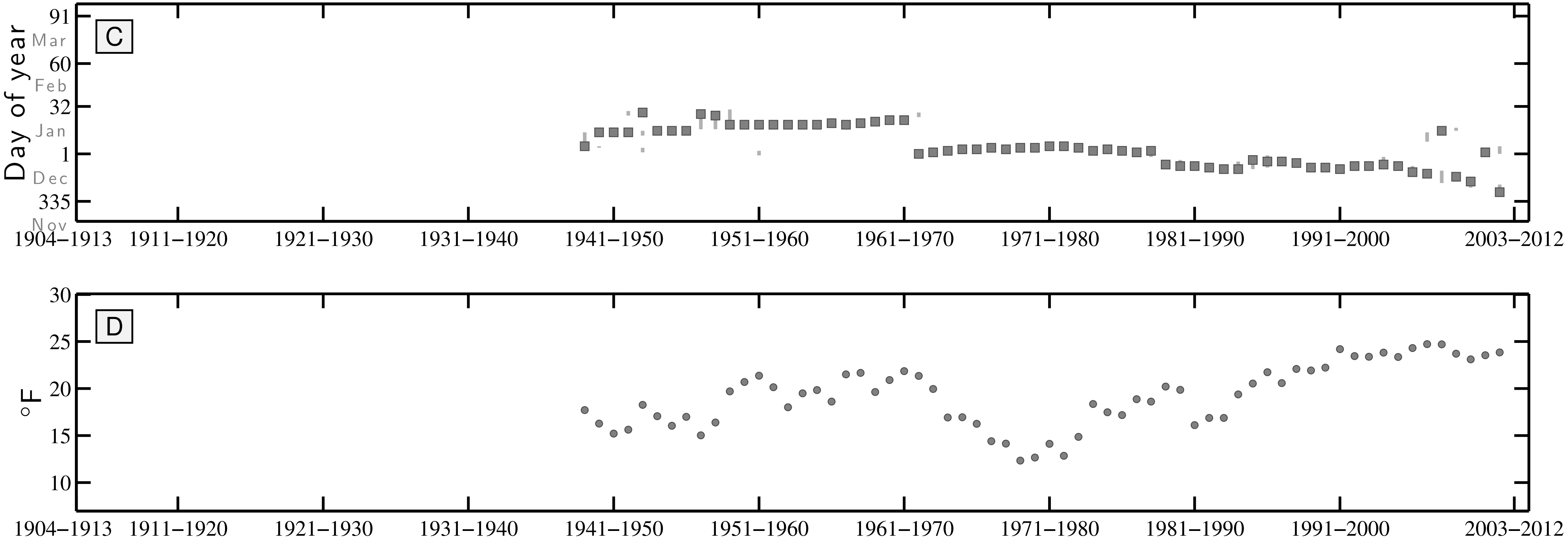

10 year Summer Teletherm dynamics for RAYMOND 2 S, WA:

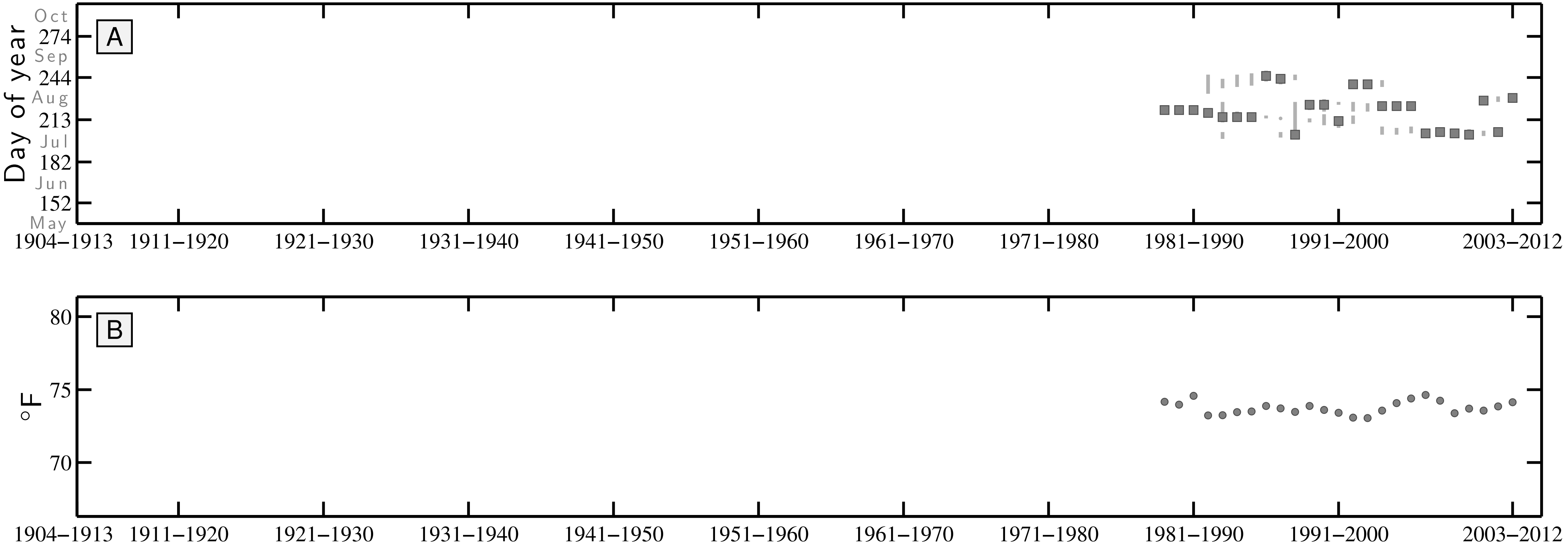

10 year Winter Teletherm dynamics for RAYMOND 2 S, WA:

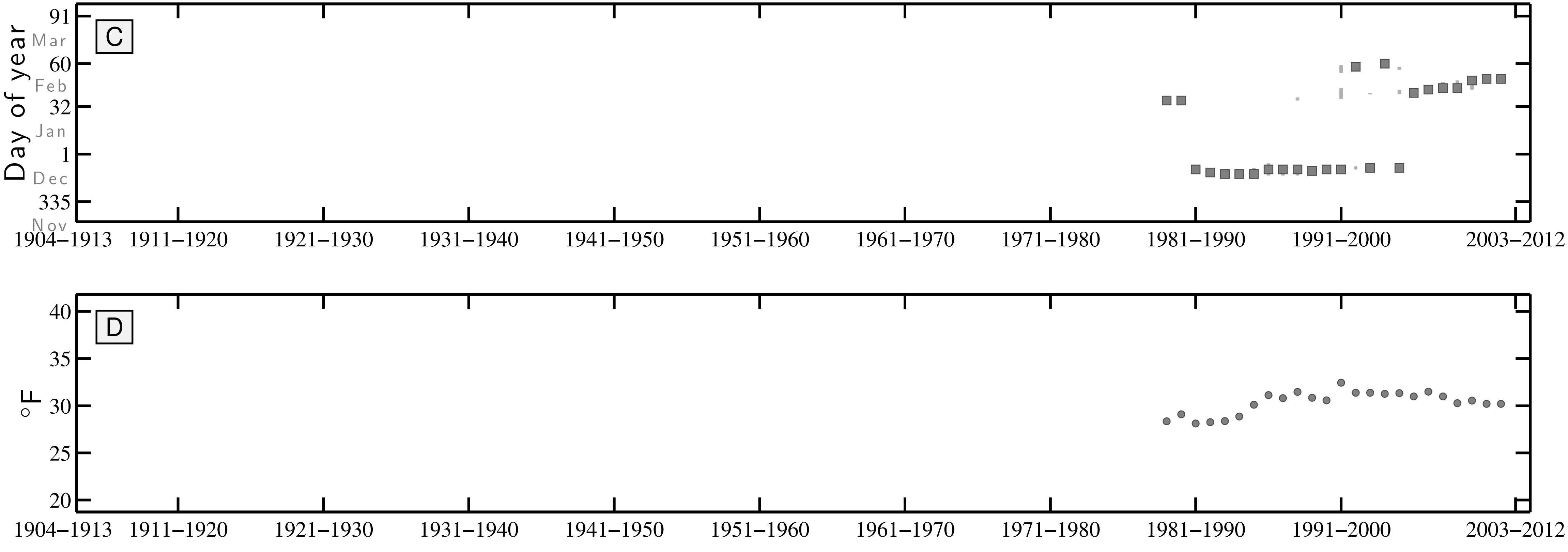

10 year Summer Teletherm dynamics for RITZVILLE 1 SSE, WA:

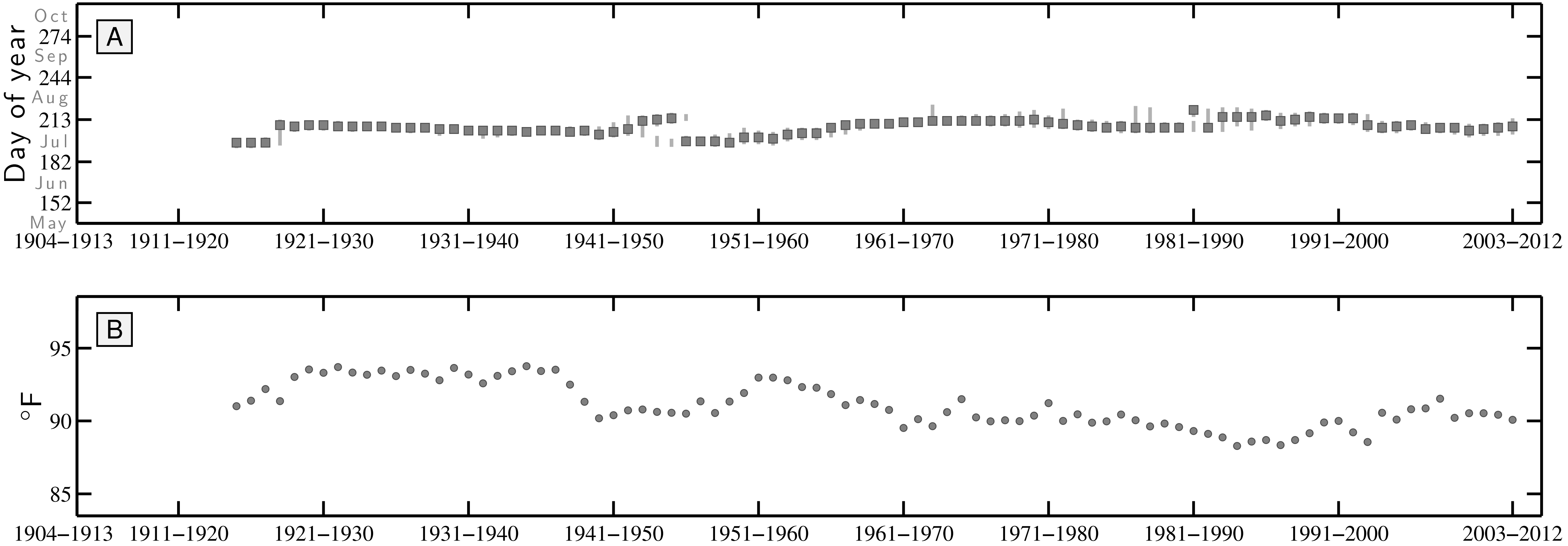

10 year Winter Teletherm dynamics for RITZVILLE 1 SSE, WA:

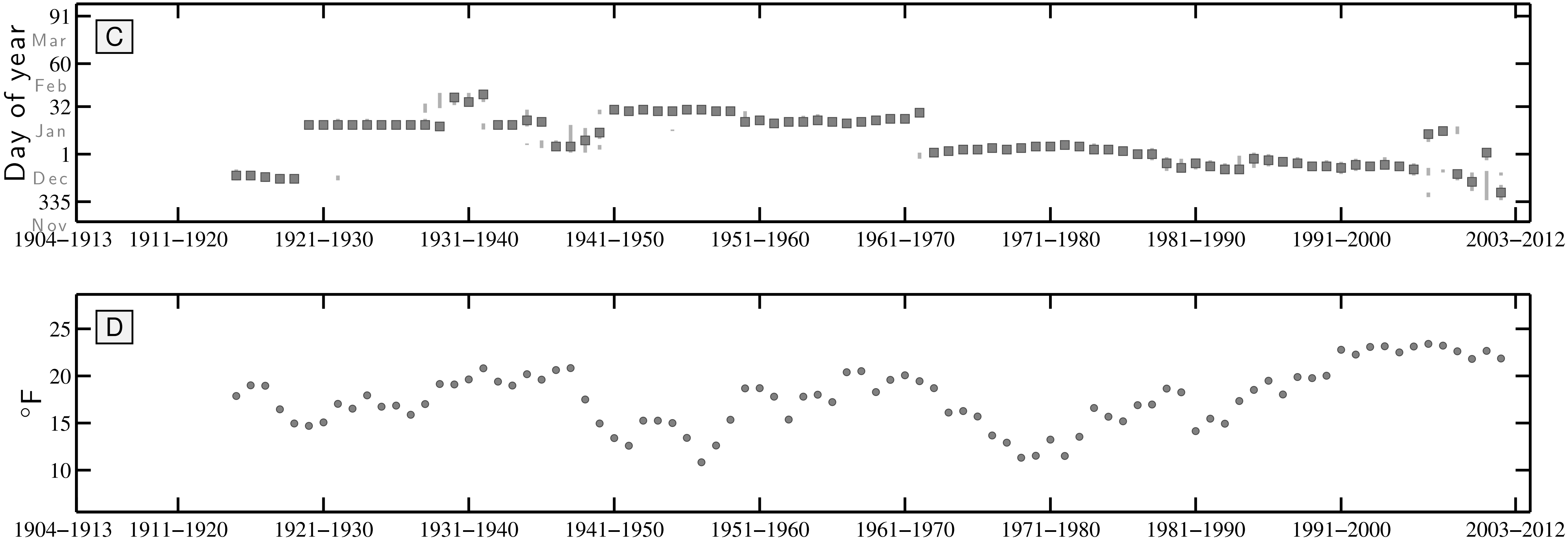

10 year Summer Teletherm dynamics for SAINT JOHN, WA:

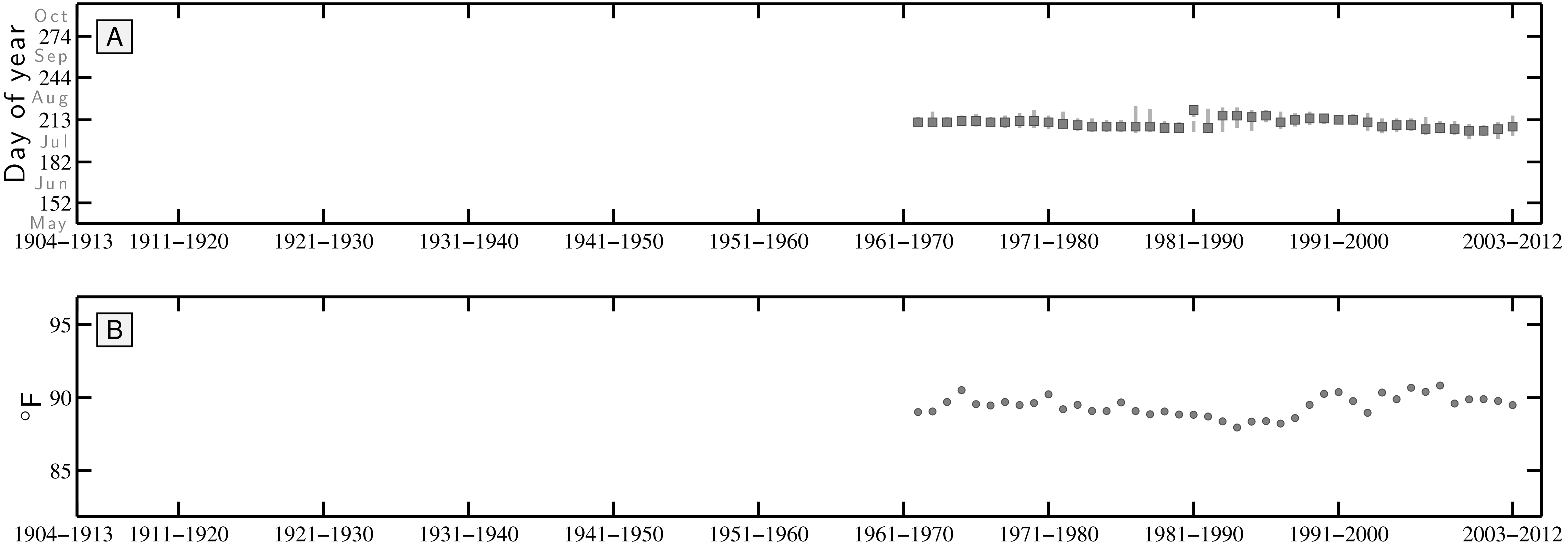

10 year Winter Teletherm dynamics for SAINT JOHN, WA:

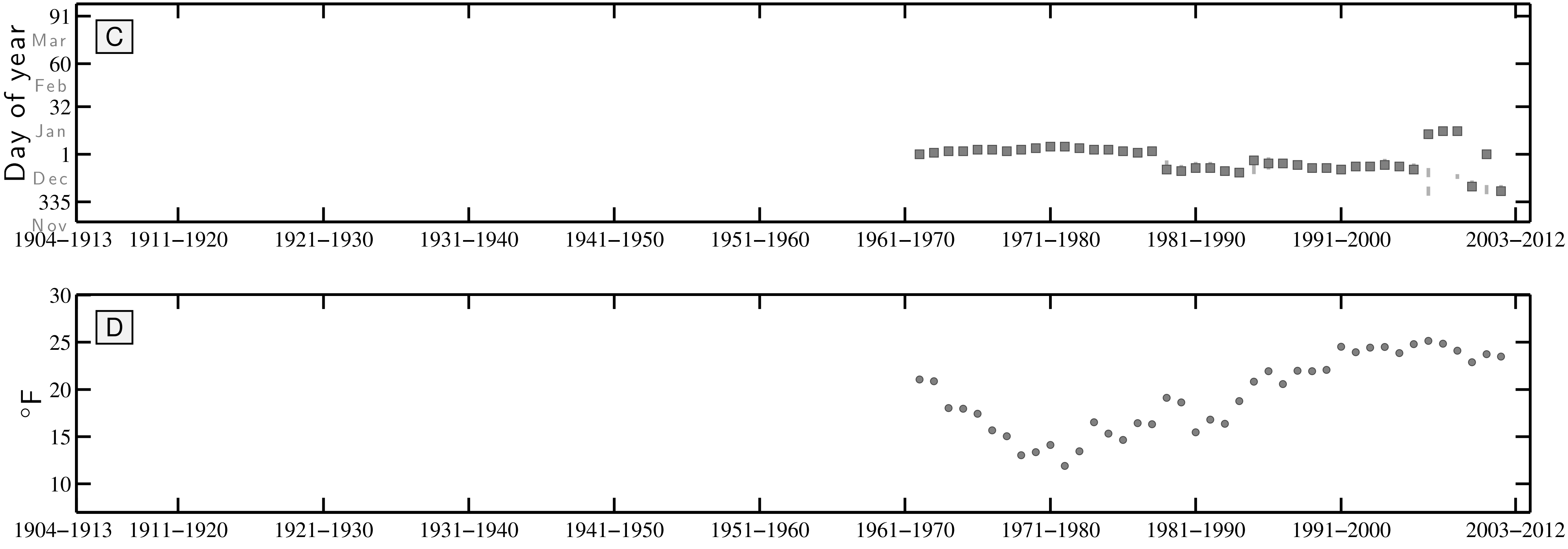

10 year Summer Teletherm dynamics for SEATTLE URBAN SITE, WA:

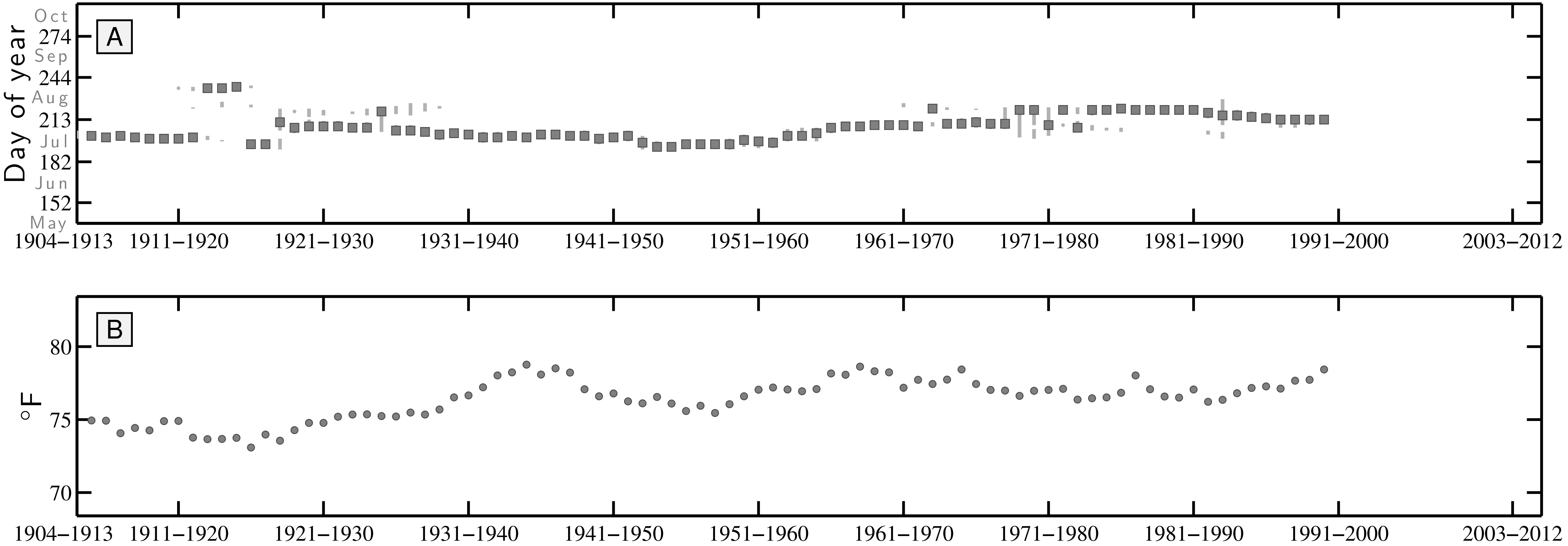

10 year Winter Teletherm dynamics for SEATTLE URBAN SITE, WA:

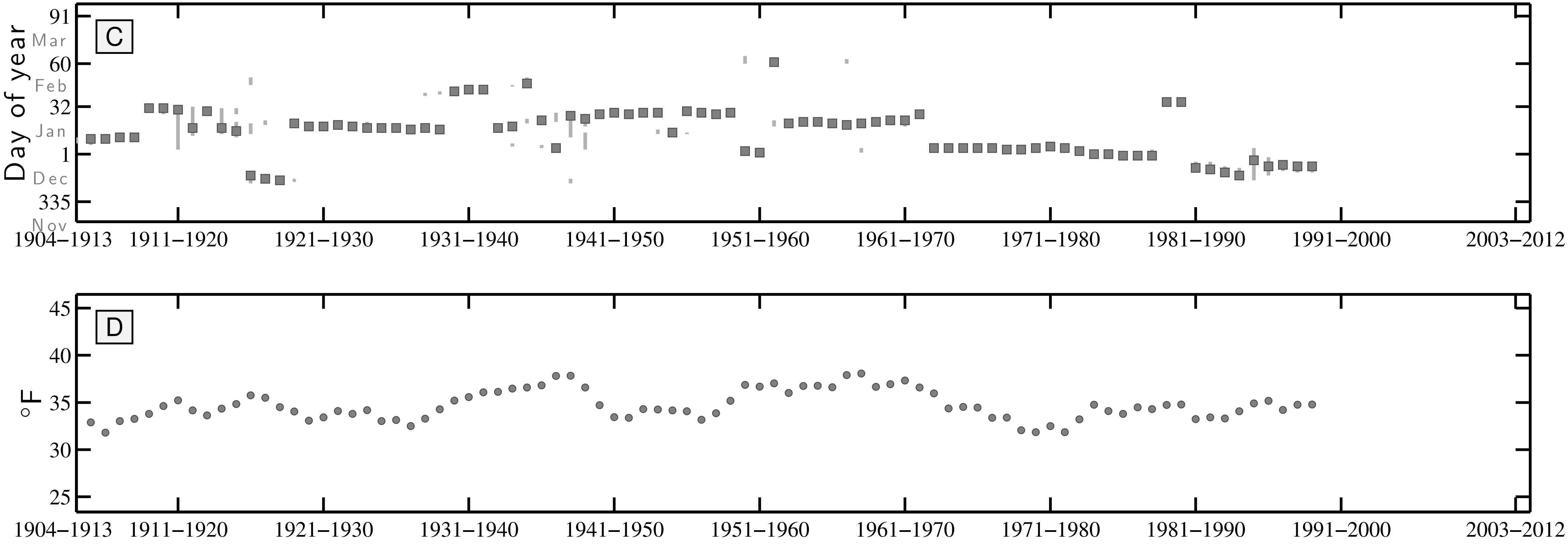

10 year Summer Teletherm dynamics for SEDRO WOOLLEY, WA:

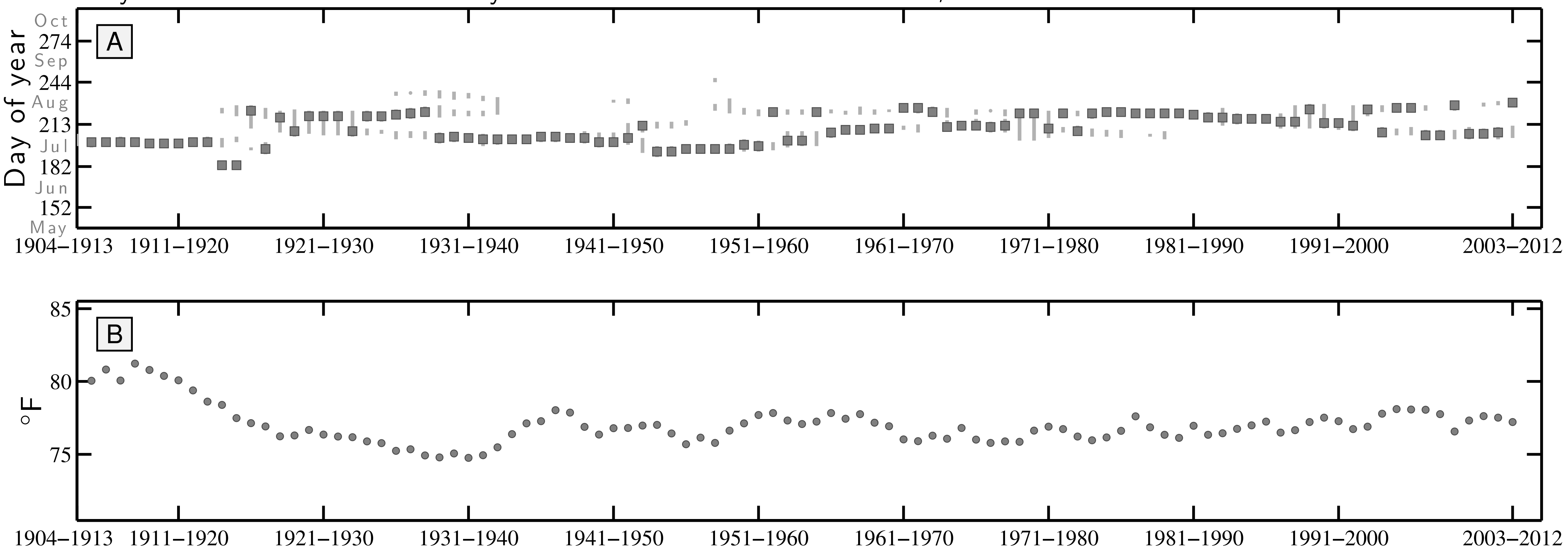

10 year Winter Teletherm dynamics for SEDRO WOOLLEY, WA:

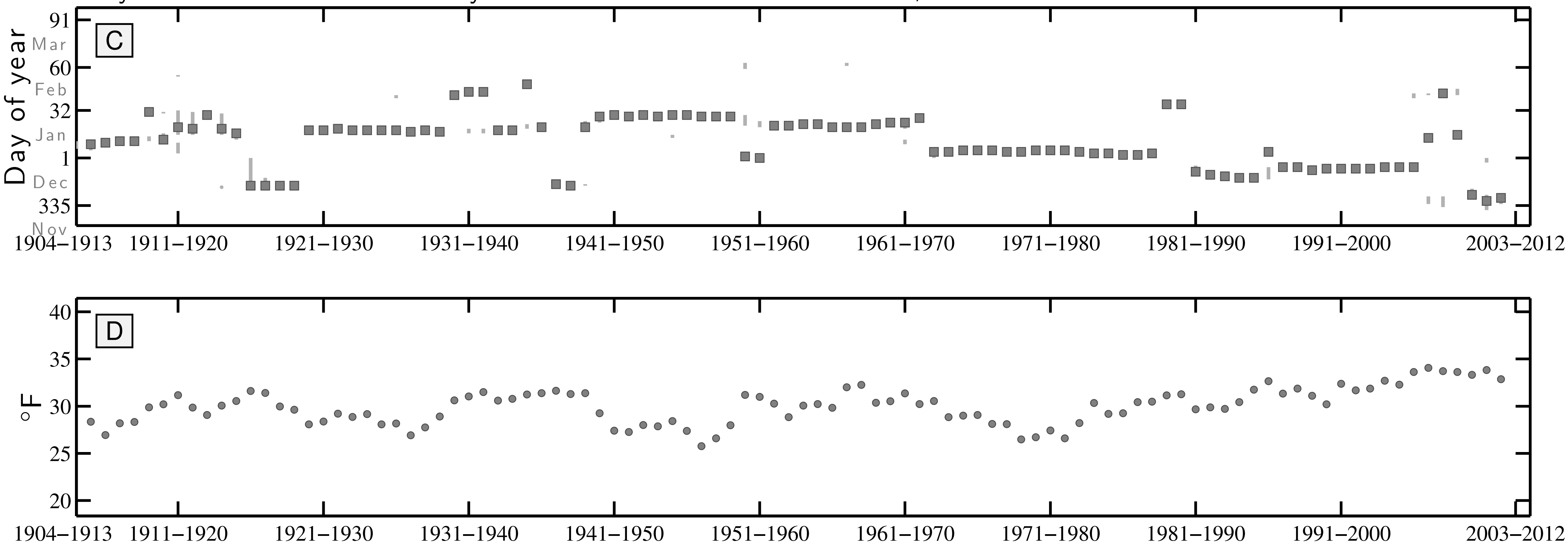

10 year Summer Teletherm dynamics for SNOQUALMIE FALLS, WA:

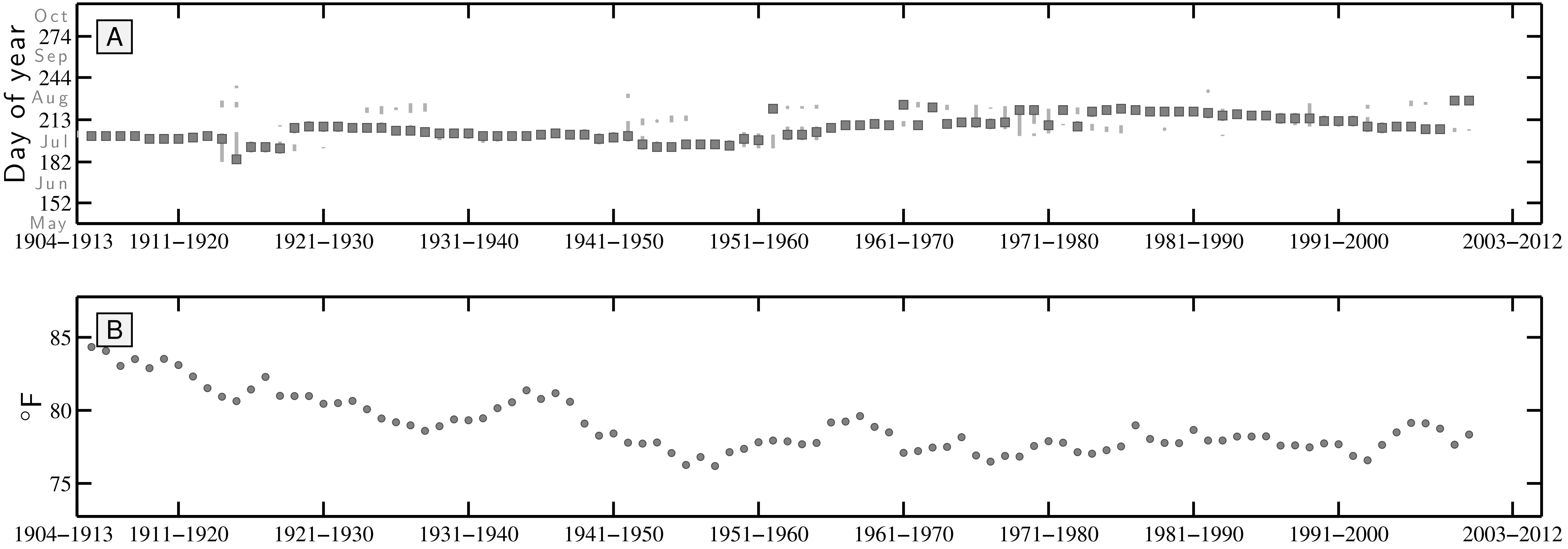

10 year Winter Teletherm dynamics for SNOQUALMIE FALLS, WA:

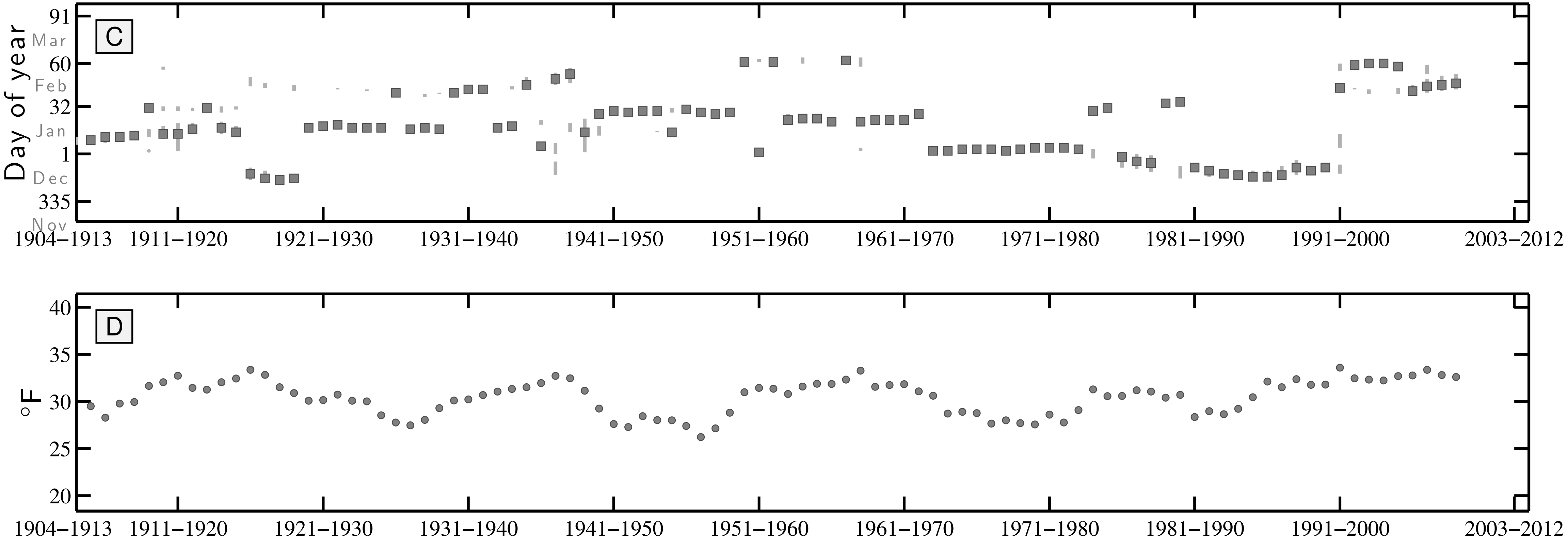

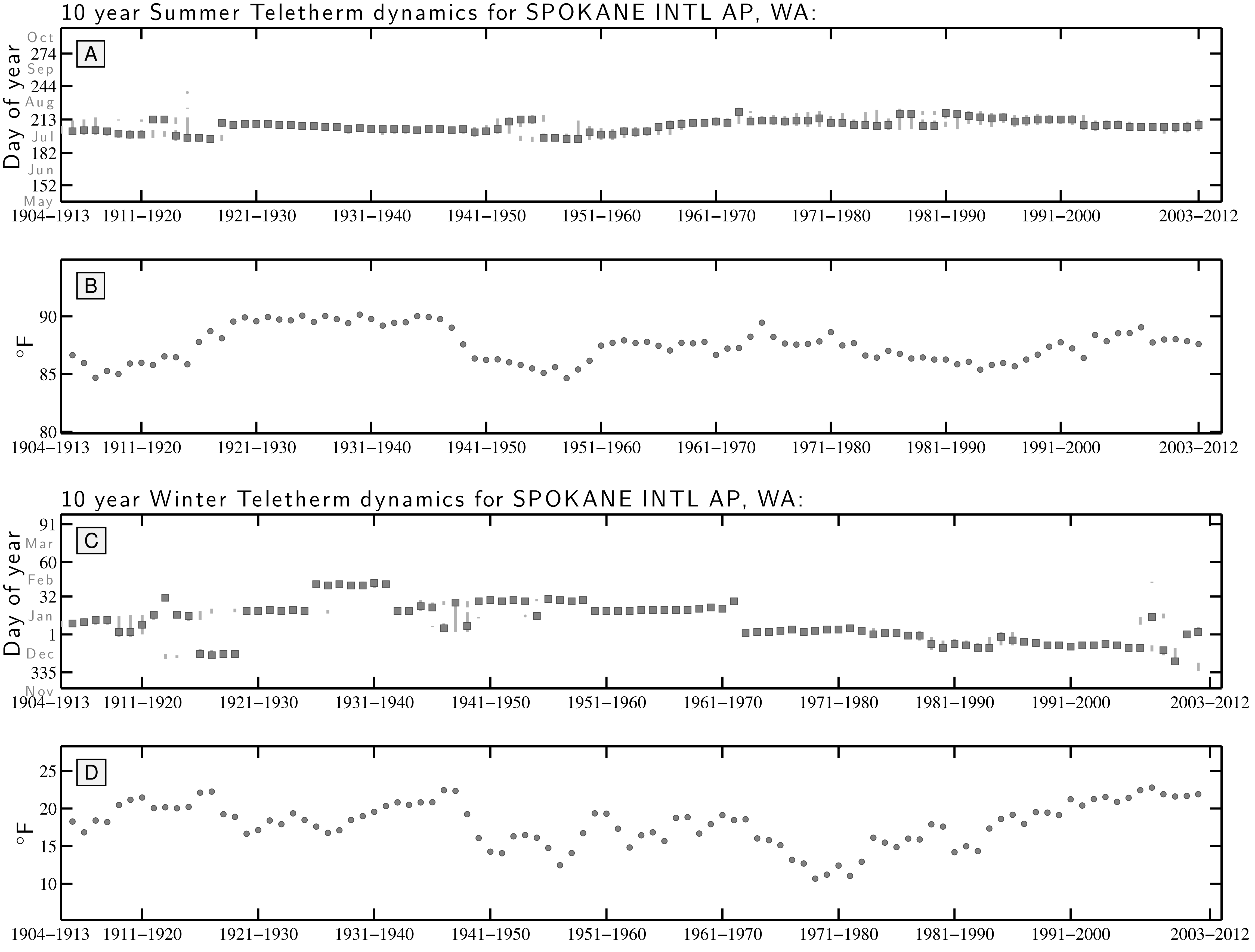

10 year Summer Teletherm dynamics for SPOKANE INTL AP, WA:
A
Day of year
Oct
274
Sep
244
Aug
213
Jul
182
Jun
152
May
1904–1913
1911–1920
1921–1930
1931–1940
1941–1950
1951–1960
1961–1970
1971–1980
1981–1990
1991–2000
2003–2012
B
°F
90
85
80
10 year Winter Teletherm dynamics for SPOKANE INTL AP, WA:
C
91
Mar
60
Feb
32
Jan
1
Dec
335
Nov
D
25
20
15
10

10 year Summer Teletherm dynamics for STEHEKIN 4 NW, WA:

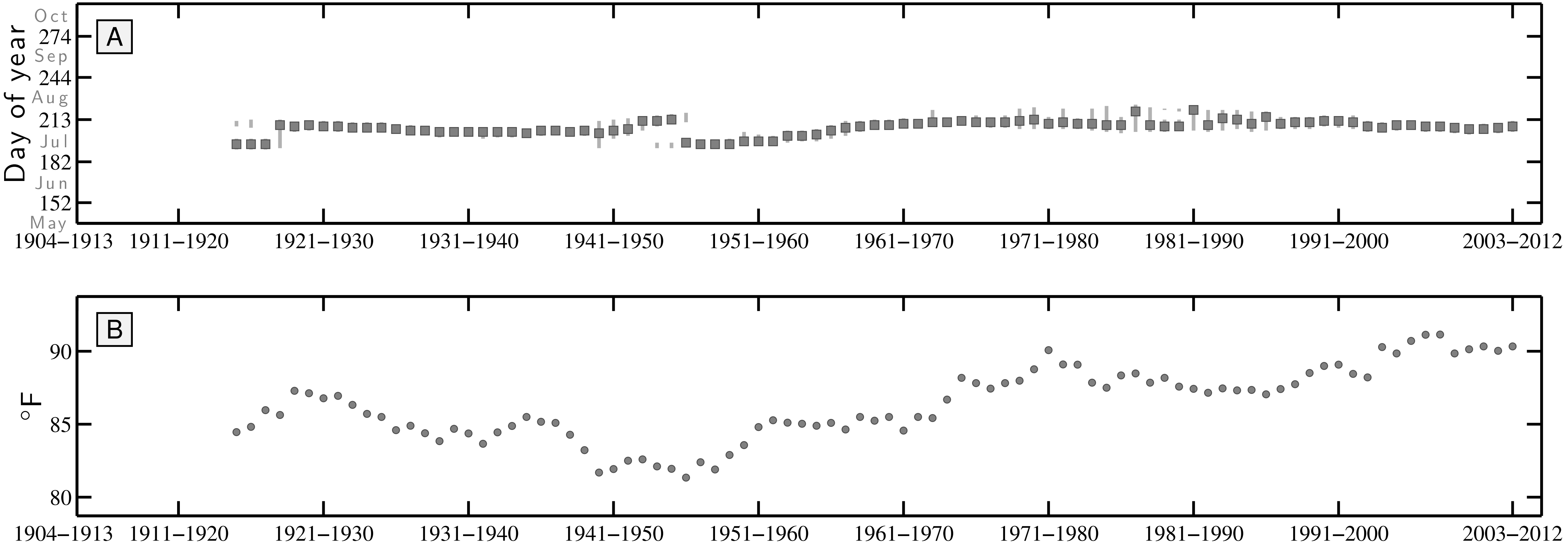

10 year Winter Teletherm dynamics for STEHEKIN 4 NW, WA:

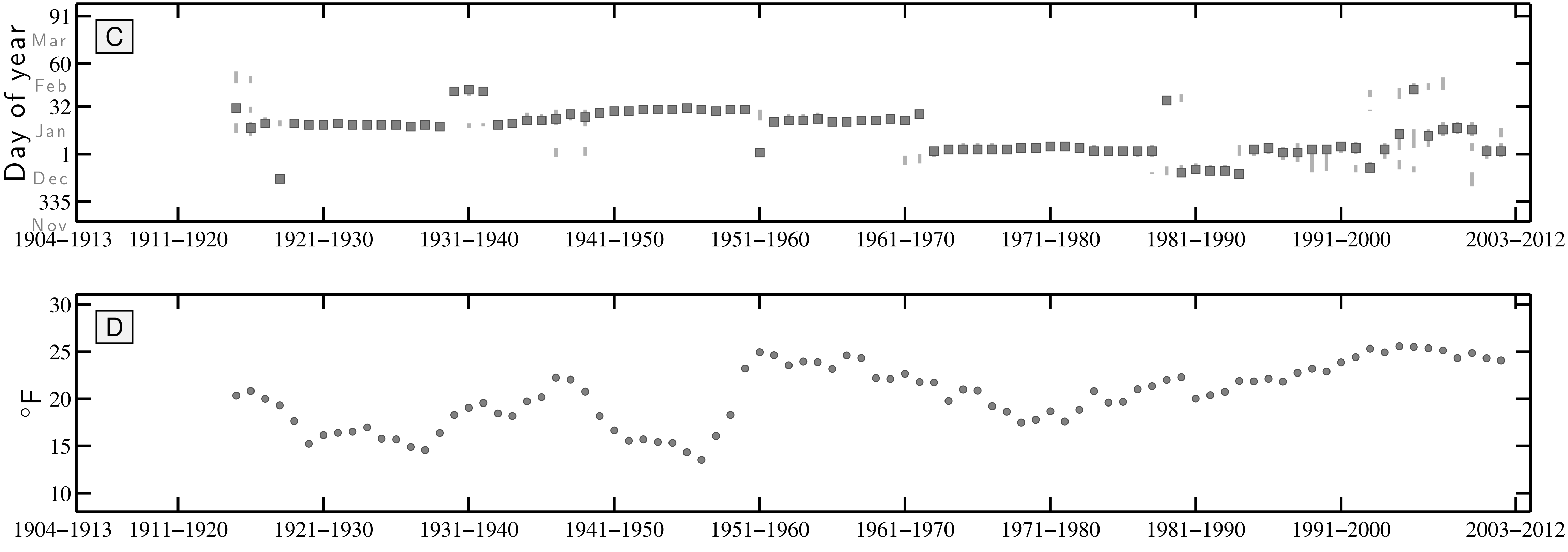

10 year Summer Teletherm dynamics for SUNNYSIDE, WA:

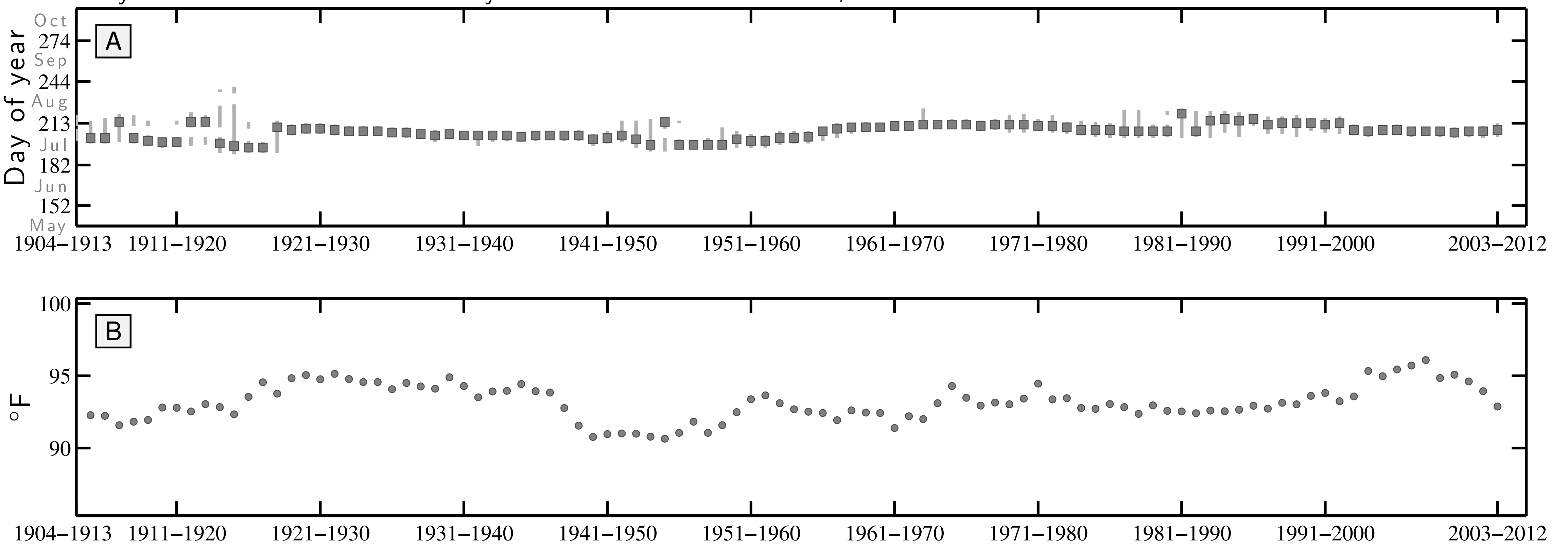

10 year Winter Teletherm dynamics for SUNNYSIDE, WA:

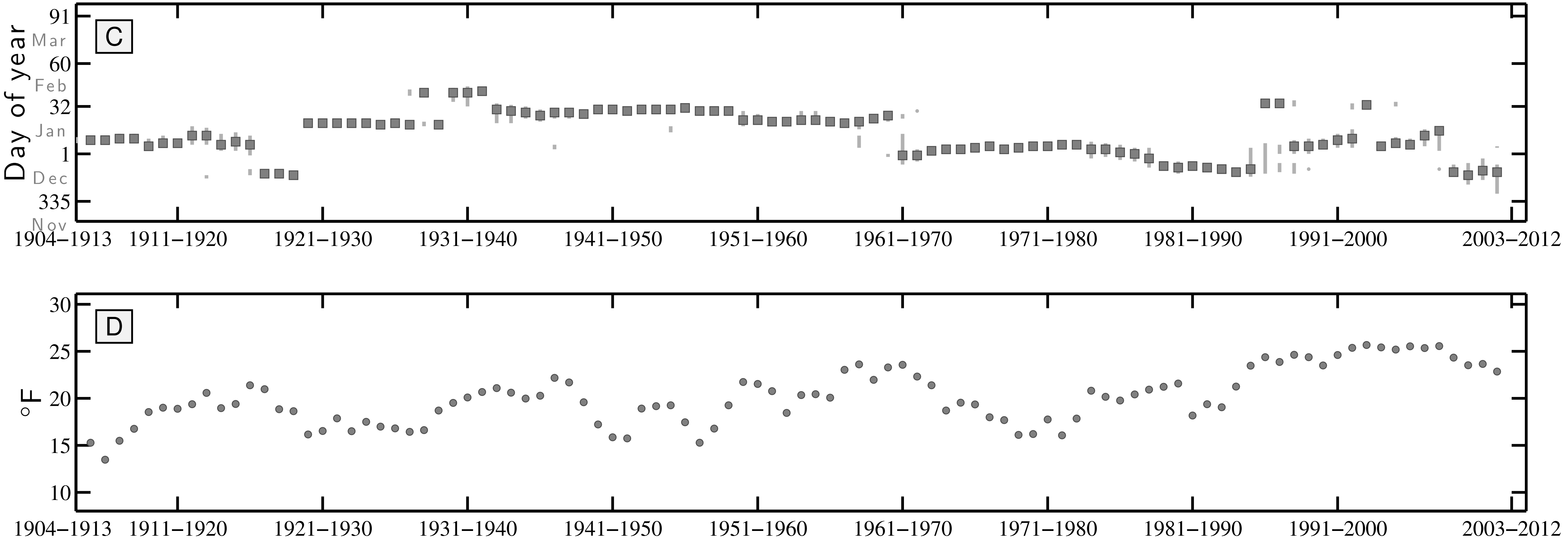

10 year Summer Teletherm dynamics for VANCOUVER 4 NNE, WA:

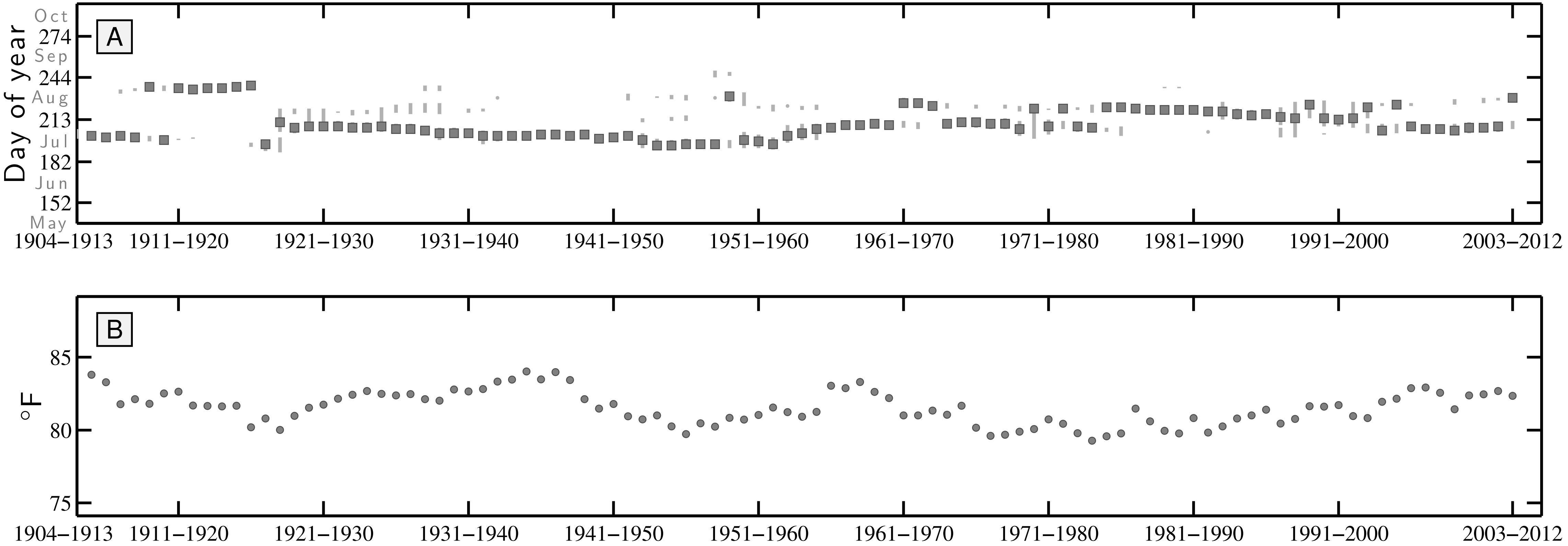

10 year Winter Teletherm dynamics for VANCOUVER 4 NNE, WA:

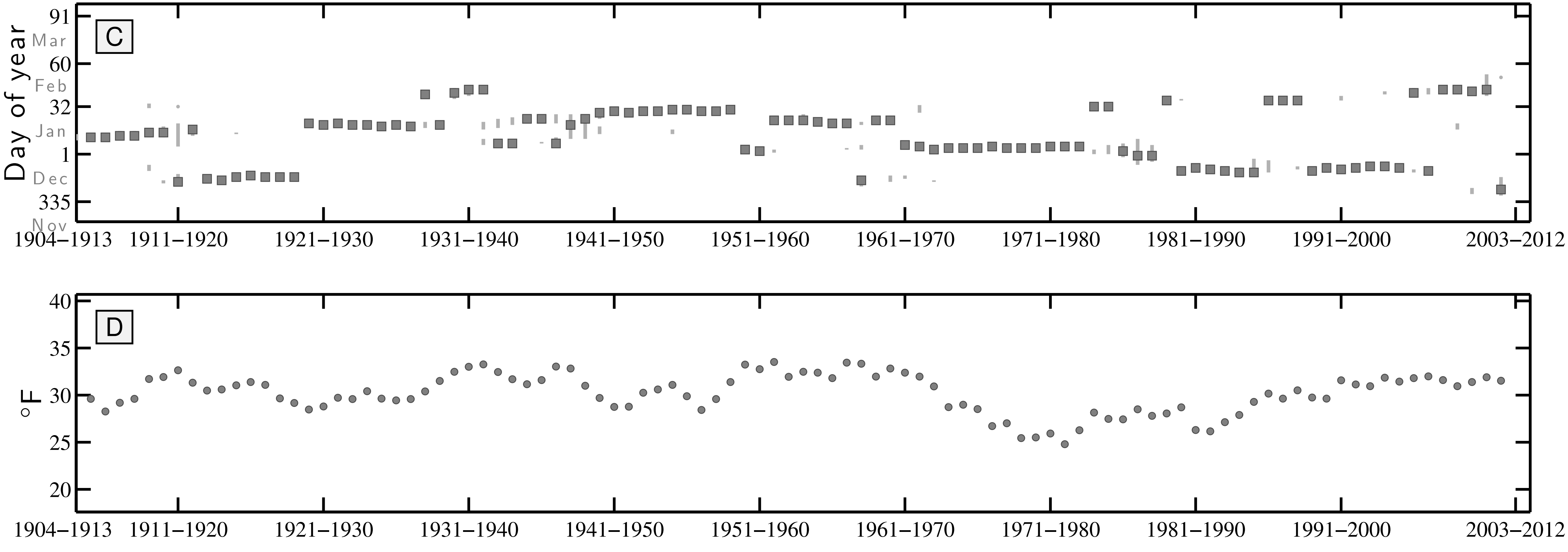

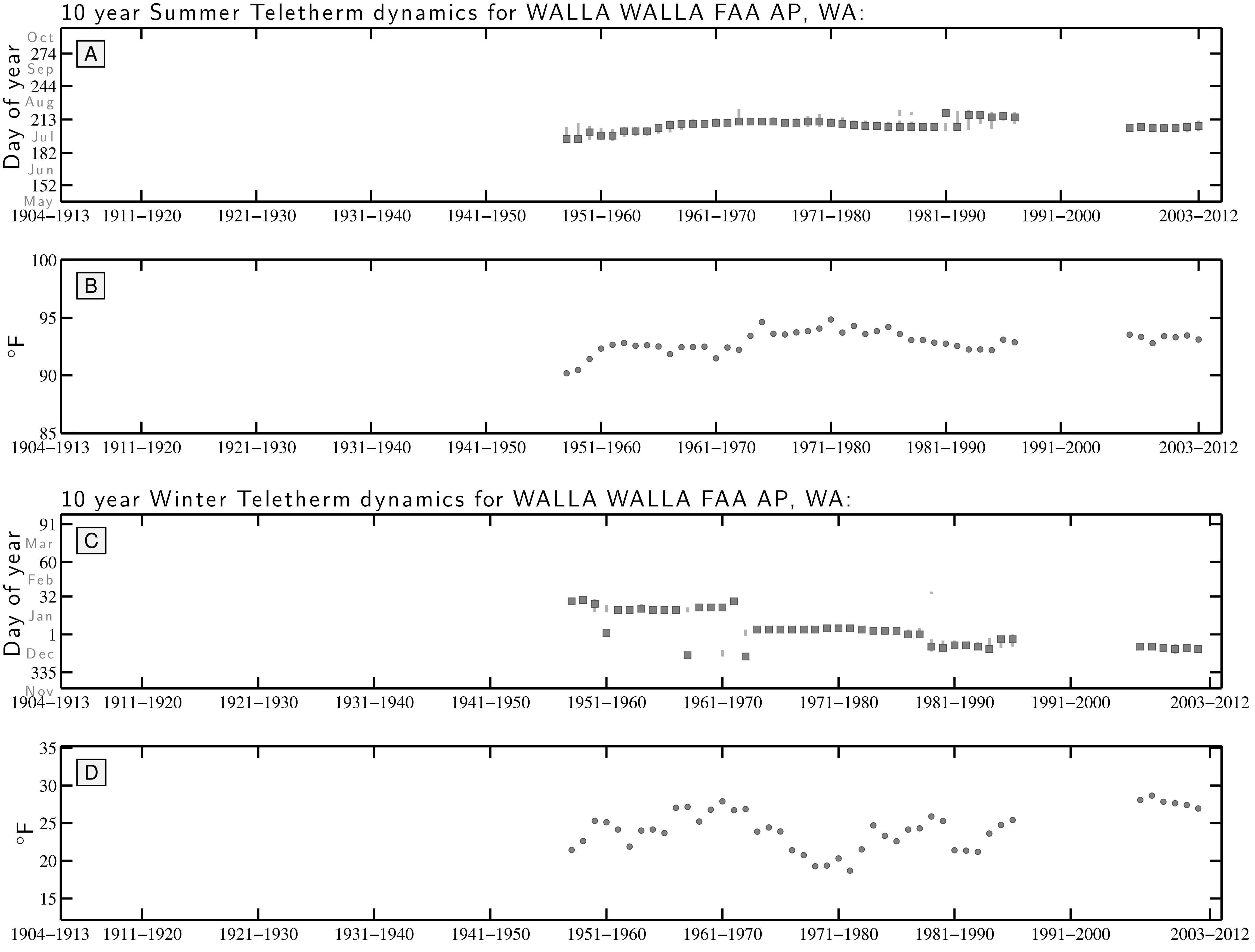
10 year Summer Teletherm dynamics for WALLA WALLA FAA AP, WA:
A
Day of year
Oct
274
Sep
244
Aug
213
Jul
182
Jun
152
May
1904–1913
1911–1920
1921–1930
1931–1940
1941–1950
1951–1960
1961–1970
1971–1980
1981–1990
1991–2000
2003–2012
B
°F
100
95
90
85
10 year Winter Teletherm dynamics for WALLA WALLA FAA AP, WA:
C
91
Mar
60
Feb
32
Jan
1
Dec
335
Nov
D
35
30
25
20
15

10 year Summer Teletherm dynamics for WATERVILLE, WA:

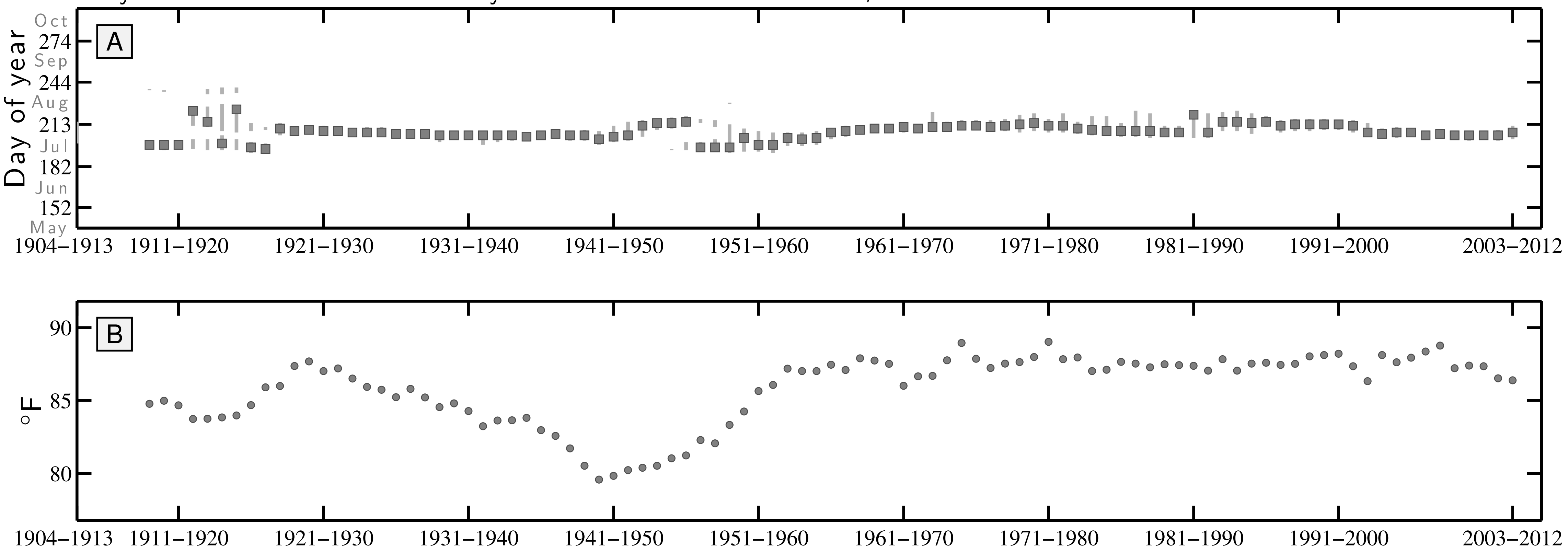

10 year Winter Teletherm dynamics for WATERVILLE, WA:

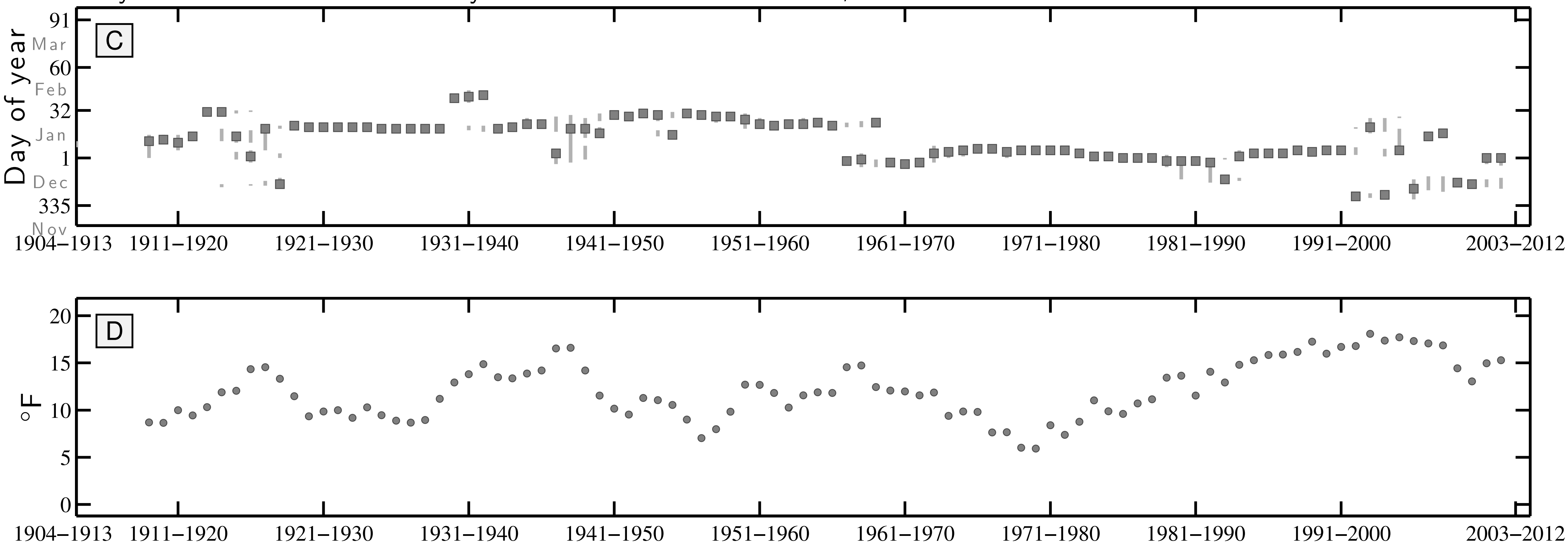

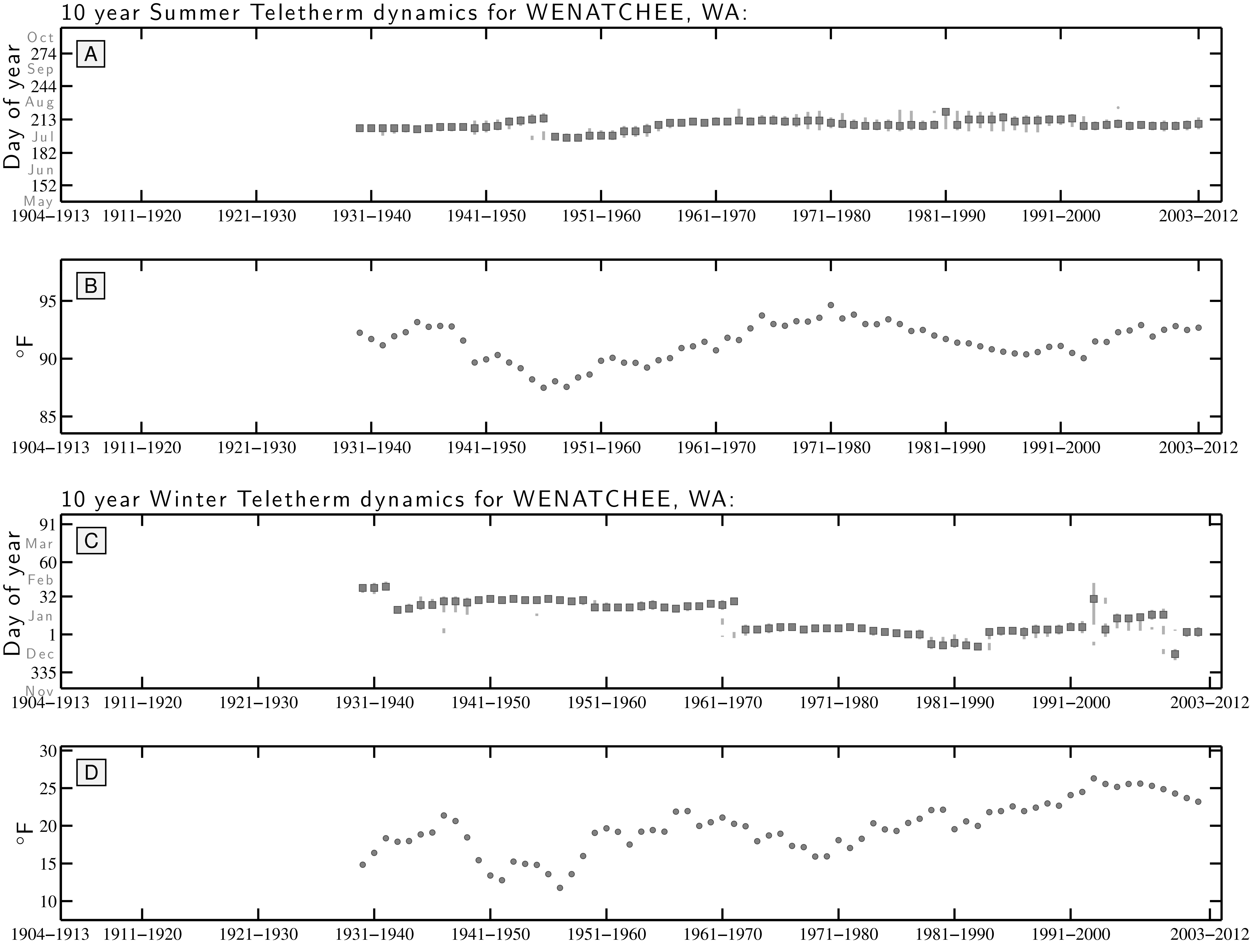
10 year Summer Teletherm dynamics for WENATCHEE, WA:
A
Day of year
Oct
274
Sep
244
Aug
213
Jul
182
Jun
152
May
B
°F
95
90
85
10 year Winter Teletherm dynamics for WENATCHEE, WA:
C
91
Mar
60
Feb
32
Jan
1
Dec
335
Nov
D
30
25
20
15
10
1904–1913
1911–1920
1921–1930
1931–1940
1941–1950
1951–1960
1961–1970
1971–1980
1981–1990
1991–2000
2003–2012

10 year Summer Teletherm dynamics for WILBUR, WA:

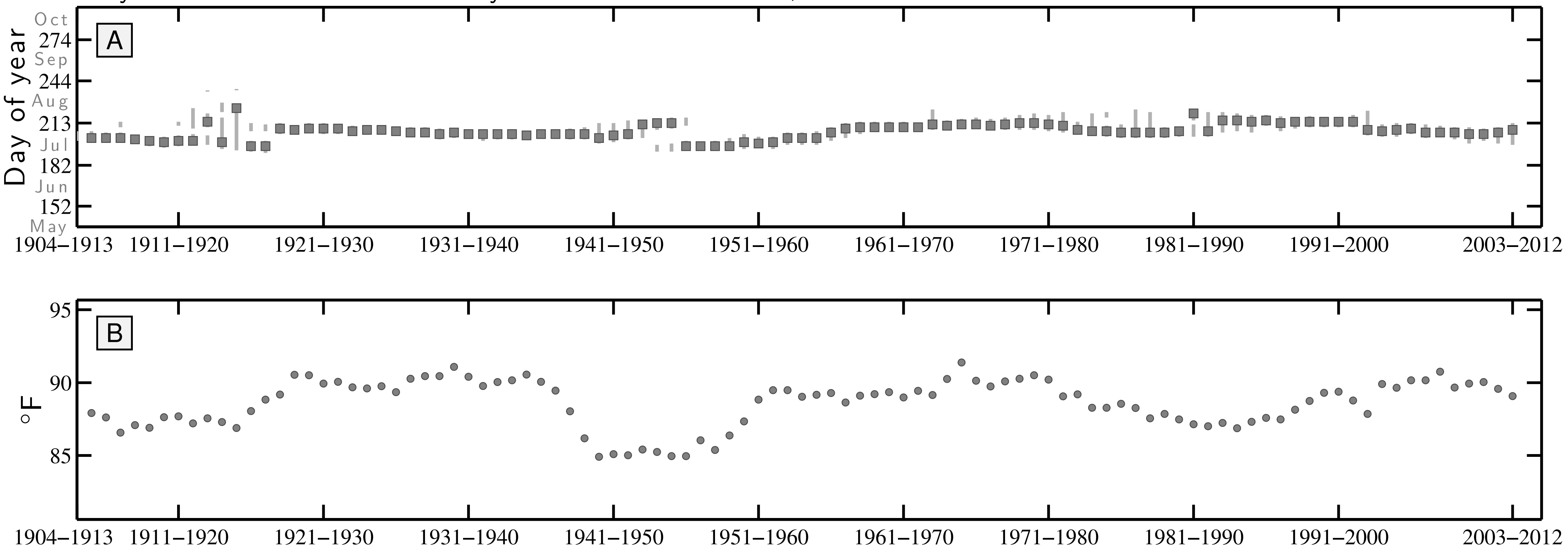

10 year Winter Teletherm dynamics for WILBUR, WA:

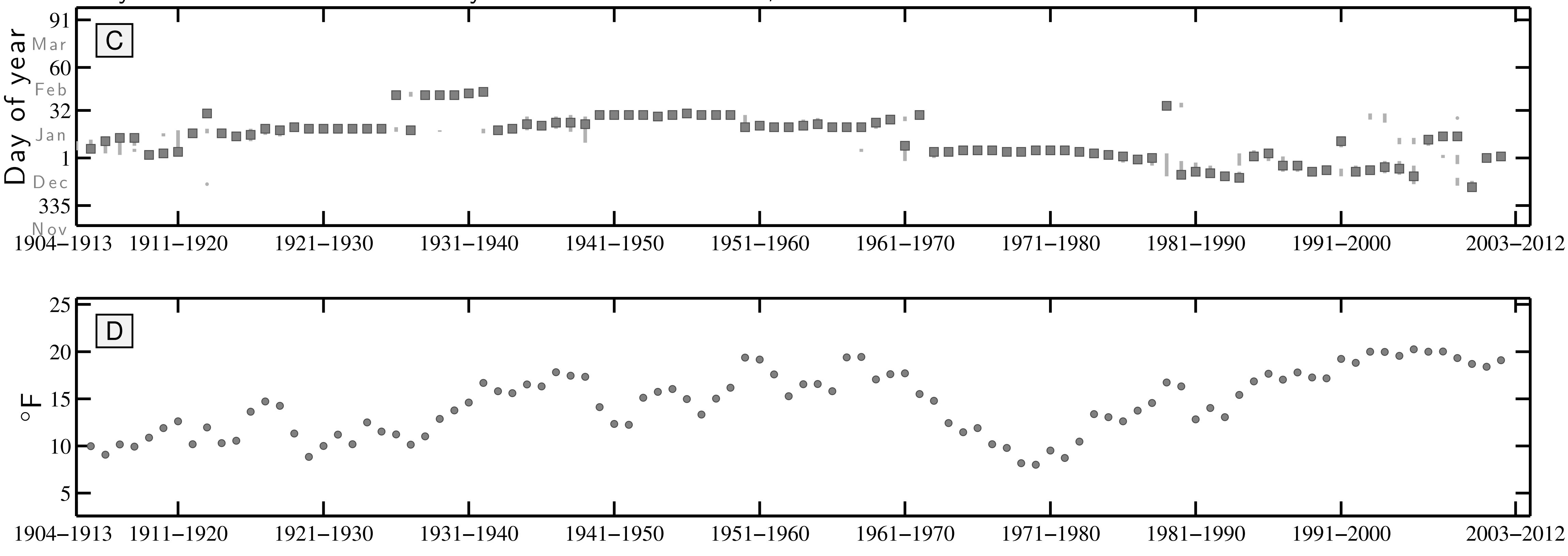

10 year Summer Teletherm dynamics for WINTHROP 1 WSW, WA:

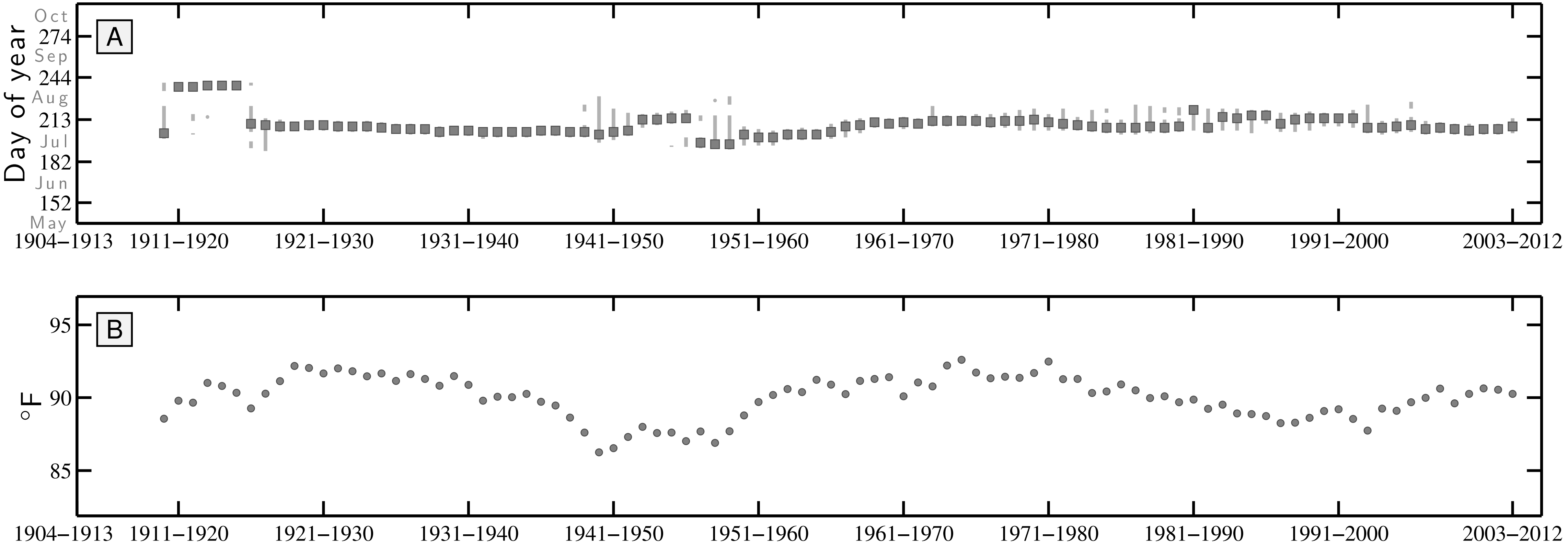

10 year Winter Teletherm dynamics for WINTHROP 1 WSW, WA:

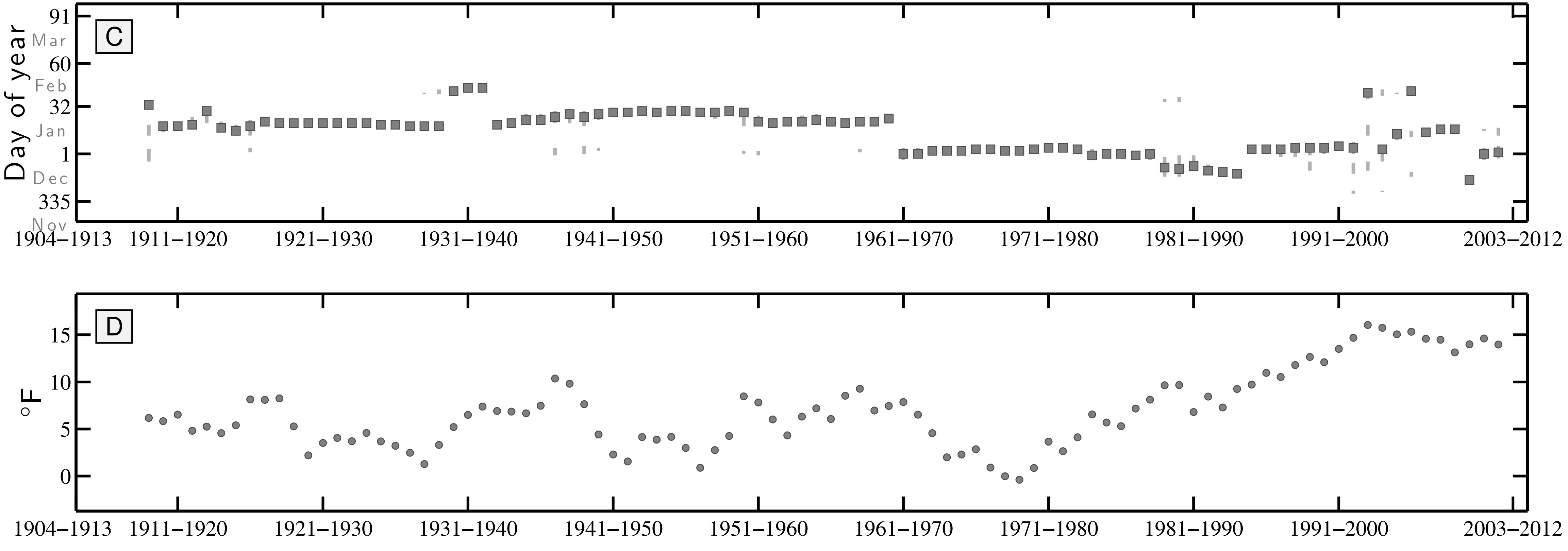

10 year Summer Teletherm dynamics for BUCKHANNON, WV:

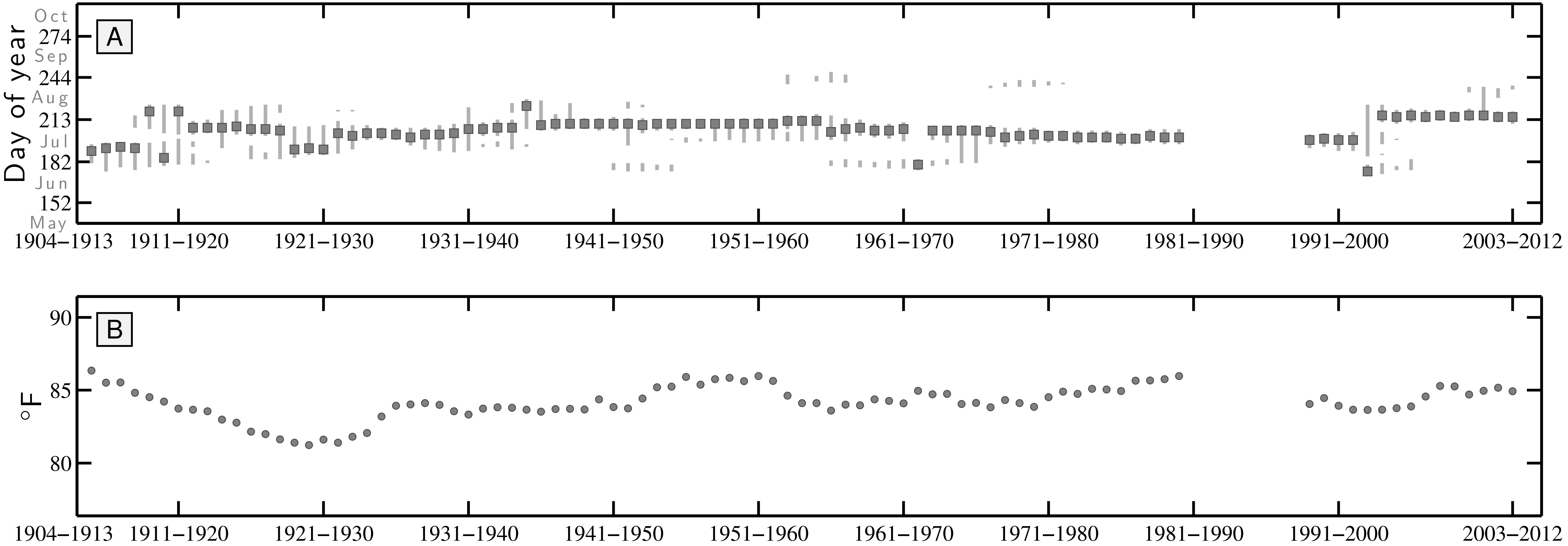

10 year Winter Teletherm dynamics for BUCKHANNON, WV:

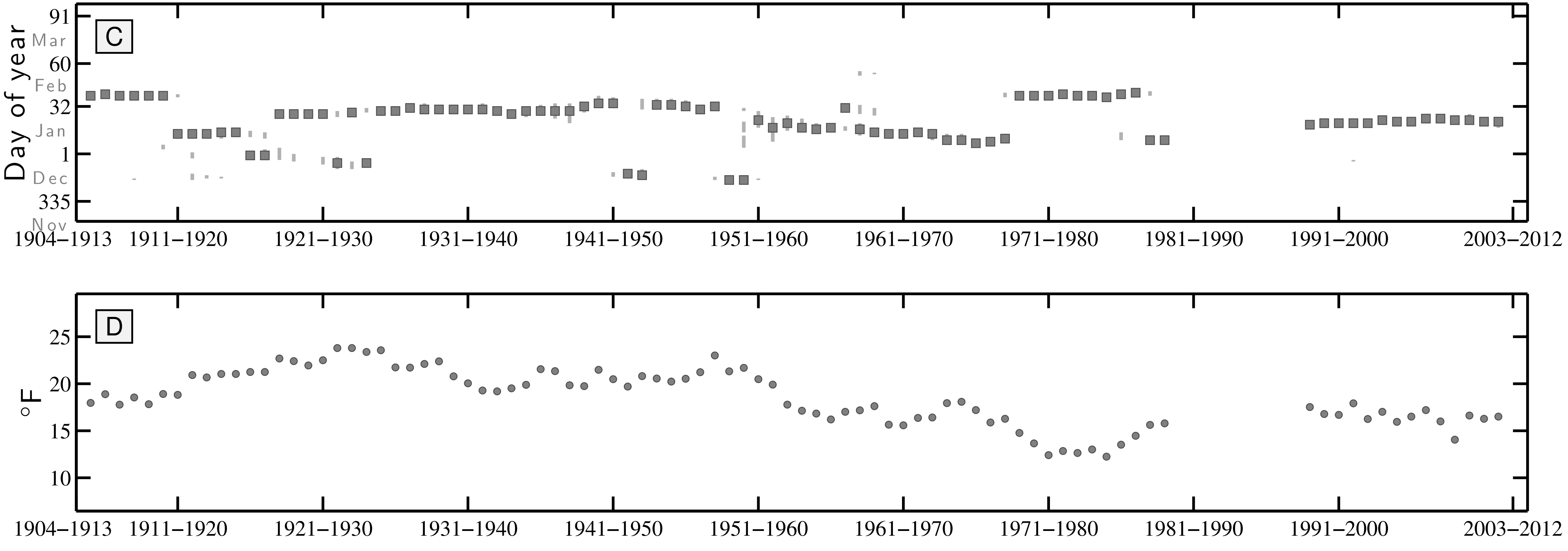

10 year Summer Teletherm dynamics for CAIRO, WV:

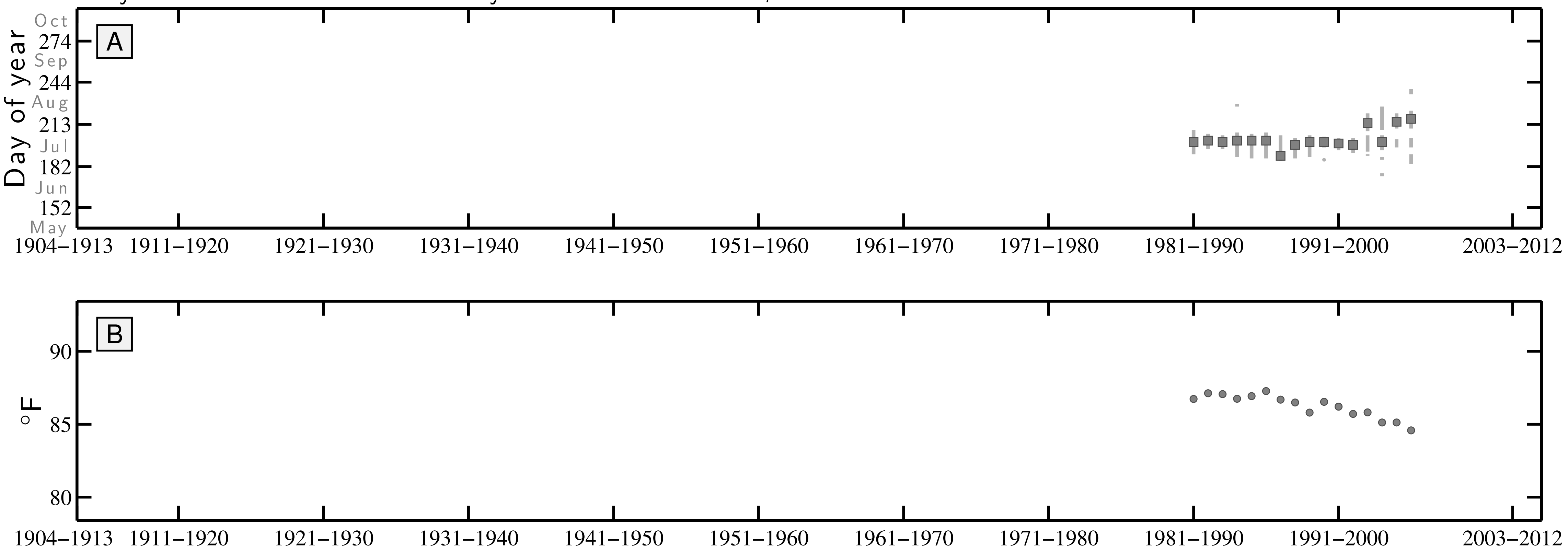

10 year Winter Teletherm dynamics for CAIRO, WV:

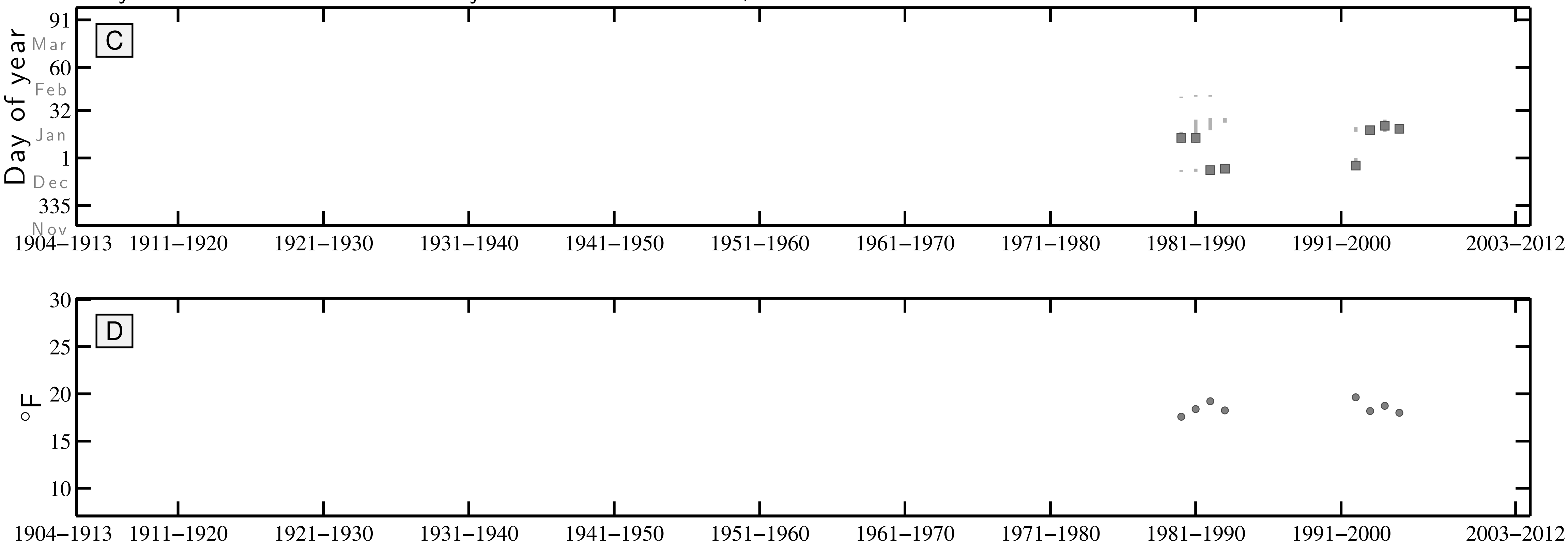

10 year Summer Teletherm dynamics for GLENVILLE, WV:

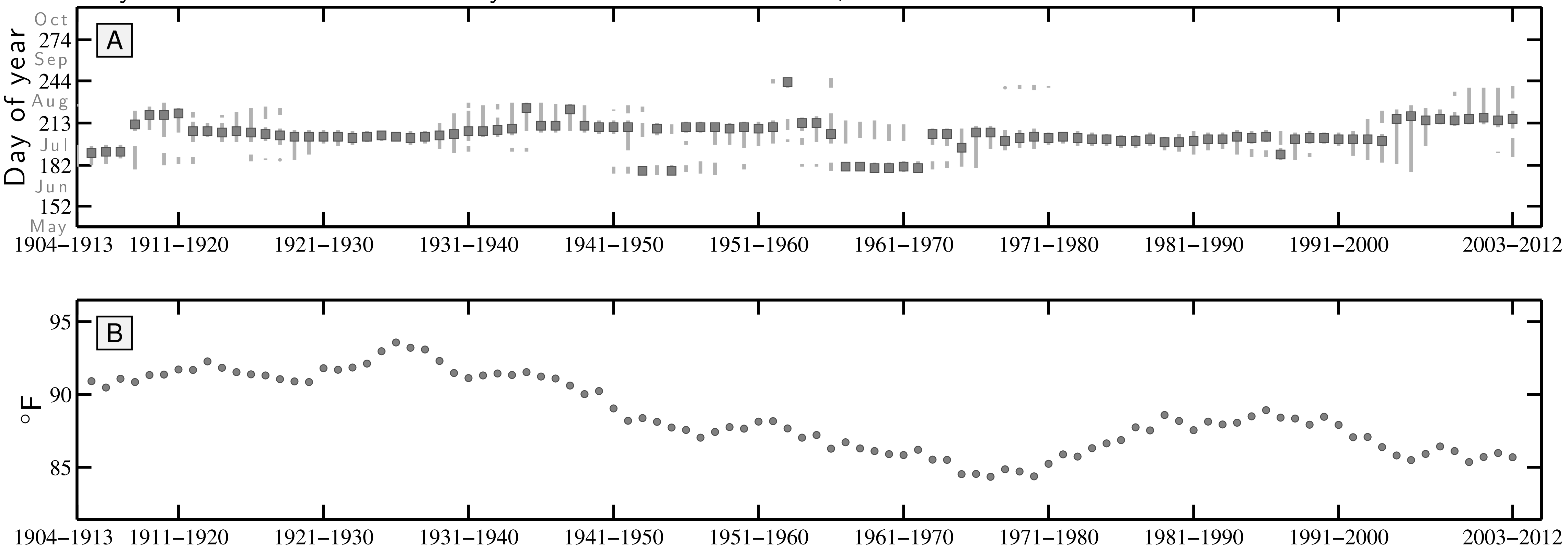

10 year Winter Teletherm dynamics for GLENVILLE, WV:

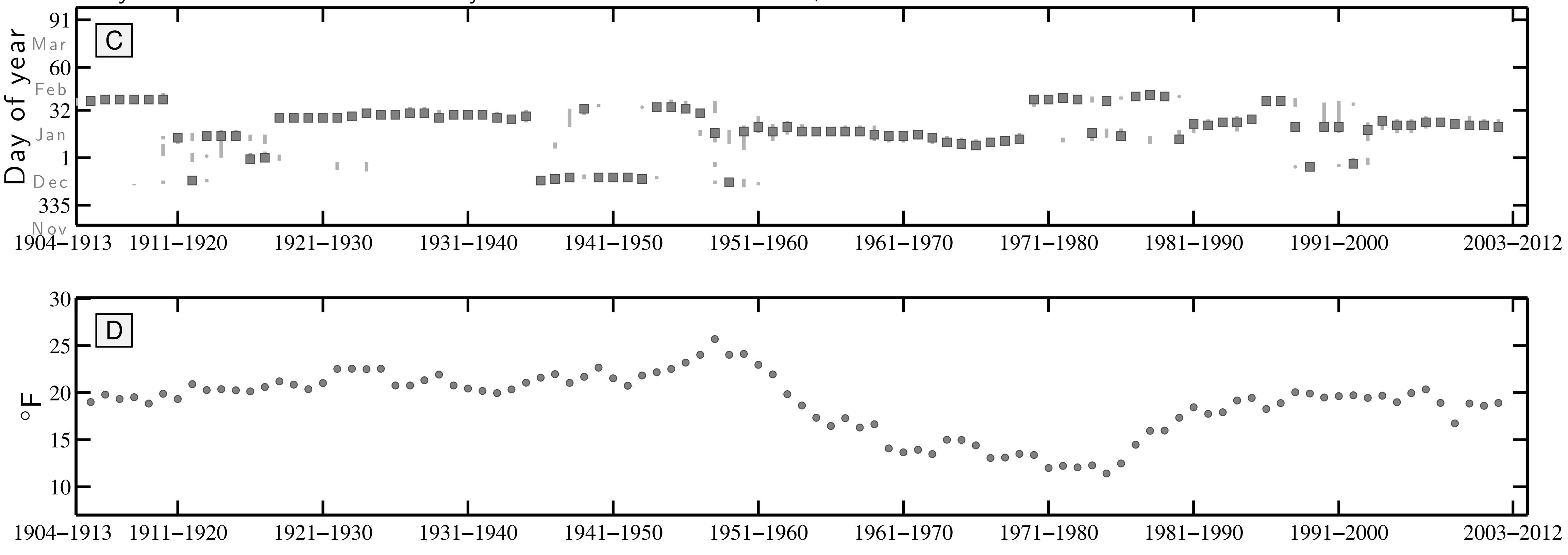

10 year Summer Teletherm dynamics for LEWISBURG 3 N, WV:

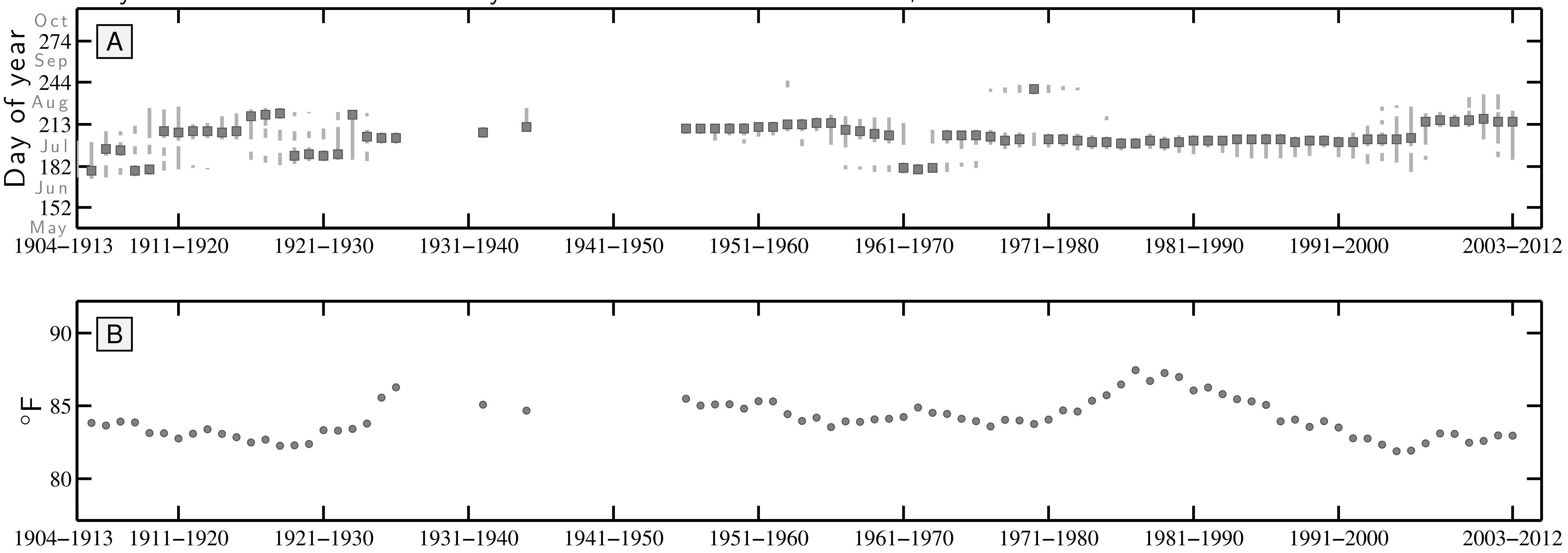

10 year Winter Teletherm dynamics for LEWISBURG 3 N, WV:

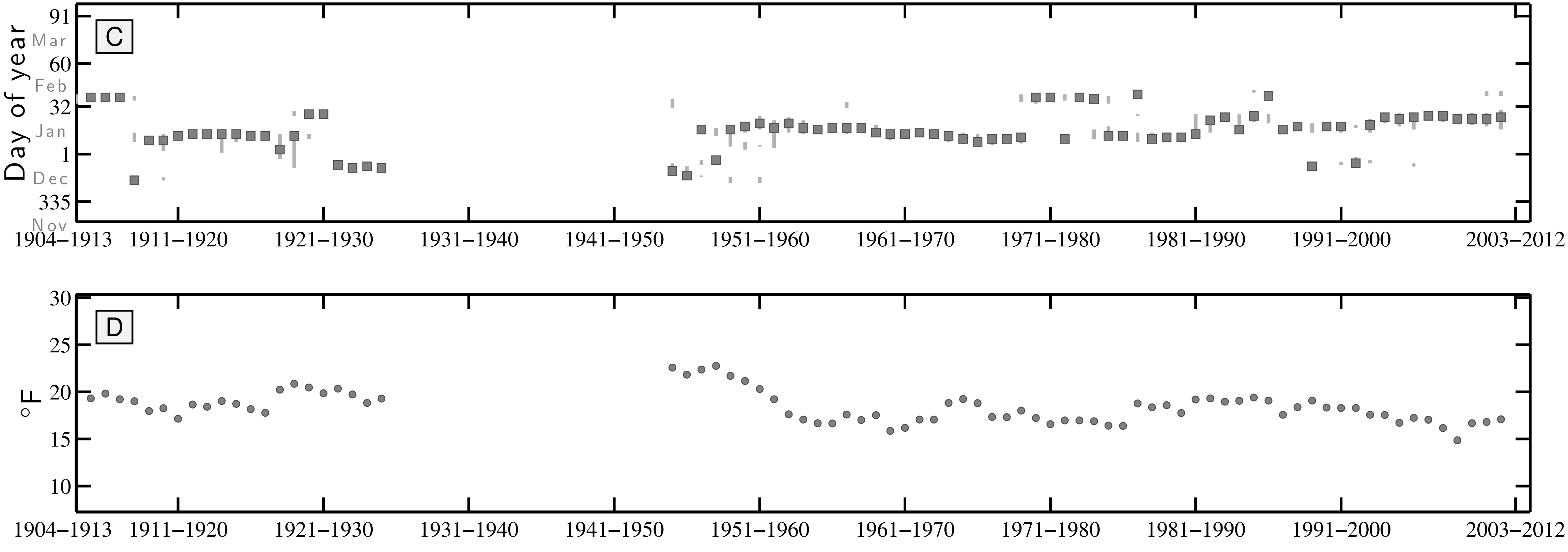

10 year Summer Teletherm dynamics for MANNINGTON 8 WNW, WV:

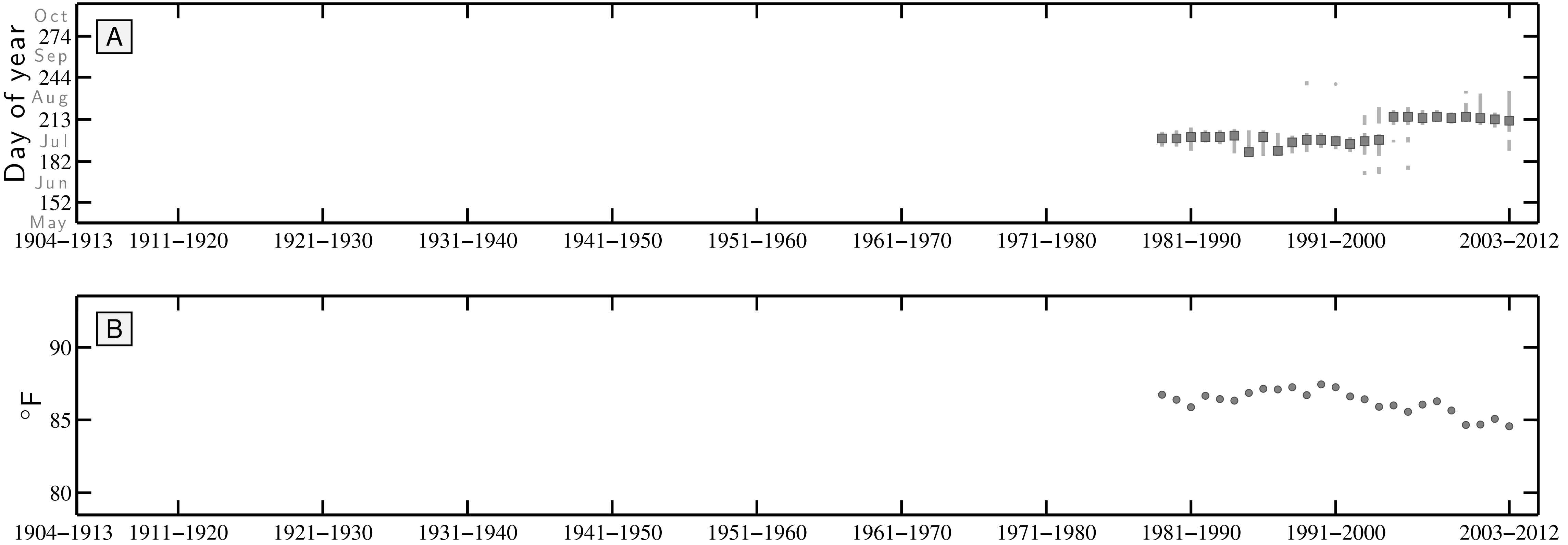

10 year Winter Teletherm dynamics for MANNINGTON 8 WNW, WV:

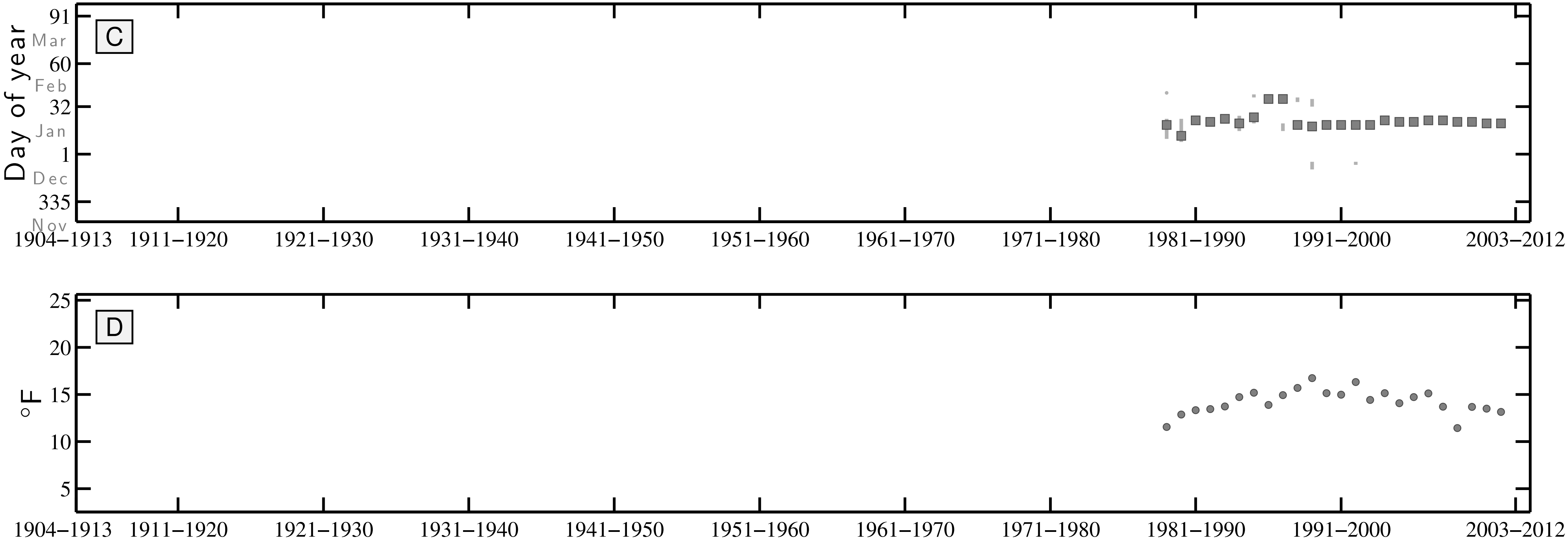

10 year Summer Teletherm dynamics for MARTINSBURG E WV RGNL, WV:

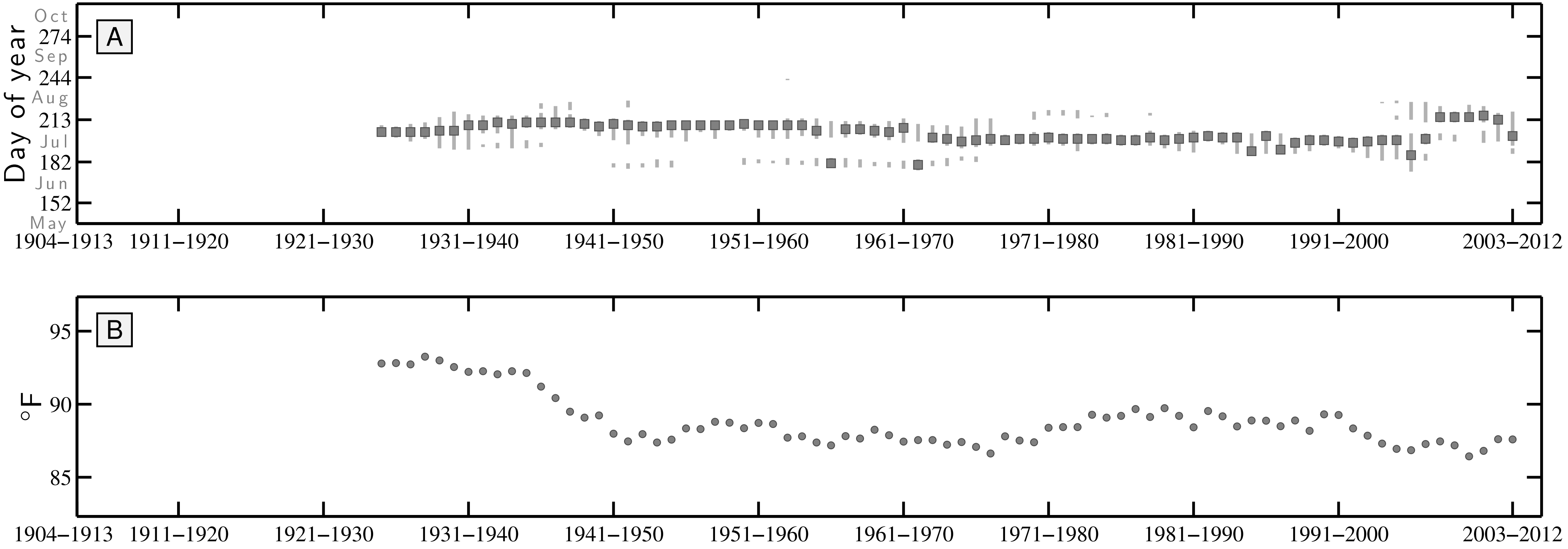

10 year Winter Teletherm dynamics for MARTINSBURG E WV RGNL, WV:

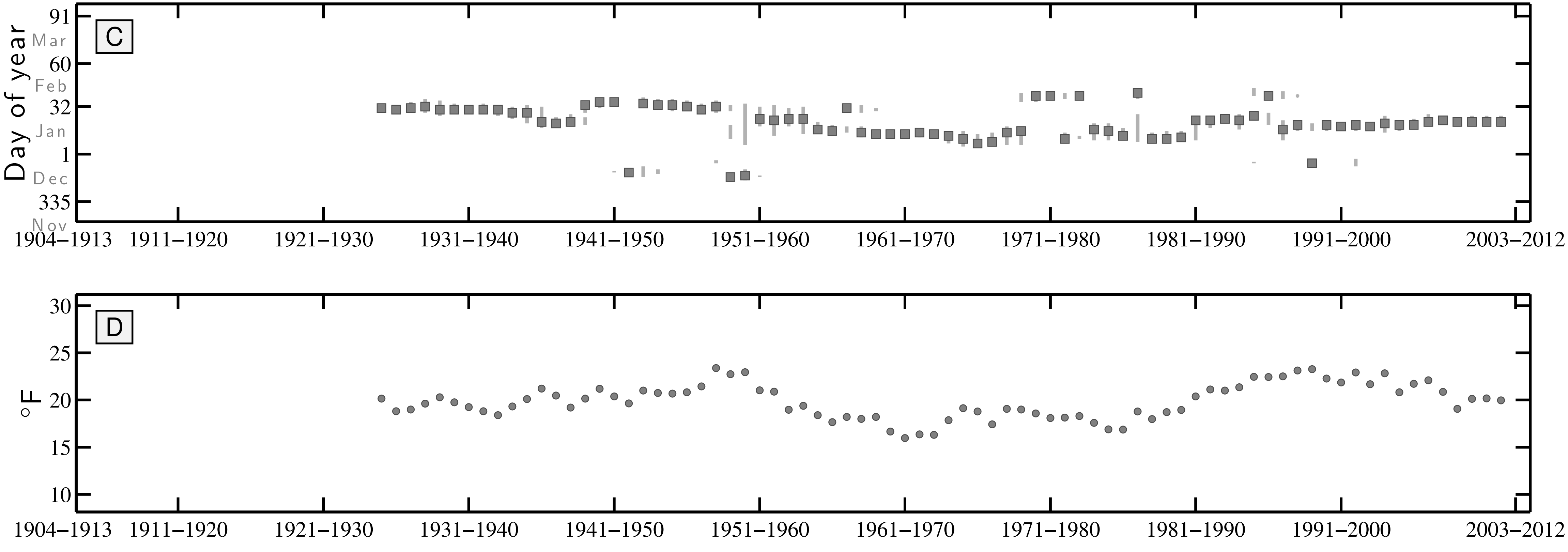

10 year Summer Teletherm dynamics for PARSONS 1 NE, WV:

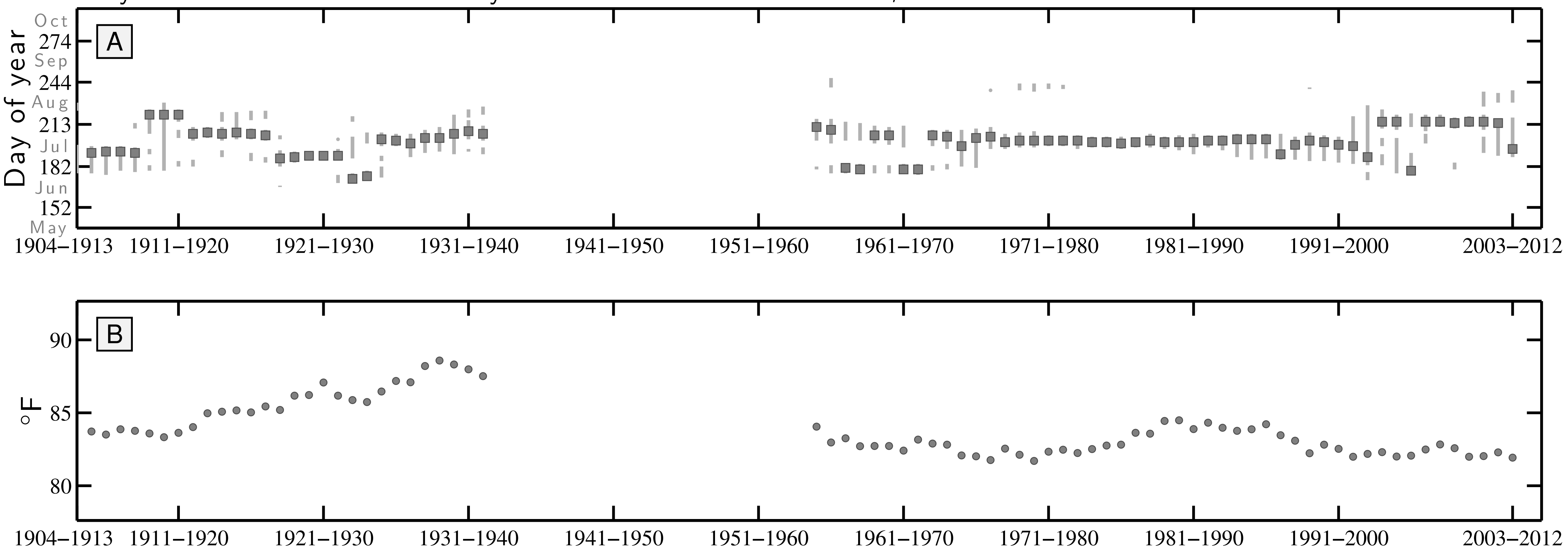

10 year Winter Teletherm dynamics for PARSONS 1 NE, WV:

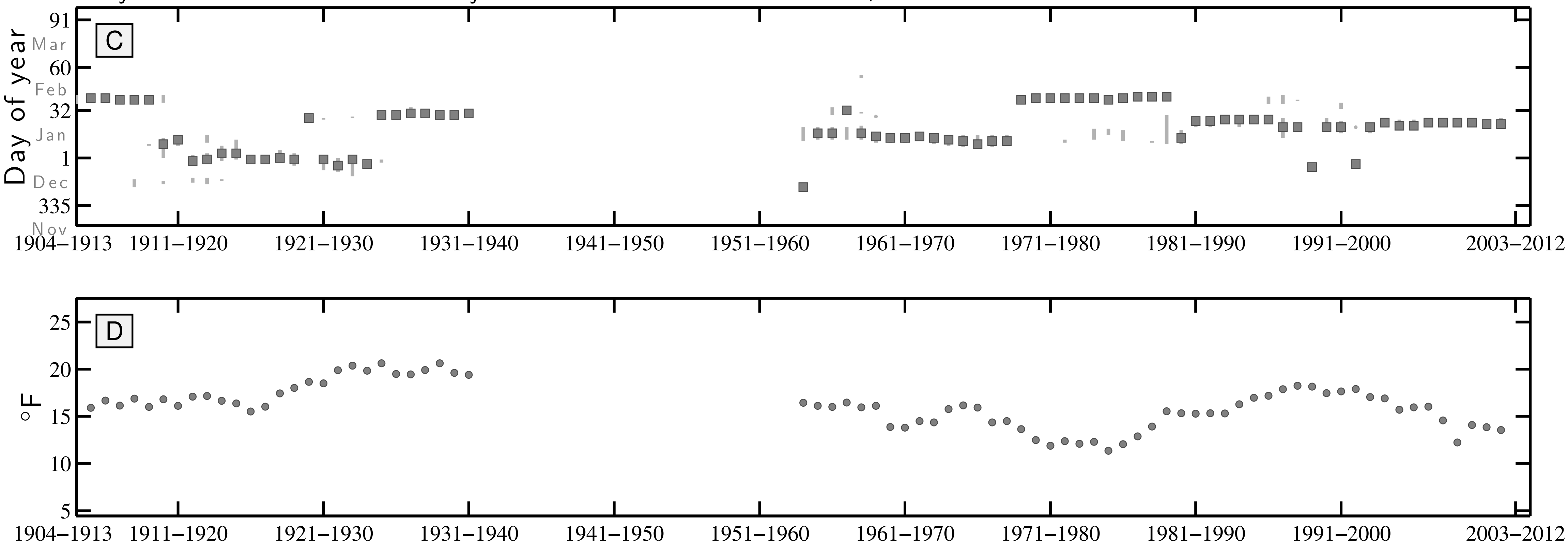

10 year Summer Teletherm dynamics for PICKENS 2 N, WV:

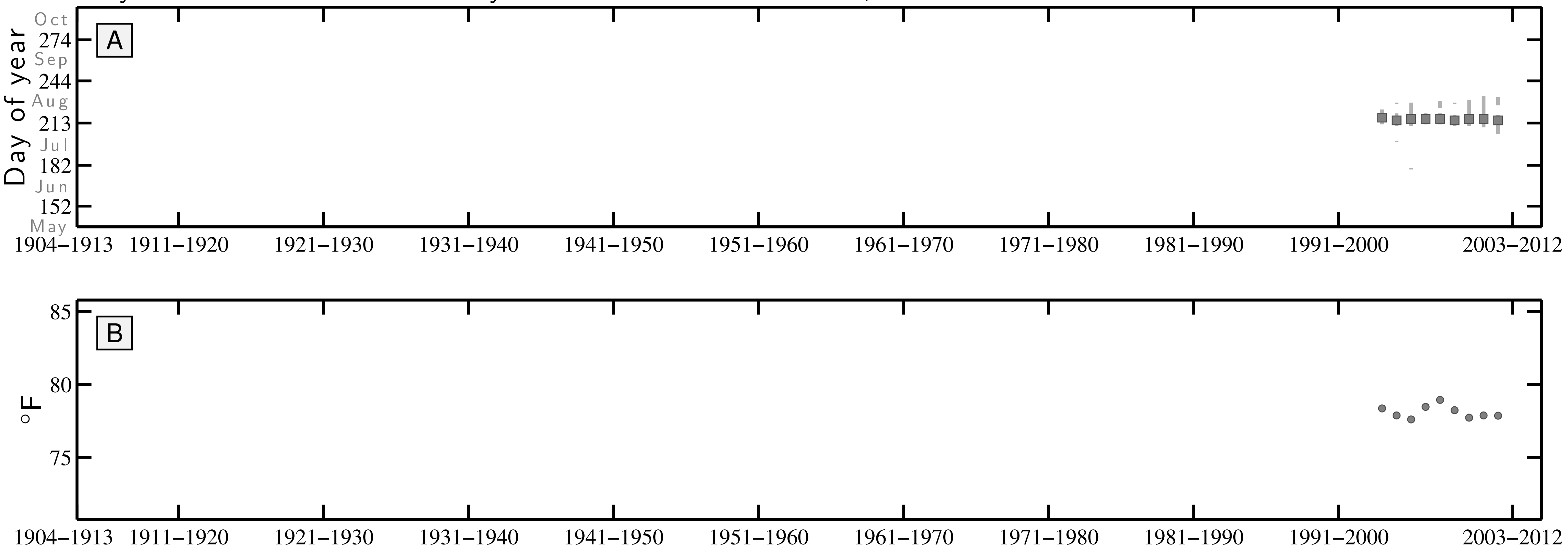

10 year Winter Teletherm dynamics for PICKENS 2 N, WV:

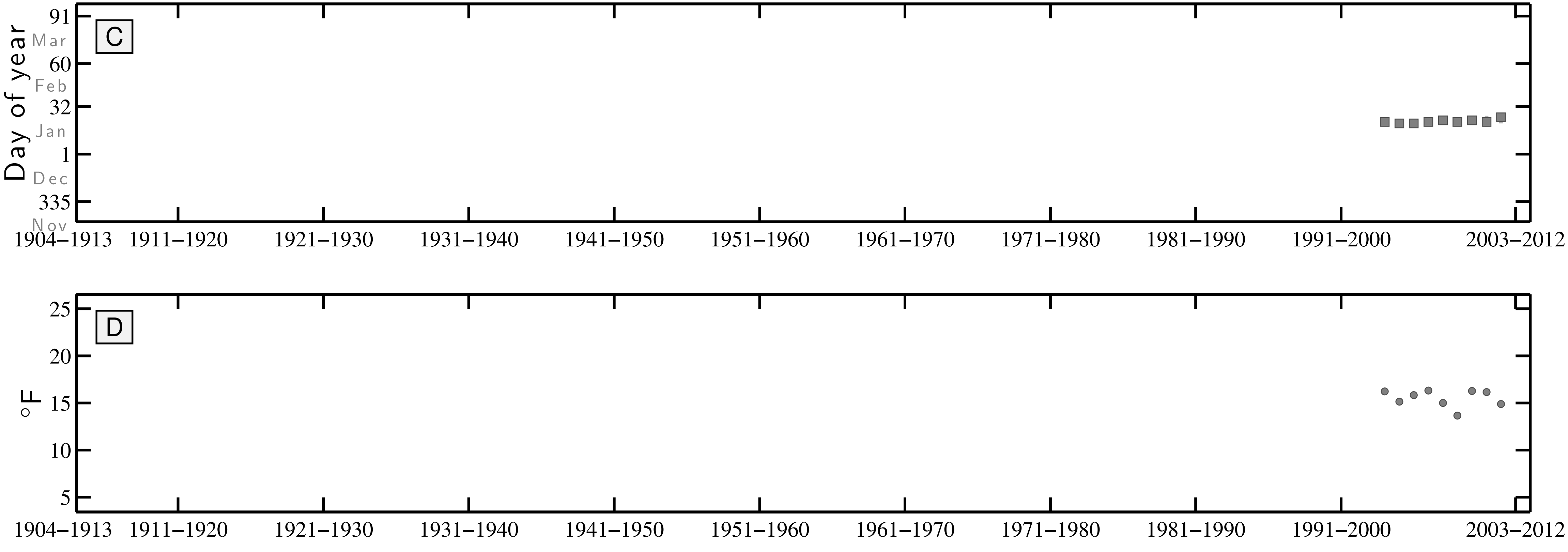

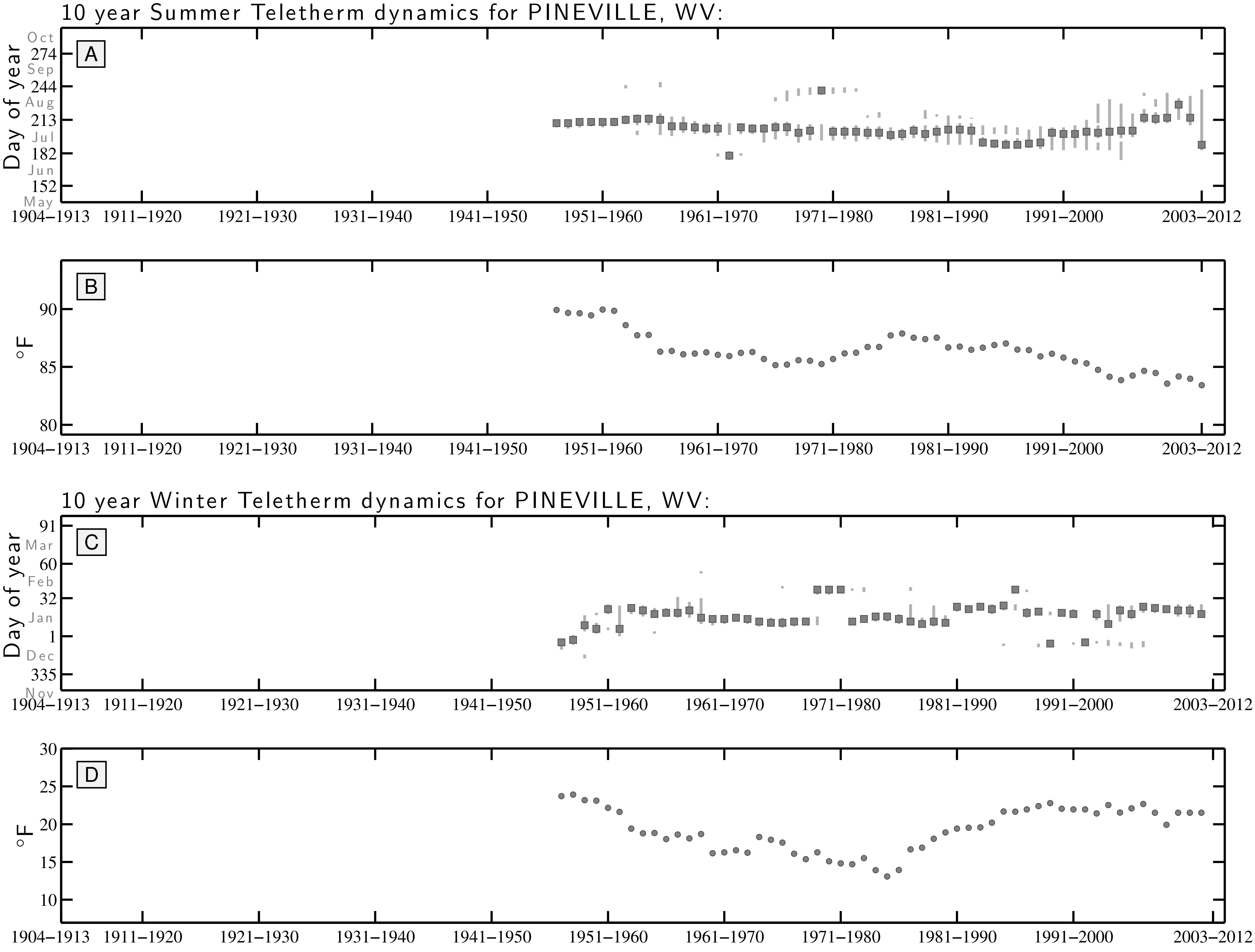
10 year Summer Teletherm dynamics for PINEVILLE, WV:
A
B
10 year Winter Teletherm dynamics for PINEVILLE, WV:
C
D
Day of year
°F
Oct
274
Sep
244
Aug
213
Jul
182
Jun
152
May
90
85
80
91
Mar
60
Feb
32
Jan
1
Dec
335
Nov
30
25
20
15
10
1904–1913
1911–1920
1921–1930
1931–1940
1941–1950
1951–1960
1961–1970
1971–1980
1981–1990
1991–2000
2003–2012

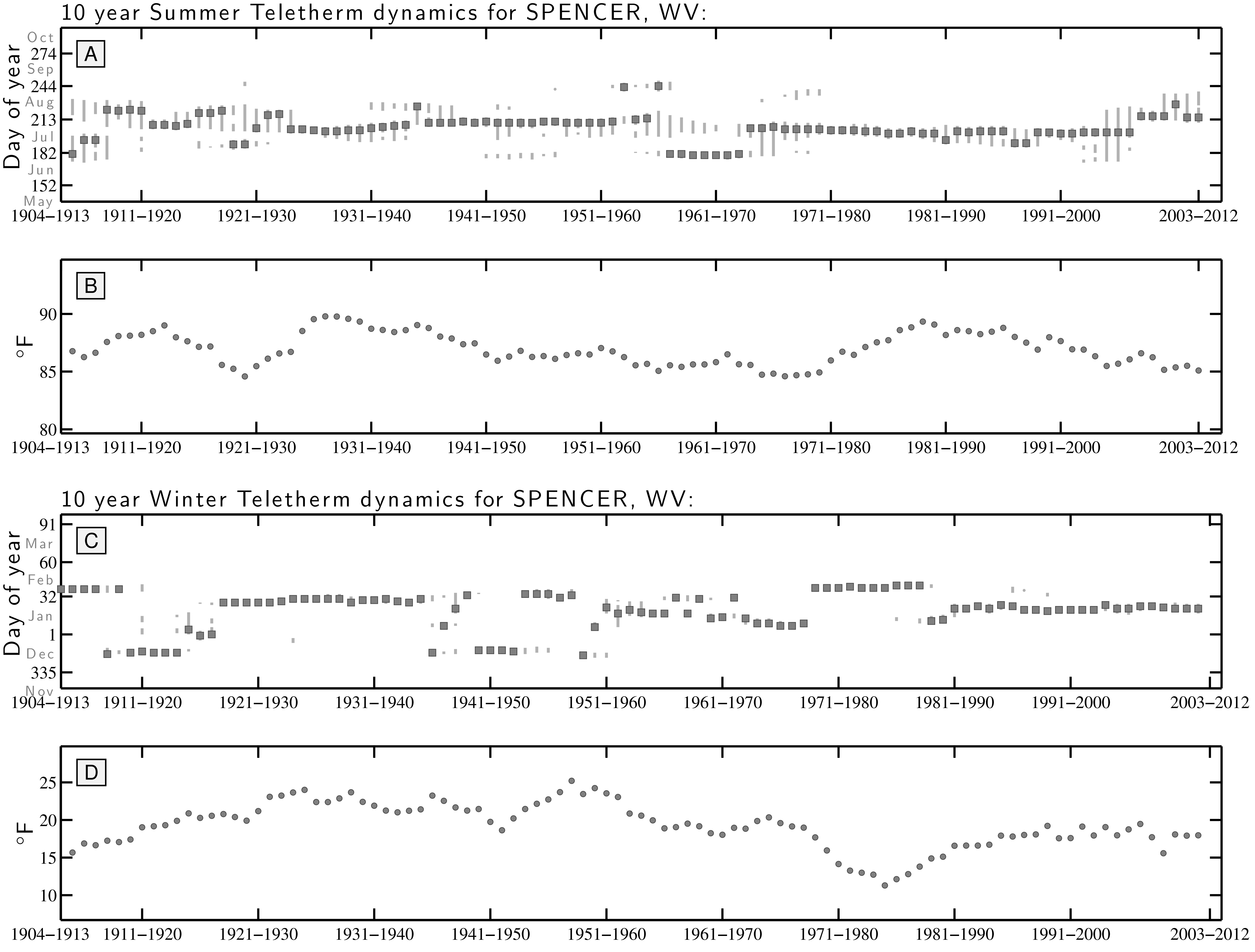
10 year Summer Teletherm dynamics for SPENCER, WV:
A
Day of year
Oct
274
Sep
244
Aug
213
Jul
182
Jun
152
May
1904–1913
1911–1920
1921–1930
1931–1940
1941–1950
1951–1960
1961–1970
1971–1980
1981–1990
1991–2000
2003–2012
B
°F
90
85
80
10 year Winter Teletherm dynamics for SPENCER, WV:
C
91
Mar
60
Feb
32
Jan
1
Dec
335
Nov
D
25
20
15
10

10 year Summer Teletherm dynamics for WELLSBURG WTR TRMT PL, WV:

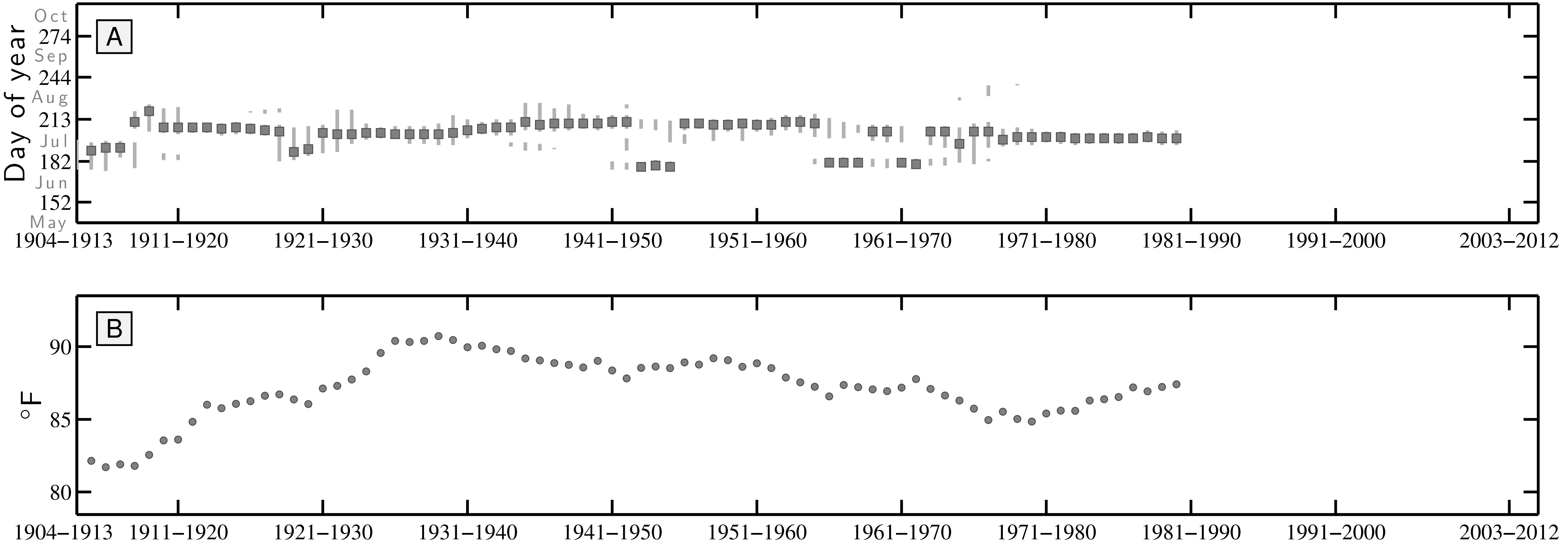

10 year Winter Teletherm dynamics for WELLSBURG WTR TRMT PL, WV:

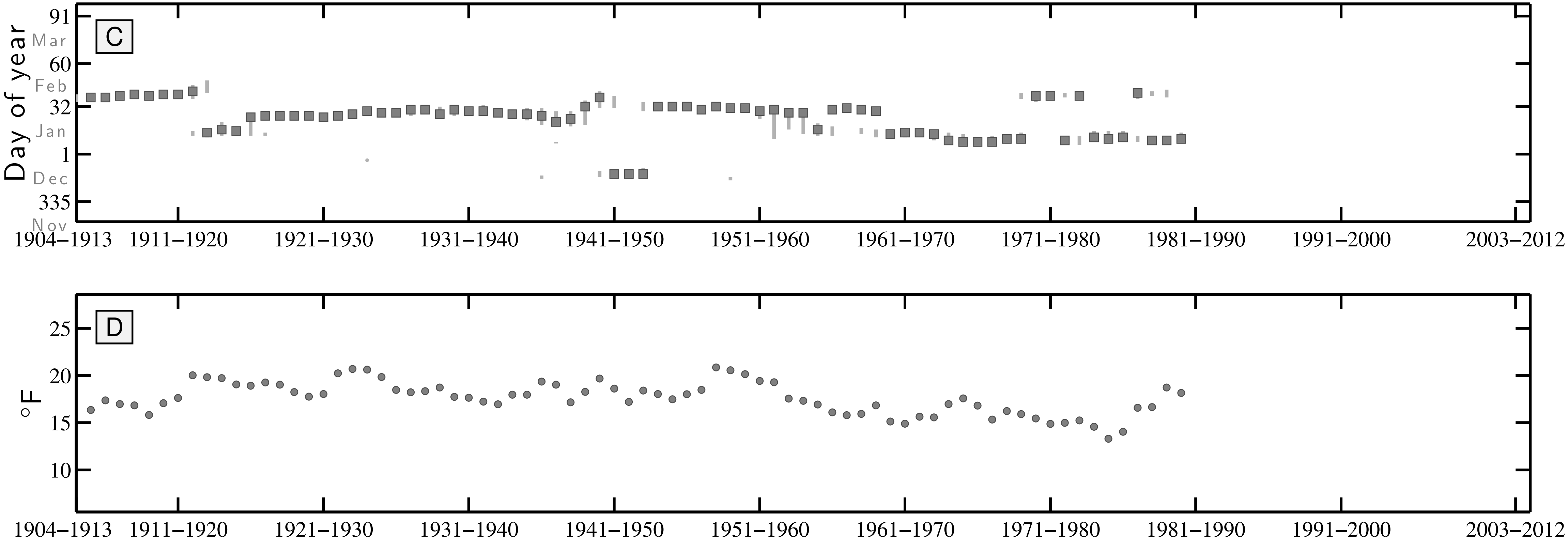

10 year Summer Teletherm dynamics for WILLIAMSON, WV:

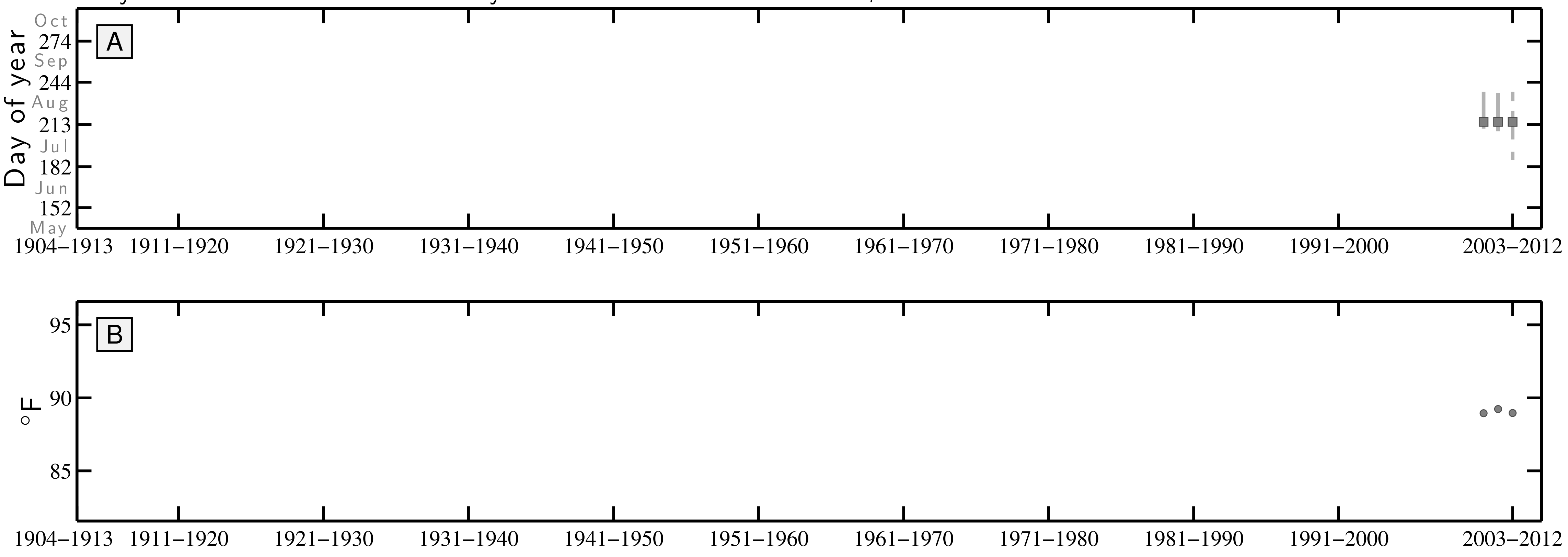

10 year Winter Teletherm dynamics for WILLIAMSON, WV:

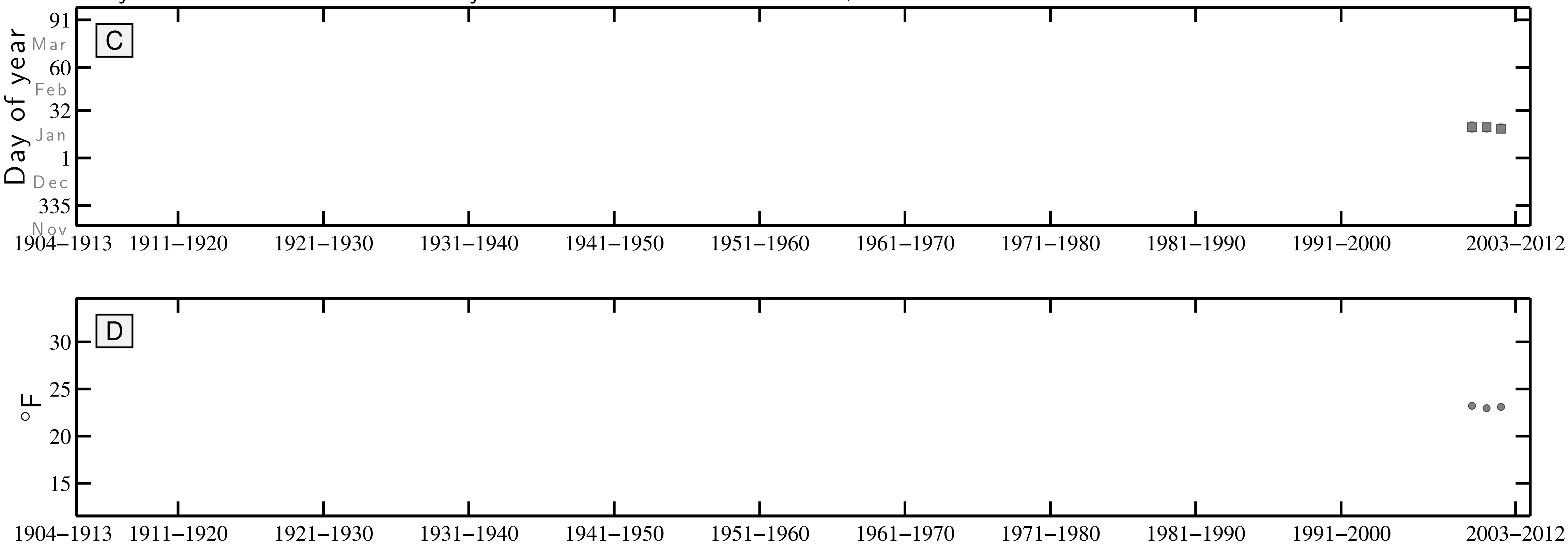

10 year Summer Teletherm dynamics for WINFIELD LOCKS, WV:

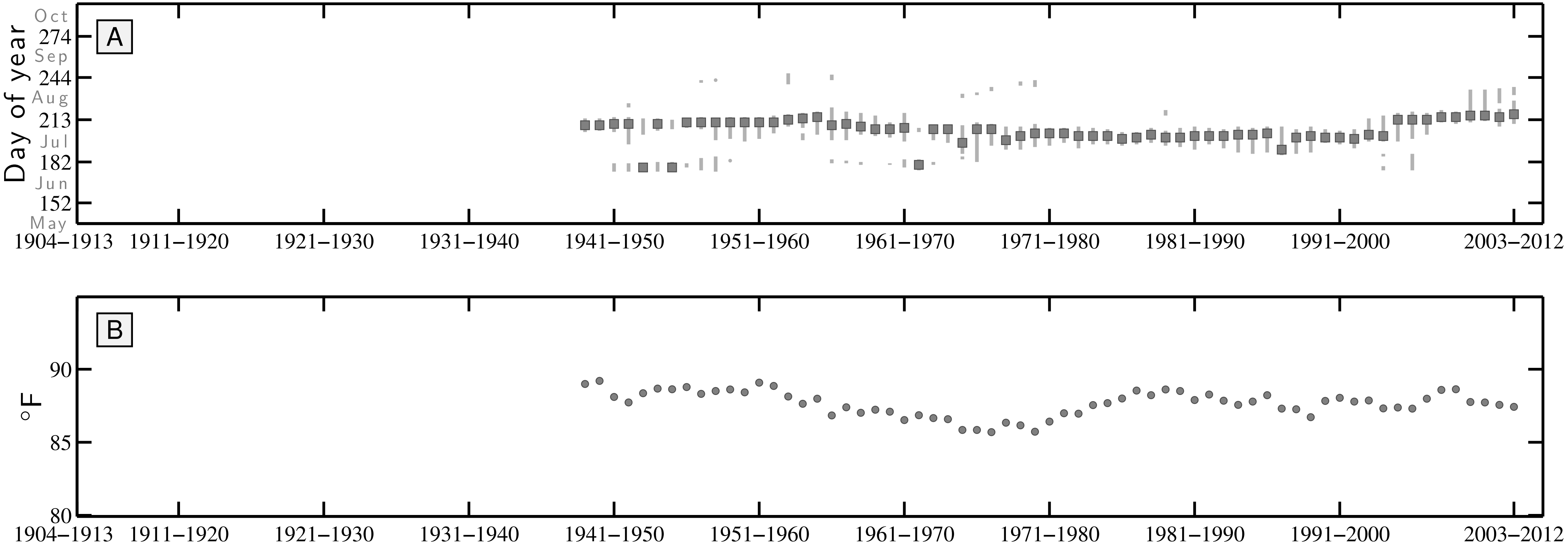

10 year Winter Teletherm dynamics for WINFIELD LOCKS, WV:

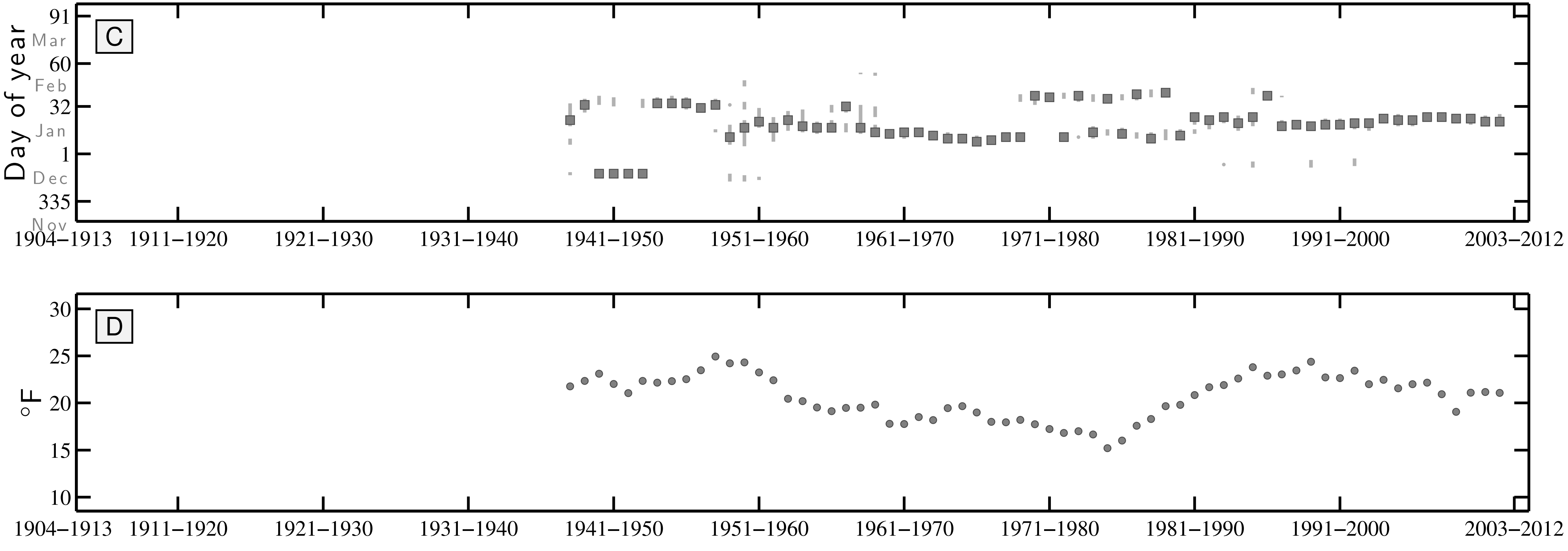

10 year Summer Teletherm dynamics for ASHLAND EXP FARM, WI:

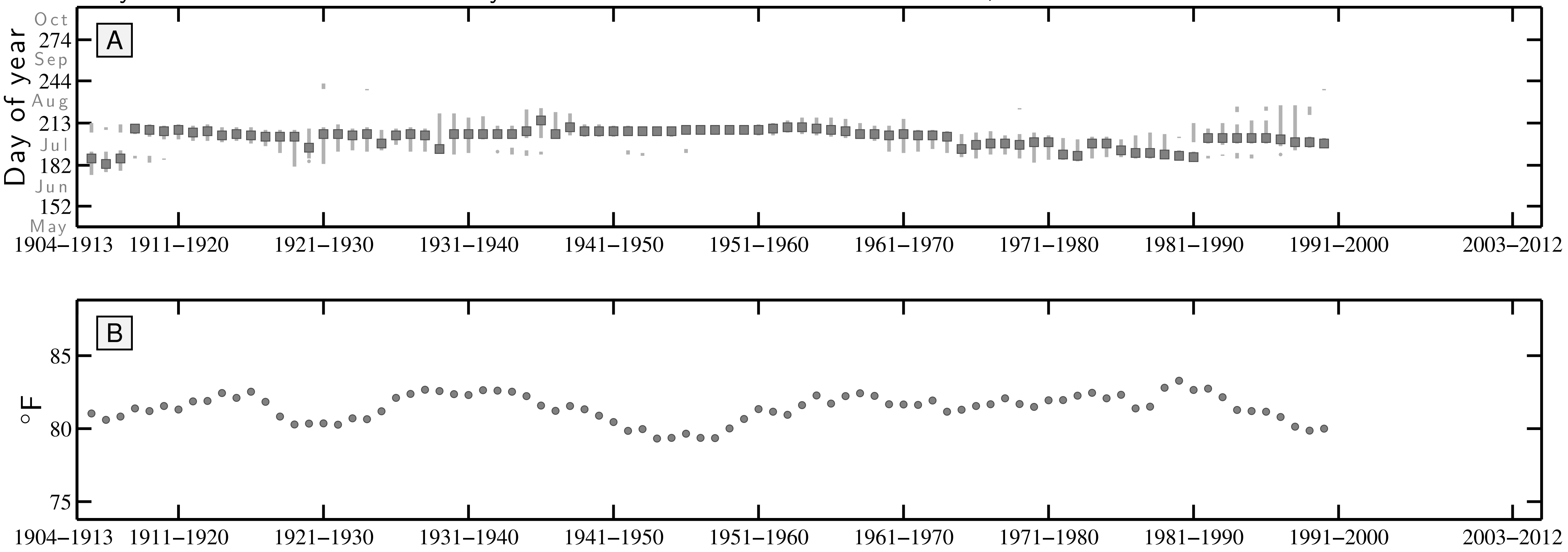

10 year Winter Teletherm dynamics for ASHLAND EXP FARM, WI:

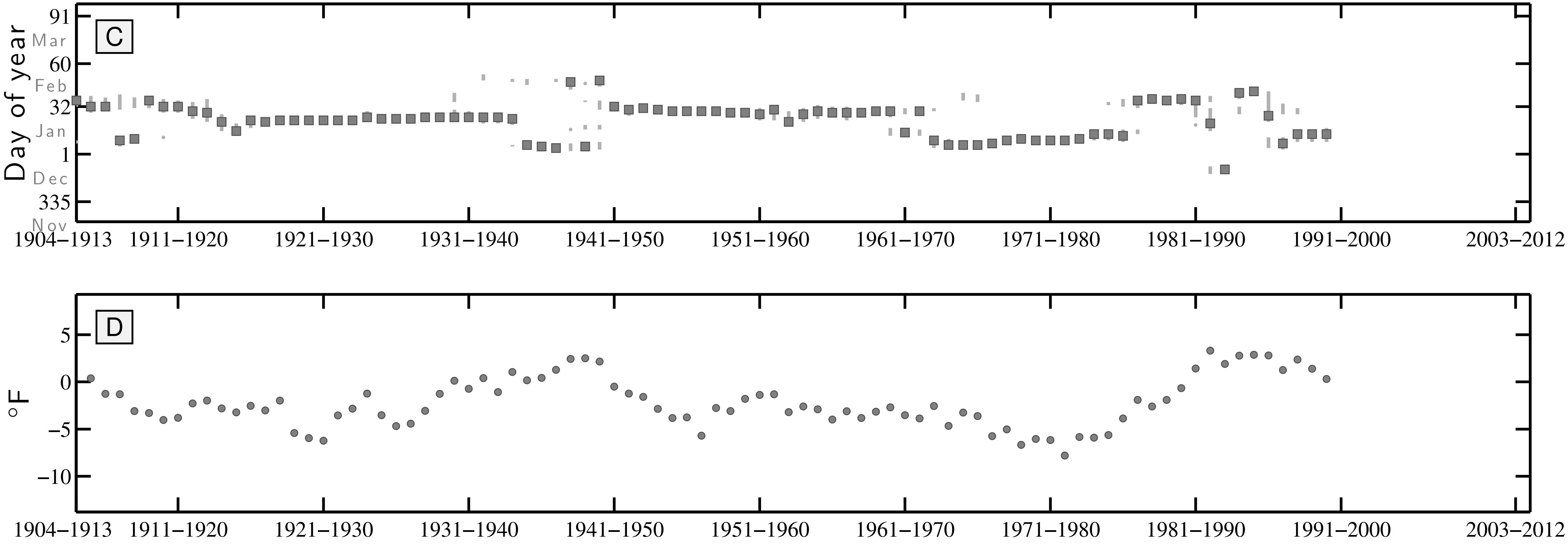

10 year Summer Teletherm dynamics for BOWLER, WI:

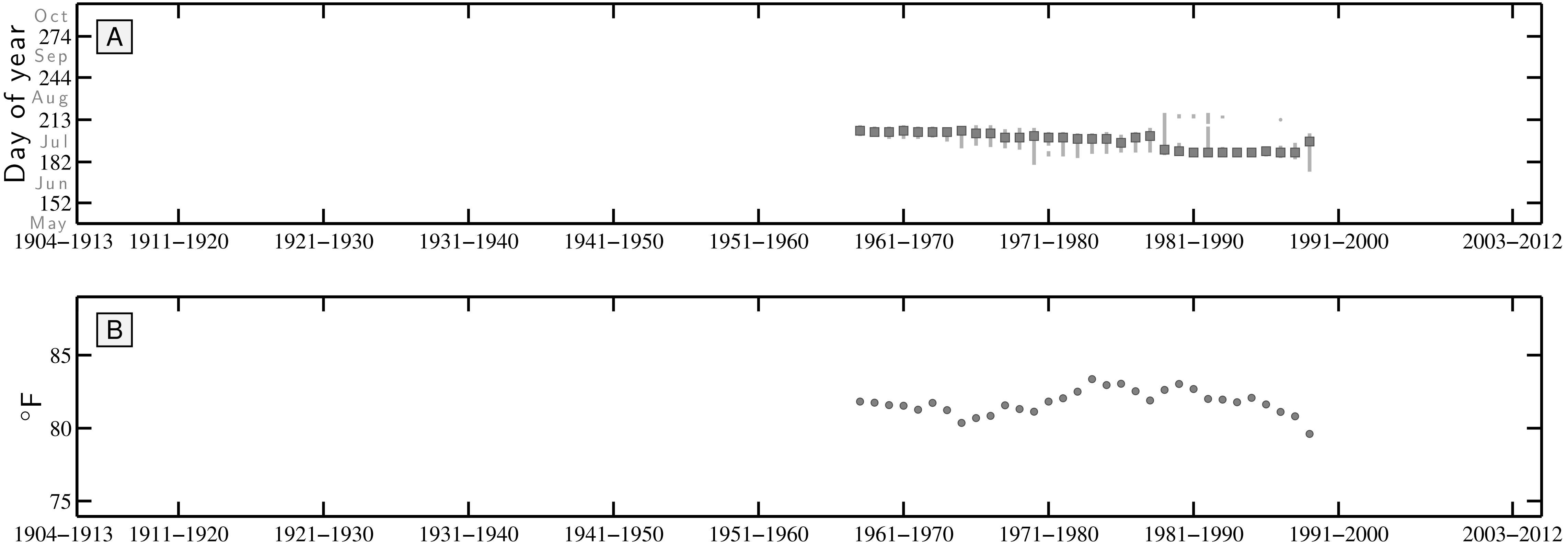

10 year Winter Teletherm dynamics for BOWLER, WI:

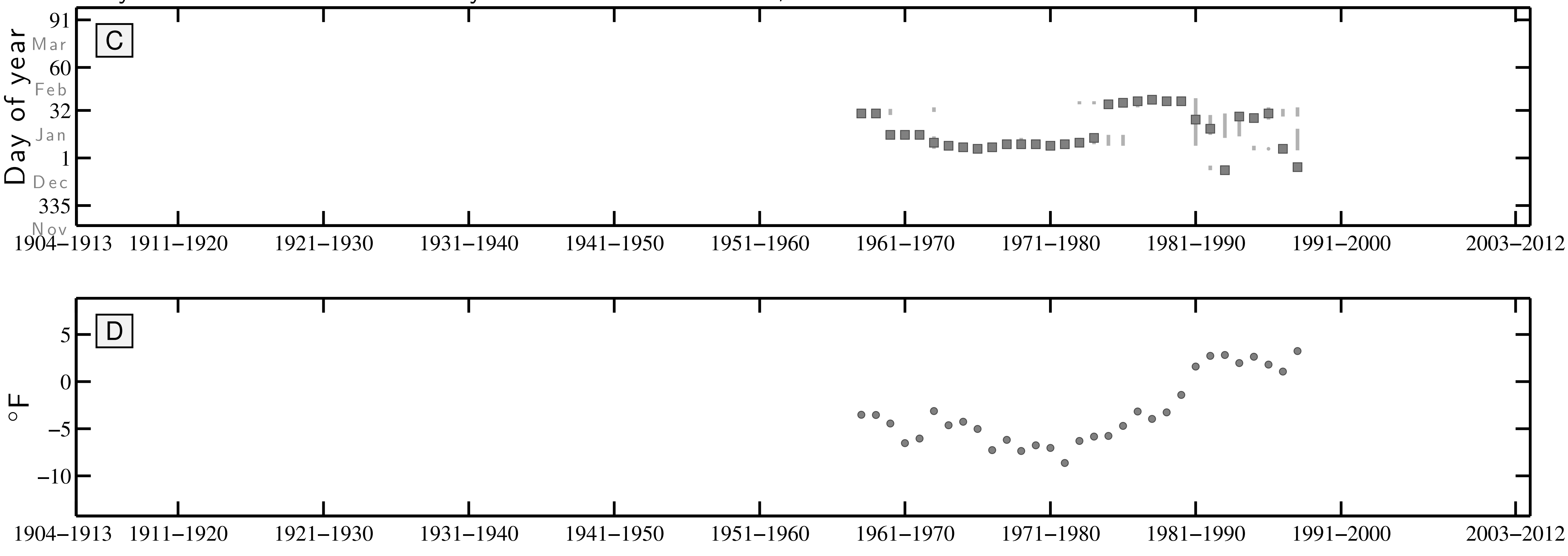

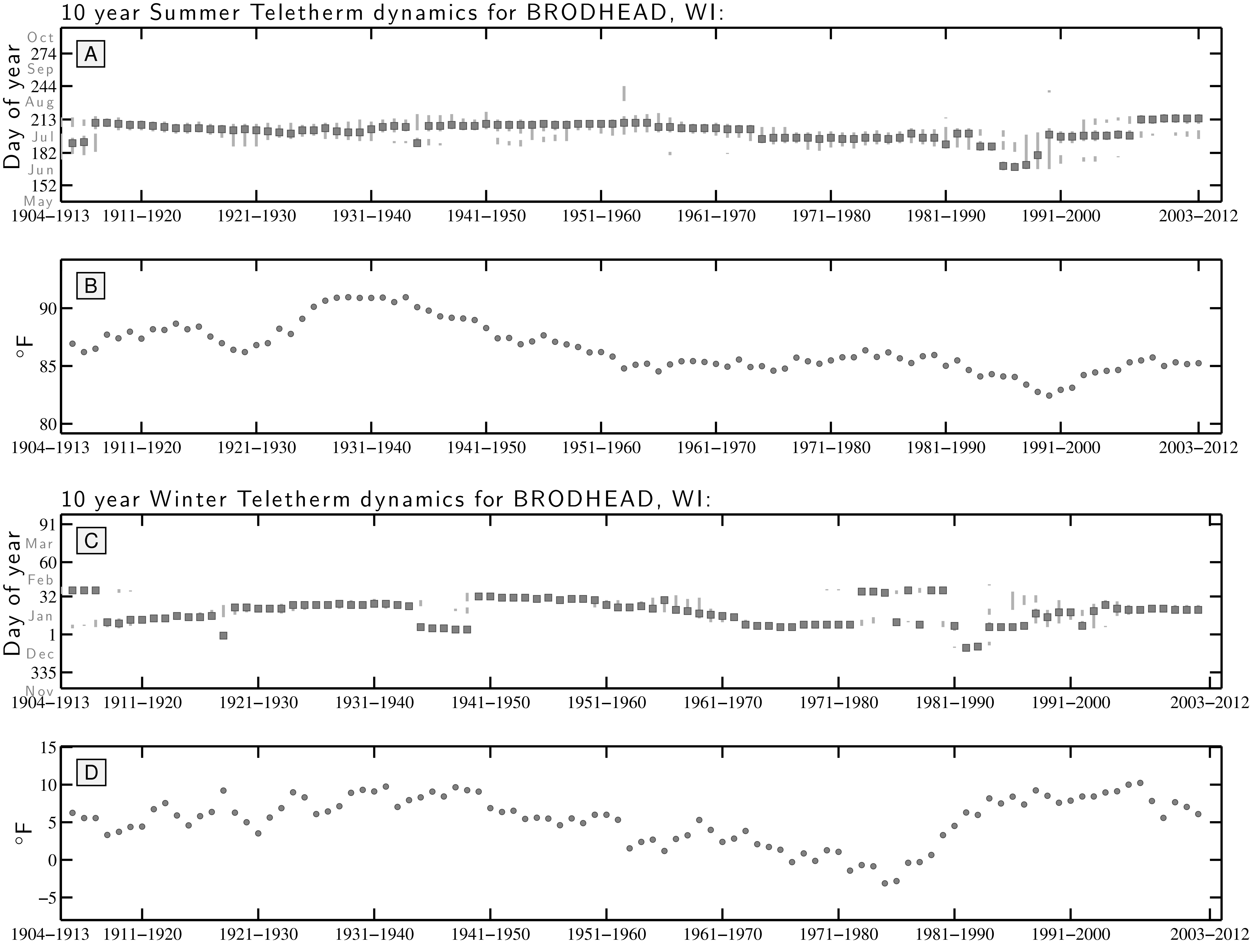
10 year Summer Teletherm dynamics for BRODHEAD, WI:
A
B
Day of year
°F
Oct
274
Sep
244
Aug
213
Jul
182
Jun
152
May
90
85
80
1904–1913
1911–1920
1921–1930
1931–1940
1941–1950
1951–1960
1961–1970
1971–1980
1981–1990
1991–2000
2003–2012
10 year Winter Teletherm dynamics for BRODHEAD, WI:
C
D
91
Mar
60
Feb
32
Jan
1
Dec
335
Nov
15
10
5
0
−5

10 year Summer Teletherm dynamics for DARLINGTON, WI:

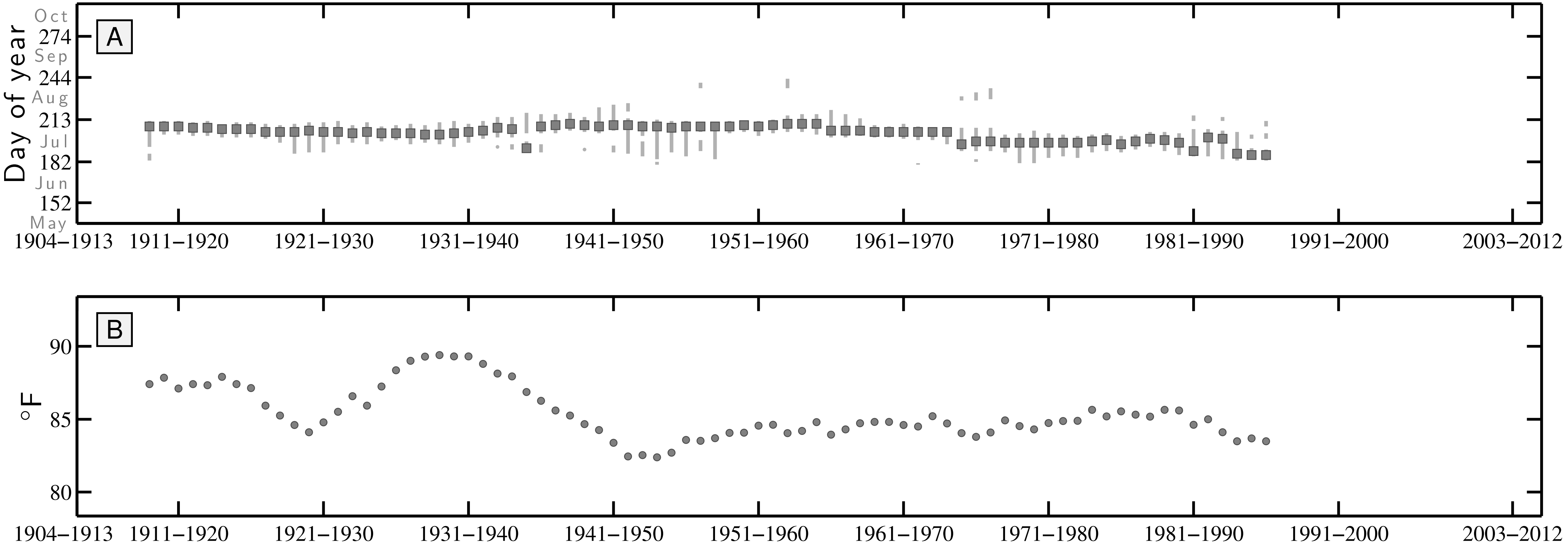

10 year Winter Teletherm dynamics for DARLINGTON, WI:

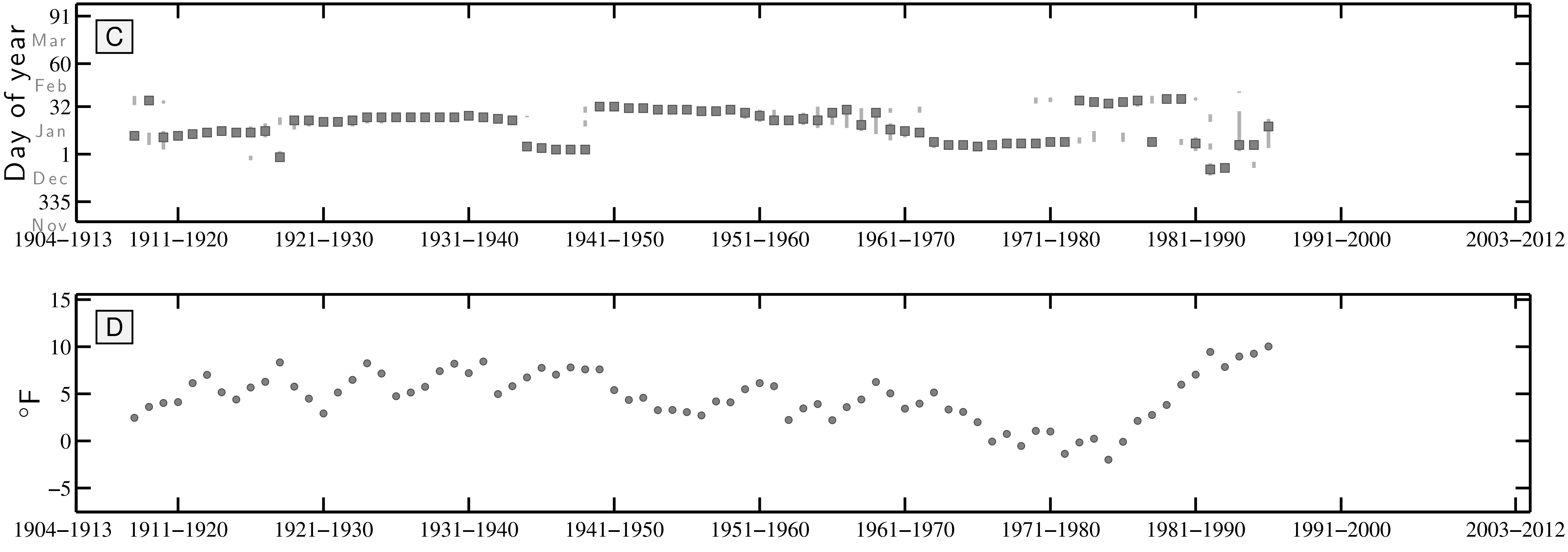

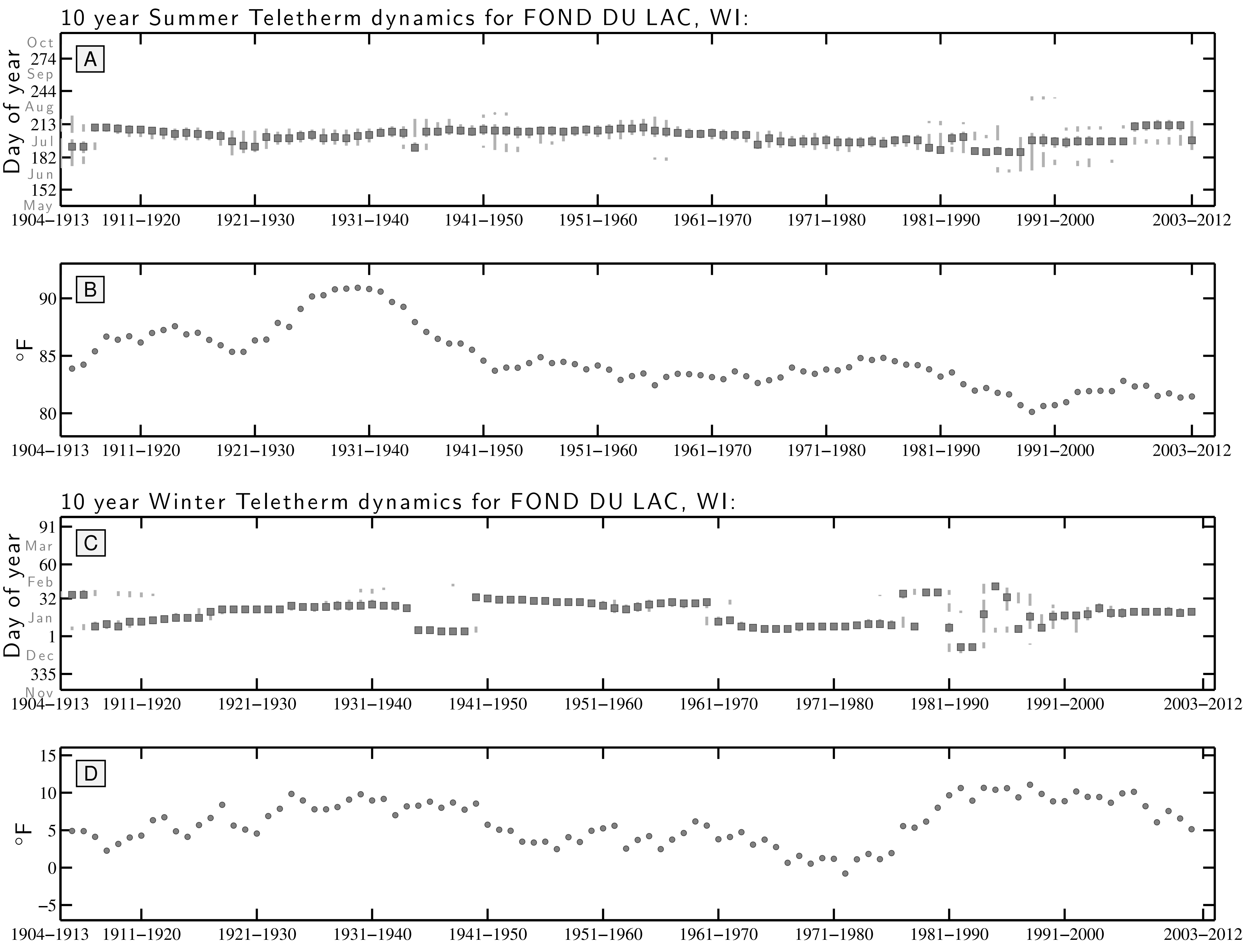
10 year Summer Teletherm dynamics for FOND DU LAC, WI:
A
Day of year
Oct
274
Sep
244
Aug
213
Jul
182
Jun
152
May
B
°F
90
85
80
1904–1913
1911–1920
1921–1930
1931–1940
1941–1950
1951–1960
1961–1970
1971–1980
1981–1990
1991–2000
2003–2012
10 year Winter Teletherm dynamics for FOND DU LAC, WI:
C
91
Mar
60
Feb
32
Jan
1
Dec
335
Nov
D
15
10
5
0
−5

10 year Summer Teletherm dynamics for HANCOCK EXP FARM, WI:

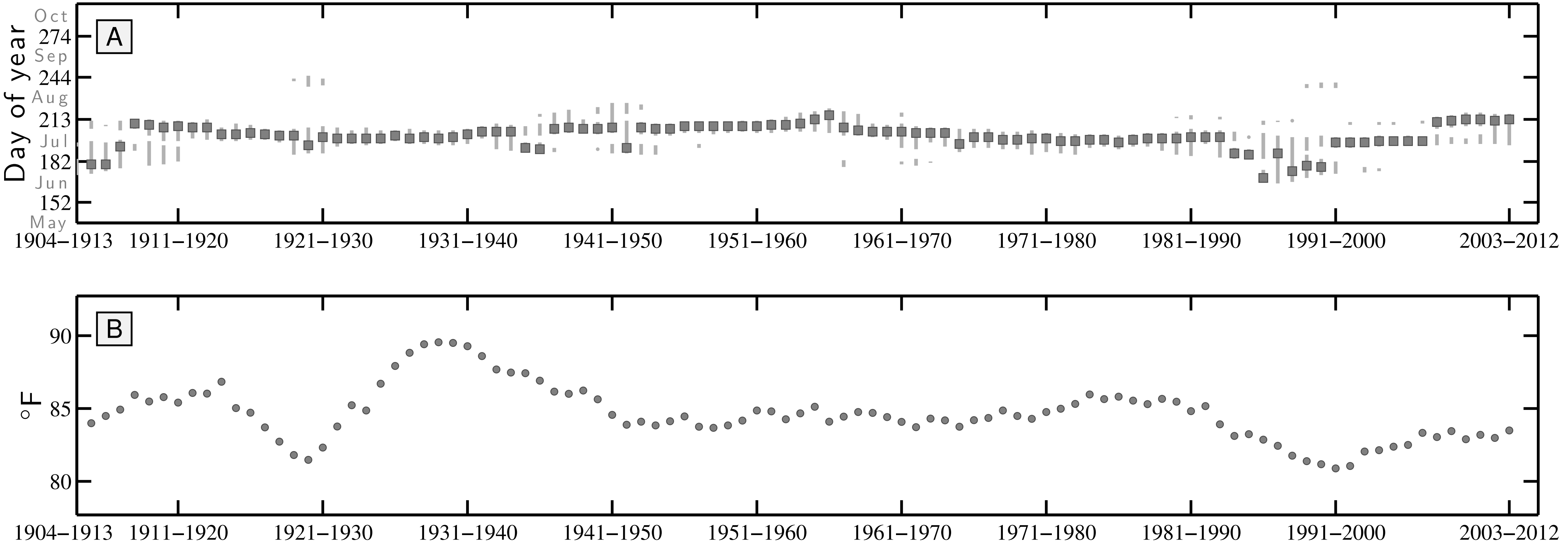

10 year Winter Teletherm dynamics for HANCOCK EXP FARM, WI:

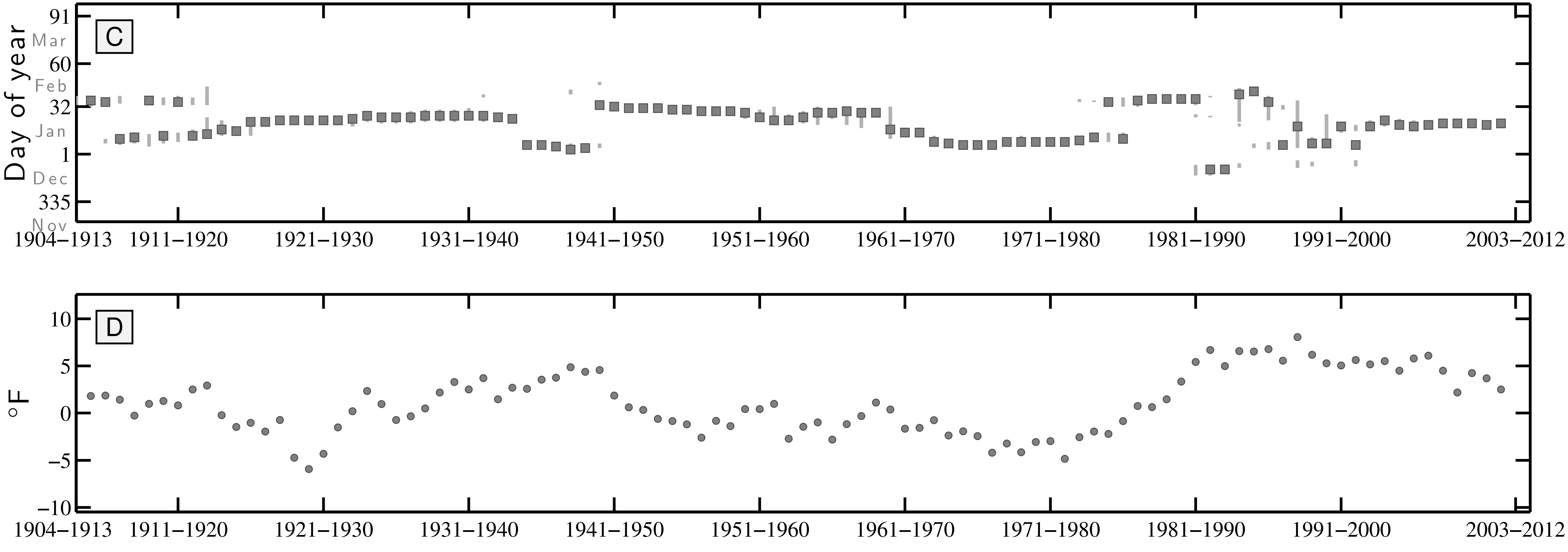

10 year Summer Teletherm dynamics for LANCASTER 4 WSW, WI:

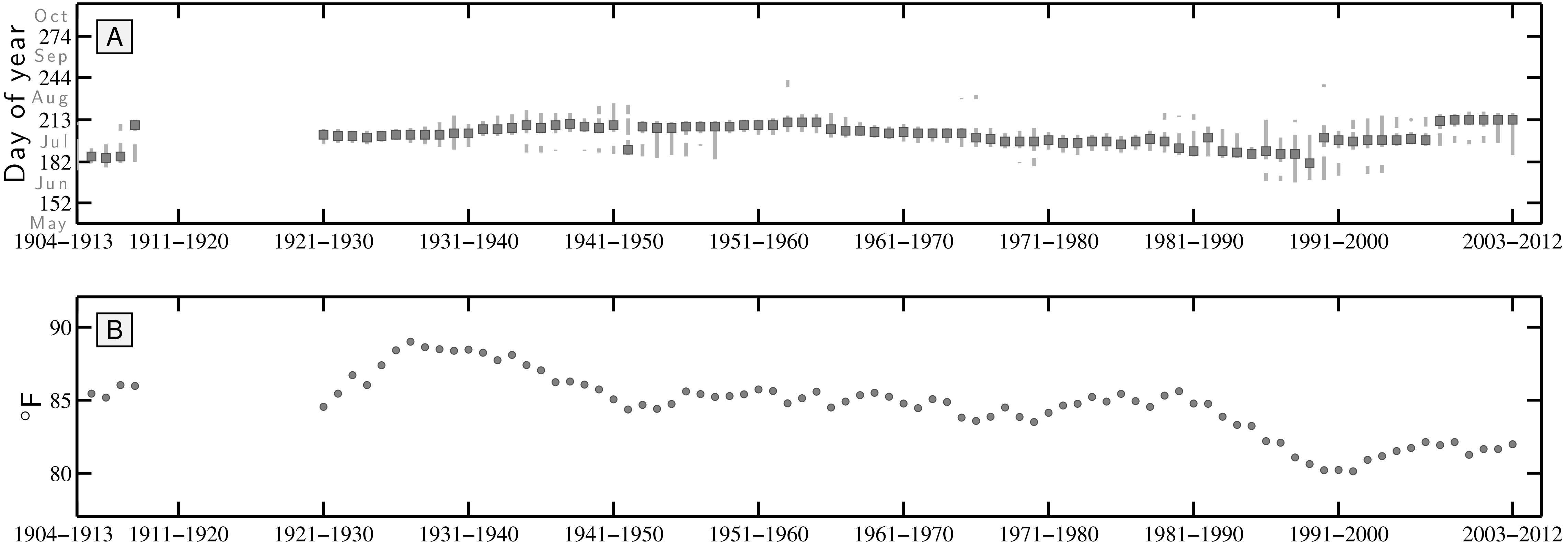

10 year Winter Teletherm dynamics for LANCASTER 4 WSW, WI:

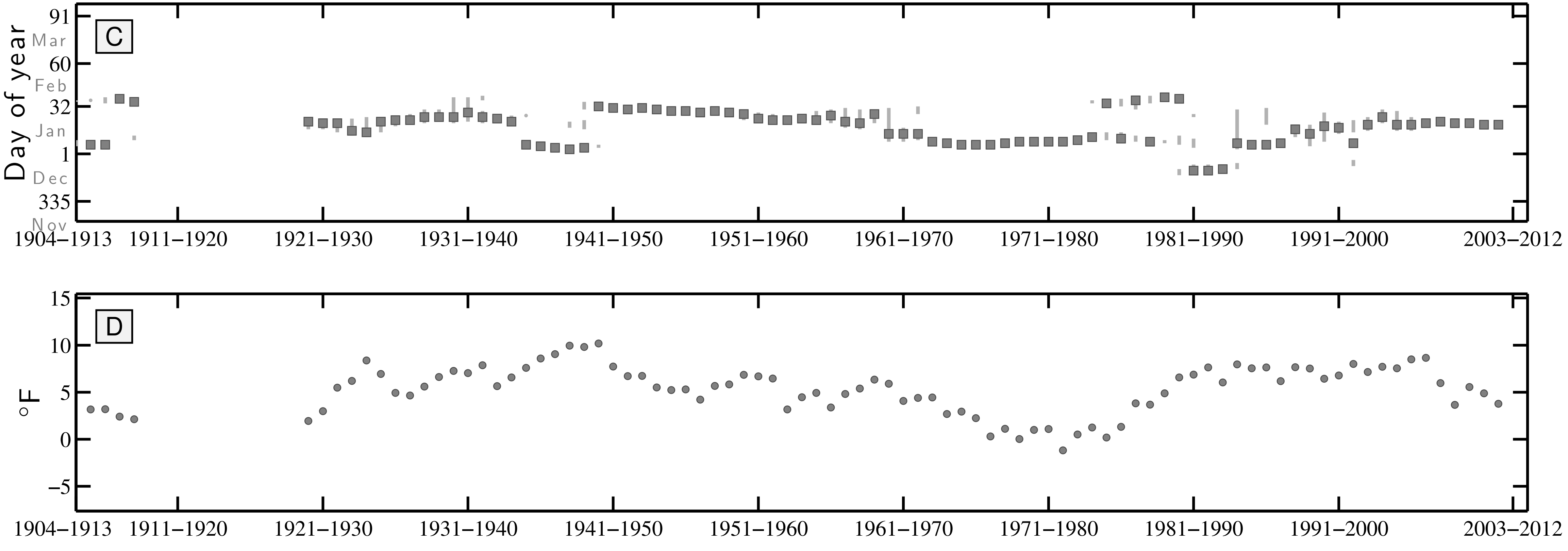

10 year Summer Teletherm dynamics for MANITOWOC, WI:

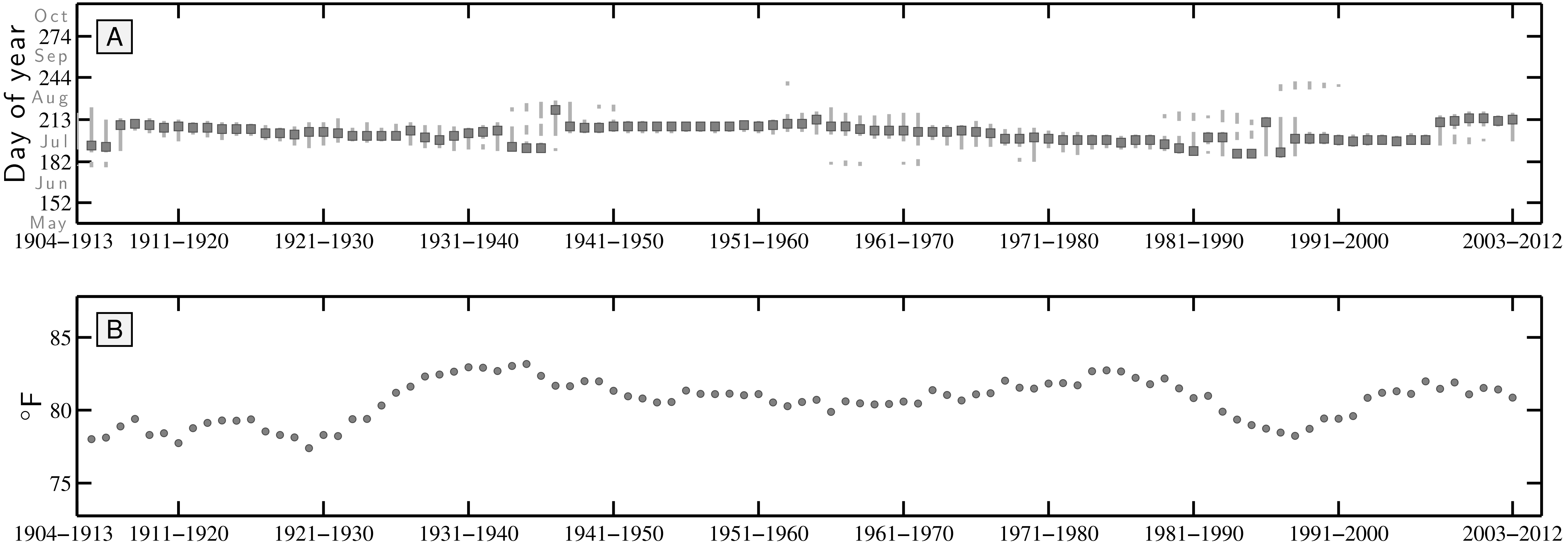

10 year Winter Teletherm dynamics for MANITOWOC, WI:

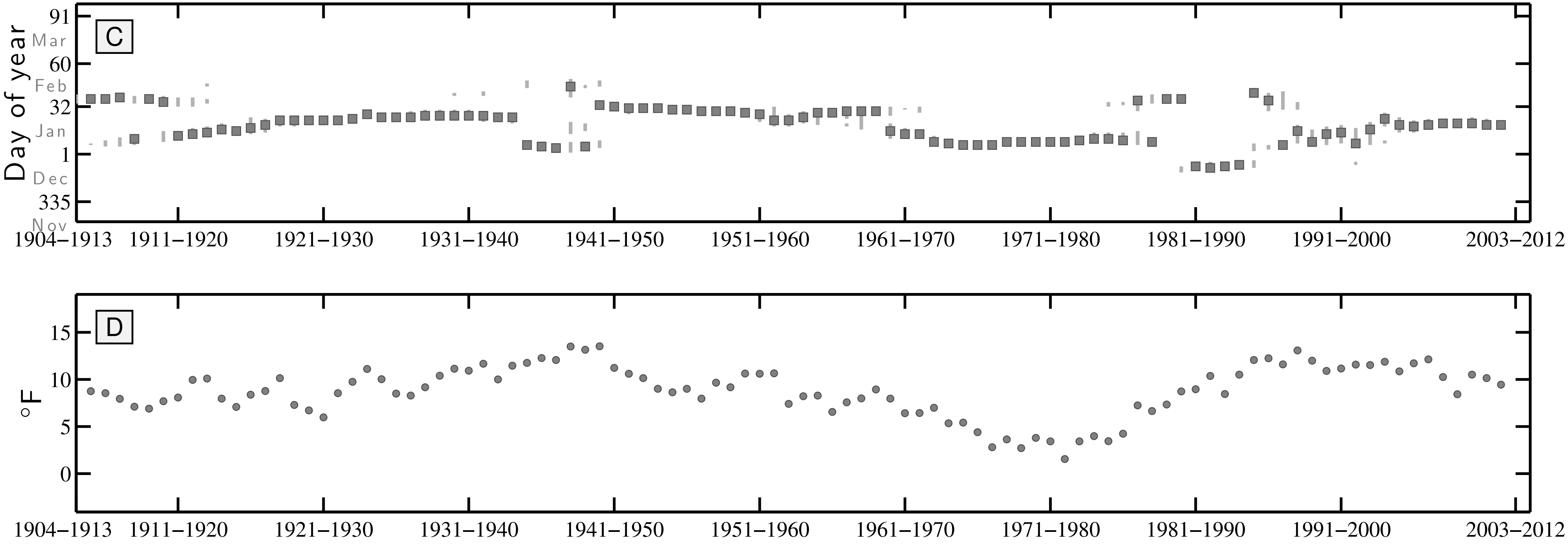

10 year Summer Teletherm dynamics for MARSHFIELD EXP FARM, WI:

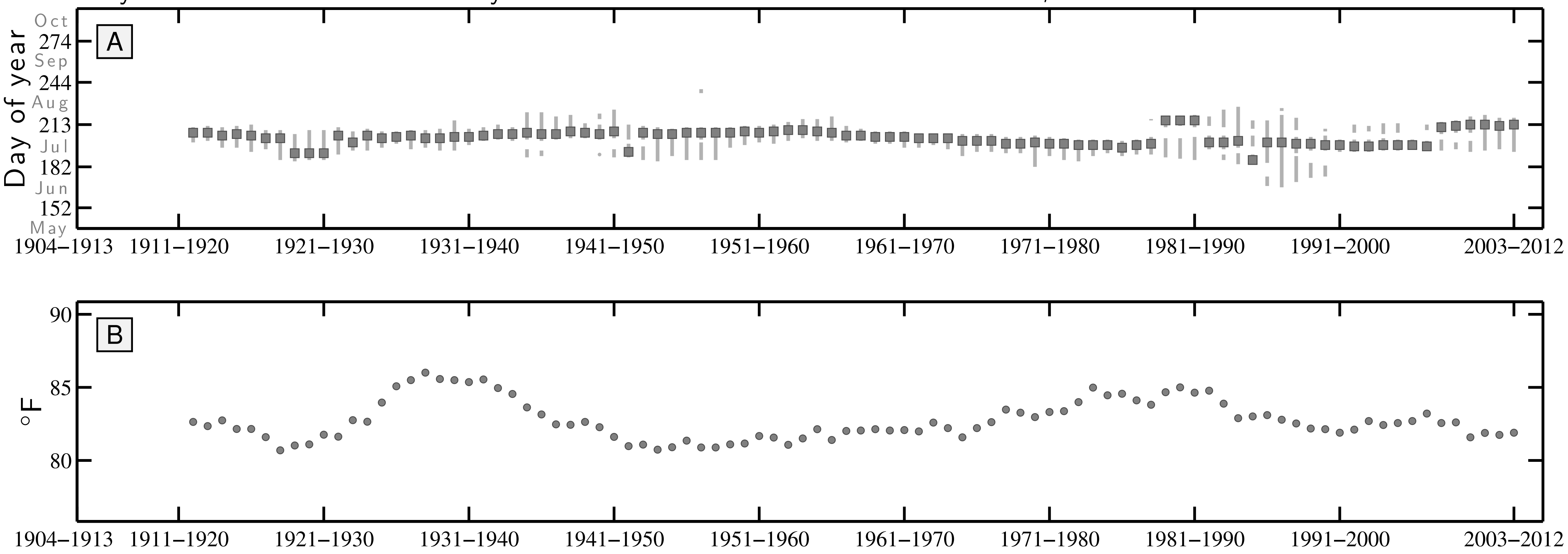

10 year Winter Teletherm dynamics for MARSHFIELD EXP FARM, WI:

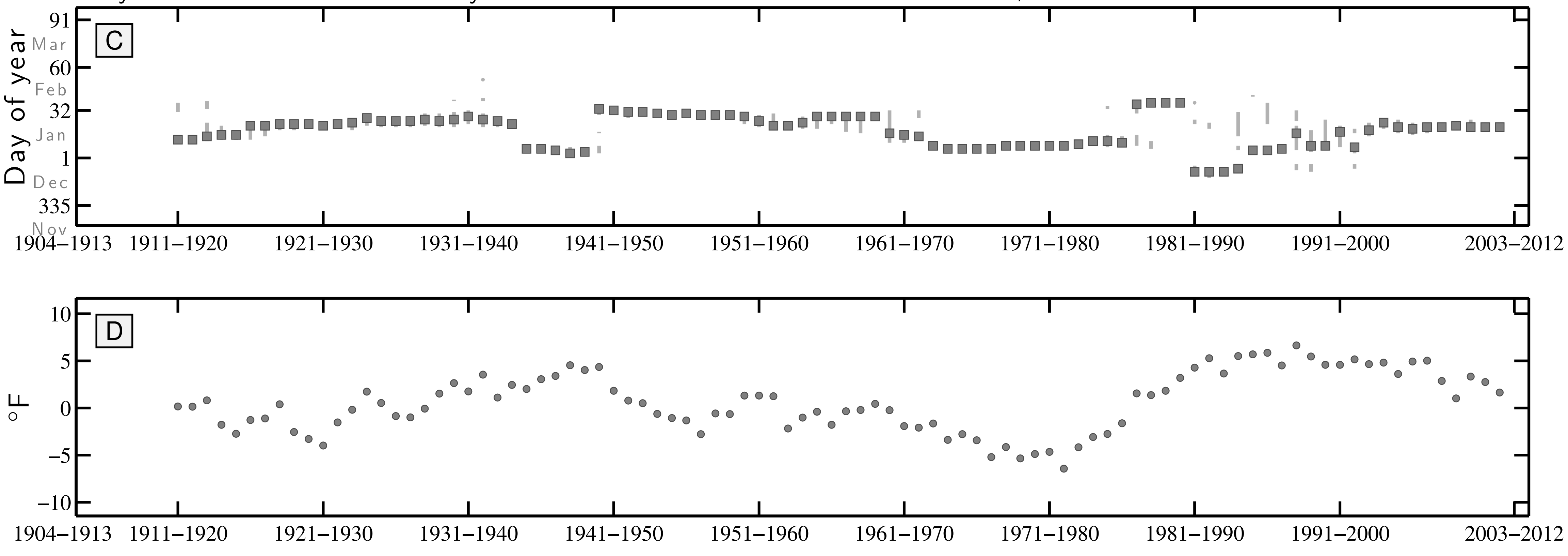

10 year Summer Teletherm dynamics for MEDFORD, WI:

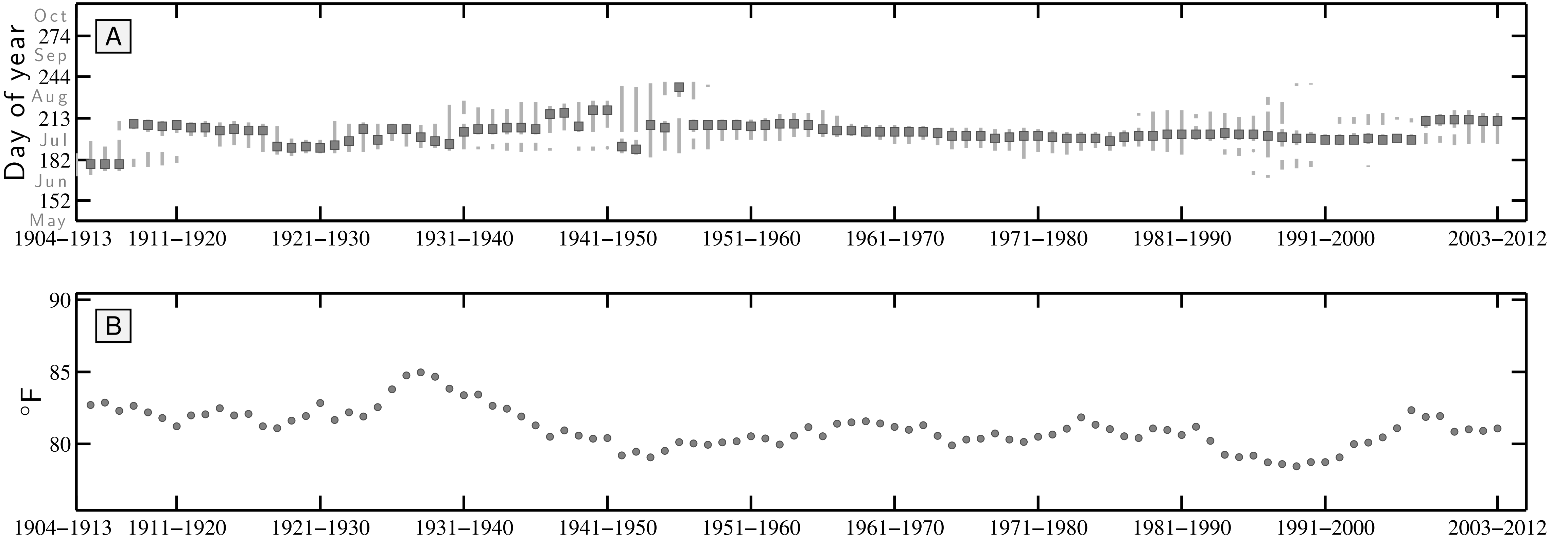

10 year Winter Teletherm dynamics for MEDFORD, WI:

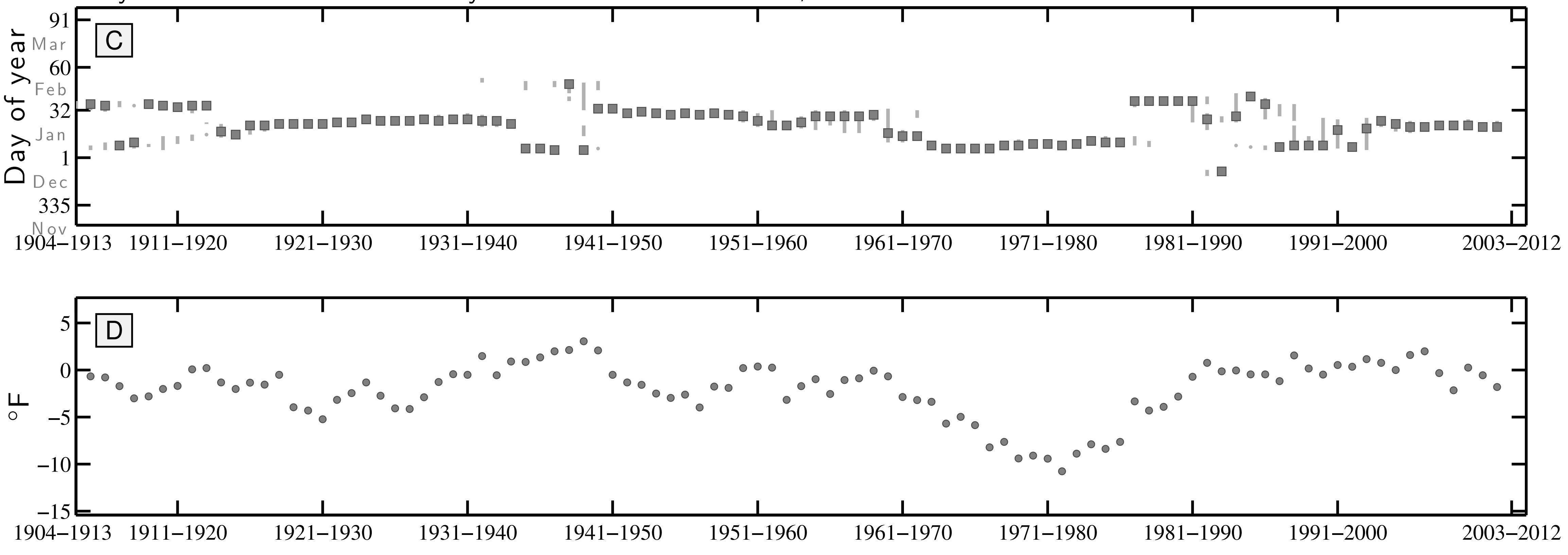

10 year Summer Teletherm dynamics for MILWAUKEE MT MARY COL, WI:

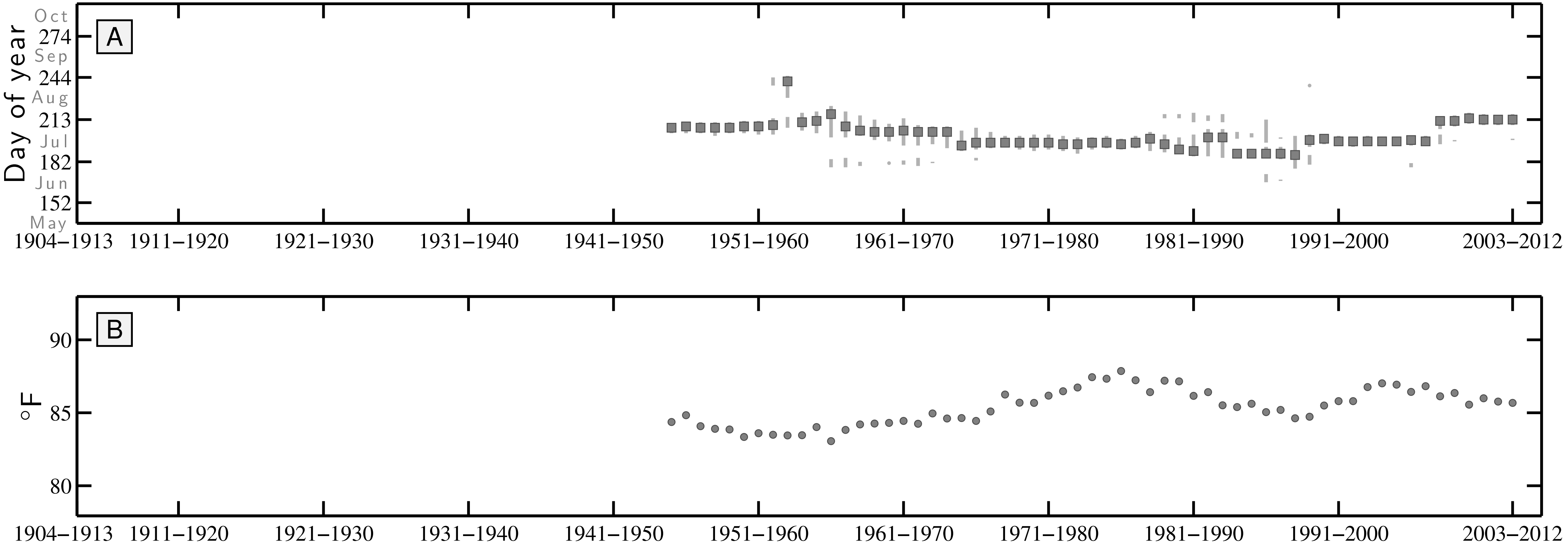

10 year Winter Teletherm dynamics for MILWAUKEE MT MARY COL, WI:

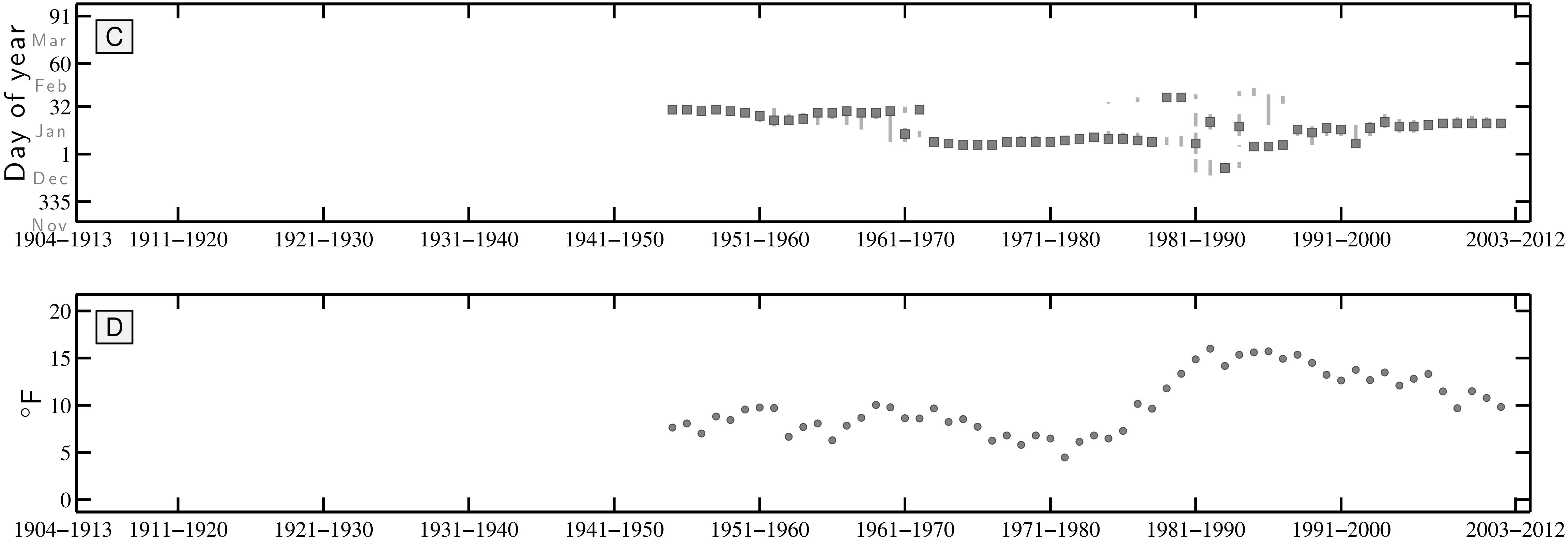

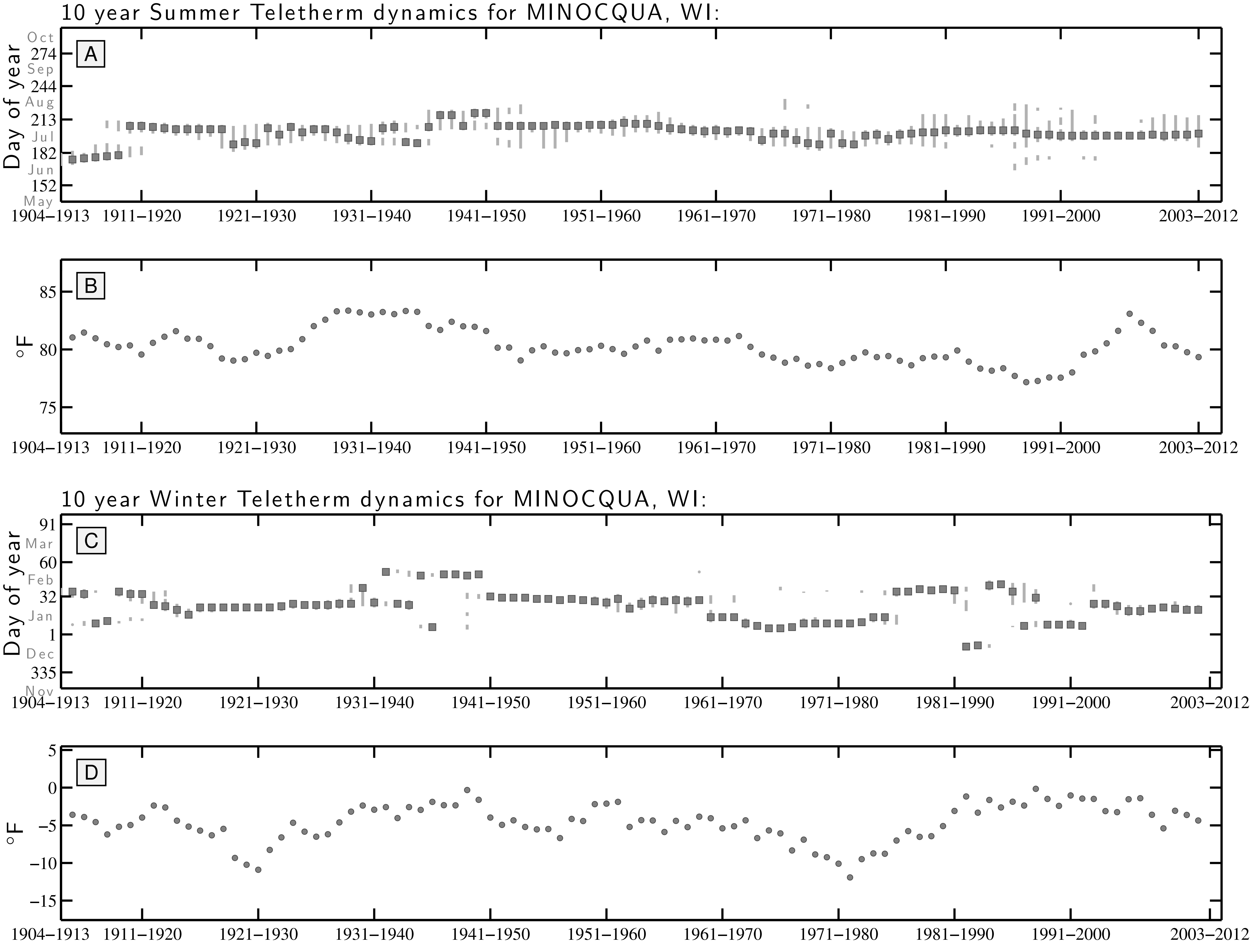
10 year Summer Teletherm dynamics for MINOCQUA, WI:
A
Day of year
Oct
274
Sep
244
Aug
213
Jul
182
Jun
152
May
1904–1913
1911–1920
1921–1930
1931–1940
1941–1950
1951–1960
1961–1970
1971–1980
1981–1990
1991–2000
2003–2012
B
°F
85
80
75
10 year Winter Teletherm dynamics for MINOCQUA, WI:
C
Day of year
91
Mar
60
Feb
32
Jan
1
Dec
335
Nov
D
°F
5
0
−5
−10
−15

10 year Summer Teletherm dynamics for NEILLSVILLE 3 SW, WI:

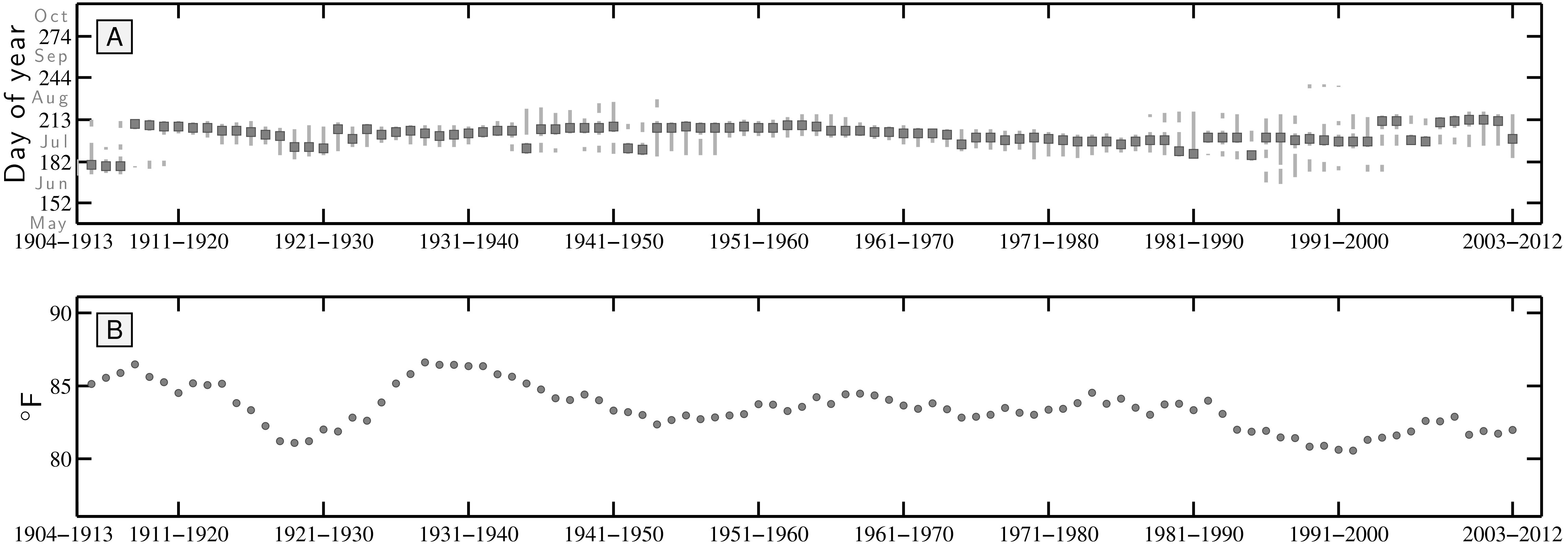

10 year Winter Teletherm dynamics for NEILLSVILLE 3 SW, WI:

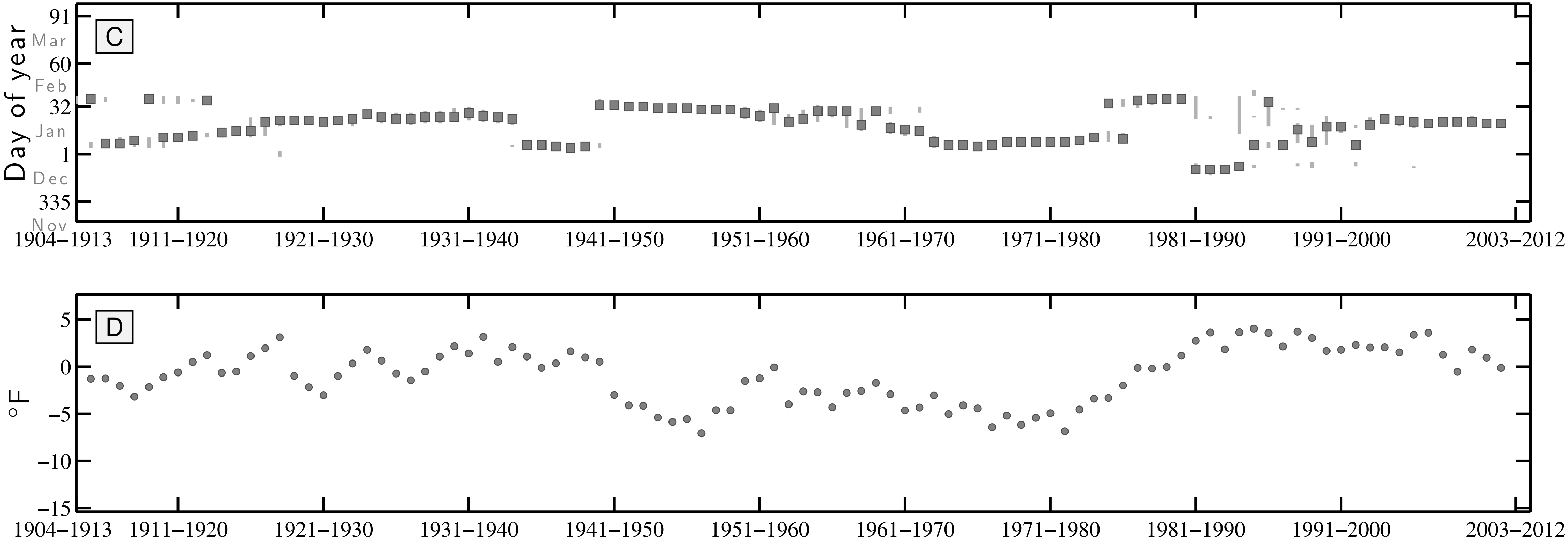

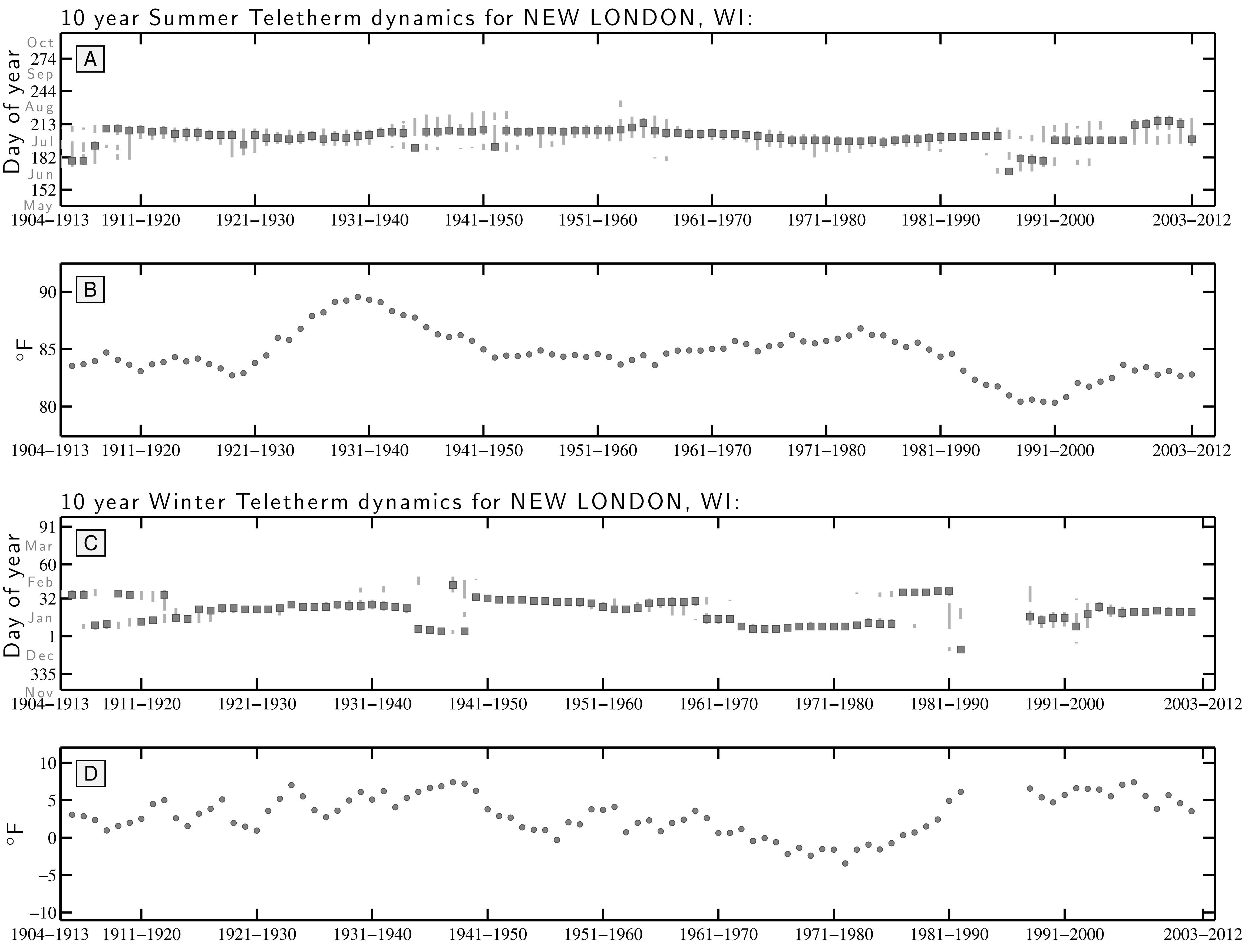
10 year Summer Teletherm dynamics for NEW LONDON, WI:
A
Day of year
Oct
274
Sep
244
Aug
213
Jul
182
Jun
152
May
B
°F
90
85
80
10 year Winter Teletherm dynamics for NEW LONDON, WI:
C
91
Mar
60
Feb
32
Jan
1
Dec
335
Nov
D
10
5
0
−5
−10
1904–1913
1911–1920
1921–1930
1931–1940
1941–1950
1951–1960
1961–1970
1971–1980
1981–1990
1991–2000
2003–2012

10 year Summer Teletherm dynamics for OCONTO 4 W, WI:

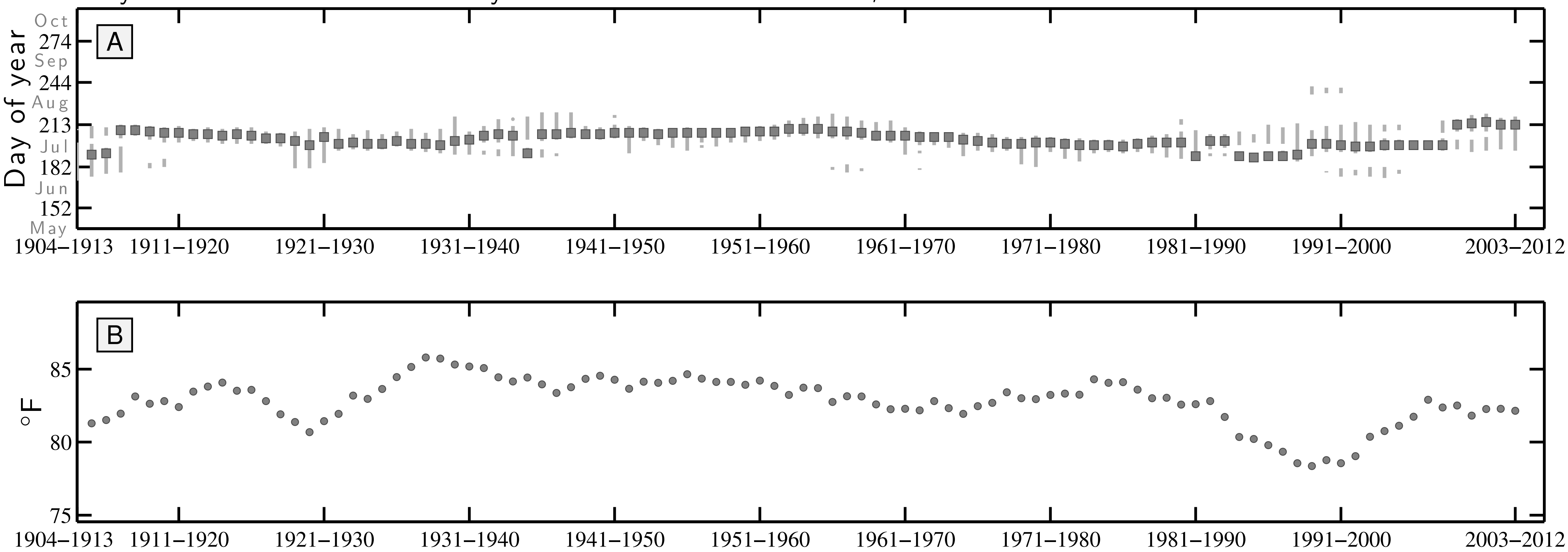

10 year Winter Teletherm dynamics for OCONTO 4 W, WI:

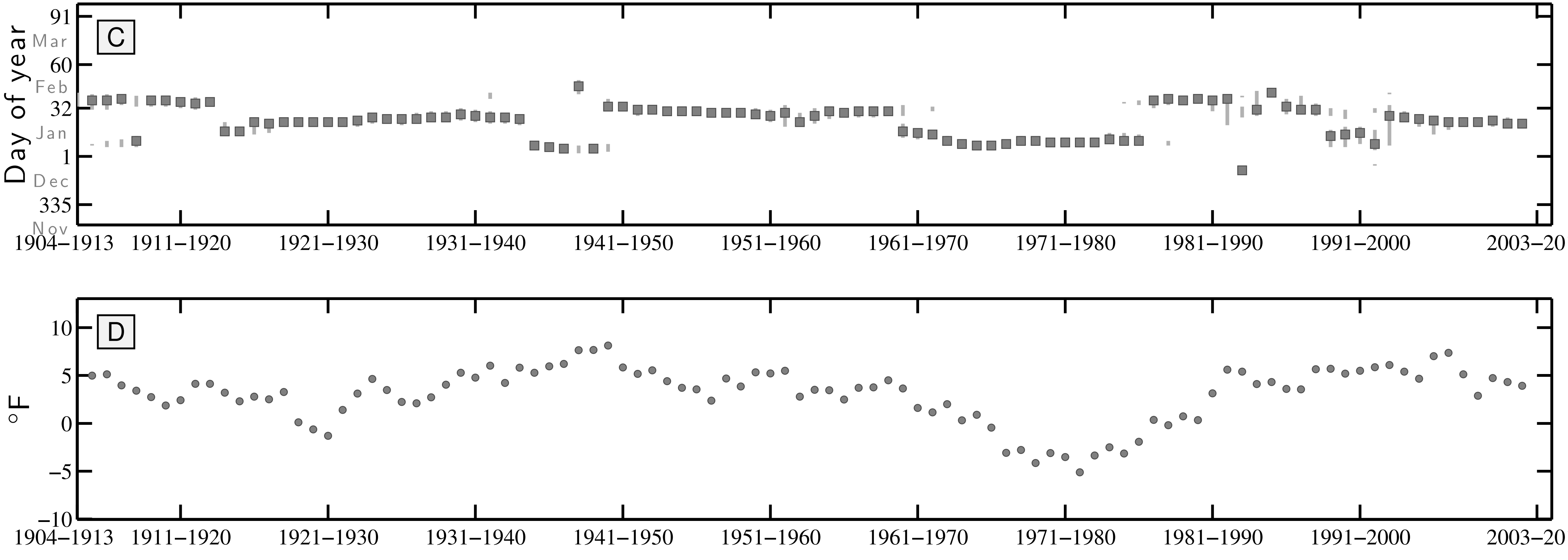

10 year Summer Teletherm dynamics for OSHKOSH, WI:

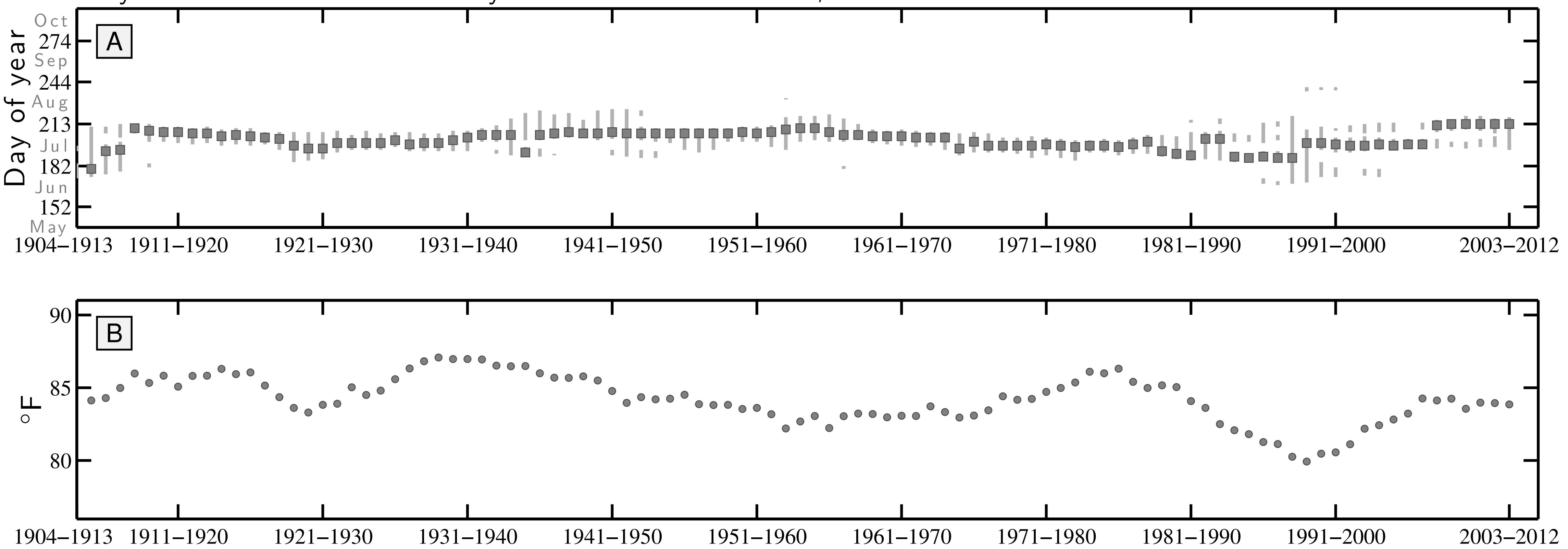

10 year Winter Teletherm dynamics for OSHKOSH, WI:

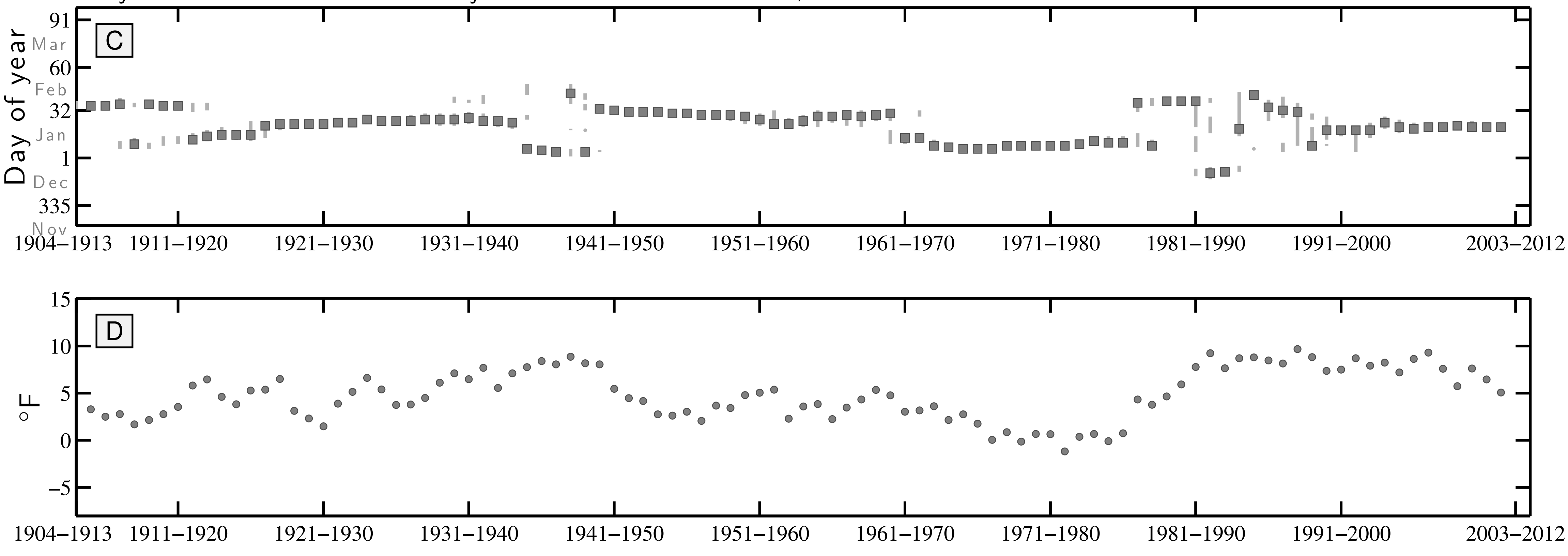

10 year Summer Teletherm dynamics for PORTAGE, WI:

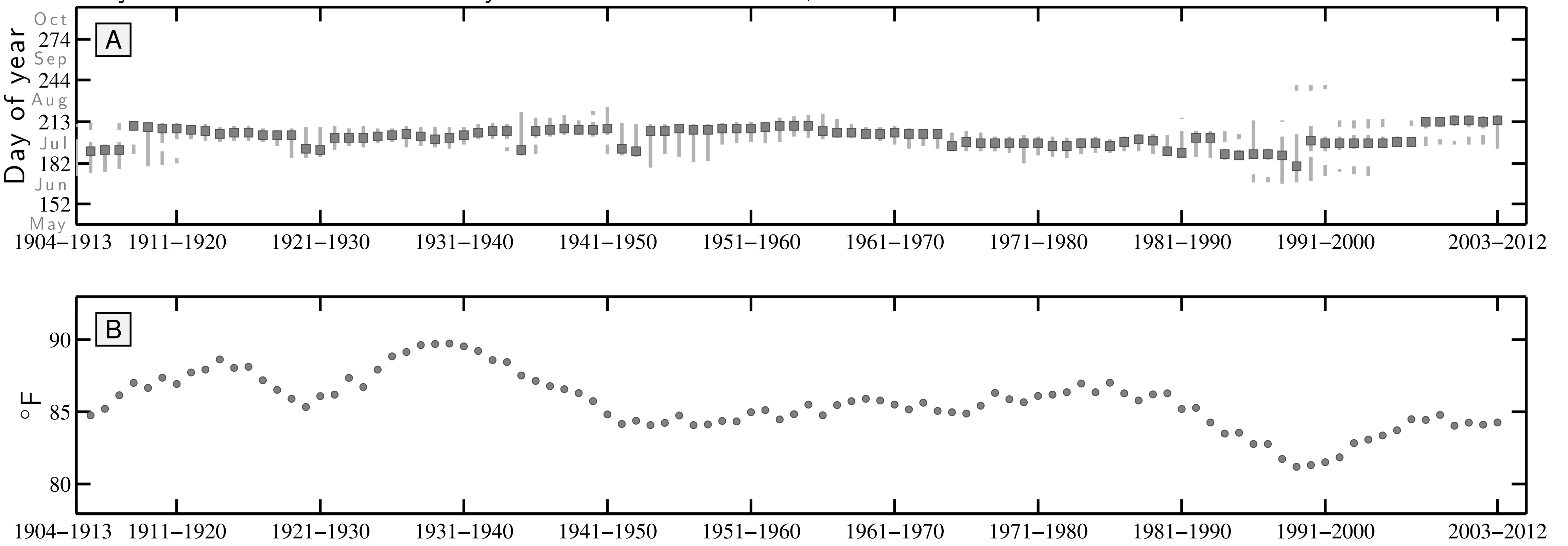

10 year Winter Teletherm dynamics for PORTAGE, WI:

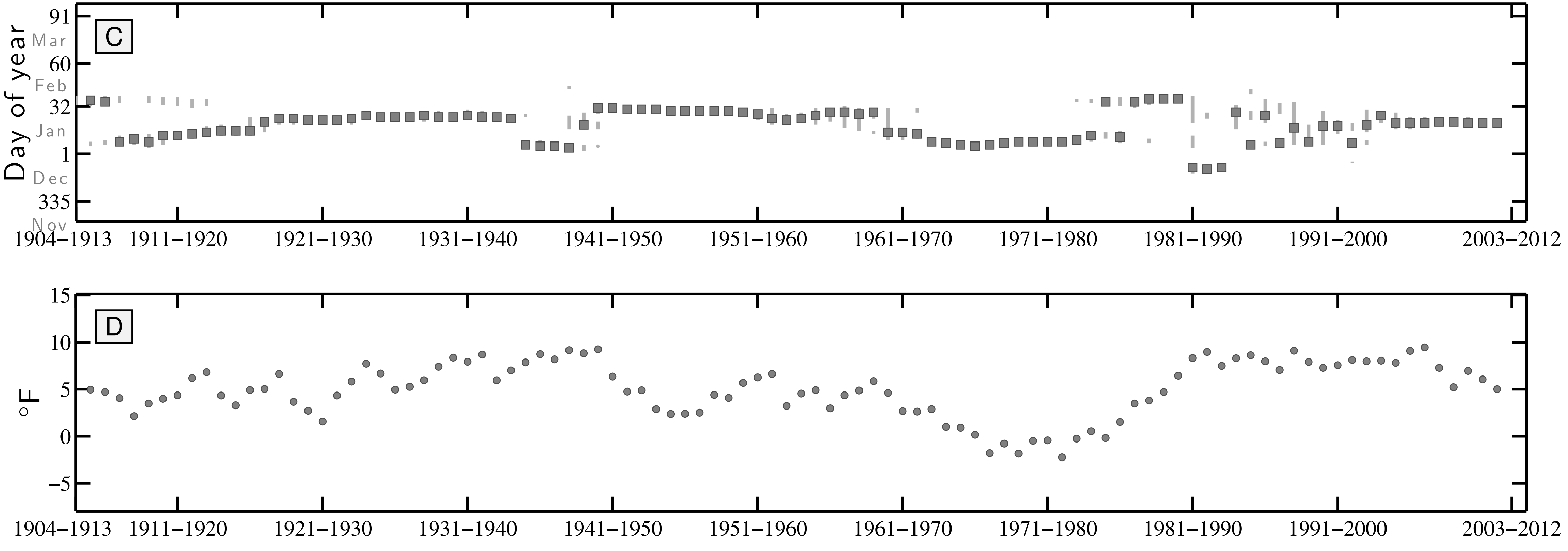

10 year Summer Teletherm dynamics for PRAIRIE DU CHIEN, WI:

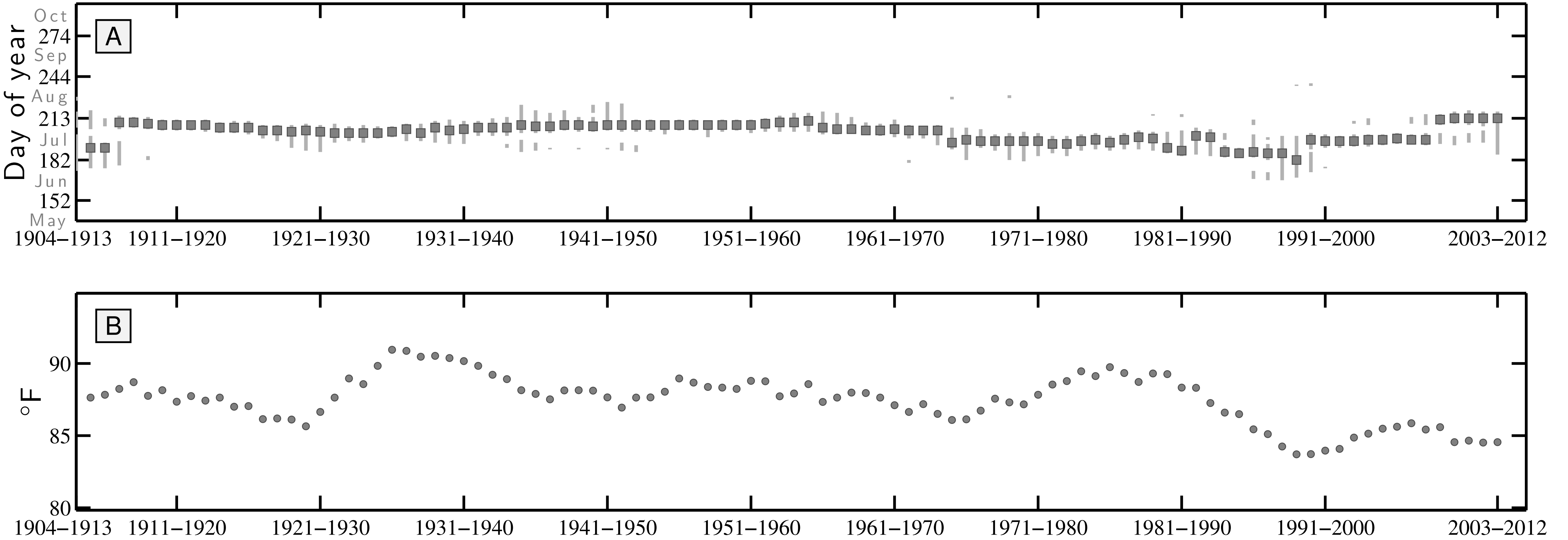

10 year Winter Teletherm dynamics for PRAIRIE DU CHIEN, WI:

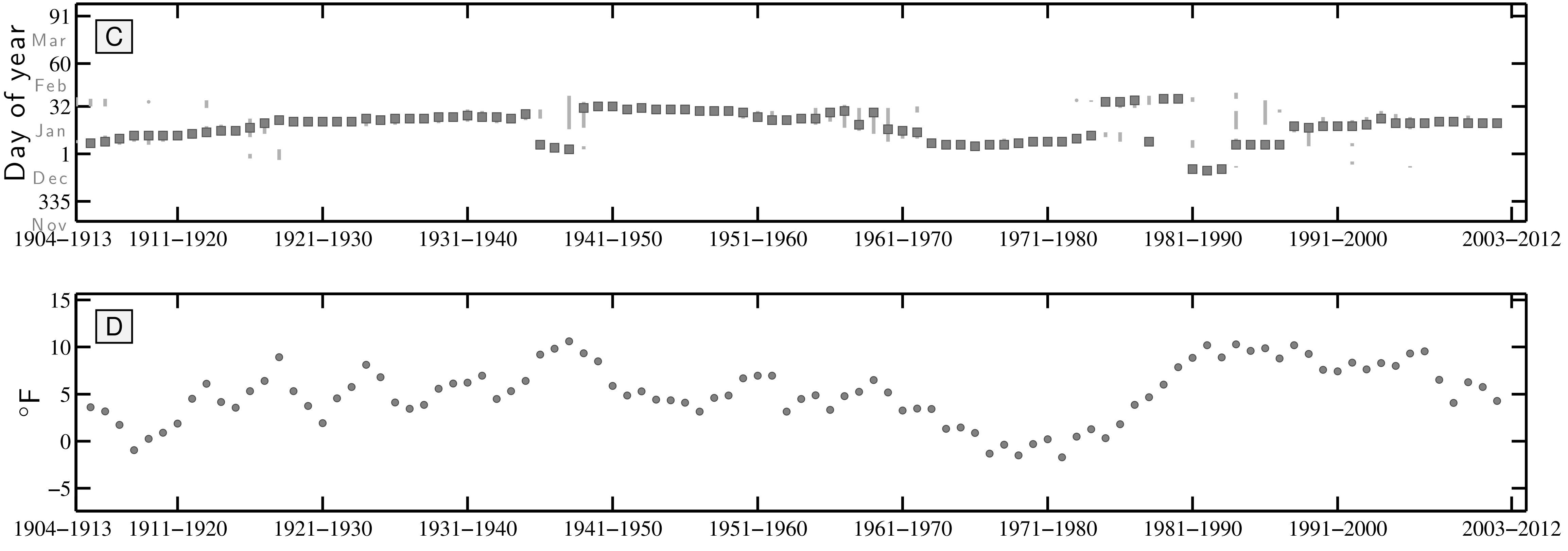

10 year Summer Teletherm dynamics for RACINE, WI:

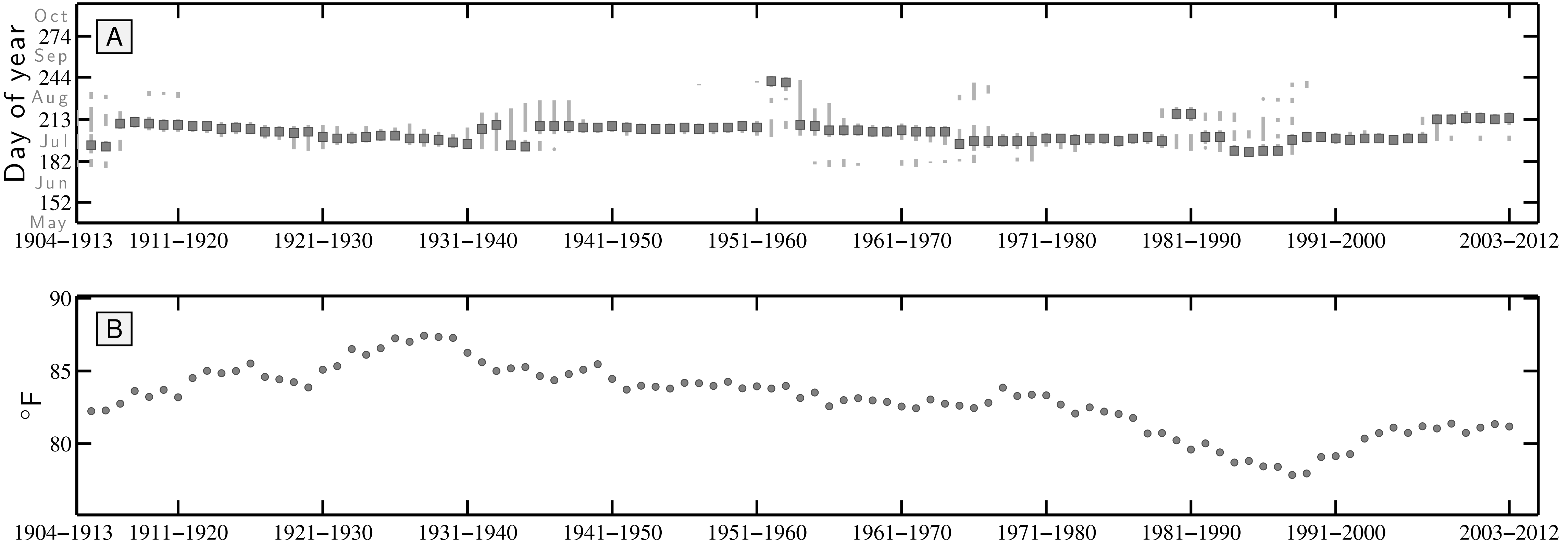

10 year Winter Teletherm dynamics for RACINE, WI:

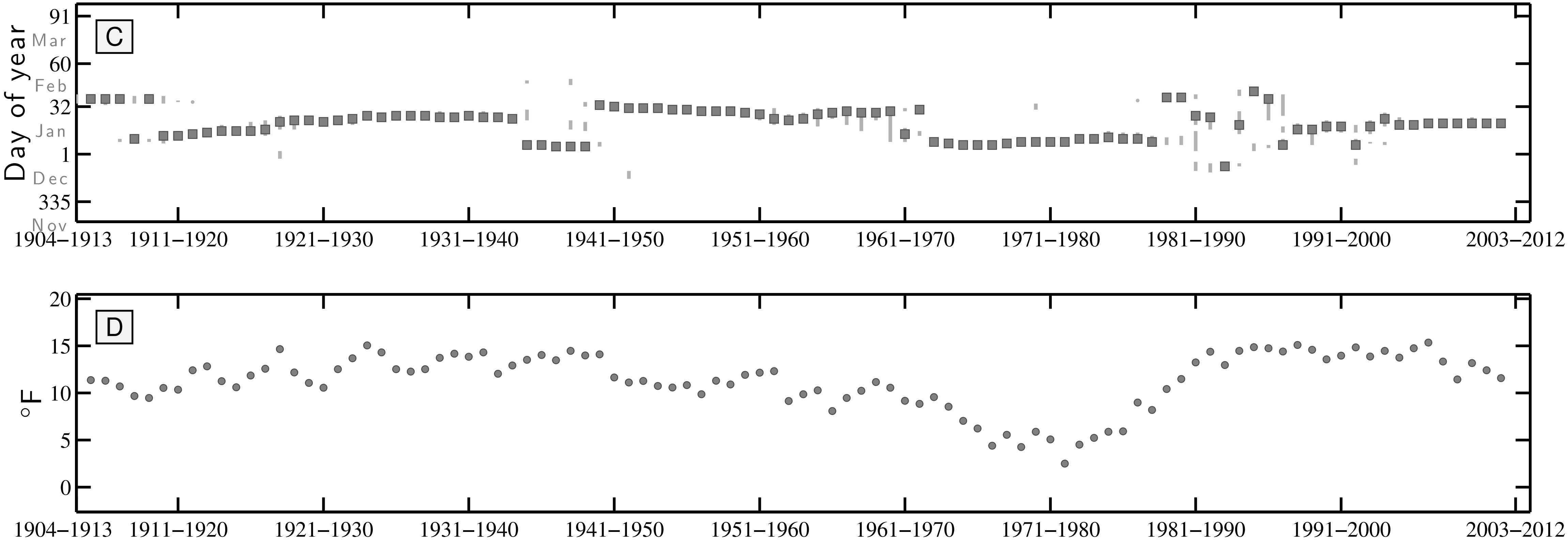

10 year Summer Teletherm dynamics for SPOONER AG RES STN, WI:

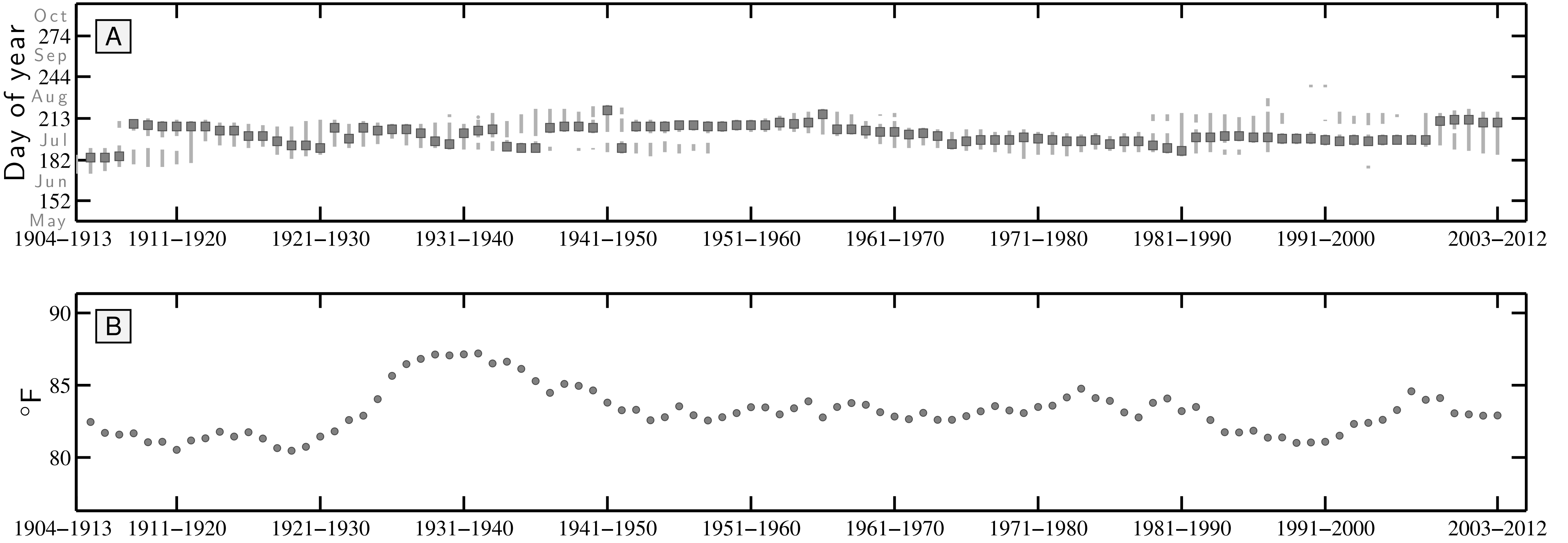

10 year Winter Teletherm dynamics for SPOONER AG RES STN, WI:

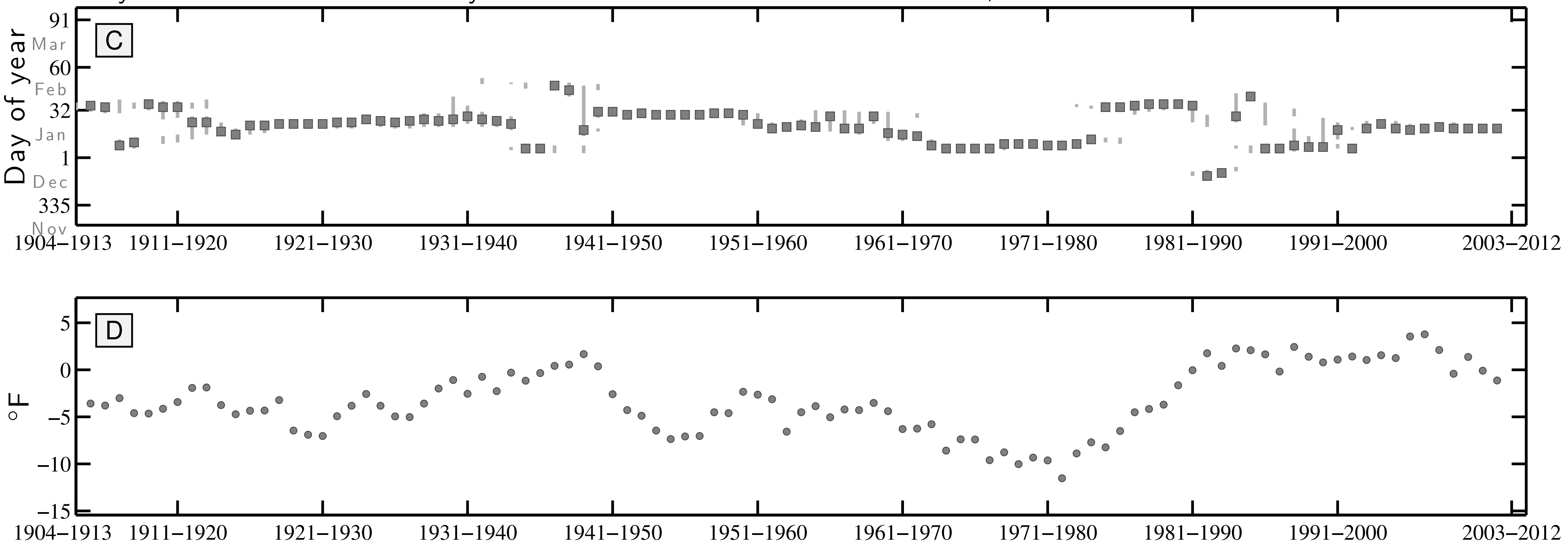

10 year Summer Teletherm dynamics for STANLEY, WI:

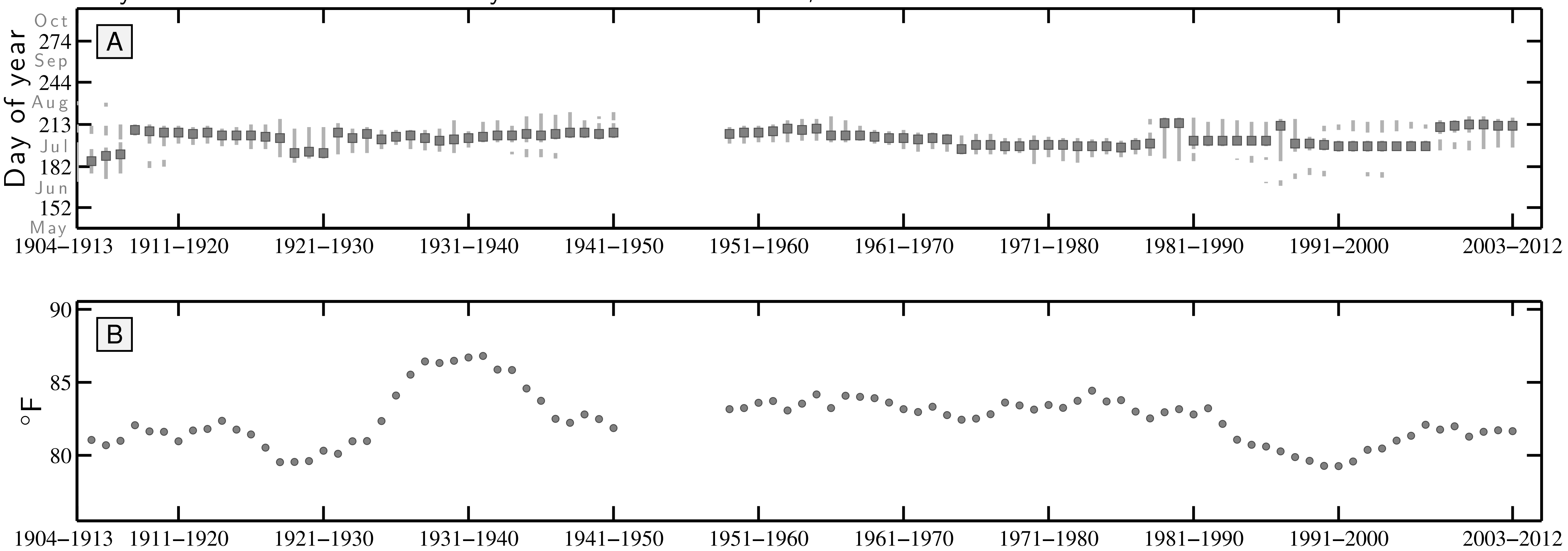

10 year Winter Teletherm dynamics for STANLEY, WI:

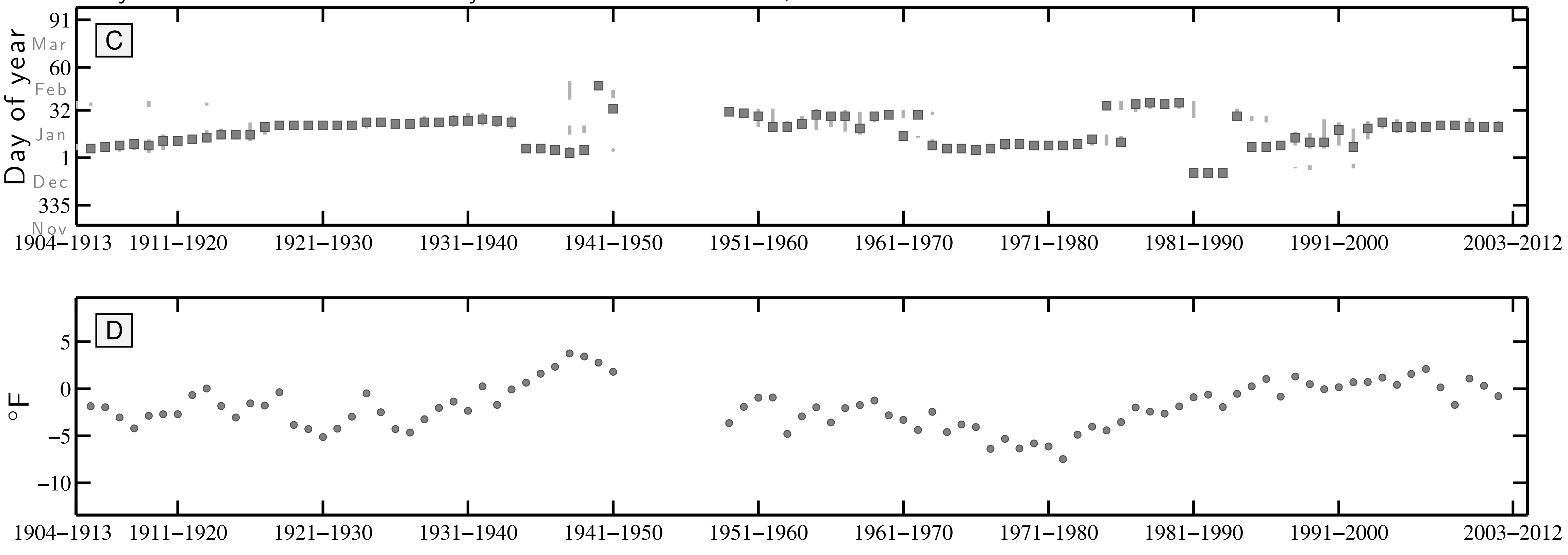

10 year Summer Teletherm dynamics for VIROQUA, WI:

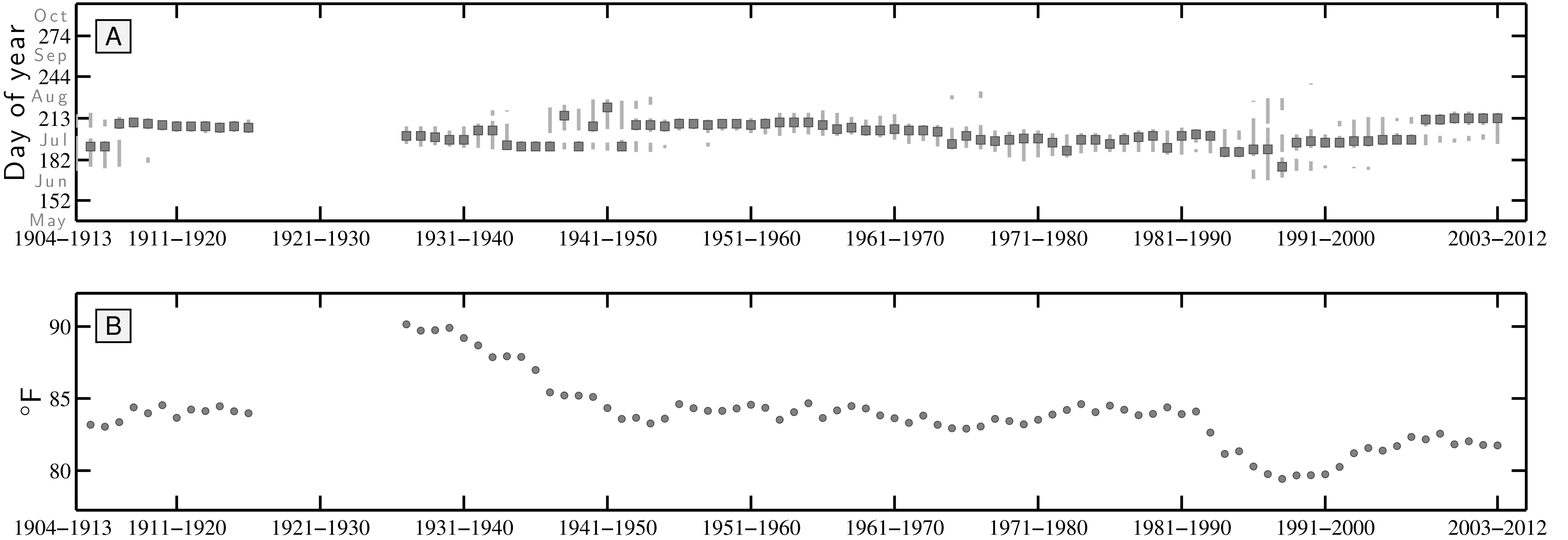

10 year Winter Teletherm dynamics for VIROQUA, WI:

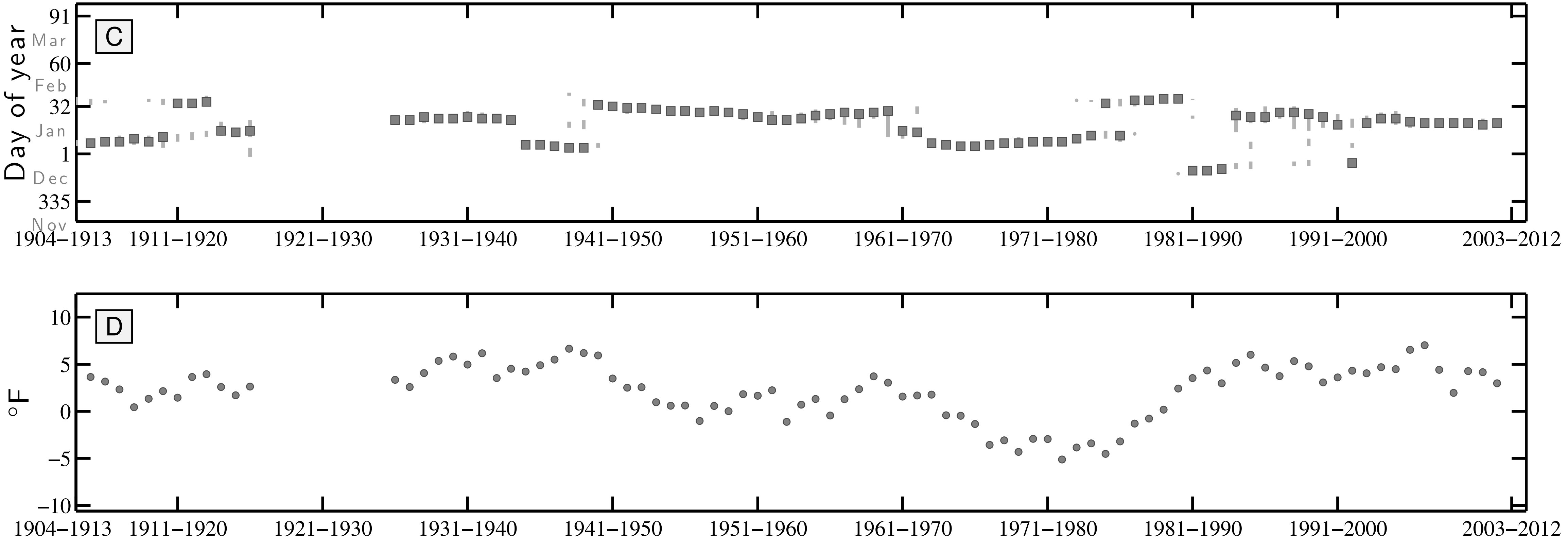

10 year Summer Teletherm dynamics for WATERTOWN, WI:

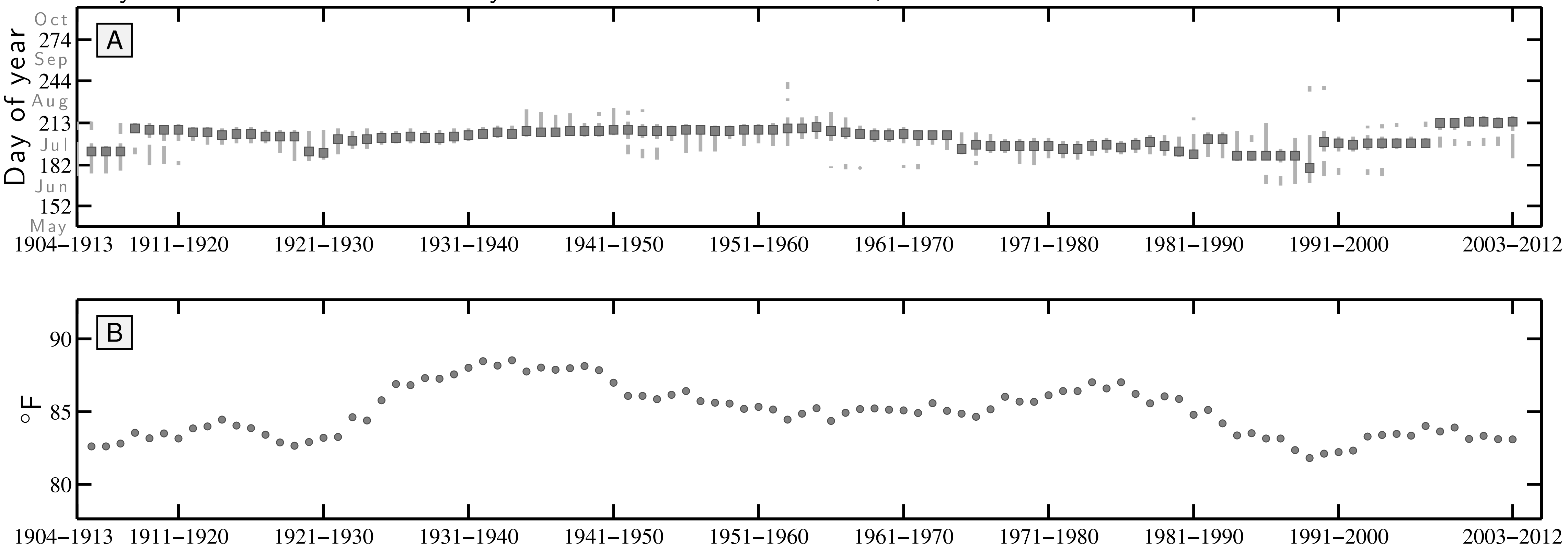

10 year Winter Teletherm dynamics for WATERTOWN, WI:

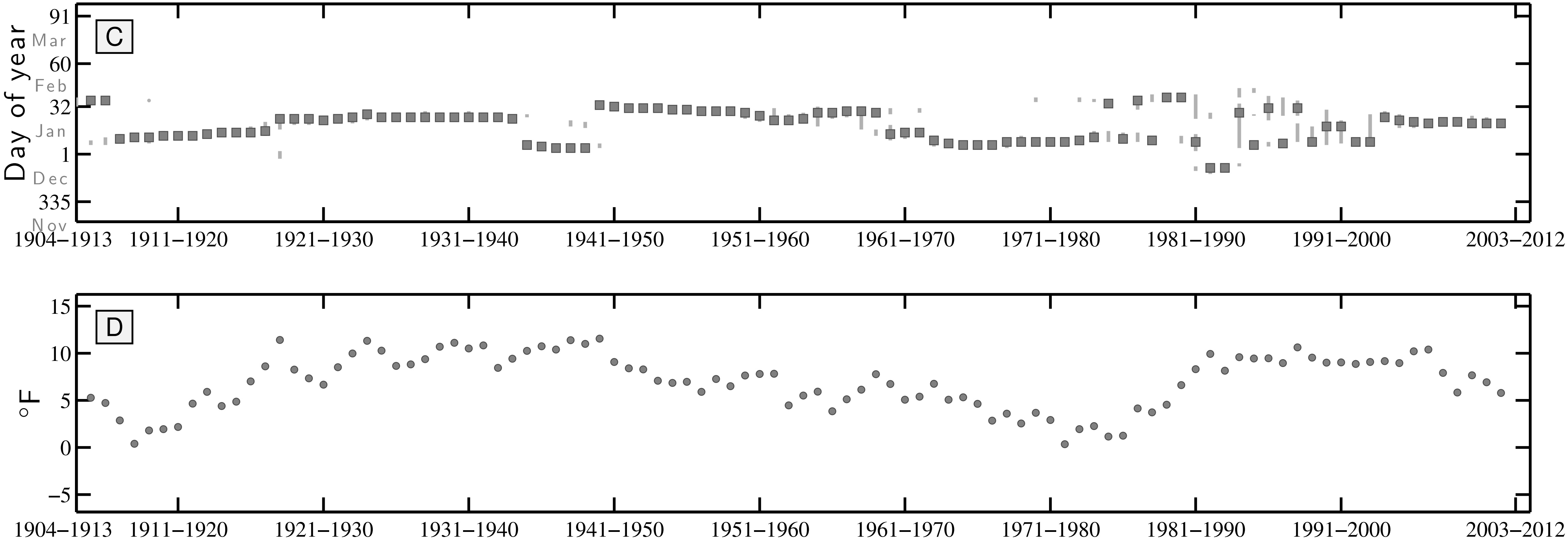

10 year Summer Teletherm dynamics for ALTA 1 NNW, WY:

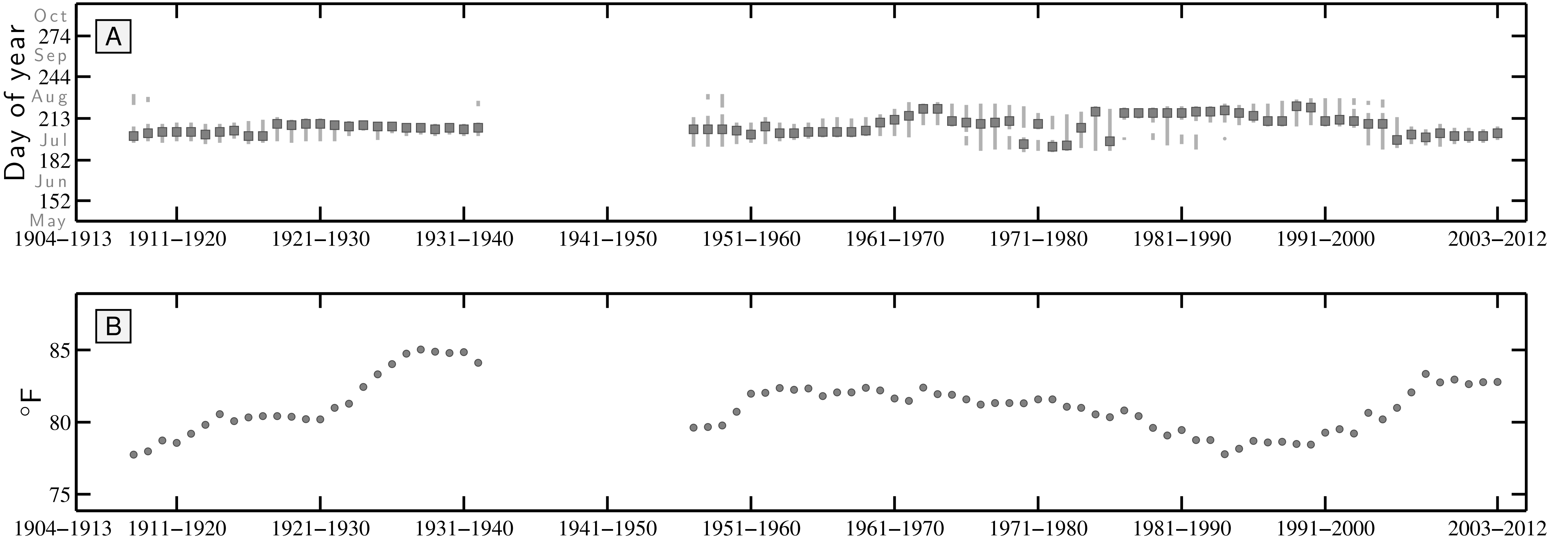

10 year Winter Teletherm dynamics for ALTA 1 NNW, WY:

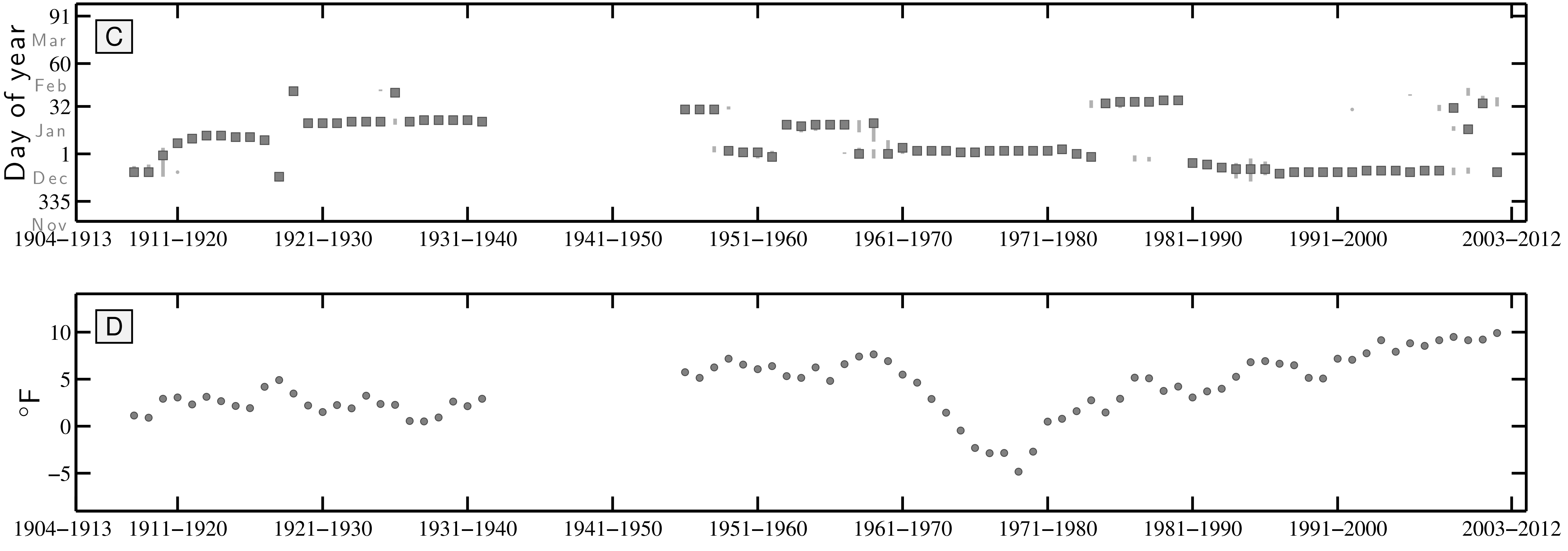

10 year Summer Teletherm dynamics for BASIN, WY:

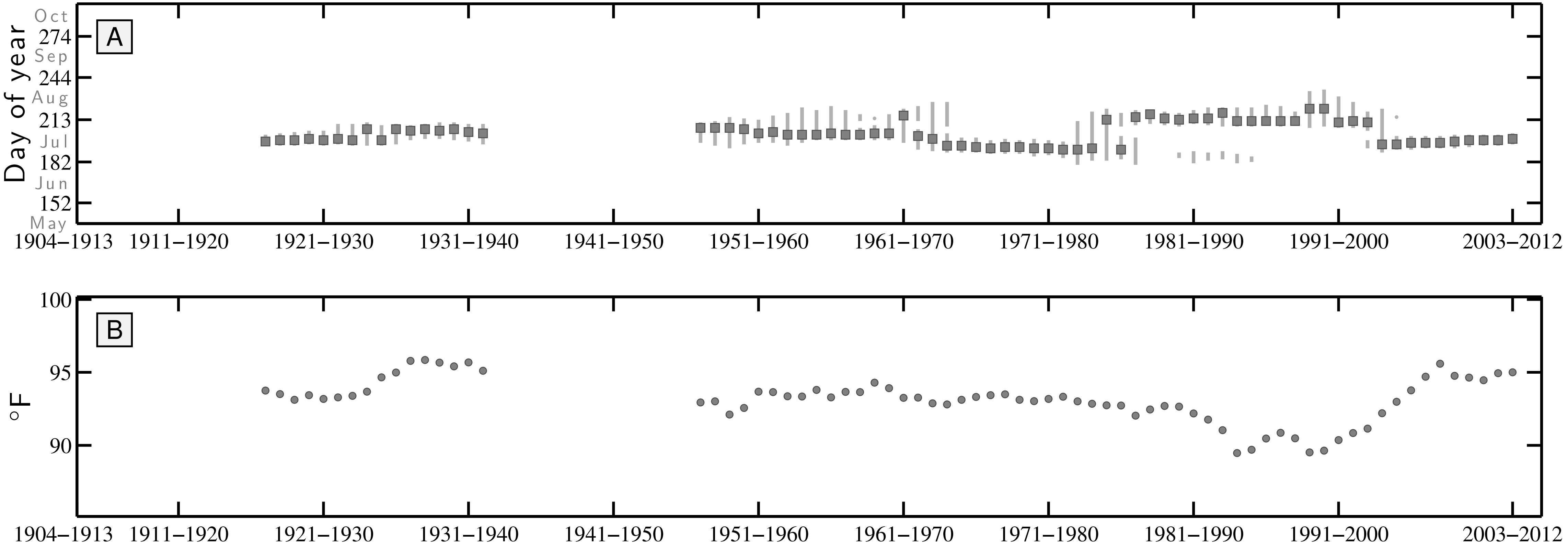

10 year Winter Teletherm dynamics for BASIN, WY:

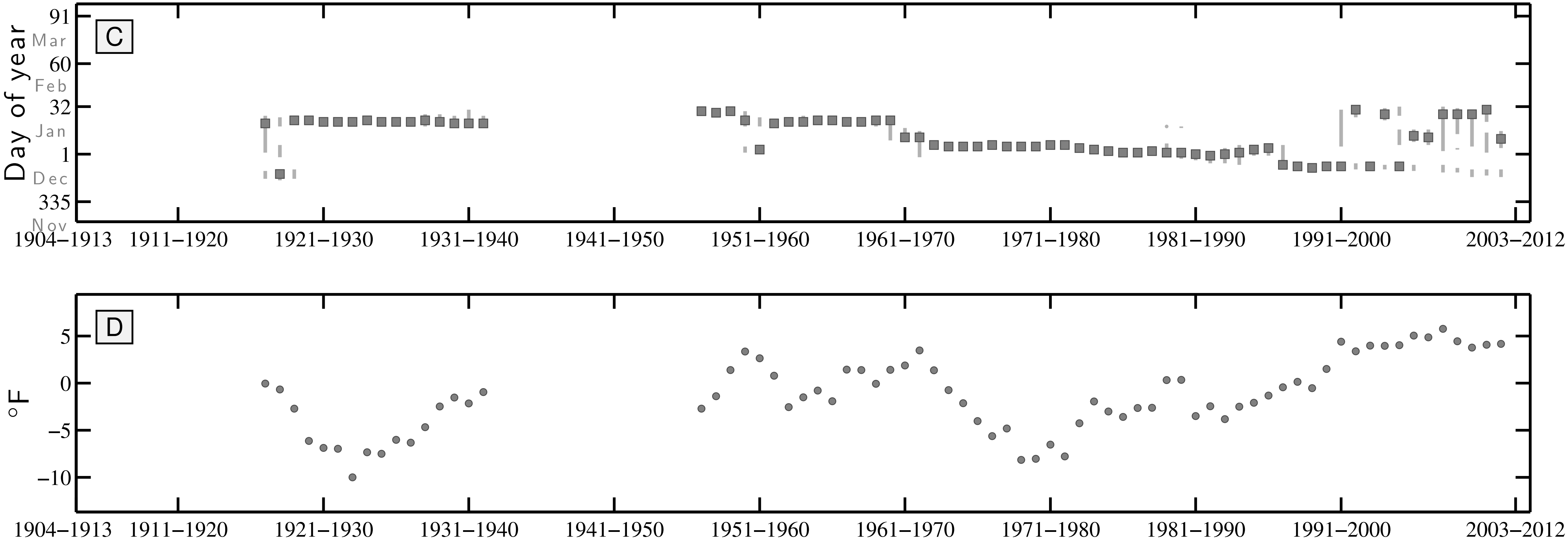

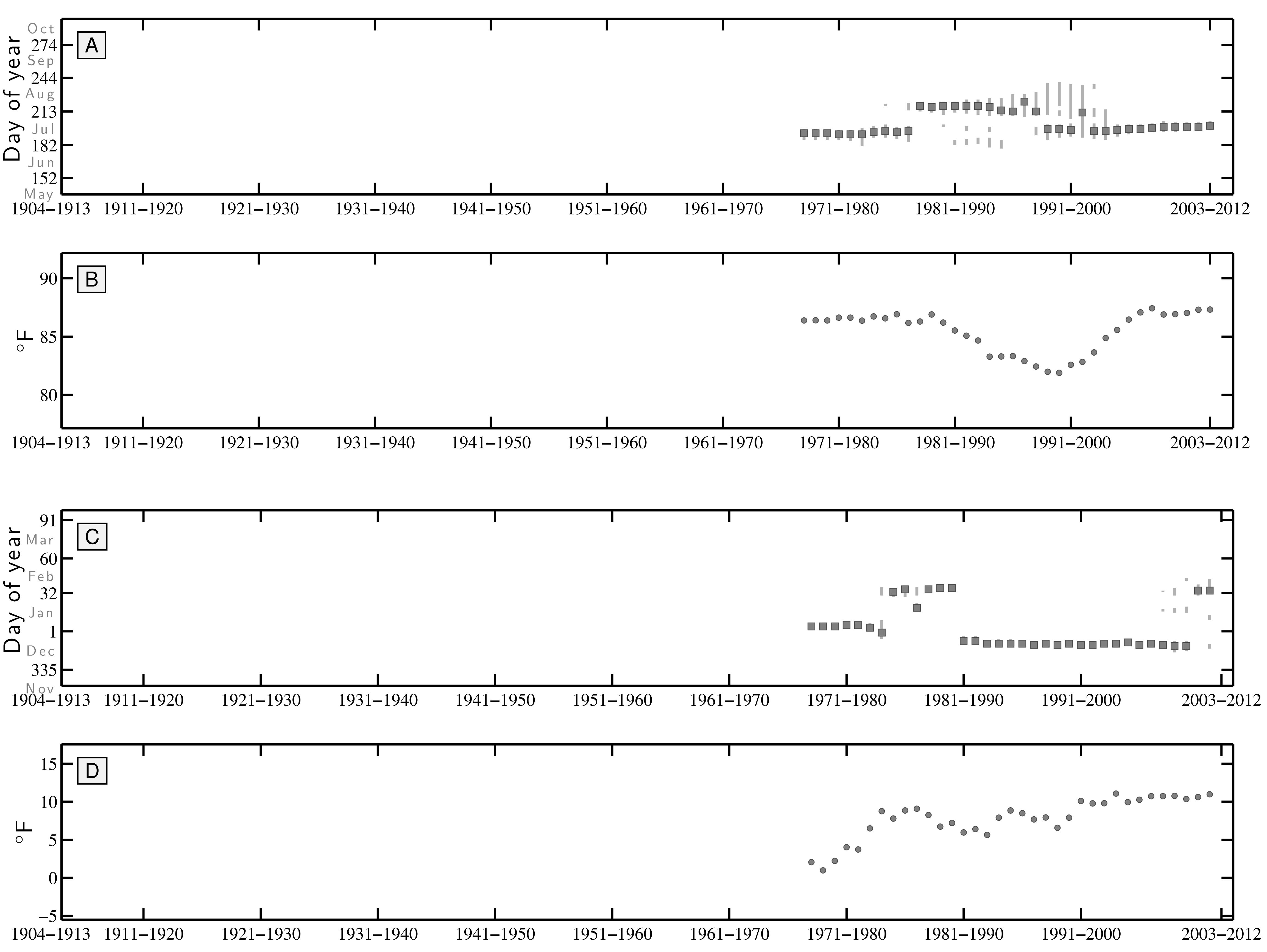

A
Day of year
Oct
274
Sep
244
Aug
213
Jul
182
Jun
152
May
1904–1913
1911–1920
1921–1930
1931–1940
1941–1950
1951–1960
1961–1970
1971–1980
1981–1990
1991–2000
2003–2012
B
°F
90
85
80
C
91
Mar
60
Feb
32
Jan
1
Dec
335
Nov
D
15
10
5
0
−5

10 year Summer Teletherm dynamics for CHEYENNE WSFO AP, WY:

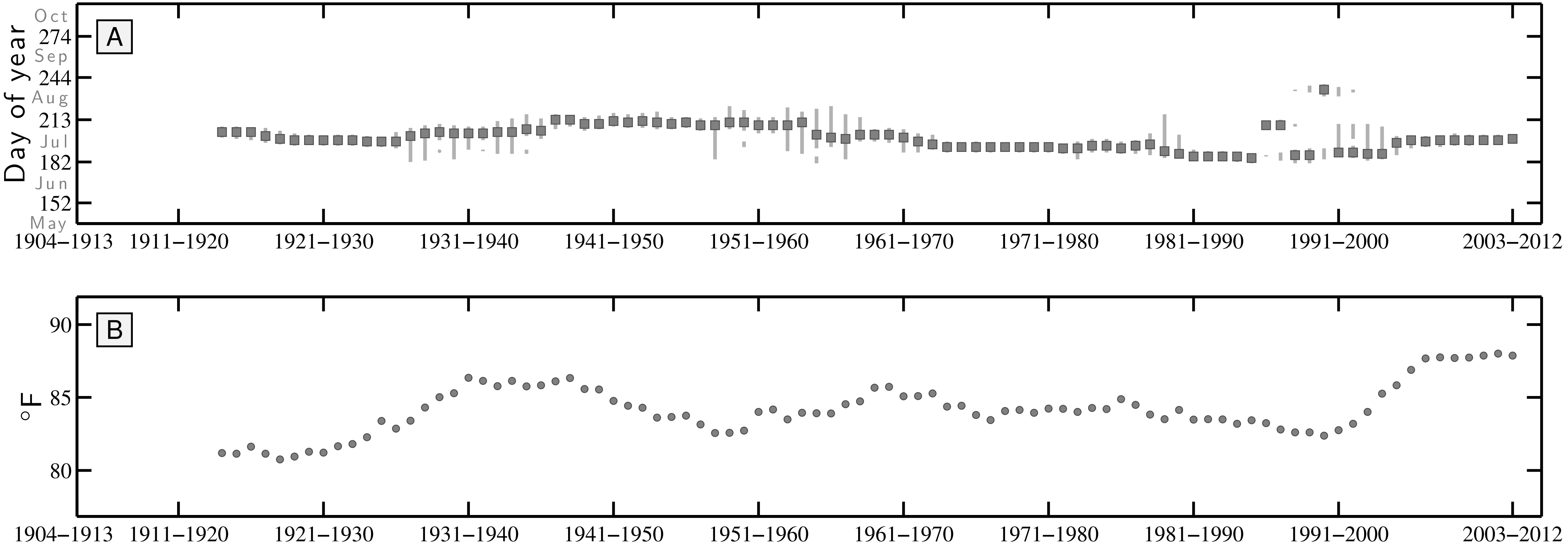

10 year Winter Teletherm dynamics for CHEYENNE WSFO AP, WY:

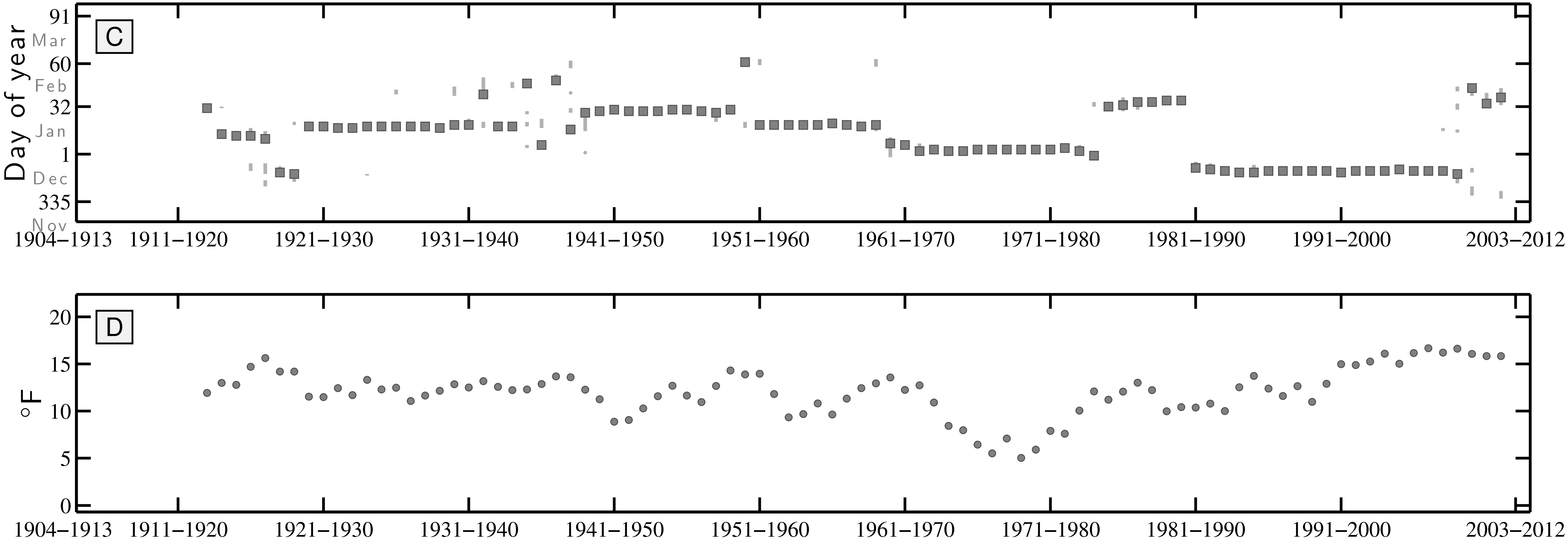

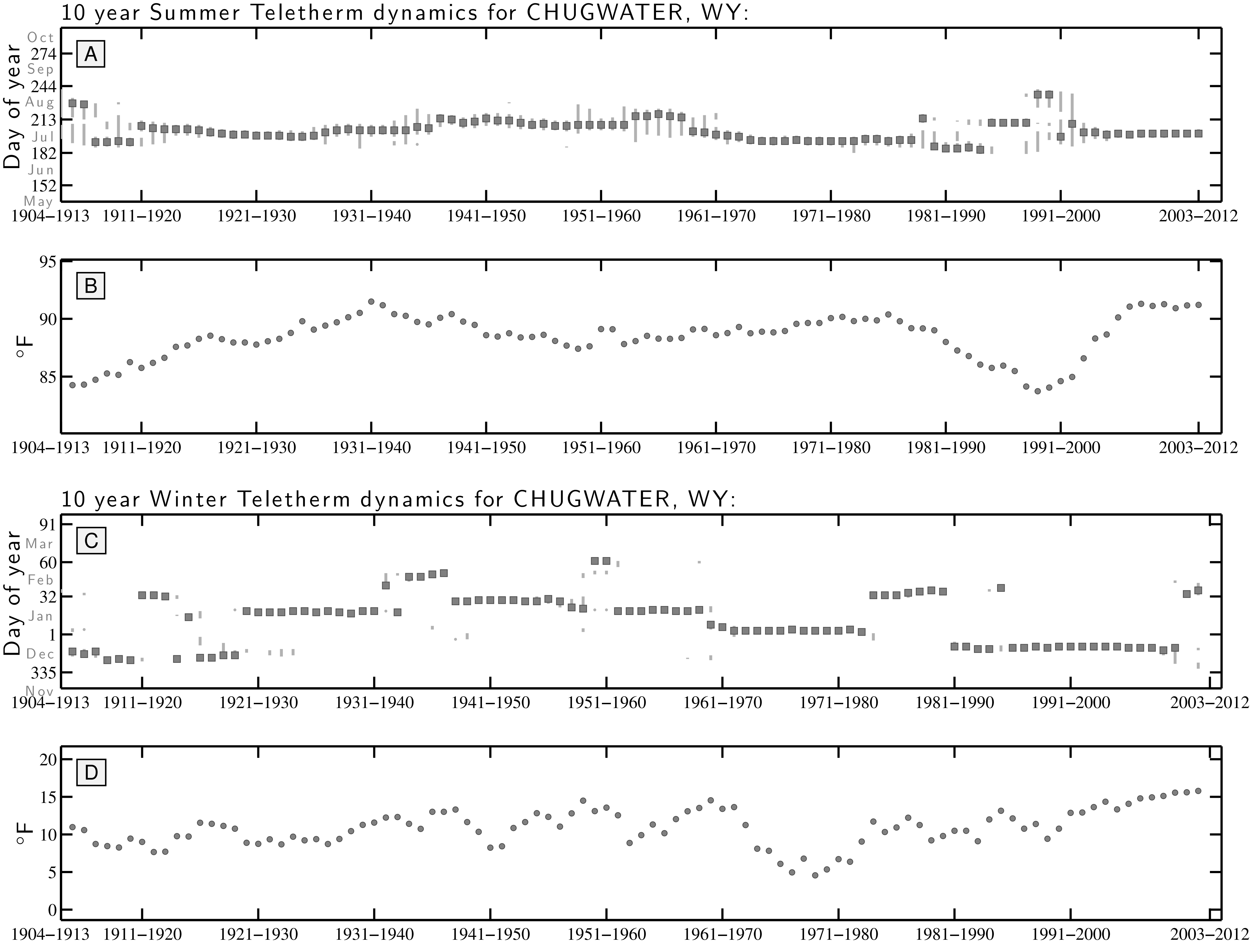
10 year Summer Teletherm dynamics for CHUGWATER, WY:
A
B
Day of year
°F
Oct
274
Sep
244
Aug
213
Jul
182
Jun
152
May
95
90
85
1904–1913
1911–1920
1921–1930
1931–1940
1941–1950
1951–1960
1961–1970
1971–1980
1981–1990
1991–2000
2003–2012
10 year Winter Teletherm dynamics for CHUGWATER, WY:
C
D
91
Mar
60
Feb
32
Jan
1
Dec
335
Nov
20
15
10
5
0

10 year Summer Teletherm dynamics for CODY, WY:

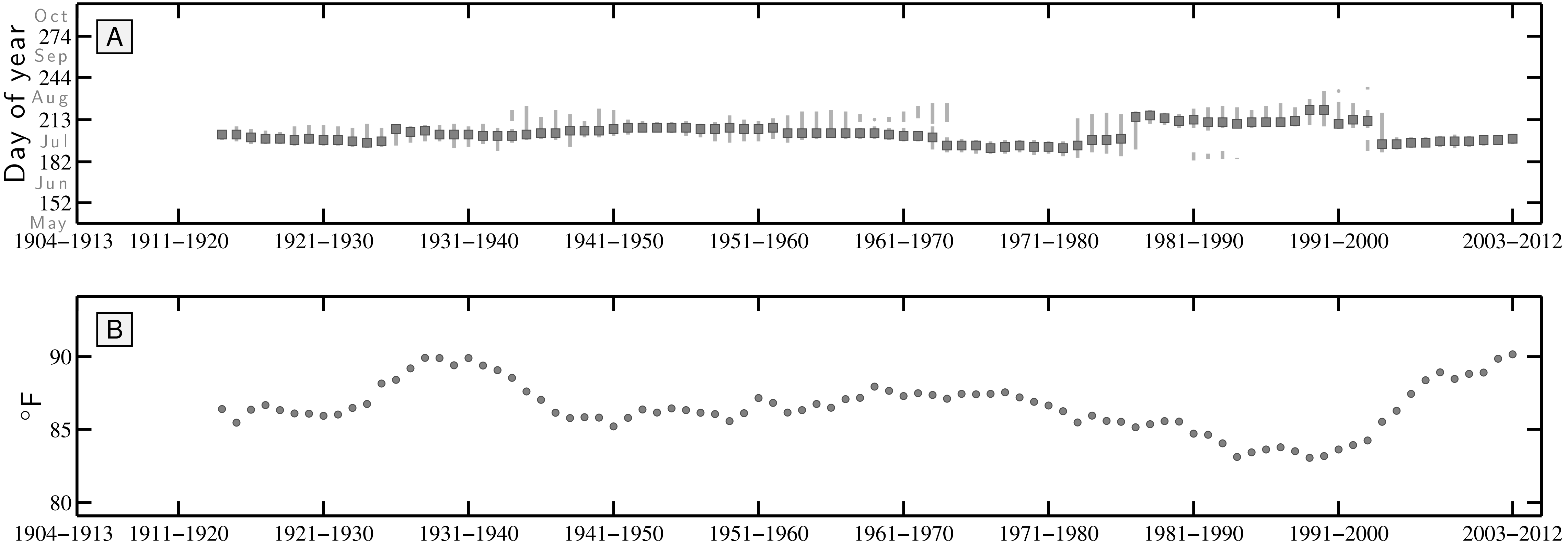

10 year Winter Teletherm dynamics for CODY, WY:

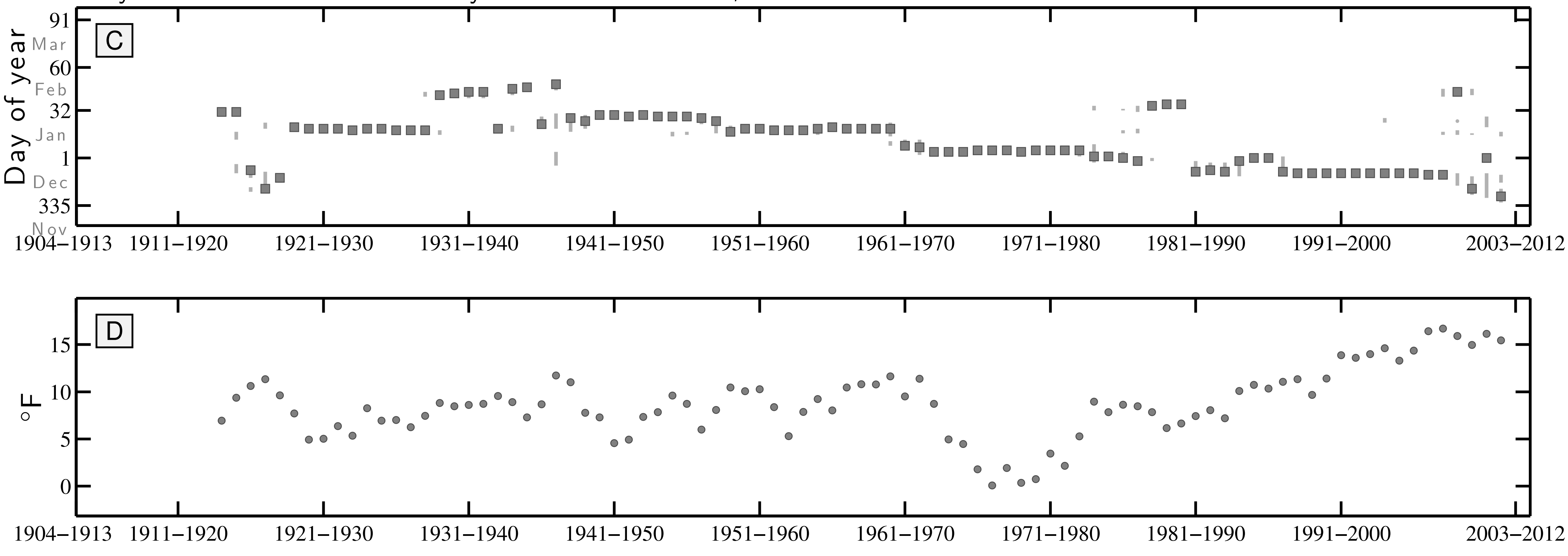

10 year Summer Teletherm dynamics for COLONY, WY:

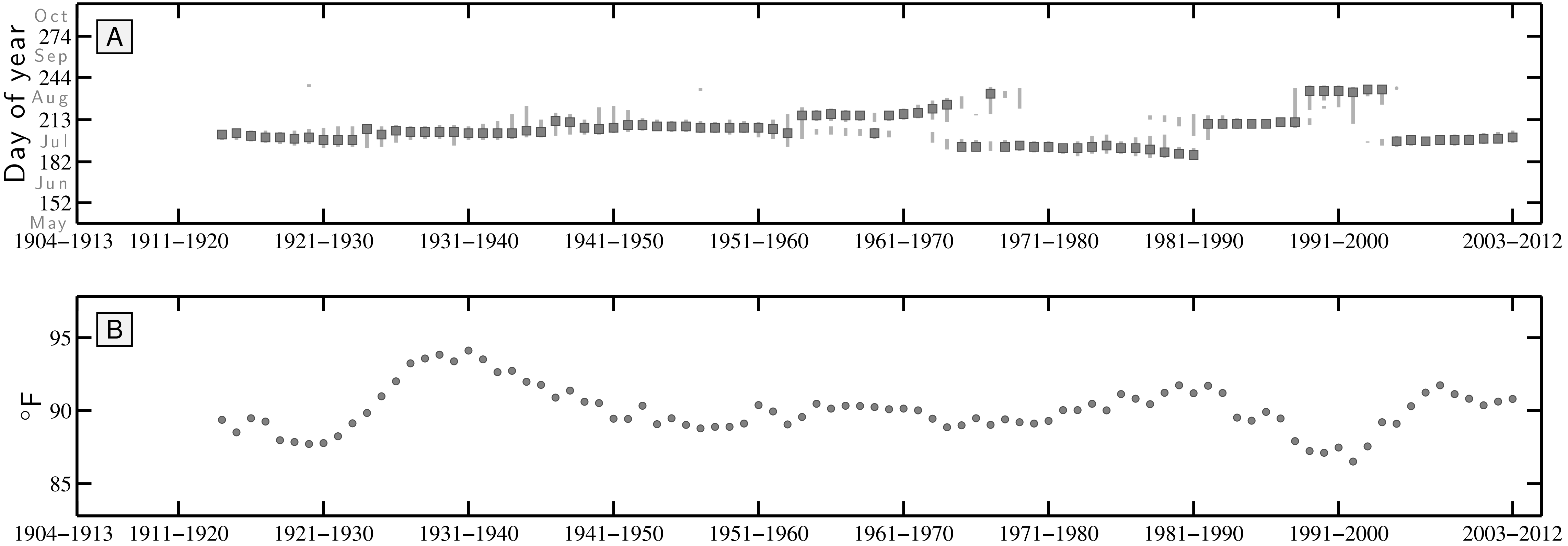

10 year Winter Teletherm dynamics for COLONY, WY:

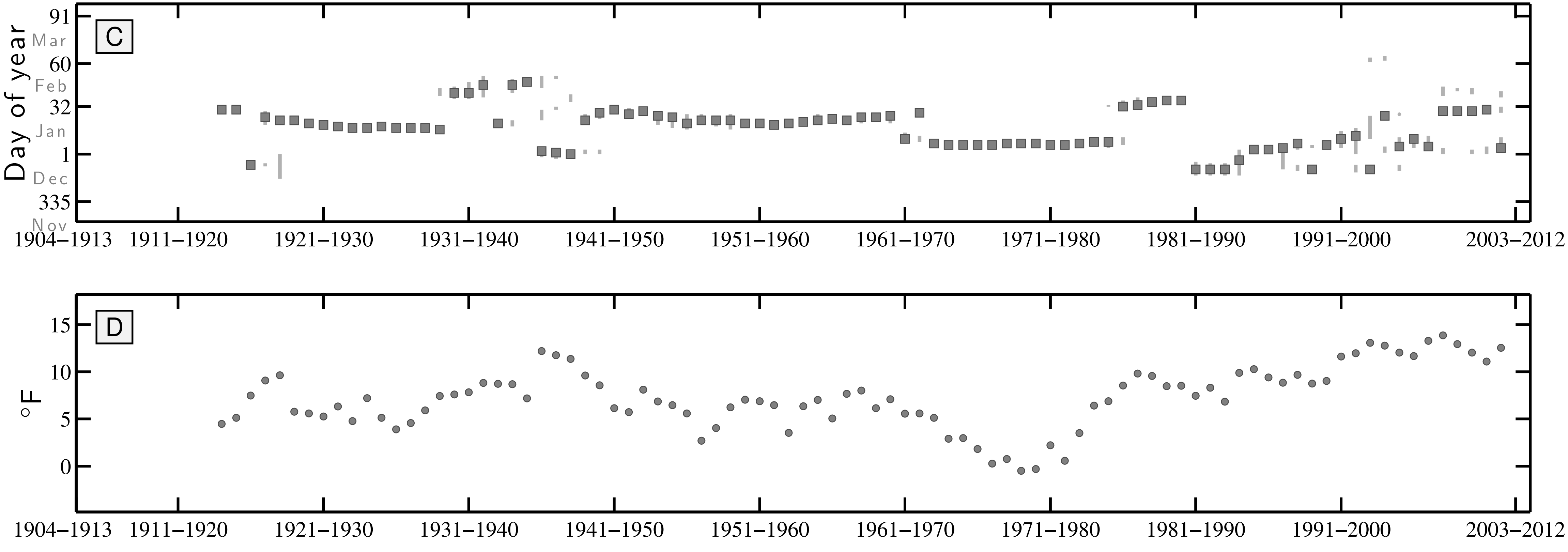

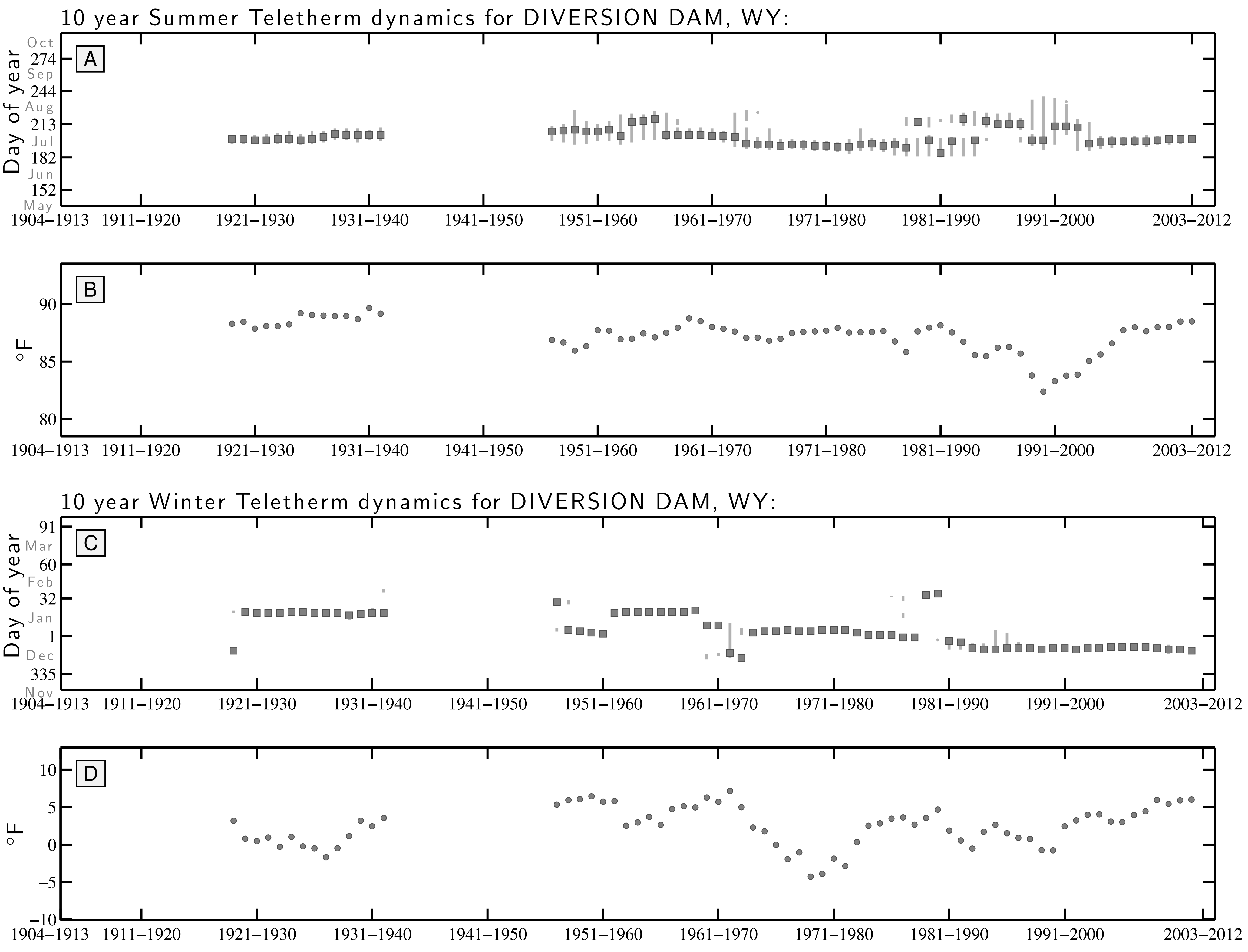
10 year Summer Teletherm dynamics for DIVERSION DAM, WY:
A
B
Day of year
°F
Oct
274
Sep
244
Aug
213
Jul
182
Jun
152
May
90
85
80
10 year Winter Teletherm dynamics for DIVERSION DAM, WY:
C
D
Day of year
°F
91
Mar
60
Feb
32
Jan
1
Dec
335
Nov
10
5
0
−5
−10
1904–1913
1911–1920
1921–1930
1931–1940
1941–1950
1951–1960
1961–1970
1971–1980
1981–1990
1991–2000
2003–2012

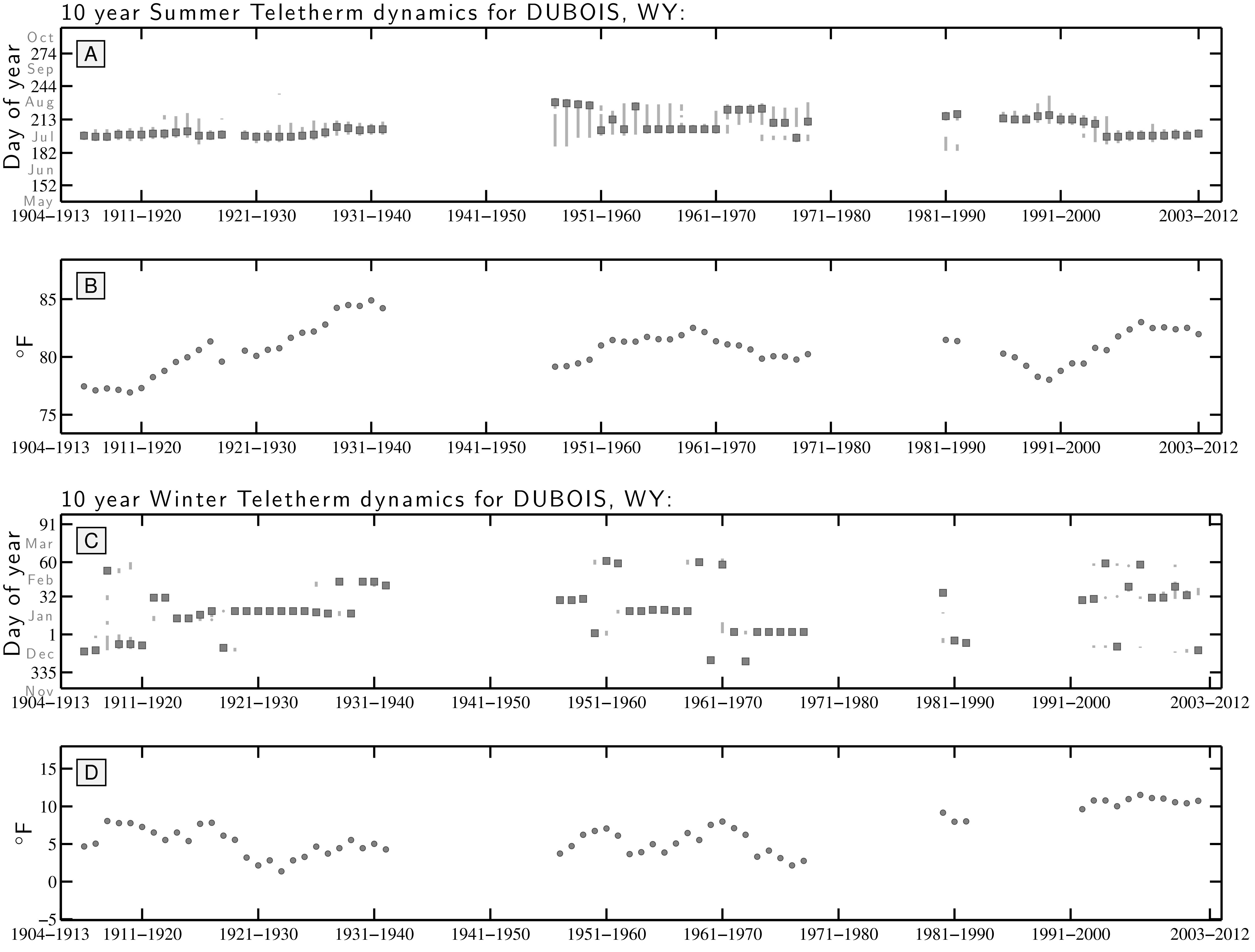

10 year Summer Teletherm dynamics for DUBOIS, WY:
A
Day of year
Oct
274
Sep
244
Aug
213
Jul
182
Jun
152
May
1904–1913
1911–1920
1921–1930
1931–1940
1941–1950
1951–1960
1961–1970
1971–1980
1981–1990
1991–2000
2003–2012
B
°F
85
80
75
10 year Winter Teletherm dynamics for DUBOIS, WY:
C
91
Mar
60
Feb
32
Jan
1
Dec
335
Nov
D
15
10
5
0
−5

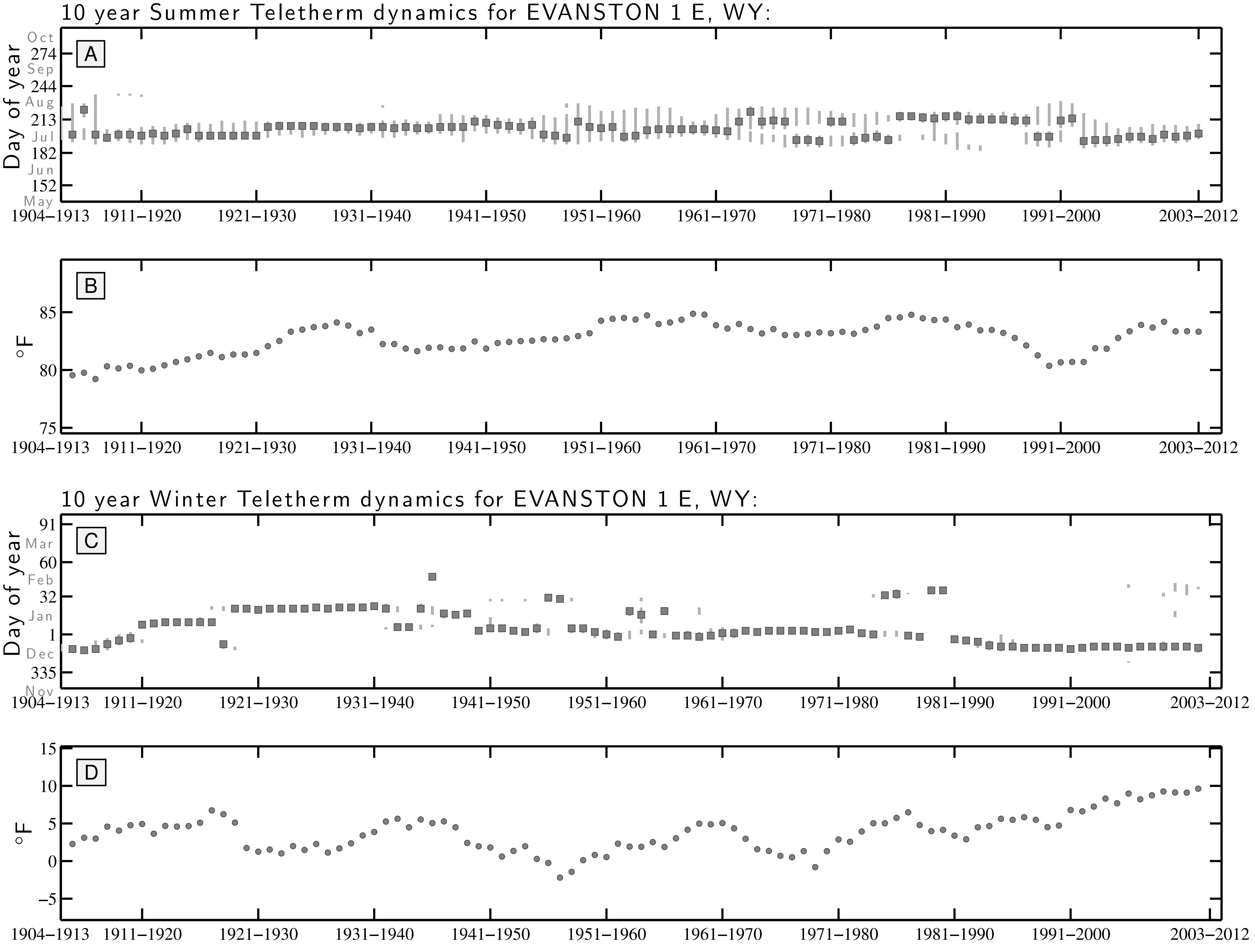

10 year Summer Teletherm dynamics for EVANSTON 1 E, WY:
A
Day of year
Oct
274
Sep
244
Aug
213
Jul
182
Jun
152
May
1904–1913
1911–1920
1921–1930
1931–1940
1941–1950
1951–1960
1961–1970
1971–1980
1981–1990
1991–2000
2003–2012
B
°F
85
80
75
1904–1913
1911–1920
1921–1930
1931–1940
1941–1950
1951–1960
1961–1970
1971–1980
1981–1990
1991–2000
2003–2012
10 year Winter Teletherm dynamics for EVANSTON 1 E, WY:
C
Day of year
91
Mar
60
Feb
32
Jan
1
Dec
335
Nov
1904–1913
1911–1920
1921–1930
1931–1940
1941–1950
1951–1960
1961–1970
1971–1980
1981–1990
1991–2000
2003–2012
D
°F
15
10
5
0
−5
1904–1913
1911–1920
1921–1930
1931–1940
1941–1950
1951–1960
1961–1970
1971–1980
1981–1990
1991–2000
2003–2012

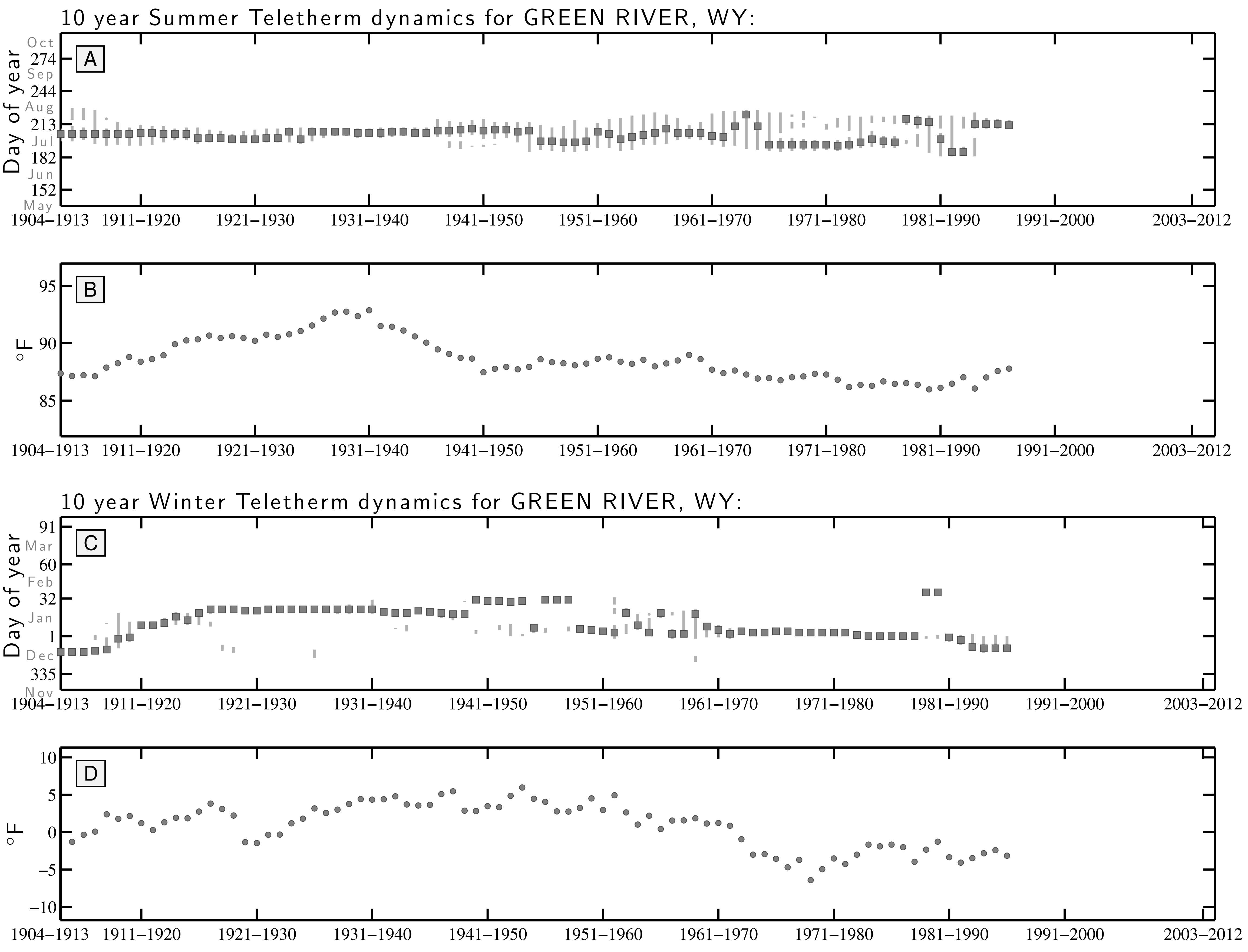
10 year Summer Teletherm dynamics for GREEN RIVER, WY:
A
Day of year
Oct
274
Sep
244
Aug
213
Jul
182
Jun
152
May
B
°F
95
90
85
10 year Winter Teletherm dynamics for GREEN RIVER, WY:
C
91
Mar
60
Feb
32
Jan
1
Dec
335
Nov
D
10
5
0
−5
−10
1904–1913
1911–1920
1921–1930
1931–1940
1941–1950
1951–1960
1961–1970
1971–1980
1981–1990
1991–2000
2003–2012

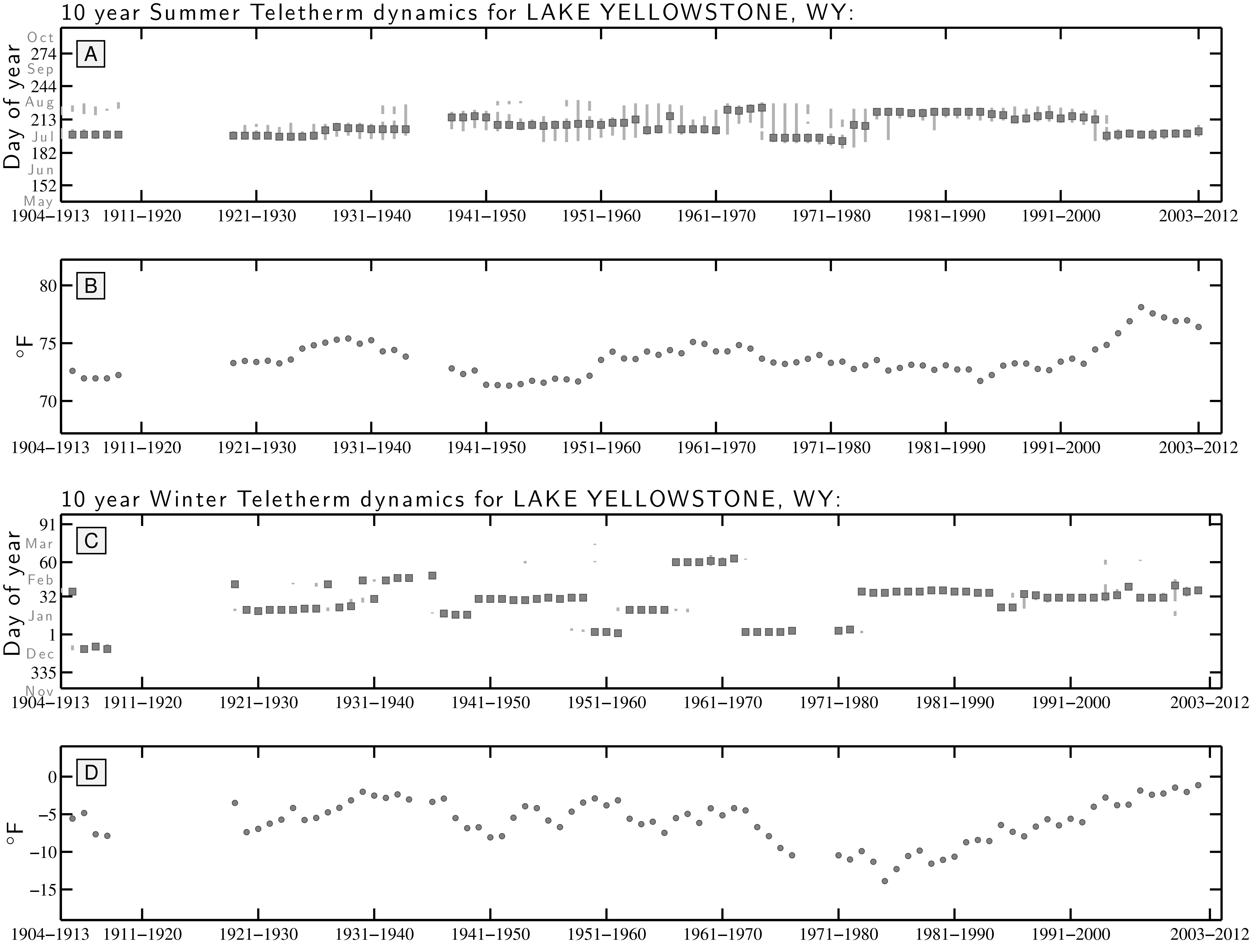
10 year Summer Teletherm dynamics for LAKE YELLOWSTONE, WY:
A
Day of year
Oct
274
Sep
244
Aug
213
Jul
182
Jun
152
May
B
°F
80
75
70
10 year Winter Teletherm dynamics for LAKE YELLOWSTONE, WY:
C
Day of year
91
Mar
60
Feb
32
Jan
1
Dec
335
Nov
D
°F
0
−5
−10
−15
1904–1913
1911–1920
1921–1930
1931–1940
1941–1950
1951–1960
1961–1970
1971–1980
1981–1990
1991–2000
2003–2012

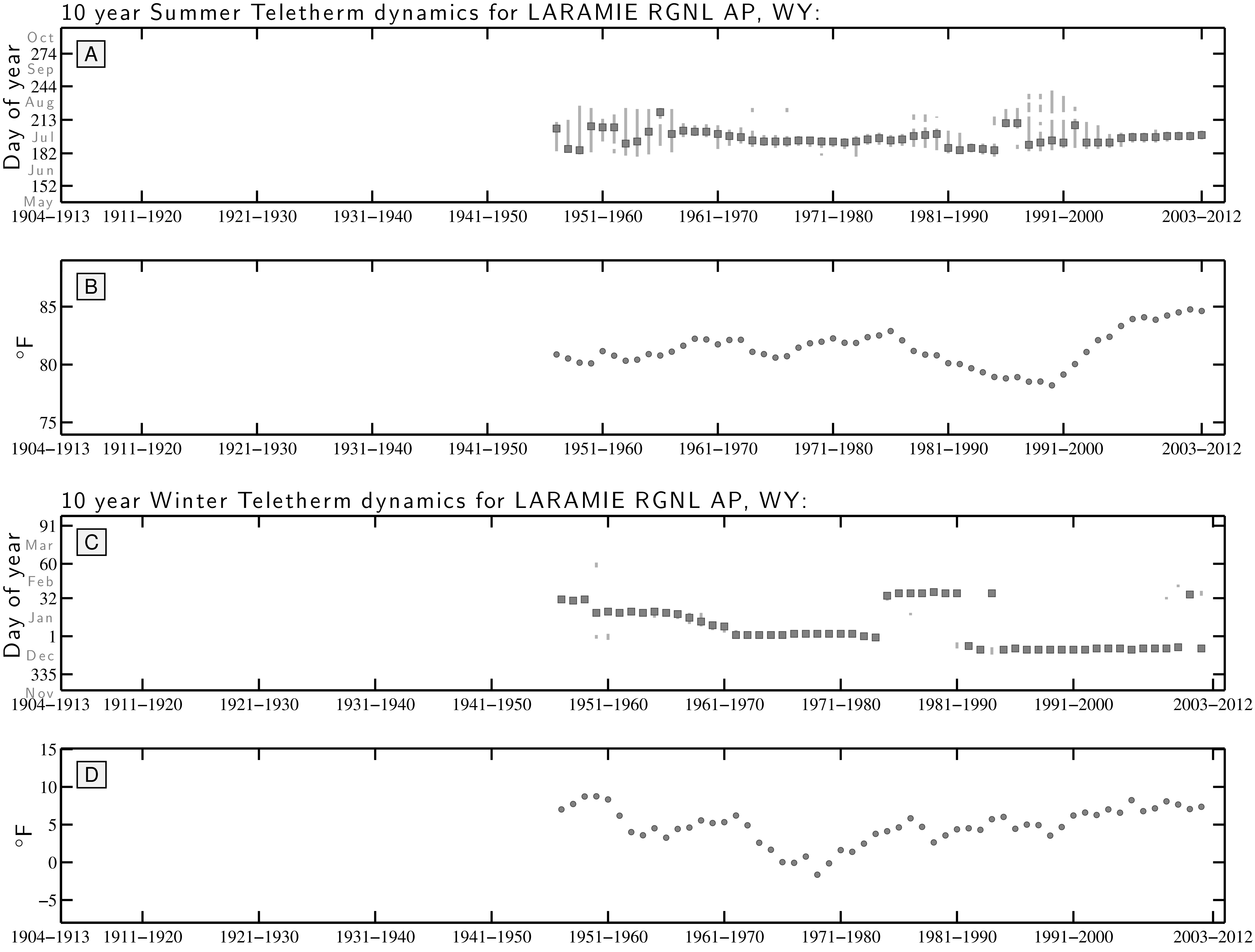
10 year Summer Teletherm dynamics for LARAMIE RGNL AP, WY:
A
B
Day of year
°F
Oct
274
Sep
244
Aug
213
Jul
182
Jun
152
May
85
80
75
1904–1913
1911–1920
1921–1930
1931–1940
1941–1950
1951–1960
1961–1970
1971–1980
1981–1990
1991–2000
2003–2012
10 year Winter Teletherm dynamics for LARAMIE RGNL AP, WY:
C
D
91
Mar
60
Feb
32
Jan
1
Dec
335
Nov
15
10
5
0
−5

10 year Summer Teletherm dynamics for LUSK 2 SW, WY:

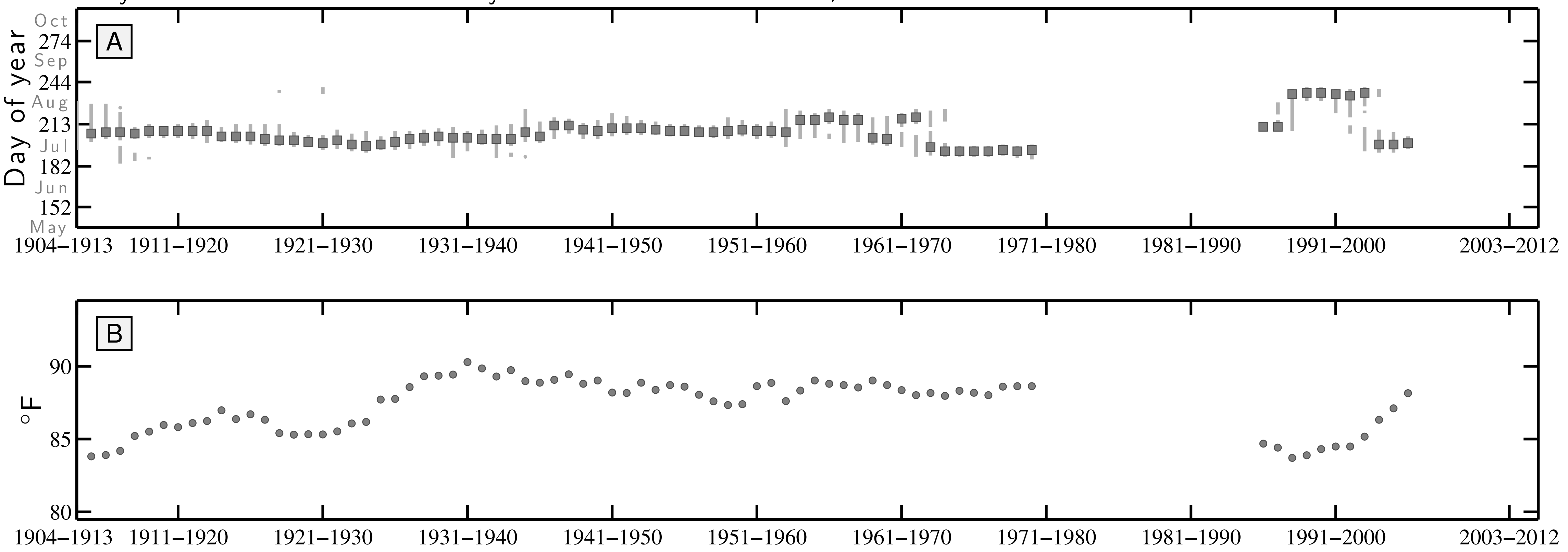

10 year Winter Teletherm dynamics for LUSK 2 SW, WY:

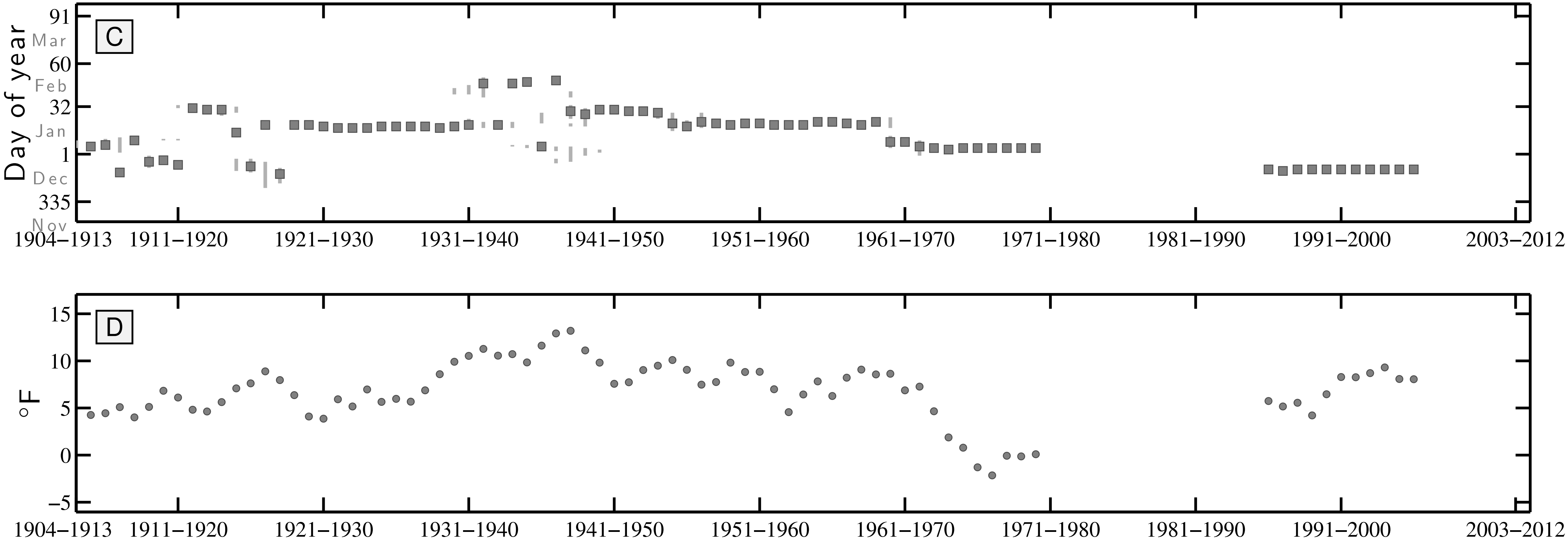

10 year Summer Teletherm dynamics for MIDWEST, WY:

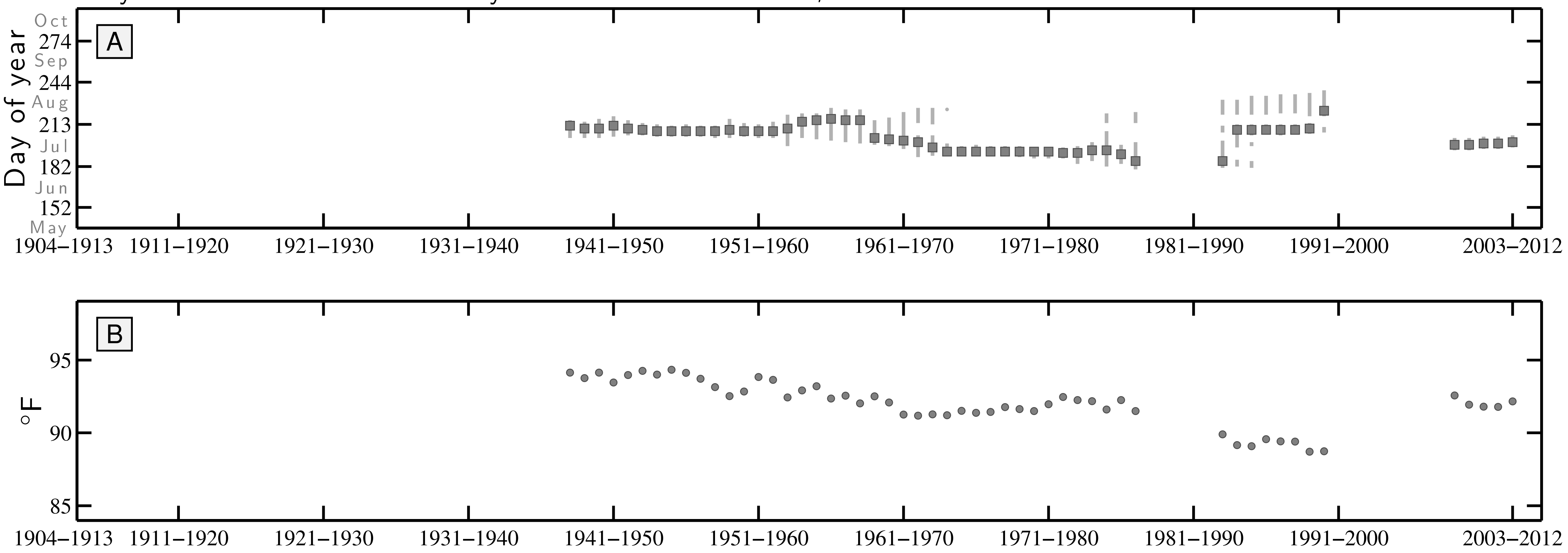

10 year Winter Teletherm dynamics for MIDWEST, WY:

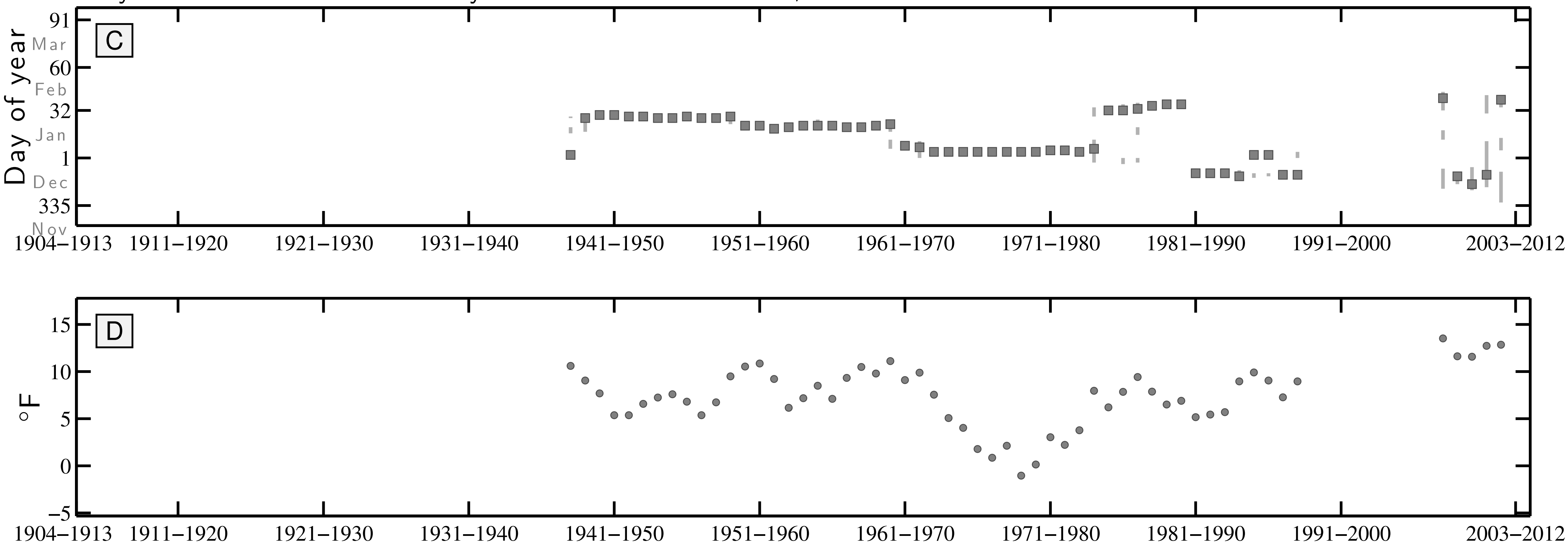

10 year Summer Teletherm dynamics for MORAN 5 WNW, WY:

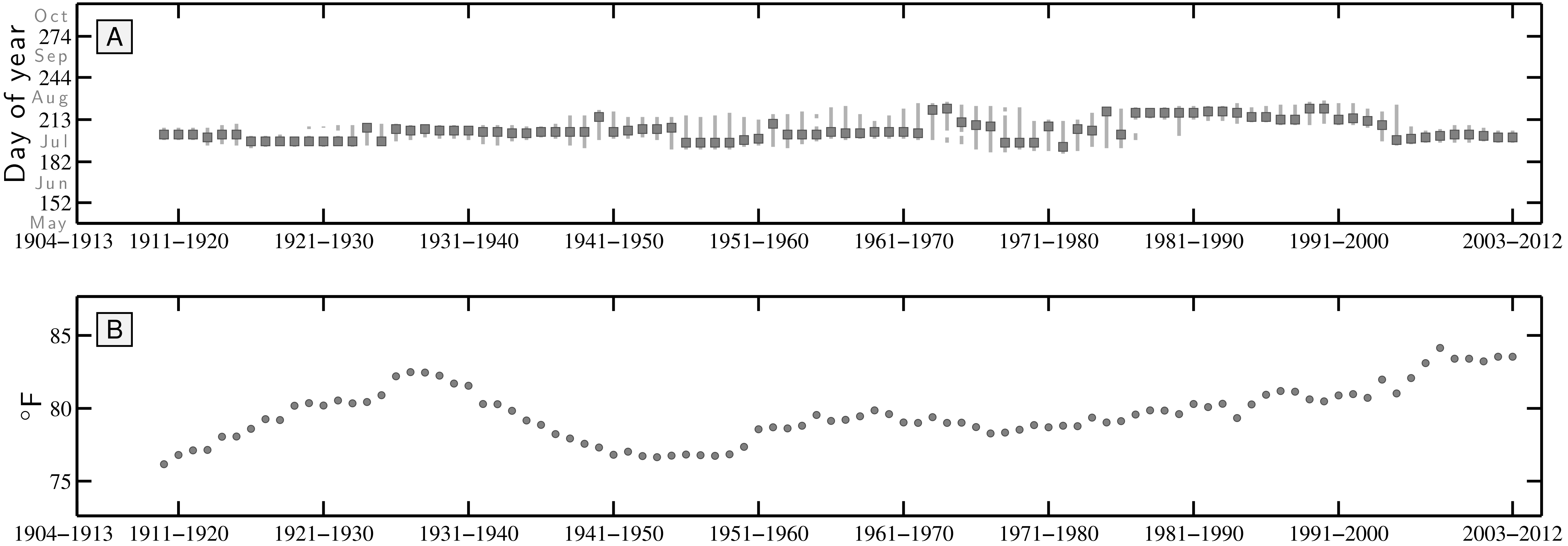

10 year Winter Teletherm dynamics for MORAN 5 WNW, WY:

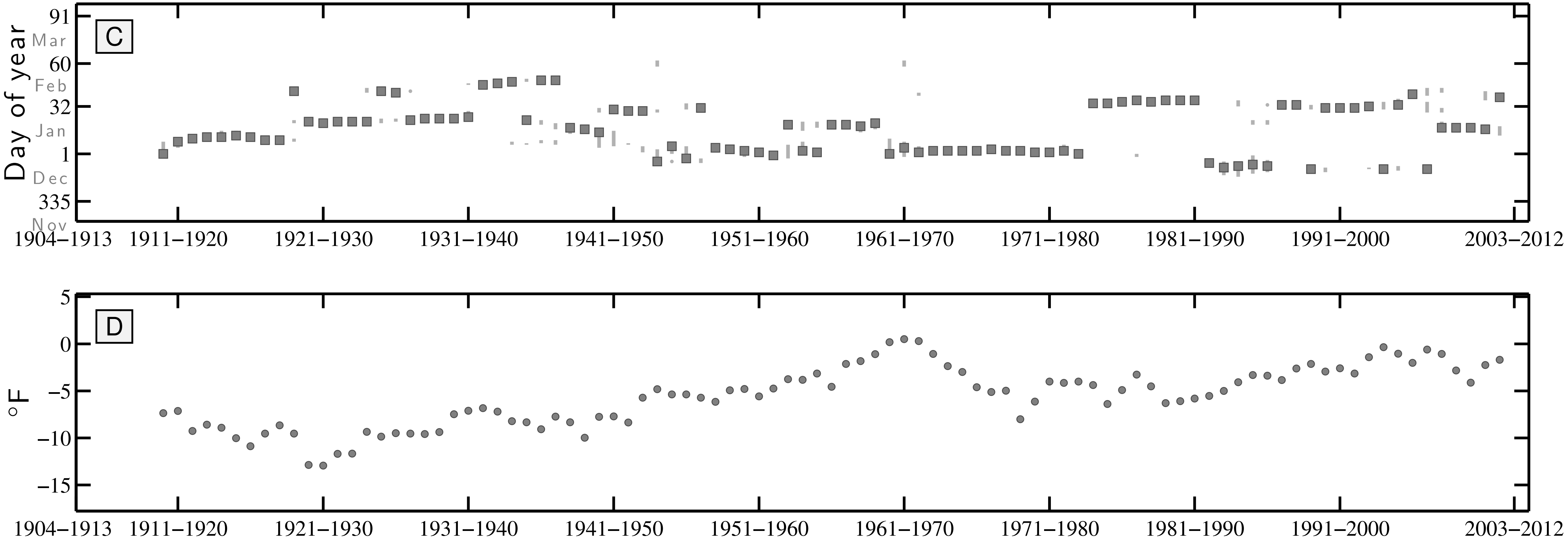

10 year Summer Teletherm dynamics for NEWCASTLE, WY:

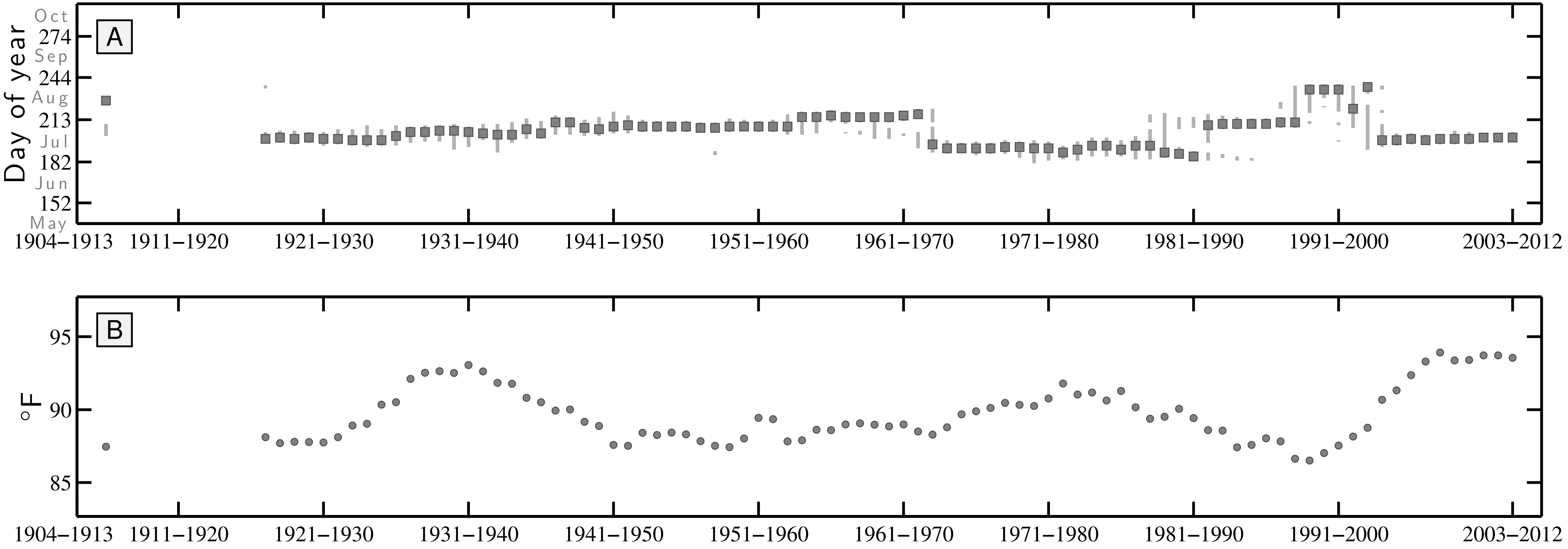

10 year Winter Teletherm dynamics for NEWCASTLE, WY:

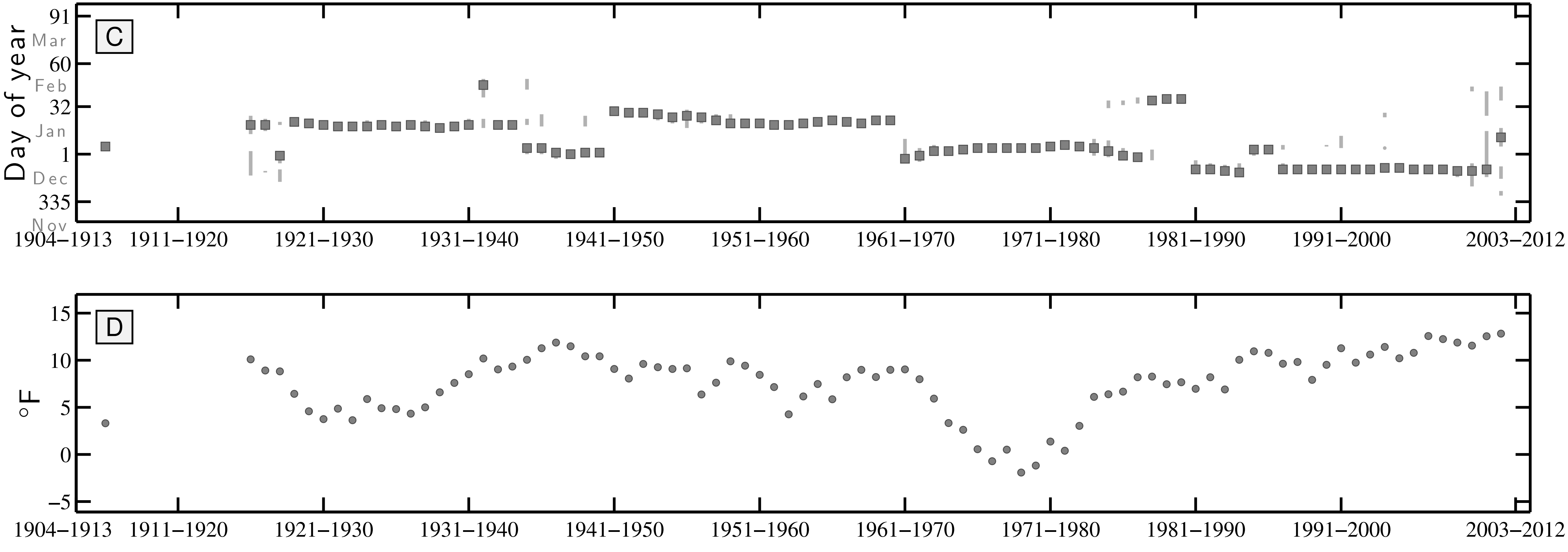

10 year Summer Teletherm dynamics for PAVILLION, WY:

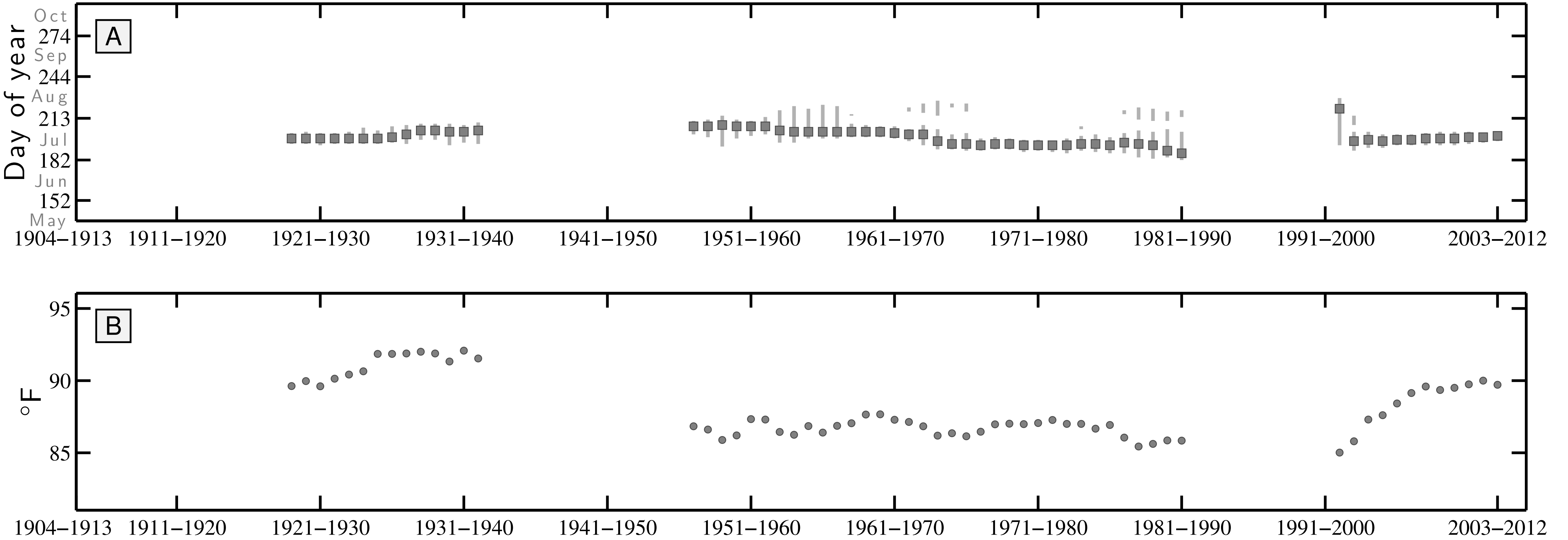

10 year Winter Teletherm dynamics for PAVILLION, WY:

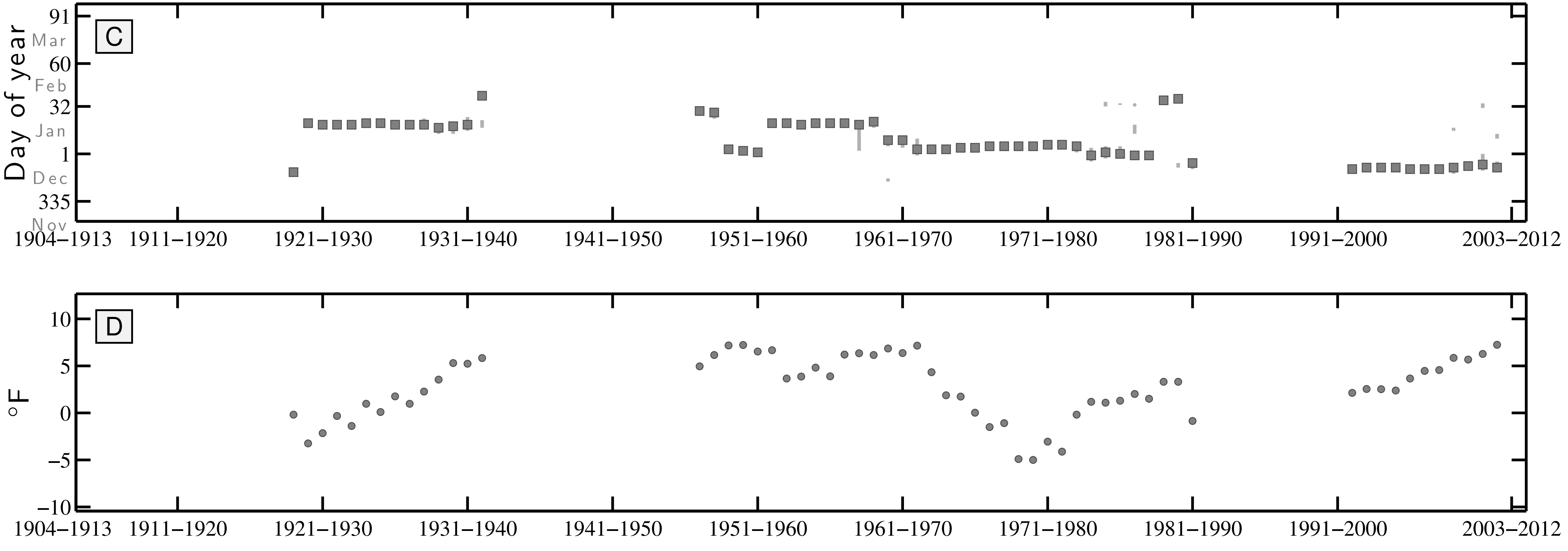

10 year Summer Teletherm dynamics for PINE BLUFFS 5W, WY:

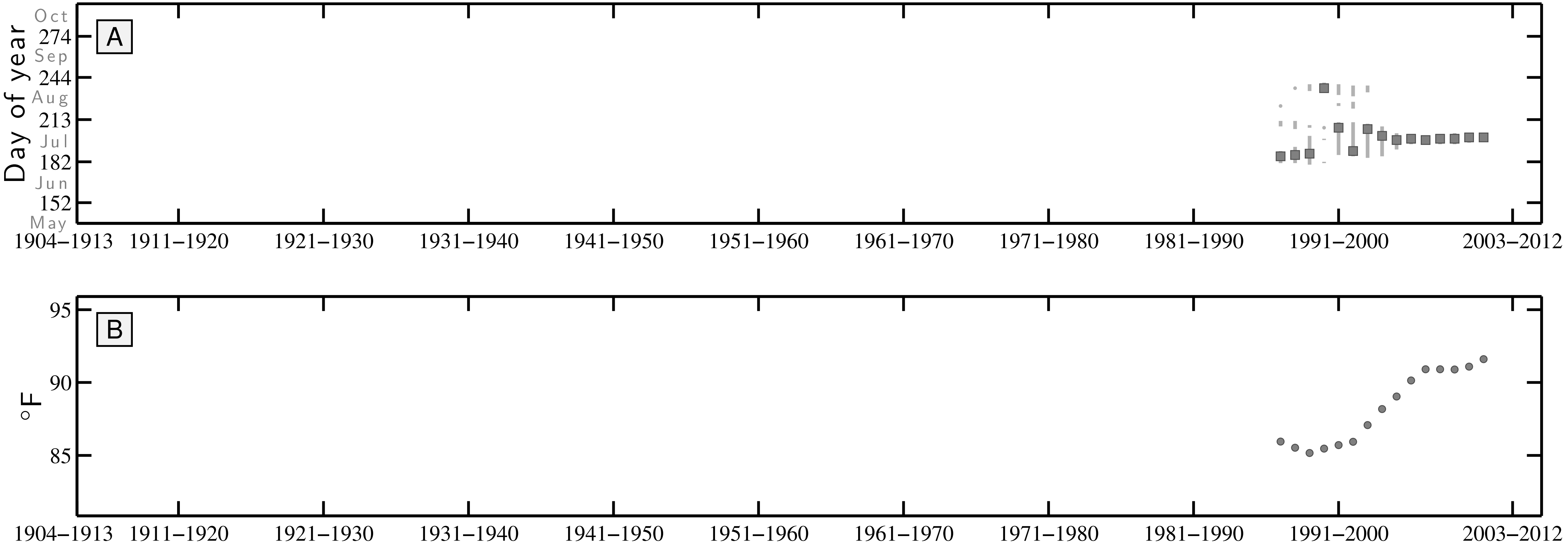

10 year Winter Teletherm dynamics for PINE BLUFFS 5W, WY:

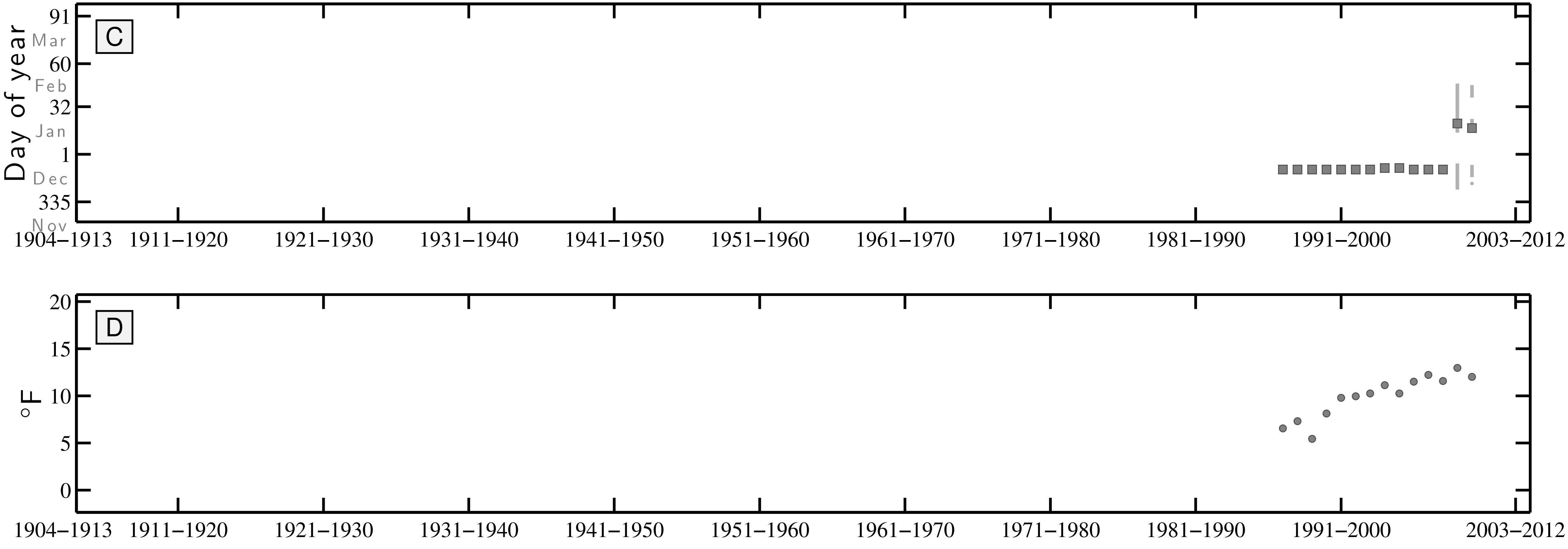

10 year Summer Teletherm dynamics for PINEDALE, WY:

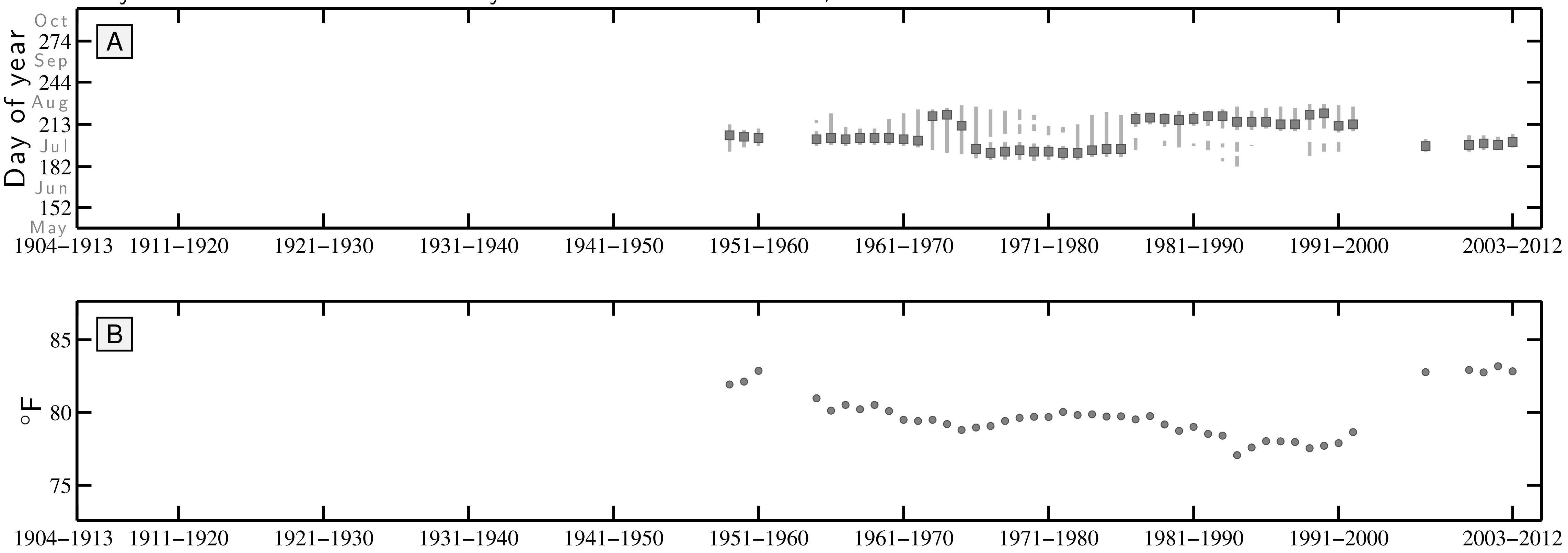

10 year Winter Teletherm dynamics for PINEDALE, WY:

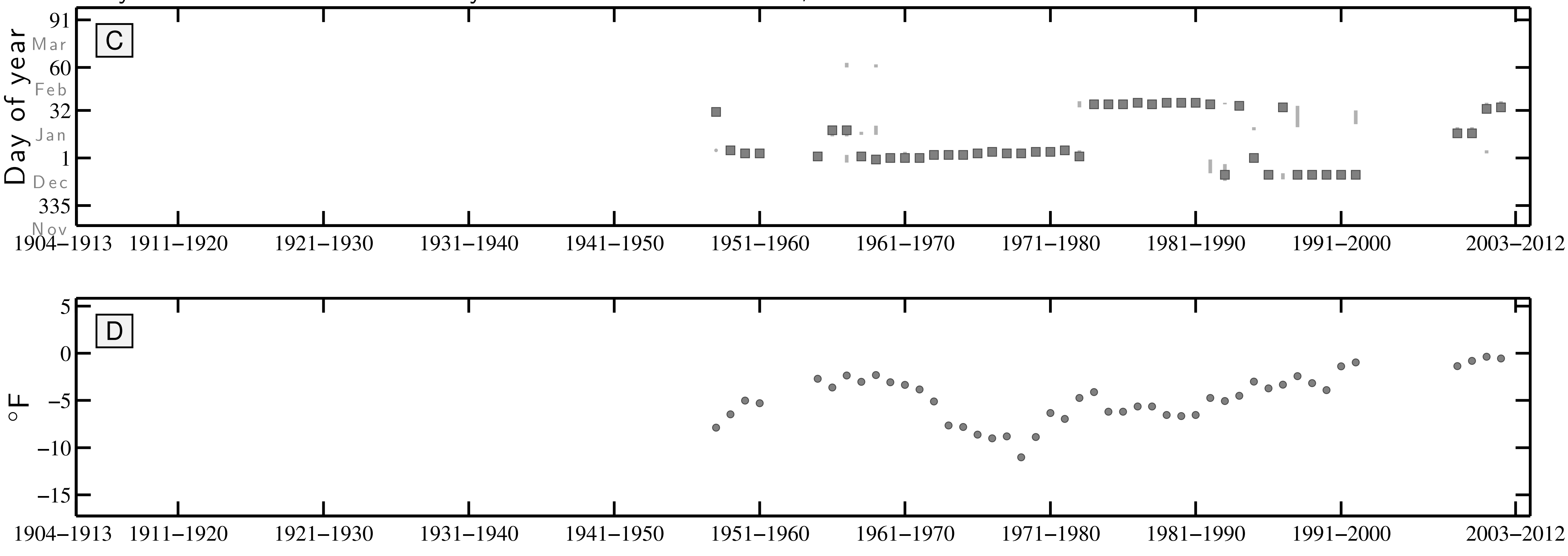

10 year Summer Teletherm dynamics for POWELL FLD STN, WY:

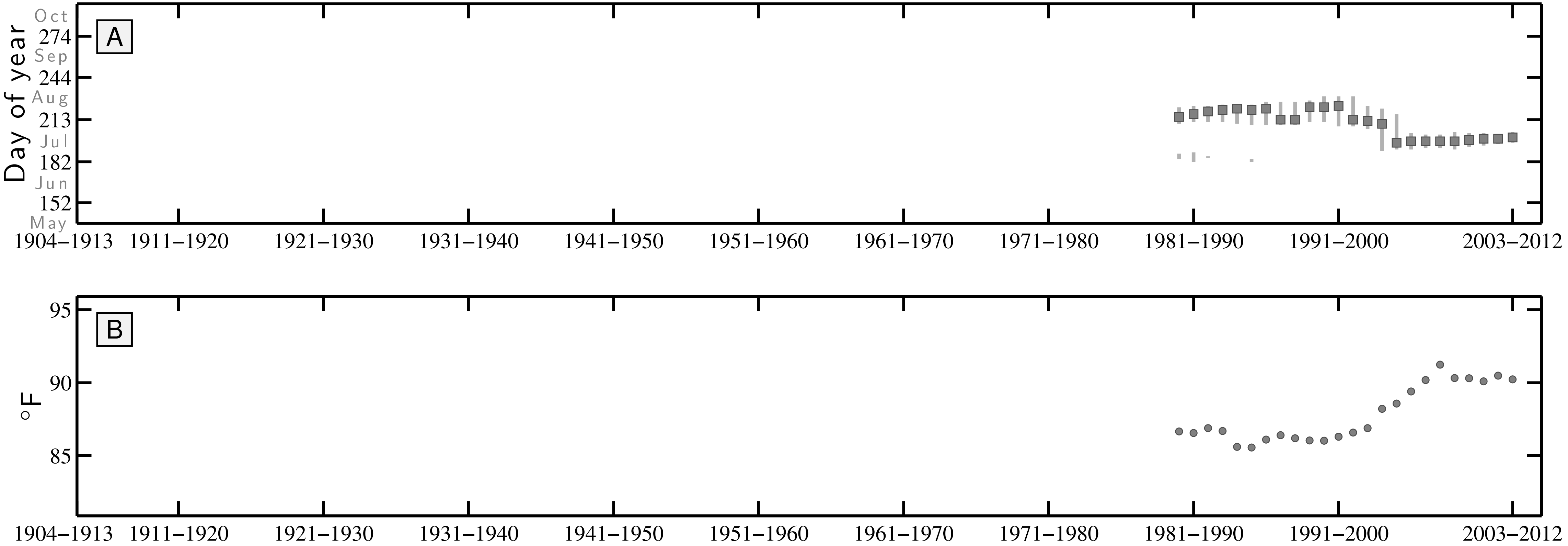

10 year Winter Teletherm dynamics for POWELL FLD STN, WY:

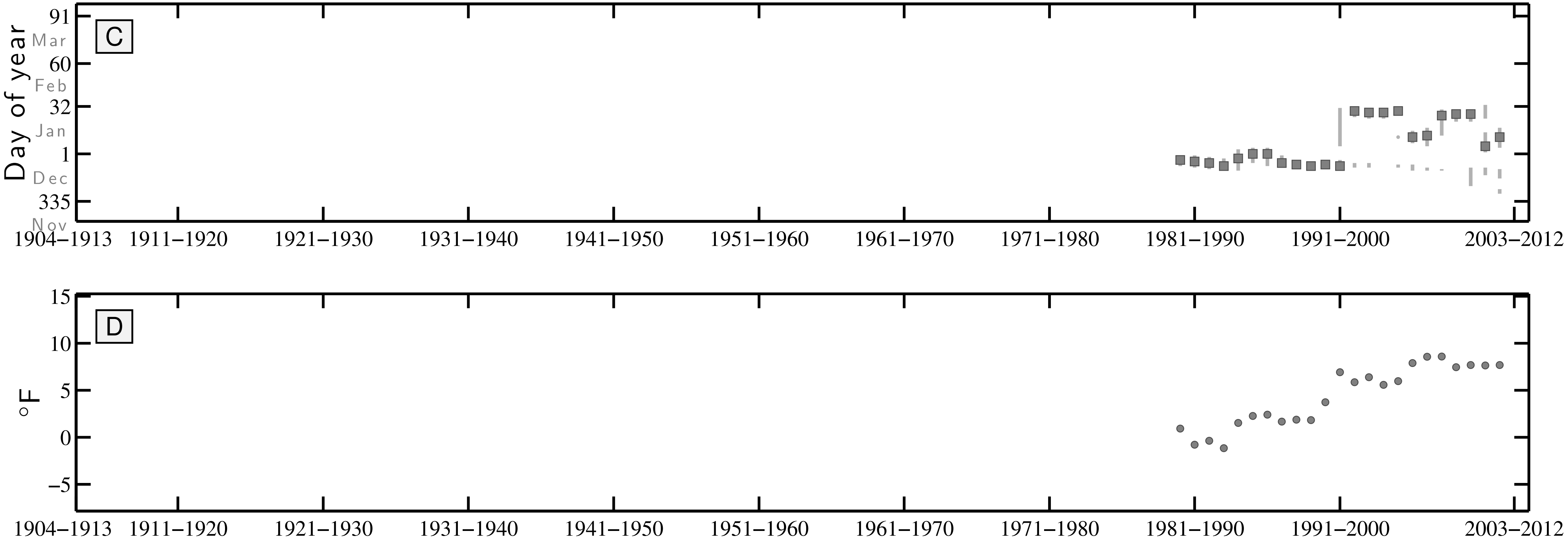

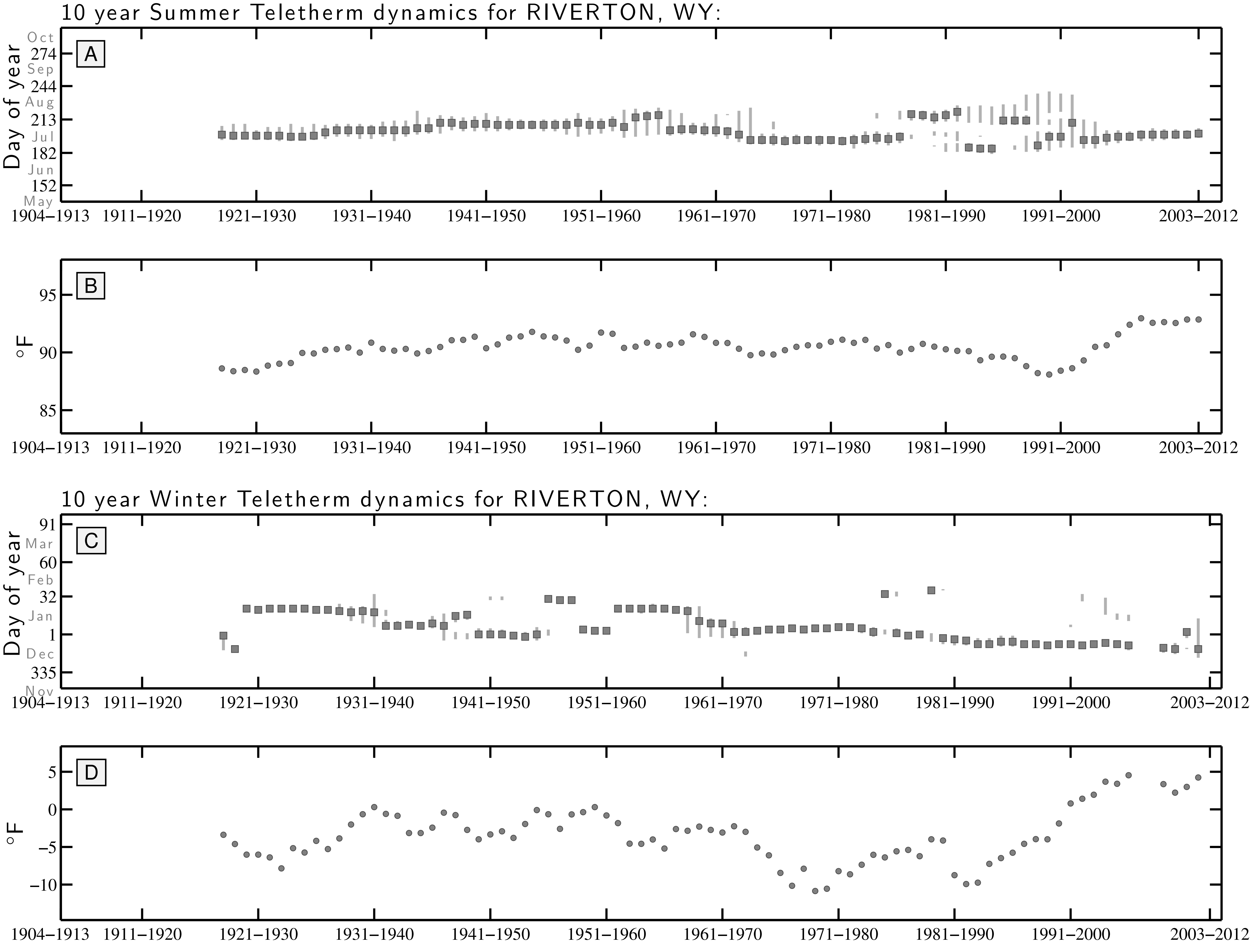
10 year Summer Teletherm dynamics for RIVERTON, WY:
A
B
Day of year
°F
Oct
274
Sep
244
Aug
213
Jul
182
Jun
152
May
95
90
85
1904–1913
1911–1920
1921–1930
1931–1940
1941–1950
1951–1960
1961–1970
1971–1980
1981–1990
1991–2000
2003–2012
10 year Winter Teletherm dynamics for RIVERTON, WY:
C
D
91
Mar
60
Feb
32
Jan
1
Dec
335
Nov
5
0
−5
−10

10 year Summer Teletherm dynamics for ROCK SPRINGS AP, WY:

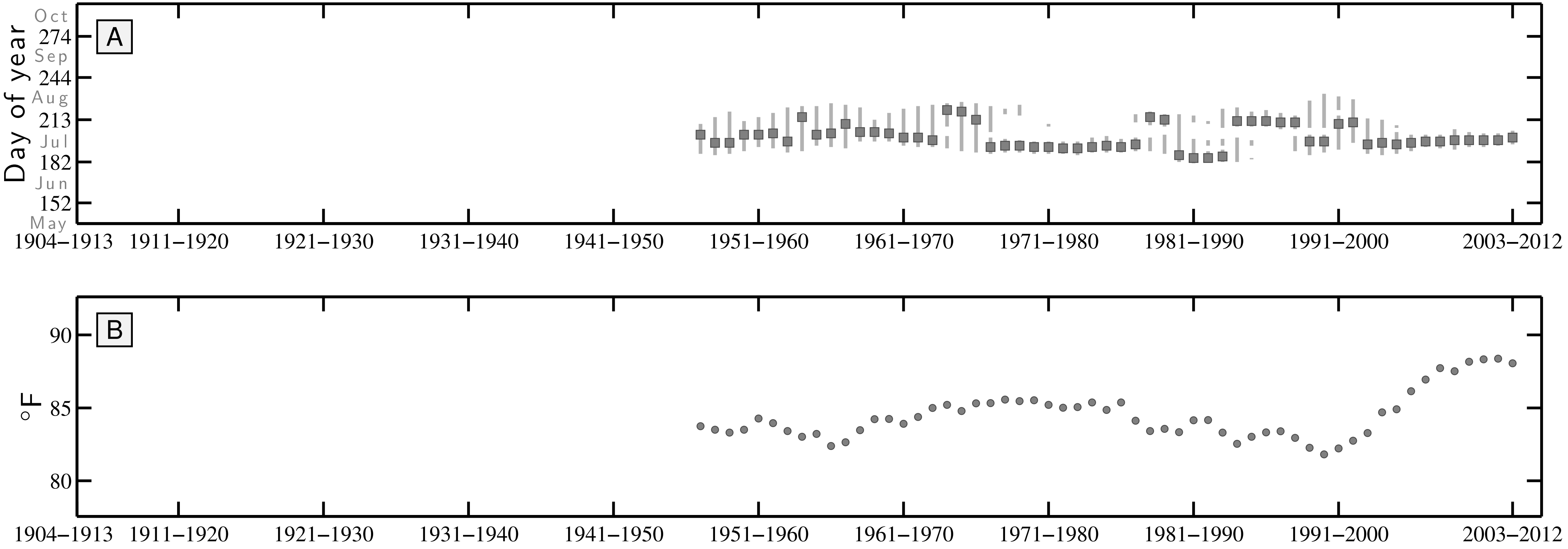

10 year Winter Teletherm dynamics for ROCK SPRINGS AP, WY:

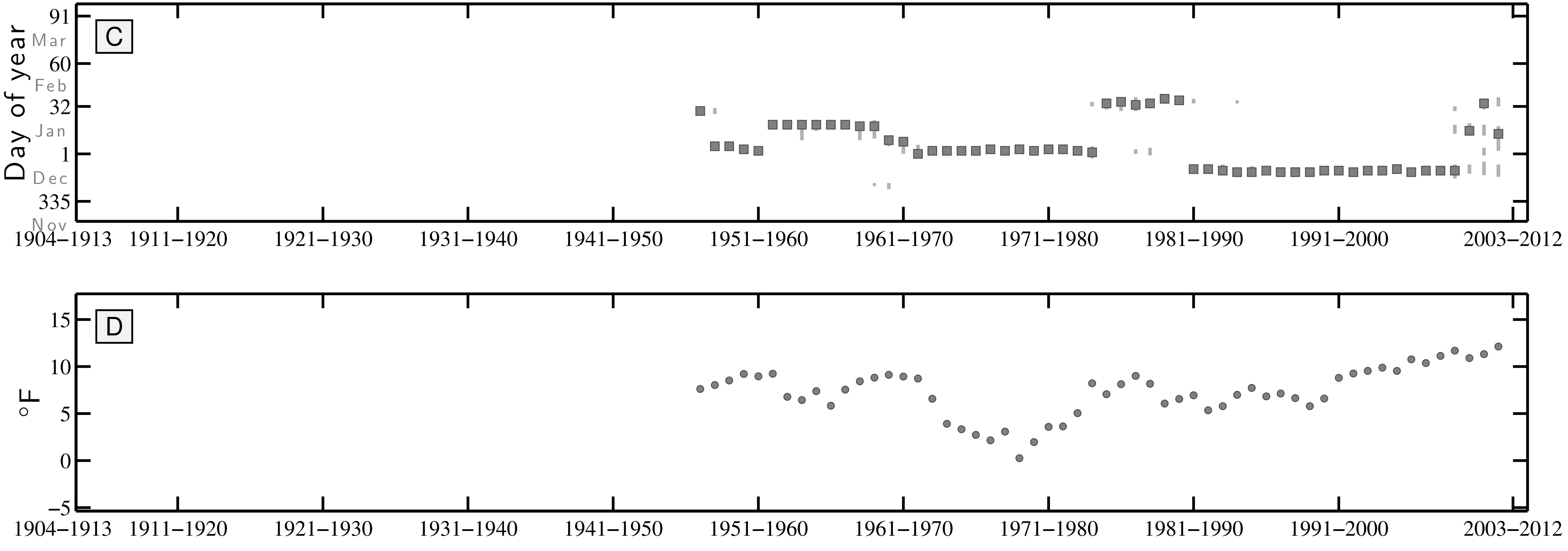

10 year Summer Teletherm dynamics for SARATOGA, WY:

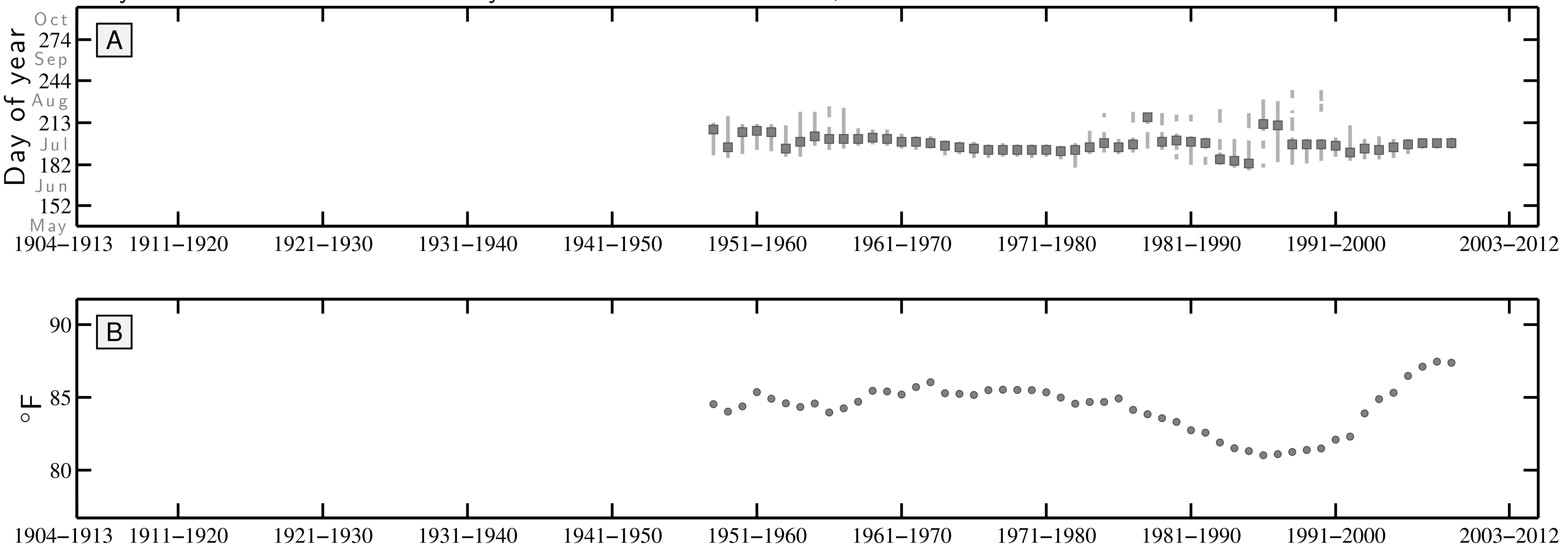

10 year Winter Teletherm dynamics for SARATOGA, WY:

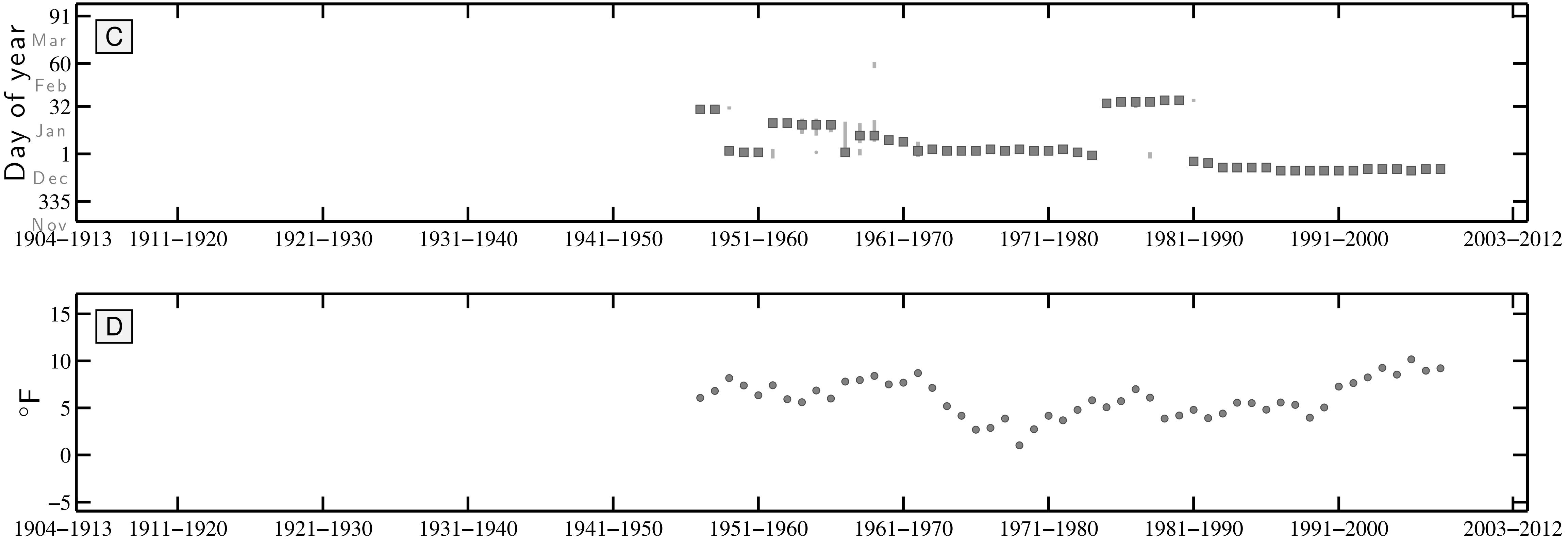

10 year Summer Teletherm dynamics for SHERIDAN FLD STN, WY:

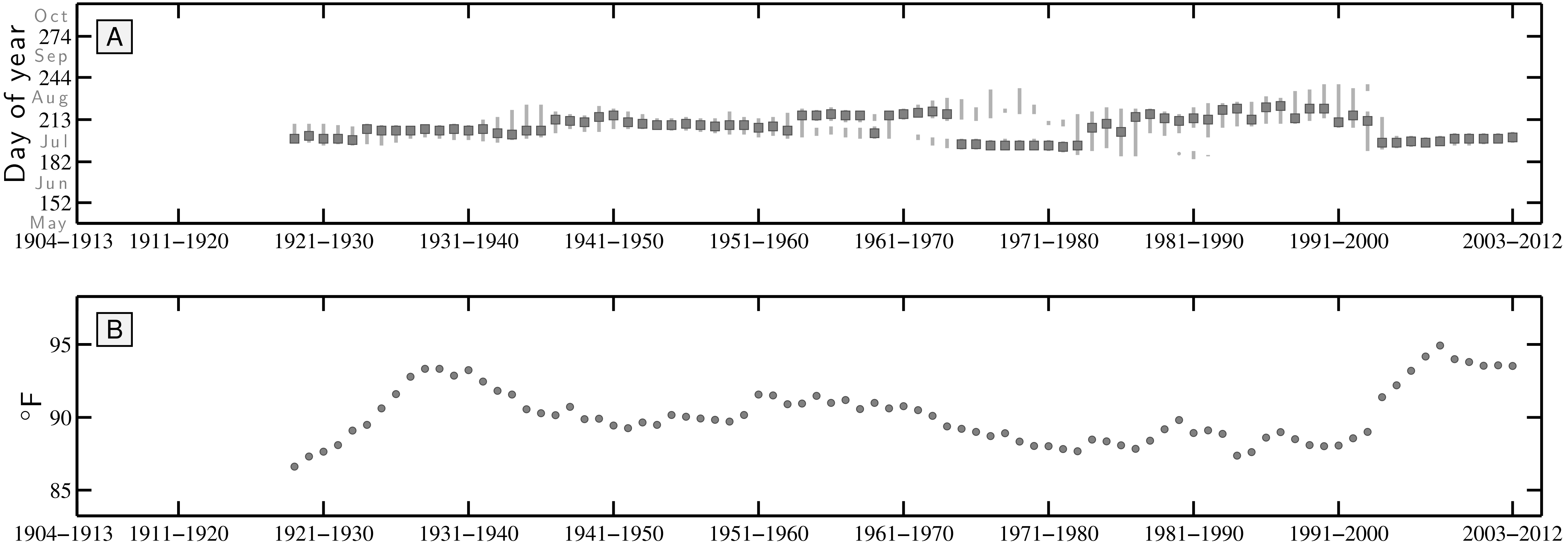

10 year Winter Teletherm dynamics for SHERIDAN FLD STN, WY:

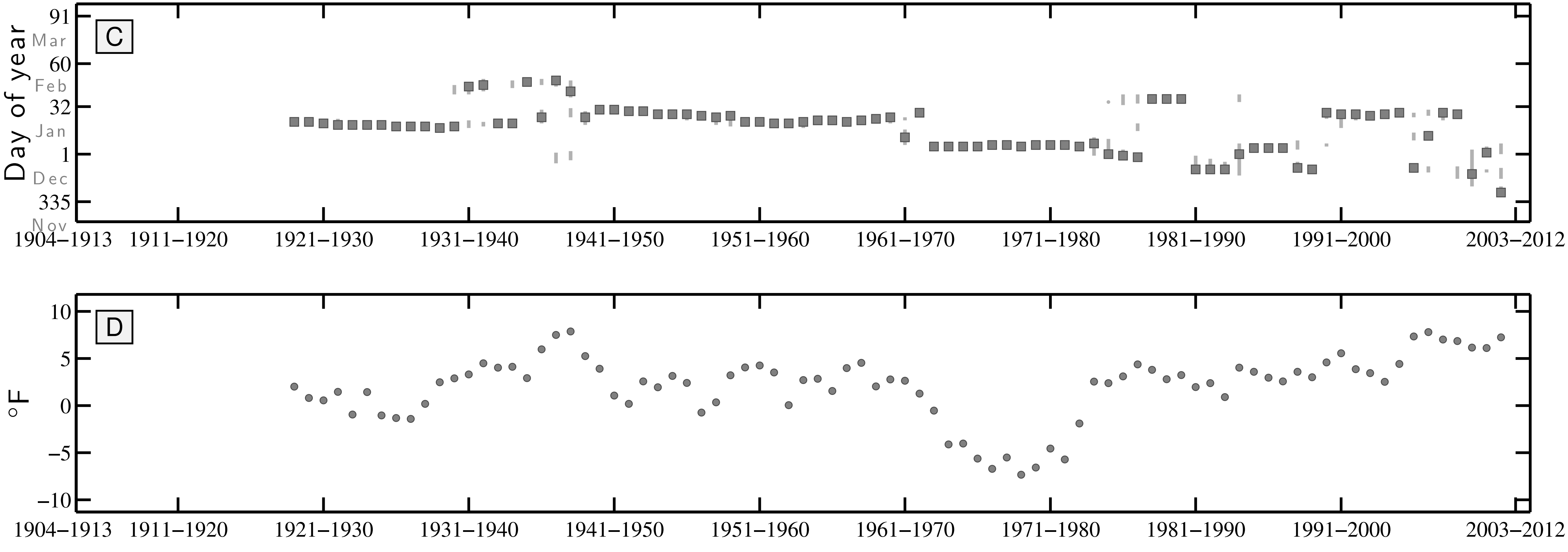

10 year Summer Teletherm dynamics for TORRINGTON EXP FARM, WY:

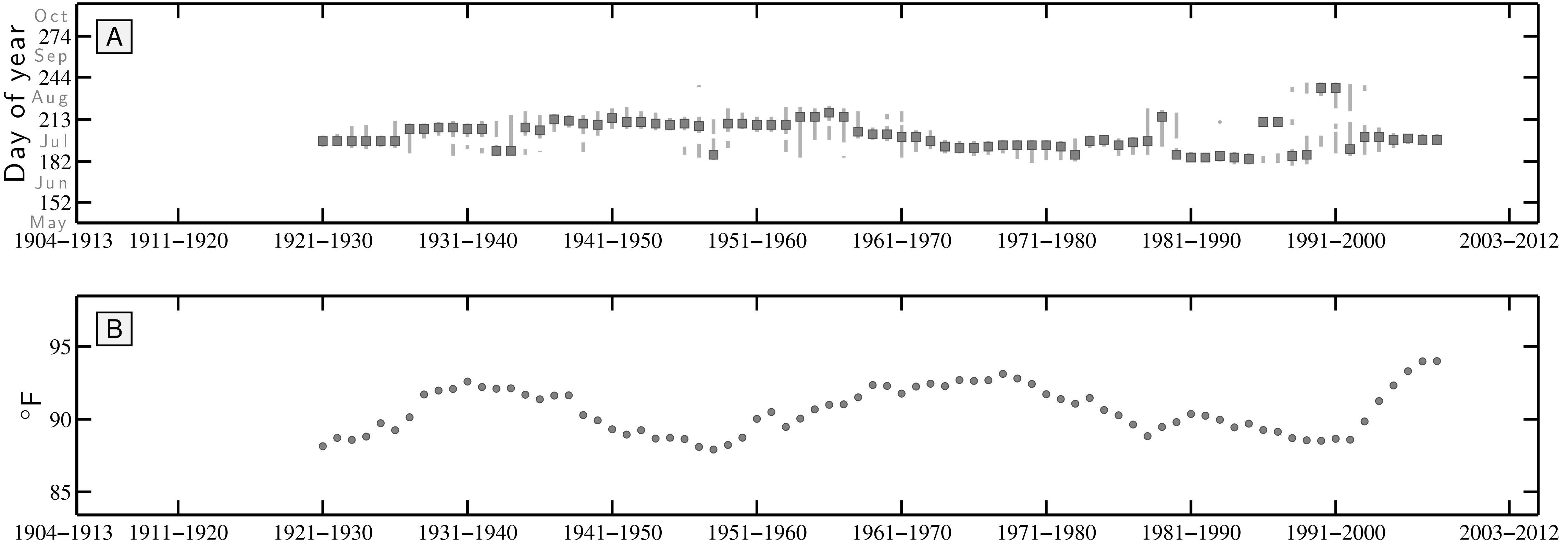

10 year Winter Teletherm dynamics for TORRINGTON EXP FARM, WY:

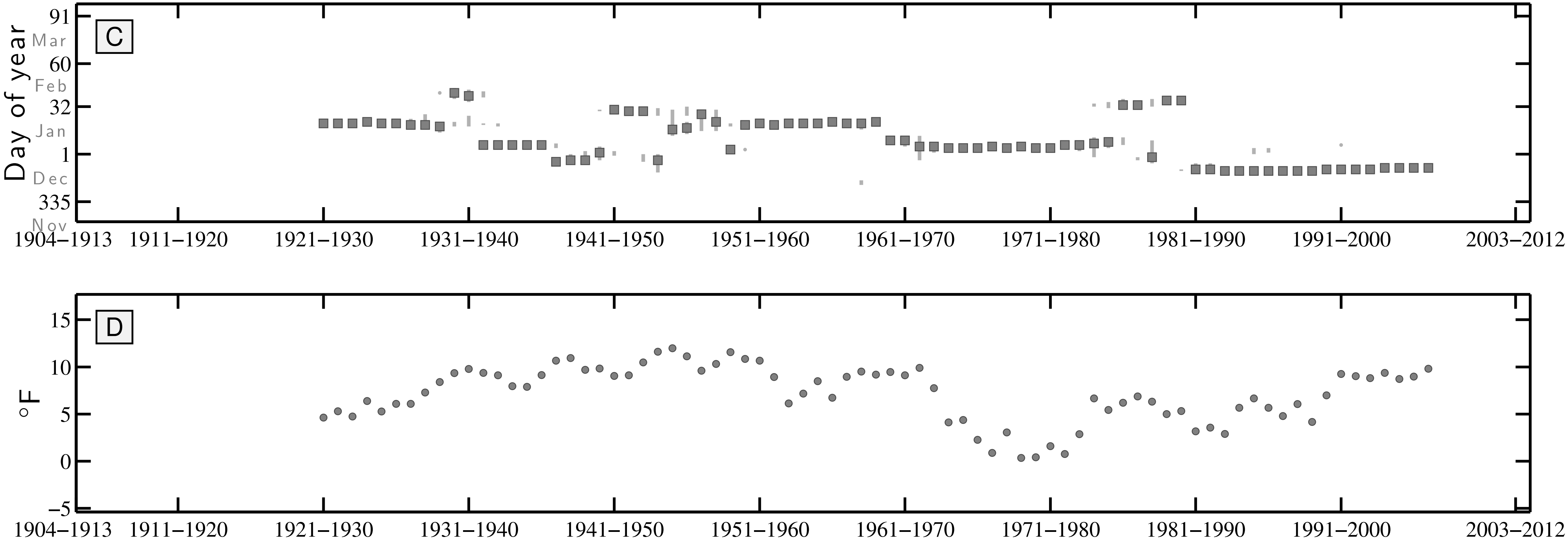

10 year Summer Teletherm dynamics for WHEATLAND 4 N, WY:

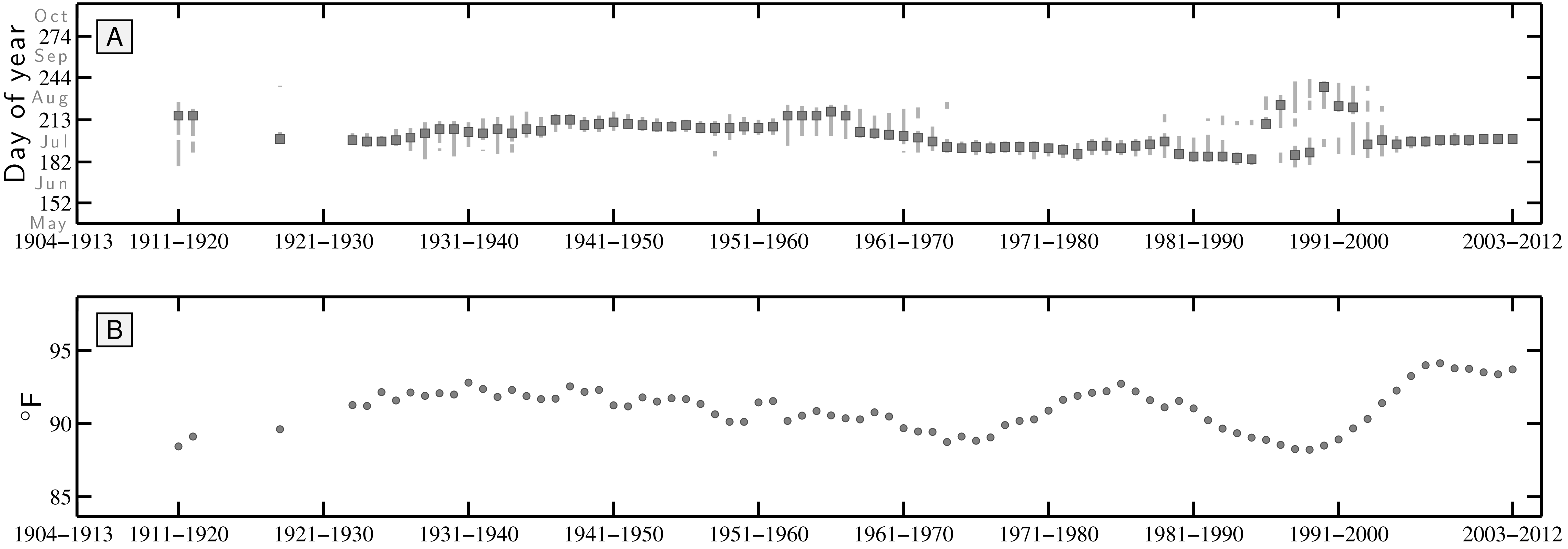

10 year Winter Teletherm dynamics for WHEATLAND 4 N, WY:

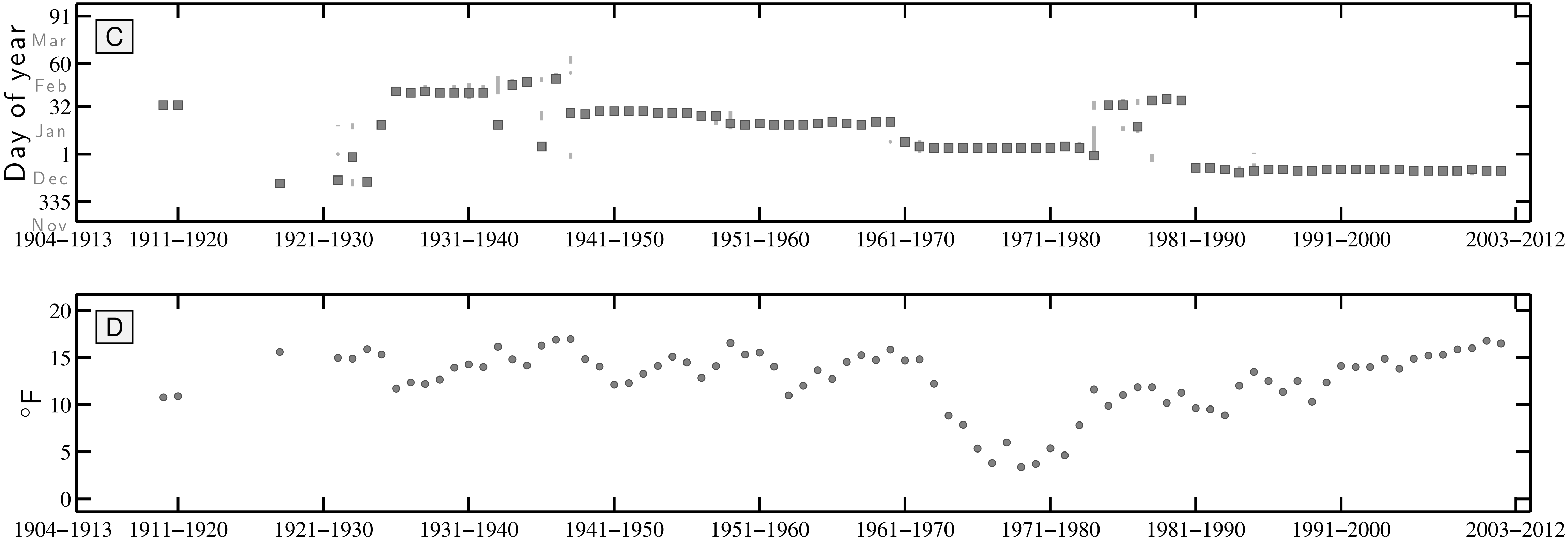

10 year Summer Teletherm dynamics for WORLAND, WY:

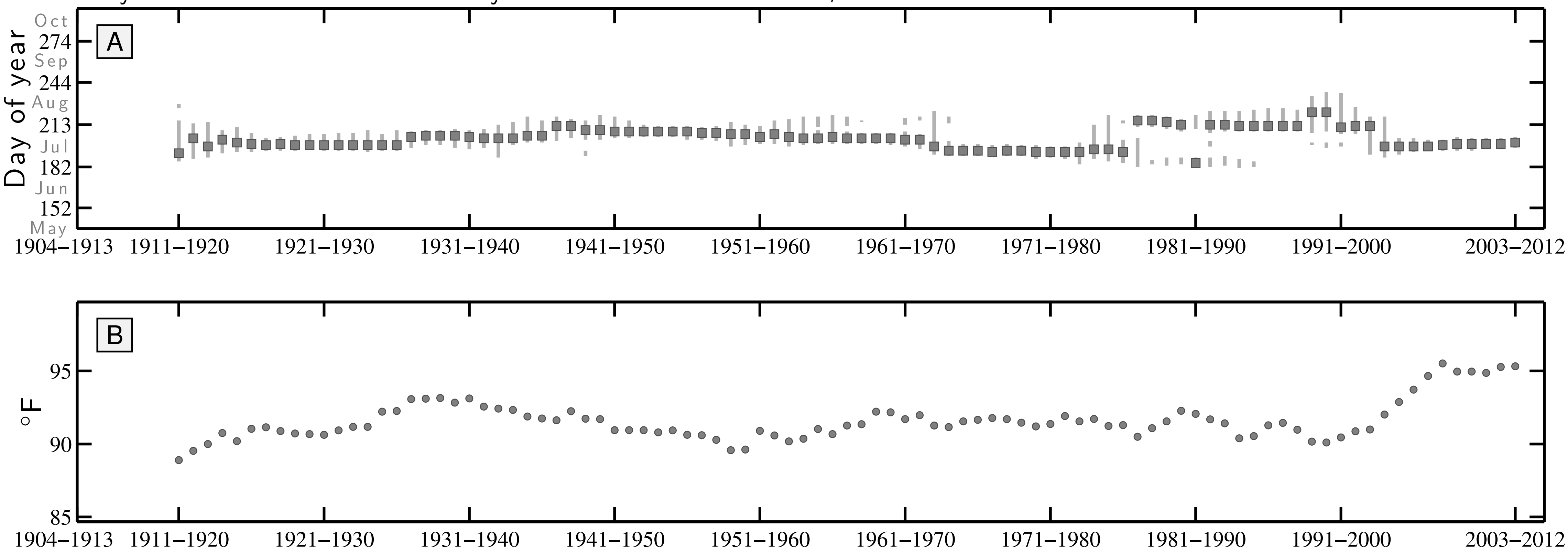

10 year Winter Teletherm dynamics for WORLAND, WY:

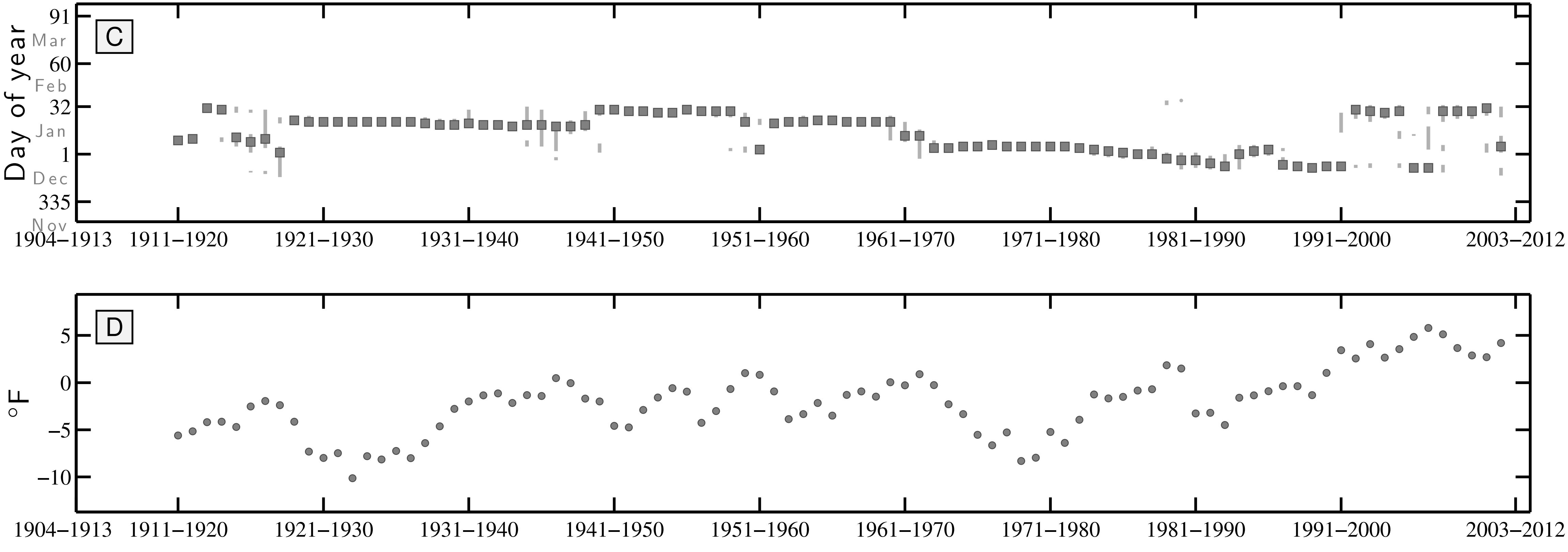

10 year Summer Teletherm dynamics for YELLOWSTONE PK MAMMOTH, WY:

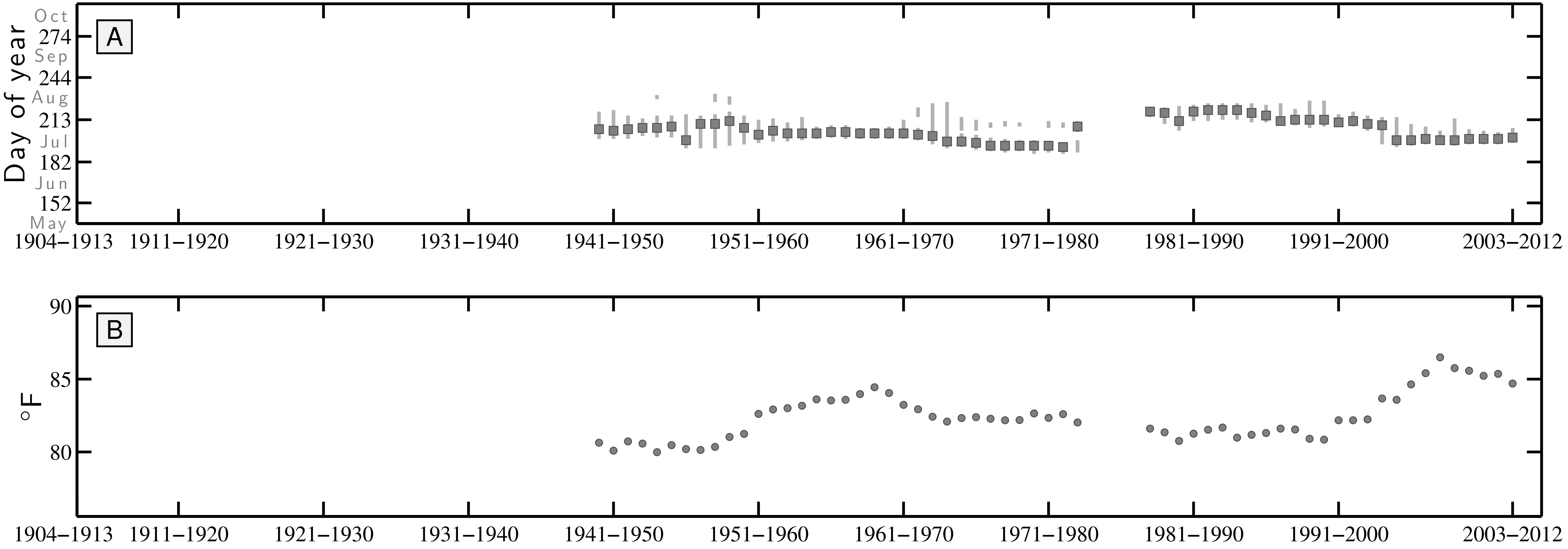

10 year Winter Teletherm dynamics for YELLOWSTONE PK MAMMOTH, WY:

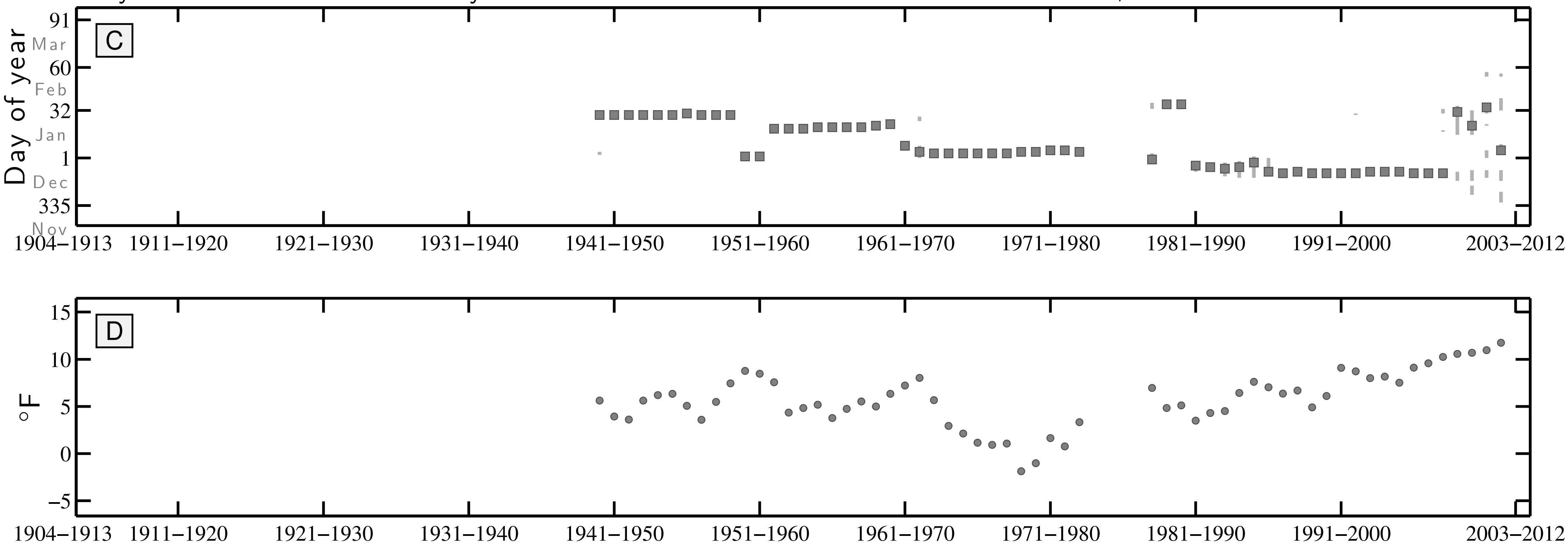

Supplement: S5 File — (PDF) [file pone.0154184.s026.pdf]
